# Quantitative profiling of protease specificity

**DOI:** 10.1371/journal.pcbi.1008101

**Published:** 2021-02-22

**Authors:** Boris I. Ratnikov, Piotr Cieplak, Albert G. Remacle, Elise Nguyen, Jeffrey W. Smith

**Affiliations:** Sanford Burnham Prebys Medical Discovery Institute, La Jolla, California, United States of America; University College London, UNITED KINGDOM

## Abstract

Proteases are an important class of enzymes, whose activity is central to many physiologic and pathologic processes. Detailed knowledge of protease specificity is key to understanding their function. Although many methods have been developed to profile specificities of proteases, few have the diversity and quantitative grasp necessary to fully define specificity of a protease, both in terms of substrate numbers and their catalytic efficiencies. We have developed a concept of “selectome”; the set of substrate amino acid sequences that uniquely represent the specificity of a protease. We applied it to two closely related members of the Matrixin family–MMP-2 and MMP-9 by using substrate phage display coupled with Next Generation Sequencing and information theory-based data analysis. We have also derived a quantitative measure of substrate specificity, which accounts for both the number of substrates and their relative catalytic efficiencies. Using these advances greatly facilitates elucidation of substrate selectivity between closely related members of a protease family. The study also provides insight into the degree to which the catalytic cleft defines substrate recognition, thus providing basis for overcoming two of the major challenges in the field of proteolysis: 1) development of highly selective activity probes for studying proteases with overlapping specificities, and 2) distinguishing targeted proteolysis from bystander proteolytic events.

## Author summary

Proteases and proteolysis are intimately involved in virtually all biological processes from embryonic development to programmed cell death and cellular protein recycling. As the only irreversible posttranslational modification, proteolysis represents a committed step in regulation of biological networks and pathways. Imbalance of proteolytic activity has catastrophic implications and is the basis of many genetic disorders as well as a multitude of pathological states of varying etiologies. To understand protease function, one must gain insight into the repertoires of substrates targeted by these enzymes. As many proteases recognize a wide variety of sequences in proteins, it is a challenge to establish if a particular cleavage represents a targeted or a bystander proteolytic event. In addition, since many proteases have overlapping specificities, especially among closely related members of the same gene families, it is a challenge to develop highly selective tools for studying or inhibition of these enzymes. In this work, we used two closely related proteases (MMP-2 and 9) as a model system for development of an information theory-based approach to quantification of substrate specificity and demonstrated its potential for distinguishing between the target and bystander proteolytic events as well as for uncovering selectivity between closely related proteases.

## Introduction

Proteases are classified according to their catalytic mechanism into serine, threonine, cysteine, aspartic, glutamic and metalloproteinases [1]. Proteases listed in the MEROPS database of proteolytic enzymes are members of 268 gene families and their number is growing as the number of sequenced genomes increases [2]. In humans, 560 unique proteases comprise approximately 3% of the protein-coding genome [3]. Proteases are involved in all aspects of biology from embryonic development to programmed cell death and cellular protein recycling and therefore are an integral part of proteolytic pathways that connect different biological processes into functional networks [3–8]. Protease activity has to be tightly regulated, as deleterious consequences of uncontrolled proteolysis can be devastating [4,9]. Thus, newly synthesized enzymes often require proenzyme activation, and the mature proteases are subject to inhibition by a variety of endogenous inhibitors.

Proteolysis is the only irreversible post-translational modification. A proteolytic cleavage is thus a committed step in the function of networks and pathways. Yet, proteases present unique features/characteristics that have made them difficult to functionally disentangle and reveal their individual roles in biology. These features include: 1) redundancy and overlap in substrate specificity between proteases belonging to the same families [4,10], 2) overlapping specificities of proteases belonging to different families and classes [4,11], 3) difficulty in distinguishing physiologically relevant cleavages from coincidental proteolytic events [3,4], 4) lack of information about selectivity due to insufficiency of the tools currently available for their study [4,12–15].

At the center of proteolysis is the recognition of substrate at the catalytic cleft. In most cases this region is the primary regulatory point for substrate recognition and selectivity. The function and specificity of the catalytic cleft of proteases has been studied with 1) synthetic peptide libraries [16,17], 2) covalent active site probes and suicide substrates [18,19], 3) substrate phage display [10,20] and 4) proteome-derived peptide libraries [21]. With the exception of phage display, these approaches are limited by the diversity of the sequence space covered by the libraries of probes used for substrate identification. Even in the case of phage display the amount of data typically collected falls far short of its true potential because most substrate sequences simply aren’t analyzed. Advances in DNA sequencing technology made it possible to take full advantage of the datasets generated by substrate phage display. Three recent studies have incorporated NGS into substrate phage profiling of the catalytic cleft specificity of proteases [22–24], but approaches for the analysis of these large data sets to gain important mechanistic insight beyond what was possible with a typical substrate phage display experiment are lacking. A quantitative view of protease specificity that incorporates both the sequence space and catalytic efficiency of substrates is required to harness the full power of the data sets afforded by phage display analysis.

Combinatoric analysis of data obtained by NGS of substrate phage selections can be used for quantification of protease specificity and catalytic efficiency. Information on position and stringency of selectivity determinants allows to reveal sequence motifs recognized by the catalytic cleft. The number of unique recognition motifs in substrate selections is a quantitative measure of substrate specificity. The number of unique substrates containing each recognition motif is a quantitative measure of individual motif’s contribution to catalytic efficiency. We used two closely related proteases of the 23-member Matrix Metalloproteinase (MMP) family (MMP-2 and 9) as a model system to demonstrate the utility of this approach for generating quantitative insight into specificity and selectivity in protease families. The S3 and S1՛ binding pockets are the main selectivity determinants in the catalytic cleft of MMPs [25–28]. Together with S2 and S1 between them they form a tetramer binding unit. Therefore, the P3-P1՛ tetramer is the primary substrate recognition motif by MMPs. To quantify the contribution of individual P3-P1՛ sequences to catalytic efficiency of substrates, we used a library of fully randomized hexapeptides (See the Methods section for details) as probes for selection of substrates of MMP-2 and 9. This approach allows for collection of information on the P3 to P1՛ tetramer in the context of adjacent interactions with P5, P4, and P2՛, P3՛. Random sequences are displayed on the PIII gene product of M13 phage with flanking sequences that disrupt secondary structure and provide an N-terminal FLAG tag [29]. The task of identifying the scissile bond with phage substrates is technically challenging, especially when one seeks to characterize millions of substrates. By aligning hexapeptide substrates containing identical tetramer sequences we were able to identify the full scope of P3-P1՛ motifs recognized by MMP-2 and 9 without the need for experimental identification of scissile bonds. The number of unique hexapeptide substrates with identical tetramer sequences is a quantitative measure of contribution of the P3-P1’ recognition motif to catalytic efficiency. The number of P3-P1’ sequences recognized by MMP-2 and 9 is a quantitative measure of specificity of MMP-2 and 9.

Enzyme specificity (defined as k_cat_/K_M_ for a given substrate relative to all others [30,31]), is an elusive concept when applied to proteases, as more than one substrate can often have similar k_cat_/K_M_ for a given protease and more than one protease can have similar k_cat_/K_M_ values for the same substrates. As a result of this basic uncertainty, proteases are difficult to study [4]. Therefore, when describing specificity of proteases, it is useful to introduce a concept of “selectome”, which implies a multiplicity of substrates selective for a given protease. The selectome of a protease can be conceptually defined as a set of amino acid sequences of the length determined by the number and positions of selectivity determinants in its catalytic cleft, that only as a whole, is unique to that protease and thereby represents its proteolytic signature. For MMPs, the selectome is defined as a set of unique tetramers recognized by the S3-S1՛ sites in the catalytic cleft and therefore overrepresented in the substrate sets relative to the library of probes used for their selection. Since we used randomized hexapeptides as probes for substrate selections, the selectomes of MMP-2 and 9 were determined using Kullback-Leibler divergence between frequency distributions of the hexapeptide sequences containing identical tetramers in the substrate and the random hexamer sets.

Combining Next Generation Sequencing (NGS) of substrate phage DNA with information theory-based data analysis allowed us to define the selectomes of MMP-2 and 9. Analysis of the overlap and distinction between selectomes of MMP-2 and 9 shows the structural basis for selectivity and for redundancy in substrate recognition between these closely related enzymes. In addition, detailed specificity profiles obtained using this approach closely represent cleavage profiles derived from protein substrates using N-terminomics and other experimental approaches. Based on the results of these analyses, we conclude that S3-S1՛ catalytic cleft specificity is the main driver of physiologic substrate recognition by MMP-2 and 9 and that other features such as exosites or auxiliary domains are modifiers of specificity. Thus, using MMP-2 and 9 as a model system, we show that quantitative analysis of specificity can be used for solving two major problems in protease research: 1) distinguishing between specificities of closely related proteases and 2) distinguishing between targeted and bystander proteolytic events in protein substrates.

## Results

### Basis for quantitative approach to defining substrate specificity using phage display analysis

#### Combinatorics of substrate recognition by MMPs provides basis for quantification of specificity and catalytic efficiency

**Fig 1:**
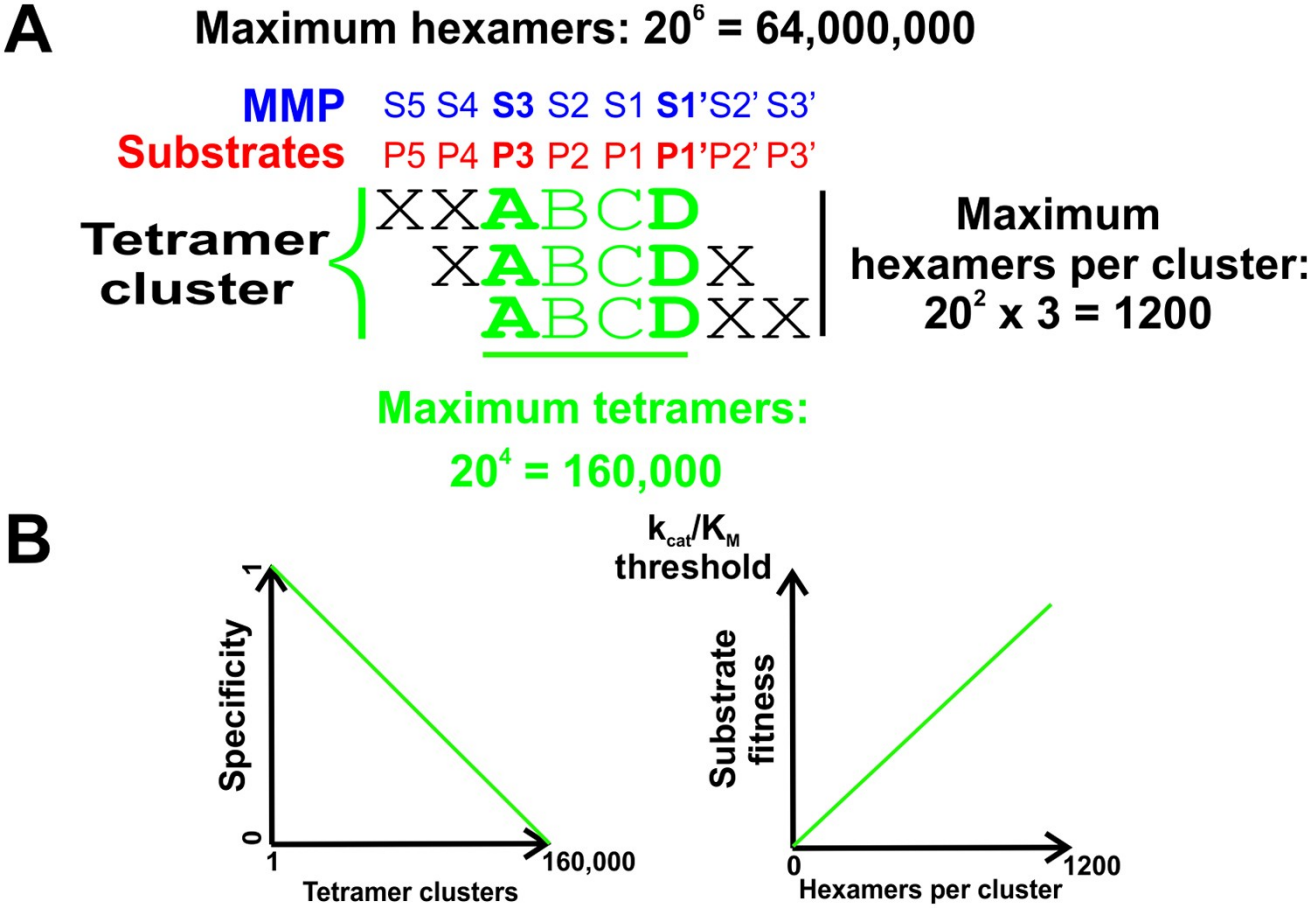
Basis for quantitative specificity profiling of the MMP catalytic cleft across S3 –S1՛. **A**. *Combinatorics of MMP***–***substrate interactions define the limits of substrate specificity and substrate fitness in experiments using hexapeptide probes*. S3 and S1՛ are the most selective binding sites in the catalytic cleft of the MMPs (bold blue lettering). Together with the S2 and S1, they interact with P3-P1՛ tetramers in substrates (red lettering). To interrogate specificity of MMP-2 and 9, we used a library of randomized hexapeptides displayed on PIII gene product of M13 phage. The theoretical maximum of hexamer combinations is 64,000,000. The theoretical maximum for the number of hexapeptides containing identical tetramers (tetramer cluster) is 1200. There are 160,000 combinations of natural amino acid residues in random tetramers. **B**. *Results of phage display analysis can be interpreted to quantify MMP specificity as well as the fitness of individual P3-P1*՛ *substrates*. The number of tetramer clusters defines the amount of specificity of proteases recognizing P3-P1՛ positions in substrates, which ranges from absolutely specific (1 tetramer cluster) to absolutely non-specific (160,000 tetramer clusters). The number of hexamers per tetramer cluster is a measure of substrate fitness of all hexamers comprising it up to the k_cat_/K_M_ threshold defined by experimental conditions.

We used MMP-2 and 9, two closely related members of the MMP family as a model system for developing a quantitative approach to defining specificity and selectivity in protease families. As probes for interrogating enzyme-substrate interactions, we used a library of fully randomized hexapeptides displayed on the PIII gene product of M13 phage. Since S3 and S1՛ are the main selectivity determinants in the catalytic cleft of MMPs, which together with S2 and S1 form a tetramer binding unit, the P3-P1՛ tetramer is the primary substrate recognition motif of these enzymes (Fig 1A). We used clustering of unique hexamer sequences containing identical tetramers (See the Methods section for details) to reveal the P3-P1՛ positions in substrates, which eliminated the need for experimental identification of scissile bonds. The number of hexamer sequences in a tetramer cluster that can range between 1 and 1200, is a quantitative measure of the contribution of a P3-P1՛ tetramer to catalytic efficiency of substrates containing it, which we called “substrate fitness” (Fig 1B). The number of unique tetramer clusters, which can range from 1 for a perfectly specific to 160,000 for a non-specific protease, is a quantitative measure of specificity.

If every possible hexamer peptide containing a given tetramer sequence can be found in the substrate set, then that tetramer cluster has a substrate fitness and correspondingly an average k_cat_/K_M_ value at or above the upper threshold determined by the conditions of the experiment (Fig 1B, see Methods for details) beyond which the number of hexamers per tetramer cluster will not increase. Conversely, tetramer clusters with fewer than maximum number of hexamers must have lower than maximum substrate fitness and lower k_cat_/K_M_. **The k**_**cat**_**/K**_**M**_ **of a tetramer cluster is defined as the average value of k**_**cat**_**/K**_**M**_ **over all hexamer substrates in it**. The k_cat_/K_M_ threshold value is an important parameter for comparing fitness levels of substrates of a given protease as well as between proteases. Choice of the k_cat_/K_M_ threshold could be tricky, as ideally, it requires to have an estimate of the range of specificity constants for the protease being studied. We based our choice on the data previously published for the substrate phage display system used in this study [10,27,29]. It should be noted that quantification of the contribution of a P3-P1՛ sequence to catalytic efficiency of MMP substrates containing it, is made possible by using probes with broader sequence coverage than the core recognition motif. Randomized hexamers provide 1200 unique contexts to the P3-P1՛ tetramers in substrates. Using just randomized tetrapeptides would eliminate that possibility entirely.

#### The ratio between probabilities of finding a P3-P1՛ tetramer sequence in the substrate and the random hexamer probe sets is a measure of its contribution to catalytic efficiency

To characterize the tetramer clusters in substrate sets in terms of contribution of the P3-P1՛ sequences to catalytic efficiency of substrates, defined by us as **substrate fitness**, we introduced the ratio between probabilities (Relative Probability or RP, see the Methods section for formal definition) of finding identical tetramers in the MMP selections and the naïve phage display library. The use of RP eliminates potential biases due to deviation from uniformity of the tetramer probability distribution in the naïve library and potential differences in sequencing depth relative to the substrate sets, which makes the direct use of the number of hexamers per tetramer cluster problematic. Importantly, RP must correlate with substrate fitness across its range of values for a given protease. To validate this assumption, we used the data obtained for a published set of 1369 phage substrates with experimentally determined scissile bonds and K_(obs)_ values (S6 Table) [10]. In order to establish if P3-P1՛ positions defined by tetramer clustering match those obtained experimentally, we performed a standard statistical binary classification test (S7 Table, see Methods for details) to determine if RP is a good predictor of a phage displayed hexamer peptide being a substrate. In this set, of all substrates containing non-redundant tetramers only 1.2% and 1.9% had no matching tetramer clusters in the MMP-2 and MMP-9 substrate selections, respectively. This observation confirms the accuracy of the P3-P1՛ assignments in tetramer clusters of the substrate selections.

Next, we performed an analysis of correlation between RP of tetramer clusters and K_(obs)_ of the hexamer substrates containing the matching P3-P1՛ tetramers. We started our analysis with plotting the plain correlation between K_(obs)_ values and the corresponding RP values for each individual tetramer cluster (S6 Table). The corresponding Pearson correlation coefficient R values for the raw data are 0.66 and 0.76 for MMP-2 and MMP-9, respectively, which already indicates well correlated data. When looking at the plots (S6 Table, last plots in the last two tabs for MMP2 and MMP9, respectively), one can see that for every range of the RP values, K_(obs)_ exhibit substantially spread-out range of values. We attributed this to the unpredictable contribution of residues located at P5-P4 and P2՛-P3՛ positions of the hexamers to catalytic efficiency and to the lack of much larger kinetic data for hexamers needed to fully characterize each tetramer cluster. Unfortunately, it is impractical and impossible to collect all needed data for all hexamers (1,200 hexamers x 160,000 tetramers = 192,000,000 individual measurements) from a phage display experiment or any other approach available today. In order to deal with observed spread of K_(obs)_ values in the absence of sufficient data, we approximated the averaged values of K_(obs)_ using groups of tetramers clusters generated according to their RP values. Since tetramer clusters were ordered according to RP values, we grouped together the neighboring tetramer clusters in evenly distributed ranges (bins). Those neighboring tetramer sequences should have similar contributions to catalytic efficiency of hexamer substrates containing them, as measured by RP. After the binning, the correlation coefficient increased by 20–30% relative to the no binning situation. Application of different bins sizes does not change much of the outcome, based on the resultant R values. The binning reduces noise appreciably. Observed increase of correlation coefficient is a result of smoothing of the data associated with averaging out of the influence of residues located at P5-P4 and P2՛-P3՛ positions of hexamer substrates, and thus reflects the expected trend toward the situation when all kinetic data are known. If there was no correlation between RP and the contribution of P3-P1՛ motifs to catalytic efficiency of substrates containing them, then the correlation coefficient would not improve upon the binning.

**Fig 2:**
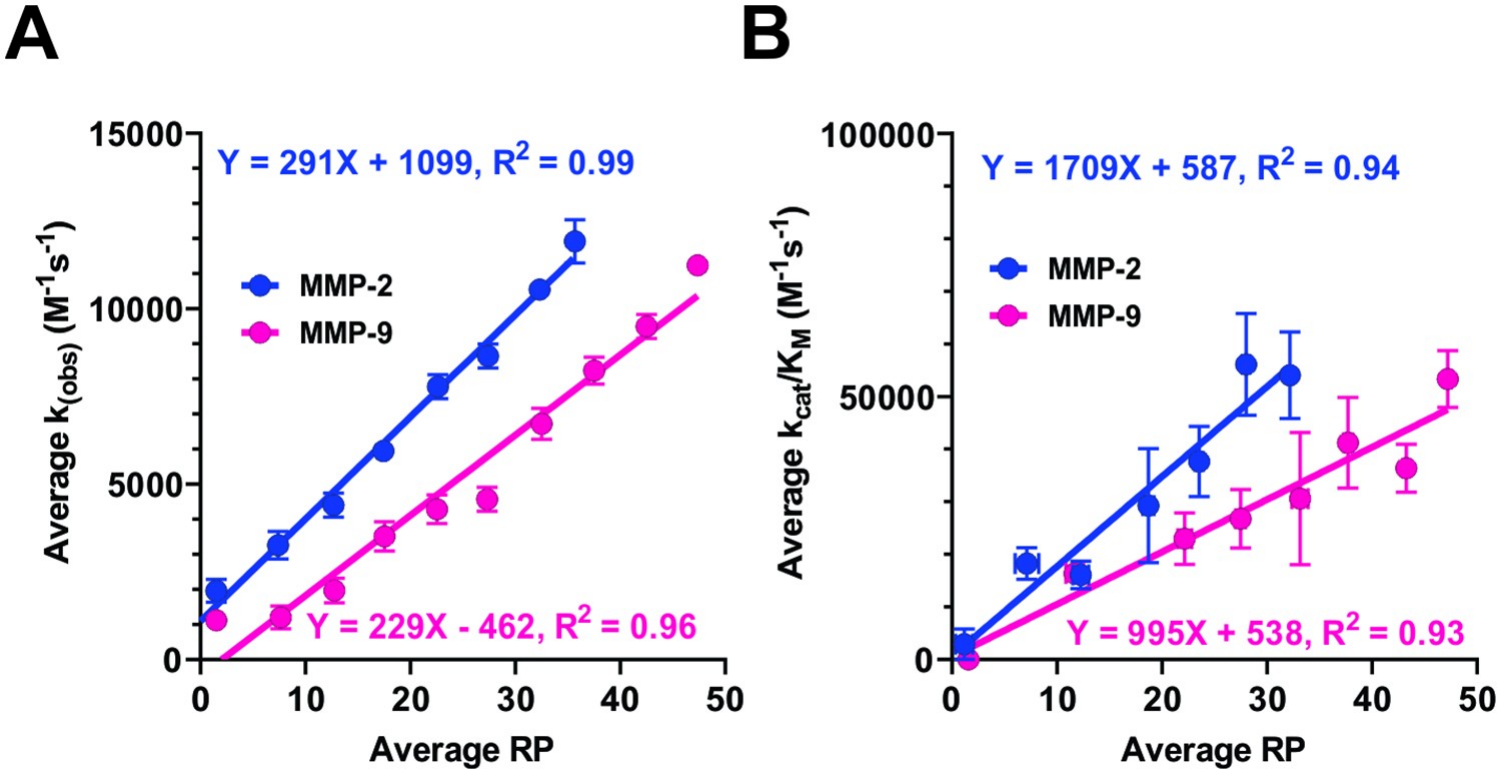
Probability of finding a tetramer cluster in substrate selections relative to the naïve library (RP) correlates with substrate fitness. K_(obs)_ values for 1369 individual phage substrates or k_cat_/K_M_ values of 100 peptides derived from substrate phage sequences were experimentally determined as described in the text. The substrates were binned into evenly distributed groups based on the RP values of the tetramer clusters corresponding to their P3-P1՛ positions in substrates. The average K_(obs)_ (**A**) or k_cat_/K_M_ (**B**) values for each bin were plotted as a function of the corresponding average RP values and the data were subjected to linear regression analysis. The equations and goodness of fit parameters (R^2^) of the linear regression analyses of the MMP-2 and 9 data are shown at the top and bottom of the graph, respectively. These results can be compared to raw, unbinned data presented in the last graphs of the S6 Table, in the corresponding MMP specific tabs. The corresponding Pearson correlation coefficient R values for the raw (unbinned) data are 0.66 and 0.76 for MMP-2 and MMP-9, respectively, indicating already well correlated data.

As expected, **averages** of the catalytic efficiency constants of hexamer substrates correlate with RP better than the individual values because although the P3-P1՛ tetramer is the principal contributor to catalytic efficiency of individual substrates, it does not control it fully (See the Methods section for details and S6 Table). As shown in Fig 2A, linear regression analysis demonstrates that average RP significantly correlates with average K_(obs)_ for phage substrates of both MMP-2 and 9. K_(obs)_ obtained for each of the 1369 substrates has a range of 0 and 12,792 (M^-1^ s^-1^) due to the experimental conditions used in the study [10]. So, all substrates with the true K_(obs)_ above this value will nevertheless have a K_(obs)_ equal to the preset maximum. With this limitation in mind, we corroborated the results using synthetic peptides, thereby extending the correlation to the entire range of k_cat_/K_M_ values for each MMP. We performed a correlation analysis using a set of 100 peptides with sequences derived from substrate phage selections and their k_cat_/K_M_ values were experimentally determined as described in [27] (S8 Table). The results are presented in Fig 2B. The analyses clearly demonstrate that RP is a quantitative measure of contribution of the P3-P1՛ tetramer sequences to catalytic efficiency of hexamer substrates containing them.

#### Tetramer clusters overrepresented in substrate selections relative to the naïve phage display library define specificity of the catalytic cleft of MMP-2 and 9

**Fig 3:**
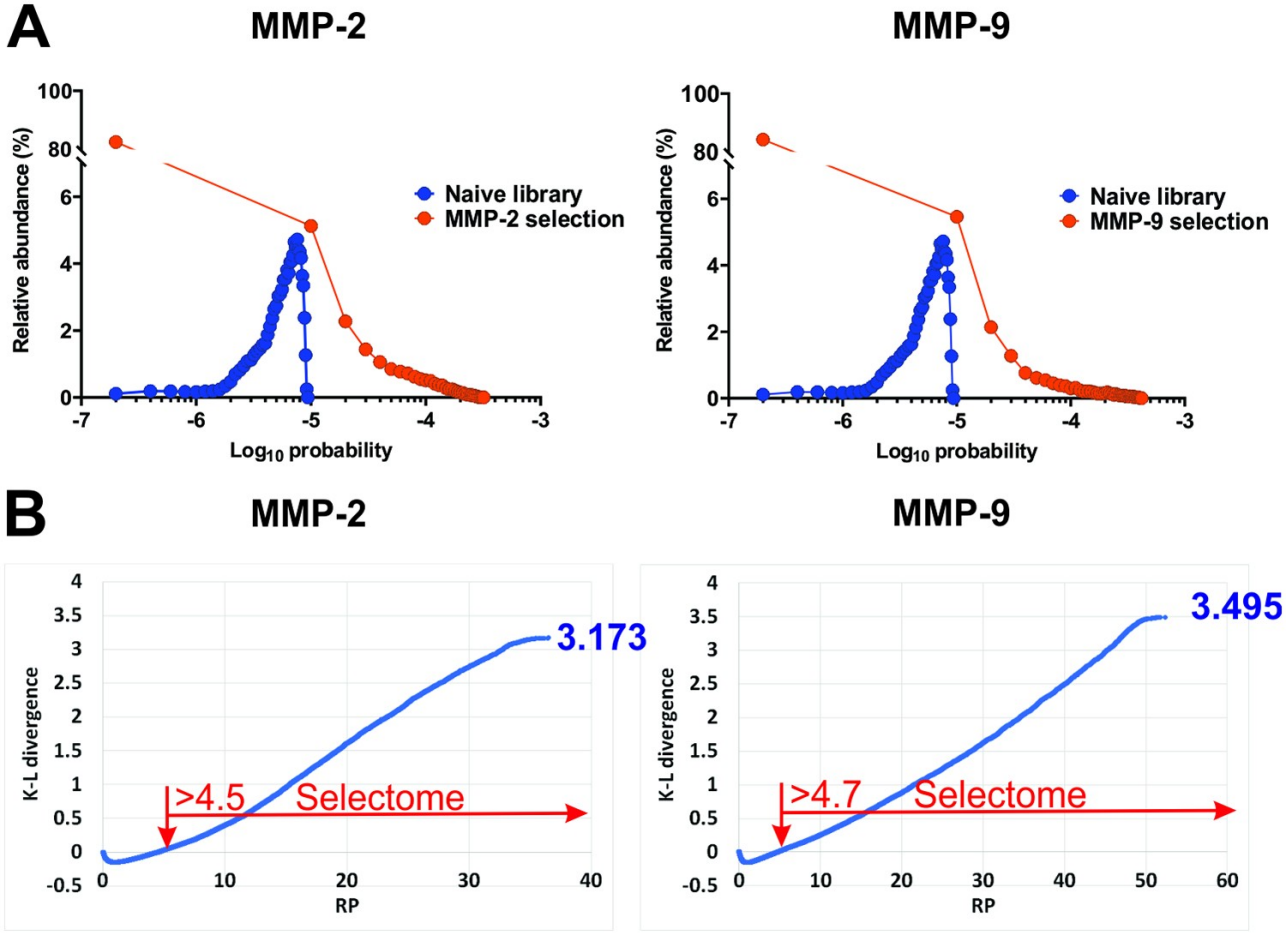
Divergence between probability distributions of tetramer clusters in substrate selections and the naïve library is a measure of substrate specificity. **A**. *Distributions of relative abundances of tetramer clusters in the MMP substrate selections are significantly different from that in the naïve phage display library*. Tetramer clusters within evenly spaced ranges of probabilities were binned together and their relative abundances were plotted as a function of log_10_ of average probabilities in the respective sets. **B**. *A subset of tetramer clusters in substrate selections with positive cumulative contribution to K-L divergence relative to the naïve library constitutes the selectome of a protease*. Cumulative contribution of individual tetramer clusters with RP values between of 0 and 4.5 for MMP-2 and 0 and 4.7 for MMP-9 to the K-L divergence relative to the naïve library is equal to 0. The K-L divergence between the probability distributions in the substrate sets of a protease with no definable specificity and the naïve library is always equal to 0. Therefore, the substrate sets with overall positive cumulative individual contributions to K-L divergences constitute the selectomes of proteases with definable specificities (MMP-2 and 9, red arrows).

We compared the distributions of relative abundances of tetramer clusters in the naïve phage display library and the substrate sets of MMP-2 and 9. As can be seen in Fig 3A, they have changed significantly following substrate selections. To quantify the degree of the observed change, we calculated Shannon entropy values for each of the distributions. The tetramer cluster distribution in the naïve phage display library has a Shannon entropy value of 17.218 (S4 and S5 Tables, see the Methods section for details of calculations), which is similar to that of a uniform distribution equal to 17.288 (log_2_ 160,000). This is an important characteristic of the library we used for substrate selections that gives an idea of its diversity relative to the maximum. Shannon entropy values of the distributions in substrate sets are 13.93 and 13.67 for MMP-2 and 9, respectively (S4 and S5 Tables), which, as expected, are significantly lower than that of the naïve library.

Substrate selections contain close to a half of the theoretically possible tetramer clusters, most of which have RP values below 1. The RP values in substrate selections of MMP-2 and 9 rise continuously over the entire range without reaching a plateau (S2 and S3 Tables). Therefore, these probability distributions must be representative of the entire ranges of the respective catalytic cleft specificities. Since the majority of the tetramer clusters in the substrate sets of MMP-2 and 9 have probabilities lower than in the naïve library and constitute rare events, they must be relatively poor substrates and therefore contribute little if at all to the specificity of the two enzymes. To select the tetramer clusters with statistically significant contribution to specificity of the catalytic cleft, we used Kullback-Leibler (K-L) divergence [32] as a measure of distinction between the probability distributions of tetramer clusters in substrate selections and the naïve phage display library. The K-L divergence, or relative entropy determines how one probability distribution is different from another, reference distribution. The K-L divergence values for a protease recognizing P3-P1՛ positions in substrates, can range from 0 for a protease with no specificity to 17.288 for a perfectly specific protease with a single tetramer substrate assuming the uniform probability distribution for the reference set. We performed K-L divergence analysis using probability distributions of tetramer clusters in the MMP selections as the test and those in the naïve library as the reference sets, respectively (See the Methods section for details). The relative entropies are 3.173 and 3.495 (S4 and S5 Tables) for MMP-2 and 9 tetramer clusters, respectively, indicating that MMP-9 has a narrower specificity than MMP-2, although not by much. The total number of tetramer clusters with non-zero probabilities and thus non-zero contributions to the values of K-L divergence, is 78,757 and 76,696 for MMP-2 and 9, respectively. Plots of the sum of individual components in the calculations of the expected value using Eq 4 (See the Methods section) as a function of RP for MMP-2 and 9 have two distinct parts: one below and the other above the zero value of K-L divergence (Fig 3B). While the former has no net contribution to the K-L divergence, the latter contributes to it entirely. The RP value at the intersection of the line in the graph with the X-axis is a useful threshold for defining the set of tetramer clusters overrepresented in substrate selections and, only as a whole, unique to a given protease, thereby constituting its “**selectome**”. These values are 4.5 and 4.7 for MMP-2 and 9, respectively (indicated by red arrows in Fig 3B). There are 7,921 and 6,094 tetramers above the RP threshold, belonging to the MMP-2 and 9 selectomes, respectively (S4 and S5 Tables). They constitute 8–10% of all tetramers with non-zero value of RP.

To corroborate the findings of the K-L divergence analysis, we looked at the distributions of tetramer clusters across the RP range in 10% increments from highest to lowest (S1A Fig). The number of tetramer clusters across the RP range shows a slow increase until it reaches the lowest 10%, where it increases dramatically. S1B Fig shows the distribution of the numbers of hexamers per tetramer cluster across the same intervals. Not surprisingly, the lowest 10% have a precipitous decline in that metric compared to the nearest neighbor. Thus, this analysis of tetramer cluster distributions agrees with the relative entropy-based analysis, showing that the top 10% of tetramer clusters are populated the highest, which is consistent with percentages of tetramer clusters in the selectomes of MMP-2 and 9. To put these data in perspective, one must keep in mind that the tetramer clusters with positive cumulative contribution to K-L divergence (the selectome) in the set of MMP-2 substrates contain 2.31 x 10^6^ hexamers substrates, while those with zero cumulative contribution to K-L divergence, (RP interval between 0 and 4.5)—only 0.56 x 10^6^. The same numbers for MMP-9 are 1.64 x 10^6^ and 0.54 x 10^6^, respectively. So, 80% of hexamer substrates of MMP-2 and 75% of MMP-9 belong to their respective selectomes. This observation provides basis for the conclusion that catalytic cleft specificity of MMP-2 and 9 is primarily defined by S3-S1՛ subsites, as expected. The poorly populated tetramer clusters are represented by sequences that, as P3-P1՛ tetramers, contribute little to the fitness of substrates, which may be modulated by exosites outside S3-S1՛ and are found in the minority (20–25%) of the hexamer substrates.

In this section of the Results we developed the concept of “selectome”, which though intuitive, needs to be defined quantitatively. To the best of our knowledge, there have been no prior reports of an approach aimed at defining the full set of substrates that indicate the substrate specificity of a protease. In the following sections, we will substantiate this concept by applying it to analyses of selectivity between MMP-2 and 9 and contribution of the catalytic cleft specificity to protein substrate recognition.

### Analysis of substrate specificity and selectivity of MMP-2 and 9

#### Substrate specificity in the selectome

**Fig 4:**
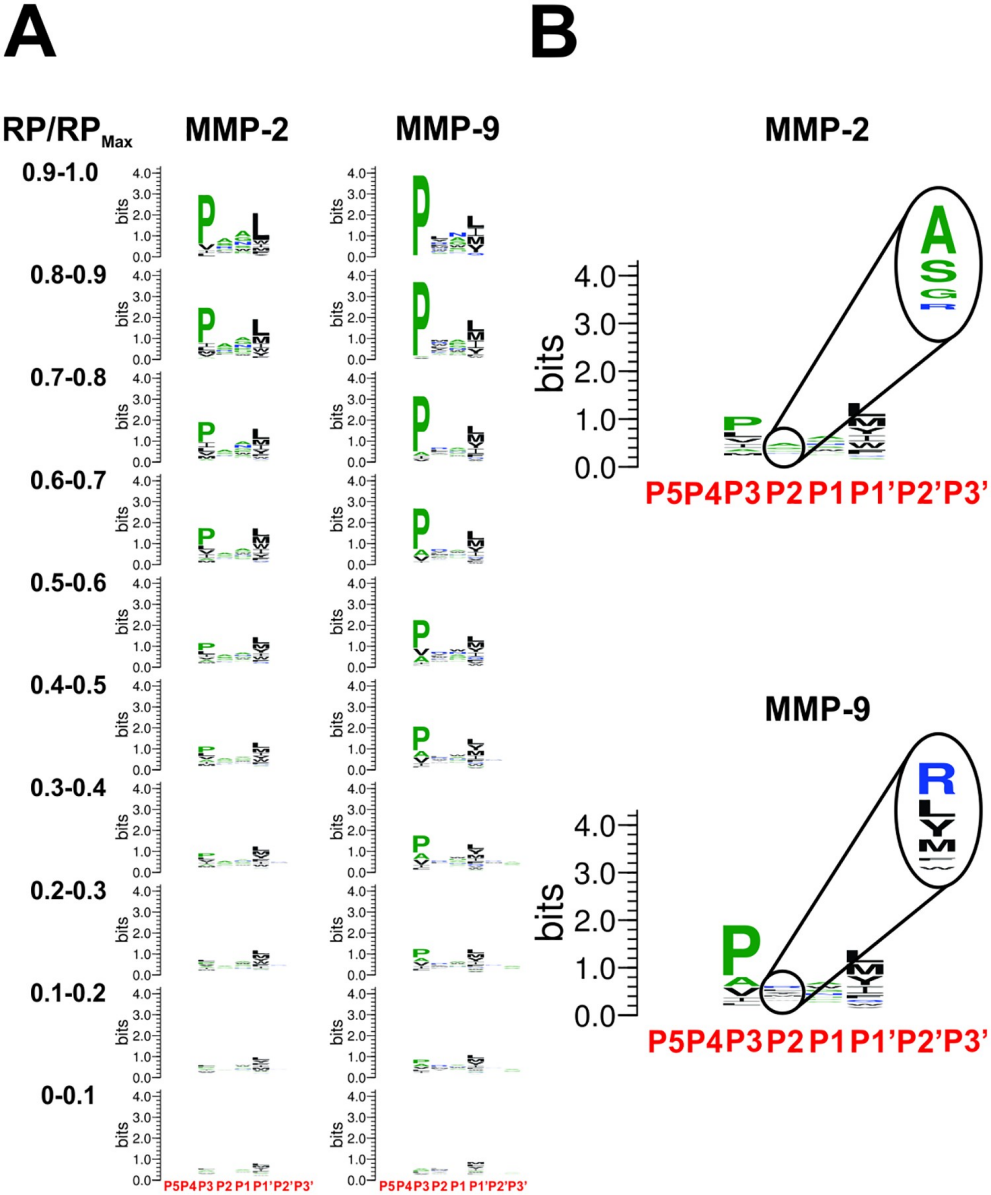
Composition of the selectomes of MMP-2 and 9 reflect differences in their specificities. **A**. *Selectome-based specificity profiles of MMP-2 and 9 reflect changes in substrate composition as a function of substrate fitness*. Peptide hexamers in the selectomes of MMP-2 and 9 were aligned along the P5-P3՛ positions based on P3-P1՛ matches in the corresponding tetramer clusters and divided into 10 groups according to their RP values relative to the maximum (RP/RP_Max_). Relative abundances of amino acid residues at each position were calculated and presented in the form of a logo plot for each of the groups. **B**. *Aggregate specificity profiles of MMP-2 and 9 reveal the major selectivity features of MMP-2 and 9*. Hexamers belonging to the tetramer clusters constituting the selectomes of MMP-2 and 9 were aligned along the P5-P3՛ positions based on P3-P1՛ matches in the corresponding tetramer clusters, and relative abundances of residues at each position were plotted in the form of a logo. Zoom-in ovals show the relative contributions of residues to the P2 specificity profile of each enzyme.

To analyze the composition of substrates in the selectomes of MMP-2 and 9, hexamer sequences were aligned along the P3-P1՛ tetramers and sequence logos were generated based on frequency of occurrence of residues at individual positions across P5-P3՛ interval using WebLogo [33]. Compositions of the selectomes of MMP-2 and 9 are shown in Fig 4A and 4B. Fig 4A shows the composition of substrates across the RP/RP_Max_ range for MMP-2 and 9. Consistent with the roles of S3 and S1՛ as primary selectivity determinants in the catalytic clefts of MMPs, P3 and P1՛ positions in substrates contribute the most to substrate specificities of both MMP-2 and 9. Logo plots of distributions of residues along the P5 -P3՛ (Fig 4A) interval show dominance of the P3 position in tetrameric clusters with highest relative probabilities, which diminishes together with RP. Relative contribution of the P1՛ position to the information content of the logo plots grows as relative probability decreases. These findings suggest that in general, the P1՛ position of MMP substrates determines whether a particular sequence will be cleaved and the P3 position primarily determines the substrate fitness of a given tetramer sequence. Patterns of amino acid distribution across P3-P1՛ positions of MMP-2 and 9 selectomes (Fig 4B) are similar in general to those published elsewhere for substrates of MMP-2 and 9 [10,26,34]. Data in Fig 4B show a clear distinction between the aggregate specificity profiles of MMP-2 and 9 at the P2 position of substrates. The P2 repertoire of MMP-9 is very different from MMP-2, with significant contributions of residues with aliphatic (Leu and Met) and aromatic (Phe, Tyr, Trp) side chains compared to MMP-2’s Ala, Ser and Gly. There is structural basis for S2 selectivity between MMP-2 and 9 which has already been reported in [35] and will be discussed further in the text.

In this section, we have derived the substrate recognition motifs of the selectomes of MMP-2 and 9. We also showed how substrate sequences change across the range of fitness, providing valuable insight into the correlation between catalytic efficiency and subsite specificity.

#### Comparative analysis of selectomes reveals distinctions between selectivity determinants of MMP-2 and 9

**Fig 5:**
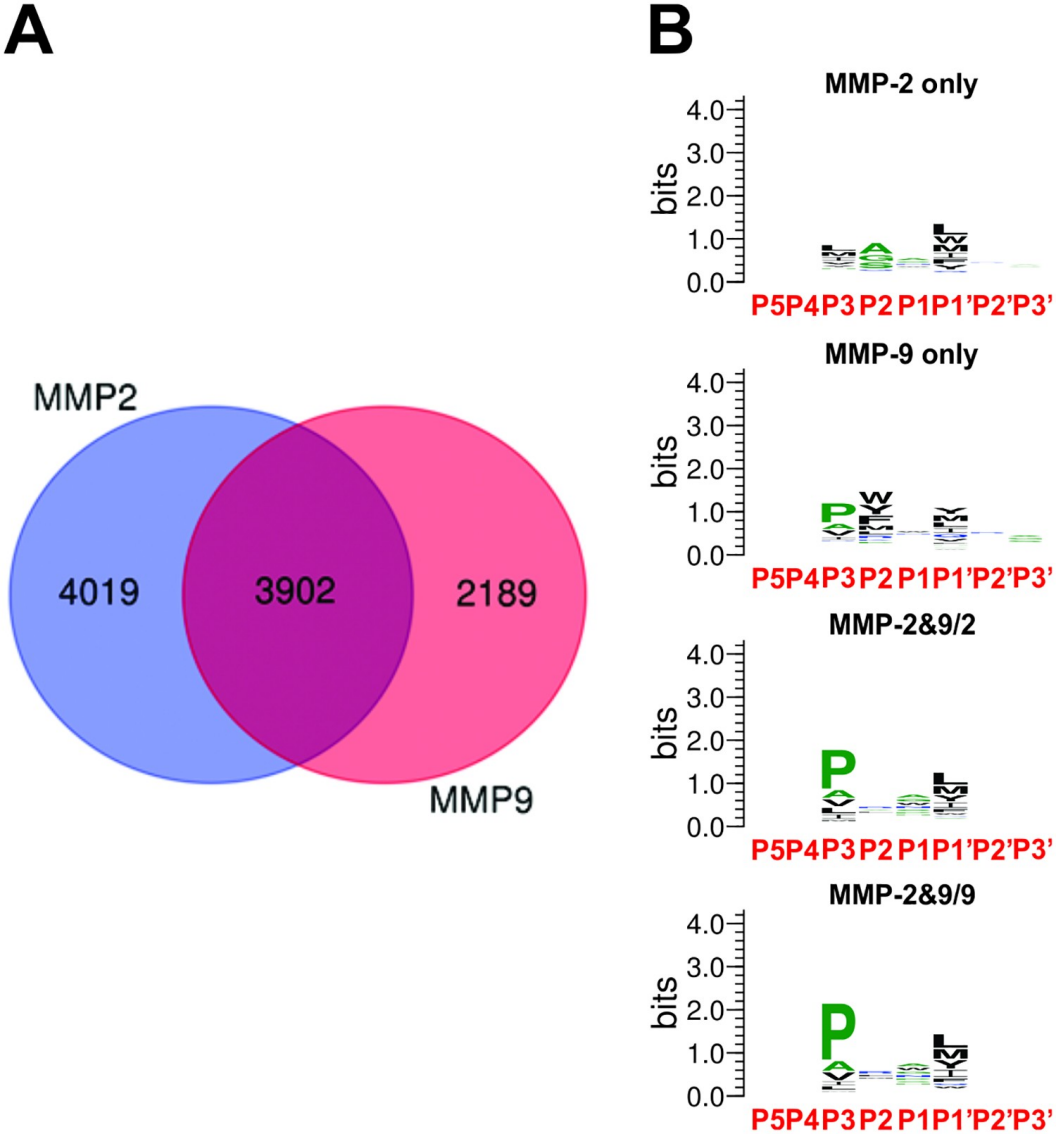
Comparative analysis of selectomes reveals divergent and conserved features in substrate recognition between MMP-2 and 9. **A**. *Venn diagram of substrate specificity overlap and distinction between the selectomes of MMP-2 and 9 shows significant selectivity between the two enzymes*. Tetramer clusters comprising the selectomes of MMP-2 and 9 were grouped based on their occurrence in the individual and overlapping substrate sets and the corresponding numbers are shown in the Venn diagram. For making Venn diagram we used on-line service at: http://bioinformatics.psb.ugent.be/webtools/Venn/. **B**. *Aggregate specificity profiles based on the unique and overlapping tetramer clusters of MMP-2 and 9 reveal the distribution of selectivity across the catalytic cleft*. Hexamer peptides belonging to the tetramer clusters constituting the selective and common substrate sets of MMP-2 and 9 were aligned along P5-P3՛ positions based on the P3-P1՛ matches and the relative abundances of residues at each position were plotted as logos. MMP-2&9/2 denotes residue frequencies based on MMP-2 RP values, while MMP-2&9/9 denotes residue frequencies based on MMP-9 RP values.

One of the central obstacles to understanding protease biology is functional redundancy and specificity overlap between proteases from the same phylogenetic groups [4]. Selectome profiling presented in this study makes it possible to determine how much overlap and distinction there is between specificities of closely related proteases. Catalytic domains of human MMP-2 and 9 are 73% identical and 81% similar in their amino acid sequences. Direct comparison reveals that out of the total of 10,110 tetramers comprising the combined selectomes, 3,902 are shared by both, and 4,019 and 2,189 are found exclusively in the respective selectomes of MMP-2 and 9 (Fig 5A and S9–S12 Tables). Thus, a pair of 73% identical proteases has only 39% of the combined selectomes in common, demonstrating a significant amount of S3-S1՛ distinction between the two MMPs. MMP-2 has the broader specificity of the pair with 40% unique tetramers, while MMP-9 has only 22%, almost two-fold less than its closest relative in the MMP family.

Logo profiles based on the selective tetramer clusters (S2 Fig), which were segregated according to the Venn diagram (Fig 5A) show consistent patterns across the RP range with decreasing information content as the RP value goes down, as expected. The logos representing all substrates from the selective tetramer clusters (Fig 5B) show the aggregate picture across the entire range of RP. Both MMP-2 and 9 display selectivity at S3, S2 and S1՛ subsites of the catalytic cleft with similar contributions from each. Very different, however, are the repertoires and relative abundances of residues in substrates at the positions reflecting selectivity of each of the subsites.

**Fig 6:**
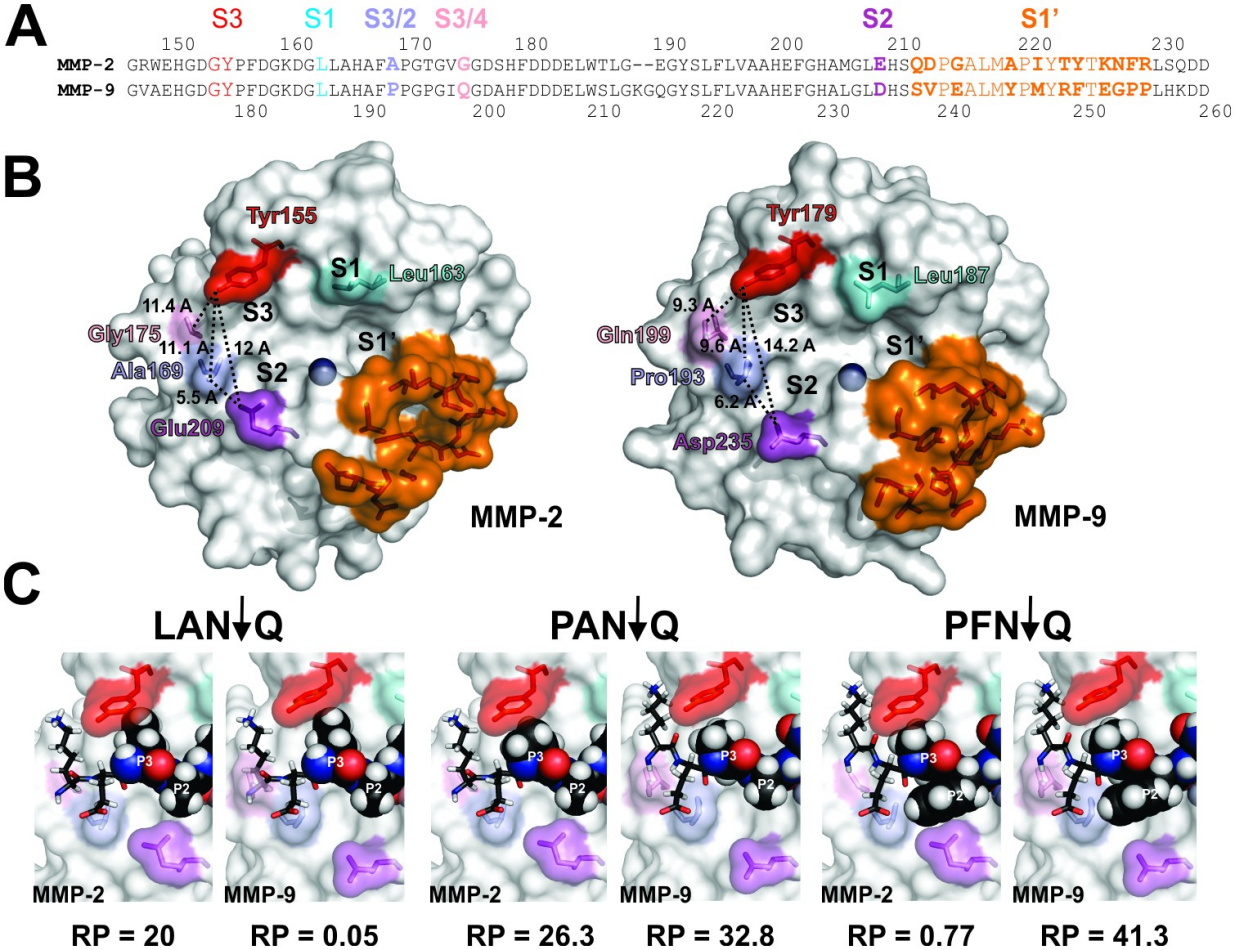
Changes in composition of selective substrates correlate with changes in selectivity determinants between MMP-2 and 9. **A**. *Distribution of the selectivity determinants across the subsites of the catalytic clefts of MMP-2 and 9*. Sequences of the catalytic domains of MMP-2 and 9 were aligned based on the crystal structures of the catalytic domains of the respective enzymes (PDB IDs: 1QID for MMP2, 1GKC for MMP9). Residue numberings from the native N-termini are shown above (MMP-2) or below (MMP-9) the sequence. Selectivity Determining Positions (SDPs) are shown in color and marked in larger font. The catalytic cleft subsites they contribute to are shown directly above each SDP. Residues marked in bold differ between the two proteases (see text for details). **B**. *Structural features of the SDPs in the catalytic clefts of MMP-2 and 9 provide basis for experimentally determined subsite selectivity*. Residues contributing to the SDPs at S3, S2, S1 and S1՛ binding pockets are shown on the surface representations of the three-dimensional structures of MMP-2 and 9 in colors matching the sequence alignments in A. See text for more details. PyMOL molecular visualization system was used for display and analysis of 3D structures. **C**. *Single substitutions in substrate tetramers illustrate distinctions in substrate recognition by MMP-2 and 9*. Substrates selective for MMP-2 (KELAN↓Q), MMP-9 (KEPFN↓Q) and in common between the two enzymes (KEPAN↓Q) were docked into the catalytic clefts of MMP-2 and MMP-9 (See the Methods section for details). The P3-P1՛ sequences are shown above the structures. The docked residues below corresponding to the P3 and P2 residues in tetramers are shown as spheres, while the rest of the sequence is shown as sticks. Positions of residues relative to the scissile bond in the docked peptides are marked by white lettering. The RP values for the corresponding P3-P1՛ tetramer clusters are shown directly below the structures of each complex.

Previously, we have mapped the selectivity determining positions (SDPs) in the catalytic cleft of the MMP family [10]. By comparing compositions of the subsites contributing to selectivity between MMP-2 and 9, one can account for distinctions observed between the unique substrate sets (Fig 6). SDPs at S4/S3 junction (Gly175 in MMP-2 and Gln199 in MMP-9) and S2/S3 junction (Ala169 in MMP-2 and Pro193 in MMP-9) control the height of the catalytic groove at the S3 binding pocket together with the conserved S3 Tyr155/179 in MMP-2 and MMP-9, respectively (Fig 6A and 6B). Distances between residues determining the height of the catalytic cleft in MMP-2 are 11.4 Å (between Tyr155 and Gly175) and 11.1 Å (between Tyr155 and Ala179). In MMP-9 the space between the corresponding residues is narrower (9.3 Å between Tyr179 and Gln199) and 9.6 Å (between Tyr179 and Pro193), which is consistent with the presence of the large aliphatic Leu, Met and Ile in the P3 positions of the MMP-2 selective substrates and their virtual absence in the MMP-9 ones (Figs 5C and S2). More compact Pro and to a lesser extent Ala and Val at the P3 of the substrates are accepted by MMP-9. To illustrate how the SDP features shown in Fig 6B contribute to selectivity with specific examples, we docked peptide substrates selective for one MMP over the other as well as those shared by both in the respective catalytic clefts (Fig 6C) and showed how single substitutions affect RP. P3 Leu is favored by MMP-2 (Fig 5C), resulting in a higher RP value over that of MMP-9, which is virtually incapable of cleaving the docked peptide (LAN↓Q). Mutating P3 Leu to Pro changes the situation dramatically for MMP-9 leading to a ~660-fold increase in RP, while increasing that of MMP-2 just by 30%.

Another notable difference between specificities of MMP-2 and 9 is evident from the repertoire of residues at the P2 position of selective substrates (Figs 5C and S2). Dominance of Ala, Gly and Ser at the P2 of substrates selective for MMP-2 is contrasted by the preponderance of bulky aromatic and to some extent aliphatic side chain residues of the MMP-9 selective substrates (Figs 5C and S2). This observation is consistent with the differences in composition of the S2 binding pocket sandwiched between the S2/S3 Ala169 and S2 Glu 209 in MMP-2 and S2/S3 Pro193 and S2 Asp 235 in MMP-9 (Fig 6A and 6B). The distance between these residues is 5.5 Å in MMP-2 A vs. 6.2 Å in MMP-9, limiting the space for P2 binding. Additionally, a bulkier Glu209 narrows the catalytic cleft in MMP-2 to 12 Å from 14.2 Å in MMP-9, that has a more compact Asp235 in that position (Fig 6B), which leaves less room for a P2 residue interaction. Correspondingly, mutating P2 Ala to Phe (Fig 6C) in the PAN↓Q non-selective tetramer causes a 34-fold decline in catalytic efficiency of MMP-2 against it but a 26% increase in that of MMP-9.

Quite remarkable is the lack of significant contribution of P1 to selectivity as expected (Figs 5C and S2) based on identical residues at the S1 SDPs of both enzymes (Leu163 in MMP-2 and Leu187 in MMP-9).

Differences in P1՛ composition of the selective tetramers are more difficult to explain structurally due to the complexity of the S1՛ binding site, formed by an allosteric hydrophobic tunnel (Fig 6B) preferentially occupied by Leu, Trp, Met and Ile residues in the selective substrates of MMP-2 (Fig 5C). In MMP-9 substrates, P1՛ Leu, the preferred residue by the S1՛ pocket of the entire MMP family, is virtually absent and becomes noticeable only in the lower (0.2–0.5) RP/RP_Max_ range of the MMP-9 tetramer clusters (S2 Fig). Out of the 18 residues comprising the S1՛ loop, 10 are different between MMP-2 and 9, with 5 non-conserved substitutions. The fact that the selective substrates of both enzymes have significant differences in the repertoires of the P1՛ residues is consistent with significant differences in SDP compositions of the S1՛ binding pocket between the two enzymes. As an example, substitution of the P1՛ Gln to Leu in the PFN↓Q MMP-9 selective tetramer enhances the catalytic efficiency toward MMP-2 40-fold (S4 Table) but leads to just a 16% improvement in that of MMP-9. This observation suggests that the MMP-2 S1՛ interaction with P1՛ L provides support for the P2 Phe fitting in the narrow S2 binding pocket, which is possibly a cooperative interaction between the two binding sites. Selectivity of MMP-9 toward P1՛ Gln can potentially be explained by the presence of the flexible Arg249 at its S1՛ pocket [36], which could support accommodating the polar side chain residues better than the corresponding Thr223 in MMP-2. Leucine is preferred by S1՛ of both MMP-2 and 9 (Fig 4C). Substituting Q for L in the P1՛ of the MMP-2 selective LAN↓Q tetramer improves the RP for MMP-2 by 75% but causes a 240-fold increase for MMP-9 (S4 and S5 Tables). Again, we see potential subsite cooperativity, this time between the S3 and S1՛ improving the chances of the P3 Leu to be bound by the narrow S3 pocket of MMP-9. Even though there was a dramatic increase in RP of MMP-9 upon mutation P1՛ Gln to Leu (LAN↓Q, RP = 0.05, LAN↓L, RP = 11.54), preference for P3 Pro over Leu is still noticeable when comparing to PAN↓L (RP = 47.2).

In this section, for the first time, we provided a quantitative measure of overlap and distinction between specificities of closely related proteases that accounts for both substrate numbers and fitness. In addition, comparative analysis of composition of the selectomes of MMP-2 and 9 presented here combined with information on location and composition of selectivity determinants provides clear structural basis for identifying SDPs responsible for selectivity between these closely related enzymes.

### Telling a target from a bystander: Contribution of the catalytic cleft specificity to protein substrate recognition

#### Selectome-based specificity is the primary mode of protein substrate recognition and distinction by MMP-2 and 9

To assess the contribution of the catalytic cleft specificity to physiologic substrate recognition by MMP-2 and 9, we used the data on protein substrate hydrolysis obtained by us and those available in the literature. The data set published in [37] was taken as a benchmark for protein cleavage site identification due to the rigor of data analysis and independent verification (S13 Table). Based on the comparison of this data set with ours, 71% of MMP-2 and 79% of MMP-9 cut sites belong to their respective selectomes (S13 Table). These numbers are not very far from the probability (86%) of unambiguous identification of cut sites of a protease with known specificity (Glu C) used by the authors for validation of the statistical model for cleavage site identification used in their study [37]. The rest of the identified cleavages (19% for MMP-2 and 16% for MMP-9) do not belong to the selectome (13.6% for MMP-2 with RP < 4.5 and 10.5% for MMP-9 with RP < 4.7) or are not found in the substrate sets identified by phage display analysis (RP = 0, 5.5% for MMP-2 and 5.3% for MMP-9). Thus, there is a very good correlation between a cut site being a part of the selectome of MMP-2 or 9 and also being a validated substrate of the same MMP.

**Fig 7:**
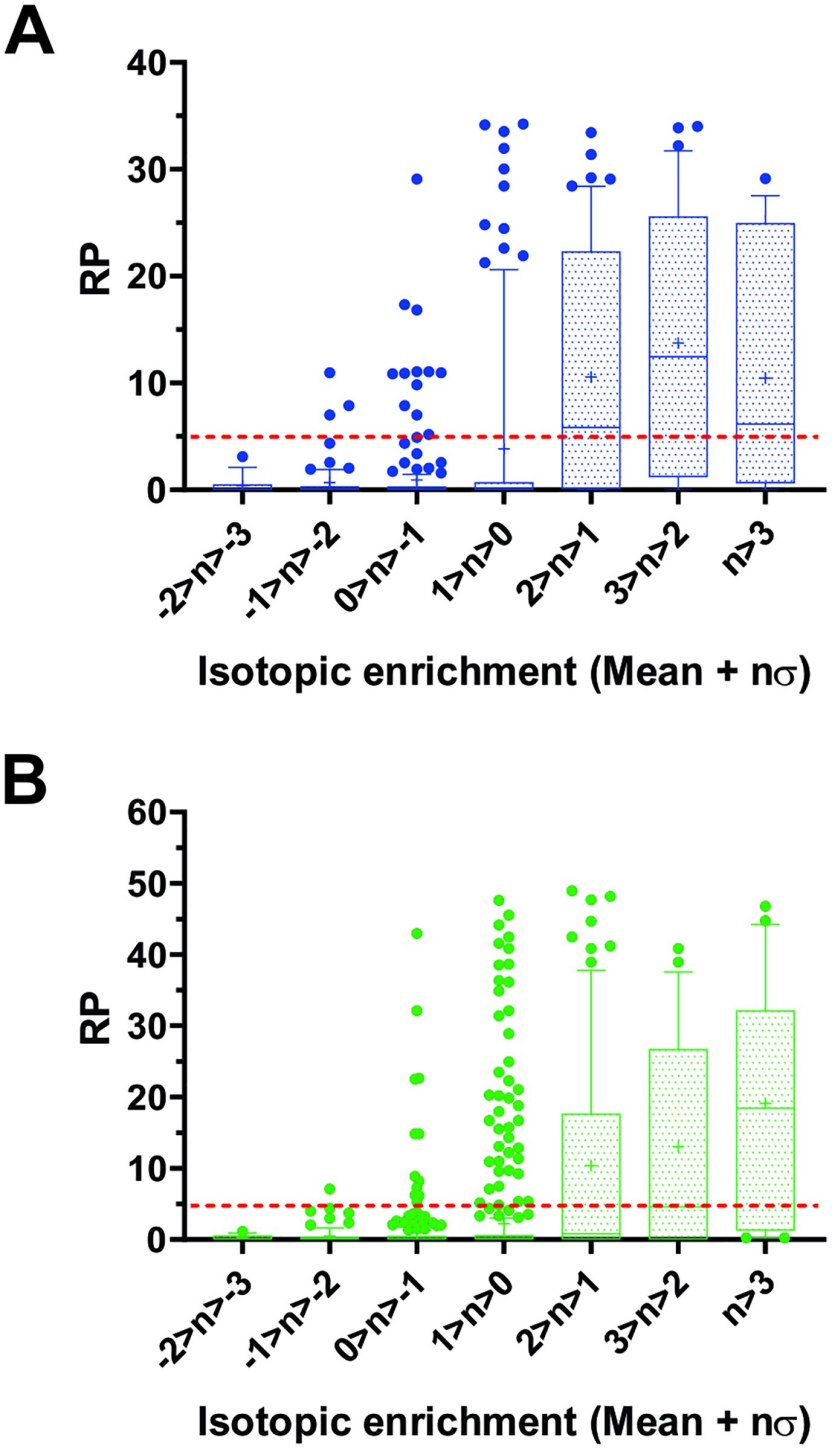
Enrichment in novel N-termini following hydrolysis by MMP-2 or 9 reflects the proportion of protein substrates in respective selectomes. Following hydrolysis with MMP-2 or 9, the secretome of HEK293 cells was labeled with TMT isobaric tags. Isotopic enrichment (IE) of the novel N-terminally labeled peptides in the MMP-treated samples relative to the untreated controls, was determined by LC/MS analysis of tryptic digests of the labeled secretomes (See text and Methods section for details). The graphs show 10-90^th^ percentile box and whiskers plots of the RP values of the P3-P1՛ sequences corresponding to cleavages in human proteins by MMP-2 (**A**) and 9 (**B**) deduced from sequences of N-terminally labeled peptides, as a function of IE expressed as multiples of standard deviation relative to the population mean. The median and mean RP values are marked in the boxes by horizontal lines and crosses, respectively. The red dotted lines mark the selectome thresholds for MMP-2 (RP = 4.5) and MMP-9 (RP = 4.7).

In the publication we used as the benchmark [37], the criteria for cleavage site identification were set very stringently, so that the ratios between the iTRAQ reporter ion intensities in the MMP-treated samples and the untreated controls had to be ≥ 10 in order for N-terminally labeled peptides to meet the statistical threshold to be considered candidate cleavage sites. This was done to achieve a reasonable compromise between the false positive and false negative rates based on the statistical model developed by the authors. To find out if the selectome-based classification can be applied to distinguishing between substrate and non-substrate N-termini, we performed N-terminomic analysis of MMP-2 and 9-treated secretomes derived from HEK293 cells [38] (See Methods for details). S14 Table shows the entire list of N-terminally labeled peptides and the corresponding positions in annotated proteins arranged according to their log_2_ of the ratios of the TMT reporter ion spectral counts relative to the untreated controls (isotopic enrichment or IE). Overall, we identified 453 N-termini in 243 proteins in the MMP-2 and 1034 N-termini in 428 proteins in the MMP-9 treated samples. Plotting relative abundances of the N-terminal peptides with different RP values as a function of IE, expressed as multiples of standard deviation away from the average values for the entire sets, shows that the N-termini with RP values above the selectome thresholds for MMP-2 (RP>4.5) and MMP-9 (RP>4.7) are predominantly found in the intervals with IE values > 1σ above the average (Fig 7). The further does the IE decline below 1σ over the population mean, the higher is the proportion of the N-termini with RP values below the selectome thresholds of both enzymes. Based on these observations, in our study, the IE cutoff for calling a labeled N-terminus a cleavage site resides one standard deviation above the population mean. To confirm that RP-based categorization is a good predictor of isotopic enrichment, we performed a binary classification analysis of the data shown in Fig 7. The results demonstrate that an RP value above the selectome threshold (RP = 4.5 for MMP-2 and 4.7 for MMP-9) is the best predictor (MCC = 0.502 and 0.435 for MMP-2 and 9, respectively) of an N-terminal peptide to have an IE value >1σ above the population mean (S15 Table). These data are consistent with what we observed using the benchmark data set discussed above and provide basis for distinguishing between the true positive and potential false positive cleavage site identifications.

We also analyzed our data to determine if categorization of cleavages as unique or shared between the two enzymes based on isotopic enrichment, correlates with that based on RP (S14 Table). Correlation between the RP/RP_MAX_ values of substrates with IE > 1σ above the mean for both MMP-2 and 9 and therefore in common between the two based on that metric, has a slope of 0.7 (R^2^ = 0.49). This means that substrates recognized well by both MMPs as determined by the N-terminomic analysis tend to have similar relative RP/RP_MAX_ values for both proteases. The same correlations performed for substrates unique to MMP-2 or 9 based on the IE threshold have slopes of 0.1 (R^2^ = 0.67) and 0.08 (R^2^ = 0.08), respectively. This means that RP values of the substrates deemed uniquely specific by N-terminomic analysis tend to reflect that selectivity as well. Thus, distinctions in substrate recognition defined using the selectome approach correlate with those obtained using N-terminomic analysis of human proteins.

#### Selectome-based analysis allows for classification of cleavage sites in whole proteins

MEROPS is a rich source of data for specificity profiling of proteases [2,11]. It is therefore of interest to compare how information on MMP-2 and 9 cleavages compiled from a wide variety of experimental studies is matched by our criteria for specificity. As can be seen in S16 Table, 55% and 36% of all cleavages belong to the selectomes of MMP-2 and MMP-9, respectively. Of the cleavages with RP values below the selectome thresholds, 33 and 46% constitute substrates with poor P3-P1՛ sequences and 12 and 18% are not found in the substrate sets of MMP-2 and 9 determined by phage display. In the published “physiologic” substrates, 44 and 31% of cleavages belong to the selectomes of MMP-2 and MMP-9, respectively. 40 and 49% of cut sites in the “physiologic” substrate category belong to the non-selectome substrates and 16 and 20% are not found in the substrate sets of MMP-2 and MMP-9 obtained by phage display. It should be noted that most cleavages reported in MEROPS have information on location of the scissile bond but not catalytic efficiency. This contrasts with the N-terminomic data that provides relative abundances of the novel N-termini between the experimental and control samples, which can be used to limit the number of poor substrates being reported.

In order to assess if selectivity between MMP-2 and 9 captured by the comparison of respective selectomes holds for physiologic substrates listed in MEROPS, we performed an analysis of correlation between the RP values of cleavages reported for both or only for one of the enzymes. S16 Table shows that the correlation between the RP/RP_MAX_ values of cleavages in the physiologic substrates reported for both MMPs is very high (Slope = 0.84, R^2^ = 0.82). In contrast, the correlations between RP/RP_MAX_ values of physiologic substrate cleavages reported for MMP-2 or MMP-9 only, are very poor (Slope = 0.08, R^2^ = 0.19 and Slope = -1.9, R^2^ = 0.135, respectively). This analysis is consistent with the view that selectivity between MMP-2 and 9 captured by phage display analysis holds for physiologic substrates reported by multiple independent studies listed in MEROPS.

In summary, based on our analysis, the P3-P1՛ substrate recognition by MMP-2 and 9 is the main determinant of substrate fitness and selectivity. If a cleavage site does not match the catalytic cleft specificity defined by the selectome, then it must be a relatively poor substrate representing a bystander proteolytic event or must have a large exosite contribution to its catalytic efficiency. This knowledge constitutes a significant advance in understanding of the mechanistic basis of MMP biology, which may need further studying. So, it is useful to know if experimentally determined cleavages can be confirmed by the selectome analysis in order to decide what category a given substrate belongs to and if additional study is needed.

## Discussion

### The concept of “selectome”

Since, as a rule, proteases cleave multiple substrates with varying specificity constants (k_cat_/K_M_), it would be advantageous to determine a set of substrates that can be used to uniquely and quantitatively define specificity of the catalytic cleft. To the best of our knowledge, we are the first to propose such a concept. A widely accepted term “degradome” refers to the repertoire of all natural substrates cleaved by a protease [39], which may or may not be reflective of the specificity of the catalytic cleft alone. In our study, we used two closely related members of the MMP family (MMP-2 and 9) and a library of fully randomized hexapeptides displayed on the PIII gene product of M13 phage to experimentally substantiate the concept of selectome. Since the enzymes of this family have two major selectivity determinants at S3 and S1՛, together with S2 and S1 between them they form a tetramer binding unit (Fig 1). Based on that, MMPs can theoretically recognize between 1 and 160,000 P3-P1՛ motifs depending on the degree of specificity they possess. For MMPs, the “selectome” is defined by the P3-P1՛ sequences with cumulative non-zero contribution to the Kullback-Leibler divergence calculated between their probability distributions in the substrate set and the randomized hexapeptide probe set used for substrate selection. We found that selectomes of MMP-2 and 9 contain 7,921 and 6,094 tetramer substrates, respectively. It is important to emphasize that the selectomes of MMP-2 and 9 constitute about 10% of all the tetramer clusters identified in the substrate sets (78,757 and 76,696, respectively) indicating that the majority of the P3-P1՛ sequences recognized by these enzymes contribute little to the catalytic efficiencies of substrates and thus contribute little, if at all, to their specificities. Currently, one of the major roadblocks to understanding protease function is the lack of sensitive approaches to distinguishing between specificities of closely related proteases from the same families [12]. Comparative analysis of the selectomes of MMP-2 and 9 in combination with structural data and prior knowledge of the composition of selectivity determinants, allowed to identify the SDPs responsible for the observed differences in specificity as shown in Fig 6. This is a very valuable aspect of the selectome-based substrate specificity profiling as it provides structural insight for developing highly selective activity probes and inhibitors.

### Quantification of substrate specificity

We developed methodology for quantification of substrate specificity based on both the number and fitness of substrates in the selectome. We introduced a probability-based quantitative metric of contribution of the recognition motif determined by the number and stringency of selectivity determinants to catalytic efficiency of substrates, we call Relative Probability or RP. For MMPs, it is defined as the ratio between probabilities of finding a unique P3-P1՛ sequence in the substrate set and the random hexamer probe set used for substrate selections (Eq 3). This quantity allows to normalize out experimental biases (i.e. differences between sequencing depths of the amplicons used for analysis) associated with direct use of the number of hexamers per tetramer cluster relative to the theoretical maximum of 1200 (Fig 1).

Studies by another group also used information theory to quantify protease specificity but purely by evaluating frequencies of occurrence of amino acid residues at different positions relative to the scissile bond [11,40]. Based on their analysis of the MEROPS database of protease substrates, specificities of most proteases are very broad and, in fact, close to the theoretical maximum. For instance, their take on the overall specificity of MMP-2 and 9 indicates that both are broadly specific, with Shannon entropies of 7.386 and 7.078 out of the theoretically maximal 8.0 for eight sub pockets covering S4 –S4՛. Shannon entropy was calculated using log_20_ so that a non-specific binding pocket accepting all residues would have a value of 1 and a totally specific binding pocket accepting a single residue would have a value of 0. Each sub pocket’s Shannon entropies are added together to obtain the overall specificity measure. In relative terms, based on their analysis, specificities of MMP-2 and 9 are 20^8^/20^7.386^ = 6.29-fold and 20^8^/20^7.078^ = 15.83-fold narrower than that of a protease with no specificity. Our data based on the Shannon entropy values of the P3-P1՛ tetramer cluster distributions in the MMP-2 and 9 substrate sets show their specificities are 2^17.288^/2^13.93^ = 10.25-fold and 2^17.288^/2^13.67^ = 12.28-fold narrower than that of a randomly specific protease. The two analyses are in a reasonably good agreement on the overall specificities of the two enzymes. These numbers imply that approximately 10% of all peptide bonds in proteins available for cleavage are substrates of MMP-2 and 9, which makes every protein in the human proteome a potential target with at least one cleavage site.

### Relevance to proteolysis of folded proteins

One of the questions central to understanding protease function is how to distinguish between targeted and coincidental proteolytic events. It stands to reason that proteases and their physiologic substrates co-evolved to be integral parts of complex physiological processes [4,41–42]. Mechanisms underlying protease-substrate recognition involve exosite, auxiliary binding domain and catalytic cleft interactions. While the K_M_ value of a proteolytic event can be affected by interactions outside the catalytic cleft, the k_cat_ value is completely dependent on the substrate fitness around the scissile bond, which is related to the rate of formation of the transition state intermediate. If that rate is close to zero, a tight interaction outside the catalytic cleft will result in inhibition of the protease. A high rate of formation of the transition state intermediate will result in faster hydrolysis if the K_M_ value is sufficiently high to allow for dissociation of the enzyme-product complex before the reverse reaction re-forms the enzyme-substrate complex. So, mechanistically, there is a fine balance that needs to be struck between the k_cat_ and K_M_ values for a physiologically relevant proteolytic event to be integrated into the larger context of underlying biology.

Using the results of a published rigorous study [37] and our own data, we determined the relevance of our selectome-based approach to identification of cleavage sites in folded proteins. Our analysis of the published results shows that from 70 to 80% of the protein substrates identified with high confidence belong to the selectomes of MMP-2 and 9. Our own analysis of cleavages in folded proteins based on enrichment of novel N-termini following MMP treatment, demonstrates that RP above the selectome threshold for the matching P3-P1՛ tetramers is the best predictor for enrichment of the corresponding N-termini >1σ above the population mean (S15 Table).

Our analysis of the MEROPS database of MMP-2 and 9 substrates shows that a significant proportion of reported cleavages (~50%) are not in the selectome. They can belong to off-target spurious proteolytic events, artifacts, or possibly constitute exosite driven proteolysis by MMP-2 and 9. This information is very useful for follow-up studies to determine if exosite participation is a significant component of substrate recognition by these and other MMPs [43]. The fact that ~80% of the P3-P1՛ tetramers in the substrate sets of MMP-2 and 9 have RP values below the selectome thresholds indicates that simple accounting for amino acid diversity at individual positions of substrates is not an accurate estimate of physiologically relevant specificity. Thus, our selectome-based analysis of cleavage events in folded proteins establishes the importance of the P3-P1՛ catalytic cleft specificity in protein substrate recognition by MMP-2 and 9.

Results of the analysis of overlap and distinction in physiologic substrate recognition between MMP-2 and 9 based on the selectome approach correlate with those based on other methodologies (S14 and S16 Tables). We would like to illustrate how selectome-based specificity analysis applies to biologically significant proteolysis with specific examples. One of the cleavages found selective for MMP-2 occurs in SERPINE2, a broad serine protease inhibitor, whose polymorphism is known to be a risk-mitigating factor in COPD [44]. The cleavage occurs in the IDN166↓L167 P3-P1՛ tetramer (S14 Table) selective for MMP-2 due to the P3 Ile preferred by S3 of MMP-2 over MMP-9 (Fig 6C). The same cleavage was reported for MMP-2 elsewhere [21]. Cleavages of this protein by MMP-9 were reported in literature but at different positions [45]. We speculate that differential proteolysis of SERPINE2 by MMP-2 and 9 may result in a synergistic increase of serine protease activity due to loss of inhibition, leading to tissue damage. SERPINE2 gene polymorphism may result in mutations inactivating some of the cleavage sites, helping to keep the serine protease activity low, thereby decreasing the risk of COPD. Another example of selectivity between MMP-2 and 9 potentially relevant to biology is observed for Semaphorin 3D (S14 Table), a member of the family of signaling proteins involved in axon guidance. Cleavage of the PFA191↓S192 P3-P1՛ tetramer is selective for MMP-9 due to the preference for P2 Phe and S1՛ Ser over MMP-2. We speculate that by affecting functionality of Semaphorin D3, MMP-9 may have a unique regulatory role in neural development. These two examples illustrate how detailed knowledge of selectivity made available by the selectome approach presented in this study, can help functionally disentangle closely related members of protease families, thus revealing their individual roles in regulation of networks and pathways controlled by them.

### Conclusion

Work presented here establishes a novel approach to studying substrate specificity of proteases and possibly other enzymes involved in posttranslational modification [46]. It is based on statistically saturated data sets and a new way of applying information theory to quantitatively defining substrate specificity of proteases by employing a novel concept of “selectome”. In practical terms, this approach can be invaluable for developing highly selective activity probes and inhibitors for closely related members of protease families. By providing a measure of catalytic efficiency, our approach can also be used to help determine which cleavages in human proteins represent physiologic and pathologic targets and which are bystander proteolytic events.

## Methods

### Expression and purification of recombinant catalytic domains and activity assays

The recombinant catalytic domains of MMP-2 and -9 were expressed in HEK293 cells stably transfected with respective constructs and purified from serum-free culture medium using Gelatin Sepharose 4B (GE Healthcare) as described in [29,35]. Following activation with APMA (Sigma-Aldrich), the amount of active enzyme was determined by active site titration using GM6001 (Sigma-Aldrich) [29]. The k_cat_/K_M_ values of 100 peptides derived from phage substrates (S6 Table) were determined in triplicate as described in [27].

### Substrate phage selections and NGS analysis

The conditions for substrate phage selection were set so that 99% of all substrates with k_cat_/K_M_ of 3,289 M^-1^s^-1^ would be cleaved. This means that it would take 200 nM protease 2 hours to digest 99% of substrates with the k_cat_/K_M_ of 3,289 M^-1^s^-1^, provided the substrate concentration is much below K_M_, which is the case in our experiment. Substrates with k_cat_/K_M_ values above the threshold are cleaved faster and those below slower.

Selection of substrate phage was performed as described in [10]. Briefly, 5 × 10^11^ phage particles were mixed with a protease at 200 nM of active enzyme in 0.5 mL of the reaction buffer and incubated for 2 h at 37°C. A reaction without addition of protease was performed as a control. Following incubation, MMP activity was halted by addition of GM6001. Phage with uncleaved FLAG-tag were removed by immunodepletion using M2 anti-FLAG monoclonal antibody (Sigma-Aldrich) coupled to epoxy-activated M-450 magnetic beads (Invitrogen). The extent of immunodepletion in the protease treated and untreated control samples was determined by ELISA. Unbound phage were propagated, purified by PEG precipitation and used in the second round of selection. Phage ssDNA from the control and protease selections was purified from 8 x 10^12^ phage particles using phenol-chloroform extraction. The region harboring the randomized hexamer and flanking constant tags was amplified from 1 μg ssDNA for 5 cycles using the Q5 Hot Start High-Fidelity PCR Master Mix (New England Biolabs) and the following primers: 5՛-ACGACGACGACAAACCCG-3՛ forward and 5՛-AACAGTTTCGGCCCCAGA-3՛ reverse. The NGS library was prepared using NEBNext Ultra II DNA Library Prep Kit for Illumina and NEBNext Multiplex Oligos for Illumina. Amplicons were cleaned up using AMPure XP beads (Beckman Coulter) and subjected to NGS analysis performed at the Genomics Core of the Sanford-Burnham-Prebys Medical Discovery Institute. In total, 324,285,851 reads were generated to sequence the naïve phage display library, and 27,221,954 and 34,786,206 to sequence the MMP-2 and MMP-9 substrate selections, respectively. We developed a series of Linux shell scripts and Fortran programs for processing and analysis of NGS data. FASTQC program (Andrews, S. (2010). FastQC: AQuality Control Tool for High Throughput Sequence Data [Online], available online at: http://www.bioinformatics.babraham.ac.uk/projects/fastqc/) was used to check for quality of sequencing data. No low-quality sequences were detected in the NGS raw fastq files. The DNA sequences were translated in forward and reverse directions using all three reading frames. Sequences of the variable hexamer region were accepted only if they were flanked by the constant tag flanking sequences. Sequences belonging to the variable hexamer region were used for all downstream analyses. Sequences of peptide hexamers found in the untreated controls were removed from the downstream analysis of substrate selections. These analyses include sorting, assigning hexamer sequences to tetramer clusters, calculating probabilities of tetramers, deriving Shannon entropy and Kullback-Leibler divergence.

### Data analysis

#### Hexamer alignment and clustering

We performed two rounds of selection of MMP-2 and 9 substrates. Sequences of hexamer peptide substrates were obtained using NGS analysis of the substrate phage DNA. Next, we grouped each of the hexamer peptides containing the same tetramer sequences into tetramer clusters (Fig 1A and S1, S2 and S3 Tables). In order to avoid influences of hexamers belonging to more than one tetramer cluster and potentially representing co-occurring cleavages in the same hexamer or positions outside of the P3-P1՛ tetramer, each hexamer MMP substrate was assigned to the most abundant tetramer cluster it can be found in. Tetramer clustering was done by assigning each hexamer to the three tetramer clusters it can be found in. The maximum possible number of tetramers clusters is 160,000. In the next step low abundance tetramer clusters containing non-unique hexamer sequences were eliminated. It was done by step-by-step removal of redundant hexamers starting with the least abundant tetramer cluster and leaving each hexamer sequence in the most abundant cluster. Finally, the hexamers in each tetramer cluster were aligned along the P3-P1՛ core sequence, yielding sequence coverage across P5-P3՛ positions in substrates. Thus, every substrate hexamer belongs to a single tetramer cluster.

In parallel, we have also performed NGS analysis of the naïve (or initial) phage display library and also grouped the hexamer peptide sequences into tetramer clusters (Fig 1B and S1 Table), but without eliminating redundancy (i.e. allowing the same hexamer to belong to more than one tetramer cluster), since no selective pressure that could influence the distribution of the tetramer clusters has been applied in generating this set.

#### Equations used for statistical analysis of the data

$$\text{H}(T)=-\sum_{t\in T}P(t){\text{log}}_{2}P(t),$$ (1)

where H*(T)* is Shannon entropy of the distribution of tetramer clusters *(T)* each with probability *P(t)*. The probability of a given tetramer can be defined as:

$$P(t)=\frac{n_{t}}{\sum_{i=1}^{N}n_{i}},$$ (2)

where *P(t)* is the probability of the *t*^*th*^ tetramer calculated as the ratio between the number of hexamers in that tetramer cluster *(n*_*t*_*)* and the number of hexamers in the entire set of tetramer clusters. The total number of all possible tetramer clusters, N, is equal to 20^4^ = 160,000.

$$RP(t)=\frac{P_{S}(t)}{P_{NL}(t)},$$ (3)

Where RP is the relative probability, *P*_*S*_*(t)* is the probability of a *t*^*th*^ tetramer cluster in substrate selection and *P*_*NL*_*(t)* is the probability of that tetramer cluster in the naïve phage display library.

The RP value for tetramer clusters in substrate sets has a theoretical range of maximum values between 1 (for a non-specific protease cleaving all tetramer substrates with equal efficiency: $1=\frac{1/160,000}{1/160,000}$) and 160,000 (for a maximally specific protease with only one tetramer substrate: $160,000=\frac{1}{1/160,000}$).

$$D_{KL}(P_{S}∥P_{NL})=\sum_{t\in T}P_{S}(t)log_{2}(\frac{P_{S}(t)}{P_{NL}(t)}),$$ (4)

Where *D*_*KL*_ *(P*_*S*_ *|| P*_*NL*_*)* is the Kullback-Leibler divergence between the tetramer probability distributions in the selections *P*_*S*_*(t)* and the naïve library *P*_*NL*_*(t)* defined on the same probability space *T*.

### Binary classification analysis of correlation between RP and catalytic efficiency constants

In this analysis, all P3-P1՛ tetramers with RP values above a certain threshold and a non-zero value of K_(obs)_ were considered as true positives (TP). All tetramers with RP values below that threshold and a K_(obs)_ equal to 0 were considered as true negatives (TN). If a value of RP was above the threshold, but the K_(obs)_ was equal to 0, then the tetramer was classified as a false positive (FP). Finally, the tetramers with RP values below a threshold but a non-zero K_(obs)_ were classified as false negatives (FN).

### Analysis of correlation between average values of RP and catalytic efficiency constants

First, we obtained the RP values for the tetramer clusters matching the P3-P1՛ positions in hexamer substrates. Next, the tetramers were grouped based on their RP values to generate an evenly spaced distribution of bins across the RP range. The average values and standard errors of the mean of the RP and corresponding K_(obs)_ have been calculated for substrates in each bin. To demonstrate that the number of substrates used for binning does not affect the correlation, the analysis was repeated using several bin sizes (S6 Table) shows that regardless of the number of substrates used in each bin, strong correlation between RP and k_cat_/K_M_ or K_(obs)_ still holds.

### Structural modeling

We docked model substrates into MMP2 and MMP9 catalytic domains using published modelled structures by Diaz et al. [47] as a guideline and crystallographic structures containing peptide-like inhibitors as templates [25]. Initial docking and follow up mutations have been done using PyMOL Molecular Graphics System (Schrodinger LLC). Initially built structures have been energy minimized (5000 steps) followed by limited molecular dynamics simulations using GBSA implicit solvent model [48]. During minimization and molecular dynamics simulations all atoms in enzyme have been retrained allowing for the movement of ligand atoms. Minimization and simulations phases were carried out by the SANDER module of AMBER 17 [49] using 99 Å cutoff for non-bonded interactions and ff14SB force field [50].

### Proteomic identification of novel N-termini in folded proteins

#### Sample preparation

HEK293 cells were seeded in 150-mm tissue culture dishes (Corning) and grown to confluence. The confluent cultures were washed with PBS several times to remove serum proteins and kept in serum-free DMEM supplemented with 1 μM GM6001 for 72 hours at 37 C, 5% CO_2_. The conditioned media was centrifuged at 10,000 x g for 30 minutes at 4 C and concentrated ~100-fold using Centricon Plus-70 (AMD Millipore) 3-kDa m.w. cut-off ultrafilter, and the buffer was exchanged to 50 mM HEPES pH 7.0 containing 150 mM NaCl and 10 mM CaCl_2_ (Reaction Buffer) using a PD-10 desalting column (GE Healthcare). Aliquots containing 240 μg total protein were incubated at 37 C in the presence of 750 nM MMP2 or 9 or the Reaction Buffer alone for 2 hours. Each reaction was carried out in duplicate. The reactions were stopped by addition of 20 mM EDTA and subjected to denaturation/reduction (4M Urea and 10 mM TCEP, 1 h, 22°C) and alkylation (20 mM iodoacetamide, 30 min, 22°C, in dark). Each sample was labeled for 2 h at 22°C with a different TMT-tag obtained from a TMT10plex kit (Thermo Fisher Scientific), and then quenched (0.27% hydroxylamine, 15 min, 22°C). The buffer control samples were labeled with TMT-127N and TMT-127C, the MMP-2 treated samples were labeled with TMT-128N and TMT-128C and the MMP-9 treated sampled were labeled with TMT-129C and TMT-130C. The TMT-labeled samples were pooled together, and subjected to TCA precipitation (20% TCA, 16 h, 4°C). Each pellet was washed with -20°C acetone, recovered in 50 mM Tris-HCl pH 8.0, and then digested with trypsin/Lys-C (Promega) for 16 h at 37°C. Digestions were stopped with 0.2% TFA and centrifuged to remove insoluble material. The supernatants were then desalted using Sep-Pak C18 cartridge (50 mg), and then lyophilized.

#### Offline fractionation

Dried pooled sample was reconstituted in 20 mM ammonium formate pH ~10 and fractionated using a Waters Acquity BEH C18 column (2.1x 15 cm, 1.7 μm pore size) mounted on an M-Class Ultra Performance Liquid Chromatography (UPLC) system (Waters). Peptides were then separated using a 35-min gradient: 5% to 18% B in 3 min, 18% to 36% B in 20 min, 36% to 46% B in 2 min, 46% to 60% B in 5 min, and 60% to 70% B in 5 min (A = 20 mM ammonium formate, pH 10; B = 100% ACN). A total of 32 fractions were collected and pooled in a non-contiguous manner into 16 total fractions. Pooled fractions were dried to completeness in a SpeedVac concentrator prior to mass spectrometry analysis.

#### LC-MS/MS analysis

Dried peptide fractions were reconstituted with 2% ACN-0.1% FA and analyzed by LC-MS/MS using a Proxeon EASY nanoLC system (Thermo Fisher Scientific) coupled to a Q-Exactive Plus mass spectrometer (Thermo Fisher Scientific). Peptides were separated using an analytical C18 Acclaim PepMap column (75μm x 250 mm, 2μm particles, Thermo Scientific) at a flow rate of 300 μl/min using a 58-min gradient: 1% to 6% B in 1 min, 6% to 23% B in 35 min, and 23% to 34% B in 22 min (A = FA, 0.1%; B = 80% ACN: 0.1% FA). The mass spectrometer was operated in positive data-dependent acquisition mode. MS1 spectra were measured with a resolution of 70,000 (AGC target: 1e6; mass range: 350–1700 m/z). Up to 12 MS2 spectra per duty cycle were triggered, fragmented by HCD, and acquired with a resolution of 17,500 (AGC target 1e5, isolation window; 1.2 m/z; normalized collision: 32) Dynamic exclusion was enabled with a duration of 25 sec.

#### Analysis of the proteomics data

Raw files were analyzed using MaxQuant software version 1.6.8.0 and MS/MS spectra were searched against the *Homo sapiens* Uniprot protein sequence database (downloaded in January 2019). The false discovery rate (FDR) filter for spectrum and protein identification was set to 1%. Carbamidomethylation of cysteines was searched as a fixed modification, while oxidation of methionines and N-terminal acetylation were searched as variable modifications. Enzyme was set to trypsin in a semispecific mode and a maximum of two missed cleavages was allowed for searching. To obtain quantification of the TMT-labeled N-termini, two independent searches were performed–one with TMT10plex MS2 N-terminal reporter quantification and the other for quantification of N-terminal and lysine sidechain. The results were combined to obtain the complete set of the N-terminally labeled peptides.

## Supporting information

S1 Fig Most hexamers in substrate selections of MMP-2 and 9 belong to few tetramer clusters. Tetramer clusters in substrate selections of MMP-2 and 9 were grouped into 10% bins based on their RP values relative to the maximum. The numbers of tetramer clusters in each bin (**A**) and the numbers of hexamers in the corresponding tetramer clusters (**B**) are plotted as a function of the RP interval they belong to.
(TIF)

S2 Fig Specificity profiles based on the unique and overlapping tetramer clusters of MMP-2 and 9 selectomes as a function of RP/RP_Max_. Logo plots demonstrate the composition of substrates across P5 to P3՛ positions as a function of RP/RP_Max_. Peptide hexamers belonging to tetramer clusters in the selectomes of MMP-2 and 9 were aligned across P3-P1՛ positions and divided into 10 groups based on their RP values relative to the maximum (RP/RP_Max_). First and second columns of logo plots correspond to unique substrates of MMP-2 and 9 selectomes, respectively. The third and fourth columns of logo plots represent the common set of MMP-2 and 9 selectomes, respectively. The RP values for the corresponding tetramer clusters have been calculated either according to MMP-2 (MMP-2&9/2) or MMP-9 (MMP-2&9/9) ranking.
(TIF)

S1 Table Information about the numbers of hexamers and resultant tetramer clusters in the naïve library and MMP-2 and 9 substrate selections. (XLSX)

S2 Table List of all tetramer clusters and corresponding hexamers aligned along P3-P1՛ positions of MMP-2 substrates. Tetramer amino acid sequence, rank and the number of hexamers in it are shown in the header for each tetramer cluster.
(PDF)

S3 Table List of all tetramer clusters and corresponding hexamers aligned along P3-P1՛ positions of MMP-9 substrates. Tetramer amino acid sequence, rank and the number of hexamers in it are shown in the header for each tetramer cluster.
(PDF)

S4 Table Statistical information about tetramer clusters for MMP-2. The table contains information about: a) the amino acid sequence of tetramer cluster, b) rank of the tetramer cluster calculated using relative probability, c) number of hexamers in a cluster from MMP set and d) number of hexamers in the corresponding cluster in naïve library, e) ratio of hexamer numbers in the MMP substrate set and the naïve library (%), f) probability of a tetramer in MMP set, g) probability of corresponding tetramer in naïve library, h) relative probability calculated as a ratio of (f) and (g) probabilities, i) individual contribution of each tetramer cluster to Kullback-Leibler (K-L) divergence, j) cumulative values of K-L divergence over tetramer clusters, k) individual contribution of each tetramer cluster to Shannon entropy, l) cumulative values of Shannon entropy over tetramer clusters. The resultant value of K-L divergence and Shannon entropy for MMP set and naïve library is provided at the end of each table.
(XLSX)

S5 Table Statistical information about tetramer clusters for MMP-9. See S4 Table for explanation of table content.
(XLSX)

S6 Table Table of 1369 peptide set derived and published previously [10] for MMP-2 and 9, for which positions of scissile bonds and K_(obs)_ values were determined experimentally. First two tabs, corresponding to MMP-2 and 9, respectively, contain information about: a) the sequence of dodecamer substrate with marked cleavage position, b) the corresponding amino acid tetramer sequences for P3-P1՛ positions, c) rank of tetramer based on RP value, d) RP value, e) measured K_(obs)_ (M^-1^s^-1^). Additional tabs contain results for averaged K_(obs)_ of substrates in evenly distributed ranges of RP values. The binning was done for different bin sizes ranging from 1 to 6 spanning the entire range of RP values between 0 and RP_max_. For every bin size the results are plotted as a function of the corresponding average RP values.
(XLSX)

S7 Table Statistical performance of tetramer approach to analysis of cleavages in set of 1369 substrates [10] for MMP-2 and MMP-9. Only nonredundant sets of peptide substrates have been selected for statistical assessment. The value of K_(obs)_ (M^-1^s^-1^) for each tetramer has been calculated as an average value over all redundant entries. The calculations have been performed for three different thresholds related to K-L divergence analysis: a) for RP above 4.5 or 4.7 for MMP-2 or 9, respectively (“selectome”) and b) for RP above 0, which includes all substrates.
(XLSX)

S8 Table Table of 100 peptide set derived from substrate phage selections of MMP-2 and 9, for which k_cat_/K_M_ values were experimentally determined. First two tabs, corresponding to MMP-2 and 9, respectively, contain information about a) the sequence of hexamer substrate, b) the corresponding amino acid tetramer sequences for P3-P1՛ positions, c) rank of tetramer based on RP value, d) relative probability (RP), e) measured k_cat_/K_M_ (M^-1^s^-1^), f-g) standard deviation and standard error for measured k_cat_/K_M_ (M^-1^s^-1^), based on triplicate experiments for each experiment. Additional tabs contain results for averaged k_cat_/K_M_ of substrates in evenly distributed ranges of RP values. The binning was done for different bin sizes ranging from 1 to 6 spanning the entire range of RP values between 0 and RP_max_. For every bin size the results are plotted as a function of the corresponding average RP values.
(XLSX)

S9 Table Analysis of combined selectomes of MMP-2 and 9. Results for MMP-2. List of unique tetramer clusters and corresponding hexamer sequences aligned across P3-P1՛ positions of substrates belonging to MMP-2 selectome. Tetramers are ranked according to MMP-2 relative probability. The number of hexamers in a tetramer cluster depends on which MMP ranking was applied. Information about the tetramer amino acids sequence, rank, number of hexamers and relative probability of a cluster is provided in the header for each tetramer cluster.
(TXT)

S10 Table Analysis of combined selectomes of MMP-2 and 9. Results for MMP-9. List of unique tetramer clusters and corresponding hexamer sequences aligned across P3-P1՛ positions of substrates belonging to MMP-9 selectome. Tetramers are ranked according to MMP-9 relative probability. Information about the tetramer amino acids sequence, rank, number of hexamers and relative probability of a cluster is provided in the header for each tetramer cluster.
(TXT)

S11 Table Analysis of combined selectomes of MMP-2 and 9. List of tetramer clusters common between the selectomes of MMP-2 and 9 together with the corresponding hexamer sequences aligned across P3-P1՛ positions of substrates. Tetramers are ranked according to MMP-2 relative probability. Information about the tetramer amino acids sequence, rank, number of hexamers and relative probability of a cluster is provided in the header of each tetramer cluster.
(PDF)

S12 Table Analysis of combined selectomes of MMP-2 and 9. List of tetramer clusters common between the selectomes of MMP-2 and 9 together with the corresponding hexamer sequences aligned across P3-P1՛ positions of substrates. Tetramers are ranked according to MMP-9 relative probability. Information about the tetramer amino acids sequence, rank, number of hexamers and relative probability of a cluster is provided in the header of each tetramer cluster.
(PDF)

S13 Table Identification of cleavage sites using tetramer projection in MMP-2 (A) and MMP-9 (B) substrates, determined by Prudova *et al*. (2010) [37]. Each table contains information about tetramer sequence projected onto the cleavage site, its rank and relative probability.
(XLSX)

S14 Table Tetramer matching to novel N-termini in proteins secreted by HEK293 cells following treatment with MMP-2 (**A**) and MMP-9 (**B**). Each table contains sequences of deduced cleavages grouped according to two thresholds: a) IE > 1σ above the mean value of isotopic enrichment of the corresponding N-terminally labeled peptides, and b) RP values of the matching tetramers above MMP specific cutoff defining the selectome. For each identified cleavage the following information has been provided: the rank and RP of the matching tetramer, isotopic enrichment (log_2_(ratio)) and p-value for each N-terminally labeled peptide based on duplicate determinations and respective protein ids. The tabs: **C, D, E, F, G** contain the same information as above, but for novel N-termini in proteins grouped according to their matching tetramers belonging to various parts of Venn’s diagram in Fig 5. In each tab the isotopic enrichment, IE, RP and p values have been specified as they were determined for both MMP-2 and 9 enzymes. (**C**)–MMP-2 cleaved proteins belonging to common set of MMP-2-9 selectomes (45 entries), (**D**)–MMP-9 cleaved proteins belonging to common set of MMP-2-9 selectomes (50 entries), (**E**)–MMP-2 cleaved proteins belonging to unique part of MMP-2 selectome (15 entries), (**F**)–MMP-9 cleaved proteins belonging to unique part of MMP-9 selectome (10 entries).
(XLSX)

S15 Table Binary classification of cleavage sites in HEK293 cell secretomes following treatment with MMP-2 (A) and MMP-9 (B). Cleavages detected in HEK293 cell secretome have been grouped according to the value of selectome-based RP thresholds 4.5 (MMP-2) or 4.7 (MMP-9), and n x σ distance away from the average value of log_2_ IE (isotopic enrichment). For each group the following binary classifiers have been used: TP—number of cases for which log_2_ IE is above a certain n x σ, and RP of associated tetramers is above a specified threshold; TN—number of cases for which log_2_ IE is below the n x σ, and RP is below the threshold; FN—number of cases for which log_2_ IE is above the n x σ, and RP is below the threshold; FP—number of cases for which log_2_ IE is below the n x σ, and RP is above the threshold. For each group, sensitivity, specificity, accuracy, FP rate, precision and Matthews correlation coefficient (MCC) have been determined.
(XLSX)

S16 Table Tetramer annotation of cleavage sites in MMP-2 and 9 substrates collected from the MEROPS database. The data have been divided into two groups–those annotated as physiologic substrates (**A**)–MMP2, (**C)**–MMP9, and all substrates (**B**)–MMP2, (**D**)–MMP9. Each table contains information about octamer sequences covering P4-P4՛ positions of substrates as reported in MEROPS, the corresponding tetramers, their ranks and relative probabilities, as well as MEROPS annotation relating the cleavage site to its position in a protein or analyzed polypeptide. Tab (E) General Statistics–contains information about the number of all and physiologic substrates found in MEROPS that have their RP values above the corresponding selectome thresholds for MMP-2 and 9 enzymes. Tab **F** contain information about the common set of MMP-2 and 9 physiologic substrates found in MEROPS. The P3-P1՛ tetramer rankings and corresponding RP values are provided for both MMP-2 and 9 together with corresponding correlation plot between RP values. The tabs **G** and **H** contain the same type of information, as in the Tab **F**, for unique physiologic substrates for MMP-2 and 9, respectively.
(XLSX)
