# Supplementary figures and images for: Quantitative profiling of protease specificity
Source: PLoS Comput Biol. 2021 Feb 22;17(2):e1008101. doi: 10.1371/journal.pcbi.1008101 (PMC7932537; doi:10.1371/journal.pcbi.1008101)

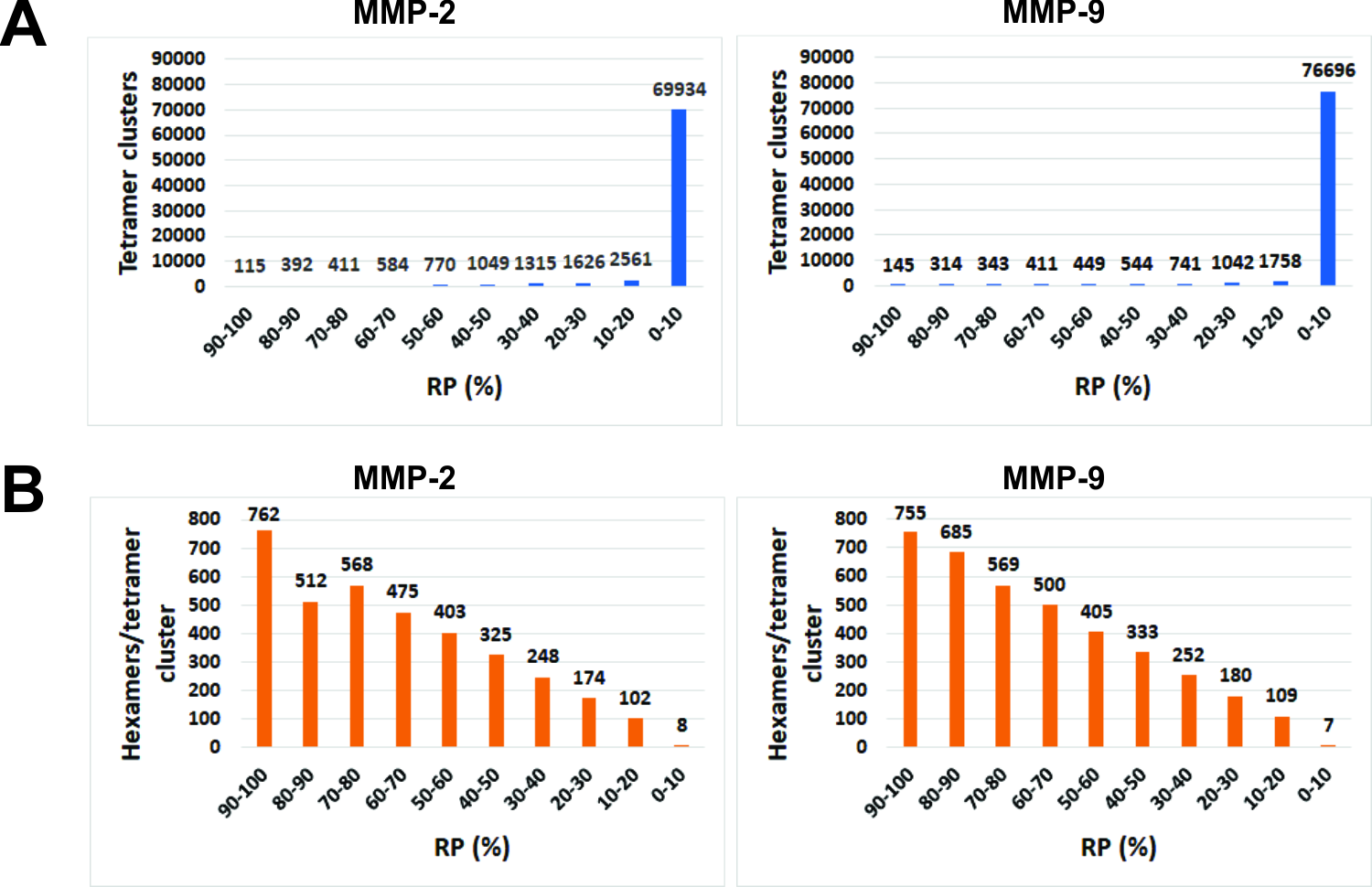

Supplement: S1 Fig — Tetramer clusters in substrate selections of MMP-2 and 9 were grouped into 10% bins based on their RP values relative to the maximum. The numbers of tetramer clusters in each bin (A) and the numbers of hexamers in the corresponding tetramer clusters (B) are plotted as a function of the RP interval they belong to. (TIF) [file pcbi.1008101.s001.tif]

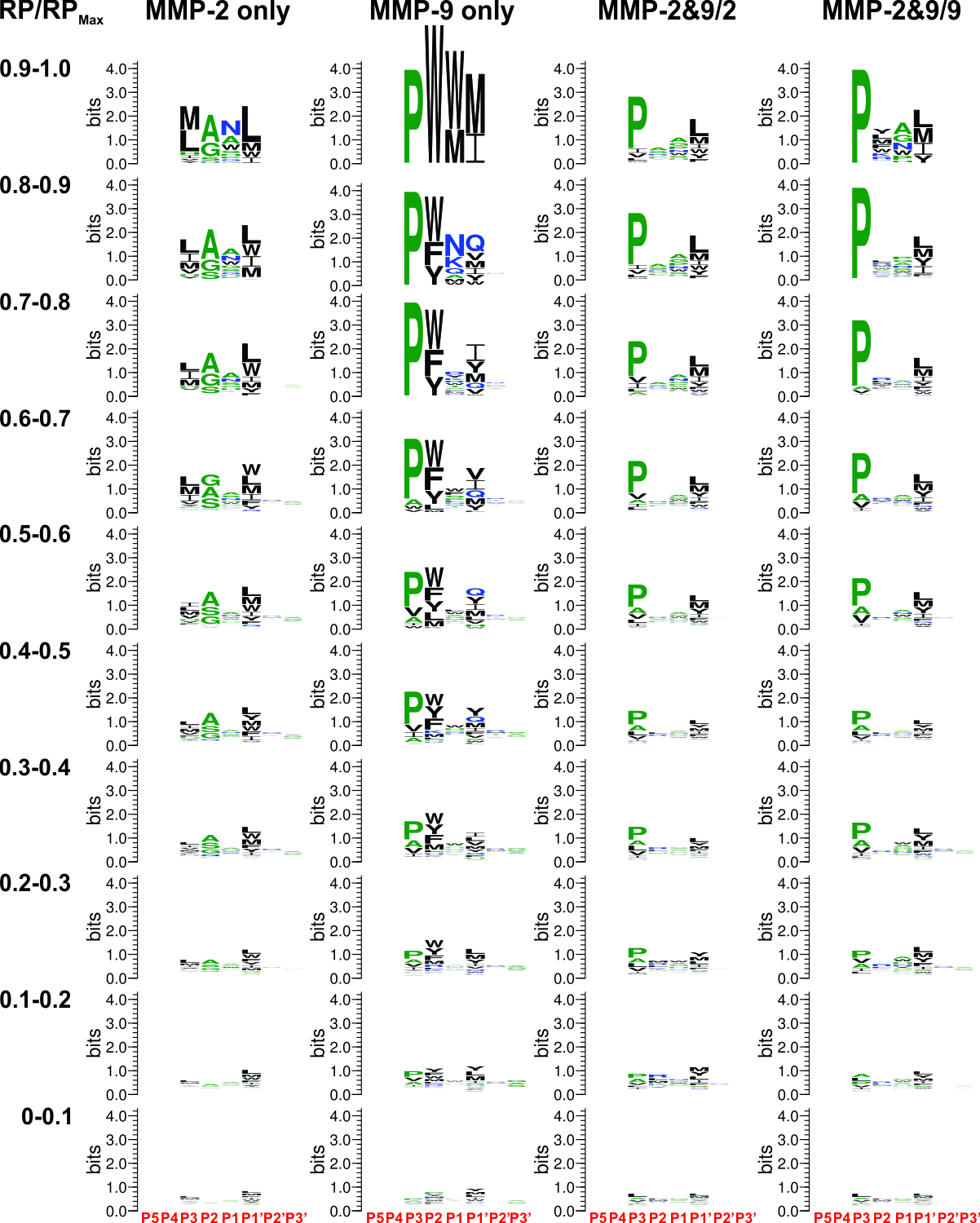

Supplement: S2 Fig — Logo plots demonstrate the composition of substrates across P5 to P3՛ positions as a function of RP/RPMax. Peptide hexamers belonging to tetramer clusters in the selectomes of MMP-2 and 9 were aligned across P3-P1՛ positions and divided into 10 groups based on their RP values relative to the maximum (RP/RPMax). First and second columns of logo plots correspond to unique substrates of MMP-2 and 9 selectomes, respectively. The third and fourth columns of logo plots represent the common set of MMP-2 and 9 selectomes, respectively. The RP values for the corresponding tetramer clusters have been calculated either according to MMP-2 (MMP-2&9/2) or MMP-9 (MMP-2&9/9) ranking. (TIF) [file pcbi.1008101.s002.tif]
