# Supplementary material for: Quantitative profiling of protease specificity
Source: PLoS Comput Biol. 2021 Feb 22;17(2):e1008101. doi: 10.1371/journal.pcbi.1008101 (PMC7932537; doi:10.1371/journal.pcbi.1008101)
Supplement: S11 Table — List of tetramer clusters common between the selectomes of MMP-2 and 9 together with the corresponding hexamer sequences aligned across P3-P1՛ positions of substrates. Tetramers are ranked according to MMP-2 relative probability. Information about the tetramer amino acids sequence, rank, number of hexamers and relative probability of a cluster is provided in the header of each tetramer cluster. (PDF) [file pcbi.1008101.s013.pdf]

S12 Table. Analysis of combined selectomes of MMP-2 and 9. Common set of MMP-2 and 9 selectomes reranked according to MMP-2 relative probability

tetramer, rank, number_of_hexamers_in_tetramer_cluster, relative_probability

PSNL 1 933 36.52

--PSNLRQ
--PSNLGD
--PSNLDM
-APSNLR-
ALPSNL--
AQPSNL--
-APSNLH-
-APSNLS-
-APSNLL-
ASPSNL--
-APSNLT-
AWPSNL--
ATPSNL--
AEPSNL--
AIPSNL--
ADPSNL--
-APSNLV-
-APSNLI-
-APSNLG-
AMPSNL--
AAPSNL--
APPSNL--
AKPSNL--
-APSNLM-
AFPSNL--
AYPSNL--
-APSNLQ-
AGPSNL--
AVPSNL--
-APSNLD-
-APSNLA-
ANPSNL--
AHPSNL--
-APSNLE-
-APSNLN-
-APSNLF-
-APSNLC-
ACPSNL--
CLPSNL--
CTPSNL--
-CPSNLA-
-CPSNLL-
-CPSNLQ-
-CPSNLG-

CIPSNL--
CSPSNL--
CCPSNL--
CVPSNL--
-CPSNLR-
CNPSNL--
CMPSNL--
CDPSNL--
CKPSNL--
CGPSNL--
-CPSNLW-
-CPSNLV-
-CPSNLT-
-CPSNLN-
-CPSNLM-
-CPSNLK-
-CPSNLI-
-CPSNLF-
CHPSNL--
CFPSNL--
CYPSNL--
CWPSNL--
CQPSNL--
-CPSNLY-
-CPSNLS-
-CPSNLH-
CEPSNL--
CAPSNL--
-DPSNLL-
-DPSNLS-
-DPSNLR-
DMPSNL--
DWPSNL--
DKPSNL--
DSPSNL--
-DPSNLY-
DLPSNL--
DFPSNL--
DRPSNL--
-DPSNLQ-
-DPSNLF-
DAPSNL--
DQPSNL--
-DPSNLV-
-DPSNLM-
-DPSNLG-
DGPSNL--
DVPSNL--
DTPSNL--
DIPSNL--

DEPSNL--
DYPSNL--
-DPSNLN-
-DPSNLK-
-DPSNLI-
-DPSNLH-
-DPSNLA-
DPPSNL--
DNPSNL--
DCPSNL--
-EPSNLR-
ELPSNL--
EMPSNL--
ERPSNL--
-EPSNLY-
ESPSNL--
-EPSNLS-
EVPSNL--
ETPSNL--
-EPSNLV-
EIPSNL--
-EPSNLW-
EHPSNL--
EFPSNL--
EWPSNL--
-EPSNLK-
-EPSNLI-
EYPSNL--
-EPSNLQ-
-EPSNLN-
EKPSNL--
EQPSNL--
-EPSNLF-
ENPSNL--
EDPSNL--
-EPSNLT-
EGPSNL--
EEPSNL--
EAPSNL--
-EPSNLM-
-EPSNLH-
-EPSNLA-
-FPSNLL-
FRPSNL--
FIPSNL--
-FPSNLT-
FVPSNL--
-FPSNLR-
FLPSNL--
FAPSNL--

FWPSNL--
-FPSNLN-
-FPSNLY-
-FPSNLV-
FMPSNL--
FGPSNL--
FFPSNL--
FEPSNL--
-FPSNLW-
-FPSNLK-
-FPSNLI-
-FPSNLH-
FHPSNL--
FTPSNL--
-FPSNLQ-
-FPSNLG-
FKPSNL--
-FPSNLE-
FQPSNL--
-FPSNLA-
FDPSNL--
FCPSNL--
-FPSNLM-
-FPSNLF-
-FPSNLD-
FNPSNL--
-GPSNLY-
GWPSNL--
GVPSNL--
GTPSNL--
-GPSNLS-
-GPSNLL-
GGPSNL--
GSPSNL--
-GPSNLV-
-GPSNLR-
-GPSNLQ-
GRPSNL--
GEPSNL--
-GPSNLK-
-GPSNLA-
GLPSNL--
-GPSNLG-
-GPSNLF-
GMPSNL--
GIPSNL--
-GPSNLW-
-GPSNLM-
-GPSNLH-
-GPSNLN-

GPPSNL--
GNPSNL--
GYPSNL--
-GPSNLD-
GKPSNL--
GAPSNL--
GQPSNL--
-GPSNLI-
GCPSNL--
-GPSNLC-
-GPSNLE-
GHPSNL--
GDPSNL--
HGPSNL--
HVPSNL--
HSPSNL--
HRPSNL--
-HPSNLL-
HYPSNL--
HWPSNL--
-HPSNLT-
-HPSNLF-
HLPSNL--
-HPSNLY-
-HPSNLR-
HQPSNL--
-HPSNLG-
HKPSNL--
-HPSNLA-
HAPSNL--
-HPSNLW-
HNPSNL--
HIPSNL--
-HPSNLM-
HHPSNL--
-HPSNLV-
-HPSNLI-
-HPSNLK-
-HPSNLH-
HFPSNL--
-HPSNLQ-
-HPSNLE-
-HPSNLD-
HPPSNL--
HEPSNL--
-IPSNLS-
IRPSNL--
ILPSNL--
IYPSNL--
-IPSNLR-

ISPSNL--
-IPSNLG-
IWPSNL--
IVPSNL--
-IPSNLT-
-IPSNLI-
-IPSNLL-
IHPSNL--
ITPSNL--
-IPSNLK-
-IPSNLY-
-IPSNLQ-
-IPSNLA-
IIPSNL--
-IPSNLV-
-IPSNLH-
IAPSNL--
INPSNL--
-IPSNLE-
IDPSNL--
-IPSNLW-
IPPSNL--
IQPSNL--
-IPSNLD-
IMPSNL--
IEPSNL--
ICPSNL--
-IPSNLN-
-IPSNLM-
-IPSNLF-
KSPSNL--
-KPSNLR-
KGPSNL--
-KPSNLY-
KNPSNL--
-KPSNLI-
-KPSNLL-
-KPSNLK-
-KPSNLF-
-KPSNLG-
KYPSNL--
-KPSNLA-
KAPSNL--
-KPSNLS-
KFPSNL--
KTPSNL--
-KPSNLW-
KMPSNL--
KHPSNL--
KDPSNL--

-KPSNLH-
KPPSNL--
KLPSNL--
KQPSNL--
-KPSNLE-
-KPSNLN-
KKPSNL--
KIPSNL--
-KPSNLM-
-KPSNLT-
KEPSNL--
KWPSNL--
KCPSNL--
-KPSNLQ-
-LPSNLR-
LSPSNL--
-LPSNLY-
LLPSNL--
-LPSNLV-
-LPSNLT-
LAPSNL--
-LPSNLS-
LIPSNL--
LGPSNL--
-LPSNLG-
LVPSNL--
-LPSNLK-
LMPSNL--
-LPSNLM-
-LPSNLH-
LFPSNL--
LDPSNL--
LYPSNL--
LKPSNL--
LTPSNL--
LQPSNL--
-LPSNLI-
LHPSNL--
-LPSNLQ-
-LPSNLA-
LEPSNL--
-LPSNLW-
-LPSNLN-
-LPSNLE-
LCPSNL--
-LPSNLD-
MLPSNL--
MRPSNL--
MAPSNL--
-MPSNLT-

-MPSNLS-
MVPSNL--
-MPSNLI-
-MPSNLG-
-MPSNLQ-
-MPSNLH-
-MPSNLA-
MSPSNL--
-MPSNLL-
-MPSNLF-
MIPSNL--
MWPSNL--
-MPSNLV-
MGPSNL--
-MPSNLN-
-MPSNLM-
-MPSNLK-
-MPSNLE-
MNPSNL--
MYPSNL--
MFPSNL--
MQPSNL--
-MPSNLY-
-MPSNLD-
MMPSNL--
-MPSNLW-
MPPSNL--
MHPSNL--
MKPSNL--
MEPSNL--
NHPSNL--
-NPSNLR-
-NPSNLI-
NLPSNL--
NGPSNL--
NSPSNL--
NIPSNL--
NWPSNL--
-NPSNLF-
NAPSNL--
-NPSNLA-
NCPSNL--
NYPSNL--
-NPSNLY-
-NPSNLG-
NPPSNL--
NMPSNL--
NVPSNL--
-NPSNLQ-
-NPSNLK-

NNPSNL--
NTPSNL--
NQPSNL--
-NPSNLT-
NEPSNL--
-NPSNLW-
-NPSNLN-
NKPSNL--
-NPSNLV-
-NPSNLH-
-NPSNLM-
-NPSNLE-
NDPSNL--
--PSNLRL
--PSNLLL
--PSNLTL
--PSNLLS
PSPSNL--
--PSNLSR
--PSNLFR
--PSNLVR
--PSNLFS
--PSNLKS
--PSNLRA
--PSNLLR
--PSNLLT
--PSNLSL
--PSNLGS
--PSNLRT
PLPSNL--
--PSNLRS
--PSNLRG
--PSNLRR
--PSNLGR
--PSNLWR
--PSNLIV
--PSNLWS
--PSNLSQ
--PSNLVI
--PSNLTI
--PSNLSS
--PSNLAG
--PSNLRY
--PSNLLK
--PSNLWT
--PSNLGG
--PSNLFL
--PSNLVG
--PSNLNL
--PSNLGM

PGPSNL--
--PSNLTS
--PSNLSY
--PSNLSV
--PSNLRI
--PSNLMR
--PSNLKY
--PSNLAS
--PSNLST
--PSNLRV
--PSNLGI
--PSNLYR
--PSNLTV
--PSNLMG
--PSNLWQ
--PSNLRK
--PSNLNR
--PSNLLN
--PSNLGV
--PSNLFN
--PSNLSW
--PSNLSK
--PSNLQW
--PSNLMH
--PSNLGF
--PSNLAQ
--PSNLYS
--PSNLVK
--PSNLVH
--PSNLTR
--PSNLTA
--PSNLSA
--PSNLQR
--PSNLLQ
--PSNLLI
--PSNLKV
--PSNLGT
--PSNLER
--PSNLVY
--PSNLTM
--PSNLRW
--PSNLNT
--PSNLIG
--PSNLHL
--PSNLFV
--PSNLFG
PAPSNL--
PVPSNL--
--PSNLVS
--PSNLKQ

--PSNLHS
--PSNLGH
--PSNLAW
--PSNLYG
--PSNLWL
--PSNLVQ
--PSNLSN
--PSNLRM
--PSNLRD
--PSNLNM
--PSNLLW
--PSNLLA
--PSNLFY
--PSNLFT
--PSNLFI
--PSNLAT
--PSNLAI
--PSNLAA
PFPSNL--
--PSNLYT
--PSNLYN
--PSNLYL
--PSNLVW
--PSNLTT
--PSNLRP
--PSNLQS
--PSNLQL
--PSNLMT
--PSNLMN
--PSNLIQ
--PSNLHR
--PSNLFK
--PSNLDS
--PSNLWM
--PSNLWG
--PSNLTK
--PSNLMA
--PSNLLH
--PSNLKN
--PSNLKG
--PSNLIT
--PSNLIS
--PSNLHQ
--PSNLAV
--PSNLTN
--PSNLTG
--PSNLSM
--PSNLSG
--PSNLNW
--PSNLMY

```
--PSNLKK
--PSNLIK
--PSNLHM
--PSNLGN
--PSNLFW
PTPSNL--
--PSNLWW
--PSNLWN
--PSNLWI
--PSNLWH
--PSNLWE
--PSNLWA
--PSNLTH
--PSNLRE
--PSNLQY
--PSNLQN
--PSNLQA
--PSNLMK
--PSNLMF
--PSNLKI
--PSNLIA
--PSNLHI
--PSNLFQ
--PSNLES
--PSNLEF
--PSNLAM
--PSNLAK
PNPSNL--
PHPSNL--
--PSNLYK
--PSNLYI
--PSNLVM
--PSNLVF
--PSNLTW
--PSNLSD
--PSNLQH
--PSNLNN
--PSNLND
--PSNLMS
--PSNLLM
--PSNLGY
--PSNLFM
--PSNLET
--PSNLDG
--PSNLAH
--PSNLYD
--PSNLTD
--PSNLNK
--PSNLNI
--PSNLNG
```

--PSNLMV
--PSNLMD
--PSNLKT
--PSNLIW
--PSNLHT
--PSNLHN
--PSNLHG
--PSNLHF
--PSNLHA
--PSNLGK
--PSNLEQ
--PSNLEL
--PSNLEA
--PSNLAN
PIPSNL--
PYPSNL--
--PSNLYY
--PSNLYF
--PSNLWV
--PSNLWK
--PSNLTY
--PSNLTF
--PSNLSH
--PSNLSE
--PSNLQI
--PSNLMQ
--PSNLMP
--PSNLMI
--PSNLII
--PSNLIH
--PSNLHK
--PSNLHH
--PSNLFH
--PSNLFD
--PSNLCS
--PSNLAF
PEPSNL--
--PSNLYA
--PSNLWD
--PSNLVD
--PSNLTQ
--PSNLRF
--PSNLQV
--PSNLQF
--PSNLLD
--PSNLIM
--PSNLHW
--PSNLHE
--PSNLFE
--PSNLAD

PQPSNL--
PKPSNL--
--PSNLYW
--PSNLYE
--PSNLVV
--PSNLVN
--PSNLQM
--PSNLQE
--PSNLPL
--PSNLNV
--PSNLNQ
--PSNLNH
--PSNLKM
--PSNLIY
--PSNLIN
--PSNLHV
--PSNLHD
--PSNLGQ
--PSNLEV
--PSNLEM
--PSNLEH
--PSNLCT
PWPSNL--
--PSNLWF
--PSNLVP
--PSNLVE
--PSNLTC
--PSNLQK
--PSNLQG
--PSNLPM
--PSNLPK
--PSNLNF
--PSNLKW
--PSNLKE
--PSNLIP
--PSNLIE
--PSNLID
--PSNLHY
--PSNLGE
--PSNLFP
--PSNLEW
--PSNLEN
--PSNLEI
--PSNLEG
--PSNLED
--PSNLEC
--PSNLDT
--PSNLDR
--PSNLDA
--PSNLCV

--PSNLCQ
--PSNLCK
--PSNLCG
--PSNLAE
-PPSNLK-
-PPSNLG-
-PPSNLF-
-PPSNLA-
PPPSNL--
PDPSNL--
-QPSNLR-
QLPSNL--
-QPSNLG-
QIPSNL--
-QPSNLK-
QRPSNL--
-QPSNLL-
-QPSNLY-
-QPSNLS-
QVPSNL--
QQPSNL--
QWPSNL--
-QPSNLT-
QPPSNL--
QKPSNL--
QAPSNL--
-QPSNLW-
QMPSNL--
QFPSNL--
QYPSNL--
-QPSNLN-
-QPSNLA-
-QPSNLV-
-QPSNLD-
QDPSNL--
-QPSNLM-
QCPSNL--
-QPSNLH-
QHPSNL--
-QPSNLQ-
-QPSNLF-
QNPSNL--
QEPSNL--
RVPSNL--
RIPSNL--
RSPSNL--
-RPSNLL-
-RPSNLS-
RMPSNL--
-RPSNLV-

RWPSNL--
-RPSNLW-
-RPSNLK-
RRPSNL--
RFPSNL--
RTPSNL--
-RPSNLA-
RGPSNL--
RYPSNL--
-RPSNLM-
-RPSNLI-
-RPSNLN-
-RPSNLH-
RAPSNL--
RKPSNL--
RHPSNL--
RNPSNL--
-RPSNLD-
RQPSNL--
RPPSNL--
-RPSNLY-
-RPSNLE-
-RPSNLQ-
RDPSNL--
RCPSNL--
-RPSNLC-
-SPSNLL-
-SPSNLW-
SRPSNL--
SMPSNL--
SSPSNL--
-SPSNLS-
SWPSNL--
STPSNL--
-SPSNLR-
-SPSNLV-
SNPSNL--
SAPSNL--
-SPSNLH-
-SPSNLK-
-SPSNLI-
SKPSNL--
SFPSNL--
-SPSNLN-
-SPSNLG-
SYPSNL--
SQPSNL--
-SPSNLF-
-SPSNLQ-
SGPSNL--

−SPSNLM−
−SPSNLA−
−SPSNLY−
−SPSNLD−
SEPSNL−−
SDPSNL−−
SCPSNL−−
SPPSNL−−
−SPSNLE−
−SPSNLC−
−TPSNLR−
TLPSNL−−
TRPSNL−−
TVPSNL−−
THPSNL−−
−TPSNLS−
−TPSNLM−
−TPSNLG−
−TPSNLL−
−TPSNLV−
TQPSNL−−
−TPSNLA−
TPPSNL−−
TMPSNL−−
TTPSNL−−
TNPSNL−−
TIPSNL−−
−TPSNLF−
−TPSNLK−
−TPSNLE−
TAPSNL−−
TWPSNL−−
−TPSNLQ−
TGPSNL−−
−TPSNLY−
−TPSNLN−
−TPSNLI−
−TPSNLH−
TKPSNL−−
TDPSNL−−
TCPSNL−−
−TPSNLW−
−TPSNLD−
VLPSNL−−
−VPSNLL−
−VPSNLI−
VRPSNL−−
VSPSNL−−
VNPSNL−−
−VPSNLN−

```
-VPSNLV-
VYPSNL--
VHPSNL--
VTPSNL--
-VPSNLT-
VWPSNL--
-VPSNLM-
VQPSNL--
-VPSNLK-
-VPSNLY-
VGPSNL--
VFPSNL--
VEPSNL--
-VPSNLG-
VPPSNL--
-VPSNLQ-
-VPSNLF-
VMPSNL--
-VPSNLH-
-VPSNLA-
VCPSNL--
-VPSNLD-
VDPSNL--
VAPSNL--
-WPSNLA-
WFPSNL--
-WPSNLL-
WSPSNL--
-WPSNLR-
-WPSNLS-
WGPSNL--
-WPSNLM-
-WPSNLV-
-WPSNLT-
WIPSNL--
WWPSNL--
-WPSNLI-
WMPSNL--
WKPSNL--
WQPSNL--
-WPSNLG-
WAPSNL--
WVPSNL--
-WPSNLN-
-WPSNLH-
WPPSNL--
WEPSNL--
-WPSNLY-
-WPSNLW-
-WPSNLQ-
```

| | | | |
|---|---|---|---|
| | WDPSNL-- | | |
| | -WPSNLF- | | |
| | -WPSNLE- | | |
| | WHPSNL-- | | |
| | -YPSNLR- | | |
| | -YPSNLI- | | |
| | YGPSNL-- | | |
| | YSPSNL-- | | |
| | YNPSNL-- | | |
| | YLPSNL-- | | |
| | -YPSNLS- | | |
| | YQPSNL-- | | |
| | YWPSNL-- | | |
| | -YPSNLT- | | |
| | -YPSNLK- | | |
| | YPPSNL-- | | |
| | YTPSNL-- | | |
| | YFPSNL-- | | |
| | YAPSNL-- | | |
| | YYPSNL-- | | |
| | YVPSNL-- | | |
| | -YPSNLH- | | |
| | -YPSNLG- | | |
| | -YPSNLW- | | |
| | -YPSNLV- | | |
| | -YPSNLQ- | | |
| | -YPSNLA- | | |
| | YIPSNL-- | | |
| | -YPSNLN- | | |
| | YHPSNL-- | | |
| | YEPSNL-- | | |
| | YCPSNL-- | | |
| | -YPSNLD- | | |
| | YMPSNL-- | | |
| | YKPSNL-- | | |
| | -YPSNLF- | | |
| | -YPSNLC- | | |
| | -YPSNLM- | | |
| | YDPSNL-- | | |
| PASL | 9 | 877 | 36.43 |
| | -DPASLY- | | |
| | -KPASLC- | | |
| | --PASLTP | | |
| | --PASLPN | | |
| | --PASLPH | | |
| | --PASLDQ | | |
| | --PASLDL | | |
| | -RPASLP- | | |
| | -SPASLD- | | |
| | -APASLR- | | |

```
-APASLW-
-APASLL-
ARPASL--
ATPASL--
-APASLG-
AIPASL--
ALPASL--
-APASLK-
-APASLS-
ASPASL--
AGPASL--
AVPASL--
AQPASL--
-APASLE-
AYPASL--
-APASLM-
-APASLV-
-APASLF-
AMPASL--
-APASLQ-
-APASLD-
APPASL--
-APASLH-
ANPASL--
AKPASL--
AWPASL--
-APASLA-
-APASLI-
AHPASL--
AAPASL--
AEPASL--
ACPASL--
CSPASL--
CRPASL--
-CPASLL-
-CPASLV-
-CPASLR-
-CPASLA-
CKPASL--
CVPASL--
-CPASLT-
-CPASLI-
CTPASL--
-CPASLS-
CIPASL--
CHPASL--
CEPASL--
-CPASLF-
CAPASL--
CPPASL--
```

−CPASLY−
−CPASLM−
CFPASL−−
CWPASL−−
CQPASL−−
−CPASLN−
−CPASLG−
CNPASL−−
CDPASL−−
DTPASL−−
−DPASLR−
DSPASL−−
DRPASL−−
−DPASLL−
−DPASLV−
DYPASL−−
−DPASLW−
−DPASLA−
DMPASL−−
DKPASL−−
DGPASL−−
DQPASL−−
DLPASL−−
DFPASL−−
DCPASL−−
DWPASL−−
DVPASL−−
−DPASLN−
−DPASLF−
−DPASLT−
−DPASLS−
−DPASLK−
−DPASLI−
−DPASLM−
DAPASL−−
−DPASLH−
−DPASLG−
DNPASL−−
DHPASL−−
−DPASLE−
DIPASL−−
DEPASL−−
−EPASLR−
ELPASL−−
−EPASLL−
−EPASLV−
−EPASLS−
EKPASL−−
ETPASL−−
EAPASL−−

```
-EPASLK-
-EPASLG-
EGPASL--
ERPASL--
EYPASL--
ESPASL--
-EPASLH-
-EPASLW-
-EPASLA-
-EPASLM-
EIPASL--
EVPASL--
EQPASL--
ENPASL--
-EPASLQ-
EHPASL--
EWPASL--
-EPASLF-
EFPASL--
-EPASLN-
-EPASLE-
EDPASL--
ECPASL--
EMPASL--
EEPASL--
-FPASLL-
-FPASLV-
-FPASLR-
-FPASLN-
-FPASLT-
-FPASLY-
FGPASL--
FSPASL--
FAPASL--
-FPASLS-
-FPASLK-
FMPASL--
FEPASL--
FWPASL--
FQPASL--
FKPASL--
FYPASL--
-FPASLQ-
-FPASLH-
FHPASL--
FPPASL--
-FPASLF-
-FPASLW-
-FPASLE-
FDPASL--
```

-FPASLM-
FCPASL--
-GPASLR-
-GPASLS-
-GPASLL-
-GPASLK-
GRPASL--
-GPASLW-
GSPASL--
GIPASL--
GVPASL--
GFPASL--
-GPASLI-
-GPASLN-
GTPASL--
GMPASL--
-GPASLV-
-GPASLE-
-GPASLH-
GEPASL--
GYPASL--
GPPASL--
-GPASLY-
GHPASL--
GAPASL--
GQPASL--
GDPASL--
-GPASLG-
GCPASL--
-GPASLD-
HRPASL--
HVPASL--
-HPASLL-
HHPASL--
-HPASLN-
HLPASL--
HYPASL--
HWPASL--
HTPASL--
-HPASLY-
-HPASLV-
HIPASL--
HGPASL--
HFPASL--
-HPASLK-
-HPASLA-
HPPASL--
HQPASL--
-HPASLH-
-HPASLG-

HAPASL--
-HPASLE-
-HPASLM-
HNPASL--
HDPASL--
HCPASL--
-HPASLQ-
-HPASLI-
-HPASLC-
HMPASL--
HEPASL--
IRPASL--
-IPASLR-
IVPASL--
-IPASLN-
ISPASL--
-IPASLG-
-IPASLQ-
ILPASL--
-IPASLT-
-IPASLS-
-IPASLH-
IGPASL--
IAPASL--
-IPASLK-
-IPASLL-
IFPASL--
-IPASLY-
-IPASLV-
IWPASL--
IPPASL--
IIPASL--
IYPASL--
-IPASLM-
INPASL--
IHPASL--
IMPASL--
-IPASLF-
-IPASLE-
IKPASL--
IEPASL--
-IPASLW-
IDPASL--
IQPASL--
-IPASLD-
KSPASL--
-KPASLG-
KLPASL--
-KPASLV-
KAPASL--

-KPASLT-
-KPASLA-
KGPASL--
KPPASL--
-KPASLM-
KIPASL--
-KPASLY-
-KPASLW-
-KPASLK-
KYPASL--
KNPASL--
KFPASL--
-KPASLS-
-KPASLF-
-KPASLQ-
-KPASLI-
KEPASL--
KMPASL--
KHPASL--
KDPASL--
KQPASL--
KWPASL--
KKPASL--
KCPASL--
-KPASLE-
-KPASLD-
LLPASL--
-LPASLS-
LSPASL--
-LPASLK-
LTPASL--
-LPASLA-
-LPASLG-
LHPASL--
-LPASLM-
LFPASL--
-LPASLN-
LMPASL--
LNPASL--
LEPASL--
LYPASL--
LQPASL--
-LPASLY-
LDPASL--
-LPASLI-
-LPASLF-
-LPASLW-
-LPASLH-
LWPASL--
-LPASLV-

-LPASLE-
LCPASL--
-MPASLR-
MSPASL--
MRPASL--
-MPASLL-
-MPASLK-
MMPASL--
MVPASL--
-MPASLN-
MTPASL--
-MPASLS-
-MPASLY-
-MPASLG-
MFPASL--
-MPASLQ-
-MPASLF-
MGPASL--
MAPASL--
MPPASL--
-MPASLM-
-MPASLI-
MNPASL--
MIPASL--
-MPASLW-
-MPASLT-
MYPASL--
-MPASLE-
-MPASLA-
MEPASL--
MDPASL--
-MPASLV-
-MPASLH-
-MPASLD-
MHPASL--
-MPASLP-
MCPASL--
-NPASLA-
NRPASL--
NGPASL--
NMPASL--
-NPASLR-
NKPASL--
NSPASL--
-NPASLW-
NAPASL--
-NPASLN-
NPPASL--
-NPASLG-
-NPASLS-

NFPASL--
NQPASL--
-NPASLK-
NIPASL--
NYPASL--
-NPASLT-
-NPASLH-
-NPASLM-
NEPASL--
-NPASLI-
NWPASL--
NTPASL--
-NPASLV-
-NPASLF-
NHPASL--
NNPASL--
-NPASLE-
NDPASL--
-NPASLQ-
NCPASL--
-NPASLD-
--PASLLR
--PASLRS
--PASLRR
--PASLLS
--PASLGL
--PASLNL
--PASLRK
--PASLFR
--PASLVR
--PASLSS
--PASLWR
--PASLLT
--PASLTH
--PASLAS
--PASLAR
--PASLYL
--PASLSI
--PASLRT
--PASLMS
--PASLKG
--PASLVL
--PASLSY
--PASLSG
--PASLKR
--PASLGS
--PASLSK
--PASLRY
--PASLIS
--PASLNR

--PASLLN
--PASLIT
--PASLYV
--PASLNS
--PASLTI
--PASLRQ
--PASLRA
--PASLLV
--PASLLH
--PASLHL
--PASLWA
--PASLRH
--PASLLG
--PASLGY
--PASLWS
--PASLWG
--PASLSA
--PASLRD
--PASLMR
--PASLKH
--PASLFA
--PASLSV
--PASLRN
--PASLVT
--PASLTL
--PASLRV
--PASLNK
--PASLHR
--PASLGT
--PASLVF
--PASLMG
--PASLKT
--PASLHS
--PASLAV
--PASLYS
--PASLWI
--PASLNM
--PASLME
--PASLLQ
--PASLIW
--PASLYY
--PASLTV
--PASLQR
--PASLNI
--PASLGF
--PASLFG
--PASLAG
PRPASL--
--PASLTN
--PASLMW

--PASLIL
--PASLHI
--PASLGV
--PASLFN
--PASLWH
--PASLTK
--PASLTF
--PASLSQ
--PASLMI
--PASLIN
--PASLFK
--PASLWT
--PASLTG
--PASLQG
--PASLKL
--PASLKI
--PASLIG
--PASLHQ
--PASLAW
--PASLAH
--PASLVM
--PASLTT
--PASLGM
--PASLFT
--PASLES
--PASLDS
--PASLYN
--PASLYI
--PASLYG
--PASLVK
--PASLVI
--PASLTY
--PASLTW
--PASLST
--PASLSM
--PASLQS
--PASLPL
--PASLMV
--PASLKF
--PASLIA
--PASLHW
--PASLAF
PSPASL--
PNPASL--
PIPASL--
--PASLYW
--PASLWD
--PASLTA
--PASLRW
--PASLQV

--PASLQN
--PASLQM
--PASLNN
--PASLLD
--PASLKA
--PASLFV
--PASLEA
--PASLAN
--PASLWN
--PASLVN
--PASLTQ
--PASLQL
--PASLQI
--PASLNT
--PASLGW
--PASLGI
--PASLER
--PASLEQ
PTPASL--
--PASLYK
--PASLRP
--PASLRM
--PASLQW
--PASLMQ
--PASLML
--PASLKY
--PASLKN
--PASLHV
--PASLHM
--PASLGQ
--PASLFS
--PASLDG
PAPASL--
PGPASL--
--PASLTE
--PASLQA
--PASLNH
--PASLLE
--PASLKM
--PASLIY
--PASLHF
--PASLET
PMPASL--
--PASLYH
--PASLVH
--PASLVE
--PASLNW
--PASLNE
--PASLMY
--PASLMK

--PASLMF
--PASLKQ
--PASLID
--PASLFI
--PASLFD
--PASLEV
--PASLEN
PYPASL--
PLPASL--
PFPASL--
--PASLYQ
--PASLWV
--PASLWL
--PASLWK
--PASLWE
--PASLVD
--PASLSD
--PASLRE
--PASLQT
--PASLMH
--PASLMA
--PASLKD
--PASLHT
--PASLHH
--PASLFH
--PASLFE
--PASLEL
--PASLAT
--PASLAE
--PASLAD
PKPASL--
PDPASL--
PCPASL--
--PASLYA
--PASLWQ
--PASLWM
--PASLQK
--PASLPM
--PASLNY
--PASLNF
--PASLNA
--PASLKE
--PASLIQ
--PASLGN
--PASLGA
--PASLEW
PWPASL--
PHPASL--
PEPASL--
--PASLYE

--PASLYD
--PASLTM
--PASLQY
--PASLMN
--PASLMD
--PASLIM
--PASLHY
--PASLHD
--PASLGK
--PASLEY
--PASLEK
--PASLAQ
--PASLAK
--PASLHN
--PASLHA
--PASLAM
--PASLWW
--PASLQD
--PASLPY
--PASLPF
--PASLND
--PASLMM
--PASLKV
--PASLHK
--PASLHE
--PASLGH
--PASLFQ
--PASLEH
--PASLEG
--PASLAI
-PPASLS-
--PASLVC
--PASLTD
--PASLQQ
--PASLQH
--PASLNG
--PASLKW
--PASLEM
--PASLEE
--PASLCT
PQPASL--
PPPASL--
-PPASLR-
-PPASLQ-
-PPASLM-
-PPASLL-
-PPASLG-
-PPASLE-
--PASLQE
--PASLNQ

--PASLNC
--PASLLC
--PASLFC
--PASLEI
--PASLEF
--PASLDY
--PASLDI
--PASLDF
--PASLCY
--PASLCS
--PASLCM
--PASLCL
--PASLCG
-QPASLT-
-QPASLR-
QFPASL--
QSPASL--
-QPASLL-
-QPASLF-
-QPASLI-
-QPASLM-
QMPASL--
QVPASL--
QYPASL--
-QPASLW-
-QPASLH-
QEPASL--
-QPASLY-
-QPASLN-
-QPASLV-
-QPASLA-
QNPASL--
QWPASL--
-QPASLD-
QPPASL--
-QPASLQ-
-QPASLK-
QHPASL--
QKPASL--
QTPASL--
QAPASL--
QDPASL--
-QPASLE-
RLPASL--
RSPASL--
-RPASLS-
RVPASL--
RIPASL--
RAPASL--
RYPASL--

RWPASL--
RTPASL--
RGPASL--
-RPASLA-
RPPASL--
-RPASLK-
RFPASL--
-RPASLH-
-RPASLM-
RHPASL--
-RPASLI-
-RPASLF-
-RPASLQ-
-RPASLY-
-RPASLT-
RNPASL--
-RPASLE-
RCPASL--
-RPASLC-
-SPASLM-
SLPASL--
-SPASLA-
SVPASL--
SHPASL--
SGPASL--
SMPASL--
SFPASL--
-SPASLW-
SYPASL--
-SPASLT-
-SPASLN-
SIPASL--
-SPASLH-
-SPASLF-
-SPASLE-
SDPASL--
-SPASLK-
SNPASL--
SEPASL--
SKPASL--
SAPASL--
SPPASL--
SQPASL--
SCPASL--
-SPASLC-
TRPASL--
TLPASL--
-TPASLT-
TSPASL--
-TPASLF-

-TPASLA-
THPASL--
-TPASLS-
-TPASLK-
TMPASL--
-TPASLH-
-TPASLG-
TPPASL--
TYPASL--
TIPASL--
-TPASLN-
-TPASLM-
TAPASL--
-TPASLY-
-TPASLQ-
TFPASL--
TQPASL--
-TPASLI-
-TPASLW-
-TPASLE-
TEPASL--
TWPASL--
TVPASL--
-TPASLV-
TNPASL--
TDPASL--
TCPASL--
-TPASLD-
-TPASLC-
-VPASLT-
VRPASL--
VLPASL--
VAPASL--
VSPASL--
VPPASL--
-VPASLG-
VGPASL--
-VPASLV-
-VPASLN-
-VPASLY-
-VPASLK-
VFPASL--
VYPASL--
VEPASL--
VTPASL--
VNPASL--
-VPASLH-
-VPASLQ-
-VPASLE-
VKPASL--

```
VHPASL--
VQPASL--
VWPASL--
-VPASLA-
-VPASLW-
-VPASLF-
-VPASLM-
VCPASL--
-VPASLD-
VDPASL--
WGPASL--
-WPASLT-
WPPASL--
-WPASLR-
-WPASLG-
WWPASL--
-WPASLY-
-WPASLS-
WNPASL--
-WPASLW-
-WPASLN-
WFPASL--
WTPASL--
WQPASL--
-WPASLL-
-WPASLK-
-WPASLE-
WEPASL--
WDPASL--
-WPASLM-
-WPASLV-
-WPASLH-
-WPASLI-
WCPASL--
-YPASLR-
YVPASL--
-YPASLS-
-YPASLL-
YSPASL--
-YPASLY-
-YPASLW-
YEPASL--
YKPASL--
YYPASL--
YQPASL--
YIPASL--
YHPASL--
YGPASL--
-YPASLQ-
YFPASL--
```

```
               -YPASLM-
               -YPASLI-
               -YPASLF-
               -YPASLN-
               -YPASLH-
               -YPASLG-
               YAPASL--
               YWPASL--
               -YPASLK-
               YDPASL--
               -YPASLA-
               YNPASL--
               -YPASLD-
               -YPASLV-
               -YPASLP-
               -YPASLE-
               YCPASL--
PSAL              6        897      36.19
               --PSALQP
               --PSALPV
               --PSALCL
               -SPSALD-
               -APSALR-
               ALPSAL--
               -APSALS-
               AVPSAL--
               -APSALL-
               -APSALT-
               AWPSAL--
               ATPSAL--
               ASPSAL--
               AIPSAL--
               AAPSAL--
               AQPSAL--
               -APSALK-
               -APSALA-
               AGPSAL--
               -APSALY-
               -APSALI-
               AYPSAL--
               ANPSAL--
               ADPSAL--
               -APSALV-
               -APSALM-
               ACPSAL--
               -APSALG-
               AMPSAL--
               AKPSAL--
               AHPSAL--
               AEPSAL--
```

-APSALE-
-APSALF-
APPSAL--
-APSALW-
-APSALN-
AFPSAL--
-APSALH-
-APSALQ-
-APSALD-
CKPSAL--
-CPSALT-
CIPSAL--
CVPSAL--
CSPSAL--
CRPSAL--
CMPSAL--
CTPSAL--
-CPSALS-
-CPSALR-
CCPSAL--
-CPSALI-
CLPSAL--
CAPSAL--
-CPSALL-
-CPSALK-
-CPSALA-
CPPSAL--
CGPSAL--
-CPSALY-
-CPSALV-
-CPSALN-
-CPSALE-
CNPSAL--
CEPSAL--
CWPSAL--
-CPSALM-
-CPSALH-
-CPSALG-
-CPSALF-
CFPSAL--
CDPSAL--
DIPSAL--
-DPSALT-
DLPSAL--
DYPSAL--
DRPSAL--
DTPSAL--
DSPSAL--
-DPSALS-
DWPSAL--

```
-DPSALL-
DFPSAL--
DQPSAL--
-DPSALW-
-DPSALF-
DMPSAL--
DNPSAL--
DAPSAL--
-DPSALI-
DHPSAL--
-DPSALV-
-DPSALR-
-DPSALH-
-DPSALG-
DGPSAL--
-DPSALY-
-DPSALN-
-DPSALM-
DKPSAL--
DEPSAL--
DVPSAL--
-DPSALQ-
-DPSALE-
-DPSALA-
EKPSAL--
ERPSAL--
-EPSALR-
EMPSAL--
ELPSAL--
ESPSAL--
-EPSALA-
EAPSAL--
EQPSAL--
-EPSALL-
-EPSALN-
-EPSALT-
-EPSALS-
-EPSALM-
-EPSALK-
-EPSALV-
EGPSAL--
-EPSALI-
-EPSALH-
-EPSALF-
-EPSALE-
EYPSAL--
-EPSALW-
-EPSALG-
ECPSAL--
EWPSAL--
```

EHPSAL--
-FPSALT-
-FPSALS-
-FPSALR-
FGPSAL--
FYPSAL--
FPPSAL--
FRPSAL--
-FPSALL-
FKPSAL--
-FPSALI-
FWPSAL--
FVPSAL--
FTPSAL--
FMPSAL--
FHPSAL--
-FPSALY-
-FPSALK-
FLPSAL--
FSPSAL--
FNPSAL--
FIPSAL--
-FPSALW-
-FPSALM-
-FPSALF-
-FPSALA-
FAPSAL--
-FPSALN-
-FPSALH-
FDPSAL--
FFPSAL--
FQPSAL--
-FPSALV-
-FPSALE-
FEPSAL--
FCPSAL--
-GPSALR-
GRPSAL--
-GPSALL-
-GPSALT-
GWPSAL--
-GPSALS-
GVPSAL--
GFPSAL--
GIPSAL--
-GPSALG-
-GPSALH-
GKPSAL--
GQPSAL--
GMPSAL--

GAPSAL--
-GPSALV-
-GPSALQ-
-GPSALF-
GGPSAL--
GNPSAL--
-GPSALN-
-GPSALM-
-GPSALY-
-GPSALW-
-GPSALI-
GPPSAL--
GHPSAL--
-GPSALE-
-GPSALK-
-GPSALA-
GEPSAL--
GDPSAL--
-GPSALC-
GCPSAL--
HRPSAL--
HGPSAL--
-HPSALI-
HVPSAL--
HLPSAL--
HAPSAL--
-HPSALR-
HTPSAL--
-HPSALK-
-HPSALG-
-HPSALY-
HKPSAL--
-HPSALL-
-HPSALV-
-HPSALT-
HYPSAL--
-HPSALQ-
-HPSALN-
HFPSAL--
-HPSALS-
HWPSAL--
HSPSAL--
-HPSALW-
-HPSALF-
HMPSAL--
-HPSALM-
-HPSALH-
HNPSAL--
HDPSAL--
HPPSAL--

HEPSAL--
-HPSALA-
-HPSALE-
-HPSALD-
HCPSAL--
-IPSALR-
IRPSAL--
ISPSAL--
IVPSAL--
-IPSALS-
-IPSALW-
-IPSALK-
IWPSAL--
-IPSALF-
IKPSAL--
IAPSAL--
IMPSAL--
IGPSAL--
IFPSAL--
IQPSAL--
-IPSALV-
-IPSALN-
INPSAL--
IIPSAL--
-IPSALY-
-IPSALE-
-IPSALL-
-IPSALH-
IHPSAL--
ITPSAL--
-IPSALM-
-IPSALG-
-IPSALA-
-IPSALQ-
IYPSAL--
IPPSAL--
IDPSAL--
ICPSAL--
KRPSAL--
-KPSALV-
KFPSAL--
KYPSAL--
KWPSAL--
-KPSALR-
-KPSALL-
KGPSAL--
KTPSAL--
-KPSALY-
-KPSALN-
KNPSAL--

KAPSAL--
-KPSALI-
-KPSALG-
KMPSAL--
-KPSALT-
KIPSAL--
-KPSALH-
-KPSALA-
KQPSAL--
-KPSALK-
KEPSAL--
-KPSALQ-
KPPSAL--
-KPSALM-
KDPSAL--
KCPSAL--
-KPSALE-
-KPSALW-
-KPSALD-
-KPSALC-
KHPSAL--
LRPSAL--
LSPSAL--
-LPSALR-
-LPSALS-
LVPSAL--
-LPSALK-
LIPSAL--
-LPSALL-
-LPSALF-
-LPSALA-
-LPSALY-
LFPSAL--
LAPSAL--
-LPSALN-
LWPSAL--
-LPSALV-
LNPSAL--
LQPSAL--
-LPSALW-
LKPSAL--
LPPSAL--
LTPSAL--
LMPSAL--
-LPSALH-
LYPSAL--
-LPSALI-
-LPSALM-
LDPSAL--
-LPSALQ-

LHPSAL--
LCPSAL--
MRPSAL--
-MPSALV-
MKPSAL--
MFPSAL--
-MPSALR-
MVPSAL--
MSPSAL--
-MPSALS-
MQPSAL--
-MPSALY-
-MPSALW-
-MPSALT-
MGPSAL--
MTPSAL--
-MPSALL-
-MPSALK-
MHPSAL--
MDPSAL--
MNPSAL--
MYPSAL--
-MPSALI-
MMPSAL--
MWPSAL--
-MPSALH-
-MPSALG-
MAPSAL--
-MPSALA-
MPPSAL--
-MPSALE-
-MPSALN-
-MPSALF-
MCPSAL--
NRPSAL--
-NPSALG-
-NPSALR-
NTPSAL--
NSPSAL--
-NPSALL-
NLPSAL--
-NPSALN-
NYPSAL--
-NPSALS-
-NPSALH-
NPPSAL--
NNPSAL--
NAPSAL--
-NPSALK-
-NPSALT-

```
-NPSALV-
-NPSALF-
NMPSAL--
NDPSAL--
-NPSALQ-
NKPSAL--
NHPSAL--
NQPSAL--
-NPSALI-
NGPSAL--
NFPSAL--
NCPSAL--
-NPSALW-
NEPSAL--
-NPSALM-
--PSALLL
--PSALLS
--PSALVR
--PSALSS
--PSALTR
--PSALVS
--PSALHR
--PSALRF
--PSALKR
--PSALWS
--PSALST
--PSALWY
--PSALLV
--PSALRV
--PSALRG
--PSALLA
--PSALSR
--PSALLT
--PSALKS
--PSALLG
--PSALLN
--PSALPL
--PSALGL
--PSALGA
--PSALSV
--PSALVK
--PSALLY
--PSALYW
--PSALMS
--PSALAS
--PSALYS
--PSALIL
--PSALYR
--PSALTS
--PSALSK
```

```
--PSALKI
--PSALIR
--PSALVG
--PSALVF
--PSALKG
--PSALGY
--PSALAY
--PSALAR
--PSALWT
--PSALVA
--PSALAL
--PSALVV
--PSALLH
--PSALIN
--PSALFT
PAPSAL--
--PSALYI
--PSALWG
--PSALKL
PSPSAL--
--PSALSI
--PSALSH
--PSALHS
--PSALGT
--PSALTV
--PSALSA
--PSALRT
--PSALGM
--PSALFI
--PSALAV
--PSALAK
--PSALAF
PLPSAL--
--PSALWQ
--PSALSE
--PSALRP
--PSALQK
--PSALWI
--PSALVT
--PSALSW
--PSALNT
--PSALNM
--PSALMT
--PSALHL
--PSALAT
PTPSAL--
--PSALTG
--PSALSQ
--PSALRQ
--PSALRH
```

--PSALNI
--PSALIT
--PSALFD
--PSALDS
PWPSAL--
--PSALWH
--PSALTT
--PSALRE
--PSALNS
--PSALMG
--PSALKV
--PSALFG
--PSALFA
--PSALAH
--PSALYP
--PSALVM
--PSALQS
--PSALMA
--PSALIV
--PSALGF
PVPSAL--
--PSALYA
--PSALWV
--PSALSN
--PSALRD
--PSALQR
--PSALQH
--PSALIQ
--PSALIG
--PSALHK
--PSALAG
--PSALAA
--PSALYM
--PSALVN
--PSALVH
--PSALSG
--PSALQG
--PSALNY
--PSALMK
--PSALMF
--PSALLQ
--PSALKN
--PSALGV
--PSALFH
--PSALEK
--PSALAW
--PSALTQ
--PSALSM
--PSALNR
--PSALNK

--PSALKW
--PSALIE
--PSALHA
--PSALET
--PSALAD
--PSALTN
--PSALTH
--PSALQY
--PSALQT
--PSALPY
--PSALMV
--PSALLD
--PSALIH
--PSALHY
--PSALHF
--PSALFE
--PSALAI
PNPSAL--
PIPSAL--
PFPSAL--
--PSALTW
--PSALTE
--PSALQN
--PSALQL
--PSALQA
--PSALMW
--PSALMN
--PSALKE
--PSALHE
--PSALGG
--PSALEL
--PSALEH
--PSALYQ
--PSALTY
--PSALTM
--PSALTA
--PSALPM
--PSALMD
--PSALLE
--PSALKY
--PSALKM
--PSALFN
--PSALEV
--PSALES
--PSALAE
--PSALYY
--PSALWK
--PSALVQ
--PSALVI
--PSALVD

--PSALQI
--PSALPI
--PSALHT
--PSALHQ
--PSALHN
--PSALHG
--PSALHD
--PSALGQ
--PSALER
PMPSAL--
--PSALYE
--PSALWN
--PSALQV
--PSALQM
--PSALQD
--PSALNV
--PSALNQ
--PSALMQ
--PSALMP
--PSALMI
--PSALKH
--PSALKA
--PSALII
--PSALGK
--PSALGH
--PSALFQ
--PSALAN
--PSALAM
PDPSAL--
PYPSAL--
--PSALYG
--PSALWM
--PSALSD
--PSALPF
--PSALNH
--PSALIY
--PSALHM
--PSALFM
--PSALEI
--PSALEG
--PSALDA
PQPSAL--
PKPSAL--
--PSALQE
--PSALND
--PSALNA
--PSALMM
--PSALMH
--PSALKP
--PSALEW

--PSALEQ
--PSALEF
--PSALEA
--PSALDL
--PSALDG
--PSALAQ
PEPSAL--
PCPSAL--
--PSALWC
--PSALMC
--PSALLM
--PSALKQ
--PSALIF
--PSALHP
--PSALHI
--PSALFP
--PSALED
--PSALCK
--PSALCG
PHPSAL--
--PSALYN
--PSALWP
--PSALWF
--PSALWD
--PSALVE
--PSALSC
--PSALRC
--PSALQW
--PSALQQ
--PSALQF
--PSALPT
--PSALNG
--PSALNE
--PSALKF
--PSALIP
--PSALID
--PSALHV
--PSALGW
--PSALGD
--PSALEY
--PSALDT
--PSALDQ
--PSALCS
--PSALCR
--PSALCN
--PSALCH
--PSALCD
--PSALCA
-PPSALW-
PPPSAL--

```
-QPSALL-
QRPSAL--
QGPSAL--
-QPSALK-
-QPSALR-
QLPSAL--
QAPSAL--
-QPSALF-
-QPSALW-
QVPSAL--
-QPSALS-
QTPSAL--
-QPSALA-
QPPSAL--
QNPSAL--
QIPSAL--
QHPSAL--
QEPSAL--
QFPSAL--
QQPSAL--
-QPSALV-
-QPSALM-
-QPSALI-
QMPSAL--
QWPSAL--
-QPSALH-
-QPSALE-
QKPSAL--
QDPSAL--
-QPSALQ-
RVPSAL--
-RPSALK-
-RPSALA-
-RPSALT-
-RPSALN-
-RPSALG-
-RPSALS-
-RPSALF-
RPPSAL--
RIPSAL--
RGPSAL--
RTPSAL--
RFPSAL--
-RPSALH-
-RPSALY-
-RPSALM-
RMPSAL--
RQPSAL--
-RPSALQ-
REPSAL--
```

-RPSALV-
RYPSAL--
RDPSAL--
-RPSALE-
-RPSALW-
-RPSALD-
RCPSAL--
-RPSALC-
SVPSAL--
SMPSAL--
-SPSALR-
SHPSAL--
STPSAL--
SYPSAL--
SLPSAL--
-SPSALN-
-SPSALK-
-SPSALV-
SKPSAL--
-SPSALQ-
SNPSAL--
SAPSAL--
-SPSALA-
-SPSALH-
SFPSAL--
-SPSALG-
-SPSALE-
SQPSAL--
-SPSALM-
-SPSALI-
-SPSALF-
SEPSAL--
SCPSAL--
-SPSALC-
TRPSAL--
TLPSAL--
-TPSALW-
TVPSAL--
-TPSALL-
-TPSALK-
-TPSALG-
TIPSAL--
-TPSALN-
TAPSAL--
-TPSALY-
TKPSAL--
TEPSAL--
TTPSAL--
-TPSALH-
TFPSAL--

TSPSAL--
TYPSAL--
TWPSAL--
TNPSAL--
-TPSALF-
-TPSALT-
-TPSALA-
TGPSAL--
TDPSAL--
TQPSAL--
THPSAL--
-TPSALQ-
-TPSALM-
TPPSAL--
-TPSALV-
-TPSALC-
TCPSAL--
-VPSALR-
VRPSAL--
-VPSALT-
-VPSALS-
VLPSAL--
VAPSAL--
-VPSALL-
VHPSAL--
VYPSAL--
-VPSALY-
-VPSALN-
-VPSALK-
-VPSALH-
VPPSAL--
VMPSAL--
VWPSAL--
VSPSAL--
-VPSALM-
-VPSALI-
VGPSAL--
VTPSAL--
-VPSALQ-
VNPSAL--
-VPSALA-
VDPSAL--
VIPSAL--
-VPSALV-
VEPSAL--
-VPSALW-
-VPSALF-
VQPSAL--
VFPSAL--
VCPSAL--

```
-VPSALD-
WLPSAL--
-WPSALR-
WGPSAL--
WAPSAL--
WNPSAL--
-WPSALI-
WIPSAL--
-WPSALT-
-WPSALG-
WWPSAL--
WVPSAL--
-WPSALY-
WKPSAL--
-WPSALA-
WDPSAL--
-WPSALN-
WFPSAL--
WEPSAL--
-WPSALM-
WTPSAL--
WPPSAL--
-WPSALQ-
-WPSALF-
-WPSALE-
WHPSAL--
WCPSAL--
-WPSALH-
-WPSALV-
-WPSALD-
-WPSALC-
YTPSAL--
-YPSALL-
YAPSAL--
YLPSAL--
-YPSALR-
YKPSAL--
-YPSALN-
YMPSAL--
YIPSAL--
YPPSAL--
YSPSAL--
YHPSAL--
-YPSALG-
-YPSALS-
YNPSAL--
YYPSAL--
-YPSALF-
YGPSAL--
YVPSAL--
```

```
          YQPSAL--
          -YPSALV-
          -YPSALT-
          -YPSALK-
          -YPSALI-
          -YPSALH-
          -YPSALY-
          -YPSALQ-
          -YPSALA-
          YEPSAL--
          -YPSALE-
          YCPSAL--
          -YPSALP-
          -YPSALM-
          YDPSAL--
PAGL         2       914     36.02
          --PAGLPL
          --PAGLGD
          -RPAGLP-
          -TPAGLD-
          VCPAGL--
          -APAGLR-
          ALPAGL--
          -APAGLS-
          -APAGLA-
          ASPAGL--
          ARPAGL--
          AFPAGL--
          -APAGLN-
          -APAGLL-
          -APAGLD-
          -APAGLT-
          -APAGLG-
          AVPAGL--
          -APAGLF-
          AHPAGL--
          -APAGLV-
          AWPAGL--
          AQPAGL--
          -APAGLH-
          AEPAGL--
          ATPAGL--
          -APAGLW-
          -APAGLQ-
          -APAGLK-
          -APAGLE-
          ADPAGL--
          AYPAGL--
          APPAGL--
          -APAGLM-
```

AMPAGL--
AAPAGL--
ACPAGL--
-CPAGLQ-
CNPAGL--
CLPAGL--
-CPAGLS-
CSPAGL--
CRPAGL--
-CPAGLF-
CGPAGL--
CQPAGL--
-CPAGLL-
-CPAGLH-
-CPAGLE-
CCPAGL--
CAPAGL--
CYPAGL--
CVPAGL--
CTPAGL--
CPPAGL--
-CPAGLT-
-CPAGLR-
-CPAGLN-
-CPAGLM-
-CPAGLK-
-CPAGLA-
CMPAGL--
CKPAGL--
CFPAGL--
CEPAGL--
-DPAGLW-
-DPAGLR-
DRPAGL--
-DPAGLQ-
DVPAGL--
DSPAGL--
-DPAGLT-
-DPAGLN-
-DPAGLF-
DWPAGL--
DGPAGL--
DEPAGL--
-DPAGLL-
DLPAGL--
DIPAGL--
DYPAGL--
-DPAGLV-
-DPAGLM-
-DPAGLK-

DMPAGL--
DHPAGL--
DAPAGL--
DTPAGL--
-DPAGLS-
DNPAGL--
-DPAGLY-
-DPAGLH-
DFPAGL--
DDPAGL--
-EPAGLR-
EKPAGL--
EHPAGL--
ESPAGL--
ERPAGL--
-EPAGLK-
ETPAGL--
-EPAGLT-
-EPAGLN-
-EPAGLL-
EVPAGL--
EWPAGL--
-EPAGLV-
-EPAGLS-
-EPAGLQ-
-EPAGLY-
-EPAGLM-
ELPAGL--
-EPAGLG-
EIPAGL--
EFPAGL--
EQPAGL--
-EPAGLI-
EGPAGL--
EAPAGL--
EPPAGL--
-EPAGLH-
-EPAGLF-
-EPAGLA-
ENPAGL--
EMPAGL--
EDPAGL--
ECPAGL--
-FPAGLS-
-FPAGLK-
FLPAGL--
FAPAGL--
FSPAGL--
FQPAGL--
-FPAGLL-

-FPAGLY-
-FPAGLG-
FNPAGL--
FYPAGL--
FVPAGL--
FTPAGL--
-FPAGLR-
-FPAGLE-
FGPAGL--
FPPAGL--
-FPAGLV-
-FPAGLQ-
-FPAGLH-
FHPAGL--
-FPAGLM-
-FPAGLN-
FMPAGL--
FFPAGL--
FDPAGL--
-FPAGLW-
-FPAGLT-
FIPAGL--
-FPAGLF-
FWPAGL--
-FPAGLI-
-FPAGLA-
FEPAGL--
GLPAGL--
GKPAGL--
GNPAGL--
GMPAGL--
-GPAGLT-
-GPAGLG-
-GPAGLK-
-GPAGLL-
GYPAGL--
-GPAGLS-
GSPAGL--
-GPAGLM-
GAPAGL--
-GPAGLY-
GHPAGL--
-GPAGLH-
GVPAGL--
GPPAGL--
-GPAGLV-
-GPAGLQ-
GEPAGL--
GTPAGL--
GQPAGL--

```
-GPAGLE-
GIPAGL--
-GPAGLW-
-GPAGLN-
GWPAGL--
-GPAGLI-
-GPAGLF-
GCPAGL--
-GPAGLA-
GDPAGL--
-GPAGLD-
HGPAGL--
-HPAGLR-
HRPAGL--
HIPAGL--
-HPAGLS-
-HPAGLL-
HFPAGL--
-HPAGLN-
-HPAGLG-
HEPAGL--
-HPAGLH-
HYPAGL--
-HPAGLW-
-HPAGLT-
HTPAGL--
-HPAGLI-
-HPAGLQ-
-HPAGLE-
HNPAGL--
HLPAGL--
HKPAGL--
HHPAGL--
HVPAGL--
-HPAGLY-
-HPAGLF-
HMPAGL--
HAPAGL--
-HPAGLV-
HDPAGL--
HCPAGL--
HQPAGL--
HPPAGL--
-HPAGLK-
-HPAGLD-
-IPAGLL-
IRPAGL--
IKPAGL--
-IPAGLY-
-IPAGLR-
```

ISPAGL--
IWPAGL--
ITPAGL--
-IPAGLV-
-IPAGLT-
IYPAGL--
IAPAGL--
-IPAGLS-
-IPAGLQ-
-IPAGLN-
-IPAGLA-
IVPAGL--
INPAGL--
IIPAGL--
IFPAGL--
-IPAGLM-
-IPAGLH-
IQPAGL--
-IPAGLI-
IDPAGL--
-IPAGLK-
-IPAGLG-
IHPAGL--
IPPAGL--
-IPAGLF-
-IPAGLE-
-IPAGLD-
IMPAGL--
KSPAGL--
KRPAGL--
-KPAGLL-
-KPAGLT-
-KPAGLV-
KTPAGL--
-KPAGLK-
-KPAGLM-
KLPAGL--
-KPAGLW-
KIPAGL--
KPPAGL--
-KPAGLH-
KWPAGL--
-KPAGLI-
KGPAGL--
KDPAGL--
KYPAGL--
-KPAGLA-
KQPAGL--
KKPAGL--
KVPAGL--

KMPAGL--
KFPAGL--
KAPAGL--
-KPAGLD-
-KPAGLY-
-KPAGLE-
KHPAGL--
-KPAGLC-
KNPAGL--
KEPAGL--
LRPAGL--
-LPAGLK-
-LPAGLN-
-LPAGLA-
LGPAGL--
LMPAGL--
LFPAGL--
LLPAGL--
LSPAGL--
LQPAGL--
LHPAGL--
-LPAGLE-
LPPAGL--
-LPAGLH-
LEPAGL--
LYPAGL--
-LPAGLW-
-LPAGLT-
-LPAGLI-
-LPAGLG-
LVPAGL--
LKPAGL--
-LPAGLQ-
LNPAGL--
LDPAGL--
LAPAGL--
-LPAGLM-
-LPAGLF-
LCPAGL--
-MPAGLR-
MRPAGL--
MNPAGL--
MSPAGL--
MGPAGL--
-MPAGLS-
MLPAGL--
MIPAGL--
-MPAGLF-
MPPAGL--
-MPAGLV-

MMPAGL--
MQPAGL--
-MPAGLY-
MWPAGL--
MVPAGL--
-MPAGLI-
-MPAGLT-
-MPAGLN-
-MPAGLH-
MYPAGL--
-MPAGLQ-
-MPAGLM-
-MPAGLG-
-MPAGLA-
MKPAGL--
-MPAGLW-
-MPAGLK-
-MPAGLD-
MEPAGL--
MCPAGL--
MTPAGL--
MHPAGL--
MFPAGL--
MDPAGL--
-NPAGLV-
-NPAGLG-
NYPAGL--
NSPAGL--
NTPAGL--
-NPAGLS-
-NPAGLN-
NRPAGL--
NLPAGL--
NGPAGL--
-NPAGLA-
NEPAGL--
NWPAGL--
NVPAGL--
-NPAGLL-
-NPAGLI-
-NPAGLR-
NNPAGL--
NMPAGL--
NQPAGL--
-NPAGLQ-
NCPAGL--
NAPAGL--
-NPAGLM-
-NPAGLF-
-NPAGLK-

```
NIPAGL--
NHPAGL--
NFPAGL--
NPPAGL--
-NPAGLC-
--PAGLVG
--PAGLRS
--PAGLLS
--PAGLRV
--PAGLLL
--PAGLIL
--PAGLVR
--PAGLSI
--PAGLYR
--PAGLRH
--PAGLIR
--PAGLGS
--PAGLSF
--PAGLRR
--PAGLGL
--PAGLTS
--PAGLSR
--PAGLRG
--PAGLTL
--PAGLLR
--PAGLHR
--PAGLAS
--PAGLSL
--PAGLRT
--PAGLQT
--PAGLRF
--PAGLML
--PAGLLT
--PAGLYT
--PAGLRI
--PAGLLK
--PAGLGT
--PAGLVT
--PAGLMR
--PAGLVY
--PAGLVL
--PAGLTR
--PAGLTA
--PAGLLG
--PAGLRA
--PAGLLH
PVPAGL--
--PAGLSH
--PAGLRW
--PAGLRQ
```

--PAGLMM
--PAGLKL
--PAGLHY
--PAGLHL
--PAGLGR
--PAGLWG
--PAGLNT
--PAGLKR
--PAGLIN
--PAGLLQ
--PAGLIW
--PAGLAG
PSPAGL--
--PAGLSV
--PAGLIQ
--PAGLVA
--PAGLRD
--PAGLNL
--PAGLKS
--PAGLKN
--PAGLGF
--PAGLAR
--PAGLYK
--PAGLVI
--PAGLTN
--PAGLST
--PAGLSN
--PAGLND
--PAGLKT
--PAGLIS
--PAGLIK
--PAGLGG
--PAGLFV
PTPAGL--
--PAGLWM
--PAGLVM
--PAGLVH
--PAGLKA
--PAGLGN
--PAGLFR
--PAGLET
--PAGLAV
--PAGLWT
--PAGLWS
--PAGLVV
--PAGLVK
--PAGLTT
--PAGLTH
--PAGLRY
--PAGLMS

--PAGLMI
--PAGLHG
--PAGLGK
--PAGLFS
--PAGLFQ
--PAGLYM
--PAGLVD
--PAGLTY
--PAGLRP
--PAGLMW
--PAGLMA
--PAGLKW
--PAGLKM
--PAGLHN
--PAGLHK
--PAGLGY
--PAGLFM
--PAGLFI
--PAGLWR
--PAGLTK
--PAGLSQ
--PAGLKG
--PAGLIG
PNPAGL--
PLPAGL--
--PAGLTI
--PAGLTG
--PAGLSG
--PAGLQQ
--PAGLQL
--PAGLMT
--PAGLMQ
--PAGLHS
--PAGLER
--PAGLDS
--PAGLAM
PWPAGL--
PQPAGL--
PKPAGL--
PGPAGL--
--PAGLWH
--PAGLWA
--PAGLVN
--PAGLVF
--PAGLTW
--PAGLSE
--PAGLSD
--PAGLRE
--PAGLNK
--PAGLLD

--PAGLIF
--PAGLHW
--PAGLGQ
--PAGLFN
--PAGLEG
--PAGLAK
--PAGLAH
--PAGLYG
--PAGLVS
--PAGLSM
--PAGLQY
--PAGLNY
--PAGLNW
--PAGLNR
--PAGLMH
--PAGLMG
--PAGLLE
--PAGLKY
--PAGLIH
--PAGLHE
PYPAGL--
--PAGLYQ
--PAGLYA
--PAGLWN
--PAGLWK
--PAGLTQ
--PAGLRN
--PAGLQN
--PAGLMY
--PAGLMF
--PAGLIM
--PAGLHT
--PAGLHI
--PAGLGW
--PAGLGV
--PAGLGH
--PAGLFK
--PAGLFH
--PAGLEH
--PAGLAY
PAPAGL--
--PAGLYV
--PAGLYN
--PAGLWW
--PAGLWQ
--PAGLTM
--PAGLQR
--PAGLQK
--PAGLNV
--PAGLNQ

--PAGLMD
--PAGLKK
--PAGLHV
--PAGLHP
--PAGLHH
--PAGLGM
--PAGLFG
--PAGLFA
--PAGLYP
--PAGLVE
--PAGLTD
--PAGLQI
--PAGLMK
--PAGLME
--PAGLKV
--PAGLKF
--PAGLIY
--PAGLEK
--PAGLDA
--PAGLAD
PIPAGL--
--PAGLVW
--PAGLQG
--PAGLQF
--PAGLQD
--PAGLQA
--PAGLMV
--PAGLLW
--PAGLLM
--PAGLKQ
--PAGLKH
--PAGLID
--PAGLHF
--PAGLFT
--PAGLEY
--PAGLEW
--PAGLDT
--PAGLAQ
--PAGLAN
--PAGLAE
--PAGLAA
PEPAGL--
--PAGLYW
--PAGLYE
--PAGLWE
--PAGLWD
--PAGLTV
--PAGLSC
--PAGLQW
--PAGLQS

--PAGLQH
--PAGLNF
--PAGLHM
--PAGLGA
--PAGLEA
PHPAGL--
--PAGLYI
--PAGLYH
--PAGLTF
--PAGLTE
--PAGLNN
--PAGLNH
--PAGLNG
--PAGLNA
--PAGLKP
--PAGLKD
--PAGLIE
--PAGLHD
--PAGLFW
--PAGLEV
--PAGLEQ
--PAGLEL
--PAGLEE
--PAGLED
--PAGLDV
--PAGLDG
--PAGLCF
--PAGLCA
--PAGLAF
-PPAGLM-
PMPAGL--
PFPAGL--
--PAGLYD
--PAGLQV
--PAGLQM
--PAGLLC
--PAGLKE
--PAGLIC
--PAGLGE
--PAGLEN
--PAGLEI
--PAGLDN
--PAGLDI
--PAGLCV
--PAGLCS
--PAGLCQ
--PAGLCN
--PAGLCH
--PAGLAC
QRPAGL--

QSPAGL--
-QPAGLT-
-QPAGLR-
-QPAGLG-
-QPAGLV-
-QPAGLL-
QLPAGL--
-QPAGLI-
QAPAGL--
-QPAGLS-
-QPAGLA-
-QPAGLH-
-QPAGLY-
-QPAGLW-
-QPAGLN-
QIPAGL--
QGPAGL--
QVPAGL--
QQPAGL--
QTPAGL--
-QPAGLF-
QMPAGL--
-QPAGLM-
-QPAGLE-
-QPAGLD-
QEPAGL--
QYPAGL--
QKPAGL--
QDPAGL--
QWPAGL--
QPPAGL--
QHPAGL--
QCPAGL--
QNPAGL--
-RPAGLS-
RVPAGL--
RSPAGL--
RLPAGL--
-RPAGLR-
-RPAGLT-
RGPAGL--
RYPAGL--
-RPAGLY-
-RPAGLK-
RWPAGL--
RPPAGL--
-RPAGLA-
-RPAGLW-
-RPAGLF-
RKPAGL--

-RPAGLN-
-RPAGLQ-
-RPAGLG-
RIPAGL--
RHPAGL--
-RPAGLI-
-RPAGLH-
RTPAGL--
RMPAGL--
RNPAGL--
-RPAGLE-
-RPAGLD-
RDPAGL--
REPAGL--
RCPAGL--
-SPAGLR-
SRPAGL--
-SPAGLK-
SLPAGL--
-SPAGLG-
STPAGL--
-SPAGLV-
-SPAGLH-
SGPAGL--
SQPAGL--
-SPAGLW-
-SPAGLL-
SNPAGL--
SVPAGL--
SSPAGL--
-SPAGLY-
SMPAGL--
-SPAGLN-
-SPAGLA-
SFPAGL--
SWPAGL--
-SPAGLF-
-SPAGLE-
SHPAGL--
-SPAGLI-
SEPAGL--
SYPAGL--
-SPAGLQ-
SKPAGL--
-SPAGLD-
SDPAGL--
-SPAGLM-
SAPAGL--
-SPAGLC-
SCPAGL--

−TPAGLL−
TSPAGL−−
−TPAGLR−
TRPAGL−−
TLPAGL−−
−TPAGLY−
TVPAGL−−
THPAGL−−
−TPAGLV−
TIPAGL−−
−TPAGLN−
−TPAGLG−
−TPAGLF−
TGPAGL−−
−TPAGLI−
TQPAGL−−
−TPAGLT−
TKPAGL−−
−TPAGLK−
−TPAGLA−
TMPAGL−−
−TPAGLQ−
TWPAGL−−
TFPAGL−−
TPPAGL−−
−TPAGLE−
−TPAGLH−
TAPAGL−−
TNPAGL−−
TDPAGL−−
TYPAGL−−
−TPAGLW−
TEPAGL−−
−TPAGLM−
−VPAGLR−
−VPAGLK−
−VPAGLG−
VSPAGL−−
−VPAGLL−
VVPAGL−−
−VPAGLV−
VYPAGL−−
VTPAGL−−
−VPAGLY−
−VPAGLT−
VLPAGL−−
VAPAGL−−
−VPAGLN−
VNPAGL−−
VFPAGL−−

VPPAGL--
VIPAGL--
VEPAGL--
VMPAGL--
VWPAGL--
-VPAGLW-
-VPAGLQ-
-VPAGLI-
-VPAGLH-
-VPAGLD-
VGPAGL--
-VPAGLE-
-VPAGLA-
-VPAGLM-
-VPAGLP-
-VPAGLC-
WLPAGL--
WSPAGL--
-WPAGLS-
WIPAGL--
WAPAGL--
WTPAGL--
WVPAGL--
WFPAGL--
-WPAGLR-
-WPAGLQ-
-WPAGLL-
-WPAGLG-
-WPAGLH-
-WPAGLA-
-WPAGLW-
-WPAGLN-
WKPAGL--
-WPAGLK-
-WPAGLY-
WPPAGL--
WMPAGL--
WDPAGL--
-WPAGLV-
-WPAGLM-
-WPAGLE-
WGPAGL--
WQPAGL--
WCPAGL--
-WPAGLF-
WNPAGL--
WEPAGL--
-YPAGLR-
-YPAGLA-
YLPAGL--

```
                YNPAGL--
                -YPAGLT-
                YVPAGL--
                YHPAGL--
                YEPAGL--
                YSPAGL--
                YQPAGL--
                YIPAGL--
                YTPAGL--
                -YPAGLQ-
                -YPAGLI-
                YFPAGL--
                -YPAGLS-
                -YPAGLL-
                YGPAGL--
                -YPAGLG-
                -YPAGLF-
                -YPAGLN-
                YMPAGL--
                YWPAGL--
                -YPAGLM-
                -YPAGLY-
                YYPAGL--
                -YPAGLW-
                -YPAGLK-
                -YPAGLV-
                -YPAGLE-
                YDPAGL--
                YAPAGL--
                -YPAGLD-
PAAL               5        898      35.80
                --PAALWP
                --PAALDI
                --PAALAT
                ASPAAL--
                AGPAAL--
                -APAALL-
                -APAALN-
                AIPAAL--
                -APAALG-
                -APAALS-
                -APAALA-
                AWPAAL--
                APPAAL--
                AAPAAL--
                -APAALK-
                ALPAAL--
                AFPAAL--
                -APAALT-
                -APAALH-
```

AMPAAL--
-APAALI-
-APAALF-
ANPAAL--
AQPAAL--
-APAALY-
-APAALW-
AHPAAL--
-APAALQ-
AKPAAL--
-APAALM-
AEPAAL--
ACPAAL--
AYPAAL--
-APAALE-
-CPAALR-
CGPAAL--
CQPAAL--
CNPAAL--
-CPAALS-
-CPAALM-
-CPAALH-
CMPAAL--
CEPAAL--
CTPAAL--
CSPAAL--
-CPAALN-
-CPAALI-
-CPAALF-
CIPAAL--
CHPAAL--
CFPAAL--
CDPAAL--
CVPAAL--
-CPAALT-
-CPAALL-
-CPAALK-
CRPAAL--
-CPAALG-
CLPAAL--
CCPAAL--
DRPAAL--
DLPAAL--
DVPAAL--
DTPAAL--
DSPAAL--
DYPAAL--
-DPAALV-
-DPAALA-
DIPAAL--

DWPAAL--
-DPAALY-
-DPAALR-
DHPAAL--
DFPAAL--
-DPAALL-
-DPAALH-
DKPAAL--
DDPAAL--
-DPAALW-
-DPAALK-
DGPAAL--
DQPAAL--
DPPAAL--
-DPAALS-
-DPAALQ-
-DPAALG-
-DPAALF-
DMPAAL--
-EPAALL-
-EPAALK-
-EPAALS-
-EPAALR-
-EPAALE-
-EPAALV-
EGPAAL--
ETPAAL--
-EPAALQ-
EWPAAL--
-EPAALT-
ELPAAL--
EYPAAL--
EVPAAL--
ERPAAL--
-EPAALG-
ENPAAL--
EAPAAL--
-EPAALW-
-EPAALN-
-EPAALM-
-EPAALI-
-EPAALF-
-EPAALD-
-EPAALA-
EFPAAL--
-EPAALH-
EIPAAL--
EEPAAL--
EDPAAL--
ECPAAL--

FSPAAL--
FRPAAL--
-FPAALR-
FGPAAL--
FNPAAL--
FLPAAL--
FVPAAL--
FTPAAL--
-FPAALS-
FHPAAL--
-FPAALI-
FDPAAL--
-FPAALH-
-FPAALE-
FFPAAL--
FWPAAL--
FAPAAL--
FYPAAL--
-FPAALN-
-FPAALV-
-FPAALL-
FMPAAL--
FCPAAL--
FQPAAL--
-FPAALT-
-FPAALM-
-FPAALK-
-FPAALD-
-FPAALQ-
-FPAALG-
-FPAALA-
-GPAALL-
GVPAAL--
GLPAAL--
GRPAAL--
GDPAAL--
-GPAALR-
-GPAALK-
-GPAALI-
GGPAAL--
-GPAALV-
GAPAAL--
GWPAAL--
GMPAAL--
-GPAALH-
GFPAAL--
GQPAAL--
GYPAAL--
-GPAALY-
-GPAALS-

```
-GPAALA-
GHPAAL--
-GPAALN-
GNPAAL--
GKPAAL--
GTPAAL--
-GPAALT-
-GPAALG-
-GPAALE-
-GPAALQ-
-GPAALM-
GIPAAL--
-GPAALW-
HRPAAL--
-HPAALL-
HVPAAL--
-HPAALD-
HEPAAL--
-HPAALI-
HGPAAL--
HAPAAL--
-HPAALN-
HQPAAL--
-HPAALR-
-HPAALH-
-HPAALA-
HLPAAL--
HFPAAL--
HYPAAL--
-HPAALT-
-HPAALQ-
-HPAALG-
HNPAAL--
HKPAAL--
HDPAAL--
-HPAALS-
HPPAAL--
-HPAALV-
-HPAALE-
HMPAAL--
HWPAAL--
-HPAALW-
-IPAALR-
-IPAALT-
ISPAAL--
ILPAAL--
IRPAAL--
-IPAALN-
-IPAALS-
-IPAALK-
```

IWPAAL--
IVPAAL--
-IPAALV-
IKPAAL--
IDPAAL--
ITPAAL--
INPAAL--
IAPAAL--
IPPAAL--
-IPAALL-
-IPAALE-
-IPAALA-
IFPAAL--
IQPAAL--
-IPAALI-
-IPAALH-
-IPAALG-
IYPAAL--
-IPAALY-
-IPAALW-
IMPAAL--
-IPAALQ-
-IPAALD-
-IPAALC-
IHPAAL--
IGPAAL--
IEPAAL--
KSPAAL--
-KPAALY-
-KPAALR-
-KPAALS-
KLPAAL--
KMPAAL--
KNPAAL--
KTPAAL--
KPPAAL--
-KPAALV-
-KPAALT-
KWPAAL--
KQPAAL--
-KPAALM-
-KPAALH-
-KPAALW-
-KPAALD-
KYPAAL--
KCPAAL--
KAPAAL--
KHPAAL--
KGPAAL--
KEPAAL--

```
-KPAALQ-
-KPAALI-
-KPAALE-
KKPAAL--
KIPAAL--
-KPAALF-
LRPAAL--
LSPAAL--
LGPAAL--
-LPAALL-
LVPAAL--
-LPAALT-
-LPAALR-
-LPAALK-
-LPAALI-
LPPAAL--
-LPAALW-
LLPAAL--
LKPAAL--
LYPAAL--
-LPAALG-
LHPAAL--
LAPAAL--
-LPAALY-
LTPAAL--
LNPAAL--
-LPAALS-
-LPAALM-
LMPAAL--
-LPAALV-
-LPAALH-
-LPAALF-
-LPAALN-
LDPAAL--
LFPAAL--
LWPAAL--
-LPAALA-
-LPAALE-
LCPAAL--
MLPAAL--
-MPAALY-
MKPAAL--
-MPAALM-
MVPAAL--
MQPAAL--
MPPAAL--
-MPAALN-
MSPAAL--
-MPAALL-
MIPAAL--
```

-MPAALA-
MHPAAL--
MAPAAL--
-MPAALT-
-MPAALH-
MTPAAL--
MNPAAL--
MGPAAL--
MFPAAL--
MWPAAL--
MDPAAL--
MCPAAL--
MYPAAL--
-MPAALV-
-MPAALS-
-MPAALQ-
-MPAALD-
-MPAALE-
MMPAAL--
MEPAAL--
-NPAALN-
NSPAAL--
NPPAAL--
NGPAAL--
-NPAALR-
-NPAALT-
-NPAALK-
NNPAAL--
NVPAAL--
NIPAAL--
NTPAAL--
NQPAAL--
NYPAAL--
-NPAALY-
-NPAALS-
-NPAALA-
NLPAAL--
NEPAAL--
-NPAALF-
-NPAALE-
NHPAAL--
NWPAAL--
-NPAALW-
-NPAALG-
NAPAAL--
-NPAALV-
-NPAALH-
-NPAALQ-
-NPAALI-
NDPAAL--

-NPAALM-
NCPAAL--
--PAALRI
--PAALYL
--PAALPY
PRPAAL--
--PAALRR
--PAALVN
--PAALVS
--PAALRK
--PAALKL
--PAALSR
PSPAAL--
--PAALVR
--PAALLS
--PAALRT
--PAALLK
--PAALTR
--PAALGR
--PAALRG
--PAALWL
--PAALTK
--PAALSV
--PAALLR
--PAALLI
--PAALKS
--PAALWM
--PAALRM
--PAALSG
--PAALNR
--PAALLN
--PAALRS
--PAALQR
--PAALGS
--PAALWS
--PAALTL
--PAALSL
--PAALSA
--PAALRA
--PAALMM
--PAALLG
--PAALTS
--PAALST
--PAALRE
--PAALHV
--PAALWT
--PAALRY
--PAALRF
--PAALPL
--PAALHK

--PAALGL
--PAALGF
--PAALYP
--PAALVT
--PAALQN
--PAALNL
--PAALLW
--PAALLT
--PAALKD
--PAALGK
--PAALFT
--PAALFK
--PAALSN
--PAALRW
--PAALRQ
--PAALRH
--PAALNS
--PAALIL
--PAALHG
--PAALFR
PLPAAL--
--PAALYT
--PAALVI
--PAALTF
--PAALSQ
--PAALSK
--PAALRP
--PAALQG
--PAALLD
--PAALFQ
--PAALEY
--PAALEN
PHPAAL--
PGPAAL--
--PAALWN
--PAALVY
--PAALVP
--PAALVK
--PAALSW
--PAALNV
--PAALMG
--PAALLV
--PAALLA
--PAALKG
--PAALHS
--PAALGN
--PAALER
--PAALAS
PMPAAL--
--PAALVM

--PAALTG
--PAALSD
--PAALQS
--PAALMT
--PAALMS
--PAALLH
--PAALIM
--PAALIA
--PAALHT
--PAALGY
--PAALGQ
PKPAAL--
PFPAAL--
--PAALYQ
--PAALYM
--PAALYK
--PAALSY
--PAALSH
--PAALQT
--PAALQL
--PAALLQ
--PAALKP
--PAALHM
--PAALGH
--PAALFG
--PAALAH
PNPAAL--
--PAALYS
--PAALYN
--PAALWV
--PAALTD
--PAALTA
--PAALSE
--PAALKI
--PAALIV
--PAALIF
--PAALHI
--PAALHH
--PAALHA
--PAALWG
--PAALVA
--PAALSM
--PAALQV
--PAALNM
--PAALNI
--PAALNH
--PAALMV
--PAALMP
--PAALML
--PAALKN

--PAALKM
--PAALKH
--PAALIW
--PAALIG
--PAALAQ
--PAALAN
PWPAAL--
PIPAAL--
--PAALYV
--PAALYH
--PAALYF
--PAALWQ
--PAALWK
--PAALVW
--PAALVH
--PAALVG
--PAALTT
--PAALSI
--PAALRV
--PAALNW
--PAALNA
--PAALMY
--PAALMN
--PAALGG
--PAALGE
--PAALFS
--PAALDS
--PAALAM
--PAALAL
--PAALAE
--PAALAA
PQPAAL--
--PAALYA
--PAALWF
--PAALTW
--PAALTE
--PAALQK
--PAALQA
--PAALPW
--PAALNY
--PAALNE
--PAALMR
--PAALMH
--PAALMF
--PAALMD
--PAALKY
--PAALKV
--PAALIQ
--PAALIN
--PAALIK

--PAALII
--PAALHR
--PAALHQ
--PAALHP
--PAALGA
--PAALEW
--PAALET
--PAALEL
--PAALEK
--PAALEF
--PAALAW
--PAALAK
--PAALAF
--PAALYG
--PAALTM
--PAALTI
--PAALTH
--PAALRD
--PAALQY
--PAALQH
--PAALNT
--PAALNK
--PAALNF
--PAALND
--PAALMQ
--PAALMK
--PAALMI
--PAALMA
--PAALKW
--PAALKQ
--PAALKF
--PAALIY
--PAALIP
--PAALID
--PAALHE
--PAALFP
--PAALFI
--PAALFD
--PAALEV
--PAALEI
--PAALDM
--PAALDG
--PAALAR
PYPAAL--
PCPAAL--
--PAALYE
--PAALVF
--PAALVE
--PAALTN
--PAALQI

--PAALPM
--PAALPF
--PAALNQ
--PAALMW
--PAALLP
--PAALKT
--PAALKA
--PAALHD
--PAALGW
--PAALES
--PAALEQ
--PAALEM
--PAALEG
--PAALEA
--PAALDV
--PAALDT
--PAALCS
--PAALCR
--PAALCI
--PAALAV
PVPAAL--
PEPAAL--
PAPAAL--
--PAALYW
--PAALWI
--PAALWH
--PAALWE
--PAALWD
--PAALQW
--PAALQP
--PAALQF
--PAALQD
--PAALPQ
--PAALPI
--PAALNP
--PAALNG
--PAALNC
--PAALMC
--PAALKE
--PAALIH
--PAALHY
--PAALHW
--PAALHC
--PAALGV
--PAALGM
--PAALGD
--PAALFW
--PAALFH
--PAALFE
--PAALDW

--PAALDQ
--PAALDN
--PAALDF
--PAALCL
--PAALAC
-QPAALR-
QRPAAL--
-QPAALL-
QGPAAL--
-QPAALT-
-QPAALN-
-QPAALH-
QSPAAL--
-QPAALS-
-QPAALW-
QKPAAL--
-QPAALK-
-QPAALA-
QTPAAL--
-QPAALQ-
QNPAAL--
QYPAAL--
QWPAAL--
QVPAAL--
-QPAALY-
-QPAALG-
QIPAAL--
QFPAAL--
QAPAAL--
-QPAALM-
QCPAAL--
QPPAAL--
QHPAAL--
-QPAALV-
-QPAALI-
QEPAAL--
QQPAAL--
-QPAALF-
QDPAAL--
RLPAAL--
-RPAALL-
RSPAAL--
RRPAAL--
RNPAAL--
RTPAAL--
-RPAALN-
-RPAALT-
-RPAALS-
RYPAAL--
-RPAALM-

-RPAALG-
RFPAAL--
-RPAALK-
RVPAAL--
RMPAAL--
RHPAAL--
-RPAALE-
RWPAAL--
RQPAAL--
-RPAALV-
RAPAAL--
RPPAAL--
RIPAAL--
REPAAL--
-RPAALD-
-RPAALA-
-RPAALF-
RCPAAL--
RDPAAL--
-RPAALQ-
-SPAALT-
SIPAAL--
-SPAALR-
SRPAAL--
-SPAALY-
-SPAALW-
SAPAAL--
SVPAAL--
STPAAL--
-SPAALQ-
-SPAALS-
-SPAALK-
-SPAALV-
-SPAALM-
-SPAALI-
-SPAALG-
SGPAAL--
SQPAAL--
-SPAALE-
SDPAAL--
SYPAAL--
-SPAALF-
SHPAAL--
-SPAALH-
SKPAAL--
SFPAAL--
SWPAAL--
-SPAALA-
SMPAAL--
SEPAAL--

-SPAALN-
SPPAAL--
-SPAALD-
-SPAALC-
SCPAAL--
TRPAAL--
TLPAAL--
-TPAALY-
TYPAAL--
-TPAALG-
-TPAALQ-
TSPAAL--
TFPAAL--
-TPAALS-
-TPAALN-
-TPAALK-
TVPAAL--
-TPAALT-
TGPAAL--
TTPAAL--
-TPAALH-
-TPAALA-
-TPAALI-
TMPAAL--
-TPAALW-
TDPAAL--
THPAAL--
TAPAAL--
-TPAALV-
TKPAAL--
TIPAAL--
TEPAAL--
-TPAALE-
TWPAAL--
TPPAAL--
-TPAALD-
TNPAAL--
-VPAALR-
VKPAAL--
-VPAALL-
VVPAAL--
VRPAAL--
-VPAALK-
-VPAALV-
-VPAALH-
VLPAAL--
VSPAAL--
-VPAALY-
-VPAALW-
VNPAAL--

VHPAAL--
-VPAALS-
-VPAALQ-
VPPAAL--
VYPAAL--
VTPAAL--
-VPAALT-
VFPAAL--
-VPAALF-
-VPAALG-
VIPAAL--
VAPAAL--
VGPAAL--
-VPAALM-
-VPAALD-
VEPAAL--
-VPAALN-
-VPAALE-
-VPAALA-
VDPAAL--
VWPAAL--
-VPAALC-
-WPAALR-
-WPAALT-
WVPAAL--
WGPAAL--
WFPAAL--
WWPAAL--
-WPAALS-
WLPAAL--
WRPAAL--
-WPAALN-
WHPAAL--
-WPAALH-
WAPAAL--
-WPAALK-
-WPAALE-
WQPAAL--
WPPAAL--
-WPAALM-
-WPAALA-
WYPAAL--
WTPAAL--
-WPAALI-
-WPAALF-
-WPAALQ-
-WPAALD-
WCPAAL--
WNPAAL--
YSPAAL--

```
                 YHPAAL--
                 YVPAAL--
                 YRPAAL--
                 -YPAALQ-
                 YWPAAL--
                 YPPAAL--
                 -YPAALS-
                 -YPAALR-
                 YLPAAL--
                 YTPAAL--
                 YNPAAL--
                 -YPAALT-
                 YGPAAL--
                 -YPAALY-
                 -YPAALI-
                 YFPAAL--
                 -YPAALM-
                 -YPAALV-
                 -YPAALL-
                 -YPAALG-
                 YMPAAL--
                 -YPAALK-
                 YQPAAL--
                 -YPAALH-
                 -YPAALD-
                 -YPAALA-
                 YIPAAL--
                 YAPAAL--
                 YCPAAL--
PSWL              110        763      35.71
                 --PSWLNR
                 --PSWLPT
                 -QPSWLP-
                 -APSWLR-
                 ASPSWL--
                 -APSWLL-
                 -APSWLQ-
                 -APSWLK-
                 AMPSWL--
                 AVPSWL--
                 AQPSWL--
                 -APSWLI-
                 -APSWLD-
                 -APSWLT-
                 -APSWLV-
                 -APSWLY-
                 -APSWLN-
                 ANPSWL--
                 AKPSWL--
                 AIPSWL--
```

ALPSWL--
AHPSWL--
AYPSWL--
AAPSWL--
-APSWLM-
ADPSWL--
ATPSWL--
-APSWLH-
-APSWLE-
AGPSWL--
APPSWL--
AFPSWL--
AEPSWL--
AWPSWL--
ACPSWL--
CRPSWL--
CSPSWL--
-CPSWLG-
CHPSWL--
CQPSWL--
CKPSWL--
CTPSWL--
-CPSWLQ-
CFPSWL--
CCPSWL--
-CPSWLY-
-CPSWLS-
-CPSWLL-
CEPSWL--
-CPSWLV-
-CPSWLI-
CPPSWL--
CNPSWL--
CGPSWL--
CDPSWL--
DSPSWL--
DVPSWL--
DTPSWL--
DRPSWL--
DGPSWL--
-DPSWLL-
DMPSWL--
-DPSWLS-
-DPSWLR-
DKPSWL--
DYPSWL--
-DPSWLY-
-DPSWLH-
-DPSWLV-
-DPSWLN-

-DPSWLK-
-DPSWLF-
DNPSWL--
-DPSWLM-
-DPSWLI-
DHPSWL--
DFPSWL--
DWPSWL--
DQPSWL--
-DPSWLT-
-DPSWLG-
-DPSWLE-
DIPSWL--
DDPSWL--
DAPSWL--
-DPSWLW-
-DPSWLD-
DEPSWL--
-EPSWLK-
ERPSWL--
ESPSWL--
EKPSWL--
-EPSWLS-
-EPSWLT-
-EPSWLR-
ETPSWL--
EVPSWL--
-EPSWLH-
EIPSWL--
EGPSWL--
EWPSWL--
-EPSWLE-
ELPSWL--
-EPSWLI-
-EPSWLM-
-EPSWLA-
-EPSWLQ-
-EPSWLF-
EAPSWL--
-EPSWLG-
EDPSWL--
EQPSWL--
-EPSWLN-
EMPSWL--
EHPSWL--
EFPSWL--
EYPSWL--
-EPSWLV-
-FPSWLS-
FTPSWL--

FGPSWL--
FKPSWL--
FHPSWL--
-FPSWLG-
FLPSWL--
FEPSWL--
FNPSWL--
FDPSWL--
FRPSWL--
FYPSWL--
FVPSWL--
FQPSWL--
-FPSWLI-
FMPSWL--
FIPSWL--
-FPSWLQ-
-FPSWLA-
FPPSWL--
-FPSWLV-
-FPSWLK-
-FPSWLN-
-FPSWLC-
FFPSWL--
FAPSWL--
-GPSWLL-
GKPSWL--
GRPSWL--
-GPSWLG-
-GPSWLK-
GLPSWL--
GHPSWL--
GVPSWL--
GYPSWL--
GGPSWL--
GSPSWL--
GEPSWL--
-GPSWLS-
GNPSWL--
-GPSWLN-
GIPSWL--
-GPSWLH-
-GPSWLE-
GTPSWL--
-GPSWLA-
GAPSWL--
-GPSWLT-
GWPSWL--
GQPSWL--
-GPSWLQ-
-GPSWLY-

-GPSWLI-
GDPSWL--
GCPSWL--
HRPSWL--
HTPSWL--
HSPSWL--
-HPSWLL-
HIPSWL--
HEPSWL--
-HPSWLW-
HPPSWL--
HQPSWL--
-HPSWLS-
-HPSWLH-
HVPSWL--
HKPSWL--
-HPSWLR-
-HPSWLI-
-HPSWLN-
-HPSWLG-
-HPSWLT-
HYPSWL--
-HPSWLY-
-HPSWLQ-
-HPSWLA-
HAPSWL--
-HPSWLE-
-HPSWLM-
HNPSWL--
HHPSWL--
HDPSWL--
HFPSWL--
-HPSWLD-
-HPSWLC-
-IPSWLS-
ITPSWL--
ILPSWL--
-IPSWLG-
IHPSWL--
IKPSWL--
INPSWL--
IGPSWL--
-IPSWLV-
-IPSWLM-
-IPSWLQ-
-IPSWLA-
-IPSWLL-
IVPSWL--
-IPSWLN-
IRPSWL--

-IPSWLT-
-IPSWLH-
IIPSWL--
IWPSWL--
-IPSWLI-
IMPSWL--
IEPSWL--
IYPSWL--
-IPSWLK-
-IPSWLE-
IDPSWL--
IQPSWL--
-IPSWLD-
IPPSWL--
IFPSWL--
ICPSWL--
IAPSWL--
KSPSWL--
-KPSWLT-
-KPSWLQ-
KLPSWL--
KVPSWL--
KTPSWL--
-KPSWLI-
-KPSWLA-
-KPSWLS-
-KPSWLG-
KNPSWL--
KHPSWL--
KYPSWL--
-KPSWLF-
KEPSWL--
-KPSWLY-
KFPSWL--
-KPSWLK-
-KPSWLV-
KAPSWL--
KPPSWL--
KMPSWL--
-KPSWLH-
KKPSWL--
KWPSWL--
-KPSWLW-
-KPSWLM-
-KPSWLE-
KDPSWL--
KCPSWL--
KIPSWL--
-KPSWLD-
LEPSWL--

-LPSWLN-
LTPSWL--
-LPSWLL-
LKPSWL--
LYPSWL--
LHPSWL--
-LPSWLK-
LAPSWL--
-LPSWLH-
LIPSWL--
LWPSWL--
LFPSWL--
LDPSWL--
LQPSWL--
-LPSWLM-
-LPSWLE-
-LPSWLD-
-MPSWLG-
-MPSWLK-
-MPSWLI-
MNPSWL--
-MPSWLM-
-MPSWLV-
MWPSWL--
MSPSWL--
-MPSWLN-
MAPSWL--
-MPSWLQ-
MKPSWL--
MGPSWL--
MEPSWL--
-MPSWLT-
MIPSWL--
MFPSWL--
-MPSWLD-
-MPSWLH-
MPPSWL--
MHPSWL--
MQPSWL--
MCPSWL--
NRPSWL--
NGPSWL--
NTPSWL--
NHPSWL--
-NPSWLG-
NYPSWL--
-NPSWLR-
NKPSWL--
NVPSWL--
-NPSWLT-

NFPSWL--
NSPSWL--
NEPSWL--
-NPSWLY-
-NPSWLN-
-NPSWLE-
NMPSWL--
-NPSWLH-
-NPSWLK-
NNPSWL--
-NPSWLS-
-NPSWLF-
NWPSWL--
-NPSWLM-
-NPSWLA-
-NPSWLW-
NDPSWL--
-NPSWLV-
NQPSWL--
NPPSWL--
-NPSWLQ-
--PSWLRN
--PSWLSK
--PSWLGS
--PSWLKA
--PSWLVA
--PSWLRH
--PSWLMS
--PSWLAR
--PSWLVS
--PSWLGT
PSPSWL--
--PSWLSN
--PSWLLT
--PSWLKT
--PSWLIQ
--PSWLMR
--PSWLRI
--PSWLLQ
--PSWLLN
--PSWLKS
--PSWLGK
--PSWLTR
--PSWLSG
--PSWLRF
--PSWLQR
--PSWLIS
--PSWLGN
PLPSWL--
--PSWLLH

--PSWLES
--PSWLTG
--PSWLTA
--PSWLSY
--PSWLVG
--PSWLIH
--PSWLTN
--PSWLSM
--PSWLNM
--PSWLYG
--PSWLWR
--PSWLQN
--PSWLNS
--PSWLHS
PTPSWL--
--PSWLSH
--PSWLSF
--PSWLIR
--PSWLSE
--PSWLMG
--PSWLLD
--PSWLKK
--PSWLIT
--PSWLAV
PVPSWL--
--PSWLQW
PRPSWL--
PAPSWL--
--PSWLVQ
--PSWLVK
--PSWLVH
--PSWLTY
--PSWLTS
--PSWLTL
--PSWLSV
--PSWLSQ
--PSWLSI
--PSWLSD
--PSWLQS
--PSWLNT
--PSWLMA
--PSWLHG
--PSWLAY
--PSWLAG
--PSWLVN
--PSWLTQ
--PSWLSA
--PSWLRG
--PSWLMK
--PSWLGV

--PSWLGG
--PSWLFN
--PSWLAN
--PSWLAK
PGPSWL--
PYPSWL--
--PSWLYQ
--PSWLTW
--PSWLQY
--PSWLNA
--PSWLHT
--PSWLHD
--PSWLED
--PSWLWF
--PSWLNN
--PSWLNK
--PSWLLK
--PSWLKV
--PSWLKG
--PSWLKE
--PSWLKD
--PSWLHA
--PSWLGD
--PSWLAI
PKPSWL--
--PSWLYK
--PSWLWN
--PSWLTT
--PSWLTD
--PSWLRW
--PSWLQF
--PSWLNW
--PSWLMN
--PSWLMH
--PSWLMD
--PSWLIE
--PSWLHN
--PSWLFT
--PSWLFE
--PSWLEN
--PSWLDA
--PSWLAT
--PSWLAQ
--PSWLAE
--PSWLAA
--PSWLTH
--PSWLNG
--PSWLKI
--PSWLIG
--PSWLHV

--PSWLGY
--PSWLGM
--PSWLFG
--PSWLEK
PQPSWL--
--PSWLYE
--PSWLYD
--PSWLWD
--PSWLVE
--PSWLSW
--PSWLQV
--PSWLIV
--PSWLEW
--PSWLEV
--PSWLER
--PSWLDS
PNPSWL--
--PSWLWE
--PSWLVV
--PSWLTE
--PSWLRD
--PSWLQQ
--PSWLQH
--PSWLPL
--PSWLNV
--PSWLNE
--PSWLMW
--PSWLMI
--PSWLME
--PSWLLW
--PSWLKQ
--PSWLKN
--PSWLKH
--PSWLHM
--PSWLHH
--PSWLGA
--PSWLEE
--PSWLEA
PIPSWL--
PEPSWL--
--PSWLWG
--PSWLQG
--PSWLNQ
--PSWLNH
--PSWLMT
--PSWLIW
--PSWLGH
--PSWLFH
--PSWLDT
--PSWLAD

PFPSWL--
--PSWLYH
--PSWLWK
--PSWLTI
--PSWLQA
--PSWLNI
--PSWLND
--PSWLHY
--PSWLHW
--PSWLHQ
--PSWLHL
--PSWLHF
--PSWLHE
--PSWLGE
--PSWLET
--PSWLEI
--PSWLAF
-PPSWLA-
PHPSWL--
PDPSWL--
PCPSWL--
--PSWLYW
--PSWLWW
--PSWLVD
--PSWLRC
--PSWLQE
--PSWLQD
--PSWLPY
--PSWLPR
--PSWLPM
--PSWLPF
--PSWLNC
--PSWLMM
--PSWLKC
--PSWLGF
--PSWLFD
--PSWLEQ
--PSWLEG
--PSWLDK
--PSWLCR
--PSWLAM
--PSWLAH
-PPSWLY-
-PPSWLP-
-PPSWLM-
PPPSWL--
-QPSWLL-
-QPSWLA-
QLPSWL--
QRPSWL--

```
-QPSWLK-
-QPSWLV-
-QPSWLN-
QFPSWL--
QYPSWL--
QGPSWL--
-QPSWLS-
QPPSWL--
QSPSWL--
QVPSWL--
-QPSWLI-
-QPSWLH-
QMPSWL--
QTPSWL--
-QPSWLT-
QQPSWL--
-QPSWLY-
-QPSWLG-
QDPSWL--
-QPSWLM-
-QPSWLD-
QNPSWL--
QKPSWL--
-QPSWLQ-
QIPSWL--
QEPSWL--
RTPSWL--
RSPSWL--
-RPSWLG-
-RPSWLA-
RRPSWL--
-RPSWLL-
-RPSWLT-
-RPSWLN-
-RPSWLK-
RHPSWL--
RYPSWL--
-RPSWLV-
-RPSWLE-
RNPSWL--
-RPSWLY-
-RPSWLH-
-RPSWLW-
-RPSWLM-
-RPSWLI-
REPSWL--
RPPSWL--
RQPSWL--
-RPSWLF-
RCPSWL--
```

-RPSWLD-
SRPSWL--
STPSWL--
-SPSWLL-
-SPSWLR-
SVPSWL--
SNPSWL--
-SPSWLM-
-SPSWLT-
SGPSWL--
SMPSWL--
-SPSWLG-
SYPSWL--
-SPSWLQ-
SKPSWL--
-SPSWLK-
-SPSWLH-
-SPSWLY-
-SPSWLA-
SFPSWL--
SQPSWL--
SPPSWL--
SDPSWL--
SAPSWL--
-SPSWLV-
-SPSWLE-
-SPSWLD-
-SPSWLN-
SHPSWL--
SEPSWL--
SCPSWL--
TRPSWL--
-TPSWLL-
TTPSWL--
TSPSWL--
TLPSWL--
TGPSWL--
-TPSWLR-
-TPSWLS-
-TPSWLH-
TDPSWL--
-TPSWLA-
-TPSWLM-
-TPSWLK-
-TPSWLG-
-TPSWLN-
TKPSWL--
THPSWL--
TPPSWL--
TAPSWL--

TQPSWL--
-TPSWLT-
TFPSWL--
-TPSWLV-
-TPSWLE-
-TPSWLI-
TEPSWL--
TIPSWL--
TWPSWL--
-TPSWLQ-
-TPSWLD-
TYPSWL--
TCPSWL--
VRPSWL--
-VPSWLM-
-VPSWLT-
VSPSWL--
-VPSWLL-
VNPSWL--
VDPSWL--
VVPSWL--
-VPSWLA-
VYPSWL--
-VPSWLQ-
-VPSWLN-
-VPSWLV-
-VPSWLH-
VHPSWL--
-VPSWLE-
VEPSWL--
VWPSWL--
VQPSWL--
-VPSWLD-
VCPSWL--
-WPSWLT-
-WPSWLK-
WTPSWL--
WKPSWL--
WRPSWL--
WDPSWL--
WAPSWL--
-WPSWLG-
WEPSWL--
WIPSWL--
-WPSWLS-
WHPSWL--
-WPSWLE-
WNPSWL--
WFPSWL--
-WPSWLQ-

```
           -WPSWLH-
           -WPSWLF-
           WYPSWL--
           WWPSWL--
           WVPSWL--
           -WPSWLM-
           -WPSWLD-
           -WPSWLA-
           WPPSWL--
           WMPSWL--
           -YPSWLG-
           YLPSWL--
           YSPSWL--
           YRPSWL--
           YTPSWL--
           -YPSWLR-
           -YPSWLH-
           -YPSWLN-
           -YPSWLQ-
           -YPSWLT-
           -YPSWLL-
           YNPSWL--
           -YPSWLI-
           YPPSWL--
           -YPSWLK-
           -YPSWLE-
           YKPSWL--
           YMPSWL--
           YVPSWL--
           -YPSWLV-
           -YPSWLA-
           YYPSWL--
           -YPSWLY-
           YGPSWL--
           YDPSWL--
           YWPSWL--
           YQPSWL--
           -YPSWLM-
           YHPSWL--
           -YPSWLS-
           YFPSWL--
           YEPSWL--
           -YPSWLF-
PSGL          8        886     35.50
           -DPSGLG-
           -NPSGLP-
           --PSGLYP
           --PSGLPR
           --PSGLMD
           --PSGLDL
```

-QPSGLP-
-TPSGLP-
-VPSGLD-
-APSGLR-
ASPSGL--
-APSGLV-
-APSGLL-
-APSGLK-
ALPSGL--
-APSGLT-
AFPSGL--
ANPSGL--
AGPSGL--
ATPSGL--
-APSGLN-
AYPSGL--
-APSGLW-
-APSGLI-
AVPSGL--
ARPSGL--
AAPSGL--
-APSGLQ-
-APSGLM-
-APSGLG-
AQPSGL--
AIPSGL--
-APSGLF-
-APSGLY-
-APSGLE-
AMPSGL--
ADPSGL--
AKPSGL--
AHPSGL--
-APSGLH-
ACPSGL--
AWPSGL--
-APSGLD-
-APSGLC-
-CPSGLR-
CLPSGL--
CTPSGL--
CRPSGL--
CIPSGL--
-CPSGLK-
CKPSGL--
CSPSGL--
CCPSGL--
-CPSGLT-
-CPSGLH-
CEPSGL--

CVPSGL--
-CPSGLF-
CPPSGL--
CAPSGL--
CGPSGL--
CDPSGL--
CYPSGL--
-CPSGLI-
CMPSGL--
CWPSGL--
-CPSGLM-
-CPSGLL-
CHPSGL--
DSPSGL--
-DPSGLR-
DRPSGL--
DVPSGL--
-DPSGLL-
-DPSGLA-
DLPSGL--
-DPSGLS-
-DPSGLK-
DWPSGL--
DKPSGL--
DGPSGL--
DFPSGL--
DQPSGL--
-DPSGLT-
-DPSGLI-
-DPSGLH-
DAPSGL--
DYPSGL--
-DPSGLF-
DIPSGL--
DTPSGL--
-DPSGLW-
-DPSGLV-
-DPSGLE-
DNPSGL--
-DPSGLN-
-DPSGLM-
DHPSGL--
DEPSGL--
-DPSGLD-
DMPSGL--
-EPSGLL-
EWPSGL--
ERPSGL--
-EPSGLR-
ESPSGL--

−EPSGLN−
ELPSGL−−
EFPSGL−−
−EPSGLV−
EQPSGL−−
−EPSGLS−
−EPSGLW−
−EPSGLM−
−EPSGLK−
−EPSGLF−
EYPSGL−−
−EPSGLY−
−EPSGLH−
EVPSGL−−
−EPSGLG−
EMPSGL−−
EHPSGL−−
EAPSGL−−
−EPSGLT−
−EPSGLI−
ETPSGL−−
−EPSGLQ−
−EPSGLA−
EIPSGL−−
EDPSGL−−
ECPSGL−−
EPPSGL−−
EEPSGL−−
−FPSGLR−
FLPSGL−−
FRPSGL−−
−FPSGLK−
FSPSGL−−
−FPSGLT−
−FPSGLS−
FFPSGL−−
FAPSGL−−
−FPSGLM−
−FPSGLL−
FIPSGL−−
FTPSGL−−
FKPSGL−−
FYPSGL−−
FWPSGL−−
FPPSGL−−
FHPSGL−−
−FPSGLN−
FGPSGL−−
−FPSGLF−
−FPSGLA−

FQPSGL--
FVPSGL--
-FPSGLI-
-FPSGLG-
-FPSGLE-
FNPSGL--
-FPSGLV-
-FPSGLY-
FEPSGL--
-FPSGLW-
FMPSGL--
-FPSGLD-
-FPSGLC-
FDPSGL--
FCPSGL--
GRPSGL--
GTPSGL--
GIPSGL--
-GPSGLR-
GSPSGL--
GVPSGL--
-GPSGLS-
-GPSGLG-
-GPSGLM-
-GPSGLL-
GYPSGL--
-GPSGLT-
-GPSGLI-
GQPSGL--
-GPSGLE-
GNPSGL--
GFPSGL--
-GPSGLW-
GMPSGL--
-GPSGLV-
-GPSGLK-
GKPSGL--
GHPSGL--
GCPSGL--
GDPSGL--
-GPSGLQ-
-GPSGLF-
-GPSGLY-
-GPSGLN-
GPPSGL--
GEPSGL--
-HPSGLR-
HLPSGL--
HRPSGL--
HTPSGL--

HSPSGL--
HKPSGL--
HGPSGL--
-HPSGLK-
-HPSGLS-
HIPSGL--
HAPSGL--
HNPSGL--
-HPSGLM-
-HPSGLL-
HYPSGL--
-HPSGLN-
-HPSGLI-
-HPSGLY-
-HPSGLG-
HFPSGL--
-HPSGLT-
HPPSGL--
-HPSGLH-
-HPSGLF-
HHPSGL--
HQPSGL--
HWPSGL--
-HPSGLA-
HDPSGL--
HVPSGL--
-HPSGLW-
HMPSGL--
HEPSGL--
-HPSGLE-
-HPSGLC-
IRPSGL--
-IPSGLT-
-IPSGLL-
ISPSGL--
IVPSGL--
-IPSGLW-
ILPSGL--
-IPSGLS-
IFPSGL--
ITPSGL--
-IPSGLN-
-IPSGLF-
IQPSGL--
-IPSGLH-
-IPSGLA-
-IPSGLR-
IGPSGL--
IAPSGL--
-IPSGLK-

IIPSGL--
IYPSGL--
-IPSGLY-
-IPSGLQ-
-IPSGLV-
-IPSGLI-
IMPSGL--
IKPSGL--
IPPSGL--
IWPSGL--
-IPSGLE-
INPSGL--
IEPSGL--
-IPSGLM-
IDPSGL--
IHPSGL--
-IPSGLD-
ICPSGL--
KSPSGL--
KRPSGL--
-KPSGLR-
-KPSGLL-
KLPSGL--
KGPSGL--
KIPSGL--
KWPSGL--
KFPSGL--
-KPSGLF-
KYPSGL--
-KPSGLS-
KPPSGL--
-KPSGLT-
-KPSGLV-
KMPSGL--
-KPSGLI-
KNPSGL--
KQPSGL--
-KPSGLG-
-KPSGLA-
KAPSGL--
KTPSGL--
-KPSGLQ-
-KPSGLH-
-KPSGLN-
-KPSGLY-
-KPSGLM-
-KPSGLK-
-KPSGLE-
-KPSGLW-
KKPSGL--

KHPSGL--
-KPSGLC-
KCPSGL--
LRPSGL--
-LPSGLL-
LVPSGL--
LSPSGL--
LPPSGL--
LKPSGL--
LAPSGL--
LNPSGL--
LLPSGL--
LGPSGL--
LTPSGL--
-LPSGLV-
LYPSGL--
LIPSGL--
-LPSGLY-
-LPSGLW-
-LPSGLN-
-LPSGLM-
LMPSGL--
-LPSGLI-
LDPSGL--
-LPSGLH-
-LPSGLF-
LHPSGL--
-LPSGLC-
-MPSGLR-
-MPSGLT-
MSPSGL--
MTPSGL--
-MPSGLS-
MRPSGL--
-MPSGLI-
-MPSGLF-
MLPSGL--
-MPSGLG-
-MPSGLE-
MAPSGL--
MGPSGL--
-MPSGLQ-
-MPSGLY-
-MPSGLH-
MMPSGL--
MYPSGL--
-MPSGLA-
-MPSGLK-
MDPSGL--
MKPSGL--

```
-MPSGLN-
MHPSGL--
MQPSGL--
MEPSGL--
MIPSGL--
-MPSGLM-
MCPSGL--
NRPSGL--
NSPSGL--
-NPSGLL-
NLPSGL--
NGPSGL--
NTPSGL--
-NPSGLY-
-NPSGLR-
NVPSGL--
NKPSGL--
-NPSGLA-
NYPSGL--
-NPSGLW-
-NPSGLS-
-NPSGLK-
NPPSGL--
-NPSGLT-
NAPSGL--
NHPSGL--
NFPSGL--
-NPSGLV-
-NPSGLM-
-NPSGLI-
NQPSGL--
NNPSGL--
NMPSGL--
NWPSGL--
-NPSGLN-
NEPSGL--
NDPSGL--
-NPSGLQ-
-NPSGLH-
-NPSGLG-
-NPSGLE-
--PSGLRS
--PSGLRG
--PSGLSR
--PSGLRL
--PSGLRT
--PSGLTL
--PSGLSL
--PSGLSS
--PSGLFL
```

--PSGLYL
--PSGLRN
--PSGLST
--PSGLRV
--PSGLIL
--PSGLHR
--PSGLGS
--PSGLSG
--PSGLYT
--PSGLRA
--PSGLMR
--PSGLLK
--PSGLHL
--PSGLSK
--PSGLYI
--PSGLVK
--PSGLRM
--PSGLFT
--PSGLYR
--PSGLYG
--PSGLWR
--PSGLLT
--PSGLVS
--PSGLVM
--PSGLVF
--PSGLTS
--PSGLTR
--PSGLLH
--PSGLIS
--PSGLVG
--PSGLSY
--PSGLML
--PSGLAR
--PSGLYS
--PSGLRH
--PSGLLY
--PSGLGA
--PSGLIR
--PSGLFS
--PSGLWN
--PSGLMK
--PSGLGF
--PSGLWS
--PSGLVI
--PSGLSF
--PSGLII
--PSGLFQ
PSPSGL--
--PSGLSH
--PSGLMA

--PSGLVV
--PSGLTI
--PSGLNS
--PSGLLV
--PSGLHY
--PSGLGT
--PSGLFG
PWPSGL--
--PSGLTG
--PSGLQS
--PSGLLW
--PSGLKW
--PSGLHT
--PSGLFM
--PSGLYA
--PSGLWK
--PSGLVD
--PSGLMW
--PSGLLA
--PSGLKG
--PSGLGV
--PSGLAS
--PSGLVY
--PSGLVT
--PSGLSN
--PSGLRF
--PSGLNI
--PSGLMH
--PSGLLE
--PSGLES
--PSGLER
PLPSGL--
--PSGLTN
--PSGLLM
--PSGLIN
--PSGLIG
--PSGLYQ
--PSGLVE
--PSGLNR
--PSGLNH
--PSGLNF
--PSGLIV
--PSGLHW
--PSGLTT
--PSGLTH
--PSGLSQ
--PSGLSD
--PSGLQR
--PSGLGY
PVPSGL--

PTPSGL--
--PSGLWG
--PSGLSM
--PSGLRP
--PSGLQA
--PSGLMS
--PSGLLD
--PSGLKI
--PSGLAH
--PSGLAF
PAPSGL--
--PSGLNV
--PSGLMV
--PSGLLQ
--PSGLKT
--PSGLIT
--PSGLEM
--PSGLAG
--PSGLTK
--PSGLNT
--PSGLNA
--PSGLMN
--PSGLKS
--PSGLKH
--PSGLIM
--PSGLGQ
--PSGLVW
--PSGLRE
--PSGLQG
--PSGLHH
--PSGLGW
--PSGLEA
--PSGLAT
PKPSGL--
--PSGLWA
--PSGLTF
--PSGLQT
--PSGLIH
--PSGLIF
--PSGLFN
--PSGLFI
--PSGLFA
--PSGLEW
--PSGLAK
--PSGLAA
PMPSGL--
PFPSGL--
--PSGLYK
--PSGLTD
--PSGLSW

--PSGLQN
--PSGLMY
--PSGLKA
--PSGLIQ
--PSGLGL
--PSGLFH
--PSGLDA
--PSGLAY
--PSGLAM
PIPSGL--
PHPSGL--
--PSGLYH
--PSGLTQ
--PSGLTA
--PSGLSE
--PSGLQH
--PSGLND
--PSGLMT
--PSGLMI
--PSGLKF
--PSGLIK
--PSGLHV
--PSGLHG
--PSGLHA
--PSGLGK
--PSGLYY
--PSGLYM
--PSGLYE
--PSGLVN
--PSGLQK
--PSGLQE
--PSGLMQ
--PSGLMM
--PSGLKV
--PSGLKN
--PSGLKK
--PSGLIY
--PSGLHF
--PSGLGH
--PSGLEV
--PSGLET
--PSGLEH
--PSGLTW
--PSGLQL
--PSGLIA
--PSGLHI
--PSGLGM
--PSGLGI
--PSGLDS
PYPSGL--

--PSGLWQ
--PSGLVQ
--PSGLQQ
--PSGLNW
--PSGLNM
--PSGLMF
--PSGLKE
--PSGLHE
--PSGLEI
--PSGLYV
--PSGLWW
--PSGLWM
--PSGLQY
--PSGLQI
--PSGLNG
--PSGLHM
--PSGLFW
--PSGLFE
--PSGLAV
PQPSGL--
--PSGLYD
--PSGLWE
--PSGLKQ
--PSGLKD
--PSGLIE
--PSGLHK
--PSGLGN
--PSGLEY
--PSGLAQ
--PSGLAE
PEPSGL--
--PSGLQV
--PSGLQM
--PSGLNN
--PSGLNE
--PSGLHN
--PSGLFD
--PSGLEK
--PSGLEF
--PSGLAN
PDPSGL--
PCPSGL--
--PSGLWH
--PSGLTE
--PSGLQW
--PSGLNK
--PSGLHC
--PSGLDG
--PSGLQF
--PSGLME

--PSGLHQ
--PSGLFC
--PSGLEN
--PSGLEL
--PSGLEG
--PSGLCT
--PSGLCH
--PSGLSC
--PSGLQD
--PSGLKC
--PSGLIC
--PSGLGE
--PSGLDQ
--PSGLCS
--PSGLCR
--PSGLCN
--PSGLCK
--PSGLCI
--PSGLCG
QVPSGL--
QRPSGL--
-QPSGLY-
-QPSGLT-
-QPSGLW-
-QPSGLI-
QSPSGL--
QGPSGL--
QTPSGL--
QNPSGL--
QWPSGL--
-QPSGLG-
QPPSGL--
-QPSGLS-
-QPSGLM-
QYPSGL--
-QPSGLA-
QLPSGL--
QFPSGL--
QAPSGL--
-QPSGLF-
-QPSGLV-
QQPSGL--
-QPSGLK-
-QPSGLQ-
QMPSGL--
QHPSGL--
-QPSGLH-
-QPSGLE-
QIPSGL--
QEPSGL--

QDPSGL--
-RPSGLS-
RSPSGL--
RLPSGL--
-RPSGLA-
RNPSGL--
-RPSGLF-
RYPSGL--
-RPSGLN-
-RPSGLY-
-RPSGLG-
-RPSGLV-
RIPSGL--
-RPSGLQ-
-RPSGLK-
RAPSGL--
RDPSGL--
RWPSGL--
RPPSGL--
-RPSGLE-
RQPSGL--
RKPSGL--
RHPSGL--
REPSGL--
RCPSGL--
SRPSGL--
-SPSGLL-
SLPSGL--
SSPSGL--
-SPSGLR-
SVPSGL--
STPSGL--
SHPSGL--
-SPSGLF-
SKPSGL--
SAPSGL--
-SPSGLQ-
-SPSGLY-
-SPSGLV-
SNPSGL--
-SPSGLN-
-SPSGLA-
-SPSGLI-
-SPSGLS-
-SPSGLH-
-SPSGLW-
SFPSGL--
SDPSGL--
SEPSGL--
SQPSGL--

-SPSGLE-
SCPSGL--
-SPSGLD-
-SPSGLC-
TRPSGL--
TPPSGL--
-TPSGLL-
-TPSGLG-
TVPSGL--
-TPSGLR-
TGPSGL--
-TPSGLT-
-TPSGLY-
-TPSGLK-
-TPSGLN-
TFPSGL--
TMPSGL--
TLPSGL--
-TPSGLH-
TAPSGL--
TYPSGL--
TTPSGL--
-TPSGLI-
TKPSGL--
TWPSGL--
-TPSGLV-
-TPSGLF-
TSPSGL--
TQPSGL--
-TPSGLE-
THPSGL--
-TPSGLW-
TEPSGL--
TDPSGL--
TCPSGL--
-TPSGLM-
-TPSGLQ-
-VPSGLL-
VRPSGL--
-VPSGLG-
-VPSGLW-
-VPSGLK-
VNPSGL--
VVPSGL--
VSPSGL--
VKPSGL--
VGPSGL--
VFPSGL--
-VPSGLS-
-VPSGLY-

```
-VPSGLN-
-VPSGLF-
VTPSGL--
-VPSGLV-
VMPSGL--
-VPSGLM-
VPPSGL--
VEPSGL--
VAPSGL--
-VPSGLH-
-VPSGLQ-
-VPSGLE-
VHPSGL--
VDPSGL--
VCPSGL--
WLPSGL--
-WPSGLK-
-WPSGLT-
-WPSGLR-
WRPSGL--
WTPSGL--
WSPSGL--
-WPSGLI-
WVPSGL--
WNPSGL--
WKPSGL--
WIPSGL--
-WPSGLA-
WGPSGL--
WWPSGL--
-WPSGLY-
-WPSGLN-
-WPSGLS-
WHPSGL--
WAPSGL--
-WPSGLQ-
-WPSGLL-
-WPSGLH-
WMPSGL--
-WPSGLV-
-WPSGLM-
WPPSGL--
-WPSGLE-
WFPSGL--
WDPSGL--
-WPSGLW-
-WPSGLG-
WQPSGL--
WEPSGL--
-WPSGLF-
```

| | | | |
|---|---|---|---|
| | -YPSGLL- | | |
| | YLPSGL-- | | |
| | -YPSGLT- | | |
| | -YPSGLR- | | |
| | YGPSGL-- | | |
| | -YPSGLS- | | |
| | -YPSGLH- | | |
| | YTPSGL-- | | |
| | -YPSGLK- | | |
| | -YPSGLV- | | |
| | YFPSGL-- | | |
| | YVPSGL-- | | |
| | -YPSGLI- | | |
| | -YPSGLY- | | |
| | YYPSGL-- | | |
| | -YPSGLQ- | | |
| | -YPSGLN- | | |
| | YKPSGL-- | | |
| | YEPSGL-- | | |
| | -YPSGLF- | | |
| | -YPSGLA- | | |
| | YHPSGL-- | | |
| | YAPSGL-- | | |
| | -YPSGLW- | | |
| | -YPSGLE- | | |
| | YQPSGL-- | | |
| | YNPSGL-- | | |
| | -YPSGLM- | | |
| | YMPSGL-- | | |
| | YDPSGL-- | | |
| PSSL | 29 | 830 | 35.49 |
| | CQPSSL-- | | |
| | EDPSSL-- | | |
| | -LPSSLG- | | |
| | -MPSSLP- | | |
| | NDPSSL-- | | |
| | --PSSLGR | | |
| | --PSSLRP | | |
| | --PSSLPT | | |
| | --PSSLPI | | |
| | --PSSLPA | | |
| | --PSSLNC | | |
| | --PSSLLP | | |
| | --PSSLGH | | |
| | --PSSLEI | | |
| | --PSSLDT | | |
| | --PSSLCT | | |
| | -APSSLL- | | |
| | -APSSLR- | | |
| | AKPSSL-- | | |

```
-APSSLG-
AVPSSL--
-APSSLS-
AFPSSL--
-APSSLW-
-APSSLQ-
AYPSSL--
-APSSLM-
AHPSSL--
-APSSLK-
-APSSLI-
-APSSLA-
AIPSSL--
AWPSSL--
-APSSLV-
AGPSSL--
AQPSSL--
-APSSLT-
-APSSLF-
AMPSSL--
AEPSSL--
ADPSSL--
-APSSLE-
APPSSL--
ACPSSL--
CRPSSL--
CAPSSL--
-CPSSLR-
CLPSSL--
CTPSSL--
CSPSSL--
-CPSSLK-
CVPSSL--
CPPSSL--
CIPSSL--
-CPSSLW-
-CPSSLS-
-CPSSLL-
CMPSSL--
-CPSSLT-
CKPSSL--
CDPSSL--
CCPSSL--
-CPSSLI-
-CPSSLF-
-CPSSLM-
CWPSSL--
-CPSSLV-
-CPSSLN-
-CPSSLA-
```

CYPSSL--
-CPSSLY-
-CPSSLQ-
-CPSSLH-
CEPSSL--
DLPSSL--
DVPSSL--
DQPSSL--
DYPSSL--
-DPSSLT-
DSPSSL--
DWPSSL--
DMPSSL--
DIPSSL--
-DPSSLY-
-DPSSLL-
-DPSSLF-
DKPSSL--
DAPSSL--
DTPSSL--
-DPSSLS-
-DPSSLV-
-DPSSLK-
DGPSSL--
DNPSSL--
-DPSSLW-
-DPSSLI-
DHPSSL--
-DPSSLQ-
-DPSSLN-
-DPSSLH-
DCPSSL--
-DPSSLE-
DFPSSL--
-DPSSLP-
-DPSSLM-
-DPSSLA-
DDPSSL--
EVPSSL--
-EPSSLR-
-EPSSLV-
EAPSSL--
-EPSSLL-
EMPSSL--
-EPSSLS-
EKPSSL--
-EPSSLK-
-EPSSLY-
ERPSSL--
-EPSSLT-

ESPSSL--
-EPSSLH-
EYPSSL--
-EPSSLN-
EIPSSL--
EFPSSL--
ETPSSL--
-EPSSLQ-
-EPSSLM-
ENPSSL--
EGPSSL--
-EPSSLE-
-EPSSLG-
EEPSSL--
-EPSSLW-
ECPSSL--
-EPSSLA-
EHPSSL--
EPPSSL--
-EPSSLI-
FLPSSL--
-FPSSLR-
-FPSSLI-
-FPSSLT-
FEPSSL--
FTPSSL--
FMPSSL--
FHPSSL--
-FPSSLQ-
FVPSSL--
-FPSSLW-
FFPSSL--
-FPSSLK-
FQPSSL--
FNPSSL--
-FPSSLV-
FIPSSL--
FDPSSL--
FWPSSL--
-FPSSLM-
-FPSSLN-
FKPSSL--
FYPSSL--
-FPSSLY-
-FPSSLE-
FAPSSL--
-FPSSLG-
-FPSSLH-
FPPSSL--
FCPSSL--

-FPSSLC-
GLPSSL--
-GPSSLR-
GRPSSL--
GTPSSL--
-GPSSLL-
GSPSSL--
-GPSSLA-
-GPSSLF-
GFPSSL--
GYPSSL--
GNPSSL--
GQPSSL--
GAPSSL--
-GPSSLK-
GGPSSL--
GVPSSL--
-GPSSLN-
-GPSSLS-
GPPSSL--
-GPSSLM-
-GPSSLH-
-GPSSLQ-
GMPSSL--
GHPSSL--
-GPSSLY-
-GPSSLW-
-GPSSLV-
-GPSSLE-
GWPSSL--
GEPSSL--
GCPSSL--
GDPSSL--
HVPSSL--
-HPSSLS-
-HPSSLL-
HAPSSL--
HYPSSL--
HSPSSL--
-HPSSLY-
HNPSSL--
HKPSSL--
HWPSSL--
-HPSSLK-
-HPSSLI-
-HPSSLR-
-HPSSLA-
-HPSSLV-
HFPSSL--
HEPSSL--

-HPSSLQ-
HQPSSL--
-HPSSLH-
-HPSSLG-
HDPSSL--
-HPSSLM-
HHPSSL--
HMPSSL--
-HPSSLW-
-HPSSLE-
HPPSSL--
HCPSSL--
-IPSSLR-
ISPSSL--
-IPSSLT-
IGPSSL--
ITPSSL--
-IPSSLL-
-IPSSLV-
ILPSSL--
IKPSSL--
IMPSSL--
-IPSSLQ-
IAPSSL--
-IPSSLM-
-IPSSLG-
-IPSSLF-
IHPSSL--
IYPSSL--
-IPSSLI-
-IPSSLE-
-IPSSLK-
INPSSL--
-IPSSLY-
IIPSSL--
IPPSSL--
IDPSSL--
IWPSSL--
IQPSSL--
-IPSSLN-
-IPSSLH-
-IPSSLW-
IEPSSL--
IFPSSL--
ICPSSL--
KSPSSL--
KLPSSL--
KVPSSL--
-KPSSLR-
KTPSSL--

KRPSSL--
-KPSSLF-
-KPSSLS-
-KPSSLN-
KWPSSL--
-KPSSLT-
-KPSSLK-
KAPSSL--
KMPSSL--
KKPSSL--
KHPSSL--
KNPSSL--
-KPSSLW-
-KPSSLA-
KDPSSL--
-KPSSLY-
-KPSSLI-
-KPSSLV-
-KPSSLQ-
-KPSSLH-
KPPSSL--
-KPSSLE-
KEPSSL--
KCPSSL--
-KPSSLC-
LRPSSL--
LLPSSL--
LSPSSL--
LVPSSL--
LFPSSL--
LKPSSL--
-LPSSLT-
LYPSSL--
-LPSSLK-
LIPSSL--
LMPSSL--
-LPSSLY-
-LPSSLH-
LPPSSL--
LNPSSL--
-LPSSLQ-
-LPSSLA-
-LPSSLI-
LTPSSL--
LHPSSL--
-LPSSLW-
LDPSSL--
LEPSSL--
-LPSSLM-
-LPSSLE-

LCPSSL--
MVPSSL--
-MPSSLR-
MSPSSL--
MGPSSL--
-MPSSLL-
MMPSSL--
MKPSSL--
-MPSSLS-
MNPSSL--
MIPSSL--
MAPSSL--
-MPSSLW-
MWPSSL--
-MPSSLH-
-MPSSLT-
-MPSSLA-
-MPSSLN-
-MPSSLY-
-MPSSLK-
-MPSSLG-
-MPSSLM-
-MPSSLI-
MEPSSL--
MPPSSL--
-MPSSLQ-
MFPSSL--
-MPSSLF-
-MPSSLE-
-MPSSLV-
MQPSSL--
MCPSSL--
MDPSSL--
-NPSSLR-
NWPSSL--
NVPSSL--
-NPSSLL-
NIPSSL--
NAPSSL--
NTPSSL--
-NPSSLT-
NFPSSL--
-NPSSLS-
NNPSSL--
-NPSSLH-
NHPSSL--
NGPSSL--
NMPSSL--
NQPSSL--
-NPSSLV-

-NPSSLA-
-NPSSLY-
-NPSSLW-
NYPSSL--
NCPSSL--
NPPSSL--
-NPSSLN-
-NPSSLI-
NEPSSL--
-NPSSLG-
--PSSLRT
--PSSLLR
--PSSLRS
--PSSLRG
PRPSSL--
PSPSSL--
--PSSLRN
--PSSLLT
--PSSLSR
--PSSLVR
--PSSLRI
--PSSLTR
--PSSLIS
--PSSLIR
--PSSLVS
--PSSLVG
--PSSLTL
--PSSLRF
--PSSLLK
--PSSLKR
--PSSLKG
--PSSLSN
--PSSLSK
--PSSLSG
--PSSLWA
--PSSLST
--PSSLFN
--PSSLHS
--PSSLVT
--PSSLLY
PLPSSL--
--PSSLTA
--PSSLSF
--PSSLAW
--PSSLWR
--PSSLTN
--PSSLTH
--PSSLQL
--PSSLNR
PGPSSL--

```
--PSSLSQ
--PSSLGG
--PSSLFS
--PSSLVM
--PSSLKT
--PSSLHL
--PSSLFG
--PSSLAG
--PSSLVN
--PSSLRY
--PSSLMA
--PSSLSA
--PSSLRE
--PSSLHG
PVPSSL--
--PSSLFH
--PSSLAI
--PSSLWG
--PSSLTW
--PSSLQR
--PSSLGL
--PSSLVK
--PSSLTI
--PSSLSI
--PSSLRH
--PSSLNA
--PSSLMI
--PSSLLI
--PSSLGS
--PSSLAR
PTPSSL--
--PSSLWS
--PSSLTT
--PSSLTF
--PSSLSH
--PSSLQS
--PSSLYM
--PSSLWK
--PSSLVY
--PSSLTG
--PSSLGA
PWPSSL--
--PSSLWV
--PSSLWH
--PSSLQT
--PSSLKA
--PSSLEG
--PSSLVW
--PSSLVH
--PSSLMS
```

```
--PSSLLH
--PSSLKI
--PSSLHF
--PSSLFT
--PSSLYK
--PSSLWW
--PSSLTV
--PSSLSY
--PSSLLQ
--PSSLAT
--PSSLYT
--PSSLNS
--PSSLLD
--PSSLFW
--PSSLES
PMPSSL--
--PSSLMM
--PSSLKK
--PSSLFY
--PSSLAM
--PSSLAF
--PSSLVQ
--PSSLVI
--PSSLTY
--PSSLTK
--PSSLPL
--PSSLNK
--PSSLMF
--PSSLMD
--PSSLIH
--PSSLAV
--PSSLYF
--PSSLSM
--PSSLQV
--PSSLMK
--PSSLGW
--PSSLAH
PQPSSL--
--PSSLTQ
--PSSLQG
--PSSLNQ
--PSSLMT
--PSSLIF
--PSSLHT
--PSSLHK
--PSSLAQ
--PSSLYN
--PSSLYH
--PSSLNH
--PSSLKQ
```

```
--PSSLIK
--PSSLHA
--PSSLAK
--PSSLYD
--PSSLWQ
--PSSLSD
--PSSLNV
--PSSLKD
--PSSLIY
--PSSLIM
--PSSLFA
PAPSSL--
--PSSLYG
--PSSLWD
--PSSLTE
--PSSLTD
--PSSLSE
--PSSLNT
--PSSLNM
--PSSLMY
--PSSLIV
--PSSLHQ
--PSSLHN
--PSSLFK
--PSSLWN
--PSSLVE
--PSSLVD
--PSSLMW
--PSSLMN
--PSSLKM
--PSSLHH
--PSSLFD
--PSSLYW
--PSSLQF
PIPSSL--
PYPSSL--
--PSSLYQ
--PSSLYE
--PSSLQK
--PSSLMQ
--PSSLFQ
--PSSLEW
--PSSLYI
--PSSLTM
--PSSLQY
--PSSLQN
--PSSLQA
--PSSLNN
--PSSLMH
--PSSLKF
```

--PSSLHM
--PSSLHE
--PSSLFE
--PSSLEY
PHPSSL--
PFPSSL--
--PSSLWT
--PSSLQQ
--PSSLME
--PSSLIQ
--PSSLGY
PNPSSL--
--PSSLQH
--PSSLNW
--PSSLNI
--PSSLID
--PSSLGM
--PSSLDS
--PSSLAD
PEPSSL--
--PSSLWI
--PSSLQI
--PSSLKE
--PSSLCS
--PSSLET
--PSSLEM
--PSSLEK
--PSSLEF
--PSSLDA
PCPSSL--
--PSSLWE
--PSSLQM
--PSSLPY
--PSSLPN
--PSSLPM
--PSSLNG
--PSSLIE
--PSSLHY
--PSSLHD
--PSSLGF
--PSSLEV
--PSSLAE
--PSSLRC
--PSSLNE
--PSSLND
--PSSLHV
--PSSLEH
--PSSLDG
-PPSSLR-
-PPSSLI-

--PSSLWC
--PSSLVC
--PSSLQE
--PSSLMC
--PSSLDH
--PSSLCN
--PSSLCH
-PPSSLS-
-PPSSLK-
-PPSSLA-
PPPSSL--
QSPSSL--
-QPSSLR-
QVPSSL--
-QPSSLT-
-QPSSLH-
-QPSSLY-
-QPSSLK-
-QPSSLF-
-QPSSLA-
QAPSSL--
QIPSSL--
QHPSSL--
QDPSSL--
QQPSSL--
-QPSSLS-
QNPSSL--
QEPSSL--
-QPSSLQ-
QFPSSL--
-QPSSLM-
-QPSSLW-
-QPSSLN-
-QPSSLV-
QPPSSL--
-QPSSLG-
-QPSSLE-
QMPSSL--
QCPSSL--
RSPSSL--
RGPSSL--
RMPSSL--
-RPSSLA-
-RPSSLS-
RAPSSL--
RTPSSL--
RWPSSL--
-RPSSLY-
-RPSSLG-
-RPSSLH-

-RPSSLT-
RYPSSL--
RIPSSL--
RFPSSL--
-RPSSLV-
-RPSSLK-
RPPSSL--
RQPSSL--
REPSSL--
-RPSSLI-
RDPSSL--
-RPSSLF-
-RPSSLQ-
-RPSSLE-
RCPSSL--
-RPSSLC-
SRPSSL--
-SPSSLL-
SSPSSL--
SFPSSL--
SIPSSL--
SMPSSL--
SYPSSL--
SKPSSL--
-SPSSLY-
-SPSSLI-
-SPSSLA-
SAPSSL--
SPPSSL--
SHPSSL--
-SPSSLV-
-SPSSLH-
-SPSSLK-
SEPSSL--
-SPSSLF-
-SPSSLM-
-SPSSLE-
SCPSSL--
-SPSSLN-
-SPSSLQ-
SQPSSL--
SDPSSL--
-SPSSLC-
TRPSSL--
-TPSSLR-
-TPSSLL-
TLPSSL--
TTPSSL--
-TPSSLT-
-TPSSLA-

TAPSSL--
TSPSSL--
TFPSSL--
-TPSSLW-
TGPSSL--
-TPSSLV-
-TPSSLS-
-TPSSLK-
-TPSSLI-
-TPSSLF-
TYPSSL--
-TPSSLY-
-TPSSLM-
TWPSSL--
TDPSSL--
TQPSSL--
-TPSSLN-
-TPSSLG-
THPSSL--
TNPSSL--
TKPSSL--
TMPSSL--
TEPSSL--
TCPSSL--
-TPSSLE-
-TPSSLC-
VSPSSL--
VYPSSL--
VIPSSL--
-VPSSLQ-
VHPSSL--
-VPSSLG-
-VPSSLK-
VNPSSL--
VTPSSL--
-VPSSLW-
-VPSSLN-
-VPSSLF-
-VPSSLH-
VEPSSL--
-VPSSLI-
-VPSSLY-
-VPSSLV-
VPPSSL--
VFPSSL--
VQPSSL--
-VPSSLM-
VWPSSL--
-VPSSLE-
VMPSSL--

VAPSSL--
VDPSSL--
VCPSSL--
-VPSSLC-
WSPSSL--
-WPSSLS-
-WPSSLI-
WGPSSL--
WDPSSL--
-WPSSLM-
-WPSSLQ-
-WPSSLR-
-WPSSLK-
-WPSSLT-
WEPSSL--
WAPSSL--
-WPSSLW-
WPPSSL--
WFPSSL--
WTPSSL--
WYPSSL--
-WPSSLH-
WQPSSL--
-WPSSLG-
WHPSSL--
-WPSSLE-
-WPSSLV-
-WPSSLA-
WNPSSL--
WCPSSL--
YLPSSL--
-YPSSLS-
YRPSSL--
YKPSSL--
YGPSSL--
YFPSSL--
-YPSSLR-
-YPSSLY-
-YPSSLT-
YYPSSL--
-YPSSLI-
YVPSSL--
YIPSSL--
YNPSSL--
-YPSSLF-
YPPSSL--
YAPSSL--
-YPSSLA-
-YPSSLK-
-YPSSLH-

| | | | | |
|---|---|---|---|---|
| | YWPSSL-- | | | |
| | -YPSSLQ- | | | |
| | -YPSSLG- | | | |
| | -YPSSLE- | | | |
| | YDPSSL-- | | | |
| | YQPSSL-- | | | |
| | -YPSSLW- | | | |
| | YMPSSL-- | | | |
| | YEPSSL-- | | | |
| | YHPSSL-- | | | |
| | YCPSSL-- | | | |
| PLGL | 43 | 812 | 35.35 | |
| | -FPLGLD- | | | |
| | -HPLGLE- | | | |
| | --PLGLTP | | | |
| | --PLGLSP | | | |
| | --PLGLPR | | | |
| | --PLGLPM | | | |
| | --PLGLPE | | | |
| | --PLGLPA | | | |
| | --PLGLDR | | | |
| | -RPLGLC- | | | |
| | -APLGLS- | | | |
| | -APLGLR- | | | |
| | -APLGLL- | | | |
| | AAPLGL-- | | | |
| | AWPLGL-- | | | |
| | ASPLGL-- | | | |
| | -APLGLT- | | | |
| | -APLGLK- | | | |
| | ARPLGL-- | | | |
| | AIPLGL-- | | | |
| | -APLGLQ- | | | |
| | -APLGLG- | | | |
| | AGPLGL-- | | | |
| | AMPLGL-- | | | |
| | ANPLGL-- | | | |
| | AHPLGL-- | | | |
| | -APLGLH- | | | |
| | -APLGLV- | | | |
| | APPLGL-- | | | |
| | -APLGLM- | | | |
| | -APLGLA- | | | |
| | AFPLGL-- | | | |
| | -APLGLI- | | | |
| | AQPLGL-- | | | |
| | -APLGLN- | | | |
| | -APLGLF- | | | |
| | -APLGLD- | | | |
| | ADPLGL-- | | | |

AEPLGL--
ACPLGL--
CRPLGL--
-CPLGLL-
CVPLGL--
-CPLGLS-
CLPLGL--
CAPLGL--
-CPLGLV-
-CPLGLT-
CIPLGL--
CGPLGL--
CFPLGL--
CWPLGL--
-CPLGLK-
CMPLGL--
-CPLGLY-
-CPLGLG-
CNPLGL--
CHPLGL--
CTPLGL--
CKPLGL--
CEPLGL--
CCPLGL--
CSPLGL--
-CPLGLQ-
-CPLGLI-
-CPLGLF-
-CPLGLE-
CDPLGL--
-DPLGLL-
-DPLGLS-
-DPLGLR-
-DPLGLK-
DTPLGL--
DSPLGL--
-DPLGLN-
DHPLGL--
DFPLGL--
-DPLGLW-
-DPLGLQ-
-DPLGLT-
-DPLGLH-
-DPLGLI-
DDPLGL--
DYPLGL--
-DPLGLG-
-DPLGLY-
-DPLGLF-
DNPLGL--

DQPLGL--
-DPLGLV-
-DPLGLE-
DMPLGL--
DAPLGL--
DWPLGL--
DKPLGL--
DIPLGL--
DCPLGL--
-DPLGLC-
-DPLGLA-
DGPLGL--
ERPLGL--
-EPLGLR-
EAPLGL--
ESPLGL--
-EPLGLY-
EHPLGL--
-EPLGLG-
-EPLGLN-
-EPLGLF-
-EPLGLM-
-EPLGLT-
-EPLGLK-
ELPLGL--
EKPLGL--
EQPLGL--
EGPLGL--
EVPLGL--
-EPLGLW-
EWPLGL--
-EPLGLS-
EMPLGL--
-EPLGLA-
ENPLGL--
EYPLGL--
EFPLGL--
-EPLGLV-
ECPLGL--
-EPLGLQ-
-EPLGLH-
-EPLGLE-
EDPLGL--
EPPLGL--
-EPLGLD-
FGPLGL--
FIPLGL--
FRPLGL--
FSPLGL--
FLPLGL--

-FPLGLH-
-FPLGLT-
-FPLGLA-
-FPLGLN-
-FPLGLG-
FAPLGL--
FVPLGL--
FNPLGL--
-FPLGLV-
FTPLGL--
-FPLGLM-
-FPLGLI-
FMPLGL--
FEPLGL--
FQPLGL--
FPPLGL--
-FPLGLW-
-FPLGLQ-
-FPLGLK-
FDPLGL--
FKPLGL--
GVPLGL--
-GPLGLS-
GSPLGL--
GFPLGL--
GLPLGL--
GTPLGL--
GKPLGL--
GGPLGL--
GQPLGL--
GYPLGL--
GWPLGL--
-GPLGLV-
GAPLGL--
-GPLGLN-
-GPLGLT-
GMPLGL--
-GPLGLA-
GPPLGL--
-GPLGLI-
GIPLGL--
GHPLGL--
-GPLGLQ-
GDPLGL--
-GPLGLM-
-GPLGLE-
-GPLGLH-
-GPLGLF-
GEPLGL--
GNPLGL--

GCPLGL--
-GPLGLC-
-HPLGLT-
HRPLGL--
-HPLGLS-
HSPLGL--
HLPLGL--
-HPLGLN-
-HPLGLA-
HFPLGL--
HYPLGL--
HGPLGL--
HWPLGL--
-HPLGLG-
HEPLGL--
-HPLGLW-
HNPLGL--
HPPLGL--
-HPLGLY-
-HPLGLV-
-HPLGLQ-
HIPLGL--
HMPLGL--
HKPLGL--
-HPLGLM-
HAPLGL--
HVPLGL--
-HPLGLH-
-HPLGLF-
HQPLGL--
-HPLGLK-
HCPLGL--
-HPLGLD-
-HPLGLI-
HHPLGL--
-HPLGLC-
-IPLGLS-
ISPLGL--
-IPLGLI-
-IPLGLL-
IGPLGL--
-IPLGLG-
-IPLGLT-
IPPLGL--
IAPLGL--
IRPLGL--
IKPLGL--
-IPLGLQ-
ILPLGL--
-IPLGLV-

```
-IPLGLE-
INPLGL--
IHPLGL--
IWPLGL--
-IPLGLH-
-IPLGLA-
IFPLGL--
IMPLGL--
-IPLGLY-
-IPLGLK-
IYPLGL--
IVPLGL--
-IPLGLD-
IEPLGL--
-IPLGLM-
-IPLGLF-
-IPLGLN-
IDPLGL--
ICPLGL--
-KPLGLR-
-KPLGLL-
-KPLGLI-
KLPLGL--
-KPLGLV-
KGPLGL--
-KPLGLK-
KTPLGL--
KVPLGL--
KPPLGL--
KWPLGL--
-KPLGLF-
KIPLGL--
KFPLGL--
-KPLGLY-
KNPLGL--
KMPLGL--
-KPLGLH-
KYPLGL--
KHPLGL--
-KPLGLW-
KQPLGL--
-KPLGLQ-
KEPLGL--
-KPLGLG-
KKPLGL--
-KPLGLM-
KDPLGL--
-KPLGLE-
-KPLGLD-
KCPLGL--
```

LRPLGL--
LSPLGL--
-LPLGLT-
LVPLGL--
-LPLGLK-
-LPLGLG-
LHPLGL--
LFPLGL--
LWPLGL--
-LPLGLI-
LAPLGL--
-LPLGLL-
LKPLGL--
LLPLGL--
LYPLGL--
-LPLGLH-
LQPLGL--
LTPLGL--
LPPLGL--
-LPLGLN-
-LPLGLA-
-LPLGLQ-
LEPLGL--
-LPLGLF-
LDPLGL--
-MPLGLR-
MSPLGL--
-MPLGLS-
-MPLGLV-
MLPLGL--
MKPLGL--
MGPLGL--
MRPLGL--
MTPLGL--
MVPLGL--
-MPLGLA-
-MPLGLH-
-MPLGLG-
MEPLGL--
MPPLGL--
-MPLGLL-
MHPLGL--
MMPLGL--
MAPLGL--
-MPLGLW-
-MPLGLQ-
MQPLGL--
-MPLGLY-
-MPLGLI-
MNPLGL--

```
-MPLGLF-
MFPLGL--
MDPLGL--
-MPLGLM-
MWPLGL--
-MPLGLE-
MCPLGL--
-MPLGLD-
-NPLGLR-
NTPLGL--
NVPLGL--
NRPLGL--
-NPLGLH-
NIPLGL--
-NPLGLL-
NGPLGL--
NWPLGL--
-NPLGLT-
NLPLGL--
-NPLGLV-
-NPLGLG-
-NPLGLF-
-NPLGLI-
-NPLGLA-
NPPLGL--
-NPLGLY-
-NPLGLM-
NFPLGL--
-NPLGLK-
NAPLGL--
NNPLGL--
NEPLGL--
-NPLGLW-
-NPLGLN-
NQPLGL--
-NPLGLQ-
NDPLGL--
--PLGLRS
--PLGLNR
--PLGLTR
--PLGLVR
--PLGLRT
--PLGLRA
--PLGLQR
--PLGLTL
--PLGLGT
--PLGLSS
--PLGLMR
--PLGLVS
--PLGLTS
```

--PLGLWS
--PLGLFS
--PLGLFR
--PLGLYR
--PLGLRN
--PLGLRV
--PLGLKR
--PLGLHR
--PLGLRP
--PLGLGL
--PLGLVG
--PLGLLA
--PLGLLK
--PLGLRQ
--PLGLST
--PLGLKH
--PLGLLV
--PLGLKG
--PLGLIS
--PLGLGS
--PLGLDS
--PLGLAS
--PLGLYK
--PLGLTT
--PLGLTA
--PLGLMA
--PLGLFK
PRPLGL--
--PLGLSA
--PLGLNT
--PLGLRI
--PLGLRH
--PLGLLN
--PLGLKT
--PLGLHS
--PLGLHM
--PLGLTG
--PLGLNS
--PLGLHF
--PLGLVN
--PLGLTI
--PLGLMK
--PLGLGH
--PLGLER
--PLGLAY
--PLGLAH
--PLGLWK
--PLGLVK
--PLGLSN
--PLGLSH

--PLGLQS
--PLGLVH
--PLGLSQ
--PLGLIY
--PLGLSI
--PLGLRK
--PLGLGV
--PLGLEY
--PLGLRD
--PLGLML
--PLGLIQ
--PLGLHT
--PLGLAW
--PLGLYT
--PLGLRM
--PLGLKM
--PLGLKA
--PLGLYN
--PLGLYH
--PLGLSM
--PLGLHI
--PLGLSF
--PLGLQG
--PLGLNV
--PLGLNM
--PLGLLD
--PLGLIN
--PLGLHY
PIPLGL--
--PLGLTK
--PLGLTH
--PLGLTE
--PLGLGY
--PLGLAV
--PLGLVI
--PLGLNQ
--PLGLNH
--PLGLGG
--PLGLYQ
--PLGLWT
--PLGLWN
--PLGLWG
--PLGLWE
--PLGLQV
--PLGLKQ
--PLGLIM
--PLGLGK
--PLGLFQ
--PLGLAA
PGPLGL--

PMPLGL--
--PLGLSW
--PLGLNW
--PLGLNN
PYPLGL--
--PLGLVW
--PLGLTV
--PLGLSD
--PLGLNI
--PLGLKN
--PLGLAT
--PLGLWR
--PLGLVA
--PLGLQT
--PLGLIE
--PLGLIA
--PLGLFT
--PLGLFD
--PLGLVY
--PLGLVM
--PLGLQY
--PLGLKK
--PLGLFH
--PLGLEG
--PLGLEF
--PLGLWQ
--PLGLTQ
--PLGLQI
--PLGLQD
--PLGLLE
--PLGLIW
--PLGLIT
--PLGLIH
--PLGLHD
--PLGLFN
--PLGLED
--PLGLAN
--PLGLYD
--PLGLTM
--PLGLQL
--PLGLMT
--PLGLKE
--PLGLIK
--PLGLHW
--PLGLHG
--PLGLGF
--PLGLAI
--PLGLAD
--PLGLVE
--PLGLTW

--PLGLND
--PLGLMQ
--PLGLMM
--PLGLLH
--PLGLHV
--PLGLHP
--PLGLHH
--PLGLGN
PQPLGL--
--PLGLYP
--PLGLVQ
--PLGLVD
--PLGLTN
--PLGLQA
--PLGLMV
--PLGLKF
--PLGLGQ
--PLGLEH
--PLGLEE
--PLGLAK
PHPLGL--
--PLGLNE
--PLGLME
--PLGLMD
--PLGLKD
--PLGLEL
--PLGLDA
--PLGLCA
PNPLGL--
--PLGLYE
--PLGLWH
--PLGLWD
--PLGLSE
--PLGLQW
--PLGLKV
--PLGLID
--PLGLGW
--PLGLEN
--PLGLEK
--PLGLAQ
--PLGLRE
--PLGLPL
--PLGLMF
--PLGLKP
--PLGLGD
--PLGLEM
--PLGLEI
--PLGLDT
--PLGLDG
--PLGLAE

--PLGLTF
--PLGLQN
--PLGLQH
--PLGLMH
--PLGLHQ
--PLGLHE
--PLGLFP
PWPLGL--
--PLGLQQ
--PLGLQM
--PLGLNG
--PLGLFM
--PLGLES
PDPLGL--
PCPLGL--
-PPLGLM-
--PLGLVP
--PLGLSC
--PLGLQF
--PLGLMP
--PLGLIP
--PLGLHN
--PLGLGE
PEPLGL--
-PPLGLG-
-PPLGLD-
--PLGLYC
--PLGLWC
--PLGLNC
--PLGLEW
--PLGLEQ
--PLGLCT
--PLGLCN
--PLGLCF
--PLGLCD
-QPLGLR-
-QPLGLL-
-QPLGLG-
-QPLGLS-
-QPLGLT-
QSPLGL--
-QPLGLY-
QVPLGL--
QFPLGL--
QLPLGL--
-QPLGLN-
QIPLGL--
QGPLGL--
QQPLGL--
QDPLGL--

-QPLGLM-
QHPLGL--
-QPLGLV-
QPPLGL--
QTPLGL--
-QPLGLQ-
QEPLGL--
-QPLGLH-
-QPLGLA-
QKPLGL--
-QPLGLI-
-QPLGLE-
-QPLGLD-
QMPLGL--
-QPLGLF-
QAPLGL--
QCPLGL--
RGPLGL--
-RPLGLK-
-RPLGLY-
-RPLGLF-
RYPLGL--
RTPLGL--
-RPLGLN-
-RPLGLA-
-RPLGLT-
RPPLGL--
-RPLGLH-
RHPLGL--
REPLGL--
RQPLGL--
-RPLGLQ-
-RPLGLE-
-RPLGLD-
RFPLGL--
RDPLGL--
RCPLGL--
-SPLGLL-
-SPLGLT-
-SPLGLS-
SSPLGL--
SYPLGL--
SVPLGL--
SMPLGL--
-SPLGLY-
STPLGL--
SNPLGL--
-SPLGLM-
SQPLGL--
SGPLGL--

```
-SPLGLI-
SIPLGL--
SWPLGL--
-SPLGLK-
-SPLGLV-
SHPLGL--
SFPLGL--
SPPLGL--
SKPLGL--
-SPLGLN-
-SPLGLQ-
-SPLGLE-
SDPLGL--
SCPLGL--
-TPLGLS-
TSPLGL--
-TPLGLT-
TTPLGL--
THPLGL--
-TPLGLG-
TGPLGL--
TWPLGL--
TKPLGL--
TVPLGL--
-TPLGLW-
-TPLGLA-
-TPLGLM-
-TPLGLK-
TAPLGL--
TQPLGL--
TFPLGL--
-TPLGLQ-
-TPLGLH-
-TPLGLN-
TMPLGL--
TPPLGL--
-TPLGLF-
-TPLGLV-
TIPLGL--
TDPLGL--
TEPLGL--
TCPLGL--
-TPLGLD-
VRPLGL--
VLPLGL--
-VPLGLR-
VKPLGL--
-VPLGLL-
VMPLGL--
VWPLGL--
```

```
-VPLGLG-
VQPLGL--
VTPLGL--
-VPLGLN-
-VPLGLA-
-VPLGLV-
VNPLGL--
VHPLGL--
-VPLGLT-
-VPLGLF-
-VPLGLH-
VVPLGL--
-VPLGLM-
VIPLGL--
-VPLGLE-
VEPLGL--
-VPLGLD-
VCPLGL--
-VPLGLC-
-WPLGLK-
WSPLGL--
WKPLGL--
-WPLGLA-
-WPLGLM-
WTPLGL--
-WPLGLT-
-WPLGLN-
-WPLGLE-
WVPLGL--
WYPLGL--
WFPLGL--
WDPLGL--
WNPLGL--
-WPLGLV-
WMPLGL--
-WPLGLF-
-WPLGLY-
WHPLGL--
-WPLGLL-
WWPLGL--
-WPLGLH-
-WPLGLG-
-WPLGLW-
-WPLGLC-
-WPLGLD-
WEPLGL--
WCPLGL--
-YPLGLR-
YSPLGL--
-YPLGLS-
```

| | | | |
|---|---|---|---|
| | YGPLGL-- | | |
| | YNPLGL-- | | |
| | YLPLGL-- | | |
| | -YPLGLL- | | |
| | YTPLGL-- | | |
| | -YPLGLG- | | |
| | -YPLGLV- | | |
| | -YPLGLQ- | | |
| | -YPLGLA- | | |
| | -YPLGLK- | | |
| | -YPLGLN- | | |
| | YYPLGL-- | | |
| | YFPLGL-- | | |
| | YPPLGL-- | | |
| | YHPLGL-- | | |
| | YDPLGL-- | | |
| | YWPLGL-- | | |
| | -YPLGLE- | | |
| | -YPLGLT- | | |
| | -YPLGLM- | | |
| | YMPLGL-- | | |
| | YQPLGL-- | | |
| | YIPLGL-- | | |
| | -YPLGLW- | | |
| PSPL | 4 | 898 | 35.35 |
| | GDPSPL-- | | |
| | -IPSPLV- | | |
| | -MPSPLT- | | |
| | -NPSPLP- | | |
| | -NPSPLN- | | |
| | --PSPLSE | | |
| | --PSPLGP | | |
| | -RPSPLD- | | |
| | ARPSPL-- | | |
| | -APSPLR- | | |
| | ALPSPL-- | | |
| | AYPSPL-- | | |
| | -APSPLY- | | |
| | -APSPLK- | | |
| | AWPSPL-- | | |
| | ASPSPL-- | | |
| | AIPSPL-- | | |
| | AHPSPL-- | | |
| | -APSPLL- | | |
| | AFPSPL-- | | |
| | AGPSPL-- | | |
| | -APSPLQ- | | |
| | AKPSPL-- | | |
| | AVPSPL-- | | |
| | ATPSPL-- | | |

```
-APSPLV-
APPSPL--
AAPSPL--
-APSPLT-
-APSPLG-
ADPSPL--
-APSPLS-
-APSPLH-
-APSPLE-
-APSPLA-
AMPSPL--
-APSPLM-
ACPSPL--
AQPSPL--
-APSPLF-
-APSPLW-
-APSPLP-
CSPSPL--
-CPSPLS-
-CPSPLI-
CKPSPL--
CTPSPL--
-CPSPLL-
CPPSPL--
CEPSPL--
-CPSPLY-
-CPSPLV-
-CPSPLR-
-CPSPLK-
-CPSPLH-
CCPSPL--
CWPSPL--
CNPSPL--
CMPSPL--
CHPSPL--
CQPSPL--
-CPSPLT-
-CPSPLA-
CIPSPL--
CFPSPL--
CAPSPL--
DTPSPL--
-DPSPLR-
DYPSPL--
-DPSPLI-
DVPSPL--
DRPSPL--
-DPSPLT-
-DPSPLS-
DSPSPL--
```

```
-DPSPLN-
DFPSPL--
-DPSPLW-
-DPSPLQ-
-DPSPLG-
DHPSPL--
DCPSPL--
DWPSPL--
-DPSPLY-
-DPSPLF-
DDPSPL--
-DPSPLK-
-DPSPLA-
DNPSPL--
DMPSPL--
DGPSPL--
ESPSPL--
EIPSPL--
-EPSPLT-
EVPSPL--
ETPSPL--
-EPSPLW-
-EPSPLG-
-EPSPLL-
-EPSPLK-
-EPSPLF-
EWPSPL--
-EPSPLQ-
-EPSPLA-
EKPSPL--
EHPSPL--
EYPSPL--
EAPSPL--
ERPSPL--
-EPSPLI-
ENPSPL--
-EPSPLY-
-EPSPLN-
EFPSPL--
EQPSPL--
-EPSPLV-
-EPSPLM-
-EPSPLH-
EGPSPL--
FVPSPL--
-FPSPLR-
FRPSPL--
FLPSPL--
FSPSPL--
-FPSPLH-
```

FEPSPL--
FTPSPL--
FHPSPL--
FAPSPL--
-FPSPLW-
FFPSPL--
-FPSPLN-
FKPSPL--
-FPSPLS-
FMPSPL--
-FPSPLF-
-FPSPLK-
FIPSPL--
FQPSPL--
-FPSPLA-
FPPSPL--
FWPSPL--
-FPSPLQ-
FGPSPL--
FYPSPL--
-FPSPLV-
-FPSPLY-
-FPSPLG-
FNPSPL--
-FPSPLP-
-FPSPLM-
FDPSPL--
FCPSPL--
GAPSPL--
GVPSPL--
GSPSPL--
-GPSPLY-
GYPSPL--
-GPSPLM-
GFPSPL--
-GPSPLK-
GNPSPL--
-GPSPLV-
GLPSPL--
GTPSPL--
-GPSPLT-
-GPSPLH-
GKPSPL--
GQPSPL--
GHPSPL--
-GPSPLL-
-GPSPLI-
-GPSPLE-
GMPSPL--
-GPSPLQ-

−GPSPLG−
−GPSPLW−
GIPSPL−−
−GPSPLN−
−GPSPLA−
GCPSPL−−
GPPSPL−−
−GPSPLF−
HSPSPL−−
HRPSPL−−
−HPSPLS−
−HPSPLL−
−HPSPLR−
HLPSPL−−
HGPSPL−−
HAPSPL−−
−HPSPLT−
HWPSPL−−
−HPSPLA−
HKPSPL−−
−HPSPLY−
−HPSPLN−
HFPSPL−−
−HPSPLF−
HVPSPL−−
HHPSPL−−
−HPSPLV−
−HPSPLQ−
−HPSPLI−
−HPSPLH−
HNPSPL−−
HQPSPL−−
−HPSPLW−
−HPSPLK−
HMPSPL−−
HDPSPL−−
−HPSPLD−
HPPSPL−−
HCPSPL−−
−IPSPLS−
−IPSPLL−
IAPSPL−−
−IPSPLR−
−IPSPLF−
IPPSPL−−
IGPSPL−−
IRPSPL−−
IQPSPL−−
−IPSPLA−
−IPSPLK−

−IPSPLG−
IKPSPL−−
ISPSPL−−
IFPSPL−−
IVPSPL−−
−IPSPLY−
IIPSPL−−
IHPSPL−−
IYPSPL−−
−IPSPLW−
ITPSPL−−
INPSPL−−
−IPSPLN−
ICPSPL−−
−IPSPLM−
−IPSPLE−
IMPSPL−−
IEPSPL−−
−IPSPLQ−
−IPSPLH−
IDPSPL−−
IWPSPL−−
KSPSPL−−
−KPSPLR−
−KPSPLL−
−KPSPLK−
KYPSPL−−
−KPSPLQ−
−KPSPLG−
−KPSPLM−
−KPSPLI−
KIPSPL−−
KLPSPL−−
KWPSPL−−
−KPSPLF−
−KPSPLE−
−KPSPLT−
KQPSPL−−
−KPSPLY−
KTPSPL−−
KNPSPL−−
−KPSPLS−
−KPSPLN−
KFPSPL−−
−KPSPLA−
−KPSPLV−
KMPSPL−−
KKPSPL−−
KDPSPL−−
KHPSPL−−

KEPSPL--
KCPSPL--
KVPSPL--
-KPSPLH-
KPPSPL--
-KPSPLD-
-KPSPLC-
LHPSPL--
LGPSPL--
-LPSPLR-
-LPSPLK-
LQPSPL--
-LPSPLT-
-LPSPLW-
-LPSPLL-
-LPSPLQ-
LLPSPL--
-LPSPLM-
LWPSPL--
LMPSPL--
LTPSPL--
LVPSPL--
LNPSPL--
LKPSPL--
-LPSPLY-
LYPSPL--
-LPSPLV-
-LPSPLH-
-LPSPLA-
LDPSPL--
LPPSPL--
LIPSPL--
LEPSPL--
-LPSPLN-
LCPSPL--
-LPSPLG-
-MPSPLK-
MSPSPL--
MRPSPL--
-MPSPLL-
MLPSPL--
MYPSPL--
MKPSPL--
MTPSPL--
-MPSPLY-
-MPSPLW-
-MPSPLH-
MQPSPL--
MHPSPL--
MMPSPL--

```
MAPSPL--
MGPSPL--
-MPSPLQ-
MVPSPL--
-MPSPLV-
MNPSPL--
MIPSPL--
-MPSPLN-
-MPSPLG-
MEPSPL--
-MPSPLI-
MPPSPL--
MFPSPL--
MCPSPL--
-MPSPLM-
-MPSPLF-
-MPSPLE-
MDPSPL--
MWPSPL--
-MPSPLA-
-NPSPLL-
NGPSPL--
-NPSPLR-
NTPSPL--
NLPSPL--
-NPSPLV-
-NPSPLS-
NMPSPL--
NSPSPL--
-NPSPLW-
NHPSPL--
-NPSPLK-
NNPSPL--
NAPSPL--
-NPSPLY-
-NPSPLH-
NDPSPL--
-NPSPLA-
NKPSPL--
NFPSPL--
NVPSPL--
NQPSPL--
-NPSPLI-
-NPSPLT-
-NPSPLQ-
-NPSPLF-
NPPSPL--
NEPSPL--
NYPSPL--
-NPSPLG-
```

-NPSPLE-
NCPSPL--
--PSPLRS
--PSPLVR
--PSPLMR
--PSPLAL
--PSPLST
--PSPLRY
--PSPLRT
--PSPLLY
--PSPLYS
--PSPLTI
--PSPLHR
--PSPLTL
--PSPLNR
--PSPLFY
--PSPLTG
--PSPLSR
--PSPLSK
--PSPLRI
--PSPLVS
--PSPLTR
--PSPLRR
--PSPLRF
--PSPLIM
--PSPLWS
--PSPLWL
--PSPLVG
--PSPLRV
--PSPLLI
--PSPLKS
--PSPLIK
--PSPLYI
--PSPLVI
--PSPLLR
--PSPLRH
--PSPLIR
--PSPLFT
--PSPLFL
--PSPLVL
--PSPLTA
--PSPLSV
--PSPLLK
--PSPLKL
--PSPLGL
--PSPLGF
--PSPLTS
--PSPLIW
--PSPLIF
--PSPLSW

--PSPLSI
--PSPLQQ
--PSPLQL
--PSPLGS
--PSPLYK
--PSPLVK
--PSPLRA
--PSPLQR
--PSPLLW
--PSPLKY
--PSPLIG
--PSPLGY
PRPSPL--
--PSPLYN
--PSPLWQ
--PSPLRQ
--PSPLRG
--PSPLMK
--PSPLKA
--PSPLGT
--PSPLYV
--PSPLYA
--PSPLWR
--PSPLVY
--PSPLQF
--PSPLPV
--PSPLNS
--PSPLGM
--PSPLGK
--PSPLGH
--PSPLFW
--PSPLAA
PLPSPL--
PSPSPL--
--PSPLWI
--PSPLTK
--PSPLRD
--PSPLNT
--PSPLLD
--PSPLKG
--PSPLIH
--PSPLVH
--PSPLTW
--PSPLSN
--PSPLMN
--PSPLHL
--PSPLAT
--PSPLAH
--PSPLAG
PMPSPL--

--PSPLSG
--PSPLLG
--PSPLKT
--PSPLKH
--PSPLIN
--PSPLGV
--PSPLFG
--PSPLAS
--PSPLWV
--PSPLWM
--PSPLTY
--PSPLSM
--PSPLSH
--PSPLQT
--PSPLNK
--PSPLLF
--PSPLKM
--PSPLHK
--PSPLFH
--PSPLAW
--PSPLYQ
--PSPLWN
--PSPLVD
--PSPLQS
--PSPLNY
--PSPLMS
--PSPLMF
--PSPLII
--PSPLHS
--PSPLHF
--PSPLFS
--PSPLFQ
PKPSPL--
PTPSPL--
--PSPLVV
--PSPLTM
--PSPLSD
--PSPLRM
--PSPLQK
--PSPLQH
--PSPLMY
--PSPLML
--PSPLLN
--PSPLIT
--PSPLIS
--PSPLIL
--PSPLHI
--PSPLHH
--PSPLGA
--PSPLAK

--PSPLAF
PGPSPL--
PVPSPL--
--PSPLYT
--PSPLYP
--PSPLYE
--PSPLYD
--PSPLWG
--PSPLWA
--PSPLVN
--PSPLQW
--PSPLQA
--PSPLPW
--PSPLNA
--PSPLMM
--PSPLHY
--PSPLHN
--PSPLFV
--PSPLEL
--PSPLDM
--PSPLAY
--PSPLAV
--PSPLAM
--PSPLYY
--PSPLYM
--PSPLYG
--PSPLWH
--PSPLVQ
--PSPLNF
--PSPLMV
--PSPLMG
--PSPLKV
--PSPLKR
--PSPLIA
--PSPLEV
--PSPLDY
--PSPLDL
--PSPLWW
--PSPLWT
--PSPLWD
--PSPLVW
--PSPLTQ
--PSPLTN
--PSPLTF
--PSPLTE
--PSPLRW
--PSPLPY
--PSPLPS
--PSPLPQ
--PSPLPH

--PSPLNM
--PSPLNI
--PSPLNH
--PSPLMT
--PSPLMH
--PSPLME
--PSPLKN
--PSPLID
--PSPLHQ
--PSPLHG
--PSPLHD
--PSPLHA
--PSPLEN
--PSPLEM
--PSPLAN
PEPSPL--
--PSPLWY
--PSPLWK
--PSPLVE
--PSPLSQ
--PSPLRP
--PSPLQD
--PSPLNV
--PSPLNN
--PSPLLQ
--PSPLLE
--PSPLKW
--PSPLKF
--PSPLIY
--PSPLIE
--PSPLHW
--PSPLHT
--PSPLGW
--PSPLGQ
--PSPLGN
--PSPLGI
--PSPLFI
--PSPLFD
--PSPLEF
--PSPLAI
--PSPLVM
--PSPLQV
--PSPLQN
--PSPLQM
--PSPLQI
--PSPLPM
--PSPLNQ
--PSPLMI
--PSPLKQ
--PSPLKK

--PSPLIQ
--PSPLEI
--PSPLEG
--PSPLDI
PHPSPL--
PAPSPL--
PYPSPL--
--PSPLWE
--PSPLVP
--PSPLPI
--PSPLPF
--PSPLMW
--PSPLMD
--PSPLKE
--PSPLHM
--PSPLHC
--PSPLGG
--PSPLFN
--PSPLFM
--PSPLFA
--PSPLET
--PSPLER
--PSPLEA
--PSPLDS
--PSPLDG
-PPSPLM-
PDPSPL--
PWPSPL--
--PSPLYF
--PSPLTC
--PSPLQY
--PSPLQG
--PSPLQE
--PSPLPC
--PSPLPA
--PSPLMA
--PSPLKI
--PSPLHV
--PSPLGE
--PSPLFC
--PSPLEW
--PSPLEQ
--PSPLDW
--PSPLDR
--PSPLDA
--PSPLCY
--PSPLCV
--PSPLCT
--PSPLCS
--PSPLCQ

```
--PSPLCL
--PSPLAP
PQPSPL--
-PPSPLY-
PNPSPL--
PFPSPL--
QWPSPL--
-QPSPLR-
QRPSPL--
-QPSPLT-
QGPSPL--
-QPSPLS-
QYPSPL--
-QPSPLG-
QQPSPL--
-QPSPLA-
QFPSPL--
QTPSPL--
QPPSPL--
-QPSPLH-
QKPSPL--
QHPSPL--
QAPSPL--
-QPSPLN-
QLPSPL--
QEPSPL--
-QPSPLY-
-QPSPLM-
-QPSPLF-
QIPSPL--
-QPSPLV-
-QPSPLQ-
-QPSPLK-
-QPSPLE-
QNPSPL--
-QPSPLW-
QMPSPL--
RSPSPL--
RYPSPL--
RAPSPL--
RQPSPL--
RIPSPL--
-RPSPLS-
-RPSPLY-
-RPSPLG-
RVPSPL--
-RPSPLI-
RTPSPL--
RHPSPL--
RRPSPL--
```

−RPSPLA−
RWPSPL−−
−RPSPLW−
−RPSPLH−
RKPSPL−−
RNPSPL−−
RMPSPL−−
RFPSPL−−
RPPSPL−−
−RPSPLT−
REPSPL−−
−RPSPLQ−
−RPSPLN−
−RPSPLF−
−RPSPLE−
RDPSPL−−
−SPSPLR−
SWPSPL−−
−SPSPLL−
SSPSPL−−
−SPSPLK−
SLPSPL−−
SAPSPL−−
SVPSPL−−
−SPSPLS−
SMPSPL−−
−SPSPLY−
SQPSPL−−
−SPSPLH−
−SPSPLW−
SKPSPL−−
SIPSPL−−
SDPSPL−−
STPSPL−−
−SPSPLT−
−SPSPLM−
SNPSPL−−
SHPSPL−−
−SPSPLI−
−SPSPLE−
−SPSPLA−
SPPSPL−−
−SPSPLV−
−SPSPLQ−
SFPSPL−−
−SPSPLC−
SCPSPL−−
−TPSPLR−
−TPSPLL−
−TPSPLG−

TNPSPL--
TLPSPL--
TVPSPL--
-TPSPLT-
TSPSPL--
THPSPL--
TEPSPL--
-TPSPLY-
-TPSPLK-
-TPSPLQ-
TAPSPL--
-TPSPLV-
TQPSPL--
TRPSPL--
TWPSPL--
-TPSPLS-
-TPSPLA-
TTPSPL--
-TPSPLN-
-TPSPLH-
TMPSPL--
TIPSPL--
-TPSPLW-
-TPSPLI-
-TPSPLF-
TFPSPL--
-TPSPLE-
TCPSPL--
-VPSPLR-
VRPSPL--
-VPSPLK-
VLPSPL--
-VPSPLS-
VYPSPL--
-VPSPLL-
VVPSPL--
-VPSPLN-
-VPSPLT-
-VPSPLA-
VKPSPL--
-VPSPLY-
-VPSPLG-
VPPSPL--
-VPSPLV-
-VPSPLI-
VFPSPL--
VDPSPL--
VIPSPL--
VEPSPL--
-VPSPLW-

VWPSPL--
-VPSPLM-
-VPSPLQ-
VGPSPL--
-VPSPLE-
VCPSPL--
VQPSPL--
VMPSPL--
-VPSPLF-
VHPSPL--
-WPSPLR-
WLPSPL--
WAPSPL--
WSPSPL--
-WPSPLL-
WTPSPL--
-WPSPLA-
-WPSPLS-
-WPSPLH-
-WPSPLK-
WKPSPL--
WVPSPL--
-WPSPLT-
-WPSPLG-
WHPSPL--
-WPSPLQ-
-WPSPLN-
-WPSPLM-
WIPSPL--
WGPSPL--
WYPSPL--
WNPSPL--
WEPSPL--
-WPSPLV-
WPPSPL--
-WPSPLW-
-WPSPLI-
-WPSPLF-
WWPSPL--
WQPSPL--
WDPSPL--
YSPSPL--
YRPSPL--
-YPSPLS-
YQPSPL--
-YPSPLT-
-YPSPLL-
YVPSPL--
YWPSPL--
-YPSPLR-

```
              -YPSPLG-
              YAPSPL--
              -YPSPLW-
              YDPSPL--
              -YPSPLM-
              YIPSPL--
              YYPSPL--
              YKPSPL--
              YCPSPL--
              -YPSPLK-
              YHPSPL--
              -YPSPLA-
              -YPSPLQ-
              -YPSPLN-
              YMPSPL--
              YTPSPL--
              -YPSPLI-
              -YPSPLE-
              YGPSPL--
              YEPSPL--
              -YPSPLV-
              YPPSPL--
              -YPSPLH-
PANL             3        907      35.33
              -APANLT-
              -APANLR-
              AFPANL--
              -APANLL-
              -APANLK-
              -APANLG-
              AEPANL--
              -APANLV-
              -APANLF-
              AIPANL--
              AYPANL--
              ARPANL--
              -APANLW-
              AKPANL--
              AGPANL--
              -APANLM-
              ASPANL--
              -APANLA-
              ANPANL--
              AVPANL--
              -APANLS-
              APPANL--
              -APANLN-
              -APANLD-
              AAPANL--
              ATPANL--
```

-APANLQ-
AMPANL--
-APANLI-
-APANLH-
-APANLE-
ADPANL--
ACPANL--
CHPANL--
CNPANL--
-CPANLV-
CCPANL--
CAPANL--
CVPANL--
CSPANL--
CRPANL--
-CPANLG-
CEPANL--
CYPANL--
-CPANLY-
-CPANLS-
-CPANLR-
-CPANLN-
-CPANLI-
-CPANLF-
CGPANL--
CTPANL--
CPPANL--
-CPANLT-
-CPANLQ-
CLPANL--
CIPANL--
CDPANL--
DAPANL--
DRPANL--
DTPANL--
-DPANLS-
-DPANLR-
DKPANL--
DSPANL--
-DPANLY-
-DPANLL-
DLPANL--
DWPANL--
-DPANLG-
DIPANL--
DEPANL--
-DPANLW-
-DPANLV-
-DPANLT-
DMPANL--

DHPANL--
DYPANL--
DVPANL--
-DPANLM-
-DPANLE-
-DPANLQ-
-DPANLN-
-DPANLK-
-DPANLI-
-DPANLH-
DNPANL--
DGPANL--
ERPANL--
ESPANL--
-EPANLR-
-EPANLL-
EIPANL--
-EPANLM-
-EPANLF-
EMPANL--
-EPANLS-
-EPANLK-
EGPANL--
EQPANL--
-EPANLY-
-EPANLW-
-EPANLG-
-EPANLT-
ENPANL--
ELPANL--
EAPANL--
EYPANL--
EWPANL--
EVPANL--
-EPANLQ-
EEPANL--
-EPANLI-
-EPANLH-
-EPANLD-
EHPANL--
FGPANL--
FSPANL--
-FPANLT-
FLPANL--
-FPANLY-
-FPANLR-
-FPANLH-
FKPANL--
-FPANLE-
FNPANL--

FYPANL--
FTPANL--
-FPANLW-
FDPANL--
-FPANLG-
-FPANLN-
-FPANLK-
FMPANL--
FIPANL--
FVPANL--
-FPANLL-
-FPANLD-
FFPANL--
FEPANL--
FAPANL--
FPPANL--
FHPANL--
-FPANLM-
-FPANLF-
-FPANLA-
-FPANLQ-
GVPANL--
GLPANL--
GMPANL--
-GPANLR-
-GPANLL-
GSPANL--
GTPANL--
-GPANLS-
GKPANL--
GAPANL--
-GPANLD-
-GPANLG-
GNPANL--
GIPANL--
GGPANL--
GWPANL--
-GPANLY-
-GPANLN-
-GPANLT-
GEPANL--
GPPANL--
-GPANLW-
-GPANLF-
-GPANLA-
GRPANL--
-GPANLH-
-GPANLE-
GHPANL--
GDPANL--

GQPANL--
-GPANLK-
GYPANL--
-GPANLQ-
-GPANLI-
-HPANLR-
HGPANL--
HVPANL--
HYPANL--
HPPANL--
-HPANLQ-
HQPANL--
-HPANLL-
HRPANL--
-HPANLY-
-HPANLS-
-HPANLI-
-HPANLF-
-HPANLT-
HWPANL--
-HPANLV-
-HPANLK-
HNPANL--
HLPANL--
HSPANL--
-HPANLG-
HMPANL--
HIPANL--
HAPANL--
HTPANL--
-HPANLW-
-HPANLN-
-HPANLE-
-HPANLD-
HHPANL--
HEPANL--
-HPANLM-
HKPANL--
IQPANL--
IKPANL--
IRPANL--
ITPANL--
IYPANL--
-IPANLI-
-IPANLS-
-IPANLR-
IVPANL--
IHPANL--
IFPANL--
INPANL--

```
-IPANLW-
-IPANLT-
-IPANLL-
ILPANL--
-IPANLY-
-IPANLG-
-IPANLE-
IMPANL--
ISPANL--
IEPANL--
IDPANL--
IAPANL--
IWPANL--
-IPANLN-
-IPANLK-
-IPANLD-
IGPANL--
-IPANLV-
-IPANLQ-
-IPANLM-
-IPANLH-
-IPANLF-
-KPANLL-
-KPANLD-
KIPANL--
-KPANLS-
-KPANLR-
KLPANL--
KGPANL--
KVPANL--
KPPANL--
-KPANLV-
-KPANLT-
-KPANLY-
KTPANL--
-KPANLQ-
KAPANL--
KNPANL--
KEPANL--
-KPANLN-
KYPANL--
-KPANLI-
KWPANL--
-KPANLW-
-KPANLG-
-KPANLA-
KMPANL--
-KPANLM-
KDPANL--
-KPANLF-
```

-KPANLE-
KKPANL--
-KPANLK-
KHPANL--
KCPANL--
LNPANL--
LGPANL--
LWPANL--
LTPANL--
-LPANLK-
LVPANL--
-LPANLT-
LQPANL--
-LPANLH-
-LPANLF-
LFPANL--
-LPANLA-
LLPANL--
-LPANLY-
-LPANLV-
LAPANL--
-LPANLW-
-LPANLI-
-LPANLE-
LKPANL--
LPPANL--
LMPANL--
-LPANLN-
-LPANLQ-
-LPANLG-
LEPANL--
-LPANLD-
LHPANL--
LIPANL--
-MPANLS-
-MPANLG-
-MPANLR-
MHPANL--
MSPANL--
MTPANL--
MKPANL--
MWPANL--
-MPANLN-
MLPANL--
-MPANLI-
MNPANL--
MQPANL--
-MPANLV-
-MPANLH-
-MPANLW-

```
-MPANLT-
MIPANL--
-MPANLY-
-MPANLL-
-MPANLE-
MEPANL--
MAPANL--
MVPANL--
MRPANL--
-MPANLM-
MDPANL--
MMPANL--
MCPANL--
MYPANL--
MPPANL--
-MPANLQ-
-MPANLK-
-MPANLF-
MFPANL--
NLPANL--
NSPANL--
-NPANLS-
-NPANLR-
-NPANLH-
-NPANLG-
NGPANL--
NRPANL--
-NPANLY-
-NPANLT-
NFPANL--
NYPANL--
-NPANLL-
-NPANLI-
NAPANL--
NTPANL--
NPPANL--
-NPANLK-
-NPANLD-
-NPANLA-
NNPANL--
NKPANL--
NQPANL--
-NPANLE-
NMPANL--
-NPANLV-
-NPANLQ-
-NPANLN-
NIPANL--
NDPANL--
NVPANL--
```

```
NWPANL--
-NPANLM-
-NPANLP-
NEPANL--
NCPANL--
--PANLVL
--PANLRS
--PANLLL
--PANLLT
--PANLVR
--PANLRL
--PANLGI
--PANLRT
--PANLSV
--PANLNR
--PANLTS
--PANLTL
--PANLLG
--PANLST
--PANLRG
--PANLLS
--PANLGR
--PANLYL
--PANLVV
--PANLAS
--PANLVA
--PANLVS
--PANLTV
--PANLSR
--PANLKS
--PANLHR
--PANLAL
--PANLTR
--PANLSL
--PANLMT
--PANLLH
--PANLFT
--PANLLM
--PANLLD
--PANLKR
--PANLGL
PGPANL--
--PANLSN
--PANLSK
--PANLRI
--PANLRA
--PANLQR
--PANLIR
--PANLGF
--PANLAF
```

--PANLSQ
--PANLAG
PRPANL--
--PANLYW
--PANLWT
--PANLVN
--PANLVI
--PANLVG
--PANLSI
--PANLSG
--PANLSA
--PANLNV
--PANLLK
--PANLHT
--PANLGS
--PANLGN
PLPANL--
--PANLWR
--PANLWQ
--PANLTI
--PANLTH
--PANLRV
--PANLMG
--PANLIQ
--PANLGG
--PANLYR
--PANLYH
--PANLVQ
--PANLVP
--PANLTY
--PANLTF
--PANLTA
--PANLRN
--PANLLA
--PANLIG
--PANLIF
--PANLFK
--PANLFH
--PANLFD
--PANLAR
PSPANL--
--PANLYV
--PANLYS
--PANLWS
--PANLVT
--PANLSS
--PANLSE
--PANLRH
--PANLQL
--PANLMR

--PANLLR
--PANLLQ
--PANLLN
--PANLKV
--PANLGW
--PANLEY
--PANLES
--PANLAK
PKPANL--
--PANLYT
--PANLYI
--PANLWN
--PANLWH
--PANLWF
--PANLVM
--PANLSY
--PANLSW
--PANLSH
--PANLRM
--PANLRK
--PANLMS
--PANLLW
--PANLLV
--PANLIT
--PANLIS
--PANLGY
--PANLGT
--PANLGK
--PANLFY
--PANLFL
PYPANL--
PWPANL--
--PANLYG
--PANLVY
--PANLVD
--PANLTN
--PANLNM
--PANLNL
--PANLKY
--PANLKQ
--PANLKI
--PANLIN
--PANLHM
--PANLHL
--PANLGE
PTPANL--
--PANLYK
--PANLWL
--PANLVK
--PANLVH

--PANLVF
--PANLTT
--PANLTG
--PANLSD
--PANLRF
--PANLQW
--PANLPL
--PANLMQ
--PANLMI
--PANLMH
--PANLLE
--PANLKN
--PANLHN
--PANLGQ
--PANLGA
--PANLEN
--PANLDS
--PANLAT
PNPANL--
PMPANL--
PAPANL--
--PANLYN
--PANLWK
--PANLTE
--PANLRW
--PANLRQ
--PANLRE
--PANLQV
--PANLNW
--PANLNI
--PANLMW
--PANLMN
--PANLMF
--PANLKT
--PANLKK
--PANLKD
--PANLIK
--PANLHS
--PANLHG
--PANLHF
--PANLFG
--PANLEW
--PANLER
--PANLEL
--PANLEH
--PANLEA
--PANLDV
--PANLAY
--PANLAA
PHPANL--

PFPANL--
--PANLYY
--PANLYM
--PANLYF
--PANLYA
--PANLWV
--PANLWG
--PANLWD
--PANLVW
--PANLQY
--PANLQQ
--PANLQH
--PANLQG
--PANLNS
--PANLMP
--PANLME
--PANLKW
--PANLKM
--PANLKL
--PANLKF
--PANLIW
--PANLHQ
--PANLGH
--PANLFQ
--PANLFN
--PANLEV
--PANLEQ
--PANLEI
--PANLAH
--PANLAE
PVPANL--
PQPANL--
--PANLYQ
--PANLWM
--PANLWI
--PANLTW
--PANLTK
--PANLQK
--PANLNF
--PANLND
--PANLMA
--PANLKH
--PANLKG
--PANLIY
--PANLIP
--PANLIH
--PANLID
--PANLHY
--PANLGM
--PANLFS

--PANLFE
--PANLEM
--PANLEG
--PANLDT
--PANLDM
--PANLAW
--PANLAQ
--PANLAI
--PANLAD
PEPANL--
PDPANL--
--PANLYD
--PANLVE
--PANLVC
--PANLTQ
--PANLTC
--PANLSM
--PANLSF
--PANLRC
--PANLQN
--PANLQI
--PANLQF
--PANLQE
--PANLQA
--PANLPY
--PANLPS
--PANLPF
--PANLNK
--PANLNE
--PANLMY
--PANLMV
--PANLMK
--PANLLC
--PANLKE
--PANLII
--PANLIA
--PANLHW
--PANLHV
--PANLHI
--PANLHH
--PANLHE
--PANLHC
--PANLHA
--PANLGP
--PANLEK
--PANLEC
--PANLDQ
--PANLDI
--PANLDG
--PANLCF

```
--PANLCE
--PANLAN
--PANLAC
QVPANL--
-QPANLL-
-QPANLS-
QYPANL--
QSPANL--
QWPANL--
QLPANL--
-QPANLR-
-QPANLF-
QHPANL--
QRPANL--
QQPANL--
-QPANLV-
-QPANLQ-
-QPANLK-
-QPANLW-
-QPANLA-
QNPANL--
QFPANL--
QTPANL--
-QPANLH-
QGPANL--
QPPANL--
-QPANLT-
-QPANLM-
-QPANLI-
-QPANLY-
-QPANLN-
-QPANLE-
-QPANLD-
QCPANL--
RLPANL--
-RPANLL-
-RPANLS-
RSPANL--
RVPANL--
-RPANLT-
-RPANLR-
-RPANLW-
-RPANLV-
-RPANLK-
RNPANL--
RGPANL--
RAPANL--
-RPANLN-
-RPANLG-
RIPANL--
```

RFPANL--
-RPANLD-
-RPANLM-
RKPANL--
RTPANL--
RHPANL--
RPPANL--
-RPANLI-
-RPANLF-
-RPANLE-
RMPANL--
RYPANL--
-RPANLA-
REPANL--
-RPANLQ-
RDPANL--
RCPANL--
-RPANLC-
SRPANL--
-SPANLS-
SWPANL--
SVPANL--
-SPANLR-
-SPANLI-
SSPANL--
SGPANL--
SKPANL--
SAPANL--
SQPANL--
-SPANLH-
STPANL--
-SPANLV-
SNPANL--
SFPANL--
SPPANL--
-SPANLY-
SIPANL--
SDPANL--
SYPANL--
-SPANLT-
SMPANL--
SEPANL--
-SPANLF-
-SPANLK-
-SPANLD-
SCPANL--
-SPANLQ-
-SPANLA-
SHPANL--
-SPANLG-

−SPANLM−
−SPANLE−
−SPANLW−
TRPANL−−
−TPANLS−
TFPANL−−
−TPANLR−
TSPANL−−
−TPANLY−
TIPANL−−
−TPANLL−
−TPANLN−
−TPANLG−
−TPANLW−
TWPANL−−
−TPANLA−
TYPANL−−
−TPANLQ−
−TPANLK−
THPANL−−
TAPANL−−
TQPANL−−
TPPANL−−
−TPANLE−
TLPANL−−
−TPANLV−
−TPANLF−
TMPANL−−
TKPANL−−
−TPANLM−
TNPANL−−
−TPANLD−
TDPANL−−
−TPANLH−
TEPANL−−
TCPANL−−
VWPANL−−
−VPANLL−
VSPANL−−
VYPANL−−
VMPANL−−
−VPANLT−
−VPANLK−
−VPANLN−
−VPANLV−
VGPANL−−
−VPANLF−
VLPANL−−
VAPANL−−
−VPANLG−

VKPANL--
VNPANL--
VIPANL--
VFPANL--
VPPANL--
VVPANL--
VQPANL--
VEPANL--
-VPANLM-
VHPANL--
VDPANL--
-VPANLE-
-VPANLI-
-VPANLA-
-VPANLD-
VCPANL--
WRPANL--
-WPANLR-
WLPANL--
WKPANL--
-WPANLL-
-WPANLH-
WAPANL--
WSPANL--
-WPANLK-
-WPANLA-
WGPANL--
-WPANLM-
WHPANL--
WPPANL--
-WPANLS-
WFPANL--
WMPANL--
-WPANLF-
WEPANL--
-WPANLY-
-WPANLV-
-WPANLN-
-WPANLE-
WDPANL--
WQPANL--
-WPANLW-
-WPANLQ-
-WPANLG-
-WPANLD-
WWPANL--
YSPANL--
-YPANLK-
YRPANL--
YAPANL--

```
              -YPANLS-
              YQPANL--
              YNPANL--
              -YPANLR-
              -YPANLL-
              YMPANL--
              YGPANL--
              YPPANL--
              -YPANLV-
              -YPANLA-
              YTPANL--
              -YPANLY-
              YIPANL--
              YDPANL--
              YVPANL--
              -YPANLT-
              -YPANLN-
              -YPANLI-
              -YPANLH-
              YLPANL--
              YEPANL--
              -YPANLG-
              YKPANL--
              YHPANL--
              -YPANLW-
              -YPANLQ-
              -YPANLF-
              -YPANLE-
              YFPANL--
              YYPANL--
              -YPANLM-
PRGL           525       599      35.25
              --PRGLQP
              --PRGLSC
              --PRGLPV
              --PRGLPS
              --PRGLPQ
              --PRGLGP
              --PRGLDR
              --PRGLCL
              ASPRGL--
              -APRGLI-
              ATPRGL--
              AQPRGL--
              -APRGLN-
              -APRGLT-
              -APRGLF-
              AGPRGL--
              -APRGLV-
              AVPRGL--
```

```
-APRGLG-
-APRGLY-
-APRGLE-
APPRGL--
-APRGLS-
-APRGLW-
-APRGLD-
-APRGLA-
-APRGLH-
ADPRGL--
-APRGLM-
AEPRGL--
-APRGLC-
-CPRGLL-
-CPRGLR-
CSPRGL--
CGPRGL--
CAPRGL--
CQPRGL--
-CPRGLW-
CLPRGL--
CIPRGL--
CCPRGL--
-CPRGLY-
CHPRGL--
CVPRGL--
-CPRGLE-
CNPRGL--
CKPRGL--
CRPRGL--
CMPRGL--
CEPRGL--
-CPRGLT-
-CPRGLA-
CFPRGL--
CYPRGL--
CWPRGL--
-CPRGLQ-
-CPRGLK-
-CPRGLF-
-DPRGLL-
DIPRGL--
-DPRGLR-
-DPRGLG-
DVPRGL--
-DPRGLT-
DLPRGL--
DYPRGL--
-DPRGLE-
DSPRGL--
```

```
-DPRGLV-
-DPRGLN-
-DPRGLI-
-DPRGLH-
DGPRGL--
DTPRGL--
-DPRGLK-
-DPRGLA-
DCPRGL--
-DPRGLY-
DNPRGL--
-DPRGLF-
DMPRGL--
DDPRGL--
DHPRGL--
DFPRGL--
DEPRGL--
-DPRGLQ-
-DPRGLP-
-DPRGLC-
ESPRGL--
-EPRGLT-
-EPRGLL-
ELPRGL--
-EPRGLS-
-EPRGLV-
-EPRGLF-
ERPRGL--
EHPRGL--
EIPRGL--
EFPRGL--
EAPRGL--
-EPRGLW-
EGPRGL--
ETPRGL--
ENPRGL--
-EPRGLH-
EMPRGL--
EDPRGL--
-EPRGLY-
-EPRGLQ-
EKPRGL--
-EPRGLK-
-EPRGLE-
EEPRGL--
EQPRGL--
-EPRGLD-
-EPRGLM-
-EPRGLI-
ECPRGL--
```

FLPRGL--
FDPRGL--
FEPRGL--
FAPRGL--
-FPRGLH-
FYPRGL--
FVPRGL--
-FPRGLY-
-FPRGLQ-
FKPRGL--
FPPRGL--
FMPRGL--
-FPRGLE-
-FPRGLD-
GLPRGL--
GSPRGL--
GYPRGL--
-GPRGLR-
GNPRGL--
-GPRGLM-
-GPRGLW-
-GPRGLI-
-GPRGLA-
-GPRGLT-
-GPRGLQ-
-GPRGLH-
-GPRGLN-
-GPRGLV-
-GPRGLF-
GKPRGL--
-GPRGLY-
-GPRGLE-
GDPRGL--
GPPRGL--
GCPRGL--
-GPRGLD-
-HPRGLS-
-HPRGLT-
HGPRGL--
-HPRGLF-
-HPRGLK-
HYPRGL--
-HPRGLY-
HVPRGL--
HKPRGL--
-HPRGLE-
HDPRGL--
HAPRGL--
-HPRGLG-
HFPRGL--

HEPRGL--
-HPRGLN-
-HPRGLM-
HNPRGL--
HQPRGL--
HPPRGL--
HHPRGL--
-HPRGLQ-
-HPRGLC-
HCPRGL--
-IPRGLS-
-IPRGLY-
ISPRGL--
INPRGL--
-IPRGLH-
-IPRGLG-
IGPRGL--
-IPRGLT-
IKPRGL--
-IPRGLA-
-IPRGLE-
IHPRGL--
-IPRGLQ-
-IPRGLN-
ITPRGL--
-IPRGLD-
IEPRGL--
-IPRGLM-
IDPRGL--
-IPRGLC-
-KPRGLK-
-KPRGLL-
-KPRGLT-
-KPRGLF-
KGPRGL--
KTPRGL--
-KPRGLI-
KIPRGL--
-KPRGLH-
-KPRGLA-
-KPRGLV-
KPPRGL--
-KPRGLE-
KQPRGL--
-KPRGLQ-
-KPRGLN-
-KPRGLM-
KNPRGL--
-KPRGLW-
KDPRGL--

–KPRGLC–
KCPRGL––
–LPRGLS–
–LPRGLH–
–LPRGLN–
–LPRGLK–
–LPRGLY–
–LPRGLF–
–LPRGLE–
–LPRGLQ–
LHPRGL––
–LPRGLC–
MMPRGL––
–MPRGLL–
MGPRGL––
MNPRGL––
–MPRGLY–
–MPRGLS–
–MPRGLN–
–MPRGLG–
–MPRGLV–
–MPRGLI–
MEPRGL––
–MPRGLQ–
MDPRGL––
MAPRGL––
–MPRGLW–
MQPRGL––
MCPRGL––
–MPRGLD–
NRPRGL––
–NPRGLT–
–NPRGLL–
–NPRGLH–
NYPRGL––
–NPRGLV–
–NPRGLK–
–NPRGLS–
–NPRGLA–
–NPRGLY–
–NPRGLN–
NKPRGL––
NAPRGL––
–NPRGLE–
–NPRGLM–
–NPRGLQ–
NEPRGL––
–NPRGLI–
–NPRGLG–
–NPRGLF–

```
NPPRGL--
NCPRGL--
-NPRGLW-
-NPRGLC-
--PRGLTL
--PRGLSK
--PRGLSS
--PRGLST
--PRGLRF
--PRGLFL
--PRGLAV
--PRGLAR
--PRGLYV
--PRGLAT
--PRGLSD
--PRGLFS
--PRGLFQ
--PRGLYP
--PRGLVK
PLPRGL--
--PRGLAL
--PRGLWG
--PRGLTT
--PRGLIG
--PRGLYS
--PRGLWT
--PRGLNL
--PRGLGM
--PRGLWS
--PRGLNR
--PRGLAI
--PRGLEL
--PRGLRD
--PRGLQG
--PRGLAQ
--PRGLSH
--PRGLAS
--PRGLTW
--PRGLSV
--PRGLGG
--PRGLFH
--PRGLLM
--PRGLYT
--PRGLSF
--PRGLFN
--PRGLWR
--PRGLLD
--PRGLWW
--PRGLWQ
--PRGLWI
```

```
--PRGLNF
--PRGLSQ
--PRGLSE
--PRGLNW
--PRGLLW
--PRGLGV
--PRGLGQ
PIPRGL--
--PRGLYA
--PRGLVV
--PRGLTK
--PRGLMT
--PRGLMA
PNPRGL--
--PRGLYD
--PRGLTM
--PRGLTH
--PRGLNQ
--PRGLNM
--PRGLIQ
--PRGLIH
--PRGLYI
--PRGLTY
--PRGLNY
--PRGLNA
--PRGLMG
--PRGLKQ
--PRGLID
--PRGLAH
PVPRGL--
--PRGLWA
--PRGLIY
--PRGLHV
--PRGLVE
--PRGLFK
--PRGLEY
PKPRGL--
PAPRGL--
PSPRGL--
--PRGLYN
--PRGLWV
--PRGLRE
--PRGLHA
--PRGLAG
--PRGLAD
--PRGLQT
--PRGLHT
--PRGLHK
--PRGLEI
--PRGLMP
```

--PRGLMK
--PRGLET
--PRGLAW
--PRGLQY
--PRGLMN
--PRGLKI
--PRGLKD
--PRGLFG
--PRGLFE
--PRGLDS
--PRGLAN
--PRGLTF
--PRGLQQ
--PRGLMW
--PRGLIN
--PRGLII
--PRGLEV
--PRGLVP
--PRGLTE
--PRGLKN
--PRGLHQ
--PRGLHF
--PRGLQV
--PRGLQE
--PRGLIM
--PRGLFA
--PRGLEW
PWPRGL--
--PRGLYY
--PRGLYQ
--PRGLYE
--PRGLTQ
--PRGLNK
--PRGLND
--PRGLMM
--PRGLME
--PRGLHP
--PRGLES
--PRGLEK
--PRGLNI
--PRGLMI
--PRGLEH
--PRGLAY
--PRGLNN
--PRGLFW
--PRGLFD
PDPRGL--
--PRGLWM
--PRGLQH
--PRGLNG

```
--PRGLKF
--PRGLEG
--PRGLEF
PQPRGL--
PMPRGL--
--PRGLWE
--PRGLQK
--PRGLIE
--PRGLHE
--PRGLFP
--PRGLEN
--PRGLVW
--PRGLQF
--PRGLMD
--PRGLHC
--PRGLEA
PFPRGL--
PCPRGL--
--PRGLTD
--PRGLQW
--PRGLQN
--PRGLQI
--PRGLED
--PRGLDG
--PRGLWD
--PRGLNH
--PRGLMQ
--PRGLIP
--PRGLAM
--PRGLWH
--PRGLVC
--PRGLLC
--PRGLKE
--PRGLHD
--PRGLEQ
--PRGLDT
--PRGLCH
--PRGLCF
--PRGLQM
--PRGLNC
--PRGLGE
--PRGLGC
--PRGLFC
--PRGLEE
--PRGLDY
--PRGLCN
-PPRGLQ-
PEPRGL--
--PRGLWC
--PRGLDE
```

```
--PRGLCW
--PRGLCT
--PRGLCS
--PRGLCR
--PRGLCM
-PPRGLK-
-PPRGLI-
-PPRGLF-
-PPRGLA-
QRPRGL--
-QPRGLL-
-QPRGLT-
QYPRGL--
QSPRGL--
-QPRGLW-
QQPRGL--
-QPRGLS-
-QPRGLK-
-QPRGLG-
QGPRGL--
-QPRGLV-
QNPRGL--
QTPRGL--
-QPRGLQ-
-QPRGLY-
-QPRGLA-
QFPRGL--
-QPRGLN-
QKPRGL--
QIPRGL--
-QPRGLH-
QAPRGL--
QEPRGL--
-QPRGLM-
QPPRGL--
QMPRGL--
QDPRGL--
-QPRGLD-
QCPRGL--
RSPRGL--
RIPRGL--
-RPRGLN-
-RPRGLM-
-RPRGLG-
REPRGL--
-RPRGLH-
RPPRGL--
-RPRGLE-
RDPRGL--
RKPRGL--
```

-RPRGLD-
RCPRGL--
-RPRGLC-
-SPRGLS-
-SPRGLA-
STPRGL--
-SPRGLY-
-SPRGLH-
-SPRGLQ-
SNPRGL--
SAPRGL--
SPPRGL--
-SPRGLM-
-SPRGLF-
-SPRGLE-
-SPRGLD-
SEPRGL--
SCPRGL--
-TPRGLT-
TLPRGL--
-TPRGLN-
-TPRGLQ-
-TPRGLA-
TQPRGL--
-TPRGLY-
-TPRGLW-
-TPRGLV-
TIPRGL--
-TPRGLE-
TNPRGL--
-TPRGLK-
TCPRGL--
TDPRGL--
-TPRGLD-
-TPRGLC-
-VPRGLG-
-VPRGLY-
-VPRGLN-
-VPRGLA-
VKPRGL--
-VPRGLQ-
-VPRGLH-
-VPRGLF-
VAPRGL--
-VPRGLE-
-VPRGLV-
-VPRGLW-
VEPRGL--
-VPRGLM-
VPPRGL--

VDPRGL--
VCPRGL--
WSPRGL--
WRPRGL--
WVPRGL--
WMPRGL--
WNPRGL--
-WPRGLN-
WPPRGL--
-WPRGLE-
WGPRGL--
-WPRGLF-
WFPRGL--
WDPRGL--
-WPRGLM-
-WPRGLI-
-WPRGLH-
-WPRGLQ-
-YPRGLI-
YSPRGL--
YIPRGL--
-YPRGLG-
YFPRGL--
-YPRGLF-
-YPRGLN-
-YPRGLM-
YKPRGL--
YEPRGL--
YDPRGL--
-YPRGLE-
YCPRGL--

| | | | |
|---|---|---|---|
| PRAL | 824 | 532 | 35.23 |

-FPRALP-
-FPRALE-
-LPRALP-
--PRALDI
--PRALPT
--PRALPS
ASPRAL--
ATPRAL--
AGPRAL--
-APRALQ-
-APRALN-
AVPRAL--
AYPRAL--
AAPRAL--
AQPRAL--
-APRALV-
-APRALE-
-APRALM-

AEPRAL--
-APRALH-
ADPRAL--
-APRALA-
-APRALD-
ACPRAL--
CLPRAL--
CVPRAL--
-CPRALS-
CSPRAL--
-CPRALW-
-CPRALL-
CKPRAL--
-CPRALT-
-CPRALI-
-CPRALH-
CYPRAL--
CRPRAL--
CMPRAL--
-CPRALV-
-CPRALK-
CPPRAL--
CGPRAL--
CCPRAL--
CNPRAL--
-CPRALQ-
-CPRALM-
-CPRALF-
CQPRAL--
-CPRALN-
-CPRALE-
CHPRAL--
CFPRAL--
CDPRAL--
DQPRAL--
DSPRAL--
-DPRALR-
-DPRALV-
DIPRAL--
DKPRAL--
DYPRAL--
DWPRAL--
-DPRALY-
-DPRALK-
DFPRAL--
DVPRAL--
DGPRAL--
-DPRALS-
-DPRALM-
-DPRALA-

DEPRAL--
-DPRALT-
-DPRALH-
-DPRALF-
DHPRAL--
DDPRAL--
-DPRALQ-
DNPRAL--
-DPRALW-
-DPRALG-
DCPRAL--
-DPRALN-
ESPRAL--
EVPRAL--
EWPRAL--
EFPRAL--
ERPRAL--
-EPRALS-
-EPRALR-
-EPRALV-
-EPRALI-
-EPRALA-
ETPRAL--
-EPRALW-
EAPRAL--
-EPRALY-
-EPRALT-
-EPRALM-
-EPRALE-
EGPRAL--
EMPRAL--
-EPRALH-
-EPRALF-
-EPRALK-
EKPRAL--
-EPRALG-
EIPRAL--
EHPRAL--
-EPRALN-
-EPRALD-
-EPRALQ-
-EPRALC-
EPPRAL--
ECPRAL--
FTPRAL--
FGPRAL--
FQPRAL--
FIPRAL--
FEPRAL--
-FPRALW-

-FPRALN-
-FPRALY-
FDPRAL--
-FPRALD-
-FPRALC-
-GPRALL-
-GPRALA-
GLPRAL--
-GPRALK-
GIPRAL--
-GPRALI-
-GPRALW-
-GPRALG-
GPPRAL--
GHPRAL--
-GPRALY-
-GPRALN-
GKPRAL--
GQPRAL--
-GPRALV-
-GPRALH-
-GPRALF-
-GPRALE-
-GPRALT-
-GPRALM-
GDPRAL--
GCPRAL--
-GPRALC-
-GPRALQ-
HIPRAL--
-HPRALN-
-HPRALV-
HTPRAL--
HSPRAL--
-HPRALQ-
HPPRAL--
-HPRALW-
-HPRALG-
-HPRALE-
HAPRAL--
HQPRAL--
-HPRALT-
HNPRAL--
-HPRALF-
-HPRALM-
HDPRAL--
HEPRAL--
HFPRAL--
IIPRAL--
-IPRALA-

INPRAL--
-IPRALE-
IHPRAL--
IQPRAL--
-IPRALN-
-IPRALV-
-IPRALI-
IMPRAL--
IGPRAL--
IDPRAL--
-IPRALW-
-IPRALH-
IPPRAL--
-IPRALM-
IEPRAL--
-IPRALQ-
-KPRALY-
-KPRALG-
KTPRAL--
-KPRALW-
-KPRALN-
-KPRALF-
-KPRALV-
-KPRALA-
KGPRAL--
-KPRALM-
KKPRAL--
KNPRAL--
-KPRALE-
KPPRAL--
KIPRAL--
KHPRAL--
KQPRAL--
-KPRALH-
-KPRALK-
KDPRAL--
KEPRAL--
KCPRAL--
-KPRALD-
LSPRAL--
LVPRAL--
-LPRALI-
LMPRAL--
LQPRAL--
-LPRALT-
-LPRALN-
LDPRAL--
-LPRALY-
-LPRALE-
LEPRAL--

LCPRAL--
-LPRALC-
MSPRAL--
-MPRALG-
-MPRALK-
-MPRALT-
-MPRALN-
-MPRALH-
MKPRAL--
MTPRAL--
-MPRALQ-
MEPRAL--
-MPRALV-
-MPRALE-
MMPRAL--
-MPRALM-
MDPRAL--
MWPRAL--
MCPRAL--
-NPRALN-
-NPRALI-
NGPRAL--
-NPRALH-
-NPRALS-
NSPRAL--
-NPRALW-
-NPRALK-
-NPRALV-
-NPRALQ-
NHPRAL--
NAPRAL--
NQPRAL--
-NPRALY-
-NPRALG-
NDPRAL--
-NPRALA-
NMPRAL--
NKPRAL--
NCPRAL--
-NPRALM-
--PRALAI
--PRALSI
--PRALSK
--PRALVV
PLPRAL--
--PRALVQ
--PRALLV
--PRALHL
--PRALNR
--PRALYT

--PRALVI
--PRALGT
--PRALYH
--PRALYA
--PRALGN
--PRALSG
--PRALGI
--PRALTH
--PRALLM
--PRALGG
PSPRAL--
--PRALWS
--PRALIS
--PRALLH
--PRALTN
--PRALNM
--PRALNL
--PRALFA
--PRALSQ
--PRALMG
--PRALHI
--PRALFM
--PRALYW
--PRALQH
--PRALKS
--PRALWG
--PRALIF
--PRALFV
--PRALFQ
--PRALAV
--PRALAM
--PRALYQ
--PRALVM
--PRALTV
--PRALPY
--PRALTK
--PRALNK
--PRALGA
--PRALER
--PRALWL
--PRALVW
--PRALNV
--PRALNT
--PRALAH
--PRALES
--PRALEL
--PRALYD
--PRALMD
--PRALIM
--PRALYN

```
--PRALSM
--PRALNA
--PRALMK
--PRALIV
--PRALFN
--PRALAY
--PRALWA
--PRALTM
--PRALIW
--PRALGV
--PRALEG
--PRALSN
--PRALQF
--PRALMP
--PRALET
--PRALAW
--PRALAQ
--PRALWK
--PRALNQ
--PRALNI
--PRALMN
--PRALII
--PRALID
--PRALHN
PHPRAL--
PEPRAL--
--PRALYE
--PRALTQ
--PRALLE
--PRALHK
--PRALFK
--PRALDL
PKPRAL--
--PRALYK
--PRALWE
--PRALTT
--PRALTD
--PRALMF
--PRALHF
--PRALMQ
--PRALEY
--PRALDS
--PRALDG
--PRALSD
--PRALQN
--PRALKN
--PRALIK
--PRALHH
--PRALFG
--PRALEN
```

```
--PRALEH
--PRALWQ
--PRALVE
--PRALQT
--PRALQD
--PRALNN
--PRALNH
--PRALHD
--PRALGF
--PRALSE
--PRALQG
--PRALND
--PRALKP
PIPRAL--
PAPRAL--
--PRALYY
--PRALVD
--PRALNE
--PRALIP
--PRALIN
--PRALFP
--PRALEI
PQPRAL--
PMPRAL--
--PRALHY
--PRALHW
--PRALFD
--PRALAE
--PRALWW
--PRALLC
--PRALIQ
--PRALFE
--PRALEV
--PRALDT
--PRALTE
--PRALNG
--PRALEF
--PRALED
--PRALQW
--PRALQE
--PRALPQ
--PRALIE
--PRALWM
--PRALTC
--PRALPV
--PRALME
--PRALGE
--PRALGD
--PRALEW
--PRALDY
```

--PRALCK
-PPRALK-
PPPRAL--
--PRALHE
--PRALHC
--PRALEE
--PRALDF
--PRALCN
--PRALCH
--PRALCF
--PRALCA
--PRALAC
-PPRALN-
PCPRAL--
-QPRALL-
-QPRALK-
-QPRALS-
QAPRAL--
-QPRALA-
QVPRAL--
-QPRALY-
QPPRAL--
-QPRALF-
QMPRAL--
QYPRAL--
QTPRAL--
-QPRALV-
-QPRALI-
-QPRALH-
-QPRALE-
QDPRAL--
-QPRALT-
QEPRAL--
-QPRALQ-
-QPRALM-
QCPRAL--
RSPRAL--
RGPRAL--
RPPRAL--
-RPRALE-
-RPRALF-
RQPRAL--
-RPRALH-
-RPRALQ-
REPRAL--
-RPRALW-
RDPRAL--
RCPRAL--
-RPRALD-
-RPRALC-

SAPRAL--
-SPRALY-
-SPRALN-
-SPRALQ-
-SPRALE-
SEPRAL--
SCPRAL--
-SPRALC-
TKPRAL--
THPRAL--
-TPRALN-
-TPRALY-
-TPRALA-
TNPRAL--
TMPRAL--
-TPRALI-
-TPRALG-
-TPRALM-
TQPRAL--
TEPRAL--
TDPRAL--
-TPRALD-
TCPRAL--
-VPRALL-
VSPRAL--
VVPRAL--
VKPRAL--
VPPRAL--
VNPRAL--
-VPRALH-
VGPRAL--
-VPRALI-
VEPRAL--
-VPRALW-
VQPRAL--
-VPRALD-
-VPRALE-
-VPRALC-
VCPRAL--
-WPRALY-
WNPRAL--
-WPRALE-
WFPRAL--
WQPRAL--
WAPRAL--
-WPRALH-
WDPRAL--
-WPRALN-
-WPRALD-
WCPRAL--

YTPRAL--
-YPRALY-
-YPRALL-
-YPRALW-
-YPRALN-
-YPRALI-
YDPRAL--
YKPRAL--
-YPRALE-
-YPRALD-
YMPRAL--
YIPRAL--
YQPRAL--
YCPRAL--

| | | | |
|---|---|---|---|
| PVGL | 21 | 844 | 35.20 |

-APVGLM-
FEPVGL--
-MPVGLG-
--PVGLYP
--PVGLPL
--PVGLPG
--PVGLPF
--PVGLMP
--PVGLED
-VPVGLD-
VAPVGL--
-APVGLR-
-APVGLG-
-APVGLK-
AVPVGL--
ATPVGL--
-APVGLS-
-APVGLN-
ASPVGL--
AEPVGL--
AMPVGL--
-APVGLW-
AKPVGL--
-APVGLD-
APPVGL--
-APVGLL-
-APVGLH-
-APVGLF-
AWPVGL--
-APVGLY-
-APVGLV-
-APVGLT-
ALPVGL--
AGPVGL--
AAPVGL--

```
AYPVGL--
-APVGLE-
AFPVGL--
AQPVGL--
ANPVGL--
AHPVGL--
-APVGLQ-
-APVGLC-
ADPVGL--
ACPVGL--
CRPVGL--
-CPVGLS-
CSPVGL--
-CPVGLT-
CCPVGL--
-CPVGLR-
-CPVGLI-
CGPVGL--
CFPVGL--
-CPVGLM-
CVPVGL--
-CPVGLY-
-CPVGLQ-
-CPVGLL-
-CPVGLK-
-CPVGLH-
CEPVGL--
CDPVGL--
CYPVGL--
CWPVGL--
-CPVGLW-
-CPVGLV-
-CPVGLF-
-CPVGLA-
CNPVGL--
CIPVGL--
CHPVGL--
CAPVGL--
DVPVGL--
-DPVGLR-
-DPVGLT-
-DPVGLL-
DMPVGL--
DLPVGL--
DKPVGL--
DSPVGL--
-DPVGLG-
DYPVGL--
DWPVGL--
DTPVGL--
```

DRPVGL--
-DPVGLV-
-DPVGLA-
DQPVGL--
DEPVGL--
-DPVGLW-
-DPVGLS-
-DPVGLK-
DGPVGL--
-DPVGLN-
DIPVGL--
DFPVGL--
DDPVGL--
-DPVGLH-
DAPVGL--
-DPVGLQ-
-DPVGLM-
-DPVGLE-
DNPVGL--
DHPVGL--
ERPVGL--
-EPVGLR-
-EPVGLY-
-EPVGLL-
ELPVGL--
-EPVGLS-
-EPVGLM-
EVPVGL--
ESPVGL--
-EPVGLI-
EGPVGL--
EAPVGL--
EWPVGL--
EYPVGL--
-EPVGLH-
EMPVGL--
-EPVGLW-
-EPVGLT-
-EPVGLK-
-EPVGLG-
-EPVGLE-
EKPVGL--
EIPVGL--
-EPVGLN-
EEPVGL--
EPPVGL--
EHPVGL--
EFPVGL--
ETPVGL--
EQPVGL--

−EPVGLQ−
−EPVGLP−
−EPVGLF−
FSPVGL−−
−FPVGLS−
−FPVGLL−
FYPVGL−−
−FPVGLV−
FGPVGL−−
−FPVGLQ−
−FPVGLT−
−FPVGLH−
FKPVGL−−
−FPVGLE−
−FPVGLI−
−FPVGLG−
FNPVGL−−
FIPVGL−−
FFPVGL−−
−FPVGLM−
−FPVGLF−
FTPVGL−−
−FPVGLK−
−FPVGLA−
FDPVGL−−
FWPVGL−−
FQPVGL−−
FPPVGL−−
−GPVGLS−
−GPVGLR−
GVPVGL−−
−GPVGLK−
−GPVGLL−
GLPVGL−−
−GPVGLA−
GKPVGL−−
−GPVGLT−
GMPVGL−−
GWPVGL−−
GYPVGL−−
−GPVGLG−
−GPVGLM−
GNPVGL−−
GAPVGL−−
−GPVGLV−
−GPVGLI−
GFPVGL−−
GSPVGL−−
−GPVGLW−
−GPVGLH−

GIPVGL--
GTPVGL--
-GPVGLE-
GGPVGL--
GCPVGL--
GQPVGL--
GEPVGL--
-GPVGLQ-
GHPVGL--
-GPVGLD-
GPPVGL--
GDPVGL--
-HPVGLQ-
HSPVGL--
-HPVGLN-
HVPVGL--
-HPVGLH-
-HPVGLF-
HLPVGL--
-HPVGLV-
HWPVGL--
-HPVGLS-
-HPVGLG-
HYPVGL--
-HPVGLY-
-HPVGLW-
-HPVGLM-
HEPVGL--
HAPVGL--
-HPVGLK-
HMPVGL--
-HPVGLA-
HHPVGL--
HPPVGL--
HKPVGL--
-HPVGLE-
HNPVGL--
HQPVGL--
HFPVGL--
HIPVGL--
-IPVGLT-
-IPVGLY-
-IPVGLL-
IRPVGL--
-IPVGLS-
-IPVGLK-
ILPVGL--
IPPVGL--
IWPVGL--
-IPVGLA-

IKPVGL--
IYPVGL--
IEPVGL--
ISPVGL--
-IPVGLQ-
IMPVGL--
IIPVGL--
IAPVGL--
INPVGL--
ITPVGL--
-IPVGLV-
-IPVGLH-
IHPVGL--
IGPVGL--
IFPVGL--
IDPVGL--
IQPVGL--
-IPVGLN-
-IPVGLE-
ICPVGL--
-IPVGLM-
-IPVGLD-
KLPVGL--
-KPVGLR-
KVPVGL--
-KPVGLW-
-KPVGLT-
KRPVGL--
KNPVGL--
KAPVGL--
-KPVGLM-
-KPVGLA-
KHPVGL--
KGPVGL--
KPPVGL--
KKPVGL--
KYPVGL--
-KPVGLV-
KFPVGL--
KEPVGL--
-KPVGLQ-
KDPVGL--
KWPVGL--
KCPVGL--
-KPVGLN-
-KPVGLD-
-KPVGLE-
LFPVGL--
LRPVGL--
LLPVGL--

-LPVGLT-
-LPVGLR-
-LPVGLV-
-LPVGLY-
LAPVGL--
LTPVGL--
-LPVGLI-
LKPVGL--
LNPVGL--
-LPVGLE-
LWPVGL--
LGPVGL--
-LPVGLM-
-LPVGLG-
LIPVGL--
LHPVGL--
LYPVGL--
-LPVGLK-
-LPVGLF-
-LPVGLN-
-LPVGLW-
-LPVGLQ-
-LPVGLH-
LEPVGL--
LPPVGL--
-LPVGLA-
MLPVGL--
-MPVGLL-
MRPVGL--
-MPVGLR-
-MPVGLK-
-MPVGLS-
-MPVGLI-
-MPVGLN-
MSPVGL--
-MPVGLY-
MPPVGL--
-MPVGLV-
MTPVGL--
MHPVGL--
MGPVGL--
MYPVGL--
-MPVGLT-
-MPVGLM-
-MPVGLA-
-MPVGLE-
MMPVGL--
MWPVGL--
MQPVGL--
MFPVGL--

```
-MPVGLF-
MKPVGL--
MAPVGL--
-MPVGLQ-
MDPVGL--
-NPVGLK-
-NPVGLR-
NWPVGL--
NLPVGL--
NPPVGL--
-NPVGLQ-
NSPVGL--
-NPVGLW-
-NPVGLV-
-NPVGLI-
NHPVGL--
-NPVGLS-
-NPVGLT-
NTPVGL--
NAPVGL--
-NPVGLN-
NMPVGL--
-NPVGLH-
NIPVGL--
NGPVGL--
-NPVGLM-
-NPVGLE-
-NPVGLA-
NNPVGL--
-NPVGLY-
-NPVGLF-
NKPVGL--
NFPVGL--
-NPVGLG-
NEPVGL--
NVPVGL--
NQPVGL--
NYPVGL--
NDPVGL--
NCPVGL--
--PVGLLR
--PVGLRS
--PVGLVR
--PVGLSL
--PVGLRR
--PVGLLS
--PVGLRT
--PVGLGT
--PVGLSS
--PVGLRK
```

--PVGLLG
--PVGLFG
--PVGLAR
--PVGLRY
--PVGLLN
--PVGLTK
--PVGLRQ
--PVGLLA
--PVGLKV
--PVGLKR
--PVGLVT
--PVGLTR
--PVGLGV
--PVGLNR
--PVGLHS
--PVGLGS
--PVGLFT
--PVGLGL
--PVGLNL
PGPVGL--
--PVGLVA
--PVGLRN
--PVGLRF
--PVGLQS
--PVGLML
--PVGLTT
--PVGLSV
--PVGLRA
--PVGLTN
--PVGLQR
--PVGLHL
--PVGLSG
--PVGLMR
--PVGLLI
--PVGLHR
--PVGLYK
--PVGLTI
--PVGLTG
--PVGLRP
--PVGLMI
--PVGLLQ
--PVGLKH
--PVGLAL
--PVGLYS
--PVGLVS
--PVGLST
--PVGLSD
--PVGLLV
--PVGLLK
--PVGLLE

--PVGLWK
--PVGLTS
--PVGLRG
--PVGLIS
--PVGLEV
--PVGLWN
--PVGLRW
--PVGLNT
--PVGLKT
--PVGLKS
--PVGLGA
--PVGLRH
--PVGLIN
--PVGLGY
--PVGLGG
--PVGLFN
--PVGLER
--PVGLNN
--PVGLMN
--PVGLMK
--PVGLKY
--PVGLIR
--PVGLIG
--PVGLHK
--PVGLFQ
--PVGLYQ
--PVGLYH
--PVGLYA
--PVGLTF
--PVGLQA
--PVGLMT
--PVGLAS
--PVGLTM
--PVGLRM
--PVGLRE
PSPVGL--
--PVGLQL
--PVGLNI
--PVGLMH
--PVGLKK
--PVGLIT
--PVGLHF
--PVGLGW
--PVGLGI
--PVGLEY
--PVGLEH
--PVGLAK
--PVGLVH
--PVGLSI
--PVGLSA

--PVGLNH
--PVGLMD
--PVGLIA
--PVGLEG
--PVGLEF
--PVGLTA
--PVGLSY
--PVGLSM
--PVGLNS
--PVGLKQ
--PVGLKG
--PVGLKF
--PVGLYD
--PVGLTQ
--PVGLSW
--PVGLSQ
--PVGLMM
--PVGLMG
--PVGLKD
PTPVGL--
PKPVGL--
--PVGLVE
--PVGLNF
--PVGLIK
--PVGLFH
--PVGLAA
PVPVGL--
--PVGLWG
--PVGLVQ
--PVGLSE
--PVGLNM
--PVGLMV
--PVGLMS
--PVGLKI
--PVGLEW
--PVGLAW
--PVGLAV
--PVGLWD
--PVGLVV
--PVGLVN
--PVGLVI
--PVGLVD
--PVGLTY
--PVGLTD
--PVGLQW
--PVGLQQ
--PVGLQK
--PVGLNW
--PVGLMY
--PVGLIQ

--PVGLHV
--PVGLHT
--PVGLHH
--PVGLAY
--PVGLAT
--PVGLAM
--PVGLAD
PNPVGL--
PAPVGL--
--PVGLYE
--PVGLWM
--PVGLVM
--PVGLTV
--PVGLQY
--PVGLQT
--PVGLQN
--PVGLFA
--PVGLEA
--PVGLAG
PLPVGL--
--PVGLWF
--PVGLWE
--PVGLWA
--PVGLVW
--PVGLRD
--PVGLQE
--PVGLMA
--PVGLKW
--PVGLKN
--PVGLKA
--PVGLHA
--PVGLGN
--PVGLGK
--PVGLAN
PDPVGL--
PYPVGL--
--PVGLQF
--PVGLNY
--PVGLHY
--PVGLHN
--PVGLHG
--PVGLEN
--PVGLAQ
--PVGLAH
PIPVGL--
--PVGLTH
--PVGLQG
--PVGLMF
--PVGLLD
--PVGLII

--PVGLHQ
--PVGLAI
PWPVGL--
--PVGLYN
--PVGLQV
--PVGLMQ
--PVGLIE
--PVGLHW
--PVGLHM
--PVGLHI
--PVGLCK
PQPVGL--
--PVGLWH
--PVGLQD
--PVGLNG
--PVGLME
--PVGLKE
--PVGLHD
--PVGLFD
--PVGLEL
PHPVGL--
PFPVGL--
--PVGLVP
--PVGLQH
--PVGLNV
--PVGLNQ
--PVGLND
--PVGLNA
--PVGLKP
--PVGLHP
--PVGLEQ
--PVGLEM
--PVGLEI
--PVGLCY
PEPVGL--
PCPVGL--
--PVGLYC
--PVGLWQ
--PVGLSC
--PVGLQC
--PVGLMW
--PVGLHE
--PVGLGE
--PVGLEK
--PVGLEE
--PVGLCL
--PVGLCG
--PVGLCD
--PVGLCA
-PPVGLR-

-PPVGLQ-
-PPVGLM-
-PPVGLG-
PPPVGL--
QRPVGL--
-QPVGLL-
-QPVGLR-
-QPVGLN-
-QPVGLS-
QHPVGL--
-QPVGLT-
QVPVGL--
QSPVGL--
-QPVGLW-
QKPVGL--
QIPVGL--
-QPVGLK-
-QPVGLA-
-QPVGLY-
-QPVGLF-
QPPVGL--
QGPVGL--
QTPVGL--
-QPVGLQ-
QLPVGL--
-QPVGLI-
-QPVGLE-
-QPVGLG-
-QPVGLV-
QNPVGL--
QQPVGL--
-QPVGLH-
QMPVGL--
QEPVGL--
QYPVGL--
QAPVGL--
-QPVGLM-
QDPVGL--
RWPVGL--
RGPVGL--
-RPVGLH-
RAPVGL--
-RPVGLN-
RYPVGL--
-RPVGLA-
-RPVGLG-
RIPVGL--
RDPVGL--
-RPVGLK-
RTPVGL--

RHPVGL--
-RPVGLM-
REPVGL--
RQPVGL--
RCPVGL--
-SPVGLA-
SLPVGL--
SGPVGL--
SVPVGL--
-SPVGLV-
-SPVGLT-
-SPVGLN-
SNPVGL--
SKPVGL--
SYPVGL--
-SPVGLG-
SFPVGL--
-SPVGLK-
STPVGL--
-SPVGLM-
-SPVGLI-
SHPVGL--
SAPVGL--
SWPVGL--
-SPVGLW-
-SPVGLF-
-SPVGLH-
-SPVGLQ-
SIPVGL--
SQPVGL--
SPPVGL--
-SPVGLE-
SEPVGL--
SDPVGL--
-SPVGLD-
SCPVGL--
-TPVGLR-
TRPVGL--
-TPVGLS-
TKPVGL--
TYPVGL--
-TPVGLL-
TGPVGL--
TNPVGL--
TAPVGL--
TLPVGL--
-TPVGLM-
TPPVGL--
-TPVGLT-
-TPVGLY-

-TPVGLN-
-TPVGLK-
-TPVGLI-
TSPVGL--
TIPVGL--
TWPVGL--
-TPVGLG-
TMPVGL--
-TPVGLA-
-TPVGLV-
-TPVGLE-
-TPVGLF-
TFPVGL--
TDPVGL--
TQPVGL--
THPVGL--
-TPVGLW-
TEPVGL--
-TPVGLQ-
-TPVGLH-
TCPVGL--
-VPVGLR-
VKPVGL--
-VPVGLL-
VTPVGL--
VIPVGL--
-VPVGLK-
VSPVGL--
VQPVGL--
VVPVGL--
-VPVGLT-
-VPVGLA-
VDPVGL--
-VPVGLV-
-VPVGLN-
-VPVGLI-
-VPVGLH-
VPPVGL--
VCPVGL--
-VPVGLY-
-VPVGLM-
VGPVGL--
VFPVGL--
-VPVGLG-
-VPVGLE-
VMPVGL--
VYPVGL--
VHPVGL--
VEPVGL--
VWPVGL--

```
-VPVGLC-
WLPVGL--
-WPVGLS-
-WPVGLK-
-WPVGLL-
WNPVGL--
-WPVGLT-
WGPVGL--
WYPVGL--
-WPVGLA-
WKPVGL--
WDPVGL--
WSPVGL--
-WPVGLR-
-WPVGLN-
-WPVGLE-
-WPVGLQ-
-WPVGLI-
WPPVGL--
WVPVGL--
-WPVGLV-
WQPVGL--
-WPVGLM-
WFPVGL--
-WPVGLH-
WEPVGL--
-WPVGLY-
-WPVGLF-
-YPVGLR-
-YPVGLT-
-YPVGLS-
YRPVGL--
-YPVGLK-
YTPVGL--
YQPVGL--
YPPVGL--
-YPVGLE-
YLPVGL--
-YPVGLA-
YGPVGL--
-YPVGLQ-
YEPVGL--
-YPVGLL-
-YPVGLH-
YNPVGL--
-YPVGLM-
YAPVGL--
-YPVGLN-
-YPVGLI-
YHPVGL--
```

```
          -YPVGLF-
          YDPVGL--
          -YPVGLV-
          YMPVGL--
          YYPVGL--
          YFPVGL--
          YWPVGL--
          YKPVGL--
          YCPVGL--
PRNL       362        647      35.13
          --PRNLGY
          --PRNLFP
          --PRNLDI
          ASPRNL--
          -APRNLS-
          -APRNLF-
          -APRNLY-
          AAPRNL--
          AGPRNL--
          -APRNLL-
          AQPRNL--
          -APRNLA-
          AHPRNL--
          -APRNLV-
          ANPRNL--
          -APRNLH-
          AKPRNL--
          AEPRNL--
          -APRNLN-
          -APRNLK-
          ADPRNL--
          -APRNLW-
          -APRNLD-
          -APRNLE-
          CSPRNL--
          CLPRNL--
          -CPRNLT-
          -CPRNLL-
          CGPRNL--
          CHPRNL--
          CCPRNL--
          CIPRNL--
          CTPRNL--
          CPPRNL--
          CNPRNL--
          -CPRNLM-
          -CPRNLE-
          CVPRNL--
          -CPRNLV-
          CEPRNL--
```

CRPRNL--
-CPRNLY-
-CPRNLQ-
-CPRNLR-
-CPRNLI-
CFPRNL--
CYPRNL--
CWPRNL--
CQPRNL--
-CPRNLN-
-CPRNLA-
CMPRNL--
CKPRNL--
CAPRNL--
-DPRNLY-
-DPRNLS-
DIPRNL--
DRPRNL--
DLPRNL--
-DPRNLR-
-DPRNLT-
DVPRNL--
-DPRNLK-
-DPRNLI-
DSPRNL--
-DPRNLW-
-DPRNLL-
DWPRNL--
DQPRNL--
DTPRNL--
DYPRNL--
DAPRNL--
-DPRNLV-
-DPRNLQ-
-DPRNLG-
-DPRNLM-
-DPRNLH-
DNPRNL--
DMPRNL--
DKPRNL--
-DPRNLN-
-DPRNLF-
-DPRNLA-
DHPRNL--
-DPRNLE-
DGPRNL--
DDPRNL--
-EPRNLL-
ELPRNL--
EQPRNL--

ETPRNL--
EEPRNL--
EVPRNL--
EWPRNL--
-EPRNLS-
-EPRNLT-
-EPRNLK-
EMPRNL--
-EPRNLE-
EYPRNL--
ENPRNL--
EIPRNL--
-EPRNLV-
-EPRNLI-
EKPRNL--
EGPRNL--
-EPRNLQ-
-EPRNLM-
EPPRNL--
EHPRNL--
EDPRNL--
EAPRNL--
-EPRNLY-
-EPRNLH-
-EPRNLN-
-EPRNLD-
FSPRNL--
FNPRNL--
-FPRNLY-
FVPRNL--
-FPRNLL-
FGPRNL--
FDPRNL--
-FPRNLM-
-FPRNLW-
-FPRNLQ-
FQPRNL--
-FPRNLF-
FMPRNL--
FPPRNL--
-FPRNLE-
FEPRNL--
-FPRNLD-
FHPRNL--
FCPRNL--
-GPRNLL-
GLPRNL--
-GPRNLV-
GDPRNL--
-GPRNLG-

-GPRNLA-
GMPRNL--
-GPRNLY-
GVPRNL--
GHPRNL--
-GPRNLW-
GNPRNL--
GGPRNL--
-GPRNLM-
-GPRNLH-
-GPRNLN-
-GPRNLD-
-GPRNLS-
-GPRNLQ-
-GPRNLE-
GEPRNL--
GQPRNL--
GAPRNL--
GPPRNL--
-HPRNLL-
HQPRNL--
HHPRNL--
-HPRNLT-
HDPRNL--
-HPRNLS-
-HPRNLW-
HIPRNL--
HGPRNL--
-HPRNLG-
-HPRNLI-
HTPRNL--
-HPRNLY-
-HPRNLF-
HNPRNL--
HYPRNL--
-HPRNLQ-
-HPRNLN-
HPPRNL--
-HPRNLV-
-HPRNLH-
HKPRNL--
HMPRNL--
-HPRNLM-
-HPRNLE-
-HPRNLC-
HFPRNL--
HCPRNL--
ISPRNL--
IPPRNL--
-IPRNLK-

-IPRNLG-
IKPRNL--
-IPRNLH-
-IPRNLW-
IIPRNL--
-IPRNLS-
IGPRNL--
-IPRNLT-
-IPRNLN-
IHPRNL--
-IPRNLM-
IQPRNL--
IEPRNL--
IDPRNL--
ICPRNL--
-IPRNLE-
-IPRNLD-
-KPRNLL-
KQPRNL--
KGPRNL--
KHPRNL--
-KPRNLY-
-KPRNLG-
KVPRNL--
-KPRNLW-
-KPRNLK-
-KPRNLN-
-KPRNLI-
KYPRNL--
-KPRNLT-
KNPRNL--
-KPRNLV-
-KPRNLQ-
KEPRNL--
KCPRNL--
-KPRNLH-
-KPRNLE-
-KPRNLD-
KDPRNL--
LSPRNL--
LKPRNL--
LGPRNL--
LIPRNL--
-LPRNLN-
-LPRNLI-
LQPRNL--
LHPRNL--
LEPRNL--
LDPRNL--
-LPRNLW-

-LPRNLV-
-LPRNLQ-
-LPRNLM-
LCPRNL--
-LPRNLE-
-LPRNLD-
MGPRNL--
MSPRNL--
-MPRNLG-
MTPRNL--
MQPRNL--
-MPRNLA-
MNPRNL--
MAPRNL--
-MPRNLQ-
-MPRNLM-
MPPRNL--
MDPRNL--
-MPRNLS-
-MPRNLH-
MFPRNL--
MHPRNL--
-MPRNLY-
-MPRNLV-
-MPRNLN-
-MPRNLK-
-MPRNLD-
MEPRNL--
MCPRNL--
-MPRNLE-
-NPRNLV-
-NPRNLL-
-NPRNLW-
-NPRNLG-
NPPRNL--
-NPRNLQ-
-NPRNLM-
NGPRNL--
-NPRNLK-
-NPRNLA-
NQPRNL--
-NPRNLY-
-NPRNLH-
-NPRNLE-
-NPRNLF-
NNPRNL--
NKPRNL--
NEPRNL--
NDPRNL--
NCPRNL--

--PRNLGL
--PRNLFV
--PRNLGV
--PRNLFK
--PRNLWG
--PRNLKT
--PRNLRT
--PRNLSW
--PRNLLY
--PRNLVS
--PRNLTV
--PRNLRA
--PRNLNL
--PRNLLQ
--PRNLYS
--PRNLGG
--PRNLYL
--PRNLFT
--PRNLST
--PRNLRI
--PRNLQL
--PRNLIV
--PRNLAF
--PRNLTA
--PRNLHT
--PRNLHG
--PRNLFM
--PRNLVD
--PRNLYM
--PRNLYI
--PRNLMK
--PRNLYR
--PRNLVQ
--PRNLVP
--PRNLTG
--PRNLQH
--PRNLVM
--PRNLNV
--PRNLGA
--PRNLFS
--PRNLFI
--PRNLAH
--PRNLYQ
--PRNLYG
--PRNLTY
--PRNLTF
--PRNLSY
--PRNLSN
--PRNLNI
--PRNLIA

--PRNLFN
--PRNLYT
--PRNLWT
--PRNLRW
--PRNLRQ
--PRNLQT
--PRNLNR
--PRNLLD
--PRNLWK
--PRNLTN
--PRNLTM
--PRNLNQ
--PRNLMT
--PRNLYE
--PRNLWN
--PRNLTQ
--PRNLMY
--PRNLMD
--PRNLIM
--PRNLIF
--PRNLAW
--PRNLAM
--PRNLYY
--PRNLNS
--PRNLKN
--PRNLHI
--PRNLFH
--PRNLAY
--PRNLVY
--PRNLTT
--PRNLQS
--PRNLMI
--PRNLKG
--PRNLHK
--PRNLAN
--PRNLWR
--PRNLVE
--PRNLIQ
--PRNLGN
--PRNLGM
--PRNLFQ
--PRNLTI
--PRNLTD
--PRNLSM
--PRNLSK
--PRNLLM
--PRNLGQ
--PRNLGH
--PRNLEL
--PRNLEI

```
--PRNLDS
--PRNLAA
--PRNLYW
--PRNLTE
--PRNLSE
--PRNLQY
--PRNLNT
--PRNLMM
--PRNLII
--PRNLEY
--PRNLEQ
--PRNLEN
PTPRNL--
--PRNLYV
--PRNLWA
--PRNLQG
--PRNLKH
--PRNLHV
--PRNLHD
--PRNLGW
--PRNLFL
--PRNLFE
--PRNLDT
PQPRNL--
PVPRNL--
--PRNLYD
--PRNLWD
--PRNLRE
--PRNLQV
--PRNLQN
--PRNLNH
--PRNLMW
--PRNLKV
--PRNLKQ
--PRNLKD
--PRNLHA
--PRNLEV
--PRNLEA
--PRNLAS
PAPRNL--
--PRNLVV
--PRNLLE
--PRNLHY
--PRNLHM
--PRNLGE
--PRNLET
--PRNLWH
--PRNLSD
--PRNLPL
--PRNLMP
```

--PRNLMH
--PRNLMF
--PRNLME
--PRNLIN
--PRNLIE
--PRNLHQ
--PRNLFA
--PRNLEW
--PRNLEM
--PRNLAD
--PRNLWY
--PRNLQQ
--PRNLQP
--PRNLQM
--PRNLQK
--PRNLNY
--PRNLNK
--PRNLNG
--PRNLMQ
--PRNLKE
--PRNLFY
--PRNLEK
--PRNLDM
PMPRNL--
PHPRNL--
PEPRNL--
--PRNLWM
--PRNLWE
--PRNLTC
--PRNLQW
--PRNLQI
--PRNLQF
--PRNLQE
--PRNLHF
--PRNLHE
--PRNLGD
--PRNLFD
--PRNLDV
--PRNLAE
PNPRNL--
--PRNLWW
--PRNLPM
--PRNLNW
--PRNLNE
--PRNLND
--PRNLEH
--PRNLED
--PRNLDA
--PRNLTP
--PRNLQD

--PRNLMC
--PRNLEP
--PRNLEE
--PRNLDP
--PRNLDG
--PRNLCV
-PPRNLP-
-PPRNLK-
-PPRNLI-
-PPRNLA-
PDPRNL--
QSPRNL--
-QPRNLH-
-QPRNLS-
-QPRNLI-
-QPRNLG-
-QPRNLF-
QQPRNL--
-QPRNLL-
-QPRNLK-
QTPRNL--
-QPRNLY-
-QPRNLT-
QPPRNL--
QNPRNL--
QHPRNL--
-QPRNLV-
-QPRNLQ-
-QPRNLA-
QAPRNL--
QGPRNL--
-QPRNLW-
-QPRNLM-
-QPRNLD-
QEPRNL--
QDPRNL--
-QPRNLE-
QIPRNL--
QCPRNL--
-QPRNLC-
-RPRNLW-
RPPRNL--
-RPRNLQ-
-RPRNLV-
-RPRNLI-
-RPRNLE-
-RPRNLM-
RNPRNL--
RDPRNL--
-RPRNLD-

```
-SPRNLR-
-SPRNLL-
SSPRNL--
-SPRNLG-
SQPRNL--
-SPRNLY-
-SPRNLK-
-SPRNLA-
STPRNL--
-SPRNLV-
SNPRNL--
-SPRNLI-
SAPRNL--
-SPRNLF-
SMPRNL--
SHPRNL--
SEPRNL--
-SPRNLQ-
-SPRNLD-
-SPRNLW-
-SPRNLM-
SPPRNL--
SCPRNL--
TSPRNL--
TQPRNL--
-TPRNLS-
-TPRNLV-
TAPRNL--
-TPRNLA-
-TPRNLM-
-TPRNLW-
-TPRNLH-
TLPRNL--
-TPRNLG-
-TPRNLT-
-TPRNLQ-
TGPRNL--
-TPRNLY-
-TPRNLN-
TPPRNL--
TNPRNL--
-TPRNLD-
TIPRNL--
TEPRNL--
THPRNL--
TKPRNL--
TDPRNL--
-TPRNLE-
TCPRNL--
-VPRNLL-
```

VPPRNL--
VKPRNL--
-VPRNLI-
VIPRNL--
-VPRNLG-
-VPRNLW-
-VPRNLM-
-VPRNLA-
VNPRNL--
-VPRNLK-
-VPRNLN-
VGPRNL--
VAPRNL--
-VPRNLQ-
-VPRNLE-
-VPRNLV-
-VPRNLD-
VEPRNL--
-VPRNLF-
VDPRNL--
VCPRNL--
WPPRNL--
-WPRNLS-
WFPRNL--
WTPRNL--
WLPRNL--
WMPRNL--
WGPRNL--
-WPRNLY-
WEPRNL--
WVPRNL--
WQPRNL--
WAPRNL--
-WPRNLL-
-WPRNLD-
-WPRNLM-
-WPRNLE-
WDPRNL--
WCPRNL--
YLPRNL--
YTPRNL--
YAPRNL--
YSPRNL--
YNPRNL--
-YPRNLW-
-YPRNLA-
YVPRNL--
YKPRNL--
YEPRNL--
YQPRNL--

| | | | |
|---|---|---|---|
| | -YPRNLD- | | |
| | YMPRNL-- | | |
| | -YPRNLE- | | |
| | YPPRNL-- | | |
| | -YPRNLY- | | |
| | YCPRNL-- | | |
| | YDPRNL-- | | |
| PLPL | 11 | 867 | 35.08 |
| | -EPLPLS- | | |
| | --PLPLQD | | |
| | --PLPLPS | | |
| | --PLPLLP | | |
| | -RPLPLD- | | |
| | -SPLPLP- | | |
| | ASPLPL-- | | |
| | -APLPLS- | | |
| | -APLPLR- | | |
| | ALPLPL-- | | |
| | AMPLPL-- | | |
| | -APLPLY- | | |
| | -APLPLH- | | |
| | AAPLPL-- | | |
| | AYPLPL-- | | |
| | APPLPL-- | | |
| | -APLPLG- | | |
| | AKPLPL-- | | |
| | AQPLPL-- | | |
| | AGPLPL-- | | |
| | -APLPLW- | | |
| | -APLPLT- | | |
| | ANPLPL-- | | |
| | AIPLPL-- | | |
| | -APLPLV- | | |
| | AFPLPL-- | | |
| | AEPLPL-- | | |
| | AWPLPL-- | | |
| | -APLPLQ- | | |
| | -APLPLI- | | |
| | -APLPLN- | | |
| | AHPLPL-- | | |
| | -APLPLM- | | |
| | -APLPLD- | | |
| | -APLPLA- | | |
| | ADPLPL-- | | |
| | -APLPLE- | | |
| | ACPLPL-- | | |
| | -CPLPLQ- | | |
| | -CPLPLH- | | |
| | -CPLPLF- | | |
| | CCPLPL-- | | |

```
-CPLPLS-
CSPLPL--
CRPLPL--
-CPLPLY-
-CPLPLR-
CNPLPL--
CHPLPL--
CGPLPL--
CEPLPL--
-CPLPLN-
-CPLPLL-
-CPLPLK-
CMPLPL--
CLPLPL--
CFPLPL--
CTPLPL--
CQPLPL--
-CPLPLW-
-CPLPLI-
-CPLPLA-
CKPLPL--
CDPLPL--
CAPLPL--
DIPLPL--
DRPLPL--
-DPLPLW-
DAPLPL--
DTPLPL--
-DPLPLR-
DGPLPL--
DFPLPL--
DEPLPL--
DQPLPL--
-DPLPLA-
DMPLPL--
DHPLPL--
-DPLPLG-
DNPLPL--
DLPLPL--
DVPLPL--
DSPLPL--
-DPLPLS-
DYPLPL--
-DPLPLY-
-DPLPLQ-
-DPLPLL-
-DPLPLI-
-DPLPLF-
DKPLPL--
DWPLPL--
```

```
-DPLPLT-
-DPLPLN-
-DPLPLK-
-DPLPLH-
EVPLPL--
ERPLPL--
-EPLPLL-
-EPLPLA-
-EPLPLR-
-EPLPLK-
EWPLPL--
ETPLPL--
-EPLPLY-
-EPLPLV-
-EPLPLF-
-EPLPLT-
-EPLPLQ-
EAPLPL--
-EPLPLH-
EYPLPL--
EPPLPL--
-EPLPLM-
-EPLPLG-
-EPLPLE-
ENPLPL--
EKPLPL--
EGPLPL--
-EPLPLW-
-EPLPLN-
EMPLPL--
EHPLPL--
FRPLPL--
FLPLPL--
-FPLPLK-
-FPLPLY-
-FPLPLL-
FNPLPL--
FIPLPL--
FVPLPL--
FEPLPL--
FPPLPL--
-FPLPLN-
FAPLPL--
-FPLPLT-
-FPLPLS-
FHPLPL--
FFPLPL--
-FPLPLG-
FSPLPL--
-FPLPLI-
```

-FPLPLF-
FYPLPL--
FQPLPL--
-FPLPLH-
FMPLPL--
FWPLPL--
FGPLPL--
FDPLPL--
-FPLPLW-
FCPLPL--
-FPLPLV-
-FPLPLD-
GRPLPL--
-GPLPLR-
GYPLPL--
GSPLPL--
-GPLPLW-
-GPLPLF-
GAPLPL--
GFPLPL--
GTPLPL--
-GPLPLS-
GLPLPL--
GGPLPL--
-GPLPLV-
-GPLPLY-
-GPLPLN-
-GPLPLH-
GNPLPL--
GHPLPL--
GWPLPL--
-GPLPLK-
GMPLPL--
-GPLPLE-
GKPLPL--
GIPLPL--
-GPLPLA-
-GPLPLT-
-GPLPLQ-
-GPLPLI-
-GPLPLG-
GEPLPL--
GCPLPL--
GQPLPL--
GPPLPL--
HRPLPL--
-HPLPLH-
HSPLPL--
HYPLPL--
HGPLPL--

HKPLPL--
-HPLPLF-
HMPLPL--
-HPLPLY-
HLPLPL--
-HPLPLR-
-HPLPLK-
-HPLPLG-
HIPLPL--
-HPLPLV-
-HPLPLT-
-HPLPLA-
HHPLPL--
HTPLPL--
HEPLPL--
HAPLPL--
-HPLPLN-
-HPLPLI-
HNPLPL--
HWPLPL--
HPPLPL--
-HPLPLL-
HFPLPL--
-HPLPLW-
-HPLPLM-
-HPLPLE-
-IPLPLA-
ILPLPL--
ISPLPL--
IGPLPL--
-IPLPLM-
ITPLPL--
INPLPL--
-IPLPLT-
-IPLPLL-
-IPLPLK-
IVPLPL--
IKPLPL--
IPPLPL--
-IPLPLS-
IEPLPL--
IWPLPL--
-IPLPLH-
IDPLPL--
IYPLPL--
-IPLPLQ-
-IPLPLF-
IIPLPL--
IFPLPL--
IAPLPL--

-IPLPLG-
-IPLPLY-
-IPLPLW-
-IPLPLN-
-IPLPLI-
-IPLPLP-
IMPLPL--
ICPLPL--
-KPLPLR-
KLPLPL--
-KPLPLL-
KMPLPL--
KGPLPL--
-KPLPLT-
-KPLPLS-
-KPLPLK-
-KPLPLN-
-KPLPLV-
KYPLPL--
-KPLPLG-
KFPLPL--
-KPLPLH-
KNPLPL--
KTPLPL--
-KPLPLY-
KIPLPL--
-KPLPLW-
-KPLPLE-
-KPLPLA-
KWPLPL--
-KPLPLQ-
KPPLPL--
KHPLPL--
-KPLPLM-
-KPLPLI-
KEPLPL--
KDPLPL--
KQPLPL--
-LPLPLK-
-LPLPLR-
LGPLPL--
LMPLPL--
LYPLPL--
LTPLPL--
LAPLPL--
-LPLPLN-
LVPLPL--
LPPLPL--
-LPLPLQ-
LFPLPL--

LEPLPL--
-LPLPLF-
LWPLPL--
-LPLPLY-
LNPLPL--
LHPLPL--
-LPLPLW-
-LPLPLV-
LIPLPL--
-LPLPLT-
LQPLPL--
LCPLPL--
-LPLPLH-
LDPLPL--
-LPLPLE-
MRPLPL--
MSPLPL--
MYPLPL--
-MPLPLT-
-MPLPLR-
-MPLPLS-
-MPLPLG-
-MPLPLN-
-MPLPLL-
MWPLPL--
MVPLPL--
-MPLPLW-
-MPLPLV-
MGPLPL--
-MPLPLQ-
-MPLPLK-
MLPLPL--
MTPLPL--
MAPLPL--
MQPLPL--
MPPLPL--
-MPLPLE-
-MPLPLY-
-MPLPLI-
-MPLPLH-
MNPLPL--
MHPLPL--
MMPLPL--
MKPLPL--
MEPLPL--
MIPLPL--
MCPLPL--
MFPLPL--
MDPLPL--
-NPLPLR-

-NPLPLF-
NAPLPL--
NLPLPL--
NWPLPL--
-NPLPLY-
NRPLPL--
-NPLPLS-
-NPLPLK-
-NPLPLN-
-NPLPLI-
NVPLPL--
-NPLPLH-
NQPLPL--
NNPLPL--
NYPLPL--
NTPLPL--
-NPLPLA-
NPPLPL--
-NPLPLT-
NKPLPL--
NMPLPL--
-NPLPLQ-
-NPLPLW-
-NPLPLV-
-NPLPLE-
NIPLPL--
NEPLPL--
-NPLPLM-
NDPLPL--
-NPLPLC-
--PLPLVL
--PLPLLR
--PLPLFR
--PLPLRL
--PLPLVS
--PLPLTR
--PLPLSL
--PLPLIT
--PLPLTL
--PLPLTD
--PLPLWR
--PLPLYQ
--PLPLSA
--PLPLRN
--PLPLPL
--PLPLYA
--PLPLSS
--PLPLRA
--PLPLYR
--PLPLSR

--PLPLSK
--PLPLRR
--PLPLLL
--PLPLGS
--PLPLRH
--PLPLLT
--PLPLYL
--PLPLQL
--PLPLHR
--PLPLAS
--PLPLAL
--PLPLRV
--PLPLRT
--PLPLRG
--PLPLLG
--PLPLGL
--PLPLVN
--PLPLTK
--PLPLQT
--PLPLEW
PRPLPL--
--PLPLTS
--PLPLYN
--PLPLVQ
--PLPLVK
--PLPLVI
--PLPLNR
--PLPLKL
--PLPLGT
--PLPLVY
--PLPLVV
--PLPLSQ
--PLPLNI
--PLPLKT
--PLPLIS
--PLPLIA
--PLPLVT
--PLPLSV
--PLPLSI
--PLPLRP
--PLPLLE
--PLPLIG
--PLPLHG
--PLPLFN
--PLPLFG
--PLPLAG
--PLPLYS
--PLPLYI
--PLPLSY
--PLPLMR

--PLPLML
--PLPLLW
--PLPLLA
--PLPLHA
--PLPLAI
PYPLPL--
--PLPLYK
--PLPLWS
--PLPLWG
--PLPLRY
--PLPLRF
--PLPLNL
--PLPLMK
--PLPLGV
--PLPLGQ
--PLPLGM
PNPLPL--
--PLPLRD
--PLPLQS
--PLPLNV
--PLPLKI
--PLPLKG
--PLPLIF
--PLPLHS
--PLPLHI
--PLPLHF
--PLPLDL
--PLPLAK
--PLPLAF
--PLPLYT
--PLPLVA
--PLPLTF
--PLPLSF
--PLPLSD
--PLPLQQ
--PLPLMT
--PLPLLV
--PLPLKR
--PLPLKK
--PLPLFA
--PLPLEM
--PLPLAY
--PLPLYH
--PLPLWH
--PLPLTY
--PLPLTQ
--PLPLTA
--PLPLSW
--PLPLQM
--PLPLQI

--PLPLPV
--PLPLNG
--PLPLKV
--PLPLKA
--PLPLIM
--PLPLHV
--PLPLGW
--PLPLFS
PHPLPL--
--PLPLYY
--PLPLYW
--PLPLYE
--PLPLVH
--PLPLRQ
--PLPLQY
--PLPLMG
--PLPLLQ
--PLPLLM
--PLPLLD
--PLPLIY
--PLPLII
--PLPLID
--PLPLHW
--PLPLHN
--PLPLGI
--PLPLGF
--PLPLFT
--PLPLFK
--PLPLAT
--PLPLAQ
--PLPLAN
--PLPLWV
--PLPLWM
--PLPLTT
--PLPLSH
--PLPLRW
--PLPLRE
--PLPLQW
--PLPLQN
--PLPLPQ
--PLPLNY
--PLPLNW
--PLPLNA
--PLPLLK
--PLPLIW
--PLPLIH
--PLPLHM
--PLPLGH
--PLPLFM
--PLPLEF

--PLPLDY
--PLPLAM
PGPLPL--
--PLPLYM
--PLPLWN
--PLPLWA
--PLPLVW
--PLPLVG
--PLPLTV
--PLPLTN
--PLPLTG
--PLPLTE
--PLPLRM
--PLPLPW
--PLPLPM
--PLPLNQ
--PLPLNN
--PLPLNH
--PLPLMV
--PLPLMN
--PLPLIV
--PLPLIE
--PLPLHT
--PLPLGY
--PLPLFQ
--PLPLFH
--PLPLEY
--PLPLAV
PQPLPL--
--PLPLWK
--PLPLTW
--PLPLTI
--PLPLSE
--PLPLQA
--PLPLPF
--PLPLNT
--PLPLMY
--PLPLMW
--PLPLLH
--PLPLKW
--PLPLKM
--PLPLGK
--PLPLES
--PLPLEG
--PLPLDM
PFPLPL--
--PLPLYG
--PLPLYD
--PLPLWI
--PLPLVE

--PLPLSM
--PLPLQR
--PLPLQH
--PLPLQF
--PLPLPY
--PLPLPH
--PLPLNS
--PLPLMF
--PLPLLN
--PLPLKY
--PLPLKQ
--PLPLKN
--PLPLKD
--PLPLIQ
--PLPLHY
--PLPLHQ
--PLPLHE
--PLPLGR
--PLPLFD
--PLPLEV
--PLPLEK
--PLPLDG
--PLPLAD
PIPLPL--
PEPLPL--
--PLPLVD
--PLPLQV
--PLPLQK
--PLPLPI
--PLPLMM
--PLPLMH
--PLPLIN
--PLPLHD
--PLPLET
--PLPLEH
--PLPLAA
PDPLPL--
-PPLPLQ-
-PPLPLF-
PMPLPL--
--PLPLYP
--PLPLWW
--PLPLWQ
--PLPLWE
--PLPLQE
--PLPLPE
--PLPLNP
--PLPLNK
--PLPLMQ
--PLPLMP

--PLPLME
--PLPLKH
--PLPLKE
--PLPLHK
--PLPLHH
--PLPLGE
--PLPLEQ
--PLPLEN
--PLPLEI
--PLPLEA
--PLPLDW
--PLPLDF
--PLPLDA
--PLPLCG
QLPLPL--
QGPLPL--
-QPLPLN-
-QPLPLI-
-QPLPLR-
QSPLPL--
QMPLPL--
QYPLPL--
QWPLPL--
-QPLPLY-
QAPLPL--
QHPLPL--
QVPLPL--
-QPLPLM-
-QPLPLG-
QQPLPL--
-QPLPLT-
-QPLPLF-
QNPLPL--
QKPLPL--
QTPLPL--
QPPLPL--
-QPLPLQ-
QFPLPL--
-QPLPLA-
QEPLPL--
-QPLPLV-
-QPLPLE-
-QPLPLC-
QCPLPL--
-RPLPLR-
RYPLPL--
-RPLPLS-
RVPLPL--
RGPLPL--
-RPLPLT-

RAPLPL--
-RPLPLN-
-RPLPLE-
-RPLPLI-
RWPLPL--
-RPLPLF-
RNPLPL--
RDPLPL--
-RPLPLQ-
-RPLPLH-
RQPLPL--
RIPLPL--
RHPLPL--
-RPLPLA-
RFPLPL--
RPPLPL--
-RPLPLW-
-RPLPLM-
RCPLPL--
SIPLPL--
-SPLPLS-
SLPLPL--
SNPLPL--
SVPLPL--
SKPLPL--
SHPLPL--
STPLPL--
-SPLPLG-
SSPLPL--
-SPLPLK-
-SPLPLH-
SGPLPL--
-SPLPLI-
SMPLPL--
SWPLPL--
-SPLPLW-
SFPLPL--
-SPLPLQ-
-SPLPLN-
SAPLPL--
SYPLPL--
SQPLPL--
-SPLPLV-
-SPLPLF-
SEPLPL--
-SPLPLA-
-SPLPLM-
-SPLPLE-
SDPLPL--
SCPLPL--

SPPLPL--
-SPLPLD-
-TPLPLR-
TAPLPL--
TLPLPL--
TSPLPL--
-TPLPLL-
TRPLPL--
TVPLPL--
-TPLPLT-
-TPLPLS-
-TPLPLF-
TIPLPL--
TFPLPL--
TWPLPL--
-TPLPLK-
TGPLPL--
-TPLPLM-
-TPLPLA-
TKPLPL--
THPLPL--
-TPLPLY-
-TPLPLG-
TPPLPL--
TNPLPL--
-TPLPLQ-
-TPLPLN-
-TPLPLI-
TQPLPL--
TMPLPL--
-TPLPLW-
-TPLPLE-
-TPLPLV-
TEPLPL--
TDPLPL--
VRPLPL--
-VPLPLS-
VWPLPL--
VVPLPL--
-VPLPLR-
VKPLPL--
VSPLPL--
VFPLPL--
-VPLPLV-
VYPLPL--
-VPLPLT-
-VPLPLA-
VIPLPL--
VQPLPL--
-VPLPLF-

VLPLPL--
VAPLPL--
-VPLPLG-
-VPLPLK-
-VPLPLH-
VHPLPL--
VDPLPL--
-VPLPLN-
VNPLPL--
VMPLPL--
-VPLPLM-
-VPLPLE-
VEPLPL--
-VPLPLQ-
VCPLPL--
WSPLPL--
-WPLPLS-
-WPLPLA-
-WPLPLT-
WAPLPL--
-WPLPLN-
WLPLPL--
WGPLPL--
-WPLPLL-
-WPLPLH-
WMPLPL--
WYPLPL--
WNPLPL--
WQPLPL--
-WPLPLV-
WFPLPL--
-WPLPLQ-
-WPLPLK-
WKPLPL--
WEPLPL--
-WPLPLM-
WTPLPL--
-WPLPLY-
-WPLPLI-
WCPLPL--
WWPLPL--
WPPLPL--
-WPLPLG-
WHPLPL--
YRPLPL--
-YPLPLL-
YTPLPL--
YKPLPL--
-YPLPLT-
YHPLPL--

```
                YAPLPL--
                YEPLPL--
                -YPLPLS-
                YMPLPL--
                YLPLPL--
                YIPLPL--
                YWPLPL--
                -YPLPLQ-
                -YPLPLN-
                -YPLPLG-
                YNPLPL--
                YGPLPL--
                YPPLPL--
                -YPLPLY-
                -YPLPLM-
                -YPLPLI-
                -YPLPLA-
                YQPLPL--
                -YPLPLH-
                -YPLPLV-
                -YPLPLK-
                -YPLPLF-
                YDPLPL--
                -YPLPLW-
                YCPLPL--
VANL              40         816       34.97
                -RVANLD-
                --VANLPL
                --VANLGN
                --VANLAG
                --VANLNK
                ARVANL--
                -AVANLT-
                AKVANL--
                ATVANL--
                -AVANLG-
                -AVANLY-
                AMVANL--
                -AVANLS-
                ASVANL--
                -AVANLK-
                AGVANL--
                AFVANL--
                AVVANL--
                AIVANL--
                AWVANL--
                -AVANLW-
                APVANL--
                AYVANL--
                -AVANLA-
```

AQVANL--
AHVANL--
AAVANL--
-AVANLI-
-AVANLQ-
-AVANLE-
ANVANL--
-AVANLH-
-AVANLN-
-AVANLM-
AEVANL--
-AVANLF-
ADVANL--
ACVANL--
-AVANLD-
CRVANL--
-CVANLL-
-CVANLV-
-CVANLS-
-CVANLG-
CGVANL--
CFVANL--
CDVANL--
-CVANLT-
-CVANLQ-
CYVANL--
-CVANLR-
-CVANLA-
CTVANL--
CSVANL--
CQVANL--
CMVANL--
-CVANLY-
-CVANLW-
-CVANLK-
-CVANLI-
-CVANLF-
-CVANLE-
CPVANL--
CLVANL--
CKVANL--
CHVANL--
-DVANLR-
-DVANLL-
DSVANL--
-DVANLY-
-DVANLF-
-DVANLW-
-DVANLT-
-DVANLK-

DGVANL--
DQVANL--
DLVANL--
DVVANL--
-DVANLE-
DPVANL--
DKVANL--
-DVANLS-
DMVANL--
DAVANL--
-DVANLV-
-DVANLG-
-DVANLA-
DTVANL--
DNVANL--
DYVANL--
-DVANLQ-
-DVANLN-
-DVANLM-
DIVANL--
DHVANL--
DDVANL--
DCVANL--
ESVANL--
EWVANL--
-EVANLR-
EKVANL--
-EVANLS-
-EVANLF-
-EVANLA-
-EVANLN-
-EVANLY-
-EVANLT-
-EVANLI-
-EVANLH-
ERVANL--
EYVANL--
-EVANLL-
-EVANLK-
-EVANLV-
EVVANL--
-EVANLM-
-EVANLE-
ETVANL--
EPVANL--
ENVANL--
EGVANL--
EAVANL--
EMVANL--
EIVANL--

EFVANL--
-EVANLQ-
EHVANL--
ECVANL--
-FVANLR-
-FVANLK-
FGVANL--
-FVANLS-
-FVANLN-
-FVANLQ-
FMVANL--
-FVANLA-
FTVANL--
-FVANLT-
-FVANLV-
-FVANLE-
FQVANL--
FVVANL--
-FVANLI-
-FVANLH-
FCVANL--
FAVANL--
-FVANLM-
FNVANL--
FHVANL--
FEVANL--
FDVANL--
FPVANL--
-GVANLR-
GKVANL--
-GVANLY-
-GVANLS-
GQVANL--
GYVANL--
GSVANL--
GPVANL--
-GVANLI-
GIVANL--
-GVANLA-
GHVANL--
-GVANLT-
-GVANLM-
-GVANLH-
GGVANL--
GVVANL--
-GVANLW-
-GVANLK-
-GVANLN-
GMVANL--
-GVANLV-

```
-GVANLE-
-GVANLQ-
-GVANLF-
GEVANL--
GDVANL--
GCVANL--
-HVANLK-
-HVANLR-
-HVANLL-
HRVANL--
-HVANLW-
HKVANL--
-HVANLA-
HVVANL--
-HVANLT-
-HVANLG-
-HVANLS-
HPVANL--
HSVANL--
-HVANLN-
HNVANL--
HYVANL--
-HVANLY-
HIVANL--
HHVANL--
HFVANL--
HWVANL--
-HVANLQ-
-HVANLM-
-HVANLV-
-HVANLH-
HAVANL--
-HVANLI-
HQVANL--
HDVANL--
-HVANLF-
HTVANL--
HMVANL--
HGVANL--
-HVANLE-
HEVANL--
-HVANLD-
-IVANLR-
ILVANL--
ISVANL--
IGVANL--
-IVANLS-
IYVANL--
-IVANLL-
IDVANL--
```

-IVANLT-
IQVANL--
IWVANL--
IVVANL--
-IVANLN-
ITVANL--
IMVANL--
-IVANLQ-
IKVANL--
IIVANL--
-IVANLM-
-IVANLE-
-IVANLV-
-IVANLH-
IFVANL--
IAVANL--
-IVANLI-
-IVANLA-
IPVANL--
-IVANLC-
IHVANL--
IEVANL--
-KVANLS-
-KVANLT-
-KVANLA-
KFVANL--
-KVANLR-
KLVANL--
-KVANLI-
KPVANL--
-KVANLN-
-KVANLF-
KAVANL--
-KVANLV-
KHVANL--
-KVANLY-
-KVANLW-
-KVANLH-
KGVANL--
KYVANL--
KEVANL--
KNVANL--
-KVANLM-
-KVANLE-
KDVANL--
KCVANL--
-LVANLS-
-LVANLA-
LNVANL--
LGVANL--

LTVANL--
LWVANL--
-LVANLL-
-LVANLK-
LYVANL--
LQVANL--
LIVANL--
LAVANL--
-LVANLT-
-LVANLQ-
LEVANL--
-LVANLN-
LMVANL--
LDVANL--
LFVANL--
-LVANLV-
-LVANLD-
LHVANL--
-LVANLH-
-LVANLF-
-MVANLS-
-MVANLL-
MLVANL--
MKVANL--
MRVANL--
-MVANLG-
MSVANL--
MYVANL--
-MVANLY-
MTVANL--
MMVANL--
-MVANLR-
-MVANLH-
MWVANL--
-MVANLN-
MNVANL--
MAVANL--
-MVANLT-
-MVANLA-
-MVANLM-
-MVANLV-
MHVANL--
-MVANLK-
MGVANL--
MFVANL--
MEVANL--
-MVANLQ-
-MVANLE-
-MVANLD-
MPVANL--

MCVANL--
-NVANLM-
-NVANLR-
-NVANLK-
-NVANLT-
NVVANL--
NTVANL--
NGVANL--
-NVANLG-
NLVANL--
-NVANLL-
-NVANLF-
-NVANLW-
-NVANLH-
-NVANLY-
NFVANL--
-NVANLN-
NMVANL--
NIVANL--
NDVANL--
-NVANLS-
-NVANLI-
NEVANL--
NAVANL--
NWVANL--
-NVANLV-
-NVANLE-
-NVANLA-
NPVANL--
NNVANL--
NHVANL--
-NVANLQ-
-NVANLD-
-NVANLC-
NQVANL--
-PVANLR-
PTVANL--
-PVANLS-
PRVANL--
PVVANL--
-PVANLK-
PSVANL--
PKVANL--
-PVANLW-
-PVANLF-
-PVANLM-
PLVANL--
PGVANL--
PYVANL--
-PVANLL-

-PVANLI-
PMVANL--
PIVANL--
PDVANL--
-PVANLN-
-PVANLE-
PWVANL--
-PVANLT-
PEVANL--
-PVANLQ-
PHVANL--
PQVANL--
-PVANLY-
-PVANLH-
-QVANLL-
-QVANLR-
QNVANL--
QLVANL--
-QVANLM-
QRVANL--
QPVANL--
-QVANLI-
QGVANL--
QQVANL--
QFVANL--
-QVANLA-
-QVANLS-
QVVANL--
-QVANLV-
QWVANL--
-QVANLT-
-QVANLG-
QIVANL--
QDVANL--
QAVANL--
QTVANL--
-QVANLN-
QMVANL--
-QVANLH-
-QVANLD-
QCVANL--
-QVANLW-
-QVANLE-
-QVANLQ-
-QVANLC-
-RVANLS-
-RVANLT-
-RVANLL-
-RVANLA-
-RVANLG-

RQVANL--
-RVANLK-
RAVANL--
-RVANLV-
-RVANLN-
RTVANL--
-RVANLQ-
RDVANL--
-RVANLI-
RPVANL--
-RVANLE-
-RVANLM-
-RVANLF-
RCVANL--
-RVANLC-
-SVANLS-
SGVANL--
SYVANL--
SVVANL--
-SVANLL-
-SVANLV-
-SVANLQ-
-SVANLG-
-SVANLA-
SKVANL--
-SVANLI-
-SVANLT-
STVANL--
SSVANL--
SMVANL--
-SVANLM-
SPVANL--
SAVANL--
SNVANL--
-SVANLF-
SIVANL--
SDVANL--
SWVANL--
-SVANLN-
SFVANL--
SEVANL--
-SVANLH-
-SVANLE-
SQVANL--
SCVANL--
TRVANL--
-TVANLL-
-TVANLT-
-TVANLG-
-TVANLS-

```
TYVANL--
TVVANL--
TGVANL--
TNVANL--
TFVANL--
TAVANL--
TWVANL--
TMVANL--
-TVANLF-
TQVANL--
-TVANLW-
-TVANLI-
TSVANL--
TKVANL--
-TVANLK-
TTVANL--
THVANL--
TDVANL--
-TVANLN-
-TVANLH-
TIVANL--
-TVANLM-
-TVANLQ-
TCVANL--
-TVANLV-
TEVANL--
-TVANLE-
-TVANLD-
--VANLRS
--VANLTL
VSVANL--
--VANLFK
-VVANLR-
-VVANLS-
VLVANL--
--VANLVA
--VANLLN
--VANLKR
--VANLAR
--VANLSG
--VANLQR
--VANLVS
--VANLVG
--VANLMS
--VANLIS
VTVANL--
--VANLSK
--VANLAL
VYVANL--
-VVANLL-
```

--VANLVR
--VANLTR
--VANLRD
-VVANLG-
--VANLYG
--VANLRQ
--VANLLG
--VANLAK
-VVANLN-
--VANLRI
--VANLKK
-VVANLK-
VMVANL--
--VANLLK
--VANLIA
-VVANLT-
VQVANL--
--VANLLE
--VANLGS
--VANLGL
VGVANL--
--VANLYL
--VANLRW
--VANLRN
--VANLRA
--VANLQY
--VANLKL
--VANLAS
VNVANL--
VHVANL--
--VANLSY
--VANLSI
--VANLRH
--VANLRG
--VANLFA
--VANLSQ
VVVANL--
--VANLYM
--VANLWG
--VANLVD
--VANLSF
--VANLNV
--VANLKT
--VANLIK
--VANLAV
--VANLVI
--VANLMR
--VANLLV
--VANLKA
--VANLGW

--VANLFD
--VANLAT
--VANLAA
VKVANL--
--VANLYD
--VANLWT
--VANLTN
--VANLTK
--VANLSV
--VANLSH
--VANLNA
--VANLFS
-VVANLH-
--VANLYQ
--VANLTV
--VANLKS
--VANLKN
--VANLKG
--VANLHR
--VANLHH
--VANLGG
-VVANLA-
--VANLYE
--VANLYA
--VANLWQ
--VANLWK
--VANLVW
--VANLVN
--VANLTY
--VANLTT
--VANLTG
--VANLTF
--VANLSN
--VANLQT
--VANLNS
--VANLMA
--VANLIG
--VANLHT
--VANLHM
--VANLFR
--VANLWR
--VANLVV
--VANLQS
--VANLMV
--VANLGT
--VANLFQ
--VANLET
-VVANLD-
VDVANL--
--VANLYK

--VANLYF
--VANLTS
--VANLTQ
--VANLTI
--VANLTE
--VANLTA
--VANLNT
--VANLNQ
--VANLMQ
--VANLMN
--VANLMK
--VANLKY
--VANLKW
--VANLHG
--VANLHA
--VANLDS
VEVANL--
--VANLYT
--VANLYH
--VANLVT
--VANLVF
--VANLSW
--VANLLD
--VANLIY
--VANLIT
--VANLIQ
--VANLHW
--VANLGA
--VANLFG
--VANLAN
--VANLWH
--VANLQL
--VANLMI
--VANLKV
--VANLKI
--VANLKF
--VANLIW
--VANLHF
-VVANLM-
--VANLRF
--VANLQK
--VANLMY
--VANLHN
--VANLGM
--VANLFW
--VANLFN
--VANLFH
--VANLEM
--VANLEG
-VVANLI-

--VANLWN
--VANLSE
--VANLQI
--VANLQE
--VANLQD
--VANLNY
--VANLNW
--VANLNL
--VANLNG
--VANLMW
--VANLMH
--VANLMD
--VANLKD
--VANLHL
--VANLER
--VANLEF
--VANLDA
--VANLAQ
-VVANLQ-
VPVANL--
VCVANL--
--VANLWW
--VANLWI
--VANLVK
--VANLVE
--VANLTW
--VANLSM
--VANLSD
--VANLQV
--VANLQQ
--VANLQN
--VANLQH
--VANLQF
--VANLNN
--VANLMT
--VANLKM
--VANLKH
--VANLID
--VANLHQ
--VANLHK
--VANLHE
--VANLEN
--VANLEK
--VANLEA
--VANLAM
--VANLAI
--VANLAD
--VANLWM
--VANLWD
--VANLWA

--VANLVQ
--VANLTM
--VANLQM
--VANLQG
--VANLQA
--VANLNI
--VANLNH
--VANLMM
--VANLMF
--VANLKE
--VANLIM
--VANLII
--VANLIH
--VANLIF
--VANLHY
--VANLHI
--VANLGI
--VANLEI
--VANLDF
--VANLAY
--VANLAW
--VANLAH
--VANLYY
--VANLYN
--VANLSC
--VANLQW
--VANLNE
--VANLHV
--VANLGE
--VANLEY
--VANLEW
--VANLEV
--VANLEQ
--VANLEP
--VANLED
--VANLDT
--VANLCW
--VANLCK
--VANLCG
--VANLAE
WRVANL--
-WVANLR-
WSVANL--
-WVANLK-
WFVANL--
-WVANLL-
-WVANLG-
WQVANL--
WGVANL--
-WVANLA-

WIVANL--
-WVANLE-
-WVANLM-
WAVANL--
-WVANLN-
WTVANL--
WPVANL--
-WVANLD-
WEVANL--
WDVANL--
-WVANLH-
WCVANL--
YEVANL--
-YVANLS-
YTVANL--
YRVANL--
YKVANL--
-YVANLN-
-YVANLL-
YLVANL--
YWVANL--
YSVANL--
-YVANLT-
YDVANL--
YYVANL--
-YVANLV-
-YVANLH-
-YVANLM-
-YVANLG-
-YVANLA-
YGVANL--
YQVANL--
YMVANL--
-YVANLI-
YAVANL--
-YVANLW-
-YVANLD-
YHVANL--
YFVANL--
YCVANL--
YPVANL--
-YVANLE-

| | | | |
|---|---|---|---|
| PARL | 146 | 741 | 34.92 |

-DPARLG-
--PARLGL
--PARLNG
--PARLLP
--PARLEL
--PARLPV
--PARLPN

--PARLPL
--PARLNR
--PARLGK
--PARLAE
ALPARL--
-APARLR-
APPARL--
-APARLL-
-APARLK-
-APARLW-
-APARLM-
ASPARL--
AIPARL--
-APARLT-
-APARLA-
AMPARL--
AKPARL--
-APARLN-
-APARLG-
-APARLI-
-APARLV-
AEPARL--
-APARLH-
AVPARL--
AAPARL--
AGPARL--
-APARLF-
-APARLQ-
ATPARL--
AQPARL--
AHPARL--
AYPARL--
ANPARL--
-APARLE-
ADPARL--
-APARLD-
ACPARL--
-APARLC-
CLPARL--
CCPARL--
CIPARL--
CSPARL--
CMPARL--
CEPARL--
CTPARL--
CRPARL--
CKPARL--
CGPARL--
CAPARL--
CPPARL--

-CPARLT-
-CPARLR-
-CPARLM-
-CPARLI-
-CPARLE-
CNPARL--
CYPARL--
-CPARLY-
-CPARLQ-
-CPARLA-
-DPARLL-
DNPARL--
DLPARL--
-DPARLK-
DRPARL--
DSPARL--
DQPARL--
-DPARLS-
-DPARLA-
DIPARL--
-DPARLV-
-DPARLW-
DGPARL--
DYPARL--
-DPARLR-
DCPARL--
DWPARL--
-DPARLT-
DVPARL--
DTPARL--
-DPARLI-
DMPARL--
-DPARLY-
-DPARLH-
-DPARLQ-
-DPARLF-
-DPARLE-
DEPARL--
-DPARLN-
DAPARL--
-EPARLR-
ERPARL--
ESPARL--
-EPARLK-
-EPARLY-
ETPARL--
-EPARLV-
ELPARL--
EAPARL--
EVPARL--

EKPARL--
-EPARLN-
-EPARLT-
-EPARLH-
-EPARLA-
ENPARL--
EGPARL--
EYPARL--
-EPARLM-
-EPARLG-
-EPARLQ-
EMPARL--
EFPARL--
EEPARL--
-EPARLF-
-EPARLE-
EHPARL--
-EPARLC-
EIPARL--
FKPARL--
-FPARLH-
-FPARLY-
FGPARL--
FQPARL--
FYPARL--
-FPARLV-
FEPARL--
-FPARLE-
FPPARL--
FTPARL--
FNPARL--
-FPARLW-
FMPARL--
FIPARL--
FHPARL--
-FPARLG-
FDPARL--
-GPARLL-
-GPARLS-
GIPARL--
GKPARL--
-GPARLT-
GVPARL--
-GPARLK-
GFPARL--
GYPARL--
-GPARLG-
-GPARLF-
GGPARL--
GAPARL--

GMPARL--
-GPARLH-
GNPARL--
-GPARLM-
GHPARL--
GWPARL--
GDPARL--
-GPARLY-
-GPARLQ-
GEPARL--
GPPARL--
-GPARLE-
-GPARLW-
GCPARL--
-HPARLL-
HLPARL--
-HPARLR-
HVPARL--
-HPARLK-
HTPARL--
HMPARL--
HNPARL--
HIPARL--
-HPARLT-
HHPARL--
-HPARLM-
-HPARLN-
-HPARLH-
-HPARLS-
HFPARL--
-HPARLV-
HDPARL--
HYPARL--
HQPARL--
-HPARLG-
HEPARL--
-HPARLI-
HAPARL--
HPPARL--
-HPARLQ-
-HPARLE-
-HPARLF-
-HPARLD-
ISPARL--
-IPARLL-
IFPARL--
-IPARLS-
INPARL--
IVPARL--
IMPARL--

IGPARL--
-IPARLY-
-IPARLV-
-IPARLT-
-IPARLF-
IAPARL--
IIPARL--
IYPARL--
-IPARLW-
-IPARLN-
ITPARL--
IKPARL--
-IPARLA-
IEPARL--
IDPARL--
IHPARL--
-IPARLQ-
-IPARLM-
-IPARLE-
-IPARLH-
-IPARLD-
IQPARL--
-IPARLC-
ICPARL--
KSPARL--
-KPARLT-
KLPARL--
-KPARLR-
KEPARL--
-KPARLV-
-KPARLW-
KYPARL--
KGPARL--
KTPARL--
-KPARLN-
KAPARL--
KQPARL--
-KPARLY-
KIPARL--
KDPARL--
-KPARLG-
KMPARL--
-KPARLF-
KNPARL--
-KPARLQ-
KKPARL--
KHPARL--
-KPARLM-
-KPARLE-
-KPARLC-

KCPARL--
LVPARL--
-LPARLR-
LGPARL--
LKPARL--
LHPARL--
-LPARLK-
LPPARL--
-LPARLH-
LDPARL--
-LPARLT-
LAPARL--
-LPARLF-
LQPARL--
-LPARLW-
-LPARLY-
-LPARLM-
-LPARLE-
LFPARL--
-LPARLQ-
-LPARLG-
-LPARLN-
LCPARL--
-MPARLR-
-MPARLL-
-MPARLT-
-MPARLS-
-MPARLK-
-MPARLV-
-MPARLI-
MVPARL--
MGPARL--
MHPARL--
MMPARL--
MPPARL--
-MPARLH-
-MPARLA-
-MPARLY-
-MPARLQ-
-MPARLN-
MKPARL--
MDPARL--
MAPARL--
-MPARLW-
-MPARLG-
-MPARLE-
MYPARL--
-MPARLM-
MNPARL--
MTPARL--

-MPARLF-
MEPARL--
MCPARL--
-MPARLD-
-MPARLC-
NLPARL--
-NPARLT-
NRPARL--
NSPARL--
-NPARLL-
NTPARL--
-NPARLS-
NNPARL--
NWPARL--
NEPARL--
-NPARLH-
NVPARL--
NMPARL--
NAPARL--
NIPARL--
NGPARL--
-NPARLA-
-NPARLF-
NKPARL--
-NPARLY-
-NPARLI-
NHPARL--
NPPARL--
-NPARLG-
-NPARLE-
-NPARLQ-
-NPARLN-
-NPARLM-
NCPARL--
NQPARL--
NDPARL--
--PARLRT
--PARLRV
--PARLYS
--PARLLS
--PARLMR
--PARLSI
--PARLSG
--PARLSA
--PARLLN
--PARLLI
--PARLTK
--PARLFS
--PARLST
--PARLSQ

--PARLGS
--PARLWS
--PARLWA
--PARLLD
--PARLIW
--PARLWR
--PARLLK
--PARLSM
--PARLSN
--PARLGG
--PARLVY
--PARLVT
--PARLTY
--PARLTA
--PARLLG
--PARLIH
--PARLFW
--PARLRG
--PARLKG
--PARLKA
--PARLIQ
--PARLVA
--PARLSW
--PARLSK
--PARLFN
--PARLWV
--PARLNS
--PARLTV
--PARLWT
--PARLWG
--PARLTN
--PARLRN
--PARLQT
--PARLKV
--PARLHA
--PARLAG
PSPARL--
PEPARL--
--PARLRQ
PAPARL--
PGPARL--
--PARLQV
--PARLMQ
--PARLIL
--PARLTG
--PARLLW
--PARLFA
--PARLTH
--PARLQY
--PARLMY

--PARLKH
--PARLKD
--PARLHH
--PARLES
--PARLAN
--PARLVM
--PARLLE
--PARLLA
--PARLFG
--PARLYY
--PARLYI
--PARLRH
--PARLHG
--PARLGT
--PARLEY
--PARLYN
--PARLNT
--PARLMM
--PARLLQ
--PARLLM
--PARLDA
PYPARL--
--PARLTE
--PARLIN
--PARLHV
--PARLFK
PNPARL--
PIPARL--
--PARLYH
--PARLYD
--PARLTF
--PARLRM
--PARLQH
--PARLKS
--PARLFE
--PARLET
--PARLEF
PMPARL--
--PARLYL
--PARLTD
--PARLSF
--PARLSE
--PARLNW
--PARLKI
--PARLAY
--PARLWN
--PARLKN
--PARLIA
--PARLHN
PFPARL--

--PARLTQ
--PARLQL
--PARLNF
--PARLMH
--PARLMF
--PARLMA
--PARLIT
--PARLTW
--PARLTM
--PARLQI
--PARLQG
--PARLII
--PARLIG
--PARLEQ
--PARLEH
--PARLAL
--PARLYQ
--PARLRY
--PARLIM
--PARLGA
--PARLEV
--PARLDG
--PARLAH
--PARLSY
--PARLNV
--PARLMI
--PARLKY
--PARLIE
--PARLHQ
--PARLHM
--PARLGI
--PARLEN
--PARLDS
--PARLYW
--PARLWI
--PARLVQ
--PARLVD
--PARLQE
--PARLQA
--PARLNY
--PARLMD
--PARLKT
--PARLIK
--PARLHT
--PARLFH
--PARLEK
--PARLEA
--PARLAQ
PQPARL--
--PARLYE

--PARLWQ
--PARLWF
--PARLQM
--PARLQK
--PARLKQ
--PARLHW
--PARLHK
--PARLGW
--PARLFV
--PARLFM
--PARLWW
--PARLWL
--PARLWH
--PARLWD
--PARLSD
--PARLQN
--PARLKL
--PARLKF
--PARLKE
--PARLGY
--PARLEI
PKPARL--
--PARLQW
--PARLNN
--PARLNM
--PARLNK
--PARLNA
--PARLMT
--PARLHI
--PARLHE
--PARLHD
--PARLGM
--PARLEG
--PARLEE
--PARLWE
--PARLRE
--PARLQQ
--PARLQF
--PARLMV
--PARLEM
--PARLAK
--PARLAD
--PARLWM
--PARLME
--PARLHY
--PARLGQ
--PARLGF
--PARLDT
--PARLCK
--PARLCG

PPPARL--
-PPARLY-
-PPARLL-
PCPARL--
--PARLYK
--PARLTC
--PARLQD
--PARLNQ
--PARLNI
--PARLMC
--PARLCY
--PARLCV
--PARLCS
--PARLCR
--PARLCN
--PARLCH
-QPARLL-
QRPARL--
-QPARLT-
-QPARLV-
QSPARL--
QTPARL--
-QPARLA-
QLPARL--
-QPARLM-
QIPARL--
QPPARL--
-QPARLQ-
-QPARLH-
-QPARLS-
-QPARLG-
QYPARL--
QQPARL--
-QPARLN-
-QPARLE-
QMPARL--
QVPARL--
-QPARLY-
-QPARLK-
QKPARL--
QDPARL--
QNPARL--
QEPARL--
-QPARLF-
-QPARLD-
QHPARL--
QCPARL--
-QPARLC-
-RPARLS-
RTPARL--

RYPARL--
RHPARL--
-RPARLY-
-RPARLM-
RQPARL--
RNPARL--
REPARL--
-RPARLT-
RKPARL--
RDPARL--
-RPARLD-
RCPARL--
-RPARLC-
-SPARLS-
SYPARL--
SLPARL--
SSPARL--
SVPARL--
SIPARL--
-SPARLN-
-SPARLG-
SGPARL--
SEPARL--
-SPARLH-
SQPARL--
-SPARLK-
STPARL--
SNPARL--
SHPARL--
-SPARLQ-
-SPARLW-
-SPARLE-
SCPARL--
-SPARLD-
-SPARLC-
TLPARL--
-TPARLV-
-TPARLH-
-TPARLS-
TVPARL--
TNPARL--
-TPARLY-
TGPARL--
-TPARLF-
TTPARL--
TYPARL--
TIPARL--
TAPARL--
-TPARLT-
TKPARL--

```
-TPARLM-
-TPARLI-
-TPARLK-
TDPARL--
TFPARL--
TEPARL--
THPARL--
TQPARL--
-TPARLW-
TCPARL--
-TPARLE-
-TPARLC-
VLPARL--
-VPARLG-
-VPARLH-
VTPARL--
VVPARL--
-VPARLS-
-VPARLN-
VKPARL--
VNPARL--
-VPARLA-
VPPARL--
-VPARLF-
VAPARL--
-VPARLY-
-VPARLE-
VQPARL--
-VPARLK-
VYPARL--
VHPARL--
VGPARL--
VDPARL--
-VPARLM-
VEPARL--
-VPARLD-
-VPARLQ-
-VPARLC-
-WPARLL-
-WPARLQ-
WLPARL--
WKPARL--
WPPARL--
-WPARLY-
WMPARL--
-WPARLI-
WIPARL--
-WPARLM-
WGPARL--
-WPARLF-
```

WAPARL--
-WPARLG-
WNPARL--
WDPARL--
WCPARL--
-WPARLW-
-WPARLN-
-YPARLL-
YRPARL--
-YPARLS-
-YPARLK-
YIPARL--
-YPARLA-
YAPARL--
YKPARL--
YSPARL--
YQPARL--
-YPARLG-
YNPARL--
-YPARLR-
-YPARLN-
YEPARL--
YVPARL--
-YPARLH-
YPPARL--
-YPARLV-
-YPARLE-
YGPARL--
-YPARLY-
-YPARLQ-
YHPARL--
YDPARL--
-YPARLW-
-YPARLM-

PAWL 134 747 34.88

AGPAWL--
-APAWLT-
-APAWLM-
AYPAWL--
-APAWLA-
ASPAWL--
-APAWLN-
AEPAWL--
ATPAWL--
-APAWLY-
-APAWLS-
-APAWLK-
-APAWLG-
ANPAWL--
-APAWLH-

-APAWLI-
ALPAWL--
AKPAWL--
AIPAWL--
AVPAWL--
-APAWLD-
AMPAWL--
AAPAWL--
AFPAWL--
AWPAWL--
-APAWLE-
-APAWLV-
AQPAWL--
APPAWL--
AHPAWL--
CIPAWL--
CRPAWL--
-CPAWLT-
-CPAWLL-
CSPAWL--
CLPAWL--
CVPAWL--
-CPAWLV-
-CPAWLS-
CNPAWL--
CFPAWL--
CEPAWL--
CCPAWL--
DLPAWL--
DMPAWL--
DQPAWL--
-DPAWLK-
DNPAWL--
DTPAWL--
-DPAWLT-
-DPAWLS-
-DPAWLL-
DVPAWL--
DRPAWL--
-DPAWLN-
DIPAWL--
-DPAWLR-
-DPAWLI-
-DPAWLH-
-DPAWLF-
DHPAWL--
DGPAWL--
DWPAWL--
DPPAWL--
-DPAWLY-

```
-DPAWLW-
DFPAWL--
DEPAWL--
DYPAWL--
DSPAWL--
-DPAWLV-
-DPAWLG-
-DPAWLE-
-DPAWLD-
-DPAWLA-
DKPAWL--
DAPAWL--
-EPAWLR-
-EPAWLL-
EVPAWL--
EKPAWL--
-EPAWLV-
-EPAWLS-
ETPAWL--
ERPAWL--
-EPAWLK-
-EPAWLA-
EAPAWL--
-EPAWLT-
-EPAWLI-
-EPAWLG-
-EPAWLE-
ELPAWL--
EGPAWL--
EEPAWL--
EYPAWL--
ESPAWL--
-EPAWLN-
EHPAWL--
EWPAWL--
-EPAWLQ-
-EPAWLF-
EFPAWL--
EPPAWL--
-EPAWLW-
ENPAWL--
EMPAWL--
FTPAWL--
-FPAWLG-
-FPAWLA-
FSPAWL--
FPPAWL--
-FPAWLQ-
-FPAWLN-
-FPAWLD-
```

FVPAWL--
-FPAWLE-
FDPAWL--
-FPAWLR-
-FPAWLK-
FLPAWL--
FKPAWL--
FHPAWL--
-FPAWLM-
FFPAWL--
FAPAWL--
-FPAWLI-
-FPAWLH-
FEPAWL--
FQPAWL--
-FPAWLV-
-FPAWLT-
-FPAWLL-
FMPAWL--
GGPAWL--
GVPAWL--
GSPAWL--
-GPAWLS-
-GPAWLT-
-GPAWLA-
GAPAWL--
-GPAWLL-
GLPAWL--
GKPAWL--
-GPAWLG-
GWPAWL--
GRPAWL--
GIPAWL--
GEPAWL--
GTPAWL--
-GPAWLN-
GMPAWL--
GQPAWL--
-GPAWLR-
-GPAWLD-
GPPAWL--
-GPAWLV-
-GPAWLM-
GNPAWL--
GDPAWL--
-GPAWLF-
-GPAWLE-
-GPAWLH-
-GPAWLW-
-GPAWLK-

HEPAWL--
HSPAWL--
-HPAWLW-
HAPAWL--
-HPAWLN-
-HPAWLD-
-HPAWLV-
-HPAWLR-
-HPAWLK-
HTPAWL--
HGPAWL--
-HPAWLI-
-HPAWLF-
HYPAWL--
-HPAWLL-
-HPAWLH-
-HPAWLG-
HNPAWL--
HLPAWL--
HDPAWL--
HPPAWL--
-HPAWLY-
HHPAWL--
HWPAWL--
HRPAWL--
-HPAWLT-
-HPAWLE-
-HPAWLA-
HIPAWL--
HFPAWL--
IGPAWL--
-IPAWLG-
-IPAWLT-
IFPAWL--
IEPAWL--
IVPAWL--
ITPAWL--
IIPAWL--
-IPAWLR-
-IPAWLS-
-IPAWLI-
IKPAWL--
IHPAWL--
-IPAWLN-
-IPAWLL-
ILPAWL--
IPPAWL--
INPAWL--
IDPAWL--
IYPAWL--

-IPAWLW-
-IPAWLQ-
IWPAWL--
ISPAWL--
-IPAWLK-
-IPAWLH-
-IPAWLE-
-IPAWLA-
IMPAWL--
IAPAWL--
KSPAWL--
-KPAWLS-
KIPAWL--
KLPAWL--
KYPAWL--
-KPAWLN-
-KPAWLH-
KRPAWL--
KNPAWL--
KVPAWL--
-KPAWLY-
-KPAWLT-
KTPAWL--
-KPAWLI-
KKPAWL--
KPPAWL--
-KPAWLG-
-KPAWLF-
KGPAWL--
-KPAWLQ-
KDPAWL--
KWPAWL--
KQPAWL--
-KPAWLK-
-KPAWLA-
KAPAWL--
-KPAWLW-
-KPAWLD-
KHPAWL--
KCPAWL--
-KPAWLE-
KEPAWL--
-LPAWLS-
LRPAWL--
-LPAWLI-
LNPAWL--
-LPAWLA-
-LPAWLG-
LVPAWL--
LLPAWL--

-LPAWLH-
LEPAWL--
LQPAWL--
-LPAWLT-
-LPAWLE-
-LPAWLD-
LDPAWL--
LWPAWL--
LPPAWL--
LFPAWL--
-LPAWLV-
-LPAWLQ-
LIPAWL--
LYPAWL--
MQPAWL--
-MPAWLN-
MWPAWL--
-MPAWLT-
-MPAWLQ-
-MPAWLR-
MVPAWL--
-MPAWLL-
MNPAWL--
MHPAWL--
MGPAWL--
-MPAWLI-
-MPAWLA-
-MPAWLY-
MLPAWL--
MTPAWL--
-MPAWLM-
-MPAWLG-
-MPAWLD-
MKPAWL--
MAPAWL--
MYPAWL--
MSPAWL--
MPPAWL--
-MPAWLW-
-MPAWLV-
-MPAWLS-
-MPAWLK-
-MPAWLH-
-MPAWLE-
MEPAWL--
MDPAWL--
NRPAWL--
-NPAWLS-
NHPAWL--
-NPAWLH-

NKPAWL--
NTPAWL--
NSPAWL--
-NPAWLT-
-NPAWLF-
NLPAWL--
NVPAWL--
NGPAWL--
NYPAWL--
-NPAWLY-
-NPAWLW-
-NPAWLR-
-NPAWLG-
NIPAWL--
NWPAWL--
-NPAWLN-
-NPAWLL-
NMPAWL--
NPPAWL--
-NPAWLK-
-NPAWLE-
-NPAWLA-
NAPAWL--
NQPAWL--
-NPAWLQ-
-NPAWLD-
-NPAWLC-
NNPAWL--
NEPAWL--
--PAWLLN
--PAWLKR
--PAWLVR
--PAWLKI
--PAWLSN
--PAWLRN
--PAWLGK
--PAWLQS
--PAWLHR
--PAWLVG
--PAWLSK
--PAWLRG
--PAWLRD
--PAWLWK
--PAWLTK
--PAWLRV
--PAWLRH
--PAWLNA
--PAWLKS
--PAWLGT
--PAWLAN

--PAWLTT
--PAWLTS
--PAWLST
--PAWLGS
--PAWLNI
--PAWLLH
--PAWLKY
--PAWLKQ
--PAWLIG
--PAWLVS
--PAWLVA
--PAWLKK
--PAWLGF
--PAWLYG
--PAWLVT
--PAWLNK
--PAWLMW
--PAWLGA
--PAWLAK
PMPAWL--
--PAWLYH
--PAWLVI
--PAWLTN
--PAWLSD
--PAWLQR
--PAWLLK
--PAWLIA
--PAWLTG
--PAWLSI
--PAWLRI
--PAWLQT
--PAWLMG
--PAWLLG
--PAWLFS
--PAWLAA
PSPAWL--
PEPAWL--
--PAWLWQ
--PAWLRA
--PAWLNH
--PAWLMT
--PAWLMS
--PAWLIR
--PAWLGV
--PAWLGR
PIPAWL--
--PAWLYS
--PAWLVH
--PAWLTW
--PAWLTD

--PAWLSH
--PAWLRM
--PAWLQV
--PAWLQA
--PAWLPL
--PAWLNQ
--PAWLMD
--PAWLKL
--PAWLHT
--PAWLFH
--PAWLET
--PAWLYW
--PAWLYK
--PAWLWH
--PAWLVQ
--PAWLVD
--PAWLSY
--PAWLSW
--PAWLMK
--PAWLLE
--PAWLKT
--PAWLKG
--PAWLIE
--PAWLGY
--PAWLES
--PAWLDT
PAPAWL--
PNPAWL--
PKPAWL--
PDPAWL--
--PAWLTI
--PAWLRF
--PAWLQH
--PAWLPY
--PAWLIS
--PAWLHY
--PAWLHN
--PAWLHM
--PAWLHG
--PAWLHE
--PAWLHA
--PAWLFE
--PAWLEW
--PAWLEA
--PAWLDS
--PAWLAS
--PAWLYN
--PAWLYD
--PAWLWN
--PAWLWE

--PAWLWD
--PAWLTY
--PAWLTH
--PAWLSE
--PAWLQW
--PAWLNW
--PAWLNV
--PAWLND
--PAWLMI
--PAWLLW
--PAWLKH
--PAWLHL
--PAWLHI
--PAWLGW
--PAWLGI
--PAWLGE
--PAWLEV
--PAWLEN
--PAWLEK
--PAWLDG
--PAWLAF
PVPAWL--
--PAWLYT
--PAWLWS
--PAWLWG
--PAWLVY
--PAWLVK
--PAWLTV
--PAWLTF
--PAWLTE
--PAWLSM
--PAWLQK
--PAWLQD
--PAWLNY
--PAWLNM
--PAWLMN
--PAWLME
--PAWLLI
--PAWLKW
--PAWLIN
--PAWLID
--PAWLHS
--PAWLHK
--PAWLHH
--PAWLGQ
--PAWLGN
--PAWLGH
--PAWLGG
--PAWLGD
--PAWLFD

--PAWLEQ
--PAWLCN
--PAWLAV
--PAWLAQ
--PAWLAM
--PAWLAH
PYPAWL--
PQPAWL--
-PPAWLN-
-PPAWLK-
PHPAWL--
PFPAWL--
--PAWLVW
--PAWLSQ
--PAWLRY
--PAWLQY
--PAWLQN
--PAWLQM
--PAWLQI
--PAWLQG
--PAWLNT
--PAWLMA
--PAWLLM
--PAWLKM
--PAWLKF
--PAWLKE
--PAWLKD
--PAWLIY
--PAWLIV
--PAWLIM
--PAWLIK
--PAWLHV
--PAWLHQ
--PAWLHD
--PAWLHC
--PAWLEH
--PAWLED
--PAWLDA
--PAWLAY
-QPAWLR-
-QPAWLS-
QTPAWL--
QRPAWL--
-QPAWLA-
-QPAWLV-
-QPAWLQ-
QNPAWL--
-QPAWLL-
-QPAWLI-
QFPAWL--

−QPAWLG−
QSPAWL−−
−QPAWLY−
−QPAWLT−
QKPAWL−−
−QPAWLE−
QMPAWL−−
QAPAWL−−
QPPAWL−−
−QPAWLN−
−QPAWLK−
QIPAWL−−
QYPAWL−−
QVPAWL−−
−QPAWLW−
−QPAWLF−
−QPAWLD−
QLPAWL−−
QHPAWL−−
QGPAWL−−
QDPAWL−−
−RPAWLK−
−RPAWLS−
−RPAWLN−
−RPAWLT−
RSPAWL−−
RGPAWL−−
RNPAWL−−
−RPAWLY−
−RPAWLE−
RLPAWL−−
RIPAWL−−
RHPAWL−−
RYPAWL−−
−RPAWLG−
RMPAWL−−
−RPAWLI−
−RPAWLH−
RAPAWL−−
RWPAWL−−
RTPAWL−−
RQPAWL−−
REPAWL−−
−RPAWLV−
RDPAWL−−
−RPAWLA−
RCPAWL−−
−RPAWLM−
−SPAWLR−
SSPAWL−−

−SPAWLG−
−SPAWLS−
SAPAWL−−
−SPAWLQ−
SVPAWL−−
−SPAWLV−
STPAWL−−
SKPAWL−−
SYPAWL−−
SRPAWL−−
SNPAWL−−
SHPAWL−−
−SPAWLM−
−SPAWLK−
SLPAWL−−
−SPAWLT−
−SPAWLL−
SIPAWL−−
−SPAWLY−
−SPAWLA−
−SPAWLD−
SQPAWL−−
SEPAWL−−
SDPAWL−−
−SPAWLI−
−SPAWLH−
SPPAWL−−
SMPAWL−−
−SPAWLE−
−SPAWLN−
SCPAWL−−
−TPAWLV−
−TPAWLG−
−TPAWLL−
−TPAWLK−
TAPAWL−−
−TPAWLH−
−TPAWLS−
−TPAWLN−
−TPAWLT−
TVPAWL−−
TNPAWL−−
TEPAWL−−
−TPAWLW−
−TPAWLQ−
−TPAWLM−
−TPAWLE−
TTPAWL−−
−TPAWLY−
−TPAWLA−

THPAWL--
TPPAWL--
-TPAWLI-
TKPAWL--
TDPAWL--
TWPAWL--
TQPAWL--
-TPAWLF-
-TPAWLD-
TIPAWL--
VSPAWL--
-VPAWLR-
VHPAWL--
-VPAWLG-
-VPAWLS-
-VPAWLA-
VNPAWL--
-VPAWLI-
-VPAWLD-
-VPAWLL-
VLPAWL--
-VPAWLV-
-VPAWLE-
VDPAWL--
VIPAWL--
VQPAWL--
VTPAWL--
VPPAWL--
-VPAWLK-
VMPAWL--
VWPAWL--
VEPAWL--
VCPAWL--
-VPAWLM-
-VPAWLH-
VGPAWL--
VFPAWL--
VAPAWL--
WTPAWL--
WAPAWL--
WNPAWL--
-WPAWLR-
WDPAWL--
WSPAWL--
WPPAWL--
-WPAWLT-
-WPAWLS-
WLPAWL--
-WPAWLV-
-WPAWLD-

| | | | |
|---|---|---|---|
| | WCPAWL-- | | |
| | WYPAWL-- | | |
| | -WPAWLK- | | |
| | -WPAWLG- | | |
| | WVPAWL-- | | |
| | WQPAWL-- | | |
| | -WPAWLN- | | |
| | -WPAWLM- | | |
| | YGPAWL-- | | |
| | -YPAWLG- | | |
| | -YPAWLK- | | |
| | YMPAWL-- | | |
| | -YPAWLQ- | | |
| | YFPAWL-- | | |
| | YPPAWL-- | | |
| | -YPAWLL- | | |
| | YNPAWL-- | | |
| | YKPAWL-- | | |
| | YIPAWL-- | | |
| | YHPAWL-- | | |
| | YVPAWL-- | | |
| | YQPAWL-- | | |
| | -YPAWLV- | | |
| | -YPAWLM- | | |
| | -YPAWLD- | | |
| | -YPAWLA- | | |
| | YEPAWL-- | | |
| | YWPAWL-- | | |
| | YTPAWL-- | | |
| | -YPAWLT- | | |
| | -YPAWLS- | | |
| | -YPAWLH- | | |
| PMGL | 13 | 858 | 34.79 |
| | -LPMGLG- | | |
| | --PMGLGM | | |
| | --PMGLEL | | |
| | -APMGLF- | | |
| | -APMGLL- | | |
| | ANPMGL-- | | |
| | AAPMGL-- | | |
| | ALPMGL-- | | |
| | AYPMGL-- | | |
| | AGPMGL-- | | |
| | ASPMGL-- | | |
| | AVPMGL-- | | |
| | ATPMGL-- | | |
| | AWPMGL-- | | |
| | ARPMGL-- | | |
| | AFPMGL-- | | |
| | APPMGL-- | | |

-APMGLH-
AKPMGL--
-APMGLK-
AEPMGL--
-APMGLI-
-APMGLR-
-APMGLQ-
-APMGLV-
-APMGLE-
-APMGLT-
-APMGLY-
-APMGLW-
-APMGLM-
AMPMGL--
AHPMGL--
ADPMGL--
-APMGLD-
AIPMGL--
ACPMGL--
CRPMGL--
CTPMGL--
CSPMGL--
-CPMGLV-
CFPMGL--
CVPMGL--
-CPMGLS-
-CPMGLN-
CAPMGL--
CPPMGL--
-CPMGLE-
CLPMGL--
CDPMGL--
CCPMGL--
-CPMGLY-
-CPMGLT-
-CPMGLI-
CNPMGL--
DRPMGL--
-DPMGLS-
DKPMGL--
DSPMGL--
-DPMGLR-
-DPMGLK-
-DPMGLT-
-DPMGLA-
DWPMGL--
DVPMGL--
-DPMGLY-
DNPMGL--
DMPMGL--

```
DLPMGL--
DGPMGL--
DFPMGL--
-DPMGLW-
DIPMGL--
DAPMGL--
DTPMGL--
-DPMGLQ-
-DPMGLI-
DHPMGL--
DEPMGL--
DYPMGL--
-DPMGLV-
-DPMGLN-
-DPMGLM-
-DPMGLG-
-EPMGLK-
-EPMGLW-
-EPMGLR-
EVPMGL--
-EPMGLL-
ESPMGL--
EHPMGL--
EFPMGL--
-EPMGLA-
-EPMGLT-
EWPMGL--
ERPMGL--
-EPMGLV-
EMPMGL--
EAPMGL--
-EPMGLY-
-EPMGLE-
-EPMGLQ-
-EPMGLG-
ENPMGL--
EIPMGL--
EDPMGL--
EYPMGL--
-EPMGLS-
-EPMGLM-
-EPMGLI-
-EPMGLH-
EGPMGL--
EEPMGL--
ECPMGL--
-FPMGLL-
FSPMGL--
FFPMGL--
-FPMGLA-
```

FLPMGL--
FNPMGL--
FGPMGL--
-FPMGLY-
-FPMGLS-
-FPMGLI-
FYPMGL--
FQPMGL--
-FPMGLK-
FVPMGL--
-FPMGLT-
FDPMGL--
FEPMGL--
-FPMGLR-
-FPMGLM-
-FPMGLG-
FIPMGL--
FRPMGL--
FPPMGL--
FHPMGL--
FWPMGL--
-FPMGLQ-
-FPMGLN-
-FPMGLE-
-FPMGLW-
FMPMGL--
FTPMGL--
-FPMGLF-
-FPMGLC-
FCPMGL--
-GPMGLR-
-GPMGLS-
GVPMGL--
GSPMGL--
GLPMGL--
GYPMGL--
GKPMGL--
GTPMGL--
GEPMGL--
-GPMGLM-
GWPMGL--
GDPMGL--
-GPMGLK-
-GPMGLH-
GNPMGL--
-GPMGLT-
-GPMGLI-
-GPMGLA-
GMPMGL--
GIPMGL--

GAPMGL--
-GPMGLY-
-GPMGLG-
-GPMGLQ-
-GPMGLN-
GQPMGL--
-GPMGLF-
GGPMGL--
GHPMGL--
-GPMGLW-
-GPMGLE-
GFPMGL--
GCPMGL--
HRPMGL--
-HPMGLS-
-HPMGLA-
-HPMGLL-
-HPMGLW-
HAPMGL--
HLPMGL--
HIPMGL--
HEPMGL--
HVPMGL--
-HPMGLR-
-HPMGLI-
HKPMGL--
HFPMGL--
HYPMGL--
-HPMGLF-
HNPMGL--
HMPMGL--
-HPMGLN-
-HPMGLV-
-HPMGLT-
-HPMGLH-
HTPMGL--
-HPMGLY-
-HPMGLQ-
-HPMGLK-
HWPMGL--
-HPMGLM-
HSPMGL--
HPPMGL--
-HPMGLG-
HHPMGL--
-HPMGLE-
HGPMGL--
HDPMGL--
HCPMGL--
IRPMGL--

```
-IPMGLY-
-IPMGLA-
IYPMGL--
IKPMGL--
-IPMGLR-
ITPMGL--
ILPMGL--
-IPMGLK-
ISPMGL--
IVPMGL--
IPPMGL--
-IPMGLT-
-IPMGLE-
-IPMGLS-
IHPMGL--
IIPMGL--
IAPMGL--
IFPMGL--
-IPMGLN-
-IPMGLM-
-IPMGLH-
IWPMGL--
IQPMGL--
INPMGL--
IGPMGL--
-IPMGLW-
-IPMGLF-
-IPMGLG-
IDPMGL--
IEPMGL--
ICPMGL--
KRPMGL--
KLPMGL--
KVPMGL--
-KPMGLG-
-KPMGLL-
-KPMGLY-
-KPMGLI-
KHPMGL--
KYPMGL--
KGPMGL--
KWPMGL--
KPPMGL--
-KPMGLM-
-KPMGLK-
KMPMGL--
-KPMGLN-
KNPMGL--
KTPMGL--
KEPMGL--
```

-KPMGLQ-
-KPMGLH-
KKPMGL--
KFPMGL--
KAPMGL--
-KPMGLT-
KDPMGL--
-KPMGLW-
-KPMGLF-
-KPMGLA-
KIPMGL--
KCPMGL--
LYPMGL--
LSPMGL--
LGPMGL--
LRPMGL--
-LPMGLR-
-LPMGLS-
LLPMGL--
LWPMGL--
-LPMGLI-
-LPMGLT-
-LPMGLK-
-LPMGLV-
LVPMGL--
-LPMGLN-
LPPMGL--
LMPMGL--
LNPMGL--
LAPMGL--
-LPMGLQ-
LTPMGL--
-LPMGLA-
-LPMGLM-
LFPMGL--
-LPMGLY-
LEPMGL--
-LPMGLH-
LDPMGL--
LQPMGL--
LCPMGL--
-LPMGLE-
-LPMGLW-
LIPMGL--
MFPMGL--
MRPMGL--
MPPMGL--
-MPMGLS-
-MPMGLH-
MSPMGL--

MWPMGL--
MLPMGL--
MVPMGL--
MTPMGL--
-MPMGLF-
-MPMGLR-
-MPMGLN-
MHPMGL--
-MPMGLI-
MGPMGL--
MAPMGL--
MQPMGL--
-MPMGLK-
-MPMGLW-
-MPMGLV-
-MPMGLE-
MDPMGL--
MYPMGL--
-MPMGLG-
MMPMGL--
MKPMGL--
-MPMGLT-
-MPMGLM-
-MPMGLD-
-MPMGLA-
MEPMGL--
MCPMGL--
NRPMGL--
NLPMGL--
NGPMGL--
-NPMGLK-
NNPMGL--
NSPMGL--
-NPMGLW-
NMPMGL--
NIPMGL--
NWPMGL--
NYPMGL--
-NPMGLI-
NKPMGL--
NQPMGL--
-NPMGLS-
-NPMGLE-
NAPMGL--
NFPMGL--
-NPMGLR-
-NPMGLN-
-NPMGLV-
NTPMGL--
NPPMGL--

```
-NPMGLY-
-NPMGLT-
-NPMGLQ-
-NPMGLH-
NHPMGL--
-NPMGLG-
-NPMGLA-
NCPMGL--
NVPMGL--
-NPMGLM-
NEPMGL--
--PMGLRG
--PMGLRA
--PMGLGS
--PMGLVR
--PMGLRV
--PMGLRL
--PMGLSS
--PMGLWR
--PMGLVL
--PMGLSR
--PMGLLS
--PMGLRS
--PMGLLK
--PMGLVV
--PMGLVS
--PMGLSL
--PMGLRH
--PMGLMT
--PMGLRT
--PMGLFS
--PMGLVF
--PMGLTK
--PMGLYG
--PMGLYR
--PMGLSV
--PMGLMS
--PMGLVA
--PMGLSD
--PMGLRN
--PMGLRM
--PMGLLT
--PMGLLD
--PMGLKA
--PMGLVG
--PMGLSA
--PMGLKL
--PMGLHL
--PMGLTM
--PMGLSF
```

--PMGLIS
--PMGLIG
--PMGLHF
--PMGLAK
--PMGLTS
--PMGLST
--PMGLRW
--PMGLNI
--PMGLMH
--PMGLLH
--PMGLLA
--PMGLTA
--PMGLSK
--PMGLSG
--PMGLKY
--PMGLAR
--PMGLWG
--PMGLVY
--PMGLTT
--PMGLRI
--PMGLNS
--PMGLTN
--PMGLSM
--PMGLQI
PHPMGL--
PAPMGL--
PSPMGL--
--PMGLLW
--PMGLKF
--PMGLII
--PMGLHY
--PMGLHG
--PMGLFA
PRPMGL--
--PMGLVQ
--PMGLTF
--PMGLRF
--PMGLRE
--PMGLNL
--PMGLMV
--PMGLID
--PMGLHS
--PMGLFN
--PMGLEG
--PMGLAS
--PMGLWT
--PMGLVI
--PMGLTV
--PMGLTL
--PMGLTG

--PMGLNA
--PMGLKS
--PMGLGL
--PMGLFK
--PMGLAY
--PMGLAM
--PMGLYS
--PMGLYK
--PMGLYE
--PMGLVW
--PMGLTR
--PMGLRD
--PMGLNK
--PMGLND
--PMGLLQ
--PMGLLM
--PMGLKV
--PMGLKI
--PMGLIH
--PMGLHM
--PMGLAA
PGPMGL--
PFPMGL--
--PMGLVT
--PMGLVD
--PMGLSN
--PMGLRQ
--PMGLQL
--PMGLNV
--PMGLIN
--PMGLIL
--PMGLHV
--PMGLHT
--PMGLGV
--PMGLGT
--PMGLET
--PMGLES
--PMGLER
--PMGLEA
--PMGLDA
--PMGLAH
--PMGLAG
--PMGLAF
--PMGLYQ
--PMGLYH
--PMGLWK
--PMGLTQ
--PMGLTH
--PMGLTE
--PMGLRY

--PMGLQS
--PMGLQA
--PMGLNY
--PMGLNM
--PMGLKR
--PMGLKN
--PMGLIK
--PMGLHN
--PMGLGA
--PMGLFQ
--PMGLFG
PVPMGL--
PTPMGL--
--PMGLVN
--PMGLVE
--PMGLTD
--PMGLSW
--PMGLSH
--PMGLQV
--PMGLNT
--PMGLMY
--PMGLMR
--PMGLMA
--PMGLKG
--PMGLIY
--PMGLHQ
--PMGLHK
--PMGLHI
--PMGLHH
--PMGLGR
--PMGLGG
--PMGLGF
--PMGLFF
--PMGLAV
--PMGLAN
PKPMGL--
PIPMGL--
--PMGLTW
--PMGLQR
--PMGLQN
--PMGLNN
--PMGLMK
--PMGLMD
--PMGLKQ
--PMGLKD
--PMGLIE
--PMGLHW
--PMGLFD
PDPMGL--
PYPMGL--

PNPMGL--
PMPMGL--
--PMGLWH
--PMGLVM
--PMGLQM
--PMGLQH
--PMGLQG
--PMGLPL
--PMGLMN
--PMGLMG
--PMGLLE
--PMGLKW
--PMGLKH
--PMGLIM
--PMGLGW
--PMGLAT
--PMGLAI
PEPMGL--
PWPMGL--
--PMGLYN
--PMGLYD
--PMGLYA
--PMGLWN
--PMGLWM
--PMGLWD
--PMGLRP
--PMGLQW
--PMGLQK
--PMGLQF
--PMGLPF
--PMGLNR
--PMGLNH
--PMGLMM
--PMGLMI
--PMGLMF
--PMGLKE
--PMGLIV
--PMGLIA
--PMGLHA
--PMGLGI
--PMGLFH
--PMGLFE
--PMGLEM
--PMGLEI
--PMGLAD
PLPMGL--
PQPMGL--
--PMGLWW
--PMGLWQ
--PMGLTC

--PMGLSQ
--PMGLSE
--PMGLRC
--PMGLQQ
--PMGLQD
--PMGLNQ
--PMGLMW
--PMGLME
--PMGLKM
--PMGLIQ
--PMGLGY
--PMGLGP
--PMGLEY
--PMGLEW
--PMGLEH
--PMGLDG
--PMGLCV
--PMGLCT
--PMGLCG
--PMGLAQ
QRPMGL--
QGPMGL--
QLPMGL--
-QPMGLN-
-QPMGLI-
-QPMGLR-
-QPMGLY-
-QPMGLW-
QPPMGL--
QTPMGL--
QSPMGL--
-QPMGLK-
QIPMGL--
QFPMGL--
QAPMGL--
QWPMGL--
-QPMGLT-
-QPMGLG-
QEPMGL--
QYPMGL--
-QPMGLS-
-QPMGLH-
-QPMGLE-
QHPMGL--
QVPMGL--
QQPMGL--
-QPMGLL-
-QPMGLV-
-QPMGLQ-
QNPMGL--

QDPMGL--
QKPMGL--
QCPMGL--
RLPMGL--
RSPMGL--
RMPMGL--
-RPMGLL-
RGPMGL--
RYPMGL--
-RPMGLM-
-RPMGLT-
RIPMGL--
RHPMGL--
RVPMGL--
-RPMGLA-
-RPMGLE-
RKPMGL--
RFPMGL--
RPPMGL--
REPMGL--
-RPMGLV-
-RPMGLS-
-RPMGLW-
RDPMGL--
-RPMGLK-
-RPMGLH-
RAPMGL--
-RPMGLI-
RWPMGL--
-RPMGLG-
-RPMGLF-
RQPMGL--
-RPMGLD-
RCPMGL--
SVPMGL--
-SPMGLS-
SSPMGL--
-SPMGLV-
SRPMGL--
SHPMGL--
-SPMGLR-
SYPMGL--
-SPMGLH-
STPMGL--
-SPMGLG-
-SPMGLM-
-SPMGLY-
SAPMGL--
SFPMGL--
SDPMGL--

SPPMGL--
-SPMGLK-
SGPMGL--
-SPMGLW-
SKPMGL--
SIPMGL--
SWPMGL--
-SPMGLQ-
SNPMGL--
SQPMGL--
SEPMGL--
-SPMGLE-
SMPMGL--
SCPMGL--
TSPMGL--
-TPMGLR-
-TPMGLS-
-TPMGLY-
TAPMGL--
TYPMGL--
-TPMGLN-
-TPMGLL-
TGPMGL--
-TPMGLG-
TPPMGL--
TLPMGL--
TWPMGL--
-TPMGLV-
THPMGL--
TIPMGL--
-TPMGLF-
-TPMGLA-
TMPMGL--
-TPMGLT-
TNPMGL--
TTPMGL--
-TPMGLQ-
-TPMGLH-
TKPMGL--
TQPMGL--
-TPMGLW-
-TPMGLK-
-TPMGLI-
TFPMGL--
-TPMGLE-
TEPMGL--
TCPMGL--
TDPMGL--
VKPMGL--
-VPMGLE-

VLPMGL--
-VPMGLL-
VSPMGL--
-VPMGLH-
-VPMGLG-
-VPMGLF-
VTPMGL--
VGPMGL--
VRPMGL--
-VPMGLW-
VIPMGL--
VMPMGL--
-VPMGLK-
VNPMGL--
VHPMGL--
VVPMGL--
-VPMGLV-
VAPMGL--
-VPMGLY-
-VPMGLM-
-VPMGLI-
VFPMGL--
VYPMGL--
-VPMGLS-
-VPMGLQ-
-VPMGLN-
-VPMGLA-
VEPMGL--
VWPMGL--
VQPMGL--
VCPMGL--
WGPMGL--
-WPMGLR-
-WPMGLK-
WLPMGL--
-WPMGLL-
WIPMGL--
WFPMGL--
-WPMGLV-
-WPMGLM-
-WPMGLH-
WRPMGL--
-WPMGLA-
-WPMGLY-
-WPMGLS-
WNPMGL--
WDPMGL--
WAPMGL--
WTPMGL--
-WPMGLQ-

| | | | | |
|---|---|---|---|---|
| | WEPMGL-- | | | |
| | WWPMGL-- | | | |
| | -WPMGLI- | | | |
| | -WPMGLE- | | | |
| | WQPMGL-- | | | |
| | -WPMGLN- | | | |
| | -WPMGLG- | | | |
| | WMPMGL-- | | | |
| | YRPMGL-- | | | |
| | -YPMGLK- | | | |
| | YTPMGL-- | | | |
| | YVPMGL-- | | | |
| | -YPMGLR- | | | |
| | -YPMGLA- | | | |
| | -YPMGLL- | | | |
| | -YPMGLV- | | | |
| | -YPMGLS- | | | |
| | YKPMGL-- | | | |
| | YEPMGL-- | | | |
| | YSPMGL-- | | | |
| | -YPMGLT- | | | |
| | -YPMGLH- | | | |
| | -YPMGLF- | | | |
| | YGPMGL-- | | | |
| | YAPMGL-- | | | |
| | YLPMGL-- | | | |
| | YHPMGL-- | | | |
| | -YPMGLG- | | | |
| | YQPMGL-- | | | |
| | YMPMGL-- | | | |
| | YYPMGL-- | | | |
| | YDPMGL-- | | | |
| | YWPMGL-- | | | |
| | -YPMGLY- | | | |
| | -YPMGLQ- | | | |
| | -YPMGLM- | | | |
| | -YPMGLE- | | | |
| | -YPMGLC- | | | |
| | YNPMGL-- | | | |
| | YIPMGL-- | | | |
| | YCPMGL-- | | | |
| PRGW | 18 | 848 | 34.73 | |
| | AQPRGW-- | | | |
| | --PRGWER | | | |
| | -SPRGWD- | | | |
| | -APRGWL- | | | |
| | -APRGWK- | | | |
| | -APRGWS- | | | |
| | -APRGWT- | | | |
| | AVPRGW-- | | | |

ASPRGW--
-APRGWR-
-APRGWI-
-APRGWH-
-APRGWQ-
-APRGWG-
-APRGWA-
-APRGWF-
AEPRGW--
AAPRGW--
AWPRGW--
-APRGWY-
ACPRGW--
APPRGW--
AIPRGW--
AGPRGW--
ARPRGW--
-APRGWV-
-APRGWE-
ANPRGW--
ADPRGW--
-APRGWM-
-APRGWC-
AFPRGW--
CVPRGW--
CGPRGW--
CAPRGW--
CQPRGW--
-CPRGWR-
-CPRGWK-
CCPRGW--
CYPRGW--
CTPRGW--
CSPRGW--
CRPRGW--
-CPRGWY-
-CPRGWS-
-CPRGWP-
-CPRGWL-
-CPRGWI-
CNPRGW--
CIPRGW--
-DPRGWT-
DIPRGW--
DQPRGW--
DWPRGW--
DTPRGW--
-DPRGWY-
-DPRGWR-
-DPRGWS-

DFPRGW--
DYPRGW--
DRPRGW--
DGPRGW--
-DPRGWN-
-DPRGWW-
DLPRGW--
-DPRGWF-
DMPRGW--
DAPRGW--
DVPRGW--
-DPRGWV-
-DPRGWK-
-DPRGWH-
-DPRGWG-
-DPRGWA-
DNPRGW--
DHPRGW--
DCPRGW--
-EPRGWS-
ESPRGW--
-EPRGWL-
-EPRGWG-
EMPRGW--
ENPRGW--
ERPRGW--
-EPRGWW-
EVPRGW--
-EPRGWI-
-EPRGWN-
EIPRGW--
EYPRGW--
-EPRGWM-
-EPRGWK-
EGPRGW--
-EPRGWY-
ECPRGW--
-EPRGWT-
-EPRGWA-
EFPRGW--
ETPRGW--
EKPRGW--
EHPRGW--
EAPRGW--
EQPRGW--
-EPRGWQ-
EDPRGW--
-FPRGWL-
-FPRGWK-
FSPRGW--

FYPRGW--
-FPRGWR-
FNPRGW--
-FPRGWY-
FLPRGW--
-FPRGWT-
FAPRGW--
FQPRGW--
-FPRGWI-
FVPRGW--
-FPRGWN-
-FPRGWA-
FGPRGW--
-FPRGWV-
FFPRGW--
FRPRGW--
FPPRGW--
FHPRGW--
FTPRGW--
-FPRGWW-
-FPRGWE-
FMPRGW--
FIPRGW--
FEPRGW--
FDPRGW--
-FPRGWQ-
-FPRGWH-
-FPRGWG-
-FPRGWF-
FKPRGW--
-GPRGWL-
-GPRGWA-
GVPRGW--
-GPRGWS-
GSPRGW--
-GPRGWR-
-GPRGWG-
GNPRGW--
GIPRGW--
-GPRGWW-
GKPRGW--
GGPRGW--
GDPRGW--
-GPRGWH-
-GPRGWQ-
-GPRGWI-
GLPRGW--
GYPRGW--
-GPRGWY-
-GPRGWV-

-GPRGWF-
-GPRGWT-
-GPRGWN-
GMPRGW--
GHPRGW--
GEPRGW--
GTPRGW--
GAPRGW--
GWPRGW--
GRPRGW--
-GPRGWK-
GPPRGW--
GCPRGW--
-GPRGWE-
-HPRGWH-
-HPRGWL-
-HPRGWI-
HGPRGW--
HLPRGW--
-HPRGWM-
-HPRGWN-
HVPRGW--
-HPRGWW-
HTPRGW--
-HPRGWR-
HSPRGW--
HQPRGW--
-HPRGWV-
-HPRGWS-
-HPRGWK-
HMPRGW--
HHPRGW--
HYPRGW--
HRPRGW--
-HPRGWQ-
HFPRGW--
HWPRGW--
-HPRGWF-
-HPRGWE-
-HPRGWA-
HEPRGW--
-HPRGWG-
HPPRGW--
HNPRGW--
HKPRGW--
HIPRGW--
-IPRGWS-
ISPRGW--
IKPRGW--
-IPRGWR-

ILPRGW--
-IPRGWL-
IQPRGW--
-IPRGWN-
IWPRGW--
-IPRGWV-
IDPRGW--
IIPRGW--
-IPRGWE-
INPRGW--
IVPRGW--
IRPRGW--
-IPRGWA-
IPPRGW--
ITPRGW--
-IPRGWF-
IMPRGW--
IGPRGW--
-IPRGWY-
IAPRGW--
-IPRGWW-
-IPRGWQ-
IHPRGW--
IEPRGW--
-KPRGWY-
-KPRGWL-
-KPRGWS-
-KPRGWR-
KGPRGW--
-KPRGWA-
KWPRGW--
KVPRGW--
-KPRGWW-
-KPRGWQ-
-KPRGWI-
KAPRGW--
-KPRGWN-
-KPRGWT-
KHPRGW--
KFPRGW--
KTPRGW--
KMPRGW--
-KPRGWV-
-KPRGWF-
KEPRGW--
KIPRGW--
KYPRGW--
-KPRGWK-
-KPRGWH-
KRPRGW--

KQPRGW--
-KPRGWM-
-KPRGWE-
KPPRGW--
KDPRGW--
-LPRGWR-
-LPRGWL-
-LPRGWS-
LTPRGW--
LKPRGW--
LRPRGW--
LSPRGW--
-LPRGWK-
LYPRGW--
-LPRGWM-
-LPRGWI-
LLPRGW--
LFPRGW--
LGPRGW--
-LPRGWT-
-LPRGWA-
LEPRGW--
LNPRGW--
-LPRGWY-
-LPRGWW-
-LPRGWQ-
LMPRGW--
-LPRGWF-
LVPRGW--
LDPRGW--
LWPRGW--
LHPRGW--
LCPRGW--
LAPRGW--
LQPRGW--
-LPRGWE-
-MPRGWR-
MVPRGW--
MTPRGW--
-MPRGWE-
MSPRGW--
MRPRGW--
-MPRGWK-
MHPRGW--
MLPRGW--
-MPRGWS-
MNPRGW--
-MPRGWT-
-MPRGWI-
MQPRGW--

```
-MPRGWY-
-MPRGWF-
-MPRGWV-
MMPRGW--
MFPRGW--
MYPRGW--
-MPRGWM-
MPPRGW--
MKPRGW--
MIPRGW--
MGPRGW--
MAPRGW--
MWPRGW--
-MPRGWW-
-MPRGWQ-
-MPRGWN-
MEPRGW--
MDPRGW--
MCPRGW--
-NPRGWL-
-NPRGWV-
-NPRGWS-
-NPRGWI-
NGPRGW--
-NPRGWK-
NVPRGW--
-NPRGWY-
NMPRGW--
NWPRGW--
NHPRGW--
-NPRGWM-
-NPRGWR-
-NPRGWF-
NFPRGW--
-NPRGWW-
NAPRGW--
NTPRGW--
NYPRGW--
NIPRGW--
NEPRGW--
NQPRGW--
-NPRGWT-
-NPRGWN-
-NPRGWH-
-NPRGWA-
NKPRGW--
NDPRGW--
--PRGWSL
--PRGWQS
--PRGWLR
```

--PRGWTL
--PRGWSS
--PRGWLN
--PRGWRS
--PRGWYR
--PRGWLT
--PRGWGS
--PRGWSN
--PRGWSF
--PRGWLV
--PRGWIL
--PRGWMS
--PRGWSV
--PRGWFW
--PRGWRH
--PRGWLI
--PRGWKF
--PRGWHV
--PRGWFT
--PRGWKT
--PRGWAS
--PRGWSM
--PRGWNL
--PRGWWR
--PRGWVL
--PRGWVV
--PRGWVS
--PRGWTK
--PRGWLH
--PRGWKL
--PRGWIS
--PRGWVT
--PRGWTA
--PRGWSA
PGPRGW--
--PRGWVA
--PRGWTY
--PRGWRT
--PRGWQT
--PRGWQI
--PRGWMI
--PRGWLM
--PRGWIT
--PRGWAY
--PRGWAI
--PRGWTT
--PRGWSW
--PRGWSK
--PRGWHL
--PRGWES

--PRGWYH
--PRGWYG
--PRGWTF
--PRGWTD
--PRGWSD
--PRGWII
--PRGWHM
--PRGWFY
--PRGWAT
--PRGWAR
PWPRGW--
PVPRGW--
PTPRGW--
--PRGWYY
--PRGWYS
--PRGWTH
--PRGWST
--PRGWSG
--PRGWRY
--PRGWRQ
--PRGWRP
--PRGWLW
--PRGWKS
--PRGWVN
--PRGWTR
--PRGWRF
--PRGWQL
--PRGWFD
--PRGWWS
--PRGWVH
--PRGWTW
--PRGWSQ
--PRGWRW
--PRGWMK
--PRGWLY
--PRGWLK
--PRGWHS
--PRGWHD
--PRGWFS
--PRGWFR
--PRGWAN
--PRGWRV
--PRGWRN
--PRGWRD
--PRGWQR
--PRGWNF
--PRGWIN
--PRGWIK
--PRGWHK
--PRGWYN

--PRGWWE
--PRGWTN
--PRGWTG
--PRGWSI
--PRGWNT
--PRGWMA
--PRGWKN
--PRGWKI
--PRGWKG
--PRGWIF
--PRGWHG
--PRGWWI
--PRGWWA
--PRGWVW
--PRGWSE
--PRGWRE
--PRGWMV
--PRGWMN
--PRGWLL
--PRGWLF
--PRGWIV
--PRGWID
--PRGWHH
--PRGWFN
--PRGWEY
--PRGWAV
PYPRGW--
--PRGWYV
--PRGWYI
--PRGWWY
--PRGWVG
--PRGWTE
--PRGWSH
--PRGWQQ
--PRGWQE
--PRGWNG
--PRGWLQ
--PRGWLD
--PRGWKM
--PRGWGI
--PRGWFQ
--PRGWFK
--PRGWFH
--PRGWEA
--PRGWYW
--PRGWYK
--PRGWYA
--PRGWWW
--PRGWWL
--PRGWVY

--PRGWVK
--PRGWTM
--PRGWRG
--PRGWQV
--PRGWQF
--PRGWNM
--PRGWNA
--PRGWMM
--PRGWKY
--PRGWKQ
--PRGWIQ
--PRGWIE
--PRGWIA
--PRGWHT
--PRGWHN
--PRGWHI
--PRGWGY
--PRGWFV
--PRGWFL
--PRGWFA
--PRGWAK
PIPRGW--
--PRGWYF
--PRGWWD
--PRGWVQ
--PRGWTQ
--PRGWTI
--PRGWRM
--PRGWQA
--PRGWNY
--PRGWNN
--PRGWMW
--PRGWMH
--PRGWKD
--PRGWKA
--PRGWHP
--PRGWGW
--PRGWGL
--PRGWEW
--PRGWDS
--PRGWAM
--PRGWAH
--PRGWAG
--PRGWAE
PNPRGW--
PLPRGW--
--PRGWYE
--PRGWWV
--PRGWWQ
--PRGWWH

--PRGWWF
--PRGWVE
--PRGWVC
--PRGWNQ
--PRGWNK
--PRGWMF
--PRGWMD
--PRGWKW
--PRGWIY
--PRGWIM
--PRGWIH
--PRGWHE
--PRGWGR
--PRGWGK
--PRGWFI
--PRGWAQ
PQPRGW--
PFPRGW--
PEPRGW--
PSPRGW--
PRPRGW--
--PRGWYT
--PRGWYP
--PRGWRA
--PRGWQY
--PRGWQN
--PRGWQG
--PRGWQD
--PRGWNH
--PRGWMT
--PRGWLE
--PRGWKE
--PRGWIW
--PRGWGN
--PRGWGA
--PRGWEV
--PRGWAF
--PRGWAD
PKPRGW--
PDPRGW--
PAPRGW--
--PRGWWK
--PRGWSY
--PRGWSP
--PRGWQW
--PRGWPS
--PRGWNW
--PRGWNI
--PRGWNE
--PRGWMQ

--PRGWMP
--PRGWMG
--PRGWLC
--PRGWKH
--PRGWHY
--PRGWHQ
--PRGWHF
--PRGWGQ
--PRGWGM
--PRGWGG
--PRGWGF
--PRGWFG
--PRGWFE
--PRGWET
--PRGWEN
--PRGWEM
--PRGWDT
--PRGWDG
--PRGWCM
--PRGWAW
-QPRGWS-
-QPRGWI-
QVPRGW--
-QPRGWL-
-QPRGWR-
-QPRGWY-
-QPRGWQ-
-QPRGWF-
QLPRGW--
QRPRGW--
-QPRGWN-
-QPRGWK-
-QPRGWV-
-QPRGWA-
QHPRGW--
-QPRGWW-
-QPRGWM-
-QPRGWG-
-QPRGWE-
QPPRGW--
QQPRGW--
QEPRGW--
QDPRGW--
QWPRGW--
QTPRGW--
-QPRGWT-
QKPRGW--
QIPRGW--
QFPRGW--
QAPRGW--

-RPRGWL-
RSPRGW--
-RPRGWM-
RTPRGW--
-RPRGWT-
-RPRGWS-
RWPRGW--
-RPRGWN-
-RPRGWE-
RKPRGW--
-RPRGWQ-
RIPRGW--
-RPRGWW-
RVPRGW--
RMPRGW--
REPRGW--
RYPRGW--
-RPRGWY-
-RPRGWH-
RQPRGW--
RLPRGW--
-RPRGWF-
RHPRGW--
-RPRGWA-
-RPRGWV-
-RPRGWR-
RPPRGW--
-RPRGWK-
-RPRGWG-
RDPRGW--
-SPRGWL-
SLPRGW--
-SPRGWS-
SYPRGW--
-SPRGWV-
SGPRGW--
SFPRGW--
-SPRGWT-
-SPRGWG-
SAPRGW--
SNPRGW--
SEPRGW--
-SPRGWN-
-SPRGWI-
SWPRGW--
-SPRGWQ-
SHPRGW--
-SPRGWK-
-SPRGWY-
SVPRGW--

SMPRGW--
SKPRGW--
SIPRGW--
SDPRGW--
-SPRGWW-
-SPRGWM-
-SPRGWH-
SQPRGW--
STPRGW--
-SPRGWE-
SPPRGW--
TSPRGW--
TFPRGW--
TAPRGW--
-TPRGWR-
-TPRGWA-
TLPRGW--
-TPRGWT-
-TPRGWS-
TGPRGW--
TRPRGW--
-TPRGWV-
-TPRGWH-
-TPRGWI-
-TPRGWL-
TMPRGW--
TNPRGW--
TVPRGW--
-TPRGWW-
-TPRGWG-
TKPRGW--
TTPRGW--
-TPRGWQ-
TIPRGW--
TYPRGW--
-TPRGWN-
-TPRGWK-
THPRGW--
-TPRGWY-
-TPRGWE-
TWPRGW--
TEPRGW--
TQPRGW--
-TPRGWM-
-TPRGWF-
TDPRGW--
-VPRGWQ-
-VPRGWT-
-VPRGWL-
-VPRGWS-

VRPRGW--
-VPRGWR-
-VPRGWN-
-VPRGWH-
VFPRGW--
VYPRGW--
VVPRGW--
VNPRGW--
VSPRGW--
VGPRGW--
VWPRGW--
-VPRGWY-
VIPRGW--
VMPRGW--
-VPRGWK-
VPPRGW--
-VPRGWV-
-VPRGWI-
-VPRGWE-
VDPRGW--
VAPRGW--
-VPRGWW-
-VPRGWM-
VKPRGW--
VQPRGW--
-VPRGWG-
VEPRGW--
-VPRGWF-
-VPRGWA-
VHPRGW--
VCPRGW--
WSPRGW--
-WPRGWV-
-WPRGWI-
WLPRGW--
-WPRGWH-
-WPRGWS-
-WPRGWQ-
WIPRGW--
WEPRGW--
-WPRGWN-
-WPRGWL-
-WPRGWT-
WHPRGW--
WPPRGW--
WNPRGW--
WDPRGW--
WRPRGW--
-WPRGWR-
-WPRGWE-

WKPRGW--
WGPRGW--
WYPRGW--
WQPRGW--
WFPRGW--
WVPRGW--
-WPRGWM-
-WPRGWK-
-WPRGWG-
-YPRGWS-
YYPRGW--
YRPRGW--
-YPRGWY-
-YPRGWK-
-YPRGWL-
YLPRGW--
-YPRGWE-
-YPRGWF-
YTPRGW--
YGPRGW--
YVPRGW--
-YPRGWN-
YAPRGW--
YMPRGW--
YSPRGW--
-YPRGWR-
-YPRGWG-
YNPRGW--
YEPRGW--
-YPRGWH-
YHPRGW--
-YPRGWV-
-YPRGWM-
-YPRGWI-
YPPRGW--
YWPRGW--
-YPRGWQ-
YIPRGW--
-YPRGWC-
YDPRGW--

PSHL 7 897 34.71

-FPSHLG-
-HPSHLG-
--PSHLQV
--PSHLPL
--PSHLGN
--PSHLFL
-VPSHLY-
-YPSHLP-
ALPSHL--

-APSHLT-
-APSHLG-
-APSHLS-
-APSHLR-
ANPSHL--
AVPSHL--
-APSHLW-
-APSHLN-
-APSHLL-
-APSHLA-
-APSHLV-
-APSHLK-
AKPSHL--
AYPSHL--
AWPSHL--
-APSHLY-
ASPSHL--
AEPSHL--
AAPSHL--
-APSHLM-
AQPSHL--
-APSHLI-
ADPSHL--
-APSHLH-
-APSHLF-
-APSHLD-
AFPSHL--
-APSHLQ-
AMPSHL--
AHPSHL--
-APSHLE-
ACPSHL--
-CPSHLR-
CSPSHL--
CRPSHL--
-CPSHLS-
CTPSHL--
-CPSHLV-
CYPSHL--
CPPSHL--
CLPSHL--
CWPSHL--
-CPSHLY-
-CPSHLT-
-CPSHLN-
-CPSHLM-
-CPSHLI-
-CPSHLH-
CMPSHL--
CIPSHL--

CGPSHL--
CFPSHL--
CCPSHL--
CAPSHL--
DMPSHL--
-DPSHLR-
-DPSHLL-
DVPSHL--
DTPSHL--
DSPSHL--
DKPSHL--
DRPSHL--
DIPSHL--
DFPSHL--
-DPSHLT-
DGPSHL--
DHPSHL--
DAPSHL--
DYPSHL--
DWPSHL--
DNPSHL--
DCPSHL--
-DPSHLE-
-DPSHLW-
-DPSHLM-
-DPSHLK-
-DPSHLH-
-DPSHLA-
DEPSHL--
-DPSHLV-
-DPSHLS-
-DPSHLF-
DDPSHL--
ERPSHL--
-EPSHLR-
-EPSHLS-
-EPSHLL-
-EPSHLY-
EIPSHL--
-EPSHLT-
ELPSHL--
EAPSHL--
-EPSHLK-
-EPSHLA-
ESPSHL--
EMPSHL--
EVPSHL--
-EPSHLW-
ENPSHL--
-EPSHLQ-

EFPSHL--
EWPSHL--
ETPSHL--
-EPSHLV-
-EPSHLI-
-EPSHLF-
EKPSHL--
EGPSHL--
-EPSHLM-
-EPSHLG-
EHPSHL--
EPPSHL--
EDPSHL--
ECPSHL--
-FPSHLL-
-FPSHLR-
FLPSHL--
FDPSHL--
FTPSHL--
FGPSHL--
-FPSHLS-
FQPSHL--
-FPSHLT-
FVPSHL--
FKPSHL--
FFPSHL--
-FPSHLY-
-FPSHLQ-
FRPSHL--
FYPSHL--
FSPSHL--
-FPSHLK-
-FPSHLH-
FWPSHL--
-FPSHLN-
FIPSHL--
FHPSHL--
-FPSHLW-
FEPSHL--
-FPSHLA-
FNPSHL--
-FPSHLM-
-FPSHLF-
FPPSHL--
FCPSHL--
FAPSHL--
-GPSHLA-
GSPSHL--
-GPSHLS-
GRPSHL--

-GPSHLR-
GLPSHL--
GWPSHL--
GIPSHL--
-GPSHLY-
GYPSHL--
-GPSHLW-
-GPSHLT-
-GPSHLL-
GMPSHL--
GTPSHL--
GNPSHL--
GKPSHL--
GVPSHL--
GEPSHL--
GFPSHL--
GAPSHL--
-GPSHLN-
-GPSHLM-
GHPSHL--
GGPSHL--
-GPSHLF-
-GPSHLQ-
GCPSHL--
-GPSHLV-
GDPSHL--
-GPSHLK-
-GPSHLI-
-GPSHLH-
-GPSHLE-
-GPSHLD-
GPPSHL--
HRPSHL--
HWPSHL--
-HPSHLY-
HGPSHL--
-HPSHLT-
-HPSHLR-
HFPSHL--
HVPSHL--
HSPSHL--
-HPSHLK-
HEPSHL--
-HPSHLH-
HLPSHL--
-HPSHLL-
HNPSHL--
HAPSHL--
HKPSHL--
HIPSHL--

```
-HPSHLQ-
-HPSHLV-
-HPSHLM-
HHPSHL--
HDPSHL--
HTPSHL--
HQPSHL--
-HPSHLN-
-HPSHLA-
HPPSHL--
HMPSHL--
-HPSHLP-
-HPSHLF-
IRPSHL--
-IPSHLR-
-IPSHLF-
ISPSHL--
IVPSHL--
INPSHL--
ITPSHL--
-IPSHLL-
-IPSHLA-
IFPSHL--
-IPSHLK-
IQPSHL--
-IPSHLS-
IPPSHL--
ILPSHL--
IEPSHL--
IAPSHL--
IWPSHL--
-IPSHLI-
-IPSHLG-
-IPSHLY-
-IPSHLV-
IKPSHL--
IYPSHL--
-IPSHLW-
IMPSHL--
IDPSHL--
-IPSHLT-
-IPSHLQ-
-IPSHLH-
-IPSHLN-
IHPSHL--
-IPSHLM-
IIPSHL--
IGPSHL--
-IPSHLE-
KSPSHL--
```

KVPSHL--
KWPSHL--
KTPSHL--
KNPSHL--
-KPSHLV-
KAPSHL--
KDPSHL--
-KPSHLY-
-KPSHLS-
-KPSHLI-
-KPSHLG-
KKPSHL--
KLPSHL--
-KPSHLL-
KIPSHL--
KEPSHL--
-KPSHLT-
-KPSHLK-
-KPSHLA-
KHPSHL--
KGPSHL--
KFPSHL--
KRPSHL--
KPPSHL--
-KPSHLW-
KYPSHL--
KQPSHL--
-KPSHLM-
-KPSHLH-
-KPSHLE-
-KPSHLQ-
-KPSHLN-
-KPSHLF-
KMPSHL--
-LPSHLR-
LSPSHL--
LLPSHL--
LKPSHL--
-LPSHLS-
LMPSHL--
LYPSHL--
LTPSHL--
LAPSHL--
-LPSHLH-
LIPSHL--
LWPSHL--
-LPSHLK-
-LPSHLQ-
-LPSHLF-
LPPSHL--

LDPSHL--
-LPSHLI-
-LPSHLA-
LFPSHL--
-LPSHLL-
LNPSHL--
-LPSHLY-
LHPSHL--
-LPSHLE-
LEPSHL--
LQPSHL--
-LPSHLM-
-LPSHLN-
LCPSHL--
-LPSHLC-
MAPSHL--
-MPSHLS-
-MPSHLL-
MSPSHL--
MLPSHL--
MGPSHL--
MFPSHL--
MTPSHL--
-MPSHLV-
-MPSHLH-
MWPSHL--
-MPSHLR-
MNPSHL--
MIPSHL--
-MPSHLY-
-MPSHLK-
MRPSHL--
-MPSHLM-
-MPSHLQ-
-MPSHLI-
-MPSHLT-
-MPSHLG-
-MPSHLA-
MHPSHL--
MYPSHL--
MPPSHL--
MEPSHL--
MQPSHL--
-MPSHLN-
MMPSHL--
MKPSHL--
-MPSHLW-
MDPSHL--
-NPSHLR-
NTPSHL--

NLPSHL--
-NPSHLS-
NVPSHL--
NSPSHL--
-NPSHLK-
-NPSHLW-
NAPSHL--
NWPSHL--
NRPSHL--
-NPSHLF-
NMPSHL--
NIPSHL--
NQPSHL--
-NPSHLY-
-NPSHLQ-
-NPSHLM-
-NPSHLH-
-NPSHLA-
NYPSHL--
NNPSHL--
NGPSHL--
NEPSHL--
-NPSHLN-
-NPSHLI-
NKPSHL--
NFPSHL--
-NPSHLT-
NPPSHL--
NHPSHL--
-NPSHLV-
-NPSHLE-
NDPSHL--
NCPSHL--
--PSHLLR
--PSHLRS
--PSHLLS
PLPSHL--
--PSHLVR
--PSHLLG
--PSHLRR
--PSHLWT
--PSHLSG
--PSHLIR
--PSHLRV
--PSHLRT
--PSHLRF
--PSHLSS
--PSHLRN
--PSHLMK
--PSHLSM

PNPSHL--
--PSHLVG
--PSHLIL
--PSHLTK
--PSHLSV
--PSHLWV
--PSHLSI
--PSHLIS
PSPSHL--
--PSHLYK
--PSHLYG
--PSHLWN
--PSHLTL
--PSHLMS
--PSHLLN
--PSHLFK
--PSHLSK
--PSHLML
--PSHLLY
--PSHLLT
--PSHLLH
--PSHLWI
--PSHLVT
--PSHLST
--PSHLSR
--PSHLSH
--PSHLKR
--PSHLWE
--PSHLVS
--PSHLVK
--PSHLVH
--PSHLSD
--PSHLRD
--PSHLHH
--PSHLSY
--PSHLNA
--PSHLMR
--PSHLLK
--PSHLHS
--PSHLFM
--PSHLAR
--PSHLYV
--PSHLYS
--PSHLWL
--PSHLVM
--PSHLTI
--PSHLLI
--PSHLKV
PVPSHL--
--PSHLYF

--PSHLVV
--PSHLVI
--PSHLTS
--PSHLSQ
--PSHLRY
--PSHLNY
--PSHLLF
--PSHLKA
--PSHLHW
--PSHLFT
--PSHLAN
--PSHLYI
--PSHLYH
--PSHLVA
--PSHLTT
--PSHLQS
--PSHLQH
--PSHLKK
--PSHLES
--PSHLYT
--PSHLWD
--PSHLVN
--PSHLTY
--PSHLTA
--PSHLSA
--PSHLKN
--PSHLKI
--PSHLHA
--PSHLEA
--PSHLAS
PMPSHL--
PGPSHL--
--PSHLWS
--PSHLTM
--PSHLQR
--PSHLQI
--PSHLMG
--PSHLLW
--PSHLKT
--PSHLKQ
--PSHLKH
--PSHLII
--PSHLGS
--PSHLFN
--PSHLFG
--PSHLAF
--PSHLWR
--PSHLVQ
--PSHLVF
--PSHLTH

--PSHLNM
--PSHLNK
--PSHLMN
--PSHLLD
--PSHLIV
--PSHLIT
--PSHLIF
--PSHLGT
--PSHLFD
--PSHLET
--PSHLAH
-PPSHLW-
PIPSHL--
--PSHLYM
--PSHLTG
--PSHLQT
--PSHLQL
--PSHLMI
--PSHLLL
--PSHLKG
--PSHLIW
--PSHLIM
--PSHLHT
--PSHLGL
--PSHLFS
--PSHLFA
--PSHLAW
--PSHLAT
--PSHLAD
PRPSHL--
PYPSHL--
--PSHLYE
--PSHLYA
--PSHLSN
--PSHLMY
--PSHLMT
--PSHLME
--PSHLKL
--PSHLIY
--PSHLIG
--PSHLHN
--PSHLGV
--PSHLFI
--PSHLAI
PAPSHL--
--PSHLYW
--PSHLTN
--PSHLTD
--PSHLQW
--PSHLQA

--PSHLNL
--PSHLFQ
--PSHLEV
--PSHLEL
--PSHLYQ
--PSHLYN
--PSHLWK
--PSHLVY
--PSHLVD
--PSHLTW
--PSHLTF
--PSHLRM
--PSHLRH
--PSHLQK
--PSHLNW
--PSHLNV
--PSHLNT
--PSHLNS
--PSHLNI
--PSHLNF
--PSHLMW
--PSHLMV
--PSHLLQ
--PSHLKE
--PSHLHR
--PSHLGM
--PSHLGI
--PSHLFV
--PSHLER
--PSHLEF
--PSHLAG
PKPSHL--
PEPSHL--
PDPSHL--
PWPSHL--
--PSHLYY
--PSHLWQ
--PSHLWM
--PSHLWA
--PSHLVE
--PSHLTQ
--PSHLSE
--PSHLQN
--PSHLQM
--PSHLIQ
--PSHLHY
--PSHLHQ
--PSHLHM
--PSHLHL
--PSHLGG

--PSHLFE
--PSHLEN
--PSHLEK
--PSHLCR
--PSHLAY
--PSHLAM
PTPSHL--
--PSHLWG
--PSHLWF
--PSHLQQ
--PSHLQG
--PSHLQF
--PSHLQD
--PSHLNR
--PSHLNH
--PSHLMQ
--PSHLMH
--PSHLMA
--PSHLKY
--PSHLKM
--PSHLKD
--PSHLIE
--PSHLHF
--PSHLHE
--PSHLGY
--PSHLGW
--PSHLGF
--PSHLFW
--PSHLEY
--PSHLEM
--PSHLDS
--PSHLAV
--PSHLAA
PCPSHL--
--PSHLYD
--PSHLWY
--PSHLWC
--PSHLTE
--PSHLRC
--PSHLQE
--PSHLPY
--PSHLNP
--PSHLNG
--PSHLND
--PSHLKW
--PSHLKC
--PSHLIN
--PSHLIK
--PSHLIH
--PSHLID

--PSHLHV
--PSHLHD
--PSHLFY
--PSHLFH
--PSHLEW
--PSHLEQ
--PSHLDM
--PSHLDG
--PSHLDA
--PSHLCL
--PSHLAQ
--PSHLAC
PQPSHL--
-PPSHLG-
-QPSHLR-
-QPSHLK-
-QPSHLQ-
QLPSHL--
QVPSHL--
QRPSHL--
-QPSHLV-
QIPSHL--
QAPSHL--
QWPSHL--
QHPSHL--
QFPSHL--
-QPSHLW-
-QPSHLT-
QCPSHL--
QTPSHL--
QPPSHL--
QNPSHL--
QEPSHL--
-QPSHLL-
QKPSHL--
-QPSHLS-
-QPSHLN-
-QPSHLG-
QGPSHL--
QMPSHL--
QDPSHL--
QYPSHL--
-QPSHLM-
-QPSHLI-
-QPSHLH-
RLPSHL--
RSPSHL--
-RPSHLS-
RGPSHL--
RAPSHL--

RWPSHL--
-RPSHLK-
-RPSHLY-
RVPSHL--
-RPSHLM-
-RPSHLA-
RIPSHL--
-RPSHLT-
RHPSHL--
RNPSHL--
RYPSHL--
-RPSHLW-
-RPSHLV-
RKPSHL--
RMPSHL--
-RPSHLG-
-RPSHLF-
RFPSHL--
-RPSHLQ-
-RPSHLI-
RDPSHL--
-RPSHLH-
RPPSHL--
-RPSHLE-
RQPSHL--
-RPSHLC-
RCPSHL--
-SPSHLL-
SLPSHL--
SSPSHL--
SRPSHL--
SGPSHL--
SVPSHL--
-SPSHLS-
-SPSHLK-
SMPSHL--
-SPSHLG-
SNPSHL--
SIPSHL--
STPSHL--
-SPSHLI-
SFPSHL--
SKPSHL--
SPPSHL--
-SPSHLA-
SYPSHL--
-SPSHLT-
SWPSHL--
SCPSHL--
-SPSHLQ-

−SPSHLN−
−SPSHLH−
SEPSHL−−
SDPSHL−−
SAPSHL−−
−SPSHLV−
SHPSHL−−
−SPSHLM−
−SPSHLY−
SQPSHL−−
−SPSHLE−
TRPSHL−−
TVPSHL−−
TFPSHL−−
−TPSHLR−
TGPSHL−−
−TPSHLL−
TTPSHL−−
−TPSHLQ−
TPPSHL−−
TLPSHL−−
−TPSHLN−
−TPSHLA−
TMPSHL−−
−TPSHLH−
TIPSHL−−
TWPSHL−−
TDPSHL−−
−TPSHLW−
TKPSHL−−
TAPSHL−−
−TPSHLM−
−TPSHLG−
−TPSHLY−
−TPSHLE−
−TPSHLV−
−TPSHLK−
−TPSHLI−
−TPSHLF−
−TPSHLT−
TQPSHL−−
−TPSHLC−
−VPSHLR−
VYPSHL−−
VPPSHL−−
VGPSHL−−
−VPSHLS−
VTPSHL−−
−VPSHLM−
−VPSHLH−

VAPSHL--
-VPSHLI-
-VPSHLQ-
-VPSHLL-
VMPSHL--
VQPSHL--
-VPSHLG-
VKPSHL--
VDPSHL--
-VPSHLT-
VNPSHL--
-VPSHLV-
-VPSHLW-
-VPSHLA-
VIPSHL--
VHPSHL--
VFPSHL--
-VPSHLN-
VEPSHL--
-VPSHLE-
-VPSHLK-
-VPSHLC-
-VPSHLD-
-WPSHLR-
WNPSHL--
WLPSHL--
WRPSHL--
WKPSHL--
WHPSHL--
WEPSHL--
WSPSHL--
-WPSHLT-
-WPSHLW-
WYPSHL--
-WPSHLS-
-WPSHLY-
WFPSHL--
WWPSHL--
WIPSHL--
-WPSHLQ-
-WPSHLV-
WDPSHL--
-WPSHLF-
WMPSHL--
WGPSHL--
-WPSHLE-
-WPSHLA-
WQPSHL--
-WPSHLL-
-WPSHLN-

| | | | |
|---|---|---|---|
| | -WPSHLK- | | |
| | -WPSHLI- | | |
| | YRPSHL-- | | |
| | -YPSHLA- | | |
| | -YPSHLR- | | |
| | YMPSHL-- | | |
| | YSPSHL-- | | |
| | YLPSHL-- | | |
| | YGPSHL-- | | |
| | YYPSHL-- | | |
| | YVPSHL-- | | |
| | YHPSHL-- | | |
| | -YPSHLY- | | |
| | -YPSHLG- | | |
| | YNPSHL-- | | |
| | YKPSHL-- | | |
| | YAPSHL-- | | |
| | YWPSHL-- | | |
| | -YPSHLL- | | |
| | YPPSHL-- | | |
| | YDPSHL-- | | |
| | YCPSHL-- | | |
| | YTPSHL-- | | |
| | -YPSHLV- | | |
| | -YPSHLT- | | |
| | -YPSHLH- | | |
| | -YPSHLS- | | |
| | -YPSHLQ- | | |
| | -YPSHLM- | | |
| | YQPSHL-- | | |
| | -YPSHLE- | | |
| | YIPSHL-- | | |
| | YEPSHL-- | | |
| | -YPSHLK- | | |
| | YFPSHL-- | | |
| | -YPSHLW- | | |
| | -YPSHLI- | | |
| | -YPSHLF- | | |
| PLNL | 118 | 757 | 34.71 |
| | -APLNLE- | | |
| | -LPLNLP- | | |
| | MNPLNL-- | | |
| | --PLNLRP | | |
| | --PLNLPA | | |
| | ASPLNL-- | | |
| | -APLNLS- | | |
| | -APLNLR- | | |
| | -APLNLT- | | |
| | -APLNLV- | | |
| | ALPLNL-- | | |

AVPLNL--
-APLNLK-
-APLNLI-
-APLNLH-
AFPLNL--
APPLNL--
-APLNLN-
-APLNLA-
-APLNLG-
AMPLNL--
ADPLNL--
ANPLNL--
AHPLNL--
AQPLNL--
-APLNLM-
AGPLNL--
AWPLNL--
-APLNLQ-
AKPLNL--
-APLNLD-
AYPLNL--
AEPLNL--
CRPLNL--
-CPLNLS-
CLPLNL--
-CPLNLV-
CFPLNL--
CCPLNL--
CSPLNL--
CQPLNL--
-CPLNLR-
-CPLNLH-
CKPLNL--
CGPLNL--
CTPLNL--
-CPLNLT-
CHPLNL--
CDPLNL--
CAPLNL--
CVPLNL--
-CPLNLY-
-CPLNLW-
-CPLNLQ-
-CPLNLL-
-CPLNLI-
CWPLNL--
-CPLNLM-
-CPLNLG-
CEPLNL--
DRPLNL--

DTPLNL--
-DPLNLR-
DNPLNL--
DSPLNL--
-DPLNLS-
-DPLNLW-
DKPLNL--
DHPLNL--
DWPLNL--
-DPLNLV-
DIPLNL--
DVPLNL--
-DPLNLK-
-DPLNLH-
-DPLNLL-
-DPLNLI-
DYPLNL--
-DPLNLT-
-DPLNLM-
-DPLNLA-
DMPLNL--
-DPLNLQ-
-DPLNLN-
-DPLNLF-
DEPLNL--
-DPLNLE-
-DPLNLC-
DFPLNL--
DDPLNL--
ETPLNL--
ESPLNL--
-EPLNLR-
EVPLNL--
-EPLNLS-
-EPLNLK-
ERPLNL--
-EPLNLY-
-EPLNLF-
-EPLNLA-
EGPLNL--
EFPLNL--
-EPLNLI-
EWPLNL--
-EPLNLV-
-EPLNLQ-
-EPLNLG-
ELPLNL--
EYPLNL--
EKPLNL--
-EPLNLT-

EMPLNL--
EPPLNL--
-EPLNLM-
-EPLNLL-
EDPLNL--
EQPLNL--
-EPLNLW-
-EPLNLP-
-EPLNLN-
-EPLNLH-
-EPLNLE-
ENPLNL--
EHPLNL--
EEPLNL--
FRPLNL--
FSPLNL--
-FPLNLT-
-FPLNLH-
FPPLNL--
-FPLNLQ-
-FPLNLE-
-FPLNLN-
FVPLNL--
FNPLNL--
-FPLNLR-
FTPLNL--
FKPLNL--
-FPLNLV-
-FPLNLS-
-FPLNLG-
-FPLNLA-
FHPLNL--
-FPLNLK-
FQPLNL--
FEPLNL--
FDPLNL--
-FPLNLD-
GTPLNL--
GRPLNL--
-GPLNLH-
GFPLNL--
GLPLNL--
-GPLNLK-
GMPLNL--
-GPLNLI-
-GPLNLM-
-GPLNLS-
-GPLNLA-
GIPLNL--
GYPLNL--

GAPLNL--
GWPLNL--
GQPLNL--
GNPLNL--
-GPLNLV-
-GPLNLN-
-GPLNLL-
-GPLNLE-
-GPLNLT-
GGPLNL--
-GPLNLG-
GEPLNL--
-GPLNLQ-
GDPLNL--
-GPLNLD-
GCPLNL--
-HPLNLG-
HRPLNL--
HSPLNL--
HLPLNL--
HTPLNL--
-HPLNLS-
HYPLNL--
HWPLNL--
-HPLNLV-
HGPLNL--
HEPLNL--
HAPLNL--
-HPLNLF-
-HPLNLA-
HDPLNL--
-HPLNLY-
HQPLNL--
-HPLNLT-
-HPLNLL-
-HPLNLH-
HKPLNL--
HFPLNL--
-HPLNLM-
-HPLNLK-
HPPLNL--
-HPLNLQ-
HIPLNL--
-HPLNLW-
HNPLNL--
HMPLNL--
-HPLNLP-
-HPLNLE-
-IPLNLR-
-IPLNLL-

IGPLNL--
-IPLNLS-
IVPLNL--
IKPLNL--
IHPLNL--
IDPLNL--
-IPLNLK-
IYPLNL--
-IPLNLT-
-IPLNLE-
IAPLNL--
IQPLNL--
-IPLNLN-
-IPLNLG-
ITPLNL--
-IPLNLH-
-IPLNLM-
ICPLNL--
IPPLNL--
-IPLNLA-
INPLNL--
IEPLNL--
-IPLNLQ-
-IPLNLD-
-IPLNLC-
IMPLNL--
KSPLNL--
-KPLNLL-
-KPLNLR-
-KPLNLV-
KAPLNL--
-KPLNLI-
-KPLNLT-
KHPLNL--
KYPLNL--
KPPLNL--
-KPLNLQ-
KGPLNL--
KEPLNL--
-KPLNLH-
-KPLNLW-
KNPLNL--
KQPLNL--
KTPLNL--
-KPLNLY-
KIPLNL--
-KPLNLA-
KMPLNL--
-KPLNLN-
KDPLNL--

-KPLNLE-
-KPLNLD-
KCPLNL--
LSPLNL--
LTPLNL--
-LPLNLR-
-LPLNLI-
LPPLNL--
-LPLNLQ-
-LPLNLT-
LKPLNL--
LQPLNL--
-LPLNLM-
LIPLNL--
LEPLNL--
LAPLNL--
-LPLNLN-
-LPLNLE-
-LPLNLV-
LWPLNL--
-LPLNLK-
LHPLNL--
LDPLNL--
-LPLNLH-
-LPLNLG-
LYPLNL--
-LPLNLA-
LCPLNL--
MEPLNL--
MVPLNL--
MIPLNL--
MSPLNL--
MQPLNL--
-MPLNLK-
-MPLNLS-
MGPLNL--
-MPLNLR-
MMPLNL--
MAPLNL--
MYPLNL--
-MPLNLV-
-MPLNLI-
-MPLNLN-
-MPLNLG-
MRPLNL--
-MPLNLT-
-MPLNLE-
MKPLNL--
MHPLNL--
MFPLNL--

```
-MPLNLQ-
-MPLNLM-
-MPLNLD-
NRPLNL--
NLPLNL--
-NPLNLS-
NEPLNL--
NGPLNL--
-NPLNLT-
-NPLNLV-
NTPLNL--
NMPLNL--
-NPLNLE-
NNPLNL--
NKPLNL--
NWPLNL--
-NPLNLM-
-NPLNLA-
-NPLNLK-
-NPLNLH-
NVPLNL--
NIPLNL--
-NPLNLY-
NFPLNL--
NQPLNL--
NPPLNL--
NAPLNL--
NSPLNL--
NHPLNL--
-NPLNLQ-
-NPLNLN-
NCPLNL--
NDPLNL--
--PLNLLG
--PLNLRR
--PLNLLS
--PLNLGL
--PLNLSR
--PLNLRT
--PLNLSS
--PLNLRA
--PLNLGS
--PLNLKS
--PLNLTR
--PLNLRQ
--PLNLYS
--PLNLQS
--PLNLTT
--PLNLTS
--PLNLAR
```

--PLNLMK
PRPLNL--
--PLNLSQ
--PLNLLQ
--PLNLLH
--PLNLKG
--PLNLST
--PLNLSN
--PLNLIS
--PLNLIR
--PLNLGT
--PLNLTL
--PLNLGG
--PLNLWN
--PLNLTV
--PLNLSG
--PLNLMR
--PLNLKA
--PLNLIN
--PLNLNS
--PLNLKR
--PLNLGV
--PLNLGA
--PLNLAW
--PLNLAT
--PLNLAL
--PLNLTY
--PLNLTI
--PLNLSH
--PLNLQL
--PLNLLK
--PLNLAI
--PLNLVT
--PLNLTG
--PLNLSK
--PLNLQT
--PLNLQQ
--PLNLQK
--PLNLLD
--PLNLHT
--PLNLGI
--PLNLVN
--PLNLKT
--PLNLGW
--PLNLAK
--PLNLTH
--PLNLTA
--PLNLSW
--PLNLQG
--PLNLKN

--PLNLHH
--PLNLEY
--PLNLSY
--PLNLSV
--PLNLNL
--PLNLMN
--PLNLME
--PLNLHA
--PLNLDS
PQPLNL--
--PLNLVK
--PLNLKW
PYPLNL--
--PLNLRH
--PLNLRE
--PLNLRD
--PLNLMY
--PLNLML
--PLNLMH
--PLNLID
--PLNLHR
--PLNLGM
--PLNLGF
--PLNLEV
--PLNLEG
--PLNLAH
PEPLNL--
--PLNLWK
--PLNLVW
--PLNLQV
--PLNLMS
--PLNLLN
--PLNLKL
--PLNLKF
--PLNLHI
--PLNLFN
--PLNLES
--PLNLAG
--PLNLAA
--PLNLYG
--PLNLQN
--PLNLQM
--PLNLNR
--PLNLNK
--PLNLHS
--PLNLHL
--PLNLFG
--PLNLDG
--PLNLAS
--PLNLAQ

--PLNLYN
--PLNLWH
--PLNLVD
--PLNLTN
--PLNLSI
--PLNLSD
--PLNLNA
--PLNLMT
--PLNLMA
--PLNLKV
--PLNLKQ
--PLNLIH
--PLNLHW
--PLNLHG
--PLNLET
--PLNLEK
--PLNLAE
--PLNLWD
--PLNLNT
--PLNLMD
--PLNLHN
--PLNLHK
--PLNLFD
--PLNLEL
--PLNLDA
--PLNLAM
--PLNLWQ
--PLNLTQ
--PLNLTE
--PLNLSM
--PLNLNV
--PLNLNM
--PLNLNG
--PLNLMQ
--PLNLMG
--PLNLLE
--PLNLKI
--PLNLHQ
--PLNLHF
--PLNLGN
--PLNLGK
--PLNLER
--PLNLAN
PHPLNL--
PWPLNL--
PNPLNL--
PMPLNL--
--PLNLYD
--PLNLYA
--PLNLWG

--PLNLWA
--PLNLTM
--PLNLTD
--PLNLSE
--PLNLQR
--PLNLQH
--PLNLMW
--PLNLMV
--PLNLKH
--PLNLKD
--PLNLGY
--PLNLYH
--PLNLVP
--PLNLQY
--PLNLQD
--PLNLPY
--PLNLPI
--PLNLPF
--PLNLNW
--PLNLND
--PLNLKY
--PLNLKK
--PLNLHY
--PLNLHM
--PLNLGH
--PLNLEH
--PLNLEF
--PLNLAD
PDPLNL--
--PLNLQI
--PLNLQF
--PLNLQE
--PLNLPM
--PLNLNY
--PLNLNN
--PLNLKM
--PLNLIE
--PLNLGD
--PLNLFQ
--PLNLEE
--PLNLED
PFPLNL--
-PPLNLH-
--PLNLVE
--PLNLTW
--PLNLRC
--PLNLQW
--PLNLQC
--PLNLNP
--PLNLMP

--PLNLKE
--PLNLHD
--PLNLEW
--PLNLEQ
--PLNLEI
--PLNLDT
--PLNLCR
QLPLNL--
QFPLNL--
-QPLNLR-
QRPLNL--
-QPLNLI-
-QPLNLQ-
-QPLNLL-
-QPLNLG-
QPPLNL--
QMPLNL--
-QPLNLT-
QGPLNL--
-QPLNLH-
QAPLNL--
-QPLNLS-
-QPLNLK-
QVPLNL--
-QPLNLA-
QEPLNL--
-QPLNLN-
-QPLNLM-
QWPLNL--
-QPLNLF-
QKPLNL--
QTPLNL--
-QPLNLV-
-QPLNLE-
QDPLNL--
QQPLNL--
QNPLNL--
QHPLNL--
-QPLNLW-
-QPLNLD-
RVPLNL--
RTPLNL--
RLPLNL--
RMPLNL--
-RPLNLT-
RWPLNL--
-RPLNLS-
REPLNL--
RIPLNL--
RGPLNL--

-RPLNLW-
-RPLNLA-
-RPLNLI-
-RPLNLM-
-RPLNLQ-
-RPLNLE-
-RPLNLD-
RDPLNL--
RHPLNL--
RQPLNL--
SRPLNL--
-SPLNLR-
SGPLNL--
-SPLNLL-
-SPLNLN-
-SPLNLV-
-SPLNLF-
SSPLNL--
SNPLNL--
-SPLNLK-
-SPLNLT-
SYPLNL--
-SPLNLI-
STPLNL--
SMPLNL--
SKPLNL--
-SPLNLA-
-SPLNLH-
SAPLNL--
SHPLNL--
SWPLNL--
-SPLNLQ-
SIPLNL--
SFPLNL--
SQPLNL--
-SPLNLM-
SEPLNL--
-SPLNLE-
SDPLNL--
SPPLNL--
-SPLNLD-
SCPLNL--
TGPLNL--
TSPLNL--
-TPLNLS-
TVPLNL--
TLPLNL--
TAPLNL--
-TPLNLK-
-TPLNLH-

TFPLNL--
TTPLNL--
-TPLNLN-
-TPLNLI-
TYPLNL--
-TPLNLA-
TWPLNL--
TKPLNL--
-TPLNLQ-
-TPLNLM-
-TPLNLE-
TPPLNL--
THPLNL--
TDPLNL--
TNPLNL--
-TPLNLD-
TCPLNL--
-TPLNLC-
TEPLNL--
-VPLNLR-
-VPLNLS-
-VPLNLL-
VSPLNL--
VLPLNL--
VRPLNL--
VIPLNL--
-VPLNLT-
-VPLNLI-
-VPLNLV-
VAPLNL--
VKPLNL--
VMPLNL--
VGPLNL--
-VPLNLN-
VHPLNL--
-VPLNLK-
VDPLNL--
-VPLNLQ-
-VPLNLG-
-VPLNLA-
-VPLNLH-
VNPLNL--
-VPLNLM-
-VPLNLE-
VFPLNL--
VEPLNL--
VCPLNL--
VPPLNL--
-VPLNLD-
-WPLNLT-

-WPLNLS-
WGPLNL--
-WPLNLQ-
WEPLNL--
WVPLNL--
WTPLNL--
-WPLNLG-
-WPLNLL-
WKPLNL--
WQPLNL--
WPPLNL--
WNPLNL--
WDPLNL--
-WPLNLM-
-WPLNLK-
-WPLNLV-
YKPLNL--
YSPLNL--
-YPLNLR-
-YPLNLK-
-YPLNLL-
YTPLNL--
-YPLNLS-
YLPLNL--
YVPLNL--
YQPLNL--
-YPLNLM-
YPPLNL--
-YPLNLT-
-YPLNLQ-
-YPLNLG-
-YPLNLV-
-YPLNLN-
-YPLNLH-
-YPLNLA-
YMPLNL--
YEPLNL--
YAPLNL--
YNPLNL--
-YPLNLI-
-YPLNLF-
YIPLNL--
YDPLNL--
YHPLNL--
-YPLNLE-
-YPLNLD-

| | | | |
|---|---|---|---|
| PAKL | 25 | 835 | 34.65 |

-EPAKLS-
--PAKLWL
--PAKLGL

--PAKLGK
-APAKLR-
-APAKLL-
ALPAKL--
-APAKLT-
AYPAKL--
AMPAKL--
AVPAKL--
ANPAKL--
-APAKLV-
-APAKLH-
AGPAKL--
AAPAKL--
ATPAKL--
ASPAKL--
-APAKLI-
AKPAKL--
AHPAKL--
AIPAKL--
AQPAKL--
APPAKL--
-APAKLY-
-APAKLW-
-APAKLQ-
-APAKLF-
-APAKLE-
-APAKLA-
AFPAKL--
ACPAKL--
-APAKLK-
-APAKLD-
-APAKLC-
AEPAKL--
CQPAKL--
-CPAKLV-
CIPAKL--
CCPAKL--
-CPAKLR-
CSPAKL--
CLPAKL--
CKPAKL--
CVPAKL--
-CPAKLL-
-CPAKLK-
CYPAKL--
-CPAKLM-
-CPAKLH-
CNPAKL--
CHPAKL--
CEPAKL--

-DPAKLL-
DTPAKL--
DLPAKL--
DRPAKL--
-DPAKLI-
-DPAKLW-
-DPAKLS-
DEPAKL--
DVPAKL--
DQPAKL--
-DPAKLV-
-DPAKLR-
DHPAKL--
DGPAKL--
DSPAKL--
-DPAKLQ-
-DPAKLK-
-DPAKLH-
DNPAKL--
DKPAKL--
DWPAKL--
-DPAKLT-
-DPAKLN-
-DPAKLG-
-DPAKLE-
DMPAKL--
DIPAKL--
DDPAKL--
DCPAKL--
ESPAKL--
EVPAKL--
EQPAKL--
EWPAKL--
-EPAKLK-
EAPAKL--
-EPAKLR-
ETPAKL--
-EPAKLI-
ELPAKL--
-EPAKLV-
-EPAKLL-
EMPAKL--
EKPAKL--
EHPAKL--
-EPAKLH-
-EPAKLG-
EIPAKL--
ERPAKL--
-EPAKLY-
-EPAKLF-

−EPAKLM−
−EPAKLE−
ENPAKL−−
−EPAKLT−
−EPAKLQ−
−EPAKLC−
EGPAKL−−
EFPAKL−−
FKPAKL−−
FGPAKL−−
−FPAKLL−
FAPAKL−−
FYPAKL−−
−FPAKLR−
FTPAKL−−
−FPAKLY−
FHPAKL−−
FFPAKL−−
FIPAKL−−
−FPAKLW−
−FPAKLQ−
−FPAKLF−
FQPAKL−−
−FPAKLN−
−FPAKLM−
FNPAKL−−
FMPAKL−−
FSPAKL−−
−FPAKLS−
−FPAKLK−
−FPAKLH−
−FPAKLA−
FWPAKL−−
−FPAKLI−
−FPAKLE−
−FPAKLT−
FDPAKL−−
FCPAKL−−
−GPAKLA−
GRPAKL−−
GMPAKL−−
−GPAKLK−
−GPAKLF−
−GPAKLG−
−GPAKLR−
GYPAKL−−
−GPAKLN−
GWPAKL−−
GPPAKL−−
−GPAKLQ−

GIPAKL--
-GPAKLS-
GLPAKL--
GKPAKL--
GAPAKL--
GFPAKL--
GTPAKL--
GQPAKL--
-GPAKLV-
-GPAKLH-
GVPAKL--
-GPAKLY-
-GPAKLW-
-GPAKLT-
GEPAKL--
GDPAKL--
-GPAKLI-
GGPAKL--
GCPAKL--
GNPAKL--
GHPAKL--
HRPAKL--
-HPAKLR-
-HPAKLS-
HMPAKL--
-HPAKLI-
HFPAKL--
HTPAKL--
HSPAKL--
-HPAKLN-
HGPAKL--
-HPAKLL-
HNPAKL--
-HPAKLG-
-HPAKLT-
-HPAKLQ-
-HPAKLM-
-HPAKLV-
-HPAKLF-
-HPAKLA-
HYPAKL--
-HPAKLH-
HLPAKL--
HEPAKL--
HDPAKL--
HPPAKL--
-HPAKLK-
-HPAKLY-
HAPAKL--
HWPAKL--

HVPAKL--
-HPAKLW-
-HPAKLE-
HIPAKL--
-IPAKLL-
-IPAKLK-
IAPAKL--
IYPAKL--
ITPAKL--
-IPAKLY-
IGPAKL--
IFPAKL--
-IPAKLM-
ILPAKL--
IPPAKL--
-IPAKLT-
-IPAKLQ-
-IPAKLA-
ISPAKL--
-IPAKLV-
-IPAKLS-
-IPAKLI-
-IPAKLH-
IMPAKL--
IIPAKL--
IHPAKL--
IVPAKL--
-IPAKLW-
-IPAKLN-
IRPAKL--
-IPAKLG-
IQPAKL--
-IPAKLF-
-IPAKLE-
INPAKL--
IKPAKL--
KSPAKL--
-KPAKLR-
-KPAKLT-
KVPAKL--
KTPAKL--
-KPAKLH-
KRPAKL--
-KPAKLK-
-KPAKLF-
-KPAKLS-
-KPAKLQ-
KAPAKL--
-KPAKLG-
-KPAKLW-

KPPAKL--
KGPAKL--
KDPAKL--
KIPAKL--
-KPAKLI-
-KPAKLV-
-KPAKLN-
-KPAKLE-
KNPAKL--
KQPAKL--
KMPAKL--
KYPAKL--
-KPAKLC-
KLPAKL--
KKPAKL--
KHPAKL--
LSPAKL--
-LPAKLQ-
LWPAKL--
LLPAKL--
LRPAKL--
LFPAKL--
LHPAKL--
LTPAKL--
-LPAKLT-
-LPAKLI-
-LPAKLS-
LNPAKL--
LMPAKL--
LIPAKL--
LAPAKL--
LCPAKL--
LYPAKL--
LPPAKL--
-LPAKLV-
-LPAKLH-
-LPAKLA-
-LPAKLY-
-LPAKLN-
LQPAKL--
-LPAKLW-
-LPAKLE-
-LPAKLG-
LDPAKL--
LEPAKL--
-MPAKLT-
-MPAKLV-
MHPAKL--
-MPAKLS-
-MPAKLK-

MVPAKL--
MSPAKL--
-MPAKLY-
-MPAKLR-
-MPAKLL-
MNPAKL--
-MPAKLI-
-MPAKLW-
-MPAKLA-
MTPAKL--
MAPAKL--
MQPAKL--
MDPAKL--
MYPAKL--
MPPAKL--
-MPAKLG-
MLPAKL--
-MPAKLM-
MIPAKL--
MEPAKL--
-MPAKLQ-
-MPAKLN-
-MPAKLH-
-MPAKLE-
MGPAKL--
MMPAKL--
MFPAKL--
MCPAKL--
-NPAKLR-
NRPAKL--
NLPAKL--
-NPAKLY-
NKPAKL--
NSPAKL--
-NPAKLI-
-NPAKLA-
NVPAKL--
-NPAKLL-
-NPAKLV-
NIPAKL--
NDPAKL--
NWPAKL--
NEPAKL--
-NPAKLW-
-NPAKLS-
NHPAKL--
NTPAKL--
-NPAKLE-
NNPAKL--
NMPAKL--

-NPAKLK-
-NPAKLF-
NAPAKL--
NQPAKL--
NPPAKL--
-NPAKLT-
-NPAKLQ-
-NPAKLH-
NCPAKL--
--PAKLSR
--PAKLQT
--PAKLVA
--PAKLAS
--PAKLVR
--PAKLRL
--PAKLSS
--PAKLRV
--PAKLLS
--PAKLHS
--PAKLSK
--PAKLMK
--PAKLGS
--PAKLVH
PSPAKL--
PRPAKL--
--PAKLRT
--PAKLRA
--PAKLIR
--PAKLRN
PLPAKL--
--PAKLVT
--PAKLRW
--PAKLLN
--PAKLIG
--PAKLYN
--PAKLVI
--PAKLLY
--PAKLVK
--PAKLSF
--PAKLLK
--PAKLYA
--PAKLWR
--PAKLTK
--PAKLRK
--PAKLYK
--PAKLVW
--PAKLSV
--PAKLRE
--PAKLMT
--PAKLEV

--PAKLTA
--PAKLSN
--PAKLRS
--PAKLRG
--PAKLNV
--PAKLKH
--PAKLWS
--PAKLWK
--PAKLVV
--PAKLVF
--PAKLTY
--PAKLTV
--PAKLWM
--PAKLSG
--PAKLRM
--PAKLMF
--PAKLIV
--PAKLIK
--PAKLYS
--PAKLYF
--PAKLWQ
--PAKLVS
--PAKLSY
--PAKLQL
--PAKLLV
--PAKLKV
--PAKLKS
--PAKLKI
--PAKLIW
--PAKLHW
--PAKLHA
--PAKLFR
--PAKLES
--PAKLER
--PAKLAG
--PAKLWN
--PAKLVN
--PAKLMR
--PAKLML
--PAKLLA
--PAKLKW
--PAKLIA
--PAKLGG
--PAKLGA
--PAKLET
PFPAKL--
--PAKLWT
--PAKLTN
--PAKLST
--PAKLSI

```
--PAKLRD
--PAKLQK
--PAKLMV
--PAKLLE
--PAKLIS
--PAKLIL
--PAKLHM
--PAKLGT
--PAKLFQ
--PAKLFN
--PAKLYG
--PAKLWH
--PAKLTI
--PAKLTG
--PAKLRH
--PAKLNL
--PAKLLW
--PAKLLM
--PAKLLD
--PAKLKG
--PAKLKE
--PAKLIT
--PAKLIF
--PAKLHV
--PAKLFA
--PAKLEH
--PAKLAL
PVPAKL--
PQPAKL--
PGPAKL--
PAPAKL--
--PAKLWV
--PAKLWG
--PAKLVE
--PAKLSD
--PAKLSA
--PAKLQE
--PAKLNW
--PAKLMY
--PAKLKQ
--PAKLHI
--PAKLFE
--PAKLDS
--PAKLAV
--PAKLYQ
--PAKLVG
--PAKLTS
--PAKLSQ
--PAKLQY
--PAKLQQ
```

--PAKLNT
--PAKLMD
--PAKLLQ
--PAKLIQ
--PAKLIM
--PAKLHY
--PAKLAK
PTPAKL--
PHPAKL--
--PAKLYT
--PAKLYE
--PAKLWI
--PAKLWF
--PAKLVD
--PAKLTQ
--PAKLTH
--PAKLSE
--PAKLQV
--PAKLQN
--PAKLQM
--PAKLNY
--PAKLLG
--PAKLKL
--PAKLKK
--PAKLII
--PAKLHR
--PAKLHN
--PAKLFH
--PAKLFG
--PAKLAT
PIPAKL--
--PAKLYW
--PAKLYL
--PAKLYH
--PAKLYD
--PAKLWY
--PAKLTT
--PAKLRF
--PAKLQW
--PAKLQS
--PAKLQI
--PAKLQG
--PAKLQF
--PAKLNS
--PAKLNR
--PAKLMQ
--PAKLLI
--PAKLKD
--PAKLIH
--PAKLID

--PAKLGV
--PAKLFK
--PAKLEI
--PAKLAM
PYPAKL--
--PAKLYM
--PAKLTF
--PAKLSW
--PAKLKY
--PAKLKN
--PAKLEQ
--PAKLEG
--PAKLAW
--PAKLAR
--PAKLAQ
--PAKLAI
--PAKLAA
PMPAKL--
--PAKLYY
--PAKLYV
--PAKLWD
--PAKLVQ
--PAKLSM
--PAKLQA
--PAKLNM
--PAKLNA
--PAKLMI
--PAKLMG
--PAKLKM
--PAKLHL
--PAKLHK
--PAKLHG
--PAKLHE
--PAKLGM
--PAKLFD
--PAKLEN
--PAKLEL
--PAKLEF
--PAKLDG
--PAKLCS
--PAKLAN
--PAKLAH
-PPAKLI-
--PAKLYI
--PAKLWW
--PAKLWE
--PAKLVM
--PAKLVC
--PAKLRP
--PAKLNQ

--PAKLNN
--PAKLNF
--PAKLMW
--PAKLMC
--PAKLMA
--PAKLHT
--PAKLHF
--PAKLGQ
--PAKLGI
--PAKLGH
--PAKLGF
--PAKLFM
--PAKLFL
--PAKLEY
--PAKLEK
--PAKLDA
--PAKLCT
--PAKLCK
--PAKLCA
--PAKLAF
-QPAKLL-
-QPAKLN-
QNPAKL--
-QPAKLK-
-QPAKLQ-
QSPAKL--
-QPAKLS-
-QPAKLR-
QAPAKL--
QVPAKL--
QRPAKL--
-QPAKLF-
-QPAKLW-
-QPAKLA-
QMPAKL--
QKPAKL--
QHPAKL--
QYPAKL--
-QPAKLE-
QWPAKL--
QIPAKL--
QFPAKL--
QTPAKL--
QQPAKL--
-QPAKLT-
-QPAKLH-
-QPAKLY-
-QPAKLI-
-QPAKLD-
QCPAKL--

-RPAKLA-
RTPAKL--
-RPAKLR-
RRPAKL--
RAPAKL--
RQPAKL--
RPPAKL--
RIPAKL--
RWPAKL--
-RPAKLF-
RGPAKL--
-RPAKLN-
REPAKL--
RYPAKL--
-RPAKLI-
-RPAKLQ-
-RPAKLH-
-RPAKLT-
RMPAKL--
-RPAKLM-
-RPAKLW-
-RPAKLE-
RHPAKL--
RFPAKL--
RDPAKL--
RCPAKL--
-SPAKLS-
SWPAKL--
-SPAKLA-
SSPAKL--
SRPAKL--
SVPAKL--
-SPAKLN-
STPAKL--
SLPAKL--
SGPAKL--
SYPAKL--
-SPAKLV-
-SPAKLK-
-SPAKLH-
SFPAKL--
-SPAKLR-
-SPAKLQ-
-SPAKLG-
SHPAKL--
SEPAKL--
SPPAKL--
-SPAKLI-
-SPAKLT-
SIPAKL--

SNPAKL--
SAPAKL--
SQPAKL--
-SPAKLF-
SMPAKL--
SDPAKL--
-SPAKLM-
-SPAKLE-
-SPAKLD-
-SPAKLC-
SCPAKL--
-TPAKLL-
-TPAKLR-
TGPAKL--
-TPAKLT-
-TPAKLS-
TIPAKL--
TTPAKL--
-TPAKLV-
TVPAKL--
TNPAKL--
-TPAKLH-
TYPAKL--
TAPAKL--
TWPAKL--
-TPAKLY-
-TPAKLW-
-TPAKLF-
THPAKL--
-TPAKLM-
TMPAKL--
-TPAKLN-
-TPAKLK-
-TPAKLE-
-TPAKLI-
TLPAKL--
TEPAKL--
TPPAKL--
-TPAKLQ-
TDPAKL--
TQPAKL--
-TPAKLA-
TFPAKL--
VVPAKL--
-VPAKLS-
VSPAKL--
-VPAKLT-
-VPAKLL-
-VPAKLF-
VTPAKL--

```
-VPAKLR-
-VPAKLK-
VIPAKL--
VYPAKL--
VHPAKL--
VEPAKL--
-VPAKLQ-
-VPAKLN-
VAPAKL--
VPPAKL--
-VPAKLG-
VMPAKL--
-VPAKLV-
VGPAKL--
-VPAKLW-
VFPAKL--
VQPAKL--
VNPAKL--
-VPAKLI-
-VPAKLE-
VDPAKL--
VKPAKL--
-VPAKLC-
-WPAKLQ-
-WPAKLL-
WMPAKL--
WGPAKL--
-WPAKLF-
WRPAKL--
-WPAKLT-
-WPAKLS-
WPPAKL--
WLPAKL--
WYPAKL--
WHPAKL--
WAPAKL--
-WPAKLH-
-WPAKLA-
-WPAKLW-
-WPAKLN-
-WPAKLM-
WEPAKL--
WCPAKL--
-WPAKLK-
-WPAKLI-
-WPAKLG-
WNPAKL--
WIPAKL--
WFPAKL--
WDPAKL--
```

| | | | |
|---|---|---|---|
| | YSPAKL-- | | |
| | -YPAKLQ- | | |
| | -YPAKLT- | | |
| | YRPAKL-- | | |
| | YMPAKL-- | | |
| | -YPAKLI- | | |
| | YDPAKL-- | | |
| | -YPAKLV- | | |
| | YFPAKL-- | | |
| | YYPAKL-- | | |
| | YVPAKL-- | | |
| | YPPAKL-- | | |
| | -YPAKLA- | | |
| | YLPAKL-- | | |
| | -YPAKLW- | | |
| | -YPAKLH- | | |
| | YNPAKL-- | | |
| | -YPAKLY- | | |
| | -YPAKLE- | | |
| | YGPAKL-- | | |
| | -YPAKLG- | | |
| | -YPAKLK- | | |
| | YAPAKL-- | | |
| | YWPAKL-- | | |
| | YTPAKL-- | | |
| | YQPAKL-- | | |
| | -YPAKLN- | | |
| | -YPAKLM- | | |
| | -YPAKLC- | | |
| | YHPAKL-- | | |
| | YEPAKL-- | | |
| | YCPAKL-- | | |
| PKGL | 105 | 767 | 34.62 |
| | -APKGLD- | | |
| | EFPKGL-- | | |
| | --PKGLPL | | |
| | -WPKGLG- | | |
| | AVPKGL-- | | |
| | -APKGLL- | | |
| | ASPKGL-- | | |
| | -APKGLS- | | |
| | -APKGLR- | | |
| | -APKGLH- | | |
| | -APKGLW- | | |
| | ALPKGL-- | | |
| | -APKGLV- | | |
| | -APKGLM- | | |
| | -APKGLI- | | |
| | AYPKGL-- | | |
| | ATPKGL-- | | |

AIPKGL--
-APKGLY-
-APKGLK-
-APKGLG-
-APKGLN-
-APKGLT-
AHPKGL--
AGPKGL--
APPKGL--
AMPKGL--
ANPKGL--
AQPKGL--
AEPKGL--
-APKGLF-
-APKGLA-
ADPKGL--
ACPKGL--
AAPKGL--
-APKGLC-
AKPKGL--
CVPKGL--
CCPKGL--
CRPKGL--
-CPKGLS-
CGPKGL--
-CPKGLH-
CSPKGL--
-CPKGLQ-
-CPKGLK-
CHPKGL--
CEPKGL--
-CPKGLY-
-CPKGLN-
-CPKGLL-
-CPKGLI-
CYPKGL--
CTPKGL--
-CPKGLT-
CFPKGL--
CWPKGL--
CPPKGL--
-CPKGLW-
-CPKGLV-
-CPKGLM-
-CPKGLF-
CLPKGL--
CIPKGL--
CDPKGL--
-DPKGLL-
DSPKGL--

-DPKGLR-
-DPKGLF-
-DPKGLA-
-DPKGLM-
-DPKGLK-
DLPKGL--
DYPKGL--
DQPKGL--
DDPKGL--
-DPKGLY-
DAPKGL--
DTPKGL--
-DPKGLV-
-DPKGLN-
-DPKGLI-
DIPKGL--
DWPKGL--
-DPKGLG-
DKPKGL--
-DPKGLT-
-DPKGLS-
-DPKGLH-
DNPKGL--
DMPKGL--
DHPKGL--
DFPKGL--
-DPKGLW-
DGPKGL--
-EPKGLR-
EVPKGL--
EMPKGL--
-EPKGLM-
EHPKGL--
ESPKGL--
ETPKGL--
ERPKGL--
-EPKGLV-
-EPKGLS-
-EPKGLL-
-EPKGLI-
-EPKGLH-
EYPKGL--
-EPKGLN-
EWPKGL--
-EPKGLW-
EEPKGL--
EAPKGL--
-EPKGLG-
ELPKGL--
EIPKGL--

-EPKGLQ-
-EPKGLD-
-EPKGLA-
EGPKGL--
FGPKGL--
FWPKGL--
FNPKGL--
FVPKGL--
FPPKGL--
FMPKGL--
-FPKGLT-
-FPKGLK-
FDPKGL--
-FPKGLY-
-FPKGLI-
-FPKGLN-
FIPKGL--
-FPKGLM-
FTPKGL--
-FPKGLF-
FKPKGL--
FLPKGL--
FQPKGL--
-FPKGLW-
GPPKGL--
-GPKGLL-
-GPKGLG-
GSPKGL--
GYPKGL--
GLPKGL--
GHPKGL--
GAPKGL--
GQPKGL--
-GPKGLT-
-GPKGLA-
-GPKGLN-
-GPKGLV-
-GPKGLE-
GNPKGL--
GGPKGL--
GWPKGL--
-GPKGLY-
-GPKGLS-
GVPKGL--
-GPKGLM-
GTPKGL--
-GPKGLQ-
GEPKGL--
-GPKGLW-
-GPKGLF-

GIPKGL--
GDPKGL--
-GPKGLK-
-GPKGLC-
GCPKGL--
-HPKGLT-
-HPKGLL-
-HPKGLS-
HYPKGL--
HQPKGL--
HDPKGL--
HPPKGL--
-HPKGLY-
-HPKGLF-
HSPKGL--
HFPKGL--
HTPKGL--
-HPKGLQ-
-HPKGLK-
HIPKGL--
HVPKGL--
HNPKGL--
-HPKGLH-
-HPKGLA-
-HPKGLG-
-HPKGLN-
-HPKGLM-
HMPKGL--
HEPKGL--
-HPKGLE-
HHPKGL--
HGPKGL--
HAPKGL--
-HPKGLC-
HCPKGL--
-IPKGLS-
IRPKGL--
-IPKGLL-
-IPKGLT-
-IPKGLG-
IYPKGL--
IIPKGL--
ISPKGL--
-IPKGLR-
IGPKGL--
-IPKGLH-
-IPKGLI-
IDPKGL--
-IPKGLY-
-IPKGLW-

−IPKGLA−
IMPKGL−−
ILPKGL−−
IHPKGL−−
IQPKGL−−
−IPKGLF−
INPKGL−−
−IPKGLK−
−IPKGLM−
IKPKGL−−
−IPKGLQ−
−IPKGLN−
−IPKGLE−
IAPKGL−−
IEPKGL−−
KSPKGL−−
−KPKGLL−
−KPKGLS−
KGPKGL−−
KVPKGL−−
−KPKGLN−
KEPKGL−−
−KPKGLY−
−KPKGLE−
KAPKGL−−
KPPKGL−−
KLPKGL−−
−KPKGLW−
−KPKGLM−
KIPKGL−−
−KPKGLT−
−KPKGLF−
−KPKGLA−
−KPKGLI−
KHPKGL−−
KTPKGL−−
−KPKGLQ−
−KPKGLG−
KKPKGL−−
KQPKGL−−
−KPKGLH−
KDPKGL−−
KNPKGL−−
−LPKGLT−
LSPKGL−−
−LPKGLY−
−LPKGLV−
LLPKGL−−
LYPKGL−−
−LPKGLF−

LVPKGL--
LMPKGL--
LHPKGL--
-LPKGLR-
-LPKGLN-
-LPKGLS-
LNPKGL--
-LPKGLQ-
LGPKGL--
LTPKGL--
-LPKGLH-
-LPKGLA-
-LPKGLE-
-LPKGLG-
LEPKGL--
LDPKGL--
LCPKGL--
LPPKGL--
-LPKGLC-
-MPKGLL-
-MPKGLS-
MIPKGL--
MKPKGL--
MSPKGL--
-MPKGLK-
-MPKGLT-
MAPKGL--
MQPKGL--
-MPKGLG-
-MPKGLQ-
-MPKGLR-
-MPKGLV-
-MPKGLY-
MNPKGL--
MGPKGL--
MYPKGL--
-MPKGLA-
MEPKGL--
-MPKGLH-
MMPKGL--
MPPKGL--
-MPKGLF-
-MPKGLC-
MDPKGL--
-MPKGLN-
-MPKGLM-
-MPKGLE-
MCPKGL--
NIPKGL--
-NPKGLL-

NSPKGL--
NGPKGL--
-NPKGLY-
NVPKGL--
-NPKGLT-
NNPKGL--
-NPKGLQ-
-NPKGLN-
NAPKGL--
-NPKGLW-
-NPKGLH-
NTPKGL--
NQPKGL--
-NPKGLV-
-NPKGLF-
NYPKGL--
-NPKGLS-
-NPKGLK-
-NPKGLI-
-NPKGLE-
NCPKGL--
-NPKGLM-
-NPKGLA-
NEPKGL--
NDPKGL--
NWPKGL--
NFPKGL--
NPPKGL--
--PKGLSL
--PKGLVL
--PKGLFL
--PKGLSW
--PKGLRT
--PKGLVG
--PKGLGV
--PKGLAS
--PKGLYL
--PKGLLH
--PKGLGA
--PKGLLK
--PKGLLG
--PKGLIL
--PKGLTS
--PKGLRG
--PKGLMK
--PKGLWQ
--PKGLSG
--PKGLMA
--PKGLLF
--PKGLKG

--PKGLSI
--PKGLGS
--PKGLYS
--PKGLKT
--PKGLVI
--PKGLFS
--PKGLYF
--PKGLNM
--PKGLMI
--PKGLHG
--PKGLVT
PLPKGL--
--PKGLYT
--PKGLHS
--PKGLGT
PVPKGL--
--PKGLYH
--PKGLWY
--PKGLVA
--PKGLTT
--PKGLTI
--PKGLTG
--PKGLSK
--PKGLKN
--PKGLHT
--PKGLYK
--PKGLNR
--PKGLNF
--PKGLGL
--PKGLYV
--PKGLKS
--PKGLIM
--PKGLFN
--PKGLFH
--PKGLAV
--PKGLAK
--PKGLWS
--PKGLSF
--PKGLML
--PKGLMG
--PKGLFI
--PKGLAI
PYPKGL--
--PKGLYN
--PKGLYM
--PKGLWG
--PKGLTY
--PKGLTN
--PKGLSN
--PKGLNW

--PKGLNV
--PKGLNS
--PKGLIV
--PKGLIS
--PKGLIK
--PKGLIA
--PKGLFV
--PKGLFK
PTPKGL--
PRPKGL--
--PKGLVN
--PKGLTF
--PKGLSV
--PKGLRQ
--PKGLMF
--PKGLAY
--PKGLYR
--PKGLWT
--PKGLWA
--PKGLSY
--PKGLSE
--PKGLSA
--PKGLRD
--PKGLQG
--PKGLMT
--PKGLLY
--PKGLLQ
--PKGLKH
--PKGLIQ
--PKGLHV
--PKGLAT
--PKGLWN
--PKGLVF
--PKGLTM
--PKGLNI
--PKGLID
--PKGLET
--PKGLWV
--PKGLQW
--PKGLQL
--PKGLII
--PKGLIH
--PKGLGG
--PKGLFF
--PKGLEF
--PKGLVD
--PKGLTQ
--PKGLTE
--PKGLNY
--PKGLNT

--PKGLNN
--PKGLMN
--PKGLKQ
--PKGLIW
--PKGLEY
--PKGLEG
--PKGLYY
--PKGLWD
--PKGLME
--PKGLLE
--PKGLIG
--PKGLAN
PGPKGL--
PAPKGL--
PKPKGL--
--PKGLYI
--PKGLYD
--PKGLYA
--PKGLVW
--PKGLVV
--PKGLVQ
--PKGLTV
--PKGLSQ
--PKGLQI
--PKGLQE
--PKGLNG
--PKGLMQ
--PKGLIT
--PKGLHQ
--PKGLHH
--PKGLGI
--PKGLEW
--PKGLAG
PQPKGL--
PMPKGL--
--PKGLYQ
--PKGLVE
--PKGLTH
--PKGLMH
--PKGLIF
--PKGLHY
--PKGLHN
--PKGLGK
--PKGLFD
--PKGLES
--PKGLER
--PKGLAW
PEPKGL--
PSPKGL--
--PKGLTK

--PKGLSM
--PKGLQV
--PKGLQT
--PKGLQS
--PKGLNH
--PKGLNE
--PKGLLD
--PKGLKK
--PKGLIN
--PKGLHI
PHPKGL--
PDPKGL--
PNPKGL--
--PKGLWM
--PKGLRE
--PKGLNA
--PKGLKM
--PKGLKF
--PKGLHW
--PKGLHE
--PKGLHD
--PKGLFY
--PKGLFQ
--PKGLEI
--PKGLDS
--PKGLAQ
--PKGLAF
--PKGLAD
--PKGLTW
--PKGLTD
--PKGLND
--PKGLMW
--PKGLMM
--PKGLFM
--PKGLEV
--PKGLEN
--PKGLEM
--PKGLDG
--PKGLDA
--PKGLCR
--PKGLAA
PCPKGL--
--PKGLQQ
--PKGLQM
--PKGLQK
--PKGLQF
--PKGLQA
--PKGLNQ
--PKGLMD
--PKGLHK

--PKGLGW
--PKGLGH
--PKGLEQ
--PKGLEK
--PKGLEH
--PKGLCS
--PKGLAE
PPPKGL--
--PKGLWW
--PKGLWE
--PKGLQD
--PKGLPN
--PKGLNK
--PKGLNC
--PKGLKP
--PKGLKE
--PKGLIE
--PKGLHM
--PKGLHF
--PKGLGQ
--PKGLGE
--PKGLEA
--PKGLCG
--PKGLCE
--PKGLCA
QSPKGL--
QIPKGL--
QTPKGL--
QAPKGL--
QVPKGL--
QGPKGL--
-QPKGLL-
QLPKGL--
QRPKGL--
-QPKGLY-
QNPKGL--
-QPKGLF-
QQPKGL--
-QPKGLS-
QPPKGL--
-QPKGLT-
QHPKGL--
QYPKGL--
-QPKGLN-
-QPKGLE-
QMPKGL--
-QPKGLH-
-QPKGLK-
-QPKGLI-
-QPKGLG-

QKPKGL--
QEPKGL--
-QPKGLA-
-QPKGLW-
-QPKGLM-
-QPKGLC-
-RPKGLV-
-RPKGLL-
RPPKGL--
RMPKGL--
-RPKGLA-
RNPKGL--
-RPKGLW-
RYPKGL--
-RPKGLN-
REPKGL--
RTPKGL--
-RPKGLI-
RCPKGL--
-RPKGLH-
-RPKGLE-
RIPKGL--
-RPKGLF-
RGPKGL--
-RPKGLQ-
RQPKGL--
RDPKGL--
SLPKGL--
-SPKGLT-
-SPKGLL-
SGPKGL--
SHPKGL--
-SPKGLN-
SMPKGL--
-SPKGLY-
-SPKGLH-
SNPKGL--
-SPKGLW-
-SPKGLV-
SQPKGL--
-SPKGLM-
-SPKGLG-
-SPKGLE-
SDPKGL--
-SPKGLQ-
SKPKGL--
SCPKGL--
SEPKGL--
-TPKGLS-
TLPKGL--

-TPKGLG-
TSPKGL--
TGPKGL--
-TPKGLI-
-TPKGLV-
TTPKGL--
-TPKGLM-
-TPKGLA-
TEPKGL--
-TPKGLY-
-TPKGLN-
-TPKGLK-
-TPKGLF-
TKPKGL--
-TPKGLQ-
TAPKGL--
TIPKGL--
TWPKGL--
TPPKGL--
TMPKGL--
TDPKGL--
-TPKGLW-
TYPKGL--
-TPKGLE-
TCPKGL--
-TPKGLC-
-VPKGLT-
-VPKGLL-
VTPKGL--
-VPKGLR-
VVPKGL--
-VPKGLK-
VIPKGL--
VPPKGL--
-VPKGLM-
VGPKGL--
VDPKGL--
VAPKGL--
-VPKGLN-
-VPKGLA-
-VPKGLV-
-VPKGLF-
VQPKGL--
-VPKGLH-
-VPKGLE-
VHPKGL--
-VPKGLY-
VEPKGL--
-VPKGLW-
-VPKGLI-

VMPKGL--
VCPKGL--
-WPKGLL-
-WPKGLY-
WVPKGL--
-WPKGLV-
WKPKGL--
WFPKGL--
WWPKGL--
-WPKGLT-
WGPKGL--
WYPKGL--
-WPKGLN-
WMPKGL--
WSPKGL--
WQPKGL--
-WPKGLM-
-WPKGLS-
WTPKGL--
-WPKGLA-
WEPKGL--
WPPKGL--
-WPKGLE-
WHPKGL--
-YPKGLY-
YLPKGL--
-YPKGLL-
YYPKGL--
YVPKGL--
YIPKGL--
-YPKGLS-
YSPKGL--
-YPKGLA-
YGPKGL--
YMPKGL--
-YPKGLV-
-YPKGLG-
YDPKGL--
-YPKGLM-
-YPKGLF-
YEPKGL--
YWPKGL--
YQPKGL--
YPPKGL--
-YPKGLW-
-YPKGLE-
YAPKGL--
-YPKGLN-
-YPKGLQ-
-YPKGLD-

PAHL 14 854 34.56

```
INPAHL--
LDPAHL--
--PAHLGY
--PAHLGR
-APAHLR-
AVPAHL--
-APAHLN-
-APAHLI-
AIPAHL--
ATPAHL--
-APAHLL-
-APAHLT-
ARPAHL--
-APAHLW-
AQPAHL--
ASPAHL--
-APAHLM-
AHPAHL--
APPAHL--
AKPAHL--
-APAHLH-
AAPAHL--
-APAHLV-
-APAHLF-
ALPAHL--
AEPAHL--
AWPAHL--
-APAHLG-
-APAHLA-
AYPAHL--
-APAHLQ-
-APAHLK-
ANPAHL--
AMPAHL--
AGPAHL--
AFPAHL--
-APAHLY-
-APAHLS-
-APAHLE-
ADPAHL--
CRPAHL--
CFPAHL--
CSPAHL--
-CPAHLE-
CYPAHL--
CMPAHL--
CNPAHL--
CVPAHL--
-CPAHLV-
```

-CPAHLL-
CGPAHL--
CWPAHL--
-CPAHLW-
-CPAHLR-
CKPAHL--
CHPAHL--
DRPAHL--
DKPAHL--
DYPAHL--
DWPAHL--
DVPAHL--
-DPAHLQ-
DNPAHL--
-DPAHLL-
DGPAHL--
DEPAHL--
DTPAHL--
DSPAHL--
-DPAHLV-
-DPAHLR-
-DPAHLK-
DQPAHL--
-DPAHLY-
-DPAHLT-
-DPAHLI-
-DPAHLG-
-DPAHLF-
DMPAHL--
DFPAHL--
-DPAHLW-
-DPAHLS-
-DPAHLN-
-DPAHLA-
DHPAHL--
DAPAHL--
EWPAHL--
-EPAHLL-
ESPAHL--
EGPAHL--
-EPAHLR-
-EPAHLG-
EIPAHL--
ERPAHL--
-EPAHLK-
ELPAHL--
ETPAHL--
-EPAHLS-
-EPAHLQ-
ENPAHL--

EVPAHL--
EQPAHL--
-EPAHLT-
-EPAHLH-
-EPAHLF-
-EPAHLV-
-EPAHLN-
-EPAHLI-
ECPAHL--
EYPAHL--
-EPAHLW-
-EPAHLE-
-EPAHLC-
-EPAHLA-
EMPAHL--
FSPAHL--
FHPAHL--
FRPAHL--
-FPAHLR-
-FPAHLS-
-FPAHLQ-
FIPAHL--
FTPAHL--
-FPAHLW-
FGPAHL--
-FPAHLT-
-FPAHLA-
FAPAHL--
FVPAHL--
FYPAHL--
FNPAHL--
-FPAHLF-
FDPAHL--
-FPAHLM-
-FPAHLL-
-FPAHLH-
-FPAHLE-
FLPAHL--
FEPAHL--
-FPAHLY-
-FPAHLV-
-FPAHLN-
-FPAHLI-
-FPAHLG-
FWPAHL--
FPPAHL--
-FPAHLK-
FMPAHL--
-GPAHLL-
GRPAHL--

GQPAHL--
-GPAHLR-
GVPAHL--
-GPAHLN-
GKPAHL--
-GPAHLV-
GSPAHL--
GTPAHL--
-GPAHLK-
-GPAHLT-
-GPAHLQ-
GYPAHL--
-GPAHLH-
GGPAHL--
GEPAHL--
GPPAHL--
-GPAHLF-
-GPAHLM-
-GPAHLI-
-GPAHLG-
-GPAHLE-
-GPAHLA-
GIPAHL--
GCPAHL--
GAPAHL--
GWPAHL--
-GPAHLY-
-GPAHLS-
GDPAHL--
HRPAHL--
HFPAHL--
-HPAHLR-
-HPAHLY-
-HPAHLS-
-HPAHLV-
HSPAHL--
-HPAHLN-
-HPAHLK-
HGPAHL--
HPPAHL--
-HPAHLL-
HWPAHL--
HVPAHL--
-HPAHLA-
HLPAHL--
HHPAHL--
HIPAHL--
HQPAHL--
-HPAHLT-
-HPAHLQ-

−HPAHLE−
HMPAHL−−
−HPAHLW−
−HPAHLP−
−HPAHLM−
−HPAHLI−
−HPAHLG−
−HPAHLF−
HNPAHL−−
IYPAHL−−
IWPAHL−−
−IPAHLY−
ILPAHL−−
ISPAHL−−
IRPAHL−−
−IPAHLR−
ITPAHL−−
−IPAHLL−
−IPAHLI−
IIPAHL−−
−IPAHLG−
IFPAHL−−
IQPAHL−−
−IPAHLA−
IMPAHL−−
−IPAHLT−
−IPAHLN−
IKPAHL−−
IGPAHL−−
IAPAHL−−
IVPAHL−−
−IPAHLK−
−IPAHLH−
ICPAHL−−
IPPAHL−−
−IPAHLV−
−IPAHLF−
IDPAHL−−
−IPAHLE−
IHPAHL−−
−IPAHLQ−
IEPAHL−−
KSPAHL−−
KTPAHL−−
−KPAHLR−
−KPAHLS−
−KPAHLQ−
KAPAHL−−
−KPAHLT−
−KPAHLI−

KGPAHL--
KWPAHL--
KRPAHL--
-KPAHLL-
-KPAHLH-
KQPAHL--
-KPAHLE-
KHPAHL--
KFPAHL--
-KPAHLM-
-KPAHLF-
-KPAHLA-
KLPAHL--
-KPAHLG-
KVPAHL--
-KPAHLW-
-KPAHLV-
KMPAHL--
-KPAHLY-
KPPAHL--
-KPAHLN-
KIPAHL--
-KPAHLK-
-LPAHLS-
LRPAHL--
LVPAHL--
LFPAHL--
LSPAHL--
-LPAHLV-
-LPAHLA-
LPPAHL--
LAPAHL--
LTPAHL--
-LPAHLL-
LGPAHL--
-LPAHLR-
-LPAHLN-
-LPAHLY-
-LPAHLK-
LKPAHL--
-LPAHLT-
-LPAHLG-
LWPAHL--
LQPAHL--
-LPAHLW-
-LPAHLI-
-LPAHLH-
LYPAHL--
-LPAHLF-
-LPAHLQ-

LNPAHL--
LIPAHL--
LEPAHL--
-LPAHLM-
LHPAHL--
LCPAHL--
MRPAHL--
MQPAHL--
-MPAHLR-
MPPAHL--
-MPAHLL-
MSPAHL--
MYPAHL--
MKPAHL--
-MPAHLW-
-MPAHLE-
MLPAHL--
-MPAHLT-
-MPAHLM-
-MPAHLH-
MGPAHL--
-MPAHLS-
-MPAHLI-
-MPAHLN-
-MPAHLA-
MNPAHL--
MDPAHL--
MWPAHL--
MTPAHL--
-MPAHLY-
-MPAHLK-
MMPAHL--
MEPAHL--
-MPAHLG-
MHPAHL--
MAPAHL--
MVPAHL--
-MPAHLV-
-MPAHLF-
MIPAHL--
NLPAHL--
-NPAHLL-
NGPAHL--
-NPAHLY-
-NPAHLG-
NSPAHL--
-NPAHLN-
-NPAHLA-
NMPAHL--
NRPAHL--

NPPAHL--
-NPAHLS-
-NPAHLF-
-NPAHLT-
-NPAHLR-
-NPAHLQ-
NNPAHL--
NHPAHL--
NDPAHL--
NYPAHL--
NTPAHL--
-NPAHLI-
NWPAHL--
-NPAHLV-
NIPAHL--
NFPAHL--
NVPAHL--
NQPAHL--
-NPAHLM-
-NPAHLE-
NKPAHL--
NEPAHL--
NCPAHL--
--PAHLSR
--PAHLLL
--PAHLRS
--PAHLLN
--PAHLRK
--PAHLLR
--PAHLSQ
--PAHLVT
--PAHLVS
--PAHLRR
PGPAHL--
--PAHLTS
--PAHLVL
--PAHLSS
--PAHLLH
--PAHLSA
--PAHLHF
--PAHLAS
--PAHLMA
--PAHLAL
--PAHLVG
--PAHLTR
--PAHLIS
--PAHLHR
--PAHLGS
PTPAHL--
--PAHLYR

--PAHLWH
--PAHLVR
--PAHLST
--PAHLRW
--PAHLRV
--PAHLGL
--PAHLIL
--PAHLGT
--PAHLFV
PVPAHL--
--PAHLYL
--PAHLWG
--PAHLTL
--PAHLSG
--PAHLRM
--PAHLLW
--PAHLFT
--PAHLSI
--PAHLMT
--PAHLMH
--PAHLKL
PWPAHL--
PLPAHL--
PKPAHL--
PIPAHL--
--PAHLYK
--PAHLVA
--PAHLTY
--PAHLTN
--PAHLSV
--PAHLSM
--PAHLRT
--PAHLRN
--PAHLQN
--PAHLNA
--PAHLMG
--PAHLLY
--PAHLLM
--PAHLKG
--PAHLIK
--PAHLGI
--PAHLAG
--PAHLVY
--PAHLRF
--PAHLKS
--PAHLKR
--PAHLKI
--PAHLIM
--PAHLGK
--PAHLGA

--PAHLFA
--PAHLEF
PRPAHL--
PAPAHL--
--PAHLWA
--PAHLVW
--PAHLVV
--PAHLVM
--PAHLTA
--PAHLSD
--PAHLRD
--PAHLQY
--PAHLMS
--PAHLML
--PAHLLQ
--PAHLKN
--PAHLKK
--PAHLHS
--PAHLHN
--PAHLHG
--PAHLGG
--PAHLAV
--PAHLAQ
PSPAHL--
PEPAHL--
--PAHLYW
--PAHLWY
--PAHLVQ
--PAHLTF
--PAHLQV
--PAHLNW
--PAHLNI
--PAHLLE
--PAHLIT
--PAHLHA
--PAHLGN
--PAHLYT
--PAHLYG
--PAHLWR
--PAHLWK
--PAHLVN
--PAHLVK
--PAHLTV
--PAHLSK
--PAHLQS
--PAHLQR
--PAHLNR
--PAHLNL
--PAHLKV
--PAHLKQ

--PAHLIN
--PAHLIH
--PAHLIG
--PAHLFS
--PAHLFR
--PAHLFI
--PAHLFG
--PAHLEN
--PAHLEL
--PAHLCT
--PAHLAY
--PAHLAT
--PAHLAK
--PAHLAI
--PAHLYY
--PAHLYF
--PAHLWN
--PAHLVH
--PAHLTT
--PAHLTM
--PAHLTG
--PAHLSE
--PAHLQL
--PAHLQI
--PAHLNT
--PAHLNS
--PAHLNN
--PAHLNK
--PAHLNG
--PAHLMV
--PAHLMR
--PAHLLI
--PAHLLF
--PAHLKA
--PAHLIW
--PAHLIE
--PAHLHE
--PAHLGQ
--PAHLFE
--PAHLFD
--PAHLET
--PAHLEK
--PAHLAA
PYPAHL--
PQPAHL--
--PAHLYV
--PAHLYH
--PAHLYD
--PAHLWL
--PAHLWE

--PAHLTH
--PAHLTE
--PAHLSH
--PAHLRQ
--PAHLRE
--PAHLQK
--PAHLMW
--PAHLMK
--PAHLLV
--PAHLLK
--PAHLKM
--PAHLKH
--PAHLHQ
--PAHLHK
--PAHLGV
--PAHLED
--PAHLAN
--PAHLAF
--PAHLAD
--PAHLYI
--PAHLWV
--PAHLWT
--PAHLWS
--PAHLWM
--PAHLWI
--PAHLWF
--PAHLVE
--PAHLTW
--PAHLTK
--PAHLQH
--PAHLQF
--PAHLQA
--PAHLNV
--PAHLNM
--PAHLNH
--PAHLNF
--PAHLMQ
--PAHLMD
--PAHLLT
--PAHLLD
--PAHLKT
--PAHLKE
--PAHLIV
--PAHLIA
--PAHLHY
--PAHLHT
--PAHLHL
--PAHLEW
--PAHLES
--PAHLER

--PAHLEQ
--PAHLDG
--PAHLAE
PHPAHL--
PDPAHL--
PCPAHL--
--PAHLYS
--PAHLYM
--PAHLYE
--PAHLYA
--PAHLWQ
--PAHLVI
--PAHLVF
--PAHLTQ
--PAHLSW
--PAHLRH
--PAHLQQ
--PAHLQG
--PAHLPT
--PAHLPI
--PAHLPA
--PAHLNP
--PAHLMY
--PAHLMN
--PAHLMM
--PAHLME
--PAHLKY
--PAHLKW
--PAHLKD
--PAHLIR
--PAHLIQ
--PAHLIF
--PAHLID
--PAHLHD
--PAHLGF
--PAHLFM
--PAHLFH
--PAHLEV
--PAHLEH
--PAHLEG
--PAHLEE
--PAHLEA
--PAHLDV
--PAHLDT
--PAHLDS
--PAHLDA
--PAHLCS
--PAHLCN
--PAHLAW
--PAHLAH

-QPAHLT-
QLPAHL--
QGPAHL--
QSPAHL--
-QPAHLH-
-QPAHLL-
QMPAHL--
QAPAHL--
-QPAHLN-
QWPAHL--
QRPAHL--
-QPAHLS-
-QPAHLW-
-QPAHLE-
QVPAHL--
-QPAHLF-
QIPAHL--
QPPAHL--
-QPAHLV-
-QPAHLR-
QNPAHL--
QKPAHL--
QFPAHL--
QQPAHL--
-QPAHLG-
-QPAHLD-
-QPAHLA-
QCPAHL--
RVPAHL--
RTPAHL--
RSPAHL--
-RPAHLG-
RPPAHL--
RMPAHL--
-RPAHLV-
RLPAHL--
RHPAHL--
RAPAHL--
RNPAHL--
RFPAHL--
-RPAHLH-
RRPAHL--
-RPAHLF-
RIPAHL--
-RPAHLE-
-RPAHLA-
RDPAHL--
-RPAHLT-
-RPAHLL-
-RPAHLK-

RWPAHL--
-RPAHLW-
-RPAHLN-
-RPAHLM-
-RPAHLQ-
-RPAHLI-
RCPAHL--
RQPAHL--
REPAHL--
-RPAHLD-
SVPAHL--
-SPAHLW-
SRPAHL--
SLPAHL--
SFPAHL--
-SPAHLT-
-SPAHLK-
SAPAHL--
SYPAHL--
SSPAHL--
SQPAHL--
-SPAHLS-
-SPAHLH-
-SPAHLG-
SNPAHL--
SWPAHL--
STPAHL--
SPPAHL--
-SPAHLF-
-SPAHLR-
-SPAHLQ-
-SPAHLN-
SEPAHL--
SHPAHL--
SIPAHL--
-SPAHLE-
SMPAHL--
-SPAHLD-
-SPAHLC-
SDPAHL--
TVPAHL--
-TPAHLS-
-TPAHLR-
TNPAHL--
TKPAHL--
-TPAHLT-
TFPAHL--
TGPAHL--
TRPAHL--
-TPAHLW-

```
-TPAHLQ-
-TPAHLG-
-TPAHLF-
-TPAHLY-
-TPAHLI-
TTPAHL--
TQPAHL--
TPPAHL--
-TPAHLV-
-TPAHLL-
TIPAHL--
-TPAHLK-
TAPAHL--
TYPAHL--
-TPAHLN-
TLPAHL--
TCPAHL--
-TPAHLE-
-TPAHLA-
THPAHL--
-VPAHLG-
VMPAHL--
VLPAHL--
VRPAHL--
-VPAHLL-
-VPAHLT-
VFPAHL--
VTPAHL--
VQPAHL--
-VPAHLK-
VKPAHL--
-VPAHLN-
VVPAHL--
-VPAHLW-
-VPAHLH-
VNPAHL--
VEPAHL--
VDPAHL--
VYPAHL--
-VPAHLR-
-VPAHLA-
-VPAHLM-
-VPAHLI-
-VPAHLF-
-VPAHLE-
VIPAHL--
VWPAHL--
VPPAHL--
-VPAHLV-
VHPAHL--
```

WVPAHL--
WLPAHL--
WKPAHL--
WHPAHL--
WRPAHL--
-WPAHLT-
WQPAHL--
-WPAHLK-
-WPAHLA-
WTPAHL--
-WPAHLG-
-WPAHLE-
WIPAHL--
WWPAHL--
-WPAHLL-
WGPAHL--
WSPAHL--
-WPAHLS-
-WPAHLN-
-WPAHLF-
WNPAHL--
WMPAHL--
WEPAHL--
WAPAHL--
-WPAHLW-
WCPAHL--
-WPAHLV-
YVPAHL--
YRPAHL--
-YPAHLV-
-YPAHLS-
YLPAHL--
-YPAHLH-
-YPAHLT-
YIPAHL--
YTPAHL--
-YPAHLQ-
YSPAHL--
-YPAHLK-
YKPAHL--
-YPAHLF-
YEPAHL--
YWPAHL--
YQPAHL--
-YPAHLE-
-YPAHLA-
YNPAHL--
-YPAHLN-
YHPAHL--
YPPAHL--

| | | | |
|---|---|---|---|
| | -YPAHLL- | | |
| | YMPAHL-- | | |
| | YFPAHL-- | | |
| | YCPAHL-- | | |
| | -YPAHLW- | | |
| PVNL | 81 | 779 | 34.51 |
| | -APVNLP- | | |
| | -EPVNLL- | | |
| | -EPVNLH- | | |
| | GHPVNL-- | | |
| | -LPVNLG- | | |
| | --PVNLGR | | |
| | --PVNLPR | | |
| | --PVNLMP | | |
| | --PVNLGP | | |
| | --PVNLGN | | |
| | -APVNLR- | | |
| | ASPVNL-- | | |
| | AKPVNL-- | | |
| | -APVNLH- | | |
| | ALPVNL-- | | |
| | -APVNLL- | | |
| | AVPVNL-- | | |
| | -APVNLG- | | |
| | -APVNLN- | | |
| | -APVNLT- | | |
| | -APVNLS- | | |
| | -APVNLI- | | |
| | ANPVNL-- | | |
| | AQPVNL-- | | |
| | -APVNLV- | | |
| | AGPVNL-- | | |
| | AYPVNL-- | | |
| | -APVNLK- | | |
| | AIPVNL-- | | |
| | AWPVNL-- | | |
| | -APVNLQ- | | |
| | ADPVNL-- | | |
| | -APVNLM- | | |
| | APPVNL-- | | |
| | AEPVNL-- | | |
| | -APVNLA- | | |
| | AMPVNL-- | | |
| | AHPVNL-- | | |
| | AFPVNL-- | | |
| | -APVNLE- | | |
| | CGPVNL-- | | |
| | -CPVNLM- | | |
| | CSPVNL-- | | |
| | -CPVNLT- | | |

CTPVNL--
-CPVNLS-
-CPVNLL-
CRPVNL--
-CPVNLY-
-CPVNLR-
-CPVNLQ-
CDPVNL--
CYPVNL--
CWPVNL--
-CPVNLK-
-CPVNLF-
CPPVNL--
CNPVNL--
CKPVNL--
CEPVNL--
CQPVNL--
-CPVNLP-
-CPVNLA-
CMPVNL--
CFPVNL--
DVPVNL--
DLPVNL--
DRPVNL--
DAPVNL--
DTPVNL--
-DPVNLS-
DQPVNL--
-DPVNLR-
-DPVNLK-
DMPVNL--
-DPVNLL-
DKPVNL--
DHPVNL--
DWPVNL--
DSPVNL--
DFPVNL--
-DPVNLW-
-DPVNLT-
-DPVNLI-
-DPVNLV-
DNPVNL--
DIPVNL--
DGPVNL--
-DPVNLY-
DCPVNL--
DYPVNL--
-DPVNLM-
-DPVNLF-
-DPVNLE-

-DPVNLA-
DPPVNL--
DEPVNL--
ERPVNL--
-EPVNLR-
EGPVNL--
ESPVNL--
-EPVNLY-
-EPVNLW-
-EPVNLS-
EFPVNL--
ETPVNL--
-EPVNLN-
-EPVNLK-
-EPVNLV-
-EPVNLI-
-EPVNLG-
-EPVNLA-
ENPVNL--
EQPVNL--
-EPVNLE-
-EPVNLQ-
-EPVNLF-
EEPVNL--
ELPVNL--
EHPVNL--
EDPVNL--
EYPVNL--
-EPVNLT-
EPPVNL--
EMPVNL--
EKPVNL--
EWPVNL--
EVPVNL--
EAPVNL--
FSPVNL--
-FPVNLA-
FLPVNL--
-FPVNLS-
FGPVNL--
-FPVNLH-
-FPVNLL-
FAPVNL--
-FPVNLK-
FMPVNL--
FHPVNL--
FEPVNL--
FQPVNL--
-FPVNLR-
FYPVNL--

FRPVNL--
FKPVNL--
-FPVNLV-
-FPVNLN-
-FPVNLE-
-FPVNLT-
FDPVNL--
-FPVNLQ-
-FPVNLM-
FPPVNL--
FCPVNL--
-FPVNLD-
-FPVNLC-
FNPVNL--
GVPVNL--
GRPVNL--
GSPVNL--
-GPVNLR-
GLPVNL--
GTPVNL--
-GPVNLS-
-GPVNLL-
GGPVNL--
-GPVNLT-
-GPVNLK-
GNPVNL--
GKPVNL--
-GPVNLN-
-GPVNLI-
GCPVNL--
GAPVNL--
GYPVNL--
-GPVNLW-
GWPVNL--
GQPVNL--
-GPVNLQ-
GMPVNL--
-GPVNLG-
-GPVNLH-
-GPVNLV-
-GPVNLM-
-GPVNLE-
GEPVNL--
-GPVNLA-
GPPVNL--
GDPVNL--
-GPVNLD-
-HPVNLS-
-HPVNLM-
-HPVNLR-

HKPVNL--
-HPVNLL-
-HPVNLG-
HAPVNL--
HYPVNL--
HSPVNL--
HGPVNL--
HLPVNL--
-HPVNLA-
HMPVNL--
-HPVNLT-
-HPVNLK-
-HPVNLF-
HVPVNL--
-HPVNLI-
HIPVNL--
-HPVNLY-
-HPVNLN-
-HPVNLH-
HTPVNL--
-HPVNLW-
-HPVNLV-
-HPVNLE-
HNPVNL--
HHPVNL--
HDPVNL--
HWPVNL--
-HPVNLP-
HPPVNL--
HFPVNL--
HCPVNL--
-IPVNLK-
INPVNL--
ITPVNL--
ISPVNL--
IYPVNL--
IVPVNL--
-IPVNLG-
IKPVNL--
IQPVNL--
IFPVNL--
-IPVNLM-
IGPVNL--
-IPVNLN-
-IPVNLV-
-IPVNLT-
-IPVNLL-
IEPVNL--
IDPVNL--
-IPVNLQ-

-IPVNLH-
IMPVNL--
IHPVNL--
IAPVNL--
-IPVNLE-
-IPVNLA-
-IPVNLI-
KSPVNL--
-KPVNLR-
-KPVNLL-
KGPVNL--
KVPVNL--
KAPVNL--
-KPVNLN-
-KPVNLM-
KMPVNL--
KIPVNL--
-KPVNLA-
KFPVNL--
-KPVNLV-
KLPVNL--
KNPVNL--
-KPVNLW-
-KPVNLH-
KPPVNL--
-KPVNLG-
-KPVNLF-
KTPVNL--
KYPVNL--
-KPVNLQ-
KEPVNL--
-KPVNLI-
-KPVNLD-
KQPVNL--
KDPVNL--
-LPVNLL-
LQPVNL--
-LPVNLV-
-LPVNLH-
-LPVNLS-
LLPVNL--
LGPVNL--
LAPVNL--
LSPVNL--
-LPVNLQ-
-LPVNLA-
LNPVNL--
LMPVNL--
-LPVNLK-
-LPVNLI-

LFPVNL--
LDPVNL--
LWPVNL--
LPPVNL--
LKPVNL--
-LPVNLN-
-LPVNLM-
-LPVNLE-
LEPVNL--
MSPVNL--
MYPVNL--
-MPVNLL-
MFPVNL--
MAPVNL--
MRPVNL--
MDPVNL--
MIPVNL--
MTPVNL--
-MPVNLT-
-MPVNLA-
MPPVNL--
-MPVNLV-
-MPVNLS-
-MPVNLQ-
-MPVNLE-
-MPVNLK-
-MPVNLG-
MQPVNL--
-MPVNLN-
-MPVNLM-
-MPVNLI-
MGPVNL--
MEPVNL--
MWPVNL--
MNPVNL--
-MPVNLD-
MCPVNL--
NSPVNL--
NIPVNL--
-NPVNLI-
-NPVNLL-
NQPVNL--
NLPVNL--
NVPVNL--
-NPVNLR-
-NPVNLT-
NWPVNL--
NHPVNL--
-NPVNLY-
-NPVNLW-

-NPVNLQ-
-NPVNLA-
NPPVNL--
NKPVNL--
NTPVNL--
-NPVNLS-
-NPVNLN-
-NPVNLF-
-NPVNLV-
NAPVNL--
-NPVNLG-
NGPVNL--
-NPVNLK-
NNPVNL--
NYPVNL--
-NPVNLM-
NDPVNL--
-NPVNLE-
NMPVNL--
NCPVNL--
-NPVNLH-
--PVNLRH
--PVNLVR
--PVNLLR
--PVNLRV
--PVNLSG
--PVNLRS
--PVNLTR
--PVNLLG
--PVNLSS
--PVNLVS
--PVNLRY
--PVNLKR
--PVNLGL
--PVNLSR
--PVNLRG
--PVNLRT
--PVNLRM
--PVNLRK
--PVNLMA
--PVNLKL
--PVNLGS
--PVNLST
--PVNLSQ
--PVNLRN
--PVNLVK
--PVNLSV
--PVNLQW
--PVNLAH
--PVNLRR

--PVNLTQ
--PVNLRA
PGPVNL--
--PVNLYN
--PVNLVA
--PVNLSY
--PVNLSA
--PVNLMN
--PVNLLD
--PVNLNS
--PVNLLN
--PVNLLH
--PVNLVG
--PVNLTL
--PVNLAA
--PVNLTG
--PVNLTA
--PVNLQR
--PVNLPL
--PVNLMR
--PVNLGA
--PVNLAR
--PVNLVN
--PVNLTT
--PVNLSF
--PVNLMG
--PVNLKI
--PVNLGV
PRPVNL--
PLPVNL--
PWPVNL--
--PVNLVT
--PVNLLK
--PVNLGI
PTPVNL--
--PVNLTS
--PVNLSN
--PVNLQE
--PVNLKY
--PVNLHG
PAPVNL--
--PVNLTN
--PVNLSM
--PVNLRF
--PVNLNT
--PVNLKT
--PVNLIG
--PVNLFG
--PVNLEK
--PVNLAM

--PVNLSI
--PVNLSH
--PVNLMY
--PVNLHL
--PVNLFQ
--PVNLEV
--PVNLAG
PSPVNL--
PYPVNL--
--PVNLYK
--PVNLYG
--PVNLVD
--PVNLTV
--PVNLTM
--PVNLSE
--PVNLQT
--PVNLQG
--PVNLNN
--PVNLMK
--PVNLKG
--PVNLHR
--PVNLHF
--PVNLGW
--PVNLES
--PVNLAS
--PVNLTI
--PVNLTH
--PVNLSW
--PVNLRD
--PVNLQS
--PVNLQI
--PVNLQH
--PVNLNW
--PVNLNF
--PVNLNA
--PVNLLM
--PVNLGG
--PVNLFH
--PVNLEL
--PVNLEG
--PVNLEA
PNPVNL--
PMPVNL--
PDPVNL--
--PVNLWG
--PVNLVH
--PVNLTY
--PVNLSK
--PVNLQK
--PVNLMW

--PVNLMI
--PVNLIH
--PVNLID
--PVNLEF
--PVNLAI
PHPVNL--
PVPVNL--
--PVNLWE
--PVNLNV
--PVNLNG
--PVNLHM
--PVNLHK
--PVNLFN
--PVNLAT
--PVNLYH
--PVNLVE
--PVNLSD
--PVNLQY
--PVNLQV
--PVNLMH
--PVNLKQ
--PVNLKF
--PVNLHT
--PVNLHH
--PVNLGM
--PVNLET
--PVNLER
--PVNLEI
PIPVNL--
PEPVNL--
--PVNLYE
--PVNLWK
--PVNLVQ
--PVNLTK
--PVNLTD
--PVNLRW
--PVNLQM
--PVNLPY
--PVNLNR
--PVNLNM
--PVNLMQ
--PVNLKW
--PVNLKM
--PVNLKH
--PVNLIN
--PVNLIK
--PVNLHA
--PVNLEQ
--PVNLEH
--PVNLDS

--PVNLYD
--PVNLWD
--PVNLTW
--PVNLQQ
--PVNLQL
--PVNLNK
--PVNLNI
--PVNLMD
--PVNLLP
--PVNLKV
--PVNLKN
--PVNLHY
--PVNLHQ
--PVNLHN
--PVNLHE
--PVNLHD
--PVNLFD
--PVNLEY
--PVNLDA
--PVNLAD
--PVNLYQ
--PVNLWQ
--PVNLWF
--PVNLWC
--PVNLVC
--PVNLTE
--PVNLSC
--PVNLQD
--PVNLNQ
--PVNLME
--PVNLKE
--PVNLKD
--PVNLIC
--PVNLGQ
--PVNLGF
--PVNLDG
--PVNLCA
--PVNLAQ
--PVNLAN
--PVNLAC
PQPVNL--
-PPVNLS-
PKPVNL--
QSPVNL--
-QPVNLL-
-QPVNLA-
QGPVNL--
QRPVNL--
-QPVNLH-
QNPVNL--

−QPVNLR−
QQPVNL−−
−QPVNLT−
−QPVNLK−
QLPVNL−−
−QPVNLS−
−QPVNLV−
QYPVNL−−
QPPVNL−−
QKPVNL−−
QTPVNL−−
−QPVNLG−
QMPVNL−−
QWPVNL−−
−QPVNLQ−
−QPVNLN−
−QPVNLM−
QDPVNL−−
QVPVNL−−
−QPVNLE−
QFPVNL−−
−QPVNLF−
QEPVNL−−
QCPVNL−−
RVPVNL−−
−RPVNLL−
RPPVNL−−
−RPVNLH−
RGPVNL−−
−RPVNLG−
RMPVNL−−
−RPVNLV−
RDPVNL−−
−RPVNLI−
−RPVNLF−
−RPVNLK−
RIPVNL−−
RTPVNL−−
−RPVNLM−
RYPVNL−−
RQPVNL−−
−RPVNLN−
−RPVNLW−
−RPVNLE−
RNPVNL−−
REPVNL−−
RHPVNL−−
−RPVNLD−
RCPVNL−−
−SPVNLR−

-SPVNLL-
SLPVNL--
-SPVNLS-
SGPVNL--
SSPVNL--
-SPVNLK-
SNPVNL--
SVPVNL--
-SPVNLA-
SFPVNL--
-SPVNLI-
STPVNL--
-SPVNLY-
-SPVNLN-
-SPVNLE-
SHPVNL--
-SPVNLH-
-SPVNLF-
SMPVNL--
SWPVNL--
-SPVNLW-
SCPVNL--
SAPVNL--
SQPVNL--
SEPVNL--
SPPVNL--
SDPVNL--
-TPVNLR-
TLPVNL--
-TPVNLS-
-TPVNLL-
-TPVNLK-
-TPVNLH-
TSPVNL--
TGPVNL--
-TPVNLT-
-TPVNLF-
-TPVNLV-
-TPVNLQ-
TMPVNL--
TVPVNL--
-TPVNLW-
TQPVNL--
-TPVNLY-
-TPVNLM-
TPPVNL--
TEPVNL--
TDPVNL--
-TPVNLG-
TNPVNL--

TIPVNL--
TFPVNL--
THPVNL--
TAPVNL--
-TPVNLE-
TCPVNL--
VRPVNL--
VSPVNL--
-VPVNLR-
VGPVNL--
VVPVNL--
-VPVNLK-
-VPVNLH-
VYPVNL--
VLPVNL--
VFPVNL--
-VPVNLG-
VNPVNL--
VTPVNL--
-VPVNLT-
-VPVNLA-
VAPVNL--
-VPVNLN-
VEPVNL--
VPPVNL--
-VPVNLQ-
VMPVNL--
VWPVNL--
VKPVNL--
VIPVNL--
VDPVNL--
-VPVNLM-
-VPVNLD-
-VPVNLE-
WSPVNL--
WTPVNL--
-WPVNLS-
WEPVNL--
WYPVNL--
-WPVNLL-
WGPVNL--
WAPVNL--
-WPVNLR-
WQPVNL--
-WPVNLN-
-WPVNLK-
WVPVNL--
WMPVNL--
-WPVNLH-
-WPVNLA-

WNPVNL--
-WPVNLM-
-WPVNLG-
-WPVNLY-
-WPVNLE-
WPPVNL--
WHPVNL--
-WPVNLT-
WDPVNL--
-YPVNLR-
YRPVNL--
YKPVNL--
-YPVNLL-
-YPVNLN-
YYPVNL--
-YPVNLS-
YGPVNL--
-YPVNLK-
YSPVNL--
YFPVNL--
YAPVNL--
-YPVNLA-
YMPVNL--
-YPVNLT-
-YPVNLM-
-YPVNLI-
-YPVNLG-
YPPVNL--
YEPVNL--
YNPVNL--
YHPVNL--
-YPVNLY-
-YPVNLQ-
YCPVNL--
YDPVNL--
PLAL 215 704 34.49
-APLALP-
KMPLAL--
--PLALGD
--PLALDT
--PLALDF
-SPLALD-
-YPLALD-
-APLALT-
ALPLAL--
ATPLAL--
-APLALS-
AKPLAL--
-APLALE-
AQPLAL--

AWPLAL--
AMPLAL--
-APLALH-
-APLALG-
-APLALA-
AGPLAL--
AEPLAL--
AIPLAL--
AFPLAL--
-APLALQ-
APPLAL--
AHPLAL--
AYPLAL--
-APLALI-
-APLALV-
-APLALM-
ADPLAL--
-APLALK-
-APLALN-
-APLALD-
ACPLAL--
CKPLAL--
CTPLAL--
-CPLALS-
CFPLAL--
CVPLAL--
CRPLAL--
CQPLAL--
CGPLAL--
CDPLAL--
CSPLAL--
-CPLALK-
-CPLALH-
CMPLAL--
CHPLAL--
CEPLAL--
CNPLAL--
CWPLAL--
-CPLALV-
-CPLALQ-
-CPLALN-
-CPLALG-
-CPLALF-
-CPLALE-
-CPLALA-
CIPLAL--
DVPLAL--
DSPLAL--
-DPLALL-
DLPLAL--

-DPLALN-
DMPLAL--
DAPLAL--
-DPLALV-
DIPLAL--
DGPLAL--
DNPLAL--
DWPLAL--
DTPLAL--
-DPLALT-
DKPLAL--
DQPLAL--
-DPLALK-
DFPLAL--
-DPLALQ-
-DPLALI-
-DPLALH-
DHPLAL--
-DPLALG-
-DPLALA-
-DPLALM-
DEPLAL--
ERPLAL--
-EPLALR-
ESPLAL--
EKPLAL--
ELPLAL--
EVPLAL--
-EPLALS-
ETPLAL--
-EPLALH-
EHPLAL--
-EPLALV-
-EPLALK-
-EPLALG-
-EPLALA-
-EPLALN-
-EPLALE-
EAPLAL--
-EPLALQ-
-EPLALI-
EMPLAL--
EQPLAL--
ENPLAL--
EGPLAL--
EWPLAL--
EFPLAL--
ECPLAL--
EDPLAL--
FSPLAL--

-FPLALT-
FYPLAL--
-FPLALN-
FGPLAL--
-FPLALG-
-FPLALQ-
-FPLALS-
FNPLAL--
FQPLAL--
-FPLALH-
FLPLAL--
FMPLAL--
FAPLAL--
FEPLAL--
-FPLALI-
-FPLALA-
-FPLALE-
FDPLAL--
-FPLALM-
-GPLALS-
GRPLAL--
GLPLAL--
-GPLALR-
-GPLALG-
GAPLAL--
GWPLAL--
GPPLAL--
-GPLALT-
-GPLALA-
GNPLAL--
-GPLALM-
GFPLAL--
-GPLALK-
-GPLALN-
GGPLAL--
GEPLAL--
-GPLALV-
GQPLAL--
GKPLAL--
-GPLALQ-
GYPLAL--
-GPLALE-
-GPLALD-
GCPLAL--
-GPLALP-
HRPLAL--
HYPLAL--
-HPLALH-
-HPLALR-
HLPLAL--

-HPLALL-
-HPLALN-
-HPLALS-
HSPLAL--
-HPLALT-
HGPLAL--
HVPLAL--
-HPLALV-
HNPLAL--
-HPLALI-
HKPLAL--
-HPLALA-
HIPLAL--
HWPLAL--
HEPLAL--
-HPLALE-
HTPLAL--
HQPLAL--
HMPLAL--
HFPLAL--
-HPLALK-
HHPLAL--
-HPLALM-
HDPLAL--
-HPLALD-
HCPLAL--
-IPLALS-
IAPLAL--
-IPLALL-
ISPLAL--
IMPLAL--
ILPLAL--
-IPLALH-
ITPLAL--
-IPLALG-
-IPLALN-
IFPLAL--
IIPLAL--
-IPLALK-
INPLAL--
-IPLALQ-
IHPLAL--
IEPLAL--
IYPLAL--
IQPLAL--
-IPLALA-
IVPLAL--
IPPLAL--
IKPLAL--
IDPLAL--

IWPLAL--
-IPLALM-
-IPLALE-
-KPLALA-
KHPLAL--
-KPLALS-
KTPLAL--
KVPLAL--
-KPLALN-
KLPLAL--
-KPLALG-
-KPLALQ-
KKPLAL--
KAPLAL--
KDPLAL--
-KPLALV-
-KPLALK-
KYPLAL--
KWPLAL--
-KPLALE-
KIPLAL--
KFPLAL--
KPPLAL--
-KPLALM-
-KPLALH-
KQPLAL--
KEPLAL--
KCPLAL--
-KPLALC-
LQPLAL--
LRPLAL--
LTPLAL--
-LPLALH-
-LPLALS-
LMPLAL--
-LPLALN-
-LPLALT-
LNPLAL--
LPPLAL--
LHPLAL--
-LPLALV-
-LPLALK-
LWPLAL--
LKPLAL--
LIPLAL--
LFPLAL--
-LPLALA-
-LPLALQ-
-LPLALM-
LDPLAL--

LCPLAL--
MRPLAL--
MFPLAL--
-MPLALV-
MVPLAL--
-MPLALH-
-MPLALA-
MKPLAL--
MGPLAL--
-MPLALG-
MTPLAL--
-MPLALL-
-MPLALK-
MLPLAL--
-MPLALS-
-MPLALN-
MMPLAL--
MIPLAL--
MHPLAL--
MQPLAL--
MNPLAL--
MWPLAL--
MEPLAL--
-MPLALQ-
-MPLALE-
MCPLAL--
NSPLAL--
-NPLALR-
NRPLAL--
-NPLALL-
NGPLAL--
-NPLALI-
-NPLALT-
-NPLALG-
NKPLAL--
NIPLAL--
-NPLALF-
-NPLALA-
-NPLALS-
-NPLALK-
NFPLAL--
NTPLAL--
NPPLAL--
-NPLALW-
-NPLALQ-
NEPLAL--
NWPLAL--
NMPLAL--
-NPLALV-
-NPLALH-

NQPLAL--
NYPLAL--
NVPLAL--
NAPLAL--
NHPLAL--
-NPLALE-
NNPLAL--
-NPLALN-
NCPLAL--
-NPLALM-
--PLALRR
--PLALRW
--PLALHR
--PLALVR
--PLALGG
--PLALAL
--PLALSH
--PLALMG
--PLALRK
--PLALRG
--PLALSS
--PLALNL
--PLALNS
--PLALWS
--PLALRP
--PLALKT
--PLALKL
--PLALHS
--PLALGY
--PLALRI
--PLALNT
--PLALTM
--PLALRA
--PLALHT
--PLALGT
--PLALAS
--PLALSN
--PLALMS
--PLALLN
--PLALTS
--PLALKK
--PLALTI
--PLALFG
--PLALYG
--PLALSV
--PLALRN
--PLALQT
--PLALHL
--PLALSE
--PLALKS

--PLALGR
--PLALRE
--PLALPL
--PLALIR
--PLALHM
--PLALHK
--PLALTA
--PLALNR
--PLALNH
--PLALIN
--PLALLQ
--PLALKV
--PLALER
PEPLAL--
--PLALSM
--PLALSK
--PLALSF
--PLALMN
--PLALLK
--PLALGI
--PLALFN
--PLALFH
PNPLAL--
--PLALMK
--PLALKN
--PLALGQ
--PLALTV
--PLALSQ
--PLALSI
--PLALQG
--PLALKM
--PLALKE
--PLALID
--PLALGW
--PLALES
--PLALDS
--PLALAH
PDPLAL--
--PLALSD
--PLALRQ
--PLALLH
--PLALKG
--PLALHG
--PLALGV
--PLALGN
--PLALEM
--PLALYP
--PLALVK
--PLALSW
--PLALQL

--PLALPW
--PLALEK
--PLALAG
--PLALWD
--PLALVE
--PLALTK
--PLALSY
--PLALST
--PLALQS
--PLALQA
--PLALNN
--PLALLD
--PLALET
--PLALYN
--PLALYH
--PLALVG
--PLALRD
--PLALQV
--PLALNV
--PLALKW
--PLALHF
--PLALGA
--PLALDL
--PLALAN
--PLALPI
--PLALKH
--PLALKF
--PLALHA
--PLALYD
--PLALTH
--PLALTD
--PLALQY
--PLALQM
--PLALNW
--PLALMQ
--PLALKY
--PLALKQ
--PLALHE
--PLALGF
--PLALAQ
--PLALAA
PWPLAL--
--PLALTW
--PLALTQ
--PLALPY
--PLALMT
--PLALMP
--PLALHN
--PLALDG
--PLALTN

--PLALNY
--PLALNM
--PLALHI
--PLALEI
--PLALQR
--PLALPM
--PLALPF
--PLALNA
--PLALKP
--PLALHV
--PLALEW
--PLALEL
--PLALEA
--PLALAE
--PLALQF
--PLALPV
--PLALNQ
--PLALNK
--PLALNF
--PLALIP
--PLALHP
--PLALEY
--PLALEG
--PLALDY
--PLALAW
--PLALAK
--PLALAD
-PPLALS-
--PLALWN
--PLALVP
--PLALQW
--PLALQN
--PLALQH
--PLALNI
--PLALNG
--PLALKD
--PLALIH
--PLALHY
--PLALHD
--PLALED
--PLALDM
--PLALDA
--PLALCT
--PLALCR
--PLALAI
PPPLAL--
-PPLALR-
--PLALTC
--PLALRC
--PLALQQ

--PLALQK
--PLALQD
--PLALPQ
--PLALNE
--PLALMW
--PLALKC
--PLALHW
--PLALGM
--PLALGE
--PLALFC
--PLALEN
--PLALEE
--PLALCV
--PLALCA
--PLALAC
-QPLALR-
-QPLALS-
-QPLALL-
QSPLAL--
QRPLAL--
-QPLALH-
QEPLAL--
QTPLAL--
-QPLALG-
QFPLAL--
QLPLAL--
-QPLALA-
QWPLAL--
-QPLALT-
-QPLALM-
QVPLAL--
QQPLAL--
QDPLAL--
-QPLALK-
QNPLAL--
QYPLAL--
QKPLAL--
-QPLALE-
QMPLAL--
QIPLAL--
QAPLAL--
QPPLAL--
-QPLALQ-
-QPLALD-
-QPLALC-
QCPLAL--
RSPLAL--
-RPLALG-
RMPLAL--
RYPLAL--

RAPLAL--
-RPLALK-
-RPLALV-
RGPLAL--
-RPLALM-
RQPLAL--
RIPLAL--
-RPLALE-
RFPLAL--
RWPLAL--
-RPLALQ-
RNPLAL--
RDPLAL--
-RPLALH-
RHPLAL--
-RPLALD-
REPLAL--
RCPLAL--
-SPLALS-
-SPLALR-
-SPLALK-
SSPLAL--
SKPLAL--
STPLAL--
SMPLAL--
SGPLAL--
SYPLAL--
SPPLAL--
-SPLALA-
SAPLAL--
-SPLALG-
-SPLALQ-
-SPLALM-
-SPLALV-
SDPLAL--
SHPLAL--
SFPLAL--
SQPLAL--
-SPLALI-
SEPLAL--
SCPLAL--
-TPLALL-
THPLAL--
TVPLAL--
-TPLALG-
-TPLALT-
TGPLAL--
-TPLALS-
TQPLAL--
TFPLAL--

```
-TPLALN-
-TPLALH-
TMPLAL--
-TPLALQ-
TTPLAL--
TSPLAL--
TAPLAL--
TNPLAL--
TIPLAL--
TEPLAL--
-TPLALM-
TWPLAL--
TDPLAL--
-TPLALE-
TCPLAL--
-TPLALK-
VSPLAL--
VRPLAL--
-VPLALS-
-VPLALL-
VWPLAL--
-VPLALT-
-VPLALR-
VKPLAL--
-VPLALA-
VTPLAL--
-VPLALH-
VNPLAL--
VEPLAL--
-VPLALI-
VMPLAL--
VIPLAL--
-VPLALN-
VDPLAL--
VYPLAL--
-VPLALQ-
VPPLAL--
VQPLAL--
-VPLALE-
VHPLAL--
-VPLALD-
-WPLALK-
-WPLALR-
WPPLAL--
WGPLAL--
WEPLAL--
WMPLAL--
WNPLAL--
WAPLAL--
WVPLAL--
```

-WPLALM-
-WPLALH-
-WPLALG-
WYPLAL--
-WPLALT-
-WPLALE-
WFPLAL--
-WPLALV-
-WPLALA-
-WPLALQ-
WDPLAL--
WIPLAL--
WHPLAL--
-YPLALV-
YRPLAL--
YTPLAL--
-YPLALK-
-YPLALR-
-YPLALT-
YFPLAL--
YAPLAL--
YSPLAL--
-YPLALN-
YLPLAL--
YIPLAL--
YQPLAL--
-YPLALG-
-YPLALS-
-YPLALH-
-YPLALQ-
YEPLAL--
YWPLAL--
YYPLAL--
-YPLALA-
YDPLAL--
YMPLAL--
-YPLALM-
YHPLAL--
-YPLALI-
YCPLAL--

PKAL 241 692 34.49

--PKALGR
--PKALER
SVPKAL--
-APKALS-
-APKALT-
ALPKAL--
AVPKAL--
-APKALR-
-APKALQ-

-APKALL-
ATPKAL--
-APKALY-
-APKALK-
AIPKAL--
-APKALN-
-APKALG-
APPKAL--
-APKALM-
-APKALH-
-APKALA-
AGPKAL--
ACPKAL--
AQPKAL--
-APKALV-
AAPKAL--
AYPKAL--
AKPKAL--
-APKALF-
ANPKAL--
AMPKAL--
AEPKAL--
ADPKAL--
-APKALW-
-APKALE-
CCPKAL--
CRPKAL--
CTPKAL--
-CPKALK-
-CPKALM-
CHPKAL--
CGPKAL--
CVPKAL--
CSPKAL--
CPPKAL--
-CPKALS-
-CPKALF-
CMPKAL--
CIPKAL--
CFPKAL--
CYPKAL--
CWPKAL--
-CPKALV-
-CPKALQ-
-CPKALL-
-CPKALH-
-CPKALE-
CNPKAL--
CKPKAL--
DLPKAL--

DMPKAL--
-DPKALI-
DNPKAL--
DVPKAL--
DWPKAL--
DQPKAL--
DAPKAL--
-DPKALV-
-DPKALK-
-DPKALH-
DTPKAL--
-DPKALY-
-DPKALG-
-DPKALA-
-DPKALS-
-DPKALR-
-DPKALM-
DKPKAL--
DFPKAL--
DSPKAL--
-DPKALW-
-DPKALQ-
DGPKAL--
DDPKAL--
DCPKAL--
-EPKALL-
-EPKALV-
-EPKALR-
ENPKAL--
ELPKAL--
-EPKALT-
-EPKALM-
-EPKALY-
EMPKAL--
EGPKAL--
ESPKAL--
-EPKALS-
EYPKAL--
EVPKAL--
ETPKAL--
-EPKALF-
EIPKAL--
EHPKAL--
-EPKALK-
-EPKALA-
EAPKAL--
EQPKAL--
-EPKALQ-
-EPKALI-
-EPKALG-

EFPKAL--
EEPKAL--
ECPKAL--
EWPKAL--
-EPKALN-
-EPKALC-
-FPKALA-
FLPKAL--
FMPKAL--
-FPKALY-
FAPKAL--
FYPKAL--
FEPKAL--
FSPKAL--
-FPKALQ-
FVPKAL--
-FPKALM-
FNPKAL--
FRPKAL--
FFPKAL--
FHPKAL--
FPPKAL--
-FPKALW-
-FPKALV-
-FPKALE-
-GPKALR-
GLPKAL--
GSPKAL--
GMPKAL--
-GPKALW-
-GPKALA-
GRPKAL--
-GPKALM-
GPPKAL--
-GPKALT-
GKPKAL--
GWPKAL--
-GPKALS-
-GPKALF-
GYPKAL--
-GPKALG-
GNPKAL--
-GPKALV-
-GPKALQ-
-GPKALK-
GHPKAL--
-GPKALN-
GEPKAL--
-GPKALE-
GTPKAL--

GDPKAL--
GAPKAL--
-GPKALI-
-GPKALH-
-GPKALC-
HVPKAL--
-HPKALS-
HRPKAL--
HLPKAL--
HWPKAL--
-HPKALW-
HNPKAL--
HSPKAL--
-HPKALV-
HPPKAL--
HYPKAL--
-HPKALL-
-HPKALK-
HGPKAL--
-HPKALE-
HKPKAL--
-HPKALY-
HMPKAL--
HIPKAL--
HDPKAL--
-HPKALM-
-HPKALH-
-HPKALA-
HEPKAL--
HQPKAL--
-HPKALT-
-HPKALQ-
-HPKALG-
-IPKALS-
ISPKAL--
-IPKALT-
IAPKAL--
IVPKAL--
IGPKAL--
ITPKAL--
-IPKALY-
-IPKALM-
-IPKALE-
-IPKALG-
-IPKALN-
-IPKALI-
IYPKAL--
INPKAL--
IMPKAL--
IHPKAL--

IDPKAL--
-IPKALW-
-IPKALV-
IQPKAL--
-IPKALQ-
-IPKALA-
-IPKALH-
KAPKAL--
KTPKAL--
-KPKALS-
-KPKALL-
KGPKAL--
-KPKALA-
KVPKAL--
-KPKALI-
-KPKALK-
KMPKAL--
-KPKALM-
-KPKALG-
-KPKALQ-
KEPKAL--
-KPKALV-
-KPKALN-
KNPKAL--
KKPKAL--
KHPKAL--
-KPKALW-
KQPKAL--
-KPKALH-
-KPKALE-
KCPKAL--
LYPKAL--
LMPKAL--
-LPKALT-
LGPKAL--
-LPKALV-
-LPKALL-
-LPKALM-
-LPKALI-
-LPKALW-
LEPKAL--
LLPKAL--
LKPKAL--
LDPKAL--
-LPKALF-
-LPKALA-
LQPKAL--
-LPKALH-
-LPKALY-
LPPKAL--

-LPKALC-
-LPKALG-
-MPKALS-
-MPKALK-
-MPKALQ-
MLPKAL--
MGPKAL--
MWPKAL--
MKPKAL--
-MPKALV-
MIPKAL--
MSPKAL--
MQPKAL--
MAPKAL--
MDPKAL--
MMPKAL--
MEPKAL--
-MPKALT-
-MPKALM-
-MPKALA-
MVPKAL--
MTPKAL--
-MPKALN-
-MPKALG-
MNPKAL--
-MPKALI-
-MPKALW-
-MPKALH-
-MPKALE-
NVPKAL--
-NPKALV-
-NPKALN-
-NPKALF-
NQPKAL--
NMPKAL--
NEPKAL--
NNPKAL--
-NPKALW-
-NPKALQ-
-NPKALM-
-NPKALA-
NCPKAL--
NHPKAL--
NGPKAL--
NDPKAL--
NSPKAL--
--PKALLN
--PKALGL
--PKALNL
--PKALAL

--PKALYH
--PKALLK
--PKALFL
PVPKAL--
--PKALSF
--PKALYK
--PKALLL
--PKALAA
--PKALNK
--PKALLA
--PKALWH
--PKALTT
--PKALST
--PKALSG
--PKALNH
--PKALQW
--PKALML
--PKALKS
--PKALFV
--PKALFK
--PKALTA
--PKALSW
--PKALMV
--PKALGY
--PKALGG
--PKALAS
--PKALAR
--PKALVT
--PKALSY
--PKALFG
--PKALSS
--PKALNF
--PKALFT
--PKALFS
--PKALAI
--PKALYM
--PKALVG
--PKALTY
--PKALGT
--PKALWT
--PKALTW
--PKALSN
--PKALNY
--PKALII
--PKALIF
PLPKAL--
--PKALVI
--PKALQQ
--PKALPF
--PKALNW

--PKALHS
--PKALFI
--PKALAF
--PKALWY
--PKALWQ
--PKALWM
--PKALLV
--PKALKT
--PKALAY
PRPKAL--
--PKALYV
--PKALWS
--PKALWG
--PKALVA
--PKALTM
--PKALRQ
--PKALRE
--PKALMY
--PKALMT
--PKALMN
--PKALLQ
--PKALIT
--PKALIL
--PKALTN
--PKALTK
--PKALTG
--PKALSK
--PKALRV
--PKALQL
--PKALHQ
--PKALGM
--PKALGA
--PKALAV
--PKALYQ
--PKALYG
--PKALWV
--PKALVD
--PKALQE
--PKALMG
--PKALKW
--PKALIH
--PKALGF
--PKALES
--PKALAH
PGPKAL--
--PKALYE
--PKALWW
--PKALWA
--PKALVM
--PKALTQ

--PKALSE
--PKALIN
--PKALIK
--PKALHG
--PKALGV
--PKALGI
--PKALEA
--PKALDS
--PKALAT
--PKALAM
PFPKAL--
PSPKAL--
PQPKAL--
--PKALYT
--PKALYN
--PKALWN
--PKALWL
--PKALWK
--PKALTH
--PKALTE
--PKALPW
--PKALNV
--PKALNA
--PKALMK
--PKALMA
--PKALKK
--PKALHW
--PKALHV
--PKALHN
--PKALHA
--PKALFN
--PKALEV
--PKALAE
PHPKAL--
PAPKAL--
PMPKAL--
--PKALYI
--PKALYF
--PKALQT
--PKALQS
--PKALQK
--PKALPY
--PKALPL
--PKALNT
--PKALNQ
--PKALNN
--PKALNM
--PKALND
--PKALKF
--PKALKD

```
--PKALIM
--PKALIG
--PKALGK
--PKALFM
--PKALFD
--PKALAQ
--PKALAK
PIPKAL--
PNPKAL--
--PKALTF
--PKALQV
--PKALQN
--PKALQG
--PKALMF
--PKALMD
--PKALLD
--PKALIQ
--PKALHI
--PKALHF
--PKALHD
--PKALGQ
--PKALFH
--PKALEH
PDPKAL--
PTPKAL--
PKPKAL--
--PKALYD
--PKALYA
--PKALWE
--PKALTI
--PKALSQ
--PKALSH
--PKALRC
--PKALQY
--PKALQD
--PKALMQ
--PKALMM
--PKALME
--PKALKQ
--PKALHM
--PKALHK
--PKALHH
--PKALFW
--PKALFP
--PKALEY
--PKALEN
--PKALAG
--PKALAD
PEPKAL--
PYPKAL--
```

--PKALYP
--PKALWD
--PKALVP
--PKALVE
--PKALSD
--PKALSC
--PKALPV
--PKALPI
--PKALNE
--PKALMI
--PKALKM
--PKALIW
--PKALIV
--PKALIE
--PKALIA
--PKALHE
--PKALGW
--PKALGH
--PKALFE
--PKALFC
--PKALDQ
--PKALDM
--PKALDG
--PKALDA
--PKALCI
--PKALCG
--PKALCF
--PKALCD
PCPKAL--
QRPKAL--
QSPKAL--
-QPKALT-
-QPKALR-
-QPKALK-
-QPKALA-
QYPKAL--
QQPKAL--
QIPKAL--
-QPKALG-
-QPKALW-
-QPKALQ-
QTPKAL--
-QPKALV-
QVPKAL--
-QPKALL-
QHPKAL--
-QPKALI-
QNPKAL--
QLPKAL--
QGPKAL--

QWPKAL--
QPPKAL--
-QPKALN-
-QPKALH-
-QPKALE-
QEPKAL--
QCPKAL--
-QPKALM-
-QPKALF-
QDPKAL--
RTPKAL--
-RPKALM-
RIPKAL--
-RPKALV-
RVPKAL--
-RPKALA-
REPKAL--
-RPKALN-
-RPKALG-
RKPKAL--
RHPKAL--
-RPKALE-
-RPKALH-
-SPKALL-
-SPKALT-
SLPKAL--
SKPKAL--
STPKAL--
-SPKALS-
SGPKAL--
SIPKAL--
-SPKALA-
-SPKALH-
-SPKALF-
SQPKAL--
-SPKALI-
SPPKAL--
SNPKAL--
SCPKAL--
-SPKALE-
-SPKALW-
-SPKALM-
-SPKALV-
-SPKALQ-
SDPKAL--
-SPKALC-
SEPKAL--
-TPKALV-
-TPKALM-
TKPKAL--

TVPKAL--
TYPKAL--
-TPKALK-
TWPKAL--
-TPKALW-
TLPKAL--
TPPKAL--
-TPKALA-
TGPKAL--
TAPKAL--
-TPKALY-
-TPKALN-
TFPKAL--
TDPKAL--
TTPKAL--
TNPKAL--
TMPKAL--
-TPKALS-
TEPKAL--
-TPKALQ-
-TPKALE-
THPKAL--
TCPKAL--
VSPKAL--
-VPKALL-
VLPKAL--
VQPKAL--
VTPKAL--
-VPKALW-
-VPKALA-
-VPKALV-
-VPKALQ-
VDPKAL--
VVPKAL--
-VPKALN-
-VPKALE-
VMPKAL--
VGPKAL--
VHPKAL--
-VPKALM-
-VPKALF-
VNPKAL--
VEPKAL--
VCPKAL--
-VPKALI-
WLPKAL--
-WPKALT-
WFPKAL--
WYPKAL--
WQPKAL--

WNPKAL--
WKPKAL--
WDPKAL--
WPPKAL--
-WPKALM-
-WPKALF-
WEPKAL--
-WPKALH-
-WPKALW-
WWPKAL--
WMPKAL--
-WPKALN-
-WPKALE-
-WPKALC-
-WPKALA-
YEPKAL--
YMPKAL--
-YPKALL-
YIPKAL--
YGPKAL--
-YPKALS-
YNPKAL--
YSPKAL--
-YPKALH-
YKPKAL--
YAPKAL--
YPPKAL--
-YPKALQ-
-YPKALI-
YYPKAL--
YTPKAL--
-YPKALE-
YFPKAL--

| | | | |
|---|---|---|---|
| LANL | 181 | 725 | 34.48 |

-DLANLG-
-ELANLV-
-ELANLM-
-LLANLP-
--LANLQF
--LANLNG
--LANLGY
--LANLGQ
-RLANLD-
-SLANLD-
-TLANLD-
ARLANL--
ASLANL--
-ALANLT-
-ALANLS-
AMLANL--

-ALANLK-
-ALANLI-
AILANL--
ATLANL--
-ALANLV-
AELANL--
-ALANLN-
-ALANLG-
-ALANLF-
AGLANL--
AALANL--
AYLANL--
AFLANL--
-ALANLA-
-ALANLM-
-ALANLE-
AHLANL--
ANLANL--
-ALANLQ-
-ALANLH-
ADLANL--
ACLANL--
AQLANL--
APLANL--
-ALANLD-
-CLANLA-
-CLANLN-
-CLANLI-
CLLANL--
-CLANLV-
-CLANLR-
-CLANLM-
-CLANLG-
CGLANL--
CVLANL--
CQLANL--
CMLANL--
-CLANLL-
-CLANLE-
CILANL--
CDLANL--
CSLANL--
CCLANL--
CYLANL--
CTLANL--
-CLANLY-
-CLANLT-
CKLANL--
CFLANL--
CALANL--

CPLANL--
CNLANL--
-CLANLW-
-CLANLK-
CHLANL--
CELANL--
-DLANLL-
DGLANL--
DSLANL--
-DLANLV-
-DLANLR-
DALANL--
-DLANLT-
DNLANL--
-DLANLS-
DQLANL--
DKLANL--
DELANL--
DPLANL--
DFLANL--
DWLANL--
DRLANL--
-DLANLI-
-DLANLF-
DHLANL--
DCLANL--
DYLANL--
-DLANLQ-
-DLANLK-
-DLANLE-
DMLANL--
-DLANLM-
DTLANL--
-DLANLH-
-DLANLY-
DDLANL--
-DLANLP-
-DLANLC-
-DLANLA-
-ELANLR-
EVLANL--
-ELANLS-
ETLANL--
-ELANLK-
EYLANL--
EGLANL--
-ELANLE-
EFLANL--
-ELANLA-
-ELANLT-

-ELANLI-
-ELANLG-
-ELANLF-
EKLANL--
EWLANL--
EPLANL--
ENLANL--
EMLANL--
-ELANLN-
EILANL--
EHLANL--
EALANL--
EELANL--
EDLANL--
ESLANL--
EQLANL--
-ELANLQ-
ECLANL--
FGLANL--
-FLANLK-
FRLANL--
FNLANL--
-FLANLT-
-FLANLH-
FALANL--
FPLANL--
-FLANLV-
-FLANLE-
FQLANL--
-FLANLN-
FDLANL--
-FLANLM-
-FLANLD-
FCLANL--
-GLANLR-
GLLANL--
GSLANL--
GRLANL--
GTLANL--
GWLANL--
GNLANL--
-GLANLG-
-GLANLS-
-GLANLA-
GPLANL--
-GLANLW-
GGLANL--
-GLANLT-
-GLANLH-
-GLANLK-

GHLANL--
-GLANLL-
GVLANL--
GILANL--
-GLANLN-
-GLANLI-
GQLANL--
GELANL--
GYLANL--
GKLANL--
-GLANLE-
GALANL--
-GLANLQ-
GCLANL--
-HLANLS-
HSLANL--
HTLANL--
-HLANLY-
HGLANL--
HWLANL--
-HLANLL-
-HLANLF-
HALANL--
-HLANLI-
-HLANLH-
HVLANL--
-HLANLV-
HNLANL--
-HLANLQ-
HMLANL--
-HLANLN-
-HLANLK-
-HLANLG-
HKLANL--
HDLANL--
HPLANL--
HILANL--
HQLANL--
-HLANLE-
-HLANLW-
HELANL--
-HLANLT-
-ILANLL-
-ILANLK-
IGLANL--
-ILANLT-
-ILANLS-
IDLANL--
-ILANLN-
IKLANL--

-ILANLE-
IQLANL--
-ILANLQ-
-ILANLA-
-ILANLV-
-ILANLM-
-ILANLH-
ICLANL--
-ILANLG-
IHLANL--
-ILANLD-
KLLANL--
KSLANL--
-KLANLV-
KVLANL--
-KLANLS-
KNLANL--
-KLANLT-
-KLANLQ-
KYLANL--
KMLANL--
KGLANL--
KTLANL--
KWLANL--
-KLANLW-
KILANL--
KPLANL--
-KLANLE-
-KLANLA-
KDLANL--
KFLANL--
-KLANLI-
-KLANLG-
KHLANL--
-KLANLK-
-KLANLM-
KELANL--
--LANLLG
-LLANLK-
--LANLRR
--LANLSL
--LANLSS
--LANLSR
LGLANL--
--LANLAL
--LANLLN
--LANLYG
--LANLVS
--LANLLA
-LLANLM-

--LANLST
--LANLRT
--LANLVR
--LANLSY
--LANLSV
--LANLMS
--LANLMG
--LANLIR
--LANLFS
LTLANL--
--LANLVA
--LANLRN
--LANLVG
--LANLRH
--LANLKV
--LANLVK
--LANLTL
--LANLKA
-LLANLH-
LDLANL--
LELANL--
--LANLTN
--LANLIT
--LANLGT
--LANLGS
--LANLQS
--LANLQA
--LANLYR
--LANLYQ
--LANLTS
--LANLSG
--LANLLQ
--LANLKL
--LANLEA
--LANLYH
--LANLVN
--LANLVD
--LANLSW
--LANLSI
--LANLSH
--LANLSA
--LANLNS
--LANLKT
--LANLFT
--LANLAY
LALANL--
--LANLYN
--LANLYK
--LANLWG
--LANLTK

--LANLTI
--LANLRQ
--LANLMA
--LANLKR
--LANLKQ
--LANLGG
--LANLAS
--LANLAI
--LANLYT
--LANLWR
--LANLHT
--LANLFH
--LANLWA
--LANLTV
--LANLSF
--LANLQG
--LANLKS
--LANLAG
LQLANL--
-LLANLI-
--LANLVH
--LANLTH
--LANLRM
--LANLQK
--LANLPL
--LANLKG
LHLANL--
--LANLVQ
--LANLTD
--LANLSQ
--LANLSM
--LANLMY
--LANLMQ
--LANLKH
--LANLHW
--LANLGV
--LANLFQ
--LANLAV
--LANLAN
--LANLAA
--LANLSN
--LANLRD
--LANLQM
--LANLMV
--LANLKF
--LANLIE
--LANLHS
--LANLGL
--LANLES
--LANLWK

```
--LANLNK
--LANLKN
--LANLFK
--LANLEI
--LANLAK
-LLANLE-
--LANLVM
--LANLTW
--LANLSK
--LANLMI
--LANLKI
--LANLHQ
--LANLHG
--LANLAT
-LLANLA-
--LANLTA
--LANLQN
--LANLQI
--LANLNA
--LANLMH
--LANLKD
--LANLIS
--LANLIK
--LANLFE
--LANLEN
--LANLDG
--LANLYD
--LANLYA
--LANLNV
--LANLIG
--LANLHL
--LANLHA
--LANLFG
--LANLFD
--LANLET
--LANLEG
--LANLAM
--LANLWE
--LANLQD
--LANLNT
--LANLMW
--LANLHF
--LANLFN
--LANLAQ
--LANLAD
LCLANL--
--LANLWH
--LANLVE
--LANLQW
--LANLNY
```

--LANLMM
--LANLKM
--LANLID
--LANLHK
--LANLGW
--LANLDS
--LANLWM
--LANLTQ
--LANLTM
--LANLTE
--LANLRE
--LANLNH
--LANLMN
--LANLME
--LANLMD
--LANLKW
--LANLIH
--LANLHY
--LANLHV
--LANLHH
--LANLYE
--LANLQH
--LANLQE
--LANLNW
--LANLHR
--LANLGI
--LANLER
--LANLEQ
--LANLEK
--LANLAH
-LLANLC-
--LANLSE
--LANLQQ
--LANLNQ
--LANLNF
--LANLKE
--LANLHN
--LANLCV
--LANLCA
--LANLYC
--LANLQV
--LANLKP
--LANLIN
--LANLIC
--LANLHE
--LANLHD
--LANLFC
--LANLEV
--LANLEH
--LANLEF

--LANLCQ
--LANLCK
--LANLCI
--LANLCG
--LANLAC
-MLANLI-
MGLANL--
-MLANLS-
MKLANL--
-MLANLL-
-MLANLH-
-MLANLV-
-MLANLT-
-MLANLG-
-MLANLA-
MHLANL--
MPLANL--
-MLANLN-
-MLANLM-
-MLANLK-
MQLANL--
-MLANLW-
MALANL--
-MLANLQ-
MTLANL--
MDLANL--
MELANL--
-MLANLD-
NRLANL--
NSLANL--
NGLANL--
-NLANLM-
-NLANLK-
-NLANLG-
-NLANLR-
NALANL--
NELANL--
NWLANL--
NMLANL--
-NLANLY-
-NLANLV-
-NLANLT-
NVLANL--
-NLANLN-
-NLANLA-
NNLANL--
NLLANL--
-NLANLL-
-NLANLF-
NILANL--

NPLANL--
-NLANLI-
-NLANLH-
NFLANL--
NYLANL--
NQLANL--
-NLANLQ-
-NLANLE-
NHLANL--
-PLANLR-
PVLANL--
-PLANLS-
PKLANL--
PNLANL--
-PLANLM-
PSLANL--
PILANL--
-PLANLA-
-PLANLQ-
PTLANL--
-PLANLI-
-PLANLV-
-PLANLF-
PFLANL--
PWLANL--
PMLANL--
PHLANL--
PALANL--
-PLANLT-
-PLANLH-
-PLANLE-
PYLANL--
PGLANL--
PDLANL--
-PLANLG-
-PLANLN-
PPLANL--
QRLANL--
-QLANLR-
-QLANLL-
-QLANLT-
QILANL--
QKLANL--
QPLANL--
QQLANL--
-QLANLV-
QDLANL--
-QLANLK-
QGLANL--
QTLANL--

QNLANL--
-QLANLW-
QVLANL--
-QLANLS-
-QLANLG-
-QLANLA-
QSLANL--
-QLANLQ-
-QLANLY-
-QLANLM-
QYLANL--
QMLANL--
-QLANLN-
-QLANLH-
-QLANLF-
-QLANLE-
QELANL--
QWLANL--
-QLANLI-
QFLANL--
QCLANL--
-QLANLD-
RALANL--
RLLANL--
RILANL--
-RLANLS-
-RLANLK-
RYLANL--
RPLANL--
-RLANLV-
RQLANL--
-RLANLQ-
RNLANL--
-RLANLI-
-RLANLG-
-RLANLY-
-RLANLH-
-RLANLE-
RDLANL--
-RLANLM-
-RLANLN-
RHLANL--
RCLANL--
-SLANLS-
SLLANL--
-SLANLT-
SNLANL--
-SLANLK-
SGLANL--
-SLANLA-

-SLANLV-
-SLANLN-
SILANL--
SALANL--
SMLANL--
-SLANLF-
SWLANL--
SQLANL--
SFLANL--
SHLANL--
SDLANL--
-SLANLH-
SCLANL--
-SLANLE-
SELANL--
TRLANL--
TSLANL--
-TLANLR-
TGLANL--
-TLANLT-
TTLANL--
-TLANLK-
TKLANL--
TPLANL--
-TLANLL-
-TLANLS-
TELANL--
TNLANL--
-TLANLG-
TQLANL--
-TLANLY-
-TLANLA-
-TLANLN-
TFLANL--
TALANL--
-TLANLW-
-TLANLE-
-TLANLI-
TDLANL--
TMLANL--
-TLANLQ-
-TLANLM-
TCLANL--
-VLANLS-
VTLANL--
VSLANL--
-VLANLA-
VKLANL--
VNLANL--
VGLANL--

VMLANL--
VQLANL--
-VLANLN-
-VLANLG-
-VLANLM-
VDLANL--
-VLANLT-
-VLANLV-
VHLANL--
VPLANL--
-VLANLH-
VELANL--
-VLANLI-
-VLANLE-
VCLANL--
-VLANLD-
-VLANLC-
WRLANL--
WSLANL--
WDLANL--
WGLANL--
WHLANL--
WWLANL--
WQLANL--
-WLANLG-
-WLANLS-
-WLANLE-
-WLANLA-
WCLANL--
-WLANLI-
WYLANL--
-WLANLQ-
-WLANLD-
WPLANL--
WNLANL--
WELANL--
-YLANLR-
-YLANLS-
YSLANL--
YTLANL--
YQLANL--
-YLANLH-
YGLANL--
-YLANLK-
YDLANL--
YPLANL--
-YLANLA-
YALANL--
-YLANLQ-
YYLANL--

-YLANLE-
YNLANL--
-YLANLM-
-YLANLD-
YELANL--
-YLANLV-
-YLANLI-
YCLANL--
YFLANL--

| | | | |
|---|---|---|---|
| PVPL | 19 | 846 | 34.47 |

APPVPL--
KIPVPL--
-LPVPLG-
--PVPLTM
--PVPLGR
-APVPLR-
-APVPLL-
-APVPLH-
ATPVPL--
ALPVPL--
-APVPLI-
-APVPLA-
AYPVPL--
ARPVPL--
-APVPLK-
ASPVPL--
AQPVPL--
-APVPLF-
AKPVPL--
AHPVPL--
AVPVPL--
-APVPLT-
AGPVPL--
-APVPLY-
-APVPLV-
-APVPLS-
AMPVPL--
AFPVPL--
-APVPLQ-
-APVPLG-
AAPVPL--
-APVPLE-
ANPVPL--
AIPVPL--
AWPVPL--
-APVPLP-
-APVPLM-
CRPVPL--
CSPVPL--
-CPVPLY-

CEPVPL--
CAPVPL--
-CPVPLL-
-CPVPLK-
CNPVPL--
CGPVPL--
-CPVPLQ-
-CPVPLM-
CPPVPL--
CIPVPL--
CYPVPL--
CWPVPL--
-CPVPLW-
-CPVPLT-
-CPVPLR-
-CPVPLN-
CCPVPL--
DRPVPL--
-DPVPLS-
-DPVPLW-
-DPVPLR-
DSPVPL--
-DPVPLL-
-DPVPLA-
DIPVPL--
DHPVPL--
DWPVPL--
DKPVPL--
DLPVPL--
DEPVPL--
-DPVPLN-
-DPVPLI-
DTPVPL--
-DPVPLV-
-DPVPLQ-
-DPVPLM-
-DPVPLH-
DGPVPL--
DFPVPL--
DAPVPL--
-EPVPLR-
-EPVPLS-
ESPVPL--
-EPVPLL-
ERPVPL--
EAPVPL--
-EPVPLT-
-EPVPLF-
-EPVPLV-
-EPVPLK-

EKPVPL--
EYPVPL--
-EPVPLM-
-EPVPLI-
-EPVPLH-
EWPVPL--
EVPVPL--
ETPVPL--
-EPVPLG-
-EPVPLA-
ELPVPL--
EGPVPL--
-EPVPLY-
-EPVPLQ-
-EPVPLN-
EIPVPL--
EDPVPL--
EQPVPL--
-EPVPLE-
FRPVPL--
-FPVPLR-
-FPVPLY-
-FPVPLI-
FYPVPL--
FEPVPL--
FVPVPL--
FSPVPL--
FQPVPL--
-FPVPLT-
-FPVPLM-
-FPVPLW-
FNPVPL--
FGPVPL--
FTPVPL--
FKPVPL--
-FPVPLS-
-FPVPLN-
FMPVPL--
-FPVPLV-
-FPVPLA-
FHPVPL--
FAPVPL--
-FPVPLK-
-FPVPLH-
-FPVPLG-
-FPVPLQ-
-FPVPLF-
FPPVPL--
FFPVPL--
-FPVPLE-

GIPVPL--
GRPVPL--
-GPVPLV-
GSPVPL--
-GPVPLG-
-GPVPLE-
-GPVPLF-
GVPVPL--
-GPVPLT-
-GPVPLS-
-GPVPLK-
-GPVPLQ-
GNPVPL--
GYPVPL--
-GPVPLN-
GTPVPL--
-GPVPLH-
GLPVPL--
GWPVPL--
GDPVPL--
-GPVPLW-
GMPVPL--
GKPVPL--
GFPVPL--
-GPVPLM-
-GPVPLA-
GQPVPL--
-GPVPLI-
GEPVPL--
GHPVPL--
GAPVPL--
-HPVPLT-
HKPVPL--
HRPVPL--
HNPVPL--
HLPVPL--
-HPVPLA-
-HPVPLW-
HFPVPL--
-HPVPLN-
HGPVPL--
-HPVPLL-
-HPVPLG-
HHPVPL--
-HPVPLS-
-HPVPLY-
HSPVPL--
HDPVPL--
HYPVPL--
-HPVPLE-

HAPVPL--
HTPVPL--
-HPVPLK-
HEPVPL--
HQPVPL--
-HPVPLV-
-HPVPLM-
-HPVPLH-
HIPVPL--
HWPVPL--
-HPVPLQ-
-HPVPLI-
-HPVPLF-
HPPVPL--
HMPVPL--
HCPVPL--
IKPVPL--
IRPVPL--
IYPVPL--
IIPVPL--
IGPVPL--
ISPVPL--
-IPVPLK-
-IPVPLL-
-IPVPLI-
ILPVPL--
-IPVPLT-
-IPVPLR-
-IPVPLH-
IAPVPL--
ITPVPL--
-IPVPLV-
-IPVPLQ-
-IPVPLM-
-IPVPLF-
IFPVPL--
-IPVPLW-
-IPVPLA-
INPVPL--
IWPVPL--
IPPVPL--
ICPVPL--
-IPVPLS-
-IPVPLN-
-IPVPLE-
IHPVPL--
IEPVPL--
-KPVPLR-
-KPVPLS-
-KPVPLN-

-KPVPLH-
-KPVPLV-
KAPVPL--
KMPVPL--
KRPVPL--
-KPVPLW-
-KPVPLG-
-KPVPLI-
KGPVPL--
KYPVPL--
-KPVPLQ-
-KPVPLA-
KEPVPL--
-KPVPLT-
-KPVPLM-
-KPVPLK-
KWPVPL--
KPPVPL--
KNPVPL--
KKPVPL--
-KPVPLC-
KCPVPL--
KQPVPL--
-KPVPLE-
-KPVPLD-
LRPVPL--
-LPVPLS-
-LPVPLR-
LGPVPL--
LLPVPL--
LVPVPL--
-LPVPLM-
-LPVPLT-
LWPVPL--
LKPVPL--
LHPVPL--
LMPVPL--
-LPVPLA-
LNPVPL--
LTPVPL--
LYPVPL--
LSPVPL--
-LPVPLN-
-LPVPLQ-
-LPVPLK-
LFPVPL--
LAPVPL--
LIPVPL--
LDPVPL--
-LPVPLV-

LQPVPL--
LCPVPL--
-LPVPLP-
-LPVPLH-
-MPVPLW-
-MPVPLR-
-MPVPLI-
-MPVPLT-
MVPVPL--
-MPVPLS-
MGPVPL--
MFPVPL--
MPPVPL--
MNPVPL--
-MPVPLL-
MSPVPL--
-MPVPLN-
-MPVPLA-
MAPVPL--
MWPVPL--
MKPVPL--
MIPVPL--
-MPVPLG-
MTPVPL--
MQPVPL--
-MPVPLK-
MMPVPL--
MDPVPL--
MYPVPL--
-MPVPLV-
-MPVPLM-
MHPVPL--
-MPVPLQ-
NRPVPL--
-NPVPLN-
-NPVPLT-
-NPVPLS-
NHPVPL--
NEPVPL--
-NPVPLF-
-NPVPLI-
NYPVPL--
NSPVPL--
NIPVPL--
NTPVPL--
-NPVPLR-
-NPVPLK-
-NPVPLY-
-NPVPLH-
-NPVPLA-

NMPVPL--
NKPVPL--
NFPVPL--
-NPVPLQ-
NNPVPL--
NVPVPL--
NPPVPL--
NLPVPL--
NAPVPL--
-NPVPLW-
NGPVPL--
NWPVPL--
NQPVPL--
-NPVPLG-
NDPVPL--
NCPVPL--
--PVPLRL
--PVPLRT
--PVPLLR
--PVPLHL
--PVPLYS
--PVPLLY
--PVPLWW
--PVPLVR
--PVPLRN
--PVPLSS
--PVPLSL
--PVPLVS
--PVPLST
--PVPLVT
--PVPLRA
--PVPLKL
--PVPLFK
--PVPLMR
--PVPLIV
--PVPLWL
--PVPLTS
PMPVPL--
--PVPLSH
--PVPLSG
--PVPLLV
--PVPLFR
--PVPLRG
--PVPLNL
--PVPLKG
--PVPLGL
PGPVPL--
--PVPLVY
--PVPLLT
--PVPLIL

--PVPLIA
--PVPLHW
--PVPLWS
--PVPLVF
--PVPLTI
--PVPLRQ
--PVPLRF
--PVPLHI
--PVPLAS
--PVPLWV
--PVPLVN
--PVPLVM
--PVPLLH
--PVPLGT
--PVPLAW
PVPVPL--
--PVPLYM
--PVPLWY
--PVPLRI
--PVPLGS
PYPVPL--
--PVPLYI
--PVPLYE
--PVPLTK
--PVPLTA
--PVPLSV
--PVPLSI
--PVPLPS
--PVPLLG
--PVPLKV
--PVPLKI
--PVPLHR
--PVPLFL
--PVPLEI
--PVPLDL
--PVPLAQ
--PVPLWE
--PVPLVD
--PVPLSM
--PVPLSA
--PVPLRY
--PVPLPH
--PVPLNN
--PVPLMK
--PVPLLN
--PVPLLM
--PVPLLI
--PVPLGG
--PVPLGF
--PVPLFA

--PVPLEL
--PVPLAK
PSPVPL--
--PVPLWF
--PVPLWA
--PVPLVI
--PVPLVH
--PVPLVE
--PVPLTT
--PVPLNI
--PVPLKT
--PVPLKR
--PVPLIS
--PVPLIR
--PVPLHK
--PVPLGN
--PVPLAL
--PVPLYV
--PVPLYR
--PVPLYN
--PVPLWT
--PVPLTN
--PVPLTE
--PVPLSY
--PVPLSN
--PVPLSK
--PVPLRK
--PVPLQS
--PVPLPQ
--PVPLLK
--PVPLLA
--PVPLII
--PVPLES
--PVPLAF
PLPVPL--
--PVPLYA
--PVPLTH
--PVPLSW
--PVPLRR
--PVPLNV
--PVPLIK
--PVPLHH
--PVPLGM
--PVPLEV
--PVPLAI
PIPVPL--
PDPVPL--
PWPVPL--
--PVPLYH
--PVPLWK

--PVPLTQ
--PVPLSF
--PVPLRM
--PVPLRE
--PVPLRD
--PVPLQV
--PVPLQE
--PVPLMD
--PVPLLW
--PVPLLF
--PVPLKK
--PVPLKA
--PVPLIG
--PVPLHD
--PVPLGH
--PVPLFW
--PVPLFG
--PVPLAY
--PVPLAV
--PVPLAA
PRPVPL--
--PVPLYF
--PVPLTV
--PVPLTF
--PVPLSD
--PVPLQR
--PVPLQK
--PVPLQH
--PVPLQG
--PVPLQA
--PVPLPF
--PVPLNH
--PVPLNA
--PVPLMY
--PVPLMT
--PVPLLE
--PVPLLD
--PVPLIW
--PVPLIQ
--PVPLIH
--PVPLHV
--PVPLHT
--PVPLGV
--PVPLFS
--PVPLFH
--PVPLEF
--PVPLAG
PTPVPL--
PKPVPL--
PFPVPL--

--PVPLYG
--PVPLTG
--PVPLRP
--PVPLRH
--PVPLQN
--PVPLQI
--PVPLPY
--PVPLNR
--PVPLNM
--PVPLNK
--PVPLMN
--PVPLLQ
--PVPLKY
--PVPLKF
--PVPLIN
--PVPLHA
--PVPLFY
--PVPLEA
--PVPLDS
--PVPLDF
--PVPLAT
PHPVPL--
--PVPLWH
--PVPLWG
--PVPLPN
--PVPLPM
--PVPLPA
--PVPLNT
--PVPLNS
--PVPLNG
--PVPLND
--PVPLMW
--PVPLMA
--PVPLKE
--PVPLKD
--PVPLIT
--PVPLHN
--PVPLHG
--PVPLGW
--PVPLFF
--PVPLEY
--PVPLAN
--PVPLYY
--PVPLYW
--PVPLYD
--PVPLWQ
--PVPLWI
--PVPLTD
--PVPLQY
--PVPLQW

--PVPLQM
--PVPLQF
--PVPLQD
--PVPLMV
--PVPLMQ
--PVPLMI
--PVPLMG
--PVPLME
--PVPLKW
--PVPLKQ
--PVPLKH
--PVPLHF
--PVPLGQ
--PVPLFN
--PVPLFD
--PVPLET
--PVPLEG
--PVPLDY
--PVPLDW
--PVPLDM
--PVPLDG
--PVPLAM
PQPVPL--
PNPVPL--
PEPVPL--
PCPVPL--
PAPVPL--
--PVPLWP
--PVPLVA
--PVPLTY
--PVPLTW
--PVPLPW
--PVPLPI
--PVPLNW
--PVPLKN
--PVPLID
--PVPLHM
--PVPLHE
--PVPLFT
--PVPLFQ
--PVPLFE
--PVPLEQ
--PVPLEM
--PVPLCN
--PVPLAH
-QPVPLR-
-QPVPLK-
QRPVPL--
QVPVPL--
QTPVPL--

QSPVPL--
-QPVPLS-
-QPVPLY-
-QPVPLN-
QWPVPL--
-QPVPLL-
-QPVPLW-
-QPVPLT-
QNPVPL--
QYPVPL--
-QPVPLA-
QMPVPL--
QLPVPL--
QQPVPL--
-QPVPLV-
-QPVPLQ-
QIPVPL--
QHPVPL--
-QPVPLG-
QFPVPL--
-QPVPLE-
QDPVPL--
QAPVPL--
-QPVPLM-
-QPVPLH-
QEPVPL--
-RPVPLR-
-RPVPLK-
-RPVPLS-
-RPVPLT-
RVPVPL--
RLPVPL--
-RPVPLN-
RRPVPL--
RQPVPL--
RNPVPL--
-RPVPLY-
-RPVPLM-
-RPVPLV-
RPPVPL--
-RPVPLQ-
RHPVPL--
REPVPL--
RAPVPL--
RIPVPL--
-RPVPLA-
RDPVPL--
-RPVPLI-
-RPVPLE-
RCPVPL--

-SPVPLS-
SRPVPL--
-SPVPLR-
-SPVPLT-
-SPVPLH-
STPVPL--
SSPVPL--
SKPVPL--
SGPVPL--
SHPVPL--
SNPVPL--
-SPVPLY-
-SPVPLV-
-SPVPLQ-
-SPVPLI-
SPPVPL--
SVPVPL--
SQPVPL--
-SPVPLW-
SWPVPL--
-SPVPLN-
SEPVPL--
SDPVPL--
SFPVPL--
-SPVPLM-
-SPVPLE-
SCPVPL--
-TPVPLK-
-TPVPLL-
TVPVPL--
TRPVPL--
TSPVPL--
TGPVPL--
TLPVPL--
-TPVPLW-
-TPVPLS-
-TPVPLA-
-TPVPLT-
-TPVPLN-
TTPVPL--
TNPVPL--
TAPVPL--
TYPVPL--
-TPVPLV-
-TPVPLH-
-TPVPLM-
-TPVPLY-
-TPVPLF-
TFPVPL--
-TPVPLI-

TEPVPL--
TWPVPL--
TCPVPL--
TQPVPL--
TMPVPL--
-TPVPLG-
THPVPL--
-TPVPLE-
TPPVPL--
VLPVPL--
-VPVPLL-
-VPVPLS-
-VPVPLI-
-VPVPLT-
VSPVPL--
-VPVPLK-
VYPVPL--
VPPVPL--
-VPVPLV-
VMPVPL--
VQPVPL--
-VPVPLQ-
VTPVPL--
-VPVPLG-
VVPVPL--
-VPVPLW-
VKPVPL--
VHPVPL--
VNPVPL--
-VPVPLN-
VFPVPL--
VGPVPL--
-VPVPLA-
-VPVPLY-
-VPVPLH-
-VPVPLF-
-VPVPLE-
VIPVPL--
VEPVPL--
VCPVPL--
-VPVPLM-
WRPVPL--
-WPVPLL-
-WPVPLK-
-WPVPLR-
WKPVPL--
-WPVPLG-
WIPVPL--
-WPVPLW-
WPPVPL--

WSPVPL--
WGPVPL--
-WPVPLM-
-WPVPLH-
WMPVPL--
WLPVPL--
-WPVPLY-
-WPVPLS-
-WPVPLN-
WNPVPL--
WQPVPL--
-WPVPLQ-
-WPVPLA-
WDPVPL--
WAPVPL--
WEPVPL--
WYPVPL--
WVPVPL--
WTPVPL--
-WPVPLV-
WHPVPL--
WCPVPL--
YSPVPL--
-YPVPLR-
YKPVPL--
-YPVPLS-
YIPVPL--
YLPVPL--
YWPVPL--
-YPVPLI-
YMPVPL--
YHPVPL--
YAPVPL--
-YPVPLY-
-YPVPLF-
YGPVPL--
YYPVPL--
-YPVPLT-
YNPVPL--
YVPVPL--
-YPVPLN-
-YPVPLK-
-YPVPLM-
YDPVPL--
YPPVPL--
YEPVPL--
-YPVPLV-
-YPVPLL-
-YPVPLG-
YTPVPL--

| | | | |
|---|---|---|---|
| | YQPVPL-- | | |
| | -YPVPLE- | | |
| | YFPVPL-- | | |
| | -YPVPLW- | | |
| | -YPVPLQ- | | |
| | -YPVPLA- | | |
| PQAL | 32 | 826 | 34.45 |
| | -LPQALA- | | |
| | --PQALPS | | |
| | -RPQALE- | | |
| | ARPQAL-- | | |
| | AVPQAL-- | | |
| | ASPQAL-- | | |
| | -APQALR- | | |
| | -APQALA- | | |
| | AAPQAL-- | | |
| | -APQALT- | | |
| | AYPQAL-- | | |
| | AMPQAL-- | | |
| | AKPQAL-- | | |
| | -APQALS- | | |
| | ALPQAL-- | | |
| | APPQAL-- | | |
| | AEPQAL-- | | |
| | -APQALY- | | |
| | -APQALK- | | |
| | AGPQAL-- | | |
| | -APQALQ- | | |
| | -APQALE- | | |
| | AIPQAL-- | | |
| | -APQALV- | | |
| | -APQALN- | | |
| | -APQALH- | | |
| | ANPQAL-- | | |
| | AFPQAL-- | | |
| | AWPQAL-- | | |
| | ATPQAL-- | | |
| | -APQALM- | | |
| | AQPQAL-- | | |
| | ADPQAL-- | | |
| | CTPQAL-- | | |
| | CQPQAL-- | | |
| | CMPQAL-- | | |
| | -CPQALK- | | |
| | -CPQALY- | | |
| | -CPQALV- | | |
| | CVPQAL-- | | |
| | CSPQAL-- | | |
| | -CPQALI- | | |
| | -CPQALF- | | |

CIPQAL--
CGPQAL--
CYPQAL--
-CPQALR-
-CPQALD-
CKPQAL--
CAPQAL--
DLPQAL--
-DPQALR-
-DPQALI-
DKPQAL--
DGPQAL--
-DPQALK-
-DPQALN-
DIPQAL--
DHPQAL--
DTPQAL--
DSPQAL--
-DPQALS-
-DPQALL-
DVPQAL--
-DPQALF-
DYPQAL--
DWPQAL--
DRPQAL--
DQPQAL--
-DPQALV-
-DPQALT-
-DPQALQ-
-DPQALH-
DMPQAL--
DAPQAL--
ETPQAL--
-EPQALK-
ERPQAL--
-EPQALL-
EKPQAL--
EYPQAL--
-EPQALY-
-EPQALV-
-EPQALS-
-EPQALQ-
-EPQALT-
-EPQALI-
ESPQAL--
EQPQAL--
EIPQAL--
EDPQAL--
-EPQALR-
-EPQALG-

```
-EPQALE-
EHPQAL--
EVPQAL--
EMPQAL--
ELPQAL--
EFPQAL--
EEPQAL--
ECPQAL--
EAPQAL--
FRPQAL--
-FPQALI-
FGPQAL--
FVPQAL--
-FPQALS-
-FPQALN-
FLPQAL--
FFPQAL--
FQPQAL--
FNPQAL--
FWPQAL--
FMPQAL--
FHPQAL--
FTPQAL--
-FPQALR-
-FPQALQ-
-FPQALL-
-FPQALH-
FPPQAL--
FDPQAL--
-FPQALY-
-FPQALW-
-FPQALT-
-FPQALK-
FIPQAL--
-FPQALV-
-FPQALF-
FKPQAL--
-FPQALM-
-FPQALA-
FEPQAL--
FCPQAL--
FAPQAL--
-GPQALM-
GYPQAL--
GAPQAL--
GRPQAL--
-GPQALW-
-GPQALG-
GFPQAL--
GSPQAL--
```

-GPQALT-
-GPQALA-
GNPQAL--
-GPQALR-
-GPQALH-
-GPQALK-
GHPQAL--
-GPQALV-
-GPQALS-
-GPQALI-
-GPQALF-
GTPQAL--
-GPQALE-
GKPQAL--
GGPQAL--
-GPQALQ-
-GPQALN-
GDPQAL--
GVPQAL--
-GPQALY-
GMPQAL--
GIPQAL--
-HPQALI-
HYPQAL--
-HPQALS-
-HPQALR-
HWPQAL--
HNPQAL--
HAPQAL--
HSPQAL--
-HPQALV-
-HPQALT-
-HPQALG-
HLPQAL--
HFPQAL--
HQPQAL--
HMPQAL--
HGPQAL--
HVPQAL--
HRPQAL--
-HPQALN-
-HPQALH-
HKPQAL--
-HPQALL-
-HPQALK-
-HPQALF-
HIPQAL--
HEPQAL--
HTPQAL--
-HPQALQ-

–HPQALM–
–HPQALE–
ILPQAL––
–IPQALH–
–IPQALR–
–IPQALS–
IVPQAL––
IPPQAL––
IKPQAL––
–IPQALT–
IYPQAL––
IGPQAL––
IRPQAL––
–IPQALI–
IMPQAL––
IWPQAL––
ISPQAL––
–IPQALW–
–IPQALN–
INPQAL––
–IPQALY–
–IPQALL–
–IPQALK–
IIPQAL––
IEPQAL––
ICPQAL––
IAPQAL––
–IPQALG–
–IPQALF–
–IPQALE–
IHPQAL––
IDPQAL––
KLPQAL––
–KPQALT–
–KPQALN–
KIPQAL––
KRPQAL––
–KPQALI–
–KPQALQ–
KQPQAL––
KPPQAL––
KTPQAL––
–KPQALF–
–KPQALV–
KGPQAL––
–KPQALE–
–KPQALA–
KWPQAL––
–KPQALS–
–KPQALH–

KFPQAL--
KEPQAL--
KYPQAL--
-KPQALW-
KMPQAL--
KAPQAL--
KVPQAL--
-KPQALY-
-KPQALM-
KNPQAL--
KDPQAL--
-LPQALR-
LRPQAL--
LYPQAL--
LLPQAL--
LHPQAL--
-LPQALS-
LAPQAL--
LKPQAL--
LGPQAL--
LMPQAL--
LQPQAL--
-LPQALV-
LNPQAL--
LCPQAL--
-LPQALI-
LTPQAL--
LEPQAL--
LDPQAL--
-LPQALW-
-LPQALT-
-LPQALQ-
-LPQALH-
-LPQALF-
-LPQALM-
-LPQALK-
-LPQALE-
LPPQAL--
LFPQAL--
-LPQALN-
LIPQAL--
-MPQALR-
MFPQAL--
MSPQAL--
MVPQAL--
-MPQALV-
MNPQAL--
MLPQAL--
MIPQAL--
-MPQALT-

```
-MPQALS-
MMPQAL--
MRPQAL--
-MPQALI-
MKPQAL--
MTPQAL--
-MPQALL-
-MPQALF-
-MPQALE-
MDPQAL--
MWPQAL--
-MPQALY-
MHPQAL--
-MPQALQ-
-MPQALH-
MPPQAL--
MGPQAL--
MYPQAL--
MQPQAL--
-MPQALN-
MAPQAL--
NRPQAL--
NTPQAL--
NLPQAL--
NPPQAL--
NSPQAL--
-NPQALK-
NYPQAL--
-NPQALT-
-NPQALI-
NNPQAL--
NMPQAL--
-NPQALW-
-NPQALV-
-NPQALS-
-NPQALN-
NCPQAL--
NVPQAL--
-NPQALH-
-NPQALF-
NKPQAL--
NGPQAL--
NDPQAL--
-NPQALL-
-NPQALG-
-NPQALA-
NWPQAL--
NQPQAL--
-NPQALY-
-NPQALR-
```

-NPQALQ-
NIPQAL--
--PQALVL
--PQALLK
--PQALRL
--PQALLA
--PQALSY
--PQALIS
--PQALSR
--PQALSF
--PQALRT
--PQALLS
--PQALSL
--PQALLR
PVPQAL--
--PQALVS
--PQALSW
--PQALSS
--PQALRF
--PQALLT
--PQALVV
--PQALNS
--PQALLL
PRPQAL--
--PQALRV
--PQALRS
--PQALML
--PQALHM
--PQALGL
--PQALFR
--PQALTT
--PQALSI
--PQALSG
--PQALRH
--PQALYN
--PQALVH
--PQALTR
--PQALTL
--PQALRY
--PQALLE
--PQALRW
--PQALRG
--PQALPL
--PQALLQ
--PQALKY
--PQALHL
--PQALGA
--PQALFT
--PQALYG
--PQALYA

--PQALWY
--PQALWT
--PQALWH
--PQALTQ
--PQALRQ
--PQALPF
--PQALNR
--PQALLI
--PQALHI
--PQALGI
--PQALWL
--PQALVR
--PQALVK
--PQALTG
--PQALST
--PQALSA
--PQALRK
--PQALRA
--PQALQL
--PQALNL
--PQALNI
--PQALIL
--PQALII
--PQALYQ
--PQALYL
--PQALWR
--PQALWA
--PQALVQ
--PQALTS
--PQALTA
--PQALSE
--PQALRN
--PQALKK
--PQALKI
--PQALIW
--PQALHV
--PQALGF
--PQALEY
PKPQAL--
PWPQAL--
--PQALVN
--PQALVI
--PQALSN
--PQALQT
--PQALMK
--PQALME
--PQALKN
--PQALKA
--PQALFM
--PQALEV

PLPQAL--
PIPQAL--
--PQALYS
--PQALYH
--PQALYE
--PQALWQ
--PQALWK
--PQALVW
--PQALTN
--PQALTI
--PQALSK
--PQALQK
--PQALNA
--PQALMR
--PQALKV
--PQALKL
--PQALHS
--PQALGV
--PQALGT
--PQALGS
--PQALAR
--PQALAL
PFPQAL--
PTPQAL--
--PQALYD
--PQALWS
--PQALVA
--PQALTW
--PQALSM
--PQALRE
--PQALQF
--PQALNN
--PQALNG
--PQALNE
--PQALMV
--PQALMS
--PQALMD
--PQALLN
--PQALLH
--PQALKW
--PQALKR
--PQALIA
--PQALHA
--PQALGM
--PQALFG
--PQALES
--PQALEG
--PQALAA
PSPQAL--
--PQALYT

--PQALWN
--PQALWM
--PQALTY
--PQALTF
--PQALTE
--PQALSH
--PQALQS
--PQALPM
--PQALNQ
--PQALNM
--PQALMQ
--PQALLM
--PQALLD
--PQALKQ
--PQALIV
--PQALIT
--PQALIN
--PQALHY
--PQALHT
--PQALHN
--PQALHF
--PQALFS
--PQALFQ
--PQALFE
--PQALEA
--PQALDL
--PQALAW
--PQALAT
--PQALAS
--PQALAQ
PGPQAL--
PYPQAL--
PQPQAL--
--PQALYI
--PQALWW
--PQALTH
--PQALTD
--PQALSD
--PQALRP
--PQALQV
--PQALQR
--PQALQA
--PQALPW
--PQALNV
--PQALNF
--PQALMP
--PQALMH
--PQALMF
--PQALLC
--PQALKE

--PQALIQ
--PQALIK
--PQALIG
--PQALIF
--PQALHR
--PQALHH
--PQALGY
--PQALGW
--PQALGQ
--PQALER
--PQALAY
--PQALAM
--PQALAK
--PQALAI
--PQALAF
--PQALAE
PMPQAL--
--PQALYK
--PQALYF
--PQALWI
--PQALWG
--PQALVF
--PQALVE
--PQALTV
--PQALTM
--PQALSV
--PQALSQ
--PQALQW
--PQALQQ
--PQALQN
--PQALQH
--PQALQG
--PQALQD
--PQALPY
--PQALNH
--PQALMW
--PQALMI
--PQALLW
--PQALKT
--PQALKP
--PQALKM
--PQALKF
--PQALIM
--PQALIE
--PQALID
--PQALHQ
--PQALGK
--PQALGH
--PQALGG
--PQALFK

--PQALFH
--PQALFD
--PQALFA
--PQALEW
--PQALET
--PQALEQ
--PQALEN
--PQALEM
--PQALEK
--PQALEH
--PQALDS
--PQALCQ
--PQALCG
--PQALAV
-PPQALA-
PHPQAL--
PEPQAL--
-QPQALQ-
-QPQALW-
-QPQALR-
QNPQAL--
QYPQAL--
QIPQAL--
-QPQALS-
QLPQAL--
QQPQAL--
-QPQALT-
-QPQALN-
-QPQALL-
-QPQALK-
-QPQALH-
QAPQAL--
QSPQAL--
QRPQAL--
-QPQALY-
-QPQALV-
QDPQAL--
QWPQAL--
QVPQAL--
QGPQAL--
QTPQAL--
-QPQALI-
-QPQALA-
QPPQAL--
QMPQAL--
QKPQAL--
QHPQAL--
RIPQAL--
RTPQAL--
-RPQALL-

RSPQAL--
-RPQALR-
-RPQALY-
-RPQALN-
RGPQAL--
RAPQAL--
RLPQAL--
RVPQAL--
-RPQALW-
-RPQALV-
RNPQAL--
-RPQALM-
-RPQALF-
RFPQAL--
RYPQAL--
-RPQALK-
-RPQALH-
RHPQAL--
-RPQALT-
REPQAL--
RQPQAL--
RWPQAL--
-RPQALQ-
RPPQAL--
RKPQAL--
RMPQAL--
RDPQAL--
RCPQAL--
-RPQALI-
-RPQALA-
-SPQALL-
SRPQAL--
SLPQAL--
-SPQALR-
STPQAL--
SHPQAL--
-SPQALN-
SKPQAL--
SGPQAL--
-SPQALK-
-SPQALA-
-SPQALY-
-SPQALI-
SSPQAL--
-SPQALW-
-SPQALH-
SFPQAL--
-SPQALV-
SPPQAL--
SYPQAL--

-SPQALF-
SNPQAL--
SWPQAL--
SQPQAL--
-SPQALT-
-SPQALS-
-SPQALG-
-SPQALQ-
SCPQAL--
-SPQALM-
SMPQAL--
SEPQAL--
-SPQALC-
SIPQAL--
SDPQAL--
-TPQALR-
TNPQAL--
TRPQAL--
TTPQAL--
TAPQAL--
TGPQAL--
-TPQALI-
TWPQAL--
-TPQALF-
-TPQALA-
THPQAL--
TDPQAL--
-TPQALW-
-TPQALL-
-TPQALN-
-TPQALH-
TPPQAL--
TSPQAL--
TQPQAL--
-TPQALV-
-TPQALK-
-TPQALE-
TVPQAL--
-TPQALY-
TKPQAL--
TEPQAL--
TYPQAL--
TIPQAL--
TFPQAL--
-TPQALG-
TMPQAL--
-TPQALS-
TCPQAL--
-VPQALR-
-VPQALW-

VRPQAL--
-VPQALL-
-VPQALS-
VSPQAL--
-VPQALK-
VLPQAL--
VHPQAL--
-VPQALT-
-VPQALE-
VYPQAL--
-VPQALN-
-VPQALG-
VWPQAL--
VKPQAL--
VEPQAL--
VFPQAL--
VCPQAL--
-VPQALM-
-VPQALH-
-VPQALA-
VMPQAL--
VIPQAL--
VGPQAL--
VDPQAL--
VPPQAL--
WVPQAL--
WSPQAL--
WLPQAL--
WRPQAL--
-WPQALH-
WNPQAL--
-WPQALS-
-WPQALK-
WAPQAL--
-WPQALQ-
WTPQAL--
WFPQAL--
-WPQALL-
-WPQALI-
WMPQAL--
WHPQAL--
WQPQAL--
-WPQALW-
-WPQALV-
WDPQAL--
-WPQALY-
-WPQALN-
-WPQALM-
WPPQAL--
WKPQAL--

| | | | |
|---|---|---|---|
| | WCPQAL-- | | |
| | WYPQAL-- | | |
| | -WPQALE- | | |
| | -YPQALS- | | |
| | YHPQAL-- | | |
| | YAPQAL-- | | |
| | -YPQALL- | | |
| | YSPQAL-- | | |
| | YRPQAL-- | | |
| | YLPQAL-- | | |
| | YVPQAL-- | | |
| | YNPQAL-- | | |
| | YTPQAL-- | | |
| | -YPQALR- | | |
| | -YPQALM- | | |
| | YMPQAL-- | | |
| | -YPQALE- | | |
| | YKPQAL-- | | |
| | YDPQAL-- | | |
| | YYPQAL-- | | |
| | YQPQAL-- | | |
| | -YPQALY- | | |
| | -YPQALH- | | |
| | -YPQALA- | | |
| | YIPQAL-- | | |
| | -YPQALN- | | |
| | -YPQALK- | | |
| | YGPQAL-- | | |
| | YWPQAL-- | | |
| | -YPQALT- | | |
| | -YPQALQ- | | |
| | YPPQAL-- | | |
| | YCPQAL-- | | |
| PRGM | 86 | 777 | 34.38 |
| | -SPRGMD- | | |
| | -WPRGMA- | | |
| | -APRGMG- | | |
| | -APRGMI- | | |
| | -APRGMY- | | |
| | AFPRGM-- | | |
| | -APRGMK- | | |
| | AGPRGM-- | | |
| | AYPRGM-- | | |
| | -APRGMS- | | |
| | -APRGML- | | |
| | ALPRGM-- | | |
| | AKPRGM-- | | |
| | -APRGMH- | | |
| | -APRGME- | | |
| | -APRGMA- | | |

-APRGMT-
-APRGMN-
ATPRGM--
AVPRGM--
-APRGMQ-
APPRGM--
AIPRGM--
-APRGMM-
-APRGMD-
AAPRGM--
ARPRGM--
-APRGMV-
-APRGMF-
ANPRGM--
AMPRGM--
AQPRGM--
-APRGMC-
AHPRGM--
AEPRGM--
ADPRGM--
CCPRGM--
-CPRGMA-
CVPRGM--
CTPRGM--
CRPRGM--
-CPRGMI-
-CPRGMT-
-CPRGMR-
-CPRGMQ-
-CPRGMN-
-CPRGML-
CPPRGM--
CGPRGM--
-CPRGMV-
-CPRGMF-
-CPRGME-
CMPRGM--
CKPRGM--
CEPRGM--
-DPRGMS-
DVPRGM--
-DPRGMF-
DSPRGM--
-DPRGMR-
DRPRGM--
-DPRGMK-
DFPRGM--
DMPRGM--
DKPRGM--
DWPRGM--

```
-DPRGMW-
-DPRGMI-
DNPRGM--
DLPRGM--
DTPRGM--
-DPRGMY-
-DPRGMG-
DGPRGM--
-DPRGMT-
-DPRGMM-
-DPRGMH-
-DPRGMA-
DIPRGM--
DQPRGM--
-DPRGMV-
-DPRGMQ-
-DPRGMN-
-DPRGME-
DHPRGM--
ERPRGM--
-EPRGMY-
-EPRGMM-
EGPRGM--
ESPRGM--
-EPRGMA-
EKPRGM--
-EPRGMW-
-EPRGMS-
-EPRGML-
-EPRGME-
ETPRGM--
-EPRGMG-
EYPRGM--
-EPRGMV-
-EPRGMN-
-EPRGMI-
ELPRGM--
EQPRGM--
-EPRGMH-
EHPRGM--
EVPRGM--
-EPRGMT-
EPPRGM--
EMPRGM--
EFPRGM--
EEPRGM--
EAPRGM--
FGPRGM--
FHPRGM--
FSPRGM--
```

FKPRGM--
-FPRGMS-
FIPRGM--
-FPRGMG-
FFPRGM--
FVPRGM--
-FPRGMV-
-FPRGMQ-
-FPRGMN-
FMPRGM--
FTPRGM--
FNPRGM--
-FPRGMA-
FEPRGM--
FDPRGM--
FAPRGM--
-FPRGME-
-FPRGMT-
FCPRGM--
-FPRGMW-
FPPRGM--
-FPRGMM-
-GPRGMR-
-GPRGMT-
-GPRGML-
GTPRGM--
GSPRGM--
GRPRGM--
GMPRGM--
-GPRGMS-
-GPRGMA-
GGPRGM--
-GPRGMW-
GEPRGM--
-GPRGMV-
-GPRGME-
GYPRGM--
GKPRGM--
GAPRGM--
-GPRGMQ-
-GPRGMH-
GNPRGM--
-GPRGMG-
-GPRGMF-
GVPRGM--
-GPRGMI-
GPPRGM--
-GPRGMM-
GQPRGM--
GIPRGM--

GHPRGM--
-GPRGMN-
-GPRGMK-
GDPRGM--
GCPRGM--
-HPRGML-
HAPRGM--
HSPRGM--
-HPRGMY-
-HPRGMG-
-HPRGMT-
HYPRGM--
-HPRGMI-
HKPRGM--
HIPRGM--
HRPRGM--
-HPRGMH-
HFPRGM--
-HPRGMA-
HHPRGM--
HQPRGM--
-HPRGMV-
HVPRGM--
-HPRGMW-
-HPRGMS-
-HPRGMN-
-HPRGMF-
HTPRGM--
-HPRGMQ-
-HPRGMC-
HMPRGM--
HGPRGM--
-IPRGML-
-IPRGMK-
-IPRGMS-
-IPRGMI-
IRPRGM--
-IPRGMT-
IMPRGM--
IGPRGM--
ITPRGM--
-IPRGMN-
-IPRGMG-
-IPRGMA-
IPPRGM--
IHPRGM--
IYPRGM--
ISPRGM--
INPRGM--
IKPRGM--

IEPRGM--
IAPRGM--
-IPRGMY-
-IPRGME-
IIPRGM--
IVPRGM--
-IPRGMW-
-IPRGMM-
-IPRGMH-
IDPRGM--
-IPRGMQ-
-KPRGML-
-KPRGMS-
KVPRGM--
KIPRGM--
-KPRGMH-
-KPRGMV-
-KPRGMI-
KYPRGM--
KQPRGM--
KGPRGM--
-KPRGMT-
KLPRGM--
-KPRGMW-
-KPRGMF-
KMPRGM--
-KPRGMN-
-KPRGMG-
KPPRGM--
KEPRGM--
-KPRGMM-
KFPRGM--
-KPRGME-
KNPRGM--
KDPRGM--
KTPRGM--
-KPRGMQ-
-KPRGMK-
KKPRGM--
KCPRGM--
LRPRGM--
LLPRGM--
-LPRGMS-
LAPRGM--
-LPRGMV-
LYPRGM--
LFPRGM--
LEPRGM--
-LPRGMY-
-LPRGMW-

LTPRGM--
-LPRGMH-
-LPRGMF-
-LPRGMI-
LPPRGM--
LIPRGM--
-LPRGMQ-
-LPRGMA-
LNPRGM--
LKPRGM--
-LPRGME-
-LPRGMM-
-LPRGMG-
-LPRGMC-
LQPRGM--
MVPRGM--
-MPRGMR-
-MPRGMW-
MNPRGM--
MGPRGM--
MSPRGM--
-MPRGMQ-
-MPRGMK-
-MPRGMI-
-MPRGMT-
-MPRGMS-
-MPRGML-
MAPRGM--
-MPRGMN-
-MPRGMF-
MPPRGM--
MHPRGM--
MEPRGM--
MTPRGM--
-MPRGMY-
-MPRGMV-
-MPRGMA-
MIPRGM--
MQPRGM--
MMPRGM--
-MPRGMM-
-MPRGMG-
-MPRGMH-
-MPRGME-
MKPRGM--
-NPRGMI-
-NPRGMR-
NYPRGM--
NGPRGM--
-NPRGML-

```
NFPRGM--
-NPRGMY-
-NPRGMV-
-NPRGMS-
-NPRGMH-
NMPRGM--
NPPRGM--
NHPRGM--
NEPRGM--
NWPRGM--
NTPRGM--
NNPRGM--
NDPRGM--
NAPRGM--
NVPRGM--
-NPRGMM-
-NPRGMK-
-NPRGME-
NKPRGM--
NQPRGM--
-NPRGMQ-
NSPRGM--
NRPRGM--
-NPRGMN-
-NPRGMG-
-NPRGMF-
NIPRGM--
NCPRGM--
-NPRGMW-
--PRGMGA
--PRGMVL
--PRGMSL
--PRGMLS
--PRGMFS
--PRGMRP
--PRGMVS
--PRGMRQ
--PRGMIL
--PRGMVK
--PRGMLY
--PRGMTL
--PRGMTT
--PRGMFL
--PRGMST
--PRGMWL
--PRGMNR
--PRGMYV
--PRGMSS
--PRGMSQ
--PRGMMV
```

--PRGMFK
PSPRGM--
--PRGMYS
--PRGMWS
--PRGMTS
--PRGMTG
--PRGMQR
--PRGMSV
--PRGMLT
--PRGMLH
--PRGMIA
--PRGMWW
--PRGMSW
--PRGMNQ
--PRGMMF
--PRGMLI
--PRGMKS
--PRGMHT
--PRGMAR
--PRGMKY
--PRGMGG
--PRGMFI
--PRGMYR
--PRGMYL
--PRGMTY
--PRGMSY
--PRGMNM
--PRGMET
--PRGMDS
--PRGMVM
--PRGMSN
--PRGMSI
--PRGMSA
--PRGMNS
--PRGMLQ
--PRGMLF
--PRGMHH
--PRGMWI
--PRGMWG
--PRGMTF
--PRGMQA
--PRGMNN
--PRGMNI
--PRGMMG
--PRGMLM
--PRGMKW
--PRGMIK
--PRGMII
--PRGMEM
--PRGMAF

--PRGMYW
--PRGMTN
--PRGMTK
--PRGMNY
--PRGMMW
--PRGMMH
--PRGMMA
--PRGMLD
--PRGMHY
--PRGMGW
PVPRGM--
--PRGMYT
--PRGMVT
--PRGMRW
--PRGMQG
--PRGMNT
--PRGMIF
--PRGMGS
--PRGMFT
--PRGMFQ
PKPRGM--
PIPRGM--
PRPRGM--
--PRGMYI
--PRGMWN
--PRGMWK
--PRGMWA
--PRGMSF
--PRGMNF
--PRGMMT
--PRGMMN
--PRGMKH
--PRGMHV
--PRGMGK
--PRGMFR
--PRGMFN
--PRGMYQ
--PRGMWE
--PRGMTQ
--PRGMTM
--PRGMTH
--PRGMSK
--PRGMQS
--PRGMQF
--PRGMHK
--PRGMFY
--PRGMAM
--PRGMYH
--PRGMVF
--PRGMTE

--PRGMQY
--PRGMQT
--PRGMQK
--PRGMNV
--PRGMMM
--PRGMMI
--PRGMLE
--PRGMKF
--PRGMKE
--PRGMHG
--PRGMHA
--PRGMGT
--PRGMFH
--PRGMEF
PHPRGM--
PYPRGM--
--PRGMWY
--PRGMWV
--PRGMVV
--PRGMTI
--PRGMSM
--PRGMSD
--PRGMQQ
--PRGMNA
--PRGMME
--PRGMKG
--PRGMIN
--PRGMHS
--PRGMHR
--PRGMHM
--PRGMGN
--PRGMAY
--PRGMAN
PWPRGM--
--PRGMYY
--PRGMYD
--PRGMWF
--PRGMTD
--PRGMQM
--PRGMQH
--PRGMNW
--PRGMNK
--PRGMKT
--PRGMKD
--PRGMKA
--PRGMIY
--PRGMHL
--PRGMGM
--PRGMFE
--PRGMFA

--PRGMES
PNPRGM--
--PRGMYM
--PRGMYA
--PRGMRE
--PRGMQN
--PRGMQC
--PRGMNE
--PRGMIQ
--PRGMHW
--PRGMHF
--PRGMHD
--PRGMGY
--PRGMGV
--PRGMGF
--PRGMFM
--PRGMFG
--PRGMDA
--PRGMCK
PEPRGM--
PDPRGM--
PAPRGM--
PTPRGM--
--PRGMYK
--PRGMYE
--PRGMWT
--PRGMWH
--PRGMVH
--PRGMVE
--PRGMVD
--PRGMQE
--PRGMNH
--PRGMNG
--PRGMKN
--PRGMIV
--PRGMHQ
--PRGMHI
--PRGMGI
--PRGMER
--PRGMDG
--PRGMCR
--PRGMAI
--PRGMAD
PQPRGM--
PMPRGM--
PGPRGM--
PFPRGM--
--PRGMWM
--PRGMVQ
--PRGMQW

--PRGMNP
--PRGMMY
--PRGMMQ
--PRGMMK
--PRGMMD
--PRGMKP
--PRGMID
--PRGMFD
--PRGMEW
--PRGMEQ
--PRGMEN
--PRGMEK
--PRGMEH
--PRGMCV
--PRGMCT
--PRGMCQ
--PRGMCG
--PRGMAK
-PPRGMK-
QVPRGM--
-QPRGMR-
QSPRGM--
-QPRGMS-
-QPRGMT-
-QPRGMI-
-QPRGMA-
QLPRGM--
QGPRGM--
-QPRGMQ-
-QPRGMN-
QTPRGM--
-QPRGMY-
-QPRGMW-
QQPRGM--
-QPRGMV-
-QPRGMG-
-QPRGMK-
-QPRGMH-
QEPRGM--
-QPRGMM-
-QPRGMF-
-QPRGME-
QNPRGM--
QMPRGM--
QHPRGM--
RLPRGM--
-RPRGMI-
-RPRGMG-
-RPRGML-
RSPRGM--

RGPRGM--
-RPRGMV-
-RPRGMQ-
-RPRGMM-
-RPRGMA-
RIPRGM--
RAPRGM--
-RPRGMY-
-RPRGME-
RPPRGM--
-RPRGMS-
RDPRGM--
-RPRGMT-
REPRGM--
RQPRGM--
-RPRGMN-
RKPRGM--
-RPRGMW-
-RPRGMK-
RMPRGM--
-RPRGMH-
-RPRGMF-
-RPRGMC-
RHPRGM--
SSPRGM--
STPRGM--
-SPRGMS-
-SPRGMY-
-SPRGMA-
-SPRGMT-
-SPRGMG-
-SPRGMK-
SRPRGM--
-SPRGMQ-
-SPRGMF-
SLPRGM--
SHPRGM--
SGPRGM--
SMPRGM--
SKPRGM--
-SPRGMV-
SQPRGM--
-SPRGMI-
SDPRGM--
SAPRGM--
-SPRGMW-
-SPRGMH-
-SPRGMM-
SNPRGM--
-SPRGME-

SPPRGM--
SCPRGM--
SEPRGM--
-TPRGML-
-TPRGMH-
-TPRGMI-
TSPRGM--
-TPRGMS-
-TPRGMV-
-TPRGMA-
TVPRGM--
TLPRGM--
-TPRGMN-
-TPRGMF-
TPPRGM--
TNPRGM--
-TPRGMT-
-TPRGMY-
-TPRGMG-
-TPRGMM-
-TPRGME-
TYPRGM--
TGPRGM--
TEPRGM--
-TPRGMQ-
TKPRGM--
-TPRGMW-
TIPRGM--
TDPRGM--
TTPRGM--
-TPRGMK-
THPRGM--
TQPRGM--
TCPRGM--
-VPRGML-
-VPRGMS-
-VPRGMV-
-VPRGME-
-VPRGMK-
-VPRGMY-
-VPRGMH-
-VPRGMF-
VNPRGM--
VKPRGM--
VYPRGM--
-VPRGMM-
VIPRGM--
VRPRGM--
-VPRGMI-
VFPRGM--

VTPRGM--
-VPRGMN-
VMPRGM--
VQPRGM--
-VPRGMG-
VHPRGM--
VEPRGM--
VPPRGM--
VAPRGM--
-VPRGMA-
VDPRGM--
-VPRGMQ-
-VPRGMD-
VGPRGM--
-VPRGMC-
WSPRGM--
WGPRGM--
-WPRGMQ-
-WPRGMG-
WEPRGM--
WNPRGM--
WDPRGM--
WAPRGM--
-WPRGMH-
-WPRGMF-
WPPRGM--
WQPRGM--
-WPRGMN-
-WPRGML-
-WPRGMK-
-WPRGME-
WKPRGM--
WMPRGM--
WIPRGM--
-WPRGMW-
-WPRGMM-
-YPRGML-
YGPRGM--
YNPRGM--
YYPRGM--
YSPRGM--
-YPRGMY-
YLPRGM--
-YPRGMM-
YVPRGM--
YRPRGM--
-YPRGMK-
-YPRGMA-
-YPRGMN-
YEPRGM--

| | | | |
|---|---|---|---|
| | -YPRGMG- | | |
| | YMPRGM-- | | |
| | YKPRGM-- | | |
| | YPPRGM-- | | |
| | YFPRGM-- | | |
| | -YPRGMV- | | |
| | -YPRGMI- | | |
| | -YPRGMF- | | |
| | YHPRGM-- | | |
| | -YPRGME- | | |
| | YDPRGM-- | | |
| PAWM | 476 | 610 | 34.36 |
| | -APAWMS- | | |
| | -APAWMR- | | |
| | AQPAWM-- | | |
| | -APAWML- | | |
| | ALPAWM-- | | |
| | ARPAWM-- | | |
| | AYPAWM-- | | |
| | -APAWMY- | | |
| | -APAWMG- | | |
| | -APAWMF- | | |
| | APPAWM-- | | |
| | -APAWMH- | | |
| | -APAWMA- | | |
| | AHPAWM-- | | |
| | AGPAWM-- | | |
| | ATPAWM-- | | |
| | ASPAWM-- | | |
| | -APAWMW- | | |
| | -APAWMT- | | |
| | -APAWMQ- | | |
| | AKPAWM-- | | |
| | AFPAWM-- | | |
| | -APAWMV- | | |
| | -APAWMN- | | |
| | -APAWMK- | | |
| | -APAWMI- | | |
| | AEPAWM-- | | |
| | -CPAWMV- | | |
| | -CPAWMT- | | |
| | -CPAWMN- | | |
| | -CPAWMK- | | |
| | -CPAWMI- | | |
| | -CPAWMH- | | |
| | -CPAWMA- | | |
| | CGPAWM-- | | |
| | CFPAWM-- | | |
| | CEPAWM-- | | |
| | DSPAWM-- | | |

DYPAWM--
-DPAWMT-
-DPAWMR-
DRPAWM--
-DPAWMY-
DWPAWM--
DVPAWM--
-DPAWML-
-DPAWMA-
DNPAWM--
DIPAWM--
DHPAWM--
DDPAWM--
-EPAWMD-
ESPAWM--
-EPAWML-
-EPAWMQ-
-EPAWMA-
ELPAWM--
EWPAWM--
-EPAWMF-
ENPAWM--
EHPAWM--
EAPAWM--
EVPAWM--
ETPAWM--
ERPAWM--
EQPAWM--
-EPAWMY-
-EPAWMV-
-EPAWMN-
-EPAWMM-
-EPAWMI-
EMPAWM--
EIPAWM--
EGPAWM--
EFPAWM--
FGPAWM--
FRPAWM--
-FPAWMR-
-FPAWML-
FHPAWM--
FLPAWM--
FSPAWM--
-FPAWMG-
FVPAWM--
-FPAWMI-
FMPAWM--
-FPAWMM-
-FPAWME-

FNPAWM--
-GPAWMR-
GNPAWM--
-GPAWMT-
-GPAWMM-
-GPAWMH-
-GPAWML-
-GPAWMS-
GSPAWM--
GRPAWM--
-GPAWMY-
-GPAWMG-
-GPAWMD-
GKPAWM--
GGPAWM--
-GPAWMN-
-GPAWME-
GTPAWM--
-GPAWMK-
-GPAWMF-
-GPAWMA-
GYPAWM--
GPPAWM--
-GPAWMW-
-GPAWMI-
GLPAWM--
GIPAWM--
GFPAWM--
GAPAWM--
-HPAWMR-
-HPAWMK-
-HPAWMS-
HTPAWM--
-HPAWMN-
-HPAWMG-
-HPAWME-
HIPAWM--
HFPAWM--
HEPAWM--
HYPAWM--
-HPAWMY-
-HPAWMM-
-HPAWML-
-HPAWMI-
HLPAWM--
HHPAWM--
-IPAWMS-
-IPAWMA-
IRPAWM--
-IPAWMG-

```
-IPAWMQ-
IFPAWM--
IVPAWM--
INPAWM--
-IPAWMR-
-IPAWMM-
IQPAWM--
-IPAWMW-
-IPAWMV-
-IPAWMN-
-IPAWMK-
-IPAWMH-
-IPAWMD-
ILPAWM--
KSPAWM--
-KPAWMN-
-KPAWMM-
-KPAWMF-
KYPAWM--
KGPAWM--
KTPAWM--
-KPAWMI-
KLPAWM--
KRPAWM--
-KPAWMH-
-KPAWMG-
-KPAWMA-
KNPAWM--
KMPAWM--
KVPAWM--
KQPAWM--
KPPAWM--
-KPAWMT-
-KPAWMS-
-KPAWMK-
-KPAWMD-
KKPAWM--
KWPAWM--
-KPAWMY-
-KPAWMV-
KEPAWM--
KAPAWM--
-LPAWMR-
LLPAWM--
LEPAWM--
-LPAWMF-
LQPAWM--
-LPAWMT-
-LPAWMM-
-LPAWML-
```

LSPAWM--
LRPAWM--
-LPAWMS-
-LPAWMN-
-LPAWMK-
-LPAWMG-
LNPAWM--
LTPAWM--
-LPAWMV-
LDPAWM--
LYPAWM--
LWPAWM--
-LPAWMD-
LMPAWM--
LVPAWM--
-LPAWMH-
LHPAWM--
LFPAWM--
-MPAWMG-
-MPAWMA-
MGPAWM--
MWPAWM--
-MPAWML-
-MPAWMS-
-MPAWMH-
MPPAWM--
MNPAWM--
-MPAWMV-
MEPAWM--
MVPAWM--
MTPAWM--
MSPAWM--
MLPAWM--
MAPAWM--
-MPAWMT-
-MPAWMQ-
-MPAWMM-
-NPAWMN-
-NPAWML-
NSPAWM--
NRPAWM--
NGPAWM--
-NPAWMS-
-NPAWMI-
NWPAWM--
-NPAWMG-
NVPAWM--
NPPAWM--
-NPAWMY-
-NPAWMW-

```
-NPAWMT-
-NPAWMD-
-NPAWMV-
-NPAWMK-
-NPAWME-
NMPAWM--
--PAWMLR
--PAWMSV
--PAWMTV
--PAWMSG
--PAWMRS
--PAWMSF
--PAWMLA
--PAWMYH
--PAWMWG
--PAWMMD
--PAWMGL
PTPAWM--
--PAWMRE
--PAWMMA
--PAWMKY
--PAWMGY
--PAWMRN
--PAWMMS
--PAWMKW
--PAWMGT
--PAWMAS
--PAWMAA
PVPAWM--
--PAWMSL
--PAWMSD
--PAWMKR
PGPAWM--
--PAWMVW
--PAWMVS
--PAWMVR
--PAWMSQ
--PAWMRV
--PAWMNM
--PAWMLT
--PAWMKL
--PAWMHR
--PAWMGG
--PAWMAR
--PAWMAL
--PAWMTK
--PAWMSS
--PAWMQS
--PAWMQL
--PAWMQI
```

--PAWMMT
--PAWMKV
--PAWMKK
--PAWMIN
--PAWMIH
--PAWMFT
--PAWMFN
PYPAWM--
--PAWMYA
--PAWMVT
--PAWMTR
--PAWMST
--PAWMRM
--PAWMNY
--PAWMLV
--PAWMLM
--PAWMIS
--PAWMIE
--PAWMGV
--PAWMFR
--PAWMAV
--PAWMAH
PQPAWM--
--PAWMYV
--PAWMYS
--PAWMYG
--PAWMWY
--PAWMVI
--PAWMTS
--PAWMTH
--PAWMTF
--PAWMSN
--PAWMSK
--PAWMSA
--PAWMRH
--PAWMRG
--PAWMQT
--PAWMNW
--PAWMNQ
--PAWMMK
--PAWMLS
--PAWMLN
--PAWMLK
--PAWMLH
--PAWMLD
--PAWMKQ
--PAWMKD
--PAWMIT
--PAWMIR
--PAWMHL

--PAWMGR
--PAWMGM
--PAWMGI
--PAWMFS
--PAWMES
--PAWMDA
PNPAWM--
--PAWMYM
--PAWMYL
--PAWMVV
--PAWMTL
--PAWMTI
--PAWMSM
--PAWMRL
--PAWMQY
--PAWMNT
--PAWMNS
--PAWMNL
--PAWMNK
--PAWMNH
--PAWMMV
--PAWMLI
--PAWMLG
--PAWMLE
--PAWMKT
--PAWMKH
--PAWMKF
--PAWMIY
--PAWMIQ
--PAWMHN
--PAWMHG
--PAWMHD
--PAWMHA
--PAWMGW
--PAWMGK
--PAWMFE
--PAWMAW
--PAWMAF
PAPAWM--
PWPAWM--
PSPAWM--
PLPAWM--
--PAWMYR
--PAWMYI
--PAWMWT
--PAWMWR
--PAWMWF
--PAWMVQ
--PAWMVN
--PAWMVK

--PAWMVH
--PAWMVE
--PAWMVD
--PAWMVA
--PAWMTG
--PAWMSY
--PAWMSI
--PAWMSH
--PAWMSE
--PAWMRY
--PAWMRW
--PAWMRQ
--PAWMRF
--PAWMQN
--PAWMQM
--PAWMQE
--PAWMQD
--PAWMPF
--PAWMNR
--PAWMMR
--PAWMLQ
--PAWMKS
--PAWMKA
--PAWMIK
--PAWMHT
--PAWMHQ
--PAWMHM
--PAWMHK
--PAWMHH
--PAWMGS
--PAWMGH
--PAWMGE
--PAWMFY
--PAWMFQ
--PAWMFK
--PAWMFA
--PAWMEV
--PAWMEQ
--PAWMEK
--PAWMEE
--PAWMCS
--PAWMCN
--PAWMCH
--PAWMCG
--PAWMAM
--PAWMAI
QTPAWM--
-QPAWMY-
-QPAWMV-
-QPAWML-

QVPAWM--
-QPAWMT-
QGPAWM--
QDPAWM--
QRPAWM--
-QPAWMS-
-QPAWMI-
QQPAWM--
-QPAWMW-
-QPAWMR-
-QPAWMQ-
-QPAWMM-
-QPAWMK-
-QPAWMH-
-QPAWMF-
QNPAWM--
QEPAWM--
-RPAWMS-
-RPAWMR-
RSPAWM--
RLPAWM--
RRPAWM--
-RPAWMK-
-RPAWMG-
-RPAWMA-
RGPAWM--
RFPAWM--
RTPAWM--
RWPAWM--
-RPAWMY-
-RPAWMH-
RKPAWM--
RAPAWM--
RVPAWM--
-RPAWMD-
-RPAWMW-
-RPAWMT-
RQPAWM--
-RPAWMN-
-RPAWMF-
RDPAWM--
-RPAWMV-
-RPAWMQ-
-RPAWMI-
RIPAWM--
-RPAWME-
-RPAWMC-
RNPAWM--
RMPAWM--
RHPAWM--

SRPAWM--
SLPAWM--
-SPAWMV-
-SPAWML-
SHPAWM--
-SPAWMS-
SYPAWM--
SEPAWM--
SVPAWM--
-SPAWMR-
SPPAWM--
-SPAWMD-
SIPAWM--
STPAWM--
-SPAWMG-
SFPAWM--
-SPAWMW-
-SPAWMT-
-SPAWMK-
-SPAWMA-
SNPAWM--
SKPAWM--
SSPAWM--
SQPAWM--
-SPAWMQ-
SGPAWM--
SWPAWM--
-SPAWMY-
-SPAWMM-
-SPAWMI-
-SPAWME-
SMPAWM--
-TPAWMQ-
TQPAWM--
-TPAWMS-
TRPAWM--
TAPAWM--
-TPAWMT-
TNPAWM--
TGPAWM--
TSPAWM--
TLPAWM--
THPAWM--
-TPAWMR-
-TPAWMA-
TTPAWM--
-TPAWMV-
-TPAWMK-
-TPAWMI-
TFPAWM--

TDPAWM--
TWPAWM--
TPPAWM--
-TPAWMW-
-TPAWMM-
TMPAWM--
-TPAWMH-
-TPAWMF-
VYPAWM--
-VPAWMS-
VFPAWM--
-VPAWMR-
VHPAWM--
VTPAWM--
-VPAWMN-
-VPAWML-
-VPAWMH-
VVPAWM--
VRPAWM--
VPPAWM--
-VPAWMT-
-VPAWMK-
-VPAWME-
VKPAWM--
-VPAWMQ-
VNPAWM--
VAPAWM--
-VPAWMI-
-VPAWMG-
-VPAWMY-
-VPAWMM-
-VPAWMF-
-VPAWMA-
VMPAWM--
VLPAWM--
VIPAWM--
VGPAWM--
WAPAWM--
-WPAWMR-
-WPAWMM-
WRPAWM--
-WPAWMY-
-WPAWMK-
-WPAWMI-
-WPAWMH-
-WPAWMG-
WHPAWM--
WLPAWM--
WIPAWM--
WFPAWM--

WDPAWM--
WSPAWM--
-WPAWMS-
YSPAWM--
-YPAWMK-
YYPAWM--
-YPAWMS-
-YPAWMG-
-YPAWMR-
YTPAWM--
-YPAWMT-
-YPAWMA-
YNPAWM--
YLPAWM--
YHPAWM--
YQPAWM--
-YPAWMQ-
-YPAWMI-
-YPAWMH-
-YPAWMD-
YMPAWM--
YAPAWM--

| | | | |
|---|---|---|---|
| PRPL | 155 | 737 | 34.33 |

-APRPLN-
-APRPLK-
--PRPLNG
-APRPLR-
-APRPLG-
AQPRPL--
AGPRPL--
-APRPLS-
-APRPLE-
ARPRPL--
-APRPLA-
-APRPLF-
AHPRPL--
AAPRPL--
-APRPLQ-
-APRPLI-
-APRPLH-
AVPRPL--
APPRPL--
AWPRPL--
AYPRPL--
-APRPLY-
AFPRPL--
AKPRPL--
-CPRPLR-
CCPRPL--
CVPRPL--

CFPRPL--
CTPRPL--
-CPRPLT-
-CPRPLA-
CSPRPL--
-CPRPLS-
-CPRPLM-
-CPRPLL-
-CPRPLI-
CPPRPL--
CNPRPL--
CMPRPL--
CHPRPL--
CYPRPL--
CQPRPL--
-CPRPLY-
-CPRPLW-
-CPRPLQ-
-CPRPLN-
-CPRPLK-
-CPRPLH-
CIPRPL--
CGPRPL--
CEPRPL--
CAPRPL--
-DPRPLH-
DQPRPL--
DVPRPL--
-DPRPLQ-
DKPRPL--
DSPRPL--
-DPRPLY-
DHPRPL--
DWPRPL--
-DPRPLV-
-DPRPLL-
-DPRPLG-
-DPRPLS-
DEPRPL--
-DPRPLR-
-DPRPLF-
-DPRPLE-
DDPRPL--
DAPRPL--
-DPRPLA-
DIPRPL--
DGPRPL--
DYPRPL--
-DPRPLK-
DCPRPL--

-DPRPLW-
DMPRPL--
DFPRPL--
ESPRPL--
-EPRPLW-
EVPRPL--
ETPRPL--
EYPRPL--
ELPRPL--
-EPRPLM-
EMPRPL--
EIPRPL--
-EPRPLI-
-EPRPLG-
ERPRPL--
-EPRPLT-
-EPRPLL-
-EPRPLK-
-EPRPLF-
-EPRPLE-
EAPRPL--
ENPRPL--
EGPRPL--
EFPRPL--
EEPRPL--
EQPRPL--
-EPRPLY-
EDPRPL--
EWPRPL--
-EPRPLR-
-EPRPLH-
-EPRPLQ-
-EPRPLD-
-EPRPLN-
-EPRPLA-
EHPRPL--
FHPRPL--
FRPRPL--
-FPRPLS-
FAPRPL--
FYPRPL--
FFPRPL--
-FPRPLF-
-FPRPLN-
-FPRPLL-
FWPRPL--
FLPRPL--
FGPRPL--
-FPRPLW-
FMPRPL--

-FPRPLH-
FDPRPL--
FQPRPL--
-FPRPLE-
-FPRPLG-
GTPRPL--
-GPRPLQ-
-GPRPLL-
-GPRPLW-
-GPRPLG-
-GPRPLF-
GYPRPL--
-GPRPLT-
GRPRPL--
GWPRPL--
GIPRPL--
GFPRPL--
GQPRPL--
-GPRPLM-
-GPRPLI-
GEPRPL--
-GPRPLY-
GHPRPL--
-GPRPLN-
GMPRPL--
-GPRPLV-
-GPRPLH-
GDPRPL--
GCPRPL--
GPPRPL--
-GPRPLE-
-HPRPLL-
HRPRPL--
HMPRPL--
-HPRPLR-
-HPRPLI-
-HPRPLV-
-HPRPLS-
HIPRPL--
HGPRPL--
HVPRPL--
-HPRPLN-
-HPRPLG-
HSPRPL--
-HPRPLY-
-HPRPLK-
HPPRPL--
HAPRPL--
-HPRPLF-
-HPRPLW-

HHPRPL--
HYPRPL--
HWPRPL--
-HPRPLM-
-HPRPLQ-
-HPRPLH-
HQPRPL--
HEPRPL--
HDPRPL--
HFPRPL--
-HPRPLD-
-IPRPLS-
ILPRPL--
-IPRPLN-
-IPRPLY-
IRPRPL--
-IPRPLV-
IGPRPL--
IVPRPL--
-IPRPLH-
ITPRPL--
-IPRPLW-
-IPRPLG-
-IPRPLT-
-IPRPLA-
INPRPL--
IEPRPL--
IYPRPL--
IAPRPL--
-IPRPLI-
IQPRPL--
IFPRPL--
-IPRPLE-
-IPRPLM-
IIPRPL--
-IPRPLF-
IMPRPL--
-IPRPLQ-
IPPRPL--
-KPRPLS-
-KPRPLN-
KGPRPL--
-KPRPLF-
-KPRPLR-
-KPRPLY-
KIPRPL--
KFPRPL--
KYPRPL--
-KPRPLM-
-KPRPLH-

-KPRPLT-
-KPRPLK-
-KPRPLA-
-KPRPLG-
KEPRPL--
-KPRPLW-
-KPRPLV-
KNPRPL--
KAPRPL--
-KPRPLI-
KHPRPL--
-KPRPLE-
KDPRPL--
LTPRPL--
LGPRPL--
LNPRPL--
LFPRPL--
LIPRPL--
LDPRPL--
LQPRPL--
-LPRPLI-
-LPRPLK-
-LPRPLM-
-LPRPLE-
LKPRPL--
LAPRPL--
-LPRPLW-
-LPRPLH-
LHPRPL--
-LPRPLF-
-LPRPLG-
LCPRPL--
MRPRPL--
-MPRPLS-
-MPRPLW-
-MPRPLI-
MVPRPL--
MTPRPL--
-MPRPLF-
MAPRPL--
-MPRPLV-
-MPRPLN-
MLPRPL--
-MPRPLQ-
MIPRPL--
-MPRPLK-
-MPRPLM-
MNPRPL--
MHPRPL--
MWPRPL--

```
MQPRPL--
-MPRPLY-
-MPRPLG-
MGPRPL--
MPPRPL--
MFPRPL--
MEPRPL--
MDPRPL--
MCPRPL--
-MPRPLE-
-NPRPLS-
NSPRPL--
NIPRPL--
-NPRPLQ-
NLPRPL--
NVPRPL--
NRPRPL--
-NPRPLL-
NAPRPL--
NGPRPL--
-NPRPLM-
NNPRPL--
-NPRPLH-
-NPRPLA-
NMPRPL--
NTPRPL--
NKPRPL--
-NPRPLN-
NHPRPL--
-NPRPLW-
NCPRPL--
-NPRPLY-
NWPRPL--
-NPRPLT-
NEPRPL--
-NPRPLK-
-NPRPLE-
-NPRPLI-
NDPRPL--
--PRPLVL
--PRPLFS
--PRPLAL
--PRPLYL
--PRPLRG
--PRPLIY
--PRPLIS
--PRPLVT
--PRPLNR
--PRPLMG
--PRPLTA
```

--PRPLSR
--PRPLLN
--PRPLSV
--PRPLYS
--PRPLSK
--PRPLWL
--PRPLFQ
--PRPLFL
--PRPLTG
--PRPLLQ
--PRPLYV
--PRPLWT
--PRPLVW
--PRPLRH
--PRPLQA
--PRPLFR
--PRPLFI
--PRPLYA
--PRPLWS
--PRPLVM
--PRPLKT
--PRPLFY
--PRPLSH
--PRPLKR
--PRPLFK
--PRPLVA
--PRPLSY
--PRPLQT
--PRPLQL
--PRPLMS
--PRPLLH
--PRPLVQ
--PRPLRY
--PRPLRF
--PRPLLE
--PRPLIV
--PRPLAT
--PRPLYF
--PRPLSQ
--PRPLSN
--PRPLSG
--PRPLPV
--PRPLNS
--PRPLKY
--PRPLKL
--PRPLWG
--PRPLRQ
--PRPLNF
--PRPLHH
--PRPLGK

--PRPLVH
--PRPLSA
--PRPLNV
--PRPLKH
--PRPLKF
--PRPLIT
--PRPLIG
PIPRPL--
--PRPLVK
--PRPLNM
--PRPLMV
--PRPLLK
--PRPLFN
--PRPLVR
--PRPLTK
--PRPLTI
--PRPLTH
--PRPLHY
--PRPLHT
--PRPLHK
--PRPLAW
--PRPLWM
--PRPLQS
--PRPLHN
--PRPLEL
PWPRPL--
--PRPLYK
--PRPLVG
--PRPLTN
--PRPLPF
--PRPLNA
--PRPLIM
--PRPLGG
--PRPLAA
--PRPLYY
--PRPLWV
--PRPLVY
--PRPLRM
--PRPLQN
--PRPLNN
--PRPLIN
--PRPLII
--PRPLHS
--PRPLGN
--PRPLDY
--PRPLAM
--PRPLAK
--PRPLAG
--PRPLYM
--PRPLYE

--PRPLQK
--PRPLPW
--PRPLKI
--PRPLHM
--PRPLEM
--PRPLTV
--PRPLSE
--PRPLQV
--PRPLQF
--PRPLLD
--PRPLIE
--PRPLFG
--PRPLET
--PRPLYI
--PRPLWQ
--PRPLWK
--PRPLWI
--PRPLVE
--PRPLVD
--PRPLTM
--PRPLTD
--PRPLLI
--PRPLKQ
--PRPLKN
--PRPLIK
--PRPLID
--PRPLFH
--PRPLEN
--PRPLDF
PRPRPL--
--PRPLTT
--PRPLTE
--PRPLRW
--PRPLRE
--PRPLQW
--PRPLQQ
--PRPLQE
--PRPLNY
--PRPLMM
--PRPLKW
--PRPLIQ
--PRPLHD
--PRPLGH
--PRPLGA
--PRPLFM
--PRPLYN
--PRPLYG
--PRPLSW
--PRPLQH
--PRPLNH

--PRPLMK
--PRPLHV
--PRPLHQ
--PRPLEK
--PRPLAE
PNPRPL--
--PRPLYH
--PRPLWW
--PRPLWA
--PRPLTF
--PRPLHA
--PRPLDI
--PRPLAH
PMPRPL--
--PRPLVP
--PRPLQG
--PRPLPN
--PRPLNT
--PRPLNE
--PRPLME
--PRPLKM
--PRPLKD
--PRPLHF
--PRPLHE
--PRPLES
--PRPLEI
--PRPLEA
--PRPLAF
PHPRPL--
--PRPLSD
--PRPLQD
--PRPLPH
--PRPLNQ
--PRPLMQ
--PRPLKE
--PRPLHI
--PRPLHG
--PRPLFW
--PRPLEY
--PRPLDS
--PRPLDM
--PRPLDA
--PRPLYD
--PRPLWD
--PRPLPA
--PRPLMY
--PRPLKA
--PRPLFD
--PRPLEV
--PRPLEH

--PRPLAQ
--PRPLAN
PEPRPL--
--PRPLYP
--PRPLWY
--PRPLWN
--PRPLQY
--PRPLQI
--PRPLPM
--PRPLMW
--PRPLMD
--PRPLEW
--PRPLER
--PRPLEG
--PRPLDV
--PRPLCY
PKPRPL--
PGPRPL--
--PRPLWP
--PRPLWF
--PRPLVC
--PRPLSC
--PRPLRD
--PRPLPT
--PRPLMN
--PRPLMF
--PRPLMC
--PRPLKK
--PRPLKC
--PRPLEQ
--PRPLCV
--PRPLCN
--PRPLCL
--PRPLCI
--PRPLAD
PQPRPL--
-PPRPLY-
-PPRPLM-
-PPRPLL-
PDPRPL--
PCPRPL--
-QPRPLL-
QIPRPL--
-QPRPLW-
QRPRPL--
QWPRPL--
QSPRPL--
-QPRPLA-
-QPRPLT-
-QPRPLN-

```
-QPRPLI-
-QPRPLH-
QNPRPL--
QFPRPL--
-QPRPLR-
QMPRPL--
QAPRPL--
-QPRPLF-
QDPRPL--
-QPRPLQ-
-QPRPLK-
QGPRPL--
QYPRPL--
-QPRPLY-
-QPRPLG-
QEPRPL--
QHPRPL--
QCPRPL--
RAPRPL--
-RPRPLT-
-RPRPLI-
RVPRPL--
-RPRPLR-
RGPRPL--
RYPRPL--
RQPRPL--
-RPRPLK-
-RPRPLV-
-RPRPLS-
RDPRPL--
-RPRPLW-
-RPRPLN-
-RPRPLM-
-RPRPLG-
RNPRPL--
-RPRPLH-
REPRPL--
RHPRPL--
RCPRPL--
-RPRPLE-
-RPRPLD-
SNPRPL--
SQPRPL--
SRPRPL--
SHPRPL--
STPRPL--
-SPRPLG-
SMPRPL--
SAPRPL--
-SPRPLA-
```

−SPRPLT−
SIPRPL−−
−SPRPLN−
−SPRPLW−
SKPRPL−−
−SPRPLQ−
−SPRPLK−
−SPRPLM−
−SPRPLE−
SEPRPL−−
SCPRPL−−
−SPRPLH−
SDPRPL−−
−SPRPLC−
−TPRPLL−
−TPRPLT−
−TPRPLG−
−TPRPLR−
TFPRPL−−
−TPRPLK−
−TPRPLA−
TGPRPL−−
TYPRPL−−
TQPRPL−−
−TPRPLN−
TIPRPL−−
−TPRPLY−
−TPRPLW−
−TPRPLS−
TPPRPL−−
TEPRPL−−
−TPRPLI−
−TPRPLF−
−TPRPLV−
−TPRPLM−
TMPRPL−−
TDPRPL−−
TAPRPL−−
−TPRPLH−
−TPRPLE−
TCPRPL−−
−VPRPLL−
VRPRPL−−
−VPRPLG−
VYPRPL−−
VGPRPL−−
−VPRPLT−
VFPRPL−−
−VPRPLV−
VHPRPL−−

```
-VPRPLQ-
VAPRPL--
VDPRPL--
-VPRPLW-
VQPRPL--
-VPRPLA-
-VPRPLK-
-VPRPLH-
VMPRPL--
VEPRPL--
-VPRPLE-
VCPRPL--
WIPRPL--
-WPRPLV-
-WPRPLL-
WGPRPL--
-WPRPLS-
-WPRPLK-
-WPRPLT-
WMPRPL--
WKPRPL--
WHPRPL--
WVPRPL--
WEPRPL--
-WPRPLY-
-WPRPLG-
WLPRPL--
-WPRPLA-
WTPRPL--
-WPRPLF-
-WPRPLI-
-WPRPLE-
WPPRPL--
WDPRPL--
WQPRPL--
-WPRPLM-
-WPRPLH-
WAPRPL--
-WPRPLQ-
-WPRPLD-
YLPRPL--
-YPRPLN-
YIPRPL--
-YPRPLF-
-YPRPLH-
YHPRPL--
YAPRPL--
-YPRPLL-
YFPRPL--
-YPRPLG-
```

YTPRPL--
-YPRPLY-
YNPRPL--
-YPRPLQ-
-YPRPLE-
-YPRPLA-
-YPRPLM-
-YPRPLI-
YEPRPL--
YMPRPL--

| | | | |
|---|---|---|---|
| PVAL | 153 | 738 | 34.30 |

-DPVALG-
-KPVALD-
-LPVALP-
--PVALDY
-APVALR-
AVPVAL--
ATPVAL--
-APVALS-
AYPVAL--
ASPVAL--
AKPVAL--
ALPVAL--
-APVALH-
AGPVAL--
AMPVAL--
-APVALV-
-APVALE-
AWPVAL--
-APVALK-
AIPVAL--
AHPVAL--
ADPVAL--
-APVALG-
AEPVAL--
APPVAL--
AQPVAL--
AFPVAL--
-APVALN-
ACPVAL--
-APVALQ-
-APVALA-
AAPVAL--
-CPVALR-
-CPVALS-
CIPVAL--
CTPVAL--
CRPVAL--
CKPVAL--
CEPVAL--

CCPVAL--
CVPVAL--
CSPVAL--
-CPVALV-
-CPVALT-
CAPVAL--
CGPVAL--
CWPVAL--
-CPVALW-
-CPVALQ-
-CPVALM-
CNPVAL--
CMPVAL--
CLPVAL--
CHPVAL--
-DPVALR-
-DPVALQ-
DFPVAL--
DRPVAL--
DTPVAL--
-DPVALS-
DIPVAL--
-DPVALN-
-DPVALI-
DGPVAL--
-DPVALV-
-DPVALT-
-DPVALK-
DLPVAL--
DYPVAL--
-DPVALY-
-DPVALW-
DMPVAL--
DKPVAL--
DEPVAL--
-DPVALL-
-DPVALA-
DAPVAL--
DQPVAL--
-DPVALH-
-DPVALF-
DDPVAL--
DVPVAL--
-EPVALL-
ERPVAL--
-EPVALI-
EKPVAL--
-EPVALR-
-EPVALH-
ETPVAL--

```
-EPVALA-
ELPVAL--
ESPVAL--
EFPVAL--
-EPVALV-
EWPVAL--
EVPVAL--
-EPVALK-
-EPVALG-
EGPVAL--
-EPVALQ-
ENPVAL--
EAPVAL--
EYPVAL--
-EPVALT-
-EPVALN-
EQPVAL--
-EPVALW-
-EPVALS-
EMPVAL--
-EPVALE-
EEPVAL--
EDPVAL--
FHPVAL--
-FPVALR-
FLPVAL--
-FPVALN-
FGPVAL--
-FPVALT-
-FPVALV-
FQPVAL--
-FPVALH-
FYPVAL--
-FPVALM-
-FPVALG-
FEPVAL--
-FPVALA-
FNPVAL--
-FPVALQ-
-FPVALK-
FPPVAL--
FDPVAL--
FAPVAL--
-FPVALE-
GKPVAL--
GVPVAL--
GIPVAL--
GLPVAL--
GSPVAL--
-GPVALT-
```

-GPVALS-
-GPVALR-
-GPVALL-
-GPVALI-
GTPVAL--
GFPVAL--
GQPVAL--
GNPVAL--
-GPVALM-
GGPVAL--
GAPVAL--
-GPVALH-
GEPVAL--
-GPVALE-
-GPVALG-
GHPVAL--
-GPVALQ-
-GPVALK-
GYPVAL--
GPPVAL--
-GPVALN-
GDPVAL--
-GPVALA-
-GPVALC-
HRPVAL--
-HPVALT-
HQPVAL--
HEPVAL--
-HPVALM-
-HPVALI-
-HPVALA-
HSPVAL--
-HPVALN-
HAPVAL--
HGPVAL--
HVPVAL--
-HPVALY-
HIPVAL--
HTPVAL--
-HPVALH-
HNPVAL--
-HPVALV-
-HPVALG-
HMPVAL--
HFPVAL--
HWPVAL--
HYPVAL--
HLPVAL--
-HPVALW-
-HPVALE-

```
-HPVALK-
-HPVALF-
HPPVAL--
HHPVAL--
-IPVALR-
ISPVAL--
-IPVALA-
IAPVAL--
IGPVAL--
-IPVALG-
ILPVAL--
-IPVALN-
IEPVAL--
IDPVAL--
-IPVALQ-
-IPVALL-
IHPVAL--
ITPVAL--
-IPVALH-
INPVAL--
IMPVAL--
IKPVAL--
IYPVAL--
IQPVAL--
-IPVALV-
-IPVALK-
-IPVALT-
-IPVALS-
-IPVALM-
-IPVALI-
IWPVAL--
IPPVAL--
ICPVAL--
KSPVAL--
-KPVALS-
KGPVAL--
KVPVAL--
KMPVAL--
KAPVAL--
-KPVALY-
-KPVALQ-
KQPVAL--
-KPVALI-
-KPVALT-
KNPVAL--
-KPVALK-
-KPVALN-
-KPVALM-
KFPVAL--
-KPVALF-
```

KHPVAL--
KTPVAL--
-KPVALR-
-KPVALG-
KEPVAL--
KDPVAL--
-KPVALV-
KIPVAL--
KWPVAL--
KPPVAL--
-KPVALC-
KCPVAL--
-LPVALR-
LHPVAL--
-LPVALQ-
-LPVALK-
LQPVAL--
LGPVAL--
-LPVALM-
-LPVALH-
-LPVALT-
LPPVAL--
LYPVAL--
-LPVALA-
LDPVAL--
-LPVALI-
LFPVAL--
-LPVALV-
-LPVALN-
LNPVAL--
-LPVALS-
-LPVALG-
-LPVALE-
MKPVAL--
MTPVAL--
MNPVAL--
-MPVALI-
-MPVALR-
-MPVALT-
-MPVALH-
MHPVAL--
-MPVALK-
-MPVALA-
MFPVAL--
MWPVAL--
-MPVALG-
MPPVAL--
MIPVAL--
MQPVAL--
-MPVALV-

MCPVAL--
-MPVALQ-
-MPVALF-
MAPVAL--
MYPVAL--
-MPVALM-
MGPVAL--
-MPVALN-
-MPVALE-
-MPVALD-
-MPVALC-
NSPVAL--
NLPVAL--
NFPVAL--
NRPVAL--
NGPVAL--
-NPVALR-
-NPVALI-
NNPVAL--
NTPVAL--
-NPVALT-
-NPVALS-
-NPVALK-
-NPVALQ-
NVPVAL--
-NPVALA-
NEPVAL--
-NPVALV-
-NPVALE-
NIPVAL--
NHPVAL--
NDPVAL--
NAPVAL--
NWPVAL--
NQPVAL--
NMPVAL--
-NPVALL-
-NPVALH-
-NPVALG-
NKPVAL--
-NPVALM-
-NPVALN-
NCPVAL--
--PVALRS
--PVALRR
--PVALRY
--PVALRG
--PVALLH
--PVALTK
--PVALSH

--PVALVR
--PVALRT
--PVALTR
--PVALLT
--PVALKL
--PVALHR
--PVALGH
--PVALRA
--PVALYG
--PVALRN
--PVALGS
--PVALGA
--PVALAR
--PVALRH
--PVALKA
--PVALSD
--PVALKR
PLPVAL--
--PVALSI
--PVALST
--PVALSG
--PVALRK
--PVALNY
--PVALLN
--PVALLK
--PVALGL
--PVALNR
--PVALHH
--PVALSA
--PVALKI
--PVALGF
--PVALAS
--PVALTA
--PVALSW
--PVALQR
--PVALNS
--PVALLG
--PVALGV
--PVALYD
--PVALRP
--PVALQL
--PVALKQ
--PVALGY
--PVALFG
--PVALRQ
--PVALNK
--PVALMS
--PVALMR
PHPVAL--
PVPVAL--

--PVALTY
--PVALRW
PRPVAL--
--PVALTL
--PVALSN
PSPVAL--
--PVALVG
--PVALTV
--PVALTT
--PVALTS
--PVALTH
--PVALQA
--PVALHK
--PVALGG
--PVALVN
--PVALSM
--PVALNW
--PVALNA
--PVALMG
--PVALKN
--PVALKF
--PVALIN
--PVALAA
PGPVAL--
PAPVAL--
--PVALTD
--PVALSY
--PVALSV
--PVALSE
--PVALQK
--PVALME
--PVALKH
--PVALKG
--PVALIG
--PVALET
--PVALVK
--PVALTW
--PVALQT
--PVALNQ
--PVALNI
--PVALLQ
--PVALKT
--PVALID
--PVALFD
--PVALES
--PVALEL
--PVALYH
--PVALTN
--PVALSF
--PVALPI

--PVALPF
--PVALMH
--PVALKE
--PVALHG
--PVALGI
PTPVAL--
PMPVAL--
PYPVAL--
--PVALTI
--PVALQF
--PVALQE
--PVALNM
--PVALHM
--PVALEY
--PVALAN
--PVALAM
--PVALAI
--PVALAH
--PVALTE
--PVALRD
--PVALQV
--PVALNV
--PVALNG
--PVALKY
--PVALHA
--PVALGT
--PVALEK
--PVALEI
--PVALEA
--PVALAG
PDPVAL--
PWPVAL--
--PVALWN
--PVALSQ
--PVALQS
--PVALQG
--PVALPV
--PVALNN
--PVALNH
--PVALMN
--PVALKM
--PVALHT
--PVALGQ
--PVALGN
--PVALER
--PVALEN
--PVALDS
--PVALAT
--PVALTM
--PVALQN

--PVALPQ
--PVALPL
--PVALNT
--PVALMA
--PVALKK
--PVALHN
--PVALGK
--PVALFH
--PVALDL
--PVALDG
--PVALAD
PQPVAL--
PIPVAL--
PEPVAL--
--PVALWD
--PVALTF
--PVALQH
--PVALQD
--PVALPM
--PVALNE
--PVALND
--PVALMW
--PVALKD
--PVALEW
--PVALEV
--PVALEQ
--PVALEF
--PVALAE
--PVALYP
--PVALTQ
--PVALQY
--PVALNF
--PVALMT
--PVALLP
--PVALHF
--PVALFN
--PVALEH
PFPVAL--
--PVALWH
--PVALWC
--PVALQW
--PVALQM
--PVALPW
--PVALMD
--PVALKC
--PVALHY
--PVALHV
--PVALHQ
--PVALHE
--PVALEG

--PVALDM
--PVALCR
--PVALAQ
-PPVALT-
PCPVAL--
QSPVAL--
-QPVALK-
-QPVALL-
QQPVAL--
QLPVAL--
QKPVAL--
QTPVAL--
-QPVALS-
-QPVALM-
-QPVALN-
QGPVAL--
QYPVAL--
QVPVAL--
-QPVALG-
-QPVALA-
-QPVALI-
-QPVALH-
QMPVAL--
-QPVALV-
QNPVAL--
QFPVAL--
QAPVAL--
QWPVAL--
-QPVALT-
QDPVAL--
-QPVALE-
QIPVAL--
QPPVAL--
QHPVAL--
RTPVAL--
-RPVALG-
-RPVALI-
RMPVAL--
RVPVAL--
RKPVAL--
RGPVAL--
-RPVALV-
RNPVAL--
RHPVAL--
-RPVALH-
-RPVALT-
RIPVAL--
RPPVAL--
RAPVAL--
-RPVALW-

RDPVAL--
RQPVAL--
-RPVALN-
REPVAL--
-RPVALM-
-RPVALD-
RCPVAL--
-RPVALE-
-SPVALR-
-SPVALL-
SLPVAL--
-SPVALT-
SAPVAL--
-SPVALS-
SEPVAL--
SIPVAL--
SSPVAL--
-SPVALV-
SGPVAL--
-SPVALI-
SHPVAL--
-SPVALG-
SMPVAL--
SPPVAL--
SQPVAL--
-SPVALN-
SVPVAL--
-SPVALQ-
SWPVAL--
SFPVAL--
-SPVALH-
SYPVAL--
-SPVALE-
-SPVALA-
SDPVAL--
-SPVALK-
SCPVAL--
TRPVAL--
-TPVALR-
TLPVAL--
TVPVAL--
TYPVAL--
TAPVAL--
TWPVAL--
TSPVAL--
-TPVALK-
TIPVAL--
THPVAL--
-TPVALL-
-TPVALS-

-TPVALH-
TFPVAL--
TPPVAL--
TTPVAL--
TGPVAL--
-TPVALQ-
TQPVAL--
TNPVAL--
TEPVAL--
-TPVALN-
-TPVALA-
-TPVALV-
-TPVALG-
-TPVALE-
TKPVAL--
TDPVAL--
-TPVALT-
TCPVAL--
-VPVALR-
VSPVAL--
-VPVALS-
VTPVAL--
VGPVAL--
VHPVAL--
-VPVALK-
VNPVAL--
-VPVALT-
VMPVAL--
VYPVAL--
-VPVALL-
VVPVAL--
VPPVAL--
VQPVAL--
-VPVALN-
-VPVALQ-
VWPVAL--
VEPVAL--
VDPVAL--
-VPVALH-
-VPVALA-
VIPVAL--
-VPVALV-
-VPVALM-
VCPVAL--
-VPVALE-
-WPVALR-
-WPVALL-
-WPVALS-
WAPVAL--
WSPVAL--

WNPVAL--
WMPVAL--
-WPVALT-
-WPVALG-
WHPVAL--
-WPVALN-
-WPVALA-
WKPVAL--
WFPVAL--
-WPVALQ-
-WPVALK-
WEPVAL--
WYPVAL--
-WPVALV-
-WPVALE-
WPPVAL--
WDPVAL--
WCPVAL--
WTPVAL--
YRPVAL--
-YPVALR-
-YPVALK-
-YPVALH-
YVPVAL--
YGPVAL--
YPPVAL--
-YPVALN-
YTPVAL--
-YPVALG-
YKPVAL--
YYPVAL--
-YPVALM-
YAPVAL--
YQPVAL--
-YPVALT-
YNPVAL--
YFPVAL--
-YPVALS-
-YPVALQ-
-YPVALL-
YHPVAL--
-YPVALA-
YIPVAL--
YLPVAL--
-YPVALE-
YEPVAL--
YDPVAL--
YWPVAL--
YCPVAL--

PIGL 35 824 34.30

AIPIGL--
FGPIGL--
-NPIGLG-
--PIGLGR
AWPIGL--
AGPIGL--
ASPIGL--
-APIGLT-
-APIGLS-
AYPIGL--
ARPIGL--
APPIGL--
-APIGLV-
-APIGLR-
-APIGLK-
AMPIGL--
ATPIGL--
-APIGLI-
-APIGLH-
-APIGLL-
ALPIGL--
-APIGLY-
-APIGLN-
-APIGLA-
ACPIGL--
AEPIGL--
AAPIGL--
AVPIGL--
-APIGLM-
-APIGLF-
AQPIGL--
-APIGLG-
-APIGLD-
ANPIGL--
AHPIGL--
AFPIGL--
-APIGLQ-
-APIGLE-
CGPIGL--
-CPIGLK-
CMPIGL--
CCPIGL--
CSPIGL--
CRPIGL--
-CPIGLV-
-CPIGLR-
-CPIGLI-
CLPIGL--
CFPIGL--
-CPIGLM-

-CPIGLL-
-CPIGLG-
-CPIGLA-
CIPIGL--
CHPIGL--
CEPIGL--
CDPIGL--
CAPIGL--
DTPIGL--
DLPIGL--
-DPIGLL-
-DPIGLR-
-DPIGLA-
DCPIGL--
DYPIGL--
DWPIGL--
DSPIGL--
-DPIGLY-
-DPIGLF-
DEPIGL--
-DPIGLS-
-DPIGLM-
DNPIGL--
DMPIGL--
DQPIGL--
-DPIGLW-
-DPIGLQ-
-DPIGLN-
-DPIGLK-
-DPIGLI-
DHPIGL--
DDPIGL--
-DPIGLT-
DKPIGL--
DIPIGL--
DGPIGL--
DAPIGL--
-EPIGLF-
ESPIGL--
-EPIGLS-
-EPIGLN-
ELPIGL--
EYPIGL--
-EPIGLL-
-EPIGLG-
ETPIGL--
ERPIGL--
-EPIGLV-
-EPIGLK-
-EPIGLE-

EGPIGL--
-EPIGLW-
-EPIGLI-
EHPIGL--
EVPIGL--
-EPIGLY-
EAPIGL--
-EPIGLQ-
-EPIGLM-
-EPIGLA-
EMPIGL--
-EPIGLH-
EKPIGL--
EIPIGL--
EFPIGL--
EWPIGL--
ENPIGL--
-EPIGLT-
EDPIGL--
-FPIGLR-
FSPIGL--
-FPIGLS-
-FPIGLM-
-FPIGLN-
-FPIGLF-
FHPIGL--
-FPIGLV-
-FPIGLE-
FMPIGL--
FYPIGL--
-FPIGLY-
-FPIGLT-
-FPIGLK-
-FPIGLI-
-FPIGLG-
-FPIGLH-
FIPIGL--
FQPIGL--
FDPIGL--
-FPIGLA-
FLPIGL--
FTPIGL--
FWPIGL--
FPPIGL--
-FPIGLW-
-GPIGLK-
GLPIGL--
GRPIGL--
-GPIGLL-
-GPIGLT-

-GPIGLS-
GWPIGL--
GTPIGL--
GSPIGL--
-GPIGLG-
GFPIGL--
-GPIGLN-
-GPIGLA-
-GPIGLQ-
-GPIGLF-
-GPIGLE-
GKPIGL--
GGPIGL--
GDPIGL--
GQPIGL--
GVPIGL--
-GPIGLM-
-GPIGLI-
GIPIGL--
GNPIGL--
GEPIGL--
GCPIGL--
GMPIGL--
GAPIGL--
GPPIGL--
-GPIGLH-
GHPIGL--
HKPIGL--
-HPIGLL-
-HPIGLR-
HRPIGL--
HLPIGL--
HQPIGL--
-HPIGLT-
HWPIGL--
-HPIGLY-
-HPIGLG-
HGPIGL--
-HPIGLN-
HVPIGL--
HFPIGL--
HEPIGL--
HTPIGL--
-HPIGLQ-
-HPIGLE-
-HPIGLA-
HIPIGL--
-HPIGLW-
-HPIGLK-
HSPIGL--

-HPIGLS-
-HPIGLM-
-HPIGLV-
-HPIGLF-
HMPIGL--
HPPIGL--
-HPIGLI-
HHPIGL--
HDPIGL--
HCPIGL--
ILPIGL--
IPPIGL--
ISPIGL--
-IPIGLQ-
-IPIGLK-
-IPIGLE-
-IPIGLR-
IRPIGL--
-IPIGLS-
-IPIGLI-
INPIGL--
IEPIGL--
-IPIGLV-
IGPIGL--
IFPIGL--
IAPIGL--
ITPIGL--
-IPIGLW-
-IPIGLG-
IKPIGL--
-IPIGLT-
IIPIGL--
IHPIGL--
IDPIGL--
IYPIGL--
-IPIGLA-
IQPIGL--
-IPIGLM-
-IPIGLH-
KSPIGL--
-KPIGLT-
KRPIGL--
KQPIGL--
KGPIGL--
KVPIGL--
KAPIGL--
KLPIGL--
KFPIGL--
-KPIGLQ-
KHPIGL--

-KPIGLY-
-KPIGLK-
-KPIGLG-
KYPIGL--
-KPIGLV-
KTPIGL--
-KPIGLN-
-KPIGLI-
-KPIGLH-
-KPIGLA-
KIPIGL--
-KPIGLM-
-KPIGLF-
-KPIGLE-
KNPIGL--
KWPIGL--
KPPIGL--
KDPIGL--
KEPIGL--
-LPIGLN-
-LPIGLR-
LTPIGL--
LSPIGL--
LLPIGL--
-LPIGLT-
LVPIGL--
-LPIGLS-
-LPIGLQ-
-LPIGLL-
LFPIGL--
LYPIGL--
LGPIGL--
-LPIGLI-
LMPIGL--
LWPIGL--
-LPIGLM-
LQPIGL--
-LPIGLA-
LHPIGL--
LPPIGL--
-LPIGLV-
LNPIGL--
-LPIGLH-
LEPIGL--
-LPIGLE-
LDPIGL--
-MPIGLS-
-MPIGLR-
MLPIGL--
MKPIGL--

-MPIGLT-
MTPIGL--
MRPIGL--
MSPIGL--
-MPIGLI-
MVPIGL--
-MPIGLG-
-MPIGLF-
MGPIGL--
MMPIGL--
MAPIGL--
MWPIGL--
-MPIGLL-
-MPIGLA-
MPPIGL--
-MPIGLY-
MFPIGL--
-MPIGLW-
-MPIGLV-
-MPIGLQ-
-MPIGLN-
MNPIGL--
MIPIGL--
MEPIGL--
MYPIGL--
-MPIGLM-
-MPIGLK-
-MPIGLE-
MDPIGL--
-MPIGLH-
-MPIGLC-
MHPIGL--
MCPIGL--
-NPIGLL-
NLPIGL--
-NPIGLR-
NVPIGL--
NYPIGL--
-NPIGLI-
-NPIGLF-
NIPIGL--
NSPIGL--
-NPIGLN-
NAPIGL--
-NPIGLV-
-NPIGLM-
NHPIGL--
NGPIGL--
-NPIGLY-
NWPIGL--

```
-NPIGLT-
-NPIGLK-
NNPIGL--
NTPIGL--
-NPIGLH-
-NPIGLE-
NKPIGL--
NEPIGL--
NQPIGL--
NDPIGL--
NPPIGL--
NFPIGL--
--PIGLVR
--PIGLRL
--PIGLVM
--PIGLSK
--PIGLRG
--PIGLTK
--PIGLVL
--PIGLRA
--PIGLLR
--PIGLGS
--PIGLTA
--PIGLLG
--PIGLST
--PIGLSS
--PIGLTS
--PIGLKL
--PIGLRV
--PIGLRY
--PIGLRS
--PIGLFT
--PIGLYT
--PIGLKG
--PIGLNF
PLPIGL--
--PIGLVS
--PIGLTN
--PIGLTL
--PIGLSI
--PIGLKQ
--PIGLHR
--PIGLFS
--PIGLRN
--PIGLRH
--PIGLRD
--PIGLLN
--PIGLKS
--PIGLGW
--PIGLVT
```

--PIGLVA
--PIGLSN
--PIGLSG
--PIGLRI
--PIGLNM
--PIGLMF
--PIGLLD
--PIGLIT
--PIGLAK
PSPIGL--
--PIGLTH
--PIGLSW
--PIGLNR
--PIGLMS
--PIGLLQ
--PIGLLK
--PIGLKY
--PIGLKI
--PIGLGT
--PIGLAR
PWPIGL--
PNPIGL--
PKPIGL--
--PIGLYS
--PIGLVV
--PIGLSF
--PIGLQT
--PIGLMR
--PIGLML
--PIGLKR
--PIGLIS
--PIGLHS
--PIGLGG
PTPIGL--
--PIGLYG
--PIGLWS
--PIGLTT
--PIGLSM
--PIGLRM
--PIGLRF
--PIGLQS
--PIGLQR
--PIGLNV
--PIGLMY
--PIGLHF
--PIGLAN
--PIGLAF
--PIGLTG
--PIGLSV
--PIGLSA

--PIGLPL
--PIGLNT
--PIGLNH
--PIGLMV
--PIGLKT
--PIGLAH
--PIGLAG
--PIGLLT
--PIGLKH
--PIGLHT
--PIGLGV
--PIGLFH
--PIGLAA
PYPIGL--
--PIGLYD
--PIGLWH
--PIGLVH
--PIGLVF
--PIGLTI
--PIGLQV
--PIGLNY
--PIGLNI
--PIGLMM
--PIGLMG
--PIGLHY
--PIGLHV
--PIGLHL
--PIGLEY
--PIGLYE
--PIGLVY
--PIGLVK
--PIGLVI
--PIGLSQ
--PIGLNN
--PIGLNL
--PIGLMW
--PIGLKD
--PIGLIK
--PIGLID
--PIGLHK
--PIGLER
--PIGLAM
PHPIGL--
PVPIGL--
PQPIGL--
--PIGLYH
--PIGLWG
--PIGLVN
--PIGLTQ
--PIGLTD

--PIGLRK
--PIGLQY
--PIGLQN
--PIGLQA
--PIGLMT
--PIGLKA
--PIGLIQ
--PIGLIA
--PIGLHH
--PIGLHG
--PIGLDG
--PIGLAV
--PIGLAI
--PIGLYK
--PIGLVE
--PIGLSD
--PIGLRQ
--PIGLQK
--PIGLNW
--PIGLMI
--PIGLLE
--PIGLII
--PIGLIG
--PIGLIE
--PIGLHN
--PIGLGY
--PIGLGL
--PIGLGI
--PIGLGA
--PIGLES
--PIGLDS
--PIGLAY
PEPIGL--
--PIGLYQ
--PIGLTW
--PIGLTM
--PIGLTE
--PIGLRW
--PIGLNG
--PIGLNA
--PIGLMH
--PIGLIW
--PIGLHW
--PIGLEV
--PIGLEK
--PIGLEG
--PIGLAW
--PIGLAL
PFPIGL--
--PIGLVQ

--PIGLTY
--PIGLQL
--PIGLQF
--PIGLPY
--PIGLNK
--PIGLKM
--PIGLIN
--PIGLIH
--PIGLGF
--PIGLFQ
PAPIGL--
PMPIGL--
--PIGLYN
--PIGLWQ
--PIGLWD
--PIGLVD
--PIGLRE
--PIGLQI
--PIGLQG
--PIGLNQ
--PIGLNC
--PIGLMN
--PIGLMA
--PIGLKW
--PIGLKN
--PIGLHQ
--PIGLHI
--PIGLHD
--PIGLFA
--PIGLEW
--PIGLET
--PIGLEQ
--PIGLEM
--PIGLEF
--PIGLEA
-PPIGLV-
--PIGLVP
--PIGLQQ
--PIGLQM
--PIGLPI
--PIGLPF
--PIGLMQ
--PIGLMK
--PIGLME
--PIGLMD
--PIGLKF
--PIGLKE
--PIGLIC
--PIGLHM
--PIGLHE

--PIGLGQ
--PIGLGM
--PIGLFD
--PIGLEN
--PIGLCT
--PIGLAQ
QTPIGL--
QFPIGL--
-QPIGLS-
QLPIGL--
-QPIGLR-
-QPIGLG-
QGPIGL--
QRPIGL--
-QPIGLY-
-QPIGLT-
QVPIGL--
-QPIGLL-
QKPIGL--
QAPIGL--
QSPIGL--
-QPIGLH-
-QPIGLI-
-QPIGLF-
QYPIGL--
QPPIGL--
-QPIGLQ-
-QPIGLA-
QNPIGL--
QIPIGL--
QWPIGL--
QQPIGL--
-QPIGLW-
-QPIGLN-
-QPIGLK-
-QPIGLE-
QMPIGL--
QDPIGL--
QCPIGL--
RLPIGL--
RIPIGL--
-RPIGLT-
RSPIGL--
RAPIGL--
-RPIGLN-
RVPIGL--
-RPIGLA-
RFPIGL--
-RPIGLH-
RGPIGL--

RRPIGL--
RWPIGL--
RQPIGL--
-RPIGLK-
-RPIGLE-
-RPIGLW-
REPIGL--
RHPIGL--
-RPIGLI-
-RPIGLV-
RMPIGL--
RDPIGL--
RNPIGL--
RCPIGL--
RPPIGL--
-SPIGLR-
SSPIGL--
SLPIGL--
-SPIGLS-
SWPIGL--
-SPIGLK-
SDPIGL--
STPIGL--
SQPIGL--
SAPIGL--
-SPIGLT-
SNPIGL--
SHPIGL--
SFPIGL--
SMPIGL--
-SPIGLN-
-SPIGLF-
-SPIGLA-
-SPIGLW-
-SPIGLV-
-SPIGLQ-
SGPIGL--
SVPIGL--
SCPIGL--
-SPIGLY-
-SPIGLG-
SKPIGL--
-SPIGLH-
SIPIGL--
-SPIGLI-
SYPIGL--
SPPIGL--
SEPIGL--
-SPIGLE-
TSPIGL--

-TPIGLS-
-TPIGLL-
-TPIGLR-
TGPIGL--
TWPIGL--
TRPIGL--
TVPIGL--
TMPIGL--
TFPIGL--
-TPIGLK-
-TPIGLT-
-TPIGLI-
-TPIGLG-
-TPIGLF-
TTPIGL--
-TPIGLY-
TYPIGL--
-TPIGLN-
TKPIGL--
-TPIGLW-
-TPIGLM-
TEPIGL--
-TPIGLV-
-TPIGLA-
TQPIGL--
TPPIGL--
-TPIGLE-
-TPIGLH-
TIPIGL--
-TPIGLQ-
TDPIGL--
THPIGL--
-VPIGLR-
VRPIGL--
VIPIGL--
VKPIGL--
VSPIGL--
VMPIGL--
-VPIGLE-
-VPIGLS-
-VPIGLH-
-VPIGLG-
VTPIGL--
VDPIGL--
-VPIGLT-
VLPIGL--
-VPIGLV-
-VPIGLN-
-VPIGLK-
VNPIGL--

-VPIGLI-
-VPIGLA-
VEPIGL--
VAPIGL--
VWPIGL--
VYPIGL--
VVPIGL--
VHPIGL--
VFPIGL--
VPPIGL--
-VPIGLQ-
VQPIGL--
VCPIGL--
-WPIGLR-
WVPIGL--
WSPIGL--
WTPIGL--
WGPIGL--
-WPIGLQ-
-WPIGLK-
-WPIGLL-
WIPIGL--
-WPIGLT-
-WPIGLE-
WNPIGL--
WLPIGL--
WPPIGL--
-WPIGLS-
-WPIGLI-
WEPIGL--
WAPIGL--
-WPIGLF-
WWPIGL--
WQPIGL--
-WPIGLV-
-WPIGLG-
WHPIGL--
WFPIGL--
-WPIGLN-
WDPIGL--
-WPIGLM-
-WPIGLH-
-WPIGLA-
YGPIGL--
YLPIGL--
-YPIGLS-
YTPIGL--
-YPIGLR-
YVPIGL--
YHPIGL--

YYPIGL--
YSPIGL--
-YPIGLQ-
-YPIGLA-
-YPIGLV-
YKPIGL--
-YPIGLW-
-YPIGLK-
YQPIGL--
-YPIGLG-
-YPIGLE-
YWPIGL--
-YPIGLI-
YMPIGL--
YEPIGL--
-YPIGLY-
-YPIGLT-
-YPIGLH-
-YPIGLF-
-YPIGLN-
YNPIGL--
YFPIGL--
YDPIGL--
YCPIGL--

| | | | |
|---|---|---|---|
| PAAW | 282 | 677 | 34.28 |

-APAAWT-
ASPAAW--
-APAAWR-
-APAAWS-
AAPAAW--
ARPAAW--
ALPAAW--
AMPAAW--
AGPAAW--
ANPAAW--
AQPAAW--
APPAAW--
-APAAWW-
-APAAWV-
-APAAWQ-
-APAAWK-
ADPAAW--
AVPAAW--
ATPAAW--
-APAAWH-
-APAAWG-
ACPAAW--
AYPAAW--
-APAAWY-
-APAAWN-

AIPAAW--
AHPAAW--
AFPAAW--
AEPAAW--
CWPAAW--
-CPAAWS-
CSPAAW--
CNPAAW--
-CPAAWY-
-CPAAWK-
-CPAAWI-
CLPAAW--
CGPAAW--
-DPAAWL-
-DPAAWK-
DMPAAW--
DDPAAW--
DWPAAW--
DTPAAW--
DVPAAW--
DRPAAW--
-DPAAWS-
-DPAAWR-
-DPAAWM-
-DPAAWI-
-DPAAWA-
DLPAAW--
DIPAAW--
DSPAAW--
-DPAAWV-
-DPAAWN-
DCPAAW--
-EPAAWF-
-EPAAWT-
-EPAAWL-
-EPAAWH-
EYPAAW--
EVPAAW--
-EPAAWR-
-EPAAWQ-
-EPAAWN-
-EPAAWK-
EKPAAW--
EHPAAW--
EWPAAW--
-EPAAWY-
-EPAAWI-
EAPAAW--
-FPAAWR-
FWPAAW--

FRPAAW--
-FPAAWS-
-FPAAWK-
FNPAAW--
-FPAAWH-
FEPAAW--
FPPAAW--
-FPAAWY-
-FPAAWT-
-FPAAWN-
-FPAAWI-
FKPAAW--
FGPAAW--
FTPAAW--
FSPAAW--
FQPAAW--
-FPAAWQ-
-FPAAWE-
-FPAAWA-
FHPAAW--
GRPAAW--
GKPAAW--
-GPAAWR-
GLPAAW--
GSPAAW--
GEPAAW--
GVPAAW--
-GPAAWM-
GNPAAW--
GPPAAW--
-GPAAWS-
-GPAAWF-
GGPAAW--
-GPAAWY-
-GPAAWT-
-GPAAWE-
GFPAAW--
-GPAAWW-
-GPAAWV-
-GPAAWN-
-GPAAWI-
GDPAAW--
GYPAAW--
GWPAAW--
GTPAAW--
-GPAAWQ-
-GPAAWL-
-GPAAWH-
-GPAAWG-
GMPAAW--

HRPAAW--
-HPAAWR-
-HPAAWG-
HLPAAW--
-HPAAWT-
-HPAAWL-
-HPAAWQ-
-HPAAWN-
-HPAAWM-
-HPAAWK-
HGPAAW--
HYPAAW--
HTPAAW--
-HPAAWE-
HHPAAW--
HAPAAW--
-HPAAWY-
-HPAAWI-
-HPAAWH-
HWPAAW--
HPPAAW--
-HPAAWS-
HIPAAW--
HFPAAW--
HEPAAW--
HDPAAW--
INPAAW--
-IPAAWI-
-IPAAWV-
-IPAAWN-
-IPAAWK-
ITPAAW--
ISPAAW--
-IPAAWQ-
-IPAAWL-
ILPAAW--
IYPAAW--
IRPAAW--
IPPAAW--
-IPAAWY-
-IPAAWT-
-IPAAWH-
-IPAAWG-
IMPAAW--
KSPAAW--
-KPAAWS-
-KPAAWM-
-KPAAWL-
KLPAAW--
-KPAAWH-

-KPAAWY-
-KPAAWQ-
-KPAAWR-
-KPAAWN-
-KPAAWI-
KDPAAW--
KVPAAW--
-KPAAWV-
-KPAAWK-
-KPAAWF-
KIPAAW--
KGPAAW--
KFPAAW--
-KPAAWT-
-KPAAWG-
KHPAAW--
KAPAAW--
KYPAAW--
KWPAAW--
KTPAAW--
KRPAAW--
KPPAAW--
KNPAAW--
KEPAAW--
-LPAAWR-
LIPAAW--
-LPAAWW-
LRPAAW--
-LPAAWS-
LMPAAW--
LYPAAW--
-LPAAWN-
-LPAAWV-
LGPAAW--
LSPAAW--
-LPAAWI-
LLPAAW--
-LPAAWT-
-LPAAWH-
-LPAAWE-
-LPAAWA-
LTPAAW--
-LPAAWQ-
-LPAAWK-
LEPAAW--
LDPAAW--
LWPAAW--
LQPAAW--
LPPAAW--
-LPAAWM-

```
LNPAAW--
LAPAAW--
LVPAAW--
LFPAAW--
LCPAAW--
MRPAAW--
MVPAAW--
MYPAAW--
-MPAAWG-
-MPAAWF-
MSPAAW--
-MPAAWK-
MLPAAW--
MGPAAW--
MPPAAW--
-MPAAWS-
-MPAAWQ-
-MPAAWN-
MIPAAW--
MTPAAW--
-MPAAWW-
-MPAAWV-
-MPAAWI-
-MPAAWH-
MNPAAW--
MHPAAW--
MFPAAW--
MEPAAW--
-NPAAWR-
-NPAAWI-
NFPAAW--
-NPAAWN-
NWPAAW--
-NPAAWV-
NLPAAW--
-NPAAWL-
NAPAAW--
NSPAAW--
-NPAAWQ-
NMPAAW--
NIPAAW--
NEPAAW--
-NPAAWY-
-NPAAWF-
NNPAAW--
NKPAAW--
NYPAAW--
NVPAAW--
NTPAAW--
-NPAAWT-
```

--PAAWSL
--PAAWSR
--PAAWSA
--PAAWRW
--PAAWST
--PAAWMR
--PAAWGL
--PAAWLR
--PAAWKL
--PAAWGA
--PAAWYT
--PAAWVS
--PAAWSG
--PAAWRN
--PAAWKV
--PAAWVL
--PAAWRL
--PAAWLS
--PAAWLG
--PAAWYR
--PAAWVM
--PAAWTR
--PAAWSN
--PAAWRK
--PAAWMA
--PAAWLI
--PAAWAT
PQPAAW--
--PAAWLN
--PAAWIT
--PAAWGF
--PAAWFS
--PAAWES
--PAAWTL
--PAAWRT
--PAAWRS
PRPAAW--
PNPAAW--
--PAAWYW
--PAAWYL
--PAAWWS
--PAAWWQ
--PAAWRV
--PAAWRQ
--PAAWQW
--PAAWNG
--PAAWKG
--PAAWHE
--PAAWGG
--PAAWFI

--PAAWAR
--PAAWAI
PDPAAW--
--PAAWYH
--PAAWVT
--PAAWVI
--PAAWVA
--PAAWTI
--PAAWTH
--PAAWRP
--PAAWPL
--PAAWNS
--PAAWNN
--PAAWMT
--PAAWML
--PAAWKI
--PAAWIN
--PAAWHA
--PAAWEV
--PAAWEG
--PAAWAS
PGPAAW--
--PAAWVY
--PAAWTW
--PAAWTS
--PAAWTQ
--PAAWTK
--PAAWSI
--PAAWNL
--PAAWNK
--PAAWMS
--PAAWLL
--PAAWLK
--PAAWKN
--PAAWKK
--PAAWIR
--PAAWHW
--PAAWHR
--PAAWGV
--PAAWFR
--PAAWFL
--PAAWAQ
--PAAWWN
--PAAWWD
--PAAWWA
--PAAWVH
--PAAWTT
--PAAWTE
--PAAWSY
--PAAWSF

--PAAWRH
--PAAWRG
--PAAWQV
--PAAWMN
--PAAWMG
--PAAWLH
--PAAWIS
--PAAWIL
--PAAWIA
--PAAWGI
--PAAWFN
--PAAWAM
PIPAAW--
PHPAAW--
PFPAAW--
--PAAWYN
--PAAWVN
--PAAWVK
--PAAWTV
--PAAWTD
--PAAWSV
--PAAWSM
--PAAWSK
--PAAWRY
--PAAWRI
--PAAWRA
--PAAWQR
--PAAWQD
--PAAWPF
--PAAWNT
--PAAWNI
--PAAWMQ
--PAAWMI
--PAAWLM
--PAAWLE
--PAAWKY
--PAAWKS
--PAAWKA
--PAAWIK
--PAAWIG
--PAAWHS
--PAAWHK
--PAAWGS
--PAAWFK
--PAAWET
--PAAWAV
--PAAWAK
PSPAAW--
--PAAWYQ
--PAAWYM

--PAAWYK
--PAAWYA
--PAAWWI
--PAAWTN
--PAAWQY
--PAAWQK
--PAAWQH
--PAAWQE
--PAAWQA
--PAAWNY
--PAAWNW
--PAAWNF
--PAAWNA
--PAAWMY
--PAAWMW
--PAAWMP
--PAAWLQ
--PAAWIM
--PAAWIH
--PAAWID
--PAAWHN
--PAAWHL
--PAAWHG
--PAAWGW
--PAAWFV
--PAAWFT
--PAAWFE
--PAAWER
--PAAWDS
--PAAWAW
--PAAWAH
-PPAAWS-
-PPAAWL-
-PPAAWG-
PMPAAW--
--PAAWYD
--PAAWWL
--PAAWWK
--PAAWWG
--PAAWWE
--PAAWVV
--PAAWVQ
--PAAWVE
--PAAWVD
--PAAWTA
--PAAWSW
--PAAWSS
--PAAWSQ
--PAAWSP
--PAAWSH

--PAAWRM
--PAAWQS
--PAAWQQ
--PAAWQG
--PAAWPM
--PAAWNH
--PAAWMH
--PAAWLD
--PAAWKQ
--PAAWKM
--PAAWKH
--PAAWKF
--PAAWKD
--PAAWIW
--PAAWIQ
--PAAWIE
--PAAWHV
--PAAWHH
--PAAWGN
--PAAWGM
--PAAWGH
--PAAWFG
--PAAWFD
--PAAWAN
--PAAWAG
--PAAWAE
--PAAWAD
--PAAWAA
-QPAAWN-
QAPAAW--
-QPAAWG-
-QPAAWS-
-QPAAWW-
-QPAAWT-
QSPAAW--
-QPAAWR-
QYPAAW--
-QPAAWL-
QHPAAW--
QEPAAW--
QTPAAW--
QQPAAW--
QPPAAW--
QMPAAW--
QGPAAW--
-QPAAWV-
QLPAAW--
QIPAAW--
RSPAAW--
RRPAAW--

RVPAAW--
-RPAAWL-
-RPAAWF-
-RPAAWI-
-RPAAWR-
-RPAAWA-
-RPAAWV-
-RPAAWG-
RQPAAW--
-RPAAWN-
-RPAAWM-
RLPAAW--
RDPAAW--
RIPAAW--
RFPAAW--
-RPAAWT-
RHPAAW--
RYPAAW--
-RPAAWH-
RNPAAW--
-RPAAWW-
-RPAAWQ-
RKPAAW--
RGPAAW--
RWPAAW--
RCPAAW--
RPPAAW--
-RPAAWY-
-RPAAWK-
REPAAW--
RAPAAW--
SSPAAW--
-SPAAWF-
-SPAAWR-
-SPAAWK-
SLPAAW--
-SPAAWI-
-SPAAWG-
STPAAW--
-SPAAWN-
-SPAAWE-
-SPAAWS-
-SPAAWL-
SVPAAW--
-SPAAWY-
-SPAAWV-
SKPAAW--
SEPAAW--
-SPAAWT-
SHPAAW--

SAPAAW--
SNPAAW--
SGPAAW--
SPPAAW--
-SPAAWW-
SMPAAW--
-SPAAWH-
-SPAAWA-
SFPAAW--
SDPAAW--
TYPAAW--
-TPAAWT-
TRPAAW--
-TPAAWS-
-TPAAWR-
-TPAAWL-
TVPAAW--
TSPAAW--
TLPAAW--
-TPAAWV-
TEPAAW--
TTPAAW--
-TPAAWK-
-TPAAWE-
THPAAW--
TAPAAW--
-TPAAWN-
-TPAAWM-
-TPAAWF-
TDPAAW--
-VPAAWT-
-VPAAWG-
VGPAAW--
VRPAAW--
-VPAAWN-
-VPAAWS-
VNPAAW--
-VPAAWY-
VLPAAW--
-VPAAWK-
VKPAAW--
VAPAAW--
VVPAAW--
VSPAAW--
VPPAAW--
-VPAAWW-
-VPAAWL-
VFPAAW--
VTPAAW--
-VPAAWR-

VIPAAW--
VWPAAW--
-VPAAWH-
VDPAAW--
-VPAAWV-
-VPAAWM-
-VPAAWI-
-VPAAWF-
VEPAAW--
WRPAAW--
WQPAAW--
-WPAAWR-
WKPAAW--
-WPAAWA-
-WPAAWS-
WEPAAW--
WYPAAW--
WTPAAW--
WSPAAW--
-WPAAWY-
-WPAAWV-
-WPAAWL-
-WPAAWK-
-WPAAWH-
-WPAAWF-
WMPAAW--
WLPAAW--
-YPAAWG-
-YPAAWT-
-YPAAWA-
YHPAAW--
-YPAAWH-
YDPAAW--
-YPAAWN-
YRPAAW--
-YPAAWF-
YKPAAW--
YWPAAW--
YVPAAW--
-YPAAWE-
YYPAAW--
YPPAAW--
-YPAAWL-
YTPAAW--
YSPAAW--
-YPAAWY-
-YPAAWV-
-YPAAWS-
-YPAAWR-
-YPAAWI-

YLPAAW--
YGPAAW--

| | | | |
|---|---|---|---|
| IGNL | 15 | 852 | 34.24 |

--IGNLTP
--IGNLNL
--IGNLGW
--IGNLGI
--IGNLGH
-KIGNLG-
VNIGNL--
-WIGNLH-
ALIGNL--
ASIGNL--
AGIGNL--
-AIGNLS-
AQIGNL--
-AIGNLR-
ARIGNL--
ANIGNL--
-AIGNLI-
-AIGNLT-
ATIGNL--
-AIGNLA-
AKIGNL--
-AIGNLG-
AFIGNL--
AVIGNL--
AAIGNL--
-AIGNLH-
APIGNL--
AYIGNL--
-AIGNLF-
AEIGNL--
AWIGNL--
-AIGNLY-
AHIGNL--
-AIGNLN-
AMIGNL--
-AIGNLK-
AIIGNL--
-AIGNLQ-
-AIGNLE-
CAIGNL--
CMIGNL--
CTIGNL--
CPIGNL--
-CIGNLR-
-CIGNLL-
CVIGNL--
CSIGNL--

```
CIIGNL--
-CIGNLI-
CHIGNL--
CCIGNL--
CRIGNL--
CQIGNL--
CNIGNL--
-CIGNLT-
-CIGNLQ-
-CIGNLK-
CGIGNL--
CEIGNL--
CLIGNL--
CKIGNL--
-CIGNLY-
-CIGNLW-
-CIGNLN-
-CIGNLM-
-CIGNLG-
CFIGNL--
-DIGNLL-
DRIGNL--
-DIGNLS-
-DIGNLR-
DKIGNL--
DSIGNL--
-DIGNLM-
DFIGNL--
DLIGNL--
-DIGNLW-
-DIGNLT-
-DIGNLI-
DTIGNL--
DNIGNL--
DIIGNL--
DVIGNL--
DQIGNL--
-DIGNLH-
-DIGNLF-
DDIGNL--
DPIGNL--
-DIGNLV-
-DIGNLK-
DAIGNL--
DYIGNL--
DMIGNL--
DGIGNL--
DWIGNL--
-DIGNLA-
EGIGNL--
```

```
EYIGNL--
ESIGNL--
EKIGNL--
-EIGNLK-
-EIGNLW-
ETIGNL--
EVIGNL--
-EIGNLT-
-EIGNLR-
-EIGNLF-
ERIGNL--
ELIGNL--
-EIGNLY-
-EIGNLS-
-EIGNLI-
EWIGNL--
EPIGNL--
EDIGNL--
ENIGNL--
-EIGNLQ-
-EIGNLM-
-EIGNLA-
EHIGNL--
EFIGNL--
EIIGNL--
-EIGNLV-
-EIGNLN-
-EIGNLH-
EEIGNL--
EAIGNL--
FRIGNL--
FGIGNL--
FSIGNL--
FVIGNL--
-FIGNLR-
FNIGNL--
FHIGNL--
FYIGNL--
FLIGNL--
-FIGNLH-
FTIGNL--
FEIGNL--
FAIGNL--
FIIGNL--
-FIGNLG-
-FIGNLL-
FWIGNL--
-FIGNLT-
-FIGNLM-
-FIGNLY-
```

-FIGNLN-
-FIGNLF-
-FIGNLV-
FDIGNL--
FPIGNL--
FMIGNL--
-FIGNLE-
FFIGNL--
FQIGNL--
FKIGNL--
-FIGNLK-
-GIGNLR-
GRIGNL--
-GIGNLS-
GTIGNL--
GLIGNL--
GSIGNL--
GVIGNL--
GWIGNL--
-GIGNLV-
GIIGNL--
-GIGNLN-
-GIGNLL-
GYIGNL--
-GIGNLY-
-GIGNLT-
-GIGNLI-
GGIGNL--
-GIGNLK-
-GIGNLF-
GAIGNL--
-GIGNLM-
-GIGNLG-
GFIGNL--
GEIGNL--
-GIGNLH-
GHIGNL--
GMIGNL--
-GIGNLQ-
GCIGNL--
GPIGNL--
GKIGNL--
GQIGNL--
GNIGNL--
GDIGNL--
-HIGNLS-
-HIGNLF-
-HIGNLR-
HKIGNL--
HAIGNL--

HVIGNL--
HGIGNL--
HIIGNL--
HSIGNL--
-HIGNLV-
-HIGNLL-
-HIGNLH-
-HIGNLG-
-HIGNLN-
-HIGNLA-
HFIGNL--
HYIGNL--
HLIGNL--
-HIGNLT-
-HIGNLM-
HWIGNL--
HTIGNL--
HPIGNL--
-HIGNLE-
HDIGNL--
HMIGNL--
-HIGNLI-
HHIGNL--
HEIGNL--
-HIGNLK-
-HIGNLQ-
HCIGNL--
--IGNLLK
--IGNLTR
--IGNLSS
--IGNLRR
--IGNLRY
--IGNLVG
--IGNLML
--IGNLLT
IYIGNL--
--IGNLYR
--IGNLVT
--IGNLSI
--IGNLLG
--IGNLRI
--IGNLAT
--IGNLSA
--IGNLLA
IAIGNL--
--IGNLTS
--IGNLSR
--IGNLGS
--IGNLSV
--IGNLYL

--IGNLVL
--IGNLVK
--IGNLRV
--IGNLRH
--IGNLRA
--IGNLQY
--IGNLMT
--IGNLKN
--IGNLIG
--IGNLGG
--IGNLAM
ISIGNL--
INIGNL--
-IIGNLI-
--IGNLYG
--IGNLVH
--IGNLQG
--IGNLMS
--IGNLIR
-IIGNLK-
--IGNLYT
--IGNLVM
--IGNLTG
--IGNLRT
--IGNLIT
--IGNLIA
--IGNLFA
--IGNLAV
IKIGNL--
--IGNLWV
--IGNLWS
--IGNLVV
--IGNLVI
--IGNLTV
--IGNLRN
--IGNLQT
--IGNLLQ
--IGNLGA
--IGNLFG
-IIGNLR-
--IGNLSN
--IGNLQS
--IGNLKY
--IGNLKT
--IGNLKK
--IGNLHG
IVIGNL--
ITIGNL--
--IGNLYS
--IGNLYH

--IGNLWG
--IGNLTF
--IGNLQR
--IGNLNV
--IGNLMG
--IGNLLN
--IGNLKS
--IGNLIY
--IGNLFM
--IGNLEF
--IGNLAS
--IGNLTL
--IGNLST
--IGNLSK
--IGNLRD
--IGNLKG
--IGNLFK
IPIGNL--
IHIGNL--
--IGNLYQ
--IGNLYK
--IGNLWA
--IGNLTT
--IGNLTK
--IGNLTI
--IGNLSG
--IGNLQK
--IGNLKV
--IGNLIM
--IGNLHL
--IGNLGT
--IGNLFT
--IGNLAI
--IGNLAG
IGIGNL--
-IIGNLN-
--IGNLYV
--IGNLYM
--IGNLVN
--IGNLTW
--IGNLTQ
--IGNLRQ
--IGNLQL
--IGNLLH
--IGNLKL
--IGNLIV
--IGNLIK
--IGNLHA
--IGNLGL
-IIGNLS-

-IIGNLE-
--IGNLYN
--IGNLYD
--IGNLVW
--IGNLVE
--IGNLSM
--IGNLRE
--IGNLMH
--IGNLMF
--IGNLMA
--IGNLFS
--IGNLFN
--IGNLES
IWIGNL--
-IIGNLW-
-IIGNLL-
-IIGNLG-
-IIGNLF-
-IIGNLA-
--IGNLYY
--IGNLTN
--IGNLSH
--IGNLQA
--IGNLNS
--IGNLMY
--IGNLLI
--IGNLKW
--IGNLKR
--IGNLKH
--IGNLID
--IGNLHY
--IGNLFW
--IGNLFD
--IGNLAR
IEIGNL--
-IIGNLV-
-IIGNLT-
--IGNLWM
--IGNLWL
--IGNLSY
--IGNLQF
--IGNLNW
--IGNLNF
--IGNLMW
--IGNLMM
--IGNLMK
--IGNLLM
--IGNLKI
--IGNLIE
--IGNLHW

```
--IGNLHS
--IGNLHQ
--IGNLHF
--IGNLGM
--IGNLFV
--IGNLAY
IFIGNL--
IDIGNL--
IQIGNL--
--IGNLYI
--IGNLYE
--IGNLWK
--IGNLWI
--IGNLVA
--IGNLSF
--IGNLQV
--IGNLNK
--IGNLNA
--IGNLMQ
--IGNLMN
--IGNLLE
--IGNLKF
--IGNLIH
--IGNLHN
--IGNLHK
--IGNLGV
--IGNLFH
--IGNLER
--IGNLEN
--IGNLEA
--IGNLDS
-IIGNLM-
--IGNLWN
--IGNLWD
--IGNLVD
--IGNLTH
--IGNLTE
--IGNLSW
--IGNLSQ
--IGNLSD
--IGNLQW
--IGNLQQ
--IGNLQN
--IGNLPL
--IGNLNY
--IGNLNR
--IGNLMV
--IGNLME
--IGNLLD
--IGNLIW
```

--IGNLIN
--IGNLIF
--IGNLHV
--IGNLHM
--IGNLHH
--IGNLGK
--IGNLFE
--IGNLEW
--IGNLET
--IGNLAW
--IGNLAQ
--IGNLAH
IMIGNL--
--IGNLYW
--IGNLWH
--IGNLSE
--IGNLQM
--IGNLQH
--IGNLNQ
--IGNLNN
--IGNLNM
--IGNLKQ
--IGNLKM
--IGNLKD
--IGNLKA
--IGNLII
--IGNLHT
--IGNLGF
--IGNLGE
--IGNLFQ
--IGNLEM
--IGNLEH
--IGNLEG
--IGNLAD
ICIGNL--
KWIGNL--
-KIGNLS-
-KIGNLL-
-KIGNLY-
KLIGNL--
KAIGNL--
-KIGNLR-
KFIGNL--
KNIGNL--
KTIGNL--
KGIGNL--
-KIGNLQ-
KHIGNL--
-KIGNLW-
KEIGNL--

-KIGNLI-
-KIGNLK-
-KIGNLF-
KVIGNL--
-KIGNLV-
-KIGNLT-
KYIGNL--
-KIGNLN-
-KIGNLA-
KPIGNL--
KIIGNL--
-KIGNLH-
KQIGNL--
KCIGNL--
-LIGNLS-
-LIGNLR-
LRIGNL--
LVIGNL--
LLIGNL--
-LIGNLT-
-LIGNLN-
-LIGNLK-
-LIGNLA-
-LIGNLG-
LTIGNL--
LGIGNL--
LIIGNL--
LNIGNL--
-LIGNLY-
LHIGNL--
-LIGNLM-
-LIGNLV-
LFIGNL--
LAIGNL--
-LIGNLH-
LYIGNL--
LPIGNL--
-LIGNLW-
LDIGNL--
LWIGNL--
-LIGNLI-
LEIGNL--
LQIGNL--
-LIGNLQ-
-LIGNLD-
LMIGNL--
-LIGNLF-
LCIGNL--
-LIGNLP-
-LIGNLE-

MLIGNL--
-MIGNLR-
-MIGNLS-
-MIGNLN-
-MIGNLG-
-MIGNLF-
MFIGNL--
MTIGNL--
MRIGNL--
MDIGNL--
-MIGNLV-
-MIGNLT-
-MIGNLQ-
-MIGNLL-
-MIGNLA-
MYIGNL--
MPIGNL--
-MIGNLY-
-MIGNLK-
MGIGNL--
MAIGNL--
-MIGNLH-
MWIGNL--
-MIGNLW-
-MIGNLI-
MEIGNL--
MNIGNL--
MIIGNL--
MHIGNL--
MKIGNL--
-MIGNLE-
MMIGNL--
MCIGNL--
-MIGNLD-
NRIGNL--
NWIGNL--
-NIGNLL-
-NIGNLN-
-NIGNLY-
NLIGNL--
-NIGNLR-
NGIGNL--
-NIGNLF-
-NIGNLA-
NSIGNL--
NAIGNL--
NPIGNL--
NTIGNL--
NKIGNL--
-NIGNLS-

-NIGNLG-
NEIGNL--
NCIGNL--
NVIGNL--
NMIGNL--
NFIGNL--
-NIGNLV-
NDIGNL--
-NIGNLW-
NYIGNL--
NQIGNL--
-NIGNLM-
-NIGNLK-
-NIGNLI-
-NIGNLH-
NNIGNL--
-NIGNLT-
-NIGNLE-
-NIGNLP-
PRIGNL--
PWIGNL--
PSIGNL--
-PIGNLG-
-PIGNLR-
PLIGNL--
PAIGNL--
PIIGNL--
-PIGNLY-
-PIGNLL-
-PIGNLF-
-PIGNLH-
-PIGNLI-
PGIGNL--
PYIGNL--
PVIGNL--
PNIGNL--
-PIGNLV-
-PIGNLT-
-PIGNLN-
PMIGNL--
PKIGNL--
-PIGNLK-
PTIGNL--
PHIGNL--
PFIGNL--
-PIGNLQ-
-PIGNLA-
PDIGNL--
PQIGNL--
PEIGNL--

-PIGNLW-
-PIGNLC-
PCIGNL--
QIIGNL--
-QIGNLL-
QVIGNL--
-QIGNLK-
QTIGNL--
QYIGNL--
QLIGNL--
QNIGNL--
-QIGNLS-
QAIGNL--
QMIGNL--
QKIGNL--
-QIGNLV-
-QIGNLT-
-QIGNLQ-
-QIGNLN-
-QIGNLF-
QRIGNL--
QPIGNL--
-QIGNLM-
-QIGNLE-
QGIGNL--
-QIGNLW-
-QIGNLI-
QEIGNL--
QWIGNL--
QQIGNL--
-QIGNLR-
-QIGNLG-
-QIGNLY-
QHIGNL--
QDIGNL--
RAIGNL--
-RIGNLR-
-RIGNLS-
-RIGNLL-
RGIGNL--
RHIGNL--
-RIGNLK-
-RIGNLF-
-RIGNLI-
-RIGNLN-
-RIGNLY-
-RIGNLW-
-RIGNLV-
RPIGNL--
-RIGNLQ-

```
RIIGNL--
-RIGNLE-
RQIGNL--
RCIGNL--
-RIGNLA-
REIGNL--
-SIGNLR-
-SIGNLL-
-SIGNLA-
SLIGNL--
SQIGNL--
-SIGNLS-
-SIGNLT-
SGIGNL--
SIIGNL--
SVIGNL--
-SIGNLN-
-SIGNLV-
SNIGNL--
-SIGNLK-
-SIGNLG-
SFIGNL--
SPIGNL--
SKIGNL--
-SIGNLF-
SHIGNL--
-SIGNLY-
SAIGNL--
SWIGNL--
STIGNL--
SEIGNL--
-SIGNLM-
SCIGNL--
-SIGNLW-
-SIGNLI-
SDIGNL--
-SIGNLH-
-SIGNLE-
TKIGNL--
-TIGNLR-
-TIGNLS-
-TIGNLL-
TNIGNL--
TLIGNL--
THIGNL--
-TIGNLI-
TAIGNL--
TYIGNL--
TVIGNL--
TPIGNL--
```

-TIGNLY-
TIIGNL--
-TIGNLN-
TFIGNL--
TGIGNL--
TWIGNL--
TMIGNL--
-TIGNLT-
-TIGNLH-
-TIGNLV-
-TIGNLQ-
-TIGNLF-
-TIGNLE-
TQIGNL--
-TIGNLA-
-TIGNLW-
TEIGNL--
-VIGNLY-
-VIGNLR-
-VIGNLN-
VSIGNL--
-VIGNLS-
VGIGNL--
-VIGNLL-
VKIGNL--
-VIGNLK-
-VIGNLG-
VLIGNL--
VVIGNL--
VRIGNL--
-VIGNLV-
-VIGNLW-
VIIGNL--
-VIGNLH-
-VIGNLF-
-VIGNLA-
VFIGNL--
VHIGNL--
VQIGNL--
VAIGNL--
VTIGNL--
VYIGNL--
VWIGNL--
-VIGNLM-
VDIGNL--
-VIGNLQ-
VCIGNL--
VPIGNL--
VEIGNL--
-WIGNLR-

WGIGNL--
WSIGNL--
WVIGNL--
-WIGNLI-
WWIGNL--
WTIGNL--
WLIGNL--
-WIGNLY-
-WIGNLV-
-WIGNLL-
-WIGNLQ-
-WIGNLG-
-WIGNLA-
WNIGNL--
WQIGNL--
WPIGNL--
WMIGNL--
WIIGNL--
WEIGNL--
WDIGNL--
-WIGNLE-
-WIGNLC-
WFIGNL--
YRIGNL--
YSIGNL--
-YIGNLT-
-YIGNLK-
-YIGNLR-
YWIGNL--
YLIGNL--
YGIGNL--
-YIGNLA-
YFIGNL--
YYIGNL--
YNIGNL--
-YIGNLL-
YTIGNL--
YKIGNL--
-YIGNLS-
-YIGNLG-
-YIGNLV-
YPIGNL--
YMIGNL--
-YIGNLF-
-YIGNLQ-
YAIGNL--
-YIGNLY-
-YIGNLM-
YDIGNL--
YQIGNL--

| | | | |
|---|---|---|---|
| | -YIGNLE- | | |
| | -YIGNLD- | | |
| | YHIGNL-- | | |
| | YEIGNL-- | | |
| | -YIGNLN- | | |
| PAML | 188 | 722 | 34.22 |
| | -APAMLL- | | |
| | -APAMLR- | | |
| | ALPAML-- | | |
| | AVPAML-- | | |
| | AKPAML-- | | |
| | AIPAML-- | | |
| | -APAMLN- | | |
| | -APAMLK- | | |
| | ASPAML-- | | |
| | -APAMLV- | | |
| | -APAMLA- | | |
| | ANPAML-- | | |
| | -APAMLH- | | |
| | AAPAML-- | | |
| | AFPAML-- | | |
| | AWPAML-- | | |
| | ATPAML-- | | |
| | -APAMLS- | | |
| | -APAMLM- | | |
| | -APAMLT- | | |
| | -APAMLI- | | |
| | AGPAML-- | | |
| | AMPAML-- | | |
| | AQPAML-- | | |
| | AHPAML-- | | |
| | AYPAML-- | | |
| | -APAMLE- | | |
| | ADPAML-- | | |
| | ACPAML-- | | |
| | CVPAML-- | | |
| | -CPAMLY- | | |
| | -CPAMLN- | | |
| | CIPAML-- | | |
| | CEPAML-- | | |
| | CTPAML-- | | |
| | CPPAML-- | | |
| | -CPAMLK- | | |
| | CKPAML-- | | |
| | CWPAML-- | | |
| | CSPAML-- | | |
| | CRPAML-- | | |
| | CQPAML-- | | |
| | -CPAMLT- | | |
| | -CPAMLS- | | |

```
-CPAMLA-
CMPAML--
CLPAML--
CAPAML--
-DPAMLR-
DTPAML--
DYPAML--
DWPAML--
-DPAMLL-
DFPAML--
DEPAML--
DVPAML--
DRPAML--
-DPAMLS-
-DPAMLN-
DSPAML--
-DPAMLQ-
-DPAMLK-
DLPAML--
-DPAMLV-
-DPAMLF-
DNPAML--
DMPAML--
DDPAML--
DQPAML--
DPPAML--
-DPAMLW-
-DPAMLI-
-DPAMLH-
-DPAMLA-
DHPAML--
DAPAML--
ERPAML--
-EPAMLS-
-EPAMLK-
-EPAMLR-
-EPAMLT-
EQPAML--
-EPAMLL-
EHPAML--
ESPAML--
-EPAMLY-
-EPAMLI-
EKPAML--
EGPAML--
EWPAML--
ELPAML--
EIPAML--
-EPAMLQ-
-EPAMLH-
```

-EPAMLF-
EVPAML--
ETPAML--
-EPAMLV-
-EPAMLM-
-EPAMLG-
EMPAML--
-EPAMLN-
-EPAMLA-
ENPAML--
FRPAML--
FLPAML--
-FPAMLT-
-FPAMLA-
-FPAMLS-
FGPAML--
FVPAML--
FTPAML--
FHPAML--
-FPAMLQ-
-FPAMLK-
-FPAMLN-
FQPAML--
-FPAMLW-
FNPAML--
FDPAML--
FWPAML--
FSPAML--
-FPAMLV-
FMPAML--
FEPAML--
FPPAML--
-FPAMLH-
FFPAML--
GGPAML--
-GPAMLG-
-GPAMLQ-
-GPAMLM-
GYPAML--
GRPAML--
GSPAML--
GNPAML--
GHPAML--
GTPAML--
GQPAML--
-GPAMLN-
-GPAMLH-
-GPAMLF-
GAPAML--
-GPAMLT-

GKPAML--
-GPAMLV-
-GPAMLR-
GFPAML--
GEPAML--
-GPAMLK-
-GPAMLI-
-GPAMLA-
GPPAML--
-HPAMLS-
-HPAMLK-
HTPAML--
-HPAMLW-
HLPAML--
HAPAML--
-HPAMLL-
HNPAML--
HEPAML--
HYPAML--
HSPAML--
-HPAMLY-
-HPAMLT-
HPPAML--
-HPAMLV-
-HPAMLA-
HVPAML--
HQPAML--
HKPAML--
HDPAML--
-HPAMLI-
-HPAMLH-
-HPAMLG-
HHPAML--
-HPAMLQ-
-HPAMLM-
HMPAML--
HIPAML--
HGPAML--
IVPAML--
IYPAML--
-IPAMLG-
IRPAML--
-IPAMLR-
-IPAMLW-
INPAML--
IQPAML--
-IPAMLT-
-IPAMLK-
ILPAML--
IKPAML--

IIPAML--
IGPAML--
IAPAML--
IHPAML--
IDPAML--
IWPAML--
-IPAMLY-
-IPAMLN-
IEPAML--
ITPAML--
ISPAML--
-IPAMLI-
ICPAML--
-IPAMLV-
-IPAMLQ-
-IPAMLM-
KSPAML--
-KPAMLR-
-KPAMLS-
-KPAMLY-
-KPAMLW-
KKPAML--
KPPAML--
-KPAMLG-
-KPAMLI-
-KPAMLH-
KIPAML--
-KPAMLQ-
KQPAML--
-KPAMLA-
KGPAML--
KCPAML--
KTPAML--
-KPAMLM-
KWPAML--
KLPAML--
KHPAML--
KVPAML--
-KPAMLF-
-KPAMLV-
-KPAMLT-
KDPAML--
KNPAML--
LSPAML--
-LPAMLK-
LNPAML--
-LPAMLN-
-LPAMLM-
LTPAML--
LGPAML--

LFPAML--
LDPAML--
LAPAML--
LEPAML--
LPPAML--
-LPAMLT-
-LPAMLH-
-LPAMLQ-
-LPAMLG-
-LPAMLI-
LIPAML--
LQPAML--
-LPAMLF-
-LPAMLC-
LCPAML--
MNPAML--
MRPAML--
MPPAML--
-MPAMLK-
-MPAMLL-
MHPAML--
-MPAMLQ-
MTPAML--
-MPAMLG-
MIPAML--
MWPAML--
-MPAMLN-
MFPAML--
MEPAML--
-MPAMLS-
MGPAML--
MYPAML--
-MPAMLV-
-MPAMLH-
-MPAMLF-
MKPAML--
-NPAMLS-
NGPAML--
NRPAML--
NTPAML--
-NPAMLL-
NLPAML--
-NPAMLR-
-NPAMLK-
-NPAMLA-
NKPAML--
NDPAML--
-NPAMLN-
NHPAML--
-NPAMLY-

```
-NPAMLQ-
NVPAML--
-NPAMLW-
-NPAMLV-
-NPAMLT-
NNPAML--
NEPAML--
-NPAMLG-
NAPAML--
NWPAML--
NQPAML--
NYPAML--
NPPAML--
-NPAMLM-
-NPAMLH-
NFPAML--
--PAMLRS
--PAMLLG
--PAMLLR
--PAMLGL
--PAMLVR
--PAMLMR
--PAMLVV
--PAMLVT
--PAMLRA
--PAMLRL
--PAMLLN
--PAMLSS
--PAMLMS
--PAMLGA
--PAMLSY
--PAMLSN
--PAMLLT
--PAMLVG
--PAMLRH
--PAMLQR
--PAMLGT
--PAMLFR
--PAMLKT
--PAMLWT
--PAMLWH
--PAMLMT
--PAMLLS
--PAMLRQ
--PAMLFN
PVPAML--
--PAMLTT
--PAMLTS
--PAMLTN
--PAMLST
```

--PAMLSI
--PAMLRT
--PAMLRF
--PAMLMK
--PAMLHA
--PAMLFH
--PAMLAK
PYPAML--
--PAMLSV
--PAMLRY
--PAMLRW
--PAMLNK
--PAMLHR
--PAMLGS
--PAMLEA
--PAMLAV
--PAMLYG
--PAMLVN
--PAMLTV
--PAMLSA
--PAMLNW
--PAMLNI
--PAMLNA
--PAMLMG
--PAMLIK
--PAMLHG
--PAMLGV
--PAMLYM
--PAMLTK
--PAMLNR
--PAMLND
--PAMLLQ
--PAMLKQ
--PAMLKK
--PAMLHS
--PAMLAT
--PAMLYR
--PAMLVQ
--PAMLVK
--PAMLRN
--PAMLRG
--PAMLLD
--PAMLKL
--PAMLIV
--PAMLFT
--PAMLAI
PHPAML--
PEPAML--
--PAMLYT
--PAMLTQ

--PAMLSE
--PAMLQS
--PAMLNT
--PAMLNM
--PAMLLK
--PAMLKI
--PAMLKF
--PAMLKA
--PAMLHN
--PAMLHH
--PAMLGF
--PAMLFG
PMPAML--
PFPAML--
--PAMLYK
--PAMLWG
--PAMLTE
--PAMLSH
--PAMLQY
--PAMLQH
--PAMLNN
--PAMLLH
--PAMLKS
--PAMLIQ
--PAMLIH
--PAMLHM
--PAMLGM
--PAMLGI
PSPAML--
PQPAML--
PGPAML--
--PAMLYE
--PAMLTY
--PAMLTW
--PAMLQV
--PAMLQK
--PAMLQG
--PAMLMH
--PAMLKN
--PAMLKE
--PAMLIT
--PAMLIN
--PAMLID
--PAMLHL
--PAMLHI
--PAMLHF
--PAMLGY
--PAMLFD
--PAMLEG
--PAMLAD

PWPAML--
PTPAML--
PNPAML--
PDPAML--
--PAMLYQ
--PAMLYH
--PAMLWK
--PAMLTG
--PAMLTA
--PAMLSQ
--PAMLSD
--PAMLQN
--PAMLQF
--PAMLPI
--PAMLMQ
--PAMLLE
--PAMLKY
--PAMLGW
--PAMLGR
--PAMLFK
--PAMLEL
--PAMLAY
--PAMLAQ
--PAMLAN
--PAMLAM
--PAMLWM
--PAMLWE
--PAMLVD
--PAMLRD
--PAMLQT
--PAMLQQ
--PAMLQD
--PAMLNV
--PAMLNQ
--PAMLNH
--PAMLMN
--PAMLMI
--PAMLIF
--PAMLFQ
--PAMLES
--PAMLEF
--PAMLCK
--PAMLAW
--PAMLAG
-PPAMLP-
-PPAMLL-
-PPAMLH-
--PAMLYD
--PAMLWD
--PAMLVW

```
--PAMLVE
--PAMLTI
--PAMLTF
--PAMLTD
--PAMLRE
--PAMLRC
--PAMLQW
--PAMLPY
--PAMLPM
--PAMLNF
--PAMLMW
--PAMLMV
--PAMLMD
--PAMLMA
--PAMLKM
--PAMLKG
--PAMLKD
--PAMLIW
--PAMLII
--PAMLHY
--PAMLHV
--PAMLHT
--PAMLHE
--PAMLHD
--PAMLGG
--PAMLEW
--PAMLEV
--PAMLER
--PAMLEI
--PAMLEH
--PAMLCT
--PAMLCN
--PAMLAH
--PAMLAE
-QPAMLY-
-QPAMLK-
-QPAMLA-
QSPAML--
QRPAML--
-QPAMLI-
-QPAMLT-
-QPAMLL-
QVPAML--
QPPAML--
-QPAMLM-
QNPAML--
QYPAML--
QWPAML--
-QPAMLN-
-QPAMLH-
```

−QPAMLQ−
−QPAMLG−
QEPAML−−
QAPAML−−
−QPAMLV−
−QPAMLS−
QDPAML−−
QCPAML−−
QTPAML−−
−QPAMLE−
QGPAML−−
QFPAML−−
−RPAMLL−
RLPAML−−
−RPAMLW−
−RPAMLN−
RAPAML−−
RSPAML−−
RGPAML−−
−RPAMLG−
−RPAMLT−
−RPAMLR−
−RPAMLY−
RPPAML−−
−RPAMLA−
RTPAML−−
RDPAML−−
−RPAMLV−
−RPAMLF−
RNPAML−−
RYPAML−−
−RPAMLM−
−RPAMLE−
−RPAMLQ−
−RPAMLI−
REPAML−−
RCPAML−−
RKPAML−−
−SPAMLT−
STPAML−−
−SPAMLK−
−SPAMLG−
SWPAML−−
SMPAML−−
SYPAML−−
SSPAML−−
−SPAMLM−
−SPAMLV−
SNPAML−−
SHPAML−−

SLPAML--
SIPAML--
SAPAML--
-SPAMLW-
-SPAMLE-
SKPAML--
SGPAML--
-SPAMLH-
SQPAML--
-SPAMLY-
-SPAMLN-
SDPAML--
-SPAMLF-
SEPAML--
SCPAML--
SPPAML--
TTPAML--
TSPAML--
-TPAMLK-
-TPAMLS-
TGPAML--
-TPAMLA-
TKPAML--
TIPAML--
TVPAML--
TMPAML--
TLPAML--
-TPAMLV-
-TPAMLT-
TYPAML--
TWPAML--
-TPAMLY-
-TPAMLQ-
-TPAMLN-
-TPAMLL-
-TPAMLH-
TPPAML--
-TPAMLG-
-TPAMLF-
TNPAML--
TAPAML--
TQPAML--
-TPAMLM-
THPAML--
TDPAML--
-VPAMLR-
VSPAML--
-VPAMLL-
-VPAMLA-
VNPAML--

VVPAML--
VGPAML--
-VPAMLT-
-VPAMLS-
VQPAML--
VEPAML--
-VPAMLK-
VIPAML--
VTPAML--
VPPAML--
-VPAMLN-
VFPAML--
-VPAMLI-
-VPAMLH-
VWPAML--
-VPAMLG-
VDPAML--
VHPAML--
-VPAMLM-
-VPAMLE-
-VPAMLW-
-VPAMLQ-
WKPAML--
WMPAML--
-WPAMLW-
WSPAML--
WRPAML--
-WPAMLA-
WPPAML--
-WPAMLT-
WGPAML--
WDPAML--
-WPAMLV-
-WPAMLH-
WHPAML--
-WPAMLQ-
-WPAMLG-
-WPAMLK-
-WPAMLI-
-WPAMLF-
-WPAMLE-
WIPAML--
WAPAML--
WQPAML--
WYPAML--
WNPAML--
YHPAML--
-YPAMLR-
-YPAMLF-
-YPAMLQ-

| | | | |
|---|---|---|---|
| | YAPAML-- | | |
| | YLPAML-- | | |
| | YNPAML-- | | |
| | YKPAML-- | | |
| | -YPAMLH- | | |
| | YMPAML-- | | |
| | YRPAML-- | | |
| | YPPAML-- | | |
| | -YPAMLS- | | |
| | -YPAMLN- | | |
| | YEPAML-- | | |
| | YTPAML-- | | |
| | YIPAML-- | | |
| | YFPAML-- | | |
| | YWPAML-- | | |
| | -YPAMLT- | | |
| | -YPAMLK- | | |
| | -YPAMLG- | | |
| | YGPAML-- | | |
| | YYPAML-- | | |
| | -YPAMLE- | | |
| | YDPAML-- | | |
| | YVPAML-- | | |
| | YSPAML-- | | |
| | YQPAML-- | | |
| | -YPAMLY- | | |
| | -YPAMLV- | | |
| | -YPAMLA- | | |
| PLHL | 45 | 807 | 34.18 |
| | -LPLHLN- | | |
| | --PLHLHK | | |
| | --PLHLDL | | |
| | -SPLHLD- | | |
| | ARPLHL-- | | |
| | ALPLHL-- | | |
| | -APLHLR- | | |
| | ATPLHL-- | | |
| | -APLHLS- | | |
| | AGPLHL-- | | |
| | ASPLHL-- | | |
| | ANPLHL-- | | |
| | AVPLHL-- | | |
| | -APLHLK- | | |
| | AYPLHL-- | | |
| | -APLHLT- | | |
| | AKPLHL-- | | |
| | ADPLHL-- | | |
| | -APLHLQ- | | |
| | -APLHLY- | | |
| | -APLHLM- | | |

-APLHLL-
-APLHLH-
-APLHLI-
AHPLHL--
AAPLHL--
APPLHL--
AWPLHL--
AQPLHL--
-APLHLW-
-APLHLE-
-APLHLN-
AEPLHL--
ACPLHL--
-APLHLV-
-APLHLF-
-APLHLP-
-APLHLD-
CTPLHL--
CSPLHL--
CRPLHL--
CLPLHL--
CFPLHL--
-CPLHLK-
CNPLHL--
CMPLHL--
CCPLHL--
CPPLHL--
-CPLHLS-
CKPLHL--
CGPLHL--
CAPLHL--
-CPLHLM-
-CPLHLI-
-CPLHLF-
CIPLHL--
CEPLHL--
CDPLHL--
DRPLHL--
DVPLHL--
DMPLHL--
DWPLHL--
-DPLHLY-
DSPLHL--
-DPLHLR-
DNPLHL--
DQPLHL--
DLPLHL--
DYPLHL--
DKPLHL--
DGPLHL--

DTPLHL--
-DPLHLK-
-DPLHLS-
DIPLHL--
-DPLHLI-
-DPLHLE-
DDPLHL--
DAPLHL--
-DPLHLL-
-DPLHLG-
DEPLHL--
-DPLHLW-
-DPLHLV-
-DPLHLT-
-DPLHLQ-
DHPLHL--
ESPLHL--
ELPLHL--
EQPLHL--
EKPLHL--
-EPLHLW-
-EPLHLV-
EEPLHL--
EYPLHL--
-EPLHLR-
ENPLHL--
EGPLHL--
-EPLHLY-
ERPLHL--
-EPLHLA-
EFPLHL--
EWPLHL--
EVPLHL--
ETPLHL--
-EPLHLS-
-EPLHLQ-
-EPLHLM-
EHPLHL--
EAPLHL--
-EPLHLG-
-EPLHLF-
EIPLHL--
-EPLHLT-
-EPLHLI-
-EPLHLH-
-EPLHLE-
-EPLHLK-
EMPLHL--
FGPLHL--
-FPLHLR-

FLPLHL--
-FPLHLS-
FKPLHL--
FNPLHL--
FYPLHL--
FTPLHL--
FRPLHL--
-FPLHLY-
FDPLHL--
FQPLHL--
-FPLHLM-
-FPLHLL-
FIPLHL--
-FPLHLG-
FHPLHL--
FAPLHL--
-FPLHLI-
FSPLHL--
-FPLHLH-
FEPLHL--
-FPLHLV-
-FPLHLF-
FMPLHL--
-FPLHLW-
FPPLHL--
-FPLHLN-
FWPLHL--
-FPLHLQ-
GRPLHL--
-GPLHLL-
GSPLHL--
-GPLHLM-
GMPLHL--
GWPLHL--
-GPLHLR-
GNPLHL--
GLPLHL--
-GPLHLY-
-GPLHLH-
GKPLHL--
GAPLHL--
-GPLHLN-
-GPLHLV-
GTPLHL--
GDPLHL--
-GPLHLT-
-GPLHLG-
GFPLHL--
-GPLHLW-
-GPLHLQ-

-GPLHLE-
GHPLHL--
GPPLHL--
-GPLHLA-
GYPLHL--
GQPLHL--
-GPLHLF-
GCPLHL--
-HPLHLR-
-HPLHLY-
HVPLHL--
-HPLHLH-
HMPLHL--
HKPLHL--
HWPLHL--
HGPLHL--
HSPLHL--
-HPLHLS-
HLPLHL--
-HPLHLV-
-HPLHLG-
-HPLHLW-
-HPLHLT-
-HPLHLA-
HAPLHL--
HNPLHL--
HFPLHL--
HCPLHL--
-HPLHLL-
HIPLHL--
HHPLHL--
HPPLHL--
HTPLHL--
-HPLHLM-
-HPLHLI-
HEPLHL--
-HPLHLQ-
-HPLHLF-
-HPLHLN-
-IPLHLM-
IRPLHL--
-IPLHLS-
IMPLHL--
IKPLHL--
-IPLHLV-
IGPLHL--
IVPLHL--
-IPLHLR-
-IPLHLL-
IHPLHL--

-IPLHLY-
IIPLHL--
ILPLHL--
IDPLHL--
ITPLHL--
IYPLHL--
-IPLHLK-
INPLHL--
ISPLHL--
IQPLHL--
-IPLHLW-
IEPLHL--
-IPLHLH-
IFPLHL--
IWPLHL--
-IPLHLG-
-IPLHLA-
IAPLHL--
-IPLHLQ-
-IPLHLE-
ICPLHL--
-IPLHLF-
-IPLHLD-
KRPLHL--
-KPLHLL-
-KPLHLS-
KLPLHL--
-KPLHLV-
-KPLHLR-
-KPLHLY-
-KPLHLT-
KGPLHL--
-KPLHLM-
-KPLHLH-
KAPLHL--
-KPLHLN-
-KPLHLA-
KIPLHL--
KEPLHL--
KPPLHL--
-KPLHLK-
-KPLHLG-
-KPLHLF-
-KPLHLI-
KNPLHL--
KWPLHL--
-KPLHLQ-
KQPLHL--
KMPLHL--
KFPLHL--

-KPLHLE-
KHPLHL--
KDPLHL--
LYPLHL--
LSPLHL--
-LPLHLR-
LQPLHL--
LLPLHL--
-LPLHLT-
LEPLHL--
LPPLHL--
LIPLHL--
LVPLHL--
-LPLHLV-
LGPLHL--
-LPLHLL-
LNPLHL--
-LPLHLS-
LFPLHL--
LDPLHL--
-LPLHLI-
-LPLHLH-
-LPLHLQ-
-LPLHLK-
-LPLHLM-
-LPLHLA-
LCPLHL--
-LPLHLW-
MAPLHL--
MHPLHL--
-MPLHLQ-
MGPLHL--
MTPLHL--
-MPLHLY-
-MPLHLL-
MIPLHL--
MYPLHL--
-MPLHLT-
-MPLHLW-
MDPLHL--
MWPLHL--
-MPLHLH-
MMPLHL--
-MPLHLS-
-MPLHLA-
-MPLHLF-
MSPLHL--
-MPLHLN-
MQPLHL--
MPPLHL--

```
-MPLHLI-
-MPLHLE-
MEPLHL--
MCPLHL--
-MPLHLK-
-MPLHLG-
MNPLHL--
-MPLHLM-
-MPLHLC-
NRPLHL--
NNPLHL--
NVPLHL--
NSPLHL--
-NPLHLT-
-NPLHLS-
-NPLHLR-
NQPLHL--
-NPLHLF-
NHPLHL--
-NPLHLK-
NYPLHL--
NTPLHL--
NMPLHL--
NGPLHL--
-NPLHLI-
NPPLHL--
NWPLHL--
-NPLHLW-
NKPLHL--
NAPLHL--
-NPLHLE-
NIPLHL--
-NPLHLQ-
NDPLHL--
-NPLHLV-
-NPLHLM-
-NPLHLH-
-NPLHLN-
NFPLHL--
NEPLHL--
NCPLHL--
-NPLHLA-
--PLHLRS
--PLHLST
--PLHLWS
--PLHLVR
--PLHLSI
--PLHLRN
--PLHLMS
--PLHLFS
```

--PLHLLS
--PLHLYR
--PLHLLA
PRPLHL--
--PLHLTR
--PLHLVG
--PLHLTG
--PLHLLG
--PLHLKT
--PLHLSV
--PLHLRT
--PLHLSG
--PLHLRD
--PLHLYK
--PLHLVK
--PLHLIS
--PLHLGL
--PLHLAR
--PLHLRH
--PLHLMR
--PLHLKA
--PLHLGS
--PLHLGG
--PLHLWG
--PLHLVD
--PLHLKR
--PLHLTT
--PLHLKY
--PLHLIR
--PLHLHS
--PLHLEV
--PLHLTS
--PLHLTN
--PLHLKV
--PLHLVM
--PLHLRM
--PLHLLM
PKPLHL--
PTPLHL--
--PLHLVT
--PLHLTL
--PLHLSA
--PLHLQL
--PLHLLH
--PLHLVH
--PLHLRE
--PLHLMN
--PLHLKK
--PLHLFH
--PLHLYA

--PLHLTM
--PLHLTF
--PLHLMM
--PLHLLN
--PLHLIH
--PLHLYH
--PLHLYG
--PLHLWA
--PLHLSQ
--PLHLSM
--PLHLQM
--PLHLPY
--PLHLNY
--PLHLKS
--PLHLIT
--PLHLFK
--PLHLAI
--PLHLWN
--PLHLTH
--PLHLKI
--PLHLAW
--PLHLAN
--PLHLYI
--PLHLVQ
--PLHLTY
--PLHLSN
--PLHLSH
--PLHLNS
--PLHLML
--PLHLKW
--PLHLHL
--PLHLAT
--PLHLAE
PMPLHL--
--PLHLYD
--PLHLVI
--PLHLSK
--PLHLSD
--PLHLRG
--PLHLQV
--PLHLQR
--PLHLKQ
--PLHLIY
--PLHLHT
--PLHLHG
--PLHLGM
--PLHLAS
--PLHLAA
PFPLHL--
--PLHLVY

--PLHLTV
--PLHLSE
--PLHLQT
--PLHLQA
--PLHLMY
--PLHLMD
--PLHLLI
--PLHLKN
--PLHLIW
--PLHLIV
--PLHLHV
--PLHLGT
--PLHLES
PIPLHL--
PYPLHL--
PNPLHL--
--PLHLYY
--PLHLYV
--PLHLYQ
--PLHLYN
--PLHLYF
--PLHLWV
--PLHLVV
--PLHLPL
--PLHLMA
--PLHLKD
--PLHLIQ
--PLHLHF
--PLHLFN
--PLHLEW
--PLHLER
--PLHLAG
--PLHLWH
--PLHLTI
--PLHLQF
--PLHLNW
--PLHLNV
--PLHLMT
--PLHLME
--PLHLIN
--PLHLHW
--PLHLHR
--PLHLHA
--PLHLGA
--PLHLFR
--PLHLFL
--PLHLET
--PLHLAD
PCPLHL--
--PLHLWT

--PLHLNF
--PLHLMV
--PLHLMK
--PLHLMI
--PLHLLE
--PLHLKM
--PLHLKE
--PLHLIM
--PLHLIK
--PLHLID
--PLHLEN
--PLHLEM
--PLHLEL
--PLHLEG
--PLHLDS
--PLHLAV
--PLHLAQ
--PLHLAH
--PLHLAF
PWPLHL--
--PLHLYT
--PLHLWI
--PLHLWD
--PLHLTD
--PLHLQN
--PLHLQK
--PLHLQH
--PLHLQE
--PLHLNN
--PLHLNM
--PLHLKG
--PLHLHY
--PLHLHQ
--PLHLGY
--PLHLGW
--PLHLGV
--PLHLGI
--PLHLFQ
--PLHLFD
--PLHLEY
--PLHLEK
--PLHLEA
PEPLHL--
PDPLHL--
PQPLHL--
--PLHLYE
--PLHLVW
--PLHLTE
--PLHLQI
--PLHLQG

--PLHLQD
--PLHLNG
--PLHLII
--PLHLIF
--PLHLIA
--PLHLHP
--PLHLHN
--PLHLEH
--PLHLED
--PLHLAK
-PPLHLP-
-PPLHLF-
--PLHLWW
--PLHLVF
--PLHLQW
--PLHLPP
--PLHLPM
--PLHLNQ
--PLHLNH
--PLHLNE
--PLHLMW
--PLHLMQ
--PLHLMG
--PLHLKH
--PLHLKF
--PLHLHM
--PLHLHI
--PLHLGN
--PLHLFW
--PLHLFE
--PLHLEQ
--PLHLEE
--PLHLDK
--PLHLDF
--PLHLDA
--PLHLCS
--PLHLCA
-QPLHLS-
-QPLHLT-
QRPLHL--
-QPLHLR-
QAPLHL--
-QPLHLL-
-QPLHLQ-
QMPLHL--
-QPLHLK-
-QPLHLV-
QPPLHL--
QWPLHL--
QVPLHL--

QTPLHL--
QSPLHL--
QLPLHL--
QNPLHL--
QDPLHL--
-QPLHLG-
-QPLHLF-
QKPLHL--
QHPLHL--
QFPLHL--
QEPLHL--
QQPLHL--
-QPLHLM-
QYPLHL--
-QPLHLW-
QIPLHL--
-QPLHLN-
-QPLHLE-
QCPLHL--
RIPLHL--
-RPLHLT-
RPPLHL--
-RPLHLS-
RAPLHL--
-RPLHLL-
RMPLHL--
RHPLHL--
-RPLHLV-
-RPLHLQ-
-RPLHLE-
-RPLHLK-
RNPLHL--
-RPLHLA-
RQPLHL--
-RPLHLM-
RYPLHL--
RWPLHL--
-RPLHLH-
RDPLHL--
RCPLHL--
SLPLHL--
SSPLHL--
-SPLHLR-
-SPLHLT-
SVPLHL--
SYPLHL--
-SPLHLK-
SAPLHL--
SGPLHL--
-SPLHLA-

−SPLHLH−
−SPLHLV−
SNPLHL−−
SPPLHL−−
SWPLHL−−
−SPLHLW−
SQPLHL−−
−SPLHLN−
SHPLHL−−
SEPLHL−−
−SPLHLF−
SFPLHL−−
−SPLHLM−
−SPLHLE−
−SPLHLG−
SDPLHL−−
SCPLHL−−
−TPLHLS−
−TPLHLR−
TSPLHL−−
TPPLHL−−
TGPLHL−−
−TPLHLH−
TIPLHL−−
TMPLHL−−
−TPLHLK−
TLPLHL−−
−TPLHLA−
TTPLHL−−
TFPLHL−−
TVPLHL−−
TQPLHL−−
−TPLHLM−
−TPLHLT−
−TPLHLL−
−TPLHLG−
−TPLHLQ−
−TPLHLN−
TKPLHL−−
TYPLHL−−
TWPLHL−−
−TPLHLY−
TAPLHL−−
−TPLHLI−
TDPLHL−−
TNPLHL−−
−TPLHLW−
TEPLHL−−
TCPLHL−−
−TPLHLP−

VSPLHL--
VRPLHL--
VVPLHL--
-VPLHLS-
-VPLHLL-
-VPLHLI-
VPPLHL--
VTPLHL--
VIPLHL--
VAPLHL--
-VPLHLK-
-VPLHLY-
-VPLHLV-
-VPLHLM-
-VPLHLH-
-VPLHLF-
-VPLHLA-
VHPLHL--
VQPLHL--
VFPLHL--
VDPLHL--
VWPLHL--
-VPLHLQ-
VYPLHL--
-VPLHLW-
VGPLHL--
VEPLHL--
-VPLHLE-
WVPLHL--
WSPLHL--
WEPLHL--
WRPLHL--
WLPLHL--
WQPLHL--
-WPLHLT-
-WPLHLS-
-WPLHLR-
-WPLHLA-
WGPLHL--
WDPLHL--
WNPLHL--
WYPLHL--
WKPLHL--
WPPLHL--
-WPLHLM-
-WPLHLG-
-WPLHLK-
-WPLHLI-
WIPLHL--
WHPLHL--

| | | | |
|---|---|---|---|
| | WWPLHL-- | | |
| | -WPLHLW- | | |
| | -WPLHLV- | | |
| | -WPLHLQ- | | |
| | WCPLHL-- | | |
| | -WPLHLE- | | |
| | WFPLHL-- | | |
| | -YPLHLT- | | |
| | -YPLHLR- | | |
| | YTPLHL-- | | |
| | -YPLHLN- | | |
| | -YPLHLI- | | |
| | YGPLHL-- | | |
| | -YPLHLK- | | |
| | YYPLHL-- | | |
| | -YPLHLV- | | |
| | -YPLHLH- | | |
| | -YPLHLY- | | |
| | YPPLHL-- | | |
| | -YPLHLS- | | |
| | -YPLHLA- | | |
| | YAPLHL-- | | |
| | -YPLHLL- | | |
| | YIPLHL-- | | |
| | YDPLHL-- | | |
| | YNPLHL-- | | |
| | YQPLHL-- | | |
| | -YPLHLM- | | |
| | -YPLHLE- | | |
| | YHPLHL-- | | |
| | YEPLHL-- | | |
| | -YPLHLG- | | |
| | YMPLHL-- | | |
| | YKPLHL-- | | |
| | YCPLHL-- | | |
| | -YPLHLP- | | |
| PRWL | 438 | 622 | 34.18 |
| | -KPRWLE- | | |
| | --PRWLNR | | |
| | --PRWLEM | | |
| | -APRWLT- | | |
| | -APRWLH- | | |
| | AGPRWL-- | | |
| | AAPRWL-- | | |
| | ANPRWL-- | | |
| | -APRWLS- | | |
| | AMPRWL-- | | |
| | ARPRWL-- | | |
| | AKPRWL-- | | |
| | AEPRWL-- | | |

```
ADPRWL--
ASPRWL--
-APRWLW-
-APRWLA-
APPRWL--
AHPRWL--
ATPRWL--
-CPRWLA-
CMPRWL--
CAPRWL--
CLPRWL--
CTPRWL--
CPPRWL--
CIPRWL--
-CPRWLV-
-CPRWLS-
-CPRWLR-
-CPRWLK-
CGPRWL--
CEPRWL--
-DPRWLS-
DSPRWL--
DYPRWL--
DAPRWL--
DIPRWL--
DRPRWL--
-DPRWLY-
DWPRWL--
DTPRWL--
DGPRWL--
-DPRWLR-
-DPRWLN-
-DPRWLI-
DMPRWL--
-DPRWLV-
DLPRWL--
DKPRWL--
DVPRWL--
-DPRWLA-
DDPRWL--
DHPRWL--
DQPRWL--
-DPRWLM-
-DPRWLG-
DEPRWL--
-DPRWLH-
-DPRWLT-
-DPRWLQ-
ESPRWL--
-EPRWLS-
```

-EPRWLI-
ERPRWL--
EYPRWL--
-EPRWLK-
ETPRWL--
EKPRWL--
EFPRWL--
-EPRWLV-
-EPRWLN-
-EPRWLL-
EMPRWL--
ELPRWL--
-EPRWLW-
-EPRWLT-
-EPRWLG-
-EPRWLA-
-EPRWLM-
-EPRWLE-
EIPRWL--
EHPRWL--
-EPRWLQ-
EEPRWL--
EDPRWL--
-EPRWLF-
EWPRWL--
EVPRWL--
ENPRWL--
EQPRWL--
-EPRWLY-
-EPRWLH-
FKPRWL--
FEPRWL--
FDPRWL--
FSPRWL--
-FPRWLV-
FPPRWL--
FNPRWL--
FLPRWL--
FIPRWL--
FMPRWL--
-FPRWLI-
-FPRWLF-
FTPRWL--
-FPRWLY-
FYPRWL--
FQPRWL--
-FPRWLN-
-FPRWLH-
-FPRWLA-
-FPRWLE-

```
-FPRWLQ-
-FPRWLD-
GSPRWL--
-GPRWLR-
GVPRWL--
-GPRWLV-
-GPRWLG-
GKPRWL--
GRPRWL--
GNPRWL--
GTPRWL--
-GPRWLM-
-GPRWLA-
GYPRWL--
GIPRWL--
-GPRWLN-
GGPRWL--
GEPRWL--
-GPRWLH-
-GPRWLD-
GDPRWL--
GHPRWL--
GWPRWL--
GQPRWL--
-GPRWLI-
-GPRWLE-
-GPRWLY-
-HPRWLS-
-HPRWLM-
-HPRWLL-
HAPRWL--
HRPRWL--
HGPRWL--
HSPRWL--
-HPRWLV-
-HPRWLH-
HVPRWL--
-HPRWLK-
HPPRWL--
-HPRWLA-
HTPRWL--
-HPRWLI-
HNPRWL--
-HPRWLQ-
-HPRWLT-
-HPRWLN-
-HPRWLF-
HDPRWL--
-HPRWLE-
-IPRWLR-
```

ITPRWL--
-IPRWLG-
IRPRWL--
-IPRWLN-
IKPRWL--
IMPRWL--
IEPRWL--
-IPRWLY-
-IPRWLH-
IPPRWL--
-IPRWLI-
-IPRWLK-
IYPRWL--
-IPRWLV-
-IPRWLL-
IVPRWL--
IDPRWL--
-IPRWLM-
-IPRWLF-
-IPRWLD-
ICPRWL--
-KPRWLS-
KLPRWL--
-KPRWLQ-
-KPRWLK-
KGPRWL--
KEPRWL--
-KPRWLV-
-KPRWLG-
-KPRWLT-
KIPRWL--
KNPRWL--
KRPRWL--
-KPRWLI-
-KPRWLH-
-KPRWLM-
KHPRWL--
-KPRWLA-
KDPRWL--
KQPRWL--
KYPRWL--
KWPRWL--
-KPRWLN-
-KPRWLD-
LVPRWL--
-LPRWLV-
-LPRWLS-
-LPRWLG-
LAPRWL--
LKPRWL--

LQPRWL--
-LPRWLQ-
LHPRWL--
LFPRWL--
-LPRWLE-
LCPRWL--
-LPRWLW-
-MPRWLK-
MVPRWL--
MHPRWL--
MDPRWL--
-MPRWLE-
-MPRWLN-
MTPRWL--
MWPRWL--
MYPRWL--
-MPRWLL-
MFPRWL--
MPPRWL--
MNPRWL--
MEPRWL--
-MPRWLY-
-MPRWLH-
-MPRWLQ-
-MPRWLA-
MMPRWL--
MAPRWL--
MQPRWL--
-MPRWLM-
-MPRWLD-
MCPRWL--
NVPRWL--
NLPRWL--
NSPRWL--
NRPRWL--
NYPRWL--
-NPRWLV-
NHPRWL--
NAPRWL--
-NPRWLS-
NNPRWL--
-NPRWLK-
-NPRWLQ-
-NPRWLA-
NGPRWL--
NFPRWL--
NDPRWL--
-NPRWLG-
NPPRWL--
NMPRWL--

NQPRWL--
-NPRWLN-
NTPRWL--
-NPRWLY-
-NPRWLH-
-NPRWLM-
--PRWLSD
--PRWLLS
--PRWLTI
--PRWLLT
--PRWLRT
PSPRWL--
--PRWLVT
--PRWLLN
--PRWLGL
--PRWLVK
--PRWLSV
--PRWLSN
--PRWLKH
--PRWLSA
--PRWLSW
--PRWLLK
--PRWLGA
--PRWLSQ
--PRWLHA
--PRWLTV
--PRWLMS
--PRWLLA
PKPRWL--
PWPRWL--
--PRWLNT
--PRWLHS
PRPRWL--
--PRWLYS
--PRWLSM
--PRWLIS
--PRWLAN
--PRWLHG
--PRWLAH
--PRWLTW
--PRWLRE
--PRWLRD
--PRWLKG
--PRWLIH
--PRWLVW
--PRWLRI
--PRWLLD
PEPRWL--
--PRWLYM
--PRWLVN

--PRWLQI
--PRWLKY
--PRWLHV
--PRWLHK
--PRWLAQ
PDPRWL--
--PRWLYV
--PRWLWG
--PRWLTD
--PRWLIG
PVPRWL--
--PRWLQG
--PRWLNY
--PRWLKT
--PRWLKQ
--PRWLGW
--PRWLGG
--PRWLAA
PTPRWL--
--PRWLYN
--PRWLYG
--PRWLQN
--PRWLQK
--PRWLLV
PYPRWL--
--PRWLYH
--PRWLTQ
--PRWLNW
--PRWLHT
--PRWLGR
--PRWLFT
PIPRWL--
PGPRWL--
--PRWLYT
--PRWLTE
--PRWLTA
--PRWLSH
--PRWLRM
--PRWLQS
--PRWLNV
--PRWLNA
--PRWLMK
--PRWLKE
--PRWLEG
--PRWLEA
--PRWLAM
--PRWLAD
--PRWLWV
--PRWLWT
--PRWLVM

--PRWLMH
--PRWLLQ
--PRWLIW
--PRWLGM
--PRWLGH
PHPRWL--
--PRWLVD
--PRWLTM
--PRWLTH
--PRWLNQ
--PRWLLI
--PRWLKD
--PRWLKA
--PRWLGK
--PRWLFE
--PRWLES
--PRWLSF
--PRWLRY
--PRWLQY
--PRWLQD
--PRWLNK
--PRWLNH
--PRWLKF
--PRWLIA
--PRWLFK
--PRWLFD
--PRWLEV
--PRWLET
PMPRWL--
PAPRWL--
--PRWLYK
--PRWLWN
--PRWLTY
--PRWLQA
--PRWLMW
--PRWLMM
--PRWLMD
--PRWLIV
--PRWLIQ
--PRWLII
--PRWLHM
--PRWLGN
--PRWLFQ
--PRWLFN
--PRWLEN
--PRWLYI
--PRWLWA
--PRWLVY
--PRWLTF
--PRWLQQ

```
--PRWLPL
--PRWLNM
--PRWLLW
--PRWLIN
--PRWLIE
--PRWLHD
--PRWLGD
--PRWLEH
--PRWLYD
--PRWLQV
--PRWLQE
--PRWLPM
--PRWLME
--PRWLKM
--PRWLHN
--PRWLHF
--PRWLAY
--PRWLWI
--PRWLWH
--PRWLQW
--PRWLQT
--PRWLQH
--PRWLPY
--PRWLNN
--PRWLMY
--PRWLFY
--PRWLEW
--PRWLER
--PRWLEQ
--PRWLEK
--PRWLEI
--PRWLDG
--PRWLYE
--PRWLWW
--PRWLWF
--PRWLPP
--PRWLNE
--PRWLND
--PRWLKW
--PRWLHQ
--PRWLHH
--PRWLHE
--PRWLEE
--PRWLCG
--PRWLCD
-PPRWLS-
PFPRWL--
-QPRWLL-
-QPRWLV-
-QPRWLA-
```

QLPRWL--
QGPRWL--
QSPRWL--
QVPRWL--
QEPRWL--
-QPRWLQ-
-QPRWLS-
-QPRWLG-
QQPRWL--
-QPRWLF-
QIPRWL--
QTPRWL--
QYPRWL--
-QPRWLT-
-QPRWLM-
QNPRWL--
-QPRWLI-
QHPRWL--
-QPRWLN-
QDPRWL--
-QPRWLH-
-QPRWLE-
QKPRWL--
QFPRWL--
QCPRWL--
-RPRWLT-
-RPRWLL-
-RPRWLS-
RHPRWL--
-RPRWLG-
REPRWL--
RPPRWL--
-RPRWLI-
-RPRWLN-
RDPRWL--
RKPRWL--
-RPRWLK-
-RPRWLF-
-RPRWLQ-
RQPRWL--
-RPRWLY-
-RPRWLE-
-RPRWLW-
SRPRWL--
SFPRWL--
-SPRWLV-
-SPRWLH-
-SPRWLA-
SGPRWL--
SNPRWL--

SEPRWL--
SAPRWL--
-SPRWLN-
SSPRWL--
SVPRWL--
STPRWL--
-SPRWLI-
-SPRWLQ-
SPPRWL--
SQPRWL--
-SPRWLK-
-SPRWLM-
SHPRWL--
-SPRWLD-
-SPRWLF-
-SPRWLE-
SCPRWL--
-TPRWLL-
TVPRWL--
-TPRWLF-
-TPRWLQ-
TKPRWL--
THPRWL--
TAPRWL--
TPPRWL--
TLPRWL--
-TPRWLS-
-TPRWLI-
TRPRWL--
-TPRWLA-
TGPRWL--
-TPRWLW-
-TPRWLT-
TTPRWL--
TQPRWL--
-TPRWLH-
-TPRWLE-
TYPRWL--
TWPRWL--
-TPRWLY-
-TPRWLG-
-TPRWLD-
TCPRWL--
-VPRWLN-
-VPRWLL-
VYPRWL--
VEPRWL--
VTPRWL--
VKPRWL--
-VPRWLE-

VIPRWL--
-VPRWLM-
-VPRWLD-
-VPRWLH-
VAPRWL--
-VPRWLW-
VVPRWL--
VQPRWL--
-VPRWLF-
-VPRWLV-
VCPRWL--
WNPRWL--
WRPRWL--
WTPRWL--
-WPRWLS-
WLPRWL--
-WPRWLA-
WGPRWL--
WKPRWL--
WFPRWL--
WWPRWL--
WVPRWL--
-WPRWLQ-
WQPRWL--
-WPRWLN-
-WPRWLG-
WIPRWL--
WEPRWL--
WYPRWL--
-WPRWLI-
WPPRWL--
-WPRWLH-
WHPRWL--
-WPRWLF-
-YPRWLT-
-YPRWLR-
YAPRWL--
YQPRWL--
-YPRWLS-
YYPRWL--
-YPRWLG-
YTPRWL--
-YPRWLV-
-YPRWLA-
YNPRWL--
YEPRWL--
YPPRWL--
-YPRWLQ-
YKPRWL--
YWPRWL--

| | | | |
|---|---|---|---|
| | -YPRWLE- | | |
| | YIPRWL-- | | |
| | -YPRWLK- | | |
| | -YPRWLH- | | |
| | -YPRWLF- | | |
| | -YPRWLM- | | |
| | YDPRWL-- | | |
| | -YPRWLP- | | |
| | -YPRWLD- | | |
| PRAW | 142 | 743 | 34.18 |
| | --PRAWFV | | |
| | ALPRAW-- | | |
| | -APRAWR- | | |
| | AKPRAW-- | | |
| | -APRAWL- | | |
| | AMPRAW-- | | |
| | ANPRAW-- | | |
| | -APRAWA- | | |
| | -APRAWH- | | |
| | AFPRAW-- | | |
| | -APRAWK- | | |
| | AGPRAW-- | | |
| | AWPRAW-- | | |
| | AEPRAW-- | | |
| | AVPRAW-- | | |
| | ATPRAW-- | | |
| | -APRAWV- | | |
| | -APRAWM- | | |
| | -APRAWG- | | |
| | ARPRAW-- | | |
| | -APRAWT- | | |
| | -APRAWN- | | |
| | -APRAWE- | | |
| | -APRAWY- | | |
| | -APRAWW- | | |
| | -APRAWF- | | |
| | APPRAW-- | | |
| | AIPRAW-- | | |
| | AHPRAW-- | | |
| | AYPRAW-- | | |
| | AQPRAW-- | | |
| | -APRAWC- | | |
| | AAPRAW-- | | |
| | -APRAWI- | | |
| | ADPRAW-- | | |
| | CRPRAW-- | | |
| | -CPRAWS- | | |
| | CSPRAW-- | | |
| | -CPRAWL- | | |
| | -CPRAWK- | | |

-CPRAWF-
CLPRAW--
CHPRAW--
CGPRAW--
CCPRAW--
DSPRAW--
DIPRAW--
-DPRAWS-
DVPRAW--
DMPRAW--
-DPRAWK-
-DPRAWI-
DNPRAW--
DTPRAW--
-DPRAWV-
-DPRAWT-
-DPRAWF-
-DPRAWA-
DYPRAW--
DQPRAW--
-DPRAWR-
-DPRAWQ-
-DPRAWH-
DLPRAW--
DAPRAW--
DWPRAW--
-DPRAWY-
-DPRAWW-
-DPRAWN-
-DPRAWM-
DGPRAW--
DEPRAW--
DDPRAW--
EIPRAW--
ESPRAW--
-EPRAWR-
-EPRAWA-
-EPRAWH-
EMPRAW--
EAPRAW--
EQPRAW--
-EPRAWW-
-EPRAWL-
EFPRAW--
ETPRAW--
-EPRAWG-
-EPRAWF-
ELPRAW--
EHPRAW--
EVPRAW--

-EPRAWT-
-EPRAWS-
-EPRAWQ-
-EPRAWI-
EKPRAW--
EEPRAW--
EYPRAW--
ERPRAW--
-EPRAWY-
EGPRAW--
-FPRAWH-
-FPRAWN-
FGPRAW--
FYPRAW--
FRPRAW--
FTPRAW--
FSPRAW--
-FPRAWK-
FIPRAW--
FEPRAW--
FVPRAW--
FPPRAW--
-FPRAWI-
FNPRAW--
-FPRAWQ-
FLPRAW--
-FPRAWT-
FAPRAW--
-FPRAWV-
FKPRAW--
FFPRAW--
-FPRAWL-
FMPRAW--
FHPRAW--
-GPRAWV-
GVPRAW--
GLPRAW--
-GPRAWR-
GKPRAW--
-GPRAWG-
GSPRAW--
GMPRAW--
GRPRAW--
-GPRAWL-
-GPRAWI-
-GPRAWK-
GEPRAW--
-GPRAWS-
GFPRAW--
GAPRAW--

GYPRAW--
GQPRAW--
-GPRAWT-
-GPRAWQ-
-GPRAWM-
GGPRAW--
-GPRAWY-
GNPRAW--
-GPRAWA-
GHPRAW--
GPPRAW--
-GPRAWW-
-GPRAWH-
-GPRAWE-
HLPRAW--
-HPRAWR-
HRPRAW--
-HPRAWL-
-HPRAWW-
HYPRAW--
-HPRAWG-
-HPRAWF-
HIPRAW--
-HPRAWS-
-HPRAWN-
-HPRAWH-
HGPRAW--
HWPRAW--
HQPRAW--
-HPRAWK-
-HPRAWA-
HFPRAW--
-HPRAWQ-
HPPRAW--
HNPRAW--
HEPRAW--
HDPRAW--
-IPRAWS-
-IPRAWL-
-IPRAWR-
ILPRAW--
IGPRAW--
IVPRAW--
ITPRAW--
-IPRAWT-
-IPRAWM-
-IPRAWW-
IHPRAW--
-IPRAWI-
-IPRAWV-

-IPRAWA-
IRPRAW--
IMPRAW--
-IPRAWY-
INPRAW--
IYPRAW--
IQPRAW--
-IPRAWK-
IKPRAW--
IEPRAW--
IAPRAW--
-IPRAWG-
-IPRAWE-
IIPRAW--
IDPRAW--
-KPRAWW-
-KPRAWS-
-KPRAWQ-
KTPRAW--
-KPRAWV-
-KPRAWT-
-KPRAWL-
-KPRAWI-
-KPRAWH-
KDPRAW--
KAPRAW--
KYPRAW--
KGPRAW--
KFPRAW--
-KPRAWY-
-KPRAWN-
KMPRAW--
-KPRAWK-
-KPRAWE-
KEPRAW--
KPPRAW--
KHPRAW--
KQPRAW--
-KPRAWM-
-KPRAWF-
LNPRAW--
LRPRAW--
-LPRAWR-
LYPRAW--
-LPRAWH-
LTPRAW--
-LPRAWY-
LIPRAW--
LWPRAW--
LAPRAW--

-LPRAWW-
-LPRAWT-
-LPRAWK-
LEPRAW--
LDPRAW--
-LPRAWA-
LHPRAW--
-LPRAWL-
LPPRAW--
-LPRAWM-
-LPRAWQ-
-LPRAWN-
-LPRAWE-
MLPRAW--
MGPRAW--
MSPRAW--
MKPRAW--
MRPRAW--
-MPRAWN-
MNPRAW--
MWPRAW--
-MPRAWL-
-MPRAWK-
MHPRAW--
-MPRAWT-
-MPRAWA-
MQPRAW--
-MPRAWY-
MDPRAW--
MAPRAW--
-MPRAWH-
-MPRAWF-
MEPRAW--
MVPRAW--
-MPRAWE-
MPPRAW--
MFPRAW--
MTPRAW--
-MPRAWW-
-MPRAWG-
-NPRAWR-
-NPRAWA-
-NPRAWV-
-NPRAWI-
NRPRAW--
-NPRAWT-
NVPRAW--
NSPRAW--
-NPRAWY-
NNPRAW--

-NPRAWM-
-NPRAWF-
NIPRAW--
NTPRAW--
-NPRAWS-
-NPRAWH-
NKPRAW--
NAPRAW--
-NPRAWN-
NMPRAW--
NLPRAW--
NHPRAW--
NDPRAW--
NYPRAW--
-NPRAWQ-
NPPRAW--
NWPRAW--
NGPRAW--
NEPRAW--
--PRAWGL
--PRAWST
--PRAWLY
--PRAWAL
--PRAWTT
--PRAWTG
--PRAWAV
--PRAWRT
--PRAWVT
--PRAWLV
--PRAWFI
--PRAWVV
--PRAWLT
--PRAWLS
--PRAWTY
--PRAWKT
--PRAWFM
--PRAWMV
--PRAWLA
--PRAWSH
--PRAWHA
--PRAWVF
--PRAWLE
--PRAWYF
--PRAWMG
--PRAWMF
--PRAWLG
--PRAWYG
--PRAWWR
--PRAWVE
--PRAWPL

--PRAWMQ
--PRAWAF
--PRAWWH
--PRAWTM
--PRAWSN
--PRAWIT
--PRAWGI
--PRAWYN
--PRAWWI
--PRAWVY
--PRAWQY
--PRAWQG
--PRAWMD
--PRAWAA
--PRAWYA
--PRAWVN
--PRAWVG
--PRAWTH
--PRAWMY
--PRAWKQ
--PRAWKA
--PRAWIN
--PRAWIM
--PRAWGS
--PRAWAW
--PRAWAS
--PRAWAG
--PRAWMN
--PRAWLH
--PRAWLD
--PRAWHS
--PRAWGG
--PRAWFG
--PRAWYK
--PRAWTE
--PRAWRF
--PRAWMS
--PRAWMR
--PRAWLK
--PRAWKS
--PRAWKN
--PRAWGT
--PRAWFQ
PVPRAW--
PTPRAW--
--PRAWYQ
--PRAWYH
--PRAWTS
--PRAWNS
--PRAWIY

--PRAWHT
--PRAWFF
--PRAWAY
--PRAWVQ
--PRAWTV
--PRAWSW
--PRAWSI
--PRAWQF
--PRAWPI
--PRAWNF
--PRAWMT
--PRAWMK
--PRAWLQ
--PRAWKE
--PRAWIG
--PRAWID
--PRAWHN
--PRAWYM
--PRAWWT
--PRAWVA
--PRAWTD
--PRAWRI
--PRAWMA
--PRAWKG
--PRAWIK
--PRAWHF
--PRAWYY
--PRAWYI
--PRAWWL
--PRAWTN
--PRAWTA
--PRAWQE
--PRAWHV
--PRAWGY
--PRAWFK
PKPRAW--
PGPRAW--
--PRAWWN
--PRAWWA
--PRAWVD
--PRAWSM
--PRAWSD
--PRAWRY
--PRAWRG
--PRAWQS
--PRAWQQ
--PRAWNK
--PRAWKI
--PRAWKH
--PRAWIQ

--PRAWHR
--PRAWHL
--PRAWHE
--PRAWEV
--PRAWEG
--PRAWEF
--PRAWAN
PFPRAW--
--PRAWWQ
--PRAWWK
--PRAWWD
--PRAWVM
--PRAWRP
--PRAWRH
--PRAWRD
--PRAWQI
--PRAWPY
--PRAWNH
--PRAWNG
--PRAWMH
--PRAWKW
--PRAWKM
--PRAWIS
--PRAWIF
--PRAWFA
--PRAWEM
PQPRAW--
PNPRAW--
PDPRAW--
--PRAWWS
--PRAWVW
--PRAWVP
--PRAWTQ
--PRAWTF
--PRAWSF
--PRAWSE
--PRAWSA
--PRAWRA
--PRAWNM
--PRAWMP
--PRAWKD
--PRAWHW
--PRAWHM
--PRAWHG
--PRAWET
--PRAWES
--PRAWER
--PRAWEN
--PRAWEL
--PRAWEK

--PRAWAT
PLPRAW--
--PRAWYT
--PRAWWY
--PRAWVK
--PRAWQV
--PRAWQR
--PRAWQA
--PRAWNT
--PRAWMW
--PRAWMM
--PRAWLM
--PRAWHQ
--PRAWHK
--PRAWHI
--PRAWGQ
--PRAWGN
--PRAWFW
--PRAWFE
--PRAWFD
--PRAWEI
--PRAWDL
--PRAWAE
--PRAWAD
PMPRAW--
PEPRAW--
PWPRAW--
--PRAWYV
--PRAWYP
--PRAWTC
--PRAWSQ
--PRAWQW
--PRAWQT
--PRAWQN
--PRAWQK
--PRAWQD
--PRAWPM
--PRAWNQ
--PRAWNN
--PRAWKP
--PRAWKF
--PRAWIP
--PRAWIH
--PRAWIA
--PRAWHY
--PRAWHH
--PRAWGV
--PRAWGM
--PRAWGH
--PRAWGF

--PRAWGA
--PRAWEY
--PRAWDA
--PRAWAQ
--PRAWAM
--PRAWAK
-PPRAWT-
PIPRAW--
QAPRAW--
-QPRAWS-
-QPRAWM-
QGPRAW--
QNPRAW--
QRPRAW--
QFPRAW--
QWPRAW--
-QPRAWN-
-QPRAWH-
-QPRAWL-
QTPRAW--
-QPRAWF-
-QPRAWT-
-QPRAWK-
-QPRAWA-
QEPRAW--
QYPRAW--
QQPRAW--
-QPRAWI-
QIPRAW--
-QPRAWY-
-QPRAWC-
QLPRAW--
QDPRAW--
RSPRAW--
-RPRAWS-
-RPRAWN-
-RPRAWA-
RVPRAW--
-RPRAWM-
-RPRAWT-
-RPRAWF-
RAPRAW--
-RPRAWY-
RWPRAW--
RIPRAW--
RTPRAW--
RPPRAW--
RFPRAW--
-RPRAWI-
-RPRAWG-

RNPRAW--
RMPRAW--
RGPRAW--
RQPRAW--
-RPRAWW-
-RPRAWH-
RDPRAW--
-RPRAWQ-
-RPRAWE-
RRPRAW--
RKPRAW--
-SPRAWV-
STPRAW--
SRPRAW--
-SPRAWG-
SMPRAW--
SDPRAW--
-SPRAWK-
SHPRAW--
-SPRAWA-
SQPRAW--
-SPRAWH-
SGPRAW--
SEPRAW--
-SPRAWQ-
-SPRAWT-
SYPRAW--
-SPRAWE-
SNPRAW--
-SPRAWF-
SVPRAW--
-SPRAWN-
SKPRAW--
-SPRAWW-
SPPRAW--
SIPRAW--
SCPRAW--
TRPRAW--
-TPRAWL-
TVPRAW--
TSPRAW--
TQPRAW--
TAPRAW--
-TPRAWT-
-TPRAWK-
TMPRAW--
TPPRAW--
TLPRAW--
-TPRAWH-
-TPRAWG-

TNPRAW--
TEPRAW--
-TPRAWM-
TKPRAW--
TYPRAW--
-TPRAWQ-
TIPRAW--
-TPRAWS-
-TPRAWF-
-TPRAWA-
TTPRAW--
-TPRAWV-
TWPRAW--
-TPRAWW-
-TPRAWD-
TGPRAW--
TDPRAW--
-VPRAWI-
VVPRAW--
VRPRAW--
-VPRAWH-
VLPRAW--
VPPRAW--
-VPRAWY-
VTPRAW--
VFPRAW--
-VPRAWV-
VNPRAW--
VDPRAW--
VAPRAW--
VSPRAW--
VIPRAW--
-VPRAWT-
-VPRAWN-
VWPRAW--
-VPRAWS-
-VPRAWA-
VHPRAW--
VYPRAW--
-VPRAWG-
VKPRAW--
-VPRAWW-
-VPRAWQ-
-VPRAWM-
-VPRAWE-
VEPRAW--
-VPRAWF-
VGPRAW--
WTPRAW--
WIPRAW--

WNPRAW--
WQPRAW--
-WPRAWT-
WGPRAW--
-WPRAWA-
WFPRAW--
-WPRAWN-
-WPRAWV-
-WPRAWH-
WLPRAW--
WDPRAW--
-WPRAWI-
-WPRAWF-
WPPRAW--
WKPRAW--
WAPRAW--
-WPRAWW-
WHPRAW--
WEPRAW--
WYPRAW--
WWPRAW--
-WPRAWQ-
-WPRAWM-
-WPRAWL-
-WPRAWE-
-YPRAWT-
-YPRAWV-
YRPRAW--
-YPRAWK-
-YPRAWH-
-YPRAWA-
YGPRAW--
YYPRAW--
-YPRAWF-
-YPRAWI-
-YPRAWY-
-YPRAWN-
-YPRAWE-
YLPRAW--
-YPRAWW-
-YPRAWM-
YNPRAW--
YCPRAW--
YAPRAW--
YWPRAW--
YVPRAW--
YQPRAW--
YPPRAW--
YKPRAW--
YIPRAW--

| | | | |
|---|---|---|---|
| | YDPRAW-- | | |
| | -YPRAWQ- | | |
| | YFPRAW-- | | |
| PMAL | 154 | 737 | 34.17 |
| | --PMALSN | | |
| | --PMALNI | | |
| | --PMALGN | | |
| | -APMALI- | | |
| | AEPMAL-- | | |
| | ATPMAL-- | | |
| | AVPMAL-- | | |
| | -APMALS- | | |
| | ALPMAL-- | | |
| | ASPMAL-- | | |
| | ADPMAL-- | | |
| | -APMALN- | | |
| | AWPMAL-- | | |
| | -APMALT- | | |
| | -APMALQ- | | |
| | AMPMAL-- | | |
| | APPMAL-- | | |
| | -APMALA- | | |
| | ANPMAL-- | | |
| | -APMALK- | | |
| | -APMALH- | | |
| | AKPMAL-- | | |
| | AHPMAL-- | | |
| | AYPMAL-- | | |
| | AQPMAL-- | | |
| | -APMALV- | | |
| | AIPMAL-- | | |
| | AFPMAL-- | | |
| | AAPMAL-- | | |
| | -APMALW- | | |
| | -APMALM- | | |
| | -APMALE- | | |
| | -APMALD- | | |
| | ACPMAL-- | | |
| | CSPMAL-- | | |
| | -CPMALN- | | |
| | CYPMAL-- | | |
| | CMPMAL-- | | |
| | CTPMAL-- | | |
| | -CPMALW- | | |
| | -CPMALR- | | |
| | -CPMALL- | | |
| | -CPMALI- | | |
| | CNPMAL-- | | |
| | CDPMAL-- | | |
| | CCPMAL-- | | |

CVPMAL--
-CPMALS-
-CPMALQ-
-CPMALA-
CKPMAL--
CGPMAL--
-DPMALR-
DWPMAL--
-DPMALS-
DRPMAL--
DKPMAL--
-DPMALV-
DFPMAL--
-DPMALQ-
-DPMALI-
-DPMALE-
DEPMAL--
DSPMAL--
-DPMALT-
DLPMAL--
DHPMAL--
DGPMAL--
DAPMAL--
DYPMAL--
DVPMAL--
DNPMAL--
DMPMAL--
DIPMAL--
-EPMALL-
-EPMALR-
EKPMAL--
ERPMAL--
ESPMAL--
EHPMAL--
EIPMAL--
EFPMAL--
EYPMAL--
EVPMAL--
EMPMAL--
-EPMALT-
-EPMALQ-
-EPMALG-
-EPMALA-
EGPMAL--
-EPMALM-
-EPMALH-
-EPMALE-
EDPMAL--
EWPMAL--
ETPMAL--

```
-EPMALV-
-EPMALK-
-EPMALI-
ELPMAL--
EEPMAL--
EAPMAL--
-EPMALY-
-EPMALS-
ECPMAL--
FRPMAL--
FGPMAL--
-FPMALS-
FMPMAL--
FIPMAL--
FSPMAL--
-FPMALN-
FLPMAL--
FQPMAL--
FDPMAL--
-FPMALW-
-FPMALV-
-FPMALQ-
FVPMAL--
-FPMALM-
-FPMALA-
FHPMAL--
FAPMAL--
-FPMALH-
FKPMAL--
FFPMAL--
FPPMAL--
-FPMALT-
-FPMALG-
FEPMAL--
-GPMALL-
GRPMAL--
-GPMALR-
GAPMAL--
GNPMAL--
GLPMAL--
GTPMAL--
-GPMALS-
GVPMAL--
GHPMAL--
-GPMALA-
-GPMALK-
GEPMAL--
-GPMALV-
-GPMALH-
GMPMAL--
```

GGPMAL--
GSPMAL--
GPPMAL--
-GPMALM-
-GPMALI-
GWPMAL--
-GPMALN-
-GPMALG-
GIPMAL--
GQPMAL--
GKPMAL--
-GPMALE-
GDPMAL--
HRPMAL--
-HPMALR-
HSPMAL--
-HPMALL-
-HPMALK-
HQPMAL--
HFPMAL--
HEPMAL--
HPPMAL--
-HPMALT-
HMPMAL--
-HPMALN-
-HPMALH-
HKPMAL--
HWPMAL--
-HPMALS-
-HPMALG-
-HPMALF-
HNPMAL--
HHPMAL--
HYPMAL--
-HPMALY-
-HPMALV-
HIPMAL--
HVPMAL--
-HPMALW-
-HPMALM-
-HPMALI-
HTPMAL--
-HPMALA-
HGPMAL--
ILPMAL--
IAPMAL--
-IPMALL-
ISPMAL--
-IPMALT-
ITPMAL--

-IPMALG-
IMPMAL--
-IPMALS-
-IPMALK-
IKPMAL--
IGPMAL--
IYPMAL--
-IPMALV-
-IPMALI-
-IPMALH-
-IPMALA-
IQPMAL--
-IPMALN-
IDPMAL--
IPPMAL--
IHPMAL--
IFPMAL--
IWPMAL--
-IPMALE-
KSPMAL--
KTPMAL--
KRPMAL--
-KPMALW-
KNPMAL--
KYPMAL--
-KPMALY-
-KPMALF-
-KPMALG-
-KPMALN-
KWPMAL--
KLPMAL--
KHPMAL--
KQPMAL--
KPPMAL--
-KPMALQ-
-KPMALA-
KGPMAL--
KEPMAL--
KAPMAL--
-KPMALK-
KMPMAL--
KDPMAL--
KVPMAL--
-KPMALV-
-KPMALI-
-KPMALE-
KCPMAL--
-LPMALT-
LGPMAL--
-LPMALR-

LLPMAL--
LIPMAL--
-LPMALL-
LKPMAL--
-LPMALV-
-LPMALS-
LTPMAL--
-LPMALN-
-LPMALH-
-LPMALK-
-LPMALQ-
LNPMAL--
LAPMAL--
LYPMAL--
LHPMAL--
LPPMAL--
LEPMAL--
LCPMAL--
-LPMALE-
-LPMALA-
-LPMALM-
LDPMAL--
-MPMALA-
MLPMAL--
-MPMALN-
MIPMAL--
MSPMAL--
MPPMAL--
MGPMAL--
-MPMALR-
MTPMAL--
MDPMAL--
MWPMAL--
-MPMALT-
MHPMAL--
-MPMALV-
-MPMALQ-
-MPMALL-
MAPMAL--
MYPMAL--
-MPMALS-
-MPMALK-
-MPMALG-
MMPMAL--
MKPMAL--
MEPMAL--
-NPMALR-
NLPMAL--
NVPMAL--
-NPMALT-

NEPMAL--
NWPMAL--
NMPMAL--
NFPMAL--
NRPMAL--
-NPMALQ-
-NPMALM-
NYPMAL--
NTPMAL--
-NPMALW-
-NPMALS-
-NPMALL-
-NPMALK-
-NPMALV-
NKPMAL--
NGPMAL--
NQPMAL--
NPPMAL--
-NPMALY-
-NPMALN-
-NPMALA-
NNPMAL--
-NPMALI-
NHPMAL--
NAPMAL--
NDPMAL--
--PMALVR
--PMALSI
--PMALKA
--PMALWN
--PMALTA
--PMALRT
--PMALLR
--PMALHW
--PMALSG
--PMALRR
--PMALRV
--PMALRH
--PMALSL
PVPMAL--
--PMALQS
--PMALTR
--PMALRI
--PMALLG
--PMALLE
--PMALIR
--PMALGS
--PMALGI
--PMALWS
--PMALPL

--PMALKK
--PMALAL
--PMALVN
--PMALRG
--PMALGL
--PMALYG
--PMALSY
--PMALSS
--PMALRE
--PMALMR
--PMALLA
--PMALHR
PAPMAL--
--PMALVD
--PMALSH
--PMALGM
PTPMAL--
--PMALWG
--PMALWA
--PMALVK
--PMALVG
--PMALTK
--PMALTI
--PMALRN
--PMALMS
--PMALAK
--PMALAI
PLPMAL--
--PMALYH
--PMALTG
--PMALSA
--PMALNT
--PMALLI
--PMALGF
PMPMAL--
--PMALRK
--PMALNF
--PMALMG
--PMALLD
--PMALIQ
--PMALID
--PMALES
--PMALAT
--PMALAS
PWPMAL--
--PMALWT
--PMALVT
--PMALTV
--PMALQV
--PMALQR

--PMALQN
--PMALNR
--PMALML
--PMALKM
--PMALIN
--PMALHV
--PMALHQ
--PMALGQ
--PMALFS
--PMALFH
--PMALAY
--PMALAV
PYPMAL--
--PMALYQ
--PMALVS
--PMALTM
--PMALQL
--PMALQK
--PMALNN
--PMALMW
--PMALMV
--PMALMI
--PMALMH
--PMALHL
--PMALHG
--PMALGV
--PMALFK
--PMALEW
--PMALAH
--PMALSM
--PMALSF
--PMALSE
--PMALRA
--PMALMQ
--PMALME
--PMALKR
--PMALIT
--PMALGY
--PMALGT
--PMALGG
--PMALEG
--PMALAW
PNPMAL--
--PMALWR
--PMALVQ
--PMALTQ
--PMALTF
--PMALRW
--PMALRD
--PMALQT

--PMALPW
--PMALNK
--PMALMD
--PMALMA
--PMALKV
--PMALIG
--PMALHS
--PMALDY
--PMALAR
--PMALYF
--PMALWM
--PMALWE
--PMALVW
--PMALSW
--PMALSK
--PMALQI
--PMALQD
--PMALNV
--PMALND
--PMALMN
--PMALMM
--PMALLQ
--PMALLH
--PMALKQ
--PMALKN
--PMALKE
--PMALHM
--PMALHK
--PMALHA
--PMALGH
--PMALEK
--PMALDS
--PMALAN
--PMALAG
PHPMAL--
PQPMAL--
--PMALVH
--PMALTT
--PMALTN
--PMALRY
--PMALRP
--PMALQY
--PMALQG
--PMALPI
--PMALPF
--PMALNW
--PMALNA
--PMALKY
--PMALKT
--PMALKH

--PMALKG
--PMALHY
--PMALHF
--PMALHE
--PMALET
--PMALER
--PMALEQ
--PMALEH
--PMALEA
--PMALCA
PKPMAL--
PCPMAL--
-PPMALF-
--PMALYD
--PMALWQ
--PMALWH
--PMALWF
--PMALVE
--PMALTE
--PMALSC
--PMALQW
--PMALQQ
--PMALQM
--PMALQF
--PMALPY
--PMALPV
--PMALPR
--PMALPQ
--PMALNH
--PMALNG
--PMALMY
--PMALMT
--PMALKF
--PMALKD
--PMALIC
--PMALHD
--PMALGA
--PMALFC
--PMALEN
--PMALEM
--PMALEF
--PMALED
--PMALDL
--PMALDG
--PMALCL
--PMALAD
--PMALAA
PIPMAL--
PFPMAL--
PEPMAL--

QRPMAL--
-QPMALS-
QIPMAL--
-QPMALL-
QLPMAL--
-QPMALR-
-QPMALI-
QWPMAL--
QSPMAL--
-QPMALA-
QHPMAL--
QYPMAL--
-QPMALT-
QAPMAL--
-QPMALN-
QNPMAL--
QTPMAL--
-QPMALV-
-QPMALQ-
-QPMALM-
-QPMALK-
-QPMALH-
-QPMALF-
QMPMAL--
QKPMAL--
QGPMAL--
QFPMAL--
QDPMAL--
RSPMAL--
-RPMALL-
-RPMALR-
RRPMAL--
RGPMAL--
-RPMALK-
RWPMAL--
-RPMALI-
RNPMAL--
RPPMAL--
RAPMAL--
RQPMAL--
-RPMALV-
-RPMALA-
-RPMALW-
-RPMALN-
RTPMAL--
-RPMALQ-
RIPMAL--
-RPMALG-
RDPMAL--
-RPMALH-

RMPMAL--
REPMAL--
-RPMALE-
RCPMAL--
SLPMAL--
SRPMAL--
-SPMALR-
-SPMALL-
SVPMAL--
SSPMAL--
-SPMALK-
-SPMALF-
STPMAL--
SWPMAL--
-SPMALQ-
-SPMALH-
SIPMAL--
SYPMAL--
-SPMALG-
-SPMALV-
SEPMAL--
SAPMAL--
SPPMAL--
-SPMALM-
SFPMAL--
-SPMALW-
-SPMALI-
SMPMAL--
SGPMAL--
-SPMALT-
-SPMALA-
SHPMAL--
SDPMAL--
SQPMAL--
-SPMALN-
SCPMAL--
-SPMALE-
TSPMAL--
-TPMALK-
-TPMALR-
-TPMALL-
TNPMAL--
TPPMAL--
TGPMAL--
-TPMALS-
-TPMALH-
TMPMAL--
TIPMAL--
TAPMAL--
-TPMALV-

-TPMALE-
TDPMAL--
-TPMALN-
TEPMAL--
TVPMAL--
TTPMAL--
TWPMAL--
-TPMALG-
-TPMALA-
TFPMAL--
TQPMAL--
-TPMALW-
-TPMALI-
THPMAL--
TYPMAL--
TKPMAL--
VAPMAL--
VTPMAL--
VSPMAL--
VEPMAL--
VYPMAL--
-VPMALS-
VWPMAL--
VPPMAL--
VGPMAL--
VKPMAL--
-VPMALL-
-VPMALQ-
-VPMALI-
-VPMALH-
VQPMAL--
-VPMALK-
-VPMALG-
VMPMAL--
VLPMAL--
VDPMAL--
-VPMALM-
-VPMALA-
VNPMAL--
-VPMALN-
VFPMAL--
-VPMALE-
VCPMAL--
-WPMALK-
WLPMAL--
WRPMAL--
WGPMAL--
WSPMAL--
WKPMAL--
WQPMAL--

```
            -WPMALR-
            WAPMAL--
            WHPMAL--
            -WPMALG-
            -WPMALS-
            -WPMALH-
            WNPMAL--
            -WPMALN-
            -WPMALE-
            WFPMAL--
            WEPMAL--
            -WPMALL-
            -WPMALA-
            WDPMAL--
            -YPMALS-
            -YPMALT-
            YRPMAL--
            -YPMALR-
            YQPMAL--
            YKPMAL--
            YGPMAL--
            -YPMALH-
            -YPMALA-
            YYPMAL--
            YWPMAL--
            YTPMAL--
            -YPMALQ-
            YFPMAL--
            -YPMALW-
            YNPMAL--
            YMPMAL--
            YIPMAL--
            YHPMAL--
            YEPMAL--
            YPPMAL--
            -YPMALK-
            -YPMALV-
            -YPMALI-
            -YPMALG-
            YDPMAL--
            YAPMAL--
PRGI         204         709      34.16
            DTPRGI--
            -HPRGIR-
            --PRGIVP
            --PRGIPL
            --PRGIFH
            --PRGIDV
            -SPRGIP-
            -APRGIL-
```

-APRGIW-
-APRGIT-
AKPRGI--
-APRGIY-
-APRGIG-
AVPRGI--
-APRGIR-
-APRGIF-
-APRGIA-
AAPRGI--
ADPRGI--
AEPRGI--
-APRGIM-
-APRGIK-
AGPRGI--
-APRGIV-
-APRGIH-
APPRGI--
AIPRGI--
AQPRGI--
-APRGIN-
AFPRGI--
-APRGIQ-
AHPRGI--
-APRGIE-
AMPRGI--
CGPRGI--
CTPRGI--
CKPRGI--
CIPRGI--
-CPRGIL-
-CPRGIA-
CHPRGI--
-CPRGIT-
-CPRGIS-
CRPRGI--
CQPRGI--
CSPRGI--
-CPRGIY-
CNPRGI--
-CPRGIQ-
CPPRGI--
CFPRGI--
CCPRGI--
CAPRGI--
-DPRGIL-
-DPRGIA-
DLPRGI--
-DPRGIS-
-DPRGIK-

```
DSPRGI--
-DPRGIR-
-DPRGII-
DKPRGI--
DRPRGI--
-DPRGIV-
-DPRGIM-
-DPRGIH-
-DPRGIG-
DMPRGI--
DGPRGI--
DDPRGI--
DAPRGI--
-DPRGIY-
-DPRGIT-
DCPRGI--
DYPRGI--
-DPRGIW-
-DPRGIF-
-DPRGIE-
DNPRGI--
DHPRGI--
DFPRGI--
-EPRGIR-
-EPRGIH-
EVPRGI--
-EPRGIG-
-EPRGIY-
-EPRGIS-
-EPRGIL-
ERPRGI--
EGPRGI--
EWPRGI--
ESPRGI--
ENPRGI--
EDPRGI--
ETPRGI--
-EPRGII-
ELPRGI--
EEPRGI--
-EPRGIQ-
-EPRGIM-
EIPRGI--
-EPRGIW-
-EPRGIT-
-EPRGIN-
EMPRGI--
EFPRGI--
EQPRGI--
-EPRGIV-
```

−EPRGIK−
−EPRGIE−
EHPRGI−−
−FPRGIS−
FGPRGI−−
−FPRGII−
FPPRGI−−
FQPRGI−−
FRPRGI−−
FKPRGI−−
FDPRGI−−
−FPRGIV−
−FPRGIN−
FIPRGI−−
−FPRGIF−
FMPRGI−−
FAPRGI−−
FYPRGI−−
FVPRGI−−
FCPRGI−−
−FPRGIK−
−FPRGIE−
FNPRGI−−
−FPRGIH−
−FPRGIM−
−FPRGIC−
FEPRGI−−
−GPRGIL−
−GPRGIS−
−GPRGIV−
−GPRGIM−
−GPRGIF−
−GPRGIT−
−GPRGIY−
−GPRGIN−
GNPRGI−−
GKPRGI−−
−GPRGIK−
GHPRGI−−
GAPRGI−−
GWPRGI−−
−GPRGIW−
−GPRGIQ−
−GPRGII−
GIPRGI−−
GDPRGI−−
GRPRGI−−
GEPRGI−−
GQPRGI−−
−GPRGIE−

−GPRGIA−
GPPRGI−−
GCPRGI−−
−HPRGIS−
HTPRGI−−
−HPRGIL−
HSPRGI−−
HIPRGI−−
−HPRGIH−
−HPRGIA−
−HPRGIM−
HLPRGI−−
HVPRGI−−
−HPRGIE−
HGPRGI−−
HQPRGI−−
−HPRGIN−
−HPRGIK−
−HPRGIT−
HKPRGI−−
HDPRGI−−
HYPRGI−−
−HPRGIW−
HMPRGI−−
HHPRGI−−
−HPRGIQ−
HPPRGI−−
HNPRGI−−
−HPRGIY−
−HPRGIV−
HEPRGI−−
IRPRGI−−
IKPRGI−−
IAPRGI−−
−IPRGIN−
ILPRGI−−
IWPRGI−−
−IPRGII−
−IPRGIT−
IPPRGI−−
IVPRGI−−
−IPRGIM−
−IPRGIG−
IIPRGI−−
IHPRGI−−
ISPRGI−−
−IPRGIH−
IQPRGI−−
−IPRGIQ−
−IPRGIA−

IGPRGI--
-IPRGIK-
IEPRGI--
-IPRGIE-
-IPRGIW-
-IPRGIY-
IMPRGI--
IDPRGI--
ICPRGI--
-KPRGIV-
-KPRGIG-
KYPRGI--
KLPRGI--
-KPRGIA-
KNPRGI--
-KPRGIF-
KEPRGI--
KVPRGI--
-KPRGIM-
-KPRGIW-
-KPRGIQ-
KPPRGI--
KTPRGI--
KQPRGI--
-KPRGIE-
KHPRGI--
KCPRGI--
-KPRGIN-
LRPRGI--
-LPRGIS-
-LPRGIV-
-LPRGII-
LAPRGI--
-LPRGIQ-
-LPRGIM-
LNPRGI--
LHPRGI--
LKPRGI--
-LPRGIN-
-LPRGIH-
-LPRGIF-
LYPRGI--
-LPRGIY-
-LPRGIE-
LQPRGI--
LPPRGI--
LIPRGI--
LEPRGI--
LCPRGI--
-LPRGIC-

−LPRGIG−
−LPRGIW−
−MPRGIT−
−MPRGII−
MHPRGI−−
−MPRGIN−
−MPRGIQ−
MPPRGI−−
MTPRGI−−
MMPRGI−−
MVPRGI−−
MQPRGI−−
−MPRGIS−
−MPRGIG−
−MPRGIH−
MGPRGI−−
MIPRGI−−
MAPRGI−−
−MPRGIK−
−MPRGIE−
MDPRGI−−
NAPRGI−−
−NPRGIF−
NRPRGI−−
−NPRGIS−
−NPRGIT−
NQPRGI−−
−NPRGIV−
NTPRGI−−
−NPRGIY−
−NPRGIW−
−NPRGIQ−
−NPRGIN−
−NPRGII−
−NPRGIA−
NEPRGI−−
−NPRGIH−
NMPRGI−−
NKPRGI−−
NFPRGI−−
−NPRGIE−
NDPRGI−−
NCPRGI−−
−NPRGIM−
−NPRGIG−
NWPRGI−−
NGPRGI−−
−−PRGIRS
−−PRGISG
−−PRGISS

--PRGILA
--PRGIVL
--PRGIRQ
--PRGIIL
--PRGIIA
--PRGIHL
--PRGIAL
--PRGIVG
--PRGITS
--PRGIRH
--PRGIGT
--PRGIAR
PLPRGI--
--PRGIYS
--PRGIWT
--PRGIAS
--PRGIYW
--PRGIFN
--PRGIKI
PSPRGI--
--PRGILQ
--PRGIGV
--PRGITK
--PRGISM
--PRGISK
--PRGISI
--PRGITM
--PRGITI
--PRGISN
--PRGIKV
--PRGIIV
--PRGITA
--PRGIRT
--PRGILH
--PRGIHS
--PRGIVV
--PRGIQK
--PRGIKS
--PRGIFQ
--PRGIAH
PAPRGI--
--PRGIVS
--PRGISF
--PRGIIN
--PRGIIK
--PRGIHW
--PRGIEV
--PRGIHR
--PRGIHQ
--PRGIDG

--PRGIAG
--PRGIYN
--PRGIWE
--PRGIWD
--PRGINT
--PRGILD
--PRGIGM
--PRGIFF
--PRGIWA
--PRGIVY
--PRGITG
--PRGIML
--PRGIKA
--PRGIGI
--PRGIFV
--PRGIFE
--PRGIYI
--PRGIWW
--PRGITV
--PRGINH
--PRGIMT
--PRGIHF
--PRGIGW
--PRGIGN
--PRGIVW
--PRGIVE
--PRGISW
--PRGINR
--PRGIHA
--PRGIGY
PIPRGI--
PGPRGI--
--PRGIYV
--PRGIYK
--PRGIWM
--PRGIWK
--PRGIWF
--PRGIVT
--PRGIVA
--PRGIRM
--PRGINY
--PRGINI
--PRGIMI
--PRGIKY
--PRGIKN
--PRGIKE
--PRGIHT
--PRGIHH
--PRGIHG
--PRGIFD

--PRGIET
--PRGIYD
--PRGIVK
--PRGIVI
--PRGIVH
--PRGITY
--PRGISH
--PRGISE
--PRGISD
--PRGIRE
--PRGIQV
--PRGIND
--PRGIMK
--PRGILM
--PRGIIM
--PRGIID
--PRGIAY
PRPRGI--
--PRGIWQ
--PRGIVM
--PRGIQT
--PRGIQA
--PRGIMW
--PRGIKD
--PRGIDS
--PRGIAT
PVPRGI--
--PRGIWN
--PRGIQS
--PRGIMH
--PRGIKQ
--PRGIKF
--PRGIIW
--PRGIFG
--PRGIFA
--PRGIEY
--PRGIES
--PRGIEN
--PRGIEH
--PRGIAF
--PRGIAA
PKPRGI--
PYPRGI--
PTPRGI--
--PRGIYQ
--PRGIYH
--PRGIYG
--PRGIQH
--PRGINQ
--PRGINM

--PRGINK
--PRGIMY
--PRGIMD
--PRGIMA
--PRGIHN
--PRGIHK
--PRGIHI
--PRGIGG
--PRGIEK
--PRGIAD
--PRGIYY
--PRGIYP
--PRGIWV
--PRGIVQ
--PRGITW
--PRGITQ
--PRGITD
--PRGIQW
--PRGIQI
--PRGING
--PRGILE
--PRGIKW
--PRGIIH
--PRGIIE
--PRGIFW
--PRGIEG
--PRGIAV
--PRGIAN
--PRGIAK
--PRGIYE
--PRGIQE
--PRGINA
--PRGIME
--PRGIKK
--PRGIKG
--PRGIIQ
--PRGIGF
--PRGIFM
--PRGIEA
--PRGIDA
--PRGIAW
--PRGIAI
--PRGIAE
--PRGIYT
--PRGIYM
--PRGIVD
--PRGIQF
--PRGIQD
--PRGIMQ
--PRGIMN

--PRGILC
--PRGIKP
--PRGIKC
--PRGIGQ
--PRGIGH
--PRGIFT
--PRGICK
--PRGICF
--PRGIAQ
PWPRGI--
--PRGITC
--PRGISC
--PRGIQG
--PRGIPH
--PRGINE
--PRGIMG
--PRGIMC
--PRGIIC
--PRGIHY
--PRGIHD
--PRGIEW
--PRGIEQ
--PRGIEP
--PRGIEM
--PRGIEF
--PRGIED
--PRGIEC
--PRGICS
--PRGICN
-PPRGIT-
PHPRGI--
PCPRGI--
QGPRGI--
-QPRGIS-
-QPRGIL-
QSPRGI--
QRPRGI--
-QPRGIN-
QTPRGI--
-QPRGIF-
-QPRGIY-
-QPRGIR-
-QPRGIW-
QLPRGI--
-QPRGII-
QNPRGI--
QIPRGI--
QFPRGI--
QAPRGI--
QQPRGI--

−QPRGIQ−
−QPRGIV−
−QPRGIT−
QHPRGI−−
−QPRGIH−
−QPRGIM−
QYPRGI−−
QVPRGI−−
−QPRGIE−
QCPRGI−−
−QPRGIA−
QPPRGI−−
RSPRGI−−
−RPRGIL−
RMPRGI−−
−RPRGIQ−
RDPRGI−−
RGPRGI−−
−RPRGIY−
−RPRGIV−
−RPRGIE−
RQPRGI−−
RPPRGI−−
−RPRGIF−
REPRGI−−
−RPRGIH−
RNPRGI−−
RCPRGI−−
RHPRGI−−
−RPRGIW−
RKPRGI−−
−SPRGIL−
STPRGI−−
−SPRGIW−
−SPRGIG−
−SPRGIS−
−SPRGIA−
SAPRGI−−
−SPRGIK−
SFPRGI−−
−SPRGIF−
SIPRGI−−
SEPRGI−−
−SPRGIV−
SPPRGI−−
SMPRGI−−
−SPRGIN−
−SPRGIY−
−SPRGIM−
SYPRGI−−

-SPRGIQ-
-SPRGIH-
SDPRGI--
-SPRGIE-
SCPRGI--
-TPRGIR-
-TPRGIW-
TRPRGI--
-TPRGIG-
-TPRGIM-
-TPRGIV-
-TPRGIA-
-TPRGIS-
TTPRGI--
-TPRGIY-
TYPRGI--
-TPRGIN-
TKPRGI--
TGPRGI--
TEPRGI--
TAPRGI--
-TPRGIK-
-TPRGIE-
TDPRGI--
TIPRGI--
-TPRGII-
-TPRGIP-
VLPRGI--
-VPRGIK-
-VPRGIL-
-VPRGIR-
-VPRGIY-
-VPRGIT-
-VPRGIM-
-VPRGIS-
-VPRGIN-
VIPRGI--
VSPRGI--
VTPRGI--
VYPRGI--
VPPRGI--
VVPRGI--
-VPRGIA-
-VPRGIQ-
VQPRGI--
VEPRGI--
-VPRGII-
VNPRGI--
VWPRGI--
-VPRGIE-

VMPRGI--
VDPRGI--
-VPRGIW-
WSPRGI--
WLPRGI--
-WPRGIS-
WTPRGI--
-WPRGIL-
-WPRGIY-
-WPRGIT-
WGPRGI--
-WPRGIE-
WIPRGI--
WQPRGI--
-WPRGII-
-WPRGIH-
WPPRGI--
WMPRGI--
WKPRGI--
-WPRGIM-
WEPRGI--
WDPRGI--
-WPRGIN-
-WPRGIF-
-YPRGIL-
-YPRGIS-
-YPRGIG-
-YPRGIM-
YYPRGI--
-YPRGIT-
YLPRGI--
YVPRGI--
-YPRGIN-
-YPRGIH-
YMPRGI--
-YPRGIF-
YTPRGI--
-YPRGIQ-
YNPRGI--
YPPRGI--
YIPRGI--
YFPRGI--
YDPRGI--
YAPRGI--
-YPRGIW-
-YPRGIA-
YCPRGI--
-YPRGII-
YEPRGI--
YQPRGI--

| | | | |
|---|---|---|---|
| | YKPRGI-- | | |
| PLGM | 42 | 812 | 34.11 |
| | IHPLGM-- | | |
| | -LPLGMG- | | |
| | --PLGMLM | | |
| | --PLGMGH | | |
| | --PLGMGF | | |
| | -SPLGMD- | | |
| | -TPLGMQ- | | |
| | -APLGMI- | | |
| | -APLGMS- | | |
| | -APLGMR- | | |
| | ARPLGM-- | | |
| | -APLGMW- | | |
| | APPLGM-- | | |
| | AAPLGM-- | | |
| | -APLGML- | | |
| | -APLGMY- | | |
| | -APLGMN- | | |
| | -APLGMG- | | |
| | ASPLGM-- | | |
| | -APLGMA- | | |
| | AVPLGM-- | | |
| | ATPLGM-- | | |
| | -APLGMV- | | |
| | AEPLGM-- | | |
| | AWPLGM-- | | |
| | -APLGMK- | | |
| | ALPLGM-- | | |
| | ADPLGM-- | | |
| | -APLGMQ- | | |
| | ANPLGM-- | | |
| | AGPLGM-- | | |
| | -APLGMH- | | |
| | AKPLGM-- | | |
| | -APLGMM- | | |
| | AIPLGM-- | | |
| | AHPLGM-- | | |
| | AFPLGM-- | | |
| | AYPLGM-- | | |
| | -APLGME- | | |
| | CRPLGM-- | | |
| | CEPLGM-- | | |
| | -CPLGML- | | |
| | CLPLGM-- | | |
| | CSPLGM-- | | |
| | -CPLGMR- | | |
| | CNPLGM-- | | |
| | CKPLGM-- | | |
| | CYPLGM-- | | |

CTPLGM--
CQPLGM--
-CPLGMV-
-CPLGMT-
-CPLGMS-
CMPLGM--
CIPLGM--
CGPLGM--
-DPLGML-
-DPLGMT-
-DPLGMR-
DAPLGM--
-DPLGMI-
DGPLGM--
-DPLGMS-
-DPLGMK-
DIPLGM--
-DPLGMY-
-DPLGMN-
-DPLGMF-
DLPLGM--
DWPLGM--
DTPLGM--
DSPLGM--
-DPLGMW-
-DPLGMV-
-DPLGMA-
DNPLGM--
DMPLGM--
DEPLGM--
DQPLGM--
DKPLGM--
DHPLGM--
-EPLGML-
ESPLGM--
-EPLGMS-
-EPLGMT-
-EPLGMG-
EAPLGM--
ERPLGM--
-EPLGMM-
-EPLGMN-
EKPLGM--
EGPLGM--
-EPLGMI-
EMPLGM--
ELPLGM--
EYPLGM--
EVPLGM--
-EPLGMW-

−EPLGMQ−
−EPLGMK−
ETPLGM−−
−EPLGMV−
EIPLGM−−
EEPLGM−−
ECPLGM−−
FRPLGM−−
−FPLGMF−
−FPLGMG−
FVPLGM−−
−FPLGMY−
−FPLGMM−
−FPLGMW−
−FPLGMT−
FKPLGM−−
FSPLGM−−
−FPLGML−
FQPLGM−−
FPPLGM−−
−FPLGMS−
−FPLGMK−
FNPLGM−−
FGPLGM−−
−FPLGMH−
FDPLGM−−
FTPLGM−−
−FPLGMV−
−FPLGMQ−
−FPLGMI−
FLPLGM−−
FIPLGM−−
FMPLGM−−
FHPLGM−−
GRPLGM−−
GLPLGM−−
−GPLGMR−
−GPLGMG−
GNPLGM−−
−GPLGMT−
GTPLGM−−
−GPLGML−
GMPLGM−−
GAPLGM−−
−GPLGMY−
−GPLGMS−
GGPLGM−−
GVPLGM−−
−GPLGMV−
−GPLGMN−

-GPLGMK-
-GPLGMI-
-GPLGMH-
GSPLGM--
-GPLGMF-
-GPLGMM-
-GPLGMA-
GWPLGM--
GQPLGM--
GKPLGM--
GIPLGM--
GEPLGM--
GCPLGM--
-GPLGMD-
GDPLGM--
GPPLGM--
-GPLGMQ-
-GPLGME-
-HPLGMK-
-HPLGMR-
HRPLGM--
-HPLGMM-
-HPLGMT-
-HPLGMG-
HKPLGM--
-HPLGMA-
HWPLGM--
HQPLGM--
HPPLGM--
-HPLGMN-
-HPLGMV-
-HPLGME-
HFPLGM--
HEPLGM--
HSPLGM--
-HPLGML-
HVPLGM--
HMPLGM--
HGPLGM--
HYPLGM--
-HPLGMS-
-HPLGMQ-
-HPLGMI-
-HPLGMH-
HDPLGM--
HAPLGM--
HTPLGM--
-HPLGMY-
-HPLGMC-
HHPLGM--

-IPLGMS-
IRPLGM--
-IPLGMR-
-IPLGML-
ISPLGM--
-IPLGMT-
IYPLGM--
IPPLGM--
-IPLGMN-
IDPLGM--
ITPLGM--
-IPLGMW-
-IPLGMK-
-IPLGME-
INPLGM--
IIPLGM--
IQPLGM--
-IPLGMY-
-IPLGMI-
IGPLGM--
IFPLGM--
IAPLGM--
IVPLGM--
IKPLGM--
ICPLGM--
IWPLGM--
-IPLGMQ-
-IPLGMM-
-IPLGMA-
IMPLGM--
KIPLGM--
KVPLGM--
-KPLGMT-
KAPLGM--
-KPLGMG-
-KPLGMK-
KYPLGM--
KWPLGM--
-KPLGMF-
KRPLGM--
-KPLGMV-
KNPLGM--
KMPLGM--
-KPLGMS-
-KPLGMI-
-KPLGMD-
-KPLGMH-
-KPLGMA-
KFPLGM--
-KPLGMY-

KLPLGM--
-KPLGMQ-
-KPLGMC-
KKPLGM--
KEPLGM--
KTPLGM--
-KPLGME-
KHPLGM--
KDPLGM--
LSPLGM--
LRPLGM--
-LPLGMK-
LGPLGM--
-LPLGMS-
-LPLGMT-
-LPLGMM-
-LPLGMI-
LNPLGM--
LKPLGM--
-LPLGMA-
LLPLGM--
LTPLGM--
-LPLGMW-
LFPLGM--
LAPLGM--
LYPLGM--
-LPLGMH-
LIPLGM--
LMPLGM--
-LPLGMY-
-LPLGMN-
LPPLGM--
-LPLGMF-
LHPLGM--
LQPLGM--
-LPLGMV-
-LPLGME-
-LPLGMQ-
MTPLGM--
-MPLGMW-
-MPLGMR-
MRPLGM--
MGPLGM--
MVPLGM--
-MPLGMT-
-MPLGMS-
-MPLGMF-
MKPLGM--
MAPLGM--
MLPLGM--

```
-MPLGMV-
-MPLGMN-
-MPLGMH-
MIPLGM--
MPPLGM--
-MPLGMY-
-MPLGMG-
MMPLGM--
MFPLGM--
MQPLGM--
-MPLGMK-
-MPLGME-
MEPLGM--
MDPLGM--
MYPLGM--
MWPLGM--
-MPLGMD-
-MPLGMA-
MNPLGM--
-NPLGML-
NLPLGM--
-NPLGMI-
-NPLGMT-
-NPLGMS-
NEPLGM--
NGPLGM--
NVPLGM--
-NPLGMK-
NNPLGM--
NRPLGM--
-NPLGMA-
NKPLGM--
NFPLGM--
NWPLGM--
NSPLGM--
-NPLGMQ-
NYPLGM--
-NPLGMN-
-NPLGMG-
NHPLGM--
-NPLGMV-
-NPLGMH-
NTPLGM--
-NPLGMF-
NIPLGM--
NCPLGM--
NAPLGM--
NQPLGM--
NPPLGM--
-NPLGMM-
```

NMPLGM--
--PLGMRL
--PLGMSR
--PLGMRG
--PLGMRA
--PLGMVR
--PLGMST
--PLGMRT
--PLGMVG
--PLGMKR
--PLGMLQ
--PLGMFR
--PLGMVW
--PLGMVL
--PLGMRN
--PLGMRH
--PLGMLI
--PLGMSD
--PLGMRW
--PLGMYR
--PLGMNR
--PLGMNL
--PLGMYG
--PLGMVK
--PLGMTL
--PLGMGA
--PLGMAS
--PLGMTA
--PLGMVM
--PLGMMR
--PLGMHL
--PLGMHH
--PLGMVN
--PLGMTW
--PLGMTR
--PLGMSH
--PLGMNI
--PLGMLH
--PLGMIR
--PLGMAA
--PLGMVS
--PLGMVH
--PLGMTS
--PLGMSG
--PLGMRK
--PLGMLV
--PLGMKY
--PLGMRQ
--PLGMKL
--PLGMKA

--PLGMIN
--PLGMHR
--PLGMFS
--PLGMVI
--PLGMTV
--PLGMQT
--PLGMQS
--PLGMQA
--PLGMNF
--PLGMML
--PLGMLN
--PLGMLK
--PLGMKT
--PLGMGV
--PLGMFD
--PLGMDG
--PLGMAL
--PLGMAK
--PLGMAI
--PLGMYT
--PLGMQV
--PLGMLT
--PLGMIS
--PLGMHS
--PLGMHA
--PLGMFA
--PLGMEF
--PLGMAY
--PLGMAF
PYPLGM--
--PLGMWR
--PLGMVY
--PLGMVA
--PLGMSV
--PLGMRV
--PLGMQR
--PLGMMN
--PLGMLD
--PLGMIY
--PLGMHT
--PLGMGS
--PLGMGI
PFPLGM--
PMPLGM--
--PLGMYN
--PLGMYM
--PLGMWS
--PLGMWN
--PLGMVT
--PLGMVD

--PLGMTY
--PLGMTE
--PLGMSW
--PLGMSM
--PLGMSE
--PLGMRE
--PLGMNV
--PLGMNQ
--PLGMMT
--PLGMMM
--PLGMMA
--PLGMKS
--PLGMKK
--PLGMIL
--PLGMII
--PLGMFT
--PLGMFN
--PLGMFM
--PLGMDS
PWPLGM--
PRPLGM--
--PLGMTT
--PLGMTK
--PLGMTH
--PLGMSQ
--PLGMSK
--PLGMSI
--PLGMSF
--PLGMRY
--PLGMRP
--PLGMQN
--PLGMNW
--PLGMMQ
--PLGMMD
--PLGMKG
--PLGMIV
--PLGMGL
--PLGMEV
--PLGMAM
--PLGMAG
--PLGMYS
--PLGMVE
--PLGMTF
--PLGMSY
--PLGMRM
--PLGMQM
--PLGMQK
--PLGMNH
--PLGMMI
--PLGMLE

--PLGMIH
--PLGMIA
--PLGMGY
--PLGMGT
--PLGMFQ
PIPLGM--
PTPLGM--
--PLGMYQ
--PLGMWI
--PLGMVQ
--PLGMTM
--PLGMTI
--PLGMTG
--PLGMRD
--PLGMQL
--PLGMNN
--PLGMKV
--PLGMKE
--PLGMIQ
--PLGMHM
--PLGMGK
--PLGMEW
--PLGMAV
--PLGMAN
--PLGMWQ
--PLGMTQ
--PLGMSN
--PLGMNS
--PLGMNM
--PLGMKN
--PLGMKM
--PLGMKI
--PLGMHW
--PLGMHN
--PLGMGW
--PLGMFW
--PLGMFK
--PLGMER
--PLGMAT
--PLGMAH
PKPLGM--
PGPLGM--
PNPLGM--
--PLGMYH
--PLGMYE
--PLGMYD
--PLGMYA
--PLGMWT
--PLGMTN
--PLGMRF

--PLGMQE
--PLGMNK
--PLGMMK
--PLGMMG
--PLGMKQ
--PLGMKH
--PLGMKF
--PLGMIG
--PLGMHV
--PLGMGR
--PLGMGN
--PLGMEY
--PLGMEN
--PLGMAW
--PLGMAQ
-PPLGMH-
--PLGMYK
--PLGMWH
--PLGMWF
--PLGMTD
--PLGMSC
--PLGMQW
--PLGMQQ
--PLGMQI
--PLGMQH
--PLGMQD
--PLGMNE
--PLGMMH
--PLGMLP
--PLGMKW
--PLGMKP
--PLGMKD
--PLGMIW
--PLGMIT
--PLGMID
--PLGMHI
--PLGMHG
--PLGMHD
--PLGMGM
--PLGMGG
--PLGMEH
--PLGMDA
--PLGMCG
--PLGMCD
--PLGMAE
PHPLGM--
PEPLGM--
PCPLGM--
-QPLGMS-
-QPLGMI-

QRPLGM--
QLPLGM--
-QPLGMG-
-QPLGMY-
QVPLGM--
QSPLGM--
-QPLGMT-
-QPLGMK-
-QPLGMR-
QTPLGM--
-QPLGMV-
-QPLGMH-
QYPLGM--
-QPLGMW-
-QPLGMM-
QQPLGM--
-QPLGMQ-
-QPLGML-
QMPLGM--
QPPLGM--
-QPLGME-
QNPLGM--
-QPLGMF-
-QPLGMA-
QIPLGM--
QGPLGM--
QAPLGM--
-RPLGMS-
-RPLGMY-
-RPLGMG-
-RPLGMI-
RGPLGM--
RAPLGM--
RVPLGM--
-RPLGMV-
-RPLGML-
-RPLGMH-
-RPLGMQ-
RHPLGM--
RYPLGM--
RMPLGM--
-RPLGMK-
-RPLGMF-
RTPLGM--
-RPLGMN-
RNPLGM--
RWPLGM--
-RPLGMW-
RPPLGM--
RKPLGM--

-RPLGMM-
RIPLGM--
RFPLGM--
RQPLGM--
REPLGM--
-RPLGME-
RDPLGM--
RCPLGM--
-RPLGMD-
-RPLGMC-
SLPLGM--
SSPLGM--
-SPLGMI-
-SPLGMW-
SFPLGM--
SRPLGM--
-SPLGMG-
SWPLGM--
-SPLGMV-
-SPLGMK-
SQPLGM--
-SPLGMF-
-SPLGMA-
SHPLGM--
-SPLGMY-
SIPLGM--
STPLGM--
-SPLGMH-
-SPLGMS-
-SPLGMN-
SAPLGM--
SPPLGM--
SEPLGM--
-SPLGMT-
SNPLGM--
-SPLGMM-
SMPLGM--
SKPLGM--
SCPLGM--
SYPLGM--
-SPLGMQ-
-SPLGME-
SGPLGM--
SDPLGM--
-TPLGMY-
-TPLGMR-
-TPLGML-
-TPLGMS-
-TPLGMT-
TVPLGM--

-TPLGMV-
-TPLGMI-
TSPLGM--
TPPLGM--
TMPLGM--
TWPLGM--
-TPLGMH-
TYPLGM--
TTPLGM--
-TPLGMF-
TIPLGM--
TEPLGM--
TQPLGM--
-TPLGMW-
-TPLGME-
TGPLGM--
-TPLGMK-
-TPLGMG-
TFPLGM--
TDPLGM--
TAPLGM--
-TPLGMA-
TNPLGM--
TCPLGM--
-TPLGMM-
VSPLGM--
VRPLGM--
VVPLGM--
VPPLGM--
-VPLGMS-
VAPLGM--
-VPLGMK-
-VPLGMR-
-VPLGMN-
-VPLGMW-
-VPLGML-
VFPLGM--
VYPLGM--
-VPLGMT-
-VPLGMG-
VHPLGM--
VWPLGM--
-VPLGMV-
-VPLGMM-
VMPLGM--
VLPLGM--
VIPLGM--
-VPLGMQ-
-VPLGMI-
-VPLGMF-

-VPLGMA-
VGPLGM--
VDPLGM--
VQPLGM--
-VPLGMY-
VEPLGM--
-VPLGMH-
-VPLGME-
VCPLGM--
WTPLGM--
WKPLGM--
-WPLGMR-
WSPLGM--
-WPLGMT-
WYPLGM--
-WPLGMM-
-WPLGMI-
-WPLGML-
-WPLGMN-
-WPLGME-
-WPLGMV-
WGPLGM--
WWPLGM--
-WPLGMA-
WNPLGM--
WFPLGM--
WRPLGM--
-WPLGMW-
-WPLGMG-
WHPLGM--
WPPLGM--
-WPLGMK-
-WPLGMF-
WLPLGM--
WIPLGM--
WDPLGM--
WAPLGM--
-YPLGMS-
YKPLGM--
YAPLGM--
-YPLGMY-
-YPLGMV-
-YPLGMN-
YLPLGM--
-YPLGMK-
-YPLGMF-
YIPLGM--
-YPLGMH-
-YPLGMT-
-YPLGMR-

| | | | |
|---|---|---|---|
| | -YPLGMQ- | | |
| | YGPLGM-- | | |
| | YDPLGM-- | | |
| | YSPLGM-- | | |
| | YHPLGM-- | | |
| | -YPLGMG- | | |
| | YNPLGM-- | | |
| | YQPLGM-- | | |
| | -YPLGMI- | | |
| | YTPLGM-- | | |
| | YYPLGM-- | | |
| | YPPLGM-- | | |
| | YMPLGM-- | | |
| | YEPLGM-- | | |
| PRAM | 299 | 667 | 34.08 |
| | --PRAMFP | | |
| | ALPRAM-- | | |
| | -APRAML- | | |
| | -APRAMA- | | |
| | -APRAMS- | | |
| | AAPRAM-- | | |
| | AFPRAM-- | | |
| | -APRAMK- | | |
| | AWPRAM-- | | |
| | AVPRAM-- | | |
| | ASPRAM-- | | |
| | AIPRAM-- | | |
| | AHPRAM-- | | |
| | AEPRAM-- | | |
| | -APRAMM- | | |
| | -APRAMF- | | |
| | AGPRAM-- | | |
| | ATPRAM-- | | |
| | -APRAMY- | | |
| | ANPRAM-- | | |
| | -APRAMT- | | |
| | -APRAMD- | | |
| | -APRAMI- | | |
| | -APRAMH- | | |
| | AMPRAM-- | | |
| | ACPRAM-- | | |
| | -CPRAMQ- | | |
| | -CPRAMY- | | |
| | -CPRAMS- | | |
| | -CPRAMH- | | |
| | CYPRAM-- | | |
| | CSPRAM-- | | |
| | -CPRAMV- | | |
| | -CPRAMK- | | |
| | -CPRAMF- | | |

-CPRAME-
CNPRAM--
CLPRAM--
CIPRAM--
DVPRAM--
-DPRAMA-
-DPRAMS-
DMPRAM--
DRPRAM--
-DPRAMI-
DNPRAM--
DLPRAM--
DYPRAM--
-DPRAMR-
-DPRAMQ-
-DPRAMF-
DIPRAM--
-DPRAMV-
-DPRAMK-
-DPRAMH-
DEPRAM--
-DPRAML-
DGPRAM--
DWPRAM--
DTPRAM--
-DPRAMY-
-DPRAMT-
-DPRAMG-
DKPRAM--
DFPRAM--
DCPRAM--
-EPRAMR-
ETPRAM--
EWPRAM--
ELPRAM--
-EPRAML-
-EPRAMA-
EEPRAM--
ERPRAM--
-EPRAMY-
ENPRAM--
-EPRAMW-
-EPRAMG-
-EPRAME-
EMPRAM--
EAPRAM--
EYPRAM--
EIPRAM--
EHPRAM--
EGPRAM--

EDPRAM--
EVPRAM--
EQPRAM--
-EPRAMV-
-EPRAMC-
EFPRAM--
-FPRAMY-
-FPRAMM-
FDPRAM--
-FPRAMK-
FYPRAM--
FQPRAM--
FMPRAM--
FKPRAM--
-FPRAMA-
FGPRAM--
FPPRAM--
FAPRAM--
FRPRAM--
-FPRAMV-
-FPRAMQ-
FIPRAM--
FHPRAM--
-FPRAMI-
-FPRAMH-
-FPRAMF-
-FPRAME-
FEPRAM--
-FPRAMW-
-GPRAMS-
GFPRAM--
-GPRAMT-
-GPRAMY-
GSPRAM--
-GPRAMM-
-GPRAMA-
-GPRAMI-
GLPRAM--
GGPRAM--
-GPRAML-
GNPRAM--
GWPRAM--
-GPRAMQ-
GMPRAM--
GVPRAM--
-GPRAMK-
-GPRAMG-
-GPRAME-
GEPRAM--
GAPRAM--

-GPRAMV-
-GPRAMW-
-GPRAMH-
GKPRAM--
GHPRAM--
GCPRAM--
GQPRAM--
GPPRAM--
-GPRAMN-
-HPRAMS-
HSPRAM--
-HPRAMV-
-HPRAMF-
-HPRAMT-
-HPRAMY-
-HPRAME-
HLPRAM--
-HPRAMA-
HKPRAM--
HFPRAM--
HDPRAM--
HYPRAM--
HQPRAM--
-HPRAMQ-
-HPRAMN-
HPPRAM--
HGPRAM--
-HPRAMW-
-HPRAMM-
HMPRAM--
HEPRAM--
HAPRAM--
-IPRAML-
ISPRAM--
-IPRAMV-
IIPRAM--
IRPRAM--
ITPRAM--
-IPRAMH-
-IPRAMG-
-IPRAMQ-
-IPRAMK-
IKPRAM--
IAPRAM--
-IPRAMY-
-IPRAMN-
-IPRAME-
-IPRAMA-
IDPRAM--
IYPRAM--

IMPRAM--
IGPRAM--
IQPRAM--
-IPRAMI-
INPRAM--
IEPRAM--
KSPRAM--
-KPRAML-
-KPRAMN-
-KPRAMT-
-KPRAMI-
KVPRAM--
-KPRAMV-
KLPRAM--
-KPRAMK-
-KPRAMA-
-KPRAMY-
-KPRAMF-
-KPRAME-
KPPRAM--
KEPRAM--
KQPRAM--
-KPRAMQ-
-KPRAMH-
KGPRAM--
-KPRAMM-
-KPRAMD-
KMPRAM--
KKPRAM--
-LPRAMI-
LGPRAM--
-LPRAMK-
LSPRAM--
LVPRAM--
-LPRAMS-
LNPRAM--
LTPRAM--
-LPRAMT-
-LPRAMH-
-LPRAMW-
-LPRAMN-
LHPRAM--
LDPRAM--
LPPRAM--
LMPRAM--
LKPRAM--
LIPRAM--
-LPRAMY-
LAPRAM--
LQPRAM--

-LPRAMQ-
-LPRAMG-
-LPRAME-
LCPRAM--
-MPRAML-
-MPRAMS-
MSPRAM--
MVPRAM--
-MPRAMT-
-MPRAMV-
MAPRAM--
-MPRAME-
-MPRAMA-
MGPRAM--
MWPRAM--
-MPRAMQ-
-MPRAMF-
MLPRAM--
MEPRAM--
-MPRAMK-
-MPRAMI-
MFPRAM--
MYPRAM--
-MPRAMY-
-MPRAMW-
-MPRAMN-
MPPRAM--
MMPRAM--
MHPRAM--
-NPRAMK-
NVPRAM--
-NPRAML-
-NPRAMY-
-NPRAMA-
-NPRAMS-
-NPRAMW-
NLPRAM--
NKPRAM--
-NPRAMM-
NTPRAM--
NSPRAM--
-NPRAMI-
NIPRAM--
NQPRAM--
-NPRAMF-
NPPRAM--
-NPRAMV-
-NPRAMN-
NMPRAM--
NGPRAM--

NDPRAM--
--PRAMLS
--PRAMLK
--PRAMVS
--PRAMVG
--PRAMLT
--PRAMIL
--PRAMVI
--PRAMMS
--PRAMGT
--PRAMFS
--PRAMAH
--PRAMSS
--PRAMIK
--PRAMAN
--PRAMSG
--PRAMYS
--PRAMMT
--PRAMLF
--PRAMIA
--PRAMGW
--PRAMSM
--PRAMSI
--PRAMIG
--PRAMGA
--PRAMAT
--PRAMSH
--PRAMMG
--PRAMLH
--PRAMII
--PRAMVA
--PRAMNK
--PRAMIS
--PRAMHR
--PRAMGG
--PRAMFA
--PRAMWL
--PRAMVY
--PRAMVQ
--PRAMVH
--PRAMSK
--PRAMNT
--PRAMLQ
--PRAMFK
--PRAMAV
--PRAMYV
--PRAMYT
--PRAMYR
--PRAMYK
--PRAMTI

--PRAMPL
--PRAMFG
--PRAMVT
--PRAMTA
--PRAMNN
--PRAMKV
--PRAMKI
--PRAMIV
--PRAMGI
--PRAMFD
--PRAMAG
PRPRAM--
--PRAMYY
--PRAMYG
--PRAMWH
--PRAMTW
--PRAMSD
--PRAMSA
--PRAMPM
--PRAMND
--PRAMKT
--PRAMKS
--PRAMIT
--PRAMAS
--PRAMAK
PLPRAM--
PIPRAM--
PTPRAM--
--PRAMYM
--PRAMTK
--PRAMTE
--PRAMQN
--PRAMNR
--PRAMMN
--PRAMGH
--PRAMFY
--PRAMFT
--PRAMEV
PFPRAM--
--PRAMVN
--PRAMTV
--PRAMTT
--PRAMTH
--PRAMPI
--PRAMIF
--PRAMHS
--PRAMHK
--PRAMER
--PRAMEI
--PRAMWQ

--PRAMVD
--PRAMPY
--PRAMLE
--PRAMLD
--PRAMKW
--PRAMHV
--PRAMHF
--PRAMGV
--PRAMGM
--PRAMGK
--PRAMEG
--PRAMAI
--PRAMAD
PAPRAM--
PVPRAM--
--PRAMWW
--PRAMWT
--PRAMWD
--PRAMVM
--PRAMVK
--PRAMVF
--PRAMRE
--PRAMQK
--PRAMQI
--PRAMPF
--PRAMNI
--PRAMMW
--PRAMMH
--PRAMLW
--PRAMKL
--PRAMHY
--PRAMHH
--PRAMHD
--PRAMAY
PWPRAM--
--PRAMYW
--PRAMYN
--PRAMWR
--PRAMWI
--PRAMWA
--PRAMQV
--PRAMQM
--PRAMQL
--PRAMQH
--PRAMQG
--PRAMNW
--PRAMNS
--PRAMMY
--PRAMMQ
--PRAMKQ

--PRAMIW
--PRAMIQ
--PRAMHG
--PRAMHA
--PRAMGY
--PRAMAA
PKPRAM--
PDPRAM--
--PRAMYQ
--PRAMWV
--PRAMWM
--PRAMWK
--PRAMSW
--PRAMQY
--PRAMQW
--PRAMNV
--PRAMNQ
--PRAMML
--PRAMMI
--PRAMME
--PRAMKN
--PRAMKD
--PRAMKA
--PRAMHT
--PRAMGN
--PRAMGF
--PRAMFI
--PRAMFH
--PRAMES
--PRAMDL
PNPRAM--
--PRAMYD
--PRAMVE
--PRAMTM
--PRAMSE
--PRAMQS
--PRAMQQ
--PRAMNY
--PRAMNA
--PRAMMP
--PRAMIP
--PRAMIM
--PRAMHI
--PRAMHE
--PRAMEN
--PRAMDA
--PRAMCG
PYPRAM--
--PRAMYE
--PRAMWN

--PRAMWE
--PRAMTQ
--PRAMSC
--PRAMQF
--PRAMPW
--PRAMNH
--PRAMNE
--PRAMLC
--PRAMIH
--PRAMIE
--PRAMID
--PRAMHM
--PRAMGD
--PRAMFQ
--PRAMFE
--PRAMEQ
--PRAMEF
--PRAMCV
--PRAMCH
--PRAMAQ
--PRAMAE
PHPRAM--
PGPRAM--
PEPRAM--
-QPRAMV-
-QPRAMR-
QFPRAM--
-QPRAMK-
-QPRAMT-
-QPRAMI-
-QPRAMG-
QSPRAM--
-QPRAMS-
-QPRAMN-
QPPRAM--
QDPRAM--
QTPRAM--
-QPRAMW-
-QPRAMH-
-QPRAMA-
QMPRAM--
QGPRAM--
-QPRAMF-
-QPRAMY-
QNPRAM--
QIPRAM--
RAPRAM--
RSPRAM--
-RPRAML-
-RPRAMQ-

−RPRAMM−
RPPRAM−−
RIPRAM−−
RNPRAM−−
−RPRAMN−
RDPRAM−−
−RPRAMY−
−RPRAMH−
−RPRAMG−
RQPRAM−−
−RPRAMK−
−RPRAMF−
−RPRAMT−
−RPRAMV−
−RPRAME−
REPRAM−−
RRPRAM−−
RKPRAM−−
−SPRAML−
−SPRAMS−
−SPRAMI−
SGPRAM−−
−SPRAME−
−SPRAMW−
SSPRAM−−
SNPRAM−−
SLPRAM−−
−SPRAMH−
−SPRAMG−
SKPRAM−−
SEPRAM−−
−SPRAMQ−
SIPRAM−−
−SPRAMY−
−SPRAMF−
STPRAM−−
−SPRAMM−
SYPRAM−−
−SPRAMT−
SPPRAM−−
SDPRAM−−
−TPRAML−
TLPRAM−−
−TPRAMS−
TSPRAM−−
TKPRAM−−
−TPRAMA−
TPPRAM−−
−TPRAMR−
TVPRAM−−

-TPRAMT-
TWPRAM--
TAPRAM--
-TPRAMG-
TGPRAM--
TRPRAM--
-TPRAMN-
TFPRAM--
TTPRAM--
TQPRAM--
-TPRAMF-
-TPRAMV-
TEPRAM--
TYPRAM--
-TPRAMM-
-TPRAME-
VNPRAM--
-VPRAMK-
VSPRAM--
VMPRAM--
VTPRAM--
VAPRAM--
-VPRAMH-
VDPRAM--
VQPRAM--
-VPRAMW-
-VPRAMN-
-VPRAMF-
VPPRAM--
VGPRAM--
VVPRAM--
-VPRAMV-
-VPRAMQ-
VYPRAM--
-VPRAMY-
VKPRAM--
-VPRAME-
VEPRAM--
-VPRAMD-
WNPRAM--
WRPRAM--
WKPRAM--
-WPRAMI-
WMPRAM--
WHPRAM--
WTPRAM--
-WPRAMY-
WEPRAM--
-WPRAMG-
WGPRAM--

WVPRAM--
-WPRAMN-
-WPRAMM-
-WPRAMH-
WDPRAM--
WYPRAM--
-WPRAME-
WCPRAM--
-YPRAMT-
-YPRAMN-
YYPRAM--
YSPRAM--
-YPRAMA-
-YPRAMH-
-YPRAMV-
YWPRAM--
YRPRAM--
YQPRAM--
YMPRAM--
-YPRAMW-
-YPRAMM-
YEPRAM--
YVPRAM--
-YPRAMY-
-YPRAMC-
YPPRAM--
YIPRAM--
YAPRAM--
-YPRAME-
YNPRAM--
YGPRAM--
YDPRAM--
PAPL 10 869 34.04
--PAPLNA
TNPAPL--
-APAPLR-
-APAPLS-
-APAPLL-
AVPAPL--
-APAPLK-
ALPAPL--
ASPAPL--
AIPAPL--
ARPAPL--
ANPAPL--
-APAPLY-
-APAPLI-
AFPAPL--
APPAPL--
AHPAPL--

AAPAPL--
AYPAPL--
-APAPLQ-
-APAPLG-
AQPAPL--
-APAPLV-
-APAPLT-
-APAPLE-
-APAPLA-
AGPAPL--
-APAPLN-
-APAPLH-
-APAPLF-
AMPAPL--
AEPAPL--
AWPAPL--
ATPAPL--
ADPAPL--
ACPAPL--
-CPAPLR-
CRPAPL--
CSPAPL--
CMPAPL--
CLPAPL--
CGPAPL--
-CPAPLW-
-CPAPLN-
-CPAPLK-
CNPAPL--
CVPAPL--
CTPAPL--
-CPAPLY-
-CPAPLT-
-CPAPLS-
-CPAPLL-
-CPAPLG-
-CPAPLF-
-CPAPLA-
CKPAPL--
CCPAPL--
CAPAPL--
-DPAPLL-
DVPAPL--
-DPAPLT-
-DPAPLR-
-DPAPLA-
DGPAPL--
DAPAPL--
DRPAPL--
-DPAPLK-

-DPAPLH-
DFPAPL--
DSPAPL--
-DPAPLW-
-DPAPLY-
-DPAPLF-
DNPAPL--
DMPAPL--
DKPAPL--
DWPAPL--
-DPAPLV-
-DPAPLG-
DLPAPL--
DHPAPL--
DQPAPL--
-DPAPLN-
-DPAPLM-
DIPAPL--
-EPAPLT-
-EPAPLL-
EGPAPL--
ELPAPL--
-EPAPLH-
ETPAPL--
-EPAPLQ-
-EPAPLY-
-EPAPLR-
ERPAPL--
-EPAPLW-
-EPAPLG-
EMPAPL--
ESPAPL--
EQPAPL--
-EPAPLS-
-EPAPLI-
-EPAPLA-
EYPAPL--
EWPAPL--
-EPAPLK-
EVPAPL--
-EPAPLN-
-EPAPLM-
EIPAPL--
EFPAPL--
-FPAPLY-
FLPAPL--
FGPAPL--
FQPAPL--
-FPAPLS-
-FPAPLK-

FSPAPL--
-FPAPLA-
FHPAPL--
FFPAPL--
FYPAPL--
FRPAPL--
FPPAPL--
-FPAPLW-
-FPAPLV-
-FPAPLR-
-FPAPLN-
-FPAPLH-
-FPAPLE-
FNPAPL--
-FPAPLQ-
-FPAPLF-
FIPAPL--
FWPAPL--
FTPAPL--
-FPAPLM-
-FPAPLL-
FEPAPL--
FVPAPL--
-FPAPLG-
-GPAPLR-
-GPAPLL-
GRPAPL--
GLPAPL--
GFPAPL--
-GPAPLM-
GKPAPL--
GYPAPL--
-GPAPLQ-
-GPAPLS-
GGPAPL--
-GPAPLT-
-GPAPLH-
GMPAPL--
GVPAPL--
-GPAPLA-
GQPAPL--
-GPAPLK-
-GPAPLI-
-GPAPLF-
GEPAPL--
GTPAPL--
-GPAPLY-
-GPAPLV-
-GPAPLE-
GIPAPL--

GHPAPL--
-GPAPLW-
-GPAPLN-
GDPAPL--
HNPAPL--
HRPAPL--
-HPAPLL-
HEPAPL--
HVPAPL--
-HPAPLT-
-HPAPLK-
HMPAPL--
HTPAPL--
-HPAPLY-
-HPAPLE-
HKPAPL--
HIPAPL--
HYPAPL--
-HPAPLA-
HGPAPL--
HFPAPL--
HWPAPL--
-HPAPLW-
-HPAPLS-
-HPAPLH-
-HPAPLF-
HPPAPL--
-HPAPLV-
-HPAPLN-
-HPAPLG-
HLPAPL--
HDPAPL--
HCPAPL--
-HPAPLM-
-HPAPLI-
HAPAPL--
-IPAPLK-
-IPAPLR-
ISPAPL--
IRPAPL--
IGPAPL--
IVPAPL--
-IPAPLN-
-IPAPLL-
-IPAPLW-
-IPAPLH-
-IPAPLE-
-IPAPLA-
ITPAPL--
-IPAPLG-

ILPAPL--
ICPAPL--
IYPAPL--
-IPAPLS-
-IPAPLQ-
IHPAPL--
IWPAPL--
-IPAPLY-
-IPAPLI-
IKPAPL--
-IPAPLV-
-IPAPLT-
IIPAPL--
IQPAPL--
-IPAPLM-
IMPAPL--
IEPAPL--
IAPAPL--
-KPAPLV-
-KPAPLT-
-KPAPLA-
KVPAPL--
-KPAPLQ-
KLPAPL--
KWPAPL--
KQPAPL--
-KPAPLY-
-KPAPLR-
-KPAPLN-
KKPAPL--
KTPAPL--
-KPAPLM-
KHPAPL--
KEPAPL--
KRPAPL--
KIPAPL--
KFPAPL--
KPPAPL--
-KPAPLK-
-KPAPLI-
KNPAPL--
KGPAPL--
KDPAPL--
KYPAPL--
-KPAPLG-
KAPAPL--
KMPAPL--
-LPAPLR-
-LPAPLL-
LRPAPL--

-LPAPLS-
LYPAPL--
LKPAPL--
-LPAPLN-
-LPAPLK-
-LPAPLA-
LTPAPL--
LMPAPL--
LGPAPL--
-LPAPLI-
-LPAPLT-
LSPAPL--
-LPAPLG-
LIPAPL--
-LPAPLY-
LEPAPL--
LWPAPL--
LQPAPL--
LPPAPL--
-LPAPLV-
-LPAPLF-
LLPAPL--
LFPAPL--
LVPAPL--
-LPAPLM-
LHPAPL--
LNPAPL--
LDPAPL--
-LPAPLH-
MRPAPL--
-MPAPLR-
-MPAPLK-
-MPAPLT-
MVPAPL--
MTPAPL--
MYPAPL--
MLPAPL--
MPPAPL--
MIPAPL--
MGPAPL--
-MPAPLS-
-MPAPLV-
-MPAPLQ-
-MPAPLI-
-MPAPLM-
-MPAPLL-
-MPAPLH-
-MPAPLF-
-MPAPLA-
MNPAPL--

MHPAPL--
MAPAPL--
-MPAPLY-
-MPAPLW-
-MPAPLG-
-MPAPLE-
-MPAPLC-
MKPAPL--
MFPAPL--
-NPAPLL-
-NPAPLS-
NVPAPL--
-NPAPLR-
NTPAPL--
NSPAPL--
NPPAPL--
-NPAPLF-
NLPAPL--
NGPAPL--
-NPAPLY-
-NPAPLQ-
NIPAPL--
-NPAPLK-
NWPAPL--
NNPAPL--
NHPAPL--
NAPAPL--
NYPAPL--
-NPAPLI-
-NPAPLA-
NKPAPL--
NDPAPL--
NRPAPL--
NQPAPL--
-NPAPLV-
-NPAPLM-
-NPAPLH-
-NPAPLG-
NMPAPL--
NFPAPL--
NCPAPL--
--PAPLVK
--PAPLRI
--PAPLRS
--PAPLVL
--PAPLLR
--PAPLMS
--PAPLYL
--PAPLAL
--PAPLVR

--PAPLHL
--PAPLTK
--PAPLSH
--PAPLMR
--PAPLRT
--PAPLLQ
--PAPLRA
--PAPLPL
--PAPLLK
--PAPLKL
--PAPLLV
--PAPLLS
--PAPLIR
PVPAPL--
--PAPLTT
--PAPLTL
--PAPLSR
--PAPLWN
--PAPLVY
--PAPLTG
--PAPLTA
--PAPLRK
--PAPLNR
--PAPLHK
--PAPLST
--PAPLSG
--PAPLRQ
--PAPLRG
--PAPLQS
--PAPLML
--PAPLFS
--PAPLEL
--PAPLYK
--PAPLTR
--PAPLSS
--PAPLQL
--PAPLIL
--PAPLFK
--PAPLYA
--PAPLWR
--PAPLWM
--PAPLRN
--PAPLPY
--PAPLNL
--PAPLMT
--PAPLHR
--PAPLFR
--PAPLAW
PKPAPL--
--PAPLYF

--PAPLMI
--PAPLLW
--PAPLLI
--PAPLLG
--PAPLKR
--PAPLHN
--PAPLGW
--PAPLGV
--PAPLYV
--PAPLYS
--PAPLVS
--PAPLVN
--PAPLVI
--PAPLRY
--PAPLQK
--PAPLNT
--PAPLMN
--PAPLGT
--PAPLGS
--PAPLGF
--PAPLFL
--PAPLET
PRPAPL--
--PAPLVT
--PAPLTM
--PAPLSY
--PAPLQA
--PAPLLT
--PAPLKG
--PAPLIV
--PAPLIT
--PAPLIK
--PAPLDL
PLPAPL--
--PAPLTS
--PAPLSM
--PAPLQG
--PAPLNN
--PAPLNF
--PAPLMA
--PAPLLF
--PAPLKQ
--PAPLKA
--PAPLIS
--PAPLII
--PAPLGK
--PAPLGI
--PAPLGG
--PAPLFG
PGPAPL--

--PAPLWW
--PAPLWV
--PAPLWQ
--PAPLVG
--PAPLTI
--PAPLSQ
--PAPLSK
--PAPLQM
--PAPLQH
--PAPLPS
--PAPLPI
--PAPLPH
--PAPLNY
--PAPLMV
--PAPLLD
--PAPLLA
--PAPLKM
--PAPLIY
--PAPLIF
--PAPLHH
--PAPLGH
--PAPLFN
--PAPLFM
--PAPLES
--PAPLEI
--PAPLDS
--PAPLAN
--PAPLAA
PSPAPL--
--PAPLYN
--PAPLYH
--PAPLYG
--PAPLYE
--PAPLWY
--PAPLVM
--PAPLSV
--PAPLRM
--PAPLRH
--PAPLQT
--PAPLNV
--PAPLLN
--PAPLKS
--PAPLIH
--PAPLFV
--PAPLEK
--PAPLAM
--PAPLAH
--PAPLYY
--PAPLYW
--PAPLYT

--PAPLYI
--PAPLWD
--PAPLTQ
--PAPLTN
--PAPLSN
--PAPLSE
--PAPLSD
--PAPLSA
--PAPLRW
--PAPLQR
--PAPLQQ
--PAPLQI
--PAPLMG
--PAPLKY
--PAPLKW
--PAPLKV
--PAPLKT
--PAPLKI
--PAPLIQ
--PAPLHY
--PAPLHS
--PAPLHM
--PAPLGA
--PAPLFA
--PAPLAR
PMPAPL--
--PAPLYD
--PAPLWK
--PAPLWG
--PAPLVW
--PAPLVQ
--PAPLVH
--PAPLVF
--PAPLVE
--PAPLSW
--PAPLRE
--PAPLPV
--PAPLLM
--PAPLKF
--PAPLKE
--PAPLIG
--PAPLHV
--PAPLHF
--PAPLHA
--PAPLGY
--PAPLGQ
--PAPLFY
--PAPLFT
--PAPLFQ
--PAPLFH

--PAPLAI
PAPAPL--
PEPAPL--
--PAPLYQ
--PAPLWA
--PAPLVV
--PAPLVD
--PAPLVA
--PAPLTW
--PAPLTV
--PAPLTF
--PAPLRD
--PAPLQD
--PAPLPA
--PAPLNH
--PAPLMQ
--PAPLMM
--PAPLMH
--PAPLME
--PAPLLH
--PAPLIW
--PAPLIP
--PAPLIM
--PAPLID
--PAPLIA
--PAPLHT
--PAPLHG
--PAPLEV
--PAPLDG
--PAPLAV
--PAPLAT
--PAPLAQ
PYPAPL--
-PPAPLA-
PHPAPL--
PFPAPL--
--PAPLYP
--PAPLYM
--PAPLWT
--PAPLWI
--PAPLWH
--PAPLWF
--PAPLWE
--PAPLTE
--PAPLTD
--PAPLSF
--PAPLRP
--PAPLQY
--PAPLQE
--PAPLPW

--PAPLPP
--PAPLPN
--PAPLNQ
--PAPLNK
--PAPLNI
--PAPLMY
--PAPLLE
--PAPLLC
--PAPLKH
--PAPLIN
--PAPLIE
--PAPLIC
--PAPLHQ
--PAPLHP
--PAPLFP
--PAPLFD
--PAPLEQ
--PAPLEN
--PAPLEF
--PAPLEE
--PAPLDY
--PAPLDV
--PAPLDM
--PAPLAP
--PAPLAK
--PAPLAG
--PAPLAF
--PAPLAE
--PAPLAD
-QPAPLR-
-QPAPLY-
QWPAPL--
-QPAPLT-
-QPAPLM-
QKPAPL--
QRPAPL--
QMPAPL--
QTPAPL--
-QPAPLW-
-QPAPLL-
-QPAPLS-
-QPAPLI-
-QPAPLE-
QPPAPL--
-QPAPLA-
-QPAPLK-
QIPAPL--
QGPAPL--
QYPAPL--
QSPAPL--

```
-QPAPLV-
-QPAPLN-
QNPAPL--
QFPAPL--
-QPAPLG-
-QPAPLF-
QHPAPL--
QDPAPL--
QAPAPL--
QQPAPL--
-QPAPLC-
QEPAPL--
-RPAPLT-
-RPAPLR-
RYPAPL--
-RPAPLS-
RIPAPL--
-RPAPLH-
RSPAPL--
RVPAPL--
RTPAPL--
-RPAPLK-
RFPAPL--
RQPAPL--
-RPAPLG-
RLPAPL--
-RPAPLV-
-RPAPLA-
-RPAPLW-
-RPAPLQ-
RHPAPL--
REPAPL--
RDPAPL--
RAPAPL--
RPPAPL--
-RPAPLE-
-RPAPLN-
RMPAPL--
-RPAPLI-
RNPAPL--
-RPAPLM-
-RPAPLD-
-SPAPLR-
-SPAPLS-
-SPAPLG-
-SPAPLW-
SRPAPL--
SSPAPL--
SAPAPL--
SYPAPL--
```

-SPAPLK-
SNPAPL--
STPAPL--
-SPAPLT-
-SPAPLI-
SLPAPL--
SKPAPL--
SVPAPL--
-SPAPLF-
-SPAPLN-
-SPAPLH-
-SPAPLV-
SIPAPL--
SPPAPL--
-SPAPLY-
SGPAPL--
-SPAPLA-
SFPAPL--
SQPAPL--
SEPAPL--
SWPAPL--
SMPAPL--
SDPAPL--
-SPAPLQ-
-SPAPLM-
SCPAPL--
TRPAPL--
-TPAPLL-
-TPAPLS-
-TPAPLR-
-TPAPLF-
TKPAPL--
TYPAPL--
-TPAPLY-
TQPAPL--
-TPAPLI-
TGPAPL--
TEPAPL--
-TPAPLT-
TVPAPL--
-TPAPLV-
-TPAPLH-
-TPAPLG-
-TPAPLA-
TSPAPL--
TTPAPL--
-TPAPLK-
-TPAPLN-
TAPAPL--
TPPAPL--

-TPAPLW-
-TPAPLQ-
TMPAPL--
THPAPL--
TWPAPL--
-TPAPLM-
-TPAPLE-
-TPAPLC-
TDPAPL--
-VPAPLR-
VRPAPL--
-VPAPLS-
-VPAPLT-
-VPAPLL-
-VPAPLK-
VTPAPL--
VLPAPL--
VKPAPL--
-VPAPLY-
-VPAPLA-
VGPAPL--
VWPAPL--
VPPAPL--
VAPAPL--
VQPAPL--
-VPAPLW-
-VPAPLQ-
-VPAPLI-
-VPAPLM-
VNPAPL--
VFPAPL--
VYPAPL--
VVPAPL--
-VPAPLV-
VIPAPL--
-VPAPLN-
-VPAPLH-
-VPAPLG-
-VPAPLF-
-VPAPLD-
VDPAPL--
VCPAPL--
WGPAPL--
WNPAPL--
WTPAPL--
-WPAPLT-
WYPAPL--
WRPAPL--
WIPAPL--
WSPAPL--

WVPAPL--
-WPAPLY-
-WPAPLR-
-WPAPLI-
-WPAPLG-
-WPAPLA-
WKPAPL--
WHPAPL--
-WPAPLV-
-WPAPLQ-
-WPAPLN-
WFPAPL--
WQPAPL--
-WPAPLS-
WPPAPL--
-WPAPLK-
-WPAPLE-
WMPAPL--
WWPAPL--
-WPAPLW-
-WPAPLD-
-YPAPLR-
YAPAPL--
YSPAPL--
YRPAPL--
YLPAPL--
-YPAPLK-
YFPAPL--
-YPAPLS-
YVPAPL--
-YPAPLT-
YHPAPL--
YYPAPL--
-YPAPLI-
YTPAPL--
-YPAPLG-
-YPAPLN-
-YPAPLE-
YNPAPL--
YPPAPL--
-YPAPLH-
-YPAPLA-
YEPAPL--
YMPAPL--
YIPAPL--
YQPAPL--
-YPAPLY-
-YPAPLF-
YKPAPL--
YCPAPL--

| | | | |
|---|---|---|---|
| | -YPAPLQ- | | |
| | -YPAPLM- | | |
| PRGY | 116 | 758 | 34.02 |
| | PTPRGY-- | | |
| | --PRGYVD | | |
| | --PRGYTP | | |
| | --PRGYGK | | |
| | VEPRGY-- | | |
| | AHPRGY-- | | |
| | -APRGYV- | | |
| | -APRGYS- | | |
| | -APRGYR- | | |
| | -APRGYY- | | |
| | AVPRGY-- | | |
| | ASPRGY-- | | |
| | -APRGYT- | | |
| | AAPRGY-- | | |
| | -APRGYG- | | |
| | -APRGYA- | | |
| | -APRGYM- | | |
| | -APRGYI- | | |
| | ATPRGY-- | | |
| | AMPRGY-- | | |
| | -APRGYW- | | |
| | AGPRGY-- | | |
| | -APRGYN- | | |
| | -APRGYQ- | | |
| | ANPRGY-- | | |
| | -APRGYK- | | |
| | -APRGYE- | | |
| | -APRGYD- | | |
| | AKPRGY-- | | |
| | ADPRGY-- | | |
| | ACPRGY-- | | |
| | -APRGYH- | | |
| | -APRGYF- | | |
| | APPRGY-- | | |
| | -CPRGYY- | | |
| | -CPRGYR- | | |
| | -CPRGYN- | | |
| | CEPRGY-- | | |
| | CCPRGY-- | | |
| | CYPRGY-- | | |
| | CTPRGY-- | | |
| | -CPRGYF- | | |
| | -CPRGYA- | | |
| | CMPRGY-- | | |
| | CHPRGY-- | | |
| | CWPRGY-- | | |
| | -CPRGYK- | | |

CPPRGY--
CNPRGY--
CDPRGY--
DRPRGY--
-DPRGYR-
-DPRGYT-
-DPRGYL-
DSPRGY--
DAPRGY--
DYPRGY--
-DPRGYI-
-DPRGYN-
-DPRGYF-
DLPRGY--
-DPRGYS-
-DPRGYQ-
DTPRGY--
-DPRGYW-
-DPRGYV-
DHPRGY--
DEPRGY--
-DPRGYK-
-DPRGYA-
DNPRGY--
DKPRGY--
-DPRGYM-
DMPRGY--
-DPRGYY-
-DPRGYP-
-DPRGYH-
-DPRGYG-
DDPRGY--
ESPRGY--
-EPRGYF-
EQPRGY--
-EPRGYL-
EVPRGY--
-EPRGYT-
ETPRGY--
-EPRGYI-
-EPRGYR-
EFPRGY--
EMPRGY--
EWPRGY--
-EPRGYV-
ELPRGY--
EAPRGY--
EYPRGY--
-EPRGYW-
-EPRGYH-

−EPRGYA−
EPPRGY−−
ENPRGY−−
−EPRGYY−
EKPRGY−−
−EPRGYS−
ERPRGY−−
−EPRGYN−
EGPRGY−−
EEPRGY−−
ECPRGY−−
FSPRGY−−
FEPRGY−−
−FPRGYS−
FKPRGY−−
FGPRGY−−
FYPRGY−−
−FPRGYN−
−FPRGYT−
−FPRGYF−
FRPRGY−−
−FPRGYW−
−FPRGYV−
FIPRGY−−
−FPRGYH−
FTPRGY−−
FAPRGY−−
FMPRGY−−
FVPRGY−−
−FPRGYE−
FHPRGY−−
−FPRGYQ−
−FPRGYC−
FPPRGY−−
FDPRGY−−
GLPRGY−−
GTPRGY−−
GWPRGY−−
GMPRGY−−
GVPRGY−−
−GPRGYS−
−GPRGYR−
−GPRGYG−
GQPRGY−−
−GPRGYM−
−GPRGYT−
−GPRGYN−
GIPRGY−−
GGPRGY−−
−GPRGYI−

−GPRGYQ−
GAPRGY−−
−GPRGYH−
GNPRGY−−
GKPRGY−−
GFPRGY−−
−GPRGYF−
GHPRGY−−
GEPRGY−−
−GPRGYY−
−GPRGYW−
−GPRGYE−
GRPRGY−−
−GPRGYA−
−GPRGYV−
GDPRGY−−
GCPRGY−−
−HPRGYL−
−HPRGYN−
−HPRGYI−
−HPRGYG−
HNPRGY−−
HAPRGY−−
HRPRGY−−
−HPRGYV−
HKPRGY−−
HDPRGY−−
HVPRGY−−
−HPRGYK−
−HPRGYA−
HGPRGY−−
HLPRGY−−
HHPRGY−−
HSPRGY−−
−HPRGYW−
−HPRGYT−
HEPRGY−−
−HPRGYM−
−HPRGYE−
HPPRGY−−
HTPRGY−−
−HPRGYS−
−HPRGYH−
HQPRGY−−
HYPRGY−−
−HPRGYY−
−HPRGYF−
−IPRGYT−
ISPRGY−−
−IPRGYR−

−IPRGYL−
−IPRGYV−
−IPRGYS−
−IPRGYK−
ILPRGY−−
IVPRGY−−
ITPRGY−−
IIPRGY−−
IYPRGY−−
IMPRGY−−
IAPRGY−−
−IPRGYM−
−IPRGYG−
IGPRGY−−
IFPRGY−−
−IPRGYY−
INPRGY−−
IEPRGY−−
−IPRGYN−
IDPRGY−−
IWPRGY−−
−IPRGYQ−
IHPRGY−−
ICPRGY−−
−IPRGYW−
IQPRGY−−
−IPRGYE−
IPPRGY−−
IKPRGY−−
−KPRGYL−
−KPRGYS−
KTPRGY−−
KFPRGY−−
KWPRGY−−
KVPRGY−−
−KPRGYT−
KLPRGY−−
KGPRGY−−
KQPRGY−−
−KPRGYA−
KPPRGY−−
−KPRGYY−
−KPRGYG−
−KPRGYF−
KYPRGY−−
−KPRGYH−
KNPRGY−−
−KPRGYV−
−KPRGYW−
KMPRGY−−

KKPRGY--
KAPRGY--
KIPRGY--
KHPRGY--
KCPRGY--
-KPRGYI-
-KPRGYE-
KDPRGY--
-LPRGYL-
LAPRGY--
LSPRGY--
LKPRGY--
LGPRGY--
-LPRGYI-
LVPRGY--
-LPRGYA-
LPPRGY--
-LPRGYQ-
-LPRGYY-
-LPRGYF-
LYPRGY--
-LPRGYN-
LNPRGY--
LEPRGY--
-LPRGYW-
LHPRGY--
LDPRGY--
-LPRGYM-
-LPRGYE-
MSPRGY--
MGPRGY--
-MPRGYS-
-MPRGYT-
-MPRGYH-
-MPRGYL-
MNPRGY--
MKPRGY--
-MPRGYV-
-MPRGYN-
MYPRGY--
-MPRGYM-
-MPRGYW-
-MPRGYI-
MPPRGY--
MMPRGY--
MHPRGY--
-MPRGYQ-
-MPRGYK-
-MPRGYF-
MIPRGY--

-MPRGYY-
-MPRGYG-
-MPRGYE-
MEPRGY--
MDPRGY--
MAPRGY--
NSPRGY--
NTPRGY--
NRPRGY--
NVPRGY--
-NPRGYT-
-NPRGYS-
-NPRGYL-
-NPRGYY-
-NPRGYV-
-NPRGYA-
NKPRGY--
NIPRGY--
-NPRGYN-
NAPRGY--
NNPRGY--
-NPRGYM-
NGPRGY--
NMPRGY--
NYPRGY--
-NPRGYW-
-NPRGYK-
-NPRGYI-
NQPRGY--
NPPRGY--
NDPRGY--
NCPRGY--
-NPRGYQ-
-NPRGYG-
--PRGYSS
--PRGYGS
--PRGYSL
--PRGYTS
--PRGYIL
--PRGYVT
--PRGYFN
--PRGYVS
--PRGYRI
--PRGYLF
--PRGYKG
--PRGYYW
--PRGYSI
--PRGYSV
--PRGYAY
--PRGYVK

--PRGYSN
--PRGYMG
--PRGYFT
--PRGYYS
--PRGYYG
--PRGYST
--PRGYGT
--PRGYVI
--PRGYSQ
--PRGYMN
--PRGYWY
--PRGYRN
--PRGYMF
--PRGYES
--PRGYYT
--PRGYWS
--PRGYWK
--PRGYIA
--PRGYVV
--PRGYVH
--PRGYIT
--PRGYVA
--PRGYTL
--PRGYLQ
--PRGYLK
--PRGYHS
--PRGYFV
--PRGYWN
--PRGYNR
--PRGYNA
--PRGYMA
--PRGYHG
--PRGYGG
--PRGYFR
--PRGYTN
--PRGYTI
--PRGYSW
PGPRGY--
--PRGYTQ
--PRGYSY
--PRGYSM
--PRGYSK
--PRGYFS
--PRGYYN
--PRGYYM
--PRGYYA
--PRGYWH
--PRGYTY
--PRGYTA
--PRGYLW

--PRGYKW
--PRGYAS
--PRGYYR
--PRGYWT
--PRGYWL
--PRGYTM
--PRGYRH
--PRGYRD
--PRGYQW
--PRGYQV
--PRGYNL
--PRGYHM
--PRGYEG
--PRGYWV
--PRGYMY
--PRGYLE
--PRGYKM
--PRGYHF
--PRGYGI
--PRGYWD
--PRGYSF
--PRGYRQ
--PRGYQI
--PRGYNV
--PRGYNQ
--PRGYNH
--PRGYMT
--PRGYMR
--PRGYMI
--PRGYHN
--PRGYFK
--PRGYAG
--PRGYAF
PRPRGY--
--PRGYYF
--PRGYVQ
--PRGYTD
--PRGYSE
--PRGYNF
--PRGYLD
--PRGYKI
--PRGYKH
--PRGYII
--PRGYIF
--PRGYHI
--PRGYFY
--PRGYAW
--PRGYAK
--PRGYAA
PSPRGY--

--PRGYWW
--PRGYWM
--PRGYVF
--PRGYRV
--PRGYQT
--PRGYQK
--PRGYMH
--PRGYIK
--PRGYHR
--PRGYFA
--PRGYEQ
--PRGYAT
--PRGYWG
--PRGYVE
--PRGYTH
--PRGYTF
--PRGYTE
--PRGYRF
--PRGYQG
--PRGYNW
--PRGYNN
--PRGYIW
--PRGYFH
--PRGYAN
--PRGYAH
PQPRGY--
PEPRGY--
PDPRGY--
PVPRGY--
--PRGYQF
--PRGYNE
--PRGYLM
--PRGYGA
--PRGYEV
--PRGYAM
--PRGYAI
PHPRGY--
--PRGYYV
--PRGYWF
--PRGYSD
--PRGYRE
--PRGYQM
--PRGYNT
--PRGYND
--PRGYMV
--PRGYIM
--PRGYHW
--PRGYHT
--PRGYGV
--PRGYFG

--PRGYFD
--PRGYEW
--PRGYET
--PRGYDS
--PRGYDA
--PRGYAE
PNPRGY--
--PRGYSH
--PRGYRW
--PRGYQL
--PRGYQD
--PRGYMD
--PRGYKF
--PRGYKE
--PRGYIV
--PRGYIQ
--PRGYIH
--PRGYHE
--PRGYEA
PKPRGY--
--PRGYWI
--PRGYTW
--PRGYQS
--PRGYQN
--PRGYMK
--PRGYKY
--PRGYIE
--PRGYHH
--PRGYHA
--PRGYGW
--PRGYGM
--PRGYFF
--PRGYFE
--PRGYEY
--PRGYEN
PCPRGY--
--PRGYYE
--PRGYRC
--PRGYQH
--PRGYNM
--PRGYME
--PRGYKD
--PRGYIG
--PRGYHQ
--PRGYGF
--PRGYEH
--PRGYEF
--PRGYYY
--PRGYYQ
--PRGYYK

--PRGYYD
--PRGYYC
--PRGYWE
--PRGYQY
--PRGYQQ
--PRGYQE
--PRGYQA
--PRGYMQ
--PRGYKN
--PRGYGY
--PRGYFI
--PRGYDL
--PRGYDK
--PRGYCM
--PRGYCF
--PRGYAV
--PRGYAQ
-PPRGYS-
-PPRGYR-
-QPRGYA-
QSPRGY--
-QPRGYL-
QEPRGY--
-QPRGYV-
-QPRGYS-
-QPRGYT-
QVPRGY--
-QPRGYI-
QRPRGY--
QLPRGY--
QFPRGY--
QTPRGY--
QPPRGY--
QMPRGY--
QGPRGY--
-QPRGYN-
-QPRGYK-
-QPRGYW-
-QPRGYG-
QDPRGY--
-QPRGYQ-
-QPRGYH-
-QPRGYF-
-QPRGYM-
QNPRGY--
-QPRGYE-
QKPRGY--
QHPRGY--
-QPRGYC-
QIPRGY--

QCPRGY--
QAPRGY--
-RPRGYL-
-RPRGYI-
-RPRGYV-
RPPRGY--
-RPRGYN-
RVPRGY--
RTPRGY--
-RPRGYM-
RAPRGY--
RHPRGY--
REPRGY--
-RPRGYW-
-RPRGYS-
-RPRGYF-
RGPRGY--
RQPRGY--
RNPRGY--
RDPRGY--
-RPRGYY-
RKPRGY--
RCPRGY--
-RPRGYE-
SSPRGY--
SLPRGY--
STPRGY--
-SPRGYR-
SGPRGY--
-SPRGYG-
SYPRGY--
-SPRGYY-
-SPRGYV-
-SPRGYW-
SIPRGY--
-SPRGYM-
-SPRGYK-
-SPRGYN-
-SPRGYE-
-SPRGYA-
SQPRGY--
-SPRGYF-
-SPRGYH-
-SPRGYQ-
SDPRGY--
SFPRGY--
SCPRGY--
SEPRGY--
SHPRGY--
SAPRGY--

SPPRGY--
-TPRGYL-
TSPRGY--
TQPRGY--
THPRGY--
-TPRGYT-
-TPRGYS-
-TPRGYY-
-TPRGYV-
-TPRGYK-
TNPRGY--
TPPRGY--
-TPRGYG-
-TPRGYM-
TWPRGY--
TTPRGY--
-TPRGYN-
TEPRGY--
TVPRGY--
-TPRGYA-
TYPRGY--
-TPRGYW-
TKPRGY--
TDPRGY--
-TPRGYQ-
TFPRGY--
TAPRGY--
-TPRGYI-
-TPRGYE-
TCPRGY--
-TPRGYF-
TMPRGY--
TIPRGY--
VGPRGY--
VSPRGY--
VKPRGY--
VFPRGY--
VRPRGY--
VQPRGY--
VTPRGY--
VNPRGY--
-VPRGYY-
VVPRGY--
-VPRGYN-
-VPRGYH-
VCPRGY--
-VPRGYQ-
-VPRGYW-
-VPRGYT-
VYPRGY--

-VPRGYR-
VAPRGY--
-VPRGYS-
-VPRGYG-
-VPRGYI-
VIPRGY--
VHPRGY--
VDPRGY--
-VPRGYF-
-VPRGYE-
VPPRGY--
WSPRGY--
WTPRGY--
-WPRGYM-
-WPRGYV-
WMPRGY--
WKPRGY--
WGPRGY--
WRPRGY--
WNPRGY--
WHPRGY--
-WPRGYF-
WYPRGY--
WDPRGY--
-WPRGYQ-
WAPRGY--
WPPRGY--
-WPRGYN-
-WPRGYG-
WFPRGY--
WWPRGY--
-WPRGYW-
WEPRGY--
-WPRGYY-
-WPRGYE-
YSPRGY--
YEPRGY--
-YPRGYM-
YMPRGY--
-YPRGYW-
-YPRGYG-
YGPRGY--
-YPRGYY-
-YPRGYS-
YDPRGY--
-YPRGYN-
-YPRGYV-
YLPRGY--
YVPRGY--
YPPRGY--

```
            YNPRGY--
            YYPRGY--
            YKPRGY--
            -YPRGYT-
            YAPRGY--
            -YPRGYQ-
            -YPRGYE-
            -YPRGYH-
            YQPRGY--
            -YPRGYF-
            -YPRGYA-
PSNW          84        778     33.98
            -LPSNWG-
            --PSNWPS
            --PSNWHL
            --PSNWGR
            --PSNWEL
            -TPSNWI-
            -APSNWT-
            -APSNWK-
            -APSNWI-
            -APSNWV-
            -APSNWR-
            ARPSNW--
            -APSNWA-
            ASPSNW--
            -APSNWG-
            ALPSNW--
            -APSNWN-
            AWPSNW--
            -APSNWY-
            -APSNWF-
            ANPSNW--
            AMPSNW--
            AQPSNW--
            AEPSNW--
            AYPSNW--
            ATPSNW--
            -APSNWQ-
            AHPSNW--
            -APSNWW-
            AKPSNW--
            -APSNWL-
            -APSNWH-
            -APSNWE-
            AIPSNW--
            ADPSNW--
            CSPSNW--
            -CPSNWL-
            CWPSNW--
```

-CPSNWF-
CLPSNW--
CHPSNW--
-CPSNWW-
-CPSNWT-
-CPSNWA-
CNPSNW--
CGPSNW--
CFPSNW--
-DPSNWK-
-DPSNWL-
DWPSNW--
-DPSNWW-
DMPSNW--
DLPSNW--
DVPSNW--
DQPSNW--
-DPSNWY-
-DPSNWT-
-DPSNWI-
DKPSNW--
DSPSNW--
-DPSNWF-
DRPSNW--
-DPSNWV-
-DPSNWS-
-DPSNWR-
-DPSNWQ-
-DPSNWM-
-DPSNWG-
DGPSNW--
DEPSNW--
DDPSNW--
-EPSNWR-
ETPSNW--
-EPSNWN-
EEPSNW--
EWPSNW--
EQPSNW--
-EPSNWW-
-EPSNWL-
EIPSNW--
EAPSNW--
EVPSNW--
ESPSNW--
-EPSNWV-
-EPSNWS-
ELPSNW--
EKPSNW--
-EPSNWM-

-EPSNWK-
-EPSNWI-
-EPSNWF-
-EPSNWA-
EFPSNW--
EYPSNW--
ERPSNW--
-EPSNWY-
-EPSNWT-
-EPSNWH-
-EPSNWG-
EHPSNW--
-FPSNWS-
FGPSNW--
FLPSNW--
-FPSNWV-
FSPSNW--
-FPSNWQ-
FVPSNW--
FAPSNW--
FTPSNW--
-FPSNWT-
-FPSNWF-
-FPSNWY-
-FPSNWR-
-FPSNWM-
-FPSNWI-
FPPSNW--
FEPSNW--
FYPSNW--
FWPSNW--
FQPSNW--
-FPSNWW-
-FPSNWK-
-FPSNWH-
-FPSNWA-
FMPSNW--
FKPSNW--
FHPSNW--
GGPSNW--
GFPSNW--
-GPSNWR-
-GPSNWL-
GKPSNW--
GLPSNW--
GSPSNW--
GTPSNW--
-GPSNWW-
-GPSNWT-
GVPSNW--

GQPSNW--
-GPSNWS-
-GPSNWI-
-GPSNWV-
GIPSNW--
-GPSNWA-
GYPSNW--
GWPSNW--
-GPSNWG-
-GPSNWF-
-GPSNWE-
GHPSNW--
GEPSNW--
GCPSNW--
-GPSNWY-
-GPSNWN-
-GPSNWM-
-GPSNWD-
GNPSNW--
GMPSNW--
GAPSNW--
-HPSNWL-
HVPSNW--
-HPSNWV-
-HPSNWA-
HIPSNW--
-HPSNWG-
HGPSNW--
HWPSNW--
-HPSNWR-
HLPSNW--
HSPSNW--
HRPSNW--
-HPSNWN-
-HPSNWM-
HKPSNW--
HDPSNW--
HCPSNW--
-HPSNWY-
-HPSNWW-
-HPSNWT-
-HPSNWS-
-HPSNWI-
-HPSNWH-
HPPSNW--
HMPSNW--
HAPSNW--
-IPSNWL-
IRPSNW--
-IPSNWR-

```
-IPSNWM-
ILPSNW--
IIPSNW--
-IPSNWD-
-IPSNWK-
IGPSNW--
ISPSNW--
-IPSNWW-
-IPSNWV-
IVPSNW--
IPPSNW--
IAPSNW--
ITPSNW--
-IPSNWG-
INPSNW--
IKPSNW--
IFPSNW--
-IPSNWT-
-IPSNWS-
-IPSNWE-
IYPSNW--
-IPSNWN-
-IPSNWI-
-IPSNWH-
-IPSNWA-
IEPSNW--
ICPSNW--
-KPSNWV-
-KPSNWL-
-KPSNWG-
KTPSNW--
-KPSNWW-
-KPSNWR-
-KPSNWM-
-KPSNWS-
-KPSNWI-
KYPSNW--
KRPSNW--
KIPSNW--
KVPSNW--
-KPSNWN-
-KPSNWA-
KGPSNW--
KQPSNW--
-KPSNWQ-
KPPSNW--
KNPSNW--
KCPSNW--
-KPSNWT-
-KPSNWF-
```

```
KMPSNW--
KLPSNW--
KKPSNW--
KHPSNW--
KFPSNW--
KAPSNW--
KWPSNW--
-KPSNWY-
KEPSNW--
KDPSNW--
LRPSNW--
-LPSNWF-
LGPSNW--
-LPSNWR-
LTPSNW--
-LPSNWL-
-LPSNWM-
LWPSNW--
-LPSNWS-
LLPSNW--
-LPSNWT-
LAPSNW--
-LPSNWY-
-LPSNWA-
-LPSNWI-
LHPSNW--
LVPSNW--
LPPSNW--
LKPSNW--
-LPSNWQ-
-LPSNWE-
LIPSNW--
LQPSNW--
-LPSNWK-
LNPSNW--
LFPSNW--
LYPSNW--
-MPSNWR-
MFPSNW--
MHPSNW--
MGPSNW--
MRPSNW--
-MPSNWI-
MNPSNW--
-MPSNWS-
MWPSNW--
MSPSNW--
-MPSNWY-
-MPSNWW-
-MPSNWT-
```

```
-MPSNWN-
-MPSNWL-
MYPSNW--
MVPSNW--
-MPSNWK-
-MPSNWG-
-MPSNWE-
MPPSNW--
MKPSNW--
MAPSNW--
MTPSNW--
-MPSNWM-
-MPSNWA-
MMPSNW--
MLPSNW--
MEPSNW--
-NPSNWR-
-NPSNWT-
-NPSNWL-
NLPSNW--
-NPSNWS-
-NPSNWH-
-NPSNWW-
NTPSNW--
NYPSNW--
NWPSNW--
NPPSNW--
NEPSNW--
NAPSNW--
NGPSNW--
NSPSNW--
NRPSNW--
-NPSNWF-
NIPSNW--
-NPSNWV-
-NPSNWQ-
-NPSNWK-
NHPSNW--
NFPSNW--
NDPSNW--
NCPSNW--
NQPSNW--
-NPSNWM-
-NPSNWG-
NNPSNW--
NMPSNW--
--PSNWVT
--PSNWTS
--PSNWRS
--PSNWFS
```

--PSNWYH
--PSNWVR
--PSNWVN
--PSNWTL
--PSNWSL
--PSNWRD
--PSNWLN
--PSNWVV
--PSNWLR
--PSNWLA
--PSNWKL
--PSNWTT
--PSNWLI
--PSNWYL
--PSNWRI
--PSNWIL
--PSNWIH
--PSNWSV
--PSNWRQ
--PSNWLG
--PSNWKS
--PSNWIW
--PSNWAL
--PSNWWN
--PSNWVI
--PSNWVG
--PSNWTR
--PSNWTI
--PSNWST
--PSNWRN
--PSNWML
--PSNWLT
--PSNWAI
--PSNWWR
--PSNWVQ
--PSNWRK
--PSNWMK
--PSNWLS
--PSNWLL
--PSNWIV
--PSNWAR
--PSNWYY
--PSNWYQ
--PSNWVH
--PSNWSS
--PSNWSN
--PSNWSH
--PSNWRH
--PSNWRA
--PSNWNA

```
--PSNWMS
--PSNWMA
--PSNWKG
--PSNWHR
--PSNWES
--PSNWAW
--PSNWAF
--PSNWWG
--PSNWWA
--PSNWTG
--PSNWSI
--PSNWSF
--PSNWRW
--PSNWQY
--PSNWLV
--PSNWLE
--PSNWAM
--PSNWYS
--PSNWYN
--PSNWWV
--PSNWWT
--PSNWVK
--PSNWVF
--PSNWTV
--PSNWTQ
--PSNWTA
--PSNWRL
--PSNWRG
--PSNWLH
--PSNWIS
--PSNWIR
--PSNWIQ
--PSNWIM
--PSNWIG
--PSNWIA
--PSNWGT
--PSNWGS
--PSNWGA
--PSNWFI
--PSNWER
--PSNWEN
--PSNWEM
PEPSNW--
PVPSNW--
--PSNWWY
--PSNWWQ
--PSNWSY
--PSNWSK
--PSNWRY
--PSNWQH
```

--PSNWMN
--PSNWMH
--PSNWMD
--PSNWLK
--PSNWKY
--PSNWHG
--PSNWAS
PNPSNW--
PLPSNW--
PTPSNW--
--PSNWVM
--PSNWVA
--PSNWTM
--PSNWTK
--PSNWRV
--PSNWRM
--PSNWRF
--PSNWKW
--PSNWKV
--PSNWIT
--PSNWIF
--PSNWID
--PSNWHK
--PSNWGI
--PSNWFR
--PSNWFK
--PSNWAV
--PSNWAH
--PSNWAG
PMPSNW--
--PSNWYD
--PSNWYA
--PSNWVE
--PSNWTY
--PSNWTN
--PSNWTF
--PSNWSR
--PSNWRT
--PSNWRE
--PSNWQM
--PSNWQG
--PSNWNV
--PSNWNG
--PSNWMW
--PSNWLQ
--PSNWLM
--PSNWIK
--PSNWHD
--PSNWGV
--PSNWFL

--PSNWET
--PSNWAT
--PSNWAK
PAPSNW--
PSPSNW--
--PSNWYK
--PSNWYE
--PSNWWL
--PSNWWK
--PSNWWF
--PSNWTW
--PSNWTD
--PSNWSW
--PSNWSQ
--PSNWQV
--PSNWQS
--PSNWQR
--PSNWQK
--PSNWQA
--PSNWNT
--PSNWNS
--PSNWMG
--PSNWKT
--PSNWKA
--PSNWIY
--PSNWIN
--PSNWGK
--PSNWFT
--PSNWFH
--PSNWFE
--PSNWEV
--PSNWAA
PWPSNW--
--PSNWYV
--PSNWYM
--PSNWYI
--PSNWWS
--PSNWWI
--PSNWVY
--PSNWVD
--PSNWTH
--PSNWTC
--PSNWSD
--PSNWSA
--PSNWQL
--PSNWQI
--PSNWQE
--PSNWQD
--PSNWNY
--PSNWNI

--PSNWNF
--PSNWMV
--PSNWMP
--PSNWMF
--PSNWLD
--PSNWKQ
--PSNWKM
--PSNWKK
--PSNWKF
--PSNWII
--PSNWIE
--PSNWHW
--PSNWHV
--PSNWHM
--PSNWHI
--PSNWGW
--PSNWGL
--PSNWGF
--PSNWFW
--PSNWFD
--PSNWEY
--PSNWEW
--PSNWEG
--PSNWEF
--PSNWAY
--PSNWAE
PKPSNW--
PIPSNW--
PGPSNW--
-QPSNWL-
QGPSNW--
-QPSNWI-
QWPSNW--
QQPSNW--
-QPSNWV-
QVPSNW--
QRPSNW--
-QPSNWT-
-QPSNWS-
QPPSNW--
QAPSNW--
QKPSNW--
-QPSNWY-
-QPSNWR-
-QPSNWN-
-QPSNWM-
-QPSNWK-
-QPSNWG-
-QPSNWE-
QNPSNW--

QMPSNW--
QIPSNW--
QDPSNW--
-RPSNWV-
-RPSNWS-
RSPSNW--
RHPSNW--
-RPSNWT-
-RPSNWL-
RGPSNW--
-RPSNWG-
-RPSNWN-
RMPSNW--
RAPSNW--
-RPSNWI-
RLPSNW--
-RPSNWM-
RWPSNW--
RTPSNW--
RVPSNW--
-RPSNWY-
-RPSNWR-
RNPSNW--
RIPSNW--
RDPSNW--
-RPSNWK-
RPPSNW--
RYPSNW--
RRPSNW--
RQPSNW--
-RPSNWQ-
RKPSNW--
RFPSNW--
-RPSNWW-
-RPSNWH-
-RPSNWE-
REPSNW--
RCPSNW--
-SPSNWV-
-SPSNWL-
-SPSNWF-
SGPSNW--
-SPSNWN-
SSPSNW--
SNPSNW--
SMPSNW--
SKPSNW--
SLPSNW--
SVPSNW--
SRPSNW--

```
-SPSNWA-
-SPSNWY-
-SPSNWW-
-SPSNWS-
SHPSNW--
SYPSNW--
-SPSNWG-
STPSNW--
SFPSNW--
SIPSNW--
-SPSNWM-
-SPSNWI-
-SPSNWE-
SPPSNW--
SWPSNW--
-SPSNWK-
-SPSNWH-
SDPSNW--
SAPSNW--
SEPSNW--
SCPSNW--
-TPSNWL-
TGPSNW--
TSPSNW--
-TPSNWR-
TIPSNW--
TTPSNW--
TAPSNW--
TYPSNW--
-TPSNWA-
-TPSNWY-
-TPSNWV-
-TPSNWN-
-TPSNWH-
-TPSNWT-
TMPSNW--
-TPSNWS-
-TPSNWM-
-TPSNWK-
-TPSNWG-
TLPSNW--
TWPSNW--
TVPSNW--
-TPSNWW-
-TPSNWF-
THPSNW--
-TPSNWE-
TNPSNW--
TFPSNW--
TQPSNW--
```

```
-TPSNWC-
TDPSNW--
-VPSNWR-
-VPSNWI-
VSPSNW--
VVPSNW--
VLPSNW--
VRPSNW--
VFPSNW--
-VPSNWK-
-VPSNWA-
VHPSNW--
VQPSNW--
-VPSNWY-
-VPSNWG-
-VPSNWF-
-VPSNWE-
VPPSNW--
-VPSNWT-
VKPSNW--
VIPSNW--
VYPSNW--
-VPSNWN-
-VPSNWM-
VMPSNW--
VGPSNW--
VEPSNW--
VWPSNW--
VTPSNW--
-VPSNWH-
VAPSNW--
WRPSNW--
-WPSNWR-
-WPSNWM-
WLPSNW--
WVPSNW--
WSPSNW--
-WPSNWV-
-WPSNWF-
WEPSNW--
WYPSNW--
-WPSNWH-
-WPSNWA-
WHPSNW--
-WPSNWK-
WGPSNW--
WTPSNW--
-WPSNWY-
-WPSNWL-
WPPSNW--
```

| | | | |
|---|---|---|---|
| | WIPSNW-- | | |
| | WFPSNW-- | | |
| | WWPSNW-- | | |
| | WQPSNW-- | | |
| | -WPSNWW- | | |
| | -WPSNWT- | | |
| | -WPSNWS- | | |
| | -WPSNWI- | | |
| | -WPSNWG- | | |
| | WDPSNW-- | | |
| | -YPSNWR- | | |
| | -YPSNWS- | | |
| | -YPSNWV- | | |
| | YLPSNW-- | | |
| | YMPSNW-- | | |
| | -YPSNWN- | | |
| | -YPSNWY- | | |
| | -YPSNWM- | | |
| | YKPSNW-- | | |
| | -YPSNWQ- | | |
| | YHPSNW-- | | |
| | -YPSNWL- | | |
| | -YPSNWK- | | |
| | YVPSNW-- | | |
| | -YPSNWT- | | |
| | YDPSNW-- | | |
| | YAPSNW-- | | |
| | YSPSNW-- | | |
| | -YPSNWH- | | |
| | -YPSNWG- | | |
| | YPPSNW-- | | |
| | YWPSNW-- | | |
| | YTPSNW-- | | |
| | -YPSNWE- | | |
| | YIPSNW-- | | |
| | YQPSNW-- | | |
| | -YPSNWA- | | |
| | YNPSNW-- | | |
| | YFPSNW-- | | |
| | YEPSNW-- | | |
| IGWL | 242 | 692 | 33.95 |
| | TMIGWL-- | | |
| | AGIGWL-- | | |
| | ASIGWL-- | | |
| | -AIGWLR- | | |
| | -AIGWLG- | | |
| | -AIGWLD- | | |
| | ANIGWL-- | | |
| | AHIGWL-- | | |
| | AVIGWL-- | | |

AKIGWL--
-AIGWLN-
-AIGWLH-
ADIGWL--
APIGWL--
AIIGWL--
-AIGWLK-
-AIGWLA-
AMIGWL--
-AIGWLT-
-AIGWLS-
-AIGWLQ-
-AIGWLM-
-AIGWLL-
AWIGWL--
AQIGWL--
-AIGWLI-
-AIGWLE-
ACIGWL--
AYIGWL--
-AIGWLW-
-AIGWLF-
AFIGWL--
AEIGWL--
CGIGWL--
-CIGWLN-
-CIGWLI-
CSIGWL--
CRIGWL--
CKIGWL--
-CIGWLT-
-CIGWLL-
-CIGWLA-
-DIGWLL-
-DIGWLR-
DYIGWL--
-DIGWLT-
DRIGWL--
DTIGWL--
DMIGWL--
-DIGWLI-
-DIGWLH-
DVIGWL--
-DIGWLS-
DNIGWL--
DLIGWL--
-DIGWLQ-
-DIGWLA-
DGIGWL--
DSIGWL--

DQIGWL--
-DIGWLY-
-DIGWLK-
-DIGWLG-
DHIGWL--
DFIGWL--
DEIGWL--
DWIGWL--
DPIGWL--
-DIGWLW-
-DIGWLV-
-DIGWLN-
DDIGWL--
-EIGWLL-
-EIGWLT-
EPIGWL--
EKIGWL--
-EIGWLR-
-EIGWLA-
-EIGWLS-
-EIGWLI-
EVIGWL--
ERIGWL--
EGIGWL--
EYIGWL--
-EIGWLG-
EDIGWL--
EWIGWL--
ESIGWL--
EMIGWL--
-EIGWLN-
EAIGWL--
EIIGWL--
-EIGWLV-
-EIGWLQ-
-EIGWLK-
ETIGWL--
EQIGWL--
-EIGWLH-
-EIGWLE-
ECIGWL--
FHIGWL--
FEIGWL--
FNIGWL--
-FIGWLQ-
-FIGWLE-
FTIGWL--
-FIGWLY-
-FIGWLR-
-FIGWLN-

-FIGWLL-
-FIGWLA-
FKIGWL--
-FIGWLT-
-FIGWLS-
FDIGWL--
-FIGWLI-
-FIGWLH-
-FIGWLD-
FMIGWL--
-FIGWLG-
FGIGWL--
FVIGWL--
FLIGWL--
-GIGWLS-
GRIGWL--
GSIGWL--
-GIGWLR-
GQIGWL--
GLIGWL--
-GIGWLN-
GGIGWL--
-GIGWLT-
-GIGWLQ-
-GIGWLK-
-GIGWLH-
-GIGWLV-
GFIGWL--
-GIGWLL-
-GIGWLG-
-GIGWLE-
GYIGWL--
GPIGWL--
-GIGWLI-
-GIGWLD-
GWIGWL--
GNIGWL--
-GIGWLM-
GEIGWL--
GDIGWL--
-GIGWLA-
GHIGWL--
GAIGWL--
GIIGWL--
HSIGWL--
-HIGWLR-
-HIGWLN-
HTIGWL--
-HIGWLL-
HHIGWL--

HEIGWL--
HAIGWL--
-HIGWLS-
-HIGWLI-
HNIGWL--
HRIGWL--
-HIGWLA-
HVIGWL--
-HIGWLT-
HMIGWL--
-HIGWLH-
-HIGWLD-
HFIGWL--
HYIGWL--
HIIGWL--
-HIGWLQ-
-HIGWLE-
HQIGWL--
HPIGWL--
HKIGWL--
-HIGWLV-
-HIGWLK-
HGIGWL--
--IGWLST
--IGWLRS
--IGWLGS
--IGWLTS
IRIGWL--
--IGWLSS
--IGWLSL
--IGWLKT
--IGWLKG
--IGWLRG
--IGWLGA
-IIGWLR-
--IGWLTK
--IGWLTT
--IGWLKS
--IGWLGR
--IGWLSG
--IGWLQA
--IGWLLN
--IGWLGT
--IGWLRT
--IGWLNT
--IGWLLR
--IGWLER
IGIGWL--
--IGWLRQ
--IGWLVA

--IGWLRV
--IGWLRD
--IGWLNY
--IGWLEG
-IIGWLH-
--IGWLSN
--IGWLRL
--IGWLRF
--IGWLNR
--IGWLLK
--IGWLHG
--IGWLGQ
--IGWLAR
--IGWLAE
--IGWLVH
--IGWLTV
--IGWLKA
--IGWLID
--IGWLHM
IEIGWL--
ISIGWL--
IHIGWL--
--IGWLYG
--IGWLTR
--IGWLTN
--IGWLSK
--IGWLSE
--IGWLRM
--IGWLQK
--IGWLNS
--IGWLNM
--IGWLLG
--IGWLIG
--IGWLHS
--IGWLGL
--IGWLGK
--IGWLGG
--IGWLGF
--IGWLFS
IAIGWL--
IPIGWL--
IKIGWL--
--IGWLVK
--IGWLSV
--IGWLSH
--IGWLRI
--IGWLQS
--IGWLQN
--IGWLKV
--IGWLKL

```
--IGWLHR
--IGWLHN
--IGWLHI
--IGWLHA
--IGWLGI
--IGWLFH
--IGWLAA
-IIGWLQ-
-IIGWLE-
--IGWLTQ
--IGWLSW
--IGWLSQ
--IGWLSA
--IGWLLD
--IGWLGV
--IGWLGH
IDIGWL--
--IGWLWG
--IGWLVS
--IGWLTG
--IGWLTD
--IGWLQR
--IGWLQH
--IGWLQG
--IGWLNA
--IGWLMG
--IGWLIH
--IGWLGN
--IGWLEW
--IGWLDT
--IGWLDA
--IGWLAS
--IGWLAN
--IGWLAK
--IGWLAD
ITIGWL--
--IGWLYN
--IGWLTH
--IGWLTF
--IGWLSM
--IGWLSI
--IGWLRW
--IGWLRN
--IGWLRE
--IGWLNV
--IGWLNK
--IGWLNG
--IGWLMR
--IGWLMI
--IGWLME
```

--IGWLKQ
--IGWLIS
--IGWLIQ
--IGWLIA
--IGWLGY
--IGWLFY
--IGWLFE
ILIGWL--
IIIGWL--
-IIGWLG-
--IGWLWQ
--IGWLTI
--IGWLSY
--IGWLRY
--IGWLQV
--IGWLNH
--IGWLMV
--IGWLMS
--IGWLMK
--IGWLLE
--IGWLKK
--IGWLIT
--IGWLGM
--IGWLGD
--IGWLFG
--IGWLEN
--IGWLEA
--IGWLAW
IQIGWL--
-IIGWLT-
-IIGWLS-
-IIGWLL-
-IIGWLK-
--IGWLYD
--IGWLWR
--IGWLWN
--IGWLWK
--IGWLWE
--IGWLVD
--IGWLTY
--IGWLQT
--IGWLQE
--IGWLNQ
--IGWLNE
--IGWLMQ
--IGWLMH
--IGWLMF
--IGWLLQ
--IGWLLH
--IGWLKY

```
--IGWLIR
--IGWLIE
--IGWLHY
--IGWLHL
--IGWLHH
--IGWLHE
--IGWLGW
--IGWLFD
--IGWLEK
--IGWLAQ
--IGWLAF
IVIGWL--
IMIGWL--
-IIGWLN-
-IIGWLI-
-IIGWLD-
-IIGWLA-
--IGWLYY
--IGWLYH
--IGWLWY
--IGWLWS
--IGWLWL
--IGWLVE
--IGWLTW
--IGWLSD
--IGWLQY
--IGWLQW
--IGWLQD
--IGWLPL
--IGWLND
--IGWLML
--IGWLLM
--IGWLKW
--IGWLIK
--IGWLIF
--IGWLHQ
--IGWLGE
--IGWLFV
--IGWLES
--IGWLEH
--IGWLDG
--IGWLCQ
--IGWLCK
--IGWLCG
--IGWLCA
--IGWLAT
--IGWLAG
-KIGWLR-
KGIGWL--
-KIGWLQ-
```

KTIGWL--
-KIGWLT-
-KIGWLN-
-KIGWLK-
KSIGWL--
-KIGWLV-
-KIGWLL-
KLIGWL--
-KIGWLS-
KEIGWL--
-KIGWLI-
KAIGWL--
KMIGWL--
-KIGWLW-
-KIGWLG-
-KIGWLD-
KCIGWL--
KYIGWL--
KPIGWL--
-KIGWLA-
LRIGWL--
LTIGWL--
LPIGWL--
LNIGWL--
-LIGWLT-
-LIGWLG-
-LIGWLV-
-LIGWLR-
LGIGWL--
LEIGWL--
LHIGWL--
LLIGWL--
-LIGWLK-
LMIGWL--
LIIGWL--
-LIGWLS-
-LIGWLD-
LAIGWL--
LVIGWL--
-LIGWLM-
-LIGWLI-
MSIGWL--
-MIGWLG-
MTIGWL--
MNIGWL--
-MIGWLS-
MPIGWL--
MKIGWL--
-MIGWLH-
-MIGWLE-

MHIGWL--
MLIGWL--
-MIGWLT-
-MIGWLV-
-MIGWLN-
MDIGWL--
MAIGWL--
-MIGWLL-
-MIGWLK-
MVIGWL--
-MIGWLR-
-MIGWLD-
MEIGWL--
NSIGWL--
NRIGWL--
-NIGWLR-
NKIGWL--
-NIGWLL-
NHIGWL--
NYIGWL--
-NIGWLV-
NAIGWL--
-NIGWLG-
NVIGWL--
-NIGWLN-
NNIGWL--
NLIGWL--
-NIGWLS-
-NIGWLQ-
-NIGWLA-
-NIGWLH-
NQIGWL--
NPIGWL--
-NIGWLK-
NDIGWL--
NWIGWL--
NTIGWL--
-NIGWLM-
-NIGWLI-
NGIGWL--
NEIGWL--
NIIGWL--
-NIGWLE-
-NIGWLD-
NFIGWL--
-PIGWLR-
-PIGWLQ-
-PIGWLG-
PTIGWL--
PEIGWL--

PLIGWL--
-PIGWLS-
PHIGWL--
-PIGWLK-
PSIGWL--
PRIGWL--
-PIGWLH-
-PIGWLN-
-PIGWLI-
PGIGWL--
PNIGWL--
-PIGWLV-
-PIGWLT-
-PIGWLD-
PYIGWL--
PQIGWL--
-PIGWLA-
PDIGWL--
PAIGWL--
PFIGWL--
-PIGWLM-
-PIGWLE-
QRIGWL--
-QIGWLS-
-QIGWLL-
-QIGWLR-
-QIGWLG-
QAIGWL--
QSIGWL--
-QIGWLV-
QTIGWL--
QLIGWL--
-QIGWLT-
-QIGWLI-
-QIGWLA-
QMIGWL--
QKIGWL--
-QIGWLH-
QGIGWL--
-QIGWLQ-
QWIGWL--
QPIGWL--
QQIGWL--
-QIGWLN-
-QIGWLD-
QYIGWL--
QIIGWL--
-QIGWLE-
QEIGWL--
QDIGWL--

QCIGWL--
-RIGWLS-
RPIGWL--
-RIGWLL-
-RIGWLV-
-RIGWLG-
-RIGWLE-
-RIGWLT-
-RIGWLI-
RDIGWL--
-RIGWLA-
REIGWL--
-RIGWLD-
-RIGWLF-
-RIGWLP-
RCIGWL--
-SIGWLR-
SRIGWL--
SDIGWL--
SVIGWL--
-SIGWLS-
-SIGWLT-
SKIGWL--
STIGWL--
-SIGWLK-
SFIGWL--
-SIGWLQ-
SGIGWL--
SQIGWL--
SPIGWL--
-SIGWLD-
SIIGWL--
-SIGWLN-
-SIGWLH-
SEIGWL--
-SIGWLV-
-SIGWLA-
SHIGWL--
SAIGWL--
SWIGWL--
SNIGWL--
-SIGWLM-
-SIGWLE-
TSIGWL--
-TIGWLN-
TQIGWL--
-TIGWLR-
-TIGWLK-
-TIGWLL-
TYIGWL--

TDIGWL--
TVIGWL--
TTIGWL--
TPIGWL--
TKIGWL--
-TIGWLM-
TGIGWL--
TEIGWL--
-TIGWLV-
-TIGWLQ-
TIIGWL--
-TIGWLE-
-TIGWLD-
THIGWL--
TFIGWL--
VNIGWL--
VTIGWL--
-VIGWLR-
-VIGWLH-
-VIGWLG-
VDIGWL--
VAIGWL--
VIIGWL--
-VIGWLS-
VMIGWL--
-VIGWLL-
-VIGWLK-
VGIGWL--
VQIGWL--
VPIGWL--
-VIGWLT-
VVIGWL--
-VIGWLV-
-VIGWLQ-
-VIGWLN-
VFIGWL--
VEIGWL--
VWIGWL--
-VIGWLE-
VHIGWL--
-VIGWLW-
-VIGWLF-
-VIGWLD-
-VIGWLA-
VCIGWL--
WNIGWL--
-WIGWLG-
WKIGWL--
WEIGWL--
WAIGWL--

| | | | |
|---|---|---|---|
| | -WIGWLS- | | |
| | WPIGWL-- | | |
| | WSIGWL-- | | |
| | -WIGWLV- | | |
| | -WIGWLN- | | |
| | -WIGWLI- | | |
| | WGIGWL-- | | |
| | WTIGWL-- | | |
| | WQIGWL-- | | |
| | -WIGWLP- | | |
| | -WIGWLL- | | |
| | -WIGWLE- | | |
| | WFIGWL-- | | |
| | -YIGWLR- | | |
| | YSIGWL-- | | |
| | YTIGWL-- | | |
| | -YIGWLA- | | |
| | -YIGWLS- | | |
| | -YIGWLK- | | |
| | YQIGWL-- | | |
| | -YIGWLQ- | | |
| | -YIGWLG- | | |
| | -YIGWLD- | | |
| | YGIGWL-- | | |
| | YVIGWL-- | | |
| | YNIGWL-- | | |
| | -YIGWLM- | | |
| | YAIGWL-- | | |
| | YMIGWL-- | | |
| | -YIGWLY- | | |
| | -YIGWLN- | | |
| | YHIGWL-- | | |
| | YEIGWL-- | | |
| PLGI | 36 | 822 | 33.94 |
| | CTPLGI-- | | |
| | IQPLGI-- | | |
| | -LPLGIG- | | |
| | --PLGITA | | |
| | --PLGIMY | | |
| | --PLGILW | | |
| | -YPLGID- | | |
| | ASPLGI-- | | |
| | -APLGIS- | | |
| | -APLGIR- | | |
| | -APLGIT- | | |
| | AYPLGI-- | | |
| | ATPLGI-- | | |
| | -APLGII- | | |
| | -APLGIV- | | |
| | ALPLGI-- | | |

AGPLGI--
-APLGIK-
-APLGIG-
AWPLGI--
ARPLGI--
-APLGIQ-
-APLGIN-
-APLGID-
APPLGI--
AMPLGI--
AEPLGI--
ADPLGI--
ANPLGI--
AHPLGI--
AFPLGI--
AQPLGI--
-APLGIH-
-APLGIF-
-APLGIC-
AIPLGI--
ACPLGI--
-CPLGIR-
CYPLGI--
CLPLGI--
-CPLGIQ-
-CPLGIK-
CAPLGI--
CWPLGI--
CSPLGI--
CRPLGI--
-CPLGIT-
-CPLGIS-
-CPLGIL-
-CPLGIF-
CFPLGI--
CDPLGI--
CCPLGI--
DLPLGI--
DSPLGI--
-DPLGIS-
DTPLGI--
DHPLGI--
DWPLGI--
-DPLGIY-
-DPLGIQ-
DFPLGI--
DDPLGI--
-DPLGIT-
-DPLGIM-
-DPLGIK-

DMPLGI--
DAPLGI--
DYPLGI--
-DPLGIL-
DIPLGI--
DQPLGI--
-DPLGIV-
-DPLGIR-
-DPLGIA-
DGPLGI--
DNPLGI--
DKPLGI--
DEPLGI--
-EPLGIR-
ESPLGI--
-EPLGIL-
ETPLGI--
EAPLGI--
EGPLGI--
-EPLGIW-
-EPLGIK-
-EPLGIG-
-EPLGIA-
EIPLGI--
-EPLGIY-
-EPLGIV-
-EPLGIT-
-EPLGIS-
EMPLGI--
EVPLGI--
-EPLGIF-
ENPLGI--
ELPLGI--
EKPLGI--
EWPLGI--
ERPLGI--
-EPLGIM-
EYPLGI--
-EPLGIN-
-EPLGII-
-EPLGIE-
EFPLGI--
FWPLGI--
FFPLGI--
-FPLGIK-
-FPLGII-
-FPLGIS-
-FPLGIM-
-FPLGIH-
-FPLGIT-

-FPLGIL-
FIPLGI--
FGPLGI--
FDPLGI--
-FPLGIG-
FKPLGI--
-FPLGIE-
FNPLGI--
FHPLGI--
FYPLGI--
FTPLGI--
FPPLGI--
FAPLGI--
FQPLGI--
-FPLGIN-
-FPLGIA-
FMPLGI--
-FPLGIQ-
-GPLGIR-
GWPLGI--
-GPLGIF-
-GPLGIT-
-GPLGIL-
GMPLGI--
-GPLGIK-
GNPLGI--
GGPLGI--
GVPLGI--
GYPLGI--
-GPLGIS-
-GPLGIQ-
GSPLGI--
-GPLGIN-
-GPLGIH-
GLPLGI--
GIPLGI--
-GPLGII-
GKPLGI--
-GPLGIW-
GPPLGI--
-GPLGIV-
GFPLGI--
-GPLGIG-
GHPLGI--
GAPLGI--
GEPLGI--
GQPLGI--
-GPLGIM-
-GPLGIA-
-GPLGIE-

GDPLGI--
HAPLGI--
HRPLGI--
-HPLGIY-
-HPLGIT-
-HPLGIL-
HGPLGI--
HVPLGI--
HKPLGI--
-HPLGIK-
-HPLGIA-
-HPLGIS-
-HPLGIH-
-HPLGIG-
-HPLGIF-
HMPLGI--
HNPLGI--
HEPLGI--
HTPLGI--
HQPLGI--
-HPLGIR-
-HPLGIM-
HDPLGI--
-HPLGIQ-
-HPLGII-
HWPLGI--
-HPLGIW-
-HPLGIV-
HYPLGI--
-HPLGIE-
-IPLGIR-
ISPLGI--
-IPLGIL-
ILPLGI--
IRPLGI--
-IPLGIT-
IVPLGI--
IKPLGI--
IIPLGI--
-IPLGIN-
IGPLGI--
IMPLGI--
IAPLGI--
-IPLGIV-
-IPLGIM-
IDPLGI--
IWPLGI--
ITPLGI--
IPPLGI--
-IPLGIS-

-IPLGIQ-
-IPLGIH-
IHPLGI--
IEPLGI--
IFPLGI--
-IPLGIG-
ICPLGI--
-IPLGIK-
-IPLGII-
-IPLGIA-
INPLGI--
IYPLGI--
-IPLGIW-
-IPLGIE-
KMPLGI--
KVPLGI--
-KPLGIL-
-KPLGIS-
-KPLGIR-
-KPLGII-
KGPLGI--
KYPLGI--
-KPLGIM-
-KPLGIA-
KPPLGI--
-KPLGIY-
-KPLGIT-
-KPLGIQ-
-KPLGIK-
-KPLGIF-
KIPLGI--
KAPLGI--
-KPLGIN-
-KPLGIH-
-KPLGIE-
KHPLGI--
KEPLGI--
KDPLGI--
KWPLGI--
KQPLGI--
-KPLGIW-
KNPLGI--
-LPLGIL-
LRPLGI--
LLPLGI--
LVPLGI--
LNPLGI--
-LPLGIM-
LGPLGI--
LMPLGI--

```
LHPLGI--
LTPLGI--
-LPLGIK-
LIPLGI--
-LPLGIR-
-LPLGIT-
LAPLGI--
-LPLGIS-
LYPLGI--
LDPLGI--
LWPLGI--
-LPLGII-
LPPLGI--
-LPLGIA-
LQPLGI--
-LPLGIQ-
-LPLGIV-
-LPLGIN-
LEPLGI--
LKPLGI--
-LPLGIE-
-LPLGIF-
LFPLGI--
LCPLGI--
MRPLGI--
-MPLGIL-
-MPLGIA-
MSPLGI--
-MPLGIH-
-MPLGIM-
-MPLGII-
MFPLGI--
-MPLGIS-
-MPLGIG-
MNPLGI--
MAPLGI--
-MPLGIT-
MIPLGI--
MEPLGI--
MTPLGI--
-MPLGIV-
-MPLGIR-
-MPLGIK-
MVPLGI--
-MPLGIN-
MYPLGI--
MPPLGI--
MKPLGI--
MGPLGI--
-MPLGIY-
```

-MPLGIE-
MWPLGI--
-MPLGIF-
MHPLGI--
MDPLGI--
-MPLGIQ-
NRPLGI--
-NPLGIL-
-NPLGIK-
NLPLGI--
-NPLGIY-
NAPLGI--
NVPLGI--
NTPLGI--
-NPLGIA-
NHPLGI--
NWPLGI--
-NPLGIM-
NMPLGI--
NGPLGI--
NQPLGI--
-NPLGII-
-NPLGIE-
NKPLGI--
NSPLGI--
-NPLGIS-
-NPLGIN-
-NPLGIH-
NYPLGI--
NPPLGI--
-NPLGIQ-
NFPLGI--
-NPLGIT-
-NPLGIG-
-NPLGIF-
NNPLGI--
NEPLGI--
--PLGILR
--PLGIWR
--PLGIMR
--PLGITR
--PLGIVR
--PLGIRL
--PLGIVS
--PLGIRM
--PLGIRN
--PLGILY
--PLGIGS
--PLGIVG
--PLGIST

--PLGIRI
--PLGISV
--PLGIRV
--PLGIRG
--PLGIRA
--PLGITL
--PLGISS
--PLGIRD
--PLGINR
--PLGILT
--PLGIKR
--PLGIKA
--PLGIGT
PKPLGI--
--PLGISN
--PLGISL
--PLGIHS
--PLGITI
--PLGISA
--PLGIMG
--PLGIFT
--PLGIFR
--PLGIFK
--PLGIYR
--PLGISK
--PLGIKT
--PLGIYT
--PLGIYN
--PLGIKV
--PLGIHR
--PLGIAH
--PLGITN
--PLGISI
--PLGISG
--PLGIRY
--PLGIRW
--PLGIQR
--PLGILK
--PLGIKG
--PLGIIG
--PLGIHL
PTPLGI--
--PLGIWT
--PLGISW
--PLGIRH
PGPLGI--
--PLGIVI
--PLGIRP
--PLGILG
--PLGIKS

--PLGIKK
--PLGIIY
--PLGIIS
--PLGIIQ
--PLGIHK
--PLGIYQ
--PLGIVT
--PLGIVM
--PLGITF
--PLGISY
--PLGIQS
--PLGINY
--PLGINQ
--PLGIML
--PLGIKY
--PLGIKH
--PLGIIT
--PLGIIK
--PLGIFN
--PLGIEF
--PLGIYK
--PLGITH
--PLGITG
--PLGINI
--PLGILH
--PLGILD
--PLGILA
--PLGIII
--PLGIHW
--PLGIFS
--PLGIEA
--PLGIAT
--PLGIAL
PIPLGI--
PHPLGI--
--PLGIYG
--PLGIVV
--PLGIVQ
--PLGISM
--PLGIQT
--PLGIMT
--PLGILV
--PLGILQ
--PLGIIA
--PLGIGI
--PLGIFH
--PLGIAV
--PLGIYE
--PLGIWG
--PLGITY

--PLGITV
--PLGITK
--PLGINM
--PLGIKM
--PLGIKE
--PLGIIR
--PLGIHF
--PLGIGG
--PLGIAG
PQPLGI--
--PLGIVW
--PLGISQ
--PLGISF
--PLGIRF
--PLGIRE
--PLGIQV
--PLGIQN
--PLGIQH
--PLGINW
--PLGINV
--PLGINA
--PLGIHT
--PLGIHQ
--PLGIHA
--PLGIGW
--PLGIFG
--PLGIAK
--PLGIYD
--PLGIVY
--PLGIVA
--PLGITT
--PLGITS
--PLGITE
--PLGISD
--PLGIQK
--PLGIQG
--PLGIQA
--PLGINS
--PLGING
--PLGILE
--PLGIKL
--PLGIHG
--PLGIGK
PMPLGI--
--PLGIYH
--PLGIWN
--PLGIVN
--PLGITQ
--PLGITM
--PLGISH

--PLGISE
--PLGINT
--PLGIMH
--PLGIKI
--PLGIHN
--PLGIHD
--PLGIFA
--PLGIEV
--PLGIET
--PLGIDS
--PLGIAM
--PLGIAI
--PLGIWE
--PLGIWD
--PLGIWA
--PLGIVE
--PLGITW
--PLGIQI
--PLGIQF
--PLGINF
--PLGIMD
--PLGIKD
--PLGIIM
--PLGIGN
--PLGIFQ
--PLGIFE
--PLGIEK
--PLGIEI
--PLGIEH
--PLGIAN
--PLGIAD
PEPLGI--
PWPLGI--
--PLGIWK
--PLGIVH
--PLGIVD
--PLGITD
--PLGIQL
--PLGIQE
--PLGINN
--PLGINK
--PLGINH
--PLGINE
--PLGIMW
--PLGIMK
--PLGIMI
--PLGIMF
--PLGIME
--PLGIIN
--PLGIIE

--PLGIHI
--PLGIGL
--PLGIEW
--PLGIDG
--PLGIAW
--PLGIAE
PFPLGI--
-PPLGIY-
-PPLGII-
--PLGIYP
--PLGIWH
--PLGIVP
--PLGIQY
--PLGIQQ
--PLGIQM
--PLGIQD
--PLGIPM
--PLGIPI
--PLGINP
--PLGIND
--PLGIMA
--PLGIKQ
--PLGIKN
--PLGIKF
--PLGIIH
--PLGIHP
--PLGIHM
--PLGIHH
--PLGIHE
--PLGIGY
--PLGIGH
--PLGIGF
--PLGIGA
--PLGIFD
--PLGIEQ
--PLGIEM
--PLGIED
--PLGIDA
--PLGICN
--PLGIAQ
QRPLGI--
-QPLGIY-
QDPLGI--
QYPLGI--
QSPLGI--
QPPLGI--
QVPLGI--
-QPLGIH-
QHPLGI--
-QPLGIV-

```
-QPLGIR-
-QPLGIM-
QGPLGI--
-QPLGIG-
-QPLGIE-
QLPLGI--
-QPLGIT-
-QPLGIK-
-QPLGII-
QNPLGI--
QMPLGI--
QKPLGI--
QIPLGI--
QEPLGI--
QQPLGI--
-QPLGIQ-
-QPLGIF-
-QPLGIA-
QTPLGI--
QCPLGI--
QAPLGI--
QWPLGI--
-QPLGIP-
-QPLGIN-
-QPLGIL-
-RPLGIL-
-RPLGIS-
-RPLGIR-
RSPLGI--
RAPLGI--
RRPLGI--
-RPLGIQ-
RVPLGI--
RPPLGI--
-RPLGIK-
-RPLGIT-
-RPLGIN-
RIPLGI--
-RPLGIG-
RMPLGI--
RLPLGI--
-RPLGIY-
-RPLGIV-
RGPLGI--
RNPLGI--
-RPLGIH-
RKPLGI--
RWPLGI--
RTPLGI--
RFPLGI--
```

RHPLGI--
RDPLGI--
RYPLGI--
RQPLGI--
REPLGI--
RCPLGI--
-RPLGIA-
SVPLGI--
-SPLGIS-
-SPLGIK-
SLPLGI--
-SPLGIT-
SKPLGI--
SGPLGI--
STPLGI--
-SPLGIQ-
-SPLGIV-
-SPLGIN-
SNPLGI--
-SPLGIM-
SDPLGI--
-SPLGIH-
SYPLGI--
SHPLGI--
SEPLGI--
SQPLGI--
-SPLGIG-
-SPLGIE-
SWPLGI--
SPPLGI--
SIPLGI--
SAPLGI--
-SPLGIP-
SCPLGI--
-TPLGIH-
TSPLGI--
TYPLGI--
-TPLGIS-
TKPLGI--
TNPLGI--
TGPLGI--
-TPLGIR-
TFPLGI--
-TPLGIG-
-TPLGIY-
-TPLGIT-
TLPLGI--
TRPLGI--
THPLGI--
TWPLGI--

-TPLGII-
TAPLGI--
TTPLGI--
-TPLGIE-
TEPLGI--
TPPLGI--
-TPLGIM-
TQPLGI--
TIPLGI--
-TPLGIV-
-TPLGIQ-
TDPLGI--
TMPLGI--
-TPLGIK-
-TPLGIF-
-TPLGIA-
VSPLGI--
VRPLGI--
-VPLGIV-
VLPLGI--
-VPLGIM-
VQPLGI--
VWPLGI--
-VPLGIS-
VFPLGI--
-VPLGIR-
VNPLGI--
VTPLGI--
-VPLGIQ-
-VPLGIG-
VEPLGI--
VYPLGI--
VPPLGI--
-VPLGIH-
-VPLGIN-
VMPLGI--
-VPLGII-
-VPLGIF-
VHPLGI--
-VPLGIE-
-VPLGIA-
VAPLGI--
VIPLGI--
VGPLGI--
VDPLGI--
VCPLGI--
-WPLGIR-
WAPLGI--
WLPLGI--
WSPLGI--

WYPLGI--
WRPLGI--
WIPLGI--
WGPLGI--
-WPLGIK-
WMPLGI--
WVPLGI--
-WPLGIT-
WKPLGI--
WDPLGI--
-WPLGIS-
-WPLGII-
WNPLGI--
WWPLGI--
-WPLGIM-
-WPLGIH-
WPPLGI--
-WPLGIF-
-WPLGIA-
WHPLGI--
WFPLGI--
WEPLGI--
YSPLGI--
YRPLGI--
YYPLGI--
-YPLGIG-
YHPLGI--
YTPLGI--
-YPLGIN-
-YPLGIH-
-YPLGIK-
-YPLGIY-
-YPLGIM-
YKPLGI--
-YPLGIR-
-YPLGIL-
YGPLGI--
-YPLGIS-
YWPLGI--
-YPLGIT-
-YPLGIA-
YLPLGI--
YNPLGI--
-YPLGIV-
YCPLGI--
YQPLGI--
-YPLGIQ-
-YPLGII-
YMPLGI--
YFPLGI--

```
            YEPLGI--
            YVPLGI--
            YIPLGI--
            -YPLGIE-
            YDPLGI--
            YPPLGI--
PRAI         423        625     33.93
            --PRAIHP
            ARPRAI--
            AVPRAI--
            ALPRAI--
            -APRAIK-
            APPRAI--
            ANPRAI--
            -APRAIL-
            -APRAIN-
            AMPRAI--
            ASPRAI--
            -APRAIW-
            -APRAIM-
            AFPRAI--
            AYPRAI--
            AHPRAI--
            -APRAIV-
            -APRAIA-
            AGPRAI--
            AEPRAI--
            ADPRAI--
            -APRAIQ-
            ACPRAI--
            CLPRAI--
            -CPRAIR-
            -CPRAIF-
            CHPRAI--
            CYPRAI--
            -CPRAIY-
            CGPRAI--
            CAPRAI--
            CRPRAI--
            CEPRAI--
            CSPRAI--
            CQPRAI--
            -CPRAIQ-
            -CPRAIM-
            -CPRAII-
            CWPRAI--
            CVPRAI--
            CTPRAI--
            -CPRAIS-
            -CPRAIK-
```

−CPRAIH−
−CPRAIA−
CPPRAI−−
CNPRAI−−
CIPRAI−−
CFPRAI−−
−DPRAIK−
DSPRAI−−
−DPRAIR−
DLPRAI−−
−DPRAIT−
−DPRAIL−
DRPRAI−−
−DPRAIA−
DVPRAI−−
−DPRAIF−
DGPRAI−−
−DPRAIS−
−DPRAIQ−
−DPRAIH−
DYPRAI−−
DTPRAI−−
−DPRAIV−
−DPRAII−
DCPRAI−−
DAPRAI−−
DQPRAI−−
−DPRAIE−
DWPRAI−−
−DPRAIY−
−DPRAIM−
−DPRAIG−
DNPRAI−−
DMPRAI−−
DKPRAI−−
DIPRAI−−
DFPRAI−−
ESPRAI−−
ERPRAI−−
ELPRAI−−
ETPRAI−−
−EPRAIN−
−EPRAIK−
−EPRAII−
EAPRAI−−
−EPRAIM−
−EPRAIG−
−EPRAIF−
EYPRAI−−
−EPRAIT−

-EPRAIL-
-EPRAIA-
EMPRAI--
EIPRAI--
EVPRAI--
EKPRAI--
EFPRAI--
EQPRAI--
-EPRAIS-
EHPRAI--
EEPRAI--
-EPRAIY-
ENPRAI--
FVPRAI--
FDPRAI--
FIPRAI--
FGPRAI--
FLPRAI--
FTPRAI--
FPPRAI--
FNPRAI--
FEPRAI--
FAPRAI--
FCPRAI--
-FPRAIV-
-FPRAIN-
-FPRAIG-
FHPRAI--
-FPRAIQ-
-FPRAIE-
-GPRAIL-
GSPRAI--
-GPRAIS-
-GPRAIT-
-GPRAIG-
GVPRAI--
-GPRAIV-
GIPRAI--
-GPRAIN-
-GPRAIM-
GMPRAI--
GQPRAI--
GNPRAI--
GKPRAI--
GAPRAI--
-GPRAII-
-GPRAIK-
-GPRAIH-
-GPRAIE-
-GPRAIF-

GEPRAI--
GYPRAI--
GTPRAI--
GDPRAI--
GPPRAI--
GCPRAI--
-HPRAIT-
HGPRAI--
HVPRAI--
HLPRAI--
HFPRAI--
HPPRAI--
-HPRAIY-
-HPRAIQ-
-HPRAIM-
HQPRAI--
HMPRAI--
HKPRAI--
HYPRAI--
-HPRAIL-
HDPRAI--
-HPRAII-
HEPRAI--
-IPRAIS-
IRPRAI--
-IPRAIH-
ISPRAI--
-IPRAIQ-
-IPRAIR-
-IPRAIK-
IPPRAI--
IGPRAI--
-IPRAIV-
IDPRAI--
-IPRAIW-
IQPRAI--
IAPRAI--
-IPRAII-
INPRAI--
IEPRAI--
IYPRAI--
-IPRAIE-
-IPRAIY-
-IPRAIG-
IWPRAI--
-IPRAIT-
-KPRAIV-
KLPRAI--
-KPRAIY-
KVPRAI--

-KPRAII-
KPPRAI--
KIPRAI--
-KPRAIQ-
KKPRAI--
-KPRAIG-
-KPRAIE-
KDPRAI--
-KPRAIH-
KCPRAI--
LKPRAI--
LPPRAI--
-LPRAIQ-
LGPRAI--
LDPRAI--
LVPRAI--
-LPRAII-
-LPRAIV-
-LPRAIH-
LQPRAI--
-LPRAIN-
LNPRAI--
-LPRAIY-
-LPRAIG-
-LPRAIM-
-LPRAIE-
-LPRAIW-
LEPRAI--
MSPRAI--
-MPRAIN-
MTPRAI--
-MPRAII-
-MPRAIY-
MHPRAI--
-MPRAIT-
MPPRAI--
MGPRAI--
-MPRAIV-
-MPRAIM-
-MPRAIF-
MEPRAI--
-MPRAIH-
MIPRAI--
MFPRAI--
MYPRAI--
-MPRAIS-
-MPRAIA-
MNPRAI--
MDPRAI--
MAPRAI--

-MPRAIQ-
-MPRAIE-
-MPRAIC-
-NPRAIL-
-NPRAIS-
NVPRAI--
NYPRAI--
-NPRAIT-
NHPRAI--
NLPRAI--
NIPRAI--
-NPRAIY-
-NPRAIW-
-NPRAIQ-
NKPRAI--
-NPRAIV-
NNPRAI--
NCPRAI--
NQPRAI--
-NPRAIK-
-NPRAIH-
-NPRAIF-
-NPRAIE-
NDPRAI--
NEPRAI--
--PRAIIN
--PRAIVS
--PRAIYF
--PRAILV
--PRAIVL
--PRAIGS
--PRAIWV
--PRAIVK
--PRAITL
--PRAIWQ
--PRAITG
--PRAILT
--PRAIFT
--PRAILI
--PRAILG
--PRAIWS
--PRAITW
--PRAISY
--PRAISH
--PRAIKG
--PRAIIT
--PRAIGK
--PRAIAF
--PRAIYW
--PRAISN

--PRAISM
--PRAILK
--PRAIIS
--PRAIYG
--PRAIVT
--PRAITV
--PRAIPY
--PRAIMY
--PRAIKH
--PRAITQ
--PRAISQ
--PRAIRV
--PRAINY
--PRAINL
--PRAIIW
--PRAIHT
--PRAISG
--PRAIMS
--PRAIMN
PLPRAI--
--PRAIYK
--PRAIVF
--PRAIVA
--PRAIQY
--PRAIQV
--PRAIGI
--PRAIEL
--PRAIAH
PMPRAI--
--PRAIYN
--PRAIWG
--PRAISD
--PRAIMG
--PRAILE
--PRAIGH
--PRAIYS
--PRAIVQ
--PRAIVM
--PRAIST
--PRAISA
--PRAILN
--PRAIIA
--PRAIHY
--PRAIGM
PQPRAI--
--PRAIYT
--PRAIYD
--PRAIYA
--PRAIWI
--PRAIVH

--PRAIMT
--PRAIHI
--PRAIGY
--PRAIAT
--PRAIWT
--PRAIVG
--PRAITH
--PRAISK
--PRAIPW
--PRAINS
--PRAIMM
--PRAIHN
--PRAIGA
--PRAIFN
--PRAIFG
--PRAIFA
--PRAIAY
PAPRAI--
--PRAIWW
--PRAIWK
--PRAIVY
--PRAIQS
--PRAIQI
--PRAIQG
--PRAINT
--PRAINF
--PRAIME
--PRAIKF
--PRAIHR
--PRAIHQ
--PRAIHK
--PRAIFE
--PRAIEV
--PRAIWE
--PRAIVV
--PRAITT
--PRAITK
--PRAIRW
--PRAIRP
--PRAINK
--PRAILD
--PRAIHS
--PRAIHG
--PRAIGF
--PRAIAQ
--PRAIAG
--PRAIWY
--PRAIQD
--PRAIPI
--PRAIPF

--PRAINV
--PRAINH
--PRAIMQ
--PRAIML
--PRAIMF
--PRAIMD
--PRAIKV
--PRAIHV
--PRAIEN
--PRAIEA
PDPRAI--
--PRAIWD
--PRAIVI
--PRAIQW
--PRAIQA
--PRAINQ
--PRAIIF
--PRAIHH
--PRAIHD
--PRAIHA
--PRAIFW
--PRAIES
PGPRAI--
PFPRAI--
PSPRAI--
--PRAIWH
--PRAITE
--PRAIQE
--PRAINA
--PRAIMW
--PRAIKM
--PRAIKA
--PRAIHW
--PRAIGQ
--PRAIGN
--PRAIGG
--PRAIFI
--PRAIFD
--PRAIEY
--PRAIET
--PRAIAV
--PRAIAA
--PRAIYE
--PRAIVN
--PRAIVE
--PRAITM
--PRAISW
--PRAISE
--PRAIQM
--PRAIQH

--PRAIPV
--PRAIII
--PRAIIH
--PRAIHE
--PRAIGD
--PRAIEH
--PRAIDF
--PRAICT
--PRAIAN
PVPRAI--
--PRAITD
--PRAIRC
--PRAIQT
--PRAIQQ
--PRAIQP
--PRAINN
--PRAINE
--PRAIND
--PRAIMP
--PRAIKQ
--PRAIKE
--PRAIIY
--PRAIIQ
--PRAIIK
--PRAIIE
--PRAIHM
--PRAIFH
--PRAIEW
--PRAIEQ
--PRAIEK
--PRAIEG
--PRAIEF
--PRAIDS
--PRAIDA
--PRAICR
--PRAICM
--PRAICF
--PRAIAI
PEPRAI--
PCPRAI--
QLPRAI--
-QPRAIS-
-QPRAIY-
-QPRAIF-
-QPRAIL-
-QPRAIK-
QIPRAI--
QGPRAI--
QSPRAI--
QKPRAI--

QFPRAI--
QNPRAI--
QEPRAI--
-QPRAIQ-
QYPRAI--
-QPRAIV-
-QPRAIM-
-QPRAIG-
-QPRAIA-
QHPRAI--
QWPRAI--
QAPRAI--
QTPRAI--
-QPRAII-
QPPRAI--
-QPRAIW-
QMPRAI--
QDPRAI--
-RPRAIT-
-RPRAIS-
RPPRAI--
RNPRAI--
-RPRAIY-
-RPRAIN-
RKPRAI--
-RPRAIM-
REPRAI--
RDPRAI--
RQPRAI--
-RPRAIW-
-RPRAIE-
RCPRAI--
-SPRAIS-
STPRAI--
-SPRAII-
-SPRAIY-
SSPRAI--
-SPRAIK-
-SPRAIN-
-SPRAIF-
-SPRAIE-
SPPRAI--
SHPRAI--
-SPRAIM-
SNPRAI--
SGPRAI--
SQPRAI--
-SPRAIV-
SEPRAI--
SCPRAI--

TLPRAI--
TVPRAI--
TSPRAI--
-TPRAIR-
-TPRAIS-
-TPRAIQ-
-TPRAIN-
-TPRAII-
-TPRAIG-
-TPRAIA-
TKPRAI--
-TPRAIV-
-TPRAIF-
TIPRAI--
TWPRAI--
TDPRAI--
THPRAI--
-TPRAIH-
TPPRAI--
-TPRAIK-
-TPRAIE-
TEPRAI--
-TPRAIC-
VLPRAI--
VKPRAI--
-VPRAIK-
VIPRAI--
-VPRAIS-
VDPRAI--
VAPRAI--
-VPRAIA-
VVPRAI--
-VPRAIG-
VNPRAI--
-VPRAII-
-VPRAIM-
VGPRAI--
VYPRAI--
VEPRAI--
VQPRAI--
-VPRAIV-
-VPRAIE-
VPPRAI--
-VPRAIY-
-VPRAIW-
-VPRAIC-
VCPRAI--
WTPRAI--
-WPRAIV-
-WPRAIL-

| | | | |
|---|---|---|---|
| | WLPRAI-- | | |
| | WKPRAI-- | | |
| | WGPRAI-- | | |
| | -WPRAIF- | | |
| | WHPRAI-- | | |
| | WEPRAI-- | | |
| | WDPRAI-- | | |
| | WPPRAI-- | | |
| | -WPRAIH- | | |
| | -WPRAIE- | | |
| | WMPRAI-- | | |
| | WCPRAI-- | | |
| | -WPRAIM- | | |
| | WNPRAI-- | | |
| | YRPRAI-- | | |
| | YTPRAI-- | | |
| | YAPRAI-- | | |
| | -YPRAIV- | | |
| | YVPRAI-- | | |
| | -YPRAIW- | | |
| | YNPRAI-- | | |
| | YKPRAI-- | | |
| | YEPRAI-- | | |
| | YYPRAI-- | | |
| | -YPRAIY- | | |
| | -YPRAIQ- | | |
| | YMPRAI-- | | |
| | -YPRAII- | | |
| | -YPRAIH- | | |
| | YPPRAI-- | | |
| | YQPRAI-- | | |
| | YDPRAI-- | | |
| PSAI | 17 | 849 | 33.91 |
| | -DPSAIS- | | |
| | -LPSAIY- | | |
| | -NPSAIG- | | |
| | --PSAIIR | | |
| | SEPSAI-- | | |
| | AYPSAI-- | | |
| | ASPSAI-- | | |
| | AMPSAI-- | | |
| | -APSAIL- | | |
| | -APSAIA- | | |
| | ALPSAI-- | | |
| | -APSAIR- | | |
| | AGPSAI-- | | |
| | -APSAIF- | | |
| | -APSAIV- | | |
| | -APSAIH- | | |
| | -APSAIG- | | |

```
AQPSAI--
-APSAIT-
AWPSAI--
-APSAIS-
AKPSAI--
AAPSAI--
AVPSAI--
ATPSAI--
-APSAIN-
-APSAIK-
AIPSAI--
-APSAIQ-
-APSAIE-
APPSAI--
AEPSAI--
-APSAIM-
ANPSAI--
ADPSAI--
-CPSAIL-
CLPSAI--
CSPSAI--
-CPSAIY-
-CPSAIV-
-CPSAIS-
-CPSAIK-
CNPSAI--
CGPSAI--
CFPSAI--
CAPSAI--
CWPSAI--
CRPSAI--
-CPSAIM-
-CPSAIF-
CMPSAI--
CKPSAI--
CHPSAI--
DVPSAI--
-DPSAIR-
DAPSAI--
DSPSAI--
DMPSAI--
DRPSAI--
-DPSAIQ-
DGPSAI--
DNPSAI--
DWPSAI--
DTPSAI--
-DPSAIL-
DQPSAI--
-DPSAIW-
```

DKPSAI--
DHPSAI--
DFPSAI--
DYPSAI--
-DPSAIY-
-DPSAIN-
-DPSAIM-
-DPSAIF-
-EPSAIK-
EFPSAI--
-EPSAIR-
-EPSAIT-
-EPSAIL-
ERPSAI--
ETPSAI--
ESPSAI--
-EPSAIG-
-EPSAIF-
-EPSAIA-
ENPSAI--
EYPSAI--
EVPSAI--
EQPSAI--
-EPSAIY-
-EPSAIW-
-EPSAIV-
-EPSAIS-
-EPSAIM-
-EPSAIH-
ELPSAI--
EGPSAI--
EEPSAI--
-EPSAIN-
EMPSAI--
EHPSAI--
EDPSAI--
EAPSAI--
FRPSAI--
-FPSAII-
-FPSAIR-
FSPSAI--
-FPSAIK-
FAPSAI--
FLPSAI--
FVPSAI--
-FPSAIM-
-FPSAIL-
-FPSAIA-
FGPSAI--
-FPSAIN-

FWPSAI--
-FPSAIS-
-FPSAIV-
-FPSAIW-
-FPSAIT-
FTPSAI--
FQPSAI--
FKPSAI--
-FPSAIF-
FMPSAI--
FIPSAI--
-FPSAIY-
-FPSAIQ-
-FPSAIG-
-FPSAIE-
FFPSAI--
-GPSAIR-
GTPSAI--
GIPSAI--
GSPSAI--
-GPSAIT-
GVPSAI--
GYPSAI--
GAPSAI--
-GPSAIM-
-GPSAIN-
-GPSAIG-
GKPSAI--
GGPSAI--
GMPSAI--
GLPSAI--
GFPSAI--
GNPSAI--
GHPSAI--
GWPSAI--
-GPSAIW-
-GPSAIV-
-GPSAIQ-
GCPSAI--
-GPSAII-
-GPSAIH-
-GPSAIF-
GDPSAI--
-GPSAIY-
-GPSAIK-
GEPSAI--
HLPSAI--
HRPSAI--
-HPSAIR-
HSPSAI--

-HPSAIF-
-HPSAIV-
-HPSAIS-
-HPSAIN-
HKPSAI--
HGPSAI--
HHPSAI--
HVPSAI--
-HPSAIY-
-HPSAIL-
HPPSAI--
HFPSAI--
HCPSAI--
HQPSAI--
-HPSAIW-
-HPSAIG-
HAPSAI--
HYPSAI--
-HPSAIQ-
-HPSAIK-
HNPSAI--
HDPSAI--
HWPSAI--
HTPSAI--
-HPSAIM-
-HPSAIH-
-HPSAID-
-HPSAIA-
HMPSAI--
HIPSAI--
-IPSAIG-
IHPSAI--
IRPSAI--
-IPSAIS-
IYPSAI--
ITPSAI--
ISPSAI--
-IPSAIW-
IMPSAI--
-IPSAIF-
IQPSAI--
-IPSAIR-
-IPSAIA-
-IPSAII-
IVPSAI--
-IPSAIV-
-IPSAIL-
-IPSAIN-
-IPSAIT-
IKPSAI--

IIPSAI--
IFPSAI--
-IPSAIY-
-IPSAIQ-
INPSAI--
ILPSAI--
IEPSAI--
IDPSAI--
-IPSAIM-
-IPSAIK-
-IPSAIH-
-IPSAIE-
IPPSAI--
IAPSAI--
ICPSAI--
KSPSAI--
KVPSAI--
-KPSAIG-
KGPSAI--
KTPSAI--
-KPSAIS-
KRPSAI--
-KPSAIW-
-KPSAIQ-
KMPSAI--
KWPSAI--
-KPSAIL-
-KPSAIK-
-KPSAII-
KNPSAI--
-KPSAIT-
KAPSAI--
-KPSAIY-
-KPSAIH-
-KPSAIM-
-KPSAIE-
KCPSAI--
KYPSAI--
KQPSAI--
-KPSAIF-
KEPSAI--
KPPSAI--
KKPSAI--
KIPSAI--
-KPSAIV-
-KPSAIN-
LSPSAI--
LVPSAI--
-LPSAIK-
LQPSAI--

LWPSAI--
-LPSAIR-
LLPSAI--
-LPSAIS-
LKPSAI--
-LPSAIV-
-LPSAIT-
-LPSAIN-
LHPSAI--
LYPSAI--
-LPSAIH-
LEPSAI--
-LPSAIW-
LPPSAI--
LTPSAI--
-LPSAII-
LIPSAI--
-LPSAIF-
LAPSAI--
-LPSAIM-
LMPSAI--
LGPSAI--
LNPSAI--
LFPSAI--
LDPSAI--
-LPSAIG-
-LPSAIA-
MRPSAI--
MVPSAI--
MLPSAI--
-MPSAIW-
-MPSAIR-
MSPSAI--
-MPSAIV-
MGPSAI--
MFPSAI--
MYPSAI--
-MPSAII-
-MPSAIF-
MPPSAI--
MWPSAI--
-MPSAIT-
-MPSAIM-
MNPSAI--
MMPSAI--
MKPSAI--
-MPSAIY-
-MPSAIA-
MIPSAI--
MAPSAI--

MTPSAI--
-MPSAIH-
MDPSAI--
-MPSAIQ-
-MPSAIN-
-MPSAIK-
-MPSAIE-
-NPSAIV-
NRPSAI--
NNPSAI--
NGPSAI--
-NPSAIA-
NLPSAI--
NWPSAI--
-NPSAIS-
-NPSAIR-
-NPSAIL-
-NPSAIM-
NIPSAI--
NAPSAI--
NYPSAI--
NVPSAI--
NSPSAI--
-NPSAIY-
-NPSAII-
NFPSAI--
NDPSAI--
NQPSAI--
-NPSAIW-
-NPSAIK-
NMPSAI--
NHPSAI--
-NPSAIN-
NTPSAI--
-NPSAIQ-
-NPSAIH-
NPPSAI--
--PSAITL
--PSAIRL
--PSAIRT
--PSAILS
--PSAILT
--PSAIAR
--PSAISK
--PSAIRM
--PSAIHL
--PSAIFR
--PSAIWR
--PSAIVS
--PSAISG

--PSAIKN
--PSAIGL
--PSAISL
--PSAIKT
--PSAIVH
--PSAIRK
--PSAIMR
--PSAILR
--PSAIFS
--PSAIYF
--PSAITR
--PSAISS
--PSAISN
--PSAIKS
--PSAIIL
--PSAIHG
--PSAIAL
--PSAIST
--PSAIVY
--PSAITI
--PSAIRQ
--PSAIRI
--PSAIRD
--PSAINV
--PSAILA
--PSAIKE
--PSAIAY
--PSAISQ
--PSAIRY
--PSAIPL
--PSAILL
--PSAILK
--PSAIGT
--PSAIYG
--PSAIRA
--PSAIKR
--PSAIGI
PGPSAI--
--PSAIYT
--PSAIYS
--PSAITG
--PSAISW
--PSAISA
--PSAIQG
--PSAINS
--PSAIMS
--PSAILN
--PSAILM
--PSAIHT
--PSAIGF

--PSAIWY
--PSAIVR
--PSAIMT
--PSAILV
--PSAIKI
--PSAIIT
--PSAIIK
--PSAIHR
--PSAIAS
--PSAIAA
PRPSAI--
--PSAIWV
--PSAIWK
--PSAIVK
--PSAITS
--PSAITH
--PSAISM
--PSAIRW
--PSAIRN
--PSAINT
--PSAINQ
--PSAIMQ
--PSAIMI
--PSAILY
--PSAIKG
--PSAIFT
--PSAIFE
--PSAIAW
--PSAIAT
--PSAIAK
--PSAIAG
PYPSAI--
--PSAIYI
--PSAIVI
--PSAIVG
--PSAITK
--PSAISH
--PSAISD
--PSAINK
--PSAIMH
--PSAILD
--PSAIKY
--PSAIKK
--PSAIIM
--PSAIGG
--PSAIFM
--PSAIFF
--PSAIEW
PLPSAI--
PFPSAI--

PTPSAI--
PSPSAI--
--PSAIYK
--PSAIVT
--PSAIVM
--PSAITN
--PSAITM
--PSAISY
--PSAIRH
--PSAIRE
--PSAIQT
--PSAIQS
--PSAINL
--PSAIKV
--PSAIKM
--PSAIHY
--PSAIHF
--PSAIFN
PIPSAI--
PVPSAI--
--PSAIYM
--PSAIYD
--PSAIWQ
--PSAIVV
--PSAITV
--PSAITA
--PSAISV
--PSAISE
--PSAIRP
--PSAIQY
--PSAIPY
--PSAIPM
--PSAINW
--PSAINI
--PSAIMY
--PSAIMV
--PSAIMN
--PSAIMG
--PSAILH
--PSAILG
--PSAILE
--PSAIIW
--PSAIIQ
--PSAIHS
--PSAIHK
--PSAIGM
--PSAIFV
--PSAIFG
--PSAIES
--PSAIEG

--PSAICA
--PSAIAV
--PSAIAI
PAPSAI--
PWPSAI--
--PSAIYH
--PSAIYE
--PSAIWT
--PSAIWI
--PSAIWH
--PSAIVF
--PSAITF
--PSAITD
--PSAISI
--PSAIRG
--PSAIRF
--PSAIQW
--PSAIQK
--PSAIPI
--PSAINY
--PSAINR
--PSAINN
--PSAIML
--PSAIMD
--PSAIKW
--PSAIKF
--PSAIIS
--PSAIIN
--PSAIHW
--PSAIHH
--PSAIAM
PDPSAI--
PCPSAI--
--PSAIYQ
--PSAIYN
--PSAIWW
--PSAIWM
--PSAIWE
--PSAIVW
--PSAIVA
--PSAITT
--PSAITE
--PSAISF
--PSAIQV
--PSAIQQ
--PSAIQM
--PSAIQL
--PSAINH
--PSAIMW
--PSAILW

--PSAILQ
--PSAIKQ
--PSAIIV
--PSAIIG
--PSAIIE
--PSAIHQ
--PSAIHI
--PSAIHE
--PSAIGY
--PSAIGV
--PSAIGQ
--PSAIGA
--PSAIFQ
--PSAIFK
--PSAIFI
--PSAIET
--PSAIAQ
--PSAIAD
PNPSAI--
--PSAIYY
--PSAIYW
--PSAIYA
--PSAIWS
--PSAIWG
--PSAIWF
--PSAIWA
--PSAIVN
--PSAIQH
--PSAIQE
--PSAIPW
--PSAIPK
--PSAINF
--PSAIND
--PSAIMM
--PSAIMK
--PSAIMF
--PSAIKH
--PSAIKD
--PSAIIH
--PSAIIA
--PSAIHP
--PSAIHD
--PSAIHA
--PSAIGW
--PSAIGH
--PSAIEY
--PSAIEV
--PSAIEA
--PSAIDA
--PSAICV

--PSAICT
--PSAICS
PQPSAI--
-PPSAIY-
-PPSAIA-
QAPSAI--
-QPSAIK-
QRPSAI--
QLPSAI--
-QPSAIT-
-QPSAIR-
QVPSAI--
-QPSAIH-
QWPSAI--
-QPSAIS-
-QPSAIN-
-QPSAIL-
QKPSAI--
-QPSAIE-
QSPSAI--
-QPSAIF-
QPPSAI--
QFPSAI--
QQPSAI--
QNPSAI--
QIPSAI--
QTPSAI--
-QPSAIV-
-QPSAIQ-
-QPSAIG-
QEPSAI--
QYPSAI--
-QPSAIY-
-QPSAIW-
-QPSAIM-
QHPSAI--
QCPSAI--
-RPSAIL-
RIPSAI--
-RPSAIS-
RLPSAI--
RYPSAI--
RVPSAI--
-RPSAIA-
RSPSAI--
RTPSAI--
RFPSAI--
RGPSAI--
-RPSAII-
-RPSAIT-

RWPSAI--
-RPSAIQ-
RMPSAI--
-RPSAIV-
-RPSAIE-
-RPSAIH-
RNPSAI--
-RPSAIY-
-RPSAIG-
RQPSAI--
-RPSAIK-
-RPSAIF-
RDPSAI--
-RPSAIW-
RPPSAI--
RHPSAI--
REPSAI--
-RPSAIM-
-RPSAID-
RCPSAI--
-RPSAIC-
SYPSAI--
-SPSAIL-
-SPSAIR-
SVPSAI--
SLPSAI--
-SPSAIS-
STPSAI--
-SPSAIW-
-SPSAIV-
-SPSAIA-
SHPSAI--
-SPSAIY-
-SPSAIT-
-SPSAIE-
SQPSAI--
-SPSAIN-
-SPSAIK-
-SPSAIG-
SIPSAI--
SMPSAI--
SFPSAI--
SGPSAI--
SAPSAI--
-SPSAIM-
SPPSAI--
-SPSAIH-
SWPSAI--
SKPSAI--
SDPSAI--

SCPSAI--
-SPSAIC-
TRPSAI--
-TPSAIS-
TLPSAI--
TSPSAI--
-TPSAIK-
TFPSAI--
-TPSAIY-
-TPSAIV-
-TPSAIQ-
-TPSAIR-
-TPSAIM-
-TPSAIG-
TYPSAI--
-TPSAIN-
TPPSAI--
TQPSAI--
TTPSAI--
TVPSAI--
-TPSAII-
TMPSAI--
THPSAI--
TGPSAI--
TEPSAI--
-TPSAIH-
TIPSAI--
TWPSAI--
TKPSAI--
-TPSAIW-
-TPSAIT-
-TPSAIP-
-TPSAIF-
-TPSAIE-
-TPSAIA-
TNPSAI--
VTPSAI--
VRPSAI--
VLPSAI--
-VPSAIL-
VNPSAI--
VPPSAI--
VVPSAI--
-VPSAIH-
VKPSAI--
VSPSAI--
-VPSAIR-
-VPSAIS-
-VPSAIN-
-VPSAIG-

-VPSAIT-
VWPSAI--
-VPSAII-
VIPSAI--
VYPSAI--
VFPSAI--
-VPSAIY-
-VPSAIM-
-VPSAIA-
VMPSAI--
VGPSAI--
VAPSAI--
-VPSAIW-
-VPSAIF-
VHPSAI--
VDPSAI--
VQPSAI--
-VPSAIQ-
VEPSAI--
VCPSAI--
-VPSAIV-
-VPSAIC-
WKPSAI--
-WPSAIR-
-WPSAIS-
-WPSAIK-
WRPSAI--
WNPSAI--
WGPSAI--
WSPSAI--
-WPSAIL-
WQPSAI--
WFPSAI--
-WPSAIQ-
-WPSAIF-
-WPSAIA-
WPPSAI--
WMPSAI--
WTPSAI--
-WPSAIV-
-WPSAII-
WEPSAI--
WAPSAI--
WVPSAI--
-WPSAIT-
-WPSAIG-
WIPSAI--
WYPSAI--
-WPSAIE-
-YPSAIA-

```
           -YPSAIK-
           -YPSAIL-
           -YPSAIR-
           YKPSAI--
           YVPSAI--
           -YPSAIG-
           YFPSAI--
           YRPSAI--
           YQPSAI--
           -YPSAIT-
           YSPSAI--
           -YPSAIM-
           -YPSAII-
           -YPSAIW-
           YNPSAI--
           YLPSAI--
           YAPSAI--
           -YPSAIS-
           -YPSAIH-
           -YPSAIE-
           YMPSAI--
           -YPSAIQ-
           YIPSAI--
           YGPSAI--
           YDPSAI--
           YYPSAI--
           YWPSAI--
           -YPSAIY-
           -YPSAIN-
           -YPSAIF-
           YHPSAI--
           YEPSAI--
IANL         93        774     33.88
           DSIANL--
           --IANLPT
           --IANLNT
           --IANLKV
           VGIANL--
           -AIANLK-
           -AIANLR-
           ALIANL--
           -AIANLY-
           ATIANL--
           ARIANL--
           -AIANLG-
           AAIANL--
           AGIANL--
           AIIANL--
           -AIANLT-
           -AIANLM-
```

```
-AIANLF-
ANIANL--
AKIANL--
-AIANLL-
AFIANL--
-AIANLS-
-AIANLN-
-AIANLH-
AHIANL--
AWIANL--
AQIANL--
-AIANLI-
ADIANL--
AYIANL--
APIANL--
AEIANL--
AVIANL--
AMIANL--
-AIANLQ-
-AIANLE-
-AIANLD-
-AIANLA-
ACIANL--
CRIANL--
-CIANLW-
CTIANL--
-CIANLK-
-CIANLG-
CAIANL--
CVIANL--
-CIANLV-
-CIANLS-
-CIANLQ-
CGIANL--
CCIANL--
CWIANL--
CSIANL--
CMIANL--
-CIANLY-
-CIANLT-
-CIANLR-
-CIANLN-
-CIANLM-
-CIANLL-
-CIANLA-
DLIANL--
-DIANLL-
DKIANL--
-DIANLR-
DTIANL--
```

DGIANL--
DRIANL--
DEIANL--
DAIANL--
DWIANL--
-DIANLS-
DFIANL--
DVIANL--
-DIANLK-
-DIANLI-
-DIANLH-
DMIANL--
-DIANLW-
-DIANLN-
-DIANLE-
-DIANLA-
DPIANL--
DNIANL--
-DIANLM-
-DIANLG-
DHIANL--
-EIANLR-
EYIANL--
-EIANLF-
-EIANLQ-
ESIANL--
-EIANLS-
-EIANLA-
EGIANL--
EPIANL--
-EIANLI-
-EIANLG-
-EIANLE-
EWIANL--
ETIANL--
EMIANL--
-EIANLT-
EVIANL--
ELIANL--
-EIANLN-
ENIANL--
EIIANL--
EFIANL--
-EIANLW-
-EIANLP-
-EIANLK-
EHIANL--
EAIANL--
-FIANLG-
-FIANLR-

FNIANL--
FLIANL--
FSIANL--
FGIANL--
-FIANLK-
FAIANL--
-FIANLV-
-FIANLH-
FKIANL--
FIIANL--
-FIANLN-
FHIANL--
FEIANL--
-FIANLL-
FTIANL--
-FIANLA-
-FIANLS-
-FIANLQ-
FDIANL--
FVIANL--
-FIANLI-
-FIANLE-
GVIANL--
GSIANL--
-GIANLT-
GRIANL--
GLIANL--
-GIANLY-
GGIANL--
-GIANLQ-
GYIANL--
-GIANLS-
-GIANLA-
GMIANL--
GIIANL--
-GIANLK-
GEIANL--
GDIANL--
GWIANL--
-GIANLH-
-GIANLG-
GAIANL--
GTIANL--
GKIANL--
-GIANLI-
GPIANL--
-GIANLV-
-GIANLE-
GHIANL--
-GIANLN-

```
-GIANLM-
-GIANLF-
GFIANL--
GQIANL--
-GIANLC-
HFIANL--
-HIANLG-
-HIANLF-
HGIANL--
-HIANLR-
HRIANL--
-HIANLT-
HSIANL--
HLIANL--
-HIANLQ-
-HIANLL-
-HIANLY-
HYIANL--
HWIANL--
HKIANL--
HTIANL--
HPIANL--
HIIANL--
-HIANLN-
-HIANLK-
-HIANLA-
HVIANL--
HMIANL--
-HIANLE-
HAIANL--
-HIANLS-
-HIANLI-
HEIANL--
HDIANL--
HQIANL--
-HIANLW-
-HIANLV-
-HIANLM-
-HIANLH-
HHIANL--
--IANLRS
--IANLTL
--IANLST
--IANLLG
--IANLLT
--IANLKL
--IANLSG
--IANLLR
--IANLSM
--IANLRA
```

--IANLMR
--IANLIR
--IANLRH
--IANLSV
--IANLGL
--IANLLA
--IANLTR
--IANLSI
--IANLVA
--IANLRT
--IANLNL
--IANLLV
--IANLLM
--IANLHK
--IANLAT
--IANLVK
--IANLTG
--IANLAR
--IANLAN
--IANLAL
ILIANL--
--IANLWG
--IANLKG
--IANLGT
--IANLYS
--IANLRI
--IANLLK
IKIANL--
--IANLWQ
--IANLTW
--IANLTK
--IANLSS
--IANLSA
--IANLHL
--IANLGG
--IANLGA
IPIANL--
-IIANLR-
--IANLVY
--IANLSW
--IANLRG
--IANLRF
--IANLMG
--IANLGY
--IANLAG
-IIANLV-
--IANLYT
--IANLYA
--IANLWS
--IANLWD

```
--IANLVS
--IANLML
--IANLLH
--IANLIW
--IANLAK
--IANLVT
--IANLTT
--IANLTN
--IANLKS
--IANLKI
--IANLIS
--IANLAV
--IANLAS
-IIANLK-
--IANLWW
--IANLWT
--IANLTE
--IANLRN
--IANLPL
--IANLMN
--IANLLD
--IANLKR
--IANLIH
--IANLIG
--IANLHS
--IANLGS
--IANLAH
--IANLAD
INIANL--
-IIANLA-
--IANLVR
--IANLVH
--IANLTY
--IANLSQ
--IANLRD
--IANLIK
--IANLHG
--IANLFS
--IANLEV
--IANLYD
--IANLVF
--IANLTQ
--IANLTD
--IANLRQ
--IANLRE
--IANLQV
--IANLQS
--IANLQR
--IANLQH
--IANLNK
```

```
--IANLMV
--IANLMK
--IANLMI
--IANLLQ
--IANLKY
--IANLHW
--IANLFH
--IANLEA
--IANLAA
ITIANL--
-IIANLG-
--IANLYN
--IANLVW
--IANLVD
--IANLTI
--IANLTH
--IANLSK
--IANLSE
--IANLRW
--IANLRM
--IANLNG
--IANLNA
--IANLKK
--IANLIT
--IANLIQ
--IANLHT
--IANLHR
--IANLHM
--IANLFQ
--IANLFN
--IANLFE
--IANLEW
--IANLES
--IANLDS
--IANLAW
--IANLAF
IMIANL--
-IIANLS-
-IIANLQ-
-IIANLI-
-IIANLF-
--IANLYF
--IANLVM
--IANLVG
--IANLTV
--IANLTM
--IANLTF
--IANLSY
--IANLSN
--IANLRY
```

--IANLQF
--IANLNY
--IANLNW
--IANLNV
--IANLNS
--IANLNQ
--IANLMT
--IANLKM
--IANLKH
--IANLFG
--IANLEK
IAIANL--
IQIANL--
-IIANLM-
IFIANL--
--IANLYW
--IANLYH
--IANLVV
--IANLVN
--IANLVI
--IANLTS
--IANLSH
--IANLQL
--IANLQG
--IANLQE
--IANLQA
--IANLNM
--IANLNI
--IANLMQ
--IANLMM
--IANLKT
--IANLKD
--IANLIY
--IANLIM
--IANLII
--IANLHQ
--IANLHN
--IANLHA
--IANLGV
--IANLGI
--IANLER
--IANLEL
--IANLAM
IVIANL--
IIIANL--
-IIANLE-
IHIANL--
IGIANL--
ICIANL--
--IANLYE

--IANLWH
--IANLVQ
--IANLVE
--IANLSD
--IANLQQ
--IANLQN
--IANLQK
--IANLPY
--IANLNF
--IANLMY
--IANLMH
--IANLMF
--IANLME
--IANLLE
--IANLKW
--IANLKQ
--IANLKN
--IANLIP
--IANLIE
--IANLHI
--IANLHF
--IANLGW
--IANLGR
--IANLGQ
--IANLGF
--IANLGE
--IANLFI
--IANLET
--IANLEQ
--IANLEI
--IANLDY
--IANLDG
--IANLDA
--IANLCT
--IANLCR
--IANLAY
--IANLAQ
--IANLAE
-KIANLS-
KVIANL--
KGIANL--
-KIANLY-
-KIANLA-
-KIANLI-
KLIANL--
KIIANL--
-KIANLG-
KAIANL--
KWIANL--
KPIANL--

```
-KIANLT-
-KIANLV-
-KIANLF-
KDIANL--
KHIANL--
-KIANLN-
KEIANL--
-KIANLE-
KFIANL--
-LIANLS-
-LIANLA-
LTIANL--
-LIANLV-
-LIANLK-
LVIANL--
-LIANLG-
-LIANLN-
LAIANL--
LPIANL--
-LIANLQ-
LDIANL--
-LIANLI-
-LIANLD-
LQIANL--
LHIANL--
LFIANL--
LEIANL--
LWIANL--
LIIANL--
LGIANL--
LNIANL--
-LIANLE-
MRIANL--
MHIANL--
MEIANL--
MSIANL--
-MIANLL-
MMIANL--
-MIANLK-
MTIANL--
-MIANLR-
MNIANL--
MGIANL--
MVIANL--
-MIANLM-
MYIANL--
-MIANLY-
-MIANLA-
MAIANL--
MPIANL--
```

MKIANL--
MFIANL--
MDIANL--
-MIANLQ-
-MIANLN-
-MIANLH-
-MIANLE-
-MIANLD-
-MIANLC-
-NIANLR-
NTIANL--
NRIANL--
-NIANLL-
NGIANL--
-NIANLY-
-NIANLA-
-NIANLS-
NSIANL--
NIIANL--
-NIANLW-
-NIANLK-
NFIANL--
NWIANL--
NVIANL--
-NIANLV-
NHIANL--
NDIANL--
NLIANL--
-NIANLE-
NPIANL--
NQIANL--
NMIANL--
-NIANLQ-
-NIANLN-
-NIANLD-
NNIANL--
NKIANL--
-NIANLM-
-NIANLF-
-NIANLI-
-NIANLC-
NEIANL--
NCIANL--
PSIANL--
-PIANLR-
PRIANL--
PLIANL--
PTIANL--
-PIANLL-
-PIANLV-

PWIANL--
PIIANL--
PQIANL--
PNIANL--
-PIANLT-
-PIANLS-
-PIANLI-
-PIANLH-
-PIANLA-
-PIANLQ-
-PIANLN-
-PIANLK-
PGIANL--
PFIANL--
PEIANL--
PDIANL--
PKIANL--
-PIANLF-
-PIANLM-
PPIANL--
-PIANLY-
-PIANLE-
-PIANLD-
PAIANL--
-QIANLS-
-QIANLR-
-QIANLT-
-QIANLW-
-QIANLN-
QSIANL--
-QIANLV-
-QIANLG-
-QIANLL-
-QIANLA-
QLIANL--
QGIANL--
QDIANL--
QWIANL--
QTIANL--
-QIANLY-
-QIANLI-
-QIANLH-
-QIANLD-
QHIANL--
QCIANL--
QAIANL--
QYIANL--
QPIANL--
QIIANL--
-QIANLQ-

-QIANLK-
-QIANLE-
QEIANL--
-RIANLS-
RAIANL--
-RIANLQ-
-RIANLI-
-RIANLG-
-RIANLN-
-RIANLM-
-RIANLY-
RHIANL--
RTIANL--
-RIANLT-
-RIANLA-
RPIANL--
-RIANLW-
RDIANL--
-RIANLE-
REIANL--
-RIANLC-
-SIANLR-
SWIANL--
-SIANLV-
SHIANL--
-SIANLL-
-SIANLG-
STIANL--
-SIANLY-
-SIANLT-
-SIANLN-
-SIANLK-
SFIANL--
SQIANL--
SYIANL--
-SIANLF-
SAIANL--
SVIANL--
-SIANLH-
SEIANL--
SDIANL--
SIIANL--
-SIANLW-
-SIANLE-
-SIANLM-
-SIANLQ-
-SIANLI-
-SIANLA-
SPIANL--
-SIANLS-

TRIANL--
-TIANLT-
-TIANLL-
TVIANL--
TLIANL--
TSIANL--
-TIANLR-
TGIANL--
TNIANL--
-TIANLV-
-TIANLM-
-TIANLQ-
-TIANLF-
TDIANL--
TYIANL--
TIIANL--
-TIANLS-
-TIANLE-
-TIANLA-
TEIANL--
TAIANL--
-TIANLK-
-TIANLI-
TKIANL--
-TIANLG-
TPIANL--
TFIANL--
-TIANLY-
-TIANLN-
TWIANL--
-TIANLH-
THIANL--
-VIANLR-
VRIANL--
VHIANL--
VLIANL--
VAIANL--
VTIANL--
-VIANLS-
VSIANL--
VVIANL--
-VIANLK-
-VIANLT-
-VIANLG-
-VIANLA-
VMIANL--
VKIANL--
VYIANL--
VNIANL--
VIIANL--

```
-VIANLN-
VQIANL--
-VIANLQ-
VDIANL--
-VIANLH-
VCIANL--
-VIANLV-
-VIANLM-
-VIANLE-
VEIANL--
-WIANLA-
-WIANLQ-
WSIANL--
WKIANL--
WPIANL--
-WIANLG-
WEIANL--
WFIANL--
WTIANL--
-WIANLT-
-WIANLM-
-WIANLL-
WAIANL--
WMIANL--
-WIANLD-
WRIANL--
-WIANLN-
-WIANLF-
WGIANL--
WCIANL--
WQIANL--
-YIANLS-
YGIANL--
-YIANLR-
YWIANL--
YTIANL--
YLIANL--
YEIANL--
YVIANL--
YKIANL--
-YIANLG-
-YIANLI-
-YIANLH-
-YIANLD-
YHIANL--
YQIANL--
-YIANLA-
YYIANL--
-YIANLT-
-YIANLQ-
```

| | | | |
|---|---|---|---|
| | -YIANLE- | | |
| | -YIANLN- | | |
| | YDIANL-- | | |
| | YNIANL-- | | |
| | -YIANLV- | | |
| | -YIANLK- | | |
| | YIIANL-- | | |
| VSNL | 163 | 734 | 33.88 |
| | -EVSNLN- | | |
| | -GVSNLG- | | |
| | -PVSNLD- | | |
| | --VSNLGV | | |
| | --VSNLVP | | |
| | --VSNLTP | | |
| | --VSNLTD | | |
| | --VSNLTC | | |
| | --VSNLSE | | |
| | --VSNLPN | | |
| | --VSNLPG | | |
| | --VSNLPF | | |
| | --VSNLNF | | |
| | --VSNLIP | | |
| | --VSNLHN | | |
| | --VSNLEM | | |
| | --VSNLAQ | | |
| | --VSNLAD | | |
| | -YVSNLD- | | |
| | ALVSNL-- | | |
| | -AVSNLL- | | |
| | ASVSNL-- | | |
| | -AVSNLR- | | |
| | ARVSNL-- | | |
| | -AVSNLA- | | |
| | AGVSNL-- | | |
| | AYVSNL-- | | |
| | -AVSNLV- | | |
| | -AVSNLT- | | |
| | ANVSNL-- | | |
| | -AVSNLI- | | |
| | ATVSNL-- | | |
| | AKVSNL-- | | |
| | -AVSNLM- | | |
| | APVSNL-- | | |
| | AHVSNL-- | | |
| | AFVSNL-- | | |
| | -AVSNLQ- | | |
| | -AVSNLF- | | |
| | -AVSNLN- | | |
| | -AVSNLE- | | |
| | ADVSNL-- | | |

```
-AVSNLH-
AQVSNL--
AEVSNL--
-AVSNLG-
-CVSNLL-
CCVSNL--
CLVSNL--
CSVSNL--
-CVSNLR-
CRVSNL--
CKVSNL--
CVVSNL--
-CVSNLV-
-CVSNLT-
-CVSNLS-
CGVSNL--
CDVSNL--
-CVSNLY-
-CVSNLQ-
-CVSNLH-
-CVSNLF-
-CVSNLA-
CQVSNL--
CMVSNL--
CAVSNL--
CTVSNL--
CPVSNL--
CIVSNL--
CYVSNL--
CWVSNL--
-CVSNLM-
-CVSNLK-
-CVSNLG-
CNVSNL--
CEVSNL--
-DVSNLL-
DLVSNL--
DWVSNL--
DVVSNL--
-DVSNLS-
DMVSNL--
DSVSNL--
DPVSNL--
-DVSNLY-
DTVSNL--
DAVSNL--
-DVSNLI-
DEVSNL--
DFVSNL--
-DVSNLK-
```

-DVSNLA-
DNVSNL--
DGVSNL--
DYVSNL--
-DVSNLF-
DKVSNL--
DHVSNL--
-DVSNLV-
-DVSNLQ-
-DVSNLE-
DIVSNL--
-DVSNLT-
-DVSNLM-
-DVSNLN-
-DVSNLH-
-DVSNLD-
DQVSNL--
-EVSNLL-
-EVSNLR-
EWVSNL--
-EVSNLS-
-EVSNLK-
-EVSNLW-
-EVSNLY-
-EVSNLQ-
-EVSNLM-
-EVSNLV-
-EVSNLE-
ESVSNL--
ECVSNL--
ETVSNL--
EGVSNL--
-EVSNLI-
-EVSNLA-
ENVSNL--
EKVSNL--
EEVSNL--
EHVSNL--
EAVSNL--
EYVSNL--
EQVSNL--
-EVSNLH-
-EVSNLG-
EPVSNL--
EDVSNL--
-EVSNLF-
FTVSNL--
-FVSNLS-
FSVSNL--
FYVSNL--

FVVSNL--
-FVSNLI-
-FVSNLQ-
FNVSNL--
FLVSNL--
FQVSNL--
FHVSNL--
-FVSNLM-
-FVSNLA-
FDVSNL--
-FVSNLK-
-FVSNLH-
FEVSNL--
-FVSNLE-
FPVSNL--
FCVSNL--
-FVSNLD-
-GVSNLL-
GSVSNL--
GVVSNL--
-GVSNLT-
-GVSNLK-
GPVSNL--
-GVSNLS-
-GVSNLH-
GYVSNL--
-GVSNLV-
GGVSNL--
GNVSNL--
-GVSNLM-
-GVSNLY-
GTVSNL--
-GVSNLQ-
GAVSNL--
GQVSNL--
-GVSNLW-
GHVSNL--
-GVSNLN-
GIVSNL--
-GVSNLI-
GFVSNL--
GEVSNL--
-GVSNLC-
GCVSNL--
HRVSNL--
HVVSNL--
HAVSNL--
-HVSNLT-
HKVSNL--
HLVSNL--

-HVSNLY-
HTVSNL--
HGVSNL--
HSVSNL--
-HVSNLI-
-HVSNLV-
-HVSNLA-
HMVSNL--
HFVSNL--
-HVSNLH-
HHVSNL--
-HVSNLW-
HQVSNL--
HNVSNL--
HYVSNL--
-HVSNLG-
HIVSNL--
HEVSNL--
HPVSNL--
-HVSNLN-
-HVSNLK-
-HVSNLE-
HCVSNL--
HDVSNL--
-IVSNLL-
IRVSNL--
IVVSNL--
IQVSNL--
IKVSNL--
ISVSNL--
ITVSNL--
-IVSNLS-
IGVSNL--
INVSNL--
-IVSNLA-
IMVSNL--
IAVSNL--
IYVSNL--
-IVSNLV-
-IVSNLN-
IPVSNL--
-IVSNLM-
-IVSNLG-
-IVSNLE-
-IVSNLQ-
IIVSNL--
-IVSNLK-
IHVSNL--
IWVSNL--
-IVSNLH-

IEVSNL--
-IVSNLI-
ICVSNL--
-IVSNLD-
-KVSNLR-
-KVSNLS-
KVVSNL--
KTVSNL--
KYVSNL--
KFVSNL--
-KVSNLT-
-KVSNLL-
KAVSNL--
-KVSNLV-
KEVSNL--
KQVSNL--
-KVSNLY-
-KVSNLI-
-KVSNLM-
KDVSNL--
-KVSNLH-
KHVSNL--
-KVSNLF-
-KVSNLQ-
-KVSNLA-
KPVSNL--
-KVSNLK-
-KVSNLN-
-KVSNLE-
KCVSNL--
-LVSNLR-
LVVSNL--
LLVSNL--
-LVSNLS-
LKVSNL--
LYVSNL--
-LVSNLK-
LEVSNL--
LIVSNL--
-LVSNLM-
-LVSNLH-
-LVSNLQ-
LQVSNL--
LDVSNL--
-LVSNLE-
LCVSNL--
-LVSNLC-
-MVSNLR-
MSVSNL--
-MVSNLF-

MKVSNL--
MGVSNL--
MQVSNL--
-MVSNLA-
MDVSNL--
MWVSNL--
-MVSNLV-
MIVSNL--
-MVSNLL-
-MVSNLE-
MAVSNL--
-MVSNLN-
MNVSNL--
-MVSNLK-
MHVSNL--
-MVSNLH-
-MVSNLM-
-MVSNLW-
-MVSNLT-
-MVSNLI-
MEVSNL--
MYVSNL--
MFVSNL--
-MVSNLQ-
-MVSNLD-
-MVSNLC-
-MVSNLP-
MCVSNL--
-NVSNLV-
NYVSNL--
NLVSNL--
-NVSNLS-
-NVSNLR-
NTVSNL--
-NVSNLL-
NAVSNL--
NSVSNL--
NKVSNL--
NGVSNL--
-NVSNLK-
-NVSNLI-
NFVSNL--
NDVSNL--
-NVSNLY-
NMVSNL--
-NVSNLA-
-NVSNLM-
-NVSNLT-
-NVSNLF-
NPVSNL--

NIVSNL--
-NVSNLH-
-NVSNLE-
NQVSNL--
-NVSNLN-
NHVSNL--
NCVSNL--
-PVSNLL-
PGVSNL--
PKVSNL--
-PVSNLR-
PLVSNL--
PVVSNL--
-PVSNLS-
-PVSNLH-
-PVSNLY-
PTVSNL--
-PVSNLW-
-PVSNLT-
PNVSNL--
PHVSNL--
-PVSNLV-
-PVSNLI-
-PVSNLN-
PYVSNL--
-PVSNLA-
PIVSNL--
PWVSNL--
-PVSNLQ-
-PVSNLG-
PMVSNL--
PAVSNL--
-PVSNLE-
-PVSNLF-
PQVSNL--
PDVSNL--
PPVSNL--
PCVSNL--
-QVSNLL-
-QVSNLN-
QGVSNL--
QIVSNL--
-QVSNLS-
-QVSNLR-
-QVSNLK-
QKVSNL--
QLVSNL--
-QVSNLT-
QSVSNL--
-QVSNLI-

QTVSNL--
QFVSNL--
QEVSNL--
QYVSNL--
-QVSNLF-
-QVSNLY-
QQVSNL--
-QVSNLG-
QAVSNL--
-QVSNLQ-
QPVSNL--
QNVSNL--
-QVSNLW-
-QVSNLH-
QDVSNL--
-QVSNLM-
QHVSNL--
-QVSNLV-
-QVSNLE-
QCVSNL--
-RVSNLR-
-RVSNLL-
-RVSNLS-
-RVSNLI-
-RVSNLH-
-RVSNLQ-
-RVSNLW-
REVSNL--
-RVSNLM-
RDVSNL--
-RVSNLN-
-RVSNLE-
RCVSNL--
SVVSNL--
-SVSNLS-
SPVSNL--
SKVSNL--
-SVSNLA-
SAVSNL--
-SVSNLY-
-SVSNLT-
SSVSNL--
SWVSNL--
-SVSNLI-
SIVSNL--
-SVSNLH-
SQVSNL--
SMVSNL--
SYVSNL--
-SVSNLN-

-SVSNLM-
SNVSNL--
-SVSNLE-
-SVSNLG-
TKVSNL--
-TVSNLL-
-TVSNLR-
-TVSNLT-
-TVSNLI-
TNVSNL--
TGVSNL--
TVVSNL--
-TVSNLF-
TPVSNL--
TMVSNL--
-TVSNLY-
TTVSNL--
TIVSNL--
TSVSNL--
TYVSNL--
-TVSNLQ-
-TVSNLN-
-TVSNLK-
TQVSNL--
TWVSNL--
-TVSNLA-
TFVSNL--
-TVSNLG-
TEVSNL--
TDVSNL--
THVSNL--
TCVSNL--
VRVSNL--
-VVSNLL-
--VSNLRG
--VSNLRR
--VSNLRT
--VSNLRN
-VVSNLS-
--VSNLLG
--VSNLMR
--VSNLGS
VGVSNL--
--VSNLKA
-VVSNLR-
--VSNLRA
--VSNLFS
VTVSNL--
VVVSNL--
--VSNLST

```
--VSNLKT
-VVSNLN-
--VSNLRY
--VSNLYK
--VSNLKS
-VVSNLV-
--VSNLRE
--VSNLSL
--VSNLMS
--VSNLVY
--VSNLLN
--VSNLRH
--VSNLRD
--VSNLWG
--VSNLTG
--VSNLIR
VAVSNL--
--VSNLYR
--VSNLVV
VMVSNL--
-VVSNLI-
--VSNLTS
--VSNLTR
--VSNLSR
--VSNLVF
--VSNLMA
--VSNLIS
--VSNLFT
-VVSNLT-
--VSNLSM
--VSNLNS
--VSNLTT
--VSNLSF
--VSNLMI
--VSNLLQ
-VVSNLQ-
--VSNLAS
VYVSNL--
--VSNLWT
--VSNLMG
--VSNLFG
--VSNLAL
VQVSNL--
--VSNLES
--VSNLTK
--VSNLMT
--VSNLFN
--VSNLYT
--VSNLRW
--VSNLMK
```

```
--VSNLLM
--VSNLAG
-VVSNLA-
--VSNLVA
--VSNLMV
--VSNLIA
VNVSNL--
-VVSNLM-
--VSNLTY
--VSNLTW
--VSNLTN
--VSNLQH
--VSNLNA
--VSNLKG
--VSNLAV
--VSNLTQ
--VSNLLK
--VSNLHS
-VVSNLH-
--VSNLVH
--VSNLTI
--VSNLTH
--VSNLTA
--VSNLKN
--VSNLIK
--VSNLIH
--VSNLGT
--VSNLFK
VIVSNL--
--VSNLKL
VDVSNL--
--VSNLWN
--VSNLVW
--VSNLVM
--VSNLQV
--VSNLQG
--VSNLMY
--VSNLLH
--VSNLIW
--VSNLIT
--VSNLAY
--VSNLYG
--VSNLYA
--VSNLWR
--VSNLSQ
--VSNLSH
--VSNLAH
--VSNLTM
--VSNLQT
--VSNLNG
```

--VSNLLE
--VSNLYN
--VSNLMQ
--VSNLKF
--VSNLYM
--VSNLYI
--VSNLQR
--VSNLNT
--VSNLIV
VHVSNL--
--VSNLWA
--VSNLKQ
--VSNLHG
--VSNLGA
--VSNLAT
--VSNLYW
--VSNLWK
--VSNLQM
--VSNLMD
--VSNLIQ
--VSNLET
--VSNLAM
--VSNLAA
--VSNLWQ
--VSNLWH
--VSNLVQ
--VSNLVE
--VSNLMM
--VSNLIE
--VSNLHY
--VSNLFQ
--VSNLEY
-VVSNLE-
--VSNLYQ
--VSNLQK
--VSNLNV
--VSNLLD
--VSNLKH
--VSNLIN
--VSNLHV
--VSNLEG
VCVSNL--
--VSNLQW
--VSNLQA
--VSNLMH
--VSNLKW
--VSNLHW
--VSNLFH
--VSNLAR
--VSNLAN

--VSNLAK
--VSNLQY
--VSNLQL
--VSNLQF
--VSNLME
--VSNLKE
--VSNLKD
--VSNLHM
--VSNLHA
--VSNLFD
--VSNLAF
-VVSNLC-
--VSNLYD
--VSNLHK
--VSNLHH
--VSNLHF
--VSNLAI
--VSNLSD
--VSNLQN
--VSNLPL
--VSNLHR
--VSNLGW
--VSNLER
--VSNLEF
--VSNLWD
--VSNLQQ
--VSNLQE
--VSNLNY
--VSNLNM
--VSNLHQ
--VSNLFE
--VSNLEI
--VSNLQD
--VSNLHI
--VSNLHD
--VSNLEK
--VSNLED
--VSNLCT
--VSNLCS
--VSNLCI
-WVSNLS-
-WVSNLV-
-WVSNLA-
WYVSNL--
WEVSNL--
-WVSNLQ-
-WVSNLK-
-WVSNLE-
-WVSNLH-
-WVSNLG-

-WVSNLN-
WLVSNL--
WKVSNL--
WHVSNL--
-WVSNLM-
WPVSNL--
WQVSNL--
WMVSNL--
WVVSNL--
-WVSNLD-
WDVSNL--
WNVSNL--
YRVSNL--
YSVSNL--
-YVSNLS-
YTVSNL--
YVVSNL--
YKVSNL--
-YVSNLV-
-YVSNLR-
YWVSNL--
-YVSNLI-
YQVSNL--
YGVSNL--
YFVSNL--
-YVSNLG-
-YVSNLH-
YHVSNL--
-YVSNLA-
YDVSNL--
-YVSNLN-
-YVSNLK-
YPVSNL--
YEVSNL--
-YVSNLE-
-YVSNLQ-
YAVSNL--
YYVSNL--
YNVSNL--
-YVSNLM-
-YVSNLW-
YCVSNL--

| | | | |
|---|---|---|---|
| LGAL | 192 | 716 | 33.86 |

-ALGALP-
-ALGALG-
-DLGALD-
-GLGALE-
-LLGALD-
--LGALRP
--LGALNR

--LGALVP
--LGALTE
--LGALTD
--LGALQN
--LGALLP
--LGALLD
--LGALKY
--LGALGN
--LGALEY
--LGALEV
--LGALDH
--LGALCA
-NLGALP-
-SLGALG-
-TLGALG-
-ALGALT-
ALLGAL--
ASLGAL--
-ALGALL-
AVLGAL--
-ALGALY-
-ALGALH-
AYLGAL--
-ALGALS-
AILGAL--
AGLGAL--
-ALGALI-
-ALGALN-
-ALGALF-
ANLGAL--
-ALGALV-
AWLGAL--
-ALGALK-
-ALGALQ-
AQLGAL--
ADLGAL--
-ALGALM-
AFLGAL--
AELGAL--
AMLGAL--
-ALGALA-
ACLGAL--
-ALGALE-
-CLGALR-
CGLGAL--
CCLGAL--
CTLGAL--
-CLGALY-
CSLGAL--
CDLGAL--

CYLGAL--
CPLGAL--
-CLGALS-
CVLGAL--
CNLGAL--
-CLGALW-
-CLGALT-
-CLGALN-
CFLGAL--
CMLGAL--
CLLGAL--
-CLGALK-
-CLGALF-
-CLGALA-
CILGAL--
CWLGAL--
-CLGALV-
-CLGALI-
CALGAL--
-DLGALR-
DKLGAL--
DRLGAL--
DLLGAL--
DALGAL--
DFLGAL--
-DLGALF-
-DLGALH-
-DLGALI-
DQLGAL--
DSLGAL--
-DLGALT-
DYLGAL--
DTLGAL--
-DLGALL-
DGLGAL--
DILGAL--
DNLGAL--
-DLGALS-
DCLGAL--
DMLGAL--
-DLGALN-
DWLGAL--
-DLGALQ-
DDLGAL--
-DLGALY-
-DLGALM-
DELGAL--
ERLGAL--
-ELGALK-
-ELGALR-

EWLGAL--
EVLGAL--
EILGAL--
EKLGAL--
-ELGALL-
-ELGALY-
EGLGAL--
EYLGAL--
EQLGAL--
-ELGALH-
-ELGALT-
-ELGALF-
ENLGAL--
-ELGALV-
-ELGALQ-
EFLGAL--
EALGAL--
ETLGAL--
ELLGAL--
EMLGAL--
EELGAL--
-ELGALA-
EDLGAL--
EPLGAL--
-ELGALN-
ECLGAL--
-FLGALR-
FKLGAL--
-FLGALA-
FGLGAL--
FLLGAL--
-FLGALT-
-FLGALL-
FHLGAL--
-FLGALN-
FQLGAL--
FNLGAL--
FILGAL--
FELGAL--
FALGAL--
-FLGALQ-
-FLGALI-
-FLGALM-
-FLGALH-
-FLGALG-
-FLGALE-
FWLGAL--
FPLGAL--
FCLGAL--
-GLGALL-

GNLGAL--
GTLGAL--
GVLGAL--
GHLGAL--
GMLGAL--
GGLGAL--
-GLGALI-
GILGAL--
GFLGAL--
-GLGALT-
GYLGAL--
GWLGAL--
GPLGAL--
-GLGALH-
-GLGALQ-
-GLGALN-
-GLGALK-
-GLGALW-
-GLGALV-
GDLGAL--
GCLGAL--
GELGAL--
-GLGALA-
-GLGALC-
HVLGAL--
-HLGALT-
HLLGAL--
HSLGAL--
HRLGAL--
HMLGAL--
-HLGALV-
-HLGALY-
HYLGAL--
HPLGAL--
-HLGALS-
-HLGALQ-
HNLGAL--
-HLGALL-
-HLGALF-
HKLGAL--
-HLGALH-
HFLGAL--
HILGAL--
HELGAL--
-HLGALA-
HALGAL--
-HLGALN-
-HLGALK-
HGLGAL--
HWLGAL--

-HLGALM-
HCLGAL--
IRLGAL--
ILLGAL--
-ILGALT-
IQLGAL--
IKLGAL--
IHLGAL--
-ILGALS-
IALGAL--
ITLGAL--
-ILGALR-
-ILGALN-
IDLGAL--
INLGAL--
-ILGALQ-
IFLGAL--
IPLGAL--
-ILGALV-
IWLGAL--
-ILGALK-
-ILGALA-
IELGAL--
-ILGALI-
-ILGALG-
-ILGALE-
-ILGALH-
ICLGAL--
-ILGALM-
-ILGALC-
KSLGAL--
-KLGALS-
KGLGAL--
-KLGALY-
KPLGAL--
-KLGALF-
KALGAL--
KLLGAL--
KDLGAL--
KNLGAL--
-KLGALI-
KILGAL--
KQLGAL--
-KLGALK-
-KLGALV-
-KLGALQ-
KMLGAL--
KWLGAL--
-KLGALW-
KFLGAL--

KCLGAL--
-KLGALM-
KELGAL--
-KLGALA-
-KLGALN-
LSLGAL--
--LGALRY
--LGALSS
--LGALRG
LTLGAL--
--LGALSL
--LGALLG
--LGALAL
--LGALHL
--LGALRV
--LGALLS
--LGALVN
--LGALSV
--LGALNS
--LGALLA
--LGALKT
LILGAL--
--LGALTT
--LGALSI
--LGALMG
--LGALHR
LALGAL--
LYLGAL--
--LGALRT
-LLGALK-
--LGALYR
--LGALTS
--LGALFS
--LGALFR
-LLGALT-
--LGALYS
--LGALSG
--LGALRH
--LGALMN
--LGALAG
LQLGAL--
LHLGAL--
--LGALWT
-LLGALG-
--LGALHG
-LLGALQ-
--LGALIS
--LGALRD
--LGALMS
--LGALSM

```
--LGALSF
--LGALPL
--LGALNT
--LGALKS
LMLGAL--
-LLGALN-
--LGALYT
--LGALVT
--LGALTG
--LGALRQ
--LGALML
--LGALLK
LWLGAL--
--LGALWI
--LGALPY
--LGALHK
-LLGALH-
--LGALWN
--LGALVY
--LGALRW
--LGALNA
--LGALGI
--LGALMA
--LGALLH
--LGALKG
--LGALHV
--LGALFK
--LGALES
--LGALSY
--LGALNM
--LGALIR
LELGAL--
--LGALLN
-LLGALP-
-LLGALE-
--LGALKV
--LGALYN
--LGALWH
--LGALRE
--LGALKF
--LGALGM
--LGALEG
--LGALAA
--LGALNY
--LGALLY
LPLGAL--
--LGALPF
--LGALMH
--LGALKN
--LGALKA
```

--LGALIN
--LGALFN
--LGALYQ
--LGALVF
--LGALVA
--LGALQG
--LGALLM
--LGALKR
--LGALKM
--LGALAV
--LGALTM
--LGALQV
--LGALQS
--LGALMD
--LGALIV
--LGALIG
--LGALHI
--LGALWK
--LGALWG
--LGALVH
--LGALTY
--LGALSN
--LGALIW
--LGALIA
--LGALHD
--LGALAI
-LLGALW-
--LGALYW
--LGALVW
--LGALQW
--LGALNV
--LGALHT
--LGALFD
--LGALAW
--LGALPI
--LGALLW
--LGALIK
--LGALHA
--LGALAM
--LGALYH
--LGALWW
--LGALVM
--LGALTW
--LGALQT
--LGALNI
--LGALIH
--LGALHN
--LGALHM
--LGALHH
-LLGALM-

--LGALSQ
--LGALQL
--LGALMM
--LGALHF
--LGALFE
--LGALYA
--LGALWQ
--LGALLQ
--LGALGS
--LGALAQ
--LGALYD
--LGALVE
--LGALVD
--LGALTN
--LGALNW
--LGALNH
--LGALME
--LGALKW
--LGALHQ
--LGALHE
--LGALAT
LCLGAL--
--LGALWE
--LGALVQ
--LGALSK
--LGALQY
--LGALQK
--LGALEA
--LGALAF
--LGALTH
--LGALQR
--LGALPM
--LGALNF
--LGALMQ
--LGALKQ
--LGALGA
--LGALFH
--LGALTQ
--LGALQI
--LGALQH
--LGALQF
--LGALNK
--LGALNG
--LGALII
-LLGALC-
--LGALQM
--LGALNQ
--LGALKD
--LGALIE
--LGALGQ

--LGALQD
--LGALPK
--LGALND
--LGALHC
--LGALGC
--LGALEF
--LGALDN
--LGALCY
--LGALCT
--LGALCS
--LGALCG
--LGALAH
MVLGAL--
-MLGALR-
MSLGAL--
-MLGALN-
-MLGALL-
-MLGALS-
MKLGAL--
-MLGALQ-
-MLGALT-
MDLGAL--
MMLGAL--
MGLGAL--
-MLGALH-
MQLGAL--
MYLGAL--
MILGAL--
MALGAL--
MWLGAL--
MELGAL--
-MLGALK-
-MLGALG-
-MLGALF-
MPLGAL--
-MLGALM-
-MLGALI-
MHLGAL--
MCLGAL--
-NLGALL-
-NLGALR-
NKLGAL--
-NLGALN-
NVLGAL--
NSLGAL--
NLLGAL--
-NLGALY-
NFLGAL--
NILGAL--
NQLGAL--

```
-NLGALI-
-NLGALH-
NHLGAL--
NGLGAL--
NWLGAL--
-NLGALS-
-NLGALA-
NALGAL--
NYLGAL--
-NLGALT-
-NLGALF-
-NLGALQ-
-NLGALV-
-NLGALK-
NELGAL--
NMLGAL--
-NLGALM-
NDLGAL--
-NLGALE-
-NLGALC-
-PLGALR-
PSLGAL--
-PLGALI-
PYLGAL--
PALGAL--
PWLGAL--
PMLGAL--
-PLGALT-
-PLGALH-
PDLGAL--
-PLGALA-
PNLGAL--
PGLGAL--
PFLGAL--
-PLGALN-
-PLGALG-
PILGAL--
-PLGALK-
-PLGALW-
PQLGAL--
-PLGALQ-
PHLGAL--
-PLGALE-
-PLGALC-
QPLGAL--
QSLGAL--
-QLGALT-
-QLGALR-
-QLGALK-
QRLGAL--
```

QTLGAL--
QKLGAL--
QNLGAL--
-QLGALY-
-QLGALV-
-QLGALS-
QELGAL--
QQLGAL--
-QLGALW-
-QLGALA-
QILGAL--
QHLGAL--
QGLGAL--
-QLGALQ-
QDLGAL--
QLLGAL--
QFLGAL--
QCLGAL--
QMLGAL--
QALGAL--
QWLGAL--
-QLGALN-
-QLGALM-
-QLGALE-
-RLGALL-
-RLGALV-
RDLGAL--
-RLGALI-
RPLGAL--
RMLGAL--
-RLGALQ-
-RLGALA-
-RLGALM-
RCLGAL--
-RLGALC-
SRLGAL--
SLLGAL--
SGLGAL--
-SLGALR-
-SLGALK-
-SLGALI-
-SLGALS-
-SLGALT-
SVLGAL--
SNLGAL--
SHLGAL--
-SLGALY-
SDLGAL--
-SLGALH-
SKLGAL--

SPLGAL--
-SLGALV-
SWLGAL--
-SLGALQ-
-SLGALM-
SQLGAL--
SMLGAL--
SELGAL--
-SLGALW-
SILGAL--
-SLGALA-
SCLGAL--
-SLGALC-
TSLGAL--
TWLGAL--
TVLGAL--
TTLGAL--
-TLGALS-
-TLGALK-
-TLGALW-
-TLGALT-
TYLGAL--
TFLGAL--
TALGAL--
TGLGAL--
-TLGALM-
-TLGALY-
TDLGAL--
TPLGAL--
-TLGALN-
TELGAL--
TKLGAL--
TNLGAL--
TQLGAL--
-TLGALQ-
-TLGALF-
TMLGAL--
TCLGAL--
THLGAL--
-TLGALE-
VLLGAL--
VSLGAL--
-VLGALS-
-VLGALA-
VMLGAL--
VNLGAL--
VGLGAL--
-VLGALL-
VFLGAL--
VYLGAL--

VHLGAL--
VQLGAL--
-VLGALI-
VPLGAL--
VDLGAL--
VILGAL--
VELGAL--
-VLGALM-
-VLGALE-
VCLGAL--
-WLGALS-
WTLGAL--
WYLGAL--
-WLGALA-
-WLGALR-
WALGAL--
-WLGALT-
-WLGALH-
WKLGAL--
WNLGAL--
-WLGALN-
WPLGAL--
-WLGALL-
WMLGAL--
-WLGALQ-
WLLGAL--
WFLGAL--
-WLGALI-
WELGAL--
-WLGALM-
WHLGAL--
WWLGAL--
WCLGAL--
-YLGALR-
YSLGAL--
-YLGALS-
YALGAL--
YLLGAL--
YTLGAL--
YFLGAL--
-YLGALK-
YHLGAL--
YNLGAL--
-YLGALI-
YGLGAL--
YKLGAL--
YQLGAL--
YPLGAL--
-YLGALQ-
-YLGALN-

```
           -YLGALW-
           YILGAL--
           YELGAL--
           -YLGALM-
           YWLGAL--
           -YLGALG-
           -YLGALE-
           YDLGAL--
           YCLGAL--
PLAI        103        768     33.76
           GSPLAI--
           --PLAILD
           AVPLAI--
           ALPLAI--
           AKPLAI--
           ASPLAI--
           ARPLAI--
           -APLAIN-
           AIPLAI--
           -APLAIT-
           -APLAIG-
           -APLAIS-
           AAPLAI--
           -APLAIA-
           ATPLAI--
           AQPLAI--
           -APLAIK-
           -APLAII-
           -APLAIY-
           -APLAIH-
           APPLAI--
           ANPLAI--
           AGPLAI--
           AEPLAI--
           ADPLAI--
           AWPLAI--
           -APLAIV-
           AFPLAI--
           -APLAIF-
           AYPLAI--
           -APLAIW-
           -APLAIM-
           -APLAIE-
           AHPLAI--
           CSPLAI--
           -CPLAIR-
           CRPLAI--
           -CPLAIT-
           CKPLAI--
           -CPLAIK-
```

```
CVPLAI--
-CPLAIY-
-CPLAIS-
-CPLAIM-
CMPLAI--
CLPLAI--
CGPLAI--
-DPLAIL-
DFPLAI--
DNPLAI--
DVPLAI--
DRPLAI--
DWPLAI--
-DPLAIS-
DTPLAI--
-DPLAII-
DIPLAI--
DHPLAI--
-DPLAIW-
-DPLAIF-
DMPLAI--
DYPLAI--
DSPLAI--
DQPLAI--
-DPLAIN-
DKPLAI--
DEPLAI--
-DPLAIT-
-DPLAIR-
-DPLAIQ-
-DPLAIK-
-DPLAIH-
DLPLAI--
DAPLAI--
ESPLAI--
ERPLAI--
-EPLAIL-
EVPLAI--
EMPLAI--
-EPLAIH-
EKPLAI--
EFPLAI--
EPPLAI--
-EPLAIF-
-EPLAIQ-
-EPLAIM-
ENPLAI--
ELPLAI--
EQPLAI--
-EPLAIS-
```

-EPLAIR-
-EPLAIG-
-EPLAIA-
EEPLAI--
EAPLAI--
EYPLAI--
EWPLAI--
ETPLAI--
-EPLAIW-
-EPLAIV-
-EPLAIT-
-EPLAIN-
EIPLAI--
EGPLAI--
FNPLAI--
FSPLAI--
FGPLAI--
FVPLAI--
-FPLAIS-
FAPLAI--
-FPLAIG-
FMPLAI--
-FPLAIE-
FKPLAI--
FWPLAI--
FQPLAI--
-FPLAIK-
FPPLAI--
-FPLAIN-
-FPLAIH-
FFPLAI--
-FPLAIT-
FHPLAI--
FDPLAI--
-GPLAIL-
-GPLAIR-
-GPLAIV-
GGPLAI--
GAPLAI--
GVPLAI--
GWPLAI--
GHPLAI--
-GPLAII-
GFPLAI--
-GPLAIS-
GMPLAI--
-GPLAIN-
-GPLAIA-
GNPLAI--
GQPLAI--

-GPLAIW-
-GPLAIT-
GEPLAI--
-GPLAIG-
GKPLAI--
-GPLAIY-
-GPLAIK-
-GPLAIH-
-GPLAIE-
GIPLAI--
GYPLAI--
GPPLAI--
-GPLAIM-
GCPLAI--
-HPLAIS-
-HPLAIL-
HRPLAI--
-HPLAIW-
-HPLAIV-
-HPLAIQ-
-HPLAIG-
HVPLAI--
-HPLAIY-
-HPLAIR-
-HPLAIA-
-HPLAIN-
HNPLAI--
HAPLAI--
HQPLAI--
-HPLAIH-
HLPLAI--
HSPLAI--
-HPLAIM-
-HPLAIK-
HHPLAI--
HEPLAI--
HCPLAI--
HWPLAI--
HTPLAI--
HPPLAI--
-HPLAII-
HKPLAI--
HFPLAI--
ISPLAI--
ILPLAI--
IVPLAI--
IPPLAI--
-IPLAIV-
IMPLAI--
-IPLAIM-

IRPLAI--
-IPLAIW-
INPLAI--
IGPLAI--
-IPLAIS-
-IPLAIL-
IHPLAI--
IWPLAI--
-IPLAIT-
-IPLAIQ-
-IPLAIN-
-IPLAIG-
IDPLAI--
IQPLAI--
-IPLAII-
IKPLAI--
IYPLAI--
-IPLAIH-
-IPLAIA-
IAPLAI--
ITPLAI--
ICPLAI--
IIPLAI--
IEPLAI--
KSPLAI--
KLPLAI--
-KPLAIR-
-KPLAIL-
KYPLAI--
-KPLAIE-
KAPLAI--
-KPLAIV-
-KPLAIG-
-KPLAIT-
-KPLAIY-
-KPLAIS-
KGPLAI--
-KPLAIN-
KIPLAI--
KFPLAI--
KQPLAI--
-KPLAII-
-KPLAIH-
KDPLAI--
-KPLAIQ-
-KPLAIK-
KVPLAI--
KPPLAI--
-KPLAIM-
-KPLAIF-

KKPLAI--
KHPLAI--
KEPLAI--
KWPLAI--
-KPLAIW-
KNPLAI--
KMPLAI--
KCPLAI--
LRPLAI--
-LPLAIR-
LWPLAI--
LPPLAI--
-LPLAIM-
-LPLAIH-
LQPLAI--
-LPLAIS-
-LPLAIF-
LGPLAI--
LLPLAI--
LKPLAI--
-LPLAIN-
LYPLAI--
-LPLAIV-
LNPLAI--
LMPLAI--
LEPLAI--
LAPLAI--
-LPLAIL-
-LPLAIT-
-LPLAIQ-
-LPLAII-
-LPLAIW-
LIPLAI--
LCPLAI--
MGPLAI--
MRPLAI--
-MPLAIG-
MWPLAI--
MQPLAI--
MSPLAI--
-MPLAIS-
-MPLAIR-
MAPLAI--
MKPLAI--
MVPLAI--
-MPLAIV-
-MPLAIL-
MLPLAI--
-MPLAIK-
-MPLAIA-

```
MNPLAI--
MIPLAI--
-MPLAIT-
-MPLAIN-
MFPLAI--
MYPLAI--
MPPLAI--
-MPLAIW-
MEPLAI--
MCPLAI--
NKPLAI--
NVPLAI--
NLPLAI--
NSPLAI--
-NPLAIR-
NRPLAI--
NQPLAI--
-NPLAIY-
-NPLAIS-
NEPLAI--
NWPLAI--
-NPLAIA-
NTPLAI--
NDPLAI--
NAPLAI--
NYPLAI--
NPPLAI--
NHPLAI--
-NPLAIV-
-NPLAIQ-
-NPLAIM-
NIPLAI--
-NPLAIK-
-NPLAIH-
-NPLAIE-
NMPLAI--
NFPLAI--
-NPLAII-
NNPLAI--
NCPLAI--
--PLAIRS
--PLAILT
--PLAILR
--PLAIMR
--PLAILS
--PLAIWK
--PLAIRR
--PLAIHG
--PLAIRA
--PLAIKG
```

--PLAIIQ
--PLAINR
--PLAIMG
--PLAILN
--PLAIKS
--PLAIWS
--PLAIKA
--PLAIHM
--PLAIAS
--PLAIAA
--PLAIKL
--PLAIIS
--PLAIHL
--PLAIVA
--PLAIRP
--PLAIRD
PHPLAI--
--PLAIVR
--PLAIRM
--PLAIFR
--PLAIST
--PLAISR
--PLAIQR
PMPLAI--
--PLAIVS
--PLAITN
--PLAISY
--PLAIQY
--PLAIIN
--PLAIIA
--PLAIHV
--PLAITY
--PLAITM
--PLAIRT
--PLAIRI
--PLAIRF
--PLAIRE
--PLAIQG
--PLAIMW
--PLAIKR
--PLAIFH
--PLAIEG
--PLAIVK
--PLAITL
--PLAITA
--PLAISS
--PLAISG
--PLAIRQ
--PLAINL
--PLAILQ

--PLAILE
--PLAIIT
--PLAIFG
--PLAIAG
--PLAIWQ
--PLAIVV
--PLAISV
--PLAISK
--PLAIRN
--PLAIPL
--PLAIIR
--PLAIHR
--PLAIHA
--PLAIGV
--PLAIGG
--PLAIAQ
PYPLAI--
--PLAIYT
--PLAIYQ
--PLAIYK
--PLAIWH
--PLAIVN
--PLAIVH
--PLAITT
--PLAITQ
--PLAITK
--PLAITG
--PLAISQ
--PLAIRW
--PLAIQV
--PLAIMH
--PLAIMA
--PLAIKH
--PLAIGT
--PLAIGS
--PLAIES
PDPLAI--
--PLAIYY
--PLAIYH
--PLAIQH
--PLAINK
--PLAING
--PLAIIY
--PLAIGF
--PLAIFK
PQPLAI--
--PLAIWT
--PLAIVY
--PLAIVG
--PLAIVE

--PLAITI
--PLAITD
--PLAISW
--PLAISM
--PLAIRH
--PLAIQW
--PLAIQT
--PLAIQS
--PLAIQA
--PLAINY
--PLAINS
--PLAINQ
--PLAIMV
--PLAIKT
--PLAIKN
--PLAIKD
--PLAIID
--PLAIHT
--PLAIHN
--PLAIGH
--PLAIGA
--PLAIFT
--PLAIFS
--PLAIAL
--PLAIYS
--PLAIYR
--PLAIYG
--PLAIWG
--PLAIVW
--PLAIVQ
--PLAIVD
--PLAITV
--PLAISN
--PLAISE
--PLAISD
--PLAIRY
--PLAIQN
--PLAIQI
--PLAIPF
--PLAINW
--PLAINV
--PLAIMS
--PLAIMK
--PLAIMI
--PLAIKQ
--PLAIKK
--PLAIHS
--PLAIHK
--PLAIGM
--PLAIGI

--PLAIFN
--PLAIAV
--PLAIAT
--PLAIAK
PNPLAI--
--PLAIYN
--PLAIYD
--PLAIWD
--PLAISH
--PLAIQQ
--PLAIQM
--PLAIQD
--PLAIPW
--PLAIPM
--PLAINT
--PLAINN
--PLAINF
--PLAINE
--PLAINA
--PLAIMQ
--PLAIMM
--PLAILH
--PLAIKY
--PLAIKV
--PLAIKI
--PLAIKE
--PLAIIV
--PLAIHY
--PLAIGY
--PLAIFE
--PLAIEW
--PLAIEM
--PLAIEK
--PLAIEA
--PLAICK
--PLAIAH
PWPLAI--
PPPLAI--
-PPLAIV-
--PLAIYP
--PLAIYM
--PLAIYE
--PLAIWY
--PLAIWV
--PLAIWE
--PLAIVT
--PLAITP
--PLAITE
--PLAISC
--PLAIPV

--PLAINP
--PLAIMY
--PLAIMD
--PLAILW
--PLAILM
--PLAIIW
--PLAIIK
--PLAIII
--PLAIIH
--PLAIHW
--PLAIHQ
--PLAIHH
--PLAIHF
--PLAIHE
--PLAIGK
--PLAIFW
--PLAIFV
--PLAIFM
--PLAIFD
--PLAIEV
--PLAIET
--PLAIER
--PLAIEN
--PLAICQ
--PLAIAY
--PLAIAW
--PLAIAN
PEPLAI--
QGPLAI--
QVPLAI--
-QPLAIR-
QSPLAI--
QTPLAI--
QIPLAI--
QAPLAI--
QRPLAI--
-QPLAIS-
QLPLAI--
-QPLAIA-
QFPLAI--
-QPLAIQ-
-QPLAIN-
-QPLAIV-
-QPLAIH-
QQPLAI--
-QPLAIT-
-QPLAIK-
QMPLAI--
QYPLAI--
QPPLAI--

-QPLAII-
-QPLAIF-
QKPLAI--
QEPLAI--
-QPLAIW-
-QPLAIL-
-QPLAIE-
QNPLAI--
QHPLAI--
RSPLAI--
RGPLAI--
-RPLAIH-
RVPLAI--
-RPLAIK-
-RPLAIM-
RWPLAI--
-RPLAII-
RNPLAI--
RIPLAI--
-RPLAIF-
RDPLAI--
-RPLAIA-
RTPLAI--
-RPLAIG-
RMPLAI--
RAPLAI--
-RPLAIQ-
REPLAI--
-RPLAIV-
-RPLAIN-
RFPLAI--
RQPLAI--
RPPLAI--
-RPLAIE-
RHPLAI--
RYPLAI--
-SPLAIR-
SSPLAI--
SLPLAI--
-SPLAIK-
SNPLAI--
STPLAI--
-SPLAIT-
-SPLAIN-
-SPLAIF-
-SPLAIM-
-SPLAIV-
SKPLAI--
SAPLAI--
-SPLAIH-

-SPLAIA-
-SPLAIQ-
SEPLAI--
SYPLAI--
-SPLAII-
-SPLAIG-
SMPLAI--
SFPLAI--
SPPLAI--
-SPLAIE-
SHPLAI--
SQPLAI--
-SPLAIW-
-TPLAIS-
TKPLAI--
TSPLAI--
-TPLAIR-
-TPLAIN-
TAPLAI--
TRPLAI--
TVPLAI--
TGPLAI--
-TPLAIH-
-TPLAIF-
-TPLAIA-
TDPLAI--
TPPLAI--
TLPLAI--
TIPLAI--
TFPLAI--
-TPLAIY-
-TPLAIQ-
TNPLAI--
TTPLAI--
TYPLAI--
-TPLAIW-
-TPLAIG-
-TPLAIE-
TQPLAI--
-TPLAIT-
TEPLAI--
-TPLAIV-
-TPLAIK-
-TPLAID-
TCPLAI--
VRPLAI--
-VPLAIR-
VWPLAI--
VQPLAI--
VVPLAI--

VNPLAI--
-VPLAII-
VHPLAI--
VSPLAI--
VTPLAI--
VEPLAI--
-VPLAIN-
VDPLAI--
-VPLAIQ-
-VPLAIK-
-VPLAIG-
VGPLAI--
-VPLAIY-
VAPLAI--
VFPLAI--
VYPLAI--
-VPLAIV-
-VPLAIT-
VPPLAI--
-VPLAIE-
-VPLAIA-
VCPLAI--
WEPLAI--
-WPLAIR-
-WPLAIQ-
WAPLAI--
WTPLAI--
-WPLAIK-
WYPLAI--
-WPLAIS-
WVPLAI--
WPPLAI--
WMPLAI--
-WPLAIN-
-WPLAIH-
-WPLAIA-
WHPLAI--
WWPLAI--
-WPLAIW-
-WPLAIE-
WNPLAI--
WLPLAI--
WQPLAI--
-WPLAIV-
-WPLAIM-
-WPLAII-
-WPLAIG-
WIPLAI--
WDPLAI--
WCPLAI--

| | | | |
|---|---|---|---|
| | YVPLAI-- | | |
| | YGPLAI-- | | |
| | YRPLAI-- | | |
| | YSPLAI-- | | |
| | -YPLAIS- | | |
| | YLPLAI-- | | |
| | YAPLAI-- | | |
| | YIPLAI-- | | |
| | YEPLAI-- | | |
| | -YPLAIW- | | |
| | YTPLAI-- | | |
| | YPPLAI-- | | |
| | -YPLAIR- | | |
| | -YPLAIN- | | |
| | -YPLAIE- | | |
| | YYPLAI-- | | |
| | YWPLAI-- | | |
| | YQPLAI-- | | |
| | -YPLAIV- | | |
| | -YPLAIK- | | |
| | YNPLAI-- | | |
| | YHPLAI-- | | |
| | YDPLAI-- | | |
| | -YPLAIT- | | |
| | -YPLAIQ- | | |
| | -YPLAIP- | | |
| | -YPLAII- | | |
| | -YPLAIA- | | |
| PIAL | 244 | 691 | 33.73 |
| | -LPIALG- | | |
| | --PIALTD | | |
| | --PIALRP | | |
| | ARPIAL-- | | |
| | ALPIAL-- | | |
| | ATPIAL-- | | |
| | -APIALS- | | |
| | AMPIAL-- | | |
| | AYPIAL-- | | |
| | -APIALV- | | |
| | -APIALN- | | |
| | AHPIAL-- | | |
| | AAPIAL-- | | |
| | -APIALT- | | |
| | -APIALM- | | |
| | -APIALG- | | |
| | AIPIAL-- | | |
| | AGPIAL-- | | |
| | AEPIAL-- | | |
| | AQPIAL-- | | |
| | -APIALK- | | |

ANPIAL--
-APIALA-
AFPIAL--
-APIALL-
AWPIAL--
AKPIAL--
ADPIAL--
ASPIAL--
APPIAL--
-APIALQ-
-APIALH-
-APIALE-
CRPIAL--
-CPIALV-
CGPIAL--
CVPIAL--
CQPIAL--
CTPIAL--
-CPIALY-
-CPIALW-
-CPIALM-
-CPIALK-
-CPIALH-
-CPIALF-
CLPIAL--
CDPIAL--
DRPIAL--
DLPIAL--
-DPIALS-
DKPIAL--
-DPIALK-
DVPIAL--
-DPIALL-
DTPIAL--
-DPIALN-
DHPIAL--
DFPIAL--
DAPIAL--
DSPIAL--
-DPIALH-
DIPIAL--
DGPIAL--
-DPIALT-
DWPIAL--
-DPIALV-
DDPIAL--
-EPIALR-
EVPIAL--
ETPIAL--
ESPIAL--

EAPIAL--
ERPIAL--
-EPIALW-
-EPIALT-
-EPIALA-
ELPIAL--
-EPIALY-
-EPIALQ-
-EPIALN-
-EPIALM-
-EPIALL-
ENPIAL--
EKPIAL--
EGPIAL--
EEPIAL--
EYPIAL--
EQPIAL--
-EPIALK-
EWPIAL--
-EPIALV-
-EPIALI-
-EPIALH-
-EPIALG-
-EPIALE-
EMPIAL--
EIPIAL--
-FPIALK-
FSPIAL--
-FPIALT-
-FPIALQ-
FTPIAL--
-FPIALR-
FGPIAL--
FAPIAL--
FHPIAL--
FEPIAL--
FKPIAL--
-FPIALM-
-FPIALH-
-FPIALE-
FNPIAL--
FMPIAL--
-FPIALN-
-FPIALG-
FQPIAL--
-FPIALC-
-FPIALA-
FFPIAL--
FDPIAL--
GKPIAL--

-GPIALS-
-GPIALR-
GYPIAL--
GLPIAL--
-GPIALA-
GEPIAL--
GAPIAL--
GSPIAL--
GIPIAL--
GGPIAL--
-GPIALT-
-GPIALG-
-GPIALH-
GWPIAL--
GTPIAL--
-GPIALM-
GHPIAL--
GNPIAL--
GDPIAL--
-GPIALL-
-GPIALK-
GQPIAL--
GPPIAL--
-GPIALQ-
-GPIALN-
-GPIALE-
-GPIALD-
-HPIALS-
-HPIALR-
-HPIALL-
HLPIAL--
HGPIAL--
HMPIAL--
HTPIAL--
-HPIALN-
-HPIALT-
HAPIAL--
HNPIAL--
HYPIAL--
-HPIALM-
-HPIALA-
-HPIALI-
-HPIALH-
-HPIALE-
HEPIAL--
-HPIALV-
HKPIAL--
HHPIAL--
HDPIAL--
HPPIAL--

−HPIALQ−
HIPIAL−−
HCPIAL−−
HVPIAL−−
HQPIAL−−
−HPIALK−
−HPIALG−
−IPIALR−
ILPIAL−−
−IPIALH−
−IPIALT−
−IPIALS−
−IPIALQ−
ISPIAL−−
−IPIALK−
−IPIALA−
−IPIALN−
IGPIAL−−
IFPIAL−−
IAPIAL−−
IIPIAL−−
IVPIAL−−
IPPIAL−−
−IPIALV−
−IPIALM−
IKPIAL−−
−IPIALL−
−IPIALG−
IYPIAL−−
IQPIAL−−
IMPIAL−−
KSPIAL−−
KFPIAL−−
−KPIALN−
−KPIALK−
KRPIAL−−
KIPIAL−−
−KPIALT−
KVPIAL−−
KQPIAL−−
KNPIAL−−
−KPIALS−
−KPIALG−
−KPIALR−
KYPIAL−−
KLPIAL−−
KGPIAL−−
−KPIALV−
KWPIAL−−
−KPIALM−

KTPIAL--
-KPIALW-
-KPIALQ-
KMPIAL--
KKPIAL--
KDPIAL--
KPPIAL--
-KPIALE-
KHPIAL--
-KPIALH-
KCPIAL--
LGPIAL--
LAPIAL--
-LPIALT-
-LPIALL-
LKPIAL--
-LPIALR-
LVPIAL--
LLPIAL--
LNPIAL--
-LPIALS-
-LPIALI-
LYPIAL--
LPPIAL--
-LPIALN-
LEPIAL--
LHPIAL--
-LPIALM-
-LPIALH-
-LPIALA-
-LPIALQ-
LDPIAL--
-LPIALE-
-LPIALD-
LCPIAL--
-MPIALR-
MRPIAL--
MSPIAL--
MMPIAL--
MHPIAL--
MTPIAL--
-MPIALN-
MQPIAL--
MYPIAL--
-MPIALS-
-MPIALL-
MVPIAL--
-MPIALH-
-MPIALA-
-MPIALQ-

```
MKPIAL--
MGPIAL--
MFPIAL--
-MPIALM-
MIPIAL--
MEPIAL--
MDPIAL--
-MPIALT-
-MPIALK-
-MPIALG-
MCPIAL--
NRPIAL--
NHPIAL--
NVPIAL--
-NPIALT-
-NPIALL-
NSPIAL--
NTPIAL--
NLPIAL--
-NPIALR-
NIPIAL--
NYPIAL--
-NPIALW-
NGPIAL--
-NPIALK-
-NPIALA-
-NPIALV-
NDPIAL--
NAPIAL--
-NPIALH-
NPPIAL--
-NPIALS-
NNPIAL--
NWPIAL--
NQPIAL--
NMPIAL--
-NPIALQ-
-NPIALP-
-NPIALM-
-NPIALF-
-NPIALC-
NKPIAL--
NCPIAL--
--PIALGM
--PIALSG
--PIALRY
--PIALRT
--PIALSQ
--PIALGS
--PIALSA
```

--PIALRH
--PIALRG
--PIALKG
--PIALGL
PGPIAL--
--PIALQS
--PIALGF
--PIALSS
--PIALKF
--PIALTR
--PIALTK
--PIALRQ
--PIALRD
--PIALQG
--PIALQH
--PIALHL
--PIALAL
--PIALVQ
--PIALTY
--PIALTS
--PIALTG
--PIALTA
--PIALSK
--PIALKL
--PIALKK
--PIALHK
--PIALFG
PIPIAL--
--PIALTH
--PIALSW
--PIALSF
--PIALLG
--PIALHS
--PIALHR
--PIALHA
--PIALGI
--PIALGA
PFPIAL--
--PIALTV
--PIALTM
--PIALTI
--PIALST
--PIALRE
--PIALPL
--PIALNV
--PIALMS
--PIALMQ
--PIALKE
--PIALGY
--PIALGT

--PIALVG
--PIALSH
--PIALQL
--PIALNL
--PIALLD
--PIALKT
--PIALIN
--PIALIG
--PIALHV
--PIALGQ
--PIALFD
--PIALEY
--PIALES
--PIALEA
--PIALAN
--PIALWH
--PIALVN
--PIALSV
--PIALSM
--PIALQK
--PIALNA
--PIALMR
--PIALMG
--PIALLN
--PIALKN
--PIALHT
--PIALGG
--PIALET
--PIALDL
--PIALWD
--PIALTQ
--PIALSE
--PIALRW
--PIALRN
--PIALNW
--PIALMA
--PIALHW
--PIALGH
PWPIAL--
PTPIAL--
--PIALQV
--PIALQT
--PIALQI
--PIALQA
--PIALMN
--PIALMH
--PIALLH
--PIALKV
--PIALKR
--PIALKH

--PIALKD
--PIALHF
--PIALGV
PMPIAL--
--PIALVM
--PIALVH
--PIALTT
--PIALTF
--PIALSD
--PIALRK
--PIALPM
--PIALPF
--PIALNR
--PIALNI
--PIALMI
--PIALKM
--PIALHQ
--PIALHN
--PIALHM
--PIALHH
--PIALHG
--PIALGN
--PIALEG
--PIALDM
PHPIAL--
PEPIAL--
PCPIAL--
PAPIAL--
PQPIAL--
PNPIAL--
--PIALVD
--PIALTE
--PIALQW
--PIALQM
--PIALPY
--PIALNN
--PIALNM
--PIALNK
--PIALNG
--PIALMF
--PIALME
--PIALMD
--PIALLE
--PIALKY
--PIALKQ
--PIALID
--PIALEN
--PIALEM
--PIALEL
--PIALAS

--PIALAQ
--PIALAI
--PIALAH
--PIALAE
--PIALAD
PDPIAL--
PPPIAL--
-PPIALL-
--PIALYA
--PIALWN
--PIALTN
--PIALSI
--PIALQF
--PIALQC
--PIALPW
--PIALPI
--PIALNT
--PIALND
--PIALMT
--PIALMK
--PIALKI
--PIALIH
--PIALIF
--PIALIA
--PIALGK
--PIALFW
--PIALFC
--PIALER
--PIALEK
--PIALDY
--PIALDF
--PIALCS
--PIALCA
--PIALAT
-QPIALR-
QVPIAL--
QGPIAL--
-QPIALT-
-QPIALN-
QSPIAL--
-QPIALI-
QLPIAL--
QTPIAL--
-QPIALL-
QMPIAL--
-QPIALH-
-QPIALA-
QNPIAL--
-QPIALQ-
QIPIAL--

```
QHPIAL--
QWPIAL--
-QPIALS-
-QPIALM-
-QPIALE-
QAPIAL--
QYPIAL--
QQPIAL--
-QPIALG-
QKPIAL--
QEPIAL--
QDPIAL--
-RPIALM-
RGPIAL--
-RPIALR-
RVPIAL--
RFPIAL--
RTPIAL--
RLPIAL--
RMPIAL--
RKPIAL--
RAPIAL--
-RPIALG-
-RPIALV-
-RPIALS-
REPIAL--
RSPIAL--
-RPIALN-
RIPIAL--
RYPIAL--
RPPIAL--
RDPIAL--
RWPIAL--
-RPIALE-
-RPIALQ-
RNPIAL--
RCPIAL--
RHPIAL--
-RPIALA-
-RPIALI-
SKPIAL--
SLPIAL--
SVPIAL--
-SPIALR-
SNPIAL--
-SPIALL-
-SPIALT-
-SPIALS-
-SPIALQ-
-SPIALN-
```

```
SIPIAL--
SAPIAL--
STPIAL--
-SPIALI-
SFPIAL--
SSPIAL--
-SPIALV-
SGPIAL--
-SPIALK-
SEPIAL--
SPPIAL--
-SPIALH-
SDPIAL--
SWPIAL--
SMPIAL--
-SPIALM-
SHPIAL--
SQPIAL--
-SPIALE-
-SPIALG-
SCPIAL--
TRPIAL--
-TPIALA-
-TPIALR-
TAPIAL--
-TPIALK-
-TPIALS-
-TPIALI-
TFPIAL--
TSPIAL--
TYPIAL--
-TPIALL-
TLPIAL--
TVPIAL--
-TPIALN-
TQPIAL--
-TPIALM-
TKPIAL--
TDPIAL--
TTPIAL--
-TPIALE-
TNPIAL--
TGPIAL--
TWPIAL--
THPIAL--
TPPIAL--
TEPIAL--
-TPIALQ-
-TPIALP-
-TPIALG-
```

VLPIAL--
-VPIALL-
VSPIAL--
-VPIALT-
VKPIAL--
-VPIALR-
VGPIAL--
VFPIAL--
-VPIALH-
VNPIAL--
-VPIALS-
VPPIAL--
VDPIAL--
-VPIALQ-
-VPIALN-
-VPIALM-
VWPIAL--
VMPIAL--
VYPIAL--
VTPIAL--
-VPIALV-
-VPIALK-
-VPIALG-
-VPIALA-
VHPIAL--
VCPIAL--
VAPIAL--
-WPIALL-
WEPIAL--
WFPIAL--
WGPIAL--
WAPIAL--
-WPIALN-
WHPIAL--
-WPIALQ-
WLPIAL--
-WPIALH-
-WPIALA-
WDPIAL--
-WPIALR-
-WPIALK-
WQPIAL--
-WPIALG-
YSPIAL--
-YPIALR-
YAPIAL--
YFPIAL--
-YPIALH-
YLPIAL--
YHPIAL--

-YPIALN-
YVPIAL--
-YPIALA-
YKPIAL--
YRPIAL--
YIPIAL--
-YPIALQ-
YYPIAL--
YTPIAL--
-YPIALW-
-YPIALV-
-YPIALT-
-YPIALK-
YDPIAL--
YQPIAL--
YPPIAL--
-YPIALM-
-YPIALE-
YMPIAL--
YGPIAL--

| | | | |
|---|---|---|---|
| PLAM | 160 | 734 | 33.73 |

-NPLAMF-
--PLAMGR
--PLAMKY
--PLAMGK
--PLAMDT
ALPLAM--
ATPLAM--
AQPLAM--
AVPLAM--
-APLAMR-
-APLAMY-
-APLAMV-
-APLAMG-
ANPLAM--
ASPLAM--
-APLAMA-
-APLAMN-
-APLAMH-
ARPLAM--
-APLAML-
-APLAMK-
-APLAMD-
AIPLAM--
-APLAMM-
AMPLAM--
AGPLAM--
AYPLAM--
-APLAMW-
AKPLAM--

AHPLAM--
AWPLAM--
APPLAM--
-APLAMT-
-APLAMF-
AEPLAM--
ADPLAM--
-CPLAMY-
CRPLAM--
CPPLAM--
CYPLAM--
-CPLAMS-
CAPLAM--
-CPLAMT-
-CPLAMR-
-CPLAMH-
CCPLAM--
CVPLAM--
-CPLAMF-
CLPLAM--
DSPLAM--
-DPLAMH-
DTPLAM--
DRPLAM--
-DPLAMI-
-DPLAMS-
-DPLAMK-
DYPLAM--
-DPLAMA-
DMPLAM--
-DPLAMN-
-DPLAML-
-DPLAMF-
DEPLAM--
DWPLAM--
-DPLAMT-
DLPLAM--
DIPLAM--
DGPLAM--
DAPLAM--
DVPLAM--
DQPLAM--
-DPLAMY-
-DPLAMW-
-DPLAMM-
DNPLAM--
DKPLAM--
DHPLAM--
-EPLAML-
ERPLAM--

ELPLAM--
-EPLAMY-
-EPLAMR-
EVPLAM--
ETPLAM--
ESPLAM--
EIPLAM--
EYPLAM--
EMPLAM--
-EPLAMN-
-EPLAMG-
EKPLAM--
EDPLAM--
EAPLAM--
EPPLAM--
-EPLAMS-
-EPLAMQ-
-EPLAMI-
-EPLAMH-
-EPLAMC-
ECPLAM--
-FPLAMR-
-FPLAMS-
FSPLAM--
-FPLAMI-
-FPLAMN-
FKPLAM--
-FPLAMQ-
FGPLAM--
FYPLAM--
-FPLAMK-
FFPLAM--
FTPLAM--
-FPLAMT-
-FPLAMH-
-FPLAMG-
FNPLAM--
FEPLAM--
FWPLAM--
FVPLAM--
-FPLAMA-
FDPLAM--
FQPLAM--
FPPLAM--
FIPLAM--
FHPLAM--
FAPLAM--
-GPLAMR-
GRPLAM--
-GPLAMS-

GYPLAM--
GVPLAM--
GTPLAM--
-GPLAMV-
-GPLAMK-
GSPLAM--
GQPLAM--
-GPLAMH-
GAPLAM--
-GPLAMW-
-GPLAMT-
-GPLAMI-
GKPLAM--
GFPLAM--
GWPLAM--
GIPLAM--
GHPLAM--
-GPLAMQ-
-GPLAMN-
-GPLAMG-
GLPLAM--
GGPLAM--
GEPLAM--
-GPLAMY-
-GPLAMM-
GMPLAM--
-GPLAMF-
-GPLAME-
-GPLAMA-
GNPLAM--
-HPLAML-
HVPLAM--
-HPLAMA-
HRPLAM--
-HPLAMR-
-HPLAMH-
HDPLAM--
HSPLAM--
HPPLAM--
-HPLAMY-
-HPLAMS-
HNPLAM--
HLPLAM--
HKPLAM--
HYPLAM--
-HPLAMI-
HIPLAM--
HEPLAM--
-HPLAMW-
-HPLAMV-

-HPLAME-
HGPLAM--
HFPLAM--
HAPLAM--
HWPLAM--
-HPLAMT-
-HPLAMK-
-HPLAMG-
HHPLAM--
ITPLAM--
IIPLAM--
ISPLAM--
-IPLAMT-
-IPLAMG-
IWPLAM--
-IPLAMQ-
-IPLAMK-
IFPLAM--
IYPLAM--
IQPLAM--
-IPLAMH-
INPLAM--
IMPLAM--
ILPLAM--
IHPLAM--
IDPLAM--
ICPLAM--
-IPLAMW-
-IPLAMI-
-IPLAMC-
-IPLAMA-
IEPLAM--
KSPLAM--
KMPLAM--
-KPLAMR-
-KPLAMF-
-KPLAMV-
KGPLAM--
KPPLAM--
-KPLAMG-
KVPLAM--
-KPLAMK-
-KPLAMA-
KYPLAM--
KQPLAM--
-KPLAMQ-
-KPLAMS-
-KPLAMI-
-KPLAMH-
KKPLAM--

KFPLAM--
KAPLAM--
KWPLAM--
-KPLAMN-
KNPLAM--
KIPLAM--
KTPLAM--
-KPLAMY-
-KPLAMM-
-KPLAME-
KLPLAM--
-KPLAMW-
KDPLAM--
KCPLAM--
LRPLAM--
LVPLAM--
-LPLAMS-
LPPLAM--
-LPLAMV-
-LPLAMN-
-LPLAMM-
LKPLAM--
-LPLAMW-
-LPLAMT-
-LPLAMK-
-LPLAMH-
LWPLAM--
LSPLAM--
LGPLAM--
LIPLAM--
LEPLAM--
LDPLAM--
-LPLAMQ-
LMPLAM--
LFPLAM--
-LPLAMG-
LHPLAM--
-LPLAME-
LCPLAM--
-MPLAMR-
-MPLAMI-
MVPLAM--
-MPLAMN-
-MPLAMH-
MDPLAM--
-MPLAMS-
MLPLAM--
MWPLAM--
MMPLAM--
MNPLAM--

MIPLAM--
MRPLAM--
-MPLAMQ-
-MPLAMG-
MHPLAM--
MEPLAM--
MSPLAM--
-MPLAMY-
-MPLAMV-
-MPLAMT-
MKPLAM--
-NPLAML-
-NPLAMS-
NVPLAM--
-NPLAMI-
NLPLAM--
-NPLAMR-
-NPLAMT-
NRPLAM--
NSPLAM--
NGPLAM--
-NPLAMY-
-NPLAMA-
NEPLAM--
-NPLAMQ-
-NPLAMH-
NHPLAM--
NFPLAM--
-NPLAMW-
-NPLAMK-
-NPLAME-
NDPLAM--
NAPLAM--
NQPLAM--
-NPLAMV-
-NPLAMM-
-NPLAMG-
NNPLAM--
NMPLAM--
NIPLAM--
--PLAMRT
--PLAMVR
--PLAMTG
--PLAMRA
--PLAMWR
--PLAMNR
--PLAMRN
--PLAMGL
--PLAMTE
--PLAMAL

--PLAMNA
--PLAMML
--PLAMMK
--PLAMAR
--PLAMKT
--PLAMTV
--PLAMSG
--PLAMLD
--PLAMRI
--PLAMNL
--PLAMTL
--PLAMSF
--PLAMNV
--PLAMNI
--PLAMMR
--PLAMMH
--PLAMTM
--PLAMSI
--PLAMRV
--PLAMMG
--PLAMLN
--PLAMIT
--PLAMAI
--PLAMAG
--PLAMRQ
--PLAMNS
--PLAMLH
--PLAMLG
--PLAMHV
--PLAMAS
--PLAMYN
--PLAMWH
--PLAMSY
--PLAMSK
--PLAMRH
--PLAMQS
--PLAMQK
--PLAMKN
--PLAMKF
--PLAMGM
--PLAMGA
--PLAMEH
--PLAMVT
--PLAMVS
--PLAMSH
--PLAMSD
--PLAMRF
--PLAMKL
--PLAMKI
--PLAMID

--PLAMHF
--PLAMYQ
--PLAMWK
--PLAMVA
--PLAMTI
--PLAMSA
--PLAMRE
--PLAMQL
--PLAMQD
--PLAMKH
--PLAMKD
--PLAMIG
--PLAMHR
--PLAMHA
--PLAMGV
--PLAMYD
--PLAMWN
--PLAMSE
--PLAMQG
--PLAMPL
--PLAMNY
--PLAMLA
--PLAMKV
--PLAMKS
--PLAMKQ
--PLAMIV
--PLAMHS
--PLAMHK
--PLAMGT
--PLAMGF
--PLAMFG
--PLAMAN
--PLAMYG
--PLAMWG
--PLAMVY
--PLAMVN
--PLAMVM
--PLAMTH
--PLAMTA
--PLAMRY
--PLAMRP
--PLAMQF
--PLAMQA
--PLAMPW
--PLAMNK
--PLAMLE
--PLAMKG
--PLAMKE
--PLAMHG
--PLAMGG

--PLAMFS
--PLAMEY
--PLAMEM
--PLAMDS
PWPLAM--
--PLAMYE
--PLAMYA
--PLAMWE
--PLAMWD
--PLAMVD
--PLAMTN
--PLAMTK
--PLAMTF
--PLAMSW
--PLAMSV
--PLAMST
--PLAMQT
--PLAMNW
--PLAMMN
--PLAMMD
--PLAMLQ
--PLAMIW
--PLAMIN
--PLAMHY
--PLAMHT
--PLAMHH
--PLAMGQ
--PLAMFQ
--PLAMEW
--PLAMET
--PLAMEI
--PLAMAM
PFPLAM--
--PLAMYK
--PLAMYH
--PLAMWF
--PLAMVH
--PLAMVG
--PLAMVE
--PLAMTD
--PLAMSQ
--PLAMSN
--PLAMRM
--PLAMQQ
--PLAMPI
--PLAMNQ
--PLAMNM
--PLAMNH
--PLAMMT
--PLAMLM

--PLAMKM
--PLAMKA
--PLAMIK
--PLAMHW
--PLAMHL
--PLAMHI
--PLAMGH
--PLAMFE
--PLAMER
--PLAMEN
--PLAMEG
--PLAMDG
--PLAMDA
--PLAMAY
--PLAMAK
--PLAMAE
-PPLAMA-
--PLAMYS
--PLAMVV
--PLAMTW
--PLAMTQ
--PLAMTP
--PLAMQW
--PLAMQN
--PLAMQI
--PLAMQH
--PLAMPY
--PLAMPP
--PLAMNT
--PLAMMQ
--PLAMMM
--PLAMMA
--PLAMIE
--PLAMIA
--PLAMHQ
--PLAMHD
--PLAMGW
--PLAMFH
--PLAMFD
--PLAMEV
--PLAMEL
--PLAMAT
--PLAMAH
--PLAMAD
--PLAMAA
PCPLAM--
-QPLAMK-
-QPLAMA-
QTPLAM--
-QPLAMR-

−QPLAML−
QRPLAM−−
−QPLAMH−
QKPLAM−−
QVPLAM−−
−QPLAMV−
−QPLAMT−
−QPLAMS−
QLPLAM−−
QGPLAM−−
QFPLAM−−
QCPLAM−−
QYPLAM−−
QWPLAM−−
−QPLAMY−
QQPLAM−−
−QPLAMW−
−QPLAMQ−
−QPLAMI−
−QPLAMG−
QMPLAM−−
QEPLAM−−
QSPLAM−−
QPPLAM−−
−QPLAMF−
QNPLAM−−
QIPLAM−−
QHPLAM−−
QAPLAM−−
−RPLAMV−
RFPLAM−−
RLPLAM−−
RGPLAM−−
RIPLAM−−
RPPLAM−−
−RPLAMW−
−RPLAMK−
RVPLAM−−
RNPLAM−−
RSPLAM−−
−RPLAMI−
RTPLAM−−
−RPLAMY−
RYPLAM−−
−RPLAMH−
RMPLAM−−
RKPLAM−−
REPLAM−−
−RPLAMG−
−RPLAME−

RAPLAM--
RWPLAM--
-RPLAMQ-
-RPLAMD-
RCPLAM--
RQPLAM--
-RPLAMM-
RDPLAM--
-RPLAMP-
-SPLAMR-
SSPLAM--
-SPLAMY-
-SPLAMV-
-SPLAMK-
SLPLAM--
SYPLAM--
SVPLAM--
-SPLAMN-
-SPLAMI-
SKPLAM--
STPLAM--
-SPLAMA-
SNPLAM--
-SPLAMF-
-SPLAMM-
-SPLAMH-
-SPLAMG-
SHPLAM--
SGPLAM--
SFPLAM--
SWPLAM--
SPPLAM--
-SPLAME-
SAPLAM--
SIPLAM--
SDPLAM--
-SPLAMW-
-SPLAMQ-
SMPLAM--
SEPLAM--
SCPLAM--
-TPLAMR-
-TPLAMS-
-TPLAMV-
TNPLAM--
TRPLAM--
-TPLAMI-
-TPLAMY-
TIPLAM--
-TPLAMA-

TPPLAM--
-TPLAMT-
-TPLAMG-
TGPLAM--
TSPLAM--
-TPLAMH-
TVPLAM--
-TPLAMW-
TQPLAM--
-TPLAMN-
-TPLAMK-
-TPLAMF-
-TPLAME-
TEPLAM--
TAPLAM--
TYPLAM--
TWPLAM--
TTPLAM--
-TPLAMM-
TDPLAM--
-TPLAMQ-
TMPLAM--
TLPLAM--
THPLAM--
-VPLAMR-
-VPLAMH-
-VPLAMQ-
VNPLAM--
VTPLAM--
VLPLAM--
VKPLAM--
VEPLAM--
-VPLAMV-
-VPLAMS-
-VPLAML-
-VPLAMK-
VFPLAM--
VDPLAM--
-VPLAMM-
VVPLAM--
-VPLAMN-
-VPLAMW-
-VPLAMG-
VIPLAM--
VHPLAM--
VQPLAM--
-VPLAMT-
-VPLAMI-
-VPLAMA-
VMPLAM--

VYPLAM--
VWPLAM--
VPPLAM--
-VPLAME-
-VPLAMD-
-WPLAMA-
WHPLAM--
-WPLAMG-
WWPLAM--
WSPLAM--
-WPLAMS-
WFPLAM--
WRPLAM--
WAPLAM--
-WPLAMY-
-WPLAMW-
-WPLAMV-
-WPLAMM-
WKPLAM--
-WPLAMT-
-WPLAMN-
WLPLAM--
WIPLAM--
WYPLAM--
-WPLAMK-
-WPLAMH-
-YPLAML-
YYPLAM--
YRPLAM--
YLPLAM--
-YPLAMA-
-YPLAMI-
-YPLAMH-
YVPLAM--
-YPLAMG-
-YPLAMF-
YKPLAM--
YPPLAM--
-YPLAMV-
-YPLAMT-
-YPLAMK-
-YPLAME-
YIPLAM--
YGPLAM--
YTPLAM--
YHPLAM--
YWPLAM--
YDPLAM--
YAPLAM--
YQPLAM--

| | | | |
|---|---|---|---|
| | -YPLAMR- | | |
| | -YPLAMN- | | |
| | -YPLAMM- | | |
| | YMPLAM-- | | |
| | YCPLAM-- | | |
| ISNL | 122 | 756 | 33.67 |
| | -AISNLE- | | |
| | -GISNLG- | | |
| | --ISNLSE | | |
| | --ISNLSD | | |
| | --ISNLAR | | |
| | --ISNLQI | | |
| | --ISNLPP | | |
| | --ISNLPA | | |
| | --ISNLNN | | |
| | --ISNLNL | | |
| | --ISNLNH | | |
| | --ISNLGY | | |
| | --ISNLGL | | |
| | --ISNLER | | |
| | --ISNLCL | | |
| | -VISNLP- | | |
| | ATISNL-- | | |
| | -AISNLL- | | |
| | -AISNLV- | | |
| | ALISNL-- | | |
| | -AISNLR- | | |
| | -AISNLS- | | |
| | -AISNLT- | | |
| | AHISNL-- | | |
| | AVISNL-- | | |
| | AQISNL-- | | |
| | AAISNL-- | | |
| | ASISNL-- | | |
| | -AISNLK- | | |
| | -AISNLI- | | |
| | AGISNL-- | | |
| | AKISNL-- | | |
| | AWISNL-- | | |
| | AYISNL-- | | |
| | APISNL-- | | |
| | -AISNLA- | | |
| | AIISNL-- | | |
| | AFISNL-- | | |
| | AMISNL-- | | |
| | ANISNL-- | | |
| | -AISNLN- | | |
| | -AISNLH- | | |
| | -AISNLQ- | | |
| | -AISNLG- | | |

ADISNL--
ACISNL--
CRISNL--
-CISNLQ-
-CISNLL-
CCISNL--
CTISNL--
-CISNLR-
-CISNLA-
CVISNL--
CLISNL--
CQISNL--
-CISNLT-
-CISNLK-
-CISNLH-
-CISNLE-
CYISNL--
CPISNL--
-CISNLV-
-CISNLN-
-CISNLM-
-CISNLG-
CGISNL--
CFISNL--
CDISNL--
DRISNL--
-DISNLL-
DTISNL--
DSISNL--
-DISNLR-
DKISNL--
-DISNLW-
-DISNLS-
-DISNLF-
-DISNLY-
-DISNLT-
-DISNLQ-
DMISNL--
-DISNLM-
DEISNL--
-DISNLN-
-DISNLH-
DIISNL--
DGISNL--
DDISNL--
DQISNL--
-DISNLI-
DFISNL--
DYISNL--
DNISNL--

-DISNLE-
DHISNL--
DPISNL--
-DISNLK-
-DISNLA-
-EISNLW-
-EISNLR-
EPISNL--
ERISNL--
ESISNL--
-EISNLI-
EVISNL--
ETISNL--
-EISNLS-
-EISNLL-
ELISNL--
-EISNLT-
EFISNL--
EWISNL--
EKISNL--
-EISNLQ-
-EISNLH-
-EISNLF-
EAISNL--
ENISNL--
-EISNLY-
-EISNLV-
-EISNLM-
EMISNL--
-EISNLE-
-EISNLA-
EYISNL--
EQISNL--
-EISNLK-
EDISNL--
-EISNLN-
-EISNLG-
EGISNL--
-FISNLG-
FTISNL--
FSISNL--
FMISNL--
-FISNLQ-
-FISNLL-
-FISNLS-
FDISNL--
-FISNLR-
FGISNL--
-FISNLA-
FNISNL--

FQISNL--
FKISNL--
FEISNL--
FPISNL--
-FISNLI-
-FISNLK-
FFISNL--
FIISNL--
FHISNL--
-FISNLP-
-FISNLM-
-FISNLD-
GSISNL--
-GISNLT-
GVISNL--
GAISNL--
GLISNL--
GWISNL--
GFISNL--
-GISNLK-
-GISNLA-
GGISNL--
GYISNL--
GKISNL--
GTISNL--
GNISNL--
-GISNLM-
GPISNL--
-GISNLY-
GHISNL--
-GISNLI-
GMISNL--
GIISNL--
GEISNL--
-GISNLQ-
-GISNLN-
GQISNL--
-GISNLE-
GCISNL--
-GISNLH-
-HISNLK-
-HISNLL-
-HISNLM-
HAISNL--
-HISNLS-
-HISNLV-
-HISNLT-
-HISNLR-
HIISNL--
-HISNLN-

HGISNL--
HQISNL--
HTISNL--
HMISNL--
-HISNLA-
HKISNL--
HPISNL--
-HISNLW-
-HISNLF-
HHISNL--
HWISNL--
HNISNL--
-HISNLG-
-HISNLY-
-HISNLQ-
-HISNLH-
HFISNL--
HEISNL--
HDISNL--
HCISNL--
-HISNLE-
--ISNLEG
--ISNLLS
--ISNLRS
--ISNLWS
--ISNLKS
--ISNLQS
--ISNLAS
--ISNLRV
--ISNLLA
--ISNLSS
--ISNLMG
--ISNLYR
--ISNLYS
--ISNLLH
--ISNLGS
--ISNLLG
IAISNL--
--ISNLYQ
--ISNLSM
--ISNLRQ
--ISNLLK
-IISNLR-
--ISNLTK
--ISNLRT
--ISNLIG
--ISNLIS
--ISNLRG
--ISNLQT
--ISNLMT

--ISNLMR
--ISNLVF
--ISNLVA
--ISNLTV
--ISNLSL
--ISNLRH
--ISNLKK
--ISNLYK
--ISNLRM
--ISNLIT
--ISNLTA
--ISNLSR
--ISNLHV
--ISNLHA
--ISNLTI
--ISNLVS
--ISNLVG
-IISNLK-
--ISNLTQ
--ISNLRW
--ISNLHS
--ISNLAW
IGISNL--
--ISNLSV
--ISNLRA
--ISNLKY
ISISNL--
-IISNLG-
--ISNLSH
--ISNLQR
--ISNLMV
--ISNLMM
--ISNLKN
--ISNLIA
--ISNLAG
--ISNLAA
IKISNL--
ITISNL--
--ISNLYH
--ISNLTN
--ISNLMN
--ISNLFG
--ISNLEA
--ISNLAY
IPISNL--
-IISNLH-
--ISNLWH
--ISNLSQ
--ISNLSI
--ISNLSF

--ISNLRF
--ISNLRD
--ISNLQG
--ISNLMS
--ISNLKL
--ISNLKI
--ISNLIQ
--ISNLAV
-IISNLV-
-IISNLT-
-IISNLL-
--ISNLYG
--ISNLWK
--ISNLVT
--ISNLVM
--ISNLTT
--ISNLKF
--ISNLHG
--ISNLES
--ISNLYT
--ISNLWT
--ISNLWR
--ISNLWD
--ISNLRE
--ISNLML
--ISNLMI
--ISNLMH
--ISNLME
--ISNLKT
--ISNLKM
--ISNLKH
--ISNLIH
--ISNLIE
--ISNLHY
--ISNLFK
INISNL--
--ISNLYN
--ISNLWQ
--ISNLVE
--ISNLTW
--ISNLQK
--ISNLPY
--ISNLMD
--ISNLMA
--ISNLKW
--ISNLYE
--ISNLYA
--ISNLVY
--ISNLVV
--ISNLTD

--ISNLQN
--ISNLPL
--ISNLNT
--ISNLNS
--ISNLHN
--ISNLFH
--ISNLFD
-IISNLA-
--ISNLTF
--ISNLKV
--ISNLKQ
--ISNLHW
--ISNLHK
--ISNLGT
--ISNLEM
--ISNLAH
--ISNLAF
IMISNL--
-IISNLM-
IYISNL--
--ISNLTY
--ISNLTL
--ISNLQW
--ISNLNA
--ISNLMK
--ISNLMF
--ISNLLD
--ISNLIW
--ISNLIK
--ISNLII
--ISNLHQ
--ISNLHM
--ISNLGV
--ISNLFA
--ISNLAK
--ISNLYD
--ISNLTM
--ISNLTE
--ISNLMQ
--ISNLID
--ISNLHT
--ISNLHL
--ISNLHI
--ISNLGA
--ISNLFQ
--ISNLFE
IQISNL--
IHISNL--
IDISNL--
--ISNLVD

--ISNLRC
--ISNLQY
--ISNLQV
--ISNLQM
--ISNLHH
--ISNLEV
--ISNLAI
--ISNLWA
--ISNLQH
--ISNLQD
--ISNLNY
--ISNLNW
--ISNLNM
--ISNLHR
--ISNLHF
--ISNLEY
--ISNLEI
--ISNLAM
ICISNL--
--ISNLQF
--ISNLQE
--ISNLQC
--ISNLNK
--ISNLNF
--ISNLKE
--ISNLKD
--ISNLGM
--ISNLGI
--ISNLEQ
--ISNLEK
--ISNLEF
--ISNLEE
--ISNLDG
--ISNLDA
--ISNLCT
--ISNLCS
--ISNLCN
-IISNLE-
-KISNLL-
KTISNL--
-KISNLS-
KGISNL--
-KISNLT-
KAISNL--
-KISNLK-
KEISNL--
KWISNL--
KMISNL--
KQISNL--
KFISNL--

-KISNLY-
-KISNLE-
KDISNL--
KPISNL--
-KISNLQ-
-KISNLF-
-KISNLA-
KCISNL--
-KISNLV-
-KISNLH-
-KISNLN-
-LISNLT-
LKISNL--
LSISNL--
-LISNLK-
-LISNLH-
-LISNLG-
LYISNL--
LMISNL--
-LISNLE-
LAISNL--
-LISNLL-
LIISNL--
-LISNLS-
LDISNL--
LPISNL--
LNISNL--
LEISNL--
-LISNLA-
-LISNLQ-
LHISNL--
LCISNL--
-LISNLM-
-LISNLD-
-LISNLP-
MSISNL--
-MISNLR-
MGISNL--
MTISNL--
MVISNL--
-MISNLS-
-MISNLL-
-MISNLI-
-MISNLA-
MMISNL--
MPISNL--
-MISNLK-
MDISNL--
MWISNL--
-MISNLV-

```
MFISNL--
-MISNLQ-
MAISNL--
-MISNLM-
-MISNLG-
MYISNL--
MKISNL--
-MISNLT-
-MISNLE-
MQISNL--
-MISNLY-
-MISNLN-
MIISNL--
MEISNL--
-MISNLD-
MHISNL--
MCISNL--
-NISNLR-
NSISNL--
NGISNL--
NYISNL--
NVISNL--
NIISNL--
NWISNL--
-NISNLS-
NPISNL--
-NISNLL-
-NISNLF-
NAISNL--
NMISNL--
NFISNL--
NDISNL--
NTISNL--
-NISNLT-
-NISNLN-
-NISNLK-
-NISNLI-
NQISNL--
NNISNL--
-NISNLV-
-NISNLM-
-NISNLG-
-NISNLA-
NEISNL--
NCISNL--
-NISNLH-
-NISNLE-
-NISNLQ-
PRISNL--
-PISNLS-
```

PVISNL--
-PISNLT-
-PISNLY-
PNISNL--
-PISNLA-
-PISNLV-
-PISNLF-
PMISNL--
-PISNLK-
PIISNL--
PGISNL--
PEISNL--
PHISNL--
PAISNL--
-PISNLH-
PWISNL--
-PISNLN-
-PISNLE-
PFISNL--
PQISNL--
PDISNL--
-PISNLQ-
-PISNLI-
-PISNLG-
-PISNLM-
PCISNL--
QRISNL--
-QISNLL-
-QISNLS-
QFISNL--
QTISNL--
QSISNL--
QKISNL--
-QISNLA-
QWISNL--
QPISNL--
QMISNL--
-QISNLT-
QGISNL--
-QISNLV-
QDISNL--
QVISNL--
-QISNLK-
QYISNL--
-QISNLQ-
-QISNLE-
QNISNL--
-QISNLM-
-QISNLY-
-QISNLW-

QEISNL--
QCISNL--
QAISNL--
-QISNLI-
-QISNLH-
-QISNLF-
RVISNL--
-RISNLS-
-RISNLT-
-RISNLA-
REISNL--
-RISNLQ-
RFISNL--
RQISNL--
RNISNL--
-RISNLM-
-RISNLK-
RDISNL--
-RISNLN-
-RISNLE-
-RISNLG-
-RISNLC-
RCISNL--
-SISNLR-
SVISNL--
-SISNLY-
-SISNLS-
STISNL--
-SISNLV-
-SISNLI-
SHISNL--
SAISNL--
SKISNL--
SFISNL--
-SISNLW-
-SISNLM-
SPISNL--
SNISNL--
-SISNLH-
SWISNL--
-SISNLQ-
-SISNLF-
-SISNLE-
SEISNL--
-SISNLN-
SCISNL--
SQISNL--
-TISNLT-
-TISNLS-
TFISNL--

TVISNL--
TTISNL--
-TISNLL-
TKISNL--
THISNL--
TLISNL--
TIISNL--
TAISNL--
TNISNL--
-TISNLI-
-TISNLK-
-TISNLV-
-TISNLQ-
-TISNLF-
TPISNL--
TQISNL--
TMISNL--
TDISNL--
TWISNL--
-TISNLG-
TEISNL--
-TISNLM-
TCISNL--
-TISNLY-
TYISNL--
-TISNLN-
-TISNLE-
VRISNL--
-VISNLL-
-VISNLR-
-VISNLS-
VKISNL--
-VISNLK-
VSISNL--
VVISNL--
VNISNL--
-VISNLG-
-VISNLT-
VAISNL--
VPISNL--
VYISNL--
VMISNL--
-VISNLV-
-VISNLQ-
VTISNL--
-VISNLH-
-VISNLA-
VHISNL--
VGISNL--
VFISNL--

```
-VISNLE-
VEISNL--
VDISNL--
-VISNLN-
-VISNLM-
VQISNL--
VCISNL--
WRISNL--
WNISNL--
WVISNL--
WSISNL--
-WISNLM-
-WISNLA-
-WISNLL-
-WISNLG-
-WISNLK-
-WISNLH-
WAISNL--
-WISNLT-
WHISNL--
WWISNL--
-WISNLQ-
WGISNL--
-WISNLE-
-WISNLV-
WPISNL--
WDISNL--
WIISNL--
WYISNL--
WKISNL--
WQISNL--
WEISNL--
-WISNLF-
YRISNL--
-YISNLR-
YSISNL--
-YISNLT-
-YISNLG-
YGISNL--
YVISNL--
-YISNLS-
YKISNL--
-YISNLM-
-YISNLK-
-YISNLL-
-YISNLA-
YHISNL--
YYISNL--
YLISNL--
YPISNL--
```

YNISNL--
-YISNLQ-
-YISNLE-
YDISNL--
YMISNL--
-YISNLV-
-YISNLW-
-YISNLD-
YIISNL--
YFISNL--
YWISNL--
YCISNL--

PANW 327 658 33.62

ANPANW--
-APANWR-
-APANWM-
-APANWI-
-APANWH-
AFPANW--
-APANWT-
ATPANW--
APPANW--
-APANWG-
-APANWA-
ADPANW--
AAPANW--
ASPANW--
-APANWF-
AMPANW--
AKPANW--
AWPANW--
ARPANW--
AQPANW--
-APANWV-
-APANWQ-
-APANWL-
ALPANW--
AHPANW--
AEPANW--
CVPANW--
CAPANW--
CTPANW--
CRPANW--
-CPANWT-
-CPANWS-
-CPANWQ-
-CPANWL-
CLPANW--
-DPANWL-
DLPANW--

```
DMPANW--
-DPANWW-
DQPANW--
-DPANWR-
-DPANWK-
-DPANWI-
DNPANW--
DVPANW--
DSPANW--
-DPANWT-
-DPANWS-
-DPANWG-
DGPANW--
DYPANW--
DRPANW--
-DPANWQ-
-DPANWD-
DKPANW--
DFPANW--
EKPANW--
-EPANWL-
-EPANWV-
ESPANW--
EMPANW--
-EPANWR-
-EPANWH-
EVPANW--
-EPANWT-
-EPANWS-
-EPANWM-
EYPANW--
EPPANW--
-EPANWN-
-EPANWG-
-EPANWF-
ENPANW--
ELPANW--
EDPANW--
FKPANW--
FFPANW--
-FPANWR-
-FPANWS-
FLPANW--
FNPANW--
-FPANWY-
-FPANWG-
FGPANW--
FYPANW--
FSPANW--
-FPANWT-
```

-FPANWN-
-FPANWI-
FQPANW--
FPPANW--
-FPANWV-
-FPANWK-
FHPANW--
-GPANWL-
GSPANW--
-GPANWI-
-GPANWF-
GRPANW--
-GPANWV-
-GPANWG-
GLPANW--
GEPANW--
-GPANWY-
-GPANWT-
-GPANWS-
-GPANWK-
-GPANWH-
GKPANW--
GFPANW--
GDPANW--
GAPANW--
-GPANWQ-
GCPANW--
GVPANW--
GTPANW--
GQPANW--
-GPANWW-
GNPANW--
GGPANW--
-GPANWN-
-GPANWM-
-GPANWA-
-HPANWE-
-HPANWW-
HYPANW--
-HPANWL-
-HPANWI-
HTPANW--
-HPANWS-
-HPANWA-
HRPANW--
-HPANWV-
-HPANWG-
HWPANW--
HVPANW--
HPPANW--

```
-HPANWH-
HLPANW--
HIPANW--
HFPANW--
-IPANWL-
-IPANWV-
-IPANWR-
IRPANW--
IAPANW--
ITPANW--
-IPANWQ-
IFPANW--
IEPANW--
-IPANWM-
IIPANW--
IWPANW--
ISPANW--
-IPANWT-
-IPANWK-
-IPANWE-
ILPANW--
IDPANW--
IYPANW--
IVPANW--
-IPANWY-
-IPANWS-
-IPANWI-
-IPANWD-
INPANW--
IGPANW--
ICPANW--
-KPANWN-
-KPANWF-
-KPANWL-
-KPANWI-
KVPANW--
KTPANW--
KRPANW--
-KPANWT-
-KPANWE-
-KPANWR-
KEPANW--
KWPANW--
-KPANWH-
KYPANW--
-KPANWA-
KIPANW--
-KPANWQ-
-KPANWK-
KMPANW--
```

KGPANW--
-LPANWL-
LVPANW--
LYPANW--
-LPANWN-
-LPANWA-
-LPANWT-
-LPANWS-
-LPANWK-
-LPANWI-
LNPANW--
LSPANW--
-LPANWW-
LAPANW--
LGPANW--
LTPANW--
LQPANW--
-LPANWY-
-LPANWV-
-LPANWF-
LMPANW--
LRPANW--
LPPANW--
-LPANWR-
-LPANWM-
LLPANW--
LKPANW--
LEPANW--
LDPANW--
-LPANWG-
LIPANW--
-MPANWI-
MRPANW--
MKPANW--
MSPANW--
MNPANW--
MMPANW--
MYPANW--
MGPANW--
MTPANW--
-MPANWR-
-MPANWK-
MAPANW--
-MPANWY-
-MPANWV-
-MPANWH-
-MPANWE-
-MPANWA-
MLPANW--
MWPANW--

```
-MPANWQ-
-MPANWM-
-MPANWL-
-MPANWG-
-MPANWD-
-NPANWS-
-NPANWV-
-NPANWI-
-NPANWA-
NWPANW--
NKPANW--
NHPANW--
NGPANW--
NSPANW--
-NPANWW-
-NPANWE-
-NPANWG-
NVPANW--
-NPANWK-
-NPANWH-
NYPANW--
NPPANW--
-NPANWL-
NLPANW--
NDPANW--
--PANWRY
--PANWVR
--PANWTL
--PANWRS
--PANWYR
--PANWRT
--PANWRG
--PANWYL
--PANWSR
PRPANW--
--PANWWS
--PANWFT
--PANWYH
--PANWVG
--PANWFR
--PANWRL
--PANWMG
--PANWLL
--PANWLG
--PANWHW
--PANWFS
PSPANW--
--PANWTW
--PANWRV
--PANWMR
```

--PANWIT
--PANWAY
--PANWVS
--PANWTM
--PANWSI
--PANWQY
--PANWLT
--PANWLK
--PANWLA
--PANWGT
--PANWFV
--PANWAM
--PANWYN
--PANWWK
--PANWVT
--PANWVN
--PANWVH
--PANWTS
--PANWTG
--PANWSA
--PANWRA
--PANWMD
--PANWLV
--PANWLN
--PANWHK
--PANWEH
PAPANW--
--PANWYG
--PANWWY
--PANWWT
--PANWWI
--PANWTK
--PANWTF
--PANWSY
--PANWSS
--PANWSN
--PANWSF
--PANWQT
--PANWNA
--PANWMT
--PANWLQ
--PANWLM
--PANWLH
--PANWKG
--PANWIS
--PANWHS
--PANWGS
--PANWGR
--PANWGK
--PANWGG

--PANWFQ
--PANWEA
--PANWAL
--PANWAI
--PANWAG
--PANWAA
PMPANW--
--PANWVA
--PANWTR
--PANWTE
--PANWSW
--PANWST
--PANWSM
--PANWSH
--PANWRW
--PANWRH
--PANWRD
--PANWQR
--PANWQK
--PANWNV
--PANWNL
--PANWMK
--PANWLS
--PANWLI
--PANWKY
--PANWKV
--PANWKR
--PANWIN
--PANWIL
--PANWII
--PANWGN
--PANWFH
--PANWFG
--PANWFA
--PANWEW
--PANWEV
--PANWAV
--PANWAE
PVPANW--
--PANWYT
--PANWYQ
--PANWYM
--PANWWM
--PANWWL
--PANWWF
--PANWWD
--PANWSQ
--PANWSE
--PANWRP
--PANWQW

```
--PANWQF
--PANWNS
--PANWNF
--PANWMY
--PANWMS
--PANWMI
--PANWMH
--PANWLD
--PANWKP
--PANWKN
--PANWIY
--PANWIR
--PANWIH
--PANWIG
--PANWID
--PANWIA
--PANWHG
--PANWGY
--PANWEY
--PANWES
--PANWEN
--PANWEL
--PANWEF
--PANWAW
--PANWAS
--PANWAQ
--PANWAN
PWPANW--
--PANWYY
--PANWYW
--PANWYS
--PANWYK
--PANWYI
--PANWYE
--PANWWQ
--PANWWN
--PANWWG
--PANWWA
--PANWVY
--PANWVV
--PANWVQ
--PANWVI
--PANWVF
--PANWTY
--PANWTT
--PANWTQ
--PANWTI
--PANWTD
--PANWSK
--PANWRR
```

--PANWRF
--PANWQS
--PANWQQ
--PANWQL
--PANWQH
--PANWQE
--PANWQA
--PANWPP
--PANWNT
--PANWNQ
--PANWMV
--PANWMP
--PANWMA
--PANWLW
--PANWKW
--PANWKT
--PANWKS
--PANWKL
--PANWKK
--PANWIM
--PANWHT
--PANWHR
--PANWHN
--PANWHL
--PANWHI
--PANWGV
--PANWGH
--PANWFY
--PANWFI
--PANWFF
--PANWFD
--PANWEG
--PANWAT
-QPANWV-
-QPANWH-
QNPANW--
-QPANWL-
QRPANW--
QIPANW--
QGPANW--
-QPANWM-
-QPANWE-
QSPANW--
-QPANWS-
QQPANW--
-QPANWT-
-QPANWI-
-QPANWF-
QKPANW--
QAPANW--

-RPANWL-
-RPANWG-
RIPANW--
RLPANW--
RSPANW--
RPPANW--
-RPANWW-
-RPANWN-
-RPANWM-
-RPANWI-
RDPANW--
RTPANW--
RRPANW--
-RPANWR-
-RPANWF-
RYPANW--
-RPANWV-
-RPANWT-
-RPANWS-
-RPANWQ-
-RPANWH-
RNPANW--
RMPANW--
RHPANW--
REPANW--
RVPANW--
-RPANWD-
RGPANW--
RQPANW--
-RPANWY-
-RPANWP-
RKPANW--
RFPANW--
RAPANW--
SPPANW--
STPANW--
-SPANWW-
-SPANWT-
-SPANWR-
SLPANW--
SKPANW--
SWPANW--
-SPANWA-
SIPANW--
-SPANWS-
-SPANWL-
-SPANWG-
SAPANW--
-SPANWN-
-SPANWF-

SVPANW--
SGPANW--
-SPANWK-
-SPANWI-
-SPANWE-
SHPANW--
SFPANW--
SEPANW--
SDPANW--
-SPANWY-
-SPANWV-
-SPANWQ-
SCPANW--
-TPANWS-
-TPANWR-
-TPANWW-
TAPANW--
-TPANWH-
TNPANW--
TTPANW--
-TPANWA-
TKPANW--
TVPANW--
-TPANWG-
TIPANW--
TYPANW--
-TPANWV-
-TPANWQ-
-TPANWN-
TGPANW--
TWPANW--
TRPANW--
-TPANWY-
-TPANWL-
-TPANWK-
TFPANW--
TSPANW--
TPPANW--
-TPANWM-
TMPANW--
TLPANW--
TEPANW--
TDPANW--
-VPANWK-
-VPANWS-
-VPANWL-
-VPANWI-
VKPANW--
-VPANWY-
VDPANW--

VAPANW--
VRPANW--
VLPANW--
VIPANW--
VVPANW--
VPPANW--
-VPANWV-
-VPANWG-
VTPANW--
-VPANWQ-
-VPANWH-
VNPANW--
VGPANW--
VSPANW--
-VPANWW-
-VPANWE-
-VPANWA-
-VPANWT-
-VPANWM-
VFPANW--
VEPANW--
-WPANWV-
-WPANWR-
WRPANW--
WPPANW--
WVPANW--
WAPANW--
WWPANW--
WTPANW--
-WPANWT-
WSPANW--
WQPANW--
-WPANWQ-
-WPANWM-
-WPANWF-
WEPANW--
WDPANW--
WYPANW--
-WPANWS-
-WPANWE-
WHPANW--
WGPANW--
-YPANWL-
YRPANW--
-YPANWS-
-YPANWN-
YLPANW--
-YPANWT-
-YPANWF-
YIPANW--

YDPANW--
YYPANW--
YSPANW--
YQPANW--
-YPANWG-
YGPANW--
YTPANW--
YPPANW--
-YPANWY-
-YPANWE-
YNPANW--
YHPANW--
YFPANW--
-YPANWR-
-YPANWQ-
-YPANWM-
-YPANWI-
-YPANWA-
YMPANW--
YCPANW--
YAPANW--
PKPL 121 756 33.56
--PKPLES
ALPKPL--
AVPKPL--
-APKPLA-
-APKPLS-
-APKPLR-
-APKPLK-
-APKPLH-
AYPKPL--
-APKPLW-
AAPKPL--
APPKPL--
-APKPLV-
-APKPLM-
AIPKPL--
ADPKPL--
AMPKPL--
AHPKPL--
-APKPLF-
ANPKPL--
AEPKPL--
-APKPLN-
AQPKPL--
-APKPLQ-
-APKPLP-
AGPKPL--
ACPKPL--
CYPKPL--

CTPKPL--
CKPKPL--
CAPKPL--
-CPKPLR-
CIPKPL--
CCPKPL--
CSPKPL--
-CPKPLQ-
CRPKPL--
-CPKPLT-
-CPKPLS-
-CPKPLL-
-CPKPLK-
-CPKPLI-
-CPKPLH-
-CPKPLF-
CLPKPL--
DYPKPL--
-DPKPLA-
-DPKPLL-
DCPKPL--
DTPKPL--
-DPKPLV-
-DPKPLR-
DMPKPL--
DSPKPL--
-DPKPLY-
DNPKPL--
DWPKPL--
DRPKPL--
-DPKPLK-
DLPKPL--
DVPKPL--
DQPKPL--
-DPKPLS-
-DPKPLI-
-DPKPLH-
DKPKPL--
DHPKPL--
DGPKPL--
DFPKPL--
DEPKPL--
DAPKPL--
-EPKPLT-
-EPKPLR-
-EPKPLL-
EWPKPL--
EVPKPL--
EQPKPL--
ESPKPL--

EAPKPL--
-EPKPLW-
-EPKPLN-
-EPKPLF-
ETPKPL--
-EPKPLS-
-EPKPLK-
-EPKPLH-
-EPKPLA-
ENPKPL--
EGPKPL--
ERPKPL--
-EPKPLY-
-EPKPLI-
-EPKPLE-
EMPKPL--
EIPKPL--
EHPKPL--
EFPKPL--
EDPKPL--
-FPKPLL-
FLPKPL--
FRPKPL--
FMPKPL--
-FPKPLY-
-FPKPLS-
-FPKPLK-
FYPKPL--
FPPKPL--
-FPKPLN-
FGPKPL--
FWPKPL--
FVPKPL--
FTPKPL--
FQPKPL--
-FPKPLI-
-FPKPLV-
-FPKPLQ-
FKPKPL--
FAPKPL--
-FPKPLR-
-FPKPLH-
-FPKPLF-
-FPKPLA-
FNPKPL--
FIPKPL--
FEPKPL--
FDPKPL--
FHPKPL--
FFPKPL--

-GPKPLI-
GLPKPL--
-GPKPLS-
-GPKPLM-
GAPKPL--
-GPKPLK-
GTPKPL--
GMPKPL--
GFPKPL--
-GPKPLL-
GRPKPL--
-GPKPLR-
-GPKPLA-
-GPKPLW-
-GPKPLV-
-GPKPLN-
GNPKPL--
GIPKPL--
GQPKPL--
GPPKPL--
-GPKPLT-
-GPKPLF-
GYPKPL--
GVPKPL--
GSPKPL--
-GPKPLQ-
-GPKPLH-
GEPKPL--
GCPKPL--
GWPKPL--
-GPKPLY-
-HPKPLL-
-HPKPLK-
HRPKPL--
-HPKPLW-
-HPKPLR-
HLPKPL--
-HPKPLA-
HYPKPL--
HGPKPL--
HFPKPL--
HDPKPL--
-HPKPLF-
-HPKPLV-
-HPKPLN-
HKPKPL--
HSPKPL--
-HPKPLS-
HAPKPL--
HWPKPL--

```
-HPKPLH-
HEPKPL--
HQPKPL--
-HPKPLY-
-HPKPLT-
-HPKPLC-
HNPKPL--
HMPKPL--
ILPKPL--
-IPKPLI-
-IPKPLR-
IRPKPL--
IVPKPL--
-IPKPLL-
INPKPL--
ISPKPL--
-IPKPLH-
IIPKPL--
IWPKPL--
IFPKPL--
IAPKPL--
-IPKPLY-
-IPKPLT-
-IPKPLQ-
-IPKPLN-
-IPKPLE-
-IPKPLF-
ITPKPL--
IQPKPL--
-IPKPLW-
-IPKPLS-
-IPKPLM-
-IPKPLK-
IKPKPL--
IEPKPL--
IDPKPL--
KSPKPL--
-KPKPLL-
-KPKPLS-
-KPKPLF-
KGPKPL--
KWPKPL--
KHPKPL--
KPPKPL--
KQPKPL--
-KPKPLM-
KIPKPL--
-KPKPLW-
-KPKPLN-
KVPKPL--
```

-KPKPLA-
KAPKPL--
-KPKPLI-
KTPKPL--
-KPKPLY-
-KPKPLK-
-KPKPLE-
KEPKPL--
-LPKPLL-
LLPKPL--
-LPKPLI-
-LPKPLS-
-LPKPLR-
LFPKPL--
-LPKPLK-
-LPKPLW-
-LPKPLT-
-LPKPLA-
LKPKPL--
LDPKPL--
LWPKPL--
LVPKPL--
LTPKPL--
LPPKPL--
-LPKPLV-
LNPKPL--
LHPKPL--
-LPKPLF-
LAPKPL--
LQPKPL--
-LPKPLN-
-LPKPLG-
-LPKPLM-
MRPKPL--
-MPKPLI-
-MPKPLR-
MLPKPL--
-MPKPLS-
-MPKPLK-
MMPKPL--
MWPKPL--
MTPKPL--
MIPKPL--
MHPKPL--
MAPKPL--
MYPKPL--
MPPKPL--
-MPKPLQ-
-MPKPLN-
-MPKPLH-

```
-MPKPLG-
-MPKPLF-
MKPKPL--
MVPKPL--
MQPKPL--
-MPKPLA-
-MPKPLY-
-MPKPLW-
MNPKPL--
MEPKPL--
MDPKPL--
-NPKPLL-
NIPKPL--
-NPKPLQ-
-NPKPLR-
NLPKPL--
NTPKPL--
NDPKPL--
-NPKPLY-
-NPKPLT-
NMPKPL--
NHPKPL--
-NPKPLS-
-NPKPLH-
NRPKPL--
-NPKPLN-
-NPKPLI-
NVPKPL--
NPPKPL--
-NPKPLV-
-NPKPLK-
-NPKPLA-
NGPKPL--
NCPKPL--
--PKPLLS
--PKPLYL
--PKPLSL
--PKPLYG
--PKPLRA
--PKPLLV
--PKPLLY
--PKPLSK
--PKPLRV
--PKPLHF
--PKPLAL
--PKPLWY
--PKPLLQ
--PKPLFV
--PKPLAV
--PKPLTR
```

--PKPLSS
--PKPLRT
--PKPLVS
--PKPLSW
--PKPLSG
--PKPLMY
--PKPLKT
--PKPLIF
--PKPLVN
--PKPLTY
--PKPLSF
--PKPLGA
PQPKPL--
--PKPLYS
--PKPLSV
--PKPLST
--PKPLQA
--PKPLMR
--PKPLFR
--PKPLYY
--PKPLYI
--PKPLVT
--PKPLVA
--PKPLTT
--PKPLTI
--PKPLTF
--PKPLRY
--PKPLGS
--PKPLFS
--PKPLYT
--PKPLWS
--PKPLWF
--PKPLVD
--PKPLPW
--PKPLIT
--PKPLHG
--PKPLFW
--PKPLYF
--PKPLWK
--PKPLVI
--PKPLVG
--PKPLRD
--PKPLLH
--PKPLKK
--PKPLIL
--PKPLGK
PWPKPL--
PMPKPL--
--PKPLYM
--PKPLWT

--PKPLVL
--PKPLVF
--PKPLTH
--PKPLTG
--PKPLRW
--PKPLRI
--PKPLLW
--PKPLLD
--PKPLKR
--PKPLHS
--PKPLGT
--PKPLFL
--PKPLAT
--PKPLAH
PVPKPL--
--PKPLTA
--PKPLSH
--PKPLSD
--PKPLRQ
--PKPLQL
--PKPLNY
--PKPLNV
--PKPLNQ
--PKPLNL
--PKPLMN
--PKPLHE
--PKPLFH
--PKPLYW
--PKPLWL
--PKPLVW
--PKPLVQ
--PKPLTV
--PKPLSA
--PKPLRN
--PKPLRK
--PKPLQS
--PKPLQI
--PKPLNT
--PKPLNI
--PKPLMH
--PKPLLI
--PKPLKF
--PKPLIH
--PKPLGQ
--PKPLEI
--PKPLYN
--PKPLWN
--PKPLTW
--PKPLSY
--PKPLRE

--PKPLQY
--PKPLQQ
--PKPLQK
--PKPLQG
--PKPLNS
--PKPLMT
--PKPLMS
--PKPLKL
--PKPLIN
--PKPLIG
--PKPLIE
--PKPLIA
--PKPLHQ
--PKPLFK
--PKPLEY
--PKPLEL
--PKPLEF
--PKPLDQ
--PKPLDL
--PKPLAN
--PKPLAK
--PKPLAA
PGPKPL--
--PKPLYK
--PKPLWG
--PKPLVY
--PKPLVK
--PKPLTK
--PKPLRM
--PKPLRH
--PKPLQT
--PKPLNM
--PKPLNA
--PKPLMW
--PKPLMV
--PKPLMA
--PKPLLM
--PKPLLK
--PKPLKV
--PKPLKN
--PKPLKI
--PKPLIQ
--PKPLIK
--PKPLII
--PKPLHT
--PKPLHR
--PKPLHK
--PKPLHH
--PKPLGH
--PKPLFY

--PKPLFM
--PKPLFA
--PKPLEG
--PKPLAW
--PKPLAR
PAPKPL--
PRPKPL--
--PKPLWV
--PKPLWM
--PKPLWE
--PKPLVV
--PKPLVM
--PKPLTQ
--PKPLQW
--PKPLPY
--PKPLPV
--PKPLPQ
--PKPLPA
--PKPLNF
--PKPLMK
--PKPLMG
--PKPLMF
--PKPLLF
--PKPLIV
--PKPLHN
--PKPLHA
--PKPLFN
--PKPLFG
--PKPLFF
--PKPLFD
--PKPLCG
PSPKPL--
--PKPLYV
--PKPLWI
--PKPLWH
--PKPLWD
--PKPLWA
--PKPLTM
--PKPLTD
--PKPLSE
--PKPLQV
--PKPLQN
--PKPLQE
--PKPLQD
--PKPLPS
--PKPLPF
--PKPLNW
--PKPLNN
--PKPLNG
--PKPLMQ

--PKPLMI
--PKPLLE
--PKPLKM
--PKPLKE
--PKPLKD
--PKPLIW
--PKPLHW
--PKPLHM
--PKPLGN
--PKPLGG
--PKPLFT
--PKPLEW
--PKPLEV
--PKPLEQ
--PKPLDY
--PKPLDV
--PKPLDF
--PKPLDA
--PKPLCV
--PKPLAQ
--PKPLAM
--PKPLAG
--PKPLAF
PKPKPL--
-QPKPLL-
QSPKPL--
QIPKPL--
QLPKPL--
-QPKPLF-
QRPKPL--
-QPKPLS-
-QPKPLM-
-QPKPLY-
-QPKPLT-
QMPKPL--
QFPKPL--
QYPKPL--
-QPKPLK-
QWPKPL--
-QPKPLV-
QEPKPL--
QHPKPL--
QVPKPL--
-QPKPLA-
QNPKPL--
QAPKPL--
QPPKPL--
-QPKPLW-
-QPKPLN-
-QPKPLH-

-QPKPLE-
QKPKPL--
QGPKPL--
-RPKPLT-
-RPKPLI-
RLPKPL--
-RPKPLK-
-RPKPLR-
-RPKPLA-
-RPKPLS-
RVPKPL--
RTPKPL--
RAPKPL--
RQPKPL--
RMPKPL--
RFPKPL--
RDPKPL--
RYPKPL--
-RPKPLN-
RWPKPL--
RSPKPL--
RPPKPL--
-RPKPLY-
-RPKPLE-
RHPKPL--
RIPKPL--
-RPKPLM-
RKPKPL--
RCPKPL--
-SPKPLS-
-SPKPLK-
SVPKPL--
-SPKPLI-
SQPKPL--
SYPKPL--
SSPKPL--
-SPKPLH-
SWPKPL--
-SPKPLW-
SLPKPL--
SIPKPL--
-SPKPLY-
SMPKPL--
STPKPL--
SRPKPL--
SEPKPL--
-SPKPLV-
-SPKPLT-
-SPKPLQ-
-SPKPLF-

```
SDPKPL--
-SPKPLN-
SCPKPL--
SAPKPL--
-SPKPLM-
-SPKPLA-
SFPKPL--
SHPKPL--
SPPKPL--
-SPKPLE-
SKPKPL--
-TPKPLR-
-TPKPLA-
TSPKPL--
TLPKPL--
-TPKPLL-
-TPKPLS-
-TPKPLH-
-TPKPLN-
TGPKPL--
TFPKPL--
-TPKPLM-
-TPKPLK-
TAPKPL--
TRPKPL--
TPPKPL--
-TPKPLY-
TNPKPL--
TKPKPL--
TVPKPL--
-TPKPLI-
-TPKPLV-
-TPKPLW-
-TPKPLQ-
TMPKPL--
TIPKPL--
TYPKPL--
THPKPL--
TEPKPL--
TQPKPL--
TDPKPL--
VRPKPL--
VLPKPL--
-VPKPLR-
VVPKPL--
VWPKPL--
-VPKPLV-
-VPKPLL-
VTPKPL--
VGPKPL--
```

VKPKPL--
VYPKPL--
-VPKPLG-
-VPKPLA-
-VPKPLT-
-VPKPLN-
VAPKPL--
VSPKPL--
-VPKPLS-
VNPKPL--
-VPKPLF-
VHPKPL--
VCPKPL--
-VPKPLY-
-VPKPLQ-
-VPKPLM-
VQPKPL--
-VPKPLI-
-VPKPLH-
VEPKPL--
VDPKPL--
-WPKPLR-
-WPKPLS-
WVPKPL--
WLPKPL--
-WPKPLK-
WHPKPL--
WYPKPL--
-WPKPLL-
-WPKPLI-
WIPKPL--
WGPKPL--
WSPKPL--
-WPKPLQ-
-WPKPLH-
-WPKPLT-
WDPKPL--
WRPKPL--
-WPKPLV-
-WPKPLG-
-WPKPLA-
WEPKPL--
WTPKPL--
WMPKPL--
WKPKPL--
WAPKPL--
WWPKPL--
WPPKPL--
-WPKPLY-
-WPKPLM-

WNPKPL--
YVPKPL--
YAPKPL--
-YPKPLI-
YTPKPL--
YNPKPL--
YPPKPL--
YMPKPL--
-YPKPLH-
YYPKPL--
YRPKPL--
YLPKPL--
YGPKPL--
YQPKPL--
-YPKPLS-
-YPKPLF-
-YPKPLY-
YIPKPL--
YEPKPL--
-YPKPLV-
-YPKPLP-
-YPKPLM-
-YPKPLK-
YHPKPL--
-YPKPLQ-
-YPKPLC-
-YPKPLA-
YFPKPL--

| | | | |
|---|---|---|---|
| PANI | 189 | 722 | 33.55 |

-APANIR-
ANPANI--
ARPANI--
AFPANI--
-APANIL-
ASPANI--
AQPANI--
-APANIY-
-APANIV-
-APANII-
-APANIH-
ATPANI--
-APANIS-
-APANIK-
AMPANI--
AYPANI--
AWPANI--
AVPANI--
APPANI--
-APANIW-
-APANID-

ALPANI--
AGPANI--
AAPANI--
-APANIE-
AKPANI--
AIPANI--
-APANIT-
-APANIN-
-CPANIV-
-CPANIS-
-CPANIA-
CVPANI--
CSPANI--
-CPANIR-
-CPANIN-
CIPANI--
CGPANI--
CEPANI--
-DPANIS-
DRPANI--
DMPANI--
DVPANI--
-DPANIR-
DLPANI--
DWPANI--
DTPANI--
-DPANIL-
-DPANIA-
DKPANI--
DYPANI--
-DPANIV-
-DPANIQ-
-DPANIN-
-DPANII-
DNPANI--
DGPANI--
DDPANI--
-EPANIR-
-EPANIW-
-EPANIF-
-EPANIH-
EYPANI--
ETPANI--
ESPANI--
-EPANIA-
-EPANIE-
-EPANIY-
-EPANIL-
ELPANI--
EKPANI--

EFPANI--
-EPANIV-
-EPANIS-
-EPANIN-
-EPANIM-
-EPANIK-
-EPANII-
ENPANI--
EGPANI--
EEPANI--
EAPANI--
FDPANI--
FMPANI--
FGPANI--
FSPANI--
-FPANIR-
FWPANI--
-FPANIQ-
-FPANIL-
-FPANII-
-FPANIA-
FQPANI--
-FPANIM-
FKPANI--
FAPANI--
-FPANIT-
FNPANI--
FRPANI--
-FPANIS-
-FPANIG-
FIPANI--
FHPANI--
FEPANI--
FPPANI--
-FPANIN-
-FPANIK-
FTPANI--
-FPANIY-
-FPANIV-
-FPANIE-
FLPANI--
FFPANI--
-GPANIL-
GLPANI--
GEPANI--
GYPANI--
GSPANI--
-GPANIW-
GQPANI--
-GPANIK-

−GPANIH−
−GPANIG−
GVPANI−−
GIPANI−−
−GPANIT−
−GPANIQ−
GGPANI−−
GAPANI−−
GTPANI−−
GRPANI−−
−GPANIV−
−GPANIR−
−GPANIF−
GFPANI−−
−GPANIY−
−GPANIN−
−GPANIM−
−GPANII−
−GPANIE−
HAPANI−−
−HPANIQ−
−HPANIE−
HRPANI−−
−HPANIR−
−HPANIM−
HVPANI−−
−HPANIH−
HTPANI−−
−HPANIS−
−HPANIN−
HMPANI−−
HLPANI−−
HHPANI−−
HYPANI−−
HSPANI−−
−HPANIY−
−HPANIW−
−HPANIA−
HNPANI−−
HIPANI−−
HFPANI−−
ISPANI−−
IEPANI−−
IRPANI−−
ILPANI−−
INPANI−−
−IPANIS−
IKPANI−−
IFPANI−−
IDPANI−−

-IPANIQ-
-IPANIN-
-IPANIL-
IQPANI--
-IPANIW-
-IPANIR-
IGPANI--
IYPANI--
IVPANI--
-IPANIV-
-IPANIM-
-IPANIK-
-IPANIH-
IIPANI--
IHPANI--
IAPANI--
KSPANI--
-KPANIL-
KFPANI--
-KPANIF-
-KPANIW-
-KPANIG-
KNPANI--
-KPANIY-
-KPANIA-
KWPANI--
-KPANIV-
KRPANI--
-KPANIS-
-KPANIH-
KMPANI--
KKPANI--
KTPANI--
KQPANI--
KPPANI--
-KPANIK-
-KPANIE-
KLPANI--
KIPANI--
KAPANI--
-KPANIP-
-KPANID-
-KPANIC-
KHPANI--
LGPANI--
-LPANIL-
LRPANI--
-LPANIY-
-LPANIT-
-LPANIR-

LNPANI--
LYPANI--
LTPANI--
LSPANI--
LQPANI--
LPPANI--
-LPANIK-
LKPANI--
-LPANIG-
LMPANI--
-LPANIS-
-LPANIQ-
-LPANIN-
LFPANI--
LWPANI--
LVPANI--
LEPANI--
-LPANIM-
-LPANIF-
LLPANI--
-LPANIW-
-LPANIV-
-LPANIH-
-LPANIE-
LIPANI--
LHPANI--
LCPANI--
-MPANIT-
-MPANIS-
-MPANIY-
-MPANIA-
MEPANI--
-MPANIW-
-MPANIK-
-MPANIH-
MLPANI--
MAPANI--
-MPANIR-
-MPANIL-
MNPANI--
MIPANI--
MVPANI--
MQPANI--
-MPANIV-
-MPANIQ-
-MPANIM-
-MPANIE-
MYPANI--
MWPANI--
MTPANI--

```
MSPANI--
MPPANI--
-MPANII-
-MPANIG-
-MPANIF-
MKPANI--
MHPANI--
MFPANI--
-NPANIL-
NLPANI--
NRPANI--
NMPANI--
-NPANIS-
-NPANIM-
NSPANI--
-NPANIG-
NAPANI--
NQPANI--
-NPANIT-
-NPANIR-
-NPANIQ-
-NPANIF-
-NPANIE-
NGPANI--
NWPANI--
-NPANIN-
NKPANI--
NIPANI--
-NPANIY-
-NPANIW-
-NPANIV-
-NPANII-
-NPANIA-
NNPANI--
--PANIRR
--PANISR
--PANIYK
--PANIRL
--PANILL
--PANILR
--PANIWK
--PANIRY
--PANIWM
--PANIMR
--PANIHR
--PANIWS
--PANINL
--PANILK
--PANIIQ
--PANIFG
```

--PANIAS
--PANIWN
--PANIWE
--PANILT
--PANIKL
--PANIHS
--PANIFR
--PANIWY
--PANISS
--PANISF
--PANIRS
--PANIKG
--PANIAR
--PANIVL
--PANISG
--PANIQR
--PANILV
--PANILS
--PANIFN
--PANIAG
PGPANI--
PAPANI--
--PANIWV
--PANIRG
--PANIRD
--PANIQQ
--PANIIY
--PANIIR
--PANIHG
--PANIWT
--PANIVS
--PANIVG
--PANIVF
--PANITS
--PANITR
--PANIST
--PANISN
--PANISM
--PANIQS
--PANINS
--PANIMK
--PANILM
--PANILE
--PANIIN
--PANIGG
--PANIEA
--PANIAA
--PANIWD
--PANIVK
--PANITT

```
--PANITL
--PANITK
--PANITI
--PANISK
--PANISA
--PANIRM
--PANIRI
--PANIRH
--PANINK
--PANIMY
--PANIKW
--PANIKR
--PANIKM
--PANIGK
--PANIFS
--PANIFK
--PANIEL
PVPANI--
PSPANI--
PRPANI--
--PANIYQ
--PANIYH
--PANIYF
--PANIWR
--PANIWH
--PANIVR
--PANIVM
--PANITQ
--PANISY
--PANISH
--PANIQI
--PANIMS
--PANIML
--PANIME
--PANILY
--PANILH
--PANIKS
--PANIHV
--PANIHL
--PANIHK
--PANIHA
--PANIGW
--PANIGN
--PANIFE
--PANIFA
--PANIAL
--PANIAF
PQPANI--
--PANIYT
--PANIYN
```

--PANIYI
--PANIYE
--PANIWQ
--PANIWF
--PANIVT
--PANIVH
--PANIVE
--PANITM
--PANITG
--PANISW
--PANISE
--PANIRV
--PANIRN
--PANIRF
--PANIRA
--PANIQM
--PANIQL
--PANINV
--PANINF
--PANIMH
--PANIMG
--PANIMD
--PANILG
--PANIKT
--PANIIG
--PANIIE
--PANIHN
--PANIHH
--PANIGL
--PANIFV
--PANIES
--PANIEG
--PANIEE
PLPANI--
PIPANI--
PHPANI--
PFPANI--
--PANIYY
--PANIYV
--PANIYS
--PANIYL
--PANIYG
--PANIYA
--PANIVW
--PANIVV
--PANIVA
--PANITN
--PANITF
--PANISQ
--PANISI

--PANISC
--PANIRW
--PANIRP
--PANIRE
--PANIQY
--PANIQE
--PANIPG
--PANINT
--PANINQ
--PANINN
--PANINM
--PANINI
--PANIMV
--PANIMT
--PANIMM
--PANILF
--PANIKY
--PANIKH
--PANIKD
--PANIIW
--PANIIV
--PANIIT
--PANIIK
--PANIIA
--PANIHY
--PANIHT
--PANIHI
--PANIHD
--PANIGV
--PANIGT
--PANIGS
--PANIGI
--PANIGH
--PANIFY
--PANIFW
--PANIFQ
--PANIFD
--PANIEV
--PANIEN
--PANIEM
--PANIEK
--PANIEI
--PANIDS
--PANICS
--PANIAT
--PANIAH
--PANIAE
QVPANI--
QLPANI--
-QPANII-

QKPANI--
-QPANIR-
QIPANI--
QRPANI--
-QPANIY-
-QPANIS-
-QPANIL-
QWPANI--
-QPANIM-
-QPANIK-
QGPANI--
QTPANI--
-QPANIQ-
QMPANI--
QYPANI--
QPPANI--
-QPANIV-
-QPANIN-
-QPANIH-
-QPANIA-
QEPANI--
QAPANI--
RQPANI--
-RPANIS-
-RPANIY-
RVPANI--
-RPANIL-
RGPANI--
RYPANI--
RSPANI--
-RPANIE-
RLPANI--
RKPANI--
-RPANIM-
RNPANI--
RTPANI--
-RPANIK-
RMPANI--
RFPANI--
-RPANIW-
-RPANIR-
-RPANIQ-
-RPANIN-
-RPANIH-
RIPANI--
RPPANI--
-RPANIV-
-RPANIT-
-RPANIA-
REPANI--

RWPANI--
-RPANII-
-RPANIF-
RDPANI--
RAPANI--
RRPANI--
-RPANID-
RHPANI--
RCPANI--
-SPANIR-
-SPANIL-
SNPANI--
SHPANI--
-SPANIS-
-SPANII-
SVPANI--
SKPANI--
SGPANI--
SQPANI--
SPPANI--
-SPANIY-
-SPANIT-
-SPANIG-
-SPANIQ-
-SPANIK-
STPANI--
-SPANIW-
-SPANIE-
SMPANI--
SAPANI--
SYPANI--
SSPANI--
-SPANIA-
SRPANI--
-SPANIV-
SLPANI--
SIPANI--
SDPANI--
TRPANI--
-TPANIL-
TIPANI--
TGPANI--
-TPANIK-
TWPANI--
-TPANIY-
-TPANIN-
-TPANIH-
-TPANIS-
-TPANIF-
TTPANI--

```
-TPANIQ-
TNPANI--
TMPANI--
TFPANI--
TYPANI--
TPPANI--
-TPANIR-
-TPANIM-
-TPANIE-
TEPANI--
TQPANI--
-TPANIV-
-TPANIT-
-TPANII-
-TPANIG-
-TPANIA-
TLPANI--
TKPANI--
TAPANI--
-VPANIL-
VLPANI--
VVPANI--
VSPANI--
VRPANI--
VGPANI--
-VPANIS-
VHPANI--
VTPANI--
-VPANIK-
-VPANIV-
VQPANI--
VPPANI--
-VPANIY-
-VPANIW-
-VPANIT-
-VPANIM-
-VPANIG-
-VPANIQ-
-VPANIN-
-VPANII-
-VPANIF-
-VPANIA-
VMPANI--
VKPANI--
VIPANI--
VFPANI--
VEPANI--
WTPANI--
-WPANIW-
-WPANIL-
```

WIPANI--
WGPANI--
-WPANIS-
WSPANI--
-WPANIR-
-WPANIK-
WMPANI--
WHPANI--
-WPANIM-
-WPANII-
WLPANI--
WEPANI--
WAPANI--
-WPANIY-
-WPANIV-
-WPANIT-
-WPANIQ-
-WPANIN-
-WPANIG-
-WPANIA-
WKPANI--
-YPANIR-
YLPANI--
YQPANI--
-YPANIS-
YEPANI--
-YPANIQ-
YPPANI--
-YPANIH-
YVPANI--
-YPANIA-
YKPANI--
-YPANII-
YIPANI--
YGPANI--
YWPANI--
YSPANI--
YRPANI--
-YPANIY-
-YPANIF-
-YPANIE-
YAPANI--
YTPANI--
-YPANIT-
-YPANIN-
-YPANIM-
-YPANIK-
YNPANI--
YHPANI--
YFPANI--

| | | | |
|---|---|---|---|
| | YCPANI-- | | |
| PVGI | 31 | 827 | 33.53 |
| | -LPVGIG- | | |
| | --PVGIPL | | |
| | --PVGIIW | | |
| | --PVGIGR | | |
| | --PVGIGK | | |
| | -APVGIR- | | |
| | -APVGII- | | |
| | ARPVGI-- | | |
| | -APVGIS- | | |
| | -APVGIY- | | |
| | -APVGIT- | | |
| | AMPVGI-- | | |
| | AWPVGI-- | | |
| | AVPVGI-- | | |
| | ASPVGI-- | | |
| | AKPVGI-- | | |
| | AIPVGI-- | | |
| | -APVGIW- | | |
| | -APVGIL- | | |
| | -APVGIA- | | |
| | -APVGIG- | | |
| | ATPVGI-- | | |
| | -APVGIH- | | |
| | AAPVGI-- | | |
| | -APVGIQ- | | |
| | AFPVGI-- | | |
| | AEPVGI-- | | |
| | AYPVGI-- | | |
| | -APVGIN- | | |
| | -APVGIM- | | |
| | -APVGIF- | | |
| | -APVGIE- | | |
| | AQPVGI-- | | |
| | APPVGI-- | | |
| | ADPVGI-- | | |
| | -APVGIV- | | |
| | -APVGIK- | | |
| | ACPVGI-- | | |
| | CAPVGI-- | | |
| | CRPVGI-- | | |
| | -CPVGIR- | | |
| | CQPVGI-- | | |
| | -CPVGIT- | | |
| | -CPVGII- | | |
| | -CPVGIH- | | |
| | CNPVGI-- | | |
| | CWPVGI-- | | |
| | CVPVGI-- | | |

CTPVGI--
CSPVGI--
-CPVGIG-
CKPVGI--
CEPVGI--
DLPVGI--
-DPVGIL-
DVPVGI--
DTPVGI--
DRPVGI--
DGPVGI--
DAPVGI--
DWPVGI--
-DPVGIY-
-DPVGIT-
-DPVGIK-
DYPVGI--
DSPVGI--
-DPVGIW-
-DPVGIV-
-DPVGIS-
-DPVGIM-
-DPVGIA-
-DPVGIQ-
-DPVGIN-
-EPVGIL-
-EPVGIS-
ESPVGI--
EWPVGI--
EIPVGI--
EVPVGI--
-EPVGIR-
EFPVGI--
ELPVGI--
-EPVGIY-
-EPVGIV-
-EPVGIH-
EHPVGI--
EEPVGI--
ERPVGI--
-EPVGIT-
-EPVGII-
EGPVGI--
EYPVGI--
-EPVGIW-
-EPVGIG-
-EPVGIF-
-EPVGIE-
-EPVGIA-
EMPVGI--

EKPVGI--
EAPVGI--
-FPVGIS-
-FPVGIN-
-FPVGIY-
FDPVGI--
-FPVGIA-
FGPVGI--
FLPVGI--
FRPVGI--
-FPVGIT-
-FPVGIQ-
-FPVGIV-
-FPVGIL-
FFPVGI--
-FPVGIR-
FKPVGI--
FTPVGI--
-FPVGII-
FVPVGI--
FEPVGI--
FSPVGI--
FMPVGI--
FYPVGI--
FNPVGI--
FQPVGI--
-FPVGIK-
-FPVGIG-
FPPVGI--
FIPVGI--
-FPVGIM-
FHPVGI--
GGPVGI--
GRPVGI--
-GPVGIR-
-GPVGIM-
-GPVGIL-
-GPVGIT-
GAPVGI--
-GPVGIK-
-GPVGIW-
-GPVGIN-
-GPVGIV-
-GPVGIQ-
GPPVGI--
GMPVGI--
GVPVGI--
GSPVGI--
-GPVGIS-
-GPVGIH-

−GPVGIA−
GEPVGI−−
GCPVGI−−
GYPVGI−−
GLPVGI−−
GQPVGI−−
−GPVGIG−
−GPVGIE−
GTPVGI−−
−GPVGII−
GIPVGI−−
GHPVGI−−
GWPVGI−−
GNPVGI−−
GFPVGI−−
GDPVGI−−
HFPVGI−−
−HPVGIL−
HLPVGI−−
−HPVGIK−
−HPVGIR−
HGPVGI−−
HYPVGI−−
−HPVGIY−
−HPVGIS−
HNPVGI−−
−HPVGIF−
HQPVGI−−
−HPVGIM−
HIPVGI−−
HEPVGI−−
HWPVGI−−
HVPVGI−−
HTPVGI−−
HSPVGI−−
−HPVGIW−
−HPVGIV−
−HPVGIN−
−HPVGII−
−HPVGIH−
−HPVGIQ−
−HPVGIG−
−HPVGIE−
HHPVGI−−
HDPVGI−−
HAPVGI−−
−IPVGIL−
IGPVGI−−
−IPVGIR−
IRPVGI−−

IPPVGI--
-IPVGIT-
ILPVGI--
IKPVGI--
IQPVGI--
INPVGI--
-IPVGIW-
IMPVGI--
-IPVGIS-
-IPVGIE-
IAPVGI--
IYPVGI--
ITPVGI--
-IPVGIQ-
-IPVGIG-
IHPVGI--
IVPVGI--
-IPVGIY-
-IPVGIV-
-IPVGIK-
-IPVGII-
-IPVGIF-
-IPVGIA-
IFPVGI--
IEPVGI--
IWPVGI--
-IPVGIM-
-IPVGIH-
IIPVGI--
IDPVGI--
KSPVGI--
-KPVGIL-
KRPVGI--
KYPVGI--
-KPVGIS-
-KPVGIR-
KVPVGI--
-KPVGIV-
-KPVGIT-
KNPVGI--
KIPVGI--
KHPVGI--
KGPVGI--
-KPVGIN-
-KPVGIK-
KLPVGI--
KDPVGI--
KTPVGI--
-KPVGIF-
KAPVGI--

-KPVGIH-
-KPVGIG-
-KPVGIY-
KMPVGI--
-KPVGIM-
-KPVGII-
-KPVGIA-
KPPVGI--
KWPVGI--
-KPVGIE-
KEPVGI--
KCPVGI--
LRPVGI--
-LPVGIR-
LLPVGI--
LTPVGI--
-LPVGIK-
-LPVGIM-
LKPVGI--
-LPVGII-
LIPVGI--
LNPVGI--
LMPVGI--
LGPVGI--
LAPVGI--
LYPVGI--
LSPVGI--
LWPVGI--
LDPVGI--
-LPVGIH-
-LPVGIY-
-LPVGIQ-
-LPVGIN-
-LPVGIA-
LVPVGI--
LQPVGI--
-LPVGIV-
LHPVGI--
LEPVGI--
-LPVGIW-
LPPVGI--
LFPVGI--
MGPVGI--
-MPVGIK-
MPPVGI--
MLPVGI--
MIPVGI--
MFPVGI--
MSPVGI--
-MPVGIR-

```
MDPVGI--
-MPVGIS-
-MPVGIL-
-MPVGIW-
-MPVGIT-
-MPVGIY-
-MPVGIG-
MQPVGI--
-MPVGIV-
-MPVGII-
MEPVGI--
MVPVGI--
-MPVGIA-
MYPVGI--
-MPVGIM-
-MPVGIF-
MNPVGI--
MWPVGI--
-MPVGIH-
-NPVGIL-
NRPVGI--
-NPVGIS-
-NPVGIV-
-NPVGIR-
-NPVGIT-
-NPVGIN-
NHPVGI--
NGPVGI--
NTPVGI--
NLPVGI--
NKPVGI--
NAPVGI--
NQPVGI--
-NPVGIM-
-NPVGIK-
-NPVGIQ-
-NPVGIH-
NNPVGI--
NIPVGI--
NSPVGI--
-NPVGIY-
-NPVGII-
-NPVGIA-
NMPVGI--
NEPVGI--
NDPVGI--
NWPVGI--
-NPVGIW-
-NPVGIG-
-NPVGIF-
```

NFPVGI--
--PVGIRL
--PVGIRV
--PVGIRS
--PVGIFR
--PVGILA
--PVGILR
--PVGISR
--PVGIRK
--PVGISG
--PVGILN
--PVGIRM
--PVGILS
--PVGIGS
--PVGIYT
--PVGIKS
--PVGITR
--PVGIIR
--PVGIWN
--PVGISK
PRPVGI--
--PVGIVQ
--PVGIRN
--PVGITG
PGPVGI--
--PVGIYS
--PVGIVS
--PVGIVN
--PVGISM
--PVGIRT
--PVGIFN
PNPVGI--
--PVGIWR
--PVGIVK
--PVGIVI
--PVGIVA
--PVGITA
--PVGIHL
--PVGIVR
--PVGITY
--PVGITQ
--PVGINR
--PVGIHE
PSPVGI--
--PVGITM
--PVGITK
--PVGISL
--PVGIRQ
--PVGIKL
--PVGIKG

--PVGIHR
PVPVGI--
--PVGIYR
--PVGIVG
--PVGITT
--PVGISY
--PVGIQR
--PVGILY
--PVGILT
--PVGIII
--PVGIFT
--PVGIFK
PMPVGI--
--PVGIWW
--PVGIVV
--PVGIVF
--PVGITL
--PVGITE
--PVGISW
--PVGIST
--PVGISS
--PVGISN
--PVGILG
--PVGIKR
--PVGIKH
--PVGIIV
--PVGIVT
--PVGITS
--PVGILK
--PVGIKK
--PVGIHG
--PVGIFH
--PVGIES
--PVGIAR
--PVGIVE
--PVGIVD
--PVGITN
--PVGIRI
--PVGINS
--PVGILD
--PVGIKT
--PVGIIQ
--PVGIHS
--PVGIGV
--PVGIGA
--PVGIFG
--PVGIAK
PQPVGI--
PLPVGI--
PKPVGI--

PAPVGI--
--PVGIWS
--PVGIWK
--PVGISF
--PVGISE
--PVGIRY
--PVGIQS
--PVGIQG
--PVGINL
--PVGINF
--PVGIMS
--PVGIMG
--PVGILW
--PVGILL
--PVGILF
--PVGIHT
--PVGIGT
--PVGIGN
--PVGIFS
--PVGIAS
--PVGIAQ
PYPVGI--
PWPVGI--
--PVGIYH
--PVGITI
--PVGISI
--PVGIRG
--PVGIRF
--PVGIRD
--PVGIRA
--PVGINV
--PVGIMQ
--PVGIMF
--PVGILM
--PVGILE
--PVGIKV
--PVGIIS
--PVGIIH
--PVGIHA
--PVGIYF
--PVGIWH
--PVGIWE
--PVGITW
--PVGITV
--PVGITH
--PVGISQ
--PVGISA
--PVGIQL
--PVGIQA
--PVGINY

```
--PVGINT
--PVGINQ
--PVGING
--PVGIMY
--PVGIMW
--PVGIMK
--PVGIMH
--PVGILQ
--PVGILH
--PVGIKW
--PVGIHV
--PVGIHH
--PVGIGW
--PVGIGG
--PVGIFQ
--PVGIAV
--PVGIAT
--PVGIAN
--PVGIAL
--PVGIAI
PTPVGI--
--PVGIYK
--PVGIYG
--PVGIYD
--PVGIWG
--PVGITF
--PVGISD
--PVGIRP
--PVGIRE
--PVGIQF
--PVGINW
--PVGINA
--PVGIMV
--PVGIMT
--PVGILV
--PVGIKF
--PVGIIE
--PVGIID
--PVGIHW
--PVGIHN
--PVGIGY
--PVGIGL
--PVGIFY
--PVGIEV
--PVGIAM
--PVGIAG
--PVGIAA
PHPVGI--
PFPVGI--
PDPVGI--
```

--PVGIYY
--PVGIWQ
--PVGIVW
--PVGIVH
--PVGISV
--PVGIRH
--PVGIQY
--PVGIQW
--PVGIQD
--PVGINN
--PVGINK
--PVGINH
--PVGIMN
--PVGIML
--PVGIKQ
--PVGIKI
--PVGIKD
--PVGIIG
--PVGIHM
--PVGIHD
--PVGIET
--PVGIEG
--PVGIEA
--PVGIAF
PIPVGI--
PEPVGI--
--PVGIYQ
--PVGIYN
--PVGIYA
--PVGIWD
--PVGIVM
--PVGITD
--PVGITC
--PVGIRW
--PVGIQT
--PVGIQQ
--PVGIQN
--PVGIQH
--PVGINI
--PVGIND
--PVGIME
--PVGIMC
--PVGIMA
--PVGIKY
--PVGIKA
--PVGIIT
--PVGIIK
--PVGIHY
--PVGIHK
--PVGIHI

--PVGIHF
--PVGIFE
--PVGIER
--PVGIEN
--PVGIEI
--PVGIEF
--PVGIDS
--PVGICS
--PVGICN
--PVGIAW
--PVGIAH
--PVGIAD
PCPVGI--
-QPVGIV-
QRPVGI--
-QPVGIS-
QPPVGI--
-QPVGIR-
-QPVGIL-
QVPVGI--
-QPVGIQ-
QLPVGI--
-QPVGIY-
-QPVGIK-
QHPVGI--
QGPVGI--
QNPVGI--
QTPVGI--
QSPVGI--
-QPVGIW-
-QPVGIF-
QYPVGI--
-QPVGIT-
-QPVGIM-
QWPVGI--
-QPVGIN-
-QPVGII-
QMPVGI--
QDPVGI--
QQPVGI--
-QPVGIG-
QIPVGI--
QFPVGI--
QAPVGI--
-RPVGIY-
RLPVGI--
RVPVGI--
-RPVGIR-
RGPVGI--
-RPVGIM-

-RPVGII-
-RPVGIA-
RYPVGI--
-RPVGIV-
RIPVGI--
RWPVGI--
REPVGI--
RAPVGI--
-RPVGIT-
-RPVGIN-
RHPVGI--
RTPVGI--
-RPVGIS-
RPPVGI--
-RPVGIK-
-RPVGIG-
-RPVGIF-
RDPVGI--
-RPVGIE-
-RPVGIQ-
RQPVGI--
RFPVGI--
RNPVGI--
-RPVGIW-
-RPVGIH-
-RPVGIC-
-SPVGIR-
SLPVGI--
SGPVGI--
SSPVGI--
-SPVGIH-
-SPVGIY-
-SPVGIN-
SAPVGI--
SMPVGI--
SRPVGI--
-SPVGIS-
SNPVGI--
-SPVGIE-
SFPVGI--
SEPVGI--
-SPVGII-
SYPVGI--
-SPVGIW-
-SPVGIV-
-SPVGIQ-
-SPVGIG-
-SPVGIF-
SQPVGI--
-SPVGIT-

SHPVGI--
SDPVGI--
-SPVGIA-
SWPVGI--
SPPVGI--
SIPVGI--
TGPVGI--
-TPVGIS-
TVPVGI--
-TPVGIR-
-TPVGIA-
TWPVGI--
-TPVGIL-
-TPVGIW-
-TPVGIT-
TFPVGI--
TTPVGI--
TDPVGI--
TSPVGI--
-TPVGIE-
TNPVGI--
TIPVGI--
TQPVGI--
-TPVGIY-
TMPVGI--
TEPVGI--
-TPVGIG-
-TPVGIF-
TAPVGI--
TYPVGI--
-TPVGIQ-
-TPVGIN-
-TPVGIK-
-TPVGIH-
THPVGI--
-TPVGIM-
TPPVGI--
-VPVGIL-
-VPVGIF-
VRPVGI--
VLPVGI--
-VPVGIK-
VVPVGI--
VSPVGI--
-VPVGIT-
-VPVGIR-
-VPVGIH-
-VPVGIY-
-VPVGIS-
VPPVGI--

VGPVGI--
VAPVGI--
VWPVGI--
-VPVGIV-
VKPVGI--
VDPVGI--
VTPVGI--
VIPVGI--
-VPVGIM-
VHPVGI--
VYPVGI--
-VPVGIG-
-VPVGIE-
VNPVGI--
VMPVGI--
VEPVGI--
VCPVGI--
-VPVGIQ-
VFPVGI--
WDPVGI--
-WPVGIR-
WLPVGI--
-WPVGIV-
WAPVGI--
WVPVGI--
-WPVGIT-
WNPVGI--
WKPVGI--
-WPVGIN-
WFPVGI--
WWPVGI--
-WPVGIH-
WPPVGI--
WGPVGI--
WEPVGI--
WSPVGI--
WIPVGI--
-WPVGIK-
WMPVGI--
WYPVGI--
WQPVGI--
-WPVGIA-
-WPVGIS-
-WPVGIQ-
-WPVGIM-
-WPVGIL-
-YPVGIS-
YLPVGI--
YVPVGI--
YSPVGI--

| | | | |
|---|---|---|---|
| | -YPVGIK- | | |
| | -YPVGIL- | | |
| | YTPVGI-- | | |
| | YKPVGI-- | | |
| | -YPVGIN- | | |
| | -YPVGIF- | | |
| | -YPVGIR- | | |
| | -YPVGII- | | |
| | -YPVGIT- | | |
| | YHPVGI-- | | |
| | YWPVGI-- | | |
| | YRPVGI-- | | |
| | -YPVGIM- | | |
| | YIPVGI-- | | |
| | YAPVGI-- | | |
| | -YPVGIV- | | |
| | YGPVGI-- | | |
| | -YPVGIW- | | |
| | -YPVGIQ- | | |
| | YPPVGI-- | | |
| | YEPVGI-- | | |
| | YQPVGI-- | | |
| | -YPVGIH- | | |
| | -YPVGIG- | | |
| | -YPVGIE- | | |
| | -YPVGIA- | | |
| | YMPVGI-- | | |
| | YDPVGI-- | | |
| | YCPVGI-- | | |
| PLSL | 495 | 606 | 33.53 |
| | -APLSLD- | | |
| | LCPLSL-- | | |
| | -NPLSLP- | | |
| | --PLSLPP | | |
| | --PLSLGH | | |
| | --PLSLEL | | |
| | --PLSLED | | |
| | --PLSLDT | | |
| | --PLSLDG | | |
| | --PLSLAP | | |
| | -RPLSLD- | | |
| | -SPLSLD- | | |
| | -VPLSLD- | | |
| | -YPLSLD- | | |
| | -APLSLL- | | |
| | ALPLSL-- | | |
| | ATPLSL-- | | |
| | -APLSLG- | | |
| | AKPLSL-- | | |
| | AWPLSL-- | | |

-APLSLA-
-APLSLQ-
-APLSLN-
AYPLSL--
-APLSLS-
AMPLSL--
-APLSLM-
-APLSLK-
-APLSLH-
AEPLSL--
-APLSLI-
AQPLSL--
APPLSL--
ADPLSL--
-APLSLE-
ACPLSL--
-APLSLC-
CLPLSL--
CRPLSL--
CQPLSL--
CGPLSL--
-CPLSLR-
CSPLSL--
-CPLSLY-
-CPLSLQ-
CYPLSL--
CTPLSL--
CCPLSL--
CAPLSL--
CDPLSL--
CVPLSL--
-CPLSLV-
-CPLSLS-
-CPLSLL-
CMPLSL--
CIPLSL--
CFPLSL--
CEPLSL--
CWPLSL--
-CPLSLT-
-CPLSLK-
-CPLSLI-
CNPLSL--
CKPLSL--
DRPLSL--
DSPLSL--
-DPLSLR-
DFPLSL--
DTPLSL--
DKPLSL--

DVPLSL--
DLPLSL--
-DPLSLN-
DIPLSL--
-DPLSLL-
-DPLSLF-
-DPLSLK-
-DPLSLH-
DAPLSL--
-DPLSLT-
-DPLSLA-
DMPLSL--
DHPLSL--
DDPLSL--
DYPLSL--
-DPLSLI-
-DPLSLV-
DGPLSL--
DWPLSL--
DEPLSL--
DQPLSL--
-DPLSLQ-
-DPLSLE-
-DPLSLC-
ESPLSL--
ERPLSL--
EVPLSL--
-EPLSLI-
-EPLSLA-
ELPLSL--
ETPLSL--
-EPLSLS-
-EPLSLV-
EKPLSL--
-EPLSLK-
EAPLSL--
-EPLSLF-
EYPLSL--
EHPLSL--
ENPLSL--
EMPLSL--
EGPLSL--
-EPLSLN-
-EPLSLH-
-EPLSLM-
-EPLSLG-
EPPLSL--
-EPLSLQ-
EQPLSL--
EIPLSL--

EFPLSL--
EDPLSL--
ECPLSL--
FGPLSL--
FKPLSL--
FSPLSL--
-FPLSLA-
-FPLSLS-
FHPLSL--
FNPLSL--
-FPLSLT-
FPPLSL--
-FPLSLQ-
-FPLSLN-
FEPLSL--
-FPLSLG-
FIPLSL--
-FPLSLI-
FDPLSL--
-FPLSLM-
-FPLSLE-
FQPLSL--
FCPLSL--
GYPLSL--
GKPLSL--
GTPLSL--
-GPLSLA-
GMPLSL--
GRPLSL--
GAPLSL--
-GPLSLK-
-GPLSLT-
GGPLSL--
GWPLSL--
GIPLSL--
-GPLSLN-
GPPLSL--
-GPLSLH-
GNPLSL--
GHPLSL--
-GPLSLE-
GFPLSL--
GEPLSL--
GDPLSL--
-GPLSLQ-
-GPLSLI-
GCPLSL--
-HPLSLT-
HSPLSL--
-HPLSLL-

HTPLSL--
-HPLSLS-
-HPLSLY-
HQPLSL--
-HPLSLV-
HGPLSL--
-HPLSLN-
HFPLSL--
-HPLSLA-
HDPLSL--
-HPLSLQ-
-HPLSLI-
HNPLSL--
-HPLSLW-
-HPLSLK-
-HPLSLH-
HPPLSL--
-HPLSLG-
HMPLSL--
HHPLSL--
-HPLSLF-
-HPLSLE-
HIPLSL--
HCPLSL--
-IPLSLR-
-IPLSLA-
IQPLSL--
IEPLSL--
-IPLSLN-
IKPLSL--
IYPLSL--
IFPLSL--
-IPLSLE-
INPLSL--
-IPLSLG-
IPPLSL--
IMPLSL--
IWPLSL--
ICPLSL--
-IPLSLM-
-KPLSLR-
KGPLSL--
KTPLSL--
KNPLSL--
-KPLSLN-
KAPLSL--
-KPLSLK-
-KPLSLI-
KFPLSL--
KEPLSL--

KYPLSL--
-KPLSLA-
KDPLSL--
KHPLSL--
-KPLSLE-
KQPLSL--
KCPLSL--
-LPLSLS-
LLPLSL--
LNPLSL--
-LPLSLL-
LYPLSL--
-LPLSLK-
-LPLSLV-
LDPLSL--
LHPLSL--
LEPLSL--
-LPLSLM-
-LPLSLQ-
-LPLSLA-
LQPLSL--
-LPLSLN-
-LPLSLE-
-MPLSLL-
-MPLSLV-
-MPLSLS-
-MPLSLM-
-MPLSLG-
-MPLSLI-
MHPLSL--
MYPLSL--
MNPLSL--
MTPLSL--
MIPLSL--
MWPLSL--
MMPLSL--
MQPLSL--
-MPLSLH-
-MPLSLQ-
-MPLSLN-
-MPLSLE-
MEPLSL--
-MPLSLC-
MCPLSL--
-NPLSLS-
NLPLSL--
NAPLSL--
NVPLSL--
-NPLSLT-
NNPLSL--

NIPLSL--
NMPLSL--
NPPLSL--
NHPLSL--
-NPLSLH-
NYPLSL--
-NPLSLQ-
-NPLSLI-
NWPLSL--
-NPLSLK-
-NPLSLA-
NFPLSL--
-NPLSLE-
-NPLSLN-
NEPLSL--
-NPLSLG-
NCPLSL--
-NPLSLF-
NDPLSL--
--PLSLGV
--PLSLSR
--PLSLRG
--PLSLSG
--PLSLRV
--PLSLTS
--PLSLSS
--PLSLGG
--PLSLLG
--PLSLRN
--PLSLPL
--PLSLTR
--PLSLSK
--PLSLKS
--PLSLHR
--PLSLST
--PLSLRA
--PLSLIR
--PLSLQS
--PLSLRQ
--PLSLGS
--PLSLYG
--PLSLNS
--PLSLSQ
--PLSLTV
--PLSLSW
--PLSLLH
--PLSLVG
--PLSLLK
--PLSLAR
--PLSLKN

--PLSLTI
--PLSLMR
--PLSLGA
--PLSLWS
--PLSLVA
--PLSLTN
--PLSLQT
--PLSLMG
--PLSLAA
--PLSLYN
--PLSLRD
--PLSLNR
--PLSLVD
--PLSLSV
--PLSLHT
--PLSLKI
--PLSLKA
--PLSLVT
--PLSLTH
--PLSLTG
--PLSLHS
--PLSLKD
--PLSLWG
--PLSLTT
--PLSLTQ
--PLSLSN
--PLSLNV
--PLSLRW
--PLSLNT
--PLSLLD
--PLSLTM
--PLSLTF
--PLSLTE
--PLSLIG
--PLSLGT
--PLSLFG
--PLSLQN
--PLSLKV
--PLSLES
--PLSLKK
--PLSLAH
--PLSLAW
--PLSLAQ
--PLSLHQ
--PLSLHL
--PLSLHK
--PLSLHG
--PLSLGY
--PLSLGM
--PLSLAT

PNPLSL--
--PLSLTW
--PLSLSE
--PLSLNK
--PLSLMN
--PLSLAM
--PLSLYQ
--PLSLPY
--PLSLKM
--PLSLID
PDPLSL--
--PLSLPM
--PLSLNQ
--PLSLLQ
--PLSLHV
--PLSLFH
PEPLSL--
--PLSLYD
--PLSLVE
--PLSLQA
--PLSLNW
--PLSLKY
--PLSLKT
--PLSLGI
--PLSLER
--PLSLEF
--PLSLAK
--PLSLNG
--PLSLIH
--PLSLQW
--PLSLMT
--PLSLHN
--PLSLHE
--PLSLWH
--PLSLQM
--PLSLHD
--PLSLEW
--PLSLET
--PLSLEA
--PLSLQI
--PLSLNN
--PLSLNM
--PLSLNH
--PLSLMM
--PLSLKE
--PLSLIQ
--PLSLHY
--PLSLEH
--PLSLTD
--PLSLPI

```
--PLSLKF
--PLSLHM
--PLSLEY
--PLSLPF
--PLSLHW
--PLSLHF
--PLSLEN
--PLSLEI
--PLSLDS
--PLSLQY
--PLSLME
--PLSLWD
--PLSLQF
--PLSLNE
--PLSLAD
PCPLSL--
PPPLSL--
-PPLSLL-
--PLSLWF
--PLSLQE
--PLSLHH
--PLSLEQ
--PLSLEE
--PLSLDA
--PLSLAE
-PPLSLV-
-PPLSLR-
-PPLSLK-
-PPLSLI-
--PLSLYC
--PLSLRC
--PLSLQH
--PLSLPW
--PLSLKC
--PLSLFC
--PLSLEK
--PLSLCY
--PLSLCW
--PLSLCV
--PLSLCN
--PLSLCH
--PLSLCG
--PLSLCE
--PLSLCA
--PLSLAC
-QPLSLR-
QSPLSL--
-QPLSLS-
-QPLSLN-
QKPLSL--
```

QLPLSL--
-QPLSLA-
QFPLSL--
QGPLSL--
-QPLSLV-
-QPLSLI-
QHPLSL--
QWPLSL--
QVPLSL--
QAPLSL--
QYPLSL--
QIPLSL--
QMPLSL--
-QPLSLG-
QDPLSL--
QNPLSL--
QQPLSL--
-QPLSLH-
QCPLSL--
-RPLSLT-
RTPLSL--
-RPLSLG-
-RPLSLA-
-RPLSLQ-
RNPLSL--
-RPLSLN-
-RPLSLM-
REPLSL--
-RPLSLE-
RCPLSL--
-RPLSLC-
SLPLSL--
SVPLSL--
SSPLSL--
-SPLSLT-
-SPLSLA-
SNPLSL--
-SPLSLK-
-SPLSLH-
SAPLSL--
-SPLSLV-
SFPLSL--
SIPLSL--
SHPLSL--
SWPLSL--
-SPLSLQ-
-SPLSLN-
SEPLSL--
-SPLSLG-
-SPLSLF-

SPPLSL--
SQPLSL--
SDPLSL--
-SPLSLI-
SCPLSL--
-SPLSLC-
-TPLSLR-
TLPLSL--
-TPLSLT-
TTPLSL--
TVPLSL--
TIPLSL--
-TPLSLS-
-TPLSLA-
-TPLSLI-
TYPLSL--
TKPLSL--
-TPLSLK-
-TPLSLH-
THPLSL--
-TPLSLQ-
TQPLSL--
TEPLSL--
-TPLSLE-
TDPLSL--
TCPLSL--
-VPLSLS-
VKPLSL--
-VPLSLK-
-VPLSLR-
VPPLSL--
VLPLSL--
-VPLSLM-
VIPLSL--
-VPLSLN-
-VPLSLE-
VNPLSL--
VQPLSL--
VYPLSL--
-VPLSLQ-
-VPLSLH-
-VPLSLG-
-VPLSLV-
VHPLSL--
VEPLSL--
VGPLSL--
VCPLSL--
WKPLSL--
WSPLSL--
WFPLSL--

| | | | |
|---|---|---|---|
| | WPPLSL-- | | |
| | WAPLSL-- | | |
| | -WPLSLV- | | |
| | WNPLSL-- | | |
| | WMPLSL-- | | |
| | WQPLSL-- | | |
| | -WPLSLN- | | |
| | WEPLSL-- | | |
| | WWPLSL-- | | |
| | -WPLSLM- | | |
| | -WPLSLH- | | |
| | WCPLSL-- | | |
| | -WPLSLE- | | |
| | -YPLSLL- | | |
| | YLPLSL-- | | |
| | -YPLSLS- | | |
| | YTPLSL-- | | |
| | -YPLSLT- | | |
| | YHPLSL-- | | |
| | -YPLSLH- | | |
| | -YPLSLG- | | |
| | YKPLSL-- | | |
| | -YPLSLI- | | |
| | YIPLSL-- | | |
| | -YPLSLN- | | |
| | -YPLSLM- | | |
| | YFPLSL-- | | |
| | -YPLSLV- | | |
| | YNPLSL-- | | |
| | YQPLSL-- | | |
| | YAPLSL-- | | |
| | YDPLSL-- | | |
| | -YPLSLQ- | | |
| | -YPLSLE- | | |
| | YPPLSL-- | | |
| | YCPLSL-- | | |
| PQGL | 20 | 845 | 33.52 |
| | --PQGLPR | | |
| | --PQGLGY | | |
| | -TPQGLG- | | |
| | AIPQGL-- | | |
| | ASPQGL-- | | |
| | -APQGLS- | | |
| | -APQGLL- | | |
| | ATPQGL-- | | |
| | AGPQGL-- | | |
| | ARPQGL-- | | |
| | -APQGLW- | | |
| | AYPQGL-- | | |
| | -APQGLR- | | |

AKPQGL--
-APQGLY-
-APQGLG-
AWPQGL--
AMPQGL--
AVPQGL--
APPQGL--
AFPQGL--
AQPQGL--
-APQGLT-
-APQGLN-
-APQGLH-
AHPQGL--
-APQGLQ-
-APQGLI-
ADPQGL--
-APQGLK-
-APQGLE-
ANPQGL--
ACPQGL--
-APQGLV-
-APQGLM-
-APQGLF-
AEPQGL--
AAPQGL--
-CPQGLW-
-CPQGLI-
CKPQGL--
-CPQGLM-
-CPQGLK-
CWPQGL--
CVPQGL--
CSPQGL--
CRPQGL--
-CPQGLH-
-CPQGLV-
CPPQGL--
CIPQGL--
CAPQGL--
-DPQGLR-
DWPQGL--
-DPQGLL-
DMPQGL--
DKPQGL--
DLPQGL--
DIPQGL--
DFPQGL--
DAPQGL--
-DPQGLI-
-DPQGLH-

DNPQGL--
DSPQGL--
-DPQGLY-
-DPQGLT-
DEPQGL--
DVPQGL--
-DPQGLV-
-DPQGLS-
DGPQGL--
DYPQGL--
DTPQGL--
DQPQGL--
-DPQGLW-
-DPQGLQ-
-DPQGLN-
DDPQGL--
ESPQGL--
EVPQGL--
ETPQGL--
EMPQGL--
-EPQGLK-
-EPQGLY-
ELPQGL--
EYPQGL--
-EPQGLS-
-EPQGLG-
EFPQGL--
ERPQGL--
-EPQGLV-
ENPQGL--
EKPQGL--
EWPQGL--
-EPQGLW-
-EPQGLR-
EDPQGL--
-FPQGLL-
FLPQGL--
-FPQGLR-
-FPQGLH-
-FPQGLK-
FGPQGL--
FTPQGL--
FSPQGL--
FPPQGL--
FFPQGL--
FRPQGL--
-FPQGLY-
-FPQGLS-
-FPQGLF-
-FPQGLA-

FAPQGL--
FVPQGL--
FNPQGL--
FMPQGL--
FQPQGL--
-FPQGLT-
FIPQGL--
FKPQGL--
-FPQGLM-
FWPQGL--
-FPQGLQ-
-FPQGLN-
FDPQGL--
-FPQGLI-
-FPQGLG-
FHPQGL--
FCPQGL--
-GPQGLR-
GFPQGL--
-GPQGLQ-
-GPQGLF-
-GPQGLT-
GKPQGL--
GIPQGL--
GRPQGL--
-GPQGLW-
-GPQGLG-
-GPQGLK-
GNPQGL--
GHPQGL--
-GPQGLL-
-GPQGLI-
GLPQGL--
GEPQGL--
-GPQGLV-
GYPQGL--
-GPQGLM-
-GPQGLN-
-GPQGLE-
GPPQGL--
GCPQGL--
GAPQGL--
GWPQGL--
GQPQGL--
-GPQGLY-
-GPQGLA-
GVPQGL--
GTPQGL--
GMPQGL--
GGPQGL--

-GPQGLH-
-GPQGLC-
HRPQGL--
-HPQGLR-
-HPQGLL-
HNPQGL--
HIPQGL--
-HPQGLS-
-HPQGLT-
HLPQGL--
HMPQGL--
HVPQGL--
HSPQGL--
-HPQGLM-
-HPQGLK-
HTPQGL--
-HPQGLW-
-HPQGLF-
-HPQGLV-
-HPQGLG-
HAPQGL--
HQPQGL--
-HPQGLY-
-HPQGLN-
HHPQGL--
HWPQGL--
-HPQGLI-
-HPQGLH-
HGPQGL--
HFPQGL--
HDPQGL--
HEPQGL--
ISPQGL--
-IPQGLV-
-IPQGLT-
-IPQGLA-
-IPQGLE-
IIPQGL--
-IPQGLM-
-IPQGLI-
-IPQGLG-
IYPQGL--
-IPQGLR-
-IPQGLH-
-IPQGLF-
INPQGL--
ILPQGL--
IFPQGL--
IKPQGL--
IRPQGL--

−IPQGLY−
−IPQGLS−
−IPQGLQ−
−IPQGLK−
IPPQGL−−
IDPQGL−−
IVPQGL−−
−IPQGLN−
IEPQGL−−
IAPQGL−−
IQPQGL−−
−IPQGLW−
IMPQGL−−
−KPQGLK−
−KPQGLR−
−KPQGLV−
−KPQGLS−
KGPQGL−−
KVPQGL−−
−KPQGLN−
−KPQGLY−
−KPQGLM−
KTPQGL−−
KLPQGL−−
−KPQGLG−
KRPQGL−−
−KPQGLI−
−KPQGLA−
KKPQGL−−
−KPQGLF−
KPPQGL−−
KIPQGL−−
KEPQGL−−
KAPQGL−−
KYPQGL−−
−KPQGLQ−
−KPQGLE−
KNPQGL−−
KHPQGL−−
KFPQGL−−
−KPQGLH−
−LPQGLS−
LGPQGL−−
−LPQGLR−
LLPQGL−−
LTPQGL−−
LMPQGL−−
−LPQGLQ−
LVPQGL−−
−LPQGLV−

-LPQGLH-
LAPQGL--
LKPQGL--
LNPQGL--
LFPQGL--
LWPQGL--
-LPQGLK-
LEPQGL--
-LPQGLY-
-LPQGLN-
-LPQGLI-
-LPQGLA-
-LPQGLW-
-LPQGLT-
LPPQGL--
LYPQGL--
LQPQGL--
LHPQGL--
-LPQGLF-
LIPQGL--
-LPQGLM-
-LPQGLG-
LDPQGL--
LCPQGL--
MTPQGL--
-MPQGLS-
-MPQGLG-
MLPQGL--
MYPQGL--
-MPQGLF-
MWPQGL--
MRPQGL--
-MPQGLH-
-MPQGLR-
-MPQGLI-
MGPQGL--
-MPQGLY-
-MPQGLN-
MQPQGL--
-MPQGLT-
-MPQGLK-
-MPQGLA-
MKPQGL--
MVPQGL--
-MPQGLW-
-MPQGLL-
MMPQGL--
MEPQGL--
MSPQGL--
MIPQGL--

```
-MPQGLM-
MNPQGL--
MDPQGL--
MCPQGL--
MAPQGL--
-MPQGLV-
-MPQGLQ-
-MPQGLE-
MPPQGL--
-NPQGLR-
-NPQGLY-
-NPQGLS-
NMPQGL--
NVPQGL--
NTPQGL--
-NPQGLL-
-NPQGLK-
-NPQGLI-
NPPQGL--
NHPQGL--
NRPQGL--
NWPQGL--
NSPQGL--
-NPQGLN-
-NPQGLM-
-NPQGLA-
-NPQGLT-
-NPQGLF-
NEPQGL--
NAPQGL--
-NPQGLQ-
NFPQGL--
NDPQGL--
NYPQGL--
-NPQGLW-
NCPQGL--
-NPQGLH-
-NPQGLG-
NNPQGL--
--PQGLRA
--PQGLFR
--PQGLTL
--PQGLRL
--PQGLST
--PQGLLR
--PQGLTG
--PQGLLQ
--PQGLVL
--PQGLTV
--PQGLTR
```

--PQGLLY
--PQGLHS
--PQGLVR
--PQGLYR
--PQGLWK
--PQGLLG
--PQGLAS
--PQGLTS
--PQGLMS
--PQGLLK
--PQGLVT
--PQGLRR
--PQGLRT
--PQGLMA
--PQGLKM
--PQGLKL
--PQGLIR
--PQGLFK
--PQGLAT
--PQGLWT
--PQGLWS
--PQGLTM
--PQGLSL
--PQGLRH
--PQGLKR
--PQGLFY
--PQGLLW
--PQGLHL
--PQGLYT
--PQGLVI
--PQGLTK
--PQGLRY
--PQGLSW
--PQGLSF
--PQGLRM
--PQGLGS
PVPQGL--
--PQGLVV
--PQGLVS
--PQGLVK
--PQGLVF
--PQGLRN
--PQGLQR
--PQGLLT
--PQGLLA
--PQGLIY
--PQGLHH
--PQGLHF
--PQGLAM
--PQGLYQ

--PQGLTF
--PQGLSR
--PQGLRS
--PQGLRK
--PQGLRI
--PQGLLF
--PQGLFL
--PQGLVA
--PQGLQT
--PQGLNT
--PQGLKF
--PQGLHR
--PQGLGA
--PQGLFN
--PQGLFA
--PQGLWL
--PQGLWH
--PQGLVE
--PQGLSV
--PQGLNN
--PQGLKH
--PQGLHN
--PQGLAH
PLPQGL--
PTPQGL--
--PQGLYV
--PQGLYA
--PQGLWR
--PQGLSN
--PQGLNM
--PQGLLV
--PQGLLS
--PQGLIS
--PQGLIN
--PQGLIG
--PQGLIF
--PQGLIA
--PQGLHT
--PQGLAI
--PQGLYS
--PQGLWG
--PQGLVY
--PQGLVG
--PQGLSS
--PQGLSH
--PQGLRF
--PQGLQV
--PQGLQK
--PQGLMH
--PQGLKI

--PQGLIM
--PQGLHE
--PQGLGV
--PQGLFT
--PQGLFM
--PQGLAY
PNPQGL--
PWPQGL--
PSPQGL--
--PQGLYW
--PQGLYI
--PQGLYH
--PQGLTH
--PQGLSQ
--PQGLSM
--PQGLNY
--PQGLNF
--PQGLMI
--PQGLMG
--PQGLLM
--PQGLKG
--PQGLIL
--PQGLGI
--PQGLAF
PHPQGL--
PGPQGL--
--PQGLYN
--PQGLVN
--PQGLVM
--PQGLVD
--PQGLTI
--PQGLSI
--PQGLSG
--PQGLRE
--PQGLQS
--PQGLQH
--PQGLQA
--PQGLNV
--PQGLNQ
--PQGLNG
--PQGLLH
--PQGLIK
--PQGLID
--PQGLFI
--PQGLFG
--PQGLET
PRPQGL--
--PQGLYM
--PQGLWW
--PQGLTA

--PQGLQI
--PQGLQD
--PQGLMV
--PQGLMQ
--PQGLMN
--PQGLMF
--PQGLLD
--PQGLKS
--PQGLKA
--PQGLII
--PQGLHK
--PQGLGW
--PQGLFW
--PQGLFD
--PQGLAW
--PQGLAL
--PQGLAG
PKPQGL--
--PQGLYG
--PQGLWV
--PQGLWM
--PQGLWE
--PQGLWD
--PQGLVQ
--PQGLTW
--PQGLTQ
--PQGLSD
--PQGLRD
--PQGLQL
--PQGLQE
--PQGLNS
--PQGLNL
--PQGLMT
--PQGLKV
--PQGLKN
--PQGLIQ
--PQGLIE
--PQGLHM
--PQGLHI
--PQGLGM
--PQGLGF
--PQGLFS
--PQGLAV
PMPQGL--
PIPQGL--
PDPQGL--
PYPQGL--
--PQGLYF
--PQGLYE
--PQGLVW

--PQGLVH
--PQGLTC
--PQGLSY
--PQGLSE
--PQGLRQ
--PQGLNI
--PQGLMW
--PQGLME
--PQGLKW
--PQGLKT
--PQGLKK
--PQGLKE
--PQGLKD
--PQGLIV
--PQGLGL
--PQGLFQ
--PQGLEW
--PQGLEV
--PQGLEM
--PQGLEI
--PQGLEF
--PQGLDG
--PQGLDA
--PQGLAN
--PQGLAK
--PQGLAA
PEPQGL--
--PQGLYY
--PQGLYK
--PQGLYD
--PQGLWQ
--PQGLWN
--PQGLWI
--PQGLWA
--PQGLQW
--PQGLQQ
--PQGLQN
--PQGLPT
--PQGLNH
--PQGLNA
--PQGLMD
--PQGLLE
--PQGLKQ
--PQGLHY
--PQGLHV
--PQGLHD
--PQGLHA
--PQGLGK
--PQGLFH
--PQGLER

--PQGLCR
--PQGLCG
--PQGLCC
--PQGLAD
PAPQGL--
-QPQGLR-
QLPQGL--
-QPQGLT-
QSPQGL--
-QPQGLS-
QVPQGL--
-QPQGLN-
-QPQGLF-
-QPQGLA-
-QPQGLG-
QKPQGL--
QFPQGL--
QCPQGL--
-QPQGLL-
-QPQGLK-
QAPQGL--
QWPQGL--
-QPQGLV-
-QPQGLI-
-QPQGLH-
QEPQGL--
QYPQGL--
QQPQGL--
QGPQGL--
QDPQGL--
QTPQGL--
-QPQGLY-
-QPQGLW-
-QPQGLQ-
RSPQGL--
-RPQGLA-
-RPQGLS-
RGPQGL--
-RPQGLN-
RRPQGL--
RYPQGL--
-RPQGLL-
RMPQGL--
RAPQGL--
-RPQGLW-
-RPQGLM-
-RPQGLG-
-RPQGLY-
RNPQGL--
RFPQGL--

RQPQGL--
-RPQGLT-
-RPQGLR-
-RPQGLK-
RWPQGL--
-RPQGLF-
RPPQGL--
-RPQGLI-
-RPQGLH-
-RPQGLE-
REPQGL--
-RPQGLQ-
RIPQGL--
RKPQGL--
RHPQGL--
-RPQGLV-
RDPQGL--
-RPQGLC-
RCPQGL--
-SPQGLR-
-SPQGLL-
SWPQGL--
-SPQGLT-
SNPQGL--
SKPQGL--
-SPQGLS-
-SPQGLG-
SRPQGL--
SHPQGL--
-SPQGLA-
SSPQGL--
-SPQGLI-
SLPQGL--
SGPQGL--
-SPQGLN-
-SPQGLY-
-SPQGLE-
SFPQGL--
-SPQGLK-
SYPQGL--
-SPQGLW-
SIPQGL--
SEPQGL--
SAPQGL--
SQPQGL--
SPPQGL--
SCPQGL--
STPQGL--
-SPQGLH-
SMPQGL--

−SPQGLV−
−SPQGLF−
SDPQGL−−
−TPQGLL−
TLPQGL−−
TAPQGL−−
TYPQGL−−
TSPQGL−−
TRPQGL−−
−TPQGLQ−
TGPQGL−−
−TPQGLA−
−TPQGLH−
TTPQGL−−
TQPQGL−−
−TPQGLF−
TNPQGL−−
TMPQGL−−
−TPQGLS−
TFPQGL−−
−TPQGLT−
TKPQGL−−
TVPQGL−−
−TPQGLW−
−TPQGLV−
−TPQGLN−
TPPQGL−−
THPQGL−−
TWPQGL−−
−TPQGLM−
−TPQGLK−
−TPQGLI−
TIPQGL−−
TEPQGL−−
TDPQGL−−
VLPQGL−−
VRPQGL−−
−VPQGLS−
−VPQGLR−
−VPQGLA−
VAPQGL−−
VMPQGL−−
VGPQGL−−
VTPQGL−−
−VPQGLL−
−VPQGLI−
VYPQGL−−
VQPQGL−−
−VPQGLH−
−VPQGLT−

```
-VPQGLF-
VVPQGL--
VSPQGL--
-VPQGLE-
-VPQGLG-
VHPQGL--
-VPQGLY-
-VPQGLQ-
-VPQGLK-
VEPQGL--
VDPQGL--
VIPQGL--
-VPQGLW-
VCPQGL--
VWPQGL--
-VPQGLN-
-VPQGLM-
VNPQGL--
VFPQGL--
-WPQGLR-
-WPQGLQ-
-WPQGLL-
-WPQGLS-
-WPQGLK-
WTPQGL--
WAPQGL--
WSPQGL--
-WPQGLF-
WFPQGL--
-WPQGLT-
-WPQGLH-
WVPQGL--
-WPQGLW-
WNPQGL--
WWPQGL--
WKPQGL--
-WPQGLY-
-WPQGLG-
WPPQGL--
WIPQGL--
WRPQGL--
-WPQGLV-
-WPQGLN-
-WPQGLI-
-WPQGLM-
WMPQGL--
WGPQGL--
WEPQGL--
WDPQGL--
WCPQGL--
```

| | | | |
|---|---|---|---|
| | YVPQGL-- | | |
| | -YPQGLV- | | |
| | -YPQGLS- | | |
| | -YPQGLR- | | |
| | YIPQGL-- | | |
| | YSPQGL-- | | |
| | YTPQGL-- | | |
| | -YPQGLT- | | |
| | YGPQGL-- | | |
| | -YPQGLN- | | |
| | -YPQGLL- | | |
| | -YPQGLK- | | |
| | YNPQGL-- | | |
| | YAPQGL-- | | |
| | -YPQGLW- | | |
| | -YPQGLA- | | |
| | YQPQGL-- | | |
| | -YPQGLI- | | |
| | YLPQGL-- | | |
| | YFPQGL-- | | |
| | YWPQGL-- | | |
| | -YPQGLM- | | |
| | -YPQGLG- | | |
| | YMPQGL-- | | |
| | YKPQGL-- | | |
| | YEPQGL-- | | |
| | -YPQGLY- | | |
| | -YPQGLQ- | | |
| | -YPQGLH- | | |
| | -YPQGLC- | | |
| | YDPQGL-- | | |
| | YCPQGL-- | | |
| PKGI | 177 | 727 | 33.52 |
| | PRPKGI-- | | |
| | -TPKGIS- | | |
| | ASPKGI-- | | |
| | -APKGIW- | | |
| | -APKGIT- | | |
| | -APKGIL- | | |
| | -APKGIS- | | |
| | AWPKGI-- | | |
| | ATPKGI-- | | |
| | -APKGIG- | | |
| | AQPKGI-- | | |
| | -APKGIA- | | |
| | -APKGIQ- | | |
| | -APKGIF- | | |
| | AVPKGI-- | | |
| | -APKGIY- | | |
| | ARPKGI-- | | |

-APKGIN-
ANPKGI--
AMPKGI--
AIPKGI--
-APKGII-
-APKGIE-
ACPKGI--
-APKGIM-
-APKGIH-
AKPKGI--
-CPKGIK-
CYPKGI--
CKPKGI--
CWPKGI--
CSPKGI--
CRPKGI--
CLPKGI--
CDPKGI--
-CPKGIY-
-CPKGIT-
CHPKGI--
CCPKGI--
CAPKGI--
-DPKGII-
DLPKGI--
DAPKGI--
-DPKGIR-
DRPKGI--
-DPKGIL-
-DPKGIK-
DFPKGI--
-DPKGIV-
-DPKGIE-
DKPKGI--
DIPKGI--
DHPKGI--
DYPKGI--
DWPKGI--
-DPKGIW-
-DPKGIS-
-DPKGIN-
-DPKGIM-
-DPKGIH-
DNPKGI--
-EPKGIS-
-EPKGIT-
EGPKGI--
EFPKGI--
-EPKGIF-
ENPKGI--

EDPKGI--
EWPKGI--
-EPKGIR-
-EPKGIL-
EVPKGI--
ERPKGI--
EQPKGI--
-EPKGIY-
-EPKGIH-
EKPKGI--
ETPKGI--
ESPKGI--
-EPKGIM-
-EPKGIK-
-EPKGIG-
-EPKGIE-
ELPKGI--
EHPKGI--
ECPKGI--
FSPKGI--
-FPKGII-
FMPKGI--
FLPKGI--
FKPKGI--
FVPKGI--
FTPKGI--
-FPKGIV-
FRPKGI--
-FPKGIS-
-FPKGIN-
-FPKGIH-
FFPKGI--
FEPKGI--
FYPKGI--
-FPKGIQ-
FAPKGI--
-FPKGIF-
FCPKGI--
GSPKGI--
-GPKGII-
-GPKGIV-
-GPKGIR-
GLPKGI--
-GPKGIS-
-GPKGIT-
GTPKGI--
GRPKGI--
GGPKGI--
GFPKGI--
GEPKGI--

GCPKGI--
GAPKGI--
-GPKGIL-
-GPKGIA-
GDPKGI--
GPPKGI--
-GPKGIY-
-GPKGIQ-
-GPKGIN-
-GPKGIM-
GKPKGI--
GIPKGI--
-GPKGIW-
-GPKGIK-
-GPKGIG-
GNPKGI--
GHPKGI--
GYPKGI--
-GPKGIP-
-GPKGIH-
-GPKGIF-
GMPKGI--
HLPKGI--
-HPKGIR-
HTPKGI--
-HPKGIV-
HKPKGI--
HGPKGI--
HIPKGI--
-HPKGIM-
HMPKGI--
HWPKGI--
HYPKGI--
HVPKGI--
HSPKGI--
-HPKGII-
-HPKGIA-
HPPKGI--
-HPKGIL-
-HPKGIH-
HQPKGI--
-HPKGIW-
-HPKGIK-
HNPKGI--
HAPKGI--
-HPKGIY-
-HPKGIN-
-HPKGIG-
-HPKGIF-
HHPKGI--

HEPKGI--
HDPKGI--
IKPKGI--
-IPKGIE-
-IPKGIS-
-IPKGIR-
INPKGI--
IWPKGI--
-IPKGIY-
ITPKGI--
-IPKGIW-
IPPKGI--
-IPKGIT-
-IPKGIA-
IAPKGI--
ISPKGI--
-IPKGIN-
-IPKGIF-
IHPKGI--
IYPKGI--
IVPKGI--
IQPKGI--
-IPKGIV-
-IPKGIM-
-IPKGII-
-IPKGIH-
-IPKGIC-
IMPKGI--
ILPKGI--
IFPKGI--
ICPKGI--
-KPKGII-
-KPKGIS-
-KPKGIK-
KFPKGI--
KYPKGI--
KHPKGI--
-KPKGIV-
-KPKGIE-
-KPKGIA-
KWPKGI--
KQPKGI--
-KPKGIM-
-KPKGIF-
KPPKGI--
-KPKGIW-
KAPKGI--
KVPKGI--
-KPKGIT-
-KPKGIQ-

KIPKGI--
-LPKGIY-
-LPKGIT-
LIPKGI--
-LPKGIR-
LTPKGI--
-LPKGIH-
LVPKGI--
LAPKGI--
-LPKGIF-
LCPKGI--
LMPKGI--
-LPKGIQ-
LNPKGI--
LQPKGI--
-LPKGIS-
-LPKGIM-
-LPKGIW-
-LPKGIA-
LDPKGI--
LPPKGI--
-LPKGIN-
-LPKGII-
LKPKGI--
LGPKGI--
-LPKGIC-
-MPKGIL-
MSPKGI--
MLPKGI--
-MPKGIN-
MRPKGI--
-MPKGIT-
MIPKGI--
-MPKGIV-
MHPKGI--
MTPKGI--
-MPKGIW-
-MPKGII-
MFPKGI--
MWPKGI--
MVPKGI--
-MPKGIR-
-MPKGIK-
MGPKGI--
MDPKGI--
-MPKGIF-
-MPKGIE-
-MPKGIY-
-MPKGIH-
MNPKGI--

```
MAPKGI--
NSPKGI--
-NPKGII-
NEPKGI--
NRPKGI--
NLPKGI--
NTPKGI--
-NPKGIK-
NMPKGI--
-NPKGIT-
-NPKGIM-
NHPKGI--
NYPKGI--
-NPKGIV-
-NPKGIF-
-NPKGIH-
NNPKGI--
NKPKGI--
NIPKGI--
NGPKGI--
NVPKGI--
NQPKGI--
NPPKGI--
-NPKGIW-
-NPKGIQ-
-NPKGIN-
NDPKGI--
NAPKGI--
--PKGITN
--PKGIRL
--PKGITR
--PKGISF
--PKGIWG
--PKGIMF
--PKGIHI
--PKGIKS
--PKGIST
--PKGISS
--PKGISR
--PKGISA
--PKGIMS
--PKGILH
--PKGIFA
--PKGIWT
--PKGITW
--PKGILT
--PKGILK
--PKGIVH
--PKGIIK
--PKGIIV
```

--PKGIYN
--PKGIYL
--PKGIWV
--PKGILW
--PKGIFI
--PKGITS
--PKGITL
--PKGIFV
--PKGIAV
PWPKGI--
--PKGITA
--PKGISQ
--PKGISI
--PKGIRG
--PKGINR
--PKGIMG
--PKGIIT
PLPKGI--
--PKGIWE
--PKGITY
--PKGISW
--PKGISM
--PKGISH
--PKGISG
--PKGIQS
--PKGIMV
--PKGIMA
--PKGILQ
--PKGILN
--PKGIKY
--PKGIKG
--PKGIHM
--PKGIHL
--PKGIAT
--PKGIWA
--PKGIVY
--PKGIVT
--PKGIVM
--PKGIVL
--PKGIRM
--PKGINS
--PKGIMT
--PKGILV
--PKGIKV
--PKGIKI
--PKGIFS
--PKGIEG
--PKGIAS
PTPKGI--
--PKGIWW

```
--PKGIWL
--PKGIVW
--PKGIVA
--PKGISY
--PKGIMY
--PKGIMQ
--PKGIKN
--PKGIFM
--PKGIYQ
--PKGIYM
--PKGIYI
--PKGIWH
--PKGITK
--PKGISV
--PKGIQY
--PKGIQI
--PKGIQG
--PKGINF
--PKGIMW
--PKGILM
--PKGILI
--PKGIIQ
--PKGIGG
--PKGIFN
--PKGIFG
--PKGIEW
--PKGIAL
PHPKGI--
PQPKGI--
--PKGIYT
--PKGIYS
--PKGIWY
--PKGIVQ
--PKGITV
--PKGITT
--PKGISL
--PKGIQR
--PKGIQK
--PKGINW
--PKGIMH
--PKGIME
--PKGILY
--PKGIIH
--PKGIIA
--PKGIHT
--PKGIHR
--PKGIHK
--PKGIGA
--PKGIAW
PIPKGI--
```

PAPKGI--
PSPKGI--
--PKGIYH
--PKGIYE
--PKGIWS
--PKGIVS
--PKGIVN
--PKGIVK
--PKGIVE
--PKGITM
--PKGITH
--PKGITE
--PKGIRT
--PKGINV
--PKGING
--PKGINA
--PKGIMI
--PKGIKM
--PKGIKH
--PKGIIM
--PKGIHV
--PKGIHS
--PKGIHF
--PKGIFK
--PKGIAH
PYPKGI--
--PKGIYY
--PKGIYW
--PKGIYK
--PKGIYF
--PKGIWI
--PKGIWD
--PKGITG
--PKGITF
--PKGISN
--PKGIRQ
--PKGIRE
--PKGIRD
--PKGIQT
--PKGIQN
--PKGIQE
--PKGINY
--PKGINM
--PKGIMM
--PKGILF
--PKGILD
--PKGIKD
--PKGIII
--PKGIHW
--PKGIHH

--PKGIHD
--PKGIGV
--PKGIGT
--PKGIGL
--PKGIFQ
--PKGIFE
--PKGIFD
--PKGIEV
--PKGIEK
--PKGIAQ
--PKGIAN
--PKGIAI
--PKGIAF
PVPKGI--
-PPKGIW-
PKPKGI--
--PKGIYV
--PKGIYR
--PKGIYD
--PKGIYA
--PKGIWN
--PKGIWF
--PKGIVV
--PKGIVG
--PKGIVD
--PKGITQ
--PKGITI
--PKGITD
--PKGISE
--PKGISD
--PKGIRI
--PKGIRH
--PKGIQD
--PKGIQA
--PKGIPI
--PKGINT
--PKGINQ
--PKGINK
--PKGINH
--PKGIMD
--PKGIMC
--PKGILE
--PKGIKW
--PKGIIY
--PKGIIE
--PKGIHY
--PKGIHG
--PKGIHA
--PKGIGY
--PKGIGW

--PKGIFY
--PKGIFT
--PKGIFH
--PKGIFF
--PKGIEY
--PKGIEM
--PKGIEH
--PKGIDA
--PKGICL
--PKGIAY
--PKGIAM
--PKGIAK
--PKGIAA
PDPKGI--
-QPKGIL-
-QPKGIF-
-QPKGIR-
QSPKGI--
-QPKGII-
QVPKGI--
QRPKGI--
-QPKGIW-
-QPKGIM-
QWPKGI--
-QPKGIT-
QEPKGI--
-QPKGIQ-
QLPKGI--
QGPKGI--
-QPKGIK-
QHPKGI--
QYPKGI--
QTPKGI--
-QPKGIY-
-QPKGIV-
-QPKGIH-
QMPKGI--
-QPKGIS-
-QPKGIG-
QIPKGI--
QFPKGI--
QCPKGI--
-RPKGIL-
-RPKGIF-
RLPKGI--
RSPKGI--
-RPKGIS-
-RPKGIQ-
-RPKGIH-
RTPKGI--

```
-RPKGII-
-RPKGIM-
RGPKGI--
RAPKGI--
-RPKGIV-
-RPKGIK-
-RPKGIY-
-RPKGIT-
-RPKGIN-
-RPKGIA-
RQPKGI--
RPPKGI--
RNPKGI--
RDPKGI--
-RPKGIE-
RKPKGI--
REPKGI--
RCPKGI--
-SPKGIS-
-SPKGIL-
-SPKGIW-
SVPKGI--
SRPKGI--
STPKGI--
-SPKGIA-
SMPKGI--
SIPKGI--
SWPKGI--
SSPKGI--
SLPKGI--
-SPKGIV-
-SPKGIK-
-SPKGIH-
-SPKGIF-
-SPKGIY-
-SPKGIR-
SGPKGI--
SEPKGI--
SAPKGI--
-SPKGIG-
SQPKGI--
-SPKGIE-
SDPKGI--
SNPKGI--
SHPKGI--
SCPKGI--
SYPKGI--
SPPKGI--
-SPKGID-
-SPKGIC-
```

```
-TPKGIV-
TSPKGI--
TVPKGI--
TGPKGI--
-TPKGII-
-TPKGIT-
-TPKGIK-
TEPKGI--
TTPKGI--
-TPKGIF-
TIPKGI--
TDPKGI--
TFPKGI--
TYPKGI--
TQPKGI--
-TPKGIN-
-TPKGIH-
THPKGI--
-TPKGIW-
-TPKGIE-
TMPKGI--
TAPKGI--
TNPKGI--
TKPKGI--
TCPKGI--
-VPKGIR-
-VPKGIK-
-VPKGIT-
-VPKGIL-
VEPKGI--
VLPKGI--
VRPKGI--
VQPKGI--
VKPKGI--
VGPKGI--
VIPKGI--
VFPKGI--
VYPKGI--
-VPKGIF-
VMPKGI--
-VPKGIY-
-VPKGII-
-VPKGIV-
VDPKGI--
VVPKGI--
VPPKGI--
-VPKGIN-
-VPKGIG-
VAPKGI--
VTPKGI--
```

-VPKGIE-
-VPKGIA-
VHPKGI--
VCPKGI--
-VPKGIH-
VWPKGI--
-VPKGIW-
WLPKGI--
-WPKGIS-
WKPKGI--
-WPKGIV-
WRPKGI--
WAPKGI--
-WPKGIL-
WVPKGI--
WHPKGI--
WTPKGI--
-WPKGIT-
WSPKGI--
WPPKGI--
-WPKGIQ-
-WPKGIM-
-WPKGIK-
-WPKGIH-
-WPKGIA-
WGPKGI--
WYPKGI--
-WPKGIW-
-WPKGII-
-WPKGIE-
WNPKGI--
WDPKGI--
YQPKGI--
-YPKGIL-
YYPKGI--
YTPKGI--
YRPKGI--
-YPKGIN-
-YPKGIF-
YLPKGI--
YNPKGI--
YDPKGI--
YPPKGI--
-YPKGII-
YGPKGI--
YWPKGI--
-YPKGIQ-
-YPKGIM-
-YPKGIA-
YEPKGI--

| | | | |
|---|---|---|---|
| | -YPKGIK- | | |
| | -YPKGIH- | | |
| | YHPKGI-- | | |
| | -YPKGIW- | | |
| | -YPKGIT- | | |
| | YVPKGI-- | | |
| | -YPKGIV- | | |
| | -YPKGIS- | | |
| | YMPKGI-- | | |
| | YCPKGI-- | | |
| PQSL | 30 | 829 | 33.51 |
| | -GPQSLG- | | |
| | --PQSLNR | | |
| | -RPQSLD- | | |
| | RCPQSL-- | | |
| | -TPQSLG- | | |
| | ALPQSL-- | | |
| | -APQSLR- | | |
| | ASPQSL-- | | |
| | -APQSLN- | | |
| | ANPQSL-- | | |
| | -APQSLL- | | |
| | -APQSLS- | | |
| | ATPQSL-- | | |
| | ARPQSL-- | | |
| | AQPQSL-- | | |
| | -APQSLM- | | |
| | -APQSLA- | | |
| | AKPQSL-- | | |
| | AVPQSL-- | | |
| | AHPQSL-- | | |
| | ACPQSL-- | | |
| | -APQSLW- | | |
| | -APQSLT- | | |
| | AIPQSL-- | | |
| | AGPQSL-- | | |
| | ADPQSL-- | | |
| | -APQSLK- | | |
| | -APQSLG- | | |
| | -APQSLF- | | |
| | -APQSLY- | | |
| | AFPQSL-- | | |
| | -APQSLV- | | |
| | AEPQSL-- | | |
| | AAPQSL-- | | |
| | -APQSLQ- | | |
| | -APQSLI- | | |
| | -APQSLE- | | |
| | AMPQSL-- | | |
| | -APQSLH- | | |

-APQSLC-
CSPQSL--
CLPQSL--
CTPQSL--
-CPQSLR-
CNPQSL--
CRPQSL--
CKPQSL--
CHPQSL--
CFPQSL--
CDPQSL--
CCPQSL--
CYPQSL--
CVPQSL--
-CPQSLT-
CMPQSL--
CEPQSL--
-CPQSLW-
-CPQSLV-
-CPQSLS-
-CPQSLL-
-CPQSLF-
CGPQSL--
DKPQSL--
DQPQSL--
DIPQSL--
-DPQSLS-
DWPQSL--
-DPQSLW-
-DPQSLI-
DGPQSL--
DYPQSL--
DSPQSL--
DRPQSL--
DMPQSL--
DVPQSL--
-DPQSLT-
-DPQSLR-
-DPQSLH-
DLPQSL--
DTPQSL--
DNPQSL--
DHPQSL--
-DPQSLL-
-DPQSLK-
-DPQSLA-
DFPQSL--
DEPQSL--
DAPQSL--
-DPQSLY-

-DPQSLQ-
DDPQSL--
DCPQSL--
ERPQSL--
EFPQSL--
EWPQSL--
ETPQSL--
ELPQSL--
-EPQSLR-
ESPQSL--
EQPQSL--
-EPQSLK-
EIPQSL--
EGPQSL--
-EPQSLY-
-EPQSLT-
EKPQSL--
EAPQSL--
EVPQSL--
-EPQSLF-
EEPQSL--
-EPQSLV-
-EPQSLQ-
-EPQSLH-
ENPQSL--
EHPQSL--
EYPQSL--
-EPQSLI-
-EPQSLE-
-EPQSLA-
EMPQSL--
FRPQSL--
-FPQSLL-
FVPQSL--
-FPQSLT-
FKPQSL--
FQPQSL--
-FPQSLH-
FLPQSL--
FYPQSL--
FPPQSL--
FNPQSL--
FMPQSL--
FSPQSL--
-FPQSLV-
FDPQSL--
FAPQSL--
-FPQSLY-
-FPQSLQ-
-FPQSLI-

FGPQSL--
FEPQSL--
FWPQSL--
-FPQSLW-
-FPQSLN-
-FPQSLA-
FHPQSL--
FIPQSL--
-FPQSLM-
-FPQSLK-
-FPQSLF-
-FPQSLS-
-FPQSLG-
-FPQSLE-
-GPQSLR-
-GPQSLL-
-GPQSLS-
GVPQSL--
-GPQSLV-
GLPQSL--
GRPQSL--
GAPQSL--
GSPQSL--
-GPQSLK-
GNPQSL--
GTPQSL--
GMPQSL--
-GPQSLY-
GWPQSL--
-GPQSLH-
GQPQSL--
GIPQSL--
-GPQSLW-
-GPQSLM-
GKPQSL--
-GPQSLT-
GPPQSL--
GHPQSL--
GGPQSL--
-GPQSLF-
-GPQSLN-
-GPQSLI-
-GPQSLA-
-GPQSLE-
GFPQSL--
GDPQSL--
HIPQSL--
HSPQSL--
HWPQSL--
HGPQSL--

```
-HPQSLL-
HYPQSL--
-HPQSLY-
-HPQSLT-
-HPQSLR-
HMPQSL--
HLPQSL--
-HPQSLV-
-HPQSLK-
-HPQSLS-
-HPQSLN-
-HPQSLF-
-HPQSLA-
HFPQSL--
HHPQSL--
HAPQSL--
-HPQSLI-
-HPQSLG-
HKPQSL--
-HPQSLQ-
HPPQSL--
-HPQSLW-
HEPQSL--
HDPQSL--
HTPQSL--
HQPQSL--
HCPQSL--
-IPQSLL-
IVPQSL--
-IPQSLR-
-IPQSLH-
ITPQSL--
ILPQSL--
-IPQSLS-
IMPQSL--
IAPQSL--
-IPQSLY-
IGPQSL--
ISPQSL--
-IPQSLV-
IRPQSL--
IIPQSL--
IQPQSL--
-IPQSLE-
IYPQSL--
INPQSL--
IHPQSL--
IFPQSL--
IEPQSL--
-IPQSLT-
```

-IPQSLN-
-IPQSLI-
-IPQSLA-
IKPQSL--
-IPQSLM-
-IPQSLQ-
-IPQSLG-
IDPQSL--
IWPQSL--
-IPQSLW-
-IPQSLK-
KLPQSL--
KGPQSL--
-KPQSLW-
-KPQSLT-
KAPQSL--
KTPQSL--
-KPQSLI-
-KPQSLA-
KYPQSL--
KWPQSL--
-KPQSLF-
KMPQSL--
-KPQSLQ-
-KPQSLN-
-KPQSLG-
KIPQSL--
KPPQSL--
KNPQSL--
KHPQSL--
KEPQSL--
-KPQSLV-
KKPQSL--
-KPQSLK-
-KPQSLH-
KQPQSL--
KFPQSL--
KDPQSL--
KCPQSL--
-KPQSLE-
-LPQSLR-
-LPQSLY-
LKPQSL--
LSPQSL--
-LPQSLS-
-LPQSLL-
LYPQSL--
LNPQSL--
LHPQSL--
-LPQSLV-

```
-LPQSLT-
LVPQSL--
LPPQSL--
-LPQSLK-
LMPQSL--
LGPQSL--
LTPQSL--
LWPQSL--
LDPQSL--
LQPQSL--
LEPQSL--
LIPQSL--
-LPQSLW-
-LPQSLI-
-LPQSLH-
-LPQSLM-
-LPQSLN-
-LPQSLF-
LCPQSL--
MSPQSL--
-MPQSLN-
MRPQSL--
-MPQSLK-
MTPQSL--
MGPQSL--
-MPQSLL-
-MPQSLR-
-MPQSLH-
MKPQSL--
MWPQSL--
MQPQSL--
-MPQSLA-
MMPQSL--
MLPQSL--
MAPQSL--
-MPQSLV-
-MPQSLS-
-MPQSLQ-
-MPQSLE-
MPPQSL--
MIPQSL--
-MPQSLT-
-MPQSLF-
MNPQSL--
MEPQSL--
-MPQSLI-
MHPQSL--
MDPQSL--
-MPQSLM-
MFPQSL--
```

-MPQSLY-
-MPQSLW-
MCPQSL--
NSPQSL--
NAPQSL--
NWPQSL--
-NPQSLL-
NRPQSL--
NLPQSL--
NGPQSL--
NTPQSL--
-NPQSLV-
-NPQSLK-
NKPQSL--
NFPQSL--
-NPQSLW-
NVPQSL--
-NPQSLQ-
NEPQSL--
NMPQSL--
-NPQSLS-
-NPQSLR-
NHPQSL--
-NPQSLT-
-NPQSLM-
NIPQSL--
NQPQSL--
-NPQSLA-
NPPQSL--
NNPQSL--
NYPQSL--
-NPQSLI-
-NPQSLH-
NCPQSL--
-NPQSLF-
-NPQSLE-
NDPQSL--
--PQSLRY
--PQSLRA
--PQSLSG
--PQSLRN
--PQSLSS
--PQSLLA
--PQSLYL
--PQSLSV
--PQSLLG
--PQSLTG
--PQSLLS
--PQSLAT
PSPQSL--

--PQSLVL
--PQSLLN
--PQSLHT
--PQSLVN
--PQSLVF
--PQSLLH
--PQSLVR
--PQSLPL
--PQSLIR
--PQSLVY
--PQSLST
PVPQSL--
--PQSLWQ
--PQSLVK
--PQSLVH
--PQSLSK
--PQSLNL
--PQSLLD
--PQSLGS
--PQSLWL
--PQSLVV
--PQSLTW
--PQSLSW
--PQSLQS
--PQSLNT
--PQSLMS
--PQSLKV
--PQSLKI
--PQSLAS
--PQSLWR
--PQSLSL
--PQSLQY
--PQSLMG
--PQSLIK
--PQSLFS
--PQSLFR
--PQSLVS
--PQSLTI
--PQSLSR
--PQSLRG
--PQSLQK
--PQSLMI
--PQSLIV
--PQSLAK
PNPQSL--
PRPQSL--
--PQSLWS
--PQSLTA
--PQSLSF
--PQSLNV

--PQSLLT
--PQSLIT
--PQSLHQ
PTPQSL--
--PQSLYR
--PQSLWH
--PQSLWA
--PQSLVI
--PQSLVA
--PQSLTT
--PQSLSI
--PQSLQG
--PQSLLV
--PQSLLK
--PQSLIW
--PQSLHG
--PQSLVM
--PQSLTN
--PQSLTL
--PQSLSM
--PQSLNA
--PQSLMQ
--PQSLKD
--PQSLHK
--PQSLGA
--PQSLFK
--PQSLFE
--PQSLVQ
--PQSLVG
--PQSLTF
--PQSLSY
--PQSLSA
--PQSLQT
--PQSLNS
--PQSLMY
--PQSLMV
--PQSLMD
--PQSLLE
--PQSLKM
--PQSLKG
--PQSLIN
--PQSLGT
--PQSLGG
--PQSLFI
--PQSLFH
--PQSLEG
--PQSLEA
--PQSLAY
--PQSLYH
--PQSLTQ

--PQSLLM
--PQSLKA
--PQSLHM
--PQSLFQ
PQPQSL--
PGPQSL--
PWPQSL--
--PQSLYT
--PQSLYG
--PQSLTR
--PQSLRD
--PQSLQR
--PQSLMK
--PQSLMH
--PQSLLI
--PQSLIH
--PQSLHY
--PQSLHS
--PQSLHR
--PQSLGL
--PQSLFT
PIPQSL--
--PQSLYS
--PQSLYQ
--PQSLYA
--PQSLWD
--PQSLTY
--PQSLSH
--PQSLRW
--PQSLRE
--PQSLNY
--PQSLMN
--PQSLKE
--PQSLIM
--PQSLHL
--PQSLFG
--PQSLFD
--PQSLES
--PQSLEM
--PQSLAW
--PQSLAM
--PQSLAG
PYPQSL--
--PQSLVW
--PQSLVE
--PQSLTV
--PQSLSN
--PQSLKY
--PQSLKT
--PQSLKN

--PQSLKF
--PQSLIQ
--PQSLIG
--PQSLHF
PMPQSL--
--PQSLYE
--PQSLWM
--PQSLWG
--PQSLTM
--PQSLRQ
--PQSLQW
--PQSLQI
--PQSLPI
--PQSLNK
--PQSLNI
--PQSLNG
--PQSLMW
--PQSLKQ
--PQSLIA
--PQSLHW
--PQSLHH
--PQSLHE
--PQSLGV
--PQSLFN
--PQSLFL
--PQSLFA
--PQSLEV
--PQSLAF
PFPQSL--
--PQSLYN
--PQSLYK
--PQSLWK
--PQSLVD
--PQSLTK
--PQSLTH
--PQSLTD
--PQSLQV
--PQSLQQ
--PQSLQN
--PQSLQH
--PQSLQD
--PQSLPM
--PQSLNW
--PQSLNQ
--PQSLNM
--PQSLNH
--PQSLMA
--PQSLLQ
--PQSLKR
--PQSLKH

--PQSLIE
--PQSLID
--PQSLHI
--PQSLHD
--PQSLGY
--PQSLGW
--PQSLAN
PAPQSL--
--PQSLYD
--PQSLWW
--PQSLWN
--PQSLWE
--PQSLRC
--PQSLQM
--PQSLQL
--PQSLPY
--PQSLPF
--PQSLNN
--PQSLMF
--PQSLKW
--PQSLHN
--PQSLHA
--PQSLGQ
--PQSLGN
--PQSLGF
--PQSLEY
--PQSLEW
--PQSLEL
--PQSLDK
--PQSLAV
-QPQSLR-
QSPQSL--
-QPQSLL-
-QPQSLY-
QGPQSL--
-QPQSLS-
-QPQSLF-
QYPQSL--
QPPQSL--
QRPQSL--
-QPQSLI-
-QPQSLH-
QAPQSL--
QVPQSL--
-QPQSLK-
QWPQSL--
-QPQSLT-
QMPQSL--
-QPQSLA-
QNPQSL--

QTPQSL--
-QPQSLW-
-QPQSLV-
-QPQSLN-
QKPQSL--
QHPQSL--
QFPQSL--
QDPQSL--
QQPQSL--
-QPQSLM-
QLPQSL--
QIPQSL--
RVPQSL--
RSPQSL--
-RPQSLS-
RAPQSL--
RFPQSL--
-RPQSLV-
RHPQSL--
RTPQSL--
-RPQSLY-
-RPQSLW-
-RPQSLF-
RDPQSL--
-RPQSLH-
REPQSL--
RYPQSL--
RNPQSL--
RPPQSL--
RKPQSL--
-RPQSLN-
-RPQSLG-
-RPQSLA-
-RPQSLM-
RQPQSL--
-RPQSLQ-
-RPQSLK-
-RPQSLE-
-RPQSLC-
-SPQSLS-
SRPQSL--
SLPQSL--
SYPQSL--
-SPQSLR-
SPPQSL--
SNPQSL--
-SPQSLV-
-SPQSLH-
SKPQSL--
SFPQSL--

SAPQSL--
-SPQSLT-
SSPQSL--
-SPQSLK-
-SPQSLI-
-SPQSLA-
SGPQSL--
-SPQSLN-
-SPQSLF-
STPQSL--
-SPQSLY-
-SPQSLQ-
SIPQSL--
-SPQSLG-
SHPQSL--
SQPQSL--
SMPQSL--
-SPQSLE-
SDPQSL--
SEPQSL--
SCPQSL--
-SPQSLW-
-TPQSLL-
TRPQSL--
TLPQSL--
TSPQSL--
TAPQSL--
-TPQSLS-
TPPQSL--
TTPQSL--
TKPQSL--
-TPQSLH-
TYPQSL--
-TPQSLK-
TGPQSL--
-TPQSLI-
-TPQSLA-
-TPQSLY-
TQPQSL--
-TPQSLQ-
-TPQSLM-
-TPQSLF-
TNPQSL--
TMPQSL--
TEPQSL--
TWPQSL--
-TPQSLV-
THPQSL--
TIPQSL--
-TPQSLE-

TDPQSL--
-VPQSLR-
VRPQSL--
VLPQSL--
VTPQSL--
VYPQSL--
VVPQSL--
-VPQSLS-
VKPQSL--
VNPQSL--
VPPQSL--
-VPQSLV-
VQPQSL--
-VPQSLA-
VDPQSL--
-VPQSLW-
-VPQSLI-
-VPQSLT-
-VPQSLQ-
VGPQSL--
-VPQSLN-
VWPQSL--
-VPQSLK-
VSPQSL--
VFPQSL--
VAPQSL--
VMPQSL--
VEPQSL--
-VPQSLE-
VHPQSL--
-VPQSLM-
-VPQSLG-
VCPQSL--
-WPQSLT-
WSPQSL--
WRPQSL--
-WPQSLL-
-WPQSLA-
WPPQSL--
-WPQSLS-
WNPQSL--
-WPQSLR-
WGPQSL--
WTPQSL--
WYPQSL--
-WPQSLM-
-WPQSLI-
WKPQSL--
WQPQSL--
-WPQSLV-

WEPQSL--
-WPQSLH-
WIPQSL--
-WPQSLN-
WFPQSL--
WWPQSL--
-WPQSLQ-
-WPQSLK-
-WPQSLY-
WDPQSL--
YLPQSL--
-YPQSLR-
-YPQSLL-
YMPQSL--
YRPQSL--
-YPQSLY-
-YPQSLS-
YTPQSL--
YGPQSL--
-YPQSLV-
-YPQSLM-
-YPQSLN-
YAPQSL--
YYPQSL--
-YPQSLK-
YQPQSL--
-YPQSLG-
YKPQSL--
-YPQSLQ-
YNPQSL--
-YPQSLT-
-YPQSLI-
-YPQSLH-
-YPQSLA-
YHPQSL--
YDPQSL--
-YPQSLW-
YPPQSL--
-YPQSLF-
-YPQSLE-

PASI 22 839 33.48

KDPASI--
-LPASIG-
--PASIRP
--PASIGR
RPPASI--
ASPASI--
-APASIS-
ARPASI--
-APASIR-

-APASIL-
AMPASI--
AKPASI--
ALPASI--
-APASIV-
-APASIT-
APPASI--
-APASIK-
-APASIE-
AAPASI--
AYPASI--
ATPASI--
-APASIW-
-APASIH-
ADPASI--
-APASII-
AGPASI--
AFPASI--
-APASIY-
-APASIF-
ANPASI--
AIPASI--
AEPASI--
AWPASI--
AQPASI--
-APASIQ-
-APASIC-
-CPASIL-
CTPASI--
CPPASI--
CMPASI--
CCPASI--
-CPASIS-
-CPASIM-
-CPASIF-
CFPASI--
CQPASI--
-CPASIY-
-CPASIV-
-CPASIQ-
CKPASI--
CDPASI--
-DPASIR-
DRPASI--
-DPASIL-
DYPASI--
DAPASI--
-DPASIQ-
DLPASI--
-DPASIV-

-DPASIS-
DMPASI--
DWPASI--
DSPASI--
DQPASI--
-DPASIW-
-DPASIK-
DIPASI--
-DPASIF-
-DPASIA-
DNPASI--
DHPASI--
-DPASIT-
-DPASIN-
-DPASIM-
DKPASI--
DEPASI--
ESPASI--
-EPASIR-
EFPASI--
EVPASI--
ELPASI--
-EPASIL-
EKPASI--
-EPASIH-
EIPASI--
EWPASI--
EYPASI--
ERPASI--
-EPASIY-
-EPASIT-
-EPASIS-
-EPASIQ-
-EPASIM-
-EPASIK-
-EPASII-
-EPASIF-
-EPASIE-
EMPASI--
ECPASI--
ETPASI--
EQPASI--
EPPASI--
-EPASIV-
EGPASI--
EAPASI--
FSPASI--
FRPASI--
FQPASI--
-FPASIL-

FVPASI--
FIPASI--
-FPASII-
FNPASI--
-FPASIW-
FWPASI--
FPPASI--
-FPASIV-
-FPASIF-
FHPASI--
-FPASIY-
-FPASIM-
FGPASI--
FDPASI--
FCPASI--
-FPASIS-
-FPASIN-
-FPASIK-
-FPASIA-
FKPASI--
FYPASI--
-FPASIQ-
FFPASI--
FEPASI--
-FPASIH-
-FPASIG-
FAPASI--
GWPASI--
GVPASI--
GEPASI--
-GPASIS-
-GPASIL-
-GPASIT-
GTPASI--
GHPASI--
-GPASII-
-GPASIV-
GIPASI--
GGPASI--
-GPASIR-
-GPASIH-
GNPASI--
-GPASIG-
GKPASI--
GFPASI--
GDPASI--
GYPASI--
GQPASI--
-GPASIQ-
GAPASI--

-GPASIF-
GLPASI--
-GPASIY-
-GPASIW-
-GPASIK-
-GPASIA-
GCPASI--
GPPASI--
-HPASIS-
HLPASI--
HQPASI--
-HPASIN-
-HPASIT-
HMPASI--
-HPASIM-
HIPASI--
HWPASI--
HVPASI--
HSPASI--
-HPASIL-
-HPASIK-
HTPASI--
-HPASII-
HHPASI--
HAPASI--
HYPASI--
HPPASI--
HKPASI--
HDPASI--
-HPASIW-
-HPASIG-
-HPASIA-
-HPASIV-
-HPASIF-
HGPASI--
HEPASI--
HCPASI--
IKPASI--
-IPASIF-
-IPASIS-
ISPASI--
IRPASI--
IAPASI--
IYPASI--
-IPASIT-
-IPASIM-
-IPASIY-
-IPASIH-
IDPASI--
INPASI--

IFPASI--
IWPASI--
ITPASI--
-IPASIW-
-IPASIV-
-IPASIA-
IGPASI--
IPPASI--
-IPASIQ-
-IPASIL-
-IPASIG-
IQPASI--
IEPASI--
-IPASIN-
-IPASIK-
-IPASII-
-IPASIE-
-IPASIC-
IHPASI--
KSPASI--
KRPASI--
-KPASIW-
KLPASI--
-KPASIR-
KKPASI--
-KPASIF-
KIPASI--
KFPASI--
-KPASIE-
KYPASI--
-KPASIQ-
KGPASI--
-KPASIN-
KNPASI--
KAPASI--
-KPASIY-
KVPASI--
-KPASIA-
-KPASIM-
KMPASI--
KPPASI--
-KPASIT-
-KPASII-
KHPASI--
KEPASI--
-KPASIH-
KCPASI--
KWPASI--
KQPASI--
-KPASIK-

LYPASI--
LSPASI--
LNPASI--
LKPASI--
-LPASIT-
LGPASI--
-LPASIW-
LHPASI--
LPPASI--
-LPASIL-
-LPASII-
LAPASI--
-LPASIM-
LTPASI--
LQPASI--
-LPASIY-
-LPASIS-
-LPASIV-
LMPASI--
LEPASI--
-LPASIQ-
-LPASIF-
LIPASI--
-LPASIK-
-LPASIH-
-LPASIE-
-LPASIA-
-LPASIN-
LFPASI--
-LPASID-
LDPASI--
LCPASI--
-MPASIR-
-MPASIS-
MRPASI--
MLPASI--
-MPASIK-
MKPASI--
MTPASI--
-MPASIW-
MSPASI--
MFPASI--
MVPASI--
MNPASI--
MMPASI--
-MPASIE-
MYPASI--
-MPASIA-
-MPASIN-
-MPASIM-

-MPASIG-
-MPASIT-
-MPASIH-
MGPASI--
MPPASI--
MIPASI--
MAPASI--
MQPASI--
-MPASIL-
MEPASI--
-MPASIY-
-MPASIV-
-MPASII-
-MPASID-
MHPASI--
MDPASI--
-NPASIL-
-NPASIY-
NLPASI--
NRPASI--
-NPASII-
-NPASIH-
NMPASI--
NVPASI--
-NPASIS-
NYPASI--
-NPASIW-
-NPASIR-
-NPASIM-
-NPASIK-
NQPASI--
NKPASI--
NTPASI--
-NPASIN-
NNPASI--
NGPASI--
-NPASIV-
NHPASI--
NPPASI--
NSPASI--
-NPASIT-
NFPASI--
NWPASI--
-NPASIG-
-NPASIF-
NEPASI--
NDPASI--
-NPASIQ-
-NPASIE-
NIPASI--

PVPASI--
--PASIRR
--PASILS
--PASIRL
--PASILH
--PASIGS
--PASILR
--PASIIR
--PASIVR
--PASIKL
--PASISR
--PASIFS
--PASITR
--PASISL
--PASILK
--PASIIT
--PASISS
--PASIRA
--PASIVS
--PASIRV
--PASIHL
--PASIRE
--PASINR
--PASIMM
--PASILQ
--PASISN
--PASIWR
--PASIRH
--PASIRK
--PASIML
--PASILY
PLPASI--
--PASIYK
--PASIWS
--PASITM
--PASISW
--PASIMA
--PASILL
--PASIIS
--PASIHT
PSPASI--
--PASIYT
--PASIWA
--PASIVH
--PASISQ
--PASIRT
--PASIQS
--PASIMV
--PASIMS
--PASIMF

--PASILN
PFPASI--
--PASIWG
--PASIVY
--PASITI
--PASINA
--PASILE
--PASIHS
PRPASI--
PIPASI--
--PASIYY
--PASIYG
--PASIWT
--PASIWN
--PASITK
--PASITD
--PASISG
--PASISF
--PASIMI
--PASIKV
--PASIKT
--PASIGG
--PASIAK
PAPASI--
--PASIYD
--PASITS
--PASIST
--PASISI
--PASISH
--PASIRY
--PASINW
--PASINK
--PASILA
--PASIIM
--PASIIK
--PASIFN
--PASIFK
--PASIEM
PEPASI--
--PASIYV
--PASIYF
--PASIVT
--PASIVI
--PASITL
--PASIQV
--PASIQR
--PASIQK
--PASIQA
--PASINS
--PASIMR

--PASIMN
--PASILD
--PASIGL
--PASIFM
--PASIFH
--PASIAY
--PASIAN
--PASIAI
--PASIYW
--PASIWK
--PASIVN
--PASIVD
--PASITW
--PASITV
--PASITT
--PASISV
--PASISM
--PASIQF
--PASINV
--PASINQ
--PASINL
--PASIMQ
--PASIMH
--PASIMG
--PASIMD
--PASILT
--PASIIV
--PASIIG
--PASIID
--PASIHQ
--PASIHG
--PASIFE
PYPASI--
PMPASI--
--PASIYM
--PASIYI
--PASIVV
--PASITN
--PASISY
--PASIRI
--PASINY
--PASINM
--PASIME
--PASILM
--PASILF
--PASIKH
--PASIIE
--PASIIA
--PASIHN
--PASIHM

--PASIGT
--PASIGF
--PASIEY
--PASIEG
--PASIAV
--PASIAR
--PASIAL
--PASIAG
PNPASI--
PGPASI--
--PASIYN
--PASIYH
--PASIYE
--PASIWD
--PASIVF
--PASITY
--PASITE
--PASISD
--PASIRF
--PASIQQ
--PASIQM
--PASIQL
--PASING
--PASIMY
--PASIMT
--PASIKM
--PASIKK
--PASIKG
--PASIKE
--PASIKA
--PASIIN
--PASIIL
--PASIHA
--PASIGI
--PASIFV
--PASIFG
--PASIAW
--PASIAF
-PPASIW-
--PASIYL
--PASIWY
--PASIWW
--PASIWQ
--PASIVL
--PASIVG
--PASIVE
--PASITH
--PASITA
--PASIRW
--PASIRQ

--PASIQT
--PASIQN
--PASIQG
--PASIQE
--PASINT
--PASINH
--PASIMK
--PASIKS
--PASIKR
--PASIKN
--PASIKI
--PASIIY
--PASIHY
--PASIHW
--PASIHK
--PASIHH
--PASIFF
--PASIFA
--PASIAD
PTPASI--
PKPASI--
PDPASI--
--PASIYQ
--PASIYC
--PASIWM
--PASIWH
--PASIWF
--PASIVW
--PASIVQ
--PASIVM
--PASIVC
--PASIVA
--PASITQ
--PASITF
--PASISP
--PASISE
--PASIQY
--PASIQW
--PASINF
--PASIMW
--PASIKY
--PASIKW
--PASIKD
--PASIIQ
--PASIIH
--PASIHV
--PASIHE
--PASIHD
--PASIGY
--PASIGV

--PASIGQ
--PASIGM
--PASIGH
--PASIFW
--PASIFT
--PASIFD
--PASIEV
--PASIET
--PASIER
--PASIEK
--PASIDY
--PASIAT
--PASIAQ
--PASIAM
--PASIAH
--PASIAA
-QPASIM-
-QPASIL-
-QPASIR-
QMPASI--
-QPASIQ-
-QPASIY-
QYPASI--
QPPASI--
-QPASIS-
-QPASIA-
QSPASI--
QGPASI--
QVPASI--
-QPASIW-
QKPASI--
QRPASI--
QQPASI--
-QPASIN-
-QPASIE-
QLPASI--
QTPASI--
-QPASIV-
-QPASIT-
QNPASI--
QWPASI--
-QPASIK-
-QPASII-
-QPASIH-
-QPASIG-
QFPASI--
QDPASI--
-QPASIF-
QIPASI--
QHPASI--

QAPASI--
RMPASI--
-RPASIL-
RTPASI--
-RPASIS-
-RPASIQ-
-RPASIR-
-RPASIH-
-RPASIK-
-RPASIM-
RQPASI--
-RPASIV-
RYPASI--
-RPASIG-
-RPASIN-
RKPASI--
RFPASI--
RDPASI--
RAPASI--
RGPASI--
-RPASIW-
RHPASI--
RCPASI--
REPASI--
SRPASI--
SIPASI--
-SPASIL-
-SPASIS-
SLPASI--
SSPASI--
-SPASIT-
-SPASII-
SQPASI--
SFPASI--
-SPASIH-
-SPASIF-
SNPASI--
SEPASI--
SYPASI--
SWPASI--
STPASI--
SVPASI--
SPPASI--
-SPASIV-
-SPASIM-
-SPASIN-
SGPASI--
-SPASIY-
-SPASIG-
SKPASI--

SMPASI--
SHPASI--
-SPASIW-
-SPASIE-
-SPASIA-
SDPASI--
-SPASIQ-
-SPASIK-
SCPASI--
-TPASIL-
TSPASI--
TKPASI--
TAPASI--
-TPASIQ-
-TPASIK-
-TPASIS-
-TPASII-
TTPASI--
TVPASI--
-TPASIR-
THPASI--
TYPASI--
-TPASIV-
TNPASI--
TLPASI--
TWPASI--
TPPASI--
TEPASI--
-TPASIY-
TGPASI--
TDPASI--
-TPASIW-
-TPASIG-
-TPASIE-
TIPASI--
-TPASIT-
TMPASI--
TFPASI--
-TPASIM-
-TPASIA-
TCPASI--
VRPASI--
VSPASI--
-VPASIR-
VKPASI--
-VPASIL-
-VPASII-
-VPASIS-
-VPASIF-
-VPASIA-

VLPASI--
VAPASI--
VQPASI--
-VPASIY-
-VPASIG-
VGPASI--
-VPASIM-
VNPASI--
VFPASI--
VWPASI--
-VPASIN-
VCPASI--
-VPASIQ-
-VPASIT-
VIPASI--
VHPASI--
VDPASI--
VVPASI--
VEPASI--
-VPASIE-
WQPASI--
WRPASI--
WNPASI--
WDPASI--
-WPASIL-
WHPASI--
WIPASI--
WPPASI--
WYPASI--
-WPASIT-
-WPASIF-
WAPASI--
-WPASIV-
-WPASIQ-
WGPASI--
WVPASI--
WTPASI--
-WPASIY-
-WPASII-
-WPASIH-
WWPASI--
-WPASIW-
-WPASIS-
-WPASIN-
-WPASIK-
-WPASIG-
-WPASIA-
YVPASI--
YWPASI--
-YPASIL-

| | | | |
|---|---|---|---|
| | -YPASIS- | | |
| | YTPASI-- | | |
| | -YPASIK- | | |
| | YGPASI-- | | |
| | YAPASI-- | | |
| | -YPASIR- | | |
| | -YPASIM- | | |
| | -YPASIF- | | |
| | -YPASIV- | | |
| | -YPASIN- | | |
| | YLPASI-- | | |
| | YQPASI-- | | |
| | -YPASIY- | | |
| | -YPASIA- | | |
| | YMPASI-- | | |
| | YIPASI-- | | |
| | YHPASI-- | | |
| | YYPASI-- | | |
| | YPPASI-- | | |
| | -YPASIG- | | |
| | YNPASI-- | | |
| | YDPASI-- | | |
| | -YPASIT- | | |
| | YKPASI-- | | |
| | YFPASI-- | | |
| | YSPASI-- | | |
| | -YPASIQ- | | |
| | -YPASIH- | | |
| | -YPASIE- | | |
| | YCPASI-- | | |
| PAGY | 58 | 797 | 33.45 |
| | -APAGYR- | | |
| | -APAGYY- | | |
| | ALPAGY-- | | |
| | ARPAGY-- | | |
| | ATPAGY-- | | |
| | -APAGYG- | | |
| | AGPAGY-- | | |
| | -APAGYS- | | |
| | -APAGYL- | | |
| | AYPAGY-- | | |
| | AVPAGY-- | | |
| | ASPAGY-- | | |
| | -APAGYN- | | |
| | -APAGYI- | | |
| | AHPAGY-- | | |
| | APPAGY-- | | |
| | AAPAGY-- | | |
| | -APAGYT- | | |
| | AEPAGY-- | | |

-APAGYQ-
AMPAGY--
AIPAGY--
-APAGYV-
-APAGYH-
AKPAGY--
AWPAGY--
-APAGYM-
ANPAGY--
AFPAGY--
ACPAGY--
CLPAGY--
CVPAGY--
-CPAGYR-
-CPAGYA-
CRPAGY--
-CPAGYS-
-CPAGYQ-
CNPAGY--
CCPAGY--
CYPAGY--
CSPAGY--
CQPAGY--
-CPAGYK-
-CPAGYI-
CKPAGY--
CGPAGY--
-DPAGYL-
DKPAGY--
-DPAGYR-
DQPAGY--
-DPAGYY-
-DPAGYS-
-DPAGYN-
-DPAGYM-
-DPAGYG-
-DPAGYA-
DSPAGY--
DNPAGY--
DLPAGY--
DEPAGY--
DAPAGY--
DVPAGY--
DTPAGY--
DRPAGY--
-DPAGYF-
DYPAGY--
DWPAGY--
-DPAGYT-
-DPAGYQ-

```
-DPAGYK-
-DPAGYI-
-DPAGYH-
DIPAGY--
DGPAGY--
-EPAGYL-
-EPAGYR-
ESPAGY--
ELPAGY--
-EPAGYA-
-EPAGYV-
-EPAGYS-
ERPAGY--
-EPAGYY-
-EPAGYQ-
ENPAGY--
EVPAGY--
-EPAGYM-
-EPAGYF-
EIPAGY--
EGPAGY--
EEPAGY--
EWPAGY--
ETPAGY--
-EPAGYT-
-EPAGYE-
EMPAGY--
EFPAGY--
-FPAGYR-
-FPAGYT-
-FPAGYV-
-FPAGYI-
-FPAGYH-
FKPAGY--
-FPAGYM-
-FPAGYF-
-FPAGYK-
FVPAGY--
-FPAGYY-
FNPAGY--
-FPAGYL-
FFPAGY--
FYPAGY--
FSPAGY--
FEPAGY--
FTPAGY--
FQPAGY--
FPPAGY--
-FPAGYE-
FMPAGY--
```

FLPAGY--
FHPAGY--
FGPAGY--
FDPAGY--
FAPAGY--
-GPAGYL-
GRPAGY--
-GPAGYH-
GLPAGY--
GSPAGY--
GMPAGY--
GVPAGY--
GPPAGY--
-GPAGYM-
-GPAGYF-
GHPAGY--
GTPAGY--
-GPAGYR-
GIPAGY--
GGPAGY--
GYPAGY--
GQPAGY--
-GPAGYT-
-GPAGYS-
GNPAGY--
GAPAGY--
-GPAGYK-
-GPAGYW-
-GPAGYE-
GKPAGY--
GEPAGY--
GWPAGY--
-GPAGYY-
-GPAGYG-
GCPAGY--
-GPAGYQ-
-GPAGYN-
-GPAGYI-
-GPAGYA-
GFPAGY--
GDPAGY--
HLPAGY--
HRPAGY--
HVPAGY--
-HPAGYT-
-HPAGYY-
-HPAGYR-
HGPAGY--
HEPAGY--
HAPAGY--

HSPAGY--
HPPAGY--
-HPAGYW-
-HPAGYK-
HIPAGY--
-HPAGYQ-
-HPAGYE-
HNPAGY--
HTPAGY--
-HPAGYV-
-HPAGYH-
-HPAGYF-
HMPAGY--
HYPAGY--
HWPAGY--
-HPAGYN-
-HPAGYM-
-HPAGYA-
HKPAGY--
HHPAGY--
-IPAGYW-
IQPAGY--
-IPAGYI-
ILPAGY--
-IPAGYT-
-IPAGYF-
-IPAGYR-
-IPAGYS-
-IPAGYN-
-IPAGYL-
-IPAGYK-
IAPAGY--
IYPAGY--
ISPAGY--
-IPAGYG-
-IPAGYA-
IWPAGY--
IVPAGY--
ITPAGY--
IPPAGY--
IMPAGY--
IHPAGY--
IFPAGY--
-IPAGYY-
-IPAGYH-
-IPAGYE-
IKPAGY--
IIPAGY--
IGPAGY--
IEPAGY--

ICPAGY--
KSPAGY--
KVPAGY--
KGPAGY--
KLPAGY--
-KPAGYL-
-KPAGYW-
-KPAGYT-
-KPAGYS-
-KPAGYR-
-KPAGYK-
-KPAGYF-
KAPAGY--
KCPAGY--
KRPAGY--
-KPAGYI-
-KPAGYM-
-KPAGYH-
-KPAGYN-
-KPAGYA-
-KPAGYG-
-KPAGYE-
KNPAGY--
KYPAGY--
KWPAGY--
KTPAGY--
KKPAGY--
KQPAGY--
KPPAGY--
KFPAGY--
KDPAGY--
-KPAGYV-
-KPAGYD-
KHPAGY--
KEPAGY--
LRPAGY--
-LPAGYR-
-LPAGYL-
LSPAGY--
-LPAGYK-
LGPAGY--
LYPAGY--
LMPAGY--
LTPAGY--
-LPAGYV-
LHPAGY--
-LPAGYF-
-LPAGYN-
-LPAGYI-
LNPAGY--

-LPAGYS-
LWPAGY--
LLPAGY--
-LPAGYM-
LPPAGY--
LIPAGY--
LAPAGY--
LQPAGY--
-LPAGYY-
-LPAGYW-
-LPAGYT-
-LPAGYA-
LDPAGY--
-LPAGYQ-
-LPAGYE-
LFPAGY--
-LPAGYH-
LEPAGY--
MVPAGY--
-MPAGYR-
MLPAGY--
MKPAGY--
MAPAGY--
-MPAGYS-
-MPAGYY-
-MPAGYQ-
MFPAGY--
MWPAGY--
MTPAGY--
-MPAGYT-
-MPAGYM-
-MPAGYG-
-MPAGYN-
-MPAGYK-
-MPAGYV-
-MPAGYL-
-MPAGYF-
MIPAGY--
MGPAGY--
MDPAGY--
MSPAGY--
MQPAGY--
NNPAGY--
-NPAGYR-
NLPAGY--
NDPAGY--
NTPAGY--
-NPAGYI-
NSPAGY--
-NPAGYV-

-NPAGYN-
-NPAGYF-
NVPAGY--
-NPAGYY-
-NPAGYW-
-NPAGYK-
-NPAGYA-
NWPAGY--
-NPAGYT-
-NPAGYS-
-NPAGYM-
NMPAGY--
NGPAGY--
NRPAGY--
NPPAGY--
-NPAGYH-
-NPAGYE-
NHPAGY--
NFPAGY--
NEPAGY--
--PAGYSR
--PAGYYT
--PAGYTR
--PAGYMS
--PAGYSV
--PAGYRI
--PAGYTT
--PAGYSL
--PAGYYL
--PAGYTS
--PAGYSS
--PAGYSA
--PAGYRL
--PAGYIS
--PAGYHL
--PAGYVN
--PAGYRH
--PAGYLS
--PAGYLN
--PAGYTI
--PAGYSK
--PAGYVT
--PAGYTH
--PAGYSF
--PAGYRG
--PAGYWL
--PAGYRS
--PAGYLR
--PAGYWT
--PAGYVK

--PAGYRT
--PAGYQA
--PAGYLM
--PAGYAG
--PAGYYN
--PAGYVR
--PAGYRD
--PAGYLW
--PAGYLK
PNPAGY--
--PAGYWI
--PAGYTY
--PAGYTG
--PAGYQG
--PAGYMA
--PAGYLY
PRPAGY--
--PAGYYQ
--PAGYSM
--PAGYRF
--PAGYMT
--PAGYLH
--PAGYIR
--PAGYGS
--PAGYVV
--PAGYVQ
--PAGYVL
--PAGYVH
--PAGYTQ
--PAGYMK
--PAGYKY
--PAGYKS
--PAGYIY
--PAGYHY
--PAGYHV
--PAGYHS
--PAGYHA
PYPAGY--
--PAGYWR
--PAGYTM
--PAGYTL
--PAGYSG
--PAGYKV
--PAGYIL
--PAGYGV
--PAGYFN
--PAGYAY
--PAGYAM
--PAGYYS
--PAGYYG

--PAGYRN
--PAGYNL
--PAGYML
--PAGYKG
--PAGYIT
--PAGYIF
--PAGYFM
--PAGYAV
--PAGYAT
PLPAGY--
PFPAGY--
PEPAGY--
--PAGYWM
--PAGYVS
--PAGYVA
--PAGYTV
--PAGYTN
--PAGYSY
--PAGYSE
--PAGYRY
--PAGYQR
--PAGYNR
--PAGYNK
--PAGYLV
--PAGYKM
--PAGYKK
--PAGYGT
--PAGYGA
--PAGYEY
--PAGYET
PQPAGY--
--PAGYYW
--PAGYYR
--PAGYYI
--PAGYTF
--PAGYSW
--PAGYRQ
--PAGYRE
--PAGYRA
--PAGYNS
--PAGYMY
--PAGYMI
--PAGYMG
--PAGYLG
--PAGYKW
--PAGYKT
--PAGYIN
--PAGYIG
--PAGYGM
--PAGYFV

```
--PAGYFL
--PAGYFK
--PAGYAW
PSPAGY--
--PAGYYM
--PAGYWS
--PAGYVG
--PAGYTK
--PAGYTA
--PAGYSI
--PAGYRV
--PAGYQT
--PAGYQM
--PAGYNT
--PAGYLD
--PAGYKQ
--PAGYIQ
--PAGYIM
--PAGYIH
--PAGYHW
--PAGYHK
--PAGYHD
--PAGYFQ
--PAGYFG
--PAGYFD
--PAGYEV
--PAGYES
--PAGYEM
--PAGYAR
--PAGYAI
PVPAGY--
PHPAGY--
PAPAGY--
--PAGYYH
--PAGYYD
--PAGYYA
--PAGYWQ
--PAGYWK
--PAGYWH
--PAGYWG
--PAGYWE
--PAGYVM
--PAGYVI
--PAGYVD
--PAGYSH
--PAGYSD
--PAGYQV
--PAGYQS
--PAGYQI
--PAGYQF
```

--PAGYNV
--PAGYNM
--PAGYNI
--PAGYNF
--PAGYMV
--PAGYMN
--PAGYLQ
--PAGYLA
--PAGYKN
--PAGYKI
--PAGYKH
--PAGYKF
--PAGYKA
--PAGYIV
--PAGYII
--PAGYIE
--PAGYHT
--PAGYHR
--PAGYHN
--PAGYHI
--PAGYHG
--PAGYGG
--PAGYFS
--PAGYFH
--PAGYFA
--PAGYDG
PTPAGY--
-PPAGYI-
PMPAGY--
PKPAGY--
PGPAGY--
--PAGYYY
--PAGYYE
--PAGYWD
--PAGYVW
--PAGYTD
--PAGYSC
--PAGYRW
--PAGYRM
--PAGYQN
--PAGYQH
--PAGYNH
--PAGYNG
--PAGYNA
--PAGYMQ
--PAGYMH
--PAGYMD
--PAGYLP
--PAGYLE
--PAGYKL

```
--PAGYKE
--PAGYIK
--PAGYID
--PAGYHM
--PAGYHH
--PAGYHF
--PAGYHE
--PAGYGY
--PAGYGI
--PAGYGF
--PAGYFE
--PAGYEW
--PAGYEN
--PAGYEG
--PAGYAQ
--PAGYAN
--PAGYAH
--PAGYAA
QVPAGY--
-QPAGYL-
QKPAGY--
-QPAGYR-
QPPAGY--
-QPAGYH-
QTPAGY--
QSPAGY--
-QPAGYY-
-QPAGYT-
-QPAGYS-
QEPAGY--
QRPAGY--
-QPAGYV-
-QPAGYN-
-QPAGYG-
QMPAGY--
QWPAGY--
-QPAGYQ-
-QPAGYE-
QIPAGY--
QFPAGY--
QCPAGY--
QYPAGY--
-QPAGYW-
-QPAGYM-
-QPAGYK-
-QPAGYI-
QNPAGY--
QLPAGY--
-RPAGYL-
-RPAGYS-
```

RLPAGY--
-RPAGYV-
RNPAGY--
-RPAGYG-
RTPAGY--
RHPAGY--
-RPAGYN-
RYPAGY--
-RPAGYI-
RKPAGY--
-RPAGYF-
RGPAGY--
RPPAGY--
-RPAGYT-
RWPAGY--
RSPAGY--
RAPAGY--
-RPAGYM-
-RPAGYE-
-RPAGYQ-
RVPAGY--
RQPAGY--
RFPAGY--
-RPAGYK-
-RPAGYH-
RIPAGY--
RDPAGY--
REPAGY--
RCPAGY--
-SPAGYF-
SSPAGY--
-SPAGYV-
-SPAGYI-
SAPAGY--
-SPAGYR-
SGPAGY--
SEPAGY--
-SPAGYT-
-SPAGYL-
SNPAGY--
STPAGY--
SRPAGY--
SLPAGY--
SYPAGY--
SWPAGY--
-SPAGYY-
SKPAGY--
-SPAGYW-
SFPAGY--
-SPAGYQ-

SVPAGY--
-SPAGYN-
-SPAGYM-
SIPAGY--
SQPAGY--
-SPAGYH-
-SPAGYG-
SPPAGY--
SMPAGY--
SHPAGY--
-TPAGYL-
TKPAGY--
TAPAGY--
-TPAGYK-
TWPAGY--
TFPAGY--
THPAGY--
-TPAGYT-
-TPAGYI-
TVPAGY--
-TPAGYH-
-TPAGYA-
TGPAGY--
TSPAGY--
TPPAGY--
-TPAGYW-
-TPAGYQ-
-TPAGYE-
TMPAGY--
-TPAGYY-
-TPAGYV-
-TPAGYN-
-TPAGYM-
-TPAGYG-
-TPAGYF-
TIPAGY--
TDPAGY--
TQPAGY--
TLPAGY--
TEPAGY--
-VPAGYW-
-VPAGYL-
VGPAGY--
-VPAGYQ-
-VPAGYF-
VWPAGY--
VQPAGY--
-VPAGYY-
VSPAGY--
VPPAGY--

-VPAGYS-
-VPAGYK-
-VPAGYA-
VVPAGY--
VTPAGY--
VRPAGY--
VHPAGY--
-VPAGYN-
VYPAGY--
VNPAGY--
-VPAGYV-
-VPAGYT-
-VPAGYM-
VIPAGY--
VDPAGY--
-VPAGYI-
-VPAGYH-
VKPAGY--
VEPAGY--
-WPAGYR-
WTPAGY--
WRPAGY--
WPPAGY--
-WPAGYI-
WAPAGY--
WWPAGY--
-WPAGYN-
-WPAGYH-
-WPAGYF-
WIPAGY--
WGPAGY--
WQPAGY--
-WPAGYM-
WLPAGY--
WHPAGY--
-WPAGYT-
-WPAGYS-
-WPAGYQ-
-WPAGYK-
-WPAGYA-
WKPAGY--
WSPAGY--
-WPAGYY-
-WPAGYW-
-WPAGYL-
WNPAGY--
WMPAGY--
WEPAGY--
YKPAGY--
-YPAGYH-

| | | | |
|---|---|---|---|
| | -YPAGYL- | | |
| | YRPAGY-- | | |
| | -YPAGYS- | | |
| | -YPAGYK- | | |
| | YYPAGY-- | | |
| | YTPAGY-- | | |
| | -YPAGYR- | | |
| | -YPAGYN- | | |
| | YVPAGY-- | | |
| | -YPAGYA- | | |
| | YNPAGY-- | | |
| | YGPAGY-- | | |
| | -YPAGYI- | | |
| | -YPAGYG- | | |
| | YWPAGY-- | | |
| | YSPAGY-- | | |
| | YQPAGY-- | | |
| | YMPAGY-- | | |
| | YHPAGY-- | | |
| | YAPAGY-- | | |
| | YPPAGY-- | | |
| | -YPAGYE- | | |
| | -YPAGYY- | | |
| | -YPAGYT- | | |
| | -YPAGYQ- | | |
| | -YPAGYM- | | |
| | YEPAGY-- | | |
| | YDPAGY-- | | |
| PRPY | 53 | 801 | 33.44 |
| | -IPRPYW- | | |
| | --PRPYEI | | |
| | -APRPYV- | | |
| | -APRPYL- | | |
| | -APRPYT- | | |
| | -APRPYI- | | |
| | AKPRPY-- | | |
| | AVPRPY-- | | |
| | AIPRPY-- | | |
| | ASPRPY-- | | |
| | -APRPYK- | | |
| | -APRPYN- | | |
| | -APRPYG- | | |
| | AAPRPY-- | | |
| | -APRPYR- | | |
| | ARPRPY-- | | |
| | APPRPY-- | | |
| | AWPRPY-- | | |
| | -APRPYW- | | |
| | -APRPYS- | | |
| | ANPRPY-- | | |

AFPRPY--
-APRPYQ-
-APRPYH-
-APRPYE-
-APRPYY-
-APRPYM-
-APRPYF-
AHPRPY--
AGPRPY--
AMPRPY--
AEPRPY--
-CPRPYS-
CLPRPY--
CVPRPY--
-CPRPYR-
CMPRPY--
CIPRPY--
CAPRPY--
CSPRPY--
-CPRPYM-
CGPRPY--
CCPRPY--
CYPRPY--
CTPRPY--
CRPRPY--
-CPRPYL-
CHPRPY--
CFPRPY--
-DPRPYL-
-DPRPYR-
-DPRPYS-
DHPRPY--
DNPRPY--
DYPRPY--
DSPRPY--
-DPRPYI-
-DPRPYF-
DTPRPY--
DRPRPY--
DQPRPY--
-DPRPYT-
DDPRPY--
DLPRPY--
DVPRPY--
-DPRPYY-
DKPRPY--
DIPRPY--
DGPRPY--
-DPRPYW-
DMPRPY--

DWPRPY--
-DPRPYV-
-DPRPYN-
-DPRPYK-
-DPRPYC-
DFPRPY--
DEPRPY--
DCPRPY--
EVPRPY--
-EPRPYY-
-EPRPYR-
EMPRPY--
ESPRPY--
-EPRPYI-
EKPRPY--
EIPRPY--
EFPRPY--
ETPRPY--
ERPRPY--
-EPRPYL-
EYPRPY--
-EPRPYK-
EWPRPY--
-EPRPYW-
ENPRPY--
ELPRPY--
EQPRPY--
-EPRPYH-
-EPRPYG-
EPPRPY--
EEPRPY--
-EPRPYT-
-EPRPYA-
EGPRPY--
EAPRPY--
-FPRPYR-
FIPRPY--
FSPRPY--
FLPRPY--
FTPRPY--
-FPRPYK-
FAPRPY--
-FPRPYL-
-FPRPYF-
-FPRPYS-
-FPRPYI-
-FPRPYA-
FYPRPY--
FVPRPY--
-FPRPYW-

FKPRPY--
FGPRPY--
FDPRPY--
-FPRPYH-
FQPRPY--
-FPRPYY-
-FPRPYN-
FNPRPY--
FMPRPY--
FEPRPY--
-FPRPYM-
FHPRPY--
-GPRPYL-
GVPRPY--
GSPRPY--
GKPRPY--
-GPRPYY-
-GPRPYS-
-GPRPYN-
GNPRPY--
-GPRPYG-
GYPRPY--
-GPRPYM-
-GPRPYA-
GIPRPY--
GWPRPY--
-GPRPYW-
-GPRPYR-
GPPRPY--
GGPRPY--
GQPRPY--
-GPRPYI-
GLPRPY--
GTPRPY--
-GPRPYK-
GHPRPY--
GEPRPY--
GRPRPY--
-GPRPYH-
-GPRPYF-
-GPRPYE-
GAPRPY--
-GPRPYV-
-GPRPYP-
GFPRPY--
-HPRPYL-
-HPRPYT-
-HPRPYS-
HAPRPY--
-HPRPYI-

HLPRPY--
HYPRPY--
HTPRPY--
HSPRPY--
-HPRPYR-
-HPRPYM-
HHPRPY--
HQPRPY--
HPPRPY--
HNPRPY--
HEPRPY--
HWPRPY--
-HPRPYW-
-HPRPYV-
HMPRPY--
HVPRPY--
HRPRPY--
-HPRPYG-
-HPRPYE-
HDPRPY--
-HPRPYQ-
-HPRPYN-
-HPRPYA-
HGPRPY--
HFPRPY--
HCPRPY--
-IPRPYL-
ILPRPY--
IVPRPY--
-IPRPYI-
ISPRPY--
IYPRPY--
IRPRPY--
-IPRPYR-
IHPRPY--
-IPRPYS-
IKPRPY--
IGPRPY--
-IPRPYM-
IPPRPY--
IIPRPY--
IDPRPY--
-IPRPYQ-
IAPRPY--
ITPRPY--
IFPRPY--
IQPRPY--
-IPRPYN-
-IPRPYA-
IMPRPY--

-IPRPYV-
-IPRPYT-
-IPRPYK-
-IPRPYG-
INPRPY--
KMPRPY--
-KPRPYL-
-KPRPYW-
KLPRPY--
KIPRPY--
-KPRPYV-
-KPRPYM-
KTPRPY--
KVPRPY--
-KPRPYN-
KNPRPY--
-KPRPYF-
KHPRPY--
-KPRPYQ-
KKPRPY--
KGPRPY--
KAPRPY--
KYPRPY--
-KPRPYK-
-KPRPYI-
KFPRPY--
KDPRPY--
KWPRPY--
KQPRPY--
-KPRPYY-
-KPRPYG-
KPPRPY--
KEPRPY--
-LPRPYL-
-LPRPYY-
LVPRPY--
LYPRPY--
LIPRPY--
-LPRPYT-
LLPRPY--
LTPRPY--
LRPRPY--
-LPRPYI-
LGPRPY--
-LPRPYS-
LHPRPY--
-LPRPYW-
LAPRPY--
-LPRPYR-
LQPRPY--

-LPRPYK-
-LPRPYM-
LEPRPY--
-LPRPYV-
-LPRPYA-
-LPRPYH-
LMPRPY--
LWPRPY--
LPPRPY--
LDPRPY--
-LPRPYQ-
-LPRPYF-
LCPRPY--
MSPRPY--
-MPRPYL-
MKPRPY--
-MPRPYR-
MGPRPY--
-MPRPYQ-
-MPRPYF-
MFPRPY--
MTPRPY--
MYPRPY--
MVPRPY--
MRPRPY--
-MPRPYN-
-MPRPYM-
-MPRPYK-
MIPRPY--
-MPRPYW-
-MPRPYV-
MNPRPY--
MEPRPY--
MMPRPY--
MDPRPY--
-MPRPYT-
-MPRPYA-
MPPRPY--
MQPRPY--
-MPRPYY-
-MPRPYI-
-MPRPYG-
-MPRPYC-
NLPRPY--
-NPRPYM-
NVPRPY--
-NPRPYR-
NYPRPY--
-NPRPYT-
-NPRPYI-

NFPRPY--
-NPRPYW-
NTPRPY--
NAPRPY--
NMPRPY--
NSPRPY--
NPPRPY--
NNPRPY--
NGPRPY--
-NPRPYV-
-NPRPYK-
-NPRPYH-
-NPRPYF-
-NPRPYA-
NHPRPY--
NWPRPY--
NQPRPY--
-NPRPYY-
NKPRPY--
NIPRPY--
NEPRPY--
NDPRPY--
--PRPYRL
--PRPYSG
--PRPYTF
--PRPYLS
--PRPYSL
--PRPYSA
--PRPYVL
--PRPYLA
--PRPYRT
--PRPYRS
--PRPYLN
--PRPYTL
--PRPYFL
--PRPYFA
--PRPYTW
--PRPYNV
--PRPYML
--PRPYLK
--PRPYTV
--PRPYSV
--PRPYST
--PRPYSI
--PRPYMS
--PRPYTT
--PRPYMM
PVPRPY--
--PRPYWA
--PRPYSR

```
--PRPYIV
--PRPYWS
--PRPYLT
--PRPYHL
--PRPYAS
--PRPYAL
--PRPYVV
--PRPYVS
--PRPYSY
--PRPYLQ
--PRPYPW
--PRPYIA
--PRPYFV
--PRPYVW
--PRPYIT
--PRPYVR
--PRPYRG
--PRPYRA
--PRPYKL
--PRPYIN
--PRPYGL
--PRPYFK
--PRPYVD
--PRPYNL
--PRPYLI
--PRPYLF
--PRPYIS
--PRPYIL
--PRPYAV
--PRPYWT
--PRPYVT
--PRPYVQ
--PRPYTS
--PRPYTQ
--PRPYNQ
--PRPYGM
--PRPYGF
--PRPYFS
--PRPYAY
--PRPYAF
PSPRPY--
--PRPYSQ
--PRPYIY
--PRPYEL
--PRPYAT
--PRPYYG
--PRPYWV
--PRPYVY
--PRPYVG
--PRPYSS
```

--PRPYSN
--PRPYSF
--PRPYRI
--PRPYRF
--PRPYQV
--PRPYQI
--PRPYNS
--PRPYMK
--PRPYGT
--PRPYGA
--PRPYFH
--PRPYDL
PYPRPY--
--PRPYVM
--PRPYVH
--PRPYVF
--PRPYTN
--PRPYRY
--PRPYQT
--PRPYMA
--PRPYLW
--PRPYLM
--PRPYGI
--PRPYFT
--PRPYFQ
--PRPYAR
PFPRPY--
--PRPYYW
--PRPYYV
--PRPYWI
--PRPYWF
--PRPYVK
--PRPYSK
--PRPYRM
--PRPYQL
--PRPYPM
--PRPYPF
--PRPYMQ
--PRPYMN
--PRPYIF
--PRPYHV
--PRPYHG
--PRPYAK
--PRPYAH
PRPRPY--
PTPRPY--
--PRPYYN
--PRPYYA
--PRPYWG
--PRPYTY

--PRPYTM
--PRPYTA
--PRPYRV
--PRPYRN
--PRPYQS
--PRPYPH
--PRPYNF
--PRPYMV
--PRPYMG
--PRPYLH
--PRPYKS
--PRPYHM
--PRPYGS
--PRPYGG
--PRPYEF
--PRPYAQ
--PRPYAI
PLPRPY--
PGPRPY--
--PRPYYM
--PRPYYK
--PRPYWW
--PRPYWM
--PRPYVA
--PRPYTG
--PRPYSW
--PRPYRH
--PRPYRD
--PRPYQA
--PRPYNY
--PRPYII
--PRPYHF
--PRPYFM
--PRPYFI
--PRPYFG
--PRPYES
--PRPYEM
--PRPYAA
PAPRPY--
--PRPYYY
--PRPYYT
--PRPYYI
--PRPYWQ
--PRPYWL
--PRPYWE
--PRPYSD
--PRPYNT
--PRPYMI
--PRPYME
--PRPYLD

--PRPYKT
--PRPYKI
--PRPYIH
--PRPYIE
--PRPYHW
--PRPYGV
--PRPYEY
--PRPYCV
--PRPYYS
--PRPYYQ
--PRPYYF
--PRPYWN
--PRPYWK
--PRPYVE
--PRPYTK
--PRPYRE
--PRPYQR
--PRPYPY
--PRPYPQ
--PRPYKQ
--PRPYKG
--PRPYKF
--PRPYHT
--PRPYHR
--PRPYHQ
--PRPYGY
--PRPYGW
--PRPYGQ
--PRPYGN
--PRPYGH
--PRPYFF
--PRPYCA
--PRPYAM
--PRPYAG
PIPRPY--
PHPRPY--
PEPRPY--
PDPRPY--
--PRPYYD
--PRPYWY
--PRPYWR
--PRPYWD
--PRPYVN
--PRPYTH
--PRPYTE
--PRPYTD
--PRPYRQ
--PRPYQM
--PRPYQG
--PRPYQD

--PRPYPS
--PRPYPI
--PRPYNN
--PRPYNK
--PRPYNA
--PRPYMT
--PRPYMF
--PRPYLC
--PRPYKV
--PRPYKN
--PRPYKM
--PRPYID
--PRPYHS
--PRPYHP
--PRPYHN
--PRPYHK
--PRPYFY
--PRPYFW
--PRPYFE
--PRPYFD
--PRPYEW
--PRPYET
--PRPYDY
--PRPYDW
--PRPYDM
PQPRPY--
-PPRPYF-
QPPRPY--
-QPRPYL-
QSPRPY--
-QPRPYS-
QLPRPY--
QWPRPY--
-QPRPYR-
QAPRPY--
QRPRPY--
QIPRPY--
-QPRPYY-
-QPRPYH-
-QPRPYF-
QGPRPY--
-QPRPYA-
QHPRPY--
QYPRPY--
QVPRPY--
-QPRPYV-
-QPRPYT-
-QPRPYN-
-QPRPYM-
-QPRPYI-

QNPRPY--
-QPRPYW-
-QPRPYK-
QMPRPY--
QFPRPY--
QQPRPY--
-QPRPYE-
QKPRPY--
QEPRPY--
-RPRPYL-
RMPRPY--
RYPRPY--
-RPRPYV-
RSPRPY--
-RPRPYS-
RGPRPY--
RAPRPY--
RNPRPY--
RTPRPY--
REPRPY--
RVPRPY--
-RPRPYA-
-RPRPYT-
-RPRPYY-
RPPRPY--
-RPRPYF-
-RPRPYN-
RIPRPY--
RQPRPY--
RRPRPY--
-RPRPYG-
RDPRPY--
RWPRPY--
-RPRPYQ-
-RPRPYH-
RFPRPY--
-RPRPYM-
-RPRPYP-
-RPRPYK-
-RPRPYE-
RCPRPY--
-SPRPYR-
-SPRPYH-
SAPRPY--
-SPRPYL-
SVPRPY--
STPRPY--
SSPRPY--
SLPRPY--
SGPRPY--

-SPRPYQ-
SPPRPY--
SMPRPY--
SRPRPY--
-SPRPYN-
SNPRPY--
-SPRPYV-
SEPRPY--
-SPRPYY-
-SPRPYW-
-SPRPYF-
-SPRPYA-
SIPRPY--
SFPRPY--
-SPRPYM-
SKPRPY--
SDPRPY--
-SPRPYI-
SHPRPY--
SQPRPY--
TLPRPY--
-TPRPYL-
-TPRPYS-
-TPRPYI-
TMPRPY--
-TPRPYR-
-TPRPYA-
TAPRPY--
TQPRPY--
THPRPY--
-TPRPYY-
TYPRPY--
-TPRPYF-
TFPRPY--
TEPRPY--
-TPRPYW-
TKPRPY--
TPPRPY--
TIPRPY--
TGPRPY--
TDPRPY--
-TPRPYT-
-TPRPYM-
TVPRPY--
-TPRPYQ-
-TPRPYN-
-TPRPYH-
-TPRPYG-
VSPRPY--
-VPRPYL-

```
-VPRPYV-
-VPRPYT-
-VPRPYQ-
VMPRPY--
VYPRPY--
VVPRPY--
-VPRPYS-
-VPRPYH-
-VPRPYA-
VAPRPY--
VRPRPY--
VWPRPY--
-VPRPYR-
VIPRPY--
VTPRPY--
-VPRPYM-
VHPRPY--
VGPRPY--
VQPRPY--
-VPRPYW-
VEPRPY--
VDPRPY--
-VPRPYN-
-VPRPYK-
-VPRPYI-
VPPRPY--
WVPRPY--
-WPRPYA-
-WPRPYG-
WHPRPY--
WMPRPY--
WAPRPY--
-WPRPYM-
-WPRPYK-
WIPRPY--
-WPRPYN-
WGPRPY--
-WPRPYV-
-WPRPYH-
WLPRPY--
WWPRPY--
WTPRPY--
WQPRPY--
-WPRPYS-
-WPRPYI-
WPPRPY--
WFPRPY--
WEPRPY--
-WPRPYT-
WDPRPY--
```

| | | | |
|---|---|---|---|
| | -YPRPYA- | | |
| | YRPRPY-- | | |
| | YFPRPY-- | | |
| | YLPRPY-- | | |
| | YAPRPY-- | | |
| | -YPRPYQ- | | |
| | YTPRPY-- | | |
| | -YPRPYT- | | |
| | -YPRPYR- | | |
| | YKPRPY-- | | |
| | YQPRPY-- | | |
| | -YPRPYS- | | |
| | -YPRPYH- | | |
| | YGPRPY-- | | |
| | -YPRPYW- | | |
| | -YPRPYF- | | |
| | YNPRPY-- | | |
| | YWPRPY-- | | |
| | -YPRPYM- | | |
| | YHPRPY-- | | |
| | YVPRPY-- | | |
| | -YPRPYV- | | |
| | -YPRPYN- | | |
| | -YPRPYK- | | |
| | YPPRPY-- | | |
| | YIPRPY-- | | |
| | -YPRPYY- | | |
| | -YPRPYE- | | |
| | YMPRPY-- | | |
| | YEPRPY-- | | |
| PRSL | 1543 | 430 | 33.37 |
| | -HPRSLP- | | |
| | -IPRSLD- | | |
| | -KPRSLD- | | |
| | -LPRSLP- | | |
| | -LPRSLC- | | |
| | -MPRSLW- | | |
| | --PRSLYP | | |
| | --PRSLSC | | |
| | --PRSLDK | | |
| | --PRSLDA | | |
| | --PRSLCG | | |
| | -RPRSLC- | | |
| | -SPRSLP- | | |
| | -SPRSLD- | | |
| | -TPRSLC- | | |
| | -WPRSLD- | | |
| | -WPRSLP- | | |
| | -APRSLT- | | |
| | -APRSLA- | | |

-APRSLG-
-APRSLS-
-APRSLF-
-APRSLV-
-APRSLN-
APPRSL--
-APRSLH-
-APRSLE-
-APRSLW-
-APRSLQ-
ADPRSL--
AEPRSL--
ACPRSL--
CFPRSL--
CCPRSL--
CKPRSL--
-CPRSLI-
CTPRSL--
CAPRSL--
CVPRSL--
CSPRSL--
-CPRSLR-
-CPRSLK-
CNPRSL--
CDPRSL--
-CPRSLT-
CGPRSL--
-CPRSLS-
-CPRSLL-
-CPRSLW-
-CPRSLQ-
-CPRSLA-
CPPRSL--
CEPRSL--
CQPRSL--
-CPRSLV-
-CPRSLM-
CHPRSL--
CYPRSL--
CWPRSL--
-CPRSLF-
CIPRSL--
DTPRSL--
-DPRSLI-
-DPRSLL-
-DPRSLV-
DAPRSL--
DIPRSL--
DYPRSL--
DMPRSL--

DHPRSL--
DGPRSL--
-DPRSLY-
-DPRSLK-
-DPRSLW-
DQPRSL--
-DPRSLT-
-DPRSLF-
-DPRSLA-
-DPRSLH-
DEPRSL--
-DPRSLN-
-DPRSLE-
DDPRSL--
-DPRSLG-
-DPRSLQ-
-EPRSLL-
-EPRSLR-
ELPRSL--
EVPRSL--
ENPRSL--
-EPRSLK-
EAPRSL--
-EPRSLS-
EYPRSL--
ERPRSL--
-EPRSLT-
-EPRSLN-
-EPRSLH-
-EPRSLG-
EMPRSL--
EQPRSL--
EIPRSL--
-EPRSLV-
EEPRSL--
-EPRSLM-
-EPRSLE-
EDPRSL--
EPPRSL--
-EPRSLQ-
-EPRSLD-
ECPRSL--
-EPRSLC-
FQPRSL--
-FPRSLY-
FEPRSL--
FTPRSL--
FDPRSL--
FMPRSL--
-FPRSLE-

−FPRSLC−
−GPRSLL−
GTPRSL−−
−GPRSLY−
−GPRSLA−
GNPRSL−−
GHPRSL−−
−GPRSLG−
−GPRSLT−
GEPRSL−−
−GPRSLW−
GQPRSL−−
−GPRSLI−
−GPRSLQ−
−GPRSLF−
−GPRSLE−
−GPRSLH−
GDPRSL−−
−GPRSLD−
−HPRSLN−
−HPRSLL−
HPPRSL−−
HAPRSL−−
−HPRSLY−
HHPRSL−−
−HPRSLM−
−HPRSLA−
−HPRSLW−
−HPRSLV−
−HPRSLH−
HEPRSL−−
HDPRSL−−
HCPRSL−−
−HPRSLE−
ISPRSL−−
INPRSL−−
−IPRSLN−
−IPRSLY−
IIPRSL−−
IEPRSL−−
IDPRSL−−
IAPRSL−−
IPPRSL−−
IHPRSL−−
−IPRSLF−
ICPRSL−−
−KPRSLA−
−KPRSLT−
−KPRSLW−
−KPRSLY−

KPPRSL--
KIPRSL--
-KPRSLV-
-KPRSLN-
KNPRSL--
KKPRSL--
KCPRSL--
KDPRSL--
-KPRSLE-
-LPRSLV-
-LPRSLN-
-LPRSLF-
-LPRSLQ-
-LPRSLE-
LCPRSL--
MDPRSL--
-MPRSLI-
-MPRSLG-
MEPRSL--
-MPRSLM-
MAPRSL--
-MPRSLF-
-MPRSLY-
-MPRSLH-
MPPRSL--
MHPRSL--
-MPRSLE-
MCPRSL--
-NPRSLY-
-NPRSLS-
NTPRSL--
-NPRSLI-
NPPRSL--
-NPRSLL-
NMPRSL--
-NPRSLF-
NHPRSL--
-NPRSLQ-
-NPRSLE-
-NPRSLM-
NDPRSL--
NCPRSL--
--PRSLLT
--PRSLGS
--PRSLNL
--PRSLYS
--PRSLSG
--PRSLSV
--PRSLWS
--PRSLFS

--PRSLVV
--PRSLWL
--PRSLFG
--PRSLIS
--PRSLGT
--PRSLAV
--PRSLKT
--PRSLYG
--PRSLTT
--PRSLSI
--PRSLFA
--PRSLNS
--PRSLAA
--PRSLTV
--PRSLTN
--PRSLVD
--PRSLWG
--PRSLER
PHPRSL--
--PRSLYA
--PRSLNI
--PRSLPL
--PRSLMN
--PRSLLH
--PRSLHT
--PRSLFF
--PRSLEW
--PRSLAT
--PRSLVQ
--PRSLFI
--PRSLIF
--PRSLYK
--PRSLSE
--PRSLGI
--PRSLQG
--PRSLWD
--PRSLTQ
--PRSLWW
--PRSLAM
PIPRSL--
--PRSLTK
--PRSLQT
PNPRSL--
--PRSLYI
--PRSLWI
--PRSLWH
--PRSLNH
--PRSLNF
--PRSLAN
--PRSLAQ

--PRSLYQ
--PRSLYM
--PRSLYH
--PRSLWF
--PRSLWE
--PRSLTE
--PRSLQA
--PRSLNV
--PRSLMQ
--PRSLIK
--PRSLHG
--PRSLSQ
--PRSLNN
--PRSLGF
--PRSLFE
PMPRSL--
--PRSLNT
--PRSLMT
--PRSLIE
--PRSLHF
--PRSLHA
--PRSLEY
--PRSLEF
--PRSLYF
--PRSLWM
--PRSLWV
--PRSLEM
--PRSLEA
--PRSLDS
--PRSLHQ
--PRSLVE
--PRSLQF
--PRSLQD
--PRSLNY
--PRSLNW
--PRSLNQ
--PRSLNK
--PRSLFW
--PRSLYD
--PRSLTD
--PRSLII
--PRSLEH
--PRSLQV
--PRSLHH
--PRSLYE
--PRSLQH
--PRSLID
--PRSLGK
--PRSLWY
--PRSLQQ

--PRSLKD
--PRSLEL
--PRSLAE
--PRSLAD
--PRSLND
--PRSLEI
--PRSLDG
--PRSLEG
PEPRSL--
--PRSLQY
--PRSLMD
--PRSLFQ
--PRSLHM
--PRSLEV
--PRSLPM
--PRSLNE
--PRSLQE
--PRSLHD
--PRSLGH
--PRSLEK
--PRSLDT
-PPRSLN-
--PRSLQC
--PRSLPF
--PRSLMP
--PRSLHY
--PRSLEQ
--PRSLCK
--PRSLCH
--PRSLCA
--PRSLWC
--PRSLVC
--PRSLFC
--PRSLEC
--PRSLCY
--PRSLAC
-PPRSLV-
-PPRSLT-
-PPRSLM-
-PPRSLL-
-PPRSLK-
-PPRSLG-
PPPRSL--
PCPRSL--
QSPRSL--
-QPRSLI-
-QPRSLQ-
-QPRSLF-
QNPRSL--
-QPRSLA-

QKPRSL--
-QPRSLH-
-QPRSLG-
QGPRSL--
-QPRSLY-
-QPRSLE-
-QPRSLW-
QDPRSL--
QPPRSL--
QTPRSL--
QCPRSL--
QEPRSL--
RPPRSL--
-RPRSLD-
-SPRSLF-
-SPRSLY-
-SPRSLA-
-SPRSLM-
-SPRSLN-
-SPRSLH-
SEPRSL--
SDPRSL--
-TPRSLL-
TGPRSL--
TNPRSL--
-TPRSLH-
TDPRSL--
-TPRSLG-
TPPRSL--
-TPRSLN-
-TPRSLE-
TEPRSL--
-VPRSLS-
VDPRSL--
VPPRSL--
-VPRSLN-
-VPRSLV-
VGPRSL--
VEPRSL--
-VPRSLE-
VCPRSL--
WTPRSL--
-WPRSLN-
WPPRSL--
WEPRSL--
WKPRSL--
WHPRSL--
WDPRSL--
WQPRSL--
-WPRSLC-

| | | | |
|---|---|---|---|
| | YVPRSL-- | | |
| | YNPRSL-- | | |
| | -YPRSLN- | | |
| | -YPRSLF- | | |
| | YTPRSL-- | | |
| | -YPRSLM- | | |
| | YPPRSL-- | | |
| | YQPRSL-- | | |
| | -YPRSLE- | | |
| | YEPRSL-- | | |
| | YCPRSL-- | | |
| LGNL | 97 | 771 | 33.35 |
| | -ALGNLG- | | |
| | -ELGNLG- | | |
| | -ILGNLD- | | |
| | -KLGNLP- | | |
| | -KLGNLG- | | |
| | -LLGNLD- | | |
| | --LGNLLP | | |
| | --LGNLWP | | |
| | --LGNLSE | | |
| | --LGNLPS | | |
| | --LGNLPL | | |
| | --LGNLNQ | | |
| | --LGNLNN | | |
| | --LGNLNM | | |
| | --LGNLNK | | |
| | --LGNLNG | | |
| | --LGNLLE | | |
| | --LGNLGQ | | |
| | --LGNLGL | | |
| | --LGNLGF | | |
| | --LGNLFE | | |
| | --LGNLER | | |
| | --LGNLCV | | |
| | --LGNLAR | | |
| | -NLGNLN- | | |
| | -SLGNLD- | | |
| | -TLGNLD- | | |
| | ARLGNL-- | | |
| | AVLGNL-- | | |
| | -ALGNLK- | | |
| | ALLGNL-- | | |
| | AKLGNL-- | | |
| | AWLGNL-- | | |
| | -ALGNLF- | | |
| | -ALGNLI- | | |
| | AFLGNL-- | | |
| | AGLGNL-- | | |
| | ANLGNL-- | | |

```
-ALGNLV-
ATLGNL--
AMLGNL--
-ALGNLW-
AYLGNL--
-ALGNLT-
-ALGNLA-
APLGNL--
-ALGNLH-
AQLGNL--
-ALGNLQ-
-ALGNLN-
AILGNL--
AELGNL--
-ALGNLM-
ADLGNL--
-ALGNLY-
AALGNL--
-ALGNLE-
CSLGNL--
-CLGNLR-
-CLGNLK-
CKLGNL--
CELGNL--
CVLGNL--
CPLGNL--
-CLGNLI-
CGLGNL--
-CLGNLT-
CHLGNL--
CWLGNL--
CLLGNL--
CFLGNL--
CYLGNL--
CRLGNL--
-CLGNLW-
-CLGNLV-
CALGNL--
CTLGNL--
CQLGNL--
-CLGNLY-
-CLGNLS-
-CLGNLH-
CCLGNL--
-CLGNLN-
-CLGNLL-
-CLGNLF-
CMLGNL--
-CLGNLQ-
-CLGNLG-
```

-CLGNLE-
-CLGNLA-
CILGNL--
DRLGNL--
-DLGNLL-
-DLGNLR-
DLLGNL--
-DLGNLV-
-DLGNLS-
DVLGNL--
DPLGNL--
-DLGNLM-
DYLGNL--
-DLGNLT-
-DLGNLF-
DTLGNL--
DSLGNL--
-DLGNLI-
DALGNL--
-DLGNLK-
DGLGNL--
DMLGNL--
DHLGNL--
DFLGNL--
DQLGNL--
DWLGNL--
-DLGNLY-
DNLGNL--
-DLGNLH-
DILGNL--
-DLGNLQ-
-DLGNLW-
-DLGNLN-
DELGNL--
DDLGNL--
ERLGNL--
EPLGNL--
-ELGNLI-
EYLGNL--
-ELGNLF-
EGLGNL--
ESLGNL--
EMLGNL--
-ELGNLS-
-ELGNLK-
ENLGNL--
-ELGNLV-
EVLGNL--
-ELGNLH-
EKLGNL--

EFLGNL--
EQLGNL--
-ELGNLL-
-ELGNLA-
EILGNL--
ETLGNL--
-ELGNLY-
ELLGNL--
EALGNL--
-ELGNLQ-
EDLGNL--
EHLGNL--
EWLGNL--
-ELGNLW-
-ELGNLT-
-ELGNLN-
EELGNL--
-ELGNLM-
-FLGNLR-
FRLGNL--
-FLGNLK-
-FLGNLL-
-FLGNLS-
-FLGNLV-
FNLGNL--
FTLGNL--
FVLGNL--
-FLGNLG-
FALGNL--
FWLGNL--
-FLGNLQ-
-FLGNLN-
-FLGNLA-
FQLGNL--
FKLGNL--
FPLGNL--
FMLGNL--
FHLGNL--
FGLGNL--
FDLGNL--
-FLGNLH-
FILGNL--
-FLGNLT-
-FLGNLW-
-FLGNLM-
FELGNL--
GKLGNL--
GSLGNL--
-GLGNLM-
GTLGNL--

-GLGNLL-
-GLGNLT-
-GLGNLK-
GLLGNL--
GYLGNL--
GPLGNL--
GALGNL--
GVLGNL--
-GLGNLV-
-GLGNLF-
GILGNL--
GWLGNL--
-GLGNLQ-
-GLGNLA-
-GLGNLW-
-GLGNLH-
GGLGNL--
GELGNL--
GNLGNL--
GMLGNL--
GHLGNL--
GQLGNL--
-GLGNLY-
-GLGNLN-
-GLGNLE-
GDLGNL--
-HLGNLR-
HRLGNL--
HVLGNL--
HWLGNL--
-HLGNLS-
-HLGNLT-
HGLGNL--
-HLGNLF-
HYLGNL--
HSLGNL--
HTLGNL--
-HLGNLK-
HKLGNL--
HFLGNL--
HNLGNL--
-HLGNLA-
-HLGNLV-
-HLGNLQ-
HPLGNL--
-HLGNLH-
HILGNL--
HHLGNL--
-HLGNLM-
-HLGNLW-

-HLGNLN-
-HLGNLI-
HMLGNL--
HELGNL--
HQLGNL--
HALGNL--
-HLGNLG-
-HLGNLE-
HDLGNL--
HCLGNL--
-ILGNLR-
IRLGNL--
ITLGNL--
-ILGNLT-
IQLGNL--
-ILGNLK-
IILGNL--
ISLGNL--
IKLGNL--
-ILGNLS-
IGLGNL--
IHLGNL--
-ILGNLV-
IMLGNL--
-ILGNLH-
-ILGNLQ-
-ILGNLN-
-ILGNLF-
-ILGNLM-
-ILGNLI-
IFLGNL--
IDLGNL--
IYLGNL--
INLGNL--
IELGNL--
-ILGNLA-
IPLGNL--
-ILGNLE-
ICLGNL--
IWLGNL--
KVLGNL--
-KLGNLS-
-KLGNLL-
-KLGNLT-
-KLGNLV-
KILGNL--
KTLGNL--
KPLGNL--
KYLGNL--
KGLGNL--

KMLGNL--
KWLGNL--
-KLGNLK-
-KLGNLI-
-KLGNLA-
KHLGNL--
KELGNL--
-KLGNLF-
KDLGNL--
KFLGNL--
-KLGNLQ-
KQLGNL--
-KLGNLN-
-KLGNLE-
-KLGNLM-
KCLGNL--
--LGNLRS
--LGNLSS
--LGNLRL
-LLGNLS-
LGLGNL--
--LGNLST
--LGNLRA
--LGNLRT
--LGNLVR
--LGNLLG
--LGNLMR
--LGNLQG
--LGNLSA
--LGNLLS
--LGNLVA
--LGNLRM
-LLGNLT-
--LGNLSG
LVLGNL--
-LLGNLG-
--LGNLVK
--LGNLFG
--LGNLSV
--LGNLLV
-LLGNLN-
--LGNLGS
--LGNLWS
--LGNLVT
--LGNLWT
--LGNLRQ
--LGNLLA
--LGNLKS
--LGNLAA
LALGNL--

-LLGNLV-
LKLGNL--
--LGNLTG
--LGNLML
--LGNLYN
--LGNLLH
--LGNLSF
--LGNLRY
--LGNLFT
--LGNLAS
--LGNLTT
LQLGNL--
-LLGNLA-
--LGNLMN
--LGNLIS
--LGNLKT
--LGNLTW
--LGNLMK
-LLGNLF-
--LGNLVI
--LGNLLM
--LGNLRE
--LGNLVW
--LGNLRW
--LGNLMM
--LGNLMG
--LGNLIT
--LGNLYG
--LGNLVV
--LGNLVH
--LGNLVF
--LGNLTN
--LGNLLN
--LGNLIQ
--LGNLYS
--LGNLYR
--LGNLVY
--LGNLSN
--LGNLSI
-LLGNLQ-
-LLGNLE-
--LGNLWR
--LGNLWM
--LGNLTM
--LGNLTH
--LGNLSM
--LGNLKY
--LGNLKN
--LGNLKH
--LGNLIN

--LGNLHT
--LGNLAT
--LGNLMS
--LGNLIH
--LGNLFM
--LGNLWG
--LGNLVQ
--LGNLRD
--LGNLMI
--LGNLIV
--LGNLAG
--LGNLQA
--LGNLMA
--LGNLKM
--LGNLHS
--LGNLHG
--LGNLFR
LWLGNL--
--LGNLYM
--LGNLKW
--LGNLHV
--LGNLFQ
--LGNLYT
--LGNLVM
--LGNLSQ
--LGNLQS
--LGNLAW
--LGNLAM
--LGNLYQ
--LGNLYH
--LGNLSK
--LGNLMQ
--LGNLES
--LGNLQM
--LGNLKQ
--LGNLII
--LGNLHA
--LGNLAV
LCLGNL--
--LGNLYL
--LGNLWQ
--LGNLWN
--LGNLTI
--LGNLQH
--LGNLIK
--LGNLFK
--LGNLTK
--LGNLTF
--LGNLMT
--LGNLIW

--LGNLHM
--LGNLFH
--LGNLEA
--LGNLTL
--LGNLMW
--LGNLHY
--LGNLHR
--LGNLHK
--LGNLGA
--LGNLFN
--LGNLEV
--LGNLWK
--LGNLSH
--LGNLNA
--LGNLMH
--LGNLMD
--LGNLKV
--LGNLKL
--LGNLIY
--LGNLHQ
--LGNLAN
--LGNLQN
--LGNLQF
--LGNLID
--LGNLHH
--LGNLET
--LGNLAY
--LGNLWH
--LGNLTQ
--LGNLQL
--LGNLQK
--LGNLQI
--LGNLQD
--LGNLNV
--LGNLLD
--LGNLKI
--LGNLHN
--LGNLAH
--LGNLYW
--LGNLVD
--LGNLQY
--LGNLQQ
--LGNLNF
--LGNLIF
--LGNLGV
--LGNLYD
--LGNLWW
--LGNLVE
--LGNLTE
--LGNLTD

--LGNLQR
--LGNLNW
--LGNLME
--LGNLKD
--LGNLIE
--LGNLHW
--LGNLHI
--LGNLGG
--LGNLEW
--LGNLEG
--LGNLWC
--LGNLIP
--LGNLEQ
--LGNLEH
--LGNLCN
MSLGNL--
MKLGNL--
-MLGNLR-
-MLGNLT-
MTLGNL--
MNLGNL--
MMLGNL--
-MLGNLV-
-MLGNLM-
-MLGNLH-
MYLGNL--
-MLGNLA-
-MLGNLS-
-MLGNLN-
MPLGNL--
MFLGNL--
-MLGNLK-
MDLGNL--
-MLGNLE-
MELGNL--
MQLGNL--
-MLGNLQ-
-MLGNLF-
-NLGNLL-
-NLGNLR-
NRLGNL--
NSLGNL--
NLLGNL--
-NLGNLS-
NILGNL--
NNLGNL--
NTLGNL--
NYLGNL--
NMLGNL--
NKLGNL--

NGLGNL--
NPLGNL--
-NLGNLH-
NQLGNL--
NELGNL--
-NLGNLI-
NFLGNL--
-NLGNLF-
NVLGNL--
-NLGNLQ-
-NLGNLA-
NWLGNL--
-NLGNLT-
-NLGNLK-
NHLGNL--
-NLGNLY-
-NLGNLE-
-NLGNLG-
-NLGNLM-
NCLGNL--
PVLGNL--
-PLGNLS-
PRLGNL--
PALGNL--
-PLGNLL-
-PLGNLW-
PSLGNL--
-PLGNLQ-
-PLGNLK-
PDLGNL--
-PLGNLI-
-PLGNLT-
-PLGNLF-
-PLGNLA-
PTLGNL--
-PLGNLG-
PGLGNL--
-PLGNLN-
PHLGNL--
PYLGNL--
PNLGNL--
-PLGNLH-
PWLGNL--
-PLGNLY-
-PLGNLE-
PILGNL--
PFLGNL--
-PLGNLM-
PQLGNL--
PELGNL--

PCLGNL--
QRLGNL--
QKLGNL--
QSLGNL--
-QLGNLL-
-QLGNLS-
-QLGNLR-
QTLGNL--
-QLGNLV-
QILGNL--
QGLGNL--
-QLGNLK-
-QLGNLI-
QYLGNL--
QELGNL--
QWLGNL--
QPLGNL--
QMLGNL--
QHLGNL--
-QLGNLH-
QALGNL--
QFLGNL--
QDLGNL--
-QLGNLE-
QCLGNL--
QQLGNL--
QNLGNL--
-QLGNLQ-
-QLGNLM-
-QLGNLG-
-QLGNLW-
-QLGNLN-
-QLGNLF-
-RLGNLL-
RWLGNL--
-RLGNLS-
RTLGNL--
RNLGNL--
-RLGNLT-
-RLGNLQ-
-RLGNLF-
RPLGNL--
RYLGNL--
-RLGNLI-
RQLGNL--
RMLGNL--
-RLGNLK-
RDLGNL--
-RLGNLE-
RCLGNL--

-SLGNLL-
-SLGNLS-
STLGNL--
SALGNL--
-SLGNLR-
SLLGNL--
SGLGNL--
-SLGNLT-
SSLGNL--
-SLGNLV-
SMLGNL--
-SLGNLI-
SNLGNL--
SFLGNL--
SPLGNL--
SQLGNL--
-SLGNLA-
-SLGNLQ-
SWLGNL--
SILGNL--
SHLGNL--
-SLGNLN-
-SLGNLH-
-SLGNLK-
SDLGNL--
SELGNL--
SCLGNL--
-SLGNLE-
TRLGNL--
-TLGNLR-
-TLGNLL-
-TLGNLS-
TLLGNL--
-TLGNLI-
TSLGNL--
TGLGNL--
-TLGNLT-
-TLGNLY-
TVLGNL--
TMLGNL--
TTLGNL--
-TLGNLF-
-TLGNLK-
-TLGNLM-
-TLGNLW-
-TLGNLQ-
TYLGNL--
TFLGNL--
-TLGNLH-
TELGNL--

TQLGNL--
TDLGNL--
THLGNL--
TPLGNL--
-TLGNLG-
TCLGNL--
-VLGNLR-
VSLGNL--
VGLGNL--
VNLGNL--
-VLGNLS-
-VLGNLK-
VHLGNL--
-VLGNLA-
-VLGNLV-
-VLGNLQ-
VYLGNL--
VMLGNL--
VILGNL--
-VLGNLM-
VWLGNL--
VELGNL--
-VLGNLN-
-VLGNLH-
VDLGNL--
-VLGNLF-
-VLGNLE-
VQLGNL--
VFLGNL--
VCLGNL--
WRLGNL--
WALGNL--
WGLGNL--
-WLGNLV-
-WLGNLN-
-WLGNLL-
-WLGNLQ-
WQLGNL--
WDLGNL--
WKLGNL--
-WLGNLI-
WYLGNL--
-WLGNLT-
-WLGNLK-
-WLGNLH-
WILGNL--
WPLGNL--
-WLGNLW-
-WLGNLG-
-WLGNLM-

-WLGNLE-
WCLGNL--
-YLGNLS-
-YLGNLR-
-YLGNLT-
-YLGNLV-
YGLGNL--
-YLGNLY-
-YLGNLK-
-YLGNLG-
-YLGNLA-
YHLGNL--
-YLGNLL-
YALGNL--
YTLGNL--
YSLGNL--
-YLGNLN-
YKLGNL--
YVLGNL--
YQLGNL--
YPLGNL--
-YLGNLF-
YDLGNL--
-YLGNLI-
YFLGNL--
-YLGNLM-
-YLGNLH-
YELGNL--
YMLGNL--
-YLGNLQ-
YYLGNL--
-YLGNLE-
YCLGNL--

| | | | |
|---|---|---|---|
| PSAW | 69 | 790 | 33.33 |

AYPSAW--
ARPSAW--
-APSAWN-
AHPSAW--
-APSAWT-
-APSAWI-
AFPSAW--
AKPSAW--
ATPSAW--
-APSAWY-
AAPSAW--
ASPSAW--
-APSAWR-
-APSAWL-
-APSAWK-
-APSAWF-

ALPSAW--
AGPSAW--
-APSAWW-
-APSAWS-
AQPSAW--
-APSAWH-
AWPSAW--
-APSAWG-
-APSAWQ-
-APSAWM-
ANPSAW--
AIPSAW--
AEPSAW--
ACPSAW--
-CPSAWV-
-CPSAWT-
-CPSAWI-
CSPSAW--
-CPSAWL-
CHPSAW--
CEPSAW--
CRPSAW--
-CPSAWY-
-CPSAWS-
-CPSAWM-
CLPSAW--
CFPSAW--
CDPSAW--
DLPSAW--
DAPSAW--
-DPSAWL-
-DPSAWV-
DWPSAW--
DSPSAW--
DTPSAW--
DRPSAW--
-DPSAWW-
-DPSAWS-
-DPSAWI-
DHPSAW--
-DPSAWT-
-DPSAWR-
-DPSAWH-
DMPSAW--
DKPSAW--
DIPSAW--
DEPSAW--
DQPSAW--
-DPSAWQ-
-DPSAWM-

```
-DPSAWF-
-DPSAWE-
-DPSAWA-
DNPSAW--
DGPSAW--
DFPSAW--
-EPSAWL-
EKPSAW--
ESPSAW--
-EPSAWR-
-EPSAWV-
ELPSAW--
ERPSAW--
-EPSAWW-
-EPSAWH-
EFPSAW--
EYPSAW--
EVPSAW--
ETPSAW--
-EPSAWT-
EMPSAW--
EWPSAW--
-EPSAWS-
-EPSAWK-
-EPSAWG-
-EPSAWE-
ENPSAW--
EIPSAW--
EHPSAW--
EGPSAW--
EEPSAW--
EAPSAW--
-FPSAWT-
FNPSAW--
FIPSAW--
FGPSAW--
FTPSAW--
-FPSAWI-
-FPSAWW-
-FPSAWQ-
-FPSAWM-
-FPSAWR-
FMPSAW--
-FPSAWS-
-FPSAWL-
-FPSAWF-
FVPSAW--
-FPSAWK-
FKPSAW--
FAPSAW--
```

FYPSAW--
-FPSAWY-
-FPSAWV-
-FPSAWN-
-FPSAWH-
-FPSAWA-
FPPSAW--
FLPSAW--
FHPSAW--
FFPSAW--
-GPSAWW-
GHPSAW--
-GPSAWL-
GAPSAW--
GVPSAW--
-GPSAWA-
GSPSAW--
-GPSAWS-
GTPSAW--
-GPSAWI-
GNPSAW--
GRPSAW--
GQPSAW--
-GPSAWV-
-GPSAWN-
GKPSAW--
-GPSAWM-
-GPSAWH-
-GPSAWF-
-GPSAWQ-
-GPSAWK-
GGPSAW--
GWPSAW--
-GPSAWT-
-GPSAWR-
GYPSAW--
-GPSAWP-
-GPSAWE-
-GPSAWC-
GPPSAW--
GIPSAW--
GFPSAW--
-HPSAWS-
-HPSAWI-
HLPSAW--
-HPSAWR-
HYPSAW--
HRPSAW--
-HPSAWN-
-HPSAWL-

HVPSAW--
-HPSAWW-
HIPSAW--
HWPSAW--
HTPSAW--
HKPSAW--
HGPSAW--
-HPSAWY-
-HPSAWT-
HHPSAW--
HQPSAW--
-HPSAWV-
-HPSAWM-
-HPSAWK-
-HPSAWD-
HPPSAW--
HDPSAW--
HCPSAW--
HAPSAW--
-IPSAWT-
-IPSAWS-
ISPSAW--
-IPSAWG-
ITPSAW--
-IPSAWN-
IIPSAW--
IDPSAW--
-IPSAWL-
-IPSAWH-
-IPSAWF-
IVPSAW--
-IPSAWA-
IAPSAW--
IEPSAW--
-IPSAWR-
-IPSAWQ-
INPSAW--
IWPSAW--
-IPSAWV-
IGPSAW--
IFPSAW--
IQPSAW--
-IPSAWK-
KSPSAW--
-KPSAWR-
KTPSAW--
KGPSAW--
-KPSAWL-
KLPSAW--
-KPSAWV-

-KPSAWN-
KFPSAW--
KHPSAW--
-KPSAWI-
-KPSAWH-
-KPSAWS-
-KPSAWE-
KYPSAW--
KMPSAW--
KAPSAW--
-KPSAWQ-
-KPSAWM-
-KPSAWK-
-KPSAWG-
-KPSAWA-
KKPSAW--
KIPSAW--
KRPSAW--
KCPSAW--
KWPSAW--
KQPSAW--
-KPSAWW-
-KPSAWT-
-KPSAWF-
KEPSAW--
KVPSAW--
KPPSAW--
KNPSAW--
KDPSAW--
LGPSAW--
-LPSAWR-
LKPSAW--
-LPSAWL-
LRPSAW--
LVPSAW--
LPPSAW--
LNPSAW--
-LPSAWT-
LTPSAW--
LQPSAW--
-LPSAWV-
LFPSAW--
LAPSAW--
-LPSAWH-
LMPSAW--
LLPSAW--
LHPSAW--
LEPSAW--
-LPSAWS-
-LPSAWW-

-LPSAWI-
-LPSAWA-
LYPSAW--
LWPSAW--
-LPSAWY-
-LPSAWN-
-LPSAWM-
-LPSAWK-
LDPSAW--
-LPSAWF-
MRPSAW--
-MPSAWL-
-MPSAWR-
MNPSAW--
-MPSAWK-
MTPSAW--
MPPSAW--
MHPSAW--
MSPSAW--
-MPSAWT-
MKPSAW--
MVPSAW--
-MPSAWY-
-MPSAWV-
-MPSAWN-
-MPSAWE-
MEPSAW--
-MPSAWS-
-MPSAWM-
-MPSAWI-
-MPSAWH-
-MPSAWF-
MGPSAW--
MFPSAW--
MAPSAW--
MWPSAW--
-MPSAWW-
-MPSAWQ-
-MPSAWG-
MMPSAW--
MIPSAW--
-NPSAWT-
-NPSAWR-
-NPSAWK-
-NPSAWL-
NRPSAW--
-NPSAWI-
NSPSAW--
-NPSAWH-
NAPSAW--

```
NYPSAW--
-NPSAWY-
NTPSAW--
-NPSAWF-
NNPSAW--
NMPSAW--
NIPSAW--
NQPSAW--
NDPSAW--
NWPSAW--
-NPSAWS-
NFPSAW--
-NPSAWM-
-NPSAWA-
NPPSAW--
-NPSAWW-
-NPSAWV-
-NPSAWN-
-NPSAWE-
NHPSAW--
NGPSAW--
NCPSAW--
--PSAWVK
--PSAWRS
--PSAWSL
--PSAWAL
--PSAWYS
--PSAWVR
--PSAWLS
--PSAWLL
--PSAWRQ
--PSAWSS
--PSAWRL
--PSAWTT
--PSAWML
--PSAWKV
--PSAWHT
--PSAWLK
--PSAWKG
--PSAWTL
--PSAWIL
--PSAWHG
--PSAWSR
--PSAWSG
--PSAWFS
--PSAWFR
--PSAWFQ
--PSAWAS
--PSAWVG
--PSAWVA
```

--PSAWTG
--PSAWRT
--PSAWRH
--PSAWRG
--PSAWGS
--PSAWMV
--PSAWMG
--PSAWLH
--PSAWFG
--PSAWWS
--PSAWVI
--PSAWTY
--PSAWSK
--PSAWSF
--PSAWRA
--PSAWLN
--PSAWIT
--PSAWHR
--PSAWFT
--PSAWAW
--PSAWWF
--PSAWRN
--PSAWNA
--PSAWIQ
--PSAWFH
PLPSAW--
--PSAWWL
--PSAWVT
--PSAWVS
--PSAWTV
--PSAWSI
--PSAWNS
--PSAWMT
--PSAWKL
--PSAWHL
--PSAWAN
--PSAWYM
--PSAWTI
--PSAWTA
--PSAWRE
--PSAWME
--PSAWIV
--PSAWHN
--PSAWGL
--PSAWFL
--PSAWAG
PAPSAW--
--PSAWYQ
--PSAWWM
--PSAWTF

--PSAWMK
--PSAWLY
--PSAWFN
--PSAWYT
--PSAWYG
--PSAWTQ
--PSAWSV
--PSAWSM
--PSAWRF
--PSAWNG
--PSAWMR
--PSAWKS
--PSAWKK
--PSAWID
--PSAWGT
--PSAWGA
--PSAWVV
--PSAWVF
--PSAWVD
--PSAWTS
--PSAWTH
--PSAWSQ
--PSAWRD
--PSAWQT
--PSAWQS
--PSAWQN
--PSAWQK
--PSAWQG
--PSAWNF
--PSAWMS
--PSAWMN
--PSAWKN
--PSAWIG
--PSAWHY
--PSAWFE
--PSAWEA
PWPSAW--
--PSAWYK
--PSAWWA
--PSAWVY
--PSAWVE
--PSAWTW
--PSAWTN
--PSAWRM
--PSAWNT
--PSAWNL
--PSAWMM
--PSAWMI
--PSAWMF
--PSAWKW

--PSAWKI
--PSAWKF
--PSAWFK
--PSAWAY
PKPSAW--
--PSAWYR
--PSAWYA
--PSAWWI
--PSAWVN
--PSAWTD
--PSAWQL
--PSAWPY
--PSAWNV
--PSAWNQ
--PSAWNN
--PSAWMY
--PSAWMH
--PSAWLQ
--PSAWKD
--PSAWIN
--PSAWIH
--PSAWIA
--PSAWHV
--PSAWHQ
--PSAWHM
--PSAWGM
--PSAWEG
--PSAWAQ
--PSAWAI
--PSAWAA
PYPSAW--
PVPSAW--
PSPSAW--
--PSAWYD
--PSAWWV
--PSAWWG
--PSAWVQ
--PSAWTK
--PSAWSN
--PSAWQV
--PSAWPI
--PSAWNW
--PSAWNK
--PSAWNI
--PSAWLM
--PSAWKE
--PSAWKA
--PSAWIS
--PSAWIK
--PSAWHS

--PSAWGW
--PSAWGV
--PSAWGI
--PSAWCV
--PSAWAT
--PSAWAM
--PSAWAH
--PSAWAF
PGPSAW--
PTPSAW--
--PSAWYY
--PSAWYN
--PSAWYH
--PSAWWY
--PSAWWH
--PSAWWE
--PSAWVH
--PSAWSH
--PSAWRP
--PSAWQR
--PSAWQQ
--PSAWQI
--PSAWQA
--PSAWNY
--PSAWNM
--PSAWNH
--PSAWNE
--PSAWMW
--PSAWMD
--PSAWLV
--PSAWLE
--PSAWLD
--PSAWLA
--PSAWKT
--PSAWIE
--PSAWHW
--PSAWHK
--PSAWHI
--PSAWHH
--PSAWHF
--PSAWGG
--PSAWGF
--PSAWFA
--PSAWEV
--PSAWET
--PSAWER
--PSAWDT
--PSAWDA
--PSAWCR
--PSAWCN

```
--PSAWCG
--PSAWAV
--PSAWAK
PQPSAW--
-PPSAWF-
-PPSAWA-
PNPSAW--
PHPSAW--
PDPSAW--
QRPSAW--
-QPSAWS-
QSPSAW--
-QPSAWA-
QYPSAW--
-QPSAWL-
-QPSAWI-
-QPSAWR-
-QPSAWM-
-QPSAWN-
-QPSAWK-
QKPSAW--
-QPSAWV-
QAPSAW--
QWPSAW--
QTPSAW--
-QPSAWY-
-QPSAWH-
-QPSAWG-
QLPSAW--
QHPSAW--
QDPSAW--
QVPSAW--
QQPSAW--
-QPSAWQ-
-QPSAWF-
-QPSAWE-
QMPSAW--
QIPSAW--
QGPSAW--
QFPSAW--
RSPSAW--
-RPSAWR-
RVPSAW--
RQPSAW--
-RPSAWA-
RLPSAW--
RYPSAW--
-RPSAWT-
RGPSAW--
-RPSAWQ-
```

-RPSAWN-
-RPSAWV-
RTPSAW--
RNPSAW--
RPPSAW--
RMPSAW--
-RPSAWK-
-RPSAWG-
RIPSAW--
-RPSAWW-
RFPSAW--
REPSAW--
-RPSAWF-
-RPSAWE-
RKPSAW--
RCPSAW--
-RPSAWY-
RHPSAW--
RDPSAW--
SRPSAW--
SSPSAW--
-SPSAWS-
-SPSAWR-
SKPSAW--
-SPSAWV-
-SPSAWT-
-SPSAWI-
SWPSAW--
-SPSAWM-
-SPSAWH-
-SPSAWA-
SLPSAW--
SVPSAW--
-SPSAWL-
STPSAW--
SIPSAW--
SHPSAW--
SGPSAW--
SEPSAW--
-SPSAWW-
-SPSAWF-
SMPSAW--
SYPSAW--
-SPSAWY-
-SPSAWG-
SPPSAW--
SAPSAW--
-SPSAWN-
SNPSAW--
SQPSAW--

-SPSAWE-
SFPSAW--
SDPSAW--
SCPSAW--
-SPSAWK-
-TPSAWR-
-TPSAWL-
TSPSAW--
-TPSAWH-
TVPSAW--
TRPSAW--
-TPSAWW-
-TPSAWK-
TAPSAW--
TDPSAW--
-TPSAWI-
TIPSAW--
TQPSAW--
-TPSAWN-
THPSAW--
-TPSAWV-
-TPSAWG-
TLPSAW--
TTPSAW--
-TPSAWS-
-TPSAWA-
TPPSAW--
TNPSAW--
TGPSAW--
TYPSAW--
TWPSAW--
-TPSAWY-
-TPSAWQ-
-TPSAWM-
-TPSAWF-
TMPSAW--
TKPSAW--
-TPSAWT-
TFPSAW--
TCPSAW--
-VPSAWR-
-VPSAWS-
VNPSAW--
VVPSAW--
-VPSAWM-
VIPSAW--
VTPSAW--
-VPSAWI-
VMPSAW--
-VPSAWT-

VFPSAW--
-VPSAWY-
-VPSAWA-
VWPSAW--
VSPSAW--
VRPSAW--
-VPSAWV-
-VPSAWK-
-VPSAWH-
VLPSAW--
-VPSAWG-
VEPSAW--
VDPSAW--
VYPSAW--
-VPSAWQ-
VPPSAW--
VAPSAW--
VQPSAW--
-VPSAWF-
WGPSAW--
WKPSAW--
WPPSAW--
WLPSAW--
WVPSAW--
WQPSAW--
-WPSAWI-
WDPSAW--
-WPSAWL-
-WPSAWH-
WNPSAW--
WIPSAW--
WAPSAW--
-WPSAWS-
-WPSAWM-
-WPSAWA-
WHPSAW--
WEPSAW--
WYPSAW--
-WPSAWY-
-WPSAWQ-
-WPSAWN-
-YPSAWA-
YRPSAW--
-YPSAWL-
-YPSAWV-
YYPSAW--
YVPSAW--
-YPSAWT-
-YPSAWS-
YKPSAW--

```
          YSPSAW--
          YPPSAW--
          YIPSAW--
          YHPSAW--
          YTPSAW--
          -YPSAWW-
          YNPSAW--
          YWPSAW--
          -YPSAWR-
          YLPSAW--
          YGPSAW--
          -YPSAWM-
          -YPSAWK-
          YMPSAW--
          YEPSAW--
          YCPSAW--
          YAPSAW--
          YQPSAW--
          -YPSAWN-
          -YPSAWH-
          -YPSAWG-
          -YPSAWF-
          YFPSAW--
          YDPSAW--
PAGI        51        803      33.32
          -GPAGIT-
          -GPAGIN-
          -MPAGIL-
          --PAGIAA
          SEPAGI--
          -APAGIF-
          -APAGIG-
          -APAGIS-
          ASPAGI--
          ARPAGI--
          -APAGIL-
          -APAGIA-
          ALPAGI--
          -APAGIH-
          -APAGIT-
          -APAGIN-
          -APAGIM-
          -APAGII-
          AFPAGI--
          AQPAGI--
          AKPAGI--
          AAPAGI--
          AYPAGI--
          ATPAGI--
          -APAGIY-
```

```
-APAGIV-
AGPAGI--
AEPAGI--
-APAGIW-
-APAGIR-
-APAGIK-
ANPAGI--
AMPAGI--
AIPAGI--
CMPAGI--
CIPAGI--
CLPAGI--
CHPAGI--
CPPAGI--
-CPAGIY-
-CPAGIN-
-CPAGIK-
-CPAGIF-
CNPAGI--
CFPAGI--
DSPAGI--
-DPAGIR-
DVPAGI--
-DPAGIV-
DMPAGI--
DDPAGI--
DTPAGI--
DRPAGI--
-DPAGIG-
-DPAGIC-
DNPAGI--
DLPAGI--
DFPAGI--
-DPAGIW-
-DPAGIS-
-DPAGIQ-
-DPAGIN-
-DPAGIM-
-DPAGIH-
-DPAGIF-
-DPAGIA-
DKPAGI--
DIPAGI--
DHPAGI--
DGPAGI--
DEPAGI--
DAPAGI--
-EPAGIQ-
-EPAGIR-
EVPAGI--
```

−EPAGIV−
−EPAGIS−
ETPAGI−−
−EPAGIY−
EAPAGI−−
−EPAGIH−
−EPAGIT−
ERPAGI−−
−EPAGIL−
−EPAGIK−
−EPAGIF−
EGPAGI−−
EWPAGI−−
EQPAGI−−
−EPAGIW−
−EPAGIN−
ENPAGI−−
EIPAGI−−
−FPAGIS−
FRPAGI−−
−FPAGIT−
−FPAGIE−
FFPAGI−−
FSPAGI−−
FGPAGI−−
FKPAGI−−
FVPAGI−−
−FPAGIY−
−FPAGIR−
FLPAGI−−
FMPAGI−−
−FPAGIL−
−FPAGIH−
−FPAGIQ−
−FPAGIK−
−FPAGII−
−FPAGIF−
FIPAGI−−
FHPAGI−−
FAPAGI−−
FWPAGI−−
−FPAGIN−
FEPAGI−−
FYPAGI−−
−FPAGIV−
−FPAGIM−
FNPAGI−−
−GPAGIK−
−GPAGII−
−GPAGIF−

GKPAGI--
GSPAGI--
-GPAGIL-
-GPAGIA-
-GPAGIH-
GLPAGI--
-GPAGIR-
GEPAGI--
-GPAGIG-
GWPAGI--
GTPAGI--
GQPAGI--
-GPAGIV-
-GPAGIM-
GIPAGI--
GHPAGI--
GGPAGI--
GPPAGI--
-GPAGIS-
GAPAGI--
-GPAGIQ-
-GPAGIE-
-GPAGIC-
GDPAGI--
-HPAGIS-
HRPAGI--
-HPAGIA-
HFPAGI--
HLPAGI--
-HPAGIL-
HIPAGI--
HQPAGI--
-HPAGIY-
HSPAGI--
HYPAGI--
-HPAGII-
HGPAGI--
-HPAGIW-
HNPAGI--
HPPAGI--
-HPAGIG-
-HPAGIR-
-HPAGIQ-
-HPAGIE-
HMPAGI--
HKPAGI--
HWPAGI--
HVPAGI--
HTPAGI--
-HPAGIV-

-HPAGIT-
-HPAGIN-
-HPAGIK-
-HPAGIF-
HAPAGI--
-IPAGIR-
IRPAGI--
IIPAGI--
ILPAGI--
-IPAGIS-
-IPAGIY-
-IPAGIW-
-IPAGIA-
-IPAGIT-
INPAGI--
IAPAGI--
ITPAGI--
IQPAGI--
IVPAGI--
-IPAGIL-
-IPAGIH-
-IPAGIG-
-IPAGIE-
IFPAGI--
IYPAGI--
IWPAGI--
ISPAGI--
-IPAGII-
IMPAGI--
IPPAGI--
-IPAGIN-
-IPAGIK-
-IPAGIF-
IHPAGI--
IGPAGI--
IEPAGI--
IDPAGI--
ICPAGI--
KSPAGI--
-KPAGIR-
-KPAGIV-
KVPAGI--
-KPAGIT-
-KPAGIY-
KQPAGI--
-KPAGIF-
KRPAGI--
-KPAGIG-
KGPAGI--
-KPAGIN-

-KPAGIK-
-KPAGIA-
KAPAGI--
KTPAGI--
-KPAGIS-
KPPAGI--
KFPAGI--
-KPAGID-
KKPAGI--
KDPAGI--
KYPAGI--
-KPAGIW-
-KPAGIQ-
-KPAGIH-
KLPAGI--
KWPAGI--
-KPAGIM-
-KPAGII-
KMPAGI--
KIPAGI--
KCPAGI--
-LPAGIL-
LRPAGI--
LWPAGI--
-LPAGIR-
LLPAGI--
LVPAGI--
LTPAGI--
-LPAGIY-
LAPAGI--
-LPAGIS-
-LPAGIA-
LNPAGI--
LHPAGI--
-LPAGIW-
LMPAGI--
-LPAGIT-
-LPAGIN-
LKPAGI--
-LPAGIK-
LYPAGI--
LEPAGI--
LQPAGI--
-LPAGIQ-
LPPAGI--
-LPAGIM-
-LPAGIF-
LCPAGI--
-LPAGIV-
-LPAGII-

```
-LPAGIH-
-LPAGIG-
-LPAGIE-
LIPAGI--
LDPAGI--
LFPAGI--
MRPAGI--
MFPAGI--
-MPAGII-
-MPAGIH-
MGPAGI--
-MPAGIR-
MMPAGI--
MLPAGI--
MEPAGI--
MTPAGI--
MSPAGI--
-MPAGIY-
-MPAGIT-
-MPAGIN-
-MPAGIK-
MAPAGI--
MYPAGI--
MPPAGI--
-MPAGIS-
-MPAGIQ-
MIPAGI--
MHPAGI--
MCPAGI--
MVPAGI--
MQPAGI--
-MPAGIW-
-MPAGIV-
-MPAGIF-
MKPAGI--
-NPAGIS-
NRPAGI--
-NPAGIW-
NVPAGI--
-NPAGIT-
-NPAGIR-
NEPAGI--
NAPAGI--
-NPAGIL-
-NPAGIA-
NHPAGI--
NSPAGI--
-NPAGIH-
NWPAGI--
NTPAGI--
```

-NPAGIF-
-NPAGIQ-
-NPAGIN-
-NPAGIK-
NLPAGI--
NQPAGI--
NPPAGI--
-NPAGIV-
-NPAGII-
-NPAGIG-
NNPAGI--
NMPAGI--
NKPAGI--
NIPAGI--
NFPAGI--
--PAGILK
--PAGIRL
--PAGIFR
--PAGILG
--PAGIAR
--PAGISL
--PAGIYS
--PAGISS
--PAGIRS
--PAGIVR
--PAGIMR
--PAGISG
--PAGISA
--PAGIRK
--PAGIRA
--PAGILL
--PAGIVG
--PAGILT
--PAGIHF
--PAGIGL
--PAGITI
--PAGISK
--PAGIRH
--PAGIRY
--PAGIRG
--PAGINS
--PAGIMY
--PAGISM
--PAGIMG
--PAGILS
--PAGIGS
--PAGIEK
--PAGIVQ
--PAGIRT
--PAGILN

--PAGILH
--PAGIKR
--PAGIIL
PNPAGI--
--PAGIVH
--PAGITL
--PAGILY
--PAGIKY
--PAGIKM
--PAGIKL
PMPAGI--
PGPAGI--
--PAGIYT
--PAGIYN
--PAGIWT
--PAGITT
--PAGITK
--PAGITG
--PAGISN
--PAGISI
--PAGIRM
--PAGINM
--PAGIAW
--PAGIWK
--PAGIVW
--PAGIVD
--PAGISV
--PAGISQ
--PAGIRN
--PAGIMS
--PAGIMH
--PAGILW
--PAGILF
--PAGILD
--PAGILA
--PAGIKG
--PAGIHW
--PAGIHS
--PAGIGT
--PAGIGA
--PAGIFA
--PAGIAQ
--PAGIAK
PLPAGI--
--PAGIYK
--PAGIWN
--PAGIVM
--PAGISR
--PAGINQ
--PAGIKT

--PAGIKS
--PAGIII
--PAGIHR
--PAGIGV
--PAGIGI
--PAGIGG
--PAGIAL
--PAGIAF
--PAGIYR
--PAGIYG
--PAGIYF
--PAGIYD
--PAGIWY
--PAGIWS
--PAGIVT
--PAGIVS
--PAGITN
--PAGITA
--PAGIST
--PAGIRQ
--PAGIQG
--PAGINV
--PAGINT
--PAGIMV
--PAGIMT
--PAGILQ
--PAGIKA
--PAGIIW
--PAGIIV
--PAGIGR
--PAGIGF
--PAGIFN
--PAGIAV
--PAGIAH
PYPAGI--
--PAGIYY
--PAGIVN
--PAGITR
--PAGISH
--PAGILM
--PAGIKW
--PAGIIR
--PAGIHH
--PAGIHE
--PAGIFT
--PAGIAT
PWPAGI--
PDPAGI--
--PAGIWD
--PAGIVA

--PAGITS
--PAGITM
--PAGISF
--PAGIRF
--PAGIQV
--PAGIQT
--PAGIQS
--PAGIQN
--PAGIQL
--PAGIQK
--PAGINY
--PAGINI
--PAGINF
--PAGIMN
--PAGIML
--PAGIKV
--PAGIKI
--PAGIIM
--PAGIIK
--PAGIIH
--PAGIIE
--PAGIID
--PAGIGK
--PAGIFS
--PAGIFM
--PAGIFF
--PAGIFD
--PAGIES
--PAGIAY
--PAGIAD
PVPAGI--
PSPAGI--
PFPAGI--
--PAGIYH
--PAGIYA
--PAGIWW
--PAGIVV
--PAGIVI
--PAGITV
--PAGITQ
--PAGITF
--PAGISY
--PAGISE
--PAGISD
--PAGIRP
--PAGIQI
--PAGINN
--PAGINL
--PAGINE
--PAGINA

--PAGIMW
--PAGIMQ
--PAGIMK
--PAGIMA
--PAGIKK
--PAGIIT
--PAGIIG
--PAGIIA
--PAGIHK
--PAGIHG
--PAGIEW
--PAGIEV
--PAGIEG
--PAGIDA
--PAGIAM
PTPAGI--
-PPAGIT-
-PPAGIH-
PKPAGI--
PHPAGI--
--PAGIYW
--PAGIYV
--PAGIYE
--PAGIVK
--PAGIVF
--PAGIVE
--PAGITY
--PAGITW
--PAGITH
--PAGITC
--PAGIQQ
--PAGIQP
--PAGIQH
--PAGINW
--PAGINK
--PAGINH
--PAGING
--PAGIND
--PAGIMM
--PAGIMI
--PAGIME
--PAGILC
--PAGIKN
--PAGIKH
--PAGIKE
--PAGIKD
--PAGIIQ
--PAGIIF
--PAGIIC
--PAGIHI

--PAGIGH
--PAGIFY
--PAGIFE
--PAGIET
--PAGIER
--PAGIEI
--PAGIEH
--PAGIEA
--PAGIDS
--PAGIDG
--PAGIAN
--PAGIAI
--PAGIAG
-QPAGIT-
QGPAGI--
-QPAGIA-
-QPAGIK-
QVPAGI--
QSPAGI--
QAPAGI--
QQPAGI--
QPPAGI--
-QPAGIV-
-QPAGIN-
QEPAGI--
-QPAGIY-
-QPAGIR-
-QPAGIM-
-QPAGII-
-QPAGIH-
-QPAGIG-
QMPAGI--
QHPAGI--
QRPAGI--
-QPAGIS-
-QPAGIQ-
-QPAGIF-
QNPAGI--
QKPAGI--
QIPAGI--
QFPAGI--
-RPAGIG-
-RPAGIR-
-RPAGIS-
-RPAGII-
RLPAGI--
-RPAGIV-
RGPAGI--
-RPAGIK-
RVPAGI--

RFPAGI--
RAPAGI--
RHPAGI--
RSPAGI--
-RPAGIH-
RPPAGI--
RIPAGI--
RYPAGI--
RWPAGI--
RNPAGI--
RMPAGI--
-RPAGIT-
RDPAGI--
-RPAGIW-
-RPAGIM-
-RPAGIA-
-RPAGIF-
-RPAGIE-
REPAGI--
-RPAGIQ-
-RPAGID-
-SPAGIT-
-SPAGIS-
-SPAGIR-
-SPAGIL-
STPAGI--
-SPAGIG-
SAPAGI--
SYPAGI--
SVPAGI--
-SPAGIY-
SNPAGI--
-SPAGII-
SHPAGI--
SLPAGI--
-SPAGIW-
-SPAGIN-
-SPAGIK-
SQPAGI--
-SPAGIM-
SGPAGI--
SCPAGI--
-SPAGIH-
-SPAGIF-
SMPAGI--
SIPAGI--
SFPAGI--
SPPAGI--
-SPAGID-
-SPAGIA-

SDPAGI--
TGPAGI--
-TPAGIR-
THPAGI--
-TPAGIM-
TVPAGI--
-TPAGIE-
TLPAGI--
-TPAGIT-
TRPAGI--
-TPAGIF-
TFPAGI--
TWPAGI--
-TPAGIN-
TKPAGI--
TIPAGI--
-TPAGIW-
-TPAGIG-
TAPAGI--
TYPAGI--
TTPAGI--
TPPAGI--
-TPAGII-
TMPAGI--
-TPAGIY-
-TPAGIS-
-TPAGIA-
-TPAGIV-
-TPAGIQ-
-TPAGIK-
TQPAGI--
-TPAGIH-
TNPAGI--
TDPAGI--
VRPAGI--
VLPAGI--
VYPAGI--
VTPAGI--
-VPAGIQ-
VSPAGI--
VWPAGI--
VMPAGI--
-VPAGIW-
VNPAGI--
VGPAGI--
-VPAGIY-
-VPAGIS-
-VPAGII-
VAPAGI--
VVPAGI--

-VPAGIR-
-VPAGIM-
-VPAGIT-
-VPAGIK-
-VPAGIF-
VIPAGI--
VHPAGI--
VQPAGI--
-VPAGIV-
-VPAGIH-
-VPAGIG-
-VPAGIE-
-VPAGIA-
VEPAGI--
-VPAGIN-
WLPAGI--
WMPAGI--
-WPAGIL-
WKPAGI--
WSPAGI--
-WPAGIS-
-WPAGIA-
WIPAGI--
WEPAGI--
WYPAGI--
WFPAGI--
WVPAGI--
WRPAGI--
-WPAGIH-
-WPAGIE-
WAPAGI--
WTPAGI--
WPPAGI--
-WPAGIY-
-WPAGIM-
-WPAGIF-
WNPAGI--
WGPAGI--
WQPAGI--
-WPAGIT-
-WPAGIR-
-WPAGIN-
-WPAGII-
WWPAGI--
-WPAGIK-
-WPAGIG-
WDPAGI--
WCPAGI--
YLPAGI--
YRPAGI--

```
          -YPAGIN-
          -YPAGIR-
          YEPAGI--
          -YPAGIQ-
          -YPAGIL-
          YYPAGI--
          YSPAGI--
          -YPAGIS-
          YPPAGI--
          -YPAGIF-
          -YPAGIA-
          YIPAGI--
          YAPAGI--
          -YPAGIW-
          -YPAGIE-
          YMPAGI--
          YDPAGI--
          YTPAGI--
          -YPAGIK-
          -YPAGIH-
          YGPAGI--
          YFPAGI--
          YWPAGI--
          YVPAGI--
          -YPAGIY-
          -YPAGIV-
          -YPAGIT-
          YHPAGI--
PAAM       268       684      33.31
          -APAAMK-
          ATPAAM--
          ASPAAM--
          ARPAAM--
          -APAAMM-
          -APAAML-
          AYPAAM--
          -APAAMG-
          ALPAAM--
          AIPAAM--
          -APAAMT-
          -APAAMA-
          AMPAAM--
          -APAAMH-
          AKPAAM--
          AWPAAM--
          -APAAMW-
          -APAAMV-
          -APAAMS-
          -APAAMN-
          -APAAMI-
```

-APAAME-
APPAAM--
-APAAMF-
-APAAMD-
AHPAAM--
AFPAAM--
AAPAAM--
-CPAAMS-
-CPAAMK-
CSPAAM--
-CPAAMY-
-DPAAMI-
DRPAAM--
DVPAAM--
-DPAAMQ-
DNPAAM--
-DPAAMY-
-DPAAMR-
-DPAAMG-
-DPAAMD-
DLPAAM--
DGPAAM--
DFPAAM--
-EPAAMI-
ERPAAM--
-EPAAML-
EIPAAM--
-EPAAMR-
-EPAAMS-
-EPAAMA-
EMPAAM--
EGPAAM--
EWPAAM--
ESPAAM--
-EPAAMW-
-EPAAMK-
-EPAAMH-
EKPAAM--
EYPAAM--
EVPAAM--
ETPAAM--
-EPAAMV-
-EPAAMT-
ENPAAM--
EHPAAM--
EFPAAM--
-FPAAMG-
-FPAAMR-
FSPAAM--
-FPAAMV-

FNPAAM--
FKPAAM--
FFPAAM--
-FPAAMT-
-FPAAMN-
-FPAAMF-
-FPAAMA-
FHPAAM--
FRPAAM--
-FPAAMS-
-FPAAMM-
-FPAAML-
-FPAAMI-
-FPAAMH-
GSPAAM--
-GPAAMA-
GLPAAM--
-GPAAML-
-GPAAMS-
GTPAAM--
GVPAAM--
GRPAAM--
-GPAAMM-
GKPAAM--
GGPAAM--
-GPAAMN-
-GPAAMK-
-GPAAMI-
-GPAAMG-
-GPAAME-
GFPAAM--
GYPAAM--
GMPAAM--
GIPAAM--
GEPAAM--
GAPAAM--
GWPAAM--
GQPAAM--
GPPAAM--
-GPAAMV-
-GPAAMR-
-GPAAMH-
-GPAAMF-
-GPAAMD-
GNPAAM--
-HPAAMR-
-HPAAMQ-
HRPAAM--
-HPAAMS-
-HPAAML-

-HPAAMK-
-HPAAMA-
-HPAAMG-
HPPAAM--
-HPAAMH-
HAPAAM--
HYPAAM--
HTPAAM--
HSPAAM--
-HPAAMY-
-HPAAMM-
-HPAAMI-
HNPAAM--
HLPAAM--
HEPAAM--
-IPAAMG-
-IPAAMR-
-IPAAMM-
-IPAAML-
-IPAAMI-
ILPAAM--
-IPAAMY-
-IPAAMS-
IFPAAM--
IWPAAM--
-IPAAMH-
-IPAAME-
IHPAAM--
ISPAAM--
IPPAAM--
-IPAAMT-
-IPAAMK-
-IPAAMF-
IGPAAM--
-KPAAMR-
KSPAAM--
-KPAAMN-
KLPAAM--
-KPAAMS-
-KPAAMT-
KYPAAM--
-KPAAML-
KGPAAM--
KAPAAM--
-KPAAMY-
KPPAAM--
-KPAAMQ-
-KPAAMF-
-KPAAMA-
-KPAAMV-

-KPAAMH-
-KPAAMD-
KNPAAM--
KHPAAM--
KFPAAM--
KVPAAM--
KTPAAM--
KRPAAM--
-KPAAMW-
-KPAAMK-
-KPAAMI-
-KPAAME-
KKPAAM--
-LPAAMR-
LVPAAM--
-LPAAMS-
-LPAAML-
LSPAAM--
LTPAAM--
LRPAAM--
LLPAAM--
-LPAAMQ-
-LPAAMI-
LNPAAM--
LFPAAM--
-LPAAMW-
-LPAAMT-
LMPAAM--
LPPAAM--
LGPAAM--
LYPAAM--
-LPAAMY-
-LPAAMN-
-LPAAMA-
LKPAAM--
LAPAAM--
LQPAAM--
-LPAAMK-
-LPAAMH-
LHPAAM--
LEPAAM--
-MPAAML-
-MPAAMH-
MRPAAM--
-MPAAMT-
MHPAAM--
-MPAAMV-
-MPAAMQ-
-MPAAMS-
MVPAAM--

MSPAAM--
-MPAAMR-
-MPAAMA-
MNPAAM--
MFPAAM--
MQPAAM--
-MPAAMY-
-MPAAMW-
-MPAAMI-
-MPAAMF-
MDPAAM--
MAPAAM--
-NPAAMR-
-NPAAMM-
-NPAAMT-
-NPAAML-
NYPAAM--
-NPAAMA-
-NPAAMS-
-NPAAMY-
-NPAAMV-
NDPAAM--
NSPAAM--
-NPAAMI-
NLPAAM--
-NPAAMW-
-NPAAMN-
NNPAAM--
NIPAAM--
NVPAAM--
NTPAAM--
-NPAAMK-
-NPAAMH-
NKPAAM--
NCPAAM--
NAPAAM--
--PAAMLR
--PAAMLL
--PAAMAW
--PAAMVR
--PAAMRR
--PAAMRN
--PAAMAK
--PAAMVT
--PAAMWS
--PAAMWR
--PAAMRK
--PAAMRH
--PAAMKR
--PAAMGL

--PAAMTR
--PAAMRV
--PAAMPL
--PAAMLS
--PAAMKN
--PAAMKG
--PAAMHL
--PAAMGA
--PAAMFR
PTPAAM--
--PAAMVN
--PAAMRF
--PAAMRA
--PAAMNL
--PAAMKS
--PAAMAI
--PAAMWQ
--PAAMSG
--PAAMRQ
--PAAMQL
--PAAMMS
--PAAMLK
--PAAMFL
--PAAMVI
--PAAMTL
--PAAMRS
--PAAMRI
--PAAMQW
--PAAMLN
--PAAMGS
--PAAMAR
--PAAMYA
--PAAMWN
--PAAMVQ
--PAAMTF
--PAAMTD
--PAAMST
--PAAMSH
--PAAMSA
--PAAMQG
--PAAMLH
--PAAMIS
--PAAMIR
--PAAMHR
--PAAMFI
--PAAMFH
--PAAMYT
--PAAMYR
--PAAMYH
--PAAMVV

--PAAMVS
--PAAMTT
--PAAMSR
--PAAMSQ
--PAAMSL
--PAAMSE
--PAAMRW
--PAAMRG
--PAAMRD
--PAAMPY
--PAAMMY
--PAAMMN
--PAAMMA
--PAAMLG
--PAAMKL
--PAAMIT
--PAAMGI
--PAAMET
--PAAMEA
--PAAMAT
--PAAMAS
--PAAMAD
PRPAAM--
--PAAMYM
--PAAMYL
--PAAMWD
--PAAMVH
--PAAMTV
--PAAMTA
--PAAMSY
--PAAMRM
--PAAMQK
--PAAMQA
--PAAMPI
--PAAMNQ
--PAAMLV
--PAAMLA
--PAAMKQ
--PAAMIN
--PAAMIG
--PAAMHS
--PAAMGY
--PAAMGV
--PAAMFW
--PAAMFQ
--PAAMER
--PAAMDS
--PAAMAG
PNPAAM--
PKPAAM--

PHPAAM--
PCPAAM--
--PAAMYW
--PAAMYN
--PAAMWT
--PAAMWK
--PAAMVA
--PAAMTS
--PAAMSS
--PAAMSN
--PAAMRY
--PAAMQQ
--PAAMQN
--PAAMPM
--PAAMNR
--PAAMNG
--PAAMLQ
--PAAMLM
--PAAMLD
--PAAMKV
--PAAMKH
--PAAMIL
--PAAMHN
--PAAMFT
--PAAMFS
--PAAMFK
--PAAMFD
--PAAMEK
--PAAMEG
--PAAMDG
--PAAMAY
--PAAMAV
--PAAMAL
--PAAMAH
PWPAAM--
PMPAAM--
PFPAAM--
--PAAMYS
--PAAMYP
--PAAMYK
--PAAMWA
--PAAMTW
--PAAMTM
--PAAMSI
--PAAMRT
--PAAMRP
--PAAMRE
--PAAMQR
--PAAMQF
--PAAMNS

--PAAMMR
--PAAMMQ
--PAAMMK
--PAAMLW
--PAAMKY
--PAAMKW
--PAAMIY
--PAAMIW
--PAAMIM
--PAAMIA
--PAAMHK
--PAAMHI
--PAAMHG
--PAAMGW
--PAAMGT
--PAAMGR
--PAAMGQ
--PAAMGK
--PAAMGF
--PAAMFG
--PAAMEW
--PAAMDT
--PAAMAN
--PAAMAA
PLPAAM--
PAPAAM--
--PAAMYV
--PAAMYQ
--PAAMYE
--PAAMWW
--PAAMWH
--PAAMWE
--PAAMVM
--PAAMVG
--PAAMVD
--PAAMTY
--PAAMTG
--PAAMSW
--PAAMSV
--PAAMSF
--PAAMRC
--PAAMQS
--PAAMQH
--PAAMQE
--PAAMPF
--PAAMNY
--PAAMNM
--PAAMNI
--PAAMNH
--PAAMNA

--PAAMMV
--PAAMMD
--PAAMKT
--PAAMKM
--PAAMKK
--PAAMKI
--PAAMKE
--PAAMII
--PAAMIH
--PAAMIF
--PAAMID
--PAAMHW
--PAAMHV
--PAAMHP
--PAAMHH
--PAAMHD
--PAAMGM
--PAAMGG
--PAAMGE
--PAAMFV
--PAAMFM
--PAAMFC
--PAAMEY
--PAAMED
--PAAMDV
--PAAMDA
--PAAMCR
--PAAMCQ
--PAAMCG
--PAAMAE
-QPAAMK-
QFPAAM--
QTPAAM--
-QPAAMI-
QLPAAM--
QGPAAM--
QRPAAM--
-QPAAMV-
-QPAAMT-
-QPAAML-
-QPAAME-
QMPAAM--
-QPAAMY-
-QPAAMS-
-QPAAMG-
QIPAAM--
QVPAAM--
-QPAAMW-
-QPAAMH-
-QPAAMD-

-RPAAML-
-RPAAMR-
RYPAAM--
-RPAAMT-
-RPAAMV-
-RPAAMW-
RRPAAM--
-RPAAMG-
RGPAAM--
RLPAAM--
-RPAAMI-
-RPAAMH-
RWPAAM--
RVPAAM--
RSPAAM--
-RPAAMN-
-RPAAMK-
RKPAAM--
-RPAAMQ-
RIPAAM--
-RPAAMS-
-RPAAMD-
-RPAAMM-
-RPAAMF-
RMPAAM--
RAPAAM--
RTPAAM--
RPPAAM--
-RPAAMA-
RNPAAM--
RFPAAM--
RQPAAM--
-RPAAME-
-RPAAMC-
RHPAAM--
RDPAAM--
-SPAAMK-
-SPAAML-
-SPAAMG-
-SPAAMR-
SHPAAM--
SVPAAM--
-SPAAMV-
-SPAAMM-
SGPAAM--
-SPAAMS-
-SPAAMF-
-SPAAMA-
SLPAAM--
SKPAAM--

SAPAAM--
SRPAAM--
-SPAAMQ-
-SPAAMN-
SIPAAM--
SFPAAM--
STPAAM--
SSPAAM--
-SPAAMY-
-SPAAMT-
SNPAAM--
SCPAAM--
SYPAAM--
SQPAAM--
SPPAAM--
-SPAAMW-
-SPAAMI-
-SPAAMH-
-SPAAMD-
SMPAAM--
SDPAAM--
-TPAAML-
-TPAAMS-
TLPAAM--
TPPAAM--
TRPAAM--
-TPAAMT-
-TPAAMA-
TIPAAM--
-TPAAMW-
-TPAAMG-
-TPAAMQ-
-TPAAMI-
TVPAAM--
-TPAAMY-
-TPAAMV-
-TPAAMR-
TNPAAM--
TAPAAM--
TYPAAM--
TTPAAM--
TSPAAM--
TKPAAM--
-TPAAMN-
-VPAAML-
VFPAAM--
-VPAAMK-
-VPAAMS-
VIPAAM--
-VPAAMY-

−VPAAMA−
−VPAAMW−
VTPAAM−−
VRPAAM−−
−VPAAMT−
−VPAAMH−
VNPAAM−−
VHPAAM−−
VVPAAM−−
VPPAAM−−
−VPAAMI−
VYPAAM−−
VSPAAM−−
−VPAAMV−
VLPAAM−−
VKPAAM−−
VGPAAM−−
VWPAAM−−
−VPAAMN−
−VPAAMF−
−VPAAME−
VMPAAM−−
VDPAAM−−
VAPAAM−−
−WPAAML−
−WPAAMM−
−WPAAMS−
−WPAAMR−
−WPAAMI−
WKPAAM−−
−WPAAMQ−
−WPAAMN−
−WPAAMH−
WFPAAM−−
WAPAAM−−
WVPAAM−−
WRPAAM−−
WPPAAM−−
−WPAAMY−
−WPAAMV−
−WPAAMF−
−YPAAMR−
YAPAAM−−
−YPAAML−
−YPAAMN−
−YPAAMG−
YHPAAM−−
YTPAAM−−
YSPAAM−−
−YPAAMV−

| | | | |
|---|---|---|---|
| | -YPAAMS- | | |
| | -YPAAMM- | | |
| | -YPAAMT- | | |
| | -YPAAMH- | | |
| | YGPAAM-- | | |
| | YVPAAM-- | | |
| | -YPAAMK- | | |
| | -YPAAMI- | | |
| | -YPAAMF- | | |
| | -YPAAMA- | | |
| | YLPAAM-- | | |
| | YFPAAM-- | | |
| | YEPAAM-- | | |
| PQWL | 429 | 623 | 33.29 |
| | -APQWLL- | | |
| | AMPQWL-- | | |
| | -APQWLR- | | |
| | AGPQWL-- | | |
| | -APQWLS- | | |
| | -APQWLK- | | |
| | AVPQWL-- | | |
| | AQPQWL-- | | |
| | AWPQWL-- | | |
| | -APQWLG- | | |
| | AHPQWL-- | | |
| | ARPQWL-- | | |
| | -APQWLN- | | |
| | -APQWLE- | | |
| | -APQWLD- | | |
| | ANPQWL-- | | |
| | ALPQWL-- | | |
| | AIPQWL-- | | |
| | AAPQWL-- | | |
| | ATPQWL-- | | |
| | -APQWLQ- | | |
| | AKPQWL-- | | |
| | -APQWLV- | | |
| | APPQWL-- | | |
| | AEPQWL-- | | |
| | -APQWLT- | | |
| | ASPQWL-- | | |
| | -APQWLM- | | |
| | -APQWLA- | | |
| | CAPQWL-- | | |
| | CRPQWL-- | | |
| | CQPQWL-- | | |
| | -CPQWLR- | | |
| | CSPQWL-- | | |
| | -CPQWLI- | | |
| | CHPQWL-- | | |

DMPQWL--
DRPQWL--
-DPQWLS-
-DPQWLL-
DYPQWL--
-DPQWLH-
-DPQWLG-
DKPQWL--
DAPQWL--
-DPQWLA-
DNPQWL--
DEPQWL--
DSPQWL--
-DPQWLY-
-DPQWLT-
-DPQWLR-
-DPQWLN-
-DPQWLI-
-DPQWLF-
ELPQWL--
EVPQWL--
-EPQWLV-
ERPQWL--
-EPQWLR-
EKPQWL--
-EPQWLS-
-EPQWLG-
EQPQWL--
-EPQWLK-
-EPQWLH-
ENPQWL--
-EPQWLN-
EIPQWL--
-EPQWLM-
EWPQWL--
-EPQWLY-
FSPQWL--
FHPQWL--
FKPQWL--
-FPQWLS-
-FPQWLL-
-FPQWLA-
FRPQWL--
-FPQWLG-
FFPQWL--
FTPQWL--
-FPQWLT-
FVPQWL--
-FPQWLE-
FGPQWL--

FYPQWL--
-FPQWLQ-
-FPQWLM-
-FPQWLK-
FNPQWL--
GSPQWL--
-GPQWLT-
-GPQWLS-
GVPQWL--
GAPQWL--
-GPQWLM-
GMPQWL--
GLPQWL--
-GPQWLL-
-GPQWLI-
GPPQWL--
GWPQWL--
-GPQWLN-
GYPQWL--
GTPQWL--
GQPQWL--
-GPQWLG-
-GPQWLA-
-GPQWLK-
GNPQWL--
GKPQWL--
GFPQWL--
-GPQWLH-
-GPQWLD-
GEPQWL--
GDPQWL--
GRPQWL--
-GPQWLV-
-GPQWLE-
HQPQWL--
HHPQWL--
-HPQWLK-
HPPQWL--
HNPQWL--
HRPQWL--
-HPQWLH-
HFPQWL--
HYPQWL--
HSPQWL--
-HPQWLY-
-HPQWLR-
-HPQWLL-
HGPQWL--
HDPQWL--
HTPQWL--

```
-HPQWLV-
-HPQWLT-
-HPQWLQ-
-HPQWLG-
-HPQWLF-
HMPQWL--
HCPQWL--
-IPQWLR-
IVPQWL--
-IPQWLT-
IGPQWL--
ILPQWL--
ITPQWL--
IRPQWL--
-IPQWLA-
ISPQWL--
-IPQWLK-
IYPQWL--
IPPQWL--
IWPQWL--
-IPQWLV-
-IPQWLQ-
IHPQWL--
IDPQWL--
IEPQWL--
KSPQWL--
KVPQWL--
-KPQWLR-
KPPQWL--
KNPQWL--
KTPQWL--
-KPQWLS-
-KPQWLA-
KAPQWL--
-KPQWLI-
KHPQWL--
-KPQWLT-
-KPQWLN-
KLPQWL--
-KPQWLL-
-KPQWLG-
KMPQWL--
KWPQWL--
KQPQWL--
-KPQWLV-
-KPQWLH-
KIPQWL--
-KPQWLW-
-KPQWLM-
-KPQWLF-
```

LRPQWL--
-LPQWLT-
-LPQWLQ-
LSPQWL--
-LPQWLK-
LKPQWL--
-LPQWLR-
LTPQWL--
LYPQWL--
LDPQWL--
-LPQWLV-
-LPQWLL-
LNPQWL--
LLPQWL--
LGPQWL--
LQPQWL--
-LPQWLH-
-LPQWLA-
LPPQWL--
LMPQWL--
LVPQWL--
LCPQWL--
LWPQWL--
-LPQWLN-
-LPQWLI-
-LPQWLM-
LIPQWL--
-MPQWLV-
MSPQWL--
MRPQWL--
MQPQWL--
-MPQWLG-
-MPQWLH-
MMPQWL--
MGPQWL--
-MPQWLQ-
MNPQWL--
MAPQWL--
-MPQWLL-
MEPQWL--
MVPQWL--
MTPQWL--
-MPQWLN-
-MPQWLK-
MLPQWL--
MKPQWL--
MFPQWL--
-MPQWLW-
-NPQWLV-
NSPQWL--

-NPQWLK-
NRPQWL--
-NPQWLM-
NGPQWL--
NKPQWL--
-NPQWLS-
NAPQWL--
NVPQWL--
NQPQWL--
NYPQWL--
NTPQWL--
-NPQWLN-
-NPQWLE-
NNPQWL--
NMPQWL--
-NPQWLR-
-NPQWLQ-
NFPQWL--
-NPQWLW-
-NPQWLT-
-NPQWLI-
-NPQWLG-
NIPQWL--
NEPQWL--
--PQWLVR
--PQWLGL
--PQWLST
--PQWLTS
--PQWLIS
--PQWLTR
--PQWLQI
--PQWLRV
--PQWLRS
--PQWLVG
--PQWLKV
--PQWLIG
--PQWLTK
--PQWLRG
--PQWLMS
--PQWLAR
--PQWLSY
--PQWLRA
--PQWLMN
--PQWLKH
--PQWLHG
--PQWLGS
--PQWLVS
--PQWLTV
--PQWLLS
--PQWLVN

--PQWLTN
--PQWLTA
--PQWLSN
--PQWLSG
--PQWLRH
--PQWLLV
--PQWLLN
--PQWLLG
--PQWLKT
--PQWLKK
PGPQWL--
PTPQWL--
--PQWLWG
--PQWLVT
--PQWLVA
--PQWLSV
--PQWLRT
--PQWLRE
--PQWLNG
--PQWLMM
--PQWLMH
--PQWLKF
--PQWLHA
--PQWLGV
--PQWLFG
PVPQWL--
--PQWLYN
--PQWLTT
--PQWLIT
--PQWLIR
--PQWLIN
--PQWLHT
--PQWLHS
--PQWLGQ
--PQWLAQ
--PQWLAF
--PQWLYG
--PQWLVD
--PQWLSI
--PQWLSD
--PQWLSA
--PQWLRQ
--PQWLRI
--PQWLQH
--PQWLNT
--PQWLNA
--PQWLMR
--PQWLLH
--PQWLKA
--PQWLII

--PQWLHM
--PQWLGN
--PQWLGM
--PQWLGI
--PQWLFN
--PQWLAY
--PQWLAK
--PQWLAE
--PQWLWS
--PQWLWH
--PQWLSH
--PQWLSF
--PQWLSE
--PQWLRW
--PQWLRN
--PQWLMK
--PQWLMI
--PQWLKG
--PQWLIK
--PQWLID
--PQWLGT
--PQWLGA
--PQWLFM
--PQWLEY
--PQWLEV
--PQWLEG
--PQWLDS
--PQWLDA
--PQWLAN
--PQWLAM
--PQWLAH
--PQWLAG
PYPQWL--
PSPQWL--
--PQWLWW
--PQWLWR
--PQWLTW
--PQWLTQ
--PQWLRM
--PQWLRD
--PQWLQG
--PQWLQA
--PQWLNN
--PQWLNF
--PQWLMY
--PQWLMW
--PQWLMF
--PQWLMA
--PQWLLW
--PQWLLD

```
--PQWLIY
--PQWLIH
--PQWLIF
--PQWLHD
--PQWLFH
--PQWLEW
--PQWLER
--PQWLAS
PNPQWL--
PLPQWL--
--PQWLYW
--PQWLYQ
--PQWLYH
--PQWLVH
--PQWLVE
--PQWLTM
--PQWLTI
--PQWLSW
--PQWLSQ
--PQWLQN
--PQWLPY
--PQWLPL
--PQWLLE
--PQWLKS
--PQWLKN
--PQWLIW
--PQWLHV
--PQWLHQ
--PQWLHK
--PQWLHI
--PQWLHF
--PQWLGY
--PQWLGG
--PQWLGD
--PQWLES
--PQWLEA
--PQWLDT
--PQWLAW
--PQWLAT
PFPQWL--
PAPQWL--
-QPQWLI-
QTPQWL--
QSPQWL--
QDPQWL--
QVPQWL--
QKPQWL--
-QPQWLT-
-QPQWLR-
-QPQWLK-
```

QLPQWL--
QYPQWL--
QRPQWL--
-QPQWLL-
QIPQWL--
-QPQWLS-
-QPQWLQ-
QHPQWL--
QFPQWL--
-QPQWLV-
-QPQWLH-
-QPQWLE-
-QPQWLA-
QNPQWL--
QMPQWL--
QGPQWL--
QEPQWL--
QCPQWL--
RSPQWL--
-RPQWLL-
RIPQWL--
RTPQWL--
RVPQWL--
-RPQWLS-
-RPQWLN-
RRPQWL--
-RPQWLG-
RMPQWL--
RDPQWL--
-RPQWLV-
RAPQWL--
-RPQWLY-
RNPQWL--
RGPQWL--
REPQWL--
RPPQWL--
RFPQWL--
RYPQWL--
RWPQWL--
RQPQWL--
-RPQWLM-
-RPQWLI-
RKPQWL--
RHPQWL--
-RPQWLW-
-RPQWLF-
-RPQWLQ-
-RPQWLH-
-RPQWLE-
RCPQWL--

-SPQWLR-
SSPQWL--
-SPQWLF-
SLPQWL--
SGPQWL--
SYPQWL--
-SPQWLA-
-SPQWLV-
-SPQWLG-
STPQWL--
-SPQWLS-
-SPQWLL-
SVPQWL--
SNPQWL--
SHPQWL--
SKPQWL--
SRPQWL--
-SPQWLE-
-SPQWLM-
-SPQWLI-
SPPQWL--
SFPQWL--
SEPQWL--
-SPQWLK-
-SPQWLQ-
-SPQWLD-
SWPQWL--
SQPQWL--
-SPQWLH-
SCPQWL--
SAPQWL--
TQPQWL--
-TPQWLL-
-TPQWLT-
-TPQWLS-
TVPQWL--
TSPQWL--
-TPQWLR-
TTPQWL--
-TPQWLG-
TMPQWL--
TLPQWL--
TFPQWL--
-TPQWLA-
TPPQWL--
TAPQWL--
-TPQWLH-
TEPQWL--
TYPQWL--
-TPQWLY-

TGPQWL--
-TPQWLK-
-TPQWLE-
TKPQWL--
TRPQWL--
TIPQWL--
THPQWL--
-TPQWLV-
TDPQWL--
-VPQWLT-
-VPQWLG-
VLPQWL--
VKPQWL--
VRPQWL--
VQPQWL--
-VPQWLK-
VFPQWL--
VAPQWL--
VSPQWL--
-VPQWLR-
-VPQWLI-
-VPQWLA-
VNPQWL--
-VPQWLL-
VGPQWL--
VYPQWL--
-VPQWLH-
-VPQWLQ-
-VPQWLE-
VMPQWL--
VHPQWL--
VEPQWL--
VVPQWL--
VTPQWL--
-VPQWLM-
VPPQWL--
VWPQWL--
VDPQWL--
WYPQWL--
-WPQWLT-
-WPQWLR-
-WPQWLL-
-WPQWLK-
WGPQWL--
WVPQWL--
-WPQWLS-
WSPQWL--
-WPQWLM-
WPPQWL--
-WPQWLV-

-WPQWLQ-
-WPQWLA-
WNPQWL--
WLPQWL--
WFPQWL--
WDPQWL--
WCPQWL--
-YPQWLA-
-YPQWLK-
-YPQWLN-
-YPQWLS-
-YPQWLR-
YNPQWL--
-YPQWLE-
YVPQWL--
YSPQWL--
YMPQWL--
YHPQWL--
-YPQWLT-
-YPQWLH-
YPPQWL--
YIPQWL--
YFPQWL--
YTPQWL--
-YPQWLQ-
-YPQWLM-
-YPQWLG-
YGPQWL--
YYPQWL--
YWPQWL--
YQPQWL--
-YPQWLW-
-YPQWLL-
-YPQWLD-
YLPQWL--
YEPQWL--
YAPQWL--

MGWL 286 674 33.26

-AMGWLS-
-HMGWLD-
--MGWLNI
AVMGWL--
ASMGWL--
ATMGWL--
-AMGWLT-
AWMGWL--
-AMGWLK-
APMGWL--
AKMGWL--
ADMGWL--

-AMGWLL-
-AMGWLG-
-AMGWLH-
AIMGWL--
ALMGWL--
AQMGWL--
AAMGWL--
AGMGWL--
-AMGWLV-
-AMGWLA-
AHMGWL--
AYMGWL--
ANMGWL--
-AMGWLW-
-AMGWLN-
-AMGWLI-
CCMGWL--
CSMGWL--
CLMGWL--
-CMGWLI-
CTMGWL--
CRMGWL--
-CMGWLL-
-CMGWLG-
CHMGWL--
CQMGWL--
CNMGWL--
CMMGWL--
-CMGWLR-
DSMGWL--
-DMGWLS-
-DMGWLL-
DGMGWL--
-DMGWLA-
-DMGWLT-
DKMGWL--
DAMGWL--
DRMGWL--
DQMGWL--
-DMGWLQ-
-DMGWLG-
DLMGWL--
DIMGWL--
DPMGWL--
-DMGWLN-
DHMGWL--
DFMGWL--
DEMGWL--
DWMGWL--
DTMGWL--

DNMGWL--
-DMGWLV-
DYMGWL--
DVMGWL--
-DMGWLK-
-DMGWLI-
-DMGWLH-
ERMGWL--
EGMGWL--
ESMGWL--
-EMGWLS-
-EMGWLA-
-EMGWLK-
EIMGWL--
ETMGWL--
EEMGWL--
EDMGWL--
EPMGWL--
ELMGWL--
EAMGWL--
EQMGWL--
-EMGWLY-
-EMGWLM-
-EMGWLL-
EYMGWL--
EWMGWL--
EVMGWL--
ENMGWL--
-EMGWLV-
-EMGWLQ-
-EMGWLT-
-EMGWLN-
-EMGWLI-
-EMGWLH-
EKMGWL--
-FMGWLT-
-FMGWLR-
-FMGWLD-
FNMGWL--
-FMGWLS-
-FMGWLK-
FYMGWL--
FVMGWL--
FPMGWL--
-FMGWLW-
FHMGWL--
FFMGWL--
-FMGWLN-
-FMGWLH-
FLMGWL--

FMMGWL--
-FMGWLI-
-FMGWLE-
FDMGWL--
FAMGWL--
FWMGWL--
FQMGWL--
-FMGWLQ-
-FMGWLM-
FGMGWL--
FEMGWL--
GSMGWL--
-GMGWLR-
-GMGWLS-
GNMGWL--
GRMGWL--
-GMGWLL-
-GMGWLK-
-GMGWLG-
-GMGWLY-
GYMGWL--
-GMGWLM-
-GMGWLD-
-GMGWLA-
GIMGWL--
GHMGWL--
-GMGWLV-
-GMGWLT-
GQMGWL--
-GMGWLH-
GLMGWL--
GDMGWL--
GTMGWL--
-GMGWLN-
-GMGWLI-
-GMGWLQ-
GFMGWL--
GEMGWL--
GCMGWL--
GMMGWL--
-GMGWLE-
HMMGWL--
HVMGWL--
HLMGWL--
HGMGWL--
HYMGWL--
-HMGWLR-
HPMGWL--
HNMGWL--
-HMGWLQ-

HSMGWL--
HTMGWL--
-HMGWLS-
-HMGWLN-
-HMGWLH-
HHMGWL--
-HMGWLY-
-HMGWLK-
-HMGWLE-
-HMGWLA-
-HMGWLT-
-HMGWLM-
-HMGWLF-
HFMGWL--
HWMGWL--
-HMGWLI-
-HMGWLG-
IPMGWL--
INMGWL--
IAMGWL--
-IMGWLQ-
IYMGWL--
-IMGWLD-
IKMGWL--
IEMGWL--
IDMGWL--
ITMGWL--
-IMGWLS-
-IMGWLT-
-IMGWLN-
IQMGWL--
-IMGWLV-
ILMGWL--
-IMGWLK-
-IMGWLH-
-IMGWLE-
IHMGWL--
IGMGWL--
IVMGWL--
IIMGWL--
-KMGWLG-
-KMGWLL-
-KMGWLY-
KVMGWL--
KSMGWL--
KAMGWL--
-KMGWLH-
-KMGWLQ-
KDMGWL--
KEMGWL--

KTMGWL--
KPMGWL--
KNMGWL--
KQMGWL--
-KMGWLV-
-KMGWLK-
-KMGWLI-
-KMGWLF-
-KMGWLA-
KMMGWL--
-KMGWLT-
KIMGWL--
KFMGWL--
KWMGWL--
-KMGWLM-
-KMGWLE-
KGMGWL--
-KMGWLW-
-KMGWLR-
-KMGWLD-
KHMGWL--
LGMGWL--
LEMGWL--
LSMGWL--
-LMGWLK-
-LMGWLG-
LDMGWL--
LLMGWL--
LNMGWL--
LYMGWL--
-LMGWLA-
LTMGWL--
-LMGWLT-
-LMGWLN-
-LMGWLD-
LQMGWL--
-LMGWLQ-
-LMGWLH-
LWMGWL--
-LMGWLP-
-LMGWLE-
LCMGWL--
--MGWLVR
--MGWLSS
--MGWLSV
--MGWLKG
--MGWLGA
--MGWLTT
--MGWLRS
--MGWLRH

MKMGWL--
--MGWLIT
--MGWLSY
--MGWLSG
--MGWLMG
--MGWLHT
--MGWLHS
--MGWLHG
--MGWLGL
--MGWLAS
MAMGWL--
--MGWLVK
--MGWLSN
--MGWLRM
--MGWLLK
--MGWLGT
--MGWLGM
MNMGWL--
--MGWLLS
--MGWLLH
--MGWLKL
--MGWLGS
--MGWLAK
--MGWLLT
--MGWLLQ
--MGWLAA
-MMGWLK-
--MGWLVH
--MGWLRW
--MGWLNS
--MGWLIA
--MGWLAR
MSMGWL--
--MGWLYG
--MGWLTR
--MGWLSI
--MGWLQR
--MGWLKS
--MGWLGI
--MGWLAT
MGMGWL--
MYMGWL--
-MMGWLT-
--MGWLYH
--MGWLVT
--MGWLTN
--MGWLRV
--MGWLQY
--MGWLLN
--MGWLEA

MPMGWL--
MIMGWL--
MHMGWL--
--MGWLVW
--MGWLTV
--MGWLTF
--MGWLSK
--MGWLRF
--MGWLQN
--MGWLQM
--MGWLMT
--MGWLMS
--MGWLLR
--MGWLLG
--MGWLKQ
--MGWLKN
--MGWLKI
--MGWLIS
--MGWLHV
--MGWLFS
--MGWLFG
--MGWLDG
-MMGWLR-
--MGWLWS
--MGWLTS
--MGWLSQ
--MGWLSE
--MGWLQT
--MGWLNV
--MGWLIN
--MGWLHI
--MGWLES
--MGWLAM
MTMGWL--
-MMGWLL-
-MMGWLG-
--MGWLYK
--MGWLVQ
--MGWLVN
--MGWLTA
--MGWLSD
--MGWLSA
--MGWLRQ
--MGWLRD
--MGWLNT
--MGWLNG
--MGWLLA
--MGWLKE
--MGWLFN
--MGWLEV

```
--MGWLAN
MFMGWL--
-MMGWLS-
--MGWLVE
--MGWLVD
--MGWLTQ
--MGWLTH
--MGWLTD
--MGWLSH
--MGWLRG
--MGWLRE
--MGWLGN
--MGWLET
--MGWLAI
--MGWLAH
-MMGWLN-
-MMGWLI-
-MMGWLH-
--MGWLYQ
--MGWLVG
--MGWLSM
--MGWLNM
--MGWLMR
--MGWLKT
--MGWLKD
--MGWLKA
--MGWLIQ
--MGWLIH
--MGWLIG
--MGWLID
--MGWLHR
--MGWLGV
--MGWLAQ
MEMGWL--
MWMGWL--
-MMGWLQ-
--MGWLYV
--MGWLWN
--MGWLWK
--MGWLTI
--MGWLTE
--MGWLSW
--MGWLRL
--MGWLQK
--MGWLQF
--MGWLQD
--MGWLNN
--MGWLMV
--MGWLMN
--MGWLMH
```

--MGWLMD
--MGWLLW
--MGWLLD
--MGWLHY
--MGWLHW
--MGWLHQ
--MGWLHN
--MGWLHA
--MGWLGW
--MGWLFH
--MGWLEG
--MGWLDT
--MGWLDS
--MGWLAV
MQMGWL--
-MMGWLV-
-MMGWLD-
--MGWLWM
--MGWLWH
--MGWLWG
--MGWLVF
--MGWLTY
--MGWLTK
--MGWLQI
--MGWLQG
--MGWLQE
--MGWLNY
--MGWLNW
--MGWLNK
--MGWLNF
--MGWLND
--MGWLMM
--MGWLMI
--MGWLKY
--MGWLKM
--MGWLII
--MGWLHK
--MGWLHH
--MGWLGQ
--MGWLGG
--MGWLER
--MGWLDA
--MGWLAW
--MGWLAE
MDMGWL--
NHMGWL--
NSMGWL--
NRMGWL--
NVMGWL--
-NMGWLT-

NGMGWL--
NNMGWL--
-NMGWLV-
-NMGWLN-
-NMGWLG-
NTMGWL--
NQMGWL--
-NMGWLI-
NEMGWL--
-NMGWLL-
NAMGWL--
-NMGWLK-
-NMGWLE-
NIMGWL--
NYMGWL--
NPMGWL--
NMMGWL--
NDMGWL--
PSMGWL--
PEMGWL--
-PMGWLG-
-PMGWLA-
-PMGWLL-
PTMGWL--
PMMGWL--
-PMGWLT-
-PMGWLR-
-PMGWLM-
PYMGWL--
PNMGWL--
-PMGWLQ-
-PMGWLH-
PFMGWL--
PKMGWL--
PGMGWL--
-PMGWLI-
PLMGWL--
PQMGWL--
-PMGWLN-
PIMGWL--
-PMGWLV-
PDMGWL--
PWMGWL--
-PMGWLK-
-PMGWLD-
-PMGWLE-
PHMGWL--
PAMGWL--
-QMGWLG-
-QMGWLL-

-QMGWLA-
QMMGWL--
QFMGWL--
QKMGWL--
QVMGWL--
QPMGWL--
-QMGWLR-
-QMGWLM-
-QMGWLI-
QIMGWL--
-QMGWLS-
-QMGWLH-
QHMGWL--
QSMGWL--
QNMGWL--
QDMGWL--
-QMGWLW-
QLMGWL--
QTMGWL--
-QMGWLV-
QEMGWL--
-QMGWLN-
-QMGWLK-
QQMGWL--
-QMGWLQ-
-QMGWLE-
QAMGWL--
-RMGWLR-
RTMGWL--
RMMGWL--
-RMGWLQ-
RAMGWL--
-RMGWLS-
-RMGWLT-
RHMGWL--
RQMGWL--
RPMGWL--
RDMGWL--
-RMGWLN-
-RMGWLK-
-RMGWLA-
-RMGWLH-
RWMGWL--
-RMGWLE-
RIMGWL--
RCMGWL--
-RMGWLM-
SAMGWL--
-SMGWLQ-
SNMGWL--

-SMGWLR-
-SMGWLM-
-SMGWLK-
-SMGWLH-
SGMGWL--
-SMGWLN-
SIMGWL--
-SMGWLA-
-SMGWLL-
-SMGWLV-
SEMGWL--
SDMGWL--
SQMGWL--
-SMGWLD-
SFMGWL--
-SMGWLY-
SYMGWL--
SWMGWL--
-SMGWLE-
-TMGWLG-
TSMGWL--
TRMGWL--
TMMGWL--
TNMGWL--
TLMGWL--
-TMGWLI-
TFMGWL--
-TMGWLK-
TKMGWL--
TTMGWL--
-TMGWLH-
-TMGWLS-
TQMGWL--
-TMGWLV-
-TMGWLT-
TYMGWL--
-TMGWLR-
-TMGWLA-
TVMGWL--
THMGWL--
TPMGWL--
-TMGWLY-
TGMGWL--
TAMGWL--
-TMGWLQ-
-TMGWLE-
TEMGWL--
TDMGWL--
VYMGWL--
VSMGWL--

VGMGWL--
-VMGWLR-
VNMGWL--
-VMGWLQ-
-VMGWLG-
-VMGWLE-
VTMGWL--
-VMGWLH-
-VMGWLV-
-VMGWLN-
VEMGWL--
-VMGWLT-
-VMGWLK-
-VMGWLA-
VDMGWL--
-VMGWLI-
VHMGWL--
VWMGWL--
-VMGWLM-
-VMGWLD-
VIMGWL--
VFMGWL--
WSMGWL--
WKMGWL--
WTMGWL--
-WMGWLG-
WNMGWL--
-WMGWLS-
WYMGWL--
-WMGWLM-
-WMGWLQ-
-WMGWLN-
WDMGWL--
WAMGWL--
-WMGWLV-
-WMGWLT-
-WMGWLR-
-WMGWLA-
WEMGWL--
WVMGWL--
WPMGWL--
-WMGWLH-
WGMGWL--
WFMGWL--
YIMGWL--
YRMGWL--
YKMGWL--
YQMGWL--
YNMGWL--
YDMGWL--

| | | | |
|---|---|---|---|
| | -YMGWLQ- | | |
| | -YMGWLI- | | |
| | -YMGWLG- | | |
| | YSMGWL-- | | |
| | -YMGWLH- | | |
| | -YMGWLD- | | |
| | YGMGWL-- | | |
| | YYMGWL-- | | |
| | -YMGWLV- | | |
| | YHMGWL-- | | |
| | YEMGWL-- | | |
| | YCMGWL-- | | |
| PSWW | 351 | 651 | 33.26 |
| | -KPSWWN- | | |
| | --PSWWNR | | |
| | --PSWWED | | |
| | -APSWWK- | | |
| | -APSWWR- | | |
| | AGPSWW-- | | |
| | -APSWWG- | | |
| | AEPSWW-- | | |
| | ASPSWW-- | | |
| | ARPSWW-- | | |
| | AIPSWW-- | | |
| | AHPSWW-- | | |
| | -APSWWY- | | |
| | -APSWWF- | | |
| | APPSWW-- | | |
| | ALPSWW-- | | |
| | AFPSWW-- | | |
| | ATPSWW-- | | |
| | AQPSWW-- | | |
| | -APSWWT- | | |
| | -APSWWN- | | |
| | -APSWWA- | | |
| | -APSWWH- | | |
| | AAPSWW-- | | |
| | AYPSWW-- | | |
| | AWPSWW-- | | |
| | AVPSWW-- | | |
| | -APSWWW- | | |
| | -APSWWQ- | | |
| | -APSWWM- | | |
| | -APSWWL- | | |
| | -APSWWI- | | |
| | ANPSWW-- | | |
| | CQPSWW-- | | |
| | CVPSWW-- | | |
| | CRPSWW-- | | |
| | -CPSWWV- | | |

-CPSWWR-
CIPSWW--
CFPSWW--
DRPSWW--
-DPSWWQ-
DHPSWW--
-DPSWWM-
-DPSWWG-
-DPSWWT-
-DPSWWE-
DEPSWW--
DWPSWW--
DTPSWW--
-DPSWWI-
-DPSWWF-
-DPSWWA-
DLPSWW--
DKPSWW--
DIPSWW--
DGPSWW--
DFPSWW--
DAPSWW--
DVPSWW--
-DPSWWW-
-DPSWWS-
-DPSWWN-
-EPSWWL-
ELPSWW--
-EPSWWR-
EYPSWW--
ESPSWW--
-EPSWWK-
-EPSWWF-
-EPSWWG-
ETPSWW--
-EPSWWM-
ERPSWW--
EGPSWW--
EVPSWW--
-EPSWWW-
-EPSWWV-
-EPSWWS-
-EPSWWI-
-EPSWWH-
EIPSWW--
EWPSWW--
-EPSWWT-
-EPSWWA-
EFPSWW--
FSPSWW--

-FPSWWR-
FYPSWW--
FRPSWW--
FIPSWW--
-FPSWWT-
FKPSWW--
FQPSWW--
FLPSWW--
FFPSWW--
FTPSWW--
-FPSWWF-
FPPSWW--
FHPSWW--
FGPSWW--
FAPSWW--
FWPSWW--
FVPSWW--
-FPSWWV-
-FPSWWL-
-FPSWWA-
FNPSWW--
GTPSWW--
-GPSWWS-
-GPSWWL-
-GPSWWI-
GRPSWW--
-GPSWWY-
GHPSWW--
GYPSWW--
GIPSWW--
-GPSWWV-
-GPSWWN-
GSPSWW--
-GPSWWH-
GNPSWW--
GKPSWW--
GAPSWW--
GPPSWW--
GGPSWW--
GFPSWW--
GDPSWW--
GVPSWW--
-GPSWWR-
-GPSWWG-
GWPSWW--
GQPSWW--
-GPSWWT-
-GPSWWK-
-GPSWWA-
GEPSWW--

HLPSWW--
HVPSWW--
HQPSWW--
HWPSWW--
-HPSWWT-
-HPSWWG-
-HPSWWA-
HKPSWW--
HIPSWW--
-HPSWWQ-
-HPSWWS-
-HPSWWL-
-HPSWWH-
-HPSWWF-
HHPSWW--
HGPSWW--
HSPSWW--
HRPSWW--
-HPSWWY-
-HPSWWW-
-HPSWWV-
-HPSWWR-
-HPSWWI-
-HPSWWE-
HNPSWW--
-IPSWWS-
ISPSWW--
-IPSWWR-
IEPSWW--
IHPSWW--
IYPSWW--
INPSWW--
IFPSWW--
IWPSWW--
-IPSWWQ-
-IPSWWA-
ILPSWW--
IGPSWW--
-IPSWWT-
IAPSWW--
ITPSWW--
IRPSWW--
-IPSWWV-
-IPSWWM-
-IPSWWL-
-IPSWWK-
-IPSWWE-
IMPSWW--
IIPSWW--
IDPSWW--

KSPSWW--
-KPSWWG-
-KPSWWR-
-KPSWWV-
-KPSWWI-
KTPSWW--
-KPSWWY-
-KPSWWE-
-KPSWWS-
-KPSWWL-
KPPSWW--
-KPSWWT-
KGPSWW--
KVPSWW--
-KPSWWQ-
-KPSWWM-
KYPSWW--
-KPSWWH-
KNPSWW--
KLPSWW--
KIPSWW--
KEPSWW--
LSPSWW--
LGPSWW--
LEPSWW--
-LPSWWQ-
LWPSWW--
LVPSWW--
-LPSWWV-
-LPSWWS-
-LPSWWT-
LYPSWW--
LIPSWW--
LDPSWW--
LRPSWW--
-LPSWWA-
-LPSWWK-
LNPSWW--
LLPSWW--
LTPSWW--
-LPSWWM-
LAPSWW--
-LPSWWW-
-LPSWWL-
-LPSWWI-
-LPSWWG-
LMPSWW--
LHPSWW--
-MPSWWR-
MRPSWW--

```
-MPSWWS-
-MPSWWA-
MNPSWW--
MSPSWW--
MGPSWW--
-MPSWWQ-
MMPSWW--
MKPSWW--
MEPSWW--
MDPSWW--
-MPSWWT-
-MPSWWI-
MYPSWW--
-MPSWWV-
MLPSWW--
MTPSWW--
-MPSWWM-
-MPSWWG-
-MPSWWE-
MPPSWW--
MIPSWW--
MFPSWW--
MAPSWW--
-NPSWWA-
NRPSWW--
NSPSWW--
NPPSWW--
NLPSWW--
NGPSWW--
-NPSWWT-
NFPSWW--
-NPSWWS-
-NPSWWI-
NKPSWW--
NHPSWW--
NYPSWW--
-NPSWWQ-
NIPSWW--
NEPSWW--
NWPSWW--
NQPSWW--
-NPSWWY-
-NPSWWW-
-NPSWWE-
NAPSWW--
--PSWWNS
--PSWWGT
--PSWWST
--PSWWTR
--PSWWSA
```

--PSWWMR
--PSWWLT
--PSWWLN
--PSWWVR
--PSWWWN
--PSWWRY
--PSWWRN
--PSWWGS
--PSWWGI
--PSWWRQ
--PSWWMG
PGPSWW--
--PSWWVG
--PSWWTV
--PSWWRI
--PSWWRG
--PSWWMN
--PSWWLK
--PSWWGV
--PSWWSS
--PSWWSL
--PSWWQI
--PSWWKT
--PSWWIK
--PSWWGL
--PSWWAH
--PSWWTT
--PSWWRD
--PSWWIT
--PSWWAS
--PSWWYS
--PSWWTN
--PSWWTG
--PSWWRT
--PSWWQH
--PSWWMK
--PSWWLG
--PSWWLE
--PSWWAW
--PSWWAT
--PSWWAL
--PSWWVT
--PSWWVK
--PSWWLD
--PSWWKI
--PSWWKD
--PSWWHR
--PSWWFK
--PSWWES
PLPSWW--

```
PTPSWW--
--PSWWWG
--PSWWVI
--PSWWTS
--PSWWTL
--PSWWSQ
--PSWWSM
--PSWWSG
--PSWWSF
--PSWWRH
--PSWWRE
--PSWWQR
--PSWWQL
--PSWWMV
--PSWWLI
--PSWWIA
--PSWWAY
--PSWWAR
PSPSWW--
PQPSWW--
--PSWWYQ
--PSWWYN
--PSWWWS
--PSWWVS
--PSWWVE
--PSWWSK
--PSWWQY
--PSWWKV
--PSWWKM
--PSWWKL
--PSWWKH
--PSWWIV
--PSWWHQ
--PSWWGA
--PSWWAQ
PKPSWW--
PIPSWW--
--PSWWYY
--PSWWYI
--PSWWVY
--PSWWVW
--PSWWVN
--PSWWVH
--PSWWVD
--PSWWTK
--PSWWTE
--PSWWSY
--PSWWSV
--PSWWSN
--PSWWSI
```

--PSWWQW
--PSWWQN
--PSWWNA
--PSWWMY
--PSWWMT
--PSWWMD
--PSWWLR
--PSWWLQ
--PSWWLM
--PSWWKQ
--PSWWIY
--PSWWIQ
--PSWWHY
--PSWWHK
--PSWWGR
--PSWWGN
--PSWWFS
--PSWWFN
--PSWWFD
--PSWWEY
--PSWWEW
--PSWWEV
--PSWWAI
--PSWWAG
--PSWWAD
--PSWWAA
PRPSWW--
PNPSWW--
PHPSWW--
PFPSWW--
--PSWWYW
--PSWWYK
--PSWWYH
--PSWWYG
--PSWWYD
--PSWWWY
--PSWWWW
--PSWWWR
--PSWWWA
--PSWWVQ
--PSWWVA
--PSWWTY
--PSWWTQ
--PSWWTI
--PSWWTH
--PSWWTD
--PSWWSH
--PSWWSD
--PSWWQV
--PSWWQT

--PSWWPR
--PSWWNT
--PSWWNQ
--PSWWNI
--PSWWMQ
--PSWWMI
--PSWWKW
--PSWWKG
--PSWWKF
--PSWWIN
--PSWWIM
--PSWWIG
--PSWWIF
--PSWWIE
--PSWWHV
--PSWWHP
--PSWWHF
--PSWWGK
--PSWWGF
--PSWWFW
--PSWWFG
--PSWWEH
--PSWWEG
--PSWWEF
--PSWWEA
--PSWWAM
--PSWWAF
-PPSWWS-
PAPSWW--
QWPSWW--
QRPSWW--
-QPSWWV-
QHPSWW--
QTPSWW--
-QPSWWS-
-QPSWWK-
-QPSWWI-
-QPSWWH-
-QPSWWY-
-QPSWWT-
QPPSWW--
QYPSWW--
QSPSWW--
-QPSWWR-
-QPSWWN-
QGPSWW--
QVPSWW--
QNPSWW--
-QPSWWM-
-QPSWWL-

-QPSWWE-
QMPSWW--
QLPSWW--
-RPSWWH-
-RPSWWV-
RTPSWW--
-RPSWWR-
RAPSWW--
-RPSWWT-
-RPSWWG-
RNPSWW--
RYPSWW--
-RPSWWW-
-RPSWWA-
RVPSWW--
-RPSWWL-
RDPSWW--
RMPSWW--
RLPSWW--
RKPSWW--
REPSWW--
-RPSWWF-
RGPSWW--
RWPSWW--
-RPSWWQ-
-RPSWWN-
-RPSWWI-
RIPSWW--
RQPSWW--
-RPSWWM-
RPPSWW--
RHPSWW--
SRPSWW--
-SPSWWL-
-SPSWWT-
-SPSWWQ-
SQPSWW--
-SPSWWK-
-SPSWWA-
SLPSWW--
-SPSWWS-
-SPSWWF-
SWPSWW--
SIPSWW--
STPSWW--
-SPSWWG-
SYPSWW--
-SPSWWW-
-SPSWWV-
SGPSWW--

SFPSWW--
SAPSWW--
SSPSWW--
-SPSWWE-
SKPSWW--
-SPSWWY-
-SPSWWR-
-SPSWWI-
SMPSWW--
-SPSWWN-
SNPSWW--
-SPSWWM-
-SPSWWH-
SHPSWW--
TRPSWW--
-TPSWWR-
-TPSWWL-
-TPSWWT-
-TPSWWG-
TLPSWW--
-TPSWWK-
-TPSWWI-
-TPSWWW-
TVPSWW--
TTPSWW--
-TPSWWQ-
-TPSWWH-
-TPSWWF-
TFPSWW--
TEPSWW--
-TPSWWS-
TPPSWW--
TGPSWW--
TWPSWW--
-TPSWWY-
-TPSWWN-
-TPSWWA-
TNPSWW--
TIPSWW--
TQPSWW--
-TPSWWM-
-TPSWWE-
VRPSWW--
VSPSWW--
-VPSWWR-
VKPSWW--
VHPSWW--
-VPSWWK-
VFPSWW--
-VPSWWV-

VGPSWW--
-VPSWWQ-
-VPSWWG-
-VPSWWA-
VQPSWW--
-VPSWWT-
-VPSWWS-
VPPSWW--
VLPSWW--
VTPSWW--
-VPSWWM-
-VPSWWI-
VEPSWW--
VWPSWW--
VVPSWW--
-VPSWWW-
-VPSWWN-
-VPSWWH-
VDPSWW--
-WPSWWS-
WTPSWW--
WQPSWW--
WLPSWW--
-WPSWWT-
-WPSWWV-
-WPSWWR-
WEPSWW--
WYPSWW--
WSPSWW--
-WPSWWQ-
-WPSWWL-
WPPSWW--
WKPSWW--
WAPSWW--
-WPSWWY-
-WPSWWK-
-WPSWWI-
-WPSWWH-
-WPSWWG-
WGPSWW--
-YPSWWR-
YRPSWW--
-YPSWWV-
YKPSWW--
YGPSWW--
YAPSWW--
YWPSWW--
YTPSWW--
-YPSWWT-
-YPSWWA-

| | | | |
|---|---|---|---|
| | YYPSWW-- | | |
| | YVPSWW-- | | |
| | YQPSWW-- | | |
| | -YPSWWY- | | |
| | YMPSWW-- | | |
| | YEPSWW-- | | |
| | -YPSWWQ- | | |
| | -YPSWWI- | | |
| | -YPSWWH- | | |
| | -YPSWWG- | | |
| | YNPSWW-- | | |
| | YIPSWW-- | | |
| | YFPSWW-- | | |
| | YCPSWW-- | | |
| PYAL | 138 | 745 | 33.26 |
| | -DPYALS- | | |
| | GTPYAL-- | | |
| | -NPYALA- | | |
| | -TPYALG- | | |
| | ALPYAL-- | | |
| | ARPYAL-- | | |
| | ATPYAL-- | | |
| | ASPYAL-- | | |
| | AKPYAL-- | | |
| | AVPYAL-- | | |
| | -APYALS- | | |
| | -APYALR- | | |
| | -APYALL- | | |
| | -APYALG- | | |
| | -APYALT- | | |
| | AGPYAL-- | | |
| | AFPYAL-- | | |
| | AIPYAL-- | | |
| | AAPYAL-- | | |
| | -APYALA- | | |
| | AYPYAL-- | | |
| | -APYALN- | | |
| | -APYALM- | | |
| | -APYALV- | | |
| | ANPYAL-- | | |
| | AHPYAL-- | | |
| | APPYAL-- | | |
| | AQPYAL-- | | |
| | -APYALQ- | | |
| | -APYALK- | | |
| | -APYALC- | | |
| | AMPYAL-- | | |
| | AEPYAL-- | | |
| | AWPYAL-- | | |
| | ACPYAL-- | | |

CVPYAL--
-CPYALR-
CLPYAL--
CTPYAL--
CSPYAL--
-CPYALL-
-CPYALF-
CHPYAL--
CEPYAL--
DRPYAL--
DKPYAL--
DTPYAL--
-DPYALL-
-DPYALR-
-DPYALM-
DFPYAL--
DYPYAL--
DQPYAL--
DIPYAL--
DDPYAL--
DSPYAL--
-DPYALV-
DNPYAL--
DMPYAL--
DGPYAL--
DAPYAL--
-DPYALW-
DLPYAL--
DHPYAL--
DCPYAL--
EAPYAL--
ESPYAL--
ELPYAL--
-EPYALL-
-EPYALR-
-EPYALA-
-EPYALY-
ETPYAL--
-EPYALW-
-EPYALN-
ENPYAL--
EKPYAL--
EEPYAL--
EWPYAL--
-EPYALK-
-EPYALI-
EMPYAL--
EIPYAL--
EGPYAL--
EYPYAL--

EVPYAL--
ERPYAL--
EQPYAL--
-EPYALH-
-EPYALV-
-EPYALM-
-EPYALF-
EFPYAL--
EDPYAL--
-FPYALR-
-FPYALS-
-FPYALQ-
FSPYAL--
-FPYALK-
-FPYALN-
FNPYAL--
FLPYAL--
FVPYAL--
FRPYAL--
-FPYALH-
FIPYAL--
FYPYAL--
FGPYAL--
FQPYAL--
-FPYALY-
-FPYALP-
-FPYALM-
FKPYAL--
FEPYAL--
-FPYALT-
-FPYALA-
FFPYAL--
FAPYAL--
-FPYALV-
FPPYAL--
GKPYAL--
GLPYAL--
GNPYAL--
GRPYAL--
-GPYALS-
-GPYALT-
GSPYAL--
-GPYALH-
-GPYALF-
GAPYAL--
GQPYAL--
-GPYALL-
GFPYAL--
GEPYAL--
GWPYAL--

```
-GPYALN-
GYPYAL--
GVPYAL--
-GPYALV-
-GPYALI-
GIPYAL--
GHPYAL--
-GPYALK-
GMPYAL--
-GPYALW-
-GPYALM-
-GPYALG-
-GPYALE-
-GPYALA-
-GPYALQ-
GPPYAL--
GCPYAL--
-HPYALT-
HSPYAL--
HKPYAL--
-HPYALA-
HLPYAL--
HRPYAL--
-HPYALQ-
HAPYAL--
HGPYAL--
HYPYAL--
-HPYALK-
-HPYALI-
-HPYALG-
HDPYAL--
HVPYAL--
-HPYALR-
-HPYALN-
HWPYAL--
-HPYALF-
HPPYAL--
HIPYAL--
HHPYAL--
-HPYALY-
-HPYALW-
-HPYALP-
HQPYAL--
-HPYALH-
-HPYALE-
HNPYAL--
IRPYAL--
IKPYAL--
IHPYAL--
-IPYALR-
```

-IPYALN-
-IPYALK-
ITPYAL--
IFPYAL--
IYPYAL--
IVPYAL--
-IPYALS-
-IPYALM-
IAPYAL--
IQPYAL--
-IPYALT-
-IPYALI-
ILPYAL--
-IPYALV-
-IPYALH-
-IPYALG-
IPPYAL--
-IPYALW-
-IPYALL-
-IPYALA-
IIPYAL--
IGPYAL--
IEPYAL--
KGPYAL--
-KPYALR-
KRPYAL--
KVPYAL--
KTPYAL--
KYPYAL--
-KPYALA-
KWPYAL--
KFPYAL--
KNPYAL--
-KPYALW-
-KPYALN-
-KPYALL-
-KPYALK-
-KPYALI-
KEPYAL--
-KPYALV-
-KPYALT-
-KPYALG-
KQPYAL--
-KPYALY-
-KPYALQ-
-KPYALM-
-KPYALF-
KPPYAL--
KDPYAL--
-KPYALH-

KIPYAL--
KHPYAL--
LRPYAL--
LSPYAL--
-LPYALI-
LMPYAL--
LTPYAL--
-LPYALR-
LGPYAL--
LQPYAL--
LAPYAL--
LVPYAL--
-LPYALT-
-LPYALH-
-LPYALS-
-LPYALL-
-LPYALK-
LWPYAL--
-LPYALV-
-LPYALA-
LDPYAL--
LPPYAL--
LLPYAL--
-LPYALW-
-LPYALQ-
LHPYAL--
-LPYALN-
LNPYAL--
LEPYAL--
-MPYALS-
-MPYALL-
MSPYAL--
-MPYALI-
MPPYAL--
MVPYAL--
-MPYALH-
-MPYALQ-
MGPYAL--
MYPYAL--
MNPYAL--
-MPYALT-
-MPYALG-
-MPYALA-
MMPYAL--
-MPYALR-
MIPYAL--
MHPYAL--
-MPYALW-
-MPYALV-
MFPYAL--

```
MEPYAL--
MDPYAL--
MAPYAL--
-MPYALN-
MKPYAL--
NGPYAL--
-NPYALK-
NAPYAL--
NRPYAL--
NFPYAL--
-NPYALS-
NKPYAL--
-NPYALV-
-NPYALQ-
-NPYALL-
NLPYAL--
NEPYAL--
NWPYAL--
NSPYAL--
-NPYALI-
NYPYAL--
-NPYALY-
-NPYALR-
NIPYAL--
-NPYALT-
NNPYAL--
-NPYALH-
NHPYAL--
NVPYAL--
NTPYAL--
-NPYALG-
NPPYAL--
NMPYAL--
NDPYAL--
NCPYAL--
--PYALRA
--PYALTR
--PYALSR
--PYALST
--PYALRS
--PYALWR
--PYALRT
--PYALLK
--PYALHR
--PYALSS
--PYALHG
--PYALVN
--PYALMN
--PYALMR
--PYALRY
```

--PYALRH
--PYALWS
--PYALWH
--PYALWG
--PYALNF
--PYALLG
--PYALGA
--PYALRN
--PYALNL
--PYALHT
--PYALGY
--PYALGS
--PYALFN
--PYALYS
--PYALLQ
--PYALHH
--PYALGL
--PYALTS
--PYALRD
--PYALQS
--PYALQH
--PYALSW
--PYALLN
--PYALVA
--PYALSN
--PYALSG
--PYALNV
--PYALKK
--PYALKI
--PYALIH
PGPYAL--
--PYALTK
--PYALTI
--PYALSM
--PYALNS
--PYALLS
--PYALIS
--PYALHN
--PYALHL
--PYALHI
--PYALAV
--PYALVS
--PYALVD
--PYALQI
--PYALPI
--PYALMS
--PYALLH
--PYALKN
--PYALGF
PVPYAL--

PKPYAL--
PAPYAL--
--PYALYN
--PYALYI
--PYALYD
--PYALVH
--PYALSY
--PYALSK
--PYALSI
--PYALQW
--PYALQL
--PYALMV
--PYALKV
--PYALKG
--PYALIR
--PYALGM
--PYALGI
--PYALFY
PTPYAL--
PNPYAL--
--PYALWN
--PYALVV
--PYALTN
--PYALTL
--PYALQM
--PYALPW
--PYALNI
--PYALNA
--PYALLA
--PYALKA
--PYALAS
--PYALYG
--PYALWT
--PYALWD
--PYALSV
--PYALSQ
--PYALSA
--PYALRW
--PYALRQ
--PYALQT
--PYALQN
--PYALQA
--PYALNK
--PYALMK
--PYALKT
--PYALKD
--PYALIA
--PYALGQ
--PYALAQ
--PYALAM

--PYALVT
--PYALQK
--PYALPM
--PYALPF
--PYALNQ
--PYALMH
--PYALKW
--PYALIG
--PYALHQ
--PYALHF
--PYALGG
--PYALAW
--PYALAL
--PYALAH
--PYALAG
PYPYAL--
--PYALYV
--PYALTY
--PYALTT
--PYALTQ
--PYALTH
--PYALTE
--PYALSH
--PYALQY
--PYALQR
--PYALQQ
--PYALPQ
--PYALNG
--PYALLD
--PYALKR
--PYALKM
--PYALKF
--PYALKE
--PYALIN
--PYALHY
--PYALHK
--PYALHE
--PYALGV
--PYALGT
--PYALFK
--PYALFG
--PYALFE
--PYALES
--PYALDM
--PYALAT
PHPYAL--
PDPYAL--
--PYALWM
--PYALWK
--PYALWE

--PYALVQ
--PYALVE
--PYALTV
--PYALTD
--PYALTA
--PYALQV
--PYALPL
--PYALNN
--PYALMT
--PYALHD
--PYALET
--PYALEG
--PYALEA
--PYALDL
--PYALAR
--PYALAK
PQPYAL--
PMPYAL--
PIPYAL--
--PYALYQ
--PYALWQ
--PYALTW
--PYALSD
--PYALRC
--PYALQG
--PYALQF
--PYALQE
--PYALQD
--PYALPV
--PYALNT
--PYALNM
--PYALMQ
--PYALMI
--PYALMG
--PYALMD
--PYALLP
--PYALLE
--PYALKY
--PYALKL
--PYALIY
--PYALHW
--PYALFQ
--PYALFD
--PYALFC
--PYALEV
--PYALEF
--PYALAF
--PYALAA
-PPYALL-
PFPYAL--

PCPYAL--
QVPYAL--
QFPYAL--
-QPYALS-
QRPYAL--
-QPYALQ-
QLPYAL--
QSPYAL--
-QPYALR-
QYPYAL--
QPPYAL--
QNPYAL--
QGPYAL--
QKPYAL--
QTPYAL--
-QPYALN-
-QPYALK-
-QPYALA-
QIPYAL--
QQPYAL--
-QPYALW-
-QPYALL-
-QPYALI-
-QPYALH-
QHPYAL--
QAPYAL--
-QPYALV-
-QPYALG-
QWPYAL--
-QPYALT-
QEPYAL--
QDPYAL--
RVPYAL--
RLPYAL--
-RPYALR-
RFPYAL--
-RPYALV-
RMPYAL--
RSPYAL--
RGPYAL--
RIPYAL--
-RPYALW-
-RPYALA-
RYPYAL--
-RPYALK-
RHPYAL--
RPPYAL--
-RPYALG-
RTPYAL--
RNPYAL--

RAPYAL--
RQPYAL--
REPYAL--
RWPYAL--
-RPYALT-
-RPYALH-
RDPYAL--
RKPYAL--
-RPYALF-
-RPYALQ-
-RPYALN-
-RPYALM-
RCPYAL--
-SPYALH-
SAPYAL--
SGPYAL--
SSPYAL--
STPYAL--
-SPYALS-
SMPYAL--
SEPYAL--
SVPYAL--
SNPYAL--
SKPYAL--
SQPYAL--
SIPYAL--
SDPYAL--
-SPYALV-
-SPYALL-
-SPYALI-
-SPYALA-
SWPYAL--
-SPYALN-
-SPYALK-
-SPYALF-
-SPYALW-
SFPYAL--
-SPYALM-
SHPYAL--
SPPYAL--
SYPYAL--
-SPYALY-
-SPYALE-
TSPYAL--
-TPYALL-
TLPYAL--
TYPYAL--
TWPYAL--
-TPYALR-
TRPYAL--

TKPYAL--
-TPYALK-
TFPYAL--
-TPYALI-
-TPYALH-
TPPYAL--
TMPYAL--
-TPYALV-
-TPYALQ-
TTPYAL--
-TPYALA-
TNPYAL--
TVPYAL--
-TPYALT-
THPYAL--
-TPYALM-
TIPYAL--
TDPYAL--
TQPYAL--
-TPYALW-
-TPYALN-
VRPYAL--
-VPYALR-
VFPYAL--
-VPYALW-
VQPYAL--
VLPYAL--
VKPYAL--
-VPYALV-
-VPYALK-
-VPYALN-
VSPYAL--
VTPYAL--
VVPYAL--
-VPYALH-
-VPYALA-
VGPYAL--
-VPYALS-
-VPYALT-
-VPYALQ-
VAPYAL--
VEPYAL--
VMPYAL--
VHPYAL--
VPPYAL--
VNPYAL--
VIPYAL--
VWPYAL--
-VPYALM-
VYPYAL--

```
-VPYALE-
VDPYAL--
-VPYALG-
VCPYAL--
WEPYAL--
WNPYAL--
-WPYALR-
WLPYAL--
WTPYAL--
-WPYALS-
-WPYALL-
WQPYAL--
-WPYALQ-
WKPYAL--
WYPYAL--
-WPYALT-
-WPYALN-
-WPYALI-
WPPYAL--
WDPYAL--
WIPYAL--
WGPYAL--
WFPYAL--
-WPYALM-
-WPYALH-
-WPYALA-
WRPYAL--
WAPYAL--
-WPYALW-
-WPYALK-
WCPYAL--
-YPYALH-
YIPYAL--
YAPYAL--
-YPYALM-
YHPYAL--
YLPYAL--
YVPYAL--
YSPYAL--
-YPYALK-
YMPYAL--
-YPYALA-
YNPYAL--
YRPYAL--
YKPYAL--
-YPYALN-
-YPYALL-
YPPYAL--
YCPYAL--
YYPYAL--
```

YQPYAL--
-YPYALW-
-YPYALQ-
YTPYAL--
-YPYALE-
YDPYAL--
YWPYAL--
-YPYALG-
YFPYAL--
YEPYAL--
VAHL 64 794 33.22
-FVAHLD-
--VAHLTL
--VAHLDL
--VAHLAR
ANVAHL--
ALVAHL--
ASVAHL--
ATVAHL--
AKVAHL--
AEVAHL--
AAVAHL--
AQVAHL--
-AVAHLS-
AGVAHL--
-AVAHLY-
-AVAHLV-
-AVAHLQ-
-AVAHLN-
-AVAHLK-
-AVAHLE-
AVVAHL--
-AVAHLW-
APVAHL--
AMVAHL--
AIVAHL--
ADVAHL--
AFVAHL--
-AVAHLI-
-AVAHLG-
-AVAHLM-
-CVAHLT-
CQVAHL--
CCVAHL--
CRVAHL--
CAVAHL--
-CVAHLM-
CSVAHL--
CIVAHL--
CGVAHL--

```
-CVAHLV-
-CVAHLS-
-CVAHLR-
CMVAHL--
CLVAHL--
CYVAHL--
-CVAHLY-
-CVAHLN-
-CVAHLK-
-CVAHLG-
-CVAHLA-
CTVAHL--
CPVAHL--
CHVAHL--
CDVAHL--
DWVAHL--
DTVAHL--
DRVAHL--
-DVAHLL-
-DVAHLK-
DFVAHL--
DVVAHL--
-DVAHLV-
-DVAHLA-
DMVAHL--
DAVAHL--
DIVAHL--
DEVAHL--
-DVAHLT-
-DVAHLS-
-DVAHLR-
-DVAHLH-
-DVAHLF-
-DVAHLE-
DLVAHL--
-DVAHLY-
-DVAHLQ-
-DVAHLI-
DSVAHL--
DYVAHL--
DPVAHL--
DHVAHL--
DDVAHL--
-DVAHLM-
DKVAHL--
ELVAHL--
-EVAHLK-
-EVAHLS-
-EVAHLH-
EGVAHL--
```

EIVAHL--
ENVAHL--
EVVAHL--
-EVAHLN-
ERVAHL--
-EVAHLT-
-EVAHLI-
EHVAHL--
ETVAHL--
EAVAHL--
-EVAHLW-
-EVAHLR-
EQVAHL--
EFVAHL--
EYVAHL--
-EVAHLA-
ESVAHL--
EKVAHL--
EEVAHL--
EWVAHL--
-EVAHLQ-
-EVAHLL-
-EVAHLF-
-EVAHLE-
EMVAHL--
-EVAHLY-
-EVAHLV-
-EVAHLM-
-EVAHLG-
EDVAHL--
ECVAHL--
-FVAHLT-
-FVAHLA-
FSVAHL--
FAVAHL--
-FVAHLH-
FHVAHL--
-FVAHLV-
-FVAHLQ-
-FVAHLK-
-FVAHLY-
FKVAHL--
FNVAHL--
-FVAHLE-
FCVAHL--
-FVAHLN-
FQVAHL--
-FVAHLI-
-FVAHLF-
FIVAHL--

FDVAHL--
FWVAHL--
-FVAHLM-
FMVAHL--
GIVAHL--
GSVAHL--
-GVAHLR-
-GVAHLT-
-GVAHLF-
GTVAHL--
GVVAHL--
-GVAHLY-
GYVAHL--
-GVAHLS-
GKVAHL--
GLVAHL--
GWVAHL--
GMVAHL--
GGVAHL--
-GVAHLW-
-GVAHLL-
GPVAHL--
-GVAHLI-
-GVAHLV-
-GVAHLQ-
-GVAHLM-
-GVAHLH-
GEVAHL--
GQVAHL--
GNVAHL--
-GVAHLN-
-GVAHLA-
GDVAHL--
GCVAHL--
-GVAHLE-
GHVAHL--
GAVAHL--
-GVAHLP-
-GVAHLG-
-GVAHLC-
HFVAHL--
HSVAHL--
-HVAHLV-
-HVAHLS-
HIVAHL--
-HVAHLY-
HPVAHL--
-HVAHLR-
HNVAHL--
-HVAHLA-

```
-HVAHLN-
-HVAHLK-
HLVAHL--
HHVAHL--
HYVAHL--
-HVAHLG-
HQVAHL--
-HVAHLI-
-HVAHLH-
HGVAHL--
HWVAHL--
HVVAHL--
-HVAHLQ-
HTVAHL--
HEVAHL--
HAVAHL--
-HVAHLT-
-HVAHLL-
-HVAHLW-
-HVAHLM-
-HVAHLF-
HMVAHL--
HDVAHL--
ISVAHL--
-IVAHLL-
ITVAHL--
-IVAHLS-
IDVAHL--
-IVAHLY-
-IVAHLH-
IFVAHL--
IHVAHL--
-IVAHLR-
-IVAHLI-
IPVAHL--
IKVAHL--
-IVAHLV-
-IVAHLN-
-IVAHLM-
INVAHL--
IGVAHL--
IYVAHL--
-IVAHLT-
-IVAHLQ-
IAVAHL--
-IVAHLA-
-IVAHLF-
-IVAHLE-
IQVAHL--
IMVAHL--
```

IIVAHL--
-IVAHLW-
-IVAHLK-
KLVAHL--
-KVAHLQ-
-KVAHLV-
KSVAHL--
-KVAHLY-
-KVAHLT-
KIVAHL--
KPVAHL--
-KVAHLM-
-KVAHLA-
-KVAHLI-
-KVAHLF-
-KVAHLE-
KDVAHL--
KVVAHL--
-KVAHLK-
KFVAHL--
KQVAHL--
KEVAHL--
KYVAHL--
LSVAHL--
-LVAHLG-
LAVAHL--
LYVAHL--
LTVAHL--
LEVAHL--
-LVAHLT-
-LVAHLV-
LFVAHL--
-LVAHLN-
LQVAHL--
-LVAHLH-
LGVAHL--
LIVAHL--
LVVAHL--
LMVAHL--
LHVAHL--
-LVAHLQ-
-LVAHLK-
-LVAHLE-
LPVAHL--
LDVAHL--
-LVAHLY-
LNVAHL--
-LVAHLM-
-LVAHLD-
-LVAHLF-

```
-LVAHLC-
MRVAHL--
MVVAHL--
MSVAHL--
MTVAHL--
-MVAHLQ-
-MVAHLL-
MYVAHL--
-MVAHLY-
-MVAHLV-
-MVAHLT-
-MVAHLK-
-MVAHLG-
MEVAHL--
-MVAHLS-
-MVAHLE-
MKVAHL--
MDVAHL--
-MVAHLA-
-MVAHLW-
-MVAHLM-
-MVAHLI-
MNVAHL--
MGVAHL--
MLVAHL--
MAVAHL--
MWVAHL--
MCVAHL--
MPVAHL--
MMVAHL--
MIVAHL--
NRVAHL--
NDVAHL--
NVVAHL--
-NVAHLR-
NQVAHL--
-NVAHLS-
NNVAHL--
NKVAHL--
NHVAHL--
-NVAHLI-
-NVAHLF-
NSVAHL--
-NVAHLN-
NLVAHL--
-NVAHLT-
-NVAHLK-
NTVAHL--
NGVAHL--
-NVAHLY-
```

NPVAHL--
NIVAHL--
NWVAHL--
-NVAHLA-
NYVAHL--
-NVAHLG-
-NVAHLE-
NEVAHL--
-NVAHLW-
-NVAHLV-
-NVAHLQ-
-NVAHLM-
NMVAHL--
-NVAHLH-
-NVAHLC-
-PVAHLL-
-PVAHLT-
-PVAHLY-
-PVAHLV-
-PVAHLR-
-PVAHLK-
PTVAHL--
PSVAHL--
PRVAHL--
PMVAHL--
PAVAHL--
-PVAHLI-
PKVAHL--
PFVAHL--
PVVAHL--
-PVAHLN-
-PVAHLM-
-PVAHLF-
PEVAHL--
-PVAHLG-
-PVAHLS-
-PVAHLH-
-PVAHLE-
PNVAHL--
PIVAHL--
PDVAHL--
PYVAHL--
-PVAHLQ-
-PVAHLA-
PHVAHL--
PCVAHL--
-QVAHLG-
-QVAHLT-
QKVAHL--
QSVAHL--

QFVAHL--
-QVAHLL-
QIVAHL--
-QVAHLW-
-QVAHLR-
-QVAHLV-
-QVAHLQ-
QQVAHL--
QWVAHL--
-QVAHLF-
-QVAHLE-
QGVAHL--
-QVAHLK-
QNVAHL--
QVVAHL--
-QVAHLI-
-QVAHLH-
-QVAHLA-
QEVAHL--
QAVAHL--
QYVAHL--
-QVAHLY-
QCVAHL--
-QVAHLN-
-QVAHLM-
QDVAHL--
QTVAHL--
QPVAHL--
QMVAHL--
-RVAHLR-
RGVAHL--
RVVAHL--
RLVAHL--
-RVAHLK-
-RVAHLY-
-RVAHLA-
-RVAHLQ-
-RVAHLN-
-RVAHLH-
REVAHL--
-RVAHLM-
-RVAHLE-
-RVAHLI-
RDVAHL--
-RVAHLG-
-SVAHLR-
SSVAHL--
-SVAHLS-
SVVAHL--
SNVAHL--

```
-SVAHLL-
SLVAHL--
SWVAHL--
SPVAHL--
SGVAHL--
-SVAHLY-
-SVAHLK-
SAVAHL--
-SVAHLT-
-SVAHLA-
SEVAHL--
-SVAHLH-
SQVAHL--
SIVAHL--
-SVAHLW-
-SVAHLV-
-SVAHLQ-
SHVAHL--
-SVAHLF-
-SVAHLN-
STVAHL--
SCVAHL--
-SVAHLM-
-SVAHLI-
-SVAHLG-
SMVAHL--
-SVAHLE-
TSVAHL--
-TVAHLS-
-TVAHLR-
TGVAHL--
TMVAHL--
TIVAHL--
-TVAHLK-
-TVAHLN-
-TVAHLH-
-TVAHLE-
-TVAHLT-
TPVAHL--
TEVAHL--
TQVAHL--
TFVAHL--
TVVAHL--
-TVAHLI-
-TVAHLL-
TTVAHL--
THVAHL--
TWVAHL--
-TVAHLG-
-TVAHLF-
```

-TVAHLW-
-TVAHLQ-
-TVAHLA-
TDVAHL--
-TVAHLY-
TCVAHL--
-TVAHLM-
VSVAHL--
--VAHLTN
--VAHLRR
--VAHLVR
VNVAHL--
--VAHLTR
--VAHLSA
--VAHLRG
VTVAHL--
--VAHLVS
--VAHLLT
VKVAHL--
--VAHLAW
-VVAHLR-
--VAHLLV
--VAHLST
--VAHLNS
--VAHLGS
VLVAHL--
--VAHLRV
--VAHLRN
--VAHLKY
--VAHLKS
--VAHLKA
--VAHLMS
--VAHLAA
VVVAHL--
VHVAHL--
--VAHLVT
--VAHLFG
--VAHLSG
--VAHLKW
--VAHLFT
VFVAHL--
--VAHLSV
--VAHLLS
--VAHLKD
--VAHLVA
--VAHLLR
--VAHLLF
--VAHLIN
-VVAHLT-
-VVAHLS-

--VAHLVV
--VAHLVG
--VAHLSY
--VAHLRD
--VAHLLH
--VAHLFS
--VAHLYS
--VAHLWS
--VAHLWG
--VAHLVK
--VAHLSF
--VAHLMI
-VVAHLI-
-VVAHLF-
--VAHLYR
--VAHLYN
--VAHLYG
--VAHLVY
--VAHLSN
--VAHLRH
--VAHLQM
--VAHLLW
--VAHLLM
--VAHLIV
--VAHLAS
VQVAHL--
VGVAHL--
--VAHLYM
--VAHLVH
--VAHLSW
--VAHLSK
--VAHLRW
--VAHLMR
--VAHLEA
VYVAHL--
-VVAHLV-
--VAHLYI
--VAHLVQ
--VAHLQR
--VAHLLK
--VAHLKM
--VAHLIS
--VAHLES
VPVAHL--
VEVAHL--
--VAHLTT
--VAHLNA
--VAHLLN
--VAHLKI
--VAHLFH

-VVAHLN-
--VAHLYT
--VAHLYA
--VAHLWL
--VAHLTF
--VAHLRY
--VAHLRM
--VAHLQS
--VAHLMW
--VAHLIT
--VAHLFN
-VVAHLQ-
-VVAHLM-
VMVAHL--
--VAHLWM
--VAHLTS
--VAHLTG
--VAHLTA
--VAHLSM
--VAHLSI
--VAHLSH
--VAHLQV
--VAHLMN
--VAHLMG
--VAHLMF
--VAHLLA
--VAHLKF
--VAHLIG
--VAHLHF
--VAHLFQ
--VAHLAV
--VAHLAT
--VAHLAN
--VAHLAG
-VVAHLK-
-VVAHLH-
VDVAHL--
--VAHLYY
--VAHLYH
--VAHLWT
--VAHLVL
--VAHLTV
--VAHLTK
--VAHLTI
--VAHLQY
--VAHLQT
--VAHLQQ
--VAHLQN
--VAHLQK
--VAHLMD

--VAHLIL
--VAHLHT
--VAHLFR
--VAHLEF
VWVAHL--
VAVAHL--
--VAHLYV
--VAHLWY
--VAHLWK
--VAHLWF
--VAHLTW
--VAHLSQ
--VAHLRE
--VAHLQA
--VAHLNM
--VAHLMM
--VAHLMH
--VAHLKE
--VAHLIH
--VAHLHV
--VAHLHG
--VAHLGA
--VAHLAL
-VVAHLY-
--VAHLYW
--VAHLYL
--VAHLWV
--VAHLVM
--VAHLVE
--VAHLVD
--VAHLQL
--VAHLNL
--VAHLNI
--VAHLMV
--VAHLML
--VAHLKN
--VAHLKK
--VAHLIK
--VAHLGW
--VAHLFK
--VAHLEV
--VAHLEM
--VAHLEG
--VAHLAY
VCVAHL--
--VAHLYQ
--VAHLYD
--VAHLWQ
--VAHLWN
--VAHLTY

--VAHLTH
--VAHLQW
--VAHLQI
--VAHLMQ
--VAHLMA
--VAHLLI
--VAHLIW
--VAHLIM
--VAHLIF
--VAHLHY
--VAHLHM
--VAHLGY
--VAHLGV
--VAHLGF
--VAHLFM
--VAHLEW
--VAHLAQ
--VAHLAH
--VAHLAF
--VAHLYE
--VAHLWR
--VAHLWI
--VAHLTQ
--VAHLTM
--VAHLTE
--VAHLTD
--VAHLSE
--VAHLQH
--VAHLNY
--VAHLNW
--VAHLMY
--VAHLMK
--VAHLME
--VAHLKH
--VAHLIY
--VAHLII
--VAHLID
--VAHLHQ
--VAHLHN
--VAHLHL
--VAHLHH
--VAHLGI
--VAHLGG
--VAHLFI
--VAHLFD
--VAHLEN
--VAHLEH
--VAHLCV
--VAHLCS
--VAHLAK

-WVAHLS-
WNVAHL--
WKVAHL--
-WVAHLT-
WPVAHL--
-WVAHLL-
-WVAHLG-
WGVAHL--
-WVAHLY-
-WVAHLW-
-WVAHLN-
WQVAHL--
WLVAHL--
-WVAHLH-
WWVAHL--
-WVAHLK-
WEVAHL--
WYVAHL--
WVVAHL--
-WVAHLE-
-WVAHLA-
-WVAHLI-
-WVAHLD-
WHVAHL--
-WVAHLM-
WDVAHL--
WAVAHL--
YSVAHL--
-YVAHLV-
-YVAHLL-
YFVAHL--
YAVAHL--
YVVAHL--
-YVAHLH-
YTVAHL--
-YVAHLS-
-YVAHLK-
YIVAHL--
-YVAHLT-
-YVAHLN-
YWVAHL--
YDVAHL--
YNVAHL--
YPVAHL--
YMVAHL--
-YVAHLW-
YEVAHL--
-YVAHLG-
YHVAHL--
YCVAHL--

```
            -YVAHLY-
            -YVAHLI-
            -YVAHLE-
            -YVAHLD-
            YQVAHL--
PRPI         175        727     33.22
            EFPRPI--
            -APRPIS-
            -APRPIY-
            ANPRPI--
            -APRPIR-
            -APRPIN-
            ALPRPI--
            -APRPIH-
            APPRPI--
            AAPRPI--
            ASPRPI--
            ARPRPI--
            AQPRPI--
            AGPRPI--
            -APRPIV-
            AHPRPI--
            AYPRPI--
            AWPRPI--
            -APRPIK-
            -APRPIG-
            -APRPIE-
            -APRPIA-
            ATPRPI--
            AEPRPI--
            ADPRPI--
            -APRPIW-
            -APRPIM-
            -APRPII-
            -APRPIF-
            -APRPIT-
            -APRPIQ-
            ACPRPI--
            CHPRPI--
            CVPRPI--
            CQPRPI--
            CTPRPI--
            CSPRPI--
            CRPRPI--
            CKPRPI--
            CDPRPI--
            -CPRPIY-
            -CPRPIV-
            -CPRPIR-
            -CPRPIF-
```

CNPRPI--
CMPRPI--
CLPRPI--
CGPRPI--
CFPRPI--
CCPRPI--
DVPRPI--
-DPRPII-
-DPRPIT-
-DPRPIW-
DLPRPI--
DKPRPI--
DYPRPI--
-DPRPIY-
-DPRPIL-
DSPRPI--
-DPRPIS-
DIPRPI--
-DPRPIR-
-DPRPIF-
DHPRPI--
DWPRPI--
-DPRPIV-
-DPRPIQ-
-DPRPIK-
-DPRPIA-
DPPRPI--
DDPRPI--
DCPRPI--
DAPRPI--
-EPRPIR-
ESPRPI--
ERPRPI--
EYPRPI--
-EPRPIF-
EWPRPI--
-EPRPIY-
-EPRPIT-
ENPRPI--
EVPRPI--
-EPRPIW-
-EPRPIV-
-EPRPIS-
-EPRPIQ-
-EPRPIM-
-EPRPIL-
-EPRPII-
-EPRPIG-
-EPRPIA-
ELPRPI--

ETPRPI--
-EPRPIN-
EMPRPI--
EKPRPI--
EHPRPI--
FLPRPI--
FKPRPI--
FMPRPI--
FSPRPI--
FPPRPI--
FIPRPI--
FYPRPI--
FTPRPI--
-FPRPIR-
-FPRPIK-
FFPRPI--
FEPRPI--
FQPRPI--
-FPRPIQ-
-FPRPIN-
-FPRPIF-
-FPRPIA-
FGPRPI--
FDPRPI--
FAPRPI--
-FPRPIW-
-FPRPIH-
-FPRPIG-
-FPRPIE-
-GPRPIR-
GSPRPI--
GNPRPI--
GWPRPI--
-GPRPII-
GAPRPI--
-GPRPIT-
GLPRPI--
GVPRPI--
-GPRPIK-
GDPRPI--
GYPRPI--
-GPRPIN-
GQPRPI--
-GPRPIS-
-GPRPIH-
GPPRPI--
-GPRPIQ-
-GPRPIF-
-GPRPIE-
GIPRPI--

GGPRPI--
GFPRPI--
-GPRPIM-
GMPRPI--
GHPRPI--
GEPRPI--
-GPRPIY-
-GPRPIV-
-GPRPIA-
GKPRPI--
HVPRPI--
HAPRPI--
HRPRPI--
-HPRPIS-
HTPRPI--
-HPRPIL-
HEPRPI--
-HPRPIY-
HDPRPI--
HWPRPI--
HSPRPI--
-HPRPIV-
HLPRPI--
-HPRPIK-
-HPRPII-
-HPRPIA-
-HPRPIT-
-HPRPIG-
HGPRPI--
HNPRPI--
HKPRPI--
HHPRPI--
HFPRPI--
HYPRPI--
-HPRPIN-
-HPRPIM-
-HPRPIH-
-IPRPIY-
-IPRPIS-
ILPRPI--
IIPRPI--
IVPRPI--
ITPRPI--
-IPRPIA-
IGPRPI--
-IPRPIT-
IPPRPI--
-IPRPIR-
-IPRPIM-
-IPRPIK-

```
-IPRPII-
-IPRPIG-
IMPRPI--
IYPRPI--
-IPRPIH-
IKPRPI--
IEPRPI--
IAPRPI--
-IPRPIN-
-IPRPIF-
IFPRPI--
IDPRPI--
KSPRPI--
KLPRPI--
KVPRPI--
-KPRPIH-
KAPRPI--
-KPRPIR-
KFPRPI--
KGPRPI--
KYPRPI--
-KPRPIY-
-KPRPIS-
-KPRPIM-
KWPRPI--
-KPRPIF-
KDPRPI--
-KPRPIK-
-KPRPIA-
KIPRPI--
-KPRPIW-
-KPRPII-
KPPRPI--
KKPRPI--
KQPRPI--
-KPRPIC-
KNPRPI--
KHPRPI--
LLPRPI--
LIPRPI--
LMPRPI--
LRPRPI--
LSPRPI--
LKPRPI--
LVPRPI--
LFPRPI--
LEPRPI--
LYPRPI--
LTPRPI--
LAPRPI--
```

LWPRPI--
LQPRPI--
-LPRPIY-
-LPRPIK-
-LPRPIW-
-LPRPIV-
LDPRPI--
-LPRPIM-
-LPRPIH-
LGPRPI--
-LPRPIA-
LPPRPI--
MLPRPI--
MVPRPI--
-MPRPIL-
MWPRPI--
MRPRPI--
-MPRPIY-
MPPRPI--
MKPRPI--
MTPRPI--
-MPRPIK-
MHPRPI--
MSPRPI--
MQPRPI--
MYPRPI--
-MPRPIW-
MEPRPI--
-MPRPIT-
-MPRPIR-
MFPRPI--
MAPRPI--
-MPRPII-
-MPRPIV-
-MPRPIN-
MMPRPI--
MIPRPI--
-MPRPIQ-
-MPRPIG-
-MPRPIF-
-MPRPIE-
NDPRPI--
NKPRPI--
NHPRPI--
-NPRPIY-
NVPRPI--
-NPRPIH-
NLPRPI--
NYPRPI--
NQPRPI--

NNPRPI--
NTPRPI--
-NPRPIF-
NRPRPI--
-NPRPIW-
-NPRPIL-
-NPRPIT-
-NPRPII-
-NPRPIA-
NGPRPI--
-NPRPIV-
-NPRPIM-
-NPRPIS-
-NPRPIQ-
-NPRPIN-
NMPRPI--
--PRPIFL
PSPRPI--
--PRPILV
--PRPITR
--PRPIRL
PYPRPI--
--PRPISS
--PRPIQT
--PRPILT
--PRPIQV
--PRPIYY
--PRPIYA
--PRPIWT
--PRPISL
--PRPIRQ
--PRPIVT
--PRPIRA
--PRPINL
--PRPIML
--PRPIHL
--PRPIWM
--PRPISW
--PRPINS
--PRPIIR
--PRPIAL
--PRPIYL
--PRPIVA
--PRPISY
--PRPISF
--PRPIRH
--PRPILK
--PRPIKY
--PRPIIT
--PRPIWN

--PRPIFM
--PRPIAF
--PRPIYI
--PRPISV
--PRPIRY
--PRPIIH
--PRPIHR
--PRPIGG
--PRPIYH
--PRPITS
--PRPITF
--PRPISN
--PRPISI
--PRPISH
--PRPISG
--PRPIRV
--PRPILF
--PRPIGH
--PRPIFS
--PRPIYV
--PRPIWF
--PRPIVR
--PRPITK
--PRPIST
--PRPISR
--PRPIRF
--PRPIQR
--PRPIQG
--PRPINW
--PRPINF
--PRPIKV
PQPRPI--
PLPRPI--
PDPRPI--
--PRPIYT
--PRPIYK
--PRPITA
--PRPIPM
--PRPINV
--PRPINI
--PRPIIV
--PRPIIS
--PRPIFD
--PRPIFA
--PRPIAI
--PRPIWW
--PRPIVY
--PRPIVK
--PRPISM
--PRPIRW

--PRPIRN
--PRPIQH
--PRPIPY
--PRPINY
--PRPIMW
--PRPIKS
--PRPIKR
--PRPIKL
--PRPIKA
--PRPIGV
--PRPIGN
--PRPIGI
--PRPIGF
--PRPIFV
--PRPIEL
--PRPIAY
--PRPIAV
--PRPIYS
--PRPIWK
--PRPIVV
--PRPIVN
--PRPITG
--PRPIRM
--PRPIRD
--PRPIQN
--PRPIQK
--PRPINT
--PRPIMT
--PRPIMS
--PRPIMK
--PRPIMH
--PRPILM
--PRPIKQ
--PRPIKM
--PRPIKG
--PRPIIK
--PRPIIG
--PRPIHI
--PRPIHA
--PRPIFW
--PRPIFI
--PRPIFH
--PRPIAT
--PRPIYW
--PRPIWY
--PRPIVM
--PRPITY
--PRPITV
--PRPITN
--PRPITE

--PRPIRR
--PRPIQI
--PRPINM
--PRPINK
--PRPINA
--PRPIMQ
--PRPIKT
--PRPIIF
--PRPIGS
--PRPIGK
--PRPIFQ
--PRPIAM
--PRPIAA
--PRPIWI
--PRPIVS
--PRPITH
--PRPIRE
--PRPIQQ
--PRPIQL
--PRPIPW
--PRPIPF
--PRPINN
--PRPINH
--PRPIMM
--PRPIMA
--PRPIHM
--PRPIGY
--PRPIGA
--PRPIFF
--PRPIFE
--PRPIEY
--PRPIES
--PRPIEI
--PRPIEF
--PRPIDY
--PRPIDI
--PRPIAS
--PRPIAQ
--PRPIAN
PMPRPI--
PHPRPI--
PGPRPI--
PCPRPI--
PAPRPI--
--PRPIYN
--PRPIYM
--PRPIYG
--PRPISQ
--PRPISE
--PRPISA

--PRPIQD
--PRPIPV
--PRPIPS
--PRPIPQ
--PRPIPH
--PRPIMF
--PRPIKK
--PRPIKI
--PRPIKF
--PRPIIY
--PRPIIW
--PRPIII
--PRPIHY
--PRPIHW
--PRPIHT
--PRPIHQ
--PRPIHN
--PRPIHH
--PRPIGR
--PRPIFY
--PRPIEW
--PRPIAK
--PRPIAD
PNPRPI--
PIPRPI--
PEPRPI--
--PRPIYF
--PRPIWQ
--PRPIWA
--PRPIVW
--PRPIVD
--PRPITW
--PRPITD
--PRPIQW
--PRPIQS
--PRPIQM
--PRPIQF
--PRPIPI
--PRPIPG
--PRPINQ
--PRPIMY
--PRPIMV
--PRPIMN
--PRPIME
--PRPIMD
--PRPILE
--PRPILC
--PRPIKN
--PRPIKH
--PRPIIA

--PRPIHV
--PRPIHK
--PRPIHE
--PRPIHC
--PRPIGW
--PRPIGM
--PRPIEV
--PRPIET
--PRPIER
--PRPIEQ
--PRPIDA
--PRPICV
--PRPICR
--PRPIAG
-PPRPIV-
QLPRPI--
QVPRPI--
-QPRPIM-
-QPRPII-
QWPRPI--
QTPRPI--
-QPRPIS-
-QPRPIL-
QGPRPI--
QRPRPI--
QDPRPI--
QIPRPI--
QMPRPI--
QKPRPI--
QSPRPI--
QQPRPI--
-QPRPIY-
-QPRPIW-
-QPRPIT-
QFPRPI--
QAPRPI--
-QPRPIK-
-QPRPIG-
-QPRPIA-
RFPRPI--
RMPRPI--
RVPRPI--
RLPRPI--
-RPRPIL-
-RPRPIF-
RNPRPI--
-RPRPIY-
-RPRPIV-
-RPRPII-
RGPRPI--

-RPRPIH-
RHPRPI--
RYPRPI--
RPPRPI--
RTPRPI--
-RPRPIS-
-RPRPIT-
RRPRPI--
RQPRPI--
REPRPI--
-RPRPIE-
RKPRPI--
RDPRPI--
-RPRPIQ-
-RPRPIN-
-RPRPIC-
RCPRPI--
SLPRPI--
-SPRPIR-
SVPRPI--
-SPRPIT-
-SPRPIQ-
SSPRPI--
SQPRPI--
SMPRPI--
SEPRPI--
-SPRPIH-
STPRPI--
-SPRPIM-
-SPRPIF-
SPPRPI--
SKPRPI--
SIPRPI--
SNPRPI--
-SPRPIA-
SWPRPI--
-SPRPIN-
SGPRPI--
SCPRPI--
TVPRPI--
-TPRPIL-
THPRPI--
-TPRPIY-
-TPRPIR-
-TPRPIK-
-TPRPIV-
-TPRPIS-
-TPRPIA-
TFPRPI--
TQPRPI--

-TPRPIM-
TGPRPI--
TAPRPI--
TWPRPI--
TMPRPI--
TEPRPI--
TDPRPI--
-TPRPIQ-
-TPRPIH-
-TPRPIG-
VQPRPI--
VLPRPI--
-VPRPIL-
VKPRPI--
VEPRPI--
VYPRPI--
VVPRPI--
VGPRPI--
VRPRPI--
VWPRPI--
-VPRPIV-
-VPRPIG-
-VPRPIQ-
-VPRPII-
VDPRPI--
VTPRPI--
-VPRPIM-
-VPRPIK-
-VPRPIF-
VMPRPI--
VSPRPI--
VNPRPI--
-VPRPIT-
VPPRPI--
VHPRPI--
VFPRPI--
-VPRPIN-
-VPRPIA-
-VPRPIE-
-WPRPIF-
WIPRPI--
WWPRPI--
WVPRPI--
WSPRPI--
-WPRPIK-
WEPRPI--
-WPRPIT-
WYPRPI--
WNPRPI--
WMPRPI--

| | | | |
|---|---|---|---|
| | WAPRPI-- | | |
| | WTPRPI-- | | |
| | WQPRPI-- | | |
| | WKPRPI-- | | |
| | WGPRPI-- | | |
| | WFPRPI-- | | |
| | WRPRPI-- | | |
| | WHPRPI-- | | |
| | -WPRPIW- | | |
| | -WPRPIN- | | |
| | -WPRPIE- | | |
| | YVPRPI-- | | |
| | -YPRPIY- | | |
| | -YPRPIS- | | |
| | YAPRPI-- | | |
| | -YPRPIQ- | | |
| | YTPRPI-- | | |
| | -YPRPIH- | | |
| | YKPRPI-- | | |
| | YNPRPI-- | | |
| | YHPRPI-- | | |
| | YDPRPI-- | | |
| | -YPRPIK- | | |
| | YWPRPI-- | | |
| | YGPRPI-- | | |
| | YEPRPI-- | | |
| | YYPRPI-- | | |
| | -YPRPIN- | | |
| | -YPRPIE- | | |
| | YPPRPI-- | | |
| | YIPRPI-- | | |
| | YFPRPI-- | | |
| | YCPRPI-- | | |
| VGNL | 71 | 789 | 33.21 |
| | ADVGNL-- | | |
| | -DVGNLN- | | |
| | -KVGNLG- | | |
| | -MVGNLH- | | |
| | -QVGNLH- | | |
| | -RVGNLC- | | |
| | --VGNLNG | | |
| | --VGNLVP | | |
| | --VGNLTD | | |
| | --VGNLSD | | |
| | --VGNLLD | | |
| | --VGNLKQ | | |
| | --VGNLGV | | |
| | --VGNLAR | | |
| | --VGNLAH | | |
| | -YVGNLD- | | |

ALVGNL--
ASVGNL--
-AVGNLR-
-AVGNLL-
AVVGNL--
AMVGNL--
-AVGNLT-
AFVGNL--
-AVGNLV-
-AVGNLA-
AIVGNL--
AGVGNL--
-AVGNLW-
-AVGNLY-
-AVGNLN-
ATVGNL--
AKVGNL--
-AVGNLE-
-AVGNLG-
APVGNL--
AYVGNL--
-AVGNLK-
-AVGNLI-
-AVGNLM-
-AVGNLF-
ACVGNL--
AWVGNL--
AEVGNL--
AQVGNL--
AHVGNL--
-AVGNLC-
-CVGNLS-
CSVGNL--
CLVGNL--
-CVGNLM-
CRVGNL--
CIVGNL--
CPVGNL--
-CVGNLV-
CYVGNL--
-CVGNLR-
-CVGNLL-
-CVGNLI-
CEVGNL--
CCVGNL--
-CVGNLH-
CQVGNL--
CWVGNL--
CVVGNL--
-CVGNLE-

-CVGNLA-
CMVGNL--
CFVGNL--
CAVGNL--
-CVGNLY-
-CVGNLT-
-CVGNLQ-
CTVGNL--
-DVGNLR-
DRVGNL--
DHVGNL--
DSVGNL--
DYVGNL--
-DVGNLY-
-DVGNLT-
DLVGNL--
-DVGNLL-
-DVGNLH-
-DVGNLV-
DIVGNL--
DGVGNL--
-DVGNLW-
-DVGNLA-
DNVGNL--
DMVGNL--
DKVGNL--
DWVGNL--
-DVGNLS-
-DVGNLM-
-DVGNLK-
-DVGNLI-
DPVGNL--
DFVGNL--
DQVGNL--
DDVGNL--
DCVGNL--
ERVGNL--
ESVGNL--
-EVGNLF-
EAVGNL--
-EVGNLL-
-EVGNLK-
EPVGNL--
ELVGNL--
EVVGNL--
-EVGNLY-
-EVGNLV-
-EVGNLT-
-EVGNLR-
-EVGNLI-

EKVGNL--
EWVGNL--
EMVGNL--
EEVGNL--
EYVGNL--
-EVGNLM-
-EVGNLW-
-EVGNLS-
ETVGNL--
EHVGNL--
EFVGNL--
-EVGNLH-
-EVGNLE-
ENVGNL--
EDVGNL--
ECVGNL--
-EVGNLQ-
-EVGNLN-
-EVGNLA-
EIVGNL--
-FVGNLR-
-FVGNLG-
FKVGNL--
-FVGNLS-
-FVGNLT-
-FVGNLM-
-FVGNLL-
-FVGNLA-
FPVGNL--
FFVGNL--
-FVGNLN-
-FVGNLH-
-FVGNLV-
-FVGNLQ-
-FVGNLW-
-FVGNLK-
-FVGNLY-
FDVGNL--
FYVGNL--
-FVGNLE-
FHVGNL--
FCVGNL--
-FVGNLD-
GPVGNL--
-GVGNLK-
-GVGNLS-
GHVGNL--
-GVGNLT-
GIVGNL--
-GVGNLR-

-GVGNLQ-
GQVGNL--
GWVGNL--
-GVGNLV-
-GVGNLI-
-GVGNLM-
GNVGNL--
GMVGNL--
GFVGNL--
GGVGNL--
-GVGNLA-
GYVGNL--
-GVGNLE-
GEVGNL--
GDVGNL--
-GVGNLF-
GCVGNL--
HRVGNL--
-HVGNLS-
HKVGNL--
HFVGNL--
-HVGNLT-
-HVGNLL-
HLVGNL--
HGVGNL--
HMVGNL--
HIVGNL--
-HVGNLY-
-HVGNLN-
-HVGNLA-
-HVGNLR-
HHVGNL--
HVVGNL--
-HVGNLQ-
HSVGNL--
HTVGNL--
-HVGNLH-
HPVGNL--
HYVGNL--
-HVGNLV-
-HVGNLI-
HQVGNL--
HEVGNL--
HAVGNL--
-HVGNLM-
-HVGNLK-
-HVGNLG-
HDVGNL--
HCVGNL--
-HVGNLW-

-HVGNLE-
ITVGNL--
-IVGNLL-
-IVGNLR-
-IVGNLK-
ISVGNL--
IPVGNL--
IGVGNL--
IVVGNL--
-IVGNLN-
-IVGNLT-
-IVGNLQ-
IAVGNL--
-IVGNLF-
IIVGNL--
IDVGNL--
IWVGNL--
-IVGNLH-
IFVGNL--
IEVGNL--
-IVGNLM-
-IVGNLI-
IHVGNL--
IYVGNL--
-IVGNLY-
-IVGNLA-
IQVGNL--
-IVGNLE-
INVGNL--
ICVGNL--
-KVGNLR-
KSVGNL--
-KVGNLS-
-KVGNLT-
-KVGNLV-
KYVGNL--
KVVGNL--
-KVGNLY-
KFVGNL--
-KVGNLK-
KPVGNL--
-KVGNLW-
-KVGNLL-
KTVGNL--
KNVGNL--
KQVGNL--
KGVGNL--
-KVGNLH-
KAVGNL--
KHVGNL--

-KVGNLA-
KDVGNL--
-KVGNLQ-
-KVGNLM-
-KVGNLI-
-KVGNLF-
KEVGNL--
-KVGNLE-
-LVGNLK-
-LVGNLS-
-LVGNLG-
-LVGNLT-
-LVGNLA-
-LVGNLN-
LKVGNL--
-LVGNLV-
LGVGNL--
LEVGNL--
-LVGNLW-
LNVGNL--
LWVGNL--
LYVGNL--
-LVGNLE-
-LVGNLF-
-LVGNLM-
-LVGNLQ-
LCVGNL--
-LVGNLP-
-LVGNLD-
-MVGNLL-
-MVGNLR-
MYVGNL--
-MVGNLS-
-MVGNLG-
MIVGNL--
MQVGNL--
-MVGNLM-
MKVGNL--
-MVGNLW-
-MVGNLA-
MDVGNL--
MLVGNL--
-MVGNLN-
-MVGNLF-
-MVGNLV-
-MVGNLQ-
-MVGNLI-
MCVGNL--
MMVGNL--
MWVGNL--

```
MNVGNL--
-MVGNLT-
MGVGNL--
MEVGNL--
-MVGNLE-
MPVGNL--
-MVGNLY-
MHVGNL--
MAVGNL--
NVVGNL--
-NVGNLR-
-NVGNLL-
-NVGNLT-
NSVGNL--
-NVGNLF-
NLVGNL--
NWVGNL--
NKVGNL--
NFVGNL--
NDVGNL--
-NVGNLM-
-NVGNLQ-
NQVGNL--
NHVGNL--
NYVGNL--
-NVGNLY-
-NVGNLV-
-NVGNLK-
-NVGNLI-
NNVGNL--
NIVGNL--
-NVGNLH-
NTVGNL--
NMVGNL--
NGVGNL--
-NVGNLS-
NPVGNL--
-NVGNLA-
-NVGNLG-
-NVGNLE-
NEVGNL--
-NVGNLW-
PRVGNL--
PYVGNL--
PGVGNL--
-PVGNLY-
PLVGNL--
-PVGNLW-
-PVGNLS-
-PVGNLT-
```

-PVGNLA-
PEVGNL--
PSVGNL--
-PVGNLG-
PFVGNL--
-PVGNLK-
PNVGNL--
-PVGNLH-
PHVGNL--
PAVGNL--
PVVGNL--
-PVGNLV-
PWVGNL--
-PVGNLQ-
-PVGNLN-
-PVGNLM-
PKVGNL--
PIVGNL--
PMVGNL--
PCVGNL--
-PVGNLE-
-PVGNLI-
-QVGNLS-
-QVGNLL-
QSVGNL--
QVVGNL--
-QVGNLA-
QRVGNL--
QYVGNL--
QLVGNL--
-QVGNLT-
-QVGNLQ-
-QVGNLF-
QIVGNL--
-QVGNLR-
-QVGNLN-
-QVGNLK-
QGVGNL--
-QVGNLW-
QFVGNL--
QAVGNL--
-QVGNLY-
-QVGNLV-
QTVGNL--
QHVGNL--
QEVGNL--
QWVGNL--
QNVGNL--
-QVGNLM-
-QVGNLG-

QMVGNL--
QCVGNL--
-QVGNLI-
-QVGNLE-
QQVGNL--
QKVGNL--
QPVGNL--
QDVGNL--
-RVGNLL-
-RVGNLK-
RVVGNL--
RTVGNL--
-RVGNLW-
RGVGNL--
RQVGNL--
-RVGNLI-
-RVGNLV-
REVGNL--
-RVGNLQ-
-RVGNLN-
RCVGNL--
-RVGNLE-
-SVGNLR-
SKVGNL--
SVVGNL--
SAVGNL--
SSVGNL--
SYVGNL--
SLVGNL--
-SVGNLV-
STVGNL--
-SVGNLL-
-SVGNLH-
SMVGNL--
SIVGNL--
SFVGNL--
-SVGNLY-
-SVGNLQ-
-SVGNLA-
-SVGNLM-
-SVGNLK-
SNVGNL--
-SVGNLN-
SEVGNL--
SQVGNL--
SPVGNL--
-SVGNLW-
-SVGNLE-
SDVGNL--
-SVGNLG-

SCVGNL--
TKVGNL--
TLVGNL--
TSVGNL--
TRVGNL--
TPVGNL--
-TVGNLS-
TAVGNL--
TNVGNL--
-TVGNLI-
-TVGNLH-
-TVGNLK-
-TVGNLT-
TTVGNL--
TGVGNL--
TVVGNL--
-TVGNLV-
TFVGNL--
-TVGNLF-
-TVGNLA-
TMVGNL--
-TVGNLY-
-TVGNLQ-
TDVGNL--
TWVGNL--
-TVGNLG-
-TVGNLN-
TQVGNL--
TCVGNL--
-TVGNLE-
-TVGNLC-
--VGNLLT
--VGNLRK
VRVGNL--
--VGNLRS
--VGNLVL
--VGNLSG
VKVGNL--
--VGNLST
--VGNLRR
--VGNLRG
--VGNLVG
--VGNLRI
--VGNLLA
--VGNLTS
-VVGNLT-
--VGNLTT
-VVGNLV-
--VGNLTF
--VGNLRA

VVVGNL--
--VGNLGS
VPVGNL--
--VGNLIG
VQVGNL--
--VGNLKS
--VGNLLH
--VGNLIS
VMVGNL--
VGVGNL--
--VGNLRT
--VGNLAV
VWVGNL--
-VVGNLA-
--VGNLYI
--VGNLTR
--VGNLRH
--VGNLMS
VTVGNL--
--VGNLVN
--VGNLRF
--VGNLQA
--VGNLSA
--VGNLMN
--VGNLIK
-VVGNLI-
--VGNLRN
--VGNLMT
--VGNLML
--VGNLLN
--VGNLKR
--VGNLFT
--VGNLFS
-VVGNLF-
--VGNLYQ
--VGNLYG
--VGNLSR
--VGNLRD
--VGNLLV
--VGNLLQ
VAVGNL--
-VVGNLN-
-VVGNLH-
--VGNLYT
--VGNLYS
--VGNLVY
--VGNLVT
--VGNLTY
--VGNLMR
--VGNLAS

VHVGNL--
--VGNLWS
--VGNLVF
--VGNLSV
--VGNLRY
--VGNLNS
--VGNLKM
--VGNLFG
-VVGNLQ-
VNVGNL--
--VGNLYH
--VGNLYA
--VGNLVI
--VGNLVA
--VGNLNA
--VGNLHY
--VGNLHG
--VGNLAT
VFVGNL--
-VVGNLW-
--VGNLVQ
--VGNLVK
--VGNLTH
--VGNLTA
--VGNLRQ
--VGNLLM
--VGNLKY
--VGNLAG
VIVGNL--
--VGNLVM
--VGNLTG
--VGNLSW
--VGNLMW
VEVGNL--
--VGNLYN
--VGNLKV
--VGNLKA
--VGNLHR
--VGNLWG
--VGNLTW
--VGNLSL
--VGNLSK
--VGNLAW
--VGNLYV
--VGNLWA
--VGNLVV
--VGNLVH
--VGNLSM
--VGNLKT
--VGNLIV

--VGNLGT
--VGNLFQ
--VGNLAY
--VGNLVW
--VGNLTI
--VGNLMK
--VGNLMH
--VGNLKG
--VGNLIM
--VGNLHW
--VGNLHQ
--VGNLGA
--VGNLFN
--VGNLEA
--VGNLAL
--VGNLRW
--VGNLKN
--VGNLIQ
--VGNLIN
--VGNLIL
--VGNLIA
--VGNLHV
--VGNLAN
--VGNLAM
--VGNLAA
-VVGNLE-
--VGNLYL
--VGNLTQ
--VGNLSQ
--VGNLSI
--VGNLQW
--VGNLMY
--VGNLMD
--VGNLMA
--VGNLKI
--VGNLIY
--VGNLHS
--VGNLHH
--VGNLFK
--VGNLFH
--VGNLET
--VGNLES
VDVGNL--
--VGNLRE
--VGNLQT
--VGNLQR
--VGNLNV
--VGNLMI
--VGNLKL
--VGNLGG

--VGNLYW
--VGNLWQ
--VGNLWL
--VGNLWI
--VGNLWH
--VGNLTN
--VGNLQG
--VGNLQF
--VGNLNT
--VGNLKH
--VGNLKF
--VGNLII
--VGNLEG
--VGNLYD
--VGNLWN
--VGNLWK
--VGNLTM
--VGNLSN
--VGNLSF
--VGNLQY
--VGNLQV
--VGNLQM
--VGNLQK
--VGNLMV
--VGNLIW
--VGNLID
--VGNLHF
--VGNLHA
--VGNLAI
--VGNLVD
--VGNLQL
--VGNLQH
--VGNLNW
--VGNLNM
--VGNLNH
--VGNLIF
--VGNLIE
--VGNLHT
--VGNLHM
--VGNLHI
--VGNLDS
--VGNLAQ
--VGNLAF
--VGNLQQ
--VGNLQP
--VGNLQI
--VGNLQE
--VGNLNF
--VGNLMQ
--VGNLKE

```
--VGNLHN
--VGNLHK
--VGNLGW
--VGNLFC
--VGNLEQ
--VGNLEN
--VGNLEI
--VGNLEH
--VGNLCS
--VGNLCL
--VGNLAK
-WVGNLR-
-WVGNLS-
-WVGNLL-
WKVGNL--
WSVGNL--
-WVGNLV-
WVVGNL--
-WVGNLK-
-WVGNLA-
WYVGNL--
-WVGNLY-
-WVGNLN-
WQVGNL--
WIVGNL--
WAVGNL--
-WVGNLW-
-WVGNLM-
WNVGNL--
-WVGNLH-
WDVGNL--
WPVGNL--
WWVGNL--
-WVGNLQ-
-WVGNLG-
WHVGNL--
WFVGNL--
WCVGNL--
-WVGNLF-
-WVGNLD-
YRVGNL--
-YVGNLR-
-YVGNLY-
-YVGNLK-
YLVGNL--
-YVGNLI-
YKVGNL--
YEVGNL--
YWVGNL--
-YVGNLS-
```

-YVGNLA-
-YVGNLV-
-YVGNLM-
-YVGNLE-
YIVGNL--
-YVGNLQ-
YNVGNL--
YMVGNL--
YGVGNL--
YVVGNL--
-YVGNLN-
YHVGNL--
-YVGNLT-
YQVGNL--
YYVGNL--
-YVGNLF-
YFVGNL--
-YVGNLH-
YPVGNL--
YDVGNL--
YTVGNL--
YCVGNL--
-YVGNLW-

| | | | |
|---|---|---|---|
| PSPM | 112 | 761 | 33.20 |

LIPSPM--
--PSPMNR
-YPSPMS-
ARPSPM--
-APSPMR-
-APSPMS-
AVPSPM--
-APSPML-
AQPSPM--
ASPSPM--
-APSPMK-
-APSPMA-
AFPSPM--
-APSPMY-
-APSPMF-
-APSPME-
AMPSPM--
AKPSPM--
AWPSPM--
ATPSPM--
-APSPMI-
ANPSPM--
-APSPMW-
-APSPMV-
-APSPMT-
-APSPMN-

-APSPMM-
-APSPMH-
AIPSPM--
AGPSPM--
-APSPMQ-
-APSPMG-
-APSPMC-
APPSPM--
ALPSPM--
CSPSPM--
-CPSPML-
-CPSPMQ-
-CPSPMR-
-CPSPMI-
-CPSPMT-
CMPSPM--
CLPSPM--
CYPSPM--
CPPSPM--
CNPSPM--
CKPSPM--
DAPSPM--
-DPSPMS-
-DPSPMV-
DHPSPM--
DSPSPM--
-DPSPMT-
DLPSPM--
DVPSPM--
DRPSPM--
DTPSPM--
-DPSPMQ-
-DPSPMN-
-DPSPMM-
-DPSPML-
-DPSPMK-
-DPSPMF-
-DPSPMA-
DNPSPM--
DMPSPM--
DIPSPM--
-EPSPML-
-EPSPMR-
EFPSPM--
ESPSPM--
-EPSPMS-
-EPSPMI-
-EPSPMM-
EVPSPM--
-EPSPMW-

-EPSPMQ-
-EPSPMK-
ETPSPM--
ERPSPM--
EQPSPM--
-EPSPMY-
-EPSPMV-
-EPSPMT-
-EPSPMF-
EWPSPM--
-EPSPMN-
ELPSPM--
EIPSPM--
EAPSPM--
-FPSPMR-
FLPSPM--
-FPSPMN-
FKPSPM--
-FPSPMA-
FAPSPM--
FNPSPM--
FHPSPM--
FGPSPM--
FRPSPM--
-FPSPMV-
-FPSPMT-
-FPSPMQ-
-FPSPMK-
FMPSPM--
FIPSPM--
FVPSPM--
-FPSPMY-
-FPSPMS-
FEPSPM--
FTPSPM--
FSPSPM--
-FPSPMI-
-FPSPMG-
-FPSPMF-
-FPSPME-
FPPSPM--
-GPSPMR-
GFPSPM--
GRPSPM--
-GPSPMV-
-GPSPMW-
GLPSPM--
GKPSPM--
GYPSPM--
GQPSPM--

-GPSPMM-
-GPSPMT-
-GPSPML-
-GPSPMK-
GNPSPM--
GSPSPM--
-GPSPMQ-
-GPSPMG-
GAPSPM--
GVPSPM--
GTPSPM--
-GPSPMF-
GHPSPM--
GWPSPM--
-GPSPMY-
-GPSPMN-
-GPSPMI-
-GPSPMH-
-GPSPME-
GMPSPM--
HRPSPM--
-HPSPMS-
-HPSPMW-
-HPSPML-
-HPSPMV-
HGPSPM--
-HPSPMI-
HNPSPM--
HSPSPM--
-HPSPMT-
-HPSPMN-
-HPSPMF-
HVPSPM--
-HPSPMA-
HMPSPM--
HAPSPM--
HTPSPM--
-HPSPMY-
-HPSPMR-
-HPSPMQ-
-HPSPMM-
-HPSPMH-
-HPSPMG-
HPPSPM--
HLPSPM--
HIPSPM--
HEPSPM--
HDPSPM--
IVPSPM--
-IPSPMR-

-IPSPML-
ISPSPM--
IPPSPM--
INPSPM--
IGPSPM--
IYPSPM--
-IPSPMW-
-IPSPMQ-
IEPSPM--
IRPSPM--
-IPSPMV-
-IPSPMS-
-IPSPMN-
-IPSPMK-
-IPSPMG-
IAPSPM--
IWPSPM--
-IPSPMI-
-IPSPMH-
IKPSPM--
IIPSPM--
IHPSPM--
IFPSPM--
KSPSPM--
-KPSPMK-
-KPSPMT-
-KPSPMR-
-KPSPML-
-KPSPMI-
-KPSPME-
KPPSPM--
KNPSPM--
KLPSPM--
KFPSPM--
KAPSPM--
KVPSPM--
KYPSPM--
KTPSPM--
KRPSPM--
-KPSPMY-
-KPSPMM-
KGPSPM--
-KPSPMW-
-KPSPMQ-
-KPSPMN-
KEPSPM--
KWPSPM--
-KPSPMV-
-KPSPMS-
KKPSPM--

KHPSPM--
KDPSPM--
LSPSPM--
-LPSPMS-
-LPSPMR-
-LPSPMK-
LGPSPM--
LFPSPM--
LRPSPM--
-LPSPMT-
LYPSPM--
LVPSPM--
LTPSPM--
-LPSPMI-
-LPSPMH-
LLPSPM--
-LPSPMV-
LDPSPM--
-LPSPMW-
LWPSPM--
LPPSPM--
LNPSPM--
LKPSPM--
-LPSPMY-
-LPSPMQ-
-LPSPMN-
LEPSPM--
LAPSPM--
-LPSPMM-
-LPSPMG-
LMPSPM--
LHPSPM--
-MPSPML-
-MPSPMR-
-MPSPMN-
-MPSPME-
MVPSPM--
-MPSPMV-
-MPSPMT-
-MPSPMS-
-MPSPMH-
-MPSPMA-
MKPSPM--
-MPSPMF-
MPPSPM--
MMPSPM--
MIPSPM--
MEPSPM--
MYPSPM--
MWPSPM--

MRPSPM--
-MPSPMY-
MAPSPM--
NYPSPM--
NSPSPM--
-NPSPMV-
-NPSPMF-
-NPSPMW-
-NPSPMT-
-NPSPMR-
NTPSPM--
NVPSPM--
NRPSPM--
NQPSPM--
-NPSPMI-
NMPSPM--
NWPSPM--
-NPSPMY-
-NPSPMS-
-NPSPMM-
-NPSPMK-
-NPSPMH-
-NPSPMA-
NPPSPM--
NLPSPM--
NEPSPM--
NCPSPM--
-NPSPMQ-
-NPSPME-
NNPSPM--
NKPSPM--
NHPSPM--
NGPSPM--
NFPSPM--
NDPSPM--
NAPSPM--
--PSPMAS
--PSPMLL
--PSPMQV
--PSPMML
--PSPMLY
--PSPMLG
--PSPMYI
--PSPMSL
PRPSPM--
--PSPMYR
--PSPMQA
--PSPMAL
--PSPMLQ
--PSPMGF

--PSPMWI
--PSPMTI
--PSPMSW
--PSPMST
--PSPMSR
--PSPMRT
--PSPMPW
--PSPMLS
--PSPMSY
--PSPMRH
--PSPMKW
--PSPMHL
--PSPMVG
--PSPMRS
--PSPMRN
--PSPMFS
--PSPMSK
--PSPMQR
--PSPMNL
--PSPMMN
--PSPMLT
--PSPMIT
--PSPMSV
--PSPMLV
--PSPMKS
--PSPMKL
--PSPMFR
--PSPMFK
--PSPMAT
PAPSPM--
--PSPMYA
--PSPMVR
--PSPMRR
--PSPMGG
PKPSPM--
--PSPMYL
--PSPMWL
--PSPMWF
--PSPMVY
--PSPMVW
--PSPMVS
--PSPMVL
--PSPMVA
--PSPMTL
--PSPMSA
--PSPMPI
--PSPMKK
--PSPMIR
--PSPMHY
--PSPMGY

--PSPMGV
--PSPMGA
--PSPMEW
--PSPMYW
--PSPMYS
--PSPMYN
--PSPMYK
--PSPMYG
--PSPMWQ
--PSPMVH
--PSPMVD
--PSPMTV
--PSPMTK
--PSPMTG
--PSPMSI
--PSPMSG
--PSPMSF
--PSPMNY
--PSPMNW
--PSPMMI
--PSPMMH
--PSPMLM
--PSPMLE
--PSPMKY
--PSPMKI
--PSPMIV
--PSPMIK
--PSPMHQ
--PSPMHE
--PSPMGW
--PSPMGS
--PSPMGH
--PSPMFI
--PSPMAW
--PSPMYM
--PSPMWT
--PSPMWS
--PSPMWA
--PSPMVQ
--PSPMTF
--PSPMRF
--PSPMRD
--PSPMPL
--PSPMNV
--PSPMNA
--PSPMMV
--PSPMLW
--PSPMLK
--PSPMLA
--PSPMKT

--PSPMKE
--PSPMKD
--PSPMHH
--PSPMFG
--PSPMEL
--PSPMDM
PGPSPM--
--PSPMYV
--PSPMYT
--PSPMVV
--PSPMVN
--PSPMVM
--PSPMVI
--PSPMTT
--PSPMSN
--PSPMRV
--PSPMRM
--PSPMRG
--PSPMRE
--PSPMRA
--PSPMQS
--PSPMNT
--PSPMNI
--PSPMMS
--PSPMMR
--PSPMMM
--PSPMMG
--PSPMLI
--PSPMKA
--PSPMIY
--PSPMIS
--PSPMIQ
--PSPMIN
--PSPMIL
--PSPMIG
--PSPMHT
--PSPMFW
--PSPMFA
--PSPMEM
--PSPMDL
--PSPMAR
--PSPMAH
PFPSPM--
PVPSPM--
--PSPMYH
--PSPMYE
--PSPMTY
--PSPMTS
--PSPMTR
--PSPMSS

--PSPMSH
--PSPMRW
--PSPMRQ
--PSPMRK
--PSPMQW
--PSPMQK
--PSPMQI
--PSPMQG
--PSPMPY
--PSPMPH
--PSPMNQ
--PSPMNH
--PSPMNG
--PSPMMY
--PSPMLH
--PSPMLD
--PSPMKG
--PSPMIF
--PSPMHS
--PSPMHG
--PSPMFQ
--PSPMET
--PSPMEQ
--PSPMAN
--PSPMAF
--PSPMAA
PTPSPM--
--PSPMYY
--PSPMWV
--PSPMWR
--PSPMWN
--PSPMWH
--PSPMWE
--PSPMWD
--PSPMVF
--PSPMVE
--PSPMTW
--PSPMTN
--PSPMTM
--PSPMTD
--PSPMSQ
--PSPMRI
--PSPMQT
--PSPMPV
--PSPMNM
--PSPMNK
--PSPMMQ
--PSPMMK
--PSPMMD
--PSPMKR

--PSPMKN
--PSPMKM
--PSPMIM
--PSPMIH
--PSPMIA
--PSPMHN
--PSPMHM
--PSPMHK
--PSPMHF
--PSPMGT
--PSPMGM
--PSPMGK
--PSPMGI
--PSPMFT
--PSPMFN
--PSPMFM
--PSPMFL
--PSPMFD
--PSPMFC
--PSPMEI
--PSPMEA
--PSPMCS
--PSPMCR
--PSPMAI
--PSPMAG
--PSPMAE
--PSPMAD
--PSPMAC
-PPSPMN-
-PPSPMA-
PNPSPM--
PMPSPM--
PLPSPM--
PIPSPM--
QRPSPM--
-QPSPMY-
-QPSPMV-
-QPSPMR-
-QPSPML-
QFPSPM--
QLPSPM--
QVPSPM--
QTPSPM--
-QPSPMI-
QIPSPM--
QGPSPM--
-QPSPMT-
-QPSPMS-
-QPSPMF-
-QPSPMA-

QPPSPM--
QYPSPM--
QSPSPM--
-QPSPMN-
-QPSPMM-
-QPSPMK-
QAPSPM--
-RPSPMR-
RYPSPM--
RFPSPM--
RSPSPM--
-RPSPMG-
RRPSPM--
-RPSPMS-
-RPSPMN-
-RPSPMI-
RMPSPM--
-RPSPMM-
RNPSPM--
-RPSPMV-
-RPSPMT-
RIPSPM--
RGPSPM--
RWPSPM--
-RPSPMH-
RHPSPM--
-RPSPMY-
-RPSPMQ-
REPSPM--
RDPSPM--
-RPSPMW-
-RPSPME-
RQPSPM--
-RPSPMF-
RPPSPM--
-RPSPMA-
RKPSPM--
SLPSPM--
-SPSPML-
SSPSPM--
-SPSPMS-
-SPSPMV-
STPSPM--
-SPSPMR-
SWPSPM--
SRPSPM--
SYPSPM--
SQPSPM--
SVPSPM--
SKPSPM--

-SPSPMQ-
SNPSPM--
SMPSPM--
-SPSPMT-
-SPSPMK-
-SPSPMA-
SFPSPM--
SEPSPM--
-SPSPMY-
-SPSPMF-
SPPSPM--
-SPSPMM-
-SPSPMH-
SGPSPM--
SCPSPM--
SAPSPM--
-SPSPMN-
SIPSPM--
TKPSPM--
-TPSPMR-
-TPSPMK-
-TPSPMH-
-TPSPMQ-
-TPSPMY-
TVPSPM--
TSPSPM--
-TPSPMW-
TAPSPM--
TTPSPM--
-TPSPMA-
TGPSPM--
TQPSPM--
-TPSPMI-
TIPSPM--
TCPSPM--
TRPSPM--
-TPSPMM-
-TPSPMS-
-TPSPMG-
TLPSPM--
-TPSPMV-
-TPSPMT-
-TPSPMN-
TPPSPM--
TMPSPM--
TWPSPM--
TNPSPM--
TFPSPM--
TEPSPM--
VDPSPM--

-VPSPML-
VRPSPM--
-VPSPMS-
-VPSPMR-
-VPSPMQ-
VLPSPM--
VPPSPM--
VSPSPM--
-VPSPMV-
VYPSPM--
-VPSPMA-
-VPSPMW-
-VPSPMT-
VGPSPM--
VQPSPM--
-VPSPMF-
VIPSPM--
VFPSPM--
-VPSPMY-
-VPSPMH-
VTPSPM--
-VPSPMK-
-VPSPMI-
VKPSPM--
VHPSPM--
-VPSPMN-
-VPSPMM-
-VPSPMG-
-VPSPME-
VNPSPM--
VMPSPM--
VEPSPM--
VCPSPM--
-WPSPMV-
WVPSPM--
-WPSPMT-
-WPSPMR-
WSPSPM--
WRPSPM--
-WPSPML-
WGPSPM--
-WPSPMI-
WNPSPM--
-WPSPMW-
-WPSPMK-
-WPSPMA-
WPPSPM--
WKPSPM--
WIPSPM--
WQPSPM--

```
              -WPSPMN-
              -WPSPMM-
              -WPSPMF-
              WMPSPM--
              WEPSPM--
              WDPSPM--
              -YPSPML-
              -YPSPMR-
              YWPSPM--
              -YPSPMH-
              YMPSPM--
              YTPSPM--
              -YPSPMT-
              YYPSPM--
              YPPSPM--
              YHPSPM--
              YAPSPM--
              YVPSPM--
              YRPSPM--
              -YPSPMA-
              -YPSPMN-
              -YPSPMM-
              -YPSPMI-
              -YPSPME-
              YNPSPM--
              YQPSPM--
              -YPSPMW-
              -YPSPMV-
              -YPSPMQ-
              -YPSPMK-
              -YPSPMG-
              -YPSPMF-
              YIPSPM--
              YFPSPM--
              YCPSPM--
PSGI            16        850      33.20
              -LPSGIG-
              --PSGIRP
              RCPSGI--
              SEPSGI--
              -TPSGIG-
              TPPSGI--
              TEPSGI--
              ARPSGI--
              AYPSGI--
              -APSGIS-
              -APSGIL-
              AFPSGI--
              -APSGIR-
              -APSGIH-
```

```
-APSGIQ-
AAPSGI--
ANPSGI--
AWPSGI--
ALPSGI--
ASPSGI--
-APSGIN-
-APSGIT-
ATPSGI--
-APSGIK-
-APSGIA-
-APSGIM-
-APSGIF-
APPSGI--
AVPSGI--
AQPSGI--
-APSGIW-
-APSGIV-
AKPSGI--
-APSGIY-
AHPSGI--
AGPSGI--
AMPSGI--
-APSGII-
-APSGIG-
AEPSGI--
ADPSGI--
ACPSGI--
CDPSGI--
CRPSGI--
-CPSGIF-
CKPSGI--
CYPSGI--
CFPSGI--
CSPSGI--
-CPSGIR-
-CPSGII-
-CPSGIH-
CLPSGI--
CGPSGI--
CEPSGI--
CTPSGI--
CQPSGI--
-CPSGIT-
-CPSGIS-
-CPSGIM-
-CPSGIL-
-CPSGIK-
CNPSGI--
CIPSGI--
```

CCPSGI--
CAPSGI--
DYPSGI--
-DPSGIL-
DSPSGI--
DIPSGI--
DAPSGI--
-DPSGIF-
DLPSGI--
DFPSGI--
DWPSGI--
DTPSGI--
DQPSGI--
-DPSGIS-
-DPSGIQ-
-DPSGIM-
DNPSGI--
DDPSGI--
DRPSGI--
-DPSGIY-
-DPSGIW-
-DPSGIV-
-DPSGIT-
-DPSGIN-
-DPSGIK-
-DPSGII-
DMPSGI--
DHPSGI--
DEPSGI--
ERPSGI--
-EPSGIM-
-EPSGIR-
EQPSGI--
EKPSGI--
-EPSGIL-
-EPSGIW-
-EPSGIQ-
-EPSGIK-
ELPSGI--
EYPSGI--
-EPSGIN-
-EPSGIT-
EVPSGI--
ETPSGI--
ESPSGI--
-EPSGIY-
-EPSGIS-
-EPSGII-
-EPSGIF-
EHPSGI--

EWPSGI--
-EPSGIV-
-EPSGIG-
EIPSGI--
EAPSGI--
-EPSGIE-
-EPSGIA-
EGPSGI--
EFPSGI--
ECPSGI--
-FPSGIL-
FSPSGI--
FLPSGI--
-FPSGIT-
FFPSGI--
FDPSGI--
-FPSGIR-
FNPSGI--
FVPSGI--
FRPSGI--
FGPSGI--
FHPSGI--
FYPSGI--
FPPSGI--
FKPSGI--
FTPSGI--
-FPSGIW-
-FPSGIF-
FQPSGI--
-FPSGIS-
-FPSGIQ-
-FPSGIA-
FIPSGI--
FAPSGI--
-FPSGIK-
-FPSGII-
-FPSGIH-
FMPSGI--
-FPSGIN-
-FPSGIE-
-FPSGIV-
-FPSGIY-
-FPSGIM-
FCPSGI--
-GPSGIR-
-GPSGIS-
GLPSGI--
GNPSGI--
-GPSGIL-
GWPSGI--

GGPSGI--
-GPSGIQ-
-GPSGIH-
-GPSGIN-
-GPSGIA-
GVPSGI--
-GPSGIT-
GQPSGI--
-GPSGII-
-GPSGIF-
-GPSGIW-
-GPSGIM-
-GPSGIK-
GYPSGI--
GTPSGI--
GSPSGI--
-GPSGIY-
-GPSGIG-
GHPSGI--
GMPSGI--
GEPSGI--
GCPSGI--
-GPSGIE-
GKPSGI--
GDPSGI--
GPPSGI--
GIPSGI--
-HPSGIL-
-HPSGIW-
HIPSGI--
-HPSGIV-
HVPSGI--
-HPSGIF-
-HPSGIM-
HMPSGI--
HLPSGI--
HQPSGI--
-HPSGIS-
HWPSGI--
-HPSGIT-
-HPSGIR-
-HPSGIN-
-HPSGIK-
HGPSGI--
-HPSGIA-
HFPSGI--
HYPSGI--
HTPSGI--
HKPSGI--
HEPSGI--

HDPSGI--
-HPSGIQ-
HNPSGI--
-HPSGIG-
-HPSGIY-
ILPSGI--
IRPSGI--
-IPSGIV-
IHPSGI--
-IPSGIR-
IPPSGI--
IAPSGI--
ISPSGI--
IMPSGI--
IGPSGI--
IFPSGI--
-IPSGIQ-
-IPSGIH-
-IPSGII-
IQPSGI--
IIPSGI--
IWPSGI--
-IPSGIT-
-IPSGIN-
IDPSGI--
IVPSGI--
-IPSGIS-
-IPSGIM-
-IPSGIK-
-IPSGIE-
-IPSGIW-
-IPSGIA-
INPSGI--
ITPSGI--
IEPSGI--
KSPSGI--
-KPSGIR-
KTPSGI--
-KPSGIG-
KGPSGI--
-KPSGIL-
KFPSGI--
-KPSGIV-
KLPSGI--
KAPSGI--
KVPSGI--
-KPSGIK-
KHPSGI--
-KPSGIY-
KIPSGI--

-KPSGIN-
-KPSGIQ-
-KPSGIM-
-KPSGIS-
-KPSGIH-
-KPSGIF-
KCPSGI--
-KPSGIA-
KMPSGI--
KYPSGI--
KPPSGI--
KWPSGI--
-KPSGII-
-KPSGIE-
KEPSGI--
-LPSGIR-
LRPSGI--
-LPSGIS-
-LPSGIT-
LYPSGI--
LSPSGI--
LFPSGI--
-LPSGIL-
LLPSGI--
LWPSGI--
LMPSGI--
-LPSGIH-
LHPSGI--
LAPSGI--
LGPSGI--
LQPSGI--
-LPSGIQ-
-LPSGIK-
-LPSGII-
LTPSGI--
-LPSGIW-
LNPSGI--
LIPSGI--
LEPSGI--
LVPSGI--
-LPSGIV-
LDPSGI--
LCPSGI--
LPPSGI--
-MPSGIL-
MSPSGI--
MRPSGI--
-MPSGIF-
MLPSGI--
MHPSGI--

-MPSGIR-
-MPSGII-
MAPSGI--
MYPSGI--
-MPSGIN-
-MPSGIM-
MNPSGI--
MKPSGI--
MGPSGI--
-MPSGIY-
-MPSGIS-
-MPSGIK-
-MPSGIE-
MCPSGI--
MWPSGI--
MVPSGI--
MTPSGI--
-MPSGIW-
-MPSGIQ-
MFPSGI--
MQPSGI--
-MPSGIV-
-MPSGIG-
-MPSGIA-
MPPSGI--
MMPSGI--
MEPSGI--
MDPSGI--
-MPSGIT-
-MPSGIH-
-MPSGIC-
-NPSGIL-
NRPSGI--
NSPSGI--
-NPSGIT-
-NPSGII-
NLPSGI--
-NPSGIR-
NAPSGI--
-NPSGIS-
-NPSGIY-
NFPSGI--
-NPSGIV-
NNPSGI--
NYPSGI--
NQPSGI--
NPPSGI--
NMPSGI--
NWPSGI--
NVPSGI--

NIPSGI--
NHPSGI--
-NPSGIK-
NCPSGI--
-NPSGIQ-
-NPSGIM-
-NPSGIF-
-NPSGIW-
-NPSGIH-
-NPSGIA-
NEPSGI--
--PSGILR
--PSGIRS
--PSGIVR
--PSGILW
--PSGIWR
--PSGILS
--PSGIRH
--PSGIRL
--PSGILG
--PSGIRI
--PSGIVL
--PSGIMR
--PSGIYG
--PSGISS
--PSGIRW
--PSGISR
--PSGIFR
--PSGIRG
--PSGIRA
--PSGIRN
--PSGIML
--PSGIMG
--PSGIFL
--PSGIST
--PSGIRF
--PSGINS
--PSGILK
--PSGIVH
--PSGIRK
--PSGIIV
--PSGIHR
--PSGIYF
--PSGITW
--PSGITK
--PSGISW
--PSGIRY
--PSGIKR
--PSGIFS
--PSGIFQ

--PSGIYA
--PSGIWS
--PSGISK
--PSGIQR
--PSGIVY
--PSGIVG
--PSGIRT
--PSGIMN
--PSGIKS
--PSGIVI
--PSGITQ
--PSGITH
--PSGIRV
--PSGINK
--PSGILQ
--PSGIIS
--PSGIGS
--PSGIFH
PLPSGI--
--PSGIYI
--PSGIWG
--PSGITS
--PSGITG
--PSGISA
--PSGIRD
--PSGINR
--PSGIMI
--PSGILE
--PSGIEA
PAPSGI--
--PSGIYR
--PSGIWY
--PSGIVN
--PSGITI
--PSGIQH
--PSGIMT
--PSGILH
--PSGIKQ
--PSGIHS
--PSGIHE
--PSGIFV
--PSGIFM
PYPSGI--
--PSGIYY
--PSGIWT
--PSGITT
--PSGISY
--PSGIMS
--PSGIKI
--PSGIIT

--PSGIYS
--PSGIYN
--PSGIYH
--PSGIWA
--PSGIVW
--PSGIVT
--PSGISV
--PSGISH
--PSGISG
--PSGIKL
--PSGIIK
--PSGIGG
--PSGIEV
--PSGIEG
--PSGIYV
--PSGIWK
--PSGIVK
--PSGIVE
--PSGITN
--PSGITM
--PSGISF
--PSGIRM
--PSGIQA
--PSGIMM
--PSGIKK
--PSGIIH
--PSGIHH
--PSGIHG
--PSGIFT
--PSGIFN
--PSGIES
PVPSGI--
--PSGIWD
--PSGISL
--PSGIRQ
--PSGIQV
--PSGIQT
--PSGINV
--PSGINT
--PSGIMA
--PSGILD
--PSGIKW
--PSGIIY
--PSGIIN
--PSGIII
--PSGIIA
--PSGIHN
--PSGIFK
--PSGIET
--PSGIAW

--PSGIAT
--PSGIYW
--PSGIVA
--PSGITV
--PSGITF
--PSGITD
--PSGISN
--PSGISM
--PSGISI
--PSGIQL
--PSGIQI
--PSGINL
--PSGINF
--PSGIKN
--PSGIKA
--PSGIHW
--PSGIHQ
--PSGIHA
--PSGIGT
--PSGIFG
--PSGIAV
PFPSGI--
PEPSGI--
--PSGIYQ
--PSGIVF
--PSGIVD
--PSGISQ
--PSGISD
--PSGIRE
--PSGIQQ
--PSGIQF
--PSGIMF
--PSGIKM
--PSGIKH
--PSGIHV
--PSGIHI
--PSGIFY
--PSGIFA
--PSGIEY
--PSGIAS
PKPSGI--
PHPSGI--
PSPSGI--
--PSGIWW
--PSGIWM
--PSGIQW
--PSGIQK
--PSGIMW
--PSGIID
--PSGIHK

--PSGIGL
--PSGIGI
--PSGIEW
--PSGIEM
--PSGIEF
--PSGIAG
PIPSGI--
--PSGIYK
--PSGIWH
--PSGIVM
--PSGITY
--PSGIQN
--PSGIQM
--PSGIQE
--PSGINY
--PSGINW
--PSGINQ
--PSGINM
--PSGINA
--PSGIMY
--PSGIMV
--PSGIMH
--PSGIME
--PSGIKV
--PSGIKF
--PSGIKE
--PSGIKD
--PSGIIQ
--PSGIHY
--PSGIHF
--PSGIGW
--PSGIEH
--PSGIAY
--PSGIAR
--PSGIAN
--PSGIAK
--PSGIAF
--PSGIAA
PDPSGI--
PTPSGI--
--PSGIYT
--PSGIYM
--PSGIYE
--PSGIWV
--PSGIWN
--PSGIWF
--PSGIVV
--PSGISE
--PSGIRC
--PSGIQY

--PSGIQD
--PSGINN
--PSGIMQ
--PSGIKT
--PSGIKC
--PSGIHM
--PSGIGY
--PSGIGV
--PSGIGH
--PSGIFF
--PSGIFE
--PSGIFD
--PSGIDY
--PSGIDG
--PSGIDA
--PSGICT
--PSGICA
--PSGIAD
PQPSGI--
-PPSGIL-
PMPSGI--
QLPSGI--
QSPSGI--
QQPSGI--
QVPSGI--
QIPSGI--
QGPSGI--
QAPSGI--
QRPSGI--
-QPSGIS-
-QPSGIN-
-QPSGIT-
-QPSGIL-
-QPSGIA-
-QPSGIV-
-QPSGIR-
-QPSGIH-
QNPSGI--
QFPSGI--
QYPSGI--
-QPSGIY-
-QPSGIQ-
-QPSGII-
-QPSGIG-
QMPSGI--
-QPSGIF-
QPPSGI--
QHPSGI--
QWPSGI--
-QPSGIW-

-RPSGIL-
RSPSGI--
RGPSGI--
-RPSGIS-
RIPSGI--
RVPSGI--
-RPSGIK-
-RPSGIV-
-RPSGIW-
REPSGI--
-RPSGIN-
-RPSGIT-
RRPSGI--
-RPSGIG-
RAPSGI--
RQPSGI--
-RPSGII-
RMPSGI--
RTPSGI--
-RPSGIQ-
-RPSGIE-
-RPSGIY-
-RPSGIF-
-RPSGIA-
RPPSGI--
RNPSGI--
RYPSGI--
RWPSGI--
RHPSGI--
RDPSGI--
SVPSGI--
SLPSGI--
-SPSGIR-
-SPSGIS-
SSPSGI--
SRPSGI--
-SPSGIL-
SIPSGI--
SKPSGI--
SAPSGI--
STPSGI--
-SPSGIM-
SYPSGI--
-SPSGIH-
SMPSGI--
-SPSGIK-
-SPSGIA-
-SPSGIF-
SWPSGI--
-SPSGII-

SPPSGI--
SGPSGI--
-SPSGIT-
SNPSGI--
-SPSGIE-
SFPSGI--
-SPSGIG-
SHPSGI--
-SPSGIN-
SDPSGI--
SCPSGI--
-SPSGIW-
SQPSGI--
-TPSGIR-
TSPSGI--
TRPSGI--
-TPSGIL-
-TPSGIT-
TWPSGI--
TFPSGI--
TTPSGI--
-TPSGIV-
-TPSGIF-
TIPSGI--
TGPSGI--
-TPSGIK-
TNPSGI--
TAPSGI--
TYPSGI--
-TPSGIQ-
-TPSGIN-
-TPSGIH-
THPSGI--
-TPSGII-
TQPSGI--
-TPSGIW-
-TPSGIY-
-TPSGIA-
TMPSGI--
-TPSGIS-
TKPSGI--
TDPSGI--
-TPSGIE-
-VPSGIR-
-VPSGIA-
VWPSGI--
VRPSGI--
-VPSGIM-
VGPSGI--
-VPSGIW-

-VPSGIL-
-VPSGII-
VQPSGI--
-VPSGIV-
-VPSGIQ-
VKPSGI--
VEPSGI--
-VPSGIE-
VVPSGI--
VMPSGI--
VYPSGI--
-VPSGIT-
VTPSGI--
-VPSGIS-
-VPSGIK-
VPPSGI--
-VPSGIN-
VDPSGI--
-VPSGIY-
-VPSGIH-
-VPSGIF-
VNPSGI--
VCPSGI--
VAPSGI--
WEPSGI--
-WPSGIR-
-WPSGIS-
WLPSGI--
WVPSGI--
WYPSGI--
WKPSGI--
WTPSGI--
-WPSGIY-
-WPSGIQ-
-WPSGII-
WMPSGI--
WSPSGI--
WRPSGI--
-WPSGIT-
WDPSGI--
WFPSGI--
-WPSGIH-
WGPSGI--
WPPSGI--
-WPSGIN-
-WPSGIF-
WIPSGI--
-WPSGIW-
-WPSGIK-
WNPSGI--

```
           -WPSGIV-
           WHPSGI--
           WWPSGI--
           -WPSGIM-
           WAPSGI--
           -YPSGIS-
           YGPSGI--
           YRPSGI--
           -YPSGIW-
           -YPSGIL-
           -YPSGIR-
           -YPSGIF-
           -YPSGIA-
           YWPSGI--
           YSPSGI--
           YLPSGI--
           YKPSGI--
           YIPSGI--
           -YPSGIN-
           YEPSGI--
           YAPSGI--
           YYPSGI--
           -YPSGIH-
           YHPSGI--
           YFPSGI--
           YVPSGI--
           -YPSGIQ-
           -YPSGIM-
           YTPSGI--
           YQPSGI--
           -YPSGIY-
           -YPSGIG-
           YMPSGI--
           -YPSGIK-
           YDPSGI--
           -YPSGIE-
PLGY         47         806      33.17
           -APLGYD-
           DLPLGY--
           -NPLGYK-
           -NPLGYG-
           --PLGYTT
           --PLGYRP
           --PLGYPG
           --PLGYLD
           --PLGYAH
           -RPLGYD-
           SQPLGY--
           -TPLGYA-
           -VPLGYA-
```

-APLGYR-
-APLGYI-
-APLGYS-
-APLGYV-
-APLGYG-
-APLGYQ-
-APLGYA-
ALPLGY--
ASPLGY--
AKPLGY--
AQPLGY--
-APLGYL-
AMPLGY--
-APLGYH-
AIPLGY--
-APLGYN-
-APLGYE-
AAPLGY--
APPLGY--
-APLGYT-
-APLGYF-
AGPLGY--
AYPLGY--
AVPLGY--
AHPLGY--
AEPLGY--
-APLGYM-
-APLGYK-
AFPLGY--
ADPLGY--
ACPLGY--
AWPLGY--
CAPLGY--
-CPLGYT-
-CPLGYK-
CVPLGY--
-CPLGYS-
-CPLGYL-
CMPLGY--
CLPLGY--
CYPLGY--
CSPLGY--
CRPLGY--
-CPLGYV-
-CPLGYI-
CKPLGY--
CGPLGY--
-DPLGYL-
DRPLGY--
-DPLGYS-

DVPLGY--
-DPLGYR-
-DPLGYI-
-DPLGYH-
DKPLGY--
-DPLGYT-
-DPLGYF-
DYPLGY--
DNPLGY--
DFPLGY--
DTPLGY--
DSPLGY--
-DPLGYY-
-DPLGYQ-
-DPLGYA-
DMPLGY--
DHPLGY--
DGPLGY--
DQPLGY--
-DPLGYK-
DWPLGY--
-DPLGYW-
-DPLGYV-
-DPLGYN-
-DPLGYM-
-DPLGYE-
-DPLGYD-
DEPLGY--
DAPLGY--
-EPLGYK-
ESPLGY--
-EPLGYV-
-EPLGYA-
ERPLGY--
EAPLGY--
-EPLGYR-
ENPLGY--
EKPLGY--
-EPLGYW-
-EPLGYG-
EIPLGY--
EEPLGY--
-EPLGYY-
ELPLGY--
ETPLGY--
EQPLGY--
-EPLGYS-
-EPLGYN-
-EPLGYM-
-EPLGYI-

EHPLGY--
EGPLGY--
EFPLGY--
EVPLGY--
-EPLGYQ-
-EPLGYF-
ECPLGY--
FSPLGY--
-FPLGYK-
FQPLGY--
FFPLGY--
-FPLGYM-
-FPLGYA-
FYPLGY--
FTPLGY--
FGPLGY--
-FPLGYS-
-FPLGYN-
FPPLGY--
-FPLGYI-
-FPLGYT-
FNPLGY--
FIPLGY--
FEPLGY--
-FPLGYG-
FDPLGY--
FAPLGY--
FVPLGY--
-FPLGYV-
-FPLGYQ-
-FPLGYW-
FWPLGY--
-FPLGYY-
-FPLGYR-
-FPLGYH-
FMPLGY--
FHPLGY--
-GPLGYL-
-GPLGYR-
-GPLGYM-
-GPLGYH-
GTPLGY--
GSPLGY--
-GPLGYS-
GGPLGY--
GAPLGY--
-GPLGYI-
GYPLGY--
-GPLGYK-
GRPLGY--

−GPLGYY−
−GPLGYN−
GQPLGY−−
−GPLGYV−
−GPLGYT−
GKPLGY−−
GIPLGY−−
GEPLGY−−
GMPLGY−−
−GPLGYQ−
−GPLGYG−
−GPLGYW−
−GPLGYA−
GFPLGY−−
GDPLGY−−
GVPLGY−−
GNPLGY−−
GPPLGY−−
−GPLGYF−
GHPLGY−−
−HPLGYL−
−HPLGYV−
HSPLGY−−
HAPLGY−−
−HPLGYG−
−HPLGYA−
−HPLGYN−
−HPLGYS−
−HPLGYM−
−HPLGYK−
HLPLGY−−
−HPLGYY−
−HPLGYI−
−HPLGYR−
HMPLGY−−
HIPLGY−−
−HPLGYT−
HTPLGY−−
HFPLGY−−
HWPLGY−−
HVPLGY−−
HQPLGY−−
HPPLGY−−
HNPLGY−−
HHPLGY−−
HEPLGY−−
HCPLGY−−
−HPLGYW−
−HPLGYH−
HDPLGY−−

HYPLGY--
-HPLGYE-
HKPLGY--
ISPLGY--
IRPLGY--
-IPLGYR-
-IPLGYI-
-IPLGYN-
-IPLGYE-
ITPLGY--
IAPLGY--
-IPLGYM-
IYPLGY--
IIPLGY--
IQPLGY--
-IPLGYS-
-IPLGYH-
-IPLGYA-
IGPLGY--
IDPLGY--
-IPLGYY-
-IPLGYG-
-IPLGYW-
-IPLGYF-
IMPLGY--
IHPLGY--
IEPLGY--
IWPLGY--
-IPLGYQ-
-IPLGYK-
INPLGY--
IKPLGY--
KSPLGY--
-KPLGYR-
KQPLGY--
KYPLGY--
KLPLGY--
-KPLGYI-
-KPLGYF-
-KPLGYK-
KVPLGY--
-KPLGYS-
KEPLGY--
-KPLGYM-
-KPLGYH-
KNPLGY--
KWPLGY--
-KPLGYN-
-KPLGYG-
KHPLGY--

-KPLGYY-
-KPLGYT-
-KPLGYE-
KMPLGY--
KGPLGY--
KAPLGY--
-KPLGYA-
KDPLGY--
KPPLGY--
-KPLGYW-
-KPLGYQ-
KCPLGY--
KFPLGY--
-LPLGYL-
LGPLGY--
-LPLGYT-
LSPLGY--
-LPLGYR-
LIPLGY--
LFPLGY--
-LPLGYS-
LAPLGY--
LDPLGY--
-LPLGYW-
-LPLGYI-
-LPLGYQ-
-LPLGYH-
LNPLGY--
LPPLGY--
LMPLGY--
-LPLGYF-
LVPLGY--
-LPLGYK-
-LPLGYA-
LQPLGY--
LTPLGY--
-LPLGYN-
LEPLGY--
LHPLGY--
LWPLGY--
-LPLGYE-
-MPLGYL-
MSPLGY--
-MPLGYG-
-MPLGYM-
MLPLGY--
MVPLGY--
MNPLGY--
MQPLGY--
MPPLGY--

-MPLGYT-
-MPLGYS-
MGPLGY--
-MPLGYH-
MAPLGY--
MKPLGY--
MIPLGY--
-MPLGYQ-
-MPLGYK-
MFPLGY--
-MPLGYA-
MTPLGY--
-MPLGYW-
-MPLGYV-
-MPLGYI-
-MPLGYE-
MHPLGY--
MEPLGY--
MYPLGY--
MMPLGY--
MDPLGY--
NSPLGY--
-NPLGYS-
-NPLGYR-
NIPLGY--
NGPLGY--
-NPLGYY-
NFPLGY--
NVPLGY--
-NPLGYI-
NEPLGY--
-NPLGYL-
NYPLGY--
-NPLGYH-
-NPLGYF-
NQPLGY--
NKPLGY--
NWPLGY--
NTPLGY--
NAPLGY--
NPPLGY--
-NPLGYN-
NHPLGY--
-NPLGYA-
NCPLGY--
-NPLGYV-
-NPLGYQ-
NMPLGY--
NNPLGY--
NLPLGY--

-NPLGYW-
--PLGYWN
--PLGYLR
--PLGYSK
--PLGYFR
--PLGYMR
--PLGYRL
--PLGYLG
--PLGYHG
--PLGYSG
--PLGYRV
--PLGYKS
--PLGYTR
--PLGYST
--PLGYSH
--PLGYRM
--PLGYRH
--PLGYYT
--PLGYRY
--PLGYAS
--PLGYVK
--PLGYTS
--PLGYSN
--PLGYRN
--PLGYFS
--PLGYTN
--PLGYRD
--PLGYRA
--PLGYIS
--PLGYFK
--PLGYRK
--PLGYKG
--PLGYAL
--PLGYTM
--PLGYSF
--PLGYRT
--PLGYNL
--PLGYLN
--PLGYHS
--PLGYHR
PRPLGY--
--PLGYYN
--PLGYWR
--PLGYVG
--PLGYVA
--PLGYMK
--PLGYMI
--PLGYMG
--PLGYLA
--PLGYKY

--PLGYKI
--PLGYKH
--PLGYIR
--PLGYFT
--PLGYFG
PWPLGY--
--PLGYWA
--PLGYVY
--PLGYVS
--PLGYSD
--PLGYQA
--PLGYMH
--PLGYLT
--PLGYGS
--PLGYAY
--PLGYYD
--PLGYSS
--PLGYSI
--PLGYQY
--PLGYNS
--PLGYLI
--PLGYLH
--PLGYKR
--PLGYKE
--PLGYHE
PQPLGY--
--PLGYVW
--PLGYVI
--PLGYTV
--PLGYRE
--PLGYQG
--PLGYNK
--PLGYNF
--PLGYML
--PLGYIH
--PLGYAK
--PLGYYK
--PLGYWG
--PLGYSV
--PLGYQN
--PLGYMS
--PLGYME
--PLGYLK
--PLGYLE
--PLGYIM
--PLGYHT
PFPLGY--
--PLGYYS
--PLGYWV
--PLGYVT

--PLGYVM
--PLGYTH
--PLGYSY
--PLGYQK
--PLGYIY
--PLGYIT
--PLGYIN
--PLGYIK
--PLGYHH
--PLGYFH
--PLGYEG
--PLGYEA
--PLGYAI
--PLGYAA
PNPLGY--
--PLGYWT
--PLGYWK
--PLGYVQ
--PLGYVN
--PLGYVH
--PLGYTY
--PLGYTE
--PLGYSM
--PLGYQW
--PLGYQV
--PLGYLQ
--PLGYKL
--PLGYID
--PLGYHK
--PLGYGV
--PLGYGF
--PLGYAT
--PLGYAN
PGPLGY--
PYPLGY--
--PLGYYM
--PLGYWD
--PLGYTA
--PLGYSQ
--PLGYSE
--PLGYSA
--PLGYQR
--PLGYNY
--PLGYNR
--PLGYNM
--PLGYNH
--PLGYMW
--PLGYMN
--PLGYMD
--PLGYII

--PLGYGG
--PLGYEV
--PLGYAW
--PLGYAV
--PLGYAF
PEPLGY--
PTPLGY--
--PLGYWE
--PLGYVF
--PLGYVD
--PLGYTF
--PLGYTD
--PLGYRQ
--PLGYQS
--PLGYQH
--PLGYNV
--PLGYNG
--PLGYKT
--PLGYKN
--PLGYKD
--PLGYIW
--PLGYHV
--PLGYHI
--PLGYFN
--PLGYEY
PKPLGY--
PMPLGY--
--PLGYTI
--PLGYTG
--PLGYQF
--PLGYNT
--PLGYNQ
--PLGYNN
--PLGYNA
--PLGYMT
--PLGYMA
--PLGYLM
--PLGYKQ
--PLGYKM
--PLGYKF
--PLGYIQ
--PLGYIA
--PLGYHM
--PLGYHD
--PLGYHA
--PLGYFM
--PLGYET
--PLGYEK
--PLGYAR
PIPLGY--

--PLGYTK
--PLGYQM
--PLGYQI
--PLGYNI
--PLGYKW
--PLGYIE
--PLGYHW
--PLGYGM
--PLGYFE
--PLGYES
--PLGYEH
--PLGYDY
--PLGYCN
PCPLGY--
-PPLGYV-
--PLGYYH
--PLGYYG
--PLGYWQ
--PLGYVE
--PLGYQQ
--PLGYNW
--PLGYNE
--PLGYMQ
--PLGYIG
--PLGYHN
--PLGYHF
--PLGYGI
--PLGYER
--PLGYEF
--PLGYDG
--PLGYDA
PHPLGY--
QTPLGY--
-QPLGYL-
-QPLGYR-
QLPLGY--
-QPLGYT-
-QPLGYY-
-QPLGYN-
QVPLGY--
QQPLGY--
QIPLGY--
-QPLGYA-
QNPLGY--
QPPLGY--
-QPLGYM-
-QPLGYH-
QFPLGY--
QYPLGY--
-QPLGYK-

QDPLGY--
QSPLGY--
-QPLGYG-
-QPLGYF-
-QPLGYQ-
QWPLGY--
-QPLGYV-
-QPLGYS-
QGPLGY--
QAPLGY--
-QPLGYI-
-QPLGYE-
QMPLGY--
QHPLGY--
-RPLGYL-
RVPLGY--
-RPLGYS-
-RPLGYG-
-RPLGYM-
RLPLGY--
-RPLGYF-
-RPLGYW-
-RPLGYT-
-RPLGYK-
RAPLGY--
RGPLGY--
RTPLGY--
-RPLGYY-
REPLGY--
RHPLGY--
RPPLGY--
-RPLGYQ-
-RPLGYN-
RIPLGY--
-RPLGYH-
RKPLGY--
RNPLGY--
RMPLGY--
RQPLGY--
RYPLGY--
-RPLGYE-
RDPLGY--
-RPLGYC-
SSPLGY--
SAPLGY--
-SPLGYS-
-SPLGYR-
SLPLGY--
-SPLGYV-
SKPLGY--

SHPLGY--
-SPLGYQ-
-SPLGYN-
-SPLGYI-
SGPLGY--
SWPLGY--
-SPLGYG-
SMPLGY--
SYPLGY--
-SPLGYA-
SNPLGY--
STPLGY--
SPPLGY--
-SPLGYY-
-SPLGYW-
SFPLGY--
SVPLGY--
-SPLGYM-
-SPLGYE-
SIPLGY--
-SPLGYH-
SDPLGY--
SEPLGY--
SCPLGY--
-TPLGYN-
-TPLGYL-
-TPLGYI-
TGPLGY--
TVPLGY--
-TPLGYV-
-TPLGYH-
-TPLGYG-
-TPLGYS-
-TPLGYK-
TIPLGY--
TFPLGY--
-TPLGYT-
-TPLGYM-
-TPLGYY-
TMPLGY--
TAPLGY--
TKPLGY--
-TPLGYW-
THPLGY--
TYPLGY--
TQPLGY--
-TPLGYF-
TTPLGY--
TNPLGY--
TWPLGY--

-TPLGYE-
TDPLGY--
TCPLGY--
-VPLGYS-
-VPLGYI-
VSPLGY--
-VPLGYL-
-VPLGYT-
VKPLGY--
-VPLGYR-
-VPLGYM-
VHPLGY--
-VPLGYN-
-VPLGYG-
-VPLGYK-
-VPLGYV-
-VPLGYF-
VMPLGY--
VFPLGY--
VYPLGY--
VQPLGY--
-VPLGYY-
VIPLGY--
-VPLGYE-
VVPLGY--
VGPLGY--
VPPLGY--
-VPLGYW-
-VPLGYQ-
VEPLGY--
VWPLGY--
-VPLGYH-
VAPLGY--
VDPLGY--
VCPLGY--
WEPLGY--
WAPLGY--
WKPLGY--
-WPLGYS-
-WPLGYR-
-WPLGYA-
WQPLGY--
WPPLGY--
WVPLGY--
-WPLGYN-
-WPLGYF-
WMPLGY--
WGPLGY--
-WPLGYQ-
WWPLGY--

| | | | |
|---|---|---|---|
| | -WPLGYM- | | |
| | WLPLGY-- | | |
| | WFPLGY-- | | |
| | WYPLGY-- | | |
| | WTPLGY-- | | |
| | -WPLGYV- | | |
| | -WPLGYT- | | |
| | WNPLGY-- | | |
| | -WPLGYK- | | |
| | -WPLGYI- | | |
| | -WPLGYY- | | |
| | -WPLGYL- | | |
| | -YPLGYR- | | |
| | YTPLGY-- | | |
| | YVPLGY-- | | |
| | -YPLGYS- | | |
| | -YPLGYN- | | |
| | YSPLGY-- | | |
| | YYPLGY-- | | |
| | YAPLGY-- | | |
| | -YPLGYY- | | |
| | YGPLGY-- | | |
| | -YPLGYT- | | |
| | -YPLGYM- | | |
| | -YPLGYF- | | |
| | -YPLGYA- | | |
| | YKPLGY-- | | |
| | YIPLGY-- | | |
| | -YPLGYH- | | |
| | -YPLGYG- | | |
| | YMPLGY-- | | |
| | YHPLGY-- | | |
| | -YPLGYQ- | | |
| | -YPLGYL- | | |
| | -YPLGYK- | | |
| | YPPLGY-- | | |
| | YQPLGY-- | | |
| | YDPLGY-- | | |
| | -YPLGYI- | | |
| | YEPLGY-- | | |
| | YWPLGY-- | | |
| | YNPLGY-- | | |
| | YFPLGY-- | | |
| PSQL | 28 | 831 | 33.16 |
| | IRPSQL-- | | |
| | -LPSQLA- | | |
| | --PSQLGY | | |
| | --PSQLNN | | |
| | -SPSQLE- | | |
| | -APSQLS- | | |

```
-APSQLI-
-APSQLR-
AVPSQL--
ARPSQL--
ALPSQL--
-APSQLA-
-APSQLK-
AFPSQL--
-APSQLL-
-APSQLF-
AYPSQL--
ASPSQL--
-APSQLV-
AKPSQL--
APPSQL--
-APSQLW-
-APSQLH-
-APSQLQ-
-APSQLM-
AHPSQL--
AGPSQL--
-APSQLY-
AIPSQL--
AWPSQL--
-APSQLN-
-APSQLG-
ANPSQL--
AEPSQL--
ADPSQL--
ACPSQL--
AAPSQL--
-APSQLT-
-CPSQLR-
CPPSQL--
CVPSQL--
CSPSQL--
CAPSQL--
CYPSQL--
CQPSQL--
-CPSQLV-
CCPSQL--
CRPSQL--
-CPSQLW-
-CPSQLS-
-CPSQLN-
-CPSQLL-
-CPSQLI-
-CPSQLH-
CMPSQL--
CHPSQL--
```

DRPSQL--
-DPSQLR-
DLPSQL--
-DPSQLS-
DYPSQL--
DVPSQL--
-DPSQLT-
-DPSQLL-
DTPSQL--
DIPSQL--
DAPSQL--
DWPSQL--
DSPSQL--
-DPSQLN-
DGPSQL--
-DPSQLV-
-DPSQLF-
DDPSQL--
DQPSQL--
-DPSQLY-
-DPSQLW-
-DPSQLK-
-DPSQLI-
-DPSQLH-
DKPSQL--
DHPSQL--
-DPSQLG-
DMPSQL--
DFPSQL--
DEPSQL--
-EPSQLW-
EYPSQL--
ERPSQL--
-EPSQLS-
EKPSQL--
ESPSQL--
-EPSQLR-
ELPSQL--
EEPSQL--
-EPSQLV-
ETPSQL--
-EPSQLK-
-EPSQLF-
-EPSQLA-
ENPSQL--
EVPSQL--
-EPSQLL-
EIPSQL--
EFPSQL--
EDPSQL--

EWPSQL--
-EPSQLY-
-EPSQLH-
-EPSQLG-
EMPSQL--
EHPSQL--
EGPSQL--
EQPSQL--
-EPSQLT-
-EPSQLM-
FRPSQL--
FSPSQL--
FLPSQL--
FYPSQL--
FVPSQL--
-FPSQLS-
-FPSQLV-
-FPSQLL-
FTPSQL--
FKPSQL--
-FPSQLR-
FGPSQL--
FNPSQL--
FMPSQL--
FFPSQL--
FDPSQL--
FAPSQL--
-FPSQLA-
FEPSQL--
FWPSQL--
FQPSQL--
-FPSQLK-
-FPSQLI-
-FPSQLF-
FPPSQL--
FCPSQL--
-FPSQLY-
-FPSQLM-
-FPSQLH-
-GPSQLL-
GLPSQL--
-GPSQLV-
GRPSQL--
-GPSQLF-
-GPSQLK-
GTPSQL--
-GPSQLS-
GDPSQL--
-GPSQLY-
-GPSQLM-

GHPSQL--
-GPSQLT-
GMPSQL--
GWPSQL--
GVPSQL--
GQPSQL--
-GPSQLR-
-GPSQLI-
GNPSQL--
GIPSQL--
GSPSQL--
GPPSQL--
-GPSQLN-
GFPSQL--
GYPSQL--
-GPSQLE-
GCPSQL--
GGPSQL--
-GPSQLW-
-GPSQLH-
-GPSQLG-
-GPSQLA-
GKPSQL--
-GPSQLQ-
-HPSQLR-
-HPSQLW-
-HPSQLL-
HRPSQL--
HTPSQL--
HLPSQL--
HNPSQL--
HGPSQL--
HSPSQL--
-HPSQLY-
-HPSQLK-
HMPSQL--
HVPSQL--
-HPSQLM-
-HPSQLH-
-HPSQLF-
HWPSQL--
-HPSQLV-
-HPSQLT-
HPPSQL--
HYPSQL--
-HPSQLQ-
-HPSQLI-
HAPSQL--
HQPSQL--
-HPSQLG-

−HPSQLA−
HIPSQL−−
HHPSQL−−
HFPSQL−−
HDPSQL−−
IVPSQL−−
−IPSQLL−
ILPSQL−−
−IPSQLS−
−IPSQLI−
−IPSQLT−
INPSQL−−
−IPSQLY−
−IPSQLR−
−IPSQLK−
−IPSQLA−
IKPSQL−−
IGPSQL−−
IFPSQL−−
ISPSQL−−
IEPSQL−−
IIPSQL−−
IHPSQL−−
IAPSQL−−
IWPSQL−−
ITPSQL−−
IQPSQL−−
−IPSQLQ−
−IPSQLN−
−IPSQLM−
−IPSQLV−
−IPSQLE−
IYPSQL−−
−IPSQLH−
IPPSQL−−
IMPSQL−−
IDPSQL−−
KSPSQL−−
−KPSQLL−
−KPSQLR−
−KPSQLS−
−KPSQLA−
−KPSQLI−
KQPSQL−−
KTPSQL−−
−KPSQLT−
KFPSQL−−
KMPSQL−−
KAPSQL−−
KVPSQL−−

KIPSQL--
KEPSQL--
KWPSQL--
-KPSQLK-
-KPSQLM-
-KPSQLH-
KNPSQL--
KDPSQL--
KYPSQL--
-KPSQLF-
-KPSQLE-
-KPSQLY-
-KPSQLQ-
-KPSQLG-
KGPSQL--
-KPSQLW-
KKPSQL--
KPPSQL--
LSPSQL--
LRPSQL--
-LPSQLR-
LVPSQL--
-LPSQLS-
LLPSQL--
LKPSQL--
LAPSQL--
LWPSQL--
LPPSQL--
LEPSQL--
LYPSQL--
-LPSQLY-
LFPSQL--
LTPSQL--
-LPSQLK-
-LPSQLH-
LNPSQL--
LIPSQL--
LDPSQL--
-LPSQLV-
-LPSQLN-
LMPSQL--
LHPSQL--
-LPSQLF-
LCPSQL--
-LPSQLG-
-LPSQLC-
-MPSQLR-
MLPSQL--
MRPSQL--
MKPSQL--

```
-MPSQLL-
MVPSQL--
MSPSQL--
-MPSQLH-
-MPSQLS-
MIPSQL--
-MPSQLV-
-MPSQLN-
MYPSQL--
MTPSQL--
MHPSQL--
-MPSQLY-
MDPSQL--
-MPSQLK-
MGPSQL--
MQPSQL--
-MPSQLT-
-MPSQLW-
-MPSQLG-
MFPSQL--
MEPSQL--
-MPSQLQ-
-MPSQLM-
MMPSQL--
-MPSQLI-
-MPSQLC-
MCPSQL--
MWPSQL--
MPPSQL--
NLPSQL--
-NPSQLT-
-NPSQLR-
-NPSQLS-
NMPSQL--
NFPSQL--
NYPSQL--
-NPSQLI-
NVPSQL--
NPPSQL--
NTPSQL--
NNPSQL--
-NPSQLV-
NKPSQL--
-NPSQLM-
-NPSQLK-
-NPSQLH-
NHPSQL--
NWPSQL--
NSPSQL--
-NPSQLN-
```

NQPSQL--
-NPSQLY-
-NPSQLA-
NAPSQL--
-NPSQLQ-
NIPSQL--
NEPSQL--
-NPSQLW-
-NPSQLP-
NDPSQL--
NCPSQL--
--PSQLRI
--PSQLRL
--PSQLVG
--PSQLAS
--PSQLLS
--PSQLWT
--PSQLLK
--PSQLRH
--PSQLRS
PRPSQL--
--PSQLRT
--PSQLFR
--PSQLSV
--PSQLRY
--PSQLTS
--PSQLSL
--PSQLWH
--PSQLVR
--PSQLSN
--PSQLRM
--PSQLVT
--PSQLVK
--PSQLSG
--PSQLRN
--PSQLLW
--PSQLLQ
--PSQLST
--PSQLKA
--PSQLIR
--PSQLGT
--PSQLAG
--PSQLVL
--PSQLSS
--PSQLRK
--PSQLIS
--PSQLFH
PSPSQL--
--PSQLYT
--PSQLRV

--PSQLLG
--PSQLKR
--PSQLIT
--PSQLGG
--PSQLTA
PFPSQL--
--PSQLYS
--PSQLWS
--PSQLWR
--PSQLTY
--PSQLTT
--PSQLPL
--PSQLNR
--PSQLMG
--PSQLFG
--PSQLWG
--PSQLWA
--PSQLTR
--PSQLSQ
--PSQLNS
--PSQLNK
--PSQLMI
--PSQLLV
--PSQLLD
--PSQLKL
PLPSQL--
--PSQLYV
--PSQLVS
--PSQLTL
--PSQLTI
--PSQLSR
--PSQLMR
--PSQLHG
--PSQLGS
--PSQLFS
--PSQLAW
--PSQLAT
PYPSQL--
--PSQLSI
--PSQLRQ
--PSQLKV
--PSQLFK
--PSQLYQ
--PSQLWI
--PSQLVW
--PSQLVE
--PSQLVA
--PSQLSH
--PSQLSA
--PSQLQR

--PSQLLT
--PSQLLE
--PSQLKT
--PSQLKG
--PSQLIK
--PSQLIH
PHPSQL--
--PSQLTG
--PSQLSM
--PSQLQN
--PSQLMN
--PSQLKS
--PSQLIV
--PSQLGL
--PSQLFM
--PSQLAR
--PSQLAI
--PSQLYG
--PSQLWW
--PSQLWQ
--PSQLVY
--PSQLVQ
--PSQLVN
--PSQLTF
--PSQLSW
--PSQLMT
--PSQLKH
--PSQLKF
--PSQLIY
--PSQLII
--PSQLHW
--PSQLFT
PMPSQL--
PEPSQL--
PCPSQL--
PWPSQL--
--PSQLYK
--PSQLYD
--PSQLWK
--PSQLWD
--PSQLVH
--PSQLTN
--PSQLTK
--PSQLRD
--PSQLQI
--PSQLNM
--PSQLML
--PSQLME
--PSQLMD
--PSQLLM

--PSQLKM
--PSQLKK
--PSQLIQ
--PSQLIL
--PSQLFQ
--PSQLAA
PAPSQL--
--PSQLYW
--PSQLYN
--PSQLYM
--PSQLWL
--PSQLTV
--PSQLTH
--PSQLSD
--PSQLRW
--PSQLQY
--PSQLNY
--PSQLMF
--PSQLMA
--PSQLKN
--PSQLHY
--PSQLHI
--PSQLAF
PKPSQL--
PGPSQL--
--PSQLYY
--PSQLYH
--PSQLYE
--PSQLYA
--PSQLVI
--PSQLTQ
--PSQLTD
--PSQLQW
--PSQLQS
--PSQLQM
--PSQLQL
--PSQLNI
--PSQLNH
--PSQLMM
--PSQLKQ
--PSQLKI
--PSQLKE
--PSQLIW
--PSQLIG
--PSQLHV
--PSQLHS
--PSQLHM
--PSQLHE
--PSQLGA
--PSQLFA

--PSQLEG
PNPSQL--
PIPSQL--
PVPSQL--
--PSQLYF
--PSQLWN
--PSQLWM
--PSQLQD
--PSQLQA
--PSQLNQ
--PSQLND
--PSQLNA
--PSQLMW
--PSQLKY
--PSQLHH
--PSQLEA
--PSQLAN
--PSQLAL
PQPSQL--
--PSQLWE
--PSQLWC
--PSQLVM
--PSQLTE
--PSQLSE
--PSQLQV
--PSQLQT
--PSQLQQ
--PSQLQH
--PSQLQF
--PSQLNG
--PSQLNF
--PSQLNE
--PSQLMK
--PSQLMH
--PSQLLH
--PSQLLC
--PSQLKD
--PSQLIN
--PSQLIC
--PSQLHT
--PSQLHR
--PSQLHK
--PSQLFY
--PSQLFE
--PSQLEY
--PSQLET
--PSQLCT
--PSQLAY
--PSQLAV
--PSQLAQ

```
-PPSQLL-
PDPSQL--
-QPSQLL-
-QPSQLR-
QTPSQL--
-QPSQLT-
QRPSQL--
QLPSQL--
QVPSQL--
-QPSQLA-
-QPSQLS-
QSPSQL--
QAPSQL--
-QPSQLH-
QYPSQL--
-QPSQLF-
-QPSQLY-
-QPSQLK-
-QPSQLI-
QPPSQL--
-QPSQLM-
QGPSQL--
QWPSQL--
-QPSQLN-
QEPSQL--
-QPSQLW-
QMPSQL--
QKPSQL--
QHPSQL--
QDPSQL--
-QPSQLV-
QNPSQL--
QIPSQL--
QFPSQL--
RGPSQL--
RVPSQL--
-RPSQLS-
RWPSQL--
RKPSQL--
RFPSQL--
RYPSQL--
RSPSQL--
RMPSQL--
-RPSQLV-
-RPSQLG-
-RPSQLQ-
RQPSQL--
RAPSQL--
-RPSQLH-
RIPSQL--
```

RTPSQL--
-RPSQLA-
REPSQL--
-RPSQLN-
-RPSQLF-
RPPSQL--
-RPSQLI-
-RPSQLY-
-RPSQLT-
-RPSQLM-
-RPSQLE-
RCPSQL--
RHPSQL--
-RPSQLD-
-SPSQLI-
SFPSQL--
SVPSQL--
SLPSQL--
SYPSQL--
-SPSQLL-
SHPSQL--
SIPSQL--
SSPSQL--
-SPSQLY-
-SPSQLQ-
SGPSQL--
STPSQL--
SQPSQL--
-SPSQLS-
SWPSQL--
SAPSQL--
-SPSQLV-
SMPSQL--
-SPSQLT-
-SPSQLH-
-SPSQLA-
-SPSQLK-
SNPSQL--
-SPSQLN-
-SPSQLF-
SEPSQL--
SDPSQL--
SPPSQL--
SCPSQL--
-TPSQLR-
TSPSQL--
TAPSQL--
TVPSQL--
THPSQL--
TMPSQL--

−TPSQLW−
TKPSQL−−
−TPSQLN−
TLPSQL−−
−TPSQLT−
−TPSQLH−
TWPSQL−−
−TPSQLK−
TTPSQL−−
TRPSQL−−
−TPSQLV−
−TPSQLM−
TFPSQL−−
−TPSQLY−
−TPSQLQ−
TGPSQL−−
TEPSQL−−
−TPSQLA−
−TPSQLF−
TNPSQL−−
TIPSQL−−
TDPSQL−−
TQPSQL−−
−TPSQLC−
TYPSQL−−
−TPSQLE−
TPPSQL−−
TCPSQL−−
−VPSQLL−
VSPSQL−−
−VPSQLR−
VVPSQL−−
VNPSQL−−
−VPSQLT−
−VPSQLI−
−VPSQLA−
−VPSQLS−
VLPSQL−−
VKPSQL−−
−VPSQLK−
VRPSQL−−
−VPSQLN−
VIPSQL−−
VGPSQL−−
VPPSQL−−
VHPSQL−−
VAPSQL−−
−VPSQLY−
−VPSQLV−
VEPSQL−−

VTPSQL--
VMPSQL--
VDPSQL--
-VPSQLH-
VWPSQL--
VQPSQL--
-VPSQLE-
VCPSQL--
-VPSQLW-
-VPSQLF-
-VPSQLQ-
VFPSQL--
WRPSQL--
-WPSQLR-
-WPSQLS-
WPPSQL--
WTPSQL--
WVPSQL--
WYPSQL--
-WPSQLT-
WIPSQL--
-WPSQLN-
WMPSQL--
WLPSQL--
WGPSQL--
-WPSQLM-
-WPSQLK-
-WPSQLA-
WDPSQL--
WSPSQL--
-WPSQLY-
-WPSQLV-
-WPSQLI-
WWPSQL--
WQPSQL--
-WPSQLL-
-WPSQLH-
WHPSQL--
-WPSQLQ-
WFPSQL--
WAPSQL--
-WPSQLF-
-WPSQLE-
WNPSQL--
WCPSQL--
YVPSQL--
-YPSQLS-
YSPSQL--
-YPSQLR-
-YPSQLL-

YYPSQL--
YTPSQL--
YRPSQL--
YQPSQL--
-YPSQLT-
-YPSQLV-
-YPSQLN-
YKPSQL--
YIPSQL--
-YPSQLA-
YWPSQL--
-YPSQLK-
-YPSQLH-
YNPSQL--
YCPSQL--
-YPSQLY-
-YPSQLW-
YPPSQL--
YFPSQL--
YEPSQL--
YAPSQL--
-YPSQLM-
-YPSQLG-
-YPSQLF-
YMPSQL--

VAGL 109 764 33.16

-IVAGLG-
-LVAGLP-
-NVAGLM-
-TVAGLG-
-VVAGLD-
--VAGLYP
--VAGLSE
--VAGLSD
--VAGLNN
--VAGLNL
--VAGLND
--VAGLGT
--VAGLGR
--VAGLEV
--VAGLDR
--VAGLAR
--VAGLAL
--VAGLAK
--VAGLAI
ALVAGL--
-AVAGLL-
-AVAGLS-
ASVAGL--
-AVAGLY-

-AVAGLT-
AVVAGL--
ATVAGL--
-AVAGLR-
-AVAGLK-
-AVAGLI-
-AVAGLA-
ANVAGL--
AAVAGL--
-AVAGLM-
AHVAGL--
AMVAGL--
AGVAGL--
-AVAGLE-
-AVAGLH-
AIVAGL--
-AVAGLN-
-AVAGLF-
AQVAGL--
AWVAGL--
-AVAGLP-
ADVAGL--
-CVAGLR-
CKVAGL--
CFVAGL--
-CVAGLW-
-CVAGLS-
-CVAGLN-
CYVAGL--
-CVAGLV-
CSVAGL--
CRVAGL--
CLVAGL--
CMVAGL--
CIVAGL--
CDVAGL--
CWVAGL--
CVVAGL--
-CVAGLY-
-CVAGLT-
-CVAGLM-
-CVAGLK-
CNVAGL--
CHVAGL--
CGVAGL--
CEVAGL--
CAVAGL--
-CVAGLQ-
-CVAGLI-
-CVAGLG-

```
-CVAGLF-
-CVAGLE-
-CVAGLA-
CTVAGL--
CQVAGL--
CCVAGL--
DRVAGL--
-DVAGLT-
DSVAGL--
-DVAGLS-
-DVAGLY-
-DVAGLF-
-DVAGLV-
DWVAGL--
DLVAGL--
DVVAGL--
-DVAGLR-
-DVAGLI-
DTVAGL--
DFVAGL--
-DVAGLQ-
-DVAGLN-
-DVAGLH-
DPVAGL--
DMVAGL--
DEVAGL--
DDVAGL--
-DVAGLW-
-DVAGLA-
DHVAGL--
DAVAGL--
DKVAGL--
DIVAGL--
DGVAGL--
DQVAGL--
ERVAGL--
-EVAGLH-
EKVAGL--
-EVAGLI-
-EVAGLL-
ELVAGL--
-EVAGLS-
EIVAGL--
ETVAGL--
-EVAGLA-
EWVAGL--
-EVAGLN-
EVVAGL--
EPVAGL--
EAVAGL--
```

-EVAGLT-
EFVAGL--
-EVAGLQ-
-EVAGLK-
ESVAGL--
-EVAGLR-
-EVAGLM-
-EVAGLF-
EHVAGL--
-EVAGLW-
EQVAGL--
EYVAGL--
EEVAGL--
ECVAGL--
-EVAGLV-
-EVAGLE-
-EVAGLD-
ENVAGL--
EMVAGL--
-FVAGLS-
FKVAGL--
FLVAGL--
FSVAGL--
-FVAGLL-
FDVAGL--
FVVAGL--
-FVAGLV-
-FVAGLK-
FMVAGL--
-FVAGLR-
-FVAGLH-
FAVAGL--
-FVAGLW-
-FVAGLQ-
-FVAGLI-
-FVAGLN-
FIVAGL--
FGVAGL--
-FVAGLA-
FQVAGL--
-FVAGLE-
-FVAGLM-
FPVAGL--
FEVAGL--
-FVAGLF-
-GVAGLR-
GIVAGL--
-GVAGLN-
GSVAGL--
-GVAGLQ-

GKVAGL--
-GVAGLK-
GYVAGL--
GTVAGL--
GQVAGL--
-GVAGLT-
-GVAGLI-
-GVAGLF-
-GVAGLY-
GFVAGL--
GPVAGL--
GHVAGL--
GCVAGL--
-GVAGLH-
-GVAGLA-
GDVAGL--
GAVAGL--
GNVAGL--
-GVAGLW-
GGVAGL--
GEVAGL--
-GVAGLM-
-GVAGLE-
-HVAGLR-
-HVAGLY-
HHVAGL--
HLVAGL--
HMVAGL--
-HVAGLS-
-HVAGLL-
HEVAGL--
-HVAGLF-
-HVAGLT-
-HVAGLQ-
HNVAGL--
HVVAGL--
-HVAGLI-
-HVAGLA-
HTVAGL--
HKVAGL--
-HVAGLG-
HIVAGL--
HFVAGL--
HCVAGL--
-HVAGLN-
HGVAGL--
-HVAGLW-
-HVAGLV-
HQVAGL--
HWVAGL--

-HVAGLH-
HPVAGL--
-HVAGLK-
-HVAGLE-
HDVAGL--
IGVAGL--
IWVAGL--
-IVAGLA-
IVVAGL--
-IVAGLR-
IHVAGL--
-IVAGLQ-
IAVAGL--
-IVAGLN-
-IVAGLF-
ITVAGL--
-IVAGLH-
IQVAGL--
-IVAGLI-
-IVAGLS-
INVAGL--
IFVAGL--
-IVAGLK-
IMVAGL--
-IVAGLT-
IIVAGL--
-IVAGLV-
-IVAGLE-
IPVAGL--
-IVAGLL-
-IVAGLY-
IKVAGL--
-IVAGLM-
-KVAGLL-
KLVAGL--
KMVAGL--
-KVAGLS-
KHVAGL--
KTVAGL--
-KVAGLY-
-KVAGLN-
-KVAGLQ-
KNVAGL--
KVVAGL--
-KVAGLH-
KGVAGL--
-KVAGLV-
-KVAGLI-
KDVAGL--
KQVAGL--

KEVAGL--
KAVAGL--
KFVAGL--
-KVAGLW-
-KVAGLM-
-KVAGLE-
LNVAGL--
-LVAGLT-
-LVAGLK-
LKVAGL--
LIVAGL--
-LVAGLH-
LYVAGL--
-LVAGLE-
LPVAGL--
-LVAGLN-
LDVAGL--
-LVAGLA-
LQVAGL--
LHVAGL--
-LVAGLM-
LMVAGL--
-LVAGLQ-
LCVAGL--
-MVAGLL-
MTVAGL--
MRVAGL--
MLVAGL--
-MVAGLG-
-MVAGLS-
-MVAGLA-
MYVAGL--
-MVAGLT-
MNVAGL--
-MVAGLV-
-MVAGLF-
MGVAGL--
-MVAGLK-
MMVAGL--
MIVAGL--
-MVAGLH-
MPVAGL--
MKVAGL--
-MVAGLQ-
-MVAGLM-
-MVAGLE-
MVVAGL--
-MVAGLI-
MFVAGL--
MHVAGL--

```
-MVAGLN-
MQVAGL--
MWVAGL--
MEVAGL--
MDVAGL--
MCVAGL--
-NVAGLI-
-NVAGLV-
-NVAGLR-
-NVAGLN-
-NVAGLL-
-NVAGLA-
NQVAGL--
NVVAGL--
NTVAGL--
-NVAGLS-
NNVAGL--
NHVAGL--
NSVAGL--
NGVAGL--
-NVAGLW-
-NVAGLY-
-NVAGLK-
NAVAGL--
-NVAGLH-
NWVAGL--
NIVAGL--
-NVAGLQ-
-NVAGLT-
-NVAGLF-
-NVAGLE-
NMVAGL--
NDVAGL--
-NVAGLG-
NCVAGL--
PRVAGL--
-PVAGLR-
-PVAGLL-
-PVAGLI-
-PVAGLV-
PNVAGL--
-PVAGLT-
-PVAGLS-
-PVAGLQ-
PFVAGL--
PGVAGL--
-PVAGLH-
-PVAGLY-
-PVAGLM-
-PVAGLK-
```

-PVAGLW-
PTVAGL--
PIVAGL--
PYVAGL--
-PVAGLA-
PDVAGL--
PAVAGL--
-PVAGLG-
PEVAGL--
-PVAGLF-
-PVAGLE-
PQVAGL--
PWVAGL--
PPVAGL--
PCVAGL--
-QVAGLS-
-QVAGLL-
QRVAGL--
-QVAGLF-
-QVAGLR-
QSVAGL--
QPVAGL--
QIVAGL--
-QVAGLT-
-QVAGLI-
QVVAGL--
-QVAGLW-
QNVAGL--
QYVAGL--
-QVAGLN-
QTVAGL--
QGVAGL--
-QVAGLQ-
-QVAGLG-
-QVAGLK-
-QVAGLH-
-QVAGLY-
QMVAGL--
QFVAGL--
-QVAGLA-
QQVAGL--
-QVAGLM-
-QVAGLE-
QEVAGL--
QAVAGL--
-QVAGLD-
QHVAGL--
QDVAGL--
RLVAGL--
-RVAGLS-

-RVAGLA-
RTVAGL--
-RVAGLH-
RNVAGL--
-RVAGLY-
RPVAGL--
RFVAGL--
RDVAGL--
-RVAGLF-
-RVAGLI-
-RVAGLN-
-RVAGLQ-
RQVAGL--
-RVAGLM-
-RVAGLE-
RCVAGL--
-RVAGLG-
-SVAGLL-
-SVAGLR-
SLVAGL--
SVVAGL--
SKVAGL--
-SVAGLH-
SIVAGL--
-SVAGLV-
-SVAGLF-
-SVAGLN-
SPVAGL--
-SVAGLW-
-SVAGLK-
SAVAGL--
-SVAGLQ-
-SVAGLY-
SYVAGL--
SQVAGL--
SWVAGL--
SMVAGL--
SDVAGL--
SFVAGL--
-SVAGLA-
SEVAGL--
SCVAGL--
-SVAGLG-
-SVAGLM-
-SVAGLC-
-TVAGLL-
-TVAGLR-
TRVAGL--
-TVAGLS-
TYVAGL--

TFVAGL--
-TVAGLA-
TPVAGL--
TVVAGL--
TGVAGL--
TWVAGL--
-TVAGLN-
THVAGL--
-TVAGLM-
-TVAGLV-
TKVAGL--
-TVAGLT-
-TVAGLF-
TMVAGL--
-TVAGLI-
-TVAGLH-
TQVAGL--
TEVAGL--
-TVAGLQ-
TTVAGL--
TIVAGL--
TAVAGL--
-TVAGLE-
-TVAGLC-
--VAGLYR
--VAGLRS
--VAGLRY
--VAGLRI
--VAGLLS
VVVAGL--
--VAGLRG
-VVAGLS-
--VAGLRN
VLVAGL--
-VVAGLH-
--VAGLSS
--VAGLNS
VRVAGL--
--VAGLYS
--VAGLTS
--VAGLRT
--VAGLRM
-VVAGLR-
-VVAGLQ-
--VAGLYK
--VAGLRE
--VAGLHR
--VAGLMR
--VAGLLG
--VAGLLA

--VAGLKG
VSVAGL--
--VAGLWK
--VAGLTG
--VAGLRH
--VAGLMA
--VAGLVK
--VAGLSA
VFVAGL--
--VAGLVG
--VAGLKS
--VAGLIA
--VAGLYG
--VAGLKK
--VAGLTV
--VAGLSV
--VAGLNV
--VAGLMG
--VAGLYT
--VAGLVS
--VAGLLR
--VAGLLN
-VVAGLA-
--VAGLTY
VQVAGL--
--VAGLWN
--VAGLNA
--VAGLFT
-VVAGLN-
--VAGLTA
--VAGLML
--VAGLKY
--VAGLHG
--VAGLRQ
--VAGLKR
--VAGLFR
--VAGLVN
--VAGLTT
--VAGLTR
--VAGLSM
--VAGLSI
--VAGLSF
--VAGLHS
--VAGLGS
--VAGLFN
--VAGLTM
--VAGLSW
--VAGLLH
--VAGLEA
VYVAGL--

VNVAGL--
--VAGLTF
--VAGLMT
--VAGLKF
--VAGLIV
--VAGLIM
--VAGLHA
--VAGLES
-VVAGLM-
VPVAGL--
--VAGLFV
--VAGLWT
--VAGLWQ
--VAGLWG
--VAGLVF
--VAGLTW
--VAGLSY
--VAGLQV
--VAGLMH
--VAGLMF
--VAGLKN
VWVAGL--
--VAGLYV
--VAGLVY
--VAGLQT
--VAGLMQ
--VAGLKI
--VAGLHL
--VAGLFQ
--VAGLAS
--VAGLYA
--VAGLSK
--VAGLSH
--VAGLNM
--VAGLHN
VAVAGL--
--VAGLQA
--VAGLIY
--VAGLHQ
--VAGLFK
--VAGLFA
-VVAGLK-
VEVAGL--
--VAGLWH
--VAGLQG
--VAGLIN
--VAGLHK
--VAGLHH
--VAGLFW
--VAGLFH

VHVAGL--
--VAGLVI
--VAGLNT
--VAGLNH
--VAGLMN
--VAGLKT
--VAGLIT
--VAGLIS
--VAGLIG
--VAGLHW
--VAGLFM
--VAGLAW
-VVAGLG-
--VAGLYN
--VAGLYH
--VAGLVH
--VAGLSL
--VAGLLW
--VAGLKA
--VAGLIW
--VAGLIH
--VAGLHM
--VAGLAM
VMVAGL--
--VAGLTQ
--VAGLSN
--VAGLQY
--VAGLNF
--VAGLMW
--VAGLMV
--VAGLMM
--VAGLMI
--VAGLID
--VAGLHV
--VAGLAV
-VVAGLE-
--VAGLYI
--VAGLWD
--VAGLTL
--VAGLQR
--VAGLQN
--VAGLQH
--VAGLNQ
--VAGLMD
--VAGLKQ
--VAGLKL
--VAGLIQ
--VAGLHY
--VAGLYW
--VAGLYM

--VAGLWM
--VAGLVQ
--VAGLQW
--VAGLQM
--VAGLQK
--VAGLNG
--VAGLLD
--VAGLKW
--VAGLII
--VAGLIE
--VAGLHF
--VAGLGG
--VAGLWA
--VAGLVE
--VAGLTK
--VAGLMK
--VAGLME
--VAGLKD
--VAGLHD
--VAGLGW
--VAGLEG
--VAGLDE
--VAGLCT
--VAGLAY
VCVAGL--
--VAGLTD
--VAGLQQ
--VAGLQF
--VAGLNY
--VAGLNW
--VAGLKE
--VAGLFE
--VAGLEY
--VAGLEP
--VAGLEM
--VAGLED
--VAGLCY
--VAGLCS
-WVAGLS-
WAVAGL--
-WVAGLK-
WKVAGL--
WMVAGL--
WNVAGL--
WLVAGL--
WDVAGL--
-WVAGLN-
-WVAGLV-
-WVAGLT-
-WVAGLE-

```
               -WVAGLA-
               -WVAGLG-
               WHVAGL--
               WPVAGL--
               WEVAGL--
               -WVAGLM-
               -WVAGLY-
               -WVAGLF-
               WCVAGL--
               YSVAGL--
               YLVAGL--
               -YVAGLN-
               -YVAGLL-
               -YVAGLR-
               YPVAGL--
               YGVAGL--
               -YVAGLH-
               YHVAGL--
               YWVAGL--
               -YVAGLY-
               -YVAGLF-
               -YVAGLW-
               -YVAGLI-
               -YVAGLT-
               -YVAGLS-
               YIVAGL--
               -YVAGLQ-
               YAVAGL--
               -YVAGLE-
               YNVAGL--
               YMVAGL--
               YYVAGL--
               YQVAGL--
               YDVAGL--
               YEVAGL--
               -YVAGLA-
               YKVAGL--
               YCVAGL--
               -YVAGLM-
               -YVAGLC-
PGAL             34          825      33.15
               -LPGALP-
               --PGALYP
               --PGALSP
               --PGALSE
               --PGALHN
               --PGALGR
               -RPGALP-
               -YPGALP-
               ARPGAL--
```

-APGALR-
-APGALG-
ATPGAL--
AVPGAL--
-APGALI-
ALPGAL--
-APGALV-
-APGALK-
-APGALT-
-APGALL-
-APGALH-
AGPGAL--
-APGALN-
APPGAL--
AIPGAL--
AWPGAL--
AHPGAL--
-APGALW-
ANPGAL--
-APGALQ-
AAPGAL--
AYPGAL--
AQPGAL--
-APGALA-
AMPGAL--
AFPGAL--
-APGALY-
-APGALM-
AEPGAL--
ADPGAL--
ACPGAL--
CRPGAL--
CKPGAL--
CTPGAL--
CLPGAL--
CIPGAL--
CHPGAL--
-CPGALY-
-CPGALT-
-CPGALS-
-CPGALQ-
-CPGALM-
CGPGAL--
CFPGAL--
CWPGAL--
-CPGALR-
CEPGAL--
CCPGAL--
CVPGAL--
CSPGAL--

-CPGALW-
-CPGALL-
CNPGAL--
-DPGALW-
DHPGAL--
-DPGALT-
DIPGAL--
DVPGAL--
DMPGAL--
-DPGALI-
DWPGAL--
DSPGAL--
-DPGALN-
DLPGAL--
DAPGAL--
DYPGAL--
-DPGALR-
DGPGAL--
DRPGAL--
DKPGAL--
-DPGALY-
-DPGALF-
DEPGAL--
DDPGAL--
DTPGAL--
-DPGALQ-
-DPGALK-
DNPGAL--
DCPGAL--
-EPGALK-
-EPGALL-
-EPGALS-
ERPGAL--
-EPGALT-
ETPGAL--
-EPGALY-
EGPGAL--
ELPGAL--
EVPGAL--
ESPGAL--
-EPGALW-
-EPGALH-
EDPGAL--
EFPGAL--
ENPGAL--
EYPGAL--
EWPGAL--
-EPGALQ-
-EPGALN-
-EPGALM-

-EPGALI-
EQPGAL--
EPPGAL--
-EPGALF-
EIPGAL--
-EPGALE-
-EPGALA-
EKPGAL--
FRPGAL--
-FPGALL-
-FPGALR-
FGPGAL--
FYPGAL--
-FPGALN-
FQPGAL--
FNPGAL--
FMPGAL--
FKPGAL--
FPPGAL--
FAPGAL--
FSPGAL--
FIPGAL--
FHPGAL--
FFPGAL--
FWPGAL--
-FPGALT-
-FPGALK-
-FPGALQ-
-FPGALI-
-FPGALV-
-FPGALF-
-FPGALH-
-FPGALA-
-FPGALM-
-FPGALE-
FEPGAL--
GWPGAL--
GKPGAL--
-GPGALL-
GTPGAL--
GLPGAL--
GRPGAL--
-GPGALM-
GVPGAL--
GYPGAL--
GSPGAL--
GQPGAL--
-GPGALK-
GIPGAL--
GEPGAL--

GAPGAL--
GMPGAL--
-GPGALY-
-GPGALQ-
-GPGALI-
-GPGALG-
GHPGAL--
GGPGAL--
-GPGALN-
-GPGALH-
-GPGALA-
GDPGAL--
GPPGAL--
-GPGALV-
GCPGAL--
-HPGALR-
-HPGALL-
HRPGAL--
HVPGAL--
HTPGAL--
-HPGALT-
-HPGALF-
HLPGAL--
HHPGAL--
HKPGAL--
HMPGAL--
HEPGAL--
HWPGAL--
HSPGAL--
-HPGALV-
-HPGALI-
-HPGALS-
HIPGAL--
HAPGAL--
-HPGALW-
-HPGALN-
HPPGAL--
-HPGALM-
-HPGALH-
HFPGAL--
HQPGAL--
HYPGAL--
-HPGALY-
-HPGALQ-
-HPGALG-
-HPGALE-
HNPGAL--
HDPGAL--
ILPGAL--
IMPGAL--

INPGAL--
ITPGAL--
IKPGAL--
-IPGALT-
-IPGALQ-
-IPGALL-
IVPGAL--
-IPGALN-
IPPGAL--
-IPGALY-
-IPGALS-
-IPGALI-
-IPGALV-
-IPGALF-
IIPGAL--
IYPGAL--
ISPGAL--
IHPGAL--
-IPGALW-
-IPGALK-
IDPGAL--
-IPGALG-
-IPGALA-
-IPGALM-
IFPGAL--
-IPGALE-
IQPGAL--
-IPGALH-
ICPGAL--
KSPGAL--
-KPGALT-
-KPGALR-
KRPGAL--
-KPGALY-
-KPGALL-
KKPGAL--
KHPGAL--
-KPGALS-
KGPGAL--
KFPGAL--
KVPGAL--
-KPGALH-
KNPGAL--
KYPGAL--
-KPGALV-
-KPGALW-
-KPGALI-
-KPGALA-
KAPGAL--
-KPGALM-

KCPGAL--
-KPGALQ-
KPPGAL--
-KPGALG-
-KPGALF-
KMPGAL--
KIPGAL--
-KPGALE-
KEPGAL--
KDPGAL--
KQPGAL--
LNPGAL--
LRPGAL--
-LPGALW-
LVPGAL--
LIPGAL--
LSPGAL--
-LPGALT-
-LPGALL-
-LPGALR-
LMPGAL--
-LPGALK-
LGPGAL--
LAPGAL--
-LPGALS-
LHPGAL--
LTPGAL--
LYPGAL--
-LPGALN-
LEPGAL--
-LPGALY-
-LPGALA-
-LPGALI-
LPPGAL--
LQPGAL--
LWPGAL--
-LPGALF-
-LPGALQ-
LDPGAL--
-MPGALW-
-MPGALR-
-MPGALQ-
MAPGAL--
MSPGAL--
MGPGAL--
MIPGAL--
MTPGAL--
-MPGALK-
MQPGAL--
-MPGALS-

MMPGAL--
-MPGALT-
MLPGAL--
MYPGAL--
MNPGAL--
-MPGALV-
-MPGALN-
-MPGALI-
MPPGAL--
-MPGALG-
-MPGALF-
MKPGAL--
MDPGAL--
-MPGALY-
-MPGALM-
MHPGAL--
MFPGAL--
MEPGAL--
NVPGAL--
-NPGALF-
-NPGALT-
NTPGAL--
NYPGAL--
NRPGAL--
-NPGALL-
NLPGAL--
NSPGAL--
-NPGALS-
-NPGALR-
-NPGALV-
-NPGALA-
NNPGAL--
-NPGALY-
NWPGAL--
-NPGALK-
NIPGAL--
NGPGAL--
NMPGAL--
NKPGAL--
-NPGALI-
NHPGAL--
NDPGAL--
NFPGAL--
NEPGAL--
NQPGAL--
-NPGALM-
-NPGALN-
NCPGAL--
-NPGALG-
-NPGALE-

--PGALGL
--PGALSL
--PGALLS
--PGALLA
--PGALLT
--PGALSR
--PGALVR
--PGALRN
--PGALLR
--PGALRS
PSPGAL--
--PGALYS
--PGALYA
--PGALRV
--PGALGS
--PGALFK
--PGALSS
--PGALSI
--PGALRT
--PGALKR
--PGALWR
--PGALTR
--PGALSA
--PGALRW
--PGALFL
--PGALYN
--PGALTL
--PGALAS
--PGALRH
--PGALMS
--PGALRQ
--PGALAG
--PGALST
--PGALHL
PWPGAL--
PLPGAL--
--PGALTM
--PGALMV
--PGALLW
--PGALLK
--PGALIR
--PGALYW
--PGALWL
--PGALTS
--PGALLG
--PGALSY
--PGALLI
--PGALKG
PEPGAL--
--PGALTI

--PGALMG
--PGALKY
--PGALHS
--PGALGI
--PGALFS
PYPGAL--
--PGALVG
--PGALTT
--PGALRY
--PGALNL
--PGALLN
--PGALKK
--PGALIG
--PGALHY
--PGALGF
--PGALFM
PMPGAL--
--PGALWV
--PGALTA
--PGALSV
--PGALSN
--PGALLH
--PGALLF
--PGALKF
--PGALII
--PGALHV
--PGALHR
PGPGAL--
--PGALYQ
--PGALYL
--PGALWG
--PGALTN
--PGALTK
--PGALTG
--PGALSK
--PGALRM
--PGALNV
--PGALMW
--PGALMH
--PGALKW
--PGALKA
--PGALIT
--PGALFW
--PGALYV
--PGALYR
--PGALYG
--PGALWN
--PGALWA
--PGALVI
--PGALSG

--PGALNR
--PGALNI
--PGALAF
PIPGAL--
--PGALYF
--PGALWY
--PGALWF
--PGALVD
--PGALTY
--PGALPF
--PGALLL
--PGALIS
--PGALHK
--PGALGM
--PGALFN
--PGALAW
PKPGAL--
--PGALVN
--PGALVM
--PGALTW
--PGALTV
--PGALSQ
--PGALQY
--PGALNW
--PGALNH
--PGALNA
--PGALMK
--PGALLE
--PGALKN
--PGALKI
--PGALIA
--PGALFA
--PGALAI
--PGALYY
--PGALWS
--PGALWH
--PGALTH
--PGALSH
--PGALMR
--PGALMN
--PGALLQ
--PGALLM
--PGALHI
--PGALGA
PAPGAL--
--PGALWE
--PGALVQ
--PGALTE
--PGALNT
--PGALMA

--PGALKV
--PGALGT
--PGALFT
--PGALFI
PNPGAL--
--PGALYK
--PGALYH
--PGALYE
--PGALWT
--PGALVW
--PGALSW
--PGALRD
--PGALPM
--PGALMY
--PGALMI
--PGALLV
--PGALIN
--PGALHW
--PGALHQ
--PGALHH
--PGALHA
--PGALGY
--PGALFG
--PGALAN
PQPGAL--
--PGALYI
--PGALRG
--PGALQV
--PGALQS
--PGALQG
--PGALNN
--PGALMM
--PGALKM
--PGALIW
--PGALIQ
--PGALIK
--PGALIF
--PGALGG
--PGALFV
PTPGAL--
--PGALYM
--PGALWQ
--PGALVF
--PGALVA
--PGALTQ
--PGALRE
--PGALQT
--PGALQM
--PGALQI
--PGALPL

--PGALMT
--PGALMF
--PGALKH
--PGALIY
--PGALIV
--PGALHG
--PGALGW
--PGALGV
--PGALEV
--PGALET
--PGALES
PFPGAL--
--PGALWW
--PGALWI
--PGALWD
--PGALVE
--PGALTP
--PGALTF
--PGALTD
--PGALQQ
--PGALQN
--PGALQA
--PGALPI
--PGALNY
--PGALNM
--PGALND
--PGALME
--PGALMD
--PGALKS
--PGALKE
--PGALHT
--PGALHF
--PGALAV
--PGALAK
--PGALAA
-PPGALR-
--PGALYD
--PGALWM
--PGALQP
--PGALQK
--PGALQD
--PGALNQ
--PGALHD
--PGALGQ
--PGALFF
--PGALEG
--PGALEA
--PGALDV
--PGALCN
--PGALCM

PDPGAL--
-QPGALL-
-QPGALR-
-QPGALH-
QFPGAL--
QKPGAL--
QRPGAL--
-QPGALM-
QPPGAL--
QHPGAL--
-QPGALS-
QLPGAL--
QIPGAL--
QYPGAL--
-QPGALV-
QDPGAL--
QWPGAL--
-QPGALN-
-QPGALG-
QNPGAL--
-QPGALY-
-QPGALF-
-QPGALQ-
QTPGAL--
-QPGALI-
-QPGALE-
QMPGAL--
-QPGALW-
QEPGAL--
QCPGAL--
QAPGAL--
QGPGAL--
-RPGALR-
-RPGALL-
RLPGAL--
-RPGALS-
RSPGAL--
RRPGAL--
-RPGALT-
-RPGALK-
RIPGAL--
RGPGAL--
-RPGALW-
-RPGALF-
RAPGAL--
RMPGAL--
RFPGAL--
RVPGAL--
-RPGALN-
-RPGALE-

RHPGAL--
RNPGAL--
-RPGALY-
RDPGAL--
-RPGALI-
RPPGAL--
-RPGALQ-
RWPGAL--
-RPGALM-
RQPGAL--
REPGAL--
-RPGALH-
RCPGAL--
SSPGAL--
STPGAL--
SIPGAL--
-SPGALL-
SLPGAL--
-SPGALR-
SFPGAL--
-SPGALA-
SWPGAL--
-SPGALT-
SKPGAL--
-SPGALN-
SVPGAL--
-SPGALH-
SAPGAL--
-SPGALV-
SNPGAL--
SMPGAL--
SGPGAL--
-SPGALK-
-SPGALI-
SYPGAL--
-SPGALF-
SEPGAL--
-SPGALQ-
-SPGALM-
-SPGALE-
SDPGAL--
SCPGAL--
-TPGALW-
TKPGAL--
TSPGAL--
TRPGAL--
-TPGALL-
TFPGAL--
TVPGAL--
TLPGAL--

```
-TPGALY-
TWPGAL--
-TPGALK-
TEPGAL--
-TPGALT-
-TPGALQ-
-TPGALH-
TIPGAL--
-TPGALM-
TYPGAL--
-TPGALV-
-TPGALA-
TTPGAL--
-TPGALS-
TNPGAL--
TQPGAL--
-TPGALN-
TAPGAL--
-TPGALG-
-TPGALF-
TMPGAL--
TDPGAL--
THPGAL--
TCPGAL--
-VPGALR-
VSPGAL--
VVPGAL--
-VPGALH-
-VPGALT-
-VPGALK-
VLPGAL--
-VPGALW-
VFPGAL--
VRPGAL--
-VPGALI-
VMPGAL--
VYPGAL--
VQPGAL--
-VPGALQ-
-VPGALN-
VTPGAL--
-VPGALE-
VHPGAL--
-VPGALV-
-VPGALG-
-VPGALA-
VNPGAL--
VWPGAL--
VPPGAL--
-VPGALM-
```

VIPGAL--
VEPGAL--
VDPGAL--
VCPGAL--
-WPGALR-
-WPGALL-
-WPGALK-
-WPGALS-
-WPGALN-
WPPGAL--
-WPGALF-
WTPGAL--
WQPGAL--
WVPGAL--
WIPGAL--
-WPGALY-
-WPGALV-
WNPGAL--
WMPGAL--
-WPGALW-
-WPGALH-
WYPGAL--
WWPGAL--
-WPGALI-
WGPGAL--
WFPGAL--
-WPGALA-
WKPGAL--
WHPGAL--
WAPGAL--
-WPGALQ-
-WPGALE-
WDPGAL--
WCPGAL--
-WPGALM-
YRPGAL--
-YPGALR-
YLPGAL--
-YPGALS-
YSPGAL--
YQPGAL--
YFPGAL--
YYPGAL--
YIPGAL--
-YPGALY-
-YPGALW-
YNPGAL--
YAPGAL--
-YPGALN-
-YPGALH-

| | | | |
|---|---|---|---|
| | YHPGAL-- | | |
| | YVPGAL-- | | |
| | -YPGALI- | | |
| | YWPGAL-- | | |
| | YPPGAL-- | | |
| | -YPGALA- | | |
| | YGPGAL-- | | |
| | -YPGALK- | | |
| | -YPGALF- | | |
| | -YPGALQ- | | |
| | -YPGALM- | | |
| | YDPGAL-- | | |
| | -YPGALV- | | |
| | -YPGALG- | | |
| | YEPGAL-- | | |
| | -YPGALE- | | |
| PGWL | 82 | 778 | 33.13 |
| | -APGWLP- | | |
| | NRPGWL-- | | |
| | --PGWLVY | | |
| | --PGWLSP | | |
| | --PGWLNR | | |
| | --PGWLGN | | |
| | --PGWLGK | | |
| | -SPGWLD- | | |
| | -YPGWLN- | | |
| | -APGWLS- | | |
| | ATPGWL-- | | |
| | ARPGWL-- | | |
| | -APGWLK- | | |
| | -APGWLG- | | |
| | ASPGWL-- | | |
| | ALPGWL-- | | |
| | -APGWLY- | | |
| | -APGWLR- | | |
| | -APGWLL- | | |
| | AFPGWL-- | | |
| | -APGWLV- | | |
| | AGPGWL-- | | |
| | -APGWLN- | | |
| | -APGWLA- | | |
| | ANPGWL-- | | |
| | AAPGWL-- | | |
| | AWPGWL-- | | |
| | -APGWLT- | | |
| | -APGWLQ- | | |
| | AIPGWL-- | | |
| | AEPGWL-- | | |
| | -APGWLM- | | |
| | -APGWLF- | | |

-APGWLE-
-APGWLD-
APPGWL--
-APGWLW-
-APGWLH-
AMPGWL--
AKPGWL--
ADPGWL--
AQPGWL--
AHPGWL--
-CPGWLR-
-CPGWLT-
CHPGWL--
CGPGWL--
CEPGWL--
CRPGWL--
-CPGWLV-
CAPGWL--
CWPGWL--
CQPGWL--
-CPGWLS-
-CPGWLK-
CLPGWL--
CKPGWL--
CCPGWL--
DIPGWL--
DRPGWL--
-DPGWLG-
DAPGWL--
DQPGWL--
DVPGWL--
-DPGWLI-
DKPGWL--
-DPGWLS-
DLPGWL--
DYPGWL--
-DPGWLR-
-DPGWLL-
-DPGWLK-
DMPGWL--
DTPGWL--
DSPGWL--
-DPGWLY-
-DPGWLT-
-DPGWLA-
DDPGWL--
-DPGWLF-
DNPGWL--
DHPGWL--
DGPGWL--

-EPGWLK-
ESPGWL--
-EPGWLY-
-EPGWLT-
EGPGWL--
-EPGWLV-
ELPGWL--
ERPGWL--
EVPGWL--
-EPGWLL-
-EPGWLG-
ENPGWL--
-EPGWLS-
ETPGWL--
-EPGWLQ-
-EPGWLM-
EQPGWL--
-EPGWLN-
-EPGWLI-
-EPGWLA-
EKPGWL--
EYPGWL--
EWPGWL--
EMPGWL--
EIPGWL--
EHPGWL--
EPPGWL--
-EPGWLH-
EFPGWL--
EEPGWL--
EAPGWL--
FLPGWL--
FSPGWL--
-FPGWLQ-
-FPGWLV-
-FPGWLK-
-FPGWLG-
FMPGWL--
FKPGWL--
-FPGWLY-
-FPGWLR-
-FPGWLA-
FNPGWL--
FYPGWL--
-FPGWLT-
FIPGWL--
-FPGWLM-
FEPGWL--
-FPGWLS-
-FPGWLH-

FHPGWL--
FDPGWL--
FAPGWL--
FWPGWL--
FRPGWL--
FPPGWL--
FFPGWL--
-GPGWLL-
GRPGWL--
GGPGWL--
-GPGWLT-
-GPGWLM-
GMPGWL--
GLPGWL--
GVPGWL--
GTPGWL--
GAPGWL--
GWPGWL--
GQPGWL--
-GPGWLV-
-GPGWLI-
-GPGWLE-
GHPGWL--
-GPGWLG-
GFPGWL--
GNPGWL--
GEPGWL--
GPPGWL--
-GPGWLK-
GKPGWL--
-GPGWLF-
GIPGWL--
GYPGWL--
-GPGWLY-
GCPGWL--
-GPGWLN-
-GPGWLH-
-GPGWLA-
-GPGWLW-
GDPGWL--
-HPGWLR-
HSPGWL--
HIPGWL--
HLPGWL--
-HPGWLV-
-HPGWLA-
-HPGWLT-
HWPGWL--
-HPGWLL-
HGPGWL--

```
-HPGWLK-
HNPGWL--
HYPGWL--
-HPGWLM-
-HPGWLQ-
HAPGWL--
HRPGWL--
HFPGWL--
HVPGWL--
-HPGWLY-
HTPGWL--
HQPGWL--
-HPGWLW-
-HPGWLS-
-HPGWLN-
-HPGWLH-
HPPGWL--
-HPGWLI-
-HPGWLG-
HHPGWL--
HDPGWL--
-HPGWLF-
HEPGWL--
HCPGWL--
IRPGWL--
-IPGWLS-
IFPGWL--
IVPGWL--
-IPGWLR-
ILPGWL--
IYPGWL--
-IPGWLA-
ISPGWL--
-IPGWLL-
IKPGWL--
-IPGWLV-
-IPGWLT-
-IPGWLY-
IMPGWL--
IQPGWL--
-IPGWLQ-
IAPGWL--
IWPGWL--
-IPGWLI-
-IPGWLG-
IIPGWL--
IGPGWL--
-IPGWLK-
-IPGWLF-
IHPGWL--
```

-IPGWLN-
-IPGWLH-
-IPGWLE-
IEPGWL--
KGPGWL--
KIPGWL--
KVPGWL--
-KPGWLT-
-KPGWLQ-
-KPGWLN-
-KPGWLV-
-KPGWLS-
-KPGWLY-
KYPGWL--
KLPGWL--
KNPGWL--
KEPGWL--
KAPGWL--
-KPGWLM-
-KPGWLI-
KMPGWL--
-KPGWLW-
-KPGWLG-
KKPGWL--
KHPGWL--
KFPGWL--
KWPGWL--
KQPGWL--
KPPGWL--
-KPGWLK-
-KPGWLF-
KDPGWL--
-KPGWLH-
-KPGWLE-
-KPGWLD-
-KPGWLA-
KCPGWL--
-LPGWLK-
-LPGWLR-
-LPGWLG-
-LPGWLL-
LAPGWL--
-LPGWLQ-
-LPGWLA-
LMPGWL--
LHPGWL--
-LPGWLH-
LKPGWL--
LVPGWL--
LNPGWL--

LTPGWL--
LEPGWL--
LGPGWL--
LWPGWL--
-LPGWLN-
-LPGWLF-
LQPGWL--
LPPGWL--
LFPGWL--
LIPGWL--
LDPGWL--
LCPGWL--
-MPGWLK-
MMPGWL--
MRPGWL--
-MPGWLR-
-MPGWLG-
MYPGWL--
MDPGWL--
-MPGWLT-
-MPGWLS-
-MPGWLM-
-MPGWLL-
MEPGWL--
MAPGWL--
MVPGWL--
MSPGWL--
MQPGWL--
-MPGWLV-
MLPGWL--
MWPGWL--
MKPGWL--
MFPGWL--
MTPGWL--
MPPGWL--
-MPGWLN-
MHPGWL--
-MPGWLQ-
-MPGWLA-
MNPGWL--
MIPGWL--
-MPGWLW-
-MPGWLI-
-MPGWLF-
-MPGWLE-
NNPGWL--
NVPGWL--
-NPGWLL-
-NPGWLR-
NKPGWL--

```
-NPGWLG-
NYPGWL--
-NPGWLY-
-NPGWLK-
-NPGWLV-
-NPGWLT-
-NPGWLS-
-NPGWLI-
NMPGWL--
NIPGWL--
NSPGWL--
NGPGWL--
NAPGWL--
NQPGWL--
NPPGWL--
-NPGWLH-
-NPGWLF-
-NPGWLN-
NWPGWL--
-NPGWLM-
NHPGWL--
NDPGWL--
NTPGWL--
-NPGWLA-
--PGWLRS
--PGWLRN
--PGWLVR
--PGWLSS
--PGWLTR
--PGWLGL
--PGWLRG
--PGWLLN
--PGWLSL
--PGWLVA
--PGWLLS
PMPGWL--
--PGWLFS
--PGWLSN
--PGWLVK
--PGWLTK
PNPGWL--
--PGWLVN
--PGWLHS
--PGWLTH
--PGWLSH
--PGWLQH
--PGWLKN
PVPGWL--
PRPGWL--
--PGWLVT
```

```
--PGWLSA
--PGWLRH
--PGWLKG
--PGWLKA
--PGWLGG
--PGWLEA
--PGWLVG
--PGWLSY
--PGWLRY
--PGWLKS
--PGWLIA
--PGWLAT
--PGWLVD
--PGWLSW
--PGWLRA
--PGWLLH
--PGWLKH
--PGWLKE
--PGWLIS
--PGWLIN
PKPGWL--
--PGWLVV
--PGWLRD
--PGWLLG
--PGWLKT
--PGWLGT
--PGWLFT
--PGWLFH
PAPGWL--
PSPGWL--
--PGWLYS
--PGWLTM
--PGWLTG
--PGWLSG
--PGWLRL
--PGWLMK
--PGWLLA
--PGWLYY
--PGWLRQ
--PGWLQV
--PGWLMR
--PGWLLT
--PGWLKF
--PGWLIR
--PGWLHV
--PGWLAN
--PGWLVS
--PGWLTI
--PGWLSI
--PGWLSF
```

--PGWLNS
--PGWLMT
--PGWLMN
--PGWLMI
--PGWLLR
--PGWLLQ
--PGWLLD
--PGWLIQ
--PGWLIG
--PGWLHK
--PGWLHF
--PGWLGQ
--PGWLAS
--PGWLAM
PQPGWL--
PLPGWL--
--PGWLYA
--PGWLWT
--PGWLWS
--PGWLTT
--PGWLTQ
--PGWLSQ
--PGWLSM
--PGWLRM
--PGWLRF
--PGWLQQ
--PGWLQK
--PGWLQI
--PGWLNA
--PGWLME
--PGWLKV
--PGWLKL
--PGWLKD
--PGWLHN
--PGWLGW
--PGWLGA
--PGWLAA
PYPGWL--
PIPGWL--
--PGWLYR
--PGWLYQ
--PGWLYK
--PGWLYH
--PGWLTY
--PGWLTW
--PGWLTF
--PGWLTD
--PGWLSV
--PGWLSE
--PGWLQY

--PGWLQS
--PGWLNY
--PGWLNT
--PGWLMM
--PGWLMG
--PGWLHT
--PGWLHA
--PGWLGM
--PGWLEF
--PGWLAV
--PGWLAR
--PGWLAK
--PGWLYV
--PGWLYI
--PGWLWN
--PGWLWG
--PGWLVF
--PGWLVE
--PGWLRW
--PGWLQT
--PGWLQM
--PGWLPL
--PGWLMV
--PGWLMS
--PGWLKQ
--PGWLIW
--PGWLIT
--PGWLIH
--PGWLID
--PGWLGV
--PGWLGI
--PGWLEV
--PGWLEG
--PGWLDS
--PGWLAH
PGPGWL--
--PGWLYM
--PGWLVW
--PGWLTN
--PGWLTE
--PGWLSK
--PGWLRE
--PGWLQR
--PGWLQA
--PGWLNW
--PGWLNN
--PGWLNM
--PGWLMQ
--PGWLMD
--PGWLKY

--PGWLKM
--PGWLIV
--PGWLIK
--PGWLIE
--PGWLHW
--PGWLHI
--PGWLHH
--PGWLHG
--PGWLHD
--PGWLFR
--PGWLFN
--PGWLFM
--PGWLFK
--PGWLET
--PGWLAI
--PGWLAG
--PGWLAF
PFPGWL--
PEPGWL--
PWPGWL--
-PPGWLS-
-PPGWLM-
PHPGWL--
--PGWLYT
--PGWLYG
--PGWLYD
--PGWLWM
--PGWLWE
--PGWLWA
--PGWLVQ
--PGWLVM
--PGWLVH
--PGWLQW
--PGWLQN
--PGWLQG
--PGWLPS
--PGWLNV
--PGWLNG
--PGWLND
--PGWLKI
--PGWLIY
--PGWLII
--PGWLHY
--PGWLHQ
--PGWLGH
--PGWLGF
--PGWLGD
--PGWLFQ
--PGWLFG
--PGWLES

--PGWLER
--PGWLEQ
--PGWLEN
--PGWLDA
--PGWLAY
--PGWLAQ
--PGWLAD
-QPGWLR-
-QPGWLL-
QRPGWL--
QSPGWL--
-QPGWLM-
QFPGWL--
QDPGWL--
QLPGWL--
QPPGWL--
-QPGWLY-
QHPGWL--
QYPGWL--
QKPGWL--
QEPGWL--
-QPGWLT-
-QPGWLV-
-QPGWLQ-
-QPGWLK-
QAPGWL--
QVPGWL--
-QPGWLS-
-QPGWLH-
-QPGWLE-
QNPGWL--
-QPGWLG-
-QPGWLA-
QGPGWL--
QWPGWL--
QTPGWL--
QIPGWL--
-QPGWLW-
-QPGWLN-
-QPGWLF-
-RPGWLL-
-RPGWLK-
-RPGWLS-
-RPGWLG-
RSPGWL--
RAPGWL--
RHPGWL--
-RPGWLF-
RDPGWL--
RGPGWL--

-RPGWLW-
-RPGWLV-
-RPGWLA-
RQPGWL--
RYPGWL--
-RPGWLM-
-RPGWLY-
-RPGWLQ-
RNPGWL--
RMPGWL--
RFPGWL--
-RPGWLI-
RPPGWL--
-RPGWLH-
REPGWL--
-RPGWLN-
-RPGWLE-
RCPGWL--
-RPGWLD-
-SPGWLL-
-SPGWLK-
-SPGWLR-
SRPGWL--
SSPGWL--
-SPGWLT-
-SPGWLV-
STPGWL--
SPPGWL--
SVPGWL--
-SPGWLN-
-SPGWLI-
SKPGWL--
SQPGWL--
SIPGWL--
-SPGWLE-
SYPGWL--
-SPGWLM-
-SPGWLG-
SWPGWL--
-SPGWLQ-
SAPGWL--
SHPGWL--
SFPGWL--
-SPGWLW-
SEPGWL--
TRPGWL--
TVPGWL--
-TPGWLS-
TTPGWL--
TAPGWL--

TQPGWL--
-TPGWLK-
TKPGWL--
-TPGWLH-
THPGWL--
TGPGWL--
TPPGWL--
TMPGWL--
-TPGWLY-
-TPGWLE-
-TPGWLV-
-TPGWLI-
-TPGWLT-
-TPGWLQ-
TIPGWL--
TEPGWL--
TYPGWL--
-TPGWLG-
TNPGWL--
TDPGWL--
TWPGWL--
-TPGWLA-
VVPGWL--
-VPGWLR-
-VPGWLT-
VKPGWL--
VGPGWL--
VAPGWL--
VRPGWL--
VEPGWL--
-VPGWLL-
-VPGWLG-
VPPGWL--
-VPGWLI-
-VPGWLM-
VHPGWL--
VTPGWL--
VIPGWL--
-VPGWLS-
VYPGWL--
-VPGWLY-
-VPGWLA-
VQPGWL--
-VPGWLN-
-VPGWLH-
VNPGWL--
VFPGWL--
-VPGWLQ-
-VPGWLE-
VDPGWL--

WNPGWL--
WHPGWL--
WRPGWL--
WTPGWL--
-WPGWLK-
WAPGWL--
WYPGWL--
WPPGWL--
-WPGWLV-
WIPGWL--
WGPGWL--
WEPGWL--
-WPGWLY-
-WPGWLR-
WLPGWL--
WWPGWL--
-WPGWLI-
-WPGWLG-
-WPGWLF-
-WPGWLA-
WFPGWL--
YGPGWL--
-YPGWLS-
-YPGWLG-
-YPGWLA-
-YPGWLM-
-YPGWLV-
YMPGWL--
YQPGWL--
-YPGWLT-
-YPGWLH-
-YPGWLF-
YNPGWL--
YAPGWL--
-YPGWLQ-
-YPGWLL-
-YPGWLK-
-YPGWLY-
YEPGWL--
YKPGWL--
YFPGWL--
YWPGWL--
YVPGWL--
YIPGWL--
YDPGWL--

| | | | |
|---|---|---|---|
| PIPL | 94 | 773 | 33.12 |

AVPIPL--
GHPIPL--
RHPIPL--
-YPIPLL-

AKPIPL--
-APIPLA-
ANPIPL--
AIPIPL--
AWPIPL--
-APIPLH-
-APIPLT-
-APIPLL-
ASPIPL--
APPIPL--
-APIPLM-
-APIPLW-
-APIPLV-
-APIPLS-
-APIPLK-
AHPIPL--
AGPIPL--
AFPIPL--
AYPIPL--
AAPIPL--
-APIPLG-
AMPIPL--
AEPIPL--
AQPIPL--
-APIPLF-
-APIPLE-
-CPIPLR-
CAPIPL--
-CPIPLS-
CLPIPL--
CFPIPL--
CVPIPL--
-CPIPLQ-
-CPIPLM-
-CPIPLL-
-CPIPLK-
-CPIPLI-
CHPIPL--
CCPIPL--
DEPIPL--
DRPIPL--
DLPIPL--
DKPIPL--
-DPIPLF-
-DPIPLT-
-DPIPLS-
-DPIPLL-
DSPIPL--
-DPIPLR-
DHPIPL--

DAPIPL--
DYPIPL--
DVPIPL--
-DPIPLV-
-DPIPLM-
DNPIPL--
DTPIPL--
-DPIPLI-
-DPIPLH-
-DPIPLG-
-DPIPLA-
DMPIPL--
DFPIPL--
-EPIPLA-
-EPIPLL-
EYPIPL--
ERPIPL--
-EPIPLH-
-EPIPLM-
-EPIPLI-
EEPIPL--
-EPIPLS-
ELPIPL--
EFPIPL--
EAPIPL--
-EPIPLY-
-EPIPLW-
-EPIPLR-
-EPIPLN-
-EPIPLK-
-EPIPLG-
EKPIPL--
EIPIPL--
EDPIPL--
-FPIPLS-
FSPIPL--
FGPIPL--
FQPIPL--
-FPIPLA-
FKPIPL--
FNPIPL--
FYPIPL--
FWPIPL--
FVPIPL--
-FPIPLT-
-FPIPLI-
FAPIPL--
-FPIPLW-
-FPIPLF-
FIPIPL--

FHPIPL--
FEPIPL--
FTPIPL--
-FPIPLQ-
-FPIPLK-
-FPIPLH-
-FPIPLY-
-FPIPLV-
-FPIPLR-
-FPIPLE-
FDPIPL--
GSPIPL--
-GPIPLT-
GIPIPL--
GNPIPL--
GMPIPL--
-GPIPLR-
GYPIPL--
GVPIPL--
GLPIPL--
GGPIPL--
GTPIPL--
GKPIPL--
GEPIPL--
GRPIPL--
-GPIPLV-
-GPIPLM-
GQPIPL--
GPPIPL--
-GPIPLW-
-GPIPLS-
-GPIPLI-
GFPIPL--
GAPIPL--
GWPIPL--
-GPIPLY-
-GPIPLN-
-GPIPLH-
-GPIPLF-
-GPIPLQ-
-GPIPLK-
-GPIPLG-
-GPIPLA-
GDPIPL--
GCPIPL--
HSPIPL--
-HPIPLR-
-HPIPLK-
-HPIPLN-
HKPIPL--

HGPIPL--
HTPIPL--
-HPIPLY-
-HPIPLQ-
-HPIPLG-
HNPIPL--
HLPIPL--
HFPIPL--
HVPIPL--
-HPIPLS-
HEPIPL--
HAPIPL--
HQPIPL--
HPPIPL--
-HPIPLM-
-HPIPLL-
-HPIPLI-
-HPIPLF-
-HPIPLA-
HMPIPL--
HIPIPL--
HHPIPL--
IGPIPL--
ISPIPL--
IRPIPL--
-IPIPLA-
IEPIPL--
INPIPL--
-IPIPLV-
ILPIPL--
IVPIPL--
-IPIPLT-
-IPIPLQ-
-IPIPLL-
IIPIPL--
ICPIPL--
-IPIPLR-
IQPIPL--
-IPIPLY-
IYPIPL--
IWPIPL--
-IPIPLS-
-IPIPLM-
IHPIPL--
IDPIPL--
IAPIPL--
IPPIPL--
-IPIPLW-
-IPIPLH-
IKPIPL--

KSPIPL--
-KPIPLL-
KGPIPL--
KYPIPL--
-KPIPLA-
-KPIPLF-
KQPIPL--
-KPIPLV-
-KPIPLQ-
KVPIPL--
KMPIPL--
KHPIPL--
KWPIPL--
KTPIPL--
-KPIPLY-
-KPIPLT-
-KPIPLS-
KLPIPL--
KFPIPL--
-KPIPLW-
-KPIPLG-
KKPIPL--
KAPIPL--
-KPIPLN-
KDPIPL--
-KPIPLM-
-KPIPLI-
KEPIPL--
-LPIPLR-
LSPIPL--
LRPIPL--
LVPIPL--
-LPIPLL-
LLPIPL--
-LPIPLK-
LAPIPL--
-LPIPLS-
LKPIPL--
LHPIPL--
-LPIPLT-
-LPIPLY-
-LPIPLQ-
-LPIPLV-
LFPIPL--
LYPIPL--
-LPIPLH-
LTPIPL--
LPPIPL--
-LPIPLW-
-LPIPLM-

-LPIPLI-
LIPIPL--
-LPIPLF-
LNPIPL--
LEPIPL--
LQPIPL--
-LPIPLA-
LDPIPL--
-MPIPLR-
-MPIPLL-
MEPIPL--
MQPIPL--
MPPIPL--
-MPIPLQ-
MYPIPL--
MVPIPL--
-MPIPLY-
-MPIPLW-
-MPIPLS-
-MPIPLN-
MNPIPL--
MLPIPL--
MAPIPL--
MWPIPL--
-MPIPLA-
MGPIPL--
MFPIPL--
-MPIPLV-
-MPIPLI-
-MPIPLH-
-MPIPLG-
MMPIPL--
MHPIPL--
-NPIPLR-
NWPIPL--
NSPIPL--
-NPIPLA-
NEPIPL--
-NPIPLS-
NHPIPL--
-NPIPLV-
-NPIPLI-
NMPIPL--
NPPIPL--
-NPIPLL-
-NPIPLF-
NGPIPL--
NYPIPL--
NVPIPL--
-NPIPLY-

-NPIPLT-
-NPIPLH-
NIPIPL--
-NPIPLW-
-NPIPLN-
NTPIPL--
NQPIPL--
-NPIPLM-
NNPIPL--
NFPIPL--
--PIPLSL
--PIPLVR
--PIPLTT
--PIPLLG
--PIPLAL
--PIPLLR
--PIPLWL
--PIPLPF
--PIPLRY
--PIPLFS
--PIPLTK
--PIPLLL
--PIPLKL
--PIPLST
--PIPLNA
--PIPLDI
PVPIPL--
--PIPLVM
--PIPLSF
--PIPLRR
--PIPLLK
--PIPLYS
--PIPLMA
--PIPLKY
--PIPLIR
--PIPLWS
--PIPLTS
--PIPLNW
--PIPLLV
--PIPLLN
--PIPLKT
--PIPLIT
--PIPLAT
--PIPLYR
--PIPLWY
--PIPLTY
--PIPLTL
--PIPLSS
--PIPLKN
--PIPLSW

--PIPLSV
--PIPLQS
--PIPLNL
--PIPLMM
--PIPLLH
--PIPLFI
--PIPLEV
PTPIPL--
--PIPLWD
--PIPLVI
--PIPLVH
--PIPLSG
--PIPLRW
--PIPLRT
--PIPLRD
--PIPLMI
--PIPLLT
--PIPLLS
--PIPLHS
--PIPLFM
PGPIPL--
--PIPLVG
--PIPLVA
--PIPLTR
--PIPLRS
--PIPLRN
--PIPLRH
--PIPLNS
--PIPLML
--PIPLLI
--PIPLFY
PNPIPL--
--PIPLWK
--PIPLSN
--PIPLRI
--PIPLQI
--PIPLNK
--PIPLKI
--PIPLIS
--PIPLHF
--PIPLGQ
--PIPLGH
--PIPLYY
--PIPLYT
--PIPLWH
--PIPLTW
--PIPLSK
--PIPLSA
--PIPLQK
--PIPLPS

--PIPLPI
--PIPLNQ
--PIPLMS
--PIPLMR
--PIPLLA
--PIPLKR
--PIPLKM
--PIPLIN
--PIPLHT
--PIPLFG
--PIPLAQ
PLPIPL--
--PIPLWT
--PIPLVQ
--PIPLTG
--PIPLTF
--PIPLRK
--PIPLRA
--PIPLQY
--PIPLQT
--PIPLQR
--PIPLQG
--PIPLQF
--PIPLPW
--PIPLPV
--PIPLNH
--PIPLMV
--PIPLMQ
--PIPLME
--PIPLLD
--PIPLKW
--PIPLKV
--PIPLKE
--PIPLIY
--PIPLIK
--PIPLHG
--PIPLGS
--PIPLGN
--PIPLGF
--PIPLDL
--PIPLAF
PFPIPL--
PMPIPL--
PKPIPL--
--PIPLYW
--PIPLYV
--PIPLYH
--PIPLYG
--PIPLWV
--PIPLWI

--PIPLVV
--PIPLTN
--PIPLTA
--PIPLRM
--PIPLRF
--PIPLQV
--PIPLNY
--PIPLMY
--PIPLMK
--PIPLMG
--PIPLLE
--PIPLKS
--PIPLKH
--PIPLKG
--PIPLHY
--PIPLHV
--PIPLHR
--PIPLHQ
--PIPLHK
--PIPLFN
--PIPLFK
--PIPLFH
--PIPLFA
--PIPLES
--PIPLAM
--PIPLAI
--PIPLAG
PYPIPL--
PWPIPL--
PQPIPL--
--PIPLYN
--PIPLYM
--PIPLYK
--PIPLWW
--PIPLWQ
--PIPLVE
--PIPLVD
--PIPLTH
--PIPLQL
--PIPLQH
--PIPLNV
--PIPLNT
--PIPLNF
--PIPLMD
--PIPLLW
--PIPLLQ
--PIPLLM
--PIPLKQ
--PIPLKA
--PIPLIG

--PIPLHM
--PIPLGV
--PIPLGK
--PIPLGA
--PIPLFL
--PIPLFE
--PIPLFD
--PIPLEQ
--PIPLEI
--PIPLEA
--PIPLDS
--PIPLDQ
--PIPLAY
--PIPLAS
--PIPLAR
PHPIPL--
-PPIPLY-
--PIPLYQ
--PIPLYI
--PIPLYC
--PIPLWN
--PIPLWM
--PIPLWE
--PIPLVW
--PIPLVT
--PIPLTM
--PIPLTI
--PIPLSH
--PIPLSC
--PIPLQQ
--PIPLQN
--PIPLQA
--PIPLPY
--PIPLPQ
--PIPLPM
--PIPLPH
--PIPLPE
--PIPLNN
--PIPLNM
--PIPLNI
--PIPLNG
--PIPLNE
--PIPLMN
--PIPLMF
--PIPLKF
--PIPLKD
--PIPLKC
--PIPLIV
--PIPLIQ
--PIPLIM

--PIPLIE
--PIPLHW
--PIPLHI
--PIPLHE
--PIPLGT
--PIPLFW
--PIPLFV
--PIPLEY
--PIPLEG
--PIPLDM
--PIPLDF
--PIPLAV
--PIPLAN
--PIPLAK
--PIPLAH
--PIPLAA
PAPIPL--
QIPIPL--
-QPIPLL-
-QPIPLI-
QRPIPL--
-QPIPLH-
QSPIPL--
QWPIPL--
QTPIPL--
QQPIPL--
-QPIPLY-
-QPIPLR-
-QPIPLF-
-QPIPLN-
QYPIPL--
-QPIPLT-
QMPIPL--
-QPIPLS-
-QPIPLM-
QFPIPL--
QAPIPL--
-QPIPLQ-
QNPIPL--
QLPIPL--
QEPIPL--
QPPIPL--
-QPIPLK-
-QPIPLA-
QKPIPL--
QHPIPL--
QGPIPL--
RVPIPL--
RTPIPL--
-RPIPLK-

-RPIPLH-
RWPIPL--
-RPIPLS-
-RPIPLG-
-RPIPLI-
-RPIPLA-
RNPIPL--
-RPIPLV-
-RPIPLT-
RAPIPL--
RYPIPL--
-RPIPLR-
RMPIPL--
-RPIPLQ-
-RPIPLN-
RDPIPL--
RIPIPL--
-RPIPLW-
-RPIPLM-
RQPIPL--
RPPIPL--
-RPIPLE-
RCPIPL--
REPIPL--
SGPIPL--
SLPIPL--
SIPIPL--
SPPIPL--
SRPIPL--
-SPIPLQ-
SFPIPL--
SYPIPL--
SSPIPL--
-SPIPLT-
-SPIPLR-
-SPIPLI-
-SPIPLW-
-SPIPLF-
SMPIPL--
SKPIPL--
SAPIPL--
-SPIPLV-
SHPIPL--
SEPIPL--
SWPIPL--
SVPIPL--
-SPIPLY-
-SPIPLK-
-SPIPLA-
STPIPL--

```
-SPIPLN-
SDPIPL--
SQPIPL--
-SPIPLM-
-SPIPLH-
-SPIPLG-
-TPIPLS-
TGPIPL--
-TPIPLR-
TYPIPL--
TMPIPL--
TSPIPL--
-TPIPLY-
TVPIPL--
-TPIPLQ-
-TPIPLN-
-TPIPLK-
TPPIPL--
-TPIPLV-
-TPIPLA-
TNPIPL--
TLPIPL--
TQPIPL--
-TPIPLL-
-TPIPLT-
-TPIPLH-
TKPIPL--
TIPIPL--
TWPIPL--
-TPIPLI-
TFPIPL--
TEPIPL--
TAPIPL--
-TPIPLW-
-TPIPLG-
-VPIPLL-
VLPIPL--
-VPIPLY-
-VPIPLV-
-VPIPLT-
VEPIPL--
VSPIPL--
-VPIPLS-
-VPIPLR-
VIPIPL--
VFPIPL--
VVPIPL--
VKPIPL--
VDPIPL--
-VPIPLF-
```

VWPIPL--
-VPIPLW-
-VPIPLM-
-VPIPLK-
VNPIPL--
VYPIPL--
VTPIPL--
VQPIPL--
VPPIPL--
VHPIPL--
VGPIPL--
-VPIPLQ-
-VPIPLG-
-VPIPLE-
-VPIPLA-
VMPIPL--
VAPIPL--
-WPIPLS-
WDPIPL--
WYPIPL--
WGPIPL--
WFPIPL--
WSPIPL--
WAPIPL--
WRPIPL--
WPPIPL--
WTPIPL--
-WPIPLR-
-WPIPLM-
-WPIPLF-
WLPIPL--
WIPIPL--
-WPIPLV-
-WPIPLT-
-WPIPLL-
-WPIPLG-
WKPIPL--
WWPIPL--
WQPIPL--
-WPIPLN-
WNPIPL--
YLPIPL--
YAPIPL--
-YPIPLM-
YVPIPL--
-YPIPLR-
-YPIPLI-
YSPIPL--
YGPIPL--
-YPIPLT-

| | | | |
|---|---|---|---|
| | YMPIPL-- | | |
| | YKPIPL-- | | |
| | YEPIPL-- | | |
| | YDPIPL-- | | |
| | YQPIPL-- | | |
| | -YPIPLW- | | |
| | -YPIPLN- | | |
| | -YPIPLH- | | |
| | -YPIPLG- | | |
| | -YPIPLA- | | |
| | YPPIPL-- | | |
| | -YPIPLY- | | |
| | -YPIPLE- | | |
| | YNPIPL-- | | |
| | YHPIPL-- | | |
| | -YPIPLV- | | |
| | -YPIPLK- | | |
| | YIPIPL-- | | |
| | YFPIPL-- | | |
| PSAY | 37 | 820 | 33.11 |
| | EGPSAY-- | | |
| | -IPSAYV- | | |
| | --PSAYDS | | |
| | -APSAYR- | | |
| | -APSAYL- | | |
| | ALPSAY-- | | |
| | AIPSAY-- | | |
| | -APSAYI- | | |
| | ATPSAY-- | | |
| | -APSAYM- | | |
| | ASPSAY-- | | |
| | -APSAYY- | | |
| | AGPSAY-- | | |
| | ARPSAY-- | | |
| | -APSAYF- | | |
| | AYPSAY-- | | |
| | -APSAYV- | | |
| | -APSAYH- | | |
| | -APSAYG- | | |
| | -APSAYT- | | |
| | -APSAYQ- | | |
| | -APSAYE- | | |
| | AKPSAY-- | | |
| | -APSAYN- | | |
| | -APSAYK- | | |
| | -APSAYA- | | |
| | AVPSAY-- | | |
| | AQPSAY-- | | |
| | ADPSAY-- | | |
| | AAPSAY-- | | |

AWPSAY--
APPSAY--
AFPSAY--
AEPSAY--
ACPSAY--
AMPSAY--
CTPSAY--
CRPSAY--
-CPSAYT-
-CPSAYR-
-CPSAYL-
CLPSAY--
CAPSAY--
CCPSAY--
CVPSAY--
CSPSAY--
-CPSAYS-
-CPSAYQ-
CNPSAY--
CHPSAY--
CGPSAY--
DVPSAY--
DQPSAY--
-DPSAYS-
-DPSAYL-
DRPSAY--
DTPSAY--
-DPSAYM-
DMPSAY--
-DPSAYI-
-DPSAYK-
DAPSAY--
DWPSAY--
-DPSAYV-
DFPSAY--
-DPSAYT-
-DPSAYQ-
-DPSAYF-
DNPSAY--
DLPSAY--
DIPSAY--
DEPSAY--
DDPSAY--
DCPSAY--
-EPSAYR-
EWPSAY--
ERPSAY--
-EPSAYL-
EMPSAY--
-EPSAYN-

ESPSAY--
EKPSAY--
-EPSAYW-
-EPSAYG-
-EPSAYA-
EDPSAY--
-EPSAYY-
ELPSAY--
EHPSAY--
EAPSAY--
EVPSAY--
ETPSAY--
-EPSAYT-
-EPSAYH-
EQPSAY--
-EPSAYQ-
-EPSAYI-
-EPSAYE-
ENPSAY--
EFPSAY--
EEPSAY--
FLPSAY--
-FPSAYR-
FSPSAY--
FNPSAY--
-FPSAYT-
-FPSAYM-
-FPSAYI-
FFPSAY--
FYPSAY--
FIPSAY--
FTPSAY--
FGPSAY--
-FPSAYV-
-FPSAYL-
-FPSAYY-
-FPSAYS-
FMPSAY--
FHPSAY--
-FPSAYK-
FKPSAY--
FQPSAY--
-FPSAYF-
FEPSAY--
FDPSAY--
-FPSAYW-
-FPSAYQ-
-FPSAYH-
-FPSAYC-
-GPSAYR-

-GPSAYG-
-GPSAYL-
GRPSAY--
-GPSAYV-
GTPSAY--
GKPSAY--
GIPSAY--
GLPSAY--
GQPSAY--
-GPSAYM-
GGPSAY--
GSPSAY--
-GPSAYS-
GNPSAY--
GMPSAY--
-GPSAYT-
-GPSAYH-
GFPSAY--
-GPSAYK-
-GPSAYI-
-GPSAYF-
-GPSAYY-
-GPSAYW-
GVPSAY--
-GPSAYE-
GDPSAY--
GWPSAY--
GPPSAY--
GYPSAY--
-GPSAYQ-
GEPSAY--
GCPSAY--
HRPSAY--
-HPSAYL-
HYPSAY--
-HPSAYA-
-HPSAYK-
-HPSAYV-
-HPSAYT-
-HPSAYR-
HNPSAY--
HSPSAY--
HLPSAY--
HTPSAY--
-HPSAYS-
HGPSAY--
HAPSAY--
HMPSAY--
HVPSAY--
-HPSAYY-

−HPSAYW−
−HPSAYQ−
−HPSAYI−
−HPSAYH−
−HPSAYG−
−HPSAYF−
HPPSAY−−
HKPSAY−−
HIPSAY−−
HHPSAY−−
HFPSAY−−
HEPSAY−−
−IPSAYR−
−IPSAYL−
IYPSAY−−
−IPSAYE−
ISPSAY−−
−IPSAYQ−
−IPSAYI−
IDPSAY−−
ITPSAY−−
−IPSAYS−
ILPSAY−−
IAPSAY−−
IVPSAY−−
IRPSAY−−
−IPSAYM−
IEPSAY−−
−IPSAYY−
−IPSAYK−
−IPSAYH−
−IPSAYF−
−IPSAYT−
INPSAY−−
IGPSAY−−
IFPSAY−−
−IPSAYG−
IIPSAY−−
−IPSAYA−
IMPSAY−−
KSPSAY−−
KRPSAY−−
−KPSAYI−
−KPSAYT−
KLPSAY−−
KVPSAY−−
−KPSAYL−
−KPSAYS−
KWPSAY−−
KHPSAY−−

-KPSAYG-
KGPSAY--
-KPSAYW-
-KPSAYF-
-KPSAYA-
KNPSAY--
KKPSAY--
KQPSAY--
-KPSAYQ-
-KPSAYM-
KEPSAY--
-KPSAYN-
-KPSAYH-
KYPSAY--
-KPSAYK-
-KPSAYC-
KPPSAY--
KCPSAY--
KAPSAY--
-KPSAYY-
-KPSAYV-
-KPSAYE-
KMPSAY--
KFPSAY--
LRPSAY--
LLPSAY--
LVPSAY--
LHPSAY--
-LPSAYY-
-LPSAYT-
LPPSAY--
LYPSAY--
LGPSAY--
LWPSAY--
LTPSAY--
-LPSAYM-
LKPSAY--
-LPSAYR-
-LPSAYI-
-LPSAYW-
-LPSAYK-
-LPSAYV-
-LPSAYS-
LIPSAY--
LEPSAY--
LQPSAY--
-LPSAYH-
LNPSAY--
LCPSAY--
-LPSAYN-

LDPSAY--
-MPSAYV-
MRPSAY--
-MPSAYL-
MTPSAY--
MMPSAY--
MGPSAY--
MVPSAY--
MKPSAY--
-MPSAYM-
-MPSAYI-
-MPSAYH-
MHPSAY--
MQPSAY--
MPPSAY--
MLPSAY--
MYPSAY--
-MPSAYS-
MAPSAY--
MWPSAY--
-MPSAYW-
MFPSAY--
MEPSAY--
MDPSAY--
-MPSAYT-
-MPSAYK-
NNPSAY--
-NPSAYL-
-NPSAYR-
NKPSAY--
NRPSAY--
NWPSAY--
NSPSAY--
-NPSAYY-
NFPSAY--
NTPSAY--
-NPSAYV-
-NPSAYW-
NIPSAY--
-NPSAYK-
-NPSAYF-
-NPSAYT-
-NPSAYI-
-NPSAYA-
NQPSAY--
-NPSAYQ-
-NPSAYN-
NVPSAY--
-NPSAYS-
NHPSAY--

NDPSAY--
NYPSAY--
-NPSAYH-
NLPSAY--
-NPSAYM-
NPPSAY--
NMPSAY--
NCPSAY--
NAPSAY--
--PSAYGL
--PSAYLT
--PSAYTR
--PSAYFS
--PSAYLS
--PSAYSA
--PSAYYR
--PSAYWQ
--PSAYSL
--PSAYHS
--PSAYWL
--PSAYSM
--PSAYRF
--PSAYMK
--PSAYHL
--PSAYLN
--PSAYVI
--PSAYRY
--PSAYRQ
--PSAYKR
--PSAYFK
--PSAYAL
--PSAYWR
--PSAYFR
--PSAYFN
--PSAYES
PRPSAY--
--PSAYLY
--PSAYLK
PNPSAY--
--PSAYYS
--PSAYYM
--PSAYVS
--PSAYSQ
--PSAYRG
--PSAYLG
--PSAYKS
--PSAYIR
--PSAYGS
PWPSAY--
--PSAYTL

--PSAYSY
--PSAYST
--PSAYSS
--PSAYRI
--PSAYNS
--PSAYLW
--PSAYLM
--PSAYEG
PGPSAY--
--PSAYYK
--PSAYVM
--PSAYTK
--PSAYSH
--PSAYQM
--PSAYNF
--PSAYMR
--PSAYGW
--PSAYGM
--PSAYVT
--PSAYSV
--PSAYRM
--PSAYRH
--PSAYMT
--PSAYLV
--PSAYLQ
--PSAYIG
--PSAYHR
--PSAYWN
--PSAYVY
--PSAYVQ
--PSAYVN
--PSAYTN
--PSAYTI
--PSAYSG
--PSAYNL
--PSAYKL
--PSAYHT
PFPSAY--
--PSAYYE
--PSAYWM
--PSAYWH
--PSAYVW
--PSAYVH
--PSAYVF
--PSAYTV
--PSAYTA
--PSAYQY
--PSAYKM
--PSAYIL
--PSAYHK

--PSAYAS
--PSAYAH
PYPSAY--
--PSAYSW
--PSAYSI
--PSAYRA
--PSAYPL
--PSAYNT
--PSAYNR
--PSAYMN
--PSAYKN
--PSAYKD
--PSAYGT
--PSAYFG
--PSAYFD
--PSAYAY
--PSAYAM
PLPSAY--
PSPSAY--
--PSAYYG
--PSAYYD
--PSAYYA
--PSAYWW
--PSAYWT
--PSAYWS
--PSAYVG
--PSAYTT
--PSAYRE
--PSAYQW
--PSAYQS
--PSAYQL
--PSAYQA
--PSAYMS
--PSAYMI
--PSAYMF
--PSAYMD
--PSAYLF
--PSAYIS
--PSAYIK
--PSAYHY
--PSAYHN
--PSAYGA
--PSAYFA
--PSAYAV
--PSAYAN
--PSAYAI
--PSAYAG
PKPSAY--
--PSAYYQ
--PSAYWG

--PSAYVK
--PSAYTM
--PSAYSF
--PSAYRT
--PSAYRD
--PSAYQK
--PSAYPI
--PSAYNI
--PSAYMV
--PSAYMH
--PSAYLH
--PSAYKA
--PSAYGF
--PSAYET
--PSAYAQ
--PSAYAF
PAPSAY--
PVPSAY--
--PSAYYT
--PSAYWF
--PSAYTH
--PSAYTD
--PSAYSD
--PSAYQR
--PSAYNV
--PSAYMM
--PSAYMG
--PSAYMA
--PSAYLE
--PSAYKV
--PSAYKQ
--PSAYIY
--PSAYIH
--PSAYID
--PSAYHH
--PSAYHG
--PSAYHA
--PSAYGY
--PSAYFM
--PSAYFH
--PSAYFE
--PSAYEW
--PSAYAR
--PSAYAA
PTPSAY--
--PSAYYN
--PSAYVV
--PSAYVE
--PSAYVD
--PSAYVA

--PSAYTY
--PSAYTW
--PSAYTF
--PSAYTE
--PSAYRW
--PSAYQQ
--PSAYQN
--PSAYQI
--PSAYQH
--PSAYQF
--PSAYNW
--PSAYNH
--PSAYMY
--PSAYMW
--PSAYKK
--PSAYKI
--PSAYKG
--PSAYIW
--PSAYIV
--PSAYII
--PSAYHV
--PSAYHQ
--PSAYHM
--PSAYHI
--PSAYGV
--PSAYFF
--PSAYEV
--PSAYEI
--PSAYEA
--PSAYAW
--PSAYAT
--PSAYAK
PQPSAY--
PEPSAY--
PCPSAY--
--PSAYYW
--PSAYWK
--PSAYWA
--PSAYTQ
--PSAYSN
--PSAYRV
--PSAYQV
--PSAYPR
--PSAYPM
--PSAYNQ
--PSAYNN
--PSAYNM
--PSAYNG
--PSAYNA
--PSAYMQ

```
--PSAYML
--PSAYME
--PSAYKY
--PSAYKW
--PSAYKT
--PSAYKH
--PSAYKF
--PSAYIT
--PSAYIQ
--PSAYIM
--PSAYIA
--PSAYHF
--PSAYGQ
--PSAYGI
--PSAYFT
--PSAYEN
--PSAYEM
--PSAYEL
--PSAYEF
--PSAYCA
-PPSAYF-
PIPSAY--
PHPSAY--
PDPSAY--
-QPSAYR-
-QPSAYL-
QRPSAY--
-QPSAYS-
QTPSAY--
QSPSAY--
-QPSAYN-
-QPSAYK-
QMPSAY--
QQPSAY--
QKPSAY--
QPPSAY--
QVPSAY--
-QPSAYM-
-QPSAYI-
-QPSAYG-
QHPSAY--
QGPSAY--
-QPSAYW-
-QPSAYF-
QFPSAY--
QYPSAY--
QWPSAY--
-QPSAYY-
QEPSAY--
QCPSAY--
```

QAPSAY--
-QPSAYT-
QIPSAY--
QDPSAY--
-RPSAYW-
-RPSAYS-
-RPSAYI-
RSPSAY--
RVPSAY--
RRPSAY--
-RPSAYT-
-RPSAYM-
-RPSAYF-
RNPSAY--
RIPSAY--
RGPSAY--
RAPSAY--
-RPSAYV-
-RPSAYN-
-RPSAYG-
-RPSAYY-
-RPSAYQ-
RFPSAY--
RHPSAY--
REPSAY--
RYPSAY--
-RPSAYH-
RCPSAY--
RWPSAY--
-RPSAYE-
RQPSAY--
RKPSAY--
RPPSAY--
-SPSAYL-
SRPSAY--
SLPSAY--
SSPSAY--
-SPSAYT-
STPSAY--
-SPSAYM-
SWPSAY--
-SPSAYS-
-SPSAYG-
SNPSAY--
SGPSAY--
SQPSAY--
SAPSAY--
SHPSAY--
-SPSAYK-
SMPSAY--

-SPSAYY-
-SPSAYF-
SKPSAY--
SEPSAY--
-SPSAYH-
SPPSAY--
SFPSAY--
SDPSAY--
-SPSAYW-
-SPSAYI-
-SPSAYA-
SIPSAY--
SYPSAY--
-SPSAYQ-
SCPSAY--
-TPSAYL-
TVPSAY--
-TPSAYT-
TAPSAY--
TNPSAY--
TLPSAY--
-TPSAYF-
-TPSAYV-
-TPSAYN-
THPSAY--
TDPSAY--
TTPSAY--
TSPSAY--
TPPSAY--
TQPSAY--
-TPSAYY-
-TPSAYK-
-TPSAYI-
-TPSAYS-
-TPSAYA-
TKPSAY--
-TPSAYW-
-TPSAYM-
TMPSAY--
TGPSAY--
TFPSAY--
TEPSAY--
-TPSAYQ-
-TPSAYH-
-TPSAYG-
TCPSAY--
-TPSAYE-
VSPSAY--
VRPSAY--
VKPSAY--

-VPSAYS-
-VPSAYA-
-VPSAYH-
VNPSAY--
-VPSAYI-
VFPSAY--
VYPSAY--
VVPSAY--
-VPSAYM-
VWPSAY--
VTPSAY--
VLPSAY--
VIPSAY--
-VPSAYN-
-VPSAYE-
VEPSAY--
-VPSAYF-
VHPSAY--
VGPSAY--
-VPSAYT-
-VPSAYK-
VMPSAY--
-VPSAYW-
-VPSAYQ-
VPPSAY--
VDPSAY--
-VPSAYY-
VAPSAY--
WRPSAY--
WKPSAY--
-WPSAYM-
WLPSAY--
WGPSAY--
WTPSAY--
-WPSAYH-
WHPSAY--
WNPSAY--
-WPSAYS-
WMPSAY--
-WPSAYY-
WAPSAY--
WPPSAY--
WIPSAY--
WYPSAY--
WSPSAY--
-WPSAYW-
-WPSAYV-
-WPSAYQ-
WDPSAY--
WWPSAY--

WQPSAY--
-WPSAYT-
-WPSAYK-
-WPSAYG-
-WPSAYF-
-WPSAYA-
WCPSAY--
YRPSAY--
-YPSAYS-
-YPSAYA-
-YPSAYL-
YHPSAY--
YKPSAY--
YYPSAY--
-YPSAYT-
-YPSAYW-
-YPSAYR-
YMPSAY--
YLPSAY--
-YPSAYN-
YNPSAY--
YIPSAY--
YFPSAY--
YAPSAY--
YVPSAY--
YSPSAY--
YTPSAY--
YGPSAY--
YQPSAY--
-YPSAYM-
-YPSAYH-
YPPSAY--
YDPSAY--
YWPSAY--
-YPSAYY-
-YPSAYK-
-YPSAYG-
-YPSAYI-
-YPSAYE-
YEPSAY--

| | | | |
|---|---|---|---|
| PTGL | 24 | 837 | 33.11 |

-CPTGLI-
-DPTGLN-
-GPTGLG-
-LPTGLG-
--PTGLIL
--PTGLPL
--PTGLSE
--PTGLSD
--PTGLQL

--PTGLGT
--PTGLGK
--PTGLEV
--PTGLDS
--PTGLAQ
-RPTGLC-
-TPTGLT-
-TPTGLG-
-APTGLS-
AVPTGL--
ARPTGL--
ALPTGL--
AKPTGL--
AFPTGL--
AYPTGL--
-APTGLI-
ASPTGL--
AQPTGL--
AIPTGL--
-APTGLV-
-APTGLR-
-APTGLQ-
-APTGLY-
ANPTGL--
AWPTGL--
-APTGLH-
AGPTGL--
-APTGLG-
AAPTGL--
ATPTGL--
-APTGLK-
-APTGLT-
AMPTGL--
-APTGLA-
ADPTGL--
-APTGLW-
-APTGLL-
-APTGLF-
-APTGLE-
-APTGLN-
-APTGLM-
-APTGLC-
AEPTGL--
ACPTGL--
CLPTGL--
CRPTGL--
-CPTGLY-
CYPTGL--
-CPTGLQ-
CKPTGL--

```
CIPTGL--
-CPTGLT-
-CPTGLF-
CVPTGL--
CSPTGL--
CNPTGL--
CGPTGL--
CQPTGL--
-CPTGLV-
-CPTGLN-
-CPTGLM-
CPPTGL--
CMPTGL--
CEPTGL--
CAPTGL--
DRPTGL--
DLPTGL--
DVPTGL--
DSPTGL--
-DPTGLV-
DKPTGL--
-DPTGLR-
DFPTGL--
DWPTGL--
-DPTGLY-
-DPTGLL-
-DPTGLK-
-DPTGLF-
DMPTGL--
DTPTGL--
-DPTGLT-
-DPTGLS-
DEPTGL--
DQPTGL--
DAPTGL--
DYPTGL--
-DPTGLW-
-DPTGLM-
-DPTGLH-
DIPTGL--
DGPTGL--
-DPTGLI-
DPPTGL--
DNPTGL--
DDPTGL--
-EPTGLT-
ESPTGL--
ELPTGL--
-EPTGLS-
EVPTGL--
```

ERPTGL--
-EPTGLY-
-EPTGLR-
-EPTGLL-
-EPTGLW-
EYPTGL--
EQPTGL--
-EPTGLV-
EFPTGL--
EGPTGL--
-EPTGLQ-
-EPTGLK-
-EPTGLM-
EKPTGL--
EHPTGL--
EAPTGL--
ENPTGL--
ETPTGL--
-EPTGLN-
-EPTGLI-
-EPTGLF-
EMPTGL--
EIPTGL--
-EPTGLH-
-EPTGLE-
EPPTGL--
FRPTGL--
FTPTGL--
-FPTGLV-
FAPTGL--
FFPTGL--
FNPTGL--
-FPTGLK-
FGPTGL--
FDPTGL--
FYPTGL--
-FPTGLR-
-FPTGLI-
-FPTGLY-
-FPTGLL-
-FPTGLS-
-FPTGLF-
FLPTGL--
FIPTGL--
-FPTGLA-
FMPTGL--
-FPTGLH-
FKPTGL--
FEPTGL--
FHPTGL--

-FPTGLT-
-FPTGLM-
FCPTGL--
FWPTGL--
-FPTGLE-
GSPTGL--
GLPTGL--
-GPTGLR-
GNPTGL--
-GPTGLS-
GHPTGL--
-GPTGLN-
GTPTGL--
-GPTGLT-
GKPTGL--
GRPTGL--
GYPTGL--
GQPTGL--
-GPTGLW-
-GPTGLV-
GWPTGL--
-GPTGLY-
-GPTGLF-
-GPTGLK-
GPPTGL--
-GPTGLH-
-GPTGLI-
GGPTGL--
-GPTGLM-
-GPTGLE-
GEPTGL--
-GPTGLA-
GDPTGL--
GCPTGL--
HRPTGL--
HLPTGL--
-HPTGLA-
HSPTGL--
HYPTGL--
-HPTGLS-
HPPTGL--
HMPTGL--
-HPTGLK-
-HPTGLT-
HQPTGL--
HGPTGL--
HWPTGL--
-HPTGLV-
-HPTGLY-
HAPTGL--

HVPTGL--
HTPTGL--
-HPTGLR-
-HPTGLH-
-HPTGLM-
HNPTGL--
HIPTGL--
-HPTGLN-
HKPTGL--
HEPTGL--
-HPTGLI-
-HPTGLQ-
HFPTGL--
HDPTGL--
-IPTGLR-
-IPTGLL-
IIPTGL--
IEPTGL--
ITPTGL--
-IPTGLQ-
IRPTGL--
IMPTGL--
-IPTGLV-
-IPTGLS-
-IPTGLT-
-IPTGLW-
-IPTGLK-
-IPTGLI-
IGPTGL--
IAPTGL--
-IPTGLH-
-IPTGLY-
-IPTGLN-
IKPTGL--
IVPTGL--
IFPTGL--
-IPTGLA-
IHPTGL--
-IPTGLF-
ILPTGL--
IWPTGL--
IDPTGL--
ICPTGL--
-IPTGLM-
-IPTGLE-
-IPTGLC-
INPTGL--
-KPTGLR-
-KPTGLL-
KTPTGL--

-KPTGLN-
-KPTGLA-
KLPTGL--
KYPTGL--
-KPTGLV-
-KPTGLK-
KIPTGL--
KGPTGL--
-KPTGLY-
-KPTGLQ-
-KPTGLM-
KFPTGL--
-KPTGLT-
-KPTGLH-
-KPTGLW-
KPPTGL--
KNPTGL--
KAPTGL--
KEPTGL--
-KPTGLI-
-KPTGLG-
KMPTGL--
KDPTGL--
-KPTGLF-
-KPTGLE-
KCPTGL--
KWPTGL--
LFPTGL--
LRPTGL--
-LPTGLR-
LWPTGL--
LLPTGL--
LVPTGL--
LTPTGL--
-LPTGLL-
-LPTGLT-
-LPTGLK-
LIPTGL--
LGPTGL--
LAPTGL--
-LPTGLS-
LHPTGL--
-LPTGLI-
-LPTGLF-
LNPTGL--
LPPTGL--
LMPTGL--
-LPTGLQ-
-LPTGLH-
LYPTGL--

LQPTGL--
LEPTGL--
-LPTGLY-
-LPTGLV-
-LPTGLM-
LDPTGL--
-LPTGLW-
LCPTGL--
-LPTGLE-
-MPTGLT-
MSPTGL--
-MPTGLR-
MTPTGL--
MPPTGL--
MVPTGL--
-MPTGLY-
-MPTGLL-
MDPTGL--
-MPTGLN-
MFPTGL--
-MPTGLK-
-MPTGLA-
-MPTGLQ-
-MPTGLI-
MGPTGL--
MIPTGL--
MAPTGL--
-MPTGLV-
MCPTGL--
MNPTGL--
MKPTGL--
-MPTGLH-
-MPTGLE-
MHPTGL--
MEPTGL--
MYPTGL--
MQPTGL--
-NPTGLK-
-NPTGLR-
NIPTGL--
NFPTGL--
-NPTGLT-
-NPTGLS-
NWPTGL--
-NPTGLL-
NTPTGL--
NAPTGL--
NVPTGL--
-NPTGLY-
-NPTGLW-

NNPTGL--
NGPTGL--
NRPTGL--
NYPTGL--
NKPTGL--
-NPTGLM-
-NPTGLH-
NPPTGL--
NQPTGL--
-NPTGLA-
NMPTGL--
NEPTGL--
-NPTGLI-
-NPTGLF-
NCPTGL--
-NPTGLV-
NDPTGL--
--PTGLRL
--PTGLMR
--PTGLRK
--PTGLRG
--PTGLMS
--PTGLLT
--PTGLLG
--PTGLTV
--PTGLRT
--PTGLSR
--PTGLYR
--PTGLVK
--PTGLVR
--PTGLRS
--PTGLIN
--PTGLTA
--PTGLVS
--PTGLHL
PRPTGL--
--PTGLYG
--PTGLSV
--PTGLLN
--PTGLLA
--PTGLFV
--PTGLGS
--PTGLYN
PYPTGL--
--PTGLYT
--PTGLRY
--PTGLRH
--PTGLIQ
--PTGLFT
--PTGLYK

--PTGLVF
--PTGLVG
--PTGLNS
--PTGLKK
--PTGLKA
--PTGLFN
--PTGLSL
--PTGLRQ
--PTGLQG
--PTGLMA
--PTGLLQ
--PTGLLM
PLPTGL--
--PTGLYS
--PTGLWT
--PTGLTL
--PTGLRD
--PTGLKR
--PTGLIV
--PTGLWR
--PTGLVM
--PTGLRV
--PTGLNN
--PTGLMM
--PTGLIR
--PTGLFA
--PTGLEG
--PTGLVT
--PTGLVI
--PTGLTT
--PTGLSA
--PTGLMF
--PTGLLW
--PTGLLR
--PTGLHY
--PTGLHE
PGPTGL--
--PTGLYE
--PTGLWS
--PTGLMT
--PTGLMK
--PTGLIM
--PTGLII
--PTGLHS
--PTGLAS
--PTGLYV
--PTGLVV
--PTGLTR
--PTGLTM
--PTGLSK

--PTGLSG
--PTGLQS
--PTGLNT
--PTGLKS
--PTGLKI
--PTGLKH
--PTGLIF
--PTGLHN
--PTGLGG
--PTGLFS
PIPTGL--
--PTGLVL
--PTGLVA
--PTGLTW
--PTGLTK
--PTGLQY
--PTGLQT
--PTGLNW
--PTGLLH
--PTGLKY
--PTGLIK
--PTGLFI
--PTGLWG
--PTGLVW
--PTGLTY
--PTGLTG
--PTGLSN
--PTGLSF
--PTGLIY
--PTGLFR
--PTGLFG
--PTGLAY
--PTGLWD
--PTGLSM
--PTGLRW
--PTGLNY
--PTGLMG
--PTGLKQ
--PTGLKG
--PTGLIH
--PTGLGA
--PTGLAG
PKPTGL--
--PTGLWN
--PTGLVY
--PTGLSI
--PTGLMW
--PTGLML
--PTGLHT
--PTGLHK

--PTGLHH
--PTGLFK
--PTGLAT
PQPTGL--
PWPTGL--
--PTGLYH
--PTGLSY
--PTGLRE
--PTGLQK
--PTGLNV
--PTGLMY
--PTGLMD
--PTGLIG
--PTGLHV
--PTGLHR
--PTGLHI
--PTGLFH
PTPTGL--
--PTGLWH
--PTGLVQ
--PTGLVN
--PTGLVD
--PTGLTH
--PTGLSQ
--PTGLRM
--PTGLNR
--PTGLNM
--PTGLLD
--PTGLKV
--PTGLKT
--PTGLIS
--PTGLYW
--PTGLYA
--PTGLQV
--PTGLMN
--PTGLME
--PTGLKN
--PTGLKL
--PTGLIT
PFPTGL--
--PTGLTF
--PTGLQW
--PTGLQR
--PTGLNA
--PTGLKW
--PTGLHQ
--PTGLFW
--PTGLEA
PMPTGL--
PEPTGL--

--PTGLYQ
--PTGLYD
--PTGLWQ
--PTGLTI
--PTGLQN
--PTGLQF
--PTGLQA
--PTGLNH
--PTGLKD
--PTGLIW
--PTGLIA
--PTGLHW
--PTGLHM
--PTGLHD
--PTGLFE
--PTGLES
--PTGLAL
PSPTGL--
PHPTGL--
PDPTGL--
PAPTGL--
--PTGLYM
--PTGLWE
--PTGLTE
--PTGLTD
--PTGLNL
--PTGLNK
--PTGLNI
--PTGLNF
--PTGLMQ
--PTGLMI
--PTGLKM
--PTGLKE
--PTGLHA
--PTGLGW
--PTGLFD
--PTGLAW
--PTGLAV
--PTGLAN
--PTGLAM
--PTGLAI
--PTGLAA
--PTGLWK
--PTGLTQ
--PTGLQQ
--PTGLNQ
--PTGLMH
--PTGLKF
--PTGLIE
--PTGLFQ

--PTGLEY
--PTGLEF
--PTGLAR
--PTGLAK
PNPTGL--
--PTGLQH
--PTGLQD
--PTGLHF
--PTGLGM
--PTGLFC
--PTGLDA
--PTGLCT
--PTGLCS
--PTGLAH
--PTGLAE
-PPTGLS-
QRPTGL--
-QPTGLS-
QNPTGL--
QLPTGL--
QIPTGL--
-QPTGLH-
QSPTGL--
-QPTGLN-
QFPTGL--
QVPTGL--
-QPTGLK-
-QPTGLE-
QKPTGL--
QGPTGL--
QYPTGL--
-QPTGLQ-
-QPTGLF-
QAPTGL--
-QPTGLM-
QWPTGL--
QTPTGL--
QQPTGL--
-QPTGLI-
-QPTGLW-
-QPTGLV-
QCPTGL--
QHPTGL--
QDPTGL--
-QPTGLD-
QMPTGL--
RLPTGL--
RFPTGL--
RVPTGL--
-RPTGLK-

RQPTGL--
-RPTGLL-
-RPTGLS-
-RPTGLY-
RIPTGL--
-RPTGLA-
RTPTGL--
-RPTGLN-
RPPTGL--
RAPTGL--
-RPTGLW-
RMPTGL--
-RPTGLF-
RHPTGL--
RNPTGL--
REPTGL--
-RPTGLI-
-RPTGLG-
-RPTGLM-
-RPTGLE-
RCPTGL--
RDPTGL--
-SPTGLR-
SRPTGL--
-SPTGLL-
SVPTGL--
SYPTGL--
SQPTGL--
SIPTGL--
-SPTGLT-
-SPTGLH-
STPTGL--
SGPTGL--
-SPTGLS-
-SPTGLF-
SMPTGL--
-SPTGLK-
-SPTGLN-
-SPTGLI-
-SPTGLW-
-SPTGLM-
SNPTGL--
SKPTGL--
SHPTGL--
-SPTGLQ-
-SPTGLA-
-SPTGLV-
SDPTGL--
SCPTGL--
SPPTGL--

−SPTGLG−
−SPTGLE−
TSPTGL−−
TRPTGL−−
−TPTGLL−
−TPTGLS−
−TPTGLY−
TAPTGL−−
−TPTGLK−
TTPTGL−−
TIPTGL−−
TFPTGL−−
TVPTGL−−
−TPTGLI−
−TPTGLF−
TGPTGL−−
TLPTGL−−
TWPTGL−−
TPPTGL−−
TEPTGL−−
−TPTGLQ−
TNPTGL−−
−TPTGLW−
TDPTGL−−
TQPTGL−−
−TPTGLA−
TMPTGL−−
TKPTGL−−
THPTGL−−
−TPTGLM−
−TPTGLH−
TCPTGL−−
−TPTGLE−
VLPTGL−−
VRPTGL−−
−VPTGLR−
VVPTGL−−
−VPTGLL−
VMPTGL−−
VYPTGL−−
VTPTGL−−
VAPTGL−−
VKPTGL−−
−VPTGLW−
−VPTGLV−
−VPTGLS−
−VPTGLN−
−VPTGLK−
VHPTGL−−
−VPTGLI−

VIPTGL--
-VPTGLA-
VPPTGL--
VFPTGL--
VGPTGL--
-VPTGLH-
VQPTGL--
-VPTGLQ-
-VPTGLG-
VDPTGL--
-VPTGLE-
VWPTGL--
-VPTGLM-
VEPTGL--
-VPTGLC-
VNPTGL--
VCPTGL--
WGPTGL--
WSPTGL--
-WPTGLL-
-WPTGLT-
WPPTGL--
WFPTGL--
WWPTGL--
WVPTGL--
WRPTGL--
WTPTGL--
WQPTGL--
-WPTGLS-
-WPTGLK-
-WPTGLQ-
WKPTGL--
WIPTGL--
WYPTGL--
WMPTGL--
WDPTGL--
-WPTGLV-
WEPTGL--
WAPTGL--
-WPTGLW-
-WPTGLN-
WNPTGL--
-WPTGLF-
-WPTGLH-
-WPTGLA-
WCPTGL--
-WPTGLM-
WHPTGL--
YRPTGL--
-YPTGLR-

```
              -YPTGLL-
              -YPTGLS-
              -YPTGLH-
              YDPTGL--
              -YPTGLK-
              -YPTGLF-
              YHPTGL--
              -YPTGLN-
              YFPTGL--
              YKPTGL--
              -YPTGLM-
              YVPTGL--
              YTPTGL--
              -YPTGLI-
              YNPTGL--
              YLPTGL--
              YQPTGL--
              -YPTGLY-
              YWPTGL--
              YGPTGL--
              -YPTGLV-
              -YPTGLT-
              -YPTGLQ-
              YPPTGL--
              YAPTGL--
              YCPTGL--
              -YPTGLW-
              YMPTGL--
PGNL            12        863      33.11
              -DPGNLG-
              EVPGNL--
              --PGNLTF
              --PGNLTE
              --PGNLSE
              --PGNLNH
              --PGNLNF
              --PGNLAN
              -APGNLR-
              ALPGNL--
              -APGNLL-
              -APGNLY-
              AMPGNL--
              -APGNLT-
              -APGNLS-
              -APGNLH-
              ARPGNL--
              AVPGNL--
              -APGNLF-
              AIPGNL--
              AYPGNL--
```

-APGNLV-
AFPGNL--
-APGNLN-
ANPGNL--
AHPGNL--
ASPGNL--
AQPGNL--
-APGNLA-
-APGNLM-
-APGNLI-
AAPGNL--
APPGNL--
-APGNLQ-
-APGNLK-
AKPGNL--
ACPGNL--
-APGNLW-
ATPGNL--
AGPGNL--
AEPGNL--
-APGNLE-
-APGNLD-
CWPGNL--
CCPGNL--
CTPGNL--
CLPGNL--
-CPGNLT-
-CPGNLR-
-CPGNLF-
CKPGNL--
CSPGNL--
CRPGNL--
CYPGNL--
CPPGNL--
-CPGNLY-
-CPGNLW-
-CPGNLL-
-CPGNLK-
-CPGNLI-
CMPGNL--
CIPGNL--
CHPGNL--
CEPGNL--
CDPGNL--
DLPGNL--
-DPGNLW-
DSPGNL--
DRPGNL--
DWPGNL--
-DPGNLL-

DVPGNL--
-DPGNLY-
-DPGNLM-
-DPGNLK-
DNPGNL--
DQPGNL--
DTPGNL--
-DPGNLV-
-DPGNLT-
-DPGNLQ-
-DPGNLA-
DFPGNL--
DYPGNL--
-DPGNLS-
-DPGNLR-
-DPGNLI-
-DPGNLE-
DMPGNL--
DIPGNL--
DHPGNL--
DEPGNL--
DAPGNL--
-EPGNLT-
EYPGNL--
-EPGNLL-
ETPGNL--
ESPGNL--
ERPGNL--
-EPGNLS-
-EPGNLN-
EIPGNL--
EQPGNL--
-EPGNLR-
-EPGNLV-
ELPGNL--
-EPGNLI-
-EPGNLH-
ENPGNL--
EHPGNL--
EAPGNL--
-EPGNLQ-
EMPGNL--
-EPGNLY-
EKPGNL--
-EPGNLM-
-EPGNLF-
-EPGNLC-
-EPGNLA-
EGPGNL--
EEPGNL--

-FPGNLR-
FKPGNL--
FRPGNL--
-FPGNLY-
FHPGNL--
-FPGNLS-
FDPGNL--
FSPGNL--
-FPGNLQ-
FVPGNL--
FIPGNL--
FTPGNL--
-FPGNLK-
-FPGNLI-
FLPGNL--
FYPGNL--
-FPGNLW-
-FPGNLM-
-FPGNLA-
FNPGNL--
FWPGNL--
-FPGNLT-
-FPGNLH-
FMPGNL--
-FPGNLN-
FFPGNL--
FPPGNL--
-FPGNLV-
FEPGNL--
-FPGNLG-
-FPGNLE-
-GPGNLL-
GVPGNL--
-GPGNLR-
GRPGNL--
GLPGNL--
-GPGNLT-
GYPGNL--
GWPGNL--
GAPGNL--
GSPGNL--
GMPGNL--
GIPGNL--
-GPGNLI-
GKPGNL--
GFPGNL--
GQPGNL--
GTPGNL--
-GPGNLV-
-GPGNLF-

GNPGNL--
GGPGNL--
-GPGNLY-
-GPGNLS-
-GPGNLA-
GHPGNL--
-GPGNLQ-
-GPGNLN-
-GPGNLG-
GEPGNL--
-GPGNLH-
GPPGNL--
-GPGNLW-
GDPGNL--
-GPGNLE-
-GPGNLD-
-HPGNLT-
HLPGNL--
HSPGNL--
-HPGNLS-
-HPGNLR-
-HPGNLF-
HAPGNL--
HWPGNL--
HRPGNL--
-HPGNLY-
-HPGNLW-
HFPGNL--
HYPGNL--
-HPGNLM-
-HPGNLA-
-HPGNLL-
HKPGNL--
HIPGNL--
HGPGNL--
HVPGNL--
-HPGNLQ-
-HPGNLN-
-HPGNLK-
-HPGNLE-
HEPGNL--
-HPGNLG-
HCPGNL--
HQPGNL--
HPPGNL--
-HPGNLV-
-HPGNLI-
-HPGNLH-
HNPGNL--
HDPGNL--

-IPGNLL-
ILPGNL--
IYPGNL--
-IPGNLR-
IRPGNL--
-IPGNLH-
-IPGNLK-
IAPGNL--
-IPGNLI-
IDPGNL--
-IPGNLY-
-IPGNLW-
-IPGNLT-
-IPGNLS-
-IPGNLN-
ISPGNL--
-IPGNLQ-
INPGNL--
-IPGNLM-
-IPGNLF-
ITPGNL--
-IPGNLE-
IKPGNL--
IFPGNL--
IQPGNL--
-IPGNLV-
IMPGNL--
IIPGNL--
IGPGNL--
-IPGNLA-
IEPGNL--
IWPGNL--
IPPGNL--
-IPGNLP-
IHPGNL--
KSPGNL--
-KPGNLS-
KRPGNL--
-KPGNLR-
KYPGNL--
-KPGNLL-
KAPGNL--
-KPGNLH-
KMPGNL--
KLPGNL--
KIPGNL--
KKPGNL--
KWPGNL--
-KPGNLK-
KGPGNL--

-KPGNLT-
KPPGNL--
-KPGNLF-
KQPGNL--
-KPGNLY-
-KPGNLN-
-KPGNLG-
KNPGNL--
KHPGNL--
KEPGNL--
KDPGNL--
-KPGNLM-
-KPGNLW-
-KPGNLV-
-KPGNLQ-
-KPGNLE-
KFPGNL--
KCPGNL--
LRPGNL--
-LPGNLR-
LLPGNL--
-LPGNLL-
-LPGNLK-
LYPGNL--
-LPGNLS-
LVPGNL--
LAPGNL--
LNPGNL--
LQPGNL--
LMPGNL--
-LPGNLM-
LTPGNL--
-LPGNLW-
-LPGNLQ-
-LPGNLV-
-LPGNLT-
LFPGNL--
-LPGNLF-
LPPGNL--
LHPGNL--
-LPGNLI-
LKPGNL--
-LPGNLN-
-LPGNLH-
-LPGNLA-
LWPGNL--
-LPGNLG-
-LPGNLE-
LEPGNL--
LDPGNL--

LCPGNL--
MKPGNL--
-MPGNLL-
-MPGNLK-
MRPGNL--
MLPGNL--
-MPGNLS-
MVPGNL--
-MPGNLM-
-MPGNLF-
MIPGNL--
MSPGNL--
MTPGNL--
-MPGNLT-
-MPGNLR-
-MPGNLA-
MPPGNL--
-MPGNLN-
MMPGNL--
MAPGNL--
-MPGNLY-
-MPGNLI-
MQPGNL--
MNPGNL--
-MPGNLW-
-MPGNLG-
-MPGNLE-
MHPGNL--
MGPGNL--
-MPGNLV-
-MPGNLQ-
MEPGNL--
NLPGNL--
-NPGNLL-
-NPGNLF-
NKPGNL--
NWPGNL--
NIPGNL--
-NPGNLM-
NYPGNL--
NRPGNL--
-NPGNLR-
-NPGNLT-
NGPGNL--
NFPGNL--
NQPGNL--
-NPGNLS-
-NPGNLI-
-NPGNLH-
NPPGNL--

NTPGNL--
-NPGNLN-
NMPGNL--
-NPGNLK-
-NPGNLA-
-NPGNLQ-
-NPGNLG-
NSPGNL--
-NPGNLW-
NHPGNL--
NEPGNL--
-NPGNLE-
NAPGNL--
--PGNLRA
--PGNLIL
--PGNLLY
--PGNLRS
--PGNLLR
--PGNLSL
--PGNLVR
--PGNLLH
--PGNLMG
--PGNLYH
--PGNLTS
--PGNLVG
--PGNLRR
--PGNLRN
--PGNLLS
--PGNLHG
PWPGNL--
PRPGNL--
--PGNLYR
--PGNLVV
--PGNLSG
--PGNLRF
--PGNLLT
--PGNLFS
--PGNLYL
--PGNLVN
--PGNLRK
--PGNLRG
--PGNLQR
--PGNLKS
--PGNLSH
--PGNLLN
--PGNLLM
--PGNLYI
--PGNLWR
--PGNLTG
--PGNLSK

--PGNLRT
--PGNLRI
--PGNLMY
--PGNLLA
--PGNLKA
--PGNLVS
--PGNLTV
--PGNLSW
--PGNLSS
--PGNLRQ
--PGNLKY
--PGNLHT
--PGNLFN
--PGNLAH
--PGNLYV
--PGNLVT
--PGNLTR
--PGNLTL
--PGNLSR
--PGNLSF
--PGNLRW
--PGNLML
--PGNLLV
--PGNLKL
--PGNLIR
--PGNLFM
PLPGNL--
--PGNLLL
--PGNLET
PKPGNL--
--PGNLTT
--PGNLNT
--PGNLLW
--PGNLIS
--PGNLFR
--PGNLES
--PGNLYF
--PGNLVM
--PGNLSV
--PGNLSN
--PGNLRY
--PGNLQI
--PGNLNA
--PGNLMK
--PGNLKV
--PGNLHS
--PGNLFK
--PGNLFA
PVPGNL--
--PGNLYM

--PGNLWG
--PGNLWF
--PGNLVF
--PGNLTN
--PGNLRH
--PGNLQV
--PGNLLQ
--PGNLLK
--PGNLLE
--PGNLKT
--PGNLIQ
--PGNLIG
--PGNLAS
--PGNLAI
PTPGNL--
PMPGNL--
--PGNLTW
--PGNLTA
--PGNLSQ
--PGNLHY
--PGNLGG
--PGNLFQ
PYPGNL--
--PGNLWW
--PGNLTQ
--PGNLTK
--PGNLSI
--PGNLMT
--PGNLMS
--PGNLKW
--PGNLIT
--PGNLGL
--PGNLFV
--PGNLFI
--PGNLFH
--PGNLAY
PSPGNL--
--PGNLYW
--PGNLYT
--PGNLYG
--PGNLWT
--PGNLWS
--PGNLTY
--PGNLTI
--PGNLSM
--PGNLRD
--PGNLQS
--PGNLQQ
--PGNLMW
--PGNLMH

--PGNLKK
--PGNLKH
--PGNLIM
--PGNLIK
--PGNLIA
--PGNLGV
--PGNLAM
--PGNLAG
PGPGNL--
--PGNLWY
--PGNLWE
--PGNLVK
--PGNLVE
--PGNLTM
--PGNLSY
--PGNLRE
--PGNLQM
--PGNLQK
--PGNLQG
--PGNLQA
--PGNLNR
--PGNLMV
--PGNLLD
--PGNLKR
--PGNLKG
--PGNLIY
--PGNLIF
--PGNLHR
--PGNLHN
--PGNLEV
PQPGNL--
PIPGNL--
PHPGNL--
--PGNLYQ
--PGNLYK
--PGNLWV
--PGNLWN
--PGNLWH
--PGNLVW
--PGNLVH
--PGNLTH
--PGNLQH
--PGNLQF
--PGNLNM
--PGNLME
--PGNLKM
--PGNLIV
--PGNLID
--PGNLHV
--PGNLHH

--PGNLHF
--PGNLHA
--PGNLGM
--PGNLGF
--PGNLFL
--PGNLEA
--PGNLAL
--PGNLAK
PAPGNL--
PNPGNL--
--PGNLYD
--PGNLVD
--PGNLQN
--PGNLQE
--PGNLNY
--PGNLNV
--PGNLNS
--PGNLMQ
--PGNLMN
--PGNLKQ
--PGNLKI
--PGNLHL
--PGNLGY
--PGNLGW
--PGNLGT
--PGNLFY
--PGNLFW
--PGNLEF
--PGNLCG
--PGNLAW
--PGNLAR
--PGNLAF
--PGNLAA
-PPGNLS-
-PPGNLL-
--PGNLYE
--PGNLWK
--PGNLWI
--PGNLTD
--PGNLQY
--PGNLQW
--PGNLQT
--PGNLNW
--PGNLNN
--PGNLNL
--PGNLMF
--PGNLMD
--PGNLKN
--PGNLIW
--PGNLIE

--PGNLHW
--PGNLHQ
--PGNLHM
--PGNLHK
--PGNLHI
--PGNLHE
--PGNLHD
--PGNLFT
--PGNLFD
--PGNLEG
--PGNLCV
--PGNLAT
--PGNLAE
PFPGNL--
QRPGNL--
-QPGNLL-
QSPGNL--
-QPGNLS-
QFPGNL--
QYPGNL--
QVPGNL--
-QPGNLY-
-QPGNLE-
QMPGNL--
-QPGNLR-
-QPGNLI-
QGPGNL--
QWPGNL--
QTPGNL--
-QPGNLG-
QKPGNL--
QEPGNL--
-QPGNLW-
-QPGNLT-
-QPGNLM-
QQPGNL--
QPPGNL--
QNPGNL--
QHPGNL--
-QPGNLA-
QIPGNL--
QAPGNL--
-QPGNLV-
-QPGNLQ-
-QPGNLK-
-QPGNLH-
RVPGNL--
-RPGNLR-
RSPGNL--
RGPGNL--

RTPGNL--
-RPGNLL-
-RPGNLI-
-RPGNLV-
-RPGNLY-
-RPGNLS-
RHPGNL--
RMPGNL--
-RPGNLW-
RKPGNL--
RWPGNL--
-RPGNLA-
RNPGNL--
-RPGNLK-
-RPGNLG-
RYPGNL--
REPGNL--
-RPGNLQ-
-RPGNLH-
RFPGNL--
-RPGNLM-
-RPGNLF-
RIPGNL--
RQPGNL--
RPPGNL--
RDPGNL--
-RPGNLE-
RCPGNL--
-SPGNLV-
SRPGNL--
-SPGNLR-
SVPGNL--
SLPGNL--
STPGNL--
-SPGNLS-
-SPGNLW-
SSPGNL--
SYPGNL--
SGPGNL--
SKPGNL--
SPPGNL--
SHPGNL--
-SPGNLY-
-SPGNLN-
-SPGNLG-
SNPGNL--
SAPGNL--
-SPGNLQ-
-SPGNLT-
SWPGNL--

SQPGNL--
-SPGNLH-
SIPGNL--
SMPGNL--
SFPGNL--
SEPGNL--
SCPGNL--
SDPGNL--
-SPGNLM-
-SPGNLA-
-SPGNLE-
-TPGNLL-
TRPGNL--
TWPGNL--
TTPGNL--
-TPGNLS-
TKPGNL--
TLPGNL--
TIPGNL--
-TPGNLR-
-TPGNLV-
-TPGNLT-
-TPGNLN-
TVPGNL--
-TPGNLY-
-TPGNLA-
-TPGNLM-
-TPGNLI-
-TPGNLQ-
TMPGNL--
TGPGNL--
TAPGNL--
TYPGNL--
-TPGNLK-
-TPGNLH-
TNPGNL--
TFPGNL--
THPGNL--
TEPGNL--
TCPGNL--
TPPGNL--
TDPGNL--
-TPGNLW-
-TPGNLG-
-TPGNLF-
-TPGNLE-
-VPGNLV-
-VPGNLR-
VSPGNL--
VVPGNL--

-VPGNLA-
-VPGNLW-
VTPGNL--
-VPGNLI-
VMPGNL--
VLPGNL--
VFPGNL--
-VPGNLT-
VHPGNL--
VAPGNL--
-VPGNLM-
-VPGNLF-
VKPGNL--
VQPGNL--
-VPGNLH-
VIPGNL--
VYPGNL--
-VPGNLN-
-VPGNLY-
-VPGNLG-
VNPGNL--
VEPGNL--
-VPGNLQ-
-VPGNLE-
VPPGNL--
VDPGNL--
VCPGNL--
VWPGNL--
WKPGNL--
WNPGNL--
WWPGNL--
WSPGNL--
WAPGNL--
-WPGNLR-
WLPGNL--
WRPGNL--
-WPGNLM-
-WPGNLT-
WIPGNL--
WPPGNL--
WYPGNL--
WTPGNL--
WGPGNL--
WFPGNL--
WMPGNL--
-WPGNLS-
-WPGNLI-
WEPGNL--
-WPGNLL-
-WPGNLF-

-WPGNLW-
-WPGNLV-
-WPGNLQ-
-WPGNLN-
-WPGNLE-
WHPGNL--
-WPGNLK-
WCPGNL--
-WPGNLY-
-YPGNLS-
-YPGNLF-
-YPGNLR-
YHPGNL--
YYPGNL--
-YPGNLV-
-YPGNLK-
YSPGNL--
YQPGNL--
-YPGNLT-
YTPGNL--
YIPGNL--
YWPGNL--
YVPGNL--
-YPGNLM-
YLPGNL--
-YPGNLY-
-YPGNLI-
-YPGNLG-
YKPGNL--
-YPGNLW-
-YPGNLH-
YGPGNL--
-YPGNLL-
YNPGNL--
-YPGNLE-
YDPGNL--
YPPGNL--
YMPGNL--
YFPGNL--
YEPGNL--
YAPGNL--
-YPGNLQ-
PAYL 165 733 33.08
-LPAYLE-
--PAYLNG
--PAYLGY
--PAYLEI
-QPAYLK-
-VPAYLA-
-APAYLH-

APPAYL--
ALPAYL--
-APAYLA-
ANPAYL--
AHPAYL--
ASPAYL--
-APAYLQ-
-APAYLM-
ATPAYL--
-APAYLN-
-APAYLL-
-APAYLG-
AKPAYL--
-APAYLV-
-APAYLK-
ADPAYL--
AFPAYL--
AQPAYL--
AIPAYL--
-APAYLW-
AYPAYL--
AEPAYL--
ACPAYL--
AAPAYL--
CKPAYL--
CVPAYL--
-CPAYLQ-
CLPAYL--
CWPAYL--
CQPAYL--
-CPAYLR-
-CPAYLL-
CGPAYL--
CCPAYL--
CYPAYL--
CSPAYL--
CRPAYL--
-CPAYLY-
-CPAYLT-
-CPAYLK-
CNPAYL--
CIPAYL--
CAPAYL--
DLPAYL--
-DPAYLR-
DFPAYL--
DSPAYL--
DQPAYL--
DIPAYL--
DRPAYL--

-DPAYLA-
DHPAYL--
DEPAYL--
DVPAYL--
DTPAYL--
-DPAYLQ-
-DPAYLL-
DKPAYL--
DWPAYL--
-DPAYLT-
DYPAYL--
-DPAYLY-
-DPAYLK-
DGPAYL--
DDPAYL--
-DPAYLS-
-DPAYLN-
-DPAYLF-
DNPAYL--
-DPAYLV-
-DPAYLM-
-DPAYLI-
DAPAYL--
ESPAYL--
-EPAYLS-
ETPAYL--
EIPAYL--
-EPAYLV-
EKPAYL--
-EPAYLL-
EGPAYL--
-EPAYLH-
EMPAYL--
EQPAYL--
EWPAYL--
ENPAYL--
-EPAYLT-
-EPAYLK-
-EPAYLA-
ELPAYL--
-EPAYLR-
-EPAYLI-
EPPAYL--
-EPAYLW-
-EPAYLQ-
-EPAYLN-
EEPAYL--
EYPAYL--
EFPAYL--
EVPAYL--

-EPAYLY-
-EPAYLG-
-EPAYLF-
EHPAYL--
EAPAYL--
FSPAYL--
FIPAYL--
-FPAYLS-
FVPAYL--
FYPAYL--
FLPAYL--
-FPAYLM-
FEPAYL--
-FPAYLR-
-FPAYLK-
FGPAYL--
FQPAYL--
FFPAYL--
FPPAYL--
-FPAYLT-
FHPAYL--
FAPAYL--
FWPAYL--
-FPAYLQ-
-FPAYLN-
FNPAYL--
-FPAYLY-
-FPAYLI-
-FPAYLH-
-FPAYLA-
-GPAYLI-
-GPAYLK-
-GPAYLR-
GFPAYL--
GKPAYL--
-GPAYLV-
GTPAYL--
-GPAYLW-
GYPAYL--
GQPAYL--
GAPAYL--
-GPAYLL-
-GPAYLG-
GGPAYL--
-GPAYLS-
-GPAYLQ-
-GPAYLA-
GNPAYL--
GWPAYL--
-GPAYLY-

-GPAYLN-
GCPAYL--
GPPAYL--
GEPAYL--
-GPAYLM-
-GPAYLE-
GMPAYL--
GLPAYL--
GIPAYL--
-GPAYLH-
HSPAYL--
HMPAYL--
-HPAYLR-
-HPAYLA-
HAPAYL--
-HPAYLV-
-HPAYLI-
HKPAYL--
-HPAYLT-
-HPAYLS-
-HPAYLK-
HGPAYL--
HVPAYL--
HPPAYL--
HNPAYL--
HIPAYL--
HTPAYL--
-HPAYLY-
-HPAYLG-
HFPAYL--
HCPAYL--
-HPAYLN-
-HPAYLM-
-HPAYLH-
HQPAYL--
-HPAYLQ-
-HPAYLE-
HDPAYL--
IKPAYL--
ISPAYL--
-IPAYLA-
INPAYL--
IVPAYL--
IGPAYL--
-IPAYLH-
IWPAYL--
IRPAYL--
IEPAYL--
IDPAYL--
-IPAYLS-

-IPAYLY-
IHPAYL--
-IPAYLN-
IIPAYL--
ITPAYL--
IFPAYL--
IPPAYL--
-IPAYLM-
-IPAYLK-
ICPAYL--
-IPAYLL-
-IPAYLG-
-IPAYLE-
IAPAYL--
-KPAYLS-
KTPAYL--
-KPAYLL-
KMPAYL--
KLPAYL--
KGPAYL--
-KPAYLY-
-KPAYLG-
KHPAYL--
KVPAYL--
-KPAYLK-
KPPAYL--
-KPAYLV-
KYPAYL--
KNPAYL--
KAPAYL--
-KPAYLI-
KEPAYL--
-KPAYLT-
-KPAYLN-
-KPAYLE-
KWPAYL--
-KPAYLM-
KQPAYL--
-KPAYLW-
-KPAYLF-
KKPAYL--
KDPAYL--
KCPAYL--
LSPAYL--
LMPAYL--
LGPAYL--
LWPAYL--
-LPAYLS-
-LPAYLH-
-LPAYLI-

LEPAYL--
LHPAYL--
-LPAYLL-
-LPAYLT-
-LPAYLK-
-LPAYLA-
LAPAYL--
LQPAYL--
LYPAYL--
LPPAYL--
-LPAYLQ-
-LPAYLM-
-LPAYLY-
LFPAYL--
LCPAYL--
-LPAYLN-
-MPAYLS-
-MPAYLK-
MIPAYL--
MLPAYL--
MWPAYL--
-MPAYLR-
MKPAYL--
MTPAYL--
-MPAYLE-
-MPAYLQ-
MFPAYL--
MVPAYL--
MSPAYL--
-MPAYLW-
MPPAYL--
-MPAYLT-
MEPAYL--
MDPAYL--
MQPAYL--
-MPAYLY-
-MPAYLM-
-MPAYLG-
-MPAYLA-
MNPAYL--
MHPAYL--
-MPAYLH-
MCPAYL--
MYPAYL--
-MPAYLV-
-MPAYLI-
-MPAYLN-
-MPAYLF-
MMPAYL--
NTPAYL--

NVPAYL--
NRPAYL--
NAPAYL--
NFPAYL--
-NPAYLK-
NLPAYL--
NQPAYL--
-NPAYLF-
-NPAYLS-
NNPAYL--
NGPAYL--
-NPAYLI-
NKPAYL--
-NPAYLL-
-NPAYLY-
NMPAYL--
NYPAYL--
-NPAYLT-
NIPAYL--
-NPAYLV-
-NPAYLN-
-NPAYLM-
NDPAYL--
NCPAYL--
-NPAYLQ-
-NPAYLA-
--PAYLRS
--PAYLSR
--PAYLGS
--PAYLVR
--PAYLLR
--PAYLSS
--PAYLYR
--PAYLAR
--PAYLLT
--PAYLKL
--PAYLVS
--PAYLLA
--PAYLIR
--PAYLVG
--PAYLRL
PGPAYL--
--PAYLKS
--PAYLTS
--PAYLRM
--PAYLWS
--PAYLSH
PKPAYL--
--PAYLTG
--PAYLFR

--PAYLAS
--PAYLVY
--PAYLMR
--PAYLMA
--PAYLSQ
--PAYLRN
--PAYLRI
--PAYLRA
--PAYLSN
--PAYLAK
--PAYLVN
--PAYLSK
--PAYLSG
--PAYLNY
--PAYLKF
--PAYLFM
--PAYLVA
--PAYLRQ
--PAYLQR
PAPAYL--
--PAYLVW
--PAYLIA
--PAYLAF
PWPAYL--
--PAYLTN
--PAYLNT
--PAYLMN
--PAYLEA
--PAYLWG
--PAYLTV
--PAYLSL
--PAYLSF
--PAYLSE
--PAYLQS
--PAYLQK
--PAYLLG
--PAYLKE
--PAYLIH
--PAYLGA
--PAYLAN
--PAYLMQ
--PAYLLN
--PAYLKV
--PAYLHN
--PAYLHA
--PAYLGV
--PAYLFH
--PAYLAM
--PAYLYA
--PAYLWR

--PAYLVQ
--PAYLVK
--PAYLTW
--PAYLSI
--PAYLQN
--PAYLQH
--PAYLMV
--PAYLLH
--PAYLLE
--PAYLHK
--PAYLGG
--PAYLAG
PQPAYL--
--PAYLWM
--PAYLSY
--PAYLRW
--PAYLNS
--PAYLNA
--PAYLMI
--PAYLLQ
--PAYLLK
--PAYLLD
--PAYLKW
--PAYLHS
--PAYLGT
--PAYLGL
--PAYLFW
--PAYLFS
--PAYLFK
--PAYLFG
--PAYLAW
PSPAYL--
PFPAYL--
--PAYLYW
--PAYLYK
--PAYLWN
--PAYLSD
--PAYLRT
--PAYLQA
--PAYLNN
--PAYLMF
--PAYLME
--PAYLIQ
--PAYLHQ
--PAYLFN
PTPAYL--
PNPAYL--
--PAYLYY
--PAYLYT
--PAYLYS

--PAYLYQ
--PAYLYH
--PAYLYD
--PAYLWT
--PAYLTI
--PAYLTD
--PAYLTA
--PAYLRD
--PAYLQV
--PAYLNR
--PAYLKN
--PAYLKG
--PAYLIT
--PAYLIS
--PAYLIN
--PAYLHT
--PAYLAA
--PAYLYN
--PAYLWQ
--PAYLWH
--PAYLSW
--PAYLSM
--PAYLSA
--PAYLRE
--PAYLMT
--PAYLKY
--PAYLHY
--PAYLAY
--PAYLAV
--PAYLAQ
--PAYLAL
--PAYLYM
--PAYLWA
--PAYLVV
--PAYLTT
--PAYLTQ
--PAYLTK
--PAYLTE
--PAYLQT
--PAYLQF
--PAYLNK
--PAYLKK
--PAYLKI
--PAYLIY
--PAYLGW
--PAYLAT
--PAYLAH
--PAYLAE
--PAYLAD
--PAYLYE

--PAYLWK
--PAYLVD
--PAYLTY
--PAYLTF
--PAYLQD
--PAYLNW
--PAYLKQ
--PAYLKM
--PAYLHV
--PAYLHF
--PAYLHD
--PAYLEY
--PAYLEG
--PAYLCK
-PPAYLR-
-PPAYLL-
-PPAYLK-
PHPAYL--
PEPAYL--
--PAYLWW
--PAYLVH
--PAYLQY
--PAYLQQ
--PAYLQI
--PAYLNV
--PAYLNM
--PAYLNF
--PAYLND
--PAYLMC
--PAYLLC
--PAYLKD
--PAYLHM
--PAYLHG
--PAYLHE
--PAYLGM
--PAYLGI
--PAYLEM
--PAYLEK
--PAYLDS
--PAYLCH
--PAYLAC
-QPAYLS-
-QPAYLR-
-QPAYLL-
-QPAYLY-
-QPAYLM-
QGPAYL--
QVPAYL--
-QPAYLI-
QEPAYL--

-QPAYLA-
QWPAYL--
QRPAYL--
-QPAYLQ-
QSPAYL--
QPPAYL--
-QPAYLN-
QFPAYL--
QQPAYL--
-QPAYLV-
QNPAYL--
QAPAYL--
-QPAYLW-
-QPAYLT-
QKPAYL--
-QPAYLH-
-QPAYLF-
QIPAYL--
QMPAYL--
QHPAYL--
QCPAYL--
RVPAYL--
RGPAYL--
RSPAYL--
-RPAYLS-
-RPAYLK-
-RPAYLI-
RPPAYL--
RNPAYL--
RLPAYL--
RYPAYL--
RDPAYL--
RQPAYL--
-RPAYLM-
-RPAYLY-
RHPAYL--
REPAYL--
RAPAYL--
-RPAYLH-
-RPAYLE-
-RPAYLQ-
RIPAYL--
-RPAYLF-
-SPAYLL-
-SPAYLK-
STPAYL--
-SPAYLS-
-SPAYLV-
SYPAYL--
-SPAYLH-

SEPAYL--
-SPAYLN-
SVPAYL--
SFPAYL--
-SPAYLM-
-SPAYLW-
SMPAYL--
SDPAYL--
-SPAYLG-
-SPAYLF-
SIPAYL--
SGPAYL--
SNPAYL--
-SPAYLQ-
-SPAYLI-
-SPAYLE-
SCPAYL--
-TPAYLR-
TVPAYL--
TMPAYL--
TWPAYL--
TSPAYL--
TFPAYL--
-TPAYLQ-
TLPAYL--
-TPAYLA-
-TPAYLS-
TPPAYL--
-TPAYLK-
TAPAYL--
-TPAYLY-
-TPAYLT-
TQPAYL--
-TPAYLW-
-TPAYLV-
-TPAYLI-
TDPAYL--
TYPAYL--
TTPAYL--
TNPAYL--
-TPAYLM-
-TPAYLG-
-TPAYLE-
TKPAYL--
TIPAYL--
TCPAYL--
-TPAYLF-
TEPAYL--
-TPAYLP-
-TPAYLD-

THPAYL--
VNPAYL--
-VPAYLS-
-VPAYLV-
-VPAYLL-
-VPAYLI-
-VPAYLR-
VLPAYL--
VAPAYL--
VKPAYL--
VEPAYL--
-VPAYLT-
-VPAYLM-
VTPAYL--
-VPAYLN-
VQPAYL--
VPPAYL--
VGPAYL--
VWPAYL--
-VPAYLG-
VIPAYL--
VCPAYL--
-VPAYLH-
-VPAYLW-
-VPAYLE-
VDPAYL--
WRPAYL--
WAPAYL--
WHPAYL--
WTPAYL--
WNPAYL--
WVPAYL--
WKPAYL--
WPPAYL--
-WPAYLS-
-WPAYLM-
-WPAYLH-
WIPAYL--
WGPAYL--
-WPAYLQ-
-WPAYLK-
-WPAYLA-
WMPAYL--
-WPAYLV-
-WPAYLN-
WLPAYL--
WFPAYL--
-YPAYLS-
-YPAYLR-
YFPAYL--

```
               YVPAYL--
               YTPAYL--
               -YPAYLT-
               YPPAYL--
               YGPAYL--
               -YPAYLL-
               YQPAYL--
               -YPAYLG-
               YAPAYL--
               YMPAYL--
               YIPAYL--
               YYPAYL--
               -YPAYLK-
               YNPAYL--
               -YPAYLV-
               -YPAYLQ-
               -YPAYLN-
               YCPAYL--
               YHPAYL--
               -YPAYLM-
               -YPAYLI-
               -YPAYLH-
               YDPAYL--
               YWPAYL--
               -YPAYLE-
               -YPAYLA-
VASL            340        654     33.08
               -DVASLG-
               -EVASLN-
               -GVASLP-
               -GVASLG-
               -LVASLP-
               -MVASLD-
               -PVASLD-
               -SVASLG-
               -SVASLE-
               -SVASLC-
               -TVASLG-
               --VASLSQ
               --VASLGT
               --VASLAI
               --VASLTP
               --VASLSP
               --VASLSD
               --VASLQQ
               --VASLNP
               --VASLNI
               --VASLIP
               --VASLHL
               --VASLGY
```

--VASLGW
--VASLGN
--VASLGM
--VASLGI
--VASLGG
--VASLGE
--VASLEY
--VASLET
--VASLDR
--VASLDL
ALVASL--
ASVASL--
-AVASLR-
-AVASLT-
AVVASL--
AWVASL--
-AVASLA-
AYVASL--
ATVASL--
ANVASL--
AIVASL--
-AVASLQ-
AHVASL--
APVASL--
AGVASL--
AQVASL--
-AVASLK-
-AVASLV-
-AVASLM-
AMVASL--
AAVASL--
-AVASLF-
ADVASL--
-AVASLW-
AEVASL--
ACVASL--
-AVASLE-
-AVASLH-
-AVASLG-
CRVASL--
CVVASL--
CKVASL--
CGVASL--
-CVASLS-
-CVASLQ-
-CVASLI-
-CVASLR-
CTVASL--
CSVASL--
CNVASL--

-CVASLY-
-CVASLV-
-CVASLK-
-CVASLF-
CQVASL--
CPVASL--
CCVASL--
-CVASLT-
-CVASLM-
-CVASLL-
CMVASL--
CWVASL--
-CVASLW-
CIVASL--
CHVASL--
CAVASL--
DSVASL--
DWVASL--
-DVASLR-
DHVASL--
-DVASLI-
DPVASL--
-DVASLK-
DRVASL--
DGVASL--
DTVASL--
DLVASL--
-DVASLY-
-DVASLH-
DKVASL--
-DVASLV-
DAVASL--
DVVASL--
-DVASLT-
-DVASLM-
-DVASLA-
DMVASL--
DEVASL--
-DVASLQ-
DNVASL--
DCVASL--
-DVASLE-
EYVASL--
-EVASLS-
EVVASL--
-EVASLR-
-EVASLL-
EMVASL--
EFVASL--
-EVASLK-

EGVASL--
-EVASLF-
ESVASL--
ENVASL--
-EVASLA-
EWVASL--
-EVASLV-
EPVASL--
ECVASL--
-EVASLT-
EHVASL--
-EVASLI-
EQVASL--
EEVASL--
-EVASLH-
-EVASLQ-
EKVASL--
EAVASL--
EIVASL--
EDVASL--
-EVASLM-
-FVASLS-
FSVASL--
-FVASLL-
FHVASL--
FGVASL--
-FVASLT-
FQVASL--
-FVASLI-
-FVASLH-
-FVASLV-
-FVASLG-
-FVASLK-
-FVASLQ-
FPVASL--
FDVASL--
-FVASLM-
FIVASL--
FCVASL--
-FVASLD-
-GVASLR-
GSVASL--
GQVASL--
GGVASL--
GTVASL--
GMVASL--
GAVASL--
-GVASLT-
-GVASLL-
-GVASLV-

−GVASLK−
−GVASLF−
GNVASL−−
−GVASLI−
−GVASLN−
GHVASL−−
−GVASLQ−
GEVASL−−
−GVASLM−
GPVASL−−
−GVASLY−
GIVASL−−
−GVASLW−
−GVASLA−
GDVASL−−
−GVASLE−
GCVASL−−
HLVASL−−
−HVASLS−
HGVASL−−
−HVASLW−
HIVASL−−
−HVASLY−
−HVASLV−
−HVASLL−
−HVASLK−
HSVASL−−
HTVASL−−
HVVASL−−
−HVASLI−
−HVASLM−
HYVASL−−
HHVASL−−
HQVASL−−
−HVASLF−
HMVASL−−
−HVASLH−
−HVASLE−
HPVASL−−
−HVASLG−
HCVASL−−
−HVASLQ−
HEVASL−−
HDVASL−−
−IVASLL−
−IVASLT−
IVVASL−−
−IVASLI−
IGVASL−−
IYVASL−−

IEVASL--
IAVASL--
IHVASL--
IQVASL--
INVASL--
IKVASL--
-IVASLK-
-IVASLA-
IIVASL--
-IVASLV-
ITVASL--
-IVASLE-
IDVASL--
ICVASL--
-IVASLN-
-IVASLM-
-IVASLH-
-KVASLR-
KPVASL--
-KVASLL-
-KVASLQ-
-KVASLT-
-KVASLV-
-KVASLY-
-KVASLW-
KEVASL--
-KVASLH-
KNVASL--
KWVASL--
KQVASL--
KDVASL--
KHVASL--
LGVASL--
LYVASL--
-LVASLT-
LQVASL--
LNVASL--
-LVASLK-
LEVASL--
-LVASLH-
-LVASLQ-
-LVASLN-
-LVASLM-
LCVASL--
-LVASLC-
-MVASLR-
-MVASLL-
MGVASL--
-MVASLV-
MNVASL--

-MVASLK-
-MVASLS-
-MVASLT-
-MVASLA-
MWVASL--
-MVASLI-
-MVASLH-
-MVASLQ-
MKVASL--
MPVASL--
MEVASL--
-MVASLN-
-MVASLM-
MMVASL--
-MVASLF-
MQVASL--
MCVASL--
MDVASL--
MYVASL--
-NVASLR-
-NVASLL-
NTVASL--
-NVASLS-
-NVASLY-
-NVASLF-
NNVASL--
NMVASL--
NAVASL--
-NVASLN-
NHVASL--
NWVASL--
-NVASLI-
NYVASL--
NGVASL--
-NVASLA-
-NVASLW-
-NVASLK-
NPVASL--
-NVASLE-
NDVASL--
-NVASLG-
NQVASL--
NCVASL--
-NVASLM-
NEVASL--
PSVASL--
-PVASLS-
PLVASL--
PGVASL--
PAVASL--

-PVASLK-
PTVASL--
-PVASLL-
-PVASLT-
-PVASLG-
PVVASL--
PEVASL--
PWVASL--
-PVASLQ-
-PVASLF-
-PVASLV-
-PVASLA-
PNVASL--
PHVASL--
-PVASLW-
-PVASLM-
PIVASL--
-PVASLY-
-PVASLN-
-PVASLH-
PDVASL--
PYVASL--
-PVASLI-
PQVASL--
-PVASLE-
PMVASL--
-PVASLC-
PPVASL--
PCVASL--
QSVASL--
-QVASLK-
QFVASL--
-QVASLT-
-QVASLQ-
QIVASL--
QLVASL--
QGVASL--
-QVASLN-
-QVASLF-
QNVASL--
-QVASLH-
QAVASL--
-QVASLY-
-QVASLA-
-QVASLW-
QHVASL--
-QVASLE-
QKVASL--
QYVASL--
-QVASLM-

−QVASLI−
QQVASL−−
QEVASL−−
QDVASL−−
QCVASL−−
−RVASLS−
REVASL−−
RDVASL−−
RPVASL−−
RCVASL−−
−RVASLE−
−RVASLC−
−SVASLL−
SNVASL−−
STVASL−−
SIVASL−−
SGVASL−−
SEVASL−−
SAVASL−−
−SVASLI−
SQVASL−−
SMVASL−−
−SVASLF−
SFVASL−−
−SVASLQ−
SPVASL−−
SHVASL−−
SDVASL−−
−SVASLM−
SCVASL−−
−SVASLD−
TLVASL−−
−TVASLS−
TSVASL−−
−TVASLK−
−TVASLV−
TAVASL−−
TNVASL−−
−TVASLH−
TQVASL−−
TFVASL−−
TWVASL−−
−TVASLM−
−TVASLI−
THVASL−−
−TVASLA−
−TVASLN−
TPVASL−−
−TVASLQ−
TEVASL−−

-TVASLF-
-TVASLE-
TCVASL--
--VASLSS
--VASLRN
--VASLMR
--VASLLG
VTVASL--
--VASLTS
VQVASL--
--VASLSG
--VASLTT
--VASLWS
--VASLST
--VASLSA
--VASLYS
--VASLVT
--VASLLA
--VASLVS
-VVASLH-
VVVASL--
--VASLIG
VYVASL--
--VASLLK
-VVASLK-
--VASLRI
--VASLNS
-VVASLT-
--VASLFG
--VASLMG
--VASLNT
--VASLIR
VWVASL--
VPVASL--
--VASLWG
--VASLMT
VHVASL--
--VASLRG
--VASLHT
--VASLAS
--VASLWN
--VASLFS
--VASLWA
VNVASL--
--VASLVG
--VASLMV
-VVASLQ-
VGVASL--
--VASLSW
--VASLKG

--VASLES
--VASLVW
--VASLVN
--VASLLM
--VASLSV
--VASLLW
--VASLMH
--VASLLN
--VASLVM
--VASLTM
--VASLRY
--VASLIT
--VASLWH
--VASLVQ
--VASLVH
--VASLQS
--VASLMQ
--VASLKT
--VASLKA
--VASLHV
--VASLVV
--VASLMS
--VASLGS
--VASLVA
--VASLTR
--VASLRM
--VASLRD
--VASLKV
--VASLTQ
--VASLTV
--VASLTN
--VASLNA
--VASLIN
--VASLIA
--VASLHK
--VASLHG
-VVASLM-
-VVASLE-
--VASLWK
--VASLQG
--VASLKM
--VASLKI
--VASLIH
--VASLAG
--VASLAA
--VASLVI
--VASLTH
--VASLRQ
--VASLRE
--VASLNL

--VASLMY
--VASLMD
--VASLLQ
--VASLIY
--VASLHA
--VASLIE
--VASLAV
VDVASL--
--VASLYM
--VASLMA
--VASLKK
-VVASLI-
VCVASL--
--VASLTF
--VASLMM
--VASLFW
--VASLEG
--VASLAT
--VASLWQ
--VASLSY
--VASLMN
--VASLHW
--VASLFD
--VASLSK
--VASLQT
--VASLLI
--VASLFH
--VASLAL
--VASLYH
--VASLVF
--VASLVD
--VASLTI
--VASLNM
--VASLLH
--VASLWE
--VASLTD
--VASLSI
--VASLQM
--VASLNV
--VASLKH
--VASLAF
-VVASLG-
--VASLQV
--VASLQN
--VASLKF
--VASLKD
--VASLHI
--VASLQK
--VASLQF
--VASLPY

--VASLNY
--VASLMK
--VASLKQ
--VASLKL
--VASLVE
--VASLQR
--VASLNQ
--VASLKE
--VASLIQ
--VASLII
--VASLID
--VASLHY
--VASLHH
--VASLHE
--VASLFQ
--VASLFE
--VASLSH
--VASLQY
--VASLQI
--VASLPM
--VASLNF
--VASLHQ
--VASLHD
--VASLCG
--VASLAY
--VASLAN
--VASLWD
--VASLQH
--VASLLE
--VASLIW
--VASLHN
--VASLEA
--VASLCA
--VASLTE
--VASLTC
--VASLQD
--VASLQC
--VASLMP
--VASLGF
--VASLEM
-WVASLR-
-WVASLS-
-WVASLL-
-WVASLT-
-WVASLH-
WDVASL--
-WVASLN-
-WVASLM-
WVVASL--
WPVASL--

```
            -WVASLA-
            -WVASLQ-
            -WVASLG-
            -WVASLI-
            WIVASL--
            WGVASL--
            -WVASLE-
            WQVASL--
            WCVASL--
            -YVASLR-
            -YVASLS-
            YGVASL--
            -YVASLH-
            YSVASL--
            YMVASL--
            -YVASLA-
            -YVASLT-
            YTVASL--
            YAVASL--
            -YVASLE-
            YQVASL--
            YPVASL--
            YEVASL--
            YWVASL--
            YDVASL--
            YIVASL--
            -YVASLM-
            -YVASLQ-
            YCVASL--
            YYVASL--
            -YVASLI-
PRAY         394        635     33.06
            AVPRAY--
            DLPRAY--
            IPPRAY--
            -MPRAYP-
            --PRAYGR
            --PRAYGK
            -APRAYY-
            ATPRAY--
            -APRAYL-
            AMPRAY--
            AGPRAY--
            -APRAYM-
            ANPRAY--
            -APRAYE-
            AQPRAY--
            AFPRAY--
            -APRAYV-
            -APRAYH-
```

```
-APRAYF-
ADPRAY--
AAPRAY--
-APRAYI-
-APRAYG-
AEPRAY--
AYPRAY--
-APRAYW-
-APRAYQ-
APPRAY--
AIPRAY--
AHPRAY--
-APRAYN-
ACPRAY--
-CPRAYL-
CTPRAY--
-CPRAYT-
CSPRAY--
-CPRAYR-
CVPRAY--
-CPRAYV-
-CPRAYS-
CEPRAY--
CRPRAY--
CLPRAY--
CKPRAY--
CIPRAY--
CFPRAY--
CQPRAY--
-CPRAYK-
-CPRAYI-
CHPRAY--
CGPRAY--
CDPRAY--
DRPRAY--
-DPRAYL-
-DPRAYI-
DSPRAY--
-DPRAYF-
DDPRAY--
-DPRAYH-
-DPRAYA-
DVPRAY--
DQPRAY--
-DPRAYV-
DMPRAY--
DAPRAY--
DTPRAY--
-DPRAYS-
DNPRAY--
```

DKPRAY--
DYPRAY--
-DPRAYT-
-DPRAYN-
-DPRAYM-
-DPRAYK-
-DPRAYQ-
DHPRAY--
DFPRAY--
-EPRAYR-
ELPRAY--
-EPRAYI-
-EPRAYA-
EYPRAY--
EVPRAY--
-EPRAYL-
ESPRAY--
-EPRAYW-
-EPRAYS-
-EPRAYF-
-EPRAYE-
EKPRAY--
ETPRAY--
-EPRAYV-
-EPRAYK-
EIPRAY--
EHPRAY--
EAPRAY--
EQPRAY--
-EPRAYQ-
-EPRAYM-
-EPRAYG-
ENPRAY--
ERPRAY--
-EPRAYT-
EMPRAY--
EGPRAY--
EFPRAY--
EEPRAY--
FRPRAY--
FDPRAY--
FGPRAY--
FQPRAY--
-FPRAYN-
FVPRAY--
-FPRAYE-
FIPRAY--
FYPRAY--
FPPRAY--
FMPRAY--

FNPRAY--
FKPRAY--
FEPRAY--
FCPRAY--
-FPRAYC-
GNPRAY--
GSPRAY--
GMPRAY--
GFPRAY--
GGPRAY--
GRPRAY--
-GPRAYL-
-GPRAYT-
GLPRAY--
GAPRAY--
-GPRAYA-
GIPRAY--
-GPRAYM-
GYPRAY--
GTPRAY--
-GPRAYS-
GDPRAY--
-GPRAYN-
GEPRAY--
-GPRAYW-
-GPRAYI-
GHPRAY--
-GPRAYQ-
GQPRAY--
-GPRAYV-
-GPRAYG-
-GPRAYY-
-GPRAYH-
-GPRAYE-
-GPRAYD-
GPPRAY--
HSPRAY--
HIPRAY--
HRPRAY--
-HPRAYN-
-HPRAYI-
-HPRAYH-
HNPRAY--
HEPRAY--
HVPRAY--
-HPRAYG-
HYPRAY--
-HPRAYL-
-HPRAYF-
HAPRAY--

```
-HPRAYW-
HMPRAY--
HGPRAY--
-HPRAYE-
HQPRAY--
HKPRAY--
HCPRAY--
ISPRAY--
-IPRAYV-
IVPRAY--
-IPRAYS-
-IPRAYQ-
-IPRAYH-
INPRAY--
IGPRAY--
IAPRAY--
-IPRAYT-
IIPRAY--
-IPRAYG-
IHPRAY--
IDPRAY--
-IPRAYE-
IMPRAY--
-KPRAYL-
-KPRAYV-
KMPRAY--
KQPRAY--
-KPRAYY-
-KPRAYM-
-KPRAYN-
-KPRAYI-
KGPRAY--
KNPRAY--
KRPRAY--
-KPRAYK-
-KPRAYG-
-KPRAYA-
KPPRAY--
-KPRAYF-
KEPRAY--
KDPRAY--
-LPRAYI-
-LPRAYS-
-LPRAYT-
LYPRAY--
LGPRAY--
LAPRAY--
-LPRAYY-
-LPRAYM-
-LPRAYF-
```

-LPRAYQ-
-LPRAYW-
-LPRAYV-
-LPRAYN-
LMPRAY--
LPPRAY--
LEPRAY--
LQPRAY--
LDPRAY--
-LPRAYE-
LHPRAY--
-MPRAYS-
-MPRAYF-
-MPRAYA-
MSPRAY--
MGPRAY--
MVPRAY--
MTPRAY--
MKPRAY--
MMPRAY--
MDPRAY--
-MPRAYY-
-MPRAYI-
MAPRAY--
MQPRAY--
-MPRAYE-
MCPRAY--
MPPRAY--
MIPRAY--
MEPRAY--
-MPRAYT-
MHPRAY--
-MPRAYM-
MFPRAY--
-MPRAYN-
-MPRAYK-
NVPRAY--
NEPRAY--
-NPRAYH-
-NPRAYF-
-NPRAYY-
NNPRAY--
-NPRAYV-
-NPRAYI-
NGPRAY--
NTPRAY--
-NPRAYS-
-NPRAYA-
NDPRAY--
NWPRAY--

NQPRAY--
NHPRAY--
-NPRAYM-
-NPRAYQ-
NPPRAY--
NIPRAY--
NFPRAY--
--PRAYFN
--PRAYTS
--PRAYTL
--PRAYSF
--PRAYKW
--PRAYIS
--PRAYSK
--PRAYYN
--PRAYWA
--PRAYFL
--PRAYYR
--PRAYYI
--PRAYTN
--PRAYSW
PGPRAY--
--PRAYWI
--PRAYNL
--PRAYLD
--PRAYGA
--PRAYQL
--PRAYPF
--PRAYLI
--PRAYDL
--PRAYYM
--PRAYLH
--PRAYTF
--PRAYSQ
--PRAYLT
PRPRAY--
--PRAYVY
--PRAYTY
--PRAYTV
--PRAYRI
--PRAYPL
--PRAYMY
--PRAYIY
--PRAYAT
--PRAYAA
PYPRAY--
--PRAYWM
--PRAYSD
--PRAYLW
--PRAYGI

--PRAYAM
--PRAYAG
--PRAYYV
--PRAYVN
--PRAYSG
--PRAYQT
--PRAYLM
--PRAYAK
PLPRAY--
--PRAYYW
--PRAYYH
--PRAYVF
--PRAYTI
--PRAYMV
--PRAYMF
--PRAYGF
--PRAYFW
--PRAYEM
PIPRAY--
--PRAYYA
--PRAYTK
--PRAYQY
--PRAYNT
--PRAYMQ
--PRAYMA
--PRAYKV
--PRAYKT
--PRAYKA
--PRAYIT
--PRAYII
--PRAYGV
--PRAYAD
PVPRAY--
--PRAYYS
--PRAYVV
--PRAYVE
--PRAYQV
--PRAYQQ
--PRAYLN
--PRAYIW
--PRAYHS
--PRAYHG
--PRAYGT
--PRAYFE
--PRAYEL
--PRAYEF
--PRAYAI
--PRAYYD
--PRAYVM
--PRAYVI

--PRAYTQ
--PRAYRE
--PRAYNM
--PRAYMN
--PRAYIL
--PRAYEG
PNPRAY--
--PRAYYY
--PRAYYQ
--PRAYYL
--PRAYSN
--PRAYSE
--PRAYRM
--PRAYPY
--PRAYLY
--PRAYKF
--PRAYIA
--PRAYHQ
--PRAYGW
--PRAYFI
--PRAYWD
--PRAYTH
--PRAYNA
--PRAYMH
--PRAYMD
--PRAYKE
--PRAYIV
--PRAYIM
--PRAYHA
PMPRAY--
PAPRAY--
--PRAYYG
--PRAYWV
--PRAYWT
--PRAYWF
--PRAYVW
--PRAYPM
--PRAYNV
--PRAYNH
--PRAYMI
--PRAYKH
--PRAYIG
--PRAYIE
--PRAYHW
--PRAYFA
--PRAYES
--PRAYEA
--PRAYAN
--PRAYAH
PEPRAY--

PTPRAY--
--PRAYWG
--PRAYNW
--PRAYNK
--PRAYNF
--PRAYKN
--PRAYHH
--PRAYHD
--PRAYGQ
--PRAYGG
--PRAYFD
--PRAYAY
--PRAYAS
--PRAYAQ
PKPRAY--
PHPRAY--
--PRAYYF
--PRAYWE
--PRAYVK
--PRAYTE
--PRAYTD
--PRAYQG
--PRAYQF
--PRAYNY
--PRAYLQ
--PRAYIF
--PRAYFM
--PRAYET
--PRAYEI
--PRAYAE
PDPRAY--
--PRAYQH
--PRAYQD
--PRAYPN
--PRAYNQ
--PRAYNN
--PRAYNI
--PRAYME
--PRAYIQ
--PRAYIH
--PRAYID
--PRAYHN
--PRAYHI
--PRAYHF
--PRAYHE
--PRAYGM
--PRAYEY
--PRAYEV
--PRAYEN
--PRAYCS

--PRAYCL
--PRAYCG
PQPRAY--
PPPRAY--
QPPRAY--
-QPRAYS-
QIPRAY--
-QPRAYH-
QGPRAY--
-QPRAYN-
-QPRAYK-
-QPRAYA-
QLPRAY--
QYPRAY--
QFPRAY--
-QPRAYT-
-QPRAYI-
QAPRAY--
QVPRAY--
-QPRAYY-
-QPRAYV-
QNPRAY--
-QPRAYW-
-QPRAYF-
QHPRAY--
QTPRAY--
-QPRAYE-
-QPRAYG-
QDPRAY--
QQPRAY--
-QPRAYQ-
QMPRAY--
QKPRAY--
QEPRAY--
-RPRAYS-
-RPRAYV-
RKPRAY--
RHPRAY--
-RPRAYT-
RAPRAY--
RTPRAY--
-RPRAYY-
-RPRAYM-
-RPRAYW-
-RPRAYG-
RNPRAY--
RPPRAY--
-RPRAYA-
RDPRAY--
-RPRAYN-

RIPRAY--
-RPRAYH-
-RPRAYE-
RGPRAY--
-RPRAYC-
RCPRAY--
SSPRAY--
-SPRAYL-
-SPRAYG-
-SPRAYT-
SKPRAY--
SVPRAY--
-SPRAYS-
STPRAY--
-SPRAYM-
SMPRAY--
SAPRAY--
-SPRAYY-
-SPRAYH-
-SPRAYA-
-SPRAYV-
SPPRAY--
SDPRAY--
-SPRAYW-
-SPRAYN-
SFPRAY--
-SPRAYQ-
SHPRAY--
-SPRAYE-
SEPRAY--
-SPRAYC-
SCPRAY--
TGPRAY--
-TPRAYG-
TLPRAY--
TSPRAY--
-TPRAYT-
-TPRAYN-
TVPRAY--
THPRAY--
-TPRAYS-
TIPRAY--
TKPRAY--
TYPRAY--
TNPRAY--
TEPRAY--
-TPRAYM-
TPPRAY--
TAPRAY--
TQPRAY--

TMPRAY--
TDPRAY--
-TPRAYE-
-TPRAYV-
-TPRAYQ-
TCPRAY--
-TPRAYH-
VSPRAY--
-VPRAYS-
-VPRAYA-
VTPRAY--
VLPRAY--
-VPRAYV-
VGPRAY--
VPPRAY--
VQPRAY--
-VPRAYY-
-VPRAYK-
VEPRAY--
VYPRAY--
-VPRAYN-
-VPRAYG-
-VPRAYF-
-VPRAYI-
-VPRAYQ-
-VPRAYE-
VDPRAY--
-VPRAYM-
-VPRAYH-
VHPRAY--
VCPRAY--
-WPRAYV-
WMPRAY--
WGPRAY--
WTPRAY--
WRPRAY--
WYPRAY--
WLPRAY--
-WPRAYN-
WKPRAY--
WEPRAY--
-WPRAYW-
WQPRAY--
-WPRAYY-
WPPRAY--
-WPRAYF-
WAPRAY--
-YPRAYN-
-YPRAYE-
-YPRAYW-

YNPRAY--
YQPRAY--
YTPRAY--
-YPRAYM-
YHPRAY--
YAPRAY--
-YPRAYI-
-YPRAYH-
YYPRAY--
-YPRAYV-
YPPRAY--
YKPRAY--
YVPRAY--
YGPRAY--
-YPRAYF-
YDPRAY--
-YPRAYA-

| | | | |
|---|---|---|---|
| PLPI | 114 | 759 | 33.05 |

DTPLPI--
--PLPIPG
--PLPIGT
-QPLPIR-
-SPLPIP-
-VPLPIG-
-APLPIF-
ATPLPI--
ARPLPI--
-APLPIT-
-APLPIS-
AVPLPI--
ASPLPI--
-APLPIL-
APPLPI--
-APLPIR-
-APLPIH-
AKPLPI--
ALPLPI--
AFPLPI--
ANPLPI--
AIPLPI--
AWPLPI--
-APLPIW-
AMPLPI--
-APLPIY-
-APLPIM-
-APLPII-
AHPLPI--
AGPLPI--
AYPLPI--
-APLPIQ-

-APLPIN-
-APLPIA-
AEPLPI--
AQPLPI--
-APLPIV-
-APLPIG-
-APLPIE-
-CPLPIH-
CSPLPI--
CVPLPI--
CRPLPI--
-CPLPIM-
-CPLPIL-
-CPLPII-
-CPLPIA-
CIPLPI--
CCPLPI--
-DPLPIL-
-DPLPIW-
DVPLPI--
DRPLPI--
-DPLPIS-
DAPLPI--
-DPLPIY-
DYPLPI--
DWPLPI--
DQPLPI--
-DPLPIR-
-DPLPIQ-
DLPLPI--
DHPLPI--
DNPLPI--
-DPLPIT-
-DPLPIN-
-DPLPIM-
-DPLPII-
DMPLPI--
DIPLPI--
DFPLPI--
-EPLPIT-
-EPLPIR-
-EPLPIV-
EVPLPI--
-EPLPIL-
ENPLPI--
ELPLPI--
EAPLPI--
EWPLPI--
ESPLPI--
-EPLPIY-

-EPLPIW-
-EPLPIS-
EIPLPI--
EHPLPI--
EGPLPI--
ETPLPI--
-EPLPIM-
-EPLPIA-
EFPLPI--
EDPLPI--
-FPLPIR-
FRPLPI--
FGPLPI--
FHPLPI--
FSPLPI--
-FPLPIW-
FMPLPI--
FDPLPI--
FWPLPI--
FTPLPI--
-FPLPIQ-
-FPLPIV-
FIPLPI--
FFPLPI--
FYPLPI--
FQPLPI--
FPPLPI--
-FPLPIS-
-FPLPIM-
-FPLPIL-
-FPLPIK-
-FPLPIH-
-FPLPIF-
-FPLPIA-
FNPLPI--
FKPLPI--
FEPLPI--
-FPLPIT-
FAPLPI--
GRPLPI--
-GPLPIW-
GFPLPI--
GTPLPI--
GGPLPI--
GYPLPI--
-GPLPIM-
GMPLPI--
GSPLPI--
GQPLPI--
-GPLPIR-

GAPLPI--
GVPLPI--
-GPLPIL-
-GPLPIK-
-GPLPIG-
-GPLPIE-
-GPLPIA-
GIPLPI--
GPPLPI--
-GPLPIT-
GKPLPI--
GWPLPI--
-GPLPIV-
-GPLPIS-
GDPLPI--
-GPLPIN-
-GPLPII-
-GPLPIH-
GEPLPI--
GCPLPI--
HRPLPI--
HSPLPI--
-HPLPIL-
-HPLPIS-
HFPLPI--
-HPLPIY-
HTPLPI--
-HPLPIM-
-HPLPIT-
-HPLPIN-
HLPLPI--
HHPLPI--
HWPLPI--
HPPLPI--
-HPLPIW-
-HPLPIV-
-HPLPIF-
-HPLPIA-
HYPLPI--
HQPLPI--
-HPLPIQ-
-HPLPII-
-HPLPIH-
HMPLPI--
HKPLPI--
HIPLPI--
HDPLPI--
IWPLPI--
ILPLPI--
-IPLPIK-

IRPLPI--
-IPLPIV-
-IPLPIA-
IYPLPI--
ISPLPI--
-IPLPIS-
IKPLPI--
IPPLPI--
-IPLPIT-
-IPLPIL-
IGPLPI--
IFPLPI--
-IPLPIN-
-IPLPIF-
IMPLPI--
IVPLPI--
-IPLPIM-
-IPLPII-
INPLPI--
IIPLPI--
ITPLPI--
IHPLPI--
-KPLPIR-
-KPLPIS-
KWPLPI--
KGPLPI--
-KPLPIN-
KTPLPI--
KQPLPI--
-KPLPII-
-KPLPIH-
KAPLPI--
KYPLPI--
KVPLPI--
-KPLPIG-
KNPLPI--
KIPLPI--
KFPLPI--
-KPLPIV-
-KPLPIK-
-KPLPIM-
-KPLPIA-
KLPLPI--
-KPLPIY-
-KPLPIQ-
-KPLPIE-
KMPLPI--
KKPLPI--
-KPLPIT-
KHPLPI--

KEPLPI--
KDPLPI--
LSPLPI--
LGPLPI--
-LPLPIL-
LVPLPI--
-LPLPIM-
LNPLPI--
LMPLPI--
LLPLPI--
LIPLPI--
LAPLPI--
LWPLPI--
-LPLPIS-
LKPLPI--
-LPLPIH-
LYPLPI--
LTPLPI--
-LPLPIV-
-LPLPIT-
-LPLPIF-
LDPLPI--
-LPLPIY-
-LPLPIA-
LEPLPI--
-LPLPIQ-
-LPLPIK-
-LPLPII-
LFPLPI--
LPPLPI--
-LPLPIN-
LHPLPI--
MSPLPI--
-MPLPIH-
MRPLPI--
MPPLPI--
-MPLPIY-
-MPLPIL-
-MPLPIA-
MLPLPI--
MKPLPI--
MQPLPI--
-MPLPII-
MEPLPI--
-MPLPIS-
MMPLPI--
MDPLPI--
MTPLPI--
-MPLPIW-
-MPLPIV-

-MPLPIR-
MFPLPI--
MAPLPI--
-MPLPIT-
-MPLPIQ-
-MPLPIM-
MNPLPI--
MGPLPI--
-MPLPIE-
MIPLPI--
MHPLPI--
-NPLPIT-
NTPLPI--
NRPLPI--
-NPLPII-
NGPLPI--
-NPLPIS-
-NPLPIV-
NIPLPI--
NSPLPI--
NKPLPI--
NFPLPI--
-NPLPIR-
-NPLPIA-
NWPLPI--
NNPLPI--
-NPLPIK-
-NPLPIH-
NVPLPI--
NPPLPI--
-NPLPIY-
-NPLPIN-
NHPLPI--
NQPLPI--
NMPLPI--
NEPLPI--
--PLPILH
--PLPIVG
--PLPIGF
--PLPIRT
--PLPITR
--PLPILS
--PLPIRG
--PLPIYL
--PLPITL
--PLPIRS
--PLPIRA
--PLPIMG
--PLPIFM
--PLPIWR

--PLPITA
--PLPIAR
--PLPIAL
--PLPIVR
--PLPIVD
--PLPISR
--PLPILN
--PLPIIR
--PLPIRI
--PLPILW
--PLPIGS
--PLPIAF
--PLPIYK
--PLPIVL
--PLPITK
--PLPITD
--PLPIMW
--PLPILG
--PLPIIS
--PLPIIL
--PLPIFT
--PLPIVK
--PLPISV
--PLPIRN
--PLPIMQ
--PLPIYS
--PLPIYQ
--PLPIWG
--PLPIVH
--PLPITS
--PLPIST
--PLPISS
--PLPISI
--PLPIRV
--PLPIRK
--PLPIRE
--PLPIRD
--PLPINL
--PLPIMT
--PLPIHN
--PLPIFH
PRPLPI--
--PLPIWT
--PLPIWS
--PLPITG
--PLPISQ
--PLPISM
--PLPIRF
--PLPILK
--PLPILA

--PLPIKV
--PLPIKD
--PLPIHS
--PLPIFS
--PLPIEL
--PLPIVS
--PLPIQM
--PLPIQG
--PLPIPW
--PLPIMK
--PLPIMD
--PLPILV
--PLPIKT
--PLPIKS
--PLPIKI
--PLPIIW
--PLPIHV
--PLPIFK
--PLPIFG
--PLPIFA
--PLPIAI
PKPLPI--
PDPLPI--
--PLPIYM
--PLPIWL
--PLPIVW
--PLPITY
--PLPITQ
--PLPISY
--PLPIRP
--PLPIQV
--PLPIQS
--PLPINS
--PLPIMR
--PLPILE
--PLPIKN
--PLPIKE
--PLPIIQ
--PLPIHL
--PLPIHG
--PLPIGG
--PLPIFR
--PLPIAT
--PLPIAN
--PLPIAA
PYPLPI--
PWPLPI--
--PLPIYY
--PLPIYG
--PLPIWM

--PLPIWH
--PLPIVT
--PLPITW
--PLPITT
--PLPITF
--PLPISF
--PLPIQR
--PLPINQ
--PLPINA
--PLPIMM
--PLPIKF
--PLPIIY
--PLPIIV
--PLPIII
--PLPIIG
--PLPIIA
--PLPIHR
--PLPIGV
--PLPIGI
--PLPIFN
--PLPIFD
--PLPIAK
--PLPIYV
--PLPIYN
--PLPIYH
--PLPIWW
--PLPIWV
--PLPIWQ
--PLPIWN
--PLPIWA
--PLPIVI
--PLPIVF
--PLPITH
--PLPISW
--PLPISN
--PLPISH
--PLPIRH
--PLPIQW
--PLPIQT
--PLPIQQ
--PLPIQK
--PLPIQE
--PLPIQD
--PLPIPV
--PLPIPM
--PLPIPI
--PLPINV
--PLPIMV
--PLPIMN
--PLPIMH

--PLPILM
--PLPIKY
--PLPIKQ
--PLPIIK
--PLPIIE
--PLPIHT
--PLPIHQ
--PLPIHH
--PLPIGM
--PLPIGH
--PLPIGA
--PLPIFY
--PLPIFV
--PLPIFE
--PLPIEW
--PLPIES
--PLPIAS
--PLPIAQ
--PLPIAD
PEPLPI--
-PPLPII-
PNPLPI--
PMPLPI--
--PLPIYA
--PLPIWY
--PLPIWK
--PLPIWI
--PLPIWE
--PLPIVV
--PLPIVQ
--PLPIVE
--PLPITV
--PLPITN
--PLPITM
--PLPISD
--PLPISA
--PLPIRY
--PLPIRQ
--PLPIQI
--PLPIQA
--PLPIPY
--PLPIPQ
--PLPINY
--PLPINW
--PLPINT
--PLPINK
--PLPIMP
--PLPIMF
--PLPIME
--PLPIMA

--PLPILY
--PLPILI
--PLPILC
--PLPIKK
--PLPIKH
--PLPIKG
--PLPIKA
--PLPIIT
--PLPIIN
--PLPIIH
--PLPIHM
--PLPIHK
--PLPIHI
--PLPIHA
--PLPIGW
--PLPIGQ
--PLPIFF
--PLPIEY
--PLPIEI
--PLPIEF
--PLPIDL
--PLPIAY
--PLPIAW
--PLPIAV
--PLPIAM
--PLPIAG
PHPLPI--
QSPLPI--
QMPLPI--
-QPLPIK-
QRPLPI--
-QPLPII-
QTPLPI--
QYPLPI--
QGPLPI--
QDPLPI--
QAPLPI--
QWPLPI--
QIPLPI--
-QPLPIL-
QEPLPI--
QQPLPI--
-QPLPIQ-
-QPLPIM-
-QPLPIH-
QLPLPI--
QVPLPI--
-QPLPIY-
-QPLPIS-
-QPLPIG-

-QPLPIF-
-QPLPIE-
-QPLPIA-
QKPLPI--
QHPLPI--
QFPLPI--
RVPLPI--
RIPLPI--
RGPLPI--
-RPLPIV-
-RPLPIK-
RLPLPI--
RSPLPI--
RQPLPI--
-RPLPIN-
-RPLPIS-
-RPLPIM-
-RPLPIQ-
RYPLPI--
RWPLPI--
RTPLPI--
-RPLPIG-
-RPLPIA-
RAPLPI--
RNPLPI--
RFPLPI--
RPPLPI--
-RPLPIE-
RHPLPI--
-RPLPII-
REPLPI--
-RPLPIH-
RDPLPI--
SRPLPI--
SVPLPI--
SQPLPI--
-SPLPIY-
-SPLPIA-
SWPLPI--
SSPLPI--
-SPLPIS-
-SPLPIT-
SKPLPI--
SGPLPI--
-SPLPIR-
SIPLPI--
SAPLPI--
SYPLPI--
SLPLPI--
-SPLPIN-

-SPLPII-
SFPLPI--
-SPLPIF-
STPLPI--
-SPLPIW-
-SPLPIM-
SNPLPI--
SDPLPI--
-SPLPIK-
-SPLPIE-
SCPLPI--
-SPLPIV-
-SPLPIQ-
SHPLPI--
SEPLPI--
TRPLPI--
-TPLPIL-
-TPLPIR-
-TPLPIW-
-TPLPIH-
TYPLPI--
TSPLPI--
TNPLPI--
TWPLPI--
-TPLPIY-
-TPLPIT-
-TPLPII-
TIPLPI--
TFPLPI--
TAPLPI--
-TPLPIV-
-TPLPIQ-
-TPLPIM-
-TPLPIF-
TMPLPI--
TKPLPI--
TEPLPI--
TPPLPI--
-TPLPIK-
TGPLPI--
TTPLPI--
-TPLPIG-
-TPLPIA-
TDPLPI--
TQPLPI--
-TPLPIN-
THPLPI--
VRPLPI--
-VPLPIL-
-VPLPIT-

VLPLPI--
VVPLPI--
-VPLPIV-
VHPLPI--
VGPLPI--
VQPLPI--
VDPLPI--
-VPLPIS-
-VPLPIR-
VNPLPI--
VIPLPI--
VYPLPI--
VPPLPI--
VMPLPI--
VSPLPI--
-VPLPIW-
-VPLPIM-
VKPLPI--
VTPLPI--
-VPLPII-
VEPLPI--
-VPLPIH-
-VPLPIF-
-VPLPIE-
-VPLPIA-
-VPLPIY-
-VPLPIN-
-VPLPIK-
VFPLPI--
-WPLPIT-
-WPLPIL-
-WPLPIK-
WLPLPI--
-WPLPIS-
WTPLPI--
WGPLPI--
WFPLPI--
WEPLPI--
WAPLPI--
WWPLPI--
-WPLPIY-
WMPLPI--
WKPLPI--
WVPLPI--
WSPLPI--
WQPLPI--
WPPLPI--
-WPLPIQ-
-WPLPII-
-WPLPIE-

| | | | |
|---|---|---|---|
| | WIPLPI-- | | |
| | WYPLPI-- | | |
| | -WPLPIH- | | |
| | WNPLPI-- | | |
| | -YPLPIR- | | |
| | YRPLPI-- | | |
| | -YPLPIF- | | |
| | YAPLPI-- | | |
| | -YPLPIL- | | |
| | YHPLPI-- | | |
| | YSPLPI-- | | |
| | -YPLPIS- | | |
| | YLPLPI-- | | |
| | -YPLPIG- | | |
| | -YPLPIK- | | |
| | YIPLPI-- | | |
| | -YPLPIV- | | |
| | -YPLPIH- | | |
| | -YPLPIA- | | |
| | YWPLPI-- | | |
| | YVPLPI-- | | |
| | YQPLPI-- | | |
| | YPPLPI-- | | |
| | -YPLPIY- | | |
| | YKPLPI-- | | |
| | -YPLPIQ- | | |
| | YDPLPI-- | | |
| PSML | 180 | 726 | 33.02 |
| | FKPSML-- | | |
| | -GPSMLG- | | |
| | -NPSMLA- | | |
| | --PSMLNR | | |
| | --PSMLEL | | |
| | --PSMLAH | | |
| | -SPSMLG- | | |
| | -YPSMLG- | | |
| | -APSMLR- | | |
| | ASPSML-- | | |
| | ARPSML-- | | |
| | -APSMLS- | | |
| | ALPSML-- | | |
| | AMPSML-- | | |
| | AGPSML-- | | |
| | AVPSML-- | | |
| | AWPSML-- | | |
| | AHPSML-- | | |
| | AQPSML-- | | |
| | ADPSML-- | | |
| | AAPSML-- | | |
| | -APSMLV- | | |

-APSMLM-
-APSMLT-
ANPSML--
AEPSML--
-APSMLY-
AYPSML--
-APSMLH-
AKPSML--
AFPSML--
-APSMLE-
APPSML--
-APSMLN-
ACPSML--
CRPSML--
CEPSML--
CAPSML--
CQPSML--
-CPSMLR-
-CPSMLT-
CPPSML--
CHPSML--
CVPSML--
CTPSML--
CSPSML--
-CPSMLL-
CKPSML--
-CPSMLQ-
-CPSMLM-
-CPSMLI-
-CPSMLH-
-CPSMLF-
CCPSML--
-CPSMLY-
-CPSMLN-
-CPSMLK-
CNPSML--
CIPSML--
CGPSML--
CDPSML--
DRPSML--
DWPSML--
DSPSML--
DMPSML--
DFPSML--
-DPSMLR-
DLPSML--
-DPSMLT-
-DPSMLQ-
-DPSMLK-
DKPSML--

DGPSML--
DAPSML--
DTPSML--
-DPSMLV-
-DPSMLN-
-DPSMLI-
DIPSML--
-DPSMLY-
DNPSML--
-DPSMLH-
-DPSMLM-
-DPSMLA-
DPPSML--
DCPSML--
-EPSMLT-
-EPSMLS-
ELPSML--
ERPSML--
EKPSML--
-EPSMLL-
EFPSML--
EYPSML--
-EPSMLF-
EEPSML--
-EPSMLV-
-EPSMLN-
-EPSMLI-
ETPSML--
-EPSMLY-
-EPSMLR-
-EPSMLM-
EGPSML--
EWPSML--
EQPSML--
-EPSMLK-
-EPSMLA-
ENPSML--
EHPSML--
-EPSMLW-
-EPSMLH-
EAPSML--
-EPSMLQ-
EIPSML--
EDPSML--
FRPSML--
FLPSML--
-FPSMLR-
-FPSMLK-
FDPSML--
-FPSMLM-

FSPSML--
-FPSMLT-
FHPSML--
FGPSML--
-FPSMLI-
-FPSMLS-
FVPSML--
FIPSML--
FQPSML--
-FPSMLQ-
-FPSMLH-
FNPSML--
FMPSML--
FCPSML--
FPPSML--
-FPSMLV-
FEPSML--
FFPSML--
FYPSML--
-GPSMLR-
-GPSMLM-
GGPSML--
-GPSMLS-
GVPSML--
-GPSMLK-
GTPSML--
GRPSML--
-GPSMLL-
GKPSML--
GAPSML--
-GPSMLF-
GHPSML--
GSPSML--
-GPSMLW-
GMPSML--
GQPSML--
-GPSMLH-
GNPSML--
GWPSML--
-GPSMLY-
GYPSML--
-GPSMLV-
GEPSML--
GPPSML--
-GPSMLI-
-GPSMLN-
-GPSMLE-
GDPSML--
GCPSML--
HRPSML--

-HPSMLL-
-HPSMLR-
HVPSML--
HNPSML--
HSPSML--
-HPSMLT-
HTPSML--
-HPSMLK-
-HPSMLH-
HFPSML--
HDPSML--
HIPSML--
-HPSMLS-
HGPSML--
-HPSMLY-
-HPSMLQ-
-HPSMLN-
HEPSML--
HYPSML--
HWPSML--
-HPSMLI-
-HPSMLM-
HKPSML--
HHPSML--
HQPSML--
-HPSMLV-
-HPSMLF-
-HPSMLW-
HPPSML--
HMPSML--
HCPSML--
-IPSMLR-
-IPSMLL-
-IPSMLV-
-IPSMLT-
IGPSML--
-IPSMLQ-
IFPSML--
IAPSML--
ISPSML--
-IPSMLK-
IKPSML--
IEPSML--
ITPSML--
-IPSMLA-
INPSML--
IYPSML--
IVPSML--
-IPSMLM-
IQPSML--

-IPSMLE-
IDPSML--
-IPSMLI-
-IPSMLH-
IPPSML--
IMPSML--
KSPSML--
KYPSML--
-KPSMLR-
KIPSML--
-KPSMLL-
-KPSMLA-
KLPSML--
KTPSML--
-KPSMLM-
KPPSML--
KWPSML--
-KPSMLY-
KVPSML--
KAPSML--
KNPSML--
-KPSMLW-
-KPSMLV-
-KPSMLT-
-KPSMLF-
KDPSML--
KCPSML--
KQPSML--
KMPSML--
-KPSMLN-
-KPSMLK-
-KPSMLI-
-KPSMLQ-
KHPSML--
KEPSML--
-KPSMLH-
-KPSMLG-
-KPSMLE-
LRPSML--
LLPSML--
LGPSML--
LSPSML--
-LPSMLS-
LVPSML--
LTPSML--
-LPSMLL-
LYPSML--
LKPSML--
-LPSMLK-
LEPSML--

−LPSMLI−
LQPSML−−
−LPSMLM−
LNPSML−−
LFPSML−−
LHPSML−−
−LPSMLT−
LPPSML−−
−LPSMLQ−
−LPSMLH−
−LPSMLN−
−LPSMLA−
LCPSML−−
−LPSMLF−
MRPSML−−
MPPSML−−
MDPSML−−
−MPSMLT−
MVPSML−−
MTPSML−−
−MPSMLV−
−MPSMLR−
MGPSML−−
MQPSML−−
−MPSMLN−
MKPSML−−
−MPSMLM−
−MPSMLF−
MHPSML−−
MFPSML−−
−MPSMLH−
MAPSML−−
MYPSML−−
MNPSML−−
MMPSML−−
MLPSML−−
MEPSML−−
−MPSMLW−
NLPSML−−
NKPSML−−
NAPSML−−
NYPSML−−
−NPSMLL−
−NPSMLS−
NSPSML−−
NVPSML−−
NNPSML−−
NFPSML−−
NEPSML−−
−NPSMLY−

NGPSML--
-NPSMLQ-
NQPSML--
-NPSMLW-
-NPSMLN-
-NPSMLH-
NCPSML--
-NPSMLT-
NDPSML--
NHPSML--
-NPSMLK-
-NPSMLI-
NMPSML--
-NPSMLV-
-NPSMLM-
-NPSMLE-
--PSMLHG
--PSMLRW
--PSMLKS
--PSMLLS
--PSMLRV
--PSMLRS
--PSMLTG
--PSMLTR
--PSMLRH
--PSMLIN
--PSMLFS
--PSMLVR
--PSMLSS
--PSMLRM
--PSMLMT
--PSMLLK
--PSMLGS
--PSMLWS
--PSMLVS
--PSMLKV
--PSMLIS
--PSMLYM
--PSMLTS
--PSMLSI
--PSMLQT
--PSMLNT
--PSMLAW
--PSMLTL
--PSMLAY
--PSMLAS
--PSMLYI
--PSMLVT
--PSMLTY
--PSMLRI

--PSMLNV
--PSMLLH
--PSMLKR
--PSMLKI
--PSMLIT
--PSMLIA
PSPSML--
--PSMLSN
--PSMLSA
--PSMLNS
--PSMLKL
--PSMLVI
--PSMLTI
--PSMLSG
--PSMLQS
--PSMLLN
--PSMLIW
--PSMLVM
--PSMLQG
--PSMLNW
--PSMLYG
--PSMLTM
--PSMLMS
--PSMLHV
--PSMLFH
--PSMLAT
--PSMLAL
PTPSML--
--PSMLYN
--PSMLWT
--PSMLWH
--PSMLTT
--PSMLKK
--PSMLGL
PVPSML--
--PSMLTH
--PSMLRF
--PSMLNL
--PSMLKF
--PSMLKD
--PSMLHL
PAPSML--
PYPSML--
--PSMLVQ
--PSMLSY
--PSMLRE
--PSMLLE
--PSMLKQ
--PSMLIK
--PSMLEA

PWPSML--
--PSMLYH
--PSMLVN
--PSMLQY
--PSMLKT
--PSMLIG
--PSMLHT
--PSMLHN
--PSMLHM
--PSMLAA
PIPSML--
PEPSML--
--PSMLYK
--PSMLTK
--PSMLTA
--PSMLQK
--PSMLNI
--PSMLME
--PSMLHF
--PSMLEG
PNPSML--
PHPSML--
--PSMLYT
--PSMLWN
--PSMLWG
--PSMLVK
--PSMLTV
--PSMLTD
--PSMLQN
--PSMLQM
--PSMLIE
--PSMLGY
--PSMLGW
--PSMLES
PRPSML--
--PSMLYD
--PSMLWK
--PSMLVH
--PSMLSH
--PSMLPF
--PSMLMQ
--PSMLMK
--PSMLMF
--PSMLMD
--PSMLMA
--PSMLIH
--PSMLHW
--PSMLHS
--PSMLHA
--PSMLAI

--PSMLYQ
--PSMLYE
--PSMLVW
--PSMLVD
--PSMLTQ
--PSMLSW
--PSMLQV
--PSMLQQ
--PSMLPM
--PSMLNG
--PSMLNF
--PSMLMI
--PSMLMG
--PSMLLD
--PSMLKY
--PSMLKH
--PSMLIQ
--PSMLGA
--PSMLFW
--PSMLFN
--PSMLFG
--PSMLNM
--PSMLMN
--PSMLKE
--PSMLGT
--PSMLAN
PMPSML--
PFPSML--
--PSMLWE
--PSMLSD
--PSMLQL
--PSMLQH
--PSMLQD
--PSMLNN
--PSMLNH
--PSMLKW
--PSMLII
--PSMLID
--PSMLHI
--PSMLGI
--PSMLFE
--PSMLFD
--PSMLAM
--PSMLYW
--PSMLWQ
--PSMLVP
--PSMLVE
--PSMLTE
--PSMLSE
--PSMLQW

```
--PSMLQI
--PSMLPY
--PSMLPW
--PSMLPT
--PSMLPI
--PSMLNQ
--PSMLNK
--PSMLMM
--PSMLKN
--PSMLKM
--PSMLIC
--PSMLHQ
--PSMLHH
--PSMLGM
--PSMLEW
--PSMLEV
--PSMLET
--PSMLEM
--PSMLCP
--PSMLCE
--PSMLAF
--PSMLAE
-PPSMLS-
-PPSMLL-
-QPSMLL-
QRPSML--
-QPSMLR-
-QPSMLT-
-QPSMLV-
-QPSMLS-
QSPSML--
QLPSML--
QIPSML--
QFPSML--
QYPSML--
-QPSMLN-
-QPSMLK-
-QPSMLH-
QNPSML--
QGPSML--
-QPSMLM-
-QPSMLI-
QPPSML--
QMPSML--
QHPSML--
QDPSML--
QAPSML--
QWPSML--
QVPSML--
QQPSML--
```

QEPSML--
QTPSML--
-QPSMLY-
-QPSMLW-
-QPSMLG-
QCPSML--
-QPSMLQ-
QKPSML--
RYPSML--
RSPSML--
RRPSML--
RGPSML--
RWPSML--
RAPSML--
-RPSMLV-
-RPSMLY-
RQPSML--
-RPSMLM-
-RPSMLF-
-RPSMLQ-
-RPSMLI-
RFPSML--
-RPSMLA-
-RPSMLK-
RPPSML--
-RPSMLH-
-RPSMLN-
RNPSML--
RHPSML--
RDPSML--
RCPSML--
-RPSMLE-
REPSML--
-RPSMLC-
-SPSMLR-
SLPSML--
-SPSMLV-
SKPSML--
SYPSML--
-SPSMLK-
-SPSMLI-
SWPSML--
SAPSML--
SQPSML--
-SPSMLN-
SEPSML--
SHPSML--
-SPSMLM-
-SPSMLQ-
SPPSML--

−SPSMLT−
−SPSMLW−
SCPSML−−
TRPSML−−
−TPSMLR−
TVPSML−−
−TPSMLN−
−TPSMLS−
−TPSMLH−
TWPSML−−
−TPSMLV−
−TPSMLY−
TSPSML−−
TAPSML−−
TDPSML−−
−TPSMLK−
TMPSML−−
TEPSML−−
−TPSMLA−
TPPSML−−
TKPSML−−
TFPSML−−
−TPSMLM−
TIPSML−−
THPSML−−
TQPSML−−
−TPSMLF−
−TPSMLQ−
TCPSML−−
VTPSML−−
−VPSMLR−
VLPSML−−
VIPSML−−
−VPSMLQ−
VEPSML−−
VAPSML−−
−VPSMLK−
VKPSML−−
VFPSML−−
VGPSML−−
VNPSML−−
−VPSMLA−
VDPSML−−
VYPSML−−
VMPSML−−
−VPSMLV−
−VPSMLM−
VPPSML−−
VHPSML−−
VVPSML−−

-VPSMLH-
VWPSML--
-VPSMLW-
-VPSMLN-
VQPSML--
-VPSMLG-
-VPSMLE-
VCPSML--
WYPSML--
WDPSML--
-WPSMLR-
-WPSMLV-
-WPSMLS-
WWPSML--
WLPSML--
WQPSML--
WIPSML--
-WPSMLT-
WGPSML--
WEPSML--
WNPSML--
-WPSMLI-
-WPSMLK-
WVPSML--
-WPSMLA-
-WPSMLM-
-WPSMLQ-
-WPSMLN-
WMPSML--
YTPSML--
-YPSMLL-
YGPSML--
YYPSML--
-YPSMLS-
YMPSML--
-YPSMLQ-
YHPSML--
YIPSML--
YFPSML--
YNPSML--
YLPSML--
YVPSML--
-YPSMLV-
-YPSMLT-
-YPSMLK-
YPPSML--
-YPSMLY-
YWPSML--
-YPSMLN-
YDPSML--

YQPSML--
-YPSMLM-
-YPSMLW-
-YPSMLE-

| | | | |
|---|---|---|---|
| PYGL | 59 | 796 | 33.00 |

FNPYGL--
-GPYGLG-
--PYGLSE
--PYGLSD
--PYGLNR
--PYGLMN
--PYGLGN
--PYGLDG
--PYGLAN
-SPYGLG-
-VPYGLD-
-WPYGLG-
AYPYGL--
-APYGLR-
AVPYGL--
-APYGLQ-
-APYGLL-
AWPYGL--
-APYGLT-
AGPYGL--
-APYGLN-
ATPYGL--
ARPYGL--
-APYGLK-
ANPYGL--
-APYGLA-
-APYGLY-
AKPYGL--
AFPYGL--
ADPYGL--
-APYGLS-
AIPYGL--
-APYGLE-
AMPYGL--
-APYGLG-
AQPYGL--
-APYGLM-
-APYGLH-
-CPYGLY-
CPPYGL--
CYPYGL--
CVPYGL--
CHPYGL--
CNPYGL--
CLPYGL--

CTPYGL--
CSPYGL--
-CPYGLV-
-CPYGLT-
-CPYGLR-
-CPYGLN-
-CPYGLL-
CFPYGL--
CCPYGL--
CAPYGL--
DIPYGL--
DSPYGL--
-DPYGLR-
DRPYGL--
DLPYGL--
DNPYGL--
DVPYGL--
-DPYGLL-
-DPYGLV-
-DPYGLS-
-DPYGLH-
DFPYGL--
DYPYGL--
DQPYGL--
-DPYGLW-
-DPYGLN-
DKPYGL--
-DPYGLY-
-DPYGLT-
DMPYGL--
DWPYGL--
DTPYGL--
-DPYGLQ-
-DPYGLM-
-DPYGLK-
DEPYGL--
EVPYGL--
-EPYGLL-
-EPYGLR-
-EPYGLF-
ERPYGL--
-EPYGLS-
EFPYGL--
-EPYGLK-
ESPYGL--
EQPYGL--
-EPYGLQ-
-EPYGLH-
EIPYGL--
EAPYGL--

```
-EPYGLY-
-EPYGLW-
ENPYGL--
ELPYGL--
EMPYGL--
EWPYGL--
ETPYGL--
-EPYGLV-
-EPYGLT-
-EPYGLM-
-EPYGLG-
-EPYGLA-
EDPYGL--
-EPYGLN-
EKPYGL--
EHPYGL--
ECPYGL--
FTPYGL--
-FPYGLR-
FSPYGL--
FRPYGL--
FGPYGL--
FHPYGL--
FYPYGL--
-FPYGLS-
-FPYGLK-
FIPYGL--
-FPYGLY-
-FPYGLN-
-FPYGLH-
FKPYGL--
FMPYGL--
-FPYGLQ-
FAPYGL--
-FPYGLT-
-FPYGLL-
FFPYGL--
FEPYGL--
FVPYGL--
-FPYGLE-
FPPYGL--
FQPYGL--
FDPYGL--
-FPYGLI-
-FPYGLG-
-FPYGLV-
-FPYGLF-
-FPYGLA-
-GPYGLR-
GVPYGL--
```

GRPYGL--
-GPYGLL-
GSPYGL--
GMPYGL--
-GPYGLV-
GLPYGL--
-GPYGLM-
-GPYGLA-
GYPYGL--
GTPYGL--
-GPYGLW-
-GPYGLK-
-GPYGLH-
GGPYGL--
-GPYGLY-
GNPYGL--
GHPYGL--
GDPYGL--
-GPYGLI-
GFPYGL--
GWPYGL--
GCPYGL--
-GPYGLQ-
-GPYGLN-
GEPYGL--
GAPYGL--
GIPYGL--
-GPYGLE-
-GPYGLF-
GPPYGL--
HIPYGL--
HLPYGL--
HNPYGL--
-HPYGLR-
HVPYGL--
-HPYGLS-
-HPYGLI-
HKPYGL--
HPPYGL--
HSPYGL--
HRPYGL--
-HPYGLT-
-HPYGLN-
-HPYGLH-
HHPYGL--
HWPYGL--
-HPYGLY-
HYPYGL--
-HPYGLG-
HQPYGL--

−HPYGLF−
−HPYGLM−
HMPYGL−−
HTPYGL−−
−HPYGLQ−
HDPYGL−−
−HPYGLK−
−HPYGLA−
HFPYGL−−
HEPYGL−−
IRPYGL−−
−IPYGLL−
−IPYGLR−
−IPYGLS−
IHPYGL−−
−IPYGLQ−
IVPYGL−−
−IPYGLK−
−IPYGLI−
IKPYGL−−
−IPYGLV−
−IPYGLT−
ILPYGL−−
IPPYGL−−
IGPYGL−−
−IPYGLF−
−IPYGLA−
IEPYGL−−
IYPYGL−−
IWPYGL−−
ITPYGL−−
−IPYGLY−
−IPYGLW−
−IPYGLH−
−IPYGLM−
IAPYGL−−
−IPYGLG−
−IPYGLE−
INPYGL−−
IIPYGL−−
IFPYGL−−
KSPYGL−−
KRPYGL−−
KTPYGL−−
KLPYGL−−
KFPYGL−−
KWPYGL−−
−KPYGLN−
−KPYGLY−
−KPYGLS−

-KPYGLR-
-KPYGLH-
-KPYGLV-
-KPYGLL-
KPPYGL--
KYPYGL--
-KPYGLW-
KNPYGL--
KGPYGL--
-KPYGLE-
KEPYGL--
-KPYGLM-
-KPYGLF-
-KPYGLQ-
KDPYGL--
KCPYGL--
KAPYGL--
KQPYGL--
-KPYGLK-
-KPYGLA-
KMPYGL--
LVPYGL--
-LPYGLS-
LAPYGL--
LTPYGL--
LQPYGL--
LGPYGL--
LWPYGL--
LFPYGL--
-LPYGLM-
-LPYGLL-
LLPYGL--
-LPYGLY-
LNPYGL--
-LPYGLK-
LIPYGL--
LYPYGL--
-LPYGLN-
LEPYGL--
-LPYGLF-
-LPYGLA-
LMPYGL--
LHPYGL--
LPPYGL--
LDPYGL--
-LPYGLI-
-LPYGLQ-
-LPYGLH-
-LPYGLE-
MKPYGL--

MRPYGL--
MIPYGL--
MNPYGL--
MHPYGL--
-MPYGLL-
-MPYGLI-
-MPYGLR-
MVPYGL--
-MPYGLK-
MSPYGL--
-MPYGLN-
-MPYGLW-
MLPYGL--
MAPYGL--
MTPYGL--
MQPYGL--
-MPYGLA-
-MPYGLT-
-MPYGLM-
MEPYGL--
-MPYGLE-
MGPYGL--
-MPYGLV-
MPPYGL--
MMPYGL--
-MPYGLG-
MFPYGL--
MDPYGL--
-NPYGLY-
-NPYGLR-
NVPYGL--
NTPYGL--
NGPYGL--
NWPYGL--
-NPYGLK-
NNPYGL--
NIPYGL--
NAPYGL--
NPPYGL--
-NPYGLL-
-NPYGLV-
-NPYGLM-
-NPYGLH-
NSPYGL--
NYPYGL--
-NPYGLI-
NDPYGL--
NRPYGL--
-NPYGLW-
-NPYGLT-

NKPYGL--
NQPYGL--
-NPYGLN-
NHPYGL--
NEPYGL--
NCPYGL--
-NPYGLS-
NLPYGL--
-NPYGLA-
NFPYGL--
-NPYGLQ-
--PYGLRS
--PYGLTS
--PYGLLR
--PYGLSS
--PYGLKR
--PYGLVS
--PYGLST
--PYGLRN
--PYGLRG
--PYGLKS
--PYGLGS
--PYGLAL
--PYGLVT
--PYGLSV
--PYGLRI
--PYGLYS
--PYGLVK
--PYGLRK
--PYGLKF
--PYGLVG
--PYGLSR
--PYGLSG
--PYGLRM
--PYGLLA
--PYGLYN
--PYGLVV
--PYGLRT
--PYGLMR
--PYGLYK
--PYGLVR
--PYGLSL
--PYGLNS
--PYGLTW
--PYGLTK
--PYGLSK
--PYGLMT
--PYGLWS
--PYGLWG
--PYGLTR

--PYGLRY
--PYGLRF
--PYGLKY
--PYGLIR
PQPYGL--
--PYGLTN
--PYGLSM
--PYGLNT
--PYGLFR
--PYGLTG
--PYGLSH
--PYGLQT
--PYGLMG
--PYGLLG
--PYGLIT
--PYGLIA
--PYGLTT
--PYGLRQ
--PYGLMD
--PYGLHT
--PYGLHR
--PYGLGG
--PYGLGA
--PYGLES
--PYGLVA
--PYGLSN
--PYGLRH
--PYGLRD
--PYGLKG
--PYGLIN
--PYGLAI
--PYGLWT
--PYGLTA
--PYGLSY
--PYGLNG
--PYGLLD
--PYGLFT
--PYGLFN
--PYGLML
--PYGLLM
--PYGLLH
--PYGLHS
--PYGLHI
PVPYGL--
--PYGLYG
--PYGLYD
--PYGLWR
--PYGLWD
--PYGLLQ
--PYGLKK

--PYGLKA
--PYGLIS
--PYGLIK
--PYGLII
--PYGLHN
--PYGLHH
--PYGLHD
--PYGLFG
PNPYGL--
--PYGLYQ
--PYGLWQ
--PYGLWN
--PYGLVW
--PYGLSA
--PYGLNF
--PYGLMM
--PYGLMK
--PYGLKN
--PYGLHV
--PYGLFQ
--PYGLFK
PTPYGL--
PDPYGL--
PYPYGL--
--PYGLVN
--PYGLTV
--PYGLTL
--PYGLNQ
--PYGLNL
--PYGLNK
--PYGLID
--PYGLHW
--PYGLHK
--PYGLFS
--PYGLEA
--PYGLAV
--PYGLYW
--PYGLRW
--PYGLQV
--PYGLQF
--PYGLQA
--PYGLNM
--PYGLMV
--PYGLMA
--PYGLLE
--PYGLKW
--PYGLKV
--PYGLIH
--PYGLIG
--PYGLIE

--PYGLHF
--PYGLYH
--PYGLVI
--PYGLVD
--PYGLTM
--PYGLRE
--PYGLQI
--PYGLQG
--PYGLNA
--PYGLMH
--PYGLKM
--PYGLKL
--PYGLIM
--PYGLGL
--PYGLAY
--PYGLAT
--PYGLAM
PHPYGL--
--PYGLYE
--PYGLWK
--PYGLWH
--PYGLTQ
--PYGLQR
--PYGLQK
--PYGLIW
--PYGLHQ
--PYGLHL
--PYGLEY
--PYGLDA
PEPYGL--
--PYGLWM
--PYGLWE
--PYGLTY
--PYGLTH
--PYGLSF
--PYGLQN
--PYGLNW
--PYGLMQ
--PYGLME
--PYGLKT
--PYGLKQ
--PYGLKD
--PYGLIY
--PYGLIV
--PYGLIQ
--PYGLHG
--PYGLHA
--PYGLGT
--PYGLFE
--PYGLAK

--PYGLAG
PMPYGL--
PFPYGL--
PCPYGL--
PAPYGL--
--PYGLVF
--PYGLTD
--PYGLSP
--PYGLQQ
--PYGLQL
--PYGLQH
--PYGLQD
--PYGLPY
--PYGLPP
--PYGLNN
--PYGLMP
--PYGLKP
--PYGLGQ
--PYGLGM
--PYGLGI
--PYGLFH
--PYGLFD
--PYGLFC
--PYGLEN
--PYGLAW
--PYGLAR
PWPYGL--
-PPYGLN-
-PPYGLI-
PKPYGL--
PIPYGL--
QLPYGL--
-QPYGLR-
QGPYGL--
QYPYGL--
-QPYGLT-
-QPYGLK-
-QPYGLS-
QVPYGL--
QPPYGL--
-QPYGLH-
-QPYGLA-
QSPYGL--
QAPYGL--
-QPYGLV-
-QPYGLE-
QRPYGL--
QTPYGL--
-QPYGLY-
QWPYGL--

QCPYGL--
-QPYGLW-
-QPYGLM-
QNPYGL--
QMPYGL--
QHPYGL--
QFPYGL--
QEPYGL--
-QPYGLF-
-QPYGLC-
QKPYGL--
-RPYGLR-
RSPYGL--
-RPYGLT-
-RPYGLV-
-RPYGLI-
RGPYGL--
-RPYGLF-
RTPYGL--
-RPYGLY-
-RPYGLH-
RFPYGL--
REPYGL--
RRPYGL--
-RPYGLG-
RYPYGL--
-RPYGLQ-
RVPYGL--
RPPYGL--
RDPYGL--
RAPYGL--
RMPYGL--
RHPYGL--
-RPYGLW-
-RPYGLK-
-RPYGLN-
-RPYGLE-
RQPYGL--
-RPYGLM-
-RPYGLA-
RWPYGL--
RCPYGL--
-RPYGLP-
SMPYGL--
-SPYGLL-
-SPYGLS-
SSPYGL--
SFPYGL--
-SPYGLV-
SKPYGL--

SAPYGL--
SIPYGL--
SVPYGL--
-SPYGLQ-
-SPYGLK-
SWPYGL--
-SPYGLF-
SGPYGL--
SYPYGL--
-SPYGLN-
SNPYGL--
-SPYGLI-
-SPYGLA-
SQPYGL--
SEPYGL--
STPYGL--
SPPYGL--
SDPYGL--
-SPYGLW-
-SPYGLM-
-SPYGLH-
-SPYGLE-
SCPYGL--
-TPYGLR-
TLPYGL--
TVPYGL--
-TPYGLT-
-TPYGLL-
TRPYGL--
TSPYGL--
-TPYGLM-
-TPYGLK-
TPPYGL--
TGPYGL--
-TPYGLH-
-TPYGLN-
TTPYGL--
-TPYGLQ-
TYPYGL--
TWPYGL--
-TPYGLV-
TNPYGL--
TMPYGL--
TEPYGL--
-TPYGLY-
THPYGL--
-TPYGLI-
-TPYGLA-
TKPYGL--
TIPYGL--

TFPYGL--
TCPYGL--
TQPYGL--
-TPYGLW-
TDPYGL--
-VPYGLR-
VTPYGL--
-VPYGLH-
-VPYGLY-
VSPYGL--
VLPYGL--
VYPYGL--
-VPYGLL-
-VPYGLF-
VPPYGL--
VIPYGL--
-VPYGLA-
VVPYGL--
VRPYGL--
-VPYGLI-
VNPYGL--
-VPYGLK-
VQPYGL--
VGPYGL--
VAPYGL--
-VPYGLT-
VKPYGL--
VDPYGL--
-VPYGLV-
-VPYGLE-
VFPYGL--
VEPYGL--
VWPYGL--
VMPYGL--
-VPYGLW-
-VPYGLQ-
VCPYGL--
-VPYGLC-
WRPYGL--
WTPYGL--
WVPYGL--
WQPYGL--
-WPYGLR-
WGPYGL--
-WPYGLF-
WSPYGL--
-WPYGLI-
WKPYGL--
-WPYGLS-
-WPYGLN-

WLPYGL--
WFPYGL--
WEPYGL--
-WPYGLQ-
-WPYGLH-
WWPYGL--
-WPYGLV-
-WPYGLK-
WNPYGL--
WMPYGL--
WYPYGL--
-WPYGLA-
-WPYGLT-
WPPYGL--
WAPYGL--
-WPYGLM-
WHPYGL--
WDPYGL--
YSPYGL--
YRPYGL--
-YPYGLS-
-YPYGLL-
YTPYGL--
-YPYGLN-
-YPYGLA-
YPPYGL--
-YPYGLT-
YNPYGL--
YLPYGL--
YGPYGL--
YVPYGL--
-YPYGLQ-
-YPYGLM-
YAPYGL--
-YPYGLK-
-YPYGLH-
YEPYGL--
-YPYGLR-
YFPYGL--
-YPYGLW-
-YPYGLF-
YMPYGL--
YQPYGL--
-YPYGLV-
YIPYGL--
YHPYGL--
YYPYGL--
-YPYGLI-
YWPYGL--
-YPYGLY-

YDPYGL--

| | | | |
|---|---|---|---|
| PLAY | 106 | 766 | 32.96 |

--PLAYTP
--PLAYLP
-APLAYL-
ASPLAY--
AKPLAY--
-APLAYH-
AVPLAY--
-APLAYR-
-APLAYI-
ATPLAY--
-APLAYF-
AWPLAY--
-APLAYS-
-APLAYK-
-APLAYG-
-APLAYY-
ANPLAY--
AQPLAY--
-APLAYW-
-APLAYT-
AGPLAY--
-APLAYV-
-APLAYA-
AHPLAY--
AEPLAY--
AYPLAY--
-APLAYQ-
-APLAYN-
-APLAYM-
-APLAYE-
AIPLAY--
AMPLAY--
AFPLAY--
AAPLAY--
CVPLAY--
-CPLAYA-
CHPLAY--
CSPLAY--
CQPLAY--
-CPLAYM-
CTPLAY--
CPPLAY--
-CPLAYY-
-CPLAYV-
-CPLAYS-
-CPLAYL-
-CPLAYH-
CKPLAY--

CGPLAY--
DSPLAY--
-DPLAYA-
-DPLAYL-
-DPLAYM-
DYPLAY--
DWPLAY--
DRPLAY--
-DPLAYT-
-DPLAYK-
-DPLAYS-
-DPLAYV-
-DPLAYR-
DLPLAY--
DHPLAY--
DTPLAY--
-DPLAYQ-
-DPLAYF-
DFPLAY--
DQPLAY--
DPPLAY--
-DPLAYY-
-DPLAYN-
-DPLAYI-
DGPLAY--
DCPLAY--
-EPLAYF-
ERPLAY--
-EPLAYI-
EYPLAY--
-EPLAYV-
-EPLAYS-
EVPLAY--
ESPLAY--
-EPLAYT-
-EPLAYL-
-EPLAYA-
EIPLAY--
EGPLAY--
-EPLAYN-
EKPLAY--
EFPLAY--
ETPLAY--
EPPLAY--
-EPLAYW-
EQPLAY--
-EPLAYQ-
-EPLAYK-
-EPLAYG-
-EPLAYE-

ENPLAY--
FTPLAY--
-FPLAYL-
FGPLAY--
-FPLAYS-
FVPLAY--
-FPLAYR-
-FPLAYI-
FLPLAY--
FHPLAY--
FSPLAY--
-FPLAYT-
FQPLAY--
FEPLAY--
FNPLAY--
FDPLAY--
-FPLAYK-
FKPLAY--
-FPLAYN-
-FPLAYA-
FMPLAY--
FFPLAY--
-FPLAYW-
-FPLAYF-
FIPLAY--
FAPLAY--
-FPLAYM-
-FPLAYH-
-GPLAYS-
-GPLAYR-
GRPLAY--
-GPLAYI-
GTPLAY--
-GPLAYT-
-GPLAYK-
-GPLAYA-
GVPLAY--
GSPLAY--
-GPLAYH-
GLPLAY--
GNPLAY--
-GPLAYY-
-GPLAYL-
-GPLAYV-
GIPLAY--
-GPLAYF-
GDPLAY--
GWPLAY--
GQPLAY--
GKPLAY--

-GPLAYM-
-GPLAYN-
-GPLAYE-
GHPLAY--
GGPLAY--
GFPLAY--
GEPLAY--
GAPLAY--
-GPLAYQ-
-GPLAYC-
GMPLAY--
-HPLAYV-
HVPLAY--
HTPLAY--
-HPLAYS-
HAPLAY--
HSPLAY--
HRPLAY--
HIPLAY--
-HPLAYH-
HNPLAY--
HMPLAY--
HKPLAY--
HGPLAY--
HWPLAY--
-HPLAYR-
-HPLAYT-
-HPLAYQ-
-HPLAYG-
-HPLAYF-
HYPLAY--
HQPLAY--
-HPLAYM-
-HPLAYE-
HLPLAY--
IRPLAY--
IQPLAY--
ISPLAY--
-IPLAYR-
IDPLAY--
-IPLAYG-
IIPLAY--
IGPLAY--
-IPLAYS-
INPLAY--
ILPLAY--
-IPLAYY-
-IPLAYN-
IEPLAY--
IWPLAY--

IVPLAY--
IMPLAY--
IKPLAY--
ITPLAY--
-IPLAYM-
-IPLAYI-
IAPLAY--
-IPLAYT-
-IPLAYE-
IHPLAY--
IYPLAY--
IPPLAY--
-IPLAYQ-
-IPLAYK-
-IPLAYH-
-IPLAYA-
ICPLAY--
KSPLAY--
-KPLAYI-
-KPLAYA-
KVPLAY--
-KPLAYV-
-KPLAYL-
KGPLAY--
-KPLAYK-
-KPLAYR-
KTPLAY--
-KPLAYW-
KNPLAY--
-KPLAYF-
KLPLAY--
-KPLAYQ-
-KPLAYN-
KFPLAY--
KEPLAY--
KQPLAY--
-KPLAYS-
-KPLAYG-
KWPLAY--
KMPLAY--
KIPLAY--
KHPLAY--
-KPLAYT-
-KPLAYH-
KDPLAY--
KAPLAY--
KYPLAY--
-KPLAYM-
-KPLAYY-
-KPLAYE-

KKPLAY--
-LPLAYL-
LSPLAY--
LLPLAY--
LTPLAY--
-LPLAYI-
LEPLAY--
LMPLAY--
LNPLAY--
-LPLAYT-
LQPLAY--
-LPLAYM-
-LPLAYK-
LAPLAY--
-LPLAYV-
-LPLAYS-
LPPLAY--
-LPLAYQ-
LIPLAY--
LGPLAY--
-LPLAYF-
LWPLAY--
-LPLAYH-
LHPLAY--
LCPLAY--
LKPLAY--
LDPLAY--
-LPLAYG-
-MPLAYT-
MKPLAY--
MHPLAY--
-MPLAYL-
-MPLAYR-
MRPLAY--
MFPLAY--
MAPLAY--
MVPLAY--
MTPLAY--
MQPLAY--
-MPLAYI-
-MPLAYH-
MNPLAY--
MIPLAY--
-MPLAYN-
MEPLAY--
-MPLAYV-
-MPLAYG-
-MPLAYA-
MMPLAY--
MPPLAY--

```
-MPLAYQ-
MDPLAY--
MYPLAY--
MGPLAY--
-MPLAYE-
MCPLAY--
-NPLAYT-
NRPLAY--
NGPLAY--
-NPLAYK-
NVPLAY--
NIPLAY--
NSPLAY--
-NPLAYY-
-NPLAYR-
-NPLAYM-
NFPLAY--
-NPLAYA-
-NPLAYN-
-NPLAYF-
NMPLAY--
NLPLAY--
NHPLAY--
NAPLAY--
NTPLAY--
NQPLAY--
NPPLAY--
NKPLAY--
NYPLAY--
-NPLAYV-
-NPLAYQ-
NNPLAY--
NWPLAY--
-NPLAYW-
-NPLAYH-
NEPLAY--
NDPLAY--
-NPLAYE-
NCPLAY--
--PLAYRL
--PLAYRS
--PLAYLT
--PLAYFR
--PLAYSL
--PLAYLR
--PLAYLK
--PLAYLS
--PLAYSS
--PLAYRT
--PLAYGL
```

--PLAYTR
--PLAYRY
--PLAYIS
--PLAYRA
PRPLAY--
--PLAYSR
--PLAYGS
--PLAYRH
--PLAYGF
--PLAYVV
--PLAYGV
--PLAYWS
--PLAYVS
--PLAYRG
--PLAYPL
--PLAYRM
--PLAYFS
--PLAYTS
--PLAYSQ
--PLAYRI
--PLAYMR
--PLAYGY
--PLAYYQ
--PLAYSN
--PLAYSF
--PLAYSA
--PLAYRD
--PLAYMN
--PLAYML
--PLAYHR
--PLAYYG
--PLAYVH
--PLAYVE
--PLAYVA
--PLAYSV
--PLAYSM
--PLAYSD
--PLAYQS
--PLAYQR
--PLAYLQ
--PLAYHI
--PLAYFH
PFPLAY--
--PLAYYL
--PLAYVM
--PLAYTW
--PLAYTL
--PLAYTI
--PLAYTF
--PLAYSK

--PLAYQM
--PLAYNV
--PLAYNA
--PLAYMA
--PLAYLA
--PLAYKG
--PLAYIV
--PLAYHV
--PLAYHM
--PLAYHL
--PLAYAH
--PLAYYE
--PLAYWR
--PLAYWA
--PLAYVW
--PLAYVQ
--PLAYVN
--PLAYVK
--PLAYVG
--PLAYLV
--PLAYIT
--PLAYIK
--PLAYGW
--PLAYGT
--PLAYAS
--PLAYAL
--PLAYAG
PYPLAY--
PQPLAY--
--PLAYYA
--PLAYVY
--PLAYVT
--PLAYTA
--PLAYSH
--PLAYRW
--PLAYRE
--PLAYNS
--PLAYMS
--PLAYKT
--PLAYKK
--PLAYHT
--PLAYHA
--PLAYGI
--PLAYAY
PNPLAY--
PMPLAY--
--PLAYWT
--PLAYVD
--PLAYTV
--PLAYSY

--PLAYSG
--PLAYRV
--PLAYRQ
--PLAYMV
--PLAYMG
--PLAYMD
--PLAYHS
--PLAYHG
--PLAYGG
--PLAYFQ
--PLAYAT
--PLAYAN
PHPLAY--
--PLAYYK
--PLAYWN
--PLAYWM
--PLAYWK
--PLAYTM
--PLAYTE
--PLAYSI
--PLAYQT
--PLAYPM
--PLAYNY
--PLAYMK
--PLAYLH
--PLAYLE
--PLAYKY
--PLAYKN
--PLAYKE
--PLAYIN
--PLAYGA
--PLAYAV
--PLAYAR
--PLAYAM
PKPLAY--
--PLAYYH
--PLAYWI
--PLAYTQ
--PLAYSE
--PLAYRF
--PLAYQL
--PLAYNR
--PLAYNF
--PLAYMI
--PLAYMF
--PLAYLF
--PLAYKS
--PLAYKF
--PLAYIQ
--PLAYIH

--PLAYID
--PLAYHE
--PLAYES
--PLAYDL
--PLAYAK
--PLAYAA
--PLAYYN
--PLAYYM
--PLAYYD
--PLAYWV
--PLAYWD
--PLAYTY
--PLAYTH
--PLAYTD
--PLAYSW
--PLAYST
--PLAYQN
--PLAYQI
--PLAYPW
--PLAYPI
--PLAYPF
--PLAYNM
--PLAYNK
--PLAYNH
--PLAYMT
--PLAYKV
--PLAYIM
--PLAYHD
--PLAYFG
--PLAYEY
--PLAYET
--PLAYDS
--PLAYAW
--PLAYAQ
--PLAYAI
PEPLAY--
-PPLAYY-
-PPLAYV-
--PLAYYF
--PLAYWQ
--PLAYWH
--PLAYWE
--PLAYQW
--PLAYQK
--PLAYQH
--PLAYQG
--PLAYPY
--PLAYNW
--PLAYMY
--PLAYMW

```
--PLAYLD
--PLAYKQ
--PLAYKD
--PLAYII
--PLAYIC
--PLAYIA
--PLAYHY
--PLAYHN
--PLAYHH
--PLAYHF
--PLAYGQ
--PLAYGM
--PLAYFK
--PLAYEW
--PLAYEV
--PLAYEQ
--PLAYEM
--PLAYEK
--PLAYCF
--PLAYAD
QRPLAY--
-QPLAYL-
-QPLAYH-
-QPLAYV-
QSPLAY--
QAPLAY--
-QPLAYA-
QGPLAY--
QQPLAY--
-QPLAYN-
QKPLAY--
QFPLAY--
-QPLAYT-
-QPLAYK-
-QPLAYG-
QNPLAY--
QLPLAY--
QVPLAY--
QTPLAY--
-QPLAYS-
-QPLAYE-
QIPLAY--
QYPLAY--
QWPLAY--
-QPLAYR-
QEPLAY--
QDPLAY--
-QPLAYY-
-QPLAYQ-
-QPLAYM-
```

RTPLAY--
RLPLAY--
RSPLAY--
-RPLAYI-
-RPLAYL-
RQPLAY--
-RPLAYS-
-RPLAYK-
-RPLAYH-
RGPLAY--
RNPLAY--
-RPLAYM-
RWPLAY--
RKPLAY--
RIPLAY--
RPPLAY--
-RPLAYF-
REPLAY--
RDPLAY--
RYPLAY--
-RPLAYV-
-RPLAYN-
-RPLAYE-
RFPLAY--
-RPLAYQ-
RHPLAY--
RCPLAY--
-SPLAYL-
SNPLAY--
STPLAY--
SQPLAY--
-SPLAYN-
-SPLAYM-
SGPLAY--
-SPLAYH-
SSPLAY--
SYPLAY--
-SPLAYY-
-SPLAYV-
-SPLAYI-
-SPLAYF-
SKPLAY--
-SPLAYA-
-SPLAYK-
SIPLAY--
SWPLAY--
SPPLAY--
-SPLAYG-
SAPLAY--
-SPLAYE-

SMPLAY--
SLPLAY--
SEPLAY--
SDPLAY--
SHPLAY--
TRPLAY--
TVPLAY--
-TPLAYL-
-TPLAYS-
-TPLAYI-
-TPLAYA-
-TPLAYT-
TPPLAY--
-TPLAYK-
TNPLAY--
THPLAY--
TGPLAY--
TAPLAY--
TYPLAY--
TFPLAY--
-TPLAYV-
-TPLAYN-
-TPLAYQ-
-TPLAYG-
TTPLAY--
TQPLAY--
-TPLAYM-
-TPLAYE-
TMPLAY--
TWPLAY--
-TPLAYW-
-TPLAYH-
-VPLAYR-
VTPLAY--
-VPLAYV-
VKPLAY--
-VPLAYL-
-VPLAYI-
-VPLAYG-
-VPLAYS-
VVPLAY--
VLPLAY--
VSPLAY--
-VPLAYQ-
VFPLAY--
VNPLAY--
VIPLAY--
VQPLAY--
-VPLAYT-
VMPLAY--

-VPLAYY-
VAPLAY--
-VPLAYW-
-VPLAYM-
-VPLAYH-
-VPLAYA-
-VPLAYK-
VYPLAY--
VEPLAY--
-VPLAYE-
VHPLAY--
VDPLAY--
VCPLAY--
WLPLAY--
WGPLAY--
-WPLAYS-
WTPLAY--
WNPLAY--
WQPLAY--
WSPLAY--
-WPLAYT-
-WPLAYE-
WMPLAY--
WYPLAY--
WWPLAY--
-WPLAYK-
WRPLAY--
-WPLAYM-
WDPLAY--
-WPLAYW-
-WPLAYQ-
-WPLAYI-
WAPLAY--
WVPLAY--
WPPLAY--
-WPLAYY-
-WPLAYH-
-WPLAYD-
-WPLAYA-
WEPLAY--
WCPLAY--
-YPLAYL-
YGPLAY--
YAPLAY--
-YPLAYH-
YTPLAY--
YSPLAY--
-YPLAYS-
-YPLAYN-
-YPLAYM-

YPPLAY--
-YPLAYT-
-YPLAYG-
YMPLAY--
YIPLAY--
-YPLAYY-
YKPLAY--
-YPLAYK-
-YPLAYE-
YHPLAY--
YEPLAY--
YVPLAY--
-YPLAYW-
-YPLAYF-
YNPLAY--
YQPLAY--
-YPLAYI-
-YPLAYA-

PSAF 99 770 32.96

--PSAFKL
--PSAFAR
-VPSAFP-
-YPSAFG-
-APSAFL-
-APSAFR-
ALPSAF--
ATPSAF--
ASPSAF--
ARPSAF--
-APSAFA-
-APSAFV-
-APSAFS-
-APSAFK-
-APSAFM-
-APSAFH-
AKPSAF--
-APSAFF-
AVPSAF--
AQPSAF--
-APSAFW-
-APSAFT-
-APSAFQ-
-APSAFN-
-APSAFG-
ANPSAF--
AFPSAF--
-APSAFY-
AMPSAF--
AEPSAF--
APPSAF--

AAPSAF--
-APSAFI-
AIPSAF--
AHPSAF--
-CPSAFY-
-CPSAFM-
CYPSAF--
CSPSAF--
CVPSAF--
-CPSAFV-
-CPSAFS-
-CPSAFL-
CNPSAF--
CEPSAF--
CDPSAF--
CRPSAF--
CQPSAF--
-CPSAFR-
-CPSAFK-
CPPSAF--
CKPSAF--
CAPSAF--
DSPSAF--
DFPSAF--
-DPSAFT-
DRPSAF--
-DPSAFS-
DTPSAF--
-DPSAFW-
-DPSAFY-
-DPSAFH-
DLPSAF--
DYPSAF--
DQPSAF--
-DPSAFQ-
DKPSAF--
DHPSAF--
-DPSAFR-
-DPSAFM-
-DPSAFL-
-DPSAFA-
DIPSAF--
DGPSAF--
DDPSAF--
DAPSAF--
ERPSAF--
-EPSAFA-
-EPSAFR-
-EPSAFI-
ELPSAF--

EKPSAF--
EVPSAF--
ESPSAF--
-EPSAFT-
-EPSAFL-
-EPSAFK-
-EPSAFH-
-EPSAFF-
ECPSAF--
EYPSAF--
ETPSAF--
EQPSAF--
-EPSAFQ-
-EPSAFM-
EEPSAF--
EWPSAF--
-EPSAFW-
-EPSAFV-
-EPSAFN-
-EPSAFE-
EPPSAF--
EAPSAF--
ENPSAF--
EMPSAF--
EIPSAF--
EGPSAF--
EFPSAF--
-FPSAFK-
-FPSAFR-
-FPSAFI-
FTPSAF--
FAPSAF--
-FPSAFS-
FKPSAF--
FQPSAF--
FIPSAF--
-FPSAFL-
FNPSAF--
FVPSAF--
-FPSAFW-
-FPSAFH-
FHPSAF--
FEPSAF--
FYPSAF--
FWPSAF--
-FPSAFV-
-FPSAFN-
FLPSAF--
FPPSAF--
FDPSAF--

FCPSAF--
-FPSAFQ-
FGPSAF--
-GPSAFS-
-GPSAFK-
-GPSAFR-
-GPSAFL-
GTPSAF--
GKPSAF--
-GPSAFH-
-GPSAFN-
GLPSAF--
GGPSAF--
GCPSAF--
-GPSAFM-
-GPSAFI-
GHPSAF--
GDPSAF--
GRPSAF--
GQPSAF--
-GPSAFV-
-GPSAFT-
-GPSAFQ-
GVPSAF--
-GPSAFY-
GAPSAF--
GWPSAF--
-GPSAFW-
-GPSAFE-
-GPSAFA-
GPPSAF--
GNPSAF--
GIPSAF--
-HPSAFR-
HRPSAF--
-HPSAFV-
-HPSAFQ-
-HPSAFS-
HDPSAF--
-HPSAFI-
HVPSAF--
-HPSAFM-
HKPSAF--
HFPSAF--
HYPSAF--
HTPSAF--
-HPSAFT-
HIPSAF--
HGPSAF--
HEPSAF--

-HPSAFK-
HLPSAF--
HAPSAF--
HWPSAF--
-HPSAFY-
-HPSAFW-
-HPSAFN-
-HPSAFF-
HNPSAF--
HSPSAF--
-HPSAFA-
HPPSAF--
HMPSAF--
-IPSAFS-
IIPSAF--
-IPSAFR-
-IPSAFI-
-IPSAFH-
-IPSAFA-
IVPSAF--
ISPSAF--
-IPSAFL-
ITPSAF--
-IPSAFN-
IKPSAF--
-IPSAFV-
-IPSAFQ-
-IPSAFK-
IMPSAF--
IQPSAF--
-IPSAFW-
ILPSAF--
IFPSAF--
-IPSAFT-
-IPSAFM-
IGPSAF--
ICPSAF--
IYPSAF--
IWPSAF--
-IPSAFG-
INPSAF--
IHPSAF--
IPPSAF--
IAPSAF--
KSPSAF--
-KPSAFL-
KAPSAF--
-KPSAFW-
KRPSAF--
-KPSAFV-

-KPSAFI-
KGPSAF--
KWPSAF--
-KPSAFS-
KPPSAF--
-KPSAFT-
KNPSAF--
KIPSAF--
KTPSAF--
KYPSAF--
-KPSAFH-
KMPSAF--
KHPSAF--
-KPSAFM-
KVPSAF--
-KPSAFQ-
-KPSAFN-
-KPSAFK-
KQPSAF--
KLPSAF--
KKPSAF--
KDPSAF--
-KPSAFG-
-KPSAFA-
KFPSAF--
-KPSAFY-
-LPSAFR-
-LPSAFL-
LGPSAF--
LLPSAF--
-LPSAFS-
-LPSAFI-
LVPSAF--
-LPSAFV-
LYPSAF--
LRPSAF--
LWPSAF--
LNPSAF--
-LPSAFN-
-LPSAFM-
LIPSAF--
LPPSAF--
LAPSAF--
LSPSAF--
LQPSAF--
LEPSAF--
LTPSAF--
-LPSAFT-
LFPSAF--
-LPSAFQ-

-LPSAFA-
-LPSAFP-
-LPSAFK-
LHPSAF--
-LPSAFE-
-MPSAFT-
-MPSAFS-
MIPSAF--
MWPSAF--
MSPSAF--
MRPSAF--
-MPSAFN-
MYPSAF--
MTPSAF--
-MPSAFV-
-MPSAFK-
-MPSAFQ-
-MPSAFL-
MLPSAF--
MCPSAF--
MVPSAF--
-MPSAFI-
MMPSAF--
MHPSAF--
MAPSAF--
-MPSAFG-
-MPSAFF-
MPPSAF--
-MPSAFM-
-MPSAFA-
MKPSAF--
MDPSAF--
MQPSAF--
-MPSAFW-
-MPSAFH-
MGPSAF--
MFPSAF--
NNPSAF--
NSPSAF--
-NPSAFI-
-NPSAFL-
NTPSAF--
-NPSAFT-
-NPSAFK-
NYPSAF--
NGPSAF--
NWPSAF--
-NPSAFW-
-NPSAFV-
-NPSAFA-

NMPSAF--
NIPSAF--
-NPSAFS-
NKPSAF--
NRPSAF--
-NPSAFY-
-NPSAFR-
-NPSAFQ-
NCPSAF--
NVPSAF--
-NPSAFH-
NPPSAF--
NFPSAF--
NDPSAF--
NQPSAF--
-NPSAFF-
NHPSAF--
NAPSAF--
-NPSAFN-
--PSAFAL
--PSAFTF
--PSAFSL
--PSAFRS
--PSAFGL
--PSAFRT
--PSAFRK
--PSAFIT
--PSAFLS
--PSAFYD
--PSAFWR
--PSAFRM
--PSAFII
--PSAFRQ
--PSAFFG
PLPSAF--
--PSAFVR
--PSAFSA
--PSAFSG
--PSAFRW
--PSAFTM
--PSAFTA
--PSAFSS
--PSAFRY
--PSAFNL
--PSAFIR
--PSAFSR
--PSAFSM
--PSAFNS
--PSAFLG
--PSAFST

--PSAFSK
--PSAFKD
--PSAFKH
--PSAFKG
--PSAFIK
--PSAFHI
PRPSAF--
--PSAFTS
--PSAFLN
--PSAFKM
--PSAFYG
--PSAFTW
--PSAFSV
--PSAFRH
--PSAFRG
--PSAFNM
--PSAFMW
--PSAFMS
--PSAFMR
--PSAFIS
PSPSAF--
--PSAFYS
--PSAFSN
--PSAFQS
--PSAFQR
--PSAFNV
--PSAFNT
--PSAFHA
--PSAFFA
PQPSAF--
PWPSAF--
--PSAFWW
--PSAFVG
--PSAFVA
--PSAFTI
--PSAFTH
--PSAFRV
--PSAFQV
--PSAFQT
--PSAFMT
--PSAFML
--PSAFLV
--PSAFKS
--PSAFHF
--PSAFGT
PMPSAF--
--PSAFWT
--PSAFWQ
--PSAFWN
--PSAFWA

--PSAFVV
--PSAFVQ
--PSAFSW
--PSAFMA
--PSAFKA
--PSAFIY
--PSAFFQ
--PSAFFK
--PSAFAY
--PSAFAV
--PSAFAS
--PSAFYN
--PSAFYM
--PSAFVT
--PSAFTT
--PSAFSY
--PSAFSF
--PSAFRE
--PSAFNW
--PSAFNA
--PSAFMV
--PSAFKQ
--PSAFIN
--PSAFHS
--PSAFHG
--PSAFFT
--PSAFFN
PKPSAF--
PEPSAF--
--PSAFWK
--PSAFVL
--PSAFVE
--PSAFTD
--PSAFRD
--PSAFQA
--PSAFPF
--PSAFMH
--PSAFLI
--PSAFKY
--PSAFKW
--PSAFKV
--PSAFKF
--PSAFIH
--PSAFHT
--PSAFGV
PGPSAF--
PVPSAF--
--PSAFYT
--PSAFYK
--PSAFYA

--PSAFWD
--PSAFVK
--PSAFVD
--PSAFTK
--PSAFSH
--PSAFQH
--PSAFQG
--PSAFNG
--PSAFMI
--PSAFLK
--PSAFLD
--PSAFKT
--PSAFKI
--PSAFIQ
--PSAFHY
--PSAFHV
--PSAFHQ
--PSAFGY
--PSAFFE
--PSAFAW
--PSAFAT
--PSAFAM
--PSAFAI
--PSAFAG
--PSAFAF
--PSAFAA
PYPSAF--
PTPSAF--
--PSAFYE
--PSAFWL
--PSAFVF
--PSAFSQ
--PSAFQY
--PSAFQM
--PSAFQK
--PSAFQI
--PSAFPL
--PSAFNI
--PSAFNH
--PSAFMD
--PSAFLM
--PSAFLH
--PSAFIG
--PSAFIE
--PSAFHN
--PSAFHM
--PSAFHH
--PSAFGI
--PSAFGG
--PSAFGA

```
--PSAFFM
PIPSAF--
--PSAFYH
--PSAFWM
--PSAFWH
--PSAFWF
--PSAFVN
--PSAFTQ
--PSAFTG
--PSAFSE
--PSAFQQ
--PSAFQN
--PSAFQF
--PSAFPK
--PSAFPI
--PSAFNN
--PSAFNK
--PSAFMQ
--PSAFMN
--PSAFMG
--PSAFME
--PSAFHR
--PSAFHE
--PSAFFW
--PSAFFH
--PSAFFD
--PSAFEW
--PSAFEV
--PSAFES
--PSAFEG
--PSAFAQ
--PSAFAN
--PSAFAH
PHPSAF--
PFPSAF--
PDPSAF--
PAPSAF--
-QPSAFF-
QLPSAF--
-QPSAFL-
QRPSAF--
QWPSAF--
-QPSAFV-
QHPSAF--
-QPSAFH-
-QPSAFN-
QGPSAF--
QSPSAF--
-QPSAFE-
-QPSAFT-
```

-QPSAFQ-
-QPSAFK-
QYPSAF--
QVPSAF--
-QPSAFY-
-QPSAFS-
QKPSAF--
QAPSAF--
-QPSAFA-
QFPSAF--
QTPSAF--
-QPSAFM-
QPPSAF--
QNPSAF--
QIPSAF--
QEPSAF--
-RPSAFL-
-RPSAFI-
-RPSAFS-
RTPSAF--
-RPSAFM-
RVPSAF--
RWPSAF--
RGPSAF--
RRPSAF--
RKPSAF--
RAPSAF--
RMPSAF--
-RPSAFT-
RYPSAF--
REPSAF--
-RPSAFY-
-RPSAFN-
-RPSAFH-
-RPSAFA-
RFPSAF--
RIPSAF--
-RPSAFQ-
-RPSAFG-
RQPSAF--
-RPSAFE-
-RPSAFW-
-RPSAFD-
-SPSAFR-
-SPSAFS-
-SPSAFF-
SWPSAF--
-SPSAFL-
SYPSAF--
SSPSAF--

-SPSAFK-
-SPSAFW-
SVPSAF--
-SPSAFY-
-SPSAFA-
-SPSAFQ-
-SPSAFH-
SLPSAF--
SGPSAF--
STPSAF--
-SPSAFN-
-SPSAFM-
SPPSAF--
SIPSAF--
-SPSAFI-
SKPSAF--
SQPSAF--
SMPSAF--
SFPSAF--
-SPSAFT-
SNPSAF--
SHPSAF--
SDPSAF--
SCPSAF--
-TPSAFL-
TTPSAF--
-TPSAFF-
TGPSAF--
-TPSAFS-
THPSAF--
TSPSAF--
-TPSAFT-
-TPSAFR-
-TPSAFK-
-TPSAFH-
TWPSAF--
-TPSAFQ-
TNPSAF--
TRPSAF--
-TPSAFV-
TLPSAF--
-TPSAFY-
TYPSAF--
-TPSAFM-
-TPSAFI-
TEPSAF--
-TPSAFW-
TAPSAF--
TVPSAF--
TPPSAF--

TFPSAF--
TDPSAF--
TCPSAF--
-TPSAFN-
-TPSAFA-
TKPSAF--
TIPSAF--
-VPSAFK-
VRPSAF--
VVPSAF--
-VPSAFI-
VYPSAF--
-VPSAFL-
VLPSAF--
-VPSAFM-
-VPSAFA-
VWPSAF--
VFPSAF--
-VPSAFV-
VMPSAF--
VAPSAF--
VKPSAF--
-VPSAFT-
-VPSAFN-
VQPSAF--
-VPSAFW-
-VPSAFS-
-VPSAFH-
VPPSAF--
VGPSAF--
-VPSAFQ-
VIPSAF--
VDPSAF--
VTPSAF--
-VPSAFF-
VHPSAF--
-VPSAFG-
VEPSAF--
VCPSAF--
WRPSAF--
WVPSAF--
-WPSAFR-
WLPSAF--
WSPSAF--
-WPSAFK-
WKPSAF--
WGPSAF--
WFPSAF--
WTPSAF--
-WPSAFS-

```
            -WPSAFQ-
            -WPSAFH-
            WQPSAF--
            WEPSAF--
            WYPSAF--
            -WPSAFW-
            -WPSAFV-
            -WPSAFM-
            -WPSAFG-
            YRPSAF--
            -YPSAFR-
            YIPSAF--
            YQPSAF--
            -YPSAFV-
            -YPSAFF-
            -YPSAFN-
            YHPSAF--
            -YPSAFQ-
            YMPSAF--
            YYPSAF--
            YTPSAF--
            -YPSAFT-
            -YPSAFS-
            -YPSAFM-
            YLPSAF--
            -YPSAFL-
            -YPSAFK-
            YGPSAF--
            YFPSAF--
            YVPSAF--
            YSPSAF--
            -YPSAFI-
            -YPSAFA-
            YPPSAF--
            YNPSAF--
            YEPSAF--
            -YPSAFW-
            -YPSAFH-
            YAPSAF--
PSNI          46         807      32.95
            --PSNIGM
            -QPSNIN-
            RPPSNI--
            TGPSNI--
            -APSNIR-
            -APSNIL-
            AQPSNI--
            AFPSNI--
            AWPSNI--
            AVPSNI--
```

-APSNIT-
ATPSNI--
-APSNIY-
-APSNIN-
-APSNIG-
-APSNIS-
ARPSNI--
ANPSNI--
AYPSNI--
-APSNIV-
APPSNI--
AMPSNI--
AKPSNI--
AIPSNI--
-APSNIW-
-APSNII-
-APSNIH-
-APSNIF-
-APSNIE-
-APSNIA-
AAPSNI--
ASPSNI--
-APSNIM-
-APSNIQ-
AGPSNI--
ACPSNI--
CCPSNI--
CDPSNI--
-CPSNIH-
CSPSNI--
-CPSNIY-
-CPSNIF-
-CPSNIE-
CPPSNI--
CNPSNI--
CLPSNI--
CIPSNI--
CGPSNI--
CYPSNI--
-CPSNIR-
-CPSNIM-
-CPSNIL-
-CPSNIK-
CHPSNI--
CFPSNI--
DLPSNI--
DKPSNI--
-DPSNIK-
DRPSNI--
-DPSNIY-

-DPSNIL-
DAPSNI--
DSPSNI--
DQPSNI--
DNPSNI--
DVPSNI--
-DPSNIW-
-DPSNIT-
-DPSNIS-
-DPSNII-
-DPSNIF-
-DPSNIA-
DYPSNI--
DWPSNI--
-DPSNIV-
DMPSNI--
DIPSNI--
DFPSNI--
EKPSNI--
-EPSNIL-
ELPSNI--
ERPSNI--
EIPSNI--
-EPSNIR-
-EPSNIH-
-EPSNIG-
-EPSNIF-
EHPSNI--
EQPSNI--
EYPSNI--
ESPSNI--
-EPSNIY-
-EPSNIT-
-EPSNIN-
-EPSNIK-
-EPSNIE-
EPPSNI--
ENPSNI--
EMPSNI--
EAPSNI--
EVPSNI--
ETPSNI--
-EPSNIV-
-EPSNIM-
-EPSNII-
EFPSNI--
EEPSNI--
-FPSNIR-
FVPSNI--
-FPSNIS-

FSPSNI--
-FPSNIT-
-FPSNIY-
-FPSNIW-
-FPSNIF-
FPPSNI--
FIPSNI--
FYPSNI--
FLPSNI--
FGPSNI--
FAPSNI--
FTPSNI--
FNPSNI--
FMPSNI--
-FPSNII-
-FPSNIH-
-FPSNIE-
FEPSNI--
FDPSNI--
-FPSNIQ-
-FPSNIA-
FHPSNI--
FFPSNI--
FWPSNI--
FRPSNI--
-FPSNIV-
-FPSNIG-
FKPSNI--
-GPSNIS-
-GPSNIK-
-GPSNIH-
GKPSNI--
GYPSNI--
GNPSNI--
GIPSNI--
GTPSNI--
-GPSNIR-
-GPSNIF-
GGPSNI--
GVPSNI--
-GPSNIV-
-GPSNIM-
GEPSNI--
GDPSNI--
GSPSNI--
-GPSNIA-
GMPSNI--
GWPSNI--
-GPSNIT-
-GPSNIG-

GLPSNI--
GAPSNI--
-GPSNIY-
-GPSNIQ-
-GPSNIN-
-GPSNII-
GPPSNI--
GHPSNI--
GFPSNI--
GCPSNI--
-HPSNIY-
HHPSNI--
-HPSNIS-
HVPSNI--
-HPSNIE-
HNPSNI--
HGPSNI--
-HPSNIW-
-HPSNIR-
-HPSNIM-
HLPSNI--
HKPSNI--
HYPSNI--
HWPSNI--
-HPSNIL-
-HPSNIF-
HPPSNI--
HIPSNI--
HFPSNI--
HTPSNI--
HQPSNI--
-HPSNIN-
-HPSNIH-
HMPSNI--
HEPSNI--
IRPSNI--
IWPSNI--
-IPSNIT-
-IPSNIL-
-IPSNIF-
ITPSNI--
-IPSNIM-
IPPSNI--
-IPSNIR-
INPSNI--
-IPSNIE-
-IPSNIA-
ISPSNI--
-IPSNIW-
-IPSNIV-

ILPSNI--
IEPSNI--
IDPSNI--
IYPSNI--
-IPSNIQ-
IIPSNI--
IGPSNI--
IVPSNI--
IQPSNI--
-IPSNII-
-IPSNIG-
IMPSNI--
IKPSNI--
IFPSNI--
IAPSNI--
-IPSNIY-
-IPSNIH-
ICPSNI--
KSPSNI--
KVPSNI--
-KPSNIA-
-KPSNIY-
-KPSNIT-
-KPSNIG-
KYPSNI--
KQPSNI--
-KPSNIK-
-KPSNIW-
-KPSNIQ-
KNPSNI--
-KPSNIH-
-KPSNIF-
-KPSNIV-
-KPSNIE-
KMPSNI--
KLPSNI--
KWPSNI--
-KPSNIS-
-KPSNIM-
-KPSNII-
KPPSNI--
KHPSNI--
KGPSNI--
-KPSNIN-
KIPSNI--
KFPSNI--
KEPSNI--
KDPSNI--
KCPSNI--
KAPSNI--

LSPSNI--
-LPSNIS-
-LPSNIL-
LRPSNI--
-LPSNIM-
LWPSNI--
-LPSNIF-
-LPSNIY-
LIPSNI--
LVPSNI--
LQPSNI--
-LPSNIR-
LKPSNI--
LTPSNI--
-LPSNIV-
-LPSNIA-
LNPSNI--
LAPSNI--
-LPSNIK-
-LPSNIH-
LLPSNI--
LPPSNI--
LHPSNI--
LFPSNI--
LEPSNI--
LDPSNI--
LYPSNI--
-LPSNIT-
-LPSNIQ-
-LPSNIE-
-LPSNIW-
LMPSNI--
LGPSNI--
-LPSNII-
-LPSNIG-
LCPSNI--
-MPSNIL-
MLPSNI--
MDPSNI--
MGPSNI--
MWPSNI--
MSPSNI--
-MPSNIK-
MVPSNI--
MTPSNI--
-MPSNIY-
-MPSNIV-
-MPSNIR-
MFPSNI--
MRPSNI--

-MPSNIW-
MIPSNI--
MYPSNI--
-MPSNIT-
-MPSNIM-
-MPSNIH-
MKPSNI--
-MPSNII-
-MPSNIG-
MQPSNI--
-MPSNIS-
-MPSNIQ-
-MPSNIF-
-MPSNIE-
-MPSNIA-
MNPSNI--
MEPSNI--
MAPSNI--
NTPSNI--
NRPSNI--
NAPSNI--
NQPSNI--
-NPSNIT-
NVPSNI--
-NPSNIV-
-NPSNIQ-
NMPSNI--
NSPSNI--
-NPSNIY-
-NPSNIL-
-NPSNIW-
-NPSNIM-
-NPSNIA-
NGPSNI--
NEPSNI--
NYPSNI--
-NPSNIK-
-NPSNII-
NPPSNI--
NIPSNI--
NFPSNI--
NDPSNI--
NNPSNI--
NLPSNI--
NHPSNI--
-NPSNIS-
-NPSNIH-
-NPSNIG-
--PSNILR
--PSNIYL

```
--PSNIMR
--PSNIRL
--PSNIRT
--PSNIRW
--PSNIST
--PSNIRR
--PSNILL
--PSNIWS
--PSNILQ
--PSNILK
--PSNIIS
--PSNIVS
--PSNISR
--PSNIRS
--PSNIRN
--PSNIGS
--PSNIFQ
--PSNIWN
--PSNITA
--PSNIKR
--PSNIWT
--PSNIWR
--PSNITF
--PSNISS
--PSNILA
--PSNIQT
--PSNISN
--PSNISG
--PSNIRK
--PSNIMT
--PSNILH
--PSNITL
--PSNISY
--PSNISV
--PSNIKG
--PSNIFN
--PSNIAV
PVPSNI--
--PSNIVF
--PSNIQS
--PSNIMA
--PSNILY
--PSNIKA
--PSNIFG
PMPSNI--
--PSNIYR
--PSNITW
--PSNIRY
--PSNIRV
--PSNIRI
```

--PSNIMW
--PSNIMG
--PSNILV
--PSNILT
--PSNILM
--PSNIFR
PLPSNI--
--PSNIYK
--PSNITS
--PSNITR
--PSNISQ
--PSNINM
--PSNILN
--PSNIHR
--PSNIFK
--PSNIAM
PFPSNI--
--PSNIWM
--PSNIWF
--PSNIWA
--PSNIVR
--PSNIVI
--PSNITG
--PSNISA
--PSNIMS
--PSNIGV
--PSNIFY
--PSNIFA
--PSNIAS
--PSNIAR
PWPSNI--
--PSNIWQ
--PSNITT
--PSNISK
--PSNIRF
--PSNIQG
--PSNIMV
--PSNIMN
--PSNIML
--PSNIKV
--PSNIKQ
--PSNIKK
--PSNIIR
--PSNIHG
--PSNIGG
--PSNIFD
--PSNIAI
--PSNIAG
PQPSNI--
--PSNIYG

--PSNIWI
--PSNIVT
--PSNIVQ
--PSNIVA
--PSNITM
--PSNITH
--PSNITD
--PSNISI
--PSNIRE
--PSNIQV
--PSNIQN
--PSNIQL
--PSNINS
--PSNILI
--PSNIKN
--PSNIIK
--PSNIIG
--PSNIGA
--PSNIFT
--PSNIFM
--PSNIFH
--PSNIAW
PRPSNI--
--PSNIYW
--PSNIYV
--PSNIYS
--PSNIWG
--PSNIWE
--PSNIVY
--PSNIVV
--PSNIVN
--PSNITY
--PSNITV
--PSNITN
--PSNIRA
--PSNINW
--PSNINT
--PSNIMH
--PSNIKY
--PSNIKM
--PSNIKF
--PSNIIF
--PSNIGW
--PSNIGT
--PSNIFF
--PSNICS
PNPSNI--
PTPSNI--
PSPSNI--
--PSNIYM

--PSNIYD
--PSNIWW
--PSNIWV
--PSNIWK
--PSNIWH
--PSNIVK
--PSNIVE
--PSNITI
--PSNITE
--PSNISW
--PSNISH
--PSNIRQ
--PSNIRM
--PSNIRH
--PSNIRD
--PSNIQR
--PSNIQQ
--PSNIQE
--PSNINV
--PSNINA
--PSNIMY
--PSNIMK
--PSNIMI
--PSNIKT
--PSNIKL
--PSNIKH
--PSNIIH
--PSNIHS
--PSNIGY
--PSNIFW
--PSNIEG
--PSNIEA
--PSNIAT
--PSNIAQ
--PSNIAD
--PSNIAA
PYPSNI--
--PSNIYQ
--PSNIYN
--PSNIYH
--PSNIYE
--PSNIYA
--PSNIWY
--PSNIVW
--PSNIVD
--PSNITQ
--PSNISM
--PSNISD
--PSNIRG
--PSNIQW

--PSNIQI
--PSNIQH
--PSNIQA
--PSNIPS
--PSNINQ
--PSNINN
--PSNINL
--PSNINI
--PSNINH
--PSNING
--PSNINF
--PSNIMQ
--PSNIMM
--PSNIMF
--PSNIMD
--PSNILE
--PSNIKI
--PSNIIY
--PSNIIW
--PSNIID
--PSNIIC
--PSNIHN
--PSNIGI
--PSNIEV
--PSNIEI
--PSNIEH
--PSNIAH
--PSNIAF
-PPSNIV-
-PPSNII-
PIPSNI--
PGPSNI--
PEPSNI--
PDPSNI--
PAPSNI--
-QPSNIV-
QRPSNI--
-QPSNIR-
-QPSNII-
-QPSNIT-
-QPSNIS-
QLPSNI--
QYPSNI--
QWPSNI--
QTPSNI--
-QPSNIG-
QGPSNI--
QFPSNI--
QEPSNI--
QDPSNI--

-QPSNIK-
-QPSNIF-
-QPSNIA-
QSPSNI--
QNPSNI--
-QPSNIY-
-QPSNIQ-
-QPSNIM-
-QPSNIL-
QMPSNI--
-RPSNIW-
-RPSNIS-
RVPSNI--
-RPSNIV-
RNPSNI--
RRPSNI--
-RPSNIL-
-RPSNIG-
RLPSNI--
-RPSNII-
-RPSNIF-
-RPSNIN-
-RPSNIM-
RSPSNI--
RHPSNI--
RYPSNI--
RWPSNI--
RQPSNI--
-RPSNIY-
-RPSNIH-
RKPSNI--
RIPSNI--
-RPSNIA-
-RPSNIT-
RTPSNI--
-RPSNIE-
RDPSNI--
-RPSNID-
SRPSNI--
SLPSNI--
SSPSNI--
SKPSNI--
SYPSNI--
-SPSNII-
SVPSNI--
-SPSNIR-
SMPSNI--
-SPSNIF-
STPSNI--
SAPSNI--

SQPSNI--
-SPSNIS-
-SPSNIM-
-SPSNIA-
SHPSNI--
-SPSNIY-
-SPSNIW-
-SPSNIK-
SNPSNI--
SPPSNI--
-SPSNIT-
-SPSNIG-
-SPSNIQ-
-SPSNIH-
-SPSNIE-
SGPSNI--
SWPSNI--
-SPSNIV-
SFPSNI--
-SPSNIN-
SEPSNI--
SDPSNI--
SCPSNI--
-TPSNIL-
TSPSNI--
-TPSNIM-
TRPSNI--
-TPSNIH-
-TPSNIF-
TKPSNI--
TVPSNI--
TMPSNI--
TCPSNI--
-TPSNIY-
TLPSNI--
TYPSNI--
TWPSNI--
TTPSNI--
-TPSNIK-
-TPSNII-
-TPSNIE-
TIPSNI--
TFPSNI--
TQPSNI--
-TPSNIV-
-TPSNIS-
-TPSNIG-
-TPSNIA-
TDPSNI--
TAPSNI--

-TPSNIW-
-TPSNIQ-
THPSNI--
TEPSNI--
-VPSNIR-
VSPSNI--
VRPSNI--
-VPSNIL-
VMPSNI--
VLPSNI--
VHPSNI--
-VPSNIS-
-VPSNII-
VTPSNI--
-VPSNIA-
VEPSNI--
-VPSNIN-
-VPSNIM-
-VPSNIH-
-VPSNIG-
VQPSNI--
-VPSNIY-
-VPSNIT-
VVPSNI--
-VPSNIK-
-VPSNIE-
VNPSNI--
VAPSNI--
VYPSNI--
VWPSNI--
-VPSNIW-
VPPSNI--
VKPSNI--
VIPSNI--
-VPSNIQ-
-WPSNIS-
WFPSNI--
WMPSNI--
-WPSNIK-
-WPSNIQ-
WAPSNI--
WPPSNI--
WLPSNI--
WVPSNI--
WRPSNI--
-WPSNIL-
-WPSNII-
WKPSNI--
WHPSNI--
WWPSNI--

WTPSNI--
-WPSNIV-
WYPSNI--
WSPSNI--
-WPSNIY-
-WPSNIT-
-WPSNIF-
WQPSNI--
-WPSNIM-
-WPSNIG-
-WPSNIE-
-WPSNIA-
WNPSNI--
WEPSNI--
WDPSNI--
-YPSNIR-
-YPSNIA-
YNPSNI--
YAPSNI--
-YPSNIS-
YTPSNI--
YGPSNI--
YRPSNI--
YLPSNI--
YVPSNI--
-YPSNIT-
YYPSNI--
YSPSNI--
-YPSNIN-
-YPSNIM-
-YPSNII-
YKPSNI--
YIPSNI--
YWPSNI--
-YPSNIW-
-YPSNIQ-
-YPSNIK-
-YPSNIG-
-YPSNIE-
YPPSNI--
YFPSNI--
-YPSNIY-
-YPSNIV-
-YPSNIH-
YHPSNI--
YEPSNI--
YDPSNI--
PRPM 136 746 32.94
--PRPMKT
-APRPMA-

AVPRPM--
ARPRPM--
ASPRPM--
AQPRPM--
-APRPMM-
-APRPMS-
-APRPMK-
-APRPMR-
-APRPMY-
-APRPMI-
ATPRPM--
AKPRPM--
AIPRPM--
AHPRPM--
AFPRPM--
ACPRPM--
AYPRPM--
-APRPMT-
-APRPMQ-
-APRPME-
AMPRPM--
AGPRPM--
AEPRPM--
AAPRPM--
-APRPMV-
-APRPMN-
-APRPMG-
-APRPMC-
ANPRPM--
-CPRPMS-
CKPRPM--
CGPRPM--
CEPRPM--
CQPRPM--
CVPRPM--
-CPRPMY-
-CPRPMW-
-CPRPMT-
-CPRPMR-
-CPRPMP-
CNPRPM--
-DPRPMS-
-DPRPMY-
-DPRPMK-
-DPRPMI-
DNPRPM--
DGPRPM--
DVPRPM--
DTPRPM--
DRPRPM--

-DPRPMA-
DLPRPM--
DCPRPM--
DYPRPM--
DWPRPM--
DSPRPM--
-DPRPMQ-
-DPRPMN-
-DPRPMF-
DFPRPM--
DEPRPM--
ECPRPM--
ELPRPM--
EMPRPM--
-EPRPMY-
-EPRPMI-
ERPRPM--
-EPRPMK-
-EPRPMS-
-EPRPMF-
-EPRPMA-
EWPRPM--
EVPRPM--
-EPRPMH-
ESPRPM--
EQPRPM--
-EPRPMM-
-EPRPML-
-EPRPME-
ENPRPM--
EIPRPM--
EGPRPM--
EFPRPM--
EAPRPM--
FTPRPM--
FRPRPM--
-FPRPMS-
FWPRPM--
-FPRPMF-
-FPRPMT-
FIPRPM--
FFPRPM--
FKPRPM--
FAPRPM--
-FPRPMA-
FLPRPM--
FEPRPM--
-FPRPMV-
-FPRPMK-
FVPRPM--

FSPRPM--
-FPRPMY-
-FPRPMW-
FQPRPM--
-FPRPMI-
-FPRPMH-
FMPRPM--
FHPRPM--
GTPRPM--
-GPRPMT-
-GPRPMV-
-GPRPMR-
GLPRPM--
-GPRPMA-
-GPRPMI-
GYPRPM--
GRPRPM--
GAPRPM--
GSPRPM--
-GPRPMY-
GHPRPM--
GMPRPM--
GEPRPM--
GVPRPM--
-GPRPMS-
GKPRPM--
-GPRPMQ-
-GPRPMK-
-GPRPMG-
GIPRPM--
GDPRPM--
-GPRPMF-
-GPRPME-
GNPRPM--
-HPRPML-
-HPRPMR-
-HPRPMS-
-HPRPMG-
-HPRPMT-
-HPRPMN-
-HPRPMH-
HLPRPM--
HVPRPM--
HTPRPM--
HRPRPM--
-HPRPMQ-
-HPRPMK-
HYPRPM--
HWPRPM--
HQPRPM--

-HPRPMY-
-HPRPMW-
-HPRPME-
HNPRPM--
HMPRPM--
HGPRPM--
HAPRPM--
-HPRPMV-
-HPRPMP-
-HPRPMM-
-IPRPML-
IFPRPM--
-IPRPMY-
-IPRPMV-
-IPRPMR-
-IPRPMK-
IPPRPM--
INPRPM--
IVPRPM--
ISPRPM--
ILPRPM--
ITPRPM--
-IPRPMI-
IMPRPM--
IGPRPM--
IRPRPM--
IQPRPM--
-IPRPMT-
-IPRPMQ-
-IPRPMN-
IHPRPM--
IDPRPM--
IAPRPM--
IYPRPM--
-IPRPMW-
-IPRPMH-
-IPRPMA-
IIPRPM--
IEPRPM--
KSPRPM--
KWPRPM--
-KPRPMS-
-KPRPMY-
-KPRPMQ-
-KPRPMI-
-KPRPMF-
KIPRPM--
KGPRPM--
-KPRPMT-
-KPRPML-

KEPRPM--
KDPRPM--
KAPRPM--
KRPRPM--
-KPRPMN-
KYPRPM--
KVPRPM--
KTPRPM--
-KPRPMW-
-KPRPMM-
-KPRPMK-
KMPRPM--
KLPRPM--
KKPRPM--
KHPRPM--
KFPRPM--
LMPRPM--
-LPRPML-
LLPRPM--
LRPRPM--
LYPRPM--
LNPRPM--
LTPRPM--
-LPRPMM-
-LPRPMI-
LIPRPM--
-LPRPMY-
LPPRPM--
LHPRPM--
LGPRPM--
-LPRPMS-
LKPRPM--
LSPRPM--
-LPRPMQ-
-LPRPMH-
LEPRPM--
LAPRPM--
LFPRPM--
LCPRPM--
-LPRPMW-
-LPRPMN-
LVPRPM--
-LPRPMG-
-LPRPME-
-LPRPMA-
LWPRPM--
LQPRPM--
-MPRPMA-
-MPRPMR-
-MPRPMY-

-MPRPMQ-
MSPRPM--
-MPRPML-
MYPRPM--
-MPRPMV-
-MPRPMS-
-MPRPMN-
MNPRPM--
MVPRPM--
MHPRPM--
MFPRPM--
-MPRPMI-
MLPRPM--
MGPRPM--
-MPRPMT-
-MPRPMF-
MEPRPM--
MDPRPM--
MAPRPM--
-MPRPMM-
-MPRPMH-
MPPRPM--
MIPRPM--
-NPRPMI-
-NPRPMS-
NVPRPM--
NRPRPM--
-NPRPMQ-
-NPRPMT-
-NPRPMR-
-NPRPML-
-NPRPMA-
NIPRPM--
NSPRPM--
-NPRPMN-
-NPRPMK-
-NPRPMW-
-NPRPMV-
-NPRPMF-
NWPRPM--
NAPRPM--
NLPRPM--
-NPRPMH-
NYPRPM--
NQPRPM--
-NPRPMY-
NNPRPM--
NHPRPM--
NFPRPM--
--PRPMNW

--PRPMLL
--PRPMSW
--PRPMTV
--PRPMSS
--PRPMRS
--PRPMRM
--PRPMYL
--PRPMVS
--PRPMQM
--PRPMIY
--PRPMFT
--PRPMVR
--PRPMLS
--PRPMTS
--PRPMKL
--PRPMVL
--PRPMSQ
--PRPMQG
--PRPMKS
--PRPMIG
PGPRPM--
--PRPMMR
--PRPMES
--PRPMAI
--PRPMYA
--PRPMRF
--PRPMMS
--PRPMLV
--PRPMIH
PVPRPM--
--PRPMYM
--PRPMSY
--PRPMRA
--PRPMLR
--PRPMLE
--PRPMGT
--PRPMYT
--PRPMYG
--PRPMTR
--PRPMTF
--PRPMSR
--PRPMSG
--PRPMSF
--PRPMRN
--PRPMLM
--PRPMLG
--PRPMLF
--PRPMFW
--PRPMAH
--PRPMYS

--PRPMWV
--PRPMVN
--PRPMVG
--PRPMTK
--PRPMSK
--PRPMQL
--PRPMPY
--PRPMPW
--PRPMNL
--PRPMLK
--PRPMHI
--PRPMFV
--PRPMWT
--PRPMVY
--PRPMVW
--PRPMTY
--PRPMTA
--PRPMSH
--PRPMSA
--PRPMNV
--PRPMMF
--PRPMIL
--PRPMIK
--PRPMIF
--PRPMGF
--PRPMFM
--PRPMFG
--PRPMFD
--PRPMDL
--PRPMAS
PSPRPM--
--PRPMYW
--PRPMWS
--PRPMWE
--PRPMRR
--PRPMRI
--PRPMQI
--PRPMPL
--PRPMNT
--PRPMNS
--PRPMMV
--PRPMMT
--PRPMKG
--PRPMIR
--PRPMIQ
--PRPMHS
--PRPMGQ
--PRPMFK
--PRPMEL
--PRPMAT

--PRPMAK
--PRPMYV
--PRPMYN
--PRPMWQ
--PRPMVT
--PRPMVD
--PRPMTL
--PRPMTI
--PRPMSI
--PRPMRD
--PRPMQR
--PRPMPF
--PRPMMY
--PRPMMN
--PRPMMI
--PRPMLQ
--PRPMLH
--PRPMIW
--PRPMIV
--PRPMIA
--PRPMHL
--PRPMGM
--PRPMGI
--PRPMFQ
--PRPMFN
--PRPMFH
--PRPMEA
--PRPMAY
--PRPMAW
--PRPMAV
--PRPMAN
--PRPMAM
--PRPMAG
--PRPMAF
PTPRPM--
PRPRPM--
--PRPMWN
--PRPMWK
--PRPMVV
--PRPMVQ
--PRPMVF
--PRPMVA
--PRPMTW
--PRPMST
--PRPMRW
--PRPMRK
--PRPMQT
--PRPMQN
--PRPMQH
--PRPMPM

--PRPMNQ
--PRPMHR
--PRPMHG
--PRPMHE
--PRPMGY
--PRPMGV
--PRPMGA
--PRPMFY
--PRPMFS
--PRPMFF
--PRPMFA
--PRPMEW
--PRPMDM
--PRPMYF
--PRPMVH
--PRPMRH
--PRPMNN
--PRPMNA
--PRPMMW
--PRPMME
--PRPMMD
--PRPMKI
--PRPMKH
--PRPMKF
--PRPMIT
--PRPMIM
--PRPMII
--PRPMID
--PRPMHY
--PRPMHW
--PRPMHV
--PRPMHD
--PRPMGG
--PRPMEG
--PRPMAR
--PRPMAQ
PLPRPM--
PYPRPM--
--PRPMYD
--PRPMWR
--PRPMWH
--PRPMWG
--PRPMWD
--PRPMVE
--PRPMTN
--PRPMTM
--PRPMQD
--PRPMPI
--PRPMNH
--PRPMMH

--PRPMLD
--PRPMKY
--PRPMKW
--PRPMKV
--PRPMHT
--PRPMHQ
--PRPMHN
--PRPMHK
--PRPMGW
--PRPMEY
--PRPMEV
PNPRPM--
--PRPMYY
--PRPMYQ
--PRPMYK
--PRPMYI
--PRPMYE
--PRPMWM
--PRPMWF
--PRPMWA
--PRPMTT
--PRPMTH
--PRPMTE
--PRPMSE
--PRPMSD
--PRPMRQ
--PRPMRE
--PRPMQS
--PRPMQC
--PRPMQA
--PRPMPS
--PRPMPE
--PRPMNR
--PRPMNM
--PRPMNI
--PRPMMM
--PRPMKQ
--PRPMKK
--PRPMIE
--PRPMHM
--PRPMHA
--PRPMGK
--PRPMFI
--PRPMET
--PRPMEN
--PRPMEM
--PRPMEI
--PRPMDY
--PRPMDW
--PRPMDS

--PRPMDQ
--PRPMDI
--PRPMCL
PQPRPM--
PKPRPM--
PDPRPM--
PCPRPM--
-QPRPML-
-QPRPMT-
QGPRPM--
-QPRPMR-
QTPRPM--
-QPRPMV-
-QPRPMW-
-QPRPMM-
-QPRPMI-
QLPRPM--
QAPRPM--
QVPRPM--
QQPRPM--
-QPRPMG-
-QPRPMF-
-QPRPME-
-QPRPMA-
QMPRPM--
QFPRPM--
QWPRPM--
-QPRPMQ-
QIPRPM--
QEPRPM--
QDPRPM--
-RPRPML-
RSPRPM--
-RPRPMT-
RVPRPM--
RLPRPM--
RAPRPM--
-RPRPMF-
RQPRPM--
-RPRPMM-
RIPRPM--
RYPRPM--
-RPRPMY-
RWPRPM--
-RPRPMI-
-RPRPMS-
-RPRPMN-
-RPRPMA-
-RPRPMH-
RDPRPM--

-RPRPMW-
-RPRPMV-
-RPRPMK-
-RPRPMG-
RCPRPM--
-RPRPME-
RPPRPM--
RMPRPM--
RRPRPM--
RHPRPM--
RFPRPM--
-SPRPMR-
SSPRPM--
-SPRPMI-
SVPRPM--
-SPRPMS-
STPRPM--
-SPRPMY-
SLPRPM--
SYPRPM--
-SPRPMH-
-SPRPMT-
SRPRPM--
-SPRPMG-
SAPRPM--
-SPRPMW-
-SPRPMV-
-SPRPMM-
-SPRPME-
-SPRPML-
SKPRPM--
-SPRPMA-
SIPRPM--
SHPRPM--
SQPRPM--
-SPRPMK-
-SPRPMF-
SMPRPM--
SFPRPM--
SDPRPM--
-SPRPMQ-
SNPRPM--
SGPRPM--
-TPRPMS-
-TPRPML-
TSPRPM--
-TPRPMR-
TIPRPM--
TTPRPM--
-TPRPMI-

TWPRPM--
TLPRPM--
TGPRPM--
-TPRPMH-
TAPRPM--
TFPRPM--
TKPRPM--
-TPRPMF-
TPPRPM--
TYPRPM--
TDPRPM--
TVPRPM--
TQPRPM--
-TPRPMQ-
-TPRPMY-
-TPRPMN-
-TPRPMM-
-TPRPME-
-TPRPMA-
TEPRPM--
-VPRPMR-
-VPRPMN-
-VPRPML-
VSPRPM--
-VPRPMG-
-VPRPMH-
-VPRPMS-
-VPRPMQ-
VTPRPM--
VRPRPM--
VLPRPM--
VIPRPM--
-VPRPMV-
VVPRPM--
-VPRPMI-
VFPRPM--
-VPRPMY-
VHPRPM--
VYPRPM--
-VPRPMA-
VAPRPM--
-VPRPMM-
VGPRPM--
VEPRPM--
VWPRPM--
VQPRPM--
VPPRPM--
VNPRPM--
VCPRPM--
-WPRPMK-

-WPRPMT-
-WPRPMM-
WVPRPM--
WTPRPM--
-WPRPMV-
WWPRPM--
-WPRPMH-
WNPRPM--
WLPRPM--
WKPRPM--
WRPRPM--
-WPRPMS-
-WPRPMN-
-WPRPMG-
WDPRPM--
WSPRPM--
WGPRPM--
WFPRPM--
-YPRPMN-
-YPRPMT-
-YPRPMR-
-YPRPMF-
YMPRPM--
YLPRPM--
-YPRPMS-
YPPRPM--
YSPRPM--
YKPRPM--
YTPRPM--
-YPRPMW-
YHPRPM--
YWPRPM--
YRPRPM--
-YPRPMV-
-YPRPMA-
YVPRPM--
-YPRPMQ-
-YPRPMM-
-YPRPMI-
-YPRPMH-
-YPRPMG-
YNPRPM--
YFPRPM--
YDPRPM--

| | | | |
|---|---|---|---|
| IAHL | 157 | 737 | 32.93 |

--IAHLTV
--IAHLTE
--IAHLSP
--IAHLNR
--IAHLLD

–QIAHLW–
–AIAHLR–
ALIAHL––
–AIAHLL–
–AIAHLG–
–AIAHLV–
–AIAHLY–
–AIAHLS–
ATIAHL––
AIIAHL––
–AIAHLF–
ASIAHL––
–AIAHLT–
–AIAHLM–
AQIAHL––
–AIAHLI–
–AIAHLH–
AEIAHL––
APIAHL––
AHIAHL––
ADIAHL––
AAIAHL––
AKIAHL––
AGIAHL––
–AIAHLQ–
–AIAHLK–
AVIAHL––
AMIAHL––
–AIAHLW–
–AIAHLE–
CCIAHL––
–CIAHLR–
CDIAHL––
CHIAHL––
CTIAHL––
CRIAHL––
–CIAHLW–
CPIAHL––
CMIAHL––
CIIAHL––
–CIAHLS–
–CIAHLL–
–CIAHLI–
CVIAHL––
CSIAHL––
CQIAHL––
CLIAHL––
–CIAHLY–
–CIAHLV–
–CIAHLH–

−CIAHLG−
−DIAHLL−
−DIAHLR−
DTIAHL−−
DSIAHL−−
DLIAHL−−
DEIAHL−−
DVIAHL−−
DRIAHL−−
DPIAHL−−
DIIAHL−−
−DIAHLQ−
DWIAHL−−
DNIAHL−−
−DIAHLV−
−DIAHLT−
DYIAHL−−
−DIAHLY−
DFIAHL−−
DCIAHL−−
DKIAHL−−
−DIAHLS−
−DIAHLE−
DHIAHL−−
DGIAHL−−
ERIAHL−−
EPIAHL−−
−EIAHLA−
EKIAHL−−
−EIAHLN−
−EIAHLL−
−EIAHLH−
EHIAHL−−
EAIAHL−−
ETIAHL−−
−EIAHLS−
−EIAHLK−
EGIAHL−−
EFIAHL−−
EYIAHL−−
−EIAHLG−
−EIAHLF−
EWIAHL−−
EQIAHL−−
ENIAHL−−
−EIAHLT−
−EIAHLR−
−EIAHLQ−
−FIAHLR−
FSIAHL−−

−FIAHLG−
FVIAHL−−
FWIAHL−−
FDIAHL−−
FMIAHL−−
−FIAHLV−
FTIAHL−−
−FIAHLY−
−FIAHLT−
−FIAHLS−
−FIAHLM−
−FIAHLW−
FPIAHL−−
−FIAHLK−
−FIAHLE−
FCIAHL−−
FAIAHL−−
FQIAHL−−
FKIAHL−−
−FIAHLQ−
−FIAHLN−
−FIAHLL−
FHIAHL−−
FGIAHL−−
FNIAHL−−
FIIAHL−−
−FIAHLF−
FEIAHL−−
GVIAHL−−
−GIAHLS−
−GIAHLR−
GTIAHL−−
−GIAHLQ−
GSIAHL−−
GNIAHL−−
−GIAHLA−
−GIAHLV−
GLIAHL−−
GFIAHL−−
GYIAHL−−
GWIAHL−−
GIIAHL−−
−GIAHLK−
−GIAHLG−
GAIAHL−−
−GIAHLH−
−GIAHLY−
−GIAHLM−
GGIAHL−−
GHIAHL−−

GEIAHL--
-GIAHLI-
GPIAHL--
-GIAHLW-
-GIAHLN-
-GIAHLF-
-GIAHLE-
GDIAHL--
GCIAHL--
-HIAHLT-
-HIAHLG-
HNIAHL--
-HIAHLS-
-HIAHLL-
HAIAHL--
HLIAHL--
HPIAHL--
HGIAHL--
HVIAHL--
HMIAHL--
-HIAHLQ-
HWIAHL--
HTIAHL--
HSIAHL--
HIIAHL--
-HIAHLV-
-HIAHLR-
HYIAHL--
HKIAHL--
-HIAHLF-
HEIAHL--
HQIAHL--
-HIAHLW-
-HIAHLM-
-HIAHLK-
-HIAHLN-
-HIAHLD-
-HIAHLA-
--IAHLRQ
--IAHLSL
--IAHLAV
--IAHLST
--IAHLRT
--IAHLRS
--IAHLGL
--IAHLAS
--IAHLRG
--IAHLVT
--IAHLVH
--IAHLTS

```
--IAHLSK
--IAHLRR
--IAHLRL
--IAHLLR
--IAHLLG
--IAHLVR
--IAHLRM
--IAHLLT
--IAHLLF
IVIAHL--
IKIAHL--
ISIAHL--
--IAHLWG
--IAHLVN
--IAHLTG
--IAHLSG
--IAHLQY
--IAHLMR
--IAHLKR
--IAHLTL
--IAHLSV
--IAHLKV
IRIAHL--
IPIAHL--
--IAHLSS
--IAHLMN
--IAHLMA
--IAHLKH
--IAHLAA
--IAHLYT
--IAHLYM
--IAHLWT
--IAHLVQ
--IAHLVK
--IAHLTR
--IAHLMT
--IAHLML
--IAHLKS
--IAHLKG
--IAHLIT
--IAHLAT
--IAHLWQ
--IAHLVL
--IAHLVG
--IAHLTI
--IAHLTH
--IAHLTA
--IAHLMV
--IAHLMD
--IAHLLY
```

```
--IAHLLV
--IAHLLH
--IAHLES
ILIAHL--
-IIAHLT-
--IAHLYV
--IAHLTM
--IAHLSA
--IAHLQA
--IAHLLW
--IAHLKD
--IAHLFN
--IAHLFA
--IAHLAI
-IIAHLW-
-IIAHLR-
-IIAHLK-
--IAHLYH
--IAHLVA
--IAHLSM
--IAHLRE
--IAHLQS
--IAHLQL
--IAHLNS
--IAHLLS
--IAHLKF
--IAHLIM
--IAHLHT
--IAHLGV
--IAHLAR
--IAHLAN
IQIAHL--
--IAHLYS
--IAHLYQ
--IAHLWS
--IAHLWN
--IAHLTQ
--IAHLSQ
--IAHLSF
--IAHLQR
--IAHLNA
--IAHLMS
--IAHLLQ
--IAHLLM
--IAHLLK
--IAHLKQ
--IAHLKN
--IAHLFY
--IAHLFS
--IAHLFQ
```

--IAHLYR
--IAHLYF
--IAHLWL
--IAHLVY
--IAHLSH
--IAHLSD
--IAHLRH
--IAHLNV
--IAHLNT
--IAHLKM
--IAHLIF
--IAHLIE
--IAHLHY
--IAHLHV
--IAHLGG
--IAHLFI
--IAHLEA
--IAHLAW
ITIAHL--
-IIAHLV-
-IIAHLS-
-IIAHLE-
-IIAHLA-
IHIAHL--
--IAHLYG
--IAHLYD
--IAHLVI
--IAHLVF
--IAHLQG
--IAHLNL
--IAHLMI
--IAHLME
--IAHLLA
--IAHLKI
--IAHLIY
--IAHLIG
--IAHLIA
--IAHLHR
--IAHLHN
--IAHLHM
--IAHLGW
--IAHLGT
--IAHLGM
--IAHLGA
--IAHLEG
--IAHLEF
--IAHLAY
--IAHLAQ
--IAHLAK
IYIAHL--

INIAHL--
-IIAHLL-
-IIAHLH-
IGIAHL--
IEIAHL--
IDIAHL--
IAIAHL--
--IAHLYY
--IAHLYK
--IAHLWY
--IAHLWH
--IAHLVM
--IAHLTN
--IAHLTC
--IAHLSY
--IAHLSW
--IAHLSN
--IAHLQT
--IAHLQF
--IAHLNM
--IAHLLN
--IAHLIR
--IAHLHQ
--IAHLHK
--IAHLHD
--IAHLHA
--IAHLFV
--IAHLFR
--IAHLFH
--IAHLFG
--IAHLEW
--IAHLET
--IAHLAM
--IAHLAG
--IAHLAF
IMIAHL--
IIIAHL--
-IIAHLN-
-IIAHLI-
-IIAHLF-
--IAHLYN
--IAHLYE
--IAHLWV
--IAHLVD
--IAHLTW
--IAHLRD
--IAHLQW
--IAHLQV
--IAHLQD
--IAHLPL

--IAHLNY
--IAHLNQ
--IAHLNN
--IAHLNG
--IAHLNF
--IAHLMY
--IAHLLE
--IAHLKW
--IAHLKK
--IAHLKE
--IAHLIV
--IAHLIQ
--IAHLIN
--IAHLID
--IAHLHI
--IAHLHH
--IAHLHG
--IAHLHE
--IAHLGP
--IAHLGF
--IAHLEY
--IAHLDS
--IAHLCV
KVIAHL--
KTIAHL--
KHIAHL--
KGIAHL--
-KIAHLQ-
KFIAHL--
KYIAHL--
-KIAHLW-
KDIAHL--
-KIAHLV-
-KIAHLL-
-KIAHLA-
KEIAHL--
-KIAHLH-
KIIAHL--
-KIAHLN-
-KIAHLG-
-KIAHLE-
KCIAHL--
-LIAHLR-
LSIAHL--
LNIAHL--
-LIAHLL-
LMIAHL--
-LIAHLT-
-LIAHLH-
LHIAHL--

-LIAHLA-
LLIAHL--
LGIAHL--
-LIAHLK-
LQIAHL--
-LIAHLV-
-LIAHLS-
LWIAHL--
-LIAHLM-
-LIAHLE-
LDIAHL--
LIIAHL--
-LIAHLY-
-LIAHLW-
-LIAHLI-
LPIAHL--
-LIAHLQ-
-LIAHLG-
LTIAHL--
LCIAHL--
-LIAHLD-
MAIAHL--
-MIAHLG-
-MIAHLN-
MNIAHL--
-MIAHLH-
MPIAHL--
-MIAHLR-
MGIAHL--
MVIAHL--
-MIAHLT-
-MIAHLI-
MYIAHL--
-MIAHLY-
-MIAHLQ-
MDIAHL--
MIIAHL--
MHIAHL--
MMIAHL--
-MIAHLS-
-MIAHLF-
MFIAHL--
MTIAHL--
-MIAHLA-
NAIAHL--
NYIAHL--
NGIAHL--
NLIAHL--
-NIAHLN-
NIIAHL--

-NIAHLS-
NVIAHL--
NPIAHL--
NKIAHL--
-NIAHLQ-
-NIAHLA-
NSIAHL--
NNIAHL--
-NIAHLY-
-NIAHLT-
NTIAHL--
-NIAHLR-
-NIAHLK-
NHIAHL--
-NIAHLV-
-NIAHLM-
-NIAHLG-
NFIAHL--
-NIAHLW-
-NIAHLI-
-NIAHLH-
NEIAHL--
PRIAHL--
-PIAHLG-
-PIAHLR-
PTIAHL--
PVIAHL--
PWIAHL--
-PIAHLL-
PNIAHL--
PMIAHL--
-PIAHLV-
-PIAHLI-
PGIAHL--
PSIAHL--
PLIAHL--
-PIAHLN-
PIIAHL--
-PIAHLY-
-PIAHLT-
-PIAHLA-
PFIAHL--
-PIAHLW-
-PIAHLQ-
-PIAHLM-
-PIAHLF-
PEIAHL--
PCIAHL--
QPIAHL--
-QIAHLS-

QSIAHL--
-QIAHLL-
QRIAHL--
-QIAHLN-
-QIAHLR-
QAIAHL--
QMIAHL--
-QIAHLV-
-QIAHLK-
QGIAHL--
QLIAHL--
-QIAHLM-
QQIAHL--
-QIAHLI-
-QIAHLG-
QTIAHL--
-QIAHLF-
QFIAHL--
QEIAHL--
QWIAHL--
QVIAHL--
QNIAHL--
QIIAHL--
-QIAHLY-
-QIAHLT-
QCIAHL--
RVIAHL--
RSIAHL--
-RIAHLT-
RTIAHL--
-RIAHLY-
-RIAHLQ-
-RIAHLN-
-RIAHLA-
-RIAHLF-
REIAHL--
RDIAHL--
RHIAHL--
-RIAHLH-
RCIAHL--
-RIAHLE-
-RIAHLM-
-SIAHLR-
-SIAHLH-
-SIAHLT-
SSIAHL--
-SIAHLY-
-SIAHLS-
-SIAHLL-
SIIAHL--

SHIAHL--
SYIAHL--
SNIAHL--
SWIAHL--
STIAHL--
-SIAHLQ-
SAIAHL--
SQIAHL--
-SIAHLA-
SGIAHL--
-SIAHLW-
-SIAHLF-
SDIAHL--
-SIAHLE-
SPIAHL--
SEIAHL--
-SIAHLM-
SCIAHL--
TYIAHL--
TMIAHL--
-TIAHLA-
-TIAHLK-
TVIAHL--
TWIAHL--
TGIAHL--
-TIAHLM-
-TIAHLG-
TNIAHL--
TKIAHL--
-TIAHLW-
-TIAHLR-
-TIAHLQ-
-TIAHLN-
-TIAHLL-
TAIAHL--
-TIAHLV-
TFIAHL--
TQIAHL--
TPIAHL--
-TIAHLY-
-TIAHLT-
-TIAHLS-
TTIAHL--
TIIAHL--
-TIAHLF-
TEIAHL--
TDIAHL--
-TIAHLH-
-TIAHLC-
VYIAHL--

```
-VIAHLQ-
VVIAHL--
-VIAHLR-
VDIAHL--
-VIAHLT-
-VIAHLL-
VAIAHL--
-VIAHLS-
VHIAHL--
VIIAHL--
VGIAHL--
-VIAHLK-
-VIAHLG-
VEIAHL--
VQIAHL--
-VIAHLW-
-VIAHLH-
VNIAHL--
-VIAHLF-
VTIAHL--
VPIAHL--
-VIAHLY-
-VIAHLM-
-VIAHLE-
-VIAHLA-
VWIAHL--
-VIAHLV-
-VIAHLN-
VCIAHL--
-WIAHLA-
WKIAHL--
-WIAHLQ-
WNIAHL--
WYIAHL--
-WIAHLS-
WDIAHL--
-WIAHLW-
-WIAHLR-
WWIAHL--
-WIAHLV-
-WIAHLT-
-WIAHLH-
-WIAHLG-
WAIAHL--
WTIAHL--
WPIAHL--
-WIAHLE-
WGIAHL--
WFIAHL--
WEIAHL--
```

WVIAHL--
-WIAHLY-
-WIAHLN-
-WIAHLL-
-WIAHLK-
WHIAHL--
YTIAHL--
-YIAHLS-
YPIAHL--
YGIAHL--
YAIAHL--
-YIAHLT-
YSIAHL--
-YIAHLN-
-YIAHLL-
YHIAHL--
YIIAHL--
-YIAHLY-
-YIAHLV-
-YIAHLQ-
-YIAHLI-
-YIAHLG-
YEIAHL--
YDIAHL--
YNIAHL--
YMIAHL--
-YIAHLR-
YYIAHL--
-YIAHLH-
YQIAHL--
-YIAHLW-
-YIAHLM-

PAAF 250 690 32.93

-TPAAFG-
-APAAFI-
-APAAFG-
AVPAAF--
ALPAAF--
AFPAAF--
-APAAFY-
-APAAFV-
-APAAFT-
-APAAFR-
-APAAFA-
-APAAFS-
-APAAFH-
-APAAFE-
AKPAAF--
-APAAFN-
-APAAFL-

```
-APAAFW-
-APAAFQ-
-APAAFK-
AGPAAF--
ATPAAF--
ASPAAF--
-APAAFF-
ANPAAF--
AMPAAF--
AHPAAF--
AQPAAF--
-APAAFP-
-APAAFM-
AIPAAF--
AEPAAF--
ADPAAF--
CRPAAF--
-CPAAFV-
CTPAAF--
-CPAAFH-
CYPAAF--
CVPAAF--
CSPAAF--
-CPAAFA-
CIPAAF--
CGPAAF--
CEPAAF--
CAPAAF--
-DPAAFR-
-DPAAFS-
-DPAAFL-
DVPAAF--
-DPAAFK-
-DPAAFI-
-DPAAFA-
DSPAAF--
-DPAAFQ-
DFPAAF--
-DPAAFH-
-DPAAFF-
DEPAAF--
DDPAAF--
DCPAAF--
DAPAAF--
DTPAAF--
DQPAAF--
-DPAAFW-
-DPAAFM-
DLPAAF--
-EPAAFW-
```

EGPAAF--
-EPAAFR-
EYPAAF--
-EPAAFS-
EPPAAF--
-EPAAFM-
EQPAAF--
-EPAAFN-
EMPAAF--
ELPAAF--
EIPAAF--
EWPAAF--
ESPAAF--
ERPAAF--
-EPAAFY-
-EPAAFV-
-EPAAFT-
-EPAAFQ-
-EPAAFK-
-EPAAFI-
-EPAAFF-
-EPAAFE-
EKPAAF--
EAPAAF--
-FPAAFL-
-FPAAFA-
FGPAAF--
-FPAAFQ-
-FPAAFI-
-FPAAFV-
-FPAAFN-
FSPAAF--
-FPAAFS-
-FPAAFK-
-FPAAFH-
FMPAAF--
FWPAAF--
-FPAAFW-
-FPAAFM-
FEPAAF--
FQPAAF--
-FPAAFG-
FKPAAF--
-GPAAFL-
GNPAAF--
GRPAAF--
GSPAAF--
-GPAAFA-
GLPAAF--
GGPAAF--

GEPAAF--
-GPAAFR-
-GPAAFG-
-GPAAFS-
-GPAAFK-
-GPAAFH-
GAPAAF--
-GPAAFT-
-GPAAFN-
-GPAAFM-
GMPAAF--
GQPAAF--
-GPAAFE-
GCPAAF--
GYPAAF--
-GPAAFV-
-GPAAFQ-
-GPAAFI-
GIPAAF--
HGPAAF--
-HPAAFL-
HTPAAF--
-HPAAFG-
HMPAAF--
HLPAAF--
HWPAAF--
HSPAAF--
-HPAAFQ-
-HPAAFN-
-HPAAFH-
-HPAAFT-
-HPAAFK-
HAPAAF--
HHPAAF--
HCPAAF--
HYPAAF--
-HPAAFS-
-HPAAFR-
-HPAAFI-
-HPAAFE-
HIPAAF--
HDPAAF--
-IPAAFS-
-IPAAFA-
IVPAAF--
ITPAAF--
IGPAAF--
ISPAAF--
IRPAAF--
-IPAAFN-

-IPAAFK-
-IPAAFH-
-IPAAFE-
IFPAAF--
IQPAAF--
-IPAAFQ-
-IPAAFM-
ILPAAF--
IHPAAF--
IAPAAF--
-IPAAFI-
INPAAF--
-IPAAFV-
-IPAAFP-
-IPAAFL-
-IPAAFG-
IEPAAF--
-KPAAFL-
KVPAAF--
-KPAAFH-
-KPAAFV-
-KPAAFG-
-KPAAFF-
KTPAAF--
KRPAAF--
-KPAAFW-
-KPAAFY-
-KPAAFS-
-KPAAFK-
KKPAAF--
KIPAAF--
KMPAAF--
KYPAAF--
KLPAAF--
KCPAAF--
-KPAAFA-
KGPAAF--
-KPAAFT-
-KPAAFI-
-KPAAFE-
KHPAAF--
KDPAAF--
LVPAAF--
-LPAAFR-
LSPAAF--
-LPAAFS-
LIPAAF--
LLPAAF--
LGPAAF--
-LPAAFH-

LHPAAF--
LPPAAF--
-LPAAFV-
LNPAAF--
LMPAAF--
-LPAAFM-
-LPAAFK-
LKPAAF--
-LPAAFW-
-LPAAFT-
LAPAAF--
LWPAAF--
-LPAAFQ-
LDPAAF--
LTPAAF--
-LPAAFP-
-LPAAFI-
-LPAAFG-
-LPAAFE-
-LPAAFA-
LEPAAF--
-MPAAFT-
-MPAAFK-
-MPAAFS-
MVPAAF--
-MPAAFR-
-MPAAFN-
-MPAAFV-
MRPAAF--
MYPAAF--
-MPAAFL-
-MPAAFI-
MNPAAF--
MTPAAF--
MSPAAF--
-MPAAFF-
MLPAAF--
MGPAAF--
MEPAAF--
MDPAAF--
-MPAAFQ-
-MPAAFE-
MMPAAF--
MKPAAF--
MPPAAF--
-MPAAFW-
-MPAAFM-
-MPAAFH-
MIPAAF--
MCPAAF--

-NPAAFR-
-NPAAFS-
-NPAAFL-
NLPAAF--
NSPAAF--
NTPAAF--
NMPAAF--
-NPAAFT-
-NPAAFQ-
NFPAAF--
NEPAAF--
NAPAAF--
NPPAAF--
-NPAAFY-
-NPAAFE-
NGPAAF--
NYPAAF--
-NPAAFW-
-NPAAFF-
-NPAAFA-
NNPAAF--
NKPAAF--
NRPAAF--
-NPAAFN-
-NPAAFM-
-NPAAFI-
NCPAAF--
--PAAFRS
--PAAFTL
--PAAFRI
--PAAFVQ
--PAAFRL
--PAAFWR
--PAAFRA
--PAAFWT
--PAAFSL
--PAAFIR
--PAAFVV
--PAAFTA
--PAAFLT
PGPAAF--
--PAAFLI
--PAAFFR
--PAAFSN
--PAAFKI
--PAAFRG
--PAAFYR
--PAAFYQ
--PAAFVW
--PAAFSS

--PAAFRQ
--PAAFLN
--PAAFKL
--PAAFKH
PAPAAF--
--PAAFNK
--PAAFKY
--PAAFHS
--PAAFAY
--PAAFVD
--PAAFTK
--PAAFTG
--PAAFSY
--PAAFSF
--PAAFMT
--PAAFKN
--PAAFGS
--PAAFGM
--PAAFFS
--PAAFWK
--PAAFVR
--PAAFTS
--PAAFLS
--PAAFLA
--PAAFIS
--PAAFGI
--PAAFAS
--PAAFYS
--PAAFVI
--PAAFTW
--PAAFTN
--PAAFTM
--PAAFSG
--PAAFRV
--PAAFQI
--PAAFMH
--PAAFKS
--PAAFIQ
--PAAFGF
PLPAAF--
--PAAFYK
--PAAFYH
--PAAFWE
--PAAFVY
--PAAFVT
--PAAFTT
--PAAFSQ
--PAAFSK
--PAAFPM
--PAAFPL

--PAAFNN
--PAAFLL
--PAAFKK
--PAAFHY
--PAAFHI
--PAAFGV
--PAAFAL
PSPAAF--
PHPAAF--
PFPAAF--
--PAAFYN
--PAAFWS
--PAAFWH
--PAAFWG
--PAAFVL
--PAAFTV
--PAAFST
--PAAFSM
--PAAFRE
--PAAFPY
--PAAFMR
--PAAFMN
--PAAFIL
--PAAFIE
--PAAFHG
--PAAFGY
--PAAFGG
--PAAFFQ
--PAAFAM
--PAAFAA
--PAAFYD
--PAAFWM
--PAAFWD
--PAAFSV
--PAAFSA
--PAAFRT
--PAAFQG
--PAAFNV
--PAAFNM
--PAAFNI
--PAAFMV
--PAAFMS
--PAAFMF
--PAAFLV
--PAAFLG
--PAAFKT
--PAAFHK
--PAAFGW
--PAAFGT
--PAAFAR

--PAAFAQ
--PAAFAK
PRPAAF--
PNPAAF--
--PAAFVK
--PAAFVE
--PAAFTY
--PAAFTR
--PAAFTI
--PAAFTH
--PAAFSE
--PAAFRY
--PAAFRW
--PAAFRN
--PAAFRH
--PAAFQT
--PAAFQA
--PAAFNR
--PAAFNF
--PAAFMM
--PAAFMG
--PAAFLQ
--PAAFLH
--PAAFKM
--PAAFKA
--PAAFIM
--PAAFIK
--PAAFIA
--PAAFHQ
--PAAFHN
--PAAFHD
--PAAFHA
--PAAFFI
--PAAFFA
--PAAFES
--PAAFEA
--PAAFAV
--PAAFAT
--PAAFAH
PYPAAF--
PQPAAF--
PMPAAF--
PKPAAF--
PIPAAF--
PDPAAF--
--PAAFYG
--PAAFWA
--PAAFVN
--PAAFVM
--PAAFTD

--PAAFRF
--PAAFRD
--PAAFQQ
--PAAFQK
--PAAFQD
--PAAFPA
--PAAFNS
--PAAFNH
--PAAFNE
--PAAFNA
--PAAFMY
--PAAFML
--PAAFMK
--PAAFKQ
--PAAFKF
--PAAFKE
--PAAFKD
--PAAFKC
--PAAFII
--PAAFIG
--PAAFID
--PAAFHW
--PAAFHL
--PAAFFY
--PAAFFV
--PAAFFN
--PAAFFH
--PAAFEW
--PAAFEV
--PAAFET
--PAAFCR
QGPAAF--
-QPAAFL-
-QPAAFN-
-QPAAFK-
-QPAAFG-
-QPAAFF-
QNPAAF--
QLPAAF--
QFPAAF--
QSPAAF--
QVPAAF--
QPPAAF--
-QPAAFS-
-QPAAFQ-
-QPAAFI-
-QPAAFA-
QHPAAF--
QWPAAF--
QQPAAF--

QMPAAF--
QKPAAF--
QEPAAF--
QCPAAF--
RLPAAF--
RRPAAF--
-RPAAFL-
-RPAAFA-
-RPAAFR-
-RPAAFM-
-RPAAFK-
RNPAAF--
RVPAAF--
RTPAAF--
RYPAAF--
RSPAAF--
RDPAAF--
-RPAAFQ-
-RPAAFH-
RGPAAF--
RPPAAF--
RMPAAF--
-RPAAFY-
-RPAAFV-
-RPAAFN-
RIPAAF--
RFPAAF--
RAPAAF--
RWPAAF--
RQPAAF--
RHPAAF--
RKPAAF--
-RPAAFG-
-RPAAFW-
-RPAAFE-
-SPAAFL-
SSPAAF--
-SPAAFT-
SLPAAF--
SRPAAF--
-SPAAFY-
-SPAAFS-
-SPAAFA-
SGPAAF--
STPAAF--
-SPAAFW-
SKPAAF--
SEPAAF--
-SPAAFG-
-SPAAFE-

−SPAAFQ−
SVPAAF−−
−SPAAFK−
−SPAAFH−
SAPAAF−−
−SPAAFV−
SMPAAF−−
SHPAAF−−
SPPAAF−−
−SPAAFR−
−SPAAFI−
SNPAAF−−
SIPAAF−−
SWPAAF−−
−SPAAFM−
SYPAAF−−
SQPAAF−−
−SPAAFN−
SFPAAF−−
TLPAAF−−
TIPAAF−−
−TPAAFK−
TYPAAF−−
−TPAAFV−
−TPAAFS−
−TPAAFT−
−TPAAFI−
TVPAAF−−
TTPAAF−−
TPPAAF−−
−TPAAFW−
−TPAAFL−
TAPAAF−−
−TPAAFE−
−TPAAFM−
TGPAAF−−
THPAAF−−
TFPAAF−−
TRPAAF−−
TQPAAF−−
−TPAAFQ−
TNPAAF−−
−TPAAFF−
TWPAAF−−
TSPAAF−−
TKPAAF−−
−TPAAFR−
−TPAAFN−
TMPAAF−−
TDPAAF−−

TCPAAF--
VRPAAF--
-VPAAFH-
-VPAAFT-
-VPAAFM-
VMPAAF--
VSPAAF--
VWPAAF--
VQPAAF--
-VPAAFV-
-VPAAFG-
VLPAAF--
VTPAAF--
-VPAAFS-
-VPAAFQ-
-VPAAFK-
VNPAAF--
VVPAAF--
VPPAAF--
-VPAAFL-
VHPAAF--
-VPAAFN-
-VPAAFA-
VIPAAF--
VEPAAF--
-VPAAFE-
VKPAAF--
VDPAAF--
VAPAAF--
WKPAAF--
WIPAAF--
-WPAAFM-
-WPAAFH-
WLPAAF--
WGPAAF--
-WPAAFR-
WEPAAF--
WYPAAF--
WPPAAF--
WQPAAF--
-WPAAFA-
WFPAAF--
-WPAAFK-
WHPAAF--
-WPAAFV-
-WPAAFQ-
WCPAAF--
-YPAAFK-
-YPAAFS-
YIPAAF--

| | | | |
|---|---|---|---|
| | -YPAAFT- | | |
| | -YPAAFR- | | |
| | YLPAAF-- | | |
| | YGPAAF-- | | |
| | YRPAAF-- | | |
| | -YPAAFV- | | |
| | YYPAAF-- | | |
| | YVPAAF-- | | |
| | YTPAAF-- | | |
| | -YPAAFW- | | |
| | YFPAAF-- | | |
| | YSPAAF-- | | |
| | -YPAAFM- | | |
| | YKPAAF-- | | |
| | YDPAAF-- | | |
| | YAPAAF-- | | |
| | -YPAAFH- | | |
| | -YPAAFA- | | |
| | YMPAAF-- | | |
| | -YPAAFQ- | | |
| | -YPAAFN- | | |
| | -YPAAFL- | | |
| | YEPAAF-- | | |
| PRGF | 399 | 633 | 32.92 |
| | -KPRGFN- | | |
| | -LPRGFG- | | |
| | --PRGFPT | | |
| | --PRGFLD | | |
| | --PRGFGM | | |
| | --PRGFCR | | |
| | -RPRGFD- | | |
| | -TPRGFE- | | |
| | ASPRGF-- | | |
| | -APRGFF- | | |
| | ARPRGF-- | | |
| | -APRGFT- | | |
| | ATPRGF-- | | |
| | -APRGFG- | | |
| | ADPRGF-- | | |
| | -APRGFQ- | | |
| | AKPRGF-- | | |
| | APPRGF-- | | |
| | -APRGFN- | | |
| | -APRGFW- | | |
| | ANPRGF-- | | |
| | AYPRGF-- | | |
| | -APRGFV- | | |
| | -APRGFA- | | |
| | AMPRGF-- | | |
| | AHPRGF-- | | |

AQPRGF--
-APRGFE-
AFPRGF--
AEPRGF--
CFPRGF--
CKPRGF--
CSPRGF--
-CPRGFY-
-CPRGFV-
CGPRGF--
-CPRGFM-
CNPRGF--
CLPRGF--
CYPRGF--
-CPRGFT-
-CPRGFQ-
-CPRGFL-
-CPRGFH-
CHPRGF--
CRPRGF--
CQPRGF--
-CPRGFS-
-CPRGFR-
-CPRGFN-
-CPRGFA-
CCPRGF--
-DPRGFR-
DSPRGF--
-DPRGFS-
-DPRGFL-
-DPRGFH-
DLPRGF--
DYPRGF--
-DPRGFK-
DKPRGF--
DHPRGF--
DFPRGF--
DRPRGF--
DGPRGF--
DAPRGF--
DQPRGF--
-DPRGFT-
-DPRGFQ-
DWPRGF--
-DPRGFF-
DMPRGF--
-DPRGFV-
DNPRGF--
-DPRGFY-
-DPRGFI-

DTPRGF--
-DPRGFW-
-DPRGFN-
-DPRGFM-
DIPRGF--
DDPRGF--
EMPRGF--
-EPRGFL-
EVPRGF--
-EPRGFS-
ELPRGF--
ESPRGF--
-EPRGFY-
-EPRGFW-
-EPRGFR-
-EPRGFK-
-EPRGFA-
EFPRGF--
-EPRGFI-
EKPRGF--
-EPRGFN-
ETPRGF--
-EPRGFQ-
-EPRGFG-
EGPRGF--
-EPRGFV-
-EPRGFM-
-EPRGFH-
-EPRGFT-
-EPRGFF-
ENPRGF--
EIPRGF--
EAPRGF--
EYPRGF--
EWPRGF--
EPPRGF--
ECPRGF--
-FPRGFL-
FEPRGF--
FKPRGF--
FIPRGF--
FSPRGF--
FRPRGF--
-FPRGFN-
FLPRGF--
FDPRGF--
FGPRGF--
-FPRGFV-
FAPRGF--
FTPRGF--

FMPRGF--
FFPRGF--
FHPRGF--
-FPRGFE-
FCPRGF--
FQPRGF--
-FPRGFY-
-FPRGFC-
FPPRGF--
GSPRGF--
-GPRGFV-
-GPRGFL-
GKPRGF--
GGPRGF--
-GPRGFY-
GLPRGF--
-GPRGFR-
-GPRGFH-
-GPRGFG-
GYPRGF--
GNPRGF--
GMPRGF--
GQPRGF--
GRPRGF--
GEPRGF--
GTPRGF--
-GPRGFA-
GPPRGF--
-GPRGFF-
GHPRGF--
GAPRGF--
-GPRGFQ-
-GPRGFE-
GIPRGF--
GCPRGF--
GWPRGF--
-HPRGFS-
HLPRGF--
HGPRGF--
-HPRGFM-
HNPRGF--
HAPRGF--
HRPRGF--
-HPRGFT-
-HPRGFN-
HMPRGF--
-HPRGFQ-
HHPRGF--
HSPRGF--
HVPRGF--

–HPRGFF–
–HPRGFY–
HKPRGF––
HTPRGF––
–HPRGFW–
–HPRGFG–
HEPRGF––
HYPRGF––
–HPRGFH–
–HPRGFC–
HPPRGF––
–IPRGFL–
INPRGF––
ISPRGF––
IRPRGF––
–IPRGFS–
–IPRGFI–
–IPRGFN–
IVPRGF––
–IPRGFG–
IYPRGF––
–IPRGFH–
IQPRGF––
ITPRGF––
–IPRGFY–
–IPRGFT–
IPPRGF––
IDPRGF––
IAPRGF––
–IPRGFM–
–IPRGFA–
–IPRGFE–
–IPRGFW–
IHPRGF––
–IPRGFC–
ICPRGF––
–KPRGFW–
–KPRGFS–
KTPRGF––
–KPRGFY–
KLPRGF––
KAPRGF––
–KPRGFL–
KIPRGF––
–KPRGFI–
–KPRGFT–
KGPRGF––
–KPRGFM–
–KPRGFH–
KDPRGF––

KYPRGF--
KNPRGF--
-KPRGFQ-
-KPRGFF-
KPPRGF--
KEPRGF--
KKPRGF--
-LPRGFI-
LNPRGF--
-LPRGFS-
-LPRGFV-
-LPRGFL-
LIPRGF--
LGPRGF--
LMPRGF--
-LPRGFW-
-LPRGFM-
-LPRGFH-
-LPRGFY-
LKPRGF--
LQPRGF--
-LPRGFF-
LDPRGF--
LEPRGF--
-LPRGFQ-
LCPRGF--
MLPRGF--
-MPRGFQ-
MEPRGF--
MKPRGF--
MMPRGF--
-MPRGFW-
-MPRGFH-
MGPRGF--
-MPRGFY-
MNPRGF--
MQPRGF--
-MPRGFT-
-MPRGFF-
-MPRGFN-
-MPRGFE-
MAPRGF--
-NPRGFS-
-NPRGFV-
NVPRGF--
NTPRGF--
NWPRGF--
-NPRGFT-
-NPRGFN-
-NPRGFI-

-NPRGFH-
NYPRGF--
-NPRGFW-
-NPRGFA-
NPPRGF--
NGPRGF--
NNPRGF--
NQPRGF--
-NPRGFQ-
NAPRGF--
-NPRGFM-
NKPRGF--
-NPRGFE-
NMPRGF--
--PRGFSL
--PRGFFK
--PRGFYS
--PRGFSS
--PRGFLT
--PRGFLK
--PRGFAA
--PRGFSY
--PRGFTL
--PRGFTS
--PRGFLH
--PRGFWN
--PRGFLY
--PRGFLN
--PRGFGT
--PRGFTH
--PRGFSV
--PRGFST
--PRGFSQ
--PRGFAG
--PRGFYT
--PRGFFR
--PRGFWI
--PRGFTG
--PRGFGV
--PRGFGS
--PRGFFL
--PRGFSK
--PRGFIT
--PRGFAV
--PRGFVV
--PRGFTF
--PRGFIY
--PRGFHY
--PRGFMI
--PRGFLF

--PRGFHN
--PRGFFQ
--PRGFTV
--PRGFSN
--PRGFIW
--PRGFYN
--PRGFSH
--PRGFIK
--PRGFHG
--PRGFAW
--PRGFVH
--PRGFVA
--PRGFTM
--PRGFSD
--PRGFMF
--PRGFFN
--PRGFWT
--PRGFVW
--PRGFNY
--PRGFMH
--PRGFIF
--PRGFHQ
--PRGFEG
--PRGFYH
--PRGFVN
--PRGFVG
--PRGFTI
--PRGFSW
--PRGFSG
--PRGFSE
--PRGFQG
--PRGFNW
--PRGFMQ
--PRGFIG
--PRGFFY
PFPRGF--
--PRGFYV
--PRGFYI
--PRGFNV
--PRGFNM
--PRGFNL
--PRGFME
--PRGFIV
--PRGFAM
--PRGFYY
--PRGFWK
--PRGFVQ
--PRGFTQ
--PRGFRE
--PRGFQT

--PRGFNI
--PRGFMG
--PRGFMD
--PRGFIN
--PRGFFE
--PRGFFA
--PRGFAQ
--PRGFAH
--PRGFYF
--PRGFTD
--PRGFMW
--PRGFHT
--PRGFHA
--PRGFFT
--PRGFET
--PRGFAY
PVPRGF--
--PRGFWQ
--PRGFVD
--PRGFQS
--PRGFQM
--PRGFQK
--PRGFHE
--PRGFGG
--PRGFFI
PRPRGF--
--PRGFWS
--PRGFVF
--PRGFQQ
--PRGFQN
--PRGFMV
--PRGFIE
--PRGFHI
--PRGFFW
--PRGFFH
--PRGFYA
--PRGFWF
--PRGFQY
--PRGFNQ
--PRGFNG
--PRGFMY
--PRGFID
--PRGFHF
--PRGFEM
--PRGFYD
--PRGFWG
--PRGFWE
--PRGFQW
--PRGFMN
--PRGFLE

--PRGFKE
--PRGFIH
--PRGFHD
--PRGFGL
PQPRGF--
PIPRGF--
PGPRGF--
PYPRGF--
--PRGFYQ
--PRGFYE
--PRGFVE
--PRGFQF
--PRGFNH
--PRGFNF
--PRGFIL
--PRGFIA
--PRGFHW
--PRGFHM
--PRGFFM
--PRGFEW
--PRGFES
--PRGFEQ
--PRGFCY
PNPRGF--
PKPRGF--
PHPRGF--
PDPRGF--
PTPRGF--
--PRGFWH
--PRGFWD
--PRGFSC
--PRGFRC
--PRGFQV
--PRGFQP
--PRGFPW
--PRGFNE
--PRGFNC
--PRGFMP
--PRGFKC
--PRGFIM
--PRGFHK
--PRGFHH
--PRGFFC
--PRGFER
--PRGFEF
--PRGFDS
--PRGFCG
--PRGFAP
--PRGFAN
-PPRGFM-

-PPRGFG-
-PPRGFF-
PAPRGF--
QAPRGF--
QSPRGF--
-QPRGFH-
QVPRGF--
-QPRGFS-
-QPRGFN-
-QPRGFQ-
QTPRGF--
-QPRGFM-
-QPRGFA-
QLPRGF--
QGPRGF--
-QPRGFV-
-QPRGFI-
-QPRGFF-
-QPRGFT-
QKPRGF--
QHPRGF--
QYPRGF--
QWPRGF--
-QPRGFY-
-QPRGFW-
-QPRGFG-
QPPRGF--
QNPRGF--
-QPRGFE-
QDPRGF--
-RPRGFL-
RSPRGF--
RVPRGF--
-RPRGFI-
-RPRGFS-
RQPRGF--
-RPRGFE-
RIPRGF--
-RPRGFN-
RNPRGF--
-RPRGFF-
RYPRGF--
-RPRGFY-
-RPRGFM-
-RPRGFH-
RDPRGF--
RTPRGF--
-RPRGFW-
RPPRGF--
RKPRGF--

RCPRGF--
-SPRGFL-
-SPRGFT-
SGPRGF--
-SPRGFR-
-SPRGFY-
-SPRGFM-
-SPRGFN-
-SPRGFQ-
-SPRGFV-
STPRGF--
SVPRGF--
SIPRGF--
SSPRGF--
SAPRGF--
SEPRGF--
SQPRGF--
SMPRGF--
-SPRGFE-
SHPRGF--
-SPRGFH-
-SPRGFF-
SDPRGF--
SCPRGF--
-TPRGFL-
-TPRGFT-
-TPRGFS-
-TPRGFN-
TGPRGF--
TSPRGF--
-TPRGFA-
TNPRGF--
-TPRGFW-
TIPRGF--
TAPRGF--
-TPRGFY-
-TPRGFQ-
TQPRGF--
-TPRGFV-
THPRGF--
TFPRGF--
-TPRGFF-
TEPRGF--
TPPRGF--
-VPRGFS-
-VPRGFF-
-VPRGFW-
VPPRGF--
-VPRGFI-
VHPRGF--

VSPRGF--
-VPRGFH-
VGPRGF--
-VPRGFV-
-VPRGFN-
-VPRGFM-
VTPRGF--
-VPRGFE-
VNPRGF--
VQPRGF--
-VPRGFG-
-VPRGFY-
VAPRGF--
VVPRGF--
VIPRGF--
VDPRGF--
-VPRGFQ-
VCPRGF--
WSPRGF--
WRPRGF--
WNPRGF--
WLPRGF--
WEPRGF--
WDPRGF--
-WPRGFM-
WMPRGF--
WGPRGF--
WAPRGF--
WIPRGF--
WYPRGF--
WPPRGF--
WFPRGF--
-WPRGFN-
-WPRGFE-
WTPRGF--
WQPRGF--
-WPRGFH-
-WPRGFQ-
WCPRGF--
-WPRGFI-
-WPRGFG-
-YPRGFT-
-YPRGFV-
YAPRGF--
-YPRGFN-
YSPRGF--
-YPRGFH-
-YPRGFF-
YYPRGF--
-YPRGFQ-

| | | | |
|---|---|---|---|
| | YKPRGF-- | | |
| | YIPRGF-- | | |
| | YQPRGF-- | | |
| | -YPRGFM- | | |
| | -YPRGFE- | | |
| | YPPRGF-- | | |
| | YCPRGF-- | | |
| PKAW | 667 | 565 | 32.92 |
| | -APKAWL- | | |
| | -APKAWF- | | |
| | -APKAWN- | | |
| | -APKAWK- | | |
| | AWPKAW-- | | |
| | AMPKAW-- | | |
| | ASPKAW-- | | |
| | -APKAWS- | | |
| | -APKAWA- | | |
| | AGPKAW-- | | |
| | AEPKAW-- | | |
| | AHPKAW-- | | |
| | -APKAWV- | | |
| | -APKAWH- | | |
| | AAPKAW-- | | |
| | AYPKAW-- | | |
| | AQPKAW-- | | |
| | -APKAWT- | | |
| | -APKAWG- | | |
| | AIPKAW-- | | |
| | AFPKAW-- | | |
| | CAPKAW-- | | |
| | CQPKAW-- | | |
| | -CPKAWR- | | |
| | CLPKAW-- | | |
| | DGPKAW-- | | |
| | -DPKAWS- | | |
| | -DPKAWR- | | |
| | -DPKAWM- | | |
| | DYPKAW-- | | |
| | -DPKAWV- | | |
| | -DPKAWI- | | |
| | DNPKAW-- | | |
| | DVPKAW-- | | |
| | DRPKAW-- | | |
| | -DPKAWA- | | |
| | DIPKAW-- | | |
| | DAPKAW-- | | |
| | DSPKAW-- | | |
| | DQPKAW-- | | |
| | -DPKAWY- | | |
| | -DPKAWK- | | |

-DPKAWH-
DMPKAW--
DLPKAW--
DKPKAW--
DFPKAW--
DEPKAW--
-EPKAWI-
EPPKAW--
-EPKAWF-
EQPKAW--
-EPKAWR-
ERPKAW--
-EPKAWY-
-EPKAWN-
EGPKAW--
-EPKAWM-
-EPKAWL-
EWPKAW--
-EPKAWV-
-EPKAWG-
-EPKAWA-
EMPKAW--
EIPKAW--
EEPKAW--
FLPKAW--
FGPKAW--
FIPKAW--
-FPKAWL-
-FPKAWV-
-FPKAWT-
-FPKAWM-
FYPKAW--
-FPKAWS-
FMPKAW--
FKPKAW--
FHPKAW--
FPPKAW--
-FPKAWF-
-FPKAWA-
FNPKAW--
FFPKAW--
GSPKAW--
-GPKAWT-
-GPKAWR-
GTPKAW--
-GPKAWA-
GLPKAW--
GAPKAW--
GPPKAW--
-GPKAWY-

-GPKAWV-
-GPKAWS-
GVPKAW--
GRPKAW--
-GPKAWN-
-GPKAWM-
-GPKAWI-
GGPKAW--
GQPKAW--
GIPKAW--
GWPKAW--
-GPKAWW-
-GPKAWQ-
-GPKAWF-
GNPKAW--
GMPKAW--
GKPKAW--
GDPKAW--
HRPKAW--
HIPKAW--
-HPKAWY-
HSPKAW--
HMPKAW--
-HPKAWR-
HWPKAW--
-HPKAWL-
-HPKAWI-
-HPKAWK-
-HPKAWH-
HLPKAW--
HHPKAW--
HVPKAW--
HPPKAW--
-HPKAWT-
-HPKAWM-
HAPKAW--
ITPKAW--
-IPKAWI-
IGPKAW--
IFPKAW--
-IPKAWR-
INPKAW--
IEPKAW--
-IPKAWT-
ISPKAW--
IAPKAW--
IWPKAW--
-IPKAWY-
-IPKAWK-
ILPKAW--

IVPKAW--
IRPKAW--
-IPKAWL-
-IPKAWA-
IHPKAW--
-KPKAWS-
-KPKAWK-
-KPKAWR-
KTPKAW--
-KPKAWL-
KVPKAW--
-KPKAWF-
KMPKAW--
KLPKAW--
KGPKAW--
KEPKAW--
KRPKAW--
KQPKAW--
-KPKAWY-
-KPKAWW-
-KPKAWV-
-KPKAWA-
KIPKAW--
KAPKAW--
-LPKAWL-
-LPKAWK-
LLPKAW--
LVPKAW--
LTPKAW--
LMPKAW--
-LPKAWR-
-LPKAWA-
-LPKAWY-
LHPKAW--
LSPKAW--
-LPKAWT-
-LPKAWF-
-LPKAWQ-
LGPKAW--
LQPKAW--
LDPKAW--
LPPKAW--
-LPKAWW-
-LPKAWS-
-LPKAWN-
-LPKAWM-
-LPKAWI-
LIPKAW--
LFPKAW--
LEPKAW--

MMPKAW--
-MPKAWL-
-MPKAWK-
MYPKAW--
-MPKAWW-
MIPKAW--
MSPKAW--
MQPKAW--
-MPKAWY-
-MPKAWT-
MPPKAW--
-MPKAWV-
-MPKAWS-
-MPKAWF-
MGPKAW--
-MPKAWH-
-MPKAWA-
MFPKAW--
MEPKAW--
MAPKAW--
NVPKAW--
NDPKAW--
NQPKAW--
-NPKAWV-
NLPKAW--
NWPKAW--
NGPKAW--
NYPKAW--
NHPKAW--
-NPKAWA-
NNPKAW--
NTPKAW--
NSPKAW--
-NPKAWS-
-NPKAWK-
NKPKAW--
NPPKAW--
-NPKAWT-
-NPKAWI-
NMPKAW--
NIPKAW--
NEPKAW--
NAPKAW--
--PKAWRT
--PKAWRL
--PKAWSY
--PKAWYR
--PKAWQG
--PKAWFA
--PKAWWN

--PKAWRS
--PKAWAL
--PKAWRH
--PKAWTH
--PKAWTF
--PKAWSI
--PKAWFR
--PKAWAI
--PKAWSL
--PKAWLV
--PKAWLN
--PKAWFT
--PKAWFL
--PKAWVN
--PKAWVA
--PKAWKK
--PKAWSS
--PKAWSN
--PKAWLH
--PKAWHS
--PKAWAT
--PKAWAK
--PKAWSA
--PKAWQV
--PKAWQA
--PKAWNS
--PKAWIN
--PKAWHF
--PKAWYG
--PKAWTT
--PKAWTS
--PKAWPY
--PKAWML
--PKAWMI
--PKAWLA
--PKAWIV
--PKAWIF
--PKAWHL
--PKAWFH
PRPKAW--
--PKAWYY
--PKAWWK
--PKAWVI
--PKAWSQ
--PKAWRQ
--PKAWMY
--PKAWLI
--PKAWHR
--PKAWAS
--PKAWAH

PSPKAW--
--PKAWYW
--PKAWYT
--PKAWWG
--PKAWWA
--PKAWTQ
--PKAWTM
--PKAWRA
--PKAWLQ
--PKAWKI
--PKAWGS
--PKAWGI
PFPKAW--
--PKAWYV
--PKAWVV
--PKAWTG
--PKAWSG
--PKAWRW
--PKAWRN
--PKAWRF
--PKAWQS
--PKAWQH
--PKAWNA
--PKAWIY
--PKAWIK
--PKAWIH
--PKAWHV
--PKAWHQ
--PKAWHM
--PKAWHG
--PKAWGM
--PKAWFV
--PKAWEL
--PKAWAA
PIPKAW--
--PKAWYS
--PKAWYH
--PKAWWW
--PKAWWF
--PKAWVW
--PKAWVQ
--PKAWVF
--PKAWVE
--PKAWTE
--PKAWTD
--PKAWSW
--PKAWST
--PKAWQL
--PKAWNM
--PKAWMV

--PKAWMN
--PKAWMG
--PKAWMF
--PKAWMA
--PKAWLK
--PKAWLG
--PKAWKA
--PKAWIT
--PKAWIM
--PKAWIL
--PKAWGT
--PKAWGF
--PKAWEW
--PKAWAQ
--PKAWAF
PVPKAW--
PTPKAW--
-PPKAWL-
PLPKAW--
--PKAWYQ
--PKAWYN
--PKAWYM
--PKAWYF
--PKAWWT
--PKAWWL
--PKAWVH
--PKAWVG
--PKAWTV
--PKAWTL
--PKAWTK
--PKAWTI
--PKAWSH
--PKAWRE
--PKAWQY
--PKAWQW
--PKAWQR
--PKAWQN
--PKAWQI
--PKAWQF
--PKAWNV
--PKAWNR
--PKAWNG
--PKAWMT
--PKAWMM
--PKAWLM
--PKAWIG
--PKAWHW
--PKAWHT
--PKAWHN
--PKAWGV

--PKAWFI
--PKAWFF
--PKAWFD
--PKAWES
--PKAWEM
--PKAWEF
--PKAWEA
--PKAWDL
--PKAWAY
QYPKAW--
QAPKAW--
QGPKAW--
-QPKAWV-
QVPKAW--
QTPKAW--
QSPKAW--
-QPKAWH-
-QPKAWA-
QMPKAW--
QLPKAW--
QDPKAW--
QQPKAW--
-QPKAWT-
-QPKAWI-
QFPKAW--
QPPKAW--
-QPKAWW-
-QPKAWN-
-QPKAWK-
-QPKAWF-
QEPKAW--
-RPKAWS-
RTPKAW--
RAPKAW--
-RPKAWT-
RVPKAW--
RLPKAW--
RQPKAW--
-RPKAWF-
RIPKAW--
RDPKAW--
RYPKAW--
RRPKAW--
-RPKAWA-
RHPKAW--
RGPKAW--
-RPKAWK-
RKPKAW--
REPKAW--
RPPKAW--

-RPKAWY-
-RPKAWQ-
-RPKAWN-
-RPKAWH-
RNPKAW--
RWPKAW--
-RPKAWM-
-SPKAWH-
SRPKAW--
-SPKAWA-
STPKAW--
SSPKAW--
-SPKAWV-
SNPKAW--
SLPKAW--
SFPKAW--
SPPKAW--
-SPKAWL-
SMPKAW--
SWPKAW--
-SPKAWQ-
-SPKAWM-
SIPKAW--
-SPKAWK-
SKPKAW--
SAPKAW--
SVPKAW--
-SPKAWN-
SGPKAW--
SEPKAW--
-SPKAWS-
-SPKAWF-
-SPKAWE-
SHPKAW--
-TPKAWR-
TVPKAW--
TTPKAW--
TRPKAW--
-TPKAWT-
-TPKAWF-
-TPKAWM-
-TPKAWL-
TLPKAW--
-TPKAWV-
-TPKAWA-
TSPKAW--
-TPKAWK-
TPPKAW--
-TPKAWI-
TMPKAW--

```
-TPKAWQ-
TNPKAW--
TYPKAW--
TWPKAW--
TQPKAW--
-TPKAWN-
-TPKAWH-
TGPKAW--
TEPKAW--
VSPKAW--
-VPKAWV-
-VPKAWR-
VKPKAW--
VVPKAW--
VQPKAW--
-VPKAWA-
-VPKAWL-
VNPKAW--
-VPKAWY-
-VPKAWH-
VMPKAW--
-VPKAWK-
VAPKAW--
VYPKAW--
VFPKAW--
VWPKAW--
-VPKAWQ-
-VPKAWN-
-VPKAWI-
-VPKAWF-
VGPKAW--
-VPKAWW-
-VPKAWG-
VLPKAW--
VIPKAW--
VHPKAW--
-WPKAWY-
-WPKAWM-
WVPKAW--
WGPKAW--
WSPKAW--
WTPKAW--
WKPKAW--
WAPKAW--
-WPKAWS-
WEPKAW--
WWPKAW--
WRPKAW--
WQPKAW--
-WPKAWV-
```

```
            -WPKAWT-
            -WPKAWI-
            -WPKAWF-
            WMPKAW--
            WLPKAW--
            WIPKAW--
            -YPKAWR-
            YAPKAW--
            YSPKAW--
            -YPKAWV-
            YYPKAW--
            YMPKAW--
            -YPKAWY-
            -YPKAWS-
            -YPKAWQ-
            YLPKAW--
            YFPKAW--
            YVPKAW--
            YTPKAW--
            -YPKAWG-
            YNPKAW--
            YIPKAW--
            YDPKAW--
IGAL          78        780     32.90
            AAIGAL--
            --IGALRM
            --IGALPV
            --IGALKP
            --IGALGV
            --IGALEL
            --IGALAN
            --IGALAK
            -KIGALA-
            -LIGALP-
            -NIGALE-
            -RIGALE-
            -SIGALP-
            -TIGALG-
            TEIGAL--
            VDIGAL--
            -AIGALR-
            -AIGALS-
            ASIGAL--
            ALIGAL--
            ATIGAL--
            -AIGALK-
            AHIGAL--
            AVIGAL--
            AWIGAL--
            AIIGAL--
```

```
-AIGALT-
-AIGALH-
ANIGAL--
-AIGALQ-
APIGAL--
-AIGALF-
-AIGALI-
AGIGAL--
-AIGALV-
-AIGALN-
ACIGAL--
AKIGAL--
-AIGALA-
AEIGAL--
-AIGALM-
AFIGAL--
-AIGALC-
AQIGAL--
-AIGALW-
-CIGALF-
CWIGAL--
CKIGAL--
-CIGALT-
-CIGALL-
CTIGAL--
-CIGALV-
-CIGALS-
-CIGALQ-
-CIGALM-
CSIGAL--
CIIGAL--
-CIGALR-
-CIGALH-
CCIGAL--
CAIGAL--
CYIGAL--
CMIGAL--
CLIGAL--
-CIGALW-
-CIGALN-
-CIGALK-
-CIGALI-
CGIGAL--
CFIGAL--
CEIGAL--
CDIGAL--
DRIGAL--
-DIGALT-
-DIGALH-
DGIGAL--
```

DFIGAL--
DQIGAL--
DLIGAL--
-DIGALR-
DSIGAL--
-DIGALK-
DYIGAL--
DPIGAL--
-DIGALS-
DHIGAL--
DAIGAL--
DVIGAL--
-DIGALN-
DIIGAL--
DMIGAL--
DKIGAL--
-DIGALY-
-DIGALM-
-DIGALF-
DWIGAL--
-DIGALW-
-DIGALI-
-DIGALA-
-EIGALR-
-EIGALS-
ESIGAL--
EFIGAL--
ETIGAL--
EKIGAL--
EPIGAL--
EMIGAL--
-EIGALL-
ELIGAL--
-EIGALM-
EGIGAL--
-EIGALH-
EVIGAL--
EQIGAL--
ENIGAL--
EIIGAL--
-EIGALY-
-EIGALT-
-EIGALF-
EAIGAL--
-EIGALV-
-EIGALQ-
-EIGALN-
-EIGALK-
-EIGALW-
-EIGALI-

-EIGALA-
EHIGAL--
FRIGAL--
FLIGAL--
FWIGAL--
-FIGALQ-
-FIGALS-
-FIGALH-
FYIGAL--
-FIGALT-
FPIGAL--
-FIGALA-
FDIGAL--
FKIGAL--
-FIGALN-
FNIGAL--
-FIGALR-
-FIGALL-
-FIGALK-
FVIGAL--
-FIGALG-
FQIGAL--
FIIGAL--
-FIGALY-
FMIGAL--
-FIGALE-
FEIGAL--
-GIGALK-
-GIGALR-
GSIGAL--
GTIGAL--
GLIGAL--
GRIGAL--
GQIGAL--
-GIGALN-
-GIGALI-
GVIGAL--
GPIGAL--
GIIGAL--
-GIGALA-
GWIGAL--
GNIGAL--
-GIGALV-
GHIGAL--
-GIGALF-
-GIGALW-
-GIGALH-
GGIGAL--
GYIGAL--
GMIGAL--

GFIGAL--
GEIGAL--
-GIGALE-
HTIGAL--
-HIGALL-
HGIGAL--
-HIGALR-
HSIGAL--
-HIGALS-
HYIGAL--
HRIGAL--
-HIGALT-
HNIGAL--
HLIGAL--
-HIGALN-
HVIGAL--
HFIGAL--
HKIGAL--
-HIGALY-
-HIGALV-
HWIGAL--
HQIGAL--
HPIGAL--
HMIGAL--
-HIGALF-
HAIGAL--
HIIGAL--
-HIGALQ-
-HIGALI-
-HIGALA-
HHIGAL--
HDIGAL--
-HIGALH-
HCIGAL--
-HIGALM-
HEIGAL--
-HIGALW-
--IGALVS
--IGALRT
--IGALRS
IRIGAL--
--IGALAA
--IGALGY
--IGALSS
--IGALVA
--IGALRN
--IGALHL
--IGALHV
--IGALRH
--IGALAL

```
--IGALTM
--IGALQA
--IGALSG
IKIGAL--
--IGALVK
--IGALTT
--IGALKT
--IGALIG
--IGALWR
--IGALST
--IGALKV
--IGALFA
--IGALAS
ITIGAL--
--IGALSW
--IGALPL
--IGALVY
--IGALVN
--IGALMK
--IGALLG
-IIGALK-
-IIGALR-
--IGALHR
--IGALHK
--IGALYA
--IGALVG
--IGALTN
--IGALSF
--IGALQS
--IGALNS
--IGALMS
--IGALLV
--IGALKY
--IGALFH
--IGALAI
--IGALYT
--IGALTA
--IGALLK
--IGALKI
--IGALTV
--IGALRF
--IGALRA
--IGALNL
IVIGAL--
INIGAL--
-IIGALN-
--IGALTK
--IGALRV
--IGALMV
--IGALLQ
```

```
--IGALGL
--IGALFT
--IGALAT
--IGALAM
IYIGAL--
--IGALYN
--IGALWS
--IGALTS
--IGALRQ
--IGALQG
--IGALNT
--IGALMI
--IGALIR
--IGALHI
ISIGAL--
-IIGALT-
-IIGALA-
--IGALWN
--IGALVT
--IGALSV
--IGALQV
--IGALMR
--IGALLY
--IGALIT
--IGALIA
--IGALGM
--IGALGF
-IIGALI-
IHIGAL--
--IGALYS
--IGALWD
--IGALTY
--IGALSN
--IGALSI
--IGALPI
--IGALNA
--IGALMN
--IGALMG
--IGALKR
--IGALKL
--IGALKF
--IGALIK
--IGALHF
--IGALGI
--IGALYE
--IGALVL
--IGALVI
--IGALVF
--IGALRW
--IGALQW
```

--IGALNY
--IGALMD
--IGALLW
--IGALHY
--IGALHW
--IGALHS
--IGALGS
--IGALFD
--IGALAW
--IGALAV
--IGALAG
IMIGAL--
-IIGALH-
--IGALTL
--IGALTH
--IGALSM
--IGALQY
--IGALNW
--IGALNR
--IGALME
--IGALKQ
--IGALKH
--IGALKA
--IGALIY
--IGALHA
--IGALFN
--IGALFG
--IGALEA
IPIGAL--
--IGALWE
--IGALTF
--IGALSY
--IGALQT
--IGALMT
--IGALIW
--IGALHH
--IGALFS
-IIGALG-
--IGALYK
--IGALWW
--IGALWT
--IGALVH
--IGALVE
--IGALTW
--IGALQI
--IGALNK
--IGALMA
--IGALLE
--IGALKN
--IGALKK

--IGALII
--IGALHQ
--IGALHM
--IGALHG
--IGALGG
--IGALFQ
--IGALES
IDIGAL--
IWIGAL--
--IGALYQ
--IGALWQ
--IGALTQ
--IGALRE
--IGALRD
--IGALQR
--IGALNM
--IGALNF
--IGALMY
--IGALMF
--IGALKG
--IGALKD
--IGALIH
--IGALID
IIIGAL--
-IIGALV-
-IIGALL-
--IGALVV
--IGALTE
--IGALQK
--IGALPF
--IGALNQ
--IGALNN
--IGALNI
--IGALMW
--IGALLM
--IGALLH
--IGALKW
--IGALIV
--IGALIQ
--IGALIE
--IGALHD
--IGALGT
--IGALFE
--IGALAF
ICIGAL--
IAIGAL--
-IIGALW-
-IIGALQ-
-IIGALE-
--IGALYP

--IGALWM
--IGALTD
--IGALQL
--IGALQF
--IGALQD
--IGALPM
--IGALPA
--IGALNV
--IGALNP
--IGALNG
--IGALIN
--IGALIF
--IGALGC
--IGALGA
--IGALEW
--IGALEV
--IGALEM
--IGALEG
KGIGAL--
-KIGALR-
-KIGALM-
KVIGAL--
KSIGAL--
-KIGALY-
-KIGALQ-
-KIGALK-
KPIGAL--
-KIGALL-
KMIGAL--
-KIGALI-
KYIGAL--
-KIGALH-
KCIGAL--
KTIGAL--
KEIGAL--
-KIGALV-
KAIGAL--
KQIGAL--
-KIGALW-
-KIGALS-
-KIGALN-
KHIGAL--
KDIGAL--
KWIGAL--
KNIGAL--
LSIGAL--
LGIGAL--
-LIGALT-
LQIGAL--
LLIGAL--

-LIGALA-
LWIGAL--
LTIGAL--
LFIGAL--
LPIGAL--
LNIGAL--
-LIGALH-
-LIGALN-
LYIGAL--
-LIGALL-
-LIGALG-
LEIGAL--
LAIGAL--
-LIGALQ-
LDIGAL--
-LIGALW-
LIIGAL--
-LIGALM-
LHIGAL--
LCIGAL--
MRIGAL--
MSIGAL--
-MIGALL-
MKIGAL--
-MIGALT-
-MIGALS-
MTIGAL--
-MIGALG-
MHIGAL--
MPIGAL--
MNIGAL--
-MIGALN-
-MIGALI-
MVIGAL--
MLIGAL--
-MIGALK-
-MIGALH-
-MIGALF-
MWIGAL--
MQIGAL--
MIIGAL--
-MIGALW-
-MIGALA-
MGIGAL--
MYIGAL--
MMIGAL--
-MIGALQ-
-MIGALM-
MDIGAL--
-MIGALE-

MEIGAL--
MCIGAL--
NSIGAL--
NRIGAL--
NAIGAL--
-NIGALK-
NVIGAL--
-NIGALN-
-NIGALL-
-NIGALH-
-NIGALV-
NPIGAL--
-NIGALS-
NNIGAL--
NKIGAL--
NTIGAL--
-NIGALF-
-NIGALT-
-NIGALQ-
NQIGAL--
NDIGAL--
NHIGAL--
-NIGALW-
NFIGAL--
NYIGAL--
-NIGALG-
NCIGAL--
NIIGAL--
-NIGALA-
NMIGAL--
-NIGALM-
PRIGAL--
PMIGAL--
PWIGAL--
-PIGALR-
-PIGALK-
PTIGAL--
PAIGAL--
PVIGAL--
-PIGALY-
PNIGAL--
-PIGALQ-
-PIGALN-
PYIGAL--
PKIGAL--
-PIGALS-
PLIGAL--
-PIGALI-
PHIGAL--
PFIGAL--

```
-PIGALV-
-PIGALG-
-PIGALH-
PCIGAL--
PQIGAL--
-PIGALF-
-PIGALA-
PDIGAL--
-PIGALW-
-PIGALM-
-PIGALT-
PPIGAL--
PEIGAL--
QSIGAL--
QLIGAL--
QRIGAL--
-QIGALA-
QVIGAL--
QFIGAL--
-QIGALT-
-QIGALK-
QGIGAL--
QTIGAL--
-QIGALF-
-QIGALR-
-QIGALN-
-QIGALH-
QAIGAL--
-QIGALS-
QPIGAL--
-QIGALM-
QQIGAL--
QIIGAL--
-QIGALW-
-QIGALI-
QNIGAL--
-QIGALY-
-QIGALV-
QEIGAL--
QYIGAL--
QWIGAL--
QHIGAL--
-QIGALQ-
-QIGALG-
RVIGAL--
-RIGALL-
-RIGALK-
-RIGALS-
-RIGALT-
-RIGALI-
```

RTIGAL--
-RIGALV-
-RIGALH-
-RIGALQ-
RAIGAL--
RPIGAL--
RDIGAL--
RMIGAL--
-RIGALN-
-RIGALF-
-RIGALW-
-RIGALM-
-RIGALA-
REIGAL--
RCIGAL--
-SIGALS-
STIGAL--
SNIGAL--
SKIGAL--
SVIGAL--
-SIGALI-
SGIGAL--
-SIGALF-
-SIGALK-
SWIGAL--
-SIGALW-
-SIGALL-
SFIGAL--
SAIGAL--
-SIGALT-
-SIGALH-
SHIGAL--
-SIGALV-
SPIGAL--
SEIGAL--
-SIGALQ-
SIIGAL--
-SIGALM-
SDIGAL--
TRIGAL--
-TIGALR-
TKIGAL--
TSIGAL--
TYIGAL--
-TIGALS-
-TIGALH-
TGIGAL--
TVIGAL--
-TIGALV-
-TIGALF-

TWIGAL--
-TIGALQ-
TIIGAL--
TAIGAL--
-TIGALW-
-TIGALN-
-TIGALY-
-TIGALL-
-TIGALK-
-TIGALA-
THIGAL--
TFIGAL--
TPIGAL--
-TIGALM-
-TIGALI-
TMIGAL--
TDIGAL--
-TIGALE-
VRIGAL--
VTIGAL--
VSIGAL--
VKIGAL--
VLIGAL--
VAIGAL--
-VIGALR-
-VIGALN-
VYIGAL--
VEIGAL--
VFIGAL--
VPIGAL--
VIIGAL--
-VIGALA-
-VIGALT-
-VIGALM-
VQIGAL--
-VIGALV-
-VIGALS-
-VIGALI-
VGIGAL--
VWIGAL--
-VIGALQ-
VCIGAL--
VMIGAL--
-VIGALH-
VHIGAL--
WGIGAL--
-WIGALK-
-WIGALR-
WRIGAL--
-WIGALQ-

```
-WIGALL-
WDIGAL--
WWIGAL--
WVIGAL--
WKIGAL--
-WIGALN-
-WIGALI-
WHIGAL--
WLIGAL--
WIIGAL--
-WIGALM-
-WIGALH-
-WIGALF-
-WIGALT-
-WIGALE-
WEIGAL--
WMIGAL--
-WIGALG-
-WIGALA-
WNIGAL--
WPIGAL--
-WIGALV-
YTIGAL--
YRIGAL--
-YIGALV-
-YIGALT-
-YIGALN-
-YIGALL-
YLIGAL--
YKIGAL--
-YIGALK-
-YIGALR-
-YIGALQ-
YQIGAL--
YAIGAL--
YVIGAL--
-YIGALS-
YGIGAL--
YSIGAL--
YPIGAL--
YIIGAL--
YEIGAL--
-YIGALI-
-YIGALH-
YNIGAL--
YMIGAL--
-YIGALW-
YHIGAL--
-YIGALM-
YYIGAL--
```

```
          YWIGAL--
          -YIGALF-
          -YIGALE-
          YCIGAL--
PHGL        23        839     32.90
          -DPHGLY-
          -GPHGLK-
          -LPHGLA-
          --PHGLNE
          --PHGLIL
          --PHGLAR
          -QPHGLG-
          -RPHGLG-
          -RPHGLD-
          -APHGLA-
          -APHGLS-
          AEPHGL--
          AIPHGL--
          AGPHGL--
          AAPHGL--
          ARPHGL--
          -APHGLQ-
          -APHGLG-
          APPHGL--
          -APHGLL-
          AKPHGL--
          -APHGLT-
          -APHGLK-
          -APHGLE-
          ATPHGL--
          ASPHGL--
          ANPHGL--
          -APHGLN-
          -APHGLH-
          ALPHGL--
          AVPHGL--
          -APHGLW-
          -APHGLV-
          AYPHGL--
          AWPHGL--
          AQPHGL--
          AFPHGL--
          ACPHGL--
          -APHGLI-
          -APHGLM-
          -APHGLF-
          AMPHGL--
          CCPHGL--
          CTPHGL--
          -CPHGLK-
```

-CPHGLH-
CYPHGL--
CVPHGL--
CRPHGL--
-CPHGLY-
-CPHGLR-
-CPHGLI-
CKPHGL--
CSPHGL--
CQPHGL--
-CPHGLW-
-CPHGLT-
-CPHGLN-
-CPHGLF-
CNPHGL--
CGPHGL--
CFPHGL--
-DPHGLR-
-DPHGLI-
-DPHGLF-
-DPHGLK-
DLPHGL--
DKPHGL--
-DPHGLN-
DVPHGL--
-DPHGLT-
DNPHGL--
DFPHGL--
DYPHGL--
DEPHGL--
DWPHGL--
-DPHGLQ-
-DPHGLH-
DIPHGL--
DHPHGL--
DTPHGL--
DSPHGL--
DRPHGL--
-DPHGLV-
-DPHGLS-
-DPHGLE-
DMPHGL--
DCPHGL--
DAPHGL--
-EPHGLK-
EMPHGL--
EVPHGL--
-EPHGLY-
ELPHGL--
ERPHGL--

EGPHGL--
-EPHGLI-
-EPHGLV-
-EPHGLQ-
-EPHGLL-
EFPHGL--
EYPHGL--
ETPHGL--
-EPHGLS-
EHPHGL--
-EPHGLA-
ENPHGL--
EDPHGL--
EAPHGL--
EWPHGL--
EQPHGL--
-EPHGLW-
-EPHGLT-
-EPHGLR-
-EPHGLM-
EIPHGL--
EEPHGL--
-FPHGLL-
FKPHGL--
-FPHGLM-
FDPHGL--
-FPHGLT-
-FPHGLS-
-FPHGLA-
FLPHGL--
FAPHGL--
-FPHGLN-
FQPHGL--
-FPHGLI-
FMPHGL--
FIPHGL--
-FPHGLV-
FGPHGL--
FSPHGL--
FFPHGL--
FEPHGL--
FPPHGL--
-FPHGLR-
-FPHGLE-
FHPHGL--
FCPHGL--
FVPHGL--
FTPHGL--
-FPHGLY-
-FPHGLK-

-FPHGLH-
-FPHGLG-
-FPHGLF-
FNPHGL--
GHPHGL--
-GPHGLW-
-GPHGLN-
GYPHGL--
GAPHGL--
-GPHGLT-
GVPHGL--
GTPHGL--
-GPHGLF-
-GPHGLS-
-GPHGLM-
GKPHGL--
GDPHGL--
-GPHGLL-
-GPHGLH-
GLPHGL--
GRPHGL--
GQPHGL--
GFPHGL--
-GPHGLY-
-GPHGLQ-
GMPHGL--
GEPHGL--
-GPHGLE-
-GPHGLA-
GIPHGL--
GWPHGL--
HAPHGL--
-HPHGLI-
HGPHGL--
HMPHGL--
-HPHGLV-
-HPHGLR-
HRPHGL--
HYPHGL--
HNPHGL--
-HPHGLL-
-HPHGLK-
HVPHGL--
HQPHGL--
HLPHGL--
HPPHGL--
-HPHGLS-
-HPHGLA-
HKPHGL--
HWPHGL--

HTPHGL--
-HPHGLF-
-HPHGLQ-
HFPHGL--
HEPHGL--
HSPHGL--
-HPHGLY-
-HPHGLH-
-HPHGLG-
HDPHGL--
HCPHGL--
ITPHGL--
-IPHGLL-
-IPHGLR-
-IPHGLT-
-IPHGLN-
-IPHGLF-
IRPHGL--
IPPHGL--
IKPHGL--
IGPHGL--
ICPHGL--
IWPHGL--
IIPHGL--
-IPHGLW-
-IPHGLA-
IFPHGL--
IAPHGL--
IVPHGL--
ISPHGL--
IQPHGL--
-IPHGLG-
-IPHGLI-
IMPHGL--
IHPHGL--
IDPHGL--
IYPHGL--
-IPHGLY-
-IPHGLV-
-IPHGLQ-
-IPHGLH-
INPHGL--
IEPHGL--
KLPHGL--
-KPHGLK-
-KPHGLV-
-KPHGLL-
KGPHGL--
KAPHGL--
KIPHGL--

-KPHGLY-
KNPHGL--
-KPHGLR-
KQPHGL--
-KPHGLT-
-KPHGLS-
KVPHGL--
KTPHGL--
-KPHGLI-
-KPHGLH-
-KPHGLG-
KPPHGL--
-KPHGLF-
KKPHGL--
KDPHGL--
-KPHGLA-
KHPHGL--
KFPHGL--
-KPHGLQ-
-KPHGLW-
-KPHGLN-
-KPHGLM-
-KPHGLE-
KEPHGL--
KCPHGL--
-LPHGLR-
LRPHGL--
LKPHGL--
-LPHGLT-
LSPHGL--
LGPHGL--
LNPHGL--
LLPHGL--
LVPHGL--
LPPHGL--
LYPHGL--
LTPHGL--
-LPHGLS-
-LPHGLL-
LMPHGL--
-LPHGLW-
LWPHGL--
LFPHGL--
-LPHGLV-
-LPHGLY-
-LPHGLI-
-LPHGLF-
-LPHGLQ-
LAPHGL--
-LPHGLN-

-LPHGLK-
LDPHGL--
LHPHGL--
LQPHGL--
-LPHGLM-
LEPHGL--
LCPHGL--
LIPHGL--
-LPHGLE-
MRPHGL--
-MPHGLS-
MSPHGL--
-MPHGLR-
-MPHGLL-
MVPHGL--
MTPHGL--
-MPHGLY-
MAPHGL--
-MPHGLH-
-MPHGLV-
-MPHGLT-
MLPHGL--
MFPHGL--
MDPHGL--
MYPHGL--
MPPHGL--
-MPHGLM-
-MPHGLI-
-MPHGLF-
-MPHGLW-
-MPHGLE-
-MPHGLQ-
-MPHGLN-
-MPHGLK-
MKPHGL--
MIPHGL--
MGPHGL--
MWPHGL--
MQPHGL--
-MPHGLA-
MNPHGL--
MMPHGL--
MHPHGL--
NMPHGL--
NVPHGL--
NLPHGL--
-NPHGLS-
-NPHGLI-
NKPHGL--
NRPHGL--

```
-NPHGLR-
NNPHGL--
NPPHGL--
-NPHGLK-
-NPHGLH-
-NPHGLW-
-NPHGLY-
-NPHGLL-
NHPHGL--
NGPHGL--
NSPHGL--
-NPHGLV-
-NPHGLT-
NFPHGL--
NEPHGL--
NYPHGL--
-NPHGLM-
-NPHGLA-
NIPHGL--
NAPHGL--
-NPHGLN-
-NPHGLF-
NWPHGL--
NTPHGL--
-NPHGLQ-
--PHGLRR
--PHGLLR
--PHGLVN
--PHGLRN
--PHGLLK
--PHGLFS
--PHGLAV
--PHGLSM
--PHGLYR
--PHGLYL
--PHGLST
--PHGLRL
--PHGLWT
--PHGLRS
--PHGLRG
--PHGLHQ
--PHGLVR
--PHGLSI
--PHGLFR
--PHGLAS
--PHGLNT
--PHGLLV
PLPHGL--
--PHGLVQ
--PHGLVK
```

--PHGLRF
--PHGLRD
--PHGLLT
--PHGLLS
--PHGLLM
--PHGLVG
--PHGLIS
PFPHGL--
--PHGLWR
--PHGLSV
--PHGLHL
--PHGLFN
--PHGLFL
PRPHGL--
--PHGLWS
--PHGLVF
--PHGLTR
--PHGLTG
--PHGLSS
--PHGLSG
--PHGLRK
--PHGLNS
--PHGLLG
--PHGLHE
PNPHGL--
--PHGLSA
--PHGLQA
--PHGLMT
--PHGLMN
--PHGLMA
--PHGLKF
--PHGLYV
--PHGLYD
--PHGLVM
--PHGLTS
--PHGLTQ
--PHGLMV
--PHGLLL
--PHGLFV
--PHGLFQ
--PHGLAF
--PHGLTN
--PHGLSL
--PHGLMG
--PHGLLN
--PHGLKT
--PHGLKS
--PHGLIF
--PHGLHN
--PHGLGG

--PHGLGA
--PHGLFK
PYPHGL--
--PHGLWY
--PHGLVT
--PHGLVS
--PHGLTW
--PHGLRY
--PHGLRW
--PHGLNV
--PHGLMS
--PHGLLW
--PHGLLQ
--PHGLKY
--PHGLIT
--PHGLHS
--PHGLFT
--PHGLFF
--PHGLFA
--PHGLQS
--PHGLLF
--PHGLKR
--PHGLKG
PMPHGL--
PKPHGL--
--PHGLYA
--PHGLWQ
--PHGLWH
--PHGLVI
--PHGLVA
--PHGLTT
--PHGLTI
--PHGLRA
--PHGLML
--PHGLMI
--PHGLMD
--PHGLLI
--PHGLKE
--PHGLIM
--PHGLFY
PAPHGL--
PVPHGL--
--PHGLYT
--PHGLWA
--PHGLVL
--PHGLTY
--PHGLSY
--PHGLSW
--PHGLSN
--PHGLRE

--PHGLQG
--PHGLNA
--PHGLMQ
--PHGLLD
--PHGLKN
--PHGLKH
--PHGLIY
--PHGLHR
--PHGLHI
--PHGLFW
--PHGLFM
--PHGLFI
--PHGLAL
PDPHGL--
PSPHGL--
--PHGLYI
--PHGLWV
--PHGLWI
--PHGLWG
--PHGLTV
--PHGLTL
--PHGLTK
--PHGLTH
--PHGLSH
--PHGLQY
--PHGLNY
--PHGLNR
--PHGLNK
--PHGLMK
--PHGLKI
--PHGLIK
--PHGLIA
--PHGLHV
--PHGLHG
--PHGLGV
--PHGLFG
--PHGLAN
PWPHGL--
--PHGLYW
--PHGLYQ
--PHGLYH
--PHGLWN
--PHGLWD
--PHGLVE
--PHGLVD
--PHGLTM
--PHGLSD
--PHGLRQ
--PHGLQT
--PHGLQR

--PHGLQF
--PHGLNQ
--PHGLMY
--PHGLMM
--PHGLMF
--PHGLKW
--PHGLKV
--PHGLKQ
--PHGLKD
--PHGLIW
--PHGLIV
--PHGLHA
--PHGLGS
--PHGLFD
--PHGLEV
PHPHGL--
--PHGLYY
--PHGLYS
--PHGLYK
--PHGLYG
--PHGLWW
--PHGLWM
--PHGLVY
--PHGLVV
--PHGLSK
--PHGLRM
--PHGLQW
--PHGLQV
--PHGLNM
--PHGLNH
--PHGLMW
--PHGLMP
--PHGLME
--PHGLKM
--PHGLKL
--PHGLIN
--PHGLID
--PHGLHM
--PHGLHH
--PHGLGT
--PHGLEY
--PHGLAW
PGPHGL--
PTPHGL--
-PPHGLY-
-PPHGLS-
PIPHGL--
--PHGLYM
--PHGLWK
--PHGLVW

--PHGLQM
--PHGLQH
--PHGLPV
--PHGLNW
--PHGLNN
--PHGLNF
--PHGLMH
--PHGLKK
--PHGLKA
--PHGLIH
--PHGLIE
--PHGLHF
--PHGLGI
--PHGLFE
--PHGLET
--PHGLES
--PHGLEM
--PHGLEG
--PHGLCG
--PHGLAT
--PHGLAQ
--PHGLAI
--PHGLAH
QRPHGL--
QNPHGL--
-QPHGLV-
QGPHGL--
QVPHGL--
-QPHGLS-
-QPHGLL-
-QPHGLA-
QAPHGL--
QPPHGL--
-QPHGLQ-
QKPHGL--
QCPHGL--
QYPHGL--
QDPHGL--
-QPHGLK-
QFPHGL--
QEPHGL--
QQPHGL--
-QPHGLY-
-QPHGLT-
-QPHGLI-
QTPHGL--
-QPHGLW-
-QPHGLN-
-QPHGLH-
-QPHGLF-

−QPHGLE−
QIPHGL−−
QHPHGL−−
−RPHGLT−
RLPHGL−−
−RPHGLS−
−RPHGLY−
−RPHGLR−
−RPHGLV−
−RPHGLH−
RVPHGL−−
RPPHGL−−
−RPHGLQ−
−RPHGLL−
−RPHGLA−
RMPHGL−−
RAPHGL−−
RWPHGL−−
RIPHGL−−
−RPHGLE−
−RPHGLK−
−RPHGLF−
RQPHGL−−
RNPHGL−−
RHPHGL−−
RDPHGL−−
−RPHGLW−
−RPHGLN−
−RPHGLM−
REPHGL−−
RGPHGL−−
RFPHGL−−
RCPHGL−−
RKPHGL−−
−SPHGLR−
SAPHGL−−
−SPHGLY−
−SPHGLL−
SRPHGL−−
SFPHGL−−
SGPHGL−−
SNPHGL−−
SLPHGL−−
SSPHGL−−
SVPHGL−−
−SPHGLV−
−SPHGLS−
−SPHGLI−
−SPHGLT−
−SPHGLH−

SYPHGL--
-SPHGLF-
STPHGL--
-SPHGLN-
SIPHGL--
SPPHGL--
-SPHGLQ-
-SPHGLM-
-SPHGLK-
-SPHGLA-
SWPHGL--
-SPHGLW-
-SPHGLE-
SHPHGL--
SQPHGL--
SKPHGL--
SCPHGL--
-TPHGLL-
-TPHGLR-
TLPHGL--
-TPHGLV-
TVPHGL--
-TPHGLW-
-TPHGLY-
-TPHGLK-
TAPHGL--
-TPHGLS-
TFPHGL--
TRPHGL--
TSPHGL--
-TPHGLT-
-TPHGLQ-
-TPHGLH-
TYPHGL--
TKPHGL--
TCPHGL--
TTPHGL--
TQPHGL--
TPPHGL--
-TPHGLI-
-TPHGLF-
TWPHGL--
-TPHGLN-
-TPHGLA-
TMPHGL--
TNPHGL--
TEPHGL--
TDPHGL--
-TPHGLG-
TIPHGL--

THPHGL--
TGPHGL--
-VPHGLS-
VKPHGL--
-VPHGLV-
VTPHGL--
VRPHGL--
VAPHGL--
-VPHGLY-
-VPHGLT-
-VPHGLN-
VGPHGL--
-VPHGLM-
VLPHGL--
-VPHGLR-
VVPHGL--
VSPHGL--
-VPHGLQ-
VNPHGL--
-VPHGLK-
-VPHGLF-
VPPHGL--
-VPHGLH-
VMPHGL--
VFPHGL--
VEPHGL--
VDPHGL--
VWPHGL--
VQPHGL--
-VPHGLW-
-VPHGLI-
-VPHGLE-
VIPHGL--
VHPHGL--
WAPHGL--
WRPHGL--
-WPHGLY-
WNPHGL--
WTPHGL--
-WPHGLN-
-WPHGLK-
-WPHGLQ-
WLPHGL--
WHPHGL--
WVPHGL--
-WPHGLF-
WIPHGL--
WQPHGL--
-WPHGLL-
WKPHGL--

```
            WFPHGL--
            WGPHGL--
            WWPHGL--
            WSPHGL--
            -WPHGLV-
            -WPHGLS-
            WDPHGL--
            -WPHGLT-
            WMPHGL--
            -WPHGLW-
            -WPHGLM-
            -WPHGLI-
            -WPHGLE-
            -YPHGLL-
            -YPHGLK-
            YIPHGL--
            YKPHGL--
            YTPHGL--
            -YPHGLH-
            YLPHGL--
            YPPHGL--
            YYPHGL--
            YSPHGL--
            -YPHGLS-
            YHPHGL--
            YGPHGL--
            -YPHGLW-
            -YPHGLF-
            YMPHGL--
            YQPHGL--
            -YPHGLQ-
            -YPHGLI-
            -YPHGLV-
            -YPHGLT-
            -YPHGLA-
            -YPHGLN-
            YAPHGL--
            YWPHGL--
            -YPHGLM-
            YFPHGL--
            YDPHGL--
            YVPHGL--
            -YPHGLY-
            -YPHGLE-
LSAL         690        559     32.90
            -ALSALP-
            -DLSALQ-
            EDLSAL--
            -FLSALP-
            -GLSALA-
```

-HLSALP-
-HLSALG-
-ILSALP-
-KLSALD-
--LSALGA
--LSALDL
--LSALPQ
--LSALNR
--LSALLQ
--LSALLP
--LSALGT
--LSALSE
--LSALQK
--LSALPV
--LSALPT
--LSALLE
--LSALLD
--LSALGQ
--LSALFP
--LSALCG
--LSALAH
-LLSALD-
-NLSALE-
-PLSALP-
-SLSALD-
-TLSALG-
-TLSALE-
-VLSALP-
-WLSALD-
-ALSALR-
-ALSALT-
-ALSALS-
ANLSAL--
AYLSAL--
AALSAL--
-ALSALH-
-ALSALK-
AVLSAL--
AKLSAL--
ADLSAL--
AQLSAL--
ATLSAL--
-ALSALQ-
AELSAL--
AWLSAL--
-ALSALA-
-ALSALM-
CRLSAL--
CYLSAL--
CQLSAL--

CTLSAL--
-CLSALF-
CCLSAL--
-CLSALR-
CKLSAL--
CGLSAL--
CVLSAL--
-CLSALS-
-CLSALH-
CALSAL--
-CLSALI-
-CLSALV-
CDLSAL--
CPLSAL--
CMLSAL--
-CLSALN-
CILSAL--
CHLSAL--
CNLSAL--
-CLSALW-
DILSAL--
DLLSAL--
-DLSALR-
DKLSAL--
DYLSAL--
DQLSAL--
DGLSAL--
DALSAL--
-DLSALF-
DDLSAL--
-DLSALL-
DWLSAL--
-DLSALI-
-DLSALT-
-DLSALW-
-DLSALV-
DMLSAL--
DHLSAL--
-DLSALS-
DELSAL--
-DLSALH-
DCLSAL--
-DLSALN-
-DLSALM-
-DLSALE-
-ELSALR-
EKLSAL--
ESLSAL--
EVLSAL--
ERLSAL--

EGLSAL--
EPLSAL--
-ELSALV-
ETLSAL--
EWLSAL--
EMLSAL--
-ELSALT-
ENLSAL--
EALSAL--
EQLSAL--
-ELSALI-
EFLSAL--
-ELSALK-
EILSAL--
EELSAL--
EHLSAL--
-ELSALM-
-ELSALN-
-ELSALA-
ECLSAL--
-FLSALK-
FSLSAL--
-FLSALN-
FPLSAL--
FNLSAL--
-FLSALG-
FALSAL--
FHLSAL--
FDLSAL--
FQLSAL--
-FLSALH-
FELSAL--
FCLSAL--
-GLSALS-
GVLSAL--
-GLSALL-
GNLSAL--
GQLSAL--
-GLSALK-
-GLSALT-
GYLSAL--
GMLSAL--
GKLSAL--
GILSAL--
GELSAL--
GALSAL--
-GLSALV-
GHLSAL--
GFLSAL--
-GLSALM-

GDLSAL--
-GLSALQ-
-GLSALN-
GCLSAL--
HRLSAL--
HSLSAL--
HYLSAL--
HKLSAL--
-HLSALR-
HTLSAL--
-HLSALS-
HGLSAL--
-HLSALH-
HPLSAL--
HWLSAL--
-HLSALQ-
HLLSAL--
HDLSAL--
HVLSAL--
-HLSALF-
HILSAL--
HHLSAL--
-HLSALA-
HFLSAL--
HELSAL--
HMLSAL--
HALSAL--
-HLSALY-
-HLSALK-
-HLSALW-
HNLSAL--
-HLSALM-
-HLSALN-
HCLSAL--
-HLSALE-
IRLSAL--
-ILSALS-
IGLSAL--
ITLSAL--
INLSAL--
-ILSALN-
-ILSALK-
IDLSAL--
IQLSAL--
IPLSAL--
-ILSALQ-
-ILSALE-
IALSAL--
-ILSALG-
ICLSAL--

-ILSALM-
KNLSAL--
KGLSAL--
-KLSALH-
KDLSAL--
-KLSALK-
KFLSAL--
KWLSAL--
-KLSALY-
KHLSAL--
-KLSALM-
-KLSALI-
KALSAL--
KELSAL--
-KLSALF-
KCLSAL--
-KLSALQ-
-KLSALC-
--LSALSL
--LSALRT
LSLSAL--
--LSALAL
--LSALNL
LNLSAL--
-LLSALK-
LALSAL--
--LSALTG
--LSALKG
--LSALRD
--LSALHL
-LLSALT-
LHLSAL--
--LSALST
--LSALTA
--LSALMR
LELSAL--
--LSALQS
--LSALNA
--LSALIS
--LSALRM
--LSALHT
--LSALRN
--LSALQG
--LSALLT
--LSALRQ
-LLSALN-
LQLSAL--
--LSALYK
--LSALTS
--LSALHV

--LSALMG
--LSALGI
-LLSALG-
-LLSALA-
--LSALSM
--LSALKT
--LSALNY
--LSALNS
--LSALFG
--LSALGM
--LSALAI
--LSALRE
--LSALGF
--LSALVA
--LSALPF
--LSALHK
--LSALMH
--LSALHF
--LSALEA
--LSALWN
--LSALIR
--LSALYH
--LSALTV
--LSALTT
--LSALTM
--LSALQA
--LSALKN
--LSALHM
--LSALMM
--LSALHR
--LSALMI
--LSALFQ
--LSALTK
--LSALHQ
--LSALAF
--LSALIN
--LSALAM
--LSALVM
--LSALMN
--LSALHG
--LSALAG
--LSALSK
--LSALPY
--LSALNI
--LSALIT
--LSALIA
--LSALWG
--LSALWE
--LSALMT
--LSALMA

```
--LSALHI
--LSALGW
--LSALSY
--LSALHN
-LLSALE-
--LSALTW
--LSALTQ
--LSALQT
--LSALMV
--LSALWH
--LSALVQ
--LSALPI
--LSALNT
--LSALLM
--LSALKW
--LSALKH
--LSALIK
-LLSALQ-
--LSALYN
--LSALQV
--LSALNF
--LSALHY
--LSALHH
--LSALES
--LSALWQ
--LSALWD
--LSALPM
--LSALLN
--LSALKL
--LSALYE
--LSALVE
--LSALSQ
--LSALNV
--LSALMQ
--LSALKQ
--LSALHW
--LSALAV
LCLSAL--
--LSALWM
--LSALWK
--LSALNW
--LSALYD
--LSALVD
--LSALLH
--LSALTH
--LSALQY
--LSALNM
--LSALME
--LSALMD
--LSALKK
```

--LSALIE
--LSALCS
--LSALSH
--LSALQM
--LSALNQ
--LSALNH
--LSALKD
--LSALID
--LSALAT
--LSALQF
--LSALKE
--LSALHE
--LSALEG
--LSALQQ
--LSALAD
--LSALWC
--LSALTC
--LSALQH
--LSALPE
--LSALCV
--LSALCM
--LSALCL
--LSALCF
--LSALAE
MSLSAL--
-MLSALR-
MELSAL--
-MLSALT-
MNLSAL--
-MLSALA-
MKLSAL--
MALSAL--
MTLSAL--
-MLSALN-
-MLSALG-
-MLSALI-
MQLSAL--
MPLSAL--
-MLSALV-
-MLSALH-
MWLSAL--
-MLSALQ-
-MLSALE-
-MLSALD-
-NLSALR-
-NLSALL-
-NLSALK-
-NLSALS-
-NLSALH-
NKLSAL--

NYLSAL--
NFLSAL--
NQLSAL--
-NLSALN-
NHLSAL--
NWLSAL--
NMLSAL--
NNLSAL--
-NLSALY-
-NLSALV-
-NLSALM-
-NLSALI-
NALSAL--
-NLSALQ-
NPLSAL--
-NLSALA-
-NLSALF-
NELSAL--
NDLSAL--
NILSAL--
-NLSALP-
-NLSALW-
PVLSAL--
-PLSALS-
PLLSAL--
PALSAL--
PNLSAL--
-PLSALQ-
-PLSALH-
PQLSAL--
-PLSALT-
PMLSAL--
PYLSAL--
-PLSALI-
PDLSAL--
PHLSAL--
PTLSAL--
PELSAL--
-PLSALF-
-PLSALN-
PCLSAL--
-QLSALR-
-QLSALS-
QLLSAL--
QVLSAL--
QKLSAL--
QNLSAL--
-QLSALH-
QSLSAL--
QTLSAL--

QGLSAL--
QHLSAL--
-QLSALA-
QPLSAL--
QQLSAL--
QWLSAL--
QYLSAL--
QELSAL--
-QLSALQ-
QALSAL--
-QLSALT-
QFLSAL--
QILSAL--
QDLSAL--
-QLSALV-
-QLSALM-
-QLSALE-
QCLSAL--
-RLSALT-
-RLSALV-
-RLSALK-
-RLSALW-
RHLSAL--
-RLSALY-
-RLSALF-
RELSAL--
RDLSAL--
RCLSAL--
SSLSAL--
-SLSALT-
-SLSALI-
SALSAL--
SHLSAL--
SNLSAL--
SELSAL--
SDLSAL--
SMLSAL--
SWLSAL--
-SLSALM-
-SLSALH-
-SLSALN-
-SLSALA-
SCLSAL--
-SLSALC-
-TLSALS-
TVLSAL--
-TLSALT-
TGLSAL--
-TLSALH-
TQLSAL--

TNLSAL--
TPLSAL--
TMLSAL--
TWLSAL--
-TLSALK-
TDLSAL--
TELSAL--
TALSAL--
-TLSALV-
-TLSALN-
TYLSAL--
-TLSALQ-
TCLSAL--
VRLSAL--
VQLSAL--
-VLSALH-
VELSAL--
VHLSAL--
VPLSAL--
-VLSALQ-
VDLSAL--
-VLSALM-
-VLSALE-
WSLSAL--
-WLSALS-
WTLSAL--
WDLSAL--
WELSAL--
WGLSAL--
WPLSAL--
-WLSALL-
-WLSALK-
-WLSALT-
-WLSALN-
-WLSALQ-
-WLSALP-
-WLSALA-
WCLSAL--
-YLSALT-
YKLSAL--
-YLSALA-
YMLSAL--
YDLSAL--
-YLSALH-
-YLSALK-
YHLSAL--
YPLSAL--
-YLSALE-
-YLSALN-
YELSAL--

| | | | |
|---|---|---|---|
| | -YLSALQ- | | |
| | YQLSAL-- | | |
| | YCLSAL-- | | |
| | -YLSALC- | | |
| IASL | 392 | 635 | 32.89 |
| | CRIASL-- | | |
| | --IASLSE | | |
| | --IASLPF | | |
| | --IASLKP | | |
| | --IASLGM | | |
| | --IASLEP | | |
| | --IASLDS | | |
| | --IASLAQ | | |
| | -PIASLG- | | |
| | -VIASLD- | | |
| | ATIASL-- | | |
| | -AIASLL- | | |
| | APIASL-- | | |
| | AYIASL-- | | |
| | -AIASLT- | | |
| | -AIASLK- | | |
| | -AIASLA- | | |
| | AWIASL-- | | |
| | -AIASLG- | | |
| | AHIASL-- | | |
| | -AIASLR- | | |
| | AQIASL-- | | |
| | AMIASL-- | | |
| | -AIASLH- | | |
| | -AIASLM- | | |
| | -AIASLV- | | |
| | AFIASL-- | | |
| | AEIASL-- | | |
| | -AIASLQ- | | |
| | ACIASL-- | | |
| | -AIASLD- | | |
| | CNIASL-- | | |
| | -CIASLT- | | |
| | CCIASL-- | | |
| | CPIASL-- | | |
| | -CIASLK- | | |
| | -CIASLA- | | |
| | CAIASL-- | | |
| | CKIASL-- | | |
| | -CIASLL- | | |
| | CTIASL-- | | |
| | CIIASL-- | | |
| | -CIASLE- | | |
| | -CIASLY- | | |
| | -CIASLR- | | |

```
-CIASLN-
-CIASLH-
-CIASLF-
-CIASLW-
-CIASLV-
-CIASLI-
-CIASLG-
CFIASL--
CEIASL--
-DIASLT-
DSIASL--
-DIASLK-
DGIASL--
DKIASL--
DVIASL--
-DIASLS-
DIIASL--
DTIASL--
DHIASL--
DMIASL--
DFIASL--
DRIASL--
DPIASL--
-DIASLN-
-DIASLF-
-DIASLA-
DAIASL--
DYIASL--
DNIASL--
-DIASLI-
DWIASL--
-DIASLY-
-DIASLV-
DEIASL--
-DIASLQ-
DDIASL--
-DIASLM-
-DIASLH-
ELIASL--
-EIASLR-
ESIASL--
-EIASLH-
-EIASLL-
EVIASL--
ENIASL--
EKIASL--
EYIASL--
-EIASLV-
EPIASL--
-EIASLT-
```

EWIASL--
-EIASLY-
-EIASLQ-
-EIASLN-
-EIASLK-
ETIASL--
EMIASL--
-EIASLW-
-EIASLS-
-EIASLI-
EEIASL--
-EIASLF-
ECIASL--
EQIASL--
-EIASLM-
EFIASL--
EDIASL--
-EIASLD-
-EIASLA-
EHIASL--
-FIASLG-
FDIASL--
FGIASL--
-FIASLT-
FNIASL--
-FIASLH-
FPIASL--
FAIASL--
-FIASLM-
-FIASLE-
-FIASLQ-
FKIASL--
-FIASLK-
FHIASL--
FCIASL--
GTIASL--
-GIASLR-
-GIASLT-
-GIASLS-
GLIASL--
GSIASL--
-GIASLV-
GNIASL--
GHIASL--
GKIASL--
-GIASLY-
-GIASLA-
GGIASL--
-GIASLN-
GAIASL--

```
GIIASL--
GWIASL--
-GIASLQ-
-GIASLK-
-GIASLF-
GDIASL--
-GIASLM-
-GIASLI-
GVIASL--
GQIASL--
GPIASL--
-GIASLE-
GCIASL--
GEIASL--
HSIASL--
HYIASL--
HVIASL--
HPIASL--
-HIASLY-
-HIASLA-
HFIASL--
-HIASLK-
HAIASL--
HNIASL--
-HIASLR-
-HIASLW-
HQIASL--
HMIASL--
-HIASLV-
HGIASL--
-HIASLG-
HKIASL--
-HIASLE-
HDIASL--
HIIASL--
-HIASLN-
-HIASLH-
-HIASLQ-
-HIASLM-
HEIASL--
HCIASL--
--IASLRK
--IASLSG
ISIASL--
--IASLTR
--IASLRA
--IASLGS
--IASLTS
--IASLTT
--IASLSA
```

--IASLRI
--IASLLR
IGIASL--
--IASLVY
--IASLVS
--IASLRM
--IASLVA
--IASLSK
--IASLRT
--IASLRQ
--IASLTN
--IASLRG
--IASLVG
--IASLKS
--IASLHT
IKIASL--
--IASLVK
--IASLTG
--IASLRY
--IASLRE
--IASLLG
--IASLKG
--IASLVN
--IASLMR
--IASLKT
--IASLHG
--IASLIN
--IASLAG
--IASLVF
--IASLRF
--IASLKV
--IASLKN
--IASLYH
--IASLNL
--IASLIR
--IASLFH
--IASLFG
IVIASL--
--IASLVD
--IASLTK
--IASLQG
--IASLMH
--IASLIS
--IASLYT
--IASLYS
--IASLMT
--IASLMF
-IIASLT-
--IASLSV
--IASLKY

```
--IASLNT
--IASLKK
--IASLES
-IIASLH-
--IASLWQ
--IASLVV
--IASLRW
--IASLRH
--IASLMS
--IASLMA
--IASLLQ
--IASLLE
--IASLIG
--IASLHK
-IIASLS-
-IIASLE-
--IASLWT
--IASLTY
--IASLTM
--IASLTI
--IASLEA
INIASL--
IHIASL--
--IASLSQ
--IASLKL
--IASLHL
--IASLFN
--IASLFK
--IASLAY
--IASLYN
--IASLQT
--IASLMQ
--IASLML
--IASLKH
--IASLGA
--IASLAT
-IIASLK-
IEIASL--
--IASLWH
--IASLVH
--IASLSI
--IASLMN
--IASLKW
--IASLHV
--IASLHQ
--IASLHI
--IASLHD
--IASLAN
-IIASLM-
--IASLWM
```

--IASLSW
--IASLNV
--IASLNM
--IASLKI
--IASLKF
--IASLHA
--IASLAV
-IIASLQ-
-IIASLI-
--IASLYQ
--IASLVW
--IASLTH
--IASLTD
--IASLQM
--IASLMY
--IASLMW
--IASLHH
--IASLFE
--IASLET
--IASLAA
IAIASL--
-IIASLV-
--IASLYE
--IASLTL
--IASLPM
--IASLNG
--IASLMI
--IASLMD
--IASLKE
--IASLIQ
--IASLII
--IASLID
--IASLHS
--IASLHM
--IASLGW
--IASLFQ
--IASLAM
--IASLAH
-IIASLN-
-IIASLG-
IDIASL--
--IASLWK
--IASLVE
--IASLTW
--IASLTF
--IASLSD
--IASLQY
--IASLQV
--IASLQK
--IASLQI

--IASLQF
--IASLNN
--IASLIH
--IASLIE
--IASLGT
--IASLEG
--IASLAR
IPIASL--
--IASLQQ
--IASLQH
--IASLQD
--IASLNI
--IASLNF
--IASLMV
--IASLMM
--IASLMK
--IASLIT
--IASLHF
--IASLEW
--IASLEV
--IASLAW
--IASLAK
--IASLNY
--IASLKD
--IASLIP
--IASLHW
--IASLHN
--IASLHE
--IASLEN
--IASLCY
--IASLCD
--IASLAI
-KIASLL-
KLIASL--
KAIASL--
KVIASL--
KGIASL--
KHIASL--
KPIASL--
KDIASL--
-KIASLT-
KNIASL--
-KIASLQ-
KWIASL--
-KIASLN-
KQIASL--
KYIASL--
-KIASLE-
-LIASLR-
LSIASL--

-LIASLN-
-LIASLS-
-LIASLT-
LAIASL--
LPIASL--
LNIASL--
LHIASL--
LEIASL--
-LIASLK-
-LIASLE-
LMIASL--
LCIASL--
-LIASLD-
MNIASL--
-MIASLS-
-MIASLR-
MTIASL--
MAIASL--
-MIASLG-
MEIASL--
-MIASLT-
MHIASL--
-MIASLK-
MPIASL--
-MIASLQ-
-MIASLI-
MGIASL--
MQIASL--
-MIASLH-
-MIASLM-
-MIASLE-
MMIASL--
-MIASLA-
MFIASL--
MDIASL--
MCIASL--
-NIASLL-
NLIASL--
-NIASLS-
NVIASL--
NGIASL--
NSIASL--
NQIASL--
-NIASLI-
NMIASL--
-NIASLY-
NYIASL--
NTIASL--
NPIASL--
NNIASL--

-NIASLW-
NIIASL--
-NIASLH-
-NIASLG-
-NIASLE-
-NIASLV-
-NIASLT-
NEIASL--
-NIASLQ-
-NIASLN-
-NIASLF-
NDIASL--
NWIASL--
-NIASLM-
-NIASLK-
-NIASLC-
-PIASLS-
PNIASL--
-PIASLV-
PSIASL--
PGIASL--
-PIASLT-
PAIASL--
PQIASL--
-PIASLM-
-PIASLL-
-PIASLI-
-PIASLH-
-PIASLQ-
-PIASLK-
PYIASL--
PIIASL--
-PIASLE-
PKIASL--
PDIASL--
PFIASL--
PMIASL--
PEIASL--
-PIASLA-
QGIASL--
QSIASL--
-QIASLR-
-QIASLI-
-QIASLL-
QAIASL--
QIIASL--
-QIASLT-
-QIASLA-
QLIASL--
QKIASL--

-QIASLW-
-QIASLY-
-QIASLN-
QWIASL--
QPIASL--
QNIASL--
-QIASLE-
QHIASL--
QCIASL--
-QIASLH-
QMIASL--
-QIASLV-
-QIASLQ-
-QIASLM-
-QIASLG-
QEIASL--
-QIASLC-
QFIASL--
RGIASL--
-RIASLY-
RPIASL--
-RIASLM-
-RIASLF-
RDIASL--
RCIASL--
STIASL--
-SIASLS-
-SIASLR-
-SIASLL-
SSIASL--
SYIASL--
SLIASL--
-SIASLH-
-SIASLV-
-SIASLW-
-SIASLM-
SWIASL--
SAIASL--
SIIASL--
SNIASL--
-SIASLA-
SQIASL--
SPIASL--
-SIASLQ-
-SIASLE-
SDIASL--
SHIASL--
SEIASL--
SCIASL--
-SIASLC-

TVIASL--
-TIASLR-
TAIASL--
TWIASL--
TIIASL--
-TIASLQ-
THIASL--
TSIASL--
-TIASLI-
TQIASL--
TYIASL--
-TIASLH-
TTIASL--
TPIASL--
TNIASL--
-TIASLV-
-TIASLN-
-TIASLT-
TGIASL--
TDIASL--
-TIASLM-
-TIASLK-
-TIASLE-
-TIASLA-
TEIASL--
TCIASL--
VGIASL--
-VIASLR-
-VIASLT-
VEIASL--
VIIASL--
-VIASLL-
VVIASL--
-VIASLS-
VQIASL--
VMIASL--
VPIASL--
-VIASLK-
-VIASLA-
VAIASL--
-VIASLN-
VHIASL--
VKIASL--
-VIASLV-
VDIASL--
VNIASL--
-VIASLH-
-VIASLM-
-VIASLE-
VCIASL--

| | | | |
|---|---|---|---|
| | -VIASLQ- | | |
| | WGIASL-- | | |
| | WKIASL-- | | |
| | -WIASLI- | | |
| | WQIASL-- | | |
| | -WIASLK- | | |
| | -WIASLN- | | |
| | -WIASLL- | | |
| | WHIASL-- | | |
| | -WIASLV- | | |
| | -WIASLS- | | |
| | -WIASLH- | | |
| | WLIASL-- | | |
| | WIIASL-- | | |
| | -WIASLQ- | | |
| | -WIASLE- | | |
| | WPIASL-- | | |
| | -WIASLY- | | |
| | -WIASLM- | | |
| | -YIASLR- | | |
| | YSIASL-- | | |
| | -YIASLG- | | |
| | YEIASL-- | | |
| | YQIASL-- | | |
| | -YIASLN- | | |
| | YHIASL-- | | |
| | YAIASL-- | | |
| | -YIASLV- | | |
| | YTIASL-- | | |
| | YMIASL-- | | |
| | -YIASLE- | | |
| | -YIASLH- | | |
| | -YIASLA- | | |
| | YPIASL-- | | |
| | -YIASLK- | | |
| | YGIASL-- | | |
| | YNIASL-- | | |
| | YIIASL-- | | |
| | -YIASLI- | | |
| | YFIASL-- | | |
| PTAL | 56 | 800 | 32.89 |
| | DDPTAL-- | | |
| | -GPTALI- | | |
| | -GPTALG- | | |
| | --PTALPS | | |
| | --PTALPR | | |
| | --PTALNR | | |
| | --PTALEG | | |
| | -TPTALG- | | |
| | WHPTAL-- | | |

−YPTALG−
−APTALN−
−APTALT−
ARPTAL−−
ALPTAL−−
−APTALS−
AVPTAL−−
−APTALV−
AYPTAL−−
−APTALY−
AFPTAL−−
AKPTAL−−
ANPTAL−−
AGPTAL−−
−APTALI−
AMPTAL−−
AIPTAL−−
AAPTAL−−
AHPTAL−−
−APTALM−
−APTALK−
−APTALH−
−APTALA−
AQPTAL−−
−APTALW−
APPTAL−−
AEPTAL−−
−APTALQ−
−APTALD−
ADPTAL−−
CAPTAL−−
CPPTAL−−
CKPTAL−−
−CPTALY−
−CPTALQ−
CYPTAL−−
CSPTAL−−
CRPTAL−−
−CPTALV−
−CPTALR−
−CPTALL−
CCPTAL−−
CTPTAL−−
CNPTAL−−
CMPTAL−−
CLPTAL−−
CIPTAL−−
CGPTAL−−
CEPTAL−−
CDPTAL−−

DRPTAL--
-DPTALM-
-DPTALY-
DLPTAL--
-DPTALK-
DKPTAL--
DIPTAL--
DAPTAL--
DTPTAL--
DQPTAL--
-DPTALL-
DFPTAL--
DVPTAL--
-DPTALW-
-DPTALT-
DHPTAL--
DGPTAL--
DEPTAL--
DYPTAL--
DWPTAL--
DSPTAL--
-DPTALV-
-DPTALS-
-DPTALR-
-DPTALQ-
-DPTALI-
-DPTALH-
DNPTAL--
DMPTAL--
ERPTAL--
EVPTAL--
ESPTAL--
ELPTAL--
-EPTALL-
EHPTAL--
EAPTAL--
-EPTALT-
-EPTALI-
ENPTAL--
EQPTAL--
-EPTALQ-
-EPTALK-
-EPTALW-
-EPTALV-
-EPTALH-
-EPTALA-
EGPTAL--
EYPTAL--
EWPTAL--
ETPTAL--

-EPTALS-
EKPTAL--
-EPTALN-
-EPTALY-
-EPTALG-
-EPTALE-
EPPTAL--
EEPTAL--
FKPTAL--
FGPTAL--
-FPTALR-
FLPTAL--
FAPTAL--
-FPTALS-
-FPTALL-
FVPTAL--
FIPTAL--
FSPTAL--
-FPTALK-
FYPTAL--
FQPTAL--
FMPTAL--
FTPTAL--
-FPTALT-
-FPTALQ-
FPPTAL--
FFPTAL--
FWPTAL--
-FPTALN-
-FPTALF-
-FPTALH-
-FPTALM-
-FPTALI-
-FPTALA-
-FPTALV-
FNPTAL--
FEPTAL--
FDPTAL--
FCPTAL--
-FPTALE-
GLPTAL--
GYPTAL--
GRPTAL--
GKPTAL--
GTPTAL--
-GPTALR-
GSPTAL--
GIPTAL--
GEPTAL--
GAPTAL--

-GPTALT-
GNPTAL--
GMPTAL--
GFPTAL--
-GPTALQ-
-GPTALN-
-GPTALK-
-GPTALA-
-GPTALM-
GPPTAL--
GGPTAL--
-GPTALW-
-GPTALV-
GCPTAL--
GWPTAL--
-GPTALF-
GHPTAL--
GQPTAL--
-GPTALH-
GDPTAL--
HRPTAL--
-HPTALT-
HWPTAL--
HLPTAL--
HVPTAL--
-HPTALL-
-HPTALS-
HQPTAL--
HAPTAL--
-HPTALQ-
-HPTALH-
-HPTALA-
HHPTAL--
HGPTAL--
-HPTALW-
HIPTAL--
HYPTAL--
-HPTALY-
HKPTAL--
HSPTAL--
-HPTALK-
HMPTAL--
HFPTAL--
HCPTAL--
-HPTALR-
-HPTALN-
-HPTALM-
-HPTALG-
-HPTALF-
-IPTALS-

ILPTAL--
IPPTAL--
IRPTAL--
ISPTAL--
-IPTALT-
IKPTAL--
-IPTALK-
IVPTAL--
-IPTALL-
IFPTAL--
IWPTAL--
IGPTAL--
IIPTAL--
INPTAL--
-IPTALY-
IQPTAL--
-IPTALV-
-IPTALH-
-IPTALN-
-IPTALI-
-IPTALA-
IMPTAL--
IHPTAL--
IEPTAL--
IYPTAL--
-IPTALQ-
-IPTALG-
IDPTAL--
KSPTAL--
KLPTAL--
KGPTAL--
-KPTALI-
-KPTALA-
KMPTAL--
KVPTAL--
-KPTALS-
-KPTALL-
KTPTAL--
KRPTAL--
-KPTALY-
KAPTAL--
KYPTAL--
-KPTALT-
KWPTAL--
-KPTALQ-
-KPTALN-
-KPTALW-
-KPTALK-
-KPTALH-
-KPTALF-

-KPTALM-
KFPTAL--
KQPTAL--
KDPTAL--
-KPTALE-
KNPTAL--
KKPTAL--
KIPTAL--
KHPTAL--
KEPTAL--
KCPTAL--
LRPTAL--
-LPTALR-
LGPTAL--
LSPTAL--
LLPTAL--
LWPTAL--
-LPTALT-
LKPTAL--
-LPTALH-
LIPTAL--
LTPTAL--
LYPTAL--
LMPTAL--
LEPTAL--
-LPTALV-
LNPTAL--
-LPTALI-
-LPTALM-
LPPTAL--
LFPTAL--
LDPTAL--
LQPTAL--
-LPTALN-
LHPTAL--
-LPTALA-
LCPTAL--
MVPTAL--
MNPTAL--
MSPTAL--
MLPTAL--
MGPTAL--
-MPTALT-
-MPTALH-
MMPTAL--
MAPTAL--
-MPTALL-
-MPTALA-
MYPTAL--
MTPTAL--

-MPTALK-
-MPTALI-
-MPTALF-
MEPTAL--
MWPTAL--
-MPTALQ-
MKPTAL--
MFPTAL--
-MPTALY-
-MPTALW-
MIPTAL--
MHPTAL--
-MPTALV-
MPPTAL--
MCPTAL--
-MPTALN-
MQPTAL--
NRPTAL--
NVPTAL--
NLPTAL--
-NPTALL-
NSPTAL--
-NPTALR-
NTPTAL--
NQPTAL--
-NPTALF-
NGPTAL--
NWPTAL--
-NPTALK-
NIPTAL--
NYPTAL--
NHPTAL--
NAPTAL--
-NPTALW-
-NPTALS-
-NPTALH-
NNPTAL--
NMPTAL--
NFPTAL--
NKPTAL--
-NPTALV-
NCPTAL--
-NPTALI-
-NPTALA-
-NPTALM-
-NPTALG-
NEPTAL--
NDPTAL--
--PTALSL
--PTALRL

--PTALRR
--PTALRS
--PTALRG
PSPTAL--
--PTALRT
--PTALLQ
--PTALKS
--PTALAL
--PTALSR
--PTALPL
--PTALVR
--PTALRK
--PTALLR
PWPTAL--
--PTALSV
--PTALHR
--PTALGY
--PTALYG
--PTALVY
--PTALRN
--PTALLS
--PTALFS
--PTALLK
--PTALAS
--PTALSG
--PTALMS
PRPTAL--
--PTALRF
--PTALKK
--PTALGI
--PTALSY
--PTALRM
--PTALRD
--PTALPY
--PTALLA
--PTALGS
--PTALAV
--PTALRI
--PTALFK
--PTALTR
--PTALNS
--PTALYS
--PTALVA
--PTALTS
--PTALNL
--PTALMK
--PTALLT
--PTALKI
--PTALHL
--PTALHE

PLPTAL--
--PTALTG
--PTALRE
--PTALQG
--PTALLN
--PTALWS
--PTALWM
--PTALSI
--PTALRY
--PTALML
--PTALHT
--PTALES
--PTALWK
--PTALTQ
--PTALSF
--PTALQY
--PTALLD
--PTALKT
--PTALHN
--PTALFA
PMPTAL--
PAPTAL--
PTPTAL--
--PTALVT
--PTALVH
--PTALSW
--PTALSN
--PTALSM
--PTALIT
--PTALIK
--PTALIA
--PTALAA
PGPTAL--
--PTALVG
--PTALTK
--PTALNM
--PTALLH
--PTALKA
--PTALWD
--PTALVV
--PTALRQ
--PTALQK
--PTALNV
--PTALMH
--PTALKV
--PTALIS
--PTALIH
--PTALHG
--PTALAW
--PTALAT

--PTALAF
PNPTAL--
PIPTAL--
PHPTAL--
PFPTAL--
PYPTAL--
--PTALYQ
--PTALWR
--PTALWA
--PTALVN
--PTALTT
--PTALTN
--PTALTI
--PTALTH
--PTALTA
--PTALSQ
--PTALQW
--PTALQV
--PTALQN
--PTALNT
--PTALMM
--PTALLE
--PTALKW
--PTALKH
--PTALKF
--PTALHA
--PTALFG
--PTALTW
--PTALNI
--PTALMN
--PTALKQ
--PTALKM
--PTALHS
--PTALHK
--PTALGF
--PTALFN
--PTALAY
--PTALAM
--PTALAG
--PTALYK
--PTALYH
--PTALVD
--PTALTY
--PTALSH
--PTALRH
--PTALQS
--PTALQH
--PTALNQ
--PTALNA
--PTALKG

--PTALKD
--PTALIM
--PTALHY
--PTALHM
--PTALHI
--PTALHH
--PTALHF
--PTALFH
--PTALFE
--PTALVW
--PTALSK
--PTALQA
--PTALNW
--PTALMV
--PTALME
--PTALMA
--PTALIW
--PTALIG
--PTALIE
--PTALHV
--PTALGW
--PTALGG
--PTALGA
--PTALFT
--PTALEA
--PTALAN
--PTALAK
--PTALTV
--PTALTM
--PTALQR
--PTALQD
--PTALPW
--PTALNY
--PTALMG
--PTALMF
--PTALMD
--PTALID
--PTALGM
--PTALFM
--PTALYN
--PTALYA
--PTALWH
--PTALWE
--PTALVQ
--PTALVM
--PTALTD
--PTALQT
--PTALQI
--PTALPI
--PTALNH

--PTALNG
--PTALKY
--PTALKE
--PTALFQ
PQPTAL--
PEPTAL--
--PTALWN
--PTALVK
--PTALTF
--PTALQF
--PTALQE
--PTALPQ
--PTALPH
--PTALPF
--PTALNK
--PTALNF
--PTALMR
--PTALMI
--PTALKN
--PTALIQ
--PTALHW
--PTALHD
--PTALGQ
--PTALEV
--PTALET
--PTALEM
--PTALCG
--PTALCA
--PTALAQ
-PPTALT-
-PPTALN-
QLPTAL--
QQPTAL--
-QPTALF-
QWPTAL--
QSPTAL--
-QPTALA-
QHPTAL--
-QPTALK-
QVPTAL--
QNPTAL--
QKPTAL--
QAPTAL--
-QPTALV-
-QPTALR-
QYPTAL--
-QPTALN-
-QPTALH-
QPPTAL--
QFPTAL--

QMPTAL--
-QPTALM-
-QPTALI-
QEPTAL--
QTPTAL--
-QPTALW-
-QPTALY-
-QPTALQ-
-QPTALG-
QIPTAL--
QGPTAL--
RVPTAL--
RSPTAL--
RGPTAL--
-RPTALL-
RAPTAL--
-RPTALA-
-RPTALS-
-RPTALT-
RHPTAL--
RNPTAL--
RTPTAL--
-RPTALV-
-RPTALW-
RFPTAL--
-RPTALQ-
-RPTALH-
-RPTALK-
-RPTALI-
RMPTAL--
RQPTAL--
RPPTAL--
-RPTALF-
RIPTAL--
RWPTAL--
-RPTALM-
REPTAL--
RDPTAL--
RCPTAL--
SRPTAL--
-SPTALS-
SYPTAL--
SLPTAL--
SAPTAL--
-SPTALT-
SSPTAL--
SVPTAL--
SHPTAL--
SFPTAL--
SGPTAL--

-SPTALQ-
-SPTALV-
-SPTALK-
SNPTAL--
SPPTAL--
SQPTAL--
-SPTALH-
SKPTAL--
-SPTALA-
-SPTALM-
-SPTALI-
-SPTALF-
SCPTAL--
-SPTALW-
-SPTALN-
SIPTAL--
STPTAL--
-SPTALG-
SDPTAL--
-SPTALE-
SEPTAL--
-SPTALC-
TSPTAL--
TLPTAL--
-TPTALL-
-TPTALR-
-TPTALW-
TRPTAL--
-TPTALS-
-TPTALK-
TGPTAL--
-TPTALA-
TDPTAL--
-TPTALQ-
TAPTAL--
TTPTAL--
TQPTAL--
-TPTALT-
-TPTALH-
TEPTAL--
-TPTALF-
TPPTAL--
TYPTAL--
TMPTAL--
TWPTAL--
-TPTALM-
THPTAL--
TCPTAL--
-TPTALN-
-TPTALY-

TNPTAL--
TIPTAL--
VRPTAL--
-VPTALR-
-VPTALL-
VVPTAL--
-VPTALS-
VLPTAL--
-VPTALK-
VHPTAL--
VSPTAL--
-VPTALA-
VKPTAL--
VGPTAL--
-VPTALT-
VMPTAL--
VIPTAL--
VQPTAL--
-VPTALM-
VTPTAL--
-VPTALQ-
-VPTALI-
VPPTAL--
VYPTAL--
-VPTALV-
VAPTAL--
VWPTAL--
VCPTAL--
-VPTALF-
-VPTALE-
VEPTAL--
VNPTAL--
VDPTAL--
-VPTALH-
WVPTAL--
WSPTAL--
WLPTAL--
WFPTAL--
-WPTALS-
WGPTAL--
WPPTAL--
-WPTALR-
WNPTAL--
WRPTAL--
-WPTALL-
WIPTAL--
WAPTAL--
WTPTAL--
WMPTAL--
-WPTALM-

| | | | |
|---|---|---|---|
| | -WPTALH- | | |
| | WYPTAL-- | | |
| | WWPTAL-- | | |
| | -WPTALV- | | |
| | -WPTALQ- | | |
| | WEPTAL-- | | |
| | -WPTALK- | | |
| | -WPTALI- | | |
| | WCPTAL-- | | |
| | WKPTAL-- | | |
| | YLPTAL-- | | |
| | -YPTALR- | | |
| | YTPTAL-- | | |
| | YRPTAL-- | | |
| | YGPTAL-- | | |
| | YKPTAL-- | | |
| | -YPTALI- | | |
| | YSPTAL-- | | |
| | YPPTAL-- | | |
| | -YPTALT- | | |
| | -YPTALQ- | | |
| | -YPTALL- | | |
| | -YPTALK- | | |
| | YMPTAL-- | | |
| | -YPTALY- | | |
| | -YPTALH- | | |
| | YYPTAL-- | | |
| | YWPTAL-- | | |
| | YIPTAL-- | | |
| | YVPTAL-- | | |
| | YAPTAL-- | | |
| | -YPTALF- | | |
| | YFPTAL-- | | |
| | YCPTAL-- | | |
| | YNPTAL-- | | |
| | -YPTALW- | | |
| | -YPTALV- | | |
| | -YPTALN- | | |
| | -YPTALM- | | |
| | YEPTAL-- | | |
| | YHPTAL-- | | |
| PSGY | 26 | 833 | 32.89 |
| | AQPSGY-- | | |
| | -EPSGYG- | | |
| | --PSGYQR | | |
| | --PSGYQK | | |
| | --PSGYGT | | |
| | --PSGYAK | | |
| | -QPSGYG- | | |
| | -RPSGYP- | | |

ALPSGY--
-APSGYR-
-APSGYL-
-APSGYY-
-APSGYF-
-APSGYS-
AIPSGY--
ASPSGY--
-APSGYK-
-APSGYT-
-APSGYG-
ARPSGY--
-APSGYV-
-APSGYA-
ADPSGY--
AWPSGY--
AVPSGY--
ANPSGY--
AGPSGY--
-APSGYI-
ATPSGY--
APPSGY--
AKPSGY--
AFPSGY--
-APSGYW-
-APSGYN-
-APSGYE-
-APSGYM-
AMPSGY--
AHPSGY--
ACPSGY--
AAPSGY--
AYPSGY--
AEPSGY--
-APSGYQ-
-CPSGYI-
-CPSGYL-
CKPSGY--
CRPSGY--
CNPSGY--
CQPSGY--
-CPSGYY-
CCPSGY--
CWPSGY--
CVPSGY--
CSPSGY--
-CPSGYT-
-CPSGYK-
CLPSGY--
CIPSGY--

CDPSGY--
CYPSGY--
-CPSGYW-
-CPSGYQ-
-CPSGYN-
-CPSGYF-
CHPSGY--
CEPSGY--
CAPSGY--
DSPSGY--
-DPSGYR-
DKPSGY--
DRPSGY--
-DPSGYL-
-DPSGYT-
DVPSGY--
DLPSGY--
DWPSGY--
DTPSGY--
-DPSGYQ-
-DPSGYH-
DYPSGY--
-DPSGYN-
DFPSGY--
-DPSGYV-
-DPSGYS-
-DPSGYM-
-DPSGYF-
DAPSGY--
DQPSGY--
-DPSGYY-
-DPSGYW-
-DPSGYA-
DMPSGY--
ERPSGY--
-EPSGYN-
ESPSGY--
ELPSGY--
-EPSGYI-
-EPSGYT-
-EPSGYK-
-EPSGYW-
-EPSGYL-
-EPSGYF-
EMPSGY--
-EPSGYS-
ENPSGY--
EFPSGY--
ETPSGY--
-EPSGYY-

```
-EPSGYA-
EKPSGY--
EIPSGY--
EAPSGY--
EYPSGY--
EVPSGY--
EQPSGY--
-EPSGYV-
-EPSGYQ-
-EPSGYH-
EGPSGY--
FSPSGY--
-FPSGYI-
-FPSGYR-
FGPSGY--
FVPSGY--
-FPSGYK-
FLPSGY--
FWPSGY--
-FPSGYY-
-FPSGYH-
FYPSGY--
FQPSGY--
-FPSGYV-
-FPSGYT-
-FPSGYF-
FIPSGY--
FAPSGY--
FFPSGY--
FTPSGY--
-FPSGYW-
-FPSGYS-
FNPSGY--
-FPSGYM-
FHPSGY--
FDPSGY--
-FPSGYQ-
-FPSGYN-
-FPSGYA-
FPPSGY--
FEPSGY--
FMPSGY--
GRPSGY--
-GPSGYR-
-GPSGYS-
-GPSGYL-
-GPSGYI-
GMPSGY--
GAPSGY--
GSPSGY--
```

GIPSGY--
GKPSGY--
GQPSGY--
GVPSGY--
GTPSGY--
GNPSGY--
-GPSGYV-
-GPSGYF-
GDPSGY--
GYPSGY--
GWPSGY--
-GPSGYN-
-GPSGYK-
-GPSGYH-
-GPSGYA-
GHPSGY--
-GPSGYQ-
GFPSGY--
-GPSGYG-
-GPSGYE-
GPPSGY--
GLPSGY--
GEPSGY--
-GPSGYW-
-GPSGYM-
GGPSGY--
HRPSGY--
HGPSGY--
HLPSGY--
-HPSGYL-
-HPSGYI-
HPPSGY--
HKPSGY--
HAPSGY--
HSPSGY--
-HPSGYW-
-HPSGYM-
HEPSGY--
HVPSGY--
-HPSGYT-
-HPSGYR-
-HPSGYA-
-HPSGYS-
HWPSGY--
HMPSGY--
HIPSGY--
-HPSGYY-
-HPSGYV-
-HPSGYF-
HNPSGY--

HHPSGY--
HQPSGY--
HFPSGY--
HYPSGY--
-HPSGYH-
-HPSGYG-
HDPSGY--
HCPSGY--
IQPSGY--
-IPSGYL-
-IPSGYH-
-IPSGYR-
-IPSGYY-
ILPSGY--
ITPSGY--
-IPSGYW-
-IPSGYV-
IKPSGY--
IYPSGY--
IDPSGY--
IRPSGY--
-IPSGYQ-
-IPSGYS-
-IPSGYM-
ISPSGY--
IAPSGY--
-IPSGYI-
IPPSGY--
IMPSGY--
IGPSGY--
-IPSGYK-
-IPSGYF-
-IPSGYA-
IEPSGY--
-IPSGYT-
-IPSGYN-
IHPSGY--
-IPSGYG-
-KPSGYL-
-KPSGYS-
-KPSGYR-
KTPSGY--
-KPSGYT-
KVPSGY--
KNPSGY--
-KPSGYN-
-KPSGYI-
-KPSGYH-
-KPSGYF-
KPPSGY--

KHPSGY--
KGPSGY--
-KPSGYV-
-KPSGYG-
KMPSGY--
KIPSGY--
-KPSGYW-
-KPSGYE-
-KPSGYA-
KKPSGY--
KFPSGY--
KEPSGY--
KYPSGY--
KCPSGY--
KAPSGY--
-KPSGYM-
-KPSGYC-
KDPSGY--
LSPSGY--
LKPSGY--
-LPSGYI-
LRPSGY--
-LPSGYR-
-LPSGYS-
LLPSGY--
LNPSGY--
LGPSGY--
-LPSGYF-
LTPSGY--
LIPSGY--
-LPSGYV-
LAPSGY--
LQPSGY--
-LPSGYT-
LVPSGY--
-LPSGYK-
LPPSGY--
LEPSGY--
-LPSGYY-
-LPSGYM-
LHPSGY--
LYPSGY--
-LPSGYH-
LFPSGY--
LWPSGY--
LDPSGY--
-LPSGYW-
-LPSGYN-
LCPSGY--
-LPSGYQ-

-LPSGYG-
-MPSGYT-
-MPSGYL-
MLPSGY--
-MPSGYW-
-MPSGYH-
MGPSGY--
-MPSGYR-
MRPSGY--
MSPSGY--
-MPSGYN-
MPPSGY--
MIPSGY--
MWPSGY--
MVPSGY--
MYPSGY--
MTPSGY--
-MPSGYY-
-MPSGYV-
-MPSGYG-
-MPSGYA-
MNPSGY--
MFPSGY--
MAPSGY--
-MPSGYQ-
-MPSGYM-
-MPSGYI-
MEPSGY--
MDPSGY--
-MPSGYF-
MHPSGY--
MQPSGY--
-MPSGYD-
NRPSGY--
NSPSGY--
-NPSGYI-
-NPSGYL-
NLPSGY--
NYPSGY--
-NPSGYF-
NIPSGY--
-NPSGYQ-
-NPSGYK-
NAPSGY--
-NPSGYT-
-NPSGYN-
NFPSGY--
-NPSGYR-
NGPSGY--
-NPSGYH-

NDPSGY--
NNPSGY--
-NPSGYY-
-NPSGYM-
-NPSGYA-
NPPSGY--
NMPSGY--
NHPSGY--
NCPSGY--
NTPSGY--
-NPSGYV-
NWPSGY--
-NPSGYD-
--PSGYFR
--PSGYRV
--PSGYRH
--PSGYLG
--PSGYSS
--PSGYRI
--PSGYLR
--PSGYST
--PSGYRL
--PSGYYA
--PSGYRN
--PSGYVF
--PSGYSR
--PSGYAS
--PSGYSA
--PSGYVS
--PSGYRG
--PSGYRF
--PSGYTR
--PSGYKH
--PSGYYS
--PSGYVR
--PSGYTY
--PSGYVT
--PSGYRA
--PSGYKS
--PSGYGS
--PSGYFK
--PSGYHL
--PSGYSN
--PSGYSI
--PSGYSH
--PSGYRM
--PSGYRD
--PSGYLH
--PSGYIA
--PSGYYV

--PSGYLV
--PSGYLA
PWPSGY--
--PSGYYG
--PSGYWR
--PSGYTI
--PSGYSY
--PSGYRQ
--PSGYME
--PSGYKF
--PSGYHT
--PSGYHS
--PSGYHK
--PSGYHD
PGPSGY--
--PSGYYR
--PSGYYH
--PSGYVG
--PSGYRY
--PSGYQY
--PSGYMG
--PSGYLY
--PSGYLK
--PSGYKW
--PSGYHR
--PSGYHA
--PSGYAG
--PSGYAF
PKPSGY--
--PSGYYL
--PSGYVN
--PSGYVA
--PSGYSV
--PSGYHH
--PSGYHG
--PSGYFT
--PSGYWT
--PSGYVI
--PSGYTT
--PSGYTS
--PSGYTL
--PSGYSK
--PSGYQA
--PSGYLM
--PSGYKN
--PSGYIS
--PSGYFQ
PLPSGY--
--PSGYWN
--PSGYWH

```
--PSGYSQ
--PSGYSL
--PSGYSG
--PSGYQS
--PSGYMW
--PSGYMA
--PSGYKV
--PSGYIK
--PSGYHI
--PSGYFM
--PSGYSF
--PSGYNS
--PSGYMS
--PSGYML
--PSGYIH
--PSGYHE
PVPSGY--
--PSGYYY
--PSGYYT
--PSGYYN
--PSGYYD
--PSGYWW
--PSGYVY
--PSGYVW
--PSGYVM
--PSGYVL
--PSGYTK
--PSGYSW
--PSGYQF
--PSGYMI
--PSGYKT
--PSGYIY
--PSGYIG
--PSGYGG
--PSGYFS
--PSGYEA
PIPSGY--
--PSGYYM
--PSGYWV
--PSGYWI
--PSGYVQ
--PSGYTV
--PSGYTN
--PSGYTH
--PSGYNF
--PSGYMK
--PSGYMF
--PSGYLN
--PSGYKQ
--PSGYIN
```

--PSGYII
--PSGYGV
--PSGYGA
--PSGYEG
--PSGYAW
PAPSGY--
--PSGYYF
--PSGYWL
--PSGYWK
--PSGYWD
--PSGYVH
--PSGYTE
--PSGYTD
--PSGYRE
--PSGYNR
--PSGYNM
--PSGYIW
--PSGYIM
--PSGYIE
--PSGYFV
--PSGYFN
--PSGYFH
--PSGYES
--PSGYAT
--PSGYAM
PTPSGY--
PSPSGY--
--PSGYYQ
--PSGYVK
--PSGYTG
--PSGYTF
--PSGYRW
--PSGYQW
--PSGYQT
--PSGYNW
--PSGYNV
--PSGYNN
--PSGYMY
--PSGYMT
--PSGYMR
--PSGYMN
--PSGYMH
--PSGYKM
--PSGYIV
--PSGYIF
--PSGYFE
--PSGYFD
--PSGYFA
PMPSGY--
PHPSGY--

PDPSGY--
PCPSGY--
--PSGYWA
--PSGYVV
--PSGYTW
--PSGYSD
--PSGYNY
--PSGYNT
--PSGYNI
--PSGYNH
--PSGYMM
--PSGYLQ
--PSGYLI
--PSGYKI
--PSGYKA
--PSGYHY
--PSGYHV
--PSGYHQ
--PSGYHM
--PSGYFW
--PSGYFG
--PSGYAI
--PSGYAA
-PPSGYM-
PYPSGY--
--PSGYYW
--PSGYYK
--PSGYYI
--PSGYWQ
--PSGYWE
--PSGYVE
--PSGYTM
--PSGYSE
--PSGYRC
--PSGYQQ
--PSGYQG
--PSGYNQ
--PSGYNG
--PSGYMD
--PSGYKY
--PSGYKD
--PSGYHW
--PSGYHN
--PSGYHF
--PSGYEW
--PSGYEV
--PSGYET
--PSGYDT
--PSGYCI
--PSGYAH

PQPSGY--
-PPSGYN-
-PPSGYL-
PNPSGY--
PFPSGY--
PEPSGY--
QRPSGY--
-QPSGYL-
-QPSGYV-
-QPSGYW-
-QPSGYS-
QYPSGY--
QVPSGY--
-QPSGYT-
QSPSGY--
-QPSGYM-
-QPSGYF-
QCPSGY--
QEPSGY--
QWPSGY--
QTPSGY--
-QPSGYH-
QLPSGY--
QAPSGY--
-QPSGYI-
QQPSGY--
-QPSGYA-
QMPSGY--
QKPSGY--
QGPSGY--
QPPSGY--
QNPSGY--
-QPSGYQ-
-QPSGYK-
-QPSGYE-
QHPSGY--
QFPSGY--
-RPSGYL-
-RPSGYT-
RSPSGY--
-RPSGYS-
-RPSGYR-
-RPSGYI-
RLPSGY--
RAPSGY--
RFPSGY--
-RPSGYY-
-RPSGYN-
RVPSGY--
RYPSGY--

−RPSGYK−
−RPSGYF−
RMPSGY−−
RTPSGY−−
−RPSGYV−
−RPSGYQ−
RWPSGY−−
−RPSGYE−
RNPSGY−−
REPSGY−−
RQPSGY−−
−RPSGYH−
RDPSGY−−
−RPSGYW−
−RPSGYA−
RIPSGY−−
RHPSGY−−
RCPSGY−−
RPPSGY−−
−RPSGYC−
SSPSGY−−
−SPSGYI−
−SPSGYA−
SGPSGY−−
SYPSGY−−
SVPSGY−−
SAPSGY−−
−SPSGYF−
SFPSGY−−
SLPSGY−−
−SPSGYR−
STPSGY−−
−SPSGYV−
SKPSGY−−
SIPSGY−−
−SPSGYQ−
SQPSGY−−
SNPSGY−−
SHPSGY−−
−SPSGYN−
−SPSGYM−
−SPSGYH−
−SPSGYK−
SWPSGY−−
−SPSGYW−
SPPSGY−−
−SPSGYG−
SCPSGY−−
TVPSGY−−
TSPSGY−−

TRPSGY--
TKPSGY--
-TPSGYL-
-TPSGYS-
-TPSGYK-
-TPSGYW-
-TPSGYV-
TTPSGY--
-TPSGYT-
-TPSGYH-
-TPSGYY-
TAPSGY--
-TPSGYM-
-TPSGYF-
TLPSGY--
-TPSGYN-
-TPSGYI-
TNPSGY--
TEPSGY--
TWPSGY--
TQPSGY--
-TPSGYQ-
TPPSGY--
TIPSGY--
TFPSGY--
TMPSGY--
TCPSGY--
TYPSGY--
-TPSGYG-
-TPSGYE-
-TPSGYA-
VRPSGY--
VPPSGY--
VLPSGY--
VSPSGY--
-VPSGYR-
-VPSGYW-
-VPSGYM-
VIPSGY--
-VPSGYQ-
-VPSGYA-
VGPSGY--
-VPSGYT-
-VPSGYF-
VFPSGY--
VTPSGY--
-VPSGYY-
-VPSGYI-
VVPSGY--
-VPSGYV-

-VPSGYG-
VMPSGY--
VAPSGY--
-VPSGYN-
-VPSGYE-
VNPSGY--
VEPSGY--
VWPSGY--
VQPSGY--
VKPSGY--
VHPSGY--
VDPSGY--
-VPSGYK-
-VPSGYH-
-WPSGYL-
WSPSGY--
-WPSGYS-
WPPSGY--
WRPSGY--
-WPSGYA-
WYPSGY--
WTPSGY--
-WPSGYK-
WVPSGY--
-WPSGYT-
WIPSGY--
WDPSGY--
WAPSGY--
WLPSGY--
WGPSGY--
WWPSGY--
-WPSGYW-
-WPSGYR-
-WPSGYN-
WQPSGY--
-WPSGYY-
-WPSGYQ-
-WPSGYM-
-WPSGYI-
WMPSGY--
WKPSGY--
-YPSGYR-
YTPSGY--
-YPSGYA-
YSPSGY--
-YPSGYH-
YVPSGY--
YAPSGY--
-YPSGYY-
-YPSGYS-

-YPSGYQ-
YKPSGY--
YGPSGY--
YFPSGY--
YMPSGY--
YLPSGY--
YYPSGY--
YWPSGY--
-YPSGYW-
-YPSGYT-
YPPSGY--
YNPSGY--
-YPSGYV-
-YPSGYK-
YQPSGY--
-YPSGYL-
-YPSGYN-
-YPSGYI-
YEPSGY--
-YPSGYE-
YIPSGY--
YDPSGY--
-YPSGYP-
-YPSGYF-
YHPSGY--

| | | | |
|---|---|---|---|
| PAQL | 65 | 794 | 32.85 |

-EPAQLS-
-LPAQLK-
--PAQLGR
QVPAQL--
VGPAQL--
-APAQLS-
ATPAQL--
-APAQLQ-
-APAQLI-
AFPAQL--
-APAQLR-
-APAQLT-
ALPAQL--
-APAQLG-
-APAQLY-
ASPAQL--
-APAQLW-
-APAQLL-
-APAQLF-
-APAQLA-
AGPAQL--
AAPAQL--
AVPAQL--
AQPAQL--

−APAQLM−
AMPAQL−−
ANPAQL−−
AKPAQL−−
AHPAQL−−
ADPAQL−−
AYPAQL−−
−APAQLN−
AIPAQL−−
APPAQL−−
−APAQLV−
−APAQLK−
−APAQLE−
ACPAQL−−
CTPAQL−−
−CPAQLN−
−CPAQLK−
CNPAQL−−
CAPAQL−−
−CPAQLY−
−CPAQLV−
−CPAQLQ−
−CPAQLL−
−CPAQLI−
−CPAQLA−
CMPAQL−−
CKPAQL−−
CGPAQL−−
−DPAQLW−
DSPAQL−−
−DPAQLS−
−DPAQLN−
−DPAQLF−
−DPAQLA−
DNPAQL−−
DWPAQL−−
DRPAQL−−
−DPAQLL−
−DPAQLK−
DLPAQL−−
DVPAQL−−
−DPAQLV−
−DPAQLM−
DKPAQL−−
DYPAQL−−
−DPAQLT−
DIPAQL−−
DHPAQL−−
DGPAQL−−
ESPAQL−−

-EPAQLR-
-EPAQLQ-
-EPAQLK-
-EPAQLG-
EGPAQL--
ERPAQL--
-EPAQLW-
-EPAQLL-
ELPAQL--
-EPAQLT-
EKPAQL--
ETPAQL--
EPPAQL--
-EPAQLV-
-EPAQLM-
-EPAQLH-
-EPAQLY-
ENPAQL--
-EPAQLN-
-EPAQLI-
-EPAQLF-
FYPAQL--
FSPAQL--
-FPAQLS-
-FPAQLR-
FMPAQL--
-FPAQLH-
-FPAQLV-
FKPAQL--
FLPAQL--
FTPAQL--
-FPAQLK-
FPPAQL--
-FPAQLN-
-FPAQLI-
-FPAQLA-
FIPAQL--
FQPAQL--
-FPAQLW-
FNPAQL--
-FPAQLY-
-FPAQLT-
-FPAQLQ-
-FPAQLL-
-FPAQLE-
FHPAQL--
FDPAQL--
FAPAQL--
GWPAQL--
GTPAQL--

-GPAQLL-
GRPAQL--
GVPAQL--
-GPAQLG-
GAPAQL--
GQPAQL--
-GPAQLT-
-GPAQLS-
GPPAQL--
GLPAQL--
GYPAQL--
-GPAQLY-
-GPAQLW-
-GPAQLV-
-GPAQLR-
-GPAQLF-
GNPAQL--
GIPAQL--
GKPAQL--
-GPAQLH-
-GPAQLE-
-GPAQLA-
GHPAQL--
GEPAQL--
GSPAQL--
-GPAQLN-
-GPAQLM-
-GPAQLQ-
-GPAQLK-
GMPAQL--
GDPAQL--
HLPAQL--
HSPAQL--
-HPAQLS-
-HPAQLV-
-HPAQLL-
-HPAQLR-
HAPAQL--
HMPAQL--
HGPAQL--
HKPAQL--
HTPAQL--
-HPAQLM-
-HPAQLG-
HNPAQL--
HQPAQL--
HRPAQL--
-HPAQLW-
-HPAQLQ-
-HPAQLN-

−HPAQLC−
HDPAQL−−
HWPAQL−−
HPPAQL−−
−HPAQLT−
−HPAQLI−
−HPAQLA−
HIPAQL−−
HCPAQL−−
IRPAQL−−
−IPAQLR−
−IPAQLL−
INPAQL−−
IVPAQL−−
ISPAQL−−
−IPAQLG−
IMPAQL−−
IGPAQL−−
−IPAQLS−
−IPAQLN−
ILPAQL−−
IPPAQL−−
−IPAQLK−
−IPAQLE−
−IPAQLI−
−IPAQLF−
IFPAQL−−
IEPAQL−−
IDPAQL−−
IAPAQL−−
IYPAQL−−
ITPAQL−−
−IPAQLV−
−IPAQLT−
−IPAQLH−
IKPAQL−−
IWPAQL−−
−IPAQLQ−
IIPAQL−−
−IPAQLW−
KSPAQL−−
−KPAQLS−
−KPAQLH−
−KPAQLR−
−KPAQLW−
KKPAQL−−
−KPAQLL−
KVPAQL−−
−KPAQLT−
−KPAQLN−

KWPAQL--
-KPAQLE-
KYPAQL--
-KPAQLV-
KGPAQL--
-KPAQLQ-
-KPAQLA-
KFPAQL--
KTPAQL--
-KPAQLY-
-KPAQLK-
-KPAQLI-
-KPAQLG-
KLPAQL--
KIPAQL--
KEPAQL--
KNPAQL--
KMPAQL--
KDPAQL--
LVPAQL--
LLPAQL--
LNPAQL--
-LPAQLY-
-LPAQLT-
LYPAQL--
LTPAQL--
-LPAQLW-
-LPAQLQ-
-LPAQLN-
LGPAQL--
LPPAQL--
LAPAQL--
-LPAQLA-
LKPAQL--
-LPAQLS-
LHPAQL--
LFPAQL--
-LPAQLM-
-LPAQLF-
-LPAQLV-
-LPAQLL-
-LPAQLI-
-LPAQLH-
-LPAQLG-
-LPAQLE-
LIPAQL--
LDPAQL--
LCPAQL--
-MPAQLL-
-MPAQLR-

-MPAQLG-
MSPAQL--
MWPAQL--
-MPAQLY-
-MPAQLV-
-MPAQLS-
-MPAQLN-
-MPAQLI-
MYPAQL--
MVPAQL--
-MPAQLM-
MAPAQL--
MMPAQL--
MGPAQL--
MTPAQL--
MPPAQL--
-MPAQLW-
MKPAQL--
MRPAQL--
-MPAQLQ-
MLPAQL--
MIPAQL--
MHPAQL--
MQPAQL--
-MPAQLK-
-MPAQLH-
-MPAQLF-
-MPAQLA-
MNPAQL--
MEPAQL--
NVPAQL--
-NPAQLL-
-NPAQLR-
NRPAQL--
-NPAQLY-
NNPAQL--
-NPAQLN-
NTPAQL--
NGPAQL--
NSPAQL--
-NPAQLQ-
-NPAQLK-
NMPAQL--
-NPAQLT-
-NPAQLH-
NHPAQL--
NEPAQL--
NPPAQL--
-NPAQLA-
NLPAQL--

NAPAQL--
-NPAQLI-
-NPAQLG-
-NPAQLF-
NQPAQL--
-NPAQLW-
-NPAQLS-
NIPAQL--
NWPAQL--
-NPAQLM-
NKPAQL--
NFPAQL--
NCPAQL--
--PAQLRL
--PAQLVR
--PAQLSG
--PAQLVL
--PAQLYG
--PAQLFT
--PAQLFR
--PAQLRT
--PAQLSL
--PAQLKS
--PAQLFA
--PAQLLK
--PAQLKR
--PAQLSS
--PAQLRN
--PAQLLT
--PAQLGL
--PAQLAS
--PAQLYS
--PAQLNL
--PAQLLD
--PAQLVS
--PAQLVG
--PAQLTL
--PAQLSR
--PAQLSM
--PAQLRS
--PAQLLA
--PAQLAM
PLPAQL--
--PAQLVY
--PAQLRK
--PAQLRF
--PAQLRA
PRPAQL--
--PAQLTR
--PAQLST

```
--PAQLSH
--PAQLLS
--PAQLKK
--PAQLIL
--PAQLWR
--PAQLVK
--PAQLTN
--PAQLSA
--PAQLRM
--PAQLRI
--PAQLKG
--PAQLAW
PGPAQL--
PFPAQL--
--PAQLYK
--PAQLTQ
--PAQLSK
--PAQLLN
--PAQLFI
PSPAQL--
--PAQLYL
--PAQLVN
--PAQLTT
--PAQLTK
--PAQLTD
--PAQLMR
--PAQLMF
--PAQLLV
--PAQLLQ
--PAQLKW
--PAQLIS
--PAQLHQ
--PAQLGY
--PAQLGI
--PAQLFW
--PAQLFN
--PAQLFL
--PAQLAY
--PAQLYA
--PAQLVV
--PAQLSY
--PAQLSE
--PAQLNR
--PAQLNK
--PAQLMW
--PAQLLR
--PAQLKF
--PAQLIY
--PAQLIR
--PAQLID
```

--PAQLHN
--PAQLHK
--PAQLFM
PVPAQL--
--PAQLYI
--PAQLVT
--PAQLVA
--PAQLTS
--PAQLSD
--PAQLQW
--PAQLNW
--PAQLMG
--PAQLMA
--PAQLIW
--PAQLIN
--PAQLHY
--PAQLFK
--PAQLEA
--PAQLAN
--PAQLYE
--PAQLWA
--PAQLVF
--PAQLTW
--PAQLTF
--PAQLTA
--PAQLRV
--PAQLRD
--PAQLMT
--PAQLLM
--PAQLKI
--PAQLIK
--PAQLIF
--PAQLHT
--PAQLGT
--PAQLGG
--PAQLAT
PAPAQL--
PTPAQL--
PNPAQL--
--PAQLYY
--PAQLYT
--PAQLWT
--PAQLVE
--PAQLTY
--PAQLTH
--PAQLSW
--PAQLSI
--PAQLRE
--PAQLQA
--PAQLNS

--PAQLMV
--PAQLMI
--PAQLKL
--PAQLIV
--PAQLII
--PAQLHF
--PAQLGS
--PAQLGM
--PAQLAQ
--PAQLAK
--PAQLAA
PYPAQL--
--PAQLYQ
--PAQLYM
--PAQLVQ
--PAQLVI
--PAQLVD
--PAQLTE
--PAQLSN
--PAQLRQ
--PAQLQS
--PAQLNA
--PAQLMD
--PAQLKT
--PAQLIA
--PAQLHL
--PAQLHE
--PAQLGW
--PAQLGF
--PAQLFE
--PAQLFD
--PAQLEV
--PAQLEN
--PAQLCS
--PAQLAG
--PAQLAF
PQPAQL--
PIPAQL--
--PAQLYN
--PAQLWV
--PAQLWK
--PAQLWE
--PAQLVM
--PAQLTV
--PAQLTM
--PAQLTI
--PAQLSV
--PAQLSF
--PAQLQY
--PAQLQQ

```
--PAQLQM
--PAQLQI
--PAQLQF
--PAQLQD
--PAQLPM
--PAQLNT
--PAQLNQ
--PAQLNM
--PAQLNG
--PAQLNE
--PAQLMQ
--PAQLMH
--PAQLKQ
--PAQLKH
--PAQLIT
--PAQLHH
--PAQLHG
--PAQLGN
--PAQLGH
--PAQLFY
--PAQLET
--PAQLEG
--PAQLAL
--PAQLAH
-PPAQLR-
PMPAQL--
PHPAQL--
PEPAQL--
--PAQLYW
--PAQLYV
--PAQLYF
--PAQLWN
--PAQLWM
--PAQLWH
--PAQLWG
--PAQLTG
--PAQLSC
--PAQLQV
--PAQLQT
--PAQLQN
--PAQLQK
--PAQLNN
--PAQLNI
--PAQLKY
--PAQLKV
--PAQLKD
--PAQLIQ
--PAQLIG
--PAQLIE
--PAQLHW
```

--PAQLHV
--PAQLHI
--PAQLHD
--PAQLFH
--PAQLES
--PAQLEM
--PAQLCM
--PAQLCG
--PAQLAV
--PAQLAD
-QPAQLS-
-QPAQLR-
-QPAQLN-
-QPAQLL-
-QPAQLF-
-QPAQLA-
QRPAQL--
QPPAQL--
QTPAQL--
-QPAQLW-
-QPAQLI-
QAPAQL--
QSPAQL--
QMPAQL--
QHPAQL--
-QPAQLT-
QFPAQL--
QEPAQL--
QYPAQL--
QIPAQL--
QGPAQL--
QDPAQL--
QCPAQL--
QWPAQL--
-QPAQLH-
QKPAQL--
RSPAQL--
RFPAQL--
RYPAQL--
-RPAQLQ-
RGPAQL--
RLPAQL--
-RPAQLL-
-RPAQLR-
RHPAQL--
RIPAQL--
RVPAQL--
-RPAQLY-
-RPAQLK-
-RPAQLI-

```
-RPAQLS-
RQPAQL--
-RPAQLT-
-RPAQLH-
-RPAQLG-
RMPAQL--
RPPAQL--
REPAQL--
-RPAQLF-
RAPAQL--
RTPAQL--
-RPAQLV-
-RPAQLE-
-RPAQLA-
-RPAQLW-
-RPAQLN-
RNPAQL--
RDPAQL--
RCPAQL--
-RPAQLD-
-SPAQLS-
SRPAQL--
SVPAQL--
STPAQL--
-SPAQLR-
-SPAQLG-
SAPAQL--
SGPAQL--
-SPAQLL-
-SPAQLH-
-SPAQLI-
SYPAQL--
-SPAQLA-
-SPAQLV-
-SPAQLN-
-SPAQLF-
SNPAQL--
SMPAQL--
-SPAQLT-
SIPAQL--
SPPAQL--
-SPAQLM-
-SPAQLK-
SWPAQL--
SLPAQL--
SQPAQL--
-SPAQLE-
-SPAQLQ-
SFPAQL--
-SPAQLY-
```

SKPAQL--
SDPAQL--
SCPAQL--
-TPAQLL-
-TPAQLR-
TRPAQL--
TLPAQL--
-TPAQLA-
-TPAQLV-
TWPAQL--
-TPAQLS-
-TPAQLY-
-TPAQLK-
-TPAQLG-
TVPAQL--
TSPAQL--
TQPAQL--
-TPAQLM-
TYPAQL--
TPPAQL--
-TPAQLW-
-TPAQLH-
-TPAQLF-
-TPAQLN-
TMPAQL--
THPAQL--
TTPAQL--
-TPAQLQ-
TEPAQL--
TAPAQL--
-TPAQLT-
-TPAQLI-
TNPAQL--
TKPAQL--
TFPAQL--
-TPAQLE-
-TPAQLC-
TIPAQL--
TCPAQL--
-VPAQLQ-
-VPAQLY-
VTPAQL--
VWPAQL--
-VPAQLR-
-VPAQLN-
-VPAQLI-
-VPAQLG-
-VPAQLA-
-VPAQLS-
-VPAQLK-

VYPAQL--
VVPAQL--
-VPAQLT-
-VPAQLM-
-VPAQLV-
VLPAQL--
VSPAQL--
VPPAQL--
VNPAQL--
VAPAQL--
VQPAQL--
-VPAQLF-
VFPAQL--
VMPAQL--
VIPAQL--
VHPAQL--
VDPAQL--
WLPAQL--
-WPAQLK-
-WPAQLR-
WIPAQL--
WKPAQL--
-WPAQLH-
WAPAQL--
WWPAQL--
WVPAQL--
WTPAQL--
WQPAQL--
WYPAQL--
-WPAQLY-
-WPAQLI-
WSPAQL--
-WPAQLV-
-WPAQLQ-
-WPAQLN-
-WPAQLA-
WMPAQL--
YVPAQL--
-YPAQLR-
-YPAQLK-
YFPAQL--
YYPAQL--
YTPAQL--
YRPAQL--
-YPAQLS-
YWPAQL--
-YPAQLY-
-YPAQLL-
YNPAQL--
YKPAQL--

```
            -YPAQLQ-
            YMPAQL--
            YGPAQL--
            YAPAQL--
            -YPAQLF-
            -YPAQLN-
            -YPAQLH-
            -YPAQLG-
            YLPAQL--
            YIPAQL--
            YSPAQL--
            YPPAQL--
            -YPAQLI-
            YHPAQL--
            YDPAQL--
            YQPAQL--
            -YPAQLW-
            -YPAQLE-
            -YPAQLA-
            YEPAQL--
VAAL         230        697      32.84
            -RVAALP-
            --VAALSQ
            --VAALGH
            --VAALGD
            --VAALEL
            AHVAAL--
            AGVAAL--
            -AVAALR-
            -AVAALL-
            -AVAALS-
            ATVAAL--
            APVAAL--
            AYVAAL--
            -AVAALK-
            -AVAALH-
            -AVAALI-
            AAVAAL--
            AVVAAL--
            ASVAAL--
            ADVAAL--
            AFVAAL--
            AEVAAL--
            -AVAALA-
            AWVAAL--
            -AVAALQ-
            ALVAAL--
            -AVAALG-
            -AVAALT-
            -AVAALM-
```

-AVAALE-
AMVAAL--
AIVAAL--
-AVAALN-
ACVAAL--
CSVAAL--
CCVAAL--
CNVAAL--
CRVAAL--
CAVAAL--
CYVAAL--
CVVAAL--
-CVAALL-
CIVAAL--
CDVAAL--
-CVAALY-
-CVAALQ-
CQVAAL--
CMVAAL--
-CVAALT-
-CVAALS-
-CVAALR-
-CVAALN-
-CVAALM-
CGVAAL--
CFVAAL--
CEVAAL--
DRVAAL--
-DVAALR-
-DVAALS-
-DVAALL-
DTVAAL--
DSVAAL--
DVVAAL--
DWVAAL--
-DVAALF-
DYVAAL--
-DVAALY-
-DVAALI-
DQVAAL--
DPVAAL--
DKVAAL--
DAVAAL--
-DVAALW-
-DVAALT-
DMVAAL--
-DVAALN-
-DVAALA-
DGVAAL--
-DVAALV-

```
-DVAALQ-
DNVAAL--
DIVAAL--
DCVAAL--
-DVAALK-
DEVAAL--
DDVAAL--
-EVAALS-
-EVAALR-
-EVAALL-
ESVAAL--
-EVAALV-
-EVAALQ-
-EVAALK-
ENVAAL--
-EVAALW-
EYVAAL--
-EVAALN-
-EVAALA-
ELVAAL--
ERVAAL--
EFVAAL--
EVVAAL--
-EVAALT-
EWVAAL--
EPVAAL--
EGVAAL--
ECVAAL--
-EVAALY-
-EVAALG-
ETVAAL--
EQVAAL--
EHVAAL--
EAVAAL--
-EVAALM-
-EVAALI-
-FVAALT-
FSVAAL--
-FVAALR-
FHVAAL--
FGVAAL--
FNVAAL--
FKVAAL--
FEVAAL--
-FVAALQ-
FPVAAL--
-FVAALH-
-FVAALA-
FAVAAL--
-FVAALE-
```

-FVAALS-
GSVAAL--
-GVAALF-
GTVAAL--
-GVAALL-
-GVAALR-
GVVAAL--
GNVAAL--
GKVAAL--
-GVAALI-
-GVAALT-
-GVAALH-
-GVAALA-
GGVAAL--
-GVAALW-
GMVAAL--
-GVAALV-
GPVAAL--
GIVAAL--
GAVAAL--
-GVAALN-
GYVAAL--
GQVAAL--
GFVAAL--
GDVAAL--
-GVAALY-
-GVAALQ-
GEVAAL--
GCVAAL--
-GVAALM-
-GVAALG-
-GVAALE-
HLVAAL--
-HVAALR-
HRVAAL--
-HVAALT-
HSVAAL--
HIVAAL--
HWVAAL--
-HVAALV-
-HVAALI-
-HVAALW-
-HVAALS-
-HVAALK-
HTVAAL--
-HVAALQ-
-HVAALH-
HMVAAL--
HPVAAL--
HAVAAL--

HYVAAL--
-HVAALG-
-HVAALE-
-HVAALA-
HNVAAL--
HHVAAL--
HFVAAL--
HDVAAL--
HCVAAL--
-HVAALN-
-HVAALM-
-HVAALD-
HQVAAL--
HEVAAL--
-IVAALS-
IKVAAL--
INVAAL--
-IVAALR-
IVVAAL--
IAVAAL--
-IVAALN-
IEVAAL--
IDVAAL--
-IVAALG-
-IVAALA-
IGVAAL--
-IVAALK-
-IVAALE-
IPVAAL--
-IVAALH-
-IVAALQ-
-IVAALD-
ICVAAL--
-KVAALH-
KHVAAL--
-KVAALW-
KVVAAL--
-KVAALS-
KFVAAL--
-KVAALY-
-KVAALK-
-KVAALF-
KLVAAL--
KGVAAL--
-KVAALT-
-KVAALQ-
-KVAALN-
-KVAALI-
KDVAAL--
KIVAAL--

-KVAALM-
KPVAAL--
-KVAALA-
KQVAAL--
-KVAALE-
KEVAAL--
-LVAALG-
-LVAALK-
LKVAAL--
-LVAALN-
-LVAALT-
-LVAALA-
LTVAAL--
LNVAAL--
LPVAAL--
-LVAALM-
-LVAALS-
LQVAAL--
LMVAAL--
LDVAAL--
-LVAALI-
LAVAAL--
LEVAAL--
-LVAALQ-
-LVAALE-
LCVAAL--
-LVAALD-
-MVAALR-
MMVAAL--
-MVAALQ-
-MVAALH-
MLVAAL--
MVVAAL--
-MVAALT-
MNVAAL--
-MVAALK-
-MVAALV-
MAVAAL--
-MVAALN-
-MVAALM-
-MVAALI-
MPVAAL--
-MVAALG-
-MVAALD-
-MVAALA-
MCVAAL--
MWVAAL--
MQVAAL--
MHVAAL--
-MVAALW-

MDVAAL--
NWVAAL--
-NVAALS-
NVVAAL--
-NVAALR-
NSVAAL--
NNVAAL--
-NVAALT-
NLVAAL--
-NVAALW-
NPVAAL--
NYVAAL--
-NVAALQ-
NTVAAL--
-NVAALM-
-NVAALI-
-NVAALG-
NAVAAL--
-NVAALY-
-NVAALF-
NEVAAL--
-NVAALN-
-NVAALH-
-NVAALA-
NGVAAL--
-NVAALV-
-NVAALE-
NQVAAL--
NDVAAL--
NCVAAL--
PSVAAL--
PGVAAL--
-PVAALN-
-PVAALT-
-PVAALL-
PKVAAL--
-PVAALH-
-PVAALI-
PHVAAL--
-PVAALQ-
PVVAAL--
-PVAALS-
PNVAAL--
PAVAAL--
PYVAAL--
-PVAALW-
PLVAAL--
PIVAAL--
-PVAALG-
PDVAAL--

-PVAALM-
PCVAAL--
-PVAALV-
PMVAAL--
PQVAAL--
PFVAAL--
PEVAAL--
-PVAALC-
-PVAALA-
QGVAAL--
-QVAALR-
-QVAALS-
QSVAAL--
-QVAALW-
QQVAAL--
QLVAAL--
QYVAAL--
QWVAAL--
-QVAALT-
-QVAALI-
-QVAALL-
-QVAALK-
-QVAALH-
QPVAAL--
QHVAAL--
QAVAAL--
QVVAAL--
-QVAALV-
-QVAALN-
-QVAALQ-
QEVAAL--
-QVAALM-
-QVAALE-
QTVAAL--
-QVAALG-
QFVAAL--
RNVAAL--
REVAAL--
-RVAALT-
-RVAALH-
RQVAAL--
RDVAAL--
RPVAAL--
-RVAALA-
-RVAALW-
-RVAALM-
RAVAAL--
RCVAAL--
-RVAALC-
SGVAAL--

SSVAAL--
-SVAALT-
-SVAALA-
SAVAAL--
-SVAALY-
-SVAALN-
SIVAAL--
SCVAAL--
STVAAL--
-SVAALK-
SMVAAL--
-SVAALI-
SPVAAL--
SNVAAL--
-SVAALQ-
SDVAAL--
SYVAAL--
SWVAAL--
SHVAAL--
SFVAAL--
-SVAALE-
-SVAALW-
-SVAALM-
-TVAALR-
TKVAAL--
TWVAAL--
-TVAALT-
-TVAALL-
-TVAALS-
TSVAAL--
-TVAALW-
TGVAAL--
-TVAALH-
-TVAALV-
-TVAALN-
THVAAL--
-TVAALQ-
-TVAALK-
-TVAALI-
-TVAALA-
TYVAAL--
-TVAALG-
TPVAAL--
TQVAAL--
-TVAALY-
TNVAAL--
TEVAAL--
TMVAAL--
TVVAAL--
TIVAAL--

-TVAALE-
-TVAALM-
TDVAAL--
--VAALRS
--VAALSS
--VAALGL
--VAALTS
VSVAAL--
--VAALRL
--VAALRG
--VAALVS
--VAALWS
--VAALRA
--VAALSR
--VAALLK
--VAALVT
--VAALTR
--VAALNW
--VAALFG
--VAALTG
--VAALGI
--VAALRN
--VAALRI
--VAALAL
--VAALTT
--VAALSA
--VAALLE
--VAALGY
VYVAAL--
VPVAAL--
--VAALTL
--VAALKT
--VAALWG
--VAALRV
--VAALIS
--VAALRW
--VAALPL
--VAALKR
--VAALIG
-VVAALL-
-VVAALK-
--VAALWA
--VAALLD
--VAALSI
--VAALRE
--VAALMI
--VAALTY
--VAALNS
--VAALKI
--VAALVY

--VAALTK
--VAALKG
--VAALHT
--VAALTV
--VAALSN
--VAALRH
--VAALQS
--VAALKN
--VAALHR
--VAALHM
--VAALHK
--VAALHI
--VAALFN
--VAALWT
--VAALTH
--VAALRM
--VAALNL
--VAALLL
--VAALLH
--VAALKS
--VAALFA
--VAALAG
-VVAALT-
-VVAALQ-
-VVAALI-
--VAALYK
--VAALWK
--VAALVQ
--VAALVN
--VAALVH
--VAALVA
--VAALTW
--VAALSM
--VAALSF
--VAALQG
--VAALMT
--VAALMS
--VAALMG
--VAALKY
VQVAAL--
--VAALTF
--VAALSK
--VAALSH
--VAALQY
--VAALQR
--VAALNT
--VAALKW
--VAALIQ
--VAALIK
--VAALIA

--VAALHS
--VAALFQ
--VAALAY
VNVAAL--
VIVAAL--
--VAALYQ
--VAALVV
--VAALTI
--VAALTD
--VAALRD
--VAALQL
--VAALNY
--VAALLV
--VAALKA
--VAALHG
--VAALAW
--VAALAM
-VVAALM-
--VAALYG
--VAALYA
--VAALTA
--VAALSY
--VAALNK
--VAALLQ
--VAALKD
--VAALES
--VAALEG
-VVAALN-
-VVAALA-
VMVAAL--
VEVAAL--
--VAALWH
--VAALWE
--VAALQN
--VAALLM
--VAALKH
--VAALIN
--VAALIH
--VAALHV
--VAALHN
--VAALHA
--VAALFT
--VAALFK
--VAALAK
--VAALAI
VHVAAL--
VDVAAL--
--VAALYD
--VAALVW
--VAALVK

--VAALVD
--VAALTN
--VAALNN
--VAALMN
--VAALMD
--VAALAT
--VAALAA
VAVAAL--
--VAALYH
--VAALWW
--VAALTE
--VAALQQ
--VAALQI
--VAALNR
--VAALMH
--VAALKF
--VAALHW
--VAALHQ
--VAALHL
--VAALGM
--VAALGA
--VAALWN
--VAALVE
--VAALRF
--VAALPW
--VAALPM
--VAALPI
--VAALMM
--VAALMK
--VAALME
--VAALIV
--VAALHY
--VAALGG
--VAALFE
--VAALEW
--VAALAV
--VAALAR
--VAALWQ
--VAALWD
--VAALQW
--VAALQV
--VAALQD
--VAALNV
--VAALNI
--VAALMY
--VAALKV
--VAALII
--VAALHD
--VAALGV
--VAALCS

--VAALAQ
--VAALAN
-VVAALG-
--VAALYE
--VAALQT
--VAALQP
--VAALNQ
--VAALNM
--VAALMW
--VAALKQ
--VAALKE
--VAALID
--VAALHF
--VAALGW
--VAALFH
--VAALCG
--VAALAH
VCVAAL--
--VAALTM
--VAALQH
--VAALPF
--VAALNE
--VAALND
--VAALMV
--VAALKP
--VAALIW
--VAALHE
--VAALFD
--VAALEY
--VAALET
--VAALEM
--VAALCK
-WVAALH-
-WVAALT-
-WVAALN-
WKVAAL--
-WVAALE-
-WVAALW-
WHVAAL--
WGVAAL--
-WVAALK-
WYVAAL--
-WVAALG-
WPVAAL--
WDVAAL--
WNVAAL--
YTVAAL--
-YVAALG-
-YVAALR-
-YVAALQ-

-YVAALT-
-YVAALH-
-YVAALN-
YSVAAL--
YGVAAL--
YNVAAL--
-YVAALS-
YPVAAL--
YDVAAL--
YAVAAL--
-YVAALI-
-YVAALM-
-YVAALD-
YWVAAL--
YEVAAL--
-YVAALV-
YHVAAL--
YCVAAL--

| | | | |
|---|---|---|---|
| VGAL | 170 | 730 | 32.84 |

-NVGALG-
-QVGALG-
-RVGALE-
--VGALLR
--VGALLD
-VVGALD-
--VGALVE
--VGALTD
--VGALSH
--VGALQL
--VGALNR
--VGALLI
--VGALII
--VGALDR
--VGALAT
--VGALAQ
-WVGALE-
-AVGALR-
-AVGALL-
ASVGAL--
ALVGAL--
AYVGAL--
-AVGALT-
-AVGALW-
AFVGAL--
ATVGAL--
AHVGAL--
AGVGAL--
-AVGALY-
-AVGALV-
-AVGALK-

-AVGALH-
AAVGAL--
AQVGAL--
ADVGAL--
-AVGALQ-
ANVGAL--
-AVGALI-
-AVGALF-
APVGAL--
-AVGALM-
AEVGAL--
ACVGAL--
-AVGALN-
-AVGALA-
-AVGALE-
-CVGALS-
-CVGALR-
CYVGAL--
-CVGALT-
-CVGALL-
CTVGAL--
CNVGAL--
CHVGAL--
CGVGAL--
-CVGALK-
CSVGAL--
CQVGAL--
CMVGAL--
CEVGAL--
-CVGALN-
CRVGAL--
CLVGAL--
CKVGAL--
CIVGAL--
CDVGAL--
CVVGAL--
-CVGALY-
-CVGALW-
-CVGALI-
-CVGALF-
CFVGAL--
CAVGAL--
DSVGAL--
DFVGAL--
-DVGALR-
DVVGAL--
-DVGALL-
DYVGAL--
DKVGAL--
-DVGALS-

```
-DVGALF-
-DVGALW-
DTVGAL--
DRVGAL--
DGVGAL--
DEVGAL--
-DVGALT-
-DVGALM-
DQVGAL--
DMVGAL--
-DVGALV-
-DVGALK-
DHVGAL--
-DVGALQ-
-DVGALI-
DAVGAL--
-DVGALH-
-DVGALN-
-DVGALA-
DPVGAL--
DDVGAL--
-EVGALR-
EQVGAL--
EVVGAL--
EHVGAL--
-EVGALL-
-EVGALF-
ESVGAL--
EPVGAL--
-EVGALI-
EWVGAL--
-EVGALT-
-EVGALY-
EMVGAL--
EEVGAL--
EYVGAL--
EFVGAL--
EAVGAL--
-EVGALQ-
ETVGAL--
EIVGAL--
EDVGAL--
ECVGAL--
-EVGALV-
-EVGALS-
-EVGALN-
-EVGALM-
-EVGALK-
-EVGALH-
-EVGALE-
```

-EVGALA-
EGVGAL--
-FVGALR-
FGVGAL--
-FVGALK-
FAVGAL--
FPVGAL--
-FVGALN-
-FVGALA-
-FVGALT-
-FVGALH-
FKVGAL--
-FVGALI-
FIVGAL--
-FVGALL-
-FVGALE-
FTVGAL--
FHVGAL--
-FVGALG-
-FVGALF-
-FVGALQ-
-FVGALC-
-GVGALK-
GHVGAL--
GIVGAL--
GTVGAL--
GSVGAL--
GVVGAL--
-GVGALS-
GFVGAL--
GLVGAL--
GKVGAL--
-GVGALT-
GDVGAL--
GEVGAL--
GPVGAL--
GAVGAL--
GWVGAL--
-GVGALY-
-GVGALM-
GNVGAL--
-GVGALW-
-GVGALH-
GGVGAL--
-GVGALN-
-GVGALF-
GCVGAL--
-HVGALR-
HFVGAL--
HYVGAL--

HTVGAL--
HKVGAL--
HLVGAL--
HPVGAL--
HWVGAL--
HGVGAL--
-HVGALL-
HRVGAL--
-HVGALT-
-HVGALK-
HNVGAL--
-HVGALF-
HAVGAL--
HMVGAL--
HIVGAL--
HEVGAL--
-HVGALI-
-HVGALH-
-HVGALA-
HQVGAL--
HDVGAL--
-HVGALW-
-HVGALV-
-HVGALQ-
-HVGALN-
-HVGALY-
-HVGALM-
-HVGALC-
HCVGAL--
IVVGAL--
ITVGAL--
-IVGALS-
-IVGALQ-
IPVGAL--
IKVGAL--
IWVGAL--
-IVGALF-
IAVGAL--
-IVGALL-
-IVGALH-
IHVGAL--
IEVGAL--
IYVGAL--
-IVGALV-
IMVGAL--
IGVGAL--
IDVGAL--
-IVGALK-
INVGAL--
IIVGAL--

-IVGALN-
-IVGALI-
-IVGALG-
-IVGALE-
IFVGAL--
ICVGAL--
KGVGAL--
KLVGAL--
-KVGALR-
KNVGAL--
KAVGAL--
-KVGALY-
-KVGALL-
-KVGALS-
-KVGALI-
KDVGAL--
-KVGALK-
-KVGALH-
KFVGAL--
-KVGALV-
KCVGAL--
-KVGALQ-
KHVGAL--
KPVGAL--
KMVGAL--
KEVGAL--
KYVGAL--
KQVGAL--
-KVGALM-
-KVGALT-
-KVGALF-
LLVGAL--
LNVGAL--
-LVGALT-
LPVGAL--
LKVGAL--
-LVGALR-
-LVGALK-
LGVGAL--
-LVGALV-
LHVGAL--
LTVGAL--
LMVGAL--
-LVGALN-
LDVGAL--
-LVGALH-
LEVGAL--
-LVGALI-
-LVGALW-
-LVGALL-

LQVGAL--
LFVGAL--
-LVGALQ-
-LVGALE-
-LVGALM-
-LVGALC-
MAVGAL--
-MVGALS-
-MVGALF-
MPVGAL--
-MVGALQ-
-MVGALI-
MVVGAL--
MYVGAL--
MWVGAL--
MKVGAL--
-MVGALV-
-MVGALT-
MLVGAL--
-MVGALL-
-MVGALY-
-MVGALN-
-MVGALK-
MMVGAL--
-MVGALH-
MDVGAL--
MNVGAL--
MTVGAL--
MHVGAL--
MEVGAL--
MGVGAL--
-MVGALM-
-MVGALE-
-NVGALR-
-NVGALK-
NYVGAL--
NVVGAL--
-NVGALI-
NSVGAL--
NIVGAL--
NMVGAL--
-NVGALA-
NKVGAL--
NFVGAL--
-NVGALT-
-NVGALS-
NNVGAL--
NDVGAL--
-NVGALW-
-NVGALN-

−NVGALM−
−NVGALH−
NGVGAL−−
NWVGAL−−
−NVGALY−
−NVGALQ−
NHVGAL−−
NAVGAL−−
NQVGAL−−
−NVGALV−
NPVGAL−−
NEVGAL−−
NCVGAL−−
−NVGALE−
PRVGAL−−
PLVGAL−−
−PVGALL−
PVVGAL−−
PWVGAL−−
PTVGAL−−
PNVGAL−−
−PVGALW−
PKVGAL−−
PIVGAL−−
PDVGAL−−
−PVGALT−
−PVGALM−
−PVGALI−
PHVGAL−−
−PVGALH−
PMVGAL−−
PAVGAL−−
−PVGALK−
−PVGALN−
PCVGAL−−
−PVGALS−
−PVGALG−
−PVGALA−
−PVGALQ−
PGVGAL−−
PFVGAL−−
PEVGAL−−
−PVGALF−
−QVGALR−
QLVGAL−−
QSVGAL−−
−QVGALT−
QRVGAL−−
−QVGALQ−
−QVGALS−

-QVGALY-
QDVGAL--
-QVGALK-
QMVGAL--
QTVGAL--
-QVGALW-
-QVGALN-
QKVGAL--
QWVGAL--
QIVGAL--
QAVGAL--
-QVGALH-
-QVGALA-
QPVGAL--
QNVGAL--
QFVGAL--
-QVGALM-
QQVGAL--
QGVGAL--
QEVGAL--
QYVGAL--
-QVGALF-
QCVGAL--
-QVGALI-
-QVGALE-
QHVGAL--
-RVGALV-
-RVGALH-
-RVGALY-
RGVGAL--
-RVGALN-
-RVGALF-
-RVGALQ-
-RVGALM-
RDVGAL--
-RVGALA-
RCVGAL--
SAVGAL--
SRVGAL--
-SVGALY-
SVVGAL--
-SVGALI-
STVGAL--
SKVGAL--
-SVGALL-
SGVGAL--
-SVGALT-
SYVGAL--
-SVGALK-
-SVGALF-

-SVGALH-
-SVGALW-
-SVGALQ-
-SVGALS-
SIVGAL--
SMVGAL--
SEVGAL--
SDVGAL--
SQVGAL--
-SVGALM-
-SVGALA-
SCVGAL--
TRVGAL--
-TVGALR-
-TVGALL-
TLVGAL--
TSVGAL--
TNVGAL--
TTVGAL--
TKVGAL--
-TVGALY-
-TVGALI-
-TVGALV-
TIVGAL--
THVGAL--
TGVGAL--
-TVGALN-
-TVGALH-
TMVGAL--
-TVGALW-
-TVGALT-
TQVGAL--
TEVGAL--
-TVGALM-
TPVGAL--
-TVGALK-
-TVGALQ-
TDVGAL--
TAVGAL--
TYVGAL--
--VGALRS
--VGALRG
--VGALSL
--VGALSS
--VGALIG
--VGALRN
--VGALGL
--VGALIS
VKVGAL--
--VGALLG

--VGALVS
--VGALVG
-VVGALV-
--VGALVR
--VGALVV
--VGALFS
--VGALVT
VLVGAL--
--VGALYI
--VGALTT
--VGALHR
VWVGAL--
--VGALRQ
--VGALPY
--VGALLT
--VGALQG
--VGALGF
-VVGALI-
VNVGAL--
--VGALSG
--VGALRH
--VGALNM
--VGALHT
--VGALSY
--VGALLA
--VGALWN
--VGALTG
--VGALGI
VAVGAL--
-VVGALS-
--VGALSV
--VGALPL
--VGALNA
--VGALLN
--VGALKV
--VGALIY
--VGALAS
-VVGALT-
-VVGALN-
-VVGALK-
VTVGAL--
VMVGAL--
--VGALST
--VGALRI
--VGALMT
--VGALKS
--VGALHL
--VGALFT
--VGALFH
--VGALAA

VYVGAL--
--VGALWS
--VGALVA
--VGALRY
--VGALKT
VGVGAL--
--VGALWV
--VGALSI
--VGALIR
-VVGALA-
--VGALYT
--VGALWH
--VGALVM
--VGALTV
--VGALTA
--VGALKG
--VGALHW
--VGALHN
--VGALWL
--VGALWA
--VGALVN
--VGALVH
--VGALKL
VVVGAL--
-VVGALM-
--VGALYG
--VGALWG
--VGALVK
--VGALTW
--VGALTH
--VGALRD
--VGALPM
--VGALMA
--VGALAV
VHVGAL--
--VGALKY
--VGALKN
--VGALHI
--VGALGM
--VGALAF
--VGALYN
--VGALYH
--VGALWF
--VGALTR
--VGALQA
--VGALIN
--VGALHK
--VGALGY
--VGALWT
--VGALWI

--VGALSM
--VGALQT
--VGALMY
--VGALMV
--VGALMR
--VGALMD
--VGALHA
--VGALAM
--VGALYK
--VGALYE
--VGALVY
--VGALVF
--VGALTY
--VGALNW
--VGALMK
--VGALKK
--VGALEG
-VVGALW-
--VGALWQ
--VGALNG
--VGALKR
--VGALKA
--VGALIQ
--VGALHF
--VGALFQ
VDVGAL--
-VVGALH-
--VGALYQ
--VGALVQ
--VGALTN
--VGALQV
--VGALNF
--VGALMG
--VGALME
--VGALIW
--VGALIF
--VGALHH
--VGALYM
--VGALNT
--VGALMI
--VGALLH
--VGALKW
--VGALIM
--VGALHV
--VGALHG
--VGALHD
--VGALES
--VGALAW
VEVGAL--
--VGALWK

--VGALTQ
--VGALTM
--VGALTK
--VGALNY
--VGALNV
--VGALKM
--VGALKI
--VGALKH
--VGALHY
--VGALFD
--VGALCG
--VGALAI
VCVGAL--
--VGALVD
--VGALRE
--VGALQQ
--VGALPW
--VGALPI
--VGALNI
--VGALKQ
--VGALGW
--VGALFE
-VVGALP-
-VVGALE-
--VGALYW
--VGALYD
--VGALWE
--VGALTI
--VGALTE
--VGALSQ
--VGALQM
--VGALPF
--VGALNQ
--VGALNK
--VGALMW
--VGALMQ
--VGALMF
--VGALIV
--VGALIH
--VGALGG
--VGALWW
--VGALWD
--VGALQY
--VGALQW
--VGALPK
--VGALHQ
--VGALDM
--VGALCS
--VGALCR
--VGALCI

```
          --VGALCF
          --VGALCA
          -WVGALK-
          WSVGAL--
          -WVGALN-
          -WVGALQ-
          WVVGAL--
          -WVGALR-
          WLVGAL--
          WKVGAL--
          WYVGAL--
          WMVGAL--
          WNVGAL--
          -WVGALI-
          WDVGAL--
          WWVGAL--
          WQVGAL--
          WEVGAL--
          WCVGAL--
          YSVGAL--
          -YVGALR-
          YRVGAL--
          YGVGAL--
          YPVGAL--
          YKVGAL--
          -YVGALA-
          -YVGALY-
          -YVGALL-
          -YVGALH-
          -YVGALF-
          YTVGAL--
          -YVGALT-
          YIVGAL--
          -YVGALN-
          YEVGAL--
          YAVGAL--
          -YVGALW-
          YNVGAL--
          -YVGALI-
          YDVGAL--
          YCVGAL--
          YYVGAL--
          YVVGAL--
          -YVGALK-
          YMVGAL--
          YHVGAL--
          -YVGALV-
          -YVGALE-
          -YVGALQ-
PSWM       270       683     32.83
```

--PSWMTG
-APSWMR-
-APSWML-
-APSWMS-
APPSWM--
ANPSWM--
AWPSWM--
ARPSWM--
-APSWMN-
-APSWMM-
ALPSWM--
AFPSWM--
-APSWMW-
-APSWMH-
AAPSWM--
AYPSWM--
ASPSWM--
-APSWMG-
-APSWMF-
-APSWMA-
AMPSWM--
AKPSWM--
AGPSWM--
ATPSWM--
-APSWMT-
AIPSWM--
AVPSWM--
-APSWMV-
-APSWMK-
AHPSWM--
AQPSWM--
ACPSWM--
CGPSWM--
CVPSWM--
CRPSWM--
-CPSWMA-
-CPSWMR-
-CPSWMN-
CHPSWM--
CCPSWM--
CAPSWM--
DSPSWM--
DRPSWM--
-DPSWMS-
-DPSWMA-
DDPSWM--
DTPSWM--
-DPSWMR-
DIPSWM--
DFPSWM--

```
-DPSWMG-
DGPSWM--
DVPSWM--
-DPSWMV-
-DPSWMI-
-DPSWMH-
DHPSWM--
DEPSWM--
-EPSWMK-
ERPSWM--
-EPSWML-
-EPSWMG-
ESPSWM--
-EPSWMN-
ELPSWM--
EMPSWM--
-EPSWMT-
EGPSWM--
-EPSWMR-
-EPSWMI-
EFPSWM--
EVPSWM--
-EPSWMV-
ECPSWM--
EAPSWM--
FRPSWM--
-FPSWMI-
-FPSWMV-
FTPSWM--
-FPSWMS-
-FPSWMR-
-FPSWMN-
-FPSWMG-
FNPSWM--
FHPSWM--
-FPSWMK-
-FPSWMT-
-FPSWMQ-
FYPSWM--
-FPSWMA-
FVPSWM--
FSPSWM--
-FPSWMY-
FKPSWM--
FIPSWM--
FEPSWM--
FDPSWM--
FAPSWM--
-GPSWMK-
-GPSWMS-
```

GVPSWM--
GSPSWM--
-GPSWML-
GYPSWM--
GLPSWM--
GTPSWM--
-GPSWMF-
GRPSWM--
-GPSWMR-
-GPSWMY-
GHPSWM--
GGPSWM--
-GPSWMN-
-GPSWMG-
GKPSWM--
GAPSWM--
GQPSWM--
GMPSWM--
GIPSWM--
-GPSWMW-
-GPSWMT-
-GPSWMI-
-GPSWMH-
GPPSWM--
GCPSWM--
-GPSWMM-
GEPSWM--
-GPSWMV-
-GPSWME-
GFPSWM--
GDPSWM--
-HPSWMK-
HRPSWM--
-HPSWMT-
-HPSWMG-
-HPSWMQ-
-HPSWMR-
HKPSWM--
HGPSWM--
HIPSWM--
HSPSWM--
-HPSWML-
HQPSWM--
-HPSWMV-
-HPSWMN-
-HPSWMI-
-HPSWMH-
HMPSWM--
HFPSWM--
HAPSWM--

HWPSWM--
HVPSWM--
HPPSWM--
HLPSWM--
HHPSWM--
HEPSWM--
HCPSWM--
-IPSWMS-
-IPSWMQ-
IAPSWM--
-IPSWMR-
-IPSWMA-
-IPSWMN-
INPSWM--
IEPSWM--
ISPSWM--
IQPSWM--
-IPSWMK-
IWPSWM--
-IPSWMI-
-IPSWMH-
IFPSWM--
IDPSWM--
IYPSWM--
ITPSWM--
-IPSWMT-
IKPSWM--
KSPSWM--
-KPSWMS-
-KPSWMR-
-KPSWML-
KMPSWM--
-KPSWMW-
KGPSWM--
KFPSWM--
-KPSWMK-
KTPSWM--
KYPSWM--
-KPSWMG-
-KPSWMF-
KNPSWM--
KQPSWM--
KKPSWM--
-KPSWMA-
KCPSWM--
KRPSWM--
-KPSWMY-
-KPSWMT-
-KPSWME-
KLPSWM--

KHPSWM--
KAPSWM--
-KPSWMN-
KIPSWM--
KWPSWM--
-KPSWMH-
-KPSWMD-
KPPSWM--
KEPSWM--
-LPSWMV-
-LPSWMK-
LLPSWM--
LGPSWM--
-LPSWMG-
LYPSWM--
LRPSWM--
-LPSWMS-
LVPSWM--
-LPSWMM-
-LPSWMI-
LSPSWM--
LHPSWM--
LDPSWM--
LTPSWM--
LKPSWM--
LIPSWM--
-LPSWMQ-
LWPSWM--
LPPSWM--
LFPSWM--
-LPSWMN-
-LPSWMA-
LQPSWM--
-LPSWMW-
LNPSWM--
-MPSWMR-
MSPSWM--
-MPSWMY-
-MPSWMA-
-MPSWMW-
MYPSWM--
MDPSWM--
MQPSWM--
-MPSWMI-
-MPSWMH-
MWPSWM--
-MPSWMS-
-MPSWMQ-
-MPSWML-
MPPSWM--

MNPSWM--
-MPSWMM-
-MPSWMK-
-MPSWMF-
MKPSWM--
MIPSWM--
MHPSWM--
MGPSWM--
MAPSWM--
-NPSWMR-
-NPSWMS-
-NPSWMT-
NGPSWM--
-NPSWML-
-NPSWMG-
NHPSWM--
-NPSWMY-
-NPSWMK-
NPPSWM--
NRPSWM--
-NPSWMQ-
-NPSWMM-
-NPSWMI-
NLPSWM--
NYPSWM--
NVPSWM--
NSPSWM--
-NPSWMV-
NNPSWM--
NKPSWM--
NWPSWM--
NTPSWM--
-NPSWMW-
-NPSWMN-
-NPSWMF-
NMPSWM--
NFPSWM--
--PSWMSL
--PSWMAS
--PSWMSV
--PSWMSG
--PSWMVS
--PSWMTT
--PSWMRV
PSPSWM--
--PSWMKT
--PSWMKS
--PSWMKL
--PSWMTS
--PSWMRT

--PSWMLI
--PSWMLH
--PSWMKA
--PSWMFS
--PSWMVV
--PSWMSK
--PSWMSA
--PSWMRS
--PSWMGS
--PSWMTY
--PSWMTL
--PSWMSM
--PSWMIN
--PSWMVK
--PSWMST
PGPSWM--
PAPSWM--
--PSWMVR
--PSWMTI
--PSWMQT
--PSWMYT
--PSWMYA
--PSWMRE
--PSWMQS
--PSWMLN
--PSWMIG
--PSWMIA
--PSWMFT
--PSWMAR
--PSWMAA
--PSWMYN
--PSWMVT
--PSWMTR
--PSWMRL
--PSWMLV
--PSWMLG
--PSWMKW
--PSWMIR
--PSWMYS
--PSWMVH
--PSWMVG
--PSWMSY
--PSWMRD
--PSWMLQ
--PSWMKV
--PSWMGT
--PSWMFN
--PSWMFE
--PSWMWA
--PSWMVA

--PSWMTW
--PSWMTQ
--PSWMTM
--PSWMSW
--PSWMSD
--PSWMQM
--PSWMQG
--PSWMKY
--PSWMKI
--PSWMKH
--PSWMKE
--PSWMGI
--PSWMFG
--PSWMAV
PYPSWM--
--PSWMYK
--PSWMYG
--PSWMWS
--PSWMVQ
--PSWMVI
--PSWMTE
--PSWMSS
--PSWMSH
--PSWMRM
--PSWMQI
--PSWMNS
--PSWMNA
--PSWMLD
--PSWMKK
--PSWMIQ
--PSWMHR
--PSWMFM
--PSWMFH
--PSWMEA
--PSWMAQ
PRPSWM--
PFPSWM--
--PSWMYE
--PSWMYD
--PSWMTA
--PSWMSN
--PSWMSE
--PSWMQY
--PSWMQN
--PSWMQH
--PSWMQA
--PSWMNT
--PSWMMQ
--PSWMLK
--PSWMKQ

--PSWMKN
--PSWMKD
--PSWMIT
--PSWMIM
--PSWMII
--PSWMHW
--PSWMHM
--PSWMHG
--PSWMHF
--PSWMET
--PSWMAT
--PSWMAN
--PSWMAM
--PSWMAK
--PSWMAF
PEPSWM--
--PSWMYY
--PSWMYM
--PSWMWN
--PSWMWG
--PSWMVE
--PSWMTN
--PSWMRY
--PSWMQR
--PSWMQE
--PSWMNY
--PSWMNG
--PSWMMT
--PSWMMA
--PSWMLE
--PSWMKM
--PSWMIY
--PSWMIV
--PSWMIH
--PSWMHN
--PSWMGV
--PSWMGN
--PSWMGL
--PSWMGK
--PSWMGH
--PSWMGG
--PSWMGA
--PSWMFV
--PSWMFD
--PSWMFA
--PSWMDT
--PSWMDS
--PSWMAY
--PSWMAD
PMPSWM--

PLPSWM--
PKPSWM--
PDPSWM--
PVPSWM--
PTPSWM--
--PSWMYW
--PSWMYQ
--PSWMWV
--PSWMVN
--PSWMVM
--PSWMVD
--PSWMTK
--PSWMSF
--PSWMRW
--PSWMRN
--PSWMQW
--PSWMQK
--PSWMQD
--PSWMNW
--PSWMNF
--PSWMMR
--PSWMMK
--PSWMMI
--PSWMME
--PSWMIW
--PSWMHY
--PSWMHL
--PSWMHI
--PSWMHH
--PSWMHA
--PSWMGY
--PSWMFK
--PSWMEY
--PSWMES
--PSWMCV
--PSWMAW
--PSWMAE
PQPSWM--
-PPSWMT-
PIPSWM--
-QPSWML-
-QPSWMG-
QVPSWM--
-QPSWMT-
-QPSWMS-
QGPSWM--
QAPSWM--
QRPSWM--
-QPSWMW-
-QPSWMR-

-QPSWME-
-QPSWMN-
-QPSWMH-
QHPSWM--
QSPSWM--
QQPSWM--
-QPSWMY-
-QPSWMQ-
-QPSWMD-
-QPSWMA-
QIPSWM--
QWPSWM--
-QPSWMV-
-QPSWMM-
-QPSWMK-
-QPSWMI-
QFPSWM--
-RPSWMI-
-RPSWMV-
-RPSWMR-
RTPSWM--
RSPSWM--
-RPSWMK-
RLPSWM--
-RPSWMM-
-RPSWMS-
RGPSWM--
RQPSWM--
-RPSWMF-
-RPSWMA-
RNPSWM--
RMPSWM--
-RPSWMG-
-RPSWMQ-
-RPSWMH-
RFPSWM--
-RPSWMN-
-RPSWME-
RIPSWM--
RAPSWM--
RWPSWM--
RDPSWM--
RCPSWM--
-RPSWMY-
-RPSWMW-
RHPSWM--
REPSWM--
-SPSWML-
-SPSWMT-
-SPSWMR-

SRPSWM--
-SPSWMG-
STPSWM--
-SPSWMS-
-SPSWMK-
SPPSWM--
-SPSWMM-
SLPSWM--
SYPSWM--
-SPSWMY-
SSPSWM--
-SPSWMA-
SGPSWM--
SAPSWM--
-SPSWMQ-
-SPSWMI-
-SPSWMF-
-SPSWME-
-SPSWMD-
SMPSWM--
SKPSWM--
SEPSWM--
SDPSWM--
SWPSWM--
-SPSWMV-
SNPSWM--
SIPSWM--
SHPSWM--
SFPSWM--
SQPSWM--
SCPSWM--
-TPSWMN-
TRPSWM--
-TPSWMR-
TGPSWM--
TNPSWM--
TEPSWM--
-TPSWMG-
TKPSWM--
TAPSWM--
TTPSWM--
-TPSWMI-
-TPSWMA-
-TPSWMT-
-TPSWML-
TYPSWM--
-TPSWMV-
TPPSWM--
TMPSWM--
TWPSWM--

-TPSWMS-
TIPSWM--
TFPSWM--
TQPSWM--
TLPSWM--
-TPSWMW-
-TPSWMQ-
-TPSWMM-
TSPSWM--
-VPSWMM-
-VPSWMS-
VIPSWM--
VHPSWM--
VFPSWM--
-VPSWMW-
VTPSWM--
-VPSWMV-
-VPSWMN-
-VPSWMK-
VKPSWM--
VRPSWM--
-VPSWMT-
VGPSWM--
VSPSWM--
-VPSWML-
-VPSWMA-
VMPSWM--
VAPSWM--
-VPSWMH-
-VPSWMG-
VEPSWM--
-VPSWMY-
-VPSWMI-
VYPSWM--
VLPSWM--
VVPSWM--
-VPSWMF-
VDPSWM--
-WPSWMI-
WNPSWM--
WRPSWM--
-WPSWMG-
WPPSWM--
WDPSWM--
-WPSWMM-
WGPSWM--
WSPSWM--
-WPSWMQ-
WAPSWM--
-WPSWMN-

| | | | |
|---|---|---|---|
| | -WPSWMR- | | |
| | -WPSWMA- | | |
| | WFPSWM-- | | |
| | WYPSWM-- | | |
| | -WPSWMY- | | |
| | -WPSWMV- | | |
| | WEPSWM-- | | |
| | -YPSWMR- | | |
| | -YPSWMS- | | |
| | YGPSWM-- | | |
| | YRPSWM-- | | |
| | -YPSWMG- | | |
| | -YPSWMY- | | |
| | -YPSWMN- | | |
| | YNPSWM-- | | |
| | YYPSWM-- | | |
| | YTPSWM-- | | |
| | -YPSWMK- | | |
| | YWPSWM-- | | |
| | YQPSWM-- | | |
| | -YPSWMQ- | | |
| | -YPSWME- | | |
| | YIPSWM-- | | |
| | YHPSWM-- | | |
| | YAPSWM-- | | |
| | -YPSWMV- | | |
| | -YPSWML- | | |
| | -YPSWMF- | | |
| | -YPSWMA- | | |
| | YPPSWM-- | | |
| | YLPSWM-- | | |
| | YKPSWM-- | | |
| | YCPSWM-- | | |
| IAGL | 115 | 758 | 32.83 |
| | EPIAGL-- | | |
| | -FIAGLT- | | |
| | -IIAGLG- | | |
| | --IAGLTL | | |
| | --IAGLTD | | |
| | --IAGLRP | | |
| | --IAGLPL | | |
| | --IAGLNR | | |
| | --IAGLNL | | |
| | --IAGLGL | | |
| | --IAGLAW | | |
| | --IAGLAR | | |
| | --IAGLAF | | |
| | KTIAGL-- | | |
| | -LIAGLP- | | |
| | -NIAGLK- | | |

−RIAGLG−
−SIAGLG−
AMIAGL−−
−AIAGLL−
ASIAGL−−
ALIAGL−−
−AIAGLN−
−AIAGLT−
AYIAGL−−
ATIAGL−−
AAIAGL−−
AVIAGL−−
−AIAGLW−
−AIAGLS−
−AIAGLK−
−AIAGLF−
−AIAGLQ−
−AIAGLH−
−AIAGLI−
ADIAGL−−
AFIAGL−−
−AIAGLY−
APIAGL−−
−AIAGLM−
−AIAGLA−
AGIAGL−−
AQIAGL−−
ANIAGL−−
AIIAGL−−
−AIAGLE−
ACIAGL−−
CCIAGL−−
CAIAGL−−
CKIAGL−−
−CIAGLV−
−CIAGLL−
CVIAGL−−
CQIAGL−−
CPIAGL−−
CNIAGL−−
−CIAGLN−
−CIAGLH−
CHIAGL−−
CYIAGL−−
CSIAGL−−
CLIAGL−−
−CIAGLY−
−CIAGLW−
−CIAGLT−
−CIAGLS−

-CIAGLR-
-CIAGLQ-
-CIAGLM-
-CIAGLF-
CEIAGL--
-DIAGLT-
-DIAGLS-
-DIAGLR-
DLIAGL--
-DIAGLK-
DAIAGL--
-DIAGLV-
-DIAGLN-
DEIAGL--
DYIAGL--
DTIAGL--
-DIAGLY-
-DIAGLW-
-DIAGLL-
DWIAGL--
DMIAGL--
DIIAGL--
-DIAGLM-
-DIAGLI-
-DIAGLA-
DFIAGL--
DSIAGL--
-DIAGLQ-
-DIAGLH-
-DIAGLE-
DPIAGL--
DNIAGL--
DHIAGL--
-EIAGLS-
ETIAGL--
EMIAGL--
-EIAGLR-
-EIAGLF-
ESIAGL--
-EIAGLV-
EQIAGL--
ERIAGL--
-EIAGLN-
-EIAGLI-
-EIAGLA-
EAIAGL--
ELIAGL--
EIIAGL--
EFIAGL--
EYIAGL--

EWIAGL--
-EIAGLW-
-EIAGLQ-
-EIAGLG-
EGIAGL--
EEIAGL--
EDIAGL--
ENIAGL--
-EIAGLY-
-EIAGLT-
-EIAGLM-
-EIAGLK-
-EIAGLE-
EHIAGL--
ECIAGL--
FGIAGL--
FSIAGL--
-FIAGLK-
FLIAGL--
-FIAGLV-
-FIAGLL-
FHIAGL--
-FIAGLG-
-FIAGLE-
-FIAGLW-
-FIAGLQ-
-FIAGLS-
-FIAGLR-
FAIAGL--
FDIAGL--
-FIAGLH-
FTIAGL--
FQIAGL--
FNIAGL--
-FIAGLI-
-FIAGLA-
-FIAGLF-
FEIAGL--
-FIAGLN-
-GIAGLR-
-GIAGLS-
GLIAGL--
GVIAGL--
-GIAGLL-
-GIAGLK-
GSIAGL--
-GIAGLT-
GWIAGL--
GTIAGL--
GEIAGL--

-GIAGLM-
GAIAGL--
-GIAGLV-
-GIAGLA-
-GIAGLN-
GHIAGL--
GDIAGL--
GCIAGL--
GPIAGL--
GIIAGL--
-GIAGLI-
GYIAGL--
GMIAGL--
-GIAGLY-
-GIAGLQ-
-GIAGLF-
GFIAGL--
-GIAGLW-
-GIAGLH-
-GIAGLG-
GQIAGL--
HSIAGL--
-HIAGLR-
-HIAGLA-
-HIAGLN-
-HIAGLT-
-HIAGLL-
HHIAGL--
HGIAGL--
HTIAGL--
-HIAGLS-
-HIAGLG-
HMIAGL--
HYIAGL--
HVIAGL--
HPIAGL--
HLIAGL--
HDIAGL--
HAIAGL--
HQIAGL--
-HIAGLF-
-HIAGLK-
HIIAGL--
-HIAGLW-
-HIAGLI-
-HIAGLH-
HNIAGL--
-HIAGLM-
HCIAGL--
--IAGLRV

--IAGLRS
--IAGLST
--IAGLSS
--IAGLLT
--IAGLRK
--IAGLMS
--IAGLHR
--IAGLHL
--IAGLYR
--IAGLSV
--IAGLRN
--IAGLYS
--IAGLNT
IPIAGL--
--IAGLRG
--IAGLRF
--IAGLHA
-IIAGLR-
--IAGLYA
--IAGLVG
--IAGLTR
--IAGLSG
--IAGLRQ
INIAGL--
--IAGLSA
--IAGLLG
--IAGLIN
--IAGLAG
--IAGLWN
--IAGLVR
--IAGLVA
--IAGLTS
--IAGLKM
--IAGLYG
--IAGLVT
--IAGLVK
--IAGLRH
--IAGLYH
--IAGLVH
--IAGLTT
--IAGLLH
--IAGLKR
--IAGLIK
--IAGLYN
--IAGLRD
--IAGLNS
--IAGLMG
--IAGLAS
--IAGLWT
--IAGLWR

--IAGLTY
--IAGLNK
--IAGLMY
--IAGLIH
--IAGLHQ
--IAGLFR
--IAGLFH
IGIAGL--
--IAGLWK
--IAGLSF
--IAGLKK
--IAGLFT
ISIAGL--
-IIAGLS-
-IIAGLQ-
-IIAGLH-
--IAGLWA
--IAGLTA
--IAGLSW
--IAGLRE
--IAGLMI
--IAGLMH
--IAGLLW
--IAGLKN
--IAGLIS
--IAGLES
IYIAGL--
IMIAGL--
--IAGLVV
--IAGLTG
--IAGLRT
--IAGLMK
--IAGLLN
--IAGLFN
--IAGLFA
--IAGLYD
--IAGLTV
--IAGLTN
--IAGLSK
--IAGLRW
--IAGLQT
--IAGLMA
--IAGLLK
--IAGLKY
--IAGLKV
--IAGLKQ
--IAGLKI
--IAGLKH
--IAGLKA
IVIAGL--

```
-IIAGLN-
--IAGLYM
--IAGLYE
--IAGLWS
--IAGLVI
--IAGLTW
--IAGLSH
--IAGLNV
--IAGLLV
--IAGLIT
--IAGLFS
--IAGLFK
--IAGLEA
--IAGLAV
ILIAGL--
IHIAGL--
--IAGLVN
--IAGLLQ
--IAGLIR
--IAGLIM
--IAGLIF
--IAGLHI
--IAGLHH
-IIAGLM-
-IIAGLA-
IDIAGL--
--IAGLWG
--IAGLVY
--IAGLSI
--IAGLQM
--IAGLMN
--IAGLIY
--IAGLII
--IAGLIG
--IAGLHT
--IAGLFQ
--IAGLFD
--IAGLAT
--IAGLAA
IKIAGL--
-IIAGLV-
IFIAGL--
--IAGLYT
--IAGLYQ
--IAGLTQ
--IAGLSN
--IAGLSM
--IAGLQY
--IAGLQS
--IAGLQR
```

```
--IAGLMQ
--IAGLMM
--IAGLML
--IAGLLM
--IAGLLF
--IAGLKT
--IAGLIE
--IAGLHK
--IAGLGS
--IAGLEV
ITIAGL--
IQIAGL--
-IIAGLY-
ICIAGL--
IAIAGL--
--IAGLWH
--IAGLWE
--IAGLVL
--IAGLVE
--IAGLTI
--IAGLTH
--IAGLQG
--IAGLMT
--IAGLKW
--IAGLID
--IAGLHV
--IAGLGG
--IAGLFI
--IAGLAY
--IAGLAN
--IAGLAH
-IIAGLT-
-IIAGLE-
--IAGLVQ
--IAGLTM
--IAGLRM
--IAGLQW
--IAGLQN
--IAGLQH
--IAGLQA
--IAGLNN
--IAGLKF
--IAGLKE
--IAGLIL
--IAGLHW
--IAGLHF
--IAGLGW
--IAGLGA
--IAGLET
--IAGLAK
```

IIIAGL--
-IIAGLK-
-IIAGLI-
--IAGLYW
--IAGLYP
--IAGLWQ
--IAGLTF
--IAGLRC
--IAGLQK
--IAGLQI
--IAGLQF
--IAGLNY
--IAGLNI
--IAGLNH
--IAGLND
--IAGLMD
--IAGLLD
--IAGLKL
--IAGLIW
--IAGLIQ
--IAGLHM
--IAGLHD
--IAGLGT
--IAGLFP
--IAGLFM
--IAGLFE
--IAGLEG
--IAGLDI
--IAGLDA
--IAGLAM
--IAGLAI
-KIAGLS-
-KIAGLL-
-KIAGLR-
-KIAGLF-
-KIAGLH-
KMIAGL--
-KIAGLN-
-KIAGLQ-
-KIAGLK-
KAIAGL--
-KIAGLY-
-KIAGLA-
KEIAGL--
KYIAGL--
-KIAGLT-
KWIAGL--
-KIAGLW-
KHIAGL--
KCIAGL--

KIIAGL--
-LIAGLR-
LSIAGL--
LGIAGL--
-LIAGLK-
-LIAGLH-
-LIAGLQ-
-LIAGLE-
-LIAGLA-
LEIAGL--
-LIAGLY-
LYIAGL--
-LIAGLG-
LMIAGL--
LTIAGL--
LQIAGL--
LPIAGL--
LHIAGL--
LCIAGL--
-LIAGLW-
-LIAGLV-
LDIAGL--
-LIAGLI-
-LIAGLF-
LFIAGL--
LAIAGL--
-LIAGLM-
-LIAGLC-
-MIAGLS-
-MIAGLT-
MLIAGL--
MVIAGL--
MTIAGL--
MYIAGL--
MGIAGL--
MSIAGL--
-MIAGLG-
-MIAGLA-
MMIAGL--
MIIAGL--
-MIAGLR-
MEIAGL--
-MIAGLV-
-MIAGLL-
MDIAGL--
-MIAGLN-
-MIAGLE-
-MIAGLF-
-MIAGLW-
-MIAGLI-

MAIAGL--
MNIAGL--
-MIAGLQ-
MHIAGL--
MQIAGL--
MPIAGL--
-MIAGLY-
-MIAGLH-
NRIAGL--
-NIAGLH-
NYIAGL--
NGIAGL--
NNIAGL--
-NIAGLF-
NTIAGL--
NSIAGL--
NKIAGL--
-NIAGLQ-
-NIAGLL-
NFIAGL--
-NIAGLA-
-NIAGLN-
-NIAGLW-
NPIAGL--
-NIAGLT-
NAIAGL--
NVIAGL--
NMIAGL--
-NIAGLV-
-NIAGLG-
NQIAGL--
-NIAGLY-
-NIAGLM-
NEIAGL--
NWIAGL--
-NIAGLE-
-PIAGLL-
-PIAGLS-
-PIAGLR-
PKIAGL--
PSIAGL--
PIIAGL--
PGIAGL--
PTIAGL--
PQIAGL--
-PIAGLY-
-PIAGLK-
-PIAGLH-
PVIAGL--
PMIAGL--

-PIAGLT-
-PIAGLF-
-PIAGLV-
-PIAGLN-
PFIAGL--
PDIAGL--
PAIAGL--
PNIAGL--
PEIAGL--
PYIAGL--
-PIAGLQ-
-PIAGLI-
PHIAGL--
PPIAGL--
-PIAGLE-
-PIAGLA-
PCIAGL--
QRIAGL--
QKIAGL--
QSIAGL--
QLIAGL--
-QIAGLY-
QVIAGL--
QPIAGL--
-QIAGLG-
QTIAGL--
-QIAGLT-
QAIAGL--
-QIAGLS-
-QIAGLR-
-QIAGLF-
QQIAGL--
QMIAGL--
-QIAGLQ-
QGIAGL--
QFIAGL--
-QIAGLH-
-QIAGLK-
-QIAGLA-
QYIAGL--
QNIAGL--
-QIAGLW-
-QIAGLM-
-QIAGLI-
QIIAGL--
-QIAGLN-
QHIAGL--
QEIAGL--
-QIAGLE-
RLIAGL--

RHIAGL--
RQIAGL--
-RIAGLT-
RNIAGL--
RPIAGL--
RWIAGL--
REIAGL--
-RIAGLQ-
RDIAGL--
RCIAGL--
-RIAGLE-
-RIAGLC-
SSIAGL--
-SIAGLL-
SLIAGL--
-SIAGLS-
-SIAGLH-
-SIAGLY-
SPIAGL--
SNIAGL--
SAIAGL--
STIAGL--
-SIAGLV-
SDIAGL--
-SIAGLN-
-SIAGLK-
SGIAGL--
SEIAGL--
-SIAGLQ-
SFIAGL--
SQIAGL--
-SIAGLA-
SWIAGL--
SIIAGL--
SYIAGL--
SCIAGL--
TVIAGL--
TSIAGL--
-TIAGLR-
-TIAGLH-
-TIAGLS-
-TIAGLI-
TPIAGL--
-TIAGLY-
-TIAGLQ-
TWIAGL--
THIAGL--
TAIAGL--
TTIAGL--
-TIAGLW-

TMIAGL--
-TIAGLM-
TDIAGL--
TIIAGL--
TYIAGL--
TNIAGL--
-TIAGLT-
TFIAGL--
-TIAGLK-
-TIAGLA-
TQIAGL--
-TIAGLV-
-TIAGLE-
TEIAGL--
-VIAGLR-
VTIAGL--
VSIAGL--
-VIAGLV-
VGIAGL--
VLIAGL--
-VIAGLH-
VPIAGL--
-VIAGLS-
-VIAGLT-
VKIAGL--
VVIAGL--
VQIAGL--
VNIAGL--
VMIAGL--
-VIAGLL-
-VIAGLQ-
VAIAGL--
-VIAGLK-
VHIAGL--
-VIAGLI-
VDIAGL--
-VIAGLM-
-VIAGLF-
-VIAGLA-
VEIAGL--
-WIAGLK-
WGIAGL--
-WIAGLS-
WSIAGL--
WLIAGL--
WEIAGL--
-WIAGLN-
WHIAGL--
-WIAGLY-
WNIAGL--

-WIAGLA-
-WIAGLE-
-WIAGLV-
WDIAGL--
WWIAGL--
WPIAGL--
WAIAGL--
WIIAGL--
-WIAGLQ-
-WIAGLM-
-WIAGLG-
-WIAGLP-
-WIAGLH-
-YIAGLI-
-YIAGLL-
-YIAGLA-
-YIAGLT-
YGIAGL--
-YIAGLS-
YSIAGL--
YYIAGL--
YTIAGL--
-YIAGLG-
YQIAGL--
-YIAGLY-
-YIAGLN-
-YIAGLV-
-YIAGLK-
-YIAGLH-
YNIAGL--
-YIAGLW-
YDIAGL--
YMIAGL--
YEIAGL--
YVIAGL--
YPIAGL--
-YIAGLQ-
-YIAGLM-
-YIAGLE-
YWIAGL--
YHIAGL--
YFIAGL--

PVAW 330 657 32.82

AGPVAW--
-APVAWN-
AVPVAW--
-APVAWG-
-APVAWH-
ASPVAW--
-APVAWW-

```
-APVAWS-
-APVAWR-
-APVAWM-
-APVAWL-
-APVAWY-
-APVAWQ-
-APVAWI-
ADPVAW--
AKPVAW--
AAPVAW--
AYPVAW--
AWPVAW--
AQPVAW--
-APVAWV-
APPVAW--
ANPVAW--
AHPVAW--
ARPVAW--
-APVAWK-
-APVAWA-
ALPVAW--
AIPVAW--
AFPVAW--
AEPVAW--
-CPVAWS-
CLPVAW--
-CPVAWV-
-CPVAWR-
CDPVAW--
CCPVAW--
CVPVAW--
CSPVAW--
-CPVAWT-
CPPVAW--
DRPVAW--
-DPVAWF-
DQPVAW--
DTPVAW--
DSPVAW--
-DPVAWI-
DAPVAW--
DWPVAW--
DVPVAW--
-DPVAWS-
-DPVAWR-
-DPVAWN-
-DPVAWL-
DLPVAW--
-DPVAWY-
-DPVAWT-
```

```
-DPVAWH-
DDPVAW--
DCPVAW--
-EPVAWR-
-EPVAWW-
ETPVAW--
-EPVAWL-
EGPVAW--
-EPVAWH-
-EPVAWV-
-EPVAWS-
-EPVAWT-
-EPVAWF-
ESPVAW--
EQPVAW--
-EPVAWN-
EPPVAW--
ENPVAW--
ELPVAW--
EAPVAW--
EYPVAW--
EVPVAW--
-EPVAWY-
-EPVAWM-
-EPVAWK-
-EPVAWI-
-EPVAWA-
-EPVAWE-
EKPVAW--
-FPVAWL-
FVPVAW--
FRPVAW--
-FPVAWT-
-FPVAWN-
-FPVAWY-
-FPVAWH-
FTPVAW--
FIPVAW--
FGPVAW--
FQPVAW--
-FPVAWV-
-FPVAWI-
-FPVAWF-
-FPVAWA-
FPPVAW--
FDPVAW--
FAPVAW--
-FPVAWW-
-FPVAWR-
-FPVAWQ-
```

FMPVAW--
FFPVAW--
FCPVAW--
-GPVAWL-
GVPVAW--
GYPVAW--
-GPVAWY-
-GPVAWT-
-GPVAWM-
GDPVAW--
-GPVAWR-
-GPVAWE-
GRPVAW--
GKPVAW--
GWPVAW--
GTPVAW--
-GPVAWH-
-GPVAWG-
-GPVAWA-
GMPVAW--
GGPVAW--
GQPVAW--
-GPVAWV-
GIPVAW--
-GPVAWW-
-GPVAWP-
-GPVAWF-
-HPVAWL-
-HPVAWA-
HPPVAW--
HMPVAW--
HDPVAW--
HYPVAW--
-HPVAWW-
HAPVAW--
-HPVAWI-
-HPVAWH-
-HPVAWE-
HIPVAW--
HTPVAW--
-HPVAWQ-
-HPVAWN-
HGPVAW--
HEPVAW--
HVPVAW--
-HPVAWY-
-HPVAWV-
-HPVAWT-
-HPVAWS-
-HPVAWM-

HNPVAW--
HLPVAW--
HHPVAW--
-IPVAWT-
-IPVAWS-
-IPVAWA-
ITPVAW--
-IPVAWV-
-IPVAWI-
-IPVAWW-
IEPVAW--
IAPVAW--
IYPVAW--
IRPVAW--
-IPVAWM-
-IPVAWK-
ILPVAW--
IHPVAW--
IWPVAW--
IVPVAW--
-IPVAWR-
-IPVAWH-
IKPVAW--
IGPVAW--
ISPVAW--
-IPVAWQ-
-IPVAWG-
-IPVAWF-
IPPVAW--
IIPVAW--
IFPVAW--
ICPVAW--
KSPVAW--
-KPVAWL-
KIPVAW--
KNPVAW--
KRPVAW--
-KPVAWI-
KMPVAW--
KVPVAW--
-KPVAWT-
-KPVAWR-
-KPVAWH-
KLPVAW--
KGPVAW--
-KPVAWN-
-KPVAWW-
-KPVAWS-
KAPVAW--
-KPVAWM-

KWPVAW--
KTPVAW--
-KPVAWY-
-KPVAWQ-
-KPVAWK-
-KPVAWG-
KHPVAW--
KEPVAW--
KDPVAW--
-LPVAWI-
LKPVAW--
LYPVAW--
-LPVAWK-
-LPVAWM-
-LPVAWS-
-LPVAWQ-
-LPVAWT-
-LPVAWY-
-LPVAWN-
-LPVAWW-
LAPVAW--
LVPVAW--
-LPVAWA-
LHPVAW--
LDPVAW--
-LPVAWE-
LPPVAW--
LNPVAW--
LQPVAW--
-LPVAWH-
LEPVAW--
-MPVAWS-
-MPVAWL-
-MPVAWF-
MHPVAW--
MTPVAW--
-MPVAWT-
-MPVAWR-
-MPVAWV-
-MPVAWN-
MKPVAW--
MVPVAW--
MSPVAW--
MRPVAW--
MIPVAW--
MGPVAW--
MEPVAW--
MYPVAW--
MQPVAW--
-MPVAWG-

```
MPPVAW--
MNPVAW--
MAPVAW--
-NPVAWL-
-NPVAWR-
NTPVAW--
NLPVAW--
NSPVAW--
NVPVAW--
NRPVAW--
NYPVAW--
-NPVAWH-
NFPVAW--
NAPVAW--
-NPVAWY-
-NPVAWN-
NNPVAW--
-NPVAWT-
-NPVAWQ-
NEPVAW--
-NPVAWF-
NKPVAW--
NGPVAW--
NQPVAW--
-NPVAWV-
-NPVAWS-
-NPVAWI-
-NPVAWE-
NPPVAW--
NMPVAW--
NHPVAW--
--PVAWSL
--PVAWHR
--PVAWSR
--PVAWQR
--PVAWWR
--PVAWVS
--PVAWWQ
--PVAWFS
--PVAWRS
--PVAWGL
--PVAWAK
--PVAWRL
--PVAWVR
--PVAWVL
--PVAWRG
--PVAWLR
--PVAWLD
--PVAWAS
--PVAWPI
```

--PVAWLG
--PVAWIR
--PVAWAY
--PVAWVG
--PVAWSV
--PVAWNI
--PVAWHI
--PVAWAR
--PVAWVA
--PVAWSS
--PVAWQL
--PVAWFH
--PVAWFG
--PVAWWS
--PVAWVQ
--PVAWVF
--PVAWTT
--PVAWSI
--PVAWLI
--PVAWHA
--PVAWAG
PHPVAW--
--PVAWWH
--PVAWWG
--PVAWVE
--PVAWTG
--PVAWSA
--PVAWRN
--PVAWNY
--PVAWNL
--PVAWMN
--PVAWIQ
--PVAWFQ
--PVAWFN
--PVAWYG
--PVAWTY
--PVAWTR
--PVAWTN
--PVAWTK
--PVAWSK
--PVAWSH
--PVAWRQ
--PVAWQT
--PVAWQS
--PVAWPL
--PVAWLK
--PVAWKV
--PVAWKI
--PVAWIS
--PVAWIG

--PVAWHK
--PVAWFR
--PVAWAV
--PVAWAI
PTPVAW--
--PVAWYR
--PVAWWM
--PVAWWK
--PVAWVN
--PVAWTM
--PVAWTI
--PVAWSN
--PVAWRT
--PVAWNR
--PVAWMD
--PVAWKY
--PVAWKL
--PVAWKK
--PVAWIM
--PVAWIL
--PVAWHL
--PVAWAN
PVPVAW--
--PVAWYS
--PVAWYI
--PVAWWD
--PVAWWA
--PVAWVV
--PVAWVH
--PVAWSY
--PVAWRV
--PVAWRA
--PVAWQK
--PVAWQG
--PVAWQA
--PVAWPY
--PVAWNV
--PVAWLQ
--PVAWLF
--PVAWKR
--PVAWKN
--PVAWKD
--PVAWIE
--PVAWHG
--PVAWGV
--PVAWGQ
--PVAWEY
--PVAWEG
--PVAWAT
--PVAWAL

--PVAWYT
--PVAWYQ
--PVAWYN
--PVAWYD
--PVAWWV
--PVAWWT
--PVAWVW
--PVAWTH
--PVAWTA
--PVAWSQ
--PVAWRH
--PVAWQW
--PVAWPF
--PVAWNF
--PVAWNA
--PVAWMV
--PVAWMS
--PVAWMR
--PVAWMK
--PVAWMH
--PVAWMG
--PVAWKW
--PVAWKG
--PVAWKF
--PVAWIT
--PVAWIN
--PVAWHV
--PVAWHT
--PVAWHF
--PVAWGW
--PVAWGA
--PVAWFE
--PVAWEW
--PVAWEF
--PVAWAM
--PVAWAA
PMPVAW--
PAPVAW--
--PVAWYW
--PVAWYK
--PVAWYF
--PVAWWN
--PVAWVK
--PVAWTV
--PVAWTS
--PVAWTQ
--PVAWTL
--PVAWTE
--PVAWSG
--PVAWRW

--PVAWRI
--PVAWQQ
--PVAWQN
--PVAWQI
--PVAWQE
--PVAWPM
--PVAWNM
--PVAWMY
--PVAWMT
--PVAWME
--PVAWMA
--PVAWLV
--PVAWLP
--PVAWLM
--PVAWKT
--PVAWHQ
--PVAWHE
--PVAWGM
--PVAWGI
--PVAWGG
--PVAWAQ
--PVAWAD
PQPVAW--
-PPVAWD-
PNPVAW--
PKPVAW--
PGPVAW--
PEPVAW--
QSPVAW--
-QPVAWI-
QVPVAW--
-QPVAWL-
-QPVAWY-
QKPVAW--
-QPVAWS-
QLPVAW--
QTPVAW--
-QPVAWV-
-QPVAWR-
-QPVAWF-
QPPVAW--
-QPVAWN-
QDPVAW--
QRPVAW--
QQPVAW--
-QPVAWE-
-QPVAWA-
QNPVAW--
QYPVAW--
-QPVAWT-

```
-QPVAWM-
-QPVAWK-
-QPVAWG-
QGPVAW--
QFPVAW--
-RPVAWS-
RHPVAW--
-RPVAWT-
RVPVAW--
RSPVAW--
-RPVAWY-
-RPVAWN-
RNPVAW--
RAPVAW--
RLPVAW--
RMPVAW--
RDPVAW--
RFPVAW--
-RPVAWW-
RKPVAW--
RQPVAW--
-RPVAWI-
RYPVAW--
-RPVAWQ-
-RPVAWA-
RGPVAW--
RTPVAW--
RPPVAW--
RIPVAW--
-SPVAWY-
SSPVAW--
SLPVAW--
SKPVAW--
-SPVAWM-
-SPVAWK-
SVPVAW--
STPVAW--
SHPVAW--
SGPVAW--
-SPVAWV-
-SPVAWR-
SWPVAW--
-SPVAWE-
SIPVAW--
SRPVAW--
SPPVAW--
SEPVAW--
SYPVAW--
-SPVAWT-
SAPVAW--
```

SQPVAW--
-SPVAWN-
-SPVAWI-
-SPVAWH-
-SPVAWG-
SNPVAW--
SMPVAW--
-SPVAWQ-
-SPVAWC-
SCPVAW--
TRPVAW--
TKPVAW--
-TPVAWY-
-TPVAWK-
-TPVAWL-
TLPVAW--
TSPVAW--
TQPVAW--
-TPVAWN-
-TPVAWW-
TNPVAW--
TYPVAW--
-TPVAWV-
-TPVAWS-
-TPVAWQ-
-TPVAWI-
TPPVAW--
THPVAW--
TGPVAW--
-TPVAWM-
-TPVAWH-
-TPVAWG-
-TPVAWE-
TMPVAW--
TDPVAW--
TAPVAW--
-VPVAWS-
VSPVAW--
-VPVAWH-
-VPVAWN-
VLPVAW--
VKPVAW--
VTPVAW--
-VPVAWT-
VYPVAW--
VQPVAW--
-VPVAWQ-
-VPVAWM-
-VPVAWA-
-VPVAWG-

VGPVAW--
-VPVAWK-
VPPVAW--
VMPVAW--
VHPVAW--
VWPVAW--
-VPVAWW-
-VPVAWF-
VFPVAW--
VDPVAW--
VAPVAW--
WVPVAW--
WNPVAW--
WTPVAW--
WSPVAW--
-WPVAWA-
WGPVAW--
WAPVAW--
-WPVAWK-
WYPVAW--
-WPVAWY-
-WPVAWN-
-WPVAWF-
-WPVAWE-
WDPVAW--
YKPVAW--
YGPVAW--
-YPVAWL-
-YPVAWT-
-YPVAWQ-
YWPVAW--
-YPVAWS-
-YPVAWG-
-YPVAWA-
YLPVAW--
YVPVAW--
YQPVAW--
-YPVAWV-
-YPVAWR-
-YPVAWN-
-YPVAWI-
-YPVAWH-
YIPVAW--
YAPVAW--
YRPVAW--
-YPVAWY-
YNPVAW--
YMPVAW--
YEPVAW--
YDPVAW--

LAHL 131 748 32.81

-ALAHLP-
-DLAHLT-
GPLAHL--
-GLAHLD-
--LAHLKL
--LAHLIL
--LAHLYL
--LAHLSI
--LAHLSD
--LAHLQR
--LAHLQL
--LAHLPL
--LAHLHL
--LAHLGM
--LAHLAD
-RLAHLD-
-ALAHLR-
ATLAHL--
-ALAHLL-
AGLAHL--
APLAHL--
AYLAHL--
-ALAHLH-
-ALAHLM-
ALLAHL--
-ALAHLY-
-ALAHLV-
-ALAHLT-
-ALAHLS-
-ALAHLK-
ADLAHL--
ANLAHL--
AHLAHL--
AMLAHL--
-ALAHLA-
AILAHL--
-ALAHLG-
-ALAHLN-
AFLAHL--
-ALAHLQ-
-ALAHLI-
-ALAHLF-
-ALAHLW-
AWLAHL--
AQLAHL--
AELAHL--
AALAHL--
-ALAHLE-
CLLAHL--

CTLAHL--
CHLAHL--
CCLAHL--
-CLAHLR-
-CLAHLV-
-CLAHLM-
CSLAHL--
-CLAHLF-
CGLAHL--
CYLAHL--
CVLAHL--
CRLAHL--
CQLAHL--
CNLAHL--
CMLAHL--
-CLAHLT-
-CLAHLQ-
-CLAHLI-
-CLAHLH-
-CLAHLG-
-CLAHLA-
CKLAHL--
CFLAHL--
CALAHL--
DVLAHL--
-DLAHLM-
-DLAHLY-
-DLAHLR-
DFLAHL--
-DLAHLW-
DKLAHL--
DSLAHL--
DMLAHL--
DALAHL--
DLLAHL--
-DLAHLH-
DYLAHL--
DWLAHL--
-DLAHLQ-
-DLAHLI-
-DLAHLV-
-DLAHLS-
-DLAHLK-
DELAHL--
DPLAHL--
DILAHL--
DGLAHL--
DTLAHL--
DNLAHL--
-DLAHLL-

-DLAHLF-
DQLAHL--
DHLAHL--
-DLAHLE-
ERLAHL--
-ELAHLR-
ETLAHL--
-ELAHLQ-
EHLAHL--
-ELAHLS-
EDLAHL--
EALAHL--
ENLAHL--
-ELAHLM-
-ELAHLK-
-ELAHLI-
EELAHL--
-ELAHLT-
EGLAHL--
EYLAHL--
EVLAHL--
-ELAHLY-
EKLAHL--
EQLAHL--
ESLAHL--
EFLAHL--
-ELAHLA-
-ELAHLE-
EWLAHL--
-ELAHLW-
-ELAHLV-
ECLAHL--
FKLAHL--
-FLAHLS-
FGLAHL--
FSLAHL--
FTLAHL--
-FLAHLT-
FPLAHL--
-FLAHLQ-
-FLAHLA-
-FLAHLM-
-FLAHLG-
-FLAHLL-
FALAHL--
-FLAHLV-
FHLAHL--
FYLAHL--
FDLAHL--
-FLAHLK-

-FLAHLE-
FWLAHL--
FMLAHL--
-FLAHLH-
-FLAHLF-
-FLAHLN-
FELAHL--
-FLAHLD-
-FLAHLI-
GRLAHL--
-GLAHLR-
GYLAHL--
GALAHL--
-GLAHLK-
GTLAHL--
GVLAHL--
GNLAHL--
GSLAHL--
GFLAHL--
GKLAHL--
-GLAHLA-
GQLAHL--
-GLAHLW-
-GLAHLT-
-GLAHLF-
GMLAHL--
GWLAHL--
-GLAHLQ-
-GLAHLV-
-GLAHLH-
GGLAHL--
GHLAHL--
GDLAHL--
-GLAHLN-
-GLAHLE-
HRLAHL--
-HLAHLS-
-HLAHLY-
-HLAHLR-
HHLAHL--
-HLAHLA-
HPLAHL--
-HLAHLG-
-HLAHLW-
HTLAHL--
-HLAHLK-
-HLAHLL-
HGLAHL--
HNLAHL--
-HLAHLN-

HYLAHL--
HMLAHL--
-HLAHLI-
-HLAHLH-
-HLAHLF-
HELAHL--
HDLAHL--
-HLAHLV-
HALAHL--
-HLAHLM-
HWLAHL--
HILAHL--
HCLAHL--
-ILAHLK-
ISLAHL--
IGLAHL--
-ILAHLS-
IKLAHL--
-ILAHLQ-
IALAHL--
ITLAHL--
-ILAHLL-
IMLAHL--
IDLAHL--
-ILAHLT-
-ILAHLI-
IELAHL--
INLAHL--
-ILAHLG-
IHLAHL--
IPLAHL--
-ILAHLV-
-ILAHLE-
-ILAHLN-
-ILAHLF-
IYLAHL--
IQLAHL--
-ILAHLA-
ICLAHL--
-ILAHLD-
KSLAHL--
-KLAHLR-
KALAHL--
KTLAHL--
-KLAHLV-
-KLAHLS-
-KLAHLA-
-KLAHLY-
-KLAHLT-
-KLAHLI-

KFLAHL--
KDLAHL--
KYLAHL--
KPLAHL--
KELAHL--
-KLAHLQ-
KNLAHL--
KWLAHL--
KMLAHL--
-KLAHLH-
-KLAHLF-
-KLAHLW-
-KLAHLM-
-KLAHLN-
-KLAHLG-
-KLAHLE-
KHLAHL--
KCLAHL--
KQLAHL--
--LAHLRS
--LAHLRT
--LAHLRA
--LAHLSA
--LAHLSS
LTLAHL--
LNLAHL--
--LAHLVK
LRLAHL--
--LAHLAS
--LAHLIH
--LAHLKS
--LAHLVF
LHLAHL--
--LAHLTT
--LAHLYS
--LAHLVN
LPLAHL--
LYLAHL--
--LAHLVA
LGLAHL--
-LLAHLN-
--LAHLVR
--LAHLRH
--LAHLQS
--LAHLKT
LELAHL--
--LAHLVG
--LAHLFS
--LAHLTV
--LAHLRN

--LAHLIS
--LAHLES
--LAHLRF
--LAHLKG
--LAHLRY
--LAHLNA
--LAHLKI
LQLAHL--
-LLAHLK-
-LLAHLG-
-LLAHLA-
--LAHLTK
--LAHLLH
--LAHLKR
--LAHLFT
--LAHLSY
--LAHLRW
--LAHLQA
--LAHLAG
-LLAHLH-
LDLAHL--
--LAHLYV
--LAHLVM
--LAHLIN
--LAHLTG
--LAHLRE
--LAHLMR
--LAHLMG
--LAHLAT
-LLAHLQ-
--LAHLSM
--LAHLRQ
--LAHLNS
--LAHLMV
--LAHLKK
--LAHLHV
--LAHLFM
--LAHLTW
--LAHLQG
--LAHLKE
--LAHLHT
--LAHLYR
--LAHLYA
--LAHLWG
--LAHLTY
--LAHLTA
--LAHLMF
--LAHLMA
--LAHLIG
--LAHLAW

--LAHLAM
--LAHLVY
--LAHLQH
--LAHLMM
--LAHLKN
--LAHLIY
--LAHLIT
--LAHLYT
--LAHLYK
--LAHLTN
--LAHLSF
--LAHLNL
--LAHLMT
--LAHLIQ
--LAHLIE
--LAHLGL
--LAHLGG
--LAHLFW
--LAHLYN
--LAHLYG
--LAHLVV
--LAHLTQ
--LAHLTH
--LAHLRD
--LAHLQY
--LAHLQT
--LAHLKY
--LAHLKV
--LAHLIV
--LAHLHS
--LAHLGA
--LAHLET
--LAHLAH
-LLAHLW-
--LAHLYY
--LAHLYH
--LAHLYF
--LAHLWT
--LAHLWA
--LAHLTL
--LAHLTI
--LAHLTD
--LAHLSQ
--LAHLQK
--LAHLMS
--LAHLMK
--LAHLMH
--LAHLKD
--LAHLKA
--LAHLHM

--LAHLAV
-LLAHLP-
--LAHLYD
--LAHLWH
--LAHLSW
--LAHLQV
--LAHLII
--LAHLHA
--LAHLGT
--LAHLAF
--LAHLYI
--LAHLWY
--LAHLWN
--LAHLWK
--LAHLVE
--LAHLVD
--LAHLTE
--LAHLSK
--LAHLSH
--LAHLNY
--LAHLNG
--LAHLMY
--LAHLMQ
--LAHLMI
--LAHLLW
--LAHLLM
--LAHLKQ
--LAHLKH
--LAHLIW
--LAHLIF
--LAHLHY
--LAHLHF
--LAHLFY
--LAHLFV
--LAHLFQ
--LAHLFF
--LAHLEV
--LAHLEM
--LAHLAN
LCLAHL--
--LAHLYW
--LAHLWQ
--LAHLWD
--LAHLVW
--LAHLVI
--LAHLQW
--LAHLQD
--LAHLNV
--LAHLNM
--LAHLME

--LAHLKM
--LAHLIK
--LAHLHW
--LAHLHQ
--LAHLGW
--LAHLFR
--LAHLFN
--LAHLEY
--LAHLEW
--LAHLEA
--LAHLCS
--LAHLAI
--LAHLAA
-LLAHLF-
--LAHLWM
--LAHLWF
--LAHLTF
--LAHLSE
--LAHLQM
--LAHLQE
--LAHLMW
--LAHLMD
--LAHLID
--LAHLHI
--LAHLFI
--LAHLFD
--LAHLEF
--LAHLDA
--LAHLAY
-MLAHLG-
MTLAHL--
MFLAHL--
MSLAHL--
-MLAHLK-
MPLAHL--
-MLAHLR-
-MLAHLH-
MNLAHL--
-MLAHLV-
-MLAHLL-
-MLAHLN-
MVLAHL--
MALAHL--
-MLAHLS-
-MLAHLE-
MKLAHL--
MQLAHL--
MMLAHL--
-MLAHLW-
-MLAHLF-

-MLAHLA-
MDLAHL--
-MLAHLT-
-MLAHLI-
MGLAHL--
-MLAHLQ-
MELAHL--
MCLAHL--
-MLAHLP-
-MLAHLM-
-NLAHLR-
NVLAHL--
-NLAHLL-
-NLAHLK-
NTLAHL--
-NLAHLH-
-NLAHLT-
-NLAHLA-
NSLAHL--
NMLAHL--
NYLAHL--
-NLAHLI-
NFLAHL--
-NLAHLV-
-NLAHLS-
NGLAHL--
NNLAHL--
NALAHL--
-NLAHLN-
-NLAHLF-
NHLAHL--
NELAHL--
-NLAHLQ-
NILAHL--
NQLAHL--
-NLAHLG-
NWLAHL--
-NLAHLW-
-NLAHLE-
NCLAHL--
PRLAHL--
-PLAHLR-
PNLAHL--
PGLAHL--
-PLAHLM-
-PLAHLL-
PSLAHL--
PTLAHL--
PMLAHL--
PLLAHL--

PYLAHL--
-PLAHLT-
PDLAHL--
PELAHL--
-PLAHLS-
-PLAHLG-
-PLAHLN-
-PLAHLI-
-PLAHLE-
PALAHL--
PWLAHL--
PVLAHL--
PQLAHL--
PFLAHL--
-PLAHLW-
-PLAHLQ-
-PLAHLH-
-PLAHLY-
-PLAHLD-
PCLAHL--
-QLAHLR-
QGLAHL--
QVLAHL--
QSLAHL--
QRLAHL--
QHLAHL--
-QLAHLH-
-QLAHLV-
-QLAHLK-
QQLAHL--
QDLAHL--
QLLAHL--
-QLAHLA-
QCLAHL--
-QLAHLW-
-QLAHLS-
QPLAHL--
QNLAHL--
-QLAHLF-
-QLAHLT-
-QLAHLM-
-QLAHLL-
-QLAHLE-
QYLAHL--
-QLAHLQ-
-QLAHLG-
QMLAHL--
QWLAHL--
-QLAHLN-
-RLAHLH-

RTLAHL--
-RLAHLA-
RPLAHL--
-RLAHLQ-
-RLAHLM-
-RLAHLW-
RDLAHL--
RQLAHL--
RWLAHL--
-RLAHLK-
RELAHL--
-RLAHLE-
-RLAHLN-
RCLAHL--
-SLAHLR-
SYLAHL--
-SLAHLL-
SILAHL--
SMLAHL--
SSLAHL--
SFLAHL--
SKLAHL--
SVLAHL--
-SLAHLV-
-SLAHLI-
-SLAHLH-
-SLAHLM-
-SLAHLA-
SQLAHL--
SNLAHL--
SGLAHL--
SWLAHL--
-SLAHLW-
SPLAHL--
SELAHL--
SDLAHL--
-SLAHLQ-
-SLAHLK-
-SLAHLG-
-SLAHLE-
-SLAHLN-
-SLAHLF-
SCLAHL--
THLAHL--
TNLAHL--
-TLAHLL-
-TLAHLR-
TALAHL--
TKLAHL--
TYLAHL--

TTLAHL--
-TLAHLQ-
TFLAHL--
-TLAHLK-
TGLAHL--
TDLAHL--
-TLAHLY-
-TLAHLA-
TMLAHL--
TELAHL--
-TLAHLW-
-TLAHLF-
TILAHL--
TQLAHL--
-TLAHLN-
-TLAHLE-
-TLAHLM-
TCLAHL--
VRLAHL--
VNLAHL--
-VLAHLG-
VLLAHL--
-VLAHLT-
-VLAHLH-
VKLAHL--
-VLAHLL-
VALAHL--
VTLAHL--
-VLAHLS-
-VLAHLV-
VDLAHL--
VYLAHL--
VPLAHL--
-VLAHLY-
-VLAHLN-
VQLAHL--
-VLAHLA-
VHLAHL--
VCLAHL--
-VLAHLF-
-VLAHLI-
-VLAHLW-
-VLAHLE-
VILAHL--
-WLAHLA-
WVLAHL--
-WLAHLS-
WRLAHL--
WALAHL--
-WLAHLQ-

WKLAHL--
WWLAHL--
-WLAHLG-
WDLAHL--
-WLAHLT-
WYLAHL--
-WLAHLN-
-WLAHLK-
-WLAHLM-
WGLAHL--
WPLAHL--
-WLAHLI-
-WLAHLL-
WCLAHL--
-WLAHLV-
-WLAHLH-
WELAHL--
-WLAHLW-
-WLAHLF-
WFLAHL--
YSLAHL--
YRLAHL--
YGLAHL--
-YLAHLS-
-YLAHLR-
-YLAHLM-
YYLAHL--
YPLAHL--
-YLAHLL-
-YLAHLQ-
-YLAHLV-
-YLAHLN-
-YLAHLK-
YKLAHL--
YTLAHL--
-YLAHLA-
YFLAHL--
YELAHL--
YQLAHL--
-YLAHLI-
YHLAHL--
-YLAHLT-
YALAHL--
YWLAHL--
-YLAHLY-
-YLAHLG-
YCLAHL--
-YLAHLH-
-YLAHLD-

PVGY 52 802 32.81

-DPVGYH-
--PVGYIK
--PVGYGL
-APVGYS-
-APVGYL-
ALPVGY--
-APVGYI-
AGPVGY--
AVPVGY--
ASPVGY--
-APVGYF-
ANPVGY--
-APVGYW-
-APVGYV-
ARPVGY--
-APVGYA-
AHPVGY--
AAPVGY--
-APVGYY-
-APVGYN-
-APVGYM-
APPVGY--
ATPVGY--
-APVGYH-
-APVGYT-
AFPVGY--
AIPVGY--
AYPVGY--
AQPVGY--
-APVGYE-
ADPVGY--
-APVGYQ-
AMPVGY--
-CPVGYS-
CGPVGY--
CYPVGY--
CVPVGY--
CMPVGY--
CCPVGY--
CRPVGY--
CTPVGY--
-CPVGYY-
-CPVGYV-
-CPVGYQ-
-CPVGYK-
-CPVGYI-
-CPVGYH-
CLPVGY--
CKPVGY--
CDPVGY--

CAPVGY--
DVPVGY--
-DPVGYW-
DFPVGY--
-DPVGYT-
-DPVGYS-
DGPVGY--
DRPVGY--
DAPVGY--
DLPVGY--
DKPVGY--
DHPVGY--
-DPVGYY-
-DPVGYI-
DWPVGY--
DSPVGY--
DQPVGY--
-DPVGYR-
-DPVGYL-
-DPVGYV-
-DPVGYQ-
-DPVGYM-
-DPVGYK-
DPPVGY--
ELPVGY--
ESPVGY--
-EPVGYN-
-EPVGYL-
EVPVGY--
-EPVGYM-
EWPVGY--
ERPVGY--
-EPVGYV-
-EPVGYS-
-EPVGYK-
-EPVGYA-
EAPVGY--
EYPVGY--
-EPVGYW-
-EPVGYF-
EIPVGY--
EQPVGY--
-EPVGYT-
-EPVGYQ-
ENPVGY--
-EPVGYY-
-EPVGYE-
EPPVGY--
EMPVGY--
EKPVGY--

```
-FPVGYR-
-FPVGYL-
-FPVGYM-
-FPVGYS-
-FPVGYY-
-FPVGYA-
FLPVGY--
FRPVGY--
FSPVGY--
FVPVGY--
-FPVGYV-
-FPVGYI-
-FPVGYH-
FNPVGY--
FYPVGY--
-FPVGYT-
FMPVGY--
FKPVGY--
FIPVGY--
FWPVGY--
FQPVGY--
-FPVGYN-
-FPVGYK-
-FPVGYW-
FGPVGY--
FFPVGY--
FTPVGY--
FPPVGY--
FAPVGY--
GRPVGY--
-GPVGYR-
-GPVGYL-
GLPVGY--
-GPVGYM-
-GPVGYI-
GMPVGY--
-GPVGYV-
GWPVGY--
-GPVGYT-
GAPVGY--
GYPVGY--
GSPVGY--
GDPVGY--
-GPVGYY-
GGPVGY--
-GPVGYW-
-GPVGYN-
GIPVGY--
-GPVGYQ-
-GPVGYA-
```

−GPVGYE−
GKPVGY−−
GVPVGY−−
−GPVGYK−
−GPVGYG−
GPPVGY−−
GHPVGY−−
GTPVGY−−
GQPVGY−−
GNPVGY−−
GEPVGY−−
−HPVGYR−
−HPVGYW−
HRPVGY−−
−HPVGYL−
−HPVGYV−
HNPVGY−−
−HPVGYS−
−HPVGYY−
−HPVGYN−
−HPVGYM−
HIPVGY−−
HEPVGY−−
HAPVGY−−
HVPVGY−−
HTPVGY−−
−HPVGYK−
−HPVGYI−
HMPVGY−−
HLPVGY−−
HFPVGY−−
HKPVGY−−
HDPVGY−−
HWPVGY−−
HQPVGY−−
−HPVGYQ−
−HPVGYF−
HPPVGY−−
HGPVGY−−
HYPVGY−−
−HPVGYT−
−HPVGYE−
HHPVGY−−
−IPVGYV−
IMPVGY−−
−IPVGYR−
−IPVGYL−
ILPVGY−−
−IPVGYT−
−IPVGYI−

IHPVGY--
IRPVGY--
ISPVGY--
-IPVGYK-
-IPVGYA-
INPVGY--
IEPVGY--
-IPVGYS-
-IPVGYM-
IGPVGY--
-IPVGYH-
-IPVGYF-
-IPVGYE-
IPPVGY--
IFPVGY--
IAPVGY--
IVPVGY--
-IPVGYY-
-IPVGYW-
-IPVGYN-
IIPVGY--
IYPVGY--
-IPVGYQ-
IDPVGY--
-KPVGYR-
KGPVGY--
-KPVGYS-
-KPVGYT-
-KPVGYF-
KPPVGY--
-KPVGYI-
-KPVGYA-
-KPVGYL-
KTPVGY--
-KPVGYG-
KLPVGY--
KVPVGY--
-KPVGYH-
KIPVGY--
KFPVGY--
KYPVGY--
KWPVGY--
-KPVGYY-
KKPVGY--
-KPVGYE-
-KPVGYQ-
KMPVGY--
KHPVGY--
KQPVGY--
-KPVGYN-

KCPVGY--
-KPVGYW-
-KPVGYV-
-KPVGYM-
-KPVGYD-
KDPVGY--
KAPVGY--
-LPVGYL-
-LPVGYF-
LGPVGY--
LRPVGY--
-LPVGYI-
LNPVGY--
LAPVGY--
LLPVGY--
LHPVGY--
-LPVGYT-
LVPVGY--
LSPVGY--
-LPVGYK-
-LPVGYV-
-LPVGYS-
LIPVGY--
-LPVGYQ-
LPPVGY--
LTPVGY--
-LPVGYH-
-LPVGYN-
-LPVGYA-
LKPVGY--
LEPVGY--
LQPVGY--
-LPVGYY-
LFPVGY--
LYPVGY--
-LPVGYW-
LDPVGY--
LWPVGY--
-MPVGYT-
-MPVGYI-
MKPVGY--
MDPVGY--
-MPVGYY-
MLPVGY--
-MPVGYS-
MFPVGY--
-MPVGYL-
-MPVGYH-
MRPVGY--
MWPVGY--

MSPVGY--
-MPVGYN-
-MPVGYK-
MNPVGY--
MGPVGY--
-MPVGYW-
-MPVGYF-
MEPVGY--
MQPVGY--
-MPVGYR-
-MPVGYM-
MVPVGY--
-MPVGYG-
MPPVGY--
MIPVGY--
MHPVGY--
MAPVGY--
-MPVGYQ-
-MPVGYE-
-MPVGYA-
MMPVGY--
-NPVGYS-
-NPVGYT-
NSPVGY--
-NPVGYV-
NAPVGY--
-NPVGYL-
NGPVGY--
NVPVGY--
NLPVGY--
NYPVGY--
NWPVGY--
-NPVGYI-
-NPVGYH-
NNPVGY--
NFPVGY--
NRPVGY--
-NPVGYY-
-NPVGYF-
NPPVGY--
NTPVGY--
NQPVGY--
-NPVGYQ-
-NPVGYN-
-NPVGYA-
-NPVGYK-
-NPVGYM-
NKPVGY--
NIPVGY--
NHPVGY--

-NPVGYW-
NMPVGY--
NEPVGY--
NDPVGY--
NCPVGY--
--PVGYLR
--PVGYRL
--PVGYLS
--PVGYLL
--PVGYLT
--PVGYRS
--PVGYRG
--PVGYML
--PVGYMH
--PVGYLK
--PVGYFS
--PVGYRN
--PVGYTR
--PVGYSS
--PVGYRV
--PVGYRA
--PVGYHR
--PVGYAS
--PVGYVK
--PVGYRI
--PVGYTN
--PVGYLQ
--PVGYHS
--PVGYWS
--PVGYMV
--PVGYVV
--PVGYSG
--PVGYHY
--PVGYAG
--PVGYLI
--PVGYHV
--PVGYVF
--PVGYSK
--PVGYRY
--PVGYLG
--PVGYLA
--PVGYYD
--PVGYVY
--PVGYSN
--PVGYMG
--PVGYKG
--PVGYHQ
--PVGYAT
--PVGYYS
--PVGYVT

--PVGYSA
--PVGYRQ
--PVGYLV
--PVGYKY
--PVGYKN
--PVGYFG
PSPVGY--
--PVGYWH
--PVGYVI
--PVGYVH
--PVGYVA
--PVGYTG
--PVGYSV
--PVGYRT
--PVGYNS
--PVGYNA
--PVGYIQ
--PVGYIG
--PVGYGG
--PVGYAL
PWPVGY--
--PVGYYG
--PVGYWR
--PVGYWK
--PVGYSM
--PVGYSI
--PVGYRK
--PVGYKS
--PVGYHI
--PVGYGS
--PVGYGA
--PVGYTL
--PVGYTK
--PVGYST
--PVGYRM
--PVGYRF
--PVGYQH
--PVGYNT
--PVGYKH
--PVGYII
--PVGYGV
--PVGYFN
--PVGYFH
--PVGYES
--PVGYWT
--PVGYVW
--PVGYVS
--PVGYTY
--PVGYTS
--PVGYTI

--PVGYTA
--PVGYSW
--PVGYSQ
--PVGYRE
--PVGYNY
--PVGYMS
--PVGYLW
--PVGYKT
--PVGYKK
--PVGYIM
--PVGYIA
--PVGYHN
--PVGYHL
--PVGYGT
--PVGYFQ
--PVGYFK
--PVGYAA
PKPVGY--
PVPVGY--
--PVGYWN
--PVGYVM
--PVGYVL
--PVGYTQ
--PVGYTF
--PVGYQR
--PVGYNW
--PVGYNG
--PVGYHE
PTPVGY--
PQPVGY--
PGPVGY--
--PVGYYN
--PVGYYE
--PVGYWA
--PVGYVN
--PVGYSY
--PVGYSF
--PVGYSE
--PVGYSD
--PVGYQM
--PVGYQK
--PVGYQI
--PVGYNR
--PVGYNM
--PVGYNL
--PVGYNF
--PVGYMQ
--PVGYMM
--PVGYMK
--PVGYLN

--PVGYLH
--PVGYKI
--PVGYHF
--PVGYAW
--PVGYAN
--PVGYAI
PMPVGY--
--PVGYVQ
--PVGYVG
--PVGYTV
--PVGYSH
--PVGYRW
--PVGYRD
--PVGYNV
--PVGYMN
--PVGYKL
--PVGYIY
--PVGYIH
--PVGYHW
--PVGYHT
--PVGYHM
--PVGYHG
--PVGYFI
--PVGYFD
--PVGYFA
--PVGYAY
PFPVGY--
PDPVGY--
--PVGYYA
--PVGYWG
--PVGYTW
--PVGYTM
--PVGYQY
--PVGYQV
--PVGYNK
--PVGYMI
--PVGYMF
--PVGYME
--PVGYMA
--PVGYLE
--PVGYKD
--PVGYIW
--PVGYHK
--PVGYHD
--PVGYFT
--PVGYFE
--PVGYEY
--PVGYAR
--PVGYAM
PRPVGY--

PEPVGY--
--PVGYYW
--PVGYYM
--PVGYYK
--PVGYYH
--PVGYWQ
--PVGYWM
--PVGYWE
--PVGYTH
--PVGYTD
--PVGYQT
--PVGYQS
--PVGYQQ
--PVGYPR
--PVGYMW
--PVGYKW
--PVGYKQ
--PVGYKM
--PVGYKE
--PVGYKA
--PVGYIS
--PVGYIR
--PVGYHH
--PVGYGI
--PVGYEW
--PVGYEV
--PVGYEG
--PVGYCG
--PVGYAQ
--PVGYAF
-PPVGYL-
-PPVGYF-
PIPVGY--
PCPVGY--
PAPVGY--
-QPVGYL-
-QPVGYT-
-QPVGYF-
-QPVGYY-
QKPVGY--
QSPVGY--
QAPVGY--
QTPVGY--
QRPVGY--
QPPVGY--
-QPVGYS-
QLPVGY--
QIPVGY--
-QPVGYG-
-QPVGYQ-

QYPVGY--
QVPVGY--
-QPVGYA-
QFPVGY--
QEPVGY--
QWPVGY--
-QPVGYW-
-QPVGYR-
-QPVGYK-
-QPVGYI-
QGPVGY--
-QPVGYV-
-QPVGYN-
QNPVGY--
QMPVGY--
QCPVGY--
-RPVGYS-
-RPVGYR-
-RPVGYH-
-RPVGYN-
RYPVGY--
-RPVGYM-
RTPVGY--
RLPVGY--
RHPVGY--
RVPVGY--
RMPVGY--
RWPVGY--
-RPVGYG-
RAPVGY--
-RPVGYE-
-RPVGYW-
RIPVGY--
RGPVGY--
RPPVGY--
RFPVGY--
RQPVGY--
-RPVGYF-
RNPVGY--
-RPVGYK-
REPVGY--
RDPVGY--
-RPVGYC-
-SPVGYL-
SSPVGY--
-SPVGYH-
SVPVGY--
-SPVGYV-
-SPVGYS-
SYPVGY--

SKPVGY--
SRPVGY--
-SPVGYF-
SWPVGY--
SQPVGY--
-SPVGYY-
SNPVGY--
SHPVGY--
-SPVGYM-
-SPVGYQ-
SIPVGY--
SDPVGY--
-SPVGYG-
SPPVGY--
STPVGY--
-SPVGYW-
-SPVGYI-
-SPVGYA-
-SPVGYK-
SGPVGY--
SFPVGY--
SMPVGY--
SAPVGY--
SEPVGY--
-SPVGYE-
SCPVGY--
-TPVGYL-
TRPVGY--
-TPVGYM-
TTPVGY--
-TPVGYT-
TVPVGY--
-TPVGYS-
-TPVGYI-
-TPVGYH-
TGPVGY--
TAPVGY--
TSPVGY--
TPPVGY--
THPVGY--
-TPVGYR-
-TPVGYA-
-TPVGYF-
TLPVGY--
-TPVGYW-
TFPVGY--
TDPVGY--
TQPVGY--
-TPVGYN-
TEPVGY--

TNPVGY--
TYPVGY--
TWPVGY--
-TPVGYY-
-TPVGYK-
-TPVGYG-
TIPVGY--
-TPVGYP-
-TPVGYE-
TMPVGY--
-VPVGYR-
VAPVGY--
-VPVGYA-
VNPVGY--
VEPVGY--
-VPVGYS-
VSPVGY--
-VPVGYV-
-VPVGYY-
VLPVGY--
VQPVGY--
-VPVGYG-
VPPVGY--
VWPVGY--
VVPVGY--
VHPVGY--
-VPVGYT-
-VPVGYQ-
VMPVGY--
VIPVGY--
VGPVGY--
VFPVGY--
VYPVGY--
-VPVGYH-
VDPVGY--
-VPVGYM-
-VPVGYI-
-VPVGYN-
-VPVGYF-
-VPVGYK-
-VPVGYE-
-VPVGYC-
WLPVGY--
-WPVGYR-
WPPVGY--
WRPVGY--
-WPVGYS-
-WPVGYV-
-WPVGYT-
-WPVGYL-

WKPVGY--
-WPVGYK-
-WPVGYI-
-WPVGYM-
WMPVGY--
WIPVGY--
WGPVGY--
WYPVGY--
WSPVGY--
WEPVGY--
WVPVGY--
-WPVGYA-
WHPVGY--
WDPVGY--
WAPVGY--
-WPVGYY-
WWPVGY--
WQPVGY--
-WPVGYF-
-WPVGYC-
WNPVGY--
WCPVGY--
-YPVGYR-
YLPVGY--
YRPVGY--
-YPVGYL-
-YPVGYA-
YYPVGY--
YAPVGY--
YVPVGY--
-YPVGYT-
-YPVGYK-
-YPVGYI-
-YPVGYH-
-YPVGYG-
YSPVGY--
-YPVGYS-
-YPVGYN-
-YPVGYQ-
YGPVGY--
YPPVGY--
YKPVGY--
-YPVGYY-
YMPVGY--
YWPVGY--
-YPVGYW-
YNPVGY--
YIPVGY--
YEPVGY--
-YPVGYE-

YHPVGY--
YDPVGY--

PAGM 143 743 32.81

--PAGMYT
--PAGMLE
--PAGMGR
-APAGMR-
-APAGMH-
ARPAGM--
-APAGML-
ASPAGM--
-APAGMK-
-APAGMT-
ANPAGM--
AMPAGM--
AIPAGM--
AFPAGM--
-APAGMS-
-APAGMG-
ALPAGM--
ATPAGM--
APPAGM--
-APAGMQ-
AHPAGM--
-APAGMI-
-APAGME-
-APAGMA-
AYPAGM--
AWPAGM--
AVPAGM--
-APAGMV-
-APAGMF-
AQPAGM--
-APAGMW-
-APAGMP-
-APAGMN-
-APAGMM-
AAPAGM--
-CPAGMR-
CYPAGM--
CSPAGM--
CRPAGM--
CQPAGM--
-CPAGMT-
-CPAGML-
-CPAGMK-
CLPAGM--
-DPAGML-
DVPAGM--
DSPAGM--

DRPAGM--
-DPAGMV-
-DPAGME-
DLPAGM--
-DPAGMS-
-DPAGMR-
-DPAGMA-
DNPAGM--
DIPAGM--
DHPAGM--
DGPAGM--
DAPAGM--
DYPAGM--
DTPAGM--
-DPAGMY-
-DPAGMN-
-DPAGMM-
-DPAGMI-
-DPAGMH-
-DPAGMF-
DKPAGM--
DFPAGM--
-EPAGMS-
-EPAGMY-
-EPAGMN-
-EPAGMI-
-EPAGMR-
EIPAGM--
ENPAGM--
-EPAGMV-
-EPAGML-
-EPAGMK-
ELPAGM--
EYPAGM--
EVPAGM--
ETPAGM--
EQPAGM--
-EPAGMM-
-EPAGMF-
-EPAGMA-
EAPAGM--
FNPAGM--
-FPAGML-
-FPAGMK-
-FPAGMR-
FRPAGM--
-FPAGMA-
FHPAGM--
-FPAGMS-
FLPAGM--

FFPAGM--
-FPAGMQ-
FSPAGM--
FIPAGM--
FVPAGM--
-FPAGMT-
-FPAGMI-
FMPAGM--
FYPAGM--
FWPAGM--
FTPAGM--
-FPAGMV-
-FPAGMC-
FGPAGM--
FDPAGM--
GRPAGM--
-GPAGMW-
-GPAGMR-
-GPAGMT-
GYPAGM--
-GPAGML-
GIPAGM--
GSPAGM--
-GPAGMS-
-GPAGMY-
-GPAGMI-
GNPAGM--
-GPAGMQ-
-GPAGMH-
GVPAGM--
GQPAGM--
-GPAGMA-
GWPAGM--
-GPAGMM-
-GPAGMF-
GLPAGM--
GTPAGM--
-GPAGMK-
GMPAGM--
GHPAGM--
GFPAGM--
GPPAGM--
-GPAGMG-
-GPAGME-
GKPAGM--
GGPAGM--
GCPAGM--
GAPAGM--
HMPAGM--
-HPAGMS-

-HPAGMR-
-HPAGMH-
-HPAGMG-
HNPAGM--
HYPAGM--
HVPAGM--
HSPAGM--
HPPAGM--
-HPAGMQ-
-HPAGMA-
-HPAGMT-
-HPAGMK-
-HPAGMF-
HLPAGM--
HAPAGM--
HWPAGM--
HRPAGM--
-HPAGMY-
-HPAGMI-
-HPAGMD-
HKPAGM--
HIPAGM--
HHPAGM--
HFPAGM--
ISPAGM--
IRPAGM--
-IPAGMT-
-IPAGMH-
INPAGM--
IPPAGM--
-IPAGMS-
-IPAGMK-
-IPAGMN-
-IPAGMA-
IMPAGM--
IGPAGM--
IFPAGM--
IWPAGM--
-IPAGMV-
-IPAGMR-
-IPAGML-
-IPAGMG-
-IPAGMF-
IYPAGM--
ITPAGM--
-IPAGMY-
-IPAGMW-
-IPAGMQ-
-IPAGME-
IIPAGM--

KSPAGM--
-KPAGMR-
-KPAGML-
KVPAGM--
-KPAGMK-
-KPAGMI-
-KPAGMG-
-KPAGMY-
KGPAGM--
-KPAGMV-
-KPAGMS-
KHPAGM--
-KPAGMT-
-KPAGMA-
-KPAGMW-
-KPAGMH-
-KPAGMF-
-KPAGMQ-
-KPAGMN-
-KPAGMM-
KLPAGM--
KIPAGM--
KPPAGM--
-KPAGME-
KFPAGM--
KDPAGM--
KCPAGM--
-LPAGMF-
-LPAGMR-
LQPAGM--
LVPAGM--
LSPAGM--
-LPAGMV-
-LPAGMA-
-LPAGMS-
-LPAGMN-
-LPAGML-
-LPAGMK-
LPPAGM--
-LPAGMQ-
LGPAGM--
LYPAGM--
-LPAGMH-
-LPAGMW-
-LPAGMM-
LNPAGM--
LLPAGM--
LFPAGM--
-LPAGMY-
-LPAGMI-

```
-LPAGMG-
-LPAGME-
LIPAGM--
LDPAGM--
LCPAGM--
LAPAGM--
LTPAGM--
-LPAGMT-
-MPAGMH-
MTPAGM--
-MPAGMV-
MMPAGM--
-MPAGMK-
MWPAGM--
-MPAGMR-
-MPAGME-
MNPAGM--
MPPAGM--
-MPAGMY-
-MPAGMT-
-MPAGMI-
-MPAGMA-
MKPAGM--
MQPAGM--
-MPAGMW-
-MPAGMS-
MLPAGM--
MSPAGM--
MRPAGM--
-MPAGMQ-
-MPAGML-
-MPAGMF-
MFPAGM--
-NPAGMI-
-NPAGMR-
-NPAGMH-
NIPAGM--
NMPAGM--
NVPAGM--
-NPAGMG-
NSPAGM--
-NPAGMY-
-NPAGMV-
-NPAGML-
-NPAGMK-
NFPAGM--
NTPAGM--
-NPAGMS-
-NPAGMQ-
-NPAGMN-
```

NRPAGM--
-NPAGMM-
NGPAGM--
NYPAGM--
-NPAGMW-
-NPAGME-
NLPAGM--
--PAGMLR
--PAGMRS
--PAGMSS
--PAGMSA
--PAGMRI
--PAGMFS
--PAGMRN
--PAGMRG
--PAGMVW
--PAGMSL
--PAGMRY
--PAGMLS
--PAGMLG
--PAGMTL
--PAGMRT
--PAGMQM
--PAGMVL
--PAGMRV
--PAGMVN
--PAGMML
--PAGMRL
--PAGMLY
--PAGMLK
--PAGMYR
--PAGMWH
--PAGMKR
--PAGMGS
--PAGMNR
--PAGMNL
--PAGMIS
--PAGMYI
--PAGMVI
--PAGMSN
--PAGMLV
--PAGMIT
--PAGMFV
--PAGMFG
--PAGMTV
--PAGMTS
--PAGMSW
--PAGMST
--PAGMSK
--PAGMRK

--PAGMRE
--PAGMQN
--PAGMIN
--PAGMHL
--PAGMGA
--PAGMFR
--PAGMFE
--PAGMEF
--PAGMYV
--PAGMWE
--PAGMVD
--PAGMTG
--PAGMTA
--PAGMSY
--PAGMSG
--PAGMRW
--PAGMRM
--PAGMRA
--PAGMKS
--PAGMKL
--PAGMKH
--PAGMHT
--PAGMHS
--PAGMHR
--PAGMGT
--PAGMFA
PFPAGM--
--PAGMYY
--PAGMWT
--PAGMWR
--PAGMVY
--PAGMVV
--PAGMVT
--PAGMVS
--PAGMVK
--PAGMTT
--PAGMSI
--PAGMSH
--PAGMRH
--PAGMLT
--PAGMLN
--PAGMLL
--PAGMLI
--PAGMLF
--PAGMLA
--PAGMKG
--PAGMIY
--PAGMIL
--PAGMFK
--PAGMFH

--PAGMAT
PMPAGM--
PLPAGM--
PHPAGM--
--PAGMYS
--PAGMWY
--PAGMWK
--PAGMVR
--PAGMTN
--PAGMTM
--PAGMTF
--PAGMTE
--PAGMSQ
--PAGMRF
--PAGMQL
--PAGMQI
--PAGMMI
--PAGMMG
--PAGMLD
--PAGMKV
--PAGMIH
--PAGMHM
--PAGMHF
--PAGMAL
--PAGMAK
PTPAGM--
PSPAGM--
PRPAGM--
--PAGMYM
--PAGMWS
--PAGMWN
--PAGMSD
--PAGMQY
--PAGMQV
--PAGMQS
--PAGMQH
--PAGMNW
--PAGMNV
--PAGMNT
--PAGMNI
--PAGMMQ
--PAGMMN
--PAGMMK
--PAGMKI
--PAGMKA
--PAGMIV
--PAGMIM
--PAGMIK
--PAGMHW
--PAGMHV

--PAGMHN
--PAGMGY
--PAGMGW
--PAGMGM
--PAGMAM
--PAGMAF
PNPAGM--
PEPAGM--
PCPAGM--
PAPAGM--
--PAGMYD
--PAGMWM
--PAGMVQ
--PAGMVF
--PAGMVA
--PAGMTI
--PAGMSF
--PAGMSE
--PAGMRQ
--PAGMQD
--PAGMNG
--PAGMNF
--PAGMNA
--PAGMMS
--PAGMMR
--PAGMMH
--PAGMMF
--PAGMMA
--PAGMLW
--PAGMKT
--PAGMKN
--PAGMKF
--PAGMKE
--PAGMIQ
--PAGMII
--PAGMIG
--PAGMHY
--PAGMHI
--PAGMHG
--PAGMHA
--PAGMGN
--PAGMGL
--PAGMFQ
--PAGMEY
--PAGMEG
--PAGMDA
--PAGMAY
--PAGMAN
--PAGMAE
--PAGMAD

PWPAGM--
PVPAGM--
PQPAGM--
-PPAGMP-
PKPAGM--
PIPAGM--
--PAGMYW
--PAGMYQ
--PAGMYH
--PAGMYE
--PAGMWW
--PAGMWQ
--PAGMWL
--PAGMWG
--PAGMVM
--PAGMVG
--PAGMVE
--PAGMTR
--PAGMTK
--PAGMTH
--PAGMTD
--PAGMQW
--PAGMQT
--PAGMQK
--PAGMQA
--PAGMNY
--PAGMNS
--PAGMNQ
--PAGMNK
--PAGMNE
--PAGMMT
--PAGMLQ
--PAGMLM
--PAGMLC
--PAGMKM
--PAGMKD
--PAGMIR
--PAGMIE
--PAGMID
--PAGMHE
--PAGMGK
--PAGMGI
--PAGMGF
--PAGMGE
--PAGMFI
--PAGMFF
--PAGMEW
--PAGMEV
--PAGMES
--PAGMCN

--PAGMAR
--PAGMAH
--PAGMAA
-QPAGMR-
-QPAGMK-
QVPAGM--
-QPAGMS-
-QPAGMH-
-QPAGML-
QAPAGM--
QRPAGM--
-QPAGMA-
QIPAGM--
-QPAGMY-
QLPAGM--
QGPAGM--
QTPAGM--
QSPAGM--
-QPAGMF-
QHPAGM--
QDPAGM--
-QPAGMW-
-QPAGMV-
-QPAGMT-
-QPAGMM-
-QPAGMI-
-QPAGMG-
-QPAGMD-
-RPAGMR-
RSPAGM--
-RPAGMF-
-RPAGMQ-
RMPAGM--
RAPAGM--
RRPAGM--
-RPAGMI-
-RPAGMH-
-RPAGMN-
RLPAGM--
RFPAGM--
-RPAGMK-
RDPAGM--
-RPAGMS-
-RPAGME-
RPPAGM--
-RPAGMA-
RKPAGM--
-RPAGMW-
RIPAGM--
RWPAGM--

RGPAGM--
-RPAGMV-
-RPAGMM-
RHPAGM--
-RPAGMG-
-RPAGMD-
REPAGM--
-SPAGMY-
SSPAGM--
-SPAGMR-
SRPAGM--
SWPAGM--
-SPAGMT-
-SPAGML-
-SPAGMI-
-SPAGMS-
SYPAGM--
-SPAGMN-
-SPAGMK-
-SPAGMH-
SKPAGM--
-SPAGMG-
-SPAGMF-
SGPAGM--
STPAGM--
SLPAGM--
SMPAGM--
SDPAGM--
SPPAGM--
-SPAGMQ-
-SPAGMA-
SNPAGM--
SQPAGM--
-SPAGMV-
SAPAGM--
-SPAGMM-
-SPAGMD-
SFPAGM--
SEPAGM--
-TPAGMS-
-TPAGMH-
-TPAGMY-
-TPAGMN-
-TPAGML-
TKPAGM--
TSPAGM--
TQPAGM--
-TPAGMT-
THPAGM--
TVPAGM--

-TPAGMR-
-TPAGMF-
TIPAGM--
TWPAGM--
-TPAGMV-
-TPAGMK-
TLPAGM--
TYPAGM--
-TPAGMQ-
-TPAGMI-
-TPAGMG-
-TPAGME-
TTPAGM--
TRPAGM--
TPPAGM--
-TPAGMW-
TNPAGM--
TGPAGM--
TCPAGM--
TAPAGM--
-VPAGML-
-VPAGMT-
-VPAGMF-
VGPAGM--
-VPAGMW-
-VPAGMK-
VYPAGM--
VRPAGM--
-VPAGMR-
-VPAGMG-
VNPAGM--
VHPAGM--
VSPAGM--
VMPAGM--
VVPAGM--
VQPAGM--
-VPAGMN-
VLPAGM--
VEPAGM--
-VPAGMI-
VDPAGM--
VTPAGM--
-VPAGME-
VPPAGM--
-VPAGMY-
-VPAGMH-
VKPAGM--
VCPAGM--
-WPAGMR-
WRPAGM--

WGPAGM--
WSPAGM--
-WPAGMT-
-WPAGMA-
WTPAGM--
WFPAGM--
-WPAGMY-
WEPAGM--
WWPAGM--
WPPAGM--
-WPAGMV-
-WPAGMN-
-WPAGML-
-WPAGMK-
-WPAGMI-
-WPAGMH-
-WPAGMF-
WYPAGM--
WVPAGM--
-WPAGMM-
-WPAGMG-
-WPAGME-
WHPAGM--
-YPAGMW-
-YPAGMS-
-YPAGMR-
-YPAGMY-
YTPAGM--
YSPAGM--
YRPAGM--
YGPAGM--
-YPAGMV-
-YPAGMF-
YHPAGM--
-YPAGMT-
-YPAGMI-
YIPAGM--
YWPAGM--
-YPAGMA-
YYPAGM--
-YPAGMQ-
-YPAGMN-
-YPAGMM-
-YPAGMD-
-YPAGMC-
YEPAGM--

PKNL 213 705 32.80

APPKNL--
--PKNLRP
--PKNLGR

SEPKNL--
-TPKNLN-
-APKNLS-
-APKNLR-
AAPKNL--
AQPKNL--
-APKNLY-
AGPKNL--
-APKNLK-
ASPKNL--
-APKNLI-
-APKNLA-
-APKNLV-
-APKNLL-
-APKNLE-
-APKNLW-
ADPKNL--
-APKNLG-
AIPKNL--
ATPKNL--
-APKNLQ-
AKPKNL--
AVPKNL--
AEPKNL--
AYPKNL--
-APKNLM-
AHPKNL--
-APKNLH-
-APKNLD-
AMPKNL--
CVPKNL--
CRPKNL--
CFPKNL--
CAPKNL--
CYPKNL--
CSPKNL--
CPPKNL--
-CPKNLS-
-CPKNLR-
-CPKNLN-
-CPKNLI-
-CPKNLE-
CMPKNL--
CLPKNL--
CIPKNL--
CGPKNL--
CCPKNL--
DTPKNL--
-DPKNLR-
-DPKNLL-

DWPKNL--
DMPKNL--
DLPKNL--
-DPKNLY-
DSPKNL--
DVPKNL--
DRPKNL--
-DPKNLF-
DGPKNL--
-DPKNLK-
DHPKNL--
DYPKNL--
DPPKNL--
-DPKNLT-
-DPKNLQ-
-DPKNLI-
-DPKNLH-
-DPKNLA-
DFPKNL--
DAPKNL--
-EPKNLR-
-EPKNLI-
-EPKNLV-
-EPKNLL-
EVPKNL--
-EPKNLT-
EIPKNL--
ESPKNL--
-EPKNLF-
-EPKNLK-
EHPKNL--
EGPKNL--
EAPKNL--
-EPKNLW-
-EPKNLQ-
EMPKNL--
EYPKNL--
EQPKNL--
EPPKNL--
-EPKNLY-
-EPKNLS-
-EPKNLG-
EFPKNL--
ETPKNL--
ERPKNL--
-EPKNLE-
ENPKNL--
ELPKNL--
EEPKNL--
EDPKNL--

FSPKNL--
FAPKNL--
FLPKNL--
-FPKNLM-
FNPKNL--
FMPKNL--
FHPKNL--
-FPKNLL-
-FPKNLF-
FGPKNL--
FTPKNL--
-FPKNLQ-
FWPKNL--
-FPKNLW-
FQPKNL--
-FPKNLV-
FEPKNL--
-FPKNLE-
FPPKNL--
FDPKNL--
FCPKNL--
GFPKNL--
-GPKNLN-
GLPKNL--
GEPKNL--
-GPKNLM-
-GPKNLA-
-GPKNLR-
-GPKNLL-
GRPKNL--
-GPKNLG-
-GPKNLV-
-GPKNLH-
GVPKNL--
GGPKNL--
-GPKNLY-
-GPKNLT-
-GPKNLK-
GNPKNL--
GMPKNL--
GYPKNL--
-GPKNLI-
GQPKNL--
-GPKNLD-
GDPKNL--
GPPKNL--
-GPKNLQ-
GKPKNL--
-GPKNLW-
GAPKNL--

−GPKNLF−
GIPKNL−−
HRPKNL−−
−HPKNLL−
HHPKNL−−
HVPKNL−−
−HPKNLR−
HYPKNL−−
HMPKNL−−
HCPKNL−−
HQPKNL−−
HPPKNL−−
−HPKNLS−
−HPKNLF−
HWPKNL−−
HTPKNL−−
−HPKNLW−
HGPKNL−−
−HPKNLY−
−HPKNLE−
−HPKNLA−
HAPKNL−−
HSPKNL−−
−HPKNLG−
−HPKNLV−
−HPKNLM−
−HPKNLI−
−IPKNLS−
IDPKNL−−
ISPKNL−−
−IPKNLI−
IIPKNL−−
−IPKNLK−
−IPKNLG−
−IPKNLF−
IKPKNL−−
−IPKNLL−
IGPKNL−−
IAPKNL−−
IWPKNL−−
IVPKNL−−
−IPKNLV−
IHPKNL−−
IPPKNL−−
INPKNL−−
−IPKNLY−
−IPKNLW−
IEPKNL−−
−IPKNLQ−
−IPKNLA−

IFPKNL--
-IPKNLM-
-IPKNLH-
-IPKNLE-
-KPKNLV-
-KPKNLL-
KVPKNL--
KTPKNL--
-KPKNLS-
KMPKNL--
KRPKNL--
-KPKNLN-
KPPKNL--
-KPKNLT-
-KPKNLM-
-KPKNLG-
KIPKNL--
-KPKNLH-
-KPKNLA-
KQPKNL--
-KPKNLY-
-KPKNLE-
KNPKNL--
KDPKNL--
KYPKNL--
-KPKNLW-
-KPKNLF-
KGPKNL--
KKPKNL--
KCPKNL--
LTPKNL--
LLPKNL--
LSPKNL--
LVPKNL--
-LPKNLQ-
LAPKNL--
-LPKNLT-
LYPKNL--
-LPKNLI-
LMPKNL--
LKPKNL--
LGPKNL--
-LPKNLA-
LHPKNL--
LFPKNL--
-LPKNLS-
-LPKNLH-
LWPKNL--
-LPKNLV-
LEPKNL--

-LPKNLY-
-LPKNLW-
LDPKNL--
-LPKNLN-
MKPKNL--
-MPKNLL-
MTPKNL--
MGPKNL--
-MPKNLM-
MVPKNL--
MPPKNL--
-MPKNLV-
-MPKNLT-
-MPKNLS-
-MPKNLN-
-MPKNLK-
-MPKNLH-
MLPKNL--
MFPKNL--
MDPKNL--
-MPKNLY-
-MPKNLQ-
-MPKNLI-
-MPKNLA-
MIPKNL--
MAPKNL--
MSPKNL--
MMPKNL--
-NPKNLS-
NVPKNL--
-NPKNLA-
-NPKNLL-
-NPKNLR-
-NPKNLI-
NTPKNL--
NSPKNL--
NYPKNL--
NNPKNL--
NKPKNL--
NHPKNL--
NCPKNL--
NWPKNL--
NPPKNL--
-NPKNLY-
-NPKNLQ-
-NPKNLK-
-NPKNLF-
NIPKNL--
NGPKNL--
NEPKNL--

-NPKNLV-
-NPKNLE-
-NPKNLM-
-NPKNLG-
NAPKNL--
--PKNLLT
--PKNLYL
--PKNLSS
--PKNLSL
--PKNLFR
--PKNLSW
--PKNLLG
PLPKNL--
--PKNLVL
--PKNLYR
--PKNLVT
PFPKNL--
--PKNLTL
--PKNLVS
--PKNLLW
--PKNLKY
--PKNLWQ
--PKNLSV
--PKNLFG
--PKNLAI
--PKNLWA
--PKNLRV
--PKNLHS
--PKNLGA
--PKNLFM
--PKNLVY
--PKNLVV
--PKNLSM
--PKNLSA
--PKNLET
--PKNLAL
--PKNLYH
--PKNLWY
--PKNLLV
--PKNLEA
--PKNLAS
--PKNLSI
--PKNLNT
--PKNLNA
--PKNLMF
--PKNLLN
--PKNLKS
--PKNLKI
--PKNLHG
--PKNLFT

--PKNLAT
--PKNLAQ
PHPKNL--
PSPKNL--
--PKNLYM
--PKNLQV
--PKNLNY
--PKNLLY
--PKNLLE
--PKNLIG
--PKNLGG
--PKNLFK
--PKNLYA
--PKNLTN
--PKNLTG
--PKNLST
--PKNLSE
--PKNLRD
--PKNLQM
--PKNLQL
--PKNLLH
--PKNLIA
--PKNLHI
--PKNLGI
--PKNLFL
--PKNLAW
--PKNLAA
PGPKNL--
--PKNLTV
--PKNLTI
--PKNLSN
--PKNLSK
--PKNLSF
--PKNLRI
--PKNLRA
--PKNLQR
--PKNLNM
--PKNLNF
--PKNLMI
--PKNLIS
--PKNLIN
--PKNLFS
--PKNLFE
--PKNLES
--PKNLEK
--PKNLAY
--PKNLAN
--PKNLAH
PNPKNL--
--PKNLYG

--PKNLWT
--PKNLWE
--PKNLTF
--PKNLTD
--PKNLSH
--PKNLQS
--PKNLQH
--PKNLQG
--PKNLNS
--PKNLLM
--PKNLLD
--PKNLKN
--PKNLHM
--PKNLHF
--PKNLGV
--PKNLFN
--PKNLFF
--PKNLER
--PKNLEF
--PKNLAF
PMPKNL--
--PKNLYN
--PKNLYE
--PKNLVQ
--PKNLVM
--PKNLVA
--PKNLTS
--PKNLTQ
--PKNLSG
--PKNLRE
--PKNLQQ
--PKNLLF
--PKNLKF
--PKNLHY
--PKNLGT
--PKNLFY
--PKNLFH
--PKNLYV
--PKNLYF
--PKNLYD
--PKNLWG
--PKNLVW
--PKNLRQ
--PKNLRM
--PKNLQF
--PKNLQE
--PKNLPL
--PKNLNN
--PKNLMK
--PKNLIQ

--PKNLGY
--PKNLFI
--PKNLEW
--PKNLEG
PVPKNL--
PQPKNL--
--PKNLYS
--PKNLYQ
--PKNLWV
--PKNLWS
--PKNLVH
--PKNLTY
--PKNLTT
--PKNLTH
--PKNLTA
--PKNLQY
--PKNLQN
--PKNLNG
--PKNLMT
--PKNLMH
--PKNLME
--PKNLLQ
--PKNLKM
--PKNLKH
--PKNLKD
--PKNLIL
--PKNLFV
--PKNLFQ
--PKNLFA
--PKNLEN
--PKNLEM
--PKNLCV
--PKNLAV
--PKNLAM
PIPKNL--
PPPKNL--
-PPKNLV-
-PPKNLF-
PKPKNL--
--PKNLYY
--PKNLYW
--PKNLYT
--PKNLYI
--PKNLWW
--PKNLWM
--PKNLWF
--PKNLVN
--PKNLVK
--PKNLTW
--PKNLQW

--PKNLQD
--PKNLPK
--PKNLNV
--PKNLNQ
--PKNLNI
--PKNLMQ
--PKNLKT
--PKNLIE
--PKNLID
--PKNLHW
--PKNLHV
--PKNLHQ
--PKNLHL
--PKNLHH
--PKNLGW
--PKNLFD
--PKNLEI
--PKNLEH
--PKNLDS
--PKNLAK
PEPKNL--
PDPKNL--
PCPKNL--
-QPKNLR-
QSPKNL--
QDPKNL--
-QPKNLA-
QFPKNL--
QTPKNL--
QMPKNL--
QAPKNL--
QPPKNL--
-QPKNLS-
-QPKNLL-
QIPKNL--
QYPKNL--
QNPKNL--
QLPKNL--
QWPKNL--
QVPKNL--
-QPKNLW-
-QPKNLT-
QKPKNL--
QGPKNL--
-QPKNLY-
-QPKNLQ-
-QPKNLN-
-QPKNLM-
-QPKNLH-
QEPKNL--

-RPKNLS-
RTPKNL--
-RPKNLQ-
RAPKNL--
RQPKNL--
RPPKNL--
-RPKNLW-
-RPKNLG-
RGPKNL--
RYPKNL--
RMPKNL--
RKPKNL--
-RPKNLN-
RNPKNL--
RDPKNL--
-RPKNLH-
-RPKNLM-
REPKNL--
RCPKNL--
-RPKNLE-
-SPKNLL-
SMPKNL--
-SPKNLN-
-SPKNLA-
STPKNL--
SAPKNL--
-SPKNLQ-
-SPKNLV-
-SPKNLK-
-SPKNLT-
SPPKNL--
SYPKNL--
SVPKNL--
SQPKNL--
SGPKNL--
-SPKNLS-
-SPKNLM-
-SPKNLY-
-SPKNLF-
-SPKNLE-
SKPKNL--
SCPKNL--
-SPKNLH-
SDPKNL--
-SPKNLW-
TQPKNL--
TNPKNL--
-TPKNLT-
-TPKNLS-
TLPKNL--

TEPKNL--
TVPKNL--
-TPKNLR-
TDPKNL--
TSPKNL--
-TPKNLI-
TWPKNL--
-TPKNLV-
-TPKNLH-
TAPKNL--
TKPKNL--
THPKNL--
TMPKNL--
TYPKNL--
TTPKNL--
TPPKNL--
-TPKNLK-
-TPKNLF-
TIPKNL--
TRPKNL--
-TPKNLM-
TGPKNL--
-TPKNLW-
-TPKNLQ-
-TPKNLD-
VSPKNL--
VWPKNL--
VLPKNL--
-VPKNLA-
-VPKNLN-
-VPKNLV-
VDPKNL--
VYPKNL--
VTPKNL--
VRPKNL--
-VPKNLR-
VVPKNL--
-VPKNLS-
-VPKNLG-
-VPKNLY-
VEPKNL--
VPPKNL--
-VPKNLF-
-VPKNLE-
VQPKNL--
VGPKNL--
-VPKNLW-
-VPKNLI-
-VPKNLH-
VNPKNL--

VCPKNL--
-VPKNLQ-
-WPKNLL-
WKPKNL--
WGPKNL--
WVPKNL--
WHPKNL--
WRPKNL--
WNPKNL--
WPPKNL--
-WPKNLW-
WMPKNL--
WQPKNL--
WLPKNL--
WIPKNL--
-WPKNLM-
-WPKNLF-
WTPKNL--
-WPKNLT-
-WPKNLQ-
WYPKNL--
-WPKNLN-
WEPKNL--
-YPKNLL-
YSPKNL--
YVPKNL--
YAPKNL--
-YPKNLV-
-YPKNLQ-
YLPKNL--
YTPKNL--
YQPKNL--
-YPKNLK-
YKPKNL--
YDPKNL--
YHPKNL--
YRPKNL--
-YPKNLI-
-YPKNLE-
YGPKNL--
YYPKNL--
-YPKNLY-
-YPKNLH-
-YPKNLF-
YFPKNL--
-YPKNLT-
-YPKNLM-
-YPKNLG-
YNPKNL--
YMPKNL--

| | | | |
|---|---|---|---|
| | YIPKNL-- | | |
| | YEPKNL-- | | |
| PRSW | 96 | 771 | 32.80 |
| | -DPRSWN- | | |
| | -FPRSWG- | | |
| | -IPRSWN- | | |
| | -MPRSWG- | | |
| | -NPRSWN- | | |
| | --PRSWSE | | |
| | --PRSWRP | | |
| | --PRSWPY | | |
| | --PRSWPR | | |
| | --PRSWGY | | |
| | --PRSWGA | | |
| | --PRSWDS | | |
| | QQPRSW-- | | |
| | TEPRSW-- | | |
| | -APRSWL- | | |
| | -APRSWV- | | |
| | ASPRSW-- | | |
| | -APRSWI- | | |
| | ATPRSW-- | | |
| | APPRSW-- | | |
| | AMPRSW-- | | |
| | AGPRSW-- | | |
| | -APRSWY- | | |
| | -APRSWK- | | |
| | AAPRSW-- | | |
| | -APRSWA- | | |
| | AYPRSW-- | | |
| | -APRSWS- | | |
| | AIPRSW-- | | |
| | ALPRSW-- | | |
| | AVPRSW-- | | |
| | -APRSWT- | | |
| | -APRSWF- | | |
| | -APRSWM- | | |
| | ARPRSW-- | | |
| | -APRSWN- | | |
| | AEPRSW-- | | |
| | -APRSWW- | | |
| | -APRSWG- | | |
| | ADPRSW-- | | |
| | -APRSWQ- | | |
| | -APRSWE- | | |
| | AQPRSW-- | | |
| | ANPRSW-- | | |
| | CGPRSW-- | | |
| | CVPRSW-- | | |
| | CTPRSW-- | | |

CLPRSW--
CAPRSW--
CYPRSW--
CSPRSW--
CRPRSW--
-CPRSWL-
CKPRSW--
-DPRSWR-
-DPRSWV-
DSPRSW--
-DPRSWY-
-DPRSWT-
-DPRSWL-
DVPRSW--
-DPRSWW-
-DPRSWF-
-DPRSWQ-
DLPRSW--
DFPRSW--
DYPRSW--
-DPRSWK-
DMPRSW--
DAPRSW--
DQPRSW--
-DPRSWI-
DKPRSW--
DHPRSW--
DGPRSW--
DTPRSW--
-DPRSWS-
-DPRSWM-
-DPRSWH-
-DPRSWA-
DIPRSW--
DEPRSW--
DDPRSW--
ERPRSW--
ELPRSW--
-EPRSWL-
-EPRSWI-
-EPRSWR-
ESPRSW--
EMPRSW--
-EPRSWQ-
EIPRSW--
-EPRSWM-
-EPRSWW-
EAPRSW--
-EPRSWK-
EVPRSW--

ETPRSW--
-EPRSWN-
EQPRSW--
-EPRSWY-
-EPRSWS-
EGPRSW--
EEPRSW--
EYPRSW--
-EPRSWT-
EHPRSW--
EFPRSW--
-EPRSWE-
EKPRSW--
-EPRSWG-
ENPRSW--
-EPRSWF-
-EPRSWA-
EPPRSW--
-FPRSWL-
-FPRSWQ-
FHPRSW--
-FPRSWI-
FDPRSW--
FQPRSW--
-FPRSWS-
-FPRSWH-
FVPRSW--
FSPRSW--
FRPRSW--
-FPRSWY-
-FPRSWT-
FNPRSW--
FEPRSW--
-FPRSWN-
FTPRSW--
-FPRSWM-
FMPRSW--
FYPRSW--
FWPRSW--
FGPRSW--
FAPRSW--
-FPRSWE-
FPPRSW--
-FPRSWF-
-FPRSWP-
-FPRSWA-
FIPRSW--
FFPRSW--
-GPRSWS-
-GPRSWR-

GVPRSW--
GRPRSW--
GKPRSW--
-GPRSWF-
GGPRSW--
GTPRSW--
GQPRSW--
-GPRSWW-
-GPRSWQ-
-GPRSWL-
-GPRSWK-
-GPRSWH-
-GPRSWI-
GMPRSW--
-GPRSWY-
GIPRSW--
GEPRSW--
GAPRSW--
-GPRSWA-
GYPRSW--
GSPRSW--
GDPRSW--
-GPRSWN-
GPPRSW--
GNPRSW--
GFPRSW--
-GPRSWM-
GWPRSW--
-GPRSWD-
GHPRSW--
-HPRSWS-
HGPRSW--
HRPRSW--
-HPRSWA-
HSPRSW--
-HPRSWY-
-HPRSWI-
HAPRSW--
HWPRSW--
-HPRSWQ-
HYPRSW--
-HPRSWK-
HIPRSW--
HVPRSW--
-HPRSWN-
HDPRSW--
HTPRSW--
HMPRSW--
HPPRSW--
HLPRSW--

HHPRSW--
-HPRSWE-
HNPRSW--
HKPRSW--
HQPRSW--
-HPRSWF-
HFPRSW--
ITPRSW--
-IPRSWT-
-IPRSWR-
IAPRSW--
-IPRSWF-
-IPRSWS-
-IPRSWQ-
IGPRSW--
-IPRSWK-
ILPRSW--
IRPRSW--
-IPRSWL-
IPPRSW--
IFPRSW--
IMPRSW--
IKPRSW--
IQPRSW--
IEPRSW--
-IPRSWI-
IHPRSW--
-IPRSWH-
IYPRSW--
-IPRSWY-
-IPRSWW-
IWPRSW--
-IPRSWG-
-IPRSWE-
IDPRSW--
KSPRSW--
-KPRSWV-
-KPRSWL-
-KPRSWI-
-KPRSWS-
-KPRSWA-
KLPRSW--
KGPRSW--
KPPRSW--
-KPRSWY-
KTPRSW--
KFPRSW--
-KPRSWF-
KQPRSW--
-KPRSWM-

KYPRSW--
KDPRSW--
KWPRSW--
-KPRSWQ-
-KPRSWH-
KVPRSW--
-KPRSWG-
KIPRSW--
KHPRSW--
-KPRSWW-
KEPRSW--
-KPRSWN-
KAPRSW--
LVPRSW--
LNPRSW--
-LPRSWR-
LSPRSW--
LRPRSW--
-LPRSWT-
LQPRSW--
LLPRSW--
LIPRSW--
LAPRSW--
LTPRSW--
-LPRSWQ-
LKPRSW--
-LPRSWV-
LMPRSW--
-LPRSWM-
LEPRSW--
LPPRSW--
LHPRSW--
-LPRSWH-
-LPRSWF-
-LPRSWY-
LDPRSW--
-LPRSWW-
LCPRSW--
-MPRSWS-
-MPRSWL-
-MPRSWW-
MSPRSW--
MRPRSW--
MGPRSW--
MAPRSW--
MVPRSW--
MLPRSW--
-MPRSWY-
MWPRSW--
-MPRSWQ-

MHPRSW--
MTPRSW--
-MPRSWI-
-MPRSWA-
MKPRSW--
MFPRSW--
MYPRSW--
MQPRSW--
-MPRSWN-
-MPRSWM-
-MPRSWF-
MNPRSW--
MMPRSW--
-MPRSWE-
MIPRSW--
MEPRSW--
MDPRSW--
NTPRSW--
NYPRSW--
NSPRSW--
-NPRSWM-
-NPRSWV-
NVPRSW--
-NPRSWI-
NHPRSW--
NGPRSW--
-NPRSWK-
NDPRSW--
NKPRSW--
NQPRSW--
-NPRSWF-
NIPRSW--
NEPRSW--
NFPRSW--
-NPRSWQ-
-NPRSWY-
-NPRSWT-
NPPRSW--
NAPRSW--
-NPRSWH-
-NPRSWS-
NMPRSW--
NWPRSW--
--PRSWTS
--PRSWRA
--PRSWVS
--PRSWSG
--PRSWKL
--PRSWLS
--PRSWHS

--PRSWMS
--PRSWLT
--PRSWSW
--PRSWST
--PRSWSN
--PRSWSF
--PRSWSA
--PRSWSV
--PRSWLY
--PRSWIG
--PRSWVR
--PRSWSI
--PRSWVY
--PRSWVT
--PRSWRH
--PRSWLQ
--PRSWIS
--PRSWMT
--PRSWYH
--PRSWLG
PGPRSW--
--PRSWWK
--PRSWSY
--PRSWSK
--PRSWWS
--PRSWVH
--PRSWTT
--PRSWLF
--PRSWFN
--PRSWYA
--PRSWVF
--PRSWTV
--PRSWTG
--PRSWRT
--PRSWNT
--PRSWGS
--PRSWFV
--PRSWYT
--PRSWVI
--PRSWRV
--PRSWIT
--PRSWFH
PMPRSW--
--PRSWVQ
--PRSWTW
--PRSWTI
--PRSWLA
--PRSWAY
--PRSWYS
--PRSWTN

--PRSWTK
--PRSWNS
--PRSWLV
--PRSWAL
--PRSWAF
--PRSWYQ
--PRSWRN
--PRSWMG
--PRSWIF
--PRSWFG
--PRSWYG
--PRSWVG
--PRSWTM
--PRSWRQ
--PRSWNA
--PRSWMV
--PRSWHA
--PRSWAI
--PRSWYN
--PRSWWG
--PRSWTR
--PRSWIH
--PRSWHR
--PRSWAM
PAPRSW--
--PRSWVW
--PRSWRD
--PRSWQN
--PRSWQG
--PRSWKS
--PRSWKG
--PRSWHY
--PRSWGT
--PRSWGL
--PRSWAV
--PRSWVV
--PRSWMQ
--PRSWKK
--PRSWKI
--PRSWII
--PRSWEV
--PRSWET
--PRSWEA
PRPRSW--
--PRSWWV
--PRSWTQ
--PRSWRF
--PRSWQS
--PRSWMN
--PRSWIM

--PRSWHT
--PRSWHN
--PRSWFQ
PSPRSW--
--PRSWTH
--PRSWSM
--PRSWSH
--PRSWMK
--PRSWKT
--PRSWFY
--PRSWFS
--PRSWFL
--PRSWFK
--PRSWYV
--PRSWYI
--PRSWTF
--PRSWQA
--PRSWMW
--PRSWIW
--PRSWHL
--PRSWGI
--PRSWFW
--PRSWEG
--PRSWAA
PIPRSW--
--PRSWYY
--PRSWWY
--PRSWWN
--PRSWWF
--PRSWVE
--PRSWTD
--PRSWSQ
--PRSWQT
--PRSWQQ
--PRSWNY
--PRSWLW
--PRSWLM
--PRSWIA
--PRSWFI
--PRSWAH
--PRSWYR
--PRSWWQ
--PRSWWI
--PRSWWH
--PRSWTY
--PRSWQW
--PRSWQV
--PRSWMF
--PRSWKY
--PRSWIQ

--PRSWIN
--PRSWES
PFPRSW--
PEPRSW--
PWPRSW--
--PRSWWT
--PRSWRM
--PRSWNF
--PRSWMY
--PRSWMI
--PRSWMD
--PRSWKN
--PRSWIV
--PRSWHV
--PRSWHQ
--PRSWHG
--PRSWFM
--PRSWAN
PVPRSW--
--PRSWWL
--PRSWVD
--PRSWTE
--PRSWRI
--PRSWNG
--PRSWLE
--PRSWKD
--PRSWKA
--PRSWIE
--PRSWHW
--PRSWHI
--PRSWFF
--PRSWFE
--PRSWAW
--PRSWAQ
PHPRSW--
PYPRSW--
--PRSWYK
--PRSWYD
--PRSWQI
--PRSWMH
--PRSWME
--PRSWLD
--PRSWKQ
--PRSWKM
--PRSWIK
--PRSWHH
--PRSWEW
--PRSWEN
PKPRSW--
--PRSWWM

--PRSWWD
--PRSWSD
--PRSWQY
--PRSWNM
--PRSWKW
--PRSWKE
--PRSWHM
--PRSWGV
--PRSWGG
--PRSWEY
--PRSWAE
PQPRSW--
PDPRSW--
--PRSWYM
--PRSWYF
--PRSWQD
--PRSWNW
--PRSWNN
--PRSWMC
--PRSWID
--PRSWHK
--PRSWGF
--PRSWEL
--PRSWEF
--PRSWDC
--PRSWCQ
PNPRSW--
-QPRSWS-
QAPRSW--
-QPRSWR-
QKPRSW--
QVPRSW--
-QPRSWT-
QIPRSW--
QTPRSW--
-QPRSWV-
-QPRSWI-
-QPRSWA-
QGPRSW--
QSPRSW--
-QPRSWG-
QRPRSW--
-QPRSWH-
QLPRSW--
QYPRSW--
-QPRSWY-
-QPRSWE-
QMPRSW--
QHPRSW--
-QPRSWW-

QEPRSW--
QWPRSW--
QNPRSW--
-QPRSWF-
-QPRSWK-
-QPRSWQ-
-QPRSWM-
QDPRSW--
-RPRSWL-
-RPRSWT-
RPPRSW--
RGPRSW--
-RPRSWY-
RTPRSW--
RQPRSW--
-RPRSWI-
-RPRSWS-
RLPRSW--
RWPRSW--
-RPRSWQ-
-RPRSWA-
-RPRSWH-
-RPRSWF-
RIPRSW--
RNPRSW--
RHPRSW--
-RPRSWN-
RKPRSW--
REPRSW--
RAPRSW--
-RPRSWW-
-RPRSWG-
RDPRSW--
-RPRSWE-
-RPRSWK-
-SPRSWS-
SHPRSW--
SRPRSW--
SLPRSW--
SKPRSW--
SVPRSW--
SGPRSW--
-SPRSWT-
-SPRSWV-
-SPRSWA-
-SPRSWM-
SEPRSW--
-SPRSWG-
SFPRSW--
SYPRSW--

-SPRSWQ-
-SPRSWI-
-SPRSWW-
-SPRSWF-
-SPRSWY-
-SPRSWN-
SMPRSW--
-SPRSWK-
-SPRSWH-
SWPRSW--
SPPRSW--
-SPRSWE-
-SPRSWD-
-TPRSWL-
TVPRSW--
-TPRSWV-
TSPRSW--
TIPRSW--
-TPRSWM-
TGPRSW--
TTPRSW--
TAPRSW--
-TPRSWH-
TQPRSW--
TFPRSW--
-TPRSWS-
-TPRSWA-
TNPRSW--
-TPRSWW-
TYPRSW--
TWPRSW--
-TPRSWQ-
-TPRSWF-
TMPRSW--
-TPRSWN-
-TPRSWG-
TDPRSW--
THPRSW--
TPPRSW--
-TPRSWE-
-VPRSWI-
-VPRSWR-
VFPRSW--
-VPRSWS-
VGPRSW--
VIPRSW--
-VPRSWV-
VHPRSW--
VLPRSW--
VRPRSW--

VVPRSW--
VEPRSW--
VNPRSW--
-VPRSWY-
VPPRSW--
-VPRSWM-
-VPRSWF-
VWPRSW--
-VPRSWQ-
-VPRSWK-
VMPRSW--
VYPRSW--
VAPRSW--
VQPRSW--
VKPRSW--
-VPRSWH-
-VPRSWE-
VDPRSW--
VCPRSW--
WLPRSW--
WSPRSW--
WQPRSW--
-WPRSWS-
WYPRSW--
WRPRSW--
-WPRSWQ-
-WPRSWA-
-WPRSWH-
WDPRSW--
WVPRSW--
-WPRSWN-
WMPRSW--
WEPRSW--
WAPRSW--
-WPRSWY-
-WPRSWV-
WPPRSW--
WHPRSW--
WGPRSW--
-WPRSWW-
-WPRSWM-
WTPRSW--
-WPRSWF-
WIPRSW--
-WPRSWC-
YRPRSW--
-YPRSWW-
YSPRSW--
-YPRSWH-
-YPRSWA-

| | | | |
|---|---|---|---|
| | -YPRSWV- | | |
| | YGPRSW-- | | |
| | -YPRSWF- | | |
| | YNPRSW-- | | |
| | YHPRSW-- | | |
| | YVPRSW-- | | |
| | -YPRSWM- | | |
| | YYPRSW-- | | |
| | -YPRSWT- | | |
| | YFPRSW-- | | |
| | YTPRSW-- | | |
| | -YPRSWI- | | |
| | YKPRSW-- | | |
| | YEPRSW-- | | |
| | YAPRSW-- | | |
| | YWPRSW-- | | |
| | YQPRSW-- | | |
| | -YPRSWN- | | |
| | YIPRSW-- | | |
| | -YPRSWG- | | |
| | YPPRSW-- | | |
| | -YPRSWY- | | |
| | -YPRSWE- | | |
| | YDPRSW-- | | |
| PQNL | 85 | 778 | 32.79 |
| | --PQNLRN | | |
| | -APQNLR- | | |
| | -APQNLL- | | |
| | -APQNLQ- | | |
| | -APQNLI- | | |
| | ASPQNL-- | | |
| | ARPQNL-- | | |
| | ANPQNL-- | | |
| | -APQNLY- | | |
| | -APQNLS- | | |
| | -APQNLN- | | |
| | AFPQNL-- | | |
| | AEPQNL-- | | |
| | AVPQNL-- | | |
| | AKPQNL-- | | |
| | AHPQNL-- | | |
| | -APQNLT- | | |
| | -APQNLK- | | |
| | -APQNLG- | | |
| | AIPQNL-- | | |
| | -APQNLH- | | |
| | -APQNLE- | | |
| | -APQNLF- | | |
| | AMPQNL-- | | |
| | AGPQNL-- | | |

AYPQNL--
AAPQNL--
-APQNLW-
-APQNLD-
-APQNLA-
ALPQNL--
CTPQNL--
CSPQNL--
-CPQNLR-
CKPQNL--
CYPQNL--
-CPQNLS-
-CPQNLQ-
CPPQNL--
CNPQNL--
CLPQNL--
CFPQNL--
CEPQNL--
DVPQNL--
-DPQNLW-
-DPQNLT-
-DPQNLR-
DAPQNL--
-DPQNLM-
-DPQNLL-
DYPQNL--
DRPQNL--
-DPQNLK-
-DPQNLA-
DLPQNL--
DGPQNL--
DFPQNL--
DWPQNL--
DSPQNL--
-DPQNLS-
-DPQNLH-
-DPQNLF-
DPPQNL--
DHPQNL--
ERPQNL--
-EPQNLL-
ELPQNL--
EKPQNL--
EYPQNL--
EVPQNL--
-EPQNLI-
ESPQNL--
-EPQNLS-
-EPQNLR-
-EPQNLK-

−EPQNLE−
ENPQNL−−
−EPQNLG−
EMPQNL−−
EIPQNL−−
EHPQNL−−
EGPQNL−−
EFPQNL−−
−FPQNLY−
−FPQNLH−
FRPQNL−−
−FPQNLT−
−FPQNLR−
FGPQNL−−
FMPQNL−−
FTPQNL−−
FSPQNL−−
FLPQNL−−
FFPQNL−−
FWPQNL−−
FDPQNL−−
FYPQNL−−
FVPQNL−−
−FPQNLM−
−FPQNLI−
−FPQNLA−
FAPQNL−−
−FPQNLL−
−FPQNLG−
−FPQNLF−
−FPQNLW−
−FPQNLQ−
−FPQNLE−
FCPQNL−−
−GPQNLR−
GRPQNL−−
−GPQNLK−
GIPQNL−−
GLPQNL−−
−GPQNLT−
−GPQNLM−
GHPQNL−−
−GPQNLG−
−GPQNLI−
GFPQNL−−
−GPQNLN−
GKPQNL−−
GYPQNL−−
GWPQNL−−
−GPQNLV−

−GPQNLQ−
GMPQNL−−
GAPQNL−−
GVPQNL−−
GTPQNL−−
−GPQNLY−
−GPQNLW−
GQPQNL−−
−GPQNLS−
−GPQNLL−
−GPQNLE−
GPPQNL−−
GNPQNL−−
GSPQNL−−
GGPQNL−−
GEPQNL−−
GDPQNL−−
−HPQNLR−
−HPQNLN−
HRPQNL−−
−HPQNLF−
HGPQNL−−
HMPQNL−−
HHPQNL−−
HFPQNL−−
HSPQNL−−
−HPQNLT−
−HPQNLH−
HTPQNL−−
−HPQNLY−
−HPQNLS−
−HPQNLI−
−HPQNLG−
HIPQNL−−
HEPQNL−−
HYPQNL−−
HVPQNL−−
HQPQNL−−
−HPQNLV−
−HPQNLM−
−HPQNLK−
−HPQNLA−
HKPQNL−−
IPPQNL−−
ILPQNL−−
ITPQNL−−
IRPQNL−−
−IPQNLK−
−IPQNLY−
−IPQNLR−

-IPQNLT-
-IPQNLQ-
IWPQNL--
ISPQNL--
IIPQNL--
-IPQNLS-
-IPQNLI-
INPQNL--
-IPQNLH-
IAPQNL--
-IPQNLL-
-IPQNLA-
IHPQNL--
IDPQNL--
IYPQNL--
IVPQNL--
IQPQNL--
-IPQNLW-
-IPQNLV-
-IPQNLN-
-IPQNLG-
-IPQNLF-
-IPQNLD-
IMPQNL--
IKPQNL--
IGPQNL--
IFPQNL--
ICPQNL--
KSPQNL--
-KPQNLL-
KLPQNL--
KEPQNL--
-KPQNLY-
-KPQNLR-
-KPQNLG-
KGPQNL--
KWPQNL--
-KPQNLA-
-KPQNLV-
KIPQNL--
KAPQNL--
KYPQNL--
KPPQNL--
-KPQNLK-
-KPQNLF-
-KPQNLE-
-KPQNLH-
KRPQNL--
-KPQNLT-
-KPQNLM-

−KPQNLI−
KMPQNL−−
KHPQNL−−
LVPQNL−−
LLPQNL−−
−LPQNLI−
LRPQNL−−
−LPQNLK−
−LPQNLR−
LKPQNL−−
LWPQNL−−
−LPQNLT−
LAPQNL−−
LYPQNL−−
LTPQNL−−
−LPQNLL−
−LPQNLY−
LMPQNL−−
LIPQNL−−
LGPQNL−−
LEPQNL−−
LQPQNL−−
−LPQNLS−
−LPQNLQ−
LPPQNL−−
−LPQNLV−
LNPQNL−−
LHPQNL−−
−LPQNLW−
−LPQNLA−
LDPQNL−−
LFPQNL−−
MSPQNL−−
MRPQNL−−
−MPQNLF−
−MPQNLQ−
MLPQNL−−
−MPQNLL−
MKPQNL−−
MVPQNL−−
−MPQNLT−
−MPQNLN−
−MPQNLI−
MFPQNL−−
MTPQNL−−
−MPQNLR−
−MPQNLK−
−MPQNLE−
MNPQNL−−
−MPQNLS−

```
-MPQNLA-
MQPQNL--
-MPQNLW-
-MPQNLV-
-MPQNLM-
-MPQNLH-
MPPQNL--
MMPQNL--
MHPQNL--
MGPQNL--
-NPQNLR-
-NPQNLL-
NLPQNL--
-NPQNLT-
NRPQNL--
-NPQNLY-
NPPQNL--
NTPQNL--
NQPQNL--
-NPQNLW-
-NPQNLS-
NFPQNL--
NSPQNL--
-NPQNLQ-
-NPQNLK-
NEPQNL--
-NPQNLV-
-NPQNLH-
-NPQNLG-
NKPQNL--
NGPQNL--
NWPQNL--
NYPQNL--
NVPQNL--
-NPQNLN-
-NPQNLM-
-NPQNLI-
-NPQNLE-
-NPQNLA-
NMPQNL--
NIPQNL--
NHPQNL--
NCPQNL--
NAPQNL--
--PQNLRL
--PQNLTR
--PQNLRV
--PQNLLW
--PQNLLR
--PQNLVR
```

--PQNLSL
--PQNLRT
--PQNLRA
--PQNLLL
--PQNLVI
--PQNLTS
--PQNLSV
--PQNLLI
--PQNLKL
--PQNLWQ
--PQNLRM
--PQNLAI
--PQNLVS
PLPQNL--
--PQNLYL
--PQNLTL
--PQNLSS
--PQNLLG
--PQNLHS
--PQNLTG
--PQNLRW
--PQNLQR
--PQNLQL
--PQNLMR
--PQNLFR
--PQNLFM
--PQNLYT
--PQNLWT
--PQNLVD
--PQNLNV
--PQNLLV
--PQNLLT
--PQNLLA
--PQNLKT
--PQNLHR
--PQNLGT
--PQNLAN
PAPQNL--
PRPQNL--
--PQNLVV
--PQNLTH
--PQNLRS
--PQNLML
--PQNLKS
--PQNLKM
--PQNLIS
--PQNLAY
--PQNLWV
--PQNLVY
--PQNLVK

--PQNLTT
--PQNLSY
--PQNLRI
--PQNLQN
--PQNLNL
--PQNLIY
--PQNLAS
PQPQNL--
--PQNLYM
--PQNLVH
--PQNLVF
--PQNLTY
--PQNLTQ
--PQNLTM
--PQNLSM
--PQNLSK
--PQNLSH
--PQNLRY
--PQNLQG
--PQNLNY
--PQNLLS
--PQNLLQ
--PQNLLK
--PQNLII
--PQNLHV
--PQNLAT
PKPQNL--
PWPQNL--
--PQNLYH
--PQNLYG
--PQNLWN
--PQNLVT
--PQNLVL
--PQNLVG
--PQNLTN
--PQNLNM
--PQNLMG
--PQNLLD
--PQNLKY
--PQNLKI
--PQNLHT
--PQNLHK
--PQNLHI
--PQNLGS
--PQNLFT
--PQNLEW
--PQNLAL
PGPQNL--
--PQNLYS
--PQNLYE

--PQNLWS
--PQNLWL
--PQNLVN
--PQNLSN
--PQNLSI
--PQNLSA
--PQNLQY
--PQNLQK
--PQNLMN
--PQNLMI
--PQNLLF
--PQNLKW
--PQNLIV
--PQNLIT
--PQNLIR
--PQNLIM
--PQNLGV
--PQNLGM
--PQNLGL
--PQNLGG
--PQNLFV
--PQNLEG
--PQNLAH
--PQNLAG
PFPQNL--
--PQNLWR
--PQNLWH
--PQNLVM
--PQNLVA
--PQNLTI
--PQNLTA
--PQNLSQ
--PQNLRH
--PQNLRF
--PQNLRE
--PQNLRD
--PQNLQV
--PQNLQT
--PQNLQI
--PQNLQF
--PQNLMW
--PQNLMV
--PQNLMK
--PQNLMH
--PQNLLM
--PQNLIQ
--PQNLHW
--PQNLHG
--PQNLHA
--PQNLGA

--PQNLFN
--PQNLFK
--PQNLER
--PQNLAD
PNPQNL--
PTPQNL--
PSPQNL--
--PQNLYW
--PQNLYN
--PQNLYF
--PQNLWM
--PQNLWG
--PQNLWA
--PQNLVW
--PQNLVQ
--PQNLTF
--PQNLSW
--PQNLST
--PQNLSF
--PQNLQQ
--PQNLQM
--PQNLPY
--PQNLNW
--PQNLNS
--PQNLNG
--PQNLMS
--PQNLKV
--PQNLKR
--PQNLKK
--PQNLKD
--PQNLKA
--PQNLIW
--PQNLIK
--PQNLHH
--PQNLFW
--PQNLFQ
--PQNLFI
--PQNLFG
--PQNLFA
--PQNLAW
--PQNLAV
--PQNLAQ
PIPQNL--
PEPQNL--
PVPQNL--
--PQNLYK
--PQNLYI
--PQNLYA
--PQNLVE
--PQNLTV

--PQNLTK
--PQNLSD
--PQNLRP
--PQNLQD
--PQNLPR
--PQNLNT
--PQNLNK
--PQNLNI
--PQNLNF
--PQNLMY
--PQNLMT
--PQNLMQ
--PQNLMA
--PQNLLN
--PQNLKQ
--PQNLKG
--PQNLIN
--PQNLIF
--PQNLID
--PQNLIA
--PQNLHY
--PQNLHF
--PQNLHD
--PQNLGW
--PQNLGI
--PQNLGF
--PQNLFS
--PQNLFF
--PQNLFE
--PQNLFD
--PQNLEV
--PQNLES
--PQNLEQ
--PQNLEM
--PQNLEI
--PQNLEH
--PQNLDS
--PQNLDG
--PQNLCL
--PQNLCD
--PQNLAM
--PQNLAK
PHPQNL--
QRPQNL--
QSPQNL--
-QPQNLS-
-QPQNLH-
QWPQNL--
QVPQNL--
-QPQNLL-

-QPQNLF-
-QPQNLR-
QIPQNL--
QHPQNL--
QQPQNL--
-QPQNLV-
-QPQNLG-
-QPQNLN-
QPPQNL--
QMPQNL--
QFPQNL--
QDPQNL--
QTPQNL--
-QPQNLW-
-QPQNLT-
-QPQNLQ-
QLPQNL--
QEPQNL--
-RPQNLR-
RIPQNL--
RLPQNL--
-RPQNLG-
RVPQNL--
-RPQNLT-
-RPQNLF-
RSPQNL--
RQPQNL--
-RPQNLI-
RRPQNL--
RMPQNL--
RYPQNL--
-RPQNLS-
-RPQNLN-
-RPQNLH-
RGPQNL--
RFPQNL--
RPPQNL--
-RPQNLW-
-RPQNLE-
-RPQNLL-
-RPQNLM-
-RPQNLA-
REPQNL--
RAPQNL--
-RPQNLV-
-RPQNLK-
RDPQNL--
-RPQNLY-
RTPQNL--
-RPQNLQ-

SLPQNL--
-SPQNLR-
-SPQNLI-
-SPQNLL-
-SPQNLN-
-SPQNLV-
SSPQNL--
SGPQNL--
SAPQNL--
SHPQNL--
SRPQNL--
-SPQNLS-
SYPQNL--
-SPQNLY-
-SPQNLT-
SPPQNL--
SNPQNL--
SMPQNL--
SFPQNL--
-SPQNLK-
-SPQNLH-
-SPQNLG-
-SPQNLE-
SQPQNL--
-SPQNLA-
SCPQNL--
SWPQNL--
-SPQNLW-
SKPQNL--
-SPQNLQ-
-SPQNLM-
-SPQNLF-
SIPQNL--
SEPQNL--
TVPQNL--
-TPQNLW-
TSPQNL--
TRPQNL--
-TPQNLY-
-TPQNLR-
-TPQNLS-
-TPQNLL-
THPQNL--
-TPQNLG-
TTPQNL--
TMPQNL--
TKPQNL--
TLPQNL--
TYPQNL--
TQPQNL--

–TPQNLT–
–TPQNLV–
–TPQNLA–
TIPQNL––
TFPQNL––
–TPQNLM–
–TPQNLH–
–TPQNLF–
TCPQNL––
TAPQNL––
–TPQNLQ–
–TPQNLN–
–TPQNLK–
–TPQNLE–
TGPQNL––
TDPQNL––
–VPQNLI–
VRPQNL––
–VPQNLL–
VKPQNL––
VIPQNL––
VYPQNL––
VSPQNL––
VLPQNL––
VWPQNL––
–VPQNLS–
–VPQNLR–
–VPQNLW–
–VPQNLE–
VPPQNL––
VVPQNL––
VDPQNL––
VAPQNL––
–VPQNLK–
VNPQNL––
VTPQNL––
–VPQNLT–
VHPQNL––
–VPQNLY–
–VPQNLM–
–VPQNLH–
VGPQNL––
–VPQNLV–
–VPQNLQ–
–VPQNLN–
–VPQNLG–
VEPQNL––
VCPQNL––
–VPQNLA–
WNPQNL––

WSPQNL--
-WPQNLQ-
-WPQNLG-
-WPQNLT-
-WPQNLS-
WIPQNL--
WVPQNL--
-WPQNLR-
-WPQNLL-
WQPQNL--
-WPQNLY-
-WPQNLF-
-WPQNLE-
-WPQNLA-
WGPQNL--
WDPQNL--
WTPQNL--
WRPQNL--
-WPQNLV-
-WPQNLH-
WEPQNL--
-WPQNLW-
-WPQNLN-
-WPQNLK-
-WPQNLI-
WMPQNL--
-YPQNLR-
YRPQNL--
YFPQNL--
YYPQNL--
-YPQNLT-
-YPQNLH-
YSPQNL--
-YPQNLI-
YEPQNL--
-YPQNLK-
-YPQNLE-
YHPQNL--
YDPQNL--
YWPQNL--
YPPQNL--
YNPQNL--
YAPQNL--
YTPQNL--
-YPQNLY-
-YPQNLG-
YMPQNL--
YKPQNL--
YCPQNL--
-YPQNLV-

| | | | |
|---|---|---|---|
| | -YPQNLQ- | | |
| | -YPQNLN- | | |
| | YGPQNL-- | | |
| PSAM | 73 | 786 | 32.78 |
| | ALPSAM-- | | |
| | ARPSAM-- | | |
| | -APSAML- | | |
| | -APSAMY- | | |
| | -APSAMM- | | |
| | -APSAMS- | | |
| | -APSAMN- | | |
| | AVPSAM-- | | |
| | AGPSAM-- | | |
| | AQPSAM-- | | |
| | -APSAMV- | | |
| | -APSAMF- | | |
| | AAPSAM-- | | |
| | -APSAMG- | | |
| | AFPSAM-- | | |
| | ASPSAM-- | | |
| | -APSAMW- | | |
| | -APSAMR- | | |
| | AIPSAM-- | | |
| | AHPSAM-- | | |
| | AYPSAM-- | | |
| | ATPSAM-- | | |
| | -APSAMK- | | |
| | AMPSAM-- | | |
| | AWPSAM-- | | |
| | -APSAMI- | | |
| | APPSAM-- | | |
| | AKPSAM-- | | |
| | -CPSAML- | | |
| | CVPSAM-- | | |
| | CYPSAM-- | | |
| | CRPSAM-- | | |
| | -CPSAMK- | | |
| | -CPSAMI- | | |
| | CMPSAM-- | | |
| | CSPSAM-- | | |
| | CCPSAM-- | | |
| | DRPSAM-- | | |
| | -DPSAMK- | | |
| | -DPSAMR- | | |
| | -DPSAMA- | | |
| | -DPSAML- | | |
| | DVPSAM-- | | |
| | -DPSAMW- | | |
| | DMPSAM-- | | |
| | DAPSAM-- | | |

DTPSAM--
DSPSAM--
-DPSAMY-
-DPSAMT-
-DPSAMH-
DLPSAM--
DKPSAM--
-DPSAMS-
-DPSAMQ-
-DPSAMN-
DHPSAM--
DYPSAM--
-DPSAMV-
-DPSAMI-
-DPSAMF-
DFPSAM--
DEPSAM--
DDPSAM--
ERPSAM--
-EPSAMR-
-EPSAMG-
EGPSAM--
ESPSAM--
-EPSAMK-
-EPSAMA-
ELPSAM--
-EPSAMY-
ENPSAM--
EFPSAM--
ETPSAM--
-EPSAMT-
-EPSAMN-
-EPSAML-
-EPSAMI-
-EPSAMF-
EAPSAM--
EYPSAM--
EQPSAM--
-EPSAMS-
-EPSAMQ-
-EPSAMM-
-EPSAME-
EHPSAM--
ECPSAM--
-FPSAMS-
FRPSAM--
FLPSAM--
-FPSAML-
-FPSAMI-
-FPSAMW-

-FPSAMK-
-FPSAMY-
FSPSAM--
-FPSAMV-
-FPSAMR-
FYPSAM--
FVPSAM--
-FPSAMQ-
-FPSAMF-
FMPSAM--
-FPSAMH-
-FPSAMA-
FGPSAM--
FFPSAM--
FWPSAM--
-FPSAMT-
-FPSAMG-
-FPSAMC-
FNPSAM--
FKPSAM--
FEPSAM--
FDPSAM--
FAPSAM--
GRPSAM--
GLPSAM--
-GPSAMR-
-GPSAMN-
-GPSAMS-
-GPSAMT-
GGPSAM--
GEPSAM--
GSPSAM--
-GPSAML-
GKPSAM--
-GPSAMI-
-GPSAMA-
GQPSAM--
-GPSAMK-
-GPSAMY-
-GPSAMV-
-GPSAMQ-
-GPSAMH-
-GPSAMG-
-GPSAMF-
GVPSAM--
GTPSAM--
-GPSAMW-
GYPSAM--
GWPSAM--
GPPSAM--

```
-GPSAMM-
-GPSAME-
GNPSAM--
GHPSAM--
GDPSAM--
HRPSAM--
-HPSAMT-
-HPSAMQ-
HSPSAM--
HWPSAM--
HGPSAM--
HVPSAM--
-HPSAMN-
-HPSAMS-
-HPSAMF-
HDPSAM--
-HPSAMV-
-HPSAMR-
HMPSAM--
HLPSAM--
HTPSAM--
-HPSAMI-
HIPSAM--
-HPSAMK-
-HPSAMH-
HNPSAM--
HKPSAM--
HAPSAM--
IRPSAM--
INPSAM--
-IPSAMM-
-IPSAMR-
IGPSAM--
ITPSAM--
-IPSAML-
IAPSAM--
IKPSAM--
-IPSAMI-
-IPSAMY-
-IPSAMS-
IVPSAM--
IQPSAM--
-IPSAMA-
ILPSAM--
IHPSAM--
-IPSAMW-
-IPSAMT-
-IPSAMN-
-IPSAMF-
-IPSAME-
```

IFPSAM--
IYPSAM--
ISPSAM--
-IPSAMV-
-IPSAMP-
-IPSAMK-
IPPSAM--
IMPSAM--
IDPSAM--
KSPSAM--
-KPSAML-
-KPSAMN-
KGPSAM--
-KPSAMV-
-KPSAMH-
-KPSAMG-
KYPSAM--
KRPSAM--
-KPSAMY-
-KPSAMW-
-KPSAMT-
-KPSAMS-
KLPSAM--
KWPSAM--
KVPSAM--
-KPSAMR-
-KPSAME-
-KPSAMA-
KNPSAM--
KMPSAM--
KEPSAM--
KTPSAM--
-KPSAMI-
-KPSAMC-
KKPSAM--
KAPSAM--
-LPSAML-
-LPSAMR-
-LPSAMS-
LLPSAM--
LYPSAM--
LMPSAM--
-LPSAMT-
-LPSAMI-
LAPSAM--
LIPSAM--
LFPSAM--
LTPSAM--
LRPSAM--
-LPSAMK-

LKPSAM--
-LPSAMV-
LSPSAM--
-LPSAMA-
-LPSAMY-
LWPSAM--
-LPSAMG-
-LPSAMF-
LGPSAM--
LVPSAM--
-LPSAMN-
-LPSAMH-
LQPSAM--
-LPSAMW-
-LPSAMQ-
-LPSAMM-
-LPSAME-
LNPSAM--
LDPSAM--
LPPSAM--
MSPSAM--
-MPSAML-
-MPSAMG-
-MPSAMS-
MMPSAM--
MHPSAM--
MRPSAM--
MTPSAM--
-MPSAMV-
-MPSAMM-
-MPSAMF-
MLPSAM--
MKPSAM--
MIPSAM--
MGPSAM--
-MPSAMK-
-MPSAMH-
MVPSAM--
-MPSAMY-
-MPSAMW-
-MPSAMT-
MYPSAM--
-MPSAMQ-
-MPSAMI-
-MPSAMA-
MPPSAM--
MDPSAM--
MAPSAM--
NRPSAM--
NFPSAM--

NVPSAM--
-NPSAMV-
-NPSAMT-
-NPSAMA-
NWPSAM--
NPPSAM--
NLPSAM--
-NPSAMS-
-NPSAML-
-NPSAMK-
-NPSAMW-
-NPSAMQ-
NGPSAM--
NTPSAM--
NSPSAM--
NQPSAM--
-NPSAMG-
NMPSAM--
NIPSAM--
NEPSAM--
NAPSAM--
NYPSAM--
-NPSAMN-
-NPSAMF-
NKPSAM--
NHPSAM--
--PSAMSR
--PSAMLM
--PSAMHW
--PSAMVS
--PSAMSL
--PSAMME
--PSAMYG
--PSAMRL
--PSAMWG
--PSAMNL
--PSAMLS
--PSAMYR
--PSAMRG
--PSAMLT
--PSAMKL
--PSAMYT
--PSAMTF
--PSAMHL
--PSAMGT
--PSAMGS
--PSAMWS
--PSAMSG
--PSAMRH
--PSAMQG

--PSAMLY
--PSAMLR
--PSAMAN
--PSAMYN
--PSAMRM
--PSAMLV
--PSAMFG
--PSAMFD
PRPSAM--
--PSAMVL
--PSAMRR
--PSAMFL
--PSAMWR
--PSAMNI
--PSAMKG
--PSAMFS
--PSAMAL
PVPSAM--
--PSAMYM
--PSAMTV
--PSAMRY
--PSAMRQ
--PSAMRN
--PSAMRI
--PSAMNT
--PSAMMT
--PSAMIR
--PSAMGV
--PSAMGL
--PSAMFN
--PSAMFA
--PSAMAS
--PSAMWL
--PSAMVM
--PSAMVG
--PSAMTW
--PSAMTL
--PSAMTI
--PSAMSS
--PSAMSQ
--PSAMSN
--PSAMSI
--PSAMQM
--PSAMKA
--PSAMHS
--PSAMHH
--PSAMFV
--PSAMAR
--PSAMAG
--PSAMYV

--PSAMVI
--PSAMTS
--PSAMTA
--PSAMSY
--PSAMQL
--PSAMQH
--PSAMNV
--PSAMML
--PSAMMG
--PSAMLN
--PSAMLK
--PSAMLH
--PSAMLG
--PSAMKS
--PSAMIL
--PSAMHR
--PSAMFT
PYPSAM--
--PSAMYQ
--PSAMYL
--PSAMVR
--PSAMVK
--PSAMVF
--PSAMSW
--PSAMST
--PSAMQY
--PSAMQA
--PSAMNH
--PSAMMR
--PSAMLW
--PSAMIV
--PSAMIS
--PSAMIG
--PSAMID
PKPSAM--
PTPSAM--
PSPSAM--
--PSAMYK
--PSAMYD
--PSAMWT
--PSAMWA
--PSAMVE
--PSAMTG
--PSAMRW
--PSAMRF
--PSAMRE
--PSAMPL
--PSAMLD
--PSAMIK
--PSAMHF

--PSAMGY
--PSAMGM
--PSAMDG
--PSAMAY
--PSAMAM
--PSAMYI
--PSAMVV
--PSAMVA
--PSAMTN
--PSAMTK
--PSAMTH
--PSAMSV
--PSAMSE
--PSAMNY
--PSAMNF
--PSAMKY
--PSAMKV
--PSAMKT
--PSAMKR
--PSAMII
--PSAMIF
--PSAMHI
--PSAMGA
--PSAMFW
--PSAMFR
--PSAMFH
--PSAMAK
--PSAMAF
PNPSAM--
PDPSAM--
--PSAMYE
--PSAMWQ
--PSAMTT
--PSAMSH
--PSAMSF
--PSAMSD
--PSAMQR
--PSAMQI
--PSAMPM
--PSAMNA
--PSAMMN
--PSAMMK
--PSAMLE
--PSAMKK
--PSAMKI
--PSAMIN
--PSAMHY
--PSAMHV
--PSAMHG
--PSAMHD

--PSAMET
--PSAMDS
--PSAMAW
--PSAMAE
--PSAMYY
--PSAMWF
--PSAMWE
--PSAMWD
--PSAMVY
--PSAMVT
--PSAMTD
--PSAMQN
--PSAMNS
--PSAMMV
--PSAMMD
--PSAMKH
--PSAMKE
--PSAMHT
--PSAMGI
--PSAMFQ
--PSAMFM
--PSAMEW
--PSAMEN
--PSAMCT
--PSAMAQ
--PSAMAI
PAPSAM--
PWPSAM--
--PSAMYW
--PSAMYH
--PSAMWH
--PSAMWC
--PSAMVQ
--PSAMTY
--PSAMTE
--PSAMSM
--PSAMQW
--PSAMQV
--PSAMQT
--PSAMQF
--PSAMNN
--PSAMNK
--PSAMNG
--PSAMMY
--PSAMMQ
--PSAMMM
--PSAMMI
--PSAMMH
--PSAMMF
--PSAMMA

--PSAMKN
--PSAMKM
--PSAMKD
--PSAMIT
--PSAMIQ
--PSAMIH
--PSAMHQ
--PSAMHK
--PSAMGW
--PSAMGQ
--PSAMGH
--PSAMGF
--PSAMFK
--PSAMFI
--PSAMEY
--PSAMDC
--PSAMCY
--PSAMCS
--PSAMCR
--PSAMAH
PIPSAM--
PGPSAM--
PFPSAM--
PEPSAM--
QRPSAM--
-QPSAMR-
QWPSAM--
-QPSAMY-
-QPSAMW-
-QPSAML-
QLPSAM--
QKPSAM--
-QPSAMS-
QSPSAM--
-QPSAMI-
-QPSAMF-
QAPSAM--
-QPSAMT-
-QPSAMN-
QGPSAM--
QFPSAM--
-QPSAMV-
-QPSAMH-
-QPSAMG-
QNPSAM--
QVPSAM--
QTPSAM--
-QPSAMK-
-QPSAMA-
QPPSAM--

QHPSAM--
RTPSAM--
-RPSAMV-
-RPSAMK-
-RPSAMI-
RGPSAM--
RYPSAM--
RWPSAM--
RSPSAM--
-RPSAMS-
RNPSAM--
RKPSAM--
-RPSAMM-
RFPSAM--
RAPSAM--
RVPSAM--
RQPSAM--
-RPSAMF-
-RPSAMY-
RHPSAM--
-RPSAMN-
-RPSAMA-
RPPSAM--
RIPSAM--
-RPSAMQ-
-RPSAME-
RMPSAM--
-RPSAMD-
-RPSAMC-
REPSAM--
RDPSAM--
RCPSAM--
-SPSAMR-
-SPSAML-
-SPSAMT-
-SPSAMV-
-SPSAMQ-
-SPSAMF-
STPSAM--
SLPSAM--
SAPSAM--
-SPSAME-
SNPSAM--
SFPSAM--
SMPSAM--
SQPSAM--
-SPSAMI-
SHPSAM--
SYPSAM--
-SPSAMM-

−SPSAMG−
SKPSAM−−
SIPSAM−−
−SPSAMW−
SPPSAM−−
SEPSAM−−
SVPSAM−−
−SPSAMN−
−SPSAMH−
SGPSAM−−
−SPSAMY−
SDPSAM−−
−TPSAML−
−TPSAMR−
−TPSAME−
TRPSAM−−
−TPSAMS−
−TPSAMQ−
−TPSAMH−
TNPSAM−−
TGPSAM−−
TVPSAM−−
−TPSAMN−
−TPSAMW−
−TPSAMA−
TTPSAM−−
TLPSAM−−
−TPSAMI−
TYPSAM−−
−TPSAMK−
TMPSAM−−
TFPSAM−−
TCPSAM−−
TWPSAM−−
−TPSAMG−
TKPSAM−−
TSPSAM−−
TQPSAM−−
−TPSAMV−
−TPSAMT−
−TPSAMM−
TIPSAM−−
THPSAM−−
−TPSAMY−
−TPSAMP−
−TPSAMF−
−TPSAMD−
TDPSAM−−
−VPSAMS−
−VPSAML−

-VPSAMK-
VFPSAM--
VWPSAM--
VVPSAM--
VTPSAM--
-VPSAMW-
VLPSAM--
VYPSAM--
VGPSAM--
-VPSAMI-
VRPSAM--
-VPSAMM-
-VPSAMF-
VIPSAM--
VSPSAM--
-VPSAMN-
VEPSAM--
VDPSAM--
-VPSAMT-
-VPSAMG-
VMPSAM--
VKPSAM--
-VPSAMQ-
-VPSAMH-
-VPSAME-
VPPSAM--
VAPSAM--
VQPSAM--
-VPSAMY-
-VPSAMV-
VNPSAM--
-WPSAMR-
-WPSAMI-
-WPSAML-
WFPSAM--
-WPSAMV-
WKPSAM--
WYPSAM--
WVPSAM--
-WPSAMT-
WGPSAM--
WPPSAM--
WWPSAM--
WSPSAM--
-WPSAMS-
-WPSAMH-
-WPSAMF-
WMPSAM--
-WPSAMN-
-WPSAMK-

-WPSAMG-
WNPSAM--
WLPSAM--
WDPSAM--
WAPSAM--
-WPSAMW-
WIPSAM--
WHPSAM--
-YPSAMS-
YTPSAM--
-YPSAMY-
-YPSAMK-
YLPSAM--
YKPSAM--
YHPSAM--
YAPSAM--
YVPSAM--
-YPSAMM-
-YPSAML-
-YPSAMH-
-YPSAMG-
YYPSAM--
YQPSAM--
-YPSAME-
YIPSAM--
YSPSAM--
-YPSAMR-
YWPSAM--
YMPSAM--
-YPSAMV-
-YPSAMQ-
-YPSAMF-
YGPSAM--
YDPSAM--
-YPSAMW-
-YPSAMP-
-YPSAMN-
YNPSAM--
YFPSAM--
YEPSAM--
PRNM 255 689 32.77
-APRNML-
ATPRNM--
ASPRNM--
-APRNMS-
AQPRNM--
-APRNMY-
AAPRNM--
-APRNMI-
AVPRNM--

-APRNMK-
AEPRNM--
-APRNMF-
ARPRNM--
-APRNMG-
ANPRNM--
AMPRNM--
-APRNMA-
-APRNMW-
-APRNMH-
AKPRNM--
AHPRNM--
AGPRNM--
-APRNMT-
-APRNMQ-
-APRNMN-
-APRNME-
-APRNMC-
AIPRNM--
ADPRNM--
CAPRNM--
CVPRNM--
CRPRNM--
CMPRNM--
-CPRNMV-
-CPRNMS-
-CPRNMR-
-CPRNMQ-
-CPRNMH-
-CPRNME-
CGPRNM--
CFPRNM--
CCPRNM--
-DPRNMR-
-DPRNML-
DVPRNM--
DSPRNM--
-DPRNMQ-
DMPRNM--
DQPRNM--
DGPRNM--
DAPRNM--
-DPRNMW-
-DPRNMG-
-DPRNMA-
DIPRNM--
DFPRNM--
-DPRNMV-
-DPRNMT-
-DPRNMS-

-DPRNMN-
-DPRNMF-
DLPRNM--
DEPRNM--
DTPRNM--
DRPRNM--
-DPRNMK-
-DPRNMI-
EIPRNM--
EHPRNM--
ESPRNM--
-EPRNMR-
-EPRNMQ-
-EPRNMI-
EYPRNM--
-EPRNML-
EMPRNM--
ETPRNM--
EFPRNM--
-EPRNMK-
ERPRNM--
EQPRNM--
-EPRNMV-
-EPRNMT-
-EPRNMS-
-EPRNMM-
-EPRNMG-
-EPRNMA-
-FPRNMS-
-FPRNML-
-FPRNMG-
FIPRNM--
-FPRNMW-
FSPRNM--
FTPRNM--
-FPRNMV-
FAPRNM--
FRPRNM--
FMPRNM--
-FPRNMN-
-FPRNMK-
-FPRNMH-
FVPRNM--
-FPRNMY-
-FPRNMA-
FLPRNM--
FHPRNM--
FNPRNM--
GVPRNM--
GLPRNM--

-GPRNMT-
-GPRNMA-
-GPRNMR-
-GPRNMM-
-GPRNMV-
GSPRNM--
GYPRNM--
GMPRNM--
GAPRNM--
GRPRNM--
GIPRNM--
-GPRNMQ-
-GPRNMH-
-GPRNMF-
GTPRNM--
-GPRNMW-
-GPRNMN-
-GPRNMS-
-GPRNMK-
-GPRNMI-
GEPRNM--
GQPRNM--
GWPRNM--
-GPRNMG-
-GPRNME-
GNPRNM--
GKPRNM--
-HPRNMR-
-HPRNMS-
-HPRNMV-
-HPRNMM-
-HPRNMY-
-HPRNMG-
HSPRNM--
-HPRNMT-
-HPRNMA-
HKPRNM--
HTPRNM--
HVPRNM--
HQPRNM--
-HPRNMH-
HPPRNM--
HGPRNM--
HDPRNM--
-HPRNML-
HMPRNM--
HYPRNM--
HRPRNM--
-HPRNMW-
HHPRNM--

```
-HPRNMN-
-HPRNMF-
HCPRNM--
ISPRNM--
-IPRNMR-
ILPRNM--
IIPRNM--
IGPRNM--
-IPRNMM-
-IPRNMF-
-IPRNMV-
-IPRNMS-
-IPRNMQ-
-IPRNMG-
INPRNM--
ITPRNM--
IQPRNM--
-IPRNMT-
-IPRNMN-
-IPRNMW-
-IPRNME-
IKPRNM--
IFPRNM--
IAPRNM--
IYPRNM--
-IPRNMK-
-IPRNMI-
-IPRNMD-
IHPRNM--
IDPRNM--
-KPRNMS-
-KPRNMY-
KLPRNM--
KEPRNM--
KWPRNM--
-KPRNML-
KGPRNM--
KTPRNM--
KRPRNM--
-KPRNMW-
-KPRNMI-
-KPRNME-
KPPRNM--
KYPRNM--
-KPRNMM-
KVPRNM--
KQPRNM--
-KPRNMQ-
-KPRNMG-
KMPRNM--
```

KDPRNM--
LNPRNM--
LGPRNM--
LLPRNM--
-LPRNMH-
LTPRNM--
-LPRNMY-
LIPRNM--
LQPRNM--
-LPRNMV-
-LPRNMG-
-LPRNMT-
-LPRNMQ-
LMPRNM--
LYPRNM--
-LPRNMW-
LVPRNM--
-LPRNMN-
-LPRNMA-
-LPRNMM-
-LPRNME-
-LPRNMF-
LKPRNM--
LEPRNM--
-MPRNMM-
-MPRNML-
MNPRNM--
-MPRNMK-
-MPRNMT-
MGPRNM--
-MPRNMH-
-MPRNMG-
-MPRNMV-
MEPRNM--
MYPRNM--
-MPRNMS-
MPPRNM--
MMPRNM--
MVPRNM--
-MPRNMW-
-MPRNME-
-MPRNMA-
MKPRNM--
MIPRNM--
MAPRNM--
-MPRNMY-
-MPRNMN-
-MPRNMI-
-MPRNMF-
MFPRNM--

MCPRNM--
-NPRNMR-
NLPRNM--
-NPRNML-
NAPRNM--
-NPRNMA-
NYPRNM--
NTPRNM--
-NPRNMT-
-NPRNMF-
NVPRNM--
NRPRNM--
-NPRNMV-
-NPRNMQ-
-NPRNMI-
NSPRNM--
-NPRNMY-
-NPRNMN-
NNPRNM--
NIPRNM--
NEPRNM--
-NPRNME-
NMPRNM--
NHPRNM--
NDPRNM--
-NPRNMG-
NPPRNM--
NKPRNM--
--PRNMYL
--PRNMFL
--PRNMIS
--PRNMLH
--PRNMTS
--PRNMGI
--PRNMFT
--PRNMVS
--PRNMMI
--PRNMTL
--PRNMRV
--PRNMRT
--PRNMRA
--PRNMHR
--PRNMAK
--PRNMLW
--PRNMLN
--PRNMKS
--PRNMIT
--PRNMYM
--PRNMVW
--PRNMSG

--PRNMNL
--PRNMHW
--PRNMGL
--PRNMSS
--PRNMSF
--PRNMNS
--PRNMNN
--PRNMLT
--PRNMGS
--PRNMVM
--PRNMVI
--PRNMMV
--PRNMLM
--PRNMLD
--PRNMKL
--PRNMIG
--PRNMGG
--PRNMFR
--PRNMYS
--PRNMVF
--PRNMTH
--PRNMRI
--PRNMNY
--PRNMLQ
--PRNMGK
--PRNMFA
--PRNMAI
PVPRNM--
--PRNMWF
--PRNMTT
--PRNMSV
--PRNMST
--PRNMSK
--PRNMRD
--PRNMMA
--PRNMLF
--PRNMHN
--PRNMEI
--PRNMEF
--PRNMAY
PMPRNM--
--PRNMYT
--PRNMYR
--PRNMYH
--PRNMYD
--PRNMWY
--PRNMWS
--PRNMTI
--PRNMTD
--PRNMSQ

--PRNMRE
--PRNMLI
--PRNMKA
--PRNMIA
--PRNMGA
--PRNMFD
--PRNMAW
--PRNMAV
--PRNMAL
--PRNMTV
--PRNMSY
--PRNMQV
--PRNMNG
--PRNMGM
--PRNMFQ
--PRNMEY
--PRNMDS
--PRNMAF
PFPRNM--
--PRNMYI
--PRNMYF
--PRNMTW
--PRNMSA
--PRNMQS
--PRNMQL
--PRNMNR
--PRNMNI
--PRNMMY
--PRNMMS
--PRNMMR
--PRNMLE
--PRNMKF
--PRNMHV
--PRNMHT
--PRNMFS
--PRNMDA
--PRNMAS
PGPRNM--
PTPRNM--
--PRNMYV
--PRNMYK
--PRNMYE
--PRNMWQ
--PRNMVT
--PRNMVP
--PRNMVN
--PRNMTQ
--PRNMTM
--PRNMSE
--PRNMRM

--PRNMQE
--PRNMNK
--PRNMNF
--PRNMMN
--PRNMMD
--PRNMLG
--PRNMKT
--PRNMIE
--PRNMGR
--PRNMFM
--PRNMFF
--PRNMEV
--PRNMEM
--PRNMEL
--PRNMAQ
--PRNMAN
PNPRNM--
PIPRNM--
PHPRNM--
PEPRNM--
PDPRNM--
PYPRNM--
PRPRNM--
--PRNMWV
--PRNMWD
--PRNMVE
--PRNMVA
--PRNMTK
--PRNMRY
--PRNMRQ
--PRNMQT
--PRNMQQ
--PRNMQI
--PRNMQG
--PRNMMH
--PRNMLY
--PRNMKV
--PRNMKQ
--PRNMKG
--PRNMIW
--PRNMIV
--PRNMIM
--PRNMHS
--PRNMHM
--PRNMHH
--PRNMHG
--PRNMGV
--PRNMGT
--PRNMGQ
--PRNMGN

--PRNMGH
--PRNMGF
--PRNMFK
--PRNMFH
--PRNMES
--PRNMEG
--PRNMAM
--PRNMAH
--PRNMAA
PWPRNM--
PSPRNM--
--PRNMYY
--PRNMYN
--PRNMYA
--PRNMWW
--PRNMWH
--PRNMWE
--PRNMTF
--PRNMSD
--PRNMRW
--PRNMQY
--PRNMQW
--PRNMQN
--PRNMQH
--PRNMQD
--PRNMQA
--PRNMNH
--PRNMND
--PRNMMQ
--PRNMME
--PRNMKN
--PRNMKM
--PRNMIR
--PRNMHE
--PRNMGW
--PRNMFW
--PRNMFG
--PRNMFE
--PRNMET
--PRNMEH
--PRNMDT
--PRNMCH
--PRNMCD
--PRNMAG
--PRNMAD
PAPRNM--
QYPRNM--
QWPRNM--
-QPRNMW-
-QPRNMT-

−QPRNML−
QIPRNM−−
QTPRNM−−
−QPRNMY−
−QPRNMQ−
QVPRNM−−
−QPRNMV−
−QPRNMN−
−QPRNMH−
−QPRNMA−
−QPRNMS−
−QPRNMR−
−QPRNMM−
−QPRNMG−
QPPRNM−−
QFPRNM−−
QSPRNM−−
QQPRNM−−
−QPRNMC−
QMPRNM−−
QDPRNM−−
−RPRNML−
RVPRNM−−
−RPRNMV−
−RPRNMS−
−RPRNMG−
REPRNM−−
RTPRNM−−
RHPRNM−−
−RPRNMI−
−RPRNMA−
RQPRNM−−
−RPRNMY−
−RPRNMH−
−RPRNMN−
−RPRNMF−
−RPRNME−
−RPRNMC−
RDPRNM−−
RAPRNM−−
−RPRNMW−
−RPRNMM−
RPPRNM−−
−SPRNML−
−SPRNMW−
SNPRNM−−
−SPRNMV−
STPRNM−−
SRPRNM−−
−SPRNMN−

```
-SPRNMY-
-SPRNMH-
-SPRNMI-
SLPRNM--
-SPRNMG-
SGPRNM--
-SPRNMA-
-SPRNMF-
SKPRNM--
SIPRNM--
SQPRNM--
-SPRNMQ-
-SPRNMM-
-SPRNMK-
SHPRNM--
-SPRNME-
SPPRNM--
SEPRNM--
SDPRNM--
-TPRNMR-
-TPRNML-
-TPRNMH-
-TPRNMN-
-TPRNMS-
TTPRNM--
-TPRNMG-
TGPRNM--
TAPRNM--
-TPRNMA-
TLPRNM--
-TPRNMY-
TVPRNM--
TIPRNM--
-TPRNMM-
TKPRNM--
TSPRNM--
-TPRNMW-
-TPRNMI-
TDPRNM--
TYPRNM--
TQPRNM--
-TPRNMK-
TMPRNM--
THPRNM--
TEPRNM--
-TPRNMV-
TPPRNM--
TNPRNM--
TWPRNM--
TCPRNM--
```

-VPRNMA-
-VPRNMI-
VLPRNM--
-VPRNMS-
-VPRNML-
VVPRNM--
VTPRNM--
-VPRNMY-
-VPRNMW-
-VPRNMG-
VKPRNM--
VHPRNM--
VGPRNM--
-VPRNMH-
VQPRNM--
-VPRNMT-
VSPRNM--
-VPRNMN-
VMPRNM--
-VPRNMM-
-VPRNMK-
VFPRNM--
VEPRNM--
-VPRNMP-
VNPRNM--
VAPRNM--
-VPRNMQ-
VPPRNM--
VDPRNM--
WVPRNM--
-WPRNMS-
WNPRNM--
-WPRNMT-
WGPRNM--
-WPRNMG-
WKPRNM--
WHPRNM--
WFPRNM--
WAPRNM--
WYPRNM--
WRPRNM--
-WPRNMN-
-WPRNML-
-WPRNMH-
WWPRNM--
WTPRNM--
WQPRNM--
WLPRNM--
WIPRNM--
WDPRNM--

```
         -WPRNMV-
         -WPRNMF-
         -WPRNME-
         -WPRNMD-
         YYPRNM--
         YSPRNM--
         -YPRNMK-
         -YPRNMA-
         YAPRNM--
         YRPRNM--
         -YPRNMV-
         -YPRNMT-
         YMPRNM--
         YLPRNM--
         -YPRNMS-
         -YPRNMQ-
         -YPRNMI-
         -YPRNMD-
         -YPRNMN-
         -YPRNMM-
         -YPRNMG-
         YVPRNM--
         YTPRNM--
         -YPRNMW-
         -YPRNMF-
         -YPRNME-
         YPPRNM--
         YDPRNM--
         -YPRNMH-
         YEPRNM--
PSGM       27       833     32.76
         -LPSGMG-
         --PSGMYM
         --PSGMNI
         --PSGMNG
         --PSGMGL
         YGPSGM--
         -APSGMR-
         -APSGML-
         ARPSGM--
         ALPSGM--
         ASPSGM--
         AWPSGM--
         -APSGMS-
         -APSGMK-
         -APSGMW-
         -APSGMV-
         -APSGME-
         AVPSGM--
         -APSGMT-
```

ANPSGM--
-APSGMY-
-APSGMH-
AAPSGM--
AYPSGM--
ATPSGM--
AIPSGM--
AGPSGM--
-APSGMM-
AMPSGM--
-APSGMN-
-APSGMG-
-APSGMF-
AKPSGM--
AFPSGM--
-APSGMQ-
-APSGMI-
AHPSGM--
AEPSGM--
AQPSGM--
APPSGM--
CSPSGM--
-CPSGMS-
-CPSGMI-
-CPSGMF-
CPPSGM--
CQPSGM--
-CPSGMV-
-CPSGMR-
CKPSGM--
CCPSGM--
CAPSGM--
CWPSGM--
CTPSGM--
CRPSGM--
-CPSGMT-
-CPSGMM-
-CPSGML-
-CPSGMK-
-CPSGME-
-CPSGMA-
CMPSGM--
CIPSGM--
DSPSGM--
DVPSGM--
DTPSGM--
-DPSGMS-
-DPSGMF-
DLPSGM--
-DPSGMR-

-DPSGMM-
-DPSGML-
DRPSGM--
-DPSGMY-
-DPSGMW-
-DPSGMV-
-DPSGMN-
-DPSGMK-
-DPSGMH-
DKPSGM--
DDPSGM--
-DPSGMT-
DMPSGM--
-EPSGMK-
ETPSGM--
-EPSGMS-
-EPSGML-
ERPSGM--
-EPSGMT-
-EPSGMG-
EGPSGM--
-EPSGMF-
EIPSGM--
EVPSGM--
ESPSGM--
-EPSGMH-
ENPSGM--
-EPSGMR-
-EPSGMM-
-EPSGMI-
EPPSGM--
EMPSGM--
EDPSGM--
EQPSGM--
-EPSGMY-
-EPSGMW-
-EPSGMV-
-EPSGMA-
ELPSGM--
EHPSGM--
EFPSGM--
EEPSGM--
ECPSGM--
FRPSGM--
-FPSGML-
FVPSGM--
-FPSGMI-
-FPSGMY-
-FPSGMV-
FLPSGM--

FWPSGM--
FGPSGM--
-FPSGMA-
FSPSGM--
-FPSGMH-
FTPSGM--
-FPSGMW-
-FPSGMN-
-FPSGMF-
FNPSGM--
FIPSGM--
FYPSGM--
FQPSGM--
-FPSGMT-
-FPSGME-
FPPSGM--
FKPSGM--
-FPSGMQ-
-FPSGMM-
-FPSGMK-
FMPSGM--
-GPSGMR-
-GPSGML-
-GPSGMK-
GYPSGM--
-GPSGMW-
GIPSGM--
-GPSGMT-
GHPSGM--
GNPSGM--
GVPSGM--
-GPSGMH-
-GPSGMG-
-GPSGMF-
GWPSGM--
-GPSGMQ-
GTPSGM--
-GPSGMV-
GDPSGM--
GSPSGM--
-GPSGMS-
-GPSGMM-
-GPSGMA-
GPPSGM--
GLPSGM--
GKPSGM--
GGPSGM--
GAPSGM--
-GPSGMI-
-GPSGMY-

-GPSGMN-
-GPSGME-
GMPSGM--
GEPSGM--
HMPSGM--
HSPSGM--
-HPSGMS-
-HPSGMR-
-HPSGMN-
-HPSGMY-
HNPSGM--
HGPSGM--
-HPSGMW-
-HPSGML-
-HPSGMA-
HIPSGM--
HFPSGM--
HRPSGM--
-HPSGMM-
-HPSGMF-
-HPSGMK-
-HPSGMI-
HLPSGM--
HHPSGM--
HAPSGM--
HWPSGM--
HTPSGM--
HQPSGM--
-HPSGMQ-
-HPSGMV-
-HPSGMH-
-HPSGME-
HPPSGM--
HKPSGM--
IRPSGM--
-IPSGMR-
-IPSGML-
ISPSGM--
ITPSGM--
IFPSGM--
-IPSGMT-
-IPSGMS-
IGPSGM--
IPPSGM--
ILPSGM--
-IPSGMG-
IEPSGM--
-IPSGMK-
IIPSGM--
IAPSGM--

IWPSGM--
IQPSGM--
-IPSGMV-
-IPSGMI-
-IPSGMH-
-IPSGMF-
-IPSGMA-
IMPSGM--
IDPSGM--
-IPSGMM-
IVPSGM--
-IPSGMW-
-IPSGMN-
IKPSGM--
IHPSGM--
KSPSGM--
-KPSGMR-
-KPSGML-
-KPSGMW-
-KPSGMS-
-KPSGMT-
KVPSGM--
-KPSGMY-
-KPSGMA-
-KPSGMF-
KGPSGM--
KWPSGM--
KIPSGM--
-KPSGMH-
KMPSGM--
KYPSGM--
KRPSGM--
-KPSGME-
KLPSGM--
KTPSGM--
-KPSGMQ-
-KPSGMK-
-KPSGMG-
KHPSGM--
KDPSGM--
KAPSGM--
KQPSGM--
-KPSGMN-
-KPSGMM-
KPPSGM--
KFPSGM--
KCPSGM--
LRPSGM--
LSPSGM--
-LPSGMS-

-LPSGMR-
-LPSGMM-
-LPSGMA-
-LPSGMH-
LAPSGM--
-LPSGMF-
LVPSGM--
-LPSGMT-
LHPSGM--
LWPSGM--
-LPSGMY-
LPPSGM--
LQPSGM--
LLPSGM--
LTPSGM--
LYPSGM--
-LPSGMW-
LKPSGM--
LMPSGM--
LGPSGM--
-LPSGMV-
-LPSGMN-
-LPSGMK-
-LPSGME-
LNPSGM--
-LPSGMQ-
LCPSGM--
-LPSGMI-
LDPSGM--
MRPSGM--
-MPSGML-
-MPSGMT-
MKPSGM--
-MPSGMQ-
-MPSGMG-
MSPSGM--
-MPSGMR-
-MPSGMH-
-MPSGMF-
-MPSGMY-
-MPSGMK-
MLPSGM--
MIPSGM--
-MPSGMS-
-MPSGMI-
-MPSGMA-
MHPSGM--
MGPSGM--
MAPSGM--
MVPSGM--

MNPSGM--
MMPSGM--
MFPSGM--
MYPSGM--
MWPSGM--
MTPSGM--
MQPSGM--
-MPSGMW-
-MPSGMV-
-MPSGMM-
-MPSGME-
MPPSGM--
-NPSGMR-
-NPSGMH-
-NPSGML-
NGPSGM--
NTPSGM--
-NPSGMW-
-NPSGMS-
-NPSGMV-
-NPSGMN-
NRPSGM--
-NPSGMK-
NVPSGM--
-NPSGMA-
NSPSGM--
-NPSGMM-
NWPSGM--
-NPSGMQ-
NIPSGM--
NHPSGM--
NFPSGM--
NDPSGM--
-NPSGMF-
NPPSGM--
NNPSGM--
NMPSGM--
NLPSGM--
NAPSGM--
-NPSGMT-
-NPSGMG-
-NPSGME-
NKPSGM--
NYPSGM--
-NPSGMY-
-NPSGMI-
NEPSGM--
--PSGMRT
--PSGMIR
--PSGMLS

--PSGMLR
--PSGMSL
--PSGMLN
--PSGMLK
--PSGMQS
--PSGMLI
--PSGMGS
--PSGMKT
--PSGMLA
--PSGMYR
--PSGMSI
--PSGMRV
--PSGMYH
--PSGMWK
--PSGMVR
--PSGMSR
--PSGMLV
--PSGMLG
--PSGMVY
--PSGMRH
--PSGMVG
--PSGMVF
--PSGMFR
--PSGMTT
--PSGMTH
--PSGMSW
--PSGMRR
--PSGMRE
--PSGMAA
PFPSGM--
--PSGMWE
--PSGMTL
--PSGMRQ
--PSGMRK
--PSGMYL
--PSGMWI
--PSGMTY
--PSGMHK
PLPSGM--
--PSGMWW
--PSGMWL
--PSGMSS
--PSGMQR
--PSGMMS
--PSGMLW
--PSGMLT
--PSGMLH
--PSGMKW
--PSGMKA
--PSGMHS

```
--PSGMFT
--PSGMYS
--PSGMWV
--PSGMVS
--PSGMTR
--PSGMTG
--PSGMSY
--PSGMRI
--PSGMQG
--PSGMMW
--PSGMMR
--PSGMLY
--PSGMHV
--PSGMFI
--PSGMYT
--PSGMYN
--PSGMSN
--PSGMSM
--PSGMSF
--PSGMRY
--PSGMRN
--PSGMNR
--PSGMMV
--PSGMMN
--PSGMIG
--PSGMFS
--PSGMFN
--PSGMFL
PQPSGM--
--PSGMYA
--PSGMWG
--PSGMTV
--PSGMSK
--PSGMSH
--PSGMMQ
--PSGMLQ
--PSGMLF
--PSGMIS
--PSGMIE
--PSGMHL
--PSGMHG
--PSGMAF
--PSGMWM
--PSGMVM
--PSGMTS
--PSGMSV
--PSGMST
--PSGMSG
--PSGMQV
--PSGMKL
```

--PSGMFM
--PSGMAV
--PSGMAS
--PSGMAR
--PSGMAN
--PSGMWN
--PSGMVH
--PSGMTW
--PSGMRW
--PSGMRM
--PSGMQW
--PSGMQT
--PSGMNL
--PSGMMG
--PSGMLM
--PSGMHT
--PSGMGT
--PSGMFA
--PSGMAY
PRPSGM--
PWPSGM--
--PSGMYW
--PSGMYV
--PSGMVT
--PSGMTN
--PSGMTM
--PSGMTK
--PSGMTI
--PSGMTF
--PSGMQY
--PSGMNT
--PSGMNM
--PSGMNF
--PSGMMT
--PSGMMK
--PSGMMI
--PSGMMF
--PSGMMA
--PSGMKN
--PSGMKK
--PSGMKG
--PSGMKD
--PSGMIW
--PSGMHI
--PSGMHH
--PSGMFK
--PSGMFH
--PSGMEG
--PSGMAI
PSPSGM--

--PSGMVW
--PSGMVD
--PSGMQI
--PSGMNS
--PSGMMY
--PSGMML
--PSGMKV
--PSGMKH
--PSGMIY
--PSGMIL
--PSGMIK
--PSGMFQ
--PSGMFD
--PSGMEA
--PSGMCH
--PSGMAK
PKPSGM--
PIPSGM--
--PSGMYQ
--PSGMYI
--PSGMVE
--PSGMSD
--PSGMRD
--PSGMQF
--PSGMQE
--PSGMNY
--PSGMNV
--PSGMNK
--PSGMHQ
--PSGMHA
--PSGMGA
--PSGMAL
--PSGMAH
PDPSGM--
PTPSGM--
--PSGMYK
--PSGMYG
--PSGMWT
--PSGMWF
--PSGMWA
--PSGMVQ
--PSGMVN
--PSGMSA
--PSGMQK
--PSGMNN
--PSGMNA
--PSGMMM
--PSGMMH
--PSGMLD
--PSGMIV

--PSGMII
--PSGMIH
--PSGMID
--PSGMIA
--PSGMHY
--PSGMGM
--PSGMGI
--PSGMGG
--PSGMEV
--PSGMET
--PSGMEF
--PSGMDS
PMPSGM--
--PSGMYE
--PSGMWD
--PSGMVA
--PSGMRC
--PSGMQQ
--PSGMQN
--PSGMQH
--PSGMQA
--PSGMPW
--PSGMPS
--PSGMNW
--PSGMNQ
--PSGMNH
--PSGMNE
--PSGMKY
--PSGMKE
--PSGMIN
--PSGMIF
--PSGMHW
--PSGMHM
--PSGMHD
--PSGMGY
--PSGMES
--PSGMDG
--PSGMAT
--PSGMAP
PCPSGM--
-QPSGMR-
-QPSGMK-
-QPSGMT-
-QPSGMF-
QWPSGM--
-QPSGMI-
QLPSGM--
QKPSGM--
QAPSGM--
QTPSGM--

-QPSGMS-
-QPSGMM-
-QPSGML-
QNPSGM--
QGPSGM--
QVPSGM--
QSPSGM--
-QPSGMW-
QIPSGM--
QYPSGM--
-QPSGMV-
-QPSGMN-
-QPSGMG-
QFPSGM--
QRPSGM--
-QPSGME-
-QPSGMA-
QPPSGM--
RSPSGM--
RRPSGM--
-RPSGMV-
-RPSGMN-
-RPSGMG-
RLPSGM--
-RPSGML-
-RPSGMH-
-RPSGMF-
RVPSGM--
RNPSGM--
RFPSGM--
RWPSGM--
RTPSGM--
RGPSGM--
-RPSGMY-
-RPSGMA-
RQPSGM--
-RPSGMK-
RDPSGM--
-RPSGMT-
RKPSGM--
-RPSGMM-
RHPSGM--
RAPSGM--
-RPSGMW-
RYPSGM--
-RPSGME-
RPPSGM--
RIPSGM--
RCPSGM--
-RPSGMQ-

REPSGM--
-SPSGMR-
SSPSGM--
SRPSGM--
-SPSGMA-
-SPSGMS-
-SPSGMM-
SVPSGM--
SYPSGM--
-SPSGMK-
SLPSGM--
SFPSGM--
SIPSGM--
-SPSGMV-
-SPSGMN-
-SPSGMF-
SNPSGM--
SAPSGM--
STPSGM--
-SPSGMT-
-SPSGMI-
SMPSGM--
-SPSGMW-
-SPSGMH-
-SPSGMG-
-SPSGME-
SWPSGM--
SGPSGM--
SEPSGM--
SQPSGM--
-SPSGMY-
-SPSGMQ-
SKPSGM--
SHPSGM--
SPPSGM--
SDPSGM--
-TPSGMR-
-TPSGML-
-TPSGMK-
TRPSGM--
-TPSGMS-
-TPSGMH-
-TPSGMM-
-TPSGMA-
-TPSGMG-
TMPSGM--
TYPSGM--
-TPSGMQ-
TKPSGM--
-TPSGMN-

TLPSGM--
TWPSGM--
TQPSGM--
-TPSGMT-
-TPSGMW-
-TPSGMI-
TPPSGM--
TFPSGM--
TDPSGM--
TAPSGM--
TVPSGM--
-TPSGMV-
-TPSGMF-
TTPSGM--
-TPSGMY-
-TPSGME-
TIPSGM--
TGPSGM--
THPSGM--
TEPSGM--
VRPSGM--
-VPSGMY-
-VPSGMW-
VSPSGM--
-VPSGMM-
VEPSGM--
-VPSGMT-
VLPSGM--
VDPSGM--
-VPSGMR-
-VPSGMS-
VKPSGM--
-VPSGMN-
-VPSGMK-
VTPSGM--
VNPSGM--
VIPSGM--
-VPSGMA-
VWPSGM--
-VPSGMQ-
VGPSGM--
VYPSGM--
VQPSGM--
-VPSGME-
VMPSGM--
VHPSGM--
-VPSGMI-
VPPSGM--
VFPSGM--
VAPSGM--

-VPSGMH-
-VPSGMG-
VCPSGM--
-WPSGMK-
-WPSGMR-
WIPSGM--
WAPSGM--
WRPSGM--
-WPSGML-
WVPSGM--
-WPSGMT-
WYPSGM--
WSPSGM--
-WPSGMY-
WLPSGM--
WKPSGM--
-WPSGMW-
WHPSGM--
WDPSGM--
WTPSGM--
WQPSGM--
-WPSGMM-
-WPSGMA-
WGPSGM--
WWPSGM--
-WPSGMS-
-WPSGMQ-
-WPSGMI-
-WPSGMV-
-WPSGMF-
WNPSGM--
WFPSGM--
WEPSGM--
-WPSGME-
YRPSGM--
YVPSGM--
-YPSGMR-
-YPSGML-
-YPSGMK-
-YPSGMM-
-YPSGMH-
YSPSGM--
-YPSGMS-
-YPSGMT-
-YPSGMA-
-YPSGMY-
YNPSGM--
YFPSGM--
-YPSGMW-
-YPSGMN-

```
               YMPSGM--
               YTPSGM--
               -YPSGMQ-
               -YPSGMG-
               YPPSGM--
               YYPSGM--
               YWPSGM--
               YLPSGM--
               -YPSGMI-
               -YPSGMF-
               YHPSGM--
               -YPSGMC-
               YIPSGM--
               YDPSGM--
PASM             55        800      32.73
               -EPASMQ-
               -IPASMS-
               --PASMWD
               --PASMGL
               SSPASM--
               -TPASMK-
               -VPASME-
               -APASMR-
               -APASML-
               -APASMK-
               -APASMY-
               -APASMS-
               -APASMT-
               -APASMI-
               ANPASM--
               -APASMG-
               AFPASM--
               -APASMH-
               AGPASM--
               -APASME-
               -APASMA-
               ALPASM--
               AAPASM--
               ATPASM--
               AYPASM--
               AQPASM--
               -APASMN-
               AKPASM--
               AWPASM--
               AVPASM--
               -APASMF-
               AIPASM--
               AHPASM--
               -APASMV-
               AMPASM--
```

AEPASM--
ACPASM--
-CPASML-
-CPASMI-
-CPASMF-
CMPASM--
-CPASMV-
-CPASMM-
CWPASM--
-CPASMY-
CHPASM--
CVPASM--
CSPASM--
CRPASM--
CQPASM--
-CPASMS-
-CPASMH-
-CPASMA-
CKPASM--
CCPASM--
CAPASM--
DRPASM--
DVPASM--
-DPASMY-
-DPASMW-
-DPASMF-
DFPASM--
-DPASML-
-DPASMH-
-DPASMV-
-DPASMS-
-DPASMQ-
-DPASMN-
DMPASM--
DHPASM--
DYPASM--
DTPASM--
DQPASM--
-DPASMT-
-DPASMM-
-DPASMA-
DNPASM--
DCPASM--
DAPASM--
-EPASML-
ERPASM--
ELPASM--
EVPASM--
-EPASMR-
EIPASM--

EGPASM--
ETPASM--
EQPASM--
-EPASMK-
-EPASMI-
EFPASM--
-EPASMW-
-EPASMG-
EKPASM--
ECPASM--
-EPASMV-
-EPASMT-
-EPASMS-
-EPASMF-
ENPASM--
-FPASMR-
FSPASM--
-FPASMS-
-FPASMN-
FWPASM--
-FPASMK-
FLPASM--
-FPASMW-
-FPASMV-
-FPASMI-
FRPASM--
-FPASML-
FNPASM--
FKPASM--
FVPASM--
FYPASM--
-FPASMY-
-FPASMQ-
-FPASME-
FFPASM--
FEPASM--
FPPASM--
-FPASMM-
-FPASMG-
-FPASMF-
FGPASM--
FCPASM--
GVPASM--
-GPASMR-
-GPASMK-
-GPASMT-
-GPASMS-
GWPASM--
GMPASM--
GTPASM--

GRPASM--
GSPASM--
GYPASM--
-GPASMA-
GLPASM--
GIPASM--
GDPASM--
-GPASMY-
-GPASMW-
-GPASMG-
GPPASM--
-GPASMI-
GNPASM--
GKPASM--
GHPASM--
GFPASM--
-GPASMQ-
-GPASMM-
GQPASM--
-GPASMV-
GGPASM--
GEPASM--
GAPASM--
-GPASMN-
-GPASMF-
-GPASMC-
-HPASMM-
-HPASML-
-HPASMR-
-HPASMS-
HKPASM--
HTPASM--
-HPASMV-
-HPASMK-
-HPASMN-
-HPASMA-
HVPASM--
-HPASMY-
HAPASM--
HRPASM--
HQPASM--
HNPASM--
HLPASM--
HEPASM--
HSPASM--
-HPASMI-
HMPASM--
HYPASM--
HWPASM--
HPPASM--

−HPASMQ−
−HPASMG−
−HPASME−
HGPASM−−
HCPASM−−
IRPASM−−
−IPASMA−
IAPASM−−
−IPASMR−
−IPASMK−
IMPASM−−
ISPASM−−
−IPASMN−
ILPASM−−
IQPASM−−
−IPASMG−
−IPASMF−
IYPASM−−
IVPASM−−
−IPASMY−
−IPASMM−
IFPASM−−
IEPASM−−
−IPASMV−
−IPASML−
−IPASMH−
IIPASM−−
IHPASM−−
IWPASM−−
−IPASMW−
−IPASMQ−
−IPASMI−
−IPASMD−
INPASM−−
IKPASM−−
IGPASM−−
KSPASM−−
−KPASML−
−KPASMG−
−KPASMY−
KAPASM−−
−KPASMA−
KVPASM−−
KFPASM−−
−KPASMW−
KWPASM−−
KRPASM−−
KDPASM−−
KTPASM−−
−KPASMN−

```
-KPASMQ-
-KPASMH-
KMPASM--
KGPASM--
KEPASM--
-KPASMT-
-KPASMS-
-KPASMM-
KLPASM--
KYPASM--
KPPASM--
-KPASME-
KIPASM--
KHPASM--
KCPASM--
-KPASMF-
-KPASMC-
KNPASM--
KKPASM--
-LPASMW-
-LPASML-
-LPASMS-
LRPASM--
LWPASM--
-LPASMT-
LLPASM--
-LPASMV-
LSPASM--
LMPASM--
-LPASMQ-
LTPASM--
-LPASMR-
-LPASMA-
-LPASMK-
-LPASMI-
LFPASM--
-LPASMM-
-LPASMH-
LNPASM--
LYPASM--
-LPASMY-
LGPASM--
LQPASM--
-LPASMN-
-LPASME-
LKPASM--
LCPASM--
LAPASM--
LPPASM--
-LPASMG-
```

LHPASM--
LEPASM--
-LPASMD-
-MPASML-
-MPASMA-
-MPASMV-
-MPASMT-
MRPASM--
-MPASMS-
-MPASMN-
MNPASM--
MKPASM--
-MPASMK-
MPPASM--
-MPASMH-
MGPASM--
MVPASM--
-MPASMY-
MAPASM--
MYPASM--
MTPASM--
-MPASMQ-
MLPASM--
MIPASM--
-MPASMM-
MMPASM--
MWPASM--
MSPASM--
MQPASM--
-MPASMW-
-MPASMI-
-MPASMG-
-MPASMF-
MFPASM--
-NPASMR-
-NPASMQ-
-NPASMT-
-NPASMV-
-NPASMY-
NTPASM--
-NPASMW-
-NPASMS-
-NPASML-
-NPASMF-
-NPASME-
NLPASM--
NFPASM--
NPPASM--
NYPASM--
NVPASM--

NSPASM--
-NPASMI-
NNPASM--
NMPASM--
NIPASM--
NHPASM--
NWPASM--
-NPASMM-
-NPASMK-
-NPASMH-
-NPASMG-
-NPASMA-
NKPASM--
NGPASM--
NDPASM--
NCPASM--
NAPASM--
--PASMVR
--PASMLR
--PASMRL
--PASMLT
--PASMYS
--PASMVS
--PASMIL
--PASMSR
--PASMVV
--PASMVL
--PASMLS
--PASMLI
--PASMWR
--PASMRV
--PASMAY
--PASMVG
--PASMTG
--PASMMT
--PASMSL
--PASMQR
--PASMKL
--PASMAG
--PASMSH
--PASMRH
--PASMRG
--PASMNF
--PASMLN
--PASMIK
--PASMFI
--PASMYT
--PASMTR
--PASMST
--PASMLH

--PASMFR
--PASMFM
--PASMWM
--PASMVT
--PASMTN
--PASMNG
--PASMHL
--PASMWN
--PASMVI
--PASMTS
--PASMTL
--PASMSI
--PASMSF
--PASMRT
--PASMKA
--PASMHR
--PASMFA
--PASMAN
PSPASM--
--PASMYV
--PASMVA
--PASMTH
--PASMSN
--PASMRA
--PASMQL
--PASMLM
--PASMHY
--PASMYG
--PASMSS
--PASMMS
--PASMLG
--PASMIR
--PASMAR
--PASMAH
PTPASM--
PQPASM--
PMPASM--
--PASMWQ
--PASMVD
--PASMRY
--PASMRW
--PASMQN
--PASMMK
--PASMLQ
--PASMKG
--PASMIV
--PASMIS
--PASMFG
PVPASM--
--PASMYN

--PASMWT
--PASMWI
--PASMVY
--PASMTI
--PASMKI
--PASMGT
--PASMGG
--PASMFT
--PASMER
--PASMAL
--PASMAI
PYPASM--
--PASMYK
--PASMWS
--PASMVM
--PASMVH
--PASMVE
--PASMTT
--PASMTK
--PASMSY
--PASMSQ
--PASMSK
--PASMRN
--PASMQM
--PASMQK
--PASMNT
--PASMNL
--PASMMF
--PASMMA
--PASMLY
--PASMLE
--PASMKE
--PASMIY
--PASMIQ
--PASMIM
--PASMIG
--PASMGA
--PASMAV
--PASMAM
PKPASM--
PGPASM--
--PASMYE
--PASMTF
--PASMTD
--PASMSW
--PASMSV
--PASMSD
--PASMRQ
--PASMQY
--PASMMI

--PASMKW
--PASMKV
--PASMKR
--PASMKM
--PASMIW
--PASMHK
--PASMHI
--PASMGS
--PASMFV
--PASMFS
--PASMET
--PASMAS
--PASMAK
PLPASM--
--PASMYQ
--PASMWK
--PASMWH
--PASMWG
--PASMVN
--PASMTV
--PASMTM
--PASMSA
--PASMRE
--PASMQI
--PASMNY
--PASMNN
--PASMNH
--PASMND
--PASMML
--PASMMG
--PASMME
--PASMKY
--PASMKS
--PASMID
--PASMHW
--PASMHT
--PASMHM
--PASMHG
--PASMFD
--PASMEW
--PASMDS
--PASMAF
--PASMAD
--PASMYH
--PASMWY
--PASMWW
--PASMWV
--PASMVQ
--PASMSE
--PASMQS

--PASMNS
--PASMNK
--PASMMH
--PASMMD
--PASMKT
--PASMKN
--PASMIT
--PASMIH
--PASMIF
--PASMHH
--PASMHE
--PASMHA
--PASMGY
--PASMGI
--PASMFQ
--PASMFN
--PASMEY
--PASMES
--PASMEQ
--PASMEM
--PASMEG
PWPASM--
PHPASM--
PDPASM--
PCPASM--
--PASMVF
--PASMTE
--PASMTA
--PASMSM
--PASMRD
--PASMQW
--PASMQV
--PASMQT
--PASMQA
--PASMNW
--PASMNM
--PASMNA
--PASMMV
--PASMMN
--PASMLA
--PASMKQ
--PASMKH
--PASMKF
--PASMKD
--PASMIA
--PASMHV
--PASMHS
--PASMHQ
--PASMHN
--PASMHD

--PASMGM
--PASMGF
--PASMGE
--PASMFK
--PASMFF
--PASMEV
--PASMEK
--PASMEH
--PASMAE
PAPASM--
-QPASML-
-QPASMV-
-QPASMA-
QSPASM--
QRPASM--
-QPASMM-
-QPASMS-
-QPASMH-
QFPASM--
-QPASMW-
-QPASMT-
-QPASMK-
-QPASMG-
-QPASMF-
QIPASM--
QWPASM--
QTPASM--
-QPASMI-
QGPASM--
QPPASM--
-QPASMY-
-QPASMR-
-QPASMN-
-QPASME-
QAPASM--
QYPASM--
QVPASM--
-QPASMQ-
-QPASMD-
QNPASM--
QHPASM--
-RPASML-
-RPASMR-
RRPASM--
-RPASMM-
-RPASMV-
RVPASM--
RLPASM--
-RPASMG-
-RPASME-

-RPASMF-
RIPASM--
RSPASM--
-RPASMI-
RMPASM--
RPPASM--
-RPASMK-
-RPASMW-
-RPASMH-
RGPASM--
-RPASMQ-
RTPASM--
RAPASM--
-RPASMY-
-RPASMT-
RFPASM--
RWPASM--
RNPASM--
REPASM--
RHPASM--
RQPASM--
-RPASMC-
RCPASM--
-SPASML-
SLPASM--
SRPASM--
-SPASMK-
-SPASMA-
-SPASMN-
-SPASMR-
-SPASMH-
SGPASM--
SQPASM--
SIPASM--
-SPASMQ-
-SPASMF-
SVPASM--
-SPASMM-
SAPASM--
-SPASME-
SFPASM--
SYPASM--
-SPASMT-
-SPASMG-
SNPASM--
SHPASM--
SMPASM--
SWPASM--
-SPASMW-
SEPASM--

STPASM--
SDPASM--
-TPASMR-
-TPASMS-
-TPASMN-
TYPASM--
-TPASMG-
-TPASMF-
-TPASMA-
-TPASMQ-
TSPASM--
-TPASMY-
-TPASMM-
-TPASMT-
TMPASM--
TLPASM--
TGPASM--
TFPASM--
TEPASM--
TPPASM--
-TPASMV-
TIPASM--
TDPASM--
TTPASM--
-TPASMH-
TNPASM--
TKPASM--
-VPASMK-
-VPASML-
-VPASMR-
-VPASMS-
-VPASMI-
VRPASM--
VWPASM--
VPPASM--
VVPASM--
-VPASMW-
-VPASMT-
VAPASM--
VSPASM--
-VPASMN-
-VPASMF-
VNPASM--
VHPASM--
VGPASM--
VFPASM--
-VPASMG-
-VPASMC-
-VPASMA-
VMPASM--

VLPASM--
VKPASM--
VTPASM--
-VPASMY-
-VPASMH-
VIPASM--
VQPASM--
-VPASMV-
VDPASM--
VCPASM--
-WPASMA-
WRPASM--
WAPASM--
-WPASMG-
WMPASM--
WKPASM--
-WPASMY-
-WPASMV-
-WPASMS-
-WPASMN-
-WPASMI-
-WPASMF-
WEPASM--
WTPASM--
WSPASM--
-WPASMR-
WHPASM--
WYPASM--
-WPASMT-
-WPASML-
-WPASMH-
WQPASM--
WPPASM--
-WPASME-
WGPASM--
WDPASM--
-YPASMG-
YTPASM--
YLPASM--
-YPASML-
-YPASMW-
-YPASMR-
-YPASMS-
YFPASM--
YYPASM--
YVPASM--
-YPASMY-
-YPASMN-
-YPASMK-
YSPASM--

YPPASM--
-YPASME-
-YPASMA-
YIPASM--
YEPASM--
YAPASM--
YQPASM--
-YPASMT-
-YPASMQ-
-YPASMM-
-YPASMI-
YHPASM--
YGPASM--
YNPASM--
YDPASM--

| | | | |
|---|---|---|---|
| IGHL | 38 | 818 | 32.71 |

--IGHLGL
KIIGHL--
-RIGHLD-
ASIGHL--
ARIGHL--
-AIGHLL-
-AIGHLR-
-AIGHLA-
-AIGHLS-
AVIGHL--
ALIGHL--
-AIGHLM-
-AIGHLV-
AHIGHL--
AYIGHL--
-AIGHLW-
ATIGHL--
AKIGHL--
-AIGHLH-
AEIGHL--
-AIGHLQ-
AGIGHL--
AQIGHL--
-AIGHLI-
AFIGHL--
-AIGHLT-
-AIGHLN-
AWIGHL--
-AIGHLK-
-AIGHLF-
AAIGHL--
APIGHL--
ANIGHL--
ADIGHL--

AMIGHL--
-AIGHLP-
CVIGHL--
CRIGHL--
-CIGHLW-
-CIGHLL-
CCIGHL--
CTIGHL--
CSIGHL--
CNIGHL--
-CIGHLS-
CHIGHL--
CPIGHL--
CKIGHL--
-CIGHLK-
CYIGHL--
CLIGHL--
CIIGHL--
-CIGHLT-
CGIGHL--
CAIGHL--
-DIGHLR-
DSIGHL--
DFIGHL--
DGIGHL--
DLIGHL--
DVIGHL--
DQIGHL--
DIIGHL--
DWIGHL--
-DIGHLL-
DYIGHL--
-DIGHLV-
-DIGHLH-
DRIGHL--
-DIGHLY-
-DIGHLS-
-DIGHLI-
DTIGHL--
-DIGHLT-
-DIGHLF-
DNIGHL--
-DIGHLK-
DAIGHL--
DMIGHL--
-DIGHLA-
DEIGHL--
DPIGHL--
DKIGHL--
DHIGHL--

DDIGHL--
DCIGHL--
EIIGHL--
-EIGHLL-
ERIGHL--
ESIGHL--
EFIGHL--
EMIGHL--
EKIGHL--
-EIGHLR-
EPIGHL--
-EIGHLK-
-EIGHLI-
EGIGHL--
EEIGHL--
EWIGHL--
-EIGHLT-
EYIGHL--
ENIGHL--
-EIGHLW-
-EIGHLA-
ELIGHL--
-EIGHLF-
ETIGHL--
EQIGHL--
-EIGHLY-
-EIGHLV-
-EIGHLS-
-EIGHLQ-
-EIGHLH-
EHIGHL--
EAIGHL--
-FIGHLR-
FKIGHL--
-FIGHLS-
FRIGHL--
FMIGHL--
FSIGHL--
FGIGHL--
FYIGHL--
FTIGHL--
-FIGHLK-
-FIGHLY-
-FIGHLT-
-FIGHLL-
-FIGHLG-
-FIGHLI-
-FIGHLV-
FHIGHL--
FFIGHL--

-FIGHLN-
FDIGHL--
FNIGHL--
-FIGHLQ-
-FIGHLW-
-FIGHLE-
FEIGHL--
FAIGHL--
FPIGHL--
FIIGHL--
FVIGHL--
-FIGHLF-
-FIGHLA-
FWIGHL--
-FIGHLM-
FCIGHL--
GSIGHL--
GNIGHL--
-GIGHLL-
-GIGHLS-
-GIGHLR-
GRIGHL--
GLIGHL--
-GIGHLA-
GFIGHL--
-GIGHLT-
GWIGHL--
GAIGHL--
-GIGHLY-
GMIGHL--
GKIGHL--
GIIGHL--
-GIGHLN-
-GIGHLE-
-GIGHLF-
GHIGHL--
GEIGHL--
-GIGHLM-
GTIGHL--
-GIGHLV-
-GIGHLQ-
-GIGHLH-
GGIGHL--
GYIGHL--
GQIGHL--
-GIGHLI-
-GIGHLG-
GCIGHL--
GPIGHL--
HRIGHL--

HSIGHL--
-HIGHLI-
HAIGHL--
HHIGHL--
HTIGHL--
HPIGHL--
HKIGHL--
-HIGHLW-
HLIGHL--
HFIGHL--
HWIGHL--
-HIGHLR-
-HIGHLS-
-HIGHLM-
HGIGHL--
HNIGHL--
-HIGHLH-
HCIGHL--
HYIGHL--
-HIGHLT-
-HIGHLG-
HEIGHL--
-HIGHLE-
HQIGHL--
HMIGHL--
HIIGHL--
-HIGHLY-
-HIGHLL-
-HIGHLF-
HDIGHL--
--IGHLRS
--IGHLRY
--IGHLHS
--IGHLRT
IRIGHL--
--IGHLLS
--IGHLSW
--IGHLSG
--IGHLIS
--IGHLAS
--IGHLST
--IGHLSM
--IGHLRA
--IGHLTS
--IGHLRF
-IIGHLS-
--IGHLVG
--IGHLRG
-IIGHLR-
--IGHLTV

--IGHLTT
--IGHLSI
--IGHLIR
--IGHLRV
--IGHLRN
IMIGHL--
--IGHLWS
--IGHLVK
--IGHLVH
--IGHLLT
IAIGHL--
--IGHLYS
--IGHLSV
IQIGHL--
--IGHLVS
--IGHLVN
--IGHLSN
--IGHLRH
--IGHLLF
--IGHLKA
--IGHLLK
--IGHLYA
--IGHLWY
--IGHLVW
--IGHLSA
--IGHLIM
--IGHLFT
--IGHLFR
--IGHLFG
IKIGHL--
--IGHLYM
--IGHLQG
--IGHLMF
--IGHLKS
--IGHLKL
--IGHLGS
--IGHLFA
--IGHLWV
--IGHLLY
--IGHLLG
IWIGHL--
--IGHLTW
--IGHLSH
--IGHLMS
--IGHLMR
--IGHLMH
--IGHLLV
--IGHLKQ
--IGHLKI
--IGHLKH

--IGHLHG
--IGHLFQ
-IIGHLK-
--IGHLYR
--IGHLWK
--IGHLSR
--IGHLSL
--IGHLRQ
--IGHLQS
--IGHLKF
--IGHLHV
--IGHLFK
--IGHLAG
IPIGHL--
-IIGHLW-
-IIGHLN-
IGIGHL--
--IGHLYW
--IGHLWI
--IGHLTI
--IGHLTH
--IGHLSY
--IGHLRD
--IGHLNT
--IGHLMK
--IGHLLH
--IGHLKV
--IGHLES
IYIGHL--
--IGHLYT
--IGHLYK
--IGHLWT
--IGHLWM
--IGHLVA
--IGHLTA
--IGHLSK
--IGHLNL
--IGHLLW
--IGHLHT
--IGHLGA
--IGHLFN
--IGHLFH
--IGHLYV
--IGHLWH
--IGHLVV
--IGHLVT
--IGHLVM
--IGHLTR
--IGHLSQ
--IGHLLR

--IGHLKN
--IGHLKG
--IGHLIV
--IGHLIE
--IGHLEA
--IGHLAY
--IGHLAV
-IIGHLL-
-IIGHLA-
--IGHLVE
--IGHLTN
--IGHLTL
--IGHLTG
--IGHLQW
--IGHLQT
--IGHLQF
--IGHLQA
--IGHLNS
--IGHLMQ
--IGHLMA
--IGHLLM
--IGHLLE
--IGHLKT
--IGHLIT
--IGHLIG
--IGHLIA
--IGHLHY
--IGHLHF
--IGHLHA
--IGHLFY
--IGHLAT
IEIGHL--
IDIGHL--
-IIGHLI-
IHIGHL--
--IGHLYQ
--IGHLYL
--IGHLYH
--IGHLYG
--IGHLVF
--IGHLTM
--IGHLSF
--IGHLRW
--IGHLRI
--IGHLRE
--IGHLQM
--IGHLMN
--IGHLMI
--IGHLMD
--IGHLLN

--IGHLLA
--IGHLKK
--IGHLKD
--IGHLIW
--IGHLII
--IGHLHN
--IGHLGT
--IGHLFF
--IGHLET
--IGHLEG
--IGHLAR
IVIGHL--
-IIGHLG-
--IGHLWQ
--IGHLVD
--IGHLTQ
--IGHLTF
--IGHLTD
--IGHLSD
--IGHLQV
--IGHLQQ
--IGHLNA
--IGHLMW
--IGHLMV
--IGHLMT
--IGHLML
--IGHLMG
--IGHLLQ
--IGHLKY
--IGHLKM
--IGHLIY
--IGHLIQ
--IGHLIH
--IGHLID
--IGHLHH
--IGHLGV
--IGHLFV
--IGHLFS
--IGHLEW
--IGHLEV
--IGHLAM
--IGHLAL
IIIGHL--
-IIGHLT-
-IIGHLQ-
-IIGHLM-
-IIGHLH-
--IGHLYN
--IGHLYE
--IGHLWW

```
--IGHLWL
--IGHLWE
--IGHLWA
--IGHLTK
--IGHLQY
--IGHLQN
--IGHLQI
--IGHLQD
--IGHLND
--IGHLMY
--IGHLLI
--IGHLKP
--IGHLKE
--IGHLIN
--IGHLIF
--IGHLHQ
--IGHLHK
--IGHLHE
--IGHLGW
--IGHLGI
--IGHLEY
--IGHLEM
--IGHLDA
ICIGHL--
KSIGHL--
-KIGHLR-
-KIGHLS-
-KIGHLL-
KQIGHL--
KLIGHL--
-KIGHLW-
-KIGHLT-
KEIGHL--
KNIGHL--
-KIGHLY-
-KIGHLF-
-KIGHLA-
KAIGHL--
KTIGHL--
KPIGHL--
-KIGHLV-
-KIGHLK-
-KIGHLI-
KVIGHL--
-KIGHLN-
-KIGHLM-
KDIGHL--
-KIGHLH-
LRIGHL--
LLIGHL--
```

LSIGHL--
-LIGHLR-
LVIGHL--
LQIGHL--
LGIGHL--
-LIGHLL-
-LIGHLY-
LPIGHL--
-LIGHLT-
-LIGHLS-
-LIGHLN-
LIIGHL--
LKIGHL--
LHIGHL--
LYIGHL--
-LIGHLK-
LDIGHL--
LNIGHL--
-LIGHLH-
-LIGHLG-
-LIGHLF-
-LIGHLA-
LWIGHL--
LTIGHL--
-LIGHLW-
-LIGHLM-
LEIGHL--
-LIGHLV-
-LIGHLE-
-LIGHLQ-
-LIGHLD-
LCIGHL--
MAIGHL--
-MIGHLS-
-MIGHLE-
MLIGHL--
MRIGHL--
MVIGHL--
MNIGHL--
-MIGHLR-
-MIGHLA-
MEIGHL--
MSIGHL--
MKIGHL--
-MIGHLV-
MGIGHL--
-MIGHLN-
-MIGHLL-
MDIGHL--
-MIGHLI-

MTIGHL--
-MIGHLW-
-MIGHLF-
MQIGHL--
MIIGHL--
-MIGHLH-
-MIGHLK-
-MIGHLG-
MFIGHL--
MYIGHL--
MWIGHL--
MPIGHL--
-MIGHLY-
MCIGHL--
NRIGHL--
NSIGHL--
-NIGHLS-
NGIGHL--
-NIGHLQ-
NKIGHL--
NTIGHL--
NFIGHL--
NVIGHL--
-NIGHLW-
-NIGHLR-
-NIGHLM-
NHIGHL--
NPIGHL--
-NIGHLT-
NDIGHL--
NQIGHL--
-NIGHLI-
NNIGHL--
NIIGHL--
-NIGHLV-
-NIGHLG-
-NIGHLE-
NEIGHL--
NCIGHL--
NYIGHL--
-NIGHLL-
-NIGHLK-
-NIGHLH-
-NIGHLA-
NAIGHL--
-NIGHLF-
-PIGHLR-
PLIGHL--
PAIGHL--
PSIGHL--

PNIGHL--
PGIGHL--
PFIGHL--
-PIGHLA-
-PIGHLK-
PYIGHL--
PVIGHL--
PTIGHL--
-PIGHLT-
-PIGHLM-
-PIGHLL-
-PIGHLS-
PWIGHL--
-PIGHLQ-
PEIGHL--
PDIGHL--
PQIGHL--
-PIGHLF-
-PIGHLW-
-PIGHLV-
PHIGHL--
PCIGHL--
PMIGHL--
PIIGHL--
-PIGHLY-
-PIGHLN-
-QIGHLR-
QRIGHL--
QWIGHL--
-QIGHLL-
QFIGHL--
-QIGHLY-
QYIGHL--
QLIGHL--
QSIGHL--
QKIGHL--
QVIGHL--
-QIGHLT-
-QIGHLS-
QTIGHL--
QAIGHL--
QQIGHL--
-QIGHLW-
-QIGHLQ-
QPIGHL--
QNIGHL--
-QIGHLF-
QHIGHL--
QGIGHL--
-QIGHLN-

```
-QIGHLK-
-QIGHLG-
-QIGHLI-
-QIGHLM-
QDIGHL--
-RIGHLR-
RHIGHL--
-RIGHLY-
-RIGHLS-
-RIGHLI-
REIGHL--
RQIGHL--
-RIGHLV-
RPIGHL--
-RIGHLM-
-RIGHLQ-
-RIGHLT-
-RIGHLH-
-RIGHLA-
-RIGHLK-
-RIGHLF-
RFIGHL--
RCIGHL--
-RIGHLW-
-RIGHLN-
-RIGHLE-
RDIGHL--
-SIGHLR-
SRIGHL--
SLIGHL--
SGIGHL--
-SIGHLL-
SYIGHL--
-SIGHLV-
-SIGHLS-
SVIGHL--
SKIGHL--
-SIGHLY-
SNIGHL--
-SIGHLW-
STIGHL--
SMIGHL--
-SIGHLT-
SEIGHL--
-SIGHLI-
-SIGHLF-
-SIGHLQ-
SSIGHL--
SPIGHL--
SAIGHL--
```

SWIGHL--
-SIGHLN-
-SIGHLH-
SFIGHL--
SDIGHL--
-SIGHLG-
SCIGHL--
-SIGHLA-
-SIGHLM-
-SIGHLK-
-SIGHLE-
SQIGHL--
TRIGHL--
-TIGHLS-
-TIGHLL-
TNIGHL--
TFIGHL--
TQIGHL--
TLIGHL--
-TIGHLY-
TTIGHL--
TPIGHL--
-TIGHLQ-
TSIGHL--
TIIGHL--
TGIGHL--
TEIGHL--
TVIGHL--
-TIGHLF-
-TIGHLT-
-TIGHLM-
-TIGHLA-
TYIGHL--
TMIGHL--
-TIGHLH-
TDIGHL--
-TIGHLK-
-TIGHLE-
TAIGHL--
-TIGHLN-
TCIGHL--
-VIGHLR-
VGIGHL--
VLIGHL--
VRIGHL--
VKIGHL--
-VIGHLI-
VSIGHL--
-VIGHLS-
VPIGHL--

-VIGHLT-
VIIGHL--
-VIGHLL-
-VIGHLH-
VTIGHL--
-VIGHLA-
-VIGHLY-
-VIGHLN-
VMIGHL--
-VIGHLV-
VHIGHL--
VFIGHL--
VDIGHL--
VQIGHL--
VYIGHL--
VNIGHL--
-VIGHLQ-
VAIGHL--
VWIGHL--
-VIGHLG-
-VIGHLE-
-VIGHLM-
-VIGHLW-
-WIGHLK-
WGIGHL--
WTIGHL--
WEIGHL--
-WIGHLR-
-WIGHLE-
WAIGHL--
WRIGHL--
-WIGHLN-
WKIGHL--
-WIGHLS-
WNIGHL--
WVIGHL--
-WIGHLV-
-WIGHLT-
-WIGHLG-
WQIGHL--
-WIGHLQ-
-WIGHLM-
-WIGHLA-
WDIGHL--
WPIGHL--
WYIGHL--
-WIGHLW-
-WIGHLI-
-WIGHLH-
WCIGHL--

-WIGHLL-
YPIGHL--
YEIGHL--
YNIGHL--
-YIGHLN-
-YIGHLG-
-YIGHLK-
YTIGHL--
YQIGHL--
-YIGHLA-
YYIGHL--
YLIGHL--
YCIGHL--
YMIGHL--
-YIGHLY-
-YIGHLT-
-YIGHLL-
YHIGHL--
-YIGHLV-
YFIGHL--
YDIGHL--
-YIGHLW-
-YIGHLQ-
-YIGHLE-
YIIGHL--
-YIGHLS-
-YIGHLD-
YAIGHL--
YWIGHL--
-YIGHLH-
-YIGHLF-
YGIGHL--
-YIGHLM-
-YIGHLI-

PAAY 178 727 32.70

-RPAAYD-
-APAAYR-
ARPAAY--
ALPAAY--
ASPAAY--
-APAAYV-
-APAAYQ-
-APAAYE-
AVPAAY--
-APAAYK-
-APAAYI-
ANPAAY--
AMPAAY--
-APAAYT-
-APAAYH-

-APAAYG-
-APAAYA-
ATPAAY--
AQPAAY--
APPAAY--
-APAAYN-
AWPAAY--
-APAAYY-
-APAAYS-
-APAAYF-
-APAAYC-
AGPAAY--
AFPAAY--
AEPAAY--
-CPAAYK-
CSPAAY--
-CPAAYS-
CYPAAY--
CRPAAY--
-CPAAYT-
-CPAAYR-
CNPAAY--
CMPAAY--
DKPAAY--
-DPAAYI-
DRPAAY--
DFPAAY--
DVPAAY--
-DPAAYA-
DLPAAY--
DYPAAY--
DQPAAY--
-DPAAYS-
-DPAAYR-
-DPAAYH-
-DPAAYK-
DTPAAY--
-DPAAYV-
-DPAAYQ-
-DPAAYM-
-DPAAYF-
DNPAAY--
DIPAAY--
DHPAAY--
DDPAAY--
DAPAAY--
-EPAAYL-
ERPAAY--
-EPAAYR-
EAPAAY--

ESPAAY--
ELPAAY--
-EPAAYF-
-EPAAYV-
EMPAAY--
EHPAAY--
EEPAAY--
EYPAAY--
-EPAAYT-
-EPAAYS-
-EPAAYI-
EVPAAY--
ETPAAY--
-EPAAYM-
-EPAAYG-
-EPAAYE-
-EPAAYA-
EIPAAY--
EFPAAY--
FSPAAY--
-FPAAYT-
-FPAAYL-
-FPAAYS-
-FPAAYK-
FLPAAY--
-FPAAYI-
-FPAAYM-
-FPAAYE-
FNPAAY--
-FPAAYR-
-FPAAYV-
FVPAAY--
FQPAAY--
-FPAAYG-
FKPAAY--
FAPAAY--
-FPAAYN-
-FPAAYA-
-GPAAYR-
GRPAAY--
-GPAAYS-
GWPAAY--
-GPAAYN-
-GPAAYL-
GTPAAY--
GIPAAY--
-GPAAYG-
GLPAAY--
-GPAAYM-
-GPAAYK-

GKPAAY--
GSPAAY--
-GPAAYE-
GHPAAY--
GFPAAY--
GAPAAY--
-GPAAYY-
-GPAAYF-
-GPAAYA-
GGPAAY--
-GPAAYV-
-GPAAYH-
GMPAAY--
GEPAAY--
GDPAAY--
GVPAAY--
GPPAAY--
-GPAAYQ-
-GPAAYI-
GNPAAY--
GCPAAY--
HRPAAY--
-HPAAYL-
HLPAAY--
-HPAAYR-
HNPAAY--
HYPAAY--
-HPAAYS-
HTPAAY--
-HPAAYT-
-HPAAYG-
HKPAAY--
HSPAAY--
-HPAAYN-
HMPAAY--
HHPAAY--
HGPAAY--
HVPAAY--
HQPAAY--
-HPAAYV-
-HPAAYK-
HIPAAY--
IVPAAY--
-IPAAYR-
IYPAAY--
-IPAAYN-
-IPAAYG-
IPPAAY--
IGPAAY--
IAPAAY--

ITPAAY--
IQPAAY--
-IPAAYV-
-IPAAYK-
ILPAAY--
IIPAAY--
IWPAAY--
-IPAAYW-
-IPAAYS-
-IPAAYL-
IKPAAY--
IFPAAY--
-IPAAYM-
-IPAAYA-
IHPAAY--
KSPAAY--
-KPAAYW-
-KPAAYR-
-KPAAYM-
KYPAAY--
-KPAAYK-
-KPAAYS-
-KPAAYQ-
KNPAAY--
KAPAAY--
KIPAAY--
KHPAAY--
KTPAAY--
-KPAAYT-
-KPAAYN-
-KPAAYE-
KLPAAY--
KWPAAY--
-KPAAYV-
-KPAAYG-
KMPAAY--
KKPAAY--
-KPAAYI-
KGPAAY--
KVPAAY--
KQPAAY--
-KPAAYA-
KFPAAY--
KEPAAY--
KDPAAY--
KCPAAY--
-LPAAYL-
LAPAAY--
LSPAAY--
-LPAAYW-

```
-LPAAYR-
LRPAAY--
-LPAAYT-
LGPAAY--
-LPAAYE-
LKPAAY--
LYPAAY--
LVPAAY--
-LPAAYS-
LWPAAY--
-LPAAYH-
LHPAAY--
LLPAAY--
LPPAAY--
-LPAAYN-
-LPAAYI-
LCPAAY--
-LPAAYK-
-LPAAYF-
LIPAAY--
LFPAAY--
LTPAAY--
LEPAAY--
LQPAAY--
-LPAAYV-
LMPAAY--
LDPAAY--
MSPAAY--
-MPAAYG-
-MPAAYM-
MKPAAY--
-MPAAYT-
MAPAAY--
MTPAAY--
-MPAAYV-
-MPAAYI-
MLPAAY--
MDPAAY--
MRPAAY--
-MPAAYH-
MMPAAY--
MVPAAY--
-MPAAYN-
-MPAAYA-
-MPAAYS-
MNPAAY--
MGPAAY--
-MPAAYY-
-MPAAYQ-
-MPAAYL-
```

```
MHPAAY--
MFPAAY--
-NPAAYL-
NGPAAY--
NRPAAY--
-NPAAYR-
NLPAAY--
NTPAAY--
NVPAAY--
-NPAAYY-
-NPAAYM-
NWPAAY--
NQPAAY--
-NPAAYG-
NCPAAY--
NYPAAY--
NSPAAY--
-NPAAYT-
-NPAAYS-
-NPAAYK-
-NPAAYI-
-NPAAYH-
NPPAAY--
-NPAAYA-
NHPAAY--
--PAAYAL
--PAAYSK
--PAAYRS
--PAAYLL
--PAAYSS
--PAAYRL
--PAAYRG
--PAAYAG
--PAAYRH
--PAAYFR
--PAAYLQ
--PAAYNR
--PAAYGS
--PAAYRF
--PAAYLM
--PAAYYG
--PAAYVS
--PAAYRE
--PAAYLI
--PAAYGL
--PAAYWS
--PAAYVK
--PAAYMR
--PAAYLS
--PAAYLH
```

--PAAYKV
--PAAYVV
--PAAYVT
--PAAYSL
--PAAYMG
--PAAYNS
--PAAYMK
--PAAYLT
--PAAYLN
PLPAAY--
--PAAYYD
--PAAYVH
--PAAYSI
--PAAYRQ
--PAAYNL
--PAAYNI
--PAAYIS
--PAAYHT
--PAAYHR
--PAAYGF
--PAAYAK
--PAAYYS
--PAAYVR
--PAAYTS
--PAAYTK
--PAAYSG
--PAAYRI
--PAAYNV
--PAAYLR
--PAAYKN
--PAAYGT
--PAAYEV
--PAAYAS
--PAAYYT
--PAAYYQ
--PAAYYH
--PAAYSW
--PAAYSV
--PAAYST
--PAAYMT
--PAAYML
--PAAYKW
--PAAYGY
--PAAYFT
PVPAAY--
PMPAAY--
--PAAYWR
--PAAYTL
--PAAYSY
--PAAYSH

--PAAYSF
--PAAYRM
--PAAYRD
--PAAYQR
--PAAYPL
--PAAYNM
--PAAYMN
--PAAYMH
--PAAYLY
--PAAYLG
--PAAYLD
--PAAYKT
--PAAYKR
--PAAYIT
--PAAYHN
--PAAYAV
--PAAYAH
PGPAAY--
--PAAYYR
--PAAYYI
--PAAYYF
--PAAYWV
--PAAYVL
--PAAYTN
--PAAYRY
--PAAYRN
--PAAYNW
--PAAYMY
--PAAYLE
--PAAYKL
--PAAYIY
--PAAYHK
--PAAYFK
--PAAYFI
--PAAYEW
--PAAYES
--PAAYAR
--PAAYAF
PYPAAY--
PWPAAY--
PRPAAY--
PIPAAY--
--PAAYYN
--PAAYYM
--PAAYWI
--PAAYWE
--PAAYWA
--PAAYVM
--PAAYTM
--PAAYTH

--PAAYTG
--PAAYSD
--PAAYQY
--PAAYQN
--PAAYNG
--PAAYMQ
--PAAYMI
--PAAYMA
--PAAYKS
--PAAYKI
--PAAYIQ
--PAAYHW
--PAAYHS
--PAAYHM
--PAAYGW
--PAAYGI
--PAAYFD
--PAAYFA
--PAAYAT
--PAAYAI
PEPAAY--
PDPAAY--
--PAAYWT
--PAAYWL
--PAAYWH
--PAAYVE
--PAAYVA
--PAAYTQ
--PAAYSN
--PAAYSM
--PAAYRA
--PAAYQW
--PAAYQI
--PAAYPY
--PAAYNY
--PAAYNN
--PAAYMV
--PAAYLV
--PAAYIV
--PAAYIK
--PAAYHY
--PAAYHI
--PAAYHH
--PAAYHA
--PAAYGV
--PAAYFS
--PAAYFM
--PAAYEY
--PAAYAD
PSPAAY--

-PPAAYS-
-PPAAYI-
PNPAAY--
PKPAAY--
PHPAAY--
PAPAAY--
--PAAYYY
--PAAYYW
--PAAYWN
--PAAYVW
--PAAYVN
--PAAYVG
--PAAYVD
--PAAYTT
--PAAYTI
--PAAYTE
--PAAYSQ
--PAAYSA
--PAAYRV
--PAAYRC
--PAAYQV
--PAAYQT
--PAAYQS
--PAAYQM
--PAAYQL
--PAAYQH
--PAAYQG
--PAAYQD
--PAAYPW
--PAAYPS
--PAAYNQ
--PAAYNK
--PAAYNF
--PAAYND
--PAAYMC
--PAAYKG
--PAAYKE
--PAAYIM
--PAAYIH
--PAAYIG
--PAAYIE
--PAAYIA
--PAAYHV
--PAAYHG
--PAAYHF
--PAAYGQ
--PAAYGN
--PAAYGM
--PAAYGE
--PAAYFN

```
--PAAYFH
--PAAYET
--PAAYER
--PAAYEQ
--PAAYEL
--PAAYEI
--PAAYEF
--PAAYEA
--PAAYDT
--PAAYDI
--PAAYCL
--PAAYAQ
--PAAYAE
--PAAYAA
-QPAAYL-
-QPAAYQ-
-QPAAYF-
QRPAAY--
-QPAAYN-
-QPAAYI-
QMPAAY--
-QPAAYA-
QFPAAY--
QYPAAY--
QTPAAY--
QSPAAY--
-QPAAYW-
-QPAAYS-
-QPAAYR-
-QPAAYM-
-QPAAYE-
QHPAAY--
QEPAAY--
QWPAAY--
QPPAAY--
-QPAAYT-
QLPAAY--
QGPAAY--
-RPAAYR-
RRPAAY--
-RPAAYS-
RQPAAY--
RLPAAY--
RPPAAY--
RSPAAY--
-RPAAYV-
-RPAAYN-
-RPAAYI-
RYPAAY--
RTPAAY--
```

```
-RPAAYQ-
RMPAAY--
RIPAAY--
RGPAAY--
RVPAAY--
-RPAAYM-
-RPAAYG-
-RPAAYY-
RFPAAY--
-RPAAYA-
RAPAAY--
-RPAAYK-
REPAAY--
-RPAAYH-
RKPAAY--
RHPAAY--
-RPAAYE-
RDPAAY--
-RPAAYW-
RCPAAY--
-SPAAYR-
-SPAAYS-
SKPAAY--
SRPAAY--
-SPAAYL-
SFPAAY--
SGPAAY--
-SPAAYV-
SMPAAY--
-SPAAYY-
SYPAAY--
-SPAAYH-
-SPAAYF-
SHPAAY--
STPAAY--
SSPAAY--
-SPAAYT-
-SPAAYN-
SVPAAY--
SPPAAY--
-SPAAYM-
-SPAAYE-
SQPAAY--
SEPAAY--
SDPAAY--
-SPAAYG-
-SPAAYW-
-SPAAYA-
SWPAAY--
-SPAAYK-
```

-SPAAYC-
SNPAAY--
SLPAAY--
SAPAAY--
TRPAAY--
TTPAAY--
-TPAAYG-
-TPAAYW-
TSPAAY--
-TPAAYT-
-TPAAYL-
TVPAAY--
-TPAAYS-
-TPAAYH-
TMPAAY--
THPAAY--
-TPAAYK-
-TPAAYA-
TGPAAY--
TAPAAY--
-TPAAYI-
TWPAAY--
-TPAAYM-
TIPAAY--
TYPAAY--
-TPAAYV-
-TPAAYR-
-TPAAYQ-
TNPAAY--
TEPAAY--
TQPAAY--
-TPAAYE-
-VPAAYR-
-VPAAYG-
-VPAAYI-
VLPAAY--
VKPAAY--
VSPAAY--
-VPAAYV-
-VPAAYL-
VYPAAY--
VVPAAY--
-VPAAYS-
VRPAAY--
-VPAAYN-
-VPAAYH-
VMPAAY--
VTPAAY--
VQPAAY--
VPPAAY--

-VPAAYT-
VGPAAY--
VWPAAY--
-VPAAYY-
-VPAAYW-
-VPAAYQ-
-VPAAYK-
-VPAAYF-
VIPAAY--
VFPAAY--
VDPAAY--
VCPAAY--
-VPAAYM-
-VPAAYE-
VNPAAY--
VHPAAY--
VAPAAY--
WSPAAY--
WLPAAY--
WTPAAY--
-WPAAYW-
-WPAAYY-
-WPAAYT-
-WPAAYK-
-WPAAYG-
-WPAAYE-
WQPAAY--
WPPAAY--
-WPAAYV-
-WPAAYN-
-WPAAYA-
WIPAAY--
WGPAAY--
WFPAAY--
WDPAAY--
WAPAAY--
YRPAAY--
YKPAAY--
YLPAAY--
-YPAAYS-
YMPAAY--
YYPAAY--
YWPAAY--
YVPAAY--
YQPAAY--
-YPAAYM-
YTPAAY--
-YPAAYL-
-YPAAYK-
-YPAAYG-

```
            -YPAAYA-
            YSPAAY--
            -YPAAYY-
            -YPAAYW-
            -YPAAYN-
            YNPAAY--
            YPPAAY--
            -YPAAYT-
            -YPAAYQ-
            -YPAAYI-
            YHPAAY--
            YAPAAY--
PLSM          76        781      32.67
            -APLSME-
            IVPLSM--
            -IPLSMV-
            NEPLSM--
            --PLSMNR
            --PLSMNK
            --PLSMHN
            --PLSMER
            --PLSMAY
            -SPLSMD-
            -TPLSMG-
            -APLSML-
            -APLSMK-
            ARPLSM--
            -APLSMT-
            AVPLSM--
            AAPLSM--
            -APLSMN-
            AHPLSM--
            ANPLSM--
            AMPLSM--
            AFPLSM--
            AEPLSM--
            AQPLSM--
            -APLSMY-
            AYPLSM--
            -APLSMH-
            ALPLSM--
            AKPLSM--
            AIPLSM--
            ACPLSM--
            -APLSMG-
            -APLSMF-
            -APLSMA-
            ADPLSM--
            -APLSMQ-
            AWPLSM--
```

APPLSM--
-APLSMM-
-APLSMW-
-APLSMI-
CVPLSM--
CKPLSM--
CRPLSM--
CLPLSM--
-CPLSMV-
CPPLSM--
-CPLSML-
CNPLSM--
CYPLSM--
CSPLSM--
-CPLSMY-
-CPLSMT-
-CPLSMS-
CIPLSM--
-DPLSML-
DRPLSM--
DLPLSM--
DFPLSM--
DSPLSM--
-DPLSMT-
-DPLSMY-
-DPLSMQ-
-DPLSMW-
DMPLSM--
DKPLSM--
DIPLSM--
DWPLSM--
DTPLSM--
DNPLSM--
-DPLSMR-
-DPLSMM-
-DPLSMK-
-DPLSMI-
-DPLSMH-
DGPLSM--
DYPLSM--
-DPLSMV-
-DPLSMF-
-DPLSMA-
DVPLSM--
DQPLSM--
-DPLSMS-
-DPLSMP-
-DPLSMN-
-DPLSMG-
-DPLSMC-

DHPLSM--
ERPLSM--
-EPLSMR-
-EPLSML-
EVPLSM--
ETPLSM--
EPPLSM--
EWPLSM--
ESPLSM--
-EPLSMV-
ENPLSM--
ELPLSM--
EKPLSM--
EGPLSM--
EYPLSM--
EIPLSM--
-EPLSMW-
-EPLSMT-
-EPLSMK-
-EPLSMG-
-EPLSMA-
EQPLSM--
-EPLSMM-
-EPLSMI-
EMPLSM--
EHPLSM--
-EPLSME-
EFPLSM--
-FPLSML-
FSPLSM--
-FPLSMK-
-FPLSMS-
-FPLSMA-
FKPLSM--
FIPLSM--
FYPLSM--
-FPLSMQ-
-FPLSMI-
FTPLSM--
FQPLSM--
FHPLSM--
FWPLSM--
-FPLSMW-
-FPLSMV-
-FPLSME-
FLPLSM--
FAPLSM--
FPPLSM--
-FPLSMM-
-FPLSMT-

-FPLSMF-
-FPLSMY-
FNPLSM--
FDPLSM--
FFPLSM--
FEPLSM--
GLPLSM--
GSPLSM--
GIPLSM--
-GPLSMA-
GRPLSM--
GAPLSM--
GVPLSM--
GTPLSM--
-GPLSMS-
-GPLSMT-
-GPLSMV-
-GPLSMI-
GKPLSM--
GYPLSM--
-GPLSMN-
GNPLSM--
-GPLSMY-
-GPLSMF-
-GPLSMK-
GMPLSM--
GHPLSM--
GGPLSM--
-GPLSMW-
-GPLSMM-
-GPLSMH-
GEPLSM--
GCPLSM--
GWPLSM--
GFPLSM--
GDPLSM--
-GPLSMG-
-GPLSME-
-GPLSMQ-
GPPLSM--
HRPLSM--
HSPLSM--
HYPLSM--
HTPLSM--
-HPLSMR-
HGPLSM--
-HPLSMS-
-HPLSML-
HHPLSM--
-HPLSMK-

-HPLSMA-
HLPLSM--
HWPLSM--
-HPLSMW-
-HPLSMH-
-HPLSMT-
-HPLSMI-
-HPLSMG-
HQPLSM--
-HPLSMV-
HNPLSM--
HIPLSM--
HAPLSM--
HVPLSM--
HPPLSM--
-HPLSMY-
-HPLSMF-
-HPLSMM-
HKPLSM--
HMPLSM--
HFPLSM--
HEPLSM--
-IPLSML-
ISPLSM--
-IPLSMS-
-IPLSMR-
-IPLSMI-
ITPLSM--
ILPLSM--
-IPLSMM-
IPPLSM--
-IPLSMT-
IYPLSM--
-IPLSMA-
IIPLSM--
IQPLSM--
-IPLSMN-
IKPLSM--
IGPLSM--
IWPLSM--
-IPLSMH-
ICPLSM--
-IPLSMK-
IAPLSM--
-IPLSMY-
-IPLSMG-
INPLSM--
IMPLSM--
IHPLSM--
IFPLSM--

```
IEPLSM--
IDPLSM--
IRPLSM--
-IPLSMF-
-IPLSME-
KRPLSM--
-KPLSML-
-KPLSMY-
-KPLSMV-
-KPLSMS-
KLPLSM--
KGPLSM--
-KPLSMK-
-KPLSMI-
KPPLSM--
KHPLSM--
KFPLSM--
-KPLSMQ-
-KPLSMA-
KVPLSM--
-KPLSMW-
KTPLSM--
KQPLSM--
-KPLSMT-
-KPLSMN-
KAPLSM--
KNPLSM--
KWPLSM--
-KPLSMM-
-KPLSMF-
KIPLSM--
-KPLSMH-
KDPLSM--
KEPLSM--
KYPLSM--
KMPLSM--
KCPLSM--
-KPLSMG-
-KPLSME-
LRPLSM--
LSPLSM--
-LPLSMY-
-LPLSMQ-
-LPLSMI-
LVPLSM--
LTPLSM--
-LPLSMS-
LEPLSM--
-LPLSMV-
-LPLSMH-
```

LLPLSM--
LYPLSM--
LWPLSM--
LQPLSM--
-LPLSMA-
LIPLSM--
-LPLSMT-
-LPLSMM-
LKPLSM--
LFPLSM--
LPPLSM--
LMPLSM--
LHPLSM--
LAPLSM--
-LPLSMN-
LCPLSM--
-LPLSMW-
-LPLSMC-
-MPLSMS-
-MPLSMR-
-MPLSMV-
-MPLSMK-
-MPLSMQ-
-MPLSMY-
-MPLSML-
-MPLSMI-
-MPLSMA-
MTPLSM--
MGPLSM--
MSPLSM--
MRPLSM--
MEPLSM--
MNPLSM--
MKPLSM--
-MPLSMT-
MWPLSM--
-MPLSMN-
-MPLSMF-
MLPLSM--
-MPLSMM-
-MPLSMH-
MFPLSM--
MVPLSM--
MPPLSM--
MAPLSM--
MDPLSM--
MCPLSM--
MYPLSM--
MMPLSM--
MHPLSM--

-NPLSMR-
-NPLSMI-
-NPLSMS-
-NPLSMV-
-NPLSMK-
NSPLSM--
-NPLSMT-
NYPLSM--
NMPLSM--
-NPLSMQ-
NQPLSM--
NWPLSM--
-NPLSMY-
NNPLSM--
NPPLSM--
-NPLSMM-
-NPLSML-
-NPLSMF-
NFPLSM--
-NPLSMW-
NGPLSM--
NKPLSM--
NDPLSM--
NVPLSM--
-NPLSME-
-NPLSMA-
NIPLSM--
NHPLSM--
--PLSMMN
--PLSMRS
--PLSMSR
--PLSMVK
--PLSMRG
--PLSMAL
--PLSMGS
--PLSMRN
--PLSMST
--PLSMAS
--PLSMRA
--PLSMMS
--PLSMLT
--PLSMKR
--PLSMTR
--PLSMFE
--PLSMSK
--PLSMTS
--PLSMSS
--PLSMML
--PLSMVQ
--PLSMSW

--PLSMQT
--PLSMIT
--PLSMWK
--PLSMSL
--PLSMFG
--PLSMYT
--PLSMSG
--PLSMRV
--PLSMMR
--PLSMGA
--PLSMTV
--PLSMLV
--PLSMIS
--PLSMHG
--PLSMTN
--PLSMTL
--PLSMSN
--PLSMNS
--PLSMLN
--PLSMIG
--PLSMHR
--PLSMFK
--PLSMWS
--PLSMTF
--PLSMMK
--PLSMLG
--PLSMEA
--PLSMAV
--PLSMYA
--PLSMWA
--PLSMVN
--PLSMVI
--PLSMVH
--PLSMSA
--PLSMRQ
--PLSMRE
--PLSMMA
--PLSMKH
--PLSMFS
--PLSMWG
--PLSMTT
--PLSMTQ
--PLSMRW
--PLSMQW
--PLSMMV
--PLSMLQ
--PLSMKA
--PLSMII
--PLSMHV
--PLSMHH

PWPLSM--
--PLSMVM
--PLSMTW
--PLSMSV
--PLSMRM
--PLSMMT
--PLSMHY
--PLSMVW
--PLSMTH
--PLSMTD
--PLSMSY
--PLSMSM
--PLSMQH
--PLSMQG
--PLSMNY
--PLSMES
--PLSMAT
PYPLSM--
--PLSMVT
--PLSMSH
--PLSMQS
--PLSMQR
--PLSMNV
--PLSMMD
--PLSMLM
--PLSMKV
--PLSMIQ
--PLSMHW
--PLSMHS
--PLSMHK
--PLSMHF
--PLSMGQ
--PLSMAW
--PLSMAA
PQPLSM--
--PLSMYS
--PLSMYG
--PLSMYD
--PLSMWN
--PLSMVA
--PLSMTY
--PLSMTK
--PLSMSE
--PLSMQV
--PLSMQL
--PLSMKT
--PLSMIN
--PLSMHM
--PLSMGT
--PLSMFD

--PLSMET
PRPLSM--
--PLSMYN
--PLSMYH
--PLSMWH
--PLSMTE
--PLSMSI
--PLSMQA
--PLSMPL
--PLSMNA
--PLSMLD
--PLSMKN
--PLSMKI
--PLSMFH
PIPLSM--
PFPLSM--
PNPLSM--
--PLSMWD
--PLSMTM
--PLSMTG
--PLSMSD
--PLSMQQ
--PLSMMI
--PLSMME
--PLSMHD
--PLSMHA
--PLSMAG
--PLSMYE
--PLSMWT
--PLSMVY
--PLSMNT
--PLSMNQ
--PLSMKQ
--PLSMKL
--PLSMIV
--PLSMIK
--PLSMIE
--PLSMID
--PLSMHL
--PLSMHE
--PLSMGG
--PLSMAQ
--PLSMAN
--PLSMAM
--PLSMAK
--PLSMAI
PHPLSM--
--PLSMTI
--PLSMSQ
--PLSMQM

--PLSMQF
--PLSMNW
--PLSMNN
--PLSMNM
--PLSMNI
--PLSMNH
--PLSMNF
--PLSMKW
--PLSMKM
--PLSMKG
--PLSMKD
--PLSMHQ
--PLSMHI
--PLSMFY
PKPLSM--
-PPLSMY-
-PPLSMV-
-PPLSMR-
-PPLSMI-
--PLSMYQ
--PLSMQN
--PLSMMM
--PLSMFN
--PLSMEY
--PLSMEV
--PLSMEM
--PLSMEG
--PLSMEF
--PLSMCS
--PLSMAH
PEPLSM--
PDPLSM--
PCPLSM--
-QPLSMR-
QGPLSM--
QAPLSM--
QYPLSM--
QSPLSM--
-QPLSMT-
-QPLSMS-
-QPLSMK-
-QPLSMF-
-QPLSMA-
QNPLSM--
QRPLSM--
QFPLSM--
-QPLSMN-
-QPLSMI-
-QPLSML-
-QPLSMH-

-QPLSMW-
QPPLSM--
QMPLSM--
QLPLSM--
QTPLSM--
QHPLSM--
QWPLSM--
-QPLSMV-
QEPLSM--
QDPLSM--
-QPLSMY-
-QPLSMM-
QKPLSM--
QQPLSM--
-RPLSML-
RVPLSM--
RLPLSM--
-RPLSMT-
RMPLSM--
RGPLSM--
RRPLSM--
-RPLSMA-
RNPLSM--
-RPLSMQ-
-RPLSMG-
-RPLSMY-
RTPLSM--
-RPLSMV-
-RPLSMM-
RYPLSM--
RAPLSM--
RPPLSM--
-RPLSMK-
-RPLSMH-
RQPLSM--
REPLSM--
-RPLSMW-
RKPLSM--
RFPLSM--
RDPLSM--
-RPLSME-
RHPLSM--
-RPLSMF-
RCPLSM--
-SPLSMK-
-SPLSML-
SVPLSM--
-SPLSMT-
-SPLSMS-
SRPLSM--

-SPLSMY-
SNPLSM--
SSPLSM--
STPLSM--
SLPLSM--
SFPLSM--
-SPLSMA-
SKPLSM--
SIPLSM--
-SPLSMV-
-SPLSMF-
SGPLSM--
SQPLSM--
SAPLSM--
-SPLSMH-
SDPLSM--
SMPLSM--
SHPLSM--
SYPLSM--
SWPLSM--
SEPLSM--
-SPLSME-
-SPLSMP-
SCPLSM--
-TPLSMR-
TRPLSM--
-TPLSMV-
-TPLSMS-
-TPLSMT-
-TPLSMF-
TSPLSM--
-TPLSMH-
TIPLSM--
TVPLSM--
TNPLSM--
THPLSM--
TTPLSM--
-TPLSMK-
TFPLSM--
-TPLSMW-
-TPLSMY-
-TPLSMQ-
TMPLSM--
TGPLSM--
TYPLSM--
TLPLSM--
TWPLSM--
TKPLSM--
TAPLSM--
TPPLSM--

TQPLSM--
-TPLSMA-
-TPLSME-
-TPLSMM-
TDPLSM--
-TPLSMI-
TEPLSM--
VGPLSM--
VSPLSM--
VTPLSM--
-VPLSML-
VLPLSM--
-VPLSMS-
VAPLSM--
VVPLSM--
VKPLSM--
VIPLSM--
-VPLSMQ-
-VPLSMK-
VNPLSM--
VQPLSM--
-VPLSMI-
-VPLSMW-
VMPLSM--
-VPLSMV-
-VPLSME-
VFPLSM--
-VPLSMN-
VDPLSM--
VWPLSM--
VEPLSM--
VPPLSM--
VYPLSM--
-VPLSMM-
-VPLSMA-
VCPLSM--
-WPLSMS-
WAPLSM--
-WPLSMR-
WPPLSM--
-WPLSMH-
WVPLSM--
WRPLSM--
-WPLSMV-
WDPLSM--
-WPLSMT-
-WPLSMK-
WKPLSM--
-WPLSMQ-
WMPLSM--

WGPLSM--
WNPLSM--
WYPLSM--
-WPLSMF-
WQPLSM--
-WPLSMW-
-WPLSMN-
-WPLSMM-
-WPLSMI-
WEPLSM--
WSPLSM--
-WPLSME-
WLPLSM--
WFPLSM--
WCPLSM--
WHPLSM--
YVPLSM--
YRPLSM--
-YPLSMR-
-YPLSMS-
YAPLSM--
YKPLSM--
-YPLSMK-
-YPLSMW-
-YPLSMH-
YDPLSM--
YPPLSM--
-YPLSMY-
YGPLSM--
-YPLSMN-
-YPLSMI-
-YPLSMG-
-YPLSMF-
-YPLSME-
YFPLSM--
-YPLSMQ-
YNPLSM--
-YPLSMV-
-YPLSMT-
-YPLSMA-
YHPLSM--
YQPLSM--
-YPLSMM-
YMPLSM--

PRSM 412 629 32.67

-NPRSMG-
--PRSMGN
--PRSMDS
YAPRSM--
ALPRSM--

-APRSMM-
-APRSMS-
-APRSMQ-
ANPRSM--
AAPRSM--
AVPRSM--
-APRSMH-
ADPRSM--
-APRSMI-
-APRSMA-
-APRSMG-
APPRSM--
AIPRSM--
AGPRSM--
-APRSMY-
-APRSMW-
-APRSMK-
-APRSMF-
AEPRSM--
AQPRSM--
-APRSMN-
-APRSMD-
AHPRSM--
-APRSME-
-CPRSMH-
CGPRSM--
CAPRSM--
CVPRSM--
CSPRSM--
-CPRSMR-
CKPRSM--
CNPRSM--
-CPRSMS-
-CPRSML-
-CPRSMA-
CTPRSM--
CRPRSM--
CQPRSM--
CPPRSM--
CIPRSM--
CDPRSM--
-DPRSMT-
-DPRSMR-
-DPRSMS-
-DPRSML-
DMPRSM--
DRPRSM--
-DPRSMI-
DVPRSM--
DNPRSM--

DIPRSM--
DTPRSM--
-DPRSMN-
-DPRSMH-
DFPRSM--
DQPRSM--
-DPRSMK-
DYPRSM--
-DPRSMW-
-DPRSMM-
DGPRSM--
DAPRSM--
DWPRSM--
-DPRSMG-
-DPRSMF-
-DPRSMA-
DKPRSM--
-EPRSMY-
-EPRSMK-
-EPRSMM-
-EPRSMH-
EKPRSM--
-EPRSMQ-
-EPRSMF-
-EPRSMS-
-EPRSMW-
EVPRSM--
ETPRSM--
-EPRSME-
ELPRSM--
-EPRSMN-
ESPRSM--
EQPRSM--
-EPRSMA-
EAPRSM--
EGPRSM--
EFPRSM--
-EPRSMT-
EHPRSM--
EYPRSM--
-EPRSMV-
EMPRSM--
EIPRSM--
EWPRSM--
-EPRSMG-
-EPRSMD-
EPPRSM--
ENPRSM--
EEPRSM--
EDPRSM--

FNPRSM--
FGPRSM--
FKPRSM--
FDPRSM--
-FPRSMH-
FHPRSM--
FVPRSM--
FAPRSM--
FSPRSM--
FIPRSM--
FQPRSM--
FMPRSM--
-FPRSME-
FEPRSM--
FCPRSM--
-GPRSML-
-GPRSMR-
-GPRSMS-
-GPRSMG-
-GPRSMV-
-GPRSMT-
-GPRSMY-
-GPRSMQ-
GKPRSM--
GTPRSM--
GVPRSM--
-GPRSMM-
GGPRSM--
GCPRSM--
GSPRSM--
GFPRSM--
GEPRSM--
GPPRSM--
-GPRSMW-
-GPRSMK-
-GPRSME-
-GPRSMH-
-GPRSMF-
GAPRSM--
-GPRSMN-
GDPRSM--
GQPRSM--
-HPRSMS-
-HPRSMK-
-HPRSML-
-HPRSMN-
HLPRSM--
-HPRSMI-
HVPRSM--
HSPRSM--

HTPRSM--
HNPRSM--
-HPRSMA-
HMPRSM--
HGPRSM--
HQPRSM--
-HPRSMQ-
-HPRSME-
HPPRSM--
HDPRSM--
-HPRSMY-
-HPRSMV-
-HPRSMF-
HKPRSM--
HFPRSM--
HWPRSM--
HHPRSM--
IPPRSM--
IAPRSM--
-IPRSMA-
ILPRSM--
-IPRSMV-
INPRSM--
-IPRSMQ-
IMPRSM--
-IPRSMS-
-IPRSMH-
-IPRSMW-
-IPRSMN-
-IPRSML-
IGPRSM--
-IPRSME-
-IPRSMY-
-IPRSMG-
IKPRSM--
IDPRSM--
IEPRSM--
-IPRSMM-
-IPRSMF-
-KPRSMI-
-KPRSMA-
KQPRSM--
-KPRSMF-
KTPRSM--
-KPRSMW-
KNPRSM--
-KPRSMY-
KMPRSM--
KGPRSM--
-KPRSMN-

KEPRSM--
-KPRSMV-
-KPRSMG-
KRPRSM--
KVPRSM--
-KPRSMM-
-KPRSMQ-
KDPRSM--
KCPRSM--
-LPRSMT-
LYPRSM--
LIPRSM--
-LPRSMW-
-LPRSMF-
LPPRSM--
LLPRSM--
-LPRSMQ-
LMPRSM--
LAPRSM--
LNPRSM--
LEPRSM--
LDPRSM--
-LPRSMN-
LQPRSM--
LKPRSM--
-LPRSMC-
LCPRSM--
-MPRSML-
-MPRSMI-
MSPRSM--
MYPRSM--
-MPRSMS-
-MPRSMK-
MIPRSM--
-MPRSMN-
MAPRSM--
MVPRSM--
MTPRSM--
-MPRSMV-
-MPRSMG-
-MPRSMF-
-MPRSMM-
-MPRSMT-
MNPRSM--
MKPRSM--
MQPRSM--
MPPRSM--
MCPRSM--
MWPRSM--
-MPRSME-

MFPRSM--
NVPRSM--
NSPRSM--
-NPRSMH-
NMPRSM--
NLPRSM--
-NPRSMM-
-NPRSMS-
-NPRSMI-
-NPRSMY-
-NPRSMK-
NPPRSM--
-NPRSMW-
NNPRSM--
-NPRSMV-
-NPRSMQ-
NTPRSM--
NGPRSM--
NHPRSM--
NDPRSM--
-NPRSMA-
NQPRSM--
NEPRSM--
-NPRSMN-
NIPRSM--
NCPRSM--
--PRSMTL
--PRSMTG
--PRSMMS
--PRSMLT
--PRSMLN
--PRSMML
--PRSMIT
--PRSMNS
--PRSMYQ
--PRSMVN
--PRSMYG
--PRSMYV
--PRSMNA
--PRSMLH
--PRSMGS
--PRSMAV
--PRSMTT
PVPRSM--
--PRSMTA
--PRSMSH
--PRSMSG
--PRSMIS
--PRSMIK
--PRSMHS

--PRSMFY
--PRSMYT
--PRSMYN
--PRSMLA
--PRSMSV
--PRSMNL
--PRSMSA
--PRSMNT
--PRSMVI
--PRSMQG
--PRSMKN
--PRSMEW
--PRSMYM
--PRSMQA
--PRSMLM
--PRSMFT
--PRSMVF
--PRSMVE
--PRSMLV
--PRSMKA
--PRSMIG
--PRSMHM
--PRSMAY
--PRSMAG
--PRSMWW
--PRSMWV
--PRSMMA
--PRSMID
--PRSMGT
--PRSMFG
--PRSMFA
--PRSMTD
--PRSMQV
--PRSMQK
--PRSMNM
--PRSMMV
--PRSMLQ
--PRSMLF
--PRSMHY
--PRSMHL
PRPRSM--
PSPRSM--
--PRSMRM
--PRSMMG
--PRSMIM
--PRSMET
--PRSMAI
--PRSMWQ
--PRSMWG
--PRSMWF

--PRSMTN
--PRSMSF
--PRSMMN
--PRSMLE
--PRSMFE
--PRSMAW
--PRSMWN
--PRSMSN
--PRSMQM
--PRSMKM
--PRSMKI
--PRSMIF
--PRSMGG
PEPRSM--
--PRSMYK
--PRSMYE
--PRSMTI
--PRSMTE
--PRSMSD
--PRSMNG
--PRSMKK
--PRSMKF
--PRSMHI
--PRSMFH
--PRSMES
--PRSMAN
PWPRSM--
--PRSMYY
--PRSMTW
--PRSMTQ
--PRSMQS
--PRSMPL
--PRSMNW
--PRSMNV
--PRSMNI
--PRSMIH
--PRSMHN
--PRSMAT
--PRSMAF
PQPRSM--
PTPRSM--
--PRSMQN
--PRSMNF
--PRSMMM
--PRSMMD
--PRSMLD
--PRSMIV
--PRSMIA
--PRSMHG
--PRSMHD

--PRSMGV
--PRSMFI
--PRSMEV
--PRSMAQ
PGPRSM--
--PRSMYD
--PRSMTH
--PRSMNR
--PRSMMQ
--PRSMMK
--PRSMMI
--PRSMKQ
--PRSMHA
--PRSMGW
--PRSMGA
--PRSMEH
--PRSMEG
--PRSMDA
--PRSMAA
PKPRSM--
PIPRSM--
--PRSMWI
--PRSMSE
--PRSMQT
--PRSMNH
--PRSMMT
--PRSMKE
--PRSMKD
--PRSMHV
--PRSMFF
--PRSMER
--PRSMEK
--PRSMEA
--PRSMAH
--PRSMAD
PHPRSM--
PCPRSM--
--PRSMYF
--PRSMWM
--PRSMWH
--PRSMQW
--PRSMQF
--PRSMNQ
--PRSMNN
--PRSMIN
--PRSMHQ
--PRSMHK
--PRSMHH
--PRSMHE
--PRSMGI

--PRSMFD
--PRSMEN
--PRSMEF
--PRSMCV
--PRSMCD
PNPRSM--
--PRSMTM
--PRSMQQ
--PRSMQI
--PRSMNY
--PRSMNK
--PRSMND
--PRSMMW
--PRSMIE
--PRSMHW
--PRSMHP
--PRSMGQ
--PRSMEM
--PRSMDW
--PRSMDG
--PRSMAC
-PPRSMV-
-PPRSMI-
-QPRSMK-
-QPRSMS-
QTPRSM--
-QPRSMW-
QSPRSM--
QYPRSM--
QVPRSM--
-QPRSMA-
QIPRSM--
-QPRSMM-
-QPRSMI-
QGPRSM--
-QPRSMH-
QNPRSM--
-QPRSMV-
-QPRSMY-
-QPRSMT-
-QPRSMF-
QAPRSM--
-QPRSMQ-
-QPRSME-
QFPRSM--
-QPRSMN-
QPPRSM--
QMPRSM--
-QPRSMG-
QEPRSM--

QQPRSM--
QDPRSM--
RTPRSM--
-RPRSMT-
-RPRSMH-
-RPRSMQ-
-RPRSMY-
-RPRSMI-
-RPRSMF-
-RPRSME-
RQPRSM--
RPPRSM--
RAPRSM--
-RPRSMW-
RCPRSM--
RRPRSM--
RDPRSM--
SLPRSM--
SIPRSM--
SNPRSM--
-SPRSMW-
SYPRSM--
SVPRSM--
-SPRSMY-
-SPRSMV-
-SPRSMI-
-SPRSMA-
SQPRSM--
SKPRSM--
SGPRSM--
-SPRSMQ-
-SPRSMF-
-SPRSME-
-SPRSMM-
SDPRSM--
STPRSM--
SPPRSM--
SCPRSM--
TVPRSM--
-TPRSMF-
-TPRSMH-
TLPRSM--
TFPRSM--
-TPRSMV-
TYPRSM--
-TPRSMW-
-TPRSMY-
TAPRSM--
-TPRSMM-
-TPRSMK-

TGPRSM--
TEPRSM--
TKPRSM--
-TPRSMN-
TPPRSM--
-TPRSMG-
THPRSM--
-TPRSMQ-
-TPRSME-
TQPRSM--
TCPRSM--
-VPRSML-
VMPRSM--
VGPRSM--
VSPRSM--
-VPRSMA-
-VPRSMI-
-VPRSMV-
-VPRSMG-
VNPRSM--
-VPRSMM-
VAPRSM--
-VPRSMT-
-VPRSMN-
-VPRSMY-
VPPRSM--
-VPRSME-
-VPRSMW-
-VPRSMQ-
VHPRSM--
VEPRSM--
VDPRSM--
-VPRSMF-
VCPRSM--
WIPRSM--
WLPRSM--
-WPRSMY-
-WPRSME-
WMPRSM--
WGPRSM--
WPPRSM--
WYPRSM--
-WPRSMN-
-WPRSMM-
WHPRSM--
-WPRSMV-
WEPRSM--
WQPRSM--
-WPRSMH-
-WPRSMF-

-WPRSMW-
WAPRSM--
WDPRSM--
-YPRSMS-
YYPRSM--
YVPRSM--
-YPRSMV-
YTPRSM--
-YPRSMN-
-YPRSMI-
YMPRSM--
YKPRSM--
-YPRSMY-
YNPRSM--
YQPRSM--
YGPRSM--
-YPRSMF-
YHPRSM--
-YPRSME-
YIPRSM--
YEPRSM--
YPPRSM--
-YPRSMM-
YDPRSM--

| | | | |
|---|---|---|---|
| MAAL | 264 | 685 | 32.66 |

--MAALGN
-SMAALG-
-VMAALD-
-AMAALT-
AGMAAL--
-AMAALI-
AFMAAL--
AHMAAL--
ANMAAL--
-AMAALS-
AKMAAL--
AVMAAL--
APMAAL--
-AMAALV-
AMMAAL--
AEMAAL--
AYMAAL--
-AMAALQ-
-AMAALK-
-AMAALG-
-AMAALF-
AQMAAL--
-AMAALE-
ACMAAL--
AAMAAL--

-AMAALM-
-AMAALH-
CTMAAL--
-CMAALT-
CVMAAL--
-CMAALQ-
-CMAALK-
-CMAALY-
-CMAALS-
-CMAALI-
-CMAALA-
CIMAAL--
CYMAAL--
CSMAAL--
-CMAALN-
-CMAALL-
CGMAAL--
CAMAAL--
CPMAAL--
-CMAALV-
CKMAAL--
CEMAAL--
DRMAAL--
DGMAAL--
-DMAALT-
DTMAAL--
-DMAALL-
DWMAAL--
-DMAALR-
DIMAAL--
DHMAAL--
DFMAAL--
DDMAAL--
DNMAAL--
DMMAAL--
-DMAALS-
-DMAALA-
-DMAALI-
-DMAALH-
-DMAALF-
DYMAAL--
DVMAAL--
DQMAAL--
-DMAALW-
-DMAALV-
-DMAALG-
-DMAALE-
DKMAAL--
DEMAAL--
DAMAAL--

ELMAAL--
ENMAAL--
-EMAALL-
EPMAAL--
ESMAAL--
-EMAALV-
-EMAALR-
ERMAAL--
EVMAAL--
EQMAAL--
-EMAALS-
EHMAAL--
-EMAALI-
-EMAALH-
EFMAAL--
EEMAAL--
EDMAAL--
EAMAAL--
EMMAAL--
EKMAAL--
EYMAAL--
ETMAAL--
-EMAALQ-
-EMAALN-
-EMAALE-
EGMAAL--
FHMAAL--
-FMAALR-
FSMAAL--
-FMAALT-
-FMAALA-
-FMAALS-
-FMAALN-
FNMAAL--
-FMAALK-
FKMAAL--
-FMAALI-
-FMAALG-
-FMAALH-
FGMAAL--
FDMAAL--
FQMAAL--
FAMAAL--
FPMAAL--
-FMAALL-
-FMAALE-
FCMAAL--
-GMAALR-
GSMAAL--
-GMAALT-

-GMAALS-
-GMAALK-
-GMAALW-
GYMAAL--
-GMAALN-
-GMAALY-
-GMAALL-
GLMAAL--
GIMAAL--
-GMAALA-
GVMAAL--
GHMAAL--
GKMAAL--
GGMAAL--
-GMAALQ-
GWMAAL--
-GMAALM-
GTMAAL--
-GMAALH-
-GMAALG-
GFMAAL--
GDMAAL--
-GMAALI-
-GMAALF-
GCMAAL--
GQMAAL--
-GMAALV-
GAMAAL--
GPMAAL--
GMMAAL--
-GMAALE-
-HMAALR-
-HMAALT-
HTMAAL--
HPMAAL--
HNMAAL--
-HMAALH-
-HMAALY-
-HMAALS-
-HMAALA-
-HMAALQ-
HDMAAL--
HQMAAL--
-HMAALK-
-HMAALE-
HVMAAL--
HMMAAL--
-HMAALL-
-HMAALN-
-HMAALM-

```
-HMAALG-
-HMAALF-
HHMAAL--
HFMAAL--
-HMAALW-
-HMAALI-
HKMAAL--
HIMAAL--
HAMAAL--
-IMAALS-
-IMAALG-
IAMAAL--
ISMAAL--
IPMAAL--
IDMAAL--
INMAAL--
IGMAAL--
-IMAALR-
-IMAALE-
-IMAALV-
-IMAALQ-
IQMAAL--
-IMAALY-
-IMAALN-
-IMAALA-
IHMAAL--
IEMAAL--
ICMAAL--
-IMAALM-
KQMAAL--
-KMAALH-
-KMAALT-
KGMAAL--
KFMAAL--
KWMAAL--
KPMAAL--
KMMAAL--
-KMAALN-
-KMAALQ-
-KMAALS-
KTMAAL--
KIMAAL--
-KMAALY-
-KMAALI-
KYMAAL--
KVMAAL--
KDMAAL--
-KMAALP-
-KMAALM-
-KMAALA-
```

KHMAAL--
KEMAAL--
KCMAAL--
-LMAALR-
LDMAAL--
LGMAAL--
LYMAAL--
-LMAALI-
LKMAAL--
-LMAALQ-
-LMAALG-
LPMAAL--
-LMAALK-
LQMAAL--
-LMAALP-
-LMAALM-
-LMAALA-
LEMAAL--
LAMAAL--
-LMAALV-
-LMAALH-
-LMAALD-
-LMAALE-
LCMAAL--
--MAALRS
--MAALYG
--MAALLS
--MAALFS
--MAALVY
--MAALRG
--MAALIR
--MAALSL
--MAALNL
--MAALVK
--MAALRY
--MAALRA
MSMAAL--
-MMAALR-
MWMAAL--
--MAALTT
--MAALRT
--MAALSG
--MAALRF
--MAALMK
--MAALLQ
--MAALLG
--MAALKN
--MAALAG
-MMAALL-
--MAALVS

--MAALVR
--MAALTG
-MMAALN-
-MMAALH-
--MAALVN
--MAALTW
--MAALSA
--MAALRK
--MAALMI
--MAALLR
--MAALKG
--MAALAS
--MAALWS
--MAALST
--MAALSK
--MAALSH
--MAALKR
--MAALYS
--MAALNS
--MAALGW
--MAALGS
--MAALVV
--MAALTV
--MAALTS
--MAALSN
--MAALSM
--MAALMT
MYMAAL--
--MAALYQ
--MAALWA
--MAALTN
--MAALTL
--MAALTF
--MAALSV
--MAALRQ
--MAALRE
--MAALMN
--MAALKI
--MAALHR
--MAALHI
--MAALGM
--MAALYD
--MAALVM
--MAALVD
--MAALRV
--MAALQR
--MAALKT
--MAALKK
--MAALIG
--MAALHS

--MAALHK
--MAALHE
--MAALAF
-MMAALA-
--MAALWG
--MAALTA
--MAALRD
--MAALQG
--MAALPL
--MAALNA
--MAALMF
--MAALKY
--MAALFA
--MAALAW
--MAALWR
--MAALQN
--MAALMV
--MAALLN
--MAALKA
--MAALHT
--MAALHA
--MAALFT
-MMAALS-
--MAALYE
--MAALWM
--MAALPW
--MAALNT
--MAALLI
--MAALLD
--MAALKS
--MAALKQ
--MAALIH
--MAALHN
--MAALHG
--MAALAA
MNMAAL--
-MMAALK-
MHMAAL--
--MAALYT
--MAALYA
--MAALWQ
--MAALVE
--MAALRM
--MAALQT
--MAALPY
--MAALPI
--MAALNF
--MAALMY
--MAALMS
--MAALMM

--MAALMA
--MAALIQ
--MAALIN
--MAALHW
--MAALFN
--MAALFD
--MAALAV
--MAALAH
MPMAAL--
-MMAALG-
MEMAAL--
MAMAAL--
--MAALVW
--MAALQL
--MAALQF
--MAALNR
--MAALNQ
--MAALMR
--MAALMH
--MAALMG
--MAALME
--MAALLW
--MAALLL
--MAALKW
--MAALHD
--MAALGV
--MAALGT
--MAALFE
--MAALAN
-MMAALV-
-MMAALM-
MGMAAL--
--MAALWK
--MAALTY
--MAALTQ
--MAALTK
--MAALTD
--MAALSF
--MAALQY
--MAALQV
--MAALQA
--MAALNI
--MAALLH
--MAALLE
--MAALKV
--MAALKE
--MAALIE
--MAALIA
--MAALHM
--MAALGQ

--MAALGI
--MAALGG
--MAALGA
--MAALFG
--MAALEA
--MAALAT
--MAALAI
MQMAAL--
-MMAALT-
-MMAALI-
--MAALYI
--MAALYH
--MAALYC
--MAALWY
--MAALWI
--MAALWH
--MAALWF
--MAALVQ
--MAALVH
--MAALTM
--MAALTI
--MAALTH
--MAALTE
--MAALSQ
--MAALSP
--MAALQQ
--MAALQM
--MAALQI
--MAALQH
--MAALQE
--MAALPV
--MAALPA
--MAALNK
--MAALMQ
--MAALMD
--MAALKM
--MAALKH
--MAALKD
--MAALIK
--MAALHV
--MAALHH
--MAALHF
--MAALGC
--MAALFQ
--MAALET
--MAALCS
NKMAAL--
-NMAALQ-
-NMAALS-
NSMAAL--

-NMAALW-
-NMAALN-
NLMAAL--
-NMAALT-
-NMAALK-
-NMAALF-
NNMAAL--
-NMAALA-
NWMAAL--
-NMAALI-
-NMAALH-
-NMAALR-
NIMAAL--
NHMAAL--
NGMAAL--
NFMAAL--
NTMAAL--
NQMAAL--
NMMAAL--
-NMAALM-
-NMAALG-
-NMAALE-
-NMAALC-
NCMAAL--
NAMAAL--
-PMAALS-
PRMAAL--
PGMAAL--
-PMAALR-
PSMAAL--
-PMAALH-
-PMAALN-
PEMAAL--
-PMAALV-
-PMAALL-
PIMAAL--
PTMAAL--
-PMAALF-
-PMAALT-
-PMAALM-
PVMAAL--
-PMAALW-
-PMAALQ-
-PMAALA-
PAMAAL--
-PMAALK-
PLMAAL--
PPMAAL--
PFMAAL--
PDMAAL--

QFMAAL--
-QMAALN-
-QMAALA-
QGMAAL--
-QMAALM-
-QMAALQ-
-QMAALV-
-QMAALK-
-QMAALL-
QIMAAL--
QPMAAL--
-QMAALR-
QLMAAL--
QTMAAL--
QWMAAL--
QSMAAL--
-QMAALG-
-QMAALT-
-QMAALH-
QNMAAL--
-QMAALS-
-QMAALI-
QDMAAL--
QYMAAL--
QVMAAL--
QRMAAL--
QMMAAL--
QHMAAL--
RLMAAL--
RGMAAL--
-RMAALV-
RNMAAL--
RFMAAL--
RPMAAL--
-RMAALY-
RWMAAL--
REMAAL--
RMMAAL--
RCMAAL--
SRMAAL--
-SMAALS-
-SMAALL-
SGMAAL--
-SMAALR-
SSMAAL--
-SMAALN-
SFMAAL--
SAMAAL--
SNMAAL--
SKMAAL--

SYMAAL--
-SMAALY-
-SMAALK-
-SMAALA-
SHMAAL--
SQMAAL--
-SMAALH-
SPMAAL--
-SMAALT-
SCMAAL--
-SMAALW-
-SMAALQ-
SWMAAL--
SMMAAL--
-SMAALV-
-SMAALF-
SEMAAL--
SDMAAL--
TGMAAL--
TNMAAL--
-TMAALI-
TVMAAL--
TKMAAL--
-TMAALV-
-TMAALR-
-TMAALT-
TQMAAL--
-TMAALH-
-TMAALG-
TLMAAL--
THMAAL--
TYMAAL--
TSMAAL--
-TMAALW-
-TMAALK-
-TMAALQ-
TEMAAL--
TMMAAL--
TPMAAL--
-TMAALF-
TFMAAL--
TCMAAL--
VSMAAL--
-VMAALS-
-VMAALG-
VAMAAL--
-VMAALA-
VHMAAL--
-VMAALV-
-VMAALQ-

```
-VMAALL-
VGMAAL--
VPMAAL--
VNMAAL--
-VMAALH-
-VMAALT-
VEMAAL--
VQMAAL--
-VMAALN-
-VMAALI-
-VMAALE-
-VMAALM-
-VMAALC-
VCMAAL--
-WMAALR-
WTMAAL--
-WMAALL-
WEMAAL--
WNMAAL--
-WMAALS-
-WMAALQ-
WRMAAL--
WAMAAL--
WQMAAL--
WPMAAL--
-WMAALK-
-WMAALG-
-WMAALE-
WFMAAL--
-WMAALT-
WYMAAL--
WWMAAL--
-WMAALH-
WHMAAL--
WGMAAL--
WDMAAL--
YSMAAL--
-YMAALL-
-YMAALG-
YWMAAL--
YKMAAL--
-YMAALS-
YGMAAL--
YHMAAL--
-YMAALH-
YFMAAL--
YMMAAL--
-YMAALR-
-YMAALN-
-YMAALK-
```

YEMAAL--
YNMAAL--
YLMAAL--
YTMAAL--
YPMAAL--
-YMAALV-
-YMAALQ-
-YMAALM-
-YMAALF-
YAMAAL--

| | | | |
|---|---|---|---|
| PTPL | 54 | 800 | 32.63 |

-GPTPLN-
--PTPLGR
--PTPLTE
--PTPLPS
--PTPLGT
--PTPLAL
-APTPLL-
AWPTPL--
ARPTPL--
-APTPLW-
-APTPLT-
-APTPLQ-
AGPTPL--
AYPTPL--
AQPTPL--
-APTPLR-
ANPTPL--
AAPTPL--
AVPTPL--
ATPTPL--
-APTPLM-
AIPTPL--
AHPTPL--
-APTPLY-
AKPTPL--
AFPTPL--
APPTPL--
ASPTPL--
-APTPLI-
ADPTPL--
-APTPLK-
-APTPLH-
-APTPLG-
AMPTPL--
AEPTPL--
ACPTPL--
CTPTPL--
CRPTPL--
CEPTPL--

CQPTPL--
CSPTPL--
-CPTPLA-
CLPTPL--
CVPTPL--
-CPTPLY-
-CPTPLF-
CPPTPL--
CMPTPL--
CIPTPL--
CDPTPL--
DSPTPL--
DAPTPL--
DMPTPL--
DYPTPL--
-DPTPLH-
DLPTPL--
DRPTPL--
-DPTPLT-
-DPTPLR-
-DPTPLL-
DTPTPL--
DQPTPL--
-DPTPLY-
-DPTPLW-
DNPTPL--
DKPTPL--
DHPTPL--
DFPTPL--
DWPTPL--
DVPTPL--
-DPTPLQ-
-DPTPLK-
-DPTPLI-
-DPTPLF-
DIPTPL--
DDPTPL--
DCPTPL--
-EPTPLW-
-EPTPLY-
ESPTPL--
EYPTPL--
ERPTPL--
-EPTPLL-
ELPTPL--
ETPTPL--
-EPTPLV-
-EPTPLR-
EQPTPL--
-EPTPLS-

EIPTPL--
EHPTPL--
ENPTPL--
EFPTPL--
EVPTPL--
-EPTPLK-
-EPTPLI-
EMPTPL--
-EPTPLT-
-EPTPLQ-
-EPTPLN-
-EPTPLM-
-EPTPLH-
-EPTPLF-
EKPTPL--
EAPTPL--
-FPTPLR-
-FPTPLS-
-FPTPLL-
FSPTPL--
FMPTPL--
-FPTPLW-
FPPTPL--
FNPTPL--
FIPTPL--
-FPTPLH-
FKPTPL--
FYPTPL--
FVPTPL--
FTPTPL--
-FPTPLT-
FEPTPL--
-FPTPLF-
FLPTPL--
FGPTPL--
FFPTPL--
FWPTPL--
FRPTPL--
-FPTPLK-
FHPTPL--
-FPTPLV-
-FPTPLI-
GRPTPL--
GMPTPL--
GKPTPL--
-GPTPLR-
-GPTPLL-
GWPTPL--
GFPTPL--
GSPTPL--

GVPTPL--
GAPTPL--
GTPTPL--
GQPTPL--
-GPTPLV-
-GPTPLS-
-GPTPLW-
-GPTPLT-
-GPTPLM-
GIPTPL--
-GPTPLY-
GLPTPL--
GHPTPL--
-GPTPLQ-
-GPTPLH-
-GPTPLA-
GYPTPL--
-GPTPLI-
GGPTPL--
-GPTPLK-
-GPTPLC-
GPPTPL--
GNPTPL--
GEPTPL--
GDPTPL--
-HPTPLR-
-HPTPLT-
-HPTPLA-
HRPTPL--
-HPTPLS-
HLPTPL--
-HPTPLI-
HSPTPL--
HFPTPL--
HWPTPL--
HNPTPL--
HMPTPL--
HAPTPL--
-HPTPLL-
-HPTPLH-
HHPTPL--
HYPTPL--
HQPTPL--
-HPTPLQ-
HIPTPL--
HDPTPL--
-HPTPLY-
-HPTPLW-
-HPTPLV-
-HPTPLN-

HKPTPL--
HVPTPL--
HPPTPL--
HGPTPL--
ILPTPL--
IRPTPL--
-IPTPLN-
-IPTPLL-
-IPTPLF-
IFPTPL--
IWPTPL--
-IPTPLI-
-IPTPLR-
ITPTPL--
-IPTPLT-
-IPTPLS-
-IPTPLY-
-IPTPLM-
IEPTPL--
IAPTPL--
IYPTPL--
-IPTPLA-
IIPTPL--
IHPTPL--
IGPTPL--
ICPTPL--
IQPTPL--
-IPTPLK-
INPTPL--
IDPTPL--
-IPTPLW-
-IPTPLV-
-IPTPLH-
IKPTPL--
KSPTPL--
-KPTPLT-
-KPTPLF-
KFPTPL--
KVPTPL--
KYPTPL--
-KPTPLY-
KAPTPL--
-KPTPLM-
KMPTPL--
KLPTPL--
-KPTPLS-
-KPTPLH-
KPPTPL--
-KPTPLV-
-KPTPLL-

-KPTPLW-
-KPTPLK-
-KPTPLI-
KQPTPL--
KKPTPL--
KEPTPL--
KTPTPL--
-KPTPLQ-
-KPTPLE-
KNPTPL--
KHPTPL--
KIPTPL--
KCPTPL--
LRPTPL--
-LPTPLR-
LVPTPL--
LLPTPL--
LYPTPL--
LSPTPL--
-LPTPLK-
LWPTPL--
-LPTPLT-
-LPTPLM-
LHPTPL--
LGPTPL--
LTPTPL--
-LPTPLV-
LMPTPL--
LIPTPL--
LEPTPL--
LDPTPL--
LAPTPL--
-LPTPLY-
LPPTPL--
LKPTPL--
-LPTPLN-
LFPTPL--
-LPTPLH-
LQPTPL--
-LPTPLQ-
-LPTPLA-
-LPTPLI-
LCPTPL--
-MPTPLR-
-MPTPLT-
MYPTPL--
MVPTPL--
MAPTPL--
MRPTPL--
-MPTPLS-

MTPTPL--
MKPTPL--
-MPTPLA-
MPPTPL--
-MPTPLL-
MLPTPL--
-MPTPLK-
MMPTPL--
MIPTPL--
MEPTPL--
MGPTPL--
MFPTPL--
MQPTPL--
-MPTPLW-
-MPTPLN-
MWPTPL--
-MPTPLV-
-MPTPLQ-
-MPTPLM-
-MPTPLG-
-MPTPLF-
MDPTPL--
NRPTPL--
NGPTPL--
NSPTPL--
NLPTPL--
-NPTPLL-
NYPTPL--
NAPTPL--
-NPTPLW-
NFPTPL--
NQPTPL--
NPPTPL--
NTPTPL--
-NPTPLM-
NCPTPL--
NVPTPL--
-NPTPLS-
-NPTPLK-
NWPTPL--
-NPTPLA-
NKPTPL--
NIPTPL--
NHPTPL--
-NPTPLY-
-NPTPLI-
-NPTPLH-
-NPTPLF-
NNPTPL--
--PTPLRL

--PTPLRS
--PTPLRN
--PTPLSS
--PTPLLL
--PTPLIR
--PTPLGH
--PTPLLS
--PTPLRY
--PTPLRA
--PTPLVS
--PTPLWT
--PTPLSL
--PTPLLT
--PTPLSH
--PTPLLN
--PTPLRH
--PTPLLR
--PTPLRV
--PTPLRT
--PTPLMR
--PTPLLK
--PTPLIT
--PTPLSV
--PTPLSA
--PTPLLQ
--PTPLYN
--PTPLWH
--PTPLLY
--PTPLLG
--PTPLKS
--PTPLYS
--PTPLWS
--PTPLWR
--PTPLWA
--PTPLTW
--PTPLRK
PVPTPL--
--PTPLYK
--PTPLVI
--PTPLNV
--PTPLMS
--PTPLML
--PTPLKV
--PTPLKG
--PTPLAG
PRPTPL--
PGPTPL--
--PTPLYM
--PTPLYI
--PTPLVV

--PTPLQW
--PTPLNL
--PTPLMY
--PTPLHS
--PTPLFT
--PTPLFR
--PTPLAW
--PTPLAV
--PTPLVY
--PTPLVG
--PTPLTS
--PTPLSG
--PTPLKA
--PTPLIH
--PTPLIF
--PTPLHA
--PTPLEL
PEPTPL--
--PTPLWV
--PTPLVR
--PTPLVM
--PTPLVF
--PTPLVA
--PTPLTK
--PTPLSM
--PTPLQN
--PTPLPY
--PTPLMF
--PTPLKY
--PTPLKM
--PTPLIS
--PTPLHT
--PTPLHH
--PTPLGA
--PTPLFI
--PTPLDL
--PTPLYV
--PTPLYG
--PTPLWL
--PTPLTT
--PTPLST
--PTPLSR
--PTPLRM
--PTPLLI
--PTPLKI
--PTPLIW
--PTPLIK
--PTPLIG
--PTPLHR
--PTPLHQ

--PTPLGV
--PTPLGQ
--PTPLFS
--PTPLAN
PMPTPL--
PLPTPL--
PKPTPL--
PIPTPL--
PYPTPL--
--PTPLYA
--PTPLVQ
--PTPLVK
--PTPLTM
--PTPLSK
--PTPLSF
--PTPLRI
--PTPLPF
--PTPLNY
--PTPLNF
--PTPLNA
--PTPLMI
--PTPLLF
--PTPLKW
--PTPLKR
--PTPLKQ
--PTPLHF
--PTPLGF
--PTPLFV
--PTPLDF
--PTPLAT
--PTPLAS
--PTPLAF
PSPTPL--
PAPTPL--
PWPTPL--
--PTPLWI
--PTPLVD
--PTPLTY
--PTPLTV
--PTPLTD
--PTPLQS
--PTPLQG
--PTPLPI
--PTPLNM
--PTPLMW
--PTPLIM
--PTPLIL
--PTPLHM
--PTPLGS
--PTPLFY

--PTPLDI
--PTPLAI
--PTPLAA
--PTPLVW
--PTPLVT
--PTPLVN
--PTPLVL
--PTPLVH
--PTPLTR
--PTPLTQ
--PTPLTI
--PTPLSI
--PTPLQA
--PTPLPQ
--PTPLNW
--PTPLNT
--PTPLNS
--PTPLNQ
--PTPLMN
--PTPLMK
--PTPLKK
--PTPLIY
--PTPLIV
--PTPLIN
--PTPLHE
--PTPLGN
--PTPLEY
--PTPLEG
--PTPLAQ
--PTPLAM
--PTPLYW
--PTPLYQ
--PTPLWM
--PTPLTN
--PTPLTG
--PTPLTA
--PTPLSN
--PTPLRF
--PTPLRE
--PTPLQQ
--PTPLQM
--PTPLQI
--PTPLPW
--PTPLPV
--PTPLNR
--PTPLNN
--PTPLNH
--PTPLMT
--PTPLMQ
--PTPLLW

--PTPLLE
--PTPLKE
--PTPLIA
--PTPLHV
--PTPLHG
--PTPLHD
--PTPLGW
--PTPLFQ
--PTPLFE
--PTPLES
--PTPLDW
--PTPLDQ
--PTPLAH
--PTPLYY
--PTPLYE
--PTPLWW
--PTPLWN
--PTPLWF
--PTPLWE
--PTPLWD
--PTPLVE
--PTPLSE
--PTPLRP
--PTPLQV
--PTPLQT
--PTPLQR
--PTPLQH
--PTPLQF
--PTPLPT
--PTPLPM
--PTPLPA
--PTPLME
--PTPLMD
--PTPLMA
--PTPLLM
--PTPLLD
--PTPLKH
--PTPLKF
--PTPLKD
--PTPLIQ
--PTPLID
--PTPLHW
--PTPLHI
--PTPLFN
--PTPLFD
--PTPLEW
--PTPLEV
--PTPLDY
-PPTPLY-
-PPTPLT-

-PPTPLR-
-PPTPLL-
-PPTPLI-
PFPTPL--
QRPTPL--
-QPTPLY-
-QPTPLR-
-QPTPLA-
-QPTPLW-
-QPTPLH-
QKPTPL--
QVPTPL--
QTPTPL--
QSPTPL--
-QPTPLT-
-QPTPLN-
-QPTPLL-
QMPTPL--
QIPTPL--
-QPTPLK-
-QPTPLI-
QYPTPL--
QNPTPL--
QEPTPL--
QAPTPL--
QWPTPL--
QQPTPL--
-QPTPLV-
-QPTPLM-
-QPTPLG-
-QPTPLE-
QPPTPL--
QHPTPL--
-RPTPLR-
-RPTPLL-
RMPTPL--
-RPTPLT-
RLPTPL--
RFPTPL--
REPTPL--
RGPTPL--
-RPTPLY-
-RPTPLM-
-RPTPLN-
-RPTPLA-
RAPTPL--
-RPTPLF-
RPPTPL--
RKPTPL--
RQPTPL--

−RPTPLV−
−RPTPLK−
−RPTPLW−
−RPTPLG−
RWPTPL−−
RCPTPL−−
RNPTPL−−
−RPTPLH−
−RPTPLE−
SRPTPL−−
SLPTPL−−
SVPTPL−−
−SPTPLV−
−SPTPLR−
SEPTPL−−
−SPTPLK−
SMPTPL−−
SYPTPL−−
−SPTPLW−
−SPTPLL−
−SPTPLI−
SNPTPL−−
SIPTPL−−
−SPTPLS−
−SPTPLF−
−SPTPLQ−
SFPTPL−−
SSPTPL−−
SQPTPL−−
SWPTPL−−
−SPTPLT−
SHPTPL−−
−SPTPLN−
SDPTPL−−
−SPTPLH−
−SPTPLA−
SPPTPL−−
−SPTPLY−
−SPTPLM−
SCPTPL−−
TLPTPL−−
TKPTPL−−
−TPTPLR−
−TPTPLL−
−TPTPLV−
TFPTPL−−
TVPTPL−−
TAPTPL−−
TTPTPL−−
−TPTPLM−

TMPTPL--
-TPTPLT-
-TPTPLK-
TGPTPL--
TDPTPL--
-TPTPLW-
-TPTPLN-
-TPTPLF-
-TPTPLI-
TYPTPL--
TWPTPL--
TQPTPL--
-TPTPLY-
-TPTPLA-
TEPTPL--
TNPTPL--
-TPTPLQ-
-TPTPLC-
TPPTPL--
TIPTPL--
THPTPL--
VRPTPL--
-VPTPLI-
-VPTPLH-
-VPTPLV-
-VPTPLL-
VSPTPL--
-VPTPLS-
VVPTPL--
VYPTPL--
VTPTPL--
VAPTPL--
-VPTPLW-
-VPTPLK-
VIPTPL--
VFPTPL--
VQPTPL--
-VPTPLY-
-VPTPLQ-
-VPTPLG-
VGPTPL--
VEPTPL--
VWPTPL--
VHPTPL--
-VPTPLA-
VNPTPL--
VMPTPL--
VDPTPL--
-VPTPLM-
VKPTPL--

VCPTPL--
-VPTPLE-
WLPTPL--
-WPTPLL-
WGPTPL--
WQPTPL--
WSPTPL--
WRPTPL--
WIPTPL--
WVPTPL--
WTPTPL--
-WPTPLA-
WFPTPL--
WEPTPL--
WAPTPL--
-WPTPLW-
-WPTPLS-
WPPTPL--
WYPTPL--
WWPTPL--
-WPTPLY-
-WPTPLV-
-WPTPLR-
WDPTPL--
-WPTPLQ-
-WPTPLK-
WMPTPL--
-YPTPLR-
YLPTPL--
YYPTPL--
YRPTPL--
-YPTPLS-
YSPTPL--
-YPTPLM-
-YPTPLT-
-YPTPLV-
YDPTPL--
YVPTPL--
YTPTPL--
YKPTPL--
YHPTPL--
-YPTPLL-
-YPTPLH-
YIPTPL--
YQPTPL--
YPPTPL--
YFPTPL--
-YPTPLA-
YNPTPL--
-YPTPLY-

-YPTPLK-
-YPTPLG-
YEPTPL--
YWPTPL--
-YPTPLQ-
-YPTPLN-
-YPTPLF-
YMPTPL--
YGPTPL--
YCPTPL--
YAPTPL--

PATL 74 783 32.62

-EPATLG-
-LPATLN-
-LPATLG-
--PATLQL
--PATLPA
--PATLNW
--PATLNG
--PATLGY
--PATLGK
--PATLGI
-TPATLG-
ASPATL--
AWPATL--
ARPATL--
-APATLK-
AVPATL--
-APATLT-
-APATLS-
-APATLR-
AAPATL--
-APATLG-
-APATLW-
AMPATL--
ALPATL--
-APATLI-
AIPATL--
-APATLH-
-APATLA-
AKPATL--
AHPATL--
-APATLY-
-APATLV-
-APATLN-
AFPATL--
-APATLF-
-APATLQ-
-APATLM-
-APATLE-

APPATL--
AEPATL--
ADPATL--
-APATLD-
ACPATL--
CRPATL--
CWPATL--
CSPATL--
CGPATL--
-CPATLR-
CLPATL--
CKPATL--
CCPATL--
-CPATLK-
CNPATL--
CIPATL--
CTPATL--
-CPATLT-
-CPATLL-
CDPATL--
CAPATL--
CYPATL--
-CPATLV-
-CPATLS-
-CPATLM-
-CPATLI-
-CPATLF-
-CPATLA-
CEPATL--
DWPATL--
DTPATL--
DYPATL--
DSPATL--
DNPATL--
-DPATLS-
-DPATLR-
-DPATLI-
-DPATLF-
DVPATL--
DIPATL--
DRPATL--
DKPATL--
DQPATL--
DFPATL--
-DPATLY-
-DPATLT-
-DPATLK-
DMPATL--
DLPATL--
-DPATLV-

```
-DPATLW-
-DPATLQ-
-DPATLN-
-DPATLM-
-DPATLH-
DDPATL--
DAPATL--
-EPATLR-
-EPATLW-
-EPATLT-
-EPATLL-
-EPATLH-
EAPATL--
EVPATL--
-EPATLF-
ELPATL--
EHPATL--
ERPATL--
-EPATLV-
-EPATLK-
EYPATL--
-EPATLS-
-EPATLI-
ETPATL--
EFPATL--
ESPATL--
-EPATLQ-
EKPATL--
EEPATL--
ECPATL--
-EPATLM-
EWPATL--
-EPATLN-
ENPATL--
EMPATL--
EIPATL--
EGPATL--
-EPATLA-
FLPATL--
-FPATLR-
FAPATL--
-FPATLY-
-FPATLW-
-FPATLV-
-FPATLS-
FGPATL--
-FPATLI-
FMPATL--
FFPATL--
-FPATLH-
```

FNPATL--
FHPATL--
FEPATL--
FYPATL--
FWPATL--
-FPATLM-
-FPATLA-
FPPATL--
FDPATL--
FTPATL--
-FPATLQ-
FKPATL--
-FPATLN-
-FPATLK-
FQPATL--
FCPATL--
GRPATL--
-GPATLM-
GGPATL--
-GPATLS-
-GPATLY-
-GPATLR-
GPPATL--
GYPATL--
-GPATLT-
GFPATL--
GWPATL--
-GPATLH-
GVPATL--
GMPATL--
GIPATL--
GQPATL--
-GPATLL-
GAPATL--
-GPATLI-
GHPATL--
GTPATL--
-GPATLQ-
GNPATL--
-GPATLV-
-GPATLE-
-GPATLK-
-GPATLG-
-GPATLF-
GEPATL--
-GPATLN-
-GPATLW-
-GPATLA-
GCPATL--
HRPATL--

-HPATLR-
-HPATLK-
HLPATL--
-HPATLL-
HIPATL--
HHPATL--
HGPATL--
HVPATL--
HSPATL--
HAPATL--
HYPATL--
-HPATLM-
-HPATLT-
HNPATL--
HKPATL--
HFPATL--
HCPATL--
HTPATL--
-HPATLW-
-HPATLV-
HMPATL--
HEPATL--
HWPATL--
-HPATLY-
-HPATLS-
-HPATLH-
-HPATLA-
-HPATLQ-
HPPATL--
-HPATLI-
-HPATLG-
-HPATLE-
ISPATL--
IRPATL--
IVPATL--
IWPATL--
ITPATL--
IQPATL--
ILPATL--
-IPATLS-
-IPATLK-
INPATL--
IYPATL--
-IPATLN-
IKPATL--
IGPATL--
IPPATL--
-IPATLW-
-IPATLA-
IAPATL--

-IPATLV-
-IPATLI-
-IPATLF-
-IPATLT-
-IPATLG-
IEPATL--
-IPATLE-
IHPATL--
-IPATLH-
ICPATL--
-IPATLY-
-IPATLQ-
-IPATLM-
IIPATL--
KSPATL--
KYPATL--
-KPATLR-
KRPATL--
-KPATLF-
KWPATL--
KGPATL--
-KPATLY-
-KPATLQ-
KTPATL--
KPPATL--
KAPATL--
-KPATLS-
KVPATL--
KQPATL--
-KPATLM-
KNPATL--
-KPATLT-
-KPATLH-
-KPATLN-
-KPATLI-
-KPATLE-
-KPATLG-
KMPATL--
-KPATLV-
KEPATL--
KCPATL--
KIPATL--
KDPATL--
-LPATLR-
LGPATL--
LVPATL--
LFPATL--
-LPATLK-
-LPATLL-
LLPATL--

LAPATL--
LQPATL--
-LPATLY-
-LPATLT-
LEPATL--
LTPATL--
-LPATLQ-
-LPATLF-
LWPATL--
LSPATL--
-LPATLS-
LMPATL--
LDPATL--
-LPATLH-
-LPATLW-
-LPATLM-
LIPATL--
LHPATL--
LYPATL--
-LPATLV-
LCPATL--
MLPATL--
MNPATL--
MQPATL--
-MPATLT-
MGPATL--
-MPATLR-
MTPATL--
MAPATL--
-MPATLL-
-MPATLY-
-MPATLV-
-MPATLS-
MMPATL--
MVPATL--
MIPATL--
MWPATL--
-MPATLK-
MFPATL--
MPPATL--
-MPATLW-
-MPATLQ-
-MPATLN-
-MPATLF-
-MPATLA-
MKPATL--
-MPATLM-
-MPATLH-
-MPATLE-
MDPATL--

MYPATL--
-MPATLI-
MHPATL--
NTPATL--
-NPATLY-
NLPATL--
-NPATLS-
NKPATL--
-NPATLV-
-NPATLI-
-NPATLL-
NVPATL--
NQPATL--
-NPATLW-
-NPATLR-
NNPATL--
NMPATL--
NPPATL--
-NPATLF-
NGPATL--
NFPATL--
NSPATL--
-NPATLT-
-NPATLM-
NIPATL--
NAPATL--
NYPATL--
-NPATLK-
NWPATL--
-NPATLH-
NDPATL--
NHPATL--
--PATLRS
--PATLMR
--PATLWG
--PATLRT
--PATLVR
--PATLLK
--PATLRV
--PATLLH
--PATLRG
--PATLHF
--PATLGS
PYPATL--
--PATLVT
--PATLRN
--PATLAS
--PATLST
--PATLKT
--PATLTA

--PATLSY
--PATLKY
--PATLIT
--PATLRM
--PATLRF
--PATLEA
--PATLYV
--PATLSW
--PATLGA
--PATLLT
PRPATL--
--PATLYH
--PATLRE
--PATLMS
--PATLHT
--PATLAL
--PATLAG
--PATLVM
--PATLQS
--PATLQG
--PATLKL
--PATLKA
--PATLHS
--PATLFS
--PATLES
PLPATL--
--PATLWR
--PATLTY
--PATLSL
PVPATL--
PTPATL--
--PATLWW
--PATLVI
--PATLTV
--PATLTS
--PATLTL
--PATLSV
--PATLRQ
--PATLRD
--PATLQV
--PATLMK
--PATLFQ
--PATLYK
--PATLVK
--PATLQR
--PATLIR
--PATLGG
--PATLYT
--PATLTM
--PATLIW

PSPATL--
--PATLWN
--PATLWD
--PATLVW
--PATLTN
--PATLSK
--PATLSA
--PATLKK
--PATLHN
--PATLFT
PWPATL--
--PATLYN
--PATLVA
--PATLSM
--PATLSH
--PATLQN
--PATLNS
--PATLNA
--PATLMG
--PATLKW
--PATLFK
--PATLYM
--PATLVG
--PATLVE
--PATLSG
--PATLNV
--PATLML
--PATLMD
--PATLKS
--PATLIQ
--PATLIA
--PATLFA
--PATLYQ
--PATLWT
--PATLWK
--PATLWH
--PATLVH
--PATLSI
--PATLQY
--PATLMW
--PATLMV
--PATLLN
--PATLKR
--PATLHA
--PATLFD
--PATLAT
PAPATL--
--PATLYG
--PATLSF
--PATLQF

--PATLQA
--PATLMQ
--PATLMI
--PATLKH
--PATLHW
--PATLHI
--PATLAM
--PATLAK
PCPATL--
--PATLTK
--PATLQH
--PATLNF
--PATLMA
--PATLLD
--PATLII
--PATLIG
--PATLHV
--PATLHM
--PATLYY
--PATLWQ
--PATLWE
--PATLVV
--PATLTH
--PATLTD
--PATLQT
--PATLQQ
--PATLQM
--PATLMT
--PATLKM
--PATLKG
--PATLID
--PATLHG
--PATLGL
--PATLFG
--PATLFE
--PATLAA
PMPATL--
PHPATL--
PDPATL--
--PATLYR
--PATLVQ
--PATLMY
--PATLME
--PATLKE
--PATLHL
--PATLHK
--PATLGV
--PATLGM
--PATLFH
--PATLET

```
--PATLAW
-PPATLY-
PFPATL--
--PATLWV
--PATLWM
--PATLVY
--PATLTW
--PATLTP
--PATLTE
--PATLNY
--PATLNT
--PATLNL
--PATLMH
--PATLKQ
--PATLKD
--PATLIV
--PATLIN
--PATLIM
--PATLIH
--PATLGF
--PATLAY
--PATLAQ
--PATLAI
-PPATLS-
-PPATLI-
--PATLYW
--PATLYE
--PATLYD
--PATLRC
--PATLQI
--PATLQE
--PATLQD
--PATLPM
--PATLMN
--PATLIY
--PATLHE
--PATLGW
--PATLFC
--PATLEM
--PATLEI
--PATLCS
--PATLAN
-QPATLW-
-QPATLL-
-QPATLS-
QWPATL--
QLPATL--
QGPATL--
QAPATL--
QVPATL--
```

```
-QPATLM-
-QPATLR-
-QPATLI-
QKPATL--
QFPATL--
-QPATLF-
-QPATLY-
QNPATL--
-QPATLT-
QHPATL--
QEPATL--
QPPATL--
-QPATLN-
-QPATLK-
QIPATL--
QSPATL--
QMPATL--
QTPATL--
-QPATLV-
-QPATLH-
QYPATL--
QQPATL--
-QPATLQ-
QDPATL--
QCPATL--
RSPATL--
RWPATL--
RVPATL--
-RPATLS-
RGPATL--
RPPATL--
-RPATLK-
RTPATL--
RFPATL--
RAPATL--
-RPATLV-
-RPATLY-
-RPATLG-
-RPATLE-
-RPATLI-
RHPATL--
RYPATL--
RIPATL--
-RPATLA-
RNPATL--
-RPATLM-
-RPATLC-
RCPATL--
RDPATL--
REPATL--
```

SGPATL--
-SPATLR-
-SPATLS-
STPATL--
-SPATLI-
-SPATLH-
SSPATL--
SLPATL--
-SPATLT-
SYPATL--
-SPATLV-
SHPATL--
SWPATL--
SQPATL--
-SPATLW-
SAPATL--
SIPATL--
SFPATL--
-SPATLM-
-SPATLF-
SMPATL--
SEPATL--
-SPATLY-
-SPATLN-
SDPATL--
-SPATLE-
SPPATL--
SCPATL--
TVPATL--
-TPATLR-
TPPATL--
TGPATL--
-TPATLK-
TSPATL--
TRPATL--
-TPATLS-
TTPATL--
-TPATLT-
-TPATLH-
TMPATL--
TFPATL--
TAPATL--
-TPATLY-
TKPATL--
TWPATL--
-TPATLW-
-TPATLF-
-TPATLE-
TNPATL--
TQPATL--

```
-TPATLV-
TDPATL--
-TPATLN-
TIPATL--
THPATL--
TEPATL--
-TPATLA-
-TPATLM-
TCPATL--
-TPATLC-
VRPATL--
VSPATL--
VKPATL--
VTPATL--
-VPATLS-
VGPATL--
VQPATL--
VEPATL--
VVPATL--
VAPATL--
-VPATLV-
VPPATL--
VWPATL--
-VPATLQ-
-VPATLH-
VMPATL--
-VPATLK-
-VPATLI-
VYPATL--
-VPATLG-
-VPATLF-
-VPATLE-
-VPATLY-
-VPATLN-
VNPATL--
VDPATL--
VCPATL--
-VPATLM-
-VPATLW-
-WPATLR-
WVPATL--
WLPATL--
WGPATL--
-WPATLK-
-WPATLY-
WTPATL--
WMPATL--
WIPATL--
WYPATL--
-WPATLQ-
```

```
             WHPATL--
             -WPATLH-
             WPPATL--
             -WPATLM-
             -WPATLS-
             -WPATLI-
             WDPATL--
             WWPATL--
             -WPATLV-
             WNPATL--
             WQPATL--
             WCPATL--
             YLPATL--
             YTPATL--
             -YPATLL-
             YSPATL--
             YQPATL--
             YNPATL--
             -YPATLY-
             -YPATLH-
             -YPATLR-
             YIPATL--
             YDPATL--
             YEPATL--
             YGPATL--
             YYPATL--
             YPPATL--
             -YPATLS-
             -YPATLM-
             -YPATLF-
             YAPATL--
             -YPATLK-
             -YPATLI-
             YKPATL--
             -YPATLW-
             YHPATL--
             -YPATLN-
             -YPATLV-
             -YPATLQ-
             -YPATLE-
             YMPATL--
             YWPATL--
             -YPATLA-
             YFPATL--
             YCPATL--
PSGW           44        809     32.60
             -LPSGWG-
             --PSGWLL
             -TPSGWG-
             -APSGWT-
```

−APSGWR−
AYPSGW−−
AWPSGW−−
AKPSGW−−
−APSGWM−
−APSGWA−
ARPSGW−−
−APSGWV−
−APSGWL−
AFPSGW−−
−APSGWI−
−APSGWF−
ALPSGW−−
AVPSGW−−
−APSGWK−
AEPSGW−−
AAPSGW−−
−APSGWY−
−APSGWS−
APPSGW−−
ANPSGW−−
AMPSGW−−
AIPSGW−−
AHPSGW−−
−APSGWE−
ATPSGW−−
ASPSGW−−
−APSGWW−
ADPSGW−−
AQPSGW−−
AGPSGW−−
CTPSGW−−
−CPSGWR−
CYPSGW−−
CSPSGW−−
−CPSGWT−
−CPSGWI−
CLPSGW−−
CVPSGW−−
−CPSGWY−
−CPSGWV−
−CPSGWK−
−CPSGWH−
CDPSGW−−
CAPSGW−−
DKPSGW−−
−DPSGWI−
DNPSGW−−
DSPSGW−−
−DPSGWV−

-DPSGWS-
-DPSGWL-
-DPSGWK-
-DPSGWH-
-DPSGWF-
DIPSGW--
DTPSGW--
-DPSGWQ-
DHPSGW--
-DPSGWR-
DLPSGW--
DAPSGW--
DYPSGW--
DVPSGW--
DRPSGW--
DQPSGW--
-DPSGWY-
-DPSGWW-
-DPSGWT-
-DPSGWN-
-DPSGWM-
DMPSGW--
DEPSGW--
DDPSGW--
ELPSGW--
-EPSGWL-
-EPSGWN-
-EPSGWV-
ERPSGW--
ESPSGW--
EGPSGW--
EFPSGW--
EQPSGW--
-EPSGWW-
-EPSGWI-
-EPSGWA-
EYPSGW--
-EPSGWT-
-EPSGWQ-
-EPSGWM-
-EPSGWK-
EMPSGW--
EVPSGW--
-EPSGWY-
EKPSGW--
EHPSGW--
FVPSGW--
-FPSGWS-
FRPSGW--
-FPSGWK-

FGPSGW--
FSPSGW--
-FPSGWL-
-FPSGWY-
-FPSGWV-
FYPSGW--
-FPSGWI-
-FPSGWH-
-FPSGWT-
FEPSGW--
FFPSGW--
FKPSGW--
FWPSGW--
FTPSGW--
-FPSGWQ-
FPPSGW--
FMPSGW--
FIPSGW--
-FPSGWF-
-FPSGWA-
FLPSGW--
-FPSGWW-
-FPSGWR-
-FPSGWN-
FHPSGW--
-GPSGWL-
GLPSGW--
-GPSGWS-
-GPSGWM-
GTPSGW--
-GPSGWA-
-GPSGWK-
-GPSGWV-
GRPSGW--
GMPSGW--
GYPSGW--
GKPSGW--
-GPSGWR-
GNPSGW--
GSPSGW--
GVPSGW--
GEPSGW--
-GPSGWT-
-GPSGWN-
-GPSGWI-
-GPSGWY-
GHPSGW--
GFPSGW--
GDPSGW--
GWPSGW--

-GPSGWW-
-GPSGWQ-
-GPSGWF-
-GPSGWG-
GCPSGW--
GAPSGW--
GQPSGW--
GPPSGW--
GGPSGW--
-GPSGWE-
GIPSGW--
HRPSGW--
HTPSGW--
-HPSGWS-
-HPSGWR-
-HPSGWW-
-HPSGWV-
-HPSGWA-
HSPSGW--
HEPSGW--
-HPSGWL-
HMPSGW--
-HPSGWF-
-HPSGWH-
HFPSGW--
-HPSGWN-
HNPSGW--
HIPSGW--
HYPSGW--
-HPSGWY-
-HPSGWM-
-HPSGWK-
-HPSGWI-
-HPSGWE-
HGPSGW--
HAPSGW--
-HPSGWQ-
HKPSGW--
-IPSGWS-
-IPSGWN-
-IPSGWL-
IRPSGW--
-IPSGWR-
-IPSGWY-
-IPSGWT-
IYPSGW--
-IPSGWV-
INPSGW--
IKPSGW--
-IPSGWM-

ILPSGW--
-IPSGWH-
IMPSGW--
IGPSGW--
IVPSGW--
IQPSGW--
-IPSGWQ-
-IPSGWK-
IIPSGW--
-IPSGWW-
-IPSGWA-
IPPSGW--
IEPSGW--
IDPSGW--
ITPSGW--
-IPSGWI-
-IPSGWF-
-IPSGWE-
IAPSGW--
ISPSGW--
-IPSGWG-
IHPSGW--
KVPSGW--
-KPSGWR-
-KPSGWS-
-KPSGWV-
-KPSGWN-
-KPSGWY-
-KPSGWL-
-KPSGWM-
-KPSGWK-
KGPSGW--
KAPSGW--
-KPSGWT-
KHPSGW--
KPPSGW--
KTPSGW--
-KPSGWA-
-KPSGWH-
KMPSGW--
KLPSGW--
KEPSGW--
-KPSGWG-
KYPSGW--
KNPSGW--
-KPSGWQ-
KIPSGW--
KWPSGW--
KQPSGW--
-KPSGWF-

−KPSGWW−
KFPSGW−−
KDPSGW−−
LRPSGW−−
LDPSGW−−
−LPSGWI−
−LPSGWY−
−LPSGWH−
−LPSGWV−
LTPSGW−−
−LPSGWR−
−LPSGWT−
LHPSGW−−
LFPSGW−−
−LPSGWS−
LPPSGW−−
LIPSGW−−
−LPSGWL−
−LPSGWA−
LYPSGW−−
LMPSGW−−
−LPSGWQ−
−LPSGWK−
LLPSGW−−
LQPSGW−−
−LPSGWW−
−LPSGWM−
LKPSGW−−
−LPSGWF−
LEPSGW−−
−MPSGWL−
−MPSGWR−
MYPSGW−−
−MPSGWW−
−MPSGWN−
−MPSGWK−
MLPSGW−−
MGPSGW−−
MQPSGW−−
MMPSGW−−
MKPSGW−−
MVPSGW−−
MTPSGW−−
MSPSGW−−
−MPSGWV−
−MPSGWM−
−MPSGWI−
MNPSGW−−
MFPSGW−−
−MPSGWF−

-MPSGWE-
-MPSGWA-
MDPSGW--
MCPSGW--
-MPSGWS-
-MPSGWQ-
-MPSGWH-
MIPSGW--
MHPSGW--
MAPSGW--
-NPSGWS-
-NPSGWI-
NSPSGW--
NLPSGW--
-NPSGWT-
NRPSGW--
-NPSGWK-
-NPSGWY-
-NPSGWR-
NYPSGW--
NGPSGW--
NWPSGW--
-NPSGWL-
NTPSGW--
-NPSGWV-
-NPSGWN-
-NPSGWA-
-NPSGWF-
NNPSGW--
NFPSGW--
NEPSGW--
-NPSGWM-
NKPSGW--
NVPSGW--
NPPSGW--
-NPSGWW-
NMPSGW--
NIPSGW--
NAPSGW--
--PSGWAY
--PSGWLT
--PSGWST
--PSGWFS
--PSGWRS
--PSGWVS
--PSGWRL
--PSGWLV
--PSGWLR
--PSGWLH
--PSGWKR

--PSGWSM
--PSGWSG
--PSGWRN
--PSGWTN
--PSGWVT
--PSGWVR
--PSGWSA
--PSGWYG
--PSGWTS
--PSGWSS
--PSGWRI
--PSGWIR
--PSGWII
--PSGWRG
--PSGWLF
--PSGWKT
--PSGWFR
--PSGWMG
--PSGWLG
--PSGWVQ
--PSGWVK
--PSGWVF
--PSGWTR
--PSGWSR
--PSGWLA
--PSGWYR
--PSGWWS
--PSGWWK
--PSGWRK
--PSGWNA
--PSGWKS
--PSGWIT
--PSGWHA
PYPSGW--
--PSGWVI
--PSGWTK
--PSGWIA
--PSGWFL
--PSGWFI
--PSGWFH
--PSGWWM
--PSGWQV
--PSGWQT
--PSGWML
--PSGWLW
--PSGWIG
--PSGWFG
--PSGWWD
--PSGWTW
--PSGWTA

--PSGWRY
--PSGWRQ
--PSGWHL
PWPSGW--
--PSGWWT
--PSGWTH
--PSGWSY
--PSGWSI
--PSGWRM
--PSGWRH
--PSGWRF
--PSGWMI
--PSGWMH
--PSGWKW
--PSGWKI
--PSGWIS
--PSGWHK
PLPSGW--
PKPSGW--
--PSGWWA
--PSGWVL
--PSGWSV
--PSGWQF
--PSGWNF
--PSGWKH
--PSGWIQ
--PSGWHS
--PSGWGV
--PSGWFT
--PSGWAA
PNPSGW--
PGPSGW--
--PSGWYD
--PSGWYA
--PSGWWL
--PSGWVY
--PSGWVV
--PSGWTQ
--PSGWTG
--PSGWTF
--PSGWTE
--PSGWNS
--PSGWMV
--PSGWMR
--PSGWMQ
--PSGWLK
--PSGWKY
--PSGWIF
--PSGWHM
--PSGWHH

--PSGWFN
--PSGWAT
PQPSGW--
PVPSGW--
--PSGWYH
--PSGWWR
--PSGWWG
--PSGWVM
--PSGWVH
--PSGWSQ
--PSGWSL
--PSGWSF
--PSGWRA
--PSGWLQ
--PSGWLN
--PSGWLI
--PSGWKA
--PSGWHT
--PSGWHR
--PSGWHI
--PSGWES
PEPSGW--
PSPSGW--
--PSGWYN
--PSGWSH
--PSGWRP
--PSGWQS
--PSGWQI
--PSGWNV
--PSGWLY
--PSGWLM
--PSGWKD
--PSGWIV
--PSGWHY
--PSGWHQ
--PSGWHG
--PSGWFK
--PSGWFD
--PSGWAV
PRPSGW--
PMPSGW--
--PSGWYQ
--PSGWVW
--PSGWVE
--PSGWVA
--PSGWTY
--PSGWTV
--PSGWTL
--PSGWTI
--PSGWQW

--PSGWQN
--PSGWQH
--PSGWQG
--PSGWQA
--PSGWNM
--PSGWNG
--PSGWMY
--PSGWMT
--PSGWMN
--PSGWMK
--PSGWMA
--PSGWKN
--PSGWKL
--PSGWIM
--PSGWIL
--PSGWIH
--PSGWHN
--PSGWFY
--PSGWFQ
--PSGWFM
--PSGWFE
--PSGWET
--PSGWAN
--PSGWAF
PTPSGW--
--PSGWYS
--PSGWYL
--PSGWYE
--PSGWWV
--PSGWWI
--PSGWWH
--PSGWWE
--PSGWTT
--PSGWSK
--PSGWRE
--PSGWQY
--PSGWQR
--PSGWNY
--PSGWMF
--PSGWLD
--PSGWKV
--PSGWKM
--PSGWKF
--PSGWIN
--PSGWIK
--PSGWHW
--PSGWHF
--PSGWGT
--PSGWGG
--PSGWFW

--PSGWFA
--PSGWCR
--PSGWAH
PHPSGW--
PDPSGW--
PAPSGW--
--PSGWYV
--PSGWYT
--PSGWYK
--PSGWTM
--PSGWTD
--PSGWSW
--PSGWQK
--PSGWQE
--PSGWNW
--PSGWNQ
--PSGWMM
--PSGWME
--PSGWMD
--PSGWLE
--PSGWKQ
--PSGWIY
--PSGWIW
--PSGWIE
--PSGWHV
--PSGWHE
--PSGWEY
--PSGWEV
--PSGWER
--PSGWEM
--PSGWEK
--PSGWEF
--PSGWAM
-PPSGWC-
PIPSGW--
QSPSGW--
QFPSGW--
QRPSGW--
-QPSGWW-
-QPSGWV-
-QPSGWR-
-QPSGWT-
-QPSGWL-
QGPSGW--
QAPSGW--
QYPSGW--
-QPSGWS-
-QPSGWK-
-QPSGWG-
QNPSGW--

-QPSGWM-
QLPSGW--
QWPSGW--
QKPSGW--
QTPSGW--
QQPSGW--
-QPSGWH-
-QPSGWA-
QDPSGW--
-QPSGWY-
-QPSGWQ-
-QPSGWF-
-QPSGWD-
QPPSGW--
QMPSGW--
QCPSGW--
-RPSGWR-
-RPSGWL-
-RPSGWN-
-RPSGWK-
-RPSGWT-
RWPSGW--
-RPSGWV-
-RPSGWF-
-RPSGWY-
RSPSGW--
RFPSGW--
RNPSGW--
RLPSGW--
RHPSGW--
RVPSGW--
-RPSGWH-
-RPSGWA-
-RPSGWM-
REPSGW--
-RPSGWQ-
RAPSGW--
RYPSGW--
RIPSGW--
RTPSGW--
RKPSGW--
-RPSGWW-
-RPSGWG-
RDPSGW--
RMPSGW--
RQPSGW--
-RPSGWE-
RCPSGW--
RPPSGW--
SLPSGW--

-SPSGWF-
-SPSGWA-
-SPSGWV-
-SPSGWY-
STPSGW--
-SPSGWS-
SNPSGW--
-SPSGWI-
SVPSGW--
SSPSGW--
-SPSGWT-
SIPSGW--
-SPSGWR-
SGPSGW--
-SPSGWL-
-SPSGWK-
-SPSGWH-
-SPSGWN-
SRPSGW--
SHPSGW--
SFPSGW--
SEPSGW--
SQPSGW--
-SPSGWW-
-SPSGWQ-
SMPSGW--
SKPSGW--
SDPSGW--
SYPSGW--
SWPSGW--
-SPSGWG-
SCPSGW--
-SPSGWM-
-SPSGWE-
SPPSGW--
-TPSGWS-
-TPSGWL-
TAPSGW--
TIPSGW--
-TPSGWK-
-TPSGWI-
TGPSGW--
-TPSGWR-
-TPSGWN-
-TPSGWF-
TWPSGW--
TTPSGW--
-TPSGWV-
TPPSGW--
-TPSGWM-

-TPSGWH-
TFPSGW--
-TPSGWY-
TRPSGW--
-TPSGWW-
-TPSGWA-
TDPSGW--
TNPSGW--
TLPSGW--
THPSGW--
TEPSGW--
TYPSGW--
-TPSGWT-
TMPSGW--
TVPSGW--
-TPSGWQ-
TKPSGW--
TCPSGW--
VRPSGW--
-VPSGWT-
-VPSGWR-
-VPSGWS-
-VPSGWL-
-VPSGWN-
-VPSGWI-
VYPSGW--
-VPSGWF-
VTPSGW--
VQPSGW--
-VPSGWV-
VGPSGW--
VAPSGW--
-VPSGWY-
-VPSGWQ-
-VPSGWH-
VNPSGW--
VLPSGW--
VIPSGW--
-VPSGWA-
VMPSGW--
VDPSGW--
VSPSGW--
-VPSGWM-
VPPSGW--
VWPSGW--
-VPSGWG-
-VPSGWE-
VEPSGW--
-VPSGWW-
VKPSGW--

```
-WPSGWL-
-WPSGWI-
WSPSGW--
-WPSGWW-
-WPSGWS-
-WPSGWK-
WQPSGW--
-WPSGWR-
WFPSGW--
-WPSGWM-
WRPSGW--
WGPSGW--
-WPSGWN-
-WPSGWA-
WPPSGW--
WKPSGW--
-WPSGWT-
-WPSGWQ-
WLPSGW--
WAPSGW--
WYPSGW--
WWPSGW--
WTPSGW--
-WPSGWV-
-WPSGWH-
WIPSGW--
-YPSGWL-
-YPSGWS-
YLPSGW--
-YPSGWR-
YRPSGW--
-YPSGWV-
-YPSGWM-
YGPSGW--
YTPSGW--
YKPSGW--
-YPSGWN-
YEPSGW--
YQPSGW--
-YPSGWF-
YIPSGW--
-YPSGWY-
-YPSGWQ-
-YPSGWK-
-YPSGWA-
YAPSGW--
YVPSGW--
-YPSGWI-
-YPSGWE-
YPPSGW--
```

```
               YHPSGW--
               YWPSGW--
               YSPSGW--
               -YPSGWW-
               YNPSGW--
PRHL            442          621         32.58
               -DPRHLF-
               DNPRHL--
               --PRHLLM
               --PRHLHI
               --PRHLGN
               --PRHLGH
               --PRHLGE
               -YPRHLF-
               -APRHLL-
               -APRHLS-
               -APRHLH-
               ATPRHL--
               AMPRHL--
               -APRHLN-
               -APRHLM-
               AAPRHL--
               AQPRHL--
               -APRHLV-
               APPRHL--
               -APRHLW-
               -APRHLQ-
               -APRHLG-
               -APRHLI-
               -APRHLD-
               AHPRHL--
               ACPRHL--
               ANPRHL--
               -APRHLE-
               CVPRHL--
               CTPRHL--
               -CPRHLL-
               -CPRHLS-
               CNPRHL--
               CMPRHL--
               CIPRHL--
               CAPRHL--
               CSPRHL--
               -CPRHLR-
               -CPRHLK-
               -CPRHLI-
               -CPRHLA-
               CKPRHL--
               CCPRHL--
               CYPRHL--
```

CWPRHL--
CRPRHL--
CQPRHL--
-CPRHLY-
-CPRHLT-
-CPRHLM-
CHPRHL--
CEPRHL--
DAPRHL--
-DPRHLR-
-DPRHLK-
DYPRHL--
DMPRHL--
DDPRHL--
-DPRHLS-
-DPRHLV-
DTPRHL--
-DPRHLL-
-DPRHLT-
-DPRHLN-
DLPRHL--
DKPRHL--
-DPRHLM-
-DPRHLA-
DSPRHL--
DGPRHL--
-DPRHLY-
-DPRHLI-
-DPRHLH-
DIPRHL--
DHPRHL--
-DPRHLW-
-DPRHLG-
-DPRHLE-
DEPRHL--
-EPRHLV-
EMPRHL--
ENPRHL--
ELPRHL--
EGPRHL--
EAPRHL--
ESPRHL--
EQPRHL--
ETPRHL--
-EPRHLS-
-EPRHLY-
-EPRHLT-
-EPRHLN-
EKPRHL--
-EPRHLG-

-EPRHLA-
EEPRHL--
-EPRHLK-
-EPRHLH-
EIPRHL--
EWPRHL--
-EPRHLW-
EHPRHL--
-EPRHLM-
EPPRHL--
-FPRHLS-
FSPRHL--
FRPRHL--
FAPRHL--
-FPRHLI-
FDPRHL--
FTPRHL--
FEPRHL--
FVPRHL--
-FPRHLF-
-FPRHLQ-
-FPRHLY-
-FPRHLM-
FPPRHL--
-FPRHLE-
-FPRHLW-
GAPRHL--
GSPRHL--
GPPRHL--
GNPRHL--
GRPRHL--
-GPRHLM-
-GPRHLT-
-GPRHLN-
-GPRHLK-
GLPRHL--
GEPRHL--
GQPRHL--
-GPRHLV-
GHPRHL--
-GPRHLL-
-GPRHLY-
-GPRHLE-
GVPRHL--
-GPRHLF-
-GPRHLS-
-GPRHLH-
GDPRHL--
-GPRHLI-
-GPRHLW-

-GPRHLQ-
-GPRHLD-
HVPRHL--
-HPRHLL-
HSPRHL--
-HPRHLR-
HNPRHL--
-HPRHLM-
-HPRHLY-
-HPRHLT-
-HPRHLN-
HDPRHL--
-HPRHLH-
HTPRHL--
-HPRHLV-
HHPRHL--
-HPRHLS-
-HPRHLK-
HYPRHL--
-HPRHLG-
-HPRHLF-
HPPRHL--
-HPRHLI-
HQPRHL--
-HPRHLQ-
HEPRHL--
-HPRHLE-
HKPRHL--
-HPRHLW-
ITPRHL--
-IPRHLK-
-IPRHLL-
-IPRHLA-
-IPRHLY-
-IPRHLI-
-IPRHLV-
IDPRHL--
IPPRHL--
-IPRHLH-
-IPRHLW-
-IPRHLQ-
IGPRHL--
-IPRHLF-
-IPRHLE-
-IPRHLN-
IKPRHL--
-IPRHLT-
-IPRHLM-
ICPRHL--
-KPRHLT-

KIPRHL--
-KPRHLE-
KPPRHL--
KGPRHL--
-KPRHLA-
-KPRHLM-
-KPRHLH-
-KPRHLI-
-KPRHLK-
KDPRHL--
-KPRHLY-
-KPRHLW-
-KPRHLQ-
-KPRHLF-
KCPRHL--
KEPRHL--
-KPRHLG-
KNPRHL--
KKPRHL--
-LPRHLS-
LDPRHL--
LSPRHL--
LGPRHL--
-LPRHLV-
LAPRHL--
-LPRHLA-
LPPRHL--
LIPRHL--
-LPRHLK-
LCPRHL--
-LPRHLH-
-LPRHLW-
-LPRHLE-
-LPRHLN-
-LPRHLG-
MSPRHL--
-MPRHLT-
MGPRHL--
-MPRHLS-
MQPRHL--
-MPRHLG-
MKPRHL--
MHPRHL--
-MPRHLQ-
-MPRHLH-
-MPRHLA-
MNPRHL--
MDPRHL--
-MPRHLI-
-MPRHLE-

```
-MPRHLY-
-MPRHLV-
-MPRHLM-
-MPRHLC-
-MPRHLF-
-NPRHLL-
-NPRHLW-
-NPRHLR-
NNPRHL--
NEPRHL--
NYPRHL--
NKPRHL--
-NPRHLV-
-NPRHLT-
-NPRHLH-
-NPRHLA-
NWPRHL--
-NPRHLQ-
NPPRHL--
-NPRHLN-
-NPRHLK-
-NPRHLS-
-NPRHLF-
NDPRHL--
-NPRHLE-
-NPRHLM-
-NPRHLG-
NCPRHL--
--PRHLLS
--PRHLVY
--PRHLSH
--PRHLSG
--PRHLLN
--PRHLYS
--PRHLSM
--PRHLML
--PRHLWS
--PRHLTV
--PRHLSY
--PRHLSI
--PRHLHM
--PRHLRM
--PRHLLV
--PRHLQI
--PRHLVE
--PRHLIA
--PRHLET
--PRHLWK
--PRHLLH
--PRHLAL
```

--PRHLAG
--PRHLRE
--PRHLHS
--PRHLFI
--PRHLYM
--PRHLSV
--PRHLMK
--PRHLWI
--PRHLRT
--PRHLLL
PYPRHL--
--PRHLVN
--PRHLNW
--PRHLGL
--PRHLFA
--PRHLAT
--PRHLYI
--PRHLWA
--PRHLVK
--PRHLTE
--PRHLIG
--PRHLGA
--PRHLVV
--PRHLTT
--PRHLQW
--PRHLMW
--PRHLIT
--PRHLGS
--PRHLEN
--PRHLAF
--PRHLYA
--PRHLNT
--PRHLNS
--PRHLIE
--PRHLGI
--PRHLEM
--PRHLAS
--PRHLAI
--PRHLYN
--PRHLWN
--PRHLVF
--PRHLTN
--PRHLTF
--PRHLNV
--PRHLNA
--PRHLMT
--PRHLKL
--PRHLAW
--PRHLAV
PQPRHL--

PHPRHL--
--PRHLTG
--PRHLSD
--PRHLKN
--PRHLHG
--PRHLGY
--PRHLFY
--PRHLFG
--PRHLEV
--PRHLYT
--PRHLVA
--PRHLQL
--PRHLMN
--PRHLMI
--PRHLLW
--PRHLKD
--PRHLIQ
--PRHLIN
--PRHLII
--PRHLHN
--PRHLEW
--PRHLAA
PNPRHL--
PGPRHL--
--PRHLYG
--PRHLVI
--PRHLSW
--PRHLQV
--PRHLQG
--PRHLLE
--PRHLKE
--PRHLFV
--PRHLEG
--PRHLAQ
--PRHLAN
--PRHLYY
--PRHLWT
--PRHLTM
--PRHLQS
--PRHLME
--PRHLHV
--PRHLHH
--PRHLFT
--PRHLEI
--PRHLYD
--PRHLWG
--PRHLHK
--PRHLFE
--PRHLAM
PAPRHL--

--PRHLYV
--PRHLTD
--PRHLQT
--PRHLNM
--PRHLNG
--PRHLMQ
--PRHLMM
--PRHLMG
--PRHLIF
--PRHLHL
--PRHLGM
--PRHLGF
--PRHLFD
--PRHLEK
PKPRHL--
PEPRHL--
--PRHLYW
--PRHLYQ
--PRHLYF
--PRHLVD
--PRHLNN
--PRHLHY
--PRHLHA
--PRHLEL
--PRHLEF
--PRHLAD
--PRHLYE
--PRHLWE
--PRHLSE
--PRHLQY
--PRHLQF
--PRHLQA
--PRHLNL
--PRHLNI
--PRHLNF
--PRHLGQ
--PRHLFQ
--PRHLFH
--PRHLEH
--PRHLEA
--PRHLDS
--PRHLDA
--PRHLYH
--PRHLWV
--PRHLWD
--PRHLQM
--PRHLQE
--PRHLPL
--PRHLLD
--PRHLIY

--PRHLIV
--PRHLHD
--PRHLGW
--PRHLFN
--PRHLEY
--PRHLAY
PDPRHL--
--PRHLWW
--PRHLWM
--PRHLRC
--PRHLQQ
--PRHLQH
--PRHLKH
--PRHLES
--PRHLEQ
--PRHLCS
PCPRHL--
--PRHLWQ
--PRHLWF
--PRHLVC
--PRHLQD
--PRHLPF
--PRHLNH
--PRHLND
--PRHLMF
--PRHLMC
--PRHLHQ
--PRHLHE
--PRHLGC
--PRHLFF
--PRHLED
-PPRHLM-
PMPRHL--
QKPRHL--
QLPRHL--
-QPRHLS-
-QPRHLL-
-QPRHLA-
QTPRHL--
QVPRHL--
QNPRHL--
-QPRHLR-
QPPRHL--
-QPRHLF-
-QPRHLI-
-QPRHLW-
QHPRHL--
-QPRHLN-
-QPRHLK-
QMPRHL--

-QPRHLM-
-QPRHLG-
-QPRHLQ-
QGPRHL--
QDPRHL--
QCPRHL--
-RPRHLR-
-RPRHLI-
-RPRHLF-
REPRHL--
RDPRHL--
-RPRHLN-
-RPRHLG-
-RPRHLH-
RPPRHL--
RQPRHL--
-SPRHLL-
-SPRHLT-
-SPRHLS-
SSPRHL--
-SPRHLA-
SNPRHL--
-SPRHLR-
-SPRHLN-
-SPRHLV-
-SPRHLK-
-SPRHLI-
SHPRHL--
-SPRHLG-
SQPRHL--
-SPRHLM-
SEPRHL--
SPPRHL--
-SPRHLQ-
-SPRHLH-
-SPRHLF-
-SPRHLE-
SDPRHL--
SCPRHL--
TKPRHL--
TSPRHL--
TGPRHL--
TTPRHL--
-TPRHLR-
-TPRHLH-
TAPRHL--
-TPRHLY-
TMPRHL--
-TPRHLV-
THPRHL--

−TPRHLN−
−TPRHLG−
−TPRHLK−
TPPRHL−−
TDPRHL−−
TIPRHL−−
−TPRHLF−
−TPRHLE−
−TPRHLT−
−TPRHLQ−
TNPRHL−−
−TPRHLM−
TCPRHL−−
−VPRHLR−
−VPRHLS−
−VPRHLL−
VSPRHL−−
−VPRHLT−
−VPRHLY−
VVPRHL−−
−VPRHLG−
VGPRHL−−
−VPRHLF−
VPPRHL−−
−VPRHLN−
−VPRHLM−
−VPRHLQ−
−VPRHLE−
VDPRHL−−
VEPRHL−−
−VPRHLI−
−VPRHLH−
−VPRHLA−
−VPRHLW−
−WPRHLM−
WSPRHL−−
WPPRHL−−
WTPRHL−−
WGPRHL−−
WMPRHL−−
WNPRHL−−
WAPRHL−−
−WPRHLY−
−WPRHLT−
WEPRHL−−
−WPRHLH−
WKPRHL−−
−WPRHLW−
−WPRHLN−
−WPRHLG−

| | | | |
|---|---|---|---|
| | -WPRHLE- | | |
| | WHPRHL-- | | |
| | WDPRHL-- | | |
| | YTPRHL-- | | |
| | -YPRHLY- | | |
| | YKPRHL-- | | |
| | -YPRHLK- | | |
| | YAPRHL-- | | |
| | -YPRHLL- | | |
| | YDPRHL-- | | |
| | -YPRHLI- | | |
| | YYPRHL-- | | |
| | -YPRHLG- | | |
| | -YPRHLA- | | |
| | YHPRHL-- | | |
| | YIPRHL-- | | |
| | YQPRHL-- | | |
| | -YPRHLQ- | | |
| | -YPRHLN- | | |
| | -YPRHLM- | | |
| | YPPRHL-- | | |
| | -YPRHLW- | | |
| | YNPRHL-- | | |
| | -YPRHLH- | | |
| | YEPRHL-- | | |
| | -YPRHLE- | | |
| | YCPRHL-- | | |
| PRHM | 339 | 656 | 32.57 |
| | GIPRHM-- | | |
| | -LPRHMN- | | |
| | --PRHMGL | | |
| | -APRHMK- | | |
| | -APRHML- | | |
| | AVPRHM-- | | |
| | AHPRHM-- | | |
| | AGPRHM-- | | |
| | ANPRHM-- | | |
| | -APRHMV- | | |
| | APPRHM-- | | |
| | ASPRHM-- | | |
| | -APRHMM- | | |
| | -APRHMI- | | |
| | -APRHMA- | | |
| | ALPRHM-- | | |
| | ATPRHM-- | | |
| | -APRHMT- | | |
| | -APRHMW- | | |
| | -APRHMN- | | |
| | -APRHMH- | | |
| | AMPRHM-- | | |

AEPRHM--
-APRHMS-
-APRHMF-
AQPRHM--
-APRHMG-
ADPRHM--
AAPRHM--
CAPRHM--
CCPRHM--
CMPRHM--
CYPRHM--
-CPRHMR-
-CPRHML-
CPPRHM--
DSPRHM--
DWPRHM--
-DPRHMR-
DYPRHM--
-DPRHMK-
-DPRHMF-
-DPRHMA-
DLPRHM--
DKPRHM--
DEPRHM--
DVPRHM--
-DPRHMY-
-DPRHMT-
-DPRHML-
DAPRHM--
DTPRHM--
-DPRHMV-
-DPRHMH-
DFPRHM--
ETPRHM--
-EPRHML-
EFPRHM--
ESPRHM--
ERPRHM--
-EPRHMY-
-EPRHMK-
-EPRHMH-
ENPRHM--
EIPRHM--
EEPRHM--
EWPRHM--
-EPRHMT-
-EPRHMM-
EYPRHM--
EVPRHM--
-EPRHMG-

-EPRHMF-
-EPRHME-
EKPRHM--
EGPRHM--
-EPRHMV-
-EPRHMS-
-EPRHMI-
EMPRHM--
EHPRHM--
EAPRHM--
FVPRHM--
FSPRHM--
FIPRHM--
-FPRHMS-
-FPRHMY-
-FPRHMN-
FQPRHM--
FGPRHM--
-FPRHMV-
-FPRHMI-
FNPRHM--
FDPRHM--
FYPRHM--
FTPRHM--
-FPRHMM-
-FPRHMG-
FPPRHM--
FHPRHM--
-FPRHMW-
-FPRHMK-
-FPRHMH-
-FPRHMF-
-FPRHMA-
FMPRHM--
-GPRHML-
GHPRHM--
GLPRHM--
GVPRHM--
GSPRHM--
-GPRHMK-
GTPRHM--
-GPRHMS-
-GPRHMR-
-GPRHMH-
GNPRHM--
GDPRHM--
GQPRHM--
-GPRHMT-
-GPRHMW-
-GPRHMI-

GGPRHM--
GYPRHM--
-GPRHMV-
-GPRHMN-
-GPRHMG-
-GPRHME-
GMPRHM--
GKPRHM--
-GPRHMQ-
-GPRHMF-
-GPRHMA-
GEPRHM--
GPPRHM--
-HPRHMA-
HMPRHM--
HVPRHM--
-HPRHMG-
-HPRHMT-
-HPRHMN-
-HPRHMY-
-HPRHMQ-
-HPRHMW-
-HPRHMV-
HLPRHM--
HHPRHM--
HDPRHM--
HYPRHM--
HTPRHM--
HSPRHM--
-HPRHMS-
-HPRHMR-
-HPRHMM-
-HPRHMK-
-HPRHMH-
-HPRHMF-
HNPRHM--
HKPRHM--
IVPRHM--
-IPRHMR-
ITPRHM--
IYPRHM--
ISPRHM--
-IPRHMF-
IAPRHM--
-IPRHML-
-IPRHMY-
-IPRHME-
INPRHM--
IPPRHM--
IKPRHM--

IHPRHM--
IEPRHM--
-IPRHMV-
-IPRHMH-
-IPRHMG-
IQPRHM--
-IPRHMT-
-IPRHMA-
IGPRHM--
IDPRHM--
-KPRHML-
-KPRHMF-
-KPRHMT-
-KPRHMY-
KVPRHM--
-KPRHMS-
KTPRHM--
KQPRHM--
-KPRHMN-
-KPRHMM-
-KPRHMH-
-KPRHMA-
KMPRHM--
-KPRHMW-
-KPRHMQ-
-KPRHMI-
KPPRHM--
KEPRHM--
-LPRHMK-
-LPRHMS-
LLPRHM--
-LPRHMT-
LGPRHM--
LDPRHM--
-LPRHML-
LPPRHM--
LAPRHM--
LQPRHM--
-LPRHMH-
LTPRHM--
-LPRHMV-
LYPRHM--
LVPRHM--
-LPRHMA-
LNPRHM--
LMPRHM--
LEPRHM--
-LPRHMY-
-LPRHMQ-
-LPRHMM-

```
-LPRHME-
-LPRHMF-
LHPRHM--
-MPRHMI-
-MPRHMS-
MSPRHM--
-MPRHMY-
-MPRHMM-
MHPRHM--
MCPRHM--
MYPRHM--
MVPRHM--
MTPRHM--
-MPRHMA-
MNPRHM--
MQPRHM--
-MPRHMT-
-MPRHMR-
-MPRHMQ-
-MPRHMN-
-MPRHMK-
MPPRHM--
MLPRHM--
MGPRHM--
MDPRHM--
MAPRHM--
NVPRHM--
-NPRHMS-
NNPRHM--
NSPRHM--
-NPRHML-
NTPRHM--
NRPRHM--
NHPRHM--
-NPRHMW-
NMPRHM--
NEPRHM--
-NPRHMR-
NQPRHM--
-NPRHMV-
-NPRHMN-
-NPRHMM-
NCPRHM--
-NPRHMY-
-NPRHMG-
-NPRHMF-
-NPRHMA-
NGPRHM--
NAPRHM--
--PRHMYQ
```

--PRHMLS
--PRHMRI
--PRHMFG
--PRHMQT
--PRHMRT
--PRHMTL
--PRHMVK
--PRHMSS
--PRHMKL
--PRHMIE
--PRHMKT
--PRHMRH
--PRHMLN
--PRHMAS
--PRHMVI
--PRHMQS
--PRHMVD
--PRHMLF
--PRHMVT
--PRHMVS
--PRHMRG
--PRHMLW
--PRHMLI
--PRHMKH
--PRHMFT
--PRHMYS
--PRHMWS
--PRHMMT
--PRHMMS
--PRHMIL
PYPRHM--
PSPRHM--
--PRHMWA
--PRHMVF
--PRHMTW
--PRHMST
--PRHMSA
--PRHMIR
--PRHMGT
--PRHMGA
PLPRHM--
--PRHMYR
--PRHMYN
--PRHMTG
--PRHMQY
--PRHMQH
--PRHMIT
--PRHMAL
--PRHMWY
--PRHMVW

--PRHMVQ
--PRHMTV
--PRHMRD
--PRHMQM
--PRHMLH
--PRHMLA
--PRHMKD
--PRHMIS
--PRHMHT
--PRHMHS
--PRHMGS
--PRHMES
--PRHMDS
--PRHMAY
--PRHMTT
--PRHMTH
--PRHMSQ
--PRHMLV
--PRHMKY
--PRHMHG
--PRHMGG
--PRHMFI
PGPRHM--
PVPRHM--
PRPRHM--
--PRHMVV
--PRHMVA
--PRHMTD
--PRHMSY
--PRHMSI
--PRHMNS
--PRHMNG
--PRHMMY
--PRHMLT
--PRHMII
--PRHMHA
--PRHMFS
--PRHMEV
--PRHMAM
--PRHMAA
--PRHMYM
--PRHMWN
--PRHMVM
--PRHMTI
--PRHMMW
--PRHMMI
--PRHMMH
--PRHMMA
--PRHMKG
--PRHMIQ

--PRHMIG
--PRHMIF
--PRHMGW
--PRHMFR
--PRHMFQ
--PRHMFK
--PRHMAV
PAPRHM--
PTPRHM--
--PRHMYW
--PRHMYG
--PRHMWV
--PRHMTY
--PRHMTK
--PRHMTF
--PRHMSK
--PRHMSG
--PRHMQV
--PRHMMN
--PRHMLQ
--PRHMLM
--PRHMKS
--PRHMKN
--PRHMKF
--PRHMKE
--PRHMID
--PRHMHH
--PRHMGV
--PRHMFY
--PRHMFV
--PRHMEY
--PRHMEA
--PRHMAT
--PRHMAK
--PRHMAI
--PRHMAG
PIPRHM--
--PRHMYT
--PRHMYI
--PRHMYH
--PRHMYF
--PRHMWL
--PRHMVY
--PRHMVN
--PRHMTQ
--PRHMTN
--PRHMSV
--PRHMSM
--PRHMSC
--PRHMQW

--PRHMQN
--PRHMNW
--PRHMNF
--PRHMMD
--PRHMIN
--PRHMHW
--PRHMHM
--PRHMHL
--PRHMFW
--PRHMFL
--PRHMFH
--PRHMFA
--PRHMET
--PRHMER
--PRHMEG
--PRHMAW
PNPRHM--
--PRHMYY
--PRHMYD
--PRHMWF
--PRHMWE
--PRHMTE
--PRHMSD
--PRHMRM
--PRHMQF
--PRHMNY
--PRHMNV
--PRHMNT
--PRHMMV
--PRHMMQ
--PRHMMM
--PRHMMF
--PRHMLE
--PRHMKW
--PRHMKM
--PRHMIY
--PRHMIW
--PRHMIV
--PRHMIM
--PRHMHV
--PRHMHN
--PRHMHK
--PRHMHF
--PRHMFD
--PRHMEW
--PRHMEQ
--PRHMEN
--PRHMDG
--PRHMCR
--PRHMCQ

PHPRHM--
-QPRHMI-
-QPRHML-
-QPRHMY-
-QPRHMA-
-QPRHMV-
QDPRHM--
QRPRHM--
QSPRHM--
-QPRHMS-
-QPRHMR-
QMPRHM--
QIPRHM--
QGPRHM--
-QPRHMH-
QPPRHM--
-QPRHMK-
-QPRHMG-
QNPRHM--
QKPRHM--
QHPRHM--
QYPRHM--
QVPRHM--
-QPRHMT-
-QPRHMQ-
-QPRHMM-
QCPRHM--
QAPRHM--
-RPRHMV-
-RPRHMQ-
RVPRHM--
-RPRHMT-
REPRHM--
-RPRHME-
RPPRHM--
RNPRHM--
-RPRHMS-
-RPRHMH-
-RPRHMF-
RDPRHM--
RQPRHM--
-RPRHMW-
-RPRHMM-
-RPRHMI-
RAPRHM--
-RPRHMY-
-RPRHMK-
RCPRHM--
-RPRHMG-
-RPRHMA-

```
-SPRHMR-
-SPRHMS-
SAPRHM--
-SPRHMA-
SSPRHM--
SPPRHM--
SVPRHM--
-SPRHMI-
SNPRHM--
SMPRHM--
-SPRHMW-
-SPRHMT-
-SPRHMY-
SLPRHM--
SQPRHM--
-SPRHMQ-
SEPRHM--
SYPRHM--
-SPRHML-
-SPRHMK-
-SPRHMV-
-SPRHMG-
STPRHM--
-SPRHMN-
-SPRHMH-
-SPRHME-
SKPRHM--
SIPRHM--
-SPRHMM-
-SPRHMF-
SCPRHM--
TVPRHM--
TYPRHM--
TAPRHM--
TSPRHM--
-TPRHMH-
TIPRHM--
TGPRHM--
-TPRHMW-
-TPRHML-
TLPRHM--
-TPRHMT-
-TPRHMQ-
-TPRHMM-
TNPRHM--
TEPRHM--
TQPRHM--
-TPRHMY-
-TPRHMN-
-TPRHMV-
```

TTPRHM--
TRPRHM--
-TPRHMS-
TPPRHM--
TDPRHM--
-TPRHMI-
-TPRHMG-
TMPRHM--
THPRHM--
-TPRHMF-
-TPRHMA-
VVPRHM--
VGPRHM--
-VPRHMR-
-VPRHMS-
-VPRHMI-
-VPRHMY-
VNPRHM--
VEPRHM--
-VPRHMM-
VQPRHM--
VYPRHM--
-VPRHMV-
VTPRHM--
-VPRHMN-
-VPRHML-
VLPRHM--
VAPRHM--
VDPRHM--
VCPRHM--
-VPRHMT-
-VPRHMK-
-VPRHMG-
-VPRHMA-
VMPRHM--
-VPRHMW-
-VPRHMQ-
-VPRHME-
-VPRHMD-
VPPRHM--
VIPRHM--
WVPRHM--
WRPRHM--
WSPRHM--
WMPRHM--
WIPRHM--
WGPRHM--
-WPRHMS-
-WPRHMT-
-WPRHMQ-

-WPRHMI-
-WPRHMM-
-WPRHME-
WNPRHM--
WAPRHM--
WQPRHM--
-WPRHMY-
-WPRHMN-
WPPRHM--
WKPRHM--
WFPRHM--
YSPRHM--
-YPRHMQ-
YTPRHM--
YAPRHM--
-YPRHMT-
-YPRHML-
-YPRHMN-
YHPRHM--
YRPRHM--
YGPRHM--
YDPRHM--
YYPRHM--
-YPRHMF-
-YPRHME-
YMPRHM--
YVPRHM--
-YPRHMS-
-YPRHMK-
-YPRHMY-
-YPRHMV-
YPPRHM--
YIPRHM--
YEPRHM--

| | | | |
|---|---|---|---|
| PMNL | 141 | 743 | 32.56 |

FAPMNL--
-IPMNLN-
--PMNLEL
--PMNLNL
-YPMNLP-
-APMNLY-
-APMNLT-
-APMNLR-
-APMNLV-
-APMNLK-
AVPMNL--
AKPMNL--
AGPMNL--
ASPMNL--
-APMNLI-

ANPMNL--
AEPMNL--
AMPMNL--
AIPMNL--
AYPMNL--
ARPMNL--
AQPMNL--
APPMNL--
ALPMNL--
ADPMNL--
-APMNLM-
AHPMNL--
-APMNLH-
-APMNLG-
-APMNLA-
-APMNLW-
-APMNLQ-
-APMNLN-
-APMNLE-
AFPMNL--
AAPMNL--
CAPMNL--
CHPMNL--
CLPMNL--
CSPMNL--
CRPMNL--
-CPMNLY-
-CPMNLI-
-CPMNLF-
CKPMNL--
DNPMNL--
DRPMNL--
-DPMNLA-
DMPMNL--
DSPMNL--
-DPMNLY-
DEPMNL--
DAPMNL--
-DPMNLV-
-DPMNLM-
DLPMNL--
-DPMNLR-
-DPMNLQ-
DHPMNL--
DQPMNL--
-DPMNLS-
-DPMNLL-
-DPMNLI-
-DPMNLH-
-DPMNLF-

DKPMNL--
DGPMNL--
DFPMNL--
ESPMNL--
-EPMNLS-
-EPMNLL-
-EPMNLM-
-EPMNLK-
-EPMNLI-
EVPMNL--
-EPMNLY-
-EPMNLR-
ERPMNL--
ELPMNL--
EMPMNL--
EIPMNL--
EWPMNL--
ETPMNL--
-EPMNLW-
-EPMNLQ-
EAPMNL--
EYPMNL--
EQPMNL--
-EPMNLV-
-EPMNLT-
-EPMNLN-
-EPMNLG-
-EPMNLA-
EKPMNL--
EFPMNL--
-FPMNLS-
FVPMNL--
FGPMNL--
-FPMNLL-
FRPMNL--
-FPMNLM-
FPPMNL--
-FPMNLN-
FNPMNL--
FQPMNL--
-FPMNLR-
-FPMNLE-
-FPMNLA-
FHPMNL--
-FPMNLH-
FLPMNL--
-FPMNLY-
-FPMNLV-
-FPMNLT-
FYPMNL--

-FPMNLQ-
-FPMNLG-
-FPMNLF-
FFPMNL--
GRPMNL--
-GPMNLR-
-GPMNLS-
GAPMNL--
GYPMNL--
GTPMNL--
-GPMNLL-
-GPMNLA-
GLPMNL--
-GPMNLF-
GKPMNL--
GSPMNL--
GQPMNL--
-GPMNLT-
-GPMNLQ-
GMPMNL--
GWPMNL--
-GPMNLM-
GGPMNL--
-GPMNLY-
-GPMNLW-
GIPMNL--
GFPMNL--
-GPMNLE-
GVPMNL--
-GPMNLH-
GEPMNL--
GPPMNL--
-GPMNLN-
-GPMNLK-
-GPMNLG-
GDPMNL--
-GPMNLV-
-GPMNLC-
HRPMNL--
-HPMNLS-
-HPMNLR-
HLPMNL--
-HPMNLF-
HIPMNL--
-HPMNLY-
-HPMNLM-
HGPMNL--
HYPMNL--
HPPMNL--
-HPMNLL-

-HPMNLI-
-HPMNLG-
-HPMNLA-
HKPMNL--
-HPMNLN-
-HPMNLH-
HNPMNL--
HMPMNL--
HHPMNL--
HSPMNL--
HQPMNL--
-HPMNLV-
-HPMNLK-
-HPMNLE-
HFPMNL--
HWPMNL--
HTPMNL--
-HPMNLT-
HAPMNL--
IYPMNL--
IHPMNL--
IMPMNL--
IVPMNL--
-IPMNLL-
-IPMNLE-
-IPMNLA-
-IPMNLT-
ISPMNL--
IRPMNL--
-IPMNLV-
-IPMNLI-
IDPMNL--
IWPMNL--
-IPMNLR-
-IPMNLH-
-IPMNLG-
IGPMNL--
IFPMNL--
IEPMNL--
IPPMNL--
IIPMNL--
IQPMNL--
INPMNL--
ILPMNL--
IKPMNL--
IAPMNL--
ITPMNL--
-IPMNLM-
-IPMNLK-
KSPMNL--

-KPMNLS-
-KPMNLY-
-KPMNLV-
-KPMNLR-
-KPMNLI-
-KPMNLA-
-KPMNLH-
-KPMNLL-
KLPMNL--
KKPMNL--
KGPMNL--
-KPMNLT-
-KPMNLG-
KVPMNL--
-KPMNLE-
KMPMNL--
KHPMNL--
KAPMNL--
-KPMNLF-
KQPMNL--
KPPMNL--
-KPMNLN-
KIPMNL--
KFPMNL--
KDPMNL--
-KPMNLW-
-KPMNLM-
KNPMNL--
KEPMNL--
KCPMNL--
LRPMNL--
-LPMNLR-
-LPMNLL-
-LPMNLK-
LSPMNL--
LLPMNL--
LAPMNL--
LGPMNL--
LKPMNL--
-LPMNLT-
-LPMNLS-
-LPMNLI-
LIPMNL--
-LPMNLV-
-LPMNLH-
LDPMNL--
-LPMNLM-
LFPMNL--
-LPMNLA-
LWPMNL--

LQPMNL--
LTPMNL--
LPPMNL--
-LPMNLQ-
LMPMNL--
LEPMNL--
-LPMNLW-
-LPMNLG-
LHPMNL--
-MPMNLS-
MSPMNL--
MRPMNL--
-MPMNLT-
-MPMNLQ-
-MPMNLN-
MLPMNL--
-MPMNLF-
-MPMNLY-
MGPMNL--
MYPMNL--
-MPMNLH-
MHPMNL--
-MPMNLI-
MDPMNL--
MWPMNL--
MFPMNL--
MEPMNL--
MAPMNL--
-MPMNLV-
MCPMNL--
MVPMNL--
MTPMNL--
-MPMNLA-
MMPMNL--
MKPMNL--
-NPMNLR-
NLPMNL--
NRPMNL--
NWPMNL--
NTPMNL--
-NPMNLL-
-NPMNLA-
NSPMNL--
NNPMNL--
-NPMNLV-
-NPMNLN-
NPPMNL--
-NPMNLY-
-NPMNLQ-
NEPMNL--

```
NKPMNL--
NGPMNL--
NYPMNL--
NQPMNL--
-NPMNLM-
NMPMNL--
NIPMNL--
-NPMNLT-
-NPMNLI-
-NPMNLF-
-NPMNLE-
NHPMNL--
NAPMNL--
--PMNLRL
--PMNLRV
--PMNLRR
--PMNLLT
--PMNLRW
--PMNLRK
--PMNLQT
--PMNLYG
--PMNLTG
--PMNLKT
--PMNLST
PVPMNL--
--PMNLRT
--PMNLNS
--PMNLPL
--PMNLAT
--PMNLSL
--PMNLIG
--PMNLGS
--PMNLYT
--PMNLWK
--PMNLVR
--PMNLRG
--PMNLQK
--PMNLML
--PMNLFR
--PMNLTS
--PMNLRY
--PMNLMD
--PMNLKS
--PMNLGV
--PMNLFK
--PMNLWA
--PMNLTH
--PMNLSG
--PMNLLH
--PMNLFS
```

--PMNLWG
--PMNLVY
--PMNLVT
--PMNLSN
--PMNLRH
--PMNLKR
--PMNLKK
--PMNLKA
--PMNLHV
--PMNLHR
--PMNLYS
--PMNLWS
--PMNLTD
--PMNLSQ
--PMNLRI
--PMNLMS
--PMNLME
--PMNLKN
--PMNLKL
--PMNLAL
--PMNLAG
--PMNLTY
--PMNLSI
--PMNLRF
--PMNLQN
--PMNLQA
--PMNLIN
--PMNLIE
--PMNLGI
--PMNLGA
--PMNLFT
PIPMNL--
PGPMNL--
PWPMNL--
--PMNLWT
--PMNLWQ
--PMNLVM
--PMNLRN
--PMNLRE
--PMNLQQ
--PMNLMG
--PMNLLQ
--PMNLKY
--PMNLIK
--PMNLAN
--PMNLYH
--PMNLVW
--PMNLVH
--PMNLTM
--PMNLTK

--PMNLSV
--PMNLSR
--PMNLRQ
--PMNLQW
--PMNLQG
--PMNLPY
--PMNLNV
--PMNLMH
--PMNLLS
--PMNLKH
--PMNLIS
--PMNLIA
--PMNLHS
--PMNLHG
--PMNLGY
--PMNLGM
--PMNLAM
PLPMNL--
--PMNLYR
--PMNLYE
--PMNLVK
--PMNLSK
--PMNLSF
--PMNLNW
--PMNLNT
--PMNLMY
--PMNLMR
--PMNLMQ
--PMNLLD
--PMNLKW
--PMNLKQ
--PMNLKI
--PMNLHQ
--PMNLHM
--PMNLGF
--PMNLFQ
--PMNLAW
--PMNLAR
--PMNLAH
PFPMNL--
PQPMNL--
PNPMNL--
--PMNLYQ
--PMNLWH
--PMNLVN
--PMNLVI
--PMNLVE
--PMNLTQ
--PMNLTN
--PMNLSH

--PMNLQM
--PMNLQD
--PMNLNK
--PMNLNA
--PMNLMN
--PMNLKD
--PMNLHY
--PMNLHF
--PMNLHA
--PMNLGG
--PMNLAI
PKPMNL--
PHPMNL--
PTPMNL--
-PPMNLE-
--PMNLYN
--PMNLWF
--PMNLVQ
--PMNLTA
--PMNLSY
--PMNLSE
--PMNLSA
--PMNLNM
--PMNLNH
--PMNLMT
--PMNLMK
--PMNLMI
--PMNLLE
--PMNLKM
--PMNLKG
--PMNLKF
--PMNLHI
--PMNLGW
--PMNLFA
--PMNLEQ
--PMNLEM
--PMNLEH
--PMNLAS
--PMNLAQ
--PMNLAK
--PMNLAA
PEPMNL--
PYPMNL--
--PMNLYK
--PMNLYA
--PMNLWW
--PMNLWN
--PMNLWM
--PMNLWI
--PMNLWE

--PMNLTI
--PMNLSM
--PMNLRD
--PMNLQY
--PMNLQV
--PMNLQF
--PMNLQE
--PMNLPV
--PMNLNR
--PMNLMM
--PMNLMA
--PMNLKV
--PMNLID
--PMNLHW
--PMNLHN
--PMNLHK
--PMNLHH
--PMNLGT
--PMNLFW
--PMNLFH
--PMNLFG
--PMNLEV
--PMNLET
--PMNLEI
--PMNLDA
--PMNLAY
PDPMNL--
QWPMNL--
QTPMNL--
QVPMNL--
QSPMNL--
-QPMNLS-
-QPMNLL-
QRPMNL--
QQPMNL--
-QPMNLT-
-QPMNLQ-
QPPMNL--
-QPMNLW-
-QPMNLR-
QHPMNL--
-QPMNLM-
-QPMNLK-
-QPMNLI-
-QPMNLH-
QNPMNL--
-QPMNLY-
-QPMNLV-
-QPMNLE-
-QPMNLA-

QKPMNL--
QFPMNL--
QEPMNL--
QAPMNL--
-QPMNLG-
-QPMNLF-
QDPMNL--
RGPMNL--
RSPMNL--
-RPMNLR-
RVPMNL--
-RPMNLI-
-RPMNLG-
RIPMNL--
-RPMNLV-
RKPMNL--
-RPMNLQ-
RHPMNL--
RPPMNL--
RNPMNL--
RYPMNL--
-RPMNLH-
REPMNL--
-RPMNLT-
RDPMNL--
RQPMNL--
-RPMNLA-
-RPMNLN-
RAPMNL--
-RPMNLY-
RTPMNL--
-RPMNLE-
-RPMNLM-
-RPMNLF-
RCPMNL--
SLPMNL--
-SPMNLN-
-SPMNLV-
-SPMNLS-
-SPMNLI-
SMPMNL--
SSPMNL--
SPPMNL--
-SPMNLY-
SGPMNL--
SWPMNL--
-SPMNLG-
SRPMNL--
-SPMNLF-
-SPMNLE-

SKPMNL--
-SPMNLA-
SIPMNL--
SVPMNL--
-SPMNLK-
SHPMNL--
STPMNL--
-SPMNLQ-
-SPMNLM-
-SPMNLH-
SDPMNL--
SAPMNL--
SYPMNL--
-SPMNLW-
SEPMNL--
TVPMNL--
TRPMNL--
-TPMNLL-
-TPMNLF-
TLPMNL--
-TPMNLS-
TNPMNL--
TDPMNL--
TYPMNL--
-TPMNLR-
TTPMNL--
-TPMNLV-
-TPMNLQ-
THPMNL--
TAPMNL--
-TPMNLM-
-TPMNLA-
TSPMNL--
-TPMNLK-
TIPMNL--
TFPMNL--
-TPMNLT-
TEPMNL--
-TPMNLY-
-TPMNLI-
-TPMNLH-
-TPMNLG-
TKPMNL--
TWPMNL--
TQPMNL--
TPPMNL--
-TPMNLN-
TMPMNL--
TCPMNL--
-VPMNLT-

VLPMNL--
-VPMNLK-
VTPMNL--
VAPMNL--
-VPMNLI-
VVPMNL--
VPPMNL--
-VPMNLS-
-VPMNLN-
-VPMNLG-
VHPMNL--
VSPMNL--
-VPMNLH-
VMPMNL--
VWPMNL--
VFPMNL--
-VPMNLE-
-VPMNLA-
VKPMNL--
VYPMNL--
-VPMNLV-
-VPMNLM-
VNPMNL--
VGPMNL--
-VPMNLQ-
VEPMNL--
VDPMNL--
VQPMNL--
-VPMNLD-
VIPMNL--
WSPMNL--
WPPMNL--
-WPMNLR-
WKPMNL--
-WPMNLL-
WVPMNL--
WRPMNL--
-WPMNLT-
-WPMNLH-
WQPMNL--
-WPMNLV-
WYPMNL--
-WPMNLS-
-WPMNLK-
-WPMNLA-
WNPMNL--
WGPMNL--
WEPMNL--
WDPMNL--
YSPMNL--

-YPMNLM-
-YPMNLS-
-YPMNLG-
-YPMNLQ-
-YPMNLK-
YAPMNL--
-YPMNLA-
YYPMNL--
YPPMNL--
-YPMNLT-
-YPMNLR-
YMPMNL--
YEPMNL--
-YPMNLV-
-YPMNLY-
-YPMNLN-
-YPMNLE-
YWPMNL--
-YPMNLI-
-YPMNLH-
YHPMNL--
YTPMNL--
YQPMNL--
YNPMNL--
YKPMNL--
YDPMNL--
YFPMNL--
YCPMNL--

| | | | |
|---|---|---|---|
| PVGM | 68 | 791 | 32.56 |

ESPVGM--
NLPVGM--
-APVGMR-
-APVGMK-
-APVGMT-
-APVGML-
-APVGMH-
-APVGMF-
AKPVGM--
ASPVGM--
ARPVGM--
-APVGMI-
-APVGMN-
AYPVGM--
-APVGMQ-
-APVGMY-
AMPVGM--
ALPVGM--
AVPVGM--
-APVGMW-
-APVGMV-

-APVGMS-
-APVGMM-
-APVGMG-
AHPVGM--
AGPVGM--
-APVGMA-
AWPVGM--
AQPVGM--
-APVGMC-
APPVGM--
ANPVGM--
ADPVGM--
CSPVGM--
CFPVGM--
-CPVGMY-
-CPVGMR-
CLPVGM--
CTPVGM--
CRPVGM--
CQPVGM--
-CPVGMV-
-CPVGML-
-CPVGMH-
CNPVGM--
CKPVGM--
CIPVGM--
CHPVGM--
CAPVGM--
-DPVGMI-
DLPVGM--
-DPVGMS-
DVPVGM--
DKPVGM--
-DPVGMY-
-DPVGMR-
-DPVGMN-
DRPVGM--
-DPVGMV-
-DPVGML-
-DPVGMK-
DFPVGM--
DWPVGM--
DSPVGM--
-DPVGMW-
-DPVGMQ-
-DPVGME-
-DPVGMA-
DNPVGM--
DMPVGM--
DIPVGM--

DEPVGM--
DDPVGM--
DAPVGM--
-EPVGMK-
-EPVGMY-
-EPVGML-
EMPVGM--
ERPVGM--
-EPVGMS-
EVPVGM--
-EPVGMW-
-EPVGMT-
-EPVGMH-
EGPVGM--
EYPVGM--
EQPVGM--
-EPVGMR-
-EPVGMM-
-EPVGMI-
-EPVGMF-
ETPVGM--
-EPVGMV-
-EPVGMQ-
-EPVGMA-
ENPVGM--
ELPVGM--
EKPVGM--
EDPVGM--
-FPVGMR-
-FPVGML-
FRPVGM--
-FPVGMY-
FQPVGM--
FNPVGM--
FDPVGM--
FTPVGM--
FLPVGM--
-FPVGMH-
-FPVGMS-
FKPVGM--
FYPVGM--
FSPVGM--
-FPVGMW-
-FPVGMQ-
-FPVGMN-
-FPVGMK-
FIPVGM--
FHPVGM--
FAPVGM--
FCPVGM--

FWPVGM--
FVPVGM--
-FPVGMV-
-FPVGMM-
-FPVGMI-
-FPVGMF-
-FPVGMA-
-FPVGMT-
-FPVGMG-
FEPVGM--
GRPVGM--
GTPVGM--
-GPVGMR-
GLPVGM--
GSPVGM--
GKPVGM--
-GPVGML-
-GPVGMH-
-GPVGMY-
-GPVGMS-
-GPVGMW-
-GPVGMI-
GWPVGM--
-GPVGMV-
-GPVGMG-
GYPVGM--
-GPVGMK-
GVPVGM--
-GPVGMA-
GQPVGM--
-GPVGMM-
GNPVGM--
GMPVGM--
GGPVGM--
-GPVGMN-
-GPVGMF-
-GPVGME-
GIPVGM--
GCPVGM--
GAPVGM--
-GPVGMQ-
GPPVGM--
GEPVGM--
GDPVGM--
-HPVGMR-
-HPVGMV-
-HPVGMS-
-HPVGML-
-HPVGMK-
-HPVGMI-

```
-HPVGMY-
HVPVGM--
HNPVGM--
HPPVGM--
HTPVGM--
-HPVGMW-
-HPVGME-
HIPVGM--
HYPVGM--
-HPVGMQ-
-HPVGMA-
HHPVGM--
HAPVGM--
HWPVGM--
HQPVGM--
-HPVGMN-
-HPVGMT-
-HPVGMH-
-HPVGMF-
HKPVGM--
HFPVGM--
HDPVGM--
-IPVGMH-
-IPVGMY-
-IPVGMI-
-IPVGMN-
-IPVGMF-
IRPVGM--
ISPVGM--
-IPVGMT-
-IPVGMG-
INPVGM--
-IPVGMS-
ITPVGM--
-IPVGMR-
-IPVGMK-
IHPVGM--
IVPVGM--
-IPVGMW-
-IPVGMQ-
-IPVGML-
-IPVGME-
-IPVGMA-
IKPVGM--
IIPVGM--
IEPVGM--
IYPVGM--
IWPVGM--
-IPVGMM-
ILPVGM--
```

IFPVGM--
IDPVGM--
IAPVGM--
KSPVGM--
-KPVGML-
KRPVGM--
-KPVGMI-
KIPVGM--
-KPVGMW-
KLPVGM--
KGPVGM--
-KPVGMR-
KYPVGM--
-KPVGMV-
KVPVGM--
KQPVGM--
-KPVGMT-
-KPVGMM-
-KPVGMH-
KPPVGM--
-KPVGMS-
-KPVGMK-
-KPVGMF-
-KPVGMA-
KNPVGM--
KDPVGM--
KWPVGM--
KTPVGM--
KHPVGM--
-LPVGML-
-LPVGMR-
LSPVGM--
-LPVGMS-
LVPVGM--
-LPVGMK-
LTPVGM--
LFPVGM--
-LPVGMQ-
LLPVGM--
LWPVGM--
LQPVGM--
-LPVGMT-
LKPVGM--
LAPVGM--
-LPVGMV-
LGPVGM--
-LPVGMW-
LIPVGM--
-LPVGMI-
LNPVGM--

-LPVGMN-
-LPVGMH-
LHPVGM--
LEPVGM--
-LPVGMY-
LMPVGM--
LCPVGM--
LYPVGM--
-LPVGMM-
-LPVGMG-
-LPVGME-
LDPVGM--
-MPVGML-
-MPVGMR-
-MPVGMM-
MLPVGM--
-MPVGMF-
MGPVGM--
MTPVGM--
MSPVGM--
-MPVGMS-
-MPVGMI-
MAPVGM--
MWPVGM--
MVPVGM--
MRPVGM--
-MPVGMW-
-MPVGMN-
MKPVGM--
MYPVGM--
-MPVGMY-
-MPVGMT-
MIPVGM--
MEPVGM--
-MPVGMV-
-MPVGMQ-
-MPVGMH-
-MPVGME-
-MPVGMA-
MNPVGM--
MHPVGM--
NRPVGM--
NVPVGM--
NFPVGM--
-NPVGMK-
-NPVGMY-
-NPVGMS-
-NPVGMT-
NKPVGM--
NGPVGM--

NSPVGM--
-NPVGMN-
-NPVGMI-
NNPVGM--
-NPVGMR-
-NPVGML-
-NPVGMA-
-NPVGMH-
-NPVGMV-
-NPVGMF-
NHPVGM--
NWPVGM--
-NPVGMW-
-NPVGMQ-
NCPVGM--
NTPVGM--
-NPVGME-
NMPVGM--
NIPVGM--
NDPVGM--
--PVGMRS
--PVGMLS
--PVGMLR
--PVGMRI
--PVGMRG
--PVGMFR
--PVGMST
--PVGMKV
--PVGMSS
--PVGMRY
--PVGMHL
--PVGMWT
--PVGMSG
--PVGMVY
--PVGMRT
--PVGMLD
--PVGMSF
--PVGMRQ
--PVGMLN
--PVGMLL
--PVGMYR
--PVGMTR
--PVGMMQ
--PVGMWS
--PVGMVQ
--PVGMTN
--PVGMSV
--PVGMNA
--PVGMMT
--PVGMLQ

--PVGMTS
--PVGMSN
--PVGMSL
--PVGMRV
--PVGMRF
--PVGMLG
--PVGMKK
--PVGMKG
--PVGMHS
--PVGMGS
--PVGMVS
--PVGMVI
--PVGMTV
--PVGMKI
--PVGMGV
--PVGMFT
--PVGMVG
--PVGMTM
--PVGMTL
--PVGMSY
--PVGMSK
--PVGMRN
--PVGMMS
PSPVGM--
PIPVGM--
--PVGMYS
--PVGMYQ
--PVGMYK
--PVGMVR
--PVGMRH
--PVGMMR
--PVGMML
--PVGMMI
--PVGMMH
--PVGMLT
--PVGMLK
PAPVGM--
--PVGMWG
--PVGMTT
--PVGMTK
--PVGMSD
--PVGMRM
--PVGMLY
--PVGMLV
--PVGMIY
--PVGMIS
--PVGMFV
--PVGMFG
--PVGMAS
--PVGMAL

--PVGMAG
--PVGMAF
PLPVGM--
PYPVGM--
--PVGMWR
--PVGMTI
--PVGMSW
--PVGMRK
--PVGMLM
--PVGMLI
--PVGMKS
--PVGMKN
--PVGMKH
--PVGMHH
--PVGMAY
--PVGMAV
PMPVGM--
--PVGMVN
--PVGMVK
--PVGMTG
--PVGMTF
--PVGMTD
--PVGMSH
--PVGMRE
--PVGMQA
--PVGMLH
--PVGMKY
--PVGMIM
--PVGMIK
--PVGMHI
--PVGMFS
--PVGMFH
--PVGMAR
--PVGMAN
--PVGMYH
--PVGMWN
--PVGMVW
--PVGMVE
--PVGMVA
--PVGMTY
--PVGMSM
--PVGMSA
--PVGMQH
--PVGMNW
--PVGMNR
--PVGMNH
--PVGMNE
--PVGMMK
--PVGMKR
--PVGMIV

--PVGMIR
--PVGMII
--PVGMHR
--PVGMHM
--PVGMHE
--PVGMGL
--PVGMES
-PPVGMV-
--PVGMYA
--PVGMVV
--PVGMVT
--PVGMVF
--PVGMTW
--PVGMSQ
--PVGMRW
--PVGMQV
--PVGMQT
--PVGMQR
--PVGMQE
--PVGMNS
--PVGMKM
--PVGMKA
--PVGMIT
--PVGMIQ
--PVGMHV
--PVGMHN
--PVGMHA
--PVGMET
--PVGMAM
PHPVGM--
PGPVGM--
PFPVGM--
PEPVGM--
PWPVGM--
PVPVGM--
--PVGMYT
--PVGMYN
--PVGMVM
--PVGMVH
--PVGMVD
--PVGMSE
--PVGMRD
--PVGMQY
--PVGMQS
--PVGMQI
--PVGMNV
--PVGMNT
--PVGMNM
--PVGMNI
--PVGMMV

--PVGMMN
--PVGMMG
--PVGMMA
--PVGMKL
--PVGMHY
--PVGMHW
--PVGMHT
--PVGMHG
--PVGMHF
--PVGMGR
--PVGMGG
--PVGMGA
--PVGMFK
--PVGMFF
--PVGMEH
--PVGMEA
--PVGMDS
--PVGMAW
--PVGMAD
PQPVGM--
--PVGMYD
--PVGMWM
--PVGMWH
--PVGMWD
--PVGMTQ
--PVGMTA
--PVGMSC
--PVGMRP
--PVGMQM
--PVGMQL
--PVGMQK
--PVGMQF
--PVGMNQ
--PVGMNK
--PVGMNG
--PVGMND
--PVGMMY
--PVGMMD
--PVGMLE
--PVGMKE
--PVGMKD
--PVGMKC
--PVGMIW
--PVGMIN
--PVGMIH
--PVGMIG
--PVGMHQ
--PVGMHK
--PVGMGQ
--PVGMGK

--PVGMGD
--PVGMFQ
--PVGMFN
--PVGMFM
--PVGMEK
--PVGMDM
--PVGMDG
--PVGMAT
--PVGMAQ
--PVGMAK
--PVGMAE
PTPVGM--
PNPVGM--
-QPVGML-
-QPVGMR-
-QPVGMS-
QVPVGM--
-QPVGMA-
QYPVGM--
-QPVGME-
QLPVGM--
QWPVGM--
-QPVGMV-
-QPVGMI-
-QPVGMH-
-QPVGMW-
-QPVGMT-
-QPVGMF-
QKPVGM--
QTPVGM--
QRPVGM--
QAPVGM--
QSPVGM--
QNPVGM--
QMPVGM--
QIPVGM--
QGPVGM--
RSPVGM--
RMPVGM--
RVPVGM--
-RPVGMN-
-RPVGMG-
-RPVGMV-
-RPVGMM-
-RPVGMH-
-RPVGMA-
RAPVGM--
RWPVGM--
RNPVGM--
RQPVGM--

−RPVGMW−
−RPVGMK−
RLPVGM−−
−RPVGMF−
RFPVGM−−
RTPVGM−−
−RPVGME−
RIPVGM−−
RHPVGM−−
−RPVGMQ−
RDPVGM−−
RCPVGM−−
REPVGM−−
−SPVGML−
−SPVGMS−
SVPVGM−−
SNPVGM−−
SRPVGM−−
SKPVGM−−
SAPVGM−−
−SPVGMV−
−SPVGMN−
−SPVGMM−
STPVGM−−
SYPVGM−−
SSPVGM−−
−SPVGMF−
−SPVGMT−
−SPVGMQ−
−SPVGMY−
−SPVGMR−
−SPVGMA−
SHPVGM−−
SLPVGM−−
SFPVGM−−
SWPVGM−−
−SPVGMG−
SQPVGM−−
−SPVGMW−
−SPVGMK−
SPPVGM−−
SEPVGM−−
−SPVGME−
SDPVGM−−
SIPVGM−−
SMPVGM−−
−SPVGMH−
−SPVGMC−
TMPVGM−−
TVPVGM−−

-TPVGMR-
TYPVGM--
-TPVGMT-
TTPVGM--
-TPVGMV-
-TPVGMA-
TRPVGM--
-TPVGMS-
-TPVGMF-
TNPVGM--
-TPVGMK-
TIPVGM--
TWPVGM--
-TPVGMY-
-TPVGMW-
TLPVGM--
-TPVGMI-
TKPVGM--
TAPVGM--
-TPVGMN-
-TPVGMM-
TGPVGM--
TSPVGM--
-TPVGMG-
-TPVGMQ-
-TPVGME-
TEPVGM--
TQPVGM--
-TPVGMH-
THPVGM--
TDPVGM--
-VPVGMS-
VRPVGM--
-VPVGML-
VSPVGM--
VTPVGM--
VVPVGM--
VEPVGM--
-VPVGMV-
-VPVGMG-
-VPVGMW-
-VPVGMR-
-VPVGMI-
-VPVGMM-
VKPVGM--
VPPVGM--
VLPVGM--
VNPVGM--
VIPVGM--
-VPVGMA-

VQPVGM--
-VPVGMH-
VFPVGM--
VWPVGM--
-VPVGMN-
-VPVGMK-
VAPVGM--
-VPVGMY-
-VPVGMF-
-VPVGME-
VMPVGM--
VHPVGM--
VGPVGM--
VCPVGM--
-WPVGMV-
WRPVGM--
WIPVGM--
WVPVGM--
WLPVGM--
WNPVGM--
-WPVGMA-
WFPVGM--
WEPVGM--
WSPVGM--
-WPVGMT-
-WPVGMK-
-WPVGMI-
-WPVGMY-
-WPVGML-
WMPVGM--
WYPVGM--
WTPVGM--
WGPVGM--
-WPVGMF-
-WPVGMM-
WKPVGM--
WHPVGM--
WDPVGM--
-YPVGMR-
-YPVGMS-
YTPVGM--
YLPVGM--
YSPVGM--
-YPVGML-
-YPVGMT-
YRPVGM--
-YPVGMH-
YFPVGM--
-YPVGMI-
-YPVGMQ-

-YPVGMG-
YVPVGM--
-YPVGMV-
-YPVGMA-
YNPVGM--
-YPVGMN-
-YPVGMF-
YIPVGM--
YGPVGM--
YWPVGM--
-YPVGMK-
-YPVGME-
YPPVGM--
YKPVGM--
YCPVGM--
YQPVGM--
-YPVGMW-
-YPVGMM-
YMPVGM--
YAPVGM--

| | | | |
|---|---|---|---|
| PRPF | 147 | 740 | 32.53 |

-GPRPFH-
--PRPFNR
--PRPFGR
-SPRPFG-
-APRPFM-
-APRPFL-
-APRPFY-
AHPRPF--
-APRPFG-
AFPRPF--
ATPRPF--
ARPRPF--
-APRPFT-
-APRPFR-
AMPRPF--
AKPRPF--
AQPRPF--
AGPRPF--
AYPRPF--
-APRPFW-
-APRPFQ-
-APRPFN-
-APRPFE-
AIPRPF--
AAPRPF--
AWPRPF--
-APRPFK-
-APRPFI-
-APRPFH-

APPRPF--
AEPRPF--
AVPRPF--
-APRPFF-
-APRPFC-
ACPRPF--
CRPRPF--
CVPRPF--
CQPRPF--
-CPRPFS-
CGPRPF--
CSPRPF--
-CPRPFT-
-CPRPFI-
CPPRPF--
CMPRPF--
CLPRPF--
CHPRPF--
CAPRPF--
DLPRPF--
-DPRPFS-
-DPRPFL-
-DPRPFK-
-DPRPFI-
-DPRPFY-
-DPRPFF-
-DPRPFA-
DYPRPF--
DVPRPF--
DSPRPF--
DRPRPF--
DMPRPF--
DTPRPF--
-DPRPFR-
DHPRPF--
DEPRPF--
-DPRPFW-
-DPRPFV-
-DPRPFH-
DNPRPF--
DKPRPF--
DGPRPF--
DFPRPF--
DAPRPF--
ESPRPF--
ERPRPF--
EGPRPF--
EYPRPF--
EMPRPF--
ELPRPF--

-EPRPFV-
-EPRPFH-
EFPRPF--
EVPRPF--
ETPRPF--
-EPRPFN-
-EPRPFA-
EQPRPF--
-EPRPFM-
-EPRPFI-
-EPRPFG-
EIPRPF--
EAPRPF--
EWPRPF--
-EPRPFW-
-EPRPFT-
-EPRPFR-
-EPRPFL-
-EPRPFF-
-FPRPFV-
FHPRPF--
FTPRPF--
FRPRPF--
-FPRPFM-
FLPRPF--
FYPRPF--
FGPRPF--
-FPRPFK-
FDPRPF--
FVPRPF--
-FPRPFT-
FPPRPF--
FIPRPF--
FSPRPF--
FKPRPF--
FWPRPF--
-FPRPFY-
-FPRPFF-
FNPRPF--
-FPRPFW-
-FPRPFN-
-FPRPFG-
-FPRPFA-
FMPRPF--
GSPRPF--
-GPRPFM-
GVPRPF--
-GPRPFV-
-GPRPFS-
-GPRPFL-

GIPRPF--
GLPRPF--
-GPRPFN-
-GPRPFR-
-GPRPFF-
GAPRPF--
GTPRPF--
-GPRPFA-
GNPRPF--
-GPRPFT-
GMPRPF--
GFPRPF--
GEPRPF--
GDPRPF--
GRPRPF--
-GPRPFY-
-GPRPFW-
-GPRPFK-
GGPRPF--
GQPRPF--
-GPRPFD-
HVPRPF--
HGPRPF--
HLPRPF--
-HPRPFS-
HRPRPF--
HQPRPF--
HNPRPF--
-HPRPFM-
-HPRPFF-
-HPRPFA-
HPPRPF--
HYPRPF--
HSPRPF--
-HPRPFL-
-HPRPFI-
HKPRPF--
-HPRPFY-
-HPRPFH-
HIPRPF--
HFPRPF--
-HPRPFW-
-HPRPFV-
-HPRPFT-
-HPRPFN-
HMPRPF--
HHPRPF--
HEPRPF--
HAPRPF--
-IPRPFR-

-IPRPFL-
ILPRPF--
-IPRPFI-
-IPRPFW-
ITPRPF--
IMPRPF--
IWPRPF--
ISPRPF--
-IPRPFT-
IPPRPF--
IEPRPF--
IAPRPF--
IVPRPF--
IQPRPF--
INPRPF--
IGPRPF--
IYPRPF--
-IPRPFY-
-IPRPFF-
-IPRPFA-
IKPRPF--
-IPRPFQ-
IRPRPF--
ICPRPF--
KIPRPF--
-KPRPFH-
KRPRPF--
-KPRPFT-
-KPRPFA-
-KPRPFW-
KAPRPF--
-KPRPFM-
-KPRPFK-
KNPRPF--
KGPRPF--
-KPRPFV-
KKPRPF--
KLPRPF--
KFPRPF--
KTPRPF--
-KPRPFF-
-KPRPFR-
-KPRPFL-
KDPRPF--
-KPRPFY-
-KPRPFS-
-KPRPFI-
KEPRPF--
-KPRPFQ-
KHPRPF--

-KPRPFN-
LAPRPF--
-LPRPFS-
-LPRPFT-
LNPRPF--
-LPRPFW-
LMPRPF--
-LPRPFY-
-LPRPFI-
LGPRPF--
LRPRPF--
LFPRPF--
LPPRPF--
LTPRPF--
-LPRPFV-
LIPRPF--
-LPRPFK-
-LPRPFQ-
LKPRPF--
LWPRPF--
-LPRPFA-
LEPRPF--
LQPRPF--
LHPRPF--
LCPRPF--
-LPRPFF-
LDPRPF--
LYPRPF--
MIPRPF--
MVPRPF--
MSPRPF--
-MPRPFK-
-MPRPFH-
MTPRPF--
MFPRPF--
MQPRPF--
MGPRPF--
MDPRPF--
-MPRPFT-
-MPRPFS-
-MPRPFQ-
MKPRPF--
-MPRPFF-
MNPRPF--
MMPRPF--
MAPRPF--
-MPRPFY-
-MPRPFV-
-MPRPFN-
MEPRPF--

–MPRPFA–
MHPRPF––
–NPRPFL–
NPPRPF––
NGPRPF––
NSPRPF––
–NPRPFT–
–NPRPFS–
NAPRPF––
NYPRPF––
–NPRPFR–
NTPRPF––
NWPRPF––
–NPRPFI–
NVPRPF––
–NPRPFY–
–NPRPFH–
NRPRPF––
NKPRPF––
–NPRPFW–
–NPRPFK–
–NPRPFF–
NMPRPF––
NNPRPF––
NFPRPF––
–NPRPFM–
NLPRPF––
NDPRPF––
NCPRPF––
––PRPFRL
––PRPFLS
––PRPFYL
––PRPFRI
––PRPFVV
––PRPFSR
––PRPFSI
––PRPFLV
––PRPFLT
––PRPFLI
––PRPFQS
––PRPFST
––PRPFHL
––PRPFTQ
––PRPFQG
––PRPFFS
––PRPFVG
––PRPFSL
––PRPFIY
––PRPFFL
––PRPFTL

--PRPFSV
--PRPFRT
--PRPFMV
--PRPFYS
--PRPFMG
--PRPFFR
--PRPFIS
--PRPFWV
--PRPFSM
--PRPFML
--PRPFLK
--PRPFKL
--PRPFHN
--PRPFYM
--PRPFYA
--PRPFRV
--PRPFQA
--PRPFLF
--PRPFIA
--PRPFHA
--PRPFWN
--PRPFLG
--PRPFKG
--PRPFAT
--PRPFAH
--PRPFTS
--PRPFSH
--PRPFRA
--PRPFMR
--PRPFKT
--PRPFHV
--PRPFTN
--PRPFSY
--PRPFSQ
--PRPFSA
--PRPFWK
--PRPFTW
--PRPFSN
--PRPFRG
--PRPFNL
--PRPFMS
--PRPFIW
--PRPFIV
--PRPFIR
--PRPFHT
--PRPFGI
--PRPFFV
--PRPFEM
--PRPFAF
--PRPFYI

--PRPFSG
--PRPFRF
--PRPFQT
--PRPFNS
--PRPFLY
--PRPFLR
--PRPFLH
--PRPFKV
--PRPFIH
--PRPFHY
--PRPFGQ
--PRPFDY
PRPRPF--
--PRPFWL
--PRPFVF
--PRPFTV
--PRPFRN
--PRPFQY
--PRPFLM
--PRPFKY
--PRPFWY
--PRPFWG
--PRPFVY
--PRPFNH
--PRPFMY
--PRPFLQ
--PRPFIT
--PRPFHS
--PRPFGY
--PRPFGV
--PRPFGM
--PRPFGG
--PRPFGF
--PRPFEL
--PRPFAQ
--PRPFYY
--PRPFYH
--PRPFVW
--PRPFVQ
--PRPFVK
--PRPFRE
--PRPFQL
--PRPFPW
--PRPFNW
--PRPFME
--PRPFLA
--PRPFKQ
--PRPFFH
--PRPFEV
--PRPFAI

PMPRPF--
PIPRPF--
--PRPFYN
--PRPFWM
--PRPFVM
--PRPFTH
--PRPFTG
--PRPFRM
--PRPFQR
--PRPFPY
--PRPFPM
--PRPFNV
--PRPFMF
--PRPFKK
--PRPFKF
--PRPFKE
--PRPFIN
--PRPFFT
--PRPFFQ
--PRPFFG
--PRPFFA
--PRPFAV
--PRPFAG
--PRPFYT
--PRPFWS
--PRPFWH
--PRPFVT
--PRPFRW
--PRPFRQ
--PRPFRK
--PRPFRH
--PRPFQN
--PRPFPV
--PRPFNI
--PRPFNF
--PRPFMW
--PRPFMN
--PRPFMD
--PRPFKI
--PRPFIQ
--PRPFHR
--PRPFHK
--PRPFGA
PVPRPF--
--PRPFYD
--PRPFVN
--PRPFTF
--PRPFSW
--PRPFPQ
--PRPFPL

--PRPFPI
--PRPFNT
--PRPFKM
--PRPFIF
--PRPFID
--PRPFFY
--PRPFFF
--PRPFFE
--PRPFEW
--PRPFES
--PRPFDW
--PRPFAA
PQPRPF--
PHPRPF--
PYPRPF--
--PRPFYV
--PRPFYQ
--PRPFVI
--PRPFVE
--PRPFTY
--PRPFTM
--PRPFQM
--PRPFPF
--PRPFNA
--PRPFMH
--PRPFIE
--PRPFHM
--PRPFHI
--PRPFGT
--PRPFGS
--PRPFGH
--PRPFFW
--PRPFDL
--PRPFDF
--PRPFAY
--PRPFAW
--PRPFAM
PNPRPF--
PDPRPF--
PAPRPF--
PWPRPF--
--PRPFYW
--PRPFYE
--PRPFWW
--PRPFWQ
--PRPFWI
--PRPFWF
--PRPFWE
--PRPFVD
--PRPFQK

--PRPFQF
--PRPFPH
--PRPFNQ
--PRPFNM
--PRPFMM
--PRPFMI
--PRPFKW
--PRPFIM
--PRPFIK
--PRPFIG
--PRPFHH
--PRPFHF
--PRPFGK
--PRPFFP
--PRPFFN
--PRPFFD
--PRPFEY
--PRPFEG
--PRPFED
--PRPFDV
--PRPFDS
--PRPFDI
--PRPFCV
--PRPFAN
PKPRPF--
PGPRPF--
-QPRPFL-
QSPRPF--
QVPRPF--
QTPRPF--
-QPRPFT-
-QPRPFK-
QRPRPF--
-QPRPFS-
QEPRPF--
QQPRPF--
-QPRPFF-
QPPRPF--
QMPRPF--
-QPRPFI-
QNPRPF--
QIPRPF--
-QPRPFQ-
QAPRPF--
QWPRPF--
-QPRPFY-
-QPRPFV-
-QPRPFM-
-QPRPFH-
-QPRPFA-

QKPRPF--
QHPRPF--
QGPRPF--
RSPRPF--
RYPRPF--
RVPRPF--
-RPRPFI-
RIPRPF--
-RPRPFT-
-RPRPFW-
RDPRPF--
RLPRPF--
-RPRPFN-
RAPRPF--
-RPRPFM-
-RPRPFA-
RNPRPF--
RWPRPF--
-RPRPFY-
RMPRPF--
RFPRPF--
RRPRPF--
RQPRPF--
RHPRPF--
-RPRPFV-
-RPRPFF-
-RPRPFE-
RCPRPF--
STPRPF--
-SPRPFH-
-SPRPFR-
-SPRPFS-
SVPRPF--
SKPRPF--
SRPRPF--
SHPRPF--
SEPRPF--
SWPRPF--
-SPRPFI-
-SPRPFN-
SIPRPF--
SFPRPF--
SQPRPF--
-SPRPFF-
SPPRPF--
SGPRPF--
SAPRPF--
-SPRPFK-
SCPRPF--
-SPRPFA-

SDPRPF--
-SPRPFW-
-SPRPFQ-
SMPRPF--
SYPRPF--
-TPRPFL-
TSPRPF--
-TPRPFR-
TIPRPF--
-TPRPFF-
-TPRPFQ-
TTPRPF--
-TPRPFH-
TVPRPF--
-TPRPFS-
-TPRPFY-
TLPRPF--
-TPRPFW-
-TPRPFT-
-TPRPFA-
TNPRPF--
TRPRPF--
-TPRPFV-
THPRPF--
TGPRPF--
TMPRPF--
TCPRPF--
TQPRPF--
-TPRPFM-
-TPRPFI-
TPPRPF--
TEPRPF--
TAPRPF--
TWPRPF--
-TPRPFN-
VVPRPF--
-VPRPFI-
-VPRPFY-
-VPRPFF-
VMPRPF--
VIPRPF--
VTPRPF--
-VPRPFA-
-VPRPFW-
-VPRPFK-
VRPRPF--
VNPRPF--
VAPRPF--
VQPRPF--
-VPRPFV-

-VPRPFN-
VDPRPF--
VGPRPF--
VEPRPF--
-VPRPFQ-
VHPRPF--
-VPRPFH-
VPPRPF--
VFPRPF--
VCPRPF--
WSPRPF--
-WPRPFW-
WRPRPF--
-WPRPFY-
WDPRPF--
WAPRPF--
-WPRPFH-
WYPRPF--
-WPRPFA-
WMPRPF--
WQPRPF--
WKPRPF--
WGPRPF--
WNPRPF--
-WPRPFT-
-WPRPFK-
-WPRPFI-
-WPRPFF-
WWPRPF--
WTPRPF--
-WPRPFV-
-WPRPFN-
-WPRPFM-
WHPRPF--
-WPRPFG-
WFPRPF--
WCPRPF--
YVPRPF--
-YPRPFL-
-YPRPFT-
-YPRPFM-
-YPRPFA-
YGPRPF--
YRPRPF--
-YPRPFN-
YNPRPF--
YFPRPF--
YTPRPF--
YSPRPF--
YDPRPF--

YYPRPF--
-YPRPFI-
-YPRPFF-
YMPRPF--
YHPRPF--
-YPRPFK-
-YPRPFV-
-YPRPFE-
YIPRPF--
YEPRPF--
YAPRPF--

| | | | |
|---|---|---|---|
| PSPI | 67 | 791 | 32.52 |

--PSPIMT
-RPSPIQ-
SDPSPI--
-WPSPIG-
-APSPIL-
ARPSPI--
-APSPIS-
AKPSPI--
AGPSPI--
ATPSPI--
-APSPII-
-APSPIH-
-APSPIN-
-APSPIM-
AHPSPI--
-APSPIV-
-APSPIG-
-APSPIK-
-APSPIF-
AIPSPI--
AEPSPI--
AYPSPI--
AWPSPI--
ASPSPI--
-APSPIT-
-APSPIA-
AMPSPI--
-APSPIY-
AFPSPI--
AAPSPI--
-APSPIR-
-APSPIQ-
AVPSPI--
-APSPIC-
ANPSPI--
ALPSPI--
ADPSPI--
CSPSPI--

−CPSPIL−
−CPSPIF−
CRPSPI−−
−CPSPIT−
CGPSPI−−
CQPSPI−−
−CPSPIR−
CCPSPI−−
CWPSPI−−
CVPSPI−−
CTPSPI−−
−CPSPIK−
CDPSPI−−
DLPSPI−−
DYPSPI−−
−DPSPIK−
DTPSPI−−
DSPSPI−−
−DPSPIW−
−DPSPIF−
−DPSPIR−
DNPSPI−−
DHPSPI−−
DEPSPI−−
DAPSPI−−
DRPSPI−−
−DPSPIL−
−DPSPII−
−DPSPIH−
DWPSPI−−
DQPSPI−−
−DPSPIV−
−DPSPIS−
−DPSPIN−
−DPSPIA−
DKPSPI−−
DIPSPI−−
DGPSPI−−
DFPSPI−−
ESPSPI−−
−EPSPIL−
ETPSPI−−
−EPSPIR−
−EPSPIQ−
−EPSPIK−
−EPSPIA−
EFPSPI−−
ERPSPI−−
−EPSPIS−
ELPSPI−−

EYPSPI--
EWPSPI--
EVPSPI--
EMPSPI--
EHPSPI--
EEPSPI--
EQPSPI--
-EPSPIT-
-EPSPIM-
-EPSPIG-
FRPSPI--
-FPSPIS-
-FPSPIR-
FTPSPI--
FKPSPI--
-FPSPIL-
FMPSPI--
-FPSPIK-
FIPSPI--
FWPSPI--
FSPSPI--
FQPSPI--
FLPSPI--
FGPSPI--
FFPSPI--
-FPSPIT-
-FPSPII-
FDPSPI--
FCPSPI--
FYPSPI--
-FPSPIH-
-FPSPIF-
FPPSPI--
FHPSPI--
FEPSPI--
-FPSPIW-
-FPSPIV-
-FPSPIQ-
-FPSPIN-
-FPSPIM-
FNPSPI--
FAPSPI--
GTPSPI--
GAPSPI--
GWPSPI--
GKPSPI--
GIPSPI--
GVPSPI--
GRPSPI--
-GPSPIV-

−GPSPIK−
GYPSPI−−
−GPSPIF−
GNPSPI−−
GLPSPI−−
−GPSPIN−
GSPSPI−−
−GPSPIP−
−GPSPIY−
−GPSPIG−
−GPSPIW−
−GPSPIQ−
−GPSPIE−
−GPSPIA−
GMPSPI−−
GHPSPI−−
GFPSPI−−
GCPSPI−−
−GPSPIH−
GEPSPI−−
HIPSPI−−
−HPSPIS−
−HPSPIL−
HSPSPI−−
HLPSPI−−
HWPSPI−−
HQPSPI−−
−HPSPIR−
−HPSPIH−
HGPSPI−−
HRPSPI−−
−HPSPIV−
HFPSPI−−
HAPSPI−−
HYPSPI−−
HTPSPI−−
−HPSPIT−
−HPSPIM−
−HPSPIY−
−HPSPII−
HMPSPI−−
HKPSPI−−
−HPSPIK−
−HPSPIF−
−HPSPIA−
HPPSPI−−
IAPSPI−−
IWPSPI−−
IRPSPI−−
−IPSPIT−

-IPSPIL-
IGPSPI--
IFPSPI--
ITPSPI--
-IPSPIA-
IYPSPI--
-IPSPIK-
-IPSPII-
INPSPI--
IKPSPI--
-IPSPIV-
-IPSPIG-
-IPSPIF-
IMPSPI--
IIPSPI--
ILPSPI--
IHPSPI--
IVPSPI--
ISPSPI--
IQPSPI--
-IPSPIS-
-IPSPIQ-
-IPSPIH-
IPPSPI--
KSPSPI--
-KPSPIL-
-KPSPIK-
-KPSPIR-
KLPSPI--
KMPSPI--
KVPSPI--
KRPSPI--
-KPSPIS-
KAPSPI--
-KPSPIQ-
-KPSPII-
KIPSPI--
-KPSPIY-
-KPSPIM-
KNPSPI--
KGPSPI--
KYPSPI--
-KPSPIA-
KQPSPI--
-KPSPIH-
KHPSPI--
KEPSPI--
KDPSPI--
KCPSPI--
KTPSPI--

KPPSPI--
LRPSPI--
LSPSPI--
LNPSPI--
-LPSPIR-
LYPSPI--
LTPSPI--
LQPSPI--
LLPSPI--
LWPSPI--
-LPSPIT-
-LPSPIF-
LAPSPI--
-LPSPIL-
-LPSPIH-
LMPSPI--
-LPSPIM-
-LPSPIV-
-LPSPIQ-
LGPSPI--
-LPSPIS-
LKPSPI--
LDPSPI--
LPPSPI--
-LPSPIY-
-LPSPII-
LVPSPI--
-LPSPIK-
-LPSPIA-
LIPSPI--
-MPSPIR-
-MPSPIT-
MRPSPI--
MYPSPI--
-MPSPIF-
MWPSPI--
-MPSPIN-
MGPSPI--
MVPSPI--
MSPSPI--
-MPSPIG-
MNPSPI--
MKPSPI--
MLPSPI--
MEPSPI--
-MPSPIY-
-MPSPIV-
-MPSPIS-
-MPSPIM-
MMPSPI--

MAPSPI--
-MPSPIW-
-MPSPIL-
-MPSPIK-
-MPSPIH-
-MPSPIA-
MHPSPI--
MFPSPI--
MCPSPI--
-NPSPIS-
NLPSPI--
NFPSPI--
-NPSPIL-
NYPSPI--
-NPSPIR-
-NPSPIF-
NHPSPI--
NRPSPI--
NAPSPI--
NSPSPI--
-NPSPIH-
NVPSPI--
NTPSPI--
-NPSPIQ-
-NPSPIK-
-NPSPIA-
-NPSPIY-
-NPSPIW-
-NPSPIV-
NIPSPI--
NDPSPI--
NWPSPI--
-NPSPIM-
-NPSPII-
NNPSPI--
NKPSPI--
--PSPIRL
--PSPILR
--PSPIRT
--PSPIRS
--PSPIVR
--PSPISL
PRPSPI--
--PSPIGS
--PSPIFL
--PSPILF
--PSPIIV
--PSPIWL
--PSPINF
--PSPIMH

--PSPIST
--PSPILL
--PSPIHL
--PSPIVV
--PSPISR
--PSPIRW
--PSPIRM
--PSPILT
--PSPILQ
--PSPILM
--PSPILE
--PSPIGF
--PSPIWM
--PSPIVL
--PSPIVF
--PSPIRI
--PSPIGV
--PSPIFR
--PSPIES
--PSPIYM
--PSPITV
--PSPISV
--PSPIRV
--PSPIRH
--PSPILH
--PSPIKL
--PSPIIQ
--PSPIGA
--PSPIFK
--PSPIYS
--PSPIWV
--PSPITR
--PSPILI
--PSPIKV
--PSPIIT
--PSPIIM
--PSPIFV
PYPSPI--
--PSPIYA
--PSPIWG
--PSPITW
--PSPITF
--PSPISF
--PSPIQY
--PSPILY
--PSPILW
--PSPILV
--PSPIKS
--PSPIKH
--PSPIGN

--PSPIFI
--PSPIEL
--PSPITS
--PSPISA
--PSPIRA
--PSPIPF
--PSPINW
--PSPILG
--PSPIIS
--PSPIIR
--PSPIIL
--PSPIII
--PSPIFS
--PSPIEW
PLPSPI--
PTPSPI--
--PSPIWT
--PSPIWI
--PSPIVT
--PSPIVK
--PSPIVA
--PSPITD
--PSPISN
--PSPISG
--PSPIRQ
--PSPIPH
--PSPINL
--PSPIML
--PSPIKN
--PSPIKM
--PSPIHY
--PSPIFY
--PSPIEI
--PSPIAM
PQPSPI--
--PSPIYW
--PSPIYF
--PSPIYD
--PSPIWK
--PSPIVN
--PSPIVH
--PSPITL
--PSPISQ
--PSPISK
--PSPIRN
--PSPIRG
--PSPIRD
--PSPIQN
--PSPINN
--PSPINI

--PSPIMV
--PSPIMK
--PSPIMF
--PSPIKW
--PSPIKT
--PSPIKR
--PSPIKE
--PSPIKA
--PSPIIK
--PSPIHR
--PSPIGQ
--PSPIGM
--PSPIFH
--PSPIFG
--PSPIFE
--PSPIFA
--PSPIAH
PVPSPI--
--PSPIYT
--PSPIYK
--PSPIWA
--PSPIVS
--PSPIVQ
--PSPIVD
--PSPITY
--PSPITI
--PSPITG
--PSPISW
--PSPISS
--PSPISH
--PSPIRY
--PSPIQV
--PSPIQR
--PSPIQA
--PSPIPM
--PSPIPL
--PSPINQ
--PSPIMS
--PSPIMN
--PSPIMI
--PSPIMG
--PSPIKQ
--PSPIIN
--PSPIHM
--PSPIHI
--PSPIFQ
--PSPIFN
--PSPIDM
--PSPIAA
PSPSPI--

--PSPIYV
--PSPIYI
--PSPIYG
--PSPIWQ
--PSPIWN
--PSPIWF
--PSPIVG
--PSPITH
--PSPITA
--PSPIRK
--PSPIRC
--PSPIQS
--PSPIQG
--PSPIPY
--PSPINT
--PSPINK
--PSPINA
--PSPIMY
--PSPIMR
--PSPIMQ
--PSPIMM
--PSPIMA
--PSPILN
--PSPIKY
--PSPIKK
--PSPIKF
--PSPIKD
--PSPIIF
--PSPIHT
--PSPIHN
--PSPIHK
--PSPIHH
--PSPIHF
--PSPIGT
--PSPIGH
--PSPIGG
--PSPIEY
--PSPIEH
--PSPIDW
--PSPIDF
--PSPIAT
--PSPIAL
--PSPIAK
--PSPIAI
--PSPIAG
PMPSPI--
PGPSPI--
PAPSPI--
PWPSPI--
--PSPIYQ

--PSPIYH
--PSPIYE
--PSPIWW
--PSPIWH
--PSPIVY
--PSPIVW
--PSPIVE
--PSPITM
--PSPITC
--PSPISP
--PSPIRP
--PSPIRF
--PSPIPV
--PSPIPS
--PSPIPI
--PSPINY
--PSPINV
--PSPINR
--PSPINM
--PSPINH
--PSPING
--PSPIMW
--PSPIMD
--PSPILC
--PSPILA
--PSPIIY
--PSPIIG
--PSPIIA
--PSPIHQ
--PSPIHG
--PSPIGW
--PSPIFT
--PSPIET
--PSPIDY
--PSPIDL
--PSPICY
--PSPICI
--PSPICG
--PSPIAW
--PSPIAQ
--PSPIAN
-PPSPIW-
-PPSPIA-
-QPSPIS-
-QPSPIR-
-QPSPIQ-
QEPSPI--
QYPSPI--
QSPSPI--
QKPSPI--

QGPSPI--
QVPSPI--
-QPSPIY-
-QPSPIV-
-QPSPII-
-QPSPIW-
-QPSPIA-
QAPSPI--
QLPSPI--
QRPSPI--
-QPSPIT-
-QPSPIK-
-QPSPIH-
-QPSPIF-
-QPSPIE-
QPPSPI--
QNPSPI--
QHPSPI--
QFPSPI--
-RPSPIL-
RSPSPI--
-RPSPIR-
RLPSPI--
-RPSPIS-
RTPSPI--
RIPSPI--
REPSPI--
-RPSPIV-
RFPSPI--
RMPSPI--
RVPSPI--
-RPSPIF-
-RPSPIT-
-RPSPIW-
-RPSPIN-
-RPSPII-
-RPSPIA-
-RPSPIK-
-RPSPIM-
-RPSPIG-
RHPSPI--
RGPSPI--
RPPSPI--
RNPSPI--
RKPSPI--
RWPSPI--
-RPSPIC-
RYPSPI--
-RPSPIH-
RCPSPI--

RQPSPI--
SHPSPI--
STPSPI--
-SPSPIF-
SLPSPI--
-SPSPIS-
-SPSPIR-
-SPSPIL-
-SPSPIW-
SSPSPI--
SQPSPI--
-SPSPIA-
SAPSPI--
-SPSPIQ-
SGPSPI--
-SPSPIK-
SPPSPI--
SMPSPI--
SFPSPI--
-SPSPIG-
SEPSPI--
SVPSPI--
-SPSPIY-
-SPSPIV-
SKPSPI--
SIPSPI--
-SPSPIM-
-SPSPIH-
-SPSPIN-
SCPSPI--
SWPSPI--
SNPSPI--
TSPSPI--
-TPSPIN-
TYPSPI--
-TPSPIS-
-TPSPIR-
TFPSPI--
TEPSPI--
TQPSPI--
-TPSPIM-
TVPSPI--
-TPSPIV-
-TPSPIT-
-TPSPIL-
TNPSPI--
-TPSPIQ-
TKPSPI--
TWPSPI--
-TPSPIF-

TIPSPI--
TAPSPI--
TTPSPI--
TRPSPI--
-TPSPIW-
-TPSPII-
-TPSPIH-
-TPSPIA-
TPPSPI--
TGPSPI--
TDPSPI--
-TPSPIG-
TMPSPI--
TCPSPI--
-VPSPIL-
-VPSPIS-
VLPSPI--
VVPSPI--
VYPSPI--
-VPSPIK-
VRPSPI--
-VPSPIR-
-VPSPIH-
-VPSPIW-
VTPSPI--
VQPSPI--
VNPSPI--
VIPSPI--
VSPSPI--
VGPSPI--
VAPSPI--
-VPSPIT-
-VPSPIM-
VMPSPI--
-VPSPIV-
-VPSPIF-
VHPSPI--
VWPSPI--
-VPSPIY-
-VPSPIN-
-VPSPII-
-VPSPIG-
VFPSPI--
VDPSPI--
-VPSPIQ-
-VPSPIE-
VPPSPI--
VEPSPI--
WKPSPI--
WGPSPI--

-WPSPIK-
WRPSPI--
WAPSPI--
-WPSPIS-
-WPSPIV-
WVPSPI--
WIPSPI--
WFPSPI--
WQPSPI--
-WPSPIT-
-WPSPII-
-WPSPIF-
WHPSPI--
WYPSPI--
WTPSPI--
-WPSPIR-
-WPSPIQ-
WNPSPI--
WWPSPI--
-WPSPIM-
-WPSPIW-
-WPSPIL-
WPPSPI--
WEPSPI--
WDPSPI--
YLPSPI--
-YPSPIL-
-YPSPIY-
YTPSPI--
YHPSPI--
YEPSPI--
-YPSPIT-
YFPSPI--
YYPSPI--
-YPSPIS-
-YPSPIR-
YSPSPI--
YGPSPI--
-YPSPIV-
YNPSPI--
-YPSPIW-
-YPSPIH-
YWPSPI--
-YPSPIK-
-YPSPIA-
YMPSPI--
YKPSPI--
YDPSPI--
YAPSPI--
YVPSPI--

| | | | |
|---|---|---|---|
| | -YPSPIM- | | |
| | YIPSPI-- | | |
| | YCPSPI-- | | |
| PLGF | 98 | 770 | 32.52 |
| | GGPLGF-- | | |
| | --PLGFQL | | |
| | --PLGFPR | | |
| | --PLGFGR | | |
| | --PLGFGP | | |
| | --PLGFDR | | |
| | --PLGFAL | | |
| | -APLGFA- | | |
| | -APLGFF- | | |
| | ARPLGF-- | | |
| | -APLGFS- | | |
| | ALPLGF-- | | |
| | -APLGFR- | | |
| | -APLGFV- | | |
| | -APLGFK- | | |
| | -APLGFI- | | |
| | -APLGFE- | | |
| | ATPLGF-- | | |
| | AIPLGF-- | | |
| | -APLGFH- | | |
| | AMPLGF-- | | |
| | AKPLGF-- | | |
| | AWPLGF-- | | |
| | AVPLGF-- | | |
| | ASPLGF-- | | |
| | AQPLGF-- | | |
| | -APLGFN- | | |
| | AYPLGF-- | | |
| | -APLGFG- | | |
| | ANPLGF-- | | |
| | AFPLGF-- | | |
| | AGPLGF-- | | |
| | -APLGFT- | | |
| | -APLGFQ- | | |
| | -APLGFM- | | |
| | AEPLGF-- | | |
| | ADPLGF-- | | |
| | AAPLGF-- | | |
| | AHPLGF-- | | |
| | ACPLGF-- | | |
| | -CPLGFF- | | |
| | CSPLGF-- | | |
| | CHPLGF-- | | |
| | CCPLGF-- | | |
| | -CPLGFS- | | |
| | CNPLGF-- | | |

CEPLGF--
CAPLGF--
CTPLGF--
-CPLGFN-
-CPLGFM-
-CPLGFI-
CIPLGF--
CGPLGF--
-DPLGFW-
-DPLGFS-
DVPLGF--
DMPLGF--
DYPLGF--
-DPLGFT-
-DPLGFL-
DHPLGF--
DWPLGF--
-DPLGFA-
DLPLGF--
DTPLGF--
-DPLGFI-
DKPLGF--
-DPLGFY-
DGPLGF--
DFPLGF--
DRPLGF--
-DPLGFQ-
-DPLGFN-
DIPLGF--
DSPLGF--
-DPLGFM-
DAPLGF--
DQPLGF--
-DPLGFK-
DNPLGF--
DCPLGF--
-EPLGFI-
ERPLGF--
-EPLGFN-
-EPLGFY-
ESPLGF--
ENPLGF--
EMPLGF--
EYPLGF--
-EPLGFT-
-EPLGFS-
-EPLGFF-
EKPLGF--
EIPLGF--
-EPLGFM-

-EPLGFK-
EFPLGF--
EWPLGF--
EVPLGF--
ETPLGF--
-EPLGFR-
-EPLGFH-
EPPLGF--
-EPLGFQ-
EEPLGF--
EAPLGF--
-EPLGFV-
-EPLGFA-
FSPLGF--
FKPLGF--
FLPLGF--
FNPLGF--
FTPLGF--
FGPLGF--
-FPLGFV-
FQPLGF--
-FPLGFK-
FVPLGF--
-FPLGFQ-
-FPLGFH-
-FPLGFG-
FDPLGF--
FWPLGF--
-FPLGFN-
-FPLGFA-
-FPLGFS-
FIPLGF--
FPPLGF--
-FPLGFY-
-FPLGFM-
FYPLGF--
-FPLGFT-
-FPLGFI-
-FPLGFF-
FMPLGF--
FEPLGF--
FCPLGF--
-GPLGFL-
GRPLGF--
-GPLGFS-
GQPLGF--
GVPLGF--
GLPLGF--
GTPLGF--
-GPLGFV-

-GPLGFM-
GPPLGF--
-GPLGFI-
GFPLGF--
-GPLGFT-
GNPLGF--
GEPLGF--
GSPLGF--
-GPLGFN-
-GPLGFE-
-GPLGFH-
GIPLGF--
GAPLGF--
GYPLGF--
-GPLGFK-
-GPLGFA-
GWPLGF--
GHPLGF--
GDPLGF--
GMPLGF--
-GPLGFW-
-GPLGFQ-
-HPLGFA-
-HPLGFR-
-HPLGFK-
HAPLGF--
HGPLGF--
-HPLGFI-
-HPLGFS-
-HPLGFT-
HLPLGF--
HSPLGF--
HWPLGF--
HQPLGF--
HFPLGF--
-HPLGFN-
-HPLGFL-
HVPLGF--
-HPLGFQ-
-HPLGFE-
HNPLGF--
HYPLGF--
-HPLGFY-
-HPLGFH-
HMPLGF--
HIPLGF--
HKPLGF--
-HPLGFM-
-HPLGFG-
HEPLGF--

-IPLGFI-
IVPLGF--
ISPLGF--
-IPLGFM-
INPLGF--
IGPLGF--
-IPLGFL-
IMPLGF--
ILPLGF--
IHPLGF--
ITPLGF--
-IPLGFS-
IKPLGF--
IYPLGF--
-IPLGFT-
-IPLGFN-
IIPLGF--
-IPLGFH-
-IPLGFK-
-IPLGFG-
IFPLGF--
-IPLGFV-
IEPLGF--
IDPLGF--
IAPLGF--
KSPLGF--
KMPLGF--
-KPLGFR-
KKPLGF--
KTPLGF--
-KPLGFY-
KFPLGF--
KGPLGF--
-KPLGFS-
-KPLGFL-
-KPLGFE-
-KPLGFN-
-KPLGFK-
-KPLGFQ-
KPPLGF--
-KPLGFT-
KWPLGF--
-KPLGFG-
-KPLGFA-
KIPLGF--
KVPLGF--
-KPLGFI-
-KPLGFF-
-KPLGFM-
KHPLGF--

KYPLGF--
-KPLGFH-
KEPLGF--
KNPLGF--
-KPLGFV-
KDPLGF--
KAPLGF--
KQPLGF--
-LPLGFI-
-LPLGFR-
-LPLGFL-
LRPLGF--
LSPLGF--
LQPLGF--
LLPLGF--
-LPLGFT-
LKPLGF--
LGPLGF--
-LPLGFQ-
LFPLGF--
LWPLGF--
-LPLGFK-
-LPLGFS-
LNPLGF--
LMPLGF--
LAPLGF--
-LPLGFN-
-LPLGFH-
LEPLGF--
LDPLGF--
LYPLGF--
-LPLGFA-
LPPLGF--
-LPLGFM-
LIPLGF--
LHPLGF--
-LPLGFE-
MLPLGF--
-MPLGFR-
-MPLGFM-
MGPLGF--
MSPLGF--
-MPLGFS-
MAPLGF--
MVPLGF--
MKPLGF--
-MPLGFK-
MQPLGF--
-MPLGFH-
MMPLGF--

```
-MPLGFT-
MNPLGF--
-MPLGFN-
-MPLGFA-
MTPLGF--
-MPLGFV-
-MPLGFG-
-MPLGFI-
MIPLGF--
MYPLGF--
MWPLGF--
MPPLGF--
MCPLGF--
-NPLGFM-
-NPLGFH-
NRPLGF--
NHPLGF--
-NPLGFL-
-NPLGFI-
NVPLGF--
NTPLGF--
NSPLGF--
-NPLGFS-
-NPLGFK-
NQPLGF--
-NPLGFQ-
-NPLGFN-
NKPLGF--
-NPLGFY-
-NPLGFA-
-NPLGFF-
NLPLGF--
NPPLGF--
NWPLGF--
NMPLGF--
-NPLGFV-
NGPLGF--
NDPLGF--
NYPLGF--
-NPLGFW-
-NPLGFE-
NFPLGF--
NAPLGF--
NNPLGF--
NEPLGF--
--PLGFRT
--PLGFRA
--PLGFLS
--PLGFRR
--PLGFLG
```

--PLGFTG
--PLGFSS
--PLGFKY
--PLGFKS
--PLGFHS
--PLGFSN
--PLGFTI
--PLGFIS
--PLGFRG
--PLGFWR
--PLGFRI
--PLGFHL
--PLGFVR
--PLGFSK
--PLGFNA
--PLGFSI
--PLGFRW
--PLGFQR
--PLGFAS
--PLGFWS
--PLGFVN
--PLGFML
--PLGFLQ
--PLGFIA
--PLGFTK
--PLGFSL
--PLGFSA
--PLGFRV
--PLGFLN
--PLGFKR
--PLGFFS
PRPLGF--
--PLGFSM
--PLGFRE
--PLGFLA
--PLGFKW
--PLGFHR
--PLGFFR
--PLGFAY
--PLGFYT
--PLGFTR
--PLGFSY
--PLGFRY
--PLGFMA
--PLGFFN
--PLGFFK
PWPLGF--
--PLGFVT
--PLGFST
--PLGFSF

--PLGFLM
--PLGFHF
--PLGFAT
--PLGFRQ
--PLGFRH
--PLGFNV
--PLGFMT
--PLGFWK
--PLGFSW
--PLGFMK
--PLGFHD
--PLGFHA
--PLGFET
--PLGFEA
--PLGFYG
--PLGFVK
--PLGFTW
--PLGFSV
--PLGFSQ
--PLGFQH
--PLGFNY
--PLGFNS
--PLGFLH
--PLGFKF
--PLGFIR
--PLGFIM
--PLGFHK
--PLGFFT
--PLGFES
--PLGFAR
--PLGFWD
--PLGFTY
--PLGFSH
--PLGFSE
--PLGFRF
--PLGFMG
--PLGFKQ
--PLGFKG
--PLGFII
--PLGFGV
--PLGFWH
--PLGFTM
--PLGFQN
--PLGFNT
--PLGFNM
--PLGFNF
--PLGFMV
--PLGFKN
--PLGFKD
--PLGFKA

--PLGFIQ
--PLGFFG
--PLGFFA
--PLGFAI
--PLGFAF
PFPLGF--
PEPLGF--
--PLGFYK
--PLGFYF
--PLGFVI
--PLGFVE
--PLGFTF
--PLGFQS
--PLGFQG
--PLGFNI
--PLGFMI
--PLGFIN
--PLGFHE
--PLGFYN
--PLGFVV
--PLGFVD
--PLGFRD
--PLGFQK
--PLGFMM
--PLGFKI
--PLGFHM
--PLGFHI
--PLGFAW
--PLGFAV
--PLGFAN
PKPLGF--
--PLGFWV
--PLGFWG
--PLGFVM
--PLGFTQ
--PLGFTN
--PLGFTE
--PLGFSD
--PLGFQY
--PLGFKT
--PLGFKM
--PLGFKH
--PLGFIG
--PLGFHY
--PLGFHT
--PLGFHN
--PLGFHH
--PLGFGA
--PLGFFE
--PLGFAK

--PLGFAG
--PLGFAA
PQPLGF--
--PLGFYH
--PLGFYE
--PLGFWT
--PLGFWE
--PLGFTH
--PLGFTD
--PLGFQW
--PLGFQM
--PLGFQA
--PLGFNW
--PLGFNL
--PLGFNK
--PLGFMW
--PLGFMQ
--PLGFME
--PLGFLD
--PLGFIV
--PLGFIK
--PLGFHW
--PLGFGT
--PLGFGG
--PLGFFD
--PLGFAM
--PLGFAH
PHPLGF--
PYPLGF--
--PLGFYQ
--PLGFWQ
--PLGFTV
--PLGFTA
--PLGFQV
--PLGFQQ
--PLGFQF
--PLGFMN
--PLGFMH
--PLGFKE
--PLGFHQ
--PLGFHG
--PLGFFH
--PLGFEW
--PLGFEG
--PLGFDG
--PLGFAQ
-PPLGFT-
-PPLGFF-
PNPLGF--
--PLGFYD

--PLGFYC
--PLGFVQ
--PLGFQI
--PLGFQE
--PLGFPN
--PLGFNN
--PLGFNG
--PLGFMY
--PLGFMD
--PLGFKL
--PLGFIE
--PLGFIC
--PLGFHV
--PLGFGY
--PLGFGH
--PLGFEY
--PLGFEV
--PLGFEF
--PLGFDA
--PLGFCD
QRPLGF--
-QPLGFV-
QSPLGF--
-QPLGFQ-
-QPLGFI-
-QPLGFA-
-QPLGFR-
-QPLGFS-
-QPLGFF-
-QPLGFK-
QAPLGF--
QVPLGF--
QPPLGF--
-QPLGFM-
QLPLGF--
QIPLGF--
-QPLGFL-
-QPLGFH-
QDPLGF--
QTPLGF--
QWPLGF--
QFPLGF--
-QPLGFY-
-QPLGFN-
QNPLGF--
QMPLGF--
QEPLGF--
QQPLGF--
-QPLGFG-
-QPLGFE-

QKPLGF--
QHPLGF--
-RPLGFL-
RSPLGF--
RVPLGF--
-RPLGFR-
-RPLGFT-
-RPLGFN-
-RPLGFM-
-RPLGFA-
-RPLGFS-
RLPLGF--
-RPLGFH-
-RPLGFK-
-RPLGFG-
RIPLGF--
RNPLGF--
RYPLGF--
RTPLGF--
-RPLGFI-
REPLGF--
RQPLGF--
RGPLGF--
RWPLGF--
RHPLGF--
RFPLGF--
-RPLGFE-
-RPLGFQ-
RCPLGF--
-SPLGFL-
-SPLGFK-
-SPLGFS-
-SPLGFI-
-SPLGFQ-
STPLGF--
SGPLGF--
SSPLGF--
-SPLGFH-
-SPLGFV-
-SPLGFM-
-SPLGFY-
-SPLGFN-
SWPLGF--
SQPLGF--
SHPLGF--
-SPLGFW-
SIPLGF--
SFPLGF--
SVPLGF--
SMPLGF--

SNPLGF--
SYPLGF--
-SPLGFG-
-SPLGFE-
SEPLGF--
SDPLGF--
SPPLGF--
SCPLGF--
-TPLGFR-
-TPLGFL-
-TPLGFT-
TLPLGF--
-TPLGFS-
TSPLGF--
TMPLGF--
-TPLGFK-
-TPLGFA-
TPPLGF--
-TPLGFV-
-TPLGFQ-
TNPLGF--
TTPLGF--
TEPLGF--
-TPLGFN-
TFPLGF--
TYPLGF--
TVPLGF--
-TPLGFF-
-TPLGFM-
TKPLGF--
TWPLGF--
TQPLGF--
-TPLGFI-
TGPLGF--
TDPLGF--
TAPLGF--
-TPLGFH-
-TPLGFG-
THPLGF--
-TPLGFY-
-TPLGFW-
-TPLGFE-
TIPLGF--
TCPLGF--
-VPLGFT-
-VPLGFS-
VSPLGF--
-VPLGFL-
VKPLGF--
VTPLGF--

VLPLGF--
VHPLGF--
-VPLGFA-
-VPLGFV-
VNPLGF--
VYPLGF--
-VPLGFI-
-VPLGFH-
VVPLGF--
VQPLGF--
VDPLGF--
-VPLGFQ-
VGPLGF--
VWPLGF--
VEPLGF--
-VPLGFK-
-VPLGFE-
VIPLGF--
VFPLGF--
-VPLGFN-
-WPLGFW-
-WPLGFS-
WLPLGF--
WKPLGF--
WPPLGF--
-WPLGFQ-
WYPLGF--
WMPLGF--
WHPLGF--
-WPLGFY-
-WPLGFT-
-WPLGFH-
WGPLGF--
WDPLGF--
WTPLGF--
WAPLGF--
WQPLGF--
-WPLGFM-
-WPLGFA-
WWPLGF--
-WPLGFV-
-WPLGFI-
-WPLGFE-
WNPLGF--
WIPLGF--
WCPLGF--
-YPLGFV-
YVPLGF--
-YPLGFK-
-YPLGFL-

-YPLGFT-
YLPLGF--
YHPLGF--
-YPLGFN-
YNPLGF--
YEPLGF--
-YPLGFY-
-YPLGFI-
YTPLGF--
-YPLGFQ-
-YPLGFG-
-YPLGFE-
YGPLGF--
-YPLGFF-
-YPLGFA-
YYPLGF--
YWPLGF--
YPPLGF--
-YPLGFW-
YFPLGF--
YQPLGF--
-YPLGFH-
YDPLGF--
YCPLGF--

| | | | |
|---|---|---|---|
| PVSL | 365 | 645 | 32.50 |

-EPVSLG-
-GPVSLD-
-LPVSLE-
-LPVSLP-
--PVSLNR
--PVSLGQ
--PVSLQE
--PVSLPT
--PVSLPG
--PVSLPA
--PVSLNK
--PVSLNE
--PVSLND
--PVSLGY
--PVSLGK
--PVSLEN
-SPVSLG-
-SPVSLC-
VEPVSL--
-WPVSLG-
-APVSLR-
ASPVSL--
ANPVSL--
AVPVSL--
ALPVSL--

-APVSLK-
AWPVSL--
-APVSLN-
-APVSLM-
-APVSLG-
AMPVSL--
-APVSLQ-
APPVSL--
ATPVSL--
AAPVSL--
-APVSLT-
AGPVSL--
-APVSLE-
ADPVSL--
ACPVSL--
CAPVSL--
CRPVSL--
-CPVSLR-
-CPVSLL-
CLPVSL--
CCPVSL--
CGPVSL--
CKPVSL--
CQPVSL--
CPPVSL--
CVPVSL--
-CPVSLT-
CWPVSL--
-CPVSLS-
CNPVSL--
CMPVSL--
CEPVSL--
CTPVSL--
CSPVSL--
-CPVSLK-
-CPVSLV-
CIPVSL--
CHPVSL--
CDPVSL--
-CPVSLQ-
-CPVSLM-
-CPVSLF-
DRPVSL--
-DPVSLL-
-DPVSLR-
DTPVSL--
DWPVSL--
-DPVSLT-
DEPVSL--
DLPVSL--

```
-DPVSLW-
DKPVSL--
-DPVSLY-
DNPVSL--
DVPVSL--
DSPVSL--
-DPVSLM-
DGPVSL--
DFPVSL--
-DPVSLI-
DHPVSL--
DAPVSL--
DYPVSL--
-DPVSLK-
-DPVSLF-
DDPVSL--
DQPVSL--
-DPVSLV-
-DPVSLQ-
DMPVSL--
-DPVSLA-
DIPVSL--
-DPVSLE-
-EPVSLR-
-EPVSLL-
ELPVSL--
ESPVSL--
-EPVSLK-
-EPVSLA-
EYPVSL--
ETPVSL--
-EPVSLS-
-EPVSLQ-
EWPVSL--
ERPVSL--
EQPVSL--
-EPVSLV-
EGPVSL--
ENPVSL--
EMPVSL--
EHPVSL--
EFPVSL--
-EPVSLN-
-EPVSLM-
-EPVSLI-
-EPVSLH-
EKPVSL--
EEPVSL--
EVPVSL--
EPPVSL--
```

-EPVSLF-
EDPVSL--
EAPVSL--
-EPVSLE-
FVPVSL--
-FPVSLS-
FGPVSL--
-FPVSLK-
FLPVSL--
FYPVSL--
-FPVSLN-
-FPVSLV-
-FPVSLI-
-FPVSLA-
FDPVSL--
-FPVSLM-
FHPVSL--
FQPVSL--
-FPVSLQ-
FPPVSL--
FFPVSL--
-FPVSLE-
GTPVSL--
GSPVSL--
-GPVSLH-
GKPVSL--
-GPVSLK-
GAPVSL--
-GPVSLS-
GLPVSL--
-GPVSLA-
GGPVSL--
-GPVSLI-
-GPVSLM-
GNPVSL--
GWPVSL--
GQPVSL--
-GPVSLV-
GEPVSL--
-GPVSLQ-
GPPVSL--
-GPVSLN-
-GPVSLG-
-GPVSLE-
GHPVSL--
GCPVSL--
GDPVSL--
-HPVSLS-
-HPVSLL-
HKPVSL--

HVPVSL--
-HPVSLY-
-HPVSLK-
HGPVSL--
HFPVSL--
HQPVSL--
-HPVSLN-
HPPVSL--
-HPVSLQ-
-HPVSLM-
-HPVSLI-
-HPVSLH-
HEPVSL--
HYPVSL--
-HPVSLW-
HAPVSL--
-HPVSLV-
HWPVSL--
-HPVSLG-
-HPVSLE-
HMPVSL--
HDPVSL--
HNPVSL--
HIPVSL--
HHPVSL--
-HPVSLA-
-HPVSLC-
IPPVSL--
-IPVSLR-
ISPVSL--
-IPVSLS-
ILPVSL--
IQPVSL--
-IPVSLA-
IAPVSL--
-IPVSLV-
-IPVSLK-
IHPVSL--
-IPVSLI-
IYPVSL--
IVPVSL--
-IPVSLN-
IKPVSL--
IWPVSL--
-IPVSLH-
IDPVSL--
IMPVSL--
IFPVSL--
-IPVSLQ-
IEPVSL--

```
-IPVSLE-
ICPVSL--
-IPVSLG-
-KPVSLK-
KLPVSL--
-KPVSLL-
-KPVSLW-
-KPVSLA-
KVPVSL--
KYPVSL--
-KPVSLT-
-KPVSLQ-
-KPVSLN-
KWPVSL--
KTPVSL--
KAPVSL--
KHPVSL--
-KPVSLY-
-KPVSLF-
KQPVSL--
KPPVSL--
KNPVSL--
KMPVSL--
KIPVSL--
-KPVSLE-
KCPVSL--
KDPVSL--
-KPVSLG-
KEPVSL--
-LPVSLR-
-LPVSLL-
-LPVSLT-
LNPVSL--
LPPVSL--
-LPVSLH-
-LPVSLK-
LAPVSL--
-LPVSLQ-
LHPVSL--
LDPVSL--
LWPVSL--
LEPVSL--
-LPVSLN-
LCPVSL--
MGPVSL--
-MPVSLT-
-MPVSLS-
-MPVSLR-
MMPVSL--
-MPVSLL-
```

```
MVPVSL--
MHPVSL--
-MPVSLV-
-MPVSLI-
MDPVSL--
MWPVSL--
MAPVSL--
MPPVSL--
MNPVSL--
-MPVSLA-
MEPVSL--
-MPVSLQ-
-MPVSLN-
MYPVSL--
-MPVSLH-
MFPVSL--
MQPVSL--
-MPVSLM-
-MPVSLE-
-MPVSLC-
MCPVSL--
-NPVSLR-
NRPVSL--
-NPVSLS-
NHPVSL--
NVPVSL--
NLPVSL--
-NPVSLV-
-NPVSLI-
NNPVSL--
-NPVSLT-
NYPVSL--
-NPVSLA-
NQPVSL--
NMPVSL--
NGPVSL--
-NPVSLM-
NIPVSL--
NFPVSL--
NAPVSL--
NWPVSL--
-NPVSLK-
NPPVSL--
NEPVSL--
-NPVSLF-
NDPVSL--
-NPVSLY-
-NPVSLH-
-NPVSLE-
NCPVSL--
```

--PVSLRT
--PVSLLS
--PVSLVQ
--PVSLSR
--PVSLGS
--PVSLVS
--PVSLIS
--PVSLRG
--PVSLLN
--PVSLLK
--PVSLKL
--PVSLTG
--PVSLSH
--PVSLRE
--PVSLSK
--PVSLRN
--PVSLNL
--PVSLMR
--PVSLMN
--PVSLVG
--PVSLAW
--PVSLYN
--PVSLTT
--PVSLAG
--PVSLRH
--PVSLGA
--PVSLTA
--PVSLKI
--PVSLGL
--PVSLAA
PWPVSL--
--PVSLWQ
--PVSLVD
--PVSLTW
--PVSLSQ
--PVSLSA
--PVSLKM
--PVSLHA
PIPVSL--
--PVSLYH
--PVSLRF
--PVSLMT
PAPVSL--
--PVSLMG
--PVSLKT
--PVSLAV
PEPVSL--
--PVSLWN
--PVSLTM
--PVSLSY

--PVSLNS
--PVSLHR
--PVSLEW
PFPVSL--
--PVSLVI
--PVSLRW
--PVSLNW
--PVSLKY
--PVSLHV
--PVSLHD
--PVSLTQ
--PVSLQS
--PVSLQR
--PVSLKN
--PVSLWG
--PVSLVH
--PVSLSW
--PVSLIH
--PVSLES
--PVSLLH
--PVSLHN
--PVSLWW
--PVSLTL
--PVSLNY
--PVSLKW
--PVSLGT
PTPVSL--
--PVSLTK
--PVSLTI
--PVSLVN
--PVSLTY
--PVSLQA
--PVSLIG
--PVSLHH
PNPVSL--
--PVSLQH
--PVSLHM
--PVSLGW
--PVSLWH
--PVSLTN
--PVSLTH
--PVSLSI
--PVSLQW
--PVSLQT
--PVSLQK
--PVSLKA
--PVSLIN
--PVSLHL
--PVSLFH
--PVSLET

PDPVSL--
--PVSLTF
--PVSLQI
--PVSLMK
--PVSLKF
--PVSLHT
--PVSLFG
--PVSLEA
--PVSLAT
--PVSLQD
--PVSLHG
--PVSLEG
--PVSLVE
--PVSLTD
--PVSLQL
--PVSLNG
--PVSLHW
--PVSLHK
--PVSLAR
--PVSLAN
--PVSLQV
--PVSLQN
--PVSLQF
--PVSLPF
--PVSLNM
--PVSLHY
--PVSLGM
PQPVSL--
--PVSLQY
--PVSLNA
--PVSLKV
--PVSLID
--PVSLGI
--PVSLAI
--PVSLAH
--PVSLWD
--PVSLMQ
--PVSLKE
--PVSLKD
--PVSLGV
--PVSLAQ
PMPVSL--
PHPVSL--
--PVSLQM
--PVSLME
--PVSLEY
--PVSLEV
--PVSLCG
--PVSLAK
--PVSLTE

--PVSLMW
--PVSLEM
--PVSLSE
--PVSLPM
--PVSLNI
--PVSLLD
--PVSLHF
--PVSLEF
PCPVSL--
--PVSLPI
--PVSLHE
--PVSLYC
--PVSLVC
--PVSLEH
--PVSLCL
--PVSLCF
-PPVSLR-
-PPVSLL-
-QPVSLR-
QRPVSL--
-QPVSLL-
-QPVSLF-
QSPVSL--
QPPVSL--
QIPVSL--
QAPVSL--
-QPVSLH-
QTPVSL--
-QPVSLK-
QGPVSL--
-QPVSLY-
-QPVSLA-
QHPVSL--
-QPVSLS-
QFPVSL--
-QPVSLV-
QQPVSL--
-QPVSLI-
QDPVSL--
QEPVSL--
QVPVSL--
QWPVSL--
-QPVSLQ-
-QPVSLM-
QMPVSL--
QYPVSL--
-QPVSLN-
QKPVSL--
-QPVSLE-
-QPVSLC-

RLPVSL--
RSPVSL--
RVPVSL--
RAPVSL--
RYPVSL--
RPPVSL--
RGPVSL--
RIPVSL--
-RPVSLK-
RFPVSL--
-RPVSLF-
-RPVSLV-
RTPVSL--
-RPVSLG-
RNPVSL--
-RPVSLQ-
RDPVSL--
RWPVSL--
RHPVSL--
REPVSL--
-RPVSLE-
RCPVSL--
SRPVSL--
SVPVSL--
-SPVSLR-
-SPVSLS-
SFPVSL--
SHPVSL--
-SPVSLK-
-SPVSLN-
-SPVSLH-
-SPVSLA-
SIPVSL--
SQPVSL--
STPVSL--
SPPVSL--
-SPVSLQ-
SNPVSL--
-SPVSLM-
SDPVSL--
SEPVSL--
-SPVSLE-
SCPVSL--
-TPVSLR-
TGPVSL--
TVPVSL--
-TPVSLL-
TSPVSL--
-TPVSLM-
TIPVSL--

−TPVSLQ−
−TPVSLA−
TTPVSL−−
TPPVSL−−
TWPVSL−−
TDPVSL−−
−TPVSLH−
THPVSL−−
TNPVSL−−
TFPVSL−−
TYPVSL−−
TEPVSL−−
−TPVSLI−
TQPVSL−−
−TPVSLE−
TCPVSL−−
−TPVSLG−
VSPVSL−−
−VPVSLR−
VTPVSL−−
VLPVSL−−
VPPVSL−−
VDPVSL−−
VVPVSL−−
−VPVSLT−
VNPVSL−−
VMPVSL−−
VIPVSL−−
−VPVSLS−
−VPVSLH−
VYPVSL−−
VAPVSL−−
VQPVSL−−
VWPVSL−−
−VPVSLG−
VFPVSL−−
VHPVSL−−
VCPVSL−−
WRPVSL−−
−WPVSLT−
−WPVSLM−
WAPVSL−−
WGPVSL−−
−WPVSLH−
WHPVSL−−
−WPVSLN−
−WPVSLA−
WPPVSL−−
WDPVSL−−
−WPVSLE−

WCPVSL--
-YPVSLR-
-YPVSLT-
YPPVSL--
YYPVSL--
-YPVSLA-
-YPVSLI-
-YPVSLS-
YKPVSL--
-YPVSLQ-
-YPVSLH-
YMPVSL--
YHPVSL--
YEPVSL--
YTPVSL--
YAPVSL--
YGPVSL--
YQPVSL--
-YPVSLE-
YDPVSL--
LSNL 456 617 32.50
-DLSNLG-
-ELSNLG-
-FLSNLD-
-ILSNLE-
-ILSNLD-
-KLSNLN-
-KLSNLG-
--LSNLTL
--LSNLSD
--LSNLGT
--LSNLTE
--LSNLSE
--LSNLQD
--LSNLPV
--LSNLPS
--LSNLPR
--LSNLNN
--LSNLNM
--LSNLNK
--LSNLGH
--LSNLGG
--LSNLER
--LSNLEH
--LSNLDY
--LSNLDA
-QLSNLD-
-RLSNLD-
-SLSNLG-
-SLSNLP-

-TLSNLG-
-TLSNLC-
-WLSNLD-
-YLSNLD-
ARLSNL--
-ALSNLT-
-ALSNLQ-
-ALSNLM-
AGLSNL--
AALSNL--
-ALSNLI-
-ALSNLA-
-ALSNLV-
-ALSNLN-
AFLSNL--
AYLSNL--
AWLSNL--
AMLSNL--
-ALSNLK-
ATLSNL--
AQLSNL--
APLSNL--
AHLSNL--
ANLSNL--
AELSNL--
-ALSNLH-
-ALSNLW-
-ALSNLG-
ACLSNL--
-CLSNLL-
CSLSNL--
CGLSNL--
-CLSNLT-
-CLSNLH-
CNLSNL--
-CLSNLN-
-CLSNLS-
CLLSNL--
CTLSNL--
CPLSNL--
-CLSNLM-
CILSNL--
CELSNL--
CDLSNL--
CALSNL--
CWLSNL--
-CLSNLY-
-CLSNLI-
CKLSNL--
CFLSNL--

CYLSNL--
-CLSNLW-
-CLSNLK-
CHLSNL--
CMLSNL--
-CLSNLG-
-CLSNLF-
DRLSNL--
DNLSNL--
DLLSNL--
DSLSNL--
DTLSNL--
-DLSNLT-
DYLSNL--
-DLSNLL-
DALSNL--
-DLSNLV-
DGLSNL--
DWLSNL--
-DLSNLY-
-DLSNLR-
-DLSNLM-
DKLSNL--
DVLSNL--
-DLSNLH-
DPLSNL--
-DLSNLW-
-DLSNLQ-
-DLSNLI-
-DLSNLF-
DDLSNL--
DHLSNL--
DFLSNL--
-DLSNLK-
-DLSNLA-
DQLSNL--
DCLSNL--
-DLSNLN-
DELSNL--
ELLSNL--
-ELSNLT-
ERLSNL--
EYLSNL--
EVLSNL--
EGLSNL--
ESLSNL--
EKLSNL--
EILSNL--
-ELSNLR-
-ELSNLK-

ETLSNL--
EFLSNL--
-ELSNLY-
-ELSNLV-
-ELSNLF-
-ELSNLM-
-ELSNLI-
EELSNL--
EPLSNL--
-ELSNLH-
-ELSNLQ-
ENLSNL--
ECLSNL--
EDLSNL--
FSLSNL--
-FLSNLR-
-FLSNLS-
FTLSNL--
-FLSNLA-
FDLSNL--
-FLSNLT-
FALSNL--
-FLSNLM-
-FLSNLQ-
-FLSNLE-
FNLSNL--
FQLSNL--
-FLSNLH-
FELSNL--
FMLSNL--
FWLSNL--
-FLSNLN-
FCLSNL--
-FLSNLC-
GSLSNL--
-GLSNLS-
GKLSNL--
GPLSNL--
-GLSNLI-
-GLSNLT-
GYLSNL--
-GLSNLY-
GALSNL--
-GLSNLV-
-GLSNLK-
GDLSNL--
-GLSNLQ-
GMLSNL--
-GLSNLH-
GELSNL--

−GLSNLA−
GQLSNL−−
−GLSNLN−
GCLSNL−−
−GLSNLE−
−HLSNLL−
−HLSNLR−
HRLSNL−−
HKLSNL−−
−HLSNLS−
−HLSNLK−
HLLSNL−−
HTLSNL−−
HQLSNL−−
−HLSNLM−
HPLSNL−−
HNLSNL−−
−HLSNLW−
−HLSNLV−
HGLSNL−−
−HLSNLF−
HMLSNL−−
−HLSNLH−
HALSNL−−
HYLSNL−−
−HLSNLA−
HILSNL−−
HDLSNL−−
HWLSNL−−
−HLSNLI−
−HLSNLE−
−HLSNLY−
HHLSNL−−
HELSNL−−
−HLSNLQ−
HCLSNL−−
INLSNL−−
−ILSNLT−
−ILSNLG−
IQLSNL−−
IFLSNL−−
IALSNL−−
−ILSNLA−
IPLSNL−−
ICLSNL−−
IMLSNL−−
−ILSNLQ−
IHLSNL−−
IELSNL−−
−ILSNLH−

IDLSNL--
KSLSNL--
KVLSNL--
KTLSNL--
KELSNL--
-KLSNLS-
-KLSNLA-
KFLSNL--
-KLSNLK-
-KLSNLV-
-KLSNLI-
KNLSNL--
KILSNL--
KDLSNL--
-KLSNLY-
KQLSNL--
-KLSNLW-
-KLSNLH-
KCLSNL--
-KLSNLE-
--LSNLRS
LGLSNL--
-LLSNLG-
--LSNLSS
--LSNLVS
--LSNLRT
--LSNLLG
--LSNLRG
--LSNLSA
-LLSNLA-
LELSNL--
LALSNL--
--LSNLSW
--LSNLTS
--LSNLLT
LTLSNL--
--LSNLSG
--LSNLKT
--LSNLRF
LQLSNL--
--LSNLTN
--LSNLMH
--LSNLFG
-LLSNLM-
--LSNLVG
--LSNLTG
--LSNLQA
--LSNLKG
LFLSNL--
--LSNLML

--LSNLVH
LHLSNL--
--LSNLKS
--LSNLFA
--LSNLES
-LLSNLH-
--LSNLNS
--LSNLSM
--LSNLYT
--LSNLTA
--LSNLRE
--LSNLHT
--LSNLMN
--LSNLLK
--LSNLTV
--LSNLMQ
--LSNLHV
--LSNLSY
--LSNLQG
--LSNLKN
--LSNLAA
-LLSNLQ-
--LSNLMY
--LSNLAT
-LLSNLE-
--LSNLYQ
--LSNLMV
LDLSNL--
--LSNLTH
--LSNLMT
--LSNLEA
--LSNLTM
--LSNLQV
--LSNLMK
--LSNLHA
--LSNLGA
--LSNLFN
--LSNLWS
--LSNLTT
--LSNLQT
--LSNLIK
--LSNLRD
--LSNLKV
--LSNLIA
--LSNLTW
--LSNLTK
--LSNLHG
--LSNLYG
--LSNLVQ
--LSNLSI

--LSNLKM
--LSNLAV
--LSNLYD
--LSNLVD
--LSNLTQ
--LSNLSN
--LSNLMI
--LSNLID
--LSNLYN
--LSNLYM
--LSNLQY
--LSNLFH
LCLSNL--
--LSNLSQ
--LSNLQW
--LSNLQK
--LSNLQF
--LSNLME
--LSNLMD
--LSNLLQ
--LSNLYH
--LSNLVE
--LSNLQM
--LSNLNG
--LSNLKQ
--LSNLIN
--LSNLFQ
--LSNLWM
--LSNLTD
--LSNLMW
--LSNLMF
--LSNLKW
--LSNLII
--LSNLHF
--LSNLFK
--LSNLET
--LSNLSK
--LSNLQR
--LSNLNA
--LSNLHN
--LSNLAW
--LSNLAN
--LSNLWD
--LSNLQQ
--LSNLQN
--LSNLKH
--LSNLIQ
--LSNLHI
--LSNLFE
--LSNLFD

--LSNLAY
--LSNLAM
--LSNLYE
--LSNLSH
--LSNLQI
--LSNLKL
--LSNLHY
--LSNLHH
--LSNLEV
--LSNLWQ
--LSNLNT
--LSNLLE
-LLSNLD-
--LSNLNV
--LSNLKD
--LSNLHK
--LSNLAQ
--LSNLAI
--LSNLQH
--LSNLHD
--LSNLEY
--LSNLAK
--LSNLWE
--LSNLMC
--LSNLHW
--LSNLEW
--LSNLEM
--LSNLEF
--LSNLCS
--LSNLCQ
-MLSNLL-
MVLSNL--
MGLSNL--
MALSNL--
-MLSNLS-
MLLSNL--
MSLSNL--
MYLSNL--
MRLSNL--
MTLSNL--
MDLSNL--
-MLSNLV-
MPLSNL--
MNLSNL--
-MLSNLT-
-MLSNLG-
-MLSNLA-
MQLSNL--
-MLSNLM-
MELSNL--

MCLSNL--
-MLSNLQ-
NRLSNL--
-NLSNLK-
NILSNL--
NLLSNL--
NTLSNL--
-NLSNLT-
NKLSNL--
-NLSNLV-
NSLSNL--
NHLSNL--
NALSNL--
-NLSNLH-
-NLSNLI-
NFLSNL--
NVLSNL--
-NLSNLY-
-NLSNLM-
NPLSNL--
NNLSNL--
-NLSNLF-
-NLSNLW-
-NLSNLA-
-NLSNLG-
NWLSNL--
NCLSNL--
NELSNL--
PILSNL--
-PLSNLS-
PTLSNL--
-PLSNLV-
PVLSNL--
-PLSNLG-
PKLSNL--
PNLSNL--
-PLSNLQ-
-PLSNLY-
PALSNL--
-PLSNLK-
PDLSNL--
PMLSNL--
-PLSNLM-
-PLSNLI-
-PLSNLF-
PWLSNL--
-PLSNLH-
-PLSNLE-
-PLSNLA-
PCLSNL--

-QLSNLR-
QVLSNL--
QSLSNL--
-QLSNLL-
-QLSNLS-
QTLSNL--
QGLSNL--
QQLSNL--
-QLSNLT-
QMLSNL--
QYLSNL--
-QLSNLA-
QNLSNL--
QFLSNL--
QKLSNL--
QHLSNL--
-QLSNLV-
-QLSNLQ-
-QLSNLN-
QALSNL--
-QLSNLW-
QDLSNL--
-QLSNLM-
-QLSNLH-
-QLSNLG-
QELSNL--
-QLSNLE-
QCLSNL--
RYLSNL--
-RLSNLV-
RNLSNL--
-RLSNLT-
RHLSNL--
RILSNL--
RPLSNL--
RWLSNL--
-RLSNLF-
-RLSNLQ-
RMLSNL--
-RLSNLN-
-RLSNLE-
RCLSNL--
-RLSNLC-
-SLSNLL-
SLLSNL--
STLSNL--
-SLSNLY-
SYLSNL--
SNLSNL--
SWLSNL--

-SLSNLQ-
SKLSNL--
SMLSNL--
-SLSNLA-
SFLSNL--
-SLSNLF-
-SLSNLM-
SELSNL--
SQLSNL--
SDLSNL--
-SLSNLE-
-SLSNLN-
SCLSNL--
-TLSNLL-
TSLSNL--
TALSNL--
TGLSNL--
TVLSNL--
TPLSNL--
-TLSNLI-
TTLSNL--
TNLSNL--
TFLSNL--
TQLSNL--
TILSNL--
TDLSNL--
-TLSNLK-
-TLSNLQ-
-TLSNLN-
-TLSNLM-
-TLSNLE-
-TLSNLV-
TCLSNL--
-VLSNLL-
VNLSNL--
VFLSNL--
VVLSNL--
-VLSNLS-
VGLSNL--
-VLSNLK-
-VLSNLN-
-VLSNLA-
VYLSNL--
-VLSNLG-
VALSNL--
VHLSNL--
-VLSNLH-
VPLSNL--
-VLSNLV-
-VLSNLM-

VMLSNL--
-VLSNLQ-
VILSNL--
VDLSNL--
-WLSNLR-
-WLSNLS-
-WLSNLK-
WALSNL--
-WLSNLQ-
WVLSNL--
WPLSNL--
WNLSNL--
-WLSNLG-
WKLSNL--
WWLSNL--
-WLSNLM-
-WLSNLE-
WQLSNL--
WHLSNL--
WCLSNL--
YVLSNL--
YPLSNL--
YGLSNL--
-YLSNLS-
-YLSNLT-
-YLSNLQ-
YHLSNL--
YMLSNL--
YYLSNL--
-YLSNLH-
-YLSNLA-
YELSNL--
YWLSNL--
YQLSNL--
-YLSNLE-
YILSNL--
-YLSNLM-
YCLSNL--

| | | | |
|---|---|---|---|
| PSNY | 62 | 796 | 32.50 |

--PSNYWL
-APSNYS-
-APSNYL-
ARPSNY--
-APSNYR-
-APSNYY-
AMPSNY--
-APSNYF-
-APSNYW-
-APSNYM-
-APSNYE-

ATPSNY--
-APSNYV-
-APSNYT-
ALPSNY--
AKPSNY--
AGPSNY--
AAPSNY--
AQPSNY--
-APSNYI-
ACPSNY--
ASPSNY--
-APSNYQ-
-APSNYN-
-APSNYG-
APPSNY--
ANPSNY--
AIPSNY--
AFPSNY--
AYPSNY--
AWPSNY--
AVPSNY--
-APSNYP-
-APSNYK-
-APSNYH-
-APSNYA-
AHPSNY--
AEPSNY--
ADPSNY--
CVPSNY--
CTPSNY--
-CPSNYF-
CCPSNY--
CWPSNY--
-CPSNYK-
CYPSNY--
CSPSNY--
CRPSNY--
-CPSNYW-
-CPSNYS-
-CPSNYR-
-CPSNYM-
CPPSNY--
CNPSNY--
CMPSNY--
CHPSNY--
CGPSNY--
CEPSNY--
CDPSNY--
-DPSNYW-
-DPSNYL-

-DPSNYS-
DQPSNY--
-DPSNYK-
DWPSNY--
-DPSNYH-
DHPSNY--
DTPSNY--
-DPSNYY-
-DPSNYV-
-DPSNYT-
-DPSNYQ-
-DPSNYE-
DGPSNY--
DVPSNY--
DRPSNY--
-DPSNYG-
-DPSNYF-
DKPSNY--
DFPSNY--
DDPSNY--
-EPSNYL-
ESPSNY--
-EPSNYR-
-EPSNYQ-
-EPSNYT-
EAPSNY--
ERPSNY--
ENPSNY--
EWPSNY--
-EPSNYW-
-EPSNYI-
ELPSNY--
EKPSNY--
EQPSNY--
-EPSNYV-
EVPSNY--
-EPSNYY-
-EPSNYM-
EPPSNY--
EMPSNY--
EIPSNY--
-FPSNYL-
FYPSNY--
FTPSNY--
FVPSNY--
FDPSNY--
-FPSNYS-
FWPSNY--
FSPSNY--
-FPSNYY-

FRPSNY--
FLPSNY--
FIPSNY--
FEPSNY--
-FPSNYT-
-FPSNYQ-
-FPSNYM-
-FPSNYK-
-FPSNYH-
-FPSNYA-
FNPSNY--
FGPSNY--
FAPSNY--
FQPSNY--
-FPSNYW-
-FPSNYV-
-FPSNYI-
-FPSNYG-
-FPSNYE-
FMPSNY--
FKPSNY--
FHPSNY--
-GPSNYL-
GWPSNY--
GKPSNY--
GYPSNY--
-GPSNYR-
GVPSNY--
GRPSNY--
GSPSNY--
GQPSNY--
-GPSNYV-
-GPSNYS-
-GPSNYM-
-GPSNYY-
GLPSNY--
GGPSNY--
GEPSNY--
GAPSNY--
-GPSNYK-
-GPSNYG-
-GPSNYF-
GTPSNY--
GMPSNY--
GIPSNY--
GFPSNY--
-GPSNYT-
-GPSNYQ-
-GPSNYN-
-GPSNYE-

GNPSNY--
GHPSNY--
HVPSNY--
-HPSNYI-
HFPSNY--
HAPSNY--
-HPSNYM-
HWPSNY--
HSPSNY--
HRPSNY--
HQPSNY--
-HPSNYH-
-HPSNYG-
HNPSNY--
-HPSNYV-
-HPSNYT-
-HPSNYS-
-HPSNYL-
-HPSNYF-
HIPSNY--
HDPSNY--
HTPSNY--
-HPSNYN-
-HPSNYE-
HKPSNY--
HEPSNY--
HYPSNY--
-HPSNYY-
-HPSNYW-
-HPSNYR-
-HPSNYQ-
HPPSNY--
HLPSNY--
HHPSNY--
HGPSNY--
HCPSNY--
-IPSNYV-
ITPSNY--
-IPSNYL-
IHPSNY--
ISPSNY--
-IPSNYR-
-IPSNYQ-
-IPSNYK-
-IPSNYG-
ILPSNY--
-IPSNYS-
IPPSNY--
IKPSNY--
IWPSNY--

IRPSNY--
IGPSNY--
IDPSNY--
-IPSNYN-
-IPSNYH-
-IPSNYF-
INPSNY--
IAPSNY--
IVPSNY--
-IPSNYT-
-IPSNYM-
-IPSNYE-
-IPSNYD-
-IPSNYA-
IEPSNY--
KSPSNY--
-KPSNYS-
KWPSNY--
KTPSNY--
-KPSNYL-
-KPSNYV-
-KPSNYR-
-KPSNYI-
-KPSNYT-
KVPSNY--
-KPSNYY-
KPPSNY--
KRPSNY--
KQPSNY--
KLPSNY--
KKPSNY--
-KPSNYK-
-KPSNYF-
KIPSNY--
-KPSNYQ-
-KPSNYE-
KGPSNY--
-KPSNYN-
-KPSNYM-
-KPSNYA-
KNPSNY--
KHPSNY--
KDPSNY--
KAPSNY--
-KPSNYG-
-KPSNYD-
KFPSNY--
KEPSNY--
-LPSNYF-
-LPSNYL-

-LPSNYA-
LSPSNY--
LKPSNY--
LFPSNY--
LVPSNY--
-LPSNYR-
LEPSNY--
LWPSNY--
-LPSNYV-
-LPSNYS-
-LPSNYI-
LGPSNY--
LHPSNY--
LRPSNY--
LNPSNY--
-LPSNYM-
-LPSNYK-
-LPSNYT-
LLPSNY--
LMPSNY--
LQPSNY--
LIPSNY--
-LPSNYQ-
-LPSNYN-
-LPSNYE-
LTPSNY--
-LPSNYY-
-LPSNYW-
LPPSNY--
-LPSNYH-
LAPSNY--
LCPSNY--
MSPSNY--
-MPSNYT-
MMPSNY--
MWPSNY--
-MPSNYR-
MRPSNY--
-MPSNYL-
-MPSNYH-
-MPSNYN-
-MPSNYF-
MVPSNY--
MFPSNY--
MYPSNY--
MTPSNY--
-MPSNYM-
MKPSNY--
MDPSNY--
MAPSNY--

```
-MPSNYK-
MIPSNY--
MGPSNY--
MEPSNY--
MCPSNY--
MQPSNY--
-MPSNYW-
-MPSNYV-
-MPSNYG-
MLPSNY--
-NPSNYS-
NRPSNY--
-NPSNYR-
NLPSNY--
-NPSNYL-
NYPSNY--
-NPSNYM-
-NPSNYI-
NFPSNY--
NSPSNY--
NEPSNY--
NTPSNY--
NQPSNY--
-NPSNYY-
-NPSNYV-
NWPSNY--
NVPSNY--
-NPSNYW-
-NPSNYA-
NHPSNY--
NGPSNY--
-NPSNYT-
-NPSNYN-
NDPSNY--
-NPSNYH-
-NPSNYF-
-NPSNYE-
NNPSNY--
NMPSNY--
NCPSNY--
NAPSNY--
--PSNYQT
--PSNYRA
--PSNYSR
--PSNYLG
--PSNYRS
--PSNYRL
--PSNYVL
--PSNYGS
--PSNYLS
```

--PSNYRG
--PSNYMR
--PSNYVI
--PSNYAW
--PSNYAR
--PSNYSL
--PSNYVS
--PSNYRW
--PSNYQN
--PSNYLI
--PSNYLA
--PSNYIA
--PSNYTR
--PSNYSN
--PSNYSM
--PSNYRN
--PSNYYR
--PSNYTS
--PSNYST
--PSNYSQ
--PSNYSF
--PSNYLV
--PSNYFR
--PSNYFI
PGPSNY--
PVPSNY--
--PSNYYM
--PSNYYK
--PSNYVA
--PSNYSV
--PSNYSS
--PSNYRV
--PSNYQV
--PSNYNT
--PSNYMG
--PSNYLW
--PSNYKI
--PSNYIT
--PSNYIL
--PSNYGT
--PSNYGG
PKPSNY--
PHPSNY--
--PSNYWS
--PSNYVY
--PSNYVT
--PSNYTT
--PSNYTA
--PSNYSK
--PSNYML

--PSNYMI
--PSNYKQ
--PSNYKL
--PSNYIV
--PSNYIH
--PSNYEG
--PSNYAL
PWPSNY--
--PSNYVH
--PSNYRF
--PSNYQR
--PSNYMN
--PSNYMD
--PSNYKA
--PSNYHW
--PSNYHG
--PSNYFM
--PSNYFA
--PSNYAV
--PSNYWR
--PSNYWG
--PSNYTL
--PSNYSG
--PSNYSA
--PSNYRI
--PSNYIR
--PSNYIQ
--PSNYFT
--PSNYFH
--PSNYFG
--PSNYWQ
--PSNYWA
--PSNYVR
--PSNYVQ
--PSNYVG
--PSNYVF
--PSNYVD
--PSNYTG
--PSNYTF
--PSNYQS
--PSNYQA
--PSNYNY
--PSNYMQ
--PSNYLY
--PSNYLE
--PSNYKE
--PSNYIM
--PSNYIE
--PSNYHY
--PSNYHT

--PSNYFL
--PSNYYT
--PSNYYG
--PSNYWT
--PSNYWH
--PSNYVW
--PSNYVN
--PSNYTN
--PSNYSW
--PSNYSD
--PSNYRT
--PSNYRQ
--PSNYQL
--PSNYQD
--PSNYNV
--PSNYNS
--PSNYMV
--PSNYMT
--PSNYMS
--PSNYLK
--PSNYLH
--PSNYLF
--PSNYLD
--PSNYKS
--PSNYKN
--PSNYKH
--PSNYIS
--PSNYIG
--PSNYHS
--PSNYHN
--PSNYFV
--PSNYFK
--PSNYER
--PSNYAS
--PSNYAK
PRPSNY--
PEPSNY--
PYPSNY--
--PSNYYY
--PSNYYV
--PSNYYQ
--PSNYYN
--PSNYYI
--PSNYYH
--PSNYWY
--PSNYWV
--PSNYWD
--PSNYVV
--PSNYSI
--PSNYRD

--PSNYQK
--PSNYQG
--PSNYNG
--PSNYNA
--PSNYMM
--PSNYMF
--PSNYMA
--PSNYLQ
--PSNYKV
--PSNYKD
--PSNYIK
--PSNYIF
--PSNYHF
--PSNYGI
--PSNYGA
--PSNYAQ
--PSNYAN
PNPSNY--
PMPSNY--
PIPSNY--
--PSNYYS
--PSNYWW
--PSNYWN
--PSNYWM
--PSNYWK
--PSNYWI
--PSNYWF
--PSNYVM
--PSNYVK
--PSNYTY
--PSNYTW
--PSNYTQ
--PSNYTE
--PSNYTD
--PSNYSH
--PSNYRY
--PSNYRM
--PSNYQW
--PSNYQI
--PSNYNC
--PSNYMY
--PSNYKY
--PSNYKW
--PSNYKT
--PSNYKG
--PSNYKF
--PSNYII
--PSNYID
--PSNYHV
--PSNYHR

--PSNYHH
--PSNYGV
--PSNYGP
--PSNYGF
--PSNYFN
--PSNYEW
--PSNYET
--PSNYEN
--PSNYEF
--PSNYEA
--PSNYDI
--PSNYDA
--PSNYAT
--PSNYAM
--PSNYAD
PQPSNY--
-PPSNYV-
PLPSNY--
PFPSNY--
QVPSNY--
QWPSNY--
-QPSNYS-
QRPSNY--
QGPSNY--
-QPSNYK-
QPPSNY--
QQPSNY--
-QPSNYG-
QLPSNY--
-QPSNYV-
-QPSNYT-
QAPSNY--
QYPSNY--
QSPSNY--
-QPSNYW-
-QPSNYQ-
-QPSNYN-
-QPSNYM-
-QPSNYI-
-QPSNYA-
QNPSNY--
QTPSNY--
-QPSNYL-
QIPSNY--
QDPSNY--
-RPSNYR-
RVPSNY--
RSPSNY--
RGPSNY--
REPSNY--

RYPSNY--
-RPSNYS-
-RPSNYT-
-RPSNYQ-
RTPSNY--
-RPSNYY-
-RPSNYG-
RKPSNY--
-RPSNYW-
-RPSNYI-
RLPSNY--
-RPSNYK-
RIPSNY--
RFPSNY--
RAPSNY--
RWPSNY--
RQPSNY--
-RPSNYM-
-RPSNYF-
-RPSNYE-
-RPSNYN-
RHPSNY--
-RPSNYV-
RNPSNY--
RMPSNY--
RDPSNY--
-RPSNYH-
-RPSNYC-
RCPSNY--
-SPSNYL-
SAPSNY--
-SPSNYV-
-SPSNYR-
SGPSNY--
SRPSNY--
-SPSNYS-
-SPSNYF-
SSPSNY--
STPSNY--
-SPSNYW-
-SPSNYM-
SKPSNY--
SVPSNY--
-SPSNYI-
SDPSNY--
-SPSNYK-
SPPSNY--
SIPSNY--
-SPSNYY-
SWPSNY--

SMPSNY--
SFPSNY--
-SPSNYA-
-SPSNYH-
SEPSNY--
SYPSNY--
SQPSNY--
-SPSNYT-
-SPSNYQ-
-SPSNYN-
SNPSNY--
SHPSNY--
SCPSNY--
-TPSNYW-
TSPSNY--
-TPSNYL-
-TPSNYF-
-TPSNYV-
-TPSNYR-
-TPSNYH-
TNPSNY--
TKPSNY--
-TPSNYT-
-TPSNYS-
TVPSNY--
TGPSNY--
TAPSNY--
-TPSNYY-
-TPSNYG-
-TPSNYE-
TTPSNY--
TRPSNY--
TQPSNY--
-TPSNYM-
TMPSNY--
TIPSNY--
TEPSNY--
-TPSNYN-
-TPSNYI-
TCPSNY--
TWPSNY--
-TPSNYQ-
TPPSNY--
TLPSNY--
THPSNY--
TFPSNY--
TYPSNY--
-TPSNYA-
-VPSNYL-
VLPSNY--

VTPSNY--
-VPSNYF-
-VPSNYS-
-VPSNYN-
VVPSNY--
-VPSNYR-
VFPSNY--
-VPSNYY-
-VPSNYG-
VGPSNY--
VAPSNY--
-VPSNYA-
-VPSNYH-
VSPSNY--
-VPSNYV-
-VPSNYI-
VKPSNY--
VYPSNY--
VRPSNY--
-VPSNYQ-
-VPSNYM-
VMPSNY--
VHPSNY--
VPPSNY--
VNPSNY--
VEPSNY--
VWPSNY--
VQPSNY--
-VPSNYE-
-VPSNYC-
VIPSNY--
WNPSNY--
-WPSNYL-
WFPSNY--
-WPSNYV-
-WPSNYR-
WLPSNY--
WAPSNY--
-WPSNYH-
-WPSNYA-
-WPSNYM-
WKPSNY--
WVPSNY--
-WPSNYS-
-WPSNYF-
WWPSNY--
WTPSNY--
WQPSNY--
WIPSNY--
WGPSNY--

-WPSNYW-
-WPSNYT-
-WPSNYQ-
-WPSNYP-
-WPSNYK-
WDPSNY--
WCPSNY--
-YPSNYL-
-YPSNYR-
-YPSNYY-
-YPSNYT-
YKPSNY--
YGPSNY--
YVPSNY--
YSPSNY--
YRPSNY--
-YPSNYV-
YMPSNY--
YTPSNY--
-YPSNYW-
-YPSNYM-
-YPSNYS-
-YPSNYQ-
-YPSNYK-
YYPSNY--
-YPSNYI-
YIPSNY--
YHPSNY--
YEPSNY--
YQPSNY--
-YPSNYE-
YPPSNY--
YFPSNY--
YDPSNY--
YAPSNY--
PVAI 123 755 32.49
--PVAIGN
WDPVAI--
AAPVAI--
ALPVAI--
-APVAIM-
-APVAIL-
-APVAII-
AIPVAI--
-APVAIY-
-APVAIN-
AGPVAI--
ARPVAI--
AQPVAI--
-APVAIK-

−APVAIF−
AMPVAI−−
AYPVAI−−
−APVAIV−
−APVAIT−
−APVAIR−
−APVAIQ−
ANPVAI−−
AFPVAI−−
ASPVAI−−
−APVAIW−
AHPVAI−−
AVPVAI−−
ATPVAI−−
−APVAIH−
−APVAIE−
−APVAIA−
APPVAI−−
AKPVAI−−
AWPVAI−−
−APVAIG−
AEPVAI−−
CSPVAI−−
CVPVAI−−
−CPVAIK−
−CPVAIL−
−CPVAIH−
−CPVAIR−
CNPVAI−−
CKPVAI−−
CGPVAI−−
CCPVAI−−
DSPVAI−−
−DPVAIT−
DRPVAI−−
−DPVAIV−
DVPVAI−−
DLPVAI−−
DQPVAI−−
DYPVAI−−
DTPVAI−−
−DPVAIS−
−DPVAIK−
DCPVAI−−
DAPVAI−−
−DPVAIR−
DIPVAI−−
DHPVAI−−
DFPVAI−−
DWPVAI−−

-DPVAIY-
-DPVAIM-
-DPVAII-
-DPVAIA-
DNPVAI--
DMPVAI--
DKPVAI--
-EPVAIL-
EMPVAI--
-EPVAIR-
-EPVAIQ-
ELPVAI--
ERPVAI--
EWPVAI--
ESPVAI--
-EPVAIN-
-EPVAIA-
-EPVAIS-
-EPVAIM-
-EPVAIK-
-EPVAIE-
EFPVAI--
EQPVAI--
-EPVAIW-
-EPVAIV-
-EPVAIT-
-EPVAIG-
ENPVAI--
EKPVAI--
EGPVAI--
EEPVAI--
EAPVAI--
FSPVAI--
FRPVAI--
FAPVAI--
FNPVAI--
FVPVAI--
FTPVAI--
FEPVAI--
FPPVAI--
FLPVAI--
-FPVAIR-
-FPVAIW-
-FPVAIT-
-FPVAIK-
-FPVAIV-
FIPVAI--
FHPVAI--
-FPVAIS-
-FPVAIQ-

-FPVAIH-
-FPVAIF-
FMPVAI--
FKPVAI--
FGPVAI--
FCPVAI--
FYPVAI--
-FPVAIN-
-FPVAIA-
FDPVAI--
GRPVAI--
-GPVAIL-
GAPVAI--
-GPVAIM-
GKPVAI--
GSPVAI--
-GPVAIV-
GFPVAI--
GVPVAI--
-GPVAII-
GNPVAI--
GWPVAI--
-GPVAIS-
-GPVAIR-
-GPVAIN-
GLPVAI--
GTPVAI--
-GPVAIY-
GQPVAI--
-GPVAIW-
-GPVAIH-
GIPVAI--
GEPVAI--
-GPVAIK-
-GPVAIG-
GMPVAI--
-GPVAIQ-
GGPVAI--
-GPVAIF-
-GPVAIE-
GHPVAI--
HLPVAI--
HNPVAI--
HKPVAI--
-HPVAIS-
-HPVAII-
-HPVAIH-
HVPVAI--
-HPVAIR-
-HPVAIL-

HIPVAI--
HAPVAI--
-HPVAIY-
-HPVAIK-
-HPVAIE-
HMPVAI--
HYPVAI--
HPPVAI--
-HPVAIW-
HFPVAI--
HTPVAI--
-HPVAIV-
-HPVAIG-
-HPVAIF-
HGPVAI--
IKPVAI--
IRPVAI--
ISPVAI--
-IPVAIV-
-IPVAIT-
-IPVAII-
-IPVAIS-
-IPVAIY-
-IPVAIR-
INPVAI--
ILPVAI--
ITPVAI--
-IPVAIK-
-IPVAIA-
IAPVAI--
-IPVAIN-
-IPVAIH-
IHPVAI--
IWPVAI--
IVPVAI--
IMPVAI--
IGPVAI--
IDPVAI--
ICPVAI--
IYPVAI--
-IPVAIW-
-IPVAIF-
IFPVAI--
KSPVAI--
KLPVAI--
KRPVAI--
-KPVAIS-
KFPVAI--
-KPVAII-
-KPVAIN-

-KPVAIH-
KMPVAI--
-KPVAIQ-
KIPVAI--
KVPVAI--
-KPVAIF-
-KPVAIA-
-KPVAIV-
KWPVAI--
-KPVAIY-
-KPVAIW-
-KPVAIT-
KYPVAI--
-KPVAIM-
KPPVAI--
KNPVAI--
KHPVAI--
KDPVAI--
KQPVAI--
-KPVAIK-
-KPVAIE-
KGPVAI--
KAPVAI--
LSPVAI--
LEPVAI--
LAPVAI--
-LPVAIR-
-LPVAIL-
LWPVAI--
LRPVAI--
-LPVAIT-
-LPVAIS-
LKPVAI--
LMPVAI--
-LPVAIY-
LVPVAI--
-LPVAIQ-
LGPVAI--
LDPVAI--
LQPVAI--
-LPVAIF-
LIPVAI--
-LPVAIW-
-LPVAIV-
-LPVAIN-
-LPVAIK-
LNPVAI--
LLPVAI--
LFPVAI--
-LPVAII-

```
-LPVAIG-
-LPVAIA-
LPPVAI--
MLPVAI--
MRPVAI--
MTPVAI--
MIPVAI--
-MPVAIT-
MSPVAI--
-MPVAIS-
-MPVAIY-
-MPVAIA-
MMPVAI--
MAPVAI--
-MPVAIN-
MKPVAI--
MGPVAI--
-MPVAIV-
-MPVAIH-
MQPVAI--
-MPVAIQ-
-MPVAIM-
-MPVAIG-
-MPVAIE-
MYPVAI--
MWPVAI--
MVPVAI--
-MPVAIW-
-MPVAIR-
-MPVAIK-
MHPVAI--
MEPVAI--
MDPVAI--
NVPVAI--
-NPVAIT-
NLPVAI--
NIPVAI--
NEPVAI--
NWPVAI--
NSPVAI--
NHPVAI--
NYPVAI--
-NPVAIY-
-NPVAIK-
-NPVAIA-
-NPVAIV-
-NPVAIS-
-NPVAIR-
-NPVAIM-
NMPVAI--
```

NAPVAI--
NTPVAI--
NQPVAI--
-NPVAIQ-
-NPVAIL-
-NPVAII-
NPPVAI--
-NPVAIW-
-NPVAIN-
-NPVAIF-
NKPVAI--
NGPVAI--
NFPVAI--
--PVAIRS
--PVAIPL
--PVAISR
--PVAIRN
--PVAIRA
--PVAILS
--PVAIRR
--PVAIFS
PKPVAI--
--PVAIFR
--PVAISQ
--PVAIGL
--PVAIVG
--PVAIRT
--PVAINL
--PVAISG
--PVAIRG
--PVAILT
--PVAIRY
--PVAIQR
--PVAILH
--PVAIGA
--PVAIVR
--PVAISS
--PVAIGY
PRPVAI--
--PVAIVS
--PVAIRD
--PVAIMG
--PVAIKL
--PVAIIR
--PVAIAS
PMPVAI--
PIPVAI--
PVPVAI--
--PVAITI
--PVAINV

--PVAIKK
--PVAIKA
--PVAIIT
--PVAIHL
--PVAIAL
PAPVAI--
--PVAIVT
--PVAITM
--PVAISI
--PVAIKI
--PVAIGW
--PVAIFK
--PVAIFG
--PVAIFA
PTPVAI--
--PVAIWY
--PVAIWK
--PVAIWG
--PVAIVK
--PVAITS
--PVAITG
--PVAISK
--PVAINT
--PVAIKS
--PVAIKE
--PVAIFH
PLPVAI--
--PVAIVN
--PVAITR
--PVAISN
--PVAIRV
--PVAIRL
--PVAIRH
--PVAINF
--PVAILK
--PVAIKW
--PVAIKM
--PVAIHR
--PVAIHN
--PVAIAA
--PVAIWT
--PVAITK
--PVAISV
--PVAISM
--PVAISL
--PVAISH
--PVAISA
--PVAIRQ
--PVAIQS
--PVAIQA

--PVAINN
--PVAILN
--PVAIIS
--PVAIGV
--PVAIGF
PSPVAI--
--PVAIYH
--PVAIVM
--PVAITL
--PVAITH
--PVAITE
--PVAIRW
--PVAIRM
--PVAIRF
--PVAIRE
--PVAIQT
--PVAIQL
--PVAIPY
--PVAIPF
--PVAINS
--PVAIMV
--PVAIMK
--PVAILD
--PVAIKG
--PVAIIN
--PVAIHV
--PVAIHT
--PVAIHS
--PVAIHG
--PVAIGI
--PVAIGG
--PVAIFD
--PVAIES
--PVAIDL
--PVAIYT
--PVAIYN
--PVAIWQ
--PVAIWM
--PVAIVV
--PVAIVI
--PVAIVE
--PVAIVA
--PVAITY
--PVAITN
--PVAISD
--PVAIRP
--PVAIQW
--PVAIQK
--PVAIPI
--PVAIMT

--PVAIMM
--PVAIME
--PVAIMA
--PVAILM
--PVAILE
--PVAILA
--PVAIKV
--PVAIKF
--PVAIIW
--PVAIHK
--PVAIHH
--PVAIFN
--PVAIFF
--PVAIAT
--PVAIAG
--PVAIYV
--PVAIYK
--PVAIYG
--PVAIWN
--PVAIWI
--PVAIWE
--PVAITQ
--PVAITF
--PVAISW
--PVAIQY
--PVAIQI
--PVAIQH
--PVAIPQ
--PVAINI
--PVAIMY
--PVAIMS
--PVAIMH
--PVAILW
--PVAIIV
--PVAIII
--PVAIIG
--PVAIIE
--PVAIGT
--PVAIGS
--PVAIGM
--PVAIFV
--PVAIFT
--PVAIEA
--PVAIAW
PEPVAI--
--PVAIYQ
--PVAIYD
--PVAIWV
--PVAIWD
--PVAIVW

--PVAIVD
--PVAITW
--PVAITV
--PVAITT
--PVAITD
--PVAIST
--PVAISE
--PVAIQV
--PVAIQG
--PVAIPW
--PVAIPM
--PVAINQ
--PVAINK
--PVAINH
--PVAING
--PVAINE
--PVAIND
--PVAIMQ
--PVAIMN
--PVAIMI
--PVAIMF
--PVAILV
--PVAIKD
--PVAIIY
--PVAIIK
--PVAIIF
--PVAIHM
--PVAIHE
--PVAIGH
--PVAIFQ
--PVAIFM
--PVAIET
--PVAIEN
--PVAIEM
--PVAIEL
--PVAIEG
--PVAIAY
--PVAIAR
--PVAIAN
--PVAIAM
--PVAIAH
--PVAIAD
PQPVAI--
-PPVAIS-
-PPVAIL-
PHPVAI--
-QPVAIR-
-QPVAIK-
QIPVAI--
QSPVAI--

QRPVAI--
QAPVAI--
QWPVAI--
-QPVAIL-
-QPVAIA-
QLPVAI--
-QPVAIS-
-QPVAII-
QGPVAI--
QDPVAI--
-QPVAIN-
QYPVAI--
QVPVAI--
QQPVAI--
-QPVAIY-
-QPVAIT-
-QPVAIF-
QNPVAI--
QFPVAI--
-QPVAIV-
-QPVAIH-
-QPVAIE-
QMPVAI--
RAPVAI--
RSPVAI--
-RPVAIF-
RLPVAI--
RIPVAI--
RVPVAI--
RGPVAI--
-RPVAIQ-
RPPVAI--
-RPVAIV-
RKPVAI--
RYPVAI--
RQPVAI--
-RPVAIA-
RTPVAI--
-RPVAIR-
-RPVAIG-
REPVAI--
-RPVAIW-
-RPVAIY-
-RPVAIK-
-RPVAIN-
RFPVAI--
-RPVAIT-
-RPVAIE-
RMPVAI--
RWPVAI--

-RPVAIM-
RNPVAI--
RDPVAI--
SKPVAI--
SLPVAI--
STPVAI--
-SPVAIS-
-SPVAIQ-
SSPVAI--
SVPVAI--
-SPVAIK-
SGPVAI--
SAPVAI--
-SPVAIW-
SWPVAI--
-SPVAIN-
-SPVAIH-
SQPVAI--
-SPVAIY-
SIPVAI--
SEPVAI--
-SPVAIT-
-SPVAIG-
SNPVAI--
SHPVAI--
SFPVAI--
SDPVAI--
SYPVAI--
-SPVAIM-
-SPVAIA-
SPPVAI--
-SPVAIP-
-SPVAIE-
TSPVAI--
TMPVAI--
TWPVAI--
TRPVAI--
-TPVAIQ-
TQPVAI--
-TPVAIY-
-TPVAIW-
-TPVAIH-
TGPVAI--
-TPVAIV-
-TPVAIA-
TLPVAI--
TYPVAI--
TTPVAI--
TFPVAI--
TDPVAI--

-TPVAIT-
-TPVAIK-
TNPVAI--
-TPVAIN-
-TPVAIF-
TIPVAI--
TCPVAI--
-TPVAIM-
VRPVAI--
VLPVAI--
VTPVAI--
-VPVAII-
-VPVAIY-
-VPVAIS-
-VPVAIG-
VWPVAI--
-VPVAIV-
-VPVAIN-
VIPVAI--
VGPVAI--
-VPVAIR-
-VPVAIQ-
VPPVAI--
-VPVAIW-
VNPVAI--
VSPVAI--
-VPVAIL-
VAPVAI--
-VPVAIT-
-VPVAIH-
VHPVAI--
-VPVAIM-
VMPVAI--
VEPVAI--
VCPVAI--
VFPVAI--
VDPVAI--
VQPVAI--
-VPVAIE-
-WPVAIR-
-WPVAIV-
WSPVAI--
WNPVAI--
WGPVAI--
-WPVAIS-
WRPVAI--
-WPVAII-
-WPVAIH-
WKPVAI--
WAPVAI--

WTPVAI--
-WPVAIG-
WIPVAI--
WYPVAI--
WWPVAI--
WQPVAI--
-WPVAIW-
-WPVAIQ-
-WPVAIN-
-WPVAIK-
-WPVAIF-
-WPVAIA-
WLPVAI--
WFPVAI--
YRPVAI--
-YPVAIR-
YHPVAI--
-YPVAIK-
-YPVAIW-
-YPVAIT-
-YPVAIS-
-YPVAIL-
YTPVAI--
YIPVAI--
YSPVAI--
YMPVAI--
YAPVAI--
YWPVAI--
-YPVAIQ-
-YPVAII-
YFPVAI--
YYPVAI--
YQPVAI--
-YPVAIY-
-YPVAIN-
-YPVAIG-
YGPVAI--
YEPVAI--
-YPVAIV-
YNPVAI--
YDPVAI--

| | | | |
|---|---|---|---|
| PMSL | 195 | 715 | 32.49 |

-EPMSLV-
-GPMSLG-
-IPMSLG-
--PMSLGT
--PMSLNP
PIPMSL--
-TPMSLP-
-VPMSLP-

-VPMSLG-
-VPMSLE-
-YPMSLG-
-APMSLR-
AVPMSL--
AKPMSL--
ASPMSL--
AFPMSL--
-APMSLT-
-APMSLA-
AIPMSL--
AGPMSL--
-APMSLM-
AHPMSL--
ATPMSL--
-APMSLS-
APPMSL--
-APMSLE-
ANPMSL--
AAPMSL--
AQPMSL--
AYPMSL--
-APMSLN-
-APMSLK-
AMPMSL--
-APMSLH-
-APMSLW-
-APMSLI-
AEPMSL--
ADPMSL--
-APMSLV-
CGPMSL--
CEPMSL--
CSPMSL--
-CPMSLL-
-CPMSLI-
CQPMSL--
CNPMSL--
CLPMSL--
CPPMSL--
-CPMSLQ-
-CPMSLK-
CMPMSL--
CHPMSL--
CCPMSL--
CAPMSL--
-DPMSLR-
DWPMSL--
-DPMSLI-
DMPMSL--

DSPMSL--
DRPMSL--
-DPMSLL-
DGPMSL--
-DPMSLT-
-DPMSLS-
DNPMSL--
DVPMSL--
DTPMSL--
DHPMSL--
DYPMSL--
DQPMSL--
DKPMSL--
DIPMSL--
-DPMSLV-
-DPMSLQ-
-DPMSLM-
DEPMSL--
DAPMSL--
-DPMSLW-
-DPMSLK-
DFPMSL--
-DPMSLH-
-DPMSLF-
-DPMSLA-
-EPMSLL-
ERPMSL--
-EPMSLK-
-EPMSLR-
-EPMSLA-
-EPMSLN-
EFPMSL--
EQPMSL--
EGPMSL--
EWPMSL--
-EPMSLY-
EKPMSL--
EAPMSL--
-EPMSLW-
-EPMSLT-
-EPMSLI-
-EPMSLH-
EYPMSL--
ETPMSL--
-EPMSLQ-
ENPMSL--
EHPMSL--
-EPMSLM-
EEPMSL--
-FPMSLT-

−FPMSLL−
FGPMSL−−
−FPMSLR−
FKPMSL−−
−FPMSLK−
−FPMSLV−
−FPMSLA−
FFPMSL−−
FDPMSL−−
FNPMSL−−
FPPMSL−−
−FPMSLS−
−FPMSLQ−
−FPMSLH−
−FPMSLM−
−FPMSLN−
FHPMSL−−
−FPMSLI−
FEPMSL−−
FCPMSL−−
FQPMSL−−
−FPMSLE−
−GPMSLL−
GAPMSL−−
GGPMSL−−
−GPMSLR−
GKPMSL−−
GSPMSL−−
−GPMSLM−
−GPMSLI−
−GPMSLV−
GHPMSL−−
GLPMSL−−
GYPMSL−−
−GPMSLS−
−GPMSLQ−
−GPMSLA−
GQPMSL−−
−GPMSLT−
−GPMSLN−
−GPMSLW−
−GPMSLK−
GFPMSL−−
GMPMSL−−
GDPMSL−−
GWPMSL−−
GIPMSL−−
−GPMSLH−
−GPMSLE−
−GPMSLC−

HHPMSL--
HGPMSL--
HNPMSL--
HLPMSL--
HVPMSL--
HKPMSL--
HYPMSL--
HSPMSL--
HEPMSL--
HWPMSL--
HAPMSL--
-HPMSLT-
HFPMSL--
-HPMSLY-
-HPMSLA-
HMPMSL--
-HPMSLL-
HTPMSL--
-HPMSLQ-
-HPMSLK-
-HPMSLS-
-HPMSLF-
HIPMSL--
HPPMSL--
-HPMSLN-
-HPMSLM-
-HPMSLI-
-HPMSLH-
-HPMSLE-
-HPMSLC-
IRPMSL--
-IPMSLR-
-IPMSLK-
IYPMSL--
ILPMSL--
INPMSL--
-IPMSLV-
IGPMSL--
ISPMSL--
IPPMSL--
IWPMSL--
IVPMSL--
-IPMSLL-
-IPMSLI-
IKPMSL--
-IPMSLS-
-IPMSLA-
IIPMSL--
IFPMSL--
-IPMSLN-

IQPMSL--
-IPMSLT-
IHPMSL--
IDPMSL--
-IPMSLM-
IEPMSL--
ICPMSL--
-IPMSLQ-
KGPMSL--
-KPMSLS-
-KPMSLR-
-KPMSLH-
-KPMSLL-
-KPMSLW-
KQPMSL--
KTPMSL--
KVPMSL--
KRPMSL--
-KPMSLM-
-KPMSLF-
-KPMSLT-
KMPMSL--
KNPMSL--
KHPMSL--
KEPMSL--
-KPMSLN-
KAPMSL--
KKPMSL--
KIPMSL--
KPPMSL--
-KPMSLY-
-KPMSLV-
-KPMSLQ-
-KPMSLI-
KYPMSL--
-KPMSLG-
-KPMSLE-
KFPMSL--
-LPMSLR-
LLPMSL--
-LPMSLK-
LNPMSL--
-LPMSLT-
-LPMSLI-
LEPMSL--
-LPMSLS-
LTPMSL--
LDPMSL--
LAPMSL--
LHPMSL--

LFPMSL--
LYPMSL--
-LPMSLV-
LMPMSL--
LPPMSL--
-LPMSLL-
-LPMSLH-
LQPMSL--
-LPMSLQ-
-LPMSLM-
LCPMSL--
-LPMSLA-
-LPMSLN-
MRPMSL--
MMPMSL--
-MPMSLS-
MLPMSL--
MSPMSL--
-MPMSLV-
-MPMSLN-
MWPMSL--
-MPMSLK-
-MPMSLI-
MYPMSL--
MVPMSL--
MIPMSL--
MQPMSL--
-MPMSLQ-
MTPMSL--
-MPMSLT-
-MPMSLG-
-MPMSLE-
MAPMSL--
MHPMSL--
-MPMSLM-
-MPMSLA-
MCPMSL--
NRPMSL--
NSPMSL--
NIPMSL--
-NPMSLT-
-NPMSLL-
-NPMSLR-
NMPMSL--
NVPMSL--
NPPMSL--
NYPMSL--
-NPMSLF-
NTPMSL--
-NPMSLV-

NFPMSL--
NGPMSL--
-NPMSLH-
NQPMSL--
-NPMSLQ-
-NPMSLI-
NKPMSL--
-NPMSLK-
-NPMSLS-
-NPMSLM-
NEPMSL--
NWPMSL--
-NPMSLW-
NCPMSL--
-NPMSLE-
-NPMSLA-
--PMSLLR
--PMSLLS
--PMSLRG
--PMSLVS
--PMSLRQ
--PMSLYG
--PMSLSG
--PMSLYT
--PMSLSA
--PMSLQT
--PMSLGL
PWPMSL--
--PMSLSS
--PMSLLA
--PMSLKV
--PMSLTK
--PMSLRI
--PMSLLG
--PMSLVN
--PMSLRN
--PMSLKG
--PMSLVT
--PMSLTA
--PMSLTG
--PMSLQG
--PMSLHG
--PMSLTR
--PMSLMR
--PMSLGY
PVPMSL--
--PMSLWN
--PMSLWG
--PMSLVH
--PMSLTV

--PMSLKQ
--PMSLFD
--PMSLWR
--PMSLVK
--PMSLSR
--PMSLRD
--PMSLMI
--PMSLIR
--PMSLHS
PHPMSL--
--PMSLVW
--PMSLTY
--PMSLTN
--PMSLNS
--PMSLMG
--PMSLKR
--PMSLIK
--PMSLMK
--PMSLKA
--PMSLGS
--PMSLAY
--PMSLAS
--PMSLTL
--PMSLST
--PMSLMT
--PMSLIG
--PMSLHR
--PMSLFS
--PMSLTH
--PMSLPL
--PMSLKH
--PMSLFN
--PMSLFH
PDPMSL--
--PMSLWD
--PMSLTQ
--PMSLSM
--PMSLQS
--PMSLMV
--PMSLMS
--PMSLKT
--PMSLIH
--PMSLHQ
PTPMSL--
--PMSLYE
--PMSLVG
--PMSLSY
--PMSLPY
--PMSLNL
--PMSLLN

--PMSLKI
--PMSLKF
--PMSLIT
--PMSLII
--PMSLHN
PAPMSL--
PNPMSL--
--PMSLWW
--PMSLTD
--PMSLSK
--PMSLPF
--PMSLNV
--PMSLMQ
--PMSLMF
--PMSLKN
--PMSLKK
--PMSLHY
--PMSLGI
--PMSLEA
--PMSLAM
--PMSLWT
--PMSLVQ
--PMSLVM
--PMSLTW
--PMSLTI
--PMSLNM
--PMSLHA
--PMSLYH
--PMSLTM
--PMSLSW
--PMSLSF
--PMSLNT
--PMSLMM
--PMSLIA
--PMSLHM
--PMSLGG
--PMSLAN
--PMSLAG
PMPMSL--
PEPMSL--
--PMSLYN
--PMSLWE
--PMSLQW
--PMSLPM
--PMSLNY
--PMSLNK
--PMSLNA
--PMSLMD
--PMSLKL
--PMSLHW

```
--PMSLHL
--PMSLHF
--PMSLHE
--PMSLAV
--PMSLWQ
--PMSLVE
--PMSLVD
--PMSLTE
--PMSLSQ
--PMSLQY
--PMSLQQ
--PMSLQN
--PMSLQI
--PMSLQH
--PMSLPW
--PMSLPI
--PMSLNN
--PMSLNG
--PMSLMY
--PMSLMW
--PMSLMH
--PMSLLH
--PMSLHV
--PMSLGW
--PMSLAR
--PMSLAA
PQPMSL--
--PMSLYW
--PMSLYD
--PMSLQR
--PMSLNQ
--PMSLKW
--PMSLKS
--PMSLKM
--PMSLIQ
--PMSLIN
--PMSLIE
--PMSLID
--PMSLHK
--PMSLHD
--PMSLGM
--PMSLEH
--PMSLEG
--PMSLCT
--PMSLAI
PKPMSL--
PFPMSL--
-PPMSLS-
-PPMSLR-
-PPMSLA-
```

--PMSLYQ
--PMSLWH
--PMSLSC
--PMSLQV
--PMSLQK
--PMSLQF
--PMSLPV
--PMSLNI
--PMSLMA
--PMSLKE
--PMSLHH
--PMSLGV
--PMSLEM
--PMSLCS
--PMSLCG
--PMSLCD
--PMSLAK
PCPMSL--
QVPMSL--
-QPMSLL-
QSPMSL--
-QPMSLR-
QGPMSL--
QTPMSL--
-QPMSLV-
-QPMSLT-
QLPMSL--
QEPMSL--
QQPMSL--
-QPMSLS-
QFPMSL--
-QPMSLH-
-QPMSLQ-
-QPMSLM-
QAPMSL--
-QPMSLF-
-QPMSLK-
QNPMSL--
QMPMSL--
QIPMSL--
QYPMSL--
QPPMSL--
QHPMSL--
QDPMSL--
-QPMSLN-
-QPMSLA-
QKPMSL--
QCPMSL--
QWPMSL--
-QPMSLW-

```
-QPMSLE-
-RPMSLT-
RSPMSL--
RTPMSL--
RFPMSL--
RHPMSL--
-RPMSLM-
RYPMSL--
RPPMSL--
RNPMSL--
-RPMSLV-
RAPMSL--
REPMSL--
RQPMSL--
-RPMSLE-
-RPMSLA-
RDPMSL--
-RPMSLI-
-RPMSLW-
-RPMSLG-
-RPMSLQ-
-RPMSLC-
SVPMSL--
-SPMSLS-
-SPMSLT-
SAPMSL--
-SPMSLL-
STPMSL--
-SPMSLV-
SMPMSL--
-SPMSLY-
SHPMSL--
-SPMSLA-
SDPMSL--
SPPMSL--
SNPMSL--
SKPMSL--
-SPMSLW-
-SPMSLN-
SIPMSL--
SFPMSL--
-SPMSLI-
SEPMSL--
-SPMSLK-
SWPMSL--
-SPMSLM-
-SPMSLE-
SQPMSL--
SCPMSL--
-SPMSLG-
```

TRPMSL--
TLPMSL--
TAPMSL--
-TPMSLL-
-TPMSLT-
TGPMSL--
-TPMSLV-
-TPMSLS-
TMPMSL--
-TPMSLW-
TIPMSL--
TYPMSL--
TNPMSL--
TQPMSL--
TKPMSL--
THPMSL--
-TPMSLA-
-TPMSLQ-
-TPMSLH-
-TPMSLF-
TFPMSL--
-TPMSLK-
-TPMSLN-
TEPMSL--
TDPMSL--
-TPMSLE-
TCPMSL--
-TPMSLM-
-VPMSLR-
VVPMSL--
VTPMSL--
VMPMSL--
-VPMSLK-
VAPMSL--
VSPMSL--
VPPMSL--
-VPMSLM-
VFPMSL--
VQPMSL--
VKPMSL--
VGPMSL--
VYPMSL--
-VPMSLH-
VDPMSL--
-VPMSLQ-
VNPMSL--
-VPMSLT-
-VPMSLN-
-VPMSLA-
VIPMSL--

VHPMSL--
-VPMSLV-
VEPMSL--
VCPMSL--
-VPMSLD-
WRPMSL--
WNPMSL--
-WPMSLS-
-WPMSLM-
-WPMSLR-
WGPMSL--
WSPMSL--
-WPMSLK-
WKPMSL--
WLPMSL--
WHPMSL--
WQPMSL--
-WPMSLV-
-WPMSLQ-
-WPMSLA-
WWPMSL--
-WPMSLH-
WFPMSL--
WCPMSL--
WMPMSL--
-WPMSLN-
WDPMSL--
-WPMSLT-
-WPMSLE-
YRPMSL--
YLPMSL--
-YPMSLR-
YKPMSL--
YGPMSL--
-YPMSLL-
-YPMSLS-
YTPMSL--
YSPMSL--
YPPMSL--
YIPMSL--
YVPMSL--
-YPMSLQ-
-YPMSLK-
YAPMSL--
-YPMSLV-
-YPMSLT-
-YPMSLN-
YDPMSL--
YQPMSL--
YNPMSL--

| | | | |
|---|---|---|---|
| | YYPMSL-- | | |
| | YWPMSL-- | | |
| | YMPMSL-- | | |
| | YEPMSL-- | | |
| | -YPMSLA- | | |
| | YHPMSL-- | | |
| | YCPMSL-- | | |
| PLGW | 75 | 781 | 32.47 |
| | --PLGWGR | | |
| | -SPLGWG- | | |
| | ARPLGW-- | | |
| | -APLGWV- | | |
| | -APLGWS- | | |
| | -APLGWL- | | |
| | -APLGWG- | | |
| | -APLGWR- | | |
| | ANPLGW-- | | |
| | ALPLGW-- | | |
| | AWPLGW-- | | |
| | AEPLGW-- | | |
| | ADPLGW-- | | |
| | AVPLGW-- | | |
| | ATPLGW-- | | |
| | ASPLGW-- | | |
| | -APLGWI- | | |
| | -APLGWA- | | |
| | AGPLGW-- | | |
| | -APLGWY- | | |
| | -APLGWT- | | |
| | -APLGWN- | | |
| | AKPLGW-- | | |
| | AHPLGW-- | | |
| | -APLGWH- | | |
| | -APLGWF- | | |
| | AQPLGW-- | | |
| | -APLGWQ- | | |
| | AYPLGW-- | | |
| | APPLGW-- | | |
| | -APLGWK- | | |
| | AFPLGW-- | | |
| | -APLGWE- | | |
| | AMPLGW-- | | |
| | AIPLGW-- | | |
| | ACPLGW-- | | |
| | -CPLGWL- | | |
| | -CPLGWS- | | |
| | CPPLGW-- | | |
| | CGPLGW-- | | |
| | CMPLGW-- | | |
| | CSPLGW-- | | |

-CPLGWV-
-CPLGWN-
-CPLGWH-
CNPLGW--
CHPLGW--
-DPLGWR-
-DPLGWM-
-DPLGWL-
DLPLGW--
DSPLGW--
-DPLGWV-
-DPLGWH-
DKPLGW--
DWPLGW--
DTPLGW--
-DPLGWA-
DNPLGW--
DIPLGW--
DAPLGW--
DVPLGW--
DGPLGW--
DYPLGW--
-DPLGWY-
-DPLGWQ-
-DPLGWN-
-DPLGWI-
-DPLGWG-
DHPLGW--
DEPLGW--
-EPLGWL-
-EPLGWI-
EYPLGW--
ESPLGW--
EPPLGW--
-EPLGWT-
-EPLGWR-
ENPLGW--
EEPLGW--
EAPLGW--
-EPLGWS-
-EPLGWM-
ELPLGW--
EHPLGW--
-EPLGWV-
-EPLGWH-
EGPLGW--
EWPLGW--
EVPLGW--
ETPLGW--
ERPLGW--

-EPLGWY-
EMPLGW--
EFPLGW--
EQPLGW--
-EPLGWK-
-EPLGWA-
-FPLGWR-
-FPLGWH-
-FPLGWA-
FSPLGW--
FRPLGW--
-FPLGWK-
FKPLGW--
-FPLGWS-
FTPLGW--
-FPLGWV-
-FPLGWT-
-FPLGWI-
-FPLGWY-
FGPLGW--
-FPLGWL-
FLPLGW--
FYPLGW--
FMPLGW--
FQPLGW--
-FPLGWW-
-FPLGWQ-
-FPLGWF-
-FPLGWE-
FPPLGW--
-FPLGWN-
-FPLGWM-
FCPLGW--
GLPLGW--
-GPLGWR-
GKPLGW--
-GPLGWL-
GTPLGW--
-GPLGWV-
GSPLGW--
-GPLGWM-
-GPLGWE-
GIPLGW--
GFPLGW--
GGPLGW--
GEPLGW--
-GPLGWT-
-GPLGWH-
-GPLGWF-
GNPLGW--

GAPLGW--
-GPLGWN-
GMPLGW--
GCPLGW--
GYPLGW--
GPPLGW--
-GPLGWS-
-GPLGWG-
-GPLGWA-
GVPLGW--
-GPLGWW-
-GPLGWQ-
GHPLGW--
-GPLGWK-
GWPLGW--
-GPLGWC-
GDPLGW--
HSPLGW--
-HPLGWN-
HLPLGW--
HPPLGW--
-HPLGWI-
-HPLGWA-
HVPLGW--
HRPLGW--
-HPLGWQ-
HMPLGW--
HGPLGW--
-HPLGWR-
-HPLGWK-
-HPLGWH-
-HPLGWG-
-HPLGWV-
HFPLGW--
HAPLGW--
HYPLGW--
-HPLGWW-
-HPLGWL-
HKPLGW--
HQPLGW--
-HPLGWT-
-HPLGWE-
HNPLGW--
HIPLGW--
HHPLGW--
IRPLGW--
-IPLGWT-
-IPLGWS-
ISPLGW--
-IPLGWR-

IAPLGW--
ILPLGW--
IYPLGW--
IVPLGW--
ITPLGW--
IPPLGW--
-IPLGWH-
IIPLGW--
-IPLGWL-
-IPLGWI-
IQPLGW--
-IPLGWY-
-IPLGWM-
-IPLGWG-
-IPLGWA-
IMPLGW--
IGPLGW--
IFPLGW--
-IPLGWF-
IKPLGW--
-IPLGWN-
IDPLGW--
-IPLGWQ-
IHPLGW--
IEPLGW--
KSPLGW--
KLPLGW--
KRPLGW--
-KPLGWV-
KVPLGW--
KNPLGW--
-KPLGWS-
-KPLGWR-
KPPLGW--
-KPLGWT-
-KPLGWW-
-KPLGWI-
-KPLGWK-
KGPLGW--
KDPLGW--
-KPLGWY-
-KPLGWM-
KEPLGW--
KHPLGW--
KTPLGW--
-KPLGWN-
KAPLGW--
KKPLGW--
KQPLGW--
-KPLGWG-

KFPLGW--
KYPLGW--
KWPLGW--
-KPLGWH-
KIPLGW--
LSPLGW--
-LPLGWL-
LKPLGW--
-LPLGWR-
LQPLGW--
LAPLGW--
LVPLGW--
-LPLGWS-
LPPLGW--
LEPLGW--
LYPLGW--
LTPLGW--
-LPLGWY-
-LPLGWH-
-LPLGWT-
-LPLGWQ-
-LPLGWN-
-LPLGWK-
LNPLGW--
LWPLGW--
-LPLGWV-
LMPLGW--
LDPLGW--
-LPLGWM-
-LPLGWI-
LHPLGW--
LFPLGW--
-LPLGWE-
MDPLGW--
-MPLGWV-
MMPLGW--
MSPLGW--
MRPLGW--
-MPLGWS-
MYPLGW--
-MPLGWW-
MVPLGW--
MNPLGW--
-MPLGWY-
-MPLGWH-
MLPLGW--
MIPLGW--
MAPLGW--
-MPLGWM-
-MPLGWT-

```
-MPLGWK-
MGPLGW--
MEPLGW--
MPPLGW--
-MPLGWI-
-MPLGWG-
-MPLGWR-
-MPLGWN-
MTPLGW--
MQPLGW--
-MPLGWQ-
-MPLGWF-
-MPLGWE-
MKPLGW--
MHPLGW--
NLPLGW--
-NPLGWL-
NSPLGW--
NRPLGW--
-NPLGWI-
-NPLGWR-
-NPLGWA-
-NPLGWS-
NTPLGW--
-NPLGWY-
-NPLGWV-
-NPLGWH-
NWPLGW--
NVPLGW--
-NPLGWW-
NGPLGW--
NAPLGW--
NQPLGW--
NPPLGW--
NKPLGW--
NNPLGW--
NMPLGW--
NHPLGW--
-NPLGWF-
NEPLGW--
NDPLGW--
NYPLGW--
-NPLGWQ-
NIPLGW--
--PLGWSR
--PLGWVR
--PLGWSN
--PLGWSF
--PLGWQR
--PLGWLL
```

--PLGWLG
--PLGWLE
--PLGWTL
--PLGWIK
--PLGWRS
--PLGWMK
--PLGWLN
--PLGWKR
--PLGWTN
--PLGWSV
--PLGWST
--PLGWSK
--PLGWLS
--PLGWLK
--PLGWKS
--PLGWHS
--PLGWSG
--PLGWMS
--PLGWTG
--PLGWIS
--PLGWVS
--PLGWQL
--PLGWIR
--PLGWYT
--PLGWYA
--PLGWSY
--PLGWRQ
--PLGWLA
--PLGWIA
--PLGWHT
--PLGWHN
--PLGWFM
--PLGWAS
--PLGWTS
--PLGWTR
--PLGWRG
--PLGWRA
--PLGWMR
--PLGWLQ
--PLGWVH
--PLGWRN
--PLGWRM
--PLGWLT
--PLGWLM
--PLGWAT
--PLGWSS
--PLGWSM
--PLGWSL
--PLGWSI
--PLGWRP

```
--PLGWML
--PLGWMG
--PLGWKI
--PLGWHA
--PLGWFT
--PLGWAV
PQPLGW--
--PLGWTT
--PLGWTK
--PLGWSH
--PLGWQV
--PLGWKL
--PLGWIV
--PLGWTQ
--PLGWRY
--PLGWRI
--PLGWNA
--PLGWIY
--PLGWIH
--PLGWHK
--PLGWFR
--PLGWFK
--PLGWYS
--PLGWWR
--PLGWVY
--PLGWVV
--PLGWVN
--PLGWRD
--PLGWQI
--PLGWLD
--PLGWKE
--PLGWIL
--PLGWGT
--PLGWGS
--PLGWEA
--PLGWAG
--PLGWAA
--PLGWYV
--PLGWYD
--PLGWVM
--PLGWQM
--PLGWNW
--PLGWNS
--PLGWNH
--PLGWMN
--PLGWEK
--PLGWYR
--PLGWYH
--PLGWWS
--PLGWWE
```

--PLGWWA
--PLGWTM
--PLGWSW
--PLGWRK
--PLGWRE
--PLGWNV
--PLGWNM
--PLGWME
--PLGWLV
--PLGWLI
--PLGWKV
--PLGWKT
--PLGWKN
--PLGWKM
--PLGWIQ
--PLGWGV
--PLGWFQ
--PLGWFE
--PLGWES
PYPLGW--
--PLGWWG
--PLGWVQ
--PLGWVG
--PLGWTV
--PLGWTA
--PLGWSQ
--PLGWQS
--PLGWNR
--PLGWMQ
--PLGWMM
--PLGWMI
--PLGWMH
--PLGWMA
--PLGWKK
--PLGWKH
--PLGWKF
--PLGWIT
--PLGWIN
--PLGWID
--PLGWHY
--PLGWHM
--PLGWGA
--PLGWFA
--PLGWAW
PKPLGW--
--PLGWYY
--PLGWVT
--PLGWVK
--PLGWVD
--PLGWSE

--PLGWQA
--PLGWNT
--PLGWNN
--PLGWNK
--PLGWLH
--PLGWKW
--PLGWHI
--PLGWHG
--PLGWHF
--PLGWGI
--PLGWGG
--PLGWFH
--PLGWAN
--PLGWAM
PFPLGW--
PMPLGW--
--PLGWYP
--PLGWYN
--PLGWYM
--PLGWYK
--PLGWWD
--PLGWVI
--PLGWVA
--PLGWRW
--PLGWQY
--PLGWQG
--PLGWQD
--PLGWNY
--PLGWNL
--PLGWNI
--PLGWMT
--PLGWKG
--PLGWKD
--PLGWKA
--PLGWHV
--PLGWHQ
--PLGWHE
--PLGWER
--PLGWAQ
PHPLGW--
PEPLGW--
PWPLGW--
--PLGWWT
--PLGWWM
--PLGWWH
--PLGWVW
--PLGWVE
--PLGWTY
--PLGWTW
--PLGWTI

--PLGWTH
--PLGWTE
--PLGWTD
--PLGWSD
--PLGWQQ
--PLGWQN
--PLGWQK
--PLGWQF
--PLGWNG
--PLGWMV
--PLGWMF
--PLGWMD
--PLGWKY
--PLGWIM
--PLGWHW
--PLGWGM
--PLGWFV
--PLGWEY
--PLGWEI
--PLGWEG
--PLGWDG
--PLGWDA
--PLGWAY
--PLGWAD
PCPLGW--
-QPLGWS-
-QPLGWR-
-QPLGWT-
-QPLGWL-
QLPLGW--
-QPLGWA-
QRPLGW--
-QPLGWI-
-QPLGWY-
QGPLGW--
QHPLGW--
QDPLGW--
-QPLGWK-
QPPLGW--
-QPLGWG-
-QPLGWF-
QFPLGW--
QYPLGW--
QSPLGW--
-QPLGWW-
-QPLGWQ-
-QPLGWN-
-QPLGWE-
QNPLGW--
QMPLGW--

QKPLGW--
QWPLGW--
QVPLGW--
QEPLGW--
QTPLGW--
-QPLGWV-
QIPLGW--
RMPLGW--
-RPLGWR-
RGPLGW--
RVPLGW--
RSPLGW--
-RPLGWV-
-RPLGWS-
RAPLGW--
-RPLGWI-
-RPLGWF-
RKPLGW--
RHPLGW--
-RPLGWK-
RTPLGW--
-RPLGWN-
REPLGW--
-RPLGWY-
RLPLGW--
RFPLGW--
RYPLGW--
-RPLGWW-
-RPLGWH-
RWPLGW--
RPPLGW--
-RPLGWG-
RIPLGW--
RNPLGW--
RQPLGW--
RDPLGW--
-RPLGWQ-
RCPLGW--
SLPLGW--
-SPLGWS-
-SPLGWA-
-SPLGWT-
-SPLGWF-
SEPLGW--
-SPLGWK-
SMPLGW--
SSPLGW--
-SPLGWN-
STPLGW--
-SPLGWM-

SNPLGW--
SGPLGW--
SQPLGW--
-SPLGWW-
-SPLGWQ-
-SPLGWI-
SWPLGW--
SYPLGW--
SHPLGW--
SPPLGW--
-SPLGWH-
SIPLGW--
SCPLGW--
SAPLGW--
SDPLGW--
SVPLGW--
SKPLGW--
-SPLGWE-
-TPLGWT-
TVPLGW--
-TPLGWR-
-TPLGWV-
TSPLGW--
TTPLGW--
-TPLGWK-
TYPLGW--
-TPLGWM-
-TPLGWI-
THPLGW--
-TPLGWN-
-TPLGWL-
TMPLGW--
TRPLGW--
TIPLGW--
TNPLGW--
TKPLGW--
TAPLGW--
-TPLGWG-
TGPLGW--
TDPLGW--
TQPLGW--
-TPLGWF-
TPPLGW--
TLPLGW--
TWPLGW--
-TPLGWW-
-TPLGWQ-
-TPLGWH-
TEPLGW--
TFPLGW--

TCPLGW--
-VPLGWR-
-VPLGWV-
VLPLGW--
-VPLGWQ-
VTPLGW--
-VPLGWT-
VVPLGW--
VIPLGW--
VWPLGW--
VPPLGW--
-VPLGWI-
-VPLGWG-
-VPLGWN-
-VPLGWF-
VSPLGW--
VQPLGW--
-VPLGWS-
-VPLGWL-
VKPLGW--
VGPLGW--
VMPLGW--
-VPLGWY-
VAPLGW--
VHPLGW--
VYPLGW--
-VPLGWH-
VNPLGW--
-VPLGWW-
-VPLGWM-
-VPLGWK-
VDPLGW--
-WPLGWR-
WRPLGW--
-WPLGWA-
-WPLGWT-
WTPLGW--
WSPLGW--
-WPLGWS-
-WPLGWL-
WLPLGW--
WKPLGW--
WYPLGW--
WDPLGW--
-WPLGWW-
-WPLGWQ-
WMPLGW--
WGPLGW--
WEPLGW--
WAPLGW--

WPPLGW--
-WPLGWP-
-WPLGWM-
-WPLGWK-
-WPLGWI-
-WPLGWH-
-WPLGWE-
-YPLGWR-
-YPLGWV-
-YPLGWQ-
-YPLGWA-
YVPLGW--
-YPLGWI-
YLPLGW--
YRPLGW--
YAPLGW--
-YPLGWY-
-YPLGWL-
YGPLGW--
YSPLGW--
-YPLGWT-
YTPLGW--
YQPLGW--
YKPLGW--
-YPLGWW-
-YPLGWN-
-YPLGWF-
-YPLGWE-
YFPLGW--
-YPLGWS-
-YPLGWM-
YDPLGW--
YIPLGW--
YCPLGW--
YYPLGW--
-YPLGWK-
-YPLGWH-
YNPLGW--
YEPLGW--
PASW 107 764 32.46
--PASWNL
--PASWAK
-APASWQ-
-APASWS-
-APASWK-
-APASWV-
-APASWT-
ASPASW--
AKPASW--
-APASWR-

-APASWM-
ALPASW--
AYPASW--
AEPASW--
AAPASW--
AVPASW--
-APASWY-
AIPASW--
-APASWF-
APPASW--
-APASWL-
-APASWG-
ANPASW--
AFPASW--
AWPASW--
-APASWN-
-APASWI-
-APASWH-
-APASWA-
AGPASW--
ADPASW--
-CPASWT-
-CPASWL-
CHPASW--
CTPASW--
-CPASWY-
-CPASWV-
CNPASW--
CEPASW--
CCPASW--
CSPASW--
-CPASWQ-
-CPASWI-
CMPASW--
CIPASW--
CDPASW--
CAPASW--
-DPASWI-
-DPASWR-
DFPASW--
DRPASW--
-DPASWS-
DKPASW--
-DPASWV-
DIPASW--
-DPASWL-
DNPASW--
DTPASW--
DSPASW--
DQPASW--

```
-DPASWQ-
DMPASW--
DLPASW--
DGPASW--
DEPASW--
DVPASW--
-DPASWK-
-DPASWH-
-DPASWF-
-DPASWA-
-EPASWR-
EVPASW--
-EPASWI-
-EPASWL-
-EPASWG-
-EPASWM-
ESPASW--
ERPASW--
-EPASWW-
-EPASWS-
-EPASWH-
EIPASW--
-EPASWY-
-EPASWV-
-EPASWN-
-EPASWF-
ENPASW--
ELPASW--
EKPASW--
EHPASW--
EFPASW--
EAPASW--
-FPASWT-
-FPASWL-
-FPASWS-
FYPASW--
FVPASW--
FTPASW--
FNPASW--
FSPASW--
-FPASWR-
-FPASWH-
-FPASWE-
FMPASW--
FEPASW--
FRPASW--
-FPASWY-
-FPASWF-
-FPASWA-
FKPASW--
```

FGPASW--
-FPASWV-
-FPASWN-
FPPASW--
-FPASWQ-
-FPASWK-
FHPASW--
GNPASW--
-GPASWR-
-GPASWQ-
GYPASW--
GRPASW--
GLPASW--
-GPASWT-
-GPASWN-
-GPASWI-
GWPASW--
-GPASWS-
-GPASWA-
GPPASW--
GFPASW--
GKPASW--
GAPASW--
GTPASW--
-GPASWV-
-GPASWM-
GHPASW--
GDPASW--
-GPASWY-
-GPASWW-
GCPASW--
GVPASW--
GQPASW--
-GPASWG-
-GPASWF-
GIPASW--
GGPASW--
GEPASW--
-GPASWK-
-GPASWH-
-GPASWE-
-HPASWS-
HTPASW--
HSPASW--
-HPASWR-
HGPASW--
-HPASWT-
-HPASWV-
-HPASWG-
HLPASW--

-HPASWI-
-HPASWL-
-HPASWE-
-HPASWW-
HAPASW--
-HPASWM-
-HPASWA-
HHPASW--
-HPASWY-
HNPASW--
HYPASW--
HVPASW--
-HPASWN-
-HPASWK-
HMPASW--
-HPASWF-
-HPASWC-
HIPASW--
HEPASW--
HDPASW--
ISPASW--
-IPASWT-
-IPASWH-
IVPASW--
-IPASWS-
-IPASWW-
ILPASW--
-IPASWL-
IMPASW--
IYPASW--
-IPASWV-
INPASW--
IEPASW--
ITPASW--
-IPASWR-
-IPASWN-
-IPASWA-
IWPASW--
IQPASW--
IPPASW--
-IPASWG-
-IPASWE-
IHPASW--
IFPASW--
IDPASW--
KSPASW--
-KPASWR-
KGPASW--
KRPASW--
KPPASW--

-KPASWH-
KVPASW--
-KPASWK-
-KPASWA-
-KPASWS-
-KPASWF-
KNPASW--
KTPASW--
-KPASWV-
-KPASWW-
-KPASWI-
KKPASW--
KFPASW--
KEPASW--
KAPASW--
-KPASWQ-
-KPASWN-
KLPASW--
KIPASW--
KDPASW--
KYPASW--
-KPASWT-
-KPASWG-
-KPASWE-
-LPASWL-
-LPASWK-
-LPASWQ-
-LPASWF-
-LPASWV-
-LPASWT-
-LPASWI-
LLPASW--
LNPASW--
-LPASWS-
-LPASWM-
LKPASW--
LGPASW--
LYPASW--
LWPASW--
-LPASWN-
LHPASW--
LPPASW--
-LPASWY-
-LPASWH-
LQPASW--
LMPASW--
LDPASW--
LAPASW--
-LPASWW-
-LPASWE-

-LPASWA-
-MPASWI-
MKPASW--
-MPASWS-
MSPASW--
MPPASW--
-MPASWA-
-MPASWT-
-MPASWQ-
MLPASW--
MRPASW--
MVPASW--
-MPASWN-
-MPASWM-
-MPASWK-
-MPASWF-
MGPASW--
-MPASWV-
MNPASW--
MHPASW--
MQPASW--
-MPASWL-
-MPASWG-
MDPASW--
MTPASW--
MMPASW--
MIPASW--
MAPASW--
MYPASW--
MFPASW--
-NPASWV-
-NPASWR-
NSPASW--
-NPASWS-
NLPASW--
NVPASW--
-NPASWK-
NMPASW--
-NPASWH-
NIPASW--
NFPASW--
NYPASW--
NTPASW--
NRPASW--
-NPASWW-
NAPASW--
-NPASWY-
-NPASWM-
-NPASWI-
NEPASW--

NPPASW--
-NPASWN-
-NPASWF-
NCPASW--
NWPASW--
NQPASW--
-NPASWQ-
-NPASWD-
-NPASWA-
NNPASW--
NGPASW--
--PASWRA
--PASWLR
--PASWVR
--PASWVK
--PASWRL
--PASWLS
--PASWFK
--PASWYR
--PASWVG
--PASWST
--PASWSG
--PASWRH
--PASWLI
--PASWWS
--PASWMS
--PASWLL
--PASWLD
--PASWIR
--PASWWR
--PASWTK
--PASWII
--PASWYA
--PASWTY
--PASWSR
--PASWLG
--PASWFT
--PASWYT
--PASWTL
--PASWRV
--PASWQN
--PASWMR
--PASWLA
--PASWKM
--PASWHK
--PASWGS
--PASWTT
--PASWTN
--PASWGT
--PASWYH

--PASWVY
--PASWSS
--PASWRT
--PASWRQ
--PASWQL
--PASWLK
PRPASW--
--PASWWK
--PASWQS
--PASWKG
--PASWFR
PNPASW--
--PASWVV
--PASWRS
--PASWRG
--PASWRD
--PASWQK
--PASWNA
--PASWLV
--PASWKK
--PASWIN
--PASWWV
--PASWVD
--PASWSA
--PASWMN
--PASWIS
--PASWHN
--PASWHM
--PASWAV
PGPASW--
--PASWWG
--PASWVQ
--PASWVI
--PASWVF
--PASWVA
--PASWTA
--PASWSY
--PASWSM
--PASWRN
--PASWQR
--PASWLT
--PASWLM
--PASWKS
--PASWKL
--PASWHI
--PASWFI
--PASWAT
PQPASW--
--PASWYS
--PASWYL

--PASWWY
--PASWWT
--PASWWL
--PASWVT
--PASWTS
--PASWRY
--PASWRW
--PASWRP
--PASWQY
--PASWQV
--PASWQG
--PASWLE
--PASWKW
--PASWKI
--PASWIM
--PASWHY
--PASWHR
--PASWGA
--PASWAY
--PASWAS
--PASWAI
PKPASW--
--PASWYW
--PASWYD
--PASWWA
--PASWVW
--PASWTQ
--PASWTF
--PASWTD
--PASWRM
--PASWRK
--PASWNS
--PASWNG
--PASWMT
--PASWMQ
--PASWKY
--PASWKT
--PASWKN
--PASWIY
--PASWIG
--PASWIE
--PASWHG
--PASWFY
--PASWFS
--PASWFM
--PASWES
--PASWAF
PDPASW--
--PASWYV
--PASWYQ

--PASWYE
--PASWWQ
--PASWWN
--PASWWE
--PASWTW
--PASWTH
--PASWSN
--PASWSE
--PASWQT
--PASWQQ
--PASWQH
--PASWNY
--PASWNM
--PASWNH
--PASWMK
--PASWMI
--PASWMD
--PASWLN
--PASWIT
--PASWIQ
--PASWIK
--PASWIA
--PASWHQ
--PASWGL
--PASWFG
--PASWFA
--PASWEG
--PASWAN
--PASWAM
--PASWAL
PVPASW--
-PPASWT-
PHPASW--
PFPASW--
--PASWYM
--PASWVM
--PASWTV
--PASWTI
--PASWSQ
--PASWSK
--PASWSI
--PASWSF
--PASWSD
--PASWNR
--PASWNK
--PASWMM
--PASWML
--PASWMA
--PASWLQ
--PASWKH

--PASWKD
--PASWIW
--PASWID
--PASWGF
--PASWFE
--PASWEY
--PASWEV
PTPASW--
-PPASWM-
-PPASWK-
PIPASW--
--PASWYP
--PASWYN
--PASWYK
--PASWYG
--PASWWI
--PASWWH
--PASWTM
--PASWSW
--PASWQF
--PASWQD
--PASWPH
--PASWNW
--PASWNV
--PASWNF
--PASWMH
--PASWMF
--PASWLH
--PASWKE
--PASWIV
--PASWHS
--PASWHL
--PASWHF
--PASWHD
--PASWGY
--PASWFQ
--PASWFN
--PASWFH
--PASWFD
--PASWEW
--PASWEN
--PASWEK
--PASWEF
--PASWEA
--PASWDS
--PASWCR
--PASWAQ
--PASWAE
-QPASWR-
-QPASWI-

```
-QPASWS-
QRPASW--
-QPASWT-
-QPASWK-
QEPASW--
QQPASW--
-QPASWY-
-QPASWQ-
QNPASW--
QKPASW--
QAPASW--
QYPASW--
QWPASW--
-QPASWV-
-QPASWN-
-QPASWL-
-QPASWG-
QLPASW--
QDPASW--
QTPASW--
-QPASWM-
-QPASWF-
-QPASWA-
QMPASW--
QIPASW--
QFPASW--
-RPASWL-
RGPASW--
-RPASWT-
-RPASWS-
RAPASW--
RNPASW--
RTPASW--
RYPASW--
-RPASWV-
-RPASWG-
RSPASW--
-RPASWM-
-RPASWR-
RQPASW--
-RPASWE-
RDPASW--
-RPASWN-
-RPASWY-
-RPASWI-
RVPASW--
-RPASWF-
-RPASWQ-
-RPASWK-
RIPASW--
```

-RPASWW-
RFPASW--
REPASW--
RKPASW--
-RPASWH-
-RPASWA-
RHPASW--
RPPASW--
RCPASW--
-SPASWS-
-SPASWL-
-SPASWK-
-SPASWT-
SLPASW--
SSPASW--
SRPASW--
-SPASWR-
STPASW--
-SPASWY-
SGPASW--
SQPASW--
SKPASW--
SVPASW--
SIPASW--
-SPASWM-
-SPASWG-
SYPASW--
-SPASWA-
SFPASW--
-SPASWW-
SPPASW--
-SPASWQ-
-SPASWF-
-SPASWN-
SMPASW--
SHPASW--
SWPASW--
SEPASW--
SCPASW--
-SPASWE-
-TPASWL-
-TPASWF-
-TPASWS-
TSPASW--
TLPASW--
TRPASW--
TGPASW--
-TPASWI-
-TPASWG-
TVPASW--

-TPASWV-
-TPASWN-
-TPASWY-
-TPASWH-
TTPASW--
TPPASW--
-TPASWR-
TNPASW--
-TPASWT-
-TPASWQ-
TAPASW--
TQPASW--
-TPASWW-
-TPASWK-
TMPASW--
TKPASW--
TEPASW--
TYPASW--
-TPASWM-
THPASW--
-VPASWL-
-VPASWR-
-VPASWA-
VQPASW--
VGPASW--
-VPASWY-
VLPASW--
VVPASW--
VSPASW--
-VPASWI-
VTPASW--
-VPASWV-
VHPASW--
-VPASWS-
-VPASWN-
-VPASWT-
-VPASWH-
VKPASW--
VDPASW--
-VPASWM-
-VPASWF-
VPPASW--
-VPASWQ-
VFPASW--
VWPASW--
-VPASWG-
-VPASWE-
VYPASW--
-VPASWW-
-VPASWK-

VCPASW--
VAPASW--
WWPASW--
-WPASWV-
WTPASW--
-WPASWW-
-WPASWR-
-WPASWK-
-WPASWI-
WFPASW--
WDPASW--
WSPASW--
-WPASWM-
-WPASWF-
-WPASWE-
WNPASW--
WAPASW--
WQPASW--
WPPASW--
-WPASWN-
WMPASW--
YSPASW--
YVPASW--
YYPASW--
-YPASWA-
YHPASW--
YFPASW--
YQPASW--
-YPASWV-
-YPASWS-
-YPASWR-
-YPASWL-
-YPASWI-
YKPASW--
YTPASW--
-YPASWT-
-YPASWQ-
-YPASWF-
YMPASW--
-YPASWN-
-YPASWK-
-YPASWH-
YWPASW--
YPPASW--
-YPASWW-
-YPASWM-
-YPASWG-
YNPASW--
YLPASW--
YGPASW--

YAPASW--
YIPASW--
YEPASW--
YDPASW--

| | | | |
|---|---|---|---|
| PANF | 261 | 686 | 32.44 |

RQPANF--
-APANFR-
AGPANF--
-APANFL-
-APANFM-
ARPANF--
-APANFH-
-APANFA-
AHPANF--
ATPANF--
-APANFK-
ALPANF--
AAPANF--
-APANFQ-
-APANFF-
AYPANF--
AWPANF--
AVPANF--
APPANF--
-APANFI-
-APANFD-
AIPANF--
AEPANF--
ACPANF--
CTPANF--
CRPANF--
CAPANF--
CSPANF--
-CPANFY-
-CPANFM-
-CPANFL-
CLPANF--
CHPANF--
-DPANFR-
DLPANF--
-DPANFF-
DWPANF--
DVPANF--
DTPANF--
-DPANFL-
DHPANF--
DAPANF--
DRPANF--
-DPANFT-
-DPANFS-

-DPANFQ-
-DPANFH-
-DPANFE-
DNPANF--
DKPANF--
DFPANF--
DEPANF--
DCPANF--
-EPANFL-
ELPANF--
-EPANFT-
EFPANF--
EWPANF--
-EPANFG-
EGPANF--
ETPANF--
-EPANFM-
EVPANF--
ESPANF--
ERPANF--
-EPANFV-
-EPANFN-
-EPANFA-
EMPANF--
EKPANF--
FYPANF--
FRPANF--
-FPANFT-
-FPANFI-
-FPANFS-
FNPANF--
FLPANF--
FTPANF--
FHPANF--
FVPANF--
FPPANF--
-FPANFY-
-FPANFV-
-FPANFM-
-FPANFK-
FMPANF--
FDPANF--
-FPANFL-
-FPANFH-
FIPANF--
FEPANF--
FSPANF--
FQPANF--
-FPANFR-
-FPANFQ-

FCPANF--
FAPANF--
-GPANFL-
-GPANFR-
GVPANF--
-GPANFA-
GLPANF--
GSPANF--
-GPANFS-
GRPANF--
-GPANFV-
GTPANF--
-GPANFN-
-GPANFF-
GIPANF--
GPPANF--
-GPANFQ-
-GPANFM-
GMPANF--
GWPANF--
-GPANFY-
GNPANF--
GHPANF--
GGPANF--
GEPANF--
GYPANF--
GQPANF--
-GPANFK-
-GPANFI-
-GPANFH-
GFPANF--
GDPANF--
GAPANF--
HVPANF--
HTPANF--
-HPANFT-
HLPANF--
-HPANFS-
-HPANFR-
HNPANF--
HWPANF--
HSPANF--
HRPANF--
-HPANFY-
HDPANF--
HPPANF--
-HPANFQ-
-HPANFK-
-HPANFG-
-HPANFE-

HKPANF--
HIPANF--
HYPANF--
-HPANFV-
-HPANFL-
-HPANFI-
-HPANFF-
-HPANFA-
HEPANF--
HCPANF--
ILPANF--
IWPANF--
-IPANFT-
IGPANF--
-IPANFY-
-IPANFK-
ISPANF--
IYPANF--
-IPANFR-
ITPANF--
IDPANF--
-IPANFH-
INPANF--
IKPANF--
IIPANF--
IAPANF--
IVPANF--
-IPANFF-
-IPANFW-
-IPANFM-
-IPANFA-
IHPANF--
KSPANF--
KWPANF--
-KPANFG-
-KPANFH-
KVPANF--
KTPANF--
KRPANF--
KPPANF--
-KPANFS-
KNPANF--
-KPANFT-
-KPANFL-
KHPANF--
KAPANF--
-KPANFW-
-KPANFV-
-KPANFR-
-KPANFN-

KGPANF--
-KPANFQ-
-KPANFM-
-KPANFF-
KLPANF--
KKPANF--
KFPANF--
-KPANFI-
-KPANFD-
LHPANF--
-LPANFR-
LRPANF--
LGPANF--
-LPANFL-
LWPANF--
LVPANF--
LPPANF--
-LPANFS-
-LPANFM-
LLPANF--
LIPANF--
LYPANF--
-LPANFI-
-LPANFK-
LSPANF--
-LPANFW-
-LPANFV-
LFPANF--
LAPANF--
LTPANF--
LQPANF--
LEPANF--
-LPANFQ-
-LPANFH-
-LPANFF-
-LPANFY-
-LPANFT-
-MPANFT-
MYPANF--
MGPANF--
MWPANF--
MSPANF--
-MPANFY-
MAPANF--
-MPANFK-
-MPANFG-
MLPANF--
MEPANF--
MRPANF--
-MPANFS-

```
-MPANFQ-
-MPANFN-
-MPANFI-
MFPANF--
MVPANF--
-MPANFH-
MHPANF--
MPPANF--
-MPANFW-
-MPANFR-
-MPANFC-
MKPANF--
MIPANF--
NSPANF--
NLPANF--
NVPANF--
NRPANF--
NKPANF--
-NPANFS-
NIPANF--
NYPANF--
-NPANFR-
-NPANFK-
-NPANFH-
-NPANFW-
-NPANFT-
-NPANFQ-
-NPANFM-
-NPANFI-
-NPANFG-
NDPANF--
NQPANF--
-NPANFE-
-NPANFA-
NHPANF--
NGPANF--
NEPANF--
--PANFRT
--PANFRR
--PANFKR
--PANFFT
--PANFRL
--PANFFS
--PANFRY
--PANFTL
--PANFSG
--PANFRN
--PANFIR
PTPANF--
--PANFRK
```

--PANFLI
--PANFGG
--PANFVS
--PANFTR
--PANFTK
--PANFRA
--PANFQL
--PANFQI
--PANFMI
--PANFLT
--PANFHG
--PANFYG
--PANFVK
--PANFST
--PANFRW
--PANFLS
--PANFKV
--PANFSS
--PANFRI
--PANFRG
--PANFMF
--PANFIT
--PANFGS
PSPANF--
PHPANF--
--PANFYI
--PANFWY
--PANFVT
--PANFTM
--PANFTI
--PANFSA
--PANFRS
--PANFQT
--PANFNS
--PANFMR
--PANFLQ
--PANFLM
--PANFAT
--PANFAL
--PANFWT
--PANFTG
--PANFSV
--PANFQR
--PANFLV
--PANFKL
--PANFIK
--PANFID
--PANFHS
--PANFGT
--PANFFA

--PANFAM
--PANFAK
--PANFWG
--PANFVR
--PANFTQ
--PANFTF
--PANFSN
--PANFRQ
--PANFNA
--PANFLN
--PANFLA
--PANFKA
--PANFHR
--PANFFG
--PANFAW
--PANFAV
--PANFAN
--PANFAI
PVPANF--
PRPANF--
--PANFYS
--PANFYR
--PANFYA
--PANFWR
--PANFWI
--PANFVN
--PANFTT
--PANFRM
--PANFNV
--PANFNQ
--PANFMM
--PANFLG
--PANFKN
--PANFII
--PANFFQ
PYPANF--
PAPANF--
--PANFYT
--PANFYN
--PANFYL
--PANFWS
--PANFWM
--PANFWA
--PANFTY
--PANFTV
--PANFSW
--PANFSH
--PANFRE
--PANFQY
--PANFQS

--PANFQM
--PANFQF
--PANFMY
--PANFMN
--PANFMD
--PANFLD
--PANFKW
--PANFKS
--PANFKK
--PANFKI
--PANFIV
--PANFIM
--PANFIG
--PANFHT
--PANFHA
--PANFFV
--PANFFH
--PANFEA
--PANFDS
--PANFAR
--PANFAF
PWPANF--
PNPANF--
PLPANF--
PGPANF--
PFPANF--
--PANFYW
--PANFYK
--PANFYH
--PANFYE
--PANFWV
--PANFWL
--PANFWH
--PANFWE
--PANFVI
--PANFVG
--PANFVF
--PANFVE
--PANFVA
--PANFTW
--PANFTS
--PANFTE
--PANFTA
--PANFSQ
--PANFSM
--PANFSK
--PANFSF
--PANFSE
--PANFRV
--PANFRH

--PANFRF
--PANFQV
--PANFQK
--PANFQA
--PANFNY
--PANFNE
--PANFMQ
--PANFMG
--PANFMA
--PANFLE
--PANFKE
--PANFIY
--PANFIW
--PANFIQ
--PANFIN
--PANFIE
--PANFIA
--PANFHW
--PANFHQ
--PANFHM
--PANFHK
--PANFHF
--PANFHE
--PANFGN
--PANFGL
--PANFGA
--PANFFY
--PANFFR
--PANFFN
--PANFFI
--PANFFF
--PANFFE
--PANFEY
--PANFEV
--PANFER
--PANFEN
--PANFEH
--PANFDR
--PANFAS
--PANFAA
-QPANFT-
QTPANF--
-QPANFH-
QRPANF--
QMPANF--
-QPANFL-
-QPANFS-
QNPANF--
QAPANF--
QWPANF--

-QPANFY-
-QPANFW-
-QPANFM-
-QPANFI-
QFPANF--
QQPANF--
-QPANFV-
QYPANF--
QVPANF--
-QPANFK-
-QPANFG-
-QPANFA-
QGPANF--
QEPANF--
RGPANF--
-RPANFK-
-RPANFS-
RAPANF--
RSPANF--
-RPANFQ-
-RPANFV-
RHPANF--
RTPANF--
-RPANFT-
-RPANFY-
RFPANF--
RYPANF--
RVPANF--
-RPANFN-
-RPANFH-
-RPANFD-
-RPANFA-
RWPANF--
-RPANFF-
RNPANF--
RLPANF--
REPANF--
-RPANFI-
-RPANFG-
RIPANF--
-RPANFE-
RKPANF--
RRPANF--
RPPANF--
-RPANFW-
-RPANFM-
RMPANF--
RDPANF--
RCPANF--
-SPANFV-

-SPANFR-
SRPANF--
-SPANFL-
-SPANFT-
SNPANF--
SAPANF--
SLPANF--
-SPANFM-
-SPANFI-
SGPANF--
-SPANFQ-
-SPANFG-
SVPANF--
SSPANF--
-SPANFW-
-SPANFA-
-SPANFE-
SYPANF--
STPANF--
-SPANFY-
-SPANFK-
-SPANFH-
-SPANFF-
SFPANF--
SPPANF--
-SPANFS-
-SPANFC-
SMPANF--
-SPANFN-
-SPANFD-
SHPANF--
-TPANFR-
TAPANF--
TRPANF--
-TPANFS-
-TPANFI-
-TPANFK-
TVPANF--
-TPANFW-
TLPANF--
TTPANF--
-TPANFF-
TGPANF--
TDPANF--
-TPANFV-
-TPANFN-
-TPANFM-
-TPANFH-
-TPANFA-
TKPANF--

TIPANF--
TYPANF--
TQPANF--
TPPANF--
-TPANFY-
-TPANFE-
TSPANF--
-TPANFQ-
-TPANFL-
TNPANF--
TMPANF--
TEPANF--
TCPANF--
-VPANFR-
VTPANF--
VRPANF--
VVPANF--
-VPANFL-
-VPANFG-
VYPANF--
VQPANF--
VSPANF--
-VPANFH-
VLPANF--
-VPANFQ-
-VPANFF-
-VPANFA-
VKPANF--
VWPANF--
-VPANFY-
-VPANFW-
-VPANFV-
-VPANFT-
-VPANFS-
-VPANFK-
-VPANFI-
VHPANF--
-VPANFN-
-VPANFM-
-VPANFD-
VNPANF--
VDPANF--
VAPANF--
-WPANFR-
-WPANFY-
WVPANF--
-WPANFL-
-WPANFE-
WWPANF--
WTPANF--

WRPANF--
-WPANFI-
-WPANFA-
WIPANF--
-WPANFT-
-WPANFS-
-WPANFM-
-WPANFH-
WSPANF--
WPPANF--
-WPANFV-
-WPANFN-
-WPANFK-
-WPANFG-
WNPANF--
WGPANF--
-YPANFL-
-YPANFY-
-YPANFM-
-YPANFE-
YIPANF--
YSPANF--
YLPANF--
-YPANFG-
-YPANFF-
YKPANF--
-YPANFS-
YGPANF--
YDPANF--
YQPANF--
-YPANFW-
YNPANF--
-YPANFR-
-YPANFN-
-YPANFH-
YAPANF--
YVPANF--
-YPANFQ-
-YPANFI-
YMPANF--
YFPANF--

PAFL 358 649 32.42

-LPAFLG-
--PAFLTT
--PAFLKV
-WPAFLG-
-APAFLR-
ALPAFL--
APPAFL--
-APAFLS-

ASPAFL--
-APAFLG-
AGPAFL--
-APAFLT-
ANPAFL--
-APAFLK-
AKPAFL--
-APAFLV-
-APAFLQ-
-APAFLA-
ADPAFL--
AAPAFL--
AQPAFL--
-APAFLL-
-APAFLI-
AEPAFL--
ACPAFL--
AWPAFL--
-APAFLH-
AFPAFL--
AYPAFL--
AMPAFL--
AIPAFL--
-APAFLE-
AHPAFL--
-APAFLC-
CCPAFL--
CQPAFL--
CVPAFL--
CSPAFL--
CEPAFL--
CRPAFL--
CLPAFL--
CHPAFL--
CPPAFL--
-CPAFLS-
-CPAFLQ-
-CPAFLA-
CNPAFL--
CKPAFL--
CAPAFL--
DRPAFL--
DMPAFL--
-DPAFLT-
-DPAFLL-
DGPAFL--
DKPAFL--
DTPAFL--
DQPAFL--
DYPAFL--

DVPAFL--
-DPAFLS-
-DPAFLR-
DFPAFL--
-DPAFLA-
DLPAFL--
-DPAFLK-
DNPAFL--
DSPAFL--
-DPAFLY-
-DPAFLQ-
DHPAFL--
-DPAFLV-
-DPAFLI-
DCPAFL--
DAPAFL--
DWPAFL--
DPPAFL--
-DPAFLW-
-DPAFLF-
DIPAFL--
DEPAFL--
DDPAFL--
EVPAFL--
-EPAFLR-
-EPAFLS-
-EPAFLL-
EHPAFL--
ERPAFL--
ETPAFL--
ESPAFL--
-EPAFLI-
-EPAFLA-
EIPAFL--
EYPAFL--
-EPAFLM-
-EPAFLK-
EAPAFL--
-EPAFLY-
-EPAFLN-
-EPAFLF-
-EPAFLE-
EQPAFL--
EPPAFL--
-EPAFLW-
-EPAFLH-
-EPAFLG-
EKPAFL--
FSPAFL--
-FPAFLT-

FEPAFL--
FVPAFL--
-FPAFLQ-
FGPAFL--
-FPAFLR-
FPPAFL--
-FPAFLS-
-FPAFLK-
-FPAFLM-
FIPAFL--
FQPAFL--
-FPAFLN-
FFPAFL--
FDPAFL--
-FPAFLH-
-FPAFLG-
FNPAFL--
GRPAFL--
GSPAFL--
GTPAFL--
-GPAFLS-
GGPAFL--
-GPAFLL-
GAPAFL--
GYPAFL--
-GPAFLI-
GEPAFL--
-GPAFLT-
-GPAFLR-
-GPAFLK-
GVPAFL--
-GPAFLW-
-GPAFLQ-
-GPAFLN-
GNPAFL--
GDPAFL--
GQPAFL--
-GPAFLY-
-GPAFLA-
-GPAFLE-
GMPAFL--
GHPAFL--
GFPAFL--
GWPAFL--
GPPAFL--
-GPAFLV-
-GPAFLM-
-GPAFLH-
-HPAFLS-
HGPAFL--

-HPAFLR-
-HPAFLV-
-HPAFLT-
-HPAFLK-
-HPAFLN-
HLPAFL--
HYPAFL--
-HPAFLA-
HSPAFL--
HAPAFL--
HTPAFL--
-HPAFLW-
HNPAFL--
HIPAFL--
HDPAFL--
-HPAFLH-
-HPAFLM-
HKPAFL--
HHPAFL--
HQPAFL--
-HPAFLY-
-HPAFLQ-
-HPAFLL-
HMPAFL--
HVPAFL--
-HPAFLF-
-HPAFLE-
HFPAFL--
IDPAFL--
IRPAFL--
IIPAFL--
ISPAFL--
INPAFL--
-IPAFLS-
-IPAFLK-
IAPAFL--
ITPAFL--
-IPAFLT-
IKPAFL--
IGPAFL--
-IPAFLR-
IEPAFL--
-IPAFLG-
-IPAFLE-
-IPAFLA-
ILPAFL--
IVPAFL--
-IPAFLM-
IWPAFL--
-IPAFLN-

IYPAFL--
IPPAFL--
-IPAFLQ-
-IPAFLD-
KSPAFL--
KVPAFL--
-KPAFLL-
KAPAFL--
-KPAFLK-
KYPAFL--
-KPAFLN-
-KPAFLT-
KLPAFL--
-KPAFLV-
-KPAFLA-
KKPAFL--
-KPAFLG-
KNPAFL--
KFPAFL--
-KPAFLQ-
-KPAFLH-
KQPAFL--
KIPAFL--
KEPAFL--
KPPAFL--
-KPAFLY-
-KPAFLE-
KHPAFL--
KCPAFL--
KWPAFL--
-KPAFLI-
KDPAFL--
-LPAFLK-
-LPAFLR-
-LPAFLI-
-LPAFLH-
LLPAFL--
-LPAFLT-
-LPAFLQ-
LEPAFL--
LIPAFL--
-LPAFLA-
LKPAFL--
LPPAFL--
LQPAFL--
LHPAFL--
LMPAFL--
MPPAFL--
MAPAFL--
MQPAFL--

```
MSPAFL--
MTPAFL--
MVPAFL--
MGPAFL--
MYPAFL--
MKPAFL--
-MPAFLQ-
-MPAFLE-
-MPAFLN-
MLPAFL--
-MPAFLW-
-MPAFLT-
-MPAFLI-
MDPAFL--
-MPAFLH-
MNPAFL--
MHPAFL--
MEPAFL--
MCPAFL--
-NPAFLR-
NTPAFL--
NPPAFL--
NRPAFL--
NIPAFL--
NSPAFL--
-NPAFLS-
NVPAFL--
NWPAFL--
-NPAFLI-
NGPAFL--
NAPAFL--
-NPAFLT-
-NPAFLQ-
-NPAFLA-
-NPAFLK-
NNPAFL--
-NPAFLV-
NKPAFL--
-NPAFLF-
NCPAFL--
NYPAFL--
NQPAFL--
-NPAFLM-
-NPAFLH-
NHPAFL--
NMPAFL--
NEPAFL--
--PAFLRS
--PAFLTS
--PAFLSG
```

--PAFLRG
--PAFLVS
--PAFLSI
--PAFLRN
--PAFLSR
PSPAFL--
--PAFLVH
--PAFLQR
--PAFLNS
--PAFLHH
--PAFLVT
--PAFLTW
--PAFLKL
--PAFLKS
--PAFLGA
PYPAFL--
--PAFLLK
--PAFLGS
PLPAFL--
--PAFLTI
--PAFLTH
--PAFLSS
--PAFLSQ
--PAFLSM
--PAFLSK
--PAFLRW
--PAFLIR
--PAFLAR
--PAFLAN
--PAFLTE
--PAFLTA
--PAFLLQ
--PAFLLE
--PAFLKR
--PAFLIT
--PAFLWS
--PAFLQK
--PAFLLH
--PAFLLD
--PAFLKE
--PAFLGG
PTPAFL--
--PAFLSV
--PAFLSE
--PAFLRT
--PAFLQA
--PAFLHA
--PAFLFN
--PAFLYN
--PAFLWA

--PAFLVD
--PAFLTG
--PAFLST
--PAFLRM
--PAFLRD
--PAFLQV
--PAFLQS
--PAFLNA
--PAFLMI
--PAFLKG
--PAFLHM
--PAFLHF
--PAFLTV
--PAFLSW
--PAFLSN
--PAFLQN
--PAFLKT
--PAFLIN
--PAFLHG
--PAFLAT
--PAFLYQ
--PAFLVK
--PAFLVG
--PAFLSA
--PAFLQD
--PAFLKY
--PAFLKW
--PAFLIE
--PAFLIA
--PAFLHT
--PAFLGW
--PAFLEA
--PAFLAV
--PAFLYT
--PAFLYG
--PAFLWN
--PAFLTR
--PAFLTQ
--PAFLTM
--PAFLRE
--PAFLMT
--PAFLMH
--PAFLIK
--PAFLID
--PAFLHV
--PAFLHK
--PAFLHI
--PAFLGT
--PAFLEM
PNPAFL--

PEPAFL--
PAPAFL--
--PAFLYK
--PAFLWG
--PAFLVV
--PAFLSF
--PAFLNY
--PAFLMN
--PAFLLN
--PAFLKH
--PAFLHQ
PQPAFL--
PIPAFL--
PHPAFL--
PCPAFL--
--PAFLYS
--PAFLWD
--PAFLRQ
--PAFLQT
--PAFLQM
--PAFLNM
--PAFLKN
--PAFLHW
--PAFLGV
--PAFLAW
--PAFLAK
--PAFLAH
PMPAFL--
--PAFLYH
--PAFLWT
--PAFLWH
--PAFLTY
--PAFLTN
--PAFLTK
--PAFLSD
--PAFLRH
--PAFLQI
--PAFLPM
--PAFLNV
--PAFLNG
--PAFLMD
--PAFLLA
--PAFLKQ
--PAFLKK
--PAFLKF
--PAFLKD
--PAFLIQ
--PAFLHS
--PAFLGC
--PAFLFG

--PAFLFD
--PAFLFA
--PAFLDS
--PAFLCT
--PAFLAG
-PPAFLY-
-PPAFLT-
--PAFLWQ
--PAFLSH
--PAFLQE
--PAFLPY
--PAFLNW
--PAFLNR
--PAFLNK
--PAFLIH
--PAFLHR
--PAFLHN
--PAFLHE
--PAFLHD
--PAFLFF
--PAFLFE
--PAFLEN
--PAFLEI
--PAFLEG
--PAFLDG
--PAFLCS
--PAFLAA
-QPAFLL-
QVPAFL--
QYPAFL--
-QPAFLR-
QGPAFL--
QSPAFL--
-QPAFLV-
-QPAFLQ-
-QPAFLT-
-QPAFLA-
QPPAFL--
-QPAFLK-
-QPAFLG-
QAPAFL--
-QPAFLM-
-QPAFLH-
QTPAFL--
-QPAFLW-
QFPAFL--
-QPAFLS-
QKPAFL--
-QPAFLY-
-QPAFLN-

```
-QPAFLI-
QHPAFL--
QQPAFL--
QDPAFL--
QCPAFL--
RLPAFL--
RTPAFL--
RSPAFL--
RPPAFL--
RNPAFL--
RIPAFL--
RWPAFL--
RQPAFL--
-RPAFLM-
RDPAFL--
-RPAFLI-
RAPAFL--
-RPAFLT-
REPAFL--
-RPAFLA-
-RPAFLH-
-RPAFLE-
-RPAFLW-
RCPAFL--
SFPAFL--
SKPAFL--
SIPAFL--
-SPAFLL-
-SPAFLA-
SGPAFL--
SNPAFL--
SMPAFL--
-SPAFLT-
SQPAFL--
SHPAFL--
SYPAFL--
STPAFL--
-SPAFLW-
-SPAFLM-
SPPAFL--
SAPAFL--
-SPAFLN-
-SPAFLK-
SDPAFL--
-SPAFLG-
-SPAFLY-
-SPAFLQ-
-SPAFLE-
SEPAFL--
SCPAFL--
```

TGPAFL--
TLPAFL--
TTPAFL--
TVPAFL--
TNPAFL--
TQPAFL--
-TPAFLR-
-TPAFLV-
-TPAFLT-
-TPAFLI-
-TPAFLA-
TIPAFL--
-TPAFLK-
TEPAFL--
TWPAFL--
-TPAFLN-
TPPAFL--
-TPAFLG-
-TPAFLE-
TAPAFL--
TYPAFL--
-TPAFLW-
TCPAFL--
THPAFL--
TDPAFL--
VRPAFL--
VKPAFL--
-VPAFLS-
VTPAFL--
-VPAFLT-
VHPAFL--
VMPAFL--
-VPAFLA-
-VPAFLQ-
VIPAFL--
VPPAFL--
VNPAFL--
VEPAFL--
VDPAFL--
-VPAFLI-
-VPAFLG-
VFPAFL--
VYPAFL--
-VPAFLK-
VQPAFL--
-VPAFLV-
-VPAFLE-
-VPAFLM-
-VPAFLH-
WSPAFL--

WYPAFL--
WGPAFL--
WQPAFL--
WPPAFL--
-WPAFLH-
-WPAFLS-
WWPAFL--
-WPAFLM-
-WPAFLK-
WKPAFL--
-WPAFLQ-
-WPAFLN-
-WPAFLF-
WDPAFL--
-WPAFLW-
WEPAFL--
WCPAFL--
-YPAFLR-
YTPAFL--
YLPAFL--
YAPAFL--
-YPAFLA-
-YPAFLS-
YNPAFL--
YIPAFL--
-YPAFLQ-
YGPAFL--
YRPAFL--
-YPAFLT-
-YPAFLI-
-YPAFLH-
YFPAFL--
-YPAFLG-
YQPAFL--
-YPAFLP-
-YPAFLN-
-YPAFLK-
YEPAFL--
YCPAFL--
YWPAFL--
-YPAFLE-

| | | | |
|---|---|---|---|
| PRNY | 228 | 698 | 32.40 |

-KPRNYG-
-TPRNYF-
-APRNYL-
ATPRNY--
AYPRNY--
ASPRNY--
AAPRNY--
-APRNYW-

-APRNYK-
AMPRNY--
AVPRNY--
-APRNYV-
-APRNYM-
AHPRNY--
-APRNYA-
AGPRNY--
AKPRNY--
AQPRNY--
-APRNYT-
-APRNYN-
-APRNYH-
-APRNYG-
AIPRNY--
-APRNYI-
AFPRNY--
ADPRNY--
-APRNYY-
APPRNY--
AEPRNY--
CKPRNY--
CLPRNY--
CIPRNY--
CPPRNY--
CMPRNY--
CCPRNY--
CVPRNY--
-CPRNYY-
-CPRNYQ-
CAPRNY--
-DPRNYM-
-DPRNYT-
-DPRNYL-
-DPRNYE-
DLPRNY--
-DPRNYR-
-DPRNYI-
-DPRNYF-
DVPRNY--
DRPRNY--
DSPRNY--
DQPRNY--
-DPRNYA-
DDPRNY--
DAPRNY--
DYPRNY--
-DPRNYY-
-DPRNYQ-
-DPRNYH-

-DPRNYG-
DIPRNY--
DHPRNY--
DGPRNY--
DEPRNY--
DCPRNY--
ELPRNY--
-EPRNYT-
-EPRNYW-
-EPRNYL-
-EPRNYI-
-EPRNYG-
EHPRNY--
EWPRNY--
EQPRNY--
-EPRNYS-
-EPRNYM-
ENPRNY--
EMPRNY--
ESPRNY--
-EPRNYV-
-EPRNYQ-
EKPRNY--
ETPRNY--
ERPRNY--
-EPRNYF-
EYPRNY--
-EPRNYY-
-EPRNYR-
EIPRNY--
EDPRNY--
EVPRNY--
-EPRNYK-
-EPRNYH-
-EPRNYA-
EGPRNY--
EFPRNY--
EAPRNY--
-FPRNYL-
FGPRNY--
FRPRNY--
FSPRNY--
FDPRNY--
FIPRNY--
FVPRNY--
-FPRNYN-
-FPRNYG-
FKPRNY--
FAPRNY--
-FPRNYW-

-FPRNYI-
FPPRNY--
FYPRNY--
FQPRNY--
-FPRNYH-
-FPRNYE-
FNPRNY--
FMPRNY--
FHPRNY--
FEPRNY--
-GPRNYL-
-GPRNYW-
GVPRNY--
-GPRNYG-
GLPRNY--
GQPRNY--
-GPRNYI-
GRPRNY--
-GPRNYK-
GAPRNY--
GWPRNY--
-GPRNYT-
-GPRNYQ-
-GPRNYN-
GTPRNY--
GKPRNY--
GYPRNY--
-GPRNYM-
GMPRNY--
-GPRNYA-
GIPRNY--
-GPRNYD-
GNPRNY--
-GPRNYY-
-GPRNYH-
-GPRNYE-
GPPRNY--
GDPRNY--
-GPRNYV-
HKPRNY--
-HPRNYT-
HVPRNY--
HSPRNY--
-HPRNYL-
-HPRNYI-
HTPRNY--
HGPRNY--
-HPRNYK-
HPPRNY--
HIPRNY--

HDPRNY--
-HPRNYY-
-HPRNYV-
-HPRNYF-
-HPRNYE-
HMPRNY--
HLPRNY--
HAPRNY--
-HPRNYW-
-HPRNYN-
-HPRNYM-
-HPRNYH-
ICPRNY--
-IPRNYS-
ITPRNY--
ISPRNY--
-IPRNYV-
IKPRNY--
-IPRNYI-
-IPRNYL-
-IPRNYA-
IEPRNY--
IYPRNY--
IQPRNY--
-IPRNYQ-
-IPRNYH-
-IPRNYG-
IMPRNY--
ILPRNY--
IAPRNY--
IVPRNY--
-IPRNYY-
-IPRNYW-
-IPRNYT-
-IPRNYE-
IPPRNY--
INPRNY--
IIPRNY--
IFPRNY--
KSPRNY--
-KPRNYL-
-KPRNYV-
KLPRNY--
KVPRNY--
-KPRNYT-
-KPRNYS-
-KPRNYF-
KAPRNY--
-KPRNYW-
-KPRNYM-

-KPRNYN-
KMPRNY--
-KPRNYI-
KIPRNY--
KRPRNY--
-KPRNYA-
KKPRNY--
-KPRNYQ-
-KPRNYH-
-KPRNYE-
KDPRNY--
-KPRNYY-
KNPRNY--
KGPRNY--
KTPRNY--
KPPRNY--
KEPRNY--
KCPRNY--
LSPRNY--
LNPRNY--
LGPRNY--
LHPRNY--
LIPRNY--
LAPRNY--
LKPRNY--
LVPRNY--
-LPRNYV-
LTPRNY--
LPPRNY--
-LPRNYS-
-LPRNYH-
LEPRNY--
LQPRNY--
-LPRNYQ-
-LPRNYM-
-LPRNYI-
-LPRNYK-
LDPRNY--
-LPRNYN-
-LPRNYE-
LCPRNY--
-MPRNYL-
MSPRNY--
MQPRNY--
MYPRNY--
-MPRNYK-
MHPRNY--
-MPRNYV-
MGPRNY--
-MPRNYH-

-MPRNYQ-
MNPRNY--
-MPRNYI-
MLPRNY--
MAPRNY--
-MPRNYN-
-MPRNYG-
MPPRNY--
MEPRNY--
-MPRNYE-
MKPRNY--
-MPRNYM-
MDPRNY--
NSPRNY--
NLPRNY--
-NPRNYV-
NVPRNY--
-NPRNYY-
-NPRNYL-
-NPRNYK-
NEPRNY--
NQPRNY--
NNPRNY--
NMPRNY--
NTPRNY--
NHPRNY--
NGPRNY--
NFPRNY--
NYPRNY--
NRPRNY--
-NPRNYM-
-NPRNYI-
-NPRNYF-
NKPRNY--
NIPRNY--
-NPRNYS-
-NPRNYW-
-NPRNYT-
-NPRNYQ-
-NPRNYH-
--PRNYRL
--PRNYSL
--PRNYSR
--PRNYWS
--PRNYWR
--PRNYFS
--PRNYIA
--PRNYGA
--PRNYTL
--PRNYLV

--PRNYST
--PRNYIT
--PRNYIG
--PRNYRY
--PRNYML
--PRNYLF
--PRNYLE
--PRNYGS
--PRNYAW
--PRNYWT
--PRNYWI
--PRNYVT
--PRNYRE
--PRNYLY
--PRNYEG
--PRNYSI
--PRNYHA
--PRNYGG
--PRNYRD
--PRNYFV
--PRNYVF
--PRNYVA
--PRNYTI
--PRNYSS
--PRNYSA
--PRNYIS
--PRNYFN
--PRNYAF
--PRNYSM
--PRNYLW
--PRNYLN
--PRNYLM
--PRNYIL
--PRNYAH
--PRNYYR
--PRNYVG
--PRNYVD
--PRNYSY
--PRNYSV
--PRNYSG
PHPRNY--
--PRNYTN
--PRNYTK
--PRNYTG
--PRNYTF
--PRNYMV
--PRNYMR
--PRNYLD
--PRNYID
--PRNYGV

--PRNYFW
--PRNYFI
--PRNYAL
PDPRNY--
--PRNYWW
--PRNYVN
--PRNYTV
--PRNYRH
--PRNYKL
PRPRNY--
--PRNYYS
--PRNYYN
--PRNYYI
--PRNYWK
--PRNYVV
--PRNYVK
--PRNYVI
--PRNYTA
--PRNYSW
--PRNYSF
--PRNYRQ
--PRNYQV
--PRNYQL
--PRNYQI
--PRNYNS
--PRNYMT
--PRNYFH
--PRNYFG
--PRNYAS
PVPRNY--
--PRNYYV
--PRNYYA
--PRNYWQ
--PRNYVY
--PRNYVQ
--PRNYSE
--PRNYSD
--PRNYRW
--PRNYQT
--PRNYNV
--PRNYNT
--PRNYNA
--PRNYKY
--PRNYKT
--PRNYKI
--PRNYIF
--PRNYGI
--PRNYES
--PRNYAV
--PRNYAT

--PRNYAI
--PRNYAG
PNPRNY--
PSPRNY--
--PRNYYQ
--PRNYYM
--PRNYYH
--PRNYWD
--PRNYVM
--PRNYTH
--PRNYSK
--PRNYQN
--PRNYNL
--PRNYNG
--PRNYMS
--PRNYMQ
--PRNYMG
--PRNYLH
--PRNYIK
--PRNYIH
--PRNYHS
--PRNYHR
--PRNYHL
--PRNYFT
--PRNYAR
PMPRNY--
PFPRNY--
PAPRNY--
--PRNYYT
--PRNYYE
--PRNYWV
--PRNYVW
--PRNYTY
--PRNYSN
--PRNYQY
--PRNYNM
--PRNYMF
--PRNYMA
--PRNYKD
--PRNYIY
--PRNYIW
--PRNYHY
--PRNYHT
--PRNYHH
--PRNYGM
--PRNYGF
--PRNYFA
--PRNYEN
--PRNYEK
--PRNYAY

--PRNYAN
--PRNYAD
PYPRNY--
PTPRNY--
--PRNYYD
--PRNYWY
--PRNYWN
--PRNYWA
--PRNYVH
--PRNYVE
--PRNYTE
--PRNYQS
--PRNYQH
--PRNYQG
--PRNYNY
--PRNYNW
--PRNYNF
--PRNYMM
--PRNYME
--PRNYKQ
--PRNYKE
--PRNYIV
--PRNYHW
--PRNYHG
--PRNYHF
--PRNYGY
--PRNYGW
--PRNYGT
--PRNYFM
--PRNYFD
--PRNYEW
--PRNYEQ
--PRNYEF
--PRNYED
--PRNYAA
PWPRNY--
--PRNYWE
--PRNYTM
--PRNYSQ
--PRNYQW
--PRNYQM
--PRNYQE
--PRNYNQ
--PRNYMW
--PRNYMN
--PRNYMK
--PRNYMI
--PRNYMH
--PRNYKG
--PRNYKF

--PRNYIQ
--PRNYII
--PRNYGN
--PRNYFQ
--PRNYFF
--PRNYEY
--PRNYET
--PRNYEI
--PRNYDT
--PRNYDG
--PRNYCY
--PRNYCT
--PRNYAK
--PRNYAE
PQPRNY--
PKPRNY--
PIPRNY--
PGPRNY--
PCPRNY--
QSPRNY--
-QPRNYI-
-QPRNYF-
-QPRNYG-
QTPRNY--
-QPRNYL-
QIPRNY--
-QPRNYN-
QYPRNY--
QPPRNY--
QAPRNY--
-QPRNYV-
-QPRNYM-
QVPRNY--
-QPRNYY-
-QPRNYE-
QKPRNY--
QQPRNY--
-QPRNYW-
-QPRNYT-
-QPRNYH-
-QPRNYA-
QHPRNY--
-RPRNYL-
RMPRNY--
-RPRNYG-
-RPRNYM-
-RPRNYV-
-RPRNYS-
-RPRNYQ-
REPRNY--

-RPRNYI-
-RPRNYE-
-RPRNYY-
RAPRNY--
RTPRNY--
RIPRNY--
RHPRNY--
RQPRNY--
RNPRNY--
RPPRNY--
RKPRNY--
RGPRNY--
RDPRNY--
-RPRNYH-
-RPRNYF-
-SPRNYL-
SLPRNY--
-SPRNYV-
-SPRNYF-
-SPRNYT-
-SPRNYY-
SGPRNY--
SKPRNY--
SIPRNY--
-SPRNYM-
STPRNY--
SQPRNY--
-SPRNYE-
-SPRNYA-
SMPRNY--
SYPRNY--
-SPRNYH-
-SPRNYG-
-SPRNYW-
-SPRNYQ-
-SPRNYD-
-SPRNYI-
SPPRNY--
-TPRNYL-
TSPRNY--
-TPRNYV-
-TPRNYS-
-TPRNYG-
TAPRNY--
TYPRNY--
TMPRNY--
-TPRNYM-
TPPRNY--
TQPRNY--
-TPRNYY-

-TPRNYW-
-TPRNYT-
-TPRNYH-
-TPRNYA-
TEPRNY--
TRPRNY--
TTPRNY--
-TPRNYR-
TGPRNY--
TIPRNY--
TNPRNY--
TKPRNY--
TFPRNY--
TCPRNY--
TWPRNY--
THPRNY--
-VPRNYL-
VSPRNY--
VLPRNY--
-VPRNYV-
-VPRNYS-
-VPRNYK-
-VPRNYG-
VVPRNY--
VYPRNY--
VHPRNY--
VEPRNY--
-VPRNYI-
VGPRNY--
-VPRNYW-
VNPRNY--
-VPRNYT-
-VPRNYN-
-VPRNYQ-
-VPRNYE-
-VPRNYA-
VPPRNY--
VQPRNY--
VAPRNY--
-VPRNYY-
-VPRNYH-
-VPRNYD-
VDPRNY--
WEPRNY--
WSPRNY--
WGPRNY--
WTPRNY--
WLPRNY--
WVPRNY--
WDPRNY--

WYPRNY--
-WPRNYV-
WQPRNY--
-WPRNYN-
WNPRNY--
WKPRNY--
WAPRNY--
-WPRNYW-
-WPRNYI-
WFPRNY--
WRPRNY--
-WPRNYM-
WMPRNY--
WIPRNY--
-YPRNYL-
YAPRNY--
-YPRNYT-
YRPRNY--
YLPRNY--
YSPRNY--
YFPRNY--
YYPRNY--
-YPRNYY-
YVPRNY--
YTPRNY--
-YPRNYI-
-YPRNYQ-
YIPRNY--
YGPRNY--
YDPRNY--
YPPRNY--
YHPRNY--
YEPRNY--
-YPRNYM-
-YPRNYG-
YNPRNY--
YMPRNY--
YQPRNY--
-YPRNYK-
-YPRNYE-

PVAY 233 696 32.38

ERPVAY--
-TPVAYG-
-APVAYL-
-APVAYR-
ASPVAY--
AQPVAY--
AMPVAY--
AYPVAY--
ATPVAY--

AKPVAY--
-APVAYN-
AHPVAY--
-APVAYS-
ALPVAY--
AIPVAY--
-APVAYA-
ADPVAY--
AAPVAY--
AVPVAY--
-APVAYG-
-APVAYV-
ANPVAY--
-APVAYQ-
APPVAY--
-APVAYK-
-APVAYI-
CKPVAY--
-CPVAYL-
-CPVAYV-
CNPVAY--
CMPVAY--
CEPVAY--
CSPVAY--
-CPVAYS-
-CPVAYA-
-DPVAYS-
DSPVAY--
DRPVAY--
-DPVAYV-
-DPVAYI-
DVPVAY--
-DPVAYW-
DAPVAY--
-DPVAYY-
-DPVAYL-
-DPVAYM-
-DPVAYK-
DWPVAY--
-DPVAYR-
-DPVAYA-
DKPVAY--
DTPVAY--
-DPVAYT-
-DPVAYN-
-DPVAYF-
DLPVAY--
DIPVAY--
-EPVAYL-
ETPVAY--

-EPVAYF-
-EPVAYT-
EVPVAY--
-EPVAYG-
ELPVAY--
EHPVAY--
-EPVAYV-
EWPVAY--
-EPVAYS-
-EPVAYM-
-EPVAYI-
EPPVAY--
ENPVAY--
EIPVAY--
EYPVAY--
ESPVAY--
-EPVAYW-
-EPVAYR-
-EPVAYK-
EMPVAY--
EKPVAY--
EGPVAY--
EFPVAY--
ECPVAY--
EAPVAY--
FHPVAY--
FVPVAY--
-FPVAYT-
-FPVAYL-
FKPVAY--
FMPVAY--
FEPVAY--
-FPVAYS-
FAPVAY--
-FPVAYR-
-FPVAYA-
FSPVAY--
-FPVAYW-
-FPVAYV-
-FPVAYH-
FNPVAY--
FIPVAY--
FWPVAY--
-FPVAYY-
-FPVAYK-
FQPVAY--
-FPVAYF-
FGPVAY--
GRPVAY--
GVPVAY--

-GPVAYV-
-GPVAYL-
-GPVAYH-
-GPVAYQ-
GTPVAY--
GSPVAY--
-GPVAYY-
GHPVAY--
-GPVAYN-
GKPVAY--
-GPVAYM-
-GPVAYK-
GEPVAY--
GAPVAY--
-GPVAYT-
GLPVAY--
GIPVAY--
-GPVAYR-
-GPVAYI-
GNPVAY--
GDPVAY--
GYPVAY--
GQPVAY--
-GPVAYG-
GGPVAY--
GWPVAY--
-GPVAYE-
GMPVAY--
GFPVAY--
-HPVAYS-
HSPVAY--
-HPVAYL-
HMPVAY--
HVPVAY--
HRPVAY--
-HPVAYY-
-HPVAYV-
-HPVAYR-
-HPVAYA-
HYPVAY--
-HPVAYT-
-HPVAYN-
HGPVAY--
HFPVAY--
HDPVAY--
HCPVAY--
-HPVAYQ-
-HPVAYK-
-HPVAYF-
HPPVAY--

HLPVAY--
HHPVAY--
HQPVAY--
-HPVAYH-
HKPVAY--
HAPVAY--
HTPVAY--
HEPVAY--
-IPVAYN-
-IPVAYL-
-IPVAYM-
ISPVAY--
IRPVAY--
INPVAY--
IWPVAY--
IVPVAY--
-IPVAYV-
-IPVAYT-
-IPVAYK-
IMPVAY--
-IPVAYY-
-IPVAYS-
-IPVAYI-
-IPVAYF-
IAPVAY--
IYPVAY--
ITPVAY--
IQPVAY--
-IPVAYH-
-IPVAYG-
-IPVAYE-
IKPVAY--
IGPVAY--
IEPVAY--
IDPVAY--
KSPVAY--
-KPVAYQ-
-KPVAYS-
-KPVAYL-
KGPVAY--
-KPVAYR-
-KPVAYI-
-KPVAYF-
KIPVAY--
KTPVAY--
-KPVAYT-
-KPVAYM-
KEPVAY--
KVPVAY--
-KPVAYY-

-KPVAYG-
KNPVAY--
KKPVAY--
KFPVAY--
KQPVAY--
-KPVAYK-
KWPVAY--
-KPVAYA-
KMPVAY--
KYPVAY--
-KPVAYP-
-KPVAYN-
LFPVAY--
-LPVAYV-
LMPVAY--
LSPVAY--
-LPVAYL-
-LPVAYQ-
LNPVAY--
LYPVAY--
LVPVAY--
LPPVAY--
LTPVAY--
-LPVAYR-
-LPVAYH-
LHPVAY--
LAPVAY--
-LPVAYW-
-LPVAYS-
LQPVAY--
LWPVAY--
-LPVAYN-
-LPVAYK-
-LPVAYA-
LEPVAY--
LDPVAY--
-MPVAYS-
-MPVAYL-
-MPVAYY-
MSPVAY--
MRPVAY--
-MPVAYR-
-MPVAYM-
MPPVAY--
MLPVAY--
-MPVAYW-
-MPVAYH-
MMPVAY--
MAPVAY--
MGPVAY--

MYPVAY--
-MPVAYG-
MNPVAY--
MFPVAY--
MEPVAY--
MQPVAY--
-MPVAYV-
-MPVAYQ-
MKPVAY--
MHPVAY--
MVPVAY--
MTPVAY--
-MPVAYF-
-MPVAYA-
MIPVAY--
-NPVAYL-
-NPVAYM-
-NPVAYR-
NFPVAY--
NSPVAY--
NNPVAY--
NVPVAY--
-NPVAYS-
NGPVAY--
-NPVAYV-
-NPVAYK-
-NPVAYH-
NYPVAY--
NWPVAY--
-NPVAYY-
NLPVAY--
NEPVAY--
NTPVAY--
NQPVAY--
-NPVAYI-
NPPVAY--
NKPVAY--
NIPVAY--
NDPVAY--
-NPVAYW-
-NPVAYF-
NMPVAY--
NAPVAY--
-NPVAYQ-
-NPVAYA-
--PVAYSR
--PVAYSL
--PVAYRA
--PVAYRT
--PVAYLM

--PVAYKL
--PVAYRN
--PVAYRI
--PVAYLH
--PVAYWR
--PVAYSI
--PVAYKG
--PVAYFG
--PVAYRL
--PVAYRG
--PVAYPI
--PVAYML
--PVAYLR
--PVAYKW
--PVAYAS
--PVAYVT
--PVAYSG
--PVAYRS
--PVAYPQ
--PVAYNS
--PVAYMR
--PVAYLT
--PVAYGV
PSPVAY--
--PVAYVS
--PVAYTH
--PVAYRF
--PVAYKA
PYPVAY--
--PVAYYV
--PVAYWS
--PVAYWQ
--PVAYTV
--PVAYLG
--PVAYHG
--PVAYYR
--PVAYVK
--PVAYSY
--PVAYSS
--PVAYRV
--PVAYRD
--PVAYKT
--PVAYSV
--PVAYLS
--PVAYLN
--PVAYKR
--PVAYIA
--PVAYGG
--PVAYEL
PTPVAY--

PRPVAY--
PEPVAY--
--PVAYST
--PVAYSN
--PVAYRH
--PVAYQR
--PVAYQL
--PVAYLA
--PVAYKV
--PVAYKS
--PVAYII
--PVAYHM
--PVAYHA
--PVAYGY
--PVAYWH
--PVAYWA
--PVAYTS
--PVAYTN
--PVAYTM
--PVAYQS
--PVAYQM
--PVAYMI
--PVAYLQ
--PVAYGS
--PVAYGL
--PVAYGI
--PVAYFN
--PVAYFK
--PVAYAM
PMPVAY--
PGPVAY--
PAPVAY--
--PVAYYW
--PVAYYK
--PVAYYD
--PVAYTR
--PVAYSK
--PVAYSA
--PVAYQN
--PVAYMK
--PVAYKQ
--PVAYIG
--PVAYHV
--PVAYHE
--PVAYET
--PVAYEA
--PVAYAW
PNPVAY--
PKPVAY--
PHPVAY--

PFPVAY--
--PVAYYQ
--PVAYYH
--PVAYWM
--PVAYWG
--PVAYVQ
--PVAYVN
--PVAYTI
--PVAYSQ
--PVAYRW
--PVAYRQ
--PVAYQI
--PVAYPY
--PVAYPW
--PVAYNL
--PVAYMW
--PVAYMN
--PVAYMH
--PVAYMG
--PVAYMF
--PVAYIS
--PVAYIN
--PVAYHN
--PVAYHI
--PVAYDL
--PVAYAV
--PVAYAL
--PVAYAI
--PVAYAF
PWPVAY--
--PVAYYM
--PVAYYG
--PVAYWN
--PVAYVG
--PVAYTT
--PVAYTD
--PVAYSH
--PVAYSD
--PVAYRY
--PVAYRM
--PVAYQY
--PVAYQK
--PVAYQA
--PVAYPM
--PVAYPF
--PVAYPE
--PVAYNV
--PVAYNT
--PVAYME
--PVAYMD

--PVAYKN
--PVAYIR
--PVAYIK
--PVAYHR
--PVAYHQ
--PVAYGW
--PVAYGT
--PVAYGQ
--PVAYGA
--PVAYFQ
--PVAYES
--PVAYEM
--PVAYAY
--PVAYAA
PLPVAY--
--PVAYYE
--PVAYVV
--PVAYVH
--PVAYVA
--PVAYTQ
--PVAYTG
--PVAYSW
--PVAYSM
--PVAYSE
--PVAYRE
--PVAYQQ
--PVAYQH
--PVAYQG
--PVAYQF
--PVAYPV
--PVAYPS
--PVAYNN
--PVAYNG
--PVAYNA
--PVAYMV
--PVAYMA
--PVAYKY
--PVAYKI
--PVAYKH
--PVAYKD
--PVAYIH
--PVAYHT
--PVAYHS
--PVAYHF
--PVAYGN
--PVAYGM
--PVAYFW
--PVAYFA
--PVAYEF
--PVAYDK

```
--PVAYCS
--PVAYAT
--PVAYAR
--PVAYAQ
--PVAYAK
-PPVAYH-
QSPVAY--
-QPVAYV-
QKPVAY--
-QPVAYS-
-QPVAYL-
QGPVAY--
-QPVAYW-
-QPVAYT-
-QPVAYK-
QDPVAY--
QRPVAY--
-QPVAYF-
QLPVAY--
QAPVAY--
-QPVAYR-
-QPVAYQ-
-QPVAYN-
QTPVAY--
-QPVAYY-
-QPVAYA-
QNPVAY--
QIPVAY--
QFPVAY--
QYPVAY--
-QPVAYH-
QMPVAY--
-RPVAYT-
RSPVAY--
-RPVAYN-
-RPVAYH-
RIPVAY--
RAPVAY--
-RPVAYL-
RMPVAY--
RYPVAY--
-RPVAYM-
RLPVAY--
-RPVAYA-
RDPVAY--
-RPVAYK-
-RPVAYV-
-RPVAYE-
RTPVAY--
-RPVAYG-
```

RNPVAY--
RHPVAY--
-RPVAYF-
RPPVAY--
REPVAY--
-RPVAYQ-
RFPVAY--
RCPVAY--
-SPVAYR-
-SPVAYL-
SLPVAY--
SSPVAY--
-SPVAYF-
-SPVAYV-
SIPVAY--
STPVAY--
-SPVAYS-
SGPVAY--
SYPVAY--
-SPVAYW-
-SPVAYY-
-SPVAYT-
SMPVAY--
SVPVAY--
-SPVAYA-
SEPVAY--
-SPVAYM-
-SPVAYI-
SNPVAY--
-SPVAYQ-
-SPVAYN-
-SPVAYK-
-SPVAYE-
SWPVAY--
-SPVAYG-
SFPVAY--
SDPVAY--
SAPVAY--
SHPVAY--
SPPVAY--
SCPVAY--
-TPVAYR-
-TPVAYL-
-TPVAYT-
-TPVAYF-
-TPVAYY-
-TPVAYV-
TTPVAY--
TAPVAY--
TYPVAY--

TVPVAY--
-TPVAYS-
-TPVAYA-
TFPVAY--
THPVAY--
TWPVAY--
-TPVAYM-
-TPVAYK-
-TPVAYH-
TPPVAY--
TIPVAY--
-TPVAYW-
TNPVAY--
TLPVAY--
-TPVAYQ-
TMPVAY--
TKPVAY--
TGPVAY--
TEPVAY--
VMPVAY--
-VPVAYW-
-VPVAYR-
VLPVAY--
VTPVAY--
VAPVAY--
VWPVAY--
VSPVAY--
-VPVAYK-
VHPVAY--
-VPVAYI-
VPPVAY--
VQPVAY--
-VPVAYH-
VKPVAY--
VEPVAY--
-VPVAYM-
-VPVAYG-
-VPVAYE-
-VPVAYA-
VVPVAY--
-VPVAYT-
-VPVAYQ-
-VPVAYN-
-VPVAYL-
VNPVAY--
VIPVAY--
VGPVAY--
VDPVAY--
WHPVAY--
WIPVAY--

```
            WGPVAY--
            WVPVAY--
            -WPVAYA-
            WRPVAY--
            -WPVAYR-
            -WPVAYN-
            WAPVAY--
            WNPVAY--
            WTPVAY--
            -WPVAYV-
            -WPVAYS-
            -WPVAYQ-
            WLPVAY--
            WQPVAY--
            -WPVAYK-
            WFPVAY--
            WDPVAY--
            -YPVAYR-
            -YPVAYH-
            -YPVAYV-
            YAPVAY--
            YVPVAY--
            -YPVAYS-
            -YPVAYA-
            YLPVAY--
            YQPVAY--
            YSPVAY--
            -YPVAYL-
            -YPVAYQ-
            YKPVAY--
            YEPVAY--
            YGPVAY--
            YDPVAY--
            -YPVAYW-
            -YPVAYK-
            YNPVAY--
            YHPVAY--
PHAL          92         774      32.38
            -KPHALT-
            -LPHALE-
            PKPHAL--
            -SPHALP-
            -APHALT-
            -APHALR-
            ARPHAL--
            AVPHAL--
            -APHALL-
            -APHALK-
            -APHALI-
            -APHALE-
```

```
-APHALS-
AIPHAL--
AAPHAL--
AYPHAL--
APPHAL--
-APHALA-
ANPHAL--
ALPHAL--
AFPHAL--
-APHALN-
AMPHAL--
-APHALM-
AWPHAL--
AQPHAL--
-APHALY-
-APHALW-
AKPHAL--
AHPHAL--
-APHALV-
AEPHAL--
-APHALQ-
-APHALH-
AGPHAL--
ADPHAL--
-CPHALV-
CKPHAL--
CVPHAL--
CLPHAL--
CAPHAL--
CQPHAL--
-CPHALW-
-CPHALT-
-CPHALR-
-CPHALF-
CNPHAL--
CEPHAL--
DRPHAL--
DWPHAL--
-DPHALR-
DKPHAL--
DYPHAL--
DSPHAL--
-DPHALY-
DVPHAL--
-DPHALI-
-DPHALT-
-DPHALH-
DCPHAL--
DAPHAL--
DQPHAL--
```

```
DPPHAL--
-DPHALV-
-DPHALN-
-DPHALL-
-DPHALK-
-DPHALA-
DMPHAL--
DGPHAL--
DFPHAL--
DEPHAL--
ERPHAL--
ESPHAL--
-EPHALL-
ELPHAL--
EKPHAL--
-EPHALY-
-EPHALR-
-EPHALS-
EWPHAL--
EVPHAL--
ETPHAL--
-EPHALK-
EYPHAL--
-EPHALW-
-EPHALQ-
EGPHAL--
EFPHAL--
EEPHAL--
ECPHAL--
EAPHAL--
-EPHALN-
-EPHALI-
-EPHALH-
-EPHALG-
EMPHAL--
EIPHAL--
EDPHAL--
FPPHAL--
FRPHAL--
-FPHALT-
FNPHAL--
FVPHAL--
-FPHALR-
FTPHAL--
FWPHAL--
-FPHALQ-
-FPHALK-
-FPHALE-
-FPHALA-
FIPHAL--
```

FYPHAL--
-FPHALL-
-FPHALF-
FMPHAL--
FLPHAL--
FKPHAL--
FFPHAL--
FQPHAL--
-FPHALW-
GVPHAL--
-GPHALR-
GSPHAL--
GPPHAL--
GRPHAL--
-GPHALL-
GIPHAL--
GLPHAL--
-GPHALT-
-GPHALI-
-GPHALV-
-GPHALQ-
-GPHALK-
GKPHAL--
GTPHAL--
GNPHAL--
GMPHAL--
-GPHALN-
-GPHALE-
-GPHALA-
GFPHAL--
GAPHAL--
-GPHALM-
-GPHALH-
GGPHAL--
GQPHAL--
-GPHALS-
GEPHAL--
-GPHALG-
GHPHAL--
GDPHAL--
GYPHAL--
HRPHAL--
HTPHAL--
-HPHALL-
-HPHALK-
HIPHAL--
HWPHAL--
HQPHAL--
-HPHALW-
HSPHAL--

−HPHALT−
−HPHALV−
HNPHAL−−
HVPHAL−−
−HPHALS−
HLPHAL−−
HHPHAL−−
HGPHAL−−
HPPHAL−−
−HPHALR−
−HPHALA−
HMPHAL−−
HDPHAL−−
HAPHAL−−
−HPHALN−
−HPHALF−
HEPHAL−−
HCPHAL−−
IRPHAL−−
ILPHAL−−
ISPHAL−−
−IPHALT−
−IPHALL−
IVPHAL−−
IWPHAL−−
IYPHAL−−
−IPHALY−
IKPHAL−−
IDPHAL−−
−IPHALS−
−IPHALR−
INPHAL−−
IGPHAL−−
IIPHAL−−
−IPHALA−
IEPHAL−−
ITPHAL−−
IQPHAL−−
−IPHALQ−
−IPHALK−
−IPHALI−
−IPHALG−
IPPHAL−−
−IPHALM−
IHPHAL−−
IAPHAL−−
KWPHAL−−
−KPHALS−
KRPHAL−−
KAPHAL−−

```
-KPHALR-
-KPHALA-
KGPHAL--
-KPHALQ-
-KPHALM-
-KPHALI-
-KPHALG-
KVPHAL--
-KPHALL-
-KPHALF-
-KPHALN-
KIPHAL--
KYPHAL--
KTPHAL--
KPPHAL--
-KPHALY-
-KPHALV-
-KPHALH-
KKPHAL--
KMPHAL--
KFPHAL--
KQPHAL--
KNPHAL--
KHPHAL--
KCPHAL--
-LPHALA-
LSPHAL--
LTPHAL--
-LPHALS-
LLPHAL--
-LPHALL-
-LPHALR-
LMPHAL--
-LPHALK-
LHPHAL--
LQPHAL--
-LPHALT-
LYPHAL--
-LPHALN-
-LPHALI-
LKPHAL--
LFPHAL--
LDPHAL--
LAPHAL--
-LPHALF-
LNPHAL--
LIPHAL--
LCPHAL--
-LPHALV-
-LPHALQ-
```

LWPHAL--
LPPHAL--
-LPHALM-
-LPHALG-
-LPHALW-
MRPHAL--
MTPHAL--
-MPHALT-
MLPHAL--
-MPHALL-
MMPHAL--
MYPHAL--
-MPHALN-
MKPHAL--
MPPHAL--
-MPHALR-
-MPHALI-
MSPHAL--
-MPHALY-
-MPHALS-
MIPHAL--
-MPHALM-
MGPHAL--
MFPHAL--
-MPHALQ-
-MPHALE-
MQPHAL--
-MPHALK-
-MPHALG-
-MPHALA-
MNPHAL--
MDPHAL--
MWPHAL--
-MPHALF-
MEPHAL--
MAPHAL--
NGPHAL--
NVPHAL--
-NPHALY-
-NPHALS-
-NPHALL-
-NPHALK-
NLPHAL--
NYPHAL--
-NPHALI-
-NPHALR-
NIPHAL--
NFPHAL--
NTPHAL--
NKPHAL--

NEPHAL--
-NPHALW-
-NPHALH-
-NPHALT-
-NPHALM-
-NPHALG-
NHPHAL--
NWPHAL--
NQPHAL--
NPPHAL--
NNPHAL--
NAPHAL--
--PHALRA
--PHALRV
--PHALLT
--PHALVA
--PHALRN
--PHALLR
--PHALRY
--PHALSS
--PHALLG
--PHALKS
--PHALVI
--PHALYS
--PHALVK
--PHALRS
--PHALRI
--PHALYA
--PHALYR
--PHALML
--PHALVS
--PHALSL
--PHALSK
--PHALRT
--PHALNL
--PHALLK
PWPHAL--
--PHALYL
--PHALSR
--PHALSN
--PHALQI
--PHALHV
PYPHAL--
--PHALYT
--PHALTL
--PHALNT
--PHALKT
PMPHAL--
--PHALWF
--PHALTV

--PHALST
--PHALSG
--PHALRD
--PHALMR
--PHALLF
--PHALIK
--PHALGS
--PHALFA
--PHALWR
--PHALWG
--PHALVG
--PHALSV
--PHALLN
--PHALEA
--PHALVY
--PHALTS
--PHALTG
--PHALSA
--PHALRF
--PHALPY
--PHALNA
--PHALLQ
--PHALKM
--PHALAN
PFPHAL--
--PHALWL
--PHALVL
--PHALNR
--PHALMI
--PHALLD
--PHALKI
--PHALIT
--PHALIM
--PHALIL
--PHALHK
--PHALGG
--PHALAY
--PHALAM
--PHALAL
PGPHAL--
PLPHAL--
--PHALYK
--PHALWT
--PHALTT
--PHALTQ
--PHALTK
--PHALSM
--PHALRQ
--PHALRE
--PHALNW

--PHALMT
--PHALMQ
--PHALMK
--PHALLH
--PHALIY
--PHALIR
--PHALIQ
--PHALHR
--PHALHF
--PHALGL
--PHALFK
--PHALEG
--PHALAH
--PHALYN
--PHALYF
--PHALWA
--PHALVT
--PHALTN
--PHALTA
--PHALSY
--PHALRM
--PHALRH
--PHALPL
--PHALNQ
--PHALMG
--PHALKY
--PHALKV
--PHALKN
--PHALKK
--PHALKA
--PHALHY
--PHALHS
--PHALHN
--PHALHM
--PHALGF
--PHALFV
--PHALAT
--PHALAI
--PHALAA
PSPHAL--
PIPHAL--
--PHALYM
--PHALWS
--PHALWH
--PHALWE
--PHALVR
--PHALVD
--PHALSQ
--PHALSI
--PHALSH

--PHALSF
--PHALRG
--PHALPM
--PHALNK
--PHALMY
--PHALMV
--PHALLE
--PHALKG
--PHALIS
--PHALIA
--PHALHI
--PHALGV
--PHALFT
--PHALFQ
--PHALFE
--PHALAF
--PHALWY
--PHALWD
--PHALVN
--PHALVM
--PHALTW
--PHALTH
--PHALSW
--PHALRW
--PHALQS
--PHALQN
--PHALNF
--PHALLV
--PHALLA
--PHALKF
--PHALIH
--PHALHT
--PHALFY
--PHALFL
--PHALAK
PTPHAL--
--PHALYG
--PHALYD
--PHALVV
--PHALVQ
--PHALVH
--PHALVF
--PHALVE
--PHALTF
--PHALTD
--PHALSE
--PHALSD
--PHALQV
--PHALQT
--PHALQR

--PHALQG
--PHALPI
--PHALNM
--PHALNH
--PHALNE
--PHALND
--PHALMS
--PHALMN
--PHALMH
--PHALMA
--PHALKQ
--PHALKH
--PHALKD
--PHALIV
--PHALIE
--PHALHW
--PHALHL
--PHALHG
--PHALFS
--PHALFI
--PHALFD
--PHALAW
--PHALAV
--PHALAR
--PHALAG
PEPHAL--
PQPHAL--
PHPHAL--
--PHALYH
--PHALYE
--PHALWN
--PHALWM
--PHALWK
--PHALWI
--PHALTM
--PHALTI
--PHALTE
--PHALSC
--PHALQQ
--PHALQL
--PHALQA
--PHALPF
--PHALMM
--PHALIG
--PHALHE
--PHALHA
--PHALGY
--PHALGQ
--PHALFM
--PHALFH

--PHALFF
--PHALET
--PHALEK
--PHALDW
PCPHAL--
-QPHALR-
QFPHAL--
-QPHALI-
QLPHAL--
QSPHAL--
QKPHAL--
QHPHAL--
QGPHAL--
QVPHAL--
-QPHALS-
QIPHAL--
-QPHALN-
-QPHALH-
QNPHAL--
QTPHAL--
QRPHAL--
-QPHALT-
-QPHALQ-
QMPHAL--
-QPHALL-
-QPHALG-
-QPHALA-
QDPHAL--
QWPHAL--
QPPHAL--
-QPHALW-
-QPHALV-
-QPHALE-
RKPHAL--
-RPHALL-
-RPHALY-
-RPHALI-
-RPHALT-
RVPHAL--
RRPHAL--
-RPHALR-
-RPHALN-
RSPHAL--
RMPHAL--
-RPHALS-
RPPHAL--
RGPHAL--
RYPHAL--
RLPHAL--
-RPHALG-

-RPHALV-
-RPHALM-
RAPHAL--
-RPHALW-
-RPHALQ-
-RPHALH-
RNPHAL--
REPHAL--
RTPHAL--
-RPHALE-
RQPHAL--
RIPHAL--
-RPHALF-
RHPHAL--
RCPHAL--
SRPHAL--
-SPHALS-
SSPHAL--
-SPHALY-
-SPHALL-
SGPHAL--
SWPHAL--
-SPHALV-
-SPHALQ-
-SPHALK-
-SPHALH-
SYPHAL--
-SPHALI-
SLPHAL--
SFPHAL--
SMPHAL--
SIPHAL--
SHPHAL--
-SPHALW-
SAPHAL--
SVPHAL--
STPHAL--
-SPHALT-
SEPHAL--
-SPHALG-
-SPHALA-
SPPHAL--
SQPHAL--
-SPHALN-
-SPHALM-
SNPHAL--
SDPHAL--
-TPHALR-
TVPHAL--
TSPHAL--

TMPHAL--
-TPHALL-
TAPHAL--
-TPHALW-
-TPHALT-
TQPHAL--
TKPHAL--
-TPHALS-
THPHAL--
-TPHALV-
-TPHALI-
-TPHALF-
-TPHALA-
TEPHAL--
-TPHALY-
TNPHAL--
TIPHAL--
TGPHAL--
TYPHAL--
TTPHAL--
-TPHALK-
-TPHALH-
TFPHAL--
TWPHAL--
TPPHAL--
-TPHALQ-
VKPHAL--
VQPHAL--
-VPHALL-
-VPHALQ-
VVPHAL--
-VPHALM-
VRPHAL--
VLPHAL--
VPPHAL--
VNPHAL--
-VPHALY-
VMPHAL--
-VPHALF-
VIPHAL--
VGPHAL--
VTPHAL--
-VPHALW-
-VPHALN-
-VPHALK-
-VPHALE-
VEPHAL--
VWPHAL--
-VPHALT-
-VPHALI-

VHPHAL--
VCPHAL--
VAPHAL--
-VPHALA-
VFPHAL--
-VPHALV-
-VPHALG-
VDPHAL--
WGPHAL--
-WPHALT-
WTPHAL--
WKPHAL--
WSPHAL--
WMPHAL--
-WPHALM-
WVPHAL--
-WPHALF-
WLPHAL--
WEPHAL--
-WPHALH-
WQPHAL--
-WPHALK-
WWPHAL--
-WPHALV-
-WPHALN-
-WPHALG-
WDPHAL--
-WPHALA-
WIPHAL--
WAPHAL--
YMPHAL--
-YPHALS-
YLPHAL--
YTPHAL--
YRPHAL--
-YPHALV-
YSPHAL--
YIPHAL--
-YPHALK-
YHPHAL--
YGPHAL--
YVPHAL--
-YPHALY-
-YPHALW-
-YPHALT-
-YPHALN-
-YPHALI-
-YPHALA-
YAPHAL--
YPPHAL--

-YPHALE-
YFPHAL--
YYPHAL--
-YPHALL-
-YPHALH-
-YPHALF-
YEPHAL--
YDPHAL--
YWPHAL--
-YPHALM-
YKPHAL--
YCPHAL--

| | | | |
|---|---|---|---|
| PSNM | 77 | 781 | 32.37 |

--PSNMSD
-APSNML-
AHPSNM--
AAPSNM--
ATPSNM--
AGPSNM--
-APSNMS-
-APSNMR-
AEPSNM--
AYPSNM--
ASPSNM--
ARPSNM--
AMPSNM--
-APSNMH-
-APSNMA-
ALPSNM--
AVPSNM--
-APSNMQ-
ANPSNM--
-APSNMV-
-APSNMK-
-APSNMG-
AWPSNM--
AQPSNM--
-APSNMY-
-APSNMW-
-APSNMT-
-APSNMN-
-APSNME-
AKPSNM--
AFPSNM--
ADPSNM--
CQPSNM--
CGPSNM--
-CPSNML-
CWPSNM--
-CPSNMS-

CRPSNM--
-CPSNMV-
-CPSNMR-
CNPSNM--
CLPSNM--
CIPSNM--
CHPSNM--
CEPSNM--
DRPSNM--
-DPSNMM-
DAPSNM--
DTPSNM--
-DPSNMS-
DNPSNM--
DIPSNM--
DSPSNM--
-DPSNMR-
-DPSNMN-
-DPSNMI-
-DPSNMF-
DMPSNM--
DFPSNM--
DYPSNM--
-DPSNMV-
-DPSNMQ-
-DPSNMK-
-DPSNMH-
-DPSNMG-
-DPSNMA-
DHPSNM--
-EPSNMH-
-EPSNML-
-EPSNMF-
EVPSNM--
-EPSNMS-
-EPSNMR-
-EPSNMM-
ERPSNM--
-EPSNMG-
EEPSNM--
EYPSNM--
-EPSNMN-
-EPSNMK-
ENPSNM--
EMPSNM--
EIPSNM--
-EPSNMW-
-EPSNMT-
-EPSNMI-
-EPSNMA-

```
-FPSNML-
FLPSNM--
FVPSNM--
FMPSNM--
FRPSNM--
-FPSNMV-
FYPSNM--
-FPSNMT-
FWPSNM--
-FPSNMQ-
-FPSNMN-
-FPSNMK-
-FPSNMA-
FGPSNM--
FAPSNM--
-FPSNMF-
-FPSNMS-
-FPSNMR-
-FPSNMI-
FKPSNM--
FFPSNM--
FSPSNM--
-FPSNMH-
-FPSNMG-
FNPSNM--
FIPSNM--
FEPSNM--
-FPSNMW-
-FPSNMM-
FHPSNM--
FDPSNM--
GWPSNM--
-GPSNML-
GVPSNM--
GQPSNM--
-GPSNMY-
-GPSNMN-
-GPSNMR-
GNPSNM--
GLPSNM--
GHPSNM--
-GPSNMT-
-GPSNMF-
GIPSNM--
GDPSNM--
GAPSNM--
-GPSNMV-
-GPSNMI-
-GPSNMA-
GMPSNM--
```

GGPSNM--
GRPSNM--
-GPSNMS-
-GPSNMK-
-GPSNMG-
GPPSNM--
GKPSNM--
GEPSNM--
GYPSNM--
GSPSNM--
-GPSNMQ-
-GPSNMM-
-GPSNME-
-GPSNMD-
GFPSNM--
HGPSNM--
-HPSNMV-
-HPSNMS-
HRPSNM--
-HPSNMQ-
-HPSNMT-
-HPSNMR-
-HPSNMA-
-HPSNML-
HYPSNM--
HSPSNM--
-HPSNMK-
-HPSNMI-
HPPSNM--
HWPSNM--
HMPSNM--
HVPSNM--
HTPSNM--
-HPSNMM-
HLPSNM--
HIPSNM--
HDPSNM--
HAPSNM--
-IPSNMR-
-IPSNML-
IKPSNM--
IAPSNM--
ITPSNM--
ISPSNM--
IYPSNM--
-IPSNMI-
IMPSNM--
-IPSNMS-
ILPSNM--
IWPSNM--

IQPSNM--
-IPSNMW-
-IPSNMH-
-IPSNMG-
-IPSNMF-
INPSNM--
-IPSNMV-
-IPSNMT-
-IPSNMK-
IHPSNM--
IVPSNM--
IRPSNM--
-IPSNMY-
-IPSNMN-
-IPSNMM-
IPPSNM--
IGPSNM--
IDPSNM--
KSPSNM--
-KPSNMS-
KFPSNM--
-KPSNMY-
-KPSNMR-
KLPSNM--
KGPSNM--
KRPSNM--
-KPSNMV-
-KPSNMN-
-KPSNML-
-KPSNMW-
KPPSNM--
KYPSNM--
KIPSNM--
KTPSNM--
-KPSNMM-
-KPSNMK-
-KPSNMD-
KMPSNM--
KCPSNM--
-KPSNMQ-
-KPSNMG-
-KPSNMF-
-KPSNME-
KDPSNM--
KAPSNM--
-LPSNMR-
-LPSNMS-
LRPSNM--
LLPSNM--
LTPSNM--

```
-LPSNMK-
LGPSNM--
-LPSNMW-
-LPSNMF-
-LPSNMA-
LAPSNM--
-LPSNMH-
-LPSNMG-
LHPSNM--
LDPSNM--
LSPSNM--
-LPSNMM-
-LPSNMI-
LIPSNM--
LWPSNM--
LVPSNM--
-LPSNMQ-
LNPSNM--
LMPSNM--
-LPSNMY-
-LPSNMT-
-LPSNMN-
LPPSNM--
LFPSNM--
LCPSNM--
-MPSNMR-
MAPSNM--
-MPSNML-
MKPSNM--
-MPSNMQ-
-MPSNMI-
-MPSNMH-
-MPSNMG-
MMPSNM--
-MPSNMV-
-MPSNMS-
-MPSNMM-
-MPSNMK-
MIPSNM--
MGPSNM--
MFPSNM--
MVPSNM--
-MPSNMT-
-MPSNMD-
-MPSNMA-
MHPSNM--
MWPSNM--
MTPSNM--
MRPSNM--
MQPSNM--
```

-MPSNMF-
-MPSNME-
MPPSNM--
MLPSNM--
MEPSNM--
NRPSNM--
-NPSNMR-
NIPSNM--
-NPSNMS-
NPPSNM--
-NPSNMI-
NWPSNM--
NSPSNM--
-NPSNMM-
-NPSNMK-
-NPSNMF-
NVPSNM--
NKPSNM--
NGPSNM--
-NPSNMT-
-NPSNMN-
-NPSNMA-
-NPSNMV-
-NPSNML-
-NPSNMG-
NMPSNM--
NAPSNM--
NYPSNM--
NTPSNM--
-NPSNMH-
NLPSNM--
NFPSNM--
-NPSNMY-
-NPSNMW-
NHPSNM--
--PSNMVL
--PSNMLR
--PSNMHR
--PSNMAR
--PSNMVS
--PSNMSS
--PSNMLT
--PSNMRY
--PSNMMS
--PSNMRA
--PSNMWL
--PSNMRW
--PSNMRR
--PSNMKG
--PSNMFN

--PSNMRV
--PSNMHK
--PSNMFS
--PSNMAI
--PSNMVG
--PSNMSV
--PSNMSH
--PSNMRH
--PSNMRF
--PSNMLK
--PSNMLA
--PSNMAS
--PSNMWR
--PSNMVR
--PSNMTR
--PSNMST
--PSNMRK
--PSNMWT
--PSNMTV
--PSNMSR
--PSNMLV
--PSNMKS
--PSNMWN
--PSNMVI
--PSNMTS
--PSNMSY
--PSNMRL
--PSNMML
--PSNMMF
--PSNMLS
--PSNMLG
--PSNMKL
--PSNMVH
--PSNMSA
--PSNMRN
--PSNMNS
--PSNMMR
--PSNMLI
--PSNMHS
--PSNMGT
--PSNMEI
--PSNMAW
--PSNMAG
PFPSNM--
PVPSNM--
--PSNMWS
--PSNMTY
--PSNMTT
--PSNMSF
--PSNMMY

--PSNMLW
--PSNMGS
--PSNMFI
--PSNMAT
--PSNMAL
--PSNMYS
--PSNMYL
--PSNMYH
--PSNMVK
--PSNMVF
--PSNMRM
--PSNMQV
--PSNMQT
--PSNMQS
--PSNMHT
--PSNMGG
--PSNMFW
--PSNMFL
--PSNMFE
--PSNMFA
--PSNMEM
PLPSNM--
PHPSNM--
PWPSNM--
--PSNMYT
--PSNMYI
--PSNMWV
--PSNMVV
--PSNMRI
--PSNMRG
--PSNMQM
--PSNMQG
--PSNMNR
--PSNMNF
--PSNMKQ
--PSNMKK
--PSNMKI
--PSNMKD
--PSNMIR
--PSNMIG
--PSNMIA
--PSNMGL
--PSNMER
--PSNMAH
PIPSNM--
--PSNMYW
--PSNMYN
--PSNMYK
--PSNMYF
--PSNMWQ

--PSNMWD
--PSNMWA
--PSNMTQ
--PSNMSG
--PSNMRQ
--PSNMQR
--PSNMQL
--PSNMQF
--PSNMNT
--PSNMMV
--PSNMLH
--PSNMLF
--PSNMKW
--PSNMKA
--PSNMIV
--PSNMHV
--PSNMHN
--PSNMGW
--PSNMFT
--PSNMFD
--PSNMEH
--PSNMDT
--PSNMDS
--PSNMAN
--PSNMAK
PCPSNM--
PAPSNM--
PSPSNM--
--PSNMYV
--PSNMYQ
--PSNMYA
--PSNMVT
--PSNMTN
--PSNMSQ
--PSNMSM
--PSNMSK
--PSNMSI
--PSNMRE
--PSNMQY
--PSNMQA
--PSNMNY
--PSNMNM
--PSNMNA
--PSNMMW
--PSNMMN
--PSNMMK
--PSNMMI
--PSNMMH
--PSNMMG
--PSNMLQ

--PSNMLN
--PSNMLD
--PSNMKY
--PSNMIN
--PSNMIL
--PSNMHY
--PSNMHA
--PSNMGV
--PSNMGN
--PSNMGA
--PSNMFR
--PSNMFM
--PSNMFK
--PSNMEK
--PSNMEG
--PSNMEA
--PSNMAF
--PSNMAD
PRPSNM--
PQPSNM--
PGPSNM--
PYPSNM--
PTPSNM--
--PSNMYG
--PSNMYE
--PSNMWH
--PSNMWG
--PSNMWE
--PSNMVQ
--PSNMVN
--PSNMVM
--PSNMVA
--PSNMTW
--PSNMTM
--PSNMTG
--PSNMTF
--PSNMTE
--PSNMSE
--PSNMRD
--PSNMQQ
--PSNMQI
--PSNMQH
--PSNMQE
--PSNMQD
--PSNMNW
--PSNMNL
--PSNMNK
--PSNMNI
--PSNMND
--PSNMME

--PSNMMD
--PSNMMC
--PSNMKV
--PSNMKT
--PSNMKM
--PSNMKH
--PSNMIY
--PSNMIT
--PSNMIM
--PSNMIE
--PSNMID
--PSNMHI
--PSNMHH
--PSNMGY
--PSNMFQ
--PSNMFH
--PSNMEY
--PSNMEW
--PSNMEV
--PSNMET
--PSNMES
--PSNMEQ
--PSNMDH
--PSNMCS
--PSNMCR
--PSNMCF
--PSNMAY
--PSNMAV
--PSNMAQ
--PSNMAA
-PPSNMS-
PNPSNM--
PMPSNM--
PEPSNM--
QYPSNM--
QVPSNM--
QRPSNM--
-QPSNMY-
QSPSNM--
-QPSNMQ-
-QPSNMG-
-QPSNMF-
QTPSNM--
QPPSNM--
QWPSNM--
-QPSNMS-
-QPSNMN-
-QPSNMA-
QKPSNM--
QGPSNM--

-QPSNMW-
-QPSNMT-
-QPSNMH-
QNPSNM--
QFPSNM--
QQPSNM--
-QPSNMV-
-QPSNMM-
-QPSNMK-
QIPSNM--
QHPSNM--
QAPSNM--
-RPSNML-
-RPSNMV-
-RPSNMI-
RWPSNM--
-RPSNMS-
-RPSNMM-
-RPSNMK-
-RPSNMF-
-RPSNMA-
RAPSNM--
-RPSNMH-
-RPSNMG-
RYPSNM--
RQPSNM--
-RPSNMY-
-RPSNMT-
-RPSNMD-
RHPSNM--
RGPSNM--
-RPSNMW-
RKPSNM--
RIPSNM--
REPSNM--
RRPSNM--
RNPSNM--
RFPSNM--
RCPSNM--
-RPSNMN-
-RPSNME-
SHPSNM--
SRPSNM--
-SPSNMV-
-SPSNMF-
-SPSNMR-
SQPSNM--
-SPSNML-
SIPSNM--
-SPSNMN-

-SPSNMA-
SLPSNM--
SVPSNM--
-SPSNMI-
SGPSNM--
SWPSNM--
-SPSNMT-
SFPSNM--
SAPSNM--
-SPSNMQ-
-SPSNMH-
-SPSNMG-
-SPSNME-
STPSNM--
SSPSNM--
-SPSNMW-
-SPSNMK-
SMPSNM--
SKPSNM--
SEPSNM--
SCPSNM--
-SPSNMM-
-SPSNMY-
SPPSNM--
SDPSNM--
-TPSNML-
TLPSNM--
-TPSNMT-
-TPSNMA-
TWPSNM--
-TPSNMK-
-TPSNMS-
-TPSNMN-
-TPSNMH-
TNPSNM--
TIPSNM--
TAPSNM--
TTPSNM--
TRPSNM--
-TPSNMM-
-TPSNMI-
THPSNM--
TGPSNM--
TVPSNM--
-TPSNMF-
TYPSNM--
TSPSNM--
TQPSNM--
-TPSNMV-
-TPSNMG-

-TPSNMW-
-TPSNME-
TPPSNM--
TMPSNM--
TKPSNM--
TFPSNM--
TEPSNM--
-VPSNML-
VRPSNM--
-VPSNMR-
-VPSNMS-
VLPSNM--
VHPSNM--
VSPSNM--
-VPSNMI-
VQPSNM--
VVPSNM--
VNPSNM--
VGPSNM--
-VPSNMF-
VMPSNM--
VYPSNM--
-VPSNMW-
VIPSNM--
-VPSNMT-
-VPSNMQ-
-VPSNMK-
-VPSNMH-
VKPSNM--
VFPSNM--
VTPSNM--
-VPSNMM-
-VPSNMG-
-VPSNMY-
-VPSNME-
-VPSNMD-
VAPSNM--
-WPSNMS-
-WPSNMM-
-WPSNMK-
-WPSNMR-
WSPSNM--
WWPSNM--
WRPSNM--
-WPSNMT-
WPPSNM--
WGPSNM--
-WPSNMG-
-WPSNMF-
WEPSNM--

| | | | |
|---|---|---|---|
| | WTPSNM-- | | |
| | WMPSNM-- | | |
| | WFPSNM-- | | |
| | -WPSNMW- | | |
| | -WPSNMV- | | |
| | -WPSNML- | | |
| | -WPSNMA- | | |
| | WKPSNM-- | | |
| | WAPSNM-- | | |
| | WYPSNM-- | | |
| | -WPSNMN- | | |
| | -WPSNMI- | | |
| | -WPSNMH- | | |
| | WDPSNM-- | | |
| | -YPSNMA- | | |
| | YSPSNM-- | | |
| | -YPSNMR- | | |
| | -YPSNMS- | | |
| | -YPSNMM- | | |
| | YRPSNM-- | | |
| | -YPSNMT- | | |
| | YKPSNM-- | | |
| | -YPSNML- | | |
| | YTPSNM-- | | |
| | YQPSNM-- | | |
| | YYPSNM-- | | |
| | YVPSNM-- | | |
| | -YPSNMG- | | |
| | YHPSNM-- | | |
| | YNPSNM-- | | |
| | YLPSNM-- | | |
| | YIPSNM-- | | |
| | YGPSNM-- | | |
| | YFPSNM-- | | |
| | YAPSNM-- | | |
| | -YPSNMV- | | |
| | -YPSNMI- | | |
| | -YPSNMH- | | |
| | -YPSNMF- | | |
| | -YPSNME- | | |
| | YMPSNM-- | | |
| | -YPSNMN- | | |
| | -YPSNMK- | | |
| | YEPSNM-- | | |
| PRWM | 388 | 636 | 32.37 |
| | -NPRWMG- | | |
| | --PRWMSD | | |
| | -TPRWMD- | | |
| | -YPRWML- | | |
| | ARPRWM-- | | |

-APRWMN-
-APRWML-
AGPRWM--
-APRWMY-
-APRWMW-
-APRWMQ-
-APRWMK-
-APRWMT-
-APRWMH-
AAPRWM--
APPRWM--
AHPRWM--
-APRWMD-
ASPRWM--
-APRWMV-
-APRWMF-
AYPRWM--
AIPRWM--
AVPRWM--
-APRWMI-
-APRWMG-
AWPRWM--
ATPRWM--
AQPRWM--
-APRWME-
AFPRWM--
CQPRWM--
CCPRWM--
CSPRWM--
DHPRWM--
-DPRWMS-
DMPRWM--
DLPRWM--
DFPRWM--
DYPRWM--
DSPRWM--
DKPRWM--
-DPRWMT-
-DPRWMH-
DGPRWM--
DEPRWM--
DRPRWM--
DQPRWM--
-DPRWMR-
-DPRWMM-
-DPRWMA-
DIPRWM--
DVPRWM--
-DPRWMV-
-DPRWMQ-

DNPRWM--
-EPRWMR-
-EPRWML-
-EPRWMK-
ESPRWM--
-EPRWMI-
EEPRWM--
EVPRWM--
ERPRWM--
EPPRWM--
ELPRWM--
EGPRWM--
EYPRWM--
-EPRWMY-
-EPRWMT-
EWPRWM--
ETPRWM--
-EPRWMV-
-EPRWMS-
-EPRWMN-
-EPRWMM-
ENPRWM--
EKPRWM--
-EPRWMF-
-EPRWMA-
EFPRWM--
EAPRWM--
FTPRWM--
FSPRWM--
FIPRWM--
-FPRWMA-
FAPRWM--
FRPRWM--
-FPRWML-
FDPRWM--
FYPRWM--
-FPRWMQ-
-FPRWMG-
FKPRWM--
FGPRWM--
FFPRWM--
FQPRWM--
-FPRWMW-
-FPRWMM-
-FPRWMI-
-FPRWMH-
FPPRWM--
FMPRWM--
-GPRWMM-
GTPRWM--

-GPRWMV-
-GPRWMT-
-GPRWMK-
GVPRWM--
-GPRWML-
-GPRWMI-
-GPRWMN-
GSPRWM--
-GPRWMF-
GMPRWM--
GRPRWM--
-GPRWMS-
-GPRWMH-
GKPRWM--
GQPRWM--
GLPRWM--
GGPRWM--
GPPRWM--
GNPRWM--
GFPRWM--
GDPRWM--
GHPRWM--
-GPRWMY-
-GPRWMW-
-GPRWMD-
-HPRWMS-
HWPRWM--
HTPRWM--
-HPRWMQ-
HQPRWM--
-HPRWMT-
-HPRWML-
-HPRWMI-
HNPRWM--
HRPRWM--
-HPRWMY-
-HPRWMR-
-HPRWMN-
-HPRWMK-
HHPRWM--
HEPRWM--
HAPRWM--
HVPRWM--
HSPRWM--
-HPRWMW-
-HPRWME-
HMPRWM--
HIPRWM--
HGPRWM--
HFPRWM--

-IPRWMS-
IRPRWM--
-IPRWML-
IKPRWM--
IVPRWM--
ITPRWM--
ISPRWM--
-IPRWMQ-
-IPRWMI-
-IPRWMG-
IGPRWM--
-IPRWMY-
-IPRWMV-
INPRWM--
ICPRWM--
IQPRWM--
-IPRWMF-
IIPRWM--
IHPRWM--
IDPRWM--
-KPRWMK-
-KPRWMR-
KDPRWM--
-KPRWMA-
-KPRWMS-
-KPRWMQ-
KWPRWM--
KEPRWM--
KVPRWM--
KNPRWM--
KAPRWM--
-KPRWMN-
-KPRWML-
-KPRWMV-
-KPRWMT-
KIPRWM--
KHPRWM--
KTPRWM--
KRPRWM--
-KPRWMH-
KLPRWM--
KYPRWM--
KQPRWM--
-KPRWMY-
-KPRWMI-
-KPRWMG-
-KPRWME-
KPPRWM--
KKPRWM--
KCPRWM--

LSPRWM--
-LPRWMV-
-LPRWMI-
LQPRWM--
LNPRWM--
LVPRWM--
-LPRWMM-
LKPRWM--
LAPRWM--
LEPRWM--
LDPRWM--
LRPRWM--
LIPRWM--
-LPRWMK-
LGPRWM--
LYPRWM--
LWPRWM--
-LPRWMT-
LPPRWM--
LTPRWM--
-LPRWMW-
-LPRWML-
-LPRWMH-
MRPRWM--
-MPRWMK-
-MPRWMA-
MPPRWM--
MLPRWM--
-MPRWMT-
-MPRWMR-
MDPRWM--
MSPRWM--
-MPRWMH-
MMPRWM--
MFPRWM--
MEPRWM--
MAPRWM--
MVPRWM--
-MPRWMS-
-MPRWMV-
-MPRWML-
MNPRWM--
NRPRWM--
NVPRWM--
NIPRWM--
NFPRWM--
-NPRWMT-
-NPRWMK-
-NPRWMY-
-NPRWMQ-

NSPRWM--
NQPRWM--
-NPRWMA-
NLPRWM--
-NPRWMS-
NEPRWM--
-NPRWML-
NHPRWM--
NGPRWM--
NYPRWM--
-NPRWMM-
-NPRWMH-
NKPRWM--
NAPRWM--
--PRWMSS
--PRWMRV
--PRWMLT
--PRWMLS
--PRWMST
--PRWMLA
--PRWMKS
--PRWMTT
--PRWMNS
--PRWMMS
--PRWMSV
--PRWMLH
--PRWMIG
--PRWMNT
--PRWMIA
--PRWMRN
--PRWMYA
--PRWMMV
--PRWMMG
--PRWMWS
--PRWMVG
--PRWMRL
--PRWMQY
--PRWMFG
--PRWMVQ
--PRWMTL
--PRWMRH
--PRWMNA
--PRWMMK
--PRWMIK
--PRWMHS
--PRWMYN
--PRWMTH
--PRWMTA
--PRWMQS
--PRWMLE

--PRWMKN
--PRWMKG
--PRWMGS
--PRWMDS
--PRWMAN
--PRWMSN
--PRWMSH
--PRWMSG
--PRWMSE
--PRWMQT
--PRWMQN
--PRWMQG
--PRWMQF
--PRWMMQ
--PRWMFS
--PRWMES
--PRWMAG
PVPRWM--
--PRWMYG
--PRWMVS
--PRWMTI
--PRWMRW
--PRWMRT
--PRWMRD
--PRWMKT
--PRWMAQ
--PRWMYE
--PRWMVM
--PRWMRI
--PRWMQA
--PRWMKQ
--PRWMIS
--PRWMHA
--PRWMAD
PNPRWM--
PGPRWM--
PSPRWM--
--PRWMYY
--PRWMYI
--PRWMYH
--PRWMVW
--PRWMVT
--PRWMTE
--PRWMSY
--PRWMSI
--PRWMQK
--PRWMMN
--PRWMLY
--PRWMLQ
--PRWMLI

--PRWMKL
--PRWMIM
--PRWMHN
--PRWMHG
--PRWMGA
--PRWMFK
--PRWMAY
--PRWMAV
--PRWMAS
--PRWMAL
--PRWMAA
PEPRWM--
--PRWMYT
--PRWMVN
--PRWMVH
--PRWMVE
--PRWMVD
--PRWMTN
--PRWMQW
--PRWMQI
--PRWMNV
--PRWMNM
--PRWMKV
--PRWMIV
--PRWMIQ
--PRWMII
--PRWMHW
--PRWMGY
--PRWMGM
--PRWMFH
PQPRWM--
PAPRWM--
PTPRWM--
--PRWMWV
--PRWMTY
--PRWMTW
--PRWMTM
--PRWMTF
--PRWMTD
--PRWMSW
--PRWMSM
--PRWMQV
--PRWMNH
--PRWMMH
--PRWMKY
--PRWMKE
--PRWMIT
--PRWMIE
--PRWMGT
--PRWMGI

--PRWMFT
--PRWMFD
--PRWMEY
--PRWMAM
--PRWMAK
--PRWMAH
--PRWMAF
--PRWMAE
PLPRWM--
PIPRWM--
PWPRWM--
--PRWMYV
--PRWMYQ
--PRWMYK
--PRWMWY
--PRWMWN
--PRWMTV
--PRWMTQ
--PRWMTG
--PRWMSQ
--PRWMSF
--PRWMRY
--PRWMQQ
--PRWMQM
--PRWMQH
--PRWMNN
--PRWMMW
--PRWMMM
--PRWMML
--PRWMKW
--PRWMKD
--PRWMIN
--PRWMIF
--PRWMHQ
--PRWMHH
--PRWMHD
--PRWMGW
--PRWMGQ
--PRWMGK
--PRWMGD
--PRWMFM
--PRWMFA
--PRWMEV
--PRWMET
--PRWMEQ
--PRWMEH
--PRWMEA
--PRWMDT
PRPRWM--
-PPRWMS-

PDPRWM--
-QPRWML-
-QPRWMV-
QVPRWM--
QSPRWM--
-QPRWMR-
-QPRWMI-
-QPRWMS-
-QPRWMN-
-QPRWMG-
-QPRWMF-
-QPRWMA-
QAPRWM--
QTPRWM--
QGPRWM--
QRPRWM--
-QPRWMY-
-QPRWMT-
-QPRWMH-
-QPRWMD-
QKPRWM--
-QPRWMM-
-QPRWMK-
QEPRWM--
RSPRWM--
-RPRWMM-
RPPRWM--
-RPRWMT-
-RPRWMS-
-RPRWMQ-
RLPRWM--
-RPRWMI-
-RPRWMN-
-RPRWML-
RVPRWM--
-RPRWMG-
RDPRWM--
-RPRWMY-
-RPRWMF-
RHPRWM--
-RPRWMW-
-RPRWMH-
RQPRWM--
-RPRWMK-
RIPRWM--
REPRWM--
-RPRWME-
-SPRWMS-
-SPRWMG-
-SPRWMI-

SVPRWM--
-SPRWML-
SYPRWM--
-SPRWMN-
-SPRWMM-
STPRWM--
-SPRWMT-
SPPRWM--
-SPRWMV-
-SPRWMA-
SWPRWM--
SSPRWM--
-SPRWMK-
-SPRWMF-
SMPRWM--
SGPRWM--
SAPRWM--
-SPRWMW-
SNPRWM--
-SPRWMQ-
-SPRWMD-
SKPRWM--
SIPRWM--
SDPRWM--
-SPRWME-
SFPRWM--
SEPRWM--
-TPRWML-
TGPRWM--
TLPRWM--
TRPRWM--
TQPRWM--
TMPRWM--
TWPRWM--
-TPRWMV-
-TPRWMI-
-TPRWMT-
TYPRWM--
TVPRWM--
-TPRWMH-
-TPRWMY-
-TPRWMK-
-TPRWMA-
TDPRWM--
-TPRWMN-
-TPRWMG-
THPRWM--
TFPRWM--
TTPRWM--
TPPRWM--

-TPRWMF-
TEPRWM--
TAPRWM--
-TPRWMQ-
TNPRWM--
TKPRWM--
VSPRWM--
VKPRWM--
VLPRWM--
VVPRWM--
VNPRWM--
-VPRWMR-
-VPRWMT-
-VPRWMK-
VPPRWM--
VRPRWM--
-VPRWMM-
-VPRWMG-
VTPRWM--
-VPRWMY-
-VPRWMQ-
VGPRWM--
-VPRWMN-
VYPRWM--
VHPRWM--
VAPRWM--
VQPRWM--
-VPRWMV-
-VPRWMD-
-VPRWMA-
VIPRWM--
VEPRWM--
WSPRWM--
WGPRWM--
WVPRWM--
WTPRWM--
WEPRWM--
-WPRWML-
WNPRWM--
-WPRWMT-
WPPRWM--
WAPRWM--
WRPRWM--
WLPRWM--
-WPRWMR-
WKPRWM--
WIPRWM--
WHPRWM--
WYPRWM--
-WPRWMY-

-WPRWMQ-
-WPRWMM-
-WPRWMH-
-WPRWMF-
WFPRWM--
WDPRWM--
-YPRWMS-
YNPRWM--
YTPRWM--
YQPRWM--
YGPRWM--
-YPRWMV-
-YPRWMM-
-YPRWMA-
YKPRWM--
YFPRWM--
-YPRWMN-
-YPRWMG-
YLPRWM--
YVPRWM--
-YPRWMK-
YMPRWM--
YHPRWM--
YDPRWM--
YYPRWM--
YWPRWM--
YRPRWM--
-YPRWMT-
-YPRWMQ-
-YPRWMI-
-YPRWMH-

| | | | |
|---|---|---|---|
| LGWL | 504 | 604 | 32.36 |

-TLGWLP-
ASLGWL--
ANLGWL--
-ALGWLG-
-ALGWLK-
AMLGWL--
AQLGWL--
-ALGWLM-
ALLGWL--
ATLGWL--
AYLGWL--
-ALGWLA-
AKLGWL--
-ALGWLQ-
AHLGWL--
-ALGWLT-
AILGWL--
-ALGWLV-

AELGWL--
ADLGWL--
-ALGWLE-
AWLGWL--
-ALGWLP-
CELGWL--
CKLGWL--
CMLGWL--
-CLGWLG-
CTLGWL--
CLLGWL--
-CLGWLS-
CCLGWL--
CVLGWL--
CSLGWL--
-CLGWLL-
-CLGWLE-
-CLGWLA-
CILGWL--
CHLGWL--
CDLGWL--
-DLGWLL-
-DLGWLS-
-DLGWLR-
DSLGWL--
DRLGWL--
-DLGWLH-
DTLGWL--
DVLGWL--
-DLGWLV-
DFLGWL--
DGLGWL--
DMLGWL--
-DLGWLQ-
-DLGWLI-
DNLGWL--
-DLGWLT-
-DLGWLA-
-DLGWLK-
DALGWL--
-DLGWLG-
DKLGWL--
-DLGWLN-
-DLGWLM-
DELGWL--
DYLGWL--
DDLGWL--
DQLGWL--
-DLGWLD-
-DLGWLC-

```
-ELGWLL-
-ELGWLR-
EVLGWL--
ERLGWL--
ELLGWL--
-ELGWLN-
ESLGWL--
-ELGWLT-
-ELGWLS-
EALGWL--
-ELGWLV-
EKLGWL--
EQLGWL--
-ELGWLA-
EILGWL--
EHLGWL--
-ELGWLQ-
EGLGWL--
EYLGWL--
-ELGWLM-
-ELGWLG-
ETLGWL--
EMLGWL--
-ELGWLH-
-ELGWLE-
EWLGWL--
-ELGWLI-
EELGWL--
ECLGWL--
EFLGWL--
EDLGWL--
FRLGWL--
FHLGWL--
FSLGWL--
FQLGWL--
-FLGWLV-
-FLGWLK-
FGLGWL--
-FLGWLT-
-FLGWLR-
-FLGWLG-
-FLGWLD-
-FLGWLA-
FVLGWL--
-FLGWLL-
FYLGWL--
-FLGWLF-
FELGWL--
FNLGWL--
FMLGWL--
```

-FLGWLE-
-FLGWLM-
-GLGWLT-
-GLGWLK-
GHLGWL--
-GLGWLM-
GVLGWL--
-GLGWLH-
-GLGWLN-
GLLGWL--
-GLGWLG-
GDLGWL--
-GLGWLE-
-GLGWLQ-
-GLGWLD-
GGLGWL--
GWLGWL--
-GLGWLV-
GQLGWL--
GELGWL--
GILGWL--
GFLGWL--
GCLGWL--
-HLGWLK-
-HLGWLS-
-HLGWLA-
-HLGWLD-
-HLGWLR-
-HLGWLG-
HHLGWL--
HRLGWL--
HTLGWL--
-HLGWLI-
-HLGWLE-
HNLGWL--
-HLGWLN-
HVLGWL--
HDLGWL--
-HLGWLQ-
-HLGWLV-
HQLGWL--
-HLGWLH-
HALGWL--
-HLGWLT-
HWLGWL--
HKLGWL--
HCLGWL--
-HLGWLY-
-HLGWLM-
HYLGWL--

```
-HLGWLF-
IRLGWL--
IKLGWL--
IDLGWL--
IGLGWL--
IYLGWL--
-ILGWLG-
-ILGWLA-
ILLGWL--
-ILGWLT-
-ILGWLH-
IHLGWL--
-ILGWLV-
-ILGWLN-
-ILGWLK-
IELGWL--
IWLGWL--
ICLGWL--
KSLGWL--
-KLGWLS-
KLLGWL--
KELGWL--
-KLGWLK-
-KLGWLG-
KALGWL--
KNLGWL--
KDLGWL--
-KLGWLH-
KTLGWL--
KHLGWL--
-KLGWLM-
KILGWL--
KMLGWL--
KYLGWL--
KVLGWL--
KFLGWL--
KQLGWL--
-KLGWLY-
-KLGWLV-
KWLGWL--
-KLGWLE-
KCLGWL--
-KLGWLD-
--LGWLSS
LKLGWL--
--LGWLSA
--LGWLVS
--LGWLST
--LGWLRY
--LGWLHK
```

--LGWLSV
--LGWLKG
LLLGWL--
--LGWLRN
--LGWLVK
--LGWLSH
--LGWLRG
--LGWLNS
--LGWLLN
--LGWLKT
--LGWLAR
--LGWLAT
-LLGWLG-
--LGWLTN
--LGWLTA
--LGWLHS
--LGWLHN
--LGWLGT
LNLGWL--
-LLGWLR-
--LGWLTK
--LGWLRE
--LGWLMH
-LLGWLK-
--LGWLTD
--LGWLSN
--LGWLGV
--LGWLEG
LYLGWL--
--LGWLTT
--LGWLQS
--LGWLHG
--LGWLGL
--LGWLGH
--LGWLGG
--LGWLGF
-LLGWLL-
--LGWLSM
--LGWLQT
--LGWLIN
--LGWLEV
--LGWLTI
--LGWLSK
--LGWLRH
--LGWLMN
--LGWLHI
--LGWLGW
--LGWLAY
--LGWLAK
-LLGWLT-

-LLGWLN-
LILGWL--
--LGWLVE
--LGWLTM
--LGWLSD
--LGWLIQ
--LGWLAV
--LGWLSW
--LGWLRD
--LGWLGQ
--LGWLAG
-LLGWLE-
--LGWLVQ
--LGWLKY
--LGWLKN
--LGWLIS
--LGWLHV
--LGWLHT
--LGWLGY
--LGWLGM
--LGWLDS
--LGWLAQ
-LLGWLH-
-LLGWLD-
--LGWLYN
--LGWLTH
--LGWLRF
--LGWLQR
--LGWLNK
--LGWLKQ
--LGWLKM
--LGWLKA
--LGWLAN
--LGWLAA
-LLGWLA-
--LGWLTY
--LGWLQA
--LGWLNW
--LGWLNR
--LGWLNQ
--LGWLNA
--LGWLMK
--LGWLKV
--LGWLKD
--LGWLIW
--LGWLHQ
--LGWLHH
--LGWLYQ
--LGWLVN
--LGWLQM

--LGWLQD
--LGWLMD
--LGWLLE
--LGWLID
LFLGWL--
-LLGWLP-
--LGWLVH
--LGWLVG
--LGWLTF
--LGWLRW
--LGWLQI
--LGWLQG
--LGWLNM
--LGWLGK
--LGWLEI
--LGWLEA
--LGWLDG
--LGWLYH
--LGWLRM
--LGWLNV
--LGWLMQ
--LGWLKE
--LGWLIH
--LGWLEY
--LGWLAH
--LGWLAE
--LGWLWN
--LGWLSQ
--LGWLNY
--LGWLNH
--LGWLNG
--LGWLLD
--LGWLIT
--LGWLHD
--LGWLFH
--LGWLET
--LGWLAF
--LGWLVD
--LGWLQW
--LGWLQV
--LGWLQN
--LGWLMV
--LGWLKI
--LGWLKF
--LGWLHM
--LGWLGE
--LGWLES
--LGWLEQ
--LGWLEM
--LGWLEH

-LLGWLV-
-LLGWLQ-
--LGWLYD
--LGWLWH
--LGWLWF
--LGWLTE
--LGWLQY
--LGWLHA
--LGWLFG
--LGWLDT
--LGWLAW
--LGWLTQ
--LGWLMW
--LGWLME
--LGWLLV
--LGWLHL
--LGWLHE
--LGWLFY
--LGWLFI
--LGWLEW
-LLGWLF-
--LGWLYG
--LGWLWW
--LGWLQH
--LGWLNE
--LGWLMM
--LGWLHY
--LGWLHF
--LGWLGD
--LGWLER
--LGWLEN
--LGWLED
--LGWLDA
--LGWLAM
--LGWLAD
MSLGWL--
MNLGWL--
MQLGWL--
-MLGWLG-
-MLGWLS-
MGLGWL--
MVLGWL--
MLLGWL--
-MLGWLT-
-MLGWLK-
MFLGWL--
MTLGWL--
-MLGWLN-
MILGWL--
MELGWL--

MMLGWL--
MHLGWL--
-MLGWLM-
-MLGWLE-
MDLGWL--
-MLGWLD-
NRLGWL--
-NLGWLR-
NSLGWL--
-NLGWLS-
NVLGWL--
NALGWL--
NKLGWL--
-NLGWLT-
-NLGWLN-
-NLGWLG-
NNLGWL--
-NLGWLQ-
-NLGWLA-
NHLGWL--
-NLGWLE-
NILGWL--
-NLGWLM-
-NLGWLI-
NDLGWL--
NWLGWL--
NTLGWL--
NMLGWL--
NYLGWL--
-NLGWLK-
NQLGWL--
NCLGWL--
PHLGWL--
PKLGWL--
PSLGWL--
PGLGWL--
PTLGWL--
PELGWL--
PALGWL--
PYLGWL--
PQLGWL--
PMLGWL--
PNLGWL--
PILGWL--
PFLGWL--
PWLGWL--
-QLGWLS-
QGLGWL--
QSLGWL--
QKLGWL--

−QLGWLG−
−QLGWLI−
−QLGWLH−
QILGWL−−
QELGWL−−
QWLGWL−−
−QLGWLK−
QDLGWL−−
QLLGWL−−
−QLGWLN−
−QLGWLE−
−QLGWLD−
−QLGWLV−
−QLGWLQ−
QALGWL−−
−QLGWLM−
−QLGWLA−
QCLGWL−−
QNLGWL−−
QQLGWL−−
QHLGWL−−
−RLGWLG−
RTLGWL−−
−RLGWLN−
RYLGWL−−
−RLGWLH−
−RLGWLI−
−RLGWLE−
−RLGWLK−
−RLGWLQ−
RHLGWL−−
RCLGWL−−
−SLGWLR−
−SLGWLG−
SGLGWL−−
SLLGWL−−
−SLGWLM−
SKLGWL−−
SVLGWL−−
−SLGWLK−
SHLGWL−−
SELGWL−−
−SLGWLD−
SILGWL−−
SQLGWL−−
−SLGWLH−
SFLGWL−−
SDLGWL−−
SWLGWL−−
−SLGWLE−

```
-SLGWLQ-
-SLGWLC-
SCLGWL--
TSLGWL--
-TLGWLL-
-TLGWLH-
-TLGWLS-
TTLGWL--
TKLGWL--
TNLGWL--
-TLGWLG-
THLGWL--
-TLGWLT-
-TLGWLE-
TYLGWL--
-TLGWLN-
TDLGWL--
TQLGWL--
-TLGWLD-
TFLGWL--
TELGWL--
-TLGWLA-
-TLGWLV-
-TLGWLQ-
TILGWL--
TCLGWL--
VALGWL--
VDLGWL--
VNLGWL--
-VLGWLE-
VELGWL--
-VLGWLL-
-VLGWLI-
-VLGWLN-
-VLGWLK-
VGLGWL--
-VLGWLQ-
-VLGWLA-
-VLGWLH-
-VLGWLD-
VCLGWL--
WKLGWL--
WELGWL--
WSLGWL--
WQLGWL--
WNLGWL--
-WLGWLD-
WTLGWL--
WGLGWL--
WDLGWL--
```

```
              -WLGWLR-
              -WLGWLQ-
              WALGWL--
              WVLGWL--
              -WLGWLT-
              -WLGWLH-
              -WLGWLE-
              -WLGWLA-
              WMLGWL--
              -WLGWLM-
              WILGWL--
              YGLGWL--
              YTLGWL--
              YSLGWL--
              YNLGWL--
              -YLGWLD-
              -YLGWLA-
              -YLGWLN-
              YYLGWL--
              YLLGWL--
              -YLGWLG-
              YKLGWL--
              YALGWL--
              YMLGWL--
              -YLGWLQ-
              YQLGWL--
              -YLGWLL-
              YELGWL--
              -YLGWLM-
              -YLGWLK-
              -YLGWLE-
              YHLGWL--
              YFLGWL--
              YDLGWL--
              -YLGWLT-
              -YLGWLI-
VSAL           366        645      32.34
              -AVSALG-
              -GVSALG-
              -GVSALC-
              -IVSALP-
              -LVSALP-
              -RVSALE-
              -SVSALE-
              -TVSALG-
              --VSALQN
              -VVSALE-
              --VSALTE
              --VSALSP
              --VSALQK
```

--VSALPW
--VSALPN
--VSALNH
--VSALLE
--VSALHW
--VSALGT
--VSALGQ
--VSALFP
--VSALEF
--VSALDL
ARVSAL--
AKVSAL--
ASVSAL--
ANVSAL--
-AVSALL-
AAVSAL--
AVVSAL--
AHVSAL--
-AVSALS-
-AVSALH-
ATVSAL--
AWVSAL--
AMVSAL--
ADVSAL--
-AVSALK-
AIVSAL--
APVSAL--
-AVSALA-
-AVSALV-
-AVSALQ-
AQVSAL--
AEVSAL--
ACVSAL--
-AVSALN-
-AVSALE-
-AVSALM-
CCVSAL--
-CVSALS-
-CVSALR-
CNVSAL--
CTVSAL--
CSVSAL--
-CVSALQ-
CMVSAL--
-CVSALK-
CDVSAL--
CWVSAL--
-CVSALL-
CGVSAL--
-CVSALY-

-CVSALN-
-CVSALM-
-CVSALT-
CIVSAL--
CAVSAL--
CYVSAL--
-CVSALW-
CKVSAL--
-CVSALV-
-CVSALI-
-CVSALH-
-CVSALF-
-CVSALA-
CQVSAL--
DLVSAL--
DRVSAL--
DFVSAL--
-DVSALL-
DGVSAL--
-DVSALH-
DKVSAL--
DVVSAL--
-DVSALK-
-DVSALF-
-DVSALV-
DIVSAL--
DAVSAL--
DHVSAL--
-DVSALM-
DYVSAL--
DWVSAL--
-DVSALW-
-DVSALT-
DPVSAL--
DQVSAL--
DNVSAL--
-DVSALQ-
DTVSAL--
DEVSAL--
DMVSAL--
DCVSAL--
-EVSALR-
EYVSAL--
EGVSAL--
-EVSALY-
EIVSAL--
-EVSALS-
ESVSAL--
EAVSAL--
-EVSALQ-

−EVSALK−
ENVSAL−−
−EVSALW−
−EVSALT−
ETVSAL−−
ECVSAL−−
EVVSAL−−
−EVSALV−
EMVSAL−−
EPVSAL−−
EHVSAL−−
EWVSAL−−
EQVSAL−−
EDVSAL−−
−EVSALH−
EEVSAL−−
−EVSALM−
FHVSAL−−
−FVSALK−
−FVSALH−
FDVSAL−−
−FVSALA−
−FVSALG−
FQVSAL−−
FPVSAL−−
FCVSAL−−
GLVSAL−−
−GVSALR−
−GVSALT−
GWVSAL−−
−GVSALL−
GTVSAL−−
GMVSAL−−
GSVSAL−−
GVVSAL−−
GKVSAL−−
GNVSAL−−
−GVSALK−
GFVSAL−−
−GVSALI−
GHVSAL−−
GPVSAL−−
GGVSAL−−
GQVSAL−−
−GVSALH−
GDVSAL−−
−GVSALY−
−GVSALV−
GCVSAL−−
−GVSALQ−

```
-GVSALM-
-GVSALN-
-GVSALA-
-GVSALE-
HRVSAL--
HVVSAL--
-HVSALL-
HKVSAL--
-HVSALS-
-HVSALR-
HTVSAL--
HGVSAL--
HLVSAL--
HHVSAL--
-HVSALW-
HDVSAL--
HNVSAL--
-HVSALH-
HMVSAL--
-HVSALQ-
-HVSALF-
-HVSALI-
HPVSAL--
-HVSALM-
-HVSALK-
-HVSALA-
-HVSALY-
HFVSAL--
-HVSALN-
-HVSALV-
HAVSAL--
HCVSAL--
HWVSAL--
ISVSAL--
IGVSAL--
-IVSALL-
-IVSALI-
IPVSAL--
INVSAL--
-IVSALN-
-IVSALA-
IHVSAL--
IEVSAL--
IMVSAL--
IDVSAL--
IIVSAL--
IAVSAL--
-IVSALQ-
-IVSALG-
-IVSALH-
```

IQVSAL--
-IVSALE-
-IVSALK-
ICVSAL--
-IVSALV-
KAVSAL--
-KVSALV-
KVVSAL--
-KVSALS-
-KVSALT-
-KVSALH-
KHVSAL--
-KVSALY-
KNVSAL--
-KVSALF-
KPVSAL--
-KVSALQ-
KQVSAL--
KMVSAL--
KEVSAL--
KIVSAL--
KDVSAL--
KTVSAL--
-KVSALK-
-KVSALN-
-KVSALW-
KFVSAL--
-KVSALM-
LSVSAL--
-LVSALS-
LVVSAL--
LDVSAL--
LAVSAL--
-LVSALH-
-LVSALK-
LTVSAL--
-LVSALN-
LEVSAL--
-LVSALI-
-LVSALT-
-LVSALQ-
LQVSAL--
-LVSALE-
LHVSAL--
-LVSALM-
-LVSALC-
MSVSAL--
MVVSAL--
-MVSALL-
-MVSALR-

```
-MVSALQ-
MPVSAL--
-MVSALK-
MGVSAL--
-MVSALT-
MQVSAL--
-MVSALI-
-MVSALS-
MEVSAL--
MDVSAL--
-MVSALG-
MLVSAL--
MHVSAL--
-MVSALN-
MMVSAL--
MIVSAL--
-MVSALE-
-MVSALA-
MCVSAL--
-MVSALC-
NRVSAL--
NSVSAL--
-NVSALR-
-NVSALL-
NAVSAL--
-NVSALK-
NVVSAL--
NKVSAL--
NYVSAL--
-NVSALW-
NFVSAL--
NTVSAL--
-NVSALT-
NGVSAL--
-NVSALF-
-NVSALS-
NIVSAL--
NWVSAL--
NQVSAL--
NNVSAL--
NHVSAL--
-NVSALV-
NPVSAL--
-NVSALY-
-NVSALI-
-NVSALQ-
-NVSALN-
-NVSALA-
NCVSAL--
-NVSALM-
```

NDVSAL--
-NVSALC-
-NVSALE-
NEVSAL--
PSVSAL--
PKVSAL--
PLVSAL--
-PVSALL-
-PVSALI-
PYVSAL--
-PVSALW-
-PVSALT-
PGVSAL--
PAVSAL--
-PVSALK-
-PVSALQ-
-PVSALS-
-PVSALA-
PDVSAL--
-PVSALH-
-PVSALM-
-PVSALF-
PNVSAL--
PEVSAL--
-PVSALN-
PHVSAL--
PCVSAL--
-QVSALR-
-QVSALS-
-QVSALT-
-QVSALW-
QAVSAL--
-QVSALK-
-QVSALL-
-QVSALY-
-QVSALV-
QQVSAL--
QTVSAL--
QSVSAL--
QFVSAL--
QDVSAL--
-QVSALF-
QKVSAL--
QLVSAL--
QHVSAL--
QYVSAL--
QWVSAL--
-QVSALI-
-QVSALA-
-QVSALN-

```
-QVSALM-
QPVSAL--
QMVSAL--
QIVSAL--
QCVSAL--
QNVSAL--
-QVSALQ-
-QVSALH-
-QVSALG-
-QVSALE-
QEVSAL--
-RVSALL-
RGVSAL--
-RVSALH-
RWVSAL--
RQVSAL--
-RVSALF-
-RVSALA-
-RVSALN-
RCVSAL--
-SVSALL-
SLVSAL--
SKVSAL--
-SVSALT-
SIVSAL--
SVVSAL--
SPVSAL--
-SVSALV-
SNVSAL--
STVSAL--
SYVSAL--
SHVSAL--
SFVSAL--
SDVSAL--
SMVSAL--
-SVSALF-
-SVSALI-
-SVSALN-
SEVSAL--
SCVSAL--
-SVSALA-
-SVSALM-
-SVSALC-
TRVSAL--
TLVSAL--
TVVSAL--
-TVSALR-
TNVSAL--
TTVSAL--
-TVSALK-
```

```
TWVSAL--
TFVSAL--
TKVSAL--
-TVSALW-
TYVSAL--
TGVSAL--
TPVSAL--
TAVSAL--
TIVSAL--
-TVSALH-
TEVSAL--
TQVSAL--
-TVSALM-
-TVSALV-
-TVSALQ-
TDVSAL--
TCVSAL--
--VSALSS
VRVSAL--
--VSALSL
VSVSAL--
VKVSAL--
--VSALVS
--VSALKS
--VSALRY
-VVSALR-
--VSALWG
VNVSAL--
--VSALRH
--VSALLT
--VSALST
--VSALTG
--VSALRD
--VSALYS
VTVSAL--
--VSALTS
--VSALSI
--VSALIT
VGVSAL--
VEVSAL--
--VSALWS
--VSALSA
--VSALKT
--VSALIS
-VVSALK-
VAVSAL--
--VSALYT
--VSALSV
VDVSAL--
--VSALWM
```

--VSALGI
-VVSALH-
--VSALTK
--VSALHY
VQVSAL--
-VVSALQ-
--VSALVN
--VSALAI
--VSALTT
--VSALNA
--VSALAG
VPVSAL--
--VSALVH
--VSALTV
--VSALLN
--VSALKG
--VSALAS
VMVSAL--
--VSALMR
--VSALFG
--VSALWR
--VSALVY
--VSALTA
--VSALSW
--VSALIN
--VSALTR
--VSALSF
--VSALIR
--VSALHN
--VSALGM
-VVSALN-
-VVSALA-
--VSALIG
--VSALHS
--VSALWQ
--VSALTW
--VSALQS
--VSALLH
--VSALIK
--VSALMW
--VSALMH
--VSALAY
VYVSAL--
--VSALQV
--VSALMG
--VSALKN
--VSALAF
--VSALAA
-VVSALI-
--VSALYK

--VSALSN
--VSALRE
--VSALMD
--VSALKR
--VSALIA
--VSALFR
--VSALAM
--VSALVF
--VSALNY
--VSALNT
--VSALKK
--VSALHR
--VSALFH
--VSALFA
--VSALTN
--VSALMY
--VSALKH
--VSALKA
--VSALFQ
--VSALES
--VSALQT
--VSALQG
--VSALGF
--VSALMT
--VSALHV
--VSALHT
--VSALYH
--VSALHK
--VSALHD
--VSALGY
--VSALAW
--VSALAT
--VSALWN
--VSALWH
--VSALYE
--VSALMQ
--VSALHI
--VSALYD
--VSALPM
--VSALNM
--VSALLK
--VSALKI
--VSALIQ
--VSALHQ
--VSALHM
--VSALGW
--VSALVQ
--VSALSQ
--VSALQW
--VSALPI

--VSALKQ
--VSALIW
--VSALHG
--VSALGS
--VSALAV
-VVSALM-
--VSALYA
--VSALNI
--VSALMK
--VSALMF
--VSALHA
--VSALFN
VCVSAL--
--VSALNG
--VSALMV
--VSALID
--VSALNV
--VSALLD
--VSALKD
--VSALEG
--VSALEA
--VSALWE
--VSALQA
--VSALMA
--VSALIE
--VSALFD
--VSALWK
--VSALTQ
--VSALTI
--VSALTD
--VSALSE
--VSALQY
--VSALQL
--VSALQH
--VSALMM
--VSALLQ
--VSALHE
--VSALFK
--VSALNW
--VSALFE
--VSALAQ
--VSALAH
--VSALNQ
--VSALDV
--VSALCS
--VSALCM
-WVSALT-
-WVSALE-
WGVSAL--
-WVSALH-

-WVSALK-
WPVSAL--
WDVSAL--
-WVSALN-
-WVSALA-
-WVSALG-
WHVSAL--
-WVSALQ-
WQVSAL--
WCVSAL--
YPVSAL--
-YVSALS-
YGVSAL--
-YVSALT-
-YVSALL-
-YVSALI-
YTVSAL--
YVVSAL--
YMVSAL--
YNVSAL--
YDVSAL--
YIVSAL--
YQVSAL--
YKVSAL--
YAVSAL--
YYVSAL--
-YVSALA-
-YVSALQ-
-YVSALE-
YHVSAL--
-YVSALV-
-YVSALG-

PMPL 194 715 32.31

AFPMPL--
-LPMPLG-
-APMPLS-
AGPMPL--
AWPMPL--
AVPMPL--
-APMPLI-
-APMPLR-
-APMPLF-
ALPMPL--
ASPMPL--
APPMPL--
-APMPLM-
ARPMPL--
-APMPLQ-
-APMPLN-
-APMPLL-

-APMPLA-
AYPMPL--
-APMPLV-
-APMPLY-
AMPMPL--
AHPMPL--
AEPMPL--
ATPMPL--
-APMPLE-
ANPMPL--
AIPMPL--
ACPMPL--
-CPMPLR-
CRPMPL--
-CPMPLH-
CNPMPL--
CCPMPL--
CSPMPL--
CPPMPL--
-CPMPLY-
-CPMPLT-
-CPMPLL-
-CPMPLF-
DVPMPL--
DRPMPL--
DLPMPL--
-DPMPLL-
-DPMPLW-
-DPMPLI-
DNPMPL--
DHPMPL--
DSPMPL--
DQPMPL--
-DPMPLT-
-DPMPLH-
DWPMPL--
DTPMPL--
-DPMPLY-
-DPMPLS-
-DPMPLR-
-DPMPLM-
DIPMPL--
DDPMPL--
-EPMPLH-
ERPMPL--
EAPMPL--
ESPMPL--
-EPMPLY-
-EPMPLS-
EYPMPL--

ELPMPL--
EIPMPL--
EEPMPL--
-EPMPLW-
-EPMPLQ-
-EPMPLM-
-EPMPLE-
EVPMPL--
EQPMPL--
EPPMPL--
-EPMPLV-
-EPMPLL-
-EPMPLA-
EHPMPL--
EFPMPL--
EDPMPL--
-FPMPLS-
FVPMPL--
FSPMPL--
-FPMPLV-
-FPMPLK-
FGPMPL--
FWPMPL--
FQPMPL--
-FPMPLL-
FYPMPL--
-FPMPLT-
-FPMPLY-
-FPMPLR-
FMPMPL--
FTPMPL--
-FPMPLQ-
-FPMPLM-
FNPMPL--
FIPMPL--
FHPMPL--
FPPMPL--
-FPMPLN-
-FPMPLG-
-FPMPLA-
FLPMPL--
FFPMPL--
FEPMPL--
GRPMPL--
-GPMPLR-
GLPMPL--
GSPMPL--
GKPMPL--
-GPMPLS-
GWPMPL--

GYPMPL--
-GPMPLK-
GGPMPL--
GIPMPL--
GFPMPL--
GTPMPL--
-GPMPLV-
GNPMPL--
GMPMPL--
GQPMPL--
-GPMPLY-
-GPMPLM-
-GPMPLI-
-GPMPLA-
GVPMPL--
GHPMPL--
GAPMPL--
GPPMPL--
-GPMPLW-
-GPMPLQ-
-GPMPLN-
-GPMPLG-
GEPMPL--
GDPMPL--
-HPMPLT-
HRPMPL--
HYPMPL--
HWPMPL--
-HPMPLS-
HLPMPL--
-HPMPLR-
HKPMPL--
HFPMPL--
HVPMPL--
HMPMPL--
-HPMPLN-
-HPMPLM-
HIPMPL--
HQPMPL--
HEPMPL--
HTPMPL--
HPPMPL--
-HPMPLL-
-HPMPLG-
HSPMPL--
-HPMPLQ-
-HPMPLA-
HNPMPL--
HAPMPL--
-IPMPLN-

-IPMPLS-
ISPMPL--
-IPMPLR-
IVPMPL--
-IPMPLY-
IKPMPL--
IRPMPL--
-IPMPLF-
IMPMPL--
IFPMPL--
ITPMPL--
IPPMPL--
-IPMPLT-
IWPMPL--
-IPMPLI-
-IPMPLA-
-IPMPLQ-
IGPMPL--
IEPMPL--
IYPMPL--
-IPMPLM-
-IPMPLL-
-IPMPLG-
INPMPL--
ILPMPL--
KLPMPL--
-KPMPLR-
-KPMPLL-
KGPMPL--
-KPMPLI-
KAPMPL--
-KPMPLA-
-KPMPLT-
-KPMPLF-
-KPMPLS-
KRPMPL--
KHPMPL--
KDPMPL--
-KPMPLW-
-KPMPLQ-
-KPMPLK-
KMPMPL--
KYPMPL--
-KPMPLN-
-KPMPLM-
-KPMPLH-
KWPMPL--
KQPMPL--
KPPMPL--
-KPMPLY-

KEPMPL--
KTPMPL--
-KPMPLE-
KNPMPL--
KIPMPL--
KCPMPL--
LVPMPL--
LRPMPL--
-LPMPLT-
-LPMPLR-
-LPMPLK-
LLPMPL--
LTPMPL--
LSPMPL--
-LPMPLV-
-LPMPLS-
LKPMPL--
-LPMPLM-
LAPMPL--
-LPMPLH-
LYPMPL--
-LPMPLQ-
LNPMPL--
LMPMPL--
LGPMPL--
LDPMPL--
LQPMPL--
-LPMPLY-
-LPMPLW-
-LPMPLA-
LFPMPL--
-LPMPLE-
-MPMPLR-
MRPMPL--
MYPMPL--
-MPMPLS-
-MPMPLF-
-MPMPLA-
MLPMPL--
MIPMPL--
-MPMPLI-
MHPMPL--
MAPMPL--
MPPMPL--
-MPMPLV-
MWPMPL--
MNPMPL--
MKPMPL--
MVPMPL--
MQPMPL--

-MPMPLY-
-MPMPLW-
-MPMPLQ-
-MPMPLK-
-MPMPLH-
MCPMPL--
-NPMPLS-
NFPMPL--
NVPMPL--
-NPMPLR-
-NPMPLH-
NGPMPL--
-NPMPLI-
-NPMPLF-
-NPMPLV-
-NPMPLT-
-NPMPLM-
NHPMPL--
NPPMPL--
NYPMPL--
NSPMPL--
-NPMPLN-
-NPMPLL-
NNPMPL--
NKPMPL--
NIPMPL--
NDPMPL--
NWPMPL--
NTPMPL--
NMPMPL--
NEPMPL--
--PMPLWG
--PMPLSK
--PMPLVR
--PMPLSR
--PMPLRL
--PMPLGS
--PMPLRT
--PMPLQL
--PMPLNV
--PMPLVF
--PMPLSG
--PMPLRS
--PMPLIV
--PMPLFS
--PMPLML
--PMPLLS
--PMPLIT
--PMPLSV
--PMPLSW

--PMPLDL
--PMPLRN
--PMPLRA
--PMPLKL
--PMPLIK
--PMPLFM
--PMPLVL
--PMPLSY
--PMPLSA
--PMPLRW
--PMPLRK
--PMPLQV
--PMPLIY
--PMPLQA
--PMPLMR
--PMPLLH
--PMPLKS
--PMPLKQ
--PMPLHM
--PMPLGA
--PMPLVK
--PMPLTS
--PMPLNS
--PMPLII
--PMPLVY
--PMPLSS
--PMPLRY
--PMPLRG
--PMPLMS
--PMPLLT
--PMPLIR
--PMPLGG
PAPMPL--
--PMPLYR
--PMPLYI
--PMPLTT
--PMPLTR
--PMPLTK
--PMPLTH
--PMPLRM
--PMPLMK
--PMPLLV
--PMPLLM
--PMPLIS
PIPMPL--
--PMPLWN
--PMPLTY
--PMPLRV
--PMPLRE
--PMPLQR

--PMPLLN
--PMPLFV
--PMPLFT
--PMPLFL
--PMPLEF
--PMPLDW
PQPMPL--
--PMPLYY
--PMPLWR
--PMPLVH
--PMPLTW
--PMPLTL
--PMPLTG
--PMPLRQ
--PMPLRI
--PMPLPS
--PMPLPF
--PMPLLG
--PMPLKT
--PMPLKM
--PMPLHS
--PMPLHF
--PMPLGT
--PMPLFR
PMPMPL--
--PMPLYE
--PMPLWQ
--PMPLWI
--PMPLVS
--PMPLVA
--PMPLTV
--PMPLNA
--PMPLMN
--PMPLMG
--PMPLMF
--PMPLLK
--PMPLHV
--PMPLHQ
--PMPLHI
--PMPLFH
--PMPLDF
--PMPLAG
--PMPLAF
PLPMPL--
PNPMPL--
--PMPLYQ
--PMPLYN
--PMPLYK
--PMPLWY
--PMPLWS

--PMPLWL
--PMPLWK
--PMPLTI
--PMPLSF
--PMPLRH
--PMPLQT
--PMPLQN
--PMPLQK
--PMPLQI
--PMPLPH
--PMPLNT
--PMPLLI
--PMPLLE
--PMPLKY
--PMPLKR
--PMPLKN
--PMPLIG
--PMPLHY
--PMPLHW
--PMPLHT
--PMPLHR
--PMPLFG
--PMPLEW
--PMPLAS
--PMPLYT
--PMPLYG
--PMPLWH
--PMPLWA
--PMPLVV
--PMPLVT
--PMPLVQ
--PMPLVN
--PMPLVD
--PMPLTN
--PMPLTD
--PMPLST
--PMPLSQ
--PMPLSH
--PMPLQF
--PMPLPQ
--PMPLNW
--PMPLNH
--PMPLNG
--PMPLMT
--PMPLMI
--PMPLMA
--PMPLLD
--PMPLKG
--PMPLKD
--PMPLIF

--PMPLHG
--PMPLHA
--PMPLFA
--PMPLEV
--PMPLET
--PMPLES
--PMPLEA
--PMPLDY
--PMPLAW
--PMPLAV
--PMPLAN
--PMPLAK
--PMPLAA
PEPMPL--
--PMPLYH
--PMPLYA
--PMPLVM
--PMPLVE
--PMPLTQ
--PMPLTE
--PMPLSD
--PMPLRD
--PMPLQY
--PMPLQS
--PMPLQP
--PMPLQM
--PMPLQG
--PMPLQE
--PMPLPA
--PMPLNN
--PMPLMQ
--PMPLMM
--PMPLMH
--PMPLKW
--PMPLKH
--PMPLKF
--PMPLKE
--PMPLKA
--PMPLIQ
--PMPLIM
--PMPLIH
--PMPLIA
--PMPLHH
--PMPLHE
--PMPLHD
--PMPLGQ
--PMPLGK
--PMPLGH
--PMPLFN
--PMPLFK

--PMPLEY
--PMPLDV
--PMPLDM
--PMPLDI
--PMPLAE
-QPMPLK-
QVPMPL--
-QPMPLW-
-QPMPLV-
QIPMPL--
QYPMPL--
-QPMPLS-
QNPMPL--
-QPMPLY-
-QPMPLN-
QTPMPL--
QSPMPL--
-QPMPLT-
QGPMPL--
QAPMPL--
-QPMPLR-
-QPMPLL-
-QPMPLF-
-QPMPLA-
QEPMPL--
RVPMPL--
-RPMPLR-
RLPMPL--
RAPMPL--
-RPMPLL-
-RPMPLI-
RGPMPL--
-RPMPLS-
RQPMPL--
-RPMPLF-
-RPMPLH-
RMPMPL--
-RPMPLN-
RHPMPL--
-RPMPLW-
RPPMPL--
-RPMPLV-
RNPMPL--
RIPMPL--
RFPMPL--
RTPMPL--
-RPMPLY-
-RPMPLT-
-RPMPLK-
-RPMPLG-

RKPMPL--
RYPMPL--
-RPMPLQ-
-RPMPLM-
-RPMPLA-
RCPMPL--
SRPMPL--
STPMPL--
SPPMPL--
SIPMPL--
-SPMPLT-
-SPMPLS-
-SPMPLR-
SVPMPL--
SFPMPL--
-SPMPLI-
SKPMPL--
SSPMPL--
-SPMPLW-
-SPMPLL-
SQPMPL--
-SPMPLH-
SGPMPL--
SAPMPL--
SWPMPL--
-SPMPLK-
-SPMPLE-
SMPMPL--
-SPMPLD-
-SPMPLA-
SHPMPL--
-SPMPLV-
-SPMPLQ-
SNPMPL--
SEPMPL--
SDPMPL--
-SPMPLM-
-TPMPLR-
TRPMPL--
TVPMPL--
TNPMPL--
TFPMPL--
TKPMPL--
TAPMPL--
TTPMPL--
-TPMPLS-
-TPMPLI-
TPPMPL--
-TPMPLN-
-TPMPLK-

-TPMPLE-
TMPMPL--
TLPMPL--
TIPMPL--
-TPMPLY-
-TPMPLQ-
-TPMPLA-
TYPMPL--
TWPMPL--
TSPMPL--
TQPMPL--
-TPMPLT-
-TPMPLF-
TDPMPL--
-TPMPLG-
TCPMPL--
THPMPL--
VRPMPL--
VSPMPL--
-VPMPLV-
VHPMPL--
-VPMPLW-
-VPMPLI-
VYPMPL--
-VPMPLL-
VKPMPL--
VGPMPL--
VTPMPL--
VPPMPL--
VLPMPL--
VFPMPL--
VVPMPL--
-VPMPLT-
-VPMPLS-
-VPMPLN-
-VPMPLA-
VAPMPL--
-VPMPLK-
VQPMPL--
-VPMPLQ-
VNPMPL--
VIPMPL--
-VPMPLH-
VEPMPL--
VDPMPL--
-VPMPLY-
VMPMPL--
-VPMPLF-
-VPMPLE-
-WPMPLR-

```
              WVPMPL--
              WRPMPL--
              -WPMPLS-
              WSPMPL--
              WEPMPL--
              -WPMPLN-
              WLPMPL--
              -WPMPLM-
              WGPMPL--
              -WPMPLW-
              -WPMPLT-
              WFPMPL--
              WDPMPL--
              WYPMPL--
              WNPMPL--
              WIPMPL--
              WQPMPL--
              -WPMPLQ-
              -WPMPLK-
              -WPMPLH-
              WWPMPL--
              -WPMPLY-
              -WPMPLI-
              -YPMPLR-
              -YPMPLH-
              YVPMPL--
              YLPMPL--
              YTPMPL--
              -YPMPLT-
              YWPMPL--
              YQPMPL--
              YNPMPL--
              -YPMPLW-
              -YPMPLN-
              -YPMPLF-
              YIPMPL--
              YGPMPL--
              YEPMPL--
              YRPMPL--
              -YPMPLS-
              -YPMPLM-
              -YPMPLI-
              YHPMPL--
              -YPMPLV-
              -YPMPLQ-
              -YPMPLE-
              YKPMPL--
              YFPMPL--
PAEL            90         776      32.30
              -GPAELT-
```

-APAELL-
AGPAEL--
-APAELR-
-APAELK-
-APAELY-
AWPAEL--
AVPAEL--
ARPAEL--
-APAELE-
AMPAEL--
-APAELT-
-APAELA-
ANPAEL--
AHPAEL--
AYPAEL--
ATPAEL--
ASPAEL--
-APAELS-
-APAELM-
AIPAEL--
AQPAEL--
-APAELQ-
-APAELN-
ALPAEL--
AKPAEL--
-APAELW-
-APAELI-
-APAELC-
AFPAEL--
AEPAEL--
AAPAEL--
CIPAEL--
CTPAEL--
-CPAELR-
CSPAEL--
-CPAELY-
-CPAELK-
CCPAEL--
-CPAELQ-
CNPAEL--
CYPAEL--
-CPAELT-
-CPAELI-
CFPAEL--
DRPAEL--
DTPAEL--
DKPAEL--
DSPAEL--
-DPAELF-
DLPAEL--

DVPAEL--
-DPAELS-
-DPAELR-
-DPAELK-
DCPAEL--
DAPAEL--
DYPAEL--
-DPAELQ-
-DPAELG-
DQPAEL--
-DPAELY-
-DPAELV-
-DPAELN-
-DPAELL-
-DPAELH-
DIPAEL--
DHPAEL--
ELPAEL--
-EPAELR-
EVPAEL--
-EPAELW-
EMPAEL--
-EPAELA-
EWPAEL--
ERPAEL--
-EPAELQ-
-EPAELN-
-EPAELM-
-EPAELH-
-EPAELG-
EYPAEL--
ESPAEL--
-EPAELL-
-EPAELE-
EFPAEL--
ETPAEL--
-EPAELT-
-EPAELF-
EKPAEL--
ECPAEL--
-FPAELR-
FGPAEL--
-FPAELV-
FTPAEL--
-FPAELI-
FKPAEL--
FWPAEL--
-FPAELG-
-FPAELF-
FFPAEL--

FSPAEL--
-FPAELQ-
-FPAELK-
FMPAEL--
-FPAELY-
FDPAEL--
-FPAELN-
-FPAELA-
FHPAEL--
-FPAELT-
FNPAEL--
FLPAEL--
FCPAEL--
FAPAEL--
GLPAEL--
GMPAEL--
GVPAEL--
-GPAELM-
GRPAEL--
-GPAELS-
-GPAELR-
-GPAELL-
GEPAEL--
-GPAELV-
GCPAEL--
GTPAEL--
GSPAEL--
-GPAELA-
GYPAEL--
GQPAEL--
GHPAEL--
-GPAELW-
-GPAELH-
GIPAEL--
GWPAEL--
-GPAELY-
-GPAELQ-
-GPAELN-
-GPAELG-
GFPAEL--
GAPAEL--
-GPAELK-
-GPAELI-
GKPAEL--
GDPAEL--
HSPAEL--
HKPAEL--
HGPAEL--
HRPAEL--
HWPAEL--

–HPAELK–
–HPAELS–
–HPAELG–
HNPAEL––
HEPAEL––
–HPAELT–
–HPAELW–
–HPAELH–
HIPAEL––
–HPAELR–
HLPAEL––
–HPAELY–
–HPAELV–
–HPAELA–
HFPAEL––
HVPAEL––
–HPAELQ–
–HPAELM–
HDPAEL––
IYPAEL––
–IPAELL–
ILPAEL––
IVPAEL––
–IPAELY–
ISPAEL––
IPPAEL––
IMPAEL––
IGPAEL––
–IPAELT–
–IPAELR–
–IPAELQ–
IQPAEL––
–IPAELI–
IKPAEL––
ITPAEL––
–IPAELN–
–IPAELG–
IWPAEL––
–IPAELW–
–IPAELV–
–IPAELS–
–IPAELP–
–IPAELF–
INPAEL––
IHPAEL––
IFPAEL––
IDPAEL––
ICPAEL––
KRPAEL––
–KPAELL–

-KPAELK-
-KPAELQ-
-KPAELT-
KTPAEL--
KGPAEL--
KVPAEL--
KIPAEL--
KWPAEL--
-KPAELR-
-KPAELG-
KLPAEL--
-KPAELW-
KPPAEL--
-KPAELS-
-KPAELN-
-KPAELI-
-KPAELF-
-KPAELA-
KHPAEL--
KYPAEL--
-KPAELY-
-KPAELV-
-KPAELH-
KNPAEL--
KCPAEL--
KDPAEL--
KAPAEL--
-LPAELR-
-LPAELS-
LMPAEL--
-LPAELT-
LRPAEL--
LAPAEL--
LYPAEL--
LPPAEL--
-LPAELN-
-LPAELK-
LKPAEL--
-LPAELM-
-LPAELL-
-LPAELY-
-LPAELV-
LVPAEL--
LEPAEL--
-LPAELF-
-LPAELQ-
-LPAELH-
LLPAEL--
LHPAEL--
LTPAEL--

LSPAEL--
LWPAEL--
-LPAELW-
-LPAELI-
LDPAEL--
LNPAEL--
LFPAEL--
-LPAELG-
-LPAELA-
LIPAEL--
MSPAEL--
MVPAEL--
-MPAELR-
MAPAEL--
-MPAELY-
-MPAELS-
-MPAELA-
MKPAEL--
-MPAELQ-
MIPAEL--
MRPAEL--
-MPAELW-
-MPAELV-
-MPAELN-
MGPAEL--
MFPAEL--
MPPAEL--
-MPAELT-
-MPAELM-
-MPAELL-
-MPAELH-
-MPAELF-
MMPAEL--
MWPAEL--
MTPAEL--
-MPAELI-
-MPAELG-
MLPAEL--
NRPAEL--
NPPAEL--
NVPAEL--
-NPAELL-
NSPAEL--
NGPAEL--
NTPAEL--
-NPAELQ-
-NPAELI-
NMPAEL--
NHPAEL--
-NPAELF-

NNPAEL--
NQPAEL--
NLPAEL--
NIPAEL--
NFPAEL--
NYPAEL--
-NPAELY-
-NPAELW-
-NPAELT-
-NPAELS-
NKPAEL--
NAPAEL--
--PAELVK
--PAELRR
--PAELLR
--PAELRM
--PAELLS
--PAELRV
--PAELML
--PAELMK
--PAELSL
--PAELRL
--PAELMT
--PAELVL
--PAELRF
--PAELMR
--PAELKW
PRPAEL--
--PAELYL
--PAELVT
--PAELAL
--PAELYE
--PAELRY
--PAELRI
--PAELLV
--PAELLH
--PAELKV
--PAELWQ
--PAELTS
--PAELSR
--PAELLN
--PAELKL
--PAELHL
--PAELGS
--PAELFR
--PAELVA
--PAELSI
--PAELWK
--PAELVV
--PAELVR

--PAELTV
--PAELTF
--PAELSW
--PAELKR
--PAELIR
PTPAEL--
--PAELYG
--PAELVF
--PAELVD
--PAELSV
--PAELSH
--PAELRN
--PAELRG
--PAELNL
--PAELMW
--PAELLG
--PAELLD
--PAELKM
--PAELIT
--PAELAF
--PAELYS
--PAELYI
--PAELWR
--PAELVY
--PAELTH
--PAELRA
--PAELMS
--PAELKS
--PAELHV
--PAELHS
PHPAEL--
--PAELYQ
--PAELWS
--PAELTY
--PAELTL
--PAELSS
--PAELSF
--PAELSA
--PAELQH
--PAELNS
--PAELLQ
--PAELKY
--PAELKI
--PAELIG
--PAELHW
--PAELHR
--PAELHG
--PAELHA
--PAELGW
--PAELFV

PYPAEL--
PAPAEL--
--PAELWT
--PAELWN
--PAELWA
--PAELVW
--PAELTR
--PAELSM
--PAELRS
--PAELRQ
--PAELRP
--PAELRD
--PAELLT
--PAELLA
--PAELKH
--PAELKA
--PAELIN
--PAELIH
--PAELIA
--PAELHT
--PAELHE
--PAELFW
--PAELFQ
PWPAEL--
PMPAEL--
PKPAEL--
PIPAEL--
--PAELYY
--PAELYT
--PAELYN
--PAELWM
--PAELWI
--PAELWG
--PAELVS
--PAELVN
--PAELTT
--PAELTI
--PAELTG
--PAELTE
--PAELQT
--PAELQS
--PAELQR
--PAELQQ
--PAELQN
--PAELQK
--PAELQG
--PAELNY
--PAELNM
--PAELMN
--PAELME

--PAELKF
--PAELIY
--PAELIW
--PAELIQ
--PAELIK
--PAELHK
--PAELHI
--PAELHH
--PAELGT
--PAELGF
--PAELGA
--PAELFA
--PAELAW
--PAELAK
--PAELAI
PLPAEL--
PFPAEL--
--PAELYK
--PAELYH
--PAELYD
--PAELWH
--PAELVI
--PAELVG
--PAELTN
--PAELTA
--PAELSY
--PAELSQ
--PAELSN
--PAELSK
--PAELSE
--PAELRE
--PAELQY
--PAELQF
--PAELNW
--PAELNV
--PAELNR
--PAELNK
--PAELMI
--PAELMG
--PAELMA
--PAELKT
--PAELKG
--PAELIV
--PAELIS
--PAELHM
--PAELGG
--PAELFT
--PAELFK
--PAELFH
--PAELET

--PAELEG
--PAELCV
--PAELAV
--PAELAS
--PAELAR
--PAELAD
PSPAEL--
-PPAELK-
PGPAEL--
--PAELYA
--PAELWW
--PAELVQ
--PAELVE
--PAELTW
--PAELTM
--PAELTD
--PAELQV
--PAELQL
--PAELPA
--PAELNT
--PAELNN
--PAELNG
--PAELND
--PAELNC
--PAELMV
--PAELMQ
--PAELMD
--PAELLK
--PAELLE
--PAELKQ
--PAELKN
--PAELKD
--PAELII
--PAELIE
--PAELHY
--PAELHN
--PAELGY
--PAELGV
--PAELGR
--PAELGH
--PAELFN
--PAELFG
--PAELFF
--PAELFE
--PAELEV
--PAELES
--PAELDG
--PAELCN
--PAELCM
--PAELAT

--PAELAM
--PAELAE
--PAELAA
QTPAEL--
-QPAELS-
QRPAEL--
QSPAEL--
QLPAEL--
-QPAELL-
-QPAELI-
QWPAEL--
QYPAEL--
-QPAELV-
QQPAEL--
QPPAEL--
-QPAELF-
-QPAELE-
QKPAEL--
QIPAEL--
QEPAEL--
QDPAEL--
QCPAEL--
QVPAEL--
-QPAELW-
-QPAELN-
-QPAELA-
QNPAEL--
QMPAEL--
QHPAEL--
QGPAEL--
QFPAEL--
RMPAEL--
-RPAELR-
RTPAEL--
-RPAELW-
RGPAEL--
-RPAELS-
RPPAEL--
-RPAELN-
RLPAEL--
RIPAEL--
-RPAELL-
RVPAEL--
-RPAELY-
RNPAEL--
RWPAEL--
RKPAEL--
-RPAELT-
RYPAEL--
-RPAELI-

−RPAELK−
RCPAEL−−
−RPAELM−
−RPAELH−
RHPAEL−−
−RPAELG−
−RPAELF−
RDPAEL−−
RFPAEL−−
−RPAELQ−
RAPAEL−−
−RPAELA−
−RPAELC−
REPAEL−−
SSPAEL−−
−SPAELT−
−SPAELS−
SLPAEL−−
−SPAELY−
SVPAEL−−
SYPAEL−−
−SPAELR−
−SPAELQ−
−SPAELA−
−SPAELV−
SAPAEL−−
−SPAELL−
SGPAEL−−
SRPAEL−−
SIPAEL−−
−SPAELF−
SWPAEL−−
STPAEL−−
SQPAEL−−
−SPAELW−
−SPAELK−
−SPAELI−
SMPAEL−−
−SPAELN−
−SPAELM−
SKPAEL−−
−SPAELG−
SNPAEL−−
SPPAEL−−
−SPAELE−
SHPAEL−−
SFPAEL−−
SCPAEL−−
−TPAELH−
TSPAEL−−

TRPAEL--
-TPAELK-
TLPAEL--
TVPAEL--
-TPAELW-
-TPAELR-
TIPAEL--
-TPAELS-
-TPAELL-
TFPAEL--
TTPAEL--
-TPAELI-
TGPAEL--
TEPAEL--
TAPAEL--
TYPAEL--
TPPAEL--
-TPAELT-
-TPAELM-
-TPAELG-
-TPAELF-
-TPAELA-
TNPAEL--
TCPAEL--
-TPAELV-
TKPAEL--
TDPAEL--
TWPAEL--
TQPAEL--
-TPAELY-
-TPAELN-
-TPAELC-
THPAEL--
VSPAEL--
-VPAELR-
-VPAELM-
-VPAELL-
-VPAELH-
VIPAEL--
VGPAEL--
-VPAELS-
-VPAELI-
VMPAEL--
VKPAEL--
VFPAEL--
VYPAEL--
-VPAELY-
-VPAELK-
VTPAEL--
VRPAEL--

VDPAEL--
VVPAEL--
VQPAEL--
-VPAELV-
-VPAELN-
-VPAELA-
VHPAEL--
VPPAEL--
-VPAELT-
VNPAEL--
VLPAEL--
VWPAEL--
-VPAELQ-
-VPAELG-
VEPAEL--
-VPAELE-
VAPAEL--
-WPAELR-
-WPAELL-
WNPAEL--
WLPAEL--
WIPAEL--
WVPAEL--
WSPAEL--
-WPAELQ-
WEPAEL--
WAPAEL--
-WPAELW-
-WPAELK-
-WPAELA-
WMPAEL--
WYPAEL--
WRPAEL--
WPPAEL--
WGPAEL--
WDPAEL--
WCPAEL--
WQPAEL--
-WPAELT-
-WPAELI-
-WPAELH-
WKPAEL--
YVPAEL--
-YPAELL-
YLPAEL--
YAPAEL--
YSPAEL--
YRPAEL--
-YPAELK-
YYPAEL--

YQPAEL--
YPPAEL--
-YPAELW-
-YPAELT-
YIPAEL--
YHPAEL--
YGPAEL--
YEPAEL--
YWPAEL--
-YPAELR-
-YPAELF-
YNPAEL--
-YPAELQ-
-YPAELN-
-YPAELH-
-YPAELG-
-YPAELE-
YTPAEL--
-YPAELY-
-YPAELS-
-YPAELI-
-YPAELA-
YMPAEL--
YFPAEL--
YCPAEL--

| | | | |
|---|---|---|---|
| PAGW | 197 | 714 | 32.30 |

-LPAGWF-
-APAGWR-
AKPAGW--
AVPAGW--
ARPAGW--
ALPAGW--
ASPAGW--
-APAGWQ-
-APAGWL-
ANPAGW--
-APAGWN-
-APAGWI-
AGPAGW--
APPAGW--
-APAGWG-
AYPAGW--
ATPAGW--
-APAGWH-
-APAGWF-
AWPAGW--
-APAGWV-
-APAGWS-
AIPAGW--
AHPAGW--

AEPAGW--
-APAGWW-
-APAGWM-
-APAGWE-
AMPAGW--
AAPAGW--
-CPAGWM-
-CPAGWS-
CCPAGW--
-DPAGWR-
DVPAGW--
DQPAGW--
-DPAGWQ-
-DPAGWW-
-DPAGWL-
DMPAGW--
DSPAGW--
-DPAGWV-
-DPAGWT-
-DPAGWH-
DNPAGW--
DYPAGW--
DRPAGW--
-DPAGWS-
-DPAGWN-
-DPAGWI-
DIPAGW--
DFPAGW--
-EPAGWE-
ERPAGW--
ETPAGW--
-EPAGWV-
-EPAGWH-
-EPAGWY-
EKPAGW--
EYPAGW--
-EPAGWS-
-EPAGWN-
EFPAGW--
-EPAGWI-
-EPAGWA-
EGPAGW--
EDPAGW--
ESPAGW--
EQPAGW--
-EPAGWW-
-EPAGWT-
-EPAGWR-
-EPAGWK-
-EPAGWF-

ENPAGW--
EEPAGW--
EAPAGW--
-FPAGWR-
-FPAGWI-
FNPAGW--
FTPAGW--
-FPAGWT-
-FPAGWM-
-FPAGWF-
FMPAGW--
FLPAGW--
FEPAGW--
FRPAGW--
FVPAGW--
FSPAGW--
-FPAGWQ-
-FPAGWL-
-FPAGWK-
-FPAGWA-
FGPAGW--
-FPAGWY-
-FPAGWV-
-FPAGWN-
-FPAGWE-
FKPAGW--
FHPAGW--
FFPAGW--
FAPAGW--
-GPAGWV-
-GPAGWR-
GFPAGW--
GVPAGW--
-GPAGWG-
GHPAGW--
GTPAGW--
-GPAGWS-
-GPAGWQ-
GSPAGW--
-GPAGWH-
-GPAGWT-
-GPAGWA-
GLPAGW--
GMPAGW--
GYPAGW--
GWPAGW--
-GPAGWW-
-GPAGWF-
GIPAGW--
GPPAGW--

```
-GPAGWM-
-GPAGWK-
-GPAGWI-
GAPAGW--
GRPAGW--
GQPAGW--
-GPAGWE-
GKPAGW--
GEPAGW--
GCPAGW--
-HPAGWV-
-HPAGWI-
-HPAGWR-
HTPAGW--
-HPAGWK-
HLPAGW--
-HPAGWL-
HEPAGW--
HAPAGW--
HDPAGW--
HYPAGW--
-HPAGWT-
HNPAGW--
HKPAGW--
HSPAGW--
-HPAGWY-
-HPAGWQ-
-HPAGWG-
-HPAGWF-
HIPAGW--
HFPAGW--
HVPAGW--
HPPAGW--
-HPAGWH-
-HPAGWE-
-HPAGWA-
HMPAGW--
HCPAGW--
-IPAGWV-
-IPAGWT-
IAPAGW--
ILPAGW--
-IPAGWS-
-IPAGWG-
-IPAGWL-
-IPAGWK-
-IPAGWW-
-IPAGWH-
IMPAGW--
-IPAGWY-
```

```
-IPAGWF-
-IPAGWE-
-IPAGWA-
IIPAGW--
IVPAGW--
ITPAGW--
ISPAGW--
IRPAGW--
IQPAGW--
-IPAGWR-
-IPAGWN-
INPAGW--
IGPAGW--
IFPAGW--
IEPAGW--
KSPAGW--
-KPAGWL-
KNPAGW--
KEPAGW--
KVPAGW--
-KPAGWY-
-KPAGWR-
KTPAGW--
-KPAGWT-
-KPAGWF-
KLPAGW--
-KPAGWQ-
KAPAGW--
KYPAGW--
-KPAGWV-
KRPAGW--
KGPAGW--
KHPAGW--
-KPAGWN-
-KPAGWK-
-KPAGWE-
KMPAGW--
KKPAGW--
KFPAGW--
KDPAGW--
-KPAGWI-
-KPAGWH-
-LPAGWR-
LVPAGW--
LMPAGW--
LYPAGW--
-LPAGWY-
LRPAGW--
-LPAGWT-
LSPAGW--
```

LIPAGW--
-LPAGWW-
LWPAGW--
LPPAGW--
LHPAGW--
LGPAGW--
LDPAGW--
-LPAGWV-
-LPAGWS-
-LPAGWN-
-LPAGWM-
-LPAGWA-
LLPAGW--
LEPAGW--
LTPAGW--
-LPAGWK-
-LPAGWI-
-LPAGWH-
-LPAGWE-
LNPAGW--
LFPAGW--
LKPAGW--
-MPAGWF-
MVPAGW--
MRPAGW--
MTPAGW--
-MPAGWS-
MQPAGW--
-MPAGWN-
-MPAGWY-
-MPAGWL-
-MPAGWM-
-MPAGWH-
-MPAGWE-
MMPAGW--
MSPAGW--
-MPAGWR-
MHPAGW--
MFPAGW--
MDPAGW--
MWPAGW--
-MPAGWW-
-MPAGWT-
-MPAGWI-
MNPAGW--
MGPAGW--
MEPAGW--
MAPAGW--
-NPAGWY-
NRPAGW--

```
-NPAGWK-
-NPAGWW-
NYPAGW--
NLPAGW--
NFPAGW--
-NPAGWS-
-NPAGWA-
NAPAGW--
-NPAGWT-
-NPAGWQ-
NTPAGW--
-NPAGWL-
NHPAGW--
NVPAGW--
-NPAGWH-
-NPAGWF-
NIPAGW--
NSPAGW--
-NPAGWM-
-NPAGWI-
NMPAGW--
NEPAGW--
--PAGWMR
--PAGWRL
--PAGWRK
--PAGWRS
--PAGWTR
--PAGWWL
--PAGWSS
--PAGWLS
--PAGWWT
--PAGWSV
--PAGWML
--PAGWLR
--PAGWLL
--PAGWWN
--PAGWVD
--PAGWKR
PKPAGW--
--PAGWVS
--PAGWTL
--PAGWTF
--PAGWSK
--PAGWRY
--PAGWRV
--PAGWRG
--PAGWMS
--PAGWTT
--PAGWSR
--PAGWRN
```

--PAGWMG
--PAGWKL
--PAGWET
PGPAGW--
--PAGWVQ
--PAGWVK
--PAGWTS
--PAGWSG
--PAGWKA
--PAGWFV
--PAGWES
PFPAGW--
--PAGWYT
--PAGWVL
--PAGWVG
--PAGWNS
--PAGWKQ
--PAGWIN
--PAGWII
--PAGWSW
--PAGWRP
--PAGWLY
--PAGWKN
--PAGWKD
--PAGWIF
--PAGWEA
--PAGWAT
--PAGWAQ
--PAGWYG
--PAGWWK
--PAGWVR
--PAGWVF
--PAGWTW
--PAGWRR
--PAGWQS
--PAGWQI
--PAGWMD
--PAGWGS
--PAGWFT
PSPAGW--
--PAGWYL
--PAGWVV
--PAGWVA
--PAGWTH
--PAGWTA
--PAGWSQ
--PAGWRE
--PAGWNF
--PAGWIS
--PAGWIM

--PAGWGA
--PAGWFM
--PAGWFI
PWPAGW--
PRPAGW--
PNPAGW--
PLPAGW--
PEPAGW--
--PAGWYS
--PAGWWG
--PAGWVY
--PAGWVT
--PAGWTV
--PAGWST
--PAGWSL
--PAGWRW
--PAGWRQ
--PAGWQT
--PAGWMW
--PAGWMI
--PAGWME
--PAGWLT
--PAGWLK
--PAGWKS
--PAGWKI
--PAGWKG
--PAGWIT
--PAGWIH
--PAGWHS
--PAGWHR
--PAGWHQ
--PAGWHI
--PAGWFA
--PAGWAV
--PAGWAS
PAPAGW--
--PAGWWW
--PAGWWS
--PAGWWA
--PAGWVN
--PAGWTN
--PAGWTM
--PAGWSM
--PAGWRI
--PAGWRF
--PAGWRA
--PAGWNR
--PAGWNK
--PAGWLI
--PAGWLH

--PAGWLA
--PAGWKT
--PAGWIV
--PAGWIR
--PAGWHL
--PAGWGV
--PAGWFS
--PAGWFH
--PAGWFE
--PAGWAL
--PAGWAK
--PAGWAG
--PAGWYV
--PAGWYR
--PAGWYK
--PAGWWI
--PAGWVI
--PAGWSY
--PAGWSN
--PAGWSA
--PAGWQV
--PAGWQR
--PAGWQN
--PAGWQF
--PAGWQA
--PAGWNY
--PAGWNN
--PAGWNL
--PAGWNH
--PAGWMQ
--PAGWLN
--PAGWLG
--PAGWKW
--PAGWKE
--PAGWIG
--PAGWHY
--PAGWHV
--PAGWHF
--PAGWGN
--PAGWAR
--PAGWAN
--PAGWAI
--PAGWAA
PVPAGW--
-PPAGWT-
-PPAGWS-
PDPAGW--
--PAGWYM
--PAGWYI
--PAGWYA

--PAGWWQ
--PAGWWF
--PAGWVW
--PAGWTQ
--PAGWSP
--PAGWSF
--PAGWSD
--PAGWRM
--PAGWRH
--PAGWRC
--PAGWQY
--PAGWQK
--PAGWNM
--PAGWNA
--PAGWMT
--PAGWMN
--PAGWLQ
--PAGWLM
--PAGWLE
--PAGWKV
--PAGWKH
--PAGWIY
--PAGWIW
--PAGWIK
--PAGWHN
--PAGWHE
--PAGWGT
--PAGWGL
--PAGWFL
--PAGWFG
--PAGWEV
--PAGWER
--PAGWEF
--PAGWDT
--PAGWDS
--PAGWDN
--PAGWCY
--PAGWCT
--PAGWAY
--PAGWAW
--PAGWAH
--PAGWAF
-QPAGWG-
-QPAGWS-
-QPAGWR-
-QPAGWI-
QYPAGW--
QLPAGW--
QWPAGW--
QAPAGW--

QPPAGW--
-QPAGWH-
-QPAGWY-
-QPAGWL-
QEPAGW--
QDPAGW--
QVPAGW--
QSPAGW--
-QPAGWV-
-QPAGWQ-
-QPAGWN-
-QPAGWE-
QFPAGW--
QRPAGW--
QQPAGW--
-QPAGWT-
-QPAGWK-
-QPAGWA-
QKPAGW--
QHPAGW--
QGPAGW--
-RPAGWS-
-RPAGWF-
-RPAGWA-
RNPAGW--
-RPAGWK-
-RPAGWL-
-RPAGWE-
RVPAGW--
-RPAGWG-
-RPAGWV-
RYPAGW--
RMPAGW--
RGPAGW--
RAPAGW--
RLPAGW--
-RPAGWY-
-RPAGWI-
-RPAGWT-
RSPAGW--
RRPAGW--
RWPAGW--
RQPAGW--
RTPAGW--
-RPAGWW-
RHPAGW--
-RPAGWM-
RPPAGW--
-RPAGWN-
-RPAGWH-

-RPAGWC-
RDPAGW--
-RPAGWQ-
RCPAGW--
-SPAGWR-
STPAGW--
-SPAGWI-
-SPAGWY-
-SPAGWL-
-SPAGWH-
SRPAGW--
-SPAGWK-
SLPAGW--
SFPAGW--
SVPAGW--
SGPAGW--
SPPAGW--
-SPAGWT-
-SPAGWN-
SAPAGW--
SYPAGW--
-SPAGWG-
SQPAGW--
-SPAGWF-
-SPAGWQ-
SHPAGW--
SDPAGW--
-SPAGWV-
SNPAGW--
SMPAGW--
SWPAGW--
-SPAGWE-
-TPAGWR-
-TPAGWI-
TRPAGW--
TNPAGW--
-TPAGWQ-
-TPAGWA-
TSPAGW--
TKPAGW--
-TPAGWN-
-TPAGWL-
-TPAGWK-
-TPAGWT-
-TPAGWW-
-TPAGWF-
-TPAGWE-
-TPAGWM-
TTPAGW--
-TPAGWH-

TFPAGW--
TQPAGW--
-TPAGWV-
TLPAGW--
TAPAGW--
TYPAGW--
TVPAGW--
-TPAGWG-
TMPAGW--
TIPAGW--
THPAGW--
TGPAGW--
TEPAGW--
VTPAGW--
-VPAGWS-
VMPAGW--
VKPAGW--
-VPAGWA-
-VPAGWN-
-VPAGWT-
VRPAGW--
-VPAGWR-
-VPAGWL-
-VPAGWF-
VNPAGW--
VQPAGW--
-VPAGWG-
VEPAGW--
VPPAGW--
-VPAGWE-
VHPAGW--
VGPAGW--
VFPAGW--
-VPAGWV-
-VPAGWK-
-VPAGWH-
VLPAGW--
VIPAGW--
VCPAGW--
VYPAGW--
VVPAGW--
-VPAGWY-
-VPAGWQ-
VDPAGW--
VAPAGW--
-WPAGWR-
-WPAGWL-
-WPAGWY-
-WPAGWH-
WYPAGW--

| | | | |
|---|---|---|---|
| | -WPAGWI- | | |
| | WHPAGW-- | | |
| | WQPAGW-- | | |
| | -WPAGWT- | | |
| | WWPAGW-- | | |
| | -WPAGWV- | | |
| | WPPAGW-- | | |
| | -WPAGWM- | | |
| | -WPAGWK- | | |
| | WMPAGW-- | | |
| | WFPAGW-- | | |
| | WAPAGW-- | | |
| | -WPAGWS- | | |
| | -WPAGWG- | | |
| | WIPAGW-- | | |
| | WEPAGW-- | | |
| | WDPAGW-- | | |
| | YNPAGW-- | | |
| | YRPAGW-- | | |
| | -YPAGWT- | | |
| | -YPAGWF- | | |
| | YSPAGW-- | | |
| | YTPAGW-- | | |
| | -YPAGWI- | | |
| | -YPAGWR- | | |
| | YMPAGW-- | | |
| | YYPAGW-- | | |
| | -YPAGWN- | | |
| | -YPAGWH- | | |
| | YFPAGW-- | | |
| | -YPAGWS- | | |
| | -YPAGWA- | | |
| | YLPAGW-- | | |
| | YWPAGW-- | | |
| | -YPAGWY- | | |
| | -YPAGWV- | | |
| | -YPAGWK- | | |
| | -YPAGWG- | | |
| | YKPAGW-- | | |
| | YAPAGW-- | | |
| PSSW | 48 | 806 | 32.29 |
| | EAPSSW-- | | |
| | -FPSSWH- | | |
| | -GPSSWA- | | |
| | -HPSSWI- | | |
| | MTPSSW-- | | |
| | -MPSSWP- | | |
| | -NPSSWY- | | |
| | NIPSSW-- | | |
| | --PSSWLL | | |

--PSSWRP
--PSSWPL
--PSSWNL
--PSSWGR
--PSSWGL
--PSSWGF
--PSSWDT
-SPSSWG-
-SPSSWE-
-TPSSWG-
-APSSWS-
ALPSSW--
ARPSSW--
ASPSSW--
-APSSWK-
-APSSWR-
-APSSWM-
-APSSWG-
ADPSSW--
AGPSSW--
-APSSWH-
AYPSSW--
-APSSWW-
-APSSWT-
AIPSSW--
ANPSSW--
AAPSSW--
AVPSSW--
-APSSWV-
-APSSWA-
AQPSSW--
ATPSSW--
-APSSWQ-
-APSSWY-
-APSSWF-
AKPSSW--
AFPSSW--
AEPSSW--
-APSSWN-
APPSSW--
AMPSSW--
-APSSWI-
-APSSWE-
AHPSSW--
ACPSSW--
CYPSSW--
CWPSSW--
CVPSSW--
CRPSSW--
-CPSSWV-

-CPSSWI-
CPPSSW--
CMPSSW--
CEPSSW--
CTPSSW--
CSPSSW--
-CPSSWR-
CQPSSW--
-CPSSWS-
-CPSSWL-
CNPSSW--
CKPSSW--
CFPSSW--
-DPSSWL-
DSPSSW--
DVPSSW--
DRPSSW--
-DPSSWK-
DAPSSW--
-DPSSWV-
DGPSSW--
DWPSSW--
-DPSSWY-
-DPSSWT-
-DPSSWS-
DYPSSW--
DLPSSW--
DEPSSW--
DTPSSW--
-DPSSWW-
-DPSSWR-
-DPSSWH-
DIPSSW--
-DPSSWM-
-DPSSWI-
DNPSSW--
DHPSSW--
DFPSSW--
DDPSSW--
-EPSSWL-
-EPSSWS-
-EPSSWR-
ETPSSW--
-EPSSWM-
ELPSSW--
-EPSSWK-
-EPSSWI-
EYPSSW--
-EPSSWQ-
-EPSSWF-

EMPSSW--
EHPSSW--
EVPSSW--
EQPSSW--
-EPSSWW-
EKPSSW--
EWPSSW--
-EPSSWY-
EGPSSW--
-EPSSWV-
-EPSSWN-
EFPSSW--
ECPSSW--
-FPSSWL-
-FPSSWV-
-FPSSWR-
-FPSSWI-
FKPSSW--
FLPSSW--
-FPSSWM-
FYPSSW--
FFPSSW--
FIPSSW--
FDPSSW--
-FPSSWS-
-FPSSWT-
FTPSSW--
FSPSSW--
-FPSSWQ-
FAPSSW--
-FPSSWY-
FVPSSW--
-FPSSWA-
FNPSSW--
-FPSSWW-
-FPSSWN-
FEPSSW--
FQPSSW--
-FPSSWE-
FGPSSW--
GTPSSW--
-GPSSWV-
-GPSSWH-
GRPSSW--
GVPSSW--
-GPSSWR-
GGPSSW--
GKPSSW--
-GPSSWT-
GYPSSW--

GWPSSW--
-GPSSWI-
-GPSSWF-
GQPSSW--
-GPSSWW-
GAPSSW--
-GPSSWQ-
GFPSSW--
GEPSSW--
-GPSSWM-
GDPSSW--
GPPSSW--
GHPSSW--
-GPSSWN-
-GPSSWE-
GNPSSW--
-GPSSWG-
GCPSSW--
HTPSSW--
HRPSSW--
-HPSSWT-
HLPSSW--
-HPSSWR-
-HPSSWW-
-HPSSWF-
HGPSSW--
HVPSSW--
HSPSSW--
-HPSSWS-
HQPSSW--
HYPSSW--
-HPSSWA-
HNPSSW--
HAPSSW--
HPPSSW--
HFPSSW--
HDPSSW--
HWPSSW--
HIPSSW--
-HPSSWY-
HMPSSW--
HHPSSW--
-HPSSWQ-
-HPSSWN-
-HPSSWM-
-HPSSWL-
-HPSSWG-
HKPSSW--
HEPSSW--
-IPSSWL-

IRPSSW--
-IPSSWR-
IEPSSW--
-IPSSWY-
-IPSSWS-
-IPSSWH-
-IPSSWA-
IIPSSW--
IMPSSW--
-IPSSWQ-
ILPSSW--
ISPSSW--
-IPSSWT-
-IPSSWM-
ITPSSW--
-IPSSWI-
IGPSSW--
-IPSSWN-
-IPSSWK-
-IPSSWW-
INPSSW--
-IPSSWV-
IKPSSW--
IFPSSW--
IDPSSW--
IAPSSW--
IYPSSW--
IWPSSW--
-IPSSWE-
IHPSSW--
IQPSSW--
-IPSSWF-
IPPSSW--
ICPSSW--
KSPSSW--
-KPSSWL-
KMPSSW--
-KPSSWR-
KKPSSW--
-KPSSWS-
KVPSSW--
-KPSSWA-
KFPSSW--
KGPSSW--
-KPSSWQ-
KYPSSW--
-KPSSWN-
KLPSSW--
KNPSSW--
-KPSSWV-

```
-KPSSWK-
-KPSSWI-
KEPSSW--
-KPSSWY-
KWPSSW--
-KPSSWH-
-KPSSWG-
-KPSSWT-
-KPSSWE-
KIPSSW--
-KPSSWM-
KDPSSW--
-KPSSWW-
KCPSSW--
LKPSSW--
-LPSSWQ-
-LPSSWM-
LNPSSW--
-LPSSWK-
LLPSSW--
-LPSSWV-
LIPSSW--
LTPSSW--
LHPSSW--
LGPSSW--
-LPSSWT-
-LPSSWI-
-LPSSWS-
LPPSSW--
LDPSSW--
LAPSSW--
LYPSSW--
-LPSSWY-
LEPSSW--
LMPSSW--
LCPSSW--
LQPSSW--
LFPSSW--
LWPSSW--
-LPSSWN-
-LPSSWH-
MAPSSW--
-MPSSWT-
-MPSSWR-
-MPSSWH-
-MPSSWA-
MLPSSW--
MVPSSW--
MQPSSW--
-MPSSWV-
```

-MPSSWM-
MDPSSW--
MGPSSW--
MSPSSW--
-MPSSWY-
-MPSSWI-
-MPSSWF-
MNPSSW--
MMPSSW--
-MPSSWS-
-MPSSWK-
MKPSSW--
MFPSSW--
MHPSSW--
MIPSSW--
MEPSSW--
MCPSSW--
NSPSSW--
NRPSSW--
NTPSSW--
-NPSSWR-
NQPSSW--
-NPSSWM-
-NPSSWL-
-NPSSWT-
-NPSSWS-
-NPSSWK-
NYPSSW--
NAPSSW--
-NPSSWV-
NMPSSW--
NLPSSW--
NGPSSW--
NWPSSW--
-NPSSWQ-
-NPSSWI-
-NPSSWF-
NPPSSW--
NEPSSW--
-NPSSWW-
NKPSSW--
NDPSSW--
-NPSSWH-
-NPSSWA-
--PSSWST
--PSSWRS
--PSSWLR
--PSSWRT
--PSSWLT
--PSSWSG

--PSSWLS
--PSSWIR
--PSSWVT
--PSSWRI
--PSSWRH
--PSSWLG
--PSSWAV
--PSSWRD
--PSSWFG
--PSSWVS
--PSSWTA
--PSSWSS
--PSSWEA
--PSSWMS
--PSSWFQ
--PSSWMR
--PSSWVY
--PSSWVK
--PSSWVF
--PSSWTS
--PSSWRV
--PSSWHS
--PSSWVR
--PSSWRY
--PSSWRK
--PSSWVA
--PSSWFT
PIPSSW--
--PSSWYK
--PSSWVV
--PSSWVG
--PSSWIH
PSPSSW--
--PSSWYI
--PSSWYG
--PSSWWT
--PSSWWR
--PSSWTT
--PSSWTR
--PSSWTG
--PSSWRM
--PSSWMV
--PSSWLK
--PSSWLH
--PSSWKV
--PSSWKK
--PSSWKG
--PSSWFA
PFPSSW--
--PSSWSR

--PSSWRE
--PSSWQF
--PSSWKS
--PSSWTY
--PSSWSV
--PSSWRQ
--PSSWMN
--PSSWLN
--PSSWIY
--PSSWAT
PRPSSW--
--PSSWWS
--PSSWSF
--PSSWRN
--PSSWNG
PTPSSW--
--PSSWWK
--PSSWSY
--PSSWQG
--PSSWNT
--PSSWMW
--PSSWMQ
--PSSWMM
--PSSWGS
--PSSWYL
--PSSWWG
--PSSWTK
--PSSWTH
--PSSWSN
--PSSWQT
--PSSWKA
--PSSWIV
--PSSWIN
--PSSWFV
--PSSWFS
PLPSSW--
PWPSSW--
--PSSWYS
--PSSWYA
--PSSWNS
--PSSWKW
--PSSWKL
--PSSWHT
--PSSWYH
--PSSWWN
--PSSWWH
--PSSWVW
--PSSWVN
--PSSWQY
--PSSWML

--PSSWMG
--PSSWKY
--PSSWIW
--PSSWIA
--PSSWAA
--PSSWWM
--PSSWTL
--PSSWSI
--PSSWRW
--PSSWQR
--PSSWQI
--PSSWMK
--PSSWMA
--PSSWKH
--PSSWIT
--PSSWEV
--PSSWAG
--PSSWAF
PGPSSW--
--PSSWYQ
--PSSWYN
--PSSWVI
--PSSWVE
--PSSWTV
--PSSWSW
--PSSWSL
--PSSWQS
--PSSWNA
--PSSWLI
--PSSWLD
--PSSWLA
--PSSWKM
--PSSWKI
--PSSWKF
--PSSWIG
--PSSWHW
--PSSWHR
--PSSWHN
--PSSWHM
--PSSWHF
--PSSWFE
--PSSWES
PVPSSW--
--PSSWTW
--PSSWTN
--PSSWTI
--PSSWTF
--PSSWSM
--PSSWSH
--PSSWQA

--PSSWMY
--PSSWMT
--PSSWMH
--PSSWKN
--PSSWKD
--PSSWIQ
--PSSWID
--PSSWHY
--PSSWHG
--PSSWFK
--PSSWAN
--PSSWAM
PQPSSW--
PMPSSW--
PKPSSW--
PEPSSW--
--PSSWYW
--PSSWWY
--PSSWWV
--PSSWWF
--PSSWTQ
--PSSWSK
--PSSWQH
--PSSWLQ
--PSSWKT
--PSSWIL
--PSSWHV
--PSSWEY
--PSSWAY
--PSSWAH
PHPSSW--
PYPSSW--
--PSSWWD
--PSSWQN
--PSSWQL
--PSSWPP
--PSSWMI
--PSSWME
--PSSWMD
--PSSWIE
--PSSWHI
--PSSWHH
--PSSWHA
--PSSWEW
--PSSWET
--PSSWEG
--PSSWAW
--PSSWAI
-PPSSWS-
--PSSWYM

--PSSWYE
--PSSWVQ
--PSSWTE
--PSSWTD
--PSSWSD
--PSSWQQ
--PSSWQM
--PSSWQK
--PSSWQD
--PSSWNW
--PSSWNV
--PSSWNH
--PSSWMF
--PSSWLE
--PSSWKE
--PSSWHQ
--PSSWHK
--PSSWHD
--PSSWFP
--PSSWFM
--PSSWER
--PSSWCT
-PPSSWR-
-PPSSWL-
-PPSSWI-
PNPSSW--
PDPSSW--
PCPSSW--
PAPSSW--
-QPSSWI-
QVPSSW--
-QPSSWS-
QAPSSW--
-QPSSWR-
QSPSSW--
-QPSSWM-
-QPSSWL-
-QPSSWQ-
-QPSSWN-
-QPSSWK-
QHPSSW--
QLPSSW--
QKPSSW--
QGPSSW--
-QPSSWV-
-QPSSWF-
QWPSSW--
-QPSSWT-
-QPSSWH-
QNPSSW--

QFPSSW--
QDPSSW--
QTPSSW--
QQPSSW--
-QPSSWY-
QMPSSW--
QYPSSW--
-QPSSWA-
QIPSSW--
-QPSSWE-
QPPSSW--
QEPSSW--
QCPSSW--
-RPSSWL-
-RPSSWR-
-RPSSWS-
RVPSSW--
-RPSSWK-
RIPSSW--
RLPSSW--
-RPSSWY-
-RPSSWA-
-RPSSWT-
-RPSSWI-
-RPSSWH-
RNPSSW--
RTPSSW--
-RPSSWN-
RFPSSW--
RQPSSW--
RAPSSW--
RKPSSW--
RMPSSW--
RDPSSW--
-RPSSWM-
-RPSSWQ-
RPPSSW--
RYPSSW--
-RPSSWG-
-RPSSWE-
RHPSSW--
-RPSSWV-
REPSSW--
-RPSSWW-
-RPSSWC-
RCPSSW--
SSPSSW--
-SPSSWL-
SRPSSW--
-SPSSWR-

SEPSSW--
SLPSSW--
SWPSSW--
STPSSW--
SFPSSW--
SAPSSW--
SHPSSW--
-SPSSWT-
-SPSSWS-
SYPSSW--
SGPSSW--
SVPSSW--
-SPSSWV-
SNPSSW--
-SPSSWI-
SIPSSW--
-SPSSWQ-
-SPSSWM-
-SPSSWK-
SMPSSW--
SPPSSW--
SKPSSW--
-SPSSWN-
SQPSSW--
-SPSSWW-
SDPSSW--
SCPSSW--
-TPSSWL-
TKPSSW--
TRPSSW--
TGPSSW--
TTPSSW--
-TPSSWR-
TEPSSW--
-TPSSWS-
-TPSSWM-
-TPSSWY-
-TPSSWT-
-TPSSWK-
-TPSSWI-
TLPSSW--
TYPSSW--
TSPSSW--
TDPSSW--
TAPSSW--
-TPSSWW-
-TPSSWV-
TFPSSW--
TQPSSW--
-TPSSWQ-

-TPSSWH-
-TPSSWF-
TPPSSW--
TIPSSW--
-TPSSWN-
TMPSSW--
TWPSSW--
-TPSSWE-
VRPSSW--
-VPSSWL-
-VPSSWT-
-VPSSWA-
VSPSSW--
VKPSSW--
-VPSSWI-
-VPSSWF-
VLPSSW--
-VPSSWS-
-VPSSWM-
VGPSSW--
-VPSSWQ-
VTPSSW--
-VPSSWN-
-VPSSWG-
VNPSSW--
VHPSSW--
VDPSSW--
VQPSSW--
-VPSSWV-
-VPSSWK-
VIPSSW--
VYPSSW--
VWPSSW--
-VPSSWY-
VPPSSW--
VMPSSW--
VFPSSW--
VAPSSW--
-VPSSWW-
-VPSSWH-
VEPSSW--
VCPSSW--
-VPSSWE-
-WPSSWL-
-WPSSWS-
-WPSSWH-
WNPSSW--
WTPSSW--
-WPSSWT-
-WPSSWK-

| | | | |
|---|---|---|---|
| | -WPSSWV- | | |
| | -WPSSWQ- | | |
| | WGPSSW-- | | |
| | WPPSSW-- | | |
| | WEPSSW-- | | |
| | WLPSSW-- | | |
| | WYPSSW-- | | |
| | WQPSSW-- | | |
| | -WPSSWY- | | |
| | -WPSSWI- | | |
| | WHPSSW-- | | |
| | -WPSSWA- | | |
| | -WPSSWW- | | |
| | -WPSSWR- | | |
| | -WPSSWN- | | |
| | YRPSSW-- | | |
| | -YPSSWK- | | |
| | -YPSSWT- | | |
| | YIPSSW-- | | |
| | YGPSSW-- | | |
| | -YPSSWS- | | |
| | YEPSSW-- | | |
| | YAPSSW-- | | |
| | -YPSSWR- | | |
| | YNPSSW-- | | |
| | YVPSSW-- | | |
| | YSPSSW-- | | |
| | YTPSSW-- | | |
| | -YPSSWL- | | |
| | -YPSSWH- | | |
| | -YPSSWQ- | | |
| | YLPSSW-- | | |
| | YDPSSW-- | | |
| | YQPSSW-- | | |
| | -YPSSWY- | | |
| | -YPSSWV- | | |
| | -YPSSWW- | | |
| | -YPSSWI- | | |
| | YYPSSW-- | | |
| | YHPSSW-- | | |
| | -YPSSWN- | | |
| | -YPSSWM- | | |
| | YMPSSW-- | | |
| | -YPSSWF- | | |
| | -YPSSWE- | | |
| | -YPSSWD- | | |
| | YKPSSW-- | | |
| PVGW | 169 | 730 | 32.27 |
| | -APVGWS- | | |
| | -APVGWK- | | |

-APVGWT-
-APVGWL-
-APVGWV-
-APVGWH-
-APVGWA-
-APVGWI-
AAPVGW--
ASPVGW--
-APVGWR-
AFPVGW--
ATPVGW--
-APVGWF-
AVPVGW--
-APVGWY-
-APVGWE-
AWPVGW--
AQPVGW--
-APVGWW-
ADPVGW--
-APVGWN-
AMPVGW--
ALPVGW--
AYPVGW--
-APVGWQ-
-APVGWG-
AKPVGW--
AGPVGW--
AEPVGW--
-CPVGWL-
CAPVGW--
-CPVGWT-
CRPVGW--
-CPVGWV-
-CPVGWA-
CCPVGW--
CWPVGW--
CVPVGW--
CQPVGW--
-CPVGWF-
CNPVGW--
CIPVGW--
DEPVGW--
-DPVGWT-
-DPVGWL-
-DPVGWS-
-DPVGWR-
-DPVGWQ-
DYPVGW--
-DPVGWK-
-DPVGWH-

-DPVGWA-
DLPVGW--
DHPVGW--
DTPVGW--
DSPVGW--
DRPVGW--
DWPVGW--
DQPVGW--
-DPVGWW-
-DPVGWV-
-DPVGWN-
-DPVGWI-
-DPVGWF-
DNPVGW--
DFPVGW--
DDPVGW--
ELPVGW--
-EPVGWS-
EYPVGW--
EWPVGW--
ESPVGW--
-EPVGWW-
-EPVGWR-
-EPVGWN-
-EPVGWI-
EKPVGW--
EAPVGW--
EQPVGW--
ERPVGW--
-EPVGWT-
-EPVGWL-
-EPVGWK-
-EPVGWF-
EMPVGW--
EVPVGW--
ETPVGW--
-EPVGWV-
-EPVGWD-
-EPVGWA-
EHPVGW--
-FPVGWV-
FYPVGW--
-FPVGWL-
-FPVGWS-
-FPVGWR-
-FPVGWN-
FSPVGW--
FTPVGW--
FRPVGW--
FQPVGW--

-FPVGWH-
FEPVGW--
FVPVGW--
-FPVGWM-
-FPVGWT-
-FPVGWK-
FAPVGW--
FNPVGW--
FKPVGW--
FHPVGW--
FFPVGW--
FDPVGW--
FCPVGW--
GLPVGW--
GRPVGW--
-GPVGWS-
GTPVGW--
-GPVGWV-
GGPVGW--
GSPVGW--
-GPVGWK-
-GPVGWI-
GYPVGW--
GQPVGW--
GIPVGW--
GHPVGW--
-GPVGWY-
GAPVGW--
-GPVGWR-
-GPVGWQ-
-GPVGWN-
-GPVGWL-
GPPVGW--
GVPVGW--
-GPVGWT-
-GPVGWH-
-GPVGWF-
-GPVGWA-
GWPVGW--
-GPVGWW-
-GPVGWM-
GNPVGW--
GKPVGW--
GMPVGW--
GFPVGW--
GDPVGW--
HTPVGW--
HRPVGW--
-HPVGWV-
-HPVGWI-

HDPVGW--
-HPVGWM-
HSPVGW--
HLPVGW--
-HPVGWK-
HHPVGW--
-HPVGWS-
-HPVGWL-
HNPVGW--
HFPVGW--
HYPVGW--
HWPVGW--
-HPVGWW-
-HPVGWT-
-HPVGWR-
-HPVGWH-
HPPVGW--
HEPVGW--
HAPVGW--
HVPVGW--
-HPVGWN-
-HPVGWG-
-HPVGWA-
HMPVGW--
-IPVGWL-
-IPVGWK-
ISPVGW--
IRPVGW--
-IPVGWY-
-IPVGWE-
-IPVGWS-
-IPVGWF-
IVPVGW--
-IPVGWQ-
IMPVGW--
ILPVGW--
ITPVGW--
-IPVGWI-
-IPVGWH-
-IPVGWR-
IFPVGW--
IDPVGW--
IAPVGW--
-IPVGWW-
-IPVGWN-
-IPVGWG-
INPVGW--
IKPVGW--
-KPVGWL-
KYPVGW--

KNPVGW--
KLPVGW--
KTPVGW--
-KPVGWS-
-KPVGWR-
-KPVGWN-
KFPVGW--
-KPVGWQ-
KVPVGW--
-KPVGWV-
-KPVGWT-
KPPVGW--
KWPVGW--
-KPVGWY-
-KPVGWW-
-KPVGWI-
KAPVGW--
-KPVGWH-
KEPVGW--
KRPVGW--
-KPVGWM-
-KPVGWK-
-KPVGWA-
KIPVGW--
KHPVGW--
KDPVGW--
LFPVGW--
LSPVGW--
-LPVGWR-
LTPVGW--
-LPVGWQ-
LRPVGW--
LLPVGW--
LAPVGW--
-LPVGWT-
-LPVGWS-
-LPVGWF-
LQPVGW--
-LPVGWY-
-LPVGWW-
-LPVGWN-
-LPVGWA-
LIPVGW--
LGPVGW--
LVPVGW--
-LPVGWM-
-LPVGWK-
LMPVGW--
LWPVGW--
LEPVGW--

-LPVGWI-
-LPVGWH-
LPPVGW--
LNPVGW--
LKPVGW--
LDPVGW--
-LPVGWD-
MGPVGW--
-MPVGWH-
-MPVGWR-
MRPVGW--
MPPVGW--
MHPVGW--
MFPVGW--
-MPVGWV-
MYPVGW--
MSPVGW--
MQPVGW--
-MPVGWM-
-MPVGWL-
MNPVGW--
MKPVGW--
MVPVGW--
-MPVGWY-
MDPVGW--
MTPVGW--
-MPVGWW-
-MPVGWS-
-MPVGWK-
-MPVGWF-
-MPVGWE-
-MPVGWA-
MIPVGW--
-NPVGWT-
NPPVGW--
NNPVGW--
NSPVGW--
NRPVGW--
-NPVGWA-
NGPVGW--
-NPVGWV-
NAPVGW--
-NPVGWY-
-NPVGWS-
NLPVGW--
NVPVGW--
NTPVGW--
NMPVGW--
-NPVGWW-
-NPVGWR-

−NPVGWN−
−NPVGWK−
−NPVGWH−
−NPVGWF−
NWPVGW−−
NHPVGW−−
NYPVGW−−
NIPVGW−−
NFPVGW−−
NDPVGW−−
−−PVGWFR
−−PVGWMR
PGPVGW−−
−−PVGWWR
−−PVGWTR
−−PVGWRT
−−PVGWLS
−−PVGWSR
−−PVGWRG
−−PVGWAS
−−PVGWMG
−−PVGWSS
−−PVGWIR
−−PVGWQH
−−PVGWFN
−−PVGWTQ
−−PVGWRK
−−PVGWLL
−−PVGWLA
−−PVGWVA
−−PVGWSV
−−PVGWLT
PRPVGW−−
−−PVGWWT
−−PVGWSI
−−PVGWRV
−−PVGWML
−−PVGWLV
−−PVGWGS
−−PVGWVI
−−PVGWST
−−PVGWSF
−−PVGWRN
−−PVGWHN
−−PVGWHM
−−PVGWAR
−−PVGWAF
−−PVGWTT
−−PVGWKV
PSPVGW−−

--PVGWYG
--PVGWVS
--PVGWTV
--PVGWTD
--PVGWRE
--PVGWQM
--PVGWLN
--PVGWHS
--PVGWHQ
--PVGWWS
--PVGWTS
--PVGWQS
--PVGWNT
--PVGWNS
--PVGWMY
--PVGWLQ
--PVGWLH
--PVGWIT
--PVGWHL
--PVGWHK
--PVGWAV
--PVGWAT
PLPVGW--
PVPVGW--
--PVGWYN
--PVGWWA
--PVGWTK
--PVGWSW
--PVGWSN
--PVGWRY
--PVGWII
--PVGWID
--PVGWHT
--PVGWFK
--PVGWES
--PVGWWE
--PVGWVK
--PVGWTY
--PVGWRQ
--PVGWQV
--PVGWMN
--PVGWMF
--PVGWMD
--PVGWLK
--PVGWKS
--PVGWHI
--PVGWFG
--PVGWAL
--PVGWAA
--PVGWYV

--PVGWWG
--PVGWVN
--PVGWVM
--PVGWVL
--PVGWVG
--PVGWTI
--PVGWTA
--PVGWSM
--PVGWRM
--PVGWRF
--PVGWRA
--PVGWNY
--PVGWNR
--PVGWNG
--PVGWMW
--PVGWMK
--PVGWLW
--PVGWKK
--PVGWIY
--PVGWIM
--PVGWHA
--PVGWGV
--PVGWAI
PWPVGW--
--PVGWYT
--PVGWYS
--PVGWYQ
--PVGWYK
--PVGWWH
--PVGWVW
--PVGWSK
--PVGWSG
--PVGWQY
--PVGWQK
--PVGWNH
--PVGWMH
--PVGWME
--PVGWLI
--PVGWLD
--PVGWKT
--PVGWKN
--PVGWKH
--PVGWKG
--PVGWIN
--PVGWHY
--PVGWHV
--PVGWGL
--PVGWFE
--PVGWFA
--PVGWAW

PKPVGW--
PIPVGW--
PDPVGW--
--PVGWYL
--PVGWYF
--PVGWYE
--PVGWWN
--PVGWVY
--PVGWVD
--PVGWTN
--PVGWTH
--PVGWTE
--PVGWSA
--PVGWQT
--PVGWNW
--PVGWNV
--PVGWNM
--PVGWNK
--PVGWNF
--PVGWMA
--PVGWLE
--PVGWKY
--PVGWKM
--PVGWKF
--PVGWIW
--PVGWFW
--PVGWFM
--PVGWFL
--PVGWFD
--PVGWAN
--PVGWAK
--PVGWAH
PFPVGW--
--PVGWYM
--PVGWYH
--PVGWYA
--PVGWWK
--PVGWWF
--PVGWVV
--PVGWVQ
--PVGWVH
--PVGWVE
--PVGWTF
--PVGWSY
--PVGWSE
--PVGWRP
--PVGWRI
--PVGWRD
--PVGWQQ
--PVGWQG

--PVGWNQ
--PVGWNC
--PVGWMV
--PVGWMI
--PVGWLM
--PVGWKW
--PVGWKQ
--PVGWKD
--PVGWKA
--PVGWIV
--PVGWIS
--PVGWIE
--PVGWIA
--PVGWHW
--PVGWHD
--PVGWGM
--PVGWFH
--PVGWEY
--PVGWEW
--PVGWEV
--PVGWEI
--PVGWEG
--PVGWAE
PTPVGW--
PHPVGW--
PEPVGW--
PAPVGW--
-QPVGWS-
QGPVGW--
-QPVGWW-
-QPVGWT-
-QPVGWR-
-QPVGWL-
QLPVGW--
QSPVGW--
QHPVGW--
QDPVGW--
QVPVGW--
-QPVGWI-
QTPVGW--
-QPVGWA-
QIPVGW--
-QPVGWM-
-QPVGWG-
QPPVGW--
QNPVGW--
QMPVGW--
QQPVGW--
-QPVGWY-
-QPVGWN-

-QPVGWH-
-QPVGWF-
-QPVGWE-
QEPVGW--
QAPVGW--
-RPVGWL-
RSPVGW--
RAPVGW--
RVPVGW--
-RPVGWW-
-RPVGWH-
-RPVGWF-
-RPVGWY-
-RPVGWV-
RMPVGW--
-RPVGWM-
-RPVGWQ-
-RPVGWN-
-RPVGWI-
RPPVGW--
RLPVGW--
RYPVGW--
RDPVGW--
-RPVGWK-
RHPVGW--
RQPVGW--
-RPVGWG-
-RPVGWE-
RFPVGW--
RWPVGW--
RKPVGW--
REPVGW--
-RPVGWD-
-RPVGWC-
RIPVGW--
-SPVGWS-
-SPVGWW-
STPVGW--
-SPVGWN-
-SPVGWY-
SQPVGW--
-SPVGWM-
-SPVGWT-
-SPVGWL-
SNPVGW--
SMPVGW--
-SPVGWH-
-SPVGWE-
-SPVGWV-
SKPVGW--

```
-SPVGWA-
SAPVGW--
SWPVGW--
-SPVGWR-
-SPVGWK-
SIPVGW--
SHPVGW--
SFPVGW--
SCPVGW--
-SPVGWQ-
SLPVGW--
SVPVGW--
SEPVGW--
SYPVGW--
-SPVGWF-
SGPVGW--
SPPVGW--
SDPVGW--
-TPVGWL-
TLPVGW--
TYPVGW--
-TPVGWR-
TRPVGW--
TFPVGW--
TTPVGW--
TSPVGW--
-TPVGWS-
TGPVGW--
TQPVGW--
TMPVGW--
-TPVGWA-
TNPVGW--
TVPVGW--
-TPVGWT-
-TPVGWN-
-TPVGWM-
-TPVGWK-
-TPVGWY-
-TPVGWI-
-TPVGWH-
-TPVGWE-
TPPVGW--
TKPVGW--
TAPVGW--
-TPVGWW-
-TPVGWQ-
-TPVGWF-
THPVGW--
TEPVGW--
TDPVGW--
```

```
-VPVGWL-
-VPVGWV-
VFPVGW--
-VPVGWS-
VQPVGW--
-VPVGWY-
-VPVGWK-
VLPVGW--
VGPVGW--
VAPVGW--
-VPVGWT-
-VPVGWQ-
-VPVGWA-
VIPVGW--
VDPVGW--
-VPVGWM-
VVPVGW--
VTPVGW--
VEPVGW--
VSPVGW--
-VPVGWN-
-VPVGWG-
VNPVGW--
VKPVGW--
VYPVGW--
-VPVGWH-
VWPVGW--
-VPVGWW-
VHPVGW--
WYPVGW--
WGPVGW--
WSPVGW--
-WPVGWM-
-WPVGWH-
-WPVGWI-
WEPVGW--
WWPVGW--
-WPVGWL-
-WPVGWA-
WNPVGW--
WRPVGW--
-WPVGWR-
-WPVGWQ-
WLPVGW--
WKPVGW--
WDPVGW--
-WPVGWN-
-WPVGWK-
-WPVGWF-
WMPVGW--
```

WCPVGW--
WAPVGW--
-YPVGWI-
-YPVGWS-
YSPVGW--
-YPVGWA-
YGPVGW--
-YPVGWV-
-YPVGWK-
YNPVGW--
-YPVGWL-
-YPVGWY-
YKPVGW--
-YPVGWQ-
-YPVGWF-
YFPVGW--
YRPVGW--
YQPVGW--
-YPVGWT-
YMPVGW--
YIPVGW--
YWPVGW--
-YPVGWR-
-YPVGWM-
YPPVGW--
YHPVGW--
YEPVGW--
YAPVGW--

PAGF 89 776 32.27

-APAGFI-
--PAGFLL
--PAGFIL
--PAGFGK
-APAGFF-
ALPAGF--
AVPAGF--
-APAGFR-
ASPAGF--
-APAGFV-
-APAGFM-
AYPAGF--
-APAGFW-
-APAGFT-
-APAGFL-
-APAGFK-
AMPAGF--
AKPAGF--
-APAGFY-
AGPAGF--
-APAGFG-

-APAGFE-
ATPAGF--
AQPAGF--
APPAGF--
-APAGFQ-
-APAGFN-
-APAGFA-
-APAGFP-
-APAGFH-
ANPAGF--
AFPAGF--
AAPAGF--
CRPAGF--
CNPAGF--
-CPAGFV-
-CPAGFQ-
-CPAGFL-
CYPAGF--
-CPAGFN-
CIPAGF--
CWPAGF--
CTPAGF--
-CPAGFW-
-CPAGFI-
-CPAGFH-
-CPAGFA-
CMPAGF--
CLPAGF--
CKPAGF--
CHPAGF--
CDPAGF--
-DPAGFY-
-DPAGFR-
DLPAGF--
-DPAGFL-
DVPAGF--
-DPAGFS-
DKPAGF--
DAPAGF--
DRPAGF--
-DPAGFV-
-DPAGFT-
-DPAGFF-
DTPAGF--
DSPAGF--
-DPAGFN-
-DPAGFI-
-DPAGFA-
DFPAGF--
DYPAGF--

DWPAGF--
-DPAGFK-
-DPAGFH-
DHPAGF--
DGPAGF--
ETPAGF--
-EPAGFY-
-EPAGFH-
EVPAGF--
-EPAGFR-
EGPAGF--
EWPAGF--
ESPAGF--
-EPAGFL-
-EPAGFI-
ELPAGF--
-EPAGFN-
-EPAGFM-
-EPAGFK-
EIPAGF--
EHPAGF--
ERPAGF--
-EPAGFW-
-EPAGFV-
-EPAGFT-
-EPAGFS-
-EPAGFG-
EMPAGF--
EAPAGF--
FSPAGF--
-FPAGFN-
FYPAGF--
-FPAGFY-
-FPAGFS-
-FPAGFR-
FFPAGF--
FDPAGF--
FGPAGF--
FEPAGF--
-FPAGFL-
-FPAGFW-
FRPAGF--
FMPAGF--
FQPAGF--
-FPAGFT-
FHPAGF--
-FPAGFI-
-FPAGFF-
-FPAGFA-
FIPAGF--

GLPAGF--
GQPAGF--
-GPAGFK-
-GPAGFS-
GKPAGF--
GWPAGF--
GRPAGF--
GGPAGF--
GEPAGF--
GTPAGF--
GPPAGF--
GMPAGF--
GFPAGF--
GVPAGF--
-GPAGFA-
GDPAGF--
GAPAGF--
-GPAGFF-
GIPAGF--
GSPAGF--
-GPAGFY-
-GPAGFT-
-GPAGFN-
-GPAGFH-
-GPAGFQ-
-GPAGFL-
-GPAGFI-
GNPAGF--
-HPAGFQ-
-HPAGFL-
-HPAGFR-
HNPAGF--
HFPAGF--
-HPAGFS-
HSPAGF--
-HPAGFT-
HVPAGF--
HWPAGF--
-HPAGFW-
-HPAGFN-
-HPAGFI-
-HPAGFH-
HMPAGF--
HKPAGF--
HAPAGF--
HTPAGF--
HPPAGF--
-HPAGFA-
HYPAGF--
HQPAGF--

```
-HPAGFY-
-HPAGFV-
-HPAGFK-
-HPAGFF-
HHPAGF--
HGPAGF--
HDPAGF--
-IPAGFV-
IRPAGF--
IYPAGF--
ISPAGF--
IVPAGF--
ILPAGF--
-IPAGFI-
IDPAGF--
-IPAGFS-
-IPAGFK-
-IPAGFA-
ITPAGF--
-IPAGFQ-
-IPAGFN-
-IPAGFM-
-IPAGFT-
-IPAGFR-
IKPAGF--
IQPAGF--
-IPAGFY-
-IPAGFW-
-IPAGFF-
IFPAGF--
ICPAGF--
IPPAGF--
INPAGF--
IMPAGF--
IHPAGF--
IGPAGF--
IEPAGF--
IAPAGF--
KSPAGF--
-KPAGFM-
-KPAGFA-
KAPAGF--
-KPAGFL-
KVPAGF--
KRPAGF--
-KPAGFV-
KGPAGF--
-KPAGFT-
-KPAGFF-
KLPAGF--
```

KPPAGF--
-KPAGFS-
-KPAGFN-
-KPAGFK-
-KPAGFE-
KEPAGF--
-KPAGFR-
-KPAGFI-
-KPAGFG-
KHPAGF--
KTPAGF--
-KPAGFQ-
-KPAGFH-
KFPAGF--
KYPAGF--
KWPAGF--
KQPAGF--
-KPAGFW-
KIPAGF--
KDPAGF--
LSPAGF--
LRPAGF--
LMPAGF--
LNPAGF--
-LPAGFS-
LTPAGF--
-LPAGFY-
-LPAGFM-
LYPAGF--
LPPAGF--
-LPAGFN-
LFPAGF--
LKPAGF--
LQPAGF--
-LPAGFW-
-LPAGFV-
-LPAGFR-
-LPAGFI-
LLPAGF--
LGPAGF--
LAPAGF--
-LPAGFT-
-LPAGFQ-
-LPAGFF-
LWPAGF--
-LPAGFA-
LIPAGF--
-LPAGFH-
-LPAGFP-
-LPAGFG-

LEPAGF--
LDPAGF--
-MPAGFL-
MLPAGF--
MRPAGF--
-MPAGFV-
MGPAGF--
MWPAGF--
MSPAGF--
MTPAGF--
MNPAGF--
-MPAGFT-
-MPAGFR-
-MPAGFI-
MIPAGF--
MFPAGF--
MDPAGF--
-MPAGFW-
-MPAGFS-
-MPAGFN-
-MPAGFM-
-MPAGFK-
-MPAGFH-
-MPAGFG-
-MPAGFA-
MCPAGF--
MAPAGF--
MYPAGF--
MVPAGF--
MPPAGF--
-MPAGFY-
-MPAGFQ-
-MPAGFF-
NVPAGF--
-NPAGFV-
-NPAGFL-
-NPAGFR-
-NPAGFS-
NLPAGF--
-NPAGFY-
NKPAGF--
NEPAGF--
NDPAGF--
NWPAGF--
-NPAGFM-
-NPAGFA-
NTPAGF--
-NPAGFT-
-NPAGFQ-
-NPAGFK-

NGPAGF--
NFPAGF--
NPPAGF--
NMPAGF--
NYPAGF--
NSPAGF--
NAPAGF--
NRPAGF--
-NPAGFW-
-NPAGFI-
NIPAGF--
--PAGFFT
--PAGFRL
--PAGFTK
--PAGFRS
--PAGFRN
--PAGFLS
--PAGFLR
--PAGFNA
--PAGFKG
--PAGFRW
--PAGFLA
--PAGFRV
--PAGFSS
--PAGFSA
--PAGFRQ
--PAGFYR
--PAGFTT
--PAGFRI
--PAGFVS
--PAGFVM
--PAGFSM
--PAGFSG
--PAGFRY
--PAGFRM
--PAGFMW
--PAGFMV
--PAGFKY
--PAGFKS
PTPAGF--
PGPAGF--
--PAGFSR
--PAGFSK
--PAGFRR
--PAGFGA
--PAGFFA
--PAGFVI
--PAGFSD
--PAGFNS
--PAGFMS

--PAGFLW
--PAGFLT
--PAGFLN
--PAGFLH
--PAGFIM
--PAGFAW
PYPAGF--
PWPAGF--
--PAGFWG
--PAGFTR
--PAGFTL
--PAGFSI
--PAGFNT
--PAGFMN
--PAGFKV
--PAGFKR
--PAGFKA
--PAGFAV
--PAGFYL
--PAGFYD
--PAGFST
--PAGFSL
--PAGFSF
--PAGFRT
--PAGFIV
--PAGFIA
--PAGFHK
--PAGFFL
--PAGFYM
--PAGFWS
--PAGFWR
--PAGFWM
--PAGFSV
--PAGFSH
--PAGFRG
--PAGFQT
--PAGFMG
--PAGFLI
--PAGFHG
--PAGFGG
--PAGFFH
--PAGFYV
--PAGFWH
--PAGFVR
--PAGFVF
--PAGFVA
--PAGFTS
--PAGFSQ
--PAGFSN
--PAGFRF

--PAGFRA
--PAGFQV
--PAGFNY
--PAGFNM
--PAGFNF
--PAGFMK
--PAGFMH
--PAGFLV
--PAGFKL
--PAGFIY
--PAGFIS
--PAGFHV
--PAGFHL
--PAGFFS
--PAGFES
PVPAGF--
--PAGFYK
--PAGFVQ
--PAGFVN
--PAGFTW
--PAGFTQ
--PAGFTN
--PAGFSW
--PAGFSE
--PAGFQW
--PAGFQR
--PAGFQK
--PAGFNH
--PAGFMR
--PAGFHY
PRPAGF--
PMPAGF--
PLPAGF--
PEPAGF--
--PAGFYN
--PAGFYH
--PAGFWT
--PAGFWK
--PAGFVK
--PAGFTV
--PAGFTM
--PAGFTG
--PAGFRH
--PAGFQY
--PAGFNW
--PAGFMY
--PAGFMT
--PAGFLK
--PAGFKW
--PAGFKK

--PAGFKH
--PAGFKF
--PAGFIK
--PAGFII
--PAGFHT
--PAGFHS
--PAGFHR
--PAGFHN
--PAGFHM
--PAGFHH
--PAGFHF
--PAGFFQ
--PAGFFG
--PAGFAS
PSPAGF--
--PAGFYG
--PAGFWQ
--PAGFWE
--PAGFVT
--PAGFTY
--PAGFTF
--PAGFTA
--PAGFSY
--PAGFRK
--PAGFRD
--PAGFQN
--PAGFQL
--PAGFQI
--PAGFQG
--PAGFNK
--PAGFNI
--PAGFMQ
--PAGFML
--PAGFMI
--PAGFME
--PAGFLG
--PAGFKM
--PAGFIW
--PAGFIN
--PAGFIH
--PAGFHD
--PAGFFN
--PAGFEY
--PAGFEG
--PAGFAT
--PAGFAI
--PAGFAG
PPPAGF--
PKPAGF--
PHPAGF--

--PAGFYA
--PAGFWN
--PAGFWA
--PAGFVW
--PAGFVG
--PAGFVE
--PAGFVD
--PAGFTI
--PAGFTD
--PAGFQS
--PAGFQF
--PAGFNN
--PAGFNG
--PAGFMM
--PAGFMD
--PAGFMA
--PAGFLQ
--PAGFLD
--PAGFKD
--PAGFHW
--PAGFHQ
--PAGFHI
--PAGFHA
--PAGFFP
--PAGFEW
--PAGFET
--PAGFEP
--PAGFEA
--PAGFCW
--PAGFCS
--PAGFCG
--PAGFAY
--PAGFAR
--PAGFAL
--PAGFAF
--PAGFAD
--PAGFAA
-QPAGFS-
-QPAGFN-
-QPAGFR-
-QPAGFI-
QSPAGF--
-QPAGFM-
QQPAGF--
-QPAGFL-
-QPAGFF-
-QPAGFV-
-QPAGFK-
-QPAGFC-
QMPAGF--

QPPAGF--
-QPAGFY-
QLPAGF--
QGPAGF--
QTPAGF--
QNPAGF--
QYPAGF--
QWPAGF--
QRPAGF--
-QPAGFT-
QHPAGF--
QDPAGF--
QAPAGF--
-RPAGFL-
RVPAGF--
-RPAGFT-
-RPAGFS-
-RPAGFR-
RRPAGF--
-RPAGFK-
RGPAGF--
RYPAGF--
-RPAGFW-
-RPAGFA-
-RPAGFY-
RTPAGF--
RSPAGF--
-RPAGFV-
RFPAGF--
RWPAGF--
RQPAGF--
RLPAGF--
RPPAGF--
-RPAGFI-
-RPAGFH-
RNPAGF--
-RPAGFQ-
-RPAGFG-
REPAGF--
RHPAGF--
RDPAGF--
-RPAGFE-
RIPAGF--
-RPAGFC-
RAPAGF--
SKPAGF--
SWPAGF--
-SPAGFL-
-SPAGFS-
SVPAGF--

-SPAGFR-
-SPAGFH-
SSPAGF--
SAPAGF--
SLPAGF--
SMPAGF--
SRPAGF--
-SPAGFK-
-SPAGFA-
-SPAGFI-
-SPAGFF-
SGPAGF--
-SPAGFN-
SHPAGF--
STPAGF--
-SPAGFY-
SNPAGF--
SQPAGF--
-SPAGFW-
-SPAGFV-
-SPAGFM-
SFPAGF--
SYPAGF--
-SPAGFG-
SPPAGF--
-SPAGFE-
SEPAGF--
SDPAGF--
SIPAGF--
SCPAGF--
-TPAGFS-
-TPAGFW-
-TPAGFT-
TSPAGF--
-TPAGFQ-
-TPAGFY-
-TPAGFF-
TLPAGF--
-TPAGFR-
TMPAGF--
TGPAGF--
-TPAGFV-
TVPAGF--
-TPAGFA-
TIPAGF--
TTPAGF--
TQPAGF--
-TPAGFM-
TKPAGF--
TEPAGF--

TWPAGF--
TRPAGF--
-TPAGFN-
TFPAGF--
TAPAGF--
TPPAGF--
-TPAGFI-
-TPAGFG-
TNPAGF--
THPAGF--
TDPAGF--
TCPAGF--
VSPAGF--
-VPAGFR-
VPPAGF--
VRPAGF--
-VPAGFL-
VEPAGF--
VAPAGF--
VVPAGF--
VTPAGF--
-VPAGFM-
-VPAGFE-
-VPAGFA-
-VPAGFY-
-VPAGFW-
-VPAGFV-
VNPAGF--
VHPAGF--
VYPAGF--
-VPAGFQ-
-VPAGFK-
-VPAGFI-
VWPAGF--
-VPAGFN-
VMPAGF--
VLPAGF--
VGPAGF--
VFPAGF--
VQPAGF--
-VPAGFT-
-VPAGFH-
-VPAGFF-
-VPAGFG-
-VPAGFC-
VCPAGF--
WQPAGF--
-WPAGFS-
WMPAGF--
WVPAGF--

WSPAGF--
WRPAGF--
-WPAGFL-
-WPAGFR-
WFPAGF--
WEPAGF--
WYPAGF--
WGPAGF--
WTPAGF--
-WPAGFI-
WLPAGF--
-WPAGFV-
-WPAGFH-
-WPAGFF-
WIPAGF--
-WPAGFT-
-WPAGFQ-
-WPAGFK-
WKPAGF--
WDPAGF--
WAPAGF--
-YPAGFN-
YRPAGF--
-YPAGFS-
-YPAGFR-
YTPAGF--
-YPAGFL-
YLPAGF--
YQPAGF--
YEPAGF--
-YPAGFY-
-YPAGFV-
-YPAGFK-
YFPAGF--
YWPAGF--
YVPAGF--
-YPAGFA-
YMPAGF--
YDPAGF--
-YPAGFT-
YNPAGF--
YAPAGF--
YSPAGF--
YPPAGF--
-YPAGFW-
-YPAGFE-
YKPAGF--
YIPAGF--
YGPAGF--
YYPAGF--

| | | | |
|---|---|---|---|
| | -YPAGFM- | | |
| | -YPAGFI- | | |
| | -YPAGFG- | | |
| | -YPAGFF- | | |
| | YHPAGF-- | | |
| PAPY | 203 | 710 | 32.26 |
| | FEPAPY-- | | |
| | KVPAPY-- | | |
| | --PAPYSG | | |
| | ALPAPY-- | | |
| | -APAPYL- | | |
| | ASPAPY-- | | |
| | -APAPYH- | | |
| | -APAPYT- | | |
| | AGPAPY-- | | |
| | -APAPYK- | | |
| | ADPAPY-- | | |
| | -APAPYS- | | |
| | -APAPYM- | | |
| | -APAPYR- | | |
| | AMPAPY-- | | |
| | -APAPYN- | | |
| | AIPAPY-- | | |
| | AAPAPY-- | | |
| | ARPAPY-- | | |
| | APPAPY-- | | |
| | -APAPYY- | | |
| | -APAPYV- | | |
| | -APAPYA- | | |
| | AFPAPY-- | | |
| | AEPAPY-- | | |
| | AWPAPY-- | | |
| | AVPAPY-- | | |
| | -APAPYI- | | |
| | -APAPYG- | | |
| | -APAPYF- | | |
| | ANPAPY-- | | |
| | CRPAPY-- | | |
| | CTPAPY-- | | |
| | -CPAPYR- | | |
| | -CPAPYW- | | |
| | -CPAPYS- | | |
| | -CPAPYF- | | |
| | CGPAPY-- | | |
| | CQPAPY-- | | |
| | -CPAPYI- | | |
| | CKPAPY-- | | |
| | CHPAPY-- | | |
| | CFPAPY-- | | |
| | -DPAPYH- | | |

DIPAPY--
-DPAPYI-
DHPAPY--
-DPAPYW-
-DPAPYM-
DWPAPY--
-DPAPYN-
-DPAPYL-
-DPAPYF-
DYPAPY--
DVPAPY--
DTPAPY--
DSPAPY--
DRPAPY--
-DPAPYY-
-DPAPYT-
-DPAPYR-
-DPAPYC-
-DPAPYA-
DLPAPY--
DFPAPY--
EFPAPY--
EWPAPY--
EKPAPY--
-EPAPYT-
-EPAPYS-
-EPAPYL-
-EPAPYF-
ESPAPY--
-EPAPYV-
-EPAPYI-
ENPAPY--
EMPAPY--
ELPAPY--
EIPAPY--
EAPAPY--
-EPAPYY-
-EPAPYN-
EEPAPY--
EDPAPY--
-FPAPYT-
FRPAPY--
FNPAPY--
-FPAPYY-
-FPAPYL-
FQPAPY--
FKPAPY--
FGPAPY--
FFPAPY--
FAPAPY--

-FPAPYW-
-FPAPYS-
-FPAPYR-
-FPAPYH-
FLPAPY--
FDPAPY--
FYPAPY--
FWPAPY--
-FPAPYV-
FCPAPY--
GVPAPY--
-GPAPYL-
GYPAPY--
GSPAPY--
-GPAPYM-
GQPAPY--
GMPAPY--
GFPAPY--
-GPAPYS-
-GPAPYQ-
-GPAPYI-
-GPAPYH-
GKPAPY--
-GPAPYT-
-GPAPYR-
-GPAPYF-
GDPAPY--
GTPAPY--
GRPAPY--
-GPAPYY-
-GPAPYV-
-GPAPYG-
-GPAPYA-
GLPAPY--
GGPAPY--
GEPAPY--
GAPAPY--
HSPAPY--
-HPAPYL-
HHPAPY--
HTPAPY--
-HPAPYY-
-HPAPYS-
-HPAPYT-
HIPAPY--
HEPAPY--
HDPAPY--
HAPAPY--
HYPAPY--
HWPAPY--

HVPAPY--
HRPAPY--
-HPAPYW-
-HPAPYV-
-HPAPYK-
-HPAPYF-
HGPAPY--
-HPAPYQ-
-HPAPYM-
-HPAPYI-
-HPAPYA-
HLPAPY--
IRPAPY--
IQPAPY--
-IPAPYF-
-IPAPYA-
-IPAPYE-
ILPAPY--
IGPAPY--
ITPAPY--
IMPAPY--
IHPAPY--
IDPAPY--
-IPAPYQ-
-IPAPYN-
-IPAPYL-
-IPAPYI-
IFPAPY--
IAPAPY--
IWPAPY--
-IPAPYY-
-IPAPYT-
-IPAPYM-
-IPAPYK-
-IPAPYH-
IIPAPY--
IEPAPY--
ICPAPY--
KSPAPY--
KGPAPY--
-KPAPYS-
KYPAPY--
-KPAPYI-
-KPAPYW-
KRPAPY--
KKPAPY--
-KPAPYV-
-KPAPYT-
-KPAPYR-
-KPAPYN-

-KPAPYK-
-KPAPYA-
KIPAPY--
KEPAPY--
KWPAPY--
KTPAPY--
KPPAPY--
-KPAPYE-
KMPAPY--
KDPAPY--
KQPAPY--
-KPAPYY-
-KPAPYQ-
-KPAPYM-
KLPAPY--
KFPAPY--
LSPAPY--
LRPAPY--
LAPAPY--
LTPAPY--
-LPAPYL-
LKPAPY--
-LPAPYV-
LLPAPY--
LGPAPY--
LMPAPY--
-LPAPYK-
-LPAPYM-
LIPAPY--
-LPAPYN-
LHPAPY--
LDPAPY--
-LPAPYW-
-LPAPYT-
-LPAPYS-
-LPAPYQ-
LYPAPY--
LVPAPY--
LQPAPY--
LPPAPY--
-LPAPYI-
-LPAPYF-
-LPAPYA-
LFPAPY--
LEPAPY--
-MPAPYR-
MNPAPY--
MRPAPY--
MVPAPY--
MLPAPY--

MSPAPY--
MGPAPY--
MAPAPY--
-MPAPYS-
-MPAPYQ-
-MPAPYA-
MYPAPY--
MWPAPY--
MQPAPY--
MPPAPY--
-MPAPYF-
-MPAPYW-
-MPAPYV-
-MPAPYN-
-MPAPYL-
-MPAPYK-
-MPAPYI-
MMPAPY--
-NPAPYF-
-NPAPYI-
NRPAPY--
-NPAPYT-
NAPAPY--
NYPAPY--
NSPAPY--
-NPAPYS-
-NPAPYN-
NVPAPY--
-NPAPYM-
-NPAPYK-
NNPAPY--
NLPAPY--
NHPAPY--
NDPAPY--
NPPAPY--
NKPAPY--
NTPAPY--
-NPAPYQ-
-NPAPYL-
-NPAPYA-
NFPAPY--
NEPAPY--
--PAPYSL
--PAPYRT
--PAPYLR
--PAPYWL
--PAPYVR
--PAPYRA
--PAPYLV
--PAPYFS

--PAPYRS
--PAPYMR
--PAPYRY
PRPAPY--
--PAPYTI
--PAPYTF
--PAPYSR
--PAPYPY
--PAPYLS
--PAPYFI
--PAPYTR
--PAPYRL
--PAPYRI
--PAPYMV
--PAPYLM
--PAPYLA
--PAPYWY
--PAPYTV
--PAPYQA
--PAPYML
--PAPYLL
--PAPYKL
--PAPYGF
--PAPYYK
--PAPYWS
--PAPYVL
--PAPYTL
--PAPYLT
--PAPYLH
--PAPYLF
--PAPYGI
--PAPYGG
--PAPYAW
--PAPYYR
--PAPYVA
--PAPYTT
--PAPYST
--PAPYSM
--PAPYHV
--PAPYGS
--PAPYFH
PGPAPY--
--PAPYWV
--PAPYVT
--PAPYSS
--PAPYQN
--PAPYMT
--PAPYLK
--PAPYLG
--PAPYKH

--PAPYKG
--PAPYET
--PAPYAV
--PAPYAS
--PAPYAI
PSPAPY--
PQPAPY--
--PAPYYY
--PAPYYV
--PAPYYN
--PAPYYL
--PAPYYI
--PAPYYG
--PAPYYF
--PAPYYA
--PAPYWQ
--PAPYVI
--PAPYVH
--PAPYSV
--PAPYSN
--PAPYMN
--PAPYMF
--PAPYLQ
--PAPYLE
--PAPYKV
--PAPYKT
--PAPYIS
--PAPYHT
--PAPYFY
--PAPYFV
--PAPYFG
--PAPYDF
--PAPYAT
--PAPYAH
PYPAPY--
PTPAPY--
PLPAPY--
--PAPYYS
--PAPYWR
--PAPYWN
--PAPYVG
--PAPYVF
--PAPYTS
--PAPYTQ
--PAPYSF
--PAPYSA
--PAPYRF
--PAPYQS
--PAPYQR
--PAPYPW

--PAPYNS
--PAPYMY
--PAPYMI
--PAPYLN
--PAPYLI
--PAPYKM
--PAPYIA
--PAPYHI
--PAPYGT
--PAPYFM
--PAPYFL
--PAPYFK
--PAPYEI
--PAPYAY
PVPAPY--
PNPAPY--
PKPAPY--
--PAPYWG
--PAPYWF
--PAPYWA
--PAPYVN
--PAPYVK
--PAPYTW
--PAPYSY
--PAPYSI
--PAPYSH
--PAPYSE
--PAPYRW
--PAPYRQ
--PAPYRN
--PAPYRE
--PAPYRD
--PAPYQV
--PAPYQK
--PAPYQE
--PAPYPM
--PAPYPF
--PAPYNY
--PAPYNN
--PAPYMQ
--PAPYMK
--PAPYMD
--PAPYLD
--PAPYKN
--PAPYKK
--PAPYIR
--PAPYIH
--PAPYHW
--PAPYHA
--PAPYGQ

--PAPYGM
--PAPYFR
--PAPYFQ
--PAPYFN
--PAPYFE
--PAPYFA
--PAPYEY
--PAPYES
--PAPYAF
PAPAPY--
PPPAPY--
PMPAPY--
--PAPYYT
--PAPYYQ
--PAPYYM
--PAPYYE
--PAPYYD
--PAPYVW
--PAPYVV
--PAPYVS
--PAPYVQ
--PAPYVM
--PAPYVE
--PAPYVD
--PAPYTY
--PAPYTN
--PAPYTH
--PAPYTD
--PAPYSQ
--PAPYSK
--PAPYSD
--PAPYRH
--PAPYQY
--PAPYQT
--PAPYQH
--PAPYNW
--PAPYNV
--PAPYNT
--PAPYNF
--PAPYMH
--PAPYMG
--PAPYME
--PAPYLY
--PAPYKW
--PAPYKS
--PAPYKA
--PAPYIT
--PAPYIQ
--PAPYIP
--PAPYIN

--PAPYIK
--PAPYIG
--PAPYHQ
--PAPYHN
--PAPYHK
--PAPYGY
--PAPYGW
--PAPYGH
--PAPYGA
--PAPYFW
--PAPYFT
--PAPYFD
--PAPYEW
--PAPYDY
--PAPYDL
--PAPYAR
--PAPYAN
--PAPYAE
-QPAPYL-
-QPAPYT-
-QPAPYS-
QSPAPY--
QRPAPY--
QPPAPY--
-QPAPYY-
-QPAPYV-
-QPAPYQ-
-QPAPYG-
QHPAPY--
-QPAPYI-
-QPAPYA-
QAPAPY--
-QPAPYW-
-QPAPYH-
-QPAPYE-
QNPAPY--
QFPAPY--
-RPAPYS-
-RPAPYL-
RMPAPY--
RSPAPY--
-RPAPYV-
RAPAPY--
RRPAPY--
-RPAPYG-
RIPAPY--
RQPAPY--
REPAPY--
-RPAPYT-
-RPAPYQ-

```
-RPAPYH-
RWPAPY--
-RPAPYI-
-RPAPYE-
RLPAPY--
RVPAPY--
-RPAPYW-
RTPAPY--
RGPAPY--
RYPAPY--
-RPAPYN-
RDPAPY--
-RPAPYM-
RNPAPY--
RPPAPY--
-RPAPYK-
RHPAPY--
-RPAPYY-
RFPAPY--
-SPAPYL-
SLPAPY--
STPAPY--
SMPAPY--
SAPAPY--
-SPAPYV-
-SPAPYT-
SEPAPY--
SVPAPY--
-SPAPYR-
SSPAPY--
-SPAPYY-
-SPAPYS-
-SPAPYN-
-SPAPYK-
-SPAPYI-
-SPAPYA-
SNPAPY--
SKPAPY--
SDPAPY--
SYPAPY--
SQPAPY--
-SPAPYW-
SFPAPY--
SWPAPY--
SRPAPY--
SGPAPY--
SPPAPY--
-SPAPYM-
-SPAPYC-
-SPAPYQ-
```

−SPAPYG−
SHPAPY−−
SCPAPY−−
−TPAPYT−
−TPAPYI−
−TPAPYV−
−TPAPYR−
−TPAPYF−
TVPAPY−−
−TPAPYM−
−TPAPYL−
TKPAPY−−
TRPAPY−−
−TPAPYW−
−TPAPYK−
TIPAPY−−
TFPAPY−−
TWPAPY−−
TNPAPY−−
TQPAPY−−
TPPAPY−−
−TPAPYQ−
−TPAPYH−
TLPAPY−−
TGPAPY−−
TAPAPY−−
TYPAPY−−
TTPAPY−−
−TPAPYA−
TMPAPY−−
VNPAPY−−
−VPAPYM−
VLPAPY−−
−VPAPYK−
VAPAPY−−
VSPAPY−−
VQPAPY−−
−VPAPYV−
VFPAPY−−
−VPAPYH−
−VPAPYF−
VTPAPY−−
−VPAPYY−
−VPAPYI−
VKPAPY−−
VVPAPY−−
−VPAPYS−
−VPAPYA−
VMPAPY−−
VGPAPY−−

VYPAPY--
-VPAPYN-
-VPAPYG-
VRPAPY--
-VPAPYT-
-VPAPYE-
VEPAPY--
VWPAPY--
VIPAPY--
VDPAPY--
WRPAPY--
WTPAPY--
WPPAPY--
WKPAPY--
WWPAPY--
-WPAPYR-
-WPAPYT-
-WPAPYK-
-WPAPYQ-
-WPAPYN-
-WPAPYH-
WQPAPY--
-WPAPYY-
-WPAPYS-
WNPAPY--
WIPAPY--
WFPAPY--
WYPAPY--
WSPAPY--
-WPAPYW-
-WPAPYM-
-WPAPYL-
-WPAPYI-
WLPAPY--
WDPAPY--
-YPAPYK-
YRPAPY--
-YPAPYR-
YLPAPY--
YKPAPY--
YPPAPY--
-YPAPYT-
-YPAPYM-
-YPAPYF-
-YPAPYS-
YNPAPY--
YFPAPY--
-YPAPYV-
YSPAPY--
YYPAPY--

```
            YVPAPY--
            YQPAPY--
            -YPAPYY-
            -YPAPYL-
            -YPAPYE-
            YIPAPY--
            YHPAPY--
            YEPAPY--
            YAPAPY--
            -YPAPYW-
            -YPAPYN-
            -YPAPYI-
            -YPAPYH-
            -YPAPYG-
            YMPAPY--
            YGPAPY--
PLSI          91        775     32.26
            GFPLSI--
            -HPLSIP-
            -HPLSIG-
            MEPLSI--
            MAPLSI--
            -NPLSIG-
            -NPLSID-
            --PLSITE
            --PLSISD
            --PLSINI
            --PLSIGV
            --PLSIAK
            -SPLSIG-
            VYPLSI--
            YPPLSI--
            -YPLSIG-
            ARPLSI--
            AQPLSI--
            -APLSIN-
            -APLSIL-
            AVPLSI--
            ATPLSI--
            -APLSIM-
            AMPLSI--
            -APLSIV-
            -APLSIK-
            -APLSIQ-
            -APLSIE-
            -APLSIA-
            AIPLSI--
            AFPLSI--
            -APLSIR-
            -APLSIG-
```

AAPLSI--
AWPLSI--
-APLSIT-
APPLSI--
-APLSIW-
AGPLSI--
AEPLSI--
-APLSIY-
-APLSIH-
-APLSII-
-APLSIF-
ANPLSI--
ADPLSI--
ACPLSI--
-APLSIC-
CAPLSI--
CQPLSI--
CEPLSI--
CTPLSI--
CSPLSI--
CIPLSI--
CCPLSI--
-CPLSIS-
CLPLSI--
CGPLSI--
CWPLSI--
-CPLSIV-
-CPLSIT-
-CPLSIR-
-CPLSIQ-
-CPLSIM-
CKPLSI--
CHPLSI--
CFPLSI--
DVPLSI--
DRPLSI--
-DPLSIR-
DIPLSI--
DWPLSI--
-DPLSIT-
DKPLSI--
DAPLSI--
DYPLSI--
-DPLSII-
-DPLSIM-
DMPLSI--
DLPLSI--
DFPLSI--
-DPLSIS-
-DPLSIL-

-DPLSIF-
DTPLSI--
-DPLSIQ-
DHPLSI--
DQPLSI--
-DPLSIV-
-DPLSIK-
-DPLSIH-
DEPLSI--
DDPLSI--
ERPLSI--
-EPLSIS-
EGPLSI--
-EPLSIY-
-EPLSIL-
EQPLSI--
EVPLSI--
-EPLSIK-
-EPLSII-
ENPLSI--
EAPLSI--
EKPLSI--
-EPLSIW-
-EPLSIR-
EFPLSI--
EWPLSI--
-EPLSIH-
EMPLSI--
ETPLSI--
-EPLSIV-
-EPLSIT-
ELPLSI--
EIPLSI--
-EPLSIM-
EYPLSI--
EPPLSI--
-EPLSIQ-
-EPLSIE-
-EPLSIA-
EDPLSI--
-FPLSIR-
FRPLSI--
-FPLSIT-
-FPLSIL-
FGPLSI--
FYPLSI--
FFPLSI--
FSPLSI--
FLPLSI--
FEPLSI--

FQPLSI--
-FPLSIY-
-FPLSIH-
FPPLSI--
-FPLSIQ-
-FPLSIK-
FIPLSI--
FHPLSI--
FDPLSI--
FVPLSI--
FTPLSI--
-FPLSIW-
FNPLSI--
FAPLSI--
-FPLSIN-
-FPLSIA-
FMPLSI--
-FPLSII-
-FPLSIG-
-FPLSIE-
-GPLSIS-
GGPLSI--
-GPLSIR-
GLPLSI--
GKPLSI--
GRPLSI--
GVPLSI--
-GPLSIK-
GNPLSI--
GIPLSI--
-GPLSIY-
-GPLSIT-
-GPLSII-
-GPLSIL-
-GPLSIW-
GTPLSI--
-GPLSIH-
GAPLSI--
GYPLSI--
-GPLSIQ-
-GPLSIA-
GEPLSI--
GMPLSI--
GDPLSI--
-GPLSIM-
-GPLSIN-
GHPLSI--
GWPLSI--
-GPLSIG-
-GPLSIE-

GPPLSI--
HLPLSI--
-HPLSIR-
-HPLSIL-
-HPLSIS-
HSPLSI--
HYPLSI--
HIPLSI--
HAPLSI--
-HPLSIA-
-HPLSIV-
-HPLSII-
HGPLSI--
HEPLSI--
HWPLSI--
HMPLSI--
HVPLSI--
HNPLSI--
HTPLSI--
-HPLSIY-
-HPLSIE-
-HPLSIM-
HFPLSI--
HPPLSI--
HDPLSI--
HQPLSI--
-HPLSIH-
HKPLSI--
HHPLSI--
IRPLSI--
IKPLSI--
-IPLSIR-
ITPLSI--
ISPLSI--
-IPLSIL-
IGPLSI--
IYPLSI--
ILPLSI--
IMPLSI--
IVPLSI--
-IPLSII-
INPLSI--
-IPLSIM-
-IPLSIK-
IIPLSI--
-IPLSIQ-
-IPLSIA-
-IPLSIT-
IEPLSI--
IAPLSI--

IPPLSI--
-IPLSIW-
IDPLSI--
-IPLSIV-
-IPLSIN-
IHPLSI--
IQPLSI--
ICPLSI--
KSPLSI--
KRPLSI--
KGPLSI--
-KPLSIL-
KVPLSI--
-KPLSIM-
-KPLSIT-
KYPLSI--
KIPLSI--
KAPLSI--
KNPLSI--
-KPLSIQ-
-KPLSIK-
-KPLSIF-
-KPLSII-
-KPLSIN-
KDPLSI--
-KPLSIY-
-KPLSIE-
-KPLSIA-
KMPLSI--
KWPLSI--
-KPLSIW-
-KPLSIS-
KFPLSI--
KEPLSI--
KQPLSI--
-KPLSIG-
KKPLSI--
KHPLSI--
KPPLSI--
LRPLSI--
LSPLSI--
LTPLSI--
-LPLSII-
-LPLSIR-
LWPLSI--
-LPLSIT-
LNPLSI--
LKPLSI--
LQPLSI--
LEPLSI--

LPPLSI--
LGPLSI--
-LPLSIY-
LMPLSI--
-LPLSIM-
LYPLSI--
-LPLSIQ-
LFPLSI--
-LPLSIV-
-LPLSIK-
-LPLSIA-
LDPLSI--
-LPLSIW-
-LPLSIH-
MRPLSI--
MSPLSI--
MGPLSI--
-MPLSIR-
-MPLSIL-
-MPLSIS-
MKPLSI--
MTPLSI--
MWPLSI--
-MPLSIV-
MNPLSI--
-MPLSII-
-MPLSIK-
MIPLSI--
MMPLSI--
MPPLSI--
-MPLSIT-
-MPLSIF-
-MPLSIA-
MQPLSI--
-MPLSIY-
-MPLSIW-
-MPLSIN-
-MPLSIM-
-MPLSIG-
MDPLSI--
MHPLSI--
-MPLSIQ-
-MPLSIH-
-MPLSIE-
NLPLSI--
-NPLSIR-
NSPLSI--
NAPLSI--
NKPLSI--
NVPLSI--

-NPLSII-
NYPLSI--
NTPLSI--
-NPLSIY-
-NPLSIT-
-NPLSIQ-
NIPLSI--
NFPLSI--
-NPLSIS-
NDPLSI--
-NPLSIL-
-NPLSIF-
NHPLSI--
-NPLSIV-
-NPLSIK-
-NPLSIH-
NPPLSI--
NQPLSI--
-NPLSIW-
-NPLSIM-
-NPLSIA-
NMPLSI--
-NPLSIN-
NEPLSI--
--PLSILS
--PLSISG
--PLSIRK
--PLSISI
--PLSIRL
--PLSIYS
--PLSIRH
--PLSIYR
--PLSIYG
--PLSIVS
--PLSISS
--PLSILN
--PLSILG
PRPLSI--
--PLSIWR
--PLSIYT
--PLSIMS
--PLSILT
PNPLSI--
--PLSIHG
--PLSIQR
--PLSIKS
--PLSIAT
--PLSIFT
--PLSIAS
--PLSITR

--PLSIRG
--PLSIRE
--PLSIMR
--PLSIIA
--PLSIAG
--PLSIRA
--PLSINS
--PLSIMT
--PLSIST
--PLSIRY
--PLSIRI
--PLSIRD
--PLSIQA
--PLSIKL
--PLSIGA
--PLSIFR
--PLSITT
--PLSITS
--PLSITN
--PLSIKV
--PLSIKM
--PLSIGS
--PLSIFK
--PLSIWS
--PLSIWK
--PLSITG
--PLSIRV
--PLSIRQ
--PLSIRF
--PLSIIG
--PLSIAV
--PLSIWT
--PLSISM
--PLSIQS
--PLSIHS
--PLSIHR
--PLSIHN
--PLSIFN
PWPLSI--
--PLSIYN
--PLSIYM
--PLSIWN
--PLSIVQ
--PLSIQY
--PLSIMG
--PLSIKQ
--PLSIIN
--PLSIHT
--PLSIYK
--PLSITQ

--PLSITM
--PLSINA
--PLSIMV
--PLSIFH
--PLSIWV
--PLSIVK
--PLSIVI
--PLSIVD
--PLSIQN
--PLSIKG
--PLSIIT
--PLSIES
--PLSIVY
--PLSIVT
--PLSIVA
--PLSISN
--PLSIMM
--PLSIMK
--PLSIKT
--PLSIIS
--PLSIID
--PLSIHK
--PLSIFG
--PLSIAN
PYPLSI--
--PLSIYQ
--PLSIYE
--PLSIWQ
--PLSIWH
--PLSIVN
--PLSITA
--PLSIQK
--PLSIML
--PLSIKK
--PLSIKF
--PLSIIQ
PKPLSI--
PHPLSI--
PQPLSI--
--PLSIVV
--PLSISW
--PLSISH
--PLSIQT
--PLSIMD
--PLSIHM
--PLSIAW
PCPLSI--
PMPLSI--
--PLSIYD
--PLSISY

```
--PLSIMW
--PLSILW
--PLSILE
--PLSILD
--PLSIKN
--PLSIKI
--PLSIFS
--PLSIFQ
--PLSIYI
--PLSITW
--PLSITF
--PLSISK
--PLSINV
--PLSING
--PLSIMH
--PLSILQ
--PLSIKW
--PLSIIY
--PLSIIH
--PLSIIE
--PLSIHV
--PLSIGW
--PLSIAR
--PLSIAH
--PLSIAA
--PLSIYH
--PLSIVE
--PLSISQ
--PLSISE
--PLSIQV
--PLSIMN
--PLSIME
--PLSIKD
--PLSIIV
--PLSIHF
--PLSIHD
--PLSIHA
--PLSIFE
--PLSIFD
--PLSIAM
PDPLSI--
--PLSIYY
--PLSIYV
--PLSIWY
--PLSIWG
--PLSIWD
--PLSIVW
--PLSITD
--PLSIQQ
--PLSIQH
```

--PLSIQG
--PLSIQF
--PLSINT
--PLSINN
--PLSIMI
--PLSIKH
--PLSIKA
--PLSIHY
--PLSIHL
--PLSIHH
--PLSIET
--PLSIEG
--PLSIEA
--PLSIAI
PFPLSI--
PEPLSI--
-PPLSIR-
-PPLSIL-
--PLSIYW
--PLSITC
--PLSIQM
--PLSIQI
--PLSIQE
--PLSIQD
--PLSIPN
--PLSIPK
--PLSINQ
--PLSIMQ
--PLSIIW
--PLSIHW
--PLSIHE
--PLSIGI
--PLSIGC
--PLSIEW
--PLSIEV
--PLSIER
--PLSIEM
--PLSIEK
--PLSIEF
--PLSICY
--PLSICK
--PLSIAQ
--PLSIAF
QPPLSI--
-QPLSIR-
-QPLSIT-
-QPLSIY-
-QPLSIH-
-QPLSIA-
QSPLSI--

```
-QPLSIS-
-QPLSIF-
QLPLSI--
QVPLSI--
QTPLSI--
QAPLSI--
-QPLSIL-
QQPLSI--
-QPLSIW-
QWPLSI--
QGPLSI--
-QPLSIK-
QIPLSI--
QYPLSI--
-QPLSIM-
-QPLSIV-
-QPLSIE-
QKPLSI--
-QPLSIQ-
-QPLSIN-
QNPLSI--
QHPLSI--
QEPLSI--
QDPLSI--
-RPLSIL-
RSPLSI--
RGPLSI--
-RPLSIR-
RVPLSI--
-RPLSIS-
-RPLSIH-
-RPLSIT-
RAPLSI--
-RPLSIK-
-RPLSIV-
RTPLSI--
REPLSI--
-RPLSIY-
RIPLSI--
RFPLSI--
-RPLSIM-
-RPLSII-
RQPLSI--
RMPLSI--
-RPLSIQ-
-RPLSIA-
-RPLSIF-
RWPLSI--
RDPLSI--
-RPLSIG-
```

-RPLSIE-
RNPLSI--
-RPLSIW-
RCPLSI--
SRPLSI--
SVPLSI--
SSPLSI--
SLPLSI--
SKPLSI--
-SPLSIV-
STPLSI--
SNPLSI--
-SPLSIL-
-SPLSII-
-SPLSIA-
SMPLSI--
-SPLSIH-
SQPLSI--
-SPLSIY-
-SPLSIW-
-SPLSIF-
SIPLSI--
SFPLSI--
SHPLSI--
SAPLSI--
-SPLSIS-
-SPLSIM-
-SPLSIK-
SWPLSI--
SEPLSI--
-SPLSIN-
SDPLSI--
SPPLSI--
-SPLSIE-
SCPLSI--
-TPLSIR-
TRPLSI--
TLPLSI--
TGPLSI--
TVPLSI--
-TPLSIS-
TTPLSI--
-TPLSIY-
-TPLSIM-
-TPLSIL-
-TPLSIK-
TAPLSI--
TPPLSI--
-TPLSIT-
TWPLSI--

THPLSI--
TYPLSI--
TQPLSI--
-TPLSIA-
TEPLSI--
TFPLSI--
TNPLSI--
-TPLSIN-
TDPLSI--
-TPLSIE-
TCPLSI--
VRPLSI--
VLPLSI--
VTPLSI--
VKPLSI--
-VPLSIL-
-VPLSIS-
-VPLSIH-
VWPLSI--
VPPLSI--
-VPLSIV-
-VPLSII-
-VPLSIK-
-VPLSIF-
-VPLSIA-
VIPLSI--
VFPLSI--
VDPLSI--
-VPLSIN-
-VPLSIE-
VMPLSI--
VAPLSI--
VEPLSI--
VCPLSI--
-VPLSIQ-
-VPLSIW-
WSPLSI--
WHPLSI--
WRPLSI--
-WPLSIR-
-WPLSIK-
WPPLSI--
-WPLSIS-
-WPLSIT-
-WPLSIQ-
WGPLSI--
-WPLSIV-
WQPLSI--
-WPLSIL-
WYPLSI--

```
              WVPLSI--
              WDPLSI--
              -WPLSIY-
              -WPLSIW-
              -WPLSIA-
              WNPLSI--
              WAPLSI--
              -WPLSII-
              WWPLSI--
              -WPLSIM-
              -WPLSIG-
              YRPLSI--
              YLPLSI--
              YSPLSI--
              -YPLSIR-
              -YPLSIL-
              -YPLSIS-
              YVPLSI--
              YTPLSI--
              -YPLSIT-
              YKPLSI--
              YMPLSI--
              -YPLSIV-
              YNPLSI--
              -YPLSIQ-
              YAPLSI--
              YWPLSI--
              -YPLSIH-
              -YPLSII-
              YFPLSI--
              -YPLSIK-
              -YPLSIE-
              YGPLSI--
              YEPLSI--
              -YPLSIY-
              -YPLSIW-
              -YPLSIN-
              -YPLSIM-
              YYPLSI--
              YQPLSI--
              YHPLSI--
              YDPLSI--
PASY            70        789     32.25
              --PASYNG
              -SPASYG-
              -APASYL-
              -APASYS-
              -APASYR-
              -APASYT-
              -APASYI-
```

AHPASY--
-APASYY-
AKPASY--
AIPASY--
AFPASY--
ACPASY--
-APASYW-
-APASYM-
-APASYH-
-APASYA-
ATPASY--
AQPASY--
-APASYV-
AGPASY--
-APASYN-
AWPASY--
AVPASY--
-APASYQ-
-APASYG-
-APASYF-
ANPASY--
ALPASY--
ADPASY--
-APASYE-
AMPASY--
-CPASYS-
-CPASYR-
CKPASY--
-CPASYL-
CAPASY--
-CPASYQ-
CHPASY--
CVPASY--
CTPASY--
CSPASY--
-CPASYV-
-CPASYP-
-CPASYM-
-CPASYI-
CLPASY--
CIPASY--
CGPASY--
-DPASYL-
-DPASYR-
DFPASY--
DRPASY--
DQPASY--
DIPASY--
DEPASY--
DSPASY--

-DPASYV-
-DPASYA-
DNPASY--
DCPASY--
-DPASYT-
-DPASYS-
-DPASYN-
-DPASYM-
DHPASY--
DYPASY--
DWPASY--
DPPASY--
-DPASYW-
-DPASYQ-
-DPASYK-
-DPASYI-
DMPASY--
DKPASY--
DGPASY--
DDPASY--
-EPASYR-
-EPASYQ-
-EPASYF-
EMPASY--
ELPASY--
EWPASY--
EVPASY--
ESPASY--
-EPASYT-
EYPASY--
ERPASY--
-EPASYL-
-EPASYH-
ENPASY--
ETPASY--
EAPASY--
-EPASYA-
EHPASY--
EFPASY--
EQPASY--
-EPASYW-
-EPASYV-
-EPASYS-
-EPASYN-
-EPASYK-
-EPASYI-
EGPASY--
-FPASYL-
FQPASY--
FKPASY--

FSPASY--
-FPASYS-
-FPASYT-
-FPASYF-
FVPASY--
-FPASYR-
FYPASY--
-FPASYK-
-FPASYV-
-FPASYQ-
-FPASYA-
FGPASY--
FWPASY--
FPPASY--
FMPASY--
FIPASY--
FHPASY--
-FPASYN-
-FPASYM-
-FPASYH-
FEPASY--
FCPASY--
-GPASYS-
GGPASY--
GVPASY--
GRPASY--
-GPASYA-
-GPASYQ-
-GPASYT-
-GPASYR-
GLPASY--
GKPASY--
GYPASY--
GAPASY--
GQPASY--
GEPASY--
-GPASYW-
-GPASYH-
GWPASY--
GNPASY--
GIPASY--
GHPASY--
-GPASYY-
-GPASYN-
-GPASYK-
-GPASYI-
-GPASYF-
GFPASY--
-GPASYV-
-GPASYG-

-GPASYE-
-GPASYM-
GDPASY--
GCPASY--
-HPASYL-
HWPASY--
-HPASYQ-
HSPASY--
-HPASYR-
-HPASYK-
HLPASY--
HTPASY--
-HPASYF-
HMPASY--
HEPASY--
-HPASYY-
-HPASYW-
-HPASYM-
-HPASYI-
HKPASY--
HDPASY--
HNPASY--
HIPASY--
HGPASY--
-HPASYT-
-HPASYN-
HHPASY--
HVPASY--
HPPASY--
-HPASYV-
HYPASY--
HQPASY--
-HPASYS-
-HPASYE-
HCPASY--
HAPASY--
-IPASYL-
-IPASYI-
INPASY--
IGPASY--
ISPASY--
IRPASY--
-IPASYR-
ITPASY--
-IPASYN-
-IPASYH-
ILPASY--
-IPASYT-
-IPASYA-
-IPASYV-

IAPASY--
IYPASY--
IPPASY--
-IPASYE-
IWPASY--
-IPASYM-
IIPASY--
IFPASY--
IEPASY--
IVPASY--
IQPASY--
-IPASYY-
-IPASYW-
-IPASYK-
-IPASYF-
IMPASY--
-KPASYS-
-KPASYF-
-KPASYA-
-KPASYI-
KAPASY--
KGPASY--
KTPASY--
KMPASY--
-KPASYK-
-KPASYH-
KVPASY--
KEPASY--
KYPASY--
-KPASYT-
KNPASY--
-KPASYW-
-KPASYG-
KLPASY--
KIPASY--
KFPASY--
-KPASYN-
KDPASY--
-KPASYE-
KKPASY--
KQPASY--
KPPASY--
-KPASYD-
KHPASY--
LRPASY--
-LPASYR-
-LPASYI-
-LPASYF-
LSPASY--
LTPASY--

```
-LPASYA-
LMPASY--
LIPASY--
-LPASYV-
-LPASYK-
LKPASY--
LHPASY--
LPPASY--
LNPASY--
LLPASY--
LYPASY--
LVPASY--
-LPASYT-
LAPASY--
-LPASYW-
-LPASYS-
LGPASY--
LWPASY--
-LPASYY-
-LPASYH-
LDPASY--
-LPASYN-
LQPASY--
-LPASYM-
LEPASY--
LFPASY--
-MPASYR-
MAPASY--
-MPASYN-
-MPASYI-
MVPASY--
-MPASYQ-
MKPASY--
-MPASYW-
-MPASYT-
MSPASY--
MRPASY--
-MPASYS-
-MPASYM-
MNPASY--
MLPASY--
MTPASY--
MGPASY--
MQPASY--
MMPASY--
MPPASY--
-MPASYV-
-MPASYK-
-MPASYE-
-MPASYA-
```

```
MFPASY--
MDPASY--
MYPASY--
-MPASYY-
-MPASYP-
-MPASYH-
-MPASYF-
MEPASY--
-NPASYV-
-NPASYR-
NRPASY--
-NPASYS-
-NPASYI-
NQPASY--
-NPASYK-
NSPASY--
-NPASYM-
-NPASYL-
NLPASY--
NKPASY--
NYPASY--
NPPASY--
-NPASYQ-
NVPASY--
-NPASYW-
-NPASYA-
NEPASY--
NAPASY--
-NPASYY-
-NPASYN-
-NPASYF-
-NPASYH-
-NPASYE-
NNPASY--
NHPASY--
NTPASY--
-NPASYT-
-NPASYG-
NMPASY--
NIPASY--
NFPASY--
--PASYGV
--PASYRL
--PASYLG
--PASYSS
--PASYTR
--PASYSG
--PASYGS
--PASYRT
--PASYSQ
```

--PASYSL
--PASYLR
--PASYYL
--PASYST
--PASYSK
--PASYMY
--PASYIL
--PASYVS
--PASYSR
--PASYMR
--PASYKV
--PASYFS
--PASYSH
--PASYRQ
--PASYLA
--PASYIS
--PASYSV
--PASYRY
--PASYRM
--PASYKS
--PASYKL
--PASYAI
--PASYWT
--PASYTM
--PASYRI
--PASYMS
--PASYKW
--PASYVR
--PASYTG
--PASYSM
--PASYRF
--PASYMQ
--PASYLN
--PASYYR
--PASYVG
--PASYTV
--PASYTI
--PASYRA
--PASYMV
--PASYLI
--PASYIQ
--PASYWR
--PASYVK
--PASYRN
--PASYRE
--PASYMN
--PASYMH
--PASYEA
--PASYAS
PGPASY--

--PASYWA
--PASYVN
--PASYVI
--PASYQR
--PASYLT
--PASYKG
--PASYFK
--PASYAM
--PASYWV
--PASYTT
--PASYTN
--PASYTA
--PASYRG
--PASYQT
--PASYQM
--PASYLQ
--PASYLM
--PASYKT
--PASYHY
--PASYFA
--PASYAV
PVPASY--
--PASYVY
--PASYTQ
--PASYTH
--PASYSY
--PASYRH
--PASYQA
--PASYLY
--PASYIV
--PASYHA
--PASYFT
--PASYFM
--PASYFG
--PASYAR
--PASYYT
--PASYWN
--PASYWM
--PASYVW
--PASYVQ
--PASYTL
--PASYTK
--PASYSF
--PASYRW
--PASYQW
--PASYNS
--PASYKH
--PASYKF
--PASYIG
--PASYHV

--PASYYQ
--PASYYN
--PASYYM
--PASYYA
--PASYVH
--PASYVE
--PASYTD
--PASYRV
--PASYRD
--PASYMA
--PASYLK
--PASYKM
PSPASY--
PQPASY--
PKPASY--
PHPASY--
--PASYYD
--PASYWQ
--PASYVV
--PASYVM
--PASYTE
--PASYKK
--PASYIK
--PASYGA
--PASYFN
--PASYFL
--PASYAG
PAPASY--
PTPASY--
PNPASY--
PMPASY--
PIPASY--
PFPASY--
--PASYYW
--PASYYG
--PASYWW
--PASYVT
--PASYSN
--PASYSE
--PASYQI
--PASYQG
--PASYKN
--PASYKE
--PASYIN
--PASYII
--PASYHN
--PASYHL
--PASYHI
--PASYHH
--PASYHG

--PASYGM
--PASYGG
--PASYAF
PWPASY--
PLPASY--
--PASYYK
--PASYYI
--PASYWL
--PASYWK
--PASYTF
--PASYSD
--PASYQY
--PASYQS
--PASYNY
--PASYNT
--PASYNM
--PASYNL
--PASYMW
--PASYMT
--PASYMG
--PASYME
--PASYLH
--PASYLD
--PASYKI
--PASYKD
--PASYID
--PASYHT
--PASYHS
--PASYHD
--PASYGN
--PASYFW
--PASYFH
--PASYEV
--PASYES
--PASYAY
--PASYAN
--PASYAH
PYPASY--
-PPASYL-
PDPASY--
--PASYYH
--PASYYE
--PASYWG
--PASYVD
--PASYTY
--PASYTW
--PASYQN
--PASYQL
--PASYQH
--PASYQE

--PASYPL
--PASYNW
--PASYNV
--PASYNF
--PASYMM
--PASYMF
--PASYMD
--PASYKQ
--PASYIW
--PASYHM
--PASYHK
--PASYHF
--PASYGY
--PASYFI
--PASYEG
--PASYEC
--PASYDA
--PASYAT
--PASYAL
--PASYAA
-QPASYV-
QSPASY--
-QPASYL-
QMPASY--
-QPASYS-
QLPASY--
QNPASY--
-QPASYY-
-QPASYK-
-QPASYF-
QKPASY--
-QPASYT-
-QPASYQ-
QAPASY--
QYPASY--
QRPASY--
-QPASYW-
-QPASYH-
-QPASYA-
QTPASY--
-QPASYI-
QHPASY--
QGPASY--
QWPASY--
-QPASYC-
QEPASY--
RSPASY--
RQPASY--
-RPASYV-
-RPASYW-

```
-RPASYK-
RAPASY--
-RPASYT-
-RPASYA-
RNPASY--
-RPASYI-
RWPASY--
-RPASYN-
RLPASY--
RTPASY--
RIPASY--
RHPASY--
-RPASYF-
-RPASYM-
RDPASY--
-RPASYQ-
RKPASY--
RYPASY--
-RPASYH-
RPPASY--
-RPASYG-
RFPASY--
REPASY--
-RPASYE-
-RPASYD-
RCPASY--
-SPASYS-
SLPASY--
-SPASYV-
STPASY--
SGPASY--
-SPASYL-
-SPASYI-
-SPASYR-
-SPASYK-
SWPASY--
-SPASYM-
SVPASY--
SIPASY--
SHPASY--
-SPASYH-
SAPASY--
-SPASYN-
SSPASY--
-SPASYY-
SNPASY--
SYPASY--
-SPASYQ-
-SPASYA-
SMPASY--
```

SDPASY--
SQPASY--
-SPASYF-
SFPASY--
SPPASY--
-SPASYW-
SKPASY--
SEPASY--
-SPASYE-
SCPASY--
-TPASYL-
TRPASY--
-TPASYS-
-TPASYR-
TLPASY--
TAPASY--
-TPASYI-
-TPASYV-
TGPASY--
TWPASY--
TVPASY--
TPPASY--
TSPASY--
-TPASYQ-
-TPASYN-
-TPASYF-
TTPASY--
-TPASYY-
-TPASYW-
-TPASYM-
-TPASYK-
TNPASY--
TQPASY--
-TPASYH-
TMPASY--
TKPASY--
THPASY--
TFPASY--
TYPASY--
TCPASY--
-TPASYT-
TEPASY--
TDPASY--
VKPASY--
VHPASY--
-VPASYS-
-VPASYK-
VLPASY--
-VPASYI-
VAPASY--

-VPASYQ-
VSPASY--
-VPASYM-
-VPASYL-
-VPASYW-
-VPASYR-
VEPASY--
VTPASY--
-VPASYT-
VYPASY--
-VPASYY-
-VPASYV-
-VPASYN-
VMPASY--
-VPASYG-
VNPASY--
VDPASY--
VVPASY--
VRPASY--
VGPASY--
-VPASYE-
-VPASYA-
VIPASY--
VCPASY--
-VPASYH-
-WPASYR-
-WPASYL-
WVPASY--
-WPASYS-
WIPASY--
WGPASY--
WRPASY--
WLPASY--
WDPASY--
WPPASY--
WNPASY--
-WPASYT-
WMPASY--
-WPASYM-
-WPASYI-
WTPASY--
-WPASYW-
-WPASYV-
-WPASYK-
WHPASY--
WAPASY--
-WPASYY-
-WPASYN-
WFPASY--
WEPASY--

YSPASY--
YTPASY--
YKPASY--
YRPASY--
-YPASYY-
YGPASY--
-YPASYK-
YQPASY--
-YPASYI-
-YPASYT-
-YPASYQ-
-YPASYN-
-YPASYM-
YLPASY--
YEPASY--
YAPASY--
YVPASY--
YMPASY--
-YPASYR-
YNPASY--
YYPASY--
YPPASY--
-YPASYH-
-YPASYF-
YIPASY--
YDPASY--
-YPASYV-
-YPASYG-
-YPASYA-
YFPASY--
YHPASY--
YCPASY--

LAAL 590 582 32.24

EDLAAL--
--LAALVD
--LAALTP
--LAALPT
--LAALDT
--LAALAR
MELAAL--
-YLAALP-
AYLAAL--
-ALAALR-
-ALAALH-
ASLAAL--
-ALAALK-
AALAAL--
-ALAALS-
-ALAALT-
AFLAAL--

ATLAAL--
AQLAAL--
-ALAALN-
AGLAAL--
AHLAAL--
AWLAAL--
AMLAAL--
-ALAALV-
-ALAALE-
-ALAALA-
-ALAALG-
ADLAAL--
ACLAAL--
CSLAAL--
CVLAAL--
-CLAALS-
CLLAAL--
CRLAAL--
-CLAALN-
CDLAAL--
CCLAAL--
CTLAAL--
CYLAAL--
-CLAALR-
-CLAALK-
-CLAALI-
CELAAL--
CALAAL--
CQLAAL--
-CLAALL-
-CLAALH-
-CLAALG-
-CLAALE-
CKLAAL--
CWLAAL--
CMLAAL--
-CLAALT-
-CLAALQ-
-CLAALM-
CILAAL--
CHLAAL--
CGLAAL--
DYLAAL--
DTLAAL--
-DLAALF-
-DLAALW-
-DLAALL-
-DLAALR-
DHLAAL--
-DLAALV-

-DLAALT-
-DLAALY-
-DLAALH-
DSLAAL--
-DLAALS-
-DLAALQ-
DFLAAL--
DALAAL--
DKLAAL--
DQLAAL--
-DLAALK-
DGLAAL--
-DLAALN-
DELAAL--
DWLAAL--
DVLAAL--
DMLAAL--
-DLAALI-
-DLAALM-
-DLAALE-
DCLAAL--
ERLAAL--
-ELAALR-
EMLAAL--
EQLAAL--
-ELAALT-
ESLAAL--
EHLAAL--
-ELAALN-
-ELAALL-
-ELAALH-
ETLAAL--
-ELAALF-
EYLAAL--
-ELAALW-
ENLAAL--
-ELAALV-
-ELAALS-
-ELAALK-
-ELAALI-
-ELAALA-
-ELAALQ-
-ELAALE-
EWLAAL--
EFLAAL--
ECLAAL--
-ELAALG-
EELAAL--
-FLAALS-
-FLAALG-

FQLAAL--
FGLAAL--
FALAAL--
FHLAAL--
-FLAALA-
-FLAALK-
FDLAAL--
-FLAALH-
-FLAALE-
-FLAALQ-
-FLAALI-
-FLAALF-
-GLAALR-
GSLAAL--
-GLAALT-
GNLAAL--
GGLAAL--
GILAAL--
-GLAALL-
-GLAALF-
GHLAAL--
-GLAALI-
GTLAAL--
-GLAALW-
-GLAALA-
-GLAALN-
GQLAAL--
-GLAALH-
GKLAAL--
-GLAALK-
GFLAAL--
GMLAAL--
-GLAALQ-
-GLAALV-
GWLAAL--
GDLAAL--
GELAAL--
-GLAALG-
-GLAALE-
-GLAALC-
GCLAAL--
HYLAAL--
HRLAAL--
HNLAAL--
HSLAAL--
-HLAALL-
HFLAAL--
HLLAAL--
-HLAALW-
-HLAALT-

HTLAAL--
-HLAALH-
-HLAALS-
-HLAALF-
-HLAALA-
HDLAAL--
-HLAALN-
HGLAAL--
-HLAALY-
-HLAALQ-
HMLAAL--
-HLAALK-
HVLAAL--
HHLAAL--
HALAAL--
-HLAALG-
HCLAAL--
HILAAL--
-HLAALV-
-HLAALM-
-HLAALE-
-ILAALR-
-ILAALH-
-ILAALA-
-ILAALG-
-ILAALK-
IDLAAL--
-ILAALE-
IKLAAL--
IGLAAL--
-ILAALM-
-ILAALD-
ICLAAL--
KTLAAL--
-KLAALS-
KNLAAL--
-KLAALN-
-KLAALK-
KYLAAL--
-KLAALL-
-KLAALW-
KALAAL--
KGLAAL--
KWLAAL--
-KLAALI-
KDLAAL--
KELAAL--
-KLAALQ-
-KLAALF-
KILAAL--

KMLAAL--
KFLAAL--
KQLAAL--
-KLAALT-
KHLAAL--
-KLAALM-
KCLAAL--
-KLAALE-
--LAALTV
--LAALIT
--LAALLS
--LAALRT
--LAALNL
--LAALRN
LGLAAL--
--LAALVS
--LAALRR
-LLAALK-
--LAALRG
--LAALYS
--LAALSA
--LAALLK
--LAALIS
--LAALTT
--LAALKR
--LAALSM
--LAALAT
--LAALSR
--LAALAV
--LAALQA
--LAALTL
--LAALSH
--LAALSF
--LAALLV
--LAALYG
--LAALKH
--LAALHR
--LAALAS
LDLAAL--
--LAALSV
--LAALGF
--LAALTR
--LAALRH
--LAALPL
--LAALVN
--LAALTN
--LAALRW
--LAALRM
--LAALNS
--LAALTK

--LAALTG
--LAALVA
--LAALST
--LAALNF
--LAALPF
--LAALIG
--LAALHL
-LLAALN-
--LAALMH
--LAALKY
--LAALYH
--LAALVV
--LAALVQ
--LAALVH
--LAALTH
--LAALSW
--LAALKW
--LAALKG
--LAALGA
--LAALAG
-LLAALQ-
--LAALTD
--LAALRQ
--LAALRF
--LAALMK
--LAALMA
--LAALLM
--LAALIQ
--LAALHF
--LAALSY
--LAALMG
-LLAALA-
--LAALMV
--LAALIN
--LAALIK
--LAALYN
--LAALKN
-LLAALE-
--LAALWK
--LAALQL
--LAALQG
--LAALNG
--LAALMI
-LLAALH-
--LAALFG
--LAALWG
--LAALQY
--LAALPY
--LAALMQ
--LAALMD

--LAALKI
--LAALKF
--LAALHG
--LAALGV
--LAALAI
--LAALWH
--LAALTQ
--LAALQH
--LAALNK
--LAALMT
--LAALHQ
--LAALHK
--LAALHE
--LAALHA
--LAALGW
--LAALFS
--LAALFK
--LAALAQ
--LAALAA
--LAALTI
--LAALTE
--LAALRD
--LAALPW
--LAALPM
--LAALPI
--LAALNY
--LAALNT
--LAALNN
--LAALMN
--LAALME
--LAALIH
--LAALHV
--LAALHD
--LAALGI
-LLAALP-
--LAALWQ
--LAALWN
--LAALTM
--LAALQM
--LAALQK
--LAALIW
--LAALGG
--LAALAH
--LAALWD
--LAALSD
--LAALNR
--LAALNM
--LAALKD
--LAALHT
--LAALHH

--LAALGM
--LAALAN
-LLAALM-
--LAALYQ
--LAALYD
--LAALQW
--LAALQQ
--LAALNH
--LAALLE
--LAALLD
--LAALKK
--LAALIA
--LAALHM
--LAALFH
--LAALDL
--LAALDG
--LAALAY
--LAALVW
--LAALQV
--LAALQT
--LAALND
--LAALIE
--LAALGT
--LAALFQ
-LLAALD-
LCLAAL--
--LAALWM
--LAALVE
--LAALSE
--LAALQD
--LAALMW
--LAALLW
--LAALGH
--LAALFD
--LAALAM
--LAALAK
-LLAALC-
--LAALNQ
--LAALNI
--LAALGQ
--LAALEW
--LAALAC
--LAALWE
--LAALVC
--LAALQF
--LAALPQ
--LAALMC
--LAALKE
--LAALID
--LAALHC

```
--LAALEI
--LAALEF
--LAALDN
MALAAL--
-MLAALS-
MKLAAL--
MSLAAL--
-MLAALR-
-MLAALH-
-MLAALG-
-MLAALA-
-MLAALI-
-MLAALV-
MDLAAL--
MCLAAL--
-MLAALM-
-MLAALE-
-NLAALR-
-NLAALT-
NSLAAL--
NHLAAL--
NGLAAL--
NKLAAL--
-NLAALS-
NTLAAL--
-NLAALL-
-NLAALA-
-NLAALQ-
-NLAALG-
NYLAAL--
-NLAALK-
-NLAALY-
NNLAAL--
NMLAAL--
-NLAALH-
NALAAL--
NDLAAL--
-NLAALI-
NELAAL--
-NLAALE-
-NLAALW-
NCLAAL--
PLLAAL--
PDLAAL--
PELAAL--
PHLAAL--
PTLAAL--
PQLAAL--
PNLAAL--
PCLAAL--
```

PALAAL--
-QLAALS-
-QLAALT-
-QLAALN-
QKLAAL--
-QLAALR-
QALAAL--
QYLAAL--
QLLAAL--
-QLAALI-
QGLAAL--
QWLAAL--
QMLAAL--
QNLAAL--
-QLAALA-
-QLAALV-
QELAAL--
-QLAALQ-
-QLAALK-
-QLAALH-
QHLAAL--
QQLAAL--
-QLAALM-
-QLAALE-
QDLAAL--
QCLAAL--
RALAAL--
-RLAALK-
-RLAALT-
RYLAAL--
-RLAALW-
RDLAAL--
RWLAAL--
-RLAALG-
-RLAALE-
RCLAAL--
-RLAALC-
-SLAALL-
STLAAL--
SLLAAL--
SKLAAL--
SFLAAL--
-SLAALK-
-SLAALA-
SQLAAL--
-SLAALT-
-SLAALH-
SWLAAL--
SDLAAL--
-SLAALV-

-SLAALI-
-SLAALM-
SHLAAL--
SCLAAL--
SELAAL--
-SLAALE-
-SLAALC-
TRLAAL--
-TLAALT-
TGLAAL--
TNLAAL--
-TLAALR-
TQLAAL--
THLAAL--
TTLAAL--
-TLAALN-
-TLAALA-
-TLAALK-
TYLAAL--
TELAAL--
TDLAAL--
-TLAALI-
-TLAALG-
TCLAAL--
TALAAL--
-TLAALQ-
-TLAALP-
-TLAALM-
-TLAALE-
-VLAALN-
VHLAAL--
-VLAALQ-
-VLAALG-
VALAAL--
-VLAALV-
VNLAAL--
-VLAALK-
-VLAALM-
VCLAAL--
WGLAAL--
-WLAALH-
-WLAALN-
-WLAALS-
WHLAAL--
-WLAALT-
-WLAALG-
-WLAALQ-
-WLAALP-
WDLAAL--
-WLAALW-

| | | | |
|---|---|---|---|
| | -YLAALR- | | |
| | -YLAALN- | | |
| | -YLAALG- | | |
| | YSLAAL-- | | |
| | -YLAALT- | | |
| | -YLAALH- | | |
| | YGLAAL-- | | |
| | -YLAALK- | | |
| | YKLAAL-- | | |
| | -YLAALI- | | |
| | YHLAAL-- | | |
| | -YLAALQ- | | |
| | -YLAALE- | | |
| | YDLAAL-- | | |
| | YCLAAL-- | | |
| PVHL | 50 | 803 | 32.24 |
| | --PVHLWR | | |
| | --PVHLGV | | |
| | --PVHLFL | | |
| | -SPVHLN- | | |
| | -SPVHLD- | | |
| | -VPVHLG- | | |
| | AVPVHL-- | | |
| | ASPVHL-- | | |
| | -APVHLS- | | |
| | APPVHL-- | | |
| | -APVHLR- | | |
| | -APVHLA- | | |
| | AKPVHL-- | | |
| | AQPVHL-- | | |
| | ANPVHL-- | | |
| | AMPVHL-- | | |
| | -APVHLQ- | | |
| | AIPVHL-- | | |
| | AYPVHL-- | | |
| | -APVHLV- | | |
| | -APVHLL- | | |
| | -APVHLI- | | |
| | -APVHLY- | | |
| | -APVHLH- | | |
| | ATPVHL-- | | |
| | -APVHLT- | | |
| | -APVHLM- | | |
| | -APVHLG- | | |
| | AHPVHL-- | | |
| | AEPVHL-- | | |
| | -APVHLN- | | |
| | AGPVHL-- | | |
| | ADPVHL-- | | |
| | -APVHLW- | | |

AAPVHL--
-APVHLK-
-APVHLE-
CRPVHL--
CAPVHL--
CTPVHL--
CHPVHL--
CCPVHL--
CVPVHL--
CSPVHL--
-CPVHLR-
CGPVHL--
CNPVHL--
CLPVHL--
CKPVHL--
CDPVHL--
CYPVHL--
CWPVHL--
-CPVHLT-
-CPVHLH-
CMPVHL--
CEPVHL--
DHPVHL--
DSPVHL--
DTPVHL--
DWPVHL--
DMPVHL--
DRPVHL--
DFPVHL--
-DPVHLY-
-DPVHLI-
-DPVHLV-
-DPVHLS-
DVPVHL--
-DPVHLT-
-DPVHLM-
DLPVHL--
DYPVHL--
-DPVHLR-
-DPVHLL-
-DPVHLK-
-DPVHLH-
DKPVHL--
DGPVHL--
DAPVHL--
DQPVHL--
-DPVHLQ-
-DPVHLF-
-DPVHLA-
DIPVHL--

ERPVHL--
-EPVHLR-
ETPVHL--
ELPVHL--
ESPVHL--
-EPVHLK-
EGPVHL--
EWPVHL--
EMPVHL--
EKPVHL--
EDPVHL--
EYPVHL--
-EPVHLA-
ENPVHL--
EHPVHL--
EVPVHL--
-EPVHLW-
-EPVHLM-
-EPVHLL-
-EPVHLI-
EPPVHL--
EAPVHL--
EQPVHL--
-EPVHLY-
-EPVHLV-
-EPVHLT-
-EPVHLF-
-EPVHLH-
-EPVHLE-
EIPVHL--
EEPVHL--
ECPVHL--
-FPVHLS-
FQPVHL--
-FPVHLR-
FSPVHL--
FIPVHL--
FAPVHL--
FTPVHL--
FYPVHL--
FGPVHL--
FRPVHL--
-FPVHLI-
-FPVHLQ-
FMPVHL--
FVPVHL--
-FPVHLT-
-FPVHLL-
FNPVHL--
-FPVHLY-

-FPVHLM-
-FPVHLK-
FKPVHL--
FPPVHL--
FHPVHL--
FEPVHL--
FDPVHL--
-FPVHLH-
-FPVHLE-
-FPVHLW-
-FPVHLV-
-FPVHLN-
-FPVHLA-
GFPVHL--
GLPVHL--
GGPVHL--
GRPVHL--
-GPVHLL-
GNPVHL--
-GPVHLS-
-GPVHLR-
-GPVHLT-
GSPVHL--
GVPVHL--
GTPVHL--
GAPVHL--
-GPVHLM-
GQPVHL--
-GPVHLV-
-GPVHLF-
-GPVHLW-
-GPVHLQ-
-GPVHLI-
GIPVHL--
GYPVHL--
-GPVHLE-
GKPVHL--
-GPVHLN-
GEPVHL--
GCPVHL--
-GPVHLK-
-GPVHLH-
GPPVHL--
-GPVHLY-
GDPVHL--
HRPVHL--
HVPVHL--
-HPVHLS-
-HPVHLL-
HEPVHL--

−HPVHLH−
HSPVHL−−
−HPVHLI−
HYPVHL−−
HWPVHL−−
−HPVHLN−
−HPVHLA−
HKPVHL−−
−HPVHLY−
−HPVHLM−
HIPVHL−−
HFPVHL−−
HTPVHL−−
−HPVHLW−
HHPVHL−−
HGPVHL−−
HAPVHL−−
−HPVHLG−
HQPVHL−−
−HPVHLK−
−HPVHLF−
−HPVHLE−
HPPVHL−−
−HPVHLV−
−HPVHLT−
HNPVHL−−
HDPVHL−−
HCPVHL−−
−IPVHLT−
IFPVHL−−
IVPVHL−−
−IPVHLR−
−IPVHLM−
IWPVHL−−
−IPVHLK−
−IPVHLI−
IKPVHL−−
IQPVHL−−
−IPVHLS−
−IPVHLL−
IAPVHL−−
IRPVHL−−
−IPVHLW−
−IPVHLA−
INPVHL−−
IIPVHL−−
IYPVHL−−
IGPVHL−−
ITPVHL−−
ISPVHL−−

−IPVHLN−
IHPVHL−−
IDPVHL−−
−IPVHLY−
−IPVHLH−
−IPVHLF−
IPPVHL−−
−IPVHLE−
KSPVHL−−
−KPVHLL−
KLPVHL−−
KGPVHL−−
KVPVHL−−
−KPVHLS−
−KPVHLY−
−KPVHLH−
KAPVHL−−
KTPVHL−−
KNPVHL−−
−KPVHLA−
−KPVHLT−
KFPVHL−−
−KPVHLV−
−KPVHLI−
KDPVHL−−
KWPVHL−−
−KPVHLN−
KIPVHL−−
KQPVHL−−
−KPVHLM−
KEPVHL−−
KMPVHL−−
KHPVHL−−
KCPVHL−−
KPPVHL−−
−KPVHLQ−
−KPVHLF−
LSPVHL−−
−LPVHLK−
LGPVHL−−
LFPVHL−−
−LPVHLR−
−LPVHLS−
LLPVHL−−
LAPVHL−−
LPPVHL−−
LMPVHL−−
LHPVHL−−
LWPVHL−−
LEPVHL−−

LNPVHL--
-LPVHLM-
LTPVHL--
-LPVHLL-
-LPVHLI-
-LPVHLV-
-LPVHLH-
LDPVHL--
-LPVHLF-
LCPVHL--
-LPVHLY-
-LPVHLT-
MRPVHL--
MSPVHL--
-MPVHLT-
-MPVHLA-
MTPVHL--
-MPVHLI-
MEPVHL--
MYPVHL--
-MPVHLL-
MLPVHL--
MVPVHL--
-MPVHLY-
-MPVHLQ-
MAPVHL--
MQPVHL--
MKPVHL--
MFPVHL--
MNPVHL--
MMPVHL--
-MPVHLK-
MWPVHL--
-MPVHLH-
-MPVHLF-
MGPVHL--
-MPVHLS-
-MPVHLE-
MHPVHL--
-MPVHLW-
-MPVHLM-
-MPVHLG-
MPPVHL--
NGPVHL--
-NPVHLV-
-NPVHLR-
NSPVHL--
-NPVHLK-
NTPVHL--
NVPVHL--

-NPVHLS-
NWPVHL--
-NPVHLT-
NAPVHL--
NQPVHL--
-NPVHLI-
NFPVHL--
-NPVHLY-
-NPVHLQ-
NHPVHL--
-NPVHLF-
-NPVHLA-
NEPVHL--
NYPVHL--
-NPVHLW-
NNPVHL--
NKPVHL--
-NPVHLM-
-NPVHLL-
NMPVHL--
NIPVHL--
NDPVHL--
-NPVHLG-
--PVHLIG
--PVHLIA
--PVHLMN
--PVHLVR
--PVHLSA
--PVHLRL
--PVHLIR
--PVHLLW
--PVHLVS
--PVHLYA
--PVHLVK
--PVHLTR
--PVHLLT
--PVHLTV
--PVHLIK
--PVHLSH
--PVHLRT
--PVHLRG
--PVHLKN
--PVHLAM
--PVHLSV
--PVHLST
PSPVHL--
--PVHLSS
--PVHLRA
--PVHLMR
--PVHLWA

--PVHLVT
--PVHLTS
--PVHLTK
--PVHLAS
PQPVHL--
--PVHLSR
--PVHLNS
--PVHLLG
--PVHLIF
--PVHLSL
--PVHLRY
--PVHLRN
--PVHLNL
--PVHLMK
--PVHLLQ
--PVHLVG
--PVHLTQ
--PVHLSM
--PVHLRQ
--PVHLRM
--PVHLLS
--PVHLKR
--PVHLGS
--PVHLFT
--PVHLEA
--PVHLSG
--PVHLQG
--PVHLLN
--PVHLLH
--PVHLAL
--PVHLYS
--PVHLVF
--PVHLTN
--PVHLTH
--PVHLRE
--PVHLML
--PVHLKM
--PVHLIQ
PVPVHL--
--PVHLYG
--PVHLVN
--PVHLVA
--PVHLTL
--PVHLTI
--PVHLRF
--PVHLIS
--PVHLIN
--PVHLVM
--PVHLTY
--PVHLTM

--PVHLTA
--PVHLSQ
--PVHLSK
--PVHLSF
--PVHLNM
--PVHLNA
--PVHLLK
--PVHLFS
--PVHLAN
PLPVHL--
--PVHLWV
--PVHLVV
--PVHLTG
--PVHLQN
--PVHLKI
--PVHLKG
--PVHLIT
--PVHLHG
--PVHLFG
--PVHLAW
--PVHLAK
PTPVHL--
PMPVHL--
--PVHLWL
--PVHLTD
--PVHLRK
--PVHLQT
--PVHLKS
--PVHLGG
--PVHLYN
--PVHLYL
--PVHLWG
--PVHLVL
--PVHLVH
--PVHLSI
--PVHLRH
--PVHLRD
--PVHLQW
--PVHLQH
--PVHLMY
--PVHLMV
--PVHLLM
--PVHLHN
--PVHLGT
--PVHLFK
--PVHLES
--PVHLAT
PYPVHL--
--PVHLYT
--PVHLYM

--PVHLYF
--PVHLWH
--PVHLVW
--PVHLTT
--PVHLSY
--PVHLQS
--PVHLQA
--PVHLMG
--PVHLMD
--PVHLKV
--PVHLKT
--PVHLKA
--PVHLIM
--PVHLHA
--PVHLGL
--PVHLEY
--PVHLET
--PVHLEG
PIPVHL--
PCPVHL--
--PVHLYK
--PVHLWY
--PVHLWN
--PVHLVY
--PVHLVI
--PVHLVE
--PVHLSN
--PVHLSD
--PVHLRW
--PVHLQV
--PVHLQR
--PVHLNY
--PVHLNQ
--PVHLNF
--PVHLMT
--PVHLMF
--PVHLII
--PVHLIE
--PVHLHV
--PVHLHS
--PVHLGA
--PVHLFR
--PVHLAH
PGPVHL--
PFPVHL--
PAPVHL--
--PVHLYY
--PVHLWS
--PVHLWM
--PVHLWI

--PVHLVQ
--PVHLQL
--PVHLNV
--PVHLNT
--PVHLMI
--PVHLMH
--PVHLLI
--PVHLKY
--PVHLKL
--PVHLIY
--PVHLFW
--PVHLFN
--PVHLFA
--PVHLEV
--PVHLAY
--PVHLAD
PDPVHL--
--PVHLYI
--PVHLYE
--PVHLYD
--PVHLWQ
--PVHLVD
--PVHLTF
--PVHLTE
--PVHLQM
--PVHLQF
--PVHLQD
--PVHLPL
--PVHLNK
--PVHLNG
--PVHLMM
--PVHLME
--PVHLLD
--PVHLKH
--PVHLKF
--PVHLKE
--PVHLKD
--PVHLIH
--PVHLHT
--PVHLHH
--PVHLGW
--PVHLGM
--PVHLFQ
--PVHLFM
--PVHLFD
--PVHLEW
--PVHLER
--PVHLEN
--PVHLEH
--PVHLDA

--PVHLCS
--PVHLCH
--PVHLCG
--PVHLAV
--PVHLAQ
--PVHLAI
--PVHLAF
--PVHLAE
PNPVHL--
QAPVHL--
-QPVHLR-
QRPVHL--
QTPVHL--
QMPVHL--
QKPVHL--
-QPVHLL-
-QPVHLV-
QLPVHL--
-QPVHLY-
QHPVHL--
QSPVHL--
-QPVHLT-
-QPVHLI-
QFPVHL--
QQPVHL--
QGPVHL--
QYPVHL--
-QPVHLS-
-QPVHLQ-
-QPVHLM-
-QPVHLK-
-QPVHLF-
-QPVHLW-
-QPVHLA-
QNPVHL--
QWPVHL--
-QPVHLH-
-QPVHLN-
QPPVHL--
QIPVHL--
RSPVHL--
RLPVHL--
RGPVHL--
RTPVHL--
-RPVHLW-
-RPVHLY-
RIPVHL--
-RPVHLT-
RAPVHL--
-RPVHLM-

RQPVHL--
-RPVHLI-
RNPVHL--
-RPVHLV-
-RPVHLF-
-RPVHLN-
RFPVHL--
RPPVHL--
RYPVHL--
RDPVHL--
-RPVHLH-
-RPVHLE-
RHPVHL--
-RPVHLQ-
-RPVHLG-
RMPVHL--
RCPVHL--
SLPVHL--
SSPVHL--
SIPVHL--
SGPVHL--
-SPVHLY-
-SPVHLS-
-SPVHLL-
STPVHL--
SWPVHL--
-SPVHLT-
SAPVHL--
-SPVHLM-
-SPVHLK-
-SPVHLQ-
SKPVHL--
SHPVHL--
SQPVHL--
-SPVHLI-
-SPVHLH-
SMPVHL--
SDPVHL--
SPPVHL--
SFPVHL--
SYPVHL--
-SPVHLF-
SEPVHL--
SCPVHL--
-SPVHLW-
-SPVHLE-
TLPVHL--
TVPVHL--
-TPVHLR-
TFPVHL--

TGPVHL--
-TPVHLT-
TAPVHL--
TWPVHL--
TTPVHL--
TIPVHL--
-TPVHLY-
-TPVHLS-
-TPVHLL-
-TPVHLK-
TNPVHL--
TMPVHL--
TSPVHL--
-TPVHLA-
THPVHL--
TQPVHL--
-TPVHLI-
TPPVHL--
-TPVHLQ-
TKPVHL--
TDPVHL--
TYPVHL--
-TPVHLV-
TEPVHL--
-TPVHLW-
-TPVHLN-
-TPVHLF-
-TPVHLE-
-TPVHLM-
-TPVHLH-
-TPVHLG-
-VPVHLR-
VLPVHL--
VQPVHL--
VPPVHL--
VAPVHL--
VWPVHL--
VKPVHL--
VMPVHL--
VDPVHL--
VTPVHL--
-VPVHLL-
-VPVHLH-
VHPVHL--
-VPVHLM-
-VPVHLI-
-VPVHLE-
-VPVHLY-
VNPVHL--
-VPVHLW-

-VPVHLA-
VEPVHL--
-VPVHLV-
-VPVHLK-
VFPVHL--
VYPVHL--
-VPVHLS-
-VPVHLQ-
VVPVHL--
-VPVHLN-
VCPVHL--
-VPVHLF-
WLPVHL--
WFPVHL--
WWPVHL--
-WPVHLR-
WSPVHL--
-WPVHLA-
WMPVHL--
WKPVHL--
WYPVHL--
WHPVHL--
-WPVHLS-
WDPVHL--
-WPVHLT-
WAPVHL--
-WPVHLQ-
-WPVHLI-
WNPVHL--
WVPVHL--
-WPVHLE-
WTPVHL--
-WPVHLH-
-WPVHLF-
WPPVHL--
WIPVHL--
WGPVHL--
WCPVHL--
YKPVHL--
YLPVHL--
YSPVHL--
-YPVHLR-
YTPVHL--
-YPVHLS-
-YPVHLM-
YYPVHL--
-YPVHLA-
YPPVHL--
YGPVHL--
YWPVHL--

| | | | |
|---|---|---|---|
| | -YPVHLK- | | |
| | YFPVHL-- | | |
| | YDPVHL-- | | |
| | YQPVHL-- | | |
| | -YPVHLT- | | |
| | -YPVHLI- | | |
| | -YPVHLW- | | |
| | YEPVHL-- | | |
| | -YPVHLV- | | |
| | -YPVHLH- | | |
| | YMPVHL-- | | |
| | -YPVHLN- | | |
| | YNPVHL-- | | |
| | YAPVHL-- | | |
| | -YPVHLQ- | | |
| | -YPVHLL- | | |
| | -YPVHLG- | | |
| | -YPVHLE- | | |
| | YHPVHL-- | | |
| PSKL | 140 | 744 | 32.22 |
| | -FPSKLD- | | |
| | -LPSKLG- | | |
| | -MPSKLG- | | |
| | --PSKLNL | | |
| | --PSKLGT | | |
| | --PSKLWR | | |
| | --PSKLDR | | |
| | --PSKLAL | | |
| | --PSKLAI | | |
| | -WPSKLN- | | |
| | -APSKLL- | | |
| | ARPSKL-- | | |
| | -APSKLS- | | |
| | -APSKLH- | | |
| | ASPSKL-- | | |
| | ANPSKL-- | | |
| | -APSKLI- | | |
| | AVPSKL-- | | |
| | ATPSKL-- | | |
| | -APSKLV- | | |
| | -APSKLF- | | |
| | -APSKLM- | | |
| | -APSKLA- | | |
| | ALPSKL-- | | |
| | AGPSKL-- | | |
| | AIPSKL-- | | |
| | AWPSKL-- | | |
| | -APSKLT- | | |
| | -APSKLQ- | | |
| | AKPSKL-- | | |

−APSKLK−
−APSKLG−
−APSKLN−
AHPSKL−−
ACPSKL−−
−APSKLE−
AAPSKL−−
AQPSKL−−
APPSKL−−
ADPSKL−−
CYPSKL−−
−CPSKLL−
CSPSKL−−
CCPSKL−−
CRPSKL−−
CMPSKL−−
CWPSKL−−
CTPSKL−−
CLPSKL−−
CEPSKL−−
−CPSKLT−
−CPSKLS−
−CPSKLR−
CHPSKL−−
−CPSKLQ−
CFPSKL−−
CAPSKL−−
CVPSKL−−
−CPSKLV−
−CPSKLM−
−CPSKLK−
CKPSKL−−
DVPSKL−−
DWPSKL−−
−DPSKLL−
DNPSKL−−
DRPSKL−−
DTPSKL−−
DYPSKL−−
DLPSKL−−
DKPSKL−−
DIPSKL−−
−DPSKLT−
−DPSKLR−
DAPSKL−−
DHPSKL−−
DSPSKL−−
−DPSKLY−
−DPSKLV−
−DPSKLS−

-DPSKLM-
DFPSKL--
DEPSKL--
DQPSKL--
-DPSKLW-
-DPSKLH-
DGPSKL--
DDPSKL--
-DPSKLK-
-DPSKLI-
-DPSKLA-
DCPSKL--
ERPSKL--
EIPSKL--
-EPSKLV-
ETPSKL--
EAPSKL--
EWPSKL--
ESPSKL--
EEPSKL--
-EPSKLY-
-EPSKLM-
EGPSKL--
EVPSKL--
-EPSKLT-
-EPSKLL-
-EPSKLK-
EMPSKL--
-EPSKLW-
-EPSKLS-
-EPSKLQ-
-EPSKLF-
EHPSKL--
EYPSKL--
EKPSKL--
-EPSKLN-
-EPSKLH-
-EPSKLG-
ELPSKL--
EFPSKL--
ECPSKL--
EQPSKL--
EPPSKL--
ENPSKL--
FVPSKL--
FHPSKL--
FFPSKL--
FTPSKL--
-FPSKLI-
FYPSKL--

-FPSKLL-
FNPSKL--
FLPSKL--
-FPSKLV-
FRPSKL--
FIPSKL--
-FPSKLQ-
FSPSKL--
FMPSKL--
FDPSKL--
FGPSKL--
FAPSKL--
FWPSKL--
-FPSKLM-
-FPSKLK-
-FPSKLH-
-FPSKLA-
FKPSKL--
-FPSKLE-
FPPSKL--
-FPSKLN-
FCPSKL--
-GPSKLR-
-GPSKLL-
-GPSKLY-
-GPSKLS-
-GPSKLA-
-GPSKLQ-
GGPSKL--
GVPSKL--
GIPSKL--
GWPSKL--
GPPSKL--
GNPSKL--
GMPSKL--
-GPSKLW-
-GPSKLT-
-GPSKLI-
GFPSKL--
GEPSKL--
-GPSKLH-
GHPSKL--
GDPSKL--
-GPSKLV-
-GPSKLN-
-GPSKLM-
GAPSKL--
GYPSKL--
GTPSKL--
GQPSKL--

-GPSKLF-
-GPSKLG-
-GPSKLE-
GCPSKL--
HRPSKL--
HVPSKL--
HGPSKL--
-HPSKLL-
HFPSKL--
HTPSKL--
-HPSKLR-
HYPSKL--
HWPSKL--
-HPSKLS-
-HPSKLK-
-HPSKLV-
-HPSKLI-
HAPSKL--
-HPSKLY-
-HPSKLW-
HNPSKL--
-HPSKLE-
HMPSKL--
HKPSKL--
-HPSKLQ-
-HPSKLA-
HPPSKL--
HEPSKL--
HCPSKL--
HQPSKL--
-HPSKLN-
-HPSKLH-
HDPSKL--
-HPSKLM-
HHPSKL--
-HPSKLF-
IGPSKL--
ISPSKL--
-IPSKLH-
IRPSKL--
ILPSKL--
IPPSKL--
IVPSKL--
-IPSKLF-
IFPSKL--
-IPSKLR-
-IPSKLS-
-IPSKLL-
-IPSKLK-
-IPSKLI-

IAPSKL--
IEPSKL--
IWPSKL--
-IPSKLW-
-IPSKLM-
-IPSKLA-
IQPSKL--
-IPSKLE-
IMPSKL--
-IPSKLN-
INPSKL--
IIPSKL--
IDPSKL--
IKPSKL--
IHPSKL--
IYPSKL--
-IPSKLG-
ICPSKL--
-IPSKLV-
-IPSKLC-
KVPSKL--
KLPSKL--
-KPSKLS-
KYPSKL--
-KPSKLK-
KIPSKL--
KWPSKL--
-KPSKLY-
-KPSKLT-
KQPSKL--
-KPSKLM-
-KPSKLF-
KPPSKL--
-KPSKLW-
-KPSKLQ-
KNPSKL--
KHPSKL--
KDPSKL--
KTPSKL--
-KPSKLN-
KMPSKL--
-KPSKLV-
KKPSKL--
-KPSKLG-
KCPSKL--
LRPSKL--
LLPSKL--
-LPSKLS-
LSPSKL--
LMPSKL--

LQPSKL--
-LPSKLL-
-LPSKLT-
LPPSKL--
LHPSKL--
LAPSKL--
-LPSKLQ-
LEPSKL--
-LPSKLK-
-LPSKLM-
LDPSKL--
LTPSKL--
-LPSKLE-
LCPSKL--
-LPSKLY-
-LPSKLH-
-LPSKLW-
-LPSKLC-
-MPSKLS-
MGPSKL--
MWPSKL--
-MPSKLV-
-MPSKLT-
-MPSKLE-
MLPSKL--
MKPSKL--
MSPSKL--
MPPSKL--
MEPSKL--
MYPSKL--
MTPSKL--
-MPSKLW-
-MPSKLH-
-MPSKLA-
MHPSKL--
-MPSKLQ-
-MPSKLI-
-MPSKLF-
-MPSKLK-
MAPSKL--
MDPSKL--
NSPSKL--
NVPSKL--
NRPSKL--
NWPSKL--
NGPSKL--
NAPSKL--
-NPSKLT-
-NPSKLK-
NKPSKL--

```
-NPSKLQ-
NMPSKL--
-NPSKLI-
NNPSKL--
NQPSKL--
NYPSKL--
-NPSKLL-
NFPSKL--
-NPSKLS-
NPPSKL--
-NPSKLM-
NIPSKL--
-NPSKLY-
-NPSKLH-
-NPSKLE-
NEPSKL--
NDPSKL--
NCPSKL--
-NPSKLP-
-NPSKLF-
--PSKLLS
--PSKLMR
--PSKLRA
--PSKLMA
--PSKLSS
--PSKLLN
PRPSKL--
--PSKLVS
--PSKLST
--PSKLYM
--PSKLSA
--PSKLNS
--PSKLLT
--PSKLVW
--PSKLSL
--PSKLMG
--PSKLLY
--PSKLSQ
--PSKLSG
--PSKLRD
--PSKLRS
--PSKLFG
--PSKLTV
--PSKLTG
--PSKLLA
--PSKLFH
--PSKLAA
--PSKLVN
--PSKLSY
--PSKLRY
```

--PSKLMH
--PSKLIH
--PSKLVQ
--PSKLVA
--PSKLSH
--PSKLRN
--PSKLML
--PSKLFS
--PSKLAS
--PSKLVI
--PSKLTS
PSPSKL--
--PSKLWH
--PSKLTA
--PSKLSI
--PSKLMT
--PSKLIQ
PEPSKL--
--PSKLYN
--PSKLWV
--PSKLVT
--PSKLTM
--PSKLSM
--PSKLKS
--PSKLVY
--PSKLTN
--PSKLRE
--PSKLMS
--PSKLMK
--PSKLIR
--PSKLFI
--PSKLYG
--PSKLVV
--PSKLVD
--PSKLTW
--PSKLTH
--PSKLSF
--PSKLLQ
--PSKLLI
--PSKLWS
--PSKLVG
--PSKLRQ
--PSKLHS
--PSKLAT
PMPSKL--
PHPSKL--
PAPSKL--
PTPSKL--
--PSKLYR
--PSKLQG

--PSKLKF
PIPSKL--
PVPSKL--
--PSKLQR
--PSKLQA
--PSKLLM
--PSKLLL
--PSKLHT
--PSKLGA
--PSKLES
--PSKLEA
--PSKLAV
PGPSKL--
--PSKLYT
--PSKLYQ
--PSKLYI
--PSKLWW
--PSKLWM
--PSKLWF
--PSKLTI
--PSKLQQ
--PSKLLH
--PSKLKQ
--PSKLIG
--PSKLFQ
--PSKLYH
--PSKLTY
--PSKLRF
--PSKLQS
--PSKLKN
--PSKLKG
--PSKLID
--PSKLHV
PYPSKL--
--PSKLYK
--PSKLYF
--PSKLYA
--PSKLWY
--PSKLVH
--PSKLQL
--PSKLMY
--PSKLMI
--PSKLLE
--PSKLKV
--PSKLKT
--PSKLIW
--PSKLHG
--PSKLGS
--PSKLFN
--PSKLEW

PQPSKL--
PDPSKL--
--PSKLRM
--PSKLQY
--PSKLQW
--PSKLQI
--PSKLIK
--PSKLSD
--PSKLRW
--PSKLQN
--PSKLQH
--PSKLNA
--PSKLMW
--PSKLMN
--PSKLMF
--PSKLMD
--PSKLKM
--PSKLKK
--PSKLHA
--PSKLFM
--PSKLFA
--PSKLET
--PSKLDS
--PSKLYW
--PSKLWK
--PSKLVM
--PSKLTE
--PSKLSV
--PSKLQM
--PSKLNT
--PSKLKR
--PSKLHM
--PSKLDL
PKPSKL--
PFPSKL--
--PSKLWQ
--PSKLTD
--PSKLQE
--PSKLNM
--PSKLMM
--PSKLHN
--PSKLGV
--PSKLGN
--PSKLEV
--PSKLEM
--PSKLAN
--PSKLYE
--PSKLYD
--PSKLRC
--PSKLQK

```
--PSKLQF
--PSKLQD
--PSKLME
--PSKLMC
--PSKLKP
--PSKLKI
--PSKLKH
--PSKLIM
--PSKLHY
--PSKLHW
--PSKLHI
--PSKLHF
--PSKLEN
--PSKLEK
--PSKLEG
--PSKLEF
--PSKLCW
--PSKLCT
--PSKLCS
--PSKLCG
--PSKLCF
--PSKLAM
-PPSKLR-
PPPSKL--
PNPSKL--
PCPSKL--
-QPSKLT-
QLPSKL--
QIPSKL--
QAPSKL--
QPPSKL--
-QPSKLS-
-QPSKLH-
QVPSKL--
QQPSKL--
QNPSKL--
QTPSKL--
-QPSKLI-
-QPSKLE-
-QPSKLF-
QHPSKL--
QFPSKL--
QEPSKL--
-QPSKLY-
-QPSKLQ-
-QPSKLA-
QDPSKL--
QGPSKL--
-QPSKLW-
-QPSKLV-
```

−QPSKLM−
−QPSKLG−
QCPSKL−−
−QPSKLN−
−QPSKLC−
RLPSKL−−
−RPSKLS−
RSPSKL−−
RGPSKL−−
RFPSKL−−
RRPSKL−−
RIPSKL−−
−RPSKLR−
−RPSKLV−
−RPSKLA−
RMPSKL−−
RAPSKL−−
−RPSKLY−
RNPSKL−−
−RPSKLI−
−RPSKLM−
−RPSKLH−
−RPSKLQ−
REPSKL−−
RKPSKL−−
RQPSKL−−
−RPSKLN−
RPPSKL−−
RHPSKL−−
−RPSKLE−
RDPSKL−−
RCPSKL−−
SLPSKL−−
−SPSKLS−
SVPSKL−−
SRPSKL−−
STPSKL−−
−SPSKLA−
SYPSKL−−
−SPSKLL−
SMPSKL−−
SNPSKL−−
SIPSKL−−
SGPSKL−−
SQPSKL−−
−SPSKLT−
−SPSKLF−
−SPSKLR−
SFPSKL−−
−SPSKLW−

−SPSKLH−
SCPSKL−−
SHPSKL−−
−SPSKLM−
SPPSKL−−
−SPSKLN−
SEPSKL−−
−SPSKLE−
SAPSKL−−
−SPSKLQ−
TLPSKL−−
TGPSKL−−
−TPSKLT−
TRPSKL−−
−TPSKLR−
TVPSKL−−
−TPSKLS−
THPSKL−−
TIPSKL−−
−TPSKLK−
−TPSKLH−
TYPSKL−−
TWPSKL−−
−TPSKLW−
−TPSKLA−
−TPSKLV−
−TPSKLQ−
TMPSKL−−
TKPSKL−−
−TPSKLY−
TNPSKL−−
TQPSKL−−
−TPSKLE−
TTPSKL−−
TPPSKL−−
TEPSKL−−
TDPSKL−−
−TPSKLN−
−TPSKLG−
−TPSKLM−
TCPSKL−−
VRPSKL−−
−VPSKLL−
−VPSKLR−
VNPSKL−−
−VPSKLS−
−VPSKLI−
−VPSKLQ−
−VPSKLY−
−VPSKLT−

VIPSKL--
VEPSKL--
-VPSKLV-
-VPSKLK-
VGPSKL--
VMPSKL--
-VPSKLW-
-VPSKLA-
-VPSKLN-
-VPSKLH-
VPPSKL--
VWPSKL--
-VPSKLG-
VHPSKL--
-VPSKLE-
-VPSKLF-
VDPSKL--
-WPSKLK-
WSPSKL--
WVPSKL--
WIPSKL--
WGPSKL--
-WPSKLV-
WLPSKL--
-WPSKLL-
-WPSKLI-
WPPSKL--
WKPSKL--
WYPSKL--
WTPSKL--
-WPSKLY-
WEPSKL--
-WPSKLQ-
WAPSKL--
WQPSKL--
-WPSKLE-
WHPSKL--
-YPSKLL-
YLPSKL--
YGPSKL--
-YPSKLV-
-YPSKLI-
YNPSKL--
YKPSKL--
YAPSKL--
-YPSKLS-
-YPSKLT-
YIPSKL--
YPPSKL--
YTPSKL--

| | | | |
|---|---|---|---|
| | -YPSKLY- | | |
| | YHPSKL-- | | |
| | YEPSKL-- | | |
| | -YPSKLM- | | |
| | YMPSKL-- | | |
| | YWPSKL-- | | |
| | -YPSKLQ- | | |
| | -YPSKLE- | | |
| | YFPSKL-- | | |
| | -YPSKLH- | | |
| | -YPSKLA- | | |
| | -YPSKLG- | | |
| | -YPSKLP- | | |
| | YCPSKL-- | | |
| VAWL | 812 | 534 | 32.22 |
| | --VAWLKP | | |
| | AHVAWL-- | | |
| | ASVAWL-- | | |
| | ATVAWL-- | | |
| | -AVAWLS- | | |
| | ALVAWL-- | | |
| | -AVAWLR- | | |
| | -AVAWLQ- | | |
| | -AVAWLD- | | |
| | AVVAWL-- | | |
| | -AVAWLN- | | |
| | -AVAWLT- | | |
| | -AVAWLK- | | |
| | -AVAWLG- | | |
| | AMVAWL-- | | |
| | AAVAWL-- | | |
| | AQVAWL-- | | |
| | AIVAWL-- | | |
| | ADVAWL-- | | |
| | -AVAWLE- | | |
| | AGVAWL-- | | |
| | AEVAWL-- | | |
| | -AVAWLA- | | |
| | -CVAWLG- | | |
| | CSVAWL-- | | |
| | -CVAWLR- | | |
| | CQVAWL-- | | |
| | -CVAWLE- | | |
| | -CVAWLA- | | |
| | CTVAWL-- | | |
| | CNVAWL-- | | |
| | CLVAWL-- | | |
| | CYVAWL-- | | |
| | CVVAWL-- | | |
| | -CVAWLY- | | |

-CVAWLT-
DRVAWL--
DLVAWL--
-DVAWLH-
-DVAWLR-
DNVAWL--
DVVAWL--
DAVAWL--
DWVAWL--
DGVAWL--
-DVAWLA-
DTVAWL--
DHVAWL--
-DVAWLG-
-DVAWLE-
DIVAWL--
DEVAWL--
DYVAWL--
-DVAWLQ-
-DVAWLN-
-DVAWLK-
-DVAWLI-
DKVAWL--
-DVAWLY-
DMVAWL--
ELVAWL--
-EVAWLQ-
ESVAWL--
EMVAWL--
EGVAWL--
-EVAWLS-
-EVAWLL-
ETVAWL--
EKVAWL--
EAVAWL--
-EVAWLE-
-EVAWLN-
-EVAWLI-
-EVAWLH-
-EVAWLA-
EQVAWL--
EVVAWL--
-EVAWLD-
EHVAWL--
EEVAWL--
-EVAWLR-
-EVAWLG-
-EVAWLF-
ENVAWL--
FQVAWL--

FGVAWL--
-FVAWLD-
FMVAWL--
-FVAWLV-
FYVAWL--
-FVAWLH-
-FVAWLG-
GMVAWL--
GTVAWL--
-GVAWLR-
-GVAWLL-
-GVAWLS-
-GVAWLN-
-GVAWLQ-
GYVAWL--
-GVAWLT-
GSVAWL--
GVVAWL--
-GVAWLK-
-GVAWLH-
GNVAWL--
-GVAWLG-
-GVAWLA-
GWVAWL--
GAVAWL--
-GVAWLV-
-GVAWLD-
GKVAWL--
GIVAWL--
GGVAWL--
GDVAWL--
-GVAWLI-
GHVAWL--
-GVAWLE-
GEVAWL--
-GVAWLM-
GQVAWL--
-HVAWLR-
HNVAWL--
-HVAWLI-
HLVAWL--
HAVAWL--
-HVAWLQ-
-HVAWLL-
-HVAWLG-
HGVAWL--
-HVAWLS-
HDVAWL--
HSVAWL--
HMVAWL--

HVVAWL--
HQVAWL--
HWVAWL--
-HVAWLV-
-HVAWLT-
-HVAWLN-
-HVAWLA-
HIVAWL--
-HVAWLK-
HTVAWL--
-HVAWLE-
HHVAWL--
-IVAWLG-
ITVAWL--
-IVAWLD-
-IVAWLN-
IMVAWL--
IHVAWL--
IAVAWL--
-IVAWLK-
IDVAWL--
-IVAWLA-
IGVAWL--
-IVAWLV-
-IVAWLQ-
-IVAWLT-
-IVAWLH-
IIVAWL--
-KVAWLR-
KMVAWL--
-KVAWLD-
KVVAWL--
-KVAWLA-
KEVAWL--
KAVAWL--
-KVAWLQ-
-KVAWLE-
LDVAWL--
LAVAWL--
LGVAWL--
-LVAWLR-
-LVAWLG-
-LVAWLT-
-LVAWLN-
-LVAWLD-
LEVAWL--
-LVAWLF-
MVVAWL--
-MVAWLR-
-MVAWLQ-

MQVAWL--
MMVAWL--
MAVAWL--
MTVAWL--
MNVAWL--
-MVAWLL-
MEVAWL--
MHVAWL--
-MVAWLT-
-MVAWLG-
-MVAWLA-
-MVAWLV-
-MVAWLN-
-MVAWLI-
-MVAWLE-
-MVAWLD-
MDVAWL--
-NVAWLR-
NDVAWL--
-NVAWLN-
NSVAWL--
-NVAWLS-
-NVAWLA-
-NVAWLT-
NTVAWL--
NVVAWL--
NGVAWL--
-NVAWLK-
-NVAWLV-
-NVAWLH-
-NVAWLQ-
NMVAWL--
NHVAWL--
-NVAWLD-
NNVAWL--
NEVAWL--
NYVAWL--
NWVAWL--
-NVAWLP-
PAVAWL--
PSVAWL--
PNVAWL--
PDVAWL--
PLVAWL--
PHVAWL--
PGVAWL--
PVVAWL--
PTVAWL--
PQVAWL--
PMVAWL--

PEVAWL--
-QVAWLS-
QSVAWL--
-QVAWLR-
QEVAWL--
-QVAWLG-
QTVAWL--
QVVAWL--
-QVAWLE-
QDVAWL--
-QVAWLL-
QGVAWL--
-QVAWLV-
-QVAWLA-
QQVAWL--
QFVAWL--
-QVAWLN-
-QVAWLI-
QYVAWL--
QNVAWL--
QMVAWL--
QAVAWL--
-QVAWLT-
QLVAWL--
QIVAWL--
QHVAWL--
RTVAWL--
RHVAWL--
RDVAWL--
RYVAWL--
-RVAWLQ-
-RVAWLE-
RWVAWL--
SSVAWL--
-SVAWLK-
-SVAWLI-
-SVAWLG-
-SVAWLS-
SVVAWL--
SHVAWL--
-SVAWLT-
-SVAWLL-
-SVAWLA-
-SVAWLD-
-SVAWLN-
STVAWL--
SIVAWL--
-SVAWLQ-
-SVAWLE-
SGVAWL--

SNVAWL--
SAVAWL--
SDVAWL--
-SVAWLH-
SEVAWL--
-SVAWLM-
SCVAWL--
-TVAWLG-
TSVAWL--
TAVAWL--
TNVAWL--
TGVAWL--
-TVAWLH-
-TVAWLS-
-TVAWLA-
TVVAWL--
-TVAWLL-
-TVAWLT-
TTVAWL--
-TVAWLV-
-TVAWLN-
THVAWL--
-TVAWLQ-
-TVAWLE-
TDVAWL--
-TVAWLK-
TEVAWL--
--VAWLRG
--VAWLGS
--VAWLSR
--VAWLTR
--VAWLST
--VAWLRT
--VAWLRN
VEVAWL--
--VAWLRS
--VAWLGI
-VVAWLS-
--VAWLSN
--VAWLLR
--VAWLTS
--VAWLKH
VDVAWL--
--VAWLNQ
--VAWLHG
--VAWLVN
--VAWLNK
VMVAWL--
--VAWLYN
--VAWLVQ

```
--VAWLGR
--VAWLGL
--VAWLGF
--VAWLAS
--VAWLSH
--VAWLNW
--VAWLMR
--VAWLHQ
--VAWLGG
--VAWLVS
--VAWLMT
--VAWLKT
--VAWLHE
VTVAWL--
--VAWLVT
--VAWLVG
--VAWLTH
--VAWLTE
--VAWLSQ
--VAWLSG
--VAWLRV
--VAWLNT
--VAWLHH
--VAWLEG
--VAWLYG
--VAWLVK
--VAWLVD
--VAWLQT
--VAWLNE
--VAWLIH
--VAWLIG
--VAWLGW
--VAWLGA
--VAWLEK
--VAWLEA
VAVAWL--
--VAWLSE
--VAWLQN
--VAWLQK
--VAWLNM
--VAWLLD
--VAWLKY
--VAWLIN
--VAWLGN
--VAWLAI
VVVAWL--
-VVAWLD-
--VAWLTV
--VAWLTT
--VAWLQS
```

--VAWLQG
--VAWLKS
--VAWLKM
--VAWLKD
--VAWLEV
--VAWLAT
-VVAWLQ-
-VVAWLN-
--VAWLTA
--VAWLRD
--VAWLQH
--VAWLNS
--VAWLNN
--VAWLMN
--VAWLLG
--VAWLKQ
--VAWLGQ
--VAWLTN
--VAWLSI
--VAWLRQ
--VAWLLQ
--VAWLLH
--VAWLKG
--VAWLKA
--VAWLHV
--VAWLHT
--VAWLGK
--VAWLGE
--VAWLER
--VAWLEH
--VAWLDT
--VAWLAV
VQVAWL--
--VAWLVA
--VAWLTM
--VAWLMD
--VAWLIE
--VAWLET
--VAWLAG
-VVAWLH-
-VVAWLA-
--VAWLYH
--VAWLWN
--VAWLTW
--VAWLSW
--VAWLQI
--VAWLQA
--VAWLMS
--VAWLMK
--VAWLMG

--VAWLKV
--VAWLHS
--VAWLGY
--VAWLGH
--VAWLAN
--VAWLAH
--VAWLAE
VIVAWL--
--VAWLYD
--VAWLWT
--VAWLVE
--VAWLTI
--VAWLRM
--VAWLNY
--VAWLNG
--VAWLND
--VAWLLE
--VAWLKI
--VAWLKE
--VAWLID
--VAWLHD
--VAWLGV
--VAWLGD
--VAWLEW
--VAWLAK
--VAWLAA
-VVAWLE-
--VAWLRY
--VAWLQV
--VAWLNV
--VAWLNR
--VAWLMY
--VAWLHM
--VAWLFQ
--VAWLEE
--VAWLAQ
--VAWLAD
VGVAWL--
--VAWLYY
--VAWLWW
--VAWLWH
--VAWLWD
--VAWLWA
--VAWLTG
--VAWLRW
--VAWLQY
--VAWLQW
--VAWLQM
--VAWLPY
--VAWLMQ

```
              --VAWLMH
              --VAWLLV
              --VAWLLI
              --VAWLKW
              --VAWLIS
              --VAWLIK
              --VAWLHL
              --VAWLHI
              --VAWLHA
              --VAWLFV
              --VAWLFH
              --VAWLEQ
              --VAWLEN
              --VAWLCN
              --VAWLCM
              WSVAWL--
              WQVAWL--
              WDVAWL--
              WAVAWL--
              WVVAWL--
              -WVAWLK-
              -WVAWLR-
              -WVAWLN-
              -WVAWLD-
              WMVAWL--
              WGVAWL--
              WHVAWL--
              WFVAWL--
              WEVAWL--
              WWVAWL--
              -WVAWLQ-
              -WVAWLG-
              YSVAWL--
              YGVAWL--
              -YVAWLD-
              -YVAWLS-
              -YVAWLN-
              YAVAWL--
              -YVAWLG-
              -YVAWLQ-
              -YVAWLK-
              -YVAWLI-
              YDVAWL--
              -YVAWLH-
              YTVAWL--
              YNVAWL--
              YHVAWL--
              YEVAWL--
              YIVAWL--
PGSL            83        778     32.22
```

−APGSLE−
−CPGSLL−
−DPGSLA−
−GPGSLG−
−IPGSLG−
−IPGSLE−
−LPGSLA−
−LPGSLN−
−LPGSLG−
−MPGSLP−
−−PGSLGS
−−PGSLSK
−−PGSLSL
−−PGSLPL
−−PGSLAV
−−PGSLWI
−−PGSLTL
−−PGSLTI
−−PGSLTE
−−PGSLTD
−−PGSLSQ
−−PGSLQR
−−PGSLPY
−−PGSLPI
−−PGSLNN
−−PGSLNL
−−PGSLLE
−−PGSLGV
−−PGSLGL
−−PGSLEK
−−PGSLDV
−−PGSLAK
−RPGSLC−
−VPGSLG−
−APGSLR−
AVPGSL−−
−APGSLT−
−APGSLA−
ALPGSL−−
−APGSLK−
AKPGSL−−
−APGSLS−
−APGSLN−
ATPGSL−−
ARPGSL−−
−APGSLW−
−APGSLH−
AIPGSL−−
AQPGSL−−
−APGSLI−

AGPGSL--
AFPGSL--
APPGSL--
-APGSLF-
AHPGSL--
AEPGSL--
AAPGSL--
-APGSLM-
ANPGSL--
AYPGSL--
-APGSLV-
-APGSLG-
AWPGSL--
ADPGSL--
ACPGSL--
-CPGSLR-
CMPGSL--
CGPGSL--
CLPGSL--
CKPGSL--
CIPGSL--
CHPGSL--
CSPGSL--
-CPGSLH-
CNPGSL--
CAPGSL--
CRPGSL--
-CPGSLI-
CVPGSL--
CFPGSL--
CEPGSL--
CYPGSL--
CWPGSL--
CQPGSL--
-CPGSLQ-
CCPGSL--
-CPGSLM-
-CPGSLK-
DRPGSL--
-DPGSLI-
DVPGSL--
DFPGSL--
-DPGSLT-
DMPGSL--
DTPGSL--
DSPGSL--
DLPGSL--
-DPGSLW-
-DPGSLV-
-DPGSLQ-

−DPGSLL−
−DPGSLF−
−DPGSLH−
DQPGSL−−
−DPGSLY−
−DPGSLS−
−DPGSLM−
DNPGSL−−
DAPGSL−−
DWPGSL−−
DEPGSL−−
−DPGSLK−
DHPGSL−−
−EPGSLL−
−EPGSLK−
ELPGSL−−
ERPGSL−−
−EPGSLY−
ENPGSL−−
−EPGSLQ−
EMPGSL−−
ESPGSL−−
−EPGSLH−
EKPGSL−−
EIPGSL−−
EVPGSL−−
−EPGSLV−
−EPGSLS−
−EPGSLI−
−EPGSLT−
EWPGSL−−
−EPGSLF−
EFPGSL−−
−EPGSLW−
EGPGSL−−
EQPGSL−−
−EPGSLN−
ETPGSL−−
−EPGSLM−
−EPGSLA−
EPPGSL−−
EAPGSL−−
−EPGSLE−
EEPGSL−−
EDPGSL−−
ECPGSL−−
−FPGSLR−
FKPGSL−−
FNPGSL−−
FVPGSL−−

-FPGSLH-
-FPGSLI-
FLPGSL--
FGPGSL--
-FPGSLS-
-FPGSLW-
-FPGSLT-
FEPGSL--
FYPGSL--
FQPGSL--
FMPGSL--
FFPGSL--
FWPGSL--
FIPGSL--
FAPGSL--
FPPGSL--
-FPGSLQ-
-FPGSLV-
FDPGSL--
-FPGSLN-
FCPGSL--
-GPGSLL-
-GPGSLR-
GLPGSL--
-GPGSLK-
GSPGSL--
GRPGSL--
GIPGSL--
GTPGSL--
GWPGSL--
-GPGSLT-
GFPGSL--
-GPGSLY-
GKPGSL--
GYPGSL--
-GPGSLH-
GVPGSL--
-GPGSLF-
GQPGSL--
GAPGSL--
GPPGSL--
-GPGSLV-
GHPGSL--
-GPGSLQ-
-GPGSLN-
-GPGSLA-
GEPGSL--
GCPGSL--
GDPGSL--
-HPGSLR-

HLPGSL--
HVPGSL--
HGPGSL--
HTPGSL--
-HPGSLQ-
HIPGSL--
-HPGSLL-
HPPGSL--
-HPGSLK-
HFPGSL--
HSPGSL--
-HPGSLT-
-HPGSLY-
-HPGSLI-
HYPGSL--
-HPGSLS-
HQPGSL--
-HPGSLV-
HMPGSL--
HEPGSL--
-HPGSLH-
HHPGSL--
HAPGSL--
-HPGSLF-
HWPGSL--
-HPGSLM-
HNPGSL--
HKPGSL--
-HPGSLW-
-HPGSLA-
HDPGSL--
-HPGSLN-
IRPGSL--
ITPGSL--
-IPGSLH-
ILPGSL--
IHPGSL--
IGPGSL--
-IPGSLT-
IKPGSL--
ISPGSL--
-IPGSLI-
IMPGSL--
-IPGSLL-
-IPGSLQ-
IFPGSL--
-IPGSLS-
IVPGSL--
IPPGSL--
IIPGSL--

IWPGSL--
-IPGSLY-
-IPGSLK-
IAPGSL--
IYPGSL--
-IPGSLM-
-IPGSLN-
IQPGSL--
-IPGSLV-
IDPGSL--
-IPGSLA-
-IPGSLW-
IEPGSL--
-KPGSLL-
-KPGSLR-
KRPGSL--
-KPGSLK-
KLPGSL--
-KPGSLI-
KGPGSL--
KIPGSL--
KFPGSL--
KVPGSL--
KAPGSL--
-KPGSLY-
-KPGSLV-
-KPGSLN-
-KPGSLS-
-KPGSLQ-
KYPGSL--
-KPGSLW-
-KPGSLM-
KEPGSL--
KPPGSL--
-KPGSLF-
KMPGSL--
KQPGSL--
KNPGSL--
KDPGSL--
KWPGSL--
KHPGSL--
-KPGSLE-
LTPGSL--
LFPGSL--
LYPGSL--
LIPGSL--
-LPGSLK-
LKPGSL--
-LPGSLQ-
-LPGSLV-

-LPGSLS-
LPPGSL--
LAPGSL--
-LPGSLH-
LQPGSL--
-LPGSLY-
-LPGSLM-
LNPGSL--
-LPGSLI-
LWPGSL--
-LPGSLF-
-LPGSLW-
LCPGSL--
-MPGSLR-
MRPGSL--
-MPGSLT-
MTPGSL--
-MPGSLS-
-MPGSLL-
MAPGSL--
-MPGSLF-
MGPGSL--
MLPGSL--
MKPGSL--
-MPGSLK-
MVPGSL--
-MPGSLW-
MYPGSL--
MEPGSL--
-MPGSLM-
-MPGSLI-
MWPGSL--
-MPGSLN-
MHPGSL--
MQPGSL--
MFPGSL--
MPPGSL--
-MPGSLA-
MDPGSL--
MMPGSL--
-MPGSLQ-
-MPGSLE-
MCPGSL--
-NPGSLR-
NSPGSL--
NRPGSL--
NKPGSL--
NVPGSL--
-NPGSLT-
-NPGSLK-

NMPGSL--
-NPGSLI-
-NPGSLF-
NFPGSL--
NGPGSL--
NQPGSL--
-NPGSLV-
-NPGSLW-
-NPGSLS-
-NPGSLL-
NEPGSL--
NPPGSL--
NIPGSL--
NHPGSL--
-NPGSLH-
-NPGSLN-
-NPGSLM-
NAPGSL--
NNPGSL--
-NPGSLQ-
-NPGSLA-
NCPGSL--
-NPGSLC-
--PGSLLS
--PGSLRG
--PGSLRA
--PGSLST
--PGSLSV
PSPGSL--
--PGSLSA
--PGSLVS
PRPGSL--
--PGSLVT
--PGSLRH
--PGSLYV
--PGSLTS
--PGSLRY
--PGSLMK
--PGSLLG
--PGSLWS
--PGSLMH
--PGSLTA
--PGSLHR
--PGSLYR
--PGSLTV
--PGSLKT
PTPGSL--
PLPGSL--
--PGSLTT
--PGSLIG

--PGSLTG
--PGSLMS
--PGSLMI
--PGSLIA
PQPGSL--
--PGSLYN
--PGSLVH
--PGSLSG
--PGSLKV
--PGSLFT
--PGSLVV
--PGSLRQ
--PGSLIT
--PGSLAG
PIPGSL--
--PGSLYY
--PGSLYW
--PGSLSW
--PGSLNS
--PGSLMA
--PGSLHV
--PGSLYM
--PGSLYG
--PGSLVI
--PGSLRW
--PGSLFS
--PGSLFM
PVPGSL--
--PGSLWT
--PGSLQG
--PGSLMY
--PGSLIK
--PGSLHW
--PGSLFV
--PGSLFN
PAPGSL--
PYPGSL--
--PGSLVA
--PGSLTF
--PGSLLM
--PGSLKG
--PGSLIY
--PGSLIV
--PGSLYS
--PGSLMR
--PGSLIN
--PGSLHM
--PGSLVM
--PGSLTY
--PGSLIR

--PGSLHA
--PGSLFG
--PGSLFA
--PGSLYI
--PGSLWN
--PGSLRM
--PGSLMW
--PGSLMG
--PGSLLN
--PGSLKF
--PGSLIS
--PGSLIH
--PGSLHS
--PGSLFR
--PGSLFI
--PGSLAA
PMPGSL--
--PGSLRD
--PGSLLW
--PGSLLK
--PGSLLH
--PGSLKN
--PGSLKI
PFPGSL--
--PGSLYQ
--PGSLWH
--PGSLLR
--PGSLIM
--PGSLFK
--PGSLEA
--PGSLYH
--PGSLWL
--PGSLMT
--PGSLIQ
--PGSLIF
--PGSLHQ
--PGSLFH
--PGSLES
PGPGSL--
PNPGSL--
--PGSLWM
--PGSLVQ
--PGSLMQ
--PGSLHT
PKPGSL--
--PGSLYA
--PGSLWW
--PGSLSY
--PGSLSM
--PGSLQV

--PGSLQA
--PGSLMN
--PGSLKY
--PGSLHK
--PGSLAL
--PGSLSH
--PGSLRE
--PGSLKW
--PGSLHY
--PGSLHF
--PGSLFD
--PGSLWV
--PGSLWK
--PGSLWF
--PGSLWD
--PGSLVD
--PGSLTM
--PGSLSI
--PGSLSF
--PGSLKR
--PGSLKQ
--PGSLKA
--PGSLII
--PGSLHG
--PGSLAY
--PGSLAW
PEPGSL--
--PGSLNT
--PGSLLQ
--PGSLKK
--PGSLHN
PHPGSL--
--PGSLTN
--PGSLQT
--PGSLNV
--PGSLMM
--PGSLHH
--PGSLHE
--PGSLHD
PCPGSL--
--PGSLTQ
--PGSLTK
--PGSLTH
--PGSLQF
--PGSLMV
--PGSLKL
--PGSLFW
-PPGSLH-
--PGSLWE
--PGSLVE

--PGSLQN
--PGSLQK
--PGSLPT
--PGSLNW
--PGSLNQ
--PGSLNM
--PGSLNG
--PGSLMD
--PGSLIE
--PGSLGG
--PGSLGA
--PGSLFE
-PPGSLS-
--PGSLWQ
--PGSLQW
--PGSLQH
--PGSLPE
--PGSLNH
--PGSLNF
--PGSLNE
--PGSLID
--PGSLIC
--PGSLGN
--PGSLEW
--PGSLEG
--PGSLCY
--PGSLCV
--PGSLCS
--PGSLCQ
--PGSLCN
--PGSLCM
--PGSLCH
--PGSLAF
-QPGSLL-
QLPGSL--
-QPGSLW-
-QPGSLR-
QRPGSL--
-QPGSLK-
QWPGSL--
-QPGSLT-
-QPGSLS-
QVPGSL--
-QPGSLI-
QYPGSL--
-QPGSLH-
QIPGSL--
QSPGSL--
QHPGSL--
QGPGSL--

QPPGSL--
-QPGSLV-
QMPGSL--
QKPGSL--
QFPGSL--
QQPGSL--
-QPGSLY-
QAPGSL--
-QPGSLQ-
-QPGSLF-
QNPGSL--
QEPGSL--
-QPGSLN-
-QPGSLM-
-QPGSLE-
RVPGSL--
RSPGSL--
-RPGSLI-
RFPGSL--
RAPGSL--
-RPGSLS-
RYPGSL--
-RPGSLM-
-RPGSLH-
-RPGSLY-
-RPGSLW-
-RPGSLN-
RMPGSL--
-RPGSLK-
RNPGSL--
-RPGSLA-
RWPGSL--
-RPGSLV-
RDPGSL--
RIPGSL--
RQPGSL--
RPPGSL--
RHPGSL--
REPGSL--
-RPGSLQ-
-RPGSLG-
-RPGSLE-
-SPGSLL-
SRPGSL--
SVPGSL--
-SPGSLT-
STPGSL--
SLPGSL--
-SPGSLV-
-SPGSLR-

```
-SPGSLK-
SGPGSL--
SAPGSL--
-SPGSLW-
SMPGSL--
SIPGSL--
SKPGSL--
SHPGSL--
SYPGSL--
SEPGSL--
-SPGSLQ-
SNPGSL--
-SPGSLY-
SWPGSL--
SQPGSL--
-SPGSLA-
-SPGSLN-
SDPGSL--
-SPGSLM-
SPPGSL--
-TPGSLK-
TRPGSL--
TAPGSL--
-TPGSLS-
TYPGSL--
TIPGSL--
TGPGSL--
-TPGSLW-
TWPGSL--
TTPGSL--
TSPGSL--
TVPGSL--
-TPGSLQ-
TKPGSL--
-TPGSLY-
-TPGSLT-
-TPGSLM-
TNPGSL--
THPGSL--
TFPGSL--
-TPGSLI-
TPPGSL--
-TPGSLN-
-TPGSLH-
TDPGSL--
TQPGSL--
TMPGSL--
-TPGSLA-
TCPGSL--
-VPGSLR-
```

VRPGSL--
VLPGSL--
VVPGSL--
-VPGSLI-
-VPGSLK-
VHPGSL--
VGPGSL--
VNPGSL--
-VPGSLW-
VMPGSL--
VIPGSL--
-VPGSLA-
VFPGSL--
VPPGSL--
-VPGSLF-
VYPGSL--
VQPGSL--
-VPGSLM-
-VPGSLH-
-VPGSLQ-
VEPGSL--
-VPGSLV-
VDPGSL--
-VPGSLE-
WRPGSL--
-WPGSLT-
WGPGSL--
-WPGSLS-
WKPGSL--
WDPGSL--
WSPGSL--
-WPGSLM-
WYPGSL--
-WPGSLW-
-WPGSLI-
WAPGSL--
-WPGSLY-
WNPGSL--
WQPGSL--
WPPGSL--
-WPGSLV-
-WPGSLQ-
-WPGSLF-
-WPGSLN-
WHPGSL--
WEPGSL--
-WPGSLA-
WCPGSL--
YRPGSL--
-YPGSLS-

YNPGSL--
-YPGSLV-
YKPGSL--
YAPGSL--
YSPGSL--
-YPGSLT-
-YPGSLQ-
YPPGSL--
-YPGSLY-
YHPGSL--
-YPGSLK-
-YPGSLI-
-YPGSLH-
YFPGSL--
YGPGSL--
YQPGSL--
YEPGSL--
YDPGSL--
YIPGSL--
YYPGSL--
-YPGSLF-
-YPGSLM-
-YPGSLA-
YMPGSL--
-YPGSLN-
-YPGSLW-
YCPGSL--
-YPGSLE-

PGQL 60 796 32.21

ESPGQL--
-GPGQLG-
--PGQLTL
--PGQLGV
--PGQLAL
-TPGQLN-
-TPGQLG-
-APGQLR-
AYPGQL--
AHPGQL--
ARPGQL--
ASPGQL--
AKPGQL--
ALPGQL--
-APGQLN-
-APGQLF-
AVPGQL--
-APGQLL-
-APGQLA-
AIPGQL--
AAPGQL--

ATPGQL--
APPGQL--
-APGQLW-
-APGQLT-
-APGQLG-
AFPGQL--
AGPGQL--
AEPGQL--
AWPGQL--
-APGQLK-
-APGQLE-
-APGQLQ-
ANPGQL--
AQPGQL--
-APGQLY-
-APGQLV-
-APGQLM-
AMPGQL--
CCPGQL--
CTPGQL--
CSPGQL--
CVPGQL--
CQPGQL--
-CPGQLR-
CLPGQL--
CYPGQL--
CRPGQL--
-CPGQLV-
-CPGQLT-
-CPGQLS-
CKPGQL--
CFPGQL--
CDPGQL--
CAPGQL--
DRPGQL--
-DPGQLL-
-DPGQLH-
DTPGQL--
DMPGQL--
DVPGQL--
DSPGQL--
DNPGQL--
DFPGQL--
DYPGQL--
DLPGQL--
-DPGQLK-
DDPGQL--
DAPGQL--
DWPGQL--
-DPGQLS-

−DPGQLM−
−DPGQLA−
DKPGQL−−
DHPGQL−−
DGPGQL−−
DEPGQL−−
−EPGQLL−
−EPGQLH−
−EPGQLK−
EVPGQL−−
−EPGQLR−
−EPGQLS−
−EPGQLQ−
EYPGQL−−
−EPGQLF−
−EPGQLA−
EAPGQL−−
ERPGQL−−
−EPGQLV−
−EPGQLN−
−EPGQLM−
EMPGQL−−
EHPGQL−−
ETPGQL−−
EQPGQL−−
−EPGQLW−
−EPGQLE−
ELPGQL−−
EKPGQL−−
EIPGQL−−
EFPGQL−−
−FPGQLR−
FVPGQL−−
FNPGQL−−
FIPGQL−−
−FPGQLL−
FLPGQL−−
FTPGQL−−
−FPGQLM−
−FPGQLV−
−FPGQLT−
FMPGQL−−
FGPGQL−−
−FPGQLY−
FHPGQL−−
FRPGQL−−
−FPGQLS−
−FPGQLK−
FAPGQL−−
FYPGQL−−

FQPGQL--
-FPGQLQ-
-FPGQLN-
-FPGQLH-
FFPGQL--
FPPGQL--
-FPGQLF-
FKPGQL--
FWPGQL--
-FPGQLI-
FEPGQL--
-GPGQLL-
-GPGQLK-
GWPGQL--
-GPGQLW-
GRPGQL--
-GPGQLR-
GSPGQL--
-GPGQLA-
GVPGQL--
GTPGQL--
-GPGQLV-
-GPGQLS-
GDPGQL--
GHPGQL--
-GPGQLT-
GKPGQL--
GNPGQL--
GAPGQL--
GPPGQL--
-GPGQLN-
GLPGQL--
GGPGQL--
-GPGQLY-
-GPGQLF-
GIPGQL--
GFPGQL--
-GPGQLM-
-GPGQLI-
-GPGQLH-
-GPGQLE-
GMPGQL--
GYPGQL--
-GPGQLQ-
-HPGQLS-
-HPGQLL-
HRPGQL--
-HPGQLR-
-HPGQLW-
HHPGQL--

−HPGQLT−
HWPGQL−−
−HPGQLN−
HLPGQL−−
HEPGQL−−
HSPGQL−−
−HPGQLK−
−HPGQLV−
HNPGQL−−
HKPGQL−−
HIPGQL−−
HYPGQL−−
−HPGQLH−
−HPGQLG−
HGPGQL−−
HCPGQL−−
−HPGQLY−
−HPGQLQ−
−HPGQLF−
−HPGQLA−
HFPGQL−−
HVPGQL−−
−HPGQLE−
HMPGQL−−
IIPGQL−−
−IPGQLS−
IFPGQL−−
ISPGQL−−
INPGQL−−
−IPGQLF−
IYPGQL−−
−IPGQLY−
−IPGQLN−
IVPGQL−−
−IPGQLL−
IKPGQL−−
ITPGQL−−
−IPGQLQ−
ILPGQL−−
IAPGQL−−
−IPGQLW−
−IPGQLV−
−IPGQLT−
−IPGQLI−
−IPGQLH−
IMPGQL−−
IPPGQL−−
−IPGQLA−
IEPGQL−−
IRPGQL−−

-IPGQLK-
-IPGQLE-
-IPGQLC-
KSPGQL--
-KPGQLR-
KGPGQL--
-KPGQLM-
KLPGQL--
-KPGQLI-
-KPGQLT-
KMPGQL--
-KPGQLS-
-KPGQLQ-
-KPGQLL-
-KPGQLN-
-KPGQLH-
-KPGQLF-
-KPGQLA-
KAPGQL--
KTPGQL--
KQPGQL--
-KPGQLY-
-KPGQLW-
-KPGQLV-
KIPGQL--
KRPGQL--
-KPGQLK-
KWPGQL--
KNPGQL--
KFPGQL--
KYPGQL--
KCPGQL--
-KPGQLE-
KKPGQL--
-LPGQLL-
-LPGQLK-
-LPGQLW-
-LPGQLR-
LWPGQL--
LLPGQL--
LHPGQL--
-LPGQLQ-
LRPGQL--
LIPGQL--
LAPGQL--
LYPGQL--
-LPGQLF-
LSPGQL--
-LPGQLV-
LGPGQL--

LNPGQL--
-LPGQLT-
LVPGQL--
-LPGQLN-
LTPGQL--
-LPGQLH-
LQPGQL--
LFPGQL--
LMPGQL--
LKPGQL--
-LPGQLS-
LEPGQL--
-LPGQLM-
LCPGQL--
-LPGQLA-
LDPGQL--
-MPGQLR-
MSPGQL--
MRPGQL--
-MPGQLQ-
-MPGQLN-
-MPGQLL-
MKPGQL--
-MPGQLS-
-MPGQLM-
MPPGQL--
MMPGQL--
MVPGQL--
MAPGQL--
-MPGQLV-
-MPGQLF-
-MPGQLA-
MHPGQL--
MWPGQL--
-MPGQLI-
-MPGQLH-
MGPGQL--
-MPGQLY-
-MPGQLW-
-MPGQLK-
MNPGQL--
MIPGQL--
MDPGQL--
MYPGQL--
MTPGQL--
MFPGQL--
-NPGQLR-
-NPGQLT-
NKPGQL--
NVPGQL--

-NPGQLK-
NFPGQL--
-NPGQLW-
-NPGQLN-
NAPGQL--
-NPGQLQ-
NPPGQL--
NLPGQL--
NIPGQL--
NTPGQL--
-NPGQLL-
-NPGQLH-
-NPGQLA-
NMPGQL--
NHPGQL--
NGPGQL--
NQPGQL--
-NPGQLS-
-NPGQLF-
NDPGQL--
NSPGQL--
-NPGQLM-
NYPGQL--
NNPGQL--
NEPGQL--
PRPGQL--
PLPGQL--
--PGQLFW
--PGQLLA
--PGQLRI
--PGQLLS
--PGQLRS
--PGQLRN
--PGQLVS
--PGQLLT
--PGQLIA
--PGQLWW
--PGQLVA
PTPGQL--
PQPGQL--
--PGQLWT
--PGQLSW
--PGQLLV
--PGQLLG
--PGQLKS
--PGQLYV
--PGQLTY
--PGQLKR
--PGQLAG
--PGQLVR

--PGQLSI
--PGQLRY
--PGQLLH
--PGQLHA
--PGQLFG
--PGQLYS
--PGQLVN
--PGQLVF
--PGQLRM
--PGQLRF
--PGQLLN
--PGQLIR
--PGQLFY
--PGQLFS
--PGQLWS
--PGQLVH
--PGQLSA
--PGQLNV
--PGQLLK
--PGQLKL
--PGQLWR
--PGQLVG
--PGQLRV
--PGQLYK
--PGQLWN
--PGQLVY
--PGQLVW
--PGQLTS
--PGQLTG
--PGQLRW
--PGQLMQ
--PGQLKN
--PGQLKM
--PGQLHV
--PGQLFV
--PGQLAS
--PGQLYT
--PGQLVD
--PGQLSN
--PGQLSG
--PGQLRQ
--PGQLRK
--PGQLRA
--PGQLQW
--PGQLQS
--PGQLMA
--PGQLKT
--PGQLIE
PFPGQL--
PNPGQL--

--PGQLVK
--PGQLTV
--PGQLTN
--PGQLNT
--PGQLNR
--PGQLMW
--PGQLMF
--PGQLLD
--PGQLIW
--PGQLFI
--PGQLES
PVPGQL--
PMPGQL--
--PGQLYL
--PGQLWV
--PGQLWE
--PGQLWA
--PGQLVV
--PGQLTW
--PGQLSY
--PGQLSR
--PGQLRD
--PGQLNW
--PGQLNS
--PGQLMT
--PGQLLQ
--PGQLKK
--PGQLKI
--PGQLKE
PYPGQL--
--PGQLYW
--PGQLWM
--PGQLWK
--PGQLTT
--PGQLTI
--PGQLSS
--PGQLSH
--PGQLRE
--PGQLMK
--PGQLLM
--PGQLKW
--PGQLKG
--PGQLIQ
--PGQLIM
--PGQLIL
--PGQLFH
PAPGQL--
--PGQLYR
--PGQLYM
--PGQLYI

--PGQLYG
--PGQLVT
--PGQLTK
--PGQLQY
--PGQLQQ
--PGQLQA
--PGQLNN
--PGQLMS
--PGQLMR
--PGQLLR
--PGQLKA
--PGQLIT
--PGQLFA
--PGQLAT
PSPGQL--
PIPGQL--
--PGQLYQ
--PGQLYH
--PGQLYF
--PGQLYA
--PGQLWL
--PGQLWG
--PGQLVQ
--PGQLVE
--PGQLTR
--PGQLTM
--PGQLRH
--PGQLQR
--PGQLQF
--PGQLNL
--PGQLNK
--PGQLNI
--PGQLMM
--PGQLKH
--PGQLKD
--PGQLIH
--PGQLIG
--PGQLHM
--PGQLHK
--PGQLGS
--PGQLGL
--PGQLFQ
--PGQLFM
--PGQLYN
--PGQLTD
--PGQLTA
--PGQLSQ
--PGQLSM
--PGQLSE
--PGQLQV

--PGQLQL
--PGQLNM
--PGQLNG
--PGQLNF
--PGQLMI
--PGQLMH
--PGQLMG
--PGQLIS
--PGQLHT
--PGQLHR
--PGQLHQ
--PGQLGA
--PGQLFT
--PGQLFN
--PGQLEG
--PGQLEA
PKPGQL--
PHPGQL--
--PGQLYE
--PGQLYD
--PGQLVM
--PGQLVC
--PGQLTH
--PGQLSK
--PGQLSF
--PGQLQT
--PGQLQG
--PGQLQE
--PGQLQD
--PGQLPM
--PGQLPL
--PGQLNY
--PGQLND
--PGQLNA
--PGQLMY
--PGQLMN
--PGQLME
--PGQLMD
--PGQLLE
--PGQLIY
--PGQLIV
--PGQLIN
--PGQLIK
--PGQLIF
--PGQLHY
--PGQLHI
--PGQLHH
--PGQLHD
--PGQLGQ
--PGQLGI

```
--PGQLFL
--PGQLFK
--PGQLFD
--PGQLAW
--PGQLAV
-QPGQLW-
QVPGQL--
-QPGQLR-
-QPGQLM-
-QPGQLA-
QRPGQL--
-QPGQLT-
-QPGQLL-
QFPGQL--
QSPGQL--
-QPGQLS-
-QPGQLQ-
-QPGQLI-
-QPGQLH-
QKPGQL--
-QPGQLY-
QLPGQL--
QGPGQL--
QYPGQL--
QMPGQL--
QIPGQL--
QHPGQL--
QDPGQL--
QAPGQL--
QTPGQL--
QQPGQL--
QPPGQL--
-QPGQLN-
-QPGQLF-
QEPGQL--
-RPGQLL-
-RPGQLR-
RGPGQL--
RSPGQL--
-RPGQLV-
RLPGQL--
RYPGQL--
REPGQL--
-RPGQLH-
RNPGQL--
-RPGQLY-
-RPGQLS-
-RPGQLI-
RAPGQL--
RWPGQL--
```

RTPGQL--
-RPGQLM-
-RPGQLT-
-RPGQLN-
-RPGQLK-
-RPGQLQ-
RFPGQL--
RDPGQL--
RQPGQL--
RPPGQL--
-RPGQLE-
-RPGQLA-
RKPGQL--
RHPGQL--
RIPGQL--
-RPGQLG-
RCPGQL--
-SPGQLR-
SVPGQL--
-SPGQLN-
-SPGQLL-
SLPGQL--
SIPGQL--
SFPGQL--
-SPGQLT-
SKPGQL--
-SPGQLA-
SRPGQL--
-SPGQLM-
-SPGQLK-
-SPGQLW-
SEPGQL--
STPGQL--
-SPGQLQ-
SGPGQL--
SYPGQL--
-SPGQLI-
-SPGQLH-
-SPGQLF-
SSPGQL--
SQPGQL--
SHPGQL--
SMPGQL--
-SPGQLY-
SAPGQL--
SWPGQL--
SPPGQL--
SDPGQL--
-SPGQLG-
-SPGQLE-

SCPGQL--
TTPGQL--
-TPGQLR-
-TPGQLQ-
-TPGQLY-
-TPGQLT-
TSPGQL--
TRPGQL--
TWPGQL--
-TPGQLV-
TKPGQL--
TIPGQL--
-TPGQLL-
TYPGQL--
TPPGQL--
-TPGQLK-
-TPGQLI-
-TPGQLH-
TFPGQL--
TQPGQL--
TMPGQL--
TEPGQL--
TAPGQL--
TVPGQL--
-TPGQLF-
-TPGQLS-
TNPGQL--
TLPGQL--
-TPGQLW-
THPGQL--
TGPGQL--
TDPGQL--
TCPGQL--
-TPGQLM-
VSPGQL--
-VPGQLR-
VNPGQL--
VFPGQL--
-VPGQLT-
VRPGQL--
-VPGQLK-
VLPGQL--
-VPGQLV-
VIPGQL--
-VPGQLQ-
-VPGQLH-
VWPGQL--
VPPGQL--
-VPGQLN-
-VPGQLS-

VYPGQL--
VVPGQL--
VMPGQL--
VHPGQL--
-VPGQLM-
VKPGQL--
-VPGQLI-
-VPGQLA-
VGPGQL--
VDPGQL--
VAPGQL--
-VPGQLW-
-VPGQLF-
VCPGQL--
VEPGQL--
-WPGQLR-
WVPGQL--
WAPGQL--
WPPGQL--
-WPGQLV-
WLPGQL--
WTPGQL--
WRPGQL--
-WPGQLK-
WNPGQL--
-WPGQLW-
-WPGQLL-
WEPGQL--
-WPGQLS-
-WPGQLN-
WMPGQL--
WKPGQL--
-WPGQLT-
WFPGQL--
-WPGQLM-
WIPGQL--
WGPGQL--
-WPGQLI-
WCPGQL--
WYPGQL--
-WPGQLY-
WQPGQL--
-WPGQLQ-
WHPGQL--
WDPGQL--
-YPGQLR-
YRPGQL--
YVPGQL--
YQPGQL--
-YPGQLW-

-YPGQLF-
YLPGQL--
YDPGQL--
-YPGQLS-
YTPGQL--
-YPGQLL-
YKPGQL--
YEPGQL--
YSPGQL--
YAPGQL--
-YPGQLH-
YMPGQL--
YHPGQL--
-YPGQLV-
-YPGQLK-
YIPGQL--
YWPGQL--
-YPGQLY-
-YPGQLQ-
YNPGQL--
YFPGQL--
YYPGQL--
YPPGQL--
-YPGQLI-
-YPGQLC-

PLAW 209 706 32.19

--PLAWSM
-APLAWR-
-APLAWG-
-APLAWN-
-APLAWL-
-APLAWK-
AWPLAW--
-APLAWH-
ATPLAW--
-APLAWT-
-APLAWM-
AMPLAW--
ANPLAW--
AHPLAW--
AFPLAW--
APPLAW--
ARPLAW--
-APLAWS-
-APLAWF-
ALPLAW--
AQPLAW--
-APLAWY-
-APLAWQ-
-APLAWA-

AYPLAW--
-APLAWE-
-APLAWD-
AGPLAW--
ADPLAW--
AKPLAW--
CQPLAW--
CAPLAW--
CVPLAW--
CTPLAW--
-CPLAWR-
CKPLAW--
CHPLAW--
CCPLAW--
DRPLAW--
-DPLAWL-
DLPLAW--
DTPLAW--
DDPLAW--
-DPLAWT-
DAPLAW--
-DPLAWH-
-DPLAWR-
-DPLAWF-
DKPLAW--
DVPLAW--
DSPLAW--
DQPLAW--
-DPLAWW-
-DPLAWN-
-DPLAWI-
DFPLAW--
DYPLAW--
-DPLAWV-
-DPLAWS-
-DPLAWK-
-DPLAWA-
DNPLAW--
DIPLAW--
DHPLAW--
ESPLAW--
ETPLAW--
ERPLAW--
-EPLAWI-
-EPLAWL-
-EPLAWV-
EKPLAW--
EEPLAW--
-EPLAWS-
-EPLAWM-

```
-EPLAWH-
-EPLAWQ-
-EPLAWN-
-EPLAWK-
ELPLAW--
EGPLAW--
EFPLAW--
EYPLAW--
EWPLAW--
-EPLAWW-
-EPLAWT-
-EPLAWA-
EIPLAW--
EAPLAW--
-FPLAWS-
-FPLAWR-
FNPLAW--
FAPLAW--
-FPLAWH-
FTPLAW--
-FPLAWV-
-FPLAWA-
FKPLAW--
FGPLAW--
-FPLAWT-
-FPLAWL-
FLPLAW--
FIPLAW--
FPPLAW--
-FPLAWW-
-FPLAWQ-
-FPLAWN-
-FPLAWG-
FDPLAW--
FQPLAW--
-FPLAWM-
-FPLAWI-
-FPLAWE-
FFPLAW--
GRPLAW--
-GPLAWQ-
GFPLAW--
-GPLAWT-
-GPLAWW-
GSPLAW--
-GPLAWI-
GKPLAW--
GPPLAW--
-GPLAWY-
GNPLAW--
```

```
-GPLAWR-
-GPLAWA-
GVPLAW--
-GPLAWL-
-GPLAWG-
GLPLAW--
GHPLAW--
GAPLAW--
GTPLAW--
-GPLAWS-
-GPLAWH-
GIPLAW--
GEPLAW--
GYPLAW--
GQPLAW--
-GPLAWN-
GMPLAW--
GDPLAW--
GCPLAW--
GWPLAW--
-GPLAWV-
-GPLAWK-
-GPLAWF-
-GPLAWE-
HRPLAW--
HGPLAW--
-HPLAWV-
HKPLAW--
HYPLAW--
HVPLAW--
-HPLAWQ-
-HPLAWN-
-HPLAWG-
HLPLAW--
-HPLAWR-
HMPLAW--
HQPLAW--
-HPLAWK-
-HPLAWF-
HCPLAW--
HTPLAW--
HSPLAW--
HPPLAW--
-HPLAWL-
-HPLAWE-
HFPLAW--
HWPLAW--
-HPLAWT-
-HPLAWH-
HNPLAW--
```

HIPLAW--
HEPLAW--
HDPLAW--
HAPLAW--
-IPLAWI-
IGPLAW--
-IPLAWH-
-IPLAWK-
IDPLAW--
IPPLAW--
-IPLAWW-
-IPLAWY-
IVPLAW--
ITPLAW--
-IPLAWL-
ISPLAW--
IRPLAW--
-IPLAWS-
-IPLAWA-
IEPLAW--
IAPLAW--
-IPLAWT-
-IPLAWN-
-IPLAWR-
-IPLAWG-
INPLAW--
IIPLAW--
IYPLAW--
IQPLAW--
-IPLAWQ-
-IPLAWE-
IMPLAW--
ILPLAW--
IFPLAW--
KSPLAW--
-KPLAWN-
KFPLAW--
-KPLAWH-
KGPLAW--
KTPLAW--
-KPLAWM-
-KPLAWS-
-KPLAWY-
-KPLAWL-
-KPLAWE-
KYPLAW--
KVPLAW--
-KPLAWK-
-KPLAWI-
-KPLAWG-

−KPLAWF−
KLPLAW−−
KWPLAW−−
KKPLAW−−
−KPLAWV−
KDPLAW−−
−KPLAWR−
−KPLAWQ−
KNPLAW−−
KQPLAW−−
KPPLAW−−
−KPLAWW−
KMPLAW−−
LSPLAW−−
−LPLAWH−
LRPLAW−−
−LPLAWN−
−LPLAWT−
−LPLAWK−
LVPLAW−−
LKPLAW−−
LGPLAW−−
LPPLAW−−
LLPLAW−−
LEPLAW−−
LTPLAW−−
LHPLAW−−
−LPLAWA−
LMPLAW−−
LFPLAW−−
−LPLAWE−
LYPLAW−−
LWPLAW−−
LQPLAW−−
−LPLAWY−
−LPLAWS−
−LPLAWQ−
LDPLAW−−
−LPLAWV−
LIPLAW−−
MNPLAW−−
−MPLAWR−
MGPLAW−−
MSPLAW−−
−MPLAWN−
−MPLAWL−
MWPLAW−−
MRPLAW−−
MFPLAW−−
−MPLAWS−

```
MDPLAW--
MTPLAW--
MPPLAW--
-MPLAWI-
-MPLAWA-
MVPLAW--
-MPLAWK-
MKPLAW--
MHPLAW--
-MPLAWM-
-MPLAWH-
-MPLAWG-
-MPLAWF-
-MPLAWE-
MEPLAW--
MAPLAW--
NSPLAW--
-NPLAWR-
NRPLAW--
-NPLAWH-
NNPLAW--
NLPLAW--
NHPLAW--
NTPLAW--
-NPLAWT-
NWPLAW--
NVPLAW--
NEPLAW--
NQPLAW--
-NPLAWW-
-NPLAWE-
-NPLAWA-
NIPLAW--
NYPLAW--
-NPLAWV-
-NPLAWK-
NGPLAW--
-NPLAWQ-
-NPLAWM-
NKPLAW--
-NPLAWG-
NMPLAW--
NFPLAW--
NAPLAW--
--PLAWRS
--PLAWLT
--PLAWLS
--PLAWRA
--PLAWRN
--PLAWKL
```

--PLAWRY
--PLAWGI
--PLAWVT
--PLAWVN
--PLAWSL
--PLAWAS
--PLAWYS
--PLAWTN
--PLAWLG
--PLAWTL
--PLAWST
--PLAWSG
--PLAWRW
--PLAWQR
--PLAWFR
--PLAWAL
--PLAWSF
--PLAWRG
--PLAWPL
--PLAWNR
--PLAWMS
--PLAWLK
--PLAWIG
--PLAWWR
--PLAWRV
--PLAWMT
--PLAWLN
--PLAWGS
--PLAWYR
--PLAWSR
--PLAWKS
--PLAWHS
--PLAWFG
--PLAWSQ
--PLAWSK
--PLAWRD
--PLAWNL
--PLAWAY
--PLAWWA
--PLAWTT
--PLAWNV
--PLAWNS
--PLAWLQ
--PLAWLE
--PLAWIT
--PLAWYN
--PLAWYA
--PLAWVS
--PLAWVI
--PLAWTR

--PLAWSN
--PLAWRH
--PLAWQF
--PLAWMR
--PLAWIA
--PLAWHG
--PLAWGT
--PLAWYD
--PLAWWN
--PLAWTW
--PLAWTK
--PLAWTA
--PLAWSW
--PLAWSV
--PLAWRQ
--PLAWQS
--PLAWML
--PLAWKF
--PLAWIN
--PLAWHQ
--PLAWFH
--PLAWVG
--PLAWTG
--PLAWTE
--PLAWSI
--PLAWSA
--PLAWMK
--PLAWLH
--PLAWKI
--PLAWIS
--PLAWIH
--PLAWHF
--PLAWFY
--PLAWAN
PNPLAW--
--PLAWWQ
--PLAWVY
--PLAWTY
--PLAWTS
--PLAWSE
--PLAWQK
--PLAWQG
--PLAWNM
--PLAWNI
--PLAWMV
--PLAWMQ
--PLAWMG
--PLAWMA
--PLAWLA
--PLAWKV

--PLAWKT
--PLAWKQ
--PLAWKN
--PLAWKM
--PLAWKD
--PLAWGV
--PLAWGL
--PLAWAV
--PLAWAR
--PLAWAM
--PLAWAA
--PLAWWM
--PLAWWL
--PLAWWG
--PLAWVK
--PLAWVE
--PLAWTM
--PLAWSH
--PLAWRT
--PLAWNY
--PLAWNT
--PLAWNA
--PLAWMN
--PLAWMH
--PLAWKK
--PLAWKH
--PLAWKA
--PLAWIF
--PLAWHN
--PLAWHH
--PLAWGY
--PLAWFM
--PLAWFD
--PLAWEV
--PLAWET
--PLAWEL
--PLAWEI
--PLAWDS
--PLAWAT
--PLAWAI
--PLAWAH
--PLAWYY
--PLAWYE
--PLAWWS
--PLAWWK
--PLAWWI
--PLAWWD
--PLAWRE
--PLAWQW
--PLAWQL

--PLAWPM
--PLAWMM
--PLAWMD
--PLAWLD
--PLAWIY
--PLAWIW
--PLAWIV
--PLAWIM
--PLAWIK
--PLAWIE
--PLAWFE
--PLAWES
--PLAWEG
PDPLAW--
PWPLAW--
--PLAWYT
--PLAWYK
--PLAWYF
--PLAWWT
--PLAWVQ
--PLAWTQ
--PLAWTI
--PLAWSY
--PLAWQI
--PLAWQH
--PLAWQD
--PLAWPV
--PLAWNW
--PLAWNN
--PLAWMP
--PLAWMI
--PLAWKW
--PLAWKG
--PLAWKC
--PLAWII
--PLAWID
--PLAWHW
--PLAWHV
--PLAWFK
--PLAWCT
--PLAWAE
-QPLAWT-
-QPLAWR-
-QPLAWL-
-QPLAWF-
-QPLAWN-
-QPLAWW-
-QPLAWA-
QSPLAW--
-QPLAWV-

QYPLAW--
QTPLAW--
QVPLAW--
-QPLAWI-
QQPLAW--
-QPLAWH-
-QPLAWG-
QMPLAW--
QEPLAW--
-QPLAWS-
-QPLAWK-
QNPLAW--
QGPLAW--
-RPLAWS-
RSPLAW--
RGPLAW--
-RPLAWR-
RIPLAW--
RVPLAW--
-RPLAWV-
RTPLAW--
-RPLAWT-
RKPLAW--
-RPLAWF-
-RPLAWE-
REPLAW--
RPPLAW--
-RPLAWN-
RAPLAW--
-RPLAWA-
RYPLAW--
RWPLAW--
-RPLAWM-
RMPLAW--
RLPLAW--
-RPLAWQ-
RFPLAW--
-RPLAWI-
RDPLAW--
-RPLAWW-
-RPLAWH-
RHPLAW--
-SPLAWS-
-SPLAWR-
STPLAW--
SEPLAW--
-SPLAWT-
-SPLAWN-
SQPLAW--
-SPLAWQ-

SWPLAW--
SHPLAW--
-SPLAWK-
SKPLAW--
SSPLAW--
-SPLAWV-
-SPLAWF-
SMPLAW--
SLPLAW--
SPPLAW--
-SPLAWG-
SNPLAW--
-SPLAWW-
-SPLAWM-
-SPLAWH-
-SPLAWI-
SYPLAW--
-SPLAWY-
SFPLAW--
SDPLAW--
-SPLAWE-
-SPLAWD-
SIPLAW--
-TPLAWL-
-TPLAWT-
TYPLAW--
TSPLAW--
TAPLAW--
-TPLAWR-
-TPLAWH-
TQPLAW--
TIPLAW--
THPLAW--
-TPLAWV-
-TPLAWI-
TLPLAW--
TVPLAW--
-TPLAWK-
TNPLAW--
TMPLAW--
-TPLAWM-
-TPLAWA-
TKPLAW--
TFPLAW--
TCPLAW--
TTPLAW--
TPPLAW--
-TPLAWY-
-TPLAWN-
-TPLAWG-

TGPLAW--
TWPLAW--
TDPLAW--
-TPLAWW-
-TPLAWQ-
-TPLAWE-
TEPLAW--
-VPLAWS-
VKPLAW--
-VPLAWV-
VLPLAW--
VWPLAW--
-VPLAWN-
VYPLAW--
VRPLAW--
VTPLAW--
VSPLAW--
VFPLAW--
-VPLAWT-
-VPLAWQ-
-VPLAWM-
VVPLAW--
-VPLAWL-
-VPLAWI-
-VPLAWA-
-VPLAWH-
VNPLAW--
VGPLAW--
VQPLAW--
-VPLAWW-
-VPLAWG-
VHPLAW--
VPPLAW--
-VPLAWF-
-VPLAWD-
-WPLAWH-
WEPLAW--
WTPLAW--
WQPLAW--
WAPLAW--
-WPLAWR-
-WPLAWS-
WNPLAW--
WMPLAW--
WWPLAW--
-WPLAWN-
-WPLAWK-
WPPLAW--
-WPLAWV-
-WPLAWL-

-WPLAWI-
-WPLAWE-
-WPLAWA-
-YPLAWR-
-YPLAWN-
YLPLAW--
-YPLAWH-
YYPLAW--
YRPLAW--
-YPLAWS-
YSPLAW--
-YPLAWA-
-YPLAWK-
YTPLAW--
YAPLAW--
-YPLAWQ-
YKPLAW--
YHPLAW--
-YPLAWW-
-YPLAWV-
-YPLAWL-
YPPLAW--
-YPLAWM-
-YPLAWI-
YQPLAW--
-YPLAWT-
-YPLAWE-
YNPLAW--
YGPLAW--
YMPLAW--
YIPLAW--
YEPLAW--

LRGY 523 599 32.18

-FLRGYE-
-FLRGYD-
-GLRGYF-
-HLRGYG-
-KLRGYY-
-KLRGYM-
--LRGYSD
--LRGYQY
--LRGYLI
--LRGYIY
--LRGYYI
--LRGYSE
--LRGYQW
--LRGYQV
--LRGYNV
--LRGYNM
--LRGYKD

--LRGYHY
--LRGYHH
--LRGYGV
--LRGYGF
--LRGYGA
--LRGYFN
--LRGYFD
--LRGYEI
-LLRGYP-
-PLRGYV-
-RLRGYI-
-RLRGYT-
-RLRGYF-
-SLRGYD-
-TLRGYS-
-TLRGYW-
-TLRGYQ-
-TLRGYP-
-ALRGYS-
-ALRGYL-
ATLRGY--
AVLRGY--
-ALRGYG-
-ALRGYF-
-ALRGYY-
-ALRGYV-
-ALRGYA-
-ALRGYM-
AFLRGY--
-ALRGYT-
-ALRGYP-
-ALRGYN-
AQLRGY--
ANLRGY--
-ALRGYQ-
AKLRGY--
ADLRGY--
-ALRGYW-
-ALRGYD-
-ALRGYE-
ACLRGY--
CVLRGY--
CSLRGY--
CQLRGY--
CNLRGY--
CLLRGY--
CTLRGY--
CRLRGY--
CGLRGY--
CELRGY--

CDLRGY--
CWLRGY--
CPLRGY--
CMLRGY--
CFLRGY--
CALRGY--
CYLRGY--
-CLRGYM-
-CLRGYI-
CKLRGY--
DKLRGY--
DLLRGY--
DSLRGY--
DRLRGY--
DYLRGY--
DVLRGY--
-DLRGYR-
DWLRGY--
DMLRGY--
DILRGY--
DELRGY--
-DLRGYQ-
DGLRGY--
DALRGY--
-DLRGYK-
-DLRGYT-
-DLRGYH-
-DLRGYS-
DDLRGY--
DQLRGY--
-DLRGYP-
-DLRGYG-
-DLRGYA-
DFLRGY--
DPLRGY--
-DLRGYY-
DNLRGY--
-DLRGYM-
-DLRGYF-
-DLRGYW-
-DLRGYN-
DCLRGY--
ELLRGY--
-ELRGYL-
ESLRGY--
-ELRGYT-
-ELRGYR-
ETLRGY--
-ELRGYS-
EALRGY--

```
-ELRGYF-
EQLRGY--
-ELRGYG-
-ELRGYY-
-ELRGYM-
-ELRGYA-
EMLRGY--
-ELRGYQ-
EYLRGY--
EPLRGY--
EWLRGY--
-ELRGYP-
-ELRGYK-
-ELRGYI-
-ELRGYH-
EHLRGY--
EDLRGY--
EVLRGY--
-ELRGYW-
EFLRGY--
ENLRGY--
EKLRGY--
EELRGY--
-ELRGYD-
ECLRGY--
-ELRGYE-
FVLRGY--
FGLRGY--
FSLRGY--
FDLRGY--
FMLRGY--
FKLRGY--
FPLRGY--
-FLRGYM-
FQLRGY--
-FLRGYQ-
-FLRGYW-
FELRGY--
FILRGY--
FCLRGY--
GVLRGY--
GQLRGY--
GMLRGY--
GPLRGY--
-GLRGYS-
GHLRGY--
GGLRGY--
GDLRGY--
-GLRGYP-
GELRGY--
```

−GLRGYT−
−GLRGYW−
−GLRGYQ−
−GLRGYI−
GCLRGY−−
−GLRGYY−
−GLRGYV−
−GLRGYG−
−GLRGYA−
−GLRGYE−
−HLRGYL−
−HLRGYS−
HVLRGY−−
HSLRGY−−
HTLRGY−−
HLLRGY−−
−HLRGYI−
HGLRGY−−
−HLRGYH−
−HLRGYM−
HMLRGY−−
HKLRGY−−
−HLRGYV−
−HLRGYK−
HQLRGY−−
HNLRGY−−
HELRGY−−
HDLRGY−−
HPLRGY−−
−HLRGYP−
−HLRGYA−
−HLRGYQ−
−HLRGYN−
−HLRGYE−
−HLRGYC−
ISLRGY−−
IQLRGY−−
IGLRGY−−
INLRGY−−
IVLRGY−−
−ILRGYL−
−ILRGYQ−
IELRGY−−
IALRGY−−
IDLRGY−−
IKLRGY−−
−ILRGYH−
IILRGY−−
−ILRGYI−
IHLRGY−−

IPLRGY--
-ILRGYY-
-ILRGYW-
-ILRGYF-
ICLRGY--
KTLRGY--
-KLRGYT-
KFLRGY--
KHLRGY--
KPLRGY--
KELRGY--
KDLRGY--
-KLRGYQ-
--LRGYLS
LSLRGY--
--LRGYTS
LYLRGY--
--LRGYSR
--LRGYMG
LALRGY--
--LRGYSA
-LLRGYI-
--LRGYSG
--LRGYYG
--LRGYVS
LVLRGY--
--LRGYPR
LNLRGY--
--LRGYVG
--LRGYPN
--LRGYMS
--LRGYLY
--LRGYWG
--LRGYRN
--LRGYHG
--LRGYMA
--LRGYFG
LDLRGY--
--LRGYSK
--LRGYIA
--LRGYLQ
-LLRGYM-
--LRGYLN
LKLRGY--
--LRGYSQ
--LRGYSL
--LRGYPK
--LRGYYA
--LRGYQS
--LRGYQR

--LRGYQG
--LRGYVA
--LRGYTR
--LRGYSN
--LRGYRQ
--LRGYLP
--LRGYTQ
--LRGYKG
--LRGYVQ
--LRGYVN
--LRGYSP
--LRGYHT
--LRGYFA
--LRGYAQ
--LRGYQA
--LRGYKT
--LRGYKN
--LRGYTK
--LRGYNG
--LRGYMN
--LRGYGI
--LRGYGH
--LRGYEQ
--LRGYAN
--LRGYYV
--LRGYSY
--LRGYST
--LRGYPQ
--LRGYPL
--LRGYMQ
--LRGYLK
--LRGYAS
--LRGYAK
--LRGYAA
--LRGYVF
--LRGYTV
--LRGYRH
--LRGYQL
--LRGYLH
--LRGYIQ
--LRGYGK
--LRGYER
-LLRGYF-
--LRGYQK
--LRGYPV
--LRGYPH
--LRGYPF
--LRGYLT
--LRGYIV
--LRGYIR

--LRGYHM
--LRGYDR
--LRGYVY
--LRGYVV
--LRGYVT
--LRGYNP
--LRGYNK
--LRGYNA
--LRGYMV
--LRGYLW
--LRGYLV
--LRGYHR
--LRGYHP
--LRGYEA
--LRGYAM
--LRGYYN
--LRGYYK
--LRGYWR
--LRGYTW
--LRGYQT
--LRGYQP
--LRGYQH
--LRGYPY
--LRGYPI
--LRGYPG
--LRGYNQ
--LRGYMY
--LRGYML
--LRGYHW
--LRGYGN
--LRGYFR
--LRGYDP
LCLRGY--
--LRGYVW
--LRGYVP
--LRGYTF
--LRGYRE
--LRGYPP
--LRGYNT
--LRGYNL
--LRGYMW
--LRGYMK
--LRGYIT
--LRGYHK
--LRGYGW
--LRGYFH
--LRGYEN
--LRGYEG
--LRGYDQ
--LRGYAY

```
--LRGYAH
-LLRGYQ-
--LRGYSM
--LRGYQN
--LRGYPW
--LRGYPA
--LRGYNI
--LRGYMF
--LRGYHQ
--LRGYFP
--LRGYEV
--LRGYDK
--LRGYWV
--LRGYTM
--LRGYNH
--LRGYMD
--LRGYKP
--LRGYKM
--LRGYIW
--LRGYIM
--LRGYIH
--LRGYHN
--LRGYHF
--LRGYEW
--LRGYEK
--LRGYDN
--LRGYAW
--LRGYYQ
--LRGYWM
--LRGYWH
--LRGYRC
--LRGYQQ
--LRGYQI
--LRGYQE
--LRGYQD
--LRGYPD
--LRGYNW
--LRGYND
--LRGYMM
--LRGYME
--LRGYKF
--LRGYIP
--LRGYHV
--LRGYHD
--LRGYDV
--LRGYDI
--LRGYDH
MNLRGY--
MALRGY--
MTLRGY--
```

MGLRGY--
MPLRGY--
MILRGY--
MDLRGY--
-MLRGYI-
-MLRGYH-
MYLRGY--
MMLRGY--
-MLRGYQ-
-MLRGYN-
MHLRGY--
-MLRGYK-
MCLRGY--
-MLRGYG-
-MLRGYW-
-MLRGYP-
MQLRGY--
NGLRGY--
-NLRGYT-
-NLRGYG-
-NLRGYI-
NPLRGY--
NTLRGY--
-NLRGYA-
NMLRGY--
NALRGY--
NFLRGY--
-NLRGYV-
-NLRGYY-
-NLRGYM-
NQLRGY--
NDLRGY--
NNLRGY--
-NLRGYW-
-NLRGYN-
NELRGY--
-NLRGYE-
NWLRGY--
-NLRGYF-
-NLRGYQ-
NKLRGY--
-NLRGYP-
-NLRGYH-
PALRGY--
PVLRGY--
PGLRGY--
-PLRGYR-
PILRGY--
PQLRGY--
-PLRGYI-

PMLRGY--
PDLRGY--
-PLRGYK-
-PLRGYS-
-PLRGYM-
-PLRGYH-
PCLRGY--
-QLRGYL-
QTLRGY--
QVLRGY--
QPLRGY--
-QLRGYY-
-QLRGYM-
QGLRGY--
QALRGY--
-QLRGYS-
QLLRGY--
QQLRGY--
-QLRGYT-
QNLRGY--
-QLRGYQ-
QILRGY--
-QLRGYP-
-QLRGYK-
QDLRGY--
-QLRGYN-
-QLRGYH-
QFLRGY--
-QLRGYV-
-QLRGYI-
-QLRGYE-
-QLRGYF-
-QLRGYG-
QELRGY--
QCLRGY--
SSLRGY--
-SLRGYR-
SRLRGY--
STLRGY--
SQLRGY--
SGLRGY--
SWLRGY--
SDLRGY--
SHLRGY--
SKLRGY--
-SLRGYS-
SPLRGY--
-SLRGYA-
-SLRGYQ-
SELRGY--

-SLRGYY-
-SLRGYI-
-SLRGYN-
-SLRGYM-
-SLRGYG-
-SLRGYV-
-SLRGYE-
SCLRGY--
TSLRGY--
THLRGY--
TALRGY--
TELRGY--
TMLRGY--
TNLRGY--
TGLRGY--
TPLRGY--
-TLRGYY-
TQLRGY--
-TLRGYK-
TKLRGY--
TDLRGY--
-TLRGYL-
-TLRGYM-
-TLRGYI-
-TLRGYH-
TILRGY--
TCLRGY--
-TLRGYV-
-TLRGYF-
-TLRGYN-
-TLRGYA-
-TLRGYC-
VVLRGY--
VALRGY--
VMLRGY--
VDLRGY--
VELRGY--
VILRGY--
VHLRGY--
-VLRGYI-
-VLRGYY-
-VLRGYH-
-VLRGYM-
-VLRGYW-
-VLRGYT-
VCLRGY--
WGLRGY--
WNLRGY--
-WLRGYP-
WQLRGY--

WLLRGY--
WPLRGY--
WDLRGY--
WHLRGY--
-WLRGYH-
-WLRGYF-
WELRGY--
WWLRGY--
-WLRGYD-
-WLRGYE-
WILRGY--
-WLRGYV-
-WLRGYY-
WCLRGY--
YPLRGY--
YSLRGY--
YGLRGY--
-YLRGYN-
YRLRGY--
-YLRGYM-
YTLRGY--
YMLRGY--
-YLRGYG-
YVLRGY--
YDLRGY--
-YLRGYP-
-YLRGYF-
-YLRGYI-
-YLRGYA-
YWLRGY--
-YLRGYE-
YCLRGY--

PSWI 272 682 32.17

-LPSWIN-
--PSWIGN
-VPSWIM-
AKPSWI--
ASPSWI--
-APSWIV-
-APSWIT-
ANPSWI--
ARPSWI--
AMPSWI--
AGPSWI--
-APSWIG-
-APSWIQ-
AYPSWI--
AVPSWI--
-APSWII-
-APSWIA-

AHPSWI--
AQPSWI--
-APSWIE-
AEPSWI--
AAPSWI--
-APSWIW-
-APSWIM-
-APSWIH-
APPSWI--
ATPSWI--
-APSWIY-
-APSWIF-
ALPSWI--
AIPSWI--
AFPSWI--
-CPSWIM-
CPPSWI--
CCPSWI--
CYPSWI--
CTPSWI--
CRPSWI--
-CPSWIT-
-CPSWIA-
CKPSWI--
CIPSWI--
CDPSWI--
CAPSWI--
DYPSWI--
DVPSWI--
DIPSWI--
DSPSWI--
-DPSWII-
DKPSWI--
-DPSWIS-
-DPSWIM-
DAPSWI--
-DPSWIV-
-DPSWIR-
-DPSWIH-
DNPSWI--
DLPSWI--
DDPSWI--
-DPSWIT-
-DPSWIL-
-DPSWIA-
DGPSWI--
DFPSWI--
DTPSWI--
DQPSWI--
-DPSWIQ-

-DPSWIG-
DEPSWI--
EGPSWI--
-EPSWIH-
EVPSWI--
ESPSWI--
EYPSWI--
-EPSWIA-
ELPSWI--
EKPSWI--
EIPSWI--
ETPSWI--
-EPSWIL-
ENPSWI--
-EPSWIY-
-EPSWIS-
-EPSWIR-
-EPSWIG-
EEPSWI--
EQPSWI--
-EPSWIW-
-EPSWIT-
ERPSWI--
-EPSWIV-
-EPSWIM-
-EPSWIK-
-EPSWII-
FYPSWI--
FSPSWI--
FKPSWI--
FPPSWI--
FTPSWI--
FQPSWI--
-FPSWII-
FHPSWI--
-FPSWIR-
FNPSWI--
FMPSWI--
-FPSWIS-
FLPSWI--
FEPSWI--
FAPSWI--
FRPSWI--
-FPSWIH-
FWPSWI--
FVPSWI--
FFPSWI--
-FPSWIW-
-FPSWIT-
-FPSWIM-

-FPSWIK-
-FPSWIA-
FDPSWI--
GSPSWI--
-GPSWIR-
-GPSWIS-
GYPSWI--
GGPSWI--
-GPSWIN-
GLPSWI--
GTPSWI--
GVPSWI--
-GPSWIG-
-GPSWIA-
GDPSWI--
-GPSWIY-
-GPSWIQ-
GQPSWI--
-GPSWIT-
-GPSWII-
GPPSWI--
GNPSWI--
GEPSWI--
-GPSWIF-
GKPSWI--
GHPSWI--
GFPSWI--
GCPSWI--
GAPSWI--
GWPSWI--
-GPSWIH-
-GPSWID-
-HPSWIL-
-HPSWIR-
HLPSWI--
HAPSWI--
HQPSWI--
HVPSWI--
-HPSWIG-
HIPSWI--
-HPSWIS-
HKPSWI--
-HPSWIA-
HHPSWI--
HTPSWI--
HRPSWI--
-HPSWII-
HFPSWI--
HWPSWI--
-HPSWIQ-

-HPSWIK-
HPPSWI--
HDPSWI--
-HPSWIN-
-HPSWIM-
-HPSWIH-
-HPSWIE-
HNPSWI--
HMPSWI--
HEPSWI--
HCPSWI--
ISPSWI--
IRPSWI--
IVPSWI--
ILPSWI--
-IPSWIS-
INPSWI--
IIPSWI--
-IPSWIR-
-IPSWIY-
IPPSWI--
IGPSWI--
IDPSWI--
IAPSWI--
-IPSWIV-
-IPSWIK-
IFPSWI--
-IPSWIQ-
IQPSWI--
-IPSWIM-
-IPSWII-
-IPSWIH-
IKPSWI--
IWPSWI--
-IPSWIW-
-IPSWIN-
-IPSWIF-
IMPSWI--
ICPSWI--
KSPSWI--
-KPSWIQ-
KTPSWI--
-KPSWIA-
KVPSWI--
KRPSWI--
-KPSWIS-
-KPSWIH-
KMPSWI--
KGPSWI--
KYPSWI--

KQPSWI--
KAPSWI--
-KPSWIR-
KLPSWI--
KWPSWI--
-KPSWII-
-KPSWIF-
KKPSWI--
-KPSWIY-
-KPSWIT-
-KPSWIN-
KNPSWI--
-KPSWIV-
-KPSWIG-
KEPSWI--
-KPSWIK-
KPPSWI--
KHPSWI--
KDPSWI--
KCPSWI--
LEPSWI--
LNPSWI--
LAPSWI--
LVPSWI--
LPPSWI--
-LPSWIM-
-LPSWIL-
LKPSWI--
LGPSWI--
-LPSWIV-
-LPSWII-
-LPSWIY-
-LPSWIK-
-LPSWIH-
-LPSWIT-
LIPSWI--
LQPSWI--
-LPSWIQ-
-LPSWIG-
-LPSWIA-
LFPSWI--
MKPSWI--
-MPSWIH-
MMPSWI--
MAPSWI--
MGPSWI--
MSPSWI--
MLPSWI--
MTPSWI--
MEPSWI--

MWPSWI--
-MPSWIQ-
MPPSWI--
-MPSWIS-
-MPSWIL-
-MPSWIG-
-MPSWIM-
-MPSWII-
-MPSWIA-
MYPSWI--
MRPSWI--
-MPSWIN-
MIPSWI--
-MPSWIY-
MHPSWI--
-NPSWIR-
NVPSWI--
NAPSWI--
NTPSWI--
-NPSWIT-
NRPSWI--
-NPSWIS-
NGPSWI--
NQPSWI--
-NPSWIG-
-NPSWII-
NSPSWI--
NDPSWI--
-NPSWIL-
NMPSWI--
NWPSWI--
-NPSWIY-
-NPSWIV-
-NPSWIM-
-NPSWIK-
-NPSWIQ-
NKPSWI--
NIPSWI--
NFPSWI--
NYPSWI--
-NPSWIW-
-NPSWIF-
-NPSWIA-
--PSWIVR
--PSWILG
--PSWIRS
--PSWIRD
--PSWISS
--PSWISK
--PSWIRI

--PSWILY
--PSWIRA
--PSWIKS
--PSWIYR
--PSWILS
--PSWIST
--PSWIRT
--PSWIRH
--PSWIQK
--PSWILK
--PSWIAN
PIPSWI--
PAPSWI--
--PSWISE
--PSWIMN
--PSWIIK
--PSWITG
--PSWITK
--PSWIRY
--PSWIRV
--PSWIRN
--PSWIEG
PNPSWI--
--PSWIYS
--PSWIRG
--PSWIKT
--PSWIFY
--PSWIFG
--PSWIAT
PYPSWI--
--PSWIVS
--PSWIVA
--PSWITT
--PSWIRM
--PSWIFH
--PSWIAH
--PSWIWS
--PSWIVN
--PSWIVG
--PSWITS
--PSWISR
--PSWIQQ
--PSWIKM
--PSWIIH
--PSWIIG
--PSWIFS
--PSWIAI
PKPSWI--
--PSWIYT
--PSWIVT

--PSWITV
--PSWISH
--PSWIQN
--PSWINS
--PSWIGS
--PSWIGL
--PSWIFN
PQPSWI--
PMPSWI--
PHPSWI--
PFPSWI--
--PSWIYA
--PSWITE
--PSWISY
--PSWISW
--PSWISA
--PSWIQW
--PSWIQS
--PSWIMK
--PSWILR
--PSWILH
--PSWIIA
--PSWIHT
--PSWIGT
--PSWIFE
--PSWIES
PEPSWI--
--PSWITA
--PSWISQ
--PSWISG
--PSWIRE
--PSWIQT
--PSWIMH
--PSWIKA
--PSWIGA
--PSWIFW
PRPSWI--
--PSWIYW
--PSWIYN
--PSWIYD
--PSWIWN
--PSWIVW
--PSWIVV
--PSWIVD
--PSWITY
--PSWITR
--PSWIRW
--PSWIQE
--PSWIMI
--PSWIMG

--PSWIKI
--PSWIKD
--PSWIIV
--PSWIHM
--PSWIHG
--PSWIDS
--PSWIAV
--PSWIAR
PDPSWI--
PWPSWI--
--PSWIYK
--PSWIVH
--PSWITN
--PSWIQY
--PSWIQG
--PSWINT
--PSWING
--PSWIMM
--PSWILI
--PSWILD
--PSWIKQ
--PSWIKG
--PSWIKF
--PSWIIQ
--PSWIID
--PSWIHA
--PSWIGY
--PSWIFQ
--PSWIAS
--PSWIAQ
--PSWIAE
PSPSWI--
PTPSWI--
--PSWIYI
--PSWIYH
--PSWIWY
--PSWIWW
--PSWIWG
--PSWIWA
--PSWIVQ
--PSWIVP
--PSWIVF
--PSWIVE
--PSWITI
--PSWITH
--PSWITD
--PSWISI
--PSWISD
--PSWIQV
--PSWIQF

--PSWIQA
--PSWINW
--PSWINA
--PSWIMY
--PSWIMT
--PSWIMA
--PSWILQ
--PSWIKY
--PSWIKV
--PSWIKE
--PSWIIY
--PSWIIN
--PSWIHH
--PSWIGV
--PSWIGQ
--PSWIGG
--PSWIGF
--PSWIFI
--PSWIFD
--PSWIEV
--PSWIEA
--PSWIAD
PGPSWI--
QLPSWI--
-QPSWIS-
QEPSWI--
QRPSWI--
-QPSWIY-
QVPSWI--
-QPSWIQ-
-QPSWIM-
-QPSWII-
-QPSWIL-
-QPSWIG-
QGPSWI--
QFPSWI--
QDPSWI--
QWPSWI--
-QPSWIW-
QTPSWI--
QSPSWI--
QYPSWI--
QQPSWI--
-QPSWIV-
-QPSWIH-
-QPSWIA-
QPPSWI--
QKPSWI--
RTPSWI--
RLPSWI--

```
-RPSWII-
-RPSWIS-
-RPSWIK-
-RPSWIY-
RGPSWI--
RIPSWI--
-RPSWIM-
REPSWI--
RRPSWI--
-RPSWIV-
-RPSWIG-
RHPSWI--
RSPSWI--
RQPSWI--
-RPSWIH-
RWPSWI--
-RPSWIT-
-RPSWIQ-
-RPSWIA-
-RPSWIN-
-RPSWIE-
RYPSWI--
-RPSWIF-
RFPSWI--
RDPSWI--
RCPSWI--
SRPSWI--
-SPSWIR-
SSPSWI--
STPSWI--
SKPSWI--
SYPSWI--
SGPSWI--
SAPSWI--
-SPSWII-
SNPSWI--
SWPSWI--
-SPSWIA-
-SPSWIG-
-SPSWIF-
-SPSWIK-
-SPSWIV-
-SPSWIT-
SVPSWI--
-SPSWIW-
SIPSWI--
SHPSWI--
SQPSWI--
-SPSWIH-
SDPSWI--
```

SCPSWI--
-SPSWIY-
-SPSWIN-
SMPSWI--
SEPSWI--
-SPSWIM-
-SPSWIE-
-TPSWIL-
-TPSWIS-
-TPSWIR-
TLPSWI--
TNPSWI--
TSPSWI--
-TPSWIT-
-TPSWIM-
TIPSWI--
TGPSWI--
TAPSWI--
-TPSWIV-
TKPSWI--
TFPSWI--
-TPSWIF-
TQPSWI--
-TPSWIY-
-TPSWII-
TPPSWI--
TTPSWI--
TWPSWI--
-TPSWIW-
-TPSWIH-
-TPSWIG-
TYPSWI--
-TPSWIQ-
THPSWI--
TEPSWI--
TDPSWI--
-TPSWIN-
-TPSWIE-
TCPSWI--
VLPSWI--
VKPSWI--
VSPSWI--
VTPSWI--
VGPSWI--
-VPSWIR-
-VPSWIK-
VVPSWI--
-VPSWIG-
VIPSWI--
VFPSWI--

VMPSWI--
VDPSWI--
VQPSWI--
-VPSWIV-
-VPSWIY-
-VPSWIT-
VNPSWI--
VYPSWI--
-VPSWIH-
-VPSWIA-
VHPSWI--
-VPSWII-
-VPSWIE-
VPPSWI--
VEPSWI--
VWPSWI--
-WPSWIG-
WRPSWI--
WKPSWI--
-WPSWIR-
WSPSWI--
WIPSWI--
WPPSWI--
WLPSWI--
WDPSWI--
WQPSWI--
-WPSWIS-
WVPSWI--
WGPSWI--
WYPSWI--
WTPSWI--
-WPSWIW-
-WPSWIT-
-WPSWIN-
-WPSWII-
WNPSWI--
WMPSWI--
WHPSWI--
WFPSWI--
YRPSWI--
-YPSWIR-
YHPSWI--
YPPSWI--
YQPSWI--
YTPSWI--
YEPSWI--
YSPSWI--
-YPSWIL-
YLPSWI--
YIPSWI--

| | | | |
|---|---|---|---|
| | -YPSWIS- | | |
| | -YPSWIM- | | |
| | -YPSWIA- | | |
| | -YPSWII- | | |
| | -YPSWIH- | | |
| | -YPSWIG- | | |
| | YFPSWI-- | | |
| | YAPSWI-- | | |
| | YWPSWI-- | | |
| | -YPSWIK- | | |
| | YMPSWI-- | | |
| | YYPSWI-- | | |
| | -YPSWIY- | | |
| | -YPSWIT- | | |
| | YGPSWI-- | | |
| PAWI | 586 | 583 | 32.17 |
| | NGPAWI-- | | |
| | AVPAWI-- | | |
| | -APAWIK- | | |
| | ARPAWI-- | | |
| | -APAWIL- | | |
| | -APAWIR- | | |
| | AKPAWI-- | | |
| | AFPAWI-- | | |
| | -APAWII- | | |
| | -APAWIE- | | |
| | -APAWID- | | |
| | ANPAWI-- | | |
| | AYPAWI-- | | |
| | -APAWIT- | | |
| | AGPAWI-- | | |
| | AWPAWI-- | | |
| | APPAWI-- | | |
| | -APAWIS- | | |
| | -APAWIA- | | |
| | ADPAWI-- | | |
| | AAPAWI-- | | |
| | ATPAWI-- | | |
| | -APAWIG- | | |
| | AIPAWI-- | | |
| | CGPAWI-- | | |
| | CTPAWI-- | | |
| | CMPAWI-- | | |
| | CPPAWI-- | | |
| | -CPAWII- | | |
| | -CPAWIH- | | |
| | CHPAWI-- | | |
| | -DPAWIL- | | |
| | DWPAWI-- | | |
| | -DPAWIR- | | |

DVPAWI--
-DPAWIY-
DGPAWI--
DRPAWI--
-DPAWIV-
DLPAWI--
-DPAWIK-
-DPAWIE-
DIPAWI--
DSPAWI--
-DPAWIS-
DNPAWI--
DHPAWI--
DFPAWI--
DEPAWI--
DAPAWI--
-EPAWIR-
EVPAWI--
EYPAWI--
ERPAWI--
-EPAWIT-
EGPAWI--
EFPAWI--
ETPAWI--
ESPAWI--
-EPAWIQ-
ENPAWI--
ELPAWI--
EIPAWI--
-EPAWIK-
-EPAWIE-
-EPAWIW-
-EPAWIN-
-EPAWID-
-EPAWIA-
EKPAWI--
EAPAWI--
FLPAWI--
FSPAWI--
-FPAWIQ-
-FPAWIR-
FNPAWI--
FRPAWI--
FTPAWI--
-FPAWIS-
-FPAWIM-
FDPAWI--
FPPAWI--
FEPAWI--
FWPAWI--

-FPAWIK-
FYPAWI--
-FPAWIW-
-FPAWIV-
-FPAWIN-
-FPAWII-
-FPAWIF-
-FPAWIC-
FIPAWI--
FHPAWI--
FGPAWI--
FFPAWI--
-GPAWIS-
GIPAWI--
GSPAWI--
GLPAWI--
-GPAWIL-
GRPAWI--
GMPAWI--
GVPAWI--
-GPAWIF-
-GPAWIQ-
GGPAWI--
GQPAWI--
GEPAWI--
GKPAWI--
GWPAWI--
-GPAWIT-
GFPAWI--
GPPAWI--
-GPAWIY-
-GPAWIW-
-GPAWIA-
GNPAWI--
GHPAWI--
-GPAWIV-
-GPAWIM-
-GPAWII-
GAPAWI--
-HPAWIL-
HRPAWI--
-HPAWIK-
HSPAWI--
HPPAWI--
HKPAWI--
HAPAWI--
HYPAWI--
HIPAWI--
HGPAWI--
HFPAWI--

```
-HPAWIQ-
HWPAWI--
-HPAWIS-
-HPAWIF-
-HPAWIE-
HEPAWI--
-IPAWIR-
IHPAWI--
IGPAWI--
IEPAWI--
IAPAWI--
ISPAWI--
IVPAWI--
ITPAWI--
IQPAWI--
INPAWI--
IIPAWI--
IRPAWI--
-IPAWIK-
-IPAWII-
ILPAWI--
-IPAWIS-
-IPAWIH-
-IPAWIF-
-IPAWIE-
-IPAWIA-
IKPAWI--
IDPAWI--
IYPAWI--
IWPAWI--
-IPAWIV-
KSPAWI--
-KPAWIH-
KIPAWI--
KFPAWI--
-KPAWIT-
-KPAWIL-
-KPAWIF-
KGPAWI--
-KPAWIA-
-KPAWIW-
-KPAWIR-
KHPAWI--
KAPAWI--
KTPAWI--
-KPAWIY-
KVPAWI--
-KPAWIQ-
-KPAWIK-
KRPAWI--
```

KPPAWI--
-KPAWII-
KNPAWI--
KEPAWI--
LFPAWI--
LHPAWI--
LPPAWI--
-LPAWIK-
LTPAWI--
-LPAWIY-
-LPAWIR-
LNPAWI--
LAPAWI--
LVPAWI--
LSPAWI--
-LPAWIQ-
LLPAWI--
LEPAWI--
LQPAWI--
-LPAWIL-
-LPAWIG-
LKPAWI--
LGPAWI--
-LPAWIM-
LDPAWI--
-LPAWIT-
-LPAWIH-
LWPAWI--
-LPAWIA-
MSPAWI--
-MPAWIR-
MVPAWI--
-MPAWIS-
-MPAWIE-
MLPAWI--
-MPAWIK-
-MPAWIV-
MTPAWI--
MNPAWI--
-MPAWIL-
-MPAWIH-
-MPAWIA-
MYPAWI--
-MPAWIY-
-MPAWIN-
MKPAWI--
MFPAWI--
MRPAWI--
-MPAWIQ-
MGPAWI--

```
-NPAWIR-
-NPAWII-
NRPAWI--
NNPAWI--
-NPAWIF-
-NPAWIK-
NIPAWI--
NPPAWI--
NFPAWI--
NSPAWI--
NLPAWI--
-NPAWIH-
NKPAWI--
NEPAWI--
-NPAWIV-
NTPAWI--
NQPAWI--
-NPAWIY-
-NPAWIT-
-NPAWIS-
-NPAWIN-
-NPAWIE-
-NPAWIA-
--PAWIRG
--PAWITA
PRPAWI--
--PAWIKG
--PAWIIR
--PAWIHR
--PAWIRN
--PAWITS
--PAWIQT
--PAWIGL
--PAWIVS
--PAWIVH
--PAWITG
--PAWILG
--PAWILS
--PAWIHS
PKPAWI--
--PAWIST
--PAWISR
--PAWISA
--PAWIQA
--PAWILY
--PAWILT
--PAWIKS
--PAWIYR
--PAWIVT
--PAWISY
```

--PAWIRS
--PAWILQ
--PAWILH
--PAWIKM
--PAWIGG
--PAWIAR
PVPAWI--
PLPAWI--
--PAWIYS
--PAWITK
--PAWISG
--PAWIMF
--PAWIGM
--PAWIWH
--PAWIVK
--PAWITT
--PAWISK
--PAWIRA
--PAWIQR
--PAWINT
--PAWIKV
--PAWIFR
--PAWIDS
--PAWIAD
--PAWIYG
--PAWIVI
--PAWIVG
--PAWITW
--PAWITI
--PAWITH
--PAWIRY
--PAWILD
--PAWIKT
--PAWIKQ
--PAWIIN
--PAWIGR
--PAWIGQ
--PAWIAH
PGPAWI--
--PAWIWN
--PAWIVW
--PAWITR
--PAWISQ
--PAWIRH
--PAWIQV
--PAWIQK
--PAWINN
--PAWILW
--PAWILE
--PAWIKY

--PAWIIH
--PAWIHT
--PAWIHF
--PAWIFK
PWPAWI--
--PAWIYV
--PAWIWY
--PAWIWS
--PAWIVE
--PAWIVD
--PAWISW
--PAWIRW
--PAWIRQ
--PAWINV
--PAWIMT
--PAWIMR
--PAWIMN
--PAWIMI
--PAWIMH
--PAWIKF
--PAWIKA
--PAWIGS
--PAWIGN
--PAWIFY
--PAWIFF
--PAWIAV
--PAWIAN
--PAWIAK
--PAWIAG
PTPAWI--
PSPAWI--
PHPAWI--
PEPAWI--
--PAWIYT
--PAWIYQ
--PAWIYK
--PAWIYI
--PAWIYE
--PAWITV
--PAWITN
--PAWISN
--PAWISE
--PAWIRF
--PAWIRD
--PAWIQI
--PAWIPL
--PAWINY
--PAWINQ
--PAWIND
--PAWIMQ

--PAWIME
--PAWIMD
--PAWIKI
--PAWIKH
--PAWIKD
--PAWIHY
--PAWIHK
--PAWIGY
--PAWIFP
--PAWIFE
--PAWIFD
--PAWIET
--PAWIEQ
--PAWIEG
--PAWIEE
--PAWIDT
--PAWICK
--PAWIAY
--PAWIAS
--PAWIAL
--PAWIAF
--PAWIAA
PAPAWI--
-QPAWIL-
-QPAWIS-
QLPAWI--
QQPAWI--
-QPAWIM-
-QPAWIF-
QGPAWI--
QFPAWI--
-QPAWIQ-
QMPAWI--
QKPAWI--
QWPAWI--
QSPAWI--
QRPAWI--
-QPAWIR-
-QPAWIK-
-QPAWIH-
QAPAWI--
QVPAWI--
-QPAWIT-
-QPAWII-
-QPAWIA-
QHPAWI--
-RPAWIS-
RVPAWI--
-RPAWIN-
RGPAWI--

RHPAWI--
-RPAWIR-
-RPAWIG-
RYPAWI--
-RPAWIK-
RLPAWI--
-RPAWIA-
RKPAWI--
RRPAWI--
-RPAWIL-
-RPAWIH-
-RPAWIV-
RIPAWI--
RAPAWI--
RPPAWI--
RFPAWI--
-RPAWIE-
RWPAWI--
-RPAWIF-
-RPAWIW-
REPAWI--
RTPAWI--
-RPAWIM-
-RPAWII-
-RPAWID-
RNPAWI--
RDPAWI--
SLPAWI--
-SPAWIR-
SSPAWI--
-SPAWIS-
SAPAWI--
-SPAWIT-
-SPAWIL-
-SPAWIG-
SVPAWI--
STPAWI--
-SPAWIA-
-SPAWIN-
SEPAWI--
SYPAWI--
-SPAWIV-
-SPAWIM-
-SPAWIH-
-SPAWIF-
SIPAWI--
SGPAWI--
-SPAWIK-
SNPAWI--
SMPAWI--

SKPAWI--
SFPAWI--
-SPAWIY-
-SPAWIQ-
-SPAWII-
-SPAWIE-
TSPAWI--
-TPAWIR-
TIPAWI--
TRPAWI--
TYPAWI--
TFPAWI--
TVPAWI--
-TPAWIS-
-TPAWIN-
-TPAWIQ-
-TPAWIM-
-TPAWIL-
-TPAWIK-
-TPAWIG-
TGPAWI--
TEPAWI--
TWPAWI--
-TPAWIW-
-TPAWIH-
THPAWI--
TKPAWI--
TDPAWI--
TPPAWI--
-TPAWIY-
-TPAWIA-
TLPAWI--
TTPAWI--
-TPAWIV-
-TPAWID-
TNPAWI--
VLPAWI--
VRPAWI--
VAPAWI--
-VPAWIQ-
-VPAWIR-
-VPAWIN-
-VPAWIA-
-VPAWIT-
-VPAWIS-
VVPAWI--
VPPAWI--
-VPAWIG-
-VPAWIE-
VNPAWI--

VDPAWI--
VQPAWI--
-VPAWIY-
-VPAWII-
VHPAWI--
VFPAWI--
VCPAWI--
VYPAWI--
VTPAWI--
VSPAWI--
-VPAWID-
VMPAWI--
VGPAWI--
-WPAWIR-
WNPAWI--
WSPAWI--
WPPAWI--
WYPAWI--
WHPAWI--
WGPAWI--
WDPAWI--
WTPAWI--
WRPAWI--
-WPAWIY-
-WPAWIS-
-WPAWIL-
-WPAWII-
-WPAWIA-
WFPAWI--
YHPAWI--
YAPAWI--
YPPAWI--
YRPAWI--
YNPAWI--
-YPAWIS-
YKPAWI--
-YPAWIL-
-YPAWIT-
-YPAWIN-
-YPAWIE-
YLPAWI--
YYPAWI--
YTPAWI--
-YPAWIM-
-YPAWIG-
-YPAWIA-
YMPAWI--
YIPAWI--
YEPAWI--

PAQW 798 537 32.16

-TPAQWR-
AVPAQW--
-APAQWV-
-APAQWS-
AWPAQW--
-APAQWR-
-APAQWK-
ASPAQW--
-APAQWE-
-APAQWA-
AAPAQW--
ARPAQW--
AKPAQW--
AMPAQW--
-APAQWT-
ALPAQW--
-APAQWL-
-APAQWI-
-APAQWG-
AEPAQW--
ATPAQW--
AQPAQW--
-APAQWQ-
AHPAQW--
-CPAQWL-
-CPAQWM-
-CPAQWV-
-CPAQWT-
-CPAQWR-
CCPAQW--
-DPAQWR-
DRPAQW--
DKPAQW--
DTPAQW--
DSPAQW--
-DPAQWL-
-DPAQWK-
DNPAQW--
DLPAQW--
-EPAQWH-
EFPAQW--
-EPAQWS-
-EPAQWF-
-EPAQWA-
ESPAQW--
-EPAQWR-
-EPAQWM-
-EPAQWK-
-EPAQWG-
EMPAQW--

EKPAQW--
EGPAQW--
EEPAQW--
-FPAQWT-
-FPAQWQ-
-FPAQWL-
FLPAQW--
FWPAQW--
-FPAQWW-
-FPAQWN-
FSPAQW--
FQPAQW--
-FPAQWV-
-FPAQWH-
FNPAQW--
GPPAQW--
-GPAQWR-
GSPAQW--
GVPAQW--
-GPAQWY-
-GPAQWS-
-GPAQWH-
-GPAQWK-
GAPAQW--
GNPAQW--
GHPAQW--
-GPAQWW-
GIPAQW--
GYPAQW--
-GPAQWT-
-GPAQWN-
-GPAQWL-
-GPAQWF-
-GPAQWA-
GFPAQW--
HNPAQW--
HRPAQW--
-HPAQWI-
-HPAQWL-
HPPAQW--
HDPAQW--
-HPAQWH-
-HPAQWA-
HLPAQW--
HKPAQW--
HHPAQW--
HFPAQW--
HWPAQW--
-HPAQWR-
-HPAQWM-

HAPAQW--
IGPAQW--
ISPAQW--
-IPAQWL-
-IPAQWI-
ILPAQW--
IRPAQW--
IWPAQW--
-IPAQWW-
-IPAQWT-
-IPAQWR-
-IPAQWM-
IVPAQW--
-IPAQWA-
IEPAQW--
IYPAQW--
IQPAQW--
INPAQW--
IMPAQW--
IFPAQW--
KSPAQW--
-KPAQWY-
-KPAQWS-
-KPAQWV-
-KPAQWR-
-KPAQWA-
KTPAQW--
KPPAQW--
KWPAQW--
-KPAQWT-
KNPAQW--
KLPAQW--
KVPAQW--
KQPAQW--
-KPAQWM-
-KPAQWK-
-KPAQWI-
-KPAQWE-
KKPAQW--
-LPAQWM-
-LPAQWS-
LSPAQW--
LRPAQW--
-LPAQWQ-
LYPAQW--
LKPAQW--
LWPAQW--
-LPAQWT-
-LPAQWI-
-LPAQWH-

LLPAQW--
LAPAQW--
LVPAQW--
LTPAQW--
LPPAQW--
-LPAQWN-
-LPAQWL-
-LPAQWK-
-LPAQWF-
LMPAQW--
-LPAQWY-
-LPAQWW-
-LPAQWR-
LIPAQW--
LQPAQW--
LHPAQW--
LFPAQW--
LEPAQW--
LDPAQW--
-MPAQWA-
MTPAQW--
-MPAQWS-
MNPAQW--
MRPAQW--
-MPAQWV-
MFPAQW--
MVPAQW--
-MPAQWY-
-MPAQWR-
MKPAQW--
MGPAQW--
MAPAQW--
MWPAQW--
MPPAQW--
-MPAQWT-
-NPAQWR-
-NPAQWK-
-NPAQWA-
NYPAQW--
NAPAQW--
-NPAQWS-
-NPAQWQ-
NPPAQW--
-NPAQWV-
-NPAQWL-
NNPAQW--
NGPAQW--
NVPAQW--
NQPAQW--
-NPAQWM-

```
-NPAQWI-
-NPAQWF-
NLPAQW--
NHPAQW--
NDPAQW--
--PAQWLG
--PAQWYW
--PAQWST
--PAQWKI
--PAQWRS
--PAQWRY
--PAQWKG
--PAQWVS
--PAQWIL
PTPAQW--
--PAQWSG
--PAQWLT
--PAQWAS
--PAQWTT
--PAQWTF
--PAQWSR
--PAQWRA
--PAQWMR
--PAQWWD
--PAQWVR
--PAQWVD
--PAQWTQ
--PAQWRI
--PAQWNY
--PAQWIY
--PAQWHS
--PAQWAA
--PAQWSM
--PAQWRL
--PAQWQG
--PAQWNA
--PAQWLS
--PAQWKH
--PAQWIM
--PAQWIF
--PAQWHV
--PAQWHI
--PAQWFR
--PAQWAN
--PAQWAF
PIPAQW--
--PAQWYI
--PAQWWM
--PAQWVV
--PAQWVM
```

--PAQWTI
--PAQWNS
--PAQWMN
--PAQWLL
--PAQWLK
--PAQWLH
--PAQWLD
--PAQWIS
--PAQWIG
--PAQWHQ
--PAQWGA
--PAQWFS
--PAQWFK
--PAQWYY
--PAQWYS
--PAQWYM
--PAQWYH
--PAQWVN
--PAQWVL
--PAQWVG
--PAQWTY
--PAQWSS
--PAQWSH
--PAQWSA
--PAQWRP
--PAQWQV
--PAQWNL
--PAQWNE
--PAQWLV
--PAQWKY
--PAQWKW
--PAQWKS
--PAQWHG
--PAQWGS
--PAQWFL
--PAQWFI
--PAQWFG
PMPAQW--
--PAQWYE
--PAQWWS
--PAQWWQ
--PAQWWK
--PAQWWI
--PAQWVK
--PAQWVF
--PAQWTS
--PAQWTR
--PAQWSW
--PAQWSV
--PAQWSN

--PAQWSD
--PAQWRT
--PAQWRQ
--PAQWRK
--PAQWRH
--PAQWRG
--PAQWRD
--PAQWQT
--PAQWQS
--PAQWQN
--PAQWQK
--PAQWNQ
--PAQWNK
--PAQWMW
--PAQWMK
--PAQWLR
--PAQWLM
--PAQWLE
--PAQWKV
--PAQWKR
--PAQWKK
--PAQWII
--PAQWFD
--PAQWAT
--PAQWAM
PWPAQW--
PRPAQW--
PKPAQW--
PHPAQW--
PFPAQW--
PEPAQW--
PDPAQW--
--PAQWYK
--PAQWYG
--PAQWYA
--PAQWVI
--PAQWVH
--PAQWVA
--PAQWTW
--PAQWTN
--PAQWTL
--PAQWTK
--PAQWTE
--PAQWSY
--PAQWSI
--PAQWSF
--PAQWSE
--PAQWRV
--PAQWRN
--PAQWRM

--PAQWRF
--PAQWQW
--PAQWQR
--PAQWQL
--PAQWQI
--PAQWQF
--PAQWNT
--PAQWNN
--PAQWNF
--PAQWMT
--PAQWMS
--PAQWMI
--PAQWME
--PAQWMA
--PAQWLN
--PAQWKT
--PAQWKL
--PAQWKD
--PAQWIV
--PAQWIR
--PAQWIN
--PAQWIA
--PAQWHR
--PAQWGV
--PAQWGT
--PAQWGI
--PAQWFQ
--PAQWFM
--PAQWAW
-QPAQWS-
QYPAQW--
QGPAQW--
QWPAQW--
QIPAQW--
-QPAQWR-
-QPAQWF-
QFPAQW--
QNPAQW--
QPPAQW--
-QPAQWT-
-QPAQWN-
QLPAQW--
QVPAQW--
QRPAQW--
-QPAQWM-
QKPAQW--
QHPAQW--
-RPAQWS-
RRPAQW--
-RPAQWL-

RYPAQW--
-RPAQWQ-
-RPAQWA-
RPPAQW--
RSPAQW--
-RPAQWY-
-RPAQWV-
RMPAQW--
RHPAQW--
-RPAQWG-
-RPAQWI-
RGPAQW--
RTPAQW--
-RPAQWW-
-RPAQWF-
RCPAQW--
-RPAQWN-
-RPAQWM-
-RPAQWH-
-RPAQWE-
RFPAQW--
REPAQW--
RWPAQW--
RVPAQW--
RQPAQW--
-RPAQWT-
-RPAQWP-
RLPAQW--
RAPAQW--
-SPAQWH-
-SPAQWI-
SAPAQW--
SRPAQW--
-SPAQWS-
-SPAQWA-
SLPAQW--
SYPAQW--
-SPAQWM-
-SPAQWY-
SKPAQW--
SGPAQW--
SEPAQW--
SPPAQW--
-SPAQWT-
SIPAQW--
SFPAQW--
SDPAQW--
SWPAQW--
STPAQW--
SQPAQW--

−SPAQWQ−
−SPAQWN−
−SPAQWF−
−SPAQWE−
SMPAQW−−
SHPAQW−−
−TPAQWL−
TRPAQW−−
−TPAQWI−
TLPAQW−−
TVPAQW−−
TQPAQW−−
−TPAQWT−
−TPAQWK−
TKPAQW−−
TAPAQW−−
TSPAQW−−
TPPAQW−−
−TPAQWV−
−TPAQWS−
−TPAQWF−
TNPAQW−−
TMPAQW−−
TIPAQW−−
TYPAQW−−
−TPAQWY−
−TPAQWQ−
−TPAQWN−
−TPAQWM−
−TPAQWH−
−TPAQWG−
TEPAQW−−
−VPAQWR−
VRPAQW−−
VVPAQW−−
VQPAQW−−
−VPAQWS−
VPPAQW−−
−VPAQWK−
VKPAQW−−
VWPAQW−−
−VPAQWT−
−VPAQWN−
VMPAQW−−
VGPAQW−−
VYPAQW−−
VTPAQW−−
−VPAQWY−
−VPAQWW−
−VPAQWI−

-VPAQWG-
VFPAQW--
VCPAQW--
WVPAQW--
WHPAQW--
-WPAQWS-
-WPAQWN-
WPPAQW--
-WPAQWR-
WWPAQW--
WGPAQW--
-WPAQWQ-
-WPAQWK-
WNPAQW--
WEPAQW--
WDPAQW--
-YPAQWV-
YLPAQW--
YWPAQW--
-YPAQWS-
-YPAQWR-
YKPAQW--
YQPAQW--
-YPAQWN-
-YPAQWF-
-YPAQWQ-
-YPAQWA-
YGPAQW--
YYPAQW--
YTPAQW--
YPPAQW--
-YPAQWY-
-YPAQWT-
-YPAQWI-
YFPAQW--
YEPAQW--
YAPAQW--
PSSM 33 826 32.14
-IPSSMP-
-KPSSMF-
--PSSMSP
--PSSMGL
--PSSMEK
--PSSMAK
-QPSSMN-
YMPSSM--
ALPSSM--
ASPSSM--
ARPSSM--
-APSSMR-

AFPSSM--
-APSSMK-
-APSSML-
-APSSMI-
-APSSMH-
-APSSMT-
-APSSMG-
ATPSSM--
AIPSSM--
AGPSSM--
-APSSMF-
APPSSM--
AWPSSM--
AYPSSM--
-APSSMM-
-APSSMA-
-APSSMV-
-APSSMN-
-APSSME-
AAPSSM--
AQPSSM--
ANPSSM--
AHPSSM--
AEPSSM--
AMPSSM--
AKPSSM--
ADPSSM--
-APSSMY-
-APSSMQ-
CVPSSM--
CNPSSM--
CRPSSM--
CQPSSM--
-CPSSMV-
CMPSSM--
CLPSSM--
CDPSSM--
CAPSSM--
-CPSSMK-
CGPSSM--
CTPSSM--
-CPSSMY-
-CPSSMR-
-CPSSMQ-
-CPSSMM-
-CPSSMH-
CKPSSM--
CIPSSM--
CFPSSM--
DRPSSM--

DWPSSM--
DSPSSM--
DFPSSM--
-DPSSMR-
-DPSSMT-
DTPSSM--
-DPSSMV-
-DPSSMK-
DYPSSM--
-DPSSMW-
DMPSSM--
DLPSSM--
DQPSSM--
-DPSSML-
DNPSSM--
DIPSSM--
DVPSSM--
-DPSSMM-
-DPSSMA-
DKPSSM--
DAPSSM--
-DPSSMN-
-DPSSMI-
-DPSSMH-
-DPSSMG-
-DPSSMF-
DHPSSM--
DCPSSM--
-EPSSMR-
-EPSSMW-
ESPSSM--
ERPSSM--
EMPSSM--
EWPSSM--
-EPSSMH-
EVPSSM--
-EPSSML-
-EPSSMS-
ELPSSM--
EIPSSM--
EYPSSM--
ETPSSM--
-EPSSMQ-
EKPSSM--
EAPSSM--
-EPSSMV-
-EPSSMG-
-EPSSMF-
EGPSSM--
-EPSSMY-

-EPSSME-
EPPSSM--
ENPSSM--
EHPSSM--
-FPSSMV-
FRPSSM--
FNPSSM--
-FPSSMW-
FHPSSM--
FGPSSM--
-FPSSMR-
-FPSSMI-
FWPSSM--
FSPSSM--
-FPSSMT-
-FPSSMK-
FPPSSM--
FFPSSM--
FAPSSM--
FYPSSM--
-FPSSMY-
FIPSSM--
-FPSSMM-
-FPSSMA-
FMPSSM--
FKPSSM--
-FPSSMN-
FDPSSM--
-FPSSMQ-
-FPSSMH-
FEPSSM--
FQPSSM--
FCPSSM--
GRPSSM--
-GPSSML-
-GPSSMV-
-GPSSMA-
GYPSSM--
GTPSSM--
-GPSSMS-
-GPSSME-
GQPSSM--
GNPSSM--
GMPSSM--
-GPSSMQ-
-GPSSMT-
-GPSSMK-
-GPSSMI-
GGPSSM--
GVPSSM--

-GPSSMW-
GIPSSM--
-GPSSMY-
-GPSSMH-
GEPSSM--
GAPSSM--
GWPSSM--
-GPSSMN-
GCPSSM--
GHPSSM--
GFPSSM--
-GPSSMG-
-GPSSMF-
GDPSSM--
GPPSSM--
-HPSSMN-
-HPSSMA-
HRPSSM--
-HPSSML-
-HPSSMF-
-HPSSMI-
-HPSSMR-
HTPSSM--
-HPSSMW-
-HPSSMV-
HLPSSM--
HKPSSM--
HVPSSM--
HMPSSM--
-HPSSMS-
HGPSSM--
HSPSSM--
HNPSSM--
HAPSSM--
-HPSSMT-
-HPSSMQ-
-HPSSMM-
-HPSSMG-
HPPSSM--
HFPSSM--
HEPSSM--
HYPSSM--
HWPSSM--
HQPSSM--
-HPSSMY-
-HPSSMK-
-HPSSMC-
HIPSSM--
HHPSSM--
IMPSSM--

-IPSSMR-
IRPSSM--
-IPSSML-
IVPSSM--
-IPSSMT-
-IPSSMV-
IGPSSM--
-IPSSMK-
IWPSSM--
ISPSSM--
-IPSSMQ-
-IPSSMN-
-IPSSMW-
-IPSSMS-
-IPSSMA-
ILPSSM--
IKPSSM--
INPSSM--
IIPSSM--
IFPSSM--
IQPSSM--
-IPSSMG-
IHPSSM--
IEPSSM--
ITPSSM--
-IPSSMY-
-IPSSMI-
-IPSSMH-
IYPSSM--
-IPSSME-
IPPSSM--
ICPSSM--
IAPSSM--
KSPSSM--
-KPSSMA-
-KPSSME-
-KPSSMV-
KFPSSM--
-KPSSML-
-KPSSMR-
-KPSSMY-
-KPSSMS-
KAPSSM--
KVPSSM--
-KPSSMI-
KWPSSM--
-KPSSMW-
-KPSSMN-
KNPSSM--
KHPSSM--

KTPSSM--
-KPSSMM-
-KPSSMG-
-KPSSMT-
KYPSSM--
-KPSSMH-
KLPSSM--
KKPSSM--
KMPSSM--
KQPSSM--
-KPSSMQ-
-KPSSMC-
KPPSSM--
KDPSSM--
-LPSSMV-
-LPSSMR-
LWPSSM--
LRPSSM--
-LPSSMS-
LNPSSM--
LGPSSM--
LLPSSM--
LYPSSM--
LHPSSM--
-LPSSMA-
LKPSSM--
-LPSSMT-
-LPSSMI-
-LPSSMH-
LIPSSM--
-LPSSMK-
-LPSSMM-
-LPSSMW-
LPPSSM--
LEPSSM--
LAPSSM--
-LPSSMQ-
-LPSSMF-
-LPSSME-
LMPSSM--
LFPSSM--
LCPSSM--
-MPSSMV-
-MPSSMR-
-MPSSML-
MLPSSM--
-MPSSMA-
MNPSSM--
-MPSSME-
-MPSSMY-

-MPSSMK-
-MPSSMI-
MAPSSM--
-MPSSMT-
-MPSSMS-
-MPSSMF-
-MPSSMN-
MYPSSM--
MWPSSM--
MVPSSM--
MTPSSM--
MPPSSM--
MKPSSM--
-MPSSMG-
MMPSSM--
-MPSSMH-
MIPSSM--
MHPSSM--
MGPSSM--
MFPSSM--
-MPSSMW-
-MPSSMQ-
MDPSSM--
MCPSSM--
-NPSSMR-
-NPSSML-
-NPSSMT-
-NPSSMS-
-NPSSMI-
NIPSSM--
NWPSSM--
NTPSSM--
NSPSSM--
NRPSSM--
-NPSSMV-
NLPSSM--
NEPSSM--
NAPSSM--
NVPSSM--
NPPSSM--
-NPSSMW-
-NPSSMM-
NGPSSM--
NFPSSM--
-NPSSMK-
NNPSSM--
NYPSSM--
-NPSSMA-
NMPSSM--
NDPSSM--

NKPSSM--
NHPSSM--
--PSSMLR
--PSSMRA
--PSSMRG
--PSSMRT
--PSSMRS
--PSSMSG
--PSSMGS
--PSSMFR
PSPSSM--
--PSSMLT
--PSSMLS
PKPSSM--
--PSSMTR
--PSSMYR
--PSSMRY
--PSSMMR
--PSSMIR
--PSSMSL
--PSSMMS
--PSSMLA
--PSSMWR
--PSSMVN
--PSSMRV
--PSSMQN
--PSSMYL
--PSSMRW
--PSSMLI
--PSSMKI
--PSSMVK
--PSSMLH
--PSSMRK
--PSSMMF
--PSSMYT
--PSSMVA
--PSSMTL
--PSSMST
--PSSMSN
--PSSMLV
--PSSMTV
--PSSMTT
--PSSMTA
--PSSMGG
--PSSMYI
--PSSMVG
--PSSMSV
--PSSMQA
--PSSMMA
--PSSMLK

--PSSMLE
--PSSMKA
--PSSMIK
--PSSMWT
--PSSMVT
--PSSMVF
--PSSMRE
--PSSMRD
--PSSMQV
--PSSMHK
PLPSSM--
--PSSMYS
--PSSMYA
--PSSMWS
--PSSMTG
--PSSMLQ
--PSSMLG
--PSSMKR
--PSSMHV
--PSSMFV
--PSSMFT
--PSSMFM
--PSSMFI
--PSSMAA
--PSSMYV
--PSSMWL
--PSSMVW
--PSSMTS
--PSSMSW
--PSSMRN
--PSSMKY
--PSSMIQ
--PSSMIA
--PSSMHG
--PSSMFQ
--PSSMFG
--PSSMEA
PAPSSM--
--PSSMYM
--PSSMWK
--PSSMSK
--PSSMRQ
--PSSMRH
--PSSMMG
--PSSMLD
--PSSMKG
--PSSMIM
--PSSMFN
--PSSMAG
--PSSMTF

--PSSMQR
--PSSMQG
--PSSMLN
--PSSMYF
--PSSMYD
--PSSMWA
--PSSMSM
--PSSMRF
--PSSMMQ
--PSSMME
--PSSMKQ
--PSSMIH
--PSSMFS
PGPSSM--
--PSSMVQ
--PSSMVM
--PSSMTQ
--PSSMTK
--PSSMSY
--PSSMQS
--PSSMMY
--PSSMLM
--PSSMLF
--PSSMKV
--PSSMKT
--PSSMKK
--PSSMIV
--PSSMHR
--PSSMFW
--PSSMFL
--PSSMFE
--PSSMEV
--PSSMEG
PIPSSM--
PYPSSM--
PTPSSM--
--PSSMYY
--PSSMYN
--PSSMVY
--PSSMVE
--PSSMSH
--PSSMSF
--PSSMQQ
--PSSMIG
--PSSMIF
--PSSMHY
--PSSMHT
--PSSMFK
--PSSMFA
--PSSMAT

--PSSMAR
--PSSMAL
PFPSSM--
PWPSSM--
--PSSMYQ
--PSSMWI
--PSSMWH
--PSSMVD
--PSSMTW
--PSSMTD
--PSSMSQ
--PSSMQW
--PSSMNY
--PSSMNW
--PSSMNS
--PSSMNF
--PSSMNA
--PSSMMN
--PSSMHW
--PSSMHS
--PSSMHN
--PSSMHF
--PSSMES
--PSSMAV
--PSSMAN
PQPSSM--
PNPSSM--
PHPSSM--
PVPSSM--
--PSSMWV
--PSSMTY
--PSSMTM
--PSSMQT
--PSSMNV
--PSSMNK
--PSSMMM
--PSSMKN
--PSSMKD
--PSSMIN
--PSSMID
--PSSMGM
--PSSMEW
--PSSMAF
PRPSSM--
PMPSSM--
--PSSMYH
--PSSMYE
--PSSMWY
--PSSMWN
--PSSMWM

--PSSMWG
--PSSMWE
--PSSMTN
--PSSMRC
--PSSMNT
--PSSMNH
--PSSMMW
--PSSMMK
--PSSMMH
--PSSMMD
--PSSMKM
--PSSMII
--PSSMHE
--PSSMHD
--PSSMGW
--PSSMGT
--PSSMGA
--PSSMEN
PEPSSM--
--PSSMWW
--PSSMWQ
--PSSMTH
--PSSMSE
--PSSMQL
--PSSMPT
--PSSMPA
--PSSMNI
--PSSMMV
--PSSMMT
--PSSMKW
--PSSMKE
--PSSMHM
--PSSMHI
--PSSMFD
--PSSMEM
--PSSMEH
--PSSMDG
--PSSMAW
--PSSMAQ
--PSSMAM
-PPSSMR-
-PPSSML-
PDPSSM--
QVPSSM--
-QPSSMA-
QRPSSM--
-QPSSMR-
-QPSSMV-
QSPSSM--
QGPSSM--

-QPSSME-
QKPSSM--
-QPSSML-
-QPSSMF-
-QPSSMS-
-QPSSMK-
QPPSSM--
QNPSSM--
QMPSSM--
QHPSSM--
QAPSSM--
QWPSSM--
-QPSSMH-
QEPSSM--
-QPSSMY-
-QPSSMQ-
QLPSSM--
QFPSSM--
-QPSSMG-
QIPSSM--
QDPSSM--
RVPSSM--
-RPSSMV-
-RPSSMA-
-RPSSMR-
-RPSSMS-
-RPSSMT-
RFPSSM--
RAPSSM--
RGPSSM--
RSPSSM--
RWPSSM--
RRPSSM--
-RPSSMW-
-RPSSMM-
-RPSSMF-
-RPSSMN-
RTPSSM--
RMPSSM--
-RPSSMG-
RIPSSM--
RYPSSM--
RPPSSM--
-RPSSMI-
-RPSSMH-
-RPSSMQ-
-RPSSME-
RKPSSM--
RQPSSM--
RHPSSM--

RNPSSM--
RDPSSM--
-RPSSMC-
RCPSSM--
-SPSSML-
-SPSSMV-
SVPSSM--
SRPSSM--
SMPSSM--
SLPSSM--
SFPSSM--
STPSSM--
SAPSSM--
SGPSSM--
SSPSSM--
SNPSSM--
SHPSSM--
SYPSSM--
-SPSSMT-
-SPSSMR-
-SPSSMI-
SWPSSM--
-SPSSMS-
SQPSSM--
-SPSSMA-
-SPSSMK-
SKPSSM--
-SPSSMN-
-SPSSMG-
-SPSSMM-
-SPSSMF-
SEPSSM--
-SPSSMY-
-SPSSMQ-
-SPSSME-
-SPSSMH-
SDPSSM--
SPPSSM--
TRPSSM--
-TPSSMA-
TKPSSM--
-TPSSMS-
TSPSSM--
TLPSSM--
-TPSSMR-
-TPSSML-
TVPSSM--
-TPSSMT-
-TPSSMF-
TGPSSM--

-TPSSMY-
TTPSSM--
-TPSSMM-
TQPSSM--
TIPSSM--
TEPSSM--
-TPSSMK-
-TPSSMI-
THPSSM--
TCPSSM--
-TPSSMW-
-TPSSMN-
TMPSSM--
-TPSSMG-
TAPSSM--
TYPSSM--
-TPSSMQ-
-TPSSME-
TFPSSM--
TWPSSM--
-TPSSMH-
-TPSSMC-
TPPSSM--
TDPSSM--
-VPSSMS-
-VPSSML-
VSPSSM--
-VPSSMA-
-VPSSMF-
VVPSSM--
VRPSSM--
-VPSSMV-
VTPSSM--
VKPSSM--
-VPSSMM-
VAPSSM--
-VPSSMR-
VNPSSM--
-VPSSMH-
-VPSSMG-
VGPSSM--
-VPSSMT-
VIPSSM--
VHPSSM--
-VPSSMW-
VFPSSM--
-VPSSMN-
-VPSSMI-
VMPSSM--
-VPSSME-

VPPSSM--
VEPSSM--
VCPSSM--
VYPSSM--
VWPSSM--
VDPSSM--
VQPSSM--
-VPSSMC-
-WPSSMV-
-WPSSMR-
-WPSSMF-
-WPSSMK-
WRPSSM--
WKPSSM--
WIPSSM--
WAPSSM--
WLPSSM--
WMPSSM--
WHPSSM--
WSPSSM--
WTPSSM--
WVPSSM--
-WPSSMY-
-WPSSMW-
-WPSSMA-
WPPSSM--
-WPSSMN-
WYPSSM--
WNPSSM--
WFPSSM--
-WPSSMQ-
-WPSSMM-
WGPSSM--
-YPSSMR-
-YPSSMV-
-YPSSMS-
-YPSSMK-
YFPSSM--
-YPSSMF-
YSPSSM--
YLPSSM--
YRPSSM--
-YPSSMN-
YQPSSM--
YIPSSM--
-YPSSMT-
-YPSSMQ-
-YPSSMA-
YYPSSM--
YPPSSM--

| | | | |
|---|---|---|---|
| | YHPSSM-- | | |
| | YGPSSM-- | | |
| | YDPSSM-- | | |
| | -YPSSME- | | |
| | YWPSSM-- | | |
| | YNPSSM-- | | |
| | -YPSSMI- | | |
| | YEPSSM-- | | |
| | YAPSSM-- | | |
| | YVPSSM-- | | |
| | -YPSSMY- | | |
| | -YPSSMG- | | |
| | YTPSSM-- | | |
| | -YPSSMH- | | |
| PRPW | 111 | 762 | 32.11 |
| | -LPRPWN- | | |
| | -LPRPWA- | | |
| | -APRPWL- | | |
| | -APRPWV- | | |
| | ALPRPW-- | | |
| | AHPRPW-- | | |
| | -APRPWK- | | |
| | -APRPWG- | | |
| | -APRPWN- | | |
| | ANPRPW-- | | |
| | AAPRPW-- | | |
| | -APRPWY- | | |
| | AIPRPW-- | | |
| | ATPRPW-- | | |
| | -APRPWR- | | |
| | -APRPWQ- | | |
| | -APRPWF- | | |
| | -APRPWS- | | |
| | -APRPWH- | | |
| | AVPRPW-- | | |
| | AQPRPW-- | | |
| | -APRPWM- | | |
| | -APRPWA- | | |
| | AFPRPW-- | | |
| | ARPRPW-- | | |
| | -APRPWW- | | |
| | AYPRPW-- | | |
| | AWPRPW-- | | |
| | AMPRPW-- | | |
| | AGPRPW-- | | |
| | AEPRPW-- | | |
| | ADPRPW-- | | |
| | -APRPWI- | | |
| | -APRPWE- | | |
| | AKPRPW-- | | |

CTPRPW--
CGPRPW--
CEPRPW--
CAPRPW--
-DPRPWS-
-DPRPWR-
-DPRPWL-
DSPRPW--
DRPRPW--
-DPRPWV-
-DPRPWT-
-DPRPWM-
-DPRPWK-
-DPRPWF-
DLPRPW--
DNPRPW--
DMPRPW--
DHPRPW--
DVPRPW--
DQPRPW--
-DPRPWY-
-DPRPWQ-
-DPRPWI-
-DPRPWH-
DGPRPW--
-EPRPWR-
ESPRPW--
-EPRPWS-
ETPRPW--
EQPRPW--
-EPRPWV-
-EPRPWI-
EHPRPW--
EWPRPW--
-EPRPWH-
-EPRPWF-
ERPRPW--
-EPRPWY-
-EPRPWN-
-EPRPWG-
ELPRPW--
EKPRPW--
EIPRPW--
-EPRPWT-
-EPRPWL-
-EPRPWK-
-EPRPWA-
EMPRPW--
EGPRPW--
EAPRPW--

-FPRPWV-
FAPRPW--
-FPRPWT-
-FPRPWL-
FNPRPW--
FHPRPW--
-FPRPWW-
FSPRPW--
-FPRPWF-
-FPRPWM-
FGPRPW--
FLPRPW--
FFPRPW--
-FPRPWA-
FRPRPW--
-FPRPWS-
FDPRPW--
-FPRPWH-
FYPRPW--
FVPRPW--
-FPRPWY-
-FPRPWN-
-FPRPWG-
FPPRPW--
-GPRPWL-
GIPRPW--
-GPRPWV-
GSPRPW--
GQPRPW--
GNPRPW--
GVPRPW--
GTPRPW--
GWPRPW--
-GPRPWS-
-GPRPWT-
-GPRPWM-
GLPRPW--
GPPRPW--
-GPRPWI-
-GPRPWY-
-GPRPWN-
-GPRPWH-
GHPRPW--
GEPRPW--
GDPRPW--
GAPRPW--
GRPRPW--
-GPRPWW-
-GPRPWG-
-GPRPWF-

```
-GPRPWA-
GGPRPW--
GYPRPW--
-GPRPWQ-
GMPRPW--
GKPRPW--
-HPRPWL-
HSPRPW--
HVPRPW--
HRPRPW--
HLPRPW--
HTPRPW--
HIPRPW--
HEPRPW--
-HPRPWV-
HKPRPW--
-HPRPWF-
-HPRPWA-
HGPRPW--
-HPRPWT-
HNPRPW--
-HPRPWY-
HDPRPW--
HYPRPW--
-HPRPWW-
HFPRPW--
HWPRPW--
HQPRPW--
-HPRPWS-
-HPRPWQ-
-HPRPWH-
HMPRPW--
HHPRPW--
HCPRPW--
-IPRPWF-
-IPRPWL-
-IPRPWV-
-IPRPWR-
IVPRPW--
ISPRPW--
-IPRPWK-
ILPRPW--
IRPRPW--
IAPRPW--
-IPRPWA-
IEPRPW--
IWPRPW--
IGPRPW--
IDPRPW--
ITPRPW--
```

```
-IPRPWT-
IHPRPW--
-IPRPWQ-
-IPRPWE-
IMPRPW--
IQPRPW--
-IPRPWN-
-IPRPWM-
-IPRPWI-
IPPRPW--
IKPRPW--
IIPRPW--
KSPRPW--
-KPRPWI-
KLPRPW--
KHPRPW--
KGPRPW--
-KPRPWY-
KVPRPW--
KTPRPW--
KIPRPW--
KAPRPW--
-KPRPWV-
-KPRPWT-
-KPRPWR-
-KPRPWL-
-KPRPWS-
KDPRPW--
KWPRPW--
-KPRPWK-
-KPRPWH-
KMPRPW--
KFPRPW--
KRPRPW--
KQPRPW--
-KPRPWF-
KPPRPW--
KEPRPW--
LSPRPW--
-LPRPWR-
LMPRPW--
-LPRPWI-
LEPRPW--
LTPRPW--
-LPRPWL-
LVPRPW--
LGPRPW--
-LPRPWV-
LIPRPW--
LYPRPW--
```

-LPRPWY-
LQPRPW--
LAPRPW--
LNPRPW--
LWPRPW--
-LPRPWM-
-LPRPWQ-
LPPRPW--
LHPRPW--
-LPRPWH-
LFPRPW--
-LPRPWW-
LDPRPW--
-MPRPWR-
MVPRPW--
-MPRPWY-
-MPRPWS-
MRPRPW--
MGPRPW--
MSPRPW--
MIPRPW--
-MPRPWT-
-MPRPWL-
-MPRPWH-
-MPRPWA-
MNPRPW--
MMPRPW--
MLPRPW--
MEPRPW--
MQPRPW--
-MPRPWV-
MAPRPW--
-MPRPWN-
-MPRPWK-
-MPRPWQ-
-MPRPWF-
MYPRPW--
MTPRPW--
-MPRPWI-
-MPRPWG-
-MPRPWE-
MPPRPW--
MHPRPW--
NSPRPW--
-NPRPWL-
NTPRPW--
NRPRPW--
-NPRPWR-
NLPRPW--
-NPRPWY-

NWPRPW--
-NPRPWI-
NAPRPW--
NIPRPW--
NEPRPW--
NYPRPW--
NFPRPW--
NNPRPW--
NGPRPW--
NDPRPW--
-NPRPWW-
-NPRPWT-
NQPRPW--
-NPRPWV-
-NPRPWQ-
-NPRPWM-
-NPRPWF-
-NPRPWA-
--PRPWVR
--PRPWIS
--PRPWYL
--PRPWSS
--PRPWLV
--PRPWMS
--PRPWLL
--PRPWSI
--PRPWRL
--PRPWAN
--PRPWVV
--PRPWTN
--PRPWSF
--PRPWIV
PSPRPW--
--PRPWYF
--PRPWVI
--PRPWSN
--PRPWIT
--PRPWGL
--PRPWAS
--PRPWYA
--PRPWTS
--PRPWRT
--PRPWNI
--PRPWGY
--PRPWVT
--PRPWTA
--PRPWML
--PRPWLA
--PRPWHA
--PRPWRI

--PRPWRA
--PRPWIN
--PRPWGQ
--PRPWFL
PFPRPW--
--PRPWVG
--PRPWTR
--PRPWNS
--PRPWLG
--PRPWHL
--PRPWHF
--PRPWAY
--PRPWWA
--PRPWVY
--PRPWTF
--PRPWSY
--PRPWSQ
--PRPWSL
--PRPWKM
--PRPWFS
--PRPWAL
--PRPWAA
--PRPWTD
--PRPWSA
--PRPWRV
--PRPWPI
--PRPWLK
--PRPWGI
--PRPWAT
PRPRPW--
PVPRPW--
--PRPWWL
--PRPWVF
--PRPWRF
--PRPWQS
--PRPWPY
--PRPWMR
--PRPWLT
--PRPWLF
--PRPWKR
--PRPWIK
--PRPWHT
--PRPWYT
--PRPWYR
--PRPWVL
--PRPWVH
--PRPWSR
--PRPWSG
--PRPWRN
--PRPWQN

--PRPWMY
--PRPWLS
--PRPWIM
--PRPWII
--PRPWFQ
--PRPWFI
--PRPWEL
PGPRPW--
--PRPWYY
--PRPWYV
--PRPWYM
--PRPWTM
--PRPWTI
--PRPWRM
--PRPWRG
--PRPWQM
--PRPWQA
--PRPWNF
--PRPWMT
--PRPWLQ
--PRPWKA
--PRPWIY
--PRPWIR
--PRPWHG
--PRPWGT
--PRPWYS
--PRPWYG
--PRPWVW
--PRPWTG
--PRPWSV
--PRPWST
--PRPWRQ
--PRPWRK
--PRPWQV
--PRPWMV
--PRPWMN
--PRPWMG
--PRPWLW
--PRPWLN
--PRPWKH
--PRPWIG
--PRPWAW
--PRPWYN
--PRPWVN
--PRPWVK
--PRPWTV
--PRPWSM
--PRPWSH
--PRPWRD
--PRPWQR

--PRPWQG
--PRPWNA
--PRPWME
--PRPWLY
--PRPWKL
--PRPWIF
--PRPWHY
--PRPWGA
--PRPWEI
--PRPWAV
PWPRPW--
--PRPWYI
--PRPWWS
--PRPWTQ
--PRPWRW
--PRPWPV
--PRPWPF
--PRPWNW
--PRPWNQ
--PRPWNL
--PRPWMA
--PRPWKS
--PRPWIL
--PRPWHM
--PRPWHI
--PRPWGS
--PRPWGN
--PRPWFF
--PRPWDF
--PRPWAH
--PRPWYW
--PRPWVQ
--PRPWTY
--PRPWTW
--PRPWRY
--PRPWRR
--PRPWRP
--PRPWQY
--PRPWQI
--PRPWNV
--PRPWMW
--PRPWMF
--PRPWLI
--PRPWKN
--PRPWHH
--PRPWGM
--PRPWFW
--PRPWFK
--PRPWFA
--PRPWEY

--PRPWEV
--PRPWES
--PRPWEM
--PRPWDY
--PRPWDL
--PRPWAQ
--PRPWAG
PKPRPW--
PAPRPW--
--PRPWYQ
--PRPWYD
--PRPWWY
--PRPWWV
--PRPWWQ
--PRPWWM
--PRPWWG
--PRPWVD
--PRPWTE
--PRPWQH
--PRPWPQ
--PRPWLM
--PRPWLE
--PRPWKV
--PRPWKF
--PRPWIH
--PRPWIA
--PRPWHN
--PRPWGH
--PRPWGG
--PRPWFV
--PRPWFT
--PRPWFN
--PRPWFG
--PRPWEA
--PRPWDW
--PRPWAM
--PRPWAI
--PRPWAF
PQPRPW--
PLPRPW--
PDPRPW--
PYPRPW--
PTPRPW--
--PRPWYK
--PRPWWT
--PRPWWH
--PRPWVM
--PRPWVA
--PRPWTK
--PRPWTH

--PRPWQT
--PRPWQK
--PRPWPS
--PRPWPA
--PRPWNY
--PRPWNT
--PRPWNG
--PRPWMI
--PRPWMD
--PRPWLD
--PRPWKT
--PRPWKQ
--PRPWIW
--PRPWIQ
--PRPWIE
--PRPWID
--PRPWIC
--PRPWHV
--PRPWHR
--PRPWGF
--PRPWFM
--PRPWFD
--PRPWET
--PRPWCY
--PRPWCG
--PRPWAP
--PRPWAK
PMPRPW--
QVPRPW--
QLPRPW--
-QPRPWL-
QWPRPW--
QHPRPW--
QTPRPW--
-QPRPWK-
QRPRPW--
-QPRPWH-
QDPRPW--
QEPRPW--
QSPRPW--
QPPRPW--
QAPRPW--
QYPRPW--
-QPRPWY-
-QPRPWV-
-QPRPWS-
QFPRPW--
-QPRPWT-
-QPRPWR-
-QPRPWN-

-QPRPWM-
-QPRPWF-
-QPRPWA-
QNPRPW--
-RPRPWL-
RSPRPW--
RVPRPW--
RLPRPW--
-RPRPWR-
-RPRPWK-
RMPRPW--
RTPRPW--
-RPRPWS-
RIPRPW--
REPRPW--
-RPRPWY-
RGPRPW--
-RPRPWI-
-RPRPWM-
-RPRPWV-
-RPRPWT-
RYPRPW--
-RPRPWA-
RHPRPW--
-RPRPWH-
-RPRPWF-
RDPRPW--
RWPRPW--
RQPRPW--
-RPRPWQ-
-RPRPWN-
-RPRPWG-
RAPRPW--
-SPRPWL-
SLPRPW--
SVPRPW--
-SPRPWS-
SKPRPW--
SRPRPW--
-SPRPWV-
-SPRPWN-
STPRPW--
-SPRPWT-
-SPRPWM-
SGPRPW--
SSPRPW--
SPPRPW--
SFPRPW--
-SPRPWA-
SQPRPW--

-SPRPWY-
-SPRPWK-
-SPRPWF-
SAPRPW--
-SPRPWW-
SMPRPW--
SIPRPW--
SYPRPW--
-SPRPWQ-
SEPRPW--
SWPRPW--
SNPRPW--
SHPRPW--
-SPRPWH-
SDPRPW--
TVPRPW--
TSPRPW--
-TPRPWS-
TAPRPW--
TTPRPW--
TRPRPW--
-TPRPWT-
TFPRPW--
TLPRPW--
TGPRPW--
TYPRPW--
-TPRPWL-
-TPRPWH-
-TPRPWF-
-TPRPWY-
-TPRPWM-
TQPRPW--
-TPRPWK-
TMPRPW--
TKPRPW--
TIPRPW--
-TPRPWA-
TWPRPW--
-TPRPWV-
-TPRPWN-
-TPRPWI-
-TPRPWG-
TPPRPW--
TNPRPW--
TEPRPW--
-TPRPWW-
THPRPW--
TCPRPW--
VRPRPW--
-VPRPWL-

VVPRPW--
VTPRPW--
VSPRPW--
VLPRPW--
VEPRPW--
-VPRPWT-
-VPRPWS-
-VPRPWV-
-VPRPWM-
VNPRPW--
-VPRPWH-
VKPRPW--
VYPRPW--
-VPRPWG-
-VPRPWQ-
-VPRPWF-
-VPRPWA-
VIPRPW--
VDPRPW--
-VPRPWY-
VPPRPW--
VHPRPW--
VAPRPW--
VQPRPW--
VWPRPW--
-VPRPWW-
VMPRPW--
WVPRPW--
WTPRPW--
WRPRPW--
-WPRPWK-
-WPRPWV-
WLPRPW--
-WPRPWS-
WGPRPW--
WYPRPW--
-WPRPWT-
-WPRPWM-
WMPRPW--
-WPRPWW-
-WPRPWQ-
-WPRPWI-
WKPRPW--
-WPRPWY-
-WPRPWA-
WIPRPW--
WFPRPW--
WCPRPW--
YAPRPW--
YVPRPW--

| | | | |
|---|---|---|---|
| | -YPRPWI- | | |
| | -YPRPWS- | | |
| | -YPRPWV- | | |
| | -YPRPWQ- | | |
| | YGPRPW-- | | |
| | YWPRPW-- | | |
| | YSPRPW-- | | |
| | -YPRPWR- | | |
| | -YPRPWT- | | |
| | YHPRPW-- | | |
| | YRPRPW-- | | |
| | -YPRPWY- | | |
| | YMPRPW-- | | |
| | YIPRPW-- | | |
| | YDPRPW-- | | |
| | YYPRPW-- | | |
| | -YPRPWA- | | |
| | YQPRPW-- | | |
| | -YPRPWH- | | |
| | -YPRPWF- | | |
| | YKPRPW-- | | |
| | YTPRPW-- | | |
| | -YPRPWW- | | |
| | -YPRPWL- | | |
| | -YPRPWG- | | |
| | YFPRPW-- | | |
| | YEPRPW-- | | |
| PAAI | 187 | 723 | 32.10 |
| | ASPAAI-- | | |
| | AQPAAI-- | | |
| | AAPAAI-- | | |
| | -APAAIS- | | |
| | APPAAI-- | | |
| | -APAAIR- | | |
| | -APAAIL- | | |
| | -APAAIA- | | |
| | -APAAIH- | | |
| | -APAAIT- | | |
| | -APAAIK- | | |
| | AWPAAI-- | | |
| | AVPAAI-- | | |
| | ATPAAI-- | | |
| | -APAAIG- | | |
| | ALPAAI-- | | |
| | AHPAAI-- | | |
| | AYPAAI-- | | |
| | -APAAIV- | | |
| | -APAAIM- | | |
| | -APAAIF- | | |
| | ANPAAI-- | | |

CRPAAI--
CTPAAI--
CVPAAI--
-CPAAIS-
CMPAAI--
CHPAAI--
CSPAAI--
CQPAAI--
CPPAAI--
-CPAAIR-
-CPAAIK-
-CPAAIE-
CLPAAI--
DKPAAI--
DSPAAI--
-DPAAIN-
DNPAAI--
DYPAAI--
-DPAAIR-
DVPAAI--
DFPAAI--
DWPAAI--
-DPAAIQ-
-DPAAIA-
DRPAAI--
-DPAAIV-
-DPAAIT-
-DPAAIS-
-DPAAII-
DEPAAI--
DCPAAI--
EYPAAI--
ELPAAI--
-EPAAIS-
-EPAAIM-
ECPAAI--
EWPAAI--
ESPAAI--
-EPAAIY-
-EPAAIE-
EMPAAI--
EVPAAI--
ERPAAI--
-EPAAIW-
-EPAAIV-
-EPAAIL-
-EPAAIG-
ENPAAI--
EHPAAI--
EGPAAI--

EFPAAI--
EAPAAI--
FVPAAI--
FIPAAI--
FHPAAI--
FSPAAI--
FLPAAI--
-FPAAII-
-FPAAIR-
-FPAAIA-
FNPAAI--
FFPAAI--
FYPAAI--
FPPAAI--
-FPAAIL-
-FPAAIW-
-FPAAIS-
FRPAAI--
-FPAAIQ-
-FPAAIN-
-FPAAIE-
FMPAAI--
FWPAAI--
FTPAAI--
FQPAAI--
-FPAAIV-
-FPAAIM-
FAPAAI--
-GPAAIL-
GVPAAI--
-GPAAIK-
-GPAAIT-
GTPAAI--
GRPAAI--
-GPAAIV-
-GPAAIW-
GNPAAI--
GFPAAI--
-GPAAIR-
-GPAAIQ-
-GPAAIE-
GMPAAI--
GIPAAI--
GAPAAI--
GQPAAI--
-GPAAIN-
-GPAAIH-
GLPAAI--
GDPAAI--
-GPAAIG-

-GPAAIC-
-GPAAIA-
GEPAAI--
-HPAAIL-
-HPAAIR-
HSPAAI--
-HPAAIG-
HIPAAI--
HGPAAI--
HPPAAI--
-HPAAIV-
HMPAAI--
HLPAAI--
HAPAAI--
HYPAAI--
HRPAAI--
HWPAAI--
-HPAAIT-
-HPAAIM-
-HPAAIH-
-HPAAIA-
HHPAAI--
-HPAAIN-
-HPAAII-
-HPAAIE-
HNPAAI--
HFPAAI--
HEPAAI--
HTPAAI--
HQPAAI--
-HPAAIW-
-HPAAIK-
HKPAAI--
IRPAAI--
-IPAAIT-
-IPAAIY-
ISPAAI--
-IPAAIR-
ILPAAI--
-IPAAIS-
IIPAAI--
-IPAAIL-
IGPAAI--
-IPAAIG-
IEPAAI--
-IPAAIW-
-IPAAII-
ICPAAI--
IQPAAI--
-IPAAIV-

-IPAAIM-
-IPAAIE-
-IPAAIA-
INPAAI--
IKPAAI--
IHPAAI--
IAPAAI--
KSPAAI--
KWPAAI--
KQPAAI--
-KPAAIL-
-KPAAIF-
KVPAAI--
-KPAAIY-
KGPAAI--
KAPAAI--
KYPAAI--
KTPAAI--
-KPAAIV-
KRPAAI--
-KPAAIT-
-KPAAIN-
-KPAAIA-
KMPAAI--
-KPAAIS-
KIPAAI--
KPPAAI--
-KPAAIH-
-KPAAIE-
-KPAAII-
-KPAAIG-
KLPAAI--
KDPAAI--
-LPAAIL-
LVPAAI--
LGPAAI--
LSPAAI--
-LPAAIG-
-LPAAIR-
LMPAAI--
LYPAAI--
-LPAAIA-
LQPAAI--
LPPAAI--
-LPAAIN-
-LPAAII-
LLPAAI--
LKPAAI--
LFPAAI--
LTPAAI--

-LPAAIK-
LAPAAI--
-LPAAIV-
-LPAAIS-
-LPAAIE-
-LPAAIQ-
-LPAAIH-
LWPAAI--
-LPAAIY-
-LPAAIT-
-LPAAIM-
-LPAAIF-
LNPAAI--
LIPAAI--
LHPAAI--
LEPAAI--
-LPAAIW-
MSPAAI--
MNPAAI--
MTPAAI--
MYPAAI--
-MPAAIW-
MWPAAI--
MHPAAI--
-MPAAIY-
MQPAAI--
-MPAAIH-
-MPAAIF-
MGPAAI--
-MPAAIT-
-MPAAIR-
-MPAAIN-
MFPAAI--
MDPAAI--
MMPAAI--
MCPAAI--
MAPAAI--
MVPAAI--
-MPAAIV-
-MPAAIP-
-MPAAII-
MIPAAI--
-NPAAIL-
NHPAAI--
-NPAAIV-
-NPAAIS-
-NPAAIH-
NTPAAI--
-NPAAIR-
NLPAAI--

NGPAAI--
NVPAAI--
NPPAAI--
-NPAAIK-
NNPAAI--
-NPAAIM-
-NPAAIG-
-NPAAIA-
NMPAAI--
NKPAAI--
NRPAAI--
-NPAAIY-
NDPAAI--
NSPAAI--
NQPAAI--
-NPAAIT-
-NPAAII-
-NPAAIF-
--PAAIQE
--PAAIRV
--PAAISL
--PAAILR
--PAAIVS
--PAAIVK
--PAAILT
--PAAIKL
PWPAAI--
--PAAISS
--PAAILQ
--PAAILL
--PAAITR
--PAAITG
--PAAIRY
--PAAILN
--PAAIRM
--PAAILH
--PAAIYK
--PAAIVL
--PAAITL
--PAAISR
--PAAISG
--PAAILM
--PAAIKV
PRPAAI--
--PAAIQL
--PAAILS
--PAAILK
--PAAIHL
--PAAIGY
--PAAIGV

--PAAIAL
--PAAIWV
--PAAIWS
--PAAIWL
--PAAITS
--PAAIRI
--PAAIQR
--PAAIPL
--PAAIML
--PAAIMA
--PAAIGS
--PAAIFR
--PAAIYT
--PAAISE
--PAAIRT
--PAAIRK
--PAAIMQ
--PAAILY
--PAAILG
--PAAIKG
--PAAIHR
--PAAIGN
--PAAIGG
--PAAIAK
PIPAAI--
--PAAIWR
--PAAIVR
--PAAISN
--PAAIRS
--PAAIRQ
--PAAIRP
--PAAIMR
--PAAILF
--PAAILA
--PAAIKR
--PAAIHY
--PAAIAT
PHPAAI--
--PAAIYR
--PAAIWT
--PAAIVT
--PAAITT
--PAAITH
--PAAIRG
--PAAIRA
--PAAING
--PAAILW
--PAAILV
--PAAIKS
--PAAIKF

--PAAIKA
--PAAIIA
--PAAIGT
--PAAIGL
--PAAIFM
--PAAIFK
--PAAIES
--PAAIAH
PGPAAI--
--PAAIYD
--PAAITV
--PAAITN
--PAAITI
--PAAITA
--PAAISK
--PAAISH
--PAAISA
--PAAIRE
--PAAIQH
--PAAINS
--PAAINE
--PAAIMY
--PAAIKT
--PAAIKK
--PAAIIS
--PAAIGK
--PAAIER
PQPAAI--
PAPAAI--
--PAAIYY
--PAAIYM
--PAAIYL
--PAAIYH
--PAAIVQ
--PAAIVH
--PAAIVG
--PAAIVE
--PAAIVD
--PAAITW
--PAAITE
--PAAISY
--PAAISQ
--PAAISM
--PAAISC
--PAAIRH
--PAAIRF
--PAAIQT
--PAAIQK
--PAAIQD
--PAAIPY

--PAAINV
--PAAINR
--PAAINM
--PAAIMS
--PAAIKW
--PAAIKQ
--PAAIKM
--PAAIIG
--PAAIHW
--PAAIHM
--PAAIHG
--PAAIGI
--PAAIFS
--PAAIFH
--PAAIFG
--PAAIEY
--PAAIEW
--PAAIEF
PVPAAI--
PLPAAI--
--PAAIYW
--PAAIYN
--PAAIYF
--PAAIWY
--PAAIWQ
--PAAIWI
--PAAIWF
--PAAIVM
--PAAIVI
--PAAITY
--PAAITQ
--PAAISW
--PAAIST
--PAAISD
--PAAIRD
--PAAIQY
--PAAIQS
--PAAIQQ
--PAAIQN
--PAAINY
--PAAINI
--PAAIMP
--PAAIMI
--PAAILI
--PAAILD
--PAAIKN
--PAAIKE
--PAAIIY
--PAAIIR
--PAAIIN

--PAAIII
--PAAIIH
--PAAIHV
--PAAIHN
--PAAIHI
--PAAIGM
--PAAIGH
--PAAIGF
--PAAIGA
--PAAIEK
--PAAIEG
--PAAIDA
--PAAIAE
PNPAAI--
PMPAAI--
PFPAAI--
--PAAIYS
--PAAIYI
--PAAIYG
--PAAIYE
--PAAIYA
--PAAIWK
--PAAIWH
--PAAIVY
--PAAIVW
--PAAITK
--PAAITD
--PAAIRW
--PAAIRN
--PAAIQV
--PAAIQG
--PAAIPQ
--PAAIPM
--PAAINQ
--PAAINN
--PAAINL
--PAAINA
--PAAIMW
--PAAIMN
--PAAIMM
--PAAIMG
--PAAIIV
--PAAIIQ
--PAAIIK
--PAAIHT
--PAAIGW
--PAAIGR
--PAAIGQ
--PAAIGD
--PAAIFY

--PAAIFF
--PAAIEV
--PAAIEA
--PAAIDS
--PAAICN
--PAAIAV
--PAAIAN
--PAAIAM
--PAAIAG
--PAAIAF
--PAAIAD
-QPAAIR-
-QPAAIN-
QFPAAI--
-QPAAIV-
-QPAAIL-
QRPAAI--
-QPAAIQ-
-QPAAII-
-QPAAIS-
-QPAAIK-
QNPAAI--
QKPAAI--
QHPAAI--
QGPAAI--
QAPAAI--
QYPAAI--
QWPAAI--
QTPAAI--
-QPAAIT-
-QPAAIM-
-QPAAIH-
-QPAAIG-
-QPAAIE-
QIPAAI--
-RPAAIG-
RLPAAI--
RSPAAI--
-RPAAIY-
-RPAAIF-
RRPAAI--
RTPAAI--
RYPAAI--
-RPAAIL-
-RPAAIK-
RAPAAI--
RMPAAI--
RWPAAI--
-RPAAIT-
RQPAAI--

```
-RPAAIW-
-RPAAIH-
RNPAAI--
-RPAAIS-
-RPAAIM-
RPPAAI--
-RPAAIR-
RVPAAI--
RHPAAI--
RKPAAI--
RFPAAI--
REPAAI--
-RPAAIN-
-RPAAIE-
RDPAAI--
-RPAAIV-
-RPAAID-
RCPAAI--
SSPAAI--
SRPAAI--
-SPAAIL-
-SPAAIT-
-SPAAIR-
-SPAAIY-
SNPAAI--
-SPAAIK-
-SPAAIF-
SMPAAI--
SGPAAI--
SVPAAI--
-SPAAIG-
SKPAAI--
-SPAAIW-
-SPAAIN-
-SPAAIM-
SLPAAI--
-SPAAIS-
-SPAAIA-
SIPAAI--
-SPAAIV-
SHPAAI--
SYPAAI--
SEPAAI--
SAPAAI--
STPAAI--
SPPAAI--
-SPAAII-
SFPAAI--
SWPAAI--
-SPAAIE-
```

SDPAAI--
-TPAAIV-
-TPAAIM-
TNPAAI--
TYPAAI--
-TPAAIG-
-TPAAIY-
TLPAAI--
TWPAAI--
TAPAAI--
TVPAAI--
-TPAAIT-
-TPAAIL-
TGPAAI--
TFPAAI--
TSPAAI--
TQPAAI--
-TPAAIH-
-TPAAIF-
-TPAAIA-
TMPAAI--
TIPAAI--
THPAAI--
VSPAAI--
VIPAAI--
VMPAAI--
VAPAAI--
VWPAAI--
VRPAAI--
VPPAAI--
-VPAAII-
-VPAAIW-
-VPAAIL-
VKPAAI--
VGPAAI--
VNPAAI--
VLPAAI--
-VPAAIV-
-VPAAIS-
-VPAAIM-
VFPAAI--
VVPAAI--
VTPAAI--
-VPAAIT-
-VPAAIQ-
-VPAAIG-
-VPAAIF-
VHPAAI--
VEPAAI--
VDPAAI--

VQPAAI--
-VPAAIH-
VCPAAI--
-WPAAIL-
WKPAAI--
-WPAAIR-
WTPAAI--
-WPAAIG-
WVPAAI--
-WPAAIK-
WPPAAI--
-WPAAIT-
-WPAAIN-
-WPAAII-
WLPAAI--
WGPAAI--
WDPAAI--
WYPAAI--
WWPAAI--
-WPAAIW-
-WPAAIV-
-WPAAIA-
WSPAAI--
-WPAAIH-
-WPAAIE-
WAPAAI--
YRPAAI--
YLPAAI--
YSPAAI--
-YPAAIR-
-YPAAIQ-
YIPAAI--
YVPAAI--
-YPAAIK-
-YPAAIS-
-YPAAIN-
-YPAAIL-
YFPAAI--
YYPAAI--
-YPAAIG-
YMPAAI--
YGPAAI--
YPPAAI--
-YPAAIW-
YKPAAI--
YTPAAI--
-YPAAIY-
-YPAAIT-
YNPAAI--
YEPAAI--

```
             YDPAAI--
PSSI           39        816      32.06
             -APSSIP-
             -DPSSII-
             --PSSIAK
             --PSSITI
             --PSSISD
             --PSSINL
             --PSSIHL
             --PSSIGN
             --PSSIAR
             -RPSSIP-
             -SPSSIP-
             -APSSIL-
             ASPSSI--
             ARPSSI--
             -APSSIS-
             ALPSSI--
             -APSSIM-
             AVPSSI--
             AFPSSI--
             -APSSIE-
             AGPSSI--
             AAPSSI--
             ANPSSI--
             -APSSIK-
             AMPSSI--
             -APSSIT-
             APPSSI--
             AEPSSI--
             AWPSSI--
             AQPSSI--
             -APSSIG-
             -APSSIA-
             AYPSSI--
             ATPSSI--
             -APSSIH-
             -APSSIQ-
             -APSSIN-
             AIPSSI--
             ACPSSI--
             -APSSIW-
             -APSSIV-
             ADPSSI--
             AHPSSI--
             CKPSSI--
             CCPSSI--
             CSPSSI--
             CRPSSI--
             -CPSSIR-
```

−CPSSIK−
CPPSSI−−
CAPSSI−−
CTPSSI−−
CLPSSI−−
CVPSSI−−
−CPSSIY−
−CPSSIV−
CFPSSI−−
CYPSSI−−
CQPSSI−−
−CPSSIW−
−CPSSIG−
CMPSSI−−
CIPSSI−−
CEPSSI−−
DKPSSI−−
DNPSSI−−
DIPSSI−−
DTPSSI−−
DSPSSI−−
−DPSSIT−
DYPSSI−−
−DPSSIS−
−DPSSIH−
DFPSSI−−
DVPSSI−−
DQPSSI−−
−DPSSIR−
−DPSSIK−
DHPSSI−−
DAPSSI−−
−DPSSIW−
−DPSSIA−
DMPSSI−−
DWPSSI−−
−DPSSIY−
−DPSSIM−
−DPSSIF−
DEPSSI−−
DDPSSI−−
−DPSSIV−
−EPSSIM−
ESPSSI−−
−EPSSIR−
−EPSSIW−
ERPSSI−−
−EPSSIL−
ELPSSI−−
EWPSSI−−

ETPSSI--
-EPSSII-
-EPSSIV-
EGPSSI--
EYPSSI--
EVPSSI--
-EPSSIS-
ENPSSI--
EFPSSI--
EAPSSI--
-EPSSIK-
-EPSSIY-
-EPSSIT-
EIPSSI--
-EPSSIH-
-EPSSIG-
-EPSSIF-
-EPSSIA-
EKPSSI--
EEPSSI--
FRPSSI--
FVPSSI--
-FPSSIS-
-FPSSIT-
-FPSSII-
FMPSSI--
FGPSSI--
-FPSSIR-
-FPSSIY-
-FPSSIV-
FDPSSI--
FKPSSI--
FEPSSI--
FNPSSI--
-FPSSIL-
FHPSSI--
FPPSSI--
FFPSSI--
FSPSSI--
-FPSSIF-
-FPSSIK-
-FPSSIH-
FIPSSI--
FYPSSI--
-FPSSIQ-
FCPSSI--
FWPSSI--
FQPSSI--
-FPSSIM-
-FPSSIA-

GRPSSI--
GTPSSI--
-GPSSIL-
GLPSSI--
-GPSSIA-
GAPSSI--
GVPSSI--
GEPSSI--
GHPSSI--
GFPSSI--
GQPSSI--
GMPSSI--
GGPSSI--
-GPSSIY-
-GPSSIS-
GIPSSI--
GWPSSI--
-GPSSIN-
-GPSSIV-
-GPSSIK-
-GPSSIH-
GDPSSI--
-GPSSIW-
-GPSSII-
-GPSSIQ-
-GPSSIM-
-GPSSIF-
GCPSSI--
GNPSSI--
-GPSSIG-
-GPSSIE-
GPPSSI--
HRPSSI--
HVPSSI--
HLPSSI--
HKPSSI--
HYPSSI--
HMPSSI--
-HPSSIS-
-HPSSIF-
HSPSSI--
-HPSSIL-
HEPSSI--
HWPSSI--
HAPSSI--
-HPSSIK-
-HPSSIV-
HGPSSI--
HDPSSI--
-HPSSIH-

HIPSSI--
-HPSSIW-
-HPSSIR-
-HPSSIN-
HNPSSI--
HTPSSI--
-HPSSII-
HHPSSI--
HQPSSI--
-HPSSIQ-
-HPSSIM-
HCPSSI--
IRPSSI--
IIPSSI--
-IPSSIL-
-IPSSII-
IQPSSI--
IYPSSI--
-IPSSIN-
IGPSSI--
-IPSSIW-
IEPSSI--
ITPSSI--
-IPSSIK-
-IPSSIH-
IFPSSI--
-IPSSIF-
IAPSSI--
-IPSSIT-
IMPSSI--
IKPSSI--
IPPSSI--
INPSSI--
IHPSSI--
-IPSSIM-
-IPSSIA-
IDPSSI--
-IPSSIY-
-IPSSIV-
IWPSSI--
-IPSSIG-
-IPSSIE-
-IPSSIQ-
ICPSSI--
KSPSSI--
KRPSSI--
KLPSSI--
-KPSSIL-
-KPSSIR-
-KPSSIS-

-KPSSIY-
-KPSSII-
-KPSSIF-
KFPSSI--
KMPSSI--
KIPSSI--
KYPSSI--
KAPSSI--
KVPSSI--
KTPSSI--
KPPSSI--
KGPSSI--
-KPSSIV-
-KPSSIT-
KEPSSI--
KWPSSI--
-KPSSIK-
KHPSSI--
-KPSSIW-
-KPSSIH-
KCPSSI--
-KPSSIA-
KNPSSI--
-KPSSIG-
KKPSSI--
KQPSSI--
-KPSSIM-
-KPSSIE-
-KPSSIN-
-KPSSID-
-LPSSIR-
LIPSSI--
LAPSSI--
LWPSSI--
LLPSSI--
-LPSSII-
LVPSSI--
LSPSSI--
LTPSSI--
LKPSSI--
LYPSSI--
-LPSSIL-
-LPSSIS-
LPPSSI--
LFPSSI--
LEPSSI--
LHPSSI--
LMPSSI--
-LPSSIW-
-LPSSIV-

-LPSSIF-
-LPSSIT-
-LPSSIQ-
-LPSSIM-
-LPSSIY-
LDPSSI--
-LPSSIA-
LCPSSI--
MVPSSI--
MLPSSI--
MRPSSI--
MGPSSI--
MMPSSI--
-MPSSIV-
-MPSSII-
MHPSSI--
MTPSSI--
-MPSSIY-
MKPSSI--
MAPSSI--
-MPSSIW-
-MPSSIS-
-MPSSIK-
MWPSSI--
MQPSSI--
-MPSSIT-
-MPSSIH-
-MPSSIM-
MYPSSI--
-MPSSIQ-
-MPSSIA-
MNPSSI--
MSPSSI--
-MPSSIF-
MPPSSI--
MEPSSI--
MDPSSI--
-MPSSIE-
-MPSSID-
NVPSSI--
NRPSSI--
-NPSSIS-
-NPSSIT-
NPPSSI--
-NPSSIM-
NFPSSI--
NTPSSI--
-NPSSIW-
NLPSSI--
NHPSSI--

-NPSSIY-
NAPSSI--
NMPSSI--
-NPSSIK-
-NPSSIF-
NIPSSI--
-NPSSIV-
-NPSSIL-
NGPSSI--
NWPSSI--
NSPSSI--
NQPSSI--
NNPSSI--
-NPSSIH-
NKPSSI--
NEPSSI--
NYPSSI--
-NPSSIQ-
-NPSSIA-
NDPSSI--
--PSSIQN
--PSSIRN
--PSSIRL
--PSSIRK
--PSSIRI
--PSSIST
--PSSIRG
--PSSILR
--PSSITA
--PSSILM
--PSSISS
--PSSIMA
--PSSILS
--PSSIMR
--PSSIIG
--PSSIRW
--PSSIFR
--PSSIYY
--PSSIRA
--PSSITG
--PSSILY
--PSSIFS
--PSSITS
--PSSISG
--PSSILA
--PSSISH
--PSSIYR
--PSSIWS
PWPSSI--
--PSSIYQ

--PSSIRH
--PSSILF
--PSSIFT
--PSSIVT
--PSSIHN
--PSSIFW
--PSSIVY
--PSSISY
--PSSINS
--PSSIIS
--PSSIYI
--PSSISV
--PSSIRD
--PSSIMS
--PSSILW
--PSSILH
--PSSIKY
--PSSIFH
--PSSIAG
--PSSIAA
--PSSIWR
--PSSIVG
--PSSIVA
--PSSISF
--PSSIKS
--PSSIIV
--PSSIWT
--PSSIMV
--PSSIFV
--PSSIYV
--PSSIYK
--PSSIVL
--PSSIRF
--PSSIKT
--PSSIIT
--PSSIGS
--PSSIYW
--PSSIVN
--PSSITQ
--PSSITF
--PSSISM
--PSSISL
--PSSILV
--PSSIKH
--PSSIHK
PRPSSI--
--PSSIWH
--PSSISR
--PSSIRY
--PSSIMT

--PSSILN
--PSSIIL
--PSSIHT
--PSSIHG
--PSSIFN
--PSSIAV
PIPSSI--
--PSSIYL
--PSSIVE
--PSSIVD
--PSSITY
--PSSITL
--PSSIRQ
--PSSIMD
--PSSIIW
--PSSIIQ
--PSSIFG
--PSSIAS
PVPSSI--
PTPSSI--
--PSSIWL
--PSSIWG
--PSSITH
--PSSISN
--PSSIQR
--PSSIMH
--PSSIKK
--PSSIIK
--PSSIII
--PSSIFM
--PSSIES
--PSSIAT
--PSSIYF
--PSSIWQ
--PSSIWM
--PSSIVW
--PSSIVK
--PSSIVI
--PSSIQY
--PSSIQS
--PSSIQG
--PSSIQA
--PSSIMG
--PSSIKV
--PSSIKN
--PSSIKM
--PSSIKF
--PSSIKA
--PSSIFQ
--PSSIFF

PQPSSI--
PEPSSI--
PAPSSI--
--PSSIWK
--PSSIVV
--PSSITM
--PSSIRE
--PSSING
--PSSIMN
--PSSIMK
--PSSIIR
--PSSIIE
--PSSIEA
PMPSSI--
PDPSSI--
--PSSIYA
--PSSIWW
--PSSIWE
--PSSIVM
--PSSIVH
--PSSIVF
--PSSITN
--PSSISK
--PSSIQV
--PSSIQT
--PSSINV
--PSSILQ
--PSSIIM
--PSSIIA
--PSSIHV
--PSSIHF
--PSSIFL
--PSSIFK
--PSSIFE
--PSSIEG
--PSSIAM
PLPSSI--
--PSSIYN
--PSSIWN
--PSSISE
--PSSIQM
--PSSIQK
--PSSIQH
--PSSINT
--PSSINA
--PSSIMQ
--PSSIMI
--PSSIMF
--PSSILE
--PSSIIN

--PSSIHY
--PSSIHS
--PSSIHD
--PSSIGI
--PSSIFA
--PSSIAL
--PSSIYD
--PSSIWD
--PSSITK
--PSSIQW
--PSSIQQ
--PSSIQF
--PSSINW
--PSSINF
--PSSIMM
--PSSIKQ
--PSSIKE
--PSSIID
--PSSIHW
--PSSIGT
--PSSIFY
--PSSIEV
--PSSIAI
-PPSSIS-
PFPSSI--
--PSSIWA
--PSSITP
--PSSITE
--PSSITD
--PSSIRC
--PSSIPW
--PSSINK
--PSSINH
--PSSIMW
--PSSIKI
--PSSIKD
--PSSIHR
--PSSIHQ
--PSSIHM
--PSSIHH
--PSSIHE
--PSSIFD
--PSSIEW
--PSSIEK
--PSSIEF
--PSSICY
--PSSICS
--PSSICN
--PSSICG
-PPSSIN-

-PPSSIL-
-PPSSIA-
PPPSSI--
-QPSSIT-
QPPSSI--
QRPSSI--
QTPSSI--
QFPSSI--
QSPSSI--
-QPSSIR-
QGPSSI--
QVPSSI--
-QPSSIS-
QWPSSI--
-QPSSIY-
-QPSSII-
QAPSSI--
-QPSSIK-
QCPSSI--
QYPSSI--
QQPSSI--
-QPSSIH-
-QPSSIF-
QIPSSI--
-QPSSIQ-
QKPSSI--
QMPSSI--
QEPSSI--
-QPSSIV-
QNPSSI--
QHPSSI--
QDPSSI--
-RPSSIR-
RGPSSI--
-RPSSIS-
RTPSSI--
RSPSSI--
RMPSSI--
-RPSSIK-
-RPSSIN-
-RPSSIA-
-RPSSIY-
RKPSSI--
RDPSSI--
RIPSSI--
RNPSSI--
-RPSSIM-
-RPSSIH-
-RPSSIQ-
-RPSSII-

RAPSSI--
RHPSSI--
REPSSI--
RYPSSI--
-RPSSIG-
-RPSSIE-
-RPSSIT-
RQPSSI--
RPPSSI--
SRPSSI--
-SPSSIL-
SLPSSI--
SKPSSI--
-SPSSIS-
SGPSSI--
SNPSSI--
-SPSSIR-
STPSSI--
SAPSSI--
SVPSSI--
-SPSSIY-
SMPSSI--
-SPSSII-
SFPSSI--
SYPSSI--
SQPSSI--
-SPSSIV-
-SPSSIT-
-SPSSIA-
-SPSSIH-
-SPSSIM-
-SPSSIK-
SIPSSI--
SHPSSI--
SEPSSI--
SWPSSI--
-SPSSIW-
-SPSSIN-
SPPSSI--
-SPSSIQ-
SCPSSI--
-SPSSIC-
TRPSSI--
-TPSSIL-
TGPSSI--
-TPSSIR-
TSPSSI--
-TPSSIH-
TWPSSI--
-TPSSIV-

TLPSSI--
TYPSSI--
TIPSSI--
TQPSSI--
TVPSSI--
TFPSSI--
TTPSSI--
-TPSSIY-
-TPSSIT-
TKPSSI--
TDPSSI--
-TPSSIA-
THPSSI--
TAPSSI--
-TPSSIN-
-TPSSIK-
TNPSSI--
TCPSSI--
TPPSSI--
TMPSSI--
-TPSSIQ-
-TPSSII-
-TPSSIF-
-TPSSIW-
-TPSSIP-
-TPSSIC-
TEPSSI--
VPPSSI--
-VPSSIN-
VYPSSI--
VSPSSI--
VLPSSI--
-VPSSIL-
VMPSSI--
VGPSSI--
-VPSSIT-
VTPSSI--
-VPSSIR-
-VPSSIQ-
-VPSSIA-
VVPSSI--
VQPSSI--
-VPSSIY-
VKPSSI--
VIPSSI--
-VPSSIM-
VAPSSI--
VEPSSI--
VCPSSI--
VDPSSI--

-VPSSII-
VNPSSI--
VHPSSI--
-VPSSIW-
-VPSSIH-
-VPSSIE-
-VPSSIV-
-VPSSIG-
-VPSSIP-
-VPSSIC-
-WPSSIR-
WLPSSI--
WTPSSI--
-WPSSIK-
-WPSSIV-
-WPSSIS-
-WPSSIF-
WIPSSI--
WRPSSI--
-WPSSIW-
WYPSSI--
-WPSSIL-
WNPSSI--
WDPSSI--
WAPSSI--
-WPSSIA-
WGPSSI--
WHPSSI--
-WPSSIT-
WQPSSI--
WPPSSI--
WKPSSI--
WWPSSI--
-WPSSIQ-
-WPSSIM-
-WPSSIH-
-WPSSIE-
YVPSSI--
-YPSSIR-
YRPSSI--
YIPSSI--
-YPSSIL-
YAPSSI--
YKPSSI--
YTPSSI--
YSPSSI--
YFPSSI--
-YPSSIH-
-YPSSIK-
YQPSSI--

| | | | |
|---|---|---|---|
| | -YPSSIA- | | |
| | YLPSSI-- | | |
| | -YPSSIY- | | |
| | -YPSSIF- | | |
| | YGPSSI-- | | |
| | YWPSSI-- | | |
| | -YPSSIW- | | |
| | YPPSSI-- | | |
| | YNPSSI-- | | |
| | YYPSSI-- | | |
| | -YPSSIN- | | |
| | -YPSSII- | | |
| | -YPSSIQ- | | |
| | -YPSSIM- | | |
| | YHPSSI-- | | |
| | YDPSSI-- | | |
| | YCPSSI-- | | |
| | -YPSSIE- | | |
| PKSL | 602 | 579 | 32.03 |
| | --PKSLTE | | |
| | --PKSLDY | | |
| | -VPKSLG- | | |
| | -VPKSLD- | | |
| | -WPKSLN- | | |
| | -APKSLL- | | |
| | -APKSLW- | | |
| | ASPKSL-- | | |
| | -APKSLY- | | |
| | AVPKSL-- | | |
| | AFPKSL-- | | |
| | -APKSLV- | | |
| | -APKSLS- | | |
| | AQPKSL-- | | |
| | AGPKSL-- | | |
| | -APKSLT- | | |
| | -APKSLE- | | |
| | AKPKSL-- | | |
| | AIPKSL-- | | |
| | ADPKSL-- | | |
| | AAPKSL-- | | |
| | APPKSL-- | | |
| | AHPKSL-- | | |
| | ACPKSL-- | | |
| | -APKSLG- | | |
| | ANPKSL-- | | |
| | AEPKSL-- | | |
| | ATPKSL-- | | |
| | -APKSLQ- | | |
| | -APKSLN- | | |
| | -APKSLF- | | |

-APKSLA-
-APKSLC-
-CPKSLR-
CVPKSL--
CRPKSL--
CHPKSL--
CNPKSL--
CTPKSL--
-CPKSLL-
CDPKSL--
CEPKSL--
CWPKSL--
CSPKSL--
-CPKSLV-
-CPKSLT-
-CPKSLQ-
-CPKSLK-
-CPKSLI-
-CPKSLF-
-CPKSLA-
CMPKSL--
CIPKSL--
CGPKSL--
CFPKSL--
DWPKSL--
DRPKSL--
DYPKSL--
DGPKSL--
-DPKSLL-
DMPKSL--
DAPKSL--
-DPKSLY-
-DPKSLT-
-DPKSLK-
DLPKSL--
-DPKSLQ-
-DPKSLI-
-DPKSLF-
DNPKSL--
-DPKSLW-
-DPKSLS-
-DPKSLM-
-DPKSLH-
DKPKSL--
DEPKSL--
DCPKSL--
DQPKSL--
-DPKSLV-
-DPKSLG-
DDPKSL--

EVPKSL--
-EPKSLL-
ELPKSL--
ESPKSL--
-EPKSLT-
EIPKSL--
ERPKSL--
-EPKSLR-
-EPKSLN-
-EPKSLM-
EFPKSL--
-EPKSLV-
-EPKSLH-
ETPKSL--
EQPKSL--
ENPKSL--
EGPKSL--
EWPKSL--
-EPKSLF-
-EPKSLA-
EAPKSL--
-EPKSLK-
-EPKSLI-
-EPKSLG-
-EPKSLY-
-EPKSLS-
-EPKSLQ-
EMPKSL--
ECPKSL--
-EPKSLE-
-EPKSLW-
EEPKSL--
EYPKSL--
EKPKSL--
FTPKSL--
FNPKSL--
FHPKSL--
FAPKSL--
FLPKSL--
FQPKSL--
FKPKSL--
FEPKSL--
FPPKSL--
-FPKSLM-
FIPKSL--
FWPKSL--
GVPKSL--
GLPKSL--
GAPKSL--
GSPKSL--

GWPKSL--
-GPKSLF-
GKPKSL--
GGPKSL--
-GPKSLT-
-GPKSLH-
GTPKSL--
-GPKSLN-
-GPKSLW-
GHPKSL--
GEPKSL--
-GPKSLS-
GPPKSL--
-GPKSLV-
-GPKSLQ-
GDPKSL--
GQPKSL--
-GPKSLI-
-GPKSLC-
GNPKSL--
GCPKSL--
-GPKSLG-
-GPKSLE-
-HPKSLL-
-HPKSLI-
HVPKSL--
-HPKSLF-
HKPKSL--
HGPKSL--
-HPKSLS-
-HPKSLM-
-HPKSLV-
-HPKSLK-
HPPKSL--
HMPKSL--
HHPKSL--
-HPKSLQ-
-HPKSLN-
HDPKSL--
-HPKSLY-
HAPKSL--
HWPKSL--
-HPKSLH-
HNPKSL--
HQPKSL--
-IPKSLV-
-IPKSLL-
IVPKSL--
-IPKSLY-
-IPKSLA-

IIPKSL--
ISPKSL--
IQPKSL--
-IPKSLI-
-IPKSLQ-
-IPKSLN-
IHPKSL--
-IPKSLM-
IKPKSL--
IGPKSL--
ITPKSL--
IPPKSL--
IEPKSL--
IAPKSL--
-IPKSLT-
ICPKSL--
-IPKSLG-
-IPKSLE-
IDPKSL--
-KPKSLL-
KGPKSL--
KPPKSL--
-KPKSLM-
-KPKSLS-
-KPKSLF-
-KPKSLV-
KHPKSL--
KIPKSL--
-KPKSLY-
-KPKSLI-
KNPKSL--
-KPKSLQ-
KEPKSL--
-KPKSLN-
-KPKSLH-
-KPKSLE-
KDPKSL--
-KPKSLG-
-KPKSLA-
LGPKSL--
-LPKSLL-
-LPKSLF-
-LPKSLH-
-LPKSLQ-
LIPKSL--
-LPKSLV-
LDPKSL--
LQPKSL--
-LPKSLI-
-LPKSLE-

-LPKSLC-
MVPKSL--
MTPKSL--
MNPKSL--
MSPKSL--
-MPKSLS-
-MPKSLF-
-MPKSLA-
MAPKSL--
-MPKSLH-
-MPKSLW-
MPPKSL--
-MPKSLM-
-MPKSLI-
MGPKSL--
-MPKSLN-
MMPKSL--
-MPKSLQ-
-MPKSLY-
MHPKSL--
MEPKSL--
-MPKSLV-
MCPKSL--
-MPKSLE-
-MPKSLC-
MDPKSL--
NTPKSL--
NSPKSL--
-NPKSLL-
-NPKSLY-
-NPKSLH-
NGPKSL--
-NPKSLM-
-NPKSLW-
NEPKSL--
-NPKSLV-
-NPKSLF-
NPPKSL--
-NPKSLS-
NNPKSL--
NQPKSL--
-NPKSLI-
-NPKSLA-
NDPKSL--
-NPKSLQ-
--PKSLTL
--PKSLTI
--PKSLGS
--PKSLWG
--PKSLSQ

--PKSLHG
--PKSLIT
PSPKSL--
--PKSLWF
--PKSLIG
--PKSLFG
--PKSLSN
--PKSLNL
PGPKSL--
--PKSLYA
--PKSLHL
PTPKSL--
--PKSLVT
--PKSLKT
--PKSLGA
--PKSLEW
--PKSLYN
PFPKSL--
--PKSLYI
--PKSLWM
--PKSLVV
--PKSLVQ
--PKSLTN
--PKSLSI
--PKSLSA
--PKSLNS
--PKSLGL
--PKSLFV
--PKSLFA
--PKSLTF
--PKSLSV
--PKSLIM
--PKSLFY
--PKSLVW
--PKSLVM
--PKSLTQ
--PKSLNA
--PKSLIA
PIPKSL--
PMPKSL--
--PKSLKV
--PKSLII
--PKSLHS
--PKSLFT
--PKSLFM
--PKSLAF
PHPKSL--
--PKSLYF
--PKSLWD
--PKSLRE

--PKSLQG
--PKSLNV
--PKSLLT
--PKSLHV
--PKSLHH
--PKSLYQ
--PKSLVE
--PKSLSM
--PKSLQW
--PKSLNT
--PKSLLV
PAPKSL--
--PKSLWK
--PKSLLF
--PKSLHY
--PKSLES
--PKSLSY
--PKSLSW
--PKSLQN
--PKSLHT
--PKSLHM
--PKSLAA
--PKSLYM
--PKSLVD
--PKSLMH
--PKSLIH
--PKSLHN
--PKSLFK
--PKSLAV
PNPKSL--
--PKSLYY
--PKSLYW
--PKSLYK
--PKSLTV
--PKSLQT
--PKSLQR
--PKSLNM
--PKSLNF
--PKSLAN
PYPKSL--
--PKSLYT
--PKSLWN
--PKSLPL
--PKSLQV
--PKSLNW
--PKSLNK
--PKSLMY
--PKSLIK
--PKSLFQ
--PKSLEG

--PKSLAT
--PKSLAM
PKPKSL--
--PKSLYG
--PKSLYD
--PKSLWV
--PKSLSE
--PKSLQF
--PKSLMV
--PKSLMQ
--PKSLMM
--PKSLHD
--PKSLEY
--PKSLEV
--PKSLAH
PEPKSL--
PDPKSL--
--PKSLYH
--PKSLWE
--PKSLTD
--PKSLIW
--PKSLHQ
--PKSLGM
--PKSLGG
--PKSLFF
--PKSLET
--PKSLAK
--PKSLYE
--PKSLWW
--PKSLWH
--PKSLQD
--PKSLNH
--PKSLME
--PKSLMA
--PKSLKQ
--PKSLIE
--PKSLHI
--PKSLHF
--PKSLFD
--PKSLQE
--PKSLPI
--PKSLNI
--PKSLMT
--PKSLMD
--PKSLHK
--PKSLEI
--PKSLDW
--PKSLCQ
--PKSLAE
-PPKSLK-

--PKSLRC
--PKSLQM
--PKSLQL
--PKSLPY
--PKSLNQ
--PKSLMF
--PKSLHE
--PKSLEN
--PKSLEA
--PKSLCV
--PKSLCS
--PKSLCF
--PKSLAD
PCPKSL--
-QPKSLH-
-QPKSLS-
QVPKSL--
-QPKSLL-
-QPKSLA-
QYPKSL--
QTPKSL--
QEPKSL--
-QPKSLW-
-QPKSLV-
-QPKSLK-
-QPKSLF-
QFPKSL--
QAPKSL--
QIPKSL--
-QPKSLM-
QKPKSL--
QPPKSL--
QGPKSL--
QQPKSL--
-QPKSLE-
-QPKSLQ-
-QPKSLN-
QCPKSL--
QHPKSL--
RVPKSL--
-RPKSLI-
-RPKSLY-
RPPKSL--
RKPKSL--
REPKSL--
-RPKSLW-
-RPKSLE-
RDPKSL--
RCPKSL--
SIPKSL--

−SPKSLW−
SNPKSL−−
−SPKSLF−
−SPKSLI−
SSPKSL−−
SKPKSL−−
−SPKSLY−
SHPKSL−−
SAPKSL−−
−SPKSLQ−
SPPKSL−−
−SPKSLM−
−SPKSLA−
SDPKSL−−
−SPKSLH−
−SPKSLV−
SQPKSL−−
SCPKSL−−
−SPKSLN−
−SPKSLE−
SEPKSL−−
−SPKSLC−
TVPKSL−−
−TPKSLS−
TLPKSL−−
TSPKSL−−
−TPKSLL−
TAPKSL−−
TTPKSL−−
TIPKSL−−
TNPKSL−−
TYPKSL−−
−TPKSLK−
−TPKSLI−
TGPKSL−−
−TPKSLW−
TEPKSL−−
TQPKSL−−
TPPKSL−−
−TPKSLM−
−TPKSLE−
TDPKSL−−
−TPKSLA−
−TPKSLN−
−TPKSLG−
−VPKSLL−
−VPKSLW−
VLPKSL−−
VGPKSL−−
−VPKSLN−

-VPKSLT-
-VPKSLQ-
VKPKSL--
-VPKSLA-
-VPKSLV-
VMPKSL--
-VPKSLY-
-VPKSLI-
-VPKSLF-
VEPKSL--
-VPKSLH-
-VPKSLM-
VPPKSL--
-VPKSLE-
-VPKSLC-
VCPKSL--
WLPKSL--
WSPKSL--
WHPKSL--
WDPKSL--
WWPKSL--
WAPKSL--
WTPKSL--
-WPKSLH-
-WPKSLF-
WEPKSL--
WNPKSL--
WCPKSL--
WPPKSL--
-WPKSLE-
YVPKSL--
YAPKSL--
YTPKSL--
-YPKSLV-
-YPKSLI-
YKPKSL--
YEPKSL--
YIPKSL--
YDPKSL--
-YPKSLL-
YMPKSL--
-YPKSLW-
YQPKSL--
-YPKSLF-
-YPKSLA-
-YPKSLQ-
-YPKSLH-
YCPKSL--

LGWM 324 658 31.97

AILGWM--

```
-GLGWMP-
--LGWMYT
--LGWMPL
--LGWMGR
--LGWMES
-RLGWMD-
AKLGWM--
-ALGWMS-
-ALGWML-
ARLGWM--
AALGWM--
-ALGWMK-
AYLGWM--
ANLGWM--
ALLGWM--
-ALGWMT-
-ALGWMA-
-ALGWMN-
AVLGWM--
AMLGWM--
-ALGWMH-
-ALGWMM-
AWLGWM--
-ALGWMD-
ATLGWM--
-ALGWMI-
AFLGWM--
AQLGWM--
-ALGWMG-
AHLGWM--
ADLGWM--
-CLGWMS-
CSLGWM--
CQLGWM--
-CLGWMR-
CMLGWM--
CVLGWM--
-CLGWMT-
-CLGWMQ-
-CLGWMK-
CHLGWM--
-DLGWMK-
DRLGWM--
-DLGWMG-
-DLGWMT-
-DLGWMF-
-DLGWMV-
-DLGWML-
-DLGWMA-
DKLGWM--
```

DYLGWM--
-DLGWMR-
-DLGWMQ-
-DLGWMM-
DILGWM--
DGLGWM--
DWLGWM--
DTLGWM--
-DLGWMY-
-DLGWMI-
DNLGWM--
DMLGWM--
DLLGWM--
-DLGWMS-
DHLGWM--
DVLGWM--
DSLGWM--
DQLGWM--
-DLGWMN-
-DLGWME-
-ELGWMR-
-ELGWMS-
-ELGWML-
ESLGWM--
EGLGWM--
-ELGWMV-
-ELGWMH-
EDLGWM--
EALGWM--
EVLGWM--
-ELGWMT-
EFLGWM--
EYLGWM--
ERLGWM--
EMLGWM--
EWLGWM--
ETLGWM--
ELLGWM--
-ELGWMI-
-ELGWMA-
-FLGWMR-
FSLGWM--
-FLGWML-
-FLGWMS-
FKLGWM--
FLLGWM--
-FLGWMN-
FALGWM--
FVLGWM--
FRLGWM--

-FLGWMQ-
-FLGWMA-
FQLGWM--
-FLGWMK-
-FLGWME-
-FLGWMD-
FYLGWM--
FWLGWM--
FTLGWM--
-FLGWMV-
-FLGWMT-
FILGWM--
FELGWM--
FDLGWM--
GKLGWM--
GALGWM--
GSLGWM--
GRLGWM--
-GLGWMS-
GDLGWM--
GYLGWM--
-GLGWMN-
-GLGWML-
GNLGWM--
-GLGWMV-
-GLGWMK-
-GLGWMG-
GHLGWM--
GTLGWM--
GLLGWM--
-GLGWMQ-
GILGWM--
-GLGWMI-
-GLGWMH-
GGLGWM--
GQLGWM--
-GLGWMA-
GFLGWM--
-GLGWMF-
-GLGWMD-
GMLGWM--
-GLGWME-
GELGWM--
GCLGWM--
HRLGWM--
-HLGWMS-
-HLGWMK-
HNLGWM--
-HLGWMR-
HHLGWM--

-HLGWMY-
HVLGWM--
HSLGWM--
HQLGWM--
HLLGWM--
-HLGWMV-
-HLGWML-
-HLGWMF-
HGLGWM--
HKLGWM--
HDLGWM--
-HLGWMG-
-HLGWMD-
HFLGWM--
HYLGWM--
-HLGWMH-
-HLGWMA-
HALGWM--
-HLGWMN-
-HLGWMM-
HWLGWM--
HILGWM--
-ILGWMG-
-ILGWMK-
-ILGWMH-
IQLGWM--
IGLGWM--
INLGWM--
-ILGWMT-
-ILGWMA-
IILGWM--
IELGWM--
-ILGWMQ-
-ILGWMN-
IKLGWM--
ITLGWM--
-ILGWMI-
-ILGWME-
-ILGWMD-
IDLGWM--
ICLGWM--
-KLGWMG-
KSLGWM--
-KLGWMK-
KWLGWM--
-KLGWMT-
KHLGWM--
-KLGWMW-
-KLGWMA-
KGLGWM--

KVLGWM--
KQLGWM--
KLLGWM--
-KLGWMY-
KYLGWM--
-KLGWMQ-
-KLGWMF-
KILGWM--
KFLGWM--
KNLGWM--
-KLGWMM-
-KLGWMH-
KMLGWM--
-KLGWMI-
KALGWM--
-KLGWMV-
KELGWM--
KDLGWM--
-LLGWMS-
--LGWMVS
--LGWMRT
--LGWMKS
--LGWMMS
LDLGWM--
--LGWMVT
--LGWMRG
--LGWMAS
--LGWMTS
--LGWMRQ
-LLGWMR-
--LGWMTT
--LGWMGS
LALGWM--
--LGWMTA
--LGWMSS
--LGWMLN
--LGWMHS
--LGWMVG
--LGWMTY
LVLGWM--
LTLGWM--
-LLGWMP-
LHLGWM--
--LGWMYK
--LGWMVD
--LGWMSV
--LGWMSH
--LGWMSG
--LGWMKN
--LGWMTN

--LGWMSI
--LGWMRE
--LGWMLK
--LGWMKL
LYLGWM--
--LGWMYS
--LGWMTV
--LGWMTR
--LGWMTH
--LGWMSD
--LGWMRY
--LGWMKT
--LGWMAH
--LGWMAG
-LLGWMM-
LILGWM--
--LGWMYQ
--LGWMYG
--LGWMVH
--LGWMNT
--LGWMNS
--LGWMMR
--LGWMKM
--LGWMIG
--LGWMIA
--LGWMHT
--LGWMAR
--LGWMAK
LELGWM--
-LLGWMV-
--LGWMWT
--LGWMWA
--LGWMVN
--LGWMST
--LGWMRN
--LGWMRH
--LGWMKQ
--LGWMKH
--LGWMAN
LGLGWM--
-LLGWMN-
--LGWMYH
--LGWMVQ
--LGWMTG
--LGWMSN
--LGWMQQ
--LGWMMG
--LGWMME
--LGWMMD
--LGWMLH

--LGWMGY
--LGWMAF
-LLGWMG-
-LLGWME-
-LLGWMA-
--LGWMWH
--LGWMVW
--LGWMVK
--LGWMRD
--LGWMQD
--LGWMNM
--LGWMNA
--LGWMMK
--LGWMLD
--LGWMKK
--LGWMKE
--LGWMHV
--LGWMEA
LNLGWM--
-LLGWMI-
-LLGWMD-
--LGWMSF
--LGWMRW
--LGWMQR
--LGWMQK
--LGWMQI
--LGWMMW
--LGWMMN
--LGWMKW
--LGWMIH
--LGWMID
--LGWMFQ
--LGWMFN
--LGWMFG
--LGWMEG
--LGWMAT
--LGWMAA
--LGWMYD
--LGWMWN
--LGWMVI
--LGWMTM
--LGWMTK
--LGWMSM
--LGWMSK
--LGWMRM
--LGWMQV
--LGWMNR
--LGWMMT
--LGWMMQ
--LGWMKY

--LGWMKG
--LGWMKF
--LGWMIK
--LGWMHN
--LGWMHL
--LGWMHI
--LGWMHG
--LGWMHA
--LGWMGW
--LGWMGL
--LGWMGI
--LGWMGG
--LGWMFH
--LGWMDS
--LGWMAY
--LGWMAW
--LGWMAV
--LGWMAE
LFLGWM--
-LLGWMW-
-LLGWMQ-
-LLGWMH-
--LGWMYW
--LGWMYN
--LGWMWK
--LGWMVY
--LGWMTQ
--LGWMTE
--LGWMSE
--LGWMRV
--LGWMQY
--LGWMQT
--LGWMQS
--LGWMQH
--LGWMQF
--LGWMQA
--LGWMNY
--LGWMNN
--LGWMNH
--LGWMND
--LGWMLW
--LGWMIT
--LGWMIN
--LGWMIM
--LGWMIE
--LGWMHY
--LGWMHQ
--LGWMHM
--LGWMHH
--LGWMGM

--LGWMGF
--LGWMFW
--LGWMFD
--LGWMEY
--LGWMET
--LGWMER
--LGWMEQ
--LGWMEF
--LGWMDA
--LGWMCQ
--LGWMAQ
MSLGWM--
-MLGWMR-
-MLGWML-
-MLGWMV-
-MLGWMA-
MVLGWM--
MKLGWM--
-MLGWMT-
MLLGWM--
MYLGWM--
-MLGWMK-
MHLGWM--
MDLGWM--
MNLGWM--
-MLGWMH-
-MLGWMG-
-MLGWMD-
MFLGWM--
MELGWM--
MILGWM--
MMLGWM--
-MLGWMY-
-MLGWMW-
-MLGWMM-
NRLGWM--
-NLGWMT-
-NLGWMR-
-NLGWMV-
NVLGWM--
NELGWM--
NSLGWM--
NTLGWM--
-NLGWMN-
NYLGWM--
NLLGWM--
-NLGWME-
-NLGWMA-
-NLGWMY-
-NLGWMF-

-NLGWMW-
-NLGWML-
NKLGWM--
NNLGWM--
-NLGWMS-
-NLGWMI-
-NLGWMH-
NALGWM--
NWLGWM--
NQLGWM--
-NLGWMD-
-NLGWMK-
NGLGWM--
NFLGWM--
NCLGWM--
PTLGWM--
PKLGWM--
PRLGWM--
PALGWM--
PLLGWM--
PVLGWM--
PDLGWM--
PYLGWM--
PILGWM--
PSLGWM--
PNLGWM--
PHLGWM--
PWLGWM--
PQLGWM--
PMLGWM--
PGLGWM--
PELGWM--
-QLGWMR-
-QLGWMG-
-QLGWMK-
QLLGWM--
QHLGWM--
QVLGWM--
QQLGWM--
-QLGWMS-
QNLGWM--
QALGWM--
-QLGWMV-
-QLGWMA-
-QLGWMD-
QTLGWM--
-QLGWMW-
-QLGWMT-
-QLGWML-
-QLGWMH-

```
-QLGWMF-
QWLGWM--
QMLGWM--
-QLGWMQ-
-QLGWMN-
-QLGWMM-
QGLGWM--
QFLGWM--
QDLGWM--
-RLGWMN-
-RLGWMS-
RGLGWM--
RELGWM--
RLLGWM--
-RLGWMG-
RILGWM--
RYLGWM--
RNLGWM--
-RLGWMQ-
RDLGWM--
RQLGWM--
-RLGWMI-
-RLGWMY-
RMLGWM--
-RLGWMM-
-RLGWMF-
RWLGWM--
-RLGWMH-
-RLGWME-
-RLGWMW-
RCLGWM--
-SLGWML-
STLGWM--
SRLGWM--
-SLGWMG-
-SLGWMN-
SSLGWM--
SKLGWM--
SLLGWM--
-SLGWMH-
SALGWM--
SFLGWM--
SVLGWM--
SNLGWM--
-SLGWMT-
-SLGWMQ-
SGLGWM--
-SLGWME-
SHLGWM--
-SLGWMA-
```

SMLGWM--
-SLGWMD-
SILGWM--
SELGWM--
SWLGWM--
SQLGWM--
-SLGWMM-
SDLGWM--
-TLGWMS-
-TLGWMA-
-TLGWMM-
-TLGWMT-
-TLGWMG-
TLLGWM--
-TLGWMQ-
TSLGWM--
-TLGWME-
TGLGWM--
TELGWM--
TVLGWM--
TTLGWM--
TRLGWM--
TNLGWM--
-TLGWMY-
-TLGWMH-
TDLGWM--
TILGWM--
TALGWM--
TWLGWM--
TFLGWM--
TYLGWM--
-TLGWMD-
TKLGWM--
-TLGWMP-
-TLGWMI-
VSLGWM--
-VLGWMS-
VNLGWM--
-VLGWMR-
-VLGWMM-
-VLGWMI-
VQLGWM--
-VLGWME-
VHLGWM--
VTLGWM--
-VLGWMH-
VRLGWM--
-VLGWMK-
-VLGWMG-
VILGWM--

-VLGWML-
-VLGWMD-
VGLGWM--
VALGWM--
-VLGWMV-
-VLGWMQ-
-VLGWMN-
VELGWM--
VDLGWM--
WRLGWM--
WKLGWM--
WVLGWM--
-WLGWMN-
-WLGWMK-
-WLGWMD-
WELGWM--
WQLGWM--
WNLGWM--
WMLGWM--
-WLGWMT-
-WLGWMR-
-WLGWMM-
WWLGWM--
-WLGWMV-
-WLGWME-
WILGWM--
WALGWM--
-WLGWMQ-
-WLGWMH-
WFLGWM--
WDLGWM--
-YLGWMR-
-YLGWMS-
YYLGWM--
YNLGWM--
-YLGWML-
YSLGWM--
YGLGWM--
YALGWM--
-YLGWMH-
YQLGWM--
-YLGWMQ-
-YLGWME-
-YLGWMD-
YFLGWM--
YTLGWM--
-YLGWMW-
-YLGWMN-
YDLGWM--
YVLGWM--

-YLGWMT-
YKLGWM--
YLLGWM--
-YLGWMM-
YHLGWM--
-YLGWMV-
-YLGWMA-

| | | | |
|---|---|---|---|
| PRNI | 384 | 639 | 31.96 |

LEPRNI--
--PRNIYD
AFPRNI--
-APRNIL-
-APRNIA-
AVPRNI--
-APRNIR-
-APRNIQ-
AKPRNI--
-APRNIT-
ALPRNI--
-APRNIW-
AYPRNI--
AQPRNI--
-APRNIF-
AGPRNI--
-APRNIE-
APPRNI--
AHPRNI--
AAPRNI--
-APRNIH-
ACPRNI--
-APRNIY-
ANPRNI--
CVPRNI--
CRPRNI--
CCPRNI--
CLPRNI--
CEPRNI--
CTPRNI--
-CPRNIL-
CKPRNI--
CYPRNI--
CSPRNI--
-CPRNIY-
-CPRNIM-
CIPRNI--
-DPRNIK-
-DPRNIL-
DTPRNI--
DIPRNI--
DAPRNI--

DSPRNI--
DLPRNI--
DVPRNI--
-DPRNIW-
DYPRNI--
DWPRNI--
DRPRNI--
-DPRNIT-
-DPRNIS-
-DPRNIR-
-DPRNIM-
-DPRNII-
DHPRNI--
-DPRNIV-
-DPRNIQ-
DNPRNI--
DKPRNI--
DEPRNI--
ERPRNI--
-EPRNIR-
-EPRNIL-
ESPRNI--
EAPRNI--
EIPRNI--
EQPRNI--
-EPRNIS-
EMPRNI--
EGPRNI--
-EPRNIM-
-EPRNIK-
-EPRNIV-
-EPRNII-
EYPRNI--
EVPRNI--
-EPRNIY-
-EPRNIA-
ENPRNI--
ELPRNI--
-EPRNIH-
-EPRNIF-
-EPRNID-
EKPRNI--
EHPRNI--
FAPRNI--
FSPRNI--
FVPRNI--
FGPRNI--
FTPRNI--
-FPRNIQ-
-FPRNIA-

-FPRNIV-
FKPRNI--
-FPRNIL-
FIPRNI--
FQPRNI--
-FPRNIY-
-FPRNIW-
-FPRNIE-
FEPRNI--
FDPRNI--
FPPRNI--
FNPRNI--
FCPRNI--
-GPRNIL-
-GPRNIR-
-GPRNIT-
-GPRNIS-
-GPRNIA-
-GPRNIY-
-GPRNIN-
-GPRNII-
-GPRNIF-
GRPRNI--
GEPRNI--
GSPRNI--
GIPRNI--
-GPRNIQ-
GKPRNI--
-GPRNIG-
GMPRNI--
GHPRNI--
GYPRNI--
GLPRNI--
GAPRNI--
GVPRNI--
-GPRNIV-
GNPRNI--
GWPRNI--
-GPRNIW-
GTPRNI--
GQPRNI--
-GPRNIM-
-GPRNIK-
-GPRNIE-
GPPRNI--
GGPRNI--
GDPRNI--
HKPRNI--
HVPRNI--
HHPRNI--

HGPRNI--
HAPRNI--
HSPRNI--
-HPRNIS-
-HPRNIG-
HNPRNI--
HTPRNI--
-HPRNIW-
-HPRNIV-
HDPRNI--
HYPRNI--
HQPRNI--
-HPRNIY-
-HPRNIH-
HMPRNI--
HLPRNI--
HIPRNI--
HEPRNI--
-IPRNIG-
-IPRNIW-
-IPRNIE-
IAPRNI--
-IPRNIH-
IVPRNI--
IQPRNI--
-IPRNII-
IHPRNI--
IGPRNI--
IYPRNI--
-IPRNIM-
IDPRNI--
-IPRNIV-
-IPRNIT-
-IPRNID-
IPPRNI--
INPRNI--
IMPRNI--
IIPRNI--
-KPRNIL-
KDPRNI--
-KPRNIY-
KPPRNI--
-KPRNIK-
-KPRNII-
KGPRNI--
-KPRNIH-
KIPRNI--
KHPRNI--
-KPRNIA-
-KPRNIV-

-KPRNIF-
-KPRNIE-
-KPRNIQ-
-KPRNIN-
-KPRNIM-
KQPRNI--
-KPRNIW-
KKPRNI--
-LPRNIL-
LDPRNI--
-LPRNIF-
LQPRNI--
LHPRNI--
LYPRNI--
-LPRNII-
-LPRNIW-
-LPRNIT-
-LPRNIQ-
-LPRNIM-
-LPRNIA-
LPPRNI--
LKPRNI--
-LPRNIN-
-LPRNIE-
-LPRNIY-
-LPRNIP-
-LPRNIH-
-MPRNIY-
-MPRNIW-
MSPRNI--
-MPRNIL-
-MPRNIV-
MRPRNI--
-MPRNII-
MMPRNI--
-MPRNIS-
MTPRNI--
MVPRNI--
-MPRNIT-
MFPRNI--
-MPRNIQ-
-MPRNIN-
-MPRNIK-
-MPRNIE-
MNPRNI--
MLPRNI--
-MPRNIH-
MGPRNI--
MDPRNI--
MCPRNI--

MQPRNI--
MHPRNI--
MAPRNI--
MYPRNI--
-MPRNIP-
MPPRNI--
MIPRNI--
MEPRNI--
NGPRNI--
NVPRNI--
-NPRNIV-
-NPRNIR-
NRPRNI--
NQPRNI--
-NPRNIM-
-NPRNII-
NNPRNI--
-NPRNIF-
-NPRNIN-
NIPRNI--
NLPRNI--
NYPRNI--
NSPRNI--
-NPRNIL-
-NPRNIH-
-NPRNIE-
-NPRNIA-
NDPRNI--
NCPRNI--
-NPRNIY-
-NPRNIQ-
NPPRNI--
NEPRNI--
--PRNISL
--PRNISS
--PRNILM
--PRNIRA
--PRNIMA
--PRNIYT
--PRNIYS
--PRNIVN
--PRNITW
--PRNISI
--PRNIRY
--PRNIFG
--PRNIES
--PRNIAL
--PRNIVS
--PRNISV
--PRNIMV

--PRNIFA
--PRNIEL
--PRNIAF
PAPRNI--
--PRNIYG
--PRNINM
PGPRNI--
--PRNIRF
--PRNIRE
--PRNINF
--PRNILY
--PRNIHG
--PRNIHF
--PRNIYM
--PRNIYF
--PRNIVI
--PRNITN
--PRNISQ
--PRNIKY
--PRNIKT
PVPRNI--
--PRNIYL
--PRNIYA
--PRNIWN
--PRNIVV
--PRNITT
--PRNIST
--PRNIRD
--PRNILV
--PRNIKS
--PRNIKI
--PRNIKE
--PRNIGW
--PRNIGG
--PRNIAT
PMPRNI--
--PRNIYI
--PRNIWS
--PRNIVE
--PRNITG
--PRNISE
--PRNIQS
--PRNIIQ
--PRNIII
--PRNIGV
--PRNIGT
--PRNIFV
--PRNIFQ
--PRNIEF
--PRNIAH

PTPRNI--
--PRNIYK
--PRNIVD
--PRNISG
--PRNIRP
--PRNIQQ
--PRNINI
--PRNINA
--PRNIKW
--PRNIKM
--PRNIIS
--PRNIGQ
--PRNIEV
--PRNIAV
--PRNIAN
--PRNIAI
--PRNIAG
--PRNIAA
PKPRNI--
PSPRNI--
--PRNIYN
--PRNIWY
--PRNIVQ
--PRNITF
--PRNITD
--PRNIRW
--PRNIQM
--PRNIQG
--PRNINT
--PRNILE
--PRNIIH
--PRNIHQ
--PRNIHK
--PRNIGY
--PRNIFY
--PRNIFE
--PRNIEK
--PRNIAY
--PRNIAQ
--PRNIAK
PNPRNI--
PEPRNI--
--PRNIYE
--PRNIWM
--PRNIVF
--PRNITA
--PRNIQY
--PRNIQW
--PRNIQL
--PRNIQI

--PRNINS
--PRNIKQ
--PRNIKA
--PRNIIT
--PRNIIA
--PRNIHW
--PRNIHN
--PRNIHI
--PRNIHE
--PRNIGN
--PRNIFF
--PRNIEH
--PRNIDS
--PRNIAW
--PRNIAR
--PRNIYY
--PRNIWV
--PRNIWK
--PRNIVW
--PRNIVM
--PRNITY
--PRNITI
--PRNISF
--PRNISA
--PRNIQN
--PRNIQK
--PRNIQH
--PRNIQF
--PRNIQA
--PRNINW
--PRNINR
--PRNINK
--PRNIMN
--PRNIMM
--PRNILD
--PRNIKG
--PRNIIN
--PRNIIF
--PRNIHY
--PRNIHV
--PRNIHD
--PRNIGI
--PRNIGH
--PRNIFN
--PRNIFH
--PRNIFD
--PRNIEW
--PRNIER
--PRNIEI
--PRNIAM

```
--PRNIAD
PDPRNI--
--PRNIYH
--PRNIWE
--PRNIVP
--PRNITH
--PRNITE
--PRNISP
--PRNINH
--PRNIMI
--PRNIMD
--PRNIKD
--PRNIIM
--PRNIHL
--PRNIHA
--PRNIGM
--PRNIFK
--PRNIET
--PRNIEQ
--PRNIEN
--PRNIEM
--PRNIEE
--PRNIED
--PRNIDV
--PRNICR
--PRNICA
-PPRNIR-
-QPRNIV-
-QPRNIS-
QSPRNI--
-QPRNIF-
-QPRNIG-
QVPRNI--
-QPRNIT-
QGPRNI--
QFPRNI--
QHPRNI--
QRPRNI--
-QPRNIE-
QKPRNI--
QEPRNI--
QTPRNI--
-QPRNIR-
-QPRNIN-
QYPRNI--
-QPRNII-
-QPRNIM-
-QPRNIA-
QPPRNI--
QIPRNI--
```

-RPRNIL-
-RPRNIS-
-RPRNII-
-RPRNIY-
RPPRNI--
REPRNI--
-RPRNIG-
-RPRNIE-
-RPRNIA-
-RPRNIV-
-RPRNIQ-
RNPRNI--
-RPRNIN-
RHPRNI--
-RPRNIT-
-RPRNIW-
RCPRNI--
-SPRNIA-
SVPRNI--
-SPRNII-
-SPRNIS-
SGPRNI--
-SPRNIV-
-SPRNIG-
STPRNI--
-SPRNIY-
-SPRNIW-
SEPRNI--
-SPRNIN-
SMPRNI--
SIPRNI--
-SPRNIK-
SYPRNI--
-SPRNIT-
SAPRNI--
-SPRNIH-
SKPRNI--
SDPRNI--
-SPRNIE-
SPPRNI--
-SPRNIQ-
SHPRNI--
-SPRNID-
-TPRNIF-
TRPRNI--
TLPRNI--
-TPRNIL-
-TPRNIG-
-TPRNIV-
TSPRNI--

−TPRNIQ−
−TPRNIR−
THPRNI−−
−TPRNIS−
TEPRNI−−
TVPRNI−−
−TPRNIK−
TGPRNI−−
TPPRNI−−
TTPRNI−−
−TPRNIT−
−TPRNIH−
−TPRNIN−
−TPRNII−
−TPRNIE−
TYPRNI−−
TQPRNI−−
−TPRNIY−
TFPRNI−−
TCPRNI−−
TNPRNI−−
TDPRNI−−
VLPRNI−−
−VPRNIW−
VSPRNI−−
−VPRNIS−
VNPRNI−−
−VPRNIM−
VYPRNI−−
−VPRNIV−
−VPRNIR−
−VPRNIK−
VEPRNI−−
VIPRNI−−
VVPRNI−−
−VPRNIT−
−VPRNIG−
−VPRNII−
VMPRNI−−
−VPRNIQ−
−VPRNIH−
−VPRNIN−
−VPRNIE−
−VPRNIA−
VPPRNI−−
VHPRNI−−
VGPRNI−−
VFPRNI−−
VDPRNI−−
−VPRNIF−

| | | | |
|---|---|---|---|
| | -VPRNID- | | |
| | WLPRNI-- | | |
| | WEPRNI-- | | |
| | WAPRNI-- | | |
| | WQPRNI-- | | |
| | -WPRNIF- | | |
| | WKPRNI-- | | |
| | WDPRNI-- | | |
| | -WPRNII- | | |
| | WMPRNI-- | | |
| | WVPRNI-- | | |
| | WSPRNI-- | | |
| | -WPRNIY- | | |
| | -WPRNIH- | | |
| | WPPRNI-- | | |
| | WGPRNI-- | | |
| | WYPRNI-- | | |
| | WNPRNI-- | | |
| | WTPRNI-- | | |
| | -WPRNIM- | | |
| | -WPRNIE- | | |
| | WHPRNI-- | | |
| | WFPRNI-- | | |
| | YKPRNI-- | | |
| | YIPRNI-- | | |
| | YVPRNI-- | | |
| | YNPRNI-- | | |
| | YSPRNI-- | | |
| | -YPRNIL- | | |
| | YTPRNI-- | | |
| | -YPRNIM- | | |
| | YLPRNI-- | | |
| | YGPRNI-- | | |
| | -YPRNIN- | | |
| | -YPRNIG- | | |
| | -YPRNIY- | | |
| | YAPRNI-- | | |
| | -YPRNIW- | | |
| | -YPRNIE- | | |
| | YMPRNI-- | | |
| | YYPRNI-- | | |
| | YPPRNI-- | | |
| | -YPRNIT- | | |
| | -YPRNIK- | | |
| | -YPRNIH- | | |
| | YEPRNI-- | | |
| | YCPRNI-- | | |
| PRDL | 41 | 815 | 31.96 |
| | -APRDLP- | | |
| | -KPRDLG- | | |

```
-LPRDLA-
-NPRDLI-
--PRDLAL
PTPRDL--
--PRDLGF
--PRDLEV
--PRDLDR
--PRDLDL
--PRDLAT
-SPRDLQ-
-APRDLR-
-APRDLL-
AVPRDL--
-APRDLF-
-APRDLV-
ARPRDL--
-APRDLT-
ATPRDL--
ASPRDL--
AKPRDL--
-APRDLY-
-APRDLI-
ALPRDL--
AAPRDL--
AMPRDL--
AFPRDL--
-APRDLQ-
AHPRDL--
AYPRDL--
-APRDLW-
-APRDLG-
-APRDLM-
AIPRDL--
-APRDLK-
ANPRDL--
AQPRDL--
-APRDLN-
-APRDLE-
APPRDL--
AGPRDL--
AEPRDL--
AWPRDL--
-APRDLH-
CAPRDL--
CHPRDL--
CSPRDL--
CRPRDL--
CPPRDL--
CEPRDL--
CTPRDL--
```

-CPRDLK-
CLPRDL--
CVPRDL--
-CPRDLR-
-CPRDLQ-
-CPRDLM-
CKPRDL--
CIPRDL--
DTPRDL--
DVPRDL--
DSPRDL--
-DPRDLM-
-DPRDLR-
DRPRDL--
DLPRDL--
DAPRDL--
DFPRDL--
DWPRDL--
DQPRDL--
DIPRDL--
DYPRDL--
-DPRDLL-
-DPRDLH-
DHPRDL--
DGPRDL--
-DPRDLT-
-DPRDLQ-
-DPRDLF-
DNPRDL--
DMPRDL--
EGPRDL--
EYPRDL--
EWPRDL--
ETPRDL--
-EPRDLL-
-EPRDLI-
-EPRDLR-
-EPRDLN-
EVPRDL--
-EPRDLS-
EFPRDL--
ERPRDL--
-EPRDLY-
-EPRDLK-
ESPRDL--
-EPRDLV-
-EPRDLF-
-EPRDLW-
-EPRDLA-
ELPRDL--

EKPRDL--
EIPRDL--
EAPRDL--
-EPRDLT-
-EPRDLQ-
-EPRDLM-
-EPRDLH-
-EPRDLC-
EHPRDL--
FRPRDL--
FGPRDL--
FKPRDL--
FFPRDL--
-FPRDLY-
-FPRDLT-
FSPRDL--
FAPRDL--
FVPRDL--
-FPRDLQ-
FNPRDL--
FWPRDL--
FTPRDL--
FQPRDL--
-FPRDLF-
FEPRDL--
-FPRDLV-
FPPRDL--
FHPRDL--
-FPRDLM-
-FPRDLH-
FMPRDL--
FDPRDL--
-FPRDLI-
FIPRDL--
-FPRDLA-
-FPRDLW-
-GPRDLS-
-GPRDLL-
-GPRDLR-
GRPRDL--
GLPRDL--
GIPRDL--
GGPRDL--
GVPRDL--
-GPRDLN-
-GPRDLM-
-GPRDLK-
GKPRDL--
GMPRDL--
GQPRDL--

-GPRDLA-
GWPRDL--
-GPRDLY-
-GPRDLW-
-GPRDLI-
GAPRDL--
-GPRDLF-
GFPRDL--
GEPRDL--
-GPRDLG-
-GPRDLE-
GPPRDL--
GHPRDL--
GNPRDL--
GDPRDL--
-GPRDLQ-
-GPRDLH-
GCPRDL--
HRPRDL--
HIPRDL--
-HPRDLT-
-HPRDLI-
-HPRDLS-
HLPRDL--
HYPRDL--
-HPRDLV-
-HPRDLK-
HFPRDL--
HVPRDL--
-HPRDLL-
-HPRDLR-
-HPRDLM-
HWPRDL--
HTPRDL--
HSPRDL--
-HPRDLQ-
-HPRDLF-
HNPRDL--
HQPRDL--
-HPRDLY-
-HPRDLH-
HPPRDL--
HHPRDL--
HGPRDL--
HEPRDL--
-HPRDLN-
HKPRDL--
HAPRDL--
-HPRDLW-
-HPRDLA-

HDPRDL--
-IPRDLR-
-IPRDLL-
-IPRDLH-
-IPRDLS-
-IPRDLT-
ILPRDL--
-IPRDLK-
IIPRDL--
IFPRDL--
IVPRDL--
ISPRDL--
-IPRDLW-
-IPRDLY-
-IPRDLM-
ITPRDL--
-IPRDLA-
INPRDL--
IQPRDL--
-IPRDLV-
IEPRDL--
-IPRDLQ-
IMPRDL--
IAPRDL--
IPPRDL--
IKPRDL--
IDPRDL--
IYPRDL--
-IPRDLI-
-IPRDLF-
IHPRDL--
-KPRDLS-
KYPRDL--
-KPRDLV-
KAPRDL--
KVPRDL--
-KPRDLA-
-KPRDLR-
-KPRDLH-
KLPRDL--
-KPRDLI-
-KPRDLY-
-KPRDLT-
KTPRDL--
KIPRDL--
-KPRDLF-
KNPRDL--
KFPRDL--
-KPRDLQ-
-KPRDLK-

KEPRDL--
-KPRDLN-
-KPRDLM-
KPPRDL--
KMPRDL--
KGPRDL--
KKPRDL--
KWPRDL--
KQPRDL--
KRPRDL--
-KPRDLW-
KHPRDL--
LRPRDL--
LMPRDL--
LGPRDL--
LFPRDL--
-LPRDLK-
-LPRDLR-
LSPRDL--
LWPRDL--
-LPRDLT-
-LPRDLV-
-LPRDLI-
LIPRDL--
LYPRDL--
-LPRDLQ-
-LPRDLH-
LNPRDL--
LEPRDL--
-LPRDLY-
-LPRDLL-
LHPRDL--
-LPRDLM-
LQPRDL--
-LPRDLN-
-LPRDLF-
LPPRDL--
-LPRDLW-
-LPRDLS-
LAPRDL--
LDPRDL--
LCPRDL--
MAPRDL--
-MPRDLR-
MRPRDL--
MTPRDL--
MSPRDL--
MNPRDL--
MVPRDL--
-MPRDLK-

MYPRDL--
MLPRDL--
MIPRDL--
-MPRDLY-
-MPRDLS-
MFPRDL--
-MPRDLN-
MGPRDL--
-MPRDLL-
-MPRDLF-
MPPRDL--
-MPRDLM-
-MPRDLA-
MWPRDL--
-MPRDLH-
MKPRDL--
MEPRDL--
-MPRDLW-
-MPRDLI-
MQPRDL--
-MPRDLV-
-MPRDLT-
-MPRDLE-
MHPRDL--
MDPRDL--
-NPRDLR-
NTPRDL--
-NPRDLT-
NHPRDL--
-NPRDLK-
-NPRDLY-
NVPRDL--
-NPRDLQ-
NEPRDL--
-NPRDLW-
-NPRDLS-
NLPRDL--
NIPRDL--
-NPRDLM-
NYPRDL--
-NPRDLL-
NAPRDL--
-NPRDLV-
NNPRDL--
NDPRDL--
NQPRDL--
-NPRDLN-
-NPRDLA-
-NPRDLH-
-NPRDLF-

--PRDLLS
--PRDLRK
--PRDLRI
--PRDLRS
--PRDLMR
--PRDLLA
--PRDLRA
--PRDLRL
--PRDLRH
--PRDLWL
--PRDLLG
--PRDLFS
--PRDLST
--PRDLRP
--PRDLTF
--PRDLRV
--PRDLLW
--PRDLLM
--PRDLIS
--PRDLSW
--PRDLRM
--PRDLLH
--PRDLKG
--PRDLWR
--PRDLVR
--PRDLTT
--PRDLRQ
--PRDLLR
--PRDLVS
--PRDLVF
--PRDLTR
--PRDLML
PLPRDL--
--PRDLYK
--PRDLWH
--PRDLSV
--PRDLSA
--PRDLLT
--PRDLIG
--PRDLWE
--PRDLVY
--PRDLVH
--PRDLSS
--PRDLSR
--PRDLSM
--PRDLNS
--PRDLKY
PIPRDL--
--PRDLYV
--PRDLWV

```
--PRDLTV
--PRDLSH
--PRDLQS
--PRDLLY
--PRDLHW
--PRDLHK
--PRDLWG
--PRDLVL
--PRDLKV
--PRDLFG
--PRDLAG
PSPRDL--
--PRDLWT
--PRDLWN
--PRDLVT
--PRDLSY
--PRDLSG
--PRDLMA
--PRDLLN
--PRDLLF
--PRDLKN
--PRDLII
--PRDLFT
--PRDLYS
--PRDLWK
--PRDLTG
--PRDLTA
--PRDLSL
--PRDLSK
--PRDLMN
--PRDLKM
--PRDLWI
--PRDLQR
--PRDLNF
--PRDLMS
--PRDLKL
--PRDLIA
--PRDLHA
--PRDLFR
--PRDLFQ
--PRDLAW
--PRDLAA
--PRDLYI
--PRDLSF
--PRDLRG
--PRDLRF
--PRDLNN
--PRDLLV
--PRDLLK
--PRDLKK
```

```
--PRDLIN
--PRDLIE
--PRDLHY
--PRDLHR
--PRDLAY
--PRDLAF
PWPRDL--
--PRDLYT
--PRDLYQ
--PRDLVW
--PRDLVK
--PRDLTM
--PRDLTK
--PRDLSN
--PRDLMV
--PRDLKD
--PRDLIY
--PRDLIT
--PRDLIK
--PRDLHV
--PRDLHN
--PRDLHI
--PRDLGA
--PRDLFK
--PRDLFA
--PRDLYM
--PRDLYF
--PRDLWQ
--PRDLVM
--PRDLVA
--PRDLTN
--PRDLTI
--PRDLSI
--PRDLRY
--PRDLRE
--PRDLMT
--PRDLKS
--PRDLKR
--PRDLIW
--PRDLHS
--PRDLHH
--PRDLHF
--PRDLGW
--PRDLFI
--PRDLYH
--PRDLYA
--PRDLWA
--PRDLVE
--PRDLTL
--PRDLTH
```

--PRDLQH
--PRDLMH
--PRDLKT
--PRDLKQ
--PRDLIM
--PRDLID
--PRDLHM
--PRDLFV
--PRDLFN
PMPRDL--
--PRDLYW
--PRDLYN
--PRDLTY
--PRDLTD
--PRDLQV
--PRDLQK
--PRDLQE
--PRDLNR
--PRDLNI
--PRDLMY
--PRDLMK
--PRDLLQ
--PRDLIH
--PRDLGT
--PRDLAN
PGPRDL--
PFPRDL--
--PRDLYD
--PRDLRD
--PRDLQW
--PRDLQT
--PRDLNM
--PRDLNL
--PRDLNH
--PRDLMF
--PRDLHQ
--PRDLHL
--PRDLHG
--PRDLHD
--PRDLGG
--PRDLFW
--PRDLFH
PQPRDL--
--PRDLYY
--PRDLTW
--PRDLSD
--PRDLQA
--PRDLNW
--PRDLLE
--PRDLKH

--PRDLIQ
--PRDLIF
--PRDLHE
--PRDLFM
--PRDLFE
--PRDLAQ
--PRDLAK
--PRDLAH
PHPRDL--
PAPRDL--
--PRDLYG
--PRDLYE
--PRDLVN
--PRDLSQ
--PRDLQI
--PRDLQG
--PRDLNY
--PRDLNV
--PRDLNT
--PRDLNE
--PRDLMW
--PRDLME
--PRDLMD
--PRDLKW
--PRDLKI
--PRDLIV
--PRDLHT
--PRDLGV
--PRDLFY
--PRDLES
--PRDLEA
--PRDLAV
PEPRDL--
PDPRDL--
PYPRDL--
--PRDLWW
--PRDLWM
--PRDLVQ
--PRDLTP
--PRDLTC
--PRDLQY
--PRDLQQ
--PRDLQN
--PRDLQD
--PRDLNQ
--PRDLMP
--PRDLMG
--PRDLKF
--PRDLGM
--PRDLEG

--PRDLDS
--PRDLAM
-PPRDLT-
PKPRDL--
-QPRDLR-
QFPRDL--
QGPRDL--
QVPRDL--
-QPRDLK-
QWPRDL--
-QPRDLM-
QSPRDL--
-QPRDLL-
QNPRDL--
QHPRDL--
QYPRDL--
-QPRDLY-
-QPRDLT-
-QPRDLI-
-QPRDLH-
-QPRDLF-
-QPRDLA-
QIPRDL--
QTPRDL--
QRPRDL--
-QPRDLQ-
-QPRDLN-
-QPRDLG-
QKPRDL--
QEPRDL--
QDPRDL--
QPPRDL--
QCPRDL--
RTPRDL--
RIPRDL--
-RPRDLA-
RLPRDL--
-RPRDLV-
-RPRDLF-
RFPRDL--
RSPRDL--
-RPRDLY-
-RPRDLI-
-RPRDLK-
-RPRDLM-
RQPRDL--
-RPRDLT-
RHPRDL--
-RPRDLN-
RPPRDL--

-RPRDLQ-
-RPRDLH-
RYPRDL--
-RPRDLW-
RNPRDL--
RAPRDL--
RRPRDL--
RDPRDL--
-RPRDLE-
RKPRDL--
-RPRDLD-
-SPRDLL-
-SPRDLS-
-SPRDLT-
SFPRDL--
-SPRDLV-
SWPRDL--
SMPRDL--
SIPRDL--
-SPRDLK-
SVPRDL--
SSPRDL--
-SPRDLH-
SQPRDL--
-SPRDLY-
-SPRDLI-
-SPRDLA-
SNPRDL--
-SPRDLF-
SHPRDL--
SEPRDL--
STPRDL--
SKPRDL--
SYPRDL--
-SPRDLN-
-SPRDLM-
SPPRDL--
SAPRDL--
-SPRDLE-
SDPRDL--
-TPRDLL-
TSPRDL--
-TPRDLR-
-TPRDLF-
-TPRDLH-
TGPRDL--
TRPRDL--
TMPRDL--
-TPRDLY-
TWPRDL--

−TPRDLV−
−TPRDLI−
TYPRDL−−
−TPRDLK−
TPPRDL−−
TTPRDL−−
−TPRDLQ−
TFPRDL−−
−TPRDLA−
−TPRDLW−
−TPRDLN−
THPRDL−−
TVPRDL−−
TIPRDL−−
TQPRDL−−
TKPRDL−−
TNPRDL−−
−TPRDLM−
TCPRDL−−
VRPRDL−−
−VPRDLA−
−VPRDLW−
VVPRDL−−
VTPRDL−−
−VPRDLT−
VLPRDL−−
VMPRDL−−
−VPRDLM−
−VPRDLF−
VAPRDL−−
−VPRDLY−
VYPRDL−−
VPPRDL−−
VFPRDL−−
−VPRDLV−
−VPRDLK−
−VPRDLI−
VKPRDL−−
VHPRDL−−
−VPRDLS−
−VPRDLN−
VWPRDL−−
VQPRDL−−
−VPRDLQ−
−VPRDLH−
VEPRDL−−
VNPRDL−−
VGPRDL−−
−VPRDLG−
−VPRDLE−

-WPRDLT-
WSPRDL--
-WPRDLS-
-WPRDLY-
WNPRDL--
WVPRDL--
WAPRDL--
-WPRDLK-
-WPRDLR-
-WPRDLI-
WIPRDL--
WYPRDL--
-WPRDLV-
-WPRDLQ-
-WPRDLL-
WFPRDL--
WTPRDL--
WRPRDL--
-WPRDLM-
WLPRDL--
WEPRDL--
WMPRDL--
WGPRDL--
WWPRDL--
-WPRDLN-
WKPRDL--
-WPRDLA-
WPPRDL--
WHPRDL--
-WPRDLW-
WDPRDL--
YLPRDL--
YSPRDL--
-YPRDLL-
-YPRDLY-
YRPRDL--
YTPRDL--
YVPRDL--
YGPRDL--
-YPRDLT-
-YPRDLN-
YAPRDL--
YYPRDL--
-YPRDLQ-
-YPRDLK-
YIPRDL--
-YPRDLW-
YKPRDL--
YHPRDL--
YDPRDL--

-YPRDLS-
-YPRDLH-
-YPRDLF-
YMPRDL--
YEPRDL--
-YPRDLV-
YWPRDL--
-YPRDLG-
-YPRDLA-
YFPRDL--
-YPRDLI-
-YPRDLE-
YCPRDL--

PREL 100 770 31.95

--PRELVP
--PRELSD
--PRELPS
PFPREL--
-VPRELP-
ALPREL--
ASPREL--
AWPREL--
-APRELV-
-APRELL-
-APRELK-
AKPREL--
AEPREL--
-APRELS-
APPREL--
AAPREL--
AVPREL--
ATPREL--
-APRELY-
ANPREL--
AQPREL--
AFPREL--
-APRELM-
-APRELI-
AGPREL--
-APRELW-
-APRELQ-
-APRELN-
-APRELA-
AIPREL--
-APRELF-
-APRELE-
ADPREL--
-CPRELR-
CPPREL--
CGPREL--

CFPREL--
CVPREL--
CTPREL--
-CPRELT-
CIPREL--
CRPREL--
CSPREL--
CQPREL--
-CPRELS-
-CPRELN-
-CPRELK-
-CPRELI-
CNPREL--
CMPREL--
CLPREL--
CKPREL--
CHPREL--
CAPREL--
DFPREL--
DVPREL--
-DPRELS-
-DPRELR-
-DPRELY-
DRPREL--
DEPREL--
-DPRELK-
DIPREL--
DGPREL--
DYPREL--
DHPREL--
-DPRELV-
DSPREL--
-DPRELL-
-DPRELI-
DLPREL--
DCPREL--
DAPREL--
-DPRELW-
-DPRELT-
-DPRELN-
-DPRELH-
-DPRELF-
-DPRELA-
DNPREL--
DKPREL--
ESPREL--
-EPRELR-
EVPREL--
ERPREL--
EEPREL--

ETPREL--
EQPREL--
EYPREL--
-EPRELS-
-EPRELK-
ELPREL--
-EPRELY-
EHPREL--
-EPRELV-
-EPRELM-
-EPRELL-
-EPRELW-
-EPRELT-
-EPRELN-
-EPRELG-
EGPREL--
EDPREL--
-EPRELF-
-EPRELA-
ENPREL--
EIPREL--
-EPRELH-
EMPREL--
EFPREL--
EAPREL--
FSPREL--
-FPRELR-
FLPREL--
FWPREL--
-FPRELA-
FEPREL--
-FPRELK-
FPPREL--
-FPRELN-
FVPREL--
-FPRELW-
-FPRELF-
FAPREL--
-FPRELY-
-FPRELV-
-FPRELH-
FKPREL--
FTPREL--
-FPRELQ-
FGPREL--
FYPREL--
FQPREL--
-FPRELS-
-FPRELM-
-FPRELI-

FHPREL--
FCPREL--
-GPRELS-
-GPRELR-
GRPREL--
-GPRELY-
GTPREL--
GSPREL--
GWPREL--
GYPREL--
-GPRELK-
GIPREL--
-GPRELW-
-GPRELI-
GNPREL--
-GPRELM-
GLPREL--
GFPREL--
-GPRELQ-
GAPREL--
-GPRELN-
GKPREL--
GHPREL--
GQPREL--
-GPRELV-
-GPRELH-
-GPRELF-
-GPRELA-
GEPREL--
GPPREL--
GCPREL--
-GPRELE-
GMPREL--
HVPREL--
-HPRELK-
-HPRELL-
-HPRELA-
HSPREL--
-HPRELV-
HFPREL--
HHPREL--
-HPRELS-
-HPRELN-
-HPRELM-
-HPRELH-
HEPREL--
HTPREL--
-HPRELI-
HRPREL--
HMPREL--

HYPREL--
HWPREL--
-HPRELY-
-HPRELQ-
HNPREL--
HKPREL--
-HPRELW-
HGPREL--
-HPRELF-
HDPREL--
HAPREL--
-HPRELG-
HPPREL--
IIPREL--
-IPRELS-
IHPREL--
-IPRELK-
-IPRELL-
IYPREL--
IRPREL--
ITPREL--
IVPREL--
-IPRELV-
INPREL--
IWPREL--
-IPRELQ-
-IPRELA-
IMPREL--
IPPREL--
IEPREL--
ISPREL--
IGPREL--
IAPREL--
IDPREL--
-IPRELR-
-IPRELT-
-IPRELN-
-IPRELH-
-IPRELG-
-IPRELI-
IQPREL--
IKPREL--
-IPRELF-
IFPREL--
-KPRELL-
KLPREL--
KIPREL--
-KPRELA-
KEPREL--
-KPRELR-

```
-KPRELW-
KQPREL--
-KPRELM-
KFPREL--
-KPRELT-
KTPREL--
-KPRELY-
KYPREL--
-KPRELK-
-KPRELV-
-KPRELS-
-KPRELN-
KDPREL--
KRPREL--
-KPRELI-
-KPRELG-
-KPRELF-
KPPREL--
KNPREL--
KKPREL--
-KPRELH-
-LPRELR-
LVPREL--
LLPREL--
LGPREL--
-LPRELT-
-LPRELL-
LIPREL--
-LPRELK-
-LPRELI-
-LPRELF-
LNPREL--
LQPREL--
-LPRELS-
LMPREL--
LFPREL--
-LPRELY-
-LPRELV-
-LPRELH-
-LPRELM-
-LPRELA-
LHPREL--
-LPRELQ-
LPPREL--
LKPREL--
LDPREL--
LEPREL--
-MPRELL-
MSPREL--
MLPREL--
```

```
-MPRELT-
MQPREL--
MGPREL--
MVPREL--
-MPRELQ-
-MPRELS-
-MPRELF-
MNPREL--
-MPRELM-
MPPREL--
MIPREL--
-MPRELK-
-MPRELY-
-MPRELN-
-MPRELA-
MEPREL--
-MPRELI-
-MPRELG-
MHPREL--
MFPREL--
MCPREL--
MWPREL--
NSPREL--
-NPRELR-
NTPREL--
NRPREL--
NPPREL--
-NPRELT-
-NPRELS-
-NPRELK-
NWPREL--
-NPRELN-
NIPREL--
NHPREL--
-NPRELV-
NMPREL--
NKPREL--
-NPRELY-
-NPRELW-
NFPREL--
-NPRELL-
-NPRELQ-
-NPRELH-
-NPRELF-
-NPRELA-
NGPREL--
-NPRELM-
NDPREL--
--PRELLS
--PRELRR
```

--PRELNV
--PRELLK
--PRELWS
--PRELVT
--PRELSS
--PRELRI
--PRELVR
--PRELVS
--PRELTW
--PRELRT
--PRELRG
--PRELRM
--PRELRW
--PRELMM
PRPREL--
--PRELVY
--PRELVA
--PRELSR
--PRELRL
--PRELRK
--PRELFI
--PRELTT
--PRELLT
--PRELIS
PVPREL--
--PRELYR
--PRELNL
--PRELTS
--PRELST
--PRELMF
--PRELKS
PGPREL--
--PRELSN
--PRELIT
--PRELAS
PLPREL--
--PRELYM
--PRELSA
--PRELMS
--PRELLG
--PRELFV
PAPREL--
--PRELYG
--PRELVF
--PRELTF
--PRELSF
--PRELRQ
--PRELFL
--PRELTV
--PRELSM

--PRELRV
--PRELNA
--PRELMW
--PRELLW
--PRELIY
--PRELIM
--PRELWR
--PRELVK
--PRELVG
--PRELSY
--PRELRP
--PRELQT
--PRELMG
--PRELMA
--PRELLA
--PRELKM
--PRELKG
--PRELVV
--PRELVI
--PRELQK
--PRELHR
--PRELGA
--PRELFK
PQPREL--
PNPREL--
PTPREL--
--PRELYS
--PRELYQ
--PRELWN
--PRELWK
--PRELWF
--PRELRF
--PRELQR
--PRELNS
--PRELKN
--PRELIN
--PRELHN
--PRELFM
--PRELFA
--PRELVD
--PRELSG
--PRELQS
--PRELNY
--PRELNR
--PRELNK
--PRELLV
--PRELHA
--PRELGG
--PRELFH
--PRELAV

--PRELAT
PMPREL--
--PRELYW
--PRELYL
--PRELTY
--PRELTL
--PRELTH
--PRELSI
--PRELKK
--PRELHW
--PRELHG
--PRELYH
--PRELWA
--PRELTQ
--PRELTN
--PRELTM
--PRELTG
--PRELQH
--PRELQG
--PRELNQ
--PRELNI
--PRELNH
--PRELLY
--PRELKV
--PRELKI
--PRELII
--PRELHY
--PRELAG
PSPREL--
--PRELYN
--PRELWL
--PRELTI
--PRELSH
--PRELQQ
--PRELQM
--PRELNW
--PRELNG
--PRELMN
--PRELLE
--PRELKW
--PRELKQ
--PRELKH
--PRELIV
--PRELIH
--PRELIF
--PRELHM
--PRELHH
--PRELFG
--PRELFD
--PRELAM

--PRELYI
--PRELYF
--PRELVN
--PRELTK
--PRELSK
--PRELQV
--PRELQA
--PRELMR
--PRELMK
--PRELMH
--PRELKD
--PRELIL
--PRELIK
--PRELHT
--PRELFQ
--PRELYY
--PRELVH
--PRELRE
--PRELQN
--PRELID
--PRELHE
--PRELES
--PRELAI
PYPREL--
--PRELYA
--PRELWH
--PRELQW
--PRELQI
--PRELNT
--PRELML
--PRELKT
--PRELIQ
--PRELHI
--PRELGW
--PRELFN
--PRELEG
PHPREL--
PEPREL--
PDPREL--
PCPREL--
--PRELYD
--PRELWI
--PRELWE
--PRELQY
--PRELQE
--PRELQD
--PRELNE
--PRELMT
--PRELKF
--PRELKE

--PRELIW
--PRELHQ
--PRELFW
--PRELEA
--PRELAW
--PRELAQ
--PRELAN
--PRELAK
--PRELAH
--PRELAA
PIPREL--
--PRELYE
--PRELWW
--PRELWV
--PRELWQ
--PRELVE
--PRELVC
--PRELSE
--PRELQL
--PRELQF
--PRELPQ
--PRELPD
--PRELNF
--PRELKP
--PRELHV
--PRELHF
--PRELHD
--PRELFE
--PRELEH
--PRELCH
QRPREL--
-QPRELA-
-QPRELL-
-QPRELN-
QAPREL--
QEPREL--
QWPREL--
-QPRELY-
-QPRELI-
QPPREL--
-QPRELS-
QLPREL--
QVPREL--
QSPREL--
-QPRELM-
QKPREL--
-QPRELV-
-QPRELH-
QHPREL--
-QPRELQ-

```
-QPRELK-
QIPREL--
QFPREL--
QDPREL--
-QPRELT-
RIPREL--
-RPRELL-
RLPREL--
RPPREL--
-RPRELR-
RMPREL--
-RPRELS-
RSPREL--
-RPRELW-
RYPREL--
RFPREL--
RTPREL--
RAPREL--
-RPRELV-
-RPRELF-
-RPRELA-
RQPREL--
-RPRELN-
-RPRELI-
-RPRELY-
-RPRELQ-
-RPRELT-
-RPRELM-
-RPRELK-
-RPRELH-
RDPREL--
REPREL--
RNPREL--
RRPREL--
RKPREL--
SYPREL--
SVPREL--
SFPREL--
-SPRELL-
-SPRELR-
SSPREL--
-SPRELS-
SRPREL--
-SPRELT-
SMPREL--
-SPRELM-
SQPREL--
-SPRELV-
-SPRELK-
-SPRELW-
```

SIPREL--
SAPREL--
SPPREL--
-SPRELQ-
-SPRELN-
-SPRELI-
-SPRELF-
SEPREL--
-SPRELH-
-SPRELE-
SKPREL--
SDPREL--
-TPRELR-
TTPREL--
TSPREL--
-TPRELS-
TIPREL--
-TPRELY-
TPPREL--
-TPRELT-
THPREL--
TVPREL--
TRPREL--
-TPRELL-
TFPREL--
TAPREL--
-TPRELN-
-TPRELI-
TKPREL--
TDPREL--
-TPRELV-
-TPRELK-
-TPRELF-
TYPREL--
-TPRELQ-
-TPRELA-
TNPREL--
TQPREL--
-TPRELH-
TGPREL--
-TPRELG-
TEPREL--
TWPREL--
-TPRELW-
-TPRELM-
-VPRELT-
VSPREL--
-VPRELQ-
-VPRELR-
VAPREL--

-VPRELN-
-VPRELI-
-VPRELK-
-VPRELV-
-VPRELM-
-VPRELL-
VMPREL--
-VPRELG-
-VPRELF-
VVPREL--
VQPREL--
-VPRELH-
VKPREL--
-VPRELA-
VNPREL--
VYPREL--
VPPREL--
VIPREL--
-VPRELW-
VTPREL--
VEPREL--
VDPREL--
VHPREL--
WTPREL--
WRPREL--
-WPRELQ-
-WPRELN-
-WPRELA-
WSPREL--
-WPRELM-
WIPREL--
WQPREL--
WMPREL--
-WPRELL-
-WPRELI-
WKPREL--
-WPRELS-
WLPREL--
WGPREL--
-WPRELH-
WAPREL--
-WPRELT-
-WPRELF-
WNPREL--
WEPREL--
WDPREL--
-WPRELV-
-WPRELG-
WPPREL--
WWPREL--

WFPREL--
YLPREL--
-YPRELG-
YSPREL--
-YPRELH-
-YPRELS-
YTPREL--
YRPREL--
YIPREL--
YWPREL--
YEPREL--
-YPRELL-
YDPREL--
-YPRELY-
-YPRELA-
YKPREL--
YQPREL--
-YPRELT-
-YPRELR-
YFPREL--
-YPRELK-
-YPRELI-
-YPRELW-
-YPRELF-
-YPRELM-
YPPREL--
-YPRELV-
YMPREL--
YYPREL--
YGPREL--
-YPRELQ-
YNPREL--
YHPREL--
-YPRELE-
PSPW 145 742 31.93
-VPSPWG-
-APSPWR-
-APSPWV-
AAPSPW--
-APSPWH-
ALPSPW--
-APSPWL-
AVPSPW--
ASPSPW--
AGPSPW--
ARPSPW--
AFPSPW--
AYPSPW--
AKPSPW--
AWPSPW--

```
-APSPWF-
ANPSPW--
-APSPWY-
-APSPWS-
-APSPWE-
-APSPWQ-
-APSPWK-
ATPSPW--
AQPSPW--
-APSPWM-
AMPSPW--
AIPSPW--
-APSPWW-
-APSPWT-
-APSPWD-
AHPSPW--
CRPSPW--
CPPSPW--
CLPSPW--
CSPSPW--
-CPSPWR-
-CPSPWQ-
CNPSPW--
CMPSPW--
CFPSPW--
CCPSPW--
-DPSPWR-
DMPSPW--
DGPSPW--
-DPSPWS-
DVPSPW--
DRPSPW--
-DPSPWV-
-DPSPWT-
-DPSPWQ-
-DPSPWI-
-DPSPWH-
DNPSPW--
DIPSPW--
-DPSPWA-
DLPSPW--
DHPSPW--
DWPSPW--
DQPSPW--
-DPSPWY-
-DPSPWL-
-DPSPWK-
ESPSPW--
-EPSPWV-
-EPSPWH-
```

-EPSPWQ-
-EPSPWL-
EVPSPW--
ERPSPW--
-EPSPWW-
-EPSPWS-
-EPSPWK-
EEPSPW--
-EPSPWT-
ENPSPW--
-EPSPWY-
-EPSPWR-
ELPSPW--
ETPSPW--
EYPSPW--
EQPSPW--
-EPSPWA-
EIPSPW--
EHPSPW--
EFPSPW--
EAPSPW--
FRPSPW--
FQPSPW--
-FPSPWR-
FNPSPW--
-FPSPWK-
FHPSPW--
FKPSPW--
-FPSPWL-
FAPSPW--
-FPSPWQ-
FPPSPW--
FGPSPW--
FYPSPW--
FVPSPW--
-FPSPWW-
FMPSPW--
-FPSPWS-
-FPSPWI-
FDPSPW--
FTPSPW--
-FPSPWY-
-FPSPWV-
-FPSPWF-
FIPSPW--
FEPSPW--
FSPSPW--
-FPSPWT-
GKPSPW--
-GPSPWY-

-GPSPWR-
-GPSPWA-
GNPSPW--
GMPSPW--
GRPSPW--
GYPSPW--
GVPSPW--
GTPSPW--
-GPSPWW-
-GPSPWQ-
-GPSPWK-
-GPSPWI-
GEPSPW--
GAPSPW--
GSPSPW--
GQPSPW--
-GPSPWT-
-GPSPWN-
GHPSPW--
-GPSPWV-
-GPSPWS-
-GPSPWL-
GFPSPW--
GDPSPW--
-GPSPWP-
-GPSPWM-
GPPSPW--
-HPSPWY-
HRPSPW--
-HPSPWV-
HSPSPW--
HWPSPW--
HFPSPW--
HVPSPW--
HQPSPW--
HYPSPW--
-HPSPWI-
HNPSPW--
HTPSPW--
-HPSPWS-
-HPSPWR-
-HPSPWM-
-HPSPWF-
-HPSPWT-
-HPSPWQ-
-HPSPWK-
HPPSPW--
HMPSPW--
HLPSPW--
HHPSPW--

HGPSPW--
HDPSPW--
HAPSPW--
IRPSPW--
-IPSPWL-
ILPSPW--
-IPSPWA-
-IPSPWI-
IGPSPW--
IAPSPW--
-IPSPWK-
-IPSPWH-
IKPSPW--
IYPSPW--
ITPSPW--
-IPSPWT-
-IPSPWR-
-IPSPWQ-
IDPSPW--
IQPSPW--
-IPSPWY-
-IPSPWV-
IPPSPW--
INPSPW--
IMPSPW--
-IPSPWS-
-IPSPWN-
-IPSPWM-
IFPSPW--
KIPSPW--
KSPSPW--
KRPSPW--
-KPSPWL-
KMPSPW--
-KPSPWM-
-KPSPWH-
KNPSPW--
KFPSPW--
-KPSPWF-
KVPSPW--
-KPSPWA-
KLPSPW--
KGPSPW--
KWPSPW--
KQPSPW--
-KPSPWS-
-KPSPWT-
-KPSPWI-
KPPSPW--
KEPSPW--

KDPSPW--
KAPSPW--
KYPSPW--
-KPSPWY-
-KPSPWN-
KHPSPW--
KTPSPW--
-KPSPWW-
KKPSPW--
LLPSPW--
LGPSPW--
LRPSPW--
LAPSPW--
LVPSPW--
LTPSPW--
LDPSPW--
-LPSPWK-
LKPSPW--
LSPSPW--
LPPSPW--
LWPSPW--
-LPSPWF-
-LPSPWY-
-LPSPWI-
LEPSPW--
-LPSPWT-
-LPSPWQ-
LMPSPW--
LHPSPW--
-LPSPWH-
-LPSPWV-
-LPSPWS-
LNPSPW--
LFPSPW--
LYPSPW--
-LPSPWW-
-LPSPWN-
-LPSPWA-
LIPSPW--
LCPSPW--
MTPSPW--
-MPSPWL-
MRPSPW--
-MPSPWA-
MNPSPW--
MLPSPW--
-MPSPWV-
MMPSPW--
MGPSPW--
-MPSPWQ-

MKPSPW--
-MPSPWF-
-MPSPWS-
-MPSPWR-
-MPSPWH-
MDPSPW--
MYPSPW--
-MPSPWN-
-MPSPWI-
MWPSPW--
MQPSPW--
-MPSPWW-
-MPSPWT-
MPPSPW--
MIPSPW--
MHPSPW--
MFPSPW--
MCPSPW--
MAPSPW--
NLPSPW--
-NPSPWT-
NTPSPW--
NVPSPW--
NWPSPW--
NRPSPW--
-NPSPWL-
NAPSPW--
NQPSPW--
-NPSPWS-
-NPSPWR-
-NPSPWF-
NYPSPW--
NNPSPW--
NHPSPW--
-NPSPWQ-
NGPSPW--
NFPSPW--
-NPSPWY-
-NPSPWK-
-NPSPWI-
-NPSPWA-
NKPSPW--
NIPSPW--
NSPSPW--
-NPSPWV-
-NPSPWM-
NEPSPW--
NDPSPW--
--PSPWLS
--PSPWRL

--PSPWTS
--PSPWSS
--PSPWSF
--PSPWRY
--PSPWRT
--PSPWRH
--PSPWML
--PSPWIR
--PSPWAL
--PSPWVT
--PSPWGL
--PSPWYT
--PSPWSL
--PSPWMR
--PSPWFS
--PSPWVY
--PSPWVG
--PSPWGF
--PSPWVS
--PSPWTL
--PSPWLT
--PSPWKT
--PSPWKR
--PSPWYR
--PSPWYL
--PSPWYG
--PSPWTM
--PSPWRI
--PSPWRA
--PSPWNL
--PSPWLN
--PSPWWS
--PSPWWR
--PSPWVK
--PSPWSA
--PSPWRQ
--PSPWRM
--PSPWRG
--PSPWQL
--PSPWQI
--PSPWLQ
--PSPWIY
--PSPWIN
--PSPWGV
--PSPWFR
--PSPWAF
--PSPWWY
--PSPWWN
--PSPWVM
--PSPWTN

```
--PSPWTA
--PSPWQV
--PSPWNY
--PSPWNS
--PSPWMN
--PSPWLA
--PSPWKK
--PSPWHL
--PSPWYS
--PSPWVV
--PSPWVI
--PSPWTI
--PSPWTH
--PSPWSY
--PSPWSW
--PSPWPY
--PSPWMH
--PSPWMA
--PSPWLF
--PSPWIT
--PSPWAI
--PSPWYV
--PSPWWA
--PSPWVN
--PSPWSM
--PSPWSG
--PSPWQK
--PSPWPL
--PSPWMT
--PSPWLR
--PSPWLL
--PSPWLH
--PSPWIL
--PSPWHN
--PSPWFL
--PSPWFF
--PSPWFA
--PSPWEM
PGPSPW--
--PSPWWT
--PSPWWK
--PSPWVL
--PSPWTW
--PSPWTR
--PSPWST
--PSPWSQ
--PSPWQR
--PSPWNM
--PSPWMS
--PSPWMM
```

--PSPWLY
--PSPWKN
--PSPWKG
--PSPWHT
--PSPWHS
--PSPWGS
--PSPWFY
--PSPWFW
--PSPWFQ
--PSPWFK
--PSPWEG
--PSPWAY
PNPSPW--
--PSPWYH
--PSPWWV
--PSPWWG
--PSPWVH
--PSPWVF
--PSPWTT
--PSPWTQ
--PSPWRD
--PSPWNT
--PSPWMK
--PSPWLW
--PSPWKQ
--PSPWKA
--PSPWIS
--PSPWHQ
--PSPWGI
--PSPWFI
--PSPWFG
--PSPWEL
--PSPWAV
--PSPWAQ
PYPSPW--
PVPSPW--
--PSPWYI
--PSPWYE
--PSPWWL
--PSPWWH
--PSPWVD
--PSPWTY
--PSPWTK
--PSPWTD
--PSPWSV
--PSPWSN
--PSPWSK
--PSPWRN
--PSPWRE
--PSPWQT

--PSPWNV
--PSPWMV
--PSPWMI
--PSPWMG
--PSPWLM
--PSPWLK
--PSPWLI
--PSPWKV
--PSPWKF
--PSPWIW
--PSPWIG
--PSPWHG
--PSPWHF
--PSPWGY
--PSPWGQ
--PSPWFT
--PSPWAG
-PPSPWT-
PLPSPW--
PKPSPW--
--PSPWYQ
--PSPWYA
--PSPWWW
--PSPWWE
--PSPWWD
--PSPWVW
--PSPWVQ
--PSPWSH
--PSPWRV
--PSPWQW
--PSPWQM
--PSPWQG
--PSPWPV
--PSPWPP
--PSPWPI
--PSPWPF
--PSPWNH
--PSPWNF
--PSPWNA
--PSPWMQ
--PSPWLD
--PSPWKY
--PSPWKS
--PSPWIV
--PSPWIM
--PSPWIK
--PSPWIH
--PSPWID
--PSPWHR
--PSPWHH

--PSPWHA
--PSPWGW
--PSPWGT
--PSPWGM
--PSPWGD
--PSPWGA
--PSPWFV
--PSPWFM
--PSPWFD
--PSPWEI
--PSPWEA
--PSPWDF
--PSPWAN
--PSPWAM
--PSPWAH
--PSPWAA
PSPSPW--
PQPSPW--
PMPSPW--
PIPSPW--
PEPSPW--
PDPSPW--
PAPSPW--
QLPSPW--
QSPSPW--
QVPSPW--
-QPSPWS-
-QPSPWR-
-QPSPWI-
QEPSPW--
QWPSPW--
-QPSPWV-
-QPSPWQ-
-QPSPWY-
-QPSPWH-
-QPSPWK-
-QPSPWA-
QNPSPW--
QRPSPW--
QQPSPW--
-QPSPWF-
QGPSPW--
QYPSPW--
-QPSPWN-
-QPSPWM-
-QPSPWL-
QFPSPW--
RGPSPW--
-RPSPWR-
RKPSPW--

-RPSPWK-
-RPSPWS-
-RPSPWV-
RNPSPW--
-RPSPWN-
RIPSPW--
-RPSPWL-
RMPSPW--
RLPSPW--
RDPSPW--
RSPSPW--
RVPSPW--
-RPSPWM-
RTPSPW--
RAPSPW--
RQPSPW--
REPSPW--
-RPSPWW-
-RPSPWT-
RHPSPW--
RYPSPW--
-RPSPWY-
-RPSPWE-
RPPSPW--
RWPSPW--
-RPSPWG-
RFPSPW--
RCPSPW--
SSPSPW--
SRPSPW--
-SPSPWV-
SGPSPW--
-SPSPWL-
SVPSPW--
-SPSPWK-
-SPSPWI-
-SPSPWT-
SKPSPW--
SWPSPW--
-SPSPWN-
-SPSPWF-
STPSPW--
SLPSPW--
SFPSPW--
SYPSPW--
SHPSPW--
-SPSPWW-
-SPSPWA-
SAPSPW--
SMPSPW--

SIPSPW--
-SPSPWY-
SDPSPW--
SQPSPW--
-SPSPWG-
SPPSPW--
SNPSPW--
-SPSPWQ-
-SPSPWE-
TTPSPW--
-TPSPWM-
-TPSPWS-
-TPSPWK-
TSPSPW--
TRPSPW--
TKPSPW--
TIPSPW--
TVPSPW--
-TPSPWV-
-TPSPWT-
TPPSPW--
TFPSPW--
-TPSPWR-
TDPSPW--
TQPSPW--
TLPSPW--
-TPSPWY-
-TPSPWF-
TAPSPW--
TYPSPW--
-TPSPWA-
THPSPW--
-TPSPWQ-
TWPSPW--
-TPSPWH-
TNPSPW--
TMPSPW--
TGPSPW--
-TPSPWP-
-TPSPWN-
TCPSPW--
VSPSPW--
VLPSPW--
VYPSPW--
VVPSPW--
-VPSPWY-
VKPSPW--
VTPSPW--
-VPSPWW-
-VPSPWV-

```
-VPSPWK-
VRPSPW--
-VPSPWS-
-VPSPWL-
VMPSPW--
-VPSPWI-
-VPSPWH-
VIPSPW--
VEPSPW--
VWPSPW--
-VPSPWM-
VDPSPW--
VAPSPW--
VNPSPW--
VQPSPW--
VHPSPW--
-VPSPWQ-
-VPSPWE-
VFPSPW--
-VPSPWT-
-VPSPWN-
-VPSPWF-
WTPSPW--
WVPSPW--
WKPSPW--
WPPSPW--
WHPSPW--
WSPSPW--
-WPSPWL-
WMPSPW--
WGPSPW--
WYPSPW--
WWPSPW--
-WPSPWR-
WNPSPW--
-WPSPWW-
-WPSPWV-
-WPSPWN-
-WPSPWK-
WDPSPW--
WAPSPW--
WRPSPW--
WQPSPW--
-WPSPWT-
-WPSPWS-
-WPSPWI-
-WPSPWA-
-YPSPWQ-
YNPSPW--
YYPSPW--
```

| | | | |
|---|---|---|---|
| | -YPSPWL- | | |
| | YLPSPW-- | | |
| | YRPSPW-- | | |
| | -YPSPWS- | | |
| | -YPSPWH- | | |
| | YKPSPW-- | | |
| | YDPSPW-- | | |
| | -YPSPWT- | | |
| | YPPSPW-- | | |
| | YFPSPW-- | | |
| | YMPSPW-- | | |
| | YEPSPW-- | | |
| | YAPSPW-- | | |
| | -YPSPWM- | | |
| | -YPSPWF- | | |
| | -YPSPWW- | | |
| | YGPSPW-- | | |
| | YVPSPW-- | | |
| | -YPSPWR- | | |
| | -YPSPWI- | | |
| | YWPSPW-- | | |
| | YSPSPW-- | | |
| | YQPSPW-- | | |
| | -YPSPWN- | | |
| | -YPSPWK- | | |
| | -YPSPWA- | | |
| | YHPSPW-- | | |
| PAMM | 822 | 533 | 31.92 |
| | -GPAMMS- | | |
| | -RPAMMP- | | |
| | -SPAMMY- | | |
| | AYPAMM-- | | |
| | -APAMMI- | | |
| | ASPAMM-- | | |
| | ARPAMM-- | | |
| | AVPAMM-- | | |
| | -APAMMT- | | |
| | -APAMMR- | | |
| | -APAMMN- | | |
| | -APAMMG- | | |
| | AIPAMM-- | | |
| | ATPAMM-- | | |
| | AQPAMM-- | | |
| | -APAMMW- | | |
| | ALPAMM-- | | |
| | AHPAMM-- | | |
| | AFPAMM-- | | |
| | ADPAMM-- | | |
| | APPAMM-- | | |
| | -APAMMV- | | |

-APAMMM-
-APAMMK-
-APAMME-
AKPAMM--
AGPAMM--
ACPAMM--
-CPAMMT-
CYPAMM--
CRPAMM--
-CPAMML-
-CPAMMF-
CGPAMM--
CFPAMM--
DKPAMM--
DQPAMM--
-DPAMMT-
-DPAMMS-
-DPAMMR-
DRPAMM--
-DPAMML-
DYPAMM--
DTPAMM--
-DPAMMY-
-DPAMMH-
DDPAMM--
-EPAMMK-
-EPAMML-
-EPAMMH-
EMPAMM--
ETPAMM--
-EPAMMY-
-EPAMMR-
EAPAMM--
-EPAMMQ-
EKPAMM--
EDPAMM--
EYPAMM--
ERPAMM--
ENPAMM--
EIPAMM--
EHPAMM--
EGPAMM--
FLPAMM--
-FPAMMQ-
-FPAMMH-
-FPAMMS-
FWPAMM--
FRPAMM--
-FPAMMT-
FAPAMM--

FYPAMM--
FTPAMM--
FSPAMM--
-FPAMMV-
-FPAMMN-
FGPAMM--
GDPAMM--
GPPAMM--
-GPAMMQ-
-GPAMMR-
GRPAMM--
-GPAMMA-
GVPAMM--
GQPAMM--
-GPAMMG-
GMPAMM--
GIPAMM--
GHPAMM--
GSPAMM--
-GPAMMV-
-GPAMMM-
-GPAMMK-
-GPAMMI-
GKPAMM--
GEPAMM--
GWPAMM--
-GPAMMY-
-GPAMMW-
-GPAMMT-
-GPAMMF-
-GPAMME-
GAPAMM--
-HPAMML-
-HPAMMR-
HRPAMM--
-HPAMMN-
-HPAMMK-
HLPAMM--
HTPAMM--
HGPAMM--
HSPAMM--
-HPAMMV-
-HPAMMS-
-HPAMMI-
HHPAMM--
HEPAMM--
HAPAMM--
-IPAMMK-
-IPAMMN-
-IPAMML-

-IPAMMS-
IYPAMM--
IGPAMM--
IRPAMM--
-IPAMMV-
-IPAMMI-
-IPAMMH-
IVPAMM--
-IPAMMA-
ILPAMM--
IHPAMM--
ISPAMM--
-IPAMMG-
-IPAMMF-
-IPAMME-
IMPAMM--
KTPAMM--
KSPAMM--
-KPAMML-
-KPAMMY-
-KPAMMM-
KYPAMM--
KVPAMM--
-KPAMMN-
-KPAMMK-
KAPAMM--
-KPAMMW-
-KPAMMS-
KMPAMM--
KGPAMM--
KQPAMM--
KPPAMM--
-KPAMMV-
-KPAMMQ-
-KPAMMH-
-KPAMMF-
KLPAMM--
KIPAMM--
-LPAMMR-
LRPAMM--
-LPAMMY-
-LPAMML-
-LPAMMS-
-LPAMMQ-
-LPAMMT-
LYPAMM--
LWPAMM--
LSPAMM--
-LPAMMM-
LTPAMM--

-LPAMMV-
LNPAMM--
LHPAMM--
LGPAMM--
LCPAMM--
LVPAMM--
-LPAMMK-
LMPAMM--
LIPAMM--
LFPAMM--
LAPAMM--
LQPAMM--
-LPAMMN-
-LPAMMG-
-LPAMMA-
LKPAMM--
LEPAMM--
LDPAMM--
MLPAMM--
MTPAMM--
-MPAMMS-
-MPAMMH-
MHPAMM--
MAPAMM--
-MPAMMN-
-MPAMML-
-MPAMMF-
MNPAMM--
MRPAMM--
-MPAMMG-
MMPAMM--
MKPAMM--
MGPAMM--
MDPAMM--
-NPAMMR-
-NPAMMV-
-NPAMML-
NCPAMM--
NRPAMM--
-NPAMMA-
-NPAMMQ-
-NPAMMH-
NFPAMM--
NYPAMM--
NVPAMM--
-NPAMMW-
-NPAMMK-
-NPAMMI-
NLPAMM--
NIPAMM--

--PAMMRR
--PAMMVR
--PAMMMR
--PAMMSS
--PAMMRG
--PAMMSG
--PAMMIK
--PAMMRY
--PAMMRL
--PAMMIR
--PAMMSV
--PAMMRT
--PAMMMA
--PAMMHL
--PAMMST
--PAMMSI
--PAMMRK
--PAMMFG
--PAMMYK
--PAMMTV
--PAMMHH
--PAMMWK
--PAMMMH
--PAMMFS
--PAMMFE
PWPAMM--
--PAMMTR
--PAMMTQ
--PAMMSN
--PAMMRW
--PAMMRV
--PAMMLS
--PAMMLQ
--PAMMGM
PLPAMM--
--PAMMVW
--PAMMVL
--PAMMVG
--PAMMVD
--PAMMTY
--PAMMPL
--PAMMMG
--PAMMLG
--PAMMKA
--PAMMID
--PAMMHK
--PAMMAQ
PYPAMM--
PQPAMM--
PFPAMM--

--PAMMYA
--PAMMWR
--PAMMVY
--PAMMVH
--PAMMTH
--PAMMTG
--PAMMTA
--PAMMSR
--PAMMRS
--PAMMRI
--PAMMRA
--PAMMQY
--PAMMNT
--PAMMNL
--PAMMNA
--PAMMMQ
--PAMMMN
--PAMMMM
--PAMMLW
--PAMMLT
--PAMMLH
--PAMMKV
--PAMMKF
--PAMMHN
--PAMMFA
--PAMMAY
--PAMMYS
--PAMMYR
--PAMMYQ
--PAMMYH
--PAMMYG
--PAMMWW
--PAMMWS
--PAMMWQ
--PAMMWG
--PAMMTW
--PAMMTN
--PAMMTK
--PAMMSM
--PAMMSL
--PAMMSH
--PAMMSA
--PAMMRH
--PAMMQI
--PAMMNV
--PAMMNS
--PAMMNI
--PAMMMT
--PAMMMS
--PAMMML

--PAMMLK
--PAMMKT
--PAMMKQ
--PAMMIM
--PAMMHA
--PAMMFT
--PAMMFN
--PAMMFD
--PAMMAA
PTPAMM--
PPPAMM--
PEPAMM--
PAPAMM--
--PAMMYV
--PAMMYT
--PAMMWD
--PAMMWA
--PAMMVN
--PAMMVK
--PAMMVI
--PAMMVE
--PAMMTS
--PAMMTM
--PAMMTE
--PAMMSQ
--PAMMSK
--PAMMRF
--PAMMRD
--PAMMQV
--PAMMQM
--PAMMQL
--PAMMQK
--PAMMQH
--PAMMNW
--PAMMNQ
--PAMMME
--PAMMLE
--PAMMLA
--PAMMKN
--PAMMKM
--PAMMKH
--PAMMKG
--PAMMIY
--PAMMIW
--PAMMIS
--PAMMHS
--PAMMHD
--PAMMGY
--PAMMGV
--PAMMGT

--PAMMGI
--PAMMGG
--PAMMES
--PAMMEL
--PAMMEA
--PAMMAS
--PAMMAN
--PAMMAE
-QPAMMR-
-QPAMMN-
-QPAMMH-
-QPAMMM-
QNPAMM--
-QPAMMV-
-QPAMML-
QIPAMM--
QSPAMM--
QRPAMM--
QPPAMM--
-QPAMMS-
-QPAMMK-
QKPAMM--
QEPAMM--
QVPAMM--
-QPAMMW-
-QPAMMF-
-QPAMMA-
QHPAMM--
QGPAMM--
-RPAMML-
-RPAMMA-
-RPAMMV-
-RPAMMK-
-RPAMMH-
RPPAMM--
RDPAMM--
RQPAMM--
RKPAMM--
-RPAMMT-
-RPAMMI-
-RPAMMM-
RRPAMM--
-RPAMMY-
-RPAMMR-
-RPAMMG-
RVPAMM--
-RPAMMF-
RFPAMM--
RIPAMM--
-RPAMMQ-

-RPAMMN-
RGPAMM--
RAPAMM--
RSPAMM--
RNPAMM--
REPAMM--
SFPAMM--
-SPAMMR-
-SPAMMH-
SLPAMM--
-SPAMMV-
-SPAMMN-
-SPAMMS-
SKPAMM--
SHPAMM--
SYPAMM--
-SPAMML-
-SPAMMK-
-SPAMMI-
-SPAMMF-
SVPAMM--
STPAMM--
SSPAMM--
SRPAMM--
SQPAMM--
SIPAMM--
SDPAMM--
SWPAMM--
SPPAMM--
-SPAMMT-
-SPAMMQ-
SNPAMM--
SGPAMM--
-TPAMMR-
TNPAMM--
-TPAMMF-
TRPAMM--
TPPAMM--
-TPAMMY-
-TPAMML-
TTPAMM--
-TPAMMK-
-TPAMMS-
-TPAMMQ-
-TPAMMN-
TDPAMM--
TYPAMM--
-TPAMMH-
TGPAMM--
TFPAMM--

TAPAMM--
TWPAMM--
TSPAMM--
-TPAMMV-
-TPAMMG-
-TPAMMA-
-VPAMMN-
-VPAMML-
-VPAMMK-
VYPAMM--
-VPAMMH-
VRPAMM--
VGPAMM--
VDPAMM--
-VPAMMS-
-VPAMMI-
-VPAMMA-
VFPAMM--
VTPAMM--
-VPAMMV-
-VPAMMT-
-VPAMMQ-
-VPAMMM-
VIPAMM--
VAPAMM--
-VPAMMW-
-VPAMMG-
-VPAMME-
WSPAMM--
WTPAMM--
WLPAMM--
WRPAMM--
WHPAMM--
-WPAMMH-
WPPAMM--
-WPAMMR-
-WPAMMA-
WDPAMM--
-WPAMMV-
-WPAMMT-
-WPAMMQ-
-WPAMML-
WMPAMM--
YRPAMM--
-YPAMMS-
YGPAMM--
YYPAMM--
YTPAMM--
-YPAMMQ-
-YPAMMK-

| | | | |
|---|---|---|---|
| | -YPAMMI- | | |
| | -YPAMMH- | | |
| | -YPAMMG- | | |
| | YMPAMM-- | | |
| | -YPAMMY- | | |
| | -YPAMMV- | | |
| | -YPAMMR- | | |
| | -YPAMMA- | | |
| | YNPAMM-- | | |
| | YLPAMM-- | | |
| | YFPAMM-- | | |
| PWAL | 275 | 679 | 31.92 |
| | -LPWALA- | | |
| | --PWALAR | | |
| | ARPWAL-- | | |
| | -APWALK- | | |
| | AAPWAL-- | | |
| | AVPWAL-- | | |
| | -APWALR- | | |
| | AKPWAL-- | | |
| | AEPWAL-- | | |
| | ATPWAL-- | | |
| | -APWALI- | | |
| | -APWALH- | | |
| | ASPWAL-- | | |
| | -APWALT- | | |
| | AGPWAL-- | | |
| | ALPWAL-- | | |
| | -APWALA- | | |
| | AYPWAL-- | | |
| | ANPWAL-- | | |
| | -APWALM- | | |
| | -APWALE- | | |
| | AMPWAL-- | | |
| | -APWALW- | | |
| | -APWALQ- | | |
| | AWPWAL-- | | |
| | AQPWAL-- | | |
| | -APWALN- | | |
| | AIPWAL-- | | |
| | AHPWAL-- | | |
| | -APWALV- | | |
| | -APWALG- | | |
| | ADPWAL-- | | |
| | CSPWAL-- | | |
| | CAPWAL-- | | |
| | CRPWAL-- | | |
| | CNPWAL-- | | |
| | CVPWAL-- | | |
| | CQPWAL-- | | |

-CPWALV-
CKPWAL--
DHPWAL--
DYPWAL--
DKPWAL--
DSPWAL--
-DPWALR-
-DPWALH-
DIPWAL--
DWPWAL--
DRPWAL--
-DPWALY-
DGPWAL--
DTPWAL--
DNPWAL--
DAPWAL--
-DPWALT-
-DPWALI-
DFPWAL--
-DPWALW-
-DPWALV-
-DPWALS-
-DPWALQ-
DEPWAL--
ERPWAL--
ELPWAL--
EVPWAL--
-EPWALL-
ESPWAL--
-EPWALR-
EHPWAL--
-EPWALV-
-EPWALQ-
-EPWALW-
-EPWALT-
-EPWALK-
ETPWAL--
-EPWALS-
-EPWALH-
EAPWAL--
EYPWAL--
EWPWAL--
-EPWALY-
EIPWAL--
EGPWAL--
-EPWALM-
-EPWALE-
EPPWAL--
ENPWAL--
EMPWAL--

EKPWAL--
EFPWAL--
EEPWAL--
-FPWALR-
FRPWAL--
FSPWAL--
-FPWALH-
-FPWALS-
-FPWALT-
FYPWAL--
FAPWAL--
FGPWAL--
FVPWAL--
-FPWALV-
FQPWAL--
FFPWAL--
FDPWAL--
-FPWALQ-
-FPWALK-
FMPWAL--
-GPWALS-
-GPWALR-
GRPWAL--
-GPWALK-
GFPWAL--
GNPWAL--
GTPWAL--
GSPWAL--
-GPWALT-
-GPWALQ-
GVPWAL--
-GPWALF-
GQPWAL--
-GPWALN-
GMPWAL--
GLPWAL--
GKPWAL--
GIPWAL--
GGPWAL--
GAPWAL--
GWPWAL--
-GPWALI-
GPPWAL--
-GPWALA-
-GPWALM-
-GPWALE-
GEPWAL--
GDPWAL--
GCPWAL--
GYPWAL--

-GPWALW-
-GPWALC-
-HPWALH-
-HPWALS-
HAPWAL--
-HPWALT-
HTPWAL--
HLPWAL--
HMPWAL--
-HPWALR-
HPPWAL--
HEPWAL--
HVPWAL--
-HPWALN-
HGPWAL--
-HPWALV-
HFPWAL--
HSPWAL--
-HPWALW-
-HPWALL-
-HPWALI-
-HPWALA-
HRPWAL--
-HPWALQ-
-HPWALM-
-HPWALK-
-HPWALE-
HKPWAL--
HHPWAL--
HDPWAL--
IRPWAL--
ISPWAL--
IPPWAL--
IWPWAL--
-IPWALH-
INPWAL--
IGPWAL--
ITPWAL--
IHPWAL--
IVPWAL--
-IPWALL-
-IPWALA-
IFPWAL--
-IPWALM-
IIPWAL--
-IPWALS-
IYPWAL--
IQPWAL--
-IPWALW-
IMPWAL--

IKPWAL--
IEPWAL--
IDPWAL--
IAPWAL--
-IPWALT-
-IPWALN-
-IPWALK-
-IPWALI-
-IPWALG-
KSPWAL--
KMPWAL--
KRPWAL--
KGPWAL--
-KPWALR-
-KPWALT-
-KPWALL-
KIPWAL--
-KPWALY-
-KPWALA-
-KPWALS-
KVPWAL--
KLPWAL--
-KPWALK-
-KPWALQ-
-KPWALH-
KEPWAL--
KAPWAL--
-KPWALE-
KNPWAL--
KQPWAL--
-KPWALN-
-KPWALI-
-KPWALF-
KHPWAL--
KWPWAL--
KPPWAL--
KFPWAL--
KYPWAL--
-KPWALW-
KKPWAL--
KCPWAL--
LHPWAL--
LTPWAL--
LGPWAL--
LLPWAL--
-LPWALK-
LKPWAL--
-LPWALT-
-LPWALH-
LVPWAL--

LNPWAL--
-LPWALQ-
LIPWAL--
-LPWALW-
LQPWAL--
-LPWALV-
LAPWAL--
-LPWALM-
-LPWALN-
-LPWALI-
LMPWAL--
LDPWAL--
LWPWAL--
LCPWAL--
MKPWAL--
-MPWALS-
MVPWAL--
MGPWAL--
MMPWAL--
MSPWAL--
-MPWALT-
MTPWAL--
-MPWALR-
MIPWAL--
MDPWAL--
MHPWAL--
MEPWAL--
-MPWALI-
-MPWALA-
MNPWAL--
-MPWALQ-
-MPWALH-
MPPWAL--
MFPWAL--
MAPWAL--
MQPWAL--
-MPWALK-
-MPWALG-
-MPWALE-
MCPWAL--
NSPWAL--
NRPWAL--
NKPWAL--
NIPWAL--
-NPWALS-
NVPWAL--
-NPWALH-
NGPWAL--
NAPWAL--
-NPWALI-

```
-NPWALT-
NTPWAL--
NYPWAL--
NQPWAL--
-NPWALY-
-NPWALK-
NMPWAL--
NFPWAL--
-NPWALW-
-NPWALV-
NDPWAL--
-NPWALN-
-NPWALA-
-NPWALQ-
NPPWAL--
NNPWAL--
NHPWAL--
NCPWAL--
--PWALLD
--PWALRN
--PWALHL
--PWALYR
--PWALVS
--PWALRT
--PWALVR
--PWALTK
--PWALVK
--PWALTS
--PWALLT
--PWALRH
--PWALKS
PYPWAL--
--PWALWS
--PWALSV
--PWALQT
--PWALPL
--PWALHR
--PWALHH
--PWALNA
--PWALMV
--PWALMR
--PWALIS
--PWALGS
--PWALAS
--PWALVH
--PWALTN
--PWALST
--PWALSM
--PWALRW
--PWALRD
```

--PWALQG
--PWALNI
--PWALMS
--PWALMG
--PWALIR
--PWALGI
--PWALTT
--PWALSI
--PWALSH
--PWALQA
--PWALLK
--PWALIK
--PWALGL
--PWALTA
--PWALNV
--PWALKR
--PWALHT
--PWALAG
--PWALYS
--PWALSK
--PWALQS
--PWALQK
--PWALLH
--PWALKM
--PWALKG
--PWALIG
--PWALHS
--PWALHK
--PWALGY
--PWALYE
--PWALRE
--PWALLN
PTPWAL--
--PWALYH
--PWALYG
--PWALSY
--PWALSQ
--PWALSG
--PWALPY
--PWALNT
--PWALLQ
--PWALKQ
--PWALAV
--PWALAM
PFPWAL--
--PWALWG
--PWALTY
--PWALTW
--PWALTR
--PWALTM

--PWALTI
--PWALTG
--PWALTD
--PWALSN
--PWALKV
--PWALIT
--PWALID
--PWALHD
--PWALGA
--PWALFQ
--PWALAQ
PVPWAL--
--PWALWK
--PWALWD
--PWALTQ
--PWALQR
--PWALPM
--PWALMY
--PWALKT
--PWALIN
--PWALHY
--PWALHN
--PWALHE
--PWALGT
--PWALGG
--PWALFV
--PWALES
--PWALAI
--PWALAH
PDPWAL--
PAPWAL--
--PWALYQ
--PWALYN
--PWALYI
--PWALVQ
--PWALPF
--PWALNM
--PWALKH
--PWALKF
--PWALHV
--PWALHG
--PWALGM
--PWALFR
--PWALFN
--PWALEG
PHPWAL--
PGPWAL--
PEPWAL--
--PWALYT
--PWALYD

--PWALWW
--PWALVY
--PWALVE
--PWALTH
--PWALRQ
--PWALQV
--PWALQN
--PWALPW
--PWALPI
--PWALNY
--PWALNN
--PWALNK
--PWALNG
--PWALMT
--PWALMN
--PWALMK
--PWALMH
--PWALME
--PWALKY
--PWALKN
--PWALKD
--PWALHQ
--PWALGV
--PWALFH
--PWALFG
--PWALAT
PQPWAL--
PMPWAL--
PWPWAL--
--PWALWR
--PWALVN
--PWALQH
--PWALQF
--PWALPQ
--PWALPC
--PWALPA
--PWALNW
--PWALNC
--PWALMQ
--PWALMD
--PWALKW
--PWALIW
--PWALIH
--PWALHI
--PWALGW
--PWALGQ
--PWALGH
--PWALFE
--PWALDA
--PWALAA

-PPWALL-
PKPWAL--
PIPWAL--
PCPWAL--
QSPWAL--
QRPWAL--
QIPWAL--
-QPWALS-
QMPWAL--
-QPWALT-
-QPWALH-
QNPWAL--
QLPWAL--
QEPWAL--
QTPWAL--
QGPWAL--
-QPWALV-
QKPWAL--
QVPWAL--
-QPWALA-
QDPWAL--
QAPWAL--
QWPWAL--
QQPWAL--
-QPWALN-
-QPWALI-
QFPWAL--
-QPWALM-
-QPWALL-
QHPWAL--
-RPWALS-
RSPWAL--
-RPWALK-
-RPWALW-
RFPWAL--
-RPWALV-
-RPWALH-
RMPWAL--
RVPWAL--
-RPWALI-
RLPWAL--
RYPWAL--
RWPWAL--
RAPWAL--
-RPWALN-
-RPWALA-
RPPWAL--
RHPWAL--
RNPWAL--
RIPWAL--

RQPWAL--
RDPWAL--
-RPWALY-
-RPWALM-
-RPWALF-
-RPWALE-
SLPWAL--
SSPWAL--
SAPWAL--
SRPWAL--
SGPWAL--
-SPWALR-
SVPWAL--
SNPWAL--
-SPWALL-
-SPWALH-
-SPWALY-
-SPWALT-
SIPWAL--
SHPWAL--
SQPWAL--
-SPWALV-
SMPWAL--
-SPWALI-
SYPWAL--
-SPWALQ-
-SPWALN-
-SPWALM-
-SPWALK-
-SPWALA-
SPPWAL--
SKPWAL--
SFPWAL--
SWPWAL--
SEPWAL--
-SPWALW-
-SPWALE-
TRPWAL--
-TPWALK-
-TPWALL-
TAPWAL--
TTPWAL--
-TPWALS-
-TPWALI-
TIPWAL--
-TPWALV-
TQPWAL--
THPWAL--
TSPWAL--
TGPWAL--

TDPWAL--
-TPWALT-
-TPWALQ-
TLPWAL--
TMPWAL--
TVPWAL--
-TPWALN-
TEPWAL--
TFPWAL--
TYPWAL--
-TPWALW-
-TPWALA-
TPPWAL--
-TPWALH-
TCPWAL--
-TPWALE-
TNPWAL--
TKPWAL--
VTPWAL--
VHPWAL--
-VPWALS-
-VPWALK-
VQPWAL--
VLPWAL--
-VPWALM-
VVPWAL--
VKPWAL--
VFPWAL--
VRPWAL--
-VPWALT-
VSPWAL--
VAPWAL--
VGPWAL--
-VPWALW-
-VPWALN-
-VPWALI-
VMPWAL--
VEPWAL--
-VPWALQ-
-VPWALA-
VNPWAL--
-VPWALH-
VPPWAL--
VIPWAL--
WSPWAL--
WKPWAL--
-WPWALR-
WYPWAL--
-WPWALT-
-WPWALK-

| | | | |
|---|---|---|---|
| | WMPWAL-- | | |
| | WWPWAL-- | | |
| | -WPWALV- | | |
| | -WPWALI- | | |
| | WLPWAL-- | | |
| | WFPWAL-- | | |
| | WDPWAL-- | | |
| | WHPWAL-- | | |
| | WAPWAL-- | | |
| | WVPWAL-- | | |
| | -WPWALQ- | | |
| | -WPWALA- | | |
| | WGPWAL-- | | |
| | WEPWAL-- | | |
| | -YPWALR- | | |
| | YTPWAL-- | | |
| | YIPWAL-- | | |
| | YRPWAL-- | | |
| | -YPWALS- | | |
| | YEPWAL-- | | |
| | -YPWALK- | | |
| | YNPWAL-- | | |
| | YKPWAL-- | | |
| | YFPWAL-- | | |
| | -YPWALV- | | |
| | -YPWALT- | | |
| | YLPWAL-- | | |
| | YGPWAL-- | | |
| | YVPWAL-- | | |
| | YQPWAL-- | | |
| | YMPWAL-- | | |
| | YYPWAL-- | | |
| | YDPWAL-- | | |
| | YAPWAL-- | | |
| | -YPWALQ- | | |
| | -YPWALI- | | |
| | -YPWALA- | | |
| | YWPWAL-- | | |
| | -YPWALW- | | |
| | -YPWALM- | | |
| | -YPWALH- | | |
| PIGY | 265 | 684 | 31.90 |
| | -RPIGYG- | | |
| | AWPIGY-- | | |
| | -APIGYW- | | |
| | -APIGYL- | | |
| | -APIGYR- | | |
| | ALPIGY-- | | |
| | ASPIGY-- | | |
| | AFPIGY-- | | |

AYPIGY--
AVPIGY--
ARPIGY--
-APIGYS-
-APIGYG-
-APIGYT-
-APIGYQ-
-APIGYV-
-APIGYN-
-APIGYM-
-APIGYI-
AMPIGY--
AAPIGY--
-APIGYY-
-APIGYK-
-APIGYH-
ANPIGY--
AHPIGY--
AEPIGY--
-CPIGYS-
CYPIGY--
CWPIGY--
CQPIGY--
CPPIGY--
-CPIGYK-
CIPIGY--
CGPIGY--
CFPIGY--
CVPIGY--
-CPIGYV-
-CPIGYM-
-CPIGYI-
-CPIGYE-
CHPIGY--
-DPIGYR-
-DPIGYL-
DRPIGY--
DIPIGY--
DTPIGY--
-DPIGYY-
-DPIGYS-
-DPIGYN-
DAPIGY--
DYPIGY--
-DPIGYH-
DLPIGY--
DFPIGY--
DSPIGY--
-DPIGYM-
-DPIGYK-

−DPIGYI−
−DPIGYG−
−DPIGYF−
DEPIGY−−
DCPIGY−−
−EPIGYL−
−EPIGYI−
−EPIGYR−
ESPIGY−−
−EPIGYT−
−EPIGYW−
−EPIGYV−
−EPIGYA−
EWPIGY−−
EVPIGY−−
ETPIGY−−
ERPIGY−−
EQPIGY−−
−EPIGYF−
EMPIGY−−
ELPIGY−−
EEPIGY−−
ECPIGY−−
FRPIGY−−
−FPIGYL−
FLPIGY−−
FTPIGY−−
−FPIGYF−
FKPIGY−−
FPPIGY−−
FSPIGY−−
−FPIGYR−
−FPIGYV−
−FPIGYS−
−FPIGYA−
FIPIGY−−
FHPIGY−−
FQPIGY−−
−FPIGYW−
−FPIGYN−
FAPIGY−−
FYPIGY−−
FVPIGY−−
−FPIGYT−
FNPIGY−−
FDPIGY−−
FWPIGY−−
−FPIGYQ−
−FPIGYK−
−FPIGYH−

FMPIGY--
FGPIGY--
FFPIGY--
FEPIGY--
-GPIGYL-
-GPIGYS-
GRPIGY--
GSPIGY--
GHPIGY--
GGPIGY--
GWPIGY--
-GPIGYH-
GDPIGY--
-GPIGYK-
-GPIGYI-
-GPIGYN-
GYPIGY--
-GPIGYT-
-GPIGYM-
GVPIGY--
GQPIGY--
-GPIGYY-
-GPIGYW-
-GPIGYF-
GLPIGY--
GFPIGY--
-GPIGYA-
GMPIGY--
-GPIGYV-
-GPIGYR-
HTPIGY--
HKPIGY--
-HPIGYL-
HGPIGY--
HAPIGY--
HWPIGY--
-HPIGYW-
-HPIGYR-
HRPIGY--
-HPIGYT-
HVPIGY--
-HPIGYA-
HHPIGY--
HFPIGY--
HSPIGY--
-HPIGYQ-
HMPIGY--
HYPIGY--
HEPIGY--
HQPIGY--

-HPIGYV-
-HPIGYM-
-HPIGYH-
-HPIGYG-
-HPIGYF-
-HPIGYE-
HNPIGY--
HLPIGY--
HIPIGY--
ISPIGY--
IRPIGY--
-IPIGYL-
IKPIGY--
-IPIGYV-
-IPIGYR-
-IPIGYK-
IEPIGY--
-IPIGYT-
IIPIGY--
IHPIGY--
ITPIGY--
-IPIGYW-
-IPIGYM-
INPIGY--
IMPIGY--
IAPIGY--
IVPIGY--
IPPIGY--
-IPIGYS-
-IPIGYQ-
-IPIGYI-
-IPIGYH-
-IPIGYG-
-IPIGYF-
-KPIGYR-
-KPIGYV-
-KPIGYM-
-KPIGYL-
-KPIGYT-
KTPIGY--
-KPIGYS-
-KPIGYA-
KMPIGY--
KRPIGY--
KPPIGY--
-KPIGYQ-
-KPIGYI-
-KPIGYY-
-KPIGYN-
KKPIGY--

KIPIGY--
-KPIGYH-
KGPIGY--
KAPIGY--
KWPIGY--
KNPIGY--
KDPIGY--
LYPIGY--
LGPIGY--
-LPIGYK-
-LPIGYV-
LSPIGY--
LRPIGY--
LVPIGY--
LAPIGY--
-LPIGYI-
LNPIGY--
LMPIGY--
LLPIGY--
-LPIGYY-
-LPIGYT-
-LPIGYH-
-LPIGYS-
LTPIGY--
LQPIGY--
-LPIGYW-
-LPIGYF-
-LPIGYA-
LHPIGY--
-LPIGYN-
LKPIGY--
LPPIGY--
-LPIGYR-
LWPIGY--
-LPIGYQ-
-LPIGYM-
LEPIGY--
-MPIGYL-
MSPIGY--
MVPIGY--
MRPIGY--
MKPIGY--
-MPIGYT-
-MPIGYM-
MWPIGY--
-MPIGYR-
-MPIGYK-
-MPIGYF-
MYPIGY--
-MPIGYY-

```
-MPIGYI-
-MPIGYH-
MIPIGY--
MQPIGY--
-MPIGYW-
-MPIGYE-
-MPIGYC-
-MPIGYA-
MGPIGY--
MDPIGY--
MAPIGY--
NTPIGY--
NRPIGY--
-NPIGYT-
NGPIGY--
-NPIGYR-
-NPIGYN-
-NPIGYK-
NAPIGY--
NYPIGY--
-NPIGYM-
-NPIGYA-
NVPIGY--
NLPIGY--
NKPIGY--
NWPIGY--
NSPIGY--
NQPIGY--
NPPIGY--
-NPIGYW-
-NPIGYI-
NIPIGY--
NHPIGY--
-NPIGYY-
-NPIGYS-
NNPIGY--
NEPIGY--
--PIGYLR
--PIGYGS
--PIGYRL
--PIGYSS
--PIGYLT
--PIGYLI
--PIGYMS
--PIGYLS
--PIGYKS
--PIGYYR
--PIGYRQ
--PIGYLL
--PIGYLG
```

--PIGYVS
--PIGYTS
--PIGYTR
--PIGYLV
--PIGYWS
--PIGYWG
--PIGYTK
--PIGYSG
--PIGYRG
--PIGYIK
--PIGYYS
--PIGYST
--PIGYSR
--PIGYRT
--PIGYRS
--PIGYMN
--PIGYMG
--PIGYIN
--PIGYRY
--PIGYIT
--PIGYVA
--PIGYRN
--PIGYLM
--PIGYKN
--PIGYIR
--PIGYIH
--PIGYIA
--PIGYWD
--PIGYVY
--PIGYVW
--PIGYVG
--PIGYSH
--PIGYRV
--PIGYGL
--PIGYFS
PKPIGY--
--PIGYWN
--PIGYTQ
--PIGYTM
--PIGYSV
--PIGYSF
--PIGYSA
--PIGYNM
--PIGYKY
--PIGYFK
--PIGYFH
--PIGYFG
--PIGYES
--PIGYAS
PAPIGY--

PIPIGY--
--PIGYVH
--PIGYTA
--PIGYRW
--PIGYNS
--PIGYLK
--PIGYKG
--PIGYIS
--PIGYHG
--PIGYGG
--PIGYFV
--PIGYAA
PNPIGY--
--PIGYVQ
--PIGYVI
--PIGYTG
--PIGYSW
--PIGYSN
--PIGYRM
--PIGYQT
--PIGYNA
--PIGYLN
--PIGYKK
--PIGYHK
--PIGYAL
--PIGYAI
PHPIGY--
--PIGYYF
--PIGYVM
--PIGYSY
--PIGYRH
--PIGYRF
--PIGYRD
--PIGYNT
--PIGYNG
--PIGYMV
--PIGYMQ
--PIGYMA
--PIGYLQ
--PIGYLA
--PIGYKT
--PIGYKA
--PIGYHT
--PIGYHI
--PIGYFA
--PIGYEA
--PIGYAN
PFPIGY--
--PIGYYN
--PIGYWK

--PIGYTY
--PIGYTN
--PIGYTL
--PIGYTI
--PIGYRI
--PIGYRE
--PIGYRA
--PIGYQW
--PIGYQR
--PIGYQK
--PIGYQG
--PIGYQA
--PIGYNL
--PIGYMW
--PIGYMT
--PIGYME
--PIGYLH
--PIGYLF
--PIGYKQ
--PIGYKF
--PIGYIM
--PIGYIE
--PIGYHN
--PIGYHM
--PIGYHL
--PIGYHH
--PIGYHF
--PIGYHA
--PIGYGT
--PIGYGA
--PIGYFR
--PIGYFM
--PIGYAW
--PIGYAT
--PIGYAK
PDPIGY--
PTPIGY--
PQPIGY--
--PIGYYT
--PIGYYK
--PIGYYH
--PIGYWW
--PIGYWR
--PIGYVN
--PIGYVK
--PIGYTW
--PIGYTV
--PIGYTT
--PIGYTF
--PIGYSQ

--PIGYSM
--PIGYSI
--PIGYQV
--PIGYQN
--PIGYQM
--PIGYQL
--PIGYNW
--PIGYMK
--PIGYMH
--PIGYMF
--PIGYLD
--PIGYKW
--PIGYKV
--PIGYKE
--PIGYIW
--PIGYIV
--PIGYIP
--PIGYII
--PIGYIG
--PIGYIF
--PIGYHW
--PIGYHV
--PIGYHR
--PIGYHQ
--PIGYFT
--PIGYFN
--PIGYEG
--PIGYEF
--PIGYDS
--PIGYAV
--PIGYAG
-QPIGYR-
-QPIGYS-
QGPIGY--
-QPIGYH-
QHPIGY--
-QPIGYQ-
-QPIGYN-
-QPIGYL-
QTPIGY--
-QPIGYV-
-QPIGYK-
QMPIGY--
QLPIGY--
QFPIGY--
QSPIGY--
-QPIGYY-
-QPIGYM-
QKPIGY--
QVPIGY--

-QPIGYF-
-QPIGYA-
QNPIGY--
QIPIGY--
QAPIGY--
-RPIGYL-
RSPIGY--
RRPIGY--
-RPIGYI-
-RPIGYN-
RNPIGY--
-RPIGYR-
RKPIGY--
RAPIGY--
RVPIGY--
RTPIGY--
RQPIGY--
-RPIGYH-
RDPIGY--
RGPIGY--
RYPIGY--
RPPIGY--
-RPIGYQ-
-RPIGYK-
RFPIGY--
-RPIGYT-
-RPIGYM-
-RPIGYF-
-RPIGYW-
RMPIGY--
REPIGY--
-RPIGYY-
-RPIGYA-
RHPIGY--
SLPIGY--
SRPIGY--
-SPIGYS-
-SPIGYK-
-SPIGYT-
SNPIGY--
SSPIGY--
-SPIGYV-
-SPIGYR-
SKPIGY--
SVPIGY--
-SPIGYL-
SMPIGY--
SIPIGY--
-SPIGYH-
SYPIGY--

```
-SPIGYY-
-SPIGYW-
-SPIGYI-
SAPIGY--
SQPIGY--
-SPIGYN-
SFPIGY--
SPPIGY--
SEPIGY--
-SPIGYA-
SHPIGY--
SGPIGY--
SDPIGY--
SWPIGY--
STPIGY--
-SPIGYF-
SCPIGY--
-TPIGYL-
TKPIGY--
-TPIGYR-
-TPIGYV-
-TPIGYI-
TVPIGY--
TTPIGY--
TSPIGY--
TRPIGY--
-TPIGYW-
TLPIGY--
TWPIGY--
-TPIGYS-
-TPIGYK-
TGPIGY--
TYPIGY--
TQPIGY--
-TPIGYQ-
-TPIGYA-
TFPIGY--
TEPIGY--
TDPIGY--
TPPIGY--
-TPIGYM-
TNPIGY--
TMPIGY--
TIPIGY--
THPIGY--
TAPIGY--
-TPIGYN-
-TPIGYH-
-TPIGYF-
-TPIGYE-
```

TCPIGY--
VLPIGY--
-VPIGYM-
VKPIGY--
-VPIGYA-
VWPIGY--
VSPIGY--
-VPIGYR-
-VPIGYW-
-VPIGYI-
VVPIGY--
VQPIGY--
VEPIGY--
VAPIGY--
-VPIGYV-
-VPIGYG-
VDPIGY--
VYPIGY--
VPPIGY--
-VPIGYT-
-VPIGYS-
-VPIGYL-
VFPIGY--
VTPIGY--
-VPIGYQ-
-VPIGYN-
-VPIGYE-
VHPIGY--
VCPIGY--
WRPIGY--
-WPIGYR-
WPPIGY--
WSPIGY--
-WPIGYI-
WNPIGY--
WKPIGY--
WWPIGY--
WTPIGY--
-WPIGYT-
WLPIGY--
WHPIGY--
WFPIGY--
-WPIGYW-
-WPIGYV-
-WPIGYN-
-WPIGYK-
-WPIGYA-
WGPIGY--
WDPIGY--
WAPIGY--

| | | | |
|---|---|---|---|
| | YYPIGY-- | | |
| | -YPIGYL- | | |
| | YRPIGY-- | | |
| | -YPIGYS- | | |
| | YTPIGY-- | | |
| | -YPIGYV- | | |
| | YLPIGY-- | | |
| | -YPIGYK- | | |
| | YHPIGY-- | | |
| | YQPIGY-- | | |
| | -YPIGYQ- | | |
| | -YPIGYN- | | |
| | -YPIGYI- | | |
| | -YPIGYF- | | |
| | YDPIGY-- | | |
| | YWPIGY-- | | |
| | YPPIGY-- | | |
| | -YPIGYW- | | |
| | -YPIGYM- | | |
| | YNPIGY-- | | |
| | -YPIGYR- | | |
| | YMPIGY-- | | |
| | YKPIGY-- | | |
| | YFPIGY-- | | |
| | YEPIGY-- | | |
| | YAPIGY-- | | |
| PRAF | 1102 | 491 | 31.90 |
| | GDPRAF-- | | |
| | -IPRAFE- | | |
| | --PRAFNM | | |
| | -RPRAFP- | | |
| | AVPRAF-- | | |
| | AAPRAF-- | | |
| | ASPRAF-- | | |
| | -APRAFN- | | |
| | -APRAFG- | | |
| | ATPRAF-- | | |
| | ALPRAF-- | | |
| | -APRAFM- | | |
| | ANPRAF-- | | |
| | AYPRAF-- | | |
| | ARPRAF-- | | |
| | AMPRAF-- | | |
| | AEPRAF-- | | |
| | AWPRAF-- | | |
| | APPRAF-- | | |
| | AQPRAF-- | | |
| | -APRAFY- | | |
| | AFPRAF-- | | |
| | -APRAFI- | | |

-APRAFA-
AHPRAF--
ADPRAF--
-APRAFF-
-APRAFE-
-CPRAFT-
CGPRAF--
-CPRAFQ-
CKPRAF--
CTPRAF--
-CPRAFV-
CAPRAF--
CYPRAF--
CWPRAF--
CVPRAF--
-CPRAFR-
CNPRAF--
CMPRAF--
CLPRAF--
CCPRAF--
CRPRAF--
CQPRAF--
-CPRAFM-
-CPRAFL-
-CPRAFI-
-CPRAFG-
CIPRAF--
CEPRAF--
DWPRAF--
DSPRAF--
-DPRAFS-
-DPRAFF-
DHPRAF--
-DPRAFL-
-DPRAFI-
-DPRAFR-
DFPRAF--
DTPRAF--
-DPRAFV-
DIPRAF--
DYPRAF--
DRPRAF--
DQPRAF--
-DPRAFN-
-DPRAFT-
DNPRAF--
DMPRAF--
DKPRAF--
DEPRAF--
DDPRAF--

-DPRAFY-
-DPRAFM-
-DPRAFK-
DGPRAF--
ESPRAF--
-EPRAFN-
-EPRAFS-
-EPRAFL-
EIPRAF--
EFPRAF--
-EPRAFV-
EGPRAF--
ETPRAF--
-EPRAFF-
ENPRAF--
ELPRAF--
EKPRAF--
EYPRAF--
-EPRAFW-
-EPRAFT-
-EPRAFM-
EAPRAF--
-EPRAFY-
-EPRAFI-
EQPRAF--
-EPRAFE-
EPPRAF--
EMPRAF--
EDPRAF--
ECPRAF--
FTPRAF--
FDPRAF--
FAPRAF--
FQPRAF--
-FPRAFY-
FGPRAF--
FHPRAF--
FCPRAF--
-FPRAFE-
GVPRAF--
-GPRAFM-
GKPRAF--
-GPRAFS-
-GPRAFL-
GIPRAF--
GPPRAF--
-GPRAFQ-
-GPRAFT-
GTPRAF--
GMPRAF--

GSPRAF--
-GPRAFN-
-GPRAFE-
-GPRAFI-
GEPRAF--
GQPRAF--
-GPRAFY-
-GPRAFF-
-GPRAFH-
GCPRAF--
-GPRAFP-
HGPRAF--
-HPRAFY-
-HPRAFS-
HAPRAF--
-HPRAFM-
HYPRAF--
-HPRAFA-
HPPRAF--
HIPRAF--
-HPRAFV-
-HPRAFQ-
-HPRAFH-
HEPRAF--
HCPRAF--
-IPRAFW-
-IPRAFH-
-IPRAFV-
-IPRAFQ-
-IPRAFY-
IDPRAF--
IMPRAF--
-IPRAFM-
IQPRAF--
-IPRAFF-
-IPRAFN-
-IPRAFI-
IIPRAF--
IEPRAF--
IAPRAF--
IGPRAF--
-IPRAFP-
ICPRAF--
KIPRAF--
-KPRAFH-
-KPRAFL-
-KPRAFA-
KAPRAF--
KVPRAF--
-KPRAFN-

-KPRAFF-
KTPRAF--
KGPRAF--
-KPRAFY-
-KPRAFM-
KQPRAF--
KPPRAF--
-KPRAFW-
KDPRAF--
KCPRAF--
-LPRAFH-
LGPRAF--
-LPRAFN-
LEPRAF--
LIPRAF--
-LPRAFF-
LDPRAF--
-LPRAFC-
-MPRAFF-
-MPRAFN-
-MPRAFL-
MSPRAF--
MMPRAF--
-MPRAFY-
MPPRAF--
-MPRAFM-
-MPRAFQ-
MGPRAF--
MEPRAF--
MAPRAF--
-MPRAFV-
-MPRAFI-
MNPRAF--
MDPRAF--
MTPRAF--
-MPRAFE-
MWPRAF--
NGPRAF--
-NPRAFT-
NYPRAF--
-NPRAFV-
NKPRAF--
-NPRAFY-
-NPRAFH-
NEPRAF--
NRPRAF--
-NPRAFQ-
NDPRAF--
-NPRAFF-
NPPRAF--

-NPRAFI-
--PRAFSN
--PRAFSG
--PRAFNT
--PRAFSE
--PRAFLY
--PRAFIS
--PRAFGI
--PRAFPL
--PRAFAS
PTPRAF--
--PRAFWM
--PRAFGS
--PRAFFK
--PRAFVN
--PRAFVQ
--PRAFSW
--PRAFFS
--PRAFPY
--PRAFWQ
--PRAFTG
--PRAFSK
--PRAFNA
--PRAFIW
--PRAFAL
--PRAFYN
--PRAFSD
--PRAFNH
--PRAFMD
--PRAFLD
--PRAFHW
--PRAFVT
--PRAFII
--PRAFGF
--PRAFYG
--PRAFVE
--PRAFTF
--PRAFTA
--PRAFMQ
--PRAFMK
--PRAFIE
PAPRAF--
--PRAFLI
--PRAFAF
--PRAFYY
--PRAFYQ
--PRAFRP
--PRAFQY
--PRAFQI
--PRAFQA

--PRAFNY
--PRAFIH
--PRAFID
--PRAFFY
--PRAFFI
--PRAFAQ
PLPRAF--
--PRAFYK
--PRAFWG
--PRAFVD
--PRAFMG
--PRAFMA
--PRAFKI
--PRAFIV
--PRAFHK
--PRAFGY
--PRAFGV
--PRAFFM
--PRAFFA
--PRAFEY
--PRAFAV
--PRAFAT
--PRAFAI
--PRAFAG
PKPRAF--
PEPRAF--
PSPRAF--
--PRAFWI
--PRAFWH
--PRAFTH
--PRAFTE
--PRAFQW
--PRAFNV
--PRAFNN
--PRAFNI
--PRAFMV
--PRAFIA
--PRAFHQ
--PRAFHN
--PRAFFV
--PRAFFQ
--PRAFEG
--PRAFAW
--PRAFWY
--PRAFWD
--PRAFVG
--PRAFQT
--PRAFQK
--PRAFQD
--PRAFPI

--PRAFMM
--PRAFHH
--PRAFHA
--PRAFFG
--PRAFFE
--PRAFET
--PRAFAM
--PRAFYM
--PRAFYI
--PRAFYH
--PRAFWN
--PRAFWF
--PRAFWE
--PRAFPF
--PRAFNR
--PRAFNK
--PRAFMW
--PRAFIN
--PRAFIK
--PRAFHD
--PRAFEF
--PRAFDY
--PRAFDS
PIPRAF--
PHPRAF--
--PRAFYA
--PRAFWT
--PRAFQV
--PRAFQM
--PRAFNG
--PRAFMH
--PRAFHS
--PRAFFH
--PRAFEL
--PRAFEA
--PRAFAH
PQPRAF--
PNPRAF--
PDPRAF--
--PRAFYD
--PRAFQQ
--PRAFQG
--PRAFPV
--PRAFNQ
--PRAFME
--PRAFIM
--PRAFGQ
--PRAFEM
--PRAFEI
--PRAFDL

--PRAFCT
--PRAFCL
-PPRAFI-
PPPRAF--
PGPRAF--
-QPRAFL-
QSPRAF--
-QPRAFS-
QYPRAF--
QKPRAF--
QTPRAF--
-QPRAFI-
QAPRAF--
-QPRAFT-
-QPRAFH-
-QPRAFW-
-QPRAFF-
QHPRAF--
-QPRAFA-
QIPRAF--
QMPRAF--
QGPRAF--
-QPRAFY-
-QPRAFV-
-QPRAFQ-
-QPRAFM-
QEPRAF--
-RPRAFF-
RSPRAF--
-RPRAFN-
-RPRAFM-
-RPRAFY-
RNPRAF--
-RPRAFG-
-RPRAFW-
RHPRAF--
RKPRAF--
-RPRAFE-
RPPRAF--
RCPRAF--
SSPRAF--
SKPRAF--
-SPRAFA-
-SPRAFH-
-SPRAFG-
-SPRAFI-
-SPRAFN-
-SPRAFV-
SDPRAF--
-SPRAFM-

SNPRAF--
SQPRAF--
SPPRAF--
STPRAF--
SIPRAF--
-SPRAFE-
-SPRAFD-
TLPRAF--
TSPRAF--
TVPRAF--
-TPRAFL-
TGPRAF--
-TPRAFA-
TAPRAF--
TQPRAF--
-TPRAFN-
-TPRAFI-
TDPRAF--
-TPRAFW-
TPPRAF--
TMPRAF--
-TPRAFQ-
-TPRAFM-
-TPRAFF-
TNPRAF--
THPRAF--
TIPRAF--
TEPRAF--
TCPRAF--
-TPRAFY-
VNPRAF--
VAPRAF--
-VPRAFN-
-VPRAFE-
VDPRAF--
VEPRAF--
-VPRAFH-
-VPRAFF-
VKPRAF--
VQPRAF--
-VPRAFC-
WLPRAF--
WDPRAF--
WSPRAF--
-WPRAFE-
WPPRAF--
WIPRAF--
WGPRAF--
WEPRAF--
WAPRAF--

WVPRAF--
-WPRAFN-
WHPRAF--
-WPRAFI-
WMPRAF--
YSPRAF--
YTPRAF--
YRPRAF--
YVPRAF--
-YPRAFH-
-YPRAFF-
YKPRAF--
YGPRAF--
YCPRAF--
-YPRAFN-
-YPRAFE-
-YPRAFA-
YDPRAF--

| | | | |
|---|---|---|---|
| PKAY | 496 | 606 | 31.88 |

ATPKAY--
AYPKAY--
-APKAYA-
ALPKAY--
-APKAYG-
AKPKAY--
AVPKAY--
-APKAYT-
-APKAYQ-
AAPKAY--
ASPKAY--
AQPKAY--
-APKAYM-
APPKAY--
-APKAYY-
-APKAYV-
-APKAYN-
-APKAYF-
-APKAYD-
CTPKAY--
CCPKAY--
CRPKAY--
CGPKAY--
-CPKAYT-
-CPKAYM-
CHPKAY--
CEPKAY--
CAPKAY--
DRPKAY--
DVPKAY--
-DPKAYT-

-DPKAYL-
-DPKAYR-
DWPKAY--
DSPKAY--
-DPKAYY-
-DPKAYK-
DEPKAY--
-DPKAYS-
-DPKAYF-
DHPKAY--
DDPKAY--
DAPKAY--
DTPKAY--
DQPKAY--
-DPKAYM-
DMPKAY--
DLPKAY--
DCPKAY--
EWPKAY--
EKPKAY--
-EPKAYI-
EYPKAY--
EVPKAY--
ESPKAY--
ELPKAY--
-EPKAYR-
-EPKAYW-
-EPKAYL-
EGPKAY--
-EPKAYS-
-EPKAYK-
-EPKAYH-
EMPKAY--
EEPKAY--
EQPKAY--
-EPKAYT-
-EPKAYN-
-EPKAYG-
-EPKAYF-
-EPKAYA-
ENPKAY--
EAPKAY--
-FPKAYI-
FIPKAY--
FEPKAY--
FLPKAY--
FYPKAY--
FSPKAY--
FRPKAY--
-FPKAYS-

FNPKAY--
FVPKAY--
-FPKAYW-
FFPKAY--
FTPKAY--
FKPKAY--
FDPKAY--
-FPKAYY-
-FPKAYM-
-FPKAYF-
FHPKAY--
FAPKAY--
GLPKAY--
-GPKAYL-
GYPKAY--
-GPKAYS-
-GPKAYV-
GFPKAY--
-GPKAYY-
GQPKAY--
GMPKAY--
GKPKAY--
GWPKAY--
-GPKAYF-
GAPKAY--
GTPKAY--
GSPKAY--
-GPKAYM-
-GPKAYK-
-GPKAYI-
-GPKAYA-
GHPKAY--
-GPKAYT-
-GPKAYQ-
GNPKAY--
GEPKAY--
-HPKAYL-
HMPKAY--
HLPKAY--
-HPKAYS-
-HPKAYK-
HRPKAY--
-HPKAYR-
HGPKAY--
HPPKAY--
HAPKAY--
HSPKAY--
-HPKAYH-
-HPKAYG-
-HPKAYA-

HNPKAY--
HYPKAY--
HVPKAY--
-HPKAYV-
-HPKAYT-
HIPKAY--
HCPKAY--
IRPKAY--
IPPKAY--
IIPKAY--
ITPKAY--
IGPKAY--
ISPKAY--
-IPKAYV-
-IPKAYA-
IVPKAY--
-IPKAYW-
-IPKAYS-
-IPKAYN-
-IPKAYH-
IMPKAY--
IQPKAY--
-IPKAYY-
-IPKAYI-
IKPKAY--
-IPKAYF-
ICPKAY--
KSPKAY--
KWPKAY--
-KPKAYL-
-KPKAYI-
-KPKAYY-
KYPKAY--
-KPKAYM-
-KPKAYH-
-KPKAYA-
KEPKAY--
KAPKAY--
-KPKAYQ-
-KPKAYF-
KVPKAY--
KTPKAY--
KRPKAY--
-KPKAYW-
-KPKAYV-
KNPKAY--
KLPKAY--
LVPKAY--
-LPKAYL-
-LPKAYS-

LGPKAY--
-LPKAYV-
-LPKAYK-
LAPKAY--
-LPKAYM-
LDPKAY--
-LPKAYH-
LIPKAY--
LHPKAY--
LSPKAY--
-LPKAYY-
LKPKAY--
-LPKAYI-
LYPKAY--
LQPKAY--
LLPKAY--
LEPKAY--
LTPKAY--
-LPKAYF-
-LPKAYA-
LMPKAY--
-LPKAYN-
-MPKAYL-
MSPKAY--
-MPKAYR-
-MPKAYH-
MLPKAY--
-MPKAYS-
-MPKAYA-
MVPKAY--
MRPKAY--
MMPKAY--
MKPKAY--
MFPKAY--
MWPKAY--
-MPKAYV-
-MPKAYM-
-MPKAYI-
-MPKAYF-
MNPKAY--
MAPKAY--
MTPKAY--
-MPKAYN-
-MPKAYG-
MDPKAY--
-NPKAYR-
NLPKAY--
NPPKAY--
NKPKAY--
-NPKAYS-

-NPKAYM-
-NPKAYL-
NNPKAY--
NAPKAY--
-NPKAYA-
NRPKAY--
-NPKAYF-
NHPKAY--
-NPKAYY-
-NPKAYQ-
NVPKAY--
-NPKAYI-
-NPKAYH-
--PKAYGY
--PKAYVS
--PKAYSY
--PKAYWG
--PKAYSN
--PKAYMN
--PKAYLT
--PKAYFT
--PKAYNA
--PKAYSF
--PKAYIS
--PKAYIA
--PKAYHT
--PKAYVM
--PKAYVG
--PKAYID
--PKAYWK
--PKAYWA
--PKAYTV
--PKAYQS
--PKAYML
--PKAYFS
--PKAYYT
--PKAYYS
--PKAYYL
--PKAYYI
--PKAYVW
--PKAYTN
--PKAYTG
--PKAYSM
--PKAYRM
--PKAYQA
--PKAYMG
--PKAYLW
--PKAYLI
--PKAYKG
--PKAYFN

PSPKAY--
--PKAYWS
--PKAYWQ
--PKAYSW
--PKAYSI
--PKAYSG
--PKAYNT
--PKAYNI
--PKAYLQ
--PKAYLN
--PKAYGL
--PKAYGF
--PKAYFR
PRPKAY--
--PKAYWW
--PKAYVK
--PKAYTQ
--PKAYRD
--PKAYQT
--PKAYMT
--PKAYLM
--PKAYIN
--PKAYHS
--PKAYHR
--PKAYGS
--PKAYFM
--PKAYFF
--PKAYES
--PKAYYN
--PKAYWY
--PKAYWT
--PKAYWN
--PKAYWH
--PKAYVV
--PKAYVQ
--PKAYVN
--PKAYVA
--PKAYTY
--PKAYTT
--PKAYTD
--PKAYTA
--PKAYSE
--PKAYMY
--PKAYMQ
--PKAYMM
--PKAYMI
--PKAYLY
--PKAYLD
--PKAYIY
--PKAYIQ

--PKAYHH
--PKAYGW
--PKAYGV
--PKAYGI
--PKAYFY
PGPKAY--
--PKAYYY
--PKAYYG
--PKAYYF
--PKAYVH
--PKAYSQ
--PKAYRV
--PKAYQK
--PKAYQF
--PKAYPV
--PKAYNS
--PKAYNM
--PKAYMW
--PKAYLH
--PKAYLG
--PKAYLA
--PKAYKN
--PKAYIK
--PKAYFW
--PKAYFQ
--PKAYFI
--PKAYAW
--PKAYAL
--PKAYAI
--PKAYAH
PTPKAY--
--PKAYWR
--PKAYWL
--PKAYWF
--PKAYVY
--PKAYTW
--PKAYSD
--PKAYSA
--PKAYQR
--PKAYQG
--PKAYNY
--PKAYNV
--PKAYNF
--PKAYLF
--PKAYKY
--PKAYIV
--PKAYIM
--PKAYIG
--PKAYHN
--PKAYHM

--PKAYHL
--PKAYHK
--PKAYHF
--PKAYGM
--PKAYFG
--PKAYEY
--PKAYEF
--PKAYDL
--PKAYAY
--PKAYAV
--PKAYAK
PFPKAY--
PVPKAY--
PQPKAY--
-PPKAYI-
PMPKAY--
--PKAYYV
--PKAYYM
--PKAYYK
--PKAYYE
--PKAYWI
--PKAYVT
--PKAYVI
--PKAYVF
--PKAYTS
--PKAYTI
--PKAYTF
--PKAYSV
--PKAYST
--PKAYSH
--PKAYRF
--PKAYRE
--PKAYQN
--PKAYQE
--PKAYQC
--PKAYPM
--PKAYPI
--PKAYNH
--PKAYMV
--PKAYMR
--PKAYME
--PKAYMD
--PKAYLE
--PKAYKI
--PKAYKH
--PKAYKD
--PKAYIF
--PKAYHY
--PKAYHW
--PKAYGQ

--PKAYFH
--PKAYEV
--PKAYEL
--PKAYEG
--PKAYEA
--PKAYCR
--PKAYCM
--PKAYCK
-QPKAYS-
-QPKAYY-
-QPKAYL-
QNPKAY--
QGPKAY--
QSPKAY--
-QPKAYM-
-QPKAYF-
-QPKAYQ-
QRPKAY--
QPPKAY--
QKPKAY--
QIPKAY--
QYPKAY--
QVPKAY--
QLPKAY--
QWPKAY--
QTPKAY--
-QPKAYV-
-QPKAYN-
-QPKAYK-
-QPKAYI-
QFPKAY--
QEPKAY--
QAPKAY--
-RPKAYV-
RVPKAY--
RSPKAY--
RGPKAY--
RWPKAY--
RPPKAY--
-RPKAYM-
-RPKAYA-
-RPKAYT-
RHPKAY--
-RPKAYH-
-RPKAYG-
RKPKAY--
RRPKAY--
-RPKAYN-
RDPKAY--
-SPKAYL-

SSPKAY--
-SPKAYY-
-SPKAYV-
SNPKAY--
-SPKAYS-
SRPKAY--
SVPKAY--
-SPKAYT-
STPKAY--
-SPKAYH-
-SPKAYF-
SHPKAY--
SWPKAY--
-SPKAYA-
SFPKAY--
SEPKAY--
-SPKAYW-
SKPKAY--
SPPKAY--
-SPKAYQ-
-SPKAYN-
SMPKAY--
SIPKAY--
SAPKAY--
-SPKAYK-
-SPKAYG-
-SPKAYD-
SCPKAY--
-TPKAYL-
TLPKAY--
-TPKAYT-
TRPKAY--
-TPKAYW-
TAPKAY--
-TPKAYY-
-TPKAYK-
TKPKAY--
TVPKAY--
TPPKAY--
TWPKAY--
-TPKAYQ-
TMPKAY--
-TPKAYS-
-TPKAYA-
TDPKAY--
-TPKAYF-
-TPKAYR-
TIPKAY--
TQPKAY--
-TPKAYM-

-TPKAYH-
THPKAY--
TGPKAY--
VRPKAY--
-VPKAYL-
-VPKAYK-
-VPKAYS-
-VPKAYI-
VAPKAY--
VYPKAY--
VGPKAY--
VWPKAY--
-VPKAYV-
-VPKAYQ-
VVPKAY--
VPPKAY--
VDPKAY--
VTPKAY--
-VPKAYW-
-VPKAYH-
VHPKAY--
-VPKAYY-
-VPKAYN-
VEPKAY--
-VPKAYT-
-VPKAYM-
-VPKAYC-
VIPKAY--
-VPKAYG-
VNPKAY--
VCPKAY--
-WPKAYL-
WHPKAY--
-WPKAYI-
WFPKAY--
WRPKAY--
WTPKAY--
WGPKAY--
WDPKAY--
WAPKAY--
WSPKAY--
-WPKAYT-
WEPKAY--
WPPKAY--
-WPKAYF-
WNPKAY--
WLPKAY--
WYPKAY--
-WPKAYY-
-WPKAYW-

-WPKAYV-
-WPKAYQ-
WMPKAY--
WKPKAY--
YRPKAY--
YAPKAY--
-YPKAYT-
-YPKAYY-
YLPKAY--
YVPKAY--
-YPKAYS-
-YPKAYR-
-YPKAYN-
-YPKAYH-
YKPKAY--
YIPKAY--
YYPKAY--
YSPKAY--
YQPKAY--
YNPKAY--
YDPKAY--
YTPKAY--
-YPKAYW-
YMPKAY--
YEPKAY--

| | | | |
|---|---|---|---|
| PSYL | 238 | 693 | 31.88 |

-APSYLD-
-KPSYLN-
-LPSYLW-
MCPSYL--
-NPSYLN-
-NPSYLG-
--PSYLPL
--PSYLWR
--PSYLTM
--PSYLRP
--PSYLPG
--PSYLNG
--PSYLHR
-SPSYLG-
-TPSYLE-
ALPSYL--
-APSYLR-
AGPSYL--
ASPSYL--
-APSYLM-
-APSYLS-
ARPSYL--
-APSYLL-
AQPSYL--

AFPSYL--
-APSYLY-
-APSYLI-
AKPSYL--
-APSYLV-
AYPSYL--
AIPSYL--
AVPSYL--
ATPSYL--
AHPSYL--
-APSYLN-
APPSYL--
-APSYLH-
ADPSYL--
-APSYLQ-
AEPSYL--
-APSYLG-
-APSYLA-
-APSYLE-
AMPSYL--
ANPSYL--
-APSYLW-
-APSYLP-
CRPSYL--
CKPSYL--
CNPSYL--
CHPSYL--
CSPSYL--
CFPSYL--
CTPSYL--
CGPSYL--
CEPSYL--
CYPSYL--
CQPSYL--
CLPSYL--
CVPSYL--
-CPSYLK-
CAPSYL--
-CPSYLR-
-CPSYLM-
-CPSYLI-
-CPSYLF-
CIPSYL--
DVPSYL--
DRPSYL--
-DPSYLS-
DLPSYL--
-DPSYLR-
DSPSYL--
-DPSYLT-

DEPSYL--
DAPSYL--
DKPSYL--
DHPSYL--
-DPSYLL-
DWPSYL--
-DPSYLI-
DFPSYL--
DTPSYL--
-DPSYLK-
-DPSYLH-
DIPSYL--
-DPSYLV-
-DPSYLQ-
DDPSYL--
DYPSYL--
DNPSYL--
DGPSYL--
DQPSYL--
-DPSYLN-
DCPSYL--
ESPSYL--
EVPSYL--
-EPSYLL-
EFPSYL--
ENPSYL--
-EPSYLR-
-EPSYLS-
EYPSYL--
-EPSYLY-
-EPSYLM-
-EPSYLA-
-EPSYLK-
-EPSYLV-
EHPSYL--
-EPSYLT-
EGPSYL--
EEPSYL--
EDPSYL--
ETPSYL--
-EPSYLW-
EKPSYL--
EAPSYL--
EQPSYL--
-EPSYLQ-
-EPSYLI-
-EPSYLH-
-EPSYLC-
EMPSYL--
FGPSYL--

FLPSYL--
FAPSYL--
FRPSYL--
FNPSYL--
FVPSYL--
-FPSYLK-
FWPSYL--
FPPSYL--
-FPSYLT-
-FPSYLQ-
FEPSYL--
-FPSYLA-
FKPSYL--
FFPSYL--
FYPSYL--
FQPSYL--
-FPSYLW-
FMPSYL--
-FPSYLN-
GRPSYL--
-GPSYLR-
-GPSYLS-
GTPSYL--
GMPSYL--
GLPSYL--
GSPSYL--
-GPSYLA-
GKPSYL--
GFPSYL--
GQPSYL--
-GPSYLL-
GNPSYL--
GVPSYL--
GYPSYL--
GGPSYL--
-GPSYLT-
-GPSYLM-
GHPSYL--
-GPSYLH-
-GPSYLV-
-GPSYLW-
-GPSYLK-
-GPSYLQ-
GCPSYL--
GPPSYL--
GDPSYL--
-GPSYLG-
-GPSYLF-
-GPSYLN-
-GPSYLE-

```
-GPSYLD-
HRPSYL--
HLPSYL--
-HPSYLM-
-HPSYLL-
HYPSYL--
-HPSYLW-
-HPSYLI-
HGPSYL--
-HPSYLR-
HKPSYL--
HSPSYL--
-HPSYLS-
HNPSYL--
HEPSYL--
-HPSYLY-
HQPSYL--
HDPSYL--
HCPSYL--
-HPSYLT-
-HPSYLN-
-HPSYLA-
HPPSYL--
HFPSYL--
HVPSYL--
HAPSYL--
-HPSYLH-
HWPSYL--
-HPSYLV-
-HPSYLQ-
-HPSYLF-
-HPSYLC-
HHPSYL--
ILPSYL--
IGPSYL--
IVPSYL--
-IPSYLT-
IFPSYL--
ISPSYL--
IMPSYL--
IHPSYL--
-IPSYLS-
ITPSYL--
INPSYL--
-IPSYLK-
-IPSYLL-
IEPSYL--
IYPSYL--
IKPSYL--
IIPSYL--
```

IDPSYL--
IAPSYL--
IRPSYL--
-IPSYLI-
IPPSYL--
IQPSYL--
-IPSYLQ-
-IPSYLM-
-IPSYLW-
ICPSYL--
-IPSYLN-
-IPSYLH-
KRPSYL--
KVPSYL--
KTPSYL--
-KPSYLL-
KGPSYL--
-KPSYLT-
KYPSYL--
-KPSYLS-
-KPSYLR-
-KPSYLF-
KWPSYL--
KFPSYL--
-KPSYLV-
KIPSYL--
-KPSYLY-
-KPSYLW-
-KPSYLQ-
-KPSYLH-
KNPSYL--
KQPSYL--
-KPSYLG-
KHPSYL--
KDPSYL--
-KPSYLE-
KCPSYL--
-KPSYLC-
LVPSYL--
LSPSYL--
LGPSYL--
LTPSYL--
LFPSYL--
LAPSYL--
LEPSYL--
-LPSYLQ-
-LPSYLK-
LHPSYL--
LNPSYL--
LPPSYL--

LYPSYL--
LQPSYL--
-LPSYLV-
LIPSYL--
-LPSYLI-
-LPSYLA-
LCPSYL--
MRPSYL--
MNPSYL--
MPPSYL--
MLPSYL--
MYPSYL--
MTPSYL--
-MPSYLT-
MFPSYL--
MMPSYL--
MQPSYL--
-MPSYLH-
-MPSYLA-
MKPSYL--
MGPSYL--
-MPSYLW-
-MPSYLF-
MIPSYL--
-MPSYLM-
MHPSYL--
MEPSYL--
MAPSYL--
MSPSYL--
-MPSYLQ-
-MPSYLV-
MDPSYL--
NPPSYL--
-NPSYLL-
NTPSYL--
NYPSYL--
NLPSYL--
NAPSYL--
NSPSYL--
NQPSYL--
NFPSYL--
-NPSYLI-
-NPSYLT-
NMPSYL--
-NPSYLR-
NWPSYL--
-NPSYLQ-
NEPSYL--
-NPSYLV-
-NPSYLH-

NNPSYL--
NCPSYL--
NHPSYL--
-NPSYLM-
-NPSYLF-
-NPSYLA-
NDPSYL--
-NPSYLW-
PSPSYL--
--PSYLTS
--PSYLRS
--PSYLSR
--PSYLSL
--PSYLAR
--PSYLQS
--PSYLRG
--PSYLYT
--PSYLRT
--PSYLRQ
--PSYLRH
--PSYLLT
--PSYLKS
PLPSYL--
--PSYLRI
PVPSYL--
--PSYLAS
--PSYLKV
--PSYLKR
--PSYLFS
--PSYLTV
--PSYLSY
--PSYLST
--PSYLYR
--PSYLTG
--PSYLSG
PRPSYL--
--PSYLVG
--PSYLSV
--PSYLSE
--PSYLIS
--PSYLSQ
--PSYLRN
--PSYLLQ
--PSYLIK
--PSYLES
--PSYLQT
--PSYLKY
--PSYLVK
--PSYLTT
--PSYLSN

--PSYLAV
--PSYLTF
--PSYLTA
--PSYLSK
--PSYLRM
--PSYLMI
--PSYLFA
--PSYLAQ
PTPSYL--
--PSYLWG
--PSYLSF
--PSYLSA
--PSYLNT
--PSYLKT
--PSYLKH
--PSYLWS
--PSYLVV
--PSYLVT
--PSYLTR
--PSYLTQ
--PSYLKA
--PSYLFG
--PSYLYM
--PSYLRV
--PSYLLD
--PSYLKQ
--PSYLKI
--PSYLHT
--PSYLAK
--PSYLAI
--PSYLAG
--PSYLYS
--PSYLVM
--PSYLVH
--PSYLSD
--PSYLNA
--PSYLMK
--PSYLID
--PSYLIA
--PSYLHS
--PSYLFK
PAPSYL--
--PSYLTN
--PSYLSM
--PSYLQG
--PSYLMT
--PSYLIR
--PSYLAW
--PSYLAT
--PSYLVA

--PSYLMN
--PSYLKN
--PSYLKM
--PSYLKL
--PSYLFH
--PSYLTE
--PSYLQV
--PSYLHV
PFPSYL--
PWPSYL--
--PSYLWN
--PSYLMV
--PSYLLH
PQPSYL--
PIPSYL--
PGPSYL--
PYPSYL--
--PSYLVQ
--PSYLTW
--PSYLTD
--PSYLMA
--PSYLII
--PSYLIH
--PSYLHQ
--PSYLHG
--PSYLAE
PKPSYL--
--PSYLVY
--PSYLQR
--PSYLMS
--PSYLMD
--PSYLKF
--PSYLFN
--PSYLAD
PHPSYL--
--PSYLYN
--PSYLTH
--PSYLNS
--PSYLMY
--PSYLGW
--PSYLGT
--PSYLGG
--PSYLEG
PEPSYL--
--PSYLYH
--PSYLYD
--PSYLVD
--PSYLQQ
--PSYLQM
--PSYLMM

```
--PSYLIW
--PSYLHY
--PSYLGL
--PSYLAH
PDPSYL--
--PSYLYW
--PSYLYQ
--PSYLYE
--PSYLWH
--PSYLTI
--PSYLQW
--PSYLQN
--PSYLNQ
--PSYLNK
--PSYLMQ
--PSYLHW
--PSYLHH
--PSYLET
--PSYLAN
--PSYLWI
--PSYLSC
--PSYLQH
--PSYLQD
--PSYLNW
--PSYLMH
--PSYLKD
--PSYLIY
--PSYLIN
--PSYLHL
--PSYLHK
--PSYLFD
--PSYLEV
--PSYLAM
PMPSYL--
--PSYLYK
--PSYLWQ
--PSYLVC
--PSYLQE
--PSYLPK
--PSYLHN
--PSYLHM
--PSYLHI
--PSYLHF
--PSYLEY
--PSYLEA
PPPSYL--
QRPSYL--
-QPSYLR-
QVPSYL--
QSPSYL--
```

QLPSYL--
-QPSYLT-
-QPSYLK-
QIPSYL--
-QPSYLY-
QWPSYL--
-QPSYLA-
QNPSYL--
QMPSYL--
QPPSYL--
-QPSYLW-
QFPSYL--
-QPSYLQ-
-QPSYLF-
QQPSYL--
-QPSYLV-
QAPSYL--
-QPSYLM-
-QPSYLH-
QHPSYL--
QEPSYL--
QDPSYL--
QTPSYL--
-QPSYLN-
-QPSYLI-
QYPSYL--
-QPSYLG-
RLPSYL--
RGPSYL--
RTPSYL--
RFPSYL--
-RPSYLT-
RYPSYL--
RKPSYL--
RAPSYL--
RQPSYL--
RIPSYL--
-RPSYLW-
-RPSYLA-
-RPSYLG-
RHPSYL--
REPSYL--
-RPSYLQ-
RDPSYL--
-RPSYLN-
RPPSYL--
-RPSYLM-
-RPSYLE-
RCPSYL--
-SPSYLL-

-SPSYLS-
-SPSYLR-
SYPSYL--
SSPSYL--
SAPSYL--
STPSYL--
-SPSYLM-
-SPSYLH-
-SPSYLK-
SHPSYL--
SGPSYL--
SMPSYL--
-SPSYLA-
SFPSYL--
SDPSYL--
SNPSYL--
SQPSYL--
-SPSYLQ-
-SPSYLN-
SCPSYL--
TRPSYL--
-TPSYLS-
TPPSYL--
TEPSYL--
TAPSYL--
TTPSYL--
TQPSYL--
-TPSYLQ-
TFPSYL--
-TPSYLV-
-TPSYLR-
TVPSYL--
-TPSYLK-
TLPSYL--
TGPSYL--
TKPSYL--
TSPSYL--
TNPSYL--
TYPSYL--
-TPSYLY-
TIPSYL--
-TPSYLM-
TDPSYL--
-TPSYLI-
-TPSYLT-
-TPSYLA-
TWPSYL--
-TPSYLW-
TCPSYL--
-TPSYLG-

−TPSYLN−
−VPSYLK−
VSPSYL−−
VTPSYL−−
−VPSYLI−
VFPSYL−−
VAPSYL−−
VLPSYL−−
VMPSYL−−
VGPSYL−−
−VPSYLT−
VPPSYL−−
VKPSYL−−
VQPSYL−−
VWPSYL−−
−VPSYLQ−
VEPSYL−−
VDPSYL−−
−VPSYLM−
VCPSYL−−
−VPSYLG−
−VPSYLW−
−VPSYLE−
−WPSYLR−
WTPSYL−−
WVPSYL−−
−WPSYLI−
WLPSYL−−
WWPSYL−−
−WPSYLS−
WKPSYL−−
WDPSYL−−
−WPSYLL−
−WPSYLK−
WIPSYL−−
WEPSYL−−
WMPSYL−−
WYPSYL−−
−WPSYLV−
WPPSYL−−
WHPSYL−−
−WPSYLQ−
WQPSYL−−
−WPSYLH−
−WPSYLA−
YVPSYL−−
YSPSYL−−
−YPSYLS−
YKPSYL−−
YRPSYL−−

| | | | |
|---|---|---|---|
| | -YPSYLR- | | |
| | YPPSYL-- | | |
| | -YPSYLA- | | |
| | YIPSYL-- | | |
| | YNPSYL-- | | |
| | -YPSYLH- | | |
| | YYPSYL-- | | |
| | YEPSYL-- | | |
| | -YPSYLT- | | |
| | YHPSYL-- | | |
| | YCPSYL-- | | |
| | YWPSYL-- | | |
| | -YPSYLI- | | |
| | -YPSYLK- | | |
| | -YPSYLQ- | | |
| | YMPSYL-- | | |
| | -YPSYLG- | | |
| | YAPSYL-- | | |
| | -YPSYLN- | | |
| PLNM | 184 | 724 | 31.86 |
| | -LPLNMG- | | |
| | ARPLNM-- | | |
| | ALPLNM-- | | |
| | -APLNMR- | | |
| | -APLNMG- | | |
| | -APLNML- | | |
| | -APLNMI- | | |
| | -APLNMF- | | |
| | AKPLNM-- | | |
| | AQPLNM-- | | |
| | AFPLNM-- | | |
| | -APLNMS- | | |
| | -APLNMN- | | |
| | -APLNMH- | | |
| | AYPLNM-- | | |
| | -APLNME- | | |
| | -APLNMV- | | |
| | AGPLNM-- | | |
| | ADPLNM-- | | |
| | AWPLNM-- | | |
| | -APLNMW- | | |
| | AIPLNM-- | | |
| | ACPLNM-- | | |
| | AVPLNM-- | | |
| | ATPLNM-- | | |
| | -APLNMY- | | |
| | -APLNMT- | | |
| | -APLNMQ- | | |
| | -APLNMC- | | |
| | -APLNMA- | | |

ANPLNM--
AEPLNM--
CLPLNM--
CMPLNM--
CVPLNM--
CRPLNM--
-CPLNML-
CTPLNM--
CSPLNM--
-CPLNMY-
-CPLNMT-
-CPLNMS-
-CPLNMR-
CNPLNM--
CFPLNM--
CAPLNM--
DYPLNM--
-DPLNMR-
DQPLNM--
DWPLNM--
DVPLNM--
DRPLNM--
-DPLNML-
DSPLNM--
-DPLNMV-
-DPLNMQ-
-DPLNMF-
DGPLNM--
DTPLNM--
-DPLNMY-
-DPLNMS-
-DPLNMK-
-DPLNMI-
-DPLNMA-
DKPLNM--
DIPLNM--
DHPLNM--
DCPLNM--
DAPLNM--
ERPLNM--
-EPLNMY-
ELPLNM--
-EPLNMA-
EKPLNM--
EWPLNM--
-EPLNMT-
-EPLNMR-
EHPLNM--
EAPLNM--
EYPLNM--

-EPLNMK-
ETPLNM--
-EPLNMS-
-EPLNMN-
-EPLNMH-
EIPLNM--
EQPLNM--
-EPLNMV-
-EPLNML-
-EPLNMG-
-EPLNMF-
EFPLNM--
FTPLNM--
FSPLNM--
-FPLNML-
-FPLNMV-
FRPLNM--
FGPLNM--
-FPLNMR-
-FPLNMA-
FLPLNM--
-FPLNMT-
-FPLNMG-
FKPLNM--
-FPLNMQ-
FFPLNM--
FVPLNM--
FPPLNM--
-FPLNMY-
-FPLNMS-
-FPLNMI-
-FPLNMH-
FMPLNM--
FAPLNM--
FWPLNM--
-FPLNMM-
-FPLNMD-
FNPLNM--
FIPLNM--
FDPLNM--
GLPLNM--
GRPLNM--
-GPLNML-
-GPLNMS-
-GPLNMI-
GWPLNM--
-GPLNMK-
-GPLNMA-
GTPLNM--
GFPLNM--

GSPLNM--
GAPLNM--
-GPLNMW-
-GPLNMV-
-GPLNMY-
-GPLNMT-
-GPLNMR-
-GPLNMG-
GYPLNM--
GQPLNM--
GPPLNM--
-GPLNMF-
GEPLNM--
GDPLNM--
GVPLNM--
-GPLNMQ-
-GPLNMN-
GNPLNM--
GMPLNM--
GKPLNM--
GHPLNM--
GGPLNM--
-HPLNML-
-HPLNMT-
HYPLNM--
HAPLNM--
HWPLNM--
HLPLNM--
HSPLNM--
HTPLNM--
HRPLNM--
-HPLNMR-
-HPLNMK-
HVPLNM--
HIPLNM--
HPPLNM--
-HPLNMV-
-HPLNMS-
-HPLNMQ-
-HPLNMN-
-HPLNMG-
HHPLNM--
HGPLNM--
-HPLNMW-
-HPLNMM-
-HPLNMF-
-HPLNME-
-HPLNMD-
-HPLNMA-
IRPLNM--

-IPLNMT-
ILPLNM--
-IPLNMW-
-IPLNML-
IGPLNM--
IAPLNM--
IYPLNM--
-IPLNMY-
-IPLNMG-
-IPLNMR-
-IPLNMN-
IWPLNM--
IKPLNM--
IHPLNM--
-IPLNMI-
-IPLNMQ-
-IPLNMA-
INPLNM--
IMPLNM--
IVPLNM--
ITPLNM--
IQPLNM--
-IPLNMH-
IIPLNM--
IFPLNM--
IEPLNM--
KSPLNM--
-KPLNMS-
-KPLNML-
KLPLNM--
-KPLNMT-
-KPLNMH-
-KPLNMA-
KVPLNM--
-KPLNMW-
-KPLNMQ-
-KPLNMM-
-KPLNMG-
-KPLNMI-
KAPLNM--
KYPLNM--
KTPLNM--
KNPLNM--
KMPLNM--
KQPLNM--
-KPLNMV-
-KPLNMR-
-KPLNMF-
-KPLNMN-
KKPLNM--

```
KIPLNM--
KGPLNM--
KFPLNM--
KCPLNM--
KPPLNM--
-KPLNME-
-KPLNMD-
KDPLNM--
LSPLNM--
LKPLNM--
-LPLNMR-
LIPLNM--
LWPLNM--
LMPLNM--
LQPLNM--
-LPLNML-
-LPLNMV-
LHPLNM--
LEPLNM--
-LPLNMI-
-LPLNMT-
-LPLNMK-
-LPLNMS-
-LPLNMQ-
LYPLNM--
-LPLNMN-
LVPLNM--
LPPLNM--
-LPLNMH-
-LPLNMA-
LDPLNM--
-LPLNMM-
-LPLNME-
MLPLNM--
MGPLNM--
-MPLNMS-
MMPLNM--
-MPLNML-
-MPLNMA-
MYPLNM--
MTPLNM--
-MPLNMT-
MSPLNM--
MRPLNM--
MVPLNM--
-MPLNMY-
-MPLNME-
MHPLNM--
MQPLNM--
MPPLNM--
```

```
MEPLNM--
MWPLNM--
-MPLNMK-
-MPLNMI-
-MPLNMG-
MNPLNM--
MDPLNM--
-MPLNMV-
-MPLNMF-
-MPLNMD-
MKPLNM--
MIPLNM--
MFPLNM--
MAPLNM--
NGPLNM--
-NPLNMS-
NRPLNM--
NYPLNM--
-NPLNML-
NLPLNM--
NAPLNM--
-NPLNMF-
-NPLNMY-
-NPLNMM-
NWPLNM--
-NPLNMR-
-NPLNMK-
-NPLNME-
-NPLNMA-
NMPLNM--
NIPLNM--
NVPLNM--
-NPLNMV-
NDPLNM--
NQPLNM--
-NPLNMT-
-NPLNMN-
-NPLNMG-
NKPLNM--
NHPLNM--
NFPLNM--
--PLNMMH
--PLNMWT
--PLNMMR
--PLNMLG
--PLNMSL
--PLNMRS
--PLNMRD
--PLNMRF
--PLNMVL
```

--PLNMSI
--PLNMRK
--PLNMML
--PLNMGT
--PLNMVS
--PLNMVA
--PLNMTR
--PLNMSM
--PLNMES
--PLNMYR
--PLNMSN
--PLNMMT
--PLNMKS
--PLNMKA
--PLNMGS
--PLNMSV
--PLNMSA
--PLNMLT
--PLNMLR
--PLNMIR
--PLNMAL
PQPLNM--
--PLNMYT
--PLNMWG
--PLNMTL
--PLNMTA
--PLNMGA
--PLNMAT
--PLNMVH
--PLNMVG
--PLNMST
--PLNMGL
--PLNMFG
PHPLNM--
--PLNMVV
--PLNMVT
--PLNMSS
--PLNMSK
--PLNMRQ
--PLNMLE
--PLNMLD
--PLNMKV
--PLNMKG
--PLNMER
--PLNMTV
--PLNMTS
--PLNMTI
--PLNMTD
--PLNMSF
--PLNMSE

--PLNMNM
--PLNMNA
--PLNMKH
--PLNMIE
--PLNMHS
--PLNMYS
--PLNMWN
--PLNMVY
--PLNMVR
--PLNMSH
--PLNMRW
--PLNMRG
--PLNMQL
--PLNMLN
--PLNMLH
--PLNMAK
--PLNMYG
--PLNMWK
--PLNMVQ
--PLNMVN
--PLNMVM
--PLNMVI
--PLNMTY
--PLNMTN
--PLNMTE
--PLNMSG
--PLNMQY
--PLNMQG
--PLNMMK
--PLNMKR
--PLNMKL
--PLNMKE
--PLNMII
--PLNMHT
--PLNMHR
--PLNMHA
--PLNMGM
--PLNMAS
--PLNMAR
--PLNMAH
PEPLNM--
--PLNMYL
--PLNMVW
--PLNMTK
--PLNMRN
--PLNMRM
--PLNMRE
--PLNMQR
--PLNMNS
--PLNMNK

--PLNMMV
--PLNMMI
--PLNMKY
--PLNMKQ
--PLNMKN
--PLNMIA
--PLNMFQ
--PLNMFD
--PLNMET
--PLNMEE
--PLNMAY
--PLNMAV
--PLNMAM
--PLNMAA
--PLNMYH
--PLNMWI
--PLNMWH
--PLNMVD
--PLNMTT
--PLNMTM
--PLNMTH
--PLNMTG
--PLNMSW
--PLNMSD
--PLNMRL
--PLNMRC
--PLNMQT
--PLNMQS
--PLNMQE
--PLNMQA
--PLNMNW
--PLNMNV
--PLNMNL
--PLNMNI
--PLNMMS
--PLNMMA
--PLNMKD
--PLNMIT
--PLNMIK
--PLNMHH
--PLNMGY
--PLNMGG
--PLNMFS
--PLNMFN
--PLNMFH
--PLNMFE
--PLNMEI
--PLNMEG
--PLNMEF
--PLNMCG

--PLNMAI
--PLNMAG
PFPLNM--
--PLNMYW
--PLNMYM
--PLNMYD
--PLNMWQ
--PLNMVK
--PLNMTQ
--PLNMTF
--PLNMSQ
--PLNMRY
--PLNMRH
--PLNMQV
--PLNMPL
--PLNMNT
--PLNMNG
--PLNMMW
--PLNMMN
--PLNMMG
--PLNMKW
--PLNMKT
--PLNMKM
--PLNMKI
--PLNMIV
--PLNMHY
--PLNMHN
--PLNMHI
--PLNMHF
--PLNMHE
--PLNMGQ
--PLNMFA
--PLNMEY
--PLNMEV
--PLNMEM
--PLNMEH
--PLNMDG
--PLNMDA
--PLNMCS
--PLNMAW
--PLNMAN
PDPLNM--
QRPLNM--
-QPLNMK-
-QPLNMY-
QIPLNM--
-QPLNMR-
-QPLNMF-
-QPLNMA-
-QPLNML-

QGPLNM--
QTPLNM--
-QPLNMM-
QNPLNM--
QVPLNM--
-QPLNMT-
-QPLNMG-
-QPLNMV-
-QPLNMH-
-QPLNME-
QHPLNM--
QQPLNM--
QPPLNM--
-QPLNMS-
-QPLNMN-
-QPLNMI-
QKPLNM--
QEPLNM--
-RPLNMR-
RGPLNM--
RVPLNM--
-RPLNMF-
-RPLNMS-
-RPLNMY-
RMPLNM--
-RPLNMM-
RDPLNM--
-RPLNMV-
-RPLNMI-
RLPLNM--
RTPLNM--
RSPLNM--
-RPLNMH-
RAPLNM--
RIPLNM--
REPLNM--
-RPLNMA-
-RPLNMN-
RFPLNM--
RKPLNM--
RQPLNM--
RHPLNM--
RPPLNM--
-RPLNMK-
RYPLNM--
-RPLNMD-
RNPLNM--
-RPLNMQ-
RCPLNM--
-SPLNMA-

-SPLNML-
-SPLNMV-
-SPLNMR-
-SPLNMN-
-SPLNMS-
-SPLNMY-
SYPLNM--
-SPLNMG-
SIPLNM--
-SPLNMQ-
-SPLNMF-
-SPLNMI-
SMPLNM--
SGPLNM--
SQPLNM--
-SPLNMT-
-SPLNMM-
SHPLNM--
SWPLNM--
SVPLNM--
STPLNM--
SPPLNM--
SKPLNM--
SFPLNM--
SEPLNM--
SAPLNM--
-SPLNMH-
-SPLNMK-
-SPLNME-
-SPLNMC-
SDPLNM--
SCPLNM--
-TPLNML-
-TPLNMV-
TAPLNM--
THPLNM--
TVPLNM--
TWPLNM--
-TPLNMY-
-TPLNMK-
-TPLNMI-
-TPLNMR-
TFPLNM--
-TPLNMT-
-TPLNMG-
-TPLNME-
TSPLNM--
TKPLNM--
TIPLNM--
TYPLNM--

-TPLNMH-
-TPLNMM-
TNPLNM--
TEPLNM--
TTPLNM--
-TPLNMQ-
TLPLNM--
TQPLNM--
TPPLNM--
TGPLNM--
TDPLNM--
VSPLNM--
VTPLNM--
VVPLNM--
-VPLNML-
VYPLNM--
-VPLNMT-
-VPLNMR-
-VPLNMN-
-VPLNMF-
VGPLNM--
VEPLNM--
VRPLNM--
VPPLNM--
-VPLNMS-
-VPLNMK-
-VPLNMG-
-VPLNMV-
VFPLNM--
-VPLNMW-
-VPLNMM-
-VPLNMI-
-VPLNMH-
VMPLNM--
VHPLNM--
VQPLNM--
VNPLNM--
VLPLNM--
-VPLNME-
VKPLNM--
VDPLNM--
VCPLNM--
-WPLNMM-
WSPLNM--
-WPLNMR-
-WPLNMI-
WRPLNM--
WKPLNM--
WDPLNM--
WWPLNM--

WGPLNM--
WFPLNM--
-WPLNMK-
WAPLNM--
WTPLNM--
-WPLNML-
-WPLNMH-
WNPLNM--
WMPLNM--
WHPLNM--
WVPLNM--
WQPLNM--
-WPLNMV-
-WPLNMT-
-WPLNMN-
-WPLNME-
-WPLNMA-
WLPLNM--
YRPLNM--
-YPLNMR-
YGPLNM--
-YPLNMK-
-YPLNMH-
YWPLNM--
YSPLNM--
YLPLNM--
YQPLNM--
YYPLNM--
-YPLNMM-
-YPLNMG-
YKPLNM--
YDPLNM--
YAPLNM--
-YPLNMW-
-YPLNMT-
-YPLNMQ-
-YPLNMI-
-YPLNMF-
-YPLNME-
-YPLNMD-
YNPLNM--
YMPLNM--
YIPLNM--
YEPLNM--

| | | | |
|---|---|---|---|
| IAAL | 444 | 620 | 31.83 |

-IIAALS-
--IAALGN
-AIAALS-
APIAAL--
-AIAALL-

ATIAAL--
-AIAALN-
AGIAAL--
-AIAALV-
-AIAALQ-
AVIAAL--
-AIAALI-
AHIAAL--
AWIAAL--
-AIAALK-
-AIAALA-
AQIAAL--
ANIAAL--
AMIAAL--
AKIAAL--
-AIAALH-
-AIAALG-
AAIAAL--
ADIAAL--
AIIAAL--
-AIAALE-
AEIAAL--
-AIAALC-
CRIAAL--
-CIAALR-
CMIAAL--
CPIAAL--
-CIAALT-
-CIAALS-
CAIAAL--
CLIAAL--
CIIAAL--
-CIAALI-
CYIAAL--
CSIAAL--
CQIAAL--
CNIAAL--
CKIAAL--
-CIAALY-
-CIAALP-
-CIAALH-
-CIAALE-
DGIAAL--
DHIAAL--
DIIAAL--
-DIAALR-
DRIAAL--
DQIAAL--
-DIAALH-
DVIAAL--

-DIAALQ-
DKIAAL--
DPIAAL--
DWIAAL--
DTIAAL--
DNIAAL--
-DIAALS-
-DIAALN-
DAIAAL--
-DIAALY-
-DIAALT-
-DIAALK-
-DIAALI-
DFIAAL--
DEIAAL--
DDIAAL--
ERIAAL--
EVIAAL--
-EIAALS-
ETIAAL--
EGIAAL--
EMIAAL--
EIIAAL--
-EIAALT-
-EIAALL-
-EIAALI-
ELIAAL--
ENIAAL--
-EIAALK-
EQIAAL--
EPIAAL--
-EIAALN-
EKIAAL--
EYIAAL--
-EIAALY-
EWIAAL--
-EIAALQ-
-EIAALM-
-FIAALN-
-FIAALG-
FGIAAL--
FDIAAL--
FPIAAL--
-FIAALQ-
FQIAAL--
-FIAALT-
FHIAAL--
-FIAALH-
FEIAAL--
FAIAAL--

GVIAAL--
GMIAAL--
GYIAAL--
-GIAALR-
GNIAAL--
-GIAALK-
-GIAALA-
GGIAAL--
GTIAAL--
-GIAALT-
-GIAALN-
-GIAALG-
-GIAALQ-
GAIAAL--
GSIAAL--
GQIAAL--
-GIAALL-
-GIAALH-
GHIAAL--
-GIAALE-
GDIAAL--
GPIAAL--
GEIAAL--
-GIAALW-
-GIAALM-
-GIAALV-
GWIAAL--
-GIAALI-
HSIAAL--
-HIAALL-
HNIAAL--
-HIAALK-
HGIAAL--
HTIAAL--
HQIAAL--
HLIAAL--
-HIAALV-
-HIAALS-
HVIAAL--
-HIAALW-
-HIAALQ-
-HIAALH-
HFIAAL--
HPIAAL--
-HIAALR-
HHIAAL--
HMIAAL--
HKIAAL--
-HIAALN-
-HIAALI-

```
HDIAAL--
HAIAAL--
HYIAAL--
HIIAAL--
-HIAALM-
-HIAALE-
--IAALLT
--IAALKA
--IAALRN
--IAALRH
--IAALSG
--IAALQN
--IAALTR
--IAALVG
--IAALKT
--IAALMN
--IAALLK
--IAALYG
--IAALTL
--IAALRW
IPIAAL--
--IAALNA
--IAALMT
--IAALTS
--IAALRD
--IAALNT
--IAALST
--IAALMK
--IAALLS
ISIAAL--
--IAALRV
--IAALIT
--IAALTV
--IAALLI
--IAALAS
--IAALVK
--IAALRE
--IAALGI
--IAALSN
--IAALRQ
--IAALQR
--IAALLN
--IAALIG
--IAALAH
--IAALYN
--IAALWT
--IAALTG
--IAALNR
--IAALLR
--IAALAY
```

-IIAALA-
--IAALRM
--IAALKR
--IAALKF
--IAALGS
--IAALFS
--IAALWR
--IAALWH
--IAALTK
--IAALSY
--IAALKY
--IAALHG
--IAALFN
--IAALEG
INIAAL--
--IAALWQ
--IAALQA
--IAALNS
--IAALMH
--IAALMD
--IAALLQ
--IAALKL
--IAALGL
--IAALWS
--IAALWG
--IAALVQ
--IAALTW
--IAALTI
--IAALSF
--IAALPL
--IAALLG
--IAALIK
--IAALHV
--IAALHT
--IAALHQ
--IAALGW
-IIAALN-
-IIAALK-
--IAALYS
--IAALVN
--IAALTM
--IAALSH
--IAALPY
--IAALPF
--IAALKW
--IAALKS
--IAALIR
--IAALGA
--IAALFK
-IIAALT-

--IAALWD
--IAALWA
--IAALTN
--IAALTH
--IAALSA
--IAALQQ
--IAALQM
--IAALQH
--IAALNN
--IAALKV
--IAALKI
--IAALIN
--IAALFG
--IAALYD
--IAALTY
--IAALTF
--IAALRF
--IAALQK
--IAALQF
--IAALLE
--IAALLD
--IAALKH
--IAALIS
--IAALIQ
--IAALHN
--IAALGG
-IIAALQ-
--IAALVE
--IAALVD
--IAALTQ
--IAALTA
--IAALSQ
--IAALSE
--IAALQY
--IAALQT
--IAALQG
--IAALPI
--IAALNK
--IAALMV
--IAALMQ
--IAALLM
--IAALKQ
--IAALKD
--IAALIH
--IAALHH
--IAALGY
--IAALGV
--IAALFH
--IAALEF
--IAALEA

--IAALAW
--IAALAT
IDIAAL--
--IAALVM
--IAALTD
--IAALQV
--IAALPM
--IAALNY
--IAALMI
--IAALLW
--IAALKE
--IAALIE
--IAALID
--IAALHY
--IAALHW
--IAALHA
--IAALGT
--IAALFA
--IAALEY
--IAALET
--IAALDG
--IAALAN
--IAALAK
IQIAAL--
-IIAALE-
-IIAALC-
IHIAAL--
IAIAAL--
--IAALYQ
--IAALYE
--IAALWN
--IAALWK
--IAALVV
--IAALTE
--IAALSW
--IAALSD
--IAALQC
--IAALPW
--IAALNV
--IAALNQ
--IAALNM
--IAALNI
--IAALMM
--IAALII
--IAALIA
--IAALHK
--IAALHI
--IAALGQ
--IAALGH
--IAALGF

```
--IAALFQ
--IAALEV
--IAALCT
--IAALCA
--IAALAV
--IAALAQ
--IAALAM
--IAALAA
-KIAALS-
KVIAAL--
KLIAAL--
-KIAALY-
-KIAALN-
-KIAALV-
KFIAAL--
KAIAAL--
KPIAAL--
KWIAAL--
KIIAAL--
KDIAAL--
-KIAALH-
-KIAALA-
KGIAAL--
KTIAAL--
KNIAAL--
-KIAALG-
KQIAAL--
-KIAALE-
KEIAAL--
KCIAAL--
LSIAAL--
-LIAALR-
LGIAAL--
LTIAAL--
LNIAAL--
LAIAAL--
-LIAALT-
-LIAALH-
-LIAALG-
LHIAAL--
LKIAAL--
LDIAAL--
LEIAAL--
-LIAALD-
-LIAALQ-
-LIAALW-
-LIAALA-
MGIAAL--
-MIAALS-
MAIAAL--
```

-MIAALG-
MTIAAL--
-MIAALR-
MNIAAL--
-MIAALQ-
-MIAALN-
-MIAALE-
MDIAAL--
MQIAAL--
MPIAAL--
MKIAAL--
-MIAALT-
-MIAALA-
MMIAAL--
-MIAALK-
MYIAAL--
-NIAALR-
NGIAAL--
NVIAAL--
NQIAAL--
NNIAAL--
-NIAALS-
NSIAAL--
NFIAAL--
NKIAAL--
NIIAAL--
-NIAALK-
-NIAALH-
-NIAALN-
NWIAAL--
NAIAAL--
NTIAAL--
-NIAALT-
-NIAALV-
-NIAALF-
-NIAALM-
-NIAALG-
-NIAALA-
NHIAAL--
NCIAAL--
NYIAAL--
NPIAAL--
-NIAALE-
-NIAALC-
PLIAAL--
-PIAALR-
PTIAAL--
-PIAALS-
-PIAALL-
-PIAALH-

-PIAALW-
-PIAALV-
-PIAALI-
PGIAAL--
PNIAAL--
-PIAALN-
-PIAALM-
-PIAALA-
PVIAAL--
-PIAALQ-
PQIAAL--
PKIAAL--
-PIAALK-
PEIAAL--
-PIAALT-
PCIAAL--
-QIAALS-
QWIAAL--
-QIAALR-
QTIAAL--
-QIAALL-
QGIAAL--
-QIAALI-
QMIAAL--
QFIAAL--
QVIAAL--
QSIAAL--
QPIAAL--
-QIAALE-
-QIAALQ-
-QIAALN-
QHIAAL--
QAIAAL--
QYIAAL--
QKIAAL--
QIIAAL--
QNIAAL--
-QIAALK-
-QIAALH-
-QIAALG-
QQIAAL--
QLIAAL--
-QIAALV-
-QIAALT-
RGIAAL--
-RIAALT-
RNIAAL--
-RIAALQ-
RWIAAL--
-RIAALA-

```
RTIAAL--
-RIAALY-
RAIAAL--
-RIAALK-
RQIAAL--
RDIAAL--
RCIAAL--
SSIAAL--
-SIAALT-
-SIAALR-
-SIAALH-
SYIAAL--
SRIAAL--
SGIAAL--
-SIAALV-
-SIAALS-
SWIAAL--
SVIAAL--
SQIAAL--
-SIAALG-
-SIAALN-
-SIAALK-
SDIAAL--
-SIAALI-
-SIAALF-
SEIAAL--
-SIAALA-
SAIAAL--
SPIAAL--
SKIAAL--
SCIAAL--
-SIAALW-
-SIAALM-
-SIAALE-
-TIAALR-
TSIAAL--
TRIAAL--
TPIAAL--
TTIAAL--
TQIAAL--
-TIAALS-
TLIAAL--
-TIAALK-
THIAAL--
TNIAAL--
-TIAALI-
-TIAALT-
TYIAAL--
TMIAAL--
TGIAAL--
```

TAIAAL--
-TIAALH-
-TIAALN-
-TIAALM-
-TIAALC-
-TIAALQ-
TEIAAL--
TCIAAL--
VSIAAL--
VVIAAL--
VNIAAL--
-VIAALR-
VTIAAL--
-VIAALN-
VGIAAL--
-VIAALK-
-VIAALT-
-VIAALQ-
VHIAAL--
-VIAALE-
VDIAAL--
VPIAAL--
-VIAALG-
VCIAAL--
VQIAAL--
VAIAAL--
VMIAAL--
-VIAALH-
WNIAAL--
-WIAALS-
WGIAAL--
WSIAAL--
WYIAAL--
WKIAAL--
-WIAALH-
-WIAALG-
WDIAAL--
WTIAAL--
WWIAAL--
WMIAAL--
WAIAAL--
WPIAAL--
WHIAAL--
YSIAAL--
YAIAAL--
-YIAALS-
-YIAALK-
YHIAAL--
YWIAAL--
-YIAALM-

-YIAALN-
YTIAAL--
-YIAALH-
YEIAAL--
YDIAAL--
YYIAAL--
-YIAALT-
YQIAAL--
-YIAALQ-
YPIAAL--
-YIAALD-
YCIAAL--
-YIAALV-
-YIAALP-
-YIAALA-
ISAL 483 609 31.83
-AISALQ-
--ISALSN
--ISALGS
--ISALAT
--ISALTQ
--ISALTI
--ISALSD
--ISALQR
--ISALQI
--ISALPR
--ISALNV
--ISALNN
--ISALIV
--ISALGG
--ISALEF
--ISALAH
-NISALG-
-RISALG-
-RISALE-
-SISALG-
-TISALP-
-TISALG-
-VISALG-
ASISAL--
-AISALR-
-AISALL-
AGISAL--
-AISALS-
AAISAL--
-AISALN-
ANISAL--
-AISALI-
-AISALV-
AHISAL--

-AISALH-
AVISAL--
AIISAL--
ADISAL--
APISAL--
-AISALA-
AQISAL--
AEISAL--
-AISALE-
CSISAL--
CMISAL--
-CISALR-
-CISALH-
-CISALQ-
-CISALK-
-CISALF-
CFISAL--
-CISALM-
CAISAL--
CYISAL--
-CISALV-
-CISALA-
CHISAL--
CGISAL--
CWISAL--
CLISAL--
CKISAL--
-CISALW-
-CISALS-
-CISALL-
-CISALI-
CEISAL--
CDISAL--
CCISAL--
DSISAL--
DHISAL--
DIISAL--
DVISAL--
DRISAL--
DLISAL--
-DISALS-
-DISALL-
DMISAL--
-DISALT-
-DISALR-
DWISAL--
DGISAL--
DEISAL--
-DISALK-
-DISALI-

DYISAL--
DKISAL--
DPISAL--
-DISALM-
-DISALF-
DCISAL--
DNISAL--
-DISALQ-
-DISALY-
-DISALW-
-DISALN-
-DISALH-
-DISALA-
EGISAL--
ESISAL--
ELISAL--
-EISALL-
EPISAL--
EYISAL--
EVISAL--
-EISALR-
EFISAL--
ETISAL--
-EISALI-
EWISAL--
-EISALV-
-EISALK-
EAISAL--
EMISAL--
-EISALS-
-EISALQ-
-EISALM-
-EISALT-
EHISAL--
EQISAL--
-EISALN-
EIISAL--
EDISAL--
ECISAL--
-EISALA-
ENISAL--
-EISALW-
-EISALH-
EEISAL--
FTISAL--
FRISAL--
FGISAL--
FNISAL--
FDISAL--
FMISAL--

FPISAL--
FAISAL--
FEISAL--
FHISAL--
-FISALN-
-FISALG-
-FISALH-
-FISALC-
FCISAL--
GGISAL--
GSISAL--
GKISAL--
GLISAL--
-GISALT-
GTISAL--
-GISALS-
-GISALQ-
GAISAL--
GMISAL--
-GISALV-
-GISALA-
GQISAL--
-GISALH-
GYISAL--
-GISALK-
GCISAL--
-GISALI-
GDISAL--
GWISAL--
GNISAL--
-GISALW-
-GISALM-
GPISAL--
HGISAL--
HSISAL--
HLISAL--
HTISAL--
-HISALK-
HPISAL--
-HISALS-
-HISALQ-
-HISALI-
-HISALW-
-HISALV-
-HISALT-
HWISAL--
-HISALN-
-HISALF-
HHISAL--
HKISAL--

HDISAL--
-HISALM-
-HISALH-
HIISAL--
HEISAL--
HYISAL--
HQISAL--
HMISAL--
HNISAL--
HFISAL--
--ISALLK
--ISALRG
--ISALRT
--ISALVS
--ISALSS
--ISALLG
--ISALYS
IRISAL--
--ISALMR
--ISALSA
--ISALRN
--ISALNL
--ISALRY
--ISALLT
--ISALVN
--ISALNS
--ISALRV
--ISALRH
--ISALMS
--ISALIS
--ISALSV
--ISALIG
--ISALFS
--ISALLA
--ISALSG
--ISALKS
--ISALFG
--ISALSW
--ISALAS
ITISAL--
--ISALPY
--ISALGY
IGISAL--
ISISAL--
--ISALYG
--ISALVA
--ISALQS
--ISALKA
--ISALFR
--ISALFN

```
--ISALTG
--ISALSH
--ISALMH
--ISALIK
--ISALYN
--ISALWH
--ISALSI
--ISALHW
--ISALES
IMISAL--
IKISAL--
-IISALT-
--ISALVK
--ISALRW
--ISALFH
--ISALLR
--ISALLH
--ISALIT
--ISALGF
IQISAL--
INISAL--
-IISALR-
--ISALWQ
--ISALTV
--ISALSY
--ISALPL
--ISALMT
--ISALKN
--ISALHK
--ISALGM
--ISALWN
--ISALTN
--ISALPF
--ISALNA
--ISALKY
--ISALIN
--ISALAF
-IISALH-
--ISALYQ
--ISALQT
--ISALMN
--ISALKH
--ISALIQ
--ISALFA
--ISALTR
--ISALLN
--ISALHN
IIISAL--
--ISALWR
--ISALWK
```

```
--ISALRE
--ISALLV
--ISALKD
--ISALHQ
--ISALHH
--ISALFQ
--ISALFD
--ISALYD
--ISALSK
--ISALQN
--ISALMY
--ISALMA
--ISALKF
--ISALHY
--ISALAV
-IISALK-
-IISALE-
--ISALVQ
--ISALSQ
--ISALRM
--ISALQA
--ISALNT
--ISALLQ
--ISALHV
--ISALHG
--ISALHE
--ISALAI
-IISALN-
-IISALI-
--ISALWG
--ISALTK
--ISALTF
--ISALQG
--ISALPM
--ISALNY
--ISALNF
--ISALMD
--ISALKQ
--ISALKM
--ISALII
--ISALID
-IISALG-
--ISALWD
--ISALVE
--ISALTM
--ISALTH
--ISALQV
--ISALPI
--ISALNW
--ISALNM
```

--ISALNI
--ISALHT
--ISALHM
--ISALGW
--ISALGA
--ISALAY
-IISALQ-
--ISALWE
--ISALVD
--ISALQY
--ISALQH
--ISALPS
--ISALNQ
--ISALNG
--ISALMQ
--ISALIW
--ISALHD
--ISALGN
--ISALAM
--ISALAA
--ISALWW
--ISALQK
--ISALQF
--ISALNH
--ISALMI
--ISALKP
--ISALKE
--ISALIC
--ISALEA
--ISALDM
--ISALCY
--ISALCQ
--ISALCL
--ISALAN
IHISAL--
KVISAL--
KDISAL--
KTISAL--
KHISAL--
-KISALY-
-KISALS-
-KISALH-
KIISAL--
KPISAL--
KYISAL--
KAISAL--
-KISALI-
-KISALV-
-KISALK-
-KISALF-

KWISAL--
KSISAL--
-KISALW-
-KISALM-
-KISALT-
-KISALQ-
KEISAL--
KQISAL--
KCISAL--
KMISAL--
LSISAL--
LTISAL--
-LISALS-
LGISAL--
LVISAL--
LHISAL--
-LISALN-
LEISAL--
LAISAL--
LPISAL--
LNISAL--
LDISAL--
-LISALG-
-LISALQ-
-LISALE-
LCISAL--
MSISAL--
MKISAL--
MVISAL--
MTISAL--
MHISAL--
-MISALT-
-MISALA-
MAISAL--
-MISALI-
MDISAL--
-MISALK-
-MISALH-
MEISAL--
MIISAL--
-MISALN-
MPISAL--
MQISAL--
-MISALG-
-MISALE-
MMISAL--
-MISALP-
NRISAL--
NLISAL--
-NISALT-

NAISAL--
-NISALS-
NWISAL--
NVISAL--
NHISAL--
NFISAL--
-NISALH-
-NISALM-
NGISAL--
-NISALN-
-NISALI-
NMISAL--
NQISAL--
NPISAL--
NNISAL--
-NISALK-
-NISALA-
NIISAL--
NKISAL--
NDISAL--
NCISAL--
-NISALQ-
PMISAL--
PSISAL--
PRISAL--
PVISAL--
PGISAL--
PEISAL--
-PISALT-
-PISALM-
-PISALK-
PKISAL--
PTISAL--
PDISAL--
-PISALN-
-PISALH-
PHISAL--
PLISAL--
PQISAL--
-PISALQ-
-PISALI-
PCISAL--
-PISALG-
QSISAL--
QRISAL--
QVISAL--
-QISALT-
QGISAL--
-QISALL-
-QISALH-

-QISALR-
QKISAL--
QYISAL--
QPISAL--
-QISALY-
QMISAL--
QLISAL--
-QISALK-
-QISALA-
QTISAL--
-QISALS-
QIISAL--
-QISALV-
-QISALQ-
-QISALN-
-QISALM-
QQISAL--
-QISALF-
QDISAL--
QAISAL--
-QISALI-
-QISALE-
QHISAL--
QCISAL--
-QISALW-
-QISALG-
-RISALS-
-RISALT-
-RISALV-
-RISALY-
-RISALI-
RHISAL--
-RISALH-
-RISALN-
-RISALF-
REISAL--
RQISAL--
-RISALQ-
SSISAL--
-SISALR-
-SISALS-
SYISAL--
SNISAL--
-SISALK-
SKISAL--
SAISAL--
SMISAL--
-SISALN-
SPISAL--
-SISALW-

STISAL--
-SISALV-
SIISAL--
SEISAL--
-SISALQ-
-SISALI-
SDISAL--
-SISALA-
SCISAL--
TGISAL--
-TISALL-
TDISAL--
TPISAL--
TTISAL--
TSISAL--
TAISAL--
-TISALT-
-TISALR-
-TISALK-
TKISAL--
TQISAL--
-TISALQ-
-TISALH-
-TISALS-
TYISAL--
TIISAL--
-TISALM-
TCISAL--
TMISAL--
-TISALN-
-TISALA-
THISAL--
VSISAL--
VDISAL--
VKISAL--
VQISAL--
-VISALQ-
VHISAL--
VGISAL--
VPISAL--
VMISAL--
VAISAL--
VIISAL--
-VISALN-
VCISAL--
WSISAL--
WMISAL--
-WISALK-
-WISALQ-
WAISAL--

```
             -WISALN-
             WDISAL--
             WKISAL--
             WGISAL--
             -WISALH-
             -YISALT-
             YSISAL--
             YKISAL--
             YHISAL--
             YTISAL--
             YNISAL--
             -YISALS-
             YAISAL--
             YRISAL--
             -YISALN-
             YGISAL--
             -YISALQ-
             -YISALH-
             YQISAL--
             YMISAL--
             YDISAL--
             -YISALG-
             -YISALK-
             YPISAL--
             YCISAL--
PANY          232        696      31.80
             --PANYRP
             -VPANYS-
             -APANYT-
             AVPANY--
             ALPANY--
             AAPANY--
             -APANYK-
             -APANYQ-
             -APANYI-
             -APANYL-
             -APANYR-
             -APANYD-
             ADPANY--
             -APANYW-
             -APANYG-
             -APANYA-
             ANPANY--
             -APANYV-
             -APANYN-
             AIPANY--
             AHPANY--
             AQPANY--
             -APANYS-
             -APANYF-
```

-APANYE-
AGPANY--
ATPANY--
ASPANY--
ARPANY--
-APANYC-
AMPANY--
CSPANY--
-CPANYV-
CVPANY--
-CPANYH-
CLPANY--
CGPANY--
CEPANY--
DAPANY--
DRPANY--
DYPANY--
DSPANY--
-DPANYF-
DQPANY--
-DPANYM-
-DPANYK-
DTPANY--
-DPANYY-
DLPANY--
DWPANY--
-DPANYW-
-DPANYT-
-DPANYG-
DNPANY--
DMPANY--
DIPANY--
DHPANY--
DGPANY--
DDPANY--
-EPANYL-
EKPANY--
EVPANY--
EGPANY--
ETPANY--
-EPANYF-
EHPANY--
EWPANY--
EQPANY--
-EPANYR-
-EPANYN-
-EPANYV-
-EPANYT-
-EPANYS-
-EPANYG-

-EPANYA-
EMPANY--
ELPANY--
EIPANY--
-FPANYK-
FMPANY--
FHPANY--
FEPANY--
-FPANYL-
-FPANYM-
-FPANYF-
FYPANY--
FTPANY--
FSPANY--
FRPANY--
FNPANY--
FAPANY--
FPPANY--
-FPANYW-
-FPANYN-
FLPANY--
FDPANY--
FWPANY--
FVPANY--
-FPANYI-
-FPANYH-
FKPANY--
FIPANY--
FGPANY--
FFPANY--
GRPANY--
GMPANY--
GFPANY--
GWPANY--
GKPANY--
-GPANYS-
-GPANYE-
-GPANYA-
-GPANYQ-
-GPANYW-
-GPANYR-
-GPANYL-
-GPANYG-
GIPANY--
GDPANY--
-GPANYH-
-GPANYF-
GLPANY--
GGPANY--
GVPANY--

-GPANYY-
-GPANYV-
-GPANYM-
-GPANYI-
GAPANY--
GYPANY--
GTPANY--
GSPANY--
GQPANY--
-GPANYK-
GHPANY--
GEPANY--
-HPANYM-
HSPANY--
-HPANYL-
HWPANY--
-HPANYT-
-HPANYS-
-HPANYR-
-HPANYN-
-HPANYG-
HGPANY--
HYPANY--
-HPANYI-
HKPANY--
HVPANY--
HTPANY--
HRPANY--
HPPANY--
-HPANYF-
-HPANYE-
HNPANY--
HMPANY--
HFPANY--
HEPANY--
HAPANY--
-IPANYF-
-IPANYI-
IYPANY--
IQPANY--
IRPANY--
-IPANYW-
-IPANYH-
-IPANYA-
ISPANY--
-IPANYK-
-IPANYG-
IGPANY--
ITPANY--
INPANY--

ILPANY--
IIPANY--
IWPANY--
-IPANYV-
-IPANYS-
-IPANYD-
IKPANY--
KSPANY--
-KPANYM-
KVPANY--
-KPANYH-
-KPANYK-
-KPANYG-
-KPANYF-
KTPANY--
-KPANYA-
-KPANYW-
-KPANYT-
-KPANYR-
-KPANYL-
KIPANY--
KAPANY--
KRPANY--
KKPANY--
KGPANY--
-KPANYY-
-KPANYQ-
KFPANY--
KEPANY--
KYPANY--
KWPANY--
KQPANY--
KPPANY--
-KPANYI-
-KPANYE-
-KPANYD-
KNPANY--
KMPANY--
KLPANY--
KHPANY--
-LPANYR-
LSPANY--
LRPANY--
LVPANY--
LTPANY--
LDPANY--
-LPANYM-
LKPANY--
-LPANYV-
-LPANYN-

-LPANYH-
LNPANY--
-LPANYY-
LLPANY--
LHPANY--
LQPANY--
-LPANYT-
-LPANYL-
-LPANYK-
-LPANYG-
LGPANY--
-LPANYW-
LEPANY--
LAPANY--
LWPANY--
-LPANYS-
-LPANYQ-
-LPANYI-
-LPANYA-
LIPANY--
LFPANY--
LCPANY--
-MPANYR-
MNPANY--
MRPANY--
-MPANYS-
-MPANYL-
MSPANY--
MPPANY--
MKPANY--
MYPANY--
MTPANY--
MQPANY--
-MPANYF-
MGPANY--
-MPANYV-
-MPANYM-
MLPANY--
MHPANY--
MWPANY--
MVPANY--
-MPANYQ-
-MPANYI-
-MPANYG-
-MPANYE-
NRPANY--
NGPANY--
-NPANYS-
-NPANYR-
-NPANYL-

NLPANY--
NSPANY--
-NPANYW-
-NPANYN-
-NPANYI-
-NPANYA-
NEPANY--
-NPANYM-
-NPANYY-
-NPANYT-
-NPANYQ-
NKPANY--
-NPANYV-
-NPANYG-
NFPANY--
NDPANY--
--PANYLR
--PANYLV
--PANYFS
--PANYRS
--PANYRL
--PANYIR
--PANYLM
--PANYLL
--PANYTR
--PANYRT
--PANYHQ
--PANYYG
--PANYSS
--PANYRM
--PANYYV
--PANYVS
--PANYVL
--PANYSL
--PANYSA
--PANYMS
--PANYLG
--PANYKS
--PANYSR
--PANYRV
--PANYRQ
--PANYQT
--PANYQS
--PANYHV
--PANYGS
--PANYWL
--PANYVR
--PANYVM
--PANYTT
--PANYSW

--PANYSN
--PANYRD
--PANYMG
--PANYER
--PANYEK
--PANYAW
--PANYWK
--PANYTN
--PANYTL
--PANYTK
--PANYRN
--PANYMY
--PANYIS
--PANYHT
PTPANY--
PAPANY--
--PANYYL
--PANYWT
--PANYWG
--PANYWA
--PANYVA
--PANYTY
--PANYQR
--PANYNV
--PANYMR
--PANYMI
--PANYLW
--PANYLS
--PANYHL
--PANYFN
--PANYFL
--PANYAR
--PANYSQ
--PANYRY
--PANYRK
--PANYRI
--PANYRF
--PANYRE
--PANYRA
--PANYQL
--PANYQI
--PANYQA
--PANYNW
--PANYLQ
--PANYLH
--PANYLE
--PANYKR
--PANYIQ
--PANYIH
--PANYEW

--PANYAV
--PANYAL
--PANYAH
--PANYAG
--PANYAF
PNPANY--
--PANYYT
--PANYYM
--PANYYH
--PANYWV
--PANYVQ
--PANYVG
--PANYVF
--PANYTI
--PANYSY
--PANYSK
--PANYSI
--PANYSG
--PANYSD
--PANYNS
--PANYMT
--PANYMF
--PANYLN
--PANYKT
--PANYKQ
--PANYIE
--PANYHY
--PANYHE
--PANYGV
--PANYFY
--PANYFR
--PANYFI
--PANYAT
PVPANY--
PSPANY--
PRPANY--
PQPANY--
PLPANY--
PHPANY--
--PANYYR
--PANYYA
--PANYWE
--PANYVW
--PANYVV
--PANYVH
--PANYTV
--PANYSM
--PANYSE
--PANYRW
--PANYRG

--PANYQQ
--PANYQK
--PANYQH
--PANYMQ
--PANYMA
--PANYLI
--PANYKW
--PANYKN
--PANYKF
--PANYKD
--PANYKA
--PANYIM
--PANYIF
--PANYIA
--PANYHS
--PANYHN
--PANYGW
--PANYGF
--PANYFV
--PANYFT
--PANYFG
--PANYDS
--PANYAK
--PANYAI
PWPANY--
-PPANYM-
PIPANY--
PGPANY--
PFPANY--
--PANYYK
--PANYWN
--PANYVY
--PANYTS
--PANYTH
--PANYTG
--PANYTF
--PANYTE
--PANYST
--PANYSH
--PANYSF
--PANYQY
--PANYQV
--PANYQN
--PANYQG
--PANYNT
--PANYNN
--PANYNI
--PANYNF
--PANYNA
--PANYMW

--PANYMM
--PANYME
--PANYLK
--PANYKL
--PANYIV
--PANYIT
--PANYIK
--PANYIG
--PANYID
--PANYGT
--PANYGH
--PANYGA
--PANYFQ
--PANYFK
--PANYFH
--PANYFF
--PANYFE
--PANYFD
--PANYEN
--PANYEI
--PANYEG
--PANYEF
--PANYED
--PANYDV
--PANYDT
--PANYCW
--PANYCF
--PANYAN
--PANYAE
--PANYAD
--PANYAA
-QPANYL-
QRPANY--
QSPANY--
QWPANY--
-QPANYT-
-QPANYR-
-QPANYM-
QLPANY--
QTPANY--
-QPANYN-
QPPANY--
-QPANYW-
-QPANYH-
-QPANYD-
QGPANY--
-QPANYS-
-QPANYA-
QNPANY--
QIPANY--

-RPANYL-
RLPANY--
RWPANY--
-RPANYY-
-RPANYS-
RKPANY--
RAPANY--
RRPANY--
-RPANYA-
REPANY--
-RPANYV-
-RPANYQ-
-RPANYG-
RNPANY--
RTPANY--
RPPANY--
-RPANYT-
-RPANYH-
-RPANYE-
RGPANY--
RYPANY--
-RPANYW-
RIPANY--
-RPANYN-
-RPANYK-
-RPANYF-
RQPANY--
-RPANYM-
-RPANYD-
RMPANY--
RDPANY--
SRPANY--
SVPANY--
-SPANYV-
-SPANYL-
-SPANYF-
SAPANY--
SLPANY--
-SPANYI-
SNPANY--
SGPANY--
-SPANYA-
SIPANY--
SWPANY--
SSPANY--
-SPANYQ-
-SPANYD-
SPPANY--
-SPANYT-
-SPANYS-

```
-SPANYM-
SMPANY--
SHPANY--
SFPANY--
SYPANY--
STPANY--
-SPANYY-
-SPANYG-
-SPANYN-
-SPANYH-
-SPANYE-
SKPANY--
SEPANY--
-SPANYW-
SDPANY--
TGPANY--
TTPANY--
TQPANY--
-TPANYL-
TSPANY--
-TPANYQ-
TLPANY--
-TPANYV-
TPPANY--
-TPANYY-
-TPANYI-
-TPANYS-
-TPANYR-
-TPANYM-
-TPANYG-
TKPANY--
TAPANY--
TVPANY--
TRPANY--
-TPANYH-
TNPANY--
TDPANY--
TYPANY--
TWPANY--
-TPANYT-
-TPANYN-
-TPANYF-
TMPANY--
TIPANY--
THPANY--
TFPANY--
-VPANYR-
VRPANY--
VWPANY--
-VPANYL-
```

-VPANYH-
-VPANYM-
VLPANY--
VAPANY--
VTPANY--
VSPANY--
VQPANY--
-VPANYV-
-VPANYI-
VIPANY--
-VPANYT-
VKPANY--
VPPANY--
-VPANYY-
-VPANYG-
-VPANYF-
-VPANYD-
VMPANY--
VHPANY--
-VPANYQ-
-VPANYN-
-VPANYK-
-VPANYE-
VGPANY--
VFPANY--
VCPANY--
-WPANYL-
WYPANY--
WWPANY--
WTPANY--
WHPANY--
-WPANYY-
-WPANYR-
-WPANYT-
-WPANYI-
-WPANYA-
WMPANY--
WLPANY--
WGPANY--
WEPANY--
-WPANYS-
-WPANYQ-
WFPANY--
WDPANY--
-YPANYR-
-YPANYL-
YTPANY--
-YPANYF-
YRPANY--
YQPANY--

```
           -YPANYQ-
           YLPANY--
           YDPANY--
           YSPANY--
           -YPANYY-
           -YPANYT-
           YKPANY--
           YAPANY--
           YWPANY--
           -YPANYE-
           -YPANYA-
           YFPANY--
           YYPANY--
           -YPANYV-
           -YPANYN-
           -YPANYM-
           -YPANYI-
           YMPANY--
           YIPANY--
           YHPANY--
           YEPANY--
           YCPANY--
PMAI        420       626     31.78
           --PMAIAR
           -APMAIS-
           -APMAIR-
           APPMAI--
           -APMAIT-
           -APMAIA-
           AFPMAI--
           -APMAIY-
           -APMAIW-
           -APMAIM-
           -APMAIF-
           ANPMAI--
           AYPMAI--
           AWPMAI--
           ARPMAI--
           ADPMAI--
           AVPMAI--
           -APMAIN-
           -APMAIL-
           -APMAII-
           ALPMAI--
           AKPMAI--
           AHPMAI--
           AEPMAI--
           ATPMAI--
           ASPMAI--
           -APMAIV-
```

-APMAIK-
AIPMAI--
-CPMAIM-
-CPMAIS-
CHPMAI--
CVPMAI--
CRPMAI--
CGPMAI--
-DPMAIR-
DYPMAI--
DRPMAI--
-DPMAIW-
DHPMAI--
-DPMAII-
DMPMAI--
DIPMAI--
DAPMAI--
DWPMAI--
DVPMAI--
DTPMAI--
-DPMAIH-
-DPMAIF-
DGPMAI--
DCPMAI--
-EPMAIK-
-EPMAIF-
ELPMAI--
ETPMAI--
-EPMAIR-
-EPMAIN-
EAPMAI--
EYPMAI--
ESPMAI--
ERPMAI--
EQPMAI--
-EPMAIY-
-EPMAII-
EEPMAI--
FRPMAI--
-FPMAIR-
-FPMAIS-
FPPMAI--
FMPMAI--
-FPMAIT-
-FPMAIK-
-FPMAIA-
FLPMAI--
FIPMAI--
FVPMAI--
FSPMAI--

-FPMAIV-
FYPMAI--
FNPMAI--
FHPMAI--
-FPMAIY-
-FPMAIW-
-FPMAIQ-
-FPMAII-
FEPMAI--
GRPMAI--
GYPMAI--
-GPMAIV-
-GPMAIL-
GLPMAI--
GGPMAI--
GSPMAI--
GPPMAI--
-GPMAIW-
-GPMAIR-
-GPMAII-
GIPMAI--
-GPMAIA-
GDPMAI--
GQPMAI--
-GPMAIT-
-GPMAIF-
GNPMAI--
GKPMAI--
GFPMAI--
GAPMAI--
GVPMAI--
GTPMAI--
-GPMAIY-
-GPMAIS-
-GPMAIN-
-GPMAIK-
-GPMAIG-
-GPMAIE-
GEPMAI--
GCPMAI--
HVPMAI--
HRPMAI--
HKPMAI--
HNPMAI--
HWPMAI--
-HPMAIL-
HHPMAI--
-HPMAIA-
-HPMAIR-
-HPMAIQ-

HTPMAI--
-HPMAIV-
-HPMAIS-
-HPMAIK-
HLPMAI--
HIPMAI--
HCPMAI--
HAPMAI--
IRPMAI--
ISPMAI--
-IPMAIL-
INPMAI--
-IPMAIV-
ILPMAI--
IQPMAI--
-IPMAIR-
-IPMAIQ-
-IPMAII-
IDPMAI--
-IPMAIT-
-IPMAIA-
IYPMAI--
IVPMAI--
-IPMAIW-
-IPMAIS-
-IPMAIH-
IMPMAI--
IHPMAI--
IFPMAI--
IAPMAI--
-KPMAIS-
KLPMAI--
-KPMAIR-
-KPMAIK-
-KPMAIY-
-KPMAIM-
-KPMAIL-
-KPMAIT-
KQPMAI--
KFPMAI--
KYPMAI--
-KPMAIF-
KVPMAI--
KTPMAI--
KRPMAI--
KPPMAI--
-KPMAIV-
-KPMAIG-
KDPMAI--
-KPMAIQ-

-KPMAIH-
-KPMAIE-
KIPMAI--
KHPMAI--
KGPMAI--
KEPMAI--
KAPMAI--
KWPMAI--
-KPMAIN-
-KPMAII-
KCPMAI--
LNPMAI--
LGPMAI--
LVPMAI--
-LPMAIY-
-LPMAIR-
-LPMAIK-
LSPMAI--
-LPMAIS-
LIPMAI--
LQPMAI--
-LPMAIV-
-LPMAIT-
LLPMAI--
LFPMAI--
LDPMAI--
LWPMAI--
LEPMAI--
-LPMAIM-
-LPMAII-
-LPMAIH-
LPPMAI--
-LPMAIW-
-LPMAIQ-
-LPMAIA-
LHPMAI--
LCPMAI--
MTPMAI--
MQPMAI--
MKPMAI--
MNPMAI--
MLPMAI--
MSPMAI--
-MPMAIR-
-MPMAIM-
MYPMAI--
-MPMAIL-
MHPMAI--
MGPMAI--
-MPMAIH-

MIPMAI--
MDPMAI--
MVPMAI--
-MPMAIS-
-MPMAIQ-
-MPMAIK-
-MPMAII-
-MPMAIE-
NGPMAI--
NLPMAI--
-NPMAIR-
NRPMAI--
NTPMAI--
NPPMAI--
-NPMAIW-
NQPMAI--
NAPMAI--
NYPMAI--
-NPMAIS-
-NPMAII-
NNPMAI--
NVPMAI--
-NPMAIY-
NDPMAI--
NSPMAI--
-NPMAIM-
-NPMAIE-
NEPMAI--
NCPMAI--
--PMAIRA
--PMAISS
--PMAIFS
--PMAINS
--PMAIWS
--PMAIAF
--PMAILR
--PMAIAT
--PMAIWG
--PMAIQM
--PMAIYK
--PMAIVS
--PMAIQH
--PMAILK
--PMAIKG
--PMAIFR
--PMAIFH
--PMAIYS
--PMAITA
--PMAISV
--PMAIRG

--PMAIQW
--PMAIQG
--PMAILS
--PMAIGY
PIPMAI--
--PMAIYG
--PMAIVE
--PMAIRY
--PMAIRF
--PMAINY
--PMAIIT
--PMAIVG
--PMAIVD
--PMAITI
--PMAISR
--PMAIRT
--PMAIRI
--PMAINL
--PMAIMK
--PMAIME
--PMAILY
--PMAIKR
--PMAIKL
--PMAIWN
--PMAITS
--PMAITN
--PMAIST
--PMAISM
--PMAISG
--PMAIRQ
--PMAIQS
--PMAINQ
--PMAILG
--PMAIKY
--PMAIKF
--PMAIKA
--PMAIGG
--PMAIFF
PEPMAI--
PTPMAI--
PQPMAI--
PNPMAI--
--PMAIYY
--PMAIWT
--PMAIWF
--PMAIVW
--PMAIVR
--PMAIVH
--PMAITD
--PMAISY

--PMAISA
--PMAIRV
--PMAIRM
--PMAIQR
--PMAIQL
--PMAINT
--PMAIMN
--PMAILT
--PMAILD
--PMAIIW
--PMAIIN
--PMAIIK
--PMAIII
--PMAIIG
--PMAIID
--PMAIHG
--PMAIHD
--PMAIGV
--PMAIGI
--PMAIES
PYPMAI--
--PMAIWH
--PMAIWE
--PMAIVV
--PMAIVI
--PMAITT
--PMAITE
--PMAISK
--PMAIRK
--PMAIRH
--PMAIRD
--PMAIQY
--PMAIPI
--PMAIPF
--PMAINW
--PMAINR
--PMAIMV
--PMAIMT
--PMAIMQ
--PMAIML
--PMAIMA
--PMAILH
--PMAILE
--PMAILA
--PMAIKV
--PMAIKT
--PMAIKI
--PMAIKE
--PMAIIY
--PMAIIS

--PMAIHT
--PMAIHS
--PMAIHL
--PMAIHK
--PMAIGW
--PMAIGS
--PMAIFW
--PMAIFK
--PMAIEG
--PMAIAG
PGPMAI--
PFPMAI--
PAPMAI--
PMPMAI--
--PMAIYV
--PMAIYM
--PMAIWV
--PMAIVK
--PMAITY
--PMAITV
--PMAITR
--PMAITQ
--PMAITM
--PMAITL
--PMAITK
--PMAISQ
--PMAISN
--PMAISH
--PMAISF
--PMAIQV
--PMAIQN
--PMAIQK
--PMAIQI
--PMAIQD
--PMAIQA
--PMAIPY
--PMAINV
--PMAINI
--PMAINF
--PMAIMR
--PMAIMH
--PMAILN
--PMAIKQ
--PMAIKM
--PMAIKD
--PMAIIV
--PMAIIM
--PMAIIL
--PMAIIE
--PMAIHM

--PMAIHI
--PMAIHH
--PMAIHA
--PMAIGM
--PMAIGH
--PMAIGA
--PMAIFT
--PMAIFQ
--PMAIFG
--PMAIFA
--PMAIEY
--PMAIEI
--PMAIEA
--PMAICT
--PMAICS
--PMAICA
--PMAIAM
PHPMAI--
PCPMAI--
QIPMAI--
QVPMAI--
QSPMAI--
-QPMAIW-
-QPMAIL-
-QPMAIK-
-QPMAIH-
-QPMAIA-
QHPMAI--
QLPMAI--
QKPMAI--
QTPMAI--
QQPMAI--
-QPMAIY-
-QPMAIV-
-QPMAIT-
-QPMAIR-
-QPMAIN-
QMPMAI--
QGPMAI--
QPPMAI--
-QPMAIS-
-QPMAII-
-QPMAIF-
-QPMAIE-
QEPMAI--
QDPMAI--
RFPMAI--
RVPMAI--
-RPMAIL-
RWPMAI--

-RPMAIS-
-RPMAIR-
-RPMAIW-
-RPMAIM-
-RPMAIT-
-RPMAIQ-
-RPMAII-
RAPMAI--
RLPMAI--
RSPMAI--
-RPMAIN-
-RPMAIK-
RHPMAI--
RQPMAI--
RPPMAI--
-RPMAIH-
-RPMAIA-
RMPMAI--
RYPMAI--
RTPMAI--
-RPMAIV-
RCPMAI--
RNPMAI--
REPMAI--
-RPMAIF-
-RPMAIG-
-RPMAIE-
-SPMAIR-
-SPMAIS-
SRPMAI--
SWPMAI--
-SPMAIL-
SYPMAI--
SQPMAI--
-SPMAIT-
SLPMAI--
-SPMAII-
SVPMAI--
-SPMAIK-
SMPMAI--
SDPMAI--
SAPMAI--
SKPMAI--
SSPMAI--
-SPMAIV-
-SPMAIA-
SNPMAI--
STPMAI--
-SPMAIF-
SIPMAI--

-SPMAIY-
-SPMAIH-
SFPMAI--
SPPMAI--
-SPMAIQ-
-SPMAIN-
-TPMAIR-
TQPMAI--
-TPMAIH-
TMPMAI--
TLPMAI--
TPPMAI--
TTPMAI--
TSPMAI--
-TPMAIV-
-TPMAIS-
-TPMAIW-
-TPMAIL-
-TPMAIA-
TNPMAI--
TFPMAI--
TVPMAI--
-TPMAII-
TIPMAI--
TGPMAI--
TDPMAI--
TYPMAI--
TRPMAI--
-TPMAIT-
-TPMAIM-
-TPMAIK-
-TPMAIE-
THPMAI--
TAPMAI--
VRPMAI--
VLPMAI--
-VPMAIS-
VQPMAI--
-VPMAIW-
-VPMAIR-
VSPMAI--
-VPMAIF-
VKPMAI--
VEPMAI--
VAPMAI--
-VPMAIA-
-VPMAIT-
VIPMAI--
VVPMAI--
VHPMAI--

VGPMAI--
VWPMAI--
-VPMAIV-
-VPMAIK-
-VPMAII-
-VPMAIG-
VTPMAI--
VPPMAI--
-VPMAIQ-
-VPMAIM-
-VPMAIH-
VMPMAI--
WQPMAI--
WLPMAI--
WWPMAI--
WTPMAI--
WSPMAI--
-WPMAIL-
-WPMAIV-
-WPMAIS-
-WPMAIR-
-WPMAIM-
-WPMAIK-
-WPMAIH-
-WPMAIA-
WMPMAI--
WHPMAI--
WDPMAI--
WCPMAI--
WAPMAI--
YWPMAI--
-YPMAIR-
YVPMAI--
YRPMAI--
-YPMAII-
YYPMAI--
YSPMAI--
YLPMAI--
YDPMAI--
YQPMAI--
-YPMAIK-
-YPMAIQ-
-YPMAIM-
-YPMAIA-
YNPMAI--
YKPMAI--
YIPMAI--
YFPMAI--
YEPMAI--

PSRL 573 586 31.77

DMPSRL--
-EPSRLD-
-HPSRLG-
-LPSRLG-
-NPSRLY-
-NPSRLA-
-NPSRLW-
--PSRLLI
--PSRLSD
--PSRLCG
--PSRLYD
--PSRLWQ
--PSRLRP
--PSRLPN
--PSRLNY
--PSRLNH
--PSRLKI
--PSRLKH
--PSRLKD
--PSRLGY
--PSRLGW
--PSRLGV
--PSRLGR
--PSRLFE
--PSRLCT
--PSRLCN
--PSRLAY
-RPSRLP-
-WPSRLG-
-YPSRLW-
-YPSRLG-
-APSRLR-
ALPSRL--
-APSRLL-
-APSRLS-
ASPSRL--
ARPSRL--
ATPSRL--
-APSRLY-
AIPSRL--
-APSRLI-
AAPSRL--
-APSRLH-
-APSRLK-
-APSRLG-
AWPSRL--
-APSRLV-
-APSRLF-
AGPSRL--
-APSRLN-

−APSRLE−
−APSRLM−
AHPSRL−−
APPSRL−−
−APSRLW−
ADPSRL−−
ACPSRL−−
CRPSRL−−
−CPSRLL−
CKPSRL−−
−CPSRLT−
CYPSRL−−
CLPSRL−−
CEPSRL−−
CAPSRL−−
CSPSRL−−
CCPSRL−−
CQPSRL−−
CMPSRL−−
−CPSRLV−
−CPSRLS−
−CPSRLR−
CPPSRL−−
CNPSRL−−
CVPSRL−−
−CPSRLM−
−CPSRLI−
−CPSRLA−
−CPSRLQ−
DVPSRL−−
DYPSRL−−
DLPSRL−−
−DPSRLT−
−DPSRLI−
DTPSRL−−
DKPSRL−−
−DPSRLS−
DNPSRL−−
DAPSRL−−
−DPSRLV−
DIPSRL−−
DGPSRL−−
−DPSRLY−
DEPSRL−−
−DPSRLF−
−DPSRLE−
DQPSRL−−
DDPSRL−−
DCPSRL−−
−DPSRLW−

-DPSRLM-
-DPSRLK-
-DPSRLH-
-DPSRLA-
-EPSRLF-
ESPSRL--
-EPSRLL-
ERPSRL--
EKPSRL--
-EPSRLS-
-EPSRLV-
EMPSRL--
EAPSRL--
EVPSRL--
ETPSRL--
-EPSRLM-
-EPSRLA-
EIPSRL--
EQPSRL--
-EPSRLT-
-EPSRLI-
-EPSRLG-
ELPSRL--
-EPSRLY-
EFPSRL--
-EPSRLH-
ECPSRL--
EWPSRL--
EEPSRL--
-EPSRLW-
-EPSRLE-
EHPSRL--
EDPSRL--
EPPSRL--
-FPSRLL-
FSPSRL--
-FPSRLT-
FVPSRL--
-FPSRLV-
FGPSRL--
FWPSRL--
-FPSRLQ-
FEPSRL--
FMPSRL--
FFPSRL--
FQPSRL--
-FPSRLH-
FDPSRL--
FYPSRL--
-FPSRLY-

−FPSRLA−
−FPSRLM−
−FPSRLE−
FCPSRL−−
−FPSRLW−
−FPSRLC−
−GPSRLR−
GFPSRL−−
GTPSRL−−
−GPSRLA−
−GPSRLV−
GWPSRL−−
GVPSRL−−
GMPSRL−−
GKPSRL−−
GQPSRL−−
GAPSRL−−
−GPSRLK−
−GPSRLS−
−GPSRLM−
GIPSRL−−
GDPSRL−−
−GPSRLH−
−GPSRLW−
−GPSRLF−
GPPSRL−−
GNPSRL−−
−GPSRLE−
GCPSRL−−
GHPSRL−−
−GPSRLN−
−GPSRLG−
−HPSRLL−
HLPSRL−−
HTPSRL−−
−HPSRLQ−
−HPSRLM−
HEPSRL−−
HSPSRL−−
−HPSRLY−
−HPSRLT−
−HPSRLK−
HYPSRL−−
−HPSRLF−
HNPSRL−−
HHPSRL−−
−HPSRLW−
HIPSRL−−
−HPSRLH−
HMPSRL−−

HDPSRL--
-HPSRLE-
-HPSRLN-
HCPSRL--
-IPSRLS-
ITPSRL--
IIPSRL--
INPSRL--
ISPSRL--
-IPSRLQ-
-IPSRLK-
IGPSRL--
-IPSRLY-
-IPSRLI-
-IPSRLV-
IDPSRL--
-IPSRLH-
IFPSRL--
IQPSRL--
-IPSRLG-
-IPSRLN-
IWPSRL--
-IPSRLM-
ICPSRL--
-IPSRLW-
-IPSRLE-
-IPSRLC-
KIPSRL--
-KPSRLY-
KFPSRL--
KMPSRL--
-KPSRLV-
-KPSRLM-
-KPSRLW-
-KPSRLQ-
KHPSRL--
KEPSRL--
KTPSRL--
-KPSRLH-
KNPSRL--
KDPSRL--
KAPSRL--
KQPSRL--
-KPSRLF-
KPPSRL--
-KPSRLN-
-KPSRLP-
LPPSRL--
LGPSRL--
-LPSRLQ-

LQPSRL--
-LPSRLF-
-LPSRLW-
-LPSRLE-
-LPSRLA-
-LPSRLH-
-LPSRLC-
MLPSRL--
-MPSRLS-
-MPSRLT-
MYPSRL--
-MPSRLQ-
-MPSRLI-
MMPSRL--
MGPSRL--
MAPSRL--
MKPSRL--
-MPSRLV-
-MPSRLE-
MNPSRL--
-MPSRLK-
MQPSRL--
MHPSRL--
-MPSRLH-
-MPSRLF-
-MPSRLA-
MIPSRL--
MCPSRL--
-MPSRLW-
MDPSRL--
-MPSRLM-
-MPSRLC-
-NPSRLL-
-NPSRLS-
NYPSRL--
-NPSRLT-
NTPSRL--
NKPSRL--
-NPSRLI-
NPPSRL--
NHPSRL--
NIPSRL--
NNPSRL--
NEPSRL--
-NPSRLK-
NQPSRL--
NCPSRL--
-NPSRLF-
-NPSRLH-
-NPSRLE-

--PSRLYS
--PSRLRT
PLPSRL--
--PSRLMS
--PSRLFS
--PSRLVQ
--PSRLLN
--PSRLTS
--PSRLSM
--PSRLSV
--PSRLVH
--PSRLQT
--PSRLGS
--PSRLTG
--PSRLAS
--PSRLYG
--PSRLSH
--PSRLMF
--PSRLVI
--PSRLTT
--PSRLLQ
--PSRLKS
--PSRLHG
--PSRLVM
--PSRLTK
--PSRLNS
--PSRLSQ
--PSRLLV
--PSRLIS
--PSRLYA
--PSRLVD
--PSRLQY
--PSRLIN
--PSRLVF
--PSRLRF
--PSRLWT
--PSRLKV
--PSRLRW
--PSRLFM
--PSRLYQ
--PSRLQS
--PSRLIH
--PSRLFA
PGPSRL--
PAPSRL--
--PSRLQM
--PSRLMG
--PSRLMA
--PSRLLM
--PSRLFG

PNPSRL--
--PSRLIF
--PSRLWY
--PSRLHS
--PSRLRD
--PSRLMM
--PSRLDS
--PSRLYF
--PSRLWN
--PSRLTE
--PSRLMN
--PSRLGA
--PSRLFW
--PSRLYN
--PSRLLD
--PSRLAW
--PSRLAT
PIPSRL--
--PSRLWH
--PSRLTI
--PSRLTH
--PSRLQW
--PSRLQR
--PSRLET
--PSRLTW
--PSRLMD
--PSRLKT
--PSRLIQ
PKPSRL--
--PSRLYT
--PSRLHA
--PSRLEA
PHPSRL--
--PSRLYI
--PSRLMK
--PSRLLE
--PSRLHY
--PSRLHT
--PSRLQK
--PSRLMW
--PSRLIM
--PSRLFQ
--PSRLEW
PYPSRL--
--PSRLQA
--PSRLEY
--PSRLEN
--PSRLDA
--PSRLME
--PSRLEF

PFPSRL--
--PSRLTD
--PSRLQQ
--PSRLNG
--PSRLMY
--PSRLKN
--PSRLSE
--PSRLKM
--PSRLEV
--PSRLEM
--PSRLWM
--PSRLWK
--PSRLNL
--PSRLKW
--PSRLIE
--PSRLAN
PEPSRL--
PDPSRL--
--PSRLWI
--PSRLQN
--PSRLQF
--PSRLHN
--PSRLEH
--PSRLDG
--PSRLWW
--PSRLNW
--PSRLHM
--PSRLHH
--PSRLGK
--PSRLEI
--PSRLQH
--PSRLEK
--PSRLCR
--PSRLCA
--PSRLQD
--PSRLNV
--PSRLHQ
--PSRLEQ
--PSRLDD
--PSRLCV
--PSRLCS
--PSRLCM
-PPSRLF-
-QPSRLL-
-QPSRLR-
-QPSRLW-
-QPSRLK-
-QPSRLT-
QAPSRL--
-QPSRLS-

QLPSRL--
QFPSRL--
QTPSRL--
-QPSRLV-
-QPSRLY-
QKPSRL--
QYPSRL--
QIPSRL--
-QPSRLF-
QGPSRL--
-QPSRLE-
QPPSRL--
-QPSRLH-
QNPSRL--
-QPSRLM-
QEPSRL--
QHPSRL--
QDPSRL--
-QPSRLA-
QQPSRL--
-QPSRLN-
RIPSRL--
-RPSRLT-
RAPSRL--
RTPSRL--
-RPSRLI-
-RPSRLM-
RDPSRL--
RNPSRL--
-RPSRLQ-
-RPSRLF-
-RPSRLH-
RQPSRL--
-RPSRLE-
-RPSRLY-
RHPSRL--
-RPSRLC-
-SPSRLR-
-SPSRLT-
SYPSRL--
-SPSRLK-
SAPSRL--
SIPSRL--
SWPSRL--
-SPSRLA-
SNPSRL--
-SPSRLQ-
-SPSRLF-
-SPSRLH-
SEPSRL--

−SPSRLE−
SCPSRL−−
−SPSRLC−
−TPSRLV−
−TPSRLT−
TKPSRL−−
TGPSRL−−
TFPSRL−−
TYPSRL−−
TWPSRL−−
TMPSRL−−
TIPSRL−−
THPSRL−−
TAPSRL−−
−TPSRLH−
−TPSRLA−
−TPSRLY−
TNPSRL−−
−TPSRLK−
−TPSRLM−
−TPSRLE−
TCPSRL−−
TQPSRL−−
−TPSRLN−
−VPSRLS−
VRPSRL−−
VWPSRL−−
−VPSRLT−
VSPSRL−−
VAPSRL−−
−VPSRLE−
VQPSRL−−
VPPSRL−−
VYPSRL−−
−VPSRLV−
VNPSRL−−
−VPSRLQ−
−VPSRLH−
−VPSRLW−
VDPSRL−−
−VPSRLM−
VHPSRL−−
−VPSRLN−
VEPSRL−−
VCPSRL−−
−VPSRLC−
−WPSRLT−
−WPSRLL−
WDPSRL−−
WTPSRL−−

WGPSRL--
WKPSRL--
WHPSRL--
-WPSRLI-
-WPSRLQ-
WAPSRL--
WPPSRL--
WQPSRL--
WWPSRL--
WEPSRL--
-WPSRLY-
-WPSRLE-
WCPSRL--
-WPSRLC-
-YPSRLK-
YVPSRL--
-YPSRLR-
YRPSRL--
-YPSRLI-
YEPSRL--
-YPSRLV-
YKPSRL--
-YPSRLM-
YHPSRL--
YTPSRL--
-YPSRLE-
YDPSRL--
YNPSRL--
YIPSRL--
YGPSRL--
YFPSRL--
YQPSRL--
-YPSRLQ-
-YPSRLA-
YCPSRL--
-YPSRLC-

| | | | |
|---|---|---|---|
| VSGL | 315 | 661 | 31.77 |

-AVSGLE-
-DVSGLN-
-DVSGLQ-
-EVSGLQ-
-EVSGLN-
-EVSGLM-
-FVSGLP-
-GVSGLC-
-IVSGLG-
-KVSGLA-
-LVSGLD-
-MVSGLD-
-NVSGLP-

-NVSGLG-
-QVSGLG-
-RVSGLE-
-RVSGLD-
-SVSGLA-
--VSGLYL
--VSGLVK
--VSGLSM
--VSGLSH
--VSGLGL
-VVSGLD-
--VSGLVE
--VSGLTY
--VSGLTM
--VSGLSQ
--VSGLSP
--VSGLNV
--VSGLNR
--VSGLNK
--VSGLLQ
--VSGLKI
--VSGLGT
-VVSGLE-
--VSGLTQ
--VSGLTH
--VSGLQK
--VSGLPT
--VSGLNQ
--VSGLNI
--VSGLML
--VSGLME
--VSGLLI
--VSGLKQ
--VSGLKD
--VSGLIR
--VSGLIP
--VSGLIK
--VSGLIH
--VSGLGV
--VSGLGM
--VSGLGA
--VSGLFL
--VSGLCS
--VSGLAV
--VSGLAF
-WVSGLD-
AVVSGL--
-AVSGLK-
ASVSGL--
AYVSGL--

ALVSGL--
AWVSGL--
-AVSGLS-
AAVSGL--
-AVSGLY-
ANVSGL--
ATVSGL--
AQVSGL--
-AVSGLV-
AGVSGL--
-AVSGLF-
AMVSGL--
-AVSGLQ-
AFVSGL--
-AVSGLH-
-AVSGLN-
ADVSGL--
AEVSGL--
-AVSGLA-
-AVSGLW-
-AVSGLC-
CRVSGL--
-CVSGLR-
-CVSGLM-
-CVSGLT-
CWVSGL--
-CVSGLH-
CTVSGL--
CAVSGL--
-CVSGLW-
-CVSGLL-
CMVSGL--
CLVSGL--
CKVSGL--
CYVSGL--
-CVSGLQ-
-CVSGLK-
CPVSGL--
CDVSGL--
-CVSGLY-
-CVSGLF-
CNVSGL--
CVVSGL--
-CVSGLI-
CQVSGL--
CFVSGL--
CEVSGL--
-CVSGLN-
CHVSGL--
DRVSGL--

DLVSGL--
DSVSGL--
-DVSGLR-
DGVSGL--
DKVSGL--
DYVSGL--
DWVSGL--
-DVSGLW-
DAVSGL--
DPVSGL--
DNVSGL--
DFVSGL--
DQVSGL--
DHVSGL--
DMVSGL--
DIVSGL--
-DVSGLS-
-DVSGLL-
-DVSGLK-
-DVSGLF-
-DVSGLH-
-DVSGLV-
DDVSGL--
-DVSGLM-
DCVSGL--
DEVSGL--
ERVSGL--
EVVSGL--
-EVSGLT-
-EVSGLW-
ESVSGL--
EMVSGL--
EGVSGL--
ENVSGL--
-EVSGLL-
EQVSGL--
EAVSGL--
EFVSGL--
-EVSGLV-
EHVSGL--
-EVSGLS-
-EVSGLI-
-EVSGLH-
EYVSGL--
-EVSGLY-
-EVSGLK-
EIVSGL--
-EVSGLF-
EDVSGL--
ECVSGL--

-FVSGLT-
FVVSGL--
FGVSGL--
-FVSGLL-
-FVSGLK-
-FVSGLV-
FTVSGL--
-FVSGLN-
FQVSGL--
-FVSGLH-
FEVSGL--
-FVSGLY-
-FVSGLQ-
-FVSGLW-
-FVSGLG-
FPVSGL--
FHVSGL--
FDVSGL--
FIVSGL--
-FVSGLE-
FCVSGL--
-FVSGLC-
GTVSGL--
GWVSGL--
-GVSGLY-
-GVSGLH-
-GVSGLS-
GPVSGL--
-GVSGLT-
GFVSGL--
GYVSGL--
GHVSGL--
GDVSGL--
-GVSGLV-
GQVSGL--
-GVSGLW-
GEVSGL--
GAVSGL--
-GVSGLF-
-GVSGLM-
-GVSGLN-
-GVSGLQ-
GCVSGL--
-HVSGLS-
-HVSGLF-
HLVSGL--
HSVSGL--
HWVSGL--
HFVSGL--
HGVSGL--

-HVSGLW-
-HVSGLV-
-HVSGLK-
HAVSGL--
HKVSGL--
-HVSGLI-
HTVSGL--
-HVSGLA-
-HVSGLT-
-HVSGLN-
HNVSGL--
HMVSGL--
HDVSGL--
-HVSGLM-
-HVSGLH-
HHVSGL--
-HVSGLQ-
HQVSGL--
HYVSGL--
HEVSGL--
HCVSGL--
-HVSGLC-
IRVSGL--
ISVSGL--
IVVSGL--
IGVSGL--
-IVSGLS-
-IVSGLL-
-IVSGLH-
-IVSGLV-
INVSGL--
-IVSGLK-
IKVSGL--
IQVSGL--
-IVSGLY-
-IVSGLM-
IIVSGL--
-IVSGLQ-
-IVSGLF-
IAVSGL--
IYVSGL--
-IVSGLW-
IPVSGL--
IMVSGL--
-IVSGLN-
IDVSGL--
IEVSGL--
IFVSGL--
-IVSGLE-
ICVSGL--

-IVSGLC-
-KVSGLR-
-KVSGLK-
-KVSGLS-
KVVSGL--
-KVSGLH-
-KVSGLV-
KPVSGL--
KYVSGL--
KWVSGL--
-KVSGLW-
KMVSGL--
-KVSGLF-
-KVSGLT-
KDVSGL--
KNVSGL--
-KVSGLN-
KHVSGL--
-KVSGLM-
-KVSGLQ-
LSVSGL--
LKVSGL--
-LVSGLS-
-LVSGLT-
-LVSGLN-
LTVSGL--
LNVSGL--
LAVSGL--
-LVSGLI-
-LVSGLK-
LYVSGL--
LIVSGL--
-LVSGLH-
LHVSGL--
LFVSGL--
LQVSGL--
LMVSGL--
LPVSGL--
-LVSGLG-
LCVSGL--
-LVSGLP-
-LVSGLC-
-MVSGLS-
-MVSGLY-
MLVSGL--
MTVSGL--
MVVSGL--
-MVSGLT-
-MVSGLK-
MGVSGL--

-MVSGLA-
MAVSGL--
-MVSGLN-
-MVSGLH-
MWVSGL--
MEVSGL--
MIVSGL--
MDVSGL--
MHVSGL--
-MVSGLG-
-MVSGLQ-
MNVSGL--
-MVSGLV-
-MVSGLM-
MQVSGL--
-MVSGLE-
-NVSGLR-
NRVSGL--
NSVSGL--
-NVSGLS-
NPVSGL--
-NVSGLK-
NGVSGL--
NNVSGL--
-NVSGLT-
-NVSGLF-
NYVSGL--
NTVSGL--
-NVSGLW-
NAVSGL--
NQVSGL--
-NVSGLH-
NLVSGL--
NVVSGL--
NKVSGL--
-NVSGLN-
-NVSGLY-
NMVSGL--
-NVSGLV-
-NVSGLI-
NWVSGL--
-NVSGLA-
-NVSGLM-
NDVSGL--
NCVSGL--
-NVSGLE-
PVVSGL--
-PVSGLS-
-PVSGLK-
-PVSGLL-

PNVSGL--
PHVSGL--
PTVSGL--
-PVSGLY-
PFVSGL--
PQVSGL--
PAVSGL--
-PVSGLW-
PGVSGL--
PWVSGL--
-PVSGLT-
PMVSGL--
-PVSGLH-
PDVSGL--
-PVSGLI-
-PVSGLF-
-PVSGLQ-
-PVSGLM-
-PVSGLV-
PIVSGL--
-PVSGLA-
PCVSGL--
PPVSGL--
-QVSGLR-
-QVSGLS-
QRVSGL--
QVVSGL--
QSVSGL--
-QVSGLT-
-QVSGLY-
-QVSGLW-
QDVSGL--
QQVSGL--
QTVSGL--
QIVSGL--
-QVSGLK-
QYVSGL--
QWVSGL--
-QVSGLF-
QMVSGL--
QPVSGL--
QHVSGL--
-QVSGLN-
QNVSGL--
QAVSGL--
-QVSGLH-
-QVSGLQ-
-QVSGLV-
QEVSGL--
QFVSGL--

−QVSGLA−
QCVSGL−−
−QVSGLC−
−RVSGLY−
−RVSGLS−
−RVSGLI−
−RVSGLH−
−RVSGLM−
RDVSGL−−
−RVSGLQ−
RCVSGL−−
STVSGL−−
SIVSGL−−
SNVSGL−−
SQVSGL−−
−SVSGLI−
−SVSGLF−
SWVSGL−−
SAVSGL−−
−SVSGLH−
−SVSGLW−
−SVSGLV−
−SVSGLN−
−SVSGLM−
SCVSGL−−
TGVSGL−−
TAVSGL−−
TKVSGL−−
TTVSGL−−
TLVSGL−−
TIVSGL−−
TQVSGL−−
TFVSGL−−
−TVSGLW−
−TVSGLK−
TYVSGL−−
TWVSGL−−
−TVSGLF−
−TVSGLV−
−TVSGLI−
−TVSGLH−
TPVSGL−−
TEVSGL−−
TDVSGL−−
−TVSGLN−
TCVSGL−−
−TVSGLQ−
THVSGL−−
−TVSGLA−
−TVSGLM−

-TVSGLC-
--VSGLRN
VSVSGL--
--VSGLRA
--VSGLMS
--VSGLRT
--VSGLFS
-VVSGLS-
VWVSGL--
VVVSGL--
--VSGLSS
--VSGLTS
--VSGLSG
--VSGLHS
VLVSGL--
VGVSGL--
--VSGLLG
--VSGLRM
-VVSGLT-
--VSGLVG
--VSGLKA
--VSGLYS
VKVSGL--
--VSGLQG
--VSGLRH
--VSGLYT
--VSGLRD
--VSGLSV
--VSGLTA
--VSGLKT
VEVSGL--
--VSGLVA
VQVSGL--
VTVSGL--
--VSGLTG
VHVSGL--
-VVSGLN-
-VVSGLV-
--VSGLWG
--VSGLKV
--VSGLKG
--VSGLYH
--VSGLIA
--VSGLFT
--VSGLST
--VSGLMT
--VSGLFG
--VSGLWS
--VSGLAA
--VSGLYA

--VSGLYG
--VSGLQS
VPVSGL--
--VSGLHK
--VSGLFA
--VSGLAS
--VSGLYN
--VSGLWT
--VSGLQA
--VSGLIG
VDVSGL--
VAVSGL--
-VVSGLH-
--VSGLHN
--VSGLES
--VSGLFW
VNVSGL--
-VVSGLQ-
--VSGLMA
-VVSGLA-
--VSGLWH
--VSGLVW
--VSGLVF
--VSGLRE
--VSGLTW
--VSGLTV
--VSGLHW
VIVSGL--
--VSGLYQ
--VSGLWN
--VSGLIW
--VSGLHV
--VSGLMY
--VSGLLT
--VSGLHH
--VSGLSW
--VSGLMR
-VVSGLW-
--VSGLVY
--VSGLKW
--VSGLIT
--VSGLAG
--VSGLWR
--VSGLMW
--VSGLHG
--VSGLVN
--VSGLVV
--VSGLNT
--VSGLHQ
--VSGLYD

```
--VSGLVR
--VSGLKN
--VSGLEA
--VSGLYI
--VSGLWK
--VSGLMI
--VSGLHI
--VSGLEG
--VSGLMK
--VSGLHD
--VSGLMQ
--VSGLMH
--VSGLIN
--VSGLCA
-VVSGLM-
--VSGLVM
--VSGLTK
--VSGLNG
--VSGLKY
--VSGLKH
VCVSGL--
--VSGLYE
--VSGLVQ
--VSGLTN
--VSGLNW
--VSGLMM
--VSGLLH
--VSGLHA
--VSGLFQ
--VSGLWQ
--VSGLII
--VSGLIF
--VSGLAT
--VSGLAN
--VSGLNY
--VSGLMN
--VSGLMD
--VSGLIQ
--VSGLCW
--VSGLAW
--VSGLWC
--VSGLQW
--VSGLPM
--VSGLIC
-WVSGLT-
-WVSGLK-
-WVSGLA-
WTVSGL--
WHVSGL--
WSVSGL--
```

WMVSGL--
-WVSGLY-
-WVSGLN-
-WVSGLH-
WYVSGL--
WVVSGL--
-WVSGLV-
-WVSGLM-
WDVSGL--
WQVSGL--
WPVSGL--
WWVSGL--
-WVSGLG-
WEVSGL--
WAVSGL--
-WVSGLE-
-WVSGLQ-
-WVSGLF-
WCVSGL--
-WVSGLC-
-YVSGLK-
-YVSGLS-
-YVSGLN-
YNVSGL--
YHVSGL--
YVVSGL--
-YVSGLT-
-YVSGLA-
YKVSGL--
YYVSGL--
YAVSGL--
-YVSGLF-
YGVSGL--
-YVSGLV-
-YVSGLI-
YIVSGL--
-YVSGLW-
-YVSGLH-
YWVSGL--
YFVSGL--
YQVSGL--
YPVSGL--
YEVSGL--
YDVSGL--
-YVSGLM-
-YVSGLE-
YCVSGL--
-YVSGLC-

| | | | |
|---|---|---|---|
| PIAY | 611 | 577 | 31.75 |
| | --PIAYYS | | |

--PIAYWS
-APIAYV-
-APIAYT-
-APIAYI-
AAPIAY--
ASPIAY--
-APIAYK-
ANPIAY--
AIPIAY--
AVPIAY--
AQPIAY--
-APIAYY-
-APIAYW-
-APIAYR-
AHPIAY--
AYPIAY--
AWPIAY--
-APIAYH-
AKPIAY--
CMPIAY--
CRPIAY--
CNPIAY--
CWPIAY--
CVPIAY--
CPPIAY--
-CPIAYY-
-CPIAYT-
-CPIAYN-
-CPIAYL-
CLPIAY--
CGPIAY--
CDPIAY--
CAPIAY--
DVPIAY--
-DPIAYV-
-DPIAYI-
DHPIAY--
DYPIAY--
-DPIAYM-
-DPIAYL-
DRPIAY--
DGPIAY--
DEPIAY--
DSPIAY--
-DPIAYK-
-DPIAYF-
DNPIAY--
DMPIAY--
DFPIAY--
DDPIAY--

DAPIAY--
-EPIAYI-
EVPIAY--
-EPIAYL-
-EPIAYV-
ERPIAY--
-EPIAYH-
ENPIAY--
EWPIAY--
EPPIAY--
-EPIAYT-
-EPIAYF-
-EPIAYA-
ELPIAY--
EDPIAY--
EAPIAY--
FLPIAY--
FIPIAY--
FFPIAY--
-FPIAYW-
FYPIAY--
FGPIAY--
FDPIAY--
FRPIAY--
FQPIAY--
-FPIAYT-
-FPIAYR-
FMPIAY--
FKPIAY--
-FPIAYM-
FSPIAY--
-FPIAYY-
-FPIAYN-
-FPIAYH-
FEPIAY--
-GPIAYR-
GQPIAY--
GWPIAY--
-GPIAYW-
GNPIAY--
GKPIAY--
-GPIAYY-
-GPIAYS-
-GPIAYK-
-GPIAYA-
GLPIAY--
GGPIAY--
GVPIAY--
GFPIAY--
GAPIAY--

GSPIAY--
GHPIAY--
GYPIAY--
GPPIAY--
-GPIAYM-
-GPIAYI-
-GPIAYH-
-GPIAYG-
GMPIAY--
GIPIAY--
GDPIAY--
HAPIAY--
-HPIAYL-
-HPIAYT-
-HPIAYK-
HNPIAY--
HWPIAY--
HQPIAY--
-HPIAYQ-
HFPIAY--
HSPIAY--
HPPIAY--
-HPIAYM-
-HPIAYF-
HLPIAY--
HGPIAY--
HYPIAY--
HTPIAY--
-HPIAYY-
-HPIAYV-
-HPIAYR-
-HPIAYI-
HDPIAY--
-IPIAYL-
ISPIAY--
-IPIAYH-
IRPIAY--
IIPIAY--
IPPIAY--
IAPIAY--
IEPIAY--
IVPIAY--
-IPIAYS-
-IPIAYR-
IHPIAY--
-IPIAYM-
IQPIAY--
-IPIAYN-
IWPIAY--
-IPIAYQ-

-IPIAYA-
INPIAY--
IKPIAY--
IGPIAY--
IDPIAY--
-KPIAYL-
-KPIAYS-
-KPIAYI-
-KPIAYV-
KMPIAY--
KDPIAY--
KVPIAY--
KLPIAY--
KCPIAY--
KTPIAY--
-KPIAYY-
-KPIAYT-
KHPIAY--
KWPIAY--
-KPIAYR-
-KPIAYH-
-KPIAYA-
KNPIAY--
KIPIAY--
KEPIAY--
LRPIAY--
LQPIAY--
LDPIAY--
-LPIAYL-
-LPIAYS-
LNPIAY--
-LPIAYM-
LTPIAY--
LSPIAY--
LPPIAY--
-LPIAYA-
-LPIAYT-
-LPIAYR-
LGPIAY--
LAPIAY--
LEPIAY--
LCPIAY--
-LPIAYK-
-LPIAYH-
LMPIAY--
LHPIAY--
-MPIAYL-
MIPIAY--
MAPIAY--
MTPIAY--

-MPIAYR-
MMPIAY--
MLPIAY--
MSPIAY--
MRPIAY--
-MPIAYM-
MNPIAY--
MPPIAY--
-MPIAYV-
-MPIAYT-
-MPIAYN-
-MPIAYK-
-MPIAYI-
-MPIAYH-
MYPIAY--
MVPIAY--
-MPIAYS-
-MPIAYA-
MKPIAY--
MHPIAY--
MGPIAY--
NWPIAY--
NRPIAY--
NLPIAY--
-NPIAYR-
NYPIAY--
NTPIAY--
-NPIAYT-
-NPIAYH-
-NPIAYF-
-NPIAYK-
NEPIAY--
NVPIAY--
NSPIAY--
-NPIAYY-
-NPIAYS-
-NPIAYM-
-NPIAYL-
NKPIAY--
NIPIAY--
NGPIAY--
-NPIAYV-
NNPIAY--
NMPIAY--
NFPIAY--
NAPIAY--
--PIAYVT
--PIAYTS
--PIAYRG
--PIAYRA

--PIAYPF
--PIAYLS
--PIAYRS
--PIAYIT
--PIAYTR
--PIAYSS
--PIAYQS
--PIAYLI
--PIAYKS
--PIAYVR
--PIAYSQ
--PIAYSG
--PIAYSA
--PIAYRY
--PIAYLR
--PIAYES
--PIAYVS
--PIAYTL
--PIAYST
--PIAYSM
--PIAYLG
--PIAYKV
--PIAYIG
--PIAYFS
--PIAYWT
--PIAYVA
--PIAYSN
--PIAYRI
--PIAYRH
--PIAYLM
--PIAYLA
--PIAYKN
--PIAYGI
--PIAYFM
--PIAYAI
--PIAYYH
--PIAYWR
--PIAYVK
--PIAYVI
--PIAYTM
--PIAYSR
--PIAYSK
--PIAYSI
--PIAYRT
--PIAYRN
--PIAYMR
--PIAYML
--PIAYMK
--PIAYLQ
--PIAYLH

--PIAYKW
--PIAYKT
--PIAYKH
--PIAYKE
--PIAYKA
--PIAYIS
--PIAYWG
--PIAYVW
--PIAYTV
--PIAYSW
--PIAYSF
--PIAYRM
--PIAYQM
--PIAYLT
--PIAYLN
--PIAYKM
--PIAYKI
--PIAYKG
--PIAYIV
--PIAYFY
--PIAYEA
--PIAYAL
--PIAYYW
--PIAYYQ
--PIAYYG
--PIAYWK
--PIAYTT
--PIAYTQ
--PIAYTG
--PIAYSL
--PIAYRV
--PIAYRE
--PIAYQV
--PIAYQN
--PIAYPY
--PIAYNT
--PIAYNS
--PIAYNI
--PIAYMT
--PIAYMN
--PIAYLK
--PIAYLE
--PIAYKY
--PIAYIR
--PIAYHN
--PIAYGS
--PIAYGM
--PIAYEM
--PIAYEL
--PIAYEG

--PIAYAG
PFPIAY--
PEPIAY--
PNPIAY--
PKPIAY--
--PIAYYT
--PIAYYN
--PIAYVQ
--PIAYVN
--PIAYVH
--PIAYVF
--PIAYTW
--PIAYTK
--PIAYTH
--PIAYRD
--PIAYQR
--PIAYQL
--PIAYQE
--PIAYPV
--PIAYPR
--PIAYNY
--PIAYNW
--PIAYNV
--PIAYNL
--PIAYMY
--PIAYMI
--PIAYMG
--PIAYMD
--PIAYMA
--PIAYLW
--PIAYKR
--PIAYKL
--PIAYIK
--PIAYIA
--PIAYHW
--PIAYHV
--PIAYHT
--PIAYHR
--PIAYHQ
--PIAYHK
--PIAYHA
--PIAYGW
--PIAYGV
--PIAYGF
--PIAYFT
--PIAYEI
--PIAYDL
--PIAYAN
QTPIAY--
-QPIAYW-

-QPIAYR-
QLPIAY--
-QPIAYY-
QSPIAY--
-QPIAYL-
-QPIAYF-
QPPIAY--
QGPIAY--
QWPIAY--
-QPIAYV-
-QPIAYT-
-QPIAYN-
-QPIAYM-
-QPIAYK-
-QPIAYI-
-QPIAYH-
-QPIAYA-
QNPIAY--
QEPIAY--
RKPIAY--
RHPIAY--
-RPIAYY-
RNPIAY--
RAPIAY--
-RPIAYS-
-RPIAYL-
RRPIAY--
RSPIAY--
-RPIAYI-
RLPIAY--
-RPIAYT-
RMPIAY--
RQPIAY--
RWPIAY--
-RPIAYM-
RPPIAY--
-RPIAYN-
RIPIAY--
RTPIAY--
-RPIAYK-
-RPIAYH-
RFPIAY--
REPIAY--
RDPIAY--
RCPIAY--
RYPIAY--
-RPIAYE-
-SPIAYT-
STPIAY--
SRPIAY--

SNPIAY--
-SPIAYS-
SFPIAY--
-SPIAYL-
-SPIAYI-
SAPIAY--
SWPIAY--
SLPIAY--
SHPIAY--
SVPIAY--
SSPIAY--
-SPIAYR-
SDPIAY--
SQPIAY--
-SPIAYF-
SYPIAY--
SPPIAY--
-SPIAYV-
-SPIAYN-
-SPIAYM-
-SPIAYK-
-SPIAYH-
SIPIAY--
SGPIAY--
SEPIAY--
SCPIAY--
-SPIAYW-
-SPIAYQ-
-SPIAYA-
SKPIAY--
-TPIAYL-
TGPIAY--
TVPIAY--
-TPIAYK-
TRPIAY--
-TPIAYI-
TKPIAY--
-TPIAYV-
TPPIAY--
-TPIAYS-
-TPIAYF-
TSPIAY--
-TPIAYR-
-TPIAYT-
-TPIAYH-
TTPIAY--
-TPIAYM-
-TPIAYG-
-TPIAYW-
-TPIAYQ-

TNPIAY--
TFPIAY--
TDPIAY--
TAPIAY--
TYPIAY--
TWPIAY--
THPIAY--
TEPIAY--
-VPIAYL-
-VPIAYA-
-VPIAYT-
VKPIAY--
VGPIAY--
VSPIAY--
-VPIAYM-
-VPIAYK-
-VPIAYI-
VTPIAY--
VRPIAY--
VMPIAY--
VFPIAY--
VWPIAY--
VYPIAY--
VIPIAY--
-VPIAYS-
-VPIAYR-
-VPIAYG-
-VPIAYE-
VNPIAY--
VLPIAY--
VDPIAY--
VQPIAY--
-VPIAYQ-
-VPIAYH-
VEPIAY--
VAPIAY--
-WPIAYA-
WLPIAY--
WKPIAY--
WGPIAY--
WYPIAY--
WTPIAY--
WQPIAY--
-WPIAYT-
-WPIAYS-
-WPIAYQ-
-WPIAYK-
WNPIAY--
WEPIAY--
WDPIAY--

```
              WWPIAY--
              -WPIAYV-
              -WPIAYR-
              WMPIAY--
              WHPIAY--
              -YPIAYA-
              -YPIAYI-
              -YPIAYS-
              -YPIAYK-
              YLPIAY--
              -YPIAYR-
              YKPIAY--
              YRPIAY--
              YDPIAY--
              YPPIAY--
              -YPIAYT-
              YFPIAY--
              YWPIAY--
              YSPIAY--
              -YPIAYV-
              YTPIAY--
              -YPIAYY-
              -YPIAYQ-
              -YPIAYN-
              -YPIAYF-
              YAPIAY--
PAPW           314        661     31.74
              -APAPWH-
              AYPAPW--
              ATPAPW--
              -APAPWN-
              -APAPWL-
              -APAPWK-
              -APAPWI-
              AMPAPW--
              -APAPWS-
              AWPAPW--
              ARPAPW--
              -APAPWR-
              -APAPWM-
              -APAPWG-
              -APAPWA-
              ALPAPW--
              AHPAPW--
              AFPAPW--
              ADPAPW--
              AVPAPW--
              ASPAPW--
              -APAPWY-
              -APAPWV-
```

-APAPWF-
ANPAPW--
AKPAPW--
AIPAPW--
AGPAPW--
CKPAPW--
CYPAPW--
CTPAPW--
CPPAPW--
CCPAPW--
CVPAPW--
CQPAPW--
-CPAPWR-
-CPAPWL-
CMPAPW--
CLPAPW--
CEPAPW--
CAPAPW--
DMPAPW--
-DPAPWR-
DRPAPW--
-DPAPWY-
-DPAPWS-
DFPAPW--
-DPAPWM-
DLPAPW--
DKPAPW--
DVPAPW--
DSPAPW--
-DPAPWT-
-DPAPWG-
DDPAPW--
DAPAPW--
ERPAPW--
-EPAPWL-
-EPAPWT-
EIPAPW--
EGPAPW--
-EPAPWA-
ELPAPW--
EYPAPW--
EVPAPW--
-EPAPWV-
-EPAPWR-
-EPAPWS-
-EPAPWM-
EHPAPW--
EWPAPW--
EQPAPW--
-EPAPWY-

-EPAPWW-
-EPAPWQ-
-EPAPWN-
-EPAPWK-
-EPAPWF-
-FPAPWK-
FSPAPW--
-FPAPWI-
-FPAPWQ-
FRPAPW--
-FPAPWR-
FTPAPW--
-FPAPWS-
FPPAPW--
FMPAPW--
-FPAPWY-
FLPAPW--
FQPAPW--
-FPAPWT-
-FPAPWL-
-FPAPWH-
FAPAPW--
-FPAPWV-
-FPAPWA-
FNPAPW--
FIPAPW--
FEPAPW--
-GPAPWR-
GRPAPW--
-GPAPWV-
GAPAPW--
GSPAPW--
GKPAPW--
-GPAPWT-
GDPAPW--
-GPAPWL-
GFPAPW--
GVPAPW--
GPPAPW--
GTPAPW--
-GPAPWM-
GLPAPW--
GWPAPW--
GQPAPW--
-GPAPWG-
GIPAPW--
GHPAPW--
-GPAPWY-
-GPAPWN-
-GPAPWK-

-GPAPWI-
GNPAPW--
GGPAPW--
GCPAPW--
HAPAPW--
-HPAPWL-
HRPAPW--
-HPAPWT-
-HPAPWI-
HNPAPW--
HVPAPW--
HKPAPW--
HSPAPW--
HDPAPW--
HYPAPW--
-HPAPWY-
-HPAPWR-
-HPAPWK-
-HPAPWG-
-HPAPWF-
-HPAPWA-
HLPAPW--
HHPAPW--
HEPAPW--
-IPAPWR-
-IPAPWG-
ISPAPW--
-IPAPWM-
ILPAPW--
IIPAPW--
IDPAPW--
ITPAPW--
IRPAPW--
-IPAPWY-
IPPAPW--
IMPAPW--
IVPAPW--
-IPAPWH-
IAPAPW--
-IPAPWS-
-IPAPWL-
-IPAPWK-
-IPAPWF-
-IPAPWE-
INPAPW--
IKPAPW--
IHPAPW--
IFPAPW--
IEPAPW--
KSPAPW--

-KPAPWL-
-KPAPWK-
KLPAPW--
KRPAPW--
KTPAPW--
-KPAPWA-
-KPAPWV-
-KPAPWI-
-KPAPWF-
KKPAPW--
-KPAPWW-
-KPAPWS-
KVPAPW--
-KPAPWH-
KHPAPW--
KGPAPW--
KFPAPW--
KDPAPW--
KAPAPW--
-LPAPWR-
LKPAPW--
-LPAPWK-
LSPAPW--
LGPAPW--
LVPAPW--
LLPAPW--
LRPAPW--
-LPAPWS-
-LPAPWL-
LTPAPW--
LPPAPW--
LIPAPW--
LYPAPW--
-LPAPWI-
LHPAPW--
LEPAPW--
LWPAPW--
-LPAPWV-
-LPAPWT-
-LPAPWM-
LNPAPW--
LAPAPW--
-LPAPWY-
-LPAPWH-
LDPAPW--
LCPAPW--
-LPAPWP-
-LPAPWN-
-LPAPWF-
-LPAPWA-

-MPAPWL-
MAPAPW--
MIPAPW--
MSPAPW--
-MPAPWT-
MEPAPW--
-MPAPWS-
-MPAPWN-
-MPAPWM-
-MPAPWI-
MMPAPW--
MYPAPW--
MWPAPW--
MTPAPW--
MPPAPW--
-MPAPWQ-
-MPAPWF-
MFPAPW--
-MPAPWY-
-MPAPWV-
-MPAPWH-
-MPAPWG-
-MPAPWE-
-MPAPWA-
MLPAPW--
MDPAPW--
NRPAPW--
NVPAPW--
-NPAPWL-
NKPAPW--
NSPAPW--
NPPAPW--
-NPAPWW-
-NPAPWR-
-NPAPWI-
NYPAPW--
NTPAPW--
-NPAPWA-
-NPAPWS-
NMPAPW--
NAPAPW--
-NPAPWV-
-NPAPWT-
NCPAPW--
NWPAPW--
-NPAPWH-
NIPAPW--
NFPAPW--
--PAPWSL
--PAPWIS

--PAPWRL
--PAPWFR
--PAPWVG
--PAPWRR
--PAPWMA
--PAPWHG
--PAPWIR
--PAPWWY
--PAPWVH
--PAPWTS
--PAPWSS
--PAPWYL
--PAPWVS
--PAPWTT
--PAPWSV
--PAPWLF
--PAPWKR
--PAPWVF
--PAPWGT
--PAPWFT
--PAPWAT
--PAPWYK
--PAPWVV
--PAPWSG
--PAPWRT
--PAPWNY
--PAPWLV
--PAPWLK
--PAPWIK
--PAPWFQ
--PAPWFL
PVPAPW--
--PAPWTY
--PAPWRN
--PAPWLS
--PAPWKV
--PAPWKL
--PAPWAY
PTPAPW--
PQPAPW--
PHPAPW--
--PAPWYT
--PAPWVT
--PAPWTR
--PAPWTK
--PAPWNT
--PAPWNQ
--PAPWNL
--PAPWLN
--PAPWLL

--PAPWLH
--PAPWGV
--PAPWAN
PRPAPW--
--PAPWWS
--PAPWWA
--PAPWTN
--PAPWTL
--PAPWSI
--PAPWQS
--PAPWPY
--PAPWNI
--PAPWNG
--PAPWMY
--PAPWMG
--PAPWKN
--PAPWAA
--PAPWVD
--PAPWSY
--PAPWST
--PAPWSH
--PAPWRF
--PAPWQG
--PAPWNS
--PAPWNM
--PAPWMH
--PAPWLW
--PAPWLT
--PAPWLG
--PAPWKY
--PAPWHW
--PAPWGS
--PAPWFV
--PAPWFM
--PAPWFI
PSPAPW--
--PAPWYV
--PAPWYR
--PAPWYN
--PAPWYG
--PAPWYD
--PAPWWT
--PAPWWR
--PAPWWK
--PAPWWH
--PAPWVI
--PAPWSN
--PAPWSF
--PAPWRY
--PAPWQV

--PAPWMQ
--PAPWML
--PAPWLQ
--PAPWLA
--PAPWKW
--PAPWIY
--PAPWHS
--PAPWHL
--PAPWHA
--PAPWFY
--PAPWFN
--PAPWFH
--PAPWFG
--PAPWAQ
--PAPWAF
PKPAPW--
PGPAPW--
--PAPWYS
--PAPWWL
--PAPWVL
--PAPWTF
--PAPWSW
--PAPWSQ
--PAPWRQ
--PAPWRH
--PAPWRA
--PAPWQT
--PAPWQQ
--PAPWQL
--PAPWPW
--PAPWPI
--PAPWNN
--PAPWMR
--PAPWMN
--PAPWMK
--PAPWMI
--PAPWLR
--PAPWKS
--PAPWKG
--PAPWIW
--PAPWIM
--PAPWIE
--PAPWID
--PAPWIA
--PAPWHV
--PAPWHR
--PAPWHM
--PAPWGG
--PAPWFW
--PAPWFS

--PAPWFA
--PAPWES
--PAPWEG
--PAPWEF
PNPAPW--
PMPAPW--
PLPAPW--
--PAPWYH
--PAPWYA
--PAPWWP
--PAPWWN
--PAPWVQ
--PAPWVM
--PAPWVK
--PAPWVE
--PAPWTV
--PAPWTI
--PAPWTE
--PAPWTD
--PAPWSK
--PAPWSA
--PAPWRW
--PAPWRM
--PAPWRG
--PAPWRE
--PAPWQR
--PAPWQN
--PAPWQH
--PAPWPV
--PAPWPQ
--PAPWPM
--PAPWNV
--PAPWNK
--PAPWNF
--PAPWMD
--PAPWKM
--PAPWKK
--PAPWKI
--PAPWIN
--PAPWIL
--PAPWIH
--PAPWIF
--PAPWHN
--PAPWHK
--PAPWGY
--PAPWGQ
--PAPWGH
--PAPWEY
--PAPWEL
--PAPWEI

--PAPWDI
--PAPWAV
--PAPWAS
--PAPWAM
--PAPWAG
PAPAPW--
-QPAPWL-
-QPAPWT-
-QPAPWS-
-QPAPWR-
QRPAPW--
QPPAPW--
QSPAPW--
-QPAPWW-
-QPAPWQ-
QLPAPW--
QIPAPW--
QGPAPW--
QDPAPW--
QYPAPW--
QWPAPW--
-QPAPWY-
-QPAPWA-
QTPAPW--
QQPAPW--
-QPAPWV-
-QPAPWF-
QMPAPW--
QKPAPW--
QFPAPW--
QAPAPW--
RIPAPW--
RYPAPW--
-RPAPWL-
-RPAPWR-
-RPAPWS-
RGPAPW--
-RPAPWV-
RLPAPW--
RTPAPW--
RSPAPW--
-RPAPWN-
RNPAPW--
-RPAPWT-
-RPAPWG-
RAPAPW--
RKPAPW--
RHPAPW--
RWPAPW--
-RPAPWH-

RFPAPW--
RDPAPW--
-RPAPWF-
RPPAPW--
-RPAPWK-
-RPAPWI-
RMPAPW--
-RPAPWY-
-RPAPWM-
-RPAPWA-
RQPAPW--
-RPAPWW-
-RPAPWQ-
-RPAPWE-
REPAPW--
SIPAPW--
SLPAPW--
SFPAPW--
SAPAPW--
SRPAPW--
-SPAPWS-
-SPAPWQ-
-SPAPWY-
-SPAPWV-
-SPAPWK-
-SPAPWI-
-SPAPWH-
-SPAPWW-
-SPAPWT-
-SPAPWR-
SDPAPW--
SYPAPW--
SWPAPW--
STPAPW--
SSPAPW--
SGPAPW--
-SPAPWG-
-SPAPWF-
SNPAPW--
SKPAPW--
-SPAPWA-
SMPAPW--
SPPAPW--
-SPAPWM-
-SPAPWC-
SEPAPW--
-TPAPWH-
TRPAPW--
-TPAPWQ-
-TPAPWS-

-TPAPWY-
-TPAPWF-
-TPAPWT-
TQPAPW--
-TPAPWL-
TNPAPW--
TLPAPW--
TYPAPW--
TTPAPW--
TSPAPW--
TKPAPW--
TIPAPW--
TEPAPW--
-TPAPWG-
-TPAPWA-
THPAPW--
TVPAPW--
TPPAPW--
-TPAPWW-
-TPAPWV-
-TPAPWP-
-TPAPWI-
-TPAPWE-
TGPAPW--
TFPAPW--
TDPAPW--
VKPAPW--
-VPAPWY-
VLPAPW--
-VPAPWS-
VRPAPW--
VSPAPW--
-VPAPWL-
VIPAPW--
-VPAPWV-
-VPAPWT-
VYPAPW--
VHPAPW--
VAPAPW--
VVPAPW--
-VPAPWQ-
VTPAPW--
VPPAPW--
-VPAPWR-
-VPAPWM-
-VPAPWK-
-VPAPWI-
-VPAPWG-
-VPAPWA-
VWPAPW--

| | | | |
|---|---|---|---|
| | -VPAPWN- | | |
| | -VPAPWH- | | |
| | VNPAPW-- | | |
| | VGPAPW-- | | |
| | VFPAPW-- | | |
| | VEPAPW-- | | |
| | WRPAPW-- | | |
| | -WPAPWL- | | |
| | WDPAPW-- | | |
| | -WPAPWS- | | |
| | -WPAPWV- | | |
| | WNPAPW-- | | |
| | WKPAPW-- | | |
| | WYPAPW-- | | |
| | -WPAPWR- | | |
| | -WPAPWN- | | |
| | -WPAPWG- | | |
| | -WPAPWF- | | |
| | -WPAPWA- | | |
| | WAPAPW-- | | |
| | YKPAPW-- | | |
| | -YPAPWK- | | |
| | YLPAPW-- | | |
| | YWPAPW-- | | |
| | YVPAPW-- | | |
| | YRPAPW-- | | |
| | -YPAPWM- | | |
| | -YPAPWF- | | |
| | YSPAPW-- | | |
| | -YPAPWS- | | |
| | -YPAPWL- | | |
| | YNPAPW-- | | |
| | YAPAPW-- | | |
| | YYPAPW-- | | |
| | YQPAPW-- | | |
| | -YPAPWH- | | |
| | YHPAPW-- | | |
| | YGPAPW-- | | |
| PVAM | 279 | 677 | 31.72 |
| | --PVAMMR | | |
| | -APVAML- | | |
| | -APVAMR- | | |
| | -APVAMW- | | |
| | ASPVAM-- | | |
| | ATPVAM-- | | |
| | AQPVAM-- | | |
| | -APVAMK- | | |
| | AVPVAM-- | | |
| | AKPVAM-- | | |
| | -APVAMT- | | |

ANPVAM--
ARPVAM--
-APVAMN-
-APVAMG-
ALPVAM--
-APVAMQ-
-APVAMH-
-APVAMA-
AFPVAM--
-APVAMS-
-APVAMI-
AWPVAM--
-APVAMV-
-APVAMM-
AMPVAM--
AIPVAM--
AHPVAM--
AAPVAM--
CMPVAM--
CQPVAM--
CRPVAM--
-CPVAMT-
-CPVAMR-
CIPVAM--
CVPVAM--
-CPVAML-
CLPVAM--
CHPVAM--
CFPVAM--
-DPVAMT-
DTPVAM--
DLPVAM--
DRPVAM--
-DPVAMS-
DHPVAM--
DGPVAM--
-DPVAML-
DFPVAM--
DSPVAM--
-DPVAMY-
-DPVAMK-
-DPVAMA-
DVPVAM--
-DPVAMH-
DIPVAM--
DAPVAM--
-EPVAMS-
EVPVAM--
-EPVAMW-
-EPVAMT-

-EPVAMF-
-EPVAML-
-EPVAMR-
-EPVAMK-
EFPVAM--
EAPVAM--
-EPVAMY-
-EPVAMA-
ENPVAM--
EWPVAM--
ESPVAM--
EQPVAM--
-EPVAMM-
ELPVAM--
EIPVAM--
EGPVAM--
EYPVAM--
ERPVAM--
-EPVAMN-
-EPVAMH-
EKPVAM--
EEPVAM--
-FPVAML-
-FPVAMK-
FTPVAM--
-FPVAMT-
-FPVAMA-
-FPVAMR-
FIPVAM--
FSPVAM--
FLPVAM--
FYPVAM--
FWPVAM--
FVPVAM--
FQPVAM--
-FPVAMN-
-FPVAMI-
-FPVAMH-
-FPVAMC-
FHPVAM--
FAPVAM--
-FPVAMV-
-FPVAMS-
-FPVAMM-
-FPVAME-
FNPVAM--
FGPVAM--
FFPVAM--
GMPVAM--
-GPVAMG-

-GPVAML-
-GPVAMT-
-GPVAMR-
GIPVAM--
GVPVAM--
GRPVAM--
-GPVAMH-
GWPVAM--
GSPVAM--
GAPVAM--
GTPVAM--
-GPVAMS-
-GPVAMA-
GNPVAM--
-GPVAMW-
-GPVAMI-
GYPVAM--
-GPVAME-
GPPVAM--
GLPVAM--
GDPVAM--
-GPVAMQ-
-GPVAMN-
-GPVAMM-
GCPVAM--
-HPVAMR-
HGPVAM--
-HPVAMW-
HRPVAM--
HAPVAM--
HWPVAM--
-HPVAMS-
-HPVAMH-
HLPVAM--
HFPVAM--
-HPVAMT-
-HPVAML-
HTPVAM--
HSPVAM--
-HPVAMY-
HIPVAM--
HYPVAM--
HVPVAM--
-HPVAMV-
-HPVAMQ-
-HPVAMA-
HPPVAM--
HEPVAM--
-IPVAML-
IRPVAM--

-IPVAMA-
IIPVAM--
-IPVAMV-
-IPVAMK-
ITPVAM--
-IPVAMS-
INPVAM--
-IPVAMY-
ILPVAM--
IVPVAM--
ISPVAM--
IGPVAM--
IEPVAM--
IDPVAM--
IWPVAM--
IQPVAM--
-IPVAMT-
-IPVAMQ-
-IPVAMN-
-IPVAMG-
-IPVAME-
IPPVAM--
IHPVAM--
IFPVAM--
KSPVAM--
-KPVAML-
KGPVAM--
-KPVAMI-
KRPVAM--
-KPVAMR-
KIPVAM--
KTPVAM--
KVPVAM--
-KPVAMT-
-KPVAMS-
-KPVAMF-
KKPVAM--
-KPVAMY-
-KPVAMN-
KFPVAM--
-KPVAMV-
-KPVAMA-
KEPVAM--
KYPVAM--
-KPVAMH-
-KPVAME-
KNPVAM--
KLPVAM--
-KPVAMW-
-KPVAMK-

-KPVAMC-
KDPVAM--
KCPVAM--
KAPVAM--
-LPVAMR-
LLPVAM--
-LPVAMT-
-LPVAMH-
LYPVAM--
LQPVAM--
-LPVAML-
LSPVAM--
LRPVAM--
-LPVAMI-
LFPVAM--
LAPVAM--
LHPVAM--
LEPVAM--
-LPVAMK-
LNPVAM--
LGPVAM--
LWPVAM--
LVPVAM--
-LPVAMS-
-LPVAMQ-
-LPVAMM-
-LPVAMA-
LPPVAM--
LDPVAM--
LCPVAM--
MRPVAM--
-MPVAMF-
MVPVAM--
-MPVAMA-
MWPVAM--
-MPVAMK-
MYPVAM--
MTPVAM--
MFPVAM--
MAPVAM--
MSPVAM--
MLPVAM--
-MPVAMW-
-MPVAMT-
-MPVAMQ-
MNPVAM--
MHPVAM--
MEPVAM--
-NPVAML-
NRPVAM--

-NPVAMR-
-NPVAMF-
-NPVAMT-
-NPVAMQ-
NNPVAM--
NLPVAM--
NVPVAM--
NQPVAM--
-NPVAMY-
-NPVAMV-
-NPVAME-
-NPVAMA-
NMPVAM--
NKPVAM--
NYPVAM--
NWPVAM--
NSPVAM--
-NPVAMN-
-NPVAMI-
-NPVAMH-
NPPVAM--
NIPVAM--
NHPVAM--
NGPVAM--
--PVAMLR
--PVAMVR
--PVAMHI
--PVAMTR
--PVAMRN
--PVAMYG
--PVAMRA
--PVAMLS
--PVAMRT
--PVAMRH
--PVAMML
--PVAMGL
--PVAMSK
--PVAMGA
--PVAMSR
--PVAMSN
--PVAMRY
--PVAMNW
--PVAMKS
--PVAMAL
--PVAMMK
--PVAMTS
--PVAMTL
--PVAMSW
--PVAMSL
--PVAMRI

--PVAMGF
--PVAMWR
--PVAMWN
--PVAMSS
--PVAMSA
--PVAMQS
--PVAMLN
--PVAMGI
PMPVAM--
--PVAMVG
--PVAMTY
--PVAMSY
--PVAMSG
--PVAMLK
--PVAMGT
--PVAMYQ
--PVAMTV
--PVAMTG
--PVAMSD
--PVAMRQ
--PVAMQI
--PVAMNK
--PVAMMD
--PVAMIT
--PVAMIM
--PVAMFR
--PVAMAT
--PVAMAQ
--PVAMWY
--PVAMVT
--PVAMVS
--PVAMTI
--PVAMRS
--PVAMPL
--PVAMNV
--PVAMNL
--PVAMLH
--PVAMIG
--PVAMGY
--PVAMGS
--PVAMES
PAPVAM--
--PVAMVK
--PVAMTH
--PVAMSV
--PVAMSF
--PVAMQR
--PVAMNS
--PVAMMY
--PVAMLM

--PVAMIN
--PVAMHL
--PVAMGM
--PVAMFN
--PVAMFK
--PVAMFG
--PVAMAI
PLPVAM--
PGPVAM--
--PVAMWS
--PVAMWF
--PVAMVN
--PVAMTN
--PVAMTK
--PVAMSI
--PVAMRK
--PVAMQT
--PVAMQK
--PVAMQD
--PVAMND
--PVAMMN
--PVAMMM
--PVAMMG
--PVAMKT
--PVAMKR
--PVAMKL
--PVAMIS
--PVAMIH
--PVAMIA
--PVAMHW
--PVAMHK
--PVAMHH
--PVAMHA
--PVAMGW
--PVAMGQ
--PVAMFT
--PVAMEG
--PVAMAS
--PVAMAA
PVPVAM--
--PVAMYS
--PVAMYN
--PVAMWH
--PVAMVW
--PVAMVQ
--PVAMTF
--PVAMSQ
--PVAMRM
--PVAMRF
--PVAMQY

--PVAMQV
--PVAMQH
--PVAMQG
--PVAMPY
--PVAMNM
--PVAMMH
--PVAMLQ
--PVAMLE
--PVAMKQ
--PVAMKE
--PVAMKA
--PVAMIQ
--PVAMHS
--PVAMHR
PIPVAM--
PYPVAM--
--PVAMYV
--PVAMYH
--PVAMWG
--PVAMVH
--PVAMVA
--PVAMTQ
--PVAMTE
--PVAMTD
--PVAMSM
--PVAMRD
--PVAMMT
--PVAMMS
--PVAMMI
--PVAMMA
--PVAMKV
--PVAMII
--PVAMHY
--PVAMHT
--PVAMHM
--PVAMGG
--PVAMFD
--PVAMDL
--PVAMAW
--PVAMAV
--PVAMAR
--PVAMAN
--PVAMAM
--PVAMAF
PTPVAM--
PNPVAM--
--PVAMYI
--PVAMWQ
--PVAMVF
--PVAMVD

--PVAMTM
--PVAMTC
--PVAMTA
--PVAMST
--PVAMRE
--PVAMQW
--PVAMQL
--PVAMPW
--PVAMPM
--PVAMNT
--PVAMNP
--PVAMNN
--PVAMNG
--PVAMNA
--PVAMMW
--PVAMME
--PVAMLW
--PVAMLT
--PVAMLD
--PVAMKY
--PVAMKM
--PVAMKG
--PVAMKF
--PVAMIE
--PVAMID
--PVAMHE
--PVAMHD
--PVAMGV
--PVAMGH
--PVAMFH
--PVAMEY
--PVAMEI
--PVAMCT
--PVAMAK
PKPVAM--
-QPVAMR-
-QPVAML-
QRPVAM--
QMPVAM--
-QPVAMI-
QAPVAM--
QSPVAM--
-QPVAMV-
-QPVAMS-
-QPVAMN-
-QPVAMK-
QVPVAM--
QNPVAM--
QLPVAM--
QIPVAM--

QFPVAM--
-QPVAMY-
-QPVAMH-
-QPVAMG-
-QPVAME-
QHPVAM--
QGPVAM--
QDPVAM--
-RPVAMS-
RLPVAM--
RYPVAM--
RAPVAM--
-RPVAMV-
-RPVAMW-
-RPVAMK-
-RPVAMA-
RTPVAM--
-RPVAMY-
-RPVAMG-
-RPVAMM-
-RPVAME-
-RPVAMN-
-RPVAMH-
RFPVAM--
-RPVAMT-
REPVAM--
RPPVAM--
RDPVAM--
RWPVAM--
RNPVAM--
-RPVAMF-
RHPVAM--
RQPVAM--
-RPVAMQ-
RIPVAM--
-SPVAML-
-SPVAMT-
-SPVAMS-
-SPVAMK-
-SPVAMY-
-SPVAMR-
-SPVAMQ-
-SPVAMI-
SVPVAM--
-SPVAMV-
SRPVAM--
SSPVAM--
STPVAM--
-SPVAMW-
SYPVAM--

-SPVAMF-
SMPVAM--
SIPVAM--
SGPVAM--
SFPVAM--
SAPVAM--
-SPVAMG-
-SPVAMA-
SDPVAM--
SWPVAM--
SQPVAM--
-SPVAMM-
-SPVAMH-
-SPVAME-
SNPVAM--
SHPVAM--
SEPVAM--
-TPVAML-
TVPVAM--
-TPVAMK-
-TPVAMS-
-TPVAMR-
-TPVAMM-
TTPVAM--
-TPVAMV-
TWPVAM--
TSPVAM--
TNPVAM--
TLPVAM--
-TPVAMG-
TQPVAM--
THPVAM--
TAPVAM--
TRPVAM--
-TPVAMH-
-TPVAMA-
-TPVAMT-
-TPVAMQ-
-TPVAMN-
-TPVAME-
TIPVAM--
TDPVAM--
-VPVAMR-
VRPVAM--
VQPVAM--
-VPVAMT-
VNPVAM--
VGPVAM--
VVPVAM--
VFPVAM--

VSPVAM--
-VPVAMS-
-VPVAME-
VIPVAM--
-VPVAMY-
-VPVAMV-
-VPVAMK-
-VPVAMH-
VPPVAM--
VDPVAM--
VTPVAM--
VYPVAM--
-VPVAMG-
-VPVAMF-
-VPVAMA-
VMPVAM--
VLPVAM--
VKPVAM--
VHPVAM--
VEPVAM--
VCPVAM--
-WPVAMK-
WRPVAM--
-WPVAMR-
-WPVAMN-
WGPVAM--
-WPVAML-
-WPVAMS-
-WPVAMG-
-WPVAMA-
WPPVAM--
WMPVAM--
WKPVAM--
WIPVAM--
WHPVAM--
WYPVAM--
WQPVAM--
-WPVAMV-
-WPVAME-
WAPVAM--
-YPVAML-
-YPVAMI-
YRPVAM--
YLPVAM--
YGPVAM--
YSPVAM--
YDPVAM--
YWPVAM--
-YPVAMR-
-YPVAMA-

| | | | |
|---|---|---|---|
| | YKPVAM-- | | |
| | YIPVAM-- | | |
| | -YPVAMS- | | |
| | -YPVAMT- | | |
| | -YPVAMH- | | |
| | YFPVAM-- | | |
| | YEPVAM-- | | |
| | -YPVAMV- | | |
| | YNPVAM-- | | |
| | YHPVAM-- | | |
| | YAPVAM-- | | |
| | YYPVAM-- | | |
| | -YPVAMQ- | | |
| | -YPVAMG- | | |
| | -YPVAME- | | |
| | YMPVAM-- | | |
| PFGL | 128 | 751 | 31.71 |
| | -GPFGLG- | | |
| | -KPFGLN- | | |
| | -KPFGLE- | | |
| | -MPFGLG- | | |
| | --PFGLPS | | |
| | --PFGLNN | | |
| | --PFGLGT | | |
| | -TPFGLG- | | |
| | -VPFGLG- | | |
| | -APFGLR- | | |
| | -APFGLS- | | |
| | ALPFGL-- | | |
| | AVPFGL-- | | |
| | ARPFGL-- | | |
| | AAPFGL-- | | |
| | AGPFGL-- | | |
| | -APFGLN- | | |
| | -APFGLH- | | |
| | AMPFGL-- | | |
| | AEPFGL-- | | |
| | AWPFGL-- | | |
| | -APFGLV- | | |
| | -APFGLL- | | |
| | AKPFGL-- | | |
| | AFPFGL-- | | |
| | -APFGLM- | | |
| | ANPFGL-- | | |
| | AIPFGL-- | | |
| | ASPFGL-- | | |
| | -APFGLY- | | |
| | -APFGLK- | | |
| | AYPFGL-- | | |
| | AQPFGL-- | | |

ATPFGL--
APPFGL--
-APFGLW-
ADPFGL--
-APFGLE-
-APFGLQ-
-APFGLA-
AHPFGL--
-APFGLG-
-APFGLT-
CSPFGL--
CGPFGL--
-CPFGLL-
CCPFGL--
-CPFGLV-
CNPFGL--
CKPFGL--
CVPFGL--
CQPFGL--
-CPFGLM-
-CPFGLF-
CLPFGL--
CHPFGL--
CAPFGL--
-DPFGLR-
-DPFGLH-
DSPFGL--
DLPFGL--
DRPFGL--
DKPFGL--
DNPFGL--
DMPFGL--
-DPFGLK-
DWPFGL--
DTPFGL--
-DPFGLT-
DFPFGL--
DQPFGL--
-DPFGLW-
DGPFGL--
DAPFGL--
-DPFGLS-
-DPFGLM-
DHPFGL--
EMPFGL--
-EPFGLS-
-EPFGLR-
-EPFGLI-
-EPFGLT-
ERPFGL--

-EPFGLK-
-EPFGLH-
-EPFGLE-
EIPFGL--
EVPFGL--
-EPFGLW-
-EPFGLV-
-EPFGLM-
-EPFGLL-
ENPFGL--
EFPFGL--
EAPFGL--
-EPFGLF-
ELPFGL--
-EPFGLY-
-EPFGLQ-
-EPFGLA-
EWPFGL--
ETPFGL--
EYPFGL--
EQPFGL--
EKPFGL--
EEPFGL--
FSPFGL--
FVPFGL--
FTPFGL--
-FPFGLS-
FLPFGL--
FGPFGL--
FQPFGL--
-FPFGLV-
-FPFGLR-
-FPFGLQ-
-FPFGLL-
-FPFGLH-
FKPFGL--
FRPFGL--
FPPFGL--
-FPFGLN-
FFPFGL--
-FPFGLK-
-FPFGLI-
FMPFGL--
-FPFGLY-
FIPFGL--
FDPFGL--
FWPFGL--
-FPFGLM-
FEPFGL--
FCPFGL--

GRPFGL--
-GPFGLR-
-GPFGLS-
GKPFGL--
GTPFGL--
GSPFGL--
GVPFGL--
GHPFGL--
-GPFGLF-
GAPFGL--
GYPFGL--
-GPFGLM-
-GPFGLA-
GLPFGL--
-GPFGLH-
GWPFGL--
GDPFGL--
-GPFGLW-
-GPFGLV-
GIPFGL--
GFPFGL--
-GPFGLT-
GNPFGL--
GEPFGL--
-GPFGLK-
-GPFGLI-
-GPFGLQ-
-GPFGLN-
GMPFGL--
GQPFGL--
GCPFGL--
HRPFGL--
HGPFGL--
HWPFGL--
HKPFGL--
-HPFGLL-
-HPFGLK-
HSPFGL--
HNPFGL--
HMPFGL--
HPPFGL--
HFPFGL--
-HPFGLR-
-HPFGLQ-
-HPFGLI-
HEPFGL--
-HPFGLW-
HTPFGL--
-HPFGLY-
-HPFGLN-

-HPFGLH-
-HPFGLF-
HLPFGL--
HIPFGL--
HHPFGL--
HAPFGL--
HVPFGL--
-HPFGLV-
-HPFGLS-
HDPFGL--
HYPFGL--
-HPFGLE-
-HPFGLA-
HCPFGL--
IVPFGL--
IEPFGL--
-IPFGLV-
IAPFGL--
-IPFGLR-
INPFGL--
-IPFGLT-
IKPFGL--
ISPFGL--
-IPFGLM-
-IPFGLK-
-IPFGLN-
-IPFGLL-
IFPFGL--
ILPFGL--
IYPFGL--
IPPFGL--
-IPFGLS-
IHPFGL--
IWPFGL--
-IPFGLF-
IQPFGL--
-IPFGLQ-
IMPFGL--
IRPFGL--
IGPFGL--
ITPFGL--
-IPFGLI-
-IPFGLE-
IIPFGL--
-IPFGLH-
IDPFGL--
ICPFGL--
KSPFGL--
-KPFGLR-
KPPFGL--

KGPFGL--
-KPFGLV-
-KPFGLS-
KLPFGL--
-KPFGLY-
KYPFGL--
-KPFGLQ-
KNPFGL--
KTPFGL--
KAPFGL--
-KPFGLK-
KMPFGL--
KIPFGL--
-KPFGLM-
-KPFGLL-
KFPFGL--
KWPFGL--
-KPFGLH-
-KPFGLI-
-KPFGLF-
KEPFGL--
KDPFGL--
KCPFGL--
KQPFGL--
-KPFGLT-
KHPFGL--
LRPFGL--
LLPFGL--
LFPFGL--
-LPFGLH-
-LPFGLS-
-LPFGLL-
-LPFGLK-
LKPFGL--
-LPFGLV-
LVPFGL--
LTPFGL--
LHPFGL--
LAPFGL--
LDPFGL--
LMPFGL--
LYPFGL--
-LPFGLQ-
LEPFGL--
-LPFGLT-
LWPFGL--
-LPFGLN-
-LPFGLF-
LCPFGL--
-LPFGLM-

```
-LPFGLA-
LIPFGL--
MRPFGL--
-MPFGLL-
-MPFGLM-
-MPFGLA-
MKPFGL--
MNPFGL--
-MPFGLR-
MFPFGL--
MPPFGL--
-MPFGLH-
MLPFGL--
MSPFGL--
-MPFGLY-
-MPFGLS-
-MPFGLQ-
MMPFGL--
MAPFGL--
MEPFGL--
MIPFGL--
MWPFGL--
-MPFGLE-
MGPFGL--
MYPFGL--
MTPFGL--
-MPFGLK-
MDPFGL--
MCPFGL--
-MPFGLV-
-MPFGLF-
-NPFGLT-
-NPFGLR-
NSPFGL--
NTPFGL--
-NPFGLL-
NNPFGL--
NRPFGL--
NLPFGL--
-NPFGLV-
-NPFGLI-
NAPFGL--
NWPFGL--
NPPFGL--
NGPFGL--
NQPFGL--
-NPFGLF-
NDPFGL--
-NPFGLQ-
-NPFGLN-
```

NIPFGL--
NHPFGL--
NCPFGL--
NVPFGL--
-NPFGLA-
NMPFGL--
NKPFGL--
--PFGLRK
--PFGLLR
--PFGLRL
--PFGLMS
--PFGLAS
--PFGLSS
--PFGLTS
--PFGLSG
--PFGLRV
--PFGLRH
PSPFGL--
--PFGLTT
--PFGLSV
--PFGLRR
--PFGLRG
--PFGLVS
--PFGLVR
--PFGLST
PIPFGL--
PFPFGL--
--PFGLIR
--PFGLIK
--PFGLRY
--PFGLKS
--PFGLHR
--PFGLHA
--PFGLES
--PFGLYS
--PFGLYR
--PFGLVA
--PFGLTG
--PFGLRQ
--PFGLLT
--PFGLLA
--PFGLKK
--PFGLIT
--PFGLHL
--PFGLYT
--PFGLYH
--PFGLTV
--PFGLKH
PLPFGL--
--PFGLVN

--PFGLLG
--PFGLHT
PVPFGL--
PRPFGL--
--PFGLVM
--PFGLSI
--PFGLRE
--PFGLKT
--PFGLGS
PNPFGL--
--PFGLVK
--PFGLSA
--PFGLRW
--PFGLRT
--PFGLRD
--PFGLGA
--PFGLAT
--PFGLAG
--PFGLVH
--PFGLTF
--PFGLRN
--PFGLQA
--PFGLMK
--PFGLLN
--PFGLIY
--PFGLFK
--PFGLFH
--PFGLEG
--PFGLAA
--PFGLWV
--PFGLWG
--PFGLVV
--PFGLNA
--PFGLMD
--PFGLIS
--PFGLHG
--PFGLFW
--PFGLAY
PDPFGL--
PKPFGL--
--PFGLWR
--PFGLVD
--PFGLRM
--PFGLRF
--PFGLNS
--PFGLLH
--PFGLKN
--PFGLKG
--PFGLHS
--PFGLHK

--PFGLFR
PAPFGL--
PQPFGL--
--PFGLYN
--PFGLYK
--PFGLTH
--PFGLSN
--PFGLMY
--PFGLMA
--PFGLLD
--PFGLIG
--PFGLFT
--PFGLFQ
--PFGLAV
PEPFGL--
PYPFGL--
PMPFGL--
PGPFGL--
--PFGLWK
--PFGLTR
--PFGLTN
--PFGLNY
--PFGLNV
--PFGLNT
--PFGLMN
--PFGLLQ
--PFGLKV
--PFGLKF
--PFGLKA
--PFGLIA
--PFGLHI
--PFGLFS
--PFGLFE
PWPFGL--
--PFGLYQ
--PFGLVW
--PFGLVQ
--PFGLVI
--PFGLTD
--PFGLSK
--PFGLSF
--PFGLQS
--PFGLQG
--PFGLMV
--PFGLMH
--PFGLII
--PFGLHF
--PFGLFD
--PFGLYE
--PFGLWY

--PFGLWE
--PFGLTA
--PFGLSR
--PFGLSH
--PFGLQV
--PFGLMT
--PFGLKW
--PFGLKQ
--PFGLHW
--PFGLHV
--PFGLHH
--PFGLFN
--PFGLEA
--PFGLAL
PTPFGL--
--PFGLYD
--PFGLWQ
--PFGLWN
--PFGLVE
--PFGLTQ
--PFGLTI
--PFGLQW
--PFGLQN
--PFGLQM
--PFGLQK
--PFGLNM
--PFGLNK
--PFGLMQ
--PFGLMM
--PFGLMG
--PFGLKY
--PFGLKM
--PFGLKD
--PFGLIM
--PFGLHN
--PFGLGG
--PFGLET
--PFGLAM
PHPFGL--
--PFGLYG
--PFGLWD
--PFGLTW
--PFGLTM
--PFGLTE
--PFGLTC
--PFGLSW
--PFGLSQ
--PFGLQR
--PFGLQQ
--PFGLQH

--PFGLQF
--PFGLPL
--PFGLNW
--PFGLNQ
--PFGLNH
--PFGLNG
--PFGLMW
--PFGLME
--PFGLLE
--PFGLKI
--PFGLIW
--PFGLIN
--PFGLIE
--PFGLID
--PFGLHY
--PFGLHQ
--PFGLHD
--PFGLCQ
-QPFGLK-
QRPFGL--
QSPFGL--
-QPFGLV-
-QPFGLT-
QGPFGL--
QWPFGL--
QLPFGL--
QKPFGL--
QVPFGL--
QPPFGL--
-QPFGLY-
-QPFGLL-
QMPFGL--
-QPFGLS-
-QPFGLQ-
-QPFGLN-
-QPFGLI-
QIPFGL--
QQPFGL--
QEPFGL--
QDPFGL--
QYPFGL--
-QPFGLW-
-QPFGLM-
-QPFGLH-
QTPFGL--
-QPFGLE-
-QPFGLC-
-QPFGLA-
QFPFGL--
QCPFGL--

QAPFGL--
-RPFGLS-
-RPFGLR-
-RPFGLA-
RIPFGL--
RLPFGL--
RGPFGL--
RVPFGL--
-RPFGLN-
-RPFGLV-
RAPFGL--
-RPFGLI-
RMPFGL--
RKPFGL--
RYPFGL--
RNPFGL--
RQPFGL--
RTPFGL--
-RPFGLF-
RHPFGL--
RDPFGL--
-RPFGLM-
-RPFGLH-
-RPFGLG-
RPPFGL--
-RPFGLE-
RFPFGL--
REPFGL--
RWPFGL--
RCPFGL--
-RPFGLQ-
-RPFGLP-
-SPFGLK-
-SPFGLL-
-SPFGLR-
SIPFGL--
SAPFGL--
SYPFGL--
SLPFGL--
-SPFGLH-
SMPFGL--
-SPFGLS-
SNPFGL--
-SPFGLN-
-SPFGLA-
-SPFGLQ-
SFPFGL--
SDPFGL--
SVPFGL--
STPFGL--

SHPFGL--
-SPFGLV-
-SPFGLF-
SKPFGL--
SWPFGL--
SQPFGL--
-SPFGLM-
SPPFGL--
SCPFGL--
SEPFGL--
-SPFGLC-
TRPFGL--
TFPFGL--
-TPFGLK-
TKPFGL--
-TPFGLS-
THPFGL--
TSPFGL--
-TPFGLT-
TPPFGL--
-TPFGLH-
-TPFGLF-
TMPFGL--
TVPFGL--
TTPFGL--
TNPFGL--
TLPFGL--
TAPFGL--
-TPFGLM-
TYPFGL--
-TPFGLN-
TGPFGL--
TDPFGL--
TWPFGL--
-TPFGLQ-
-TPFGLI-
TCPFGL--
-TPFGLA-
TIPFGL--
-VPFGLA-
VRPFGL--
VSPFGL--
-VPFGLT-
-VPFGLK-
VYPFGL--
-VPFGLI-
VKPFGL--
VHPFGL--
-VPFGLY-
VFPFGL--

VWPFGL--
-VPFGLN-
-VPFGLH-
VMPFGL--
VIPFGL--
VTPFGL--
-VPFGLS-
VGPFGL--
VAPFGL--
-VPFGLQ-
VQPFGL--
-VPFGLV-
VPPFGL--
VNPFGL--
-VPFGLM-
VDPFGL--
-VPFGLE-
VEPFGL--
VCPFGL--
WRPFGL--
WSPFGL--
WQPFGL--
-WPFGLR-
-WPFGLV-
-WPFGLT-
-WPFGLS-
-WPFGLK-
WGPFGL--
-WPFGLY-
WPPFGL--
WNPFGL--
WKPFGL--
WTPFGL--
WHPFGL--
-WPFGLM-
WMPFGL--
WIPFGL--
WFPFGL--
WEPFGL--
-WPFGLN-
-WPFGLH-
-WPFGLA-
WAPFGL--
YRPFGL--
-YPFGLS-
YVPFGL--
-YPFGLT-
-YPFGLL-
YQPFGL--
YLPFGL--

YFPFGL--
-YPFGLR-
YIPFGL--
-YPFGLK-
YKPFGL--
-YPFGLI-
YGPFGL--
YMPFGL--
-YPFGLA-
YWPFGL--
YSPFGL--
YHPFGL--
YYPFGL--
YPPFGL--
-YPFGLQ-
-YPFGLN-
-YPFGLE-
YCPFGL--

PKAI 479 609 31.71

PGPKAI--
-APKAIW-
-APKAIS-
AWPKAI--
-APKAIN-
AYPKAI--
AVPKAI--
-APKAIV-
-APKAIT-
AEPKAI--
ASPKAI--
-APKAIG-
-APKAIF-
ANPKAI--
ALPKAI--
-APKAII-
ATPKAI--
APPKAI--
-APKAIK-
AGPKAI--
AKPKAI--
ADPKAI--
CRPKAI--
CLPKAI--
CKPKAI--
-CPKAIT-
-CPKAIQ-
-CPKAIS-
-CPKAII-
CDPKAI--
-DPKAIL-

DYPKAI--
-DPKAIA-
-DPKAIW-
-DPKAIS-
-DPKAIK-
DAPKAI--
DSPKAI--
-DPKAIV-
-DPKAIR-
-DPKAII-
DMPKAI--
DIPKAI--
DVPKAI--
-DPKAIQ-
-DPKAIF-
EQPKAI--
EYPKAI--
ERPKAI--
ESPKAI--
-EPKAIT-
ENPKAI--
-EPKAIR-
EVPKAI--
-EPKAIL-
EGPKAI--
EFPKAI--
ECPKAI--
EAPKAI--
-EPKAIY-
-EPKAIW-
-EPKAIQ-
EIPKAI--
-FPKAIV-
-FPKAIM-
-FPKAIL-
FSPKAI--
FIPKAI--
FLPKAI--
-FPKAIK-
FFPKAI--
FEPKAI--
-FPKAIY-
-FPKAIS-
FGPKAI--
FVPKAI--
FPPKAI--
-FPKAIH-
-FPKAIA-
FAPKAI--
GMPKAI--

GLPKAI--
-GPKAIQ-
-GPKAIY-
GWPKAI--
GSPKAI--
-GPKAIW-
-GPKAIT-
-GPKAIL-
-GPKAIH-
GKPKAI--
GPPKAI--
-GPKAIV-
GTPKAI--
-GPKAIN-
-GPKAIK-
-GPKAII-
GHPKAI--
-GPKAIA-
GNPKAI--
GDPKAI--
GQPKAI--
-GPKAIF-
GIPKAI--
GGPKAI--
GCPKAI--
GYPKAI--
-GPKAIG-
GFPKAI--
GEPKAI--
HSPKAI--
-HPKAIL-
-HPKAIW-
HAPKAI--
HRPKAI--
HKPKAI--
-HPKAIY-
HTPKAI--
-HPKAIA-
HVPKAI--
HLPKAI--
HQPKAI--
-HPKAII-
HNPKAI--
HEPKAI--
HCPKAI--
HYPKAI--
HPPKAI--
-HPKAIK-
HMPKAI--
HGPKAI--

ITPKAI--
-IPKAIL-
IKPKAI--
IFPKAI--
ISPKAI--
-IPKAIK-
-IPKAII-
-IPKAIH-
INPKAI--
IMPKAI--
-IPKAIA-
IVPKAI--
-IPKAIF-
IDPKAI--
IAPKAI--
-IPKAIW-
-IPKAIG-
-IPKAIE-
IIPKAI--
IEPKAI--
-KPKAIA-
KAPKAI--
KRPKAI--
-KPKAIH-
KIPKAI--
-KPKAIW-
KLPKAI--
-KPKAII-
KPPKAI--
-KPKAIK-
KFPKAI--
KEPKAI--
KGPKAI--
KVPKAI--
-KPKAIV-
-KPKAIT-
-KPKAIS-
-KPKAIQ-
-KPKAIN-
KNPKAI--
KMPKAI--
KDPKAI--
KYPKAI--
-KPKAIM-
-KPKAIF-
KHPKAI--
LTPKAI--
-LPKAIL-
LRPKAI--
-LPKAIT-

LLPKAI--
LFPKAI--
LSPKAI--
LWPKAI--
LPPKAI--
LHPKAI--
LAPKAI--
LVPKAI--
-LPKAIW-
-LPKAIV-
LMPKAI--
LEPKAI--
LKPKAI--
-LPKAIM-
-LPKAIF-
LNPKAI--
LIPKAI--
LGPKAI--
LDPKAI--
-LPKAIQ-
LCPKAI--
-LPKAIH-
MVPKAI--
-MPKAIS-
-MPKAIV-
MQPKAI--
MNPKAI--
MIPKAI--
-MPKAIW-
MDPKAI--
MAPKAI--
MPPKAI--
-MPKAIN-
-MPKAIH-
-MPKAIL-
-MPKAIK-
MGPKAI--
MYPKAI--
MSPKAI--
-MPKAIQ-
-MPKAIG-
-MPKAIA-
MLPKAI--
MKPKAI--
-NPKAIT-
NRPKAI--
NHPKAI--
NFPKAI--
NDPKAI--
NSPKAI--

NVPKAI--
-NPKAIS-
-NPKAIF-
-NPKAIR-
-NPKAIK-
NGPKAI--
NAPKAI--
NPPKAI--
NMPKAI--
NTPKAI--
NQPKAI--
-NPKAIL-
-NPKAII-
NLPKAI--
NKPKAI--
NIPKAI--
NEPKAI--
-NPKAIY-
-NPKAIW-
-NPKAIA-
NNPKAI--
--PKAIRS
--PKAILI
--PKAIVS
--PKAISL
--PKAIFM
--PKAIVN
--PKAILT
--PKAIFT
PVPKAI--
--PKAITV
--PKAIKL
--PKAILK
--PKAISW
PLPKAI--
--PKAIYK
--PKAIRI
--PKAIQV
--PKAIMS
--PKAIMK
--PKAILL
--PKAIIK
--PKAIGG
--PKAIFE
--PKAIYF
--PKAIHL
--PKAIGS
--PKAIAL
--PKAIVT
--PKAISA

--PKAIPM
--PKAILS
--PKAILG
--PKAIKA
--PKAIHF
--PKAIAM
--PKAIYW
--PKAIWN
--PKAIWG
--PKAIVW
--PKAIVH
--PKAISV
--PKAISF
--PKAIRN
--PKAIRD
--PKAIIF
--PKAIWS
--PKAIWK
--PKAIVY
--PKAIVV
--PKAIVK
--PKAITI
--PKAITA
--PKAISY
--PKAISM
--PKAIRE
--PKAIQS
--PKAIQL
--PKAIQF
--PKAINL
--PKAINA
--PKAIMF
--PKAIMA
--PKAILF
--PKAILE
--PKAIKT
--PKAIKH
--PKAIKG
--PKAIKD
--PKAIHS
--PKAIAY
--PKAIAV
--PKAIAI
--PKAIAF
PFPKAI--
PAPKAI--
PSPKAI--
PNPKAI--
PMPKAI--
--PKAIYY

--PKAIYT
--PKAIYM
--PKAIYH
--PKAIWT
--PKAIVL
--PKAITT
--PKAITS
--PKAITQ
--PKAITM
--PKAISK
--PKAIQM
--PKAIPF
--PKAINY
--PKAINS
--PKAINF
--PKAILV
--PKAIKQ
--PKAIKN
--PKAIKF
--PKAIKE
--PKAIIW
--PKAIID
--PKAIGA
--PKAIFQ
--PKAIFN
--PKAIAS
--PKAIAN
--PKAIAD
--PKAIAA
PEPKAI--
--PKAIYS
--PKAIWV
--PKAIWI
--PKAIWA
--PKAITW
--PKAISN
--PKAISI
--PKAISH
--PKAISD
--PKAIRM
--PKAIQW
--PKAIQI
--PKAIQH
--PKAIQA
--PKAINT
--PKAINR
--PKAINH
--PKAIMV
--PKAIKS
--PKAIIQ

--PKAIII
--PKAIIG
--PKAIIE
--PKAIHW
--PKAIHV
--PKAIHQ
--PKAIHK
--PKAIHD
--PKAIHA
--PKAIGY
--PKAIFV
--PKAIFK
--PKAIFI
--PKAIET
--PKAIEL
--PKAIEG
--PKAIAW
--PKAIAK
PRPKAI--
PKPKAI--
--PKAIYN
--PKAIYD
--PKAIWM
--PKAIWL
--PKAIWF
--PKAIWE
--PKAIWD
--PKAITY
--PKAITF
--PKAITE
--PKAISE
--PKAIRV
--PKAIQQ
--PKAIQG
--PKAIQD
--PKAINW
--PKAINM
--PKAINK
--PKAIMY
--PKAIMT
--PKAIMN
--PKAIMM
--PKAIMH
--PKAIMG
--PKAILC
--PKAIKY
--PKAIKM
--PKAIIS
--PKAIIH
--PKAIHI

--PKAIHG
--PKAIHE
--PKAIGT
--PKAIGM
--PKAIFW
--PKAIFH
--PKAIFG
--PKAIFF
--PKAIFD
--PKAIDS
--PKAIAG
PDPKAI--
PCPKAI--
-QPKAIF-
QGPKAI--
QMPKAI--
QFPKAI--
QPPKAI--
QRPKAI--
-QPKAIY-
-QPKAIW-
QVPKAI--
QSPKAI--
-QPKAIV-
-QPKAIT-
-QPKAII-
QLPKAI--
QAPKAI--
QYPKAI--
QWPKAI--
-QPKAIE-
QIPKAI--
QDPKAI--
-RPKAIV-
RIPKAI--
RMPKAI--
RAPKAI--
-RPKAIY-
-RPKAIF-
RRPKAI--
-RPKAIT-
RGPKAI--
RTPKAI--
RPPKAI--
-RPKAIH-
RDPKAI--
RFPKAI--
-RPKAIW-
-RPKAIQ-
RHPKAI--

```
-RPKAIA-
RNPKAI--
-RPKAIN-
-RPKAIC-
RQPKAI--
-RPKAIK-
REPKAI--
SRPKAI--
-SPKAIA-
SSPKAI--
-SPKAIT-
-SPKAIS-
SGPKAI--
SYPKAI--
-SPKAIF-
SFPKAI--
-SPKAIW-
-SPKAIE-
SIPKAI--
-SPKAIG-
-SPKAIN-
SKPKAI--
SDPKAI--
-SPKAIV-
SNPKAI--
SEPKAI--
STPKAI--
SQPKAI--
SPPKAI--
-SPKAIY-
-SPKAIM-
-SPKAII-
SCPKAI--
TLPKAI--
TYPKAI--
-TPKAIS-
TWPKAI--
TVPKAI--
-TPKAIW-
-TPKAIT-
TTPKAI--
TSPKAI--
TRPKAI--
-TPKAIM-
TQPKAI--
-TPKAIV-
THPKAI--
-TPKAIN-
TMPKAI--
TFPKAI--
```

-TPKAIF-
TKPKAI--
TGPKAI--
TPPKAI--
-TPKAIQ-
-TPKAII-
-TPKAIH-
-TPKAIA-
TEPKAI--
TAPKAI--
-VPKAIL-
-VPKAIT-
VRPKAI--
-VPKAIS-
VSPKAI--
VQPKAI--
-VPKAIR-
VLPKAI--
-VPKAIN-
VMPKAI--
VGPKAI--
VVPKAI--
VNPKAI--
VFPKAI--
-VPKAIW-
-VPKAII-
-VPKAIQ-
-VPKAIF-
VHPKAI--
VPPKAI--
-VPKAIM-
-VPKAIH-
-VPKAIA-
VKPKAI--
VAPKAI--
VYPKAI--
VTPKAI--
-VPKAIY-
-VPKAIK-
VIPKAI--
VEPKAI--
WRPKAI--
-WPKAIF-
-WPKAII-
WVPKAI--
WMPKAI--
WYPKAI--
WKPKAI--
WQPKAI--
-WPKAIM-

| | | | |
|---|---|---|---|
| | WAPKAI-- | | |
| | WWPKAI-- | | |
| | -WPKAIY- | | |
| | WEPKAI-- | | |
| | WSPKAI-- | | |
| | WNPKAI-- | | |
| | WFPKAI-- | | |
| | -YPKAIF- | | |
| | YGPKAI-- | | |
| | YYPKAI-- | | |
| | YSPKAI-- | | |
| | -YPKAIS- | | |
| | -YPKAIQ- | | |
| | -YPKAIM- | | |
| | YKPKAI-- | | |
| | YVPKAI-- | | |
| | YRPKAI-- | | |
| | -YPKAII- | | |
| | YIPKAI-- | | |
| | YHPKAI-- | | |
| | YQPKAI-- | | |
| | -YPKAIV- | | |
| | -YPKAIK- | | |
| | YNPKAI-- | | |
| | YWPKAI-- | | |
| | YPPKAI-- | | |
| | -YPKAIT- | | |
| | YDPKAI-- | | |
| PHSL | 66 | 794 | 31.69 |
| | -GPHSLG- | | |
| | -HPHSLI- | | |
| | -LPHSLN- | | |
| | --PHSLLL | | |
| | --PHSLSH | | |
| | --PHSLPI | | |
| | --PHSLNM | | |
| | --PHSLGY | | |
| | --PHSLGV | | |
| | --PHSLGT | | |
| | --PHSLGS | | |
| | --PHSLAT | | |
| | --PHSLAI | | |
| | -SPHSLP- | | |
| | -TPHSLE- | | |
| | -YPHSLG- | | |
| | -YPHSLA- | | |
| | -APHSLR- | | |
| | AWPHSL-- | | |
| | ALPHSL-- | | |
| | -APHSLL- | | |

ATPHSL--
-APHSLG-
AGPHSL--
ASPHSL--
AMPHSL--
-APHSLY-
AVPHSL--
-APHSLT-
-APHSLS-
AIPHSL--
AQPHSL--
-APHSLA-
-APHSLH-
ANPHSL--
-APHSLV-
AKPHSL--
AAPHSL--
-APHSLQ-
AHPHSL--
-APHSLM-
-APHSLK-
-APHSLI-
AYPHSL--
-APHSLE-
AFPHSL--
-APHSLW-
AEPHSL--
ADPHSL--
ACPHSL--
CFPHSL--
CSPHSL--
-CPHSLI-
CIPHSL--
CTPHSL--
CNPHSL--
CLPHSL--
CVPHSL--
CQPHSL--
-CPHSLM-
-CPHSLF-
CKPHSL--
CYPHSL--
CPPHSL--
-CPHSLT-
CMPHSL--
CGPHSL--
CAPHSL--
DVPHSL--
DTPHSL--
DWPHSL--

DRPHSL--
-DPHSLR-
DSPHSL--
-DPHSLL-
DKPHSL--
DGPHSL--
DQPHSL--
-DPHSLH-
-DPHSLF-
DFPHSL--
DAPHSL--
DLPHSL--
-DPHSLV-
-DPHSLK-
-DPHSLI-
DEPHSL--
-DPHSLY-
-DPHSLW-
-DPHSLT-
-DPHSLS-
-DPHSLM-
-DPHSLE-
DMPHSL--
DHPHSL--
ESPHSL--
EGPHSL--
ERPHSL--
-EPHSLI-
-EPHSLK-
-EPHSLR-
EKPHSL--
ETPHSL--
ELPHSL--
EIPHSL--
-EPHSLS-
-EPHSLM-
-EPHSLF-
EMPHSL--
EAPHSL--
EQPHSL--
-EPHSLV-
EHPHSL--
EDPHSL--
EYPHSL--
EPPHSL--
-EPHSLT-
EFPHSL--
-EPHSLY-
-EPHSLW-
-EPHSLQ-

-EPHSLN-
-EPHSLA-
ENPHSL--
EEPHSL--
FLPHSL--
FRPHSL--
-FPHSLM-
FDPHSL--
FGPHSL--
FAPHSL--
FWPHSL--
FKPHSL--
-FPHSLT-
-FPHSLL-
FHPHSL--
FYPHSL--
FQPHSL--
-FPHSLR-
-FPHSLK-
-FPHSLH-
-FPHSLN-
-FPHSLI-
FEPHSL--
-FPHSLY-
-FPHSLW-
-FPHSLV-
-FPHSLA-
FPPHSL--
-FPHSLQ-
FMPHSL--
GLPHSL--
-GPHSLR-
GAPHSL--
GSPHSL--
GKPHSL--
GRPHSL--
-GPHSLL-
GHPHSL--
-GPHSLN-
GFPHSL--
-GPHSLI-
GIPHSL--
GGPHSL--
GVPHSL--
-GPHSLW-
-GPHSLV-
-GPHSLQ-
-GPHSLT-
-GPHSLM-
-GPHSLA-

−GPHSLS−
−GPHSLK−
GYPHSL−−
GQPHSL−−
GPPHSL−−
−GPHSLH−
−GPHSLF−
GNPHSL−−
GEPHSL−−
GMPHSL−−
−GPHSLY−
−GPHSLE−
−HPHSLR−
HVPHSL−−
HSPHSL−−
HRPHSL−−
HLPHSL−−
HGPHSL−−
HWPHSL−−
HAPHSL−−
HKPHSL−−
HIPHSL−−
HYPHSL−−
−HPHSLA−
HQPHSL−−
−HPHSLH−
−HPHSLW−
−HPHSLQ−
−HPHSLL−
−HPHSLF−
HHPHSL−−
HEPHSL−−
HDPHSL−−
−HPHSLY−
−HPHSLT−
−HPHSLS−
−HPHSLM−
HNPHSL−−
−HPHSLK−
−HPHSLV−
−HPHSLP−
−HPHSLN−
−HPHSLG−
HCPHSL−−
−IPHSLA−
−IPHSLT−
IRPHSL−−
ISPHSL−−
−IPHSLM−
ILPHSL−−

ITPHSL--
-IPHSLS-
IAPHSL--
IVPHSL--
-IPHSLL-
-IPHSLF-
INPHSL--
IFPHSL--
IYPHSL--
-IPHSLR-
IEPHSL--
IWPHSL--
-IPHSLN-
-IPHSLH-
IIPHSL--
IQPHSL--
-IPHSLW-
-IPHSLQ-
IHPHSL--
-IPHSLE-
IMPHSL--
IKPHSL--
-IPHSLI-
IDPHSL--
ICPHSL--
KSPHSL--
KRPHSL--
-KPHSLS-
-KPHSLL-
-KPHSLT-
-KPHSLI-
-KPHSLR-
-KPHSLK-
KLPHSL--
KVPHSL--
-KPHSLV-
KFPHSL--
KYPHSL--
KAPHSL--
-KPHSLF-
KIPHSL--
-KPHSLW-
KTPHSL--
-KPHSLQ-
KKPHSL--
KQPHSL--
KPPHSL--
-KPHSLN-
-KPHSLE-
KNPHSL--

-KPHSLY-
KEPHSL--
-KPHSLM-
-KPHSLG-
KDPHSL--
KCPHSL--
LSPHSL--
LMPHSL--
LLPHSL--
LIPHSL--
-LPHSLR-
-LPHSLK-
LKPHSL--
-LPHSLV-
LVPHSL--
LAPHSL--
LYPHSL--
LPPHSL--
-LPHSLM-
LTPHSL--
LFPHSL--
LQPHSL--
-LPHSLW-
-LPHSLT-
-LPHSLI-
-LPHSLF-
LHPHSL--
-LPHSLH-
LDPHSL--
-LPHSLQ-
LEPHSL--
LCPHSL--
MRPHSL--
-MPHSLY-
-MPHSLS-
MHPHSL--
-MPHSLV-
MKPHSL--
MWPHSL--
MVPHSL--
MFPHSL--
MPPHSL--
-MPHSLK-
MGPHSL--
MLPHSL--
MIPHSL--
MAPHSL--
MTPHSL--
MQPHSL--
-MPHSLI-

MMPHSL--
MEPHSL--
-MPHSLT-
-MPHSLQ-
-MPHSLN-
-MPHSLM-
-MPHSLA-
MNPHSL--
MDPHSL--
-MPHSLW-
-MPHSLH-
MYPHSL--
-MPHSLF-
NGPHSL--
NWPHSL--
NIPHSL--
-NPHSLF-
NAPHSL--
NVPHSL--
-NPHSLY-
NPPHSL--
NLPHSL--
NYPHSL--
-NPHSLW-
NHPHSL--
-NPHSLS-
NFPHSL--
NEPHSL--
-NPHSLT-
-NPHSLR-
-NPHSLK-
NMPHSL--
-NPHSLH-
NKPHSL--
NCPHSL--
-NPHSLM-
-NPHSLQ-
NDPHSL--
NQPHSL--
-NPHSLV-
NNPHSL--
--PHSLRA
--PHSLSA
--PHSLLS
--PHSLRH
--PHSLVR
--PHSLST
--PHSLVS
--PHSLSS
PLPHSL--

--PHSLLT
--PHSLFR
--PHSLLG
--PHSLTS
--PHSLRG
PVPHSL--
--PHSLSW
--PHSLLK
--PHSLRQ
--PHSLWG
--PHSLRD
PSPHSL--
--PHSLVG
--PHSLTA
--PHSLRN
--PHSLQA
--PHSLKQ
--PHSLIH
--PHSLFG
--PHSLAG
PNPHSL--
--PHSLTK
--PHSLNS
--PHSLLQ
--PHSLLA
--PHSLIS
--PHSLIR
PQPHSL--
PIPHSL--
--PHSLYD
--PHSLVV
--PHSLTW
--PHSLSY
--PHSLRT
--PHSLNA
--PHSLHG
PRPHSL--
--PHSLYT
--PHSLYS
--PHSLVL
--PHSLHT
--PHSLHN
--PHSLYW
--PHSLYI
--PHSLVA
--PHSLTR
--PHSLKR
--PHSLIG
--PHSLIA
--PHSLFH

--PHSLFA
PHPHSL--
PGPHSL--
--PHSLYY
--PHSLYM
--PHSLVT
--PHSLVQ
--PHSLVK
--PHSLVF
--PHSLTY
--PHSLMN
--PHSLLY
--PHSLFT
--PHSLYG
--PHSLYA
--PHSLWT
--PHSLWL
--PHSLTQ
--PHSLRM
--PHSLMT
--PHSLKW
--PHSLKN
--PHSLKG
--PHSLHR
PAPHSL--
PTPHSL--
--PHSLYQ
--PHSLWV
--PHSLVN
--PHSLVM
--PHSLTT
--PHSLSV
--PHSLSN
--PHSLSK
--PHSLRI
--PHSLMW
--PHSLMI
--PHSLLF
--PHSLIT
--PHSLIK
--PHSLHV
--PHSLVY
--PHSLSL
--PHSLSF
--PHSLQF
--PHSLMK
--PHSLMD
--PHSLKM
--PHSLIY
--PHSLIN

--PHSLIM
--PHSLFF
--PHSLVW
--PHSLVE
--PHSLTV
--PHSLTI
--PHSLQS
--PHSLNV
--PHSLMH
--PHSLMG
--PHSLLR
--PHSLLM
--PHSLKY
--PHSLHS
--PHSLHL
--PHSLHF
--PHSLHD
--PHSLFI
--PHSLAF
--PHSLWI
--PHSLVI
--PHSLVH
--PHSLTN
--PHSLTM
--PHSLTH
--PHSLTG
--PHSLSI
--PHSLMY
--PHSLMM
--PHSLME
--PHSLGL
--PHSLFN
--PHSLAS
PEPHSL--
PYPHSL--
PMPHSL--
--PHSLWM
--PHSLWF
--PHSLRF
--PHSLQG
--PHSLPY
--PHSLNT
--PHSLNL
--PHSLMS
--PHSLLW
--PHSLKS
--PHSLKH
--PHSLIF
--PHSLHY
--PHSLHW

--PHSLHQ
--PHSLHA
--PHSLFV
--PHSLAW
--PHSLAM
--PHSLYF
--PHSLWN
--PHSLWK
--PHSLWH
--PHSLRY
--PHSLQI
--PHSLLI
--PHSLLH
--PHSLKT
--PHSLKL
--PHSLKK
--PHSLKA
--PHSLIW
--PHSLIQ
--PHSLHK
--PHSLGA
--PHSLFQ
--PHSLEG
--PHSLEA
--PHSLAV
PKPHSL--
--PHSLYN
--PHSLYK
--PHSLWE
--PHSLTF
--PHSLTD
--PHSLSM
--PHSLRE
--PHSLQY
--PHSLQW
--PHSLQT
--PHSLQR
--PHSLQN
--PHSLQM
--PHSLNY
--PHSLNG
--PHSLMF
--PHSLLE
--PHSLKV
--PHSLIV
--PHSLIE
--PHSLID
--PHSLHH
--PHSLGG
--PHSLFE

--PHSLES
PDPHSL--
PCPHSL--
--PHSLYH
--PHSLYE
--PHSLWY
--PHSLWW
--PHSLWQ
--PHSLVD
--PHSLQV
--PHSLQQ
--PHSLPM
--PHSLNW
--PHSLNI
--PHSLNH
--PHSLNF
--PHSLLC
--PHSLKD
--PHSLHM
--PHSLHI
--PHSLCL
--PHSLAA
PFPHSL--
-QPHSLT-
QLPHSL--
QGPHSL--
QQPHSL--
QRPHSL--
QSPHSL--
-QPHSLK-
-QPHSLR-
-QPHSLL-
-QPHSLF-
-QPHSLA-
QIPHSL--
QNPHSL--
QTPHSL--
-QPHSLY-
-QPHSLV-
QYPHSL--
-QPHSLM-
QKPHSL--
QVPHSL--
QHPHSL--
-QPHSLS-
QPPHSL--
-QPHSLW-
-QPHSLN-
QAPHSL--
-QPHSLH-

QMPHSL--
QFPHSL--
QEPHSL--
-QPHSLQ-
-QPHSLI-
-RPHSLL-
RMPHSL--
-RPHSLI-
RLPHSL--
RSPHSL--
-RPHSLS-
RYPHSL--
RGPHSL--
RVPHSL--
RHPHSL--
-RPHSLW-
-RPHSLT-
RAPHSL--
-RPHSLN-
RNPHSL--
-RPHSLY-
RWPHSL--
RPPHSL--
-RPHSLF-
-RPHSLH-
RQPHSL--
-RPHSLM-
-RPHSLG-
-RPHSLE-
REPHSL--
RDPHSL--
RCPHSL--
SVPHSL--
-SPHSLL-
SAPHSL--
SGPHSL--
-SPHSLS-
SMPHSL--
STPHSL--
SPPHSL--
-SPHSLY-
-SPHSLT-
SHPHSL--
SKPHSL--
-SPHSLI-
-SPHSLK-
SNPHSL--
-SPHSLF-
-SPHSLH-
SQPHSL--

-SPHSLN-
-SPHSLV-
-SPHSLQ-
SEPHSL--
SCPHSL--
SYPHSL--
SWPHSL--
-SPHSLE-
SDPHSL--
-TPHSLR-
-TPHSLL-
TKPHSL--
TWPHSL--
-TPHSLS-
TFPHSL--
TAPHSL--
-TPHSLH-
TIPHSL--
TNPHSL--
-TPHSLV-
-TPHSLM-
-TPHSLF-
-TPHSLT-
-TPHSLQ-
TEPHSL--
TYPHSL--
-TPHSLW-
TMPHSL--
TPPHSL--
-TPHSLY-
THPHSL--
TDPHSL--
TTPHSL--
TQPHSL--
-TPHSLI-
-TPHSLN-
TCPHSL--
VRPHSL--
VTPHSL--
VVPHSL--
VLPHSL--
-VPHSLR-
VSPHSL--
-VPHSLL-
VYPHSL--
-VPHSLT-
-VPHSLK-
-VPHSLV-
-VPHSLS-
-VPHSLI-

VPPHSL--
-VPHSLQ-
VMPHSL--
VKPHSL--
VHPHSL--
VFPHSL--
VEPHSL--
VAPHSL--
-VPHSLN-
-VPHSLM-
VQPHSL--
-VPHSLF-
VNPHSL--
-VPHSLH-
VWPHSL--
-VPHSLA-
VDPHSL--
WMPHSL--
WVPHSL--
WRPHSL--
-WPHSLR-
-WPHSLH-
WLPHSL--
WGPHSL--
WPPHSL--
-WPHSLM-
WWPHSL--
-WPHSLL-
WAPHSL--
-WPHSLQ-
WIPHSL--
WHPHSL--
WYPHSL--
-WPHSLV-
WKPHSL--
WDPHSL--
-WPHSLF-
-WPHSLA-
WFPHSL--
-WPHSLY-
-WPHSLW-
-WPHSLN-
WEPHSL--
WCPHSL--
YTPHSL--
YRPHSL--
-YPHSLR-
-YPHSLL-
-YPHSLT-
YIPHSL--

```
         -YPHSLV-
         -YPHSLK-
         -YPHSLI-
         YVPHSL--
         YSPHSL--
         YAPHSL--
         YYPHSL--
         -YPHSLS-
         YNPHSL--
         YFPHSL--
         YDPHSL--
         YMPHSL--
         YHPHSL--
         -YPHSLY-
         -YPHSLW-
         -YPHSLQ-
         YQPHSL--
         -YPHSLF-
         YWPHSL--
         YPPHSL--
         -YPHSLH-
         -YPHSLM-
         YGPHSL--
PAPF      280       677     31.68
         --PAPFNN
         -APAPFS-
         ALPAPF--
         -APAPFL-
         ASPAPF--
         -APAPFR-
         AYPAPF--
         AVPAPF--
         -APAPFY-
         -APAPFN-
         -APAPFK-
         AMPAPF--
         ARPAPF--
         AGPAPF--
         AEPAPF--
         ADPAPF--
         -APAPFV-
         -APAPFT-
         -APAPFF-
         ANPAPF--
         AWPAPF--
         ATPAPF--
         -APAPFW-
         -APAPFQ-
         -APAPFI-
         AFPAPF--
```

ACPAPF--
AAPAPF--
-CPAPFR-
CMPAPF--
CKPAPF--
-CPAPFL-
CCPAPF--
-CPAPFV-
-CPAPFG-
CLPAPF--
CHPAPF--
DAPAPF--
-DPAPFQ-
DLPAPF--
DRPAPF--
-DPAPFY-
DVPAPF--
DSPAPF--
DKPAPF--
DYPAPF--
DTPAPF--
-DPAPFS-
-DPAPFI-
-DPAPFF-
DCPAPF--
-DPAPFW-
-DPAPFL-
-DPAPFH-
-DPAPFA-
DNPAPF--
EGPAPF--
-EPAPFT-
ESPAPF--
EQPAPF--
-EPAPFV-
-EPAPFY-
-EPAPFK-
-EPAPFI-
ELPAPF--
ERPAPF--
-EPAPFW-
-EPAPFS-
-EPAPFN-
-EPAPFA-
EKPAPF--
EHPAPF--
EEPAPF--
FSPAPF--
FRPAPF--
-FPAPFL-

-FPAPFR-
-FPAPFA-
FKPAPF--
FHPAPF--
FLPAPF--
-FPAPFH-
FNPAPF--
FYPAPF--
-FPAPFM-
-FPAPFI-
FEPAPF--
FWPAPF--
FVPAPF--
-FPAPFK-
FIPAPF--
FPPAPF--
-FPAPFY-
-FPAPFW-
-FPAPFT-
-FPAPFS-
-FPAPFF-
FMPAPF--
FFPAPF--
GVPAPF--
-GPAPFV-
GRPAPF--
GNPAPF--
-GPAPFS-
-GPAPFR-
GIPAPF--
GYPAPF--
-GPAPFK-
GGPAPF--
GWPAPF--
GLPAPF--
GAPAPF--
GSPAPF--
-GPAPFY-
-GPAPFT-
-GPAPFL-
-GPAPFI-
GMPAPF--
GKPAPF--
-GPAPFQ-
-GPAPFM-
-GPAPFG-
-GPAPFF-
-GPAPFC-
-GPAPFA-
GEPAPF--

GCPAPF--
HFPAPF--
HRPAPF--
HPPAPF--
HVPAPF--
HWPAPF--
HGPAPF--
-HPAPFM-
-HPAPFA-
HMPAPF--
HLPAPF--
HSPAPF--
-HPAPFW-
-HPAPFS-
-HPAPFI-
HQPAPF--
-HPAPFY-
-HPAPFV-
-HPAPFT-
-HPAPFF-
HNPAPF--
HAPAPF--
-IPAPFK-
ILPAPF--
IPPAPF--
-IPAPFL-
IRPAPF--
INPAPF--
IKPAPF--
IEPAPF--
ITPAPF--
IGPAPF--
ISPAPF--
-IPAPFY-
-IPAPFW-
IVPAPF--
IQPAPF--
IYPAPF--
-IPAPFT-
-IPAPFS-
-IPAPFQ-
-IPAPFN-
-IPAPFM-
-IPAPFH-
IIPAPF--
KSPAPF--
-KPAPFS-
-KPAPFT-
KIPAPF--
-KPAPFI-

-KPAPFF-
KGPAPF--
-KPAPFL-
-KPAPFK-
KAPAPF--
-KPAPFH-
KYPAPF--
-KPAPFR-
-KPAPFW-
-KPAPFV-
KKPAPF--
KEPAPF--
KRPAPF--
-KPAPFY-
-KPAPFQ-
-KPAPFN-
KMPAPF--
KLPAPF--
KNPAPF--
KFPAPF--
KWPAPF--
KVPAPF--
KTPAPF--
KPPAPF--
-KPAPFG-
-KPAPFE-
LMPAPF--
-LPAPFF-
LLPAPF--
LRPAPF--
LVPAPF--
-LPAPFK-
LGPAPF--
LYPAPF--
LPPAPF--
-LPAPFT-
-LPAPFS-
LNPAPF--
LFPAPF--
LEPAPF--
LHPAPF--
-LPAPFY-
LKPAPF--
LIPAPF--
LSPAPF--
LQPAPF--
-LPAPFQ-
-LPAPFN-
-LPAPFH-
LCPAPF--

-MPAPFN-
-MPAPFI-
-MPAPFL-
-MPAPFR-
-MPAPFT-
-MPAPFK-
MKPAPF--
MPPAPF--
MSPAPF--
-MPAPFA-
MFPAPF--
MTPAPF--
MRPAPF--
-MPAPFQ-
-MPAPFH-
MEPAPF--
MDPAPF--
MWPAPF--
MQPAPF--
MMPAPF--
MIPAPF--
MGPAPF--
MYPAPF--
MVPAPF--
-MPAPFY-
-MPAPFV-
MNPAPF--
MLPAPF--
NSPAPF--
-NPAPFR-
NNPAPF--
-NPAPFV-
NWPAPF--
NVPAPF--
NRPAPF--
-NPAPFI-
-NPAPFA-
NLPAPF--
NPPAPF--
-NPAPFT-
-NPAPFM-
NYPAPF--
-NPAPFL-
NKPAPF--
NDPAPF--
NAPAPF--
NTPAPF--
NQPAPF--
-NPAPFS-
-NPAPFK-

-NPAPFF-
NMPAPF--
NIPAPF--
NFPAPF--
NEPAPF--
--PAPFRQ
--PAPFSR
--PAPFRS
--PAPFRY
--PAPFLL
--PAPFWW
--PAPFTS
--PAPFTR
--PAPFRK
--PAPFRA
--PAPFRG
--PAPFLK
--PAPFRL
--PAPFQG
--PAPFWS
--PAPFSL
--PAPFSG
--PAPFIV
PVPAPF--
--PAPFTI
--PAPFSV
--PAPFRT
--PAPFLH
--PAPFWH
--PAPFVS
--PAPFTN
--PAPFSW
--PAPFSS
--PAPFRE
--PAPFLI
--PAPFLA
--PAPFKQ
--PAPFIK
--PAPFWT
--PAPFVA
--PAPFMS
--PAPFLT
--PAPFTV
--PAPFTL
--PAPFQV
--PAPFLG
--PAPFKV
--PAPFGM
--PAPFGI
--PAPFEA

--PAPFAT
--PAPFYG
--PAPFWI
--PAPFVN
--PAPFVM
--PAPFST
--PAPFSQ
--PAPFPM
--PAPFMY
--PAPFKR
--PAPFGS
--PAPFFN
--PAPFAL
--PAPFAI
PYPAPF--
PWPAPF--
PSPAPF--
PIPAPF--
PDPAPF--
--PAPFTA
--PAPFSM
--PAPFSK
--PAPFRV
--PAPFRD
--PAPFPY
--PAPFNT
--PAPFMM
--PAPFLY
--PAPFLW
--PAPFLN
--PAPFKL
--PAPFHN
--PAPFHG
--PAPFGF
--PAPFEL
--PAPFDY
PRPAPF--
PQPAPF--
PGPAPF--
PEPAPF--
--PAPFYH
--PAPFYF
--PAPFVY
--PAPFVQ
--PAPFVL
--PAPFVK
--PAPFTQ
--PAPFTH
--PAPFSA
--PAPFRM

--PAPFRF
--PAPFQY
--PAPFQS
--PAPFLF
--PAPFKS
--PAPFKH
--PAPFIW
--PAPFGA
PTPAPF--
PNPAPF--
PHPAPF--
--PAPFYM
--PAPFYI
--PAPFWV
--PAPFWM
--PAPFWL
--PAPFWK
--PAPFVI
--PAPFVG
--PAPFTW
--PAPFTT
--PAPFSN
--PAPFRN
--PAPFRI
--PAPFQT
--PAPFQR
--PAPFQL
--PAPFQH
--PAPFQF
--PAPFQE
--PAPFPI
--PAPFNV
--PAPFNI
--PAPFMN
--PAPFML
--PAPFME
--PAPFMA
--PAPFLS
--PAPFLR
--PAPFLM
--PAPFLE
--PAPFKT
--PAPFIR
--PAPFIM
--PAPFIG
--PAPFID
--PAPFIA
--PAPFHT
--PAPFHS
--PAPFHQ

--PAPFHF
--PAPFGY
--PAPFGV
--PAPFGG
--PAPFFT
--PAPFFM
--PAPFFK
--PAPFDF
--PAPFAW
--PAPFYQ
--PAPFWN
--PAPFVW
--PAPFVF
--PAPFVE
--PAPFTY
--PAPFTF
--PAPFSP
--PAPFSI
--PAPFSE
--PAPFRW
--PAPFQN
--PAPFQI
--PAPFPW
--PAPFNY
--PAPFNW
--PAPFNS
--PAPFNM
--PAPFMV
--PAPFMK
--PAPFLV
--PAPFLQ
--PAPFKY
--PAPFKA
--PAPFIY
--PAPFIT
--PAPFIS
--PAPFIL
--PAPFHV
--PAPFHR
--PAPFHK
--PAPFHI
--PAPFHH
--PAPFHA
--PAPFFW
--PAPFFS
--PAPFFQ
--PAPFFL
--PAPFFG
--PAPFFE
--PAPFFA

--PAPFEP
--PAPFEN
--PAPFEM
--PAPFEF
--PAPFDI
--PAPFAY
--PAPFAS
--PAPFAG
-QPAPFH-
-QPAPFW-
QAPAPF--
QYPAPF--
QWPAPF--
-QPAPFS-
-QPAPFL-
QGPAPF--
QVPAPF--
QQPAPF--
-QPAPFF-
QEPAPF--
-QPAPFN-
-QPAPFA-
QLPAPF--
QKPAPF--
-QPAPFV-
-QPAPFT-
-QPAPFK-
QFPAPF--
QDPAPF--
-RPAPFL-
RTPAPF--
RSPAPF--
RLPAPF--
-RPAPFW-
-RPAPFV-
RYPAPF--
RKPAPF--
RIPAPF--
RRPAPF--
-RPAPFS-
RGPAPF--
RFPAPF--
-RPAPFR-
RHPAPF--
RWPAPF--
-RPAPFI-
-RPAPFH-
-RPAPFA-
RNPAPF--
RDPAPF--

-RPAPFK-
RAPAPF--
-RPAPFY-
-RPAPFN-
-RPAPFF-
RPPAPF--
-RPAPFQ-
-RPAPFM-
RMPAPF--
-RPAPFT-
-RPAPFG-
-RPAPFE-
RCPAPF--
-SPAPFL-
SAPAPF--
SSPAPF--
SRPAPF--
SPPAPF--
-SPAPFI-
-SPAPFV-
-SPAPFR-
SMPAPF--
-SPAPFN-
-SPAPFY-
SLPAPF--
SYPAPF--
-SPAPFM-
-SPAPFK-
-SPAPFS-
SKPAPF--
SVPAPF--
STPAPF--
-SPAPFT-
-SPAPFF-
SIPAPF--
-SPAPFH-
SHPAPF--
SCPAPF--
SQPAPF--
-SPAPFW-
-SPAPFQ-
-SPAPFG-
-SPAPFA-
SNPAPF--
SGPAPF--
-TPAPFL-
TSPAPF--
-TPAPFR-
TYPAPF--
-TPAPFS-

```
-TPAPFK-
-TPAPFF-
TLPAPF--
TIPAPF--
TAPAPF--
-TPAPFQ-
TTPAPF--
-TPAPFT-
-TPAPFM-
TWPAPF--
-TPAPFV-
-TPAPFA-
TKPAPF--
TVPAPF--
TPPAPF--
-TPAPFW-
TNPAPF--
TMPAPF--
THPAPF--
TGPAPF--
TFPAPF--
TEPAPF--
TCPAPF--
-VPAPFL-
VSPAPF--
VQPAPF--
VRPAPF--
-VPAPFR-
-VPAPFS-
VKPAPF--
-VPAPFN-
VLPAPF--
VTPAPF--
VPPAPF--
-VPAPFH-
VNPAPF--
-VPAPFY-
-VPAPFT-
VIPAPF--
VYPAPF--
VWPAPF--
VVPAPF--
-VPAPFW-
-VPAPFK-
-VPAPFG-
-VPAPFA-
VHPAPF--
VGPAPF--
VFPAPF--
-VPAPFV-
```

-VPAPFF-
VEPAPF--
VDPAPF--
VCPAPF--
VAPAPF--
-WPAPFT-
WSPAPF--
WNPAPF--
-WPAPFY-
-WPAPFL-
-WPAPFK-
WLPAPF--
WIPAPF--
WVPAPF--
WRPAPF--
WEPAPF--
WPPAPF--
-WPAPFQ-
-WPAPFI-
WTPAPF--
-WPAPFS-
-WPAPFR-
-WPAPFC-
-WPAPFA-
WGPAPF--
WDPAPF--
YRPAPF--
YSPAPF--
YNPAPF--
-YPAPFT-
YKPAPF--
-YPAPFM-
-YPAPFH-
YLPAPF--
YVPAPF--
-YPAPFA-
YYPAPF--
-YPAPFL-
YHPAPF--
YGPAPF--
YDPAPF--
-YPAPFY-
-YPAPFS-
-YPAPFV-
-YPAPFR-
-YPAPFQ-
-YPAPFN-
-YPAPFK-
-YPAPFF-
YMPAPF--

| | | | |
|---|---|---|---|
| | YCPAPF-- | | |
| PLPY | 126 | 751 | 31.68 |
| | -APLPYT- | | |
| | AYPLPY-- | | |
| | ASPLPY-- | | |
| | -APLPYS- | | |
| | AVPLPY-- | | |
| | AQPLPY-- | | |
| | ARPLPY-- | | |
| | AWPLPY-- | | |
| | -APLPYK- | | |
| | ALPLPY-- | | |
| | -APLPYL- | | |
| | AGPLPY-- | | |
| | ATPLPY-- | | |
| | -APLPYY- | | |
| | -APLPYV- | | |
| | -APLPYM- | | |
| | -APLPYI- | | |
| | AHPLPY-- | | |
| | -APLPYW- | | |
| | -APLPYG- | | |
| | ANPLPY-- | | |
| | AKPLPY-- | | |
| | AIPLPY-- | | |
| | ACPLPY-- | | |
| | -APLPYE- | | |
| | -APLPYC- | | |
| | AMPLPY-- | | |
| | AEPLPY-- | | |
| | ADPLPY-- | | |
| | -APLPYH- | | |
| | -APLPYF- | | |
| | CLPLPY-- | | |
| | CGPLPY-- | | |
| | CVPLPY-- | | |
| | CNPLPY-- | | |
| | CYPLPY-- | | |
| | CSPLPY-- | | |
| | -CPLPYY- | | |
| | -CPLPYK- | | |
| | -CPLPYI- | | |
| | CDPLPY-- | | |
| | DQPLPY-- | | |
| | DGPLPY-- | | |
| | DVPLPY-- | | |
| | DTPLPY-- | | |
| | DRPLPY-- | | |
| | -DPLPYR- | | |
| | DYPLPY-- | | |

-DPLPYV-
-DPLPYS-
DNPLPY--
DMPLPY--
DLPLPY--
DSPLPY--
-DPLPYL-
-DPLPYI-
DFPLPY--
-DPLPYF-
DKPLPY--
-DPLPYT-
-DPLPYA-
DIPLPY--
DEPLPY--
DCPLPY--
ELPLPY--
ERPLPY--
-EPLPYQ-
ESPLPY--
-EPLPYL-
EIPLPY--
-EPLPYW-
-EPLPYR-
-EPLPYF-
EAPLPY--
ETPLPY--
EQPLPY--
-EPLPYA-
ENPLPY--
EMPLPY--
EYPLPY--
EVPLPY--
-EPLPYT-
-EPLPYN-
EKPLPY--
EHPLPY--
EFPLPY--
-FPLPYR-
FRPLPY--
-FPLPYK-
FYPLPY--
-FPLPYF-
-FPLPYA-
-FPLPYI-
FLPLPY--
FVPLPY--
FSPLPY--
-FPLPYG-
FNPLPY--

FIPLPY--
FAPLPY--
-FPLPYW-
-FPLPYT-
-FPLPYS-
-FPLPYM-
FKPLPY--
FEPLPY--
FWPLPY--
FPPLPY--
FGPLPY--
FFPLPY--
FTPLPY--
-FPLPYQ-
-FPLPYP-
-FPLPYH-
FMPLPY--
-GPLPYR-
-GPLPYL-
GKPLPY--
GVPLPY--
GRPLPY--
-GPLPYH-
GNPLPY--
GTPLPY--
GIPLPY--
GFPLPY--
-GPLPYQ-
-GPLPYF-
-GPLPYA-
GSPLPY--
-GPLPYW-
-GPLPYT-
-GPLPYN-
GAPLPY--
GYPLPY--
GQPLPY--
-GPLPYS-
-GPLPYM-
GHPLPY--
GDPLPY--
-GPLPYK-
GEPLPY--
GWPLPY--
-GPLPYP-
-GPLPYG-
GMPLPY--
GCPLPY--
HTPLPY--
HMPLPY--

-HPLPYL-
HSPLPY--
-HPLPYS-
-HPLPYH-
HVPLPY--
-HPLPYT-
HRPLPY--
-HPLPYY-
HQPLPY--
-HPLPYA-
HNPLPY--
-HPLPYI-
-HPLPYF-
HFPLPY--
HAPLPY--
-HPLPYV-
-HPLPYQ-
-HPLPYN-
HIPLPY--
HGPLPY--
HYPLPY--
HWPLPY--
HPPLPY--
-HPLPYK-
HKPLPY--
IVPLPY--
-IPLPYT-
IFPLPY--
ITPLPY--
-IPLPYY-
IKPLPY--
ILPLPY--
-IPLPYK-
-IPLPYF-
IQPLPY--
-IPLPYR-
-IPLPYL-
IHPLPY--
-IPLPYW-
-IPLPYQ-
-IPLPYG-
INPLPY--
-IPLPYN-
-IPLPYM-
ISPLPY--
IPPLPY--
-IPLPYI-
-IPLPYH-
IMPLPY--
IGPLPY--

ICPLPY--
-IPLPYV-
-IPLPYS-
-IPLPYE-
IEPLPY--
IDPLPY--
KRPLPY--
KTPLPY--
KLPLPY--
KNPLPY--
-KPLPYY-
KYPLPY--
-KPLPYS-
-KPLPYA-
KMPLPY--
-KPLPYQ-
KVPLPY--
-KPLPYH-
-KPLPYE-
KKPLPY--
KIPLPY--
KEPLPY--
-KPLPYW-
-KPLPYV-
-KPLPYK-
KAPLPY--
KQPLPY--
-KPLPYI-
KFPLPY--
LRPLPY--
LLPLPY--
-LPLPYL-
-LPLPYT-
-LPLPYI-
LGPLPY--
-LPLPYV-
LYPLPY--
-LPLPYS-
LIPLPY--
LSPLPY--
LQPLPY--
-LPLPYR-
LNPLPY--
-LPLPYA-
LHPLPY--
-LPLPYK-
LKPLPY--
LFPLPY--
LEPLPY--
LPPLPY--

-LPLPYQ-
-LPLPYH-
LWPLPY--
-LPLPYM-
-LPLPYF-
MRPLPY--
-MPLPYR-
-MPLPYK-
-MPLPYH-
MHPLPY--
MAPLPY--
MTPLPY--
MGPLPY--
MPPLPY--
-MPLPYI-
MMPLPY--
MLPLPY--
-MPLPYW-
-MPLPYT-
-MPLPYN-
-MPLPYA-
MKPLPY--
MIPLPY--
MQPLPY--
-MPLPYY-
-MPLPYS-
-MPLPYF-
MNPLPY--
MFPLPY--
MYPLPY--
MSPLPY--
-MPLPYG-
MEPLPY--
-NPLPYL-
-NPLPYH-
-NPLPYS-
-NPLPYV-
NKPLPY--
-NPLPYY-
-NPLPYR-
NQPLPY--
NVPLPY--
-NPLPYF-
-NPLPYK-
NMPLPY--
-NPLPYI-
NRPLPY--
-NPLPYW-
NLPLPY--
NAPLPY--

NPPLPY--
-NPLPYA-
NIPLPY--
NEPLPY--
NYPLPY--
NWPLPY--
-NPLPYQ-
-NPLPYN-
NHPLPY--
NGPLPY--
--PLPYLT
--PLPYLS
--PLPYRS
--PLPYRL
--PLPYWI
--PLPYYL
--PLPYLG
--PLPYVS
--PLPYRV
--PLPYML
--PLPYES
--PLPYRG
--PLPYNL
--PLPYMK
--PLPYLY
--PLPYLV
--PLPYIR
--PLPYGY
--PLPYAY
--PLPYYH
--PLPYSW
--PLPYSS
--PLPYRI
--PLPYRA
--PLPYKT
--PLPYIS
--PLPYFA
--PLPYYS
--PLPYYA
--PLPYVL
--PLPYPY
--PLPYPM
--PLPYLM
--PLPYKR
--PLPYHL
--PLPYFL
PRPLPY--
--PLPYYQ
--PLPYYM
--PLPYYI

```
--PLPYVV
--PLPYSA
--PLPYMY
--PLPYFS
--PLPYAL
PFPLPY--
--PLPYYT
--PLPYYN
--PLPYVQ
--PLPYTA
--PLPYLN
--PLPYLD
--PLPYHG
--PLPYGA
--PLPYAA
--PLPYTV
--PLPYTR
--PLPYRY
--PLPYRE
--PLPYQT
--PLPYHS
--PLPYAG
--PLPYYG
--PLPYWR
--PLPYVR
--PLPYVG
--PLPYQL
--PLPYPL
--PLPYMR
--PLPYMG
--PLPYLW
--PLPYLQ
--PLPYLL
--PLPYLH
--PLPYGS
--PLPYFM
--PLPYYF
--PLPYWT
--PLPYVF
--PLPYTS
--PLPYTL
--PLPYSK
--PLPYSF
--PLPYNI
--PLPYME
--PLPYLA
--PLPYKN
--PLPYGG
--PLPYFV
--PLPYAH
```

PMPLPY--
--PLPYYV
--PLPYWK
--PLPYVA
--PLPYTY
--PLPYTM
--PLPYTI
--PLPYTG
--PLPYSY
--PLPYSR
--PLPYSG
--PLPYRN
--PLPYQN
--PLPYQG
--PLPYNS
--PLPYMN
--PLPYLK
--PLPYKY
--PLPYIT
--PLPYIL
--PLPYIA
--PLPYGW
--PLPYGM
--PLPYFY
--PLPYFH
--PLPYEA
--PLPYAW
--PLPYAS
PWPLPY--
--PLPYYD
--PLPYWL
--PLPYWH
--PLPYWA
--PLPYVY
--PLPYVM
--PLPYVI
--PLPYVH
--PLPYVD
--PLPYTN
--PLPYSV
--PLPYSQ
--PLPYRH
--PLPYQV
--PLPYQH
--PLPYMV
--PLPYKV
--PLPYKS
--PLPYKL
--PLPYKG
--PLPYKF

--PLPYIY
--PLPYIQ
--PLPYIK
--PLPYII
--PLPYHR
--PLPYFQ
--PLPYFN
--PLPYFG
--PLPYEG
--PLPYYW
--PLPYYR
--PLPYWY
--PLPYWS
--PLPYWG
--PLPYTT
--PLPYTH
--PLPYTD
--PLPYSI
--PLPYSH
--PLPYRW
--PLPYQS
--PLPYQQ
--PLPYQA
--PLPYPQ
--PLPYNW
--PLPYNM
--PLPYNF
--PLPYNA
--PLPYMW
--PLPYMD
--PLPYLF
--PLPYKW
--PLPYKQ
--PLPYIW
--PLPYIM
--PLPYIH
--PLPYIF
--PLPYHY
--PLPYHW
--PLPYGT
--PLPYGF
--PLPYFI
--PLPYAV
--PLPYAR
PYPLPY--
PNPLPY--
--PLPYYY
--PLPYYK
--PLPYWV
--PLPYWQ

--PLPYWN
--PLPYWM
--PLPYWF
--PLPYWD
--PLPYVW
--PLPYTQ
--PLPYTF
--PLPYST
--PLPYSN
--PLPYSM
--PLPYRM
--PLPYQY
--PLPYQW
--PLPYQK
--PLPYQI
--PLPYQF
--PLPYPW
--PLPYPV
--PLPYNT
--PLPYNH
--PLPYMT
--PLPYMQ
--PLPYMM
--PLPYMI
--PLPYMH
--PLPYMA
--PLPYLC
--PLPYKM
--PLPYKH
--PLPYKD
--PLPYIN
--PLPYHT
--PLPYHN
--PLPYHK
--PLPYHA
--PLPYGV
--PLPYGQ
--PLPYGN
--PLPYGD
--PLPYFK
--PLPYFE
--PLPYFD
--PLPYEY
--PLPYEL
--PLPYEI
--PLPYEF
--PLPYDS
--PLPYDM
--PLPYAQ
--PLPYAN

--PLPYAI
PHPLPY--
PEPLPY--
QNPLPY--
QRPLPY--
-QPLPYV-
-QPLPYK-
QAPLPY--
QYPLPY--
QSPLPY--
-QPLPYS-
-QPLPYR-
QGPLPY--
QCPLPY--
-QPLPYQ-
-QPLPYL-
-QPLPYH-
QLPLPY--
QKPLPY--
QTPLPY--
-QPLPYI-
QVPLPY--
-QPLPYY-
-QPLPYN-
-QPLPYM-
-QPLPYG-
-QPLPYA-
QFPLPY--
RAPLPY--
RVPLPY--
-RPLPYW-
-RPLPYK-
RSPLPY--
RNPLPY--
-RPLPYV-
-RPLPYR-
RIPLPY--
-RPLPYY-
-RPLPYN-
-RPLPYF-
RMPLPY--
RYPLPY--
-RPLPYS-
RGPLPY--
RDPLPY--
-RPLPYM-
-RPLPYT-
RLPLPY--
RTPLPY--
RPPLPY--

-RPLPYA-
RFPLPY--
RWPLPY--
-RPLPYG-
RQPLPY--
-RPLPYE-
REPLPY--
-RPLPYD-
-SPLPYL-
SAPLPY--
-SPLPYR-
SSPLPY--
-SPLPYV-
-SPLPYA-
SQPLPY--
SVPLPY--
-SPLPYS-
-SPLPYI-
-SPLPYT-
STPLPY--
SYPLPY--
SRPLPY--
-SPLPYK-
SGPLPY--
SMPLPY--
SLPLPY--
SDPLPY--
-SPLPYY-
SFPLPY--
SNPLPY--
SKPLPY--
SHPLPY--
SPPLPY--
-SPLPYM-
-SPLPYW-
-SPLPYQ-
-SPLPYG-
SEPLPY--
SCPLPY--
-SPLPYN-
-SPLPYH-
TLPLPY--
TMPLPY--
TKPLPY--
-TPLPYY-
-TPLPYL-
-TPLPYK-
TPPLPY--
TSPLPY--
THPLPY--

TTPLPY--
TGPLPY--
-TPLPYR-
-TPLPYI-
TFPLPY--
TAPLPY--
-TPLPYS-
TVPLPY--
TEPLPY--
-TPLPYQ-
TNPLPY--
TIPLPY--
TYPLPY--
-TPLPYV-
-TPLPYT-
TQPLPY--
-TPLPYN-
-TPLPYH-
TDPLPY--
-TPLPYA-
-VPLPYS-
VLPLPY--
VNPLPY--
VSPLPY--
VRPLPY--
VGPLPY--
VVPLPY--
-VPLPYR-
-VPLPYT-
VQPLPY--
-VPLPYL-
VHPLPY--
VYPLPY--
VIPLPY--
VWPLPY--
VTPLPY--
-VPLPYK-
-VPLPYF-
VPPLPY--
-VPLPYE-
VEPLPY--
-VPLPYN-
-VPLPYH-
VFPLPY--
-VPLPYY-
-VPLPYV-
-VPLPYQ-
-VPLPYI-
-VPLPYA-
VMPLPY--

VCPLPY--
WSPLPY--
WIPLPY--
WKPLPY--
-WPLPYR-
-WPLPYW-
WLPLPY--
WHPLPY--
WGPLPY--
-WPLPYS-
-WPLPYQ-
-WPLPYL-
WEPLPY--
WRPLPY--
WQPLPY--
WPPLPY--
-WPLPYK-
-WPLPYI-
WMPLPY--
WWPLPY--
WVPLPY--
-WPLPYY-
-WPLPYV-
-WPLPYT-
WFPLPY--
YSPLPY--
YAPLPY--
-YPLPYL-
-YPLPYA-
YRPLPY--
-YPLPYI-
YPPLPY--
-YPLPYS-
YFPLPY--
-YPLPYV-
-YPLPYR-
-YPLPYT-
YWPLPY--
YTPLPY--
YVPLPY--
-YPLPYH-
YLPLPY--
YKPLPY--
YHPLPY--
YGPLPY--
YDPLPY--
YQPLPY--
-YPLPYQ-
-YPLPYK-
-YPLPYG-

YNPLPY--
YEPLPY--
YCPLPY--

| | | | |
|---|---|---|---|
| VGHL | 80 | 779 | 31.68 |

-DVGHLS-
-RVGHLN-
-SVGHLD-
--VGHLNL
--VGHLSI
--VGHLRE
--VGHLLK
-VVGHLP-
--VGHLYD
--VGHLWN
--VGHLTL
--VGHLRP
--VGHLQH
--VGHLPL
--VGHLNN
--VGHLNG
--VGHLLV
--VGHLLQ
--VGHLLM
--VGHLLL
--VGHLLI
--VGHLLH
--VGHLIL
--VGHLGW
--VGHLGT
--VGHLGS
--VGHLGI
--VGHLFR
--VGHLET
--VGHLAP
--VGHLAF
AVVGHL--
AKVGHL--
AQVGHL--
-AVGHLV-
-AVGHLR-
-AVGHLL-
-AVGHLK-
-AVGHLF-
ANVGHL--
AGVGHL--
-AVGHLY-
-AVGHLS-
ALVGHL--
ATVGHL--
-AVGHLI-

-AVGHLH-
AMVGHL--
AHVGHL--
AEVGHL--
-AVGHLM-
APVGHL--
AIVGHL--
AFVGHL--
ACVGHL--
AAVGHL--
-AVGHLN-
-AVGHLG-
-AVGHLE-
ADVGHL--
CSVGHL--
CAVGHL--
CVVGHL--
-CVGHLK-
CLVGHL--
-CVGHLM-
-CVGHLH-
-CVGHLA-
CRVGHL--
CYVGHL--
CTVGHL--
CNVGHL--
CMVGHL--
CKVGHL--
CCVGHL--
CWVGHL--
-CVGHLR-
CEVGHL--
-CVGHLY-
-CVGHLS-
CPVGHL--
CIVGHL--
CHVGHL--
CFVGHL--
CDVGHL--
-DVGHLR-
DSVGHL--
DRVGHL--
DGVGHL--
DMVGHL--
DIVGHL--
DLVGHL--
-DVGHLI-
-DVGHLT-
DHVGHL--
-DVGHLY-

-DVGHLV-
DAVGHL--
DWVGHL--
DKVGHL--
-DVGHLK-
DQVGHL--
DPVGHL--
DNVGHL--
DFVGHL--
DVVGHL--
-DVGHLL-
DTVGHL--
DCVGHL--
-DVGHLW-
-DVGHLM-
DDVGHL--
-DVGHLQ-
DEVGHL--
-EVGHLR-
ELVGHL--
EVVGHL--
ETVGHL--
ENVGHL--
EHVGHL--
ESVGHL--
-EVGHLT-
-EVGHLS-
-EVGHLQ-
-EVGHLK-
EWVGHL--
-EVGHLM-
-EVGHLL-
EKVGHL--
EEVGHL--
-EVGHLF-
-EVGHLY-
EIVGHL--
EGVGHL--
EFVGHL--
EYVGHL--
-EVGHLV-
-EVGHLI-
-EVGHLA-
EPVGHL--
-EVGHLN-
-EVGHLH-
-EVGHLE-
EMVGHL--
EDVGHL--
ECVGHL--

FRVGHL--
FDVGHL--
-FVGHLA-
FSVGHL--
-FVGHLV-
FMVGHL--
-FVGHLT-
-FVGHLI-
-FVGHLL-
-FVGHLH-
FHVGHL--
FAVGHL--
-FVGHLY-
FGVGHL--
FEVGHL--
-FVGHLN-
-FVGHLM-
FPVGHL--
FQVGHL--
-FVGHLK-
FYVGHL--
-FVGHLG-
-FVGHLF-
-FVGHLQ-
-FVGHLE-
FCVGHL--
-GVGHLR-
GSVGHL--
GLVGHL--
GNVGHL--
-GVGHLV-
-GVGHLS-
-GVGHLK-
GAVGHL--
-GVGHLT-
GVVGHL--
GQVGHL--
-GVGHLL-
GFVGHL--
GWVGHL--
GIVGHL--
-GVGHLY-
GTVGHL--
GPVGHL--
GGVGHL--
-GVGHLM-
-GVGHLW-
GDVGHL--
-GVGHLI-
-GVGHLF-

-GVGHLA-
-GVGHLH-
GHVGHL--
GCVGHL--
GEVGHL--
-GVGHLE-
-HVGHLR-
HLVGHL--
-HVGHLV-
-HVGHLI-
-HVGHLL-
HMVGHL--
HHVGHL--
HVVGHL--
HPVGHL--
HYVGHL--
-HVGHLY-
HTVGHL--
HWVGHL--
-HVGHLM-
-HVGHLK-
HNVGHL--
HKVGHL--
HGVGHL--
-HVGHLT-
-HVGHLQ-
HQVGHL--
HDVGHL--
HAVGHL--
-HVGHLF-
-HVGHLA-
HFVGHL--
-HVGHLS-
-HVGHLN-
-HVGHLH-
HCVGHL--
HIVGHL--
-HVGHLG-
-HVGHLE-
IRVGHL--
IIVGHL--
-IVGHLS-
ISVGHL--
IGVGHL--
IYVGHL--
ITVGHL--
-IVGHLA-
IVVGHL--
-IVGHLQ-
-IVGHLM-

IMVGHL--
-IVGHLT-
-IVGHLL-
IAVGHL--
-IVGHLY-
IPVGHL--
INVGHL--
IDVGHL--
-IVGHLF-
-IVGHLH-
IFVGHL--
IWVGHL--
-IVGHLI-
-IVGHLG-
-IVGHLN-
IQVGHL--
IHVGHL--
IEVGHL--
-IVGHLW-
-IVGHLV-
ICVGHL--
-KVGHLR-
KLVGHL--
KTVGHL--
-KVGHLI-
-KVGHLL-
-KVGHLK-
-KVGHLV-
KQVGHL--
KPVGHL--
-KVGHLM-
KVVGHL--
KNVGHL--
-KVGHLT-
KMVGHL--
KAVGHL--
-KVGHLF-
-KVGHLQ-
KDVGHL--
-KVGHLH-
-KVGHLC-
-LVGHLL-
LSVGHL--
LGVGHL--
-LVGHLR-
LRVGHL--
LNVGHL--
LKVGHL--
-LVGHLK-
-LVGHLT-

LDVGHL--
-LVGHLQ-
-LVGHLI-
LQVGHL--
-LVGHLN-
-LVGHLG-
-LVGHLY-
-LVGHLV-
-LVGHLM-
LMVGHL--
LFVGHL--
-LVGHLW-
-LVGHLF-
LEVGHL--
LYVGHL--
-LVGHLE-
LCVGHL--
-MVGHLV-
MLVGHL--
MAVGHL--
-MVGHLL-
-MVGHLR-
MHVGHL--
MPVGHL--
-MVGHLK-
-MVGHLF-
MGVGHL--
MVVGHL--
-MVGHLT-
MFVGHL--
MYVGHL--
MMVGHL--
-MVGHLS-
MSVGHL--
MQVGHL--
-MVGHLW-
MEVGHL--
-MVGHLH-
-MVGHLG-
-MVGHLA-
MDVGHL--
-MVGHLY-
-MVGHLQ-
-MVGHLM-
MCVGHL--
MWVGHL--
-MVGHLE-
-MVGHLI-
-MVGHLD-
-NVGHLL-

-NVGHLK-
NWVGHL--
NLVGHL--
NSVGHL--
NKVGHL--
-NVGHLM-
NHVGHL--
NGVGHL--
NAVGHL--
NQVGHL--
NIVGHL--
-NVGHLV-
NPVGHL--
NNVGHL--
-NVGHLQ-
-NVGHLH-
-NVGHLY-
NTVGHL--
-NVGHLS-
NMVGHL--
NFVGHL--
-NVGHLW-
-NVGHLF-
NDVGHL--
-NVGHLI-
NEVGHL--
NCVGHL--
-NVGHLP-
-NVGHLE-
PLVGHL--
PSVGHL--
PRVGHL--
-PVGHLG-
-PVGHLR-
PVVGHL--
PTVGHL--
PKVGHL--
PFVGHL--
-PVGHLY-
-PVGHLW-
PNVGHL--
PIVGHL--
PGVGHL--
PWVGHL--
-PVGHLH-
-PVGHLI-
-PVGHLF-
PEVGHL--
PMVGHL--
PAVGHL--

–PVGHLL–
–PVGHLV–
–PVGHLQ–
–PVGHLM–
–PVGHLK–
PYVGHL––
–PVGHLA–
–PVGHLE–
PCVGHL––
–PVGHLT–
–PVGHLN–
PHVGHL––
PDVGHL––
–QVGHLH–
QRVGHL––
–QVGHLL–
QAVGHL––
–QVGHLK–
–QVGHLS–
–QVGHLR–
QKVGHL––
–QVGHLQ–
–QVGHLT–
–QVGHLW–
QPVGHL––
–QVGHLY–
–QVGHLA–
QSVGHL––
–QVGHLN–
QMVGHL––
QLVGHL––
QWVGHL––
QGVGHL––
QYVGHL––
–QVGHLI–
QTVGHL––
QQVGHL––
QDVGHL––
–QVGHLM–
QEVGHL––
–QVGHLF–
QIVGHL––
–QVGHLV–
–QVGHLG–
QNVGHL––
QHVGHL––
–QVGHLE–
QCVGHL––
–RVGHLS–
–RVGHLV–

RPVGHL--
-RVGHLI-
-RVGHLT-
-RVGHLL-
-RVGHLK-
-RVGHLY-
-RVGHLM-
RDVGHL--
REVGHL--
-RVGHLQ-
RHVGHL--
-RVGHLA-
-RVGHLW-
-RVGHLH-
-RVGHLF-
RCVGHL--
SRVGHL--
SSVGHL--
-SVGHLR-
SNVGHL--
-SVGHLK-
-SVGHLL-
-SVGHLT-
SVVGHL--
-SVGHLI-
STVGHL--
SAVGHL--
SGVGHL--
-SVGHLS-
SYVGHL--
SIVGHL--
SFVGHL--
SWVGHL--
SPVGHL--
SEVGHL--
-SVGHLQ-
SMVGHL--
-SVGHLV-
SQVGHL--
-SVGHLW-
SCVGHL--
SDVGHL--
-SVGHLY-
-SVGHLM-
-SVGHLF-
-SVGHLN-
-SVGHLE-
TRVGHL--
TLVGHL--
TKVGHL--

TGVGHL--
TVVGHL--
TSVGHL--
TTVGHL--
TQVGHL--
-TVGHLK-
-TVGHLW-
TWVGHL--
-TVGHLR-
TYVGHL--
-TVGHLY-
TMVGHL--
THVGHL--
TFVGHL--
TAVGHL--
-TVGHLL-
TPVGHL--
-TVGHLQ-
-TVGHLF-
TNVGHL--
-TVGHLI-
-TVGHLH-
TIVGHL--
-TVGHLT-
TEVGHL--
-TVGHLM-
-TVGHLA-
TCVGHL--
TDVGHL--
--VGHLRS
--VGHLRH
VLVGHL--
--VGHLYG
--VGHLVS
--VGHLTG
--VGHLRV
--VGHLSA
--VGHLAS
-VVGHLR-
--VGHLRG
--VGHLRM
VTVGHL--
VKVGHL--
--VGHLTA
--VGHLRA
--VGHLLG
-VVGHLT-
-VVGHLL-
--VGHLKG
--VGHLTS

--VGHLRQ
--VGHLMN
VIVGHL--
--VGHLFS
VAVGHL--
--VGHLNS
--VGHLIV
-VVGHLS-
VNVGHL--
--VGHLVT
--VGHLMS
VPVGHL--
VMVGHL--
--VGHLTT
--VGHLQS
--VGHLAT
--VGHLWT
--VGHLSG
--VGHLRN
--VGHLQG
--VGHLHS
VWVGHL--
-VVGHLQ-
VQVGHL--
--VGHLWS
--VGHLHT
--VGHLHG
VFVGHL--
--VGHLVA
--VGHLRD
--VGHLKA
--VGHLFF
--VGHLYT
--VGHLMT
--VGHLLT
--VGHLIY
--VGHLIT
--VGHLIA
VYVGHL--
--VGHLVN
VEVGHL--
-VVGHLA-
--VGHLYV
--VGHLVW
--VGHLMQ
--VGHLFV
--VGHLAW
-VVGHLH-
-VVGHLF-
VGVGHL--

--VGHLWA
--VGHLVV
--VGHLTV
--VGHLRW
--VGHLKT
--VGHLKS
--VGHLIR
--VGHLIG
VHVGHL--
--VGHLYY
--VGHLYH
--VGHLVM
--VGHLTN
--VGHLQA
--VGHLKM
--VGHLIH
-VVGHLN-
--VGHLYW
--VGHLWG
--VGHLVY
--VGHLMR
--VGHLKV
--VGHLIS
--VGHLES
--VGHLWF
--VGHLSL
--VGHLQY
--VGHLQW
--VGHLNA
--VGHLMY
--VGHLMK
VDVGHL--
-VVGHLV-
--VGHLYA
--VGHLTY
--VGHLTK
--VGHLQV
--VGHLQT
--VGHLMI
--VGHLMH
--VGHLIW
--VGHLIF
--VGHLHV
--VGHLFN
--VGHLFA
--VGHLYM
--VGHLYK
--VGHLWV
--VGHLWM
--VGHLWL

--VGHLVK
--VGHLVD
--VGHLTW
--VGHLTI
--VGHLTF
--VGHLSW
--VGHLSM
--VGHLMW
--VGHLKY
--VGHLKI
--VGHLKF
--VGHLKD
--VGHLIM
--VGHLFY
--VGHLFT
--VGHLAV
--VGHLAL
-VVGHLG-
--VGHLYQ
--VGHLYI
--VGHLYF
--VGHLTH
--VGHLSY
--VGHLSN
--VGHLQN
--VGHLMF
--VGHLLY
--VGHLKR
--VGHLKL
--VGHLKK
--VGHLIQ
--VGHLIK
--VGHLHW
--VGHLHH
--VGHLHA
--VGHLGA
--VGHLFQ
--VGHLAG
-VVGHLM-
--VGHLYR
--VGHLWY
--VGHLWW
--VGHLVE
--VGHLSK
--VGHLSF
--VGHLQF
--VGHLNY
--VGHLNM
--VGHLMD
--VGHLLF

--VGHLKW
--VGHLIN
--VGHLGM
--VGHLFM
--VGHLFG
--VGHLAM
--VGHLWH
--VGHLTQ
--VGHLQQ
--VGHLNV
--VGHLNT
--VGHLNF
--VGHLME
--VGHLKN
--VGHLKH
--VGHLHI
--VGHLGE
--VGHLFW
--VGHLFI
--VGHLFH
--VGHLAQ
WKVGHL--
WGVGHL--
WTVGHL--
-WVGHLY-
-WVGHLL-
-WVGHLK-
-WVGHLT-
WNVGHL--
-WVGHLI-
-WVGHLA-
WMVGHL--
WWVGHL--
WFVGHL--
-WVGHLM-
-WVGHLH-
WDVGHL--
WHVGHL--
WEVGHL--
-WVGHLQ-
-WVGHLG-
-WVGHLW-
-WVGHLV-
-WVGHLF-
WPVGHL--
WIVGHL--
WQVGHL--
-WVGHLE-
WAVGHL--
YTVGHL--

| | | | |
|---|---|---|---|
| | YLVGHL-- | | |
| | YIVGHL-- | | |
| | YYVGHL-- | | |
| | -YVGHLR- | | |
| | YSVGHL-- | | |
| | -YVGHLT- | | |
| | -YVGHLL- | | |
| | YPVGHL-- | | |
| | YMVGHL-- | | |
| | YGVGHL-- | | |
| | YVVGHL-- | | |
| | -YVGHLY- | | |
| | -YVGHLK- | | |
| | -YVGHLG- | | |
| | YNVGHL-- | | |
| | -YVGHLE- | | |
| | YQVGHL-- | | |
| | -YVGHLV- | | |
| | -YVGHLN- | | |
| | -YVGHLA- | | |
| | YKVGHL-- | | |
| | YAVGHL-- | | |
| | -YVGHLS- | | |
| | YFVGHL-- | | |
| | YWVGHL-- | | |
| | -YVGHLI- | | |
| | YHVGHL-- | | |
| | YCVGHL-- | | |
| | -YVGHLH- | | |
| | YEVGHL-- | | |
| | -YVGHLQ- | | |
| | YDVGHL-- | | |
| | -YVGHLM- | | |
| PKGM | 326 | 658 | 31.67 |
| | --PKGMLG | | |
| | --PKGMGL | | |
| | RTPKGM-- | | |
| | -TPKGMG- | | |
| | -APKGMR- | | |
| | -APKGMT- | | |
| | -APKGMY- | | |
| | -APKGMS- | | |
| | -APKGMG- | | |
| | ATPKGM-- | | |
| | -APKGMV- | | |
| | ADPKGM-- | | |
| | AYPKGM-- | | |
| | ASPKGM-- | | |
| | ARPKGM-- | | |
| | -APKGMI- | | |

-APKGME-
AGPKGM--
AAPKGM--
AVPKGM--
AQPKGM--
-APKGMW-
-APKGMF-
AKPKGM--
AHPKGM--
APPKGM--
-APKGMN-
-APKGMM-
-APKGMK-
AMPKGM--
AFPKGM--
CDPKGM--
CMPKGM--
CVPKGM--
CTPKGM--
CHPKGM--
CRPKGM--
CQPKGM--
CKPKGM--
CCPKGM--
DWPKGM--
-DPKGMN-
-DPKGMH-
-DPKGMR-
-DPKGMY-
-DPKGMV-
-DPKGMS-
-DPKGMI-
DLPKGM--
DGPKGM--
DYPKGM--
-DPKGMT-
-DPKGML-
DIPKGM--
DCPKGM--
DAPKGM--
-EPKGMW-
-EPKGMV-
-EPKGMT-
-EPKGMQ-
-EPKGMS-
-EPKGMR-
EIPKGM--
EWPKGM--
ESPKGM--
ERPKGM--

-EPKGML-
-EPKGMF-
-EPKGMA-
ELPKGM--
EEPKGM--
-EPKGMM-
-EPKGMK-
EGPKGM--
ECPKGM--
FTPKGM--
FNPKGM--
FVPKGM--
FLPKGM--
FSPKGM--
FPPKGM--
FEPKGM--
-FPKGMW-
-FPKGMV-
-FPKGMS-
-FPKGMM-
-FPKGML-
-FPKGMA-
FGPKGM--
-FPKGMY-
FMPKGM--
FQPKGM--
-FPKGMI-
FKPKGM--
FHPKGM--
FDPKGM--
FAPKGM--
GLPKGM--
-GPKGML-
GSPKGM--
-GPKGMR-
GGPKGM--
GYPKGM--
GFPKGM--
GVPKGM--
-GPKGMT-
-GPKGMS-
-GPKGMI-
-GPKGMW-
-GPKGMN-
-GPKGMK-
GDPKGM--
-GPKGMF-
GMPKGM--
GHPKGM--
GAPKGM--

GWPKGM--
GRPKGM--
GQPKGM--
-GPKGMQ-
-GPKGMM-
-GPKGMA-
GNPKGM--
GIPKGM--
GCPKGM--
-HPKGML-
HGPKGM--
HSPKGM--
-HPKGMY-
-HPKGMS-
-HPKGMV-
HLPKGM--
HDPKGM--
HVPKGM--
-HPKGMW-
-HPKGMN-
HIPKGM--
-HPKGMQ-
HNPKGM--
HFPKGM--
HEPKGM--
HCPKGM--
HTPKGM--
HQPKGM--
HPPKGM--
-HPKGME-
HMPKGM--
HHPKGM--
IKPKGM--
-IPKGMR-
ISPKGM--
-IPKGML-
ILPKGM--
IRPKGM--
-IPKGMY-
-IPKGMT-
-IPKGMA-
IIPKGM--
IGPKGM--
-IPKGMW-
-IPKGMF-
IHPKGM--
IDPKGM--
IVPKGM--
ITPKGM--
-IPKGMS-

```
-IPKGMN-
IEPKGM--
-IPKGMQ-
-IPKGMM-
-IPKGMH-
IQPKGM--
-IPKGMV-
IMPKGM--
IAPKGM--
KSPKGM--
-KPKGML-
-KPKGMW-
-KPKGMV-
-KPKGMR-
KEPKGM--
-KPKGMS-
-KPKGMF-
-KPKGMY-
-KPKGMT-
-KPKGMM-
KGPKGM--
KFPKGM--
KPPKGM--
-KPKGMA-
KNPKGM--
KMPKGM--
KYPKGM--
KRPKGM--
-KPKGMN-
-KPKGMI-
-KPKGMG-
KLPKGM--
KIPKGM--
KHPKGM--
KDPKGM--
KAPKGM--
-LPKGML-
LHPKGM--
-LPKGMS-
LTPKGM--
LSPKGM--
-LPKGMF-
LLPKGM--
-LPKGMR-
LGPKGM--
-LPKGMI-
-LPKGMH-
LPPKGM--
-LPKGMT-
-LPKGMN-
```

LKPKGM--
LIPKGM--
LVPKGM--
-LPKGMY-
-LPKGMQ-
-LPKGMM-
LMPKGM--
LDPKGM--
LAPKGM--
LYPKGM--
LWPKGM--
LQPKGM--
-LPKGMW-
LNPKGM--
MSPKGM--
-MPKGMV-
MTPKGM--
MGPKGM--
-MPKGMT-
-MPKGMH-
MAPKGM--
MYPKGM--
-MPKGMS-
MWPKGM--
-MPKGMF-
-MPKGMA-
MLPKGM--
MQPKGM--
-MPKGMN-
-MPKGMK-
-MPKGMG-
MKPKGM--
MDPKGM--
NYPKGM--
-NPKGMS-
-NPKGMK-
-NPKGMV-
NLPKGM--
NMPKGM--
NFPKGM--
NTPKGM--
-NPKGMW-
-NPKGML-
-NPKGMI-
-NPKGMA-
NHPKGM--
NAPKGM--
-NPKGMY-
-NPKGMQ-
-NPKGMM-

NIPKGM--
NGPKGM--
--PKGMVA
--PKGMLY
--PKGMST
--PKGMLA
--PKGMSS
--PKGMRT
--PKGMLT
--PKGMYM
--PKGMWS
--PKGMRN
--PKGMVV
--PKGMSR
--PKGMRI
--PKGMQF
--PKGMII
--PKGMMT
--PKGMLW
--PKGMAG
--PKGMYL
--PKGMTK
--PKGMRG
--PKGMQT
--PKGMIH
--PKGMFR
--PKGMSQ
--PKGMLE
--PKGMKV
--PKGMHM
--PKGMGG
--PKGMFN
--PKGMFM
PYPKGM--
PVPKGM--
--PKGMYY
--PKGMVW
--PKGMSW
--PKGMQS
--PKGMMV
--PKGMMR
--PKGMLV
--PKGMIS
--PKGMWL
--PKGMRA
--PKGMLN
--PKGMIA
--PKGMHA
--PKGMFL
--PKGMFI

--PKGMES
--PKGMYD
--PKGMWA
--PKGMTV
--PKGMTS
--PKGMTL
--PKGMSF
--PKGMRF
--PKGMNA
--PKGMMQ
--PKGMLF
--PKGMLD
--PKGMKQ
--PKGMKH
--PKGMIQ
--PKGMHT
--PKGMFQ
PTPKGM--
--PKGMWG
--PKGMVF
--PKGMVE
--PKGMSG
--PKGMRQ
--PKGMMN
--PKGMMI
--PKGMMD
--PKGMLL
--PKGMGT
--PKGMFS
--PKGMEG
--PKGMAR
--PKGMYN
--PKGMYG
--PKGMWK
--PKGMVT
--PKGMVI
--PKGMVG
--PKGMSN
--PKGMSM
--PKGMRV
--PKGMRD
--PKGMQA
--PKGMNM
--PKGMMK
--PKGMKG
--PKGMHV
--PKGMHL
--PKGMHK
--PKGMGV
--PKGMFH

--PKGMAQ
PSPKGM--
--PKGMYV
--PKGMYF
--PKGMVN
--PKGMSK
--PKGMSH
--PKGMRE
--PKGMNY
--PKGMNR
--PKGMNN
--PKGMNL
--PKGMNG
--PKGMMY
--PKGMLQ
--PKGMKL
--PKGMKA
--PKGMIV
--PKGMHW
--PKGMHR
--PKGMGA
--PKGMFV
--PKGMFG
PWPKGM--
--PKGMYQ
--PKGMYE
--PKGMYA
--PKGMWT
--PKGMWN
--PKGMWM
--PKGMWF
--PKGMTY
--PKGMTQ
--PKGMTM
--PKGMTI
--PKGMTF
--PKGMSA
--PKGMQY
--PKGMQH
--PKGMNW
--PKGMNV
--PKGMNQ
--PKGMNF
--PKGMLH
--PKGMKY
--PKGMKT
--PKGMKM
--PKGMIF
--PKGMHQ
--PKGMHD

--PKGMFW
--PKGMFE
--PKGMFA
--PKGMEV
--PKGMCT
--PKGMAL
PRPKGM--
-PPKGMR-
PMPKGM--
PLPKGM--
PKPKGM--
--PKGMYI
--PKGMYH
--PKGMWY
--PKGMWW
--PKGMWI
--PKGMVQ
--PKGMVD
--PKGMTH
--PKGMTE
--PKGMTD
--PKGMTA
--PKGMRC
--PKGMQW
--PKGMQN
--PKGMQD
--PKGMNT
--PKGMNH
--PKGMMW
--PKGMMS
--PKGMMH
--PKGMMA
--PKGMKN
--PKGMKE
--PKGMIT
--PKGMIN
--PKGMIM
--PKGMIK
--PKGMHY
--PKGMHI
--PKGMHH
--PKGMHF
--PKGMFT
--PKGMFK
--PKGMFD
--PKGMFC
--PKGMET
--PKGMEH
--PKGMEA
--PKGMDG

--PKGMDA
--PKGMAV
--PKGMAT
--PKGMAN
--PKGMAM
--PKGMAI
--PKGMAH
--PKGMAF
--PKGMAA
-QPKGMV-
-QPKGML-
-QPKGMH-
QLPKGM--
-QPKGMS-
QAPKGM--
-QPKGMW-
-QPKGMF-
QYPKGM--
-QPKGMM-
-QPKGMA-
QIPKGM--
QGPKGM--
QVPKGM--
QQPKGM--
-QPKGMN-
-QPKGMI-
-QPKGME-
QHPKGM--
QFPKGM--
QWPKGM--
QTPKGM--
QPPKGM--
-QPKGMY-
-QPKGMR-
-QPKGMQ-
-QPKGMG-
QDPKGM--
-RPKGMY-
RFPKGM--
RVPKGM--
-RPKGMN-
RIPKGM--
-RPKGMV-
-RPKGMF-
-RPKGMK-
RYPKGM--
-RPKGMW-
-RPKGMI-
RHPKGM--
RQPKGM--

RPPKGM--
-RPKGMM-
-RPKGME-
RGPKGM--
REPKGM--
-RPKGMT-
RNPKGM--
-RPKGMQ-
-RPKGMG-
RMPKGM--
RCPKGM--
-SPKGML-
-SPKGMS-
SLPKGM--
-SPKGMR-
-SPKGMV-
SNPKGM--
-SPKGMA-
-SPKGMT-
SYPKGM--
SKPKGM--
-SPKGMW-
SSPKGM--
-SPKGMN-
-SPKGMH-
SDPKGM--
SAPKGM--
-SPKGMY-
-SPKGMF-
-SPKGMK-
SIPKGM--
SWPKGM--
STPKGM--
-SPKGMQ-
-SPKGMI-
-SPKGMG-
-SPKGME-
SHPKGM--
SPPKGM--
SGPKGM--
SFPKGM--
-SPKGMM-
TTPKGM--
-TPKGMW-
-TPKGMR-
TRPKGM--
-TPKGML-
TLPKGM--
TVPKGM--
-TPKGMS-

-TPKGMK-
-TPKGMT-
-TPKGMQ-
TIPKGM--
TSPKGM--
-TPKGMM-
TPPKGM--
-TPKGMI-
-TPKGMH-
-TPKGMF-
-TPKGMA-
TKPKGM--
TEPKGM--
TDPKGM--
-TPKGMY-
THPKGM--
TGPKGM--
TAPKGM--
VLPKGM--
VHPKGM--
VPPKGM--
-VPKGMS-
-VPKGMN-
VFPKGM--
VVPKGM--
VKPKGM--
VYPKGM--
VWPKGM--
VTPKGM--
VSPKGM--
VQPKGM--
-VPKGMW-
-VPKGML-
-VPKGMV-
-VPKGMG-
VGPKGM--
-VPKGMF-
-VPKGMK-
-VPKGME-
-VPKGMY-
-VPKGMQ-
-VPKGMA-
VNPKGM--
VMPKGM--
VEPKGM--
VDPKGM--
VCPKGM--
-WPKGMI-
WSPKGM--
-WPKGML-

| | | | |
|---|---|---|---|
| | WYPKGM-- | | |
| | WVPKGM-- | | |
| | WRPKGM-- | | |
| | WQPKGM-- | | |
| | -WPKGMV- | | |
| | -WPKGMM- | | |
| | WIPKGM-- | | |
| | -WPKGMY- | | |
| | -WPKGMS- | | |
| | -WPKGMA- | | |
| | WGPKGM-- | | |
| | WFPKGM-- | | |
| | WLPKGM-- | | |
| | WPPKGM-- | | |
| | WMPKGM-- | | |
| | WTPKGM-- | | |
| | -WPKGMF- | | |
| | -WPKGME- | | |
| | WNPKGM-- | | |
| | WKPKGM-- | | |
| | WHPKGM-- | | |
| | YKPKGM-- | | |
| | YSPKGM-- | | |
| | -YPKGMK- | | |
| | YRPKGM-- | | |
| | -YPKGMM- | | |
| | YVPKGM-- | | |
| | -YPKGMQ- | | |
| | -YPKGMT- | | |
| | -YPKGMS- | | |
| | -YPKGMR- | | |
| | -YPKGMN- | | |
| | -YPKGMH- | | |
| | -YPKGMA- | | |
| | YIPKGM-- | | |
| | YTPKGM-- | | |
| | YQPKGM-- | | |
| | -YPKGMW- | | |
| | -YPKGMV- | | |
| | -YPKGMF- | | |
| | -YPKGME- | | |
| | YNPKGM-- | | |
| PINL | 321 | 659 | 31.64 |
| | -APINLG- | | |
| | -LPINLG- | | |
| | --PINLSH | | |
| | --PINLSQ | | |
| | --PINLGR | | |
| | RFPINL-- | | |
| | -SPINLP- | | |

−SPINLG−
AVPINL−−
AKPINL−−
ASPINL−−
−APINLL−
ANPINL−−
AMPINL−−
−APINLQ−
−APINLK−
ALPINL−−
AFPINL−−
−APINLR−
AGPINL−−
ATPINL−−
−APINLW−
−APINLH−
AQPINL−−
AEPINL−−
AYPINL−−
AAPINL−−
AWPINL−−
−APINLT−
−APINLN−
APPINL−−
−APINLS−
AIPINL−−
−CPINLI−
CMPINL−−
CLPINL−−
CYPINL−−
CSPINL−−
CRPINL−−
CQPINL−−
−CPINLV−
CKPINL−−
CGPINL−−
CFPINL−−
CCPINL−−
DRPINL−−
−DPINLR−
DLPINL−−
DQPINL−−
DMPINL−−
DWPINL−−
DYPINL−−
DAPINL−−
DVPINL−−
−DPINLI−
−DPINLT−
−DPINLS−

-DPINLV-
DSPINL--
DTPINL--
-DPINLQ-
-DPINLL-
-DPINLE-
-DPINLA-
DNPINL--
DGPINL--
DFPINL--
DDPINL--
ESPINL--
ERPINL--
EFPINL--
-EPINLV-
-EPINLK-
EGPINL--
EAPINL--
-EPINLY-
-EPINLT-
-EPINLR-
ENPINL--
EVPINL--
-EPINLA-
EKPINL--
-EPINLF-
EQPINL--
-EPINLN-
-EPINLM-
ELPINL--
EYPINL--
EWPINL--
-EPINLS-
ETPINL--
EPPINL--
-EPINLW-
-EPINLQ-
EMPINL--
EIPINL--
-FPINLR-
FRPINL--
-FPINLK-
FSPINL--
-FPINLL-
-FPINLS-
FQPINL--
-FPINLT-
-FPINLV-
-FPINLE-
FHPINL--

FKPINL--
-FPINLA-
FEPINL--
FTPINL--
-FPINLM-
FGPINL--
-FPINLN-
FNPINL--
-GPINLR-
GVPINL--
-GPINLL-
-GPINLK-
GTPINL--
GHPINL--
-GPINLQ-
GNPINL--
-GPINLH-
GRPINL--
-GPINLT-
GLPINL--
GYPINL--
GWPINL--
-GPINLA-
GFPINL--
-GPINLE-
GKPINL--
GIPINL--
-GPINLS-
GEPINL--
GDPINL--
-GPINLN-
-GPINLM-
-GPINLG-
GQPINL--
-GPINLY-
-GPINLF-
GAPINL--
GGPINL--
-GPINLI-
GMPINL--
GCPINL--
-HPINLL-
-HPINLV-
HTPINL--
-HPINLR-
-HPINLA-
HLPINL--
HGPINL--
HSPINL--
-HPINLQ-

HQPINL--
-HPINLS-
-HPINLK-
HNPINL--
HMPINL--
HRPINL--
HFPINL--
HAPINL--
HWPINL--
HVPINL--
-HPINLT-
HPPINL--
-HPINLI-
-HPINLF-
-HPINLE-
HHPINL--
HEPINL--
HYPINL--
-HPINLH-
-HPINLY-
-HPINLG-
HKPINL--
HDPINL--
IRPINL--
-IPINLR-
IQPINL--
ITPINL--
ISPINL--
IKPINL--
-IPINLS-
-IPINLN-
IIPINL--
IFPINL--
-IPINLH-
-IPINLA-
IGPINL--
IPPINL--
-IPINLT-
-IPINLL-
INPINL--
-IPINLW-
-IPINLK-
IYPINL--
-IPINLV-
-IPINLM-
IMPINL--
IWPINL--
-IPINLQ-
-IPINLI-
-IPINLG-

```
-IPINLF-
KSPINL--
KVPINL--
-KPINLR-
KTPINL--
-KPINLT-
-KPINLQ-
-KPINLL-
KGPINL--
-KPINLW-
KYPINL--
-KPINLS-
-KPINLM-
KEPINL--
KDPINL--
KWPINL--
-KPINLH-
-KPINLN-
KLPINL--
-KPINLI-
-KPINLE-
KMPINL--
KHPINL--
KAPINL--
KQPINL--
KNPINL--
KIPINL--
-KPINLK-
-KPINLG-
LRPINL--
-LPINLL-
LGPINL--
-LPINLT-
-LPINLS-
LWPINL--
LQPINL--
LTPINL--
LSPINL--
-LPINLE-
LAPINL--
LPPINL--
LLPINL--
-LPINLK-
LNPINL--
LHPINL--
LCPINL--
LYPINL--
-LPINLV-
LDPINL--
-LPINLQ-
```

```
-LPINLP-
-LPINLN-
-LPINLD-
-MPINLS-
-MPINLR-
-MPINLT-
-MPINLV-
MGPINL--
MWPINL--
MQPINL--
-MPINLQ-
MPPINL--
-MPINLY-
-MPINLM-
MEPINL--
MSPINL--
-MPINLK-
-MPINLA-
MKPINL--
MHPINL--
MLPINL--
MDPINL--
MAPINL--
-MPINLE-
MMPINL--
-MPINLH-
MNPINL--
MIPINL--
MCPINL--
-NPINLS-
NLPINL--
NHPINL--
NRPINL--
-NPINLR-
-NPINLQ-
NVPINL--
-NPINLI-
NQPINL--
NWPINL--
NTPINL--
-NPINLA-
-NPINLF-
NNPINL--
NGPINL--
NEPINL--
-NPINLW-
-NPINLH-
NFPINL--
NDPINL--
NYPINL--
```

-NPINLT-
NMPINL--
NAPINL--
--PINLRF
--PINLSA
--PINLKS
--PINLRV
--PINLVN
--PINLNS
--PINLRY
--PINLRS
--PINLRQ
--PINLRW
--PINLLG
--PINLRN
--PINLRA
--PINLFA
--PINLET
--PINLVH
--PINLST
--PINLSS
--PINLQR
--PINLTT
--PINLSG
--PINLAA
--PINLSF
--PINLKG
--PINLWN
--PINLVK
--PINLKH
--PINLTR
--PINLSR
--PINLHS
--PINLEG
--PINLAL
PWPINL--
--PINLYG
--PINLTK
--PINLNT
--PINLMA
--PINLAT
PQPINL--
PKPINL--
--PINLVG
--PINLTS
--PINLTH
--PINLQG
--PINLQF
--PINLMS
--PINLMQ

--PINLKT
--PINLHG
--PINLGA
--PINLFN
PGPINL--
--PINLTY
--PINLTN
--PINLTA
--PINLSM
--PINLRR
--PINLQS
--PINLQI
--PINLPL
--PINLNA
--PINLKN
--PINLGW
--PINLGT
--PINLES
PYPINL--
--PINLWA
--PINLSY
--PINLSV
--PINLQV
--PINLKW
--PINLKL
--PINLHV
--PINLGI
PNPINL--
PMPINL--
--PINLVT
--PINLTG
--PINLSW
--PINLRH
--PINLQA
--PINLKV
--PINLIG
--PINLID
--PINLHN
--PINLGY
--PINLGS
--PINLFM
--PINLFH
--PINLFG
PEPINL--
--PINLYW
--PINLYN
--PINLWS
--PINLWH
--PINLWG
--PINLWF

--PINLVQ
--PINLVD
--PINLSN
--PINLSI
--PINLRD
--PINLQD
--PINLPY
--PINLPI
--PINLNG
--PINLMR
--PINLME
--PINLLN
--PINLLH
--PINLKD
--PINLIN
--PINLHQ
--PINLHM
--PINLHI
--PINLHF
--PINLHA
PHPINL--
PFPINL--
PDPINL--
PTPINL--
PPPINL--
-PPINLQ-
-PPINLL-
-PPINLH-
--PINLWQ
--PINLWD
--PINLTQ
--PINLTF
--PINLTE
--PINLSK
--PINLSE
--PINLSD
--PINLQT
--PINLQQ
--PINLQL
--PINLQH
--PINLPF
--PINLNN
--PINLNM
--PINLMN
--PINLMH
--PINLMD
--PINLLD
--PINLKY
--PINLKF
--PINLKE

--PINLKA
--PINLIH
--PINLHY
--PINLHT
--PINLHR
--PINLHH
--PINLHD
--PINLGM
--PINLFD
--PINLEV
--PINLEI
--PINLEA
--PINLDL
--PINLDF
--PINLCT
--PINLAK
QRPINL--
-QPINLR-
-QPINLL-
-QPINLY-
QTPINL--
-QPINLF-
QEPINL--
-QPINLS-
-QPINLQ-
-QPINLM-
-QPINLH-
QIPINL--
QGPINL--
QSPINL--
QQPINL--
-QPINLV-
-QPINLK-
QMPINL--
QHPINL--
QWPINL--
-QPINLT-
-QPINLE-
QFPINL--
-QPINLN-
-QPINLI-
-QPINLG-
QNPINL--
QLPINL--
QKPINL--
QAPINL--
RVPINL--
-RPINLL-
RQPINL--
RGPINL--

-RPINLT-
REPINL--
RAPINL--
-RPINLM-
RYPINL--
RWPINL--
RMPINL--
-RPINLY-
RNPINL--
RHPINL--
-RPINLN-
-RPINLF-
RIPINL--
RDPINL--
-RPINLV-
-RPINLA-
-RPINLH-
-RPINLW-
-RPINLQ-
-RPINLG-
RPPINL--
-RPINLE-
STPINL--
-SPINLL-
SIPINL--
-SPINLR-
SLPINL--
SVPINL--
-SPINLS-
-SPINLH-
SRPINL--
SDPINL--
SSPINL--
-SPINLA-
SYPINL--
-SPINLI-
-SPINLV-
SGPINL--
-SPINLQ-
-SPINLK-
SMPINL--
SEPINL--
SNPINL--
SWPINL--
-SPINLN-
-SPINLM-
SHPINL--
SPPINL--
SCPINL--
-SPINLE-

TRPINL--
-TPINLR-
TMPINL--
TYPINL--
TTPINL--
-TPINLK-
TAPINL--
-TPINLV-
-TPINLI-
TLPINL--
TWPINL--
TKPINL--
TSPINL--
TQPINL--
THPINL--
-TPINLS-
-TPINLA-
TGPINL--
TVPINL--
TPPINL--
-TPINLM-
-TPINLH-
TNPINL--
-TPINLW-
-TPINLQ-
-TPINLN-
TEPINL--
-VPINLH-
VKPINL--
VLPINL--
-VPINLM-
VSPINL--
-VPINLK-
VHPINL--
VVPINL--
-VPINLR-
VFPINL--
VWPINL--
VQPINL--
-VPINLQ-
-VPINLA-
VAPINL--
-VPINLL-
VTPINL--
VMPINL--
VEPINL--
-VPINLN-
-VPINLG-
VNPINL--
VGPINL--

-VPINLV-
-VPINLS-
VYPINL--
-VPINLE-
VIPINL--
VDPINL--
VPPINL--
-WPINLR-
WNPINL--
WSPINL--
-WPINLT-
-WPINLK-
WGPINL--
WAPINL--
WRPINL--
-WPINLA-
WKPINL--
-WPINLQ-
-WPINLM-
-WPINLH-
-WPINLS-
-WPINLN-
-WPINLG-
-WPINLE-
YVPINL--
-YPINLR-
-YPINLQ-
-YPINLK-
-YPINLL-
YYPINL--
-YPINLY-
YWPINL--
YQPINL--
-YPINLT-
YGPINL--
-YPINLV-
-YPINLE-
YEPINL--
YFPINL--
-YPINLI-
YAPINL--
-YPINLH-
-YPINLG-
YDPINL--
YTPINL--
YPPINL--
-YPINLM-
-YPINLD-
-YPINLA-
YNPINL--

| | | | |
|---|---|---|---|
| | YKPINL-- | | |
| | YHPINL-- | | |
| VGWL | 320 | 659 | 31.64 |
| | -EVGWLN- | | |
| | --VGWLPR | | |
| | --VGWLNE | | |
| | --VGWLKI | | |
| | --VGWLGR | | |
| | -AVGWLR- | | |
| | ARVGWL-- | | |
| | -AVGWLG- | | |
| | -AVGWLL- | | |
| | AGVGWL-- | | |
| | AVVGWL-- | | |
| | AIVGWL-- | | |
| | -AVGWLH- | | |
| | -AVGWLT- | | |
| | AKVGWL-- | | |
| | AMVGWL-- | | |
| | -AVGWLK- | | |
| | ADVGWL-- | | |
| | -AVGWLA- | | |
| | AEVGWL-- | | |
| | ANVGWL-- | | |
| | AYVGWL-- | | |
| | -AVGWLS- | | |
| | AWVGWL-- | | |
| | -AVGWLM- | | |
| | -AVGWLE- | | |
| | ALVGWL-- | | |
| | AHVGWL-- | | |
| | -AVGWLQ- | | |
| | -AVGWLN- | | |
| | -AVGWLD- | | |
| | -AVGWLI- | | |
| | AFVGWL-- | | |
| | ACVGWL-- | | |
| | CSVGWL-- | | |
| | CNVGWL-- | | |
| | -CVGWLR- | | |
| | CRVGWL-- | | |
| | CQVGWL-- | | |
| | CGVGWL-- | | |
| | -CVGWLT- | | |
| | CKVGWL-- | | |
| | CEVGWL-- | | |
| | CYVGWL-- | | |
| | -CVGWLS- | | |
| | -CVGWLQ- | | |
| | -CVGWLM- | | |

-CVGWLH-
-CVGWLG-
-CVGWLA-
CMVGWL--
CLVGWL--
CIVGWL--
CHVGWL--
DRVGWL--
-DVGWLI-
-DVGWLR-
DVVGWL--
DQVGWL--
-DVGWLY-
-DVGWLL-
DAVGWL--
DSVGWL--
-DVGWLS-
DNVGWL--
-DVGWLT-
-DVGWLQ-
-DVGWLH-
DEVGWL--
-DVGWLK-
DMVGWL--
DLVGWL--
DWVGWL--
-DVGWLV-
DKVGWL--
DCVGWL--
DYVGWL--
-DVGWLN-
-DVGWLA-
-DVGWLM-
-DVGWLG-
DHVGWL--
DFVGWL--
DDVGWL--
ELVGWL--
-EVGWLR-
ERVGWL--
ESVGWL--
ETVGWL--
EVVGWL--
EMVGWL--
EAVGWL--
EYVGWL--
-EVGWLK-
-EVGWLS-
-EVGWLQ-
EKVGWL--

EFVGWL--
-EVGWLW-
ENVGWL--
EGVGWL--
EEVGWL--
-EVGWLT-
-EVGWLI-
EIVGWL--
-EVGWLG-
EHVGWL--
EDVGWL--
-EVGWLM-
-EVGWLA-
EQVGWL--
EWVGWL--
-FVGWLS-
-FVGWLK-
FGVGWL--
-FVGWLV-
FAVGWL--
-FVGWLQ-
FDVGWL--
-FVGWLL-
-FVGWLD-
-FVGWLR-
-FVGWLH-
FMVGWL--
-FVGWLN-
-FVGWLI-
-FVGWLA-
FIVGWL--
FCVGWL--
-GVGWLS-
GSVGWL--
GVVGWL--
-GVGWLK-
GYVGWL--
GLVGWL--
-GVGWLT-
GGVGWL--
GDVGWL--
-GVGWLV-
-GVGWLA-
-GVGWLQ-
-GVGWLH-
GFVGWL--
GIVGWL--
GHVGWL--
GQVGWL--
GWVGWL--

−GVGWLM−
−GVGWLD−
−GVGWLN−
HRVGWL−−
−HVGWLS−
HSVGWL−−
HVVGWL−−
HTVGWL−−
−HVGWLR−
−HVGWLV−
HFVGWL−−
HKVGWL−−
HLVGWL−−
−HVGWLL−
HAVGWL−−
−HVGWLN−
−HVGWLG−
−HVGWLD−
−HVGWLT−
−HVGWLM−
HYVGWL−−
−HVGWLI−
HHVGWL−−
−HVGWLK−
−HVGWLH−
HQVGWL−−
HDVGWL−−
−HVGWLE−
HNVGWL−−
HEVGWL−−
HWVGWL−−
−HVGWLW−
−HVGWLQ−
−HVGWLF−
−HVGWLA−
HMVGWL−−
−IVGWLG−
−IVGWLS−
ITVGWL−−
IRVGWL−−
−IVGWLK−
−IVGWLN−
IGVGWL−−
IEVGWL−−
−IVGWLV−
−IVGWLT−
−IVGWLH−
−IVGWLA−
IHVGWL−−
−IVGWLL−

−IVGWLD−
IDVGWL−−
−IVGWLY−
ICVGWL−−
IAVGWL−−
−KVGWLS−
−KVGWLL−
−KVGWLN−
KDVGWL−−
−KVGWLT−
−KVGWLA−
KTVGWL−−
−KVGWLK−
−KVGWLG−
KWVGWL−−
−KVGWLF−
KFVGWL−−
−KVGWLH−
−KVGWLV−
KHVGWL−−
KAVGWL−−
−KVGWLQ−
−KVGWLI−
KIVGWL−−
−KVGWLD−
−KVGWLW−
−KVGWLE−
KEVGWL−−
−KVGWLC−
LRVGWL−−
LAVGWL−−
LVVGWL−−
LNVGWL−−
−LVGWLS−
LYVGWL−−
−LVGWLG−
LIVGWL−−
−LVGWLA−
LTVGWL−−
−LVGWLE−
LHVGWL−−
−LVGWLD−
−LVGWLQ−
LLVGWL−−
LEVGWL−−
LGVGWL−−
LDVGWL−−
−LVGWLV−
LWVGWL−−
−LVGWLM−

-LVGWLI-
-LVGWLP-
LCVGWL--
MSVGWL--
MNVGWL--
MGVGWL--
-MVGWLL-
MQVGWL--
MDVGWL--
-MVGWLR-
-MVGWLN-
MRVGWL--
MHVGWL--
-MVGWLS-
-MVGWLV-
MVVGWL--
-MVGWLA-
MEVGWL--
MYVGWL--
-MVGWLT-
MWVGWL--
-MVGWLQ-
-MVGWLK-
-MVGWLI-
-MVGWLD-
MIVGWL--
MFVGWL--
MAVGWL--
-MVGWLW-
-MVGWLH-
-NVGWLR-
NSVGWL--
-NVGWLL-
NGVGWL--
NFVGWL--
-NVGWLS-
-NVGWLH-
NAVGWL--
-NVGWLN-
-NVGWLG-
NTVGWL--
-NVGWLY-
-NVGWLT-
NHVGWL--
NYVGWL--
NWVGWL--
-NVGWLA-
NKVGWL--
NQVGWL--
-NVGWLQ-

-NVGWLI-
NNVGWL--
NMVGWL--
NEVGWL--
-NVGWLW-
-NVGWLE-
NDVGWL--
NVVGWL--
-NVGWLM-
-NVGWLK-
NIVGWL--
PRVGWL--
PGVGWL--
PKVGWL--
PVVGWL--
PMVGWL--
PHVGWL--
PLVGWL--
PYVGWL--
PTVGWL--
PNVGWL--
PAVGWL--
PFVGWL--
PEVGWL--
PSVGWL--
PIVGWL--
PDVGWL--
PWVGWL--
-QVGWLK-
QRVGWL--
QSVGWL--
QVVGWL--
-QVGWLT-
-QVGWLL-
-QVGWLR-
QTVGWL--
QNVGWL--
QAVGWL--
-QVGWLQ-
-QVGWLH-
QLVGWL--
-QVGWLS-
-QVGWLV-
-QVGWLA-
QEVGWL--
QYVGWL--
-QVGWLE-
QIVGWL--
QHVGWL--
QGVGWL--

-QVGWLW-
-QVGWLN-
-QVGWLG-
QMVGWL--
QDVGWL--
-QVGWLP-
QFVGWL--
-RVGWLR-
-RVGWLT-
-RVGWLY-
RDVGWL--
-RVGWLV-
REVGWL--
-RVGWLK-
-RVGWLG-
-RVGWLM-
-RVGWLE-
-RVGWLD-
-SVGWLR-
SRVGWL--
SSVGWL--
-SVGWLL-
-SVGWLG-
-SVGWLQ-
STVGWL--
SQVGWL--
-SVGWLA-
SFVGWL--
-SVGWLN-
SLVGWL--
SGVGWL--
-SVGWLH-
SKVGWL--
-SVGWLK-
-SVGWLV-
SNVGWL--
SIVGWL--
SEVGWL--
SYVGWL--
-SVGWLE-
-SVGWLM-
SDVGWL--
SMVGWL--
SWVGWL--
-SVGWLC-
SCVGWL--
TRVGWL--
TYVGWL--
TKVGWL--
TTVGWL--

−TVGWLG−
TVVGWL−−
−TVGWLL−
TQVGWL−−
−TVGWLS−
TNVGWL−−
−TVGWLQ−
−TVGWLH−
TIVGWL−−
−TVGWLT−
TEVGWL−−
TDVGWL−−
−TVGWLN−
−TVGWLK−
−TVGWLI−
TGVGWL−−
−TVGWLA−
THVGWL−−
−TVGWLE−
−TVGWLD−
TWVGWL−−
−TVGWLM−
TCVGWL−−
VTVGWL−−
VRVGWL−−
VAVGWL−−
−VVGWLT−
VSVGWL−−
−−VGWLAG
−−VGWLSH
−−VGWLNS
−−VGWLVS
−−VGWLSK
−−VGWLRT
VYVGWL−−
−−VGWLSG
VKVGWL−−
−−VGWLSA
−VVGWLR−
−−VGWLTN
−−VGWLLG
−−VGWLGL
−−VGWLSS
−−VGWLRI
−−VGWLGS
VQVGWL−−
−−VGWLKL
−−VGWLTS
−−VGWLRV
−−VGWLRG

--VGWLQR
--VGWLNA
--VGWLLR
--VGWLKS
VNVGWL--
--VGWLVG
--VGWLSR
--VGWLRS
--VGWLRD
--VGWLMA
--VGWLAS
--VGWLAK
VDVGWL--
VVVGWL--
-VVGWLN-
-VVGWLE-
--VGWLVK
--VGWLTK
--VGWLTA
--VGWLSY
--VGWLSV
--VGWLSD
--VGWLKH
--VGWLGI
--VGWLES
-VVGWLA-
--VGWLWK
--VGWLSI
--VGWLLA
--VGWLKY
--VGWLIR
--VGWLGM
--VGWLTW
--VGWLTR
--VGWLSN
--VGWLQS
--VGWLKT
--VGWLAT
VEVGWL--
--VGWLTI
--VGWLTH
--VGWLRQ
--VGWLRN
--VGWLRM
--VGWLMG
--VGWLIN
-VVGWLG-
--VGWLNT
--VGWLNQ
--VGWLMR

--VGWLMQ
--VGWLLK
--VGWLLD
--VGWLKG
--VGWLIA
--VGWLHT
--VGWLHG
--VGWLGN
--VGWLAF
VFVGWL--
-VVGWLH-
-VVGWLD-
VMVGWL--
--VGWLYA
--VGWLVA
--VGWLTT
--VGWLTQ
--VGWLTG
--VGWLRE
--VGWLQT
--VGWLQH
--VGWLQD
--VGWLMD
--VGWLLN
--VGWLLH
--VGWLLE
--VGWLKK
--VGWLKA
--VGWLHS
--VGWLAV
--VGWLAR
-VVGWLI-
--VGWLYS
--VGWLYE
--VGWLWN
--VGWLVW
--VGWLVH
--VGWLTV
--VGWLSW
--VGWLSE
--VGWLNR
--VGWLMT
--VGWLKN
--VGWLIK
--VGWLHN
--VGWLHH
--VGWLGY
--VGWLGK
--VGWLGG
--VGWLGA

```
--VGWLFH
--VGWLFG
--VGWLAA
-VVGWLQ-
--VGWLWI
--VGWLTF
--VGWLTD
--VGWLQN
--VGWLQI
--VGWLQG
--VGWLPL
--VGWLNM
--VGWLMS
--VGWLKQ
--VGWLKM
--VGWLKE
--VGWLIG
--VGWLHM
--VGWLHF
--VGWLHA
--VGWLGH
--VGWLEY
--VGWLDS
--VGWLAI
--VGWLYY
--VGWLYN
--VGWLYM
--VGWLYK
--VGWLYH
--VGWLWV
--VGWLWS
--VGWLWH
--VGWLWF
--VGWLVN
--VGWLTM
--VGWLTE
--VGWLRC
--VGWLPF
--VGWLNW
--VGWLNV
--VGWLNN
--VGWLMW
--VGWLMV
--VGWLMN
--VGWLMK
--VGWLMH
--VGWLMF
--VGWLLM
--VGWLIF
--VGWLID
```

--VGWLHR
--VGWLHK
--VGWLGD
--VGWLFN
--VGWLEW
--VGWLEV
--VGWLET
--VGWLEQ
--VGWLEN
--VGWLEH
--VGWLEG
--VGWLEA
--VGWLDT
--VGWLDA
--VGWLCR
--VGWLAW
--VGWLAQ
--VGWLAN
--VGWLAM
--VGWLAH
--VGWLAE
--VGWLAD
-WVGWLT-
WHVGWL--
WGVGWL--
WYVGWL--
-WVGWLR-
-WVGWLH-
WRVGWL--
-WVGWLK-
WMVGWL--
-WVGWLS-
-WVGWLN-
WFVGWL--
-WVGWLQ-
-WVGWLM-
-WVGWLE-
WLVGWL--
WIVGWL--
WQVGWL--
WAVGWL--
-YVGWLA-
-YVGWLR-
YQVGWL--
-YVGWLN-
-YVGWLG-
YFVGWL--
YGVGWL--
YNVGWL--
-YVGWLS-

YDVGWL--
-YVGWLT-
-YVGWLQ-
-YVGWLK-
YLVGWL--
YIVGWL--
YCVGWL--
-YVGWLH-
-YVGWLE-
YEVGWL--
-YVGWLD-
YAVGWL--

PAWY 561 589 31.60

ASPAWY--
ANPAWY--
ARPAWY--
AKPAWY--
AVPAWY--
-APAWYK-
AEPAWY--
-APAWYY-
-APAWYG-
-APAWYS-
-APAWYF-
-APAWYA-
AAPAWY--
-APAWYV-
ADPAWY--
AYPAWY--
AQPAWY--
-APAWYT-
-APAWYM-
-APAWYI-
-APAWYD-
AMPAWY--
AIPAWY--
CRPAWY--
CQPAWY--
-CPAWYS-
CFPAWY--
CTPAWY--
-CPAWYI-
DTPAWY--
-DPAWYS-
DKPAWY--
DNPAWY--
DVPAWY--
DSPAWY--
DFPAWY--
DCPAWY--

DWPAWY--
DQPAWY--
-DPAWYY-
-DPAWYR-
-DPAWYL-
-DPAWYG-
-DPAWYF-
-DPAWYE-
DAPAWY--
-EPAWYL-
-EPAWYS-
ETPAWY--
ESPAWY--
EKPAWY--
EVPAWY--
-EPAWYT-
-EPAWYI-
-EPAWYH-
-EPAWYG-
-EPAWYF-
ELPAWY--
EQPAWY--
-EPAWYY-
-EPAWYW-
-EPAWYV-
-EPAWYN-
-EPAWYK-
-EPAWYA-
EGPAWY--
EEPAWY--
EDPAWY--
FTPAWY--
FVPAWY--
-FPAWYV-
-FPAWYI-
-FPAWYS-
FRPAWY--
FAPAWY--
FKPAWY--
FHPAWY--
-FPAWYK-
-FPAWYA-
FQPAWY--
FPPAWY--
-FPAWYL-
-FPAWYF-
FIPAWY--
FGPAWY--
GFPAWY--
-GPAWYI-

GRPAWY--
-GPAWYA-
GAPAWY--
-GPAWYR-
GVPAWY--
GIPAWY--
GGPAWY--
GSPAWY--
-GPAWYY-
-GPAWYD-
GEPAWY--
-GPAWYT-
GTPAWY--
GQPAWY--
GPPAWY--
-GPAWYL-
-GPAWYK-
-GPAWYE-
GNPAWY--
GMPAWY--
-GPAWYV-
-GPAWYQ-
-GPAWYN-
-GPAWYM-
-GPAWYH-
HSPAWY--
HLPAWY--
-HPAWYT-
HIPAWY--
HRPAWY--
-HPAWYA-
-HPAWYS-
-HPAWYI-
HKPAWY--
HAPAWY--
HVPAWY--
-HPAWYF-
HTPAWY--
-HPAWYW-
-HPAWYV-
-HPAWYR-
-HPAWYK-
-HPAWYH-
HNPAWY--
HHPAWY--
HFPAWY--
HEPAWY--
-IPAWYR-
-IPAWYH-
-IPAWYY-

-IPAWYS-
ILPAWY--
ISPAWY--
IRPAWY--
IIPAWY--
IGPAWY--
IVPAWY--
-IPAWYQ-
-IPAWYM-
INPAWY--
IKPAWY--
ITPAWY--
IPPAWY--
-IPAWYV-
-IPAWYL-
IMPAWY--
IAPAWY--
IYPAWY--
-IPAWYK-
-IPAWYG-
IEPAWY--
ICPAWY--
KSPAWY--
-KPAWYR-
-KPAWYT-
KRPAWY--
-KPAWYY-
KPPAWY--
KHPAWY--
-KPAWYK-
-KPAWYS-
KLPAWY--
KGPAWY--
-KPAWYN-
KMPAWY--
KFPAWY--
KAPAWY--
KVPAWY--
-KPAWYI-
-KPAWYH-
-KPAWYG-
KTPAWY--
KQPAWY--
-KPAWYQ-
-KPAWYF-
-KPAWYA-
KNPAWY--
KEPAWY--
LSPAWY--
-LPAWYQ-

LEPAWY--
-LPAWYK-
-LPAWYA-
LNPAWY--
LKPAWY--
-LPAWYV-
LIPAWY--
LTPAWY--
-LPAWYT-
LGPAWY--
LDPAWY--
LLPAWY--
LVPAWY--
-LPAWYH-
-LPAWYG-
LQPAWY--
LPPAWY--
-LPAWYR-
LYPAWY--
-LPAWYM-
-LPAWYI-
LCPAWY--
-MPAWYQ-
-MPAWYR-
MAPAWY--
MTPAWY--
MGPAWY--
-MPAWYF-
MSPAWY--
-MPAWYN-
-MPAWYL-
MHPAWY--
-MPAWYW-
MKPAWY--
MNPAWY--
MLPAWY--
MPPAWY--
-MPAWYY-
-MPAWYS-
-MPAWYM-
MIPAWY--
MEPAWY--
MDPAWY--
-NPAWYY-
-NPAWYS-
NFPAWY--
NYPAWY--
NTPAWY--
NAPAWY--
NPPAWY--

```
-NPAWYR-
-NPAWYL-
-NPAWYI-
NVPAWY--
-NPAWYW-
NSPAWY--
NRPAWY--
-NPAWYV-
-NPAWYA-
NNPAWY--
NLPAWY--
NWPAWY--
NQPAWY--
-NPAWYM-
NHPAWY--
NDPAWY--
--PAWYRE
--PAWYSR
--PAWYRS
--PAWYHA
--PAWYLT
--PAWYRA
--PAWYRT
--PAWYRL
--PAWYAS
--PAWYKT
--PAWYKL
--PAWYGS
--PAWYRV
--PAWYRH
--PAWYNS
--PAWYFN
PTPAWY--
--PAWYTA
--PAWYRQ
--PAWYRD
PLPAWY--
--PAWYVA
--PAWYSV
--PAWYSN
--PAWYSE
--PAWYQT
--PAWYQA
--PAWYLQ
PHPAWY--
--PAWYYG
--PAWYVR
--PAWYST
--PAWYSL
--PAWYRN
```

--PAWYKN
--PAWYIG
--PAWYHQ
--PAWYGV
--PAWYEG
--PAWYAN
--PAWYAG
PRPAWY--
PEPAWY--
--PAWYYK
--PAWYVW
--PAWYVK
--PAWYTR
--PAWYSY
--PAWYSG
--PAWYRM
--PAWYRG
--PAWYMG
--PAWYME
--PAWYLH
--PAWYKA
--PAWYGL
--PAWYFM
--PAWYFK
--PAWYEA
--PAWYAW
PKPAWY--
--PAWYYS
--PAWYTM
--PAWYSI
--PAWYRY
--PAWYQV
--PAWYMV
--PAWYMF
--PAWYKF
--PAWYHW
--PAWYHG
--PAWYGA
--PAWYFT
--PAWYFH
PYPAWY--
PVPAWY--
PMPAWY--
--PAWYWS
--PAWYWG
--PAWYVY
--PAWYVT
--PAWYVQ
--PAWYVH
--PAWYTT

--PAWYSD
--PAWYRF
--PAWYQY
--PAWYQI
--PAWYMS
--PAWYLV
--PAWYLM
--PAWYKY
--PAWYKH
--PAWYGT
--PAWYET
PSPAWY--
PFPAWY--
--PAWYYE
--PAWYWM
--PAWYVV
--PAWYVS
--PAWYTV
--PAWYSS
--PAWYSQ
--PAWYSK
--PAWYQS
--PAWYQG
--PAWYNY
--PAWYNG
--PAWYMH
--PAWYLR
--PAWYLI
--PAWYKW
--PAWYKI
--PAWYIS
--PAWYIR
--PAWYIK
--PAWYID
--PAWYHV
--PAWYHT
--PAWYHF
--PAWYGI
--PAWYFQ
--PAWYFA
--PAWYEW
--PAWYAE
--PAWYAD
PWPAWY--
PQPAWY--
--PAWYYN
--PAWYYA
--PAWYWT
--PAWYWA
--PAWYVD

--PAWYTY
--PAWYTQ
--PAWYTK
--PAWYTH
--PAWYTE
--PAWYSW
--PAWYSM
--PAWYSH
--PAWYQK
--PAWYNL
--PAWYMT
--PAWYMD
--PAWYLW
--PAWYLN
--PAWYLE
--PAWYLA
--PAWYKV
--PAWYKG
--PAWYKD
--PAWYIT
--PAWYIQ
--PAWYIN
--PAWYIM
--PAWYHN
--PAWYHI
--PAWYHH
--PAWYGY
--PAWYFG
--PAWYAY
--PAWYAV
--PAWYAL
QSPAWY--
-QPAWYR-
QHPAWY--
-QPAWYL-
-QPAWYS-
-QPAWYW-
QYPAWY--
QPPAWY--
-QPAWYV-
-QPAWYK-
QAPAWY--
QVPAWY--
-QPAWYT-
-QPAWYY-
-QPAWYN-
QNPAWY--
QLPAWY--
QKPAWY--
QIPAWY--

QWPAWY--
QTPAWY--
QRPAWY--
-QPAWYH-
-QPAWYE-
QMPAWY--
-RPAWYG-
-RPAWYL-
RFPAWY--
RPPAWY--
-RPAWYK-
-RPAWYV-
-RPAWYF-
RNPAWY--
-RPAWYW-
RYPAWY--
RWPAWY--
-RPAWYI-
RMPAWY--
RHPAWY--
-RPAWYH-
-RPAWYE-
RIPAWY--
REPAWY--
RAPAWY--
-RPAWYT-
RTPAWY--
RQPAWY--
-RPAWYY-
-RPAWYM-
RDPAWY--
-RPAWYQ-
-RPAWYA-
-SPAWYT-
STPAWY--
-SPAWYL-
SLPAWY--
-SPAWYV-
-SPAWYR-
SFPAWY--
SSPAWY--
-SPAWYI-
-SPAWYG-
SWPAWY--
SRPAWY--
SIPAWY--
-SPAWYY-
-SPAWYE-
SKPAWY--
SDPAWY--

```
-SPAWYS-
-SPAWYQ-
-SPAWYH-
-SPAWYA-
SNPAWY--
SMPAWY--
SVPAWY--
SPPAWY--
-SPAWYN-
-SPAWYM-
SHPAWY--
SEPAWY--
SYPAWY--
SQPAWY--
-SPAWYW-
-SPAWYF-
-TPAWYR-
TRPAWY--
TVPAWY--
TSPAWY--
-TPAWYL-
-TPAWYQ-
TTPAWY--
-TPAWYM-
-TPAWYV-
-TPAWYK-
-TPAWYG-
TNPAWY--
TKPAWY--
THPAWY--
TMPAWY--
TIPAWY--
TGPAWY--
TWPAWY--
TPPAWY--
-TPAWYA-
-TPAWYN-
-TPAWYI-
TDPAWY--
TAPAWY--
TEPAWY--
VRPAWY--
-VPAWYN-
-VPAWYT-
-VPAWYS-
VTPAWY--
-VPAWYL-
VGPAWY--
VVPAWY--
VQPAWY--
```

VLPAWY--
VPPAWY--
-VPAWYQ-
-VPAWYH-
VEPAWY--
VDPAWY--
-VPAWYK-
-VPAWYI-
VMPAWY--
VKPAWY--
VAPAWY--
VYPAWY--
-VPAWYM-
VIPAWY--
VHPAWY--
VCPAWY--
WDPAWY--
WVPAWY--
-WPAWYS-
WGPAWY--
WEPAWY--
WSPAWY--
WPPAWY--
-WPAWYH-
-WPAWYQ-
-WPAWYK-
WYPAWY--
-WPAWYN-
WMPAWY--
WLPAWY--
WIPAWY--
-YPAWYS-
YTPAWY--
-YPAWYK-
YMPAWY--
-YPAWYI-
YAPAWY--
YQPAWY--
YLPAWY--
YGPAWY--
YRPAWY--
-YPAWYA-
-YPAWYY-
-YPAWYQ-
-YPAWYM-
YVPAWY--
-YPAWYT-
-YPAWYN-
-YPAWYG-
-YPAWYD-

| | | | |
|---|---|---|---|
| | YKPAWY-- | | |
| | YEPAWY-- | | |
| MAWL | 1031 | 501 | 31.59 |
| | AVMAWL-- | | |
| | AGMAWL-- | | |
| | ASMAWL-- | | |
| | -AMAWLK- | | |
| | -AMAWLG- | | |
| | -AMAWLD- | | |
| | -AMAWLS- | | |
| | -AMAWLI- | | |
| | -AMAWLT- | | |
| | AAMAWL-- | | |
| | ATMAWL-- | | |
| | -AMAWLQ- | | |
| | ANMAWL-- | | |
| | AMMAWL-- | | |
| | -AMAWLE- | | |
| | ADMAWL-- | | |
| | AQMAWL-- | | |
| | AHMAWL-- | | |
| | -AMAWLW- | | |
| | -AMAWLM- | | |
| | -AMAWLA- | | |
| | -AMAWLH- | | |
| | AFMAWL-- | | |
| | AEMAWL-- | | |
| | CSMAWL-- | | |
| | CRMAWL-- | | |
| | CTMAWL-- | | |
| | CNMAWL-- | | |
| | -CMAWLT- | | |
| | -CMAWLR- | | |
| | -CMAWLN- | | |
| | -CMAWLM- | | |
| | -CMAWLH- | | |
| | -CMAWLA- | | |
| | CHMAWL-- | | |
| | -DMAWLR- | | |
| | -DMAWLG- | | |
| | DQMAWL-- | | |
| | DTMAWL-- | | |
| | DHMAWL-- | | |
| | DEMAWL-- | | |
| | DAMAWL-- | | |
| | -DMAWLT- | | |
| | -DMAWLQ- | | |
| | -DMAWLL- | | |
| | DSMAWL-- | | |
| | -DMAWLW- | | |

```
-DMAWLK-
-DMAWLA-
DVMAWL--
DRMAWL--
DNMAWL--
-DMAWLN-
DKMAWL--
DDMAWL--
DMMAWL--
-DMAWLV-
-DMAWLS-
-DMAWLE-
-DMAWLD-
-DMAWLC-
DLMAWL--
ENMAWL--
ESMAWL--
-EMAWLR-
ERMAWL--
-EMAWLS-
-EMAWLI-
EQMAWL--
EIMAWL--
EKMAWL--
EYMAWL--
ETMAWL--
EWMAWL--
-EMAWLN-
-EMAWLL-
-EMAWLK-
EMMAWL--
-EMAWLT-
-EMAWLQ-
-EMAWLM-
-EMAWLE-
EDMAWL--
FHMAWL--
FMMAWL--
-FMAWLT-
-FMAWLM-
FGMAWL--
-FMAWLK-
FLMAWL--
FWMAWL--
-FMAWLY-
-FMAWLH-
-FMAWLA-
GSMAWL--
-GMAWLS-
-GMAWLL-
```

GYMAWL--
GTMAWL--
GLMAWL--
GNMAWL--
GFMAWL--
-GMAWLT-
-GMAWLN-
-GMAWLA-
GGMAWL--
GDMAWL--
-GMAWLH-
GMMAWL--
GKMAWL--
-GMAWLV-
-GMAWLM-
-GMAWLE-
GEMAWL--
GAMAWL--
GWMAWL--
-GMAWLI-
GQMAWL--
-GMAWLQ-
-GMAWLK-
-HMAWLS-
HHMAWL--
-HMAWLR-
HTMAWL--
-HMAWLL-
HSMAWL--
-HMAWLH-
-HMAWLG-
HDMAWL--
HNMAWL--
-HMAWLN-
HYMAWL--
HVMAWL--
-HMAWLV-
-HMAWLK-
HAMAWL--
HQMAWL--
-HMAWLE-
-HMAWLD-
-HMAWLA-
HGMAWL--
HEMAWL--
-HMAWLT-
-HMAWLM-
-HMAWLF-
HLMAWL--
HIMAWL--

IDMAWL--
ISMAWL--
IAMAWL--
-IMAWLK-
IEMAWL--
IQMAWL--
-IMAWLH-
IVMAWL--
INMAWL--
-IMAWLA-
-IMAWLY-
-IMAWLR-
-IMAWLQ-
-IMAWLL-
-IMAWLD-
IGMAWL--
KDMAWL--
KAMAWL--
KNMAWL--
-KMAWLS-
KVMAWL--
KHMAWL--
KQMAWL--
KMMAWL--
-KMAWLN-
KYMAWL--
KTMAWL--
-KMAWLM-
-KMAWLL-
-KMAWLK-
KEMAWL--
-KMAWLY-
-KMAWLD-
KLMAWL--
-LMAWLP-
-LMAWLR-
LDMAWL--
LGMAWL--
LNMAWL--
-LMAWLS-
-LMAWLH-
-LMAWLA-
LIMAWL--
LHMAWL--
-LMAWLT-
LEMAWL--
-LMAWLF-
LFMAWL--
LQMAWL--
-LMAWLK-

--MAWLST
--MAWLSK
--MAWLLR
--MAWLTK
--MAWLRH
--MAWLSR
--MAWLAK
--MAWLTA
--MAWLTT
--MAWLNN
--MAWLKT
--MAWLTR
--MAWLKS
--MAWLTG
--MAWLRQ
--MAWLGR
--MAWLAN
--MAWLTQ
--MAWLQR
--MAWLGV
--MAWLSG
--MAWLRG
--MAWLGQ
MHMAWL--
--MAWLVS
--MAWLSS
--MAWLSH
--MAWLSA
--MAWLLD
--MAWLGW
--MAWLES
--MAWLTV
--MAWLRN
--MAWLRE
--MAWLRA
--MAWLQS
--MAWLNS
--MAWLHR
--MAWLGT
--MAWLGL
--MAWLRM
--MAWLRF
--MAWLQK
--MAWLNT
--MAWLNG
--MAWLLH
--MAWLIT
--MAWLHI
--MAWLGN
MKMAWL--

```
--MAWLYG
--MAWLWG
--MAWLTN
--MAWLSV
--MAWLHK
--MAWLGI
--MAWLEN
--MAWLAH
MSMAWL--
-MMAWLN-
-MMAWLK-
MGMAWL--
--MAWLVR
--MAWLVG
--MAWLSN
--MAWLQN
--MAWLPL
--MAWLNA
--MAWLMN
--MAWLMA
--MAWLKF
--MAWLKA
--MAWLHH
--MAWLGS
--MAWLGA
--MAWLAQ
--MAWLYD
--MAWLVN
--MAWLRY
--MAWLRW
--MAWLKK
--MAWLKE
--MAWLHT
--MAWLGM
--MAWLEH
MEMAWL--
MDMAWL--
--MAWLTD
--MAWLQW
--MAWLNH
--MAWLND
--MAWLMG
--MAWLLG
--MAWLHA
--MAWLEK
--MAWLDA
--MAWLAS
--MAWLAD
--MAWLYE
--MAWLVK
```

--MAWLVH
--MAWLRD
--MAWLQA
--MAWLNK
--MAWLLK
--MAWLLE
--MAWLKY
--MAWLKQ
--MAWLKG
--MAWLHM
--MAWLHE
--MAWLFH
--MAWLDS
--MAWLAR
MYMAWL--
MQMAWL--
-MMAWLT-
-MMAWLR-
-MMAWLQ-
-MMAWLE-
MCMAWL--
--MAWLYH
--MAWLYF
--MAWLWN
--MAWLWH
--MAWLWD
--MAWLSE
--MAWLSC
--MAWLQV
--MAWLQG
--MAWLPY
--MAWLPI
--MAWLNV
--MAWLNQ
--MAWLMW
--MAWLMD
--MAWLKW
--MAWLKL
--MAWLKI
--MAWLIV
--MAWLIN
--MAWLIH
--MAWLID
--MAWLHV
--MAWLHS
--MAWLHQ
--MAWLHL
--MAWLHG
--MAWLHD
--MAWLGD

```
--MAWLFG
--MAWLEG
--MAWLDT
--MAWLAW
--MAWLAT
--MAWLAA
-NMAWLR-
-NMAWLV-
-NMAWLS-
-NMAWLL-
NLMAWL--
NGMAWL--
NEMAWL--
NMMAWL--
-NMAWLQ-
-NMAWLN-
-NMAWLK-
NIMAWL--
NVMAWL--
-NMAWLH-
-NMAWLG-
-NMAWLA-
NAMAWL--
-NMAWLT-
NTMAWL--
-NMAWLI-
-NMAWLD-
-NMAWLM-
NHMAWL--
PSMAWL--
PRMAWL--
PVMAWL--
PTMAWL--
PQMAWL--
PLMAWL--
PGMAWL--
PNMAWL--
PHMAWL--
PEMAWL--
PAMAWL--
-QMAWLR-
QTMAWL--
-QMAWLL-
-QMAWLG-
QSMAWL--
QHMAWL--
-QMAWLQ-
-QMAWLN-
QIMAWL--
-QMAWLT-
```

QDMAWL--
QAMAWL--
QMMAWL--
QKMAWL--
QEMAWL--
RDMAWL--
RHMAWL--
-RMAWLQ-
RWMAWL--
RMMAWL--
-RMAWLE-
-RMAWLW-
-SMAWLG-
SGMAWL--
-SMAWLA-
SQMAWL--
-SMAWLS-
-SMAWLL-
SNMAWL--
-SMAWLT-
-SMAWLH-
SVMAWL--
-SMAWLR-
-SMAWLK-
SMMAWL--
SEMAWL--
SWMAWL--
-SMAWLI-
SAMAWL--
-SMAWLN-
SHMAWL--
-SMAWLE-
-SMAWLD-
SDMAWL--
STMAWL--
SKMAWL--
SCMAWL--
TSMAWL--
-TMAWLR-
TTMAWL--
-TMAWLS-
-TMAWLT-
-TMAWLK-
-TMAWLH-
TDMAWL--
TVMAWL--
-TMAWLM-
TAMAWL--
-TMAWLW-
TGMAWL--

TNMAWL--
-TMAWLG-
-TMAWLE-
TKMAWL--
TMMAWL--
-TMAWLQ-
THMAWL--
-TMAWLN-
-TMAWLI-
TEMAWL--
TQMAWL--
-TMAWLD-
-VMAWLK-
VEMAWL--
-VMAWLN-
-VMAWLD-
VTMAWL--
-VMAWLR-
-VMAWLQ-
VSMAWL--
-VMAWLS-
VNMAWL--
-VMAWLA-
-VMAWLI-
VQMAWL--
-VMAWLE-
VFMAWL--
VDMAWL--
WQMAWL--
WAMAWL--
-WMAWLS-
-WMAWLK-
WGMAWL--
-WMAWLN-
-WMAWLH-
WEMAWL--
WLMAWL--
WIMAWL--
WHMAWL--
YHMAWL--
-YMAWLS-
YTMAWL--
YAMAWL--
-YMAWLT-
YNMAWL--
-YMAWLQ-
-YMAWLN-
-YMAWLK-
-YMAWLG-
-YMAWLD-

YDMAWL--
-YMAWLR-
-YMAWLH-
YLMAWL--

| | | | |
|---|---|---|---|
| PLNI | 127 | 751 | 31.58 |

-DPLNIL-
-GPLNIT-
-LPLNIN-
-APLNIT-
AVPLNI--
-APLNIN-
-APLNIM-
-APLNIG-
-APLNIS-
AMPLNI--
-APLNIR-
-APLNIQ-
ASPLNI--
APPLNI--
-APLNIK-
AIPLNI--
AGPLNI--
AKPLNI--
AHPLNI--
ATPLNI--
AQPLNI--
-APLNII-
-APLNIH-
-APLNIF-
ANPLNI--
AFPLNI--
-APLNIY-
-APLNIW-
-APLNIE-
CYPLNI--
CVPLNI--
CLPLNI--
CFPLNI--
CSPLNI--
-CPLNIQ-
-CPLNIM-
-CPLNIL-
-CPLNII-
CMPLNI--
CKPLNI--
CHPLNI--
CGPLNI--
CAPLNI--
DRPLNI--
-DPLNIT-

DGPLNI--
DYPLNI--
-DPLNII-
DFPLNI--
DSPLNI--
-DPLNIV-
-DPLNIA-
DAPLNI--
DVPLNI--
DTPLNI--
-DPLNIY-
-DPLNIS-
-DPLNIR-
DNPLNI--
-DPLNIW-
-DPLNIM-
-DPLNIK-
-DPLNIE-
DMPLNI--
DLPLNI--
DHPLNI--
ESPLNI--
ETPLNI--
-EPLNIL-
-EPLNIM-
-EPLNIR-
EVPLNI--
ERPLNI--
-EPLNIS-
-EPLNIA-
ELPLNI--
-EPLNIV-
-EPLNIK-
-EPLNIF-
EWPLNI--
ENPLNI--
EKPLNI--
EGPLNI--
EAPLNI--
EYPLNI--
-EPLNIW-
-EPLNIN-
-EPLNIH-
-FPLNIR-
-FPLNIQ-
FRPLNI--
-FPLNII-
FLPLNI--
FVPLNI--
-FPLNIV-

-FPLNIS-
FGPLNI--
FTPLNI--
FPPLNI--
FMPLNI--
-FPLNIM-
-FPLNIG-
FIPLNI--
FFPLNI--
FEPLNI--
FNPLNI--
FSPLNI--
-FPLNIL-
FDPLNI--
FYPLNI--
-FPLNIY-
FKPLNI--
-FPLNIW-
-FPLNIN-
-FPLNIK-
-FPLNIE-
-FPLNIA-
FHPLNI--
FCPLNI--
FAPLNI--
-GPLNIR-
-GPLNIM-
GSPLNI--
GLPLNI--
GVPLNI--
-GPLNIL-
GKPLNI--
GIPLNI--
GTPLNI--
-GPLNIY-
-GPLNIS-
GQPLNI--
-GPLNIV-
-GPLNIG-
-GPLNIF-
GMPLNI--
GHPLNI--
GEPLNI--
GYPLNI--
-GPLNII-
GGPLNI--
GAPLNI--
-GPLNIW-
-GPLNIN-
-GPLNIK-

GWPLNI--
-GPLNIA-
-GPLNIH-
GNPLNI--
GFPLNI--
-HPLNIW-
-HPLNIL-
HSPLNI--
-HPLNIS-
-HPLNIR-
-HPLNIA-
HLPLNI--
HVPLNI--
HRPLNI--
-HPLNIY-
-HPLNIK-
HHPLNI--
HQPLNI--
-HPLNII-
HFPLNI--
HAPLNI--
-HPLNIV-
HYPLNI--
-HPLNIN-
-HPLNIG-
HNPLNI--
HMPLNI--
HIPLNI--
HDPLNI--
HTPLNI--
HPPLNI--
-HPLNIT-
-HPLNIQ-
-HPLNIH-
HKPLNI--
HEPLNI--
ILPLNI--
-IPLNIS-
-IPLNIM-
-IPLNIL-
IFPLNI--
IVPLNI--
IGPLNI--
-IPLNIR-
IKPLNI--
IHPLNI--
IRPLNI--
-IPLNIA-
-IPLNIW-
-IPLNIT-

-IPLNIK-
-IPLNIG-
IDPLNI--
IPPLNI--
-IPLNIV-
-IPLNIN-
-IPLNIH-
ITPLNI--
-IPLNIY-
INPLNI--
IMPLNI--
IWPLNI--
-IPLNIQ-
IIPLNI--
IEPLNI--
KSPLNI--
KGPLNI--
-KPLNIN-
-KPLNIV-
-KPLNIR-
-KPLNIL-
KTPLNI--
KPPLNI--
-KPLNIM-
-KPLNIA-
KRPLNI--
KQPLNI--
-KPLNIT-
KVPLNI--
-KPLNIQ-
KMPLNI--
-KPLNIF-
KLPLNI--
KWPLNI--
-KPLNIE-
KNPLNI--
KHPLNI--
KFPLNI--
KYPLNI--
-KPLNIW-
-KPLNIH-
-KPLNIG-
KKPLNI--
KIPLNI--
KDPLNI--
KCPLNI--
LRPLNI--
LMPLNI--
LYPLNI--
LNPLNI--

LGPLNI--
-LPLNIK-
LSPLNI--
-LPLNIV-
-LPLNIT-
-LPLNIS-
-LPLNIL-
-LPLNII-
-LPLNIW-
-LPLNIA-
LFPLNI--
LWPLNI--
LQPLNI--
LHPLNI--
LEPLNI--
-LPLNIQ-
LDPLNI--
MLPLNI--
-MPLNIS-
MSPLNI--
-MPLNIR-
-MPLNIV-
-MPLNII-
MKPLNI--
MFPLNI--
MMPLNI--
MGPLNI--
MDPLNI--
MTPLNI--
-MPLNIL-
-MPLNIH-
MQPLNI--
MPPLNI--
-MPLNIN-
-MPLNIT-
-MPLNIK-
-MPLNIE-
MNPLNI--
MHPLNI--
NPPLNI--
NWPLNI--
NKPLNI--
-NPLNIK-
NSPLNI--
-NPLNIR-
-NPLNIQ-
NVPLNI--
NFPLNI--
NYPLNI--
-NPLNIA-

NLPLNI--
NQPLNI--
-NPLNIV-
-NPLNIT-
-NPLNIM-
-NPLNIL-
-NPLNII-
NGPLNI--
-NPLNIY-
-NPLNIS-
NMPLNI--
-NPLNIG-
NNPLNI--
NIPLNI--
--PLNILT
--PLNIRR
--PLNIRL
--PLNILN
--PLNIYR
--PLNIVA
--PLNISR
--PLNIIR
PRPLNI--
--PLNIVY
--PLNIRY
--PLNIVT
--PLNISL
--PLNITL
--PLNISS
--PLNIRQ
--PLNIRI
--PLNIRG
--PLNITR
--PLNILH
--PLNIVR
--PLNISY
--PLNIRV
--PLNIVS
--PLNIMV
--PLNILR
--PLNIFT
--PLNIYH
--PLNIVG
--PLNIST
--PLNIMR
--PLNIHG
--PLNITT
--PLNITS
--PLNISM
--PLNISK

--PLNISA
--PLNIRF
--PLNIRA
--PLNIMG
--PLNIGS
--PLNIFR
--PLNIAI
--PLNIRK
--PLNIMN
--PLNIMA
--PLNILG
--PLNIKW
--PLNIKR
--PLNIKQ
--PLNIFS
--PLNIAG
--PLNIYF
--PLNIWR
--PLNISH
--PLNILD
--PLNIIS
--PLNIIQ
--PLNIID
--PLNIGA
--PLNIET
--PLNIAS
--PLNIAL
--PLNIAF
--PLNIVE
--PLNITG
--PLNISV
--PLNISN
--PLNISG
--PLNIRH
--PLNIQQ
--PLNING
--PLNIMT
--PLNIKY
--PLNIKV
--PLNIKS
--PLNIKM
--PLNIIK
--PLNIEW
--PLNIYT
--PLNIYI
--PLNIYG
--PLNIWD
--PLNIVW
--PLNIVI
--PLNITM

--PLNITK
--PLNISI
--PLNINS
--PLNIMS
--PLNILK
--PLNIIG
--PLNIHN
--PLNIEY
PYPLNI--
PWPLNI--
--PLNIWK
--PLNIVN
--PLNIVK
--PLNITQ
--PLNISD
--PLNIQH
--PLNIQG
--PLNINV
--PLNIMY
--PLNIIA
--PLNIHR
--PLNIGG
--PLNIFM
--PLNIFK
--PLNIES
--PLNIAM
--PLNIAH
--PLNIYW
--PLNIYK
--PLNIYA
--PLNIWQ
--PLNIWG
--PLNIVQ
--PLNISQ
--PLNIRD
--PLNIQY
--PLNIQS
--PLNIQR
--PLNIMQ
--PLNIML
--PLNIMI
--PLNIMD
--PLNIIY
--PLNIHH
--PLNIGV
--PLNIGT
--PLNIFN
--PLNIER
--PLNIEG
--PLNIAN

--PLNIAK
--PLNIAA
PFPLNI--
PNPLNI--
--PLNIYS
--PLNIYQ
--PLNIYE
--PLNIWS
--PLNIVV
--PLNIVM
--PLNIVD
--PLNITW
--PLNITV
--PLNITN
--PLNITE
--PLNITA
--PLNIRM
--PLNIQT
--PLNIQD
--PLNIQA
--PLNINY
--PLNIMM
--PLNIMH
--PLNILW
--PLNILQ
--PLNIKT
--PLNIKA
--PLNIIV
--PLNIIT
--PLNIHY
--PLNIHK
--PLNIGY
--PLNIGL
--PLNIFY
--PLNIFH
--PLNIAR
PEPLNI--
PQPLNI--
PMPLNI--
--PLNIYN
--PLNIYM
--PLNIWT
--PLNIWN
--PLNIWH
--PLNIWF
--PLNITH
--PLNITF
--PLNIQN
--PLNIPL
--PLNIPF

--PLNINT
--PLNINK
--PLNINA
--PLNIMW
--PLNIMK
--PLNILE
--PLNIKN
--PLNIKK
--PLNIIN
--PLNIII
--PLNIHV
--PLNIHT
--PLNIHQ
--PLNIHF
--PLNIHE
--PLNIHD
--PLNIGW
--PLNIGM
--PLNIFQ
--PLNIFE
--PLNIFD
--PLNIEQ
--PLNIEN
--PLNIEL
--PLNIEA
--PLNICI
--PLNICF
--PLNIAW
--PLNIAV
--PLNIAT
--PLNIAQ
--PLNIAE
PHPLNI--
QRPLNI--
QSPLNI--
-QPLNIK-
-QPLNIS-
QVPLNI--
QLPLNI--
QWPLNI--
-QPLNII-
-QPLNIF-
-QPLNIA-
QAPLNI--
QQPLNI--
-QPLNIR-
-QPLNIM-
QMPLNI--
QYPLNI--
-QPLNIH-

```
-QPLNIG-
QKPLNI--
QHPLNI--
-QPLNIT-
-QPLNIQ-
-QPLNIE-
QNPLNI--
QGPLNI--
QEPLNI--
QDPLNI--
QTPLNI--
QPPLNI--
-QPLNIY-
-QPLNIW-
-QPLNIL-
-RPLNIS-
RVPLNI--
-RPLNIR-
RSPLNI--
RLPLNI--
-RPLNIL-
-RPLNIW-
-RPLNIM-
RRPLNI--
-RPLNII-
-RPLNIV-
-RPLNIT-
RPPLNI--
-RPLNIA-
RAPLNI--
-RPLNIG-
RKPLNI--
RGPLNI--
-RPLNIF-
RTPLNI--
-RPLNIY-
RQPLNI--
-RPLNIH-
RHPLNI--
RDPLNI--
-RPLNIQ-
-RPLNIN-
-RPLNIE-
RIPLNI--
RMPLNI--
REPLNI--
RWPLNI--
-RPLNIK-
RYPLNI--
-RPLNID-
```

RCPLNI--
SVPLNI--
-SPLNIS-
-SPLNII-
-SPLNIR-
SKPLNI--
SRPLNI--
SLPLNI--
SIPLNI--
SFPLNI--
-SPLNIL-
-SPLNIM-
-SPLNIA-
SNPLNI--
SYPLNI--
SWPLNI--
-SPLNIY-
SSPLNI--
-SPLNIF-
-SPLNIW-
SPPLNI--
-SPLNIV-
-SPLNIG-
SMPLNI--
SGPLNI--
-SPLNIN-
-SPLNIH-
SHPLNI--
-SPLNIQ-
SQPLNI--
-SPLNIK-
SAPLNI--
-SPLNIT-
-SPLNIE-
SDPLNI--
SCPLNI--
-SPLNID-
SEPLNI--
-TPLNIL-
TLPLNI--
TRPLNI--
-TPLNIR-
TNPLNI--
-TPLNIS-
-TPLNIY-
-TPLNIH-
TKPLNI--
TAPLNI--
TWPLNI--
-TPLNIE-

-TPLNIA-
TMPLNI--
THPLNI--
TGPLNI--
TSPLNI--
TIPLNI--
TYPLNI--
-TPLNIT-
-TPLNIK-
-TPLNII-
TFPLNI--
TEPLNI--
-TPLNIF-
TTPLNI--
TDPLNI--
TQPLNI--
-TPLNIM-
TPPLNI--
-TPLNIQ-
-TPLNIG-
VLPLNI--
-VPLNII-
-VPLNIA-
VRPLNI--
-VPLNIT-
VYPLNI--
VWPLNI--
VPPLNI--
-VPLNIR-
VVPLNI--
VTPLNI--
VSPLNI--
VNPLNI--
-VPLNIQ-
-VPLNIY-
-VPLNIS-
-VPLNIG-
VFPLNI--
-VPLNIW-
-VPLNIV-
-VPLNIK-
-VPLNIF-
VMPLNI--
VEPLNI--
VAPLNI--
-VPLNIM-
VGPLNI--
-VPLNIN-
-VPLNIH-
VIPLNI--

VQPLNI--
-WPLNIR-
-WPLNIY-
-WPLNIS-
WTPLNI--
WRPLNI--
-WPLNIF-
-WPLNIQ-
WMPLNI--
WGPLNI--
WEPLNI--
-WPLNIT-
-WPLNIM-
-WPLNIK-
WLPLNI--
WKPLNI--
WDPLNI--
WAPLNI--
-WPLNIA-
WSPLNI--
WQPLNI--
-WPLNIN-
-WPLNII-
WNPLNI--
WFPLNI--
YSPLNI--
-YPLNII-
-YPLNIS-
YLPLNI--
YAPLNI--
YFPLNI--
YWPLNI--
-YPLNIR-
-YPLNIL-
-YPLNIG-
YPPLNI--
-YPLNIA-
YNPLNI--
YYPLNI--
-YPLNIW-
-YPLNIM-
-YPLNIK-
-YPLNIE-
YKPLNI--
YIPLNI--
YHPLNI--
YDPLNI--
YTPLNI--
-YPLNIY-
-YPLNIT-

| | | | |
|---|---|---|---|
| | -YPLNIH- | | |
| | YVPLNI-- | | |
| | YQPLNI-- | | |
| | -YPLNIQ- | | |
| | -YPLNIN- | | |
| | YEPLNI-- | | |
| PRNW | 179 | 726 | 31.58 |
| | -LPRNWG- | | |
| | VRPRNW-- | | |
| | AGPRNW-- | | |
| | ASPRNW-- | | |
| | -APRNWT- | | |
| | -APRNWS- | | |
| | -APRNWM- | | |
| | -APRNWL- | | |
| | AAPRNW-- | | |
| | -APRNWV- | | |
| | -APRNWQ- | | |
| | -APRNWI- | | |
| | ARPRNW-- | | |
| | -APRNWR- | | |
| | -APRNWK- | | |
| | -APRNWH- | | |
| | -APRNWG- | | |
| | AMPRNW-- | | |
| | ALPRNW-- | | |
| | ATPRNW-- | | |
| | -APRNWW- | | |
| | AHPRNW-- | | |
| | AYPRNW-- | | |
| | -APRNWY- | | |
| | APPRNW-- | | |
| | AIPRNW-- | | |
| | AWPRNW-- | | |
| | -APRNWE- | | |
| | ANPRNW-- | | |
| | AFPRNW-- | | |
| | -APRNWD- | | |
| | -APRNWA- | | |
| | CVPRNW-- | | |
| | CAPRNW-- | | |
| | CRPRNW-- | | |
| | CLPRNW-- | | |
| | DVPRNW-- | | |
| | DSPRNW-- | | |
| | DAPRNW-- | | |
| | DRPRNW-- | | |
| | -DPRNWY- | | |
| | DMPRNW-- | | |
| | DTPRNW-- | | |

-DPRNWR-
-DPRNWK-
DFPRNW--
DYPRNW--
DQPRNW--
-DPRNWT-
-DPRNWS-
-DPRNWM-
-DPRNWL-
DLPRNW--
DIPRNW--
DHPRNW--
DGPRNW--
-EPRNWL-
-EPRNWI-
-EPRNWY-
-EPRNWV-
-EPRNWA-
ERPRNW--
EQPRNW--
-EPRNWS-
-EPRNWR-
-EPRNWF-
ETPRNW--
ESPRNW--
-EPRNWN-
-EPRNWK-
-EPRNWH-
-EPRNWG-
EHPRNW--
EFPRNW--
EEPRNW--
EAPRNW--
EYPRNW--
-EPRNWW-
EIPRNW--
EGPRNW--
FAPRNW--
FSPRNW--
FMPRNW--
-FPRNWI-
FLPRNW--
FTPRNW--
-FPRNWS-
-FPRNWA-
FKPRNW--
-FPRNWG-
FGPRNW--
FFPRNW--
FYPRNW--

-FPRNWT-
-FPRNWL-
FNPRNW--
FVPRNW--
-FPRNWY-
-FPRNWV-
-FPRNWQ-
-FPRNWM-
-FPRNWE-
FIPRNW--
FEPRNW--
-GPRNWV-
-GPRNWR-
-GPRNWL-
-GPRNWK-
GYPRNW--
GVPRNW--
GTPRNW--
GSPRNW--
-GPRNWY-
-GPRNWN-
-GPRNWH-
GGPRNW--
GAPRNW--
GNPRNW--
GLPRNW--
GIPRNW--
GWPRNW--
GQPRNW--
-GPRNWW-
-GPRNWT-
-GPRNWE-
GEPRNW--
-GPRNWF-
GHPRNW--
HSPRNW--
HPPRNW--
HRPRNW--
HGPRNW--
-HPRNWS-
HHPRNW--
HFPRNW--
-HPRNWR-
-HPRNWK-
HWPRNW--
-HPRNWL-
-HPRNWH-
-HPRNWG-
HIPRNW--
HYPRNW--

HTPRNW--
-HPRNWW-
-HPRNWI-
-HPRNWF-
-HPRNWA-
HNPRNW--
-HPRNWY-
-HPRNWV-
-HPRNWM-
HLPRNW--
ISPRNW--
-IPRNWL-
-IPRNWR-
IRPRNW--
-IPRNWQ-
-IPRNWT-
-IPRNWI-
IQPRNW--
IPPRNW--
-IPRNWS-
-IPRNWM-
-IPRNWH-
IHPRNW--
IAPRNW--
IVPRNW--
-IPRNWN-
-IPRNWF-
-IPRNWA-
ILPRNW--
IKPRNW--
IGPRNW--
IFPRNW--
IYPRNW--
ITPRNW--
-IPRNWY-
-IPRNWV-
-IPRNWK-
-IPRNWG-
INPRNW--
IIPRNW--
-IPRNWW-
IDPRNW--
KSPRNW--
-KPRNWQ-
KAPRNW--
KVPRNW--
-KPRNWY-
-KPRNWV-
-KPRNWA-
-KPRNWT-

-KPRNWS-
-KPRNWF-
KMPRNW--
KLPRNW--
KKPRNW--
KIPRNW--
KGPRNW--
KWPRNW--
-KPRNWW-
-KPRNWM-
-KPRNWL-
-KPRNWE-
KYPRNW--
KTPRNW--
KQPRNW--
-KPRNWR-
-KPRNWI-
KNPRNW--
LHPRNW--
LSPRNW--
LFPRNW--
LGPRNW--
-LPRNWT-
LEPRNW--
LYPRNW--
-LPRNWL-
LLPRNW--
LPPRNW--
LKPRNW--
LWPRNW--
-LPRNWM-
-LPRNWF-
LIPRNW--
-LPRNWS-
-LPRNWK-
LMPRNW--
LTPRNW--
-LPRNWA-
LAPRNW--
LQPRNW--
-LPRNWQ-
-LPRNWI-
-LPRNWY-
-LPRNWV-
-LPRNWR-
-LPRNWH-
LNPRNW--
LDPRNW--
-MPRNWL-
-MPRNWS-

```
MKPRNW--
MRPRNW--
MSPRNW--
MEPRNW--
MQPRNW--
-MPRNWY-
-MPRNWN-
-MPRNWI-
-MPRNWG-
-MPRNWR-
-MPRNWK-
MPPRNW--
MAPRNW--
MYPRNW--
-MPRNWW-
-MPRNWV-
-MPRNWT-
-MPRNWM-
-MPRNWA-
MIPRNW--
-MPRNWQ-
-MPRNWH-
MWPRNW--
MVPRNW--
-MPRNWE-
MGPRNW--
MFPRNW--
NSPRNW--
NRPRNW--
NTPRNW--
NQPRNW--
NPPRNW--
-NPRNWY-
-NPRNWT-
-NPRNWF-
NMPRNW--
NWPRNW--
NVPRNW--
-NPRNWS-
-NPRNWR-
NNPRNW--
NLPRNW--
NYPRNW--
-NPRNWM-
-NPRNWK-
NDPRNW--
NAPRNW--
-NPRNWI-
NFPRNW--
--PRNWSL
```

--PRNWLS
--PRNWSS
--PRNWVR
--PRNWRL
--PRNWRY
--PRNWLI
--PRNWLQ
--PRNWKL
--PRNWLG
--PRNWKI
--PRNWHS
--PRNWST
--PRNWRI
--PRNWRA
--PRNWLT
--PRNWIL
--PRNWFT
--PRNWSV
--PRNWRV
--PRNWTS
--PRNWIS
--PRNWGS
--PRNWFS
PVPRNW--
--PRNWVV
--PRNWTW
--PRNWSQ
--PRNWIH
--PRNWFL
--PRNWAK
--PRNWAI
--PRNWYI
--PRNWYD
--PRNWLY
--PRNWKG
--PRNWIF
--PRNWYG
--PRNWSK
--PRNWKH
--PRNWIR
--PRNWHG
--PRNWAS
--PRNWWA
--PRNWVT
--PRNWVN
--PRNWRT
--PRNWRN
--PRNWMK
--PRNWLH
--PRNWGT

--PRNWAV
--PRNWYV
--PRNWWR
--PRNWVA
--PRNWTH
--PRNWMA
--PRNWLA
--PRNWII
--PRNWFR
--PRNWAN
PSPRNW--
--PRNWYR
--PRNWYL
--PRNWWS
--PRNWWH
--PRNWTM
--PRNWSN
--PRNWSH
--PRNWSF
--PRNWSD
--PRNWNV
--PRNWNG
--PRNWMF
--PRNWIA
--PRNWGA
--PRNWES
PAPRNW--
--PRNWYS
--PRNWWL
--PRNWVM
--PRNWVK
--PRNWVI
--PRNWVG
--PRNWTT
--PRNWTQ
--PRNWRM
--PRNWRK
--PRNWRF
--PRNWNT
--PRNWKS
--PRNWGL
--PRNWAW
PLPRNW--
--PRNWYA
--PRNWVH
--PRNWTF
--PRNWSW
--PRNWRQ
--PRNWQK
--PRNWGV

--PRNWFY
--PRNWET
--PRNWEL
--PRNWYY
--PRNWYF
--PRNWSI
--PRNWSG
--PRNWRE
--PRNWQL
--PRNWNF
--PRNWML
--PRNWMG
--PRNWKT
--PRNWIY
--PRNWIG
--PRNWHN
--PRNWGI
--PRNWFN
--PRNWFK
--PRNWFG
--PRNWFD
--PRNWAG
--PRNWWY
--PRNWWK
--PRNWVY
--PRNWTY
--PRNWTV
--PRNWTR
--PRNWTN
--PRNWTA
--PRNWRH
--PRNWRD
--PRNWQW
--PRNWNY
--PRNWMT
--PRNWMR
--PRNWMN
--PRNWMI
--PRNWLM
--PRNWLF
--PRNWKW
--PRNWKF
--PRNWIT
--PRNWIK
--PRNWHV
--PRNWHL
--PRNWHH
--PRNWFE
--PRNWFA
--PRNWEW

--PRNWEV
--PRNWEM
--PRNWAQ
--PRNWAH
PGPRNW--
PRPRNW--
--PRNWYW
--PRNWYQ
--PRNWYM
--PRNWWV
--PRNWWN
--PRNWWI
--PRNWWG
--PRNWWF
--PRNWVD
--PRNWTP
--PRNWTK
--PRNWTI
--PRNWTD
--PRNWSM
--PRNWSA
--PRNWRP
--PRNWQT
--PRNWQR
--PRNWQF
--PRNWQA
--PRNWNM
--PRNWNL
--PRNWNK
--PRNWMH
--PRNWMD
--PRNWLW
--PRNWLN
--PRNWLD
--PRNWKY
--PRNWKV
--PRNWKD
--PRNWIM
--PRNWIE
--PRNWHY
--PRNWGY
--PRNWEY
--PRNWEI
--PRNWEF
--PRNWAY
--PRNWAE
--PRNWAA
PHPRNW--
PTPRNW--
--PRNWYT

--PRNWYN
--PRNWYK
--PRNWYH
--PRNWWW
--PRNWVW
--PRNWVQ
--PRNWVP
--PRNWRW
--PRNWQS
--PRNWQQ
--PRNWQP
--PRNWQM
--PRNWQE
--PRNWQD
--PRNWPR
--PRNWNS
--PRNWNN
--PRNWNI
--PRNWMW
--PRNWMS
--PRNWMQ
--PRNWLE
--PRNWKQ
--PRNWKE
--PRNWKA
--PRNWIW
--PRNWIQ
--PRNWIP
--PRNWID
--PRNWHQ
--PRNWHK
--PRNWHF
--PRNWGN
--PRNWFV
--PRNWFQ
--PRNWFM
--PRNWFI
--PRNWEK
--PRNWAT
--PRNWAD
PNPRNW--
-QPRNWR-
QGPRNW--
QYPRNW--
QRPRNW--
-QPRNWS-
QMPRNW--
QTPRNW--
-QPRNWW-
-QPRNWI-

−QPRNWF−
−QPRNWE−
QSPRNW−−
−QPRNWQ−
−QPRNWL−
QLPRNW−−
QVPRNW−−
−QPRNWH−
QQPRNW−−
QWPRNW−−
−QPRNWV−
−QPRNWT−
−QPRNWA−
−QPRNWY−
QHPRNW−−
−RPRNWI−
−RPRNWS−
−RPRNWL−
−RPRNWG−
RSPRNW−−
−RPRNWV−
RVPRNW−−
RTPRNW−−
−RPRNWY−
RMPRNW−−
REPRNW−−
RYPRNW−−
−RPRNWT−
RAPRNW−−
RPPRNW−−
RIPRNW−−
RQPRNW−−
−RPRNWQ−
−RPRNWF−
RLPRNW−−
RHPRNW−−
RFPRNW−−
−RPRNWW−
−RPRNWN−
−RPRNWE−
RKPRNW−−
RDPRNW−−
−SPRNWR−
−SPRNWL−
SAPRNW−−
−SPRNWA−
−SPRNWS−
SVPRNW−−
STPRNW−−
SIPRNW−−

SSPRNW--
-SPRNWF-
SRPRNW--
-SPRNWV-
-SPRNWQ-
-SPRNWG-
SLPRNW--
-SPRNWT-
-SPRNWI-
SGPRNW--
SKPRNW--
SEPRNW--
-SPRNWN-
-SPRNWM-
SMPRNW--
SFPRNW--
-SPRNWW-
-SPRNWH-
-SPRNWE-
SHPRNW--
SYPRNW--
SWPRNW--
SPPRNW--
SNPRNW--
-TPRNWR-
TKPRNW--
-TPRNWM-
-TPRNWL-
-TPRNWK-
-TPRNWG-
TMPRNW--
TFPRNW--
TVPRNW--
TSPRNW--
-TPRNWY-
-TPRNWI-
-TPRNWT-
THPRNW--
TAPRNW--
TWPRNW--
-TPRNWF-
TGPRNW--
-TPRNWV-
-TPRNWS-
-TPRNWQ-
-TPRNWN-
TPPRNW--
TLPRNW--
TIPRNW--
TRPRNW--

TNPRNW--
TTPRNW--
-TPRNWA-
TEPRNW--
TYPRNW--
-TPRNWE-
VSPRNW--
-VPRNWL-
-VPRNWT-
-VPRNWS-
VKPRNW--
-VPRNWR-
VGPRNW--
VWPRNW--
-VPRNWN-
VAPRNW--
-VPRNWM-
VYPRNW--
VFPRNW--
-VPRNWQ-
VLPRNW--
VQPRNW--
-VPRNWW-
-VPRNWF-
VTPRNW--
-VPRNWE-
VVPRNW--
-VPRNWG-
-VPRNWV-
-VPRNWH-
-VPRNWA-
VNPRNW--
VMPRNW--
VHPRNW--
-VPRNWY-
-VPRNWI-
VPPRNW--
WIPRNW--
WGPRNW--
WMPRNW--
-WPRNWT-
-WPRNWS-
-WPRNWA-
WYPRNW--
-WPRNWL-
-WPRNWK-
-WPRNWG-
WTPRNW--
WQPRNW--
-WPRNWY-

-WPRNWN-
WAPRNW--
WVPRNW--
WPPRNW--
WKPRNW--
WHPRNW--
YVPRNW--
YTPRNW--
YQPRNW--
YIPRNW--
YPPRNW--
YEPRNW--
YRPRNW--
YLPRNW--
YSPRNW--
-YPRNWL-
YMPRNW--
YHPRNW--
-YPRNWK-
YKPRNW--
YAPRNW--
-YPRNWV-
-YPRNWS-
YYPRNW--
-YPRNWY-
-YPRNWQ-
-YPRNWN-
-YPRNWI-
-YPRNWT-
-YPRNWG-
-YPRNWF-
-YPRNWE-
YFPRNW--

IGML 357 649 31.57

-EIGMLL-
-GIGMLG-
--IGMLNR
--IGMLLK
--IGMLGI
--IGMLGH
-NIGMLG-
-AIGMLK-
-AIGMLS-
-AIGMLR-
-AIGMLG-
-AIGMLA-
-AIGMLL-
AGIGML--
ATIGML--
-AIGMLQ-

-AIGMLH-
AQIGML--
ANIGML--
-AIGMLT-
ASIGML--
AVIGML--
ADIGML--
AHIGML--
AAIGML--
APIGML--
-AIGMLV-
-AIGMLN-
-AIGMLM-
AEIGML--
AMIGML--
CRIGML--
CPIGML--
CTIGML--
-CIGMLN-
CSIGML--
-CIGMLR-
CWIGML--
CVIGML--
CMIGML--
-CIGMLY-
-CIGMLT-
CHIGML--
DRIGML--
DLIGML--
-DIGMLK-
-DIGMLH-
DVIGML--
DSIGML--
-DIGMLR-
DGIGML--
-DIGMLT-
-DIGMLI-
DMIGML--
DTIGML--
DKIGML--
DIIGML--
-DIGMLM-
DAIGML--
-DIGMLV-
DFIGML--
DDIGML--
DYIGML--
DWIGML--
DPIGML--
DNIGML--

-DIGMLQ-
-EIGMLR-
ETIGML--
EHIGML--
EAIGML--
EVIGML--
-EIGMLK-
-EIGMLI-
-EIGMLS-
ERIGML--
EWIGML--
-EIGMLW-
-EIGMLV-
EYIGML--
ELIGML--
EEIGML--
EQIGML--
EIIGML--
-EIGMLT-
-EIGMLN-
-EIGMLM-
EGIGML--
FGIGML--
FTIGML--
FAIGML--
FEIGML--
FKIGML--
FRIGML--
FNIGML--
FIIGML--
-FIGMLG-
FPIGML--
FMIGML--
-FIGMLL-
-FIGMLH-
-FIGMLA-
FHIGML--
FFIGML--
-GIGMLR-
GVIGML--
GAIGML--
-GIGMLS-
-GIGMLK-
-GIGMLQ-
GLIGML--
GTIGML--
GMIGML--
-GIGMLT-
GNIGML--
GQIGML--

GHIGML--
GWIGML--
-GIGMLM-
GPIGML--
GYIGML--
-GIGMLN-
GIIGML--
-GIGMLW-
-GIGMLV-
-GIGMLH-
GFIGML--
-GIGMLC-
GCIGML--
HKIGML--
HGIGML--
-HIGMLL-
HRIGML--
-HIGMLK-
HLIGML--
HNIGML--
-HIGMLS-
-HIGMLW-
HEIGML--
-HIGMLV-
HSIGML--
HPIGML--
HHIGML--
-HIGMLT-
HDIGML--
HTIGML--
HIIGML--
HCIGML--
HQIGML--
-HIGMLY-
-HIGMLI-
HVIGML--
-HIGMLH-
-HIGMLN-
-HIGMLF-
HAIGML--
HMIGML--
-HIGMLM-
-HIGMLE-
--IGMLSS
--IGMLSA
--IGMLLT
--IGMLRG
--IGMLLS
--IGMLLG
--IGMLST

--IGMLSL
--IGMLVT
--IGMLVS
--IGMLRA
--IGMLHS
--IGMLVH
--IGMLTN
--IGMLIS
IKIGML--
--IGMLWK
--IGMLTV
--IGMLTG
--IGMLHG
--IGMLGL
--IGMLRN
--IGMLKS
--IGMLHL
IRIGML--
-IIGMLS-
--IGMLVA
--IGMLRI
--IGMLKR
--IGMLIN
ISIGML--
--IGMLMS
--IGMLKG
--IGMLFN
--IGMLAS
--IGMLVG
--IGMLQV
--IGMLEG
--IGMLYS
--IGMLRH
--IGMLMA
--IGMLAI
--IGMLYR
--IGMLSN
--IGMLQL
--IGMLNL
--IGMLIT
--IGMLGA
-IIGMLR-
-IIGMLK-
--IGMLYH
--IGMLRW
--IGMLQY
--IGMLMT
--IGMLLA
--IGMLKN
--IGMLVK

--IGMLTS
--IGMLTM
--IGMLSF
--IGMLMH
--IGMLGW
--IGMLAL
--IGMLAA
IEIGML--
IAIGML--
--IGMLVD
--IGMLSV
--IGMLRV
--IGMLRR
--IGMLQK
--IGMLPL
--IGMLMR
--IGMLKI
--IGMLIK
--IGMLIH
--IGMLHK
--IGMLFG
IDIGML--
ILIGML--
-IIGMLT-
--IGMLYN
--IGMLVV
--IGMLTH
--IGMLSH
--IGMLRE
--IGMLQH
--IGMLNS
--IGMLLN
--IGMLKF
--IGMLAT
IPIGML--
-IIGMLG-
-IIGMLA-
--IGMLYG
--IGMLWQ
--IGMLVF
--IGMLTY
--IGMLTR
--IGMLTA
--IGMLSY
--IGMLLQ
--IGMLFK
ITIGML--
-IIGMLQ-
--IGMLWN
--IGMLVN

```
--IGMLVI
--IGMLSW
--IGMLQS
--IGMLQG
--IGMLNM
--IGMLME
--IGMLMD
--IGMLLH
--IGMLKY
--IGMLKM
--IGMLKH
--IGMLHR
--IGMLES
--IGMLAV
--IGMLAR
--IGMLAG
INIGML--
IIIGML--
--IGMLYT
--IGMLYD
--IGMLWT
--IGMLWG
--IGMLTW
--IGMLSM
--IGMLSI
--IGMLRQ
--IGMLRM
--IGMLQQ
--IGMLQN
--IGMLQI
--IGMLMW
--IGMLMG
--IGMLKV
--IGMLIQ
--IGMLID
--IGMLHW
--IGMLHT
--IGMLHQ
--IGMLHI
--IGMLHH
--IGMLHF
--IGMLHA
--IGMLGT
--IGMLFW
--IGMLFT
--IGMLAW
--IGMLAQ
--IGMLAN
IMIGML--
IHIGML--
```

--IGMLYQ
--IGMLYE
--IGMLYA
--IGMLWH
--IGMLTQ
--IGMLTI
--IGMLSQ
--IGMLSK
--IGMLSD
--IGMLQT
--IGMLNG
--IGMLNF
--IGMLMV
--IGMLMN
--IGMLMF
--IGMLLD
--IGMLKE
--IGMLHN
--IGMLFD
IGIGML--
-IIGMLN-
-IIGMLE-
--IGMLWS
--IGMLWE
--IGMLWA
--IGMLQW
--IGMLQR
--IGMLQM
--IGMLQD
--IGMLQA
--IGMLPY
--IGMLPI
--IGMLPF
--IGMLNY
--IGMLNW
--IGMLNT
--IGMLNI
--IGMLMM
--IGMLMK
--IGMLLE
--IGMLKQ
--IGMLKA
--IGMLHV
--IGMLHM
--IGMLHE
--IGMLHD
--IGMLGY
--IGMLGG
--IGMLFY
--IGMLEV

```
--IGMLDY
--IGMLCS
KYIGML--
-KIGMLK-
KGIGML--
KEIGML--
-KIGMLT-
KMIGML--
-KIGMLR-
KVIGML--
KLIGML--
-KIGMLV-
KFIGML--
KIIGML--
-KIGMLS-
-KIGMLQ-
-KIGMLW-
-KIGMLH-
KNIGML--
-KIGMLL-
KPIGML--
KDIGML--
-KIGMLN-
-KIGMLA-
KCIGML--
LAIGML--
LHIGML--
LVIGML--
LKIGML--
LMIGML--
-LIGMLQ-
LNIGML--
LIIGML--
-LIGMLT-
-LIGMLE-
LGIGML--
LTIGML--
LPIGML--
-LIGMLH-
-LIGMLA-
LEIGML--
-LIGMLK-
LDIGML--
-LIGMLG-
LQIGML--
-LIGMLN-
-LIGMLP-
LCIGML--
MSIGML--
-MIGMLR-
```

MHIGML--
-MIGMLS-
-MIGMLA-
MAIGML--
-MIGMLL-
MPIGML--
-MIGMLK-
MVIGML--
-MIGMLT-
MQIGML--
-MIGMLY-
-MIGMLQ-
-MIGMLN-
MEIGML--
MTIGML--
MKIGML--
MIIGML--
MDIGML--
-NIGMLR-
-NIGMLL-
NRIGML--
-NIGMLK-
-NIGMLS-
NAIGML--
-NIGMLT-
-NIGMLN-
NSIGML--
NYIGML--
NLIGML--
NMIGML--
-NIGMLV-
-NIGMLI-
-NIGMLF-
NGIGML--
NIIGML--
-NIGMLA-
NHIGML--
NFIGML--
NVIGML--
NNIGML--
-NIGMLW-
-NIGMLY-
NEIGML--
NQIGML--
NKIGML--
-NIGMLQ-
-NIGMLM-
-NIGMLH-
NCIGML--
NPIGML--

–NIGMLE–
–PIGMLL–
–PIGMLS–
PQIGML––
PLIGML––
–PIGMLT–
–PIGMLR–
PEIGML––
PSIGML––
PRIGML––
–PIGMLE–
PNIGML––
PMIGML––
–PIGMLN–
–PIGMLH–
PGIGML––
PAIGML––
–PIGMLQ–
–PIGMLA–
–PIGMLG–
–PIGMLD–
PHIGML––
PVIGML––
PTIGML––
–PIGMLK–
–PIGMLI–
–PIGMLV–
PDIGML––
PCIGML––
–QIGMLS–
QNIGML––
QLIGML––
–QIGMLR–
QIIGML––
QYIGML––
–QIGMLQ–
–QIGMLM–
QAIGML––
QTIGML––
–QIGMLY–
QDIGML––
QPIGML––
QMIGML––
QGIGML––
QVIGML––
–QIGMLI–
–QIGMLA–
–QIGMLV–
–QIGMLK–
–QIGMLT–

-QIGMLN-
QWIGML--
-QIGMLH-
QEIGML--
-QIGMLW-
-QIGMLL-
RTIGML--
-RIGMLT-
-RIGMLS-
RHIGML--
-RIGMLV-
RAIGML--
RMIGML--
-RIGMLI-
RDIGML--
RPIGML--
RIIGML--
-RIGMLN-
-RIGMLM-
-RIGMLG-
-SIGMLR-
SAIGML--
STIGML--
-SIGMLN-
-SIGMLQ-
SGIGML--
SQIGML--
SHIGML--
-SIGMLK-
SNIGML--
-SIGMLY-
-SIGMLV-
-SIGMLA-
SYIGML--
SKIGML--
SVIGML--
-SIGMLW-
-SIGMLM-
-SIGMLG-
-SIGMLH-
SDIGML--
SPIGML--
SCIGML--
TAIGML--
TSIGML--
-TIGMLI-
TGIGML--
TVIGML--
-TIGMLQ-
TQIGML--

THIGML--
TTIGML--
-TIGMLL-
TYIGML--
TPIGML--
TEIGML--
TWIGML--
TNIGML--
TKIGML--
-TIGMLH-
-TIGMLA-
-TIGMLY-
TCIGML--
TIIGML--
TDIGML--
TMIGML--
-TIGMLN-
-TIGMLM-
VRIGML--
VSIGML--
VGIGML--
VTIGML--
VAIGML--
VKIGML--
-VIGMLS-
VVIGML--
VPIGML--
VMIGML--
-VIGMLH-
VNIGML--
-VIGMLQ-
-VIGMLN-
-VIGMLA-
-VIGMLI-
VDIGML--
VQIGML--
-VIGMLE-
VIIGML--
WQIGML--
-WIGMLS-
WNIGML--
WKIGML--
WTIGML--
WAIGML--
-WIGMLH-
-WIGMLG-
-WIGMLQ-
WGIGML--
WSIGML--
WDIGML--

| | | | |
|---|---|---|---|
| | WVIGML-- | | |
| | WRIGML-- | | |
| | WYIGML-- | | |
| | WWIGML-- | | |
| | -WIGMLK- | | |
| | WEIGML-- | | |
| | WPIGML-- | | |
| | WLIGML-- | | |
| | WHIGML-- | | |
| | WMIGML-- | | |
| | YRIGML-- | | |
| | YLIGML-- | | |
| | -YIGMLR- | | |
| | -YIGMLN- | | |
| | YGIGML-- | | |
| | YAIGML-- | | |
| | YQIGML-- | | |
| | -YIGMLT- | | |
| | -YIGMLS- | | |
| | -YIGMLG- | | |
| | YHIGML-- | | |
| | YPIGML-- | | |
| | YNIGML-- | | |
| | YKIGML-- | | |
| | -YIGMLV- | | |
| | YYIGML-- | | |
| | YTIGML-- | | |
| | -YIGMLK- | | |
| | -YIGMLI- | | |
| | -YIGMLE- | | |
| | YMIGML-- | | |
| | -YIGMLH- | | |
| | YDIGML-- | | |
| PSPY | 104 | 767 | 31.57 |
| | --PSPYSK | | |
| | --PSPYIR | | |
| | --PSPYGS | | |
| | --PSPYDL | | |
| | SYPSPY-- | | |
| | -APSPYR- | | |
| | ALPSPY-- | | |
| | AMPSPY-- | | |
| | AKPSPY-- | | |
| | ATPSPY-- | | |
| | -APSPYE- | | |
| | AVPSPY-- | | |
| | -APSPYS- | | |
| | -APSPYL- | | |
| | ARPSPY-- | | |
| | -APSPYY- | | |

AYPSPY--
-APSPYK-
-APSPYI-
-APSPYF-
AEPSPY--
ASPSPY--
AQPSPY--
-APSPYN-
-APSPYM-
AIPSPY--
AGPSPY--
-APSPYG-
AFPSPY--
ADPSPY--
AAPSPY--
-APSPYA-
ANPSPY--
AHPSPY--
ACPSPY--
-APSPYQ-
-APSPYH-
-APSPYW-
-APSPYD-
CLPSPY--
CHPSPY--
CRPSPY--
-CPSPYL-
CKPSPY--
CGPSPY--
CYPSPY--
CVPSPY--
-CPSPYR-
CWPSPY--
-CPSPYM-
CIPSPY--
CCPSPY--
-DPSPYR-
-DPSPYS-
DFPSPY--
-DPSPYY-
DYPSPY--
-DPSPYT-
-DPSPYL-
-DPSPYA-
DNPSPY--
DSPSPY--
DRPSPY--
DCPSPY--
DWPSPY--
DVPSPY--

DTPSPY--
DEPSPY--
-EPSPYR-
ERPSPY--
-EPSPYT-
-EPSPYN-
EVPSPY--
ESPSPY--
-EPSPYL-
ELPSPY--
EKPSPY--
EAPSPY--
ETPSPY--
-EPSPYI-
EEPSPY--
EYPSPY--
ENPSPY--
-EPSPYK-
-EPSPYF-
-EPSPYA-
EMPSPY--
-EPSPYY-
-EPSPYS-
-EPSPYQ-
-EPSPYM-
-EPSPYH-
EHPSPY--
EFPSPY--
FRPSPY--
-FPSPYR-
-FPSPYQ-
-FPSPYI-
-FPSPYT-
FTPSPY--
-FPSPYS-
-FPSPYH-
-FPSPYW-
-FPSPYV-
FWPSPY--
FLPSPY--
FYPSPY--
-FPSPYK-
FKPSPY--
FGPSPY--
FVPSPY--
FSPSPY--
FQPSPY--
-FPSPYN-
-FPSPYF-
FPPSPY--

FIPSPY--
-GPSPYL-
GLPSPY--
GWPSPY--
GHPSPY--
GRPSPY--
GSPSPY--
GYPSPY--
GTPSPY--
-GPSPYR-
GVPSPY--
-GPSPYH-
GAPSPY--
-GPSPYV-
-GPSPYI-
-GPSPYK-
-GPSPYF-
GIPSPY--
GGPSPY--
GQPSPY--
-GPSPYY-
-GPSPYA-
GNPSPY--
GMPSPY--
GFPSPY--
GKPSPY--
-GPSPYW-
-GPSPYS-
-GPSPYQ-
GEPSPY--
HRPSPY--
HFPSPY--
-HPSPYS-
-HPSPYK-
HLPSPY--
-HPSPYT-
HVPSPY--
HSPSPY--
-HPSPYL-
-HPSPYR-
-HPSPYF-
HHPSPY--
-HPSPYW-
-HPSPYQ-
-HPSPYM-
-HPSPYA-
HMPSPY--
HIPSPY--
HEPSPY--
HYPSPY--

HWPSPY--
HQPSPY--
-HPSPYG-
HDPSPY--
HCPSPY--
-IPSPYR-
IVPSPY--
ILPSPY--
IHPSPY--
IAPSPY--
IRPSPY--
IFPSPY--
IDPSPY--
ISPSPY--
INPSPY--
IIPSPY--
-IPSPYG-
IKPSPY--
ITPSPY--
IQPSPY--
-IPSPYY-
-IPSPYV-
-IPSPYQ-
-IPSPYM-
-IPSPYK-
IPPSPY--
IGPSPY--
IYPSPY--
IWPSPY--
-IPSPYW-
-IPSPYA-
-KPSPYL-
-KPSPYK-
-KPSPYV-
-KPSPYT-
KLPSPY--
KAPSPY--
KPPSPY--
-KPSPYR-
-KPSPYH-
-KPSPYF-
-KPSPYA-
KYPSPY--
-KPSPYS-
KGPSPY--
KWPSPY--
KQPSPY--
-KPSPYY-
-KPSPYW-
-KPSPYQ-

-KPSPYI-
KKPSPY--
KFPSPY--
-KPSPYN-
-KPSPYG-
-KPSPYE-
KMPSPY--
KEPSPY--
LWPSPY--
-LPSPYS-
LVPSPY--
-LPSPYR-
LPPSPY--
LSPSPY--
LRPSPY--
LLPSPY--
LIPSPY--
LEPSPY--
LQPSPY--
LYPSPY--
-LPSPYL-
LGPSPY--
-LPSPYW-
-LPSPYM-
LFPSPY--
-LPSPYH-
LKPSPY--
LAPSPY--
LTPSPY--
-LPSPYV-
-LPSPYQ-
-LPSPYY-
-LPSPYT-
-LPSPYN-
-LPSPYK-
-LPSPYI-
LMPSPY--
LNPSPY--
LDPSPY--
-MPSPYR-
-MPSPYA-
MKPSPY--
MSPSPY--
-MPSPYV-
-MPSPYF-
MIPSPY--
MVPSPY--
MRPSPY--
MMPSPY--
-MPSPYI-

MWPSPY--
MTPSPY--
-MPSPYW-
-MPSPYS-
MLPSPY--
MEPSPY--
-MPSPYT-
-MPSPYN-
-MPSPYK-
-MPSPYH-
MHPSPY--
MGPSPY--
MYPSPY--
MQPSPY--
-MPSPYY-
-MPSPYQ-
-MPSPYM-
MNPSPY--
MFPSPY--
MDPSPY--
NYPSPY--
NVPSPY--
-NPSPYQ-
NPPSPY--
NRPSPY--
-NPSPYR-
NNPSPY--
NSPSPY--
NQPSPY--
-NPSPYA-
-NPSPYS-
-NPSPYI-
NHPSPY--
NTPSPY--
-NPSPYT-
-NPSPYH-
-NPSPYL-
-NPSPYK-
NKPSPY--
NWPSPY--
-NPSPYF-
NLPSPY--
NAPSPY--
-NPSPYY-
-NPSPYW-
-NPSPYV-
NMPSPY--
NIPSPY--
NFPSPY--
--PSPYRS

--PSPYHW
--PSPYTL
--PSPYWS
--PSPYVN
--PSPYLG
--PSPYMF
--PSPYLV
--PSPYLS
--PSPYLR
--PSPYVF
--PSPYRV
--PSPYYL
--PSPYSV
--PSPYSR
--PSPYPL
--PSPYFA
--PSPYTR
--PSPYMR
--PSPYVI
--PSPYSM
--PSPYRT
--PSPYRG
--PSPYLT
--PSPYLK
--PSPYFG
PRPSPY--
--PSPYYR
--PSPYVV
--PSPYTT
--PSPYSS
--PSPYSH
--PSPYRW
--PSPYQW
--PSPYQI
--PSPYML
--PSPYMG
--PSPYIW
--PSPYIS
--PSPYAG
PWPSPY--
--PSPYYY
--PSPYTA
--PSPYRH
--PSPYRD
--PSPYPI
--PSPYMV
--PSPYLQ
--PSPYLH
--PSPYLA
--PSPYKS

--PSPYGF
--PSPYAL
--PSPYYW
--PSPYYS
--PSPYWY
--PSPYVY
--PSPYVQ
--PSPYTI
--PSPYRQ
--PSPYQF
--PSPYMK
--PSPYLY
--PSPYKY
--PSPYKL
--PSPYIA
--PSPYFI
--PSPYAM
--PSPYWT
--PSPYVM
--PSPYTW
--PSPYTF
--PSPYSW
--PSPYSL
--PSPYRF
--PSPYNL
--PSPYMT
--PSPYMM
--PSPYKW
--PSPYIM
--PSPYGV
--PSPYGI
--PSPYFL
--PSPYAQ
PQPSPY--
PHPSPY--
--PSPYYF
--PSPYWL
--PSPYWF
--PSPYVT
--PSPYVG
--PSPYVA
--PSPYTS
--PSPYST
--PSPYSF
--PSPYMS
--PSPYKG
--PSPYFS
--PSPYFH
--PSPYAV
PGPSPY--

PFPSPY--
PTPSPY--
--PSPYYM
--PSPYYK
--PSPYYA
--PSPYVK
--PSPYSA
--PSPYRI
--PSPYQV
--PSPYPV
--PSPYNI
--PSPYLW
--PSPYLN
--PSPYLF
--PSPYKT
--PSPYIT
--PSPYIG
--PSPYHT
--PSPYHA
--PSPYGY
--PSPYGA
--PSPYFN
--PSPYFM
--PSPYAA
PNPSPY--
--PSPYYH
--PSPYWH
--PSPYVL
--PSPYVH
--PSPYVE
--PSPYTV
--PSPYRM
--PSPYRA
--PSPYQK
--PSPYQA
--PSPYNS
--PSPYNF
--PSPYMI
--PSPYKV
--PSPYKI
--PSPYKH
--PSPYIN
--PSPYIH
--PSPYHS
--PSPYGQ
--PSPYFY
--PSPYFR
--PSPYFQ
--PSPYFF
--PSPYEM

--PSPYDY
--PSPYAY
PVPSPY--
--PSPYYG
--PSPYWV
--PSPYWR
--PSPYWN
--PSPYWM
--PSPYVS
--PSPYTN
--PSPYTK
--PSPYSY
--PSPYSQ
--PSPYSG
--PSPYPY
--PSPYNV
--PSPYNT
--PSPYMY
--PSPYMW
--PSPYKF
--PSPYKD
--PSPYIY
--PSPYIL
--PSPYIK
--PSPYHY
--PSPYHQ
--PSPYHI
--PSPYHF
--PSPYGM
--PSPYEF
--PSPYCS
--PSPYAR
--PSPYAF
PMPSPY--
PYPSPY--
--PSPYYV
--PSPYYN
--PSPYYI
--PSPYYE
--PSPYWW
--PSPYWP
--PSPYWI
--PSPYWG
--PSPYTQ
--PSPYTE
--PSPYSE
--PSPYQT
--PSPYQQ
--PSPYQD
--PSPYPQ

--PSPYPF
--PSPYNY
--PSPYNN
--PSPYNM
--PSPYNG
--PSPYNA
--PSPYMQ
--PSPYMN
--PSPYME
--PSPYKR
--PSPYKQ
--PSPYKN
--PSPYIV
--PSPYII
--PSPYIE
--PSPYID
--PSPYHV
--PSPYHN
--PSPYHM
--PSPYHH
--PSPYGT
--PSPYFW
--PSPYFT
--PSPYFK
--PSPYFE
--PSPYES
--PSPYEL
--PSPYEI
--PSPYEA
--PSPYDM
--PSPYDF
--PSPYCI
--PSPYAH
PAPSPY--
QYPSPY--
-QPSPYR-
QRPSPY--
-QPSPYL-
QMPSPY--
QTPSPY--
QSPSPY--
QAPSPY--
-QPSPYA-
QLPSPY--
-QPSPYM-
-QPSPYH-
QCPSPY--
-QPSPYW-
QQPSPY--
-QPSPYY-

−QPSPYK−
−QPSPYG−
QPPSPY−−
QKPSPY−−
QDPSPY−−
QVPSPY−−
−QPSPYV−
−QPSPYT−
−QPSPYS−
−QPSPYP−
−QPSPYF−
QIPSPY−−
QHPSPY−−
QFPSPY−−
RVPSPY−−
−RPSPYV−
RAPSPY−−
RIPSPY−−
RRPSPY−−
RMPSPY−−
−RPSPYL−
RSPSPY−−
RYPSPY−−
−RPSPYW−
−RPSPYA−
RCPSPY−−
−RPSPYI−
REPSPY−−
−RPSPYS−
−RPSPYQ−
−RPSPYF−
RWPSPY−−
RQPSPY−−
−RPSPYY−
−RPSPYG−
RGPSPY−−
−RPSPYM−
−RPSPYE−
RNPSPY−−
−RPSPYN−
−RPSPYK−
−RPSPYH−
RHPSPY−−
RPPSPY−−
RDPSPY−−
−SPSPYL−
−SPSPYT−
STPSPY−−
−SPSPYF−
−SPSPYM−

-SPSPYV-
-SPSPYS-
-SPSPYQ-
SIPSPY--
SHPSPY--
SVPSPY--
SKPSPY--
SRPSPY--
-SPSPYW-
-SPSPYI-
SMPSPY--
SGPSPY--
-SPSPYK-
SNPSPY--
SWPSPY--
-SPSPYR-
-SPSPYN-
SEPSPY--
-SPSPYA-
SPPSPY--
SFPSPY--
-SPSPYY-
SAPSPY--
-SPSPYH-
SDPSPY--
-SPSPYG-
-SPSPYE-
TYPSPY--
TRPSPY--
-TPSPYW-
TVPSPY--
-TPSPYR-
TLPSPY--
TSPSPY--
-TPSPYK-
-TPSPYI-
-TPSPYV-
-TPSPYS-
TMPSPY--
TKPSPY--
-TPSPYL-
-TPSPYF-
THPSPY--
TEPSPY--
-TPSPYM-
TWPSPY--
TTPSPY--
-TPSPYY-
-TPSPYH-
-TPSPYA-

TAPSPY--
-TPSPYN-
TNPSPY--
TIPSPY--
TFPSPY--
TDPSPY--
-TPSPYQ-
TPPSPY--
TGPSPY--
TCPSPY--
VSPSPY--
VKPSPY--
-VPSPYL-
VLPSPY--
VNPSPY--
VRPSPY--
VFPSPY--
VYPSPY--
-VPSPYV-
VHPSPY--
-VPSPYF-
-VPSPYS-
-VPSPYH-
-VPSPYR-
VVPSPY--
VTPSPY--
VQPSPY--
-VPSPYQ-
VMPSPY--
VGPSPY--
VEPSPY--
VDPSPY--
-VPSPYN-
-VPSPYA-
-VPSPYY-
-VPSPYW-
-VPSPYM-
-VPSPYK-
-VPSPYG-
VWPSPY--
-VPSPYI-
-VPSPYE-
WSPSPY--
WPPSPY--
WEPSPY--
WGPSPY--
WRPSPY--
WKPSPY--
-WPSPYS-
-WPSPYA-

WCPSPY--
WLPSPY--
-WPSPYR-
-WPSPYQ-
-WPSPYM-
-WPSPYL-
WNPSPY--
-WPSPYT-
WYPSPY--
WVPSPY--
-WPSPYY-
-WPSPYK-
-WPSPYI-
WIPSPY--
WFPSPY--
WAPSPY--
WWPSPY--
WTPSPY--
-WPSPYW-
WMPSPY--
WDPSPY--
-YPSPYR-
YSPSPY--
YMPSPY--
YWPSPY--
YRPSPY--
YGPSPY--
-YPSPYT-
YTPSPY--
-YPSPYL-
YKPSPY--
YAPSPY--
YPPSPY--
YIPSPY--
-YPSPYN-
-YPSPYF-
YHPSPY--
YFPSPY--
YYPSPY--
YQPSPY--
-YPSPYW-
-YPSPYV-
-YPSPYH-
YLPSPY--
YVPSPY--
-YPSPYS-
YEPSPY--
-YPSPYY-
-YPSPYQ-
-YPSPYK-

| | | | |
|---|---|---|---|
| | YNPSPY-- | | |
| PKAM | 694 | 558 | 31.56 |
| | -APKAMV- | | |
| | -APKAML- | | |
| | -APKAMT- | | |
| | AGPKAM-- | | |
| | AVPKAM-- | | |
| | -APKAMK- | | |
| | -APKAMG- | | |
| | ALPKAM-- | | |
| | AHPKAM-- | | |
| | -APKAMR- | | |
| | -APKAMQ- | | |
| | -APKAMM- | | |
| | -APKAME- | | |
| | AKPKAM-- | | |
| | ASPKAM-- | | |
| | -APKAMF- | | |
| | -APKAMY- | | |
| | -APKAMS- | | |
| | -APKAMN- | | |
| | -APKAMI- | | |
| | -APKAMA- | | |
| | CSPKAM-- | | |
| | CHPKAM-- | | |
| | CRPKAM-- | | |
| | -CPKAMR- | | |
| | CLPKAM-- | | |
| | CYPKAM-- | | |
| | -CPKAMW- | | |
| | -CPKAMS- | | |
| | -CPKAMI- | | |
| | CNPKAM-- | | |
| | CCPKAM-- | | |
| | DWPKAM-- | | |
| | -DPKAML- | | |
| | -DPKAMK- | | |
| | -DPKAMQ- | | |
| | DEPKAM-- | | |
| | DLPKAM-- | | |
| | DFPKAM-- | | |
| | -DPKAMW- | | |
| | -DPKAMT- | | |
| | -DPKAMS- | | |
| | -DPKAMR- | | |
| | -DPKAMM- | | |
| | DKPKAM-- | | |
| | DCPKAM-- | | |
| | -EPKAMV- | | |
| | -EPKAMK- | | |

-EPKAMY-
-EPKAMQ-
-EPKAMI-
-EPKAME-
-EPKAMN-
-EPKAMG-
EVPKAM--
ERPKAM--
-EPKAMS-
-EPKAMM-
-EPKAML-
ENPKAM--
EMPKAM--
EHPKAM--
EGPKAM--
EEPKAM--
EAPKAM--
FFPKAM--
FMPKAM--
-FPKAMK-
FPPKAM--
FSPKAM--
-FPKAMF-
FIPKAM--
FQPKAM--
-FPKAMY-
-FPKAMS-
-FPKAMN-
FNPKAM--
FLPKAM--
FKPKAM--
-GPKAML-
-GPKAMH-
GSPKAM--
-GPKAMY-
-GPKAMS-
GIPKAM--
GWPKAM--
-GPKAMT-
GTPKAM--
-GPKAMV-
-GPKAMN-
-GPKAMK-
GLPKAM--
GKPKAM--
GYPKAM--
GRPKAM--
-GPKAMW-
-GPKAMQ-
-GPKAMI-

-GPKAMF-
GMPKAM--
GGPKAM--
GCPKAM--
GQPKAM--
GEPKAM--
GAPKAM--
GVPKAM--
-GPKAME-
GHPKAM--
HSPKAM--
HRPKAM--
HGPKAM--
-HPKAMT-
HFPKAM--
-HPKAMS-
-HPKAMN-
-HPKAMF-
-HPKAMW-
-HPKAMQ-
-HPKAMI-
-HPKAMH-
HMPKAM--
HIPKAM--
IWPKAM--
-IPKAMA-
-IPKAMH-
IIPKAM--
IAPKAM--
IQPKAM--
-IPKAMR-
IPPKAM--
-IPKAML-
-IPKAMI-
ILPKAM--
-IPKAMV-
IGPKAM--
KSPKAM--
KVPKAM--
KAPKAM--
-KPKAMS-
-KPKAMM-
KFPKAM--
-KPKAMI-
KTPKAM--
-KPKAMK-
KPPKAM--
-KPKAMQ-
-KPKAMP-
KLPKAM--

KWPKAM--
-KPKAMT-
-KPKAMH-
-KPKAMF-
KEPKAM--
KDPKAM--
LSPKAM--
LYPKAM--
-LPKAMW-
-LPKAMH-
LVPKAM--
-LPKAML-
-LPKAMF-
LTPKAM--
-LPKAMM-
LWPKAM--
LKPKAM--
-LPKAMS-
-LPKAMQ-
-LPKAMK-
LPPKAM--
-LPKAMY-
LNPKAM--
LGPKAM--
-LPKAMI-
LMPKAM--
LHPKAM--
LQPKAM--
-LPKAMN-
-MPKAMS-
MVPKAM--
-MPKAMW-
MSPKAM--
-MPKAMN-
-MPKAMH-
MMPKAM--
MYPKAM--
MPPKAM--
-MPKAMQ-
MTPKAM--
MQPKAM--
-MPKAMY-
-MPKAMG-
-MPKAML-
MIPKAM--
NWPKAM--
-NPKAMR-
-NPKAMW-
-NPKAMF-
-NPKAMV-

NEPKAM--
NTPKAM--
-NPKAMN-
-NPKAMK-
-NPKAMA-
-NPKAMI-
NMPKAM--
NGPKAM--
--PKAMSL
--PKAMLS
--PKAMAS
--PKAMAL
--PKAMWM
--PKAMSF
--PKAMSS
--PKAMHS
--PKAMYG
--PKAMTL
--PKAMNL
--PKAMTR
--PKAMVW
--PKAMSR
--PKAMKV
--PKAMWL
--PKAMIY
--PKAMIR
--PKAMVG
--PKAMRI
--PKAMFS
--PKAMVI
--PKAMSM
--PKAMRQ
--PKAMQT
--PKAMMS
--PKAMLG
--PKAMKW
--PKAMIK
--PKAMGT
--PKAMFL
--PKAMAY
--PKAMAM
--PKAMVL
--PKAMTY
--PKAMST
--PKAMSQ
--PKAMSN
--PKAMSK
--PKAMSA
--PKAMRG
--PKAMNW

--PKAMLD
--PKAMHH
--PKAMAV
--PKAMAG
--PKAMYS
--PKAMTG
--PKAMMK
--PKAMLH
--PKAMGS
--PKAMGM
--PKAMGG
--PKAMFD
--PKAMYT
--PKAMYA
--PKAMVA
--PKAMTT
--PKAMTS
--PKAMTK
--PKAMTH
--PKAMQW
--PKAMPY
--PKAMLI
--PKAMIV
--PKAMGW
--PKAMFT
--PKAMFK
--PKAMFH
--PKAMEA
--PKAMAQ
--PKAMAA
PTPKAM--
PRPKAM--
--PKAMYY
--PKAMYV
--PKAMYF
--PKAMYE
--PKAMYD
--PKAMWN
--PKAMWE
--PKAMSW
--PKAMSE
--PKAMRW
--PKAMRD
--PKAMQR
--PKAMQN
--PKAMQA
--PKAMNT
--PKAMNR
--PKAMNQ
--PKAMMR

--PKAMMQ
--PKAMLK
--PKAMKT
--PKAMKS
--PKAMKL
--PKAMIG
--PKAMHR
--PKAMHN
--PKAMGI
--PKAMFY
--PKAMFW
--PKAMFN
--PKAMFM
--PKAMFE
--PKAMAR
PSPKAM--
PQPKAM--
--PKAMWV
--PKAMWD
--PKAMVT
--PKAMVQ
--PKAMVD
--PKAMTM
--PKAMTI
--PKAMSH
--PKAMSG
--PKAMSD
--PKAMQG
--PKAMNS
--PKAMNK
--PKAMNI
--PKAMNF
--PKAMMH
--PKAMLM
--PKAMKE
--PKAMIH
--PKAMIA
--PKAMHW
--PKAMHV
--PKAMHT
--PKAMHK
--PKAMHI
--PKAMHF
--PKAMFV
--PKAMEY
--PKAMES
--PKAMEK
--PKAMAN
--PKAMAI
--PKAMAD

PGPKAM--
PEPKAM--
PLPKAM--
--PKAMYW
--PKAMYQ
--PKAMYI
--PKAMWI
--PKAMVY
--PKAMVM
--PKAMVH
--PKAMVF
--PKAMVE
--PKAMSY
--PKAMRM
--PKAMRC
--PKAMQV
--PKAMQS
--PKAMQL
--PKAMQH
--PKAMPM
--PKAMPL
--PKAMPI
--PKAMNM
--PKAMMI
--PKAMKQ
--PKAMKN
--PKAMKM
--PKAMKI
--PKAMKD
--PKAMIN
--PKAMIF
--PKAMHQ
--PKAMHD
--PKAMHA
--PKAMGF
--PKAMGC
--PKAMFA
--PKAMET
--PKAMDR
--PKAMDL
--PKAMAT
PIPKAM--
PFPKAM--
-QPKAMR-
QRPKAM--
QGPKAM--
-QPKAMA-
-QPKAMM-
-QPKAMK-
-QPKAMI-

QTPKAM--
-QPKAMQ-
QVPKAM--
-QPKAMW-
-QPKAMS-
QSPKAM--
-QPKAMH-
QMPKAM--
-QPKAMV-
-QPKAML-
-QPKAMG-
-QPKAMF-
QEPKAM--
-RPKAMT-
-RPKAMW-
-RPKAMH-
RTPKAM--
RNPKAM--
-RPKAMS-
-RPKAMI-
RLPKAM--
-RPKAMK-
RVPKAM--
RIPKAM--
-RPKAMV-
RMPKAM--
RGPKAM--
RAPKAM--
RQPKAM--
RFPKAM--
RPPKAM--
-RPKAMY-
-RPKAMG-
RHPKAM--
-RPKAMN-
RYPKAM--
-RPKAMQ-
-RPKAME-
RDPKAM--
SMPKAM--
-SPKAMY-
STPKAM--
SSPKAM--
SQPKAM--
-SPKAMR-
-SPKAMW-
-SPKAMI-
-SPKAMF-
SPPKAM--
SWPKAM--

-SPKAMV-
-SPKAMH-
SGPKAM--
SEPKAM--
-SPKAMQ-
SDPKAM--
SYPKAM--
-SPKAMS-
SAPKAM--
SVPKAM--
-SPKAME-
SKPKAM--
SHPKAM--
-SPKAMN-
-SPKAMM-
-TPKAMR-
TLPKAM--
TTPKAM--
-TPKAMY-
-TPKAMS-
TWPKAM--
TRPKAM--
TAPKAM--
-TPKAMA-
TIPKAM--
TGPKAM--
-TPKAMF-
TFPKAM--
TYPKAM--
TVPKAM--
-TPKAMN-
TSPKAM--
TQPKAM--
-TPKAMH-
THPKAM--
TCPKAM--
-TPKAMW-
-TPKAMT-
-TPKAMI-
TNPKAM--
TKPKAM--
TEPKAM--
-VPKAMR-
-VPKAMK-
-VPKAML-
-VPKAMT-
VRPKAM--
-VPKAMW-
-VPKAMQ-
VYPKAM--

VSPKAM--
-VPKAMS-
-VPKAMH-
-VPKAMA-
VKPKAM--
VIPKAM--
VWPKAM--
VVPKAM--
VPPKAM--
VDPKAM--
-VPKAMY-
-VPKAMM-
VQPKAM--
-VPKAMN-
-VPKAMC-
VGPKAM--
VCPKAM--
WRPKAM--
WLPKAM--
-WPKAML-
WFPKAM--
WEPKAM--
WDPKAM--
-WPKAMV-
-WPKAMT-
-WPKAMF-
-WPKAMA-
WMPKAM--
WGPKAM--
WAPKAM--
WWPKAM--
WVPKAM--
-WPKAMM-
WNPKAM--
WIPKAM--
YVPKAM--
YLPKAM--
YRPKAM--
YKPKAM--
-YPKAML-
YDPKAM--
-YPKAMI-
YSPKAM--
-YPKAMW-
-YPKAMT-
-YPKAMA-
YGPKAM--
YPPKAM--
-YPKAMF-
YEPKAM--

| | | | |
|---|---|---|---|
| | YYPKAM-- | | |
| | YWPKAM-- | | |
| | -YPKAMS- | | |
| | -YPKAMQ- | | |
| | -YPKAMM- | | |
| | YNPKAM-- | | |
| | YMPKAM-- | | |
| | YIPKAM-- | | |
| | YFPKAM-- | | |
| | YAPKAM-- | | |
| PANM | 348 | 651 | 31.55 |
| | -APANMR- | | |
| | ARPANM-- | | |
| | AVPANM-- | | |
| | ASPANM-- | | |
| | -APANMY- | | |
| | AFPANM-- | | |
| | AWPANM-- | | |
| | -APANMS- | | |
| | -APANML- | | |
| | -APANMG- | | |
| | AGPANM-- | | |
| | APPANM-- | | |
| | -APANMA- | | |
| | AMPANM-- | | |
| | ALPANM-- | | |
| | ADPANM-- | | |
| | ATPANM-- | | |
| | AQPANM-- | | |
| | -APANMW- | | |
| | -APANMV- | | |
| | -APANMN- | | |
| | -APANMK- | | |
| | -APANMD- | | |
| | AKPANM-- | | |
| | CRPANM-- | | |
| | -CPANML- | | |
| | -CPANMK- | | |
| | -CPANMV- | | |
| | -CPANMT- | | |
| | -CPANMS- | | |
| | -CPANMR- | | |
| | -CPANMQ- | | |
| | -CPANMM- | | |
| | -CPANMF- | | |
| | CHPANM-- | | |
| | DWPANM-- | | |
| | -DPANMW- | | |
| | DVPANM-- | | |
| | -DPANMR- | | |

-DPANMA-
DNPANM--
DFPANM--
DAPANM--
DSPANM--
DRPANM--
-DPANMT-
-DPANMY-
-DPANMV-
-DPANMS-
-DPANMQ-
-DPANMN-
-DPANMK-
-DPANMI-
-EPANMK-
-EPANMT-
-EPANMI-
EKPANM--
EAPANM--
-EPANMR-
EYPANM--
-EPANMS-
-EPANMM-
ELPANM--
EWPANM--
ETPANM--
ERPANM--
EPPANM--
-EPANML-
-EPANMH-
EMPANM--
EEPANM--
EDPANM--
FTPANM--
-FPANMY-
-FPANMR-
FHPANM--
FRPANM--
-FPANMS-
-FPANMA-
-FPANML-
FAPANM--
-FPANMG-
-FPANMV-
-FPANMM-
FYPANM--
FQPANM--
-FPANMT-
-FPANMI-
-FPANMH-

-FPANMF-
-FPANME-
FLPANM--
FFPANM--
FVPANM--
FPPANM--
-FPANMN-
-FPANMD-
FNPANM--
FIPANM--
FGPANM--
-GPANML-
GSPANM--
GFPANM--
-GPANMR-
GNPANM--
-GPANMY-
GRPANM--
-GPANMS-
-GPANMK-
GMPANM--
GLPANM--
GYPANM--
GVPANM--
-GPANMW-
-GPANMQ-
-GPANMG-
GKPANM--
GGPANM--
GDPANM--
GQPANM--
-GPANMM-
-GPANMH-
-GPANMD-
GIPANM--
GHPANM--
GAPANM--
HLPANM--
HSPANM--
-HPANMR-
HFPANM--
HYPANM--
-HPANMY-
-HPANMS-
-HPANMQ-
-HPANMN-
-HPANMG-
-HPANMD-
HKPANM--
HIPANM--

HAPANM--
HWPANM--
HTPANM--
HPPANM--
-HPANMT-
-HPANMM-
-HPANMI-
-HPANMH-
-HPANME-
HNPANM--
HHPANM--
-IPANMD-
-IPANMR-
-IPANML-
-IPANMV-
-IPANMS-
-IPANMF-
-IPANMA-
IGPANM--
IAPANM--
ISPANM--
IRPANM--
-IPANMY-
-IPANMT-
-IPANMI-
IDPANM--
IYPANM--
IWPANM--
-IPANMW-
-IPANMK-
-IPANMG-
-IPANMC-
-KPANML-
-KPANMM-
-KPANMR-
-KPANMD-
-KPANMS-
KIPANM--
-KPANMV-
-KPANMN-
KLPANM--
KKPANM--
KRPANM--
-KPANMY-
-KPANMT-
-KPANMG-
KGPANM--
KAPANM--
KTPANM--
KSPANM--

KPPANM--
-KPANMI-
-KPANMF-
-KPANME-
KHPANM--
LRPANM--
LPPANM--
LYPANM--
LSPANM--
-LPANMY-
-LPANMT-
LVPANM--
LTPANM--
LQPANM--
-LPANMI-
LNPANM--
-LPANMV-
-LPANMR-
-LPANMN-
-LPANMA-
-LPANMG-
LMPANM--
LWPANM--
-LPANML-
-LPANMH-
-LPANMD-
LLPANM--
LKPANM--
-LPANMW-
-LPANMQ-
LHPANM--
LEPANM--
LDPANM--
LCPANM--
LAPANM--
-MPANML-
-MPANMR-
MDPANM--
MRPANM--
MSPANM--
MPPANM--
MVPANM--
-MPANMH-
-MPANMF-
MNPANM--
MGPANM--
MYPANM--
MWPANM--
-MPANMT-
-MPANMS-

```
-MPANMQ-
-MPANMM-
-MPANMD-
-MPANMA-
MKPANM--
MIPANM--
NGPANM--
-NPANMR-
NNPANM--
NHPANM--
NFPANM--
NDPANM--
NLPANM--
NYPANM--
NRPANM--
-NPANMW-
NAPANM--
NWPANM--
NTPANM--
-NPANMY-
-NPANMS-
-NPANMQ-
-NPANML-
-NPANMF-
NVPANM--
NSPANM--
-NPANMV-
-NPANMI-
-NPANMH-
-NPANMG-
-NPANMA-
NEPANM--
NCPANM--
--PANMRF
--PANMTL
--PANMTR
--PANMGL
--PANMYQ
--PANMLL
--PANMTN
--PANMLS
--PANMKR
--PANMWG
--PANMRG
--PANMIL
PLPANM--
--PANMVS
--PANMTV
--PANMRK
--PANMLG
```

--PANMIS
--PANMGS
--PANMTI
--PANMSV
--PANMRY
--PANMHS
--PANMHA
--PANMYS
--PANMRS
--PANMRI
--PANMNY
--PANMNL
--PANMKL
--PANMFL
--PANMWT
--PANMVK
--PANMNN
--PANMKF
--PANMHL
--PANMFT
PYPANM--
PAPANM--
--PANMWM
--PANMWL
--PANMWK
--PANMVR
--PANMTS
--PANMRR
--PANMRN
--PANMRH
--PANMLR
--PANMIT
--PANMHH
--PANMGY
--PANMGV
--PANMFG
--PANMFA
--PANMVV
--PANMVG
--PANMTK
--PANMTG
--PANMSI
--PANMSF
--PANMML
--PANMLH
--PANMIY
--PANMIF
--PANMGT
--PANMFN
--PANMAL

--PANMAI
--PANMAG
PSPANM--
PRPANM--
PNPANM--
--PANMYG
--PANMVF
--PANMVD
--PANMTY
--PANMRT
--PANMRM
--PANMQG
--PANMQF
--PANMNR
--PANMLV
--PANMLK
--PANMLI
--PANMKY
--PANMIR
--PANMGM
--PANMGF
--PANMGA
--PANMFS
--PANMFQ
--PANMEL
--PANMAA
PWPANM--
--PANMYW
--PANMYR
--PANMWW
--PANMWR
--PANMWN
--PANMTT
--PANMSW
--PANMST
--PANMSS
--PANMSQ
--PANMSH
--PANMRE
--PANMQD
--PANMMS
--PANMMR
--PANMLA
--PANMKS
--PANMKN
--PANMKA
--PANMIV
--PANMIP
--PANMIM
--PANMIH

--PANMIE
--PANMHV
--PANMHI
--PANMGH
--PANMFK
--PANMFE
--PANMER
--PANMEK
--PANMDS
--PANMAW
--PANMAQ
--PANMAH
PGPANM--
PFPANM--
--PANMWY
--PANMWH
--PANMWE
--PANMWA
--PANMVW
--PANMVP
--PANMVI
--PANMVA
--PANMTW
--PANMTQ
--PANMTM
--PANMSM
--PANMSK
--PANMSG
--PANMSE
--PANMSD
--PANMRW
--PANMRV
--PANMRD
--PANMRA
--PANMQV
--PANMQR
--PANMQN
--PANMQA
--PANMPN
--PANMNT
--PANMNS
--PANMNK
--PANMNH
--PANMNF
--PANMNA
--PANMMN
--PANMMI
--PANMME
--PANMLQ
--PANMLN

--PANMLC
--PANMKI
--PANMKH
--PANMIW
--PANMID
--PANMIA
--PANMGW
--PANMGN
--PANMGK
--PANMGG
--PANMFV
--PANMFI
--PANMFH
--PANMFD
--PANMEM
--PANMDG
--PANMDA
--PANMCR
--PANMAT
--PANMAS
--PANMAN
--PANMAM
--PANMAK
--PANMAF
--PANMAE
--PANMAD
QRPANM--
-QPANMI-
QWPANM--
-QPANMR-
-QPANML-
QSPANM--
-QPANMT-
QVPANM--
QTPANM--
-QPANMS-
-QPANMM-
-QPANME-
QYPANM--
-QPANMY-
-QPANMW-
-QPANMH-
QLPANM--
-RPANMR-
-RPANMS-
RLPANM--
-RPANMA-
RIPANM--
RSPANM--
-RPANMH-

RRPANM--
-RPANMI-
-RPANMG-
RGPANM--
RAPANM--
RVPANM--
-RPANMK-
-RPANMV-
-RPANML-
RMPANM--
RFPANM--
RTPANM--
RKPANM--
RDPANM--
-RPANMY-
-RPANMM-
-RPANMT-
-RPANMQ-
RNPANM--
RPPANM--
-RPANMN-
-RPANME-
-RPANMD-
REPANM--
RYPANM--
RQPANM--
RHPANM--
SRPANM--
-SPANMT-
-SPANML-
SSPANM--
SWPANM--
-SPANMS-
SVPANM--
-SPANMY-
-SPANMW-
-SPANMI-
-SPANMD-
SKPANM--
-SPANMV-
-SPANMK-
-SPANMH-
SAPANM--
STPANM--
-SPANMQ-
SFPANM--
-SPANMF-
SLPANM--
SIPANM--
SGPANM--

SEPANM--
SCPANM--
-SPANMR-
-SPANMP-
-SPANMM-
-SPANME-
-SPANMA-
SMPANM--
SHPANM--
SDPANM--
-TPANML-
-TPANMR-
TWPANM--
-TPANMN-
TRPANM--
-TPANMV-
THPANM--
TTPANM--
-TPANMH-
-TPANMA-
TNPANM--
TFPANM--
TPPANM--
-TPANMY-
-TPANMM-
TMPANM--
TIPANM--
TAPANM--
-TPANMW-
-TPANMI-
TLPANM--
TKPANM--
TYPANM--
-TPANMT-
-TPANMG-
TEPANM--
-VPANML-
VKPANM--
-VPANMA-
VVPANM--
VNPANM--
VWPANM--
VSPANM--
-VPANMY-
-VPANMS-
VMPANM--
VIPANM--
VYPANM--
VRPANM--
VPPANM--

-VPANMV-
VTPANM--
-VPANMT-
VLPANM--
VQPANM--
-VPANMR-
-VPANMM-
-VPANMI-
-VPANMG-
VAPANM--
-VPANMN-
-VPANMK-
-VPANMH-
VFPANM--
VDPANM--
WRPANM--
-WPANMN-
-WPANML-
WHPANM--
WYPANM--
WTPANM--
WPPANM--
-WPANMQ-
-WPANMM-
-WPANMA-
WLPANM--
WEPANM--
-WPANMT-
-WPANMI-
-WPANMG-
WAPANM--
WVPANM--
WQPANM--
-WPANMS-
-WPANMR-
-WPANMH-
WKPANM--
WGPANM--
WDPANM--
-YPANMR-
YRPANM--
YAPANM--
YLPANM--
YFPANM--
YVPANM--
-YPANML-
-YPANMG-
YNPANM--
YTPANM--
-YPANMS-

```
            -YPANMA-
            -YPANMT-
            -YPANMD-
            YKPANM--
            YEPANM--
            YYPANM--
            YSPANM--
            -YPANMY-
            -YPANMV-
            -YPANMN-
            -YPANMC-
            YDPANM--
PSEL          49        806      31.52
            CLPSEL--
            --PSELLR
            --PSELTW
            --PSELRP
            --PSELGL
            --PSELAI
            --PSELAH
            TTPSEL--
            -YPSELL-
            ARPSEL--
            -APSELH-
            AWPSEL--
            ATPSEL--
            AFPSEL--
            ALPSEL--
            -APSELS-
            -APSELR-
            -APSELV-
            -APSELF-
            AAPSEL--
            -APSELL-
            -APSELG-
            AMPSEL--
            AKPSEL--
            AIPSEL--
            -APSELM-
            AGPSEL--
            AQPSEL--
            -APSELW-
            -APSELK-
            -APSELI-
            APPSEL--
            ANPSEL--
            -APSELY-
            -APSELT-
            -APSELN-
            -APSELA-
```

AEPSEL--
ACPSEL--
AVPSEL--
-APSELQ-
AHPSEL--
-CPSELN-
CNPSEL--
CRPSEL--
-CPSELR-
-CPSELK-
CFPSEL--
CKPSEL--
CYPSEL--
-CPSELT-
CGPSEL--
CVPSEL--
-CPSELW-
-CPSELS-
CCPSEL--
CAPSEL--
DRPSEL--
DLPSEL--
-DPSELS-
DHPSEL--
DFPSEL--
DYPSEL--
DWPSEL--
-DPSELW-
-DPSELR-
-DPSELI-
DTPSEL--
DSPSEL--
DMPSEL--
-DPSELY-
-DPSELV-
-DPSELM-
-DPSELF-
DNPSEL--
DVPSEL--
-DPSELT-
-DPSELN-
-DPSELL-
DKPSEL--
DAPSEL--
DQPSEL--
-DPSELP-
-DPSELA-
DGPSEL--
DCPSEL--
EIPSEL--

EVPSEL--
-EPSELS-
-EPSELR-
ETPSEL--
ERPSEL--
-EPSELT-
-EPSELF-
ESPSEL--
EGPSEL--
EWPSEL--
EQPSEL--
-EPSELL-
EPPSEL--
ELPSEL--
EEPSEL--
-EPSELM-
EMPSEL--
EHPSEL--
EFPSEL--
EAPSEL--
-EPSELQ-
-EPSELK-
-EPSELH-
ENPSEL--
ECPSEL--
FVPSEL--
FRPSEL--
FSPSEL--
-FPSELR-
-FPSELV-
FMPSEL--
FEPSEL--
FAPSEL--
FYPSEL--
-FPSELQ-
-FPSELF-
FHPSEL--
-FPSELW-
FLPSEL--
FFPSEL--
FDPSEL--
-FPSELS-
-FPSELN-
-FPSELI-
FQPSEL--
-FPSELL-
FNPSEL--
FIPSEL--
FTPSEL--
FGPSEL--

FWPSEL--
-FPSELK-
-FPSELH-
FPPSEL--
FKPSEL--
-FPSELY-
-FPSELG-
FCPSEL--
GVPSEL--
GAPSEL--
GWPSEL--
GPPSEL--
GYPSEL--
GTPSEL--
GQPSEL--
-GPSELS-
-GPSELW-
-GPSELL-
-GPSELY-
-GPSELV-
GMPSEL--
-GPSELT-
-GPSELQ-
GLPSEL--
GHPSEL--
GIPSEL--
-GPSELI-
GFPSEL--
GEPSEL--
GCPSEL--
GSPSEL--
-GPSELK-
-GPSELH-
-GPSELG-
GDPSEL--
-GPSELA-
GGPSEL--
-GPSELN-
-HPSELR-
HRPSEL--
HLPSEL--
HVPSEL--
-HPSELQ-
HWPSEL--
-HPSELT-
-HPSELK-
HSPSEL--
-HPSELS-
-HPSELH-
HIPSEL--

HAPSEL--
HNPSEL--
HHPSEL--
HYPSEL--
HTPSEL--
-HPSELF-
-HPSELY-
-HPSELN-
HQPSEL--
-HPSELV-
HGPSEL--
HFPSEL--
-HPSELL-
-HPSELI-
-HPSELA-
HMPSEL--
HEPSEL--
HDPSEL--
-IPSELR-
IRPSEL--
IIPSEL--
IYPSEL--
IVPSEL--
-IPSELT-
-IPSELA-
INPSEL--
IAPSEL--
IWPSEL--
ISPSEL--
IQPSEL--
-IPSELH-
-IPSELY-
-IPSELS-
-IPSELL-
-IPSELI-
IEPSEL--
IDPSEL--
ITPSEL--
-IPSELQ-
-IPSELN-
IKPSEL--
IHPSEL--
IGPSEL--
-IPSELV-
IFPSEL--
-IPSELM-
-IPSELK-
-IPSELG-
KSPSEL--
-KPSELR-

KFPSEL--
-KPSELL-
-KPSELA-
KIPSEL--
-KPSELK-
-KPSELI-
KLPSEL--
KHPSEL--
KVPSEL--
KQPSEL--
-KPSELY-
-KPSELW-
KPPSEL--
-KPSELN-
-KPSELG-
KAPSEL--
KTPSEL--
-KPSELV-
-KPSELS-
-KPSELH-
KKPSEL--
KYPSEL--
-KPSELF-
KGPSEL--
-KPSELM-
KWPSEL--
KMPSEL--
KEPSEL--
KDPSEL--
KCPSEL--
-LPSELR-
LRPSEL--
LSPSEL--
LNPSEL--
LYPSEL--
LMPSEL--
LLPSEL--
-LPSELK-
LFPSEL--
LEPSEL--
-LPSELV-
-LPSELN-
LTPSEL--
LVPSEL--
LIPSEL--
-LPSELS-
LQPSEL--
-LPSELQ-
-LPSELW-
-LPSELL-

LWPSEL--
-LPSELI-
-LPSELF-
LKPSEL--
LAPSEL--
LHPSEL--
LCPSEL--
-LPSELY-
-LPSELM-
-LPSELA-
MSPSEL--
MFPSEL--
-MPSELS-
MAPSEL--
-MPSELN-
MGPSEL--
MQPSEL--
MHPSEL--
MRPSEL--
-MPSELR-
-MPSELM-
MLPSEL--
MKPSEL--
-MPSELT-
-MPSELH-
MWPSEL--
MVPSEL--
-MPSELI-
-MPSELW-
-MPSELG-
MNPSEL--
MTPSEL--
MYPSEL--
-MPSELQ-
-MPSELK-
MIPSEL--
-MPSELY-
-MPSELF-
-MPSELE-
-MPSELA-
MPPSEL--
MMPSEL--
MCPSEL--
NFPSEL--
-NPSELV-
NSPSEL--
NRPSEL--
-NPSELT-
-NPSELY-
-NPSELW-

NHPSEL--
NTPSEL--
-NPSELK-
NNPSEL--
NVPSEL--
NIPSEL--
NYPSEL--
-NPSELS-
-NPSELQ-
NWPSEL--
-NPSELN-
-NPSELH-
-NPSELG-
-NPSELF-
-NPSELA-
NKPSEL--
NGPSEL--
NDPSEL--
NAPSEL--
NQPSEL--
-NPSELM-
-NPSELL-
NPPSEL--
NMPSEL--
NEPSEL--
NCPSEL--
--PSELRL
--PSELRR
--PSELKR
--PSELKS
--PSELRF
--PSELRN
--PSELVS
--PSELRG
--PSELVV
--PSELHS
PLPSEL--
--PSELRK
PTPSEL--
--PSELRA
--PSELLW
--PSELSG
--PSELLA
--PSELVY
--PSELGS
--PSELWG
--PSELVN
--PSELSF
--PSELRM
--PSELYW

--PSELYS
--PSELWN
--PSELVR
--PSELSN
--PSELRI
--PSELMY
--PSELLV
--PSELIV
--PSELFN
--PSELAV
PRPSEL--
PSPSEL--
--PSELWK
--PSELWA
--PSELVD
--PSELTT
--PSELTS
--PSELSS
--PSELNH
--PSELMS
--PSELMH
--PSELKG
--PSELAA
--PSELYT
--PSELYR
--PSELYK
--PSELYG
--PSELWR
--PSELVI
--PSELTG
--PSELSW
--PSELQN
--PSELNT
--PSELNA
--PSELLS
--PSELKL
--PSELHR
--PSELFS
--PSELFH
--PSELFG
--PSELWT
--PSELTR
--PSELSY
--PSELSR
--PSELSA
--PSELMA
--PSELKA
--PSELIR
--PSELHA
PNPSEL--

--PSELWH
--PSELVT
--PSELVH
--PSELTN
--PSELNS
--PSELMW
--PSELMT
--PSELML
--PSELMG
--PSELLK
--PSELIS
--PSELIQ
--PSELFQ
--PSELEA
--PSELAG
PYPSEL--
PVPSEL--
--PSELYN
--PSELYI
--PSELWE
--PSELVQ
--PSELVM
--PSELVL
--PSELVF
--PSELSM
--PSELSI
--PSELRH
--PSELQK
--PSELQG
--PSELNW
--PSELNV
--PSELNK
--PSELMR
--PSELLT
--PSELLQ
--PSELLH
--PSELKV
--PSELKH
--PSELIN
--PSELHV
--PSELHH
PKPSEL--
PGPSEL--
PAPSEL--
--PSELYQ
--PSELYD
--PSELWS
--PSELVK
--PSELVG
--PSELVE

--PSELTV
--PSELTH
--PSELTF
--PSELSV
--PSELSL
--PSELSH
--PSELRY
--PSELRQ
--PSELRE
--PSELQV
--PSELQA
--PSELNR
--PSELNI
--PSELNF
--PSELLM
--PSELKY
--PSELKQ
--PSELKE
--PSELIT
--PSELIK
--PSELII
--PSELIG
--PSELIA
--PSELGT
--PSELFT
--PSELFR
PMPSEL--
--PSELYM
--PSELYA
--PSELWF
--PSELTY
--PSELTQ
--PSELRW
--PSELQW
--PSELQT
--PSELQF
--PSELNN
--PSELMV
--PSELHN
--PSELHM
--PSELES
--PSELAM
PIPSEL--
PFPSEL--
PEPSEL--
--PSELYE
--PSELWW
--PSELWL
--PSELWI
--PSELVW

--PSELTD
--PSELTA
--PSELSQ
--PSELSK
--PSELSD
--PSELRV
--PSELQS
--PSELQR
--PSELQI
--PSELQH
--PSELMN
--PSELMM
--PSELMK
--PSELLD
--PSELKT
--PSELIM
--PSELIH
--PSELIE
--PSELHT
--PSELHQ
--PSELHK
--PSELHI
--PSELGV
--PSELGA
--PSELFA
--PSELAT
PQPSEL--
PHPSEL--
--PSELTM
--PSELTL
--PSELTK
--PSELTE
--PSELQY
--PSELQQ
--PSELQM
--PSELQE
--PSELND
--PSELMQ
--PSELMF
--PSELLN
--PSELKN
--PSELKM
--PSELKF
--PSELHY
--PSELHF
--PSELGY
--PSELFK
--PSELFD
--PSELET
--PSELEC

--PSELCM
--PSELAW
--PSELAN
--PSELAF
-PPSELW-
-PPSELR-
QVPSEL--
-QPSELR-
QPPSEL--
-QPSELK-
QRPSEL--
-QPSELW-
QYPSEL--
-QPSELT-
-QPSELH-
QKPSEL--
QIPSEL--
QGPSEL--
-QPSELS-
QAPSEL--
QQPSEL--
-QPSELV-
-QPSELM-
QFPSEL--
-QPSELY-
-QPSELI-
-QPSELL-
-QPSELA-
QNPSEL--
QWPSEL--
QTPSEL--
-QPSELQ-
QLPSEL--
QDPSEL--
QCPSEL--
RVPSEL--
-RPSELR-
RSPSEL--
RWPSEL--
-RPSELK-
RTPSEL--
-RPSELW-
RYPSEL--
-RPSELV-
-RPSELF-
-RPSELS-
RAPSEL--
-RPSELY-
-RPSELH-
-RPSELM-

−RPSELT−
RNPSEL−−
−RPSELA−
RFPSEL−−
−RPSELQ−
RKPSEL−−
−RPSELI−
−RPSELN−
−RPSELG−
RIPSEL−−
RCPSEL−−
RDPSEL−−
−RPSELE−
REPSEL−−
−RPSELC−
SRPSEL−−
−SPSELR−
−SPSELL−
SLPSEL−−
−SPSELY−
−SPSELT−
SWPSEL−−
−SPSELS−
SVPSEL−−
−SPSELA−
SPPSEL−−
−SPSELK−
SYPSEL−−
STPSEL−−
SQPSEL−−
SNPSEL−−
SMPSEL−−
SHPSEL−−
−SPSELI−
−SPSELH−
SGPSEL−−
SFPSEL−−
−SPSELN−
−SPSELM−
SIPSEL−−
−SPSELV−
SAPSEL−−
−SPSELF−
SEPSEL−−
−SPSELG−
−SPSELQ−
SKPSEL−−
SDPSEL−−
SCPSEL−−
TWPSEL−−

TIPSEL--
TRPSEL--
-TPSELR-
TAPSEL--
-TPSELL-
-TPSELF-
-TPSELV-
TSPSEL--
-TPSELT-
TGPSEL--
-TPSELK-
TLPSEL--
TFPSEL--
-TPSELM-
-TPSELH-
TPPSEL--
TMPSEL--
-TPSELY-
THPSEL--
TEPSEL--
-TPSELQ-
-TPSELG-
TNPSEL--
TYPSEL--
TVPSEL--
-TPSELW-
-TPSELN-
TCPSEL--
-TPSELE-
TDPSEL--
VLPSEL--
-VPSELR-
VRPSEL--
-VPSELY-
VVPSEL--
VMPSEL--
VKPSEL--
-VPSELS-
VFPSEL--
VSPSEL--
VTPSEL--
VAPSEL--
-VPSELT-
-VPSELI-
VIPSEL--
VGPSEL--
-VPSELK-
-VPSELL-
-VPSELG-
VPPSEL--

VHPSEL--
VYPSEL--
VQPSEL--
-VPSELQ-
-VPSELN-
VNPSEL--
VEPSEL--
-VPSELH-
-VPSELF-
-VPSELA-
-VPSELV-
-VPSELM-
VDPSEL--
WRPSEL--
-WPSELS-
WLPSEL--
WIPSEL--
WYPSEL--
-WPSELY-
-WPSELT-
WQPSEL--
-WPSELR-
WKPSEL--
-WPSELK-
WGPSEL--
WAPSEL--
-WPSELV-
WWPSEL--
WVPSEL--
-WPSELN-
WPPSEL--
WDPSEL--
WTPSEL--
WHPSEL--
WEPSEL--
-WPSELQ-
-WPSELP-
-WPSELG-
YSPSEL--
YLPSEL--
-YPSELT-
YRPSEL--
YWPSEL--
YAPSEL--
YYPSEL--
-YPSELK-
-YPSELI-
YTPSEL--
YPPSEL--
YNPSEL--

YVPSEL--
YQPSEL--
-YPSELS-
-YPSELQ-
YKPSEL--
YDPSEL--
-YPSELY-
-YPSELW-
-YPSELV-
YIPSEL--
YFPSEL--
-YPSELH-
YMPSEL--
YGPSEL--
-YPSELN-
-YPSELM-
-YPSELA-
YHPSEL--
YEPSEL--

PAHI 407 630 31.51

--PAHIST
ANPAHI--
-APAHIY-
-APAHIT-
-APAHIS-
AKPAHI--
-APAHIL-
AAPAHI--
-APAHIR-
ATPAHI--
-APAHIN-
-APAHIM-
ARPAHI--
AFPAHI--
AMPAHI--
AYPAHI--
-APAHIK-
-APAHII-
-APAHIF-
-APAHIA-
AIPAHI--
AEPAHI--
ADPAHI--
CAPAHI--
CMPAHI--
CRPAHI--
-CPAHIV-
CKPAHI--
CVPAHI--
-CPAHII-

CGPAHI--
-DPAHIK-
DVPAHI--
DTPAHI--
DSPAHI--
-DPAHIR-
-DPAHIL-
-DPAHII-
DNPAHI--
DKPAHI--
DAPAHI--
DRPAHI--
DYPAHI--
-DPAHIY-
-DPAHIW-
-DPAHIQ-
-DPAHIM-
DLPAHI--
DIPAHI--
DFPAHI--
DEPAHI--
ESPAHI--
-EPAHIM-
-EPAHIY-
-EPAHIL-
-EPAHII-
EQPAHI--
ELPAHI--
EKPAHI--
-EPAHIR-
-EPAHIK-
EFPAHI--
-EPAHIH-
EMPAHI--
EEPAHI--
EAPAHI--
FVPAHI--
-FPAHIM-
FLPAHI--
-FPAHIS-
FRPAHI--
-FPAHIW-
-FPAHIV-
FGPAHI--
FSPAHI--
-FPAHIY-
-FPAHIT-
FYPAHI--
FTPAHI--
-FPAHIL-

-FPAHIK-
-FPAHIF-
FKPAHI--
FEPAHI--
FDPAHI--
-GPAHIR-
GTPAHI--
-GPAHIS-
-GPAHIG-
-GPAHIF-
-GPAHIA-
GAPAHI--
GVPAHI--
-GPAHIY-
-GPAHIL-
GNPAHI--
GLPAHI--
GGPAHI--
GRPAHI--
-GPAHIQ-
GMPAHI--
GFPAHI--
GYPAHI--
GWPAHI--
GSPAHI--
GQPAHI--
-GPAHIV-
-GPAHIT-
-GPAHIH-
GKPAHI--
GDPAHI--
-HPAHIQ-
-HPAHIT-
-HPAHIH-
-HPAHIS-
-HPAHIR-
HHPAHI--
HFPAHI--
HWPAHI--
HTPAHI--
-HPAHIL-
HNPAHI--
HMPAHI--
HLPAHI--
-HPAHIV-
-HPAHIM-
-HPAHIK-
-HPAHIE-
HKPAHI--
HGPAHI--

HEPAHI--
HDPAHI--
HAPAHI--
-IPAHIK-
-IPAHIR-
-IPAHIS-
IYPAHI--
ISPAHI--
IQPAHI--
IPPAHI--
ILPAHI--
ITPAHI--
-IPAHIQ-
-IPAHIH-
IGPAHI--
IDPAHI--
IRPAHI--
-IPAHIV-
-IPAHIN-
-IPAHIM-
-IPAHIE-
IMPAHI--
IIPAHI--
ICPAHI--
-KPAHIL-
-KPAHII-
KTPAHI--
KHPAHI--
KWPAHI--
KRPAHI--
-KPAHIR-
KVPAHI--
KPPAHI--
KIPAHI--
-KPAHIV-
-KPAHIT-
KYPAHI--
-KPAHIM-
KEPAHI--
-KPAHIS-
-KPAHIQ-
-KPAHIE-
KMPAHI--
KLPAHI--
KDPAHI--
LLPAHI--
LWPAHI--
LSPAHI--
-LPAHIF-
LRPAHI--

-LPAHIT-
LNPAHI--
LAPAHI--
LVPAHI--
-LPAHIR-
-LPAHIV-
LTPAHI--
-LPAHIK-
-LPAHII-
LEPAHI--
LDPAHI--
LCPAHI--
LYPAHI--
-LPAHIL-
-LPAHIA-
LMPAHI--
LGPAHI--
-LPAHIW-
LIPAHI--
LFPAHI--
LPPAHI--
-LPAHIY-
-LPAHIN-
-LPAHIE-
LKPAHI--
LHPAHI--
MTPAHI--
MGPAHI--
-MPAHIS-
-MPAHIA-
MSPAHI--
-MPAHIT-
MVPAHI--
-MPAHII-
MMPAHI--
MHPAHI--
MYPAHI--
-MPAHIF-
MKPAHI--
MEPAHI--
MWPAHI--
MRPAHI--
MPPAHI--
-MPAHIY-
-MPAHIR-
-MPAHIN-
-MPAHIH-
-MPAHIE-
MAPAHI--
-NPAHIR-

NWPAHI--
NRPAHI--
-NPAHIT-
-NPAHIK-
NFPAHI--
NSPAHI--
-NPAHIA-
NKPAHI--
NVPAHI--
-NPAHIV-
NEPAHI--
NYPAHI--
-NPAHIG-
NMPAHI--
NLPAHI--
NTPAHI--
NPPAHI--
-NPAHIW-
-NPAHIM-
-NPAHIL-
NNPAHI--
NIPAHI--
--PAHILT
--PAHIRR
--PAHISS
--PAHIRN
--PAHIVK
--PAHIRY
--PAHIRA
--PAHIRL
--PAHILS
--PAHIHW
--PAHIRW
--PAHISG
--PAHIRH
--PAHIMI
--PAHIET
--PAHIYG
--PAHIYA
--PAHIRI
--PAHIKS
--PAHIYI
--PAHITH
--PAHISV
--PAHIMR
--PAHIIR
--PAHIGT
--PAHIGA
--PAHIFT
PWPAHI--

--PAHIVR
--PAHITK
--PAHIRV
--PAHIML
--PAHIKN
--PAHIIE
--PAHIHS
PFPAHI--
--PAHIYW
--PAHIVL
--PAHISW
--PAHISH
--PAHISF
--PAHIRS
--PAHINT
--PAHIMT
--PAHILR
--PAHILN
--PAHILH
--PAHILE
--PAHIKG
--PAHIFK
--PAHIAS
--PAHIYY
--PAHIWA
--PAHIVY
--PAHIRT
--PAHIRF
--PAHIMN
--PAHIMM
--PAHILV
--PAHILK
--PAHIKK
--PAHIKI
--PAHIIT
--PAHIIK
--PAHIIH
--PAHIIG
--PAHIHG
--PAHIFA
--PAHIEY
--PAHIEM
--PAHIEA
--PAHIYR
--PAHIWT
--PAHIWN
--PAHIVT
--PAHITE
--PAHISM
--PAHISI

--PAHIRK
--PAHIQS
--PAHIQI
--PAHIQA
--PAHIMV
--PAHIMK
--PAHILW
--PAHILM
--PAHILF
--PAHIIL
--PAHIIF
--PAHIHV
--PAHIHR
--PAHIHM
--PAHIAN
--PAHIAL
--PAHIAK
--PAHIAA
PTPAHI--
--PAHIYT
--PAHIYS
--PAHIYK
--PAHIVM
--PAHIVI
--PAHIVF
--PAHIVD
--PAHITW
--PAHITR
--PAHITM
--PAHITL
--PAHITI
--PAHISK
--PAHISA
--PAHIRM
--PAHIRE
--PAHIQW
--PAHIQR
--PAHIQN
--PAHIQL
--PAHIQG
--PAHINW
--PAHINV
--PAHINS
--PAHINA
--PAHIMG
--PAHILY
--PAHILD
--PAHILA
--PAHIKR
--PAHIKQ

--PAHIKE
--PAHIIW
--PAHIIM
--PAHIII
--PAHIGV
--PAHIFR
--PAHIFE
--PAHIAW
--PAHIAI
PSPAHI--
PQPAHI--
PLPAHI--
PIPAHI--
PEPAHI--
PCPAHI--
PAPAHI--
--PAHIYV
--PAHIYQ
--PAHIYF
--PAHIYE
--PAHIWW
--PAHIWK
--PAHIWI
--PAHIVV
--PAHIVS
--PAHIVN
--PAHIVA
--PAHITY
--PAHITT
--PAHITS
--PAHITG
--PAHISQ
--PAHISD
--PAHIRQ
--PAHIRD
--PAHIQT
--PAHIQM
--PAHIQF
--PAHINY
--PAHING
--PAHIMS
--PAHIMQ
--PAHIMH
--PAHIMF
--PAHILL
--PAHIKV
--PAHIKT
--PAHIKL
--PAHIKF
--PAHIKD

--PAHIIS
--PAHIIN
--PAHIID
--PAHIHY
--PAHIHL
--PAHIHK
--PAHIHI
--PAHIHH
--PAHIGS
--PAHIGM
--PAHIFY
--PAHIFN
--PAHIFM
--PAHIFI
--PAHIFH
--PAHIES
--PAHIEN
--PAHIEK
--PAHICS
--PAHICI
--PAHIAR
--PAHIAQ
--PAHIAG
QPPAHI--
QLPAHI--
QRPAHI--
QSPAHI--
-QPAHIL-
-QPAHIH-
-QPAHIA-
QVPAHI--
QMPAHI--
QQPAHI--
QGPAHI--
QYPAHI--
QKPAHI--
-QPAHIY-
-QPAHIT-
-QPAHIS-
-QPAHIR-
-QPAHIK-
-RPAHIR-
RGPAHI--
-RPAHIL-
RAPAHI--
-RPAHIS-
-RPAHII-
RLPAHI--
-RPAHIW-
RRPAHI--

RFPAHI--
-RPAHIF-
RVPAHI--
RPPAHI--
-RPAHIT-
RSPAHI--
-RPAHIN-
-RPAHIG-
RNPAHI--
RMPAHI--
RIPAHI--
-RPAHIM-
RYPAHI--
RTPAHI--
-RPAHIK-
-RPAHIH-
-RPAHIA-
-RPAHIY-
RHPAHI--
RWPAHI--
RQPAHI--
-RPAHIE-
RKPAHI--
STPAHI--
-SPAHIR-
SRPAHI--
SFPAHI--
-SPAHIL-
-SPAHIW-
SNPAHI--
SLPAHI--
SAPAHI--
-SPAHIV-
-SPAHIA-
SMPAHI--
SVPAHI--
-SPAHII-
SGPAHI--
SWPAHI--
-SPAHIS-
-SPAHIQ-
-SPAHIH-
-SPAHIF-
SIPAHI--
SPPAHI--
-SPAHIY-
-SPAHIK-
-SPAHIE-
SHPAHI--
SCPAHI--

SYPAHI--
-SPAHIN-
-SPAHIM-
-TPAHIR-
-TPAHIT-
-TPAHIS-
-TPAHIL-
TWPAHI--
-TPAHIQ-
-TPAHIA-
TQPAHI--
TFPAHI--
TVPAHI--
TLPAHI--
TGPAHI--
TEPAHI--
TNPAHI--
TAPAHI--
TSPAHI--
TRPAHI--
-TPAHIH-
TMPAHI--
TTPAHI--
TPPAHI--
-TPAHIY-
-TPAHIK-
-TPAHIW-
-TPAHIV-
-TPAHIG-
TKPAHI--
THPAHI--
VRPAHI--
VSPAHI--
-VPAHIS-
-VPAHIF-
-VPAHIL-
-VPAHII-
VMPAHI--
VDPAHI--
-VPAHIV-
-VPAHIK-
VKPAHI--
VWPAHI--
VTPAHI--
VQPAHI--
-VPAHIT-
-VPAHIQ-
VLPAHI--
VGPAHI--
VVPAHI--

-VPAHIY-
-VPAHIH-
VNPAHI--
VCPAHI--
VPPAHI--
-VPAHIN-
VHPAHI--
VFPAHI--
WVPAHI--
WIPAHI--
WYPAHI--
WAPAHI--
-WPAHIY-
WWPAHI--
-WPAHIR-
-WPAHIQ-
-WPAHIK-
WMPAHI--
WLPAHI--
WGPAHI--
WDPAHI--
WQPAHI--
-WPAHIT-
-WPAHIE-
-WPAHIA-
WNPAHI--
WHPAHI--
YPPAHI--
YSPAHI--
-YPAHIR-
YLPAHI--
YTPAHI--
-YPAHIS-
YGPAHI--
YAPAHI--
YWPAHI--
YVPAHI--
-YPAHIQ-
-YPAHIL-
-YPAHIF-
YNPAHI--
-YPAHIK-
YYPAHI--
YRPAHI--
-YPAHIN-
-YPAHII-
-YPAHIG-
-YPAHIE-
YKPAHI--
YHPAHI--

```
PLPM       202       710     31.49
           --PLPMLT
           SSPLPM--
           YLPLPM--
           AEPLPM--
           -APLPMS-
           -APLPMR-
           -APLPML-
           AKPLPM--
           ARPLPM--
           AQPLPM--
           AWPLPM--
           AVPLPM--
           AYPLPM--
           -APLPMY-
           -APLPMV-
           -APLPMK-
           ATPLPM--
           APPLPM--
           -APLPMM-
           -APLPMW-
           -APLPMI-
           ANPLPM--
           AIPLPM--
           -APLPMT-
           -APLPMN-
           AMPLPM--
           ALPLPM--
           -APLPMQ-
           -APLPMG-
           -APLPMA-
           CYPLPM--
           CVPLPM--
           CIPLPM--
           CAPLPM--
           -CPLPMV-
           -CPLPMR-
           CCPLPM--
           CSPLPM--
           -CPLPMS-
           -CPLPMQ-
           CMPLPM--
           CKPLPM--
           DRPLPM--
           DSPLPM--
           -DPLPMQ-
           -DPLPMH-
           DYPLPM--
           -DPLPMY-
           -DPLPMW-
```

-DPLPMT-
-DPLPMK-
DLPLPM--
DGPLPM--
-EPLPMM-
EFPLPM--
EVPLPM--
ETPLPM--
-EPLPMF-
-EPLPMR-
-EPLPML-
EGPLPM--
EIPLPM--
ESPLPM--
ERPLPM--
-EPLPMN-
-EPLPMK-
-EPLPMI-
EAPLPM--
EWPLPM--
EPPLPM--
-EPLPMY-
-EPLPMS-
EMPLPM--
EKPLPM--
EHPLPM--
EDPLPM--
ECPLPM--
-FPLPMT-
FRPLPM--
-FPLPMR-
FMPLPM--
FSPLPM--
FNPLPM--
FWPLPM--
FEPLPM--
FYPLPM--
-FPLPMV-
-FPLPMS-
FLPLPM--
-FPLPMH-
-FPLPMA-
FKPLPM--
FIPLPM--
FFPLPM--
FVPLPM--
-FPLPMI-
FTPLPM--
FPPLPM--
-FPLPMW-

-FPLPMQ-
-FPLPMK-
-FPLPME-
FGPLPM--
FCPLPM--
FAPLPM--
-GPLPMR-
GTPLPM--
GVPLPM--
GSPLPM--
GQPLPM--
-GPLPMW-
-GPLPMQ-
GYPLPM--
GNPLPM--
-GPLPMT-
-GPLPMG-
-GPLPMK-
GKPLPM--
GGPLPM--
GRPLPM--
-GPLPMY-
-GPLPMN-
-GPLPME-
GLPLPM--
GHPLPM--
GAPLPM--
GWPLPM--
GPPLPM--
-GPLPMI-
-GPLPMH-
-GPLPMF-
-GPLPMC-
-GPLPMA-
GIPLPM--
GDPLPM--
GCPLPM--
-GPLPMV-
-GPLPMS-
GFPLPM--
-HPLPMN-
HRPLPM--
-HPLPML-
HGPLPM--
HLPLPM--
HWPLPM--
HPPLPM--
HFPLPM--
-HPLPMW-
-HPLPMK-

HMPLPM--
HVPLPM--
HSPLPM--
-HPLPMT-
-HPLPMR-
HTPLPM--
-HPLPMI-
HNPLPM--
-HPLPMV-
-HPLPMQ-
-HPLPMH-
-HPLPMG-
-HPLPME-
-HPLPMA-
HEPLPM--
IRPLPM--
IAPLPM--
ISPLPM--
-IPLPMR-
-IPLPMY-
-IPLPMK-
-IPLPMS-
IVPLPM--
-IPLPMT-
INPLPM--
IMPLPM--
-IPLPMQ-
-IPLPMI-
ILPLPM--
IKPLPM--
IHPLPM--
IFPLPM--
IEPLPM--
IWPLPM--
-IPLPMW-
-IPLPMV-
-IPLPMH-
-IPLPME-
IDPLPM--
KSPLPM--
-KPLPML-
KGPLPM--
KLPLPM--
KTPLPM--
-KPLPMT-
-KPLPMQ-
KWPLPM--
KVPLPM--
-KPLPMS-
KHPLPM--

-KPLPMV-
-KPLPMY-
KRPLPM--
KQPLPM--
-KPLPMI-
-KPLPMH-
-KPLPMF-
KIPLPM--
KPPLPM--
-KPLPMM-
-KPLPMA-
KFPLPM--
KEPLPM--
KAPLPM--
KYPLPM--
-KPLPMN-
-KPLPMG-
KKPLPM--
-LPLPMR-
LRPLPM--
-LPLPML-
-LPLPMY-
-LPLPMT-
-LPLPMS-
LTPLPM--
LSPLPM--
LQPLPM--
-LPLPMV-
LYPLPM--
-LPLPMW-
LMPLPM--
LGPLPM--
LNPLPM--
LIPLPM--
LFPLPM--
LPPLPM--
LEPLPM--
LVPLPM--
-LPLPMQ-
-LPLPMK-
-LPLPMH-
LLPLPM--
LWPLPM--
-LPLPMN-
-LPLPMM-
LHPLPM--
LDPLPM--
MRPLPM--
-MPLPML-
MLPLPM--

MAPLPM--
MWPLPM--
-MPLPMS-
-MPLPMR-
MMPLPM--
MVPLPM--
MYPLPM--
MTPLPM--
-MPLPMV-
MNPLPM--
MPPLPM--
MGPLPM--
MFPLPM--
-MPLPMY-
-MPLPMI-
-MPLPMH-
-MPLPMG-
MEPLPM--
MDPLPM--
-MPLPMQ-
-MPLPMK-
-MPLPME-
-MPLPMA-
MKPLPM--
MHPLPM--
MCPLPM--
NRPLPM--
-NPLPMN-
-NPLPML-
-NPLPMY-
-NPLPMS-
-NPLPMR-
NLPLPM--
NYPLPM--
-NPLPMK-
NGPLPM--
NKPLPM--
NWPLPM--
-NPLPMV-
-NPLPMT-
-NPLPMA-
NIPLPM--
NTPLPM--
-NPLPMQ-
NMPLPM--
NVPLPM--
NSPLPM--
NCPLPM--
NQPLPM--
-NPLPMM-

-NPLPMI-
-NPLPMF-
NEPLPM--
--PLPMRV
--PLPMLG
--PLPMRT
--PLPMQM
--PLPMIL
--PLPMWL
--PLPMYI
--PLPMTG
--PLPMSL
--PLPMRF
--PLPMMS
--PLPMLM
--PLPMLA
--PLPMVT
--PLPMTL
--PLPMSS
--PLPMRL
--PLPMTD
--PLPMRS
--PLPMLV
--PLPMLR
--PLPMAT
--PLPMVL
--PLPMSK
--PLPMRY
--PLPMMA
--PLPMIS
--PLPMII
--PLPMGS
--PLPMDL
--PLPMAF
--PLPMWQ
--PLPMWG
--PLPMVS
--PLPMVR
--PLPMNT
PFPLPM--
--PLPMYG
--PLPMTY
--PLPMRW
--PLPMIR
--PLPMGM
--PLPMWK
--PLPMWH
--PLPMVV
--PLPMRK
--PLPMPW

--PLPMKA
--PLPMHL
--PLPMFV
--PLPMFT
--PLPMFS
--PLPMFQ
--PLPMFM
--PLPMAR
--PLPMYS
--PLPMWD
--PLPMVN
--PLPMVA
--PLPMTS
--PLPMTR
--PLPMSV
--PLPMSQ
--PLPMSN
--PLPMRN
--PLPMRG
--PLPMRA
--PLPMPY
--PLPMNI
--PLPMLQ
--PLPMKV
--PLPMKR
--PLPMKD
--PLPMIF
--PLPMFG
--PLPMES
--PLPMYW
--PLPMYM
--PLPMVM
--PLPMVI
--PLPMTT
--PLPMTF
--PLPMTE
--PLPMQS
--PLPMQR
--PLPMQN
--PLPMNV
--PLPMNM
--PLPMMY
--PLPMMT
--PLPMMG
--PLPMLD
--PLPMKL
--PLPMHV
--PLPMHI
--PLPMHA
--PLPMGQ

--PLPMFR
--PLPMFI
--PLPMAS
--PLPMAM
--PLPMAK
PWPLPM--
--PLPMWW
--PLPMWN
--PLPMVQ
--PLPMVG
--PLPMVF
--PLPMTQ
--PLPMTA
--PLPMSM
--PLPMSH
--PLPMSA
--PLPMRP
--PLPMQE
--PLPMPI
--PLPMMR
--PLPMML
--PLPMLE
--PLPMKW
--PLPMKG
--PLPMIV
--PLPMIK
--PLPMIH
--PLPMGV
--PLPMDY
--PLPMAI
--PLPMYR
--PLPMYD
--PLPMWY
--PLPMWF
--PLPMVK
--PLPMTW
--PLPMTK
--PLPMSY
--PLPMST
--PLPMSI
--PLPMSD
--PLPMRD
--PLPMQT
--PLPMQL
--PLPMQK
--PLPMPM
--PLPMPF
--PLPMNY
--PLPMNW
--PLPMNF

--PLPMLK
--PLPMKT
--PLPMKM
--PLPMIY
--PLPMIQ
--PLPMID
--PLPMHR
--PLPMHQ
--PLPMGH
--PLPMGG
--PLPMGD
--PLPMFN
--PLPMEI
--PLPMAY
--PLPMAW
--PLPMAV
--PLPMAG
--PLPMAA
-PPLPMS-
--PLPMYQ
--PLPMYN
--PLPMYE
--PLPMYA
--PLPMWV
--PLPMWR
--PLPMVW
--PLPMVH
--PLPMVD
--PLPMTV
--PLPMTM
--PLPMTI
--PLPMTH
--PLPMRQ
--PLPMQV
--PLPMQI
--PLPMQH
--PLPMQF
--PLPMQA
--PLPMNS
--PLPMNH
--PLPMMQ
--PLPMLP
--PLPMLH
--PLPMKY
--PLPMKQ
--PLPMKN
--PLPMKF
--PLPMKE
--PLPMIW
--PLPMHY

--PLPMHK
--PLPMHE
--PLPMGY
--PLPMGW
--PLPMGT
--PLPMGA
--PLPMFD
--PLPMEW
--PLPMEV
--PLPMET
--PLPMEN
--PLPMEM
--PLPMEA
-QPLPMW-
QAPLPM--
QYPLPM--
QWPLPM--
QRPLPM--
QKPLPM--
QGPLPM--
QVPLPM--
-QPLPMR-
QIPLPM--
QEPLPM--
QSPLPM--
-QPLPMQ-
-QPLPMN-
-QPLPMF-
-QPLPMA-
QFPLPM--
QTPLPM--
QPPLPM--
-QPLPMY-
-QPLPMV-
-QPLPMS-
-QPLPMH-
-QPLPME-
QMPLPM--
RLPLPM--
-RPLPMQ-
RSPLPM--
-RPLPMY-
RKPLPM--
-RPLPMV-
-RPLPMI-
RYPLPM--
RVPLPM--
RTPLPM--
-RPLPMN-
-RPLPMG-

-RPLPMF-
RIPLPM--
REPLPM--
RDPLPM--
-RPLPMT-
RFPLPM--
RMPLPM--
RHPLPM--
RAPLPM--
RWPLPM--
RPPLPM--
-RPLPMS-
RNPLPM--
RGPLPM--
RCPLPM--
-RPLPMK-
-RPLPME-
-RPLPMW-
-SPLPMK-
SRPLPM--
-SPLPMW-
-SPLPML-
STPLPM--
-SPLPMR-
-SPLPMY-
-SPLPMI-
-SPLPMF-
-SPLPMV-
SQPLPM--
-SPLPMH-
SGPLPM--
SHPLPM--
SYPLPM--
-SPLPMN-
SLPLPM--
SAPLPM--
-SPLPMA-
SKPLPM--
SEPLPM--
SDPLPM--
-SPLPMQ-
SMPLPM--
SNPLPM--
SIPLPM--
SFPLPM--
-SPLPME-
SCPLPM--
SWPLPM--
SVPLPM--
-SPLPMM-

-SPLPMG-
-TPLPML-
-TPLPMQ-
-TPLPMV-
-TPLPMR-
-TPLPMI-
TIPLPM--
TSPLPM--
TYPLPM--
TVPLPM--
-TPLPMS-
-TPLPMA-
TLPLPM--
TAPLPM--
TFPLPM--
-TPLPMH-
TRPLPM--
-TPLPMY-
-TPLPMN-
-TPLPME-
TNPLPM--
TKPLPM--
TGPLPM--
TTPLPM--
TPPLPM--
-TPLPMG-
VRPLPM--
VSPLPM--
-VPLPMN-
VLPLPM--
VHPLPM--
-VPLPMS-
-VPLPMR-
VIPLPM--
VGPLPM--
VPPLPM--
-VPLPMG-
VVPLPM--
VQPLPM--
-VPLPMW-
-VPLPMQ-
VMPLPM--
VTPLPM--
-VPLPMV-
-VPLPMK-
-VPLPMI-
-VPLPMH-
-VPLPMA-
VNPLPM--
VKPLPM--

VYPLPM--
VFPLPM--
VDPLPM--
VWPLPM--
-VPLPMM-
-VPLPME-
-VPLPMD-
VEPLPM--
-WPLPML-
-WPLPMS-
WGPLPM--
WIPLPM--
WMPLPM--
WAPLPM--
WTPLPM--
-WPLPMV-
-WPLPMQ-
WKPLPM--
WYPLPM--
WVPLPM--
WPPLPM--
WDPLPM--
WSPLPM--
-WPLPMT-
-WPLPMR-
-WPLPMG-
WNPLPM--
WWPLPM--
WRPLPM--
WQPLPM--
-WPLPMK-
-WPLPMH-
-WPLPMF-
-WPLPME-
WHPLPM--
WFPLPM--
WEPLPM--
-YPLPMR-
YRPLPM--
YHPLPM--
YGPLPM--
-YPLPME-
YSPLPM--
-YPLPMW-
-YPLPMT-
YWPLPM--
YVPLPM--
-YPLPMG-
-YPLPMA-
YTPLPM--

| | | | |
|---|---|---|---|
| | -YPLPMS- | | |
| | YAPLPM-- | | |
| | YPPLPM-- | | |
| | -YPLPMV- | | |
| | -YPLPMQ- | | |
| | -YPLPMH- | | |
| | -YPLPMF- | | |
| | YKPLPM-- | | |
| | -YPLPMI- | | |
| | -YPLPMC- | | |
| | YEPLPM-- | | |
| PRSI | 879 | 524 | 31.43 |
| | -EPRSIS- | | |
| | --PRSIYP | | |
| | --PRSIWP | | |
| | --PRSINQ | | |
| | --PRSIEH | | |
| | -VPRSIP- | | |
| | -APRSIY- | | |
| | AVPRSI-- | | |
| | -APRSIV- | | |
| | -APRSIH- | | |
| | ASPRSI-- | | |
| | ANPRSI-- | | |
| | -APRSIT- | | |
| | -APRSIA- | | |
| | -APRSIN- | | |
| | AMPRSI-- | | |
| | AKPRSI-- | | |
| | AIPRSI-- | | |
| | -APRSIM- | | |
| | -APRSII- | | |
| | AQPRSI-- | | |
| | -APRSIE- | | |
| | APPRSI-- | | |
| | AEPRSI-- | | |
| | ADPRSI-- | | |
| | -CPRSIR- | | |
| | CQPRSI-- | | |
| | CVPRSI-- | | |
| | CSPRSI-- | | |
| | CGPRSI-- | | |
| | CCPRSI-- | | |
| | CRPRSI-- | | |
| | CKPRSI-- | | |
| | CTPRSI-- | | |
| | CIPRSI-- | | |
| | CFPRSI-- | | |
| | CWPRSI-- | | |
| | -CPRSIH- | | |

CAPRSI--
-CPRSIV-
-CPRSIK-
-CPRSII-
CPPRSI--
CMPRSI--
CLPRSI--
CHPRSI--
CDPRSI--
DTPRSI--
DLPRSI--
DVPRSI--
-DPRSIL-
DRPRSI--
-DPRSIS-
-DPRSIR-
DSPRSI--
-DPRSIW-
-DPRSIT-
DQPRSI--
DMPRSI--
DKPRSI--
-DPRSIY-
-DPRSIQ-
-DPRSIA-
DAPRSI--
DYPRSI--
-DPRSIV-
-DPRSIH-
-DPRSIF-
DFPRSI--
-DPRSIK-
-DPRSIE-
DDPRSI--
-DPRSIN-
DHPRSI--
DEPRSI--
-EPRSIR-
ERPRSI--
-EPRSIY-
EQPRSI--
-EPRSIT-
-EPRSIK-
-EPRSIG-
EKPRSI--
EIPRSI--
-EPRSIF-
EPPRSI--
EGPRSI--
EAPRSI--

ELPRSI--
EEPRSI--
-EPRSIV-
-EPRSIM-
EFPRSI--
EYPRSI--
-EPRSIW-
-EPRSIN-
ENPRSI--
EWPRSI--
EHPRSI--
EDPRSI--
-EPRSIQ-
-EPRSIE-
-EPRSIA-
EMPRSI--
FNPRSI--
-FPRSIM-
FTPRSI--
FSPRSI--
FGPRSI--
FAPRSI--
FMPRSI--
FQPRSI--
FDPRSI--
FEPRSI--
FYPRSI--
FPPRSI--
FCPRSI--
-GPRSIR-
GSPRSI--
GYPRSI--
-GPRSIV-
-GPRSIF-
-GPRSIY-
-GPRSIL-
GVPRSI--
-GPRSIG-
-GPRSIS-
-GPRSIK-
-GPRSII-
GGPRSI--
-GPRSIH-
GMPRSI--
-GPRSIW-
GAPRSI--
-GPRSIN-
-GPRSIA-
GNPRSI--
GPPRSI--

GCPRSI--
-GPRSIE-
GDPRSI--
-GPRSIQ-
HSPRSI--
HAPRSI--
HCPRSI--
HIPRSI--
-HPRSIH-
-HPRSIV-
HGPRSI--
-HPRSII-
HPPRSI--
HTPRSI--
-HPRSIW-
HMPRSI--
HEPRSI--
HQPRSI--
-HPRSIK-
-HPRSIE-
HNPRSI--
HKPRSI--
HDPRSI--
-HPRSIQ-
ISPRSI--
-IPRSII-
-IPRSIY-
IGPRSI--
INPRSI--
-IPRSIV-
-IPRSIM-
IKPRSI--
IDPRSI--
-IPRSIH-
IMPRSI--
IPPRSI--
IQPRSI--
IEPRSI--
-IPRSIF-
IIPRSI--
-IPRSIS-
-IPRSIQ-
-IPRSIE-
ICPRSI--
-KPRSIL-
-KPRSIV-
KGPRSI--
-KPRSIY-
KPPRSI--
-KPRSIW-

KDPRSI--
-KPRSIH-
KEPRSI--
-KPRSIQ-
-KPRSIA-
KQPRSI--
KRPRSI--
-KPRSIG-
-KPRSIN-
KCPRSI--
-LPRSIT-
LDPRSI--
-LPRSIF-
LPPRSI--
-LPRSIV-
-LPRSIQ-
LHPRSI--
-LPRSIW-
-LPRSII-
LEPRSI--
-LPRSIH-
-LPRSIA-
-LPRSIE-
LCPRSI--
MSPRSI--
MAPRSI--
MDPRSI--
MRPRSI--
MNPRSI--
MYPRSI--
-MPRSIN-
-MPRSIA-
MIPRSI--
MGPRSI--
-MPRSIQ-
MKPRSI--
-MPRSIG-
MCPRSI--
MPPRSI--
-NPRSIL-
-NPRSIV-
NKPRSI--
NGPRSI--
NYPRSI--
-NPRSIF-
-NPRSIS-
-NPRSIM-
-NPRSIH-
NIPRSI--
NEPRSI--

-NPRSIK-
-NPRSIA-
NQPRSI--
-NPRSIW-
NVPRSI--
NCPRSI--
--PRSIYS
--PRSISG
--PRSILN
--PRSIVT
--PRSISA
--PRSIAV
--PRSIFG
--PRSIFV
--PRSIVV
--PRSISV
--PRSIIS
--PRSIYQ
--PRSISI
--PRSINS
--PRSIIV
--PRSIHG
--PRSIVS
--PRSIKQ
--PRSIGS
--PRSIAS
--PRSITT
--PRSIFI
--PRSIAL
--PRSISQ
--PRSIYY
--PRSIWN
PGPRSI--
--PRSIYN
--PRSITH
--PRSIMN
--PRSILM
--PRSIKI
--PRSINL
--PRSIHA
PRPRSI--
--PRSIYW
--PRSISM
--PRSIRD
--PRSIQH
--PRSIMK
--PRSIIH
--PRSIGL
PTPRSI--
--PRSIYI

--PRSIYE
--PRSIWF
--PRSISN
--PRSINA
--PRSILE
--PRSILD
--PRSIHT
--PRSIHS
--PRSIHI
--PRSIAF
--PRSITI
--PRSIQS
--PRSINT
--PRSIHF
--PRSIFA
--PRSIVA
--PRSIRE
--PRSIFQ
--PRSIVF
--PRSIAN
--PRSIYM
--PRSIYD
--PRSIVK
--PRSINY
--PRSIIQ
--PRSIFT
PMPRSI--
--PRSIQT
--PRSIMT
--PRSIMQ
--PRSIIM
--PRSIFH
--PRSIYT
--PRSIKG
--PRSIHW
--PRSIHN
--PRSIHE
--PRSIHD
--PRSIVH
--PRSITD
--PRSIQY
--PRSIIE
--PRSIAT
--PRSIAH
--PRSIYG
--PRSIWW
--PRSITV
--PRSITQ
--PRSIQI
--PRSIQG

--PRSIKM
--PRSIHQ
--PRSIFY
PKPRSI--
PVPRSI--
--PRSIYH
--PRSIWK
--PRSITW
--PRSIQW
--PRSIQV
--PRSINV
--PRSINF
--PRSIMD
--PRSIID
--PRSIYF
--PRSIVQ
--PRSIVE
--PRSISD
--PRSINW
--PRSIKV
--PRSIHV
--PRSIHM
--PRSIGI
--PRSIFM
--PRSIFE
--PRSIEG
--PRSIAD
PHPRSI--
PAPRSI--
--PRSIWQ
--PRSIQN
--PRSIKF
PEPRSI--
--PRSIWI
--PRSISE
--PRSIME
--PRSIKY
--PRSIFD
--PRSIEY
--PRSIET
--PRSIEI
PQPRSI--
--PRSIWH
--PRSINH
--PRSIIT
--PRSIHK
--PRSIHH
--PRSIEW
--PRSIEN
PNPRSI--

--PRSIQQ
--PRSIQF
--PRSIQE
--PRSIQA
--PRSIII
--PRSIIA
--PRSICG
--PRSIAG
--PRSIWM
--PRSIVC
--PRSIQM
--PRSIQD
--PRSIPM
--PRSIPI
--PRSING
--PRSILC
--PRSIIC
--PRSIGF
--PRSIFC
--PRSIEV
--PRSIEA
--PRSIDN
--PRSICL
--PRSICF
--PRSIAM
-PPRSIR-
-PPRSIN-
PCPRSI--
QIPRSI--
-QPRSIR-
-QPRSII-
-QPRSIQ-
QKPRSI--
QAPRSI--
-QPRSIT-
QHPRSI--
QGPRSI--
QNPRSI--
-QPRSIV-
-QPRSIS-
QYPRSI--
QPPRSI--
-QPRSIA-
QQPRSI--
-QPRSIY-
-QPRSIN-
-QPRSIF-
-QPRSIH-
-QPRSIW-
-QPRSIE-

QDPRSI--
-QPRSIM-
-RPRSIT-
-RPRSIY-
REPRSI--
-RPRSIQ-
RPPRSI--
-RPRSIN-
-RPRSIE-
RCPRSI--
-SPRSIH-
-SPRSIQ-
-SPRSIF-
-SPRSIK-
SNPRSI--
-SPRSIN-
-SPRSIA-
SPPRSI--
SDPRSI--
-SPRSIG-
SCPRSI--
-SPRSIE-
TVPRSI--
-TPRSIQ-
TAPRSI--
TNPRSI--
TGPRSI--
-TPRSIY-
-TPRSIA-
TMPRSI--
TTPRSI--
-TPRSIH-
-TPRSIN-
TEPRSI--
-TPRSIE-
TQPRSI--
VSPRSI--
-VPRSIV-
VKPRSI--
VQPRSI--
VYPRSI--
VPPRSI--
VAPRSI--
VGPRSI--
VNPRSI--
-VPRSIQ-
-VPRSIN-
-VPRSIA-
VEPRSI--
VDPRSI--

-VPRSIG-
-VPRSIE-
-VPRSIC-
VCPRSI--
WGPRSI--
WLPRSI--
WVPRSI--
WQPRSI--
WTPRSI--
WEPRSI--
WRPRSI--
-WPRSIE-
-WPRSIV-
-WPRSIQ-
WKPRSI--
-WPRSIF-
WCPRSI--
WAPRSI--
YSPRSI--
YGPRSI--
YMPRSI--
YNPRSI--
-YPRSIY-
-YPRSIF-
YTPRSI--
-YPRSII-
YKPRSI--
YEPRSI--
YPPRSI--
-YPRSIQ-
-YPRSIM-
YQPRSI--
-YPRSIG-
-YPRSIH-
YDPRSI--
YCPRSI--

| | | | |
|---|---|---|---|
| PLAF | 390 | 635 | 31.41 |

DIPLAF--
--PLAFRP
--PLAFAR
-APLAFT-
ALPLAF--
ATPLAF--
-APLAFS-
-APLAFR-
AGPLAF--
-APLAFV-
-APLAFA-
AQPLAF--
APPLAF--

AHPLAF--
AFPLAF--
AYPLAF--
-APLAFI-
-APLAFE-
AEPLAF--
AWPLAF--
-APLAFN-
ANPLAF--
AKPLAF--
-APLAFM-
-APLAFG-
ADPLAF--
-APLAFH-
AMPLAF--
AIPLAF--
ACPLAF--
-CPLAFR-
-CPLAFT-
-CPLAFL-
CMPLAF--
CGPLAF--
CRPLAF--
CAPLAF--
CVPLAF--
-CPLAFW-
-CPLAFV-
-CPLAFK-
-CPLAFH-
CKPLAF--
CCPLAF--
DRPLAF--
DYPLAF--
DNPLAF--
DLPLAF--
-DPLAFT-
-DPLAFR-
DAPLAF--
DWPLAF--
DGPLAF--
DSPLAF--
-DPLAFV-
-DPLAFM-
DKPLAF--
-DPLAFS-
-DPLAFA-
DMPLAF--
DVPLAF--
DTPLAF--
-DPLAFQ-

DEPLAF--
DCPLAF--
-EPLAFR-
ESPLAF--
EFPLAF--
EKPLAF--
-EPLAFM-
ELPLAF--
EWPLAF--
-EPLAFT-
EVPLAF--
-EPLAFQ-
-EPLAFL-
EHPLAF--
EYPLAF--
ETPLAF--
EEPLAF--
EAPLAF--
EQPLAF--
-EPLAFS-
-EPLAFE-
EMPLAF--
EIPLAF--
EGPLAF--
-FPLAFR-
FSPLAF--
FRPLAF--
FGPLAF--
FQPLAF--
FAPLAF--
FYPLAF--
-FPLAFN-
-FPLAFH-
-FPLAFT-
FEPLAF--
-FPLAFS-
-FPLAFQ-
FHPLAF--
-FPLAFA-
-FPLAFV-
-FPLAFK-
FDPLAF--
-FPLAFG-
FNPLAF--
GVPLAF--
-GPLAFR-
GAPLAF--
GYPLAF--
GEPLAF--
-GPLAFM-

-GPLAFS-
GHPLAF--
-GPLAFK-
-GPLAFH-
-GPLAFG-
-GPLAFA-
GSPLAF--
GGPLAF--
GFPLAF--
-GPLAFV-
GNPLAF--
-GPLAFT-
-GPLAFN-
-GPLAFY-
GKPLAF--
GCPLAF--
GWPLAF--
GQPLAF--
GPPLAF--
-GPLAFQ-
-GPLAFC-
HRPLAF--
HGPLAF--
-HPLAFH-
HNPLAF--
-HPLAFR-
HKPLAF--
HAPLAF--
-HPLAFK-
-HPLAFI-
HFPLAF--
-HPLAFT-
-HPLAFL-
-HPLAFA-
HMPLAF--
HHPLAF--
HVPLAF--
HTPLAF--
HLPLAF--
HWPLAF--
-HPLAFV-
HYPLAF--
HQPLAF--
HPPLAF--
-HPLAFW-
-HPLAFS-
-HPLAFM-
-HPLAFG-
-HPLAFC-
HIPLAF--

HEPLAF--
HDPLAF--
-IPLAFT-
-IPLAFN-
IRPLAF--
IPPLAF--
-IPLAFK-
-IPLAFV-
-IPLAFR-
-IPLAFQ-
-IPLAFM-
IGPLAF--
-IPLAFA-
IIPLAF--
ITPLAF--
-IPLAFH-
-IPLAFE-
IKPLAF--
IHPLAF--
IYPLAF--
IEPLAF--
-KPLAFV-
KNPLAF--
-KPLAFM-
KLPLAF--
KFPLAF--
-KPLAFK-
KRPLAF--
KVPLAF--
KGPLAF--
-KPLAFS-
-KPLAFQ-
KMPLAF--
KIPLAF--
KHPLAF--
KYPLAF--
KCPLAF--
KQPLAF--
-KPLAFN-
KWPLAF--
-KPLAFF-
-KPLAFW-
-KPLAFI-
KKPLAF--
KDPLAF--
KTPLAF--
KAPLAF--
KPPLAF--
-KPLAFP-
-KPLAFH-

LKPLAF--
LSPLAF--
-LPLAFR-
-LPLAFQ-
LYPLAF--
LTPLAF--
-LPLAFL-
-LPLAFS-
-LPLAFM-
-LPLAFA-
LQPLAF--
LWPLAF--
-LPLAFN-
LNPLAF--
LEPLAF--
LDPLAF--
-MPLAFS-
-MPLAFR-
-MPLAFL-
-MPLAFA-
-MPLAFI-
-MPLAFK-
-MPLAFM-
MMPLAF--
MYPLAF--
MVPLAF--
-MPLAFQ-
MLPLAF--
MKPLAF--
MIPLAF--
MHPLAF--
MGPLAF--
MEPLAF--
MWPLAF--
-MPLAFV-
MNPLAF--
MQPLAF--
-MPLAFN-
-MPLAFE-
MCPLAF--
-NPLAFT-
-NPLAFR-
-NPLAFH-
-NPLAFL-
NGPLAF--
NYPLAF--
NRPLAF--
-NPLAFS-
-NPLAFA-
NWPLAF--

```
-NPLAFW-
NSPLAF--
-NPLAFV-
NHPLAF--
NAPLAF--
-NPLAFQ-
NKPLAF--
-NPLAFK-
NMPLAF--
NTPLAF--
NIPLAF--
NVPLAF--
NQPLAF--
-NPLAFN-
-NPLAFM-
-NPLAFF-
NEPLAF--
NLPLAF--
NFPLAF--
--PLAFVR
--PLAFSS
--PLAFRT
--PLAFQR
--PLAFLT
--PLAFLS
--PLAFLH
--PLAFAG
--PLAFVN
--PLAFRV
--PLAFHL
--PLAFSK
--PLAFRN
--PLAFAS
--PLAFRG
--PLAFLR
--PLAFLN
--PLAFGL
--PLAFTL
--PLAFRA
--PLAFIG
--PLAFSM
--PLAFRM
--PLAFRK
--PLAFMQ
--PLAFMK
--PLAFYG
--PLAFTM
--PLAFSA
--PLAFRQ
--PLAFNL
```

--PLAFLG
--PLAFGS
--PLAFWK
--PLAFTK
--PLAFST
--PLAFRE
--PLAFMR
--PLAFKG
--PLAFIN
--PLAFVK
--PLAFTV
--PLAFTG
--PLAFSY
--PLAFSW
--PLAFSN
--PLAFSL
--PLAFRI
--PLAFPY
--PLAFNT
--PLAFME
--PLAFWS
--PLAFTR
--PLAFMH
--PLAFKR
--PLAFKA
--PLAFFN
--PLAFTN
--PLAFQS
--PLAFQH
--PLAFLQ
--PLAFKS
--PLAFKM
--PLAFHA
--PLAFEA
--PLAFTY
--PLAFTT
--PLAFQT
--PLAFQI
--PLAFLD
--PLAFHW
--PLAFHV
--PLAFGY
--PLAFAA
--PLAFYH
--PLAFTD
--PLAFTA
--PLAFSH
--PLAFSD
--PLAFNY
--PLAFNM

--PLAFHF
--PLAFGI
--PLAFAI
PEPLAF--
--PLAFWD
--PLAFVA
--PLAFSI
--PLAFSE
--PLAFQK
--PLAFPL
--PLAFMG
--PLAFLK
--PLAFKL
--PLAFIK
--PLAFIE
--PLAFHQ
--PLAFHK
--PLAFHI
--PLAFGG
--PLAFES
PNPLAF--
--PLAFYQ
--PLAFYN
--PLAFWN
--PLAFQV
--PLAFQN
--PLAFQM
--PLAFQA
--PLAFPM
--PLAFNV
--PLAFNS
--PLAFNQ
--PLAFKW
--PLAFKT
--PLAFKQ
--PLAFKK
--PLAFKE
--PLAFIR
--PLAFIH
--PLAFHS
--PLAFHG
--PLAFGV
--PLAFGT
--PLAFYD
--PLAFWR
--PLAFWQ
--PLAFTH
--PLAFTE
--PLAFSQ
--PLAFRW

--PLAFRF
--PLAFQW
--PLAFQG
--PLAFNI
--PLAFNG
--PLAFNF
--PLAFNA
--PLAFMN
--PLAFKV
--PLAFKI
--PLAFKH
--PLAFIW
--PLAFHY
--PLAFHT
--PLAFFI
--PLAFEG
--PLAFAY
--PLAFAM
--PLAFAK
-PPLAFY-
-PPLAFH-
--PLAFWT
--PLAFWM
--PLAFVH
--PLAFVD
--PLAFRH
--PLAFQQ
--PLAFQF
--PLAFPI
--PLAFPF
--PLAFMD
--PLAFMA
--PLAFKY
--PLAFKN
--PLAFKD
--PLAFIV
--PLAFHR
--PLAFHN
--PLAFGA
--PLAFFP
--PLAFFH
--PLAFFD
--PLAFEV
--PLAFET
--PLAFAV
PCPLAF--
QRPLAF--
-QPLAFF-
QTPLAF--
QMPLAF--

−QPLAFL−
QLPLAF−−
QGPLAF−−
QSPLAF−−
QNPLAF−−
QYPLAF−−
−QPLAFV−
−QPLAFS−
−QPLAFR−
QVPLAF−−
−QPLAFY−
−QPLAFN−
−QPLAFI−
−QPLAFM−
QKPLAF−−
QPPLAF−−
QIPLAF−−
−QPLAFQ−
−QPLAFG−
QHPLAF−−
QEPLAF−−
QAPLAF−−
−RPLAFS−
RGPLAF−−
RSPLAF−−
−RPLAFR−
RRPLAF−−
−RPLAFL−
RTPLAF−−
−RPLAFH−
−RPLAFQ−
RLPLAF−−
RKPLAF−−
RMPLAF−−
−RPLAFA−
−RPLAFF−
−RPLAFW−
−RPLAFT−
−RPLAFN−
−RPLAFI−
RHPLAF−−
RPPLAF−−
RNPLAF−−
REPLAF−−
RDPLAF−−
RQPLAF−−
−RPLAFC−
SGPLAF−−
−SPLAFL−
−SPLAFV−

SVPLAF--
SHPLAF--
SSPLAF--
SMPLAF--
SKPLAF--
SYPLAF--
STPLAF--
SLPLAF--
SAPLAF--
-SPLAFI-
SNPLAF--
-SPLAFH-
SEPLAF--
-SPLAFQ-
-SPLAFK-
SWPLAF--
SIPLAF--
SPPLAF--
SQPLAF--
-SPLAFG-
SDPLAF--
SCPLAF--
-SPLAFP-
-SPLAFE-
TRPLAF--
-TPLAFT-
TFPLAF--
-TPLAFR-
TPPLAF--
TIPLAF--
-TPLAFS-
-TPLAFN-
-TPLAFM-
-TPLAFA-
TLPLAF--
TAPLAF--
TVPLAF--
TTPLAF--
-TPLAFV-
TKPLAF--
TSPLAF--
-TPLAFH-
TYPLAF--
-TPLAFW-
TEPLAF--
-TPLAFG-
TNPLAF--
TDPLAF--
TWPLAF--
-TPLAFQ-

TCPLAF--
VRPLAF--
-VPLAFL-
-VPLAFH-
-VPLAFV-
-VPLAFR-
VNPLAF--
VDPLAF--
VIPLAF--
-VPLAFT-
VYPLAF--
VVPLAF--
-VPLAFS-
VFPLAF--
-VPLAFA-
VMPLAF--
VTPLAF--
VPPLAF--
-VPLAFN-
-VPLAFM-
-VPLAFK-
-VPLAFG-
-VPLAFE-
VHPLAF--
VCPLAF--
VEPLAF--
WGPLAF--
WSPLAF--
WRPLAF--
WTPLAF--
-WPLAFS-
WNPLAF--
WPPLAF--
-WPLAFQ-
WWPLAF--
-WPLAFM-
WIPLAF--
WQPLAF--
-WPLAFK-
-WPLAFH-
-WPLAFG-
WEPLAF--
WAPLAF--
-WPLAFW-
-WPLAFN-
WCPLAF--
YIPLAF--
-YPLAFR-
YHPLAF--
YLPLAF--

```
          -YPLAFI-
          YRPLAF--
          -YPLAFT-
          -YPLAFS-
          -YPLAFA-
          YPPLAF--
          YYPLAF--
          YWPLAF--
          -YPLAFM-
          -YPLAFE-
          YKPLAF--
          YQPLAF--
          -YPLAFV-
          -YPLAFQ-
          -YPLAFF-
          YNPLAF--
          YMPLAF--
          YDPLAF--
          -YPLAFN-
          -YPLAFK-
          -YPLAFH-
          YEPLAF--
LANM       186       723      31.41
          --LANMNL
          --LANMGT
          --LANMFR
          -SLANMH-
          -ALANML-
          -ALANMR-
          ARLANM--
          -ALANMT-
          ALLANM--
          -ALANMV-
          AYLANM--
          -ALANMW-
          -ALANMK-
          AALANM--
          AMLANM--
          -ALANMM-
          -ALANMF-
          -ALANMA-
          AGLANM--
          AWLANM--
          ASLANM--
          ANLANM--
          -ALANMS-
          AHLANM--
          -ALANMY-
          -ALANMN-
          -ALANME-
```

AILANM--
AFLANM--
AVLANM--
ATLANM--
-ALANMI-
-ALANMH-
-ALANMG-
ADLANM--
AQLANM--
APLANM--
-ALANMQ-
AKLANM--
-CLANMR-
CSLANM--
-CLANMA-
CCLANM--
CTLANM--
CRLANM--
-CLANMY-
-CLANMS-
CALANM--
CVLANM--
CPLANM--
-CLANMW-
-CLANML-
-CLANMG-
-CLANMF-
-CLANME-
CHLANM--
-DLANMT-
DSLANM--
DLLANM--
-DLANMY-
-DLANML-
DVLANM--
DPLANM--
DFLANM--
-DLANMV-
DHLANM--
-DLANMS-
-DLANMF-
-DLANMI-
DCLANM--
DALANM--
DYLANM--
DWLANM--
DNLANM--
-DLANMR-
-DLANMQ-
-DLANMN-

-DLANMM-
-DLANMH-
-DLANMA-
DKLANM--
DILANM--
DGLANM--
DELANM--
ELLANM--
ESLANM--
-ELANML-
-ELANMR-
-ELANMK-
EVLANM--
EPLANM--
-ELANMV-
EKLANM--
-ELANMT-
-ELANMS-
-ELANMI-
EQLANM--
EALANM--
EYLANM--
EWLANM--
ENLANM--
EMLANM--
-ELANMQ-
-ELANMN-
-ELANMH-
-ELANME-
EHLANM--
ECLANM--
-FLANMS-
FRLANM--
-FLANML-
-FLANMR-
FALANM--
FSLANM--
FNLANM--
-FLANMY-
FGLANM--
FQLANM--
-FLANMT-
FHLANM--
-FLANMV-
-FLANMQ-
FWLANM--
-FLANMN-
-FLANME-
-FLANMM-
FELANM--

FTLANM--
FPLANM--
FDLANM--
FMLANM--
-FLANMK-
GRLANM--
GWLANM--
GSLANM--
-GLANMR-
GLLANM--
-GLANMA-
-GLANMY-
-GLANMV-
GYLANM--
-GLANMS-
-GLANMM-
GILANM--
GVLANM--
GTLANM--
GMLANM--
-GLANMQ-
GNLANM--
-GLANMW-
-GLANMH-
GELANM--
GPLANM--
-GLANMN-
-GLANMG-
GFLANM--
GDLANM--
-GLANME-
-GLANMD-
GCLANM--
HLLANM--
-HLANMI-
-HLANMR-
-HLANMS-
-HLANMY-
HRLANM--
HFLANM--
HKLANM--
HALANM--
HGLANM--
HMLANM--
HTLANM--
HQLANM--
-HLANMT-
-HLANMH-
HYLANM--
HVLANM--

HPLANM--
-HLANME-
HDLANM--
HWLANM--
-HLANMQ-
-HLANMN-
-HLANMM-
-HLANMK-
-HLANMA-
HHLANM--
-ILANMT-
IRLANM--
IKLANM--
ISLANM--
IQLANM--
IPLANM--
IMLANM--
-ILANMV-
-ILANMM-
-ILANMG-
-ILANMQ-
IGLANM--
IWLANM--
ITLANM--
-ILANMN-
-ILANMH-
IDLANM--
-ILANMK-
-ILANMA-
IHLANM--
-ILANMY-
-ILANMW-
KLLANM--
KGLANM--
-KLANMT-
-KLANMS-
-KLANMA-
KPLANM--
-KLANMY-
-KLANML-
-KLANMI-
KTLANM--
-KLANMR-
-KLANMM-
-KLANMK-
KHLANM--
KWLANM--
KQLANM--
KNLANM--
KILANM--

-KLANMV-
-KLANME-
KALANM--
KYLANM--
-KLANMW-
-KLANMQ-
-KLANMH-
KVLANM--
KMLANM--
-KLANMN-
--LANMFT
--LANMVI
--LANMRV
--LANMRL
--LANMLS
-LLANMR-
--LANMRT
--LANMLQ
--LANMTF
--LANMRN
--LANMVR
--LANMRR
--LANMIR
LPLANM--
-LLANMV-
--LANMYK
--LANMRK
--LANMRG
--LANMST
LSLANM--
LNLANM--
LGLANM--
--LANMSW
LHLANM--
--LANMMS
--LANMLK
--LANMKW
--LANMKA
-LLANMT-
--LANMYR
--LANMRF
--LANMMR
--LANMMI
--LANMKS
-LLANMN-
--LANMYA
--LANMVA
--LANMTH
--LANMSL
--LANMRD

--LANMNS
--LANMLN
--LANMIS
--LANMAS
-LLANMI-
--LANMWS
--LANMWG
--LANMVD
--LANMTM
--LANMRH
--LANMML
--LANMLD
--LANMIA
--LANMHM
--LANMFQ
--LANMEG
--LANMAA
-LLANMK-
--LANMWR
--LANMVE
--LANMTT
--LANMTL
--LANMSM
--LANMSG
--LANMRQ
--LANMQS
--LANMMQ
--LANMMM
--LANMLI
--LANMLH
--LANMKN
--LANMFG
--LANMFD
--LANMFA
--LANMES
--LANMEA
--LANMAL
LALANM--
-LLANMQ-
-LLANMA-
--LANMYQ
--LANMVW
--LANMVS
--LANMVQ
--LANMVM
--LANMTG
--LANMSQ
--LANMRY
--LANMQV
--LANMQG

--LANMMH
--LANMLG
--LANMIT
--LANMIK
--LANMHT
--LANMGS
LYLANM--
-LLANMS-
LKLANM--
--LANMVN
--LANMTY
--LANMTK
--LANMSY
--LANMSK
--LANMSH
--LANMSA
--LANMRE
--LANMQT
--LANMNT
--LANMMK
--LANMKY
--LANMKH
--LANMGG
-LLANMH-
LDLANM--
--LANMYT
--LANMYI
--LANMYE
--LANMWQ
--LANMWK
--LANMWD
--LANMWA
--LANMVV
--LANMVT
--LANMTN
--LANMSI
--LANMQR
--LANMQH
--LANMQF
--LANMQA
--LANMMG
--LANMMD
--LANMMA
--LANMKG
--LANMII
--LANMID
--LANMFH
--LANMET
LWLANM--
-LLANMM-

-LLANME-
LELANM--
--LANMYH
--LANMWM
--LANMVY
--LANMVF
--LANMTE
--LANMSV
--LANMSE
--LANMQK
--LANMQI
--LANMNA
--LANMMT
--LANMME
--LANMKQ
--LANMKF
--LANMIY
--LANMIV
--LANMIG
--LANMHY
--LANMHW
--LANMHL
--LANMHF
--LANMGV
--LANMFN
--LANMEW
--LANMEV
--LANMAG
LQLANM--
-LLANMD-
LCLANM--
--LANMYN
--LANMYM
--LANMYG
--LANMYD
--LANMWV
--LANMWT
--LANMWH
--LANMTW
--LANMTQ
--LANMTD
--LANMTA
--LANMSD
--LANMRI
--LANMQY
--LANMQN
--LANMQL
--LANMNW
--LANMNV
--LANMNG

--LANMMY
--LANMLM
--LANMKV
--LANMKK
--LANMKD
--LANMIQ
--LANMIH
--LANMIF
--LANMHR
--LANMHI
--LANMHH
--LANMGY
--LANMGA
--LANMFK
--LANMEY
--LANMEF
--LANMAY
--LANMAW
--LANMAV
--LANMAN
--LANMAM
-MLANMR-
-MLANML-
MSLANM--
MRLANM--
-MLANMS-
-MLANMK-
-MLANMY-
-MLANMV-
MTLANM--
MGLANM--
MWLANM--
MALANM--
MQLANM--
MMLANM--
-MLANMN-
MYLANM--
MPLANM--
-MLANMW-
-MLANMT-
MKLANM--
MNLANM--
-MLANMQ-
MELANM--
NLLANM--
NVLANM--
NTLANM--
NKLANM--
-NLANMY-
-NLANMS-

-NLANMQ-
NPLANM--
-NLANMH-
NYLANM--
NNLANM--
-NLANMV-
NDLANM--
NMLANM--
-NLANMT-
-NLANMR-
-NLANMK-
-NLANMG-
-NLANMF-
NILANM--
NHLANM--
NGLANM--
NSLANM--
NQLANM--
-NLANME-
NFLANM--
NALANM--
-NLANMN-
-NLANMM-
NELANM--
NCLANM--
PTLANM--
PMLANM--
PRLANM--
-PLANML-
-PLANMG-
PNLANM--
-PLANMK-
PSLANM--
-PLANMR-
PDLANM--
-PLANMS-
-PLANMW-
-PLANMV-
PCLANM--
PALANM--
PLLANM--
-PLANMT-
PKLANM--
PVLANM--
-PLANMY-
-PLANMA-
PILANM--
PGLANM--
-PLANMQ-
-PLANMN-

```
-PLANMI-
-PLANMH-
PELANM--
-PLANME-
PHLANM--
QKLANM--
-QLANML-
QPLANM--
QMLANM--
QYLANM--
QSLANM--
-QLANMT-
-QLANMF-
-QLANMR-
-QLANMY-
-QLANMS-
QGLANM--
QLLANM--
QNLANM--
QRLANM--
-QLANMA-
QQLANM--
-QLANMK-
-QLANMI-
QILANM--
QDLANM--
QALANM--
QWLANM--
QTLANM--
-QLANMG-
-QLANME-
QHLANM--
QFLANM--
RGLANM--
-RLANMV-
-RLANME-
RTLANM--
-RLANMS-
RHLANM--
RILANM--
RVLANM--
RALANM--
RNLANM--
-RLANMT-
-RLANMK-
RMLANM--
-RLANMH-
RPLANM--
-RLANMW-
RWLANM--
```

```
-RLANMG-
RDLANM--
RFLANM--
RCLANM--
RQLANM--
RELANM--
-SLANMR-
SWLANM--
SVLANM--
SLLANM--
-SLANMS-
-SLANML-
SYLANM--
SQLANM--
-SLANMY-
SRLANM--
-SLANMK-
STLANM--
SMLANM--
-SLANMW-
-SLANMV-
-SLANMM-
SILANM--
SGLANM--
SPLANM--
SKLANM--
SALANM--
SSLANM--
-SLANMN-
-SLANMT-
SELANM--
SCLANM--
SNLANM--
-SLANMQ-
-SLANME-
-SLANMA-
SDLANM--
-SLANMC-
TRLANM--
TNLANM--
TGLANM--
TALANM--
-TLANMQ-
-TLANMS-
-TLANMR-
TLLANM--
THLANM--
-TLANMY-
-TLANMK-
-TLANMF-
```

TFLANM--
-TLANMW-
TILANM--
TVLANM--
TTLANM--
TPLANM--
-TLANMV-
-TLANMT-
-TLANMI-
-TLANME-
-TLANMA-
TKLANM--
-TLANMM-
-TLANMH-
TDLANM--
TYLANM--
TWLANM--
TQLANM--
-TLANMN-
-VLANMV-
VNLANM--
-VLANML-
VRLANM--
-VLANMT-
-VLANMH-
-VLANMR-
VSLANM--
VMLANM--
VQLANM--
VLLANM--
-VLANMS-
-VLANMK-
VGLANM--
VCLANM--
-VLANMW-
-VLANMG-
VDLANM--
VKLANM--
VYLANM--
VHLANM--
-VLANMY-
-VLANMN-
VILANM--
VELANM--
VALANM--
VPLANM--
-VLANMQ-
-VLANMM-
-WLANMR-
WDLANM--

| | | | |
|---|---|---|---|
| | WRLANM-- | | |
| | WSLANM-- | | |
| | WQLANM-- | | |
| | WNLANM-- | | |
| | -WLANMT- | | |
| | -WLANMQ- | | |
| | -WLANMN- | | |
| | WELANM-- | | |
| | WTLANM-- | | |
| | -WLANMS- | | |
| | WKLANM-- | | |
| | WGLANM-- | | |
| | WYLANM-- | | |
| | -WLANMK- | | |
| | -WLANMF- | | |
| | WHLANM-- | | |
| | -WLANMV- | | |
| | WPLANM-- | | |
| | -WLANMW- | | |
| | -WLANME- | | |
| | -WLANMA- | | |
| | WALANM-- | | |
| | -YLANMR- | | |
| | YRLANM-- | | |
| | YLLANM-- | | |
| | -YLANMT- | | |
| | -YLANMH- | | |
| | -YLANMS- | | |
| | -YLANMK- | | |
| | -YLANMG- | | |
| | -YLANMV- | | |
| | -YLANMN- | | |
| | -YLANMI- | | |
| | YHLANM-- | | |
| | YALANM-- | | |
| | -YLANMM- | | |
| | -YLANMA- | | |
| | YKLANM-- | | |
| | YYLANM-- | | |
| | YWLANM-- | | |
| | YQLANM-- | | |
| | YPLANM-- | | |
| | YNLANM-- | | |
| | YMLANM-- | | |
| | -YLANMW- | | |
| | -YLANMD- | | |
| PVGF | 226 | 698 | 31.40 |
| | -HPVGFG- | | |
| | HLPVGF-- | | |
| | --PVGFKL | | |

ARPVGF--
-APVGFS-
ASPVGF--
AWPVGF--
AVPVGF--
-APVGFV-
AMPVGF--
ALPVGF--
ATPVGF--
-APVGFA-
-APVGFT-
-APVGFG-
-APVGFE-
AIPVGF--
-APVGFM-
AKPVGF--
AGPVGF--
AYPVGF--
AQPVGF--
-APVGFN-
-APVGFL-
ANPVGF--
-APVGFQ-
-APVGFH-
AHPVGF--
AAPVGF--
-APVGFK-
-APVGFC-
ACPVGF--
CSPVGF--
CGPVGF--
CPPVGF--
-CPVGFH-
-CPVGFR-
-CPVGFL-
CYPVGF--
CTPVGF--
CRPVGF--
DIPVGF--
-DPVGFS-
-DPVGFL-
DLPVGF--
DWPVGF--
DVPVGF--
DRPVGF--
-DPVGFR-
DKPVGF--
DSPVGF--
-DPVGFT-
-DPVGFK-

```
-DPVGFA-
DMPVGF--
-DPVGFQ-
-DPVGFM-
-DPVGFY-
-DPVGFN-
-DPVGFF-
DNPVGF--
DGPVGF--
DFPVGF--
DEPVGF--
DDPVGF--
ESPVGF--
ERPVGF--
-EPVGFH-
ELPVGF--
EEPVGF--
-EPVGFS-
-EPVGFY-
-EPVGFR-
-EPVGFV-
EKPVGF--
EIPVGF--
EVPVGF--
-EPVGFI-
-EPVGFT-
-EPVGFA-
EDPVGF--
EAPVGF--
EWPVGF--
EQPVGF--
-EPVGFW-
-EPVGFN-
-EPVGFG-
ENPVGF--
EMPVGF--
EHPVGF--
EGPVGF--
FHPVGF--
-FPVGFL-
FYPVGF--
FVPVGF--
FQPVGF--
FMPVGF--
FGPVGF--
FSPVGF--
-FPVGFS-
-FPVGFH-
FRPVGF--
FAPVGF--
```

-FPVGFT-
-FPVGFR-
FTPVGF--
-FPVGFV-
-FPVGFQ-
-FPVGFA-
-FPVGFN-
-FPVGFK-
FNPVGF--
-FPVGFI-
FKPVGF--
FPPVGF--
FEPVGF--
FDPVGF--
FWPVGF--
FLPVGF--
GTPVGF--
GYPVGF--
-GPVGFR-
-GPVGFL-
GRPVGF--
GQPVGF--
-GPVGFN-
-GPVGFS-
GSPVGF--
-GPVGFI-
GLPVGF--
GDPVGF--
-GPVGFV-
-GPVGFT-
-GPVGFA-
GVPVGF--
-GPVGFM-
GGPVGF--
GFPVGF--
GWPVGF--
-GPVGFQ-
-GPVGFK-
GAPVGF--
-GPVGFG-
GIPVGF--
GHPVGF--
GPPVGF--
GNPVGF--
GMPVGF--
GEPVGF--
-HPVGFS-
-HPVGFR-
HVPVGF--
HPPVGF--

-HPVGFN-
-HPVGFL-
-HPVGFQ-
-HPVGFK-
-HPVGFH-
HNPVGF--
-HPVGFA-
HCPVGF--
HYPVGF--
HRPVGF--
-HPVGFY-
-HPVGFT-
-HPVGFI-
HHPVGF--
HDPVGF--
HKPVGF--
HGPVGF--
HAPVGF--
HTPVGF--
HSPVGF--
-HPVGFM-
-HPVGFC-
-IPVGFT-
ILPVGF--
IYPVGF--
-IPVGFV-
INPVGF--
ISPVGF--
-IPVGFN-
IAPVGF--
ITPVGF--
IQPVGF--
-IPVGFS-
-IPVGFA-
IVPVGF--
IRPVGF--
-IPVGFM-
-IPVGFK-
-IPVGFI-
-IPVGFR-
-IPVGFH-
IKPVGF--
IDPVGF--
ICPVGF--
IPPVGF--
IMPVGF--
IHPVGF--
IEPVGF--
-IPVGFG-
-IPVGFE-

-IPVGFC-
KSPVGF--
-KPVGFS-
-KPVGFY-
-KPVGFR-
KLPVGF--
-KPVGFL-
KGPVGF--
-KPVGFV-
KYPVGF--
KRPVGF--
-KPVGFN-
-KPVGFF-
KVPVGF--
KTPVGF--
-KPVGFK-
KEPVGF--
-KPVGFM-
-KPVGFG-
KFPVGF--
KAPVGF--
KKPVGF--
KIPVGF--
KHPVGF--
-KPVGFW-
-KPVGFA-
KCPVGF--
-KPVGFQ-
-KPVGFE-
KDPVGF--
-LPVGFL-
-LPVGFT-
LQPVGF--
-LPVGFS-
-LPVGFV-
LYPVGF--
-LPVGFK-
LPPVGF--
LWPVGF--
LSPVGF--
-LPVGFQ-
-LPVGFN-
-LPVGFH-
LVPVGF--
LGPVGF--
LNPVGF--
-LPVGFM-
-LPVGFI-
LHPVGF--
LFPVGF--

LCPVGF--
-LPVGFC-
LDPVGF--
-MPVGFR-
-MPVGFS-
MGPVGF--
-MPVGFK-
MSPVGF--
MAPVGF--
MYPVGF--
-MPVGFV-
-MPVGFA-
MKPVGF--
MTPVGF--
MQPVGF--
-MPVGFI-
-MPVGFH-
MLPVGF--
MWPVGF--
-MPVGFN-
MIPVGF--
MHPVGF--
MFPVGF--
-MPVGFQ-
-MPVGFM-
-MPVGFG-
NRPVGF--
-NPVGFK-
-NPVGFS-
NTPVGF--
NNPVGF--
NAPVGF--
NFPVGF--
-NPVGFR-
-NPVGFY-
-NPVGFV-
-NPVGFM-
NMPVGF--
NHPVGF--
NSPVGF--
-NPVGFI-
-NPVGFF-
NGPVGF--
NEPVGF--
-NPVGFT-
-NPVGFN-
NLPVGF--
NWPVGF--
-NPVGFL-
-NPVGFH-

NPPVGF--
NYPVGF--
-NPVGFW-
-NPVGFQ-
-NPVGFA-
NIPVGF--
NDPVGF--
NCPVGF--
--PVGFRT
--PVGFRS
--PVGFVG
--PVGFLT
--PVGFKR
--PVGFLN
--PVGFVR
--PVGFSR
--PVGFMR
--PVGFVS
--PVGFRY
--PVGFRR
--PVGFRL
--PVGFMA
--PVGFSV
--PVGFRW
--PVGFLG
--PVGFIR
--PVGFSF
--PVGFGS
--PVGFVN
--PVGFTW
--PVGFTT
--PVGFLA
--PVGFHT
PVPVGF--
--PVGFSI
--PVGFSG
--PVGFLH
--PVGFKA
--PVGFRF
--PVGFIN
--PVGFLQ
--PVGFVA
--PVGFTR
--PVGFSM
--PVGFHM
--PVGFAS
PKPVGF--
--PVGFRK
--PVGFRD
--PVGFQA

--PVGFNV
--PVGFNS
--PVGFMM
--PVGFIQ
--PVGFHR
PSPVGF--
PWPVGF--
--PVGFTN
--PVGFSL
--PVGFSA
--PVGFRV
--PVGFRM
--PVGFKG
--PVGFIK
--PVGFIH
--PVGFIA
--PVGFAG
--PVGFVW
--PVGFTA
--PVGFQM
--PVGFMT
--PVGFMS
--PVGFIS
--PVGFHH
--PVGFFG
--PVGFYD
--PVGFWG
--PVGFVK
--PVGFTK
--PVGFTI
--PVGFSY
--PVGFSH
--PVGFRE
--PVGFNT
--PVGFHY
--PVGFHQ
--PVGFFN
PLPVGF--
PGPVGF--
PFPVGF--
--PVGFYW
--PVGFYG
--PVGFTG
--PVGFRI
--PVGFRH
--PVGFQW
--PVGFQT
--PVGFQH
--PVGFNA
--PVGFMQ

--PVGFMH
--PVGFKS
--PVGFIW
--PVGFES
--PVGFEG
--PVGFAW
--PVGFAT
PTPVGF--
PYPVGF--
--PVGFTH
--PVGFSQ
--PVGFRA
--PVGFQS
--PVGFNN
--PVGFKM
--PVGFHN
--PVGFHI
--PVGFFH
--PVGFWN
--PVGFWI
--PVGFWD
--PVGFTY
--PVGFTV
--PVGFTE
--PVGFSE
--PVGFRQ
--PVGFNY
--PVGFML
--PVGFMG
--PVGFKY
--PVGFKF
--PVGFHS
--PVGFHG
--PVGFCS
--PVGFAV
--PVGFAI
PDPVGF--
--PVGFVM
--PVGFVI
--PVGFTF
--PVGFTD
--PVGFSN
--PVGFQV
--PVGFQQ
--PVGFQG
--PVGFQF
--PVGFNK
--PVGFNH
--PVGFMW
--PVGFMD

--PVGFLE
--PVGFKW
--PVGFKT
--PVGFKQ
--PVGFKI
--PVGFHA
--PVGFGL
--PVGFGG
--PVGFGA
--PVGFFD
--PVGFFA
--PVGFER
--PVGFCT
--PVGFAY
--PVGFAR
--PVGFAQ
--PVGFVQ
--PVGFVH
--PVGFVD
--PVGFSK
--PVGFQY
--PVGFQN
--PVGFQL
--PVGFQK
--PVGFQI
--PVGFPQ
--PVGFPM
--PVGFNM
--PVGFLD
--PVGFKH
--PVGFKD
--PVGFIT
--PVGFHK
--PVGFHF
--PVGFGR
--PVGFGK
--PVGFFI
--PVGFFE
--PVGFDG
--PVGFCH
--PVGFCD
-PPVGFS-
-PPVGFR-
-PPVGFN-
-PPVGFM-
-PPVGFF-
-PPVGFA-
PPPVGF--
PNPVGF--
PHPVGF--

PEPVGF--
PCPVGF--
PAPVGF--
-QPVGFL-
QRPVGF--
-QPVGFR-
QTPVGF--
-QPVGFT-
-QPVGFK-
-QPVGFN-
-QPVGFS-
QIPVGF--
QPPVGF--
QMPVGF--
QFPVGF--
QYPVGF--
-QPVGFQ-
-QPVGFE-
QKPVGF--
QWPVGF--
-QPVGFF-
QLPVGF--
QGPVGF--
QSPVGF--
-QPVGFM-
QCPVGF--
-RPVGFL-
-RPVGFS-
RGPVGF--
-RPVGFT-
-RPVGFY-
RTPVGF--
-RPVGFH-
-RPVGFA-
RAPVGF--
-RPVGFK-
RPPVGF--
-RPVGFV-
-RPVGFI-
RLPVGF--
-RPVGFQ-
-RPVGFN-
RNPVGF--
RHPVGF--
RYPVGF--
RWPVGF--
-RPVGFW-
RDPVGF--
RIPVGF--
RQPVGF--

RFPVGF--
-RPVGFE-
RMPVGF--
RKPVGF--
RCPVGF--
-SPVGFR-
SSPVGF--
SNPVGF--
SRPVGF--
-SPVGFT-
-SPVGFM-
SLPVGF--
-SPVGFS-
-SPVGFA-
SHPVGF--
-SPVGFV-
-SPVGFQ-
-SPVGFN-
SMPVGF--
SGPVGF--
SYPVGF--
-SPVGFK-
SIPVGF--
SDPVGF--
SAPVGF--
-SPVGFE-
SKPVGF--
SFPVGF--
-SPVGFH-
SEPVGF--
-SPVGFP-
-SPVGFG-
-TPVGFR-
TVPVGF--
-TPVGFL-
TLPVGF--
TRPVGF--
-TPVGFI-
-TPVGFN-
THPVGF--
TTPVGF--
-TPVGFS-
TIPVGF--
TAPVGF--
-TPVGFM-
TMPVGF--
TQPVGF--
TCPVGF--
-TPVGFH-
TGPVGF--

TEPVGF--
-TPVGFT-
TNPVGF--
TYPVGF--
TSPVGF--
-TPVGFY-
-TPVGFK-
-TPVGFA-
-TPVGFQ-
TFPVGF--
TDPVGF--
TWPVGF--
-TPVGFE-
TKPVGF--
VRPVGF--
-VPVGFL-
VSPVGF--
VYPVGF--
VVPVGF--
-VPVGFS-
VAPVGF--
VLPVGF--
VWPVGF--
-VPVGFK-
-VPVGFH-
VNPVGF--
-VPVGFQ-
-VPVGFM-
VKPVGF--
VIPVGF--
VQPVGF--
-VPVGFV-
-VPVGFR-
VGPVGF--
-VPVGFG-
VEPVGF--
VPPVGF--
VTPVGF--
-VPVGFN-
-VPVGFE-
VHPVGF--
VFPVGF--
WSPVGF--
WIPVGF--
-WPVGFT-
-WPVGFR-
WWPVGF--
-WPVGFS-
-WPVGFL-
-WPVGFK-

WGPVGF--
WYPVGF--
-WPVGFQ-
-WPVGFN-
WDPVGF--
WTPVGF--
-WPVGFH-
WPPVGF--
-WPVGFI-
WMPVGF--
WFPVGF--
WVPVGF--
-WPVGFV-
-WPVGFG-
-WPVGFA-
WNPVGF--
WAPVGF--
YDPVGF--
YRPVGF--
-YPVGFS-
-YPVGFT-
YAPVGF--
-YPVGFQ-
-YPVGFK-
YIPVGF--
-YPVGFL-
YLPVGF--
YYPVGF--
-YPVGFI-
YEPVGF--
YWPVGF--
YSPVGF--
-YPVGFW-
-YPVGFM-
-YPVGFF-
YPPVGF--
YGPVGF--
-YPVGFH-
YNPVGF--
YMPVGF--
YFPVGF--
YVPVGF--
YQPVGF--
-YPVGFA-
YHPVGF--
AAAL 102 769 31.40
--AAALTL
--AAALSR
--AAALQR
--AAALVQ

--AAALTD
--AAALLD
--AAALDR
--AAALAP
GEAAAL--
-GAAALA-
-KAAALP-
-RAAALP-
-SAAALP-
--AAALRN
--AAALYR
--AAALTH
--AAALRA
--AAALSV
--AAALLR
--AAALRK
--AAALHL
--AAALSL
--AAALIR
ASAAAL--
--AAALRY
--AAALWW
--AAALRE
--AAALRD
--AAALLK
--AAALIS
--AAALTS
--AAALSG
--AAALPM
--AAALNL
--AAALLQ
--AAALGY
--AAALVT
--AAALPI
-AAAALT-
-AAAALS-
ALAAAL--
--AAALLW
-AAAALF-
ANAAAL--
--AAALYS
--AAALVF
--AAALSA
--AAALRS
--AAALLV
AMAAAL--
--AAALTG
--AAALSF
--AAALRM
--AAALQS

--AAALPL
--AAALPF
--AAALMS
--AAALMA
--AAALGI
--AAALFS
AVAAAL--
--AAALWS
--AAALVN
--AAALSY
--AAALSM
--AAALRT
--AAALNS
--AAALLI
--AAALKV
--AAALKS
--AAALIG
--AAALAS
--AAALAG
-AAAALW-
ARAAAL--
--AAALYT
--AAALYG
--AAALVV
--AAALTY
--AAALTF
--AAALQW
--AAALKT
--AAALHA
--AAALFL
--AAALAY
-AAAALA-
ATAAAL--
AKAAAL--
--AAALYY
--AAALYQ
--AAALWN
--AAALVR
--AAALRW
--AAALRI
--AAALNA
--AAALMK
--AAALLA
--AAALKL
--AAALHK
--AAALYM
--AAALYK
--AAALYH
--AAALVW
--AAALTV

--AAALLN
--AAALKN
--AAALKA
--AAALHQ
--AAALFT
--AAALAM
--AAALAI
--AAALYN
--AAALMG
--AAALIK
-AAAALL-
AGAAAL--
--AAALYV
--AAALYA
--AAALVM
--AAALQM
--AAALQA
--AAALMV
--AAALLT
--AAALLM
--AAALIV
--AAALHT
--AAALFA
--AAALAF
-AAAALK-
-AAAALH-
AQAAAL--
AIAAAL--
AFAAAL--
--AAALVY
--AAALVH
--AAALVA
--AAALTT
--AAALTR
--AAALSK
--AAALMR
--AAALKR
--AAALIN
--AAALIF
--AAALHS
--AAALFG
--AAALAT
-AAAALY-
-AAAALN-
-AAAALI-
APAAAL--
--AAALWV
--AAALWT
--AAALWF
--AAALVI

--AAALPY
--AAALMI
--AAALKY
--AAALKG
--AAALIM
--AAALHH
--AAALFN
--AAALEA
AEAAAL--
--AAALWY
--AAALWR
--AAALWA
--AAALSQ
--AAALSI
--AAALRQ
--AAALNY
--AAALMQ
--AAALLH
--AAALKF
--AAALHW
--AAALHF
--AAALGS
--AAALGM
--AAALFW
--AAALFM
-AAAALQ-
AYAAAL--
--AAALYE
--AAALWM
--AAALWL
--AAALTI
--AAALSW
--AAALSN
--AAALSH
--AAALQG
--AAALNI
--AAALMY
--AAALMM
--AAALMH
--AAALLG
--AAALKK
--AAALIA
--AAALHV
--AAALHM
--AAALHI
--AAALFK
--AAALFD
--AAALEG
--AAALAW
--AAALAH

ADAAAL--
--AAALYW
--AAALYD
--AAALWG
--AAALWD
--AAALVE
--AAALVD
--AAALTN
--AAALTM
--AAALNT
--AAALNG
--AAALMW
--AAALMT
--AAALML
--AAALKW
--AAALKM
--AAALIH
--AAALID
--AAALHN
--AAALHD
--AAALGG
--AAALCG
--AAALAA
AWAAAL--
AHAAAL--
--AAALWI
--AAALQK
--AAALQI
--AAALNW
--AAALNN
--AAALNK
--AAALMN
--AAALMD
--AAALKQ
--AAALKE
--AAALIW
--AAALIE
--AAALHY
--AAALHE
--AAALGW
--AAALEW
--AAALCF
-AAAALM-
-CAAALL-
CKAAAL--
CFAAAL--
CIAAAL--
CCAAAL--
CVAAAL--
CTAAAL--

CSAAAL--
-CAAALY-
-CAAALK-
-CAAALH-
CRAAAL--
CMAAAL--
CLAAAL--
CHAAAL--
CGAAAL--
-CAAALT-
-CAAALS-
-CAAALR-
DRAAAL--
DNAAAL--
DLAAAL--
-DAAALI-
DWAAAL--
DVAAAL--
-DAAALH-
DTAAAL--
DKAAAL--
DHAAAL--
DSAAAL--
DMAAAL--
-DAAALM-
-DAAALL-
-DAAALF-
DQAAAL--
DPAAAL--
DIAAAL--
DEAAAL--
-DAAALW-
-DAAALS-
-DAAALR-
DGAAAL--
DFAAAL--
-DAAALN-
ERAAAL--
-EAAALL-
EFAAAL--
-EAAALW-
EWAAAL--
EPAAAL--
EGAAAL--
ESAAAL--
EMAAAL--
-EAAALV-
-EAAALT-
EVAAAL--
-EAAALY-

-EAAALQ-
-EAAALF-
ENAAAL--
EKAAAL--
EHAAAL--
ECAAAL--
-EAAALH-
ETAAAL--
EEAAAL--
-EAAALS-
-EAAALN-
-EAAALI-
-EAAALR-
-EAAALM-
-EAAALA-
-FAAALR-
FLAAAL--
-FAAALT-
FTAAAL--
FMAAAL--
-FAAALS-
-FAAALH-
FQAAAL--
-FAAALV-
-FAAALA-
FSAAAL--
FAAAAL--
-FAAALI-
FNAAAL--
-FAAALL-
-FAAALK-
FEAAAL--
-FAAALQ-
FIAAAL--
FGAAAL--
FFAAAL--
FCAAAL--
-FAAALM-
FYAAAL--
FWAAAL--
FKAAAL--
FPAAAL--
-FAAALN-
-FAAALF-
FVAAAL--
GRAAAL--
GSAAAL--
GNAAAL--
-GAAALW-
-GAAALS-

-GAAALH-
-GAAALM-
GHAAAL--
-GAAALI-
GVAAAL--
GDAAAL--
-GAAALT-
GTAAAL--
GGAAAL--
GYAAAL--
GWAAAL--
-GAAALL-
GAAAAL--
GIAAAL--
-GAAALK-
GFAAAL--
-GAAALY-
GPAAAL--
GCAAAL--
-GAAALN-
-GAAALG-
-GAAALF-
GQAAAL--
-GAAALD-
-HAAALR-
-HAAALL-
HGAAAL--
-HAAALT-
HTAAAL--
HYAAAL--
HIAAAL--
HQAAAL--
-HAAALI-
-HAAALH-
-HAAALF-
-HAAALA-
HAAAAL--
-HAAALV-
-HAAALQ-
HHAAAL--
-HAAALS-
-HAAALK-
HWAAAL--
-HAAALN-
HVAAAL--
HNAAAL--
HLAAAL--
HMAAAL--
HEAAAL--
-IAAALR-

IRAAAL--
ISAAAL--
ITAAAL--
INAAAL--
-IAAALN-
-IAAALL-
-IAAALK-
IAAAAL--
IEAAAL--
IMAAAL--
IKAAAL--
-IAAALS-
-IAAALG-
IPAAAL--
-IAAALH-
IHAAAL--
-IAAALY-
-IAAALV-
-IAAALQ-
IGAAAL--
IDAAAL--
IVAAAL--
IYAAAL--
IFAAAL--
-IAAALT-
-IAAALI-
-IAAALF-
IQAAAL--
ICAAAL--
-IAAALW-
-KAAALR-
KTAAAL--
KQAAAL--
KSAAAL--
KVAAAL--
KNAAAL--
KEAAAL--
-KAAALW-
KLAAAL--
-KAAALN-
KMAAAL--
-KAAALI-
KPAAAL--
KGAAAL--
-KAAALT-
KAAAAL--
-KAAALQ-
-KAAALF-
KHAAAL--
KIAAAL--

KDAAAL--
-KAAALV-
KFAAAL--
KCAAAL--
-KAAALM-
-KAAALC-
-KAAALA-
LSAAAL--
-LAAALL-
-LAAALH-
-LAAALS-
LTAAAL--
LAAAAL--
LLAAAL--
-LAAALA-
-LAAALY-
-LAAALW-
LPAAAL--
LGAAAL--
-LAAALV-
LQAAAL--
LFAAAL--
-LAAALQ-
LNAAAL--
LHAAAL--
-LAAALI-
-LAAALF-
LEAAAL--
-LAAALT-
-LAAALM-
-LAAALK-
-LAAALG-
LDAAAL--
LMAAAL--
MVAAAL--
-MAAALS-
-MAAALT-
MYAAAL--
MKAAAL--
MGAAAL--
-MAAALL-
MPAAAL--
-MAAALR-
MTAAAL--
MWAAAL--
-MAAALF-
-MAAALK-
-MAAALH-
MDAAAL--
-MAAALA-

MQAAAL--
-MAAALY-
MAAAAL--
MIAAAL--
MEAAAL--
-MAAALQ-
MNAAAL--
MHAAAL--
-MAAALW-
MFAAAL--
MCAAAL--
-MAAALN-
-MAAALM-
MMAAAL--
-MAAALI-
NTAAAL--
-NAAALR-
NSAAAL--
NLAAAL--
NGAAAL--
-NAAALI-
NVAAAL--
-NAAALW-
-NAAALK-
-NAAALQ-
NIAAAL--
NEAAAL--
-NAAALH-
NAAAAL--
NWAAAL--
-NAAALS-
-NAAALL-
NKAAAL--
NHAAAL--
NFAAAL--
-NAAALY-
NNAAAL--
-NAAALT-
-NAAALN-
NPAAAL--
-NAAALM-
-NAAALA-
NMAAAL--
-NAAALF-
-NAAALC-
-PAAALV-
-PAAALL-
-PAAALK-
PHAAAL--
-PAAALN-

PAAAAL--
PQAAAL--
-PAAALS-
-PAAALR-
-PAAALG-
PSAAAL--
-PAAALT-
-PAAALW-
PTAAAL--
PLAAAL--
PKAAAL--
PGAAAL--
-PAAALI-
PVAAAL--
-PAAALM-
-PAAALF-
PNAAAL--
PDAAAL--
PCAAAL--
PYAAAL--
PWAAAL--
PMAAAL--
-PAAALY-
-PAAALH-
-PAAALA-
PEAAAL--
-PAAALE-
-QAAALF-
-QAAALR-
-QAAALY-
-QAAALS-
-QAAALN-
QRAAAL--
QTAAAL--
QPAAAL--
-QAAALT-
-QAAALH-
QMAAAL--
QDAAAL--
-QAAALV-
-QAAALI-
QNAAAL--
QKAAAL--
-QAAALK-
-QAAALA-
QAAAAL--
QWAAAL--
QSAAAL--
QYAAAL--
QQAAAL--

QFAAAL--
QEAAAL--
-QAAALQ-
-QAAALM-
QVAAAL--
QHAAAL--
RVAAAL--
RLAAAL--
-RAAALT-
-RAAALV-
-RAAALK-
RNAAAL--
RTAAAL--
RDAAAL--
RIAAAL--
-RAAALQ-
RHAAAL--
-RAAALN-
RWAAAL--
REAAAL--
RGAAAL--
RFAAAL--
-RAAALC-
STAAAL--
-SAAALR-
SLAAAL--
-SAAALL-
SIAAAL--
SHAAAL--
SDAAAL--
SPAAAL--
-SAAALF-
-SAAALY-
-SAAALT-
-SAAALS-
SVAAAL--
-SAAALW-
-SAAALV-
SGAAAL--
-SAAALQ-
-SAAALH-
SMAAAL--
-SAAALI-
SKAAAL--
-SAAALK-
-SAAALA-
SNAAAL--
SEAAAL--
SCAAAL--
-SAAALN-

TRAAAL--
-TAAALR-
TFAAAL--
TVAAAL--
-TAAALF-
TPAAAL--
-TAAALM-
-TAAALY-
-TAAALI-
TLAAAL--
-TAAALV-
TKAAAL--
THAAAL--
TAAAAL--
TTAAAL--
TIAAAL--
-TAAALQ-
TMAAAL--
-TAAALW-
TWAAAL--
TNAAAL--
TGAAAL--
-TAAALK-
-TAAALA-
TEAAAL--
TQAAAL--
TDAAAL--
-TAAALN-
-VAAALR-
-VAAALT-
VSAAAL--
-VAAALV-
-VAAALS-
VHAAAL--
-VAAALK-
VWAAAL--
VNAAAL--
-VAAALY-
-VAAALN-
VKAAAL--
-VAAALL-
VAAAAL--
VYAAAL--
VTAAAL--
-VAAALF-
VLAAAL--
VVAAAL--
VMAAAL--
VIAAAL--
-VAAALE-

VEAAAL--
-VAAALW-
-VAAALA-
VQAAAL--
VPAAAL--
-VAAALH-
VDAAAL--
VCAAAL--
-VAAALG-
-VAAALQ-
-WAAALS-
-WAAALK-
-WAAALQ-
WRAAAL--
WVAAAL--
-WAAALR-
WTAAAL--
WFAAAL--
WGAAAL--
-WAAALI-
-WAAALA-
WDAAAL--
-WAAALT-
-WAAALH-
WAAAAL--
WNAAAL--
WIAAAL--
WYAAAL--
WPAAAL--
WKAAAL--
WWAAAL--
WEAAAL--
WCAAAL--
-WAAALM-
YKAAAL--
-YAAALS-
-YAAALQ-
-YAAALR-
YYAAAL--
YNAAAL--
-YAAALK-
-YAAALA-
YPAAAL--
-YAAALL-
-YAAALF-
YTAAAL--
-YAAALH-
YSAAAL--
YMAAAL--
YEAAAL--

```
           -YAAALG-
           -YAAALN-
           YAAAAL--
           YWAAAL--
           YIAAAL--
           YGAAAL--
           -YAAALT-
           YVAAAL--
           -YAAALI-
           YQAAAL--
           -YAAALY-
           -YAAALV-
           YDAAAL--
           -YAAALM-
           -YAAALE-
PFAL        303       666     31.35
           --PFALSP
           ARPFAL--
           -APFALT-
           -APFALS-
           ALPFAL--
           -APFALI-
           -APFALK-
           -APFALL-
           AAPFAL--
           ATPFAL--
           AWPFAL--
           -APFALW-
           -APFALH-
           AMPFAL--
           AFPFAL--
           -APFALV-
           ANPFAL--
           AGPFAL--
           AYPFAL--
           AQPFAL--
           -APFALG-
           AIPFAL--
           AVPFAL--
           -APFALN-
           -APFALQ-
           -APFALE-
           AHPFAL--
           APPFAL--
           ACPFAL--
           CKPFAL--
           CTPFAL--
           CRPFAL--
           -CPFALM-
           CIPFAL--
```

CEPFAL--
CSPFAL--
-CPFALT-
-CPFALR-
-CPFALK-
CGPFAL--
CDPFAL--
DLPFAL--
DTPFAL--
DSPFAL--
-DPFALR-
DVPFAL--
DQPFAL--
DNPFAL--
DYPFAL--
DRPFAL--
-DPFALW-
-DPFALL-
DWPFAL--
DAPFAL--
-DPFALV-
-DPFALT-
-DPFALS-
DHPFAL--
-DPFALF-
DGPFAL--
-DPFALK-
-DPFALH-
DKPFAL--
DEPFAL--
ESPFAL--
EKPFAL--
EVPFAL--
-EPFALL-
-EPFALT-
ELPFAL--
-EPFALK-
EQPFAL--
-EPFALS-
-EPFALR-
-EPFALI-
EYPFAL--
ERPFAL--
ENPFAL--
EPPFAL--
-EPFALW-
-EPFALN-
EGPFAL--
ETPFAL--
-EPFALQ-

-EPFALA-
EHPFAL--
-EPFALH-
EAPFAL--
FTPFAL--
FSPFAL--
FEPFAL--
FNPFAL--
FLPFAL--
FQPFAL--
-FPFALT-
FVPFAL--
-FPFALK-
FDPFAL--
-FPFALH-
FGPFAL--
-FPFALV-
FYPFAL--
-FPFALQ-
-FPFALA-
-FPFALN-
-FPFALF-
FHPFAL--
FAPFAL--
GVPFAL--
-GPFALH-
GSPFAL--
GNPFAL--
GIPFAL--
-GPFALS-
GLPFAL--
GQPFAL--
-GPFALN-
GKPFAL--
GGPFAL--
-GPFALL-
-GPFALK-
GAPFAL--
-GPFALI-
GFPFAL--
GEPFAL--
GWPFAL--
GHPFAL--
GMPFAL--
GDPFAL--
-GPFALV-
-GPFALT-
-GPFALR-
-GPFALM-
-GPFALG-

-GPFALF-
HRPFAL--
HSPFAL--
HLPFAL--
HDPFAL--
-HPFALR-
HMPFAL--
HHPFAL--
HTPFAL--
HPPFAL--
-HPFALM-
HAPFAL--
-HPFALI-
-HPFALT-
-HPFALL-
HKPFAL--
HIPFAL--
HWPFAL--
HVPFAL--
-HPFALS-
-HPFALQ-
-HPFALK-
-HPFALH-
HNPFAL--
HFPFAL--
HEPFAL--
HCPFAL--
HYPFAL--
-HPFALY-
-HPFALV-
-HPFALA-
IRPFAL--
-IPFALI-
-IPFALS-
-IPFALK-
IDPFAL--
IHPFAL--
-IPFALT-
-IPFALL-
IMPFAL--
-IPFALR-
-IPFALN-
-IPFALA-
IVPFAL--
-IPFALH-
IKPFAL--
ITPFAL--
-IPFALQ-
IIPFAL--
IQPFAL--

-IPFALV-
-IPFALM-
IGPFAL--
-IPFALY-
-IPFALG-
INPFAL--
IFPFAL--
IWPFAL--
-IPFALE-
ILPFAL--
IEPFAL--
KSPFAL--
KVPFAL--
-KPFALY-
KAPFAL--
KPPFAL--
-KPFALS-
-KPFALR-
KRPFAL--
-KPFALL-
-KPFALH-
KIPFAL--
KGPFAL--
KYPFAL--
-KPFALV-
-KPFALK-
KKPFAL--
-KPFALW-
-KPFALT-
KLPFAL--
KQPFAL--
KWPFAL--
-KPFALQ-
KNPFAL--
KEPFAL--
KTPFAL--
-KPFALI-
-KPFALE-
KFPFAL--
-KPFALN-
KHPFAL--
-LPFALR-
LGPFAL--
LTPFAL--
LLPFAL--
LYPFAL--
LIPFAL--
-LPFALV-
LWPFAL--
-LPFALS-

LMPFAL--
LQPFAL--
-LPFALH-
LEPFAL--
LDPFAL--
LVPFAL--
LKPFAL--
-LPFALI-
LHPFAL--
LNPFAL--
MLPFAL--
MSPFAL--
MKPFAL--
MFPFAL--
-MPFALI-
MNPFAL--
MEPFAL--
-MPFALQ-
-MPFALS-
-MPFALR-
MRPFAL--
MVPFAL--
-MPFALH-
MHPFAL--
MPPFAL--
-MPFALT-
MAPFAL--
MQPFAL--
-MPFALN-
-MPFALA-
MTPFAL--
-MPFALY-
-MPFALW-
-MPFALV-
-MPFALK-
-MPFALG-
MCPFAL--
-NPFALR-
NRPFAL--
NEPFAL--
NVPFAL--
-NPFALK-
-NPFALV-
NLPFAL--
NIPFAL--
NHPFAL--
-NPFALW-
NKPFAL--
-NPFALS-
NGPFAL--

NTPFAL--
NSPFAL--
NAPFAL--
NYPFAL--
NQPFAL--
NFPFAL--
NPPFAL--
-NPFALH-
NNPFAL--
-NPFALT-
-NPFALA-
NDPFAL--
--PFALKR
--PFALRQ
--PFALLT
--PFALSG
--PFALSL
PGPFAL--
--PFALLS
--PFALHR
PKPFAL--
--PFALTS
--PFALRA
--PFALRT
--PFALRM
--PFALYS
--PFALRS
--PFALQS
--PFALMS
--PFALLG
--PFALHT
--PFALYN
--PFALTR
--PFALRR
--PFALWG
--PFALVS
--PFALRN
--PFALFD
--PFALST
--PFALAV
--PFALLN
--PFALKY
--PFALKI
--PFALKH
--PFALYK
--PFALWR
--PFALVG
--PFALSV
--PFALRY
--PFALHG

--PFALWS
--PFALVD
--PFALTV
--PFALKM
--PFALKL
--PFALGM
--PFALAS
PMPFAL--
--PFALYG
--PFALVA
--PFALSW
--PFALSN
--PFALSA
--PFALNV
--PFALMR
--PFALKS
--PFALGS
--PFALAM
--PFALVT
--PFALTH
--PFALSQ
--PFALSI
--PFALLH
--PFALKQ
--PFALKF
--PFALIT
--PFALGY
--PFALAT
--PFALYH
--PFALVK
--PFALTT
--PFALTG
--PFALSM
--PFALQY
--PFALPY
--PFALPL
--PFALKV
--PFALHN
--PFALHF
--PFALGA
--PFALAG
PNPFAL--
--PFALYQ
--PFALTQ
--PFALTD
--PFALSY
--PFALRW
--PFALPF
--PFALLK
--PFALKK

--PFALIH
--PFALHS
--PFALHL
--PFALES
PWPFAL--
PHPFAL--
--PFALWQ
--PFALVQ
--PFALVN
--PFALVI
--PFALSH
--PFALRD
--PFALQK
--PFALNT
--PFALMA
--PFALLE
--PFALKG
--PFALKA
--PFALIS
--PFALIR
--PFALIG
--PFALHM
--PFALFH
PYPFAL--
PQPFAL--
PFPFAL--
--PFALWT
--PFALWK
--PFALWD
--PFALVW
--PFALVH
--PFALVE
--PFALTM
--PFALTK
--PFALTI
--PFALSD
--PFALRE
--PFALQN
--PFALQG
--PFALPW
--PFALNY
--PFALMT
--PFALMQ
--PFALMH
--PFALME
--PFALLQ
--PFALKD
--PFALIV
--PFALIK
--PFALII

--PFALHW
--PFALHV
--PFALHK
--PFALHA
--PFALFN
--PFALFK
--PFALFF
--PFALEG
--PFALAA
-PPFALY-
PIPFAL--
--PFALYF
--PFALYE
--PFALYD
--PFALWY
--PFALWN
--PFALWA
--PFALTY
--PFALTW
--PFALTF
--PFALQV
--PFALQT
--PFALQQ
--PFALQL
--PFALQI
--PFALPM
--PFALPI
--PFALNK
--PFALNI
--PFALNA
--PFALMN
--PFALMD
--PFALLD
--PFALKT
--PFALKE
--PFALIN
--PFALHY
--PFALHI
--PFALHH
--PFALHD
--PFALGT
--PFALGQ
--PFALFQ
--PFALEY
--PFALEV
--PFALEL
--PFALEI
--PFALDR
--PFALAN
--PFALAH

PEPPFAL--
PDPPFAL--
PCPPFAL--
QTPPFAL--
QFPPFAL--
QAPPFAL--
QRPPFAL--
QSPPFAL--
-QPPFALT-
-QPPFALH-
-QPPFALA-
QWPPFAL--
-QPPFALW-
-QPPFALI-
QKPPFAL--
QIPPFAL--
-QPPFALV-
-QPPFALS-
QLPPFAL--
QQPPFAL--
-QPPFALR-
QGPPFAL--
QPPPFAL--
-QPPFALQ-
-QPPFALM-
-QPPFALL-
QVPPFAL--
-QPPFALN-
QMPPFAL--
QEPPFAL--
RVPPFAL--
RLPPFAL--
-RPPFALS-
RRPPFAL--
-RPPFALH-
-RPPFALM-
RHPPFAL--
RGPPFAL--
-RPPFALV-
RSPPFAL--
RIPPFAL--
RWPPFAL--
RPPPFAL--
-RPPFALQ-
RFPPFAL--
-RPPFALT-
RQPPFAL--
RYPPFAL--
-RPPFALN-
-RPPFALE-

REPFAL--
RNPFAL--
RDPFAL--
RAPFAL--
-RPFALG-
-SPFALR-
SKPFAL--
SSPFAL--
SGPFAL--
SVPFAL--
-SPFALS-
SYPFAL--
-SPFALT-
-SPFALW-
SMPFAL--
SNPFAL--
SIPFAL--
SFPFAL--
SDPFAL--
-SPFALQ-
-SPFALM-
SHPFAL--
-SPFALI-
STPFAL--
-SPFALV-
-SPFALK-
-SPFALA-
-SPFALH-
-SPFALE-
-TPFALR-
TLPFAL--
-TPFALK-
-TPFALV-
TYPFAL--
TRPFAL--
TGPFAL--
TNPFAL--
TEPFAL--
-TPFALS-
TSPFAL--
-TPFALQ-
-TPFALT-
-TPFALI-
TMPFAL--
TWPFAL--
-TPFALN-
TTPFAL--
TIPFAL--
-TPFALH-
-TPFALA-

THPFAL--
TKPFAL--
TPPFAL--
-TPFALM-
TCPFAL--
-VPFALL-
-VPFALN-
VTPFAL--
VRPFAL--
VVPFAL--
VPPFAL--
-VPFALM-
VNPFAL--
VHPFAL--
-VPFALR-
VWPFAL--
VEPFAL--
-VPFALH-
VIPFAL--
VGPFAL--
VAPFAL--
-VPFALV-
-VPFALT-
VKPFAL--
-VPFALK-
-VPFALA-
VFPFAL--
-VPFALI-
VYPFAL--
VMPFAL--
VDPFAL--
VCPFAL--
-VPFALE-
WKPFAL--
WSPFAL--
WYPFAL--
WVPFAL--
-WPFALH-
WGPFAL--
-WPFALS-
WIPFAL--
WPPFAL--
WAPFAL--
-WPFALQ-
WNPFAL--
WFPFAL--
-WPFALR-
-WPFALA-
WMPFAL--
WTPFAL--

| | | | |
|---|---|---|---|
| | WQPFAL-- | | |
| | -WPFALM- | | |
| | -WPFALI- | | |
| | WHPFAL-- | | |
| | WEPFAL-- | | |
| | WCPFAL-- | | |
| | YYPFAL-- | | |
| | YRPFAL-- | | |
| | -YPFALK- | | |
| | -YPFALQ- | | |
| | YLPFAL-- | | |
| | YSPFAL-- | | |
| | -YPFALS- | | |
| | YPPFAL-- | | |
| | YNPFAL-- | | |
| | YAPFAL-- | | |
| | YGPFAL-- | | |
| | -YPFALR- | | |
| | -YPFALI- | | |
| | -YPFALH- | | |
| | YHPFAL-- | | |
| | YVPFAL-- | | |
| | YTPFAL-- | | |
| | YIPFAL-- | | |
| | -YPFALT- | | |
| | -YPFALA- | | |
| | YDPFAL-- | | |
| | -YPFALN- | | |
| | YFPFAL-- | | |
| | YCPFAL-- | | |
| | -YPFALM- | | |
| | YMPFAL-- | | |
| PVPM | 276 | 678 | 31.33 |
| | -APVPMW- | | |
| | -APVPML- | | |
| | AWPVPM-- | | |
| | -APVPMK- | | |
| | ARPVPM-- | | |
| | -APVPMI- | | |
| | AVPVPM-- | | |
| | -APVPMG- | | |
| | ALPVPM-- | | |
| | -APVPMA- | | |
| | APPVPM-- | | |
| | -APVPMR- | | |
| | AGPVPM-- | | |
| | -APVPMY- | | |
| | -APVPMS- | | |
| | -APVPMQ- | | |
| | AIPVPM-- | | |

AEPVPM--
AAPVPM--
ASPVPM--
-APVPMV-
-APVPMT-
-APVPME-
AYPVPM--
ATPVPM--
AQPVPM--
-APVPMP-
-APVPMN-
-APVPMF-
ANPVPM--
AMPVPM--
CYPVPM--
-CPVPMM-
-CPVPMW-
-CPVPML-
CGPVPM--
CCPVPM--
-DPVPMR-
-DPVPMK-
DYPVPM--
-DPVPMT-
DTPVPM--
DRPVPM--
DIPVPM--
-DPVPMY-
-DPVPMW-
-DPVPMI-
DLPVPM--
DAPVPM--
DWPVPM--
DSPVPM--
-DPVPMS-
-DPVPMH-
-DPVPMA-
-EPVPML-
-EPVPMI-
-EPVPMF-
-EPVPMS-
ERPVPM--
-EPVPMQ-
-EPVPMR-
-EPVPMM-
-EPVPMH-
-EPVPMY-
EMPVPM--
EWPVPM--
ETPVPM--

ESPVPM--
-EPVPMV-
-EPVPMT-
-EPVPMN-
-EPVPMK-
-EPVPMG-
EKPVPM--
EEPVPM--
ECPVPM--
EAPVPM--
-FPVPMR-
FSPVPM--
-FPVPMT-
-FPVPMS-
-FPVPMW-
FNPVPM--
FAPVPM--
FQPVPM--
FWPVPM--
-FPVPMV-
-FPVPMN-
-FPVPMI-
-FPVPMH-
FVPVPM--
-FPVPMY-
-FPVPML-
FMPVPM--
FKPVPM--
FEPVPM--
FRPVPM--
-FPVPMM-
FPPVPM--
FIPVPM--
FFPVPM--
-GPVPMR-
GRPVPM--
GSPVPM--
GAPVPM--
GVPVPM--
-GPVPMW-
GIPVPM--
-GPVPMT-
-GPVPMS-
-GPVPML-
-GPVPMI-
GLPVPM--
GFPVPM--
-GPVPMN-
GKPVPM--
GWPVPM--

GTPVPM--
-GPVPMF-
GMPVPM--
GGPVPM--
GQPVPM--
-GPVPMV-
-GPVPMQ-
-GPVPMK-
-GPVPMG-
-GPVPMA-
GPPVPM--
GHPVPM--
GDPVPM--
-HPVPMS-
HRPVPM--
-HPVPML-
-HPVPMK-
HQPVPM--
-HPVPMY-
-HPVPMR-
HAPVPM--
HSPVPM--
-HPVPMT-
-HPVPMQ-
-HPVPMA-
HLPVPM--
HFPVPM--
HWPVPM--
HTPVPM--
-HPVPMN-
HKPVPM--
HIPVPM--
-IPVPMR-
IIPVPM--
-IPVPMH-
-IPVPMS-
-IPVPMK-
ITPVPM--
-IPVPMV-
IVPVPM--
IPPVPM--
IKPVPM--
IGPVPM--
IYPVPM--
IWPVPM--
-IPVPMW-
-IPVPMT-
-IPVPMQ-
-IPVPMN-
-IPVPML-

-IPVPMG-
INPVPM--
ILPVPM--
IDPVPM--
KSPVPM--
-KPVPMR-
-KPVPMQ-
-KPVPMT-
-KPVPMS-
-KPVPMI-
-KPVPMW-
KVPVPM--
-KPVPMK-
-KPVPMN-
KRPVPM--
-KPVPME-
KNPVPM--
KHPVPM--
KGPVPM--
KWPVPM--
KQPVPM--
-KPVPMH-
KLPVPM--
KFPVPM--
-KPVPMG-
KMPVPM--
KDPVPM--
-KPVPMY-
-KPVPMV-
-KPVPMF-
-KPVPMA-
KIPVPM--
KAPVPM--
-LPVPML-
-LPVPMK-
LHPVPM--
LWPVPM--
-LPVPMV-
-LPVPMH-
LYPVPM--
LQPVPM--
LKPVPM--
LSPVPM--
-LPVPMF-
LPPVPM--
LLPVPM--
LGPVPM--
LAPVPM--
-LPVPMQ-
LNPVPM--

-LPVPMW-
-LPVPMT-
-LPVPMM-
-LPVPMI-
LIPVPM--
-LPVPMN-
LFPVPM--
MSPVPM--
-MPVPML-
-MPVPMR-
-MPVPMQ-
MNPVPM--
MGPVPM--
-MPVPMT-
-MPVPMK-
-MPVPMI-
MKPVPM--
MTPVPM--
-MPVPMS-
MMPVPM--
MHPVPM--
MRPVPM--
-MPVPMY-
-MPVPMF-
MFPVPM--
NRPVPM--
-NPVPMR-
-NPVPML-
-NPVPMY-
-NPVPMS-
NGPVPM--
NVPVPM--
-NPVPMT-
-NPVPMN-
-NPVPMM-
NQPVPM--
-NPVPMK-
-NPVPMI-
NLPVPM--
NYPVPM--
-NPVPMW-
-NPVPMQ-
NNPVPM--
NKPVPM--
NDPVPM--
NAPVPM--
NWPVPM--
NSPVPM--
-NPVPMV-
-NPVPMF-

NIPVPM--
NFPVPM--
NEPVPM--
--PVPMTL
--PVPMYS
--PVPMLL
--PVPMYR
--PVPMSS
--PVPMVR
--PVPMRA
--PVPMLY
--PVPMKS
--PVPMVL
--PVPMSR
--PVPMRR
--PVPMRY
--PVPMLR
--PVPMIM
--PVPMFD
--PVPMHT
--PVPMGY
--PVPMFL
--PVPMVN
--PVPMTV
--PVPMTF
--PVPMLG
--PVPMFK
--PVPMVG
--PVPMST
--PVPMSI
--PVPMNW
--PVPMKG
--PVPMFN
--PVPMSY
--PVPMSH
--PVPMRT
--PVPMRK
--PVPMTS
--PVPMSV
--PVPMLI
--PVPMGS
--PVPMYK
--PVPMWQ
--PVPMWI
--PVPMVT
--PVPMSA
--PVPMRQ
--PVPMRH
--PVPMRF
--PVPMNY

--PVPMIN
--PVPMIL
--PVPMAT
--PVPMYQ
--PVPMYI
--PVPMWY
--PVPMWG
--PVPMVH
--PVPMRI
--PVPMLS
--PVPMLN
--PVPMLM
--PVPMKR
--PVPMGM
--PVPMFS
--PVPMAN
PSPVPM--
PLPVPM--
PVPVPM--
--PVPMWS
--PVPMVS
--PVPMVA
--PVPMTW
--PVPMSG
--PVPMQG
--PVPMMS
--PVPMLV
--PVPMLK
--PVPMLE
--PVPMLA
--PVPMKK
--PVPMKH
--PVPMIG
--PVPMGI
--PVPMFV
--PVPMFI
--PVPMAS
--PVPMYT
--PVPMYA
--PVPMWR
--PVPMWM
--PVPMVI
--PVPMVF
--PVPMTR
--PVPMTI
--PVPMTG
--PVPMQR
--PVPMQK
--PVPMPW
--PVPMPV

--PVPMNQ
--PVPMMQ
--PVPMMA
--PVPMLT
--PVPMLF
--PVPMKY
--PVPMKM
--PVPMKI
--PVPMKE
--PVPMIV
--PVPMHQ
--PVPMHG
--PVPMHA
--PVPMGW
--PVPMFT
--PVPMFR
--PVPMFH
--PVPMEV
--PVPMDL
--PVPMDF
--PVPMAA
PQPVPM--
PGPVPM--
--PVPMYH
--PVPMYG
--PVPMYE
--PVPMWW
--PVPMWT
--PVPMWN
--PVPMWD
--PVPMVY
--PVPMVV
--PVPMTY
--PVPMTD
--PVPMSW
--PVPMSK
--PVPMRW
--PVPMRN
--PVPMQY
--PVPMNT
--PVPMNI
--PVPMML
--PVPMLQ
--PVPMLD
--PVPMKQ
--PVPMKN
--PVPMIT
--PVPMIS
--PVPMIF
--PVPMID

--PVPMHS
--PVPMGN
--PVPMGG
--PVPMFG
--PVPMFE
--PVPMFA
--PVPMAV
--PVPMAQ
--PVPMAI
--PVPMAG
--PVPMAF
--PVPMYW
--PVPMYF
--PVPMYD
--PVPMWV
--PVPMWE
--PVPMVQ
--PVPMVD
--PVPMTT
--PVPMTQ
--PVPMTP
--PVPMTK
--PVPMTH
--PVPMTC
--PVPMSQ
--PVPMSN
--PVPMSE
--PVPMRE
--PVPMQW
--PVPMQQ
--PVPMQM
--PVPMQD
--PVPMPY
--PVPMPQ
--PVPMPH
--PVPMPF
--PVPMPA
--PVPMNR
--PVPMNN
--PVPMNH
--PVPMNG
--PVPMNA
--PVPMMW
--PVPMMT
--PVPMMN
--PVPMMG
--PVPMMF
--PVPMKW
--PVPMKT
--PVPMKD

--PVPMKA
--PVPMIA
--PVPMHW
--PVPMHV
--PVPMHD
--PVPMGV
--PVPMGT
--PVPMGF
--PVPMFY
--PVPMFW
--PVPMET
--PVPMES
--PVPMEL
--PVPMEI
--PVPMDY
--PVPMAR
PNPVPM--
PKPVPM--
PHPVPM--
-QPVPMR-
QRPVPM--
-QPVPML-
-QPVPMI-
-QPVPMF-
QDPVPM--
QWPVPM--
QVPVPM--
QTPVPM--
QIPVPM--
-QPVPMY-
-QPVPMT-
-QPVPMA-
QMPVPM--
-QPVPMV-
-QPVPMS-
-QPVPMM-
-QPVPMH-
QNPVPM--
QGPVPM--
QFPVPM--
QEPVPM--
-RPVPMI-
-RPVPMY-
-RPVPMH-
-RPVPMR-
RSPVPM--
RKPVPM--
-RPVPMW-
RVPVPM--
RNPVPM--

-RPVPMQ-
RTPVPM--
-RPVPMT-
RLPVPM--
-RPVPMK-
RQPVPM--
-RPVPMM-
RMPVPM--
-RPVPMG-
RYPVPM--
-RPVPMF-
-RPVPME-
REPVPM--
RAPVPM--
-RPVPMV-
-RPVPMN-
-RPVPMA-
RPPVPM--
RIPVPM--
RFPVPM--
RCPVPM--
RWPVPM--
-SPVPML-
-SPVPMW-
-SPVPMR-
-SPVPMS-
-SPVPMA-
SWPVPM--
SLPVPM--
SAPVPM--
SEPVPM--
-SPVPMK-
SVPVPM--
-SPVPMQ-
SYPVPM--
SRPVPM--
-SPVPMH-
SFPVPM--
SIPVPM--
-SPVPMY-
-SPVPMI-
SHPVPM--
SQPVPM--
-SPVPMM-
-SPVPMF-
SKPVPM--
STPVPM--
-SPVPMV-
-SPVPMT-
-SPVPMN-

SDPVPM--
SSPVPM--
SMPVPM--
SGPVPM--
-TPVPMR-
-TPVPMA-
TIPVPM--
TGPVPM--
-TPVPMK-
TSPVPM--
-TPVPMV-
TDPVPM--
TVPVPM--
-TPVPMI-
TRPVPM--
-TPVPMT-
-TPVPMH-
TMPVPM--
THPVPM--
TFPVPM--
-TPVPMY-
-TPVPMW-
-TPVPMQ-
-TPVPMN-
-TPVPMF-
TYPVPM--
TWPVPM--
TTPVPM--
-TPVPMS-
-TPVPMG-
-TPVPME-
TPPVPM--
TLPVPM--
-VPVPMY-
-VPVPMR-
VVPVPM--
VSPVPM--
-VPVPMQ-
VKPVPM--
VRPVPM--
-VPVPMF-
-VPVPMS-
-VPVPMK-
-VPVPMA-
VTPVPM--
VQPVPM--
-VPVPML-
-VPVPMM-
-VPVPMG-
VGPVPM--

VAPVPM--
VWPVPM--
-VPVPMW-
VNPVPM--
VLPVPM--
VIPVPM--
-VPVPMT-
-VPVPMH-
-VPVPME-
VDPVPM--
-WPVPML-
-WPVPMR-
-WPVPMN-
-WPVPMI-
-WPVPMY-
-WPVPMT-
WPPVPM--
-WPVPMQ-
WNPVPM--
WKPVPM--
WVPVPM--
WSPVPM--
-WPVPMV-
-WPVPMS-
-WPVPMA-
WAPVPM--
WWPVPM--
WQPVPM--
-WPVPMM-
-WPVPMF-
-WPVPME-
-WPVPMC-
WDPVPM--
YRPVPM--
-YPVPMR-
YIPVPM--
YSPVPM--
-YPVPMS-
-YPVPMN-
-YPVPMK-
-YPVPML-
-YPVPMI-
YQPVPM--
-YPVPMM-
YHPVPM--
-YPVPMV-
-YPVPMF-
-YPVPMA-
YNPVPM--
-YPVPMY-

-YPVPMT-
YPPVPM--
YYPVPM--
YVPVPM--
YTPVPM--
-YPVPME-
YMPVPM--
YGPVPM--
YEPVPM--
YDPVPM--
YAPVPM--

| | | | |
|---|---|---|---|
| PWGL | 159 | 734 | 31.32 |

--PWGLTL
--PWGLPS
--PWGLLE
--PWGLGT
ALPWGL--
AVPWGL--
-APWGLR-
-APWGLS-
-APWGLA-
AKPWGL--
ARPWGL--
ANPWGL--
ASPWGL--
-APWGLL-
-APWGLG-
-APWGLT-
AHPWGL--
-APWGLN-
-APWGLK-
AGPWGL--
-APWGLQ-
APPWGL--
AIPWGL--
AFPWGL--
-APWGLF-
AMPWGL--
AAPWGL--
-APWGLM-
-APWGLH-
-APWGLI-
AEPWGL--
ADPWGL--
CRPWGL--
CTPWGL--
CVPWGL--
CKPWGL--
-CPWGLS-
-CPWGLM-

CIPWGL--
-CPWGLT-
CLPWGL--
CHPWGL--
CSPWGL--
CQPWGL--
-CPWGLY-
-CPWGLR-
CGPWGL--
DSPWGL--
DLPWGL--
DRPWGL--
-DPWGLR-
DVPWGL--
-DPWGLV-
DWPWGL--
-DPWGLL-
DYPWGL--
-DPWGLY-
-DPWGLT-
-DPWGLM-
DTPWGL--
DIPWGL--
DGPWGL--
DFPWGL--
-DPWGLS-
-DPWGLN-
-DPWGLH-
DNPWGL--
DHPWGL--
DAPWGL--
-DPWGLK-
-DPWGLA-
DMPWGL--
DDPWGL--
-EPWGLR-
-EPWGLK-
-EPWGLN-
EHPWGL--
EQPWGL--
-EPWGLT-
-EPWGLQ-
-EPWGLH-
ENPWGL--
EVPWGL--
ELPWGL--
ETPWGL--
-EPWGLL-
EAPWGL--
ESPWGL--

-EPWGLS-
-EPWGLI-
-EPWGLF-
-EPWGLA-
EIPWGL--
EYPWGL--
EWPWGL--
-EPWGLW-
EKPWGL--
EGPWGL--
EEPWGL--
EDPWGL--
FGPWGL--
FIPWGL--
FVPWGL--
-FPWGLL-
-FPWGLH-
FLPWGL--
FQPWGL--
-FPWGLS-
FWPWGL--
FTPWGL--
FRPWGL--
FSPWGL--
-FPWGLA-
FKPWGL--
-FPWGLR-
FNPWGL--
FFPWGL--
-FPWGLI-
FCPWGL--
FAPWGL--
FYPWGL--
-FPWGLY-
-FPWGLT-
FMPWGL--
FHPWGL--
-FPWGLV-
-FPWGLN-
FEPWGL--
-FPWGLK-
FPPWGL--
FDPWGL--
GVPWGL--
GAPWGL--
GRPWGL--
GIPWGL--
-GPWGLK-
GQPWGL--
-GPWGLT-

```
-GPWGLH-
GKPWGL--
GGPWGL--
-GPWGLY-
-GPWGLM-
-GPWGLI-
GMPWGL--
GSPWGL--
GLPWGL--
GHPWGL--
GNPWGL--
GYPWGL--
GTPWGL--
-GPWGLF-
-GPWGLA-
-GPWGLW-
GFPWGL--
-GPWGLN-
-GPWGLV-
-GPWGLQ-
-GPWGLE-
GWPWGL--
-GPWGLD-
GCPWGL--
-HPWGLN-
HQPWGL--
-HPWGLF-
-HPWGLA-
-HPWGLL-
HIPWGL--
HVPWGL--
HFPWGL--
-HPWGLT-
-HPWGLR-
HLPWGL--
-HPWGLS-
-HPWGLI-
HAPWGL--
HGPWGL--
HTPWGL--
HKPWGL--
HSPWGL--
-HPWGLY-
HNPWGL--
HMPWGL--
HDPWGL--
HWPWGL--
-HPWGLV-
-HPWGLQ-
-HPWGLK-
```

-HPWGLE-
HEPWGL--
-HPWGLP-
-HPWGLM-
-HPWGLH-
HPPWGL--
HHPWGL--
IRPWGL--
-IPWGLL-
-IPWGLR-
IGPWGL--
IIPWGL--
IMPWGL--
-IPWGLT-
IVPWGL--
ITPWGL--
IKPWGL--
-IPWGLY-
-IPWGLV-
-IPWGLN-
-IPWGLK-
-IPWGLF-
IQPWGL--
-IPWGLA-
-IPWGLE-
INPWGL--
-IPWGLW-
IDPWGL--
IWPWGL--
-IPWGLM-
IFPWGL--
IEPWGL--
IYPWGL--
IPPWGL--
-IPWGLI-
-IPWGLH-
IAPWGL--
KSPWGL--
-KPWGLS-
KWPWGL--
KGPWGL--
-KPWGLL-
KFPWGL--
-KPWGLH-
KYPWGL--
KVPWGL--
-KPWGLW-
-KPWGLI-
KIPWGL--
-KPWGLT-

-KPWGLF-
KNPWGL--
KHPWGL--
KEPWGL--
-KPWGLV-
KDPWGL--
-KPWGLY-
-KPWGLR-
-KPWGLG-
-KPWGLE-
KPPWGL--
-KPWGLQ-
-KPWGLA-
KMPWGL--
KKPWGL--
-KPWGLM-
-KPWGLK-
KCPWGL--
KQPWGL--
-KPWGLN-
LSPWGL--
LTPWGL--
-LPWGLV-
-LPWGLK-
LNPWGL--
LKPWGL--
LAPWGL--
-LPWGLL-
-LPWGLN-
LHPWGL--
LVPWGL--
LQPWGL--
LMPWGL--
LGPWGL--
LPPWGL--
LYPWGL--
-LPWGLI-
-LPWGLH-
-LPWGLA-
-LPWGLM-
LWPWGL--
LEPWGL--
-LPWGLQ-
LCPWGL--
MRPWGL--
MGPWGL--
-MPWGLL-
MKPWGL--
MQPWGL--
MTPWGL--

−MPWGLS−
MLPWGL−−
MAPWGL−−
MYPWGL−−
−MPWGLA−
MPPWGL−−
MVPWGL−−
−MPWGLH−
−MPWGLF−
−MPWGLI−
MIPWGL−−
MSPWGL−−
−MPWGLW−
−MPWGLV−
−MPWGLT−
−MPWGLN−
MMPWGL−−
−MPWGLK−
−MPWGLM−
−MPWGLG−
−MPWGLE−
MNPWGL−−
MDPWGL−−
MCPWGL−−
NRPWGL−−
−NPWGLL−
NNPWGL−−
NAPWGL−−
NPPWGL−−
NIPWGL−−
−NPWGLS−
−NPWGLT−
−NPWGLM−
NGPWGL−−
−NPWGLY−
−NPWGLK−
NEPWGL−−
−NPWGLN−
−NPWGLF−
NQPWGL−−
NMPWGL−−
NVPWGL−−
NWPWGL−−
−NPWGLI−
−NPWGLV−
NHPWGL−−
NFPWGL−−
NDPWGL−−
−NPWGLQ−
−NPWGLA−

NKPWGL--
--PWGLRT
--PWGLLT
--PWGLAS
--PWGLTS
--PWGLSS
--PWGLRN
--PWGLMA
PMPWGL--
--PWGLTT
--PWGLHR
--PWGLRS
--PWGLLA
--PWGLHK
PLPWGL--
--PWGLYR
--PWGLVA
--PWGLRQ
--PWGLRA
--PWGLYH
--PWGLVG
--PWGLTG
--PWGLHF
--PWGLAW
--PWGLKT
--PWGLIS
--PWGLHG
--PWGLLH
--PWGLKS
--PWGLHV
--PWGLGS
--PWGLYG
--PWGLVK
--PWGLTV
--PWGLTF
--PWGLRR
--PWGLKK
--PWGLYT
--PWGLVR
--PWGLMR
PTPWGL--
PNPWGL--
--PWGLVH
--PWGLTA
--PWGLSW
--PWGLSA
--PWGLRH
--PWGLMH
--PWGLIN
--PWGLIG

--PWGLHH
--PWGLFH
PVPWGL--
PQPWGL--
PGPWGL--
PWPWGL--
--PWGLTK
--PWGLRE
--PWGLLN
--PWGLKY
--PWGLKR
--PWGLHS
--PWGLAG
--PWGLAA
PYPWGL--
--PWGLTR
--PWGLST
--PWGLRW
--PWGLKV
--PWGLIT
--PWGLIM
--PWGLHT
--PWGLFK
PAPWGL--
--PWGLWE
--PWGLVM
--PWGLVI
--PWGLSN
--PWGLQS
--PWGLNT
--PWGLMI
--PWGLIY
--PWGLYS
--PWGLVV
--PWGLTM
--PWGLSR
--PWGLRD
--PWGLMF
--PWGLKG
--PWGLIH
--PWGLYQ
--PWGLWT
--PWGLWH
--PWGLTN
--PWGLRM
--PWGLQA
--PWGLNM
--PWGLNA
--PWGLML
--PWGLKI

```
--PWGLHI
--PWGLFT
PHPWGL--
PDPWGL--
--PWGLYN
--PWGLWK
--PWGLVQ
--PWGLVN
--PWGLSK
--PWGLQG
--PWGLMV
--PWGLMG
--PWGLKW
--PWGLIV
--PWGLHM
--PWGLHD
--PWGLFD
--PWGLES
--PWGLWR
--PWGLWN
--PWGLVY
--PWGLVT
--PWGLTH
--PWGLTD
--PWGLSV
--PWGLSI
--PWGLSH
--PWGLSF
--PWGLQT
--PWGLQH
--PWGLNV
--PWGLNL
--PWGLNH
--PWGLME
--PWGLMD
--PWGLLD
--PWGLKA
--PWGLIA
--PWGLHL
--PWGLFS
--PWGLFR
--PWGLFN
--PWGLAV
PEPWGL--
--PWGLTY
--PWGLSY
--PWGLQW
--PWGLQM
--PWGLNS
--PWGLMQ
```

--PWGLMM
--PWGLKM
--PWGLKH
--PWGLIK
--PWGLID
--PWGLHW
--PWGLHQ
--PWGLGL
--PWGLEG
--PWGLAN
PIPWGL--
--PWGLYK
--PWGLYF
--PWGLYD
--PWGLVE
--PWGLVD
--PWGLTW
--PWGLTE
--PWGLQY
--PWGLQK
--PWGLPW
--PWGLNK
--PWGLNG
--PWGLLQ
--PWGLKN
--PWGLKF
--PWGLIW
--PWGLII
--PWGLHA
--PWGLGA
--PWGLFQ
--PWGLFG
--PWGLFF
--PWGLEV
--PWGLET
--PWGLEM
--PWGLEA
--PWGLDS
--PWGLCR
--PWGLAT
--PWGLAI
-PPWGLR-
-PPWGLN-
PKPWGL--
QVPWGL--
QRPWGL--
-QPWGLR-
-QPWGLQ-
QAPWGL--
QYPWGL--

QTPWGL--
-QPWGLH-
-QPWGLF-
-QPWGLA-
QNPWGL--
QQPWGL--
-QPWGLM-
-QPWGLY-
-QPWGLK-
QSPWGL--
-QPWGLN-
QGPWGL--
QFPWGL--
QEPWGL--
-QPWGLV-
-QPWGLT-
-QPWGLE-
QPPWGL--
QMPWGL--
QLPWGL--
QKPWGL--
QHPWGL--
-QPWGLG-
QIPWGL--
QDPWGL--
-RPWGLL-
RSPWGL--
RLPWGL--
-RPWGLV-
RTPWGL--
-RPWGLN-
-RPWGLA-
-RPWGLY-
RAPWGL--
-RPWGLS-
-RPWGLR-
-RPWGLI-
-RPWGLH-
-RPWGLF-
-RPWGLM-
RFPWGL--
-RPWGLE-
RIPWGL--
RGPWGL--
RKPWGL--
RWPWGL--
RHPWGL--
REPWGL--
-RPWGLQ-
RYPWGL--

−RPWGLG−
RQPWGL−−
RDPWGL−−
RMPWGL−−
−RPWGLK−
RCPWGL−−
SLPWGL−−
SSPWGL−−
−SPWGLL−
−SPWGLR−
−SPWGLS−
−SPWGLK−
SKPWGL−−
−SPWGLI−
SYPWGL−−
SRPWGL−−
SWPWGL−−
SHPWGL−−
STPWGL−−
−SPWGLV−
−SPWGLN−
−SPWGLW−
SIPWGL−−
−SPWGLA−
SEPWGL−−
SVPWGL−−
−SPWGLH−
SQPWGL−−
SAPWGL−−
SMPWGL−−
SCPWGL−−
−SPWGLQ−
−SPWGLG−
−SPWGLE−
−SPWGLM−
TSPWGL−−
TTPWGL−−
−TPWGLR−
−TPWGLK−
TLPWGL−−
TGPWGL−−
TRPWGL−−
TKPWGL−−
TYPWGL−−
−TPWGLT−
TMPWGL−−
−TPWGLV−
−TPWGLS−
TNPWGL−−
−TPWGLH−

```
TIPWGL--
TEPWGL--
-TPWGLL-
-TPWGLN-
THPWGL--
TWPWGL--
-TPWGLM-
-TPWGLQ-
-TPWGLI-
-TPWGLA-
TAPWGL--
-TPWGLY-
TDPWGL--
TFPWGL--
TPPWGL--
-TPWGLP-
VRPWGL--
VGPWGL--
VVPWGL--
-VPWGLL-
-VPWGLV-
-VPWGLR-
VQPWGL--
VKPWGL--
VSPWGL--
VIPWGL--
-VPWGLT-
-VPWGLI-
-VPWGLH-
VHPWGL--
VAPWGL--
VYPWGL--
VTPWGL--
-VPWGLA-
VLPWGL--
VFPWGL--
VPPWGL--
VNPWGL--
VMPWGL--
-VPWGLK-
-VPWGLQ-
-VPWGLF-
-VPWGLE-
VEPWGL--
-VPWGLM-
VDPWGL--
WSPWGL--
WQPWGL--
-WPWGLT-
-WPWGLR-
```

| | | | |
|---|---|---|---|
| | WFPWGL-- | | |
| | WYPWGL-- | | |
| | WTPWGL-- | | |
| | WRPWGL-- | | |
| | WVPWGL-- | | |
| | WKPWGL-- | | |
| | WAPWGL-- | | |
| | WHPWGL-- | | |
| | -WPWGLV- | | |
| | -WPWGLS- | | |
| | -WPWGLM- | | |
| | -WPWGLI- | | |
| | -WPWGLH- | | |
| | -WPWGLF- | | |
| | -WPWGLQ- | | |
| | -WPWGLL- | | |
| | -WPWGLK- | | |
| | -WPWGLN- | | |
| | -WPWGLA- | | |
| | YRPWGL-- | | |
| | YTPWGL-- | | |
| | YKPWGL-- | | |
| | -YPWGLK- | | |
| | -YPWGLI- | | |
| | -YPWGLH- | | |
| | YDPWGL-- | | |
| | -YPWGLT- | | |
| | -YPWGLV- | | |
| | YMPWGL-- | | |
| | YAPWGL-- | | |
| | YSPWGL-- | | |
| | -YPWGLQ- | | |
| | -YPWGLL- | | |
| | YQPWGL-- | | |
| | YEPWGL-- | | |
| | YNPWGL-- | | |
| | YIPWGL-- | | |
| | YVPWGL-- | | |
| | YHPWGL-- | | |
| | YGPWGL-- | | |
| | -YPWGLR- | | |
| | -YPWGLM- | | |
| | YYPWGL-- | | |
| | YWPWGL-- | | |
| | -YPWGLA- | | |
| | YPPWGL-- | | |
| PKAF | 924 | 516 | 31.32 |
| | -APKAFA- | | |
| | -APKAFI- | | |
| | ANPKAF-- | | |

-APKAFS-
-APKAFL-
ARPKAF--
-APKAFQ-
-APKAFV-
-APKAFN-
ATPKAF--
AQPKAF--
AMPKAF--
AHPKAF--
AGPKAF--
-APKAFW-
AYPKAF--
-APKAFY-
-APKAFK-
-APKAFH-
-APKAFF-
AIPKAF--
AKPKAF--
ACPKAF--
CGPKAF--
CLPKAF--
-CPKAFI-
CYPKAF--
CRPKAF--
-CPKAFR-
-CPKAFM-
CHPKAF--
-CPKAFL-
CEPKAF--
CCPKAF--
DIPKAF--
DVPKAF--
-DPKAFR-
DRPKAF--
-DPKAFW-
DWPKAF--
-DPKAFT-
-DPKAFS-
-DPKAFH-
DHPKAF--
DGPKAF--
DTPKAF--
-DPKAFV-
-DPKAFI-
DEPKAF--
DSPKAF--
-DPKAFY-
-DPKAFM-
-DPKAFL-

−DPKAFF−
DMPKAF−−
DLPKAF−−
DKPKAF−−
−EPKAFS−
ESPKAF−−
−EPKAFN−
−EPKAFI−
EMPKAF−−
EWPKAF−−
EVPKAF−−
ELPKAF−−
EAPKAF−−
−EPKAFR−
−EPKAFL−
−EPKAFK−
−EPKAFA−
EYPKAF−−
ETPKAF−−
ERPKAF−−
−EPKAFH−
EFPKAF−−
EDPKAF−−
EPPKAF−−
−EPKAFY−
−EPKAFV−
−EPKAFT−
−EPKAFQ−
−EPKAFF−
FDPKAF−−
FSPKAF−−
FVPKAF−−
−FPKAFV−
FMPKAF−−
−FPKAFH−
FAPKAF−−
FWPKAF−−
−FPKAFI−
−FPKAFA−
FHPKAF−−
FRPKAF−−
FPPKAF−−
FNPKAF−−
FKPKAF−−
FIPKAF−−
GQPKAF−−
GHPKAF−−
−GPKAFS−
−GPKAFL−
−GPKAFY−

```
-GPKAFK-
GAPKAF--
GIPKAF--
-GPKAFN-
-GPKAFF-
GMPKAF--
GKPKAF--
GDPKAF--
GTPKAF--
GRPKAF--
-GPKAFM-
-GPKAFI-
-GPKAFA-
GNPKAF--
GLPKAF--
GSPKAF--
-GPKAFT-
-GPKAFQ-
-HPKAFS-
-HPKAFR-
HYPKAF--
HWPKAF--
HLPKAF--
HGPKAF--
HRPKAF--
HIPKAF--
-HPKAFK-
-HPKAFH-
HNPKAF--
HVPKAF--
HEPKAF--
-HPKAFW-
-HPKAFQ-
-HPKAFA-
-HPKAFM-
HMPKAF--
HDPKAF--
-IPKAFT-
IMPKAF--
IIPKAF--
IRPKAF--
INPKAF--
ILPKAF--
IGPKAF--
-IPKAFM-
-IPKAFI-
-IPKAFF-
-IPKAFA-
IKPKAF--
IWPKAF--
```

–IPKAFY–
–IPKAFW–
–IPKAFN–
IAPKAF––
KSPKAF––
–KPKAFL–
–KPKAFS–
KMPKAF––
KVPKAF––
KFPKAF––
KWPKAF––
–KPKAFV–
–KPKAFR–
KCPKAF––
KQPKAF––
KPPKAF––
–KPKAFA–
KAPKAF––
KTPKAF––
KRPKAF––
–KPKAFY–
–KPKAFQ–
–KPKAFN–
–KPKAFI–
KIPKAF––
LRPKAF––
–LPKAFI–
LVPKAF––
–LPKAFL–
LGPKAF––
LNPKAF––
LPPKAF––
–LPKAFY–
–LPKAFT–
LMPKAF––
LHPKAF––
LEPKAF––
LYPKAF––
–LPKAFV–
LKPKAF––
LIPKAF––
–LPKAFH–
LCPKAF––
MLPKAF––
–MPKAFL–
–MPKAFS–
MTPKAF––
MIPKAF––
MGPKAF––
–MPKAFV–

-MPKAFM-
MVPKAF--
-MPKAFY-
MSPKAF--
-MPKAFI-
-MPKAFH-
-MPKAFA-
MQPKAF--
-MPKAFQ-
MPPKAF--
-MPKAFF-
MNPKAF--
MAPKAF--
MWPKAF--
-MPKAFW-
MEPKAF--
NYPKAF--
NPPKAF--
NTPKAF--
NSPKAF--
-NPKAFH-
NNPKAF--
-NPKAFS-
NMPKAF--
NLPKAF--
NGPKAF--
NDPKAF--
NVPKAF--
-NPKAFT-
NKPKAF--
-NPKAFY-
-NPKAFV-
-NPKAFF-
NFPKAF--
NEPKAF--
NAPKAF--
--PKAFTK
--PKAFTV
--PKAFYT
--PKAFVN
--PKAFVS
--PKAFGI
--PKAFYI
--PKAFQA
--PKAFYV
--PKAFNL
--PKAFMI
--PKAFKS
--PKAFGL
--PKAFFS

--PKAFLN
--PKAFLG
--PKAFVH
--PKAFVA
--PKAFSY
--PKAFLY
--PKAFLA
--PKAFHA
--PKAFYQ
--PKAFYL
--PKAFVT
--PKAFLT
--PKAFLK
--PKAFFT
--PKAFYY
--PKAFSI
--PKAFQV
--PKAFIK
--PKAFFR
--PKAFVW
--PKAFQT
--PKAFMM
--PKAFMK
--PKAFFI
--PKAFFA
--PKAFES
PSPKAF--
--PKAFWY
--PKAFWI
--PKAFWG
--PKAFVM
--PKAFVF
--PKAFTG
--PKAFPI
--PKAFNT
--PKAFNA
--PKAFMG
--PKAFIV
--PKAFIF
--PKAFHW
--PKAFAF
PQPKAF--
--PKAFYS
--PKAFYD
--PKAFYA
--PKAFVV
--PKAFTI
--PKAFSQ
--PKAFSM
--PKAFPM

--PKAFPL
--PKAFNV
--PKAFME
--PKAFMA
--PKAFLM
--PKAFIY
--PKAFIH
--PKAFHS
--PKAFGY
--PKAFGA
--PKAFFH
--PKAFAA
PYPKAF--
PVPKAF--
PTPKAF--
PRPKAF--
--PKAFYW
--PKAFYN
--PKAFWW
--PKAFWM
--PKAFWF
--PKAFVG
--PKAFVD
--PKAFSW
--PKAFSN
--PKAFRQ
--PKAFRE
--PKAFPF
--PKAFLF
--PKAFIQ
--PKAFII
--PKAFIA
--PKAFHK
--PKAFGW
--PKAFGM
--PKAFFD
--PKAFEA
--PKAFAT
--PKAFAQ
--PKAFAI
PHPKAF--
PDPKAF--
PKPKAF--
--PKAFYH
--PKAFYG
--PKAFWV
--PKAFWR
--PKAFWH
--PKAFVQ
--PKAFQY

--PKAFQP
--PKAFQL
--PKAFQK
--PKAFNP
--PKAFMV
--PKAFMF
--PKAFLV
--PKAFKA
--PKAFIN
--PKAFIE
--PKAFHY
--PKAFHN
--PKAFHM
--PKAFHH
--PKAFGS
--PKAFGG
--PKAFGF
--PKAFFV
--PKAFFM
--PKAFFC
--PKAFEG
--PKAFDM
--PKAFCA
--PKAFAV
--PKAFAS
QIPKAF--
-QPKAFW-
-QPKAFS-
-QPKAFV-
QGPKAF--
QWPKAF--
QVPKAF--
QSPKAF--
-QPKAFR-
-QPKAFI-
-QPKAFF-
-QPKAFY-
-QPKAFM-
-QPKAFH-
QMPKAF--
QYPKAF--
QRPKAF--
QQPKAF--
-QPKAFN-
-QPKAFA-
QNPKAF--
QHPKAF--
QDPKAF--
RSPKAF--
RGPKAF--

-RPKAFT-
RTPKAF--
RAPKAF--
-RPKAFQ-
RNPKAF--
REPKAF--
-RPKAFM-
-RPKAFA-
RFPKAF--
-RPKAFF-
RRPKAF--
RPPKAF--
RHPKAF--
RDPKAF--
SLPKAF--
-SPKAFL-
-SPKAFM-
SIPKAF--
-SPKAFK-
STPKAF--
-SPKAFY-
-SPKAFS-
-SPKAFF-
SCPKAF--
SVPKAF--
SPPKAF--
-SPKAFT-
SAPKAF--
SSPKAF--
-SPKAFW-
SKPKAF--
-SPKAFN-
SQPKAF--
-SPKAFA-
TGPKAF--
-TPKAFS-
-TPKAFH-
TLPKAF--
TTPKAF--
TVPKAF--
-TPKAFW-
-TPKAFY-
-TPKAFA-
TIPKAF--
TAPKAF--
-TPKAFV-
TSPKAF--
-TPKAFQ-
-TPKAFI-
TQPKAF--

TPPKAF--
-TPKAFT-
TNPKAF--
TFPKAF--
TWPKAF--
-TPKAFF-
TEPKAF--
-VPKAFA-
VGPKAF--
VIPKAF--
VSPKAF--
-VPKAFM-
VRPKAF--
VHPKAF--
VTPKAF--
-VPKAFW-
-VPKAFL-
-VPKAFI-
VYPKAF--
-VPKAFT-
-VPKAFN-
-VPKAFF-
-VPKAFV-
VAPKAF--
VQPKAF--
-VPKAFY-
VNPKAF--
VKPKAF--
-VPKAFK-
VEPKAF--
-WPKAFF-
WVPKAF--
WAPKAF--
WTPKAF--
WRPKAF--
-WPKAFT-
-WPKAFS-
WMPKAF--
WKPKAF--
WSPKAF--
-WPKAFY-
WNPKAF--
WQPKAF--
-WPKAFL-
WLPKAF--
WEPKAF--
YHPKAF--
YSPKAF--
YLPKAF--
YQPKAF--

YIPKAF--
YEPKAF--
YPPKAF--
-YPKAFT-
-YPKAFM-
-YPKAFA-
YYPKAF--
YTPKAF--
-YPKAFW-
-YPKAFQ-
-YPKAFH-
-YPKAFF-
YMPKAF--

| | | | |
|---|---|---|---|
| PARM | 256 | 689 | 31.31 |

-LPARMG-
--PARMNL
-RPARMF-
-APARML-
-APARMR-
-APARMG-
-APARMH-
ARPARM--
-APARMT-
ALPARM--
ASPARM--
-APARMQ-
AWPARM--
ATPARM--
-APARMA-
-APARMW-
-APARMS-
-APARMK-
AGPARM--
AFPARM--
AAPARM--
AIPARM--
APPARM--
-APARMN-
-APARME-
ANPARM--
AMPARM--
AEPARM--
ADPARM--
AYPARM--
AVPARM--
-APARMP-
-APARMM-
-APARMF-
-APARMC-
ACPARM--

-CPARMS-
-CPARML-
CLPARM--
CRPARM--
-CPARMT-
CDPARM--
CYPARM--
CTPARM--
CSPARM--
-CPARMV-
-CPARMM-
-CPARMI-
CKPARM--
CFPARM--
CEPARM--
CCPARM--
-DPARMV-
DSPARM--
-DPARMT-
DKPARM--
DIPARM--
DYPARM--
-DPARMS-
-DPARML-
DVPARM--
DTPARM--
-DPARMW-
-DPARMA-
DMPARM--
DRPARM--
-DPARMI-
-DPARMH-
DAPARM--
DWPARM--
-DPARMF-
DNPARM--
DFPARM--
DDPARM--
DCPARM--
ESPARM--
-EPARMS-
-EPARML-
-EPARMV-
-EPARMM-
EVPARM--
-EPARMW-
-EPARMF-
EMPARM--
EGPARM--
EFPARM--

ENPARM--
ELPARM--
EKPARM--
EQPARM--
-EPARMN-
-EPARMK-
-EPARMD-
-EPARMA-
EIPARM--
-FPARML-
-FPARMR-
-FPARMS-
FTPARM--
FSPARM--
FEPARM--
-FPARMW-
-FPARMT-
-FPARME-
FFPARM--
FRPARM--
-FPARMH-
-FPARMF-
FHPARM--
FAPARM--
FYPARM--
FPPARM--
FNPARM--
FKPARM--
FGPARM--
-GPARMY-
-GPARMR-
-GPARML-
-GPARMA-
GLPARM--
-GPARMW-
-GPARMT-
-GPARMK-
GVPARM--
GAPARM--
GRPARM--
GWPARM--
-GPARMH-
GIPARM--
GSPARM--
-GPARME-
GKPARM--
-GPARMM-
GNPARM--
GYPARM--
GTPARM--

GQPARM--
-GPARMN-
GEPARM--
-GPARMV-
GGPARM--
-GPARMQ-
-GPARMI-
-GPARMG-
GHPARM--
GDPARM--
GPPARM--
-HPARML-
-HPARMH-
HFPARM--
HYPARM--
HSPARM--
-HPARMV-
-HPARMR-
HWPARM--
HTPARM--
HRPARM--
-HPARMT-
HKPARM--
-HPARMA-
HGPARM--
HAPARM--
HVPARM--
-HPARMY-
-HPARMW-
-HPARMQ-
-HPARMI-
HNPARM--
HLPARM--
-HPARMS-
-HPARMM-
-HPARMF-
HHPARM--
HPPARM--
-HPARMG-
-HPARME-
HMPARM--
-IPARML-
IKPARM--
-IPARMR-
-IPARMT-
-IPARMS-
IRPARM--
-IPARMW-
ISPARM--
INPARM--

IYPARM--
ITPARM--
IQPARM--
-IPARMN-
-IPARMG-
-IPARMF-
IMPARM--
IPPARM--
-IPARMQ-
-IPARMI-
-IPARMH-
-IPARME-
ILPARM--
IIPARM--
IEPARM--
KSPARM--
KVPARM--
-KPARMR-
-KPARMT-
KAPARM--
-KPARMF-
-KPARMQ-
-KPARMH-
KIPARM--
-KPARMW-
-KPARMV-
KNPARM--
KRPARM--
KWPARM--
KTPARM--
-KPARMM-
-KPARMK-
KLPARM--
KMPARM--
KYPARM--
KHPARM--
KPPARM--
KEPARM--
KDPARM--
-KPARMN-
KGPARM--
-LPARML-
-LPARMR-
LKPARM--
LRPARM--
-LPARMI-
-LPARMS-
LLPARM--
LDPARM--
LFPARM--

LSPARM--
LPPARM--
-LPARMK-
LGPARM--
LYPARM--
LIPARM--
LTPARM--
-LPARMM-
LNPARM--
-LPARMT-
LWPARM--
LQPARM--
-LPARMF-
-LPARME-
LMPARM--
LHPARM--
-MPARMS-
MTPARM--
MSPARM--
-MPARMH-
-MPARMR-
MRPARM--
-MPARMT-
MHPARM--
MMPARM--
MYPARM--
-MPARMA-
MLPARM--
MEPARM--
MWPARM--
-MPARMY-
-MPARML-
MIPARM--
MFPARM--
MGPARM--
MVPARM--
MAPARM--
MNPARM--
MPPARM--
-MPARMQ-
-MPARMN-
-MPARMM-
-MPARMG-
-NPARML-
NAPARM--
NSPARM--
NRPARM--
-NPARMT-
-NPARMS-
NHPARM--

NYPARM--
NVPARM--
-NPARMV-
-NPARMH-
NWPARM--
-NPARMY-
NNPARM--
NLPARM--
NPPARM--
-NPARMM-
-NPARMK-
-NPARME-
NIPARM--
-NPARMQ-
-NPARMW-
-NPARMA-
NMPARM--
NEPARM--
--PARMSR
--PARMML
--PARMLT
--PARMRT
--PARMVK
--PARMTV
--PARMRS
--PARMSY
--PARMMG
--PARMLA
PLPARM--
--PARMTA
--PARMNS
--PARMLN
--PARMLG
--PARMVQ
--PARMSI
--PARMWY
--PARMTS
--PARMSK
--PARMRV
--PARMQT
--PARMKG
--PARMDG
--PARMSV
--PARMQI
PRPARM--
--PARMTN
--PARMMS
--PARMLH
--PARMHV
--PARMVG

--PARMSG
--PARMRA
--PARMWG
--PARMSA
--PARMRN
--PARMLK
--PARMKA
--PARMFN
PVPARM--
--PARMWT
--PARMTM
--PARMSL
--PARMSH
--PARMRY
--PARMRI
--PARMGA
--PARMFG
--PARMDS
--PARMTL
--PARMTK
--PARMTF
--PARMRE
--PARMLW
--PARMLQ
--PARMKS
--PARMIV
--PARMEW
--PARMYG
--PARMYA
--PARMWW
--PARMWK
--PARMVW
--PARMTW
--PARMSQ
--PARMSM
--PARMSD
--PARMNA
--PARMMT
--PARMKW
--PARMKT
--PARMIT
--PARMEV
--PARMEI
--PARMAT
--PARMYQ
--PARMVD
--PARMTY
--PARMRH
--PARMRF
--PARMQH

--PARMQF
--PARMMR
--PARMMI
--PARMLE
--PARMID
--PARMHA
--PARMEY
--PARMEN
PTPARM--
--PARMYH
--PARMWV
--PARMTQ
--PARMTI
--PARMTE
--PARMQS
--PARMQQ
--PARMMY
--PARMMW
--PARMMQ
--PARMMN
--PARMLM
--PARMLD
--PARMKV
--PARMIY
--PARMIK
--PARMHS
--PARMFQ
--PARMEL
--PARMAV
PNPARM--
PMPARM--
PHPARM--
--PARMYN
--PARMWN
--PARMWH
--PARMTT
--PARMTD
--PARMRM
--PARMQG
--PARMMA
--PARMIQ
--PARMIN
--PARMIE
--PARMHY
PSPARM--
PGPARM--
PDPARM--
--PARMWM
--PARMVA
--PARMTG

--PARMSE
--PARMRD
--PARMQW
--PARMQR
--PARMQA
--PARMMF
--PARMLI
--PARMKY
--PARMKM
--PARMHF
--PARMGG
--PARMFT
--PARMET
--PARMEM
--PARMEH
--PARMEF
--PARMCS
--PARMCR
PYPARM--
PEPARM--
--PARMWS
--PARMWQ
--PARMWI
--PARMWC
--PARMWA
--PARMVE
--PARMRW
--PARMRQ
--PARMQV
--PARMQD
--PARMNY
--PARMNW
--PARMNT
--PARMNG
--PARMMD
--PARMKF
--PARMII
--PARMHW
--PARMHN
--PARMHM
--PARMHK
--PARMHH
--PARMHG
--PARMHE
--PARMGV
--PARMGT
--PARMGE
--PARMFH
--PARMES
--PARMER

--PARMEQ
--PARMEE
--PARMDW
--PARMDI
--PARMCN
--PARMAI
--PARMAH
PAPARM--
-QPARML-
-QPARMR-
-QPARMS-
QTPARM--
QSPARM--
QRPARM--
-QPARMT-
QVPARM--
QPPARM--
-QPARMI-
-QPARME-
QIPARM--
QGPARM--
-QPARMV-
-QPARMA-
QMPARM--
QLPARM--
QHPARM--
QYPARM--
QQPARM--
-QPARMF-
QWPARM--
-QPARMY-
-QPARMW-
QNPARM--
QKPARM--
QFPARM--
QAPARM--
-RPARMR-
-RPARMT-
RVPARM--
-RPARMQ-
-RPARMM-
-RPARML-
-RPARMY-
-RPARMH-
-RPARMK-
RTPARM--
RLPARM--
REPARM--
-RPARMI-
-RPARMG-

RMPARM--
RIPARM--
RPPARM--
RYPARM--
RHPARM--
RAPARM--
RSPARM--
RGPARM--
-RPARMN-
-RPARME-
RQPARM--
RKPARM--
RDPARM--
-SPARMW-
-SPARMR-
SAPARM--
-SPARMV-
-SPARMS-
SSPARM--
SHPARM--
SYPARM--
STPARM--
SLPARM--
SIPARM--
SVPARM--
-SPARMA-
-SPARMG-
SNPARM--
SMPARM--
-SPARMT-
-SPARMK-
SGPARM--
-SPARMH-
-SPARMQ-
-SPARMM-
-SPARMN-
SFPARM--
SQPARM--
SPPARM--
SWPARM--
-SPARMD-
SEPARM--
SCPARM--
-TPARMR-
-TPARMM-
-TPARMK-
THPARM--
TSPARM--
TLPARM--
-TPARMH-

```
-TPARMA-
-TPARMY-
-TPARMT-
TYPARM--
TWPARM--
-TPARMW-
TMPARM--
TKPARM--
TGPARM--
TRPARM--
TQPARM--
-TPARMF-
-TPARME-
-TPARMN-
-TPARMI-
-TPARMG-
TNPARM--
TAPARM--
-TPARMQ-
TCPARM--
-TPARMD-
TIPARM--
TFPARM--
TEPARM--
-VPARML-
-VPARMR-
-VPARMS-
-VPARMM-
-VPARMY-
-VPARMI-
-VPARMG-
VAPARM--
-VPARMF-
VDPARM--
-VPARMV-
VKPARM--
VLPARM--
VFPARM--
VTPARM--
VSPARM--
-VPARMT-
VWPARM--
-VPARME-
VNPARM--
VVPARM--
VGPARM--
VIPARM--
-VPARMQ-
-VPARMH-
VEPARM--
```

VPPARM--
-VPARMW-
-VPARMN-
-VPARMD-
VMPARM--
VCPARM--
-WPARMR-
-WPARMV-
-WPARMM-
WLPARM--
WIPARM--
WNPARM--
WRPARM--
-WPARMS-
-WPARMQ-
-WPARME-
WKPARM--
WHPARM--
WAPARM--
WSPARM--
WGPARM--
WQPARM--
-WPARMK-
WMPARM--
WDPARM--
-YPARMR-
YRPARM--
-YPARMT-
-YPARML-
YSPARM--
YGPARM--
YPPARM--
-YPARMS-
YIPARM--
YFPARM--
-YPARME-
-YPARMA-
YVPARM--
YQPARM--
YTPARM--
-YPARMW-
-YPARMQ-
-YPARMN-
-YPARMI-
-YPARMH-
YLPARM--
YHPARM--
YEPARM--
YAPARM--
YNPARM--

| | | | |
|---|---|---|---|
| | YCPARM-- | | |
| | -YPARMY- | | |
| | -YPARMK- | | |
| PLSW | 191 | 718 | 31.29 |
| | DTPLSW-- | | |
| | -GPLSWP- | | |
| | -GPLSWG- | | |
| | -HPLSWG- | | |
| | --PLSWNL | | |
| | --PLSWKL | | |
| | --PLSWAN | | |
| | --PLSWAL | | |
| | -APLSWS- | | |
| | -APLSWL- | | |
| | ARPLSW-- | | |
| | AVPLSW-- | | |
| | -APLSWR- | | |
| | -APLSWA- | | |
| | -APLSWY- | | |
| | -APLSWM- | | |
| | AWPLSW-- | | |
| | -APLSWF- | | |
| | -APLSWH- | | |
| | AGPLSW-- | | |
| | AYPLSW-- | | |
| | ATPLSW-- | | |
| | -APLSWG- | | |
| | ANPLSW-- | | |
| | AKPLSW-- | | |
| | AHPLSW-- | | |
| | AFPLSW-- | | |
| | ACPLSW-- | | |
| | -APLSWT- | | |
| | -APLSWN- | | |
| | AEPLSW-- | | |
| | -APLSWE- | | |
| | APPLSW-- | | |
| | -APLSWW- | | |
| | AMPLSW-- | | |
| | -APLSWQ- | | |
| | AIPLSW-- | | |
| | ADPLSW-- | | |
| | CFPLSW-- | | |
| | CRPLSW-- | | |
| | CIPLSW-- | | |
| | CGPLSW-- | | |
| | CCPLSW-- | | |
| | -CPLSWS- | | |
| | -CPLSWL- | | |
| | -CPLSWA- | | |

CKPLSW--
CTPLSW--
CSPLSW--
-CPLSWY-
-CPLSWV-
-CPLSWN-
-CPLSWF-
CMPLSW--
DWPLSW--
-DPLSWL-
-DPLSWA-
DRPLSW--
-DPLSWH-
DGPLSW--
DFPLSW--
-DPLSWS-
-DPLSWR-
DEPLSW--
-DPLSWY-
DLPLSW--
DVPLSW--
-DPLSWV-
DMPLSW--
DYPLSW--
-DPLSWI-
-DPLSWF-
-DPLSWQ-
-DPLSWK-
DKPLSW--
DIPLSW--
DAPLSW--
DQPLSW--
DPPLSW--
-DPLSWT-
DNPLSW--
DHPLSW--
DCPLSW--
-EPLSWI-
ESPLSW--
ERPLSW--
-EPLSWV-
-EPLSWL-
-EPLSWQ-
-EPLSWM-
EIPLSW--
EFPLSW--
-EPLSWY-
-EPLSWT-
-EPLSWH-
EVPLSW--

-EPLSWS-
EEPLSW--
ETPLSW--
EQPLSW--
-EPLSWW-
EMPLSW--
ELPLSW--
EGPLSW--
-EPLSWF-
-EPLSWE-
ENPLSW--
EKPLSW--
EAPLSW--
EYPLSW--
-EPLSWN-
FSPLSW--
-FPLSWT-
FRPLSW--
-FPLSWR-
-FPLSWV-
FVPLSW--
FKPLSW--
-FPLSWS-
-FPLSWL-
FTPLSW--
FAPLSW--
-FPLSWK-
-FPLSWI-
FEPLSW--
-FPLSWH-
-FPLSWG-
FMPLSW--
-FPLSWF-
-FPLSWY-
-FPLSWW-
-FPLSWN-
FHPLSW--
FCPLSW--
FWPLSW--
-FPLSWQ-
FNPLSW--
FGPLSW--
FDPLSW--
-GPLSWR-
-GPLSWA-
GTPLSW--
GFPLSW--
-GPLSWS-
-GPLSWI-
GAPLSW--

GSPLSW--
-GPLSWM-
-GPLSWK-
GIPLSW--
GYPLSW--
GRPLSW--
-GPLSWN-
-GPLSWY-
-GPLSWW-
-GPLSWQ-
GLPLSW--
GQPLSW--
-GPLSWF-
GPPLSW--
GHPLSW--
GEPLSW--
GMPLSW--
-GPLSWV-
GDPLSW--
-HPLSWR-
HRPLSW--
-HPLSWI-
-HPLSWH-
HNPLSW--
HLPLSW--
-HPLSWL-
HMPLSW--
HEPLSW--
-HPLSWT-
HPPLSW--
-HPLSWK-
HSPLSW--
-HPLSWV-
-HPLSWS-
-HPLSWQ-
-HPLSWA-
HWPLSW--
HTPLSW--
-HPLSWW-
HIPLSW--
-HPLSWN-
-HPLSWF-
-HPLSWE-
HKPLSW--
HDPLSW--
-HPLSWM-
HQPLSW--
HHPLSW--
HGPLSW--
-IPLSWR-

-IPLSWT-
-IPLSWL-
ISPLSW--
-IPLSWQ-
IGPLSW--
-IPLSWA-
IAPLSW--
-IPLSWV-
-IPLSWM-
IMPLSW--
IYPLSW--
IRPLSW--
ILPLSW--
IKPLSW--
ITPLSW--
-IPLSWF-
-IPLSWK-
IIPLSW--
IFPLSW--
INPLSW--
IDPLSW--
IVPLSW--
-IPLSWI-
-IPLSWG-
-IPLSWE-
IHPLSW--
IEPLSW--
IWPLSW--
-IPLSWW-
-IPLSWH-
KSPLSW--
-KPLSWL-
-KPLSWS-
-KPLSWY-
KAPLSW--
-KPLSWM-
-KPLSWA-
-KPLSWR-
KGPLSW--
KVPLSW--
KTPLSW--
-KPLSWT-
KIPLSW--
-KPLSWW-
KPPLSW--
-KPLSWH-
KKPLSW--
KWPLSW--
-KPLSWK-
-KPLSWF-

KNPLSW--
KMPLSW--
KLPLSW--
KEPLSW--
KYPLSW--
-KPLSWQ-
-KPLSWE-
-KPLSWG-
KHPLSW--
KFPLSW--
KDPLSW--
LSPLSW--
LRPLSW--
LKPLSW--
LGPLSW--
-LPLSWV-
-LPLSWL-
LFPLSW--
-LPLSWT-
-LPLSWM-
-LPLSWH-
-LPLSWK-
-LPLSWQ-
LPPLSW--
LNPLSW--
LEPLSW--
-LPLSWW-
LDPLSW--
LQPLSW--
-LPLSWA-
LHPLSW--
-LPLSWN-
LYPLSW--
-LPLSWY-
-LPLSWF-
LCPLSW--
-MPLSWR-
-MPLSWS-
-MPLSWN-
MLPLSW--
MRPLSW--
MVPLSW--
MTPLSW--
-MPLSWT-
-MPLSWI-
-MPLSWK-
MNPLSW--
MDPLSW--
MGPLSW--
MAPLSW--

MSPLSW--
-MPLSWQ-
MHPLSW--
MYPLSW--
MPPLSW--
MEPLSW--
-MPLSWH-
-MPLSWY-
-MPLSWM-
MMPLSW--
-MPLSWE-
MWPLSW--
-MPLSWF-
MKPLSW--
-NPLSWR-
-NPLSWQ-
NVPLSW--
-NPLSWL-
-NPLSWS-
NRPLSW--
NQPLSW--
NNPLSW--
NLPLSW--
-NPLSWF-
NYPLSW--
-NPLSWN-
-NPLSWW-
-NPLSWA-
NKPLSW--
NGPLSW--
NSPLSW--
-NPLSWY-
NEPLSW--
NAPLSW--
NTPLSW--
NPPLSW--
-NPLSWV-
-NPLSWK-
NMPLSW--
NWPLSW--
-NPLSWI-
-NPLSWH-
NHPLSW--
NDPLSW--
NIPLSW--
-NPLSWE-
NFPLSW--
--PLSWMK
--PLSWRS
--PLSWRG

--PLSWLK
--PLSWMT
--PLSWLS
--PLSWVR
--PLSWSG
--PLSWNS
--PLSWLH
--PLSWVT
--PLSWRQ
--PLSWAG
--PLSWLW
--PLSWLG
--PLSWKV
--PLSWRI
--PLSWQK
--PLSWMI
--PLSWLT
--PLSWYG
--PLSWSV
--PLSWSK
--PLSWRL
--PLSWFN
--PLSWTV
--PLSWTH
--PLSWRY
--PLSWQR
--PLSWKT
--PLSWWR
--PLSWVI
--PLSWTA
--PLSWQV
--PLSWLD
--PLSWAS
--PLSWVK
--PLSWVN
--PLSWVM
--PLSWSW
--PLSWRN
--PLSWKA
--PLSWIN
--PLSWFQ
--PLSWWK
--PLSWSN
--PLSWRM
--PLSWRD
--PLSWQQ
--PLSWMA
--PLSWHK
--PLSWSF
--PLSWKS

--PLSWIG
--PLSWIE
--PLSWAV
--PLSWYS
--PLSWYE
--PLSWTG
--PLSWSI
--PLSWQT
--PLSWMQ
--PLSWIT
--PLSWYA
--PLSWVY
--PLSWTT
--PLSWTN
--PLSWLE
--PLSWFH
--PLSWFG
--PLSWAI
--PLSWYN
--PLSWVW
--PLSWVE
--PLSWTR
--PLSWTK
--PLSWSH
--PLSWSA
--PLSWNT
--PLSWIY
--PLSWHA
--PLSWGS
--PLSWAT
PNPLSW--
--PLSWYD
--PLSWWT
--PLSWSQ
--PLSWRW
--PLSWQI
--PLSWNA
--PLSWLN
--PLSWIV
--PLSWIK
--PLSWHS
--PLSWFT
--PLSWFM
--PLSWES
--PLSWAK
PFPLSW--
PDPLSW--
PWPLSW--
--PLSWYT
--PLSWWL

--PLSWWG
--PLSWVQ
--PLSWVD
--PLSWVA
--PLSWRE
--PLSWQA
--PLSWKF
--PLSWHT
--PLSWHM
--PLSWHH
--PLSWGT
--PLSWEV
--PLSWET
--PLSWAM
--PLSWWV
--PLSWVH
--PLSWTW
--PLSWTI
--PLSWRA
--PLSWQY
--PLSWNV
--PLSWMN
--PLSWLQ
--PLSWKY
--PLSWKI
--PLSWIQ
--PLSWHR
--PLSWFS
--PLSWFK
--PLSWEA
--PLSWAA
--PLSWYQ
--PLSWWM
--PLSWWA
--PLSWTY
--PLSWQL
--PLSWQH
--PLSWQG
--PLSWNM
--PLSWNG
--PLSWME
--PLSWKN
--PLSWKM
--PLSWIH
--PLSWIF
--PLSWID
--PLSWHV
--PLSWHI
--PLSWGI
--PLSWEW

PCPLSW--
-PPLSWM-
--PLSWWH
--PLSWWE
--PLSWWD
--PLSWTM
--PLSWTE
--PLSWQW
--PLSWQN
--PLSWQE
--PLSWMW
--PLSWMV
--PLSWML
--PLSWMH
--PLSWMD
--PLSWKQ
--PLSWKH
--PLSWKC
--PLSWHN
--PLSWHE
--PLSWGW
--PLSWFF
--PLSWDS
--PLSWAH
QSPLSW--
QRPLSW--
-QPLSWI-
-QPLSWL-
-QPLSWR-
QQPLSW--
QGPLSW--
-QPLSWN-
QLPLSW--
QYPLSW--
QPPLSW--
-QPLSWF-
QNPLSW--
QWPLSW--
QTPLSW--
-QPLSWQ-
-QPLSWY-
-QPLSWW-
-QPLSWV-
-QPLSWT-
-QPLSWM-
-QPLSWE-
QKPLSW--
QHPLSW--
QDPLSW--
QVPLSW--

QMPLSW--
QEPLSW--
QAPLSW--
-QPLSWK-
QIPLSW--
RVPLSW--
-RPLSWR-
-RPLSWA-
-RPLSWM-
-RPLSWI-
RTPLSW--
RAPLSW--
RNPLSW--
RSPLSW--
-RPLSWF-
-RPLSWV-
-RPLSWW-
RFPLSW--
-RPLSWK-
-RPLSWN-
RHPLSW--
-RPLSWE-
RYPLSW--
RPPLSW--
-RPLSWY-
RMPLSW--
RWPLSW--
RDPLSW--
RQPLSW--
-RPLSWG-
-RPLSWQ-
-SPLSWR-
SLPLSW--
-SPLSWN-
SSPLSW--
-SPLSWV-
SVPLSW--
-SPLSWI-
STPLSW--
-SPLSWK-
-SPLSWA-
-SPLSWQ-
-SPLSWT-
SKPLSW--
SFPLSW--
-SPLSWH-
-SPLSWY-
-SPLSWM-
SNPLSW--
SWPLSW--

-SPLSWW-
SYPLSW--
SGPLSW--
SQPLSW--
SPPLSW--
-SPLSWF-
-SPLSWE-
SIPLSW--
SEPLSW--
TSPLSW--
TRPLSW--
-TPLSWV-
-TPLSWQ-
TNPLSW--
TVPLSW--
-TPLSWS-
TLPLSW--
-TPLSWW-
-TPLSWY-
-TPLSWN-
TPPLSW--
THPLSW--
-TPLSWM-
-TPLSWI-
TAPLSW--
-TPLSWA-
-TPLSWK-
TEPLSW--
TYPLSW--
-TPLSWH-
TGPLSW--
TFPLSW--
TKPLSW--
TTPLSW--
-TPLSWT-
TDPLSW--
TWPLSW--
TMPLSW--
TQPLSW--
-VPLSWV-
VSPLSW--
-VPLSWS-
VTPLSW--
VYPLSW--
VMPLSW--
VRPLSW--
-VPLSWR-
-VPLSWW-
VQPLSW--
-VPLSWT-

VNPLSW--
VLPLSW--
VVPLSW--
-VPLSWM-
VPPLSW--
-VPLSWI-
VGPLSW--
VEPLSW--
-VPLSWH-
-VPLSWK-
-VPLSWE-
-VPLSWA-
VIPLSW--
-VPLSWQ-
-VPLSWF-
VWPLSW--
VHPLSW--
VCPLSW--
WSPLSW--
-WPLSWR-
-WPLSWK-
WAPLSW--
WPPLSW--
-WPLSWQ-
WRPLSW--
-WPLSWH-
WMPLSW--
WGPLSW--
WDPLSW--
WYPLSW--
-WPLSWI-
WNPLSW--
-WPLSWW-
-WPLSWV-
-WPLSWN-
-WPLSWM-
-WPLSWF-
WIPLSW--
WTPLSW--
WQPLSW--
-WPLSWE-
-WPLSWA-
WFPLSW--
-YPLSWR-
YSPLSW--
-YPLSWM-
YDPLSW--
-YPLSWT-
-YPLSWL-
-YPLSWK-

YNPLSW--
-YPLSWY-
YLPLSW--
-YPLSWV-
YYPLSW--
YRPLSW--
-YPLSWI-
YAPLSW--
YMPLSW--
YPPLSW--
-YPLSWW-
YGPLSW--
YFPLSW--
YTPLSW--
-YPLSWH-
YCPLSW--
-YPLSWN-
-YPLSWF-
YHPLSW--
YIPLSW--
YEPLSW--
-YPLSWG-

| | | | |
|---|---|---|---|
| PIGI | 221 | 700 | 31.28 |

FNPIGI--
-SPIGIE-
ALPIGI--
-APIGIR-
ASPIGI--
-APIGIL-
-APIGIS-
AYPIGI--
ARPIGI--
-APIGIV-
AHPIGI--
AVPIGI--
-APIGIM-
AKPIGI--
-APIGIW-
AGPIGI--
AQPIGI--
-APIGIT-
AMPIGI--
AEPIGI--
AAPIGI--
-APIGIQ-
AIPIGI--
AWPIGI--
ATPIGI--
APPIGI--
-APIGIN-

-APIGIG-
-APIGIF-
ANPIGI--
AFPIGI--
ACPIGI--
-APIGIK-
-APIGIA-
CSPIGI--
CCPIGI--
CEPIGI--
CYPIGI--
-CPIGIN-
CKPIGI--
CFPIGI--
CTPIGI--
CPPIGI--
-CPIGIL-
-CPIGIK-
CNPIGI--
CMPIGI--
DYPIGI--
DQPIGI--
-DPIGII-
DRPIGI--
-DPIGIY-
-DPIGIV-
-DPIGIR-
-DPIGIM-
-DPIGIL-
-DPIGIF-
DLPIGI--
DIPIGI--
DWPIGI--
-DPIGIS-
-DPIGIH-
-DPIGIA-
DNPIGI--
DMPIGI--
DFPIGI--
DAPIGI--
-EPIGIL-
-EPIGIR-
ESPIGI--
ERPIGI--
-EPIGIW-
-EPIGIS-
ELPIGI--
EGPIGI--
ETPIGI--
-EPIGIY-

-EPIGIK-
-EPIGII-
EHPIGI--
EYPIGI--
-EPIGIV-
-EPIGIT-
-EPIGIQ-
EIPIGI--
FVPIGI--
FRPIGI--
FKPIGI--
FWPIGI--
FPPIGI--
FYPIGI--
FHPIGI--
FAPIGI--
FTPIGI--
-FPIGIV-
FLPIGI--
-FPIGIH-
FMPIGI--
-FPIGIR-
-FPIGIQ-
FDPIGI--
FSPIGI--
-FPIGIT-
-FPIGIN-
-FPIGIK-
-FPIGII-
-FPIGIG-
-FPIGIE-
-FPIGIA-
FIPIGI--
-GPIGIY-
-GPIGIL-
-GPIGIS-
GYPIGI--
GRPIGI--
-GPIGIV-
-GPIGIR-
-GPIGIM-
GVPIGI--
GLPIGI--
GAPIGI--
-GPIGIN-
GIPIGI--
GSPIGI--
-GPIGIT-
-GPIGIF-
GKPIGI--

GDPIGI--
GCPIGI--
GTPIGI--
-GPIGIW-
-GPIGIK-
-GPIGIE-
-GPIGIA-
GMPIGI--
GHPIGI--
GEPIGI--
GWPIGI--
-GPIGII-
GGPIGI--
GFPIGI--
HRPIGI--
HLPIGI--
-HPIGIW-
-HPIGIL-
HNPIGI--
HYPIGI--
HWPIGI--
HVPIGI--
HSPIGI--
HAPIGI--
HQPIGI--
-HPIGIT-
-HPIGIN-
-HPIGIF-
HTPIGI--
-HPIGIS-
HKPIGI--
HHPIGI--
HGPIGI--
HPPIGI--
-HPIGIV-
-HPIGIK-
-HPIGII-
-HPIGIH-
-HPIGIG-
HEPIGI--
HDPIGI--
-IPIGIL-
-IPIGIR-
ILPIGI--
IVPIGI--
-IPIGIQ-
IPPIGI--
IEPIGI--
IRPIGI--
-IPIGIY-

INPIGI--
IWPIGI--
-IPIGIA-
IGPIGI--
IAPIGI--
IQPIGI--
-IPIGIK-
-IPIGII-
IYPIGI--
-IPIGIM-
IIPIGI--
ISPIGI--
-IPIGIV-
-IPIGIH-
IMPIGI--
KSPIGI--
-KPIGIT-
KRPIGI--
-KPIGIY-
KGPIGI--
KDPIGI--
-KPIGIW-
-KPIGIF-
-KPIGII-
-KPIGIQ-
KWPIGI--
KQPIGI--
KPPIGI--
-KPIGIM-
KFPIGI--
-KPIGIS-
-KPIGIH-
-KPIGIN-
-KPIGIG-
KMPIGI--
KIPIGI--
KHPIGI--
KAPIGI--
LGPIGI--
-LPIGIR-
LIPIGI--
LYPIGI--
LHPIGI--
-LPIGII-
LPPIGI--
LRPIGI--
-LPIGIV-
-LPIGIS-
LKPIGI--
LSPIGI--

LFPIGI--
LTPIGI--
LQPIGI--
-LPIGIQ-
LMPIGI--
-LPIGIT-
LVPIGI--
-LPIGIY-
LDPIGI--
LAPIGI--
-LPIGIN-
-LPIGIF-
LNPIGI--
-LPIGIM-
-LPIGIK-
-LPIGIH-
-LPIGIA-
-MPIGIF-
MHPIGI--
MRPIGI--
MKPIGI--
MTPIGI--
MGPIGI--
-MPIGIT-
-MPIGIL-
MFPIGI--
MSPIGI--
-MPIGIR-
-MPIGII-
-MPIGIA-
MLPIGI--
MIPIGI--
MDPIGI--
MYPIGI--
-MPIGIG-
MAPIGI--
-MPIGIS-
-MPIGIQ-
-MPIGIM-
-MPIGIK-
-MPIGIH-
MMPIGI--
MCPIGI--
NMPIGI--
NYPIGI--
NKPIGI--
NRPIGI--
-NPIGIK-
-NPIGIS-
NQPIGI--

```
-NPIGIY-
NCPIGI--
NTPIGI--
NSPIGI--
NPPIGI--
-NPIGIR-
-NPIGIN-
-NPIGIM-
NNPIGI--
NGPIGI--
-NPIGIL-
-NPIGIH-
NLPIGI--
NIPIGI--
NWPIGI--
NVPIGI--
-NPIGIV-
-NPIGIQ-
-NPIGII-
-NPIGIE-
NHPIGI--
NFPIGI--
NEPIGI--
--PIGIRG
--PIGITR
--PIGISI
--PIGILN
--PIGIYE
--PIGIRL
--PIGITG
--PIGIRS
--PIGISR
--PIGIHV
--PIGIYG
--PIGIWD
--PIGIVR
--PIGIKR
--PIGIYT
--PIGIML
--PIGILS
PNPIGI--
--PIGIYS
--PIGIRE
--PIGIKS
--PIGIIR
--PIGIST
--PIGITS
--PIGISL
--PIGIRY
--PIGILT
```

--PIGILR
--PIGIHL
--PIGITL
--PIGISS
--PIGIRK
--PIGILH
--PIGIKW
--PIGIIS
--PIGIHR
--PIGIFS
--PIGIFR
--PIGIYD
--PIGITY
--PIGISN
--PIGISF
--PIGIRR
--PIGIMT
--PIGIMG
--PIGILA
--PIGIKA
--PIGIIA
--PIGIAG
--PIGIVS
--PIGIVM
--PIGITM
--PIGITI
--PIGISY
--PIGISM
--PIGIRA
--PIGILM
--PIGILD
--PIGIIG
--PIGIHN
--PIGIHG
--PIGIGW
--PIGIVI
--PIGIRQ
--PIGIQA
--PIGIMN
--PIGILQ
--PIGILK
--PIGILF
--PIGIKY
--PIGIIW
--PIGIIK
--PIGIHY
--PIGIFM
--PIGIYR
--PIGIVW
--PIGIVG

--PIGIVE
--PIGITV
--PIGITT
--PIGITA
--PIGIRW
--PIGIRM
--PIGINS
--PIGIMR
--PIGIMQ
--PIGIMK
--PIGIMD
--PIGIKI
--PIGIKD
--PIGIID
--PIGIHA
--PIGIAV
PWPIGI--
PTPIGI--
--PIGIYH
--PIGIVV
--PIGIVT
--PIGIVQ
--PIGIVN
--PIGIVK
--PIGITF
--PIGIRH
--PIGIRF
--PIGIQN
--PIGIQH
--PIGIQD
--PIGINW
--PIGINF
--PIGIMF
--PIGILG
--PIGIKQ
--PIGIKK
--PIGIKF
--PIGIIV
--PIGIIM
--PIGIHS
--PIGIHQ
--PIGIGG
--PIGIGA
--PIGIAS
--PIGIAQ
--PIGIAF
--PIGIYW
--PIGIYN
--PIGIYK
--PIGIWR

--PIGIWN
--PIGIWG
--PIGIWE
--PIGIWC
--PIGIVF
--PIGIVA
--PIGITQ
--PIGITK
--PIGISW
--PIGISV
--PIGISQ
--PIGISG
--PIGIRT
--PIGIRD
--PIGIQY
--PIGIQM
--PIGIQG
--PIGINY
--PIGINK
--PIGING
--PIGILY
--PIGILW
--PIGILV
--PIGIKV
--PIGIKT
--PIGIKL
--PIGIKG
--PIGIIY
--PIGIIH
--PIGIHM
--PIGIHK
--PIGIHH
--PIGIHF
--PIGIGV
--PIGIGL
--PIGIGI
--PIGIFT
--PIGIFH
--PIGIFG
--PIGIFD
--PIGIEV
--PIGIEG
--PIGIEA
--PIGICW
--PIGIAY
--PIGIAW
--PIGIAR
--PIGIAL
PQPIGI--
PKPIGI--

PIPIGI--
--PIGIYQ
--PIGIWQ
--PIGIWH
--PIGIVD
--PIGITW
--PIGISD
--PIGIRV
--PIGIRC
--PIGIPS
--PIGIPR
--PIGINV
--PIGINT
--PIGINL
--PIGINI
--PIGIMV
--PIGILE
--PIGIKN
--PIGIIT
--PIGIII
--PIGIIE
--PIGIHW
--PIGIHE
--PIGIGY
--PIGIGT
--PIGIGP
--PIGIFK
--PIGIFI
--PIGIEY
--PIGIES
--PIGIEM
--PIGIDT
--PIGIAI
PHPIGI--
PGPIGI--
PFPIGI--
PAPIGI--
QVPIGI--
QGPIGI--
-QPIGIL-
QSPIGI--
-QPIGIV-
QYPIGI--
-QPIGIR-
-QPIGIH-
-QPIGIA-
QLPIGI--
QTPIGI--
-QPIGIN-
QMPIGI--

-QPIGIW-
-QPIGIS-
-QPIGIQ-
-QPIGIF-
QNPIGI--
QWPIGI--
QQPIGI--
QPPIGI--
-QPIGIG-
QKPIGI--
QIPIGI--
QHPIGI--
RFPIGI--
RGPIGI--
RSPIGI--
RNPIGI--
RLPIGI--
RTPIGI--
RIPIGI--
RVPIGI--
-RPIGIN-
-RPIGIK-
RRPIGI--
-RPIGIV-
-RPIGIQ-
RAPIGI--
-RPIGIT-
-RPIGIA-
RMPIGI--
RDPIGI--
RQPIGI--
-RPIGIM-
RHPIGI--
RPPIGI--
-RPIGIS-
-RPIGIR-
-RPIGIY-
-RPIGIH-
RCPIGI--
RWPIGI--
-RPIGIG-
-SPIGIS-
-SPIGIR-
SYPIGI--
SLPIGI--
STPIGI--
-SPIGIL-
-SPIGIN-
-SPIGIH-
-SPIGIA-

SMPIGI--
SSPIGI--
-SPIGIT-
SGPIGI--
SAPIGI--
SVPIGI--
-SPIGIQ-
SIPIGI--
SEPIGI--
-SPIGIM-
-SPIGIK-
SNPIGI--
SPPIGI--
-SPIGIV-
SKPIGI--
SFPIGI--
-SPIGIW-
-SPIGIF-
SCPIGI--
-TPIGIL-
TGPIGI--
TRPIGI--
-TPIGIS-
TQPIGI--
TYPIGI--
TKPIGI--
TPPIGI--
TLPIGI--
-TPIGIH-
-TPIGIY-
-TPIGIT-
TIPIGI--
TEPIGI--
TTPIGI--
-TPIGIR-
-TPIGIA-
-TPIGIK-
-TPIGII-
TMPIGI--
TAPIGI--
TWPIGI--
-TPIGIV-
-TPIGIN-
TFPIGI--
TNPIGI--
THPIGI--
VIPIGI--
VRPIGI--
-VPIGIH-
-VPIGIR-

VNPIGI--
VVPIGI--
-VPIGIL-
-VPIGIA-
-VPIGIV-
-VPIGIS-
-VPIGIT-
VGPIGI--
-VPIGIK-
VKPIGI--
VSPIGI--
VPPIGI--
-VPIGIY-
-VPIGIW-
-VPIGIQ-
VHPIGI--
VYPIGI--
-VPIGIN-
-VPIGIM-
VMPIGI--
VFPIGI--
VDPIGI--
VCPIGI--
VAPIGI--
VTPIGI--
VQPIGI--
WRPIGI--
-WPIGIV-
-WPIGIT-
WGPIGI--
WVPIGI--
-WPIGIL-
WLPIGI--
WKPIGI--
WIPIGI--
WEPIGI--
WYPIGI--
WPPIGI--
-WPIGIQ-
-WPIGIK-
WAPIGI--
WSPIGI--
-WPIGIN-
-WPIGIM-
-WPIGII-
WHPIGI--
WFPIGI--
-YPIGIR-
YFPIGI--
-YPIGIL-

| | | | |
|---|---|---|---|
| | -YPIGIV- | | |
| | -YPIGIS- | | |
| | YSPIGI-- | | |
| | YTPIGI-- | | |
| | YRPIGI-- | | |
| | -YPIGIN- | | |
| | -YPIGIK- | | |
| | YLPIGI-- | | |
| | YVPIGI-- | | |
| | -YPIGII- | | |
| | YEPIGI-- | | |
| | -YPIGIF- | | |
| | YMPIGI-- | | |
| | -YPIGIA- | | |
| | -YPIGIQ- | | |
| | -YPIGIH- | | |
| | YAPIGI-- | | |
| | YWPIGI-- | | |
| | YPPIGI-- | | |
| | -YPIGIW- | | |
| | YKPIGI-- | | |
| | YGPIGI-- | | |
| | YDPIGI-- | | |
| PLSY | 190 | 720 | 31.25 |
| | DKPLSY-- | | |
| | -NPLSYN- | | |
| | --PLSYVD | | |
| | --PLSYTP | | |
| | --PLSYNI | | |
| | --PLSYLE | | |
| | --PLSYHL | | |
| | --PLSYGT | | |
| | --PLSYAN | | |
| | -RPLSYP- | | |
| | -SPLSYG- | | |
| | AYPLSY-- | | |
| | AVPLSY-- | | |
| | -APLSYL- | | |
| | -APLSYM- | | |
| | -APLSYF- | | |
| | ATPLSY-- | | |
| | ARPLSY-- | | |
| | ANPLSY-- | | |
| | AKPLSY-- | | |
| | ALPLSY-- | | |
| | AMPLSY-- | | |
| | AHPLSY-- | | |
| | APPLSY-- | | |
| | -APLSYS- | | |
| | -APLSYQ- | | |

AGPLSY--
AWPLSY--
AFPLSY--
-APLSYY-
-APLSYI-
-APLSYK-
AIPLSY--
-APLSYW-
-APLSYN-
-APLSYH-
-APLSYG-
-APLSYA-
ACPLSY--
-APLSYE-
ADPLSY--
CRPLSY--
-CPLSYR-
-CPLSYV-
-CPLSYI-
-CPLSYT-
CNPLSY--
CYPLSY--
CVPLSY--
CLPLSY--
CKPLSY--
CGPLSY--
CTPLSY--
CSPLSY--
CQPLSY--
CPPLSY--
-CPLSYW-
-CPLSYQ-
-CPLSYL-
-CPLSYF-
CMPLSY--
CIPLSY--
CEPLSY--
CDPLSY--
CCPLSY--
DSPLSY--
-DPLSYI-
-DPLSYQ-
DLPLSY--
DIPLSY--
-DPLSYL-
DQPLSY--
-DPLSYR-
-DPLSYM-
DTPLSY--
DRPLSY--

-DPLSYV-
-DPLSYS-
-DPLSYK-
DHPLSY--
DAPLSY--
DVPLSY--
-DPLSYW-
DYPLSY--
-DPLSYF-
DMPLSY--
DFPLSY--
DDPLSY--
-DPLSYY-
DWPLSY--
-DPLSYT-
-DPLSYA-
DGPLSY--
DEPLSY--
ESPLSY--
ELPLSY--
-EPLSYI-
EGPLSY--
ERPLSY--
EFPLSY--
EYPLSY--
-EPLSYR-
-EPLSYL-
-EPLSYQ-
EMPLSY--
-EPLSYK-
-EPLSYA-
EIPLSY--
EVPLSY--
-EPLSYV-
-EPLSYS-
EEPLSY--
ETPLSY--
EKPLSY--
-EPLSYY-
-EPLSYM-
-EPLSYH-
-EPLSYF-
EWPLSY--
EQPLSY--
EPPLSY--
-EPLSYP-
EHPLSY--
ECPLSY--
FRPLSY--
-FPLSYV-

-FPLSYT-
FAPLSY--
FIPLSY--
FPPLSY--
FTPLSY--
FLPLSY--
-FPLSYS-
-FPLSYH-
FFPLSY--
FSPLSY--
FKPLSY--
-FPLSYK-
FVPLSY--
-FPLSYY-
-FPLSYA-
FNPLSY--
-FPLSYN-
-FPLSYF-
FWPLSY--
-FPLSYQ-
FEPLSY--
-FPLSYM-
-GPLSYL-
-GPLSYQ-
GKPLSY--
GTPLSY--
GGPLSY--
GSPLSY--
GRPLSY--
-GPLSYS-
GVPLSY--
-GPLSYM-
GNPLSY--
GFPLSY--
-GPLSYR-
-GPLSYW-
-GPLSYA-
-GPLSYV-
-GPLSYT-
-GPLSYN-
GWPLSY--
GMPLSY--
GQPLSY--
GHPLSY--
GDPLSY--
GYPLSY--
GPPLSY--
GEPLSY--
GAPLSY--
-GPLSYY-

```
-GPLSYK-
-GPLSYE-
-GPLSYG-
GIPLSY--
-HPLSYR-
HTPLSY--
-HPLSYS-
HSPLSY--
-HPLSYH-
HGPLSY--
-HPLSYL-
HFPLSY--
HPPLSY--
-HPLSYV-
HMPLSY--
HVPLSY--
HQPLSY--
-HPLSYA-
-HPLSYY-
-HPLSYI-
HNPLSY--
HKPLSY--
HEPLSY--
-HPLSYQ-
-HPLSYK-
-HPLSYT-
HIPLSY--
HYPLSY--
-HPLSYM-
HHPLSY--
HDPLSY--
HAPLSY--
HWPLSY--
-HPLSYN-
-HPLSYG-
HCPLSY--
IRPLSY--
ITPLSY--
ISPLSY--
-IPLSYR-
IQPLSY--
INPLSY--
IGPLSY--
IFPLSY--
-IPLSYS-
IVPLSY--
-IPLSYV-
-IPLSYN-
-IPLSYA-
ILPLSY--
```

IKPLSY--
IDPLSY--
-IPLSYH-
-IPLSYK-
IEPLSY--
IYPLSY--
-IPLSYM-
-IPLSYF-
IMPLSY--
IIPLSY--
IWPLSY--
-IPLSYT-
-IPLSYQ-
IPPLSY--
-IPLSYW-
IHPLSY--
ICPLSY--
KSPLSY--
KTPLSY--
-KPLSYI-
KGPLSY--
-KPLSYF-
KAPLSY--
KKPLSY--
KLPLSY--
KYPLSY--
-KPLSYW-
KFPLSY--
KNPLSY--
KIPLSY--
KWPLSY--
-KPLSYQ-
KHPLSY--
KVPLSY--
-KPLSYY-
-KPLSYN-
-KPLSYA-
KEPLSY--
-KPLSYT-
KQPLSY--
KDPLSY--
KMPLSY--
KCPLSY--
-LPLSYT-
LGPLSY--
LVPLSY--
LNPLSY--
-LPLSYV-
LHPLSY--
-LPLSYR-

LMPLSY--
-LPLSYY-
LQPLSY--
LPPLSY--
-LPLSYI-
LIPLSY--
LKPLSY--
-LPLSYW-
LYPLSY--
-LPLSYF-
-LPLSYK-
-LPLSYQ-
LDPLSY--
-LPLSYH-
LCPLSY--
-LPLSYN-
-MPLSYL-
-MPLSYR-
MNPLSY--
-MPLSYV-
MLPLSY--
-MPLSYK-
MKPLSY--
MGPLSY--
MVPLSY--
MPPLSY--
MHPLSY--
MMPLSY--
MFPLSY--
MTPLSY--
-MPLSYM-
MWPLSY--
MAPLSY--
MQPLSY--
-MPLSYQ-
-MPLSYN-
-MPLSYH-
-MPLSYI-
-MPLSYA-
MDPLSY--
-MPLSYP-
MCPLSY--
-NPLSYS-
NRPLSY--
-NPLSYL-
-NPLSYR-
NFPLSY--
NAPLSY--
-NPLSYT-
NVPLSY--

NSPLSY--
NTPLSY--
NPPLSY--
NYPLSY--
NQPLSY--
NKPLSY--
-NPLSYY-
-NPLSYI-
NWPLSY--
-NPLSYQ-
NDPLSY--
NGPLSY--
-NPLSYA-
NNPLSY--
NMPLSY--
-NPLSYM-
NCPLSY--
-NPLSYW-
NLPLSY--
-NPLSYF-
NEPLSY--
-NPLSYE-
--PLSYLK
--PLSYLG
--PLSYRS
--PLSYIR
--PLSYTR
--PLSYSV
--PLSYFG
--PLSYMR
--PLSYTG
--PLSYRG
--PLSYTS
--PLSYRA
--PLSYMK
--PLSYKS
--PLSYST
--PLSYRN
--PLSYYS
--PLSYES
--PLSYRW
--PLSYRV
--PLSYVV
--PLSYTM
--PLSYSM
--PLSYSF
--PLSYMS
--PLSYKT
--PLSYWV
--PLSYVG

--PLSYRY
--PLSYVR
--PLSYVN
--PLSYVK
--PLSYRH
--PLSYMT
--PLSYFS
--PLSYWL
--PLSYYA
--PLSYVT
--PLSYVS
--PLSYTV
--PLSYTN
--PLSYYT
--PLSYTL
--PLSYTF
--PLSYSG
--PLSYRQ
--PLSYQT
--PLSYIT
--PLSYIS
--PLSYAT
--PLSYYG
--PLSYVW
--PLSYTT
--PLSYNS
--PLSYMQ
--PLSYMM
--PLSYMG
--PLSYKW
--PLSYIQ
--PLSYHS
--PLSYWA
--PLSYVH
--PLSYRM
--PLSYRF
--PLSYRE
--PLSYQG
--PLSYMW
--PLSYMH
--PLSYKN
PHPLSY--
--PLSYQW
--PLSYMV
--PLSYMA
--PLSYKV
--PLSYIN
--PLSYIH
--PLSYGL
--PLSYFM

--PLSYAL
--PLSYAI
--PLSYWK
--PLSYVA
--PLSYTQ
--PLSYTK
--PLSYII
--PLSYIA
--PLSYHG
--PLSYGS
--PLSYYH
--PLSYWN
--PLSYQR
--PLSYMY
--PLSYFN
--PLSYAG
PNPLSY--
--PLSYYQ
--PLSYWS
--PLSYWM
--PLSYVI
--PLSYTY
--PLSYTW
--PLSYQS
--PLSYNA
--PLSYKY
--PLSYKL
--PLSYHT
--PLSYYM
--PLSYWG
--PLSYNV
--PLSYNF
--PLSYKF
--PLSYIM
--PLSYHA
--PLSYEA
--PLSYVE
--PLSYTH
--PLSYSN
--PLSYSK
--PLSYQF
--PLSYNG
--PLSYMI
--PLSYLN
--PLSYHF
--PLSYGG
--PLSYGA
--PLSYFQ
--PLSYEG
--PLSYAV

--PLSYYK
--PLSYYE
--PLSYTE
--PLSYSQ
--PLSYQY
--PLSYQV
--PLSYQN
--PLSYQH
--PLSYQA
--PLSYNW
--PLSYMN
--PLSYKI
--PLSYKH
--PLSYKD
--PLSYHV
--PLSYHM
--PLSYAQ
--PLSYAM
PFPLSY--
PEPLSY--
PWPLSY--
PQPLSY--
-PPLSYT-
--PLSYWQ
--PLSYWH
--PLSYVQ
--PLSYTI
--PLSYTD
--PLSYSP
--PLSYQQ
--PLSYQM
--PLSYQL
--PLSYNY
--PLSYNM
--PLSYME
--PLSYKQ
--PLSYIE
--PLSYID
--PLSYHQ
--PLSYHI
--PLSYGK
--PLSYGI
--PLSYEV
--PLSYET
--PLSYDN
--PLSYDI
--PLSYCT
--PLSYAH
PDPLSY--
QTPLSY--

-QPLSYR-
QNPLSY--
QSPLSY--
-QPLSYF-
QGPLSY--
-QPLSYL-
QLPLSY--
QAPLSY--
QVPLSY--
QYPLSY--
QRPLSY--
QPPLSY--
-QPLSYV-
QKPLSY--
-QPLSYY-
-QPLSYS-
-QPLSYM-
-QPLSYK-
-QPLSYH-
-QPLSYT-
-QPLSYQ-
-QPLSYI-
-QPLSYW-
QWPLSY--
QIPLSY--
QHPLSY--
QMPLSY--
QFPLSY--
QQPLSY--
-QPLSYA-
QEPLSY--
RLPLSY--
-RPLSYS-
-RPLSYA-
RGPLSY--
-RPLSYM-
RFPLSY--
-RPLSYW-
RWPLSY--
RAPLSY--
-RPLSYK-
-RPLSYQ-
-RPLSYN-
RTPLSY--
RYPLSY--
RPPLSY--
-RPLSYF-
-RPLSYY-
-RPLSYH-
RNPLSY--

REPLSY--
RHPLSY--
RDPLSY--
RCPLSY--
-RPLSYG-
-SPLSYS-
SLPLSY--
SKPLSY--
SNPLSY--
SVPLSY--
SSPLSY--
SGPLSY--
STPLSY--
-SPLSYY-
-SPLSYT-
-SPLSYF-
-SPLSYM-
-SPLSYI-
SMPLSY--
SIPLSY--
-SPLSYA-
SAPLSY--
SQPLSY--
SHPLSY--
-SPLSYH-
-SPLSYW-
SEPLSY--
SPPLSY--
SWPLSY--
-SPLSYN-
-SPLSYE-
SDPLSY--
-TPLSYI-
TRPLSY--
-TPLSYV-
-TPLSYL-
TSPLSY--
-TPLSYT-
TTPLSY--
-TPLSYS-
-TPLSYM-
-TPLSYQ-
TAPLSY--
TKPLSY--
TIPLSY--
TWPLSY--
TPPLSY--
THPLSY--
TQPLSY--
-TPLSYA-

TLPLSY--
TVPLSY--
TNPLSY--
TMPLSY--
TGPLSY--
TEPLSY--
-TPLSYK-
TFPLSY--
-TPLSYN-
TYPLSY--
TDPLSY--
-TPLSYH-
-VPLSYL-
VRPLSY--
VLPLSY--
VFPLSY--
VVPLSY--
VQPLSY--
-VPLSYA-
VGPLSY--
VMPLSY--
VDPLSY--
VTPLSY--
-VPLSYV-
-VPLSYT-
-VPLSYS-
-VPLSYR-
VNPLSY--
-VPLSYM-
VEPLSY--
-VPLSYI-
-VPLSYN-
VPPLSY--
-VPLSYH-
VHPLSY--
-VPLSYW-
-VPLSYQ-
-VPLSYK-
VIPLSY--
VWPLSY--
WYPLSY--
WSPLSY--
-WPLSYI-
WKPLSY--
WFPLSY--
-WPLSYK-
WHPLSY--
-WPLSYQ-
-WPLSYL-
WNPLSY--

WAPLSY--
-WPLSYY-
-WPLSYV-
WDPLSY--
WIPLSY--
WWPLSY--
WQPLSY--
-WPLSYT-
-WPLSYM-
-WPLSYE-
-WPLSYA-
WEPLSY--
YLPLSY--
-YPLSYR-
YRPLSY--
-YPLSYS-
-YPLSYH-
YSPLSY--
-YPLSYA-
YKPLSY--
YVPLSY--
YTPLSY--
-YPLSYT-
YHPLSY--
YWPLSY--
-YPLSYK-
YGPLSY--
YQPLSY--
YNPLSY--
YEPLSY--
YMPLSY--
YIPLSY--
-YPLSYY-
-YPLSYM-
YDPLSY--
-YPLSYQ-
-YPLSYF-
YYPLSY--
YPPLSY--
-YPLSYV-
YAPLSY--
-YPLSYN-
-YPLSYG-
YCPLSY--

| | | | |
|---|---|---|---|
| PSGF | 61 | 796 | 31.24 |

DFPSGF--
-IPSGFG-
-LPSGFG-
-NPSGFA-
--PSGFVI

```
--PSGFTL
--PSGFLI
--PSGFKH
--PSGFHT
--PSGFGT
--PSGFFQ
--PSGFAV
--PSGFAI
-QPSGFG-
-RPSGFG-
-SPSGFD-
-APSGFL-
-APSGFT-
ASPSGF--
-APSGFS-
-APSGFR-
AFPSGF--
AVPSGF--
APPSGF--
AYPSGF--
-APSGFF-
AKPSGF--
ATPSGF--
-APSGFN-
-APSGFH-
ACPSGF--
-APSGFY-
AAPSGF--
ARPSGF--
ANPSGF--
AGPSGF--
AWPSGF--
AQPSGF--
-APSGFE-
-APSGFA-
AIPSGF--
ADPSGF--
AEPSGF--
-APSGFQ-
-APSGFM-
-APSGFG-
AMPSGF--
AHPSGF--
-APSGFW-
-APSGFC-
CRPSGF--
-CPSGFL-
CHPSGF--
CVPSGF--
CTPSGF--
```

-CPSGFY-
CLPSGF--
CSPSGF--
CGPSGF--
CAPSGF--
CWPSGF--
-CPSGFW-
-CPSGFR-
CMPSGF--
CKPSGF--
CIPSGF--
DRPSGF--
-DPSGFS-
-DPSGFM-
DWPSGF--
-DPSGFI-
DHPSGF--
DVPSGF--
DQPSGF--
-DPSGFY-
-DPSGFR-
DGPSGF--
DTPSGF--
-DPSGFT-
-DPSGFN-
-DPSGFL-
DYPSGF--
-DPSGFF-
DLPSGF--
DKPSGF--
DIPSGF--
DEPSGF--
DCPSGF--
DAPSGF--
-EPSGFT-
-EPSGFS-
ERPSGF--
EKPSGF--
ESPSGF--
-EPSGFL-
-EPSGFK-
EWPSGF--
ETPSGF--
-EPSGFR-
-EPSGFM-
-EPSGFH-
EVPSGF--
EHPSGF--
-EPSGFY-
-EPSGFI-

ENPSGF--
ELPSGF--
EGPSGF--
EYPSGF--
EPPSGF--
EMPSGF--
EFPSGF--
ECPSGF--
EAPSGF--
EQPSGF--
-EPSGFW-
-EPSGFN-
EIPSGF--
EEPSGF--
EDPSGF--
-FPSGFK-
FRPSGF--
-FPSGFL-
-FPSGFS-
-FPSGFR-
FTPSGF--
FSPSGF--
-FPSGFH-
-FPSGFY-
-FPSGFM-
FDPSGF--
-FPSGFV-
FYPSGF--
FLPSGF--
FGPSGF--
-FPSGFT-
FMPSGF--
FFPSGF--
-FPSGFW-
-FPSGFI-
FNPSGF--
FKPSGF--
FHPSGF--
FQPSGF--
FVPSGF--
-FPSGFN-
-FPSGFF-
FIPSGF--
FAPSGF--
-FPSGFQ-
-FPSGFA-
FEPSGF--
-GPSGFT-
-GPSGFK-
GSPSGF--

-GPSGFN-
GYPSGF--
-GPSGFL-
-GPSGFF-
GFPSGF--
GVPSGF--
-GPSGFM-
GLPSGF--
-GPSGFH-
GKPSGF--
GPPSGF--
GTPSGF--
-GPSGFA-
GHPSGF--
-GPSGFY-
-GPSGFW-
-GPSGFV-
GWPSGF--
-GPSGFI-
GNPSGF--
GQPSGF--
-GPSGFQ-
-GPSGFS-
-GPSGFE-
GMPSGF--
GIPSGF--
GGPSGF--
GEPSGF--
-GPSGFG-
GDPSGF--
GAPSGF--
HSPSGF--
HTPSGF--
-HPSGFI-
-HPSGFT-
-HPSGFM-
-HPSGFV-
-HPSGFR-
HYPSGF--
HVPSGF--
HGPSGF--
HWPSGF--
-HPSGFY-
-HPSGFL-
-HPSGFA-
HFPSGF--
HMPSGF--
HAPSGF--
HHPSGF--
HQPSGF--

−HPSGFN−
HNPSGF−−
HDPSGF−−
−HPSGFH−
HPPSGF−−
HEPSGF−−
−HPSGFQ−
IRPSGF−−
−IPSGFS−
INPSGF−−
−IPSGFT−
ISPSGF−−
IIPSGF−−
−IPSGFR−
IGPSGF−−
−IPSGFY−
ILPSGF−−
IFPSGF−−
IYPSGF−−
IQPSGF−−
ICPSGF−−
−IPSGFM−
IKPSGF−−
IHPSGF−−
−IPSGFV−
−IPSGFN−
−IPSGFK−
−IPSGFI−
−IPSGFF−
−IPSGFA−
IWPSGF−−
IVPSGF−−
−IPSGFH−
IPPSGF−−
−IPSGFQ−
IEPSGF−−
IDPSGF−−
IAPSGF−−
ITPSGF−−
−IPSGFW−
−IPSGFC−
IMPSGF−−
KSPSGF−−
KEPSGF−−
−KPSGFV−
KVPSGF−−
KIPSGF−−
KQPSGF−−
KGPSGF−−
−KPSGFK−

KCPSGF--
-KPSGFM-
-KPSGFQ-
KNPSGF--
-KPSGFW-
-KPSGFT-
-KPSGFR-
-KPSGFS-
-KPSGFH-
KLPSGF--
KFPSGF--
KYPSGF--
KTPSGF--
-KPSGFY-
KMPSGF--
-KPSGFI-
-KPSGFF-
KAPSGF--
KKPSGF--
KHPSGF--
-KPSGFE-
KPPSGF--
KDPSGF--
LSPSGF--
-LPSGFR-
-LPSGFL-
LVPSGF--
LPPSGF--
LWPSGF--
-LPSGFF-
-LPSGFS-
-LPSGFI-
LLPSGF--
LNPSGF--
-LPSGFK-
LIPSGF--
LKPSGF--
LYPSGF--
-LPSGFT-
-LPSGFH-
LTPSGF--
LAPSGF--
-LPSGFW-
LMPSGF--
LGPSGF--
LFPSGF--
LEPSGF--
-LPSGFQ-
-LPSGFV-
LQPSGF--

```
-LPSGFM-
-LPSGFN-
LDPSGF--
LCPSGF--
MTPSGF--
MRPSGF--
-MPSGFH-
MWPSGF--
-MPSGFT-
-MPSGFF-
MSPSGF--
-MPSGFN-
MPPSGF--
MKPSGF--
-MPSGFR-
-MPSGFL-
-MPSGFK-
-MPSGFA-
MLPSGF--
MAPSGF--
MGPSGF--
MFPSGF--
MYPSGF--
MVPSGF--
MQPSGF--
-MPSGFY-
MMPSGF--
MEPSGF--
MCPSGF--
-MPSGFW-
-MPSGFS-
MDPSGF--
-MPSGFI-
NSPSGF--
-NPSGFL-
-NPSGFY-
-NPSGFS-
-NPSGFM-
NTPSGF--
NRPSGF--
NQPSGF--
NLPSGF--
-NPSGFR-
NFPSGF--
-NPSGFI-
NAPSGF--
-NPSGFF-
NYPSGF--
NHPSGF--
NGPSGF--
```

NVPSGF--
NIPSGF--
NNPSGF--
NWPSGF--
-NPSGFW-
-NPSGFK-
NMPSGF--
NDPSGF--
-NPSGFT-
-NPSGFQ-
-NPSGFN-
NPPSGF--
NKPSGF--
--PSGFRR
--PSGFRS
--PSGFSG
--PSGFKS
--PSGFRL
--PSGFRT
--PSGFLS
--PSGFLA
--PSGFSS
--PSGFSN
--PSGFYR
--PSGFWG
--PSGFRG
--PSGFLG
--PSGFTK
--PSGFIS
--PSGFFR
--PSGFAT
--PSGFAS
--PSGFYK
--PSGFVT
--PSGFYG
--PSGFVG
--PSGFRN
--PSGFMR
--PSGFHS
--PSGFTW
--PSGFSA
--PSGFRI
--PSGFAA
--PSGFTV
--PSGFRD
--PSGFML
--PSGFFG
--PSGFSY
--PSGFKG
--PSGFIR

--PSGFTN
--PSGFTF
--PSGFMT
--PSGFIW
--PSGFES
--PSGFWS
--PSGFTG
--PSGFSR
--PSGFRY
--PSGFRW
--PSGFRF
--PSGFMW
--PSGFLW
--PSGFLF
--PSGFFK
PGPSGF--
--PSGFWT
--PSGFWK
--PSGFVV
--PSGFVR
--PSGFTS
--PSGFSK
--PSGFLK
--PSGFFV
--PSGFFH
--PSGFST
--PSGFNT
--PSGFMH
--PSGFHW
--PSGFHA
--PSGFEG
PRPSGF--
--PSGFYH
--PSGFTA
--PSGFSW
--PSGFSV
--PSGFSH
--PSGFRM
--PSGFRE
--PSGFRA
--PSGFQA
--PSGFNV
--PSGFNS
--PSGFLY
--PSGFKW
--PSGFKT
--PSGFKR
--PSGFKK
--PSGFHV
--PSGFHR

PAPSGF--
--PSGFYS
--PSGFYQ
--PSGFYA
--PSGFVY
--PSGFSL
--PSGFRK
--PSGFMV
--PSGFIG
--PSGFIF
--PSGFIA
--PSGFHY
--PSGFHM
--PSGFHL
--PSGFFM
PLPSGF--
--PSGFWW
--PSGFVW
--PSGFVN
--PSGFVM
--PSGFTY
--PSGFTH
--PSGFRV
--PSGFNF
--PSGFMN
--PSGFKV
--PSGFKM
--PSGFGG
PMPSGF--
PYPSGF--
PVPSGF--
--PSGFYI
--PSGFTT
--PSGFTI
--PSGFNG
--PSGFMQ
--PSGFMG
--PSGFLN
--PSGFKN
--PSGFKA
--PSGFIM
--PSGFHI
--PSGFFN
--PSGFAG
PSPSGF--
--PSGFQT
--PSGFQM
--PSGFQG
--PSGFQF
--PSGFMI

--PSGFKY
--PSGFIY
--PSGFIV
--PSGFIN
--PSGFHN
--PSGFFW
--PSGFFT
PHPSGF--
PTPSGF--
--PSGFYT
--PSGFWI
--PSGFWA
--PSGFVK
--PSGFSI
--PSGFRH
--PSGFQY
--PSGFQS
--PSGFNW
--PSGFMS
--PSGFMK
--PSGFMF
--PSGFLQ
--PSGFHH
--PSGFHG
--PSGFHD
--PSGFGS
--PSGFAF
PWPSGF--
--PSGFYM
--PSGFYF
--PSGFWN
--PSGFWL
--PSGFWH
--PSGFTQ
--PSGFSQ
--PSGFSM
--PSGFNN
--PSGFNH
--PSGFNA
--PSGFLM
--PSGFKF
--PSGFIT
--PSGFHF
--PSGFEW
PQPSGF--
PNPSGF--
--PSGFYD
--PSGFWQ
--PSGFTE
--PSGFTD

--PSGFQV
--PSGFQI
--PSGFQH
--PSGFNR
--PSGFNQ
--PSGFMM
--PSGFLH
--PSGFKQ
--PSGFIK
--PSGFID
--PSGFHQ
--PSGFHP
--PSGFFL
--PSGFFD
--PSGFDN
--PSGFAW
--PSGFAH
-PPSGFV-
-PPSGFT-
PIPSGF--
PDPSGF--
PCPSGF--
-QPSGFR-
QWPSGF--
-QPSGFT-
-QPSGFS-
QRPSGF--
-QPSGFY-
-QPSGFI-
QTPSGF--
QFPSGF--
-QPSGFH-
QYPSGF--
QSPSGF--
-QPSGFM-
QGPSGF--
-QPSGFW-
-QPSGFV-
-QPSGFK-
-QPSGFF-
-QPSGFA-
QNPSGF--
QIPSGF--
QQPSGF--
QHPSGF--
QDPSGF--
QAPSGF--
QVPSGF--
-QPSGFN-
QPPSGF--

QCPSGF--
RWPSGF--
RSPSGF--
-RPSGFL-
-RPSGFR-
-RPSGFS-
RGPSGF--
RTPSGF--
-RPSGFY-
RLPSGF--
-RPSGFF-
-RPSGFK-
RFPSGF--
-RPSGFM-
RMPSGF--
RKPSGF--
-RPSGFQ-
RIPSGF--
-RPSGFA-
RNPSGF--
-RPSGFI-
RAPSGF--
RPPSGF--
-RPSGFN-
RDPSGF--
RHPSGF--
-RPSGFH-
REPSGF--
-RPSGFE-
RYPSGF--
-RPSGFW-
RCPSGF--
-RPSGFC-
-SPSGFR-
SRPSGF--
-SPSGFI-
-SPSGFN-
-SPSGFV-
STPSGF--
-SPSGFW-
-SPSGFQ-
SAPSGF--
-SPSGFA-
SKPSGF--
-SPSGFF-
-SPSGFY-
-SPSGFM-
SYPSGF--
SSPSGF--
SMPSGF--

SFPSGF--
-SPSGFK-
SEPSGF--
-SPSGFT-
-SPSGFH-
SQPSGF--
SPPSGF--
SGPSGF--
SVPSGF--
SNPSGF--
SIPSGF--
SCPSGF--
-SPSGFG-
-SPSGFC-
-TPSGFL-
-TPSGFR-
TLPSGF--
-TPSGFW-
-TPSGFT-
TPPSGF--
TRPSGF--
-TPSGFS-
TGPSGF--
TSPSGF--
TEPSGF--
TTPSGF--
-TPSGFV-
TVPSGF--
-TPSGFQ-
-TPSGFH-
TMPSGF--
TDPSGF--
-TPSGFY-
-TPSGFA-
TKPSGF--
TFPSGF--
-TPSGFM-
TWPSGF--
TNPSGF--
TQPSGF--
-TPSGFN-
TYPSGF--
-TPSGFK-
-TPSGFF-
THPSGF--
TAPSGF--
TIPSGF--
TCPSGF--
-VPSGFR-
VLPSGF--

VTPSGF--
-VPSGFT-
-VPSGFV-
VWPSGF--
VQPSGF--
-VPSGFM-
-VPSGFL-
VKPSGF--
VRPSGF--
VVPSGF--
VSPSGF--
-VPSGFN-
-VPSGFK-
VGPSGF--
-VPSGFA-
VMPSGF--
VHPSGF--
-VPSGFI-
-VPSGFH-
VNPSGF--
-VPSGFY-
-VPSGFW-
VYPSGF--
VPPSGF--
-VPSGFF-
VIPSGF--
VEPSGF--
VDPSGF--
-VPSGFG-
VFPSGF--
VAPSGF--
WGPSGF--
-WPSGFT-
WSPSGF--
WKPSGF--
WWPSGF--
WRPSGF--
-WPSGFL-
-WPSGFY-
WNPSGF--
-WPSGFR-
WFPSGF--
-WPSGFH-
WMPSGF--
WIPSGF--
WEPSGF--
WYPSGF--
WTPSGF--
WQPSGF--
-WPSGFQ-

-WPSGFM-
-WPSGFK-
-WPSGFI-
-WPSGFF-
WDPSGF--
WAPSGF--
-WPSGFS-
-WPSGFE-
WPPSGF--
-WPSGFN-
WHPSGF--
-YPSGFR-
-YPSGFI-
-YPSGFL-
-YPSGFV-
YQPSGF--
YSPSGF--
-YPSGFN-
-YPSGFT-
-YPSGFK-
YVPSGF--
YTPSGF--
-YPSGFH-
-YPSGFS-
YRPSGF--
-YPSGFY-
YNPSGF--
YMPSGF--
YKPSGF--
YHPSGF--
YYPSGF--
-YPSGFM-
-YPSGFF-
YPPSGF--
YLPSGF--
YFPSGF--
YWPSGF--
YIPSGF--
YEPSGF--
YDPSGF--
-YPSGFQ-
PRNF 595 582 31.22
--PRNFWL
--PRNFGW
--PRNFFE
ASPRNF--
-APRNFG-
-APRNFT-
-APRNFM-
-APRNFL-

AMPRNF--
-APRNFF-
AAPRNF--
AYPRNF--
ALPRNF--
-APRNFY-
-APRNFV-
-APRNFI-
-APRNFD-
APPRNF--
AQPRNF--
-APRNFQ-
-APRNFN-
ANPRNF--
AGPRNF--
-APRNFA-
AKPRNF--
AEPRNF--
ADPRNF--
AVPRNF--
-APRNFH-
AHPRNF--
ACPRNF--
CSPRNF--
CVPRNF--
-CPRNFV-
CAPRNF--
CRPRNF--
CIPRNF--
CHPRNF--
CYPRNF--
CTPRNF--
-CPRNFT-
-CPRNFR-
-DPRNFR-
-DPRNFL-
-DPRNFS-
-DPRNFW-
-DPRNFA-
-DPRNFY-
-DPRNFF-
DSPRNF--
DRPRNF--
DQPRNF--
-DPRNFT-
DLPRNF--
DHPRNF--
DGPRNF--
DWPRNF--
DIPRNF--

DFPRNF--
DVPRNF--
-DPRNFQ-
-DPRNFM-
-DPRNFE-
DKPRNF--
DAPRNF--
-DPRNFI-
-DPRNFH-
DEPRNF--
ESPRNF--
-EPRNFV-
-EPRNFL-
EVPRNF--
EQPRNF--
-EPRNFY-
-EPRNFT-
-EPRNFS-
-EPRNFK-
-EPRNFF-
-EPRNFE-
EDPRNF--
-EPRNFN-
-EPRNFM-
-EPRNFG-
ELPRNF--
EIPRNF--
EGPRNF--
ETPRNF--
EEPRNF--
EAPRNF--
EYPRNF--
-EPRNFR-
-EPRNFI-
-EPRNFD-
ENPRNF--
EKPRNF--
EHPRNF--
FDPRNF--
FVPRNF--
FNPRNF--
FAPRNF--
FTPRNF--
FYPRNF--
FSPRNF--
FPPRNF--
FHPRNF--
-FPRNFH-
-FPRNFE-
-FPRNFF-

-FPRNFW-
FIPRNF--
FGPRNF--
FQPRNF--
FEPRNF--
GLPRNF--
-GPRNFT-
-GPRNFA-
-GPRNFS-
-GPRNFL-
-GPRNFH-
GTPRNF--
-GPRNFI-
GIPRNF--
-GPRNFM-
-GPRNFW-
GYPRNF--
GRPRNF--
-GPRNFY-
GVPRNF--
-GPRNFE-
-GPRNFV-
-GPRNFF-
GKPRNF--
GAPRNF--
-GPRNFQ-
-GPRNFG-
GNPRNF--
GCPRNF--
GQPRNF--
-GPRNFK-
GGPRNF--
GEPRNF--
HGPRNF--
-HPRNFE-
-HPRNFL-
-HPRNFI-
-HPRNFV-
-HPRNFT-
HQPRNF--
-HPRNFQ-
HAPRNF--
-HPRNFY-
-HPRNFS-
-HPRNFN-
-HPRNFM-
HVPRNF--
HTPRNF--
HSPRNF--
-HPRNFA-

HIPRNF--
-HPRNFF-
HKPRNF--
-HPRNFG-
HPPRNF--
HNPRNF--
ILPRNF--
IAPRNF--
-IPRNFY-
-IPRNFV-
-IPRNFG-
IMPRNF--
-IPRNFS-
IIPRNF--
-IPRNFF-
IVPRNF--
-IPRNFW-
IQPRNF--
-IPRNFQ-
-IPRNFH-
-IPRNFE-
-IPRNFN-
IPPRNF--
INPRNF--
IHPRNF--
IDPRNF--
ICPRNF--
-KPRNFI-
-KPRNFY-
-KPRNFM-
KGPRNF--
-KPRNFT-
-KPRNFH-
-KPRNFF-
KTPRNF--
-KPRNFV-
KIPRNF--
-KPRNFQ-
-KPRNFE-
KLPRNF--
-KPRNFR-
KEPRNF--
-KPRNFW-
KKPRNF--
KAPRNF--
-KPRNFN-
-KPRNFK-
-KPRNFG-
KHPRNF--
KDPRNF--

-LPRNFV-
LNPRNF--
LGPRNF--
LSPRNF--
LIPRNF--
-LPRNFM-
LQPRNF--
LAPRNF--
LHPRNF--
LKPRNF--
LDPRNF--
-LPRNFF-
LCPRNF--
-LPRNFW-
-LPRNFE-
-LPRNFY-
-LPRNFH-
MSPRNF--
-MPRNFW-
MKPRNF--
MDPRNF--
-MPRNFV-
MHPRNF--
-MPRNFA-
MNPRNF--
-MPRNFY-
-MPRNFN-
-MPRNFF-
MGPRNF--
-MPRNFH-
MEPRNF--
MQPRNF--
-MPRNFQ-
-MPRNFP-
MPPRNF--
-NPRNFS-
NTPRNF--
-NPRNFL-
-NPRNFT-
NIPRNF--
-NPRNFI-
NYPRNF--
NSPRNF--
-NPRNFA-
NHPRNF--
-NPRNFW-
-NPRNFE-
NPPRNF--
NGPRNF--
NQPRNF--

```
-NPRNFY-
-NPRNFF-
NEPRNF--
NDPRNF--
--PRNFSL
--PRNFFT
--PRNFLK
--PRNFSF
--PRNFLM
--PRNFGS
--PRNFAG
--PRNFRD
--PRNFTL
--PRNFLT
--PRNFSI
--PRNFFH
PWPRNF--
--PRNFYS
--PRNFTA
--PRNFMT
--PRNFWV
--PRNFTI
--PRNFML
--PRNFIS
--PRNFIG
--PRNFWI
--PRNFVG
--PRNFLH
--PRNFHN
--PRNFFR
--PRNFVY
--PRNFVH
--PRNFTE
--PRNFLN
PGPRNF--
--PRNFYV
--PRNFVI
--PRNFSQ
--PRNFQW
--PRNFMS
--PRNFME
PNPRNF--
--PRNFVV
--PRNFTF
--PRNFIV
--PRNFYT
--PRNFWS
--PRNFVN
--PRNFTV
--PRNFTT
```

--PRNFTG
--PRNFSY
--PRNFSA
--PRNFQN
--PRNFGG
--PRNFFA
--PRNFYL
--PRNFSM
--PRNFMQ
--PRNFGT
--PRNFFM
PSPRNF--
--PRNFYW
--PRNFVW
--PRNFTK
--PRNFNV
--PRNFMI
--PRNFFQ
--PRNFYG
--PRNFTM
--PRNFQG
--PRNFMG
--PRNFMA
--PRNFLA
--PRNFIW
--PRNFID
--PRNFAA
PVPRNF--
--PRNFYA
--PRNFWG
--PRNFRE
--PRNFQI
--PRNFMY
--PRNFII
--PRNFHT
--PRNFHA
--PRNFFN
--PRNFFG
--PRNFER
--PRNFAW
--PRNFAI
PAPRNF--
--PRNFYM
--PRNFYK
--PRNFWW
--PRNFWA
--PRNFVM
--PRNFSN
--PRNFQK
--PRNFNT

--PRNFMW
--PRNFMV
--PRNFLI
--PRNFLD
--PRNFIQ
--PRNFHY
--PRNFHM
--PRNFFY
--PRNFEV
--PRNFET
--PRNFES
PLPRNF--
PKPRNF--
--PRNFYQ
--PRNFYN
--PRNFWM
--PRNFVT
--PRNFVE
--PRNFVD
--PRNFTD
--PRNFQV
--PRNFQS
--PRNFQF
--PRNFQE
--PRNFNS
--PRNFNA
--PRNFIF
--PRNFEW
--PRNFAV
--PRNFAT
PEPRNF--
PDPRNF--
--PRNFYI
--PRNFWK
--PRNFWH
--PRNFSD
--PRNFQY
--PRNFQQ
--PRNFNG
--PRNFNF
--PRNFHF
--PRNFFL
--PRNFFD
--PRNFEN
--PRNFEK
--PRNFEI
--PRNFEA
--PRNFAN
PIPRNF--
--PRNFYH

--PRNFYF
--PRNFYE
--PRNFYD
--PRNFWF
--PRNFTW
--PRNFRQ
--PRNFQM
--PRNFQH
--PRNFQA
--PRNFNR
--PRNFNN
--PRNFNM
--PRNFNK
--PRNFNH
--PRNFMK
--PRNFMH
--PRNFMD
--PRNFLE
--PRNFIT
--PRNFIN
--PRNFIM
--PRNFIK
--PRNFIE
--PRNFIA
--PRNFHV
--PRNFHQ
--PRNFHI
--PRNFFV
--PRNFEY
--PRNFEG
--PRNFDS
--PRNFCD
--PRNFAY
--PRNFAQ
--PRNFAD
-QPRNFV-
-QPRNFY-
-QPRNFM-
QGPRNF--
QYPRNF--
-QPRNFK-
QAPRNF--
-QPRNFL-
-QPRNFI-
-QPRNFG-
-QPRNFF-
-QPRNFE-
QNPRNF--
QIPRNF--
QTPRNF--

QQPRNF--
-QPRNFQ-
-QPRNFH-
QKPRNF--
QVPRNF--
QPPRNF--
-QPRNFN-
QFPRNF--
-RPRNFM-
-RPRNFA-
RSPRNF--
-RPRNFY-
RTPRNF--
RGPRNF--
RNPRNF--
REPRNF--
-RPRNFN-
-RPRNFF-
RPPRNF--
-RPRNFH-
-RPRNFW-
-RPRNFE-
RKPRNF--
-RPRNFD-
RDPRNF--
-SPRNFL-
-SPRNFW-
STPRNF--
-SPRNFF-
-SPRNFE-
SNPRNF--
-SPRNFM-
-SPRNFI-
-SPRNFY-
-SPRNFK-
-SPRNFQ-
-SPRNFN-
SGPRNF--
SPPRNF--
SEPRNF--
-SPRNFG-
SKPRNF--
SQPRNF--
SDPRNF--
TLPRNF--
-TPRNFS-
-TPRNFL-
-TPRNFW-
-TPRNFV-
-TPRNFF-

-TPRNFY-
TAPRNF--
-TPRNFT-
-TPRNFI-
-TPRNFN-
-TPRNFM-
TNPRNF--
-TPRNFH-
TPPRNF--
TGPRNF--
-TPRNFE-
TKPRNF--
THPRNF--
TEPRNF--
TCPRNF--
TQPRNF--
-TPRNFG-
VLPRNF--
VGPRNF--
-VPRNFI-
VKPRNF--
-VPRNFV-
VYPRNF--
VSPRNF--
-VPRNFL-
VVPRNF--
-VPRNFA-
VHPRNF--
-VPRNFY-
VAPRNF--
-VPRNFK-
VNPRNF--
-VPRNFN-
-VPRNFG-
VEPRNF--
VDPRNF--
-VPRNFM-
-VPRNFE-
-VPRNFD-
WGPRNF--
WSPRNF--
-WPRNFF-
WAPRNF--
WRPRNF--
-WPRNFW-
WEPRNF--
WPPRNF--
WDPRNF--
-WPRNFV-
WIPRNF--

WCPRNF--
WNPRNF--
WMPRNF--
-YPRNFY-
-YPRNFL-
-YPRNFG-
YQPRNF--
YVPRNF--
YDPRNF--
YSPRNF--
-YPRNFA-
-YPRNFI-
YIPRNF--
YYPRNF--
-YPRNFW-
-YPRNFQ-
-YPRNFH-
YMPRNF--
YCPRNF--
YAPRNF--
YPPRNF--
YNPRNF--
YHPRNF--
YEPRNF--

| | | | |
|---|---|---|---|
| PQHL | 217 | 704 | 31.22 |

-EPQHLN-
--PQHLGF
-APQHLV-
-APQHLR-
-APQHLL-
-APQHLT-
-APQHLG-
AKPQHL--
AEPQHL--
ARPQHL--
ALPQHL--
AVPQHL--
-APQHLK-
-APQHLA-
AAPQHL--
-APQHLS-
AHPQHL--
AFPQHL--
-APQHLN-
AIPQHL--
-APQHLH-
AMPQHL--
ATPQHL--
APPQHL--
ACPQHL--

AYPQHL--
AWPQHL--
-APQHLY-
-APQHLQ-
-APQHLM-
-APQHLI-
-APQHLF-
CAPQHL--
CIPQHL--
CGPQHL--
CCPQHL--
CSPQHL--
-CPQHLT-
CTPQHL--
CRPQHL--
CQPQHL--
DQPQHL--
-DPQHLT-
DSPQHL--
-DPQHLK-
DAPQHL--
DRPQHL--
-DPQHLA-
DLPQHL--
DIPQHL--
DVPQHL--
-DPQHLR-
DGPQHL--
DYPQHL--
DWPQHL--
DTPQHL--
-DPQHLV-
-DPQHLS-
-DPQHLQ-
-DPQHLL-
DNPQHL--
DHPQHL--
-EPQHLL-
ESPQHL--
EVPQHL--
ENPQHL--
EIPQHL--
EGPQHL--
-EPQHLI-
-EPQHLS-
EFPQHL--
-EPQHLF-
EYPQHL--
-EPQHLA-
EMPQHL--

EAPQHL--
ETPQHL--
-EPQHLT-
-EPQHLR-
-EPQHLH-
-EPQHLC-
FGPQHL--
FRPQHL--
-FPQHLL-
FTPQHL--
FSPQHL--
-FPQHLY-
FPPQHL--
FVPQHL--
-FPQHLT-
-FPQHLS-
-FPQHLA-
FYPQHL--
-FPQHLV-
-FPQHLR-
-FPQHLK-
-FPQHLF-
FFPQHL--
FAPQHL--
-FPQHLQ-
FNPQHL--
FWPQHL--
FEPQHL--
-FPQHLN-
-FPQHLM-
FIPQHL--
FHPQHL--
GMPQHL--
GLPQHL--
GSPQHL--
-GPQHLY-
GGPQHL--
-GPQHLW-
-GPQHLS-
-GPQHLR-
-GPQHLN-
-GPQHLM-
GKPQHL--
GFPQHL--
-GPQHLV-
-GPQHLT-
-GPQHLK-
GNPQHL--
-GPQHLQ-
GWPQHL--

–GPQHLF–
GIPQHL––
GVPQHL––
GTPQHL––
GRPQHL––
–GPQHLH–
–GPQHLA–
GEPQHL––
GCPQHL––
GAPQHL––
GYPQHL––
GPPQHL––
GHPQHL––
HRPQHL––
HYPQHL––
HMPQHL––
HVPQHL––
HLPQHL––
–HPQHLS–
–HPQHLR–
–HPQHLK–
–HPQHLI–
HTPQHL––
–HPQHLL–
HWPQHL––
HSPQHL––
HPPQHL––
–HPQHLF–
–HPQHLA–
HIPQHL––
HGPQHL––
HEPQHL––
HAPQHL––
ISPQHL––
IRPQHL––
IIPQHL––
ITPQHL––
ILPQHL––
–IPQHLK–
IGPQHL––
–IPQHLL–
IMPQHL––
IFPQHL––
–IPQHLH–
IEPQHL––
IYPQHL––
–IPQHLY–
–IPQHLE–
IVPQHL––
–IPQHLW–

-IPQHLV-
-IPQHLM-
-IPQHLS-
-IPQHLR-
-IPQHLI-
-IPQHLA-
IPPQHL--
INPQHL--
IDPQHL--
KSPQHL--
KVPQHL--
KLPQHL--
KGPQHL--
-KPQHLS-
-KPQHLR-
-KPQHLA-
KRPQHL--
-KPQHLV-
-KPQHLL-
-KPQHLW-
-KPQHLT-
KNPQHL--
KMPQHL--
KYPQHL--
-KPQHLY-
-KPQHLH-
KPPQHL--
KEPQHL--
KDPQHL--
KQPQHL--
-KPQHLQ-
-KPQHLN-
-KPQHLK-
-KPQHLI-
-KPQHLF-
-KPQHLE-
KKPQHL--
KIPQHL--
KFPQHL--
LVPQHL--
LSPQHL--
LRPQHL--
LGPQHL--
-LPQHLR-
-LPQHLM-
LPPQHL--
-LPQHLT-
LFPQHL--
LKPQHL--
LTPQHL--

−LPQHLS−
LIPQHL−−
LAPQHL−−
LDPQHL−−
LYPQHL−−
LHPQHL−−
−LPQHLL−
LNPQHL−−
LMPQHL−−
LWPQHL−−
LQPQHL−−
−LPQHLY−
−LPQHLQ−
−LPQHLI−
−LPQHLV−
−LPQHLK−
LCPQHL−−
MRPQHL−−
MWPQHL−−
MLPQHL−−
−MPQHLR−
−MPQHLM−
MQPQHL−−
MHPQHL−−
MTPQHL−−
−MPQHLK−
−MPQHLI−
MMPQHL−−
MKPQHL−−
−MPQHLY−
−MPQHLS−
MDPQHL−−
−MPQHLQ−
MGPQHL−−
MCPQHL−−
MVPQHL−−
−MPQHLV−
−MPQHLT−
−MPQHLN−
MPPQHL−−
MNPQHL−−
MAPQHL−−
NVPQHL−−
NFPQHL−−
−NPQHLT−
NNPQHL−−
NYPQHL−−
NKPQHL−−
−NPQHLR−
NPPQHL−−

NMPQHL--
NAPQHL--
NTPQHL--
NRPQHL--
NIPQHL--
NGPQHL--
NEPQHL--
NSPQHL--
-NPQHLY-
-NPQHLS-
-NPQHLK-
-NPQHLI-
-NPQHLH-
NLPQHL--
NHPQHL--
NCPQHL--
PVPQHL--
--PQHLRL
--PQHLLT
--PQHLRR
--PQHLRG
--PQHLRA
PRPQHL--
--PQHLVR
PLPQHL--
--PQHLMT
--PQHLLA
--PQHLKH
--PQHLTI
--PQHLLG
--PQHLFG
--PQHLAW
--PQHLWS
--PQHLTR
--PQHLYH
--PQHLVS
--PQHLQR
--PQHLMS
--PQHLFT
PNPQHL--
PSPQHL--
--PQHLWM
--PQHLVW
--PQHLVQ
--PQHLSL
--PQHLRY
--PQHLRS
--PQHLYD
--PQHLWN
--PQHLVY

--PQHLVM
--PQHLVD
--PQHLTS
--PQHLSG
--PQHLRD
--PQHLMF
--PQHLMA
--PQHLLK
--PQHLKS
--PQHLKA
--PQHLHV
--PQHLGW
PKPQHL--
PIPQHL--
--PQHLYM
--PQHLTW
--PQHLST
--PQHLSS
--PQHLSR
--PQHLRM
--PQHLRH
--PQHLNS
--PQHLLW
--PQHLKV
--PQHLHG
--PQHLAM
--PQHLAL
--PQHLYN
--PQHLYF
--PQHLWY
--PQHLVT
--PQHLVA
--PQHLSY
--PQHLSW
--PQHLSN
--PQHLQW
--PQHLNF
--PQHLMQ
--PQHLKQ
--PQHLKG
--PQHLKF
--PQHLIV
--PQHLIS
--PQHLIK
--PQHLII
--PQHLGT
--PQHLGS
--PQHLGM
--PQHLFN
--PQHLES

--PQHLYW
--PQHLYA
--PQHLWH
--PQHLVN
--PQHLVF
--PQHLTA
--PQHLRQ
--PQHLRN
--PQHLQK
--PQHLNT
--PQHLNN
--PQHLMG
--PQHLLV
--PQHLLH
--PQHLKI
--PQHLIE
--PQHLGL
--PQHLGG
--PQHLGA
--PQHLFS
--PQHLAV
--PQHLAS
--PQHLAG
PGPQHL--
PAPQHL--
--PQHLYT
--PQHLYS
--PQHLYK
--PQHLWW
--PQHLWL
--PQHLWF
--PQHLVG
--PQHLTM
--PQHLTL
--PQHLTF
--PQHLSM
--PQHLSH
--PQHLRT
--PQHLQT
--PQHLQS
--PQHLQA
--PQHLPY
--PQHLPM
--PQHLMV
--PQHLMK
--PQHLMH
--PQHLME
--PQHLLR
--PQHLLN
--PQHLKK

--PQHLIY
--PQHLIQ
--PQHLIH
--PQHLHY
--PQHLHS
--PQHLHN
--PQHLFV
--PQHLFK
--PQHLAY
--PQHLAN
--PQHLAF
--PQHLYY
--PQHLYV
--PQHLYR
--PQHLYL
--PQHLYG
--PQHLWR
--PQHLWI
--PQHLWA
--PQHLVV
--PQHLVK
--PQHLVI
--PQHLTY
--PQHLTV
--PQHLTN
--PQHLTK
--PQHLSV
--PQHLSI
--PQHLSF
--PQHLSE
--PQHLSA
--PQHLRV
--PQHLRI
--PQHLRE
--PQHLQY
--PQHLQN
--PQHLQM
--PQHLQG
--PQHLQF
--PQHLPH
--PQHLNY
--PQHLNL
--PQHLNI
--PQHLNG
--PQHLMN
--PQHLMD
--PQHLLM
--PQHLLF
--PQHLLE
--PQHLLD

--PQHLKY
--PQHLKL
--PQHLIW
--PQHLIN
--PQHLIG
--PQHLID
--PQHLHW
--PQHLHR
--PQHLGV
--PQHLGI
--PQHLFY
--PQHLFW
--PQHLFQ
--PQHLFM
--PQHLFH
--PQHLFA
--PQHLEL
--PQHLEI
--PQHLEG
--PQHLCS
--PQHLAI
--PQHLAA
PHPQHL--
PEPQHL--
QPPQHL--
QAPQHL--
-QPQHLM-
-QPQHLF-
QVPQHL--
QTPQHL--
QSPQHL--
-QPQHLR-
-QPQHLI-
-QPQHLY-
-QPQHLV-
-QPQHLT-
-QPQHLS-
-QPQHLL-
-QPQHLK-
-QPQHLG-
QNPQHL--
QLPQHL--
QEPQHL--
-QPQHLH-
-QPQHLA-
QMPQHL--
QGPQHL--
-QPQHLW-
QIPQHL--
QFPQHL--

QDPQHL--
RSPQHL--
-RPQHLL-
-RPQHLR-
-RPQHLT-
RLPQHL--
RIPQHL--
RTPQHL--
-RPQHLH-
RRPQHL--
-RPQHLK-
RVPQHL--
-RPQHLS-
RYPQHL--
RHPQHL--
-RPQHLV-
-RPQHLW-
RNPQHL--
RFPQHL--
-RPQHLI-
-RPQHLA-
RPPQHL--
-RPQHLM-
RWPQHL--
-RPQHLY-
RGPQHL--
RDPQHL--
-RPQHLN-
-RPQHLF-
-RPQHLE-
-RPQHLQ-
RCPQHL--
-RPQHLG-
SRPQHL--
SVPQHL--
SSPQHL--
-SPQHLR-
SYPQHL--
STPQHL--
SAPQHL--
-SPQHLW-
SPPQHL--
SNPQHL--
-SPQHLV-
SKPQHL--
-SPQHLK-
-SPQHLH-
-SPQHLY-
-SPQHLS-
-SPQHLL-

```
-SPQHLA-
SGPQHL--
SQPQHL--
-SPQHLT-
SFPQHL--
-SPQHLE-
SMPQHL--
SEPQHL--
SWPQHL--
-SPQHLI-
SHPQHL--
SDPQHL--
-SPQHLM-
-SPQHLF-
SIPQHL--
SCPQHL--
-TPQHLR-
-TPQHLY-
-TPQHLV-
TSPQHL--
TVPQHL--
TRPQHL--
-TPQHLQ-
TMPQHL--
TTPQHL--
-TPQHLF-
TLPQHL--
-TPQHLM-
-TPQHLK-
-TPQHLH-
TKPQHL--
-TPQHLT-
TGPQHL--
-TPQHLN-
-TPQHLL-
TPPQHL--
TIPQHL--
-TPQHLI-
TNPQHL--
TFPQHL--
TEPQHL--
TAPQHL--
VLPQHL--
VIPQHL--
VVPQHL--
-VPQHLL-
VSPQHL--
VRPQHL--
-VPQHLS-
VGPQHL--
```

VYPQHL--
VFPQHL--
VQPQHL--
-VPQHLW-
-VPQHLA-
VWPQHL--
-VPQHLT-
-VPQHLR-
VAPQHL--
VTPQHL--
-VPQHLY-
-VPQHLM-
-VPQHLI-
-VPQHLH-
VHPQHL--
-VPQHLE-
-VPQHLV-
-VPQHLN-
-VPQHLK-
-VPQHLF-
VKPQHL--
VCPQHL--
WGPQHL--
WQPQHL--
-WPQHLS-
WLPQHL--
-WPQHLT-
-WPQHLR-
-WPQHLL-
WFPQHL--
-WPQHLK-
-WPQHLF-
WMPQHL--
WAPQHL--
WRPQHL--
WYPQHL--
-WPQHLI-
WKPQHL--
WEPQHL--
WTPQHL--
-WPQHLV-
-WPQHLN-
-WPQHLA-
WIPQHL--
WHPQHL--
WDPQHL--
WCPQHL--
-YPQHLL-
YMPQHL--
-YPQHLS-

| | | | |
|---|---|---|---|
| | YRPQHL-- | | |
| | -YPQHLI- | | |
| | -YPQHLY- | | |
| | YGPQHL-- | | |
| | YEPQHL-- | | |
| | -YPQHLQ- | | |
| | -YPQHLA- | | |
| | YIPQHL-- | | |
| | YFPQHL-- | | |
| | YLPQHL-- | | |
| | YDPQHL-- | | |
| | YYPQHL-- | | |
| | YTPQHL-- | | |
| | YSPQHL-- | | |
| | -YPQHLR- | | |
| | -YPQHLT- | | |
| | -YPQHLH- | | |
| | -YPQHLE- | | |
| | YNPQHL-- | | |
| | YVPQHL-- | | |
| | YQPQHL-- | | |
| | -YPQHLV- | | |
| | -YPQHLK- | | |
| | -YPQHLG- | | |
| | -YPQHLF- | | |
| | YPPQHL-- | | |
| | YKPQHL-- | | |
| | YHPQHL-- | | |
| | YAPQHL-- | | |
| PVPI | 164 | 733 | 31.21 |
| | GIPVPI-- | | |
| | PKPVPI-- | | |
| | AHPVPI-- | | |
| | -APVPIK- | | |
| | ATPVPI-- | | |
| | -APVPIG- | | |
| | -APVPIN- | | |
| | AVPVPI-- | | |
| | -APVPIR- | | |
| | -APVPIM- | | |
| | ADPVPI-- | | |
| | ACPVPI-- | | |
| | AYPVPI-- | | |
| | ARPVPI-- | | |
| | -APVPIY- | | |
| | AMPVPI-- | | |
| | AGPVPI-- | | |
| | ALPVPI-- | | |
| | AIPVPI-- | | |
| | AFPVPI-- | | |

-APVPIT-
AKPVPI--
AEPVPI--
AAPVPI--
AQPVPI--
-APVPIS-
-APVPIQ-
-APVPIH-
ANPVPI--
CMPVPI--
CLPVPI--
CTPVPI--
CGPVPI--
-CPVPIR-
-CPVPIL-
CPPVPI--
CFPVPI--
CEPVPI--
CAPVPI--
-DPVPIY-
DRPVPI--
DSPVPI--
DVPVPI--
DQPVPI--
-DPVPIT-
-DPVPIQ-
-DPVPIL-
DYPVPI--
DWPVPI--
DTPVPI--
-DPVPIV-
-DPVPIS-
-DPVPIR-
-DPVPII-
-DPVPIF-
DNPVPI--
DLPVPI--
-EPVPIR-
ETPVPI--
-EPVPII-
EYPVPI--
-EPVPIT-
EVPVPI--
ERPVPI--
-EPVPIS-
-EPVPIW-
EAPVPI--
-EPVPIL-
-EPVPIA-
EIPVPI--

EWPVPI--
ESPVPI--
-EPVPIY-
-EPVPIV-
-EPVPIM-
-EPVPIH-
-EPVPIF-
EMPVPI--
ELPVPI--
EHPVPI--
-FPVPIA-
FSPVPI--
-FPVPIY-
FTPVPI--
FIPVPI--
FDPVPI--
FQPVPI--
-FPVPIS-
FAPVPI--
FYPVPI--
FLPVPI--
FVPVPI--
-FPVPII-
FGPVPI--
FWPVPI--
-FPVPIT-
-FPVPIR-
FPPVPI--
FNPVPI--
FHPVPI--
-FPVPIW-
-FPVPIQ-
-FPVPIM-
-FPVPIE-
GRPVPI--
GSPVPI--
GLPVPI--
GTPVPI--
-GPVPIR-
GVPVPI--
-GPVPIY-
-GPVPIS-
-GPVPII-
GFPVPI--
-GPVPIW-
-GPVPIL-
GWPVPI--
-GPVPIT-
-GPVPIM-
-GPVPIG-

GHPVPI--
GAPVPI--
GQPVPI--
-GPVPIV-
-GPVPIQ-
GKPVPI--
GEPVPI--
-GPVPIH-
-GPVPIA-
GNPVPI--
GCPVPI--
HRPVPI--
-HPVPIW-
-HPVPIL-
HYPVPI--
HVPVPI--
-HPVPIM-
HQPVPI--
HMPVPI--
-HPVPIT-
-HPVPIF-
HWPVPI--
-HPVPIS-
-HPVPIR-
-HPVPIQ-
-HPVPII-
-HPVPIA-
HIPVPI--
HHPVPI--
HEPVPI--
HTPVPI--
-HPVPIV-
HPPVPI--
HKPVPI--
HDPVPI--
HAPVPI--
-IPVPIL-
IVPVPI--
-IPVPIR-
-IPVPIT-
IMPVPI--
IRPVPI--
-IPVPII-
INPVPI--
IWPVPI--
ITPVPI--
ISPVPI--
IPPVPI--
-IPVPIW-
-IPVPIV-

-IPVPIS-
IQPVPI--
-IPVPIY-
-IPVPIN-
-IPVPIH-
-IPVPIF-
ILPVPI--
IKPVPI--
IIPVPI--
IDPVPI--
IYPVPI--
-IPVPIA-
IGPVPI--
IEPVPI--
IAPVPI--
-KPVPIR-
-KPVPIK-
KKPVPI--
KAPVPI--
KVPVPI--
KLPVPI--
KFPVPI--
KQPVPI--
-KPVPIV-
-KPVPIW-
-KPVPII-
-KPVPIH-
KWPVPI--
-KPVPIY-
KMPVPI--
KIPVPI--
KGPVPI--
KRPVPI--
-KPVPIM-
-KPVPIL-
-KPVPIF-
KNPVPI--
KEPVPI--
KDPVPI--
KYPVPI--
KTPVPI--
KHPVPI--
-KPVPIT-
-KPVPIQ-
-KPVPIG-
-KPVPIE-
-KPVPIA-
KPPVPI--
LRPVPI--
LTPVPI--

LGPVPI--
LSPVPI--
-LPVPIR-
LAPVPI--
LPPVPI--
-LPVPIM-
LMPVPI--
-LPVPIY-
LQPVPI--
-LPVPIW-
LLPVPI--
-LPVPIT-
-LPVPIK-
LFPVPI--
LVPVPI--
LKPVPI--
LIPVPI--
LYPVPI--
-LPVPIV-
-LPVPIS-
-LPVPIL-
-LPVPIH-
LEPVPI--
-LPVPIQ-
LDPVPI--
MSPVPI--
MRPVPI--
-MPVPIV-
-MPVPIQ-
MVPVPI--
MQPVPI--
-MPVPIR-
-MPVPIK-
MIPVPI--
MAPVPI--
MYPVPI--
MTPVPI--
-MPVPIS-
-MPVPIL-
-MPVPIF-
-MPVPIY-
MKPVPI--
MHPVPI--
MFPVPI--
-MPVPIW-
-MPVPIT-
-MPVPIN-
-MPVPIM-
-MPVPII-
MLPVPI--

NRPVPI--
NSPVPI--
NDPVPI--
-NPVPII-
NPPVPI--
NLPVPI--
NHPVPI--
-NPVPIY-
-NPVPIL-
-NPVPIK-
NAPVPI--
-NPVPIS-
-NPVPIR-
-NPVPIF-
NKPVPI--
NGPVPI--
NVPVPI--
-NPVPIW-
-NPVPIV-
NYPVPI--
NWPVPI--
NQPVPI--
-NPVPIT-
-NPVPIA-
NNPVPI--
NMPVPI--
--PVPIRR
--PVPILS
--PVPIVR
--PVPIRL
--PVPILL
--PVPITG
--PVPIFS
--PVPILH
--PVPILG
--PVPIMR
--PVPIFK
--PVPITS
--PVPIRS
--PVPIML
--PVPIAW
--PVPITL
--PVPIKL
--PVPISV
--PVPIRI
--PVPIRA
--PVPIQA
--PVPILY
--PVPIKT
--PVPIIR

--PVPIAL
--PVPITI
--PVPIRH
--PVPIQT
--PVPIVL
--PVPISS
--PVPIQW
--PVPINL
--PVPILN
--PVPIIT
--PVPIGV
--PVPIYL
--PVPIVS
--PVPIRW
--PVPIRT
--PVPIQL
--PVPINW
--PVPINS
--PVPILW
--PVPILT
--PVPILA
--PVPIKK
--PVPIGI
--PVPIGA
--PVPIFR
--PVPIAM
--PVPIAH
--PVPIYH
--PVPIYG
--PVPIWT
--PVPIWS
--PVPIWK
--PVPITR
--PVPIRN
--PVPIPW
--PVPILR
--PVPIIS
--PVPIHV
--PVPIGT
--PVPIGS
--PVPIFT
PSPVPI--
PHPVPI--
PAPVPI--
--PVPIVA
--PVPIST
--PVPISK
--PVPIRV
--PVPIRG
--PVPIQH

--PVPIPH
--PVPIMH
--PVPILI
--PVPIKM
--PVPIKG
--PVPIIA
--PVPIHQ
--PVPIGW
--PVPIGQ
--PVPIFG
--PVPIYS
--PVPIYR
--PVPIYQ
--PVPIVM
--PVPIVK
--PVPITY
--PVPISR
--PVPISG
--PVPISA
--PVPIRK
--PVPINN
--PVPINI
--PVPIMW
--PVPIMS
--PVPILQ
--PVPILK
--PVPIKY
--PVPIKR
--PVPIKN
--PVPIIM
--PVPIIF
--PVPIHM
--PVPIHK
--PVPIHA
--PVPIAS
--PVPIAA
--PVPIYT
--PVPIYN
--PVPIYK
--PVPIWR
--PVPIWG
--PVPITV
--PVPITQ
--PVPITM
--PVPITK
--PVPITD
--PVPISI
--PVPIRY
--PVPIQS
--PVPIQF

--PVPIPY
--PVPIPI
--PVPING
--PVPIME
--PVPILV
--PVPILM
--PVPIKI
--PVPIIG
--PVPIHY
--PVPIGY
--PVPIGK
--PVPIFQ
--PVPIEG
--PVPIEF
--PVPIAG
PRPVPI--
PMPVPI--
PLPVPI--
PDPVPI--
--PVPIYI
--PVPIWV
--PVPIWL
--PVPIWA
--PVPIVT
--PVPIVN
--PVPIVH
--PVPIVE
--PVPITW
--PVPITT
--PVPITN
--PVPITH
--PVPITE
--PVPISY
--PVPISW
--PVPISN
--PVPIRF
--PVPIRE
--PVPIPV
--PVPIPS
--PVPIPQ
--PVPIPA
--PVPINV
--PVPINT
--PVPINM
--PVPIMI
--PVPIKW
--PVPIKH
--PVPIKD
--PVPIIY
--PVPIIV

--PVPIIE
--PVPIHS
--PVPIHR
--PVPIHN
--PVPIHF
--PVPIFY
--PVPIFN
--PVPIFE
--PVPIEW
--PVPIEL
--PVPIEH
--PVPIDY
--PVPIDL
--PVPIDI
--PVPIAR
--PVPIAN
PTPVPI--
PNPVPI--
PVPVPI--
--PVPIYY
--PVPIYW
--PVPIYM
--PVPIYA
--PVPIWM
--PVPIWI
--PVPIVY
--PVPIVW
--PVPIVQ
--PVPIVI
--PVPIVG
--PVPIVD
--PVPITA
--PVPISQ
--PVPISP
--PVPISH
--PVPIRM
--PVPIRD
--PVPIRC
--PVPIQG
--PVPINH
--PVPIMT
--PVPIMN
--PVPIMG
--PVPIMC
--PVPIKA
--PVPIIQ
--PVPIIN
--PVPIIL
--PVPIID
--PVPIHW

--PVPIHT
--PVPIHL
--PVPIHI
--PVPIHG
--PVPIGN
--PVPIGG
--PVPIFV
--PVPIFM
--PVPIFD
--PVPIFA
--PVPIEY
--PVPIDT
--PVPIDM
--PVPIDF
--PVPIAT
--PVPIAQ
-PPVPIF-
PIPVPI--
PGPVPI--
PCPVPI--
-QPVPIL-
QVPVPI--
-QPVPIR-
-QPVPIW-
-QPVPII-
QMPVPI--
QGPVPI--
QQPVPI--
QRPVPI--
QPPVPI--
QNPVPI--
QAPVPI--
QSPVPI--
-QPVPIS-
QKPVPI--
QYPVPI--
QWPVPI--
QTPVPI--
-QPVPIT-
-QPVPIM-
-QPVPIH-
-QPVPIF-
QIPVPI--
QDPVPI--
-RPVPIR-
RLPVPI--
RYPVPI--
-RPVPIV-
-RPVPIK-
RRPVPI--

RTPVPI--
RNPVPI--
RIPVPI--
RSPVPI--
-RPVPIS-
-RPVPIN-
RPPVPI--
RHPVPI--
RMPVPI--
-RPVPIH-
RGPVPI--
RAPVPI--
RQPVPI--
-RPVPIY-
-RPVPII-
-RPVPIT-
-RPVPIQ-
-RPVPIA-
-RPVPIG-
-RPVPIE-
RWPVPI--
REPVPI--
-SPVPIR-
-SPVPIL-
-SPVPIY-
SSPVPI--
SMPVPI--
SRPVPI--
SLPVPI--
-SPVPIF-
SIPVPI--
STPVPI--
-SPVPIS-
-SPVPIW-
-SPVPIT-
-SPVPIH-
SPPVPI--
SKPVPI--
SAPVPI--
-SPVPIM-
SVPVPI--
-SPVPIK-
SFPVPI--
SYPVPI--
SWPVPI--
SQPVPI--
SHPVPI--
SDPVPI--
SCPVPI--
-SPVPIV-

-SPVPIQ-
-SPVPII-
-SPVPIA-
TGPVPI--
TRPVPI--
TTPVPI--
TLPVPI--
-TPVPIT-
-TPVPIS-
TWPVPI--
-TPVPIR-
-TPVPIK-
TNPVPI--
-TPVPIW-
TSPVPI--
TYPVPI--
-TPVPIA-
TKPVPI--
TEPVPI--
TVPVPI--
-TPVPIM-
-TPVPIF-
TMPVPI--
TDPVPI--
TAPVPI--
TQPVPI--
-TPVPII-
TPPVPI--
TIPVPI--
TFPVPI--
-VPVPIL-
VRPVPI--
-VPVPIA-
VSPVPI--
-VPVPIR-
-VPVPIS-
VWPVPI--
VLPVPI--
VTPVPI--
-VPVPIK-
VQPVPI--
-VPVPIY-
-VPVPIM-
VNPVPI--
VKPVPI--
VVPVPI--
-VPVPIT-
-VPVPIF-
VGPVPI--
-VPVPIH-

VIPVPI--
VYPVPI--
VMPVPI--
VHPVPI--
VFPVPI--
VEPVPI--
VCPVPI--
-VPVPIW-
-VPVPII-
-VPVPIG-
-VPVPIE-
VPPVPI--
VDPVPI--
VAPVPI--
WTPVPI--
WIPVPI--
WEPVPI--
WGPVPI--
WNPVPI--
WSPVPI--
WRPVPI--
-WPVPIL-
WKPVPI--
-WPVPIR-
-WPVPIA-
WVPVPI--
WQPVPI--
-WPVPIV-
-WPVPIM-
WHPVPI--
-WPVPIS-
-WPVPIN-
-WPVPII-
-WPVPIF-
WDPVPI--
WCPVPI--
YRPVPI--
YAPVPI--
-YPVPIL-
YLPVPI--
-YPVPIS-
YMPVPI--
YTPVPI--
YSPVPI--
YDPVPI--
YQPVPI--
YYPVPI--
-YPVPIR-
-YPVPIA-
YGPVPI--

| | | | |
|---|---|---|---|
| | -YPVPIN- | | |
| | -YPVPIG- | | |
| | -YPVPIF- | | |
| | -YPVPIE- | | |
| | YKPVPI-- | | |
| | YIPVPI-- | | |
| | YHPVPI-- | | |
| | YFPVPI-- | | |
| | YEPVPI-- | | |
| | YWPVPI-- | | |
| | -YPVPIQ- | | |
| | -YPVPIM- | | |
| | YNPVPI-- | | |
| LSWM | 638 | 571 | 31.21 |
| | DSLSWM-- | | |
| | -DLSWML- | | |
| | -GLSWMD- | | |
| | --LSWMSD | | |
| | --LSWMLK | | |
| | --LSWMHI | | |
| | --LSWMEV | | |
| | PYLSWM-- | | |
| | -SLSWMD- | | |
| | -TLSWMP- | | |
| | -ALSWML- | | |
| | ASLSWM-- | | |
| | AFLSWM-- | | |
| | AHLSWM-- | | |
| | ALLSWM-- | | |
| | ATLSWM-- | | |
| | AWLSWM-- | | |
| | -ALSWMI- | | |
| | AKLSWM-- | | |
| | -ALSWMR- | | |
| | -ALSWMS- | | |
| | -ALSWMG- | | |
| | ANLSWM-- | | |
| | AALSWM-- | | |
| | AVLSWM-- | | |
| | -ALSWMQ- | | |
| | -ALSWMK- | | |
| | AGLSWM-- | | |
| | AQLSWM-- | | |
| | AELSWM-- | | |
| | AMLSWM-- | | |
| | -ALSWMM- | | |
| | ADLSWM-- | | |
| | AYLSWM-- | | |
| | -ALSWMH- | | |
| | -ALSWME- | | |

ACLSWM--
CRLSWM--
-CLSWML-
-CLSWMH-
-CLSWMS-
CGLSWM--
CSLSWM--
CMLSWM--
-CLSWMK-
-CLSWMA-
CVLSWM--
-CLSWMY-
-CLSWMN-
-CLSWMD-
CLLSWM--
CKLSWM--
CHLSWM--
CFLSWM--
-DLSWMT-
DLLSWM--
-DLSWMK-
DRLSWM--
-DLSWMS-
-DLSWMG-
DMLSWM--
-DLSWMQ-
-DLSWMI-
DVLSWM--
-DLSWMN-
-DLSWMA-
DILSWM--
DALSWM--
DTLSWM--
DHLSWM--
DQLSWM--
-DLSWMV-
-DLSWMM-
DCLSWM--
DYLSWM--
-DLSWMH-
DGLSWM--
DELSWM--
DDLSWM--
-ELSWMR-
-ELSWMK-
-ELSWML-
-ELSWMT-
EALSWM--
EHLSWM--
EYLSWM--

ERLSWM--
EFLSWM--
EDLSWM--
ENLSWM--
-ELSWMS-
-ELSWMI-
EILSWM--
EVLSWM--
ELLSWM--
EWLSWM--
-ELSWMA-
EKLSWM--
EQLSWM--
-ELSWMG-
-ELSWMM-
-ELSWME-
ETLSWM--
EMLSWM--
-ELSWMQ-
-ELSWMN-
EGLSWM--
EELSWM--
FTLSWM--
FNLSWM--
-FLSWMQ-
-FLSWMG-
FELSWM--
-FLSWMS-
-FLSWMN-
FKLSWM--
-FLSWMA-
FGLSWM--
-FLSWMI-
-FLSWMH-
FQLSWM--
FMLSWM--
-FLSWMT-
FDLSWM--
FALSWM--
-GLSWMS-
GSLSWM--
-GLSWMK-
-GLSWMT-
GNLSWM--
-GLSWMG-
GELSWM--
GALSWM--
-GLSWMV-
GYLSWM--
GKLSWM--

-GLSWMH-
GMLSWM--
GDLSWM--
GVLSWM--
-GLSWMY-
-GLSWMM-
-GLSWMI-
-GLSWMQ-
GHLSWM--
GCLSWM--
-HLSWMN-
-HLSWMA-
HLLSWM--
-HLSWMH-
-HLSWMT-
HVLSWM--
HNLSWM--
-HLSWMR-
-HLSWMI-
HRLSWM--
-HLSWMS-
-HLSWMG-
HMLSWM--
-HLSWML-
HILSWM--
HHLSWM--
HALSWM--
HTLSWM--
-HLSWMY-
HKLSWM--
HYLSWM--
HQLSWM--
-HLSWMQ-
-HLSWMK-
-HLSWME-
-HLSWMD-
HELSWM--
HDLSWM--
-HLSWMC-
HGLSWM--
-ILSWMN-
IKLSWM--
-ILSWMK-
ITLSWM--
-ILSWMM-
INLSWM--
IGLSWM--
IILSWM--
ISLSWM--
IMLSWM--

-ILSWMG-
-ILSWMA-
IDLSWM--
-ILSWMT-
IQLSWM--
IHLSWM--
-ILSWMQ-
IWLSWM--
-ILSWMH-
-ILSWMF-
KVLSWM--
KTLSWM--
-KLSWMK-
-KLSWMA-
KMLSWM--
-KLSWMS-
-KLSWMQ-
-KLSWMG-
KLLSWM--
-KLSWMH-
-KLSWMI-
KGLSWM--
-KLSWMM-
KHLSWM--
KILSWM--
KNLSWM--
KELSWM--
--LSWMRS
--LSWMRQ
--LSWMRN
--LSWMGT
--LSWMVA
-LLSWMT-
--LSWMTA
--LSWMSN
--LSWMKG
--LSWMNS
--LSWMSA
-LLSWME-
LELSWM--
--LSWMYH
--LSWMYG
--LSWMRF
--LSWMVG
--LSWMTF
--LSWMSK
--LSWMRA
-LLSWMG-
--LSWMSW
--LSWMIS

--LSWMAG
--LSWMTV
--LSWMRH
--LSWMRD
--LSWMAV
--LSWMYS
--LSWMVK
--LSWMSV
--LSWMSM
--LSWMMG
--LSWMAK
LNLSWM--
--LSWMVN
--LSWMTQ
--LSWMTE
--LSWMQV
--LSWMGG
-LLSWMQ-
--LSWMTD
--LSWMQG
--LSWMAW
--LSWMKM
--LSWMKD
--LSWMKA
LRLSWM--
LHLSWM--
--LSWMVD
--LSWMTN
--LSWMRY
--LSWMKY
--LSWMKV
--LSWMIH
--LSWMGL
--LSWMEG
--LSWMAN
LMLSWM--
-LLSWMN-
--LSWMWG
--LSWMVM
--LSWMTG
--LSWMSI
--LSWMRW
--LSWMAA
--LSWMVT
--LSWMQT
--LSWMQE
--LSWMQA
--LSWMNT
--LSWMMT
--LSWMKW

--LSWMIN
--LSWMIE
--LSWMHA
--LSWMAT
--LSWMAS
LTLSWM--
--LSWMWA
--LSWMVH
--LSWMTY
--LSWMSQ
--LSWMNA
--LSWMKT
--LSWMKF
--LSWMAQ
--LSWMAM
-LLSWMP-
--LSWMTM
--LSWMTI
--LSWMRE
--LSWMQW
--LSWMQS
--LSWMQM
--LSWMNG
--LSWMMQ
--LSWMMH
--LSWMMF
--LSWMKL
--LSWMHV
--LSWMGV
--LSWMGI
--LSWMFN
--LSWMFG
-LLSWMM-
-LLSWMI-
LDLSWM--
--LSWMVW
--LSWMVE
--LSWMSH
--LSWMSE
--LSWMQI
--LSWMQD
--LSWMMM
--LSWMLE
--LSWMKQ
--LSWMIQ
--LSWMIG
--LSWMHT
--LSWMHM
--LSWMHH
--LSWMGM

--LSWMFF
--LSWMDS
--LSWMYQ
--LSWMYN
--LSWMWM
--LSWMWF
--LSWMQY
--LSWMQK
--LSWMPY
--LSWMNQ
--LSWMNP
--LSWMMW
--LSWMKH
--LSWMHW
--LSWMHF
--LSWMHE
--LSWMGW
--LSWMET
--LSWMEA
--LSWMDT
-LLSWMW-
-LLSWMD-
MELSWM--
MSLSWM--
MRLSWM--
MNLSWM--
MGLSWM--
-MLSWMI-
MDLSWM--
-MLSWMQ-
-MLSWMK-
MKLSWM--
MTLSWM--
-MLSWMD-
MALSWM--
-MLSWMV-
-MLSWMN-
-MLSWMM-
-MLSWMH-
-MLSWMG-
MHLSWM--
MVLSWM--
MQLSWM--
-MLSWMW-
-NLSWMK-
NSLSWM--
NRLSWM--
-NLSWMR-
NTLSWM--
NGLSWM--

-NLSWMS-
-NLSWMI-
-NLSWMN-
-NLSWMH-
-NLSWMG-
NDLSWM--
NKLSWM--
NWLSWM--
-NLSWMV-
-NLSWMM-
-NLSWME-
NYLSWM--
-NLSWMQ-
NILSWM--
NALSWM--
NQLSWM--
NNLSWM--
-NLSWMF-
-NLSWMD-
NELSWM--
-NLSWMW-
PDLSWM--
PSLSWM--
PILSWM--
PTLSWM--
PKLSWM--
PMLSWM--
PHLSWM--
PALSWM--
PVLSWM--
PNLSWM--
PQLSWM--
PFLSWM--
PWLSWM--
PELSWM--
PCLSWM--
-QLSWMN-
-QLSWMI-
-QLSWMS-
QLLSWM--
QTLSWM--
QGLSWM--
QYLSWM--
-QLSWMT-
-QLSWMW-
QDLSWM--
QALSWM--
-QLSWMG-
QMLSWM--
QHLSWM--

QELSWM--
QVLSWM--
-QLSWMM-
-QLSWMK-
-QLSWME-
-QLSWMD-
-QLSWMA-
QFLSWM--
RTLSWM--
-RLSWMV-
-RLSWMG-
RYLSWM--
RMLSWM--
-RLSWMN-
RQLSWM--
-RLSWMM-
-RLSWMH-
-RLSWMI-
RELSWM--
RWLSWM--
RFLSWM--
-RLSWMC-
-SLSWMK-
-SLSWMT-
SSLSWM--
SVLSWM--
-SLSWMI-
-SLSWMA-
SGLSWM--
-SLSWMG-
-SLSWMQ-
SNLSWM--
-SLSWMH-
STLSWM--
SELSWM--
SQLSWM--
SDLSWM--
SMLSWM--
SHLSWM--
-SLSWMM-
SALSWM--
-SLSWME-
-TLSWMR-
TTLSWM--
-TLSWMV-
-TLSWMH-
TVLSWM--
TRLSWM--
-TLSWMK-
-TLSWMG-

-TLSWMT-
TQLSWM--
-TLSWMN-
TSLSWM--
-TLSWMI-
TLLSWM--
TGLSWM--
TNLSWM--
-TLSWMM-
TALSWM--
-TLSWMD-
-TLSWMQ-
THLSWM--
-TLSWMA-
TELSWM--
TDLSWM--
-TLSWME-
TFLSWM--
TCLSWM--
-VLSWMS-
-VLSWMM-
-VLSWMN-
VGLSWM--
VRLSWM--
-VLSWMK-
VNLSWM--
VKLSWM--
-VLSWMI-
VELSWM--
-VLSWMQ-
VSLSWM--
-VLSWMV-
VALSWM--
VVLSWM--
VQLSWM--
-VLSWMH-
-VLSWMD-
VDLSWM--
-VLSWMG-
VFLSWM--
-WLSWMS-
WNLSWM--
-WLSWMQ-
WSLSWM--
WQLSWM--
-WLSWMT-
-WLSWMR-
WKLSWM--
WALSWM--
WYLSWM--

| | | | |
|---|---|---|---|
| | WTLSWM-- | | |
| | -WLSWMN- | | |
| | -WLSWMA- | | |
| | WELSWM-- | | |
| | -WLSWMH- | | |
| | WDLSWM-- | | |
| | -WLSWML- | | |
| | -WLSWMI- | | |
| | -WLSWME- | | |
| | -WLSWMD- | | |
| | WHLSWM-- | | |
| | WGLSWM-- | | |
| | YGLSWM-- | | |
| | -YLSWMS- | | |
| | YHLSWM-- | | |
| | YVLSWM-- | | |
| | -YLSWMT- | | |
| | YQLSWM-- | | |
| | YTLSWM-- | | |
| | YSLSWM-- | | |
| | YNLSWM-- | | |
| | -YLSWMQ- | | |
| | -YLSWMG- | | |
| | -YLSWMN- | | |
| | -YLSWMA- | | |
| | -YLSWMH- | | |
| | -YLSWMD- | | |
| | -YLSWMI- | | |
| | YLLSWM-- | | |
| | YKLSWM-- | | |
| | YDLSWM-- | | |
| | YWLSWM-- | | |
| | -YLSWMM- | | |
| | -YLSWMK- | | |
| | -YLSWME- | | |
| PGGL | 72 | 787 | 31.16 |
| | -LPGGLA- | | |
| | --PGGLSQ | | |
| | --PGGLLQ | | |
| | --PGGLYD | | |
| | --PGGLVI | | |
| | --PGGLTM | | |
| | --PGGLSL | | |
| | --PGGLNI | | |
| | --PGGLKL | | |
| | --PGGLGR | | |
| | -TPGGLE- | | |
| | -WPGGLG- | | |
| | -YPGGLG- | | |
| | AYPGGL-- | | |

-APGGLI-
ASPGGL--
-APGGLY-
AGPGGL--
-APGGLS-
-APGGLL-
-APGGLK-
-APGGLW-
-APGGLR-
ANPGGL--
AEPGGL--
ALPGGL--
-APGGLT-
-APGGLN-
-APGGLF-
AIPGGL--
AVPGGL--
AKPGGL--
-APGGLH-
-APGGLA-
AHPGGL--
-APGGLG-
-APGGLE-
AMPGGL--
AQPGGL--
APPGGL--
AWPGGL--
-APGGLQ-
CTPGGL--
CVPGGL--
CRPGGL--
CYPGGL--
CQPGGL--
-CPGGLM-
CWPGGL--
-CPGGLR-
CLPGGL--
CHPGGL--
-CPGGLV-
-CPGGLT-
CMPGGL--
CIPGGL--
CSPGGL--
-CPGGLL-
-CPGGLI-
-CPGGLH-
CKPGGL--
CEPGGL--
CAPGGL--
-DPGGLV-

DVPGGL--
DFPGGL--
DAPGGL--
-DPGGLM-
DIPGGL--
-DPGGLW-
-DPGGLT-
-DPGGLR-
DSPGGL--
DRPGGL--
-DPGGLY-
-DPGGLQ-
DLPGGL--
DYPGGL--
DWPGGL--
-DPGGLF-
DNPGGL--
DMPGGL--
DGPGGL--
DKPGGL--
DQPGGL--
-DPGGLH-
DHPGGL--
DEPGGL--
DDPGGL--
-EPGGLR-
EWPGGL--
ELPGGL--
-EPGGLL-
EGPGGL--
ESPGGL--
-EPGGLV-
-EPGGLT-
-EPGGLS-
-EPGGLH-
-EPGGLQ-
EMPGGL--
EQPGGL--
-EPGGLY-
EYPGGL--
-EPGGLM-
-EPGGLF-
EIPGGL--
-EPGGLW-
ERPGGL--
-EPGGLK-
-EPGGLI-
ENPGGL--
EKPGGL--
EFPGGL--

EVPGGL--
-EPGGLN-
-EPGGLA-
ETPGGL--
EPPGGL--
FLPGGL--
-FPGGLK-
FAPGGL--
FTPGGL--
FRPGGL--
-FPGGLR-
FIPGGL--
-FPGGLY-
-FPGGLV-
-FPGGLT-
-FPGGLS-
-FPGGLH-
FGPGGL--
FVPGGL--
-FPGGLF-
FNPGGL--
-FPGGLI-
FKPGGL--
FFPGGL--
-FPGGLQ-
-FPGGLM-
FYPGGL--
FWPGGL--
FMPGGL--
FHPGGL--
FEPGGL--
FDPGGL--
FPPGGL--
-FPGGLW-
-FPGGLG-
-FPGGLD-
FCPGGL--
-GPGGLL-
-GPGGLR-
-GPGGLK-
-GPGGLT-
-GPGGLS-
GSPGGL--
GMPGGL--
GFPGGL--
-GPGGLV-
-GPGGLI-
GAPGGL--
GTPGGL--
GKPGGL--

GIPGGL--
-GPGGLN-
GLPGGL--
-GPGGLW-
-GPGGLH-
GNPGGL--
-GPGGLY-
-GPGGLF-
GEPGGL--
-GPGGLA-
GQPGGL--
-GPGGLQ-
-GPGGLM-
GHPGGL--
GGPGGL--
GWPGGL--
-GPGGLG-
GPPGGL--
GDPGGL--
-GPGGLE-
HYPGGL--
-HPGGLK-
HVPGGL--
HGPGGL--
HTPGGL--
-HPGGLW-
-HPGGLS-
-HPGGLY-
-HPGGLR-
HMPGGL--
HSPGGL--
-HPGGLL-
-HPGGLF-
HLPGGL--
HWPGGL--
HNPGGL--
-HPGGLT-
-HPGGLN-
HKPGGL--
HAPGGL--
HQPGGL--
HFPGGL--
HEPGGL--
HCPGGL--
-HPGGLV-
-HPGGLA-
HIPGGL--
-HPGGLI-
HDPGGL--
IRPGGL--

IIPGGL--
IGPGGL--
ISPGGL--
IHPGGL--
-IPGGLW-
IWPGGL--
IVPGGL--
IQPGGL--
-IPGGLS-
IAPGGL--
-IPGGLY-
-IPGGLV-
-IPGGLN-
-IPGGLF-
ILPGGL--
ITPGGL--
IMPGGL--
IFPGGL--
-IPGGLQ-
-IPGGLM-
-IPGGLA-
IKPGGL--
IYPGGL--
-IPGGLK-
IDPGGL--
IPPGGL--
INPGGL--
-IPGGLD-
-IPGGLC-
ICPGGL--
-KPGGLR-
KTPGGL--
-KPGGLF-
KVPGGL--
KNPGGL--
-KPGGLT-
-KPGGLM-
-KPGGLI-
-KPGGLW-
-KPGGLS-
KAPGGL--
-KPGGLY-
KIPGGL--
-KPGGLV-
KKPGGL--
KGPGGL--
KFPGGL--
KQPGGL--
-KPGGLK-
KHPGGL--

KWPGGL--
-KPGGLN-
KEPGGL--
-KPGGLH-
KDPGGL--
-KPGGLG-
-KPGGLA-
KMPGGL--
KPPGGL--
-KPGGLE-
-LPGGLK-
LRPGGL--
LVPGGL--
LWPGGL--
LMPGGL--
LLPGGL--
LGPGGL--
-LPGGLR-
-LPGGLI-
LTPGGL--
-LPGGLW-
-LPGGLT-
LAPGGL--
-LPGGLF-
LQPGGL--
LHPGGL--
-LPGGLS-
-LPGGLV-
-LPGGLM-
LYPGGL--
LPPGGL--
-LPGGLY-
LKPGGL--
LIPGGL--
-LPGGLN-
LCPGGL--
-LPGGLQ-
-MPGGLL-
MRPGGL--
-MPGGLK-
MSPGGL--
-MPGGLW-
MFPGGL--
MGPGGL--
MKPGGL--
MYPGGL--
MTPGGL--
-MPGGLY-
-MPGGLR-
MMPGGL--

```
-MPGGLQ-
-MPGGLF-
MLPGGL--
MIPGGL--
-MPGGLS-
-MPGGLI-
-MPGGLT-
MAPGGL--
MVPGGL--
MEPGGL--
-MPGGLN-
-MPGGLH-
-MPGGLA-
-MPGGLM-
MDPGGL--
MWPGGL--
MQPGGL--
MPPGGL--
MNPGGL--
-MPGGLV-
-MPGGLE-
MHPGGL--
NSPGGL--
NRPGGL--
-NPGGLH-
NGPGGL--
-NPGGLL-
-NPGGLF-
-NPGGLY-
-NPGGLT-
NYPGGL--
-NPGGLV-
NLPGGL--
NIPGGL--
NVPGGL--
-NPGGLI-
NKPGGL--
NAPGGL--
-NPGGLK-
NTPGGL--
-NPGGLS-
NMPGGL--
-NPGGLM-
NNPGGL--
NHPGGL--
NWPGGL--
NPPGGL--
-NPGGLQ-
NQPGGL--
NEPGGL--
```

NDPGGL--
NCPGGL--
--PGGLRS
--PGGLRG
--PGGLWT
--PGGLRR
--PGGLRY
--PGGLIN
--PGGLSS
--PGGLYS
--PGGLLA
--PGGLRV
--PGGLRM
--PGGLTV
--PGGLMS
--PGGLLT
--PGGLWS
--PGGLIS
--PGGLME
--PGGLTS
--PGGLRI
--PGGLRF
--PGGLVS
--PGGLTG
--PGGLRT
--PGGLRN
--PGGLFG
--PGGLYY
--PGGLWA
--PGGLRW
--PGGLMG
--PGGLFY
--PGGLQS
--PGGLKN
--PGGLHL
--PGGLVV
--PGGLVT
--PGGLVA
--PGGLST
--PGGLHH
--PGGLYW
--PGGLYM
--PGGLYG
--PGGLVY
--PGGLTT
--PGGLTR
--PGGLSA
--PGGLIT
--PGGLHG
--PGGLHA

--PGGLFS
--PGGLVG
--PGGLRH
--PGGLFA
--PGGLYV
--PGGLWR
--PGGLVR
--PGGLMH
--PGGLKV
--PGGLHR
--PGGLFR
PEPGGL--
PWPGGL--
--PGGLSG
--PGGLMV
--PGGLMR
--PGGLMN
--PGGLMI
--PGGLIG
--PGGLHS
--PGGLFN
PGPGGL--
--PGGLYR
--PGGLLF
--PGGLKY
--PGGLKG
--PGGLIM
--PGGLIA
--PGGLHF
--PGGLFH
--PGGLAA
PVPGGL--
PIPGGL--
--PGGLWG
--PGGLTN
--PGGLSH
--PGGLMY
--PGGLKA
--PGGLHT
--PGGLHD
PLPGGL--
--PGGLYA
--PGGLRK
--PGGLMK
--PGGLES
--PGGLAT
PRPGGL--
--PGGLYT
--PGGLWD
--PGGLTA

--PGGLSV
--PGGLSK
--PGGLRE
--PGGLNA
--PGGLMT
--PGGLLV
--PGGLKM
--PGGLAV
--PGGLAG
PFPGGL--
PHPGGL--
--PGGLYQ
--PGGLYN
--PGGLVK
--PGGLTK
--PGGLSY
--PGGLQT
--PGGLMD
--PGGLKK
--PGGLIY
PSPGGL--
--PGGLYI
--PGGLWV
--PGGLVW
--PGGLVH
--PGGLTF
--PGGLSR
--PGGLSM
--PGGLMQ
--PGGLLN
--PGGLKF
--PGGLIW
--PGGLIF
--PGGLHM
--PGGLHK
PQPGGL--
-PPGGLH-
--PGGLYH
--PGGLWM
--PGGLVM
--PGGLSN
--PGGLRD
--PGGLQA
--PGGLNV
--PGGLNS
--PGGLLY
--PGGLLW
--PGGLLH
--PGGLKW
--PGGLKT

--PGGLKH
--PGGLIQ
--PGGLAY
PYPGGL--
--PGGLWW
--PGGLWN
--PGGLWK
--PGGLWE
--PGGLVQ
--PGGLRQ
--PGGLQV
--PGGLQF
--PGGLNT
--PGGLMA
--PGGLKR
--PGGLIK
--PGGLHW
--PGGLHV
--PGGLHE
--PGGLGS
--PGGLFT
--PGGLFE
--PGGLFD
--PGGLEA
--PGGLAF
PNPGGL--
PMPGGL--
PKPGGL--
--PGGLWQ
--PGGLTW
--PGGLTI
--PGGLTH
--PGGLQM
--PGGLQH
--PGGLNY
--PGGLMW
--PGGLLD
--PGGLHI
--PGGLFM
--PGGLAS
-PPGGLW-
-PPGGLR-
-PPGGLL-
-PPGGLI-
--PGGLTC
--PGGLQW
--PGGLQI
--PGGLQG
--PGGLNW
--PGGLNN

--PGGLNM
--PGGLNH
--PGGLLM
--PGGLKQ
--PGGLKI
--PGGLKE
--PGGLKD
--PGGLHN
--PGGLGA
--PGGLEV
--PGGLEF
--PGGLAH
-QPGGLL-
-QPGGLR-
-QPGGLM-
QSPGGL--
QRPGGL--
QVPGGL--
-QPGGLV-
-QPGGLK-
QLPGGL--
-QPGGLY-
QIPGGL--
-QPGGLN-
-QPGGLF-
QPPGGL--
-QPGGLT-
QDPGGL--
QWPGGL--
QTPGGL--
-QPGGLQ-
QGPGGL--
-QPGGLW-
-QPGGLI-
-QPGGLH-
QFPGGL--
QKPGGL--
QHPGGL--
QCPGGL--
QAPGGL--
QQPGGL--
-QPGGLA-
QNPGGL--
QMPGGL--
QYPGGL--
-QPGGLG-
-RPGGLR-
-RPGGLK-
RSPGGL--
RLPGGL--

RVPGGL--
RRPGGL--
-RPGGLT-
-RPGGLS-
-RPGGLY-
RTPGGL--
-RPGGLV-
-RPGGLQ-
RFPGGL--
-RPGGLA-
RYPGGL--
RIPGGL--
-RPGGLH-
RHPGGL--
-RPGGLI-
-RPGGLN-
RGPGGL--
RPPGGL--
RAPGGL--
-RPGGLF-
RMPGGL--
RCPGGL--
RDPGGL--
-RPGGLW-
REPGGL--
-SPGGLL-
SLPGGL--
-SPGGLK-
SKPGGL--
-SPGGLI-
STPGGL--
SYPGGL--
-SPGGLF-
SVPGGL--
SQPGGL--
-SPGGLN-
SAPGGL--
-SPGGLH-
SSPGGL--
-SPGGLS-
SIPGGL--
-SPGGLY-
SNPGGL--
SGPGGL--
SWPGGL--
SPPGGL--
-SPGGLQ-
-SPGGLA-
SMPGGL--
SFPGGL--

SHPGGL--
-SPGGLW-
SDPGGL--
SCPGGL--
SEPGGL--
-TPGGLR-
-TPGGLY-
-TPGGLK-
-TPGGLS-
TWPGGL--
TGPGGL--
-TPGGLV-
-TPGGLF-
TIPGGL--
TLPGGL--
TKPGGL--
-TPGGLH-
TNPGGL--
TFPGGL--
-TPGGLN-
THPGGL--
TAPGGL--
-TPGGLI-
TYPGGL--
-TPGGLW-
TMPGGL--
TEPGGL--
TDPGGL--
TQPGGL--
-TPGGLQ-
TPPGGL--
-TPGGLC-
TCPGGL--
-VPGGLL-
VRPGGL--
-VPGGLS-
-VPGGLQ-
VPPGGL--
-VPGGLI-
VWPGGL--
VTPGGL--
VIPGGL--
VAPGGL--
VKPGGL--
-VPGGLV-
VVPGGL--
VMPGGL--
-VPGGLM-
-VPGGLA-
VSPGGL--

VNPGGL--
VHPGGL--
-VPGGLN-
-VPGGLF-
VQPGGL--
VYPGGL--
-VPGGLH-
VDPGGL--
VFPGGL--
-VPGGLD-
VEPGGL--
VCPGGL--
WLPGGL--
WGPGGL--
WTPGGL--
-WPGGLT-
-WPGGLR-
-WPGGLY-
WVPGGL--
WSPGGL--
-WPGGLL-
WIPGGL--
WFPGGL--
-WPGGLS-
-WPGGLM-
WNPGGL--
WPPGGL--
-WPGGLI-
WYPGGL--
WQPGGL--
WAPGGL--
-WPGGLV-
-WPGGLA-
-WPGGLH-
WKPGGL--
-WPGGLN-
-WPGGLQ-
-WPGGLF-
WHPGGL--
WEPGGL--
WDPGGL--
-WPGGLW-
WMPGGL--
-YPGGLR-
YSPGGL--
YRPGGL--
YTPGGL--
-YPGGLS-
-YPGGLK-
-YPGGLF-

```
          YLPGGL--
          YYPGGL--
          -YPGGLT-
          -YPGGLN-
          -YPGGLI-
          YMPGGL--
          YGPGGL--
          YPPGGL--
          -YPGGLY-
          -YPGGLH-
          YKPGGL--
          YIPGGL--
          YHPGGL--
          YAPGGL--
          YNPGGL--
          YFPGGL--
          YEPGGL--
          -YPGGLV-
          -YPGGLQ-
          YDPGGL--
          YQPGGL--
          -YPGGLM-
          YCPGGL--
PIGW       459       616     31.16
          -EPIGWN-
          --PIGWTD
          --PIGWKL
          -APIGWH-
          ATPIGW--
          -APIGWK-
          -APIGWI-
          -APIGWA-
          ARPIGW--
          AEPIGW--
          AAPIGW--
          AYPIGW--
          AWPIGW--
          -APIGWV-
          -APIGWM-
          AMPIGW--
          ALPIGW--
          AHPIGW--
          ASPIGW--
          -APIGWS-
          -APIGWQ-
          -APIGWN-
          AFPIGW--
          -APIGWY-
          -APIGWT-
          -APIGWR-
```

AIPIGW--
CSPIGW--
CIPIGW--
CEPIGW--
CRPIGW--
CCPIGW--
CTPIGW--
-CPIGWK-
-DPIGWS-
DRPIGW--
-DPIGWR-
DLPIGW--
DWPIGW--
-DPIGWH-
DVPIGW--
-DPIGWQ-
-DPIGWW-
DQPIGW--
-DPIGWY-
-DPIGWK-
-DPIGWG-
-DPIGWA-
DKPIGW--
ERPIGW--
EMPIGW--
-EPIGWV-
-EPIGWA-
-EPIGWH-
ETPIGW--
-EPIGWT-
EAPIGW--
-EPIGWS-
-EPIGWR-
EHPIGW--
EYPIGW--
EWPIGW--
-EPIGWI-
-EPIGWF-
-EPIGWD-
-FPIGWH-
FRPIGW--
-FPIGWY-
-FPIGWK-
-FPIGWA-
-FPIGWI-
FWPIGW--
FNPIGW--
FDPIGW--
FSPIGW--
-FPIGWV-

-FPIGWT-
-FPIGWR-
-FPIGWN-
-FPIGWF-
FKPIGW--
FHPIGW--
FGPIGW--
FFPIGW--
FAPIGW--
GFPIGW--
-GPIGWT-
-GPIGWV-
-GPIGWW-
-GPIGWI-
GVPIGW--
-GPIGWH-
-GPIGWA-
GYPIGW--
GTPIGW--
-GPIGWR-
-GPIGWE-
GSPIGW--
GRPIGW--
-GPIGWM-
GGPIGW--
-GPIGWY-
-GPIGWN-
GNPIGW--
GHPIGW--
GWPIGW--
-GPIGWS-
-GPIGWK-
-GPIGWF-
GLPIGW--
GDPIGW--
GAPIGW--
-HPIGWT-
HRPIGW--
-HPIGWV-
-HPIGWR-
HKPIGW--
HTPIGW--
HNPIGW--
HEPIGW--
HYPIGW--
HLPIGW--
HDPIGW--
HWPIGW--
HSPIGW--
-HPIGWQ-

-HPIGWG-
-HPIGWY-
-HPIGWW-
-HPIGWS-
-HPIGWM-
-HPIGWI-
-HPIGWE-
HMPIGW--
-IPIGWK-
-IPIGWV-
IWPIGW--
ILPIGW--
IGPIGW--
IPPIGW--
-IPIGWA-
IFPIGW--
IEPIGW--
IRPIGW--
-IPIGWT-
-IPIGWM-
-IPIGWI-
INPIGW--
IIPIGW--
ITPIGW--
-IPIGWR-
-IPIGWN-
IKPIGW--
-IPIGWS-
IAPIGW--
ISPIGW--
-IPIGWQ-
-IPIGWG-
-IPIGWF-
IHPIGW--
KSPIGW--
-KPIGWY-
KLPIGW--
KGPIGW--
KTPIGW--
-KPIGWM-
-KPIGWH-
KNPIGW--
KFPIGW--
KAPIGW--
KRPIGW--
-KPIGWR-
KVPIGW--
-KPIGWW-
-KPIGWV-
-KPIGWT-

```
-KPIGWI-
KWPIGW--
KQPIGW--
KPPIGW--
-KPIGWF-
-KPIGWA-
KIPIGW--
KHPIGW--
KEPIGW--
KDPIGW--
LRPIGW--
-LPIGWR-
LKPIGW--
-LPIGWS-
-LPIGWN-
LIPIGW--
LVPIGW--
LSPIGW--
LTPIGW--
-LPIGWY-
-LPIGWA-
-LPIGWM-
LGPIGW--
LYPIGW--
LPPIGW--
-LPIGWT-
LAPIGW--
-LPIGWV-
-LPIGWH-
LHPIGW--
LQPIGW--
-LPIGWQ-
-LPIGWK-
-LPIGWI-
LMPIGW--
LLPIGW--
LFPIGW--
LEPIGW--
MTPIGW--
MEPIGW--
MFPIGW--
MAPIGW--
MRPIGW--
-MPIGWW-
-MPIGWM-
MGPIGW--
MSPIGW--
-MPIGWI-
-MPIGWY-
-MPIGWG-
```

MLPIGW--
MWPIGW--
-MPIGWT-
-MPIGWS-
-MPIGWR-
-MPIGWN-
-MPIGWK-
-MPIGWH-
-MPIGWF-
-MPIGWE-
-MPIGWA-
MMPIGW--
MKPIGW--
MHPIGW--
MDPIGW--
MCPIGW--
NYPIGW--
NVPIGW--
-NPIGWS-
NNPIGW--
NTPIGW--
NRPIGW--
-NPIGWH-
NPPIGW--
-NPIGWQ-
-NPIGWN-
-NPIGWK-
-NPIGWM-
-NPIGWF-
NIPIGW--
NFPIGW--
-NPIGWI-
NQPIGW--
-NPIGWV-
-NPIGWE-
-NPIGWA-
NKPIGW--
NGPIGW--
NEPIGW--
NDPIGW--
--PIGWTS
--PIGWVT
--PIGWWH
--PIGWIR
--PIGWRV
--PIGWRA
--PIGWFH
--PIGWWQ
--PIGWTA
--PIGWSR

--PIGWRG
--PIGWNA
--PIGWTY
--PIGWTR
--PIGWSW
--PIGWRY
--PIGWQK
--PIGWYL
--PIGWWS
--PIGWTH
--PIGWSS
--PIGWRR
--PIGWRI
--PIGWRE
--PIGWNG
--PIGWFS
--PIGWYS
--PIGWYK
--PIGWWT
--PIGWVK
--PIGWVA
--PIGWTN
--PIGWSV
--PIGWRQ
--PIGWNV
--PIGWHS
--PIGWAS
PMPIGW--
--PIGWYM
--PIGWSA
--PIGWRT
--PIGWKH
--PIGWIS
--PIGWIG
--PIGWVG
--PIGWTF
--PIGWST
--PIGWRS
--PIGWRL
--PIGWKM
--PIGWKG
--PIGWIV
--PIGWFG
--PIGWAG
--PIGWYV
--PIGWYT
--PIGWYN
--PIGWWV
--PIGWSM
--PIGWSG

--PIGWRN
--PIGWKY
--PIGWHK
--PIGWHI
--PIGWAT
PGPIGW--
PYPIGW--
--PIGWVW
--PIGWVV
--PIGWVQ
--PIGWVI
--PIGWTT
--PIGWSL
--PIGWSI
--PIGWSH
--PIGWQQ
--PIGWMG
--PIGWME
--PIGWMD
--PIGWIW
--PIGWIT
--PIGWIK
--PIGWHY
--PIGWHL
--PIGWFT
--PIGWYR
--PIGWYG
--PIGWVF
--PIGWTW
--PIGWTV
--PIGWTK
--PIGWSQ
--PIGWNT
--PIGWMY
--PIGWKV
--PIGWKS
--PIGWKF
--PIGWKA
--PIGWIQ
--PIGWHR
--PIGWHE
--PIGWFR
--PIGWFK
--PIGWFI
--PIGWAV
--PIGWAK
--PIGWAA
PNPIGW--
PKPIGW--
PIPIGW--

--PIGWYA
--PIGWWG
--PIGWWF
--PIGWVS
--PIGWVR
--PIGWVN
--PIGWVH
--PIGWTL
--PIGWTG
--PIGWSY
--PIGWSN
--PIGWSK
--PIGWSF
--PIGWRM
--PIGWRK
--PIGWRH
--PIGWRD
--PIGWQY
--PIGWQV
--PIGWQT
--PIGWQR
--PIGWQH
--PIGWQG
--PIGWMT
--PIGWMR
--PIGWMN
--PIGWML
--PIGWMK
--PIGWKW
--PIGWKT
--PIGWKR
--PIGWKK
--PIGWIY
--PIGWIH
--PIGWIE
--PIGWID
--PIGWIA
--PIGWHT
--PIGWHH
--PIGWHG
--PIGWHF
--PIGWGL
--PIGWGK
--PIGWGA
--PIGWFN
--PIGWFF
--PIGWFA
--PIGWET
--PIGWES
--PIGWEG

--PIGWAR
--PIGWAQ
--PIGWAN
--PIGWAM
--PIGWAL
--PIGWAI
--PIGWAF
PFPIGW--
-QPIGWY-
QSPIGW--
QWPIGW--
-QPIGWK-
QGPIGW--
QYPIGW--
-QPIGWM-
-QPIGWI-
QFPIGW--
QRPIGW--
QQPIGW--
-QPIGWA-
QHPIGW--
QVPIGW--
QTPIGW--
-QPIGWV-
-QPIGWS-
-QPIGWR-
-QPIGWN-
-QPIGWF-
-QPIGWE-
-QPIGWC-
QNPIGW--
QEPIGW--
QAPIGW--
-RPIGWI-
RHPIGW--
-RPIGWS-
RGPIGW--
RSPIGW--
-RPIGWA-
RPPIGW--
-RPIGWK-
RFPIGW--
RQPIGW--
-RPIGWY-
-RPIGWN-
-RPIGWQ-
RKPIGW--
-RPIGWR-
RLPIGW--
RYPIGW--

RTPIGW--
-RPIGWM-
REPIGW--
RRPIGW--
-RPIGWH-
RIPIGW--
RAPIGW--
RWPIGW--
-RPIGWF-
SRPIGW--
SSPIGW--
SGPIGW--
SMPIGW--
-SPIGWR-
SVPIGW--
-SPIGWI-
SAPIGW--
SYPIGW--
-SPIGWM-
-SPIGWT-
-SPIGWK-
-SPIGWV-
-SPIGWQ-
-SPIGWN-
-SPIGWA-
SDPIGW--
-SPIGWW-
SNPIGW--
SWPIGW--
SQPIGW--
-SPIGWS-
-SPIGWH-
SIPIGW--
SHPIGW--
-SPIGWY-
-SPIGWF-
SLPIGW--
SKPIGW--
SFPIGW--
STPIGW--
SPPIGW--
-SPIGWP-
-SPIGWE-
SEPIGW--
TRPIGW--
TGPIGW--
-TPIGWI-
TVPIGW--
TSPIGW--
-TPIGWR-

```
-TPIGWQ-
-TPIGWK-
TFPIGW--
TYPIGW--
-TPIGWV-
-TPIGWT-
TKPIGW--
-TPIGWS-
TIPIGW--
-TPIGWA-
TNPIGW--
TAPIGW--
-TPIGWW-
-TPIGWN-
TDPIGW--
TTPIGW--
-TPIGWY-
-TPIGWM-
-TPIGWG-
-TPIGWF-
TLPIGW--
TEPIGW--
-VPIGWV-
-VPIGWS-
-VPIGWA-
VVPIGW--
-VPIGWK-
-VPIGWR-
VKPIGW--
VPPIGW--
VNPIGW--
VRPIGW--
VSPIGW--
-VPIGWT-
-VPIGWI-
VLPIGW--
VHPIGW--
VYPIGW--
VWPIGW--
-VPIGWH-
-VPIGWG-
-VPIGWE-
VGPIGW--
VDPIGW--
VAPIGW--
-VPIGWY-
-VPIGWQ-
-VPIGWN-
VIPIGW--
-WPIGWS-
```

| | | | |
|---|---|---|---|
| | WHPIGW-- | | |
| | WVPIGW-- | | |
| | WYPIGW-- | | |
| | -WPIGWA- | | |
| | WGPIGW-- | | |
| | WRPIGW-- | | |
| | -WPIGWV- | | |
| | -WPIGWM- | | |
| | WSPIGW-- | | |
| | WQPIGW-- | | |
| | -WPIGWR- | | |
| | -WPIGWN- | | |
| | WIPIGW-- | | |
| | WWPIGW-- | | |
| | -WPIGWT- | | |
| | -WPIGWK- | | |
| | WLPIGW-- | | |
| | YRPIGW-- | | |
| | YWPIGW-- | | |
| | YSPIGW-- | | |
| | YTPIGW-- | | |
| | -YPIGWQ- | | |
| | YQPIGW-- | | |
| | -YPIGWS- | | |
| | -YPIGWI- | | |
| | -YPIGWR- | | |
| | -YPIGWV- | | |
| | -YPIGWT- | | |
| | -YPIGWN- | | |
| | -YPIGWH- | | |
| | -YPIGWF- | | |
| | YFPIGW-- | | |
| | YVPIGW-- | | |
| | -YPIGWY- | | |
| | -YPIGWW- | | |
| | -YPIGWM- | | |
| | YNPIGW-- | | |
| | YMPIGW-- | | |
| | YHPIGW-- | | |
| | YDPIGW-- | | |
| PGPL | 57 | 799 | 31.15 |
| | -FPGPLN- | | |
| | -GPGPLG- | | |
| | -IPGPLN- | | |
| | -LPGPLN- | | |
| | -LPGPLG- | | |
| | --PGPLLI | | |
| | --PGPLWR | | |
| | --PGPLVV | | |
| | --PGPLSN | | |

```
--PGPLSE
--PGPLNR
--PGPLIN
--PGPLII
--PGPLFR
--PGPLAT
PAPGPL--
-RPGPLP-
-RPGPLG-
-TPGPLN-
TIPGPL--
-VPGPLG-
-VPGPLA-
ALPGPL--
-APGPLR-
AVPGPL--
ARPGPL--
-APGPLL-
-APGPLS-
-APGPLM-
-APGPLV-
-APGPLY-
AHPGPL--
AYPGPL--
AWPGPL--
ATPGPL--
-APGPLQ-
AIPGPL--
-APGPLI-
-APGPLH-
AAPGPL--
ASPGPL--
-APGPLT-
ANPGPL--
AFPGPL--
AKPGPL--
-APGPLW-
-APGPLN-
-APGPLF-
-APGPLA-
AQPGPL--
-APGPLK-
AMPGPL--
AGPGPL--
AEPGPL--
-APGPLP-
-APGPLG-
ADPGPL--
CAPGPL--
CGPGPL--
```

CMPGPL--
CTPGPL--
CSPGPL--
CRPGPL--
-CPGPLY-
-CPGPLK-
-CPGPLF-
CNPGPL--
CLPGPL--
CKPGPL--
DMPGPL--
-DPGPLW-
DRPGPL--
DQPGPL--
DIPGPL--
-DPGPLR-
-DPGPLI-
DHPGPL--
-DPGPLY-
DTPGPL--
DLPGPL--
DKPGPL--
DGPGPL--
DFPGPL--
DSPGPL--
-DPGPLL-
-DPGPLF-
DYPGPL--
DVPGPL--
-DPGPLV-
-DPGPLT-
-DPGPLQ-
-DPGPLM-
-DPGPLK-
-DPGPLH-
DNPGPL--
DEPGPL--
DAPGPL--
-EPGPLT-
-EPGPLR-
-EPGPLK-
EYPGPL--
EHPGPL--
ERPGPL--
-EPGPLF-
ENPGPL--
-EPGPLV-
-EPGPLS-
EGPGPL--
EAPGPL--

ESPGPL--
-EPGPLW-
ETPGPL--
EQPGPL--
-EPGPLI-
EMPGPL--
EIPGPL--
EWPGPL--
-EPGPLY-
-EPGPLQ-
-EPGPLN-
-EPGPLM-
-EPGPLA-
EKPGPL--
FKPGPL--
FTPGPL--
FIPGPL--
FRPGPL--
FGPGPL--
FQPGPL--
FHPGPL--
-FPGPLH-
FYPGPL--
FMPGPL--
FSPGPL--
FFPGPL--
FVPGPL--
-FPGPLY-
FNPGPL--
-FPGPLT-
FAPGPL--
FPPGPL--
-FPGPLW-
-FPGPLV-
-FPGPLK-
FWPGPL--
-FPGPLS-
-FPGPLL-
-FPGPLI-
-FPGPLF-
FDPGPL--
-FPGPLP-
-FPGPLA-
-GPGPLR-
GRPGPL--
-GPGPLK-
GSPGPL--
GAPGPL--
-GPGPLW-
-GPGPLI-

GTPGPL--
GKPGPL--
GYPGPL--
-GPGPLT-
-GPGPLM-
-GPGPLY-
-GPGPLH-
-GPGPLF-
GWPGPL--
GVPGPL--
GLPGPL--
GQPGPL--
GNPGPL--
GMPGPL--
-GPGPLQ-
GEPGPL--
GPPGPL--
-GPGPLV-
GGPGPL--
-GPGPLS-
GIPGPL--
GFPGPL--
-GPGPLN-
GHPGPL--
GCPGPL--
-GPGPLA-
HGPGPL--
HRPGPL--
-HPGPLF-
HWPGPL--
HSPGPL--
-HPGPLT-
-HPGPLI-
HVPGPL--
HLPGPL--
HIPGPL--
HTPGPL--
HMPGPL--
HAPGPL--
HYPGPL--
HPPGPL--
HFPGPL--
HQPGPL--
-HPGPLY-
-HPGPLW-
-HPGPLK-
-HPGPLH-
HNPGPL--
-HPGPLV-
-HPGPLS-

−HPGPLM−
−HPGPLL−
HKPGPL−−
HEPGPL−−
ILPGPL−−
IRPGPL−−
IAPGPL−−
−IPGPLR−
IHPGPL−−
−IPGPLL−
−IPGPLI−
−IPGPLF−
IGPGPL−−
ISPGPL−−
−IPGPLH−
IMPGPL−−
IIPGPL−−
−IPGPLM−
IKPGPL−−
IFPGPL−−
IVPGPL−−
ITPGPL−−
INPGPL−−
IEPGPL−−
IYPGPL−−
−IPGPLK−
IPPGPL−−
−IPGPLT−
−IPGPLQ−
−IPGPLA−
IWPGPL−−
−IPGPLY−
−IPGPLW−
ICPGPL−−
KSPGPL−−
−KPGPLR−
−KPGPLT−
−KPGPLS−
−KPGPLK−
KAPGPL−−
KYPGPL−−
KTPGPL−−
KVPGPL−−
KGPGPL−−
−KPGPLV−
−KPGPLL−
KFPGPL−−
−KPGPLQ−
−KPGPLI−
−KPGPLH−

KIPGPL--
-KPGPLY-
-KPGPLF-
KWPGPL--
KQPGPL--
-KPGPLM-
KHPGPL--
-KPGPLW-
KNPGPL--
KLPGPL--
KDPGPL--
KKPGPL--
KEPGPL--
LRPGPL--
-LPGPLW-
LNPGPL--
LYPGPL--
LKPGPL--
LLPGPL--
LQPGPL--
-LPGPLV-
LGPGPL--
LMPGPL--
LWPGPL--
LTPGPL--
-LPGPLL-
-LPGPLK-
LHPGPL--
-LPGPLY-
LFPGPL--
-LPGPLH-
-LPGPLF-
LPPGPL--
-LPGPLI-
LEPGPL--
-LPGPLM-
LDPGPL--
MRPGPL--
-MPGPLR-
MSPGPL--
-MPGPLL-
-MPGPLV-
-MPGPLI-
-MPGPLK-
MNPGPL--
MGPGPL--
-MPGPLT-
-MPGPLH-
MFPGPL--
-MPGPLA-

MVPGPL--
-MPGPLQ-
-MPGPLF-
MEPGPL--
MYPGPL--
-MPGPLW-
MKPGPL--
MIPGPL--
MAPGPL--
MWPGPL--
MPPGPL--
-MPGPLY-
-MPGPLS-
-MPGPLM-
MMPGPL--
MDPGPL--
MTPGPL--
MQPGPL--
NTPGPL--
NMPGPL--
-NPGPLR-
NFPGPL--
NLPGPL--
-NPGPLK-
-NPGPLF-
NKPGPL--
NGPGPL--
NQPGPL--
NPPGPL--
-NPGPLW-
-NPGPLV-
NIPGPL--
NVPGPL--
-NPGPLT-
-NPGPLM-
-NPGPLI-
-NPGPLH-
NNPGPL--
NHPGPL--
NYPGPL--
-NPGPLS-
-NPGPLN-
-NPGPLL-
NRPGPL--
-NPGPLY-
-NPGPLQ-
-NPGPLE-
NEPGPL--
NAPGPL--
--PGPLRL

--PGPLTL
--PGPLKL
--PGPLAL
--PGPLIS
--PGPLSL
--PGPLLL
--PGPLRA
--PGPLYL
--PGPLSS
--PGPLLG
--PGPLLA
--PGPLSI
--PGPLVS
--PGPLRV
--PGPLNA
--PGPLKI
--PGPLFM
--PGPLSV
--PGPLRI
--PGPLML
--PGPLLM
PLPGPL--
--PGPLSH
--PGPLNL
--PGPLMS
--PGPLYG
--PGPLSG
--PGPLRR
--PGPLRM
--PGPLMG
--PGPLVG
--PGPLSF
--PGPLFW
--PGPLEY
--PGPLWI
--PGPLWA
--PGPLGV
--PGPLYT
--PGPLTS
--PGPLRW
--PGPLRK
--PGPLLY
--PGPLKV
--PGPLKS
--PGPLAW
--PGPLTY
--PGPLTI
--PGPLSY
--PGPLSA
--PGPLRY

--PGPLRF
--PGPLIT
--PGPLEL
PQPGPL--
--PGPLYW
--PGPLWM
--PGPLSW
--PGPLPM
--PGPLFV
--PGPLAS
--PGPLTA
--PGPLRN
--PGPLKY
--PGPLHS
--PGPLHM
--PGPLAQ
--PGPLYQ
--PGPLRH
--PGPLQI
--PGPLNS
--PGPLNI
--PGPLMV
--PGPLMQ
--PGPLLW
--PGPLLV
--PGPLHG
--PGPLHF
--PGPLGS
--PGPLFL
--PGPLDI
PYPGPL--
--PGPLWL
--PGPLWH
--PGPLVN
--PGPLTG
--PGPLRD
--PGPLQS
--PGPLQL
--PGPLNV
--PGPLNQ
--PGPLMR
--PGPLKQ
--PGPLKG
--PGPLIK
--PGPLIF
--PGPLHY
--PGPLGW
--PGPLDL
PVPGPL--
PIPGPL--

--PGPLVT
--PGPLVL
--PGPLVK
--PGPLTW
--PGPLQY
--PGPLQA
--PGPLNY
--PGPLMA
--PGPLHI
--PGPLFA
--PGPLDF
--PGPLAV
--PGPLYY
--PGPLYR
--PGPLYA
--PGPLVW
--PGPLVF
--PGPLQG
--PGPLPY
--PGPLNM
--PGPLMW
--PGPLMN
--PGPLMK
--PGPLLF
--PGPLIY
--PGPLIH
--PGPLGN
--PGPLFY
--PGPLFF
--PGPLDW
--PGPLAY
--PGPLAM
--PGPLAA
PSPGPL--
--PGPLYD
--PGPLWF
--PGPLTV
--PGPLSM
--PGPLRG
--PGPLPW
--PGPLPS
--PGPLPQ
--PGPLPF
--PGPLMT
--PGPLKW
--PGPLKN
--PGPLKM
--PGPLHW
--PGPLHV
--PGPLHA

--PGPLGF
--PGPLFT
--PGPLFN
--PGPLDY
--PGPLAI
PEPGPL--
PTPGPL--
PKPGPL--
--PGPLYK
--PGPLVY
--PGPLTM
--PGPLST
--PGPLSQ
--PGPLRE
--PGPLQV
--PGPLQQ
--PGPLNW
--PGPLNT
--PGPLKF
--PGPLIW
--PGPLIR
--PGPLHT
--PGPLHR
--PGPLHH
--PGPLFK
--PGPLFI
--PGPLFG
--PGPLAN
PWPGPL--
PMPGPL--
--PGPLYI
--PGPLYH
--PGPLWW
--PGPLWQ
--PGPLWD
--PGPLVH
--PGPLTT
--PGPLTF
--PGPLQT
--PGPLQF
--PGPLMY
--PGPLMI
--PGPLMH
--PGPLMF
--PGPLLN
--PGPLKH
--PGPLEW
--PGPLEQ
--PGPLEM
--PGPLEI

--PGPLEF
--PGPLAG
PGPGPL--
--PGPLYM
--PGPLYF
--PGPLWY
--PGPLWN
--PGPLWK
--PGPLVM
--PGPLTQ
--PGPLQM
--PGPLPV
--PGPLPI
--PGPLPD
--PGPLNG
--PGPLNF
--PGPLMM
--PGPLLQ
--PGPLIQ
--PGPLHQ
--PGPLHK
--PGPLGQ
--PGPLGH
--PGPLFQ
--PGPLFD
--PGPLEA
--PGPLDV
--PGPLDM
--PGPLCN
--PGPLCL
--PGPLAK
--PGPLAF
PFPGPL--
QRPGPL--
-QPGPLW-
-QPGPLK-
QAPGPL--
QSPGPL--
-QPGPLL-
QGPGPL--
-QPGPLY-
-QPGPLI-
QTPGPL--
-QPGPLT-
-QPGPLR-
QMPGPL--
QLPGPL--
-QPGPLV-
-QPGPLS-
-QPGPLQ-

-QPGPLN-
-QPGPLG-
QIPGPL--
QWPGPL--
QPPGPL--
-QPGPLF-
QNPGPL--
QHPGPL--
QYPGPL--
QQPGPL--
-QPGPLM-
-QPGPLH-
QKPGPL--
QFPGPL--
QDPGPL--
-RPGPLL-
RSPGPL--
-RPGPLQ-
RMPGPL--
-RPGPLI-
RIPGPL--
RTPGPL--
-RPGPLM-
RGPGPL--
RYPGPL--
-RPGPLV-
-RPGPLA-
RKPGPL--
REPGPL--
-RPGPLT-
-RPGPLH-
RAPGPL--
RPPGPL--
RHPGPL--
RNPGPL--
-RPGPLN-
RLPGPL--
RFPGPL--
-RPGPLW-
-RPGPLK-
-RPGPLF-
RDPGPL--
-RPGPLE-
RWPGPL--
SRPGPL--
-SPGPLL-
-SPGPLR-
SLPGPL--
SSPGPL--
-SPGPLK-

-SPGPLS-
-SPGPLW-
-SPGPLI-
SFPGPL--
SEPGPL--
SYPGPL--
SVPGPL--
-SPGPLV-
-SPGPLQ-
-SPGPLM-
SNPGPL--
STPGPL--
SPPGPL--
-SPGPLF-
SMPGPL--
SHPGPL--
SGPGPL--
-SPGPLN-
-SPGPLH-
SDPGPL--
-SPGPLT-
SAPGPL--
SQPGPL--
-SPGPLA-
SWPGPL--
-SPGPLE-
-TPGPLR-
TSPGPL--
TRPGPL--
-TPGPLL-
-TPGPLY-
TVPGPL--
TQPGPL--
TLPGPL--
TTPGPL--
-TPGPLT-
-TPGPLK-
-TPGPLH-
TYPGPL--
-TPGPLS-
TFPGPL--
TPPGPL--
-TPGPLW-
-TPGPLV-
-TPGPLF-
TNPGPL--
TWPGPL--
TEPGPL--
TAPGPL--
TGPGPL--

-TPGPLQ-
-TPGPLA-
THPGPL--
-VPGPLR-
VRPGPL--
VLPGPL--
VTPGPL--
-VPGPLY-
VVPGPL--
VSPGPL--
-VPGPLH-
-VPGPLF-
-VPGPLM-
VYPGPL--
-VPGPLS-
VQPGPL--
VFPGPL--
VMPGPL--
VAPGPL--
-VPGPLW-
VKPGPL--
VNPGPL--
VGPGPL--
VWPGPL--
-VPGPLV-
-VPGPLQ-
VIPGPL--
VHPGPL--
VEPGPL--
VPPGPL--
-VPGPLN-
VDPGPL--
-WPGPLR-
WGPGPL--
WYPGPL--
WLPGPL--
WRPGPL--
WVPGPL--
-WPGPLM-
WSPGPL--
WTPGPL--
WKPGPL--
WDPGPL--
-WPGPLK-
WIPGPL--
-WPGPLV-
WMPGPL--
WHPGPL--
WPPGPL--
-WPGPLW-

-WPGPLL-
WQPGPL--
-WPGPLT-
-WPGPLS-
WEPGPL--
WAPGPL--
WWPGPL--
-WPGPLQ-
-WPGPLH-
WNPGPL--
YVPGPL--
-YPGPLY-
-YPGPLL-
YFPGPL--
YSPGPL--
YRPGPL--
YLPGPL--
YHPGPL--
YGPGPL--
YIPGPL--
-YPGPLH-
YYPGPL--
YQPGPL--
YNPGPL--
YAPGPL--
-YPGPLS-
-YPGPLV-
-YPGPLT-
YWPGPL--
-YPGPLM-
-YPGPLA-
YMPGPL--
YTPGPL--
-YPGPLN-
-YPGPLK-
YKPGPL--
YDPGPL--
YPPGPL--
-YPGPLF-
-YPGPLQ-
VAAM 301 667 31.14
LQVAAM--
--VAAMQR
-AVAAML-
AVVAAM--
-AVAAMR-
ALVAAM--
-AVAAMY-
ANVAAM--
AAVAAM--

AWVAAM--
-AVAAMH-
ARVAAM--
AGVAAM--
AYVAAM--
-AVAAMK-
APVAAM--
AIVAAM--
-AVAAMQ-
-AVAAMA-
ATVAAM--
ASVAAM--
-AVAAMW-
-AVAAMT-
-AVAAMS-
-AVAAMN-
-AVAAMM-
-AVAAMI-
AKVAAM--
AEVAAM--
-CVAAMS-
-CVAAML-
-CVAAMK-
CSVAAM--
CYVAAM--
-CVAAMY-
-CVAAMT-
CMVAAM--
CLVAAM--
CFVAAM--
DSVAAM--
-DVAAMS-
-DVAAMR-
-DVAAMI-
-DVAAMN-
DGVAAM--
DNVAAM--
DRVAAM--
DQVAAM--
-DVAAMY-
-DVAAMT-
-DVAAMQ-
DDVAAM--
-DVAAMW-
-DVAAML-
-DVAAMA-
DYVAAM--
DWVAAM--
-DVAAMK-
-DVAAMF-

DLVAAM--
DFVAAM--
DEVAAM--
DAVAAM--
-EVAAMR-
-EVAAMY-
EYVAAM--
-EVAAMV-
-EVAAMS-
-EVAAMN-
-EVAAML-
-EVAAMW-
-EVAAMQ-
-EVAAMK-
-EVAAMH-
-EVAAMF-
-EVAAMA-
ETVAAM--
ENVAAM--
ELVAAM--
EGVAAM--
EAVAAM--
-FVAAMR-
-FVAAMG-
FSVAAM--
-FVAAMV-
-FVAAMT-
-FVAAMN-
FHVAAM--
FGVAAM--
FYVAAM--
-FVAAMW-
-FVAAMQ-
-FVAAMI-
-FVAAMA-
FKVAAM--
FVVAAM--
FRVAAM--
FPVAAM--
-FVAAMK-
FEVAAM--
FDVAAM--
GRVAAM--
-GVAAML-
GYVAAM--
GVVAAM--
-GVAAMR-
-GVAAMI-
GMVAAM--
GIVAAM--

GLVAAM--
-GVAAMT-
GSVAAM--
-GVAAMY-
-GVAAMW-
-GVAAMV-
-GVAAMF-
GTVAAM--
GFVAAM--
-GVAAMN-
-GVAAMK-
GNVAAM--
-GVAAMC-
GPVAAM--
GKVAAM--
GDVAAM--
GWVAAM--
-GVAAMM-
-GVAAMH-
GHVAAM--
GAVAAM--
-HVAAMR-
-HVAAMN-
-HVAAMT-
HGVAAM--
HDVAAM--
-HVAAMY-
-HVAAMS-
HTVAAM--
HSVAAM--
HKVAAM--
-HVAAMQ-
-HVAAMF-
HNVAAM--
HMVAAM--
HLVAAM--
HFVAAM--
HAVAAM--
HVVAAM--
-HVAAMK-
-HVAAMH-
HRVAAM--
HQVAAM--
HIVAAM--
-IVAAMA-
-IVAAMH-
-IVAAMG-
IAVAAM--
-IVAAMY-
-IVAAMQ-

IRVAAM--
IFVAAM--
IVVAAM--
-IVAAMT-
IMVAAM--
ILVAAM--
-IVAAMV-
-IVAAMM-
-IVAAMK-
-IVAAMI-
ISVAAM--
IEVAAM--
-KVAAMW-
-KVAAML-
-KVAAMF-
-KVAAMI-
-KVAAMR-
-KVAAMH-
KVVAAM--
-KVAAMS-
KQVAAM--
KHVAAM--
KGVAAM--
KTVAAM--
KFVAAM--
-KVAAMQ-
KLVAAM--
KIVAAM--
KCVAAM--
-KVAAMA-
KSVAAM--
KMVAAM--
KWVAAM--
-KVAAMY-
-KVAAMM-
-KVAAMK-
KNVAAM--
KDVAAM--
-LVAAML-
-LVAAMR-
-LVAAMK-
-LVAAMV-
LFVAAM--
-LVAAMT-
-LVAAMN-
-LVAAMG-
-LVAAMF-
LKVAAM--
LHVAAM--
LGVAAM--

```
-LVAAMM-
-LVAAMQ-
LSVAAM--
LYVAAM--
LMVAAM--
LVVAAM--
-LVAAMS-
-LVAAMY-
LTVAAM--
-LVAAMH-
LDVAAM--
LAVAAM--
-MVAAMR-
-MVAAML-
-MVAAMH-
MMVAAM--
MAVAAM--
-MVAAMK-
MYVAAM--
-MVAAMY-
-MVAAMV-
-MVAAMQ-
-MVAAMA-
MGVAAM--
MFVAAM--
-MVAAMW-
-MVAAMS-
-MVAAMN-
-MVAAMG-
-MVAAME-
MSVAAM--
MPVAAM--
MLVAAM--
MEVAAM--
NRVAAM--
-NVAAMR-
-NVAAML-
-NVAAMA-
NSVAAM--
NYVAAM--
NVVAAM--
-NVAAMT-
-NVAAMI-
NWVAAM--
-NVAAMY-
-NVAAMW-
-NVAAMV-
-NVAAMM-
NTVAAM--
NPVAAM--
```

NAVAAM--
-NVAAMS-
-NVAAMQ-
-NVAAMK-
-NVAAMG-
-NVAAME-
NQVAAM--
NIVAAM--
-PVAAMR-
-PVAAML-
PSVAAM--
PRVAAM--
PYVAAM--
-PVAAMW-
-PVAAMV-
-PVAAMS-
-PVAAMH-
PKVAAM--
PAVAAM--
PVVAAM--
-PVAAMN-
-PVAAMA-
PLVAAM--
-PVAAMY-
-PVAAMT-
-PVAAMQ-
-PVAAMK-
-PVAAMI-
PQVAAM--
PEVAAM--
-QVAAML-
-QVAAMY-
-QVAAMA-
-QVAAMS-
QQVAAM--
-QVAAMR-
QSVAAM--
QYVAAM--
-QVAAMW-
-QVAAMN-
-QVAAMK-
QLVAAM--
QFVAAM--
QEVAAM--
-QVAAMV-
-QVAAMI-
QTVAAM--
QRVAAM--
-RVAAML-
-RVAAMY-

-RVAAMR-
RFVAAM--
-RVAAMK-
RVVAAM--
-RVAAMQ-
-RVAAMF-
-RVAAMS-
-RVAAMN-
RNVAAM--
REVAAM--
RYVAAM--
-RVAAMI-
-RVAAMH-
RQVAAM--
RGVAAM--
-RVAAMW-
-RVAAMM-
RAVAAM--
-RVAAMG-
-RVAAME-
RWVAAM--
-RVAAMT-
RMVAAM--
RDVAAM--
-SVAAML-
SLVAAM--
SKVAAM--
-SVAAMS-
SYVAAM--
-SVAAMI-
SRVAAM--
-SVAAMT-
SMVAAM--
SWVAAM--
-SVAAMQ-
-SVAAMK-
-SVAAMF-
SPVAAM--
SFVAAM--
STVAAM--
SGVAAM--
SEVAAM--
-SVAAMM-
-SVAAMA-
SHVAAM--
-SVAAMV-
-SVAAMH-
-SVAAME-
-SVAAMW-
-SVAAMN-

SDVAAM--
SNVAAM--
SCVAAM--
SAVAAM--
-TVAAMS-
-TVAAMK-
TSVAAM--
-TVAAMT-
-TVAAMR-
-TVAAMN-
-TVAAMA-
-TVAAMQ-
-TVAAMF-
TVVAAM--
-TVAAMV-
-TVAAML-
-TVAAMH-
TTVAAM--
TPVAAM--
TLVAAM--
TKVAAM--
TEVAAM--
TAVAAM--
-TVAAMW-
TGVAAM--
TFVAAM--
--VAAMSS
--VAAMRY
--VAAMSG
--VAAMSL
--VAAMRS
--VAAMLR
--VAAMKT
--VAAMLG
-VVAAMR-
--VAAMLS
--VAAMLH
-VVAAML-
--VAAMNL
--VAAMHT
--VAAMRW
--VAAMYR
--VAAMQA
-VVAAMV-
-VVAAMS-
--VAAMYG
--VAAMVN
--VAAMRT
--VAAMRE
--VAAMLI

--VAAMRQ
--VAAMHG
--VAAMGL
--VAAMMR
--VAAMKG
--VAAMIS
--VAAMIR
-VVAAMK-
--VAAMRG
--VAAMML
--VAAMLA
--VAAMHS
VGVAAM--
--VAAMVK
--VAAMVG
--VAAMVF
--VAAMTT
--VAAMTS
--VAAMRV
--VAAMRH
--VAAMNM
--VAAMMN
--VAAMKY
--VAAMII
--VAAMAY
-VVAAMF-
VRVAAM--
--VAAMYQ
--VAAMYI
--VAAMWR
--VAAMVI
--VAAMVE
--VAAMTY
--VAAMSY
--VAAMSM
--VAAMSF
--VAAMRN
--VAAMRD
--VAAMMM
--VAAMLQ
--VAAMHN
--VAAMFR
-VVAAMA-
VSVAAM--
--VAAMRA
--VAAMQS
--VAAMPL
--VAAMNA
--VAAMMG
--VAAMLV

--VAAMHA
--VAAMFV
--VAAMFN
--VAAMFK
--VAAMES
--VAAMYV
--VAAMYT
--VAAMYH
--VAAMVD
--VAAMTV
--VAAMTR
--VAAMTN
--VAAMTM
--VAAMSV
--VAAMSR
--VAAMSK
--VAAMSA
--VAAMQT
--VAAMPY
--VAAMNW
--VAAMMD
--VAAMIT
--VAAMIG
--VAAMGW
--VAAMFW
--VAAMAW
VYVAAM--
-VVAAMQ-
-VVAAMH-
VQVAAM--
VNVAAM--
VLVAAM--
VKVAAM--
VHVAAM--
--VAAMYS
--VAAMYN
--VAAMYK
--VAAMWT
--VAAMWL
--VAAMVV
--VAAMVT
--VAAMVS
--VAAMTK
--VAAMTH
--VAAMSI
--VAAMSH
--VAAMRF
--VAAMQG
--VAAMNF
--VAAMMH

--VAAMMF
--VAAMME
--VAAMLM
--VAAMLE
--VAAMKM
--VAAMIQ
--VAAMIF
--VAAMHV
--VAAMHI
--VAAMHF
--VAAMGY
--VAAMFT
--VAAMFH
--VAAMEG
--VAAMAS
--VAAMAL
--VAAMAI
VVVAAM--
-VVAAMI-
--VAAMYA
--VAAMWQ
--VAAMWN
--VAAMWI
--VAAMWH
--VAAMWA
--VAAMVQ
--VAAMVL
--VAAMTL
--VAAMTG
--VAAMTF
--VAAMST
--VAAMQW
--VAAMNV
--VAAMNS
--VAAMMV
--VAAMMT
--VAAMMS
--VAAMLT
--VAAMLD
--VAAMKW
--VAAMKV
--VAAMKR
--VAAMKN
--VAAMKL
--VAAMKK
--VAAMKH
--VAAMKF
--VAAMIY
--VAAMID
--VAAMIA

--VAAMHY
--VAAMHW
--VAAMHR
--VAAMHL
--VAAMHK
--VAAMGV
--VAAMGS
--VAAMGA
--VAAMFQ
--VAAMFG
--VAAMFA
--VAAMAF
-VVAAMN-
VTVAAM--
VPVAAM--
VIVAAM--
VDVAAM--
VAVAAM--
--VAAMWK
--VAAMWF
--VAAMWD
--VAAMVW
--VAAMVM
--VAAMVA
--VAAMTW
--VAAMTQ
--VAAMTI
--VAAMSQ
--VAAMSN
--VAAMSE
--VAAMQN
--VAAMQL
--VAAMQH
--VAAMPI
--VAAMPF
--VAAMNY
--VAAMNT
--VAAMNG
--VAAMMW
--VAAMMQ
--VAAMMK
--VAAMMA
--VAAMKQ
--VAAMIW
--VAAMIV
--VAAMIN
--VAAMIH
--VAAMIE
--VAAMHH
--VAAMGN

--VAAMGM
--VAAMGI
--VAAMFS
--VAAMFD
--VAAMEA
--VAAMCA
--VAAMAV
--VAAMAN
--VAAMAM
-WVAAMY-
-WVAAMT-
WYVAAM--
-WVAAMH-
-WVAAMG-
-WVAAMS-
-WVAAMR-
-WVAAMA-
-WVAAMQ-
-WVAAML-
-WVAAMK-
WTVAAM--
WSVAAM--
WNVAAM--
WIVAAM--
WGVAAM--
WEVAAM--
-WVAAMV-
WQVAAM--
WPVAAM--
WVVAAM--
-WVAAMW-
-WVAAMN-
YQVAAM--
-YVAAMN-
-YVAAMS-
-YVAAMR-
YHVAAM--
YGVAAM--
-YVAAMK-
-YVAAMH-
YNVAAM--
-YVAAMV-
-YVAAMA-
-YVAAMT-
-YVAAMQ-
-YVAAMM-
-YVAAML-
YMVAAM--
YLVAAM--
YAVAAM--

| | | | |
|---|---|---|---|
| | YTVAAM-- | | |
| | YSVAAM-- | | |
| | YKVAAM-- | | |
| | YVVAAM-- | | |
| | -YVAAMY- | | |
| | -YVAAMG- | | |
| | -YVAAME- | | |
| | YFVAAM-- | | |
| PVPF | 235 | 694 | 31.12 |
| | -MPVPFN- | | |
| | -NPVPFT- | | |
| | --PVPFLR | | |
| | --PVPFGS | | |
| | --PVPFGH | | |
| | AYPVPF-- | | |
| | ASPVPF-- | | |
| | -APVPFS- | | |
| | -APVPFH- | | |
| | ARPVPF-- | | |
| | -APVPFV- | | |
| | ALPVPF-- | | |
| | AHPVPF-- | | |
| | AKPVPF-- | | |
| | AIPVPF-- | | |
| | -APVPFT- | | |
| | -APVPFI- | | |
| | -APVPFG- | | |
| | AFPVPF-- | | |
| | AEPVPF-- | | |
| | AWPVPF-- | | |
| | AVPVPF-- | | |
| | ATPVPF-- | | |
| | -APVPFR- | | |
| | -APVPFQ- | | |
| | -APVPFN- | | |
| | -APVPFM- | | |
| | AMPVPF-- | | |
| | AAPVPF-- | | |
| | AQPVPF-- | | |
| | -APVPFY- | | |
| | -APVPFW- | | |
| | -APVPFK- | | |
| | -APVPFA- | | |
| | AGPVPF-- | | |
| | -CPVPFT- | | |
| | CTPVPF-- | | |
| | CSPVPF-- | | |
| | CRPVPF-- | | |
| | -CPVPFL- | | |
| | -CPVPFF- | | |

CMPVPF--
CIPVPF--
CGPVPF--
CAPVPF--
DRPVPF--
DLPVPF--
-DPVPFQ-
DMPVPF--
DDPVPF--
DQPVPF--
-DPVPFR-
-DPVPFI-
DKPVPF--
DHPVPF--
DGPVPF--
DEPVPF--
DYPVPF--
DWPVPF--
DVPVPF--
DFPVPF--
DTPVPF--
-DPVPFY-
DAPVPF--
-EPVPFR-
ESPVPF--
ERPVPF--
EQPVPF--
-EPVPFS-
EWPVPF--
EVPVPF--
ETPVPF--
ELPVPF--
EAPVPF--
-EPVPFL-
-EPVPFK-
-EPVPFI-
-EPVPFH-
-EPVPFA-
EMPVPF--
EKPVPF--
-FPVPFR-
-FPVPFI-
FRPVPF--
FKPVPF--
-FPVPFV-
-FPVPFK-
FPPVPF--
FIPVPF--
FTPVPF--
-FPVPFT-

FLPVPF--
FQPVPF--
-FPVPFQ-
-FPVPFL-
FHPVPF--
FSPVPF--
-FPVPFY-
-FPVPFM-
FNPVPF--
FGPVPF--
FEPVPF--
FVPVPF--
-FPVPFW-
-FPVPFA-
FAPVPF--
GRPVPF--
-GPVPFL-
-GPVPFS-
-GPVPFV-
-GPVPFT-
-GPVPFQ-
GIPVPF--
GAPVPF--
GWPVPF--
GTPVPF--
GSPVPF--
GLPVPF--
GHPVPF--
-GPVPFR-
GYPVPF--
GVPVPF--
-GPVPFN-
-GPVPFK-
-GPVPFI-
GMPVPF--
GKPVPF--
GEPVPF--
-GPVPFM-
-GPVPFF-
-GPVPFA-
GPPVPF--
GDPVPF--
GCPVPF--
HKPVPF--
-HPVPFR-
HSPVPF--
HRPVPF--
HVPVPF--
-HPVPFS-
-HPVPFM-

HDPVPF--
-HPVPFY-
HNPVPF--
HMPVPF--
HHPVPF--
-HPVPFQ-
-HPVPFL-
HFPVPF--
HAPVPF--
HYPVPF--
-HPVPFT-
-HPVPFW-
-HPVPFV-
-HPVPFI-
-HPVPFH-
-HPVPFF-
HEPVPF--
ILPVPF--
-IPVPFL-
-IPVPFS-
-IPVPFI-
-IPVPFH-
ITPVPF--
IWPVPF--
ISPVPF--
IMPVPF--
IFPVPF--
-IPVPFY-
-IPVPFV-
-IPVPFK-
IGPVPF--
IYPVPF--
IVPVPF--
IQPVPF--
-IPVPFW-
-IPVPFR-
IPPVPF--
INPVPF--
IKPVPF--
IAPVPF--
KSPVPF--
-KPVPFT-
-KPVPFS-
-KPVPFV-
KPPVPF--
-KPVPFF-
KVPVPF--
-KPVPFY-
KNPVPF--
KRPVPF--

```
-KPVPFK-
KMPVPF--
KQPVPF--
KHPVPF--
KFPVPF--
KTPVPF--
-KPVPFW-
-KPVPFH-
KLPVPF--
KAPVPF--
KWPVPF--
-KPVPFN-
-KPVPFM-
-KPVPFA-
KKPVPF--
KGPVPF--
KEPVPF--
KDPVPF--
-LPVPFR-
LSPVPF--
-LPVPFT-
-LPVPFS-
LYPVPF--
-LPVPFI-
LTPVPF--
-LPVPFM-
-LPVPFL-
-LPVPFK-
LLPVPF--
LGPVPF--
LDPVPF--
-LPVPFY-
-LPVPFV-
LPPVPF--
LNPVPF--
LIPVPF--
LHPVPF--
LFPVPF--
LEPVPF--
LWPVPF--
LQPVPF--
-LPVPFQ-
LMPVPF--
LKPVPF--
MSPVPF--
MVPVPF--
MAPVPF--
-MPVPFL-
MTPVPF--
MKPVPF--
```

MRPVPF--
-MPVPFM-
MWPVPF--
-MPVPFA-
MLPVPF--
MGPVPF--
MEPVPF--
MQPVPF--
-MPVPFY-
-MPVPFV-
-MPVPFR-
-MPVPFK-
-MPVPFI-
MPPVPF--
MNPVPF--
MMPVPF--
-NPVPFR-
-NPVPFL-
-NPVPFK-
-NPVPFS-
NHPVPF--
-NPVPFI-
NAPVPF--
NVPVPF--
NTPVPF--
NSPVPF--
-NPVPFM-
NYPVPF--
NWPVPF--
-NPVPFY-
NMPVPF--
NIPVPF--
NEPVPF--
-NPVPFH-
NPPVPF--
NNPVPF--
NKPVPF--
NGPVPF--
NFPVPF--
--PVPFLY
--PVPFTT
--PVPFQL
--PVPFRA
--PVPFLT
--PVPFRS
--PVPFPL
--PVPFLG
--PVPFHL
--PVPFSM
--PVPFSV

--PVPFRG
--PVPFYL
--PVPFWS
--PVPFSS
--PVPFMT
--PVPFLS
--PVPFTY
--PVPFSW
--PVPFRL
--PVPFRH
--PVPFMY
PSPVPF--
PAPVPF--
--PVPFYR
--PVPFSL
--PVPFLV
--PVPFLH
--PVPFLN
--PVPFIR
--PVPFYS
--PVPFYG
--PVPFRY
--PVPFMS
--PVPFMR
--PVPFVT
--PVPFVH
--PVPFTS
--PVPFSI
--PVPFHG
--PVPFFM
PLPVPF--
--PVPFYV
--PVPFYN
--PVPFWN
--PVPFWL
--PVPFVG
--PVPFTG
--PVPFST
--PVPFSG
--PVPFSF
--PVPFSA
--PVPFRV
--PVPFRK
--PVPFRF
--PVPFRD
--PVPFKW
--PVPFYT
--PVPFYK
--PVPFYI
--PVPFTF

--PVPFSQ
--PVPFQH
--PVPFPY
--PVPFMQ
--PVPFMI
--PVPFIY
--PVPFIK
--PVPFFG
--PVPFDY
--PVPFAS
--PVPFSY
--PVPFSN
--PVPFRT
--PVPFRN
--PVPFRI
--PVPFQS
--PVPFLA
--PVPFKY
--PVPFKR
--PVPFKI
--PVPFKF
--PVPFIF
--PVPFGY
--PVPFFL
PTPVPF--
PEPVPF--
PVPVPF--
--PVPFYY
--PVPFWY
--PVPFWF
--PVPFVW
--PVPFVV
--PVPFVR
--PVPFVN
--PVPFVA
--PVPFTW
--PVPFTH
--PVPFTA
--PVPFSK
--PVPFRW
--PVPFQR
--PVPFPQ
--PVPFNT
--PVPFMN
--PVPFMG
--PVPFMA
--PVPFLM
--PVPFLL
--PVPFLF
--PVPFKS

--PVPFKN
--PVPFIW
--PVPFIM
--PVPFIH
--PVPFHY
--PVPFHI
--PVPFGW
--PVPFGV
--PVPFGM
--PVPFGI
--PVPFGF
--PVPFFI
--PVPFES
--PVPFAV
--PVPFAQ
--PVPFAM
--PVPFAH
--PVPFAG
PWPVPF--
--PVPFWK
--PVPFWG
--PVPFWA
--PVPFVI
--PVPFTQ
--PVPFTL
--PVPFTI
--PVPFSH
--PVPFRM
--PVPFRE
--PVPFQQ
--PVPFPW
--PVPFPM
--PVPFPI
--PVPFPF
--PVPFNY
--PVPFNG
--PVPFML
--PVPFMH
--PVPFMF
--PVPFLK
--PVPFKT
--PVPFKK
--PVPFHQ
--PVPFHH
--PVPFGQ
--PVPFGN
--PVPFFT
--PVPFFS
--PVPFFQ
--PVPFFH

--PVPFEG
--PVPFDF
--PVPFAI
PRPVPF--
PNPVPF--
PIPVPF--
PHPVPF--
PFPVPF--
PYPVPF--
--PVPFYH
--PVPFYF
--PVPFYA
--PVPFWW
--PVPFWQ
--PVPFWM
--PVPFWI
--PVPFWH
--PVPFVY
--PVPFVL
--PVPFTM
--PVPFTK
--PVPFTE
--PVPFRQ
--PVPFQW
--PVPFQV
--PVPFQT
--PVPFQM
--PVPFQK
--PVPFQF
--PVPFPV
--PVPFPH
--PVPFNV
--PVPFNM
--PVPFNH
--PVPFNF
--PVPFNA
--PVPFKV
--PVPFKM
--PVPFKH
--PVPFKG
--PVPFKE
--PVPFIV
--PVPFIQ
--PVPFIG
--PVPFIA
--PVPFHW
--PVPFHT
--PVPFHM
--PVPFFV
--PVPFFN

--PVPFFK
--PVPFFA
--PVPFEV
--PVPFEI
--PVPFEF
--PVPFDL
--PVPFDI
--PVPFCS
PGPVPF--
PCPVPF--
QGPVPF--
-QPVPFR-
QQPVPF--
QAPVPF--
QRPVPF--
-QPVPFS-
-QPVPFI-
QVPVPF--
-QPVPFT-
-QPVPFM-
-QPVPFK-
QLPVPF--
QKPVPF--
QYPVPF--
QWPVPF--
-QPVPFA-
QPPVPF--
QMPVPF--
QSPVPF--
-QPVPFH-
-QPVPFF-
QNPVPF--
QIPVPF--
QHPVPF--
-RPVPFI-
-RPVPFS-
-RPVPFR-
RIPVPF--
RLPVPF--
RPPVPF--
RWPVPF--
RSPVPF--
RRPVPF--
RKPVPF--
RGPVPF--
RFPVPF--
RMPVPF--
RHPVPF--
RTPVPF--
-RPVPFW-

-RPVPFK-
RQPVPF--
-RPVPFT-
RAPVPF--
-RPVPFV-
-RPVPFQ-
REPVPF--
RYPVPF--
-RPVPFM-
-RPVPFA-
RDPVPF--
-RPVPFY-
-RPVPFN-
-RPVPFF-
-RPVPFE-
RNPVPF--
RCPVPF--
-SPVPFF-
SSPVPF--
-SPVPFV-
SRPVPF--
SWPVPF--
-SPVPFL-
SVPVPF--
-SPVPFR-
SMPVPF--
SHPVPF--
-SPVPFM-
-SPVPFS-
SGPVPF--
-SPVPFI-
SLPVPF--
SNPVPF--
-SPVPFH-
SIPVPF--
SYPVPF--
-SPVPFY-
-SPVPFW-
-SPVPFA-
SDPVPF--
SQPVPF--
-SPVPFK-
SPPVPF--
SFPVPF--
SEPVPF--
STPVPF--
-SPVPFT-
-SPVPFQ-
SKPVPF--
-SPVPFN-

SCPVPF--
-TPVPFS-
TRPVPF--
-TPVPFR-
-TPVPFL-
TLPVPF--
TGPVPF--
-TPVPFY-
TTPVPF--
TSPVPF--
-TPVPFV-
TNPVPF--
TYPVPF--
TMPVPF--
TAPVPF--
-TPVPFQ-
-TPVPFK-
TIPVPF--
-TPVPFF-
TKPVPF--
-TPVPFI-
THPVPF--
TWPVPF--
TVPVPF--
TQPVPF--
-TPVPFW-
-TPVPFT-
-TPVPFN-
-TPVPFA-
TEPVPF--
TCPVPF--
VTPVPF--
-VPVPFY-
-VPVPFS-
-VPVPFR-
-VPVPFV-
-VPVPFT-
-VPVPFL-
VYPVPF--
VRPVPF--
-VPVPFI-
VLPVPF--
VSPVPF--
-VPVPFK-
VVPVPF--
-VPVPFM-
VIPVPF--
VFPVPF--
VWPVPF--
VMPVPF--

VDPVPF--
VCPVPF--
VAPVPF--
-VPVPFH-
VKPVPF--
VHPVPF--
VEPVPF--
VQPVPF--
-VPVPFW-
-VPVPFF-
VPPVPF--
-WPVPFR-
WNPVPF--
-WPVPFT-
WLPVPF--
WQPVPF--
WEPVPF--
WSPVPF--
-WPVPFQ-
WYPVPF--
-WPVPFF-
WWPVPF--
-WPVPFY-
-WPVPFL-
-WPVPFA-
WKPVPF--
WFPVPF--
WDPVPF--
WAPVPF--
YAPVPF--
YGPVPF--
-YPVPFR-
-YPVPFT-
-YPVPFF-
-YPVPFK-
YEPVPF--
YSPVPF--
-YPVPFI-
-YPVPFY-
-YPVPFW-
YYPVPF--
YWPVPF--
-YPVPFV-
-YPVPFQ-
-YPVPFH-
YRPVPF--
YQPVPF--
-YPVPFM-
-YPVPFA-
YMPVPF--

| | | | |
|---|---|---|---|
| | YLPVPF-- | | |
| | YIPVPF-- | | |
| | YHPVPF-- | | |
| PHPL | 200 | 711 | 31.06 |
| | -FPHPLG- | | |
| | HAPHPL-- | | |
| | --PHPLAV | | |
| | ARPHPL-- | | |
| | -APHPLL- | | |
| | ALPHPL-- | | |
| | -APHPLR- | | |
| | -APHPLG- | | |
| | AFPHPL-- | | |
| | AAPHPL-- | | |
| | -APHPLN- | | |
| | AKPHPL-- | | |
| | AYPHPL-- | | |
| | -APHPLY- | | |
| | -APHPLS- | | |
| | -APHPLM- | | |
| | -APHPLI- | | |
| | ANPHPL-- | | |
| | ATPHPL-- | | |
| | -APHPLT- | | |
| | -APHPLQ- | | |
| | -APHPLH- | | |
| | -APHPLE- | | |
| | AIPHPL-- | | |
| | AGPHPL-- | | |
| | -CPHPLL- | | |
| | CSPHPL-- | | |
| | CHPHPL-- | | |
| | CYPHPL-- | | |
| | -CPHPLV- | | |
| | -CPHPLR- | | |
| | -CPHPLI- | | |
| | CLPHPL-- | | |
| | CKPHPL-- | | |
| | CGPHPL-- | | |
| | CDPHPL-- | | |
| | DRPHPL-- | | |
| | DLPHPL-- | | |
| | DWPHPL-- | | |
| | DYPHPL-- | | |
| | DVPHPL-- | | |
| | DSPHPL-- | | |
| | -DPHPLI- | | |
| | -DPHPLF- | | |
| | DKPHPL-- | | |
| | DAPHPL-- | | |

-DPHPLV-
-DPHPLL-
DIPHPL--
-DPHPLY-
-DPHPLR-
-DPHPLK-
DNPHPL--
DGPHPL--
DFPHPL--
-EPHPLR-
EIPHPL--
EAPHPL--
EWPHPL--
ERPHPL--
-EPHPLY-
ETPHPL--
-EPHPLL-
-EPHPLV-
-EPHPLS-
-EPHPLQ-
ELPHPL--
EVPHPL--
ESPHPL--
EQPHPL--
-EPHPLT-
-EPHPLF-
EKPHPL--
FSPHPL--
-FPHPLL-
-FPHPLS-
FVPHPL--
FTPHPL--
-FPHPLK-
FHPHPL--
-FPHPLW-
FKPHPL--
FFPHPL--
FAPHPL--
FRPHPL--
FQPHPL--
-FPHPLQ-
-FPHPLF-
FPPHPL--
FIPHPL--
FGPHPL--
FWPHPL--
-FPHPLY-
-FPHPLV-
-FPHPLR-
-FPHPLM-

−FPHPLI−
−FPHPLH−
−FPHPLA−
FNPHPL−−
FMPHPL−−
−GPHPLR−
GLPHPL−−
GYPHPL−−
GWPHPL−−
−GPHPLL−
GFPHPL−−
GQPHPL−−
−GPHPLT−
−GPHPLN−
−GPHPLI−
−GPHPLM−
GEPHPL−−
GTPHPL−−
−GPHPLV−
GVPHPL−−
GSPHPL−−
−GPHPLY−
GKPHPL−−
GAPHPL−−
−GPHPLW−
−GPHPLH−
GIPHPL−−
GNPHPL−−
GHPHPL−−
GGPHPL−−
GDPHPL−−
HIPHPL−−
HGPHPL−−
−HPHPLT−
−HPHPLS−
HKPHPL−−
HLPHPL−−
HRPHPL−−
−HPHPLY−
−HPHPLR−
HYPHPL−−
HPPHPL−−
HFPHPL−−
−HPHPLV−
−HPHPLL−
−HPHPLF−
HSPHPL−−
−HPHPLW−
−HPHPLN−
−HPHPLI−

HEPHPL--
HWPHPL--
HVPHPL--
HTPHPL--
-HPHPLM-
-HPHPLH-
HHPHPL--
IQPHPL--
-IPHPLN-
IRPHPL--
-IPHPLS-
-IPHPLR-
-IPHPLT-
-IPHPLL-
IIPHPL--
ITPHPL--
-IPHPLV-
ILPHPL--
IYPHPL--
-IPHPLY-
-IPHPLW-
-IPHPLA-
INPHPL--
IMPHPL--
IGPHPL--
IFPHPL--
IVPHPL--
-IPHPLM-
-IPHPLI-
IKPHPL--
IHPHPL--
IDPHPL--
KSPHPL--
-KPHPLR-
-KPHPLL-
-KPHPLT-
-KPHPLF-
KVPHPL--
-KPHPLM-
-KPHPLV-
-KPHPLS-
KMPHPL--
KHPHPL--
-KPHPLY-
-KPHPLN-
KAPHPL--
KWPHPL--
KQPHPL--
-KPHPLH-
-KPHPLA-

KGPHPL--
KRPHPL--
KNPHPL--
KLPHPL--
KKPHPL--
KFPHPL--
KDPHPL--
-KPHPLQ-
-KPHPLK-
KEPHPL--
-LPHPLR-
LTPHPL--
LSPHPL--
LLPHPL--
LKPHPL--
LWPHPL--
LRPHPL--
-LPHPLK-
LYPHPL--
-LPHPLT-
LNPHPL--
LFPHPL--
LEPHPL--
-LPHPLS-
LGPHPL--
LDPHPL--
LAPHPL--
-LPHPLI-
-LPHPLM-
LMPHPL--
LQPHPL--
LPPHPL--
-LPHPLY-
-LPHPLV-
-LPHPLH-
-LPHPLA-
-LPHPLL-
-LPHPLW-
-LPHPLQ-
-LPHPLN-
LIPHPL--
-MPHPLF-
MMPHPL--
-MPHPLR-
MRPHPL--
MNPHPL--
MFPHPL--
-MPHPLS-
MSPHPL--
MQPHPL--

MPPHPL--
-MPHPLL-
-MPHPLK-
-MPHPLA-
MAPHPL--
MYPHPL--
-MPHPLT-
-MPHPLI-
MKPHPL--
-MPHPLV-
MHPHPL--
MTPHPL--
-MPHPLW-
-MPHPLQ-
-MPHPLN-
-MPHPLG-
MIPHPL--
MGPHPL--
MCPHPL--
NSPHPL--
-NPHPLL-
-NPHPLI-
NGPHPL--
NTPHPL--
NLPHPL--
NYPHPL--
-NPHPLT-
-NPHPLR-
-NPHPLK-
-NPHPLF-
-NPHPLA-
NIPHPL--
-NPHPLQ-
NNPHPL--
NMPHPL--
NKPHPL--
NEPHPL--
-NPHPLM-
-NPHPLH-
NFPHPL--
--PHPLLT
--PHPLLM
--PHPLSF
--PHPLYL
--PHPLVH
--PHPLRN
PRPHPL--
--PHPLYV
--PHPLRS
--PHPLRM

--PHPLRA
--PHPLMW
--PHPLLN
--PHPLLG
--PHPLLA
--PHPLYF
--PHPLVY
--PHPLSS
--PHPLRR
--PHPLRQ
--PHPLHS
--PHPLWS
--PHPLWR
--PHPLWK
--PHPLTL
--PHPLRG
--PHPLKL
--PHPLFL
--PHPLFK
--PHPLAL
--PHPLWA
--PHPLVS
--PHPLVG
--PHPLIY
--PHPLFF
PSPHPL--
PNPHPL--
--PHPLYG
--PHPLTH
--PHPLST
--PHPLMT
--PHPLMN
--PHPLLS
--PHPLIS
--PHPLEI
--PHPLYY
--PHPLYS
--PHPLYR
--PHPLVA
--PHPLTS
--PHPLTG
--PHPLRT
--PHPLRE
--PHPLMS
--PHPLLY
--PHPLLW
--PHPLLQ
--PHPLLL
--PHPLIG
--PHPLDM

--PHPLYK
--PHPLVV
--PHPLVT
--PHPLTA
--PHPLQL
--PHPLNF
--PHPLMY
--PHPLMR
--PHPLLR
--PHPLLI
--PHPLLF
--PHPLIW
--PHPLIR
--PHPLHF
--PHPLFI
--PHPLEL
--PHPLAG
--PHPLVK
--PHPLTR
--PHPLSV
--PHPLSG
--PHPLRY
--PHPLRK
--PHPLRI
--PHPLRH
--PHPLPW
--PHPLNT
--PHPLNS
--PHPLMI
--PHPLKT
--PHPLKF
--PHPLIN
--PHPLHY
--PHPLHW
--PHPLHI
--PHPLFE
--PHPLAH
PYPHPL--
PVPHPL--
PKPHPL--
--PHPLWY
--PHPLWN
--PHPLWI
--PHPLVL
--PHPLTT
--PHPLTN
--PHPLTF
--PHPLRW
--PHPLMG
--PHPLKS

--PHPLKK
--PHPLKG
--PHPLIV
--PHPLIQ
--PHPLIM
--PHPLGT
--PHPLGH
--PHPLFS
--PHPLFR
--PHPLFG
--PHPLEW
--PHPLEM
--PHPLEG
--PHPLEA
--PHPLDL
--PHPLDI
--PHPLDF
--PHPLAN
PTPHPL--
PLPHPL--
--PHPLYM
--PHPLYE
--PHPLYA
--PHPLWT
--PHPLVR
--PHPLVM
--PHPLVD
--PHPLTK
--PHPLTI
--PHPLTE
--PHPLSN
--PHPLSA
--PHPLQM
--PHPLPH
--PHPLPA
--PHPLNQ
--PHPLNK
--PHPLMM
--PHPLMK
--PHPLMH
--PHPLMA
--PHPLLK
--PHPLLH
--PHPLLD
--PHPLKV
--PHPLKM
--PHPLKI
--PHPLKH
--PHPLIE
--PHPLHR

--PHPLHN
--PHPLHM
--PHPLGN
--PHPLGA
--PHPLFV
--PHPLFQ
--PHPLFA
--PHPLAW
--PHPLAS
PGPHPL--
PIPHPL--
--PHPLYQ
--PHPLYN
--PHPLYI
--PHPLYC
--PHPLWW
--PHPLWV
--PHPLWH
--PHPLWG
--PHPLVN
--PHPLVI
--PHPLVF
--PHPLVE
--PHPLTW
--PHPLTM
--PHPLSH
--PHPLQY
--PHPLQT
--PHPLQS
--PHPLQR
--PHPLQQ
--PHPLQH
--PHPLQG
--PHPLQF
--PHPLPV
--PHPLPQ
--PHPLPM
--PHPLPF
--PHPLNW
--PHPLNV
--PHPLMV
--PHPLMQ
--PHPLME
--PHPLLE
--PHPLKY
--PHPLKN
--PHPLKD
--PHPLKA
--PHPLID
--PHPLIA

--PHPLHA
--PHPLFT
--PHPLES
--PHPLEQ
--PHPLEF
--PHPLDY
--PHPLAT
--PHPLAQ
PHPHPL--
PFPHPL--
PAPHPL--
QSPHPL--
QFPHPL--
-QPHPLS-
-QPHPLI-
QYPHPL--
-QPHPLV-
QIPHPL--
-QPHPLT-
-QPHPLR-
-QPHPLL-
-QPHPLF-
QWPHPL--
QTPHPL--
-QPHPLY-
-QPHPLN-
QPPHPL--
-QPHPLH-
QDPHPL--
QCPHPL--
QRPHPL--
-QPHPLW-
QNPHPL--
QMPHPL--
QLPHPL--
QKPHPL--
QGPHPL--
QEPHPL--
-RPHPLT-
RLPHPL--
RVPHPL--
-RPHPLQ-
RGPHPL--
RFPHPL--
-RPHPLS-
RNPHPL--
RWPHPL--
-RPHPLV-
RMPHPL--
RYPHPL--

−RPHPLR−
−RPHPLM−
−RPHPLH−
−RPHPLF−
RQPHPL−−
−RPHPLW−
−RPHPLK−
RRPHPL−−
RIPHPL−−
RTPHPL−−
−RPHPLY−
−RPHPLA−
RHPHPL−−
REPHPL−−
−RPHPLI−
−RPHPLG−
RKPHPL−−
RPPHPL−−
RDPHPL−−
SAPHPL−−
−SPHPLR−
−SPHPLL−
SRPHPL−−
−SPHPLT−
SKPHPL−−
SYPHPL−−
SVPHPL−−
−SPHPLM−
SIPHPL−−
SGPHPL−−
SWPHPL−−
−SPHPLW−
−SPHPLS−
−SPHPLI−
−SPHPLA−
SQPHPL−−
−SPHPLQ−
SMPHPL−−
SFPHPL−−
−SPHPLK−
−SPHPLF−
SEPHPL−−
−SPHPLV−
SNPHPL−−
−SPHPLY−
−SPHPLH−
SDPHPL−−
−TPHPLL−
−TPHPLI−
TSPHPL−−

TLPHPL--
-TPHPLR-
-TPHPLM-
TVPHPL--
-TPHPLS-
-TPHPLY-
-TPHPLN-
TYPHPL--
TRPHPL--
-TPHPLH-
TFPHPL--
TPPHPL--
-TPHPLK-
TQPHPL--
-TPHPLA-
TMPHPL--
TGPHPL--
TTPHPL--
TNPHPL--
THPHPL--
TEPHPL--
TDPHPL--
TWPHPL--
-TPHPLV-
-TPHPLT-
-TPHPLF-
TKPHPL--
-VPHPLR-
VAPHPL--
VSPHPL--
VRPHPL--
-VPHPLH-
-VPHPLL-
-VPHPLI-
-VPHPLS-
VVPHPL--
-VPHPLF-
VLPHPL--
VGPHPL--
VTPHPL--
VKPHPL--
VIPHPL--
-VPHPLW-
-VPHPLN-
-VPHPLA-
VFPHPL--
VWPHPL--
VQPHPL--
VPPHPL--
-VPHPLY-

-VPHPLV-
-VPHPLT-
-VPHPLM-
-VPHPLK-
VNPHPL--
VEPHPL--
-VPHPLQ-
-VPHPLG-
VDPHPL--
VCPHPL--
WLPHPL--
WRPHPL--
-WPHPLA-
WPPHPL--
-WPHPLM-
-WPHPLL-
WNPHPL--
WYPHPL--
WFPHPL--
WAPHPL--
-WPHPLV-
WGPHPL--
WQPHPL--
-WPHPLW-
-WPHPLR-
-WPHPLN-
-WPHPLK-
-WPHPLG-
WMPHPL--
WKPHPL--
WTPHPL--
-WPHPLY-
-WPHPLT-
-WPHPLS-
-WPHPLQ-
WDPHPL--
YEPHPL--
YKPHPL--
YPPHPL--
YGPHPL--
-YPHPLR-
YHPHPL--
-YPHPLL-
YIPHPL--
-YPHPLV-
-YPHPLS-
YSPHPL--
YRPHPL--
-YPHPLY-
YLPHPL--

| | | | |
|---|---|---|---|
| | -YPHPLK- | | |
| | -YPHPLI- | | |
| | -YPHPLA- | | |
| | YFPHPL-- | | |
| | -YPHPLT- | | |
| | YAPHPL-- | | |
| | YVPHPL-- | | |
| | YTPHPL-- | | |
| | -YPHPLN- | | |
| | YMPHPL-- | | |
| | YQPHPL-- | | |
| | -YPHPLW- | | |
| | -YPHPLM- | | |
| | -YPHPLH- | | |
| | -YPHPLF- | | |
| IAWL | 1039 | 500 | 31.04 |
| | PTIAWL-- | | |
| | -AIAWLD- | | |
| | -AIAWLH- | | |
| | -AIAWLR- | | |
| | AEIAWL-- | | |
| | -AIAWLA- | | |
| | AVIAWL-- | | |
| | ATIAWL-- | | |
| | -AIAWLY- | | |
| | -AIAWLN- | | |
| | -AIAWLG- | | |
| | AYIAWL-- | | |
| | AWIAWL-- | | |
| | APIAWL-- | | |
| | ANIAWL-- | | |
| | AMIAWL-- | | |
| | ALIAWL-- | | |
| | -AIAWLQ- | | |
| | ADIAWL-- | | |
| | AQIAWL-- | | |
| | -AIAWLV- | | |
| | -AIAWLT- | | |
| | -AIAWLK- | | |
| | -CIAWLR- | | |
| | CVIAWL-- | | |
| | CTIAWL-- | | |
| | CPIAWL-- | | |
| | CNIAWL-- | | |
| | CKIAWL-- | | |
| | -CIAWLN- | | |
| | -CIAWLG- | | |
| | -DIAWLL- | | |
| | -DIAWLQ- | | |
| | DGIAWL-- | | |

DRIAWL--
DLIAWL--
DIIAWL--
-DIAWLR-
-DIAWLA-
DTIAWL--
DPIAWL--
DNIAWL--
-DIAWLN-
-DIAWLI-
-DIAWLG-
-DIAWLF-
DAIAWL--
DQIAWL--
DKIAWL--
-DIAWLW-
-DIAWLK-
-DIAWLT-
-DIAWLS-
-DIAWLM-
-DIAWLH-
DEIAWL--
DDIAWL--
-EIAWLL-
-EIAWLS-
EVIAWL--
EGIAWL--
EKIAWL--
-EIAWLH-
-EIAWLE-
-EIAWLD-
EWIAWL--
EPIAWL--
ENIAWL--
-EIAWLN-
-EIAWLA-
EHIAWL--
EDIAWL--
EQIAWL--
EMIAWL--
-EIAWLY-
-EIAWLQ-
-EIAWLG-
-EIAWLF-
FNIAWL--
FPIAWL--
FVIAWL--
FGIAWL--
FDIAWL--
FMIAWL--

FEIAWL--
FTIAWL--
-FIAWLW-
FHIAWL--
-GIAWLG-
GAIAWL--
-GIAWLR-
-GIAWLQ-
GTIAWL--
-GIAWLT-
GGIAWL--
-GIAWLL-
GPIAWL--
GLIAWL--
GNIAWL--
-GIAWLK-
-GIAWLS-
-GIAWLM-
GQIAWL--
-GIAWLH-
-GIAWLN-
-GIAWLD-
GYIAWL--
-GIAWLV-
-GIAWLA-
GFIAWL--
GEIAWL--
GMIAWL--
GHIAWL--
-HIAWLK-
HHIAWL--
HYIAWL--
HQIAWL--
HNIAWL--
-HIAWLT-
-HIAWLR-
-HIAWLH-
HDIAWL--
HAIAWL--
HTIAWL--
-HIAWLL-
-HIAWLD-
HPIAWL--
HMIAWL--
-HIAWLW-
-HIAWLQ-
-HIAWLM-
-HIAWLE-
-HIAWLA-
HVIAWL--

HIIAWL--
-HIAWLV-
-HIAWLS-
-HIAWLN-
-HIAWLC-
HGIAWL--
HFIAWL--
HCIAWL--
--IAWLRG
--IAWLSR
--IAWLTS
--IAWLSN
--IAWLRQ
--IAWLRH
--IAWLGI
--IAWLST
--IAWLGV
--IAWLGS
--IAWLER
--IAWLQE
--IAWLTT
--IAWLVD
--IAWLSG
--IAWLHS
--IAWLRL
--IAWLLK
--IAWLKS
--IAWLSK
--IAWLRN
--IAWLQS
--IAWLMA
--IAWLGR
--IAWLVG
--IAWLNT
--IAWLNS
--IAWLAS
--IAWLVS
--IAWLSS
--IAWLIS
IPIAWL--
-IIAWLS-
--IAWLSQ
--IAWLRF
--IAWLLR
--IAWLLD
--IAWLGF
--IAWLEA
--IAWLYD
--IAWLTE
--IAWLTA

--IAWLRA
--IAWLQY
--IAWLQH
--IAWLQG
--IAWLLT
--IAWLGY
--IAWLGL
--IAWLGG
--IAWLVH
--IAWLTG
--IAWLSH
--IAWLQV
--IAWLNQ
--IAWLNN
--IAWLNH
--IAWLLH
--IAWLHT
--IAWLHG
--IAWLGW
--IAWLGH
--IAWLGA
--IAWLEK
-IIAWLG-
IGIAWL--
--IAWLWH
--IAWLTN
--IAWLSM
--IAWLRV
--IAWLRM
--IAWLQT
--IAWLQQ
--IAWLNR
--IAWLNK
--IAWLLG
--IAWLLA
--IAWLKT
--IAWLKN
--IAWLGK
--IAWLAT
--IAWLTW
--IAWLTK
--IAWLSI
--IAWLRI
--IAWLMN
--IAWLMG
--IAWLLN
--IAWLKY
--IAWLKL
--IAWLKA
--IAWLIN

--IAWLHR
--IAWLGQ
--IAWLGN
--IAWLGE
--IAWLEY
--IAWLES
--IAWLDS
--IAWLAN
--IAWLAH
--IAWLWE
--IAWLVT
--IAWLVL
--IAWLVE
--IAWLVA
--IAWLSD
--IAWLRE
--IAWLQN
--IAWLQI
--IAWLNY
--IAWLNM
--IAWLNE
--IAWLMH
--IAWLKQ
--IAWLKH
--IAWLKG
--IAWLHQ
--IAWLHM
--IAWLHH
--IAWLHF
--IAWLHE
--IAWLET
--IAWLEN
--IAWLEH
--IAWLEG
--IAWLAV
--IAWLAQ
INIAWL--
-IIAWLT-
-IIAWLE-
IDIAWL--
--IAWLYM
--IAWLYI
--IAWLWD
--IAWLVQ
--IAWLTQ
--IAWLTM
--IAWLTD
--IAWLSY
--IAWLSW
--IAWLQR

--IAWLPY
--IAWLNG
--IAWLNF
--IAWLND
--IAWLMT
--IAWLMF
--IAWLME
--IAWLLY
--IAWLLL
--IAWLKM
--IAWLKK
--IAWLIL
--IAWLIH
--IAWLHV
--IAWLHL
--IAWLHK
--IAWLHI
--IAWLFL
--IAWLEE
--IAWLAK
--IAWLAD
--IAWLAA
-KIAWLH-
KNIAWL--
-KIAWLS-
KTIAWL--
KYIAWL--
KPIAWL--
KVIAWL--
-KIAWLI-
KHIAWL--
KMIAWL--
KEIAWL--
-KIAWLT-
LEIAWL--
LDIAWL--
LNIAWL--
LVIAWL--
-LIAWLH-
LGIAWL--
-LIAWLG-
-LIAWLY-
-LIAWLK-
-LIAWLE-
-LIAWLD-
-LIAWLM-
-LIAWLR-
MNIAWL--
MTIAWL--
-MIAWLR-

MVIAWL--
MDIAWL--
MPIAWL--
-MIAWLQ-
-MIAWLT-
-MIAWLK-
MHIAWL--
MFIAWL--
MMIAWL--
-MIAWLS-
-MIAWLG-
MGIAWL--
-MIAWLY-
-MIAWLA-
NGIAWL--
NEIAWL--
NVIAWL--
NMIAWL--
NHIAWL--
NPIAWL--
-NIAWLG-
-NIAWLV-
-NIAWLT-
-NIAWLS-
-NIAWLL-
-NIAWLA-
NDIAWL--
NTIAWL--
NKIAWL--
NIIAWL--
-NIAWLR-
-NIAWLN-
-NIAWLQ-
-NIAWLK-
-NIAWLD-
-PIAWLT-
-PIAWLG-
PIIAWL--
-PIAWLS-
-PIAWLK-
PDIAWL--
PAIAWL--
PQIAWL--
-PIAWLL-
PHIAWL--
PGIAWL--
PYIAWL--
-PIAWLD-
PVIAWL--
PNIAWL--

PEIAWL--
PCIAWL--
PMIAWL--
PLIAWL--
-PIAWLV-
-PIAWLQ-
-PIAWLA-
QAIAWL--
QLIAWL--
-QIAWLG-
-QIAWLA-
QTIAWL--
QVIAWL--
-QIAWLT-
QNIAWL--
-QIAWLN-
-QIAWLD-
QEIAWL--
-QIAWLH-
QKIAWL--
QWIAWL--
QMIAWL--
-QIAWLV-
-QIAWLR-
-QIAWLQ-
RTIAWL--
RMIAWL--
-RIAWLE-
RAIAWL--
RLIAWL--
RVIAWL--
REIAWL--
RDIAWL--
-RIAWLM-
RHIAWL--
SMIAWL--
SQIAWL--
SVIAWL--
SNIAWL--
SAIAWL--
SPIAWL--
SHIAWL--
SDIAWL--
SGIAWL--
SCIAWL--
SEIAWL--
TAIAWL--
TNIAWL--
TGIAWL--
-TIAWLR-

TPIAWL--
TQIAWL--
-TIAWLL-
-TIAWLT-
-TIAWLQ-
-TIAWLM-
-TIAWLK-
-TIAWLG-
TDIAWL--
TVIAWL--
TTIAWL--
TMIAWL--
-TIAWLE-
THIAWL--
TIIAWL--
-TIAWLN-
-VIAWLA-
VGIAWL--
VEIAWL--
VDIAWL--
-VIAWLD-
VPIAWL--
-VIAWLR-
VNIAWL--
VAIAWL--
-VIAWLQ-
-VIAWLN-
VHIAWL--
VQIAWL--
-VIAWLI-
-VIAWLG-
-VIAWLE-
WNIAWL--
-WIAWLK-
WEIAWL--
WMIAWL--
-WIAWLR-
-WIAWLN-
-WIAWLH-
WTIAWL--
-WIAWLS-
-WIAWLG-
WVIAWL--
-WIAWLM-
-WIAWLL-
-WIAWLE-
WDIAWL--
WAIAWL--
-YIAWLR-
YDIAWL--

YGIAWL--
YNIAWL--
-YIAWLG-
-YIAWLN-
YHIAWL--
YVIAWL--
YPIAWL--
-YIAWLV-
-YIAWLT-
-YIAWLK-
-YIAWLH-
YYIAWL--
YQIAWL--
YLIAWL--
-YIAWLQ-
YAIAWL--

| | | | |
|---|---|---|---|
| PTNL | 88 | 776 | 31.03 |

ELPTNL--
--PTNLLL
--PTNLVF
--PTNLNL
--PTNLNF
--PTNLHI
-RPTNLG-
-APTNLR-
AAPTNL--
ATPTNL--
-APTNLL-
-APTNLS-
AKPTNL--
AVPTNL--
ASPTNL--
ANPTNL--
AGPTNL--
-APTNLN-
AIPTNL--
ARPTNL--
-APTNLT-
-APTNLI-
-APTNLW-
AMPTNL--
ADPTNL--
ACPTNL--
-APTNLF-
AQPTNL--
-APTNLV-
-APTNLQ-
AEPTNL--
-APTNLM-
-APTNLG-

−APTNLA−
APPTNL−−
AFPTNL−−
AHPTNL−−
CEPTNL−−
CTPTNL−−
−CPTNLR−
−CPTNLM−
CLPTNL−−
CSPTNL−−
CMPTNL−−
CKPTNL−−
CCPTNL−−
CVPTNL−−
CRPTNL−−
−CPTNLT−
−CPTNLI−
CPPTNL−−
CIPTNL−−
CGPTNL−−
CFPTNL−−
CDPTNL−−
DYPTNL−−
DVPTNL−−
−DPTNLV−
−DPTNLR−
DLPTNL−−
DRPTNL−−
−DPTNLI−
DQPTNL−−
DEPTNL−−
DSPTNL−−
−DPTNLK−
DGPTNL−−
DAPTNL−−
−DPTNLY−
−DPTNLT−
−DPTNLQ−
DNPTNL−−
DIPTNL−−
DHPTNL−−
DFPTNL−−
ESPTNL−−
−EPTNLV−
−EPTNLR−
EVPTNL−−
−EPTNLL−
ERPTNL−−
−EPTNLY−
−EPTNLS−

-EPTNLK-
-EPTNLW-
-EPTNLI-
EMPTNL--
EKPTNL--
EGPTNL--
-EPTNLT-
ENPTNL--
EEPTNL--
EAPTNL--
EWPTNL--
ETPTNL--
-EPTNLH-
-EPTNLF-
-EPTNLE-
-EPTNLA-
EPPTNL--
EYPTNL--
EQPTNL--
-EPTNLN-
-EPTNLM-
EIPTNL--
FTPTNL--
-FPTNLL-
-FPTNLI-
FSPTNL--
FGPTNL--
-FPTNLY-
FAPTNL--
-FPTNLR-
-FPTNLK-
-FPTNLF-
FFPTNL--
-FPTNLS-
-FPTNLM-
FYPTNL--
-FPTNLV-
FMPTNL--
-FPTNLW-
-FPTNLE-
-FPTNLA-
FPPTNL--
FEPTNL--
FDPTNL--
FWPTNL--
FQPTNL--
-FPTNLQ-
-FPTNLG-
FNPTNL--
FIPTNL--

FCPTNL--
GSPTNL--
GLPTNL--
GYPTNL--
GKPTNL--
-GPTNLL-
GQPTNL--
GRPTNL--
GAPTNL--
GFPTNL--
GTPTNL--
GIPTNL--
-GPTNLT-
-GPTNLI-
-GPTNLQ-
-GPTNLA-
-GPTNLY-
GNPTNL--
GEPTNL--
GMPTNL--
GWPTNL--
GPPTNL--
-GPTNLK-
-GPTNLE-
GDPTNL--
-GPTNLV-
-GPTNLN-
-GPTNLF-
GHPTNL--
-GPTNLW-
-GPTNLP-
-GPTNLG-
-HPTNLK-
HRPTNL--
-HPTNLW-
-HPTNLL-
HTPTNL--
-HPTNLR-
-HPTNLA-
HLPTNL--
-HPTNLM-
HVPTNL--
HKPTNL--
HYPTNL--
-HPTNLI-
HSPTNL--
-HPTNLS-
-HPTNLH-
HNPTNL--
-HPTNLQ-

HGPTNL--
HFPTNL--
HWPTNL--
-HPTNLY-
-HPTNLF-
HIPTNL--
HAPTNL--
-HPTNLT-
HMPTNL--
HEPTNL--
HQPTNL--
-HPTNLV-
HPPTNL--
HHPTNL--
ILPTNL--
-IPTNLL-
IMPTNL--
IVPTNL--
-IPTNLT-
IRPTNL--
IPPTNL--
IWPTNL--
IGPTNL--
-IPTNLK-
IAPTNL--
IYPTNL--
IHPTNL--
ITPTNL--
-IPTNLQ-
-IPTNLG-
-IPTNLF-
-IPTNLA-
-IPTNLI-
ISPTNL--
-IPTNLH-
IKPTNL--
-IPTNLY-
-IPTNLV-
-IPTNLS-
-IPTNLE-
INPTNL--
IEPTNL--
IDPTNL--
IIPTNL--
ICPTNL--
KSPTNL--
KVPTNL--
KLPTNL--
KMPTNL--
-KPTNLL-

KFPTNL--
-KPTNLR-
KGPTNL--
KTPTNL--
KDPTNL--
-KPTNLW-
-KPTNLS-
KRPTNL--
-KPTNLH-
KYPTNL--
-KPTNLK-
KPPTNL--
KIPTNL--
KAPTNL--
KWPTNL--
-KPTNLY-
-KPTNLM-
-KPTNLF-
KHPTNL--
-KPTNLT-
-KPTNLN-
-KPTNLA-
KNPTNL--
KKPTNL--
KCPTNL--
-KPTNLQ-
-KPTNLG-
KEPTNL--
LSPTNL--
LRPTNL--
LYPTNL--
LDPTNL--
LLPTNL--
LKPTNL--
-LPTNLL-
LWPTNL--
-LPTNLS-
LIPTNL--
LVPTNL--
-LPTNLT-
LAPTNL--
-LPTNLM-
LGPTNL--
LTPTNL--
-LPTNLV-
LFPTNL--
-LPTNLI-
LPPTNL--
-LPTNLY-
-LPTNLR-

```
-LPTNLQ-
LEPTNL--
-LPTNLH-
LMPTNL--
LQPTNL--
LNPTNL--
LHPTNL--
LCPTNL--
-LPTNLW-
-LPTNLK-
-LPTNLA-
-MPTNLT-
-MPTNLS-
MKPTNL--
-MPTNLR-
MHPTNL--
MFPTNL--
MTPTNL--
-MPTNLW-
-MPTNLL-
-MPTNLV-
-MPTNLK-
MMPTNL--
MSPTNL--
MPPTNL--
MLPTNL--
MIPTNL--
MAPTNL--
MQPTNL--
-MPTNLY-
-MPTNLF-
-MPTNLE-
MYPTNL--
MVPTNL--
-MPTNLN-
-MPTNLA-
MDPTNL--
MWPTNL--
-MPTNLI-
-MPTNLH-
MNPTNL--
MGPTNL--
MEPTNL--
NRPTNL--
NLPTNL--
NSPTNL--
NWPTNL--
-NPTNLR-
-NPTNLS-
-NPTNLV-
```

NDPTNL--
NPPTNL--
NAPTNL--
NVPTNL--
-NPTNLQ-
-NPTNLM-
NKPTNL--
NFPTNL--
NNPTNL--
NIPTNL--
NYPTNL--
-NPTNLT-
NTPTNL--
-NPTNLW-
NMPTNL--
-NPTNLY-
-NPTNLI-
NHPTNL--
NGPTNL--
NQPTNL--
-NPTNLA-
NEPTNL--
NCPTNL--
--PTNLRL
--PTNLRA
--PTNLRK
--PTNLVR
--PTNLFG
--PTNLLA
--PTNLRS
--PTNLAL
--PTNLVA
--PTNLSM
--PTNLSG
--PTNLRQ
--PTNLRM
--PTNLRE
--PTNLYG
--PTNLLW
--PTNLWT
--PTNLRH
--PTNLLK
--PTNLIR
--PTNLVK
--PTNLSS
--PTNLRN
--PTNLWH
--PTNLSV
--PTNLNS
PWPTNL--

--PTNLYA
--PTNLVS
--PTNLVN
--PTNLMA
PVPTNL--
--PTNLYT
--PTNLYR
--PTNLVW
--PTNLTK
--PTNLSW
--PTNLQS
--PTNLMK
--PTNLLR
--PTNLLG
--PTNLII
--PTNLAS
--PTNLVI
--PTNLTR
--PTNLTG
--PTNLTA
--PTNLRD
--PTNLAA
--PTNLVY
--PTNLTT
--PTNLTS
--PTNLSY
--PTNLMG
--PTNLLQ
--PTNLLH
--PTNLIW
--PTNLFK
--PTNLEV
PSPTNL--
--PTNLMV
--PTNLMD
--PTNLKM
--PTNLHT
--PTNLFA
--PTNLES
--PTNLAV
PFPTNL--
--PTNLYS
--PTNLVG
--PTNLVE
--PTNLRW
--PTNLRF
--PTNLQV
--PTNLNA
--PTNLKT
--PTNLIH

--PTNLFS
PRPTNL--
PLPTNL--
PEPTNL--
--PTNLWW
--PTNLWS
--PTNLVD
--PTNLTF
--PTNLSH
--PTNLRV
--PTNLQA
--PTNLMS
--PTNLMM
--PTNLLD
--PTNLIV
--PTNLIM
--PTNLIK
--PTNLGL
--PTNLFR
--PTNLAT
PMPTNL--
--PTNLYL
--PTNLVT
--PTNLVM
--PTNLTY
--PTNLTV
--PTNLSL
--PTNLQT
--PTNLKN
--PTNLIQ
--PTNLIA
--PTNLHV
--PTNLFT
--PTNLAW
PQPTNL--
PGPTNL--
--PTNLYM
--PTNLWN
--PTNLWG
--PTNLVH
--PTNLTN
--PTNLTM
--PTNLTL
--PTNLTD
--PTNLSR
--PTNLSQ
--PTNLSN
--PTNLSF
--PTNLQF
--PTNLMY

--PTNLMH
--PTNLKR
--PTNLFM
--PTNLFH
--PTNLEA
--PTNLAN
--PTNLAI
PYPTNL--
PTPTNL--
--PTNLYN
--PTNLVQ
--PTNLTW
--PTNLTH
--PTNLSA
--PTNLRY
--PTNLQW
--PTNLQR
--PTNLQG
--PTNLPL
--PTNLNT
--PTNLMT
--PTNLKV
--PTNLKL
--PTNLKI
--PTNLKG
--PTNLKA
--PTNLIG
--PTNLHS
--PTNLHG
--PTNLGW
--PTNLFN
--PTNLAG
--PTNLYH
--PTNLYE
--PTNLWM
--PTNLWK
--PTNLTI
--PTNLSK
--PTNLSI
--PTNLSD
--PTNLME
--PTNLLM
--PTNLKY
--PTNLKQ
--PTNLKH
--PTNLKF
--PTNLHW
--PTNLGA
--PTNLFW
--PTNLFV

--PTNLFQ
--PTNLEG
PNPTNL--
PIPTNL--
PDPTNL--
PAPTNL--
--PTNLYW
--PTNLYK
--PTNLWR
--PTNLWE
--PTNLWA
--PTNLQK
--PTNLQD
--PTNLPN
--PTNLNY
--PTNLND
--PTNLIN
--PTNLIE
--PTNLHH
--PTNLHD
--PTNLHA
--PTNLEW
--PTNLEQ
--PTNLEM
--PTNLCW
--PTNLAK
--PTNLAH
--PTNLAF
PKPTNL--
QLPTNL--
QSPTNL--
QTPTNL--
QRPTNL--
-QPTNLR-
-QPTNLL-
-QPTNLT-
-QPTNLY-
QYPTNL--
-QPTNLV-
QMPTNL--
QIPTNL--
QHPTNL--
-QPTNLW-
QWPTNL--
-QPTNLM-
-QPTNLK-
-QPTNLA-
QPPTNL--
QKPTNL--
QGPTNL--

-QPTNLS-
-QPTNLI-
-QPTNLF-
QFPTNL--
QAPTNL--
QVPTNL--
-QPTNLH-
QEPTNL--
QCPTNL--
RSPTNL--
RTPTNL--
RMPTNL--
RGPTNL--
-RPTNLV-
-RPTNLS-
RNPTNL--
-RPTNLW-
-RPTNLI-
RFPTNL--
RPPTNL--
RIPTNL--
RYPTNL--
-RPTNLR-
-RPTNLT-
-RPTNLK-
-RPTNLA-
-RPTNLH-
REPTNL--
-RPTNLY-
-RPTNLQ-
-RPTNLN-
RHPTNL--
-RPTNLE-
RQPTNL--
-RPTNLM-
RAPTNL--
RDPTNL--
RCPTNL--
-SPTNLV-
SFPTNL--
SRPTNL--
SAPTNL--
SLPTNL--
SKPTNL--
-SPTNLM-
SYPTNL--
-SPTNLK-
SGPTNL--
-SPTNLH-
SWPTNL--

```
-SPTNLW-
-SPTNLT-
SMPTNL--
SIPTNL--
SDPTNL--
SQPTNL--
-SPTNLF-
-SPTNLA-
-SPTNLN-
SEPTNL--
-SPTNLI-
SHPTNL--
-SPTNLS-
-SPTNLQ-
-SPTNLE-
SPPTNL--
SNPTNL--
-TPTNLR-
-TPTNLL-
-TPTNLK-
TQPTNL--
TVPTNL--
TSPTNL--
TWPTNL--
TRPTNL--
-TPTNLT-
THPTNL--
TPPTNL--
TLPTNL--
TTPTNL--
-TPTNLS-
-TPTNLA-
TKPTNL--
-TPTNLW-
-TPTNLI-
TFPTNL--
TYPTNL--
TIPTNL--
-TPTNLM-
TMPTNL--
TDPTNL--
-TPTNLV-
-TPTNLQ-
-TPTNLH-
TCPTNL--
-TPTNLY-
-TPTNLF-
-TPTNLE-
TEPTNL--
-TPTNLG-
```

TGPTNL--
-VPTNLR-
VLPTNL--
-VPTNLT-
VSPTNL--
VVPTNL--
-VPTNLY-
VTPTNL--
VRPTNL--
-VPTNLS-
VAPTNL--
VYPTNL--
-VPTNLW-
VHPTNL--
-VPTNLA-
VNPTNL--
VMPTNL--
VKPTNL--
VPPTNL--
VDPTNL--
-VPTNLV-
-VPTNLI-
-VPTNLE-
VQPTNL--
-VPTNLN-
-VPTNLL-
-VPTNLF-
VGPTNL--
-VPTNLH-
VFPTNL--
-VPTNLC-
VEPTNL--
-WPTNLS-
-WPTNLL-
WLPTNL--
WRPTNL--
-WPTNLM-
WAPTNL--
WNPTNL--
-WPTNLK-
-WPTNLR-
-WPTNLF-
WSPTNL--
WVPTNL--
-WPTNLQ-
-WPTNLI-
WIPTNL--
WWPTNL--
-WPTNLV-
WGPTNL--

WQPTNL--
WMPTNL--
WKPTNL--
WEPTNL--
WYPTNL--
WTPTNL--
WHPTNL--
WPPTNL--
WDPTNL--
WCPTNL--
-WPTNLW-
-WPTNLH-
-WPTNLA-
YLPTNL--
-YPTNLT-
-YPTNLL-
-YPTNLS-
YTPTNL--
YRPTNL--
-YPTNLY-
YQPTNL--
YKPTNL--
YFPTNL--
YDPTNL--
YMPTNL--
YGPTNL--
-YPTNLW-
-YPTNLV-
-YPTNLE-
YSPTNL--
-YPTNLI-
-YPTNLA-
YPPTNL--
-YPTNLQ-
-YPTNLF-
YHPTNL--
-YPTNLK-
YIPTNL--
YAPTNL--
-YPTNLM-
-YPTNLH-
YNPTNL--
-YPTNLN-

| | | | |
|---|---|---|---|
| PGML | 161 | 734 | 31.03 |

-LPGMLG-
--PGMLSG
--PGMLIM
--PGMLGS
--PGMLFR
--PGMLAV

--PGMLAH
-RPGMLG-
-APGMLR-
ARPGML--
-APGMLI-
-APGMLS-
-APGMLT-
ALPGML--
AYPGML--
-APGMLQ-
ANPGML--
-APGMLW-
-APGMLV-
-APGMLA-
AWPGML--
AVPGML--
ATPGML--
-APGMLG-
AGPGML--
AFPGML--
AHPGML--
-APGMLK-
AEPGML--
AIPGML--
APPGML--
-APGMLC-
AKPGML--
AAPGML--
-APGMLY-
-APGMLH-
AMPGML--
AQPGML--
-APGMLN-
CLPGML--
CVPGML--
-CPGMLR-
-CPGMLI-
CKPGML--
CRPGML--
CMPGML--
CFPGML--
CTPGML--
CQPGML--
CEPGML--
CAPGML--
CPPGML--
-CPGMLL-
CIPGML--
CHPGML--
CCPGML--

```
-DPGMLR-
-DPGMLL-
DLPGML--
DRPGML--
DYPGML--
DSPGML--
DGPGML--
-DPGMLF-
DIPGML--
DVPGML--
-DPGMLT-
-DPGMLQ-
-DPGMLV-
-DPGMLI-
DWPGML--
-DPGMLW-
-DPGMLS-
-DPGMLM-
DKPGML--
DFPGML--
DEPGML--
DPPGML--
-DPGMLY-
-DPGMLK-
DHPGML--
DAPGML--
-EPGMLK-
-EPGMLF-
EVPGML--
EAPGML--
EIPGML--
ESPGML--
ERPGML--
-EPGMLW-
-EPGMLH-
-EPGMLL-
ELPGML--
EPPGML--
-EPGMLR-
EWPGML--
-EPGMLT-
-EPGMLM-
EHPGML--
EYPGML--
ETPGML--
-EPGMLQ-
-EPGMLN-
EEPGML--
EQPGML--
-EPGMLY-
```

−EPGMLS−
EGPGML−−
−EPGMLI−
−EPGMLA−
EKPGML−−
ENPGML−−
EMPGML−−
EFPGML−−
EDPGML−−
FSPGML−−
FRPGML−−
−FPGMLN−
FNPGML−−
FWPGML−−
FVPGML−−
FIPGML−−
FFPGML−−
−FPGMLK−
−FPGMLW−
FLPGML−−
−FPGMLV−
−FPGMLT−
FGPGML−−
FPPGML−−
−FPGMLM−
FEPGML−−
FQPGML−−
FKPGML−−
FHPGML−−
−FPGMLS−
−FPGMLH−
FYPGML−−
−FPGMLY−
−FPGMLL−
−GPGMLR−
GRPGML−−
GAPGML−−
GMPGML−−
GYPGML−−
−GPGMLT−
−GPGMLM−
GLPGML−−
−GPGMLV−
GTPGML−−
−GPGMLW−
−GPGMLI−
−GPGMLH−
GFPGML−−
−GPGMLS−
GIPGML−−

GHPGML--
GEPGML--
GWPGML--
GKPGML--
GQPGML--
-GPGMLY-
-GPGMLN-
-GPGMLA-
GGPGML--
GDPGML--
-GPGMLQ-
-GPGMLF-
HRPGML--
HVPGML--
-HPGMLR-
HAPGML--
-HPGMLL-
HQPGML--
HTPGML--
HFPGML--
-HPGMLN-
-HPGMLY-
-HPGMLW-
HIPGML--
HYPGML--
-HPGMLT-
HNPGML--
HSPGML--
-HPGMLS-
-HPGMLM-
-HPGMLK-
-HPGMLF-
HGPGML--
HEPGML--
HDPGML--
-HPGMLH-
-HPGMLA-
HLPGML--
HKPGML--
HCPGML--
HWPGML--
HPPGML--
-HPGMLQ-
HMPGML--
-IPGMLR-
IVPGML--
-IPGMLL-
IMPGML--
IPPGML--
-IPGMLS-

IWPGML--
IRPGML--
-IPGMLT-
IGPGML--
ISPGML--
-IPGMLK-
-IPGMLV-
IFPGML--
INPGML--
IIPGML--
ITPGML--
-IPGMLI-
-IPGMLF-
-IPGMLY-
-IPGMLQ-
IYPGML--
IQPGML--
-IPGMLM-
-IPGMLA-
IKPGML--
IHPGML--
IEPGML--
IAPGML--
IDPGML--
KSPGML--
-KPGMLS-
KVPGML--
KGPGML--
-KPGMLY-
-KPGMLH-
-KPGMLF-
-KPGMLA-
-KPGMLT-
KAPGML--
KLPGML--
KTPGML--
-KPGMLW-
-KPGMLR-
KKPGML--
KFPGML--
KWPGML--
KPPGML--
-KPGMLV-
-KPGMLQ-
KNPGML--
KHPGML--
-KPGMLM-
KQPGML--
-KPGMLP-
-KPGMLN-

−KPGMLK−
LRPGML−−
−LPGMLM−
LPPGML−−
LGPGML−−
LSPGML−−
LTPGML−−
−LPGMLI−
LVPGML−−
−LPGMLT−
LMPGML−−
LLPGML−−
LQPGML−−
LYPGML−−
−LPGMLR−
LFPGML−−
LNPGML−−
LKPGML−−
−LPGMLY−
LIPGML−−
−LPGMLW−
−LPGMLN−
LEPGML−−
LAPGML−−
−LPGMLF−
−LPGMLQ−
−LPGMLH−
LDPGML−−
LCPGML−−
MTPGML−−
MKPGML−−
MIPGML−−
−MPGMLR−
MLPGML−−
MYPGML−−
MAPGML−−
MRPGML−−
−MPGMLS−
−MPGMLA−
MFPGML−−
−MPGMLY−
−MPGMLW−
−MPGMLT−
MSPGML−−
−MPGMLH−
MNPGML−−
MVPGML−−
MPPGML−−
MHPGML−−
−MPGMLV−

MDPGML--
MWPGML--
-MPGMLI-
MMPGML--
-MPGMLQ-
-MPGMLM-
MEPGML--
NLPGML--
NGPGML--
NRPGML--
-NPGMLR-
NDPGML--
NVPGML--
-NPGMLV-
NNPGML--
NPPGML--
-NPGMLS-
NFPGML--
-NPGMLL-
-NPGMLI-
-NPGMLQ-
-NPGMLH-
NHPGML--
NTPGML--
-NPGMLN-
-NPGMLK-
NIPGML--
NEPGML--
NAPGML--
-NPGMLM-
NSPGML--
NQPGML--
-NPGMLT-
NMPGML--
NYPGML--
-NPGMLW-
-NPGMLF-
NKPGML--
NWPGML--
--PGMLRL
--PGMLRG
PRPGML--
--PGMLRY
--PGMLRT
--PGMLWG
--PGMLRF
--PGMLSA
--PGMLSS
--PGMLAS
--PGMLHG

PAPGML--
--PGMLVK
--PGMLTS
--PGMLRA
--PGMLMG
--PGMLTT
--PGMLTA
PQPGML--
PLPGML--
PIPGML--
--PGMLRV
--PGMLIV
--PGMLHS
--PGMLTY
--PGMLLT
--PGMLYS
--PGMLWI
--PGMLVH
--PGMLVG
--PGMLSL
--PGMLMT
--PGMLIS
--PGMLFK
--PGMLAL
PTPGML--
PMPGML--
--PGMLVV
--PGMLRM
--PGMLNT
--PGMLLM
--PGMLHL
PYPGML--
PNPGML--
--PGMLVM
--PGMLSM
--PGMLRD
--PGMLKH
--PGMLIN
--PGMLVL
--PGMLRQ
--PGMLQW
--PGMLQG
--PGMLLG
--PGMLKS
--PGMLKR
--PGMLKK
--PGMLKI
--PGMLGW
PWPGML--
PVPGML--

--PGMLYQ
--PGMLVT
--PGMLQN
--PGMLNV
--PGMLMV
--PGMLLH
--PGMLKA
--PGMLII
--PGMLIH
--PGMLIG
--PGMLHI
--PGMLGL
PEPGML--
PKPGML--
PGPGML--
--PGMLYM
--PGMLYH
--PGMLWS
--PGMLWQ
--PGMLWK
--PGMLVQ
--PGMLVF
--PGMLSY
--PGMLSI
--PGMLRW
--PGMLQT
--PGMLQA
--PGMLPL
--PGMLNA
--PGMLMH
--PGMLMA
--PGMLIA
PFPGML--
--PGMLYN
--PGMLYA
--PGMLWT
--PGMLTR
--PGMLTN
--PGMLTI
--PGMLSV
--PGMLSF
--PGMLPY
--PGMLNQ
--PGMLNL
--PGMLKV
--PGMLKT
--PGMLKL
--PGMLHV
--PGMLHQ
--PGMLYT

--PGMLYG
--PGMLTK
--PGMLTG
--PGMLRE
--PGMLQR
--PGMLQQ
--PGMLPM
--PGMLNM
--PGMLNF
--PGMLMQ
--PGMLME
--PGMLLK
--PGMLLA
--PGMLKQ
--PGMLKG
--PGMLKD
--PGMLIY
--PGMLHM
--PGMLHF
--PGMLFM
PSPGML--
--PGMLYK
--PGMLYF
--PGMLYE
--PGMLWW
--PGMLWD
--PGMLVW
--PGMLVI
--PGMLVE
--PGMLTV
--PGMLTH
--PGMLTF
--PGMLSQ
--PGMLSN
--PGMLQV
--PGMLQL
--PGMLQH
--PGMLPF
--PGMLNI
--PGMLNH
--PGMLNG
--PGMLML
--PGMLMK
--PGMLMI
--PGMLLD
--PGMLKM
--PGMLKE
--PGMLHY
--PGMLHT
--PGMLHH

--PGMLGF
--PGMLEA
--PGMLCA
PCPGML--
-PPGMLG-
PHPGML--
--PGMLYI
--PGMLWY
--PGMLWN
--PGMLWM
--PGMLVD
--PGMLTW
--PGMLTD
--PGMLSP
--PGMLQY
--PGMLPC
--PGMLNY
--PGMLNR
--PGMLNN
--PGMLMN
--PGMLMM
--PGMLKY
--PGMLID
--PGMLHW
--PGMLHK
--PGMLGY
--PGMLGM
--PGMLGC
--PGMLFN
--PGMLFG
--PGMLAY
--PGMLAN
--PGMLAG
--PGMLAA
QRPGML--
QIPGML--
-QPGMLH-
QSPGML--
-QPGMLW-
-QPGMLN-
-QPGMLY-
-QPGMLT-
-QPGMLL-
QYPGML--
-QPGMLS-
QKPGML--
QTPGML--
-QPGMLV-
QHPGML--
QAPGML--

QVPGML--
QPPGML--
-QPGMLA-
-QPGMLK-
QWPGML--
QMPGML--
QFPGML--
-QPGMLM-
-QPGMLI-
QGPGML--
QDPGML--
QNPGML--
RHPGML--
RGPGML--
RWPGML--
-RPGMLF-
-RPGMLN-
-RPGMLS-
-RPGMLH-
-RPGMLM-
RYPGML--
-RPGMLA-
-RPGMLK-
-RPGMLY-
-RPGMLI-
RPPGML--
-RPGMLQ-
RQPGML--
RNPGML--
RFPGML--
RKPGML--
RIPGML--
REPGML--
-RPGMLW-
RDPGML--
RCPGML--
SRPGML--
SLPGML--
-SPGMLR-
SWPGML--
SVPGML--
-SPGMLT-
SSPGML--
-SPGMLS-
-SPGMLI-
SMPGML--
SYPGML--
-SPGMLM-
SAPGML--
-SPGMLN-

SIPGML--
SQPGML--
-SPGMLK-
SGPGML--
STPGML--
SEPGML--
-SPGMLV-
-SPGMLA-
SFPGML--
-SPGMLQ-
SNPGML--
SHPGML--
-SPGMLY-
SDPGML--
SCPGML--
TRPGML--
TLPGML--
TSPGML--
-TPGMLS-
THPGML--
-TPGMLT-
TFPGML--
TPPGML--
TKPGML--
TWPGML--
-TPGMLF-
TQPGML--
-TPGMLN-
-TPGMLV-
-TPGMLQ-
-TPGMLK-
-TPGMLI-
TGPGML--
TYPGML--
TMPGML--
-TPGMLM-
TAPGML--
-TPGMLY-
-TPGMLW-
-TPGMLH-
-TPGMLA-
VIPGML--
-VPGMLM-
VRPGML--
VTPGML--
VYPGML--
-VPGMLW-
-VPGMLV-
VGPGML--
VPPGML--

-VPGMLA-
VHPGML--
VEPGML--
-VPGMLQ-
VWPGML--
VKPGML--
-VPGMLK-
VNPGML--
VAPGML--
-VPGMLN-
VVPGML--
VDPGML--
VQPGML--
VMPGML--
-VPGMLH-
-VPGMLR-
-VPGMLY-
-VPGMLT-
-VPGMLI-
-VPGMLF-
-VPGMLE-
VCPGML--
WSPGML--
WTPGML--
WAPGML--
WFPGML--
WRPGML--
-WPGMLI-
WGPGML--
WHPGML--
WQPGML--
-WPGMLS-
WNPGML--
-WPGMLW-
-WPGMLV-
WMPGML--
WDPGML--
WPPGML--
-WPGMLM-
WLPGML--
WIPGML--
WEPGML--
-WPGMLT-
-WPGMLQ-
-WPGMLN-
-YPGMLT-
YRPGML--
-YPGMLR-
-YPGMLL-
YTPGML--

```
              -YPGMLV-
              YNPGML--
              YSPGML--
              -YPGMLS-
              -YPGMLQ-
              YKPGML--
              YLPGML--
              YHPGML--
              -YPGMLN-
              YYPGML--
              YVPGML--
              -YPGMLI-
              YQPGML--
              YPPGML--
              -YPGMLK-
              YFPGML--
              -YPGMLW-
              YGPGML--
              YDPGML--
              YAPGML--
              YWPGML--
              -YPGMLM-
              -YPGMLH-
              YIPGML--
              -YPGMLY-
              YMPGML--
              -YPGMLF-
              -YPGMLA-
PKGW           311        663     31.03
              --PKGWSL
              --PKGWKL
              --PKGWIL
              -APKGWS-
              AGPKGW--
              -APKGWL-
              AIPKGW--
              AHPKGW--
              -APKGWK-
              -APKGWH-
              ASPKGW--
              -APKGWR-
              AFPKGW--
              ATPKGW--
              -APKGWA-
              AWPKGW--
              AVPKGW--
              ARPKGW--
              -APKGWT-
              -APKGWN-
              ALPKGW--
```

ADPKGW--
AYPKGW--
-APKGWY-
-APKGWV-
-APKGWQ-
-APKGWM-
-APKGWG-
AEPKGW--
CIPKGW--
CVPKGW--
CSPKGW--
-CPKGWR-
-CPKGWM-
-CPKGWL-
CNPKGW--
-DPKGWR-
-DPKGWF-
DVPKGW--
DMPKGW--
DSPKGW--
DYPKGW--
DRPKGW--
-DPKGWL-
DTPKGW--
-DPKGWQ-
DWPKGW--
-DPKGWT-
-DPKGWS-
DFPKGW--
DEPKGW--
DAPKGW--
-EPKGWK-
ESPKGW--
-EPKGWW-
-EPKGWV-
-EPKGWL-
-EPKGWM-
-EPKGWI-
-EPKGWY-
EFPKGW--
EQPKGW--
-EPKGWN-
-EPKGWH-
EEPKGW--
ETPKGW--
-EPKGWE-
-EPKGWA-
EKPKGW--
EDPKGW--
-FPKGWR-

FRPKGW--
FIPKGW--
-FPKGWL-
-FPKGWI-
FAPKGW--
FLPKGW--
FGPKGW--
-FPKGWH-
FFPKGW--
FVPKGW--
FTPKGW--
FHPKGW--
FSPKGW--
FPPKGW--
-FPKGWT-
FNPKGW--
FWPKGW--
FQPKGW--
FKPKGW--
FEPKGW--
GVPKGW--
-GPKGWR-
-GPKGWN-
GLPKGW--
GTPKGW--
GSPKGW--
GNPKGW--
-GPKGWT-
-GPKGWS-
GAPKGW--
GWPKGW--
GGPKGW--
GYPKGW--
-GPKGWE-
GRPKGW--
-GPKGWM-
-GPKGWF-
GKPKGW--
GIPKGW--
-GPKGWW-
-GPKGWV-
-GPKGWQ-
-GPKGWL-
-GPKGWA-
GCPKGW--
GPPKGW--
-GPKGWI-
-GPKGWH-
GMPKGW--
-HPKGWR-

HSPKGW--
-HPKGWI-
HTPKGW--
HRPKGW--
-HPKGWS-
-HPKGWK-
HWPKGW--
HLPKGW--
-HPKGWV-
-HPKGWA-
HVPKGW--
-HPKGWL-
HGPKGW--
-HPKGWQ-
HMPKGW--
-HPKGWM-
HNPKGW--
HIPKGW--
HDPKGW--
HAPKGW--
-HPKGWY-
-HPKGWT-
-HPKGWF-
HFPKGW--
HEPKGW--
-IPKGWL-
-IPKGWA-
-IPKGWK-
ITPKGW--
IPPKGW--
-IPKGWM-
IAPKGW--
IWPKGW--
IHPKGW--
IGPKGW--
IFPKGW--
IYPKGW--
IRPKGW--
-IPKGWR-
-IPKGWI-
IIPKGW--
ISPKGW--
INPKGW--
ILPKGW--
IKPKGW--
IDPKGW--
-IPKGWY-
-IPKGWV-
-IPKGWS-
-IPKGWN-

IMPKGW--
KSPKGW--
-KPKGWL-
KVPKGW--
KPPKGW--
-KPKGWV-
KLPKGW--
-KPKGWS-
KGPKGW--
KTPKGW--
-KPKGWQ-
-KPKGWI-
KFPKGW--
-KPKGWW-
-KPKGWR-
-KPKGWH-
-KPKGWT-
KYPKGW--
KRPKGW--
KQPKGW--
-KPKGWN-
-KPKGWA-
KNPKGW--
KAPKGW--
LSPKGW--
-LPKGWL-
LRPKGW--
-LPKGWR-
LHPKGW--
LAPKGW--
-LPKGWV-
LEPKGW--
-LPKGWS-
LTPKGW--
LFPKGW--
LMPKGW--
LLPKGW--
LYPKGW--
-LPKGWI-
LNPKGW--
LKPKGW--
-LPKGWY-
-LPKGWM-
LIPKGW--
-LPKGWT-
-LPKGWH-
LPPKGW--
-LPKGWK-
LGPKGW--
LWPKGW--

-LPKGWW-
LDPKGW--
LQPKGW--
-LPKGWQ-
-LPKGWN-
-LPKGWF-
MVPKGW--
-MPKGWS-
-MPKGWL-
MSPKGW--
-MPKGWT-
-MPKGWI-
MNPKGW--
MFPKGW--
MYPKGW--
-MPKGWK-
-MPKGWH-
MLPKGW--
MDPKGW--
MWPKGW--
MTPKGW--
-MPKGWV-
-MPKGWR-
-MPKGWN-
-MPKGWA-
MIPKGW--
MCPKGW--
MAPKGW--
NSPKGW--
-NPKGWL-
NTPKGW--
-NPKGWT-
-NPKGWS-
NVPKGW--
NQPKGW--
-NPKGWY-
-NPKGWV-
-NPKGWR-
-NPKGWF-
NIPKGW--
NCPKGW--
-NPKGWW-
-NPKGWK-
-NPKGWI-
NWPKGW--
NPPKGW--
-NPKGWA-
NLPKGW--
NKPKGW--
NFPKGW--

NYPKGW--
-NPKGWM-
-NPKGWH-
NNPKGW--
NGPKGW--
--PKGWRS
--PKGWFT
--PKGWRA
--PKGWMS
--PKGWWR
--PKGWVN
--PKGWRL
--PKGWRV
--PKGWVT
--PKGWWN
--PKGWTF
--PKGWSA
--PKGWLS
--PKGWFR
--PKGWLV
--PKGWKS
--PKGWWK
--PKGWVS
--PKGWRI
--PKGWYS
--PKGWTT
--PKGWIT
--PKGWVK
--PKGWRF
--PKGWYR
--PKGWTS
--PKGWRY
--PKGWST
--PKGWFS
--PKGWFA
PSPKGW--
--PKGWYG
--PKGWYF
--PKGWTK
--PKGWSN
--PKGWSM
--PKGWRD
--PKGWLT
--PKGWKT
--PKGWHR
--PKGWHG
--PKGWWI
--PKGWTY
--PKGWSK
--PKGWRH

--PKGWQW
--PKGWLN
--PKGWLG
--PKGWKV
--PKGWKA
--PKGWHW
--PKGWHS
--PKGWFN
--PKGWWT
--PKGWVV
--PKGWMA
--PKGWLF
--PKGWIW
PGPKGW--
--PKGWWS
--PKGWVH
--PKGWTI
--PKGWSV
--PKGWSS
--PKGWNS
--PKGWMV
--PKGWMR
--PKGWML
--PKGWLA
--PKGWKH
--PKGWIV
--PKGWHH
--PKGWFL
--PKGWFF
--PKGWYV
--PKGWVF
--PKGWTW
--PKGWSW
--PKGWRW
--PKGWRG
--PKGWRE
--PKGWMT
--PKGWMH
--PKGWLY
--PKGWLM
--PKGWLK
--PKGWIS
--PKGWIK
--PKGWGV
--PKGWGS
--PKGWAY
PAPKGW--
PQPKGW--
--PKGWVY
--PKGWVL

--PKGWVG
--PKGWVA
--PKGWTV
--PKGWTA
--PKGWSR
--PKGWRT
--PKGWQH
--PKGWQA
--PKGWNT
--PKGWMG
--PKGWLQ
--PKGWKI
--PKGWHL
--PKGWFI
--PKGWEW
--PKGWEA
--PKGWAV
--PKGWAA
--PKGWYT
--PKGWYM
--PKGWYE
--PKGWWF
--PKGWWD
--PKGWWA
--PKGWVM
--PKGWVI
--PKGWVD
--PKGWTN
--PKGWTH
--PKGWTD
--PKGWSQ
--PKGWSI
--PKGWSH
--PKGWRP
--PKGWRN
--PKGWRK
--PKGWQV
--PKGWNL
--PKGWNA
--PKGWMN
--PKGWMD
--PKGWKY
--PKGWKN
--PKGWFV
--PKGWFM
--PKGWEG
--PKGWAT
PYPKGW--
PRPKGW--
-PPKGWT-

-PPKGWR-
PNPKGW--
PMPKGW--
--PKGWYW
--PKGWYQ
--PKGWWY
--PKGWWW
--PKGWWV
--PKGWWH
--PKGWVQ
--PKGWTM
--PKGWTG
--PKGWTE
--PKGWSY
--PKGWSF
--PKGWRM
--PKGWQT
--PKGWQS
--PKGWQR
--PKGWQQ
--PKGWQL
--PKGWQK
--PKGWNM
--PKGWNG
--PKGWNF
--PKGWMK
--PKGWMF
--PKGWLH
--PKGWLE
--PKGWKG
--PKGWKF
--PKGWIY
--PKGWIR
--PKGWIQ
--PKGWIN
--PKGWII
--PKGWHT
--PKGWHQ
--PKGWHA
--PKGWFQ
--PKGWFH
--PKGWFD
--PKGWCI
--PKGWCG
--PKGWCA
--PKGWAW
--PKGWAL
--PKGWAG
--PKGWAF
PIPKGW--

PHPKGW--
PFPKGW--
QGPKGW--
-QPKGWH-
-QPKGWK-
-QPKGWS-
QLPKGW--
QFPKGW--
QYPKGW--
-QPKGWL-
-QPKGWI-
QSPKGW--
-QPKGWM-
-QPKGWF-
-QPKGWA-
QIPKGW--
QDPKGW--
QRPKGW--
-QPKGWW-
QNPKGW--
QKPKGW--
QHPKGW--
QEPKGW--
QCPKGW--
QAPKGW--
RSPKGW--
-RPKGWV-
RLPKGW--
-RPKGWI-
RYPKGW--
-RPKGWM-
RTPKGW--
RGPKGW--
RDPKGW--
-RPKGWS-
-RPKGWK-
RMPKGW--
RAPKGW--
RVPKGW--
-RPKGWL-
-RPKGWA-
-RPKGWY-
-RPKGWR-
-RPKGWQ-
RNPKGW--
RFPKGW--
-RPKGWW-
RHPKGW--
REPKGW--
RRPKGW--

RPPKGW--
RKPKGW--
RIPKGW--
-SPKGWL-
-SPKGWV-
SLPKGW--
-SPKGWI-
SSPKGW--
SQPKGW--
-SPKGWR-
-SPKGWA-
-SPKGWN-
-SPKGWK-
SNPKGW--
SFPKGW--
-SPKGWH-
SGPKGW--
SDPKGW--
SRPKGW--
-SPKGWY-
SVPKGW--
STPKGW--
-SPKGWQ-
SMPKGW--
-SPKGWS-
SWPKGW--
-SPKGWM-
-SPKGWF-
SYPKGW--
SPPKGW--
-SPKGWW-
-SPKGWG-
-SPKGWE-
SIPKGW--
SEPKGW--
SAPKGW--
-TPKGWL-
-TPKGWW-
-TPKGWI-
-TPKGWY-
TVPKGW--
TLPKGW--
TGPKGW--
-TPKGWR-
-TPKGWA-
-TPKGWT-
-TPKGWS-
TMPKGW--
TWPKGW--
THPKGW--

TSPKGW--
TTPKGW--
-TPKGWQ-
-TPKGWK-
TKPKGW--
TPPKGW--
-TPKGWN-
-TPKGWE-
TNPKGW--
TFPKGW--
TEPKGW--
TDPKGW--
-TPKGWM-
-TPKGWH-
TIPKGW--
TAPKGW--
-VPKGWL-
VTPKGW--
VVPKGW--
-VPKGWT-
VWPKGW--
VHPKGW--
VPPKGW--
VSPKGW--
VIPKGW--
VGPKGW--
VAPKGW--
-VPKGWV-
-VPKGWS-
-VPKGWK-
-VPKGWH-
VLPKGW--
VEPKGW--
-VPKGWN-
-VPKGWI-
-VPKGWF-
VFPKGW--
VDPKGW--
VMPKGW--
VRPKGW--
-VPKGWQ-
-VPKGWA-
VKPKGW--
VYPKGW--
-VPKGWG-
-VPKGWC-
VNPKGW--
-WPKGWK-
-WPKGWR-
WMPKGW--

WAPKGW--
WLPKGW--
WSPKGW--
WRPKGW--
-WPKGWM-
-WPKGWH-
WIPKGW--
WFPKGW--
WEPKGW--
-WPKGWT-
WYPKGW--
WWPKGW--
WVPKGW--
WQPKGW--
-WPKGWL-
WHPKGW--
YNPKGW--
-YPKGWT-
YFPKGW--
YDPKGW--
YTPKGW--
YMPKGW--
YVPKGW--
-YPKGWI-
-YPKGWW-
YLPKGW--
YHPKGW--
-YPKGWH-
-YPKGWR-
YIPKGW--
YEPKGW--
YSPKGW--
YKPKGW--
-YPKGWF-
YQPKGW--
-YPKGWY-
-YPKGWV-
-YPKGWS-
-YPKGWM-
-YPKGWL-
-YPKGWK-
YAPKGW--
PASF 158 735 31.01
--PASFLD
--PASFKF
--PASFID
-SPASFG-
-TPASFM-
TFPASF--
-APASFR-

-APASFS-
ALPASF--
AKPASF--
ARPASF--
-APASFY-
AYPASF--
AVPASF--
-APASFT-
ATPASF--
APPASF--
-APASFI-
-APASFA-
ASPASF--
-APASFQ-
-APASFL-
-APASFK-
-APASFF-
AQPASF--
-APASFW-
-APASFV-
AIPASF--
AFPASF--
AWPASF--
-APASFM-
-APASFH-
-APASFE-
ANPASF--
AGPASF--
-APASFN-
AEPASF--
ADPASF--
AAPASF--
CRPASF--
CTPASF--
-CPASFT-
CKPASF--
CSPASF--
-CPASFY-
-CPASFM-
-CPASFK-
CLPASF--
CHPASF--
CFPASF--
CEPASF--
CDPASF--
CYPASF--
CVPASF--
CNPASF--
-DPASFR-
DRPASF--

-DPASFY-
-DPASFI-
DSPASF--
-DPASFS-
-DPASFF-
DAPASF--
DVPASF--
DTPASF--
DLPASF--
-DPASFW-
-DPASFV-
DKPASF--
DCPASF--
DYPASF--
-DPASFN-
-DPASFM-
-DPASFK-
-DPASFH-
-DPASFA-
DDPASF--
-EPASFI-
EMPASF--
ELPASF--
ESPASF--
EQPASF--
EPPASF--
EVPASF--
ETPASF--
ERPASF--
-EPASFY-
-EPASFS-
-EPASFR-
EHPASF--
EFPASF--
-EPASFM-
-EPASFK-
-EPASFA-
EAPASF--
-EPASFW-
-EPASFV-
-EPASFN-
-EPASFE-
ENPASF--
EKPASF--
EIPASF--
EGPASF--
EEPASF--
EDPASF--
ECPASF--
FSPASF--

−FPASFS−
−FPASFV−
FKPASF−−
FVPASF−−
FIPASF−−
−FPASFR−
−FPASFQ−
FLPASF−−
FGPASF−−
FEPASF−−
−FPASFF−
−FPASFM−
FWPASF−−
FNPASF−−
FHPASF−−
FDPASF−−
FYPASF−−
−FPASFY−
−FPASFH−
−FPASFA−
FPPASF−−
−FPASFT−
FMPASF−−
−FPASFN−
−FPASFK−
FCPASF−−
−GPASFV−
GYPASF−−
−GPASFR−
−GPASFL−
GKPASF−−
−GPASFQ−
GQPASF−−
−GPASFW−
−GPASFM−
GLPASF−−
GVPASF−−
−GPASFT−
GNPASF−−
GSPASF−−
GRPASF−−
GEPASF−−
GMPASF−−
GAPASF−−
GTPASF−−
−GPASFS−
−GPASFF−
GPPASF−−
−GPASFK−
−GPASFA−

GFPASF--
GDPASF--
-GPASFN-
GCPASF--
GWPASF--
-GPASFC-
GIPASF--
GHPASF--
GGPASF--
HRPASF--
HGPASF--
-HPASFR-
HLPASF--
-HPASFT-
-HPASFM-
HVPASF--
HPPASF--
-HPASFW-
-HPASFL-
HTPASF--
HIPASF--
HMPASF--
-HPASFS-
HYPASF--
-HPASFK-
-HPASFI-
HAPASF--
HSPASF--
-HPASFQ-
-HPASFF-
HNPASF--
HWPASF--
-HPASFN-
-HPASFY-
-HPASFH-
-HPASFE-
HKPASF--
HHPASF--
HFPASF--
-IPASFR-
IRPASF--
-IPASFL-
IFPASF--
-IPASFS-
-IPASFN-
ISPASF--
IQPASF--
INPASF--
-IPASFW-
-IPASFH-

IVPASF--
ITPASF--
-IPASFY-
-IPASFQ-
ILPASF--
IAPASF--
IYPASF--
-IPASFT-
-IPASFM-
-IPASFK-
IGPASF--
IDPASF--
IPPASF--
-IPASFV-
-IPASFI-
-IPASFF-
IMPASF--
KSPASF--
-KPASFN-
KVPASF--
-KPASFL-
-KPASFH-
KTPASF--
-KPASFR-
-KPASFK-
KGPASF--
-KPASFS-
KIPASF--
KYPASF--
-KPASFY-
-KPASFI-
-KPASFV-
KFPASF--
KPPASF--
-KPASFG-
KLPASF--
KKPASF--
KWPASF--
-KPASFW-
KAPASF--
-KPASFT-
-KPASFE-
-KPASFA-
-KPASFQ-
-KPASFM-
KMPASF--
KHPASF--
KEPASF--
-LPASFR-
-LPASFL-

LLPASF--
LRPASF--
-LPASFK-
-LPASFV-
-LPASFS-
LSPASF--
-LPASFM-
LYPASF--
-LPASFT-
-LPASFI-
-LPASFF-
LPPASF--
-LPASFQ-
LAPASF--
LNPASF--
LMPASF--
LIPASF--
LCPASF--
LTPASF--
LHPASF--
-LPASFA-
LFPASF--
LDPASF--
LQPASF--
-LPASFH-
LEPASF--
-MPASFR-
-MPASFL-
-MPASFH-
-MPASFS-
MSPASF--
-MPASFV-
-MPASFQ-
-MPASFI-
-MPASFY-
MMPASF--
MTPASF--
MNPASF--
MHPASF--
MEPASF--
MPPASF--
-MPASFT-
-MPASFM-
-MPASFN-
MDPASF--
MWPASF--
MVPASF--
-MPASFK-
MFPASF--
-MPASFW-

```
-MPASFC-
-MPASFA-
MCPASF--
MAPASF--
-NPASFR-
NLPASF--
NVPASF--
NNPASF--
-NPASFS-
-NPASFF-
-NPASFL-
NKPASF--
NRPASF--
-NPASFV-
-NPASFK-
-NPASFM-
NSPASF--
NGPASF--
NFPASF--
NTPASF--
-NPASFQ-
-NPASFA-
NPPASF--
NYPASF--
-NPASFW-
-NPASFN-
NAPASF--
-NPASFY-
-NPASFI-
-NPASFH-
-NPASFE-
NDPASF--
NCPASF--
--PASFRS
--PASFRR
--PASFRL
--PASFFT
--PASFFR
--PASFWA
--PASFYT
--PASFRG
--PASFRY
--PASFRI
--PASFNS
--PASFST
--PASFLG
--PASFII
--PASFWH
--PASFLT
--PASFLK
```

PRPASF--
--PASFRD
--PASFSQ
--PASFRN
--PASFRM
--PASFLS
PNPASF--
PGPASF--
--PASFMR
--PASFKV
--PASFKH
--PASFHS
--PASFAS
--PASFWS
--PASFTH
--PASFLM
--PASFKG
--PASFIH
PLPASF--
PIPASF--
--PASFVM
--PASFTT
--PASFSI
--PASFLN
--PASFKW
--PASFHG
--PASFFN
--PASFFM
PEPASF--
--PASFYS
--PASFVA
--PASFTS
--PASFTQ
--PASFRV
--PASFRQ
--PASFMH
--PASFKA
--PASFHT
--PASFVW
--PASFVT
--PASFVS
--PASFVN
--PASFTM
--PASFSL
--PASFNT
--PASFMM
--PASFMF
--PASFIR
--PASFIK
--PASFFS

--PASFYG
--PASFWN
--PASFVI
--PASFTV
--PASFTN
--PASFRF
--PASFKI
--PASFIY
--PASFIW
--PASFIT
--PASFFK
--PASFYM
--PASFWK
--PASFTA
--PASFRA
--PASFQT
--PASFQM
--PASFQG
--PASFQA
--PASFNV
--PASFNA
--PASFMS
--PASFLQ
--PASFLH
--PASFAV
--PASFAT
--PASFAI
PWPASF--
PQPASF--
PFPASF--
--PASFWR
--PASFWI
--PASFVH
--PASFVG
--PASFSY
--PASFSV
--PASFSR
--PASFSK
--PASFQY
--PASFQW
--PASFQR
--PASFNY
--PASFMY
--PASFKY
--PASFKS
--PASFKQ
--PASFKM
--PASFFA
--PASFEA
PVPASF--

--PASFYV
--PASFYN
--PASFWG
--PASFVV
--PASFVQ
--PASFTY
--PASFTI
--PASFTF
--PASFTD
--PASFSM
--PASFSA
--PASFRW
--PASFQS
--PASFQK
--PASFNW
--PASFMW
--PASFMN
--PASFML
--PASFMK
--PASFMG
--PASFME
--PASFLA
--PASFKD
--PASFHV
--PASFHA
--PASFGS
--PASFFI
PTPASF--
-PPASFS-
--PASFYQ
--PASFYH
--PASFWT
--PASFTW
--PASFTK
--PASFTE
--PASFQN
--PASFNG
--PASFNF
--PASFMT
--PASFMD
--PASFMA
--PASFKT
--PASFKK
--PASFIQ
--PASFIG
--PASFHY
--PASFHW
--PASFHR
--PASFAW
--PASFAG

--PASFAF
--PASFAA
PAPASF--
PYPASF--
-PPASFL-
PKPASF--
--PASFYA
--PASFWW
--PASFWQ
--PASFVE
--PASFVD
--PASFRE
--PASFQL
--PASFQF
--PASFQD
--PASFMQ
--PASFKL
--PASFIN
--PASFIE
--PASFIA
--PASFHF
--PASFHE
--PASFGE
--PASFFH
--PASFEW
--PASFET
--PASFEG
--PASFAY
--PASFAM
-QPASFR-
QRPASF--
-QPASFV-
-QPASFL-
QAPASF--
QVPASF--
-QPASFT-
QTPASF--
QPPASF--
QNPASF--
QQPASF--
-QPASFW-
QMPASF--
QIPASF--
QSPASF--
-QPASFI-
QYPASF--
-QPASFS-
QFPASF--
-QPASFQ-
-QPASFN-

-QPASFM-
-QPASFH-
-QPASFA-
QLPASF--
QGPASF--
QEPASF--
QDPASF--
-QPASFK-
-QPASFE-
QKPASF--
QHPASF--
RMPASF--
-RPASFI-
-RPASFT-
RSPASF--
-RPASFR-
RVPASF--
-RPASFS-
RGPASF--
RNPASF--
RPPASF--
-RPASFE-
RIPASF--
-RPASFF-
-RPASFW-
RTPASF--
RKPASF--
RFPASF--
-RPASFV-
-RPASFM-
REPASF--
RWPASF--
RQPASF--
RHPASF--
RDPASF--
RCPASF--
-SPASFR-
SRPASF--
SLPASF--
-SPASFL-
STPASF--
SSPASF--
-SPASFQ-
SGPASF--
SFPASF--
SMPASF--
SWPASF--
-SPASFT-
-SPASFM-
SKPASF--

SHPASF--
SDPASF--
-SPASFV-
-SPASFY-
-SPASFK-
SAPASF--
-SPASFN-
SEPASF--
SVPASF--
-SPASFH-
-SPASFE-
-SPASFA-
SNPASF--
SQPASF--
-SPASFW-
SIPASF--
SPPASF--
SCPASF--
-TPASFR-
-TPASFL-
TVPASF--
-TPASFS-
-TPASFK-
TMPASF--
-TPASFV-
TLPASF--
TRPASF--
-TPASFT-
TGPASF--
TSPASF--
-TPASFW-
-TPASFA-
TAPASF--
-TPASFH-
-TPASFF-
-TPASFI-
-TPASFY-
TIPASF--
TTPASF--
-TPASFN-
TDPASF--
TPPASF--
TWPASF--
TQPASF--
TNPASF--
TCPASF--
VSPASF--
-VPASFR-
VWPASF--
-VPASFS-

```
-VPASFM-
VGPASF--
-VPASFI-
VVPASF--
VTPASF--
-VPASFF-
-VPASFA-
VYPASF--
-VPASFN-
-VPASFK-
VLPASF--
VKPASF--
VHPASF--
VEPASF--
VNPASF--
VAPASF--
-VPASFV-
-VPASFH-
VQPASF--
-VPASFY-
-VPASFW-
-VPASFT-
-VPASFQ-
VPPASF--
VIPASF--
-VPASFE-
VCPASF--
WSPASF--
WRPASF--
-WPASFR-
-WPASFK-
WGPASF--
-WPASFL-
WLPASF--
-WPASFA-
-WPASFW-
WDPASF--
WPPASF--
-WPASFT-
-WPASFS-
WHPASF--
WFPASF--
-WPASFI-
WEPASF--
WAPASF--
WYPASF--
WWPASF--
WQPASF--
-WPASFM-
-WPASFF-
```

YLPASF--
YKPASF--
-YPASFV-
YHPASF--
YSPASF--
-YPASFR-
-YPASFQ-
-YPASFA-
-YPASFT-
-YPASFS-
YVPASF--
YDPASF--
YQPASF--
-YPASFK-
-YPASFM-
-YPASFI-
YGPASF--
YAPASF--
YWPASF--
YRPASF--
-YPASFW-
YNPASF--
YYPASF--
-YPASFF-
YFPASF--
YPPASF--
YIPASF--
YEPASF--

PHNL 125 754 31.00

-NPHNLN-
-NPHNLG-
AVPHNL--
ASPHNL--
-APHNLT-
-APHNLR-
ARPHNL--
AHPHNL--
-APHNLK-
ANPHNL--
AMPHNL--
AIPHNL--
ATPHNL--
AQPHNL--
-APHNLL-
AKPHNL--
AFPHNL--
-APHNLS-
-APHNLF-
ALPHNL--
AGPHNL--

ADPHNL--
AAPHNL--
AYPHNL--
APPHNL--
-APHNLW-
-APHNLV-
-APHNLM-
-APHNLQ-
-APHNLN-
-APHNLI-
-APHNLG-
-APHNLE-
-APHNLA-
AEPHNL--
CYPHNL--
CSPHNL--
CAPHNL--
CGPHNL--
DRPHNL--
-DPHNLT-
DVPHNL--
DTPHNL--
-DPHNLK-
-DPHNLI-
DSPHNL--
DFPHNL--
DYPHNL--
-DPHNLS-
-DPHNLR-
-DPHNLL-
-DPHNLA-
DGPHNL--
DDPHNL--
DAPHNL--
ESPHNL--
ERPHNL--
-EPHNLV-
-EPHNLY-
EKPHNL--
EMPHNL--
-EPHNLS-
ETPHNL--
-EPHNLR-
ELPHNL--
-EPHNLT-
-EPHNLH-
EFPHNL--
EWPHNL--
-EPHNLQ-
-EPHNLK-

-EPHNLE-
-EPHNLA-
EHPHNL--
EGPHNL--
EAPHNL--
FLPHNL--
-FPHNLR-
FWPHNL--
-FPHNLS-
-FPHNLQ-
FQPHNL--
-FPHNLY-
FSPHNL--
FPPHNL--
-FPHNLT-
-FPHNLA-
FGPHNL--
FTPHNL--
FRPHNL--
-FPHNLG-
FNPHNL--
FMPHNL--
FFPHNL--
-FPHNLV-
-FPHNLN-
FKPHNL--
FHPHNL--
FAPHNL--
FYPHNL--
-FPHNLL-
-FPHNLK-
-FPHNLF-
-FPHNLE-
FIPHNL--
FCPHNL--
-GPHNLR-
GTPHNL--
-GPHNLS-
GGPHNL--
GSPHNL--
GNPHNL--
-GPHNLW-
GMPHNL--
GAPHNL--
GLPHNL--
GWPHNL--
GVPHNL--
GRPHNL--
GPPHNL--
-GPHNLH-

-GPHNLG-
-GPHNLF-
GIPHNL--
GFPHNL--
GYPHNL--
-GPHNLY-
-GPHNLV-
-GPHNLL-
-GPHNLE-
GKPHNL--
-GPHNLM-
-GPHNLK-
-GPHNLI-
GHPHNL--
GQPHNL--
-GPHNLN-
-HPHNLI-
HAPHNL--
HRPHNL--
-HPHNLK-
-HPHNLR-
HSPHNL--
HVPHNL--
HMPHNL--
HGPHNL--
HQPHNL--
-HPHNLW-
-HPHNLV-
-HPHNLL-
-HPHNLH-
-HPHNLF-
HLPHNL--
HFPHNL--
-HPHNLS-
-HPHNLN-
-HPHNLG-
HNPHNL--
HYPHNL--
-HPHNLY-
-HPHNLM-
-HPHNLA-
HEPHNL--
IRPHNL--
-IPHNLV-
IMPHNL--
IIPHNL--
-IPHNLR-
IFPHNL--
-IPHNLS-
ISPHNL--

```
-IPHNLL-
-IPHNLT-
-IPHNLY-
IVPHNL--
-IPHNLK-
ILPHNL--
IAPHNL--
ITPHNL--
IHPHNL--
IGPHNL--
-IPHNLN-
-IPHNLH-
-IPHNLF-
-IPHNLA-
IKPHNL--
IWPHNL--
IPPHNL--
-IPHNLQ-
-IPHNLM-
-IPHNLI-
INPHNL--
IEPHNL--
-KPHNLT-
-KPHNLL-
KGPHNL--
KAPHNL--
-KPHNLR-
-KPHNLG-
KFPHNL--
KRPHNL--
KHPHNL--
-KPHNLS-
KIPHNL--
KMPHNL--
KYPHNL--
-KPHNLY-
-KPHNLV-
-KPHNLI-
KQPHNL--
-KPHNLH-
KNPHNL--
KKPHNL--
KWPHNL--
KVPHNL--
KTPHNL--
-KPHNLW-
-KPHNLQ-
-KPHNLF-
KLPHNL--
KPPHNL--
```

-KPHNLM-
KDPHNL--
LRPHNL--
-LPHNLM-
LGPHNL--
-LPHNLR-
LWPHNL--
LSPHNL--
LLPHNL--
-LPHNLV-
LTPHNL--
-LPHNLS-
LNPHNL--
-LPHNLQ-
-LPHNLA-
LDPHNL--
-LPHNLL-
-LPHNLT-
LMPHNL--
-LPHNLI-
LAPHNL--
LYPHNL--
LVPHNL--
LPPHNL--
-LPHNLY-
-LPHNLK-
LKPHNL--
LEPHNL--
LFPHNL--
-LPHNLH-
-LPHNLE-
LIPHNL--
LHPHNL--
LQPHNL--
-LPHNLF-
MSPHNL--
MVPHNL--
-MPHNLR-
MRPHNL--
MIPHNL--
MQPHNL--
MLPHNL--
-MPHNLI-
MHPHNL--
MGPHNL--
-MPHNLS-
-MPHNLY-
MDPHNL--
MAPHNL--
-MPHNLH-

-MPHNLA-
MMPHNL--
MKPHNL--
MYPHNL--
MWPHNL--
MTPHNL--
-MPHNLT-
-MPHNLQ-
-MPHNLK-
-MPHNLE-
NRPHNL--
NVPHNL--
NAPHNL--
-NPHNLT-
-NPHNLK-
NLPHNL--
NGPHNL--
NTPHNL--
NQPHNL--
NPPHNL--
-NPHNLS-
-NPHNLR-
-NPHNLM-
-NPHNLY-
-NPHNLW-
-NPHNLV-
-NPHNLH-
-NPHNLE-
NIPHNL--
-NPHNLF-
NFPHNL--
NWPHNL--
-NPHNLQ-
-NPHNLL-
-NPHNLI-
-NPHNLA-
NNPHNL--
NMPHNL--
NHPHNL--
--PHNLWS
--PHNLRG
--PHNLIG
--PHNLYL
--PHNLWH
--PHNLTT
--PHNLAS
--PHNLYG
--PHNLRK
--PHNLVL
--PHNLVK

--PHNLRN
--PHNLSY
--PHNLKG
--PHNLAY
--PHNLSG
--PHNLRT
--PHNLKA
--PHNLES
--PHNLYD
--PHNLVR
--PHNLRQ
--PHNLHS
--PHNLGW
PVPHNL--
--PHNLYS
--PHNLTR
--PHNLTL
--PHNLRW
--PHNLMH
--PHNLLT
--PHNLLQ
--PHNLLN
--PHNLLH
--PHNLKY
--PHNLAV
PRPHNL--
--PHNLSL
--PHNLSK
--PHNLSA
--PHNLRA
--PHNLLS
--PHNLKL
--PHNLIR
--PHNLIA
--PHNLWL
--PHNLWF
--PHNLVI
--PHNLSS
--PHNLSM
--PHNLSI
--PHNLMR
--PHNLLI
--PHNLHQ
--PHNLHA
--PHNLGT
--PHNLGS
--PHNLFM
PLPHNL--
PIPHNL--
--PHNLYW

--PHNLYR
--PHNLYN
--PHNLTN
--PHNLTI
--PHNLSV
--PHNLRI
--PHNLMW
--PHNLMV
--PHNLMT
--PHNLML
--PHNLMK
--PHNLLR
--PHNLLG
--PHNLLF
--PHNLIY
--PHNLGY
--PHNLFR
PMPHNL--
--PHNLYY
--PHNLYK
--PHNLVY
--PHNLVT
--PHNLVS
--PHNLVQ
--PHNLSN
--PHNLQT
--PHNLQA
--PHNLNL
--PHNLMG
--PHNLLL
--PHNLIL
--PHNLHV
--PHNLGV
--PHNLGA
--PHNLFH
--PHNLAN
PYPHNL--
PWPHNL--
PTPHNL--
--PHNLYT
--PHNLYQ
--PHNLYM
--PHNLWR
--PHNLVN
--PHNLVM
--PHNLTM
--PHNLTK
--PHNLTG
--PHNLTD
--PHNLSQ

--PHNLRE
--PHNLRD
--PHNLQW
--PHNLQL
--PHNLNT
--PHNLNS
--PHNLLY
--PHNLLV
--PHNLLM
--PHNLLD
--PHNLLA
--PHNLKW
--PHNLIT
--PHNLIQ
--PHNLIK
--PHNLIE
--PHNLHG
--PHNLGL
--PHNLGG
--PHNLFW
--PHNLFS
--PHNLFQ
--PHNLFI
--PHNLEV
--PHNLAL
--PHNLAI
--PHNLAF
PFPHNL--
PQPHNL--
--PHNLYF
--PHNLWV
--PHNLWQ
--PHNLWN
--PHNLWK
--PHNLVH
--PHNLVA
--PHNLTF
--PHNLSH
--PHNLSF
--PHNLRH
--PHNLQY
--PHNLQR
--PHNLQN
--PHNLQM
--PHNLQG
--PHNLPY
--PHNLNR
--PHNLNN
--PHNLNF
--PHNLNA

--PHNLMS
--PHNLMN
--PHNLMD
--PHNLMA
--PHNLKV
--PHNLKM
--PHNLIV
--PHNLIS
--PHNLII
--PHNLIH
--PHNLHT
--PHNLFY
--PHNLFV
--PHNLEY
--PHNLAM
--PHNLAH
--PHNLAA
PAPHNL--
PSPHNL--
-PPHNLF-
PKPHNL--
PHPHNL--
--PHNLYV
--PHNLYE
--PHNLYA
--PHNLVG
--PHNLTQ
--PHNLTH
--PHNLSW
--PHNLST
--PHNLSD
--PHNLRM
--PHNLRF
--PHNLQV
--PHNLQS
--PHNLQQ
--PHNLQH
--PHNLQF
--PHNLPF
--PHNLNV
--PHNLNH
--PHNLNE
--PHNLMI
--PHNLLW
--PHNLKK
--PHNLKF
--PHNLKE
--PHNLIN
--PHNLIF
--PHNLHM

--PHNLHL
--PHNLHF
--PHNLHD
--PHNLFT
--PHNLFL
--PHNLFG
--PHNLFF
--PHNLFD
--PHNLER
--PHNLEL
--PHNLCI
--PHNLAT
--PHNLAK
--PHNLAG
PEPHNL--
QVPHNL--
-QPHNLK-
QIPHNL--
QLPHNL--
QSPHNL--
-QPHNLL-
QTPHNL--
-QPHNLR-
QAPHNL--
QRPHNL--
QPPHNL--
-QPHNLS-
QHPHNL--
-QPHNLW-
-QPHNLI-
QYPHNL--
QQPHNL--
-QPHNLH-
-QPHNLE-
QKPHNL--
QWPHNL--
-QPHNLV-
-QPHNLF-
-QPHNLT-
-QPHNLQ-
-QPHNLM-
-QPHNLA-
QNPHNL--
QMPHNL--
QFPHNL--
-RPHNLS-
-RPHNLM-
-RPHNLV-
RGPHNL--
RYPHNL--

RTPHNL--
-RPHNLL-
RMPHNL--
REPHNL--
RIPHNL--
-RPHNLI-
RVPHNL--
RRPHNL--
-RPHNLY-
RWPHNL--
-RPHNLG-
RAPHNL--
-RPHNLQ-
-RPHNLF-
-RPHNLA-
-RPHNLT-
-RPHNLW-
RPPHNL--
-RPHNLN-
RNPHNL--
RKPHNL--
RFPHNL--
RHPHNL--
RQPHNL--
-RPHNLH-
RDPHNL--
-RPHNLP-
-RPHNLD-
RCPHNL--
SRPHNL--
-SPHNLR-
-SPHNLL-
-SPHNLT-
SLPHNL--
SSPHNL--
SAPHNL--
SMPHNL--
STPHNL--
SFPHNL--
-SPHNLY-
-SPHNLS-
-SPHNLH-
SWPHNL--
SVPHNL--
SQPHNL--
-SPHNLF-
SKPHNL--
SIPHNL--
-SPHNLA-
SYPHNL--

−SPHNLG−
SGPHNL−−
SEPHNL−−
−SPHNLV−
−SPHNLQ−
−SPHNLN−
−SPHNLI−
−SPHNLM−
SNPHNL−−
SDPHNL−−
SCPHNL−−
SPPHNL−−
−SPHNLE−
SHPHNL−−
−TPHNLL−
TRPHNL−−
TLPHNL−−
−TPHNLR−
TFPHNL−−
−TPHNLS−
−TPHNLT−
−TPHNLA−
TVPHNL−−
TNPHNL−−
TKPHNL−−
TIPHNL−−
TSPHNL−−
−TPHNLF−
TMPHNL−−
TGPHNL−−
−TPHNLQ−
−TPHNLI−
TQPHNL−−
TYPHNL−−
TWPHNL−−
−TPHNLY−
−TPHNLV−
TEPHNL−−
TPPHNL−−
TAPHNL−−
−TPHNLW−
−TPHNLM−
−TPHNLG−
−TPHNLE−
TDPHNL−−
−VPHNLS−
VRPHNL−−
−VPHNLA−
VTPHNL−−
−VPHNLQ−

VNPHNL--
VMPHNL--
VSPHNL--
-VPHNLF-
VKPHNL--
-VPHNLL-
VLPHNL--
-VPHNLT-
VIPHNL--
-VPHNLV-
-VPHNLR-
VVPHNL--
VGPHNL--
VYPHNL--
-VPHNLE-
VHPHNL--
VEPHNL--
VAPHNL--
-VPHNLI-
VCPHNL--
VWPHNL--
-VPHNLM-
-VPHNLK-
-VPHNLG-
VDPHNL--
VPPHNL--
-VPHNLY-
-VPHNLW-
-VPHNLH-
VFPHNL--
WRPHNL--
-WPHNLR-
WMPHNL--
WQPHNL--
WVPHNL--
WTPHNL--
WIPHNL--
-WPHNLV-
-WPHNLL-
-WPHNLA-
WKPHNL--
WSPHNL--
-WPHNLT-
-WPHNLI-
WGPHNL--
WWPHNL--
-WPHNLQ-
-WPHNLK-
-WPHNLF-
WLPHNL--

| | | | |
|---|---|---|---|
| | WFPHNL-- | | |
| | WPPHNL-- | | |
| | -WPHNLS- | | |
| | WEPHNL-- | | |
| | WAPHNL-- | | |
| | YRPHNL-- | | |
| | -YPHNLS- | | |
| | YKPHNL-- | | |
| | YTPHNL-- | | |
| | -YPHNLL- | | |
| | -YPHNLA- | | |
| | YMPHNL-- | | |
| | YQPHNL-- | | |
| | YVPHNL-- | | |
| | YSPHNL-- | | |
| | YIPHNL-- | | |
| | YYPHNL-- | | |
| | -YPHNLV- | | |
| | -YPHNLM- | | |
| | -YPHNLK- | | |
| | YEPHNL-- | | |
| | -YPHNLF- | | |
| | YGPHNL-- | | |
| | YPPHNL-- | | |
| | YNPHNL-- | | |
| | YHPHNL-- | | |
| | -YPHNLY- | | |
| | -YPHNLQ- | | |
| | -YPHNLN- | | |
| | -YPHNLH- | | |
| | -YPHNLE- | | |
| | YLPHNL-- | | |
| | YCPHNL-- | | |
| PVNI | 185 | 724 | 30.99 |
| | -NPVNIL- | | |
| | --PVNIRP | | |
| | --PVNIHR | | |
| | -RPVNIN- | | |
| | ALPVNI-- | | |
| | ARPVNI-- | | |
| | AMPVNI-- | | |
| | -APVNIY- | | |
| | -APVNIW- | | |
| | ASPVNI-- | | |
| | AIPVNI-- | | |
| | -APVNII- | | |
| | AWPVNI-- | | |
| | -APVNIV- | | |
| | -APVNIF- | | |
| | AVPVNI-- | | |

-APVNIK-
-APVNIG-
-APVNIE-
AGPVNI--
AEPVNI--
AYPVNI--
AQPVNI--
-APVNIN-
-APVNIH-
ANPVNI--
AKPVNI--
-APVNIQ-
-APVNIA-
AFPVNI--
ACPVNI--
AAPVNI--
CWPVNI--
CCPVNI--
-CPVNIK-
CNPVNI--
CFPVNI--
CDPVNI--
CTPVNI--
CSPVNI--
-CPVNII-
CLPVNI--
CKPVNI--
CGPVNI--
CAPVNI--
DIPVNI--
DTPVNI--
DRPVNI--
-DPVNIL-
DHPVNI--
DFPVNI--
DSPVNI--
-DPVNIW-
-DPVNIS-
DYPVNI--
DWPVNI--
DVPVNI--
-DPVNIV-
-DPVNIR-
-DPVNIM-
-DPVNII-
-DPVNIF-
DLPVNI--
DKPVNI--
DGPVNI--
DEPVNI--

DAPVNI--
-EPVNII-
ERPVNI--
-EPVNIR-
-EPVNIL-
-EPVNIT-
-EPVNIS-
EFPVNI--
-EPVNIW-
-EPVNIA-
EKPVNI--
EQPVNI--
-EPVNIH-
EMPVNI--
EHPVNI--
EAPVNI--
EYPVNI--
EWPVNI--
EVPVNI--
ESPVNI--
-EPVNIY-
-EPVNIQ-
-EPVNIM-
-EPVNIK-
-EPVNIF-
ENPVNI--
EGPVNI--
EEPVNI--
FVPVNI--
FKPVNI--
-FPVNIL-
FTPVNI--
-FPVNIT-
-FPVNIS-
FSPVNI--
FRPVNI--
-FPVNIW-
-FPVNIF-
FIPVNI--
-FPVNIR-
FNPVNI--
FGPVNI--
FYPVNI--
-FPVNIY-
-FPVNIQ-
-FPVNIM-
FWPVNI--
-FPVNIK-
-FPVNIH-
FMPVNI--

FCPVNI--
FQPVNI--
-FPVNIV-
FLPVNI--
FDPVNI--
-GPVNIR-
GVPVNI--
GIPVNI--
GTPVNI--
-GPVNIS-
-GPVNIL-
-GPVNIA-
GMPVNI--
-GPVNIK-
GQPVNI--
-GPVNIV-
GLPVNI--
GHPVNI--
GGPVNI--
GSPVNI--
GRPVNI--
-GPVNIT-
-GPVNIQ-
GNPVNI--
-GPVNIH-
GKPVNI--
GDPVNI--
-GPVNII-
GPPVNI--
GFPVNI--
GYPVNI--
-GPVNIY-
-GPVNIF-
-GPVNIE-
GCPVNI--
GAPVNI--
-HPVNIT-
-HPVNIS-
-HPVNIK-
HWPVNI--
-HPVNIR-
HAPVNI--
HTPVNI--
HRPVNI--
-HPVNIW-
-HPVNIE-
HSPVNI--
-HPVNIF-
-HPVNIA-
HDPVNI--

-HPVNIY-
-HPVNIV-
-HPVNIM-
-HPVNII-
-HPVNIH-
HNPVNI--
HMPVNI--
HLPVNI--
HIPVNI--
HGPVNI--
HFPVNI--
HEPVNI--
ILPVNI--
-IPVNIA-
IRPVNI--
-IPVNIL-
IWPVNI--
IMPVNI--
IVPVNI--
-IPVNIS-
IGPVNI--
-IPVNIV-
-IPVNIM-
-IPVNIE-
IYPVNI--
ISPVNI--
-IPVNIW-
-IPVNIR-
-IPVNIH-
-IPVNIY-
-IPVNIT-
-IPVNIF-
IPPVNI--
-IPVNIQ-
INPVNI--
IFPVNI--
-IPVNIN-
-IPVNIK-
-IPVNII-
-IPVNIG-
-IPVNID-
IAPVNI--
KSPVNI--
KLPVNI--
-KPVNIR-
KIPVNI--
KRPVNI--
-KPVNIF-
KMPVNI--
KTPVNI--

-KPVNIL-
-KPVNIH-
-KPVNIT-
KPPVNI--
-KPVNIW-
-KPVNIS-
KGPVNI--
KYPVNI--
-KPVNIV-
-KPVNIN-
-KPVNIM-
-KPVNIK-
-KPVNIG-
-KPVNIE-
KNPVNI--
KFPVNI--
KDPVNI--
KWPVNI--
-KPVNII-
-KPVNIC-
KKPVNI--
KCPVNI--
KAPVNI--
LRPVNI--
LLPVNI--
LSPVNI--
LIPVNI--
-LPVNIL-
-LPVNIS-
LVPVNI--
-LPVNIM-
LTPVNI--
-LPVNIQ-
LNPVNI--
LGPVNI--
LQPVNI--
LMPVNI--
LEPVNI--
LWPVNI--
-LPVNII-
-LPVNIA-
LPPVNI--
LFPVNI--
-LPVNIY-
-LPVNIT-
-LPVNIF-
LKPVNI--
-LPVNIW-
-LPVNIV-
LDPVNI--

LCPVNI--
LAPVNI--
-MPVNIS-
-MPVNIM-
MSPVNI--
MGPVNI--
MVPVNI--
MEPVNI--
MQPVNI--
MRPVNI--
-MPVNIR-
-MPVNIL-
-MPVNIE-
MIPVNI--
MYPVNI--
-MPVNIA-
MAPVNI--
-MPVNIT-
-MPVNIQ-
-MPVNII-
-MPVNIH-
-MPVNIW-
MTPVNI--
-MPVNIY-
MPPVNI--
MNPVNI--
MMPVNI--
-MPVNIV-
MHPVNI--
MDPVNI--
-NPVNIR-
NAPVNI--
NPPVNI--
NSPVNI--
NVPVNI--
NRPVNI--
NHPVNI--
NGPVNI--
-NPVNIT-
-NPVNIS-
-NPVNIK-
-NPVNIH-
NDPVNI--
NWPVNI--
-NPVNIM-
-NPVNIF-
-NPVNIA-
NLPVNI--
NIPVNI--
NYPVNI--

NTPVNI--
-NPVNIY-
-NPVNII-
NKPVNI--
NEPVNI--
--PVNILA
--PVNIRS
--PVNIWM
--PVNIFS
--PVNIYS
--PVNIVT
--PVNILR
--PVNIMA
--PVNISI
--PVNIIR
--PVNIRT
--PVNIRG
--PVNIYG
--PVNIVG
--PVNISG
--PVNISA
PLPVNI--
--PVNIYR
--PVNISR
--PVNIRN
--PVNIRL
--PVNILT
--PVNIFR
--PVNIWS
--PVNITA
--PVNISV
--PVNIRW
--PVNIRF
--PVNIFH
--PVNIYH
--PVNIRA
--PVNIMS
--PVNIAH
--PVNIYK
--PVNIWK
--PVNIVA
--PVNITY
--PVNITN
--PVNITK
--PVNISM
--PVNIRV
--PVNIRH
--PVNINS
--PVNIMV
--PVNILM

```
--PVNILK
--PVNILG
--PVNIGG
--PVNIET
--PVNIAA
PGPVNI--
--PVNIYI
--PVNIYD
--PVNIWV
--PVNIVL
--PVNITT
--PVNISY
--PVNIRM
--PVNILH
--PVNIKG
--PVNIEY
PAPVNI--
--PVNIWA
--PVNITR
--PVNISW
--PVNISN
--PVNIQN
--PVNIQD
--PVNINA
--PVNIMT
--PVNIMQ
--PVNILS
--PVNILQ
--PVNIKV
--PVNIKH
--PVNIKA
--PVNIIG
--PVNIGS
--PVNIAS
--PVNIYQ
--PVNIVQ
--PVNIVK
--PVNITW
--PVNITQ
--PVNITL
--PVNITD
--PVNISS
--PVNISH
--PVNISF
--PVNIRQ
--PVNIRK
--PVNIRD
--PVNIQG
--PVNIMR
--PVNIMI
```

--PVNIIT
--PVNIHT
--PVNICS
--PVNIAY
--PVNIYL
--PVNIWW
--PVNIWT
--PVNIVY
--PVNIVH
--PVNITV
--PVNIQW
--PVNIQV
--PVNIQQ
--PVNING
--PVNIMK
--PVNILY
--PVNILV
--PVNIKS
--PVNIKE
--PVNIIQ
--PVNIIH
--PVNIGW
--PVNIFV
--PVNIFM
--PVNIFG
PTPVNI--
PKPVNI--
PIPVNI--
PHPVNI--
PDPVNI--
--PVNIYV
--PVNIWN
--PVNIVW
--PVNIVR
--PVNIVN
--PVNITH
--PVNISQ
--PVNIRI
--PVNIQS
--PVNIQM
--PVNIQK
--PVNIQA
--PVNIME
--PVNILN
--PVNILD
--PVNIKR
--PVNIKN
--PVNIIV
--PVNIIE
--PVNIHS

--PVNIHG
--PVNIGL
--PVNIFT
--PVNIES
--PVNIAR
--PVNIAN
--PVNIAL
PSPVNI--
PRPVNI--
PNPVNI--
PCPVNI--
PWPVNI--
--PVNIYY
--PVNIYW
--PVNIYT
--PVNIYN
--PVNIYM
--PVNIYE
--PVNIWY
--PVNIWD
--PVNIVV
--PVNIVM
--PVNITS
--PVNITM
--PVNISL
--PVNISK
--PVNIQL
--PVNIQH
--PVNIQE
--PVNINL
--PVNIMY
--PVNIMM
--PVNIMH
--PVNIMG
--PVNILW
--PVNILE
--PVNIKT
--PVNIKQ
--PVNIKL
--PVNIKI
--PVNIIN
--PVNIII
--PVNIIA
--PVNIHY
--PVNIHV
--PVNIHQ
--PVNIHF
--PVNIFY
--PVNIFQ
--PVNIFI

--PVNIFD
--PVNIEK
--PVNIEG
--PVNIEF
--PVNIAQ
--PVNIAM
PQPVNI--
-QPVNIS-
-QPVNIR-
-QPVNIL-
QSPVNI--
QPPVNI--
QGPVNI--
QFPVNI--
-QPVNIW-
-QPVNIH-
-QPVNIF-
-QPVNIE-
QHPVNI--
QVPVNI--
-QPVNIA-
QNPVNI--
QRPVNI--
-QPVNIT-
-QPVNIQ-
QLPVNI--
QWPVNI--
QTPVNI--
-QPVNIY-
-QPVNIV-
-QPVNIK-
-QPVNII-
-QPVNIG-
QKPVNI--
QEPVNI--
RAPVNI--
RSPVNI--
RRPVNI--
RIPVNI--
-RPVNIH-
-RPVNIA-
RMPVNI--
-RPVNIS-
-RPVNIR-
-RPVNIW-
RGPVNI--
-RPVNIK-
RWPVNI--
RTPVNI--
-RPVNIM-

RYPVNI--
-RPVNIE-
RFPVNI--
-RPVNIY-
-RPVNIG-
-RPVNII-
REPVNI--
-RPVNIV-
-RPVNIL-
RKPVNI--
RQPVNI--
RHPVNI--
-RPVNIQ-
RPPVNI--
RDPVNI--
RNPVNI--
-RPVNIC-
RCPVNI--
SRPVNI--
-SPVNIL-
SLPVNI--
-SPVNIF-
SNPVNI--
SVPVNI--
SSPVNI--
SKPVNI--
-SPVNIH-
SMPVNI--
-SPVNIM-
SGPVNI--
SWPVNI--
-SPVNIY-
-SPVNIS-
-SPVNIK-
-SPVNII-
SQPVNI--
SEPVNI--
-SPVNIV-
-SPVNIT-
-SPVNIQ-
SYPVNI--
-SPVNIE-
-SPVNIA-
SHPVNI--
SAPVNI--
-SPVNIW-
-SPVNIG-
SIPVNI--
SFPVNI--
SDPVNI--

SCPVNI--
-TPVNIT-
-TPVNIR-
TTPVNI--
TNPVNI--
-TPVNIM-
-TPVNIA-
TGPVNI--
TYPVNI--
TRPVNI--
-TPVNIQ-
-TPVNIL-
TLPVNI--
TAPVNI--
TWPVNI--
-TPVNIK-
TPPVNI--
-TPVNIF-
-TPVNIE-
TFPVNI--
TSPVNI--
-TPVNIW-
TKPVNI--
TEPVNI--
-TPVNIN-
-TPVNIC-
TMPVNI--
VLPVNI--
-VPVNIK-
-VPVNIY-
-VPVNIF-
-VPVNIL-
VMPVNI--
VSPVNI--
-VPVNIV-
-VPVNIA-
VEPVNI--
VRPVNI--
-VPVNIR-
VYPVNI--
-VPVNIM-
VPPVNI--
VGPVNI--
-VPVNIS-
-VPVNII-
VIPVNI--
-VPVNIT-
VTPVNI--
VHPVNI--
VWPVNI--

VKPVNI--
VDPVNI--
VQPVNI--
-VPVNIQ-
-VPVNIG-
VNPVNI--
VCPVNI--
-WPVNII-
WSPVNI--
-WPVNIV-
WIPVNI--
WVPVNI--
-WPVNIT-
-WPVNIR-
-WPVNIK-
WKPVNI--
WYPVNI--
WPPVNI--
WNPVNI--
WHPVNI--
WFPVNI--
-WPVNIY-
WQPVNI--
-WPVNIL-
-WPVNIW-
-WPVNIF-
-WPVNIA-
WLPVNI--
WEPVNI--
WDPVNI--
WAPVNI--
-YPVNIA-
YAPVNI--
-YPVNIL-
-YPVNIK-
YSPVNI--
-YPVNIT-
-YPVNIR-
YEPVNI--
YYPVNI--
YRPVNI--
-YPVNIM-
YTPVNI--
-YPVNIS-
-YPVNIH-
-YPVNIE-
YLPVNI--
YGPVNI--
YFPVNI--
YVPVNI--

YQPVNI--
-YPVNIW-
-YPVNIF-
YHPVNI--
-YPVNIV-
YPPVNI--
YMPVNI--
YKPVNI--

| | | | |
|---|---|---|---|
| PMWL | 894 | 522 | 30.99 |

--PMWLGK
ATPMWL--
ARPMWL--
AEPMWL--
-APMWLK-
AVPMWL--
ASPMWL--
-APMWLA-
ANPMWL--
AKPMWL--
APPMWL--
-APMWLT-
-APMWLR-
-APMWLG-
AIPMWL--
-APMWLQ-
-APMWLE-
ALPMWL--
AHPMWL--
AYPMWL--
-APMWLV-
-APMWLS-
-APMWLN-
ACPMWL--
-APMWLM-
-APMWLH-
AFPMWL--
CQPMWL--
CRPMWL--
CFPMWL--
-CPMWLS-
-CPMWLK-
CMPMWL--
CKPMWL--
CIPMWL--
CGPMWL--
CEPMWL--
DVPMWL--
DGPMWL--
DQPMWL--
DHPMWL--

-DPMWLK-
-DPMWLG-
DSPMWL--
DRPMWL--
-DPMWLV-
-DPMWLS-
-DPMWLL-
-DPMWLA-
DEPMWL--
DWPMWL--
DTPMWL--
-DPMWLM-
-DPMWLI-
DMPMWL--
DIPMWL--
DFPMWL--
-DPMWLY-
DNPMWL--
DDPMWL--
-EPMWLR-
-EPMWLH-
-EPMWLI-
EIPMWL--
EAPMWL--
ESPMWL--
ENPMWL--
EKPMWL--
EWPMWL--
EVPMWL--
ERPMWL--
-EPMWLK-
-EPMWLA-
ELPMWL--
-EPMWLQ-
-EPMWLE-
EHPMWL--
ETPMWL--
EQPMWL--
-EPMWLT-
-EPMWLM-
EPPMWL--
-EPMWLS-
-EPMWLG-
FNPMWL--
FAPMWL--
-FPMWLQ-
FKPMWL--
FTPMWL--
FGPMWL--
FSPMWL--

-FPMWLK-
FHPMWL--
FYPMWL--
-FPMWLT-
-FPMWLS-
FDPMWL--
-FPMWLG-
FMPMWL--
GKPMWL--
GYPMWL--
-GPMWLR-
-GPMWLK-
GRPMWL--
GSPMWL--
GQPMWL--
-GPMWLQ-
GFPMWL--
GDPMWL--
GVPMWL--
-GPMWLS-
-GPMWLA-
GLPMWL--
GHPMWL--
GGPMWL--
GAPMWL--
GTPMWL--
-GPMWLV-
-GPMWLT-
-GPMWLM-
-GPMWLG-
GMPMWL--
GIPMWL--
GWPMWL--
-GPMWLN-
GEPMWL--
HSPMWL--
HRPMWL--
-HPMWLR-
-HPMWLL-
HVPMWL--
-HPMWLV-
-HPMWLS-
-HPMWLQ-
-HPMWLT-
-HPMWLG-
-HPMWLA-
HHPMWL--
HQPMWL--
HPPMWL--
-HPMWLH-

HNPMWL--
HKPMWL--
HIPMWL--
-HPMWLI-
HGPMWL--
HEPMWL--
IFPMWL--
IRPMWL--
-IPMWLG-
-IPMWLA-
ILPMWL--
IAPMWL--
IVPMWL--
IGPMWL--
IWPMWL--
ITPMWL--
-IPMWLQ-
INPMWL--
-IPMWLS-
-IPMWLR-
IMPMWL--
IIPMWL--
IPPMWL--
-IPMWLV-
-IPMWLT-
IKPMWL--
IEPMWL--
ICPMWL--
KSPMWL--
KRPMWL--
-KPMWLT-
-KPMWLS-
-KPMWLL-
KFPMWL--
KVPMWL--
KGPMWL--
-KPMWLM-
-KPMWLG-
KIPMWL--
KEPMWL--
KAPMWL--
-KPMWLV-
-KPMWLR-
-KPMWLN-
-KPMWLI-
KKPMWL--
KYPMWL--
KWPMWL--
KQPMWL--
-KPMWLH-

KNPMWL--
KHPMWL--
LNPMWL--
LQPMWL--
-LPMWLK-
-LPMWLR-
LWPMWL--
LKPMWL--
LVPMWL--
LPPMWL--
LLPMWL--
-LPMWLQ-
-LPMWLA-
LGPMWL--
LDPMWL--
LHPMWL--
-LPMWLT-
LCPMWL--
MHPMWL--
MSPMWL--
-MPMWLT-
-MPMWLR-
-MPMWLI-
MKPMWL--
MIPMWL--
MGPMWL--
MWPMWL--
-MPMWLS-
-MPMWLN-
-MPMWLM-
-MPMWLK-
MQPMWL--
MPPMWL--
-MPMWLG-
MNPMWL--
MMPMWL--
MEPMWL--
NAPMWL--
-NPMWLT-
NSPMWL--
NLPMWL--
NGPMWL--
-NPMWLR-
-NPMWLK-
NPPMWL--
-NPMWLA-
NDPMWL--
NYPMWL--
NNPMWL--
NHPMWL--

NFPMWL--
NEPMWL--
NRPMWL--
-NPMWLQ-
NWPMWL--
-NPMWLL-
-NPMWLI-
-NPMWLH-
-NPMWLG-
NMPMWL--
--PMWLGS
PMPMWL--
--PMWLRS
--PMWLRG
--PMWLHS
--PMWLSS
--PMWLVG
--PMWLTR
--PMWLTG
--PMWLVS
--PMWLRN
--PMWLHV
--PMWLRW
--PMWLRY
--PMWLRE
--PMWLNS
--PMWLMS
--PMWLAS
PKPMWL--
--PMWLYG
--PMWLSQ
--PMWLMH
--PMWLLN
--PMWLKY
--PMWLFG
PTPMWL--
--PMWLTS
--PMWLTK
--PMWLST
--PMWLSG
--PMWLRQ
--PMWLQT
--PMWLPL
--PMWLIN
--PMWLIH
--PMWLTN
--PMWLTH
--PMWLSW
--PMWLMD
--PMWLKL

--PMWLGT
--PMWLVH
--PMWLSV
--PMWLSH
--PMWLRD
--PMWLMQ
--PMWLLW
--PMWLLH
--PMWLKS
--PMWLGA
PQPMWL--
--PMWLYQ
--PMWLVR
--PMWLVA
--PMWLTA
--PMWLSD
--PMWLQN
--PMWLQK
--PMWLQD
--PMWLQA
--PMWLNT
--PMWLMG
--PMWLKM
--PMWLKG
--PMWLKD
--PMWLHT
--PMWLGG
--PMWLAR
PHPMWL--
--PMWLVK
--PMWLTI
--PMWLSA
--PMWLQQ
--PMWLQI
--PMWLNG
--PMWLNA
--PMWLLM
--PMWLKN
--PMWLKI
--PMWLHW
--PMWLHN
--PMWLGY
--PMWLGQ
--PMWLGM
--PMWLFV
--PMWLES
--PMWLEA
--PMWLAG
--PMWLAE
PGPMWL--

PDPMWL--
PWPMWL--
--PMWLWG
--PMWLWA
--PMWLVF
--PMWLTY
--PMWLSR
--PMWLSN
--PMWLSM
--PMWLSF
--PMWLSE
--PMWLQR
--PMWLQM
--PMWLQH
--PMWLQG
--PMWLQF
--PMWLPM
--PMWLPF
--PMWLNC
--PMWLMK
--PMWLKW
--PMWLKQ
--PMWLKH
--PMWLKF
--PMWLIY
--PMWLIT
--PMWLHY
--PMWLHG
--PMWLHF
--PMWLGI
--PMWLGF
--PMWLET
--PMWLAV
--PMWLAH
--PMWLAA
QRPMWL--
QSPMWL--
QTPMWL--
QVPMWL--
QMPMWL--
QIPMWL--
-QPMWLI-
QKPMWL--
QYPMWL--
-QPMWLK-
QGPMWL--
-QPMWLS-
QWPMWL--
QPPMWL--
QHPMWL--

-QPMWLH-
-QPMWLA-
-QPMWLR-
-QPMWLQ-
-QPMWLL-
-QPMWLE-
QNPMWL--
QLPMWL--
QDPMWL--
QCPMWL--
-RPMWLK-
RGPMWL--
-RPMWLS-
-RPMWLI-
RYPMWL--
-RPMWLT-
RAPMWL--
RWPMWL--
-RPMWLV-
RDPMWL--
RVPMWL--
RNPMWL--
-RPMWLA-
RMPMWL--
RHPMWL--
RPPMWL--
-RPMWLN-
RQPMWL--
-RPMWLW-
-RPMWLE-
REPMWL--
SSPMWL--
-SPMWLS-
SVPMWL--
-SPMWLR-
-SPMWLT-
SYPMWL--
SLPMWL--
-SPMWLA-
-SPMWLH-
-SPMWLV-
SIPMWL--
SKPMWL--
SPPMWL--
SFPMWL--
SRPMWL--
-SPMWLQ-
SGPMWL--
SMPMWL--
-SPMWLK-

-SPMWLG-
SEPMWL--
SDPMWL--
SHPMWL--
-SPMWLN-
-SPMWLE-
SNPMWL--
SQPMWL--
-TPMWLA-
TVPMWL--
TGPMWL--
TYPMWL--
TLPMWL--
TMPMWL--
-TPMWLR-
-TPMWLM-
TSPMWL--
TPPMWL--
-TPMWLK-
-TPMWLT-
-TPMWLQ-
-TPMWLG-
TNPMWL--
-TPMWLV-
-TPMWLS-
TAPMWL--
TWPMWL--
-TPMWLH-
TIPMWL--
TEPMWL--
-TPMWLN-
-TPMWLE-
THPMWL--
VHPMWL--
-VPMWLT-
VNPMWL--
VTPMWL--
-VPMWLA-
VRPMWL--
VVPMWL--
VLPMWL--
VWPMWL--
VPPMWL--
-VPMWLH-
-VPMWLG-
VKPMWL--
VFPMWL--
VYPMWL--
VQPMWL--
-VPMWLK-

VGPMWL--
VEPMWL--
-VPMWLS-
-VPMWLN-
-VPMWLF-
VDPMWL--
VAPMWL--
WKPMWL--
WPPMWL--
WGPMWL--
-WPMWLR-
WAPMWL--
WFPMWL--
WEPMWL--
WLPMWL--
-YPMWLR-
YTPMWL--
YKPMWL--
YRPMWL--
-YPMWLQ-
YFPMWL--
YAPMWL--
YHPMWL--
YSPMWL--
YQPMWL--
YPPMWL--
-YPMWLN-
YMPMWL--
-YPMWLA-
YIPMWL--
YGPMWL--
ISWL 877 524 30.97
-RISWLD-
-AISWLN-
AGISWL--
ASISWL--
-AISWLS-
-AISWLR-
-AISWLG-
AFISWL--
-AISWLT-
-AISWLQ-
AQISWL--
AVISWL--
AMISWL--
AKISWL--
AHISWL--
ADISWL--
-AISWLM-
-AISWLK-

-AISWLH-
ANISWL--
AIISWL--
-AISWLE-
AEISWL--
ACISWL--
CNISWL--
-CISWLN-
-CISWLG-
-CISWLF-
CHISWL--
CDISWL--
CAISWL--
CVISWL--
CTISWL--
CSISWL--
CRISWL--
CMISWL--
-CISWLV-
-CISWLS-
-CISWLL-
CGISWL--
CCISWL--
DAISWL--
-DISWLR-
DRISWL--
-DISWLQ-
-DISWLK-
DIISWL--
-DISWLM-
-DISWLH-
-DISWLE-
DYISWL--
-DISWLN-
DHISWL--
DFISWL--
-DISWLG-
-DISWLA-
-DISWLV-
DVISWL--
-DISWLI-
DGISWL--
DEISWL--
-EISWLS-
EMISWL--
-EISWLN-
ETISWL--
-EISWLR-
-EISWLV-
EIISWL--

ESISWL--
EKISWL--
-EISWLG-
ELISWL--
-EISWLT-
EVISWL--
-EISWLA-
EHISWL--
EEISWL--
EQISWL--
ENISWL--
-EISWLQ-
-EISWLM-
-EISWLK-
-EISWLH-
EDISWL--
EAISWL--
FTISWL--
FNISWL--
-FISWLH-
-FISWLN-
FQISWL--
-FISWLT-
FDISWL--
-FISWLQ-
FAISWL--
FWISWL--
FMISWL--
GVISWL--
GSISWL--
GGISWL--
GTISWL--
-GISWLA-
GNISWL--
-GISWLV-
-GISWLN-
GHISWL--
-GISWLT-
-GISWLQ-
GAISWL--
-GISWLK-
-GISWLH-
-GISWLG-
GDISWL--
GWISWL--
-GISWLE-
-GISWLD-
GYISWL--
-GISWLM-
GCISWL--

-HISWLS-
HAISWL--
HTISWL--
-HISWLR-
-HISWLL-
HSISWL--
HKISWL--
HVISWL--
HQISWL--
HNISWL--
HHISWL--
-HISWLQ-
HMISWL--
-HISWLN-
-HISWLM-
-HISWLH-
HEISWL--
-HISWLE-
-HISWLA-
HYISWL--
HWISWL--
--ISWLKT
--ISWLTK
--ISWLLG
--ISWLTR
--ISWLSS
--ISWLVR
--ISWLTS
--ISWLSH
--ISWLHG
--ISWLKN
--ISWLKA
--ISWLRG
--ISWLKS
--ISWLKQ
--ISWLGI
--ISWLVN
--ISWLTN
--ISWLNA
--ISWLMS
--ISWLLH
--ISWLAR
--ISWLVS
--ISWLAQ
-IISWLG-
--ISWLRE
--ISWLKK
--ISWLES
--ISWLDS
--ISWLTA

--ISWLRH
--ISWLQG
--ISWLSG
--ISWLRD
--ISWLKF
-IISWLS-
--ISWLVT
--ISWLVK
--ISWLTG
--ISWLKM
--ISWLIG
--ISWLGT
--ISWLAA
IEISWL--
--ISWLSQ
--ISWLRQ
--ISWLNT
--ISWLHS
--ISWLTH
--ISWLQT
--ISWLQA
INISWL--
IGISWL--
--ISWLVQ
--ISWLSE
--ISWLRN
--ISWLQE
--ISWLKD
--ISWLHA
--ISWLFQ
--ISWLTV
--ISWLQV
--ISWLQN
--ISWLNS
--ISWLNG
--ISWLMH
--ISWLKY
--ISWLKI
--ISWLKE
--ISWLHV
--ISWLHK
--ISWLHF
--ISWLGN
--ISWLAH
-IISWLH-
-IISWLD-
--ISWLTD
--ISWLMF
--ISWLIH
--ISWLHY

--ISWLHR
--ISWLGM
--ISWLGA
--ISWLEG
--ISWLAM
-IISWLR-
-IISWLK-
IHISWL--
IAISWL--
--ISWLWG
--ISWLVD
--ISWLTW
--ISWLRW
--ISWLRF
--ISWLQM
--ISWLMT
--ISWLME
--ISWLKV
--ISWLKH
--ISWLEA
--ISWLAN
-IISWLQ-
--ISWLTQ
--ISWLNR
--ISWLMD
--ISWLKL
--ISWLHM
--ISWLGY
--ISWLGW
--ISWLGV
--ISWLGR
--ISWLGQ
--ISWLGK
--ISWLDT
--ISWLAD
-IISWLV-
--ISWLYI
--ISWLWN
--ISWLTY
--ISWLTM
--ISWLTI
--ISWLQK
--ISWLQH
--ISWLQD
--ISWLPL
--ISWLNW
--ISWLMW
--ISWLLD
--ISWLKW
--ISWLIQ

--ISWLIN
--ISWLIM
--ISWLID
--ISWLHQ
--ISWLHN
--ISWLFY
--ISWLEV
--ISWLEQ
IQISWL--
-IISWLW-
--ISWLYD
--ISWLYC
--ISWLSW
--ISWLRC
--ISWLPK
--ISWLNQ
--ISWLNH
--ISWLMQ
--ISWLMC
--ISWLHW
--ISWLHL
--ISWLHE
--ISWLGD
--ISWLFV
--ISWLEH
--ISWLAC
-IISWLM-
-IISWLI-
-KISWLQ-
KMISWL--
-KISWLT-
-KISWLK-
-KISWLH-
KGISWL--
KNISWL--
-KISWLG-
-KISWLN-
KFISWL--
-KISWLE-
KTISWL--
KDISWL--
KHISWL--
KYISWL--
KWISWL--
KQISWL--
-KISWLM-
KEISWL--
-KISWLD-
LNISWL--
-LISWLR-

LGISWL--
LDISWL--
LEISWL--
-LISWLD-
LMISWL--
-LISWLE-
-LISWLW-
-LISWLM-
LCISWL--
MSISWL--
MGISWL--
MKISWL--
MEISWL--
-MISWLS-
MTISWL--
-MISWLQ-
MQISWL--
MNISWL--
-MISWLN-
MDISWL--
-MISWLD-
MIISWL--
MHISWL--
MAISWL--
-MISWLG-
-NISWLS-
NSISWL--
-NISWLR-
NGISWL--
NDISWL--
NMISWL--
-NISWLN-
NTISWL--
-NISWLL-
-NISWLQ-
-NISWLA-
NFISWL--
NNISWL--
-NISWLV-
NYISWL--
-NISWLK-
-NISWLD-
NIISWL--
-NISWLG-
NHISWL--
NAISWL--
-NISWLI-
NVISWL--
NQISWL--
NEISWL--

-NISWLM-
-NISWLE-
NCISWL--
PGISWL--
PSISWL--
PVISWL--
PTISWL--
PHISWL--
PNISWL--
PQISWL--
PEISWL--
PIISWL--
PAISWL--
PMISWL--
PDISWL--
-QISWLS-
-QISWLR-
QSISWL--
-QISWLL-
-QISWLT-
-QISWLG-
QAISWL--
QMISWL--
-QISWLA-
-QISWLI-
QHISWL--
-QISWLK-
QGISWL--
QEISWL--
QKISWL--
QDISWL--
QYISWL--
QQISWL--
-QISWLN-
-QISWLH-
-QISWLD-
-QISWLW-
-QISWLV-
-QISWLE-
QIISWL--
-RISWLG-
-RISWLR-
RAISWL--
RFISWL--
-RISWLH-
-RISWLQ-
-RISWLK-
RNISWL--
-RISWLE-
-RISWLI-

RDISWL--
RHISWL--
-RISWLC-
-SISWLR-
-SISWLL-
SAISWL--
SHISWL--
-SISWLK-
-SISWLS-
SMISWL--
-SISWLH-
SGISWL--
-SISWLN-
-SISWLG-
SQISWL--
-SISWLI-
-SISWLT-
SEISWL--
SNISWL--
-SISWLV-
SDISWL--
-SISWLM-
-SISWLE-
-SISWLD-
SCISWL--
TGISWL--
TTISWL--
-TISWLT-
TAISWL--
TSISWL--
-TISWLG-
TQISWL--
TKISWL--
-TISWLA-
TMISWL--
-TISWLH-
TIISWL--
-TISWLQ-
-TISWLV-
-TISWLE-
TEISWL--
-TISWLM-
-TISWLL-
-TISWLK-
-TISWLD-
VSISWL--
VQISWL--
-VISWLG-
-VISWLR-
VIISWL--

-VISWLK-
-VISWLD-
-VISWLN-
VGISWL--
VTISWL--
VDISWL--
VEISWL--
-VISWLH-
-VISWLE-
-VISWLM-
VCISWL--
WSISWL--
WEISWL--
WRISWL--
WKISWL--
WQISWL--
-WISWLN-
-WISWLA-
WDISWL--
-WISWLS-
WGISWL--
WLISWL--
-WISWLV-
-WISWLQ-
-WISWLK-
WYISWL--
-WISWLW-
-WISWLT-
-WISWLR-
-WISWLI-
-WISWLH-
-WISWLE-
WIISWL--
WHISWL--
YSISWL--
YTISWL--
YQISWL--
YHISWL--
YAISWL--
YMISWL--
-YISWLH-
-YISWLS-
-YISWLK-
-YISWLT-
-YISWLR-
YYISWL--
-YISWLD-
YDISWL--
-YISWLY-
-YISWLM-

-YISWLE-
-YISWLA-
YIISWL--
-YISWLW-
-YISWLQ-
YEISWL--

| | | | |
|---|---|---|---|
| IGSL | 307 | 666 | 30.95 |

DMIGSL--
-GIGSLA-
--IGSLVL
--IGSLWI
--IGSLTL
--IGSLSI
--IGSLGL
--IGSLTQ
--IGSLNQ
--IGSLMI
--IGSLLP
--IGSLDG
--IGSLAM
--IGSLAF
-LIGSLD-
-SIGSLE-
-VIGSLE-
-VIGSLD-
-YIGSLD-
ALIGSL--
-AIGSLS-
ASIGSL--
-AIGSLT-
-AIGSLR-
-AIGSLL-
AMIGSL--
-AIGSLH-
AAIGSL--
AVIGSL--
AGIGSL--
ATIGSL--
-AIGSLV-
ANIGSL--
AYIGSL--
APIGSL--
AEIGSL--
AIIGSL--
-AIGSLQ-
-AIGSLY-
-AIGSLN-
-AIGSLM-
-AIGSLA-
AHIGSL--

AQIGSL--
-AIGSLI-
ACIGSL--
-CIGSLR-
CYIGSL--
CHIGSL--
CCIGSL--
CSIGSL--
CRIGSL--
-CIGSLK-
CLIGSL--
CNIGSL--
CVIGSL--
-CIGSLL-
CGIGSL--
CPIGSL--
-CIGSLV-
CTIGSL--
-CIGSLY-
-CIGSLW-
CQIGSL--
CMIGSL--
CIIGSL--
-CIGSLS-
-CIGSLM-
-CIGSLI-
-CIGSLF-
CAIGSL--
CWIGSL--
-CIGSLN-
CDIGSL--
DSIGSL--
-DIGSLR-
DGIGSL--
DLIGSL--
DKIGSL--
-DIGSLK-
-DIGSLY-
DYIGSL--
DVIGSL--
DNIGSL--
-DIGSLV-
DTIGSL--
DIIGSL--
-DIGSLL-
-DIGSLT-
DEIGSL--
DFIGSL--
-DIGSLH-
DDIGSL--

DAIGSL--
DWIGSL--
DQIGSL--
-DIGSLW-
-DIGSLS-
-DIGSLF-
DCIGSL--
DHIGSL--
DPIGSL--
-DIGSLA-
-EIGSLR-
ERIGSL--
ELIGSL--
EIIGSL--
-EIGSLV-
EVIGSL--
EPIGSL--
EMIGSL--
EKIGSL--
ESIGSL--
-EIGSLY-
-EIGSLK-
-EIGSLI-
EFIGSL--
EEIGSL--
EWIGSL--
ETIGSL--
-EIGSLQ-
EHIGSL--
EGIGSL--
EDIGSL--
EQIGSL--
ENIGSL--
-EIGSLS-
-EIGSLF-
-EIGSLN-
-EIGSLM-
-EIGSLL-
FLIGSL--
-FIGSLT-
-FIGSLK-
-FIGSLS-
-FIGSLI-
FAIGSL--
-FIGSLA-
-FIGSLQ-
FYIGSL--
-FIGSLH-
FDIGSL--
FWIGSL--

-FIGSLY-
-FIGSLN-
FPIGSL--
-FIGSLM-
-FIGSLE-
-GIGSLR-
GKIGSL--
-GIGSLT-
-GIGSLL-
GVIGSL--
GAIGSL--
GFIGSL--
GPIGSL--
-GIGSLK-
GQIGSL--
GNIGSL--
-GIGSLI-
GHIGSL--
-GIGSLQ-
-GIGSLF-
-GIGSLW-
-GIGSLN-
GEIGSL--
GDIGSL--
GMIGSL--
GCIGSL--
-GIGSLC-
-HIGSLS-
-HIGSLL-
HTIGSL--
-HIGSLY-
HVIGSL--
-HIGSLK-
-HIGSLW-
HAIGSL--
HLIGSL--
-HIGSLV-
-HIGSLR-
HGIGSL--
-HIGSLT-
HPIGSL--
-HIGSLF-
HNIGSL--
HIIGSL--
HYIGSL--
-HIGSLQ-
-HIGSLM-
-HIGSLI-
-HIGSLN-
-HIGSLH-

HEIGSL--
HCIGSL--
HMIGSL--
-HIGSLG-
-HIGSLE-
HDIGSL--
--IGSLKS
--IGSLRT
--IGSLSS
-IIGSLR-
--IGSLRG
ISIGSL--
--IGSLSA
IRIGSL--
--IGSLTG
--IGSLRW
--IGSLRH
--IGSLRY
--IGSLHR
--IGSLYL
--IGSLYS
--IGSLSG
--IGSLVA
--IGSLRI
INIGSL--
--IGSLWT
--IGSLRA
--IGSLVV
--IGSLIS
--IGSLHS
--IGSLAS
--IGSLVR
--IGSLWG
--IGSLRV
--IGSLRD
--IGSLMT
-IIGSLS-
--IGSLMS
--IGSLVG
--IGSLKT
--IGSLIG
IVIGSL--
--IGSLVT
--IGSLSV
--IGSLRQ
--IGSLVM
--IGSLHK
-IIGSLQ-
--IGSLKW
--IGSLKG

--IGSLYG
--IGSLMM
--IGSLIN
-IIGSLT-
--IGSLVH
--IGSLVF
--IGSLST
--IGSLKA
IKIGSL--
--IGSLWN
--IGSLTW
--IGSLMV
--IGSLIR
--IGSLHA
IDIGSL--
--IGSLVN
--IGSLQG
--IGSLKH
--IGSLIY
--IGSLFG
--IGSLAA
--IGSLWK
--IGSLVQ
--IGSLVK
--IGSLMN
--IGSLHV
--IGSLHF
--IGSLWS
--IGSLNT
--IGSLMK
--IGSLIV
--IGSLHG
--IGSLES
--IGSLYT
--IGSLYN
--IGSLWR
--IGSLQS
IQIGSL--
--IGSLYQ
--IGSLYK
--IGSLLN
--IGSLHY
--IGSLHN
--IGSLAG
--IGSLTV
--IGSLQA
--IGSLMR
--IGSLIT
--IGSLIA
--IGSLFR

```
-IIGSLA-
IHIGSL--
--IGSLYW
--IGSLYD
--IGSLWA
--IGSLRE
--IGSLLH
--IGSLKN
--IGSLHH
IAIGSL--
--IGSLMH
--IGSLLV
--IGSLHW
--IGSLHM
--IGSLFK
IIIGSL--
--IGSLWW
--IGSLQT
--IGSLLQ
--IGSLKV
--IGSLKI
--IGSLII
--IGSLIE
--IGSLHQ
--IGSLHD
--IGSLGS
--IGSLFD
--IGSLEA
--IGSLTN
--IGSLPL
--IGSLMY
--IGSLML
IEIGSL--
-IIGSLW-
-IIGSLF-
--IGSLYM
--IGSLYE
--IGSLWQ
--IGSLWH
--IGSLMQ
--IGSLKM
--IGSLHL
--IGSLHE
--IGSLFA
--IGSLAT
IYIGSL--
--IGSLYI
--IGSLTF
--IGSLQV
--IGSLKR
```

```
--IGSLKK
--IGSLIW
--IGSLIQ
--IGSLCS
--IGSLAW
--IGSLTH
--IGSLQW
--IGSLNN
--IGSLLM
--IGSLIH
--IGSLHI
--IGSLFM
-IIGSLH-
--IGSLVE
--IGSLVD
--IGSLTY
--IGSLSQ
--IGSLSM
--IGSLSH
--IGSLNG
--IGSLME
--IGSLKD
--IGSLAV
IWIGSL--
-IIGSLE-
--IGSLTK
--IGSLTI
--IGSLNK
--IGSLND
--IGSLLD
--IGSLIM
--IGSLID
--IGSLFQ
--IGSLEP
--IGSLEG
ICIGSL--
--IGSLWD
--IGSLQQ
--IGSLQM
--IGSLQH
--IGSLNW
--IGSLCL
KYIGSL--
KVIGSL--
KTIGSL--
KAIGSL--
-KIGSLR-
-KIGSLK-
-KIGSLI-
KNIGSL--
```

-KIGSLY-
KGIGSL--
-KIGSLT-
-KIGSLW-
-KIGSLV-
KEIGSL--
KQIGSL--
-KIGSLS-
-KIGSLF-
KHIGSL--
-KIGSLQ-
-KIGSLM-
KCIGSL--
-KIGSLN-
-LIGSLR-
-LIGSLS-
LTIGSL--
LLIGSL--
LNIGSL--
LAIGSL--
LKIGSL--
-LIGSLN-
LWIGSL--
-LIGSLA-
LDIGSL--
-LIGSLH-
LQIGSL--
LHIGSL--
-LIGSLM-
-LIGSLE-
-LIGSLG-
-LIGSLF-
LEIGSL--
LPIGSL--
LCIGSL--
-MIGSLT-
MGIGSL--
MLIGSL--
MAIGSL--
MPIGSL--
-MIGSLS-
-MIGSLK-
-MIGSLN-
MYIGSL--
-MIGSLA-
-MIGSLW-
-MIGSLV-
-MIGSLI-
-MIGSLF-
-MIGSLH-

-MIGSLQ-
MDIGSL--
MMIGSL--
-MIGSLG-
MHIGSL--
-MIGSLE-
MQIGSL--
-MIGSLM-
MCIGSL--
NRIGSL--
-NIGSLV-
-NIGSLT-
NMIGSL--
-NIGSLK-
NLIGSL--
NSIGSL--
NVIGSL--
NYIGSL--
NTIGSL--
NWIGSL--
-NIGSLI-
NPIGSL--
NAIGSL--
-NIGSLY-
NGIGSL--
-NIGSLQ-
-NIGSLH-
NFIGSL--
NQIGSL--
NNIGSL--
NIIGSL--
-NIGSLM-
NHIGSL--
NDIGSL--
-NIGSLW-
NEIGSL--
-NIGSLA-
-NIGSLE-
-PIGSLS-
PRIGSL--
PLIGSL--
PFIGSL--
PNIGSL--
-PIGSLR-
-PIGSLK-
-PIGSLL-
-PIGSLQ-
PAIGSL--
PDIGSL--
PIIGSL--

-PIGSLW-
PHIGSL--
-PIGSLH-
PMIGSL--
PTIGSL--
-PIGSLN-
PQIGSL--
-PIGSLV-
-PIGSLM-
PEIGSL--
-PIGSLI-
PCIGSL--
-PIGSLE-
QRIGSL--
QAIGSL--
QVIGSL--
QQIGSL--
QHIGSL--
-QIGSLL-
-QIGSLR-
-QIGSLW-
-QIGSLN-
QPIGSL--
-QIGSLS-
-QIGSLA-
QLIGSL--
QGIGSL--
QSIGSL--
QMIGSL--
QWIGSL--
-QIGSLY-
-QIGSLT-
-QIGSLQ-
-QIGSLI-
-QIGSLH-
QFIGSL--
QEIGSL--
QKIGSL--
QDIGSL--
-QIGSLV-
-QIGSLF-
-QIGSLK-
QCIGSL--
-QIGSLD-
-RIGSLT-
-RIGSLK-
-RIGSLS-
-RIGSLV-
-RIGSLF-
-RIGSLI-

```
-RIGSLM-
RDIGSL--
RNIGSL--
-RIGSLW-
RGIGSL--
-RIGSLQ-
REIGSL--
-RIGSLN-
-RIGSLA-
-RIGSLC-
RCIGSL--
-SIGSLR-
SLIGSL--
SGIGSL--
STIGSL--
-SIGSLT-
-SIGSLK-
-SIGSLS-
SWIGSL--
SIIGSL--
SHIGSL--
SAIGSL--
SFIGSL--
-SIGSLM-
SQIGSL--
SEIGSL--
SCIGSL--
-SIGSLQ-
TLIGSL--
TSIGSL--
-TIGSLY-
-TIGSLR-
-TIGSLT-
-TIGSLH-
TVIGSL--
-TIGSLV-
TTIGSL--
-TIGSLK-
THIGSL--
TNIGSL--
TMIGSL--
-TIGSLL-
TEIGSL--
-TIGSLF-
TFIGSL--
TDIGSL--
TAIGSL--
-TIGSLA-
-TIGSLW-
-TIGSLN-
```

TCIGSL--
-TIGSLM-
-VIGSLR-
VGIGSL--
VKIGSL--
-VIGSLS-
-VIGSLK-
-VIGSLT-
VWIGSL--
VAIGSL--
VQIGSL--
-VIGSLQ-
VHIGSL--
VFIGSL--
VCIGSL--
-VIGSLM-
VIIGSL--
-VIGSLW-
-VIGSLN-
-VIGSLF-
VEIGSL--
VDIGSL--
-WIGSLS-
-WIGSLL-
-WIGSLV-
WIIGSL--
WDIGSL--
WNIGSL--
WLIGSL--
-WIGSLQ-
-WIGSLM-
WWIGSL--
-WIGSLH-
-WIGSLI-
WFIGSL--
WPIGSL--
-WIGSLF-
WEIGSL--
WYIGSL--
WQIGSL--
WCIGSL--
-WIGSLG-
YSIGSL--
YRIGSL--
-YIGSLR-
YGIGSL--
-YIGSLT-
-YIGSLS-
YKIGSL--
YHIGSL--

YVIGSL--
-YIGSLY-
YPIGSL--
YNIGSL--
-YIGSLN-
YTIGSL--
-YIGSLK-
YQIGSL--
-YIGSLH-
-YIGSLV-
YEIGSL--
-YIGSLI-
-YIGSLQ-
-YIGSLE-
-YIGSLA-
YMIGSL--
YAIGSL--
YFIGSL--
-YIGSLM-
-YIGSLW-
YYIGSL--
YCIGSL--
YDIGSL--
PSPF 148 740 30.93
-APSPFL-
ALPSPF--
ARPSPF--
AWPSPF--
ATPSPF--
-APSPFF-
AFPSPF--
AVPSPF--
-APSPFT-
AAPSPF--
AYPSPF--
AEPSPF--
-APSPFW-
AMPSPF--
-APSPFK-
AIPSPF--
AGPSPF--
-APSPFS-
-APSPFM-
-APSPFV-
-APSPFA-
ADPSPF--
ASPSPF--
AQPSPF--
-APSPFI-
AKPSPF--

–APSPFQ–
–APSPFN–
–APSPFG–
–APSPFE–
–APSPFD–
APPSPF––
AHPSPF––
CWPSPF––
–CPSPFM–
CSPSPF––
–CPSPFR–
CQPSPF––
–CPSPFK–
CTPSPF––
–CPSPFY–
CMPSPF––
CLPSPF––
CIPSPF––
CEPSPF––
–DPSPFL–
DFPSPF––
–DPSPFI–
DKPSPF––
DVPSPF––
–DPSPFS–
–DPSPFM–
–DPSPFQ–
DLPSPF––
DGPSPF––
DTPSPF––
DRPSPF––
DQPSPF––
–DPSPFV–
–DPSPFT–
–DPSPFF–
DMPSPF––
DSPSPF––
–DPSPFR–
DNPSPF––
DIPSPF––
DCPSPF––
DAPSPF––
–EPSPFS–
–EPSPFR–
EWPSPF––
ERPSPF––
EVPSPF––
–EPSPFT–
EKPSPF––
–EPSPFV–

−EPSPFL−
−EPSPFF−
ELPSPF−−
EYPSPF−−
−EPSPFY−
−EPSPFW−
−EPSPFK−
EFPSPF−−
ETPSPF−−
−EPSPFM−
−EPSPFI−
ENPSPF−−
EMPSPF−−
EIPSPF−−
EEPSPF−−
−FPSPFR−
FYPSPF−−
FLPSPF−−
FRPSPF−−
−FPSPFS−
FFPSPF−−
FSPSPF−−
−FPSPFI−
FNPSPF−−
FMPSPF−−
FHPSPF−−
FEPSPF−−
FAPSPF−−
FQPSPF−−
−FPSPFT−
−FPSPFK−
FTPSPF−−
−FPSPFA−
FGPSPF−−
FVPSPF−−
−FPSPFV−
−FPSPFL−
−FPSPFQ−
−FPSPFN−
−FPSPFH−
FPPSPF−−
FKPSPF−−
FDPSPF−−
GKPSPF−−
GIPSPF−−
−GPSPFL−
GLPSPF−−
GVPSPF−−
−GPSPFF−
GYPSPF−−

-GPSPFM-
-GPSPFH-
GGPSPF--
GQPSPF--
-GPSPFV-
-GPSPFA-
GDPSPF--
GSPSPF--
-GPSPFY-
-GPSPFK-
GNPSPF--
GTPSPF--
-GPSPFT-
-GPSPFN-
-GPSPFI-
GMPSPF--
GEPSPF--
GCPSPF--
GWPSPF--
-GPSPFW-
-GPSPFP-
GPPSPF--
GFPSPF--
GAPSPF--
HRPSPF--
HKPSPF--
-HPSPFT-
HWPSPF--
HVPSPF--
-HPSPFV-
-HPSPFL-
-HPSPFK-
-HPSPFY-
-HPSPFH-
HTPSPF--
HQPSPF--
-HPSPFS-
HLPSPF--
HIPSPF--
HAPSPF--
-HPSPFI-
-HPSPFF-
HMPSPF--
HYPSPF--
-HPSPFQ-
-HPSPFM-
HHPSPF--
HGPSPF--
HFPSPF--
HCPSPF--

-IPSPFA-
ISPSPF--
-IPSPFS-
-IPSPFR-
INPSPF--
ILPSPF--
ITPSPF--
-IPSPFK-
IFPSPF--
IAPSPF--
-IPSPFN-
IMPSPF--
IIPSPF--
IGPSPF--
-IPSPFV-
-IPSPFT-
-IPSPFI-
-IPSPFQ-
-IPSPFM-
-IPSPFL-
IPPSPF--
IEPSPF--
IYPSPF--
IVPSPF--
IQPSPF--
-IPSPFY-
-IPSPFW-
-IPSPFH-
-IPSPFF-
IKPSPF--
KSPSPF--
-KPSPFK-
-KPSPFL-
KVPSPF--
KTPSPF--
-KPSPFR-
KGPSPF--
-KPSPFS-
-KPSPFI-
-KPSPFF-
KLPSPF--
KAPSPF--
-KPSPFW-
-KPSPFT-
-KPSPFN-
KPPSPF--
-KPSPFA-
KNPSPF--
KWPSPF--
-KPSPFV-

KHPSPF--
KDPSPF--
-KPSPFY-
-KPSPFQ-
-KPSPFM-
-KPSPFH-
KMPSPF--
LRPSPF--
-LPSPFR-
LAPSPF--
-LPSPFS-
-LPSPFY-
-LPSPFK-
LQPSPF--
LHPSPF--
-LPSPFT-
LLPSPF--
LCPSPF--
LMPSPF--
LIPSPF--
LYPSPF--
LWPSPF--
-LPSPFL-
-LPSPFM-
LFPSPF--
LTPSPF--
-LPSPFQ-
LKPSPF--
LGPSPF--
-LPSPFI-
LNPSPF--
-LPSPFF-
-LPSPFW-
LPPSPF--
LEPSPF--
-LPSPFV-
-LPSPFH-
MLPSPF--
MNPSPF--
MMPSPF--
-MPSPFS-
MIPSPF--
-MPSPFT-
-MPSPFM-
-MPSPFA-
MYPSPF--
MQPSPF--
-MPSPFW-
-MPSPFH-
MFPSPF--

MVPSPF--
MTPSPF--
MSPSPF--
MRPSPF--
-MPSPFY-
-MPSPFV-
-MPSPFR-
-MPSPFN-
-MPSPFI-
MKPSPF--
MWPSPF--
-MPSPFQ-
-MPSPFF-
-MPSPFC-
MHPSPF--
MGPSPF--
MEPSPF--
MDPSPF--
NRPSPF--
NTPSPF--
NKPSPF--
-NPSPFR-
NNPSPF--
-NPSPFT-
-NPSPFK-
-NPSPFS-
-NPSPFL-
-NPSPFI-
NYPSPF--
NHPSPF--
NQPSPF--
-NPSPFY-
-NPSPFQ-
-NPSPFH-
NMPSPF--
NLPSPF--
-NPSPFF-
NGPSPF--
NFPSPF--
NSPSPF--
-NPSPFM-
NPPSPF--
NIPSPF--
-NPSPFV-
-NPSPFA-
NDPSPF--
NAPSPF--
--PSPFDL
--PSPFYS
--PSPFRS

--PSPFSL
--PSPFSS
--PSPFRV
--PSPFRT
--PSPFKV
--PSPFRH
--PSPFVS
--PSPFSI
--PSPFAF
PQPSPF--
--PSPFVT
--PSPFTS
--PSPFRL
--PSPFTR
--PSPFKA
--PSPFEG
--PSPFAL
--PSPFWL
--PSPFSV
--PSPFST
--PSPFSM
--PSPFPL
--PSPFKL
--PSPFWS
--PSPFFR
--PSPFFM
--PSPFVN
--PSPFTL
--PSPFSH
--PSPFKR
--PSPFIY
--PSPFFV
--PSPFFL
--PSPFYG
--PSPFWH
--PSPFVY
--PSPFRG
--PSPFNS
--PSPFNF
--PSPFMH
--PSPFLY
--PSPFLV
--PSPFLT
--PSPFIH
--PSPFFG
PIPSPF--
--PSPFTG
--PSPFTA
--PSPFRI
--PSPFQA

--PSPFLF
--PSPFIA
--PSPFSK
--PSPFRW
--PSPFRE
--PSPFQF
--PSPFNY
--PSPFMN
--PSPFML
--PSPFMF
--PSPFMA
--PSPFLQ
--PSPFLN
--PSPFKN
--PSPFIK
--PSPFIG
--PSPFHW
--PSPFHQ
--PSPFGQ
PWPSPF--
--PSPFVW
--PSPFVR
--PSPFVG
--PSPFTV
--PSPFTT
--PSPFTK
--PSPFSY
--PSPFSQ
--PSPFRY
--PSPFRQ
--PSPFPY
--PSPFMY
--PSPFKW
--PSPFKT
--PSPFIT
--PSPFIN
--PSPFFH
--PSPFAT
--PSPFAQ
PRPSPF--
--PSPFYW
--PSPFYM
--PSPFYH
--PSPFVA
--PSPFTY
--PSPFSR
--PSPFSG
--PSPFRN
--PSPFRD
--PSPFQT

--PSPFPM
--PSPFIW
--PSPFIS
--PSPFIR
--PSPFHS
--PSPFHI
--PSPFHA
--PSPFAW
PKPSPF--
PGPSPF--
PAPSPF--
PYPSPF--
--PSPFYT
--PSPFYD
--PSPFWY
--PSPFTF
--PSPFSF
--PSPFSA
--PSPFRF
--PSPFRA
--PSPFQW
--PSPFPI
--PSPFNG
--PSPFMT
--PSPFMK
--PSPFLI
--PSPFLH
--PSPFKH
--PSPFHT
--PSPFGW
--PSPFGG
--PSPFGF
--PSPFFK
--PSPFAI
PNPSPF--
PEPSPF--
PTPSPF--
--PSPFYV
--PSPFYI
--PSPFVI
--PSPFVH
--PSPFVF
--PSPFTQ
--PSPFSN
--PSPFQS
--PSPFPF
--PSPFNL
--PSPFME
--PSPFKM
--PSPFKG

--PSPFKE
--PSPFIQ
--PSPFHM
--PSPFGI
--PSPFFY
--PSPFEW
--PSPFAS
--PSPFAM
--PSPFAH
--PSPFAA
--PSPFYY
--PSPFYQ
--PSPFYK
--PSPFYA
--PSPFWV
--PSPFWQ
--PSPFWN
--PSPFWG
--PSPFWF
--PSPFTW
--PSPFTP
--PSPFTN
--PSPFTM
--PSPFTI
--PSPFTD
--PSPFSP
--PSPFSD
--PSPFQY
--PSPFQV
--PSPFQL
--PSPFQG
--PSPFPW
--PSPFNT
--PSPFNM
--PSPFNI
--PSPFNA
--PSPFMW
--PSPFMV
--PSPFMM
--PSPFMD
--PSPFKS
--PSPFKQ
--PSPFKI
--PSPFIF
--PSPFHN
--PSPFHL
--PSPFHK
--PSPFGV
--PSPFGT
--PSPFGH

--PSPFFT
--PSPFFQ
--PSPFFN
--PSPFFF
--PSPFFD
--PSPFES
--PSPFEL
--PSPFDY
--PSPFAV
PHPSPF--
PDPSPF--
QTPSPF--
QRPSPF--
-QPSPFR-
-QPSPFF-
-QPSPFV-
QGPSPF--
-QPSPFH-
QVPSPF--
-QPSPFA-
QPPSPF--
QKPSPF--
-QPSPFY-
-QPSPFT-
-QPSPFS-
-QPSPFL-
QQPSPF--
QNPSPF--
QLPSPF--
QEPSPF--
QYPSPF--
-QPSPFN-
QHPSPF--
QSPSPF--
-QPSPFM-
-QPSPFK-
QMPSPF--
QFPSPF--
QDPSPF--
QAPSPF--
-RPSPFR-
-RPSPFL-
RLPSPF--
-RPSPFV-
RVPSPF--
-RPSPFH-
RSPSPF--
RNPSPF--
RQPSPF--
-RPSPFW-

RFPSPF--
-RPSPFT-
REPSPF--
RHPSPF--
RMPSPF--
RWPSPF--
-RPSPFM-
-RPSPFS-
RKPSPF--
-RPSPFF-
RGPSPF--
-RPSPFQ-
-RPSPFN-
-RPSPFK-
RIPSPF--
RAPSPF--
RTPSPF--
-RPSPFG-
-RPSPFA-
RCPSPF--
RPPSPF--
RDPSPF--
-SPSPFR-
-SPSPFS-
SNPSPF--
SKPSPF--
-SPSPFL-
SRPSPF--
SVPSPF--
-SPSPFY-
-SPSPFV-
-SPSPFF-
SLPSPF--
-SPSPFQ-
-SPSPFA-
SAPSPF--
SSPSPF--
-SPSPFK-
-SPSPFT-
-SPSPFI-
SYPSPF--
SEPSPF--
SQPSPF--
SIPSPF--
STPSPF--
-SPSPFH-
SMPSPF--
SGPSPF--
SDPSPF--
-SPSPFM-

SCPSPF--
-SPSPFW-
-SPSPFN-
SFPSPF--
SPPSPF--
-TPSPFR-
-TPSPFK-
TQPSPF--
-TPSPFL-
-TPSPFA-
TLPSPF--
TIPSPF--
-TPSPFV-
TFPSPF--
-TPSPFY-
-TPSPFT-
-TPSPFS-
TKPSPF--
TVPSPF--
-TPSPFN-
-TPSPFI-
-TPSPFF-
-TPSPFQ-
-TPSPFM-
TPPSPF--
TNPSPF--
TMPSPF--
TEPSPF--
TDPSPF--
-TPSPFW-
-TPSPFC-
TAPSPF--
TSPSPF--
-TPSPFH-
VLPSPF--
-VPSPFK-
-VPSPFH-
-VPSPFS-
VVPSPF--
VSPSPF--
-VPSPFM-
-VPSPFV-
-VPSPFT-
-VPSPFW-
VNPSPF--
VFPSPF--
VAPSPF--
VYPSPF--
VTPSPF--
-VPSPFI-

VIPSPF--
VWPSPF--
-VPSPFY-
-VPSPFQ-
-VPSPFF-
VKPSPF--
VEPSPF--
-VPSPFA-
VPPSPF--
VMPSPF--
VGPSPF--
VQPSPF--
WKPSPF--
WAPSPF--
WRPSPF--
-WPSPFM-
WLPSPF--
WSPSPF--
-WPSPFI-
-WPSPFT-
WMPSPF--
WTPSPF--
-WPSPFR-
-WPSPFK-
WPPSPF--
WFPSPF--
WVPSPF--
WQPSPF--
-WPSPFV-
WEPSPF--
-WPSPFQ-
-WPSPFF-
WIPSPF--
YSPSPF--
YTPSPF--
YGPSPF--
YLPSPF--
YRPSPF--
YHPSPF--
YVPSPF--
-YPSPFA-
-YPSPFQ-
-YPSPFM-
YNPSPF--
YQPSPF--
-YPSPFV-
YKPSPF--
-YPSPFY-
-YPSPFS-
-YPSPFR-

```
            -YPSPFF-
            YPPSPF--
            -YPSPFT-
            -YPSPFL-
            -YPSPFH-
            YYPSPF--
            -YPSPFK-
            YMPSPF--
            -YPSPFN-
            -YPSPFI-
            YIPSPF--
            YEPSPF--
            YDPSPF--
            YCPSPF--
VAWM         843        530     30.92
            -AVAWML-
            AHVAWM--
            -AVAWMI-
            -AVAWMV-
            ADVAWM--
            -AVAWMR-
            -AVAWMH-
            -AVAWMY-
            -AVAWMS-
            ATVAWM--
            -AVAWME-
            AGVAWM--
            -AVAWMG-
            -AVAWMA-
            -AVAWMT-
            -AVAWMQ-
            -AVAWMN-
            -AVAWMK-
            ASVAWM--
            AQVAWM--
            ANVAWM--
            AAVAWM--
            AVVAWM--
            -AVAWMW-
            ALVAWM--
            AKVAWM--
            CVVAWM--
            CSVAWM--
            CMVAWM--
            -CVAWML-
            -CVAWMD-
            CIVAWM--
            CFVAWM--
            CDVAWM--
            CAVAWM--
```

-DVAWMR-
-DVAWMG-
DTVAWM--
-DVAWMM-
-DVAWMD-
-DVAWMA-
DRVAWM--
DKVAWM--
DDVAWM--
DVVAWM--
DWVAWM--
-DVAWMV-
-DVAWMS-
-DVAWMQ-
DSVAWM--
DQVAWM--
DHVAWM--
DGVAWM--
DEVAWM--
DCVAWM--
-EVAWMR-
-EVAWML-
ELVAWM--
-EVAWMT-
-EVAWMQ-
EQVAWM--
-EVAWMI-
-EVAWMG-
EYVAWM--
-EVAWMK-
-EVAWMV-
-EVAWMS-
-EVAWMN-
-EVAWME-
-EVAWMD-
ERVAWM--
EMVAWM--
EDVAWM--
EAVAWM--
-FVAWMD-
-FVAWMA-
-FVAWMQ-
-FVAWMF-
FGVAWM--
-FVAWMS-
-FVAWMN-
FTVAWM--
FKVAWM--
FFVAWM--
FDVAWM--

−GVAWMS−
GYVAWM−−
GSVAWM−−
−GVAWMA−
−GVAWMQ−
GDVAWM−−
GWVAWM−−
−GVAWMV−
−GVAWML−
GLVAWM−−
−GVAWMF−
−GVAWME−
GGVAWM−−
GEVAWM−−
−GVAWMH−
−GVAWMG−
GIVAWM−−
GHVAWM−−
GAVAWM−−
GTVAWM−−
GKVAWM−−
−GVAWMY−
−GVAWMW−
−GVAWMT−
−GVAWMD−
GQVAWM−−
GCVAWM−−
−HVAWML−
−HVAWMR−
HGVAWM−−
−HVAWMT−
−HVAWMG−
HLVAWM−−
−HVAWMY−
HQVAWM−−
−HVAWMQ−
HNVAWM−−
HFVAWM−−
−HVAWMM−
−HVAWMK−
−HVAWMD−
−HVAWMA−
HMVAWM−−
IEVAWM−−
−IVAWMT−
IVVAWM−−
IRVAWM−−
−IVAWMD−
IAVAWM−−
−IVAWMR−

```
-IVAWMQ-
IQVAWM--
INVAWM--
-IVAWML-
-IVAWMK-
-IVAWMG-
ISVAWM--
IHVAWM--
IDVAWM--
-KVAWMR-
KLVAWM--
KSVAWM--
-KVAWMK-
KTVAWM--
-KVAWMD-
-KVAWMN-
-KVAWMM-
KHVAWM--
-KVAWMF-
KVVAWM--
-KVAWMV-
-KVAWMA-
KQVAWM--
KAVAWM--
KYVAWM--
-KVAWMT-
-KVAWMI-
KGVAWM--
KDVAWM--
-LVAWMS-
LGVAWM--
-LVAWMG-
LMVAWM--
LRVAWM--
-LVAWMT-
-LVAWMA-
LQVAWM--
LKVAWM--
LYVAWM--
-LVAWMN-
LDVAWM--
-LVAWMM-
-LVAWMH-
-LVAWMF-
LNVAWM--
LWVAWM--
-LVAWMP-
-LVAWME-
LTVAWM--
-MVAWMR-
```

MAVAWM--
-MVAWMT-
MKVAWM--
MNVAWM--
-MVAWMS-
-MVAWMI-
-MVAWMA-
MGVAWM--
MYVAWM--
MVVAWM--
-MVAWMV-
-MVAWMQ-
-MVAWMH-
MTVAWM--
MQVAWM--
MFVAWM--
-NVAWMS-
-NVAWMY-
NSVAWM--
-NVAWMR-
NQVAWM--
NKVAWM--
-NVAWMM-
NTVAWM--
NLVAWM--
NIVAWM--
-NVAWMT-
-NVAWMN-
-NVAWML-
-NVAWMC-
-NVAWMA-
NNVAWM--
NYVAWM--
-NVAWMV-
-NVAWMK-
-NVAWMI-
-NVAWME-
NHVAWM--
PAVAWM--
PDVAWM--
PSVAWM--
PKVAWM--
PHVAWM--
PVVAWM--
PEVAWM--
PMVAWM--
PRVAWM--
PWVAWM--
PQVAWM--
PTVAWM--

PLVAWM--
PIVAWM--
PCVAWM--
QGVAWM--
-QVAWML-
QKVAWM--
-QVAWMK-
-QVAWMY-
-QVAWME-
-QVAWMA-
QMVAWM--
-QVAWMT-
-QVAWMS-
-QVAWMG-
QYVAWM--
QWVAWM--
-QVAWMR-
-QVAWMI-
-QVAWMF-
QNVAWM--
RLVAWM--
-RVAWMK-
-RVAWMN-
RGVAWM--
-RVAWMQ-
RYVAWM--
-RVAWMT-
RQVAWM--
RTVAWM--
REVAWM--
RDVAWM--
RNVAWM--
-RVAWMD-
RFVAWM--
-RVAWMY-
-RVAWMH-
-RVAWMI-
-RVAWMM-
SAVAWM--
SHVAWM--
-SVAWMS-
SIVAWM--
SVVAWM--
-SVAWMD-
-SVAWMH-
SNVAWM--
SEVAWM--
-SVAWMI-
SSVAWM--
-SVAWMV-

-SVAWMQ-
-SVAWMK-
-SVAWMA-
SGVAWM--
-SVAWMT-
SQVAWM--
SMVAWM--
SDVAWM--
-SVAWML-
-SVAWMF-
-SVAWME-
SCVAWM--
-SVAWMG-
SLVAWM--
-TVAWMV-
TSVAWM--
THVAWM--
-TVAWMS-
-TVAWME-
-TVAWMA-
-TVAWML-
TEVAWM--
TVVAWM--
-TVAWMR-
-TVAWMK-
-TVAWMH-
-TVAWMG-
-TVAWMD-
TQVAWM--
TNVAWM--
TDVAWM--
-TVAWMN-
-TVAWMM-
-TVAWMI-
TTVAWM--
TAVAWM--
-VVAWMR-
--VAWMSG
--VAWMLR
--VAWMSK
--VAWMTR
--VAWMQN
-VVAWMT-
--VAWMSM
--VAWMSL
--VAWMSI
VSVAWM--
--VAWMSH
--VAWMRQ
--VAWMLT

--VAWMKY
VQVAWM--
--VAWMSS
--VAWMLG
--VAWMLE
--VAWMLA
--VAWMGT
--VAWMYR
--VAWMMR
--VAWMKV
--VAWMGS
--VAWMAK
--VAWMAA
--VAWMTH
--VAWMTD
--VAWMTA
--VAWMSY
--VAWMMT
--VAWMLN
--VAWMKS
--VAWMGY
--VAWMGW
--VAWMGR
--VAWMGI
-VVAWME-
--VAWMYK
--VAWMSW
--VAWMST
--VAWMRG
--VAWMQI
--VAWMQA
--VAWMME
--VAWMLQ
--VAWMLK
--VAWMKQ
--VAWMKL
--VAWMIT
--VAWMAY
--VAWMAH
-VVAWMS-
-VVAWMQ-
-VVAWMD-
VRVAWM--
VDVAWM--
--VAWMVY
--VAWMVQ
--VAWMTY
--VAWMSD
--VAWMRA
--VAWMQS

--VAWMQR
--VAWMNV
--VAWMKG
--VAWMIR
--VAWMHT
--VAWMGV
--VAWMGM
--VAWMGK
VVVAWM--
-VVAWMV-
-VVAWMI-
--VAWMYN
--VAWMYH
--VAWMYG
--VAWMWG
--VAWMVV
--VAWMVH
--VAWMVG
--VAWMTN
--VAWMTK
--VAWMRW
--VAWMQG
--VAWMMW
--VAWMKT
--VAWMKA
--VAWMIN
--VAWMHS
--VAWMHE
--VAWMGN
--VAWMGG
--VAWMDS
--VAWMAT
--VAWMAS
--VAWMAM
VAVAWM--
-VVAWMN-
-VVAWMA-
VGVAWM--
--VAWMYA
--VAWMVT
--VAWMVD
--VAWMVA
--VAWMTV
--VAWMTG
--VAWMSQ
--VAWMRN
--VAWMRE
--VAWMNH
--VAWMMQ
--VAWMLV

--VAWMKN
--VAWMKF
--VAWMIY
--VAWMIS
--VAWMIK
--VAWMIH
--VAWMIG
--VAWMIA
--VAWMHR
--VAWMHH
--VAWMHG
--VAWMGF
--VAWMFG
--VAWMEY
--VAWMEW
--VAWMET
--VAWMAV
--VAWMAG
VNVAWM--
VIVAWM--
VEVAWM--
--VAWMYT
--VAWMYQ
--VAWMYI
--VAWMWT
--VAWMWS
--VAWMWK
--VAWMWH
--VAWMTT
--VAWMTS
--VAWMTQ
--VAWMTM
--VAWMTI
--VAWMTE
--VAWMSN
--VAWMSC
--VAWMSA
--VAWMRL
--VAWMRH
--VAWMRF
--VAWMRD
--VAWMQY
--VAWMQL
--VAWMPL
--VAWMNQ
--VAWMNM
--VAWMNG
--VAWMMV
--VAWMMN
--VAWMMK

--VAWMMI
--VAWMLP
--VAWMLH
--VAWMKW
--VAWMKM
--VAWMKI
--VAWMKD
--VAWMIQ
--VAWMIF
--VAWMID
--VAWMGQ
--VAWMGE
--VAWMGA
--VAWMFN
--VAWMFI
--VAWMFF
--VAWMFD
--VAWMER
--VAWMEN
--VAWMEG
--VAWMEA
--VAWMAW
--VAWMAQ
--VAWMAD
-WVAWMT-
-WVAWMG-
-WVAWMD-
-WVAWMS-
WTVAWM--
WGVAWM--
WAVAWM--
WSVAWM--
WEVAWM--
-YVAWMA-
-YVAWMN-
YGVAWM--
YTVAWM--
-YVAWMS-
YRVAWM--
-YVAWMQ-
-YVAWMH-
YNVAWM--
-YVAWMT-
-YVAWMI-
YDVAWM--

PSNF 124 755 30.92

ASPSNF--
-GPSNFT-
-GPSNFN-
--PSNFYL

--PSNFQL
-TPSNFW-
-VPSNFT-
AEPSNF--
AVPSNF--
AKPSNF--
-APSNFR-
-APSNFG-
-APSNFV-
AWPSNF--
-APSNFM-
ATPSNF--
-APSNFW-
-APSNFK-
AHPSNF--
AAPSNF--
-APSNFQ-
-APSNFE-
-APSNFY-
-APSNFT-
-APSNFS-
-APSNFN-
-APSNFI-
AMPSNF--
ADPSNF--
AYPSNF--
ARPSNF--
-APSNFH-
-APSNFD-
-APSNFA-
AFPSNF--
AQPSNF--
AIPSNF--
-CPSNFL-
-CPSNFR-
CQPSNF--
CCPSNF--
CSPSNF--
CPPSNF--
CNPSNF--
CGPSNF--
CMPSNF--
CHPSNF--
CRPSNF--
-CPSNFS-
-CPSNFF-
-CPSNFE-
-CPSNFA-
CAPSNF--
-DPSNFL-

-DPSNFI-
DRPSNF--
DMPSNF--
DWPSNF--
-DPSNFY-
DVPSNF--
-DPSNFA-
DSPSNF--
-DPSNFT-
DLPSNF--
DKPSNF--
DFPSNF--
DAPSNF--
DYPSNF--
-DPSNFQ-
DNPSNF--
DIPSNF--
DEPSNF--
DDPSNF--
ELPSNF--
-EPSNFL-
-EPSNFA-
EWPSNF--
ETPSNF--
-EPSNFV-
-EPSNFF-
EEPSNF--
ERPSNF--
EQPSNF--
ESPSNF--
-EPSNFR-
-EPSNFQ-
-EPSNFI-
-EPSNFH-
-EPSNFE-
-EPSNFY-
-EPSNFW-
-EPSNFT-
-EPSNFS-
-EPSNFN-
ENPSNF--
EMPSNF--
EKPSNF--
EIPSNF--
EHPSNF--
EGPSNF--
EAPSNF--
FQPSNF--
FRPSNF--
FSPSNF--

-FPSNFI-
FAPSNF--
-FPSNFY-
-FPSNFM-
-FPSNFK-
FGPSNF--
-FPSNFS-
FHPSNF--
FEPSNF--
FYPSNF--
FVPSNF--
FTPSNF--
FKPSNF--
FWPSNF--
-FPSNFQ-
-FPSNFF-
-FPSNFL-
FMPSNF--
-FPSNFT-
FCPSNF--
-FPSNFV-
-FPSNFE-
-GPSNFL-
GNPSNF--
-GPSNFS-
GRPSNF--
-GPSNFI-
GFPSNF--
GGPSNF--
GAPSNF--
GSPSNF--
-GPSNFV-
-GPSNFK-
GVPSNF--
-GPSNFW-
GYPSNF--
-GPSNFM-
-GPSNFG-
-GPSNFF-
GWPSNF--
GQPSNF--
-GPSNFE-
GLPSNF--
-GPSNFR-
-GPSNFH-
-GPSNFA-
GKPSNF--
GIPSNF--
GHPSNF--
GTPSNF--

-GPSNFQ-
GPPSNF--
GMPSNF--
GCPSNF--
-HPSNFM-
HSPSNF--
HRPSNF--
-HPSNFQ-
HLPSNF--
HFPSNF--
-HPSNFY-
HHPSNF--
HYPSNF--
HWPSNF--
-HPSNFV-
-HPSNFT-
-HPSNFS-
HMPSNF--
HGPSNF--
-HPSNFN-
-HPSNFL-
-HPSNFI-
-HPSNFA-
HKPSNF--
-HPSNFG-
HNPSNF--
HEPSNF--
HAPSNF--
HTPSNF--
-HPSNFH-
-HPSNFF-
HPPSNF--
IRPSNF--
IGPSNF--
ILPSNF--
-IPSNFR-
ISPSNF--
-IPSNFT-
IMPSNF--
IYPSNF--
IWPSNF--
IKPSNF--
-IPSNFY-
-IPSNFH-
IQPSNF--
-IPSNFQ-
-IPSNFM-
IHPSNF--
ITPSNF--
-IPSNFS-

-IPSNFN-
-IPSNFK-
IPPSNF--
-IPSNFV-
INPSNF--
IDPSNF--
IAPSNF--
KSPSNF--
-KPSNFV-
-KPSNFL-
KTPSNF--
KVPSNF--
-KPSNFR-
KYPSNF--
-KPSNFS-
KFPSNF--
KPPSNF--
KNPSNF--
KIPSNF--
KHPSNF--
-KPSNFW-
-KPSNFF-
KLPSNF--
KAPSNF--
KWPSNF--
-KPSNFH-
-KPSNFA-
-KPSNFT-
-KPSNFM-
-KPSNFG-
-KPSNFQ-
-KPSNFI-
KMPSNF--
KGPSNF--
KEPSNF--
-KPSNFY-
-KPSNFN-
KKPSNF--
LVPSNF--
-LPSNFL-
-LPSNFR-
LRPSNF--
LLPSNF--
LSPSNF--
-LPSNFY-
-LPSNFS-
-LPSNFW-
-LPSNFK-
LNPSNF--
LWPSNF--

LFPSNF--
LYPSNF--
LKPSNF--
LGPSNF--
LPPSNF--
-LPSNFT-
-LPSNFQ-
-LPSNFM-
-LPSNFH-
-LPSNFF-
LMPSNF--
LEPSNF--
LTPSNF--
LAPSNF--
LIPSNF--
-LPSNFI-
LDPSNF--
-LPSNFV-
-LPSNFN-
-LPSNFA-
LHPSNF--
LCPSNF--
MFPSNF--
-MPSNFS-
MIPSNF--
MSPSNF--
MRPSNF--
-MPSNFL-
MQPSNF--
-MPSNFF-
-MPSNFA-
MTPSNF--
-MPSNFV-
MMPSNF--
MYPSNF--
-MPSNFY-
-MPSNFT-
-MPSNFI-
MLPSNF--
MAPSNF--
-MPSNFW-
-MPSNFK-
MPPSNF--
MWPSNF--
-MPSNFR-
-MPSNFQ-
-MPSNFH-
MKPSNF--
MEPSNF--
MVPSNF--

-MPSNFG-
MGPSNF--
NSPSNF--
-NPSNFR-
NLPSNF--
NAPSNF--
NVPSNF--
-NPSNFV-
-NPSNFS-
-NPSNFK-
NNPSNF--
NRPSNF--
-NPSNFY-
-NPSNFT-
-NPSNFM-
-NPSNFI-
NQPSNF--
-NPSNFW-
-NPSNFL-
NGPSNF--
NWPSNF--
NTPSNF--
-NPSNFQ-
-NPSNFH-
NHPSNF--
-NPSNFA-
NMPSNF--
NKPSNF--
NIPSNF--
NDPSNF--
--PSNFLR
--PSNFIR
--PSNFRT
--PSNFRL
--PSNFRS
--PSNFRD
--PSNFTR
--PSNFSV
--PSNFRV
--PSNFWA
--PSNFSS
--PSNFLK
PSPSNF--
--PSNFSG
--PSNFIT
--PSNFVM
--PSNFAY
--PSNFWG
--PSNFSI
--PSNFSA

--PSNFME
--PSNFIW
--PSNFSH
--PSNFRM
--PSNFRI
--PSNFLS
--PSNFYW
--PSNFYR
--PSNFTA
--PSNFSF
--PSNFRF
--PSNFMT
--PSNFMR
--PSNFKK
--PSNFWR
--PSNFWK
--PSNFVS
--PSNFVA
--PSNFRW
--PSNFVK
--PSNFSM
--PSNFML
--PSNFLG
--PSNFKV
--PSNFGS
--PSNFFG
--PSNFWW
--PSNFVV
--PSNFVR
--PSNFTG
--PSNFRR
--PSNFRA
--PSNFLH
--PSNFLA
--PSNFIG
--PSNFFR
--PSNFAW
--PSNFAF
--PSNFVW
--PSNFVN
--PSNFTF
--PSNFRQ
--PSNFRE
--PSNFKS
--PSNFIQ
--PSNFIF
--PSNFGA
--PSNFAA
PQPSNF--
PWPSNF--

PVPSNF--
--PSNFYS
--PSNFVY
--PSNFTK
--PSNFRN
--PSNFMS
PEPSNF--
--PSNFYA
--PSNFWM
--PSNFTW
--PSNFTS
--PSNFSY
--PSNFSN
--PSNFSK
--PSNFRY
--PSNFRH
--PSNFQH
--PSNFKQ
--PSNFFT
--PSNFFI
--PSNFAT
PFPSNF--
--PSNFVT
--PSNFTV
--PSNFTQ
--PSNFTN
--PSNFST
--PSNFQT
--PSNFQS
--PSNFQG
--PSNFMV
--PSNFMH
--PSNFMG
--PSNFLQ
--PSNFKW
--PSNFAQ
--PSNFYY
--PSNFVL
--PSNFVH
--PSNFVG
--PSNFVE
--PSNFTM
--PSNFTI
--PSNFSW
--PSNFQV
--PSNFNS
--PSNFNA
--PSNFMW
--PSNFMQ
--PSNFMI

--PSNFMA
--PSNFLN
--PSNFKL
--PSNFKH
--PSNFIM
--PSNFHH
--PSNFFN
--PSNFFH
--PSNFFA
--PSNFES
--PSNFAR
--PSNFYT
--PSNFYI
--PSNFYE
--PSNFWT
--PSNFWN
--PSNFVD
--PSNFSQ
--PSNFQY
--PSNFQK
--PSNFQI
--PSNFNT
--PSNFNG
--PSNFMY
--PSNFMN
--PSNFMD
--PSNFLM
--PSNFKY
--PSNFKT
--PSNFKN
--PSNFKG
--PSNFIS
--PSNFIK
--PSNFIE
--PSNFHT
--PSNFHI
--PSNFGR
--PSNFFK
--PSNFET
--PSNFAN
PLPSNF--
PTPSNF--
--PSNFYQ
--PSNFYN
--PSNFWH
--PSNFTY
--PSNFTH
--PSNFTD
--PSNFQR
--PSNFQQ

--PSNFQN
--PSNFQA
--PSNFNV
--PSNFMK
--PSNFMF
--PSNFKI
--PSNFKD
--PSNFKA
--PSNFIN
--PSNFIA
--PSNFHV
--PSNFHS
--PSNFHN
--PSNFHM
--PSNFHK
--PSNFHG
--PSNFHF
--PSNFHA
--PSNFGT
--PSNFGM
--PSNFGG
--PSNFFW
--PSNFFE
--PSNFFD
--PSNFEY
--PSNFEV
--PSNFEK
--PSNFEI
--PSNFDI
--PSNFCI
--PSNFCF
--PSNFAM
--PSNFAL
--PSNFAK
--PSNFAI
--PSNFAH
PNPSNF--
PKPSNF--
PIPSNF--
PHPSNF--
QRPSNF--
QVPSNF--
-QPSNFI-
-QPSNFL-
-QPSNFQ-
QSPSNF--
QGPSNF--
-QPSNFS-
-QPSNFR-
-QPSNFH-

QNPSNF--
QQPSNF--
-QPSNFT-
-QPSNFE-
QWPSNF--
-QPSNFW-
-QPSNFM-
-QPSNFG-
-QPSNFA-
QMPSNF--
QHPSNF--
QEPSNF--
QTPSNF--
-QPSNFY-
-QPSNFV-
QLPSNF--
QIPSNF--
RTPSNF--
-RPSNFL-
RYPSNF--
RLPSNF--
-RPSNFM-
-RPSNFI-
RFPSNF--
-RPSNFR-
-RPSNFG-
RIPSNF--
-RPSNFK-
-RPSNFS-
-RPSNFQ-
RMPSNF--
-RPSNFV-
-RPSNFT-
-RPSNFH-
RGPSNF--
RWPSNF--
RRPSNF--
-RPSNFF-
RVPSNF--
-RPSNFY-
-RPSNFW-
-RPSNFN-
RNPSNF--
REPSNF--
RDPSNF--
RAPSNF--
RQPSNF--
RPPSNF--
RCPSNF--
-RPSNFE-

-SPSNFL-
SRPSNF--
SKPSNF--
SYPSNF--
-SPSNFV-
STPSNF--
-SPSNFS-
-SPSNFR-
SLPSNF--
SAPSNF--
SGPSNF--
-SPSNFA-
SNPSNF--
SVPSNF--
-SPSNFM-
-SPSNFI-
-SPSNFH-
SMPSNF--
SFPSNF--
-SPSNFN-
-SPSNFK-
SQPSNF--
-SPSNFY-
SIPSNF--
-SPSNFT-
-SPSNFQ-
-SPSNFE-
-SPSNFG-
-SPSNFF-
SEPSNF--
SHPSNF--
SCPSNF--
SDPSNF--
-TPSNFL-
-TPSNFS-
-TPSNFI-
TDPSNF--
TAPSNF--
TTPSNF--
-TPSNFY-
TRPSNF--
TSPSNF--
-TPSNFT-
TKPSNF--
TEPSNF--
TQPSNF--
-TPSNFM-
TPPSNF--
TIPSNF--
TGPSNF--

−TPSNFV−
TYPSNF−−
TWPSNF−−
−TPSNFH−
−TPSNFE−
TFPSNF−−
−TPSNFR−
−TPSNFQ−
TMPSNF−−
THPSNF−−
−TPSNFA−
TNPSNF−−
TCPSNF−−
−VPSNFR−
−VPSNFF−
VYPSNF−−
−VPSNFK−
VLPSNF−−
−VPSNFA−
VSPSNF−−
−VPSNFS−
−VPSNFL−
−VPSNFG−
VRPSNF−−
−VPSNFW−
VAPSNF−−
VMPSNF−−
−VPSNFV−
VVPSNF−−
−VPSNFQ−
VNPSNF−−
−VPSNFY−
VPPSNF−−
VIPSNF−−
VFPSNF−−
−VPSNFI−
VDPSNF−−
−VPSNFN−
−VPSNFE−
VHPSNF−−
VEPSNF−−
VTPSNF−−
VQPSNF−−
−VPSNFH−
VKPSNF−−
VCPSNF−−
−WPSNFR−
WFPSNF−−
−WPSNFL−
−WPSNFS−

WQPSNF--
WSPSNF--
WVPSNF--
-WPSNFF-
-WPSNFQ-
WKPSNF--
-WPSNFY-
-WPSNFH-
WGPSNF--
WAPSNF--
-WPSNFV-
WYPSNF--
WRPSNF--
-WPSNFW-
-WPSNFT-
-WPSNFK-
-WPSNFI-
-WPSNFA-
WPPSNF--
WNPSNF--
WIPSNF--
WCPSNF--
-YPSNFR-
YWPSNF--
-YPSNFL-
YLPSNF--
YSPSNF--
YRPSNF--
-YPSNFY-
YFPSNF--
-YPSNFS-
-YPSNFE-
-YPSNFM-
-YPSNFH-
-YPSNFW-
-YPSNFV-
-YPSNFI-
YKPSNF--
-YPSNFK-
-YPSNFF-
YHPSNF--
YGPSNF--
YPPSNF--
YYPSNF--
-YPSNFT-
-YPSNFA-
YNPSNF--
YMPSNF--
YEPSNF--
YDPSNF--

-YPSNFN-

| | | | |
|---|---|---|---|
| PRWI | 707 | 556 | 30.89 |

QEPRWI--
ASPRWI--
-APRWIL-
AQPRWI--
-APRWIK-
-APRWIF-
ANPRWI--
ALPRWI--
AEPRWI--
-APRWIY-
-APRWIR-
-APRWII-
AVPRWI--
-APRWIN-
-APRWIG-
-APRWIV-
-APRWIE-
AFPRWI--
AKPRWI--
ADPRWI--
AYPRWI--
-APRWIW-
-APRWID-
-APRWIQ-
AHPRWI--
CVPRWI--
CNPRWI--
CLPRWI--
CIPRWI--
CGPRWI--
CAPRWI--
DRPRWI--
DTPRWI--
DHPRWI--
-DPRWIR-
DMPRWI--
DAPRWI--
-DPRWIT-
DVPRWI--
DIPRWI--
DSPRWI--
-DPRWIS-
DLPRWI--
-DPRWIV-
-DPRWII-
DQPRWI--
-DPRWIQ-
-DPRWIK-

DFPRWI--
DDPRWI--
DWPRWI--
-DPRWIY-
-DPRWIM-
-DPRWIA-
DNPRWI--
DKPRWI--
DGPRWI--
ESPRWI--
ERPRWI--
EGPRWI--
-EPRWIY-
-EPRWIA-
EAPRWI--
ETPRWI--
ELPRWI--
EKPRWI--
-EPRWIR-
-EPRWIQ-
-EPRWIM-
-EPRWII-
EHPRWI--
EWPRWI--
EVPRWI--
EQPRWI--
-EPRWIT-
-EPRWIN-
-EPRWIH-
-EPRWIG-
ENPRWI--
EIPRWI--
EFPRWI--
EEPRWI--
FEPRWI--
FSPRWI--
FMPRWI--
FGPRWI--
-FPRWIQ-
-FPRWIK-
FNPRWI--
FHPRWI--
FFPRWI--
-FPRWIG-
FDPRWI--
FTPRWI--
-FPRWII-
FLPRWI--
FIPRWI--
FWPRWI--

FVPRWI--
-FPRWIV-
-GPRWIL-
GTPRWI--
GVPRWI--
GGPRWI--
-GPRWIS-
GRPRWI--
-GPRWIQ-
-GPRWIR-
GNPRWI--
GSPRWI--
GQPRWI--
-GPRWIG-
GKPRWI--
GIPRWI--
GHPRWI--
GDPRWI--
-GPRWIH-
-GPRWIV-
-GPRWIK-
-GPRWIE-
GEPRWI--
-GPRWIY-
-GPRWIM-
-GPRWIF-
-GPRWIW-
-GPRWIP-
-GPRWIN-
GPPRWI--
HSPRWI--
HTPRWI--
HNPRWI--
HEPRWI--
HGPRWI--
-HPRWIQ-
-HPRWIL-
HDPRWI--
HQPRWI--
-HPRWIV-
-HPRWII-
HPPRWI--
-HPRWIY-
-HPRWIT-
-HPRWIS-
-HPRWIN-
HKPRWI--
IPPRWI--
INPRWI--
IGPRWI--

IQPRWI--
-IPRWIS-
ITPRWI--
-IPRWIT-
-IPRWIV-
IVPRWI--
-IPRWIR-
IIPRWI--
ISPRWI--
IYPRWI--
-IPRWIF-
-IPRWIL-
IMPRWI--
ILPRWI--
IWPRWI--
-IPRWIM-
-IPRWIK-
-IPRWIA-
-IPRWIY-
-IPRWIG-
IHPRWI--
IFPRWI--
IDPRWI--
ICPRWI--
KVPRWI--
-KPRWIR-
-KPRWIM-
-KPRWIQ-
KQPRWI--
-KPRWIT-
-KPRWII-
-KPRWIV-
-KPRWIS-
-KPRWIH-
KIPRWI--
-KPRWIG-
KDPRWI--
-KPRWIN-
-KPRWIF-
KPPRWI--
KEPRWI--
LNPRWI--
LEPRWI--
-LPRWIK-
-LPRWIT-
LPPRWI--
LGPRWI--
-LPRWIS-
-LPRWIG-
-LPRWIH-

LDPRWI--
LYPRWI--
-LPRWIL-
-LPRWIF-
LIPRWI--
-LPRWII-
MSPRWI--
MRPRWI--
-MPRWIV-
MPPRWI--
MVPRWI--
MYPRWI--
MTPRWI--
-MPRWIA-
MKPRWI--
MIPRWI--
MGPRWI--
-MPRWIG-
MAPRWI--
MDPRWI--
MQPRWI--
-MPRWIQ-
-MPRWIK-
MLPRWI--
MEPRWI--
MWPRWI--
-MPRWIH-
MHPRWI--
NGPRWI--
NMPRWI--
NHPRWI--
NTPRWI--
NIPRWI--
NRPRWI--
NKPRWI--
-NPRWIT-
NVPRWI--
NNPRWI--
NQPRWI--
NEPRWI--
-NPRWIR-
NPPRWI--
-NPRWIY-
-NPRWIF-
-NPRWIA-
NYPRWI--
-NPRWIS-
-NPRWIN-
-NPRWIM-
-NPRWIK-

```
-NPRWII-
--PRWITD
--PRWISN
--PRWITT
--PRWISW
PRPRWI--
PYPRWI--
--PRWISG
--PRWIQG
--PRWIAI
PSPRWI--
--PRWIVY
--PRWISV
--PRWIIS
PTPRWI--
--PRWIVT
--PRWIRN
--PRWIRI
--PRWILQ
--PRWISK
--PRWISH
--PRWIQS
--PRWIAG
--PRWIYK
--PRWIWG
--PRWITS
--PRWILN
--PRWIKA
--PRWIYS
--PRWIVE
--PRWIMD
--PRWIIT
--PRWIFT
PAPRWI--
--PRWIYH
--PRWILY
--PRWIHM
--PRWIHG
--PRWIGL
--PRWIFG
--PRWIAV
PQPRWI--
PVPRWI--
--PRWIVV
--PRWITN
--PRWIMV
--PRWIME
--PRWIKG
--PRWIIY
--PRWIIV
```

PMPRWI--
PWPRWI--
--PRWIYE
--PRWIRQ
--PRWIRA
--PRWIMT
--PRWIHS
PLPRWI--
--PRWIVS
--PRWITW
--PRWITH
--PRWIRD
--PRWIFS
--PRWIAT
--PRWIAH
PEPRWI--
--PRWIYN
--PRWIYA
--PRWIWS
--PRWIVQ
--PRWITG
--PRWISY
--PRWINT
--PRWINS
--PRWINA
--PRWIKQ
--PRWIKN
--PRWIIK
--PRWIEW
--PRWIAL
--PRWITV
--PRWISM
--PRWISA
--PRWIRY
--PRWIQV
--PRWIKD
--PRWIFH
--PRWIEV
--PRWIEA
--PRWIAQ
--PRWIYV
--PRWIYD
--PRWIWV
--PRWIWT
--PRWIVW
--PRWISQ
--PRWISE
--PRWIRW
--PRWING
--PRWILE

--PRWIKY
--PRWIKE
--PRWIIW
--PRWIIH
--PRWIHT
--PRWIGM
--PRWIGF
--PRWIDS
--PRWIAY
--PRWIAN
PNPRWI--
PDPRWI--
--PRWIWH
--PRWIVD
--PRWIQI
--PRWIQF
--PRWIQA
--PRWILW
--PRWILD
--PRWIKW
--PRWIKH
--PRWIIM
--PRWIID
--PRWIHY
--PRWIFV
--PRWIFD
--PRWIYY
--PRWIWN
--PRWITE
--PRWITA
--PRWIRH
--PRWIQW
--PRWIQN
--PRWIQM
--PRWIQE
--PRWIQD
--PRWIPA
--PRWIMW
--PRWIMN
--PRWIMM
--PRWIII
--PRWIIE
--PRWIIA
--PRWIHQ
--PRWIHK
--PRWIHI
--PRWIHH
--PRWIGY
--PRWIGW
--PRWIGA

--PRWIFW
--PRWIFN
--PRWIFE
--PRWIFA
--PRWIEY
--PRWIEH
PIPRWI--
-QPRWIR-
-QPRWIF-
QGPRWI--
QRPRWI--
-QPRWIA-
QVPRWI--
QLPRWI--
QTPRWI--
QQPRWI--
-QPRWIM-
QNPRWI--
-QPRWIS-
QFPRWI--
QYPRWI--
-QPRWIV-
-QPRWIG-
QPPRWI--
QMPRWI--
QAPRWI--
-QPRWIQ-
QKPRWI--
-QPRWIT-
QHPRWI--
-QPRWIK-
-QPRWIH-
RNPRWI--
REPRWI--
RQPRWI--
-RPRWIY-
-RPRWIQ-
-RPRWIG-
-RPRWIM-
-RPRWIH-
RPPRWI--
RIPRWI--
RDPRWI--
-RPRWIN-
-RPRWII-
-RPRWIK-
RRPRWI--
-RPRWIW-
-SPRWIR-
-SPRWIM-

STPRWI--
-SPRWIS-
-SPRWIQ-
-SPRWIY-
SYPRWI--
-SPRWIV-
SPPRWI--
-SPRWIH-
-SPRWIG-
SKPRWI--
SIPRWI--
-SPRWIF-
-SPRWIE-
SAPRWI--
SDPRWI--
-SPRWIN-
-SPRWII-
TRPRWI--
-TPRWIR-
TQPRWI--
TIPRWI--
TKPRWI--
-TPRWIT-
-TPRWIK-
TAPRWI--
TYPRWI--
-TPRWIS-
-TPRWII-
TPPRWI--
TVPRWI--
-TPRWIM-
-TPRWIH-
-TPRWIF-
TFPRWI--
TTPRWI--
-TPRWIA-
TNPRWI--
TGPRWI--
TEPRWI--
-TPRWIG-
-TPRWIN-
THPRWI--
TDPRWI--
-TPRWIW-
-TPRWIV-
VRPRWI--
VQPRWI--
VSPRWI--
VHPRWI--
-VPRWIT-

-VPRWIR-
VEPRWI--
-VPRWIA-
VVPRWI--
VLPRWI--
VGPRWI--
-VPRWIM-
-VPRWIG-
VFPRWI--
VYPRWI--
-VPRWII-
-VPRWIF-
VNPRWI--
-VPRWIQ-
VPPRWI--
-VPRWIE-
-VPRWIN-
-VPRWIH-
VDPRWI--
WHPRWI--
WTPRWI--
WNPRWI--
WAPRWI--
WSPRWI--
WEPRWI--
WVPRWI--
-WPRWIY-
-WPRWIL-
WIPRWI--
WGPRWI--
WDPRWI--
WWPRWI--
YSPRWI--
-YPRWIS-
YVPRWI--
YRPRWI--
YTPRWI--
YKPRWI--
-YPRWIR-
-YPRWIK-
YYPRWI--
YNPRWI--
YLPRWI--
YIPRWI--
-YPRWIM-
YWPRWI--
-YPRWIV-
YEPRWI--
YGPRWI--
YAPRWI--

YHPRWI--
YFPRWI--
YQPRWI--
-YPRWIQ-
-YPRWII-
YPPRWI--
YMPRWI--
YDPRWI--

| | | | |
|---|---|---|---|
| VANM | 214 | 705 | 30.83 |

--VANMLL
ASVANM--
-AVANML-
-AVANMM-
ARVANM--
AVVANM--
-AVANMW-
-AVANMV-
-AVANMR-
AFVANM--
-AVANMQ-
-AVANMN-
-AVANMI-
ANVANM--
ALVANM--
AYVANM--
-AVANMT-
-AVANME-
-AVANMA-
ATVANM--
APVANM--
AKVANM--
AEVANM--
-AVANMY-
-AVANMS-
-AVANMK-
-AVANMH-
-AVANMF-
-AVANMD-
AQVANM--
AMVANM--
AGVANM--
-CVANML-
-CVANMK-
-CVANMV-
-CVANMT-
-CVANMI-
-CVANMQ-
CYVANM--
CWVANM--
-CVANMY-

-CVANMR-
-CVANMA-
CRVANM--
CPVANM--
CMVANM--
CKVANM--
CIVANM--
-DVANMS-
-DVANMR-
DRVANM--
-DVANMF-
DQVANM--
DAVANM--
DLVANM--
-DVANMT-
-DVANML-
-DVANMI-
-DVANMH-
DFVANM--
DYVANM--
DVVANM--
-DVANMV-
-DVANMA-
DSVANM--
DPVANM--
DKVANM--
DHVANM--
-EVANMR-
-EVANML-
ESVANM--
ERVANM--
-EVANMV-
EHVANM--
-EVANMQ-
-EVANMK-
-EVANMG-
-EVANMY-
-EVANMS-
EKVANM--
EIVANM--
ECVANM--
EYVANM--
EVVANM--
-EVANMH-
-EVANMF-
ETVANM--
EPVANM--
ENVANM--
EFVANM--
-FVANMV-

FTVANM--
-FVANMR-
FRVANM--
FHVANM--
FVVANM--
-FVANML-
-FVANMG-
-FVANMS-
-FVANMF-
FQVANM--
FIVANM--
FDVANM--
-FVANMT-
-FVANMQ-
-FVANMM-
-FVANMK-
-FVANMI-
FNVANM--
FLVANM--
FKVANM--
-FVANMY-
-FVANMW-
FSVANM--
FPVANM--
FMVANM--
-FVANMD-
-FVANMA-
FAVANM--
-GVANML-
GKVANM--
-GVANMT-
-GVANMS-
GGVANM--
-GVANMW-
GTVANM--
GLVANM--
-GVANMA-
GIVANM--
-GVANMV-
-GVANMK-
-GVANMH-
-GVANMY-
-GVANMQ-
-GVANMI-
-GVANMG-
GWVANM--
GSVANM--
GPVANM--
GMVANM--
GYVANM--

GVVANM--
-GVANMF-
GQVANM--
GNVANM--
GDVANM--
GAVANM--
-HVANMR-
-HVANMY-
-HVANMS-
-HVANMG-
HRVANM--
HKVANM--
HNVANM--
HMVANM--
HAVANM--
HVVANM--
HQVANM--
HPVANM--
HFVANM--
HYVANM--
HWVANM--
-HVANML-
-HVANMI-
HEVANM--
-HVANMQ-
-HVANMN-
-HVANMM-
-HVANMH-
HTVANM--
HSVANM--
HHVANM--
-IVANMK-
-IVANML-
IVVANM--
-IVANMS-
ISVANM--
IRVANM--
IIVANM--
-IVANMG-
-IVANMA-
IGVANM--
IWVANM--
ITVANM--
IQVANM--
-IVANMR-
-IVANMQ-
-IVANMF-
IHVANM--
-IVANMT-
-IVANMN-

−IVANME−
IPVANM−−
IFVANM−−
IDVANM−−
−KVANMR−
−KVANMF−
KVVANM−−
−KVANMW−
−KVANMT−
−KVANMH−
−KVANMS−
−KVANMQ−
−KVANMI−
−KVANMA−
KIVANM−−
KAVANM−−
−KVANMV−
−KVANMK−
KPVANM−−
KHVANM−−
KWVANM−−
−KVANMN−
−KVANMM−
−KVANME−
KQVANM−−
KNVANM−−
KMVANM−−
KGVANM−−
LSVANM−−
−LVANMS−
−LVANMR−
−LVANMV−
LKVANM−−
−LVANMG−
LRVANM−−
−LVANMF−
LNVANM−−
−LVANMT−
LPVANM−−
LLVANM−−
−LVANMM−
−LVANML−
LTVANM−−
LQVANM−−
LMVANM−−
LGVANM−−
LYVANM−−
LFVANM−−
−LVANMK−
−LVANMD−

LWVANM--
-LVANMY-
-LVANMH-
LIVANM--
LHVANM--
-LVANMI-
-LVANME-
-LVANMA-
LEVANM--
-LVANMN-
LDVANM--
MVVANM--
-MVANMS-
MGVANM--
MHVANM--
-MVANML-
MTVANM--
-MVANMY-
-MVANMR-
-MVANMH-
-MVANMF-
MRVANM--
MWVANM--
-MVANMT-
MSVANM--
MKVANM--
MDVANM--
MAVANM--
MYVANM--
-MVANMN-
-MVANMI-
-MVANMA-
MNVANM--
MLVANM--
MIVANM--
-MVANMV-
-MVANME-
MQVANM--
MPVANM--
MEVANM--
NSVANM--
NFVANM--
-NVANMH-
NAVANM--
-NVANMA-
NWVANM--
-NVANMR-
-NVANMI-
NYVANM--
-NVANMT-

```
-NVANMN-
-NVANML-
-NVANMF-
NQVANM--
NPVANM--
NGVANM--
-NVANMY-
-NVANMW-
-NVANMS-
-NVANMQ-
-NVANMM-
-NVANME-
NNVANM--
NMVANM--
NKVANM--
NHVANM--
-PVANMR-
-PVANMS-
-PVANML-
PGVANM--
-PVANMG-
PIVANM--
-PVANMF-
PSVANM--
PKVANM--
-PVANMV-
-PVANMT-
-PVANMN-
-PVANMI-
PVVANM--
-PVANMA-
PTVANM--
PRVANM--
PMVANM--
-PVANMK-
PQVANM--
PHVANM--
PEVANM--
PAVANM--
PYVANM--
-PVANMW-
-PVANMQ-
-PVANMM-
-QVANMV-
-QVANMT-
-QVANML-
-QVANMF-
QGVANM--
-QVANMW-
-QVANMS-
```

QQVANM--
QVVANM--
-QVANMR-
QRVANM--
QIVANM--
-QVANMG-
-QVANMA-
QTVANM--
-QVANMY-
-QVANMN-
-QVANMI-
QSVANM--
QLVANM--
QKVANM--
-RVANMH-
-RVANML-
RAVANM--
-RVANMK-
-RVANMA-
RTVANM--
-RVANMV-
-RVANMS-
RWVANM--
-RVANMY-
RQVANM--
REVANM--
RYVANM--
-RVANMT-
-RVANMR-
RPVANM--
RLVANM--
RHVANM--
-RVANMG-
RVVANM--
-RVANMN-
-RVANME-
-RVANMQ-
RIVANM--
RCVANM--
-RVANMW-
-RVANMF-
-SVANML-
SRVANM--
-SVANMQ-
SLVANM--
-SVANMK-
-SVANMH-
-SVANMT-
-SVANMS-
SYVANM--

SGVANM--
SVVANM--
-SVANMW-
STVANM--
-SVANMA-
SPVANM--
SAVANM--
SWVANM--
-SVANMM-
-SVANMI-
SSVANM--
SMVANM--
-SVANMG-
-SVANMY-
-SVANMF-
-SVANME-
SNVANM--
SKVANM--
SCVANM--
-SVANMV-
SQVANM--
SIVANM--
SHVANM--
SDVANM--
-TVANMR-
TLVANM--
-TVANMQ-
-TVANMS-
TEVANM--
TAVANM--
TVVANM--
TQVANM--
-TVANMY-
THVANM--
TYVANM--
TWVANM--
TKVANM--
TFVANM--
-TVANMW-
-TVANMH-
TMVANM--
-TVANMN-
-TVANMM-
TTVANM--
TPVANM--
TCVANM--
-TVANML-
-TVANMK-
-TVANMI-
-TVANMF-

-TVANME-
TGVANM--
--VANMLR
--VANMSR
--VANMIR
VRVANM--
--VANMRG
--VANMRT
--VANMLS
--VANMSL
--VANMGS
--VANMIN
--VANMVL
--VANMSS
--VANMWS
--VANMWR
--VANMRN
VTVANM--
VNVANM--
--VANMVT
--VANMSN
--VANMRW
--VANMLV
--VANMFK
--VANMTS
--VANMKE
-VVANMI-
--VANMVG
--VANMVA
--VANMQH
--VANMIL
VVVANM--
--VANMWQ
--VANMSW
--VANMRY
--VANMRV
--VANMRH
--VANMMQ
-VVANMW-
VGVANM--
--VANMVN
--VANMVH
--VANMMR
--VANMLA
--VANMIG
--VANMHT
--VANMFR
--VANMFG
-VVANMY-
-VVANMG-

-VVANMA-
--VANMTW
--VANMSV
--VANMST
--VANMSI
--VANMSH
--VANMSD
--VANMRI
--VANMQY
--VANMNS
--VANMMK
--VANMMD
--VANMLQ
--VANMKV
--VANMKR
--VANMKA
--VANMIT
--VANMIQ
--VANMHA
--VANMAS
-VVANMT-
-VVANMQ-
-VVANMM-
VDVANM--
--VANMWG
--VANMVQ
--VANMTM
--VANMTK
--VANMTH
--VANMSQ
--VANMRF
--VANMRD
--VANMNT
--VANMMN
--VANMMH
--VANMMG
--VANMLN
--VANMKL
--VANMKG
--VANMIK
--VANMIH
--VANMIF
--VANMGG
--VANMFT
--VANMAL
--VANMAA
VYVANM--
VWVANM--
-VVANMR-
-VVANMN-

-VVANMF-
VSVANM--
VKVANM--
VFVANM--
--VANMYD
--VANMWN
--VANMWM
--VANMWD
--VANMVS
--VANMRQ
--VANMQW
--VANMQL
--VANMNG
--VANMMS
--VANMMI
--VANMMA
--VANMHS
--VANMHN
--VANMFS
--VANMFI
--VANMEG
--VANMEA
--VANMAF
-VVANMS-
-VVANMK-
-VVANMH-
--VANMYT
--VANMYN
--VANMYH
--VANMYG
--VANMWI
--VANMWH
--VANMVV
--VANMVI
--VANMTV
--VANMTR
--VANMSM
--VANMSK
--VANMQS
--VANMQN
--VANMNM
--VANMMW
--VANMLT
--VANMLD
--VANMKN
--VANMKF
--VANMIV
--VANMHM
--VANMHI
--VANMHF

--VANMGL
--VANMFA
--VANMET
--VANMAQ
-VVANMV-
-VVANMD-
VPVANM--
VIVANM--
VEVANM--
VAVANM--
--VANMYM
--VANMYK
--VANMYI
--VANMWL
--VANMWF
--VANMVK
--VANMVE
--VANMTY
--VANMTT
--VANMTQ
--VANMTN
--VANMTI
--VANMTG
--VANMTE
--VANMTD
--VANMTA
--VANMSG
--VANMSE
--VANMRE
--VANMQT
--VANMQR
--VANMQG
--VANMQD
--VANMQA
--VANMNV
--VANMNE
--VANMMY
--VANMMV
--VANMML
--VANMLI
--VANMLH
--VANMLE
--VANMKW
--VANMKK
--VANMIS
--VANMIM
--VANMII
--VANMIA
--VANMHV
--VANMHG

--VANMGV
--VANMGT
--VANMGA
--VANMFV
--VANMFF
--VANMFE
--VANMEV
--VANMEH
--VANMDS
--VANMCA
--VANMAV
--VANMAM
--VANMAK
-WVANMT-
-WVANMA-
WHVANM--
-WVANMR-
-WVANML-
WQVANM--
-WVANMV-
-WVANMK-
WYVANM--
-WVANMI-
-WVANMF-
-WVANME-
-WVANMD-
WKVANM--
-WVANMW-
-WVANMG-
WLVANM--
WGVANM--
WEVANM--
-WVANMQ-
-WVANMH-
WTVANM--
WMVANM--
WDVANM--
YSVANM--
-YVANMR-
-YVANMW-
-YVANMS-
YTVANM--
-YVANMK-
-YVANMT-
YLVANM--
YWVANM--
YVVANM--
-YVANMV-
-YVANMN-
YRVANM--

YQVANM--
-YVANMM-
-YVANMA-
YFVANM--
YAVANM--
YYVANM--
YIVANM--
YGVANM--
-YVANMI-
-YVANMH-
-YVANMG-
-YVANMD-
YMVANM--
YCVANM--
PYNL 168 730 30.83
AWPYNL--
DNPYNL--
-LPYNLG-
--PYNLQY
--PYNLGV
--PYNLEL
-TPYNLE-
YLPYNL--
-APYNLR-
-APYNLS-
-APYNLV-
-APYNLT-
ALPYNL--
-APYNLK-
AVPYNL--
ATPYNL--
ARPYNL--
-APYNLQ-
AAPYNL--
ASPYNL--
AKPYNL--
ADPYNL--
-APYNLG-
AMPYNL--
AEPYNL--
AQPYNL--
-APYNLW-
AHPYNL--
-APYNLY-
-APYNLE-
AYPYNL--
-APYNLN-
-APYNLM-
-APYNLI-
-APYNLH-

ANPYNL--
AIPYNL--
-APYNLF-
-APYNLA-
CNPYNL--
CCPYNL--
CAPYNL--
CRPYNL--
-CPYNLR-
CSPYNL--
CQPYNL--
-CPYNLA-
CHPYNL--
DSPYNL--
DRPYNL--
DGPYNL--
DFPYNL--
-DPYNLK-
DIPYNL--
DAPYNL--
DHPYNL--
DWPYNL--
DVPYNL--
-DPYNLY-
-DPYNLL-
DMPYNL--
-EPYNLR-
EKPYNL--
EVPYNL--
ESPYNL--
ERPYNL--
EQPYNL--
-EPYNLL-
ELPYNL--
EGPYNL--
-EPYNLV-
-EPYNLS-
EYPYNL--
-EPYNLT-
EMPYNL--
EAPYNL--
ETPYNL--
-EPYNLY-
-EPYNLW-
-EPYNLM-
-EPYNLI-
-EPYNLH-
-EPYNLA-
EIPYNL--
FQPYNL--

FMPYNL--
FSPYNL--
-FPYNLS-
-FPYNLR-
FGPYNL--
-FPYNLL-
FTPYNL--
-FPYNLK-
-FPYNLH-
FAPYNL--
-FPYNLT-
FLPYNL--
FHPYNL--
-FPYNLV-
-FPYNLQ-
FDPYNL--
FPPYNL--
FVPYNL--
FKPYNL--
FIPYNL--
FWPYNL--
-FPYNLY-
-FPYNLN-
-FPYNLG-
-FPYNLA-
FNPYNL--
FEPYNL--
GAPYNL--
GRPYNL--
-GPYNLT-
GSPYNL--
-GPYNLL-
-GPYNLS-
-GPYNLA-
GNPYNL--
GFPYNL--
-GPYNLR-
-GPYNLQ-
GIPYNL--
GWPYNL--
GLPYNL--
GTPYNL--
-GPYNLW-
-GPYNLN-
-GPYNLK-
-GPYNLH-
-GPYNLG-
-GPYNLE-
GQPYNL--
-GPYNLV-

−GPYNLI−
GPPYNL−−
GMPYNL−−
GYPYNL−−
GKPYNL−−
GEPYNL−−
−GPYNLM−
GHPYNL−−
GGPYNL−−
HLPYNL−−
HRPYNL−−
−HPYNLS−
HAPYNL−−
HNPYNL−−
HSPYNL−−
HGPYNL−−
HVPYNL−−
−HPYNLV−
HTPYNL−−
−HPYNLI−
HMPYNL−−
HYPYNL−−
−HPYNLT−
−HPYNLR−
−HPYNLF−
HKPYNL−−
HIPYNL−−
HHPYNL−−
HWPYNL−−
−HPYNLY−
−HPYNLL−
HDPYNL−−
HQPYNL−−
−HPYNLQ−
−HPYNLN−
−HPYNLM−
−HPYNLH−
−HPYNLG−
−HPYNLE−
HFPYNL−−
HEPYNL−−
−IPYNLR−
−IPYNLM−
−IPYNLL−
IRPYNL−−
−IPYNLH−
IGPYNL−−
ISPYNL−−
IQPYNL−−
−IPYNLS−

ITPYNL--
-IPYNLV-
IPPYNL--
IHPYNL--
IEPYNL--
IDPYNL--
IYPYNL--
IWPYNL--
-IPYNLY-
INPYNL--
ILPYNL--
-IPYNLQ-
IIPYNL--
-IPYNLA-
IMPYNL--
IKPYNL--
KSPYNL--
KGPYNL--
KRPYNL--
-KPYNLS-
-KPYNLK-
KYPYNL--
KVPYNL--
-KPYNLL-
-KPYNLI-
KNPYNL--
KWPYNL--
-KPYNLR-
KLPYNL--
KTPYNL--
-KPYNLN-
KAPYNL--
-KPYNLM-
-KPYNLE-
KQPYNL--
-KPYNLG-
KFPYNL--
-KPYNLY-
-KPYNLV-
-KPYNLT-
-KPYNLH-
-KPYNLA-
KPPYNL--
KMPYNL--
KKPYNL--
-KPYNLW-
KEPYNL--
KCPYNL--
LRPYNL--
LKPYNL--

```
-LPYNLR-
LGPYNL--
LSPYNL--
LMPYNL--
LVPYNL--
LWPYNL--
LIPYNL--
LQPYNL--
-LPYNLT-
-LPYNLY-
-LPYNLK-
LPPYNL--
LDPYNL--
-LPYNLS-
-LPYNLI-
LYPYNL--
-LPYNLL-
LNPYNL--
-LPYNLQ-
LFPYNL--
-LPYNLA-
LHPYNL--
LCPYNL--
-LPYNLV-
-LPYNLH-
-LPYNLW-
MRPYNL--
MGPYNL--
MSPYNL--
MLPYNL--
MVPYNL--
-MPYNLS-
MAPYNL--
MTPYNL--
-MPYNLV-
-MPYNLR-
-MPYNLM-
-MPYNLH-
MMPYNL--
MPPYNL--
MNPYNL--
MKPYNL--
MIPYNL--
-MPYNLQ-
-MPYNLI-
MHPYNL--
MQPYNL--
-MPYNLY-
-MPYNLN-
-MPYNLK-
```

-MPYNLF-
MFPYNL--
NSPYNL--
-NPYNLR-
NTPYNL--
NRPYNL--
-NPYNLA-
NLPYNL--
-NPYNLL-
-NPYNLI-
NYPYNL--
NPPYNL--
NMPYNL--
NIPYNL--
NFPYNL--
NWPYNL--
NQPYNL--
-NPYNLY-
-NPYNLW-
-NPYNLT-
-NPYNLK-
NHPYNL--
NAPYNL--
NVPYNL--
NNPYNL--
NGPYNL--
NDPYNL--
--PYNLRG
--PYNLRT
--PYNLGS
--PYNLRS
--PYNLST
--PYNLLT
--PYNLGY
--PYNLTT
--PYNLSL
--PYNLRH
--PYNLLR
--PYNLAL
--PYNLWS
--PYNLKG
--PYNLGL
--PYNLAY
--PYNLVV
--PYNLSR
--PYNLRN
--PYNLLS
--PYNLVS
--PYNLQR
--PYNLIK

--PYNLEA
--PYNLAS
--PYNLAF
--PYNLWT
--PYNLVT
--PYNLTR
--PYNLQW
--PYNLLK
--PYNLIA
--PYNLWN
--PYNLWA
--PYNLSN
--PYNLMS
--PYNLLG
--PYNLIM
--PYNLTF
--PYNLSM
--PYNLSF
--PYNLRA
--PYNLLH
--PYNLKA
--PYNLIS
--PYNLGW
--PYNLAA
--PYNLWK
--PYNLVG
--PYNLTI
--PYNLSY
--PYNLSW
--PYNLSG
--PYNLRE
--PYNLPY
--PYNLHY
--PYNLFT
PKPYNL--
PAPYNL--
--PYNLYR
--PYNLYQ
--PYNLTS
--PYNLTK
--PYNLSK
--PYNLSH
--PYNLRY
--PYNLRF
--PYNLQN
--PYNLHG
--PYNLGG
--PYNLFR
PIPYNL--
PGPYNL--

PFPYNL--
PYPYNL--
--PYNLYT
--PYNLWR
--PYNLVQ
--PYNLVH
--PYNLTH
--PYNLSV
--PYNLRW
--PYNLPF
--PYNLNT
--PYNLMR
--PYNLMG
--PYNLLN
--PYNLKV
--PYNLKR
--PYNLKQ
--PYNLIW
--PYNLIV
--PYNLIG
--PYNLHS
--PYNLFN
--PYNLFK
PVPYNL--
PNPYNL--
--PYNLYS
--PYNLVN
--PYNLTM
--PYNLTG
--PYNLTA
--PYNLSQ
--PYNLRQ
--PYNLNY
--PYNLNL
--PYNLNA
--PYNLKS
--PYNLIN
--PYNLIH
--PYNLHA
--PYNLGM
--PYNLGI
--PYNLFY
--PYNLFS
PMPYNL--
--PYNLYI
--PYNLYH
--PYNLYF
--PYNLYD
--PYNLWH
--PYNLVD

--PYNLVA
--PYNLTV
--PYNLTN
--PYNLTE
--PYNLRM
--PYNLQT
--PYNLQK
--PYNLQG
--PYNLPL
--PYNLNM
--PYNLMT
--PYNLMH
--PYNLLQ
--PYNLIT
--PYNLII
--PYNLHN
--PYNLFG
--PYNLES
--PYNLEG
--PYNLAW
--PYNLAN
--PYNLAM
--PYNLAD
PTPYNL--
PQPYNL--
PEPYNL--
--PYNLWQ
--PYNLTY
--PYNLTW
--PYNLTQ
--PYNLTD
--PYNLRK
--PYNLQL
--PYNLPI
--PYNLMQ
--PYNLMN
--PYNLMA
--PYNLKN
--PYNLKF
--PYNLIQ
--PYNLIE
--PYNLHV
--PYNLHK
--PYNLHD
--PYNLFH
--PYNLEY
--PYNLEW
--PYNLEI
--PYNLAV
--PYNLAR

--PYNLAQ
--PYNLAK
PHPYNL--
--PYNLYE
--PYNLWG
--PYNLWF
--PYNLWE
--PYNLWD
--PYNLVE
--PYNLSI
--PYNLRD
--PYNLQV
--PYNLQQ
--PYNLQF
--PYNLQA
--PYNLPM
--PYNLNN
--PYNLNI
--PYNLMV
--PYNLMM
--PYNLMK
--PYNLMD
--PYNLLP
--PYNLLD
--PYNLKY
--PYNLKT
--PYNLKH
--PYNLHM
--PYNLHL
--PYNLHH
--PYNLGT
--PYNLGH
--PYNLGA
--PYNLFD
--PYNLEN
--PYNLEK
--PYNLEH
--PYNLDA
--PYNLAT
--PYNLAH
PWPYNL--
-QPYNLL-
QKPYNL--
QGPYNL--
QRPYNL--
-QPYNLV-
QMPYNL--
QHPYNL--
-QPYNLS-
-QPYNLA-

QTPYNL--
-QPYNLI-
QSPYNL--
QQPYNL--
-QPYNLR-
QAPYNL--
-QPYNLT-
-QPYNLF-
QEPYNL--
QYPYNL--
QWPYNL--
-QPYNLW-
-QPYNLG-
-QPYNLE-
QFPYNL--
-RPYNLM-
RVPYNL--
RGPYNL--
RSPYNL--
-RPYNLS-
-RPYNLL-
-RPYNLK-
RRPYNL--
RLPYNL--
RWPYNL--
RMPYNL--
RAPYNL--
RQPYNL--
RTPYNL--
-RPYNLT-
RYPYNL--
-RPYNLQ-
-RPYNLH-
RKPYNL--
RHPYNL--
-RPYNLI-
-RPYNLY-
RPPYNL--
RFPYNL--
-RPYNLE-
-RPYNLN-
-RPYNLG-
RIPYNL--
RNPYNL--
RDPYNL--
-RPYNLF-
REPYNL--
RCPYNL--
-SPYNLR-
SLPYNL--

-SPYNLL-
-SPYNLT-
SMPYNL--
SSPYNL--
SGPYNL--
SFPYNL--
SQPYNL--
-SPYNLS-
STPYNL--
SEPYNL--
SVPYNL--
SIPYNL--
-SPYNLY-
SKPYNL--
-SPYNLA-
-SPYNLW-
-SPYNLQ-
-SPYNLM-
-SPYNLK-
SHPYNL--
SWPYNL--
-SPYNLV-
-SPYNLF-
SPPYNL--
SDPYNL--
SYPYNL--
-SPYNLN-
SAPYNL--
-SPYNLI-
-SPYNLH-
-SPYNLE-
SNPYNL--
-TPYNLR-
-TPYNLL-
TLPYNL--
-TPYNLT-
TKPYNL--
TSPYNL--
-TPYNLS-
-TPYNLA-
TRPYNL--
TAPYNL--
TMPYNL--
TVPYNL--
TIPYNL--
-TPYNLM-
-TPYNLW-
-TPYNLV-
TGPYNL--
TYPYNL--

-TPYNLI-
-TPYNLF-
TNPYNL--
TPPYNL--
TWPYNL--
-TPYNLQ-
-TPYNLN-
TFPYNL--
TDPYNL--
TTPYNL--
VSPYNL--
-VPYNLA-
-VPYNLI-
VWPYNL--
-VPYNLL-
VLPYNL--
VRPYNL--
-VPYNLS-
VVPYNL--
VIPYNL--
VYPYNL--
-VPYNLG-
VDPYNL--
-VPYNLT-
-VPYNLQ-
-VPYNLY-
-VPYNLV-
VMPYNL--
VGPYNL--
VFPYNL--
VKPYNL--
VTPYNL--
VQPYNL--
-VPYNLH-
-VPYNLW-
-VPYNLR-
VNPYNL--
VHPYNL--
VAPYNL--
-VPYNLM-
-VPYNLK-
-VPYNLE-
VPPYNL--
-WPYNLR-
WVPYNL--
WRPYNL--
WSPYNL--
WAPYNL--
WKPYNL--
WTPYNL--

| | | | |
|---|---|---|---|
| | -WPYNLV- | | |
| | -WPYNLK- | | |
| | WMPYNL-- | | |
| | -WPYNLM- | | |
| | -WPYNLL- | | |
| | -WPYNLI- | | |
| | WNPYNL-- | | |
| | WHPYNL-- | | |
| | WFPYNL-- | | |
| | WEPYNL-- | | |
| | WWPYNL-- | | |
| | -WPYNLY- | | |
| | -WPYNLT- | | |
| | -WPYNLQ- | | |
| | -WPYNLA- | | |
| | WIPYNL-- | | |
| | WGPYNL-- | | |
| | -YPYNLR- | | |
| | YRPYNL-- | | |
| | YVPYNL-- | | |
| | YSPYNL-- | | |
| | YFPYNL-- | | |
| | YAPYNL-- | | |
| | -YPYNLT- | | |
| | YIPYNL-- | | |
| | YTPYNL-- | | |
| | YKPYNL-- | | |
| | YNPYNL-- | | |
| | YWPYNL-- | | |
| | -YPYNLL- | | |
| | YPPYNL-- | | |
| | YGPYNL-- | | |
| | -YPYNLQ- | | |
| | -YPYNLK- | | |
| | -YPYNLI- | | |
| | YYPYNL-- | | |
| | YQPYNL-- | | |
| | -YPYNLV- | | |
| | -YPYNLM- | | |
| | -YPYNLG- | | |
| | -YPYNLA- | | |
| | YEPYNL-- | | |
| | YDPYNL-- | | |
| | -YPYNLY- | | |
| | -YPYNLE- | | |
| PVPY | 219 | 701 | 30.82 |
| | -APVPYL- | | |
| | -APVPYR- | | |
| | ARPVPY-- | | |
| | -APVPYF- | | |

ANPVPY--
AMPVPY--
AIPVPY--
AAPVPY--
ATPVPY--
-APVPYM-
AKPVPY--
-APVPYT-
AYPVPY--
ASPVPY--
-APVPYI-
-APVPYH-
-APVPYG-
APPVPY--
ALPVPY--
AHPVPY--
ADPVPY--
-APVPYY-
AWPVPY--
-APVPYV-
-APVPYN-
AVPVPY--
-APVPYK-
-APVPYA-
AFPVPY--
-CPVPYR-
CSPVPY--
CTPVPY--
-CPVPYK-
CFPVPY--
CCPVPY--
-DPVPYQ-
DVPVPY--
DRPVPY--
-DPVPYI-
-DPVPYT-
DFPVPY--
DTPVPY--
DLPVPY--
DDPVPY--
DAPVPY--
DYPVPY--
DWPVPY--
DQPVPY--
-DPVPYW-
-DPVPYV-
-DPVPYS-
-DPVPYL-
-DPVPYK-
DNPVPY--

DMPVPY--
DGPVPY--
ESPVPY--
-EPVPYM-
-EPVPYF-
-EPVPYA-
EVPVPY--
-EPVPYT-
EQPVPY--
-EPVPYY-
-EPVPYK-
ELPVPY--
EAPVPY--
ETPVPY--
ERPVPY--
EKPVPY--
EHPVPY--
EYPVPY--
-EPVPYS-
-EPVPYI-
-EPVPYH-
-EPVPYG-
ENPVPY--
EMPVPY--
EDPVPY--
FRPVPY--
-FPVPYL-
-FPVPYS-
FMPVPY--
-FPVPYH-
FTPVPY--
-FPVPYV-
FLPVPY--
FDPVPY--
FVPVPY--
FSPVPY--
-FPVPYM-
-FPVPYK-
-FPVPYI-
FGPVPY--
FFPVPY--
-FPVPYT-
-FPVPYR-
-FPVPYA-
FNPVPY--
-GPVPYL-
GAPVPY--
-GPVPYR-
-GPVPYV-
GVPVPY--

GSPVPY--
GIPVPY--
-GPVPYS-
-GPVPYI-
GNPVPY--
GGPVPY--
GYPVPY--
-GPVPYH-
GHPVPY--
GWPVPY--
GTPVPY--
-GPVPYW-
-GPVPYT-
-GPVPYN-
-GPVPYF-
GKPVPY--
GQPVPY--
-GPVPYY-
-GPVPYQ-
-GPVPYG-
-GPVPYE-
-GPVPYA-
GPPVPY--
GDPVPY--
GCPVPY--
-HPVPYR-
-HPVPYM-
-HPVPYL-
HYPVPY--
HVPVPY--
HAPVPY--
HSPVPY--
HRPVPY--
-HPVPYK-
HLPVPY--
HIPVPY--
HTPVPY--
-HPVPYS-
HMPVPY--
HKPVPY--
-HPVPYT-
-HPVPYI-
-HPVPYF-
-HPVPYA-
HHPVPY--
HGPVPY--
HEPVPY--
-IPVPYR-
IRPVPY--
-IPVPYA-

IWPVPY--
ISPVPY--
IKPVPY--
-IPVPYS-
ILPVPY--
-IPVPYW-
-IPVPYI-
IIPVPY--
-IPVPYY-
IPPVPY--
IMPVPY--
IVPVPY--
ITPVPY--
-IPVPYV-
-IPVPYT-
-IPVPYN-
-IPVPYM-
-IPVPYK-
-IPVPYG-
-IPVPYF-
IGPVPY--
ICPVPY--
KSPVPY--
KGPVPY--
-KPVPYV-
-KPVPYL-
KKPVPY--
-KPVPYS-
KWPVPY--
KPPVPY--
-KPVPYR-
-KPVPYA-
KLPVPY--
KIPVPY--
KAPVPY--
KVPVPY--
-KPVPYI-
-KPVPYH-
KRPVPY--
-KPVPYQ-
KMPVPY--
KEPVPY--
-KPVPYW-
-KPVPYT-
-KPVPYG-
-KPVPYF-
KHPVPY--
KFPVPY--
KQPVPY--
-KPVPYM-

```
-KPVPYE-
KNPVPY--
KDPVPY--
KCPVPY--
LRPVPY--
LSPVPY--
LLPVPY--
-LPVPYL-
LGPVPY--
LVPVPY--
-LPVPYT-
-LPVPYK-
-LPVPYS-
-LPVPYR-
LPPVPY--
LDPVPY--
LAPVPY--
-LPVPYV-
-LPVPYA-
LEPVPY--
LQPVPY--
LYPVPY--
-LPVPYW-
-LPVPYQ-
LHPVPY--
LTPVPY--
-LPVPYY-
-LPVPYI-
LKPVPY--
-LPVPYF-
LFPVPY--
-MPVPYT-
-MPVPYS-
MSPVPY--
-MPVPYR-
-MPVPYQ-
MVPVPY--
-MPVPYY-
MIPVPY--
MTPVPY--
MLPVPY--
MKPVPY--
MWPVPY--
-MPVPYF-
-MPVPYA-
MEPVPY--
-MPVPYW-
-MPVPYV-
-MPVPYM-
-MPVPYL-
```

MMPVPY--
MYPVPY--
-MPVPYN-
-MPVPYI-
MPPVPY--
MGPVPY--
MAPVPY--
NRPVPY--
-NPVPYS-
-NPVPYK-
NWPVPY--
NTPVPY--
NGPVPY--
-NPVPYV-
-NPVPYT-
-NPVPYR-
NNPVPY--
NIPVPY--
NQPVPY--
-NPVPYM-
-NPVPYI-
NEPVPY--
NCPVPY--
NAPVPY--
NVPVPY--
-NPVPYW-
NLPVPY--
-NPVPYQ-
-NPVPYN-
NKPVPY--
NHPVPY--
--PVPYRI
--PVPYLT
--PVPYLS
--PVPYRR
--PVPYRL
--PVPYFS
--PVPYTV
--PVPYYV
--PVPYRT
--PVPYKS
--PVPYFA
--PVPYQL
--PVPYGS
--PVPYYL
--PVPYIQ
--PVPYGI
--PVPYEL
--PVPYAS
--PVPYSG

--PVPYRK
--PVPYMH
--PVPYHL
--PVPYWA
--PVPYTI
--PVPYMQ
--PVPYIL
--PVPYYI
--PVPYVM
--PVPYRN
--PVPYQM
--PVPYML
--PVPYMD
--PVPYKL
--PVPYIS
--PVPYAY
PWPVPY--
--PVPYYM
--PVPYYA
--PVPYWT
--PVPYWF
--PVPYTS
--PVPYSK
--PVPYRS
--PVPYRG
--PVPYPM
--PVPYLW
--PVPYLQ
--PVPYIR
--PVPYHF
--PVPYFL
--PVPYFI
--PVPYAI
--PVPYYY
--PVPYWS
--PVPYTR
--PVPYSI
--PVPYRW
--PVPYRV
--PVPYRE
--PVPYRD
--PVPYRA
--PVPYMK
--PVPYMF
--PVPYLY
--PVPYLR
--PVPYLN
--PVPYKK
--PVPYHG
--PVPYFG

PAPVPY--
--PVPYYT
--PVPYWH
--PVPYVL
--PVPYTG
--PVPYSW
--PVPYSA
--PVPYKG
--PVPYIT
--PVPYIG
--PVPYFY
--PVPYFK
--PVPYAF
PKPVPY--
PYPVPY--
--PVPYYS
--PVPYWY
--PVPYVY
--PVPYVS
--PVPYVH
--PVPYVE
--PVPYTK
--PVPYSS
--PVPYSR
--PVPYSM
--PVPYSH
--PVPYQY
--PVPYQS
--PVPYNT
--PVPYNI
--PVPYMM
--PVPYMG
--PVPYLM
--PVPYLK
--PVPYKY
--PVPYKN
--PVPYIY
--PVPYIF
--PVPYGM
--PVPYAG
--PVPYYW
--PVPYWR
--PVPYWL
--PVPYWG
--PVPYVT
--PVPYVR
--PVPYVN
--PVPYVF
--PVPYTY
--PVPYSF

--PVPYRY
--PVPYRQ
--PVPYPI
--PVPYNF
--PVPYMN
--PVPYLI
--PVPYLA
--PVPYKQ
--PVPYKI
--PVPYKA
--PVPYHV
--PVPYHT
--PVPYHS
--PVPYHM
--PVPYFR
--PVPYFH
--PVPYEI
--PVPYDL
--PVPYAH
PVPVPY--
PLPVPY--
--PVPYYN
--PVPYYH
--PVPYYF
--PVPYWQ
--PVPYWN
--PVPYWM
--PVPYTT
--PVPYTN
--PVPYTF
--PVPYTA
--PVPYSV
--PVPYRH
--PVPYRF
--PVPYQV
--PVPYQT
--PVPYQR
--PVPYQA
--PVPYPQ
--PVPYPH
--PVPYNM
--PVPYNA
--PVPYMY
--PVPYMT
--PVPYLV
--PVPYKT
--PVPYKR
--PVPYKH
--PVPYKF
--PVPYKE

--PVPYIW
--PVPYHI
--PVPYGW
--PVPYGT
--PVPYGG
--PVPYGF
--PVPYFM
--PVPYFF
--PVPYAV
--PVPYAQ
PSPVPY--
PRPVPY--
PFPVPY--
PEPVPY--
PDPVPY--
--PVPYYD
--PVPYWK
--PVPYWI
--PVPYVQ
--PVPYVK
--PVPYVG
--PVPYVD
--PVPYTW
--PVPYTQ
--PVPYTH
--PVPYQG
--PVPYPY
--PVPYPV
--PVPYNY
--PVPYNV
--PVPYNN
--PVPYMI
--PVPYMA
--PVPYLP
--PVPYLH
--PVPYLF
--PVPYLD
--PVPYKW
--PVPYKM
--PVPYIM
--PVPYIK
--PVPYIH
--PVPYIC
--PVPYIA
--PVPYHR
--PVPYHH
--PVPYGY
--PVPYGQ
--PVPYGN
--PVPYGH

--PVPYGA
--PVPYFW
--PVPYFQ
--PVPYET
--PVPYEQ
--PVPYEM
--PVPYEA
--PVPYAT
--PVPYAK
PTPVPY--
-PPVPYR-
PNPVPY--
QSPVPY--
-QPVPYL-
QLPVPY--
QWPVPY--
-QPVPYS-
QTPVPY--
QYPVPY--
QVPVPY--
QRPVPY--
-QPVPYW-
-QPVPYV-
-QPVPYK-
-QPVPYA-
QGPVPY--
QAPVPY--
QPPVPY--
QKPVPY--
QIPVPY--
QQPVPY--
-QPVPYQ-
-QPVPYF-
QMPVPY--
QFPVPY--
QEPVPY--
-RPVPYV-
RIPVPY--
RVPVPY--
RTPVPY--
-RPVPYA-
RMPVPY--
RLPVPY--
RWPVPY--
RPPVPY--
RRPVPY--
RYPVPY--
-RPVPYH-
RFPVPY--
RSPVPY--

−RPVPYW−
RNPVPY−−
−RPVPYR−
RQPVPY−−
−RPVPYT−
RGPVPY−−
RAPVPY−−
−RPVPYY−
−RPVPYI−
−RPVPYG−
−RPVPYF−
−RPVPYK−
−RPVPYQ−
−RPVPYN−
RHPVPY−−
−RPVPYM−
REPVPY−−
RDPVPY−−
RCPVPY−−
SSPVPY−−
SRPVPY−−
SVPVPY−−
−SPVPYW−
−SPVPYS−
−SPVPYI−
SLPVPY−−
−SPVPYR−
−SPVPYF−
−SPVPYL−
SIPVPY−−
−SPVPYA−
SDPVPY−−
SYPVPY−−
−SPVPYT−
−SPVPYM−
−SPVPYK−
SGPVPY−−
SQPVPY−−
−SPVPYY−
−SPVPYV−
−SPVPYH−
SMPVPY−−
SEPVPY−−
STPVPY−−
SNPVPY−−
SHPVPY−−
−SPVPYQ−
SFPVPY−−
TRPVPY−−
−TPVPYS−

TQPVPY--
TVPVPY--
TSPVPY--
-TPVPYK-
THPVPY--
-TPVPYR-
TFPVPY--
-TPVPYY-
-TPVPYL-
TGPVPY--
TAPVPY--
-TPVPYV-
-TPVPYI-
-TPVPYT-
-TPVPYH-
TKPVPY--
TEPVPY--
-TPVPYN-
TTPVPY--
-TPVPYM-
-TPVPYF-
TMPVPY--
TYPVPY--
TWPVPY--
-TPVPYQ-
-TPVPYA-
TNPVPY--
TCPVPY--
VSPVPY--
VHPVPY--
VGPVPY--
-VPVPYS-
VLPVPY--
-VPVPYA-
VKPVPY--
VAPVPY--
-VPVPYY-
-VPVPYR-
-VPVPYI-
VPPVPY--
-VPVPYT-
VMPVPY--
VTPVPY--
-VPVPYW-
VIPVPY--
VYPVPY--
VVPVPY--
-VPVPYM-
-VPVPYK-
VNPVPY--

VDPVPY--
VRPVPY--
-VPVPYV-
VQPVPY--
-VPVPYQ-
-VPVPYG-
VCPVPY--
WRPVPY--
-WPVPYS-
-WPVPYI-
-WPVPYF-
WLPVPY--
WSPVPY--
-WPVPYA-
WFPVPY--
-WPVPYR-
-WPVPYL-
WEPVPY--
WTPVPY--
-WPVPYV-
-WPVPYT-
WGPVPY--
WYPVPY--
WVPVPY--
WNPVPY--
YVPVPY--
YSPVPY--
YRPVPY--
-YPVPYA-
-YPVPYM-
-YPVPYS-
-YPVPYT-
YKPVPY--
YIPVPY--
-YPVPYY-
-YPVPYI-
-YPVPYF-
YNPVPY--
YLPVPY--
YQPVPY--
YHPVPY--
YGPVPY--
YFPVPY--
YEPVPY--
YDPVPY--
-YPVPYG-
YCPVPY--

PALL 461 616 30.81

-GPALLG-
-GPALLN-

```
-GPALLE-
-HPALLA-
-IPALLG-
-KPALLG-
-LPALLE-
--PALLGV
--PALLTE
--PALLSD
--PALLRP
--PALLPV
--PALLPR
--PALLNV
--PALLNT
--PALLGT
--PALLGG
--PALLFE
--PALLDT
--PALLDS
--PALLAR
-RPALLP-
-TPALLN-
AGPALL--
ASPALL--
-APALLK-
ALPALL--
-APALLW-
-APALLV-
AAPALL--
ATPALL--
AHPALL--
AEPALL--
-APALLY-
-APALLG-
-APALLA-
AFPALL--
-APALLH-
AIPALL--
AYPALL--
APPALL--
-APALLN-
ADPALL--
ANPALL--
AQPALL--
-APALLQ-
-APALLE-
-APALLC-
ACPALL--
CVPALL--
CRPALL--
CIPALL--
```

CTPALL--
CSPALL--
CPPALL--
-CPALLK-
CKPALL--
CEPALL--
CMPALL--
CCPALL--
CWPALL--
-CPALLT-
CNPALL--
CLPALL--
CGPALL--
CFPALL--
CAPALL--
CYPALL--
-CPALLW-
-CPALLS-
-CPALLI-
CHPALL--
CDPALL--
DKPALL--
-DPALLL-
DLPALL--
DWPALL--
DRPALL--
-DPALLR-
DIPALL--
DGPALL--
DEPALL--
DVPALL--
DSPALL--
DAPALL--
-DPALLV-
-DPALLM-
-DPALLK-
DYPALL--
DQPALL--
DFPALL--
DHPALL--
-DPALLY-
DMPALL--
-DPALLF-
-DPALLT-
-DPALLQ-
ERPALL--
-EPALLR-
ESPALL--
-EPALLQ-
EVPALL--

EIPALL--
-EPALLM-
ELPALL--
EQPALL--
-EPALLS-
EMPALL--
EKPALL--
EGPALL--
EEPALL--
EAPALL--
-EPALLH-
-EPALLT-
-EPALLV-
ENPALL--
EYPALL--
-EPALLW-
-EPALLF-
EWPALL--
-EPALLY-
-EPALLN-
-EPALLK-
-EPALLI-
-EPALLA-
EFPALL--
-EPALLG-
EHPALL--
-FPALLK-
FKPALL--
FDPALL--
FPPALL--
FMPALL--
-FPALLQ-
FAPALL--
FWPALL--
-FPALLV-
FGPALL--
FNPALL--
FLPALL--
FTPALL--
FHPALL--
-FPALLH-
GVPALL--
-GPALLT-
GSPALL--
GQPALL--
GAPALL--
GTPALL--
-GPALLK-
GMPALL--
GEPALL--

GHPALL--
-GPALLY-
GGPALL--
-GPALLL-
-GPALLA-
-GPALLF-
-GPALLW-
-GPALLH-
-GPALLI-
GPPALL--
GWPALL--
-GPALLQ-
GCPALL--
-HPALLT-
-HPALLS-
-HPALLK-
HLPALL--
-HPALLR-
HEPALL--
HAPALL--
HTPALL--
-HPALLL-
HKPALL--
HSPALL--
-HPALLI-
-HPALLH-
HVPALL--
HMPALL--
HPPALL--
-HPALLY-
HHPALL--
-HPALLQ-
HCPALL--
-HPALLM-
-IPALLR-
IRPALL--
-IPALLK-
IYPALL--
-IPALLT-
IFPALL--
-IPALLS-
IGPALL--
IEPALL--
ISPALL--
IAPALL--
-IPALLI-
IDPALL--
IVPALL--
IPPALL--
-IPALLM-

INPALL--
IIPALL--
IHPALL--
-IPALLQ-
-IPALLH-
-IPALLA-
IMPALL--
IKPALL--
ICPALL--
-IPALLV-
-KPALLY-
KFPALL--
KWPALL--
KKPALL--
KTPALL--
KMPALL--
KQPALL--
KNPALL--
-KPALLH-
KEPALL--
KHPALL--
-KPALLQ-
KCPALL--
KIPALL--
-KPALLW-
-KPALLE-
KDPALL--
-KPALLN-
-KPALLM-
LSPALL--
-LPALLR-
LGPALL--
LKPALL--
LQPALL--
LYPALL--
LHPALL--
LDPALL--
-LPALLI-
-LPALLT-
LPPALL--
-LPALLM-
LWPALL--
LEPALL--
LCPALL--
MRPALL--
MKPALL--
MGPALL--
-MPALLV-
-MPALLR-
MNPALL--

MSPALL--
-MPALLK-
-MPALLH-
MEPALL--
MQPALL--
-MPALLM-
MDPALL--
MVPALL--
-MPALLI-
MPPALL--
MHPALL--
MCPALL--
MYPALL--
-MPALLQ-
-MPALLE-
MIPALL--
MTPALL--
-MPALLA-
-NPALLR-
NTPALL--
NKPALL--
NGPALL--
NAPALL--
-NPALLS-
NEPALL--
NNPALL--
-NPALLM-
-NPALLV-
NIPALL--
-NPALLT-
NSPALL--
NQPALL--
-NPALLH-
-NPALLI-
NYPALL--
NWPALL--
NHPALL--
-NPALLQ-
NMPALL--
-NPALLY-
-NPALLA-
NDPALL--
NCPALL--
--PALLSS
--PALLYS
--PALLVS
--PALLRS
--PALLKR
--PALLRG
--PALLLG

--PALLGL
--PALLSG
--PALLTG
--PALLSY
--PALLFS
--PALLIS
--PALLRY
--PALLYG
--PALLTS
--PALLRI
--PALLRD
--PALLSM
--PALLQT
--PALLGI
--PALLAS
--PALLMT
--PALLTL
--PALLSA
--PALLLQ
--PALLKS
PWPALL--
--PALLRV
--PALLWS
--PALLSI
--PALLRH
--PALLTR
--PALLSV
--PALLRW
--PALLLN
--PALLVH
--PALLTI
--PALLLD
--PALLRT
--PALLRM
--PALLRE
--PALLQM
--PALLKF
--PALLVG
--PALLPI
--PALLVA
--PALLRF
--PALLKT
--PALLKQ
--PALLKG
--PALLIR
--PALLAI
PSPALL--
PFPALL--
--PALLVK
--PALLRQ

--PALLKA
--PALLAA
PYPALL--
PEPALL--
PVPALL--
--PALLTT
--PALLPL
PAPALL--
--PALLYT
--PALLTY
--PALLTN
--PALLQN
--PALLLH
--PALLKK
--PALLHA
--PALLNS
--PALLMG
--PALLKY
--PALLKN
--PALLIG
--PALLHF
--PALLGW
--PALLGF
--PALLYW
--PALLYH
--PALLQV
--PALLPY
--PALLMV
--PALLKV
--PALLYA
--PALLTV
--PALLNM
--PALLNA
--PALLMN
--PALLIA
--PALLHG
--PALLAG
--PALLTW
--PALLTA
--PALLSN
--PALLNY
--PALLNF
--PALLHS
--PALLES
--PALLAQ
--PALLTQ
--PALLTK
--PALLQS
--PALLQK
--PALLQG

--PALLQA
--PALLMQ
--PALLMK
--PALLIN
--PALLIE
--PALLWQ
--PALLVD
--PALLIH
--PALLFG
--PALLYK
--PALLSR
--PALLQQ
--PALLIT
--PALLGM
--PALLGA
--PALLAT
PQPALL--
PNPALL--
--PALLVE
--PALLTD
--PALLSQ
--PALLSH
--PALLQH
--PALLNG
--PALLME
--PALLKM
--PALLKI
--PALLIK
--PALLID
--PALLHY
--PALLHT
--PALLHQ
--PALLHM
--PALLHI
--PALLAV
--PALLAH
--PALLWR
--PALLQW
--PALLPW
--PALLPF
--PALLNQ
--PALLAN
PIPALL--
--PALLYQ
--PALLTH
--PALLQY
--PALLQD
--PALLKW
--PALLII
--PALLFW

--PALLFQ
--PALLEM
--PALLWM
--PALLVM
--PALLPM
--PALLNW
--PALLKH
--PALLKE
--PALLKD
--PALLIQ
--PALLHK
--PALLHH
--PALLGN
-PPALLQ-
-PPALLL-
-PPALLE-
-PPALLA-
PCPALL--
--PALLWW
--PALLSE
--PALLQF
--PALLQE
--PALLFD
--PALLEN
--PALLEA
--PALLCS
--PALLCG
-QPALLR-
QSPALL--
QLPALL--
QKPALL--
-QPALLS-
-QPALLK-
QFPALL--
QPPALL--
-QPALLF-
QYPALL--
QHPALL--
QTPALL--
-QPALLA-
QMPALL--
QAPALL--
QQPALL--
QWPALL--
QNPALL--
QGPALL--
-QPALLI-
-QPALLW-
-QPALLQ-
-QPALLM-

-QPALLL-
-QPALLH-
QDPALL--
-QPALLV-
QCPALL--
RGPALL--
-RPALLK-
-RPALLM-
RFPALL--
RQPALL--
-RPALLI-
RDPALL--
-RPALLW-
-RPALLE-
RCPALL--
SSPALL--
-SPALLL-
SAPALL--
SLPALL--
-SPALLQ-
-SPALLA-
-SPALLM-
SMPALL--
SQPALL--
SFPALL--
SYPALL--
-SPALLH-
SHPALL--
SEPALL--
SPPALL--
-SPALLF-
SWPALL--
-SPALLI-
SIPALL--
-SPALLW-
-SPALLN-
SCPALL--
-SPALLE-
-TPALLR-
-TPALLQ-
TFPALL--
-TPALLT-
-TPALLK-
TNPALL--
TPPALL--
TTPALL--
TQPALL--
TGPALL--
-TPALLY-
TAPALL--

-TPALLS-
TMPALL--
-TPALLI-
-TPALLM-
TEPALL--
TYPALL--
THPALL--
TWPALL--
-TPALLA-
-TPALLE-
-VPALLT-
-VPALLK-
VLPALL--
VWPALL--
-VPALLQ-
VQPALL--
VFPALL--
VPPALL--
VAPALL--
-VPALLW-
-VPALLM-
-VPALLH-
-VPALLI-
VNPALL--
VHPALL--
VGPALL--
-VPALLA-
-VPALLG-
VEPALL--
WPPALL--
WEPALL--
WVPALL--
WLPALL--
-WPALLQ-
-WPALLM-
-WPALLI-
WHPALL--
WDPALL--
WWPALL--
WCPALL--
-WPALLH-
-WPALLA-
YGPALL--
YLPALL--
-YPALLT-
YVPALL--
YEPALL--
YSPALL--
-YPALLI-
YNPALL--

```
              YFPALL--
              YQPALL--
              -YPALLH-
              YHPALL--
              YCPALL--
              YIPALL--
              YDPALL--
              YPPALL--
              -YPALLK-
              -YPALLY-
              -YPALLV-
              YMPALL--
              -YPALLN-
              -YPALLW-
PYPL           173        728     30.81
              MGPYPL--
              --PYPLSR
              --PYPLPA
              -TPYPLF-
              -APYPLR-
              -APYPLI-
              ALPYPL--
              AFPYPL--
              AMPYPL--
              -APYPLM-
              AAPYPL--
              ARPYPL--
              -APYPLS-
              AIPYPL--
              -APYPLY-
              -APYPLL-
              AVPYPL--
              AWPYPL--
              -APYPLK-
              -APYPLH-
              AKPYPL--
              AGPYPL--
              ATPYPL--
              -APYPLW-
              -APYPLA-
              APPYPL--
              -APYPLN-
              -APYPLF-
              AEPYPL--
              ACPYPL--
              AYPYPL--
              AQPYPL--
              -APYPLG-
              ADPYPL--
              CSPYPL--
```

CRPYPL--
-CPYPLL-
CPPYPL--
CFPYPL--
DMPYPL--
DRPYPL--
DLPYPL--
DQPYPL--
DKPYPL--
DGPYPL--
DFPYPL--
DTPYPL--
-DPYPLK-
DYPYPL--
DVPYPL--
-DPYPLL-
-DPYPLI-
-DPYPLF-
DNPYPL--
DHPYPL--
DWPYPL--
DSPYPL--
-DPYPLY-
-DPYPLW-
-DPYPLV-
-DPYPLA-
DPPYPL--
DEPYPL--
DAPYPL--
-EPYPLR-
EKPYPL--
EVPYPL--
-EPYPLK-
-EPYPLI-
ERPYPL--
-EPYPLS-
ENPYPL--
EYPYPL--
-EPYPLY-
-EPYPLW-
-EPYPLT-
-EPYPLH-
EHPYPL--
EEPYPL--
ETPYPL--
EQPYPL--
-EPYPLV-
EMPYPL--
EIPYPL--
EGPYPL--

EFPYPL--
FSPYPL--
-FPYPLN-
FKPYPL--
FRPYPL--
FNPYPL--
FHPYPL--
FAPYPL--
FYPYPL--
-FPYPLT-
-FPYPLV-
-FPYPLA-
FPPYPL--
FMPYPL--
-FPYPLY-
-FPYPLR-
-FPYPLI-
-FPYPLQ-
FEPYPL--
FVPYPL--
FTPYPL--
FQPYPL--
-FPYPLG-
-FPYPLF-
FFPYPL--
FWPYPL--
-FPYPLE-
FGPYPL--
GLPYPL--
GRPYPL--
GKPYPL--
GIPYPL--
GGPYPL--
GAPYPL--
GTPYPL--
-GPYPLS-
-GPYPLA-
GNPYPL--
GYPYPL--
-GPYPLL-
-GPYPLI-
GMPYPL--
GVPYPL--
-GPYPLN-
-GPYPLM-
GWPYPL--
-GPYPLY-
-GPYPLR-
GFPYPL--
GQPYPL--

-GPYPLW-
-GPYPLV-
-GPYPLQ-
GPPYPL--
GEPYPL--
HRPYPL--
HTPYPL--
-HPYPLQ-
HLPYPL--
HYPYPL--
HSPYPL--
HQPYPL--
-HPYPLN-
HNPYPL--
-HPYPLR-
HGPYPL--
-HPYPLF-
HEPYPL--
-HPYPLM-
-HPYPLL-
-HPYPLI-
HWPYPL--
HVPYPL--
-HPYPLV-
-HPYPLS-
HCPYPL--
-IPYPLL-
IAPYPL--
-IPYPLT-
-IPYPLR-
IMPYPL--
ISPYPL--
-IPYPLN-
-IPYPLI-
ILPYPL--
IYPYPL--
IWPYPL--
IVPYPL--
IRPYPL--
-IPYPLS-
-IPYPLK-
IFPYPL--
-IPYPLY-
-IPYPLV-
-IPYPLQ-
-IPYPLA-
ITPYPL--
IQPYPL--
-IPYPLW-
-IPYPLM-

IPPYPL--
INPYPL--
ICPYPL--
-IPYPLH-
-IPYPLG-
-IPYPLF-
IKPYPL--
IIPYPL--
IGPYPL--
-KPYPLR-
-KPYPLL-
KLPYPL--
KGPYPL--
KVPYPL--
KAPYPL--
KIPYPL--
-KPYPLF-
KMPYPL--
KHPYPL--
KRPYPL--
-KPYPLI-
KPPYPL--
-KPYPLK-
-KPYPLH-
-KPYPLA-
KDPYPL--
KYPYPL--
-KPYPLT-
-KPYPLQ-
KQPYPL--
-KPYPLW-
-KPYPLV-
-KPYPLS-
-KPYPLM-
-KPYPLG-
-KPYPLE-
KNPYPL--
KKPYPL--
KEPYPL--
LTPYPL--
LKPYPL--
LPPYPL--
-LPYPLK-
LLPYPL--
LQPYPL--
-LPYPLM-
LHPYPL--
LGPYPL--
LYPYPL--
LVPYPL--

-LPYPLT-
LFPYPL--
-LPYPLV-
-LPYPLH-
LWPYPL--
-LPYPLS-
-LPYPLQ-
-LPYPLI-
-LPYPLA-
LNPYPL--
-LPYPLY-
LAPYPL--
-LPYPLW-
-LPYPLN-
LIPYPL--
LDPYPL--
-MPYPLR-
MRPYPL--
MDPYPL--
-MPYPLK-
MTPYPL--
MLPYPL--
MMPYPL--
-MPYPLL-
MFPYPL--
-MPYPLV-
-MPYPLI-
MIPYPL--
MEPYPL--
-MPYPLF-
MQPYPL--
MAPYPL--
-MPYPLT-
MNPYPL--
MSPYPL--
-MPYPLH-
MHPYPL--
MVPYPL--
-MPYPLW-
-MPYPLQ-
-MPYPLG-
-MPYPLA-
MPPYPL--
NRPYPL--
NSPYPL--
NLPYPL--
-NPYPLY-
NGPYPL--
-NPYPLF-
NKPYPL--

-NPYPLT-
NNPYPL--
-NPYPLV-
-NPYPLM-
NWPYPL--
NVPYPL--
NTPYPL--
NQPYPL--
-NPYPLS-
-NPYPLR-
-NPYPLL-
-NPYPLI-
-NPYPLH-
NIPYPL--
NFPYPL--
NEPYPL--
NDPYPL--
NAPYPL--
NYPYPL--
-NPYPLK-
NPPYPL--
NMPYPL--
--PYPLLG
--PYPLSW
--PYPLRS
--PYPLLA
--PYPLKL
--PYPLTL
--PYPLSV
--PYPLSG
--PYPLLT
--PYPLWV
--PYPLRK
--PYPLRY
--PYPLRR
--PYPLRQ
--PYPLRH
--PYPLYH
--PYPLWL
--PYPLWG
--PYPLVS
--PYPLVQ
--PYPLLS
--PYPLYK
--PYPLWQ
--PYPLVI
--PYPLSA
--PYPLRN
--PYPLRI
--PYPLQS

--PYPLLN
--PYPLIR
--PYPLEI
--PYPLYA
--PYPLTV
--PYPLSY
--PYPLMT
--PYPLLR
--PYPLGT
--PYPLFS
--PYPLWS
--PYPLST
--PYPLSS
--PYPLQY
--PYPLQW
--PYPLQN
--PYPLLK
--PYPLKA
--PYPLAT
--PYPLYE
--PYPLVT
--PYPLVM
--PYPLVF
--PYPLQL
--PYPLMV
--PYPLKR
--PYPLIS
--PYPLIK
--PYPLIF
--PYPLGA
--PYPLFW
--PYPLFR
--PYPLAS
--PYPLAL
--PYPLAG
--PYPLYN
--PYPLTS
--PYPLSH
--PYPLPV
--PYPLPF
--PYPLKS
--PYPLIG
--PYPLGS
--PYPLFD
--PYPLDL
PDPYPL--
--PYPLYS
--PYPLSN
--PYPLRD
--PYPLRA

--PYPLNS
--PYPLNH
--PYPLMM
--PYPLMK
--PYPLLM
--PYPLKY
--PYPLKT
--PYPLIT
--PYPLHY
--PYPLFH
--PYPLAQ
PTPYPL--
--PYPLYW
--PYPLYR
--PYPLYI
--PYPLVY
--PYPLVR
--PYPLVN
--PYPLTQ
--PYPLTN
--PYPLTM
--PYPLSK
--PYPLPS
--PYPLMY
--PYPLMW
--PYPLME
--PYPLLH
--PYPLKH
--PYPLKG
--PYPLIQ
--PYPLIH
--PYPLIA
--PYPLHR
--PYPLHN
--PYPLGN
PQPYPL--
PGPYPL--
PAPYPL--
--PYPLYG
--PYPLYD
--PYPLWY
--PYPLWN
--PYPLWK
--PYPLWH
--PYPLVW
--PYPLTT
--PYPLTK
--PYPLTI
--PYPLTH
--PYPLTG

--PYPLRE
--PYPLPQ
--PYPLPN
--PYPLNT
--PYPLNQ
--PYPLNF
--PYPLMS
--PYPLML
--PYPLKW
--PYPLKK
--PYPLKI
--PYPLIN
--PYPLHW
--PYPLHF
--PYPLFT
--PYPLFQ
--PYPLFG
--PYPLFA
--PYPLDY
PMPYPL--
--PYPLYT
--PYPLYL
--PYPLWM
--PYPLWI
--PYPLWD
--PYPLWA
--PYPLVK
--PYPLVH
--PYPLVA
--PYPLTY
--PYPLTW
--PYPLTF
--PYPLSF
--PYPLRW
--PYPLQM
--PYPLPW
--PYPLPM
--PYPLNV
--PYPLNK
--PYPLNA
--PYPLKN
--PYPLIV
--PYPLHT
--PYPLGQ
--PYPLGK
--PYPLFM
--PYPLFK
--PYPLEL
--PYPLEF
PHPYPL--

PEPYPL--
--PYPLYM
--PYPLWR
--PYPLWE
--PYPLVV
--PYPLVD
--PYPLTR
--PYPLTA
--PYPLQV
--PYPLQT
--PYPLQA
--PYPLPG
--PYPLNY
--PYPLNG
--PYPLMQ
--PYPLMN
--PYPLMI
--PYPLMG
--PYPLLE
--PYPLLD
--PYPLKM
--PYPLKF
--PYPLIY
--PYPLII
--PYPLHV
--PYPLHS
--PYPLHQ
--PYPLHH
--PYPLHA
--PYPLGH
--PYPLFN
--PYPLFF
--PYPLFE
--PYPLEW
--PYPLEV
--PYPLES
--PYPLEQ
--PYPLDW
--PYPLDM
--PYPLCA
--PYPLAV
--PYPLAN
PWPYPL--
PVPYPL--
PIPYPL--
-QPYPLL-
QRPYPL--
QAPYPL--
-QPYPLS-
QGPYPL--

```
QFPYPL--
-QPYPLY-
-QPYPLW-
-QPYPLQ-
-QPYPLA-
QMPYPL--
QHPYPL--
QSPYPL--
-QPYPLT-
-QPYPLN-
-QPYPLK-
QLPYPL--
QWPYPL--
-QPYPLH-
-QPYPLF-
QDPYPL--
QYPYPL--
QTPYPL--
QQPYPL--
-QPYPLR-
-QPYPLE-
QNPYPL--
RRPYPL--
RFPYPL--
RPPYPL--
RNPYPL--
RYPYPL--
RSPYPL--
-RPYPLW-
-RPYPLI-
RVPYPL--
RWPYPL--
-RPYPLK-
RAPYPL--
RGPYPL--
-RPYPLS-
-RPYPLN-
RIPYPL--
RMPYPL--
-RPYPLV-
-RPYPLQ-
-RPYPLL-
-RPYPLH-
-RPYPLT-
-RPYPLF-
REPYPL--
-RPYPLY-
-RPYPLG-
RTPYPL--
RQPYPL--
```

RHPYPL--
STPYPL--
SRPYPL--
SMPYPL--
SFPYPL--
-SPYPLR-
SIPYPL--
SAPYPL--
-SPYPLY-
SGPYPL--
SSPYPL--
-SPYPLA-
SNPYPL--
-SPYPLF-
-SPYPLK-
SYPYPL--
SQPYPL--
-SPYPLS-
-SPYPLL-
SVPYPL--
-SPYPLM-
SWPYPL--
SKPYPL--
-SPYPLV-
-SPYPLQ-
-SPYPLH-
SEPYPL--
SHPYPL--
SCPYPL--
-SPYPLW-
-SPYPLN-
SDPYPL--
-SPYPLG-
TRPYPL--
TVPYPL--
TSPYPL--
-TPYPLR-
TTPYPL--
TEPYPL--
-TPYPLM-
TLPYPL--
-TPYPLS-
-TPYPLL-
TYPYPL--
-TPYPLI-
TFPYPL--
-TPYPLY-
-TPYPLW-
-TPYPLT-
-TPYPLK-

TKPYPL--
THPYPL--
-TPYPLV-
TQPYPL--
-TPYPLN-
-TPYPLH-
-TPYPLA-
TPPYPL--
TMPYPL--
-TPYPLQ-
TIPYPL--
TDPYPL--
VAPYPL--
-VPYPLR-
VRPYPL--
VGPYPL--
VSPYPL--
-VPYPLS-
VLPYPL--
VKPYPL--
-VPYPLV-
-VPYPLI-
VIPYPL--
-VPYPLY-
VPPYPL--
VFPYPL--
VDPYPL--
VYPYPL--
VTPYPL--
-VPYPLT-
-VPYPLA-
-VPYPLQ-
-VPYPLK-
VHPYPL--
VVPYPL--
VQPYPL--
-VPYPLP-
-VPYPLL-
VMPYPL--
VWPYPL--
-VPYPLW-
VEPYPL--
-VPYPLN-
-VPYPLM-
-VPYPLH-
WTPYPL--
WVPYPL--
WEPYPL--
WYPYPL--
WSPYPL--

WLPYPL--
WKPYPL--
WAPYPL--
WRPYPL--
-WPYPLA-
-WPYPLK-
WNPYPL--
-WPYPLW-
WFPYPL--
-WPYPLT-
-WPYPLS-
-WPYPLI-
WPPYPL--
WGPYPL--
-WPYPLQ-
-WPYPLL-
WIPYPL--
WDPYPL--
YLPYPL--
YIPYPL--
-YPYPLT-
-YPYPLF-
YNPYPL--
-YPYPLM-
-YPYPLH-
YPPYPL--
YKPYPL--
YFPYPL--
-YPYPLS-
YGPYPL--
YSPYPL--
YHPYPL--
YEPYPL--
YYPYPL--
YWPYPL--
-YPYPLQ-
-YPYPLI-
YAPYPL--
-YPYPLV-
-YPYPLL-
-YPYPLA-
YMPYPL--
YDPYPL--

PSSY 87 777 30.80

-CPSSYL-
-DPSSYS-
-LPSSYA-
--PSSYGS
--PSSYGG
--PSSYAR

--PSSYAL
-SPSSYG-
ATPSSY--
-APSSYT-
-APSSYL-
AYPSSY--
AKPSSY--
-APSSYR-
ALPSSY--
-APSSYV-
AEPSSY--
AHPSSY--
AVPSSY--
-APSSYF-
AMPSSY--
AQPSSY--
-APSSYY-
-APSSYS-
AGPSSY--
AAPSSY--
-APSSYM-
-APSSYK-
-APSSYA-
AFPSSY--
AWPSSY--
-APSSYN-
-APSSYH-
-APSSYE-
ANPSSY--
ADPSSY--
-APSSYQ-
AIPSSY--
-APSSYD-
-APSSYC-
CSPSSY--
CLPSSY--
CGPSSY--
CCPSSY--
CMPSSY--
CKPSSY--
CHPSSY--
CNPSSY--
CVPSSY--
CRPSSY--
CQPSSY--
-CPSSYR-
CDPSSY--
CAPSSY--
CTPSSY--
-CPSSYV-

-CPSSYS-
CPPSSY--
CFPSSY--
DRPSSY--
-DPSSYL-
DTPSSY--
DQPSSY--
DKPSSY--
-DPSSYF-
DSPSSY--
DNPSSY--
-DPSSYR-
-DPSSYM-
DVPSSY--
-DPSSYW-
DMPSSY--
DAPSSY--
-DPSSYV-
-DPSSYT-
-DPSSYI-
DGPSSY--
-DPSSYY-
DIPSSY--
DEPSSY--
-EPSSYL-
-EPSSYM-
-EPSSYH-
EIPSSY--
-EPSSYV-
-EPSSYR-
ESPSSY--
ERPSSY--
EWPSSY--
-EPSSYT-
EVPSSY--
ETPSSY--
-EPSSYY-
EKPSSY--
EGPSSY--
-EPSSYQ-
EYPSSY--
EQPSSY--
EDPSSY--
-EPSSYW-
-EPSSYS-
-EPSSYN-
ENPSSY--
EMPSSY--
EFPSSY--
EEPSSY--

EAPSSY--
-EPSSYP-
-EPSSYI-
EPPSSY--
ECPSSY--
FRPSSY--
-FPSSYM-
FSPSSY--
FVPSSY--
-FPSSYT-
-FPSSYQ-
-FPSSYR-
FLPSSY--
-FPSSYF-
FFPSSY--
FEPSSY--
-FPSSYS-
-FPSSYA-
-FPSSYI-
FAPSSY--
FHPSSY--
FWPSSY--
-FPSSYW-
-FPSSYH-
FPPSSY--
FNPSSY--
FIPSSY--
FGPSSY--
-FPSSYY-
-FPSSYK-
-FPSSYV-
FQPSSY--
FKPSSY--
-GPSSYL-
-GPSSYR-
GRPSSY--
-GPSSYV-
GTPSSY--
GKPSSY--
GYPSSY--
-GPSSYS-
GGPSSY--
GSPSSY--
-GPSSYW-
-GPSSYY-
GAPSSY--
GLPSSY--
GWPSSY--
-GPSSYK-
-GPSSYA-

−GPSSYM−
GMPSSY−−
GFPSSY−−
GQPSSY−−
GHPSSY−−
GEPSSY−−
−GPSSYH−
GIPSSY−−
GCPSSY−−
−GPSSYQ−
−GPSSYG−
GPPSSY−−
GDPSSY−−
−GPSSYP−
−GPSSYN−
−HPSSYV−
HRPSSY−−
HGPSSY−−
−HPSSYS−
HTPSSY−−
HSPSSY−−
−HPSSYA−
HYPSSY−−
−HPSSYR−
−HPSSYL−
HWPSSY−−
−HPSSYT−
−HPSSYM−
HLPSSY−−
HAPSSY−−
−HPSSYY−
HKPSSY−−
HIPSSY−−
HEPSSY−−
−HPSSYF−
HNPSSY−−
HFPSSY−−
−HPSSYK−
−HPSSYI−
−HPSSYH−
HPPSSY−−
HMPSSY−−
HHPSSY−−
−HPSSYQ−
HQPSSY−−
−HPSSYN−
HCPSSY−−
IRPSSY−−
−IPSSYV−
ILPSSY−−

-IPSSYS-
-IPSSYI-
IPPSSY--
-IPSSYT-
IVPSSY--
-IPSSYR-
-IPSSYM-
IGPSSY--
IFPSSY--
-IPSSYK-
INPSSY--
IWPSSY--
IKPSSY--
IAPSSY--
ISPSSY--
-IPSSYF-
-IPSSYN-
-IPSSYA-
IMPSSY--
IIPSSY--
IEPSSY--
-IPSSYH-
-IPSSYE-
IHPSSY--
IDPSSY--
KSPSSY--
-KPSSYL-
-KPSSYA-
KGPSSY--
-KPSSYR-
-KPSSYV-
KQPSSY--
KLPSSY--
KTPSSY--
KPPSSY--
-KPSSYI-
KAPSSY--
KYPSSY--
KVPSSY--
-KPSSYT-
-KPSSYF-
KFPSSY--
KIPSSY--
-KPSSYQ-
-KPSSYM-
KWPSSY--
-KPSSYY-
-KPSSYW-
-KPSSYK-
-KPSSYH-

KEPSSY--
-KPSSYN-
KDPSSY--
-KPSSYG-
KNPSSY--
KHPSSY--
-KPSSYP-
-KPSSYE-
KCPSSY--
LRPSSY--
LVPSSY--
LSPSSY--
LKPSSY--
-LPSSYR-
-LPSSYF-
-LPSSYQ-
LGPSSY--
-LPSSYS-
LTPSSY--
LPPSSY--
-LPSSYT-
-LPSSYI-
LLPSSY--
LAPSSY--
-LPSSYW-
-LPSSYV-
-LPSSYK-
LFPSSY--
LQPSSY--
LNPSSY--
LMPSSY--
-LPSSYY-
-LPSSYM-
LHPSSY--
LEPSSY--
LDPSSY--
-LPSSYH-
-LPSSYN-
LCPSSY--
-MPSSYR-
MGPSSY--
-MPSSYL-
MNPSSY--
-MPSSYF-
MRPSSY--
MAPSSY--
-MPSSYK-
MLPSSY--
MWPSSY--
-MPSSYV-

```
-MPSSYT-
-MPSSYS-
-MPSSYM-
-MPSSYA-
MPPSSY--
MVPSSY--
MTPSSY--
MQPSSY--
-MPSSYY-
-MPSSYQ-
MKPSSY--
MHPSSY--
-MPSSYH-
MFPSSY--
MYPSSY--
-MPSSYW-
MMPSSY--
MIPSSY--
MDPSSY--
-MPSSYN-
-MPSSYI-
MCPSSY--
-NPSSYR-
NRPSSY--
NSPSSY--
-NPSSYL-
NLPSSY--
NTPSSY--
NGPSSY--
-NPSSYK-
NDPSSY--
NPPSSY--
-NPSSYV-
-NPSSYT-
-NPSSYI-
NWPSSY--
-NPSSYQ-
-NPSSYY-
-NPSSYW-
-NPSSYH-
NAPSSY--
NHPSSY--
NFPSSY--
-NPSSYM-
NMPSSY--
NKPSSY--
NEPSSY--
NCPSSY--
NQPSSY--
--PSSYLR
```

--PSSYRN
--PSSYLS
--PSSYRL
--PSSYFR
--PSSYTA
--PSSYRV
--PSSYFS
--PSSYWS
--PSSYTS
--PSSYRA
--PSSYSG
--PSSYLA
--PSSYTR
--PSSYLG
--PSSYVS
--PSSYSS
PLPSSY--
--PSSYRH
--PSSYRG
--PSSYSW
--PSSYRT
--PSSYRF
--PSSYVA
--PSSYTQ
--PSSYSM
--PSSYSA
--PSSYLK
--PSSYIT
--PSSYVF
--PSSYSN
--PSSYQS
--PSSYLV
--PSSYAV
--PSSYAT
PVPSSY--
--PSSYYS
--PSSYTY
--PSSYTG
--PSSYSL
--PSSYRE
--PSSYMH
--PSSYKV
--PSSYAA
--PSSYYT
--PSSYYA
--PSSYVV
--PSSYQT
--PSSYMM
--PSSYKG
--PSSYHT

--PSSYWG
--PSSYWA
--PSSYRY
--PSSYMQ
--PSSYMG
--PSSYIS
PSPSSY--
PRPSSY--
PQPSSY--
--PSSYYY
--PSSYYN
--PSSYWN
--PSSYWM
--PSSYVY
--PSSYVT
--PSSYVN
--PSSYVM
--PSSYTN
--PSSYRM
--PSSYQA
--PSSYKS
--PSSYIV
--PSSYIM
--PSSYHA
--PSSYFV
--PSSYFM
--PSSYYL
--PSSYTL
--PSSYTH
--PSSYRI
--PSSYLQ
--PSSYHG
--PSSYEG
--PSSYYG
--PSSYTT
--PSSYSI
--PSSYRD
--PSSYNG
--PSSYNA
--PSSYMS
--PSSYMN
--PSSYLH
--PSSYIR
--PSSYFG
PKPSSY--
--PSSYRW
--PSSYRQ
--PSSYQW
--PSSYQG
--PSSYNS

--PSSYKY
--PSSYKM
--PSSYFY
--PSSYFT
--PSSYAM
--PSSYAF
PIPSSY--
--PSSYWR
--PSSYWH
--PSSYVK
--PSSYTF
--PSSYSR
--PSSYMK
--PSSYLN
--PSSYFW
--PSSYES
--PSSYEA
--PSSYYK
--PSSYWY
--PSSYVI
--PSSYTK
--PSSYSY
--PSSYQM
--PSSYNT
--PSSYNL
--PSSYML
--PSSYKF
--PSSYIY
--PSSYIA
--PSSYAI
PAPSSY--
PYPSSY--
PTPSSY--
--PSSYYQ
--PSSYYF
--PSSYWL
--PSSYTV
--PSSYQV
--PSSYMD
--PSSYMA
--PSSYKK
--PSSYKA
--PSSYIN
--PSSYIK
--PSSYII
--PSSYAG
PMPSSY--
PGPSSY--
PFPSSY--
--PSSYYM

--PSSYVW
--PSSYVH
--PSSYTD
--PSSYSQ
--PSSYSK
--PSSYMY
--PSSYMI
--PSSYMF
--PSSYKI
--PSSYIH
--PSSYHY
--PSSYHS
--PSSYHM
--PSSYAY
--PSSYAS
PHPSSY--
--PSSYYI
--PSSYWW
--PSSYWI
--PSSYVD
--PSSYTW
--PSSYTM
--PSSYSH
--PSSYNY
--PSSYNV
--PSSYMT
--PSSYKQ
--PSSYKN
--PSSYKH
--PSSYIW
--PSSYIQ
--PSSYHV
--PSSYFQ
--PSSYFK
--PSSYCA
-PPSSYL-
PWPSSY--
--PSSYYW
--PSSYYE
--PSSYWV
--PSSYWF
--PSSYTP
--PSSYTI
--PSSYQY
--PSSYQK
--PSSYNW
--PSSYNK
--PSSYMW
--PSSYMV
--PSSYKW

--PSSYID
--PSSYHW
--PSSYHN
--PSSYHF
--PSSYFI
--PSSYFD
--PSSYEI
--PSSYCQ
-PPSSYY-
-PPSSYV-
-PPSSYM-
PNPSSY--
PEPSSY--
QSPSSY--
QGPSSY--
QRPSSY--
-QPSSYY-
QKPSSY--
-QPSSYL-
-QPSSYR-
-QPSSYI-
QVPSSY--
-QPSSYS-
QWPSSY--
-QPSSYK-
QFPSSY--
QMPSSY--
QEPSSY--
-QPSSYW-
QNPSSY--
QLPSSY--
QTPSSY--
-QPSSYH-
QPPSSY--
QIPSSY--
-QPSSYF-
-QPSSYT-
-QPSSYQ-
-QPSSYM-
QDPSSY--
QAPSSY--
QYPSSY--
QQPSSY--
-QPSSYV-
QHPSSY--
QCPSSY--
-RPSSYL-
-RPSSYF-
RSPSSY--
RLPSSY--

RGPSSY--
-RPSSYK-
-RPSSYI-
RAPSSY--
-RPSSYS-
RQPSSY--
-RPSSYY-
RTPSSY--
-RPSSYV-
RFPSSY--
RMPSSY--
-RPSSYW-
-RPSSYN-
RNPSSY--
-RPSSYH-
-RPSSYE-
RPPSSY--
RYPSSY--
RWPSSY--
REPSSY--
RDPSSY--
-RPSSYA-
RIPSSY--
-RPSSYM-
RHPSSY--
-RPSSYG-
RCPSSY--
-RPSSYC-
SVPSSY--
SLPSSY--
STPSSY--
SQPSSY--
SNPSSY--
SKPSSY--
-SPSSYA-
-SPSSYI-
SSPSSY--
-SPSSYS-
SAPSSY--
SIPSSY--
-SPSSYY-
-SPSSYQ-
-SPSSYM-
SYPSSY--
SMPSSY--
SWPSSY--
-SPSSYK-
SGPSSY--
SFPSSY--
-SPSSYF-

SEPSSY--
SHPSSY--
-SPSSYH-
SDPSSY--
SCPSSY--
SPPSSY--
-SPSSYW-
-SPSSYN-
-SPSSYE-
TSPSSY--
TLPSSY--
-TPSSYL-
-TPSSYR-
-TPSSYI-
-TPSSYV-
TGPSSY--
-TPSSYH-
TNPSSY--
-TPSSYS-
-TPSSYK-
TWPSSY--
-TPSSYT-
-TPSSYY-
-TPSSYW-
-TPSSYM-
TKPSSY--
TFPSSY--
TYPSSY--
TTPSSY--
-TPSSYF-
TPPSSY--
TVPSSY--
-TPSSYA-
-TPSSYN-
-TPSSYQ-
TEPSSY--
TIPSSY--
TDPSSY--
-TPSSYE-
-VPSSYL-
VSPSSY--
-VPSSYS-
VRPSSY--
-VPSSYT-
VGPSSY--
-VPSSYV-
-VPSSYI-
VEPSSY--
-VPSSYM-
VLPSSY--

VAPSSY--
-VPSSYH-
VDPSSY--
-VPSSYA-
VNPSSY--
VQPSSY--
VVPSSY--
-VPSSYK-
VMPSSY--
VKPSSY--
VFPSSY--
-VPSSYQ-
VWPSSY--
-VPSSYW-
-VPSSYN-
VPPSSY--
-VPSSYF-
-VPSSYY-
-VPSSYE-
WSPSSY--
WRPSSY--
-WPSSYL-
WGPSSY--
WHPSSY--
WNPSSY--
WQPSSY--
-WPSSYF-
WAPSSY--
WWPSSY--
-WPSSYT-
WDPSSY--
WMPSSY--
WIPSSY--
WYPSSY--
-WPSSYV-
WFPSSY--
-WPSSYQ-
-WPSSYK-
-WPSSYI-
-WPSSYY-
WPPSSY--
-YPSSYT-
YLPSSY--
YSPSSY--
YPPSSY--
YFPSSY--
-YPSSYY-
-YPSSYM-
-YPSSYI-
YWPSSY--

YKPSSY--
YVPSSY--
YTPSSY--
-YPSSYV-
-YPSSYR-
YMPSSY--
YIPSSY--
-YPSSYW-
YQPSSY--
YEPSSY--
YDPSSY--
-YPSSYK-
-YPSSYF-
-YPSSYA-
YHPSSY--
YYPSSY--
YAPSSY--
-YPSSYQ-
-YPSSYN-
YNPSSY--
YCPSSY--

PISL 516 601 30.78

-EPISLN-
-EPISLG-
-FPISLM-
-IPISLG-
-NPISLM-
-NPISLA-
--PISLSR
--PISLSN
--PISLTE
--PISLPN
--PISLNG
--PISLHK
--PISLGT
--PISLEY
-RPISLP-
-VPISLG-
-YPISLN-
-APISLR-
-APISLS-
ASPISL--
-APISLL-
-APISLT-
-APISLI-
ALPISL--
APPISL--
AIPISL--
AEPISL--
AMPISL--

AHPISL--
AQPISL--
AYPISL--
-APISLN-
AFPISL--
ADPISL--
AVPISL--
ATPISL--
-APISLQ-
-APISLK-
AWPISL--
-APISLG-
-APISLM-
-APISLH-
-APISLE-
ANPISL--
ACPISL--
CYPISL--
CRPISL--
CWPISL--
CVPISL--
CSPISL--
CKPISL--
CAPISL--
CPPISL--
CIPISL--
CHPISL--
CGPISL--
-CPISLV-
-CPISLR-
CMPISL--
CTPISL--
CQPISL--
-CPISLY-
-CPISLI-
CNPISL--
CFPISL--
CCPISL--
-DPISLK-
-DPISLL-
DLPISL--
DKPISL--
DAPISL--
DMPISL--
DTPISL--
DIPISL--
DWPISL--
DGPISL--
DQPISL--
-DPISLS-

DDPISL--
DSPISL--
-DPISLV-
DNPISL--
DYPISL--
DFPISL--
DEPISL--
-DPISLT-
ESPISL--
EVPISL--
-EPISLR-
-EPISLL-
-EPISLQ-
ERPISL--
-EPISLS-
ELPISL--
-EPISLW-
-EPISLM-
-EPISLI-
EIPISL--
EQPISL--
-EPISLH-
EMPISL--
EYPISL--
EKPISL--
EGPISL--
ETPISL--
EPPISL--
-EPISLK-
-EPISLA-
EHPISL--
EFPISL--
-EPISLV-
EEPISL--
EDPISL--
-FPISLR-
-FPISLL-
-FPISLK-
FHPISL--
FPPISL--
FDPISL--
FGPISL--
FSPISL--
-FPISLT-
-FPISLV-
-FPISLS-
FNPISL--
FEPISL--
-FPISLH-
-FPISLQ-

-FPISLN-
FAPISL--
FCPISL--
FQPISL--
GKPISL--
GSPISL--
-GPISLR-
-GPISLL-
-GPISLT-
-GPISLS-
GFPISL--
GWPISL--
-GPISLM-
-GPISLA-
GMPISL--
GVPISL--
-GPISLQ-
GIPISL--
GGPISL--
GPPISL--
-GPISLK-
GHPISL--
GYPISL--
GQPISL--
GAPISL--
-GPISLV-
GDPISL--
-GPISLN-
-GPISLH-
-GPISLE-
-HPISLR-
HLPISL--
HSPISL--
HFPISL--
-HPISLS-
-HPISLL-
HGPISL--
HVPISL--
-HPISLV-
HTPISL--
-HPISLM-
HNPISL--
-HPISLK-
HAPISL--
-HPISLT-
-HPISLI-
HQPISL--
-HPISLH-
-HPISLN-
-HPISLA-

HEPISL--
HYPISL--
HPPISL--
HIPISL--
-HPISLQ-
HMPISL--
HKPISL--
HHPISL--
-HPISLE-
ILPISL--
IRPISL--
IVPISL--
-IPISLS-
-IPISLT-
-IPISLK-
IHPISL--
IGPISL--
IYPISL--
IPPISL--
IQPISL--
-IPISLH-
IIPISL--
IEPISL--
-IPISLQ-
-IPISLN-
IKPISL--
IFPISL--
IAPISL--
-IPISLE-
-IPISLC-
KSPISL--
-KPISLR-
KLPISL--
-KPISLH-
-KPISLS-
-KPISLY-
KTPISL--
-KPISLT-
KHPISL--
KGPISL--
-KPISLI-
KEPISL--
KYPISL--
KFPISL--
-KPISLK-
KNPISL--
KWPISL--
-KPISLA-
KCPISL--
-KPISLV-

KIPISL--
-KPISLW-
KDPISL--
-KPISLE-
LSPISL--
-LPISLL-
-LPISLR-
-LPISLT-
LNPISL--
LYPISL--
LWPISL--
-LPISLK-
LPPISL--
-LPISLS-
LQPISL--
LDPISL--
-LPISLM-
-LPISLQ-
-LPISLH-
LCPISL--
-LPISLN-
MLPISL--
MNPISL--
MHPISL--
MMPISL--
MVPISL--
-MPISLS-
-MPISLL-
MFPISL--
-MPISLT-
MEPISL--
-MPISLV-
MDPISL--
MKPISL--
MGPISL--
MYPISL--
MPPISL--
-MPISLQ-
-MPISLH-
-MPISLK-
-MPISLA-
MAPISL--
-MPISLN-
-MPISLM-
-MPISLG-
-MPISLE-
-NPISLR-
NSPISL--
NRPISL--
NLPISL--

-NPISLS-
NYPISL--
NKPISL--
NGPISL--
NAPISL--
NPPISL--
NIPISL--
-NPISLK-
-NPISLI-
NMPISL--
NHPISL--
NWPISL--
NTPISL--
NNPISL--
NDPISL--
NQPISL--
-NPISLF-
-NPISLT-
-NPISLQ-
-NPISLH-
NFPISL--
NCPISL--
-NPISLV-
-NPISLE-
--PISLRR
--PISLVS
--PISLRG
--PISLSS
PGPISL--
--PISLVG
--PISLSL
--PISLWG
--PISLRN
--PISLRA
--PISLKS
--PISLAS
--PISLLG
--PISLAA
--PISLTA
--PISLSA
--PISLSW
--PISLVN
--PISLRQ
--PISLQA
--PISLVH
--PISLTT
--PISLRL
--PISLRH
--PISLKG
--PISLSV

--PISLST
--PISLRE
--PISLMK
--PISLHM
--PISLRI
--PISLMS
--PISLNS
--PISLAL
--PISLTH
--PISLRW
--PISLPM
--PISLMA
--PISLHA
--PISLAT
--PISLTQ
--PISLKM
--PISLKA
PDPISL--
--PISLTM
--PISLNL
--PISLKF
--PISLLH
--PISLHG
--PISLES
--PISLAG
--PISLTN
--PISLRF
--PISLKH
PIPISL--
PNPISL--
--PISLYN
--PISLWI
--PISLKY
--PISLKV
--PISLIG
--PISLSY
--PISLKQ
--PISLHN
--PISLAW
PEPISL--
--PISLTL
--PISLQG
--PISLPL
--PISLPF
--PISLNA
--PISLKN
--PISLKI
--PISLIN
--PISLHL
--PISLWH

--PISLVD
--PISLSK
--PISLKW
--PISLKT
--PISLKR
--PISLIE
PMPISL--
--PISLYG
--PISLTK
--PISLTF
--PISLQN
--PISLPW
--PISLIS
--PISLHW
PYPISL--
PQPISL--
--PISLSI
--PISLQY
--PISLQV
--PISLQM
--PISLMN
--PISLMG
--PISLMD
--PISLHT
--PISLGS
PCPISL--
--PISLWN
--PISLSH
--PISLQW
--PISLNY
--PISLNF
--PISLID
--PISLHY
--PISLGY
--PISLEA
PHPISL--
-PPISLR-
--PISLTW
--PISLSQ
--PISLMQ
--PISLKD
--PISLHQ
--PISLHH
--PISLHF
--PISLGI
--PISLFD
--PISLAI
-PPISLN-
--PISLQL
--PISLQK

--PISLQF
--PISLQC
--PISLPY
--PISLPI
--PISLNT
--PISLNM
--PISLNI
--PISLMI
--PISLHR
--PISLFP
--PISLFE
--PISLEW
--PISLET
--PISLEF
--PISLDV
-QPISLR-
QRPISL--
QLPISL--
QSPISL--
-QPISLL-
QGPISL--
QWPISL--
-QPISLS-
QAPISL--
QFPISL--
QVPISL--
-QPISLK-
-QPISLQ-
QPPISL--
QNPISL--
QMPISL--
QHPISL--
QEPISL--
QYPISL--
QTPISL--
-QPISLA-
-QPISLT-
QQPISL--
-QPISLV-
-QPISLI-
QIPISL--
QDPISL--
-QPISLN-
QCPISL--
RGPISL--
-RPISLS-
RTPISL--
RVPISL--
RYPISL--
RFPISL--

-RPISLA-
RPPISL--
-RPISLI-
RAPISL--
RWPISL--
-RPISLV-
RQPISL--
RIPISL--
-RPISLQ-
REPISL--
-RPISLM-
RDPISL--
-RPISLE-
RHPISL--
-RPISLN-
-RPISLG-
RCPISL--
-RPISLC-
SSPISL--
SYPISL--
STPISL--
-SPISLT-
SLPISL--
SVPISL--
SGPISL--
-SPISLL-
-SPISLK-
SHPISL--
-SPISLS-
SNPISL--
SIPISL--
-SPISLM-
SMPISL--
SWPISL--
-SPISLH-
SQPISL--
-SPISLV-
-SPISLI-
-SPISLN-
-SPISLQ-
SDPISL--
-SPISLE-
SCPISL--
TRPISL--
-TPISLS-
-TPISLM-
TGPISL--
TAPISL--
-TPISLV-
TMPISL--

TSPISL--
-TPISLL-
TIPISL--
-TPISLT-
TEPISL--
TKPISL--
TLPISL--
TFPISL--
-TPISLH-
TVPISL--
TQPISL--
-TPISLQ-
-TPISLK-
-TPISLA-
TNPISL--
TPPISL--
TWPISL--
TYPISL--
-TPISLN-
TCPISL--
-VPISLR-
VAPISL--
VLPISL--
-VPISLS-
-VPISLK-
-VPISLV-
-VPISLT-
-VPISLM-
VSPISL--
VPPISL--
VHPISL--
VQPISL--
VMPISL--
VKPISL--
-VPISLN-
VGPISL--
VDPISL--
VNPISL--
VFPISL--
-VPISLQ-
VEPISL--
-VPISLE-
VCPISL--
-WPISLR-
WEPISL--
WGPISL--
-WPISLK-
-WPISLS-
WPPISL--
-WPISLN-

| | | | |
|---|---|---|---|
| | -WPISLH- | | |
| | -YPISLR- | | |
| | -YPISLS- | | |
| | YSPISL-- | | |
| | -YPISLL- | | |
| | -YPISLT- | | |
| | -YPISLK- | | |
| | YMPISL-- | | |
| | YAPISL-- | | |
| | YVPISL-- | | |
| | YTPISL-- | | |
| | YNPISL-- | | |
| | -YPISLV- | | |
| | -YPISLH- | | |
| | YQPISL-- | | |
| | YYPISL-- | | |
| | -YPISLQ- | | |
| | YDPISL-- | | |
| | -YPISLA- | | |
| | -YPISLM- | | |
| | YEPISL-- | | |
| | YPPISL-- | | |
| | -YPISLP- | | |
| PFNL | 198 | 712 | 30.77 |
| | IEPFNL-- | | |
| | -SPFNLG- | | |
| | -TPFNLG- | | |
| | -WPFNLA- | | |
| | ASPFNL-- | | |
| | -APFNLL- | | |
| | AWPFNL-- | | |
| | ARPFNL-- | | |
| | ANPFNL-- | | |
| | AQPFNL-- | | |
| | AVPFNL-- | | |
| | -APFNLT- | | |
| | AYPFNL-- | | |
| | ALPFNL-- | | |
| | AKPFNL-- | | |
| | AIPFNL-- | | |
| | -APFNLY- | | |
| | -APFNLR- | | |
| | -APFNLK- | | |
| | AMPFNL-- | | |
| | AAPFNL-- | | |
| | -APFNLW- | | |
| | -APFNLM- | | |
| | ATPFNL-- | | |
| | -APFNLI- | | |
| | -APFNLH- | | |

-APFNLG-
-APFNLF-
-APFNLE-
AGPFNL--
AFPFNL--
APPFNL--
-APFNLQ-
-APFNLA-
AHPFNL--
-APFNLV-
AEPFNL--
-APFNLN-
ADPFNL--
CAPFNL--
CLPFNL--
CHPFNL--
CEPFNL--
CVPFNL--
CSPFNL--
CRPFNL--
-CPFNLT-
CNPFNL--
CIPFNL--
CGPFNL--
DRPFNL--
DNPFNL--
DLPFNL--
DAPFNL--
DTPFNL--
-DPFNLR-
DEPFNL--
DQPFNL--
DHPFNL--
DYPFNL--
-DPFNLS-
DIPFNL--
DFPFNL--
DWPFNL--
DVPFNL--
DPPFNL--
-DPFNLM-
-DPFNLL-
-DPFNLK-
-DPFNLI-
DGPFNL--
ERPFNL--
ESPFNL--
-EPFNLH-
-EPFNLW-
ELPFNL--

ETPFNL--
EQPFNL--
-EPFNLT-
EEPFNL--
EYPFNL--
EVPFNL--
-EPFNLK-
EWPFNL--
-EPFNLV-
-EPFNLS-
-EPFNLR-
-EPFNLI-
-EPFNLA-
EMPFNL--
EAPFNL--
EPPFNL--
-EPFNLQ-
-EPFNLM-
-EPFNLF-
EIPFNL--
EFPFNL--
FIPFNL--
-FPFNLK-
FTPFNL--
-FPFNLR-
FKPFNL--
FHPFNL--
FVPFNL--
FRPFNL--
-FPFNLA-
-FPFNLT-
FQPFNL--
-FPFNLV-
-FPFNLS-
-FPFNLQ-
FPPFNL--
FMPFNL--
FGPFNL--
FWPFNL--
FEPFNL--
FNPFNL--
FDPFNL--
-FPFNLY-
-FPFNLM-
-FPFNLH-
-FPFNLF-
FFPFNL--
GNPFNL--
-GPFNLS-
-GPFNLR-

-GPFNLQ-
GSPFNL--
GVPFNL--
GTPFNL--
GFPFNL--
GYPFNL--
-GPFNLK-
GLPFNL--
GIPFNL--
-GPFNLM-
-GPFNLA-
-GPFNLV-
-GPFNLH-
-GPFNLF-
GKPFNL--
GGPFNL--
-GPFNLY-
GEPFNL--
GDPFNL--
-GPFNLT-
GQPFNL--
-GPFNLI-
-GPFNLN-
-GPFNLE-
GMPFNL--
GHPFNL--
GWPFNL--
-GPFNLG-
HRPFNL--
HVPFNL--
HHPFNL--
HGPFNL--
HTPFNL--
-HPFNLS-
-HPFNLR-
-HPFNLL-
-HPFNLI-
-HPFNLT-
HEPFNL--
HYPFNL--
HSPFNL--
HQPFNL--
HNPFNL--
HKPFNL--
-HPFNLY-
-HPFNLK-
-HPFNLG-
HFPFNL--
HAPFNL--
HWPFNL--

HPPFNL--
-HPFNLW-
-HPFNLV-
-HPFNLQ-
-HPFNLN-
-HPFNLM-
-HPFNLE-
-HPFNLA-
IGPFNL--
ITPFNL--
-IPFNLR-
-IPFNLK-
INPFNL--
IVPFNL--
ISPFNL--
IYPFNL--
IWPFNL--
IAPFNL--
-IPFNLS-
-IPFNLF-
IIPFNL--
-IPFNLH-
IKPFNL--
-IPFNLV-
-IPFNLQ-
IHPFNL--
-IPFNLY-
-IPFNLT-
-IPFNLN-
-IPFNLM-
-IPFNLA-
IMPFNL--
IFPFNL--
IDPFNL--
IQPFNL--
-IPFNLI-
-IPFNLE-
-KPFNLI-
-KPFNLL-
KLPFNL--
-KPFNLH-
KNPFNL--
-KPFNLS-
-KPFNLK-
KGPFNL--
KRPFNL--
KVPFNL--
KFPFNL--
KEPFNL--
KWPFNL--

-KPFNLV-
KMPFNL--
KAPFNL--
KTPFNL--
-KPFNLY-
KPPFNL--
-KPFNLW-
-KPFNLQ-
-KPFNLN-
KIPFNL--
KQPFNL--
-KPFNLF-
-KPFNLA-
LKPFNL--
-LPFNLR-
LTPFNL--
LRPFNL--
LGPFNL--
LNPFNL--
LEPFNL--
LYPFNL--
LSPFNL--
-LPFNLV-
-LPFNLK-
-LPFNLS-
-LPFNLH-
-LPFNLM-
LFPFNL--
LAPFNL--
LWPFNL--
-LPFNLT-
-LPFNLI-
LMPFNL--
LHPFNL--
LQPFNL--
-LPFNLY-
-LPFNLA-
LDPFNL--
-LPFNLW-
LLPFNL--
LPPFNL--
-LPFNLQ-
-LPFNLE-
-MPFNLR-
MAPFNL--
MVPFNL--
MRPFNL--
MQPFNL--
-MPFNLS-
MSPFNL--

-MPFNLT-
-MPFNLQ-
-MPFNLA-
MHPFNL--
MEPFNL--
MYPFNL--
MPPFNL--
-MPFNLK-
MWPFNL--
-MPFNLV-
MDPFNL--
MTPFNL--
-MPFNLG-
-MPFNLF-
MKPFNL--
MFPFNL--
-MPFNLN-
MNPFNL--
MMPFNL--
-NPFNLR-
NSPFNL--
-NPFNLK-
NYPFNL--
NRPFNL--
-NPFNLT-
NKPFNL--
NGPFNL--
NPPFNL--
-NPFNLQ-
-NPFNLV-
-NPFNLL-
NNPFNL--
-NPFNLH-
NIPFNL--
NEPFNL--
NAPFNL--
-NPFNLS-
NFPFNL--
-NPFNLY-
NHPFNL--
NWPFNL--
NVPFNL--
NTPFNL--
NQPFNL--
-NPFNLW-
-NPFNLM-
-NPFNLI-
-NPFNLF-
-NPFNLE-
-NPFNLA-

NLPFNL--
NDPFNL--
--PFNLVS
--PFNLYA
--PFNLVW
--PFNLWS
--PFNLTV
--PFNLRA
--PFNLVK
--PFNLRT
--PFNLVR
--PFNLSY
--PFNLSS
--PFNLTT
--PFNLRV
--PFNLRN
PWPFNL--
--PFNLSF
--PFNLRH
--PFNLMS
--PFNLLN
--PFNLGL
PVPFNL--
--PFNLTF
--PFNLRR
--PFNLMT
--PFNLMM
PYPFNL--
--PFNLVT
--PFNLSG
--PFNLRK
--PFNLKG
--PFNLIR
--PFNLIG
--PFNLHG
--PFNLGW
--PFNLVN
--PFNLTR
--PFNLSM
--PFNLFT
--PFNLAS
PQPFNL--
--PFNLVM
--PFNLTQ
--PFNLST
--PFNLSI
--PFNLMG
PMPFNL--
PGPFNL--
--PFNLSR

--PFNLSN
--PFNLSK
--PFNLSA
--PFNLRF
--PFNLLM
--PFNLKA
--PFNLAV
--PFNLAH
--PFNLAG
PNPFNL--
PKPFNL--
PFPFNL--
--PFNLVG
--PFNLTI
--PFNLSH
--PFNLQV
--PFNLQH
--PFNLNW
--PFNLNS
--PFNLMR
--PFNLMN
--PFNLLT
--PFNLLH
--PFNLKT
--PFNLIE
--PFNLHT
--PFNLHS
--PFNLGM
PTPFNL--
PLPFNL--
--PFNLYT
--PFNLYN
--PFNLYG
--PFNLWG
--PFNLTA
--PFNLSV
--PFNLSL
--PFNLQK
--PFNLPY
--PFNLPL
--PFNLNL
--PFNLNA
--PFNLLQ
--PFNLLG
--PFNLKQ
--PFNLIY
--PFNLIT
--PFNLGS
--PFNLFS
--PFNLAT

--PFNLAF
--PFNLAA
--PFNLYR
--PFNLWN
--PFNLVY
--PFNLVV
--PFNLVI
--PFNLTW
--PFNLTK
--PFNLTH
--PFNLSQ
--PFNLRQ
--PFNLRE
--PFNLQS
--PFNLQM
--PFNLKN
--PFNLIV
--PFNLGY
--PFNLFW
--PFNLFH
--PFNLES
PDPFNL--
PHPFNL--
--PFNLYQ
--PFNLWV
--PFNLWM
--PFNLVA
--PFNLTL
--PFNLQY
--PFNLQW
--PFNLQN
--PFNLQI
--PFNLQF
--PFNLMH
--PFNLMD
--PFNLLK
--PFNLKV
--PFNLKK
--PFNLHF
--PFNLHA
--PFNLGA
--PFNLFQ
--PFNLFN
--PFNLFK
--PFNLAQ
-PPFNLR-
--PFNLYM
--PFNLWR
--PFNLWE
--PFNLVH

--PFNLVD
--PFNLTN
--PFNLTE
--PFNLSP
--PFNLSD
--PFNLRW
--PFNLRD
--PFNLQR
--PFNLQG
--PFNLPS
--PFNLPM
--PFNLNY
--PFNLNT
--PFNLNM
--PFNLND
--PFNLMA
--PFNLLF
--PFNLLE
--PFNLKR
--PFNLKH
--PFNLKE
--PFNLKD
--PFNLIK
--PFNLII
--PFNLIH
--PFNLHV
--PFNLHR
--PFNLHN
--PFNLGV
--PFNLGT
--PFNLGG
--PFNLGF
--PFNLFG
--PFNLFD
--PFNLEY
--PFNLEV
--PFNLET
--PFNLER
--PFNLEN
--PFNLEA
--PFNLAD
PEPFNL--
QRPFNL--
QLPFNL--
QVPFNL--
QHPFNL--
-QPFNLT-
-QPFNLR-
-QPFNLL-
QSPFNL--

-QPFNLS-
-QPFNLV-
-QPFNLY-
-QPFNLI-
QMPFNL--
QTPFNL--
-QPFNLM-
-QPFNLK-
-QPFNLW-
-QPFNLG-
-QPFNLA-
QQPFNL--
QIPFNL--
QEPFNL--
QYPFNL--
-QPFNLQ-
-QPFNLH-
QKPFNL--
QFPFNL--
-RPFNLL-
-RPFNLR-
RIPFNL--
RGPFNL--
-RPFNLY-
RVPFNL--
-RPFNLI-
RAPFNL--
RHPFNL--
RYPFNL--
-RPFNLM-
RLPFNL--
REPFNL--
-RPFNLF-
RTPFNL--
-RPFNLK-
-RPFNLN-
-RPFNLV-
RMPFNL--
RFPFNL--
RWPFNL--
RQPFNL--
-RPFNLH-
RNPFNL--
RDPFNL--
-RPFNLW-
-RPFNLE-
-SPFNLR-
SSPFNL--
SLPFNL--
SGPFNL--

SYPFNL--
-SPFNLL-
-SPFNLT-
SVPFNL--
-SPFNLK-
STPFNL--
SNPFNL--
-SPFNLS-
SPPFNL--
-SPFNLY-
-SPFNLA-
SIPFNL--
SHPFNL--
-SPFNLQ-
-SPFNLI-
-SPFNLH-
SAPFNL--
SEPFNL--
SDPFNL--
-SPFNLM-
SWPFNL--
-SPFNLE-
-TPFNLR-
TVPFNL--
TLPFNL--
TSPFNL--
-TPFNLT-
TMPFNL--
TFPFNL--
TAPFNL--
-TPFNLS-
TGPFNL--
-TPFNLL-
TNPFNL--
TYPFNL--
TRPFNL--
-TPFNLN-
-TPFNLH-
-TPFNLE-
TIPFNL--
TWPFNL--
TPPFNL--
-TPFNLK-
-TPFNLA-
TTPFNL--
-TPFNLW-
TDPFNL--
-TPFNLV-
THPFNL--
TQPFNL--

-TPFNLY-
-TPFNLQ-
-TPFNLM-
-TPFNLI-
TEPFNL--
TCPFNL--
VSPFNL--
VRPFNL--
VNPFNL--
VGPFNL--
VTPFNL--
-VPFNLQ-
-VPFNLV-
-VPFNLK-
VMPFNL--
-VPFNLY-
-VPFNLR-
VKPFNL--
-VPFNLL-
-VPFNLS-
-VPFNLM-
-VPFNLI-
VLPFNL--
VEPFNL--
VYPFNL--
-VPFNLW-
VIPFNL--
VHPFNL--
VQPFNL--
VVPFNL--
VFPFNL--
VPPFNL--
-VPFNLN-
VAPFNL--
-VPFNLT-
-VPFNLG-
-VPFNLF-
-VPFNLH-
VWPFNL--
-VPFNLE-
-VPFNLA-
VDPFNL--
WSPFNL--
-WPFNLR-
WRPFNL--
-WPFNLM-
-WPFNLT-
-WPFNLV-
WNPFNL--
WKPFNL--

WDPFNL--
WTPFNL--
WQPFNL--
-WPFNLK-
WYPFNL--
WPPFNL--
-WPFNLY-
-WPFNLI-
WIPFNL--
WGPFNL--
WEPFNL--
YRPFNL--
YGPFNL--
-YPFNLL-
YLPFNL--
-YPFNLM-
-YPFNLK-
YAPFNL--
-YPFNLV-
YHPFNL--
YKPFNL--
YSPFNL--
-YPFNLT-
YYPFNL--
YTPFNL--
-YPFNLA-
YDPFNL--
-YPFNLY-
-YPFNLI-
YIPFNL--
YFPFNL--
YEPFNL--
YWPFNL--
YQPFNL--
-YPFNLQ-
YMPFNL--

PMGI 284 674 30.76

--PMGIRP
-APMGIR-
-APMGII-
ASPMGI--
AWPMGI--
-APMGIK-
-APMGIY-
-APMGIG-
ALPMGI--
AHPMGI--
-APMGIN-
-APMGIL-
ANPMGI--

AYPMGI--
-APMGIS-
AKPMGI--
AAPMGI--
AVPMGI--
ARPMGI--
AQPMGI--
AFPMGI--
ADPMGI--
-APMGIH-
-APMGIF-
AIPMGI--
AEPMGI--
-CPMGIR-
CDPMGI--
CRPMGI--
CQPMGI--
-CPMGII-
CGPMGI--
CEPMGI--
DKPMGI--
DNPMGI--
DRPMGI--
DAPMGI--
-DPMGIT-
DFPMGI--
DYPMGI--
DTPMGI--
-DPMGIS-
-DPMGIR-
-DPMGIM-
-DPMGII-
DLPMGI--
DIPMGI--
DSPMGI--
-DPMGIY-
-DPMGIV-
-DPMGIL-
DGPMGI--
DDPMGI--
DCPMGI--
EVPMGI--
EKPMGI--
-EPMGIR-
-EPMGIM-
-EPMGIL-
EMPMGI--
EIPMGI--
ETPMGI--
-EPMGIW-

ENPMGI--
EHPMGI--
EEPMGI--
ERPMGI--
-EPMGIS-
-EPMGIH-
ECPMGI--
-FPMGIV-
-FPMGII-
FLPMGI--
FRPMGI--
-FPMGIL-
FNPMGI--
FVPMGI--
FQPMGI--
-FPMGIM-
-FPMGIS-
-FPMGIR-
FMPMGI--
FYPMGI--
FHPMGI--
FFPMGI--
FWPMGI--
FTPMGI--
-FPMGIA-
FSPMGI--
FPPMGI--
-FPMGIH-
FIPMGI--
FAPMGI--
GRPMGI--
-GPMGIL-
-GPMGIR-
GSPMGI--
GKPMGI--
GWPMGI--
GYPMGI--
-GPMGIH-
-GPMGIS-
-GPMGIK-
GMPMGI--
-GPMGIY-
-GPMGIN-
GGPMGI--
GPPMGI--
-GPMGIW-
-GPMGIM-
-GPMGII-
GIPMGI--
GVPMGI--

-GPMGIV-
-GPMGIF-
-GPMGIT-
-GPMGIQ-
-GPMGIG-
-GPMGIE-
GHPMGI--
GDPMGI--
GAPMGI--
HLPMGI--
-HPMGIV-
HVPMGI--
HMPMGI--
HKPMGI--
-HPMGIT-
HYPMGI--
HSPMGI--
-HPMGIR-
-HPMGIS-
-HPMGIM-
-HPMGIL-
-HPMGIA-
HGPMGI--
HAPMGI--
HWPMGI--
-HPMGIK-
HIPMGI--
HTPMGI--
-HPMGIY-
-HPMGII-
-HPMGIG-
-HPMGIF-
HHPMGI--
HFPMGI--
HDPMGI--
-IPMGIR-
-IPMGIF-
IRPMGI--
-IPMGIL-
-IPMGIS-
ISPMGI--
-IPMGII-
INPMGI--
ITPMGI--
-IPMGIY-
ILPMGI--
IIPMGI--
IQPMGI--
-IPMGIQ-
IPPMGI--

```
-IPMGIT-
-IPMGIN-
-IPMGIK-
IMPMGI--
IKPMGI--
IFPMGI--
IWPMGI--
-IPMGIE-
IHPMGI--
IEPMGI--
ICPMGI--
KSPMGI--
-KPMGIR-
-KPMGIK-
KTPMGI--
-KPMGIT-
-KPMGIL-
-KPMGIV-
KFPMGI--
KYPMGI--
KVPMGI--
KQPMGI--
-KPMGIQ-
KDPMGI--
KAPMGI--
KRPMGI--
-KPMGIY-
-KPMGIH-
KIPMGI--
-KPMGII-
-KPMGIA-
KGPMGI--
-KPMGIM-
-KPMGIG-
-KPMGIF-
KMPMGI--
KKPMGI--
KEPMGI--
-LPMGII-
LGPMGI--
LAPMGI--
LVPMGI--
-LPMGIT-
-LPMGIK-
LKPMGI--
LRPMGI--
-LPMGIR-
LEPMGI--
LMPMGI--
-LPMGIS-
```

LNPMGI--
LIPMGI--
LFPMGI--
-LPMGIM-
-LPMGIL-
-LPMGIY-
LHPMGI--
-LPMGIF-
LTPMGI--
LSPMGI--
LPPMGI--
-LPMGIV-
-LPMGIH-
LYPMGI--
LWPMGI--
-LPMGIW-
-LPMGIA-
LDPMGI--
-MPMGIR-
MPPMGI--
-MPMGIY-
MTPMGI--
MHPMGI--
MSPMGI--
MIPMGI--
MAPMGI--
MRPMGI--
-MPMGIL-
MKPMGI--
-MPMGIK-
MNPMGI--
-MPMGIG-
MMPMGI--
MLPMGI--
MDPMGI--
MVPMGI--
-MPMGIV-
-MPMGIT-
-MPMGIS-
-MPMGII-
-MPMGIH-
MGPMGI--
MEPMGI--
MCPMGI--
-NPMGIR-
NLPMGI--
NQPMGI--
-NPMGIF-
-NPMGIS-
-NPMGII-

NKPMGI--
NEPMGI--
NVPMGI--
NHPMGI--
NPPMGI--
NFPMGI--
NYPMGI--
-NPMGIT-
-NPMGIL-
NIPMGI--
NTPMGI--
-NPMGIY-
-NPMGIV-
-NPMGIM-
NWPMGI--
-NPMGIQ-
-NPMGIN-
-NPMGIH-
NNPMGI--
NMPMGI--
NAPMGI--
--PMGILG
--PMGILS
--PMGIST
--PMGITM
--PMGIRG
--PMGIMS
--PMGIYR
--PMGIVH
--PMGISG
--PMGIRW
--PMGIWS
--PMGITG
--PMGISA
--PMGILY
--PMGISM
--PMGILV
--PMGIHR
--PMGIHL
--PMGIVR
--PMGITK
--PMGILH
--PMGIWQ
--PMGITS
--PMGIRM
--PMGILK
--PMGIIN
--PMGIHG
--PMGIFK
PYPMGI--

--PMGIVG
--PMGIVD
--PMGIRE
--PMGINA
--PMGIMY
--PMGIMR
--PMGIMQ
--PMGIMG
--PMGILT
--PMGIIR
--PMGIHW
--PMGIFQ
--PMGIAA
--PMGIYN
--PMGIWG
--PMGIVL
--PMGISY
--PMGISN
--PMGISL
--PMGISI
--PMGIRT
--PMGIRR
--PMGIRI
--PMGIRF
--PMGIQA
--PMGINV
--PMGIMT
--PMGIMD
--PMGIKT
--PMGIGG
--PMGIFR
--PMGIFG
--PMGIFD
PHPMGI--
PTPMGI--
PMPMGI--
--PMGIYF
--PMGIWR
--PMGIWN
--PMGIVT
--PMGITT
--PMGISW
--PMGISV
--PMGIRY
--PMGIRK
--PMGILQ
--PMGIKI
--PMGIGS
--PMGIFT
--PMGIFS

--PMGIDA
--PMGIAW
--PMGIAS
PKPMGI--
PIPMGI--
--PMGIYT
--PMGIYG
--PMGIVK
--PMGITD
--PMGISR
--PMGISH
--PMGIRQ
--PMGIQS
--PMGINS
--PMGIMV
--PMGIMF
--PMGILW
--PMGIKW
--PMGIKF
--PMGIIS
--PMGIIF
--PMGIHS
--PMGIHK
PEPMGI--
--PMGIYS
--PMGIYK
--PMGIYH
--PMGIWK
--PMGIVW
--PMGIVS
--PMGIVP
--PMGIVM
--PMGIVA
--PMGITR
--PMGITA
--PMGISK
--PMGIRH
--PMGIQV
--PMGIQF
--PMGINY
--PMGING
--PMGIMK
--PMGIMI
--PMGILN
--PMGIKR
--PMGIIM
--PMGIIL
--PMGIIG
--PMGIHF
--PMGIHD

--PMGIGH
--PMGIFF
--PMGIEY
--PMGIES
--PMGIAY
--PMGIAV
--PMGIAQ
PAPMGI--
PWPMGI--
PNPMGI--
--PMGIYA
--PMGIWL
--PMGIWE
--PMGIWD
--PMGIVN
--PMGIVF
--PMGITV
--PMGITE
--PMGISQ
--PMGIRD
--PMGIRA
--PMGIQY
--PMGIQT
--PMGIQG
--PMGIPR
--PMGIPL
--PMGINT
--PMGINR
--PMGINQ
--PMGINM
--PMGIND
--PMGIMN
--PMGIMH
--PMGILR
--PMGIKV
--PMGIKQ
--PMGIKL
--PMGIKK
--PMGIKH
--PMGIKG
--PMGIIY
--PMGIIW
--PMGIIQ
--PMGIII
--PMGIIH
--PMGIIE
--PMGIID
--PMGIHT
--PMGIHN
--PMGIHM

--PMGIGT
--PMGIGA
--PMGIFV
--PMGIFN
--PMGIFH
--PMGIFC
--PMGIFA
--PMGIEW
--PMGIET
--PMGIEG
--PMGIDH
--PMGICS
--PMGIAT
--PMGIAG
--PMGIAF
PGPMGI--
QRPMGI--
-QPMGIL-
QSPMGI--
QQPMGI--
-QPMGIR-
-QPMGIT-
QKPMGI--
QAPMGI--
QWPMGI--
QTPMGI--
-QPMGIA-
QLPMGI--
QVPMGI--
-QPMGIY-
-QPMGIS-
-QPMGIQ-
-QPMGIH-
QNPMGI--
QHPMGI--
QGPMGI--
RLPMGI--
-RPMGIL-
RMPMGI--
RAPMGI--
-RPMGIS-
-RPMGIT-
RKPMGI--
RVPMGI--
RSPMGI--
RRPMGI--
-RPMGIN-
RIPMGI--
RQPMGI--
-RPMGIQ-

-RPMGIM-
-RPMGII-
RNPMGI--
RTPMGI--
-RPMGIY-
-RPMGIH-
-RPMGIK-
RFPMGI--
RWPMGI--
RPPMGI--
-RPMGIW-
-RPMGIV-
RGPMGI--
RDPMGI--
RCPMGI--
-RPMGIG-
REPMGI--
-RPMGIA-
SRPMGI--
SLPMGI--
-SPMGIV-
-SPMGIR-
-SPMGIF-
SMPMGI--
SSPMGI--
-SPMGIL-
-SPMGIN-
-SPMGII-
-SPMGIA-
SFPMGI--
SKPMGI--
SGPMGI--
STPMGI--
-SPMGIK-
-SPMGIY-
-SPMGIS-
SIPMGI--
-SPMGIQ-
SDPMGI--
SNPMGI--
SAPMGI--
SYPMGI--
SWPMGI--
SVPMGI--
-SPMGIH-
SHPMGI--
SEPMGI--
SQPMGI--
-SPMGIW-
-SPMGIT-

TRPMGI--
-TPMGIM-
-TPMGIL-
TMPMGI--
TGPMGI--
TTPMGI--
TLPMGI--
-TPMGIS-
TSPMGI--
TIPMGI--
TFPMGI--
TVPMGI--
TPPMGI--
-TPMGIY-
-TPMGIN-
-TPMGII-
-TPMGIH-
-TPMGIW-
-TPMGIV-
-TPMGIT-
TYPMGI--
TQPMGI--
TNPMGI--
TEPMGI--
TAPMGI--
-TPMGIK-
-TPMGIF-
TKPMGI--
THPMGI--
TCPMGI--
-VPMGIF-
VKPMGI--
VVPMGI--
-VPMGIS-
-VPMGIT-
-VPMGIL-
-VPMGIR-
-VPMGIV-
-VPMGIA-
VLPMGI--
VWPMGI--
-VPMGIY-
VGPMGI--
VDPMGI--
VSPMGI--
VQPMGI--
-VPMGIW-
VMPMGI--
VTPMGI--
VYPMGI--

VPPMGI--
-VPMGIQ-
VAPMGI--
VRPMGI--
-VPMGIH-
VHPMGI--
VFPMGI--
VEPMGI--
-VPMGIN-
-VPMGII-
-WPMGIT-
WVPMGI--
-WPMGIL-
WLPMGI--
WYPMGI--
WHPMGI--
-WPMGIN-
WKPMGI--
WSPMGI--
-WPMGIA-
WMPMGI--
WTPMGI--
WRPMGI--
-WPMGIV-
WGPMGI--
WDPMGI--
WWPMGI--
-WPMGIS-
-WPMGIR-
-WPMGIQ-
-WPMGIK-
-WPMGIE-
WNPMGI--
WEPMGI--
YRPMGI--
-YPMGIL-
YPPMGI--
YLPMGI--
-YPMGIH-
YNPMGI--
-YPMGIK-
YAPMGI--
YYPMGI--
YVPMGI--
-YPMGIY-
-YPMGIS-
YSPMGI--
-YPMGIT-
-YPMGIR-
YFPMGI--

| | | | |
|---|---|---|---|
| | YEPMGI-- | | |
| | YCPMGI-- | | |
| | YTPMGI-- | | |
| | -YPMGIW- | | |
| | -YPMGIV- | | |
| | YMPMGI-- | | |
| | YKPMGI-- | | |
| | YWPMGI-- | | |
| | YQPMGI-- | | |
| | -YPMGIE- | | |
| | YIPMGI-- | | |
| VSNM | 108 | 764 | 30.76 |
| | DLVSNM-- | | |
| | -GVSNMK- | | |
| | -GVSNMG- | | |
| | -PVSNMT- | | |
| | -PVSNMG- | | |
| | -QVSNMG- | | |
| | -TVSNMG- | | |
| | -VVSNMG- | | |
| | --VSNMSL | | |
| | --VSNMWR | | |
| | --VSNMVP | | |
| | --VSNMTN | | |
| | --VSNMSR | | |
| | --VSNMSI | | |
| | --VSNMLH | | |
| | --VSNMAR | | |
| | --VSNMAM | | |
| | --VSNMAF | | |
| | ARVSNM-- | | |
| | -AVSNMS- | | |
| | AVVSNM-- | | |
| | -AVSNMW- | | |
| | -AVSNML- | | |
| | APVSNM-- | | |
| | -AVSNMI- | | |
| | AHVSNM-- | | |
| | AWVSNM-- | | |
| | ASVSNM-- | | |
| | ALVSNM-- | | |
| | AFVSNM-- | | |
| | AQVSNM-- | | |
| | -AVSNMQ- | | |
| | -AVSNMK- | | |
| | ATVSNM-- | | |
| | ANVSNM-- | | |
| | -AVSNMT- | | |
| | AKVSNM-- | | |
| | AGVSNM-- | | |

-AVSNMV-
AEVSNM--
AYVSNM--
-AVSNMY-
-AVSNMH-
AAVSNM--
-AVSNMM-
-AVSNME-
AMVSNM--
AIVSNM--
ACVSNM--
CCVSNM--
-CVSNMS-
CWVSNM--
-CVSNMI-
CRVSNM--
CSVSNM--
CMVSNM--
CIVSNM--
CGVSNM--
-CVSNMY-
-CVSNMR-
-CVSNMK-
-CVSNMF-
CLVSNM--
CKVSNM--
CFVSNM--
CAVSNM--
DRVSNM--
DFVSNM--
-DVSNMV-
DVVSNM--
-DVSNML-
DYVSNM--
DWVSNM--
-DVSNMT-
-DVSNMY-
-DVSNMS-
DAVSNM--
-DVSNMF-
DNVSNM--
DIVSNM--
DTVSNM--
DSVSNM--
DKVSNM--
DGVSNM--
-DVSNMW-
-DVSNMR-
-DVSNMK-
-DVSNMI-

DPVSNM--
DMVSNM--
DEVSNM--
DCVSNM--
-DVSNME-
DDVSNM--
-EVSNML-
-EVSNMR-
-EVSNMQ-
EGVSNM--
EFVSNM--
-EVSNMW-
ELVSNM--
-EVSNMI-
-EVSNMF-
ESVSNM--
EHVSNM--
EVVSNM--
-EVSNMY-
-EVSNMV-
-EVSNMT-
EPVSNM--
EIVSNM--
EWVSNM--
-EVSNMH-
-EVSNMA-
ERVSNM--
EMVSNM--
ECVSNM--
EAVSNM--
FSVSNM--
-FVSNMK-
-FVSNMI-
FGVSNM--
-FVSNML-
FRVSNM--
FLVSNM--
-FVSNMN-
FAVSNM--
-FVSNMV-
-FVSNMS-
-FVSNMR-
-FVSNMF-
FHVSNM--
-FVSNMH-
FMVSNM--
-FVSNMQ-
-FVSNMG-
FNVSNM--
FWVSNM--

FFVSNM--
-FVSNMY-
-FVSNMT-
-FVSNMM-
FQVSNM--
FIVSNM--
FPVSNM--
FKVSNM--
FCVSNM--
FDVSNM--
-GVSNML-
GRVSNM--
GTVSNM--
GLVSNM--
-GVSNMR-
GFVSNM--
GVVSNM--
GSVSNM--
-GVSNMS-
-GVSNMI-
GMVSNM--
-GVSNMM-
GGVSNM--
GWVSNM--
-GVSNMW-
GIVSNM--
GYVSNM--
-GVSNMY-
-GVSNMH-
GPVSNM--
GNVSNM--
-GVSNMT-
-GVSNMA-
GQVSNM--
GHVSNM--
GAVSNM--
-GVSNMQ-
-GVSNME-
GEVSNM--
-GVSNMF-
GDVSNM--
-HVSNMR-
-HVSNMQ-
HYVSNM--
HAVSNM--
HSVSNM--
-HVSNMV-
-HVSNML-
HRVSNM--
HKVSNM--

HTVSNM--
-HVSNMA-
-HVSNMT-
HHVSNM--
-HVSNMK-
-HVSNMI-
HPVSNM--
HNVSNM--
-HVSNMN-
HMVSNM--
-HVSNMM-
HGVSNM--
HFVSNM--
HDVSNM--
HWVSNM--
-HVSNMY-
-HVSNMW-
-HVSNMH-
-HVSNME-
HQVSNM--
HIVSNM--
IKVSNM--
IRVSNM--
-IVSNML-
-IVSNMR-
IVVSNM--
INVSNM--
IFVSNM--
IEVSNM--
-IVSNMV-
-IVSNMS-
ISVSNM--
IAVSNM--
-IVSNMN-
IPVSNM--
IWVSNM--
-IVSNMW-
-IVSNMG-
ITVSNM--
-IVSNMK-
-IVSNMF-
IMVSNM--
IYVSNM--
-IVSNMY-
-IVSNMT-
-IVSNMQ-
-IVSNMH-
-IVSNMA-
IQVSNM--
IHVSNM--

IDVSNM--
ICVSNM--
KYVSNM--
KVVSNM--
-KVSNMY-
KIVSNM--
-KVSNMT-
-KVSNMI-
KLVSNM--
KMVSNM--
-KVSNMK-
KGVSNM--
-KVSNMW-
-KVSNMA-
KWVSNM--
KNVSNM--
KHVSNM--
KFVSNM--
-KVSNME-
KTVSNM--
KQVSNM--
KEVSNM--
-KVSNMM-
-KVSNMQ-
-KVSNMH-
-KVSNMF-
KPVSNM--
-KVSNMN-
KDVSNM--
KCVSNM--
LLVSNM--
LSVSNM--
-LVSNMS-
LRVSNM--
-LVSNMV-
-LVSNMI-
LVVSNM--
-LVSNMR-
LNVSNM--
-LVSNML-
-LVSNMA-
LMVSNM--
LTVSNM--
LIVSNM--
LPVSNM--
LWVSNM--
-LVSNMW-
-LVSNMM-
LYVSNM--
-LVSNMQ-

-LVSNMG-
LAVSNM--
-LVSNMN-
-LVSNMH-
-LVSNMY-
-LVSNMK-
LDVSNM--
LEVSNM--
LHVSNM--
-LVSNME-
LFVSNM--
-LVSNMD-
-LVSNMC-
MWVSNM--
-MVSNMV-
MSVSNM--
MLVSNM--
MEVSNM--
MNVSNM--
MKVSNM--
-MVSNMF-
-MVSNMY-
-MVSNMS-
MVVSNM--
MGVSNM--
-MVSNMT-
-MVSNMQ-
-MVSNMK-
-MVSNMG-
-MVSNMH-
MMVSNM--
MFVSNM--
MAVSNM--
-MVSNMN-
MHVSNM--
MYVSNM--
-MVSNMW-
-MVSNMD-
MTVSNM--
MIVSNM--
-MVSNMA-
MPVSNM--
-NVSNMR-
NKVSNM--
-NVSNMS-
-NVSNML-
-NVSNMV-
NLVSNM--
NTVSNM--
NRVSNM--

NFVSNM--
-NVSNMA-
NIVSNM--
NGVSNM--
NWVSNM--
NVVSNM--
-NVSNMW-
-NVSNMT-
NNVSNM--
NHVSNM--
-NVSNMQ-
-NVSNME-
-NVSNMF-
NAVSNM--
-NVSNMK-
-NVSNMI-
-NVSNMH-
NSVSNM--
NEVSNM--
NQVSNM--
NDVSNM--
-PVSNMR-
PKVSNM--
-PVSNML-
PGVSNM--
PVVSNM--
-PVSNMY-
-PVSNMW-
-PVSNMH-
PFVSNM--
PTVSNM--
PMVSNM--
-PVSNMK-
PLVSNM--
-PVSNMQ-
PWVSNM--
PNVSNM--
PIVSNM--
PYVSNM--
-PVSNMF-
PSVSNM--
-PVSNMV-
-PVSNMA-
PDVSNM--
PAVSNM--
-PVSNMI-
-PVSNMN-
-PVSNMM-
PEVSNM--
PCVSNM--

−QVSNMR−
−QVSNMT−
−QVSNMI−
QYVSNM−−
QRVSNM−−
−QVSNMY−
QSVSNM−−
QNVSNM−−
−QVSNML−
−QVSNMH−
QLVSNM−−
−QVSNMV−
−QVSNMS−
QAVSNM−−
QTVSNM−−
QWVSNM−−
−QVSNMW−
−QVSNMM−
QQVSNM−−
QVVSNM−−
−QVSNMQ−
−QVSNMF−
QIVSNM−−
QDVSNM−−
−QVSNMN−
−QVSNMK−
−QVSNME−
QPVSNM−−
QCVSNM−−
RLVSNM−−
−RVSNML−
−RVSNMV−
−RVSNMS−
RTVSNM−−
−RVSNMA−
RPVSNM−−
−RVSNMI−
RNVSNM−−
RGVSNM−−
−RVSNMQ−
RVVSNM−−
RIVSNM−−
RYVSNM−−
−RVSNMH−
REVSNM−−
−RVSNMK−
−RVSNME−
RFVSNM−−
−RVSNMW−
−RVSNMM−

RHVSNM--
RCVSNM--
-RVSNMG-
SRVSNM--
-SVSNML-
SSVSNM--
SLVSNM--
-SVSNMT-
SYVSNM--
-SVSNMR-
-SVSNMA-
SAVSNM--
-SVSNMV-
SQVSNM--
SFVSNM--
STVSNM--
-SVSNMK-
SKVSNM--
-SVSNMW-
SGVSNM--
SMVSNM--
-SVSNMM-
-SVSNMH-
-SVSNMF-
SNVSNM--
SHVSNM--
SDVSNM--
-SVSNMY-
-SVSNME-
SPVSNM--
-SVSNMN-
TSVSNM--
TNVSNM--
TLVSNM--
TWVSNM--
-TVSNMT-
-TVSNMS-
-TVSNMF-
-TVSNMR-
TPVSNM--
-TVSNMY-
TTVSNM--
TFVSNM--
TVVSNM--
-TVSNMV-
-TVSNMQ-
TKVSNM--
THVSNM--
TEVSNM--
-TVSNMK-

-TVSNMH-
TYVSNM--
-TVSNMA-
TAVSNM--
-TVSNMW-
-TVSNMM-
-TVSNMI-
TIVSNM--
TDVSNM--
-TVSNME-
TQVSNM--
TMVSNM--
TCVSNM--
--VSNMLG
VSVSNM--
--VSNMLT
--VSNMLA
VLVSNM--
--VSNMRK
-VVSNMS-
--VSNMRS
--VSNMMS
--VSNMSS
-VVSNMR-
--VSNMTS
--VSNMRT
--VSNMAG
VYVSNM--
VVVSNM--
--VSNMRV
--VSNMRA
--VSNMKS
--VSNMIR
--VSNMIQ
VGVSNM--
-VVSNML-
--VSNMVV
--VSNMVG
--VSNMTT
--VSNMRW
--VSNMQA
--VSNMYT
--VSNMYM
--VSNMWS
--VSNMVS
--VSNMVF
--VSNMLV
--VSNMHS
--VSNMFM
--VSNMES

-VVSNMY-
--VSNMWA
--VSNMVT
--VSNMVA
--VSNMMR
--VSNMIS
--VSNMFT
VFVSNM--
-VVSNMM-
--VSNMTG
--VSNMTA
--VSNMSG
--VSNMSA
--VSNMRQ
--VSNMRN
--VSNMRG
--VSNMFG
VNVSNM--
-VVSNMK-
-VVSNMI-
--VSNMYS
--VSNMYR
--VSNMNS
--VSNMIA
-VVSNMV-
--VSNMYH
--VSNMVL
--VSNMTY
--VSNMTV
--VSNMRE
--VSNMML
--VSNMMF
--VSNMKV
--VSNMKT
--VSNMWG
--VSNMVY
--VSNMVW
--VSNMVN
--VSNMTK
--VSNMTF
--VSNMSY
--VSNMQG
--VSNMMK
--VSNMMA
-VVSNMA-
--VSNMYV
--VSNMYN
--VSNMYI
--VSNMVH
--VSNMSW

--VSNMST
--VSNMRF
--VSNMMW
--VSNMKG
--VSNMIM
--VSNMHV
--VSNMFA
--VSNMEG
VMVSNM--
VIVSNM--
VEVSNM--
-VVSNMH-
-VVSNMF-
--VSNMYA
--VSNMWV
--VSNMWT
--VSNMMT
--VSNMMH
--VSNMKI
--VSNMIV
--VSNMIH
--VSNMHA
--VSNMGS
--VSNMFS
--VSNMEA
--VSNMAT
VPVSNM--
VHVSNM--
VWVSNM--
-VVSNMW-
--VSNMYK
--VSNMVQ
--VSNMSN
--VSNMSK
--VSNMRH
--VSNMQT
--VSNMNA
--VSNMMQ
--VSNMLQ
--VSNMLN
--VSNMLK
--VSNMKM
--VSNMKK
--VSNMIN
--VSNMIF
--VSNMHT
--VSNMAS
VQVSNM--
--VSNMYQ
--VSNMWK

--VSNMTQ
--VSNMTH
--VSNMSV
--VSNMSM
--VSNMMV
--VSNMMG
--VSNMLM
--VSNMKW
--VSNMKA
--VSNMIY
--VSNMIT
--VSNMET
--VSNMAA
VCVSNM--
--VSNMWQ
--VSNMWN
--VSNMVE
--VSNMTI
--VSNMSQ
--VSNMSP
--VSNMSF
--VSNMQV
--VSNMQK
--VSNMMM
--VSNMMI
--VSNMME
--VSNMLE
--VSNMKY
--VSNMKD
--VSNMIE
--VSNMFN
VKVSNM--
VAVSNM--
-VVSNMQ-
--VSNMYW
--VSNMYE
--VSNMWM
--VSNMWH
--VSNMVM
--VSNMVI
--VSNMTM
--VSNMSH
--VSNMQQ
--VSNMQE
--VSNMNM
--VSNMNG
--VSNMLD
--VSNMKQ
--VSNMKN
--VSNMKH

--VSNMKF
--VSNMIW
--VSNMHN
--VSNMHG
--VSNMHF
--VSNMGA
--VSNMFQ
--VSNMFK
--VSNMEW
--VSNMEV
--VSNMCS
--VSNMCG
--VSNMAY
--VSNMAV
--VSNMAN
--VSNMAI
WIVSNM--
WRVSNM--
WSVSNM--
-WVSNMK-
-WVSNMY-
WLVSNM--
-WVSNMG-
-WVSNMI-
WGVSNM--
-WVSNMN-
-WVSNMM-
WTVSNM--
-WVSNMV-
-WVSNML-
WQVSNM--
WNVSNM--
WKVSNM--
-WVSNMT-
-WVSNMH-
-WVSNMA-
WEVSNM--
WWVSNM--
-WVSNME-
WMVSNM--
WDVSNM--
-WVSNMS-
-WVSNMC-
WPVSNM--
WAVSNM--
YRVSNM--
-YVSNMR-
YLVSNM--
-YVSNMI-
-YVSNMK-

```
          -YVSNMT-
          -YVSNMV-
          YGVSNM--
          YYVSNM--
          -YVSNMG-
          -YVSNME-
          YSVSNM--
          -YVSNMA-
          YFVSNM--
          -YVSNMQ-
          YHVSNM--
          YAVSNM--
          YVVSNM--
          -YVSNMN-
          -YVSNML-
          YMVSNM--
          YEVSNM--
          YPVSNM--
          YKVSNM--
          YIVSNM--
          -YVSNMW-
          YNVSNM--
          -YVSNMM-
          YTVSNM--
          YQVSNM--
          YDVSNM--
PAHW       686       560      30.73
          ARPAHW--
          -APAHWL-
          AVPAHW--
          -APAHWR-
          AAPAHW--
          -APAHWM-
          -APAHWT-
          -APAHWE-
          -APAHWS-
          AKPAHW--
          ASPAHW--
          -APAHWG-
          AIPAHW--
          AHPAHW--
          AFPAHW--
          AEPAHW--
          ATPAHW--
          AQPAHW--
          -APAHWY-
          AGPAHW--
          -CPAHWR-
          CVPAHW--
          CTPAHW--
```

CSPAHW--
-CPAHWT-
-CPAHWS-
CIPAHW--
CEPAHW--
-DPAHWR-
DRPAHW--
-DPAHWT-
-DPAHWS-
-DPAHWL-
DNPAHW--
DFPAHW--
DSPAHW--
-DPAHWY-
-DPAHWQ-
-DPAHWI-
DEPAHW--
EKPAHW--
EFPAHW--
-EPAHWK-
ETPAHW--
-EPAHWV-
-EPAHWI-
ESPAHW--
ELPAHW--
EMPAHW--
-EPAHWW-
-EPAHWS-
-EPAHWE-
FHPAHW--
FLPAHW--
FYPAHW--
-FPAHWS-
-FPAHWA-
FMPAHW--
FPPAHW--
-FPAHWR-
-FPAHWL-
-FPAHWH-
FTPAHW--
FSPAHW--
FRPAHW--
-FPAHWY-
-FPAHWV-
-FPAHWQ-
-FPAHWI-
-FPAHWF-
-GPAHWL-
GLPAHW--
GAPAHW--

-GPAHWA-
GFPAHW--
GVPAHW--
GTPAHW--
-GPAHWY-
-GPAHWS-
-GPAHWR-
GRPAHW--
GYPAHW--
GSPAHW--
-GPAHWT-
-GPAHWI-
-GPAHWF-
GNPAHW--
GIPAHW--
GPPAHW--
-GPAHWM-
-GPAHWK-
GKPAHW--
GDPAHW--
-HPAHWS-
HLPAHW--
-HPAHWL-
HYPAHW--
-HPAHWT-
-HPAHWH-
HNPAHW--
HFPAHW--
HRPAHW--
-HPAHWY-
HEPAHW--
HAPAHW--
IRPAHW--
-IPAHWK-
IQPAHW--
IPPAHW--
-IPAHWR-
IIPAHW--
IEPAHW--
-IPAHWS-
-IPAHWM-
INPAHW--
IMPAHW--
ILPAHW--
IHPAHW--
IGPAHW--
IFPAHW--
KSPAHW--
KTPAHW--
KVPAHW--

-KPAHWT-
KMPAHW--
-KPAHWS-
-KPAHWN-
-KPAHWY-
KGPAHW--
KWPAHW--
-KPAHWV-
-KPAHWL-
-KPAHWK-
-KPAHWI-
-KPAHWH-
-KPAHWF-
KNPAHW--
KEPAHW--
-KPAHWR-
-KPAHWE-
KKPAHW--
-KPAHWQ-
-KPAHWA-
KLPAHW--
-LPAHWF-
LWPAHW--
LTPAHW--
-LPAHWK-
LGPAHW--
-LPAHWQ-
LYPAHW--
LSPAHW--
-LPAHWR-
LFPAHW--
LRPAHW--
-LPAHWS-
LMPAHW--
LDPAHW--
LNPAHW--
LQPAHW--
-LPAHWY-
-LPAHWV-
-LPAHWM-
LEPAHW--
LAPAHW--
LPPAHW--
-LPAHWT-
-LPAHWI-
-LPAHWH-
LLPAHW--
LHPAHW--
-MPAHWR-
MNPAHW--

```
MYPAHW--
-MPAHWL-
MMPAHW--
MKPAHW--
MAPAHW--
MWPAHW--
MPPAHW--
-MPAHWV-
-MPAHWI-
-MPAHWA-
MHPAHW--
-MPAHWQ-
MDPAHW--
NLPAHW--
NYPAHW--
NRPAHW--
NTPAHW--
-NPAHWF-
NWPAHW--
NQPAHW--
NHPAHW--
-NPAHWV-
-NPAHWM-
NDPAHW--
NPPAHW--
-NPAHWS-
NSPAHW--
-NPAHWW-
-NPAHWR-
-NPAHWQ-
-NPAHWL-
-NPAHWI-
NMPAHW--
NGPAHW--
NFPAHW--
NAPAHW--
--PAHWST
--PAHWLR
--PAHWRG
--PAHWLS
--PAHWSY
--PAHWLN
--PAHWFS
--PAHWRS
--PAHWRM
--PAHWAS
--PAHWSL
--PAHWFK
--PAHWWA
--PAHWSR
```

--PAHWML
--PAHWTI
--PAHWSS
--PAHWRT
--PAHWRN
--PAHWRH
--PAHWLI
--PAHWLE
--PAHWKL
--PAHWKD
--PAHWFT
--PAHWFA
PRPAHW--
--PAHWVR
--PAHWSK
--PAHWRA
--PAHWMK
--PAHWLQ
--PAHWKT
--PAHWKR
--PAHWEN
--PAHWTV
--PAHWSV
--PAHWSI
--PAHWSA
--PAHWRQ
--PAHWLV
--PAHWKS
--PAHWKN
--PAHWKF
--PAHWIQ
--PAHWIG
--PAHWHV
--PAHWHI
--PAHWFY
--PAHWFG
--PAHWAI
--PAHWAG
PTPAHW--
PMPAHW--
--PAHWYG
--PAHWWV
--PAHWWR
--PAHWVN
--PAHWVH
--PAHWTT
--PAHWTS
--PAHWTE
--PAHWSH
--PAHWRK

--PAHWQY
--PAHWLL
--PAHWLA
--PAHWKI
--PAHWIL
--PAHWID
--PAHWFH
--PAHWFE
--PAHWYA
--PAHWWS
--PAHWVW
--PAHWVI
--PAHWTL
--PAHWTG
--PAHWTF
--PAHWSG
--PAHWRV
--PAHWRF
--PAHWMG
--PAHWKG
--PAHWIW
--PAHWGV
--PAHWGT
--PAHWFL
--PAHWER
--PAHWEI
--PAHWEH
--PAHWEA
--PAHWAW
--PAHWAV
--PAHWAK
PYPAHW--
PSPAHW--
PLPAHW--
PGPAHW--
PEPAHW--
--PAHWYN
--PAHWVS
--PAHWTY
--PAHWTK
--PAHWSW
--PAHWRE
--PAHWNA
--PAHWLW
--PAHWLK
--PAHWLG
--PAHWIT
--PAHWIR
--PAHWIF
--PAHWHF

--PAHWGS
--PAHWFI
--PAHWET
--PAHWAY
--PAHWAQ
--PAHWAL
--PAHWYV
--PAHWYL
--PAHWYI
--PAHWWT
--PAHWWI
--PAHWWG
--PAHWVV
--PAHWVM
--PAHWVL
--PAHWVK
--PAHWVG
--PAHWVF
--PAHWVA
--PAHWTW
--PAHWTM
--PAHWTA
--PAHWSM
--PAHWSF
--PAHWSD
--PAHWRY
--PAHWQV
--PAHWQT
--PAHWQR
--PAHWQG
--PAHWQA
--PAHWNY
--PAHWNS
--PAHWNL
--PAHWNG
--PAHWMV
--PAHWMT
--PAHWMS
--PAHWMF
--PAHWLT
--PAHWLM
--PAHWLH
--PAHWLD
--PAHWKV
--PAHWKM
--PAHWKK
--PAHWIN
--PAHWII
--PAHWIH
--PAHWHY

--PAHWHQ
--PAHWHN
--PAHWHM
--PAHWHL
--PAHWHG
--PAHWGA
--PAHWFW
--PAHWES
--PAHWEG
--PAHWEF
--PAHWAT
--PAHWAH
--PAHWAF
--PAHWAA
-QPAHWR-
-QPAHWS-
-QPAHWM-
-QPAHWF-
QMPAHW--
QIPAHW--
QSPAHW--
QRPAHW--
QPPAHW--
-QPAHWK-
-QPAHWH-
-QPAHWE-
QEPAHW--
-QPAHWY-
-QPAHWT-
-QPAHWQ-
-QPAHWI-
QLPAHW--
QKPAHW--
QGPAHW--
QAPAHW--
-RPAHWS-
RYPAHW--
-RPAHWL-
-RPAHWR-
RTPAHW--
-RPAHWM-
RGPAHW--
-RPAHWW-
-RPAHWG-
REPAHW--
-RPAHWQ-
-RPAHWN-
-RPAHWI-
RNPAHW--
RSPAHW--

RVPAHW--
RPPAHW--
-RPAHWV-
RCPAHW--
RQPAHW--
-RPAHWF-
-RPAHWH-
RMPAHW--
RLPAHW--
RIPAHW--
-RPAHWA-
RFPAHW--
-RPAHWK-
RHPAHW--
RAPAHW--
SEPAHW--
SRPAHW--
SVPAHW--
-SPAHWY-
SAPAHW--
SGPAHW--
SYPAHW--
-SPAHWR-
-SPAHWF-
-SPAHWA-
SQPAHW--
-SPAHWT-
-SPAHWS-
-SPAHWI-
SFPAHW--
SDPAHW--
SWPAHW--
SSPAHW--
SPPAHW--
-SPAHWK-
SKPAHW--
SHPAHW--
STPAHW--
-SPAHWV-
-SPAHWQ-
-SPAHWM-
-SPAHWL-
-SPAHWH-
-SPAHWG-
-SPAHWE-
SMPAHW--
-TPAHWS-
TLPAHW--
TSPAHW--
TFPAHW--

-TPAHWL-
TTPAHW--
-TPAHWR-
-TPAHWQ-
-TPAHWM-
-TPAHWK-
-TPAHWH-
TGPAHW--
TRPAHW--
-TPAHWY-
TEPAHW--
TKPAHW--
TYPAHW--
-TPAHWN-
-TPAHWI-
-TPAHWG-
-TPAHWA-
THPAHW--
TDPAHW--
VTPAHW--
VRPAHW--
-VPAHWS-
VEPAHW--
VDPAHW--
-VPAHWR-
-VPAHWI-
VGPAHW--
-VPAHWK-
VWPAHW--
-VPAHWV-
-VPAHWE-
VSPAHW--
VQPAHW--
-VPAHWT-
-VPAHWL-
-VPAHWG-
-VPAHWA-
VFPAHW--
VPPAHW--
VKPAHW--
VAPAHW--
-WPAHWL-
-WPAHWK-
-WPAHWA-
WLPAHW--
-WPAHWR-
-WPAHWQ-
WDPAHW--
WMPAHW--
WGPAHW--

| | | | |
|---|---|---|---|
| | WTPAHW-- | | |
| | WRPAHW-- | | |
| | WQPAHW-- | | |
| | -WPAHWY- | | |
| | -WPAHWW- | | |
| | -WPAHWS- | | |
| | WNPAHW-- | | |
| | WHPAHW-- | | |
| | WEPAHW-- | | |
| | YRPAHW-- | | |
| | YYPAHW-- | | |
| | -YPAHWY- | | |
| | -YPAHWR- | | |
| | YFPAHW-- | | |
| | YWPAHW-- | | |
| | -YPAHWF- | | |
| | YNPAHW-- | | |
| | YAPAHW-- | | |
| | -YPAHWT- | | |
| | -YPAHWL- | | |
| | YVPAHW-- | | |
| | -YPAHWW- | | |
| | -YPAHWS- | | |
| | -YPAHWN- | | |
| | -YPAHWK- | | |
| | -YPAHWI- | | |
| | -YPAHWH- | | |
| | -YPAHWG- | | |
| | -YPAHWE- | | |
| | YLPAHW-- | | |
| | YSPAHW-- | | |
| | -YPAHWV- | | |
| | -YPAHWQ- | | |
| | YKPAHW-- | | |
| | YHPAHW-- | | |
| | YEPAHW-- | | |
| | YDPAHW-- | | |
| PYSL | 199 | 711 | 30.73 |
| | -GPYSLN- | | |
| | NDPYSL-- | | |
| | --PYSLSI | | |
| | --PYSLPS | | |
| | --PYSLNT | | |
| | --PYSLNK | | |
| | --PYSLLE | | |
| | --PYSLET | | |
| | -SPYSLA- | | |
| | -TPYSLG- | | |
| | -APYSLL- | | |
| | ARPYSL-- | | |

-APYSLS-
-APYSLA-
AKPYSL--
AQPYSL--
ALPYSL--
ASPYSL--
AVPYSL--
ATPYSL--
ANPYSL--
-APYSLK-
-APYSLN-
AAPYSL--
-APYSLT-
-APYSLH-
AWPYSL--
-APYSLQ-
AHPYSL--
AFPYSL--
ADPYSL--
AYPYSL--
APPYSL--
-APYSLV-
-APYSLM-
-APYSLW-
-APYSLE-
-APYSLG-
CVPYSL--
CSPYSL--
-CPYSLR-
CGPYSL--
CQPYSL--
-CPYSLL-
CLPYSL--
CKPYSL--
CEPYSL--
CCPYSL--
-CPYSLW-
-CPYSLV-
-CPYSLA-
CMPYSL--
CHPYSL--
DLPYSL--
DRPYSL--
DFPYSL--
DTPYSL--
DKPYSL--
DYPYSL--
-DPYSLR-
-DPYSLM-
DGPYSL--

DWPYSL--
DSPYSL--
DQPYSL--
DMPYSL--
DIPYSL--
-DPYSLW-
DAPYSL--
-DPYSLV-
DHPYSL--
DEPYSL--
ELPYSL--
-EPYSLR-
ERPYSL--
EYPYSL--
-EPYSLL-
EVPYSL--
ETPYSL--
EQPYSL--
-EPYSLK-
EHPYSL--
EAPYSL--
-EPYSLS-
ENPYSL--
ESPYSL--
-EPYSLM-
EFPYSL--
-EPYSLY-
-EPYSLQ-
-EPYSLV-
EKPYSL--
-EPYSLW-
EPPYSL--
FTPYSL--
-FPYSLS-
-FPYSLV-
-FPYSLR-
FGPYSL--
-FPYSLL-
FNPYSL--
FSPYSL--
-FPYSLK-
FYPYSL--
FMPYSL--
-FPYSLT-
-FPYSLQ-
-FPYSLI-
FHPYSL--
FPPYSL--
FEPYSL--
FDPYSL--

FAPYSL--
FQPYSL--
FIPYSL--
GRPYSL--
GSPYSL--
-GPYSLK-
-GPYSLS-
-GPYSLW-
GKPYSL--
GTPYSL--
GGPYSL--
GVPYSL--
GNPYSL--
GIPYSL--
-GPYSLY-
GMPYSL--
-GPYSLH-
-GPYSLA-
GFPYSL--
-GPYSLT-
-GPYSLI-
GYPYSL--
GQPYSL--
-GPYSLF-
GEPYSL--
-GPYSLQ-
-GPYSLM-
GHPYSL--
GWPYSL--
-GPYSLG-
HSPYSL--
HKPYSL--
HGPYSL--
HWPYSL--
-HPYSLS-
HIPYSL--
HVPYSL--
HYPYSL--
HPPYSL--
HAPYSL--
HQPYSL--
-HPYSLL-
-HPYSLH-
-HPYSLV-
HNPYSL--
-HPYSLW-
-HPYSLK-
HHPYSL--
HFPYSL--
-HPYSLQ-

```
-HPYSLN-
HEPYSL--
-HPYSLM-
-HPYSLI-
-HPYSLG-
HDPYSL--
HTPYSL--
-HPYSLF-
-HPYSLE-
-HPYSLA-
-IPYSLS-
-IPYSLK-
IMPYSL--
-IPYSLR-
-IPYSLM-
IVPYSL--
IEPYSL--
IGPYSL--
IWPYSL--
ITPYSL--
IQPYSL--
IIPYSL--
-IPYSLT-
IPPYSL--
IKPYSL--
-IPYSLV-
-IPYSLQ-
IFPYSL--
IAPYSL--
-IPYSLN-
-IPYSLH-
IDPYSL--
-IPYSLA-
IHPYSL--
ICPYSL--
-IPYSLI-
KSPYSL--
-KPYSLR-
-KPYSLL-
-KPYSLS-
KLPYSL--
KTPYSL--
KAPYSL--
KWPYSL--
KGPYSL--
KVPYSL--
KKPYSL--
KHPYSL--
KNPYSL--
KIPYSL--
```

KYPYSL--
-KPYSLT-
-KPYSLN-
KPPYSL--
KFPYSL--
-KPYSLV-
-KPYSLQ-
-KPYSLK-
-KPYSLH-
KMPYSL--
-KPYSLA-
KEPYSL--
KQPYSL--
-KPYSLY-
-KPYSLE-
LSPYSL--
LKPYSL--
LTPYSL--
LGPYSL--
LNPYSL--
LHPYSL--
LWPYSL--
LAPYSL--
LYPYSL--
-LPYSLH-
LVPYSL--
LPPYSL--
-LPYSLY-
-LPYSLT-
LEPYSL--
LMPYSL--
-LPYSLV-
-LPYSLK-
-LPYSLQ-
LDPYSL--
-LPYSLI-
-LPYSLM-
LQPYSL--
-LPYSLS-
-LPYSLN-
LCPYSL--
-MPYSLL-
MRPYSL--
MVPYSL--
MKPYSL--
-MPYSLR-
MFPYSL--
MHPYSL--
MMPYSL--
MAPYSL--

MYPYSL--
MSPYSL--
-MPYSLS-
-MPYSLI-
-MPYSLA-
MGPYSL--
-MPYSLN-
-MPYSLM-
MIPYSL--
MTPYSL--
MDPYSL--
MLPYSL--
MWPYSL--
-MPYSLV-
MPPYSL--
-MPYSLT-
-MPYSLQ-
MQPYSL--
-MPYSLW-
-MPYSLH-
MEPYSL--
-MPYSLK-
MCPYSL--
-MPYSLF-
-MPYSLE-
MNPYSL--
NAPYSL--
-NPYSLR-
NTPYSL--
NHPYSL--
NVPYSL--
NGPYSL--
-NPYSLL-
NIPYSL--
NRPYSL--
-NPYSLK-
NYPYSL--
NPPYSL--
NNPYSL--
-NPYSLY-
NWPYSL--
-NPYSLH-
-NPYSLF-
NKPYSL--
NQPYSL--
NFPYSL--
-NPYSLI-
NEPYSL--
-NPYSLV-
-NPYSLT-

NMPYSL--
-NPYSLN-
--PYSLRK
--PYSLVA
--PYSLRT
--PYSLTS
--PYSLSS
--PYSLPL
--PYSLRA
--PYSLLS
--PYSLRG
--PYSLAL
--PYSLGL
PTPYSL--
--PYSLVH
--PYSLLR
--PYSLMR
--PYSLYG
--PYSLSL
--PYSLIG
PMPYSL--
--PYSLTG
--PYSLMG
--PYSLKS
--PYSLYS
--PYSLVV
--PYSLRH
--PYSLKR
--PYSLSY
--PYSLNS
--PYSLVY
--PYSLRE
--PYSLKT
--PYSLKA
--PYSLIR
--PYSLVQ
--PYSLVG
--PYSLTA
--PYSLSV
--PYSLRQ
--PYSLRN
--PYSLLT
--PYSLHN
--PYSLFH
--PYSLTT
--PYSLTH
--PYSLIT
--PYSLFG
PGPYSL--
--PYSLWK

--PYSLRF
--PYSLNL
--PYSLMV
--PYSLLH
--PYSLGS
--PYSLAG
--PYSLYT
--PYSLWS
--PYSLVT
--PYSLVK
--PYSLTM
--PYSLMS
--PYSLMK
--PYSLIH
--PYSLHH
--PYSLES
--PYSLTK
--PYSLMN
--PYSLKY
--PYSLWR
--PYSLSM
--PYSLRW
--PYSLMQ
--PYSLMH
--PYSLLN
--PYSLKM
--PYSLHS
--PYSLVN
--PYSLVI
--PYSLTR
--PYSLTN
--PYSLSN
--PYSLRD
--PYSLMI
--PYSLLK
--PYSLHW
--PYSLHA
--PYSLGA
--PYSLAS
PIPYSL--
--PYSLYN
--PYSLYH
--PYSLSG
--PYSLSF
--PYSLNY
--PYSLIW
--PYSLHG
--PYSLWH
--PYSLTY
--PYSLTW

--PYSLTV
--PYSLQV
--PYSLHQ
--PYSLFR
--PYSLAT
PHPYSL--
--PYSLYE
--PYSLYD
--PYSLVD
--PYSLQS
--PYSLII
--PYSLHD
--PYSLGY
--PYSLFN
--PYSLEA
--PYSLAA
PKPYSL--
--PYSLWM
--PYSLWG
--PYSLPM
--PYSLPI
--PYSLNA
--PYSLKH
--PYSLHR
--PYSLHL
--PYSLGI
--PYSLFQ
--PYSLFK
--PYSLFI
--PYSLAI
PYPYSL--
PEPYSL--
--PYSLVW
--PYSLVM
--PYSLSH
--PYSLQY
--PYSLQW
--PYSLQK
--PYSLNM
--PYSLMD
--PYSLLQ
--PYSLHM
--PYSLGW
--PYSLGM
--PYSLEG
PNPYSL--
--PYSLYQ
--PYSLWN
--PYSLTL
--PYSLTF

--PYSLTE
--PYSLSR
--PYSLSK
--PYSLQN
--PYSLPY
--PYSLKG
--PYSLIN
--PYSLGG
--PYSLAV
--PYSLAM
PWPYSL--
--PYSLWA
--PYSLVE
--PYSLTQ
--PYSLTI
--PYSLQT
--PYSLQR
--PYSLQM
--PYSLQA
--PYSLPF
--PYSLNQ
--PYSLNG
--PYSLNF
--PYSLME
--PYSLKF
--PYSLIQ
--PYSLIE
--PYSLID
--PYSLHY
--PYSLHI
--PYSLHF
--PYSLFD
--PYSLAN
PFPYSL--
--PYSLWW
--PYSLWQ
--PYSLWE
--PYSLWD
--PYSLQI
--PYSLNW
--PYSLKW
--PYSLKQ
--PYSLKI
--PYSLKD
--PYSLHE
--PYSLEY
--PYSLEK
--PYSLDF
--PYSLCS
--PYSLAW

-PPYSLV-
-PPYSLI-
-PPYSLG-
-QPYSLR-
QSPYSL--
QTPYSL--
-QPYSLL-
QLPYSL--
QVPYSL--
QMPYSL--
-QPYSLH-
QFPYSL--
QYPYSL--
QGPYSL--
QWPYSL--
-QPYSLT-
QNPYSL--
-QPYSLN-
-QPYSLK-
QEPYSL--
QAPYSL--
-QPYSLI-
QKPYSL--
-QPYSLS-
-QPYSLW-
QPPYSL--
QIPYSL--
QCPYSL--
QQPYSL--
-QPYSLM-
QHPYSL--
RIPYSL--
RLPYSL--
-RPYSLR-
-RPYSLA-
-RPYSLT-
RTPYSL--
RGPYSL--
-RPYSLH-
RAPYSL--
-RPYSLW-
-RPYSLS-
-RPYSLM-
RQPYSL--
-RPYSLY-
REPYSL--
RYPYSL--
RWPYSL--
-RPYSLF-
-RPYSLQ-

-RPYSLK-
RDPYSL--
-RPYSLE-
RHPYSL--
-RPYSLG-
-RPYSLC-
SRPYSL--
SSPYSL--
SNPYSL--
-SPYSLH-
SLPYSL--
-SPYSLT-
-SPYSLL-
SMPYSL--
SHPYSL--
SIPYSL--
SKPYSL--
SYPYSL--
SGPYSL--
STPYSL--
SFPYSL--
SAPYSL--
-SPYSLK-
-SPYSLI-
-SPYSLQ-
SQPYSL--
SPPYSL--
-SPYSLV-
-SPYSLW-
-SPYSLN-
-SPYSLM-
SEPYSL--
SDPYSL--
TGPYSL--
TVPYSL--
TLPYSL--
TKPYSL--
TSPYSL--
TTPYSL--
-TPYSLW-
TPPYSL--
TMPYSL--
-TPYSLQ-
-TPYSLL-
TAPYSL--
-TPYSLY-
-TPYSLV-
THPYSL--
-TPYSLS-
TNPYSL--

TIPYSL--
-TPYSLT-
TYPYSL--
-TPYSLI-
TEPYSL--
-TPYSLH-
-TPYSLM-
-TPYSLK-
TFPYSL--
TWPYSL--
TQPYSL--
-TPYSLA-
-TPYSLN-
TDPYSL--
TCPYSL--
VSPYSL--
-VPYSLL-
VLPYSL--
VVPYSL--
VYPYSL--
VWPYSL--
VGPYSL--
-VPYSLV-
VKPYSL--
VTPYSL--
VQPYSL--
-VPYSLT-
-VPYSLM-
-VPYSLR-
VEPYSL--
-VPYSLW-
VPPYSL--
-VPYSLN-
-VPYSLF-
VNPYSL--
VFPYSL--
-VPYSLQ-
VIPYSL--
VAPYSL--
VHPYSL--
-VPYSLK-
-VPYSLH-
-VPYSLE-
VDPYSL--
-WPYSLR-
WLPYSL--
WAPYSL--
WTPYSL--
WSPYSL--
-WPYSLK-

```
        -WPYSLT-
        WPPYSL--
        WKPYSL--
        WIPYSL--
        WGPYSL--
        WQPYSL--
        WEPYSL--
        WMPYSL--
        -WPYSLS-
        WDPYSL--
        WWPYSL--
        -WPYSLQ-
        -WPYSLV-
        -WPYSLN-
        -WPYSLM-
        -WPYSLH-
        -WPYSLI-
        -WPYSLF-
        WHPYSL--
        YSPYSL--
        YLPYSL--
        -YPYSLR-
        YMPYSL--
        -YPYSLK-
        YAPYSL--
        -YPYSLL-
        YRPYSL--
        -YPYSLI-
        YPPYSL--
        YWPYSL--
        YFPYSL--
        YNPYSL--
        YHPYSL--
        YVPYSL--
        YGPYSL--
        YYPYSL--
        YTPYSL--
        -YPYSLH-
        -YPYSLM-
        YQPYSL--
        YEPYSL--
        YKPYSL--
        YIPYSL--
        -YPYSLW-
        -YPYSLV-
        -YPYSLN-
        -YPYSLY-
        -YPYSLA-
        -YPYSLE-
PAAV     166     732     30.72
```

AGPAAV--
--PAAVTL
--PAAVTK
--PAAVLL
--PAAVGQ
ARPAAV--
-APAAVT-
AIPAAV--
-APAAVR-
-APAAVH-
AKPAAV--
ATPAAV--
-APAAVI-
AYPAAV--
-APAAVV-
-APAAVS-
-APAAVL-
AFPAAV--
AVPAAV--
-APAAVK-
-APAAVF-
-APAAVA-
ANPAAV--
AEPAAV--
ASPAAV--
-APAAVY-
AMPAAV--
AWPAAV--
-APAAVM-
-APAAVE-
ALPAAV--
AQPAAV--
-APAAVW-
-APAAVN-
-APAAVG-
ADPAAV--
AAPAAV--
CSPAAV--
-CPAAVI-
CHPAAV--
CEPAAV--
DTPAAV--
DVPAAV--
-DPAAVR-
-DPAAVH-
DYPAAV--
-DPAAVY-
-DPAAVW-
-DPAAVT-
DSPAAV--

-DPAAVQ-
-DPAAVK-
DLPAAV--
DKPAAV--
DEPAAV--
DRPAAV--
-DPAAVV-
-DPAAVG-
-DPAAVF-
DIPAAV--
DGPAAV--
DDPAAV--
DAPAAV--
ERPAAV--
-EPAAVI-
-EPAAVF-
-EPAAVS-
-EPAAVL-
EAPAAV--
-EPAAVY-
-EPAAVR-
EQPAAV--
-EPAAVV-
EGPAAV--
ETPAAV--
-EPAAVW-
-EPAAVT-
EKPAAV--
-EPAAVM-
EVPAAV--
ESPAAV--
-EPAAVP-
-EPAAVH-
ENPAAV--
EMPAAV--
ELPAAV--
ECPAAV--
-FPAAVR-
-FPAAVL-
FFPAAV--
-FPAAVM-
FLPAAV--
FAPAAV--
FPPAAV--
-FPAAVW-
-FPAAVV-
-FPAAVI-
-FPAAVH-
FGPAAV--
FYPAAV--

FVPAAV--
FTPAAV--
-FPAAVK-
-FPAAVF-
FIPAAV--
FHPAAV--
-FPAAVQ-
FMPAAV--
FEPAAV--
-GPAAVR-
-GPAAVL-
GSPAAV--
GKPAAV--
GRPAAV--
-GPAAVK-
-GPAAVA-
GMPAAV--
GGPAAV--
GFPAAV--
GYPAAV--
-GPAAVV-
GVPAAV--
-GPAAVY-
-GPAAVS-
GIPAAV--
GEPAAV--
GTPAAV--
-GPAAVH-
GQPAAV--
GPPAAV--
-GPAAVW-
-GPAAVT-
-GPAAVQ-
-GPAAVM-
-GPAAVI-
-GPAAVF-
GNPAAV--
GHPAAV--
GDPAAV--
-HPAAVL-
-HPAAVS-
HGPAAV--
HHPAAV--
-HPAAVF-
HMPAAV--
HLPAAV--
HKPAAV--
HVPAAV--
HQPAAV--
-HPAAVT-

−HPAAVK−
−HPAAVH−
HAPAAV−−
HTPAAV−−
−HPAAVV−
−HPAAVM−
HFPAAV−−
HSPAAV−−
HRPAAV−−
−HPAAVY−
−HPAAVN−
−HPAAVA−
HNPAAV−−
HEPAAV−−
IYPAAV−−
IRPAAV−−
IQPAAV−−
−IPAAVV−
−IPAAVL−
−IPAAVS−
IFPAAV−−
ISPAAV−−
−IPAAVY−
−IPAAVW−
−IPAAVR−
−IPAAVK−
−IPAAVF−
INPAAV−−
−IPAAVT−
−IPAAVM−
−IPAAVI−
IKPAAV−−
IVPAAV−−
ITPAAV−−
−IPAAVQ−
−IPAAVH−
−IPAAVA−
IMPAAV−−
ILPAAV−−
IIPAAV−−
IDPAAV−−
IAPAAV−−
KSPAAV−−
−KPAAVV−
−KPAAVT−
KVPAAV−−
KGPAAV−−
KRPAAV−−
−KPAAVS−
−KPAAVH−

KTPAAV--
-KPAAVL-
-KPAAVR-
-KPAAVQ-
-KPAAVY-
-KPAAVK-
KLPAAV--
KAPAAV--
KQPAAV--
-KPAAVM-
-KPAAVI-
KMPAAV--
KKPAAV--
-KPAAVN-
-KPAAVC-
KIPAAV--
-KPAAVW-
-KPAAVA-
KNPAAV--
KHPAAV--
KEPAAV--
-LPAAVV-
LKPAAV--
LGPAAV--
-LPAAVW-
-LPAAVL-
LYPAAV--
-LPAAVY-
LEPAAV--
LVPAAV--
-LPAAVH-
LMPAAV--
LHPAAV--
LTPAAV--
LSPAAV--
-LPAAVT-
-LPAAVK-
-LPAAVF-
LLPAAV--
LDPAAV--
LWPAAV--
LQPAAV--
-LPAAVS-
-LPAAVA-
LCPAAV--
-LPAAVM-
LNPAAV--
LIPAAV--
LAPAAV--
-MPAAVI-

MTPAAV--
MSPAAV--
MWPAAV--
-MPAAVM-
MLPAAV--
MKPAAV--
MIPAAV--
MAPAAV--
-MPAAVH-
-MPAAVA-
MDPAAV--
-MPAAVV-
-MPAAVF-
MHPAAV--
MGPAAV--
MYPAAV--
MRPAAV--
MQPAAV--
MPPAAV--
-MPAAVW-
-MPAAVS-
-MPAAVK-
-NPAAVR-
-NPAAVT-
-NPAAVV-
NSPAAV--
NRPAAV--
-NPAAVS-
NKPAAV--
NIPAAV--
NFPAAV--
-NPAAVK-
NGPAAV--
NEPAAV--
NVPAAV--
NTPAAV--
-NPAAVQ-
-NPAAVI-
-NPAAVF-
NLPAAV--
-NPAAVL-
-NPAAVA-
NCPAAV--
NAPAAV--
--PAAVRL
--PAAVMT
--PAAVRN
--PAAVLG
--PAAVST
--PAAVRS

--PAAVRR
--PAAVGY
--PAAVMS
--PAAVFR
--PAAVYL
--PAAVVR
--PAAVRV
--PAAVFS
--PAAVRG
--PAAVRA
--PAAVRM
--PAAVPF
--PAAVKS
--PAAVHR
--PAAVYR
--PAAVLW
--PAAVNA
--PAAVGI
--PAAVWS
--PAAVVK
--PAAVVG
--PAAVTR
--PAAVSV
--PAAVRY
--PAAVPL
--PAAVFD
PGPAAV--
--PAAVYS
--PAAVYN
--PAAVWG
--PAAVVS
--PAAVSR
--PAAVRH
--PAAVLA
--PAAVIM
--PAAVFG
--PAAVYG
--PAAVWN
--PAAVVT
--PAAVTS
--PAAVSL
--PAAVSI
--PAAVQY
--PAAVMW
--PAAVLV
--PAAVLS
--PAAVLN
--PAAVKI
--PAAVKA
--PAAVIS

--PAAVIR
--PAAVIL
--PAAVHS
--PAAVAL
--PAAVYF
--PAAVTG
--PAAVRT
--PAAVQW
--PAAVMY
--PAAVKW
--PAAVHL
--PAAVYH
--PAAVVM
--PAAVTH
--PAAVSW
--PAAVSH
--PAAVRK
--PAAVQT
--PAAVNL
--PAAVLR
--PAAVLQ
--PAAVKT
--PAAVFK
--PAAVAG
PVPAAV--
--PAAVYV
--PAAVYM
--PAAVSY
--PAAVSS
--PAAVSN
--PAAVSG
--PAAVSA
--PAAVRF
--PAAVNY
--PAAVMK
--PAAVMH
--PAAVKV
--PAAVKR
--PAAVKH
--PAAVKD
--PAAVHI
--PAAVFT
--PAAVYT
--PAAVWL
--PAAVWI
--PAAVVV
--PAAVSQ
--PAAVSM
--PAAVSK
--PAAVQR

--PAAVNS
--PAAVNG
--PAAVMV
--PAAVMN
--PAAVLK
--PAAVLI
--PAAVLH
--PAAVKF
--PAAVIK
--PAAVIH
--PAAVHM
--PAAVGA
--PAAVFY
--PAAVFN
--PAAVFH
--PAAVFE
--PAAVES
--PAAVEA
--PAAVAF
PRPAAV--
PNPAAV--
PMPAAV--
PKPAAV--
--PAAVYA
--PAAVVN
--PAAVRQ
--PAAVRD
--PAAVQS
--PAAVQM
--PAAVQL
--PAAVQI
--PAAVPW
--PAAVPI
--PAAVLT
--PAAVKG
--PAAVIY
--PAAVIV
--PAAVII
--PAAVFM
--PAAVFA
--PAAVAW
--PAAVAV
--PAAVAI
PQPAAV--
PLPAAV--
PIPAAV--
PHPAAV--
--PAAVYE
--PAAVWR
--PAAVWM

--PAAVWH
--PAAVWA
--PAAVVY
--PAAVVH
--PAAVTY
--PAAVTV
--PAAVTT
--PAAVTQ
--PAAVTD
--PAAVRE
--PAAVQV
--PAAVQN
--PAAVNM
--PAAVMM
--PAAVMG
--PAAVMF
--PAAVME
--PAAVMA
--PAAVLD
--PAAVKQ
--PAAVKL
--PAAVIN
--PAAVHT
--PAAVHQ
--PAAVHG
--PAAVHA
--PAAVGW
--PAAVGM
--PAAVGF
--PAAVCS
--PAAVAY
--PAAVAS
--PAAVAQ
--PAAVAH
PYPAAV--
PWPAAV--
PSPAAV--
-PPAAVR-
PEPAAV--
PAPAAV--
--PAAVYY
--PAAVYQ
--PAAVWW
--PAAVWV
--PAAVWT
--PAAVWK
--PAAVWE
--PAAVVW
--PAAVVD
--PAAVVA

--PAAVTI
--PAAVTF
--PAAVSD
--PAAVRI
--PAAVQK
--PAAVQG
--PAAVQE
--PAAVQA
--PAAVPY
--PAAVNT
--PAAVNF
--PAAVMQ
--PAAVKY
--PAAVKM
--PAAVIW
--PAAVIG
--PAAVIE
--PAAVHV
--PAAVHK
--PAAVHH
--PAAVHF
--PAAVHE
--PAAVGV
--PAAVFW
--PAAVFQ
--PAAVFP
--PAAVEW
--PAAVEL
--PAAVEI
--PAAVDM
--PAAVDI
--PAAVCV
--PAAVAK
--PAAVAA
-QPAAVF-
QRPAAV--
QKPAAV--
QSPAAV--
-QPAAVI-
QTPAAV--
-QPAAVK-
QVPAAV--
-QPAAVY-
-QPAAVV-
-QPAAVQ-
QAPAAV--
-QPAAVM-
QEPAAV--
QYPAAV--
QQPAAV--

QPPAAV--
-QPAAVT-
-QPAAVS-
-QPAAVH-
QNPAAV--
QIPAAV--
QGPAAV--
RSPAAV--
RVPAAV--
RTPAAV--
-RPAAVH-
RIPAAV--
-RPAAVR-
-RPAAVW-
RPPAAV--
-RPAAVY-
-RPAAVM-
-RPAAVS-
RKPAAV--
RYPAAV--
RWPAAV--
RQPAAV--
RNPAAV--
-RPAAVK-
RMPAAV--
RLPAAV--
REPAAV--
-RPAAVF-
RFPAAV--
-RPAAVN-
-RPAAVG-
-RPAAVE-
RHPAAV--
RGPAAV--
RCPAAV--
RDPAAV--
SRPAAV--
SSPAAV--
-SPAAVW-
-SPAAVR-
-SPAAVI-
-SPAAVT-
-SPAAVK-
-SPAAVQ-
SKPAAV--
-SPAAVY-
-SPAAVS-
SLPAAV--
SMPAAV--
SGPAAV--

SEPAAV--
-SPAAVF-
STPAAV--
SQPAAV--
-SPAAVV-
-SPAAVA-
SIPAAV--
SPPAAV--
-SPAAVN-
-SPAAVM-
SFPAAV--
SVPAAV--
SAPAAV--
SWPAAV--
-SPAAVH-
-SPAAVE-
SDPAAV--
TSPAAV--
-TPAAVW-
-TPAAVR-
TFPAAV--
TQPAAV--
-TPAAVT-
-TPAAVS-
TRPAAV--
-TPAAVF-
TVPAAV--
-TPAAVV-
-TPAAVI-
-TPAAVQ-
TKPAAV--
TTPAAV--
TPPAAV--
TLPAAV--
TEPAAV--
-TPAAVM-
TMPAAV--
TDPAAV--
TWPAAV--
-TPAAVH-
-TPAAVE-
-TPAAVA-
THPAAV--
TGPAAV--
-VPAAVS-
-VPAAVL-
VRPAAV--
-VPAAVR-
VIPAAV--
VTPAAV--

VSPAAV--
-VPAAVW-
-VPAAVI-
VLPAAV--
VDPAAV--
VAPAAV--
VWPAAV--
VQPAAV--
-VPAAVT-
-VPAAVM-
VPPAAV--
-VPAAVY-
VNPAAV--
VYPAAV--
-VPAAVV-
-VPAAVQ-
-VPAAVN-
-VPAAVH-
-VPAAVA-
VMPAAV--
VKPAAV--
VHPAAV--
VFPAAV--
VEPAAV--
VVPAAV--
-VPAAVK-
VGPAAV--
VCPAAV--
-WPAAVL-
WSPAAV--
WRPAAV--
-WPAAVR-
-WPAAVK-
-WPAAVI-
-WPAAVY-
WPPAAV--
-WPAAVV-
-WPAAVT-
WMPAAV--
WKPAAV--
WDPAAV--
WTPAAV--
WQPAAV--
-WPAAVF-
WNPAAV--
WLPAAV--
WFPAAV--
WEPAAV--
WAPAAV--
WYPAAV--

WVPAAV--
-WPAAVS-
-YPAAVV-
YRPAAV--
-YPAAVS-
YEPAAV--
YSPAAV--
-YPAAVL-
YPPAAV--
-YPAAVY-
YKPAAV--
YHPAAV--
YTPAAV--
-YPAAVK-
-YPAAVA-
YVPAAV--
-YPAAVF-
YGPAAV--
-YPAAVW-
-YPAAVR-
-YPAAVN-
YAPAAV--
YWPAAV--
YQPAAV--
-YPAAVT-
-YPAAVI-
-YPAAVH-
YNPAAV--
YMPAAV--
YLPAAV--
YIPAAV--
YCPAAV--

MSAL 304 666 30.71

-AMSALP-
-AMSALG-
-GMSALI-
-GMSALA-
--MSALGS
--MSALWR
--MSALTK
--MSALRP
--MSALVK
--MSALTR
--MSALTH
--MSALSP
--MSALNT
--MSALNP
--MSALNG
--MSALLR
--MSALLE

--MSALHK
--MSALGR
--MSALAT
--MSALAP
-QMSALG-
-RMSALE-
-SMSALG-
-VMSALE-
-AMSALL-
AVMSAL--
ASMSAL--
AAMSAL--
-AMSALH-
-AMSALT-
-AMSALW-
-AMSALR-
AKMSAL--
AMMSAL--
ATMSAL--
ANMSAL--
-AMSALQ-
AGMSAL--
AQMSAL--
APMSAL--
-AMSALK-
ADMSAL--
AFMSAL--
-AMSALM-
AYMSAL--
-AMSALI-
-AMSALS-
AHMSAL--
AEMSAL--
-AMSALV-
-AMSALA-
-AMSALN-
-AMSALC-
ACMSAL--
CVMSAL--
-CMSALV-
-CMSALR-
-CMSALK-
-CMSALH-
CCMSAL--
-CMSALM-
CAMSAL--
CSMSAL--
CRMSAL--
-CMSALS-
-CMSALQ-

-CMSALI-
CGMSAL--
CFMSAL--
CPMSAL--
-CMSALT-
-CMSALN-
-CMSALL-
-CMSALF-
CDMSAL--
DHMSAL--
-DMSALR-
DWMSAL--
DRMSAL--
-DMSALY-
-DMSALV-
DIMSAL--
DLMSAL--
DGMSAL--
DCMSAL--
DVMSAL--
DSMSAL--
DYMSAL--
DPMSAL--
DNMSAL--
-DMSALT-
-DMSALL-
-DMSALH-
DEMSAL--
-DMSALS-
-DMSALQ-
DTMSAL--
DQMSAL--
DFMSAL--
-DMSALF-
DMMSAL--
DDMSAL--
ESMSAL--
ETMSAL--
EMMSAL--
EKMSAL--
EGMSAL--
-EMSALK-
-EMSALI-
EPMSAL--
ELMSAL--
EHMSAL--
-EMSALW-
-EMSALH-
EYMSAL--
ENMSAL--

-EMSALR-
-EMSALL-
EQMSAL--
-EMSALQ-
-EMSALY-
-EMSALV-
-EMSALT-
-EMSALS-
EDMSAL--
EEMSAL--
EAMSAL--
-EMSALN-
-EMSALA-
EFMSAL--
-EMSALM-
FTMSAL--
FLMSAL--
FSMSAL--
FVMSAL--
FNMSAL--
-FMSALS-
-FMSALH-
FQMSAL--
-FMSALN-
FKMSAL--
FAMSAL--
FEMSAL--
-FMSALG-
FPMSAL--
FYMSAL--
FHMSAL--
-FMSALQ-
-FMSALE-
FDMSAL--
-GMSALR-
GGMSAL--
GMMSAL--
GAMSAL--
GSMSAL--
GTMSAL--
GVMSAL--
-GMSALV-
-GMSALT-
GWMSAL--
-GMSALS-
GDMSAL--
GKMSAL--
-GMSALH-
GNMSAL--
GEMSAL--

```
-GMSALK-
GQMSAL--
GPMSAL--
GCMSAL--
-GMSALQ-
-GMSALM-
-GMSALE-
HHMSAL--
-HMSALL-
HSMSAL--
HLMSAL--
HAMSAL--
HTMSAL--
-HMSALY-
-HMSALS-
HNMSAL--
HFMSAL--
HEMSAL--
HWMSAL--
-HMSALR-
-HMSALN-
HMMSAL--
HGMSAL--
-HMSALT-
-HMSALK-
HYMSAL--
HQMSAL--
-HMSALQ-
-HMSALM-
-HMSALF-
-HMSALV-
HCMSAL--
HPMSAL--
-HMSALH-
HIMSAL--
-HMSALI-
HDMSAL--
-HMSALW-
-HMSALE-
-IMSALA-
-IMSALS-
IRMSAL--
ITMSAL--
-IMSALR-
-IMSALN-
IVMSAL--
ISMSAL--
-IMSALL-
-IMSALT-
-IMSALH-
```

IDMSAL--
IMMSAL--
IQMSAL--
INMSAL--
IPMSAL--
-IMSALI-
IHMSAL--
IEMSAL--
-IMSALW-
-IMSALM-
-IMSALE-
-IMSALK-
ICMSAL--
-KMSALR-
KGMSAL--
KLMSAL--
KTMSAL--
KWMSAL--
KYMSAL--
KDMSAL--
-KMSALI-
-KMSALT-
-KMSALM-
KAMSAL--
-KMSALY-
-KMSALK-
KCMSAL--
KPMSAL--
-KMSALS-
-KMSALQ-
KIMSAL--
KMMSAL--
KEMSAL--
KQMSAL--
KFMSAL--
KNMSAL--
-KMSALW-
KHMSAL--
-KMSALN-
LAMSAL--
LVMSAL--
-LMSALN-
-LMSALL-
LTMSAL--
-LMSALS-
-LMSALP-
-LMSALK-
LFMSAL--
-LMSALE-
LDMSAL--

LNMSAL--
LPMSAL--
LYMSAL--
-LMSALG-
LMMSAL--
-LMSALD-
LCMSAL--
--MSALNM
--MSALSL
--MSALKS
--MSALGL
-MMSALK-
--MSALKG
--MSALLG
--MSALYS
--MSALRM
--MSALLA
--MSALVS
--MSALRG
--MSALLT
--MSALSA
--MSALNS
--MSALGI
--MSALRH
--MSALKT
MKMSAL--
--MSALTS
--MSALFT
--MSALMA
--MSALHA
--MSALWT
--MSALYA
--MSALYG
--MSALSV
--MSALRE
--MSALNY
--MSALSM
--MSALSI
--MSALIS
--MSALGY
--MSALAS
MNMSAL--
--MSALRK
--MSALPL
-MMSALI-
--MSALVA
--MSALSY
--MSALRW
--MSALRD
--MSALHV

```
--MSALGM
--MSALWM
--MSALVG
--MSALSG
--MSALRY
--MSALNA
--MSALHG
-MMSALT-
-MMSALR-
--MSALTY
--MSALST
MQMSAL--
-MMSALG-
MTMSAL--
--MSALYT
--MSALYH
--MSALTV
--MSALTT
--MSALTG
--MSALRV
--MSALMV
--MSALMH
--MSALGF
MPMSAL--
-MMSALY-
-MMSALS-
--MSALWN
--MSALIA
MGMSAL--
--MSALYM
--MSALWG
--MSALVH
--MSALNI
--MSALFN
MAMSAL--
--MSALQV
--MSALQT
--MSALQG
--MSALKW
--MSALKV
--MSALHW
--MSALYD
--MSALMF
--MSALLW
--MSALIT
--MSALHT
--MSALHR
--MSALAW
-MMSALV-
MDMSAL--
```

--MSALYQ
--MSALWA
--MSALVF
--MSALPY
--MSALMR
--MSALMM
--MSALLV
--MSALKK
--MSALKA
--MSALIV
--MSALIG
--MSALHY
--MSALHM
--MSALAM
-MMSALH-
--MSALVQ
--MSALTN
--MSALTM
--MSALTF
--MSALPI
--MSALMW
--MSALKH
--MSALIY
--MSALIR
--MSALIH
--MSALHN
--MSALHF
--MSALAF
--MSALAA
-MMSALQ-
-MMSALN-
MHMSAL--
--MSALYN
--MSALWQ
--MSALPM
--MSALMQ
--MSALLN
--MSALKI
--MSALIM
--MSALIF
--MSALHQ
--MSALEG
--MSALEA
--MSALAG
-MMSALP-
MEMSAL--
--MSALYK
--MSALQA
--MSALPW
--MSALME

```
--MSALKQ
--MSALIQ
--MSALHD
--MSALFQ
--MSALFD
--MSALFA
--MSALCS
--MSALWH
--MSALVE
--MSALSC
--MSALKF
--MSALKD
--MSALII
--MSALHH
--MSALHE
--MSALCY
--MSALCG
--MSALAQ
-MMSALE-
MCMSAL--
NRMSAL--
NAMSAL--
NTMSAL--
-NMSALT-
-NMSALL-
NGMSAL--
NKMSAL--
NPMSAL--
-NMSALK-
-NMSALI-
NFMSAL--
NYMSAL--
NNMSAL--
NEMSAL--
NHMSAL--
NDMSAL--
NWMSAL--
NSMSAL--
-NMSALQ-
-NMSALH-
NIMSAL--
-NMSALW-
-NMSALA-
NMMSAL--
NQMSAL--
-NMSALF-
-PMSALR-
PLMSAL--
PSMSAL--
PRMSAL--
```

-PMSALL-
-PMSALT-
PKMSAL--
PHMSAL--
PGMSAL--
PFMSAL--
-PMSALI-
PTMSAL--
PNMSAL--
PDMSAL--
-PMSALS-
PIMSAL--
-PMSALM-
-PMSALQ-
-PMSALH-
PWMSAL--
PQMSAL--
-PMSALV-
-PMSALA-
PEMSAL--
PCMSAL--
QRMSAL--
QDMSAL--
QTMSAL--
QVMSAL--
QLMSAL--
QGMSAL--
-QMSALL-
-QMSALS-
QYMSAL--
-QMSALR-
QNMSAL--
-QMSALK-
QWMSAL--
QSMSAL--
QPMSAL--
QQMSAL--
-QMSALV-
-QMSALQ-
QKMSAL--
QMMSAL--
QIMSAL--
QCMSAL--
-QMSALT-
-QMSALM-
-QMSALH-
QAMSAL--
-QMSALN-
-QMSALI-
-QMSALF-

RSMSAL--
RYMSAL--
RMMSAL--
RNMSAL--
RGMSAL--
RHMSAL--
RDMSAL--
RVMSAL--
RAMSAL--
-RMSALT-
-RMSALY-
-RMSALK-
-RMSALI-
-RMSALS-
RPMSAL--
-RMSALW-
REMSAL--
-RMSALM-
-RMSALN-
SSMSAL--
SKMSAL--
SAMSAL--
STMSAL--
SLMSAL--
SFMSAL--
-SMSALS-
SNMSAL--
SGMSAL--
SVMSAL--
-SMSALW-
SHMSAL--
-SMSALT-
SIMSAL--
SMMSAL--
SEMSAL--
-SMSALK-
-SMSALI-
SDMSAL--
SQMSAL--
SPMSAL--
-SMSALY-
-SMSALQ-
-SMSALN-
-SMSALM-
SCMSAL--
-TMSALK-
TVMSAL--
TTMSAL--
TPMSAL--
TQMSAL--

```
-TMSALV-
-TMSALS-
TAMSAL--
-TMSALM-
-TMSALF-
TNMSAL--
TWMSAL--
-TMSALH-
TFMSAL--
TIMSAL--
-TMSALI-
TDMSAL--
TEMSAL--
-TMSALA-
TMMSAL--
THMSAL--
TCMSAL--
TYMSAL--
-TMSALN-
-VMSALS-
VVMSAL--
VYMSAL--
-VMSALI-
VLMSAL--
-VMSALK-
-VMSALH-
-VMSALL-
VNMSAL--
-VMSALT-
-VMSALQ-
VDMSAL--
VIMSAL--
VEMSAL--
VHMSAL--
VMMSAL--
VGMSAL--
VQMSAL--
VPMSAL--
-VMSALN-
VWMSAL--
-VMSALM-
VAMSAL--
VFMSAL--
VCMSAL--
WRMSAL--
WTMSAL--
WVMSAL--
WYMSAL--
WGMSAL--
WEMSAL--
```

WNMSAL--
-WMSALS-
-WMSALN-
WKMSAL--
WIMSAL--
-WMSALA-
WMMSAL--
-WMSALQ-
WAMSAL--
WWMSAL--
-WMSALH-
WFMSAL--
WPMSAL--
-WMSALK-
-WMSALT-
-WMSALE-
WLMSAL--
WDMSAL--
WCMSAL--
WQMSAL--
-WMSALI-
-WMSALP-
YVMSAL--
-YMSALL-
-YMSALA-
-YMSALN-
YDMSAL--
YSMSAL--
-YMSALS-
YGMSAL--
-YMSALK-
YTMSAL--
YQMSAL--
-YMSALW-
YAMSAL--
YMMSAL--
YLMSAL--
YIMSAL--
YHMSAL--
YPMSAL--
-YMSALE-
YFMSAL--
-YMSALQ-
-YMSALM-
-YMSALH-
YKMSAL--
YEMSAL--
-YMSALG-
-YMSALV-

PKGY 291 672 30.70

--PKGYTK
--PKGYNI
--PKGYLI
--PKGYGF
-SPKGYG-
AAPKGY--
-APKGYH-
ALPKGY--
-APKGYF-
AWPKGY--
-APKGYL-
ANPKGY--
AHPKGY--
ADPKGY--
ASPKGY--
-APKGYQ-
-APKGYM-
AFPKGY--
AEPKGY--
-APKGYW-
-APKGYV-
-APKGYT-
AYPKGY--
-APKGYY-
-APKGYS-
-APKGYA-
AIPKGY--
ARPKGY--
-APKGYN-
-APKGYK-
-APKGYI-
-APKGYG-
-APKGYE-
AGPKGY--
CIPKGY--
CEPKGY--
CRPKGY--
-CPKGYM-
CLPKGY--
-CPKGYR-
CCPKGY--
CAPKGY--
-CPKGYY-
-CPKGYL-
-CPKGYK-
DQPKGY--
DHPKGY--
DFPKGY--
DRPKGY--
-DPKGYR-

DMPKGY--
DLPKGY--
DWPKGY--
-DPKGYY-
DNPKGY--
DGPKGY--
DSPKGY--
-DPKGYV-
-DPKGYM-
-DPKGYL-
-DPKGYF-
DKPKGY--
-DPKGYT-
-DPKGYS-
-DPKGYI-
DEPKGY--
DAPKGY--
ENPKGY--
-EPKGYL-
ERPKGY--
ESPKGY--
-EPKGYF-
EKPKGY--
EGPKGY--
EVPKGY--
EQPKGY--
-EPKGYS-
-EPKGYK-
ELPKGY--
EFPKGY--
-EPKGYY-
-EPKGYV-
-EPKGYQ-
-EPKGYM-
-EPKGYI-
-EPKGYH-
EMPKGY--
EDPKGY--
EAPKGY--
FGPKGY--
-FPKGYY-
FSPKGY--
-FPKGYV-
FRPKGY--
FLPKGY--
FWPKGY--
FTPKGY--
FKPKGY--
FQPKGY--
-FPKGYF-

-FPKGYA-
FNPKGY--
FYPKGY--
FPPKGY--
-FPKGYW-
-FPKGYN-
FHPKGY--
FFPKGY--
-FPKGYT-
FCPKGY--
FAPKGY--
-GPKGYL-
-GPKGYW-
GAPKGY--
GWPKGY--
GRPKGY--
-GPKGYV-
-GPKGYQ-
-GPKGYM-
-GPKGYI-
GYPKGY--
-GPKGYS-
-GPKGYF-
GLPKGY--
-GPKGYH-
GMPKGY--
-GPKGYY-
-GPKGYT-
GNPKGY--
GTPKGY--
GVPKGY--
GSPKGY--
-GPKGYG-
GGPKGY--
GEPKGY--
GDPKGY--
GPPKGY--
-GPKGYK-
GKPKGY--
GHPKGY--
-HPKGYS-
HSPKGY--
-HPKGYK-
-HPKGYT-
HFPKGY--
-HPKGYL-
-HPKGYH-
HMPKGY--
HGPKGY--
HAPKGY--

HTPKGY--
-HPKGYW-
-HPKGYG-
HWPKGY--
-HPKGYP-
HEPKGY--
-HPKGYV-
-HPKGYN-
-HPKGYI-
HKPKGY--
HIPKGY--
HVPKGY--
HPPKGY--
-HPKGYY-
-IPKGYL-
IMPKGY--
IRPKGY--
ILPKGY--
ISPKGY--
-IPKGYV-
IYPKGY--
-IPKGYM-
IPPKGY--
-IPKGYH-
-IPKGYA-
-IPKGYK-
IKPKGY--
IVPKGY--
-IPKGYT-
-IPKGYQ-
-IPKGYN-
INPKGY--
IWPKGY--
IQPKGY--
-IPKGYY-
ICPKGY--
KSPKGY--
KAPKGY--
-KPKGYT-
-KPKGYL-
KQPKGY--
KDPKGY--
KYPKGY--
-KPKGYS-
-KPKGYN-
-KPKGYM-
KLPKGY--
KVPKGY--
-KPKGYK-
-KPKGYA-

KEPKGY--
KWPKGY--
KRPKGY--
-KPKGYV-
KKPKGY--
KGPKGY--
KCPKGY--
-KPKGYF-
KMPKGY--
KIPKGY--
LSPKGY--
LLPKGY--
LMPKGY--
LVPKGY--
-LPKGYK-
LGPKGY--
LDPKGY--
LEPKGY--
LPPKGY--
LIPKGY--
-LPKGYQ-
-LPKGYY-
-LPKGYS-
-LPKGYF-
LHPKGY--
LQPKGY--
-LPKGYV-
-LPKGYM-
-LPKGYH-
-LPKGYA-
LFPKGY--
-LPKGYW-
-LPKGYN-
LYPKGY--
LTPKGY--
LAPKGY--
-MPKGYL-
MAPKGY--
-MPKGYM-
-MPKGYT-
-MPKGYS-
MTPKGY--
-MPKGYW-
MGPKGY--
MYPKGY--
-MPKGYV-
MIPKGY--
-MPKGYY-
-MPKGYN-
-MPKGYK-

-MPKGYF-
MNPKGY--
MDPKGY--
MVPKGY--
MSPKGY--
MEPKGY--
-MPKGYQ-
-MPKGYH-
-MPKGYA-
MPPKGY--
MKPKGY--
MCPKGY--
-NPKGYL-
-NPKGYT-
NLPKGY--
NRPKGY--
NFPKGY--
NSPKGY--
-NPKGYS-
-NPKGYM-
NNPKGY--
-NPKGYR-
NWPKGY--
-NPKGYV-
NMPKGY--
NGPKGY--
NAPKGY--
NYPKGY--
NVPKGY--
-NPKGYH-
NTPKGY--
NQPKGY--
-NPKGYY-
-NPKGYQ-
-NPKGYF-
-NPKGYA-
NHPKGY--
--PKGYSM
--PKGYLS
--PKGYYS
--PKGYMW
--PKGYWR
--PKGYYV
--PKGYWS
--PKGYVG
--PKGYSL
--PKGYSK
--PKGYSA
--PKGYLA
--PKGYVH

--PKGYSV
--PKGYSR
--PKGYRN
--PKGYLH
--PKGYKY
--PKGYVV
--PKGYSS
--PKGYSF
--PKGYIV
--PKGYYM
--PKGYSW
--PKGYMF
--PKGYIS
--PKGYFA
--PKGYVT
--PKGYRI
--PKGYMG
--PKGYKW
--PKGYHS
PAPKGY--
--PKGYYY
--PKGYTS
--PKGYTM
--PKGYRQ
--PKGYQY
--PKGYKS
--PKGYHI
--PKGYYR
--PKGYYL
--PKGYYF
--PKGYYA
--PKGYWN
--PKGYVF
--PKGYTY
--PKGYTA
--PKGYSY
--PKGYSG
--PKGYLV
--PKGYIT
--PKGYIK
--PKGYGS
--PKGYGA
--PKGYVY
--PKGYTH
--PKGYST
--PKGYSN
--PKGYMS
--PKGYMI
--PKGYHN
--PKGYFD

--PKGYAI
PNPKGY--
--PKGYVN
--PKGYTV
--PKGYTR
--PKGYSQ
--PKGYQW
--PKGYQN
--PKGYNT
--PKGYNS
--PKGYML
--PKGYLK
--PKGYII
--PKGYFH
--PKGYAG
PFPKGY--
--PKGYWY
--PKGYWK
--PKGYRF
--PKGYNY
--PKGYMK
--PKGYLW
--PKGYKF
--PKGYIR
--PKGYHV
--PKGYHT
--PKGYHK
--PKGYHF
--PKGYGT
--PKGYFG
PWPKGY--
PVPKGY--
PSPKGY--
--PKGYYK
--PKGYWW
--PKGYWH
--PKGYVW
--PKGYTT
--PKGYTN
--PKGYTI
--PKGYRD
--PKGYQK
--PKGYQI
--PKGYNW
--PKGYNF
--PKGYLF
--PKGYIQ
--PKGYIM
--PKGYHR
--PKGYHG

--PKGYHE
--PKGYEG
--PKGYAY
--PKGYAW
--PKGYAL
PGPKGY--
--PKGYYT
--PKGYYH
--PKGYYG
--PKGYWT
--PKGYWI
--PKGYVQ
--PKGYTL
--PKGYTF
--PKGYRE
--PKGYNV
--PKGYNM
--PKGYMV
--PKGYLY
--PKGYKV
--PKGYIN
--PKGYIE
--PKGYHW
--PKGYHQ
--PKGYFN
--PKGYFM
--PKGYFK
--PKGYFI
--PKGYAV
--PKGYAA
PEPKGY--
PYPKGY--
PRPKGY--
--PKGYYD
--PKGYWG
--PKGYTG
--PKGYQT
--PKGYQG
--PKGYQF
--PKGYMT
--PKGYMM
--PKGYMD
--PKGYIG
--PKGYHL
--PKGYHH
--PKGYGV
--PKGYFY
--PKGYFS
--PKGYFQ
--PKGYES

--PKGYAT
--PKGYAM
--PKGYAF
PIPKGY--
PHPKGY--
PLPKGY--
--PKGYYW
--PKGYYQ
--PKGYYN
--PKGYYI
--PKGYWL
--PKGYWA
--PKGYVK
--PKGYVI
--PKGYTQ
--PKGYTE
--PKGYRC
--PKGYQV
--PKGYQA
--PKGYNQ
--PKGYNA
--PKGYME
--PKGYLQ
--PKGYLN
--PKGYKM
--PKGYFV
--PKGYET
--PKGYCS
--PKGYAK
--PKGYAH
-QPKGYH-
-QPKGYN-
QMPKGY--
QAPKGY--
QSPKGY--
QRPKGY--
QLPKGY--
QPPKGY--
-QPKGYS-
-QPKGYA-
-QPKGYW-
QYPKGY--
QTPKGY--
QQPKGY--
-QPKGYY-
QIPKGY--
QFPKGY--
QWPKGY--
QVPKGY--
-QPKGYT-

-QPKGYM-
-QPKGYL-
-QPKGYK-
-QPKGYE-
QNPKGY--
QKPKGY--
QGPKGY--
QEPKGY--
QDPKGY--
RVPKGY--
-RPKGYY-
RMPKGY--
RSPKGY--
-RPKGYL-
RYPKGY--
-RPKGYF-
-RPKGYW-
-RPKGYS-
RTPKGY--
RQPKGY--
-RPKGYI-
RHPKGY--
RGPKGY--
-RPKGYV-
-RPKGYH-
-RPKGYA-
RAPKGY--
RRPKGY--
-RPKGYT-
RPPKGY--
-RPKGYN-
RNPKGY--
RIPKGY--
-RPKGYM-
REPKGY--
-RPKGYQ-
RDPKGY--
-SPKGYL-
-SPKGYV-
SLPKGY--
SRPKGY--
SSPKGY--
SKPKGY--
SVPKGY--
-SPKGYI-
SNPKGY--
-SPKGYW-
-SPKGYH-
-SPKGYF-
SYPKGY--

SQPKGY--
-SPKGYQ-
SIPKGY--
SHPKGY--
-SPKGYN-
SMPKGY--
SAPKGY--
SPPKGY--
-SPKGYY-
-SPKGYA-
-SPKGYM-
-SPKGYE-
-TPKGYM-
-TPKGYS-
TSPKGY--
TTPKGY--
TRPKGY--
-TPKGYQ-
-TPKGYY-
-TPKGYV-
TQPKGY--
TLPKGY--
TAPKGY--
TVPKGY--
-TPKGYT-
-TPKGYA-
-TPKGYW-
-TPKGYI-
-TPKGYH-
TWPKGY--
THPKGY--
-TPKGYN-
TNPKGY--
TMPKGY--
TIPKGY--
TEPKGY--
TCPKGY--
TYPKGY--
TPPKGY--
-TPKGYK-
-TPKGYE-
TGPKGY--
VWPKGY--
VSPKGY--
-VPKGYR-
VNPKGY--
VPPKGY--
-VPKGYL-
VVPKGY--
-VPKGYV-

-VPKGYK-
VLPKGY--
VAPKGY--
VTPKGY--
VYPKGY--
-VPKGYI-
-VPKGYQ-
VQPKGY--
-VPKGYT-
-VPKGYM-
-VPKGYF-
VEPKGY--
-VPKGYY-
VHPKGY--
-VPKGYN-
-VPKGYH-
-VPKGYA-
VIPKGY--
VDPKGY--
-VPKGYW-
-VPKGYG-
VMPKGY--
VKPKGY--
WRPKGY--
-WPKGYT-
WAPKGY--
-WPKGYH-
WQPKGY--
WPPKGY--
-WPKGYQ-
WMPKGY--
WLPKGY--
-WPKGYY-
-WPKGYV-
-WPKGYM-
WTPKGY--
WNPKGY--
WSPKGY--
-WPKGYL-
-WPKGYI-
WGPKGY--
WYPKGY--
WWPKGY--
WVPKGY--
-WPKGYW-
-WPKGYS-
WIPKGY--
WHPKGY--
-WPKGYN-
-WPKGYE-

-YPKGYL-
YLPKGY--
YQPKGY--
YSPKGY--
YGPKGY--
YFPKGY--
YYPKGY--
-YPKGYV-
YTPKGY--
YRPKGY--
-YPKGYT-
YEPKGY--
YVPKGY--
-YPKGYY-
YMPKGY--
-YPKGYN-
-YPKGYA-
YHPKGY--
YWPKGY--
-YPKGYW-
-YPKGYQ-
-YPKGYM-

| | | | |
|---|---|---|---|
| ISHL | 117 | 758 | 30.70 |

-AISHLG-
-GISHLN-
--ISHLWY
--ISHLVQ
--ISHLNN
--ISHLLQ
--ISHLLN
--ISHLLL
--ISHLLK
--ISHLKL
--ISHLHN
--ISHLHL
--ISHLGY
--ISHLGG
--ISHLFN
--ISHLFK
--ISHLEG
-PISHLG-
-RISHLN-
-RISHLG-
-RISHLE-
-SISHLE-
-TISHLN-
-TISHLG-
-AISHLS-
ALISHL--
ASISHL--

ATISHL--
-AISHLK-
AYISHL--
-AISHLL-
AMISHL--
APISHL--
AGISHL--
AKISHL--
-AISHLT-
-AISHLA-
AVISHL--
AHISHL--
AAISHL--
-AISHLR-
-AISHLM-
-AISHLI-
-AISHLH-
-AISHLV-
AQISHL--
ADISHL--
AEISHL--
ACISHL--
AWISHL--
AIISHL--
-AISHLF-
-AISHLE-
AFISHL--
-AISHLW-
CRISHL--
CLISHL--
CHISHL--
CCISHL--
CSISHL--
CAISHL--
-CISHLS-
CPISHL--
-CISHLT-
CWISHL--
CKISHL--
-CISHLV-
-CISHLR-
-CISHLL-
-CISHLK-
-CISHLH-
CYISHL--
CVISHL--
CTISHL--
-CISHLP-
-CISHLF-
CIISHL--

CDISHL--
DRISHL--
DWISHL--
DSISHL--
DAISHL--
-DISHLS-
DGISHL--
-DISHLR-
DVISHL--
DLISHL--
-DISHLM-
DYISHL--
DNISHL--
DMISHL--
DFISHL--
DTISHL--
DDISHL--
-DISHLK-
DHISHL--
DQISHL--
DPISHL--
-DISHLT-
DIISHL--
DKISHL--
-DISHLV-
-DISHLQ-
-DISHLF-
DCISHL--
-EISHLR-
ESISHL--
EGISHL--
EVISHL--
EKISHL--
ERISHL--
ELISHL--
-EISHLI-
EAISHL--
EYISHL--
EMISHL--
-EISHLK-
EPISHL--
ENISHL--
-EISHLM-
-EISHLL-
EDISHL--
EWISHL--
-EISHLS-
-EISHLH-
EIISHL--
EFISHL--

-EISHLY-
ECISHL--
-EISHLW-
-EISHLV-
-EISHLT-
-EISHLQ-
-EISHLF-
-EISHLA-
EHISHL--
-FISHLR-
-FISHLS-
FKISHL--
FHISHL--
-FISHLT-
FSISHL--
FMISHL--
-FISHLH-
FEISHL--
FGISHL--
FNISHL--
FVISHL--
-FISHLG-
-FISHLV-
-FISHLK-
FDISHL--
FAISHL--
FYISHL--
FQISHL--
FCISHL--
-FISHLQ-
-FISHLA-
-FISHLY-
-FISHLW-
-FISHLE-
-FISHLC-
-GISHLR-
GRISHL--
GSISHL--
GGISHL--
GAISHL--
-GISHLM-
-GISHLY-
GVISHL--
-GISHLI-
-GISHLQ-
-GISHLL-
-GISHLK-
GMISHL--
-GISHLV-
-GISHLT-

-GISHLA-
GPISHL--
GEISHL--
GTISHL--
GIISHL--
GHISHL--
GFISHL--
-GISHLH-
-GISHLF-
-GISHLW-
GWISHL--
GDISHL--
-GISHLE-
GCISHL--
HTISHL--
HLISHL--
HVISHL--
-HISHLR-
HPISHL--
HNISHL--
-HISHLQ-
-HISHLY-
-HISHLS-
-HISHLI-
HGISHL--
HFISHL--
HWISHL--
-HISHLT-
HEISHL--
HDISHL--
HQISHL--
-HISHLV-
-HISHLM-
-HISHLL-
HAISHL--
-HISHLE-
HHISHL--
HMISHL--
-HISHLN-
-HISHLK-
HCISHL--
HYISHL--
-HISHLG-
HIISHL--
IRISHL--
--ISHLRS
--ISHLRG
--ISHLSS
--ISHLRT
--ISHLRN

IPISHL--
--ISHLSG
--ISHLRF
--ISHLRA
--ISHLFS
--ISHLTT
--ISHLIS
IKISHL--
--ISHLYS
--ISHLRV
--ISHLWS
--ISHLRY
--ISHLMS
--ISHLLA
--ISHLTS
--ISHLSW
--ISHLMG
--ISHLFG
ISISHL--
--ISHLST
--ISHLNS
-IISHLS-
--ISHLVV
--ISHLTG
--ISHLRL
--ISHLRD
--ISHLKA
ITISHL--
--ISHLNL
IQISHL--
IDISHL--
IAISHL--
--ISHLVG
--ISHLVA
--ISHLSA
--ISHLRH
--ISHLGL
--ISHLYG
--ISHLYA
--ISHLQG
--ISHLLT
--ISHLIN
--ISHLAL
-IISHLA-
IVISHL--
--ISHLWG
--ISHLSV
--ISHLIG
--ISHLWL
--ISHLVT

--ISHLMF
--ISHLIT
--ISHLHA
IIISHL--
--ISHLYV
--ISHLVD
--ISHLTA
--ISHLSL
--ISHLMV
--ISHLKH
--ISHLAT
-IISHLI-
--ISHLTV
--ISHLYY
--ISHLYT
--ISHLVM
--ISHLMN
--ISHLMK
--ISHLMI
--ISHLIW
--ISHLAV
--ISHLAS
IMISHL--
-IISHLR-
-IISHLK-
--ISHLTN
--ISHLSY
--ISHLRQ
--ISHLQS
--ISHLKT
--ISHLIY
--ISHLIR
IHISHL--
--ISHLYN
--ISHLVN
--ISHLTQ
--ISHLTK
--ISHLQT
--ISHLMT
--ISHLKY
--ISHLIA
--ISHLHS
--ISHLFW
--ISHLFT
ILISHL--
-IISHLV-
IEISHL--
--ISHLTM
--ISHLSK
--ISHLMQ

--ISHLMH
--ISHLKF
--ISHLIK
--ISHLIH
--ISHLHT
--ISHLHG
--ISHLGA
--ISHLAA
-IISHLG-
--ISHLVY
--ISHLTW
--ISHLNW
--ISHLNA
--ISHLMY
--ISHLMM
--ISHLME
--ISHLLV
--ISHLIV
--ISHLII
--ISHLHV
--ISHLFY
--ISHLFA
--ISHLET
--ISHLAM
INISHL--
-IISHLL-
IFISHL--
IYISHL--
--ISHLYM
--ISHLYH
--ISHLYF
--ISHLWT
--ISHLWN
--ISHLVW
--ISHLVF
--ISHLTD
--ISHLSR
--ISHLSM
--ISHLSH
--ISHLRE
--ISHLQY
--ISHLNM
--ISHLNG
--ISHLLM
--ISHLKK
--ISHLIQ
--ISHLID
--ISHLEA
--ISHLAW
-IISHLQ-

-IISHLN-
IWISHL--
--ISHLYQ
--ISHLYK
--ISHLWW
--ISHLWV
--ISHLWF
--ISHLVK
--ISHLVH
--ISHLTY
--ISHLTF
--ISHLSI
--ISHLQH
--ISHLQF
--ISHLNT
--ISHLNF
--ISHLKD
--ISHLIM
--ISHLIF
--ISHLIE
--ISHLFH
--ISHLES
--ISHLAI
-IISHLW-
-IISHLH-
-IISHLF-
-IISHLE-
ICISHL--
--ISHLYW
--ISHLYI
--ISHLWP
--ISHLWK
--ISHLWH
--ISHLWE
--ISHLQW
--ISHLQV
--ISHLQM
--ISHLQI
--ISHLQA
--ISHLPP
--ISHLPF
--ISHLNY
--ISHLMW
--ISHLML
--ISHLKW
--ISHLKV
--ISHLKQ
--ISHLKI
--ISHLHW
--ISHLGW

--ISHLFQ
--ISHLEV
--ISHLEL
--ISHLCS
--ISHLCM
--ISHLCA
KLISHL--
-KISHLR-
-KISHLS-
-KISHLY-
KQISHL--
-KISHLK-
KYISHL--
KAISHL--
-KISHLT-
-KISHLA-
KDISHL--
-KISHLV-
KHISHL--
KPISHL--
-KISHLH-
KEISHL--
-KISHLW-
-KISHLM-
-KISHLF-
KCISHL--
-LISHLS-
-LISHLR-
LTISHL--
LGISHL--
LSISHL--
-LISHLT-
LAISHL--
LNISHL--
-LISHLL-
-LISHLH-
LEISHL--
-LISHLM-
LDISHL--
LMISHL--
-LISHLA-
LQISHL--
-LISHLG-
LFISHL--
LIISHL--
-LISHLQ-
LPISHL--
-LISHLK-
-LISHLV-
-LISHLN-

-LISHLI-
-LISHLE-
-LISHLY-
-LISHLW-
-LISHLP-
-LISHLC-
-LISHLD-
LCISHL--
MSISHL--
MFISHL--
MLISHL--
-MISHLL-
MTISHL--
-MISHLS-
-MISHLA-
MAISHL--
MWISHL--
-MISHLM-
-MISHLH-
MYISHL--
MKISHL--
MNISHL--
-MISHLY-
-MISHLT-
-MISHLK-
-MISHLF-
-MISHLE-
MHISHL--
MDISHL--
MGISHL--
MEISHL--
MMISHL--
-MISHLN-
-MISHLI-
-MISHLG-
MCISHL--
MIISHL--
-MISHLW-
NTISHL--
NHISHL--
-NISHLR-
-NISHLA-
NSISHL--
NRISHL--
NQISHL--
NLISHL--
-NISHLT-
NDISHL--
-NISHLS-
-NISHLI-

NAISHL--
-NISHLK-
-NISHLY-
NPISHL--
NNISHL--
NGISHL--
NFISHL--
-NISHLV-
NYISHL--
NWISHL--
NKISHL--
-NISHLM-
NCISHL--
-NISHLH-
-NISHLF-
-NISHLW-
-NISHLQ-
-NISHLN-
NEISHL--
PRISHL--
-PISHLR-
PSISHL--
PYISHL--
-PISHLS-
PAISHL--
-PISHLT-
PEISHL--
PLISHL--
PHISHL--
PGISHL--
PVISHL--
-PISHLM-
-PISHLI-
PTISHL--
PMISHL--
-PISHLK-
-PISHLH-
PNISHL--
PWISHL--
-PISHLL-
-PISHLA-
PFISHL--
PQISHL--
PKISHL--
-PISHLW-
-PISHLY-
-PISHLQ-
PIISHL--
-PISHLV-
-PISHLE-

−QISHLI−
−QISHLT−
QRISHL−−
−QISHLR−
−QISHLA−
QLISHL−−
−QISHLL−
QTISHL−−
−QISHLS−
−QISHLE−
QNISHL−−
QIISHL−−
QQISHL−−
QGISHL−−
QMISHL−−
−QISHLQ−
QDISHL−−
QPISHL−−
−QISHLY−
−QISHLK−
−QISHLH−
QHISHL−−
QFISHL−−
−QISHLV−
QCISHL−−
QAISHL−−
−QISHLM−
−QISHLF−
QEISHL−−
RVISHL−−
−RISHLR−
−RISHLV−
−RISHLI−
−RISHLH−
RPISHL−−
RAISHL−−
−RISHLT−
RDISHL−−
−RISHLQ−
−RISHLM−
−RISHLW−
REISHL−−
RQISHL−−
RCISHL−−
−SISHLS−
SIISHL−−
SKISHL−−
−SISHLL−
−SISHLR−
SFISHL−−

```
-SISHLI-
-SISHLW-
SMISHL--
SYISHL--
-SISHLY-
-SISHLH-
SHISHL--
SNISHL--
-SISHLK-
-SISHLA-
SAISHL--
SQISHL--
SEISHL--
-SISHLM-
SPISHL--
-SISHLQ-
SDISHL--
-SISHLV-
SCISHL--
TYISHL--
-TISHLR-
-TISHLL-
-TISHLT-
TSISHL--
TQISHL--
TTISHL--
TGISHL--
TVISHL--
-TISHLY-
TPISHL--
TMISHL--
-TISHLS-
TAISHL--
-TISHLA-
TKISHL--
TEISHL--
TLISHL--
TIISHL--
-TISHLW-
-TISHLI-
-TISHLF-
TDISHL--
TWISHL--
THISHL--
-TISHLV-
-TISHLQ-
TCISHL--
-TISHLK-
-TISHLH-
-TISHLM-
```

VVISHL--
VRISHL--
VTISHL--
-VISHLT-
-VISHLS-
VNISHL--
-VISHLI-
-VISHLL-
-VISHLK-
-VISHLM-
VGISHL--
VHISHL--
VFISHL--
VYISHL--
VQISHL--
-VISHLF-
VAISHL--
-VISHLY-
VEISHL--
-VISHLA-
VPISHL--
VWISHL--
-VISHLW-
VCISHL--
-VISHLN-
VDISHL--
-VISHLQ-
-VISHLG-
WRISHL--
-WISHLS-
WEISHL--
WGISHL--
-WISHLA-
WSISHL--
WHISHL--
WYISHL--
-WISHLE-
WVISHL--
-WISHLH-
WPISHL--
WNISHL--
-WISHLK-
-WISHLV-
-WISHLL-
-WISHLY-
-WISHLG-
WWISHL--
WQISHL--
-WISHLT-
WAISHL--

WKISHL--
-WISHLQ-
-WISHLM-
-WISHLF-
YAISHL--
-YISHLL-
YTISHL--
YPISHL--
YSISHL--
YMISHL--
YKISHL--
YIISHL--
-YISHLA-
YVISHL--
YGISHL--
-YISHLS-
-YISHLE-
YEISHL--
-YISHLK-
-YISHLN-
YHISHL--
YFISHL--
YDISHL--
-YISHLY-
-YISHLM-
YNISHL--
-YISHLW-
YQISHL--
-YISHLQ-
YCISHL--
-YISHLH-

| | | | |
|---|---|---|---|
| LRGM | 581 | 584 | 30.68 |

-DLRGMN-
-GLRGMW-
-GLRGMQ-
-KLRGMS-
-KLRGMN-
-KLRGMM-
--LRGMGT
--LRGMLI
--LRGMGP
--LRGMGA
--LRGMYE
--LRGMVE
--LRGMTW
--LRGMSY
--LRGMSD
--LRGMQR
--LRGMNV
--LRGMMH

```
--LRGMKW
--LRGMKT
--LRGMII
--LRGMID
--LRGMGS
--LRGMGG
--LRGMFT
--LRGMEK
--LRGMAY
-MLRGMA-
-PLRGMG-
-QLRGMN-
-RLRGMS-
-RLRGMV-
-RLRGMH-
-SLRGMD-
AILRGM--
-ALRGMW-
-ALRGMT-
-ALRGMK-
AWLRGM--
ARLRGM--
ASLRGM--
AMLRGM--
ANLRGM--
-ALRGMV-
-ALRGMP-
-ALRGMF-
-ALRGMA-
AGLRGM--
AFLRGM--
-ALRGMY-
-ALRGMI-
AALRGM--
AQLRGM--
AHLRGM--
-ALRGMH-
ADLRGM--
-ALRGMM-
AYLRGM--
-ALRGMQ-
-ALRGMG-
-ALRGMN-
-ALRGMD-
CPLRGM--
CTLRGM--
CGLRGM--
CCLRGM--
CALRGM--
CLLRGM--
```

CILRGM--
CSLRGM--
CNLRGM--
CYLRGM--
CQLRGM--
CHLRGM--
CFLRGM--
-DLRGMR-
DYLRGM--
DVLRGM--
DILRGM--
DALRGM--
DWLRGM--
-DLRGMK-
-DLRGMF-
-DLRGMT-
DMLRGM--
-DLRGMM-
DQLRGM--
-DLRGMH-
DTLRGM--
DPLRGM--
-DLRGMY-
-DLRGMG-
DHLRGM--
DELRGM--
-DLRGMS-
DDLRGM--
DNLRGM--
-DLRGMW-
-DLRGMP-
-DLRGMD-
-DLRGMQ-
-ELRGMR-
-ELRGML-
-ELRGMS-
-ELRGMK-
EELRGM--
EVLRGM--
-ELRGMT-
-ELRGMN-
-ELRGMG-
EYLRGM--
ESLRGM--
-ELRGMW-
-ELRGMI-
-ELRGMH-
-ELRGMM-
EFLRGM--
EPLRGM--

-ELRGMA-
EWLRGM--
-ELRGMF-
EKLRGM--
EILRGM--
ETLRGM--
-ELRGMY-
-ELRGMP-
EALRGM--
EMLRGM--
-ELRGMQ-
-ELRGMD-
EQLRGM--
ENLRGM--
-ELRGME-
ECLRGM--
FRLRGM--
FTLRGM--
FSLRGM--
FVLRGM--
FNLRGM--
-FLRGMP-
FILRGM--
FELRGM--
FWLRGM--
-FLRGMY-
FMLRGM--
-FLRGMM-
-FLRGMF-
FPLRGM--
FQLRGM--
FFLRGM--
FALRGM--
-FLRGMV-
-FLRGMD-
-FLRGMW-
-FLRGMH-
-FLRGME-
FDLRGM--
GYLRGM--
GVLRGM--
GILRGM--
GTLRGM--
GMLRGM--
GGLRGM--
GQLRGM--
-GLRGMY-
-GLRGML-
GDLRGM--
-GLRGMV-

```
-GLRGMT-
-GLRGMP-
-GLRGMI-
GELRGM--
-GLRGMN-
-GLRGMH-
GCLRGM--
HSLRGM--
-HLRGMT-
HLLRGM--
HMLRGM--
HGLRGM--
HKLRGM--
HYLRGM--
HTLRGM--
-HLRGMR-
HILRGM--
HQLRGM--
-HLRGMS-
HNLRGM--
-HLRGMH-
HPLRGM--
-HLRGMK-
HALRGM--
-HLRGMF-
-HLRGMW-
-HLRGMI-
HHLRGM--
HELRGM--
-HLRGMP-
HDLRGM--
HCLRGM--
ISLRGM--
ILLRGM--
INLRGM--
IWLRGM--
IMLRGM--
-ILRGML-
-ILRGMM-
IYLRGM--
IPLRGM--
-ILRGMT-
IHLRGM--
-ILRGMI-
-ILRGMH-
IQLRGM--
IALRGM--
IFLRGM--
IELRGM--
-ILRGMN-
```

-ILRGMK-
KNLRGM--
KELRGM--
KGLRGM--
KPLRGM--
KDLRGM--
-KLRGMY-
-KLRGMI-
-KLRGMH-
KCLRGM--
LGLRGM--
--LRGMTS
--LRGMLS
-LLRGMT-
--LRGMSS
LMLRGM--
--LRGMIS
--LRGMYS
--LRGMWS
--LRGMNS
--LRGMSR
-LLRGMI-
--LRGMVQ
--LRGMSN
--LRGMPK
--LRGMHS
--LRGMYG
--LRGMRH
--LRGMLA
-LLRGMV-
LWLRGM--
LDLRGM--
--LRGMQG
--LRGMNL
--LRGMNA
--LRGMKG
--LRGMAS
--LRGMHG
-LLRGMH-
--LRGMYT
--LRGMPQ
--LRGMMN
--LRGMWA
--LRGMVK
--LRGMQA
--LRGMPR
--LRGMRP
--LRGMMT
--LRGMHV
--LRGMFA

--LRGMTK
--LRGMHT
--LRGMAQ
LPLRGM--
-LLRGMW-
--LRGMVT
--LRGMSK
--LRGMQS
--LRGMPH
--LRGMNR
--LRGMYQ
--LRGMSQ
--LRGMMI
--LRGMER
--LRGMSP
--LRGMMV
--LRGMYA
--LRGMST
--LRGMPY
--LRGMHN
--LRGMDN
--LRGMAL
--LRGMVN
--LRGMPV
--LRGMHY
--LRGMFQ
--LRGMYR
--LRGMYN
--LRGMVF
--LRGMKQ
--LRGMHQ
--LRGMDQ
--LRGMVV
--LRGMTM
--LRGMPT
--LRGMLM
--LRGMGY
--LRGMGK
--LRGMGI
--LRGMDR
LELRGM--
--LRGMYP
--LRGMYH
--LRGMVP
--LRGMTV
--LRGMTL
--LRGMSH
--LRGMQV
--LRGMQQ
--LRGMQF

--LRGMPM
--LRGMPA
--LRGMNY
--LRGMNF
--LRGMMY
--LRGMMQ
--LRGMKH
--LRGMHW
--LRGMHR
--LRGMFK
--LRGMEQ
--LRGMEP
-LLRGME-
LCLRGM--
--LRGMYM
--LRGMWT
--LRGMWL
--LRGMVH
--LRGMTY
--LRGMTF
--LRGMPI
--LRGMNT
--LRGMNQ
--LRGMNK
--LRGMIK
--LRGMHK
--LRGMGV
--LRGMEN
--LRGMDP
--LRGMAT
--LRGMYL
--LRGMYI
--LRGMWP
--LRGMWI
--LRGMTP
--LRGMPF
--LRGMNW
--LRGMNP
--LRGMKP
--LRGMIV
--LRGMIN
--LRGMIH
--LRGMIF
--LRGMHP
--LRGMFN
--LRGMFM
--LRGMEV
--LRGMEG
--LRGMDK
--LRGMAW

```
--LRGMAM
--LRGMWQ
--LRGMQP
--LRGMQN
--LRGMQK
--LRGMQI
--LRGMNI
--LRGMMC
--LRGMFH
--LRGMDT
--LRGMDG
MGLRGM--
MMLRGM--
MSLRGM--
-MLRGMV-
-MLRGMS-
MILRGM--
MNLRGM--
-MLRGMM-
-MLRGMW-
MQLRGM--
MALRGM--
MTLRGM--
-MLRGMT-
MHLRGM--
MPLRGM--
MDLRGM--
-MLRGMQ-
MWLRGM--
-MLRGMN-
MELRGM--
-MLRGMF-
-MLRGMD-
MCLRGM--
-NLRGMK-
-NLRGMS-
-NLRGMW-
NTLRGM--
NPLRGM--
NSLRGM--
-NLRGMH-
-NLRGMA-
-NLRGMF-
-NLRGMP-
NILRGM--
NGLRGM--
NFLRGM--
-NLRGMN-
-NLRGMI-
NQLRGM--
```

-NLRGMQ-
NMLRGM--
-NLRGME-
-NLRGMD-
NELRGM--
-NLRGMM-
-NLRGMC-
NDLRGM--
NCLRGM--
-PLRGMR-
-PLRGML-
PILRGM--
PTLRGM--
PSLRGM--
-PLRGMT-
-PLRGMK-
-PLRGMI-
PYLRGM--
PELRGM--
PALRGM--
PDLRGM--
PVLRGM--
PHLRGM--
PNLRGM--
-PLRGMV-
PWLRGM--
-PLRGMM-
-PLRGMF-
-PLRGMW-
-PLRGMH-
PKLRGM--
PCLRGM--
PQLRGM--
QQLRGM--
QALRGM--
QPLRGM--
-QLRGMY-
-QLRGMV-
-QLRGMS-
QLLRGM--
QSLRGM--
-QLRGMF-
QFLRGM--
QGLRGM--
QTLRGM--
-QLRGMM-
-QLRGMW-
-QLRGMH-
QILRGM--
QYLRGM--

-QLRGMT-
QELRGM--
QDLRGM--
QNLRGM--
-QLRGMQ-
-QLRGME-
RPLRGM--
RWLRGM--
-RLRGMM-
RDLRGM--
-RLRGML-
-RLRGMY-
-RLRGMW-
SALRGM--
-SLRGMI-
SPLRGM--
-SLRGMH-
-SLRGMK-
SHLRGM--
-SLRGMP-
-SLRGMV-
SILRGM--
SWLRGM--
SQLRGM--
-SLRGMW-
-SLRGMA-
SDLRGM--
SELRGM--
-SLRGMN-
-SLRGMQ-
-SLRGMG-
-SLRGME-
SCLRGM--
TSLRGM--
TPLRGM--
TILRGM--
TTLRGM--
TGLRGM--
TYLRGM--
-TLRGMV-
-TLRGMT-
TNLRGM--
-TLRGMY-
-TLRGMM-
TFLRGM--
TELRGM--
TQLRGM--
THLRGM--
-TLRGMS-
-TLRGMW-

TDLRGM--
TCLRGM--
VTLRGM--
VNLRGM--
VFLRGM--
-VLRGMY-
VYLRGM--
-VLRGMH-
-VLRGMT-
VMLRGM--
-VLRGMI-
VALRGM--
-VLRGMM-
VQLRGM--
VELRGM--
-VLRGMF-
VDLRGM--
-VLRGMV-
-VLRGMS-
VCLRGM--
-WLRGMA-
WSLRGM--
WNLRGM--
-WLRGMP-
WTLRGM--
-WLRGMV-
WGLRGM--
-WLRGMK-
WMLRGM--
-WLRGMS-
WHLRGM--
WQLRGM--
-WLRGME-
WELRGM--
WWLRGM--
WKLRGM--
-WLRGMW-
-WLRGMI-
WYLRGM--
WFLRGM--
WDLRGM--
-WLRGMM-
-WLRGMD-
YSLRGM--
-YLRGMN-
-YLRGMM-
YDLRGM--
YRLRGM--
YMLRGM--
YNLRGM--

| | | | |
|---|---|---|---|
| | YGLRGM-- | | |
| | -YLRGMH- | | |
| | -YLRGMA- | | |
| | YPLRGM-- | | |
| | -YLRGMW- | | |
| | -YLRGMG- | | |
| | YVLRGM-- | | |
| | YHLRGM-- | | |
| | YQLRGM-- | | |
| | -YLRGMF- | | |
| | YILRGM-- | | |
| | YALRGM-- | | |
| | -YLRGMQ- | | |
| | -YLRGMD- | | |
| | -YLRGMC- | | |
| | YELRGM-- | | |
| VSWL | 1127 | 487 | 30.66 |
| | --VSWLNH | | |
| | --VSWLNN | | |
| | --VSWLNK | | |
| | --VSWLEQ | | |
| | --VSWLEH | | |
| | -AVSWLR- | | |
| | AVVSWL-- | | |
| | -AVSWLN- | | |
| | -AVSWLT- | | |
| | AAVSWL-- | | |
| | ANVSWL-- | | |
| | -AVSWLQ- | | |
| | AGVSWL-- | | |
| | -AVSWLA- | | |
| | ADVSWL-- | | |
| | -AVSWLV- | | |
| | ASVSWL-- | | |
| | AHVSWL-- | | |
| | AKVSWL-- | | |
| | AEVSWL-- | | |
| | AQVSWL-- | | |
| | AWVSWL-- | | |
| | -AVSWLH- | | |
| | -AVSWLG- | | |
| | -AVSWLD- | | |
| | ACVSWL-- | | |
| | CKVSWL-- | | |
| | -CVSWLT- | | |
| | -CVSWLR- | | |
| | CTVSWL-- | | |
| | CMVSWL-- | | |
| | CAVSWL-- | | |
| | -CVSWLE- | | |

CLVSWL--
CHVSWL--
CGVSWL--
-CVSWLH-
-CVSWLG-
-CVSWLA-
CSVSWL--
CRVSWL--
CQVSWL--
CEVSWL--
CVVSWL--
-CVSWLY-
-CVSWLV-
-CVSWLS-
-CVSWLQ-
-CVSWLL-
CNVSWL--
CIVSWL--
CFVSWL--
CDVSWL--
DMVSWL--
DLVSWL--
DVVSWL--
-DVSWLT-
-DVSWLS-
-DVSWLG-
DGVSWL--
-DVSWLA-
DYVSWL--
-DVSWLH-
-DVSWLQ-
DTVSWL--
DSVSWL--
DKVSWL--
DIVSWL--
-DVSWLL-
DEVSWL--
DWVSWL--
-DVSWLK-
DNVSWL--
DDVSWL--
DCVSWL--
DHVSWL--
DAVSWL--
-DVSWLM-
DQVSWL--
ERVSWL--
-EVSWLR-
ELVSWL--
EKVSWL--

-EVSWLT-
ESVSWL--
-EVSWLK-
-EVSWLI-
-EVSWLS-
-EVSWLG-
EGVSWL--
EAVSWL--
-EVSWLA-
EQVSWL--
EEVSWL--
-EVSWLQ-
EIVSWL--
-EVSWLH-
-EVSWLV-
ETVSWL--
ENVSWL--
EHVSWL--
EDVSWL--
ECVSWL--
-EVSWLM-
EMVSWL--
EWVSWL--
-EVSWLN-
-EVSWLE-
-EVSWLD-
FAVSWL--
FSVSWL--
FNVSWL--
FHVSWL--
FDVSWL--
-FVSWLG-
-FVSWLE-
-FVSWLN-
-FVSWLH-
FGVSWL--
FQVSWL--
-FVSWLF-
GTVSWL--
-GVSWLT-
-GVSWLQ-
-GVSWLG-
-GVSWLS-
GGVSWL--
GVVSWL--
GNVSWL--
GIVSWL--
GAVSWL--
-GVSWLA-
GWVSWL--

GMVSWL--
GHVSWL--
-GVSWLI-
-GVSWLE-
-GVSWLN-
GDVSWL--
-GVSWLH-
GCVSWL--
-HVSWLK-
-HVSWLS-
HGVSWL--
HSVSWL--
-HVSWLT-
HVVSWL--
-HVSWLM-
-HVSWLG-
HTVSWL--
HAVSWL--
-HVSWLL-
HDVSWL--
-HVSWLN-
HFVSWL--
HWVSWL--
HIVSWL--
HEVSWL--
-HVSWLH-
-HVSWLA-
HHVSWL--
-HVSWLI-
HMVSWL--
-HVSWLE-
-HVSWLD-
HYVSWL--
-IVSWLN-
IKVSWL--
ITVSWL--
IEVSWL--
IDVSWL--
INVSWL--
-IVSWLG-
-IVSWLH-
-IVSWLD-
-KVSWLR-
-KVSWLL-
KVVSWL--
-KVSWLS-
-KVSWLH-
-KVSWLI-
-KVSWLN-
KEVSWL--

KFVSWL--
-KVSWLQ-
-KVSWLE-
KWVSWL--
-KVSWLG-
-KVSWLY-
KNVSWL--
-KVSWLM-
KDVSWL--
LNVSWL--
LEVSWL--
LAVSWL--
LDVSWL--
-LVSWLH-
-LVSWLP-
MVVSWL--
MGVSWL--
MAVSWL--
MHVSWL--
-MVSWLG-
-MVSWLR-
MEVSWL--
-MVSWLQ-
-MVSWLN-
MNVSWL--
-MVSWLE-
-MVSWLD-
MTVSWL--
-MVSWLH-
MFVSWL--
MCVSWL--
-NVSWLR-
NAVSWL--
-NVSWLS-
NHVSWL--
NGVSWL--
NVVSWL--
-NVSWLT-
-NVSWLQ-
NFVSWL--
NEVSWL--
-NVSWLH-
-NVSWLV-
-NVSWLK-
-NVSWLG-
-NVSWLA-
NSVSWL--
NDVSWL--
-NVSWLI-
-NVSWLE-

NYVSWL--
-NVSWLN-
NKVSWL--
NQVSWL--
NNVSWL--
PRVSWL--
PSVSWL--
PAVSWL--
PGVSWL--
PKVSWL--
PVVSWL--
PDVSWL--
PIVSWL--
PQVSWL--
PEVSWL--
PCVSWL--
-QVSWLS-
QGVSWL--
QAVSWL--
QRVSWL--
QVVSWL--
-QVSWLR-
QKVSWL--
-QVSWLT-
QTVSWL--
-QVSWLG-
-QVSWLK-
-QVSWLH-
-QVSWLI-
-QVSWLN-
-QVSWLE-
QQVSWL--
QYVSWL--
QHVSWL--
-QVSWLV-
QDVSWL--
-QVSWLD-
-QVSWLQ-
QIVSWL--
-QVSWLM-
QCVSWL--
-RVSWLS-
-RVSWLT-
-RVSWLK-
-RVSWLG-
RDVSWL--
-RVSWLE-
RYVSWL--
-RVSWLM-
-SVSWLR-

–SVSWLS–
SKVSWL––
STVSWL––
–SVSWLG–
–SVSWLV–
SIVSWL––
SGVSWL––
SAVSWL––
SYVSWL––
SMVSWL––
SHVSWL––
–SVSWLA–
SVVSWL––
SDVSWL––
–SVSWLH–
–SVSWLK–
SQVSWL––
–SVSWLN–
–SVSWLQ–
SEVSWL––
–SVSWLI–
–SVSWLE–
SWVSWL––
–SVSWLM–
–SVSWLD–
SCVSWL––
TRVSWL––
TKVSWL––
–TVSWLS–
TVVSWL––
–TVSWLK–
TDVSWL––
TGVSWL––
–TVSWLA–
TNVSWL––
TQVSWL––
–TVSWLN–
TMVSWL––
TWVSWL––
–TVSWLV–
–TVSWLQ–
TCVSWL––
TEVSWL––
TIVSWL––
TYVSWL––
–TVSWLE–
THVSWL––
––VSWLGS
––VSWLRG
––VSWLTS

--VSWLRT
-VVSWLR-
-VVSWLN-
--VSWLSN
--VSWLST
--VSWLSQ
--VSWLSK
--VSWLKS
--VSWLTG
--VSWLKN
--VSWLAS
--VSWLTT
--VSWLSH
--VSWLRN
VAVSWL--
--VSWLLS
--VSWLKV
--VSWLGT
VDVSWL--
--VSWLVR
--VSWLSV
--VSWLSD
--VSWLNS
--VSWLKT
--VSWLTK
--VSWLTD
--VSWLSM
--VSWLRD
--VSWLGG
--VSWLVD
--VSWLQG
--VSWLKG
--VSWLTN
--VSWLTM
--VSWLGV
--VSWLES
--VSWLNT
--VSWLMG
--VSWLTA
--VSWLQT
--VSWLQK
--VSWLRE
--VSWLQR
--VSWLAN
VQVSWL--
-VVSWLG-
--VSWLTQ
--VSWLAK
-VVSWLA-
--VSWLVQ

--VSWLTH
--VSWLMA
--VSWLHN
--VSWLHM
--VSWLGA
--VSWLSI
--VSWLEG
--VSWLAQ
--VSWLPL
--VSWLLN
--VSWLHG
--VSWLHE
--VSWLGI
--VSWLFN
-VVSWLQ-
--VSWLYG
--VSWLQV
--VSWLLK
--VSWLLH
--VSWLQM
--VSWLQD
--VSWLMN
--VSWLKY
--VSWLKF
--VSWLHV
--VSWLDS
--VSWLAA
-VVSWLD-
--VSWLSE
--VSWLQN
--VSWLLW
--VSWLIQ
--VSWLGM
--VSWLAM
--VSWLNY
--VSWLKQ
--VSWLIY
--VSWLVF
--VSWLQA
--VSWLKE
--VSWLKD
--VSWLIN
--VSWLIH
--VSWLID
--VSWLGF
--VSWLAE
--VSWLAD
--VSWLWN
--VSWLWH
--VSWLNG

--VSWLMQ
--VSWLHT
--VSWLHH
--VSWLHF
--VSWLGW
--VSWLEW
--VSWLRW
--VSWLQQ
--VSWLNW
--VSWLLD
--VSWLHY
--VSWLGN
--VSWLTE
--VSWLNV
--VSWLMD
--VSWLHW
--VSWLHQ
--VSWLHA
--VSWLGH
--VSWLEV
--VSWLEN
--VSWLTC
--VSWLQE
--VSWLNQ
--VSWLLC
--VSWLHI
--VSWLFD
--VSWLCT
--VSWLCR
--VSWLCG
-WVSWLR-
WKVSWL--
WEVSWL--
WQVSWL--
WAVSWL--
-WVSWLH-
WGVSWL--
WHVSWL--
-WVSWLF-
-WVSWLD-
-WVSWLC-
YGVSWL--
YTVSWL--
-YVSWLS-
YEVSWL--
YSVSWL--
YMVSWL--
-YVSWLG-
YKVSWL--
-YVSWLK-

```
          -YVSWLN-
          -YVSWLH-
          YHVSWL--
          YDVSWL--
PARW       251        690      30.63
          HHPARW--
          -IPARWG-
          -KPARWG-
          -LPARWA-
          --PARWSL
          --PARWYL
          --PARWSE
          --PARWNG
          -APARWL-
          -APARWS-
          ARPARW--
          -APARWQ-
          -APARWG-
          -APARWR-
          -APARWI-
          AHPARW--
          ATPARW--
          -APARWY-
          -APARWN-
          -APARWA-
          AVPARW--
          -APARWK-
          AKPARW--
          -APARWM-
          -APARWF-
          AIPARW--
          ASPARW--
          -APARWV-
          AAPARW--
          AYPARW--
          AWPARW--
          APPARW--
          -APARWW-
          -APARWT-
          AEPARW--
          -APARWH-
          -APARWE-
          AQPARW--
          ACPARW--
          CRPARW--
          CPPARW--
          -CPARWV-
          CKPARW--
          CTPARW--
          -CPARWI-
```

-CPARWF-
CMPARW--
CHPARW--
CCPARW--
-DPARWR-
-DPARWL-
DFPARW--
DMPARW--
DTPARW--
-DPARWT-
-DPARWS-
-DPARWF-
DGPARW--
DRPARW--
-DPARWI-
DKPARW--
-DPARWY-
-DPARWE-
DIPARW--
DAPARW--
DYPARW--
-DPARWA-
DWPARW--
DVPARW--
DQPARW--
-DPARWH-
DNPARW--
DLPARW--
-EPARWS-
-EPARWL-
-EPARWR-
EYPARW--
-EPARWM-
EVPARW--
ENPARW--
-EPARWW-
-EPARWN-
-EPARWF-
-EPARWA-
EAPARW--
-EPARWV-
-EPARWH-
-EPARWG-
EHPARW--
-EPARWY-
-EPARWK-
EMPARW--
EKPARW--
EIPARW--
EFPARW--

EQPARW--
-EPARWT-
-EPARWQ-
-EPARWI-
-EPARWE-
ELPARW--
ERPARW--
EEPARW--
-FPARWV-
FTPARW--
FSPARW--
-FPARWY-
-FPARWT-
-FPARWS-
FKPARW--
-FPARWA-
FDPARW--
FVPARW--
-FPARWR-
-FPARWQ-
-FPARWL-
FAPARW--
FPPARW--
-FPARWW-
-FPARWM-
-FPARWF-
FWPARW--
-FPARWE-
FLPARW--
FHPARW--
-FPARWN-
-FPARWK-
-FPARWH-
FEPARW--
-GPARWI-
GSPARW--
-GPARWY-
GQPARW--
-GPARWS-
GFPARW--
-GPARWR-
-GPARWT-
-GPARWA-
GAPARW--
-GPARWW-
-GPARWF-
GPPARW--
GLPARW--
GGPARW--
GNPARW--

GTPARW--
-GPARWV-
GKPARW--
GEPARW--
-GPARWM-
GYPARW--
-GPARWK-
-GPARWE-
GMPARW--
GRPARW--
GIPARW--
GDPARW--
GWPARW--
-GPARWG-
GHPARW--
-HPARWR-
HGPARW--
HFPARW--
-HPARWT-
HVPARW--
HWPARW--
HTPARW--
HSPARW--
HRPARW--
-HPARWV-
-HPARWM-
HKPARW--
HPPARW--
-HPARWY-
-HPARWQ-
-HPARWL-
HNPARW--
HMPARW--
HLPARW--
HDPARW--
-HPARWN-
-HPARWI-
-HPARWF-
-HPARWE-
ISPARW--
ITPARW--
-IPARWL-
-IPARWS-
IIPARW--
IGPARW--
IEPARW--
-IPARWQ-
-IPARWR-
-IPARWI-
-IPARWA-

INPARW--
-IPARWV-
-IPARWN-
ILPARW--
-IPARWK-
IYPARW--
IQPARW--
-IPARWT-
-IPARWM-
IFPARW--
IVPARW--
IPPARW--
-IPARWE-
IKPARW--
IHPARW--
IAPARW--
IWPARW--
-IPARWF-
IMPARW--
KSPARW--
-KPARWS-
-KPARWT-
-KPARWL-
-KPARWA-
KTPARW--
KMPARW--
KHPARW--
KLPARW--
-KPARWQ-
-KPARWE-
KGPARW--
KDPARW--
KEPARW--
-KPARWM-
-KPARWH-
KIPARW--
KAPARW--
KKPARW--
KVPARW--
KPPARW--
-KPARWN-
-KPARWK-
KYPARW--
KQPARW--
-KPARWW-
KNPARW--
LVPARW--
LLPARW--
LAPARW--
LGPARW--

LTPARW--
-LPARWY-
-LPARWR-
LSPARW--
-LPARWT-
-LPARWV-
LDPARW--
LHPARW--
-LPARWK-
LNPARW--
LIPARW--
LPPARW--
LMPARW--
LQPARW--
-LPARWM-
-LPARWF-
LEPARW--
LCPARW--
-LPARWH-
-LPARWE-
-MPARWV-
MSPARW--
MMPARW--
MAPARW--
-MPARWK-
-MPARWF-
-MPARWT-
MLPARW--
MIPARW--
MHPARW--
MYPARW--
MTPARW--
-MPARWQ-
-MPARWH-
MEPARW--
-MPARWL-
-MPARWI-
MNPARW--
MFPARW--
MQPARW--
-MPARWS-
MWPARW--
-MPARWG-
-MPARWE-
-NPARWQ-
-NPARWL-
NPPARW--
-NPARWT-
-NPARWI-
-NPARWR-

-NPARWS-
NLPARW--
NNPARW--
NYPARW--
-NPARWV-
-NPARWF-
NVPARW--
NQPARW--
NGPARW--
NFPARW--
NRPARW--
-NPARWY-
NKPARW--
NIPARW--
NAPARW--
NWPARW--
-NPARWE-
NMPARW--
NDPARW--
NTPARW--
-NPARWK-
-NPARWA-
--PARWLK
--PARWRT
--PARWSS
--PARWRG
--PARWST
--PARWRS
--PARWLV
--PARWLS
--PARWAS
--PARWMK
--PARWTS
--PARWLG
--PARWLT
PSPARW--
--PARWTV
--PARWWT
--PARWSI
--PARWLN
--PARWIR
--PARWFN
--PARWFA
--PARWTQ
--PARWRM
--PARWIY
--PARWIK
PMPARW--
--PARWVE
--PARWSA

--PARWLI
--PARWLA
--PARWHS
PRPARW--
--PARWTY
--PARWSG
--PARWES
PVPARW--
--PARWYT
--PARWWS
--PARWVY
--PARWTA
--PARWSV
--PARWSN
--PARWRN
--PARWMV
--PARWLQ
PYPARW--
--PARWVV
--PARWSM
--PARWQF
--PARWLH
--PARWGV
--PARWFS
--PARWVH
--PARWTM
--PARWSY
--PARWRA
--PARWQM
--PARWMM
--PARWMH
--PARWHG
--PARWAA
--PARWYS
--PARWYG
--PARWVQ
--PARWTG
--PARWSW
--PARWMY
--PARWMQ
--PARWMI
--PARWMF
--PARWKS
--PARWKG
--PARWII
--PARWGS
--PARWEV
--PARWVD
--PARWVA
--PARWTI

--PARWTF
--PARWMT
--PARWLL
--PARWIN
--PARWFT
--PARWYQ
--PARWRI
--PARWMG
--PARWME
--PARWMD
--PARWKT
--PARWIV
--PARWFM
PWPARW--
PNPARW--
PGPARW--
--PARWYH
--PARWWA
--PARWVM
--PARWTT
--PARWSH
--PARWQV
--PARWQL
--PARWLM
--PARWIQ
--PARWID
--PARWGT
--PARWAG
PTPARW--
PLPARW--
PKPARW--
--PARWYM
--PARWWW
--PARWWG
--PARWTN
--PARWSF
--PARWRY
--PARWNA
--PARWMA
--PARWLE
--PARWIM
--PARWEI
--PARWDA
--PARWAN
PAPARW--
--PARWWV
--PARWWQ
--PARWRQ
--PARWRF
--PARWRE

--PARWQN
--PARWNV
--PARWNS
--PARWMN
--PARWKV
--PARWKN
--PARWKM
--PARWHN
--PARWHM
--PARWFW
--PARWET
--PARWEN
--PARWDS
--PARWAT
--PARWAF
-PPARWY-
PEPARW--
--PARWYN
--PARWWY
--PARWVW
--PARWTH
--PARWTE
--PARWRW
--PARWRR
--PARWRH
--PARWQR
--PARWQQ
--PARWQK
--PARWQH
--PARWQA
--PARWMS
--PARWKY
--PARWKK
--PARWKF
--PARWIE
--PARWHY
--PARWHV
--PARWHT
--PARWHH
--PARWHF
--PARWHA
--PARWEM
--PARWEK
--PARWAK
QSPARW--
-QPARWT-
-QPARWS-
QIPARW--
-QPARWW-
-QPARWL-

```
-QPARWA-
-QPARWY-
QFPARW--
QTPARW--
-QPARWR-
-QPARWM-
-QPARWF-
QYPARW--
QWPARW--
QQPARW--
QPPARW--
-QPARWQ-
QAPARW--
QRPARW--
-QPARWH-
QGPARW--
QEPARW--
QVPARW--
-QPARWE-
-QPARWN-
-QPARWK-
-QPARWI-
QLPARW--
QKPARW--
QHPARW--
RSPARW--
-RPARWH-
-RPARWA-
RTPARW--
-RPARWR-
-RPARWT-
-RPARWQ-
RVPARW--
RAPARW--
RNPARW--
-RPARWS-
RRPARW--
-RPARWK-
-RPARWM-
RGPARW--
RMPARW--
RHPARW--
RWPARW--
-RPARWW-
-RPARWE-
RQPARW--
RDPARW--
RFPARW--
REPARW--
RCPARW--
```

-SPARWS-
SSPARW--
-SPARWV-
SLPARW--
STPARW--
SFPARW--
-SPARWQ-
-SPARWT-
SVPARW--
SKPARW--
SNPARW--
-SPARWG-
SYPARW--
-SPARWF-
SMPARW--
-SPARWW-
-SPARWK-
-SPARWA-
SQPARW--
SAPARW--
-SPARWH-
SPPARW--
SIPARW--
SHPARW--
SDPARW--
-SPARWY-
-SPARWN-
SGPARW--
SCPARW--
SWPARW--
-SPARWM-
-SPARWE-
SEPARW--
-TPARWS-
TMPARW--
TAPARW--
-TPARWR-
TTPARW--
-TPARWY-
-TPARWL-
-TPARWI-
TLPARW--
TRPARW--
TKPARW--
-TPARWM-
TIPARW--
TPPARW--
-TPARWK-
-TPARWF-
-TPARWA-

-TPARWW-
-TPARWH-
TFPARW--
TEPARW--
TWPARW--
-TPARWV-
TGPARW--
TDPARW--
TYPARW--
-TPARWQ-
-TPARWG-
TQPARW--
TNPARW--
TCPARW--
-VPARWF-
-VPARWL-
VNPARW--
VKPARW--
VTPARW--
-VPARWW-
VVPARW--
VSPARW--
VAPARW--
-VPARWY-
-VPARWS-
-VPARWV-
-VPARWQ-
-VPARWA-
VLPARW--
-VPARWI-
-VPARWE-
VGPARW--
VFPARW--
VDPARW--
-VPARWK-
VIPARW--
-VPARWG-
VYPARW--
VHPARW--
VEPARW--
VQPARW--
-VPARWT-
VMPARW--
-VPARWN-
-VPARWH-
-WPARWI-
WGPARW--
-WPARWM-
-WPARWF-
-WPARWY-

WNPARW--
WMPARW--
WDPARW--
WKPARW--
WVPARW--
-WPARWA-
WTPARW--
-WPARWR-
-WPARWQ-
-WPARWK-
-WPARWH-
WEPARW--
WWPARW--
WRPARW--
WPPARW--
WHPARW--
YPPARW--
YVPARW--
YTPARW--
-YPARWS-
YRPARW--
-YPARWI-
-YPARWR-
YKPARW--
-YPARWL-
YIPARW--
YAPARW--
-YPARWK-
YLPARW--
YFPARW--
YGPARW--
-YPARWT-
YQPARW--
YSPARW--
YHPARW--
-YPARWY-
-YPARWW-
-YPARWE-
YNPARW--
YDPARW--
YYPARW--
-YPARWG-
YWPARW--
-YPARWF-
YEPARW--

PQAI 509 602 30.60

--PQAIVL
-RPQAIS-
-APQAIM-
ARPQAI--

-APQAIS-
AWPQAI--
-APQAIY-
-APQAIL-
ALPQAI--
AAPQAI--
ATPQAI--
-APQAIG-
-APQAIE-
AHPQAI--
-APQAIR-
-APQAIH-
-APQAIF-
AFPQAI--
ASPQAI--
APPQAI--
AIPQAI--
AYPQAI--
-APQAII-
AGPQAI--
ADPQAI--
ACPQAI--
-CPQAIV-
CKPQAI--
CGPQAI--
CVPQAI--
-CPQAIT-
CMPQAI--
CWPQAI--
CQPQAI--
-CPQAIS-
CLPQAI--
CEPQAI--
DMPQAI--
-DPQAIR-
-DPQAIW-
DRPQAI--
DQPQAI--
DLPQAI--
DDPQAI--
DTPQAI--
-DPQAIT-
-DPQAIA-
DIPQAI--
DHPQAI--
EMPQAI--
-EPQAIR-
EYPQAI--
ESPQAI--
-EPQAIL-

EPPQAI--
EVPQAI--
-EPQAIW-
-EPQAIT-
-EPQAIM-
-EPQAIK-
-EPQAII-
ENPQAI--
ELPQAI--
EFPQAI--
FFPQAI--
-FPQAIA-
FNPQAI--
FKPQAI--
FIPQAI--
FRPQAI--
-FPQAII-
FEPQAI--
FYPQAI--
FVPQAI--
FSPQAI--
-FPQAIT-
-FPQAIL-
FMPQAI--
FGPQAI--
GRPQAI--
GLPQAI--
GTPQAI--
-GPQAIM-
-GPQAIQ-
-GPQAIT-
-GPQAIV-
GSPQAI--
GKPQAI--
GIPQAI--
GEPQAI--
GWPQAI--
GQPQAI--
-GPQAIS-
-GPQAIF-
-GPQAIE-
GAPQAI--
-GPQAIL-
-GPQAIH-
GMPQAI--
HVPQAI--
HWPQAI--
-HPQAIH-
HGPQAI--
-HPQAIV-

HLPQAI--
HTPQAI--
HRPQAI--
-HPQAIW-
-HPQAIN-
-HPQAII-
-HPQAIF-
-HPQAIA-
HMPQAI--
HSPQAI--
-HPQAIR-
-HPQAIM-
HPPQAI--
HKPQAI--
HIPQAI--
HHPQAI--
IDPQAI--
IRPQAI--
-IPQAIT-
-IPQAIR-
IQPQAI--
ISPQAI--
-IPQAIA-
ILPQAI--
IVPQAI--
-IPQAIY-
-IPQAII-
INPQAI--
IKPQAI--
IYPQAI--
-IPQAIV-
-IPQAIS-
-IPQAIQ-
-IPQAIL-
-IPQAIH-
IGPQAI--
-IPQAIW-
-IPQAIE-
IPPQAI--
IMPQAI--
IIPQAI--
IFPQAI--
KSPQAI--
KIPQAI--
KNPQAI--
KLPQAI--
KVPQAI--
KWPQAI--
-KPQAIL-
-KPQAIA-

KKPQAI--
KYPQAI--
-KPQAII-
-KPQAIQ-
-KPQAIM-
-KPQAIK-
KPPQAI--
KGPQAI--
-KPQAIT-
-KPQAIS-
-KPQAIR-
-KPQAIF-
KFPQAI--
KEPQAI--
KRPQAI--
KQPQAI--
-KPQAIY-
-KPQAIH-
-KPQAIG-
KMPQAI--
KHPQAI--
KAPQAI--
LSPQAI--
LRPQAI--
-LPQAIR-
LYPQAI--
LVPQAI--
LLPQAI--
LQPQAI--
-LPQAIY-
-LPQAIM-
LDPQAI--
LTPQAI--
-LPQAIV-
-LPQAIT-
LIPQAI--
LHPQAI--
LWPQAI--
-LPQAIS-
-LPQAIQ-
-LPQAIH-
-LPQAIF-
LNPQAI--
LMPQAI--
LKPQAI--
LGPQAI--
-MPQAIK-
MWPQAI--
MSPQAI--
-MPQAIL-

-MPQAIY-
-MPQAIH-
MMPQAI--
MYPQAI--
MKPQAI--
MTPQAI--
MQPQAI--
-MPQAIV-
-MPQAIR-
-MPQAII-
MPPQAI--
MCPQAI--
MAPQAI--
NVPQAI--
NRPQAI--
-NPQAIW-
-NPQAIL-
NPPQAI--
NNPQAI--
NIPQAI--
NWPQAI--
NTPQAI--
-NPQAIS-
-NPQAII-
NSPQAI--
-NPQAIQ-
-NPQAIF-
NKPQAI--
NYPQAI--
-NPQAIY-
-NPQAIR-
NGPQAI--
--PQAISL
--PQAILG
--PQAIRS
--PQAILA
--PQAIRM
--PQAIRK
--PQAINA
--PQAIVR
--PQAITI
--PQAILS
PWPQAI--
--PQAISW
--PQAISV
--PQAIQR
--PQAIIR
--PQAIHL
--PQAIYL
--PQAIRY

--PQAIRQ
--PQAILT
--PQAIHS
--PQAIAA
--PQAIWN
--PQAIVG
--PQAIRD
--PQAILR
--PQAILM
PKPQAI--
--PQAIVT
--PQAIST
--PQAIQT
--PQAINL
--PQAIIL
--PQAIIG
--PQAIFY
--PQAIFS
--PQAIAT
--PQAIYR
--PQAIYI
--PQAIVI
--PQAIVH
--PQAITL
--PQAIRL
--PQAIRI
--PQAIQL
--PQAINS
--PQAILQ
--PQAIKK
--PQAIGY
--PQAIFQ
--PQAIFD
--PQAIWL
--PQAIVQ
--PQAIVA
--PQAITK
--PQAITG
--PQAISS
--PQAISF
--PQAIRT
--PQAILY
--PQAIKW
--PQAIKR
--PQAIHF
--PQAIGI
--PQAIGG
--PQAIFV
--PQAIAR
--PQAIAL

PMPQAI--
--PQAIYM
--PQAIWR
--PQAIWA
--PQAIVN
--PQAIVM
--PQAITS
--PQAISG
--PQAIRW
--PQAIRN
--PQAIQN
--PQAIQA
--PQAIPY
--PQAIPM
--PQAINM
--PQAING
--PQAIMV
--PQAILV
--PQAILI
--PQAILH
--PQAIKV
--PQAIKT
--PQAIKN
--PQAIKF
--PQAIHA
--PQAIGS
--PQAIFR
--PQAIEM
PIPQAI--
--PQAIYS
--PQAIVY
--PQAIVK
--PQAIVD
--PQAITD
--PQAISY
--PQAISM
--PQAISI
--PQAIRR
--PQAIRH
--PQAIRG
--PQAIRE
--PQAIRA
--PQAIQQ
--PQAIQD
--PQAINY
--PQAINT
--PQAIMR
--PQAIMH
--PQAIKS
--PQAIKL

--PQAIKH
--PQAIIY
--PQAIHV
--PQAIHG
--PQAIHE
--PQAIGM
--PQAIGA
--PQAIFW
--PQAIFK
--PQAIFH
--PQAIFA
--PQAIAY
--PQAIAS
PNPQAI--
PLPQAI--
PDPQAI--
--PQAIYK
--PQAIYG
--PQAIWV
--PQAIWK
--PQAIWH
--PQAIWF
--PQAIVS
--PQAIVF
--PQAIVE
--PQAITY
--PQAITV
--PQAITQ
--PQAITN
--PQAITM
--PQAITH
--PQAITF
--PQAISQ
--PQAISK
--PQAISH
--PQAIRV
--PQAIRF
--PQAIQW
--PQAIQH
--PQAIPR
--PQAIPI
--PQAIPF
--PQAINR
--PQAINI
--PQAINH
--PQAINF
--PQAIND
--PQAIMY
--PQAIMT
--PQAIMS

--PQAIMN
--PQAIMK
--PQAIMG
--PQAIMD
--PQAILK
--PQAILE
--PQAIKY
--PQAIKQ
--PQAIKM
--PQAIKA
--PQAIIV
--PQAIIT
--PQAIIS
--PQAIII
--PQAIID
--PQAIHY
--PQAIHT
--PQAIHQ
--PQAIGW
--PQAIGR
--PQAIGQ
--PQAIFT
--PQAIFL
--PQAIEY
--PQAIEV
--PQAIEN
--PQAIAI
--PQAIAH
--PQAIAG
-PPQAIR-
PHPQAI--
PGPQAI--
-QPQAIR-
QYPQAI--
QWPQAI--
QRPQAI--
QQPQAI--
QLPQAI--
QEPQAI--
QNPQAI--
QDPQAI--
-QPQAIK-
QKPQAI--
QTPQAI--
-QPQAIY-
-QPQAIQ-
-QPQAIM-
-QPQAII-
-QPQAIH-
QPPQAI--

QIPQAI--
RAPQAI--
-RPQAII-
RYPQAI--
-RPQAIL-
-RPQAIF-
RGPQAI--
-RPQAIR-
-RPQAIW-
-RPQAIQ-
-RPQAIN-
-RPQAIA-
RVPQAI--
RFPQAI--
-RPQAIK-
-RPQAIE-
-RPQAIT-
-RPQAIM-
RRPQAI--
-RPQAIG-
RKPQAI--
RIPQAI--
-RPQAIH-
REPQAI--
RQPQAI--
-RPQAIV-
RMPQAI--
RHPQAI--
RDPQAI--
-SPQAIS-
SGPQAI--
SFPQAI--
SMPQAI--
SQPQAI--
-SPQAIR-
STPQAI--
SRPQAI--
SKPQAI--
-SPQAIY-
-SPQAIL-
-SPQAIH-
SWPQAI--
SSPQAI--
-SPQAIT-
-SPQAIM-
-SPQAII-
SYPQAI--
-SPQAIW-
-SPQAIV-
-SPQAIN-

-SPQAIF-
-SPQAIA-
SLPQAI--
SIPQAI--
SAPQAI--
SVPQAI--
-SPQAIQ-
SNPQAI--
SEPQAI--
TVPQAI--
TSPQAI--
TNPQAI--
-TPQAIS-
-TPQAIR-
-TPQAIF-
TLPQAI--
-TPQAIN-
TMPQAI--
TKPQAI--
TRPQAI--
TIPQAI--
THPQAI--
TDPQAI--
TYPQAI--
-TPQAIV-
-TPQAIT-
-TPQAIQ-
-TPQAIH-
-TPQAIG-
TAPQAI--
VVPQAI--
VTPQAI--
-VPQAIR-
VAPQAI--
VWPQAI--
-VPQAIS-
-VPQAIK-
VMPQAI--
-VPQAIF-
VGPQAI--
VDPQAI--
VSPQAI--
-VPQAIV-
VNPQAI--
VLPQAI--
VRPQAI--
-VPQAIW-
-VPQAIH-
VKPQAI--
VQPQAI--

VIPQAI--
VFPQAI--
-VPQAIY-
-VPQAIT-
-VPQAIN-
-VPQAIL-
VEPQAI--
-WPQAIS-
WTPQAI--
WNPQAI--
WYPQAI--
-WPQAIK-
WWPQAI--
-WPQAIY-
-WPQAIQ-
-WPQAIM-
-WPQAIL-
-WPQAIA-
WLPQAI--
WGPQAI--
WDPQAI--
WAPQAI--
YTPQAI--
-YPQAIL-
-YPQAIR-
-YPQAIF-
YVPQAI--
-YPQAIS-
YLPQAI--
YAPQAI--
YQPQAI--
-YPQAIT-
-YPQAII-
YFPQAI--
YWPQAI--
-YPQAIQ-
-YPQAIM-
-YPQAIK-
-YPQAIH-
YNPQAI--
YMPQAI--
YKPQAI--
YRPQAI--
-YPQAIV-
-YPQAIG-
-YPQAIA-
YIPQAI--
YGPQAI--

| | | | |
|---|---|---|---|
| PRML | 1938 | 389 | 30.60 |
| | -EPRMLG- | | |

```
-GPRMLG-
--PRMLGQ
--PRMLEL
-TPRMLG-
ATPRML--
AGPRML--
AAPRML--
ADPRML--
-APRMLT-
ASPRML--
-APRMLW-
-APRMLM-
-APRMLF-
APPRML--
AQPRML--
-APRMLE-
AHPRML--
ACPRML--
CIPRML--
CVPRML--
CLPRML--
CSPRML--
-CPRMLV-
CMPRML--
CFPRML--
CGPRML--
-CPRMLT-
CHPRML--
CTPRML--
-CPRMLQ-
-CPRMLN-
-CPRMLK-
-CPRMLC-
CPPRML--
CNPRML--
CKPRML--
CEPRML--
CAPRML--
DMPRML--
-DPRMLV-
-DPRMLR-
DIPRML--
-DPRMLK-
DLPRML--
-DPRMLL-
-DPRMLH-
DDPRML--
DNPRML--
DGPRML--
DAPRML--
```

```
-DPRMLY-
-DPRMLQ-
DHPRML--
DEPRML--
DQPRML--
-DPRMLT-
DPPRML--
DYPRML--
-DPRMLW-
-DPRMLE-
DCPRML--
-EPRMLR-
-EPRMLS-
ETPRML--
-EPRMLL-
-EPRMLV-
EKPRML--
-EPRMLW-
EFPRML--
-EPRMLK-
EAPRML--
EWPRML--
EYPRML--
-EPRMLA-
-EPRMLT-
-EPRMLH-
ENPRML--
EIPRML--
EEPRML--
-EPRMLI-
EPPRML--
-EPRMLY-
-EPRMLN-
-EPRMLF-
-EPRMLE-
EHPRML--
EDPRML--
FNPRML--
FTPRML--
FGPRML--
-FPRMLN-
-GPRMLS-
GSPRML--
GTPRML--
-GPRMLT-
GGPRML--
GQPRML--
GEPRML--
-GPRMLY-
GNPRML--
```

GDPRML--
-GPRMLQ-
GPPRML--
-GPRMLN-
-GPRMLI-
-GPRMLA-
-GPRMLH-
GCPRML--
-GPRMLE-
HVPRML--
HHPRML--
HEPRML--
HNPRML--
-HPRMLW-
-HPRMLY-
-HPRMLV-
-HPRMLK-
HPPRML--
HKPRML--
HIPRML--
HCPRML--
-HPRMLM-
-HPRMLG-
-HPRMLE-
IPPRML--
ITPRML--
IYPRML--
-IPRMLS-
IQPRML--
-IPRMLT-
IMPRML--
IHPRML--
IEPRML--
INPRML--
IGPRML--
-IPRMLG-
IDPRML--
-KPRMLS-
-KPRMLQ-
-KPRMLK-
-KPRMLY-
KPPRML--
-KPRMLI-
-KPRMLT-
KNPRML--
-KPRMLA-
KKPRML--
KHPRML--
-KPRMLN-
-KPRMLW-

−KPRMLE−
KDPRML−−
KCPRML−−
LGPRML−−
−LPRMLT−
LPPRML−−
LDPRML−−
−LPRMLY−
LHPRML−−
MVPRML−−
MGPRML−−
MNPRML−−
−MPRMLH−
MDPRML−−
−MPRMLN−
MKPRML−−
−MPRMLG−
MCPRML−−
−NPRMLL−
NSPRML−−
−NPRMLY−
NTPRML−−
−NPRMLM−
−NPRMLV−
−NPRMLT−
−NPRMLH−
NNPRML−−
NKPRML−−
−NPRMLF−
NDPRML−−
NCPRML−−
PLPRML−−
−−PRMLTH
−−PRMLSA
−−PRMLQS
−−PRMLAS
−−PRMLYG
PVPRML−−
−−PRMLSK
−−PRMLFK
−−PRMLVI
−−PRMLHV
−−PRMLFM
−−PRMLVQ
−−PRMLNS
−−PRMLMA
−−PRMLIG
−−PRMLYM
−−PRMLSQ
−−PRMLQA

--PRMLIT
--PRMLAY
--PRMLWM
--PRMLHM
--PRMLGI
--PRMLYW
--PRMLVE
--PRMLTF
--PRMLMK
--PRMLLE
--PRMLHN
--PRMLFE
--PRMLAN
--PRMLWN
--PRMLVD
--PRMLNY
--PRMLNT
--PRMLGG
PKPRML--
PGPRML--
PAPRML--
--PRMLTQ
--PRMLPY
PEPRML--
--PRMLWH
--PRMLWG
--PRMLWA
--PRMLVA
--PRMLNW
--PRMLWT
--PRMLQT
--PRMLQM
--PRMLQI
--PRMLKD
--PRMLIW
--PRMLFI
--PRMLES
--PRMLEA
--PRMLYH
--PRMLYF
--PRMLNA
--PRMLMF
--PRMLKV
--PRMLII
--PRMLHT
--PRMLHI
--PRMLEG
--PRMLAT
--PRMLAG
PHPRML--

PTPRML--
PSPRML--
--PRMLYY
--PRMLWK
--PRMLWI
--PRMLNF
--PRMLGM
--PRMLYN
--PRMLTM
--PRMLQV
--PRMLNI
--PRMLMY
--PRMLIF
--PRMLHH
--PRMLFA
--PRMLTK
--PRMLID
--PRMLHW
--PRMLHQ
--PRMLHE
PQPRML--
PMPRML--
--PRMLYE
--PRMLWQ
--PRMLSD
--PRMLNG
--PRMLMM
--PRMLMH
--PRMLHF
--PRMLGF
--PRMLFW
--PRMLEY
PDPRML--
--PRMLTD
--PRMLQQ
--PRMLQK
--PRMLNV
--PRMLNQ
--PRMLNE
--PRMLIE
--PRMLFQ
--PRMLAQ
PCPRML--
--PRMLYK
--PRMLYD
--PRMLQH
--PRMLQD
--PRMLNK
--PRMLKE
--PRMLHD

```
--PRMLHA
--PRMLGV
--PRMLGK
--PRMLET
--PRMLEI
--PRMLEF
--PRMLCY
--PRMLCS
--PRMLCL
--PRMLCI
-PPRMLN-
-PPRMLA-
-QPRMLL-
-QPRMLF-
-QPRMLV-
QGPRML--
-QPRMLN-
QMPRML--
QEPRML--
-QPRMLQ-
QYPRML--
-QPRMLY-
-QPRMLS-
QHPRML--
QDPRML--
QAPRML--
-QPRMLM-
QNPRML--
-QPRMLG-
QPPRML--
-QPRMLW-
QCPRML--
QQPRML--
-RPRMLF-
RCPRML--
SGPRML--
SEPRML--
-SPRMLH-
-SPRMLI-
SDPRML--
-SPRMLG-
SCPRML--
TGPRML--
-TPRMLT-
-TPRMLQ-
-TPRMLN-
-TPRMLY-
-TPRMLL-
TQPRML--
TDPRML--
```

-TPRMLF-
TNPRML--
TMPRML--
TPPRML--
-TPRMLW-
-VPRMLW-
VSPRML--
VNPRML--
-VPRMLM-
VGPRML--
VDPRML--
VHPRML--
VCPRML--
-VPRMLG-
-VPRMLC-
WGPRML--
WSPRML--
WLPRML--
WDPRML--
WHPRML--
WPPRML--
WKPRML--
WMPRML--
WIPRML--
WCPRML--
WTPRML--
-WPRMLA-
WYPRML--
-WPRMLN-
YNPRML--
YKPRML--
YDPRML--
-YPRMLY-
YPPRML--
YEPRML--
YQPRML--
-YPRMLW-
YCPRML--

| | | | |
|---|---|---|---|
| PGHL | 79 | 780 | 30.59 |

-APGHLH-
-FPGHLF-
-LPGHLL-
--PGHLLR
--PGHLTL
--PGHLGS
--PGHLLY
--PGHLLK
--PGHLLI
--PGHLGG
--PGHLFL

--PGHLAN
-SPGHLE-
TPPGHL--
-TPGHLN-
AVPGHL--
ALPGHL--
ATPGHL--
AMPGHL--
-APGHLW-
-APGHLL-
AIPGHL--
-APGHLR-
-APGHLQ-
-APGHLS-
AWPGHL--
-APGHLK-
ANPGHL--
AHPGHL--
-APGHLV-
AFPGHL--
AYPGHL--
APPGHL--
-APGHLF-
AKPGHL--
ADPGHL--
AAPGHL--
ASPGHL--
AQPGHL--
-APGHLT-
-APGHLI-
-APGHLY-
AGPGHL--
AEPGHL--
ARPGHL--
-APGHLM-
-APGHLG-
-APGHLA-
-APGHLN-
-APGHLC-
CMPGHL--
CAPGHL--
CQPGHL--
CHPGHL--
CRPGHL--
-CPGHLR-
-CPGHLS-
-CPGHLM-
CNPGHL--
CKPGHL--
CIPGHL--

CGPGHL--
CEPGHL--
DRPGHL--
-DPGHLR-
DLPGHL--
DVPGHL--
DNPGHL--
DKPGHL--
DAPGHL--
DIPGHL--
DYPGHL--
DSPGHL--
DQPGHL--
-DPGHLT-
-DPGHLY-
DWPGHL--
-DPGHLS-
-DPGHLK-
-DPGHLI-
DMPGHL--
-DPGHLM-
DHPGHL--
DGPGHL--
DFPGHL--
DEPGHL--
-DPGHLE-
DCPGHL--
EVPGHL--
EYPGHL--
ELPGHL--
-EPGHLR-
ERPGHL--
-EPGHLL-
-EPGHLH-
EGPGHL--
-EPGHLV-
-EPGHLS-
-EPGHLQ-
ENPGHL--
ESPGHL--
-EPGHLK-
-EPGHLI-
EHPGHL--
EFPGHL--
EWPGHL--
ETPGHL--
EQPGHL--
-EPGHLY-
-EPGHLT-
-EPGHLM-

EIPGHL--
EEPGHL--
EAPGHL--
FRPGHL--
-FPGHLR-
FHPGHL--
FGPGHL--
FSPGHL--
-FPGHLI-
FNPGHL--
-FPGHLM-
FIPGHL--
FQPGHL--
FLPGHL--
FDPGHL--
-FPGHLT-
FAPGHL--
FWPGHL--
-FPGHLK-
FEPGHL--
FPPGHL--
-FPGHLS-
FFPGHL--
-FPGHLV-
-FPGHLL-
FYPGHL--
-FPGHLY-
-FPGHLW-
-FPGHLH-
-FPGHLA-
FCPGHL--
-GPGHLR-
GRPGHL--
GTPGHL--
GWPGHL--
-GPGHLL-
GKPGHL--
GSPGHL--
GLPGHL--
-GPGHLM-
GMPGHL--
-GPGHLI-
GIPGHL--
GGPGHL--
GAPGHL--
GYPGHL--
GVPGHL--
GFPGHL--
GQPGHL--
-GPGHLS-

-GPGHLA-
-GPGHLV-
-GPGHLK-
-GPGHLF-
-GPGHLY-
GEPGHL--
-GPGHLH-
GPPGHL--
-GPGHLQ-
GNPGHL--
GDPGHL--
-GPGHLN-
-GPGHLG-
-GPGHLE-
-HPGHLR-
HRPGHL--
-HPGHLS-
HYPGHL--
-HPGHLL-
HLPGHL--
HWPGHL--
HTPGHL--
HMPGHL--
HVPGHL--
HEPGHL--
-HPGHLT-
HGPGHL--
HAPGHL--
HKPGHL--
HPPGHL--
-HPGHLI-
HNPGHL--
HQPGHL--
-HPGHLK-
-HPGHLH-
-HPGHLY-
-HPGHLM-
-HPGHLE-
HIPGHL--
-HPGHLQ-
-HPGHLP-
-HPGHLN-
-HPGHLG-
-HPGHLA-
HHPGHL--
ILPGHL--
IRPGHL--
-IPGHLR-
ISPGHL--
IYPGHL--

-IPGHLY-
IVPGHL--
IPPGHL--
-IPGHLI-
-IPGHLQ-
-IPGHLK-
IFPGHL--
INPGHL--
-IPGHLL-
-IPGHLA-
ITPGHL--
IQPGHL--
-IPGHLV-
-IPGHLF-
IIPGHL--
IMPGHL--
IGPGHL--
IWPGHL--
-IPGHLS-
IAPGHL--
-IPGHLM-
-IPGHLE-
IKPGHL--
IHPGHL--
IEPGHL--
-IPGHLW-
-IPGHLH-
-KPGHLL-
KRPGHL--
KLPGHL--
-KPGHLW-
KGPGHL--
KFPGHL--
KTPGHL--
KMPGHL--
-KPGHLI-
KAPGHL--
-KPGHLV-
-KPGHLK-
-KPGHLT-
-KPGHLR-
KNPGHL--
KVPGHL--
-KPGHLS-
KIPGHL--
KEPGHL--
KDPGHL--
-KPGHLY-
-KPGHLQ-
-KPGHLF-

−KPGHLH−
KYPGHL−−
−KPGHLN−
−KPGHLA−
KWPGHL−−
KPPGHL−−
KKPGHL−−
−KPGHLG−
KCPGHL−−
LRPGHL−−
LNPGHL−−
−LPGHLR−
LYPGHL−−
LWPGHL−−
−LPGHLK−
LSPGHL−−
LIPGHL−−
LQPGHL−−
−LPGHLI−
LAPGHL−−
−LPGHLT−
−LPGHLQ−
LFPGHL−−
LPPGHL−−
−LPGHLV−
LKPGHL−−
−LPGHLS−
LLPGHL−−
LHPGHL−−
−LPGHLW−
LMPGHL−−
−LPGHLH−
LCPGHL−−
−LPGHLE−
−LPGHLY−
LEPGHL−−
−MPGHLK−
MRPGHL−−
MVPGHL−−
−MPGHLW−
−MPGHLS−
−MPGHLL−
MGPGHL−−
MTPGHL−−
−MPGHLQ−
MAPGHL−−
−MPGHLI−
MSPGHL−−
−MPGHLH−
MNPGHL−−

```
MKPGHL--
-MPGHLV-
-MPGHLT-
MFPGHL--
MYPGHL--
-MPGHLM-
MHPGHL--
MEPGHL--
-MPGHLA-
MMPGHL--
MPPGHL--
-MPGHLN-
-MPGHLF-
-MPGHLE-
MQPGHL--
MIPGHL--
MCPGHL--
NLPGHL--
NIPGHL--
-NPGHLL-
-NPGHLS-
NRPGHL--
-NPGHLK-
NMPGHL--
NGPGHL--
NSPGHL--
NHPGHL--
-NPGHLV-
NAPGHL--
-NPGHLR-
NVPGHL--
NYPGHL--
-NPGHLI-
NWPGHL--
NTPGHL--
-NPGHLA-
-NPGHLF-
NNPGHL--
NFPGHL--
-NPGHLW-
NPPGHL--
-NPGHLY-
-NPGHLT-
-NPGHLM-
-NPGHLH-
NEPGHL--
NDPGHL--
NCPGHL--
PRPGHL--
--PGHLSS
```

--PGHLLT
--PGHLRN
--PGHLWS
--PGHLRY
--PGHLLA
--PGHLRS
--PGHLLS
--PGHLRQ
--PGHLRF
--PGHLRH
--PGHLTA
--PGHLSG
--PGHLRV
--PGHLMG
--PGHLIT
PLPGHL--
--PGHLYT
--PGHLVS
--PGHLST
--PGHLRG
--PGHLMF
--PGHLKA
--PGHLIR
--PGHLAT
--PGHLTV
--PGHLTG
--PGHLRA
--PGHLLV
--PGHLHG
--PGHLAS
--PGHLVT
--PGHLTT
--PGHLSA
--PGHLMM
PKPGHL--
--PGHLYV
--PGHLTS
--PGHLWG
--PGHLVQ
--PGHLVK
--PGHLSM
--PGHLRW
--PGHLRL
--PGHLRI
--PGHLMY
--PGHLKG
--PGHLHS
--PGHLYS
--PGHLVM
--PGHLMA

--PGHLKS
--PGHLIQ
--PGHLHA
--PGHLFN
PYPGHL--
PTPGHL--
--PGHLWV
--PGHLVG
--PGHLRD
--PGHLFT
PAPGHL--
PGPGHL--
--PGHLYM
--PGHLTN
--PGHLSV
--PGHLMI
--PGHLKV
--PGHLFS
--PGHLYI
--PGHLYA
--PGHLWA
--PGHLRT
--PGHLMS
--PGHLMR
--PGHLLH
--PGHLIM
--PGHLIG
--PGHLFG
--PGHLFA
PFPGHL--
--PGHLYQ
--PGHLYH
--PGHLVF
--PGHLMW
--PGHLMT
--PGHLET
--PGHLEA
--PGHLAL
--PGHLAG
PNPGHL--
--PGHLYY
--PGHLYN
--PGHLWT
--PGHLVE
--PGHLTR
--PGHLTF
--PGHLSY
--PGHLSF
--PGHLRE
--PGHLQT

--PGHLNS
--PGHLNA
--PGHLLF
--PGHLKT
--PGHLIF
--PGHLHT
--PGHLFW
--PGHLES
--PGHLEG
PWPGHL--
PSPGHL--
PQPGHL--
PHPGHL--
--PGHLYW
--PGHLYK
--PGHLYG
--PGHLVY
--PGHLVN
--PGHLQS
--PGHLNM
--PGHLLQ
--PGHLLM
--PGHLIS
--PGHLHV
--PGHLHK
--PGHLFH
PIPGHL--
--PGHLYF
--PGHLWW
--PGHLWQ
--PGHLWF
--PGHLWE
--PGHLVV
--PGHLVI
--PGHLVH
--PGHLTW
--PGHLTM
--PGHLSW
--PGHLQQ
--PGHLNL
--PGHLMV
--PGHLKM
--PGHLKE
--PGHLIV
--PGHLFY
--PGHLFR
--PGHLFQ
--PGHLAY
--PGHLAA
PMPGHL--

--PGHLYE
--PGHLWY
--PGHLWN
--PGHLVL
--PGHLSN
--PGHLSH
--PGHLQY
--PGHLQV
--PGHLQR
--PGHLQM
--PGHLQI
--PGHLQH
--PGHLNY
--PGHLMK
--PGHLMD
--PGHLKY
--PGHLKW
--PGHLKR
--PGHLKQ
--PGHLKH
--PGHLIW
--PGHLII
--PGHLIH
--PGHLIE
--PGHLHN
--PGHLHF
--PGHLAM
-PPGHLR-
--PGHLYD
--PGHLWR
--PGHLWM
--PGHLWL
--PGHLWK
--PGHLWI
--PGHLWD
--PGHLTQ
--PGHLTK
--PGHLSI
--PGHLSD
--PGHLQW
--PGHLNF
--PGHLMN
--PGHLMH
--PGHLME
--PGHLLW
--PGHLKI
--PGHLKD
--PGHLHW
--PGHLHM
--PGHLGT

```
--PGHLGA
--PGHLEV
--PGHLEN
--PGHLAW
--PGHLAV
--PGHLAK
PEPGHL--
QVPGHL--
-QPGHLR-
QWPGHL--
-QPGHLL-
-QPGHLH-
QTPGHL--
QSPGHL--
-QPGHLS-
-QPGHLW-
-QPGHLM-
QRPGHL--
-QPGHLV-
-QPGHLT-
QMPGHL--
QAPGHL--
-QPGHLI-
QLPGHL--
QKPGHL--
QHPGHL--
QGPGHL--
QPPGHL--
-QPGHLY-
-QPGHLE-
QNPGHL--
QIPGHL--
QEPGHL--
QCPGHL--
-QPGHLQ-
-QPGHLK-
-QPGHLF-
QFPGHL--
RSPGHL--
RLPGHL--
-RPGHLL-
-RPGHLR-
RVPGHL--
-RPGHLY-
RRPGHL--
-RPGHLS-
RTPGHL--
RYPGHL--
RIPGHL--
-RPGHLK-
```

RNPGHL--
RFPGHL--
-RPGHLA-
-RPGHLT-
-RPGHLQ-
-RPGHLM-
RQPGHL--
-RPGHLW-
-RPGHLI-
RHPGHL--
-RPGHLH-
RMPGHL--
RAPGHL--
-RPGHLE-
RWPGHL--
-RPGHLV-
-RPGHLF-
RKPGHL--
REPGHL--
RPPGHL--
-RPGHLG-
RDPGHL--
RCPGHL--
-RPGHLC-
-SPGHLR-
SLPGHL--
-SPGHLS-
SSPGHL--
SIPGHL--
SVPGHL--
-SPGHLL-
-SPGHLW-
-SPGHLV-
-SPGHLA-
SPPGHL--
SHPGHL--
SWPGHL--
SQPGHL--
-SPGHLY-
-SPGHLH-
SKPGHL--
-SPGHLF-
SNPGHL--
SMPGHL--
SYPGHL--
-SPGHLQ-
SEPGHL--
-SPGHLK-
-SPGHLI-
-SPGHLN-

```
-SPGHLM-
SDPGHL--
-SPGHLG-
SCPGHL--
-TPGHLR-
TLPGHL--
TSPGHL--
TIPGHL--
-TPGHLS-
TRPGHL--
TMPGHL--
TKPGHL--
-TPGHLV-
TNPGHL--
-TPGHLT-
TWPGHL--
-TPGHLL-
-TPGHLK-
-TPGHLH-
TGPGHL--
-TPGHLI-
THPGHL--
TEPGHL--
-TPGHLQ-
TFPGHL--
-TPGHLM-
-TPGHLA-
TAPGHL--
TQPGHL--
-TPGHLY-
-TPGHLW-
-TPGHLE-
TCPGHL--
-TPGHLF-
VRPGHL--
VSPGHL--
VLPGHL--
-VPGHLR-
VWPGHL--
VVPGHL--
-VPGHLL-
VTPGHL--
-VPGHLS-
VMPGHL--
VGPGHL--
VAPGHL--
VYPGHL--
-VPGHLV-
-VPGHLW-
VNPGHL--
```

-VPGHLY-
VQPGHL--
-VPGHLT-
VFPGHL--
-VPGHLH-
-VPGHLF-
VEPGHL--
VDPGHL--
-VPGHLM-
-VPGHLK-
-VPGHLE-
-VPGHLA-
-VPGHLI-
VPPGHL--
-VPGHLC-
-WPGHLR-
WGPGHL--
WWPGHL--
WRPGHL--
-WPGHLV-
-WPGHLT-
-WPGHLS-
WYPGHL--
-WPGHLY-
WFPGHL--
WAPGHL--
WTPGHL--
-WPGHLI-
-WPGHLK-
WLPGHL--
WKPGHL--
WHPGHL--
WMPGHL--
WDPGHL--
-WPGHLH-
WPPGHL--
-WPGHLW-
-WPGHLA-
WNPGHL--
WEPGHL--
-WPGHLQ-
YLPGHL--
YAPGHL--
YSPGHL--
-YPGHLT-
YGPGHL--
YRPGHL--
YPPGHL--
YMPGHL--
YFPGHL--

YTPGHL--
-YPGHLA-
YEPGHL--
YVPGHL--
YQPGHL--
YHPGHL--
-YPGHLV-
YKPGHL--
-YPGHLI-
-YPGHLL-
-YPGHLK-
YDPGHL--
YYPGHL--
-YPGHLQ-
-YPGHLM-
-YPGHLH-
-YPGHLE-
YNPGHL--
-YPGHLN-

LGWI 403 631 30.58

--LGWING
--LGWILD
-SLGWID-
AVLGWI--
AGLGWI--
ADLGWI--
ANLGWI--
-ALGWIS-
-ALGWIN-
-ALGWIL-
AQLGWI--
-ALGWIQ-
ALLGWI--
AHLGWI--
-ALGWIK-
AKLGWI--
AELGWI--
AALGWI--
AMLGWI--
-ALGWII-
-ALGWIH-
AILGWI--
-ALGWIR-
-ALGWIA-
ACLGWI--
AYLGWI--
-ALGWIT-
ATLGWI--
-ALGWIV-
AWLGWI--

-ALGWIM-
-ALGWIG-
-ALGWIW-
-ALGWIE-
CTLGWI--
-CLGWIR-
CSLGWI--
-CLGWIM-
CQLGWI--
CNLGWI--
-CLGWIY-
-CLGWIT-
-CLGWIN-
-CLGWIK-
-CLGWIG-
-CLGWIE-
CILGWI--
CDLGWI--
CCLGWI--
DRLGWI--
DALGWI--
DSLGWI--
DNLGWI--
DQLGWI--
DHLGWI--
-DLGWIV-
-DLGWIS-
-DLGWIR-
-DLGWIA-
DDLGWI--
DTLGWI--
DILGWI--
-DLGWIY-
-DLGWIT-
-DLGWIQ-
DGLGWI--
DVLGWI--
-DLGWII-
DKLGWI--
DYLGWI--
DWLGWI--
DLLGWI--
-DLGWIW-
-DLGWIK-
DFLGWI--
-DLGWIM-
-ELGWIG-
-ELGWIR-
EALGWI--
ERLGWI--

```
-ELGWIM-
ESLGWI--
EHLGWI--
EQLGWI--
EVLGWI--
ELLGWI--
ETLGWI--
-ELGWIW-
-ELGWIV-
-ELGWIK-
EKLGWI--
-ELGWIN-
EFLGWI--
EYLGWI--
-ELGWIT-
-ELGWIQ-
EELGWI--
ENLGWI--
EMLGWI--
-ELGWIS-
-ELGWII-
-ELGWIH-
-ELGWIF-
-ELGWIA-
EDLGWI--
-FLGWIR-
FNLGWI--
FHLGWI--
-FLGWID-
FGLGWI--
FTLGWI--
-FLGWIN-
FYLGWI--
-FLGWIG-
FILGWI--
FQLGWI--
-FLGWIK-
FELGWI--
FMLGWI--
-FLGWIV-
-FLGWIT-
-FLGWIE-
FDLGWI--
-GLGWIR-
GTLGWI--
GSLGWI--
-GLGWIT-
-GLGWIA-
GELGWI--
GQLGWI--
```

GVLGWI--
-GLGWIS-
-GLGWIV-
GNLGWI--
GLLGWI--
GALGWI--
GYLGWI--
-GLGWIW-
GFLGWI--
GDLGWI--
GMLGWI--
GHLGWI--
-GLGWIY-
-GLGWIQ-
-GLGWIM-
-GLGWIN-
-GLGWII-
-GLGWIE-
-GLGWID-
HSLGWI--
HTLGWI--
HMLGWI--
HHLGWI--
HVLGWI--
HNLGWI--
-HLGWIY-
-HLGWIL-
HRLGWI--
-HLGWIQ-
HELGWI--
HLLGWI--
-HLGWIN-
HYLGWI--
-HLGWIT-
HGLGWI--
HFLGWI--
-HLGWIV-
-HLGWIG-
-HLGWIW-
-HLGWIM-
HKLGWI--
HCLGWI--
HQLGWI--
-HLGWIK-
-HLGWIE-
-HLGWIA-
HWLGWI--
-HLGWII-
-HLGWIH-
HDLGWI--

ISLGWI--
ITLGWI--
-ILGWIR-
-ILGWIQ-
-ILGWIK-
IQLGWI--
-ILGWIT-
-ILGWIN-
-ILGWIH-
IKLGWI--
IGLGWI--
-ILGWIV-
IWLGWI--
-ILGWIG-
-ILGWIE-
IELGWI--
IALGWI--
-KLGWIL-
-KLGWIT-
KSLGWI--
KILGWI--
KALGWI--
KHLGWI--
KNLGWI--
-KLGWIS-
KVLGWI--
-KLGWIV-
KDLGWI--
KMLGWI--
-KLGWIG-
KGLGWI--
KLLGWI--
KELGWI--
KTLGWI--
-KLGWIQ-
-KLGWIN-
-KLGWIH-
-KLGWII-
KYLGWI--
-KLGWIW-
-KLGWIK-
KFLGWI--
KCLGWI--
KQLGWI--
-KLGWIY-
-KLGWIA-
LSLGWI--
--LGWIRG
LVLGWI--
-LLGWIT-

```
--LGWISG
--LGWIRE
LQLGWI--
--LGWISV
--LGWIVA
--LGWIKG
-LLGWIN-
--LGWIYR
--LGWITN
LGLGWI--
--LGWIVS
--LGWITG
--LGWISN
--LGWIRD
--LGWIKS
--LGWISW
--LGWIKK
--LGWIKD
LELGWI--
LTLGWI--
--LGWIRT
--LGWIQK
--LGWIHS
--LGWIEG
-LLGWIR-
--LGWIVQ
--LGWIVK
--LGWITI
--LGWIKL
--LGWIIR
-LLGWIG-
--LGWIST
--LGWIQS
--LGWINT
--LGWIKR
--LGWIFS
--LGWIAT
LHLGWI--
--LGWITD
--LGWITA
--LGWISA
--LGWIMD
LDLGWI--
-LLGWIA-
--LGWIVH
--LGWITR
--LGWITK
--LGWITH
--LGWIQT
--LGWIQN
```

--LGWIIN
--LGWIIK
--LGWIIG
--LGWIFN
--LGWIAK
LALGWI--
-LLGWIM-
--LGWIYY
--LGWIYE
--LGWIWS
--LGWIKY
--LGWIGA
--LGWIFQ
--LGWIES
--LGWIYG
--LGWIYA
--LGWIWG
--LGWIRQ
--LGWINA
--LGWIKQ
--LGWIKE
--LGWIHM
--LGWIHA
--LGWIGI
--LGWIEA
--LGWIAV
--LGWIAH
-LLGWIQ-
-LLGWIP-
-LLGWIK-
--LGWISY
--LGWISQ
--LGWISM
--LGWISE
--LGWIRW
--LGWIQV
--LGWINS
--LGWIMK
--LGWIKF
--LGWIID
--LGWIGV
--LGWIFH
--LGWIEY
--LGWIAS
--LGWIAQ
--LGWIAN
-LLGWIV-
-LLGWIH-
--LGWIVM
--LGWITS

--LGWIRM
--LGWIQM
--LGWIQH
--LGWILN
--LGWIKM
--LGWIIE
--LGWIHQ
--LGWIHI
--LGWIFT
--LGWIFK
--LGWIEF
--LGWIAD
LILGWI--
--LGWIYT
--LGWITE
--LGWISD
--LGWIQG
--LGWINY
--LGWIMQ
--LGWIMH
--LGWIME
--LGWIKW
--LGWIIH
--LGWIHY
--LGWIGM
--LGWIGG
--LGWIGF
--LGWIFG
--LGWIFE
--LGWIEW
--LGWIDS
--LGWIAY
--LGWIAM
--LGWIAG
-LLGWIY-
-LLGWIW-
--LGWIYN
--LGWIYF
--LGWIYD
--LGWIWV
--LGWIWN
--LGWIWH
--LGWIVV
--LGWIVD
--LGWITV
--LGWISI
--LGWIQA
--LGWINQ
--LGWIMT
--LGWIMA

--LGWILW
--LGWILE
--LGWIKI
--LGWIHR
--LGWIHK
--LGWIHF
--LGWIHD
--LGWIGY
--LGWIGQ
--LGWIGN
--LGWIFR
--LGWIED
--LGWIAL
-MLGWIY-
MMLGWI--
-MLGWIS-
-MLGWII-
MTLGWI--
-MLGWIN-
-MLGWIL-
MQLGWI--
MKLGWI--
MELGWI--
MNLGWI--
MHLGWI--
-MLGWIR-
-MLGWIK-
MFLGWI--
MVLGWI--
-MLGWID-
MILGWI--
MWLGWI--
-MLGWIW-
-MLGWIV-
-MLGWIE-
-MLGWIQ-
-MLGWIH-
NVLGWI--
-NLGWIG-
NGLGWI--
NALGWI--
NSLGWI--
-NLGWIS-
-NLGWIT-
-NLGWII-
-NLGWIL-
NTLGWI--
NNLGWI--
NLLGWI--
-NLGWIR-

```
-NLGWIM-
-NLGWIH-
NKLGWI--
NDLGWI--
-NLGWIQ-
-NLGWIA-
-NLGWIV-
-NLGWIK-
NILGWI--
NELGWI--
NQLGWI--
NMLGWI--
NFLGWI--
NYLGWI--
-NLGWIN-
NWLGWI--
-NLGWIE-
PNLGWI--
PALGWI--
PTLGWI--
PSLGWI--
PGLGWI--
PHLGWI--
PKLGWI--
PELGWI--
PDLGWI--
PMLGWI--
PFLGWI--
PWLGWI--
PQLGWI--
PLLGWI--
PCLGWI--
-QLGWIS-
QKLGWI--
QELGWI--
QSLGWI--
-QLGWIT-
QGLGWI--
QQLGWI--
QALGWI--
-QLGWIR-
QYLGWI--
-QLGWII-
-QLGWIN-
-QLGWIA-
QHLGWI--
QNLGWI--
-QLGWIW-
-QLGWIK-
-QLGWIH-
```

QDLGWI--
-QLGWIV-
-QLGWIQ-
-QLGWIE-
QWLGWI--
-QLGWIM-
QFLGWI--
QCLGWI--
RELGWI--
RTLGWI--
-RLGWII-
RQLGWI--
RLLGWI--
-RLGWIT-
RDLGWI--
RWLGWI--
-RLGWIH-
RHLGWI--
-RLGWIQ-
RFLGWI--
-RLGWIK-
-RLGWIV-
-RLGWIN-
RNLGWI--
-RLGWIM-
RGLGWI--
-RLGWIW-
RCLGWI--
-RLGWIE-
SRLGWI--
-SLGWIS-
-SLGWIL-
SELGWI--
SKLGWI--
SYLGWI--
SQLGWI--
SLLGWI--
-SLGWIQ-
-SLGWIG-
-SLGWIA-
STLGWI--
-SLGWIY-
SGLGWI--
SMLGWI--
-SLGWIW-
-SLGWIK-
SCLGWI--
SFLGWI--
-SLGWIN-
-SLGWII-

SALGWI--
SVLGWI--
-SLGWIV-
SNLGWI--
SDLGWI--
-SLGWIE-
-SLGWIF-
-TLGWIS-
-TLGWIH-
TGLGWI--
TELGWI--
TNLGWI--
TTLGWI--
-TLGWIM-
TVLGWI--
TQLGWI--
-TLGWIV-
-TLGWIQ-
-TLGWII-
-TLGWIK-
TALGWI--
TDLGWI--
-TLGWIG-
-TLGWIF-
TMLGWI--
-TLGWIY-
TFLGWI--
-TLGWIW-
THLGWI--
TILGWI--
-TLGWIE-
-TLGWID-
TKLGWI--
VQLGWI--
VSLGWI--
-VLGWIR-
VKLGWI--
VMLGWI--
VHLGWI--
VDLGWI--
-VLGWIW-
VELGWI--
-VLGWIT-
-VLGWIG-
VTLGWI--
-VLGWIM-
-VLGWIE-
VNLGWI--
-VLGWIY-
-VLGWII-

-VLGWIA-
VCLGWI--
-VLGWIH-
-VLGWIF-
WNLGWI--
WSLGWI--
WELGWI--
-WLGWIL-
-WLGWIE-
WKLGWI--
WTLGWI--
-WLGWIR-
-WLGWIQ-
WHLGWI--
WALGWI--
-WLGWIK-
-WLGWIY-
WGLGWI--
WRLGWI--
-WLGWIN-
-WLGWII-
-WLGWIF-
YRLGWI--
YGLGWI--
-YLGWIS-
YSLGWI--
YALGWI--
YNLGWI--
-YLGWIA-
YQLGWI--
YVLGWI--
YHLGWI--
YELGWI--
-YLGWIQ-
YILGWI--
-YLGWIW-
-YLGWIT-
-YLGWIK-
-YLGWID-
YWLGWI--
-YLGWIM-
-YLGWII-
YKLGWI--
YCLGWI--
YYLGWI--
-YLGWIV-
-YLGWIN-
-YLGWIH-
-YLGWIF-
-YLGWIE-

| | | | |
|---|---|---|---|
| | YFLGWI-- | | |
| VQWL | 1078 | 494 | 30.58 |
| | --VQWLAR | | |
| | YPVQWL-- | | |
| | ARVQWL-- | | |
| | ATVQWL-- | | |
| | -AVQWLR- | | |
| | -AVQWLY- | | |
| | -AVQWLK- | | |
| | ANVQWL-- | | |
| | AAVQWL-- | | |
| | AVVQWL-- | | |
| | ASVQWL-- | | |
| | AIVQWL-- | | |
| | AGVQWL-- | | |
| | -AVQWLT- | | |
| | -AVQWLN- | | |
| | AKVQWL-- | | |
| | -AVQWLH- | | |
| | AYVQWL-- | | |
| | -AVQWLG- | | |
| | APVQWL-- | | |
| | AMVQWL-- | | |
| | -AVQWLQ- | | |
| | -AVQWLE- | | |
| | AQVQWL-- | | |
| | -AVQWLA- | | |
| | CRVQWL-- | | |
| | -CVQWLT- | | |
| | CNVQWL-- | | |
| | CCVQWL-- | | |
| | -CVQWLR- | | |
| | -CVQWLL- | | |
| | -CVQWLG- | | |
| | CSVQWL-- | | |
| | CIVQWL-- | | |
| | DVVQWL-- | | |
| | DRVQWL-- | | |
| | DSVQWL-- | | |
| | DNVQWL-- | | |
| | DDVQWL-- | | |
| | DKVQWL-- | | |
| | -DVQWLH- | | |
| | DMVQWL-- | | |
| | DLVQWL-- | | |
| | -DVQWLR- | | |
| | -DVQWLN- | | |
| | DIVQWL-- | | |
| | -DVQWLY- | | |
| | -DVQWLW- | | |

-DVQWLS-
-DVQWLQ-
-DVQWLK-
DQVQWL--
DYVQWL--
DWVQWL--
DHVQWL--
DGVQWL--
DEVQWL--
ERVQWL--
-EVQWLK-
EVVQWL--
ESVQWL--
-EVQWLT-
-EVQWLG-
EHVQWL--
-EVQWLR-
EMVQWL--
-EVQWLS-
ENVQWL--
EGVQWL--
-EVQWLV-
-EVQWLA-
EPVQWL--
EEVQWL--
EAVQWL--
-EVQWLW-
-EVQWLN-
ETVQWL--
-FVQWLN-
FTVQWL--
-FVQWLL-
-FVQWLG-
FGVQWL--
-FVQWLH-
FVVQWL--
FPVQWL--
-FVQWLY-
-FVQWLS-
-FVQWLV-
FEVQWL--
FDVQWL--
GSVQWL--
GRVQWL--
GKVQWL--
-GVQWLS-
-GVQWLR-
GIVQWL--
GAVQWL--
GGVQWL--

```
-GVQWLA-
GTVQWL--
GLVQWL--
GDVQWL--
-GVQWLV-
GNVQWL--
-GVQWLQ-
-GVQWLN-
GHVQWL--
GQVQWL--
GEVQWL--
GPVQWL--
GWVQWL--
GMVQWL--
GYVQWL--
-GVQWLM-
HRVQWL--
HKVQWL--
-HVQWLS-
HLVQWL--
HGVQWL--
-HVQWLR-
HPVQWL--
-HVQWLL-
HMVQWL--
HAVQWL--
HYVQWL--
HVVQWL--
-HVQWLV-
-HVQWLT-
-HVQWLN-
-HVQWLG-
HHVQWL--
HWVQWL--
-HVQWLK-
-HVQWLA-
HQVQWL--
HNVQWL--
HIVQWL--
-HVQWLF-
-HVQWLD-
-HVQWLQ-
-HVQWLH-
-HVQWLE-
HEVQWL--
HDVQWL--
IKVQWL--
ISVQWL--
IQVQWL--
INVQWL--
```

```
-IVQWLF-
IHVQWL--
IDVQWL--
ITVQWL--
IGVQWL--
IAVQWL--
-IVQWLR-
-IVQWLG-
IRVQWL--
-IVQWLT-
-IVQWLN-
-IVQWLI-
IMVQWL--
-KVQWLS-
KAVQWL--
KTVQWL--
KLVQWL--
KIVQWL--
-KVQWLV-
-KVQWLT-
-KVQWLA-
KPVQWL--
KDVQWL--
KWVQWL--
KVVQWL--
KSVQWL--
KFVQWL--
KHVQWL--
KYVQWL--
-KVQWLN-
-KVQWLL-
-KVQWLG-
-KVQWLF-
-KVQWLY-
-KVQWLH-
KQVQWL--
KEVQWL--
-LVQWLK-
LAVQWL--
LEVQWL--
LRVQWL--
LGVQWL--
LTVQWL--
-LVQWLG-
LHVQWL--
LDVQWL--
-LVQWLQ-
LQVQWL--
-LVQWLE-
LNVQWL--
```

```
-LVQWLN-
-LVQWLH-
-LVQWLD-
-MVQWLT-
MSVQWL--
MQVQWL--
-MVQWLK-
MPVQWL--
MEVQWL--
MNVQWL--
-MVQWLH-
-MVQWLD-
-MVQWLV-
-MVQWLG-
MTVQWL--
MHVQWL--
MDVQWL--
-NVQWLR-
NMVQWL--
NVVQWL--
-NVQWLA-
-NVQWLS-
NNVQWL--
NEVQWL--
NIVQWL--
-NVQWLN-
-NVQWLT-
NLVQWL--
NFVQWL--
-NVQWLL-
-NVQWLK-
-NVQWLG-
NSVQWL--
-NVQWLV-
-NVQWLQ-
NDVQWL--
NYVQWL--
NWVQWL--
-NVQWLE-
NQVQWL--
NKVQWL--
NAVQWL--
PRVQWL--
PGVQWL--
PSVQWL--
-PVQWLS-
PVVQWL--
PMVQWL--
PLVQWL--
PAVQWL--
```

```
-PVQWLH-
PQVQWL--
PDVQWL--
-PVQWLR-
-PVQWLV-
-PVQWLK-
-PVQWLT-
PTVQWL--
PYVQWL--
-PVQWLQ-
-PVQWLA-
PIVQWL--
PEVQWL--
-PVQWLE-
PHVQWL--
-QVQWLI-
QTVQWL--
QPVQWL--
QLVQWL--
QGVQWL--
-QVQWLK-
QSVQWL--
QRVQWL--
-QVQWLV-
-QVQWLG-
QKVQWL--
QWVQWL--
-QVQWLA-
QMVQWL--
QVVQWL--
-QVQWLS-
-QVQWLH-
QNVQWL--
QIVQWL--
QHVQWL--
QAVQWL--
-QVQWLN-
-QVQWLM-
QDVQWL--
-QVQWLE-
-QVQWLD-
-RVQWLA-
RLVQWL--
-RVQWLQ-
-RVQWLT-
-RVQWLH-
-RVQWLG-
-RVQWLM-
RFVQWL--
RWVQWL--
```

RHVQWL--
RDVQWL--
REVQWL--
-RVQWLD-
RCVQWL--
SGVQWL--
SSVQWL--
SRVQWL--
-SVQWLQ-
STVQWL--
SEVQWL--
SVVQWL--
SHVQWL--
-SVQWLT-
SPVQWL--
-SVQWLA-
SNVQWL--
SLVQWL--
SDVQWL--
-SVQWLR-
SQVQWL--
SWVQWL--
-SVQWLK-
SAVQWL--
-SVQWLV-
-SVQWLS-
-SVQWLH-
-SVQWLG-
SKVQWL--
-SVQWLE-
SCVQWL--
TRVQWL--
TMVQWL--
TAVQWL--
TPVQWL--
TSVQWL--
-TVQWLK-
-TVQWLN-
-TVQWLH-
-TVQWLT-
-TVQWLS-
-TVQWLQ-
TVVQWL--
-TVQWLW-
TQVQWL--
TEVQWL--
TKVQWL--
TDVQWL--
-TVQWLG-
-TVQWLE-

-TVQWLA-
--VQWLGL
--VQWLVG
--VQWLST
VQVQWL--
VRVQWL--
--VQWLTT
--VQWLRH
--VQWLSN
--VQWLTN
VSVQWL--
--VQWLTG
--VQWLSS
--VQWLAT
--VQWLTA
VGVQWL--
--VQWLRQ
--VQWLKA
--VQWLAS
VAVQWL--
--VQWLRF
--VQWLLH
--VQWLGA
VNVQWL--
--VQWLTR
--VQWLTK
--VQWLRA
--VQWLGS
--VQWLSG
--VQWLQT
--VQWLQA
--VQWLHA
VDVQWL--
-VVQWLH-
VTVQWL--
--VQWLTH
--VQWLSK
--VQWLPL
VPVQWL--
-VVQWLG-
--VQWLRW
--VQWLRN
--VQWLQG
--VQWLNA
--VQWLKT
--VQWLKH
VHVQWL--
--VQWLTW
--VQWLTM
--VQWLSI

--VQWLRG
--VQWLKW
--VQWLKV
--VQWLHV
--VQWLGY
--VQWLAG
--VQWLAA
VMVQWL--
--VQWLSR
--VQWLGV
-VVQWLY-
--VQWLWA
--VQWLVH
--VQWLVD
--VQWLTS
--VQWLTD
--VQWLSW
--VQWLSD
--VQWLRD
--VQWLQY
--VQWLQV
--VQWLQK
--VQWLNG
--VQWLMG
--VQWLHS
--VQWLHG
--VQWLGF
--VQWLDS
VKVQWL--
VEVQWL--
-VVQWLR-
--VQWLWG
--VQWLWD
--VQWLSH
--VQWLRE
--VQWLMN
--VQWLKQ
--VQWLKE
--VQWLIH
--VQWLHT
--VQWLHN
--VQWLGW
--VQWLGI
--VQWLGG
--VQWLES
--VQWLAN
-VVQWLQ-
-VVQWLK-
--VQWLYK
--VQWLYF

| | | | |
|---|---|---|---|
| | --VQWLWY | | |
| | --VQWLWH | | |
| | --VQWLVW | | |
| | --VQWLVF | | |
| | --VQWLSQ | | |
| | --VQWLSE | | |
| | --VQWLQN | | |
| | --VQWLQD | | |
| | --VQWLNN | | |
| | --VQWLNH | | |
| | --VQWLMY | | |
| | --VQWLMW | | |
| | --VQWLMH | | |
| | --VQWLKD | | |
| | --VQWLIE | | |
| | --VQWLHQ | | |
| | --VQWLGM | | |
| | --VQWLAK | | |
| | --VQWLAD | | |
| | WTVQWL-- | | |
| | -WVQWLR- | | |
| | WLVQWL-- | | |
| | WSVQWL-- | | |
| | WEVQWL-- | | |
| | WGVQWL-- | | |
| | -WVQWLY- | | |
| | WDVQWL-- | | |
| | -WVQWLS- | | |
| | -WVQWLG- | | |
| | -WVQWLD- | | |
| | WPVQWL-- | | |
| | WCVQWL-- | | |
| | -WVQWLF- | | |
| | YTVQWL-- | | |
| | YGVQWL-- | | |
| | -YVQWLR- | | |
| | YNVQWL-- | | |
| | -YVQWLT- | | |
| | -YVQWLN- | | |
| | YEVQWL-- | | |
| | YSVQWL-- | | |
| | YDVQWL-- | | |
| | YWVQWL-- | | |
| | YRVQWL-- | | |
| | -YVQWLG- | | |
| | YQVQWL-- | | |
| PMAW | 896 | 521 | 30.57 |
| | -APMAWL- | | |
| | -APMAWR- | | |
| | -APMAWT- | | |

AQPMAW--
-APMAWG-
ASPMAW--
-APMAWS-
ALPMAW--
APPMAW--
-APMAWV-
AGPMAW--
-APMAWY-
-APMAWQ-
-APMAWI-
ANPMAW--
AIPMAW--
AWPMAW--
ARPMAW--
-APMAWN-
CSPMAW--
CTPMAW--
-CPMAWT-
-CPMAWS-
-CPMAWL-
-CPMAWH-
CFPMAW--
CEPMAW--
CAPMAW--
-DPMAWL-
-DPMAWS-
DYPMAW--
-DPMAWI-
-DPMAWA-
DLPMAW--
DHPMAW--
DGPMAW--
DSPMAW--
-DPMAWV-
-DPMAWQ-
DMPMAW--
DTPMAW--
-DPMAWY-
DNPMAW--
-EPMAWI-
-EPMAWV-
-EPMAWL-
-EPMAWA-
EGPMAW--
EVPMAW--
ERPMAW--
-EPMAWY-
-EPMAWR-
-EPMAWT-

-EPMAWQ-
-EPMAWN-
-EPMAWM-
EMPMAW--
ELPMAW--
EIPMAW--
-FPMAWL-
-FPMAWH-
FVPMAW--
FTPMAW--
FRPMAW--
FGPMAW--
FSPMAW--
-FPMAWV-
-FPMAWT-
FNPMAW--
FMPMAW--
FQPMAW--
-FPMAWR-
-FPMAWI-
-FPMAWG-
-FPMAWA-
FLPMAW--
FAPMAW--
-GPMAWL-
-GPMAWS-
GSPMAW--
-GPMAWA-
GHPMAW--
GVPMAW--
GNPMAW--
GKPMAW--
GRPMAW--
-GPMAWV-
-GPMAWN-
GWPMAW--
GAPMAW--
GYPMAW--
GQPMAW--
-GPMAWW-
-GPMAWT-
-GPMAWR-
-GPMAWK-
GDPMAW--
GTPMAW--
-GPMAWQ-
GLPMAW--
GIPMAW--
-HPMAWL-
HQPMAW--

-HPMAWS-
-HPMAWR-
-HPMAWN-
-HPMAWV-
-HPMAWM-
-HPMAWH-
-HPMAWA-
HDPMAW--
HSPMAW--
-HPMAWW-
HMPMAW--
HKPMAW--
HHPMAW--
HVPMAW--
HTPMAW--
-HPMAWT-
HGPMAW--
IRPMAW--
-IPMAWW-
ISPMAW--
INPMAW--
-IPMAWK-
IAPMAW--
ITPMAW--
-IPMAWR-
IKPMAW--
IVPMAW--
-IPMAWQ-
-IPMAWH-
IFPMAW--
-IPMAWV-
-IPMAWL-
IIPMAW--
IGPMAW--
KSPMAW--
KGPMAW--
KQPMAW--
KVPMAW--
-KPMAWK-
-KPMAWF-
KPPMAW--
-KPMAWT-
KLPMAW--
KHPMAW--
KAPMAW--
-KPMAWY-
KTPMAW--
-KPMAWS-
-KPMAWN-
-KPMAWL-

-KPMAWH-
-KPMAWA-
KFPMAW--
LEPMAW--
LPPMAW--
-LPMAWN-
-LPMAWR-
LTPMAW--
-LPMAWS-
-LPMAWQ-
LNPMAW--
LGPMAW--
-LPMAWT-
-LPMAWL-
-LPMAWK-
-LPMAWI-
LQPMAW--
LKPMAW--
-LPMAWW-
-LPMAWE-
LIPMAW--
MTPMAW--
-MPMAWL-
-MPMAWV-
MVPMAW--
-MPMAWW-
-MPMAWN-
MWPMAW--
MPPMAW--
-MPMAWF-
MMPMAW--
-MPMAWI-
MHPMAW--
MFPMAW--
-NPMAWF-
NTPMAW--
NRPMAW--
-NPMAWQ-
-NPMAWA-
-NPMAWN-
NGPMAW--
NPPMAW--
-NPMAWV-
NMPMAW--
NVPMAW--
NSPMAW--
-NPMAWY-
-NPMAWR-
-NPMAWK-
-NPMAWI-

NNPMAW--
NYPMAW--
NIPMAW--
-NPMAWH-
NKPMAW--
NFPMAW--
NEPMAW--
--PMAWSV
--PMAWLT
--PMAWYS
--PMAWRG
--PMAWLS
--PMAWAL
--PMAWNL
--PMAWMR
--PMAWTQ
--PMAWLH
--PMAWAA
--PMAWYG
--PMAWVQ
--PMAWVN
--PMAWVH
--PMAWSG
--PMAWRY
--PMAWRA
--PMAWMS
--PMAWHS
--PMAWWN
--PMAWTT
--PMAWTS
--PMAWSR
--PMAWSL
--PMAWSK
--PMAWRT
--PMAWLN
--PMAWKV
--PMAWKR
--PMAWAR
--PMAWTR
--PMAWTA
--PMAWRR
--PMAWRH
--PMAWLQ
--PMAWKG
--PMAWHA
--PMAWGI
--PMAWFK
--PMAWAI
--PMAWYN
--PMAWVS

--PMAWRS
--PMAWQG
--PMAWPY
--PMAWNS
--PMAWMI
--PMAWIT
--PMAWAV
--PMAWAF
PEPMAW--
--PMAWVG
--PMAWTV
--PMAWTN
--PMAWRW
--PMAWRF
--PMAWNY
--PMAWNV
--PMAWNM
--PMAWMY
--PMAWMK
--PMAWLV
--PMAWKW
--PMAWKS
--PMAWIN
--PMAWIA
--PMAWFS
--PMAWFN
--PMAWFL
--PMAWFE
--PMAWAQ
--PMAWAK
--PMAWAG
PYPMAW--
--PMAWYK
--PMAWYA
--PMAWWS
--PMAWWR
--PMAWWL
--PMAWWK
--PMAWWG
--PMAWVA
--PMAWTM
--PMAWTI
--PMAWTG
--PMAWTF
--PMAWSH
--PMAWRQ
--PMAWRK
--PMAWRE
--PMAWQR
--PMAWPL

--PMAWNW
--PMAWNT
--PMAWNR
--PMAWMT
--PMAWMF
--PMAWME
--PMAWLG
--PMAWIV
--PMAWII
--PMAWIG
--PMAWHR
--PMAWHQ
--PMAWHM
--PMAWGY
--PMAWEL
--PMAWEG
--PMAWAY
--PMAWAW
--PMAWAM
PTPMAW--
-PPMAWI-
--PMAWYM
--PMAWYH
--PMAWYD
--PMAWWT
--PMAWWQ
--PMAWWF
--PMAWWE
--PMAWWA
--PMAWVY
--PMAWVV
--PMAWVT
--PMAWVK
--PMAWTW
--PMAWSY
--PMAWST
--PMAWSN
--PMAWSM
--PMAWSF
--PMAWSA
--PMAWRN
--PMAWRD
--PMAWQY
--PMAWQV
--PMAWQQ
--PMAWQF
--PMAWPM
--PMAWNI
--PMAWNG
--PMAWMV

--PMAWMN
--PMAWMG
--PMAWLD
--PMAWKN
--PMAWIW
--PMAWIK
--PMAWHN
--PMAWHH
--PMAWHF
--PMAWGM
--PMAWGL
--PMAWGA
--PMAWFQ
--PMAWFG
--PMAWFD
--PMAWEY
--PMAWEW
PGPMAW--
PFPMAW--
QRPMAW--
-QPMAWL-
QSPMAW--
-QPMAWY-
QMPMAW--
-QPMAWT-
QQPMAW--
QKPMAW--
QWPMAW--
QTPMAW--
-QPMAWW-
-QPMAWS-
-QPMAWM-
QHPMAW--
QVPMAW--
QPPMAW--
-QPMAWV-
-QPMAWR-
-QPMAWQ-
-QPMAWH-
-QPMAWF-
-QPMAWA-
QGPMAW--
QFPMAW--
-RPMAWI-
-RPMAWT-
RTPMAW--
-RPMAWL-
RDPMAW--
-RPMAWV-
-RPMAWN-

-RPMAWM-
RYPMAW--
RVPMAW--
-RPMAWW-
-RPMAWS-
-RPMAWR-
-RPMAWK-
-RPMAWA-
-RPMAWQ-
RFPMAW--
RMPMAW--
RGPMAW--
RPPMAW--
RHPMAW--
RAPMAW--
-RPMAWG-
-RPMAWF-
SRPMAW--
SAPMAW--
SYPMAW--
SSPMAW--
-SPMAWY-
-SPMAWV-
-SPMAWL-
-SPMAWK-
-SPMAWI-
SGPMAW--
-SPMAWW-
-SPMAWF-
SPPMAW--
SMPMAW--
-SPMAWT-
-SPMAWH-
-SPMAWA-
SEPMAW--
STPMAW--
-SPMAWM-
SIPMAW--
SVPMAW--
SQPMAW--
-SPMAWN-
SKPMAW--
SDPMAW--
TRPMAW--
-TPMAWK-
TKPMAW--
-TPMAWW-
TGPMAW--
TPPMAW--
-TPMAWN-

TAPMAW--
-TPMAWV-
TSPMAW--
-TPMAWT-
-TPMAWS-
-TPMAWL-
-TPMAWH-
-TPMAWA-
-TPMAWR-
-TPMAWQ-
-TPMAWM-
-TPMAWI-
-TPMAWG-
-TPMAWE-
TNPMAW--
TIPMAW--
TFPMAW--
-VPMAWR-
VAPMAW--
-VPMAWS-
-VPMAWV-
VQPMAW--
-VPMAWL-
VGPMAW--
-VPMAWI-
VMPMAW--
VPPMAW--
-VPMAWT-
-VPMAWN-
-VPMAWH-
VIPMAW--
VHPMAW--
VEPMAW--
VTPMAW--
-VPMAWK-
-VPMAWG-
-VPMAWE-
-VPMAWA-
WVPMAW--
WRPMAW--
WNPMAW--
WKPMAW--
WTPMAW--
-WPMAWI-
WDPMAW--
-WPMAWV-
-WPMAWQ-
-WPMAWM-
-WPMAWK-
WEPMAW--

YRPMAW--
-YPMAWL-
YGPMAW--
YPPMAW--
-YPMAWW-
-YPMAWN-
YDPMAW--
YSPMAW--
YQPMAW--
-YPMAWT-
-YPMAWR-
-YPMAWQ-
-YPMAWI-
-YPMAWG-
-YPMAWA-
YNPMAW--
YIPMAW--
YAPMAW--

LRSQ 271 683 30.55

CLLRSQ--
DTLRSQ--
EGLRSQ--
EVLRSQ--
ESLRSQ--
FDLRSQ--
GPLRSQ--
-HLRSQS-
-ILRSQR-
-ILRSQY-
IDLRSQ--
KPLRSQ--
--LRSQGN
--LRSQGF
--LRSQDW
--LRSQDT
-LLRSQK-
LDLRSQ--
--LRSQTE
--LRSQNT
--LRSQNP
--LRSQNH
--LRSQMF
--LRSQHW
--LRSQEW
--LRSQET
--LRSQES
--LRSQDR
--LRSQDN
-LLRSQN-
-PLRSQY-

-PLRSQT-
-PLRSQS-
QVLRSQ--
RCLRSQ--
-TLRSQL-
-TLRSQK-
-TLRSQS-
-TLRSQD-
-VLRSQR-
VRLRSQ--
-VLRSQM-
VKLRSQ--
ASLRSQ--
ANLRSQ--
-ALRSQR-
AYLRSQ--
-ALRSQS-
AALRSQ--
AFLRSQ--
-ALRSQL-
-ALRSQV-
AWLRSQ--
-ALRSQF-
-ALRSQK-
-ALRSQG-
ATLRSQ--
-ALRSQY-
AILRSQ--
-ALRSQI-
AQLRSQ--
-ALRSQM-
ADLRSQ--
-ALRSQN-
AVLRSQ--
-ALRSQP-
-ALRSQE-
-ALRSQH-
-ALRSQT-
APLRSQ--
-ALRSQQ-
AELRSQ--
AKLRSQ--
ACLRSQ--
CHLRSQ--
CFLRSQ--
CWLRSQ--
CSLRSQ--
CPLRSQ--
CNLRSQ--
-CLRSQL-

CGLRSQ--
-DLRSQS-
DWLRSQ--
-DLRSQW-
-DLRSQL-
-DLRSQA-
-DLRSQI-
DYLRSQ--
-DLRSQV-
DMLRSQ--
-DLRSQY-
-DLRSQG-
DLLRSQ--
-DLRSQQ-
DFLRSQ--
-DLRSQP-
-DLRSQK-
DILRSQ--
-DLRSQT-
-DLRSQH-
-DLRSQF-
DHLRSQ--
DVLRSQ--
-DLRSQN-
-DLRSQM-
DNLRSQ--
-ELRSQL-
-ELRSQR-
-ELRSQG-
-ELRSQY-
-ELRSQF-
-ELRSQK-
-ELRSQS-
-ELRSQQ-
-ELRSQA-
EWLRSQ--
-ELRSQV-
-ELRSQT-
ELLRSQ--
-ELRSQN-
-ELRSQE-
EFLRSQ--
-ELRSQP-
-ELRSQM-
ETLRSQ--
EILRSQ--
ENLRSQ--
EMLRSQ--
-ELRSQH-
-ELRSQI-

EHLRSQ--
-ELRSQW-
-ELRSQC-
-FLRSQT-
-FLRSQV-
FLLRSQ--
-FLRSQI-
FYLRSQ--
-FLRSQS-
-FLRSQY-
-FLRSQQ-
-FLRSQM-
FNLRSQ--
-FLRSQK-
FILRSQ--
FVLRSQ--
FFLRSQ--
FPLRSQ--
FMLRSQ--
FHLRSQ--
FTLRSQ--
-FLRSQN-
FELRSQ--
-FLRSQH-
FWLRSQ--
-FLRSQE-
-FLRSQD-
FQLRSQ--
-FLRSQP-
FCLRSQ--
-GLRSQR-
-GLRSQI-
GLLRSQ--
GELRSQ--
GHLRSQ--
GFLRSQ--
-GLRSQT-
GALRSQ--
GYLRSQ--
-GLRSQK-
-GLRSQM-
-GLRSQW-
-GLRSQS-
GQLRSQ--
GGLRSQ--
GDLRSQ--
-GLRSQV-
-GLRSQH-
-GLRSQF-
-GLRSQA-

GVLRSQ--
-GLRSQG-
GILRSQ--
GTLRSQ--
GRLRSQ--
-GLRSQP-
-GLRSQQ-
GCLRSQ--
-HLRSQR-
-HLRSQL-
HVLRSQ--
HALRSQ--
HYLRSQ--
HHLRSQ--
HFLRSQ--
HNLRSQ--
HTLRSQ--
HMLRSQ--
-HLRSQV-
HILRSQ--
HWLRSQ--
HPLRSQ--
-HLRSQK-
HGLRSQ--
HQLRSQ--
-HLRSQF-
-HLRSQT-
-HLRSQA-
-HLRSQQ-
-HLRSQM-
-HLRSQI-
HELRSQ--
HDLRSQ--
IALRSQ--
IHLRSQ--
IGLRSQ--
IYLRSQ--
IVLRSQ--
IILRSQ--
INLRSQ--
IELRSQ--
IMLRSQ--
-ILRSQL-
IRLRSQ--
IPLRSQ--
-ILRSQW-
KFLRSQ--
KNLRSQ--
KHLRSQ--
-KLRSQW-

--LRSQRS
--LRSQVS
--LRSQRG
--LRSQAL
--LRSQIR
--LRSQIS
--LRSQVR
--LRSQTR
LYLRSQ--
--LRSQYS
LLLRSQ--
LELRSQ--
--LRSQVG
LVLRSQ--
--LRSQYL
--LRSQWL
--LRSQGL
--LRSQVP
LGLRSQ--
LWLRSQ--
--LRSQVL
--LRSQLM
--LRSQRN
LNLRSQ--
--LRSQSV
--LRSQSS
--LRSQAR
--LRSQSP
--LRSQYK
--LRSQIK
--LRSQWS
--LRSQTG
--LRSQRV
--LRSQLA
--LRSQIG
--LRSQYI
--LRSQLQ
--LRSQWP
--LRSQTS
--LRSQTA
--LRSQKG
--LRSQAW
--LRSQYN
--LRSQYG
--LRSQFG
--LRSQMK
--LRSQHR
--LRSQFS
--LRSQFA
-LLRSQR-

--LRSQYM
--LRSQVT
--LRSQST
--LRSQMT
--LRSQLV
LHLRSQ--
--LRSQYT
--LRSQYP
--LRSQVI
--LRSQSN
--LRSQRH
--LRSQHG
--LRSQVM
--LRSQLE
--LRSQFP
--LRSQAY
--LRSQWF
--LRSQRM
--LRSQNI
--LRSQIQ
--LRSQIP
--LRSQMY
--LRSQHL
--LRSQAI
--LRSQYW
--LRSQWV
--LRSQWI
--LRSQPV
--LRSQNK
--LRSQIN
--LRSQIH
--LRSQIA
--LRSQHP
--LRSQAS
--LRSQWA
--LRSQSI
--LRSQSA
--LRSQLW
--LRSQKS
--LRSQHS
--LRSQGI
--LRSQAF
LQLRSQ--
--LRSQVY
--LRSQVN
--LRSQVA
--LRSQRE
--LRSQKP
--LRSQAP
-LLRSQL-

--LRSQWE
--LRSQVH
--LRSQSH
--LRSQPG
--LRSQMA
--LRSQGS
--LRSQGR
--LRSQFQ
--LRSQAM
--LRSQAA
--LRSQYH
--LRSQWK
--LRSQVV
--LRSQTW
--LRSQTK
--LRSQSG
--LRSQPI
--LRSQKT
--LRSQHK
--LRSQFT
--LRSQFF
--LRSQAQ
--LRSQAG
-LLRSQI-
--LRSQYA
--LRSQWG
--LRSQVW
--LRSQTV
--LRSQTN
--LRSQSW
--LRSQSF
--LRSQQA
--LRSQPK
--LRSQMP
--LRSQMH
--LRSQKY
--LRSQGV
--LRSQAK
--LRSQWN
--LRSQWM
--LRSQWH
--LRSQTQ
--LRSQSM
--LRSQQP
--LRSQMI
--LRSQKW
--LRSQFV
--LRSQTY
--LRSQQT
--LRSQQS

--LRSQNY
--LRSQNV
--LRSQNQ
--LRSQMG
--LRSQKD
--LRSQGH
--LRSQGA
--LRSQAN
-LLRSQT-
-LLRSQM-
--LRSQRD
--LRSQQW
--LRSQQL
--LRSQPN
--LRSQKV
--LRSQIM
--LRSQHI
--LRSQGP
--LRSQGK
--LRSQFI
--LRSQEF
--LRSQEA
--LRSQDL
--LRSQYQ
--LRSQYD
--LRSQWW
--LRSQWD
--LRSQTT
--LRSQTF
--LRSQQV
--LRSQQQ
--LRSQQI
--LRSQQF
--LRSQPY
--LRSQPT
--LRSQPS
--LRSQMQ
--LRSQKH
--LRSQIW
--LRSQID
--LRSQHV
--LRSQHQ
--LRSQHN
--LRSQHA
--LRSQGT
--LRSQGG
--LRSQFH
--LRSQEN
--LRSQEG
--LRSQDA

--LRSQYE
--LRSQWQ
--LRSQVE
--LRSQTM
--LRSQTD
--LRSQSQ
--LRSQQN
--LRSQPW
--LRSQPE
--LRSQLD
--LRSQKM
--LRSQHD
--LRSQGQ
--LRSQAT
--LRSQAH
--LRSQVD
--LRSQQY
--LRSQQD
--LRSQPM
--LRSQNN
--LRSQMD
--LRSQKQ
--LRSQKF
--LRSQKE
--LRSQIF
--LRSQIE
--LRSQHT
--LRSQHM
--LRSQGM
--LRSQEL
--LRSQDY
--LRSQAE
--LRSQAD
--LRSQQH
--LRSQQG
--LRSQQE
--LRSQPH
--LRSQPD
--LRSQMW
--LRSQMM
--LRSQME
--LRSQHH
--LRSQFN
--LRSQFD
--LRSQEQ
--LRSQEP
--LRSQEH
--LRSQEE
--LRSQED
--LRSQDG

-MLRSQR-
-MLRSQI-
MMLRSQ--
-MLRSQY-
MYLRSQ--
MSLRSQ--
MALRSQ--
-MLRSQF-
MGLRSQ--
MTLRSQ--
-MLRSQT-
-MLRSQH-
MPLRSQ--
-MLRSQN-
-MLRSQA-
MILRSQ--
MQLRSQ--
-MLRSQQ-
-MLRSQP-
-MLRSQG-
MELRSQ--
-MLRSQM-
-MLRSQD-
-NLRSQR-
-NLRSQN-
NFLRSQ--
-NLRSQT-
-NLRSQQ-
NHLRSQ--
NSLRSQ--
-NLRSQK-
NALRSQ--
-NLRSQI-
NQLRSQ--
-NLRSQH-
NNLRSQ--
-NLRSQA-
-NLRSQY-
-NLRSQW-
-NLRSQV-
NPLRSQ--
-NLRSQP-
-NLRSQE-
NILRSQ--
NMLRSQ--
-NLRSQF-
-NLRSQD-
NDLRSQ--
NTLRSQ--
-NLRSQM-

NGLRSQ--
PFLRSQ--
PWLRSQ--
PYLRSQ--
-PLRSQL-
PILRSQ--
PTLRSQ--
-PLRSQR-
PHLRSQ--
PMLRSQ--
PELRSQ--
-PLRSQI-
PRLRSQ--
PKLRSQ--
PDLRSQ--
-QLRSQR-
-QLRSQL-
QSLRSQ--
QYLRSQ--
-QLRSQK-
QALRSQ--
-QLRSQI-
-QLRSQH-
-QLRSQS-
QELRSQ--
-QLRSQV-
-QLRSQF-
-QLRSQQ-
QNLRSQ--
QILRSQ--
QTLRSQ--
-QLRSQW-
QHLRSQ--
-QLRSQG-
-QLRSQP-
-QLRSQN-
QDLRSQ--
QMLRSQ--
-QLRSQM-
-QLRSQE-
-QLRSQA-
RELRSQ--
RDLRSQ--
SSLRSQ--
-SLRSQI-
-SLRSQK-
-SLRSQT-
-SLRSQV-
-SLRSQA-
-SLRSQY-

SHLRSQ--
SYLRSQ--
SGLRSQ--
-SLRSQM-
-SLRSQF-
SPLRSQ--
-SLRSQH-
-SLRSQS-
STLRSQ--
-SLRSQW-
SFLRSQ--
SELRSQ--
-SLRSQQ-
-SLRSQG-
SQLRSQ--
-SLRSQN-
-SLRSQE-
SVLRSQ--
-SLRSQD-
SCLRSQ--
TFLRSQ--
TWLRSQ--
TMLRSQ--
-TLRSQR-
TALRSQ--
TVLRSQ--
TILRSQ--
TSLRSQ--
THLRSQ--
TGLRSQ--
TNLRSQ--
TELRSQ--
TTLRSQ--
TQLRSQ--
-TLRSQY-
VLLRSQ--
VSLRSQ--
VHLRSQ--
VMLRSQ--
VQLRSQ--
VGLRSQ--
VTLRSQ--
VILRSQ--
VWLRSQ--
VALRSQ--
VNLRSQ--
VELRSQ--
VPLRSQ--
-VLRSQL-
VCLRSQ--

WYLRSQ--
-WLRSQG-
-WLRSQP-
-WLRSQI-
-WLRSQK-
WMLRSQ--
WLLRSQ--
WSLRSQ--
-WLRSQA-
WILRSQ--
WNLRSQ--
-WLRSQH-
-WLRSQQ-
WPLRSQ--
-WLRSQE-
WWLRSQ--
-WLRSQD-
WHLRSQ--
WGLRSQ--
WELRSQ--
WQLRSQ--
-WLRSQF-
WDLRSQ--
-WLRSQC-
-YLRSQR-
YLLRSQ--
-YLRSQH-
YWLRSQ--
-YLRSQN-
-YLRSQS-
YNLRSQ--
-YLRSQW-
YFLRSQ--
-YLRSQF-
-YLRSQA-
YTLRSQ--
-YLRSQG-
-YLRSQT-
YHLRSQ--
YELRSQ--
YMLRSQ--
-YLRSQY-
YPLRSQ--
YILRSQ--
YGLRSQ--
-YLRSQP-
YQLRSQ--
-YLRSQQ-
-YLRSQM-
-YLRSQD-

| | | | |
|---|---|---|---|
| | YYLRSQ-- | | |
| | -YLRSQE- | | |
| PAHM | 614 | 577 | 30.55 |
| | -APAHML- | | |
| | -APAHMS- | | |
| | ASPAHM-- | | |
| | ALPAHM-- | | |
| | ATPAHM-- | | |
| | ANPAHM-- | | |
| | AIPAHM-- | | |
| | ARPAHM-- | | |
| | -APAHMR- | | |
| | -APAHMK- | | |
| | AYPAHM-- | | |
| | -APAHMQ- | | |
| | AHPAHM-- | | |
| | AAPAHM-- | | |
| | -APAHMI- | | |
| | -APAHMW- | | |
| | -APAHMT- | | |
| | -APAHMN- | | |
| | -APAHMM- | | |
| | -APAHMG- | | |
| | -APAHMF- | | |
| | -APAHMA- | | |
| | AKPAHM-- | | |
| | AFPAHM-- | | |
| | AEPAHM-- | | |
| | CSPAHM-- | | |
| | CQPAHM-- | | |
| | -CPAHMA- | | |
| | CLPAHM-- | | |
| | DAPAHM-- | | |
| | DYPAHM-- | | |
| | -DPAHMR- | | |
| | DKPAHM-- | | |
| | DSPAHM-- | | |
| | DRPAHM-- | | |
| | -DPAHMF- | | |
| | DQPAHM-- | | |
| | -DPAHMY- | | |
| | -DPAHMW- | | |
| | -DPAHMK- | | |
| | DIPAHM-- | | |
| | DGPAHM-- | | |
| | DEPAHM-- | | |
| | ELPAHM-- | | |
| | ERPAHM-- | | |
| | -EPAHMW- | | |
| | -EPAHMV- | | |

-EPAHMT-
-EPAHMS-
-EPAHMF-
EYPAHM--
ESPAHM--
-EPAHMR-
-EPAHMQ-
-EPAHMN-
-EPAHMK-
-EPAHMI-
ENPAHM--
EMPAHM--
EGPAHM--
FLPAHM--
-FPAHMS-
-FPAHMV-
FKPAHM--
FWPAHM--
-FPAHMW-
-FPAHMK-
FSPAHM--
-FPAHMR-
-FPAHMM-
-FPAHMA-
FFPAHM--
FTPAHM--
-FPAHMY-
-FPAHMT-
-FPAHML-
-FPAHMI-
-FPAHMH-
FNPAHM--
FAPAHM--
-GPAHML-
GIPAHM--
-GPAHMV-
GRPAHM--
-GPAHMM-
-GPAHMH-
GNPAHM--
-GPAHME-
GAPAHM--
-GPAHMG-
GVPAHM--
-GPAHMS-
-GPAHMI-
GKPAHM--
GWPAHM--
GQPAHM--
-GPAHMR-

-GPAHMN-
-GPAHMK-
-GPAHMA-
GHPAHM--
-HPAHMR-
-HPAHMT-
HRPAHM--
-HPAHMI-
-HPAHMY-
HFPAHM--
-HPAHMA-
HNPAHM--
HTPAHM--
HQPAHM--
-HPAHMS-
-HPAHMM-
-HPAHMG-
HMPAHM--
HLPAHM--
-IPAHMR-
IRPAHM--
ILPAHM--
-IPAHMS-
IWPAHM--
ISPAHM--
-IPAHMN-
-IPAHMG-
IKPAHM--
-IPAHMT-
ITPAHM--
IPPAHM--
-IPAHMY-
-IPAHMV-
-IPAHMQ-
-IPAHMM-
-IPAHML-
-IPAHMA-
IIPAHM--
IDPAHM--
IAPAHM--
-KPAHMR-
-KPAHMA-
KPPAHM--
-KPAHMV-
-KPAHMS-
-KPAHMK-
-KPAHMG-
KLPAHM--
KSPAHM--
-KPAHML-

-KPAHMF-
KNPAHM--
KWPAHM--
KRPAHM--
-KPAHMT-
-KPAHMQ-
KCPAHM--
-LPAHML-
LRPAHM--
-LPAHMT-
LVPAHM--
LPPAHM--
-LPAHMY-
-LPAHMV-
-LPAHMR-
LAPAHM--
-LPAHMS-
-LPAHMQ-
-LPAHMI-
LNPAHM--
LIPAHM--
LWPAHM--
-LPAHMM-
LKPAHM--
LGPAHM--
LFPAHM--
LTPAHM--
LSPAHM--
LQPAHM--
-LPAHMW-
-LPAHMH-
-LPAHMF-
-LPAHMA-
LLPAHM--
-MPAHML-
MSPAHM--
-MPAHMT-
MQPAHM--
-MPAHMH-
MKPAHM--
MHPAHM--
-MPAHMR-
-MPAHMG-
MNPAHM--
MYPAHM--
MTPAHM--
-MPAHMS-
-MPAHMQ-
-MPAHMM-
MGPAHM--

MDPAHM--
NSPAHM--
-NPAHMS-
NGPAHM--
-NPAHMQ-
NMPAHM--
NYPAHM--
NTPAHM--
-NPAHMI-
-NPAHME-
-NPAHMY-
NNPAHM--
NHPAHM--
NRPAHM--
-NPAHMT-
-NPAHMR-
-NPAHMK-
-NPAHMH-
-NPAHMG-
-NPAHMF-
NLPAHM--
NIPAHM--
--PAHMLR
--PAHMLA
--PAHMSL
--PAHMRY
--PAHMRS
--PAHMVS
--PAHMRA
--PAHMWD
--PAHMRQ
--PAHMRL
--PAHMRE
--PAHMLQ
--PAHMLL
--PAHMLG
PRPAHM--
--PAHMST
--PAHMIR
--PAHMSV
--PAHMSN
--PAHMRT
--PAHMIL
--PAHMAS
--PAHMAR
PSPAHM--
--PAHMYL
--PAHMVI
--PAHMIS
--PAHMHR

--PAHMFS
--PAHMYS
--PAHMYI
--PAHMWS
--PAHMVM
--PAHMVA
--PAHMTY
--PAHMTW
--PAHMSM
--PAHMSK
--PAHMRH
--PAHMMN
--PAHMLS
--PAHMFT
--PAHMAA
--PAHMWI
--PAHMTR
--PAHMSQ
--PAHMSA
--PAHMRV
--PAHMRG
--PAHMQW
--PAHMMV
--PAHMHS
--PAHMVW
--PAHMVT
--PAHMVL
--PAHMSS
--PAHMRN
--PAHMRI
--PAHMNS
--PAHMMS
--PAHMLV
--PAHMLN
--PAHMKL
--PAHMKF
--PAHMIY
--PAHMIM
--PAHMGS
--PAHMEN
--PAHMDG
--PAHMYE
--PAHMWL
--PAHMWH
--PAHMVK
--PAHMVF
--PAHMVE
--PAHMTL
--PAHMTG
--PAHMSI

--PAHMSF
--PAHMRD
--PAHMQY
--PAHMQI
--PAHMNV
--PAHMNG
--PAHMMW
--PAHMMH
--PAHMLT
--PAHMLD
--PAHMKW
--PAHMKR
--PAHMKG
--PAHMKD
--PAHMIV
--PAHMIT
--PAHMIQ
--PAHMIA
--PAHMHW
--PAHMHK
--PAHMGV
--PAHMFA
--PAHMER
--PAHMAL
PVPAHM--
PFPAHM--
--PAHMYW
--PAHMYR
--PAHMYN
--PAHMYF
--PAHMWR
--PAHMVG
--PAHMSY
--PAHMSW
--PAHMSR
--PAHMSG
--PAHMRW
--PAHMRM
--PAHMRC
--PAHMQQ
--PAHMQN
--PAHMQL
--PAHMQG
--PAHMMR
--PAHMKT
--PAHMIH
--PAHMHQ
--PAHMHI
--PAHMHF
--PAHMHA

--PAHMGT
--PAHMGK
--PAHMFW
--PAHMFV
--PAHMFR
--PAHMFI
--PAHMFE
--PAHMEY
--PAHMEW
--PAHMET
--PAHMAF
--PAHMAD
PTPAHM--
-PPAHMP-
PLPAHM--
PIPAHM--
PEPAHM--
PDPAHM--
--PAHMYT
--PAHMYA
--PAHMWQ
--PAHMWP
--PAHMWN
--PAHMWE
--PAHMWA
--PAHMVH
--PAHMTV
--PAHMTE
--PAHMTD
--PAHMTA
--PAHMSH
--PAHMSE
--PAHMSD
--PAHMRF
--PAHMQT
--PAHMNT
--PAHMNR
[truncated: 242,915 more chars]
