# Supplementary material for: Quantitative profiling of protease specificity
Source: PLoS Comput Biol. 2021 Feb 22;17(2):e1008101. doi: 10.1371/journal.pcbi.1008101 (PMC7932537; doi:10.1371/journal.pcbi.1008101)
Supplement: S12 Table — List of tetramer clusters common between the selectomes of MMP-2 and 9 together with the corresponding hexamer sequences aligned across P3-P1՛ positions of substrates. Tetramers are ranked according to MMP-9 relative probability. Information about the tetramer amino acids sequence, rank, number of hexamers and relative probability of a cluster is provided in the header of each tetramer cluster. (PDF) [file pcbi.1008101.s014.pdf]

S13 Table. Analysis of combined selectomes of MMP-2 and 9. Common set of MMP-2 and 9 selectomes reranked according to MMP-9 relative probability
tetramer, rank, number_of_hexamers_in_tetramer_cluster, relative_probability

PMWM	727	555	52.38
	-APMWML-		
	AFPMWM--		
	AIPMWM--		
	-APMWMG-		
	AEPMWM--		
	-APMWMQ-		
	-APMWMT-		
	-APMWMR-		
	ATPMWM--		
	-APMWMS-		
	-APMWMI-		
	-APMWMY-		
	-APMWMN-		
	-APMWMD-		
	AHPMWM--		
	AAPMWM--		
	-APMWME-		
	ADPMWM--		
	AVPMWM--		
	AMPMWM--		
	ACPMWM--		
	CTPMWM--		
	CCPMWM--		
	CQPMWM--		
	DGPMWM--		
	DAPMWM--		
	DRPMWM--		
	DNPMWM--		
	-DPMWMT-		
	-DPMWMF-		
	DQPMWM--		
	-DPMWMV-		
	DKPMWM--		
	-DPMWMY-		
	-DPMWML-		
	-DPMWMI-		
	-DPMWMD-		
	DLPMWM--		
	DSPMWM--		
	-DPMWMG-		
	-EPMWMV-		
	-EPMWMI-		
	-EPMWMG-		
	ENPMWM--		

-EPMWML-
ERPMWM--
EVPMWM--
-EPMWMT-
-EPMWMR-
-EPMWMA-
ELPMWM--
ESPMWM--
-EPMWMS-
EQPMWM--
-EPMWMN-
EPPMWM--
FKPMWM--
FRPMWM--
-FPMWMR-
FVPMWM--
FAPMWM--
-FPMWMS-
FHPMWM--
FWPMWM--
FDPMWM--
FTPMWM--
-FPMWMN-
FGPMWM--
FNPMWM--
FLPMWM--
-FPMWMK-
FMPMWM--
-FPMWMT-
-FPMWMI-
GYPMWM--
-GPMWMR-
-GPMWMK-
-GPMWML-
GRPMWM--
GWPMWM--
-GPMWMT-
GAPMWM--
GQPMWM--
-GPMWMI-
-GPMWMG-
-GPMWME-
-GPMWMN-
-GPMWMH-
GSPMWM--
GNPMWM--
GPPMWM--
-GPMWMS-
-GPMWMY-
-GPMWMA-

-GPMWMV-
GHPMWM--
GFPMWM--
-HPMWMN-
HSPMWM--
-HPMWMA-
-HPMWMT-
-HPMWMR-
-HPMWMM-
HTPMWM--
-HPMWMS-
-HPMWMH-
HKPMWM--
HAPMWM--
-HPMWMI-
HDPMWM--
HEPMWM--
-HPMWMF-
HYPMWM--
HRPMWM--
-HPMWMY-
HGPMWM--
-HPMWML-
-HPMWMK-
HHPMWM--
IQPMWM--
IYPMWM--
IHPMWM--
IVPMWM--
-IPMWMT-
INPMWM--
-IPMWMV-
-IPMWMR-
IKPMWM--
-IPMWME-
IIPMWM--
-IPMWMG-
-IPMWMA-
IEPMWM--
IRPMWM--
-IPMWMS-
IFPMWM--
IDPMWM--
KRPMWM--
-KPMWMR-
-KPMWMS-
-KPMWMY-
-KPMWMT-
-KPMWMV-
-KPMWMH-

-KPMWMF-
-KPMWME-
KIPMWM--
KPPMWM--
KMPMWM--
KDPMWM--
-KPMWMI-
KVPMWM--
KNPMWM--
KFPMWM--
-KPMWMN-
-KPMWMK-
-KPMWMD-
KWPMWM--
KTPMWM--
-KPMWMQ-
-KPMWMG-
KLPMWM--
KEPMWM--
KAPMWM--
-LPMWMS-
-LPMWMK-
LWPMWM--
LSPMWM--
LRPMWM--
-LPMWMG-
LVPMWM--
-LPMWMV-
-LPMWMQ-
-LPMWMN-
LAPMWM--
LTPMWM--
LNPMWM--
-LPMWME-
LYPMWM--
LKPMWM--
LEPMWM--
LPPMWM--
LMPMWM--
LLPMWM--
LIPMWM--
MFPMWM--
-MPMWMR-
MYPMWM--
-MPMWML-
MIPMWM--
MLPMWM--
MKPMWM--
MVPMWM--
MAPMWM--

MNPMWM--
-MPMWMA-
-MPMWMN-
-MPMWMQ-
-MPMWMH-
MHPMWM--
-MPMWMT-
-MPMWMI-
-MPMWME-
MSPMWM--
-MPMWMW-
-NPMWMV-
NRPMWM--
NNPMWM--
NFPMWM--
-NPMWML-
NIPMWM--
NLPMWM--
-NPMWMR-
NAPMWM--
NVPMWM--
-NPMWMY-
-NPMWMQ-
-NPMWMI-
-NPMWMA-
NEPMWM--
-NPMWMM-
NPPMWM--
NHPMWM--
--PMWMRS
--PMWMNS
--PMWMLT
--PMWMRG
--PMWMYS
--PMWMRT
--PMWMVS
--PMWMTH
--PMWMMR
--PMWMIR
--PMWMKV
--PMWMTR
--PMWMSS
--PMWMVN
--PMWMRN
--PMWMVG
--PMWMVA
--PMWMMG
--PMWMTG
--PMWMKN
--PMWMVR

--PMWMTW
--PMWMSQ
--PMWMRQ
--PMWMNA
--PMWMAK
--PMWMTN
--PMWMLS
--PMWMIE
--PMWMHS
--PMWMEH
--PMWMWS
--PMWMSR
--PMWMQR
--PMWMHM
--PMWMGN
--PMWMEM
--PMWMYL
--PMWMTE
--PMWMSH
--PMWMQS
--PMWMLK
--PMWMKS
--PMWMKE
--PMWMFG
--PMWMAL
--PMWMWG
--PMWMRW
--PMWMLQ
--PMWMGL
--PMWMAF
--PMWMAE
--PMWMYN
--PMWMRH
--PMWMMV
--PMWMLR
--PMWMLN
--PMWMIN
--PMWMIH
--PMWMGW
PGPMWM--
PNPMWM--
--PMWMVV
--PMWMTT
--PMWMSI
--PMWMSE
--PMWMNH
--PMWMLM
--PMWMKI
--PMWMGT
--PMWMYH

--PMWMWH
--PMWMTY
--PMWMMM
--PMWMMI
--PMWMHY
--PMWMHT
--PMWMGS
--PMWMGM
--PMWMGG
--PMWMGF
--PMWMES
--PMWMAT
--PMWMAI
PDPMWM--
--PMWMYG
--PMWMWN
--PMWMVH
--PMWMSY
--PMWMSK
--PMWMQV
--PMWMQG
--PMWMNM
--PMWMKY
--PMWMHQ
--PMWMHN
--PMWMHL
--PMWMGD
--PMWMGA
--PMWMFD
--PMWMDG
--PMWMAD
PTPMWM--
--PMWMWD
--PMWMWA
--PMWMTA
--PMWMSM
--PMWMSD
--PMWMSA
--PMWMQY
--PMWMQA
--PMWMNT
--PMWMNQ
--PMWMMT
--PMWMLH
--PMWMKG
--PMWMIK
--PMWMGR
--PMWMAM
--PMWMAG
--PMWMYQ

--PMWMRE
--PMWMRD
--PMWMQI
--PMWMNN
--PMWMLD
--PMWMIT
--PMWMHA
--PMWMGY
--PMWMGV
--PMWMFS
--PMWMEN
--PMWMAQ
-PPMWMN-
--PMWMWR
--PMWMVK
--PMWMVD
--PMWMTV
--PMWMTS
--PMWMSN
--PMWMSL
--PMWMSG
--PMWMRV
--PMWMRM
--PMWMRF
--PMWMQK
--PMWMNY
--PMWMNW
--PMWMNV
--PMWMNR
--PMWMNI
--PMWMME
--PMWMMA
--PMWMLI
--PMWMKL
--PMWMIV
--PMWMIQ
--PMWMID
--PMWMGH
--PMWMFY
--PMWMFA
--PMWMET
--PMWMEQ
--PMWMAR
QIPMWM--
-QPMWMR-
-QPMWMI-
QGPMWM--
QSPMWM--
-QPMWMV-
QNPMWM--

QYPMWM--
-QPMWMW-
-QPMWMK-
-QPMWML-
-QPMWMN-
-QPMWMF-
QPPMWM--
-QPMWMY-
-QPMWMS-
QAPMWM--
RLPMWM--
RVPMWM--
-RPMWMS-
RQPMWM--
-RPMWMR-
-RPMWMH-
-RPMWMK-
-RPMWMG-
REPMWM--
RTPMWM--
-RPMWMT-
-RPMWMY-
RWPMWM--
RPPMWM--
-RPMWML-
RAPMWM--
-RPMWMN-
-RPMWMI-
RIPMWM--
-RPMWMQ-
-RPMWMM-
RHPMWM--
-RPMWMA-
RNPMWM--
RGPMWM--
-RPMWMV-
RSPMWM--
RYPMWM--
RKPMWM--
-RPMWMW-
RCPMWM--
-RPMWME-
-RPMWMD-
RFPMWM--
SNPMWM--
SVPMWM--
SIPMWM--
SWPMWM--
SKPMWM--
SAPMWM--

SYPMWM--
SSPMWM--
-SPMWMV-
STPMWM--
-SPMWMI-
-SPMWMR-
-SPMWMM-
-SPMWML-
-SPMWMK-
-SPMWMA-
-SPMWMG-
-SPMWMT-
SHPMWM--
-SPMWMY-
SFPMWM--
-SPMWMS-
SLPMWM--
SDPMWM--
SQPMWM--
-SPMWMN-
SMPMWM--
SGPMWM--
SEPMWM--
TSPMWM--
TKPMWM--
TEPMWM--
TRPMWM--
-TPMWMT-
-TPMWMN-
TLPMWM--
-TPMWMR-
TGPMWM--
TTPMWM--
-TPMWMS-
-TPMWML-
TNPMWM--
-TPMWMI-
-TPMWMH-
-TPMWMY-
-TPMWMW-
-TPMWMA-
TQPMWM--
TPPMWM--
-TPMWMG-
-TPMWMD-
TIPMWM--
TDPMWM--
VRPMWM--
-VPMWMR-
VVPMWM--

VSPMWM--
VAPMWM--
VNPMWM--
VGPMWM--
-VPMWML-
-VPMWMI-
-VPMWMY-
VDPMWM--
VKPMWM--
VIPMWM--
VPPMWM--
-VPMWMK-
-VPMWMT-
-VPMWMN-
VLPMWM--
-VPMWMA-
VEPMWM--
-VPMWMS-
-VPMWMM-
-VPMWMD-
VMPMWM--
VHPMWM--
VYPMWM--
VTPMWM--
-VPMWME-
WQPMWM--
WRPMWM--
WSPMWM--
-WPMWMR-
-WPMWMS-
-WPMWMK-
WLPMWM--
WIPMWM--
WEPMWM--
WAPMWM--
-WPMWMT-
WGPMWM--
YGPMWM--
YNPMWM--
YQPMWM--
YHPMWM--
YFPMWM--
YRPMWM--
YPPMWM--
-YPMWMK-
-YPMWMM-
YAPMWM--
YTPMWM--
YSPMWM--
-YPMWMV-

	-YPMWMT-		
	-YPMWMH-		
	YEPMWM--		
	YDPMWM--		
	-YPMWMQ-		
	-YPMWMN-		
	-YPMWMG-		
	-YPMWMD-		
	-YPMWMW-		
	-YPMWMF-		
	-YPMWMA-		
PYAI	184	757	51.75
	AKPYAI--		
	AAPYAI--		
	-APYAIM-		
	ALPYAI--		
	AGPYAI--		
	ASPYAI--		
	-APYAIR-		
	-APYAIF-		
	ARPYAI--		
	-APYAIG-		
	ANPYAI--		
	-APYAIL-		
	-APYAIK-		
	-APYAIS-		
	AWPYAI--		
	AMPYAI--		
	AIPYAI--		
	-APYAIT-		
	-APYAIH-		
	ATPYAI--		
	-APYAIY-		
	-APYAIV-		
	AQPYAI--		
	-APYAII-		
	-APYAIC-		
	-APYAIA-		
	ADPYAI--		
	APPYAI--		
	CKPYAI--		
	CGPYAI--		
	-CPYAIK-		
	CNPYAI--		
	-CPYAIW-		
	-CPYAIR-		
	CVPYAI--		
	CSPYAI--		
	CRPYAI--		
	-CPYAIT-		

```
-CPYAIS-
-CPYAII-
-CPYAIF-
CPPYAI--
CMPYAI--
DRPYAI--
DWPYAI--
DLPYAI--
-DPYAIS-
DTPYAI--
-DPYAIW-
DHPYAI--
DYPYAI--
-DPYAIY-
-DPYAIM-
-DPYAIL-
-DPYAII-
DAPYAI--
DVPYAI--
DSPYAI--
-DPYAIT-
-DPYAIV-
DGPYAI--
DEPYAI--
DKPYAI--
DFPYAI--
ERPYAI--
EGPYAI--
ETPYAI--
ESPYAI--
-EPYAIY-
-EPYAIH-
-EPYAII-
EVPYAI--
-EPYAIW-
EPPYAI--
EIPYAI--
EHPYAI--
EYPYAI--
-EPYAIV-
-EPYAIS-
-EPYAIR-
-EPYAIN-
-EPYAIL-
ELPYAI--
-EPYAIT-
-EPYAIM-
-EPYAIK-
-EPYAIG-
EMPYAI--
```

FSPYAI--
FRPYAI--
-FPYAIA-
FLPYAI--
FNPYAI--
FFPYAI--
-FPYAIS-
-FPYAIQ-
-FPYAIM-
FIPYAI--
FHPYAI--
FVPYAI--
-FPYAIV-
FPPYAI--
FQPYAI--
-FPYAII-
-FPYAIT-
FGPYAI--
-FPYAIR-
-FPYAIN-
-FPYAIK-
FKPYAI--
FYPYAI--
FTPYAI--
-FPYAIH-
-FPYAIG-
-FPYAIF-
FEPYAI--
FDPYAI--
-FPYAIE-
FMPYAI--
-GPYAIR-
-GPYAIK-
GLPYAI--
GQPYAI--
-GPYAIF-
GMPYAI--
GNPYAI--
-GPYAIS-
GTPYAI--
GRPYAI--
GSPYAI--
-GPYAIE-
-GPYAIL-
GIPYAI--
-GPYAIQ-
-GPYAIW-
-GPYAIG-
GGPYAI--
GFPYAI--

GAPYAI--
GEPYAI--
GVPYAI--
-GPYAII-
-GPYAIH-
-GPYAIV-
-GPYAIT-
-GPYAIN-
-GPYAIM-
GDPYAI--
GKPYAI--
GHPYAI--
GYPYAI--
HRPYAI--
-HPYAIN-
HKPYAI--
-HPYAIL-
HFPYAI--
-HPYAIV-
-HPYAIS-
HSPYAI--
HYPYAI--
HEPYAI--
HWPYAI--
-HPYAIR-
-HPYAII-
HIPYAI--
HGPYAI--
-HPYAIK-
-HPYAIF-
HLPYAI--
HQPYAI--
-HPYAIW-
-HPYAIM-
HPPYAI--
HNPYAI--
HHPYAI--
-HPYAIY-
-HPYAIG-
-HPYAIC-
HCPYAI--
ITPYAI--
IVPYAI--
-IPYAIS-
ISPYAI--
IKPYAI--
IRPYAI--
-IPYAIW-
-IPYAIT-
-IPYAIY-

-IPYAII-
IEPYAI--
-IPYAIQ-
-IPYAIM-
IFPYAI--
IGPYAI--
IHPYAI--
-IPYAIA-
ILPYAI--
IIPYAI--
-IPYAIN-
IAPYAI--
-IPYAIV-
-IPYAIR-
-IPYAIG-
IWPYAI--
-IPYAIH-
IMPYAI--
KSPYAI--
-KPYAIL-
KGPYAI--
-KPYAIT-
-KPYAIM-
-KPYAIW-
-KPYAIS-
KNPYAI--
-KPYAIH-
KEPYAI--
KLPYAI--
KWPYAI--
KYPYAI--
KTPYAI--
KMPYAI--
KPPYAI--
KQPYAI--
KVPYAI--
-KPYAIN-
-KPYAIK-
KKPYAI--
-KPYAII-
-KPYAIV-
KIPYAI--
KHPYAI--
-KPYAIE-
-KPYAIR-
-KPYAIA-
KAPYAI--
-KPYAIG-
LRPYAI--
LTPYAI--

-LPYAIR-
LKPYAI--
LLPYAI--
LVPYAI--
-LPYAIK-
LNPYAI--
LHPYAI--
LYPYAI--
LAPYAI--
-LPYAIS-
-LPYAIQ-
LIPYAI--
-LPYAIV-
LFPYAI--
-LPYAIM-
LDPYAI--
-LPYAIW-
LSPYAI--
LPPYAI--
LWPYAI--
-LPYAIT-
LMPYAI--
-LPYAIY-
LQPYAI--
-LPYAIG-
-LPYAIA-
-LPYAIN-
-LPYAII-
-LPYAIH-
LCPYAI--
-MPYAIL-
MSPYAI--
MFPYAI--
-MPYAIR-
MLPYAI--
MPPYAI--
-MPYAIS-
-MPYAIM-
MNPYAI--
MVPYAI--
-MPYAIK-
-MPYAIV-
MKPYAI--
MTPYAI--
MAPYAI--
-MPYAII-
-MPYAIT-
-MPYAIH-
-MPYAIG-
MDPYAI--

MWPYAI--
MRPYAI--
MQPYAI--
-MPYAIW-
-MPYAIA-
-MPYAIQ-
-MPYAID-
MGPYAI--
-NPYAIR-
-NPYAIK-
NRPYAI--
NLPYAI--
NMPYAI--
-NPYAIH-
-NPYAIS-
-NPYAIL-
NTPYAI--
NNPYAI--
NGPYAI--
-NPYAIW-
-NPYAIV-
NSPYAI--
-NPYAII-
NKPYAI--
NVPYAI--
-NPYAIY-
NFPYAI--
NEPYAI--
-NPYAIT-
NYPYAI--
-NPYAIE-
NPPYAI--
NHPYAI--
--PYAILR
--PYAIRL
--PYAIRA
--PYAIRR
--PYAIRT
--PYAILT
--PYAISS
--PYAIVT
--PYAIRS
--PYAIGS
--PYAISL
--PYAISA
--PYAIVS
--PYAIWL
--PYAILD
--PYAITA
--PYAIRK

--PYAILG
--PYAIWR
--PYAIVG
--PYAIHT
PGPYAI--
--PYAITV
--PYAITL
--PYAIKQ
--PYAIKK
--PYAIYR
--PYAIQT
--PYAIKV
--PYAIGG
--PYAIYL
--PYAIQN
--PYAIGL
--PYAITS
--PYAISY
--PYAIFS
--PYAIVA
--PYAIRW
--PYAISH
--PYAIRG
--PYAILN
--PYAITT
--PYAIMG
--PYAIAS
--PYAIYD
--PYAIWG
--PYAITN
--PYAITK
--PYAITH
--PYAINV
--PYAIMH
--PYAIKH
--PYAIGA
--PYAIWK
--PYAIST
--PYAIPF
PMPYAI--
--PYAIPL
--PYAINR
--PYAIHG
--PYAIFN
--PYAIVK
--PYAISV
--PYAIRN
--PYAIRD
--PYAIQR
--PYAIQE

--PYAIMK
--PYAIKT
--PYAIKN
--PYAIKG
--PYAIIN
--PYAIHL
--PYAIHK
--PYAIGV
--PYAIFM
--PYAIVQ
--PYAIKR
--PYAIKI
--PYAIIR
--PYAIIK
--PYAIIH
PHPYAI--
--PYAIYH
--PYAIVY
--PYAIVV
--PYAITI
--PYAITE
--PYAISW
--PYAINT
--PYAIMR
--PYAIHQ
--PYAIEG
--PYAIWM
--PYAISN
--PYAISI
--PYAINW
--PYAIKM
--PYAIFD
--PYAIEN
--PYAIAR
--PYAISM
--PYAIMY
--PYAIHI
--PYAIGY
--PYAIFG
--PYAIAF
PKPYAI--
--PYAIYG
--PYAIYE
--PYAIWN
--PYAITG
--PYAISG
--PYAIRM
--PYAIRE
--PYAING
--PYAIMT

--PYAIMN
--PYAIMD
--PYAIKS
--PYAIYT
--PYAIYQ
--PYAIVI
--PYAITM
--PYAINS
--PYAINN
--PYAIMA
--PYAILQ
--PYAIKD
--PYAIKA
--PYAIIS
--PYAIIG
--PYAIHR
--PYAIES
--PYAICN
--PYAIAG
PTPYAI--
PNPYAI--
PEPYAI--
PYPYAI--
--PYAIVN
--PYAIQY
--PYAIQK
--PYAIPY
--PYAINI
--PYAINE
--PYAIMV
--PYAIKL
--PYAIKF
--PYAIIY
--PYAIHN
--PYAIGT
--PYAIAY
--PYAIAV
--PYAIAL
--PYAIYY
--PYAITY
--PYAISQ
--PYAISE
--PYAIRH
--PYAIRF
--PYAIQV
--PYAIPM
--PYAIMS
--PYAIII
--PYAIHY
--PYAIGH

--PYAIFQ
--PYAIFH
--PYAIEH
--PYAIAH
PWPYAI--
PFPYAI--
--PYAIYN
--PYAIWQ
--PYAISC
--PYAIRP
--PYAINQ
--PYAIKC
--PYAIIT
--PYAIID
--PYAIHH
--PYAIHD
--PYAIGF
PQPYAI--
PIPYAI--
--PYAIYV
--PYAIYP
--PYAIWV
--PYAIWD
--PYAIVH
--PYAITW
--PYAITQ
--PYAISK
--PYAIRQ
--PYAIRC
--PYAIQH
--PYAIQF
--PYAIQD
--PYAIPS
--PYAIPI
--PYAINA
--PYAILP
--PYAILK
--PYAILI
--PYAILH
--PYAILE
--PYAIKY
--PYAIIC
--PYAIHW
--PYAIHV
--PYAIHM
--PYAIHE
--PYAIHA
--PYAIGM
--PYAIGK
--PYAIGD

--PYAIEV
--PYAIER
--PYAIEQ
--PYAIEF
--PYAIDH
--PYAICY
--PYAICT
--PYAIAW
--PYAIAQ
--PYAIAN
QLPYAI--
QWPYAI--
QEPYAI--
-QPYAIY-
-QPYAII-
QRPYAI--
-QPYAIA-
QPPYAI--
-QPYAIS-
-QPYAIM-
QDPYAI--
QYPYAI--
QTPYAI--
QVPYAI--
-QPYAIL-
-QPYAIK-
-QPYAIH-
-QPYAIE-
QMPYAI--
-QPYAIN-
QKPYAI--
QSPYAI--
-QPYAIV-
-QPYAIR-
QNPYAI--
QHPYAI--
RKPYAI--
-RPYAIS-
RGPYAI--
RTPYAI--
RLPYAI--
-RPYAIN-
-RPYAIK-
RAPYAI--
-RPYAIR-
-RPYAIH-
-RPYAIG-
RSPYAI--
RRPYAI--
-RPYAIW-

```
-RPYAIF-
RNPYAI--
RIPYAI--
-RPYAIV-
REPYAI--
-RPYAIM-
RVPYAI--
-RPYAIT-
RMPYAI--
RPPYAI--
RWPYAI--
RFPYAI--
-RPYAIQ-
-RPYAIA-
RHPYAI--
RDPYAI--
RCPYAI--
-RPYAIY-
-RPYAIE-
-RPYAID-
SSPYAI--
-SPYAIR-
SRPYAI--
SLPYAI--
SAPYAI--
SEPYAI--
SYPYAI--
SWPYAI--
SKPYAI--
-SPYAIS-
SVPYAI--
-SPYAIK-
SGPYAI--
-SPYAIM-
-SPYAII-
-SPYAIV-
SDPYAI--
SQPYAI--
-SPYAIT-
-SPYAIH-
-SPYAIG-
-SPYAIA-
SPPYAI--
SNPYAI--
SMPYAI--
SIPYAI--
SHPYAI--
STPYAI--
-SPYAIN-
-SPYAIE-
```

SFPYAI--
SCPYAI--
-SPYAIW-
TRPYAI--
-TPYAIR-
-TPYAIT-
TSPYAI--
TYPYAI--
TKPYAI--
-TPYAIQ-
TLPYAI--
TVPYAI--
-TPYAIY-
-TPYAIL-
-TPYAIK-
-TPYAIG-
THPYAI--
-TPYAIE-
TIPYAI--
TGPYAI--
-TPYAIV-
TMPYAI--
TFPYAI--
TQPYAI--
TAPYAI--
-TPYAIN-
-TPYAII-
TEPYAI--
TWPYAI--
-TPYAIW-
-TPYAIH-
TNPYAI--
-TPYAIA-
-TPYAIM-
TPPYAI--
TDPYAI--
TCPYAI--
VLPYAI--
-VPYAIQ-
VAPYAI--
VRPYAI--
VTPYAI--
-VPYAIR-
VHPYAI--
VVPYAI--
-VPYAIA-
-VPYAIS-
-VPYAIG-
VMPYAI--
VGPYAI--

VPPYAI--
VSPYAI--
-VPYAIK-
-VPYAIM-
-VPYAIT-
VKPYAI--
VEPYAI--
VNPYAI--
VQPYAI--
-VPYAIW-
-VPYAIE-
VFPYAI--
-VPYAIN-
-VPYAIH-
VIPYAI--
VYPYAI--
-VPYAIV-
VDPYAI--
VCPYAI--
WLPYAI--
WFPYAI--
-WPYAIS-
WGPYAI--
WSPYAI--
WRPYAI--
WQPYAI--
-WPYAIT-
-WPYAII-
-WPYAIM-
-WPYAIE-
WPPYAI--
WYPYAI--
WKPYAI--
WAPYAI--
WTPYAI--
-WPYAIV-
-WPYAIL-
-WPYAIF-
WNPYAI--
WHPYAI--
-WPYAIY-
-WPYAIG-
-WPYAIA-
WIPYAI--
-YPYAIT-
-YPYAIR-
-YPYAIL-
YKPYAI--
YSPYAI--
-YPYAIK-

YYPYAI--
-YPYAIS-
YAPYAI--
YQPYAI--
YHPYAI--
YMPYAI--
YLPYAI--
YTPYAI--
-YPYAII-
-YPYAIQ-
YDPYAI--
-YPYAIG-
-YPYAID-
-YPYAIC-
YIPYAI--
-YPYAIW-
-YPYAIV-
-YPYAIE-
YCPYAI--

PKWM 562 599 51.52

--PKWMKP
WKPKWM--
-APKWMA-
-APKWML-
ANPKWM--
ARPKWM--
-APKWMY-
-APKWMS-
AVPKWM--
-APKWMT-
-APKWMK-
-APKWMG-
AMPKWM--
APPKWM--
-APKWMH-
AKPKWM--
-APKWMV-
-APKWME-
AEPKWM--
ACPKWM--
ASPKWM--
AQPKWM--
-APKWMR-
-APKWMN-
-APKWMI-
-APKWMF-
AYPKWM--
AAPKWM--
-APKWMQ-
CTPKWM--

CSPKWM--
CRPKWM--
CWPKWM--
-CPKWMR-
CNPKWM--
CVPKWM--
CLPKWM--
-DPKWMS-
-DPKWMR-
DQPKWM--
DNPKWM--
DLPKWM--
DVPKWM--
DRPKWM--
-DPKWMY-
-DPKWMK-
DDPKWM--
-DPKWMN-
-DPKWML-
-DPKWMG-
DMPKWM--
DEPKWM--
DTPKWM--
DSPKWM--
-DPKWMV-
-DPKWMH-
-DPKWMF-
-EPKWMQ-
EAPKWM--
-EPKWMW-
-EPKWMI-
EFPKWM--
ETPKWM--
EQPKWM--
-EPKWMV-
-EPKWMM-
ELPKWM--
EVPKWM--
EGPKWM--
ESPKWM--
-EPKWMR-
-EPKWMG-
-EPKWMS-
-EPKWMA-
-EPKWMY-
-EPKWMT-
EDPKWM--
FTPKWM--
-FPKWMV-
FRPKWM--

-FPKWMY-
FPPKWM--
-FPKWMT-
-FPKWMA-
FGPKWM--
-FPKWMK-
-FPKWMS-
-FPKWMH-
-FPKWME-
FNPKWM--
FKPKWM--
FIPKWM--
FHPKWM--
FWPKWM--
-FPKWMI-
-FPKWMG-
FYPKWM--
-FPKWMW-
-FPKWMF-
-GPKWMT-
GQPKWM--
-GPKWMI-
-GPKWMN-
-GPKWMG-
GTPKWM--
-GPKWMQ-
-GPKWMV-
GAPKWM--
GIPKWM--
GRPKWM--
-GPKWMK-
GGPKWM--
-GPKWMF-
-GPKWMA-
GMPKWM--
-GPKWMH-
GKPKWM--
-GPKWMD-
GVPKWM--
GSPKWM--
-GPKWMM-
GNPKWM--
-HPKWMR-
HSPKWM--
HFPKWM--
-HPKWMS-
HTPKWM--
-HPKWMV-
HEPKWM--
-HPKWMK-

-HPKWMI-
HHPKWM--
HYPKWM--
HVPKWM--
HRPKWM--
HQPKWM--
-HPKWMG-
-HPKWMA-
HNPKWM--
HGPKWM--
HDPKWM--
HCPKWM--
HAPKWM--
IWPKWM--
-IPKWMR-
IKPKWM--
-IPKWMS-
-IPKWMT-
-IPKWMI-
ITPKWM--
IFPKWM--
-IPKWMG-
ISPKWM--
-IPKWMH-
IAPKWM--
-IPKWMV-
-IPKWME-
IEPKWM--
IDPKWM--
IVPKWM--
-IPKWMW-
IIPKWM--
IGPKWM--
KSPKWM--
-KPKWMT-
KLPKWM--
KMPKWM--
-KPKWMF-
KNPKWM--
-KPKWMS-
-KPKWMG-
KGPKWM--
KCPKWM--
-KPKWML-
KEPKWM--
-KPKWMK-
KFPKWM--
-KPKWMW-
-KPKWMM-
LVPKWM--

LRPKWM--
-LPKWMI-
LSPKWM--
LQPKWM--
-LPKWMS-
LHPKWM--
-LPKWMM-
-LPKWML-
-LPKWMF-
-LPKWMA-
LLPKWM--
LPPKWM--
-LPKWMV-
-LPKWMQ-
LDPKWM--
-LPKWMW-
-LPKWMT-
LIPKWM--
-LPKWMN-
LFPKWM--
LYPKWM--
LWPKWM--
LGPKWM--
-MPKWMR-
-MPKWME-
MVPKWM--
MAPKWM--
MMPKWM--
MWPKWM--
-MPKWMF-
-MPKWMK-
-MPKWMI-
MIPKWM--
MRPKWM--
MPPKWM--
-MPKWMT-
MLPKWM--
MYPKWM--
MNPKWM--
NLPKWM--
NRPKWM--
-NPKWMF-
NPPKWM--
-NPKWMY-
-NPKWMQ-
-NPKWMR-
-NPKWMV-
NVPKWM--
-NPKWML-
NTPKWM--

-NPKWMK-
-NPKWMI-
-NPKWMT-
-NPKWMA-
NGPKWM--
-NPKWMG-
NMPKWM--
-NPKWMN-
NKPKWM--
--PKWMLG
--PKWMSA
--PKWMKG
--PKWMLS
--PKWMEA
--PKWMRH
--PKWMIS
--PKWMHG
--PKWMGT
--PKWMYR
--PKWMWV
--PKWMTT
--PKWMQA
--PKWMNS
--PKWMES
--PKWMFT
--PKWMVS
--PKWMVA
--PKWMGV
--PKWMGI
--PKWMRI
--PKWMAQ
--PKWMGL
--PKWMSG
--PKWMST
--PKWMLY
--PKWMLT
--PKWMGK
--PKWMEG
--PKWMSS
--PKWMAS
PVPKWM--
--PKWMYT
--PKWMYG
--PKWMWS
--PKWMVR
--PKWMSI
--PKWMNG
--PKWMLQ
--PKWMIN
PGPKWM--

--PKWMSQ
--PKWMQR
--PKWMMW
--PKWMMV
--PKWMVH
--PKWMSK
--PKWMMN
--PKWMKA
PSPKWM--
--PKWMYS
--PKWMTW
--PKWMSY
--PKWMMH
--PKWMIR
--PKWMFW
--PKWMAK
--PKWMAG
--PKWMVN
--PKWMQH
--PKWMMI
--PKWMLH
--PKWMIV
--PKWMGS
--PKWMEQ
--PKWMAF
PKPKWM--
--PKWMSM
--PKWMQK
--PKWMKY
--PKWMKD
--PKWMGG
PNPKWM--
PLPKWM--
--PKWMYM
--PKWMWH
--PKWMVQ
--PKWMVG
--PKWMSW
--PKWMSH
--PKWMRQ
--PKWMLV
--PKWMIK
--PKWMGY
--PKWMFV
--PKWMFG
--PKWMAM
PQPKWM--
--PKWMYW
--PKWMYI
--PKWMRY

```
--PKWMMY
--PKWMGF
--PKWMCG
PHPKWM--
--PKWMYV
--PKWMWN
--PKWMTV
--PKWMTL
--PKWMTI
--PKWMTE
--PKWMRL
--PKWMRE
--PKWMQN
--PKWMQM
--PKWMNY
--PKWMIW
--PKWMIM
--PKWMHY
--PKWMHD
--PKWMHA
--PKWMAI
--PKWMAA
--PKWMVY
--PKWMVI
--PKWMVD
--PKWMTR
--PKWMTG
--PKWMSV
--PKWMSN
--PKWMSL
--PKWMSD
--PKWMRW
--PKWMQG
--PKWMMS
--PKWMMD
--PKWMIH
--PKWMGQ
--PKWMFN
--PKWMFH
--PKWMEY
--PKWMEK
--PKWMTQ
--PKWMTN
--PKWMNR
--PKWMNA
--PKWMME
--PKWMLL
--PKWMKV
--PKWMKT
--PKWMKL
```

--PKWMIG
--PKWMID
--PKWMHK
--PKWMHI
--PKWMHH
--PKWMEV
--PKWMAL
--PKWMVE
--PKWMTS
--PKWMSF
--PKWMNH
--PKWMMG
--PKWMLM
--PKWMIQ
--PKWMHT
--PKWMHR
--PKWMGA
--PKWMER
--PKWMAW
--PKWMAH
--PKWMAE
PFPKWM--
-PPKWMI-
-PPKWMG-
--PKWMYY
--PKWMYH
--PKWMWQ
--PKWMWG
--PKWMVT
--PKWMTY
--PKWMTM
--PKWMSE
--PKWMRN
--PKWMRK
--PKWMQS
--PKWMQE
--PKWMQD
--PKWMNE
--PKWMML
--PKWMLW
--PKWMLI
--PKWMLC
--PKWMKN
--PKWMKF
--PKWMKC
--PKWMIP
--PKWMIA
--PKWMHV
--PKWMGW
--PKWMFE

--PKWMEH
--PKWMEF
--PKWMCW
PDPKWM--
QSPKWM--
QMPKWM--
QAPKWM--
QNPKWM--
-QPKWMT-
QTPKWM--
-QPKWMR-
-QPKWMK-
-QPKWMF-
QLPKWM--
QEPKWM--
-QPKWMY-
-QPKWMS-
QPPKWM--
-QPKWMI-
-QPKWMV-
-QPKWMN-
-QPKWME-
QHPKWM--
QWPKWM--
QVPKWM--
-QPKWML-
-QPKWMG-
RSPKWM--
RGPKWM--
RLPKWM--
-RPKWMS-
RMPKWM--
-RPKWMI-
-RPKWMH-
RVPKWM--
-RPKWMT-
RYPKWM--
-RPKWMR-
-RPKWML-
-RPKWMF-
RAPKWM--
RNPKWM--
-RPKWMM-
-RPKWMA-
-RPKWMN-
RIPKWM--
RDPKWM--
-RPKWMY-
RRPKWM--
-RPKWMV-

RKPKWM--
RQPKWM--
RPPKWM--
-SPKWML-
-SPKWMS-
STPKWM--
-SPKWMR-
SSPKWM--
SPPKWM--
-SPKWMT-
SRPKWM--
-SPKWMI-
SKPKWM--
SVPKWM--
SYPKWM--
-SPKWMV-
SQPKWM--
-SPKWMA-
SMPKWM--
-SPKWMK-
SIPKWM--
SDPKWM--
-SPKWMY-
-SPKWMN-
SFPKWM--
SEPKWM--
SAPKWM--
-SPKWMG-
-SPKWMQ-
SLPKWM--
SCPKWM--
-SPKWMM-
-SPKWMF-
SNPKWM--
TGPKWM--
-TPKWMS-
TWPKWM--
TRPKWM--
TAPKWM--
-TPKWMI-
TYPKWM--
-TPKWMT-
-TPKWMH-
TPPKWM--
TIPKWM--
TVPKWM--
-TPKWMK-
TDPKWM--
-TPKWMQ-
-TPKWMA-

THPKWM--
-TPKWML-
-TPKWMR-
-TPKWMY-
-TPKWMV-
-TPKWMN-
-TPKWME-
-TPKWMD-
TCPKWM--
-VPKWMA-
VVPKWM--
-VPKWML-
-VPKWMY-
VEPKWM--
VSPKWM--
VQPKWM--
VRPKWM--
-VPKWMS-
VKPKWM--
-VPKWMM-
VPPKWM--
VLPKWM--
VIPKWM--
VWPKWM--
VFPKWM--
-VPKWMK-
-VPKWMT-
-VPKWMW-
-VPKWMG-
-VPKWMN-
VTPKWM--
-VPKWMQ-
VNPKWM--
-VPKWMI-
-VPKWMH-
VHPKWM--
VDPKWM--
-VPKWME-
WGPKWM--
-WPKWML-
WDPKWM--
WAPKWM--
-WPKWMW-
-WPKWMK-
WYPKWM--
WSPKWM--
-WPKWMG-
WPPKWM--
WIPKWM--
-WPKWMA-

YSPKWM--
YRPKWM--
-YPKWMI-
YFPKWM--
YAPKWM--
YNPKWM--
-YPKWMS-
YEPKWM--
-YPKWMA-
YKPKWM--
YHPKWM--
-YPKWMR-
-YPKWMF-
YPPKWM--
-YPKWMV-
YMPKWM--
YYPKWM--
-YPKWMQ-
-YPKWMN-

PMPL 33 865 51.25

--PMPLIM
-APMPLL-
-APMPLI-
AYPMPL--
AHPMPL--
ARPMPL--
AVPMPL--
AGPMPL--
ASPMPL--
AWPMPL--
-APMPLS-
-APMPLM-
ANPMPL--
ALPMPL--
-APMPLN-
ATPMPL--
-APMPLA-
AFPMPL--
-APMPLH-
AEPMPL--
-APMPLY-
-APMPLQ-
APPMPL--
-APMPLV-
-APMPLR-
AKPMPL--
-APMPLC-
AIPMPL--
-APMPLW-
-APMPLG-

-APMPLE-
AMPMPL--
CYPMPL--
CQPMPL--
CGPMPL--
CSPMPL--
CLPMPL--
-CPMPLI-
CNPMPL--
-CPMPLQ-
CTPMPL--
CRPMPL--
CEPMPL--
CVPMPL--
-CPMPLY-
-CPMPLV-
-CPMPLT-
-CPMPLS-
-CPMPLR-
-CPMPLM-
-CPMPLK-
-CPMPLA-
DLPMPL--
-DPMPLR-
-DPMPLW-
DRPMPL--
-DPMPLK-
-DPMPLH-
DSPMPL--
DIPMPL--
DQPMPL--
DVPMPL--
-DPMPLF-
DMPMPL--
DHPMPL--
-DPMPLT-
-DPMPLL-
DKPMPL--
DAPMPL--
DWPMPL--
DTPMPL--
-DPMPLY-
-DPMPLS-
-DPMPLN-
-DPMPLI-
-DPMPLV-
-DPMPLG-
-DPMPLC-
-DPMPLA-
DGPMPL--

DFPMPL--
ERPMPL--
ESPMPL--
ELPMPL--
-EPMPLY-
-EPMPLQ-
-EPMPLV-
-EPMPLW-
EYPMPL--
-EPMPLL-
-EPMPLK-
-EPMPLH-
-EPMPLF-
EIPMPL--
EEPMPL--
-EPMPLN-
EWPMPL--
-EPMPLT-
-EPMPLS-
-EPMPLM-
EGPMPL--
EFPMPL--
-EPMPLG-
EAPMPL--
EVPMPL--
EQPMPL--
EPPMPL--
EHPMPL--
-FPMPLS-
FQPMPL--
-FPMPLR-
FTPMPL--
-FPMPLL-
FYPMPL--
FGPMPL--
-FPMPLY-
-FPMPLK-
FIPMPL--
FHPMPL--
FSPMPL--
FFPMPL--
-FPMPLA-
-FPMPLT-
FVPMPL--
-FPMPLQ-
-FPMPLG-
FLPMPL--
FAPMPL--
FPPMPL--
-FPMPLH-

FNPMPL--
FWPMPL--
-FPMPLM-
FDPMPL--
-FPMPLV-
-FPMPLE-
FMPMPL--
FEPMPL--
GRPMPL--
-GPMPLR-
GLPMPL--
GKPMPL--
GVPMPL--
GMPMPL--
GSPMPL--
GFPMPL--
GHPMPL--
GAPMPL--
-GPMPLV-
-GPMPLS-
GIPMPL--
GTPMPL--
-GPMPLQ-
-GPMPLM-
GQPMPL--
GPPMPL--
-GPMPLK-
-GPMPLE-
GGPMPL--
GYPMPL--
-GPMPLN-
-GPMPLA-
-GPMPLY-
-GPMPLH-
GCPMPL--
GWPMPL--
-GPMPLW-
GNPMPL--
-GPMPLI-
GDPMPL--
-GPMPLG-
HKPMPL--
HLPMPL--
HRPMPL--
-HPMPLT-
HFPMPL--
-HPMPLR-
-HPMPLM-
-HPMPLL-
HMPMPL--

HWPMPL--
-HPMPLN-
-HPMPLS-
HIPMPL--
HTPMPL--
HPPMPL--
HVPMPL--
-HPMPLW-
-HPMPLH-
-HPMPLF-
HAPMPL--
-HPMPLG-
HSPMPL--
-HPMPLV-
-HPMPLE-
HYPMPL--
HQPMPL--
HCPMPL--
-HPMPLY-
-HPMPLA-
HNPMPL--
HHPMPL--
HEPMPL--
IVPMPL--
IRPMPL--
-IPMPLL-
IGPMPL--
-IPMPLN-
ISPMPL--
ITPMPL--
-IPMPLR-
-IPMPLA-
-IPMPLQ-
-IPMPLS-
IQPMPL--
IMPMPL--
IKPMPL--
IHPMPL--
-IPMPLF-
IFPMPL--
-IPMPLG-
ILPMPL--
IWPMPL--
-IPMPLV-
-IPMPLT-
-IPMPLI-
IPPMPL--
-IPMPLH-
IDPMPL--
IYPMPL--

-IPMPLM-
-IPMPLY-
-IPMPLE-
-IPMPLD-
ICPMPL--
-KPMPLR-
-KPMPLL-
-KPMPLA-
KPPMPL--
KYPMPL--
-KPMPLW-
KLPMPL--
KGPMPL--
KTPMPL--
KHPMPL--
-KPMPLF-
KRPMPL--
-KPMPLK-
-KPMPLT-
KIPMPL--
-KPMPLV-
KWPMPL--
-KPMPLN-
KMPMPL--
KEPMPL--
-KPMPLI-
KDPMPL--
-KPMPLY-
-KPMPLQ-
KCPMPL--
KAPMPL--
-KPMPLG-
KQPMPL--
KNPMPL--
-KPMPLE-
-KPMPLC-
LRPMPL--
LNPMPL--
LYPMPL--
LSPMPL--
LKPMPL--
-LPMPLS-
LTPMPL--
-LPMPLK-
LMPMPL--
-LPMPLT-
-LPMPLY-
-LPMPLR-
LLPMPL--
LVPMPL--

```
-LPMPLM-
-LPMPLH-
-LPMPLI-
-LPMPLG-
LFPMPL--
-LPMPLQ-
LQPMPL--
-LPMPLV-
LAPMPL--
LGPMPL--
LEPMPL--
LWPMPL--
-LPMPLE-
-LPMPLW-
-LPMPLA-
LDPMPL--
MPPMPL--
MLPMPL--
MIPMPL--
MRPMPL--
-MPMPLR-
-MPMPLS-
-MPMPLF-
MTPMPL--
-MPMPLW-
MVPMPL--
MYPMPL--
-MPMPLI-
MAPMPL--
-MPMPLY-
MQPMPL--
MKPMPL--
MEPMPL--
MNPMPL--
-MPMPLT-
-MPMPLQ-
MHPMPL--
MSPMPL--
-MPMPLV-
-MPMPLK-
-MPMPLA-
MFPMPL--
MWPMPL--
-MPMPLN-
MMPMPL--
-MPMPLH-
-MPMPLG-
MDPMPL--
-NPMPLR-
-NPMPLM-
```

NGPMPL--
NFPMPL--
NVPMPL--
NKPMPL--
NIPMPL--
-NPMPLY-
NHPMPL--
NAPMPL--
NPPMPL--
NSPMPL--
NDPMPL--
-NPMPLH-
NYPMPL--
NWPMPL--
-NPMPLV-
NMPMPL--
NNPMPL--
NTPMPL--
-NPMPLS-
-NPMPLT-
-NPMPLI-
NEPMPL--
-NPMPLN-
-NPMPLF-
-NPMPLL-
-NPMPLK-
NCPMPL--
--PMPLRS
--PMPLRV
--PMPLRL
--PMPLSK
--PMPLVY
--PMPLWL
--PMPLRN
--PMPLVR
--PMPLTT
--PMPLTS
--PMPLTI
--PMPLSR
--PMPLSI
--PMPLSG
--PMPLRA
--PMPLLN
--PMPLAV
--PMPLFR
--PMPLFH
--PMPLLI
--PMPLYG
--PMPLKL
PFPMPL--

PRPMPL--
--PMPLVK
--PMPLTL
--PMPLML
--PMPLKT
--PMPLIS
--PMPLRG
--PMPLLV
--PMPLIV
--PMPLGL
--PMPLFS
--PMPLWR
--PMPLVN
--PMPLRM
--PMPLNS
--PMPLRW
--PMPLLH
--PMPLVL
--PMPLQA
--PMPLMG
--PMPLAW
--PMPLSF
--PMPLQV
--PMPLLS
--PMPLGS
--PMPLGA
--PMPLFV
--PMPLEY
--PMPLYY
--PMPLRT
--PMPLRK
--PMPLIY
--PMPLIT
--PMPLAT
--PMPLVT
--PMPLNL
--PMPLLK
--PMPLWF
--PMPLQL
--PMPLLT
--PMPLHR
PDPMPL--
--PMPLSV
--PMPLSA
--PMPLKA
--PMPLIR
--PMPLIQ
--PMPLYT
--PMPLYR
--PMPLTR

--PMPLST
--PMPLRY
--PMPLQK
--PMPLKS
--PMPLKQ
--PMPLHL
--PMPLAF
PVPMPL--
--PMPLMR
--PMPLKR
--PMPLEF
--PMPLYA
--PMPLWA
--PMPLVS
--PMPLTY
--PMPLTH
--PMPLSM
--PMPLMV
--PMPLMN
--PMPLKH
--PMPLHQ
--PMPLAS
PYPMPL--
--PMPLVW
--PMPLSY
--PMPLSW
--PMPLRQ
--PMPLQS
--PMPLQM
--PMPLLG
--PMPLDW
PLPMPL--
--PMPLRH
--PMPLMM
--PMPLHV
--PMPLFG
--PMPLAI
PIPMPL--
PNPMPL--
--PMPLYI
--PMPLTG
--PMPLTF
--PMPLRI
--PMPLLM
--PMPLKN
--PMPLHW
--PMPLHF
--PMPLEW
--PMPLEV
--PMPLDL

--PMPLAN
PMPMPL--
--PMPLYW
--PMPLYS
--PMPLYF
--PMPLWI
--PMPLWG
--PMPLQY
--PMPLQF
--PMPLIK
--PMPLHM
--PMPLGV
--PMPLFN
--PMPLAM
--PMPLAG
--PMPLAA
PAPMPL--
--PMPLWN
--PMPLVA
--PMPLSQ
--PMPLMK
--PMPLHI
--PMPLYE
--PMPLVH
--PMPLVF
--PMPLTW
--PMPLTM
--PMPLSH
--PMPLRD
--PMPLNT
--PMPLKW
--PMPLKK
--PMPLII
--PMPLIG
--PMPLHT
--PMPLHS
--PMPLHE
--PMPLGF
--PMPLFL
--PMPLET
--PMPLDH
PGPMPL--
PEPMPL--
PWPMPL--
--PMPLYK
--PMPLQW
--PMPLQG
--PMPLNN
--PMPLMT
--PMPLMS

--PMPLKG
--PMPLIW
--PMPLGT
--PMPLYH
--PMPLWH
--PMPLVD
--PMPLTV
--PMPLQI
--PMPLPV
--PMPLPF
--PMPLNW
--PMPLNH
--PMPLNG
--PMPLMH
--PMPLLD
--PMPLKE
--PMPLHH
--PMPLHG
--PMPLFM
--PMPLAR
--PMPLYQ
--PMPLYD
--PMPLWS
--PMPLWM
--PMPLVQ
--PMPLSD
--PMPLQN
--PMPLPQ
--PMPLNK
--PMPLKF
--PMPLIH
--PMPLHN
--PMPLGN
--PMPLGG
--PMPLDV
--PMPLYN
--PMPLWY
--PMPLVG
--PMPLTK
--PMPLQR
--PMPLPS
--PMPLPM
--PMPLNV
--PMPLMF
--PMPLME
--PMPLLE
--PMPLLC
--PMPLIF
--PMPLIE
--PMPLID

--PMPLIA
--PMPLHK
--PMPLGW
--PMPLES
--PMPLVV
--PMPLVM
--PMPLVE
--PMPLTQ
--PMPLSS
--PMPLRP
--PMPLPT
--PMPLPH
--PMPLNA
--PMPLMY
--PMPLMA
--PMPLGH
--PMPLFA
--PMPLEN
--PMPLDI
--PMPLCL
--PMPLCG
PSPMPL--
PQPMPL--
--PMPLWV
--PMPLTN
--PMPLTA
--PMPLQT
--PMPLNQ
--PMPLMD
--PMPLFT
--PMPLFQ
--PMPLEQ
--PMPLEM
--PMPLEL
--PMPLEH
--PMPLDS
--PMPLDA
--PMPLAK
--PMPLAE
-PPMPLR-
--PMPLVP
--PMPLTE
--PMPLTD
--PMPLSE
--PMPLRE
--PMPLQH
--PMPLQD
--PMPLPR
--PMPLPI
--PMPLNM

```
--PMPLMQ
--PMPLMI
--PMPLKY
--PMPLKM
--PMPLHY
--PMPLHD
--PMPLGD
--PMPLFK
--PMPLER
--PMPLEK
--PMPLEI
--PMPLED
--PMPLCY
--PMPLCF
--PMPLAH
-QPMPLW-
-QPMPLT-
-QPMPLK-
QVPMPL--
-QPMPLL-
-QPMPLF-
QAPMPL--
-QPMPLA-
-QPMPLY-
QHPMPL--
QTPMPL--
QLPMPL--
QKPMPL--
-QPMPLS-
-QPMPLN-
-QPMPLI-
-QPMPLR-
QNPMPL--
QYPMPL--
-QPMPLG-
-QPMPLE-
QQPMPL--
-QPMPLV-
-QPMPLQ-
-QPMPLH-
QIPMPL--
QDPMPL--
QGPMPL--
QSPMPL--
QMPMPL--
QEPMPL--
QCPMPL--
-RPMPLL-
-RPMPLS-
-RPMPLR-
```

RVPMPL--
-RPMPLY-
RGPMPL--
-RPMPLH-
RLPMPL--
-RPMPLN-
RFPMPL--
RQPMPL--
RMPMPL--
RTPMPL--
-RPMPLF-
RNPMPL--
-RPMPLI-
RYPMPL--
RHPMPL--
-RPMPLV-
RKPMPL--
-RPMPLW-
RIPMPL--
RAPMPL--
-RPMPLT-
-RPMPLA-
-RPMPLG-
-RPMPLM-
-RPMPLK-
-RPMPLQ-
-RPMPLE-
RPPMPL--
SAPMPL--
SRPMPL--
SVPMPL--
SSPMPL--
-SPMPLS-
-SPMPLR-
SFPMPL--
-SPMPLL-
SIPMPL--
SGPMPL--
STPMPL--
-SPMPLK-
SHPMPL--
SMPMPL--
SPPMPL--
-SPMPLV-
SKPMPL--
SWPMPL--
SQPMPL--
-SPMPLT-
-SPMPLH-
-SPMPLA-

```
-SPMPLM-
-SPMPLI-
SNPMPL--
SEPMPL--
SDPMPL--
-SPMPLW-
-SPMPLE-
-SPMPLD-
-SPMPLQ-
-TPMPLR-
TRPMPL--
TIPMPL--
-TPMPLQ-
-TPMPLE-
TPPMPL--
-TPMPLI-
TLPMPL--
TVPMPL--
TSPMPL--
TAPMPL--
TYPMPL--
TKPMPL--
THPMPL--
TTPMPL--
-TPMPLY-
-TPMPLT-
TNPMPL--
TQPMPL--
-TPMPLW-
-TPMPLS-
-TPMPLN-
TFPMPL--
TDPMPL--
-TPMPLK-
-TPMPLF-
TWPMPL--
-TPMPLV-
-TPMPLM-
TEPMPL--
-TPMPLG-
TCPMPL--
-TPMPLC-
TMPMPL--
-VPMPLV-
-VPMPLW-
VRPMPL--
VKPMPL--
-VPMPLS-
-VPMPLK-
-VPMPLA-
```

VPPMPL--
VHPMPL--
VYPMPL--
VTPMPL--
VSPMPL--
-VPMPLL-
VDPMPL--
VIPMPL--
VEPMPL--
-VPMPLF-
VNPMPL--
VLPMPL--
VGPMPL--
VFPMPL--
VVPMPL--
VQPMPL--
-VPMPLY-
-VPMPLN-
-VPMPLE-
-VPMPLQ-
VMPMPL--
VAPMPL--
-VPMPLH-
VCPMPL--
-VPMPLT-
-VPMPLI-
-VPMPLM-
-VPMPLG-
WEPMPL--
WRPMPL--
WDPMPL--
-WPMPLS-
WPPMPL--
-WPMPLT-
WGPMPL--
WVPMPL--
WNPMPL--
-WPMPLM-
-WPMPLN-
WQPMPL--
WMPMPL--
WTPMPL--
WFPMPL--
-WPMPLW-
-WPMPLR-
WLPMPL--
WIPMPL--
-WPMPLH-
WYPMPL--
WSPMPL--

-WPMPLK-
-WPMPLY-
-WPMPLE-
-WPMPLI-
-WPMPLG-
-WPMPLA-
WCPMPL--
WAPMPL--
-YPMPLR-
YTPMPL--
YRPMPL--
YLPMPL--
YAPMPL--
YVPMPL--
YIPMPL--
-YPMPLN-
YEPMPL--
YWPMPL--
-YPMPLT-
YGPMPL--
YYPMPL--
YKPMPL--
YQPMPL--
-YPMPLM-
YFPMPL--
YPPMPL--
-YPMPLS-
-YPMPLF-
-YPMPLW-
YNPMPL--
YMPMPL--
-YPMPLV-
-YPMPLQ-
-YPMPLH-
-YPMPLG-

PMAM 260 717 51.18

-APMAML-
AMPMAM--
-APMAMS-
-APMAMT-
ATPMAM--
AQPMAM--
APPMAM--
-APMAMN-
-APMAMK-
ALPMAM--
AAPMAM--
ASPMAM--
-APMAMM-
AIPMAM--

−APMAMV−
−APMAME−
ARPMAM−−
−APMAMQ−
−APMAMG−
AKPMAM−−
AVPMAM−−
−APMAMW−
ANPMAM−−
AYPMAM−−
AHPMAM−−
−APMAMD−
CPPMAM−−
CTPMAM−−
CLPMAM−−
−CPMAMV−
−CPMAMR−
−CPMAMS−
CYPMAM−−
CQPMAM−−
−CPMAMQ−
−CPMAMG−
DIPMAM−−
DTPMAM−−
DRPMAM−−
DSPMAM−−
−DPMAMR−
DVPMAM−−
DQPMAM−−
DFPMAM−−
−DPMAMW−
−DPMAMS−
−DPMAMH−
−DPMAMG−
−DPMAMF−
DKPMAM−−
DDPMAM−−
−DPMAMI−
DLPMAM−−
DAPMAM−−
DYPMAM−−
−DPMAMK−
−EPMAMV−
EKPMAM−−
−EPMAMR−
−EPMAMH−
EQPMAM−−
−EPMAMQ−
−EPMAML−
−EPMAMK−

EYPMAM--
-EPMAMT-
-EPMAMA-
EPPMAM--
-EPMAMN-
-EPMAMG-
-EPMAMF-
ESPMAM--
-EPMAMY-
EMPMAM--
EGPMAM--
ETPMAM--
-EPMAMI-
-EPMAMC-
-FPMAMR-
FDPMAM--
FVPMAM--
-FPMAMN-
FTPMAM--
FAPMAM--
FSPMAM--
FWPMAM--
-FPMAMT-
-FPMAMS-
FRPMAM--
-FPMAMA-
-FPMAMY-
-FPMAMQ-
-FPMAMI-
FGPMAM--
FYPMAM--
FKPMAM--
FLPMAM--
-FPMAMK-
FNPMAM--
FIPMAM--
GKPMAM--
GVPMAM--
GSPMAM--
GTPMAM--
-GPMAMN-
-GPMAMK-
-GPMAMH-
-GPMAML-
GNPMAM--
-GPMAMV-
-GPMAMQ-
-GPMAMI-
GYPMAM--
GWPMAM--

-GPMAMY-
GIPMAM--
GRPMAM--
-GPMAMT-
GEPMAM--
GPPMAM--
-GPMAMG-
-GPMAMA-
-GPMAMD-
GGPMAM--
GFPMAM--
GLPMAM--
GHPMAM--
GQPMAM--
-GPMAMP-
-GPMAMF-
-HPMAMV-
-HPMAMS-
HNPMAM--
-HPMAMA-
HRPMAM--
-HPMAMK-
HPPMAM--
-HPMAMG-
-HPMAMR-
-HPMAMQ-
-HPMAMH-
HVPMAM--
HKPMAM--
HHPMAM--
-HPMAMW-
-HPMAMF-
HFPMAM--
-HPMAMY-
HLPMAM--
HMPMAM--
HIPMAM--
HGPMAM--
HWPMAM--
-HPMAMT-
-HPMAMM-
-IPMAMS-
-IPMAMA-
-IPMAMF-
-IPMAMV-
IMPMAM--
IKPMAM--
ITPMAM--
-IPMAML-
-IPMAMN-

```
-IPMAMQ-
-IPMAMY-
ISPMAM--
INPMAM--
IYPMAM--
IRPMAM--
IIPMAM--
IFPMAM--
IVPMAM--
-IPMAMR-
-IPMAMK-
-IPMAMG-
IGPMAM--
IDPMAM--
-IPMAMM-
-KPMAMR-
KTPMAM--
-KPMAMN-
KNPMAM--
KWPMAM--
-KPMAMI-
KGPMAM--
KVPMAM--
-KPMAMW-
-KPMAMF-
-KPMAMY-
-KPMAMT-
KYPMAM--
KSPMAM--
-KPMAMS-
KIPMAM--
-KPMAMM-
-KPMAMH-
-KPMAMD-
-LPMAMR-
LRPMAM--
LLPMAM--
-LPMAML-
LNPMAM--
-LPMAMW-
-LPMAMT-
-LPMAMS-
LKPMAM--
LIPMAM--
LYPMAM--
LVPMAM--
-LPMAMV-
-LPMAMN-
LAPMAM--
-LPMAMF-
```

LWPMAM--
LPPMAM--
-LPMAMG-
-LPMAME-
LFPMAM--
LTPMAM--
LQPMAM--
-LPMAMA-
-LPMAMY-
-LPMAMM-
-LPMAMI-
LGPMAM--
LCPMAM--
-LPMAMQ-
-LPMAMK-
LMPMAM--
LHPMAM--
MSPMAM--
-MPMAMT-
MKPMAM--
-MPMAMS-
MGPMAM--
MFPMAM--
-MPMAMN-
MVPMAM--
MRPMAM--
-MPMAML-
MQPMAM--
-MPMAMR-
-MPMAMQ-
-MPMAMK-
-MPMAMG-
MTPMAM--
-MPMAMH-
MIPMAM--
-MPMAMW-
-MPMAMV-
-MPMAMI-
-MPMAMF-
-MPMAME-
NRPMAM--
-NPMAMH-
NVPMAM--
NAPMAM--
-NPMAMS-
-NPMAMI-
-NPMAMR-
NGPMAM--
NSPMAM--
-NPMAMQ-

-NPMAML-
-NPMAMY-
-NPMAMA-
-NPMAMK-
NNPMAM--
-NPMAMW-
NMPMAM--
NTPMAM--
NLPMAM--
-NPMAMF-
NPPMAM--
NEPMAM--
--PMAMRL
--PMAMSL
--PMAMLT
--PMAMLG
--PMAMSF
--PMAMSR
--PMAMSH
--PMAMLS
--PMAMVW
PGPMAM--
--PMAMYR
--PMAMFS
--PMAMVG
--PMAMRS
--PMAMRH
--PMAMSS
--PMAMRA
--PMAMRR
--PMAMTM
--PMAMVR
--PMAMSY
--PMAMRD
--PMAMES
--PMAMVK
--PMAMLN
--PMAMGS
--PMAMTT
--PMAMYK
--PMAMTF
--PMAMSV
--PMAMSN
--PMAMSG
--PMAMHV
--PMAMST
--PMAMSQ
--PMAMLL
--PMAMKH
--PMAMHS

--PMAMPL
--PMAMGM
--PMAMEN
--PMAMAQ
--PMAMRE
--PMAMQR
--PMAMNF
--PMAMLM
--PMAMLE
--PMAMLA
--PMAMVY
--PMAMKG
--PMAMIR
--PMAMAV
--PMAMVQ
--PMAMRY
--PMAMNL
--PMAMKL
--PMAMIT
--PMAMIG
--PMAMAA
--PMAMWT
--PMAMWL
--PMAMVS
--PMAMTS
--PMAMTR
--PMAMKS
--PMAMHL
--PMAMFR
-PPMAMA-
--PMAMTW
--PMAMQS
--PMAMNN
--PMAMMV
--PMAMMR
--PMAMKM
--PMAMHR
PAPMAM--
--PMAMWR
--PMAMVT
--PMAMTL
--PMAMNI
--PMAMML
--PMAMMH
--PMAMLD
--PMAMKD
--PMAMIA
--PMAMHF
--PMAMET
--PMAMAS

PHPMAM--
--PMAMYS
--PMAMVL
--PMAMVD
--PMAMRI
--PMAMQT
--PMAMQG
--PMAMMW
--PMAMKY
--PMAMIQ
--PMAMYT
--PMAMWQ
--PMAMVN
--PMAMSI
--PMAMRP
--PMAMQY
--PMAMNK
--PMAMIM
--PMAMHE
--PMAMGV
--PMAMEL
--PMAMAK
PYPMAM--
--PMAMWK
--PMAMVI
--PMAMSE
--PMAMRV
--PMAMRN
--PMAMQL
--PMAMQH
--PMAMPI
--PMAMMI
--PMAMMG
--PMAMLR
--PMAMKN
--PMAMIF
--PMAMGQ
--PMAMGL
--PMAMFG
--PMAMEF
--PMAMAH
--PMAMAG
PWPMAM--
--PMAMWD
--PMAMSK
--PMAMNW
--PMAMMQ
--PMAMLV
--PMAMLH
--PMAMKR

--PMAMKA
--PMAMGF
--PMAMFE
PIPMAM--
PNPMAM--
--PMAMYA
--PMAMTQ
--PMAMTK
--PMAMSW
--PMAMSD
--PMAMRF
--PMAMQK
--PMAMNR
--PMAMNA
--PMAMKT
--PMAMIV
--PMAMHK
--PMAMYE
--PMAMWW
--PMAMWS
--PMAMWG
--PMAMWA
--PMAMVM
--PMAMVH
--PMAMTD
--PMAMSM
--PMAMRT
--PMAMRM
--PMAMNE
--PMAMMN
--PMAMLI
--PMAMKE
--PMAMIS
--PMAMIN
--PMAMII
--PMAMGY
--PMAMGW
--PMAMGN
--PMAMGG
--PMAMFQ
--PMAMFL
--PMAMEI
--PMAMDT
--PMAMCT
--PMAMAR
--PMAMYG
--PMAMWN
--PMAMWH
--PMAMVA
--PMAMTV

--PMAMTI
--PMAMTH
--PMAMTE
--PMAMQN
--PMAMQA
--PMAMNY
--PMAMNT
--PMAMNS
--PMAMNH
--PMAMGR
--PMAMFP
--PMAMEY
--PMAMEQ
--PMAMDY
--PMAMCA
PFPMAM--
--PMAMYV
--PMAMYH
--PMAMWV
--PMAMWI
--PMAMWE
--PMAMTY
--PMAMTG
--PMAMTA
--PMAMSC
--PMAMSA
--PMAMRW
--PMAMQM
--PMAMPY
--PMAMNG
--PMAMMK
--PMAMMA
--PMAMKW
--PMAMKV
--PMAMKQ
--PMAMKI
--PMAMIP
--PMAMHT
--PMAMHI
--PMAMGH
--PMAMGA
--PMAMFN
--PMAMEV
--PMAMEK
--PMAMEA
--PMAMDM
--PMAMDL
--PMAMDG
--PMAMCL
--PMAMAE

PKPMAM--
PDPMAM--
-QPMAMR-
QTPMAM--
-QPMAMQ-
-QPMAMS-
QWPMAM--
QSPMAM--
QNPMAM--
QLPMAM--
QQPMAM--
QKPMAM--
-QPMAML-
-QPMAMN-
-QPMAMH-
-QPMAMI-
-QPMAMG-
QGPMAM--
QYPMAM--
QPPMAM--
-QPMAMV-
-QPMAMT-
-QPMAME-
QRPMAM--
-QPMAMD-
-QPMAMA-
QFPMAM--
-QPMAMM-
-QPMAMF-
-RPMAML-
RQPMAM--
-RPMAMK-
-RPMAMS-
RYPMAM--
RTPMAM--
RVPMAM--
-RPMAMF-
RGPMAM--
-RPMAMY-
-RPMAMM-
-RPMAMH-
-RPMAMV-
-RPMAMI-
-RPMAMA-
-RPMAMQ-
-RPMAMW-
-RPMAMT-
RIPMAM--
RFPMAM--
RSPMAM--

-RPMAMN-
RWPMAM--
-RPMAMR-
REPMAM--
RAPMAM--
RPPMAM--
RHPMAM--
RKPMAM--
RDPMAM--
-RPMAME-
-RPMAMD-
RMPMAM--
-SPMAML-
SVPMAM--
-SPMAMY-
SNPMAM--
-SPMAMR-
-SPMAMK-
SRPMAM--
-SPMAMH-
SIPMAM--
-SPMAMV-
STPMAM--
SGPMAM--
SSPMAM--
SPPMAM--
-SPMAMI-
-SPMAMA-
-SPMAMN-
SAPMAM--
SMPMAM--
SFPMAM--
-SPMAMG-
-SPMAMW-
-SPMAMT-
-SPMAMF-
SCPMAM--
-SPMAMM-
SQPMAM--
-SPMAMQ-
-SPMAMD-
SLPMAM--
SDPMAM--
TSPMAM--
TGPMAM--
-TPMAML-
-TPMAMS-
TVPMAM--
TTPMAM--
TNPMAM--

-TPMAMI-
-TPMAMV-
TFPMAM--
TYPMAM--
TQPMAM--
-TPMAMW-
-TPMAMR-
TRPMAM--
-TPMAMN-
-TPMAMT-
TMPMAM--
TAPMAM--
-TPMAMY-
-TPMAMQ-
TIPMAM--
TPPMAM--
THPMAM--
-TPMAMK-
-TPMAMG-
-TPMAME-
TLPMAM--
-VPMAMY-
-VPMAMR-
-VPMAMK-
-VPMAMS-
VRPMAM--
-VPMAMG-
VSPMAM--
-VPMAMT-
-VPMAMI-
VMPMAM--
VEPMAM--
-VPMAMW-
-VPMAML-
VWPMAM--
-VPMAMH-
VGPMAM--
-VPMAMV-
VNPMAM--
VAPMAM--
VKPMAM--
VHPMAM--
VTPMAM--
-VPMAMQ-
VFPMAM--
-VPMAMF-
-VPMAMD-
VCPMAM--
-VPMAMM-
-VPMAME-

```
-VPMAMA-
WTPMAM--
WNPMAM--
WLPMAM--
WHPMAM--
WRPMAM--
-WPMAMW-
WIPMAM--
WPPMAM--
-WPMAMS-
-WPMAMQ-
-WPMAMN-
WSPMAM--
-WPMAMR-
-WPMAML-
-WPMAMG-
-WPMAMT-
-WPMAMM-
-WPMAME-
-WPMAMD-
-WPMAMK-
-WPMAMA-
WKPMAM--
-YPMAMY-
-YPMAMI-
-YPMAMK-
YTPMAM--
YPPMAM--
YWPMAM--
-YPMAML-
YYPMAM--
-YPMAMS-
-YPMAMT-
YKPMAM--
YEPMAM--
YDPMAM--
-YPMAME-
-YPMAMA-
YQPMAM--
-YPMAMF-
YNPMAM--
YLPMAM--
YHPMAM--
YRPMAM--
-YPMAMV-
-YPMAMH-
YIPMAM--
YFPMAM--
YAPMAM--
-YPMAMR-
```

	-YPMAMP-		
	-YPMAMG-		
	YGPMAM--		
PFAI	256	720	51.12
	AGPFAI--		
	APPFAI--		
	ARPFAI--		
	-APFAIY-		
	-APFAIE-		
	AIPFAI--		
	AEPFAI--		
	AAPFAI--		
	AWPFAI--		
	ATPFAI--		
	-APFAIW-		
	-APFAIK-		
	-APFAIA-		
	ASPFAI--		
	AQPFAI--		
	-APFAIR-		
	-APFAIT-		
	AKPFAI--		
	AYPFAI--		
	-APFAIG-		
	ANPFAI--		
	-APFAIS-		
	-APFAII-		
	AHPFAI--		
	-APFAIV-		
	-APFAIQ-		
	-APFAIN-		
	-APFAIF-		
	AMPFAI--		
	AFPFAI--		
	ADPFAI--		
	CRPFAI--		
	-CPFAIR-		
	CWPFAI--		
	CTPFAI--		
	CPPFAI--		
	-CPFAIT-		
	CNPFAI--		
	CFPFAI--		
	CVPFAI--		
	CSPFAI--		
	-CPFAIS-		
	CGPFAI--		
	CDPFAI--		
	DRPFAI--		
	-DPFAIS-		

DHPFAI--
DLPFAI--
DTPFAI--
DAPFAI--
DFPFAI--
DYPFAI--
DVPFAI--
-DPFAIQ-
DWPFAI--
-DPFAIH-
-DPFAIV-
-DPFAIR-
DGPFAI--
DSPFAI--
DQPFAI--
-DPFAIW-
-DPFAIT-
-DPFAIM-
-DPFAIL-
-EPFAIR-
ERPFAI--
EWPFAI--
-EPFAIL-
EKPFAI--
EYPFAI--
EVPFAI--
ESPFAI--
ENPFAI--
EMPFAI--
-EPFAIV-
-EPFAIN-
-EPFAIH-
-EPFAIS-
EFPFAI--
-EPFAIK-
EHPFAI--
EGPFAI--
-EPFAIT-
EQPFAI--
-EPFAIY-
EAPFAI--
FRPFAI--
-FPFAII-
FKPFAI--
FSPFAI--
FYPFAI--
-FPFAIH-
FEPFAI--
FDPFAI--
-FPFAIR-

FLPFAI--
-FPFAIV-
-FPFAIQ-
FNPFAI--
FVPFAI--
-FPFAIS-
FAPFAI--
FTPFAI--
-FPFAIT-
FPPFAI--
-FPFAIE-
FIPFAI--
FGPFAI--
FFPFAI--
-GPFAIH-
GRPFAI--
-GPFAIR-
GYPFAI--
GWPFAI--
-GPFAIQ-
GTPFAI--
GQPFAI--
GKPFAI--
GHPFAI--
-GPFAIS-
-GPFAIV-
GIPFAI--
-GPFAIY-
-GPFAII-
GMPFAI--
GVPFAI--
-GPFAIA-
GSPFAI--
-GPFAIE-
GNPFAI--
GGPFAI--
-GPFAIT-
GFPFAI--
-GPFAIW-
-GPFAIN-
GEPFAI--
GDPFAI--
GAPFAI--
-HPFAIT-
HVPFAI--
HSPFAI--
HFPFAI--
-HPFAIS-
HEPFAI--
HAPFAI--

-HPFAIW-
HYPFAI--
HRPFAI--
HGPFAI--
-HPFAIF-
-HPFAIN-
HIPFAI--
-HPFAII-
HNPFAI--
HMPFAI--
HWPFAI--
HLPFAI--
HKPFAI--
HTPFAI--
-HPFAIA-
HHPFAI--
-HPFAIK-
HPPFAI--
-HPFAIL-
-HPFAIG-
IAPFAI--
-IPFAIR-
-IPFAIS-
-IPFAIA-
ITPFAI--
IKPFAI--
IHPFAI--
IYPFAI--
-IPFAIW-
IGPFAI--
-IPFAIN-
-IPFAIQ-
INPFAI--
IMPFAI--
IIPFAI--
-IPFAIV-
ILPFAI--
IEPFAI--
ICPFAI--
IWPFAI--
-IPFAIT-
-IPFAIH-
-IPFAIF-
IQPFAI--
-IPFAIG-
-IPFAIE-
IFPFAI--
IDPFAI--
KSPFAI--
-KPFAIV-

-KPFAIS-
-KPFAIL-
-KPFAIT-
KYPFAI--
KVPFAI--
-KPFAIK-
KRPFAI--
KNPFAI--
KAPFAI--
KIPFAI--
KEPFAI--
-KPFAIH-
-KPFAIR-
KKPFAI--
-KPFAIG-
-KPFAIA-
-KPFAIE-
-KPFAIQ-
KHPFAI--
KWPFAI--
KTPFAI--
KQPFAI--
-KPFAIY-
LRPFAI--
LAPFAI--
LVPFAI--
LYPFAI--
LEPFAI--
-LPFAIR-
LLPFAI--
-LPFAIY-
LGPFAI--
LTPFAI--
-LPFAIS-
LNPFAI--
LFPFAI--
-LPFAIT-
-LPFAII-
LKPFAI--
LDPFAI--
LMPFAI--
LWPFAI--
LQPFAI--
LPPFAI--
-LPFAIV-
-LPFAIW-
-LPFAIM-
-LPFAIQ-
-LPFAIK-
LSPFAI--

```
-LPFAIE-
LHPFAI--
MRPFAI--
MSPFAI--
-MPFAIK-
-MPFAIS-
-MPFAIV-
-MPFAIT-
MVPFAI--
MPPFAI--
-MPFAIM-
MLPFAI--
MWPFAI--
-MPFAIW-
MAPFAI--
MFPFAI--
-MPFAIH-
MMPFAI--
MNPFAI--
MKPFAI--
MGPFAI--
MEPFAI--
-MPFAIG-
-MPFAIA-
MQPFAI--
-MPFAII-
MDPFAI--
-NPFAIR-
NSPFAI--
NLPFAI--
NNPFAI--
NMPFAI--
-NPFAIW-
NWPFAI--
NGPFAI--
-NPFAIM-
NIPFAI--
-NPFAIS-
-NPFAIH-
NFPFAI--
-NPFAIQ-
-NPFAII-
NTPFAI--
NEPFAI--
-NPFAIA-
NYPFAI--
-NPFAIT-
NRPFAI--
NPPFAI--
--PFAIRS
```

--PFAILN
--PFAIRE
PAPFAI--
--PFAILS
--PFAIKT
--PFAITQ
--PFAISV
--PFAIWS
--PFAISS
--PFAIVA
--PFAILK
--PFAIGS
--PFAIGG
PGPFAI--
--PFAIIR
--PFAIFQ
--PFAIVT
--PFAILG
--PFAIKR
--PFAIGL
PSPFAI--
--PFAISG
--PFAIRL
--PFAIRA
--PFAIKY
--PFAIKG
--PFAIAG
--PFAIVH
--PFAISR
PNPFAI--
--PFAIVL
--PFAISQ
--PFAIKM
--PFAIRV
--PFAILD
--PFAIKN
--PFAISH
--PFAIRY
--PFAIRR
--PFAIKL
--PFAIIG
--PFAIYQ
--PFAISY
--PFAIFK
--PFAIAV
PEPFAI--
PQPFAI--
--PFAIVN
--PFAITH
--PFAISI

--PFAIRQ
PTPFAI--
--PFAITN
--PFAIIH
--PFAIFR
--PFAIWK
--PFAIVS
--PFAITR
--PFAISL
--PFAIRK
--PFAIQA
--PFAIMR
--PFAIYT
--PFAIVQ
--PFAISE
--PFAIRH
--PFAINK
--PFAIMN
--PFAIMA
--PFAIGV
--PFAIFS
--PFAIFH
--PFAIAT
--PFAIAA
--PFAIYK
--PFAIYA
--PFAIVK
--PFAINV
--PFAILQ
--PFAIKS
--PFAIIQ
--PFAIWV
--PFAIWH
--PFAITI
--PFAITG
--PFAISW
--PFAIST
--PFAISA
--PFAIRN
--PFAIRM
--PFAIQV
--PFAIMV
--PFAIMD
--PFAIHL
--PFAIGA
--PFAIFT
--PFAIVV
--PFAITS
--PFAIMS
--PFAILY

--PFAILE
--PFAIID
--PFAIHM
--PFAIGM
--PFAIGF
--PFAIFW
--PFAIAY
--PFAIAN
PDPFAI--
PVPFAI--
--PFAIWD
--PFAIVR
--PFAIQF
--PFAIQE
--PFAINT
--PFAIMW
--PFAILR
--PFAIFG
--PFAIAQ
--PFAIYS
--PFAIWY
--PFAISM
--PFAIQM
--PFAING
--PFAILW
--PFAIKQ
--PFAIKA
--PFAIIK
--PFAIIA
--PFAIHE
--PFAIGW
--PFAIGT
--PFAIGK
--PFAIFA
--PFAIEG
PIPFAI--
--PFAIYR
--PFAIYD
--PFAIWE
--PFAIVF
--PFAITW
--PFAITL
--PFAISK
--PFAIRT
--PFAIQT
--PFAIQG
--PFAIPY
--PFAIMT
--PFAIHD
--PFAIFE

```
--PFAIAS
--PFAIYP
--PFAIWW
--PFAIWR
--PFAITE
--PFAIRP
--PFAIQS
--PFAIQI
--PFAIPI
--PFAINY
--PFAINS
--PFAIMH
--PFAIKV
--PFAIIP
--PFAIHT
--PFAIHR
--PFAIHA
--PFAIFY
--PFAIEY
--PFAIER
--PFAIAH
-PPFAIA-
PMPFAI--
--PFAITY
--PFAIQH
--PFAIPV
--PFAIPF
--PFAINH
--PFAIMK
--PFAIMG
--PFAIKF
--PFAIIT
--PFAIIS
--PFAIIN
--PFAIHW
--PFAIHN
--PFAIHK
--PFAIHI
--PFAIGY
--PFAIGI
--PFAIGH
--PFAIFV
--PFAIEL
--PFAIEF
--PFAIAR
PKPFAI--
--PFAIWT
--PFAIWI
--PFAITD
--PFAITC
```

```
--PFAIRD
--PFAIQY
--PFAIQW
--PFAIQK
--PFAIQD
--PFAINF
--PFAIND
--PFAINA
--PFAIKI
--PFAIKE
--PFAIIE
--PFAIHS
--PFAIHP
--PFAIHH
--PFAIET
--PFAIEQ
--PFAIEN
--PFAIEK
--PFAIDL
--PFAIDG
--PFAICV
--PFAICS
--PFAICI
--PFAIAF
QHPFAI--
QKPFAI--
-QPFAIM-
-QPFAII-
-QPFAIH-
QVPFAI--
QRPFAI--
QAPFAI--
QTPFAI--
-QPFAIS-
-QPFAIN-
QLPFAI--
QSPFAI--
-QPFAIR-
-QPFAIQ-
QNPFAI--
QIPFAI--
-QPFAIK-
QFPFAI--
QQPFAI--
QEPFAI--
-QPFAIF-
-QPFAIE-
-QPFAIT-
RSPFAI--
-RPFAIT-
```

RLPFAI--
-RPFAIR-
-RPFAIQ-
-RPFAIH-
RGPFAI--
-RPFAIY-
-RPFAIG-
-RPFAIA-
RRPFAI--
-RPFAIV-
RMPFAI--
RPPFAI--
RAPFAI--
-RPFAIS-
RQPFAI--
-RPFAIN-
RTPFAI--
-RPFAIM-
RFPFAI--
RDPFAI--
-RPFAIW-
-RPFAIK-
RVPFAI--
RYPFAI--
RIPFAI--
RNPFAI--
REPFAI--
-RPFAIE-
-RPFAII-
RCPFAI--
SYPFAI--
SLPFAI--
-SPFAIR-
SVPFAI--
-SPFAIV-
SRPFAI--
SSPFAI--
-SPFAIK-
SFPFAI--
-SPFAIA-
SGPFAI--
SAPFAI--
SEPFAI--
-SPFAIT-
SNPFAI--
-SPFAIY-
SKPFAI--
SIPFAI--
SWPFAI--
-SPFAIQ-

```
-SPFAIN-
SPPFAI--
-SPFAIM-
SHPFAI--
SMPFAI--
SQPFAI--
-SPFAIE-
-SPFAIW-
-SPFAIH-
-SPFAIG-
SCPFAI--
STPFAI--
TSPFAI--
-TPFAIR-
TQPFAI--
TEPFAI--
TGPFAI--
TVPFAI--
-TPFAIS-
TMPFAI--
TKPFAI--
TIPFAI--
THPFAI--
-TPFAIY-
TNPFAI--
TLPFAI--
TTPFAI--
-TPFAIT-
TAPFAI--
-TPFAII-
-TPFAIA-
TFPFAI--
-TPFAIK-
-TPFAIQ-
TPPFAI--
-TPFAIW-
-TPFAIM-
-TPFAIV-
-TPFAIH-
TDPFAI--
-TPFAIC-
-TPFAIE-
TCPFAI--
VFPFAI--
VSPFAI--
VHPFAI--
VVPFAI--
VRPFAI--
VKPFAI--
-VPFAIK-
```

-VPFAIG-
VGPFAI--
VTPFAI--
-VPFAIH-
-VPFAIS-
-VPFAIR-
-VPFAIT-
VWPFAI--
-VPFAIL-
VPPFAI--
-VPFAIQ-
VEPFAI--
VYPFAI--
-VPFAIV-
VLPFAI--
VIPFAI--
VAPFAI--
-VPFAII-
-VPFAIY-
-VPFAIW-
-VPFAIA-
VMPFAI--
-VPFAIE-
-VPFAIM-
-VPFAIC-
VDPFAI--
WKPFAI--
WAPFAI--
WRPFAI--
WVPFAI--
-WPFAIR-
WPPFAI--
WIPFAI--
WGPFAI--
-WPFAIQ-
WSPFAI--
-WPFAIA-
WMPFAI--
WLPFAI--
WHPFAI--
WQPFAI--
-WPFAIT-
-WPFAIS-
-WPFAIG-
-WPFAIV-
-WPFAIM-
-WPFAII-
-WPFAIE-
WDPFAI--
-YPFAIQ-

-YPFAIR-
-YPFAIT-
-YPFAIM-
-YPFAII-
YVPFAI--
YRPFAI--
YPPFAI--
-YPFAIS-
YWPFAI--
-YPFAIW-
-YPFAIV-
YMPFAI--
YSPFAI--
YDPFAI--
-YPFAIN-
-YPFAIK-
YNPFAI--
YHPFAI--
YAPFAI--
-YPFAIA-
YGPFAI--
YTPFAI--
YQPFAI--
YLPFAI--

PMAI	167	765	50.93

DTPMAI--
-LPMAIS-
ANPMAI--
AAPMAI--
-APMAIW-
AWPMAI--
-APMAIY-
ARPMAI--
-APMAIR-
-APMAIM-
ALPMAI--
-APMAIA-
AKPMAI--
-APMAII-
ADPMAI--
ATPMAI--
AFPMAI--
AEPMAI--
AYPMAI--
-APMAIS-
-APMAIL-
-APMAIT-
-APMAIK-
-APMAIF-
-APMAIE-

AIPMAI--
AVPMAI--
ASPMAI--
-APMAIV-
-APMAIH-
AHPMAI--
AGPMAI--
-APMAIN-
-APMAIG-
CVPMAI--
-CPMAIR-
-CPMAIH-
-CPMAIV-
CGPMAI--
CRPMAI--
-CPMAIK-
CKPMAI--
-CPMAIY-
-CPMAIS-
-CPMAIM-
-CPMAIL-
CMPMAI--
CFPMAI--
CDPMAI--
DWPMAI--
-DPMAIF-
-DPMAIS-
DVPMAI--
-DPMAIY-
-DPMAIL-
DIPMAI--
DCPMAI--
-DPMAIK-
DMPMAI--
DRPMAI--
-DPMAIV-
-DPMAIR-
DKPMAI--
DSPMAI--
DHPMAI--
DAPMAI--
DYPMAI--
-DPMAIW-
-DPMAIT-
-DPMAII-
DGPMAI--
DEPMAI--
ELPMAI--
-EPMAIY-
-EPMAIK-

```
-EPMAIR-
ESPMAI--
EKPMAI--
-EPMAIM-
ERPMAI--
EYPMAI--
-EPMAIT-
EWPMAI--
EMPMAI--
-EPMAIH-
-EPMAIF-
EVPMAI--
ETPMAI--
-EPMAIV-
-EPMAID-
-EPMAIA-
EGPMAI--
ECPMAI--
FRPMAI--
-FPMAIT-
FPPMAI--
-FPMAIQ-
FHPMAI--
FSPMAI--
FLPMAI--
-FPMAIH-
-FPMAIS-
-FPMAIR-
FAPMAI--
FTPMAI--
FFPMAI--
FNPMAI--
FIPMAI--
FCPMAI--
-FPMAIV-
FYPMAI--
-FPMAIW-
-FPMAIG-
-FPMAIE-
GRPMAI--
-GPMAIL-
GAPMAI--
GSPMAI--
-GPMAIR-
GLPMAI--
GVPMAI--
GEPMAI--
GWPMAI--
-GPMAIA-
GTPMAI--
```

GPPMAI--
GNPMAI--
GDPMAI--
-GPMAIV-
-GPMAIG-
GGPMAI--
GHPMAI--
GIPMAI--
GQPMAI--
GFPMAI--
-GPMAIY-
-GPMAIN-
-GPMAIK-
-GPMAIH-
-GPMAIF-
GYPMAI--
-GPMAIW-
GMPMAI--
GCPMAI--
HRPMAI--
-HPMAIL-
HLPMAI--
HVPMAI--
HEPMAI--
-HPMAIV-
-HPMAIR-
HAPMAI--
-HPMAII-
-HPMAIY-
HKPMAI--
-HPMAIN-
HMPMAI--
HTPMAI--
-HPMAIS-
-HPMAIM-
HFPMAI--
HSPMAI--
-HPMAIT-
-HPMAIF-
-HPMAIA-
HGPMAI--
HCPMAI--
INPMAI--
IRPMAI--
IKPMAI--
IQPMAI--
-IPMAIQ-
-IPMAIL-
-IPMAIV-
-IPMAIH-

IYPMAI--
IWPMAI--
ISPMAI--
IIPMAI--
ITPMAI--
-IPMAIS-
-IPMAIM-
ILPMAI--
-IPMAIT-
-IPMAIR-
IAPMAI--
IHPMAI--
IGPMAI--
-IPMAIN-
-IPMAIA-
IMPMAI--
IVPMAI--
-IPMAIG-
-IPMAII-
-IPMAIF-
-IPMAIE-
IDPMAI--
IEPMAI--
-KPMAIS-
-KPMAIL-
-KPMAIM-
KLPMAI--
KMPMAI--
KRPMAI--
KTPMAI--
KQPMAI--
-KPMAIV-
KDPMAI--
KVPMAI--
KYPMAI--
-KPMAIW-
-KPMAIG-
KIPMAI--
KPPMAI--
-KPMAIY-
-KPMAIT-
-KPMAIN-
-KPMAII-
KEPMAI--
-KPMAIR-
-KPMAIF-
KHPMAI--
KGPMAI--
KCPMAI--
KAPMAI--

```
-KPMAIH-
-KPMAIA-
KWPMAI--
-KPMAIE-
-KPMAID-
KFPMAI--
-LPMAIR-
LGPMAI--
LVPMAI--
LNPMAI--
LIPMAI--
LDPMAI--
-LPMAIK-
-LPMAII-
LLPMAI--
-LPMAIM-
LPPMAI--
-LPMAIA-
LFPMAI--
LSPMAI--
LQPMAI--
-LPMAIT-
LWPMAI--
LEPMAI--
-LPMAIV-
-LPMAIH-
-LPMAIQ-
LYPMAI--
-LPMAIE-
-LPMAIY-
-LPMAIN-
LHPMAI--
-LPMAIW-
-LPMAIG-
-LPMAIP-
MTPMAI--
MSPMAI--
MYPMAI--
-MPMAIM-
-MPMAIL-
-MPMAIH-
MKPMAI--
-MPMAIT-
-MPMAIQ-
MLPMAI--
MQPMAI--
MGPMAI--
-MPMAIK-
MVPMAI--
-MPMAIN-
```

MHPMAI--
-MPMAIS-
MIPMAI--
-MPMAIR-
-MPMAIE-
MNPMAI--
MMPMAI--
-MPMAIV-
-MPMAII-
MWPMAI--
MPPMAI--
MAPMAI--
-NPMAII-
NRPMAI--
NYPMAI--
NVPMAI--
-NPMAIR-
NMPMAI--
NHPMAI--
NTPMAI--
NSPMAI--
NGPMAI--
-NPMAIY-
NIPMAI--
NEPMAI--
NPPMAI--
-NPMAIS-
NLPMAI--
-NPMAIH-
NDPMAI--
NAPMAI--
-NPMAIF-
-NPMAIA-
-NPMAIQ-
-NPMAIN-
NKPMAI--
-NPMAIW-
-NPMAIV-
-NPMAIM-
-NPMAIK-
NNPMAI--
--PMAISS
--PMAIGL
--PMAILR
--PMAISR
--PMAIRI
--PMAIMK
--PMAILS
--PMAIGI
--PMAILK

--PMAIVK
--PMAINR
--PMAILT
--PMAIKI
--PMAIGS
--PMAIRH
--PMAILG
--PMAIKG
--PMAIAG
--PMAIFR
--PMAITK
--PMAINL
--PMAILH
--PMAIWR
--PMAISY
--PMAIKA
--PMAIGY
--PMAIAT
--PMAIRF
--PMAIQR
--PMAILY
--PMAILN
--PMAIAR
--PMAIVN
--PMAISV
--PMAISG
--PMAIRA
--PMAIYT
--PMAIRG
--PMAIWS
--PMAIGF
--PMAIEG
--PMAIAV
--PMAIYA
--PMAIWG
--PMAIVS
--PMAISK
--PMAIRT
--PMAIQT
--PMAIIN
--PMAIIG
--PMAIFT
--PMAIFS
--PMAIYH
--PMAIVT
--PMAITA
--PMAIRN
--PMAIRK
--PMAIQS
--PMAIHW

PNPMAI--
--PMAIYV
--PMAIYS
--PMAIRV
--PMAIQM
--PMAIKS
--PMAIKR
--PMAIIV
PGPMAI--
--PMAITG
--PMAIMR
--PMAIMH
--PMAIKL
--PMAIII
--PMAIFQ
--PMAIAY
--PMAIAH
--PMAIYG
--PMAIQG
--PMAINM
--PMAIIS
--PMAIIL
--PMAIHS
--PMAIHN
--PMAIHD
--PMAITN
--PMAIRM
--PMAIIK
--PMAIWT
--PMAIWK
--PMAIVV
--PMAIVQ
--PMAIVG
--PMAIVA
--PMAISA
--PMAIQN
--PMAIML
--PMAIMA
--PMAIGM
--PMAIAS
PIPMAI--
--PMAIWN
--PMAITV
--PMAIRY
--PMAIRE
--PMAIPL
--PMAINQ
--PMAINF
--PMAIMT
--PMAIKF

--PMAIKE
--PMAIHR
--PMAIFK
--PMAIFF
--PMAIET
--PMAIER
PQPMAI--
--PMAIYM
--PMAIVH
--PMAITR
--PMAITQ
--PMAITL
--PMAITI
--PMAIQV
--PMAINY
--PMAIMI
--PMAILQ
--PMAILA
--PMAIKT
--PMAIKK
--PMAIIQ
--PMAIFN
--PMAIAI
PHPMAI--
--PMAIWY
--PMAIWQ
--PMAIVW
--PMAIVF
--PMAIVD
--PMAIST
--PMAIQL
--PMAIIW
--PMAIIF
--PMAIHL
--PMAIGV
--PMAIGH
--PMAIES
PYPMAI--
--PMAIYY
--PMAIYP
--PMAIWD
--PMAITS
--PMAISW
--PMAISD
--PMAIRQ
--PMAIRD
--PMAIQW
--PMAIKQ
--PMAIKH
--PMAIID

--PMAIFG
--PMAIEW
PAPMAI--
PTPMAI--
--PMAIYD
--PMAIVE
--PMAISM
--PMAIRP
--PMAIPY
--PMAIPF
--PMAINS
--PMAIMY
--PMAIMN
--PMAIME
--PMAIMD
--PMAIKV
--PMAIIE
--PMAIHK
--PMAIHH
--PMAIEN
--PMAIEH
--PMAIAW
--PMAIYQ
--PMAIYK
--PMAIWV
--PMAIVY
--PMAIVI
--PMAITE
--PMAISN
--PMAISH
--PMAISE
--PMAIQY
--PMAIQK
--PMAIQH
--PMAIPI
--PMAIMQ
--PMAILE
--PMAILD
--PMAIIY
--PMAIHT
--PMAIFD
--PMAIEY
--PMAIEA
--PMAIAF
PDPMAI--
PWPMAI--
-PPMAIY-
--PMAIWW
--PMAIVR
--PMAITM

--PMAITD
--PMAISQ
--PMAISF
--PMAIQQ
--PMAIQI
--PMAIQF
--PMAIQC
--PMAINK
--PMAINH
--PMAINA
--PMAIMV
--PMAILW
--PMAIKY
--PMAIKM
--PMAIIT
--PMAIIA
--PMAIHM
--PMAIHI
--PMAIHG
--PMAIHA
--PMAIGR
--PMAIGK
--PMAIFH
--PMAIFA
--PMAIEF
--PMAIDS
--PMAIDM
--PMAIDG
--PMAICV
--PMAICN
--PMAICI
--PMAIAQ
--PMAIAM
--PMAIAK
PEPMAI--
PCPMAI--
QTPMAI--
QIPMAI--
-QPMAIR-
-QPMAIH-
QVPMAI--
QSPMAI--
QRPMAI--
QKPMAI--
QHPMAI--
QGPMAI--
-QPMAIT-
-QPMAIG-
-QPMAIF-
QDPMAI--

-QPMAII-
-QPMAIY-
QLPMAI--
-QPMAIE-
-QPMAIA-
-QPMAIS-
-QPMAIL-
-QPMAIK-
QMPMAI--
QFPMAI--
RSPMAI--
RVPMAI--
-RPMAIL-
RPPMAI--
-RPMAIT-
-RPMAIS-
RQPMAI--
-RPMAIR-
-RPMAIQ-
RTPMAI--
-RPMAIM-
RFPMAI--
RWPMAI--
-RPMAII-
RAPMAI--
-RPMAIA-
RHPMAI--
-RPMAIW-
RMPMAI--
RYPMAI--
-RPMAIG-
-RPMAIK-
RNPMAI--
RLPMAI--
-RPMAIF-
RDPMAI--
-RPMAIY-
RCPMAI--
-RPMAIV-
-RPMAIN-
-RPMAIH-
REPMAI--
-RPMAIE-
RIPMAI--
SRPMAI--
SQPMAI--
-SPMAIS-
-SPMAIL-
-SPMAII-
-SPMAIH-

-SPMAIR-
SLPMAI--
SSPMAI--
-SPMAIY-
SIPMAI--
-SPMAIK-
SMPMAI--
SYPMAI--
-SPMAIQ-
SVPMAI--
SKPMAI--
SEPMAI--
SWPMAI--
STPMAI--
-SPMAIV-
SFPMAI--
SDPMAI--
SAPMAI--
-SPMAIT-
-SPMAIF-
-SPMAIG-
SNPMAI--
SHPMAI--
SGPMAI--
-SPMAIN-
-SPMAIE-
SPPMAI--
-SPMAIC-
TQPMAI--
-TPMAIR-
TTPMAI--
TLPMAI--
-TPMAIQ-
-TPMAIV-
TYPMAI--
-TPMAIT-
TMPMAI--
TRPMAI--
-TPMAIW-
-TPMAIS-
-TPMAIA-
-TPMAIY-
TVPMAI--
-TPMAIL-
TSPMAI--
-TPMAIG-
TGPMAI--
TPPMAI--
-TPMAII-
TFPMAI--

THPMAI--
-TPMAIK-
TIPMAI--
TKPMAI--
TWPMAI--
-TPMAIE-
TAPMAI--
TDPMAI--
-TPMAIN-
-TPMAIM-
TNPMAI--
TEPMAI--
TCPMAI--
VLPMAI--
-VPMAIR-
VRPMAI--
VSPMAI--
-VPMAIT-
-VPMAIW-
-VPMAIA-
VQPMAI--
-VPMAIG-
VIPMAI--
VFPMAI--
-VPMAIV-
-VPMAIQ-
-VPMAIF-
VPPMAI--
-VPMAIH-
VMPMAI--
VKPMAI--
VGPMAI--
VVPMAI--
VTPMAI--
VEPMAI--
-VPMAIS-
VAPMAI--
-VPMAIK-
-VPMAII-
-VPMAIY-
-VPMAIM-
VNPMAI--
VWPMAI--
WPPMAI--
WAPMAI--
-WPMAIL-
WSPMAI--
-WPMAIR-
-WPMAIM-
-WPMAIG-

	-WPMAIE-		
	WCPMAI--		
	WHPMAI--		
	-WPMAIS-		
	-WPMAIK-		
	WLPMAI--		
	WKPMAI--		
	WYPMAI--		
	WTPMAI--		
	-WPMAIV-		
	-WPMAIN-		
	WGPMAI--		
	WQPMAI--		
	WMPMAI--		
	-YPMAIR-		
	YSPMAI--		
	YFPMAI--		
	YYPMAI--		
	-YPMAIA-		
	YWPMAI--		
	-YPMAII-		
	YRPMAI--		
	YKPMAI--		
	YGPMAI--		
	YVPMAI--		
	YAPMAI--		
	YQPMAI--		
	-YPMAIM-		
	YMPMAI--		
	YEPMAI--		
	YDPMAI--		
	-YPMAIQ-		
	-YPMAIN-		
	-YPMAIK-		
	-YPMAIW-		
	-YPMAIV-		
	-YPMAID-		
	YNPMAI--		
	YLPMAI--		
	YIPMAI--		
PWAI	246	726	50.87
	-APWAIT-		
	ANPWAI--		
	-APWAIG-		
	AKPWAI--		
	-APWAIR-		
	ASPWAI--		
	APPWAI--		
	-APWAIS-		
	AVPWAI--		

```
-APWAIH-
ALPWAI--
AAPWAI--
-APWAII-
AFPWAI--
-APWAIV-
AWPWAI--
AQPWAI--
-APWAIM-
AGPWAI--
ATPWAI--
ARPWAI--
-APWAIQ-
-APWAIN-
-APWAIA-
AHPWAI--
-APWAIW-
-APWAIK-
-APWAIE-
-APWAIY-
CMPWAI--
CRPWAI--
CHPWAI--
CTPWAI--
CSPWAI--
-CPWAIT-
CPPWAI--
CFPWAI--
DRPWAI--
DTPWAI--
DLPWAI--
DVPWAI--
-DPWAIT-
DYPWAI--
-DPWAIW-
-DPWAIM-
-DPWAIH-
DNPWAI--
DHPWAI--
DAPWAI--
-DPWAIA-
DSPWAI--
-DPWAIR-
-DPWAIK-
DEPWAI--
-DPWAIY-
-DPWAIQ-
-DPWAIN-
DQPWAI--
-DPWAIS-
```

```
-DPWAII-
DKPWAI--
ESPWAI--
EKPWAI--
EWPWAI--
EMPWAI--
EEPWAI--
EVPWAI--
-EPWAIM-
EGPWAI--
-EPWAIY-
EYPWAI--
-EPWAIS-
ENPWAI--
ERPWAI--
-EPWAIV-
-EPWAIL-
-EPWAIK-
-EPWAIG-
EFPWAI--
-EPWAIH-
ELPWAI--
EIPWAI--
ETPWAI--
-EPWAIN-
-EPWAIE-
-FPWAIS-
FQPWAI--
FLPWAI--
-FPWAIN-
-FPWAIT-
-FPWAIV-
-FPWAIK-
FYPWAI--
FSPWAI--
FPPWAI--
-FPWAIQ-
FIPWAI--
FGPWAI--
FAPWAI--
-FPWAIL-
-FPWAIA-
FFPWAI--
FCPWAI--
-FPWAIH-
-GPWAIH-
GSPWAI--
-GPWAIV-
GNPWAI--
GIPWAI--
```

GYPWAI--
GVPWAI--
-GPWAIS-
-GPWAII-
-GPWAIT-
GRPWAI--
GQPWAI--
-GPWAIN-
GFPWAI--
-GPWAIL-
GLPWAI--
GTPWAI--
-GPWAIA-
-GPWAIG-
GAPWAI--
GKPWAI--
GDPWAI--
-GPWAIQ-
-GPWAIK-
-GPWAIW-
-GPWAIM-
GPPWAI--
GMPWAI--
GWPWAI--
HWPWAI--
-HPWAIR-
HVPWAI--
HSPWAI--
-HPWAIL-
-HPWAIS-
-HPWAIT-
HMPWAI--
HNPWAI--
HTPWAI--
-HPWAIM-
HLPWAI--
HRPWAI--
-HPWAIN-
HGPWAI--
-HPWAII-
-HPWAIH-
HPPWAI--
HHPWAI--
HQPWAI--
-HPWAIW-
-HPWAIG-
-HPWAIE-
HKPWAI--
HFPWAI--
HDPWAI--

IRPWAI--
ISPWAI--
-IPWAIV-
INPWAI--
IMPWAI--
-IPWAIG-
-IPWAIK-
-IPWAIA-
-IPWAIS-
IVPWAI--
-IPWAIR-
-IPWAIF-
IGPWAI--
IQPWAI--
IIPWAI--
IYPWAI--
ITPWAI--
-IPWAIH-
IHPWAI--
-IPWAIT-
-IPWAIQ-
IDPWAI--
-IPWAIE-
IPPWAI--
IFPWAI--
KSPWAI--
KRPWAI--
KGPWAI--
-KPWAIM-
-KPWAIQ-
-KPWAIH-
KVPWAI--
KTPWAI--
KMPWAI--
-KPWAIS-
-KPWAIR-
KKPWAI--
KLPWAI--
-KPWAIL-
-KPWAIG-
KWPWAI--
-KPWAIF-
-KPWAIE-
KAPWAI--
KYPWAI--
-KPWAIN-
KNPWAI--
KCPWAI--
-KPWAIT-
KPPWAI--

KEPWAI--
KQPWAI--
-KPWAIV-
LGPWAI--
LSPWAI--
LHPWAI--
LVPWAI--
LLPWAI--
LYPWAI--
-LPWAIT-
-LPWAIH-
LFPWAI--
-LPWAIK-
LIPWAI--
LMPWAI--
-LPWAIM-
-LPWAIV-
-LPWAIS-
LTPWAI--
-LPWAIR-
LKPWAI--
LDPWAI--
-LPWAIE-
LAPWAI--
LNPWAI--
-LPWAII-
LQPWAI--
-LPWAIY-
LEPWAI--
-LPWAIW-
-LPWAIG-
-LPWAIA-
MRPWAI--
MEPWAI--
MSPWAI--
-MPWAIY-
-MPWAIR-
MNPWAI--
MYPWAI--
-MPWAIG-
MPPWAI--
MMPWAI--
-MPWAIN-
MAPWAI--
MVPWAI--
MWPWAI--
-MPWAIV-
MDPWAI--
MTPWAI--
-MPWAIT-

```
-MPWAIS-
-MPWAIE-
-MPWAIM-
-MPWAIH-
MCPWAI--
-MPWAIQ-
-MPWAII-
MLPWAI--
MHPWAI--
-NPWAIV-
NAPWAI--
NKPWAI--
NVPWAI--
-NPWAII-
NLPWAI--
NGPWAI--
NTPWAI--
-NPWAIT-
NDPWAI--
NWPWAI--
-NPWAIR-
NPPWAI--
NQPWAI--
NIPWAI--
NSPWAI--
NFPWAI--
-NPWAIH-
-NPWAIE-
-NPWAIY-
-NPWAIW-
-NPWAIK-
NNPWAI--
-NPWAIG-
-NPWAIN-
-NPWAIM-
-NPWAID-
-NPWAIA-
--PWAIRT
PVPWAI--
--PWAISG
--PWAIKS
PSPWAI--
--PWAIRR
--PWAIIR
--PWAIRS
--PWAIRA
--PWAINR
--PWAILG
--PWAIWD
--PWAILR
```

--PWAINQ
--PWAIKG
--PWAILN
--PWAIVR
--PWAISV
--PWAIQS
--PWAIGS
--PWAIYH
--PWAISH
--PWAIST
--PWAIMR
--PWAIHG
--PWAIVD
--PWAISK
--PWAIRG
--PWAIMK
PGPWAI--
--PWAITS
--PWAIAS
PDPWAI--
--PWAIVK
--PWAITV
--PWAISY
--PWAING
--PWAIIS
--PWAIHI
PLPWAI--
--PWAIYK
--PWAITA
--PWAISA
--PWAIHQ
--PWAIFK
--PWAIEF
PHPWAI--
--PWAIVN
--PWAISQ
--PWAILK
--PWAIIT
--PWAIWT
--PWAIMM
--PWAIHS
--PWAIHR
--PWAIVT
--PWAIVQ
--PWAITK
--PWAIRQ
--PWAINV
--PWAINS
--PWAILE
--PWAIKH

--PWAIKD
--PWAIGY
--PWAIYD
--PWAIVS
--PWAISS
--PWAIQH
--PWAIQG
--PWAINT
--PWAILD
--PWAIIK
--PWAIHL
--PWAIGI
--PWAIGA
PEPWAI--
PYPWAI--
--PWAIYR
--PWAITG
--PWAISF
--PWAIRI
--PWAIRH
--PWAIRF
--PWAIQI
--PWAIMS
PTPWAI--
--PWAIKL
--PWAIKI
--PWAIIV
--PWAIFR
--PWAIFQ
--PWAIAK
--PWAIAH
--PWAIAF
--PWAIVA
--PWAITR
--PWAIRK
--PWAIQT
--PWAILH
--PWAIHN
--PWAIGL
--PWAIFN
--PWAIAV
--PWAIYV
--PWAIWS
--PWAIWM
--PWAIWG
--PWAIVG
--PWAITY
--PWAISR
--PWAIQK
--PWAIKK

--PWAIIQ
--PWAIIN
--PWAIHE
PFPWAI--
--PWAIWR
--PWAIWN
--PWAIVH
--PWAIVF
--PWAISM
--PWAISD
--PWAIRN
--PWAIRD
--PWAIPY
--PWAIND
--PWAIMV
--PWAIKN
--PWAIKM
--PWAIIH
--PWAIFT
--PWAIFG
--PWAIYI
--PWAISN
--PWAIQD
--PWAINN
--PWAIMN
--PWAILF
--PWAIIA
--PWAIHT
--PWAIHD
--PWAIAY
PWPWAI--
--PWAIVE
--PWAIQV
--PWAIQA
--PWAIME
--PWAIKA
--PWAIIY
--PWAIID
--PWAIGM
--PWAIFV
--PWAIAM
--PWAIAI
--PWAIAA
--PWAIYT
--PWAIWA
--PWAITN
--PWAISE
--PWAIPF
--PWAINY
--PWAINA

--PWAILI
--PWAIIW
--PWAIIF
--PWAIIE
--PWAIGQ
--PWAIGG
PIPWAI--
--PWAIVI
--PWAITH
--PWAISI
--PWAIRE
--PWAIQQ
--PWAIQE
--PWAIMG
--PWAIMF
--PWAIMD
--PWAIKV
--PWAIKR
--PWAIKQ
--PWAIKF
--PWAIHY
--PWAIHW
--PWAIHV
--PWAIGV
--PWAIGR
--PWAIGN
--PWAIGH
--PWAIES
--PWAIEM
--PWAIAG
-PPWAIL-
PKPWAI--
--PWAIYN
--PWAIYG
--PWAIWV
--PWAIWI
--PWAIWH
--PWAIWE
--PWAITQ
--PWAITI
--PWAITE
--PWAITD
--PWAITC
--PWAIQM
--PWAIQL
--PWAINW
--PWAINI
--PWAIMW
--PWAIMQ
--PWAILM

--PWAIKY
--PWAIIP
--PWAIIG
--PWAIHM
--PWAIHH
--PWAIHF
--PWAIGK
--PWAIFY
--PWAIFM
--PWAIFD
--PWAIER
--PWAIEQ
--PWAIEN
--PWAIEK
--PWAIEI
--PWAIEE
--PWAIEA
--PWAIDK
--PWAIDG
--PWAICH
--PWAIAR
--PWAIAN
--PWAIAE
PNPWAI--
PCPWAI--
QTPWAI--
QLPWAI--
-QPWAIS-
-QPWAIY-
-QPWAIR-
QRPWAI--
-QPWAIV-
QSPWAI--
-QPWAIK-
QGPWAI--
QIPWAI--
-QPWAIN-
-QPWAIM-
QAPWAI--
QNPWAI--
QWPWAI--
QQPWAI--
-QPWAIH-
-QPWAIG-
QFPWAI--
-QPWAIQ-
QKPWAI--
QVPWAI--
-QPWAIW-
-QPWAIC-

-QPWAIT-
-RPWAIK-
-RPWAIT-
RTPWAI--
RKPWAI--
RRPWAI--
RVPWAI--
-RPWAIA-
RQPWAI--
RNPWAI--
RLPWAI--
REPWAI--
-RPWAIF-
RSPWAI--
-RPWAIH-
RGPWAI--
-RPWAIQ-
-RPWAIS-
RHPWAI--
RFPWAI--
-RPWAIY-
-RPWAII-
-RPWAIG-
RDPWAI--
-RPWAIW-
-RPWAIR-
RIPWAI--
RAPWAI--
RWPWAI--
-RPWAIM-
RPPWAI--
RYPWAI--
-RPWAIV-
-RPWAIN-
-SPWAIR-
-SPWAIL-
SRPWAI--
SKPWAI--
SIPWAI--
SSPWAI--
SMPWAI--
SGPWAI--
SAPWAI--
-SPWAIH-
-SPWAII-
SLPWAI--
SQPWAI--
-SPWAIN-
-SPWAIK-
SNPWAI--

SPPWAI--
SYPWAI--
STPWAI--
-SPWAIS-
-SPWAIV-
SFPWAI--
-SPWAIY-
-SPWAIQ-
SDPWAI--
SWPWAI--
-SPWAIT-
SEPWAI--
-SPWAIG-
-SPWAIA-
SHPWAI--
SCPWAI--
-SPWAIW-
-SPWAIM-
TRPWAI--
TMPWAI--
-TPWAIR-
TSPWAI--
-TPWAIK-
TVPWAI--
TTPWAI--
TAPWAI--
TGPWAI--
-TPWAIT-
-TPWAIG-
-TPWAIW-
TEPWAI--
-TPWAIN-
TKPWAI--
TDPWAI--
-TPWAIV-
-TPWAIS-
TYPWAI--
-TPWAIE-
-TPWAIQ-
-TPWAIH-
-TPWAII-
TPPWAI--
TNPWAI--
-TPWAIM-
TLPWAI--
TQPWAI--
VTPWAI--
VPPWAI--
VRPWAI--
VQPWAI--

```
-VPWAIV-
-VPWAIS-
VGPWAI--
-VPWAIT-
VLPWAI--
-VPWAIR-
VVPWAI--
VNPWAI--
VWPWAI--
VIPWAI--
-VPWAIH-
VHPWAI--
VYPWAI--
-VPWAIN-
-VPWAII-
VEPWAI--
VSPWAI--
-VPWAIG-
VDPWAI--
-VPWAIQ-
-VPWAIE-
VFPWAI--
VAPWAI--
WRPWAI--
WIPWAI--
-WPWAIS-
-WPWAIA-
-WPWAIT-
WSPWAI--
WNPWAI--
WMPWAI--
WFPWAI--
WAPWAI--
-WPWAIK-
-WPWAIY-
-WPWAIN-
-WPWAIM-
-WPWAIH-
WKPWAI--
WYPWAI--
-WPWAIQ-
WEPWAI--
YGPWAI--
YSPWAI--
YRPWAI--
-YPWAIR-
-YPWAIS-
YEPWAI--
YKPWAI--
-YPWAIV-
```

YHPWAI--
-YPWAIT-
-YPWAIK-
YQPWAI--
YPPWAI--
YNPWAI--
YTPWAI--
-YPWAIN-
YWPWAI--
-YPWAII-
YAPWAI--
-YPWAIW-
-YPWAIQ-
-YPWAIE-
-YPWAIH-
YIPWAI--
YFPWAI--
PMNI 237 728 50.75
-GPMNIS-
AGPMNI--
ARPMNI--
-APMNIL-
-APMNIK-
-APMNIF-
ADPMNI--
AWPMNI--
ASPMNI--
-APMNIS-
-APMNIH-
-APMNIV-
-APMNIN-
ALPMNI--
AYPMNI--
-APMNIT-
-APMNIE-
AVPMNI--
-APMNIW-
AMPMNI--
AIPMNI--
AHPMNI--
AFPMNI--
APPMNI--
ATPMNI--
AQPMNI--
-APMNIQ-
-APMNII-
AKPMNI--
AAPMNI--
CSPMNI--
CCPMNI--

```
-CPMNIR-
-CPMNII-
CFPMNI--
-CPMNIE-
CKPMNI--
CWPMNI--
CRPMNI--
-CPMNIQ-
CIPMNI--
CHPMNI--
CGPMNI--
DVPMNI--
-DPMNIS-
-DPMNII-
DSPMNI--
-DPMNIR-
-DPMNIL-
-DPMNIH-
-DPMNIK-
DLPMNI--
DKPMNI--
DTPMNI--
DRPMNI--
-DPMNIT-
DHPMNI--
DEPMNI--
DAPMNI--
DWPMNI--
-DPMNIV-
DGPMNI--
DYPMNI--
-DPMNIF-
-DPMNIE-
DIPMNI--
-EPMNIR-
-EPMNIM-
ELPMNI--
EAPMNI--
-EPMNIL-
-EPMNIS-
-EPMNIA-
ESPMNI--
ERPMNI--
EYPMNI--
EVPMNI--
-EPMNIY-
-EPMNIV-
EGPMNI--
EQPMNI--
-EPMNIK-
```

-EPMNIH-
-EPMNIF-
-EPMNIC-
EMPMNI--
EKPMNI--
EEPMNI--
FRPMNI--
FLPMNI--
FNPMNI--
-FPMNIS-
FKPMNI--
FTPMNI--
FVPMNI--
FGPMNI--
-FPMNIK-
-FPMNIG-
-FPMNII-
FSPMNI--
FEPMNI--
FQPMNI--
-FPMNIN-
FAPMNI--
FPPMNI--
-FPMNIV-
-FPMNIR-
FDPMNI--
FCPMNI--
GMPMNI--
GVPMNI--
-GPMNIR-
-GPMNIL-
GYPMNI--
-GPMNIV-
GGPMNI--
GKPMNI--
GRPMNI--
GWPMNI--
GPPMNI--
-GPMNIK-
GAPMNI--
GTPMNI--
GSPMNI--
-GPMNIT-
-GPMNIY-
-GPMNIM-
-GPMNIE-
GQPMNI--
-GPMNIG-
-GPMNIF-
GFPMNI--

-GPMNIH-
GLPMNI--
-GPMNIN-
-GPMNIA-
GNPMNI--
GHPMNI--
GEPMNI--
HAPMNI--
HRPMNI--
-HPMNIR-
-HPMNIY-
-HPMNIL-
-HPMNIS-
-HPMNIA-
HSPMNI--
HTPMNI--
HIPMNI--
HMPMNI--
-HPMNIW-
-HPMNIM-
-HPMNIQ-
-HPMNIE-
HNPMNI--
HKPMNI--
HHPMNI--
HYPMNI--
HVPMNI--
-HPMNII-
-HPMNIH-
HWPMNI--
-HPMNIF-
IEPMNI--
IRPMNI--
IHPMNI--
-IPMNIY-
INPMNI--
-IPMNIT-
-IPMNIS-
IFPMNI--
IWPMNI--
IMPMNI--
-IPMNIR-
IVPMNI--
-IPMNIG-
ITPMNI--
ISPMNI--
-IPMNIQ-
-IPMNIA-
ILPMNI--
IGPMNI--

```
-IPMNIL-
IPPMNI--
-IPMNIN-
-IPMNIW-
-IPMNII-
IKPMNI--
IDPMNI--
-IPMNIV-
-IPMNIH-
-IPMNIF-
IIPMNI--
KSPMNI--
-KPMNIY-
KEPMNI--
-KPMNIR-
-KPMNIF-
-KPMNIS-
KAPMNI--
KIPMNI--
KPPMNI--
KLPMNI--
-KPMNIH-
-KPMNIA-
-KPMNIQ-
-KPMNIN-
-KPMNII-
KFPMNI--
-KPMNIW-
-KPMNIG-
KVPMNI--
KTPMNI--
KMPMNI--
KRPMNI--
KNPMNI--
KYPMNI--
-KPMNIM-
-KPMNIL-
-KPMNIE-
KDPMNI--
KCPMNI--
LSPMNI--
LRPMNI--
-LPMNIV-
-LPMNIM-
LGPMNI--
-LPMNIS-
LAPMNI--
LIPMNI--
LQPMNI--
LPPMNI--
```

LNPMNI--
LMPMNI--
LKPMNI--
LWPMNI--
-LPMNIG-
LYPMNI--
-LPMNIN-
-LPMNIK-
-LPMNII-
LLPMNI--
LEPMNI--
LDPMNI--
-LPMNIF-
LFPMNI--
-LPMNIY-
-LPMNIH-
-LPMNIQ-
-LPMNIA-
LHPMNI--
LCPMNI--
-MPMNIL-
-MPMNIK-
MVPMNI--
MMPMNI--
MGPMNI--
-MPMNIY-
-MPMNIV-
MRPMNI--
-MPMNIT-
-MPMNIS-
-MPMNII-
-MPMNIG-
-MPMNIF-
MLPMNI--
MAPMNI--
MIPMNI--
MHPMNI--
MTPMNI--
MSPMNI--
-MPMNIW-
-MPMNIA-
MEPMNI--
MCPMNI--
MWPMNI--
-MPMNIR-
-MPMNIN-
MFPMNI--
MDPMNI--
NRPMNI--
-NPMNIR-

```
-NPMNIY-
NVPMNI--
-NPMNIV-
NPPMNI--
NLPMNI--
NGPMNI--
NTPMNI--
NSPMNI--
-NPMNIL-
NMPMNI--
NAPMNI--
NWPMNI--
NIPMNI--
NYPMNI--
-NPMNIS-
-NPMNIQ-
-NPMNIK-
-NPMNII-
-NPMNIH-
NQPMNI--
-NPMNIG-
NHPMNI--
-NPMNIM-
-NPMNIE-
NNPMNI--
NKPMNI--
NFPMNI--
--PMNIRA
--PMNISR
--PMNILS
--PMNILR
--PMNILQ
--PMNIYI
--PMNIVA
--PMNIKN
--PMNIRL
--PMNISF
--PMNIKM
--PMNITL
--PMNITT
--PMNITI
--PMNISS
--PMNIRS
--PMNIKR
--PMNIFM
PWPMNI--
--PMNIKG
PEPMNI--
--PMNITF
--PMNIRM
```

--PMNIIS
--PMNIIR
--PMNIYV
--PMNIYG
--PMNIST
--PMNISM
--PMNISA
--PMNILN
--PMNILG
--PMNISV
--PMNIQL
--PMNIMY
--PMNIMG
--PMNILK
--PMNIKS
--PMNIHL
--PMNIGT
--PMNIFD
--PMNIAH
--PMNIYS
--PMNIVF
--PMNIRG
--PMNITV
--PMNIRK
--PMNINF
--PMNIGR
--PMNIFS
--PMNIAV
--PMNIWT
--PMNIWK
--PMNIVI
--PMNIQY
--PMNIQW
--PMNILD
--PMNIGS
--PMNIES
--PMNIVL
--PMNIRT
--PMNIMS
--PMNILW
--PMNIIL
--PMNIVV
--PMNIVM
--PMNITM
--PMNITG
--PMNISQ
--PMNISL
--PMNISH
--PMNIFT
--PMNIVR

--PMNISW
--PMNIRQ
--PMNIMN
--PMNIYM
--PMNIYF
--PMNIWN
--PMNIWG
--PMNITK
--PMNISG
--PMNIRW
--PMNIRV
--PMNIRD
--PMNIQI
--PMNIML
--PMNIMI
--PMNIKT
--PMNIHS
--PMNIFR
--PMNIEH
--PMNIAF
--PMNIAA
PAPMNI--
PTPMNI--
--PMNIWH
--PMNIWE
--PMNIVY
--PMNITW
--PMNILH
--PMNILE
--PMNIGV
--PMNIGA
--PMNIEV
--PMNIAR
--PMNIAQ
--PMNIWS
--PMNITR
--PMNISY
--PMNISN
--PMNIRN
--PMNIQA
--PMNINH
--PMNIMQ
--PMNIII
--PMNIER
--PMNIEF
--PMNIAT
--PMNIAI
PIPMNI--
PFPMNI--
PYPMNI--

--PMNIWV
--PMNIVT
--PMNIVS
--PMNIVK
--PMNIRY
--PMNIQS
--PMNIQF
--PMNIMK
--PMNILM
--PMNIKA
--PMNIIT
--PMNIGY
--PMNIGM
--PMNIEA
--PMNIAW
PMPMNI--
--PMNIYY
--PMNIYR
--PMNIWR
--PMNIWD
--PMNIWA
--PMNIVG
--PMNISK
--PMNIRH
--PMNINR
--PMNIMM
--PMNIKY
--PMNIIV
--PMNIIK
--PMNIHY
--PMNIHA
--PMNIGF
--PMNIFK
--PMNIFI
--PMNIEY
--PMNIEN
--PMNIEG
--PMNIAL
PNPMNI--
--PMNIYQ
--PMNIVE
--PMNITN
--PMNIQV
--PMNIQR
--PMNIQK
--PMNIQG
--PMNINN
--PMNIMD
--PMNILA
--PMNIKL

```
--PMNIKI
--PMNIKE
--PMNIKD
--PMNIIG
--PMNIHR
--PMNIGL
--PMNIEW
--PMNIDS
--PMNICL
--PMNIAY
--PMNIAS
-PPMNIY-
-PPMNIR-
--PMNIYN
--PMNIYD
--PMNIWL
--PMNIWF
--PMNIVQ
--PMNITD
--PMNISD
--PMNIQT
--PMNIPA
--PMNINW
--PMNINT
--PMNINM
--PMNINK
--PMNILP
--PMNIKK
--PMNIIN
--PMNIID
--PMNIIA
--PMNIHV
--PMNIHT
--PMNIHM
--PMNIHE
--PMNIGN
--PMNIGK
--PMNIGH
--PMNIGD
--PMNIEQ
--PMNIEM
--PMNIEK
--PMNIEI
--PMNIDR
--PMNIDL
--PMNIDA
--PMNICN
--PMNICK
--PMNIAP
--PMNIAN
```

```
--PMNIAK
PKPMNI--
PCPMNI--
QPPMNI--
-QPMNIL-
QIPMNI--
QGPMNI--
-QPMNIN-
-QPMNIW-
-QPMNIQ-
-QPMNIR-
-QPMNIA-
QTPMNI--
QSPMNI--
-QPMNIS-
QEPMNI--
-QPMNIV-
QAPMNI--
QRPMNI--
-QPMNIY-
QMPMNI--
QDPMNI--
-RPMNIL-
RRPMNI--
RSPMNI--
-RPMNIG-
-RPMNIT-
-RPMNIQ-
RVPMNI--
-RPMNIR-
RPPMNI--
RNPMNI--
-RPMNIM-
RKPMNI--
RYPMNI--
RFPMNI--
-RPMNIK-
RLPMNI--
-RPMNIN-
RGPMNI--
-RPMNIH-
RTPMNI--
-RPMNIV-
-RPMNII-
RHPMNI--
-RPMNIY-
-RPMNIE-
-RPMNIA-
RDPMNI--
RAPMNI--
```

-RPMNIF-
RIPMNI--
REPMNI--
-RPMNIW-
RWPMNI--
SLPMNI--
SFPMNI--
-SPMNIY-
SYPMNI--
-SPMNIR-
SAPMNI--
-SPMNIL-
SRPMNI--
-SPMNIT-
SSPMNI--
SHPMNI--
STPMNI--
SMPMNI--
SGPMNI--
SWPMNI--
-SPMNIK-
SIPMNI--
-SPMNIF-
SKPMNI--
SQPMNI--
-SPMNIV-
-SPMNIS-
-SPMNIN-
-SPMNII-
-SPMNIW-
-SPMNIM-
-SPMNIH-
-SPMNIG-
SPPMNI--
-SPMNIQ-
SEPMNI--
SNPMNI--
SDPMNI--
TLPMNI--
-TPMNII-
-TPMNIL-
TAPMNI--
TSPMNI--
-TPMNIF-
TMPMNI--
TRPMNI--
TIPMNI--
-TPMNIV-
TVPMNI--
-TPMNIY-

-TPMNIT-
-TPMNIR-
-TPMNIM-
TNPMNI--
-TPMNIS-
-TPMNIH-
TTPMNI--
-TPMNIQ-
-TPMNIK-
-TPMNIG-
TKPMNI--
THPMNI--
-TPMNIW-
-TPMNIN-
-TPMNIE-
TWPMNI--
-TPMNIA-
TFPMNI--
VKPMNI--
VVPMNI--
VYPMNI--
-VPMNIL-
-VPMNIS-
-VPMNIR-
VSPMNI--
-VPMNIY-
VRPMNI--
VAPMNI--
-VPMNIN-
-VPMNII-
VMPMNI--
VTPMNI--
VEPMNI--
-VPMNIQ-
VFPMNI--
VGPMNI--
-VPMNIF-
VQPMNI--
-VPMNIK-
VLPMNI--
-VPMNIV-
-VPMNIM-
-VPMNIH-
-VPMNIE-
-VPMNIA-
-VPMNIW-
VNPMNI--
VIPMNI--
WSPMNI--
-WPMNIL-

	WLPMNI--		
	WAPMNI--		
	-WPMNIY-		
	-WPMNIM-		
	WVPMNI--		
	WQPMNI--		
	-WPMNIS-		
	WRPMNI--		
	WKPMNI--		
	WPPMNI--		
	-WPMNIV-		
	WGPMNI--		
	-WPMNIQ-		
	-WPMNIW-		
	-WPMNIG-		
	-WPMNIA-		
	WHPMNI--		
	WFPMNI--		
	-YPMNIL-		
	YRPMNI--		
	-YPMNIR-		
	YDPMNI--		
	-YPMNIS-		
	YLPMNI--		
	YMPMNI--		
	YYPMNI--		
	-YPMNII-		
	YFPMNI--		
	YSPMNI--		
	YGPMNI--		
	-YPMNIK-		
	YAPMNI--		
	YTPMNI--		
	-YPMNIY-		
	YNPMNI--		
	YVPMNI--		
	-YPMNIF-		
	-YPMNIE-		
	-YPMNIA-		
	-YPMNIV-		
	-YPMNIN-		
	YKPMNI--		
	YHPMNI--		
	YEPMNI--		
	-YPMNIW-		
	-YPMNIG-		
PYNM	306	694	50.69
	-GPYNMP-		
	ATPYNM--		
	-APYNMR-		

-APYNML-
-APYNMI-
-APYNMG-
ALPYNM--
AYPYNM--
AVPYNM--
AKPYNM--
-APYNMY-
ANPYNM--
APPYNM--
-APYNMM-
AGPYNM--
-APYNMW-
-APYNMV-
ARPYNM--
AEPYNM--
ASPYNM--
-APYNMK-
-APYNMH-
AMPYNM--
-APYNMT-
-APYNMA-
AWPYNM--
AQPYNM--
-APYNMQ-
-APYNMN-
-APYNMF-
-APYNME-
AIPYNM--
ACPYNM--
-CPYNMR-
CGPYNM--
CCPYNM--
CFPYNM--
CYPYNM--
-CPYNMS-
-CPYNMK-
CIPYNM--
CEPYNM--
DGPYNM--
DVPYNM--
-DPYNMT-
-DPYNMS-
-DPYNMM-
-DPYNMI-
DEPYNM--
DWPYNM--
-DPYNMK-
DNPYNM--
DTPYNM--

```
-DPYNMF-
-DPYNMA-
DLPYNM--
-EPYNML-
ETPYNM--
-EPYNMY-
EFPYNM--
EVPYNM--
-EPYNMI-
-EPYNMH-
EKPYNM--
-EPYNMG-
-EPYNMF-
EWPYNM--
-EPYNMT-
-EPYNMR-
EPPYNM--
ELPYNM--
EIPYNM--
ERPYNM--
EQPYNM--
-EPYNMA-
-FPYNMR-
-FPYNMS-
FEPYNM--
FSPYNM--
-FPYNMA-
FLPYNM--
FAPYNM--
FTPYNM--
FNPYNM--
-FPYNMT-
-FPYNMY-
FIPYNM--
FYPYNM--
-FPYNML-
FMPYNM--
FKPYNM--
FFPYNM--
FRPYNM--
-FPYNME-
-FPYNMD-
FGPYNM--
FDPYNM--
FVPYNM--
-FPYNMV-
-FPYNMN-
-FPYNMK-
-FPYNMG-
-GPYNML-
```

GKPYNM--
-GPYNMT-
GLPYNM--
-GPYNMF-
GTPYNM--
GRPYNM--
GVPYNM--
-GPYNMQ-
GSPYNM--
GYPYNM--
-GPYNMS-
-GPYNMK-
-GPYNMG-
GNPYNM--
-GPYNMV-
GIPYNM--
-GPYNMR-
-GPYNME-
-GPYNMA-
GMPYNM--
GGPYNM--
GQPYNM--
GEPYNM--
GHPYNM--
HRPYNM--
HMPYNM--
HSPYNM--
-HPYNML-
-HPYNMV-
HQPYNM--
-HPYNMR-
-HPYNMH-
-HPYNMG-
-HPYNME-
HPPYNM--
HHPYNM--
HCPYNM--
-HPYNMN-
HNPYNM--
HFPYNM--
HTPYNM--
-HPYNMW-
-HPYNMS-
HLPYNM--
-HPYNMM-
-HPYNMA-
HGPYNM--
IYPYNM--
IKPYNM--
-IPYNMY-

```
INPYNM--
ILPYNM--
-IPYNMM-
-IPYNML-
-IPYNMV-
IGPYNM--
-IPYNMR-
-IPYNMI-
IFPYNM--
IRPYNM--
-IPYNMT-
IQPYNM--
IMPYNM--
IEPYNM--
-IPYNMG-
IAPYNM--
-IPYNMS-
-IPYNME-
IIPYNM--
IVPYNM--
ITPYNM--
-IPYNMK-
-IPYNMD-
ICPYNM--
KSPYNM--
-KPYNMA-
KWPYNM--
KGPYNM--
-KPYNMS-
-KPYNMH-
KVPYNM--
-KPYNMT-
KYPYNM--
KMPYNM--
KLPYNM--
-KPYNMY-
-KPYNMI-
KIPYNM--
KTPYNM--
-KPYNMR-
KPPYNM--
KHPYNM--
KAPYNM--
-KPYNMW-
-KPYNMD-
-KPYNMN-
-KPYNMK-
-KPYNME-
KFPYNM--
KEPYNM--
```

KQPYNM--
-KPYNMQ-
-KPYNMM-
-KPYNML-
-KPYNMF-
LRPYNM--
LGPYNM--
-LPYNMR-
LWPYNM--
LSPYNM--
-LPYNML-
LVPYNM--
LYPYNM--
LLPYNM--
LQPYNM--
LMPYNM--
-LPYNMF-
LEPYNM--
-LPYNMH-
-LPYNMM-
-LPYNMG-
LPPYNM--
-LPYNMS-
-LPYNMI-
LTPYNM--
-LPYNMA-
LIPYNM--
-LPYNME-
LNPYNM--
-LPYNMT-
-LPYNMQ-
-LPYNMV-
MGPYNM--
MAPYNM--
-MPYNMR-
MLPYNM--
-MPYNMS-
MMPYNM--
MSPYNM--
-MPYNMA-
MIPYNM--
-MPYNMG-
MEPYNM--
MRPYNM--
-MPYNMF-
MHPYNM--
MQPYNM--
-MPYNMK-
MVPYNM--
-MPYNMV-

```
-MPYNML-
-MPYNMI-
MKPYNM--
MFPYNM--
NRPYNM--
-NPYNML-
-NPYNMY-
NMPYNM--
NLPYNM--
-NPYNMT-
-NPYNMI-
-NPYNMR-
NNPYNM--
NAPYNM--
-NPYNMS-
-NPYNMF-
NGPYNM--
NSPYNM--
NYPYNM--
NQPYNM--
-NPYNMQ-
NIPYNM--
-NPYNMV-
-NPYNME-
-NPYNMA-
NCPYNM--
-NPYNMW-
-NPYNMN-
-NPYNMG-
NFPYNM--
--PYNMKT
--PYNMTS
--PYNMLS
--PYNMVR
--PYNMAS
--PYNMTR
--PYNMSG
--PYNMLK
--PYNMKS
--PYNMTL
--PYNMSR
--PYNMMS
--PYNMLN
--PYNMSS
--PYNMKL
--PYNMGS
--PYNMWS
--PYNMIH
--PYNMAK
--PYNMTW
```

--PYNMRL
--PYNMII
--PYNMGV
--PYNMWQ
--PYNMRN
--PYNMAN
--PYNMIY
--PYNMWL
--PYNMVG
--PYNMVQ
--PYNMQG
--PYNMLT
--PYNMHS
--PYNMTV
--PYNMRS
--PYNMRF
--PYNMKG
--PYNMIS
--PYNMIL
--PYNMWR
--PYNMVH
--PYNMNA
--PYNMLV
--PYNMLG
--PYNMEL
--PYNMWG
--PYNMAQ
--PYNMVS
--PYNMRE
--PYNMMT
--PYNMFK
--PYNMAL
--PYNMAG
--PYNMWW
--PYNMVW
--PYNMRH
--PYNMRG
--PYNMKR
--PYNMEA
--PYNMYQ
--PYNMVD
--PYNMSM
--PYNMSL
--PYNMSI
--PYNMSH
--PYNMLQ
--PYNMKK
--PYNMIR
--PYNMHD
--PYNMDA

--PYNMAR
--PYNMVM
--PYNMSW
--PYNMSN
--PYNMSF
--PYNMSA
--PYNMQK
--PYNMMV
--PYNMMD
--PYNMHI
--PYNMFH
--PYNMES
PGPYNM--
--PYNMYN
--PYNMWM
--PYNMRQ
--PYNMRD
--PYNMQV
--PYNMQN
--PYNMQI
--PYNMIA
--PYNMFQ
--PYNMYW
--PYNMYA
--PYNMWA
--PYNMTN
--PYNMTI
--PYNMSD
--PYNMRM
--PYNMMG
--PYNMLE
--PYNMKD
--PYNMHH
--PYNMFT
--PYNMFR
--PYNMER
--PYNMEI
--PYNMAF
--PYNMYL
--PYNMYD
--PYNMWN
--PYNMWE
--PYNMVA
--PYNMTY
--PYNMSQ
--PYNMRW
--PYNMRV
--PYNMQF
--PYNMNM
--PYNMMR

--PYNMKH
--PYNMID
--PYNMHA
--PYNMGT
--PYNMGL
--PYNMGK
--PYNMGG
--PYNMEQ
--PYNMEG
--PYNMTK
--PYNMTF
--PYNMQW
--PYNMQT
--PYNMQR
--PYNMQE
--PYNMNT
--PYNMNN
--PYNMNG
--PYNMKY
--PYNMIQ
--PYNMIN
--PYNMHK
--PYNMHG
--PYNMGI
--PYNMGH
--PYNMFN
--PYNMFA
--PYNMAW
PEPYNM--
--PYNMYT
--PYNMTT
--PYNMTE
--PYNMSY
--PYNMMA
--PYNMLF
--PYNMLA
--PYNMKQ
--PYNMKA
--PYNMHF
--PYNMFF
--PYNMEW
--PYNMDT
--PYNMAI
--PYNMTQ
--PYNMTM
--PYNMSE
--PYNMQS
--PYNMNV
--PYNMNQ
--PYNMMF

--PYNMKW
--PYNMKV
--PYNMIT
--PYNMIG
--PYNMHT
--PYNMHR
--PYNMHE
--PYNMGF
--PYNMEM
--PYNMCS
--PYNMCR
--PYNMAV
PFPYNM--
--PYNMYS
--PYNMYH
--PYNMVI
--PYNMQY
--PYNMQH
--PYNMNS
--PYNMNR
--PYNMNI
--PYNMNE
--PYNMLH
--PYNMLD
--PYNMHQ
--PYNMGQ
--PYNMGD
--PYNMFY
--PYNMFE
--PYNMEH
--PYNMEF
--PYNMDI
--PYNMCV
--PYNMAY
--PYNMAM
PTPYNM--
-PPYNMW-
PPPYNM--
PNPYNM--
QRPYNM--
QHPYNM--
QMPYNM--
QYPYNM--
QAPYNM--
QGPYNM--
-QPYNMV-
-QPYNMT-
-QPYNML-
-QPYNMG-
QLPYNM--

```
-QPYNMQ-
-QPYNMS-
-QPYNMN-
QKPYNM--
-QPYNMI-
-QPYNMF-
QQPYNM--
-QPYNMW-
-QPYNMD-
-QPYNMA-
QPPYNM--
QNPYNM--
QFPYNM--
-RPYNMY-
-RPYNMS-
RLPYNM--
-RPYNML-
RGPYNM--
-RPYNMI-
RTPYNM--
-RPYNMR-
-RPYNMF-
-RPYNMK-
RAPYNM--
-RPYNMV-
-RPYNMG-
-RPYNMN-
RNPYNM--
RYPYNM--
RSPYNM--
-RPYNMT-
-RPYNMH-
RFPYNM--
RVPYNM--
-RPYNMQ-
RPPYNM--
RQPYNM--
-RPYNMA-
RHPYNM--
RIPYNM--
REPYNM--
RCPYNM--
-RPYNME-
-SPYNMR-
SRPYNM--
SMPYNM--
STPYNM--
-SPYNMS-
SQPYNM--
-SPYNMY-
```

SVPYNM--
-SPYNMV-
SLPYNM--
-SPYNMK-
SWPYNM--
SYPYNM--
SEPYNM--
-SPYNMA-
SCPYNM--
-SPYNMG-
-SPYNMT-
-SPYNMN-
SHPYNM--
-SPYNMF-
SSPYNM--
-SPYNME-
-SPYNMW-
-SPYNMM-
-SPYNMI-
SAPYNM--
-SPYNMD-
SFPYNM--
-SPYNMH-
-TPYNMS-
TTPYNM--
TGPYNM--
TYPYNM--
-TPYNMK-
TRPYNM--
-TPYNMH-
-TPYNMG-
TIPYNM--
-TPYNMT-
-TPYNML-
TPPYNM--
TSPYNM--
TMPYNM--
TVPYNM--
TLPYNM--
-TPYNMY-
-TPYNMF-
-TPYNMR-
TKPYNM--
TFPYNM--
TWPYNM--
TAPYNM--
-TPYNMQ-
-TPYNMN-
-TPYNME-
-TPYNMW-

TCPYNM--
TQPYNM--
-TPYNMV-
-TPYNMM-
-TPYNMA-
TDPYNM--
-VPYNMS-
VSPYNM--
VTPYNM--
VVPYNM--
-VPYNML-
-VPYNMA-
VLPYNM--
VNPYNM--
-VPYNMW-
-VPYNMG-
VWPYNM--
VGPYNM--
VFPYNM--
VYPYNM--
-VPYNMN-
-VPYNMY-
VKPYNM--
-VPYNMR-
-VPYNMI-
VIPYNM--
VEPYNM--
-VPYNMV-
-VPYNMK-
VPPYNM--
-VPYNMM-
VHPYNM--
VRPYNM--
-VPYNMH-
-VPYNME-
VCPYNM--
WRPYNM--
WSPYNM--
WPPYNM--
WMPYNM--
-WPYNMT-
-WPYNMW-
-WPYNMA-
-WPYNMS-
-WPYNME-
WIPYNM--
-WPYNMR-
WWPYNM--
WEPYNM--
WVPYNM--

	-WPYNML-		
	WNPYNM--		
	WLPYNM--		
	WGPYNM--		
	WFPYNM--		
	WAPYNM--		
	WTPYNM--		
	WQPYNM--		
	-WPYNMK-		
	-WPYNMI-		
	-WPYNMF-		
	WDPYNM--		
	-YPYNMS-		
	-YPYNMK-		
	YRPYNM--		
	YDPYNM--		
	YSPYNM--		
	-YPYNME-		
	YAPYNM--		
	YWPYNM--		
	YPPYNM--		
	YKPYNM--		
	-YPYNMT-		
	YLPYNM--		
	YNPYNM--		
	YTPYNM--		
	-YPYNMI-		
	YQPYNM--		
	-YPYNMY-		
	-YPYNMW-		
	-YPYNMR-		
	-YPYNML-		
	-YPYNMH-		
	-YPYNMG-		
	-YPYNMA-		
	-YPYNMN-		
	YIPYNM--		
	YGPYNM--		
	-YPYNMV-		
	-YPYNMF-		
	YHPYNM--		
PYWM	809	533	50.66
	ASPYWM--		
	AKPYWM--		
	AVPYWM--		
	-APYWMG-		
	-APYWMV-		
	-APYWMR-		
	AEPYWM--		
	AAPYWM--		

-APYWML-
-APYWMA-
ANPYWM--
AYPYWM--
-APYWMN-
-APYWMM-
-APYWMH-
-APYWMD-
-APYWMI-
AGPYWM--
-APYWMS-
AHPYWM--
ATPYWM--
-APYWMQ-
CRPYWM--
-CPYWMS-
-CPYWMM-
CCPYWM--
DVPYWM--
DSPYWM--
DMPYWM--
DTPYWM--
DKPYWM--
-DPYWMA-
DIPYWM--
-DPYWMV-
-DPYWML-
DHPYWM--
DGPYWM--
-DPYWMW-
-DPYWMR-
-DPYWMI-
DRPYWM--
-DPYWMG-
DEPYWM--
-EPYWMR-
-EPYWML-
-EPYWMS-
EVPYWM--
-EPYWMN-
ERPYWM--
-EPYWMG-
EAPYWM--
EYPYWM--
-EPYWMV-
-EPYWMF-
ELPYWM--
-EPYWMM-
-FPYWMS-
FSPYWM--

-FPYWMI-
FNPYWM--
FHPYWM--
-FPYWMM-
-FPYWMV-
-FPYWMH-
FMPYWM--
FGPYWM--
FWPYWM--
-FPYWMQ-
FEPYWM--
FYPYWM--
FQPYWM--
-FPYWMT-
-FPYWMN-
-FPYWME-
-FPYWMA-
FLPYWM--
GTPYWM--
GRPYWM--
-GPYWMV-
-GPYWMD-
-GPYWMS-
-GPYWML-
GPPYWM--
GIPYWM--
-GPYWMK-
-GPYWMM-
-GPYWMG-
-GPYWMI-
-GPYWMT-
GNPYWM--
-GPYWMR-
GMPYWM--
GEPYWM--
GAPYWM--
GSPYWM--
-GPYWME-
-GPYWMA-
-GPYWMY-
GLPYWM--
GYPYWM--
GVPYWM--
-GPYWMW-
-GPYWMQ-
GHPYWM--
GFPYWM--
HMPYWM--
-HPYWMR-
-HPYWMV-

```
-HPYWMG-
-HPYWMA-
HIPYWM--
-HPYWMS-
-HPYWMN-
HLPYWM--
HKPYWM--
HRPYWM--
-HPYWMF-
HSPYWM--
-HPYWMT-
-HPYWMH-
HHPYWM--
HFPYWM--
HAPYWM--
ILPYWM--
IIPYWM--
IVPYWM--
-IPYWMV-
ISPYWM--
IPPYWM--
-IPYWMY-
-IPYWMM-
IAPYWM--
-IPYWML-
-IPYWMH-
-IPYWME-
IHPYWM--
IYPYWM--
IRPYWM--
-KPYWMS-
KVPYWM--
-KPYWMT-
KSPYWM--
KLPYWM--
KGPYWM--
KPPYWM--
KRPYWM--
KFPYWM--
-KPYWMQ-
-KPYWMI-
-KPYWMG-
-KPYWMF-
-KPYWMD-
KAPYWM--
KYPYWM--
KQPYWM--
-KPYWME-
KWPYWM--
KNPYWM--
```

KMPYWM--
KDPYWM--
-KPYWMW-
-KPYWMV-
-KPYWMA-
KKPYWM--
LDPYWM--
LMPYWM--
-LPYWMR-
LGPYWM--
-LPYWMT-
-LPYWMS-
LRPYWM--
LYPYWM--
LVPYWM--
LTPYWM--
-LPYWMV-
LPPYWM--
LHPYWM--
LSPYWM--
LIPYWM--
LAPYWM--
LCPYWM--
-LPYWMY-
-LPYWMI-
-LPYWME-
-LPYWML-
-LPYWMH-
LNPYWM--
LKPYWM--
LFPYWM--
-MPYWMS-
-MPYWMR-
-MPYWMI-
MAPYWM--
MVPYWM--
-MPYWMK-
MEPYWM--
MQPYWM--
-MPYWMH-
MHPYWM--
MDPYWM--
MYPYWM--
MSPYWM--
-MPYWMW-
-MPYWMY-
-MPYWMA-
MNPYWM--
MKPYWM--
MGPYWM--

NTPYWM--
NSPYWM--
-NPYWMG-
NRPYWM--
-NPYWMY-
NIPYWM--
NYPYWM--
NVPYWM--
NGPYWM--
-NPYWMV-
-NPYWMQ-
-NPYWMI-
-NPYWMT-
-NPYWMK-
-NPYWMD-
--PYWMRS
--PYWMLH
--PYWMHT
--PYWMNT
--PYWMVA
--PYWMRV
--PYWMLS
--PYWMKT
--PYWMKG
--PYWMTN
--PYWMRT
--PYWMQS
--PYWMHS
--PYWMVG
--PYWMTY
--PYWMAA
--PYWMVS
--PYWMLG
PNPYWM--
--PYWMTV
--PYWMRW
--PYWMKN
--PYWMGG
--PYWMTI
--PYWMSG
--PYWMSE
--PYWMDS
--PYWMYH
--PYWMTT
--PYWMSH
--PYWMIS
--PYWMFN
--PYWMSI
--PYWMRE
--PYWMMV

--PYWMVR
--PYWMMK
--PYWMEV
--PYWMYS
--PYWMSN
--PYWMQI
--PYWMMA
--PYWMLE
--PYWMIG
--PYWMHN
--PYWMAR
--PYWMTS
--PYWMTR
--PYWMRA
--PYWMNQ
--PYWMMS
--PYWMWN
--PYWMTK
--PYWMSV
--PYWMSR
--PYWMQQ
--PYWMLN
--PYWMGS
--PYWMYT
--PYWMWS
--PYWMVN
--PYWMVH
--PYWMTH
--PYWMLQ
--PYWMKM
--PYWMIE
--PYWMGW
--PYWMYM
--PYWMSS
--PYWMNN
--PYWMNA
--PYWMMM
--PYWMKF
--PYWMGT
--PYWMES
--PYWMYG
--PYWMYD
--PYWMVY
--PYWMSW
--PYWMRG
--PYWMQN
--PYWMQA
--PYWMGL
--PYWMGA
--PYWMDT

--PYWMSK
--PYWMRQ
--PYWMRM
--PYWMRH
--PYWMNW
--PYWMMR
--PYWMMH
--PYWMIR
--PYWMHG
--PYWMFD
--PYWMAN
--PYWMAH
--PYWMVD
--PYWMTG
--PYWMSL
--PYWMRY
--PYWMQK
--PYWMNY
--PYWMNV
--PYWMNM
--PYWMLT
--PYWMLK
--PYWMKL
--PYWMIT
--PYWMHD
--PYWMGY
--PYWMGR
--PYWMGN
--PYWMGK
--PYWMGF
--PYWMGE
--PYWMFS
--PYWMFK
--PYWMFG
--PYWMAL
--PYWMVW
--PYWMVT
--PYWMTQ
--PYWMSY
--PYWMST
--PYWMRI
--PYWMQR
--PYWMQH
--PYWMQD
--PYWMPG
--PYWMNK
--PYWMNI
--PYWMNE
--PYWMND
--PYWMLW

```
--PYWMKV
--PYWMKE
--PYWMIN
--PYWMIK
--PYWMGV
--PYWMFY
--PYWMER
--PYWMEN
--PYWMEK
--PYWMEA
--PYWMAT
-QPYWMR-
QRPYWM--
QTPYWM--
-QPYWMV-
-QPYWMM-
-QPYWML-
-QPYWMG-
QMPYWM--
QLPYWM--
-QPYWMT-
-QPYWMQ-
QYPYWM--
-QPYWMH-
QAPYWM--
-QPYWMK-
-QPYWMD-
QPPYWM--
QDPYWM--
-QPYWMY-
QVPYWM--
-QPYWMN-
QIPYWM--
RIPYWM--
RKPYWM--
RGPYWM--
-RPYWMK-
RFPYWM--
-RPYWMG-
RYPYWM--
RVPYWM--
RNPYWM--
-RPYWML-
RMPYWM--
-RPYWMH-
-RPYWMI-
-RPYWMV-
-RPYWMR-
RSPYWM--
RPPYWM--
```

```
-RPYWMA-
-RPYWMT-
RDPYWM--
-RPYWMY-
RLPYWM--
RAPYWM--
-RPYWMW-
REPYWM--
-RPYWMS-
-RPYWMN-
-RPYWMF-
RRPYWM--
-RPYWMQ-
RCPYWM--
-RPYWMM-
-RPYWME-
-RPYWMD-
-SPYWMR-
SNPYWM--
SLPYWM--
-SPYWMA-
SKPYWM--
SDPYWM--
SYPYWM--
-SPYWMY-
-SPYWMV-
-SPYWMS-
SHPYWM--
STPYWM--
SMPYWM--
SVPYWM--
-SPYWMQ-
-SPYWMK-
SIPYWM--
SRPYWM--
-SPYWMH-
-SPYWME-
SGPYWM--
-SPYWMN-
SPPYWM--
SWPYWM--
SSPYWM--
SEPYWM--
-SPYWMM-
-SPYWMG-
-SPYWMD-
TRPYWM--
-TPYWMR-
-TPYWML-
-TPYWMT-
```

```
-TPYWMI-
TVPYWM--
-TPYWMA-
-TPYWMS-
TPPYWM--
TNPYWM--
TTPYWM--
TSPYWM--
-TPYWMK-
TLPYWM--
-TPYWMN-
-TPYWMH-
-TPYWMG-
TMPYWM--
TIPYWM--
-TPYWMQ-
TKPYWM--
-TPYWMV-
-TPYWMM-
-TPYWMF-
TDPYWM--
TCPYWM--
TAPYWM--
VSPYWM--
-VPYWMR-
VLPYWM--
-VPYWMI-
-VPYWMA-
VPPYWM--
-VPYWMV-
VTPYWM--
VQPYWM--
-VPYWMW-
VYPYWM--
-VPYWMH-
-VPYWMG-
-VPYWMT-
VGPYWM--
VWPYWM--
-VPYWMS-
-VPYWML-
VNPYWM--
VEPYWM--
VDPYWM--
-VPYWMY-
-VPYWMN-
VFPYWM--
VVPYWM--
-VPYWMK-
WAPYWM--
```

WTPYWM--
WNPYWM--
WRPYWM--
-WPYWMV-
-WPYWMK-
-WPYWMM-
WSPYWM--
-WPYWMT-
-WPYWMS-
WMPYWM--
-YPYWML-
YTPYWM--
-YPYWMH-
-YPYWMS-
YSPYWM--
YRPYWM--
-YPYWMK-
YEPYWM--
-YPYWMG-
-YPYWMA-
YNPYWM--
YGPYWM--
YWPYWM--
-YPYWMT-
-YPYWMF-

PRAM 196 751 50.31

--PRAMPS
--PRAMHQ
--PRAMFP
--PRAMDP
-SPRAMD-
-APRAMS-
ALPRAM--
AAPRAM--
-APRAMK-
-APRAMT-
ASPRAM--
-APRAMY-
AFPRAM--
ANPRAM--
ATPRAM--
AEPRAM--
-APRAML-
AIPRAM--
-APRAMA-
AWPRAM--
AHPRAM--
AGPRAM--
ADPRAM--
-APRAMM-

APPRAM--
-APRAMI-
AMPRAM--
ACPRAM--
-APRAMW-
-APRAMH-
-APRAMF-
-APRAMD-
AVPRAM--
-APRAMP-
CSPRAM--
CLPRAM--
CEPRAM--
-CPRAMS-
-CPRAMY-
-CPRAMT-
CMPRAM--
-CPRAML-
-CPRAMK-
-CPRAMN-
-CPRAMG-
CHPRAM--
CAPRAM--
CRPRAM--
CQPRAM--
CPPRAM--
CNPRAM--
CKPRAM--
CGPRAM--
-DPRAMI-
DMPRAM--
DTPRAM--
DRPRAM--
-DPRAMV-
-DPRAMR-
-DPRAMF-
DFPRAM--
-DPRAMS-
-DPRAMT-
DYPRAM--
DIPRAM--
DVPRAM--
DWPRAM--
DGPRAM--
DSPRAM--
-DPRAML-
-DPRAMA-
DLPRAM--
DHPRAM--
DQPRAM--

-DPRAMY-
-DPRAMQ-
-DPRAMH-
DEPRAM--
DNPRAM--
DKPRAM--
DCPRAM--
-DPRAMW-
-DPRAMK-
-DPRAME-
-DPRAMG-
-EPRAMA-
-EPRAML-
-EPRAMQ-
ERPRAM--
-EPRAMR-
ELPRAM--
EMPRAM--
EHPRAM--
EGPRAM--
ETPRAM--
-EPRAMM-
EIPRAM--
EVPRAM--
-EPRAMW-
-EPRAMN-
-EPRAMK-
EDPRAM--
-EPRAMI-
-EPRAMG-
EKPRAM--
-EPRAMV-
-EPRAMT-
EYPRAM--
-EPRAMY-
-EPRAMC-
EAPRAM--
-EPRAME-
EPPRAM--
EFPRAM--
EEPRAM--
FGPRAM--
FRPRAM--
FYPRAM--
-FPRAMV-
-FPRAMA-
-FPRAMQ-
-FPRAMK-
FDPRAM--
FQPRAM--

−FPRAMY−
FMPRAM−−
−FPRAMI−
−FPRAMH−
FPPRAM−−
−FPRAME−
FEPRAM−−
−FPRAMM−
FNPRAM−−
FAPRAM−−
−FPRAMF−
FKPRAM−−
FIPRAM−−
FCPRAM−−
GSPRAM−−
GGPRAM−−
GAPRAM−−
−GPRAMA−
−GPRAMS−
GHPRAM−−
−GPRAMM−
−GPRAML−
−GPRAMT−
GFPRAM−−
GWPRAM−−
−GPRAMW−
−GPRAMI−
−GPRAMG−
GLPRAM−−
−GPRAMV−
GNPRAM−−
GVPRAM−−
−GPRAMH−
−GPRAMK−
−GPRAMY−
GCPRAM−−
−GPRAMQ−
GKPRAM−−
−GPRAME−
−GPRAMN−
−GPRAMD−
GMPRAM−−
GEPRAM−−
GQPRAM−−
GPPRAM−−
−HPRAMY−
−HPRAMS−
−HPRAMF−
HYPRAM−−
−HPRAMV−

HSPRAM--
-HPRAMI-
HIPRAM--
HAPRAM--
-HPRAMT-
HNPRAM--
HGPRAM--
HLPRAM--
-HPRAME-
HQPRAM--
-HPRAMN-
-HPRAMA-
-HPRAMM-
-HPRAMH-
HDPRAM--
HPPRAM--
HEPRAM--
-HPRAMW-
HMPRAM--
HFPRAM--
-IPRAML-
ISPRAM--
IRPRAM--
-IPRAMN-
IGPRAM--
ITPRAM--
-IPRAMH-
-IPRAMY-
-IPRAMI-
-IPRAMG-
-IPRAME-
IIPRAM--
IDPRAM--
-IPRAMV-
INPRAM--
IMPRAM--
-IPRAMK-
IKPRAM--
-IPRAMA-
ICPRAM--
IQPRAM--
IEPRAM--
IAPRAM--
IYPRAM--
-IPRAMQ-
-IPRAMC-
KSPRAM--
-KPRAML-
-KPRAMK-
-KPRAMT-

KVPRAM--
-KPRAMI-
-KPRAMY-
-KPRAMA-
-KPRAMF-
KGPRAM--
KMPRAM--
KEPRAM--
-KPRAMN-
KAPRAM--
-KPRAMH-
KPPRAM--
-KPRAMG-
KLPRAM--
KKPRAM--
-KPRAMW-
-KPRAMM-
KTPRAM--
-KPRAMV-
KYPRAM--
KIPRAM--
KNPRAM--
KHPRAM--
KQPRAM--
-KPRAME-
-KPRAMC-
LVPRAM--
LNPRAM--
-LPRAMT-
LGPRAM--
LHPRAM--
-LPRAMI-
-LPRAMK-
LDPRAM--
LPPRAM--
LIPRAM--
LTPRAM--
-LPRAMW-
LAPRAM--
-LPRAMY-
-LPRAMQ-
LSPRAM--
-LPRAMS-
-LPRAMH-
-LPRAMG-
LMPRAM--
LQPRAM--
-LPRAME-
LKPRAM--
-LPRAMN-

LCPRAM--
-MPRAML-
MSPRAM--
MPPRAM--
-MPRAMI-
-MPRAMS-
-MPRAMM-
MVPRAM--
-MPRAMY-
-MPRAMT-
MHPRAM--
-MPRAMK-
MWPRAM--
MLPRAM--
MFPRAM--
MQPRAM--
-MPRAMQ-
-MPRAME-
MGPRAM--
MMPRAM--
MAPRAM--
-MPRAMW-
-MPRAMV-
-MPRAMF-
MCPRAM--
-MPRAMA-
MKPRAM--
MIPRAM--
MEPRAM--
MDPRAM--
-MPRAMP-
-MPRAMN-
NLPRAM--
-NPRAML-
NSPRAM--
NPPRAM--
-NPRAMW-
-NPRAMS-
NVPRAM--
-NPRAMY-
NGPRAM--
NTPRAM--
-NPRAMM-
NIPRAM--
-NPRAMV-
NMPRAM--
NDPRAM--
NQPRAM--
-NPRAMI-
-NPRAMA-

–NPRAMK–
–NPRAME–
NKPRAM––
–NPRAMQ–
–NPRAMC–
––PRAMLS
––PRAMVG
––PRAMTI
––PRAMIS
––PRAMFS
––PRAMVN
––PRAMLK
––PRAMLT
––PRAMVS
––PRAMVI
––PRAMSI
––PRAMSA
PTPRAM––
––PRAMKL
––PRAMYS
––PRAMYK
––PRAMSG
––PRAMVK
––PRAMMN
––PRAMGW
––PRAMYH
––PRAMYR
––PRAMSS
––PRAMVF
––PRAMKS
––PRAMMS
––PRAMFI
––PRAMWL
––PRAMTH
––PRAMVY
––PRAMSK
––PRAMIG
––PRAMAV
––PRAMHR
––PRAMVQ
––PRAMNS
––PRAMKN
––PRAMFK
––PRAMYG
––PRAMTV
––PRAMME
––PRAMLF
––PRAMIL
––PRAMFD
––PRAMAH

PIPRAM--
--PRAMWT
--PRAMLH
--PRAMAI
--PRAMWA
--PRAMVH
--PRAMTA
--PRAMFY
--PRAMML
--PRAMLQ
--PRAMKQ
--PRAMGI
--PRAMAT
PRPRAM--
--PRAMYV
--PRAMGG
--PRAMWV
--PRAMII
PLPRAM--
--PRAMYQ
--PRAMQI
--PRAMGT
--PRAMIK
--PRAMAY
--PRAMWK
--PRAMSM
--PRAMSH
--PRAMQG
--PRAMHH
--PRAMGN
--PRAMEG
--PRAMAN
--PRAMAA
--PRAMQN
--PRAMQL
--PRAMPY
--PRAMNT
--PRAMIA
--PRAMGA
--PRAMYN
--PRAMVD
--PRAMKI
PVPRAM--
--PRAMWR
--PRAMNA
--PRAMIV
--PRAMYT
--PRAMWD
--PRAMRE
--PRAMPI

--PRAMMY
--PRAMLE
--PRAMYD
--PRAMLD
--PRAMKD
--PRAMIF
--PRAMFQ
--PRAMDL
--PRAMAG
--PRAMWH
--PRAMSD
--PRAMQM
--PRAMVT
--PRAMTW
--PRAMTD
--PRAMNR
--PRAMMT
--PRAMHA
--PRAMGV
--PRAMYW
--PRAMPM
--PRAMNM
--PRAMMV
--PRAMKV
--PRAMHV
--PRAMHK
PFPRAM--
--PRAMQS
--PRAMNW
--PRAMEV
--PRAMDS
PQPRAM--
--PRAMVA
--PRAMQW
--PRAMQE
--PRAMPL
--PRAMNV
--PRAMKA
--PRAMHG
--PRAMHF
--PRAMGY
--PRAMES
--PRAMWN
--PRAMVM
--PRAMVE
--PRAMNY
--PRAMNN
--PRAMIM
--PRAMFT
--PRAMYM

--PRAMYE
--PRAMWM
--PRAMTT
--PRAMTE
--PRAMNQ
--PRAMMM
--PRAMMG
--PRAMKT
--PRAMFA
--PRAMDM
--PRAMVP
--PRAMSF
--PRAMMI
--PRAMIT
--PRAMFH
--PRAMDY
PWPRAM--
--PRAMWQ
--PRAMTM
--PRAMPF
--PRAMNH
--PRAMHN
--PRAMGK
--PRAMGH
--PRAMFG
--PRAMEI
PAPRAM--
--PRAMYY
--PRAMWI
--PRAMSC
--PRAMQY
--PRAMQF
--PRAMIW
--PRAMIE
--PRAMHY
--PRAMHM
--PRAMGM
--PRAMGF
--PRAMFN
--PRAMFE
--PRAMER
--PRAMDG
--PRAMAS
--PRAMTK
--PRAMSW
--PRAMLW
--PRAMIH
--PRAMHI
--PRAMQD
--PRAMMP

--PRAMMH
--PRAMIP
--PRAMHE
--PRAMEF
PGPRAM--
--PRAMWE
--PRAMSE
--PRAMQV
--PRAMNI
--PRAMMW
--PRAMHS
--PRAMEA
--PRAMAD
PNPRAM--
PKPRAM--
--PRAMYP
--PRAMWF
--PRAMVC
--PRAMTQ
--PRAMQQ
--PRAMNK
--PRAMLC
--PRAMIQ
--PRAMEN
--PRAMEM
--PRAMDI
PCPRAM--
--PRAMRC
--PRAMQH
--PRAMPV
--PRAMHD
--PRAMEY
--PRAMEQ
--PRAMED
--PRAMDA
--PRAMCD
--PRAMAQ
--PRAMAE
-PPRAMQ-
--PRAMWW
--PRAMID
--PRAMHP
--PRAMGP
--PRAMCS
--PRAMCG
--PRAMAK
-PPRAMY-
PPPRAM--
PHPRAM--
-QPRAMR-

QMPRAM--
QFPRAM--
QSPRAM--
QGPRAM--
-QPRAMT-
-QPRAMS-
-QPRAMK-
-QPRAMV-
QPPRAM--
-QPRAMH-
-QPRAMF-
-QPRAMY-
-QPRAMI-
-QPRAMA-
QVPRAM--
-QPRAMM-
QLPRAM--
-QPRAMG-
QAPRAM--
QKPRAM--
-QPRAMQ-
QIPRAM--
QDPRAM--
-QPRAMN-
QEPRAM--
QTPRAM--
RSPRAM--
-RPRAML-
RPPRAM--
RAPRAM--
-RPRAMQ-
-RPRAMM-
-RPRAMT-
-RPRAMN-
RNPRAM--
-RPRAMY-
-RPRAMV-
-RPRAMK-
-RPRAMF-
RDPRAM--
REPRAM--
RRPRAM--
-RPRAMH-
-RPRAMG-
-RPRAME-
RCPRAM--
RIPRAM--
RKPRAM--
RQPRAM--
-RPRAMC-

-SPRAML-
-SPRAMS-
-SPRAMT-
-SPRAMI-
SSPRAM--
SGPRAM--
-SPRAMQ-
-SPRAMY-
-SPRAME-
SLPRAM--
STPRAM--
-SPRAMH-
SNPRAM--
-SPRAMW-
-SPRAMM-
SEPRAM--
SQPRAM--
SIPRAM--
SYPRAM--
SPPRAM--
SKPRAM--
-SPRAMF-
-SPRAMG-
SDPRAM--
SCPRAM--
TLPRAM--
-TPRAML-
TSPRAM--
-TPRAMT-
-TPRAMA-
-TPRAMV-
TKPRAM--
-TPRAMS-
TTPRAM--
TYPRAM--
-TPRAMR-
-TPRAMF-
TQPRAM--
-TPRAMG-
-TPRAME-
TWPRAM--
TAPRAM--
-TPRAMM-
TGPRAM--
TRPRAM--
TVPRAM--
-TPRAMN-
TFPRAM--
TPPRAM--
TEPRAM--

TDPRAM--
-TPRAMC-
-TPRAMD-
TCPRAM--
VSPRAM--
VTPRAM--
VAPRAM--
-VPRAMK-
-VPRAMY-
VGPRAM--
VYPRAM--
VQPRAM--
-VPRAMQ-
-VPRAMH-
VMPRAM--
VVPRAM--
-VPRAMV-
-VPRAMW-
VPPRAM--
-VPRAMF-
VNPRAM--
VHPRAM--
VDPRAM--
-VPRAME-
VKPRAM--
VEPRAM--
-VPRAMN-
-VPRAMD-
-VPRAMP-
-WPRAME-
-WPRAMY-
WRPRAM--
-WPRAMG-
WDPRAM--
-WPRAMH-
WNPRAM--
WMPRAM--
WTPRAM--
WYPRAM--
WKPRAM--
WGPRAM--
-WPRAMN-
-WPRAMM-
-WPRAMV-
-WPRAMI-
-WPRAMF-
WEPRAM--
WVPRAM--
WHPRAM--
WQPRAM--

WCPRAM--
-WPRAMP-
-YPRAMT-
-YPRAMV-
-YPRAMY-
-YPRAMA-
YSPRAM--
YRPRAM--
-YPRAMW-
-YPRAMN-
-YPRAMM-
YMPRAM--
YVPRAM--
YPPRAM--
YIPRAM--
YYPRAM--
YNPRAM--
-YPRAME-
YGPRAM--
YWPRAM--
-YPRAMH-
YKPRAM--
YDPRAM--
-YPRAMF-
YAPRAM--
YEPRAM--
-YPRAMD-

PWNL 54 849 50.19

-DPWNLG-
--PWNLRP
--PWNLPL
--PWNLGP
-SPWNLD-
AVPWNL--
AAPWNL--
-APWNLR-
-APWNLT-
-APWNLS-
AGPWNL--
ANPWNL--
AKPWNL--
ALPWNL--
AIPWNL--
-APWNLL-
ARPWNL--
-APWNLM-
-APWNLA-
AFPWNL--
-APWNLI-
-APWNLF-

-APWNLE-
AYPWNL--
-APWNLV-
-APWNLQ-
AWPWNL--
-APWNLY-
-APWNLG-
AMPWNL--
AHPWNL--
-APWNLH-
AQPWNL--
-APWNLK-
-APWNLN-
-APWNLD-
CAPWNL--
CSPWNL--
-CPWNLI-
CVPWNL--
CQPWNL--
CRPWNL--
-CPWNLY-
-CPWNLV-
-CPWNLF-
CIPWNL--
CGPWNL--
CEPWNL--
CDPWNL--
CCPWNL--
-CPWNLN-
-CPWNLM-
-CPWNLL-
-CPWNLK-
-CPWNLA-
DRPWNL--
DSPWNL--
DHPWNL--
DTPWNL--
-DPWNLF-
DAPWNL--
-DPWNLK-
DGPWNL--
DVPWNL--
-DPWNLR-
-DPWNLL-
-DPWNLV-
-DPWNLS-
DIPWNL--
DFPWNL--
DQPWNL--
-DPWNLM-

DLPWNL--
DNPWNL--
DKPWNL--
-DPWNLI-
DDPWNL--
DYPWNL--
-DPWNLY-
-DPWNLW-
-DPWNLQ-
-DPWNLT-
DEPWNL--
-DPWNLE-
DWPWNL--
-DPWNLH-
-DPWNLA-
-EPWNLL-
ETPWNL--
EFPWNL--
-EPWNLR-
EWPWNL--
-EPWNLM-
ESPWNL--
-EPWNLV-
ERPWNL--
EAPWNL--
ENPWNL--
EQPWNL--
EGPWNL--
-EPWNLY-
ELPWNL--
EYPWNL--
-EPWNLT-
-EPWNLQ-
-EPWNLK-
-EPWNLG-
-EPWNLH-
-EPWNLE-
-EPWNLS-
-EPWNLI-
-EPWNLN-
EHPWNL--
EVPWNL--
-EPWNLW-
-EPWNLC-
-EPWNLA-
EIPWNL--
ECPWNL--
-FPWNLS-
FNPWNL--
-FPWNLM-

-FPWNLE-
FQPWNL--
FPPWNL--
FTPWNL--
-FPWNLT-
FVPWNL--
FGPWNL--
-FPWNLA-
FSPWNL--
FIPWNL--
FHPWNL--
-FPWNLH-
FLPWNL--
FEPWNL--
-FPWNLN-
FYPWNL--
-FPWNLR-
-FPWNLF-
-FPWNLK-
FFPWNL--
-FPWNLW-
-FPWNLV-
-FPWNLY-
FKPWNL--
-FPWNLI-
GRPWNL--
-GPWNLS-
GVPWNL--
GTPWNL--
GAPWNL--
GLPWNL--
-GPWNLA-
GSPWNL--
-GPWNLR-
-GPWNLQ-
GEPWNL--
GGPWNL--
-GPWNLT-
-GPWNLI-
-GPWNLW-
-GPWNLN-
GPPWNL--
GHPWNL--
GQPWNL--
-GPWNLY-
-GPWNLV-
GWPWNL--
GIPWNL--
-GPWNLG-
GYPWNL--

GFPWNL--
GMPWNL--
GDPWNL--
-GPWNLH-
GNPWNL--
-GPWNLK-
-GPWNLD-
-GPWNLE-
HRPWNL--
-HPWNLT-
HSPWNL--
HGPWNL--
-HPWNLR-
HLPWNL--
HVPWNL--
HYPWNL--
HTPWNL--
-HPWNLS-
HMPWNL--
HIPWNL--
-HPWNLN-
-HPWNLH-
HKPWNL--
HFPWNL--
-HPWNLM-
-HPWNLK-
HWPWNL--
-HPWNLV-
-HPWNLI-
-HPWNLF-
HHPWNL--
-HPWNLY-
-HPWNLW-
HAPWNL--
-HPWNLA-
HEPWNL--
-HPWNLQ-
-HPWNLG-
-HPWNLE-
-IPWNLS-
-IPWNLT-
IVPWNL--
-IPWNLR-
-IPWNLQ-
-IPWNLY-
IRPWNL--
-IPWNLN-
IGPWNL--
-IPWNLA-
IEPWNL--

-IPWNLV-
-IPWNLL-
-IPWNLE-
-IPWNLK-
-IPWNLH-
INPWNL--
-IPWNLG-
IMPWNL--
IDPWNL--
IYPWNL--
ISPWNL--
IQPWNL--
IFPWNL--
ITPWNL--
IPPWNL--
IKPWNL--
IWPWNL--
IIPWNL--
IHPWNL--
-IPWNLI-
-KPWNLR-
KRPWNL--
-KPWNLS-
KNPWNL--
KAPWNL--
-KPWNLK-
KMPWNL--
-KPWNLV-
-KPWNLH-
-KPWNLF-
KLPWNL--
KVPWNL--
KQPWNL--
-KPWNLN-
-KPWNLL-
-KPWNLY-
KTPWNL--
-KPWNLG-
-KPWNLA-
KIPWNL--
KGPWNL--
-KPWNLQ-
-KPWNLE-
KYPWNL--
-KPWNLI-
-KPWNLW-
KHPWNL--
KFPWNL--
-KPWNLM-
KPPWNL--

KDPWNL--
LRPWNL--
LLPWNL--
LMPWNL--
LSPWNL--
LTPWNL--
LVPWNL--
LGPWNL--
LKPWNL--
LYPWNL--
LEPWNL--
LPPWNL--
LIPWNL--
LFPWNL--
-LPWNLQ-
-LPWNLV-
-LPWNLT-
LAPWNL--
-LPWNLK-
LNPWNL--
-LPWNLY-
-LPWNLS-
LHPWNL--
-LPWNLA-
-LPWNLG-
-LPWNLN-
-LPWNLM-
-LPWNLF-
-LPWNLW-
LCPWNL--
-LPWNLH-
LWPWNL--
-LPWNLI-
MLPWNL--
-MPWNLT-
-MPWNLA-
MGPWNL--
MVPWNL--
MRPWNL--
-MPWNLR-
-MPWNLN-
MSPWNL--
MEPWNL--
MQPWNL--
-MPWNLV-
-MPWNLL-
MKPWNL--
MIPWNL--
-MPWNLQ-
MNPWNL--

```
-MPWNLG-
-MPWNLS-
-MPWNLE-
-MPWNLM-
-MPWNLK-
-MPWNLI-
MFPWNL--
MWPWNL--
-MPWNLY-
-MPWNLH-
MPPWNL--
MDPWNL--
MYPWNL--
-MPWNLW-
-MPWNLF-
MCPWNL--
NSPWNL--
-NPWNLL-
NLPWNL--
NKPWNL--
NWPWNL--
NTPWNL--
-NPWNLR-
-NPWNLA-
-NPWNLK-
NPPWNL--
NRPWNL--
NHPWNL--
NMPWNL--
NIPWNL--
NVPWNL--
-NPWNLV-
-NPWNLY-
-NPWNLS-
-NPWNLI-
-NPWNLH-
NFPWNL--
NEPWNL--
-NPWNLN-
NDPWNL--
-NPWNLF-
-NPWNLT-
NQPWNL--
-NPWNLE-
-NPWNLG-
NGPWNL--
NCPWNL--
NYPWNL--
-NPWNLW-
NAPWNL--
```

NNPWNL--
-NPWNLM-
--PWNLLN
--PWNLRT
--PWNLLS
--PWNLSL
--PWNLTR
PLPWNL--
--PWNLVS
--PWNLKN
--PWNLGS
--PWNLRS
--PWNLVG
--PWNLRH
--PWNLLQ
--PWNLGT
--PWNLLG
--PWNLAS
--PWNLLH
--PWNLHR
--PWNLVM
--PWNLSV
--PWNLSR
--PWNLYS
--PWNLQS
PSPWNL--
--PWNLTA
--PWNLKS
--PWNLHA
--PWNLGV
--PWNLEG
PRPWNL--
--PWNLYG
--PWNLRG
--PWNLIA
--PWNLAG
--PWNLTS
PVPWNL--
--PWNLTK
--PWNLLK
--PWNLHT
--PWNLFS
--PWNLSE
--PWNLKA
PTPWNL--
--PWNLSN
--PWNLNV
--PWNLWS
--PWNLGA
--PWNLVN

--PWNLAT
--PWNLVD
--PWNLST
--PWNLQA
--PWNLMG
--PWNLKR
--PWNLIG
--PWNLYR
--PWNLVV
--PWNLLT
--PWNLHQ
--PWNLEV
--PWNLVH
--PWNLRE
--PWNLNG
--PWNLHS
--PWNLAQ
PAPWNL--
--PWNLWA
--PWNLVK
--PWNLTV
--PWNLSF
--PWNLKT
--PWNLIS
--PWNLFQ
PQPWNL--
PKPWNL--
PEPWNL--
--PWNLTY
--PWNLTF
--PWNLSA
--PWNLNS
--PWNLIR
--PWNLVT
--PWNLTG
--PWNLVW
--PWNLMR
--PWNLAR
--PWNLYH
--PWNLTL
--PWNLQT
--PWNLLE
--PWNLKV
--PWNLVA
--PWNLSW
--PWNLHG
--PWNLSQ
--PWNLKK
--PWNLKF
--PWNLDS

PGPWNL--
--PWNLYM
--PWNLTM
--PWNLSK
--PWNLRD
--PWNLME
--PWNLKI
--PWNLIN
--PWNLHL
--PWNLHD
--PWNLFG
--PWNLAI
PNPWNL--
--PWNLTI
--PWNLTH
--PWNLSD
--PWNLQM
--PWNLNA
--PWNLHF
--PWNLEL
--PWNLEK
--PWNLAN
--PWNLTW
--PWNLQY
--PWNLMD
--PWNLKE
--PWNLYD
--PWNLWK
--PWNLVQ
--PWNLTT
--PWNLNY
--PWNLNT
--PWNLKW
--PWNLKL
--PWNLIK
--PWNLGW
--PWNLFK
--PWNLEA
--PWNLDA
PYPWNL--
--PWNLQG
--PWNLNQ
--PWNLKY
--PWNLKH
--PWNLGQ
--PWNLET
--PWNLEI
--PWNLDG
--PWNLYN
--PWNLYK

--PWNLWY
--PWNLWH
--PWNLTQ
--PWNLTE
--PWNLSM
--PWNLQR
--PWNLQI
--PWNLIT
--PWNLGG
--PWNLGF
PDPWNL--
--PWNLTD
--PWNLRY
--PWNLRQ
--PWNLMH
--PWNLKD
--PWNLGR
--PWNLAV
--PWNLWD
--PWNLSH
--PWNLQF
--PWNLQD
--PWNLNH
--PWNLMQ
--PWNLHY
--PWNLGI
--PWNLES
--PWNLEF
--PWNLAD
PWPWNL--
--PWNLWN
--PWNLNI
--PWNLMN
--PWNLHV
--PWNLHN
--PWNLHK
--PWNLHH
--PWNLDT
--PWNLAM
--PWNLYT
--PWNLTN
--PWNLQV
--PWNLNR
--PWNLNN
--PWNLMV
--PWNLMM
--PWNLMA
--PWNLIY
--PWNLID
--PWNLHI

```
--PWNLHE
--PWNLGY
--PWNLGM
--PWNLGL
--PWNLFI
--PWNLFH
--PWNLEN
--PWNLEH
--PWNLAY
PMPWNL--
PFPWNL--
--PWNLWR
--PWNLQQ
--PWNLNM
--PWNLMT
--PWNLIW
--PWNLGK
--PWNLGE
--PWNLGD
--PWNLFD
--PWNLED
--PWNLDW
--PWNLAA
--PWNLYA
--PWNLWQ
--PWNLRW
--PWNLQN
--PWNLQH
--PWNLND
--PWNLKQ
--PWNLKM
--PWNLIH
--PWNLGH
--PWNLEE
--PWNLAK
--PWNLAE
-PPWNLL-
PPPWNL--
--PWNLYE
--PWNLRC
--PWNLPT
--PWNLPF
--PWNLNW
--PWNLMS
--PWNLHW
--PWNLDV
--PWNLDM
-PPWNLS-
-PPWNLK-
-PPWNLG-
```

PIPWNL--
PHPWNL--
QSPWNL--
-QPWNLR-
QRPWNL--
-QPWNLG-
QYPWNL--
QNPWNL--
QKPWNL--
-QPWNLW-
-QPWNLH-
-QPWNLT-
QGPWNL--
QTPWNL--
QAPWNL--
-QPWNLA-
QHPWNL--
-QPWNLS-
-QPWNLE-
-QPWNLK-
QDPWNL--
-QPWNLQ-
QVPWNL--
QQPWNL--
-QPWNLL-
-QPWNLN-
QEPWNL--
QWPWNL--
QLPWNL--
-QPWNLY-
-QPWNLI-
-QPWNLF-
QFPWNL--
QPPWNL--
QMPWNL--
QCPWNL--
-QPWNLV-
-RPWNLL-
RRPWNL--
-RPWNLA-
-RPWNLT-
RLPWNL--
RWPWNL--
RSPWNL--
RAPWNL--
RIPWNL--
-RPWNLG-
RNPWNL--
RPPWNL--
-RPWNLR-

RHPWNL--
-RPWNLN-
-RPWNLQ-
RTPWNL--
RYPWNL--
-RPWNLI-
RKPWNL--
-RPWNLV-
REPWNL--
-RPWNLY-
-RPWNLE-
RQPWNL--
-RPWNLW-
-RPWNLK-
-RPWNLH-
-RPWNLD-
RDPWNL--
-RPWNLC-
RFPWNL--
SRPWNL--
-SPWNLR-
-SPWNLY-
SNPWNL--
SSPWNL--
SLPWNL--
-SPWNLK-
-SPWNLI-
STPWNL--
SKPWNL--
-SPWNLS-
SQPWNL--
SHPWNL--
-SPWNLF-
SEPWNL--
SFPWNL--
SGPWNL--
-SPWNLE-
SMPWNL--
-SPWNLV-
-SPWNLQ-
-SPWNLT-
-SPWNLM-
-SPWNLG-
SDPWNL--
-SPWNLN-
SAPWNL--
-SPWNLH-
SVPWNL--
SPPWNL--
SYPWNL--

SWPWNL--
-SPWNLA-
SCPWNL--
TRPWNL--
TLPWNL--
-TPWNLS-
-TPWNLY-
TGPWNL--
TTPWNL--
TPPWNL--
TAPWNL--
TKPWNL--
-TPWNLK-
-TPWNLL-
-TPWNLH-
-TPWNLT-
-TPWNLA-
TSPWNL--
TEPWNL--
TVPWNL--
-TPWNLV-
-TPWNLR-
-TPWNLN-
-TPWNLI-
-TPWNLM-
TQPWNL--
TMPWNL--
TIPWNL--
TFPWNL--
TWPWNL--
TNPWNL--
-TPWNLQ-
THPWNL--
-TPWNLF-
-TPWNLG-
-TPWNLE-
TYPWNL--
-TPWNLD-
-TPWNLW-
TCPWNL--
-VPWNLL-
VKPWNL--
-VPWNLS-
-VPWNLR-
-VPWNLW-
VSPWNL--
-VPWNLK-
VAPWNL--
-VPWNLT-
VYPWNL--

-VPWNLG-
VPPWNL--
-VPWNLH-
VQPWNL--
VNPWNL--
-VPWNLN-
-VPWNLA-
VIPWNL--
VDPWNL--
-VPWNLE-
-VPWNLD-
-VPWNLM-
-VPWNLI-
VVPWNL--
VHPWNL--
VGPWNL--
VFPWNL--
VWPWNL--
-VPWNLV-
VTPWNL--
-VPWNLQ-
-VPWNLF-
-VPWNLC-
VCPWNL--
WQPWNL--
WLPWNL--
WSPWNL--
-WPWNLN-
WGPWNL--
-WPWNLK-
WMPWNL--
WRPWNL--
WTPWNL--
-WPWNLR-
-WPWNLQ-
WKPWNL--
-WPWNLT-
-WPWNLV-
-WPWNLH-
WDPWNL--
WVPWNL--
-WPWNLW-
-WPWNLA-
WIPWNL--
-WPWNLM-
-WPWNLD-
WHPWNL--
YSPWNL--
YKPWNL--
YRPWNL--

```
            -YPWNLK-
            -YPWNLI-
            -YPWNLA-
            YVPWNL--
            YTPWNL--
            -YPWNLS-
            -YPWNLR-
            YGPWNL--
            -YPWNLN-
            YLPWNL--
            -YPWNLG-
            YAPWNL--
            -YPWNLQ-
            -YPWNLM-
            YEPWNL--
            YYPWNL--
            YDPWNL--
            YWPWNL--
            YNPWNL--
            YHPWNL--
            YFPWNL--
            -YPWNLV-
            -YPWNLT-
            -YPWNLH-
            -YPWNLE-
            YMPWNL--
            YPPWNL--
PYAL          44         856      50.11
            --PYALPS
            --PYALPR
            --PYALDV
            ASPYAL--
            ALPYAL--
            ATPYAL--
            -APYALT-
            AKPYAL--
            AVPYAL--
            -APYALL-
            ARPYAL--
            -APYALR-
            -APYALG-
            -APYALS-
            ANPYAL--
            AYPYAL--
            -APYALN-
            -APYALA-
            AHPYAL--
            AAPYAL--
            AQPYAL--
            APPYAL--
```

AFPYAL--
AEPYAL--
AMPYAL--
-APYALV-
AGPYAL--
AIPYAL--
AWPYAL--
-APYALK-
-APYALW-
-APYALQ-
-APYALM-
-APYALD-
CYPYAL--
CGPYAL--
CQPYAL--
CFPYAL--
-CPYALT-
CSPYAL--
-CPYALV-
CCPYAL--
-CPYALR-
-CPYALL-
-CPYALK-
CHPYAL--
-CPYALS-
-CPYALN-
-CPYALM-
-CPYALG-
CLPYAL--
-DPYALV-
DRPYAL--
DYPYAL--
DKPYAL--
DSPYAL--
-DPYALW-
DMPYAL--
DIPYAL--
DFPYAL--
DQPYAL--
-DPYALS-
DLPYAL--
DTPYAL--
DNPYAL--
DHPYAL--
-DPYALR-
DDPYAL--
DAPYAL--
DWPYAL--
-DPYALM-
-DPYALL-

-DPYALT-
DVPYAL--
-DPYALF-
-DPYALN-
-EPYALR-
-EPYALT-
ESPYAL--
ELPYAL--
-EPYALS-
ERPYAL--
-EPYALL-
EGPYAL--
EWPYAL--
ENPYAL--
-EPYALN-
-EPYALM-
-EPYALI-
EAPYAL--
EVPYAL--
ETPYAL--
-EPYALW-
EQPYAL--
-EPYALQ-
EKPYAL--
EYPYAL--
-EPYALK-
EMPYAL--
-EPYALV-
-EPYALY-
-EPYALF-
EHPYAL--
EEPYAL--
EIPYAL--
EFPYAL--
-EPYALE-
-EPYALH-
-EPYALA-
-EPYALD-
FGPYAL--
FLPYAL--
FFPYAL--
FAPYAL--
FSPYAL--
-FPYALM-
FRPYAL--
-FPYALR-
-FPYALH-
-FPYALN-
-FPYALA-
FVPYAL--

```
-FPYALS-
FPPYAL--
FNPYAL--
-FPYALQ-
FKPYAL--
FIPYAL--
FEPYAL--
-FPYALK-
FQPYAL--
-FPYALY-
-FPYALT-
-FPYALG-
FWPYAL--
-FPYALV-
FHPYAL--
-FPYALP-
-FPYALE-
FYPYAL--
GRPYAL--
GSPYAL--
-GPYALS-
GFPYAL--
-GPYALL-
GKPYAL--
-GPYALI-
GLPYAL--
-GPYALH-
GQPYAL--
-GPYALT-
GIPYAL--
GNPYAL--
GMPYAL--
GVPYAL--
-GPYALF-
-GPYALN-
GWPYAL--
GTPYAL--
GEPYAL--
GHPYAL--
GAPYAL--
-GPYALM-
-GPYALG-
GYPYAL--
-GPYALA-
-GPYALQ-
-GPYALV-
GDPYAL--
GPPYAL--
-GPYALK-
-GPYALE-
```

-GPYALW-
-HPYALR-
HRPYAL--
HYPYAL--
-HPYALT-
-HPYALQ-
HAPYAL--
HNPYAL--
HWPYAL--
-HPYALK-
HGPYAL--
HSPYAL--
-HPYALH-
HKPYAL--
HVPYAL--
-HPYALG-
HHPYAL--
HIPYAL--
-HPYALE-
HLPYAL--
-HPYALN-
-HPYALI-
-HPYALA-
HDPYAL--
HQPYAL--
-HPYALW-
-HPYALM-
HFPYAL--
-HPYALY-
HPPYAL--
-HPYALV-
-HPYALP-
HEPYAL--
-IPYALT-
ILPYAL--
IHPYAL--
IKPYAL--
IRPYAL--
-IPYALR-
-IPYALN-
-IPYALM-
IVPYAL--
IQPYAL--
IPPYAL--
IFPYAL--
IGPYAL--
-IPYALI-
-IPYALG-
INPYAL--
-IPYALQ-

-IPYALA-
IEPYAL--
IAPYAL--
-IPYALL-
ITPYAL--
-IPYALE-
-IPYALH-
IIPYAL--
IYPYAL--
IWPYAL--
-IPYALV-
-IPYALS-
IDPYAL--
-IPYALW-
-IPYALK-
-IPYALD-
-KPYALM-
KGPYAL--
-KPYALL-
KTPYAL--
-KPYALR-
KRPYAL--
-KPYALA-
-KPYALY-
KFPYAL--
KVPYAL--
KWPYAL--
-KPYALI-
KYPYAL--
-KPYALG-
KEPYAL--
-KPYALT-
-KPYALV-
-KPYALN-
-KPYALK-
-KPYALH-
-KPYALE-
-KPYALW-
KIPYAL--
KDPYAL--
-KPYALQ-
KQPYAL--
KNPYAL--
KHPYAL--
KPPYAL--
KCPYAL--
-KPYALD-
LRPYAL--
LGPYAL--
LVPYAL--

LHPYAL--
LMPYAL--
-LPYALI-
-LPYALL-
-LPYALR-
LSPYAL--
-LPYALT-
LAPYAL--
LLPYAL--
-LPYALS-
LTPYAL--
LEPYAL--
-LPYALA-
LWPYAL--
-LPYALQ-
LQPYAL--
-LPYALV-
-LPYALK-
LNPYAL--
LPPYAL--
-LPYALW-
-LPYALH-
-LPYALG-
-LPYALM-
-LPYALN-
LDPYAL--
-LPYALE-
LCPYAL--
-LPYALC-
MVPYAL--
-MPYALS-
MAPYAL--
MSPYAL--
-MPYALL-
MKPYAL--
-MPYALH-
MLPYAL--
-MPYALG-
MGPYAL--
MDPYAL--
-MPYALT-
-MPYALW-
-MPYALN-
-MPYALI-
MMPYAL--
-MPYALV-
-MPYALK-
MNPYAL--
-MPYALA-
-MPYALR-

```
MEPYAL--
MYPYAL--
-MPYALF-
MIPYAL--
-MPYALQ-
-MPYALE-
MHPYAL--
MFPYAL--
MCPYAL--
MPPYAL--
-MPYALD-
-MPYALM-
NRPYAL--
-NPYALL-
NSPYAL--
NGPYAL--
-NPYALS-
NYPYAL--
NTPYAL--
-NPYALT-
-NPYALK-
NLPYAL--
-NPYALQ-
-NPYALA-
NWPYAL--
-NPYALY-
NKPYAL--
-NPYALI-
NFPYAL--
NVPYAL--
-NPYALR-
NAPYAL--
-NPYALV-
NIPYAL--
-NPYALH-
-NPYALE-
NPPYAL--
NDPYAL--
NQPYAL--
-NPYALW-
-NPYALG-
-NPYALN-
NNPYAL--
NEPYAL--
NHPYAL--
NMPYAL--
-NPYALM-
NCPYAL--
--PYALSR
--PYALSS
```

--PYALRS
--PYALLS
--PYALKR
--PYALTR
--PYALRT
--PYALLK
--PYALVS
--PYALRN
--PYALYG
--PYALAR
--PYALLN
--PYALLA
--PYALWR
--PYALSG
--PYALNL
--PYALIR
--PYALYS
--PYALHT
--PYALRA
--PYALVN
--PYALSK
PSPYAL--
--PYALLG
--PYALGL
--PYALPL
--PYALGS
--PYALLH
PAPYAL--
--PYALQD
--PYALSV
--PYALRQ
--PYALRD
--PYALKT
--PYALHR
--PYALSH
--PYALMK
--PYALKA
--PYALAV
--PYALRY
--PYALGT
--PYALGG
--PYALQL
--PYALNR
--PYALGA
--PYALAS
--PYALSI
--PYALMS
--PYALKV
--PYALHL
--PYALER

--PYALTS
--PYALSQ
--PYALNT
--PYALNF
--PYALGY
--PYALAL
--PYALTE
--PYALLE
--PYALHG
--PYALSN
--PYALKK
--PYALWN
--PYALRH
--PYALNV
--PYALTT
--PYALTL
--PYALST
--PYALQR
--PYALMR
PGPYAL--
--PYALWS
--PYALTK
--PYALQS
--PYALIS
--PYALRW
--PYALNN
--PYALNA
--PYALMH
--PYALKL
--PYALHN
PTPYAL--
--PYALWM
--PYALTQ
--PYALKG
PHPYAL--
--PYALSY
--PYALNK
--PYALMN
PIPYAL--
--PYALVV
--PYALHK
--PYALGI
--PYALTV
--PYALTD
--PYALSA
--PYALRP
--PYALFK
--PYALET
--PYALEM
--PYALDS

--PYALAN
--PYALAA
--PYALWE
--PYALFN
--PYALFE
--PYALAK
--PYALNG
--PYALMV
--PYALMT
--PYALLQ
--PYALLD
--PYALFH
--PYALDG
PKPYAL--
--PYALYN
--PYALWH
--PYALWG
--PYALVT
--PYALQA
--PYALHA
--PYALFY
PVPYAL--
PNPYAL--
--PYALTH
--PYALQN
--PYALPY
--PYALPI
--PYALAM
--PYALYI
--PYALWD
--PYALTA
--PYALQK
--PYALQE
--PYALNS
--PYALIY
--PYALHH
--PYALGF
--PYALWT
--PYALSW
--PYALQW
--PYALQM
--PYALGV
--PYALGN
PMPYAL--
PDPYAL--
--PYALVG
--PYALTY
--PYALTM
--PYALSD
--PYALNQ

--PYALKF
--PYALIQ
--PYALDL
PYPYAL--
--PYALTI
--PYALQT
--PYALMG
--PYALKM
--PYALHY
--PYALHF
--PYALGQ
--PYALDM
--PYALTN
--PYALQQ
--PYALQI
--PYALMP
--PYALKI
--PYALHD
--PYALGM
--PYALED
--PYALAW
--PYALAQ
--PYALYP
--PYALSM
--PYALQV
--PYALMW
--PYALKN
--PYALKH
--PYALIA
--PYALHV
--PYALFQ
--PYALFD
--PYALAH
--PYALAG
--PYALYD
--PYALWQ
--PYALVM
--PYALQH
--PYALEH
--PYALEF
--PYALEA
--PYALAF
--PYALPM
--PYALMQ
--PYALMD
--PYALKW
--PYALKE
--PYALIP
--PYALHE
--PYALFP

--PYALEG
--PYALAT
--PYALYV
--PYALVE
--PYALPF
--PYALLP
--PYALKP
--PYALHQ
--PYALGD
--PYALEQ
--PYALEK
--PYALYQ
--PYALWK
--PYALVP
--PYALVD
--PYALQY
--PYALNE
--PYALLC
--PYALIH
--PYALHP
--PYALGK
--PYALGH
--PYALFG
--PYALEV
--PYALDY
--PYALDA
PFPYAL--
PEPYAL--
--PYALVA
--PYALTW
--PYALQG
--PYALQF
--PYALIG
--PYALID
--PYALEE
--PYALCK
--PYALAE
--PYALVQ
--PYALTC
--PYALSP
--PYALRC
--PYALPW
--PYALPA
--PYALNM
--PYALNI
--PYALND
--PYALMI
--PYALME
--PYALKY
--PYALKC

--PYALHW
--PYALGP
--PYALGE
--PYALGC
--PYALFC
--PYALEY
--PYALEN
--PYALEI
--PYALDF
--PYALCR
--PYALCD
-PPYALH-
PPPYAL--
QSPYAL--
QRPYAL--
-QPYALS-
-QPYALL-
-QPYALH-
QPPYAL--
QHPYAL--
-QPYALR-
-QPYALA-
-QPYALI-
QVPYAL--
-QPYALQ-
-QPYALK-
-QPYALG-
QAPYAL--
QYPYAL--
QWPYAL--
QTPYAL--
QFPYAL--
-QPYALY-
QLPYAL--
QGPYAL--
QQPYAL--
-QPYALN-
QMPYAL--
QIPYAL--
-QPYALW-
-QPYALM-
-QPYALT-
QNPYAL--
QDPYAL--
QKPYAL--
QEPYAL--
-QPYALD-
RGPYAL--
-RPYALR-
RTPYAL--

```
-RPYALK-
RVPYAL--
RLPYAL--
-RPYALG-
RAPYAL--
RFPYAL--
-RPYALA-
RMPYAL--
RSPYAL--
RIPYAL--
RPPYAL--
RHPYAL--
RYPYAL--
RWPYAL--
-RPYALW-
-RPYALV-
-RPYALN-
RQPYAL--
RNPYAL--
REPYAL--
-RPYALM-
RKPYAL--
-RPYALH-
RDPYAL--
-RPYALT-
-RPYALQ-
-RPYALF-
RCPYAL--
SSPYAL--
STPYAL--
-SPYALV-
SEPYAL--
-SPYALA-
SGPYAL--
-SPYALL-
-SPYALH-
SAPYAL--
-SPYALS-
SMPYAL--
SVPYAL--
SIPYAL--
SFPYAL--
-SPYALK-
SNPYAL--
SHPYAL--
-SPYALI-
SKPYAL--
-SPYALG-
SQPYAL--
-SPYALW-
```

```
-SPYALN-
-SPYALF-
SWPYAL--
-SPYALY-
SPPYAL--
SYPYAL--
-SPYALM-
SDPYAL--
-SPYALE-
-SPYALC-
-SPYALD-
SCPYAL--
TKPYAL--
TSPYAL--
TYPYAL--
-TPYALW-
TRPYAL--
TLPYAL--
-TPYALR-
-TPYALG-
TFPYAL--
-TPYALM-
-TPYALL-
-TPYALK-
TPPYAL--
-TPYALI-
TTPYAL--
-TPYALN-
THPYAL--
TIPYAL--
-TPYALV-
-TPYALT-
-TPYALA-
TNPYAL--
TMPYAL--
TWPYAL--
-TPYALQ-
TQPYAL--
TVPYAL--
-TPYALH-
-TPYALE-
TDPYAL--
-TPYALC-
TCPYAL--
-VPYALR-
VRPYAL--
VSPYAL--
VVPYAL--
-VPYALW-
VLPYAL--
```

VKPYAL--
VYPYAL--
VTPYAL--
-VPYALS-
VIPYAL--
VGPYAL--
-VPYALT-
-VPYALA-
VFPYAL--
VEPYAL--
VHPYAL--
VQPYAL--
-VPYALK-
VDPYAL--
-VPYALN-
VPPYAL--
-VPYALH-
-VPYALM-
VAPYAL--
-VPYALV-
-VPYALG-
VMPYAL--
VCPYAL--
VWPYAL--
-VPYALQ-
VNPYAL--
-VPYALD-
WKPYAL--
WYPYAL--
WTPYAL--
WPPYAL--
WNPYAL--
WEPYAL--
WRPYAL--
-WPYALL-
WDPYAL--
WAPYAL--
-WPYALN-
WGPYAL--
-WPYALM-
-WPYALF-
WFPYAL--
-WPYALS-
-WPYALG-
-WPYALE-
-WPYALR-
WLPYAL--
-WPYALQ-
-WPYALK-
-WPYALA-

```
              -WPYALV-
              -WPYALT-
              -WPYALI-
              WIPYAL--
              WHPYAL--
              -WPYALW-
              -WPYALH-
              YVPYAL--
              YIPYAL--
              -YPYALL-
              YSPYAL--
              YRPYAL--
              YHPYAL--
              YQPYAL--
              -YPYALN-
              YAPYAL--
              YLPYAL--
              YPPYAL--
              YTPYAL--
              YMPYAL--
              YNPYAL--
              YFPYAL--
              YDPYAL--
              -YPYALK-
              -YPYALM-
              -YPYALA-
              -YPYALH-
              -YPYALG-
              YYPYAL--
              -YPYALW-
              YEPYAL--
              -YPYALQ-
              YKPYAL--
              YCPYAL--
PLGM             9        909      50.07
              -LPLGMD-
              --PLGMPS
              --PLGMPK
              --PLGMFP
              --PLGMDN
              -SPLGMP-
              YDPLGM--
              -APLGMR-
              ARPLGM--
              -APLGMI-
              ATPLGM--
              -APLGMS-
              -APLGMQ-
              -APLGML-
              -APLGMK-
```

-APLGMN-
AKPLGM--
ASPLGM--
ALPLGM--
-APLGMG-
-APLGMY-
-APLGMW-
-APLGMV-
AGPLGM--
AFPLGM--
AEPLGM--
AAPLGM--
AIPLGM--
AWPLGM--
APPLGM--
-APLGME-
AVPLGM--
-APLGMA-
AYPLGM--
-APLGMM-
ANPLGM--
AHPLGM--
ADPLGM--
-APLGMC-
ACPLGM--
-APLGMH-
CKPLGM--
-CPLGMR-
CRPLGM--
-CPLGMS-
-CPLGMK-
-CPLGMM-
CGPLGM--
CVPLGM--
-CPLGMT-
CTPLGM--
-CPLGMW-
CNPLGM--
CLPLGM--
CSPLGM--
-CPLGML-
CEPLGM--
CDPLGM--
CQPLGM--
CMPLGM--
-CPLGMQ-
-CPLGMN-
-CPLGMI-
-CPLGMG-
-CPLGME-

CIPLGM--
CHPLGM--
CCPLGM--
CAPLGM--
-DPLGMV-
-DPLGMW-
DHPLGM--
-DPLGMS-
-DPLGMN-
-DPLGMR-
-DPLGMF-
-DPLGMY-
DWPLGM--
-DPLGMA-
DNPLGM--
DTPLGM--
DSPLGM--
DYPLGM--
DVPLGM--
-DPLGML-
-DPLGMK-
-DPLGMG-
DDPLGM--
DAPLGM--
DFPLGM--
DEPLGM--
-DPLGMT-
-DPLGMQ-
DMPLGM--
DLPLGM--
DGPLGM--
DKPLGM--
-DPLGMH-
DCPLGM--
-DPLGMI-
-DPLGME-
-EPLGML-
-EPLGMS-
ERPLGM--
ESPLGM--
EVPLGM--
-EPLGMG-
-EPLGMY-
-EPLGMN-
-EPLGMM-
ETPLGM--
-EPLGMA-
EMPLGM--
ELPLGM--
-EPLGMV-

-EPLGMT-
EGPLGM--
-EPLGMF-
-EPLGMW-
-EPLGME-
EIPLGM--
ENPLGM--
EQPLGM--
-EPLGMQ-
-EPLGMH-
EFPLGM--
EYPLGM--
-EPLGMK-
-EPLGMC-
EEPLGM--
ECPLGM--
EAPLGM--
FSPLGM--
-FPLGMS-
FRPLGM--
FVPLGM--
-FPLGMT-
FKPLGM--
FMPLGM--
-FPLGML-
-FPLGMQ-
-FPLGMG-
-FPLGMW-
FNPLGM--
-FPLGMM-
-FPLGMA-
FIPLGM--
FPPLGM--
FTPLGM--
FLPLGM--
-FPLGMV-
-FPLGMK-
-FPLGMH-
FGPLGM--
FQPLGM--
FHPLGM--
-FPLGMY-
FDPLGM--
FWPLGM--
-FPLGMF-
-FPLGME-
-FPLGMD-
FCPLGM--
-GPLGML-
GRPLGM--

-GPLGMS-
GLPLGM--
-GPLGMV-
-GPLGMR-
GAPLGM--
GTPLGM--
-GPLGMM-
-GPLGMF-
GNPLGM--
-GPLGMI-
-GPLGMT-
GGPLGM--
-GPLGMA-
GMPLGM--
-GPLGMG-
GIPLGM--
GVPLGM--
GSPLGM--
-GPLGMY-
-GPLGME-
GKPLGM--
GWPLGM--
-GPLGMK-
-GPLGMH-
-GPLGMN-
GDPLGM--
GCPLGM--
GPPLGM--
GEPLGM--
GQPLGM--
-GPLGMQ-
HDPLGM--
-HPLGMK-
-HPLGMS-
HRPLGM--
-HPLGMR-
HKPLGM--
-HPLGMM-
-HPLGMQ-
HEPLGM--
-HPLGMH-
HGPLGM--
HFPLGM--
-HPLGMG-
-HPLGMT-
HMPLGM--
HHPLGM--
HWPLGM--
HLPLGM--
HVPLGM--

HYPLGM--
HQPLGM--
-HPLGML-
-HPLGMI-
-HPLGMF-
HAPLGM--
HSPLGM--
-HPLGMA-
-HPLGMN-
HTPLGM--
HIPLGM--
-HPLGME-
HPPLGM--
-HPLGMY-
-HPLGMV-
-IPLGMR-
-IPLGMS-
-IPLGML-
IRPLGM--
IYPLGM--
ISPLGM--
IVPLGM--
-IPLGMK-
-IPLGMM-
-IPLGMA-
-IPLGMY-
-IPLGMW-
-IPLGMN-
IHPLGM--
ITPLGM--
IAPLGM--
IPPLGM--
-IPLGMT-
INPLGM--
IMPLGM--
IFPLGM--
IIPLGM--
IGPLGM--
-IPLGMQ-
-IPLGME-
IQPLGM--
-IPLGMI-
IEPLGM--
IKPLGM--
IDPLGM--
IWPLGM--
-IPLGMC-
KVPLGM--
-KPLGMI-
-KPLGMT-

KEPLGM--
KRPLGM--
-KPLGMS-
-KPLGMY-
-KPLGMV-
KLPLGM--
-KPLGMA-
KAPLGM--
KYPLGM--
KMPLGM--
-KPLGMK-
-KPLGMQ-
KWPLGM--
KTPLGM--
KHPLGM--
KIPLGM--
KQPLGM--
-KPLGMF-
KNPLGM--
KFPLGM--
-KPLGMG-
-KPLGME-
-KPLGMD-
-KPLGMP-
-KPLGMH-
KDPLGM--
LRPLGM--
-LPLGMI-
LYPLGM--
LGPLGM--
LTPLGM--
LSPLGM--
LPPLGM--
LAPLGM--
-LPLGMT-
LIPLGM--
-LPLGMS-
LLPLGM--
LMPLGM--
LFPLGM--
-LPLGMY-
-LPLGMN-
-LPLGMW-
LNPLGM--
-LPLGMK-
-LPLGMA-
LQPLGM--
-LPLGMV-
-LPLGMH-
-LPLGMF-

LKPLGM--
-LPLGME-
-LPLGMQ-
-LPLGMG-
-LPLGMM-
LHPLGM--
-MPLGMS-
MRPLGM--
-MPLGMG-
MGPLGM--
-MPLGMN-
-MPLGMR-
MLPLGM--
-MPLGMT-
-MPLGMY-
MKPLGM--
MHPLGM--
MIPLGM--
MYPLGM--
MVPLGM--
-MPLGMV-
-MPLGMK-
-MPLGMH-
MNPLGM--
MTPLGM--
-MPLGMW-
MFPLGM--
-MPLGMQ-
-MPLGME-
MEPLGM--
MWPLGM--
MQPLGM--
-MPLGMF-
MPPLGM--
-MPLGMD-
MAPLGM--
-NPLGMI-
NVPLGM--
-NPLGMS-
NLPLGM--
NEPLGM--
NNPLGM--
-NPLGML-
NGPLGM--
NFPLGM--
NHPLGM--
NSPLGM--
NQPLGM--
NWPLGM--
-NPLGMT-

NKPLGM--
NTPLGM--
-NPLGMW-
-NPLGMN-
NYPLGM--
-NPLGMM-
-NPLGMQ-
NRPLGM--
-NPLGMK-
-NPLGMA-
NMPLGM--
NIPLGM--
-NPLGMG-
-NPLGMF-
NCPLGM--
NAPLGM--
NPPLGM--
-NPLGMH-
NDPLGM--
--PLGMRL
--PLGMVR
--PLGMSR
--PLGMRA
--PLGMLT
--PLGMGS
--PLGMSY
--PLGMYR
--PLGMTR
--PLGMVA
--PLGMVL
--PLGMFR
--PLGMST
--PLGMLI
--PLGMSV
--PLGMRH
--PLGMIL
--PLGMAG
--PLGMTI
--PLGMSG
--PLGMRN
--PLGMKR
--PLGMTS
--PLGMSF
--PLGMSI
--PLGMVY
--PLGMTY
--PLGMTL
--PLGMKA
--PLGMWS
--PLGMVS

--PLGMRG
--PLGMHL
--PLGMVM
--PLGMSH
--PLGMGA
--PLGMTG
--PLGMQG
--PLGMVG
--PLGMLN
--PLGMLQ
--PLGMLK
--PLGMRM
PGPLGM--
--PLGMLV
--PLGMHR
--PLGMRV
--PLGMRT
--PLGMIN
--PLGMHH
--PLGMII
--PLGMVN
--PLGMRQ
--PLGMNL
--PLGMTT
--PLGMKS
--PLGMHM
--PLGMSQ
--PLGMRW
--PLGMML
--PLGMGR
--PLGMGI
--PLGMGF
--PLGMAS
PWPLGM--
PRPLGM--
--PLGMYS
--PLGMYG
--PLGMRY
--PLGMYD
--PLGMTF
--PLGMFS
--PLGMEV
--PLGMQL
--PLGMIR
--PLGMGV
--PLGMWN
--PLGMVI
--PLGMRD
--PLGMNR
--PLGMKV

--PLGMEW
--PLGMSK
--PLGMRK
--PLGMIV
PIPLGM--
--PLGMVT
--PLGMKY
--PLGMGW
--PLGMER
PVPLGM--
--PLGMWT
--PLGMTK
--PLGMSE
--PLGMMT
--PLGMLD
--PLGMHS
--PLGMGY
--PLGMFA
--PLGMAL
--PLGMYQ
--PLGMWI
--PLGMSW
--PLGMMR
--PLGMFK
--PLGMRP
--PLGMKK
--PLGMIS
--PLGMGL
--PLGMAN
--PLGMYT
--PLGMVK
--PLGMSN
--PLGMGT
--PLGMEY
--PLGMYH
--PLGMWR
--PLGMWH
--PLGMQR
--PLGMNV
--PLGMKM
--PLGMKG
--PLGMFQ
--PLGMAV
--PLGMWK
--PLGMTA
--PLGMFD
--PLGMDS
--PLGMYM
--PLGMRF
--PLGMMM

--PLGMMH
--PLGMLH
--PLGMKL
--PLGMIQ
--PLGMGK
--PLGMFT
--PLGMAY
--PLGMAF
--PLGMYN
--PLGMTH
--PLGMSM
--PLGMIH
--PLGMHT
--PLGMHN
--PLGMHA
--PLGMVH
--PLGMNI
--PLGMKT
--PLGMKE
--PLGMAW
PTPLGM--
--PLGMTW
--PLGMTQ
--PLGMMI
--PLGMEF
PFPLGM--
--PLGMYA
--PLGMMQ
--PLGMKH
--PLGMIY
--PLGMIW
--PLGMDG
--PLGMSD
--PLGMQS
--PLGMLE
--PLGMFW
--PLGMYK
--PLGMVE
--PLGMVD
--PLGMTV
--PLGMQM
--PLGMNH
--PLGMHW
--PLGMHV
--PLGMGG
--PLGMFN
--PLGMEG
--PLGMWQ
--PLGMVW
--PLGMTD

--PLGMQN
--PLGMQK
--PLGMQA
--PLGMNQ
--PLGMMV
--PLGMMD
--PLGMKN
--PLGMIK
--PLGMIE
--PLGMID
--PLGMHK
--PLGMAT
--PLGMAA
--PLGMRE
--PLGMME
--PLGMAI
PEPLGM--
--PLGMSC
--PLGMMN
--PLGMHE
--PLGMGQ
--PLGMDT
--PLGMAM
PNPLGM--
--PLGMQT
--PLGMNM
--PLGMMG
--PLGMFM
--PLGMAK
--PLGMYP
--PLGMVQ
--PLGMTM
--PLGMTE
--PLGMMW
--PLGMMA
--PLGMKD
--PLGMIG
--PLGMGN
--PLGMEL
PYPLGM--
PMPLGM--
--PLGMLM
--PLGMGM
--PLGMTN
--PLGMQV
--PLGMNK
--PLGMNG
--PLGMNF
--PLGMND
--PLGMIT

--PLGMGH
--PLGMEQ
--PLGMEM
--PLGMDV
PKPLGM--
--PLGMYE
--PLGMQI
--PLGMQF
--PLGMPG
--PLGMNW
--PLGMKF
--PLGMHD
--PLGMEH
--PLGMEA
--PLGMDY
--PLGMDM
--PLGMAH
--PLGMAC
--PLGMNS
--PLGMNN
--PLGMNE
--PLGMMP
--PLGMGE
--PLGMGD
--PLGMDA
--PLGMAE
PCPLGM--
--PLGMMK
--PLGMKW
--PLGMKQ
--PLGMHI
--PLGMHF
--PLGMFE
--PLGMEK
--PLGMEE
--PLGMED
--PLGMDW
--PLGMCY
--PLGMCA
--PLGMAQ
PHPLGM--
PDPLGM--
-PPLGMT-
-PPLGMN-
-PPLGML-
-PPLGMH-
--PLGMYC
--PLGMWF
--PLGMWD
--PLGMSP

--PLGMQQ
--PLGMPN
--PLGMKI
--PLGMHG
--PLGMDH
--PLGMDF
--PLGMCS
--PLGMCG
--PLGMCD
-QPLGMS-
-QPLGMR-
QSPLGM--
QLPLGM--
QRPLGM--
-QPLGMG-
-QPLGMI-
QMPLGM--
-QPLGMV-
-QPLGMK-
-QPLGMH-
QVPLGM--
QNPLGM--
-QPLGML-
-QPLGMQ-
-QPLGMM-
QIPLGM--
-QPLGMW-
QAPLGM--
-QPLGME-
-QPLGMY-
QTPLGM--
QKPLGM--
QQPLGM--
-QPLGMT-
-QPLGMN-
QYPLGM--
QPPLGM--
-QPLGMF-
-QPLGMA-
QHPLGM--
QGPLGM--
QFPLGM--
QDPLGM--
-RPLGMS-
RVPLGM--
-RPLGML-
RGPLGM--
-RPLGMI-
-RPLGMF-
RMPLGM--

-RPLGMY-
-RPLGMV-
-RPLGMN-
-RPLGMH-
RAPLGM--
RTPLGM--
RWPLGM--
-RPLGMQ-
RYPLGM--
-RPLGMG-
-RPLGMM-
RIPLGM--
-RPLGMK-
RPPLGM--
RNPLGM--
RKPLGM--
RHPLGM--
RFPLGM--
RDPLGM--
-RPLGMW-
REPLGM--
-RPLGME-
RQPLGM--
-RPLGMD-
RCPLGM--
SRPLGM--
STPLGM--
SLPLGM--
-SPLGMV-
-SPLGMK-
-SPLGMY-
SSPLGM--
SQPLGM--
SWPLGM--
-SPLGMS-
-SPLGMH-
SGPLGM--
SFPLGM--
SKPLGM--
SIPLGM--
-SPLGMI-
SMPLGM--
-SPLGMT-
-SPLGMM-
-SPLGMG-
-SPLGMN-
-SPLGMA-
SYPLGM--
-SPLGMQ-
SNPLGM--

SAPLGM--
-SPLGMF-
SHPLGM--
SDPLGM--
SPPLGM--
-SPLGMW-
-SPLGME-
SEPLGM--
-SPLGMD-
SCPLGM--
-TPLGML-
-TPLGMR-
-TPLGMY-
TTPLGM--
TSPLGM--
-TPLGMI-
-TPLGMF-
-TPLGMH-
-TPLGMS-
TAPLGM--
-TPLGMV-
TGPLGM--
-TPLGMA-
TVPLGM--
TMPLGM--
-TPLGMG-
TIPLGM--
TYPLGM--
-TPLGMW-
-TPLGME-
-TPLGMT-
-TPLGMQ-
TPPLGM--
TLPLGM--
-TPLGMK-
TFPLGM--
TWPLGM--
-TPLGMM-
TQPLGM--
TNPLGM--
TEPLGM--
TDPLGM--
-TPLGMD-
TKPLGM--
TCPLGM--
-VPLGMR-
VAPLGM--
VSPLGM--
VVPLGM--
-VPLGMS-

-VPLGMW-
-VPLGML-
-VPLGMH-
VHPLGM--
VPPLGM--
VRPLGM--
-VPLGMN-
-VPLGMK-
-VPLGMG-
VLPLGM--
-VPLGMY-
-VPLGMM-
VGPLGM--
VEPLGM--
-VPLGMT-
VYPLGM--
-VPLGMV-
VDPLGM--
-VPLGMI-
VQPLGM--
-VPLGMQ-
VFPLGM--
VMPLGM--
VWPLGM--
VIPLGM--
-VPLGME-
-VPLGMF-
-VPLGMD-
-VPLGMA-
WRPLGM--
WTPLGM--
-WPLGML-
-WPLGMR-
WGPLGM--
WLPLGM--
WWPLGM--
-WPLGMT-
WSPLGM--
WDPLGM--
-WPLGMV-
WEPLGM--
-WPLGMK-
WNPLGM--
-WPLGMY-
-WPLGMW-
-WPLGMG-
-WPLGMI-
WFPLGM--
WYPLGM--
-WPLGMQ-

-WPLGMA-
-WPLGMM-
-WPLGMF-
-WPLGMN-
WKPLGM--
WCPLGM--
WAPLGM--
WHPLGM--
WQPLGM--
-WPLGME-
WIPLGM--
-YPLGMK-
YTPLGM--
-YPLGMR-
-YPLGMT-
-YPLGMS-
YAPLGM--
YLPLGM--
-YPLGMY-
-YPLGMV-
YIPLGM--
YHPLGM--
YSPLGM--
-YPLGMQ-
YKPLGM--
-YPLGMI-
-YPLGMG-
YGPLGM--
YQPLGM--
-YPLGMN-
YMPLGM--
YEPLGM--
YNPLGM--
YYPLGM--
-YPLGME-
-YPLGMF-
-YPLGMA-
YFPLGM--
-YPLGMH-
-YPLGMD-
YPPLGM--
-YPLGMM-
-YPLGMC-
YCPLGM--

PMWI 733 554 50.07

--PMWISM
AVPMWI--
-APMWIK-
-APMWIA-
AEPMWI--

AHPMWI--
AFPMWI--
-APMWIR-
AQPMWI--
AWPMWI--
-APMWIT-
-APMWID-
AYPMWI--
-APMWIW-
-APMWIV-
-APMWIM-
-APMWIE-
ANPMWI--
AIPMWI--
ATPMWI--
APPMWI--
CMPMWI--
CSPMWI--
CVPMWI--
-CPMWIT-
-CPMWIL-
CKPMWI--
DGPMWI--
DSPMWI--
DKPMWI--
DRPMWI--
-DPMWIK-
DTPMWI--
-DPMWIS-
-DPMWIL-
DYPMWI--
DMPMWI--
DEPMWI--
DVPMWI--
DNPMWI--
-DPMWIT-
-DPMWIR-
-DPMWIA-
ESPMWI--
-EPMWIK-
-EPMWIR-
-EPMWIS-
EMPMWI--
EQPMWI--
-EPMWIL-
ELPMWI--
EAPMWI--
EHPMWI--
EVPMWI--
ETPMWI--

```
-EPMWIT-
-EPMWII-
-EPMWIA-
EKPMWI--
ERPMWI--
-EPMWIN-
-EPMWIQ-
-EPMWIH-
-EPMWIE-
-EPMWID-
ENPMWI--
FSPMWI--
FLPMWI--
FDPMWI--
FNPMWI--
FGPMWI--
FYPMWI--
-FPMWIW-
FAPMWI--
FPPMWI--
-FPMWIR-
-FPMWIM-
-FPMWIL-
FKPMWI--
FEPMWI--
FCPMWI--
-FPMWIG-
FHPMWI--
GSPMWI--
-GPMWIQ-
GKPMWI--
-GPMWIR-
GNPMWI--
GTPMWI--
GFPMWI--
GPPMWI--
-GPMWIV-
GAPMWI--
GYPMWI--
GMPMWI--
-GPMWIT-
-GPMWIS-
-GPMWIW-
GVPMWI--
GGPMWI--
-GPMWIN-
-GPMWIH-
-GPMWIG-
-GPMWIF-
GHPMWI--
```

GEPMWI--
GDPMWI--
-HPMWIT-
-HPMWIK-
HKPMWI--
-HPMWIR-
-HPMWIL-
HTPMWI--
-HPMWIA-
HRPMWI--
HHPMWI--
HQPMWI--
HFPMWI--
-HPMWII-
HGPMWI--
HDPMWI--
-HPMWIQ-
-HPMWIH-
HNPMWI--
HYPMWI--
HPPMWI--
-HPMWIY-
-HPMWIN-
HLPMWI--
HAPMWI--
ISPMWI--
IRPMWI--
IAPMWI--
-IPMWIN-
-IPMWIR-
ITPMWI--
IEPMWI--
-IPMWIT-
INPMWI--
IGPMWI--
-IPMWIV-
-IPMWIK-
IIPMWI--
IVPMWI--
-IPMWIM-
-IPMWIL-
IPPMWI--
-IPMWIS-
-IPMWIF-
ILPMWI--
KSPMWI--
KAPMWI--
-KPMWIY-
-KPMWIM-
KIPMWI--

```
-KPMWIG-
KLPMWI--
KVPMWI--
KGPMWI--
KKPMWI--
KTPMWI--
KRPMWI--
KYPMWI--
-KPMWIV-
-KPMWIS-
KEPMWI--
-KPMWIQ-
-KPMWIF-
-KPMWIA-
KPPMWI--
-KPMWIT-
-KPMWIK-
-KPMWID-
KDPMWI--
-KPMWIR-
-KPMWIN-
-KPMWIE-
LGPMWI--
LRPMWI--
LVPMWI--
LKPMWI--
LPPMWI--
-LPMWIS-
LQPMWI--
-LPMWIR-
LEPMWI--
LDPMWI--
-LPMWIQ-
-LPMWIM-
LTPMWI--
-LPMWIG-
LMPMWI--
LHPMWI--
LAPMWI--
-LPMWII-
-LPMWIE-
MNPMWI--
-MPMWIR-
MRPMWI--
-MPMWIN-
MKPMWI--
MTPMWI--
-MPMWIH-
MVPMWI--
MPPMWI--
```

```
-MPMWIT-
MLPMWI--
-MPMWIV-
MGPMWI--
-MPMWIS-
-MPMWIG-
MAPMWI--
NGPMWI--
NSPMWI--
NHPMWI--
NRPMWI--
NNPMWI--
NFPMWI--
-NPMWIR-
-NPMWII-
NVPMWI--
NAPMWI--
-NPMWIS-
NLPMWI--
NDPMWI--
-NPMWIQ-
-NPMWIM-
-NPMWIG-
NEPMWI--
NQPMWI--
NPPMWI--
NIPMWI--
-NPMWIT-
-NPMWIK-
NMPMWI--
NYPMWI--
NTPMWI--
-NPMWIF-
NKPMWI--
--PMWISG
--PMWIRS
--PMWIVS
--PMWIHG
--PMWIFT
--PMWIRT
--PMWILG
--PMWIHA
--PMWISS
--PMWISR
--PMWIGT
--PMWIYS
--PMWILH
--PMWITN
--PMWISH
--PMWIRN
```

--PMWIQK
--PMWIKG
--PMWING
--PMWIQS
--PMWIGG
--PMWIAR
--PMWIVN
--PMWISV
--PMWIIQ
--PMWIVR
--PMWITS
--PMWIMS
--PMWIGF
--PMWIFN
--PMWITG
--PMWILR
--PMWIAG
--PMWIKS
--PMWIGY
--PMWIGA
--PMWIRD
--PMWILQ
--PMWIHK
--PMWIVD
--PMWISQ
--PMWIQR
--PMWINS
--PMWIIS
--PMWIIG
--PMWIGV
--PMWIEW
--PMWIEV
--PMWIYN
--PMWIWS
--PMWIVH
--PMWIST
--PMWIKM
--PMWIAK
--PMWIWT
--PMWIVL
--PMWITA
--PMWISA
--PMWIID
--PMWIGI
--PMWIFQ
--PMWIAT
--PMWIAS
--PMWIWH
--PMWIVQ
--PMWITR

--PMWISD
--PMWIRV
--PMWIRA
--PMWIQG
--PMWIMK
--PMWIMA
--PMWILA
--PMWIKT
--PMWIKA
--PMWIGN
--PMWIGM
--PMWIEH
PTPMWI--
--PMWITI
--PMWIRH
--PMWIQT
--PMWINT
--PMWIMF
--PMWIKD
--PMWIIR
--PMWIGW
--PMWIGS
--PMWIFG
--PMWIYT
--PMWIYG
--PMWIWK
--PMWITT
--PMWIRY
--PMWIRW
--PMWIRK
--PMWINH
--PMWINA
--PMWIMG
--PMWILL
--PMWILK
--PMWIIH
--PMWIIE
--PMWIGD
--PMWIFR
--PMWIES
--PMWIAI
PEPMWI--
--PMWIVK
--PMWITV
--PMWITL
--PMWISY
--PMWIRM
--PMWIQY
--PMWIQL
--PMWIMR

--PMWILD
--PMWIKQ
--PMWIKI
--PMWIKE
--PMWIIM
--PMWIHR
--PMWIGR
--PMWIGE
--PMWIFF
--PMWIFA
--PMWIEE
--PMWIDA
--PMWIAN
PGPMWI--
-PPMWIV-
-PPMWIL-
--PMWIYI
--PMWIWE
--PMWIWA
--PMWIQW
--PMWIQQ
--PMWIQA
--PMWIPG
--PMWIPA
--PMWINI
--PMWIMH
--PMWIME
--PMWILV
--PMWILF
--PMWIKY
--PMWIKV
--PMWIIK
--PMWIHV
--PMWIHT
--PMWIHN
--PMWIHM
--PMWIGL
--PMWIFY
--PMWIFK
--PMWIFH
--PMWIDS
--PMWIAH
--PMWIAD
-QPMWIL-
-QPMWIR-
QSPMWI--
QKPMWI--
QAPMWI--
QTPMWI--
QNPMWI--

QGPMWI--
QYPMWI--
-QPMWIF-
-QPMWIV-
-QPMWIS-
QHPMWI--
-QPMWIY-
QRPMWI--
-QPMWII-
-QPMWIE-
QIPMWI--
-RPMWIR-
RRPMWI--
RSPMWI--
RGPMWI--
-RPMWIG-
-RPMWIV-
RAPMWI--
-RPMWII-
RHPMWI--
RPPMWI--
-RPMWIF-
RKPMWI--
RYPMWI--
RDPMWI--
REPMWI--
-RPMWIM-
RIPMWI--
RTPMWI--
-RPMWIN-
-RPMWIK-
-RPMWIA-
RNPMWI--
-RPMWIQ-
RWPMWI--
-RPMWIW-
-RPMWIY-
-RPMWID-
SRPMWI--
SVPMWI--
SGPMWI--
SHPMWI--
SLPMWI--
-SPMWIR-
SNPMWI--
-SPMWIQ-
-SPMWIM-
-SPMWIT-
SWPMWI--
STPMWI--

SFPMWI--
SQPMWI--
SIPMWI--
SAPMWI--
-SPMWIG-
SKPMWI--
-SPMWII-
SPPMWI--
-SPMWID-
-SPMWIK-
SDPMWI--
TRPMWI--
TKPMWI--
TYPMWI--
TVPMWI--
TSPMWI--
TNPMWI--
TGPMWI--
TIPMWI--
-TPMWIK-
-TPMWIR-
TWPMWI--
TPPMWI--
TFPMWI--
TEPMWI--
-TPMWIM-
THPMWI--
-TPMWIT-
-TPMWIG-
TDPMWI--
-TPMWII-
-TPMWIH-
TQPMWI--
-TPMWIA-
TMPMWI--
TCPMWI--
VSPMWI--
VQPMWI--
-VPMWIG-
VKPMWI--
VVPMWI--
-VPMWIR-
-VPMWIK-
VHPMWI--
VYPMWI--
-VPMWIV-
VLPMWI--
-VPMWIM-
VMPMWI--
VFPMWI--

VAPMWI--
VRPMWI--
-VPMWIT-
VEPMWI--
VWPMWI--
-VPMWII-
-VPMWIH-
-VPMWIE-
-VPMWIY-
VNPMWI--
VTPMWI--
VPPMWI--
-VPMWIS-
-VPMWIQ-
VGPMWI--
VCPMWI--
-VPMWIN-
VIPMWI--
WKPMWI--
-WPMWIY-
WDPMWI--
WQPMWI--
-WPMWIN-
WMPMWI--
WGPMWI--
WAPMWI--
WPPMWI--
-WPMWIR-
-WPMWIG-
WLPMWI--
-WPMWIQ-
YPPMWI--
YSPMWI--
-YPMWIS-
YGPMWI--
YVPMWI--
YKPMWI--
-YPMWIT-
YAPMWI--
-YPMWIY-
-YPMWIH-
YEPMWI--
YRPMWI--
YQPMWI--
-YPMWIA-
-YPMWIG-
YLPMWI--
YWPMWI--
-YPMWIV-

PKNM 375 669 50.03

-EPKNMY-
-APKNMI-
ALPKNM--
-APKNMS-
AKPKNM--
-APKNML-
-APKNMR-
-APKNMY-
AGPKNM--
AVPKNM--
AQPKNM--
-APKNMA-
ARPKNM--
-APKNMV-
-APKNMH-
-APKNMT-
AHPKNM--
AAPKNM--
AWPKNM--
-APKNMF-
AYPKNM--
AEPKNM--
-APKNMN-
-APKNMK-
ANPKNM--
AMPKNM--
APPKNM--
-APKNMM-
-APKNMD-
ACPKNM--
CIPKNM--
CQPKNM--
CVPKNM--
CTPKNM--
-CPKNMR-
-CPKNMI-
CNPKNM--
CFPKNM--
-CPKNME-
CWPKNM--
CPPKNM--
-CPKNMS-
CAPKNM--
-DPKNMI-
-DPKNMV-
DNPKNM--
DYPKNM--
DRPKNM--
DLPKNM--
DHPKNM--

DSPKNM--
-DPKNMT-
-DPKNMS-
-DPKNMR-
-DPKNMK-
DWPKNM--
DQPKNM--
-DPKNMY-
-DPKNME-
DAPKNM--
-EPKNMH-
EVPKNM--
ESPKNM--
-EPKNMR-
-EPKNMI-
ERPKNM--
EGPKNM--
-EPKNMV-
-EPKNMA-
EDPKNM--
-EPKNMS-
-EPKNMM-
EMPKNM--
EYPKNM--
EQPKNM--
EPPKNM--
-EPKNMG-
ELPKNM--
EAPKNM--
FRPKNM--
FLPKNM--
FSPKNM--
FWPKNM--
-FPKNMA-
FNPKNM--
FTPKNM--
-FPKNMW-
FGPKNM--
FVPKNM--
-FPKNMM-
-FPKNML-
-FPKNME-
FFPKNM--
FEPKNM--
-FPKNMH-
-FPKNMV-
-FPKNMS-
FIPKNM--
FPPKNM--
-FPKNMQ-

-FPKNMD-
-GPKNMR-
-GPKNML-
GVPKNM--
-GPKNMQ-
GEPKNM--
GTPKNM--
-GPKNMI-
GIPKNM--
GSPKNM--
-GPKNMS-
-GPKNMA-
-GPKNMW-
-GPKNMN-
GNPKNM--
GWPKNM--
-GPKNMT-
GYPKNM--
GRPKNM--
-GPKNMV-
-GPKNMM-
-GPKNMK-
GGPKNM--
GQPKNM--
-GPKNMD-
GMPKNM--
GLPKNM--
GKPKNM--
GCPKNM--
-HPKNMF-
HRPKNM--
HIPKNM--
HVPKNM--
-HPKNMQ-
-HPKNML-
-HPKNMI-
HMPKNM--
HLPKNM--
HAPKNM--
-HPKNMV-
-HPKNMS-
-HPKNMR-
-HPKNMM-
-HPKNMK-
-HPKNMN-
HHPKNM--
HFPKNM--
HDPKNM--
HCPKNM--
-IPKNMS-

IYPKNM--
-IPKNML-
-IPKNMT-
-IPKNMG-
IFPKNM--
-IPKNMV-
-IPKNMF-
IDPKNM--
ITPKNM--
-IPKNMH-
IPPKNM--
-IPKNMR-
IIPKNM--
-IPKNMY-
ILPKNM--
IVPKNM--
ISPKNM--
-IPKNMW-
IGPKNM--
-IPKNMD-
-IPKNMA-
INPKNM--
IWPKNM--
IQPKNM--
-IPKNMP-
KGPKNM--
KMPKNM--
-KPKNMI-
KVPKNM--
-KPKNML-
-KPKNMV-
KWPKNM--
-KPKNMM-
-KPKNMA-
KIPKNM--
KEPKNM--
KRPKNM--
-KPKNMY-
-KPKNMW-
KPPKNM--
-KPKNMT-
KNPKNM--
KLPKNM--
KDPKNM--
-LPKNML-
LLPKNM--
LVPKNM--
-LPKNMK-
LGPKNM--
LSPKNM--

LQPKNM--
-LPKNMV-
-LPKNMS-
-LPKNMI-
LPPKNM--
-LPKNMQ-
-LPKNMN-
LMPKNM--
LDPKNM--
LAPKNM--
-LPKNME-
-LPKNMA-
LKPKNM--
-LPKNMT-
-LPKNMM-
-LPKNMF-
-LPKNMY-
-LPKNMR-
-LPKNMW-
LIPKNM--
LFPKNM--
LEPKNM--
MWPKNM--
MRPKNM--
-MPKNMA-
MGPKNM--
MTPKNM--
-MPKNMY-
-MPKNMM-
MPPKNM--
-MPKNML-
MFPKNM--
-MPKNMR-
MIPKNM--
MQPKNM--
-MPKNMV-
-MPKNMF-
MLPKNM--
-MPKNMN-
MSPKNM--
-MPKNME-
-MPKNMD-
NTPKNM--
NGPKNM--
NLPKNM--
-NPKNMR-
NAPKNM--
-NPKNMF-
NNPKNM--
NKPKNM--

NSPKNM--
NPPKNM--
-NPKNMS-
-NPKNML-
-NPKNMY-
-NPKNMK-
-NPKNMI-
NCPKNM--
NYPKNM--
NRPKNM--
-NPKNMM-
NHPKNM--
-NPKNMW-
-NPKNMQ-
NIPKNM--
-NPKNME-
NMPKNM--
--PKNMTL
--PKNMIL
--PKNMLI
--PKNMTV
--PKNMLY
--PKNMLQ
--PKNMFK
--PKNMWL
--PKNMRL
--PKNMVL
--PKNMSS
--PKNMKI
--PKNMTT
--PKNMVT
--PKNMRA
--PKNMYT
--PKNMQF
--PKNMIS
--PKNMLN
--PKNMTR
--PKNMRY
--PKNMFG
--PKNMMG
--PKNMLH
--PKNMGG
--PKNMSW
--PKNMSA
--PKNMYW
--PKNMSI
--PKNMMT
--PKNMMS
--PKNMFS
--PKNMYI

--PKNMWW
--PKNMLV
--PKNMLF
--PKNMIW
--PKNMHT
--PKNMFF
--PKNMWV
--PKNMVG
--PKNMVD
--PKNMSV
--PKNMSQ
--PKNMRW
--PKNMQH
--PKNMNW
--PKNMNS
--PKNMNK
--PKNMMA
--PKNMKL
--PKNMAS
--PKNMAG
--PKNMYG
--PKNMYF
--PKNMST
--PKNMQT
--PKNMMD
--PKNMLT
--PKNMKS
--PKNMKG
--PKNMFD
--PKNMET
--PKNMYK
--PKNMWD
--PKNMVS
--PKNMTM
--PKNMQS
--PKNMKY
--PKNMKV
--PKNMKM
--PKNMKA
--PKNMIM
--PKNMHH
--PKNMGS
--PKNMFL
--PKNMWT
--PKNMTY
--PKNMSK
--PKNMHF
--PKNMFW
--PKNMTF
--PKNMSF

--PKNMMV
--PKNMIR
--PKNMIF
--PKNMHV
--PKNMHS
--PKNMFR
--PKNMFN
--PKNMAY
PDPKNM--
PMPKNM--
--PKNMYA
--PKNMWN
--PKNMVI
--PKNMTH
--PKNMRQ
--PKNMNF
--PKNMMW
--PKNMMH
--PKNMIV
--PKNMIH
--PKNMIA
--PKNMHN
--PKNMHG
--PKNMGV
PFPKNM--
PAPKNM--
PSPKNM--
--PKNMYS
--PKNMTN
--PKNMSG
--PKNMRG
--PKNMRE
--PKNMML
--PKNMLE
--PKNMKE
--PKNMIG
--PKNMHY
--PKNMAK
--PKNMAH
PRPKNM--
--PKNMVW
--PKNMVR
--PKNMVQ
--PKNMVK
--PKNMTG
--PKNMSM
--PKNMQI
--PKNMNT
--PKNMMN
--PKNMIE

--PKNMHI
--PKNMGT
--PKNMFI
--PKNMFA
--PKNMES
--PKNMAW
--PKNMAV
--PKNMAT
--PKNMAQ
--PKNMAF
PGPKNM--
PEPKNM--
PLPKNM--
--PKNMYV
--PKNMWF
--PKNMTK
--PKNMRN
--PKNMQV
--PKNMQL
--PKNMNL
--PKNMMY
--PKNMMI
--PKNMLM
--PKNMLD
--PKNMKN
--PKNMIT
--PKNMIK
--PKNMHR
--PKNMGN
--PKNMGK
--PKNMFQ
--PKNMEY
--PKNMDT
--PKNMAA
PKPKNM--
--PKNMYN
--PKNMVN
--PKNMTA
--PKNMSE
--PKNMQM
--PKNMQG
--PKNMNV
--PKNMNM
--PKNMMC
--PKNMLA
--PKNMKH
--PKNMHK
--PKNMHE
--PKNMGR
--PKNMGL

--PKNMGH
--PKNMFH
--PKNMAM
PIPKNM--
PTPKNM--
--PKNMYQ
--PKNMYE
--PKNMYD
--PKNMWY
--PKNMWG
--PKNMWE
--PKNMVV
--PKNMVE
--PKNMTQ
--PKNMTI
--PKNMSD
--PKNMRT
--PKNMPK
--PKNMNH
--PKNMNA
--PKNMLC
--PKNMKF
--PKNMIY
--PKNMIN
--PKNMGY
--PKNMFY
--PKNMFT
--PKNMEM
--PKNMEA
--PKNMDV
--PKNMCF
--PKNMAI
--PKNMAD
PHPKNM--
-QPKNMQ-
-QPKNMF-
-QPKNML-
QRPKNM--
QWPKNM--
QLPKNM--
-QPKNMV-
-QPKNMS-
-QPKNMT-
QAPKNM--
-QPKNMY-
-QPKNMM-
QSPKNM--
QMPKNM--
QVPKNM--
QQPKNM--

QPPKNM--
-QPKNMH-
-QPKNMC-
QNPKNM--
QYPKNM--
-QPKNMI-
-QPKNMA-
QKPKNM--
QDPKNM--
RWPKNM--
-RPKNMT-
RQPKNM--
-RPKNMM-
RVPKNM--
-RPKNMI-
-RPKNMS-
RIPKNM--
RGPKNM--
RYPKNM--
RTPKNM--
RPPKNM--
-RPKNMY-
-RPKNMG-
-RPKNMF-
-RPKNME-
REPKNM--
-RPKNMR-
-RPKNMN-
-RPKNMA-
-RPKNMW-
-RPKNMQ-
-RPKNMH-
RCPKNM--
RAPKNM--
-SPKNMF-
SIPKNM--
-SPKNMS-
SEPKNM--
-SPKNMK-
-SPKNMV-
SYPKNM--
SVPKNM--
-SPKNMM-
SSPKNM--
SRPKNM--
-SPKNMQ-
-SPKNMN-
SHPKNM--
SAPKNM--
-SPKNMH-

```
-SPKNMY-
SQPKNM--
-SPKNMI-
-SPKNMG-
-SPKNMW-
SGPKNM--
STPKNM--
-SPKNMR-
SMPKNM--
SFPKNM--
-SPKNMA-
SNPKNM--
SLPKNM--
SCPKNM--
-TPKNMH-
-TPKNMS-
THPKNM--
TPPKNM--
-TPKNMA-
TWPKNM--
-TPKNMM-
-TPKNMK-
-TPKNMG-
TGPKNM--
-TPKNMW-
TNPKNM--
-TPKNMI-
TFPKNM--
TSPKNM--
TRPKNM--
-TPKNMV-
-TPKNMN-
TLPKNM--
-TPKNMF-
TEPKNM--
TDPKNM--
-TPKNMR-
TVPKNM--
TAPKNM--
-TPKNML-
TYPKNM--
-VPKNMK-
-VPKNML-
VTPKNM--
-VPKNMH-
VMPKNM--
VGPKNM--
VYPKNM--
VRPKNM--
-VPKNMY-
```

-VPKNMV-
VPPKNM--
-VPKNMT-
-VPKNMW-
VSPKNM--
VQPKNM--
VLPKNM--
VFPKNM--
-VPKNMM-
VVPKNM--
-VPKNMS-
-VPKNMQ-
-VPKNMN-
-VPKNME-
VEPKNM--
-VPKNMI-
VNPKNM--
VKPKNM--
VDPKNM--
VCPKNM--
VAPKNM--
-WPKNMD-
WMPKNM--
WLPKNM--
WFPKNM--
-WPKNMQ-
WSPKNM--
-WPKNMI-
WGPKNM--
WRPKNM--
WYPKNM--
WVPKNM--
WWPKNM--
-WPKNMY-
-WPKNMA-
WPPKNM--
WAPKNM--
-WPKNMW-
-WPKNMV-
WIPKNM--
WDPKNM--
YVPKNM--
YQPKNM--
-YPKNMT-
-YPKNMI-
-YPKNMF-
YLPKNM--
-YPKNMV-
YWPKNM--
-YPKNMY-

```
           YGPKNM--
           YPPKNM--
           YFPKNM--
           YTPKNM--
           -YPKNMH-
           YAPKNM--
           YSPKNM--
           YRPKNM--
           -YPKNMA-
           -YPKNMK-
           -YPKNMG-
           YEPKNM--
           -YPKNMW-
           -YPKNML-
           -YPKNMS-
           -YPKNMN-
           -YPKNME-
           YIPKNM--
           YDPKNM--
PYWI        600        587      49.84
           -NPYWIE-
           ASPYWI--
           -APYWIA-
           AKPYWI--
           -APYWIR-
           AQPYWI--
           AIPYWI--
           APPYWI--
           -APYWIM-
           -APYWIQ-
           -APYWIG-
           ATPYWI--
           -APYWIT-
           -APYWIN-
           ANPYWI--
           AHPYWI--
           AGPYWI--
           -APYWIY-
           -APYWIE-
           AWPYWI--
           AFPYWI--
           AEPYWI--
           AVPYWI--
           -APYWIS-
           ADPYWI--
           -APYWII-
           AMPYWI--
           AAPYWI--
           CTPYWI--
           CSPYWI--
```

CGPYWI--
DLPYWI--
-DPYWIK-
DRPYWI--
-DPYWIS-
DAPYWI--
DTPYWI--
DSPYWI--
-DPYWIR-
DNPYWI--
DMPYWI--
DFPYWI--
DEPYWI--
DVPYWI--
-DPYWIQ-
-DPYWIH-
DGPYWI--
DQPYWI--
-DPYWIW-
ESPYWI--
ERPYWI--
EGPYWI--
-EPYWIR-
EAPYWI--
-EPYWIS-
EQPYWI--
EKPYWI--
-EPYWIT-
-EPYWIM-
-EPYWIQ-
-EPYWII-
ELPYWI--
ETPYWI--
-EPYWIH-
-EPYWIG-
-EPYWIA-
EDPYWI--
-EPYWIY-
-EPYWIN-
-EPYWIK-
-EPYWIF-
-EPYWIE-
EVPYWI--
-EPYWIW-
FSPYWI--
FGPYWI--
FVPYWI--
FRPYWI--
-FPYWIS-
FKPYWI--

-FPYWIY-
-FPYWIQ-
-FPYWIK-
FEPYWI--
FTPYWI--
-FPYWIA-
FLPYWI--
FWPYWI--
-FPYWIW-
-FPYWIH-
FHPYWI--
FDPYWI--
-FPYWIN-
FNPYWI--
FFPYWI--
FAPYWI--
-GPYWIS-
GIPYWI--
GTPYWI--
-GPYWIG-
GHPYWI--
-GPYWIT-
-GPYWIH-
GMPYWI--
GNPYWI--
GRPYWI--
GKPYWI--
-GPYWIA-
GSPYWI--
GQPYWI--
-GPYWIK-
GAPYWI--
GGPYWI--
-GPYWIR-
-GPYWII-
GDPYWI--
-GPYWIN-
-GPYWID-
GLPYWI--
GYPYWI--
-GPYWIY-
-GPYWIV-
HLPYWI--
HAPYWI--
-HPYWIW-
HSPYWI--
HRPYWI--
HFPYWI--
-HPYWIT-
-HPYWII-

```
-HPYWIA-
HPPYWI--
-HPYWIY-
-HPYWIS-
-HPYWIL-
HDPYWI--
-HPYWIR-
-HPYWIQ-
-HPYWIF-
-HPYWIE-
HIPYWI--
HHPYWI--
-HPYWIV-
-HPYWIM-
HNPYWI--
-HPYWIN-
HMPYWI--
IRPYWI--
ISPYWI--
IFPYWI--
ILPYWI--
-IPYWIT-
-IPYWIR-
IHPYWI--
IGPYWI--
-IPYWIG-
IKPYWI--
IMPYWI--
-IPYWIL-
INPYWI--
ITPYWI--
-IPYWIS-
-IPYWIK-
IIPYWI--
IDPYWI--
IAPYWI--
-IPYWIY-
-IPYWII-
KSPYWI--
-KPYWIR-
-KPYWIG-
KYPYWI--
-KPYWIQ-
KGPYWI--
-KPYWIT-
-KPYWIH-
KIPYWI--
KMPYWI--
KLPYWI--
KTPYWI--
```

```
KRPYWI--
KWPYWI--
-KPYWIM-
-KPYWIF-
KQPYWI--
-KPYWII-
KPPYWI--
KAPYWI--
-KPYWIN-
-KPYWIA-
KHPYWI--
-KPYWID-
KNPYWI--
LLPYWI--
LKPYWI--
LNPYWI--
LIPYWI--
LTPYWI--
LSPYWI--
-LPYWIR-
-LPYWIS-
LRPYWI--
LHPYWI--
LDPYWI--
-LPYWIQ-
LGPYWI--
-LPYWIV-
LMPYWI--
-LPYWIK-
-LPYWIY-
LCPYWI--
LAPYWI--
LPPYWI--
LWPYWI--
-LPYWII-
-LPYWIH-
LEPYWI--
MSPYWI--
MTPYWI--
MGPYWI--
-MPYWIV-
-MPYWIR-
MRPYWI--
MMPYWI--
-MPYWIS-
MKPYWI--
MEPYWI--
MIPYWI--
MPPYWI--
MYPYWI--
```

```
-MPYWII-
MNPYWI--
MHPYWI--
MDPYWI--
MWPYWI--
MVPYWI--
-MPYWIT-
-MPYWIE-
MAPYWI--
MQPYWI--
-MPYWIL-
MFPYWI--
NTPYWI--
-NPYWIS-
-NPYWIT-
-NPYWIK-
NIPYWI--
-NPYWIG-
NKPYWI--
NQPYWI--
-NPYWIR-
NEPYWI--
NAPYWI--
NVPYWI--
-NPYWIV-
NHPYWI--
NYPYWI--
NWPYWI--
NSPYWI--
NMPYWI--
NGPYWI--
NDPYWI--
-NPYWIM-
NNPYWI--
NLPYWI--
-NPYWIW-
-NPYWIA-
NPPYWI--
--PYWIGM
--PYWIRS
--PYWIRV
--PYWIAQ
--PYWISR
--PYWIAT
--PYWIHS
--PYWIGT
PNPYWI--
--PYWITT
--PYWISS
--PYWIRG
```

--PYWIST
--PYWIAV
--PYWIRD
--PYWIMK
--PYWIMD
--PYWIGI
--PYWIEA
--PYWIVR
--PYWISD
--PYWIRQ
--PYWIIS
--PYWIYA
--PYWISA
--PYWIRT
--PYWIRI
--PYWILG
--PYWIVG
--PYWIRH
--PYWINV
--PYWINT
--PYWIHA
--PYWIGV
--PYWIGN
--PYWIES
--PYWIAG
--PYWIQI
--PYWIIK
--PYWIAA
--PYWITS
--PYWIVV
--PYWITM
--PYWIQA
--PYWIKL
--PYWITK
--PYWITE
--PYWISI
--PYWIKM
--PYWIVW
--PYWITQ
--PYWITN
--PYWISN
--PYWISK
--PYWIRN
--PYWIKH
--PYWIGG
--PYWIGE
--PYWIAK
--PYWIYF
--PYWIVD
--PYWITD

--PYWISW
--PYWISG
--PYWIRY
--PYWIMR
--PYWILK
--PYWILI
--PYWIKG
--PYWIHT
--PYWIHK
--PYWIWS
--PYWIVS
--PYWIVQ
--PYWIVM
--PYWINS
--PYWING
--PYWIMT
--PYWIMS
--PYWIMN
--PYWIKN
--PYWIWY
--PYWIWG
--PYWITV
--PYWIRE
--PYWIMQ
--PYWIKQ
--PYWIKD
--PYWIIN
--PYWIFG
--PYWIET
--PYWIAF
--PYWITG
--PYWITA
--PYWISH
--PYWIQT
--PYWILW
--PYWIKV
--PYWIHM
--PYWIGK
--PYWIFN
--PYWIEQ
--PYWIEH
--PYWIEG
--PYWIAD
--PYWIVK
--PYWISV
--PYWIRW
--PYWIQS
--PYWIQG
--PYWILY
--PYWIKW

--PYWIHR
--PYWIHF
--PYWIGS
--PYWIGR
--PYWIFL
--PYWIFA
--PYWIEK
--PYWIDT
--PYWIAN
--PYWIAH
--PYWIYS
--PYWIVE
--PYWITL
--PYWITF
--PYWIQQ
--PYWIQK
--PYWINN
--PYWIMM
--PYWIMI
--PYWIMG
--PYWILH
--PYWIKK
--PYWIKI
--PYWIIE
--PYWIHV
--PYWIHN
--PYWIHL
--PYWIGY
--PYWIGL
--PYWIGD
--PYWIGA
--PYWIER
--PYWIAW
--PYWIAS
--PYWIAR
PGPYWI--
PDPYWI--
QRPYWI--
-QPYWIS-
-QPYWIG-
QSPYWI--
QHPYWI--
QLPYWI--
QPPYWI--
QWPYWI--
-QPYWIT-
-QPYWIR-
-QPYWIM-
-QPYWIW-
-QPYWIQ-

-QPYWIN-
-QPYWII-
QGPYWI--
QNPYWI--
QVPYWI--
QTPYWI--
QYPYWI--
RAPYWI--
RRPYWI--
RSPYWI--
RVPYWI--
RWPYWI--
-RPYWIR-
RKPYWI--
-RPYWIS-
REPYWI--
-RPYWIQ-
-RPYWIK-
RLPYWI--
-RPYWIH-
RTPYWI--
-RPYWIA-
RMPYWI--
-RPYWIN-
-RPYWIV-
-RPYWIG-
-RPYWIY-
RHPYWI--
RDPYWI--
-RPYWIT-
-RPYWII-
RPPYWI--
RNPYWI--
RFPYWI--
RYPYWI--
-RPYWIW-
RQPYWI--
-RPYWID-
RIPYWI--
-RPYWIM-
-RPYWIF-
RGPYWI--
SLPYWI--
SSPYWI--
SRPYWI--
-SPYWIR-
SVPYWI--
SKPYWI--
-SPYWIV-
STPYWI--

SIPYWI--
SYPYWI--
SWPYWI--
SPPYWI--
SEPYWI--
SAPYWI--
-SPYWII-
-SPYWIH-
SNPYWI--
-SPYWIM-
SGPYWI--
-SPYWIF-
-SPYWIK-
-SPYWIG-
SHPYWI--
-SPYWIA-
-SPYWIN-
SMPYWI--
SFPYWI--
-SPYWIT-
-SPYWIQ-
-SPYWIE-
-SPYWID-
-TPYWIR-
-TPYWII-
TVPYWI--
TSPYWI--
-TPYWIT-
TTPYWI--
TGPYWI--
TAPYWI--
TMPYWI--
-TPYWIV-
TFPYWI--
-TPYWIY-
TLPYWI--
THPYWI--
TYPYWI--
TRPYWI--
TQPYWI--
-TPYWIA-
TWPYWI--
-TPYWIS-
-TPYWIH-
TDPYWI--
-TPYWIG-
-TPYWID-
TPPYWI--
TNPYWI--
TEPYWI--

VSPYWI--
-VPYWIS-
VKPYWI--
-VPYWIY-
-VPYWIR-
-VPYWIV-
VPPYWI--
-VPYWIT-
VNPYWI--
VLPYWI--
VIPYWI--
VGPYWI--
-VPYWIM-
VHPYWI--
VAPYWI--
VVPYWI--
-VPYWIG-
-VPYWIA-
VMPYWI--
VQPYWI--
VTPYWI--
-VPYWIK-
VFPYWI--
VEPYWI--
WKPYWI--
WSPYWI--
-WPYWIV-
WRPYWI--
-WPYWIW-
-WPYWIE-
-WPYWIY-
-WPYWIG-
-WPYWIS-
-WPYWIR-
-WPYWIA-
WHPYWI--
YEPYWI--
YAPYWI--
YTPYWI--
YRPYWI--
YSPYWI--
YNPYWI--
YMPYWI--
YKPYWI--
YVPYWI--
-YPYWIL-
-YPYWIG-
YWPYWI--
-YPYWIS-
-YPYWIR-

	YIPYWI--		
	YQPYWI--		
	YYPYWI--		
	-YPYWIQ-		
	-YPYWIM-		
	-YPYWIE-		
	YFPYWI--		
PWGM	239	728	49.83
	-EPWGMG-		
	MMPWGM--		
	--PWGMRP		
	-APWGMS-		
	-APWGMR-		
	-APWGML-		
	ARPWGM--		
	-APWGMM-		
	ATPWGM--		
	AQPWGM--		
	-APWGMH-		
	ALPWGM--		
	AEPWGM--		
	-APWGMA-		
	AFPWGM--		
	AKPWGM--		
	-APWGME-		
	AMPWGM--		
	AHPWGM--		
	AYPWGM--		
	-APWGMY-		
	-APWGMW-		
	-APWGMK-		
	-APWGMG-		
	ANPWGM--		
	AGPWGM--		
	ASPWGM--		
	-APWGMV-		
	-APWGMN-		
	-APWGMI-		
	APPWGM--		
	ADPWGM--		
	-CPWGMH-		
	CGPWGM--		
	-CPWGMS-		
	-CPWGMQ-		
	CNPWGM--		
	-CPWGMV-		
	CKPWGM--		
	CIPWGM--		
	CYPWGM--		
	CVPWGM--		

```
CRPWGM--
-CPWGMM-
-CPWGMK-
-CPWGMF-
-DPWGMH-
DVPWGM--
-DPWGMY-
DGPWGM--
DSPWGM--
-DPWGMV-
-DPWGML-
-DPWGMS-
DTPWGM--
DNPWGM--
DIPWGM--
DWPWGM--
-DPWGMW-
-DPWGMR-
DMPWGM--
DFPWGM--
DQPWGM--
DLPWGM--
DAPWGM--
-DPWGMT-
-DPWGMQ-
-DPWGMF-
-DPWGMA-
DKPWGM--
DCPWGM--
-EPWGMW-
-EPWGMY-
-EPWGML-
EGPWGM--
-EPWGMT-
EWPWGM--
EIPWGM--
-EPWGMK-
EYPWGM--
ESPWGM--
-EPWGMH-
-EPWGMS-
EQPWGM--
-EPWGMM-
-EPWGMI-
-EPWGMR-
-EPWGMA-
EVPWGM--
EPPWGM--
EKPWGM--
EEPWGM--
```

EFPWGM--
-EPWGMQ-
-EPWGMN-
ENPWGM--
ELPWGM--
ETPWGM--
ERPWGM--
EDPWGM--
-FPWGMT-
FYPWGM--
-FPWGMQ-
FNPWGM--
FHPWGM--
-FPWGMA-
-FPWGMK-
FVPWGM--
FTPWGM--
-FPWGMS-
FLPWGM--
FFPWGM--
-FPWGML-
FGPWGM--
FKPWGM--
FPPWGM--
FSPWGM--
FRPWGM--
-FPWGMW-
-FPWGMN-
FAPWGM--
-FPWGMF-
FDPWGM--
GYPWGM--
-GPWGML-
GMPWGM--
-GPWGMT-
GPPWGM--
-GPWGMV-
-GPWGMS-
-GPWGMQ-
GNPWGM--
GRPWGM--
GAPWGM--
GSPWGM--
-GPWGMI-
GKPWGM--
-GPWGMY-
-GPWGMW-
GHPWGM--
GEPWGM--
-GPWGMM-

-GPWGMK-
GFPWGM--
GVPWGM--
-GPWGMN-
GDPWGM--
-GPWGMR-
-GPWGMA-
-GPWGMG-
GIPWGM--
-GPWGMF-
GGPWGM--
-HPWGMY-
-HPWGML-
HVPWGM--
HKPWGM--
HIPWGM--
HGPWGM--
HSPWGM--
HWPWGM--
HLPWGM--
-HPWGMH-
HDPWGM--
HRPWGM--
-HPWGMK-
-HPWGMI-
-HPWGMT-
-HPWGMR-
HTPWGM--
-HPWGMN-
HYPWGM--
HCPWGM--
HAPWGM--
ITPWGM--
-IPWGMT-
IGPWGM--
-IPWGMI-
-IPWGMA-
-IPWGMK-
ISPWGM--
-IPWGMH-
-IPWGMF-
IAPWGM--
IMPWGM--
-IPWGMV-
IVPWGM--
IQPWGM--
-IPWGMS-
IDPWGM--
-IPWGMQ-
IPPWGM--

```
-IPWGMN-
IHPWGM--
IEPWGM--
-IPWGMM-
INPWGM--
ILPWGM--
IYPWGM--
-IPWGMY-
-IPWGMG-
KSPWGM--
-KPWGML-
KLPWGM--
-KPWGMW-
-KPWGMS-
-KPWGMV-
KNPWGM--
KVPWGM--
-KPWGMT-
-KPWGMQ-
KIPWGM--
KYPWGM--
-KPWGMK-
KEPWGM--
-KPWGMR-
-KPWGMN-
-KPWGMY-
KHPWGM--
KDPWGM--
KQPWGM--
-KPWGMH-
-KPWGMG-
-KPWGMF-
KKPWGM--
KFPWGM--
KRPWGM--
-KPWGME-
-LPWGMR-
-LPWGMS-
LKPWGM--
-LPWGMM-
LYPWGM--
LLPWGM--
-LPWGMN-
LRPWGM--
-LPWGML-
LTPWGM--
LSPWGM--
-LPWGMH-
LIPWGM--
LFPWGM--
```

LMPWGM--
LHPWGM--
LDPWGM--
LVPWGM--
LNPWGM--
-LPWGMY-
-LPWGMW-
-LPWGMQ-
LGPWGM--
-LPWGMI-
-LPWGMA-
LEPWGM--
LWPWGM--
-LPWGMT-
-LPWGMK-
LPPWGM--
LCPWGM--
-LPWGMG-
MRPWGM--
MPPWGM--
MSPWGM--
MKPWGM--
-MPWGMK-
-MPWGMG-
-MPWGML-
MEPWGM--
-MPWGMW-
-MPWGMI-
MTPWGM--
-MPWGMS-
MAPWGM--
-MPWGMY-
MIPWGM--
-MPWGMV-
MDPWGM--
MCPWGM--
MQPWGM--
-MPWGME-
-NPWGMR-
-NPWGMV-
NGPWGM--
NRPWGM--
NHPWGM--
-NPWGMI-
-NPWGMH-
-NPWGML-
-NPWGMY-
-NPWGMW-
NMPWGM--
NLPWGM--

NSPWGM--
-NPWGMT-
NTPWGM--
-NPWGMS-
NIPWGM--
NNPWGM--
NDPWGM--
NVPWGM--
-NPWGMQ-
-NPWGMN-
-NPWGMK-
NFPWGM--
NEPWGM--
NCPWGM--
--PWGMLS
--PWGMSG
--PWGMYH
--PWGMTI
--PWGMLR
--PWGMRV
--PWGMLT
--PWGMMV
--PWGMYS
--PWGMST
--PWGMHS
--PWGMVI
--PWGMRT
--PWGMMA
--PWGMVR
--PWGMSR
--PWGMRR
--PWGMTM
--PWGMVY
--PWGMSS
--PWGMRY
--PWGMLN
--PWGMVV
--PWGMRN
--PWGMRK
--PWGMHF
--PWGMWR
--PWGMTV
--PWGMSN
--PWGMMQ
--PWGMAF
--PWGMVK
--PWGMTS
--PWGMTL
--PWGMSL
--PWGMNA

--PWGMAG
--PWGMTN
--PWGMSA
--PWGMRD
--PWGMLL
--PWGMIK
--PWGMIH
--PWGMWS
--PWGMTW
--PWGMRG
--PWGMHG
--PWGMAV
--PWGMWT
--PWGMTD
--PWGMLQ
PYPWGM--
--PWGMVT
--PWGMTK
--PWGMTH
--PWGMSF
PKPWGM--
--PWGMMF
--PWGMYN
--PWGMYG
--PWGMWW
--PWGMTT
--PWGMSV
--PWGMSM
--PWGMRE
--PWGMHT
--PWGMGG
--PWGMAS
--PWGMWN
--PWGMWH
--PWGMQS
--PWGMNV
--PWGMNH
--PWGMFR
--PWGMFG
--PWGMVS
--PWGMSW
--PWGMIR
--PWGMHR
PSPWGM--
PGPWGM--
PFPWGM--
--PWGMYQ
--PWGMVN
--PWGMVH
--PWGMNS

--PWGMKD
--PWGMHK
--PWGMFK
--PWGMAM
--PWGMAI
--PWGMYK
--PWGMTR
--PWGMSK
--PWGMQG
--PWGMMI
--PWGMGS
--PWGMGI
--PWGMWQ
--PWGMWD
--PWGMVD
--PWGMVA
--PWGMQM
--PWGMMG
--PWGMKN
--PWGMHH
--PWGMEA
--PWGMAT
--PWGMAL
--PWGMWE
--PWGMTQ
--PWGMTF
--PWGMSH
--PWGMNQ
--PWGMND
--PWGMMN
--PWGMKS
--PWGMKG
--PWGMKF
--PWGMIF
--PWGMID
--PWGMFH
--PWGMEI
--PWGMAR
--PWGMRW
--PWGMQW
--PWGMQA
--PWGMNF
--PWGMMS
--PWGMMR
--PWGMMK
--PWGMME
--PWGMLD
--PWGMLA
--PWGMHI
--PWGMET

--PWGMAW
--PWGMAA
PEPWGM--
--PWGMWM
--PWGMVW
--PWGMRH
--PWGMQR
--PWGMIT
--PWGMIA
--PWGMEV
PTPWGM--
PLPWGM--
PWPWGM--
--PWGMYY
--PWGMYR
--PWGMYE
--PWGMWY
--PWGMVQ
--PWGMVE
--PWGMSQ
--PWGMSD
--PWGMQV
--PWGMQT
--PWGMQF
--PWGMNI
--PWGMMD
--PWGMIN
--PWGMGV
--PWGMGQ
--PWGMFS
--PWGMFE
--PWGMAK
PVPWGM--
PNPWGM--
--PWGMYW
--PWGMYD
--PWGMWK
--PWGMSY
--PWGMLE
--PWGMKK
--PWGMKE
--PWGMIS
--PWGMHW
--PWGMHV
--PWGMHQ
--PWGMGT
--PWGMGN
--PWGMGM
--PWGMFT
--PWGMFQ

--PWGMES
--PWGMER
--PWGMEK
--PWGMDS
--PWGMDG
PIPWGM--
--PWGMWF
--PWGMVC
--PWGMSE
--PWGMRQ
--PWGMRF
--PWGMQY
--PWGMQK
--PWGMQH
--PWGMQD
--PWGMNW
--PWGMNT
--PWGMMM
--PWGMLH
--PWGMKQ
--PWGMKL
--PWGMIM
--PWGMII
--PWGMGY
--PWGMGR
--PWGMGL
--PWGMGK
--PWGMGH
--PWGMGA
--PWGMAY
--PWGMAN
-PPWGMS-
-PPWGMM-
PHPWGM--
-QPWGMS-
-QPWGMW-
-QPWGMQ-
-QPWGMR-
-QPWGML-
QSPWGM--
-QPWGMH-
QAPWGM--
-QPWGMI-
QMPWGM--
-QPWGMY-
QRPWGM--
-QPWGMT-
QYPWGM--
-QPWGMV-
-QPWGMK-

-QPWGMF-
-QPWGMA-
QHPWGM--
-QPWGMN-
QWPWGM--
QVPWGM--
QFPWGM--
QDPWGM--
-RPWGMR-
-RPWGML-
RSPWGM--
-RPWGMS-
-RPWGMM-
-RPWGMF-
RTPWGM--
RDPWGM--
RFPWGM--
RKPWGM--
-RPWGMG-
RHPWGM--
-RPWGMW-
REPWGM--
-RPWGMK-
-RPWGMI-
-RPWGMA-
RLPWGM--
RAPWGM--
-RPWGMQ-
-RPWGMN-
-RPWGMH-
RGPWGM--
RMPWGM--
RRPWGM--
RIPWGM--
-RPWGMY-
RPPWGM--
RYPWGM--
RWPWGM--
-RPWGME-
RNPWGM--
-RPWGMV-
-SPWGMS-
SLPWGM--
-SPWGMR-
-SPWGMT-
SRPWGM--
-SPWGML-
-SPWGMK-
SKPWGM--
SSPWGM--

SQPWGM--
-SPWGMN-
SYPWGM--
-SPWGMI-
SGPWGM--
STPWGM--
SIPWGM--
SVPWGM--
SMPWGM--
SAPWGM--
SNPWGM--
SDPWGM--
-SPWGMQ-
-SPWGMM-
-SPWGME-
SFPWGM--
SCPWGM--
-SPWGMG-
-SPWGMW-
-SPWGMV-
SHPWGM--
SEPWGM--
SPPWGM--
TGPWGM--
TTPWGM--
TKPWGM--
-TPWGML-
-TPWGMI-
TIPWGM--
-TPWGMS-
TSPWGM--
-TPWGMK-
-TPWGMF-
TRPWGM--
TEPWGM--
-TPWGMV-
TLPWGM--
THPWGM--
-TPWGMT-
-TPWGMN-
TPPWGM--
-TPWGME-
TNPWGM--
TDPWGM--
TQPWGM--
-TPWGMR-
TVPWGM--
-TPWGMG-
TAPWGM--
TYPWGM--

-TPWGMM-
-TPWGMH-
TMPWGM--
TWPWGM--
-TPWGMQ-
TCPWGM--
VRPWGM--
-VPWGMN-
-VPWGMH-
VWPWGM--
-VPWGMT-
VYPWGM--
VSPWGM--
VNPWGM--
VVPWGM--
VKPWGM--
VHPWGM--
-VPWGME-
VQPWGM--
VMPWGM--
VGPWGM--
-VPWGML-
-VPWGMG-
-VPWGMS-
VPPWGM--
VTPWGM--
-VPWGMK-
VIPWGM--
VDPWGM--
-VPWGMY-
-VPWGMA-
-VPWGMQ-
-VPWGMI-
-VPWGMD-
-VPWGMC-
WKPWGM--
WSPWGM--
WLPWGM--
WEPWGM--
WTPWGM--
-WPWGMR-
WGPWGM--
WYPWGM--
WRPWGM--
-WPWGMH-
WAPWGM--
-WPWGMS-
-WPWGMK-
-WPWGMQ-
WVPWGM--

-WPWGMT-
WNPWGM--
-WPWGML-
-WPWGMI-
WWPWGM--
WQPWGM--
-WPWGMY-
-WPWGMV-
-WPWGMG-
-WPWGMF-
-YPWGMR-
YHPWGM--
YDPWGM--
-YPWGMI-
YTPWGM--
-YPWGMS-
-YPWGMN-
YGPWGM--
YEPWGM--
-YPWGMH-
YIPWGM--
YYPWGM--
YWPWGM--
-YPWGMT-
YVPWGM--
-YPWGML-
-YPWGMG-
YFPWGM--
-YPWGMV-
-YPWGME-
-YPWGMA-
YSPWGM--
YRPWGM--
-YPWGMQ-
-YPWGMK-
YPPWGM--

PYGM 145 779 49.75

-WPYGMR-
AVPYGM--
-APYGML-
-APYGMF-
-APYGMS-
AAPYGM--
AHPYGM--
ASPYGM--
ALPYGM--
-APYGMM-
-APYGMH-
AFPYGM--
AYPYGM--

ANPYGM--
AEPYGM--
-APYGMQ-
AQPYGM--
AWPYGM--
ATPYGM--
-APYGMN-
AGPYGM--
-APYGMK-
-APYGMY-
-APYGMA-
APPYGM--
-APYGMT-
-APYGMG-
-APYGMI-
AMPYGM--
ACPYGM--
-APYGMV-
-APYGMC-
CMPYGM--
CKPYGM--
CCPYGM--
CRPYGM--
CVPYGM--
-CPYGMT-
CSPYGM--
-CPYGMV-
-CPYGMS-
-CPYGMH-
-CPYGME-
CPPYGM--
-DPYGMY-
-DPYGMI-
-DPYGMR-
DLPYGM--
-DPYGMV-
DNPYGM--
DYPYGM--
-DPYGMF-
DRPYGM--
-DPYGMW-
DMPYGM--
DSPYGM--
-DPYGML-
-DPYGMK-
-DPYGMH-
DKPYGM--
-DPYGMA-
DHPYGM--
DFPYGM--

DVPYGM--
-DPYGMT-
-DPYGMS-
DIPYGM--
DAPYGM--
-EPYGMR-
-EPYGMT-
ELPYGM--
ERPYGM--
EHPYGM--
-EPYGMS-
-EPYGMV-
EFPYGM--
EIPYGM--
EVPYGM--
EQPYGM--
-EPYGMM-
-EPYGMQ-
-EPYGMN-
-EPYGMK-
EPPYGM--
EMPYGM--
ETPYGM--
-EPYGML-
-EPYGMG-
EKPYGM--
-FPYGMT-
FNPYGM--
-FPYGML-
FVPYGM--
-FPYGMQ-
-FPYGMR-
FLPYGM--
-FPYGMY-
FDPYGM--
-FPYGMV-
FPPYGM--
FTPYGM--
FSPYGM--
-FPYGMF-
FKPYGM--
FGPYGM--
-FPYGMS-
-FPYGMI-
FIPYGM--
FEPYGM--
-FPYGMH-
FRPYGM--
-FPYGMK-
-FPYGMG-

FWPYGM--
-FPYGMN-
-FPYGME-
-FPYGMA-
-FPYGMM-
FMPYGM--
FHPYGM--
FYPYGM--
GLPYGM--
-GPYGML-
GRPYGM--
-GPYGMS-
-GPYGMK-
-GPYGMM-
-GPYGMI-
GQPYGM--
-GPYGMR-
GAPYGM--
GVPYGM--
-GPYGMT-
-GPYGMV-
-GPYGMH-
-GPYGMG-
GMPYGM--
GIPYGM--
GGPYGM--
GFPYGM--
GEPYGM--
-GPYGMN-
-GPYGMF-
-GPYGMY-
GWPYGM--
GTPYGM--
GNPYGM--
GYPYGM--
-GPYGMA-
GPPYGM--
GHPYGM--
GKPYGM--
GDPYGM--
GCPYGM--
-GPYGME-
-GPYGMD-
-HPYGMS-
HYPYGM--
-HPYGMI-
HLPYGM--
HHPYGM--
-HPYGMV-
-HPYGMH-

HGPYGM--
-HPYGMG-
HPPYGM--
HRPYGM--
HMPYGM--
HIPYGM--
-HPYGMY-
-HPYGMR-
-HPYGML-
-HPYGMF-
HVPYGM--
-HPYGMT-
-HPYGMK-
HTPYGM--
-HPYGMQ-
HQPYGM--
HNPYGM--
HAPYGM--
HWPYGM--
-HPYGMW-
-HPYGMN-
ILPYGM--
-IPYGME-
IRPYGM--
-IPYGMI-
IHPYGM--
-IPYGMS-
-IPYGML-
INPYGM--
ISPYGM--
IKPYGM--
IQPYGM--
-IPYGMF-
-IPYGMM-
ITPYGM--
-IPYGMY-
-IPYGMR-
-IPYGMH-
IVPYGM--
-IPYGMG-
IMPYGM--
IDPYGM--
-IPYGMV-
IPPYGM--
IIPYGM--
IFPYGM--
IEPYGM--
-IPYGMW-
-IPYGMT-
-IPYGMN-

-IPYGMA-
IGPYGM--
IYPYGM--
IWPYGM--
-IPYGMK-
KSPYGM--
-KPYGMS-
-KPYGMQ-
KFPYGM--
-KPYGMK-
-KPYGML-
-KPYGMH-
-KPYGMA-
KPPYGM--
-KPYGMI-
-KPYGMG-
KIPYGM--
KGPYGM--
-KPYGMN-
-KPYGME-
KYPYGM--
KAPYGM--
KNPYGM--
KRPYGM--
KQPYGM--
KHPYGM--
KEPYGM--
KDPYGM--
-KPYGMW-
-KPYGMF-
KTPYGM--
LGPYGM--
-LPYGMR-
LTPYGM--
LVPYGM--
LLPYGM--
-LPYGMV-
LPPYGM--
LIPYGM--
-LPYGMQ-
-LPYGMN-
LNPYGM--
LSPYGM--
-LPYGMY-
LKPYGM--
LRPYGM--
-LPYGMS-
-LPYGMM-
-LPYGMK-
LEPYGM--

```
-LPYGML-
-LPYGMT-
-LPYGMH-
LAPYGM--
LWPYGM--
LDPYGM--
-LPYGMW-
-LPYGMG-
-LPYGMA-
LYPYGM--
-LPYGMI-
LHPYGM--
LFPYGM--
MRPYGM--
MYPYGM--
MLPYGM--
MVPYGM--
MSPYGM--
-MPYGMR-
MIPYGM--
-MPYGMK-
MGPYGM--
-MPYGMS-
-MPYGMV-
-MPYGML-
MPPYGM--
MEPYGM--
-MPYGMH-
-MPYGMY-
-MPYGMQ-
MKPYGM--
-MPYGMN-
-MPYGMG-
-MPYGMF-
MNPYGM--
MWPYGM--
-MPYGME-
NSPYGM--
NQPYGM--
-NPYGMS-
-NPYGMR-
NTPYGM--
-NPYGMI-
-NPYGMT-
-NPYGMM-
NLPYGM--
-NPYGMY-
-NPYGML-
-NPYGMH-
NAPYGM--
```

NVPYGM--
-NPYGMV-
-NPYGMA-
NGPYGM--
NDPYGM--
NRPYGM--
NIPYGM--
NHPYGM--
-NPYGME-
NNPYGM--
--PYGMRL
--PYGMGS
--PYGMRT
--PYGMLT
--PYGMSS
--PYGMVY
--PYGMSR
--PYGMRS
--PYGMRG
--PYGMTA
--PYGMRI
--PYGMNS
--PYGMRK
--PYGMKG
--PYGMHL
--PYGMSN
--PYGMQK
--PYGMMT
--PYGMTS
--PYGMTK
--PYGMIQ
--PYGMMR
--PYGMGA
--PYGMNI
--PYGMHR
--PYGMTN
--PYGMSY
--PYGMSK
--PYGMRH
--PYGMLA
--PYGMVT
--PYGMFH
--PYGMSG
--PYGMFQ
PNPYGM--
--PYGMKR
--PYGMYG
--PYGMWG
--PYGMRQ
--PYGMMI

--PYGMKN
--PYGMAN
--PYGMVQ
PTPYGM--
--PYGMWH
--PYGMSI
--PYGMMK
PAPYGM--
--PYGMYR
--PYGMVI
--PYGMTR
--PYGMSH
--PYGMIS
--PYGMFS
--PYGMWN
--PYGMSA
--PYGMTI
--PYGMSQ
--PYGMRN
--PYGMNW
--PYGMMF
--PYGMKS
--PYGMWS
--PYGMVD
--PYGMTY
--PYGMTD
--PYGMNT
--PYGMFE
--PYGMAT
--PYGMAF
--PYGMTE
--PYGMQS
--PYGMMV
--PYGMMS
--PYGMKK
--PYGMFD
--PYGMAI
--PYGMVH
--PYGMVG
--PYGMRY
--PYGMNK
--PYGMMD
--PYGMLN
--PYGMKW
--PYGMKF
--PYGMHK
--PYGMFR
--PYGMAV
--PYGMAA
--PYGMVL

--PYGMTQ
--PYGMRF
--PYGMQA
--PYGMMN
--PYGMKQ
--PYGMAG
--PYGMYW
--PYGMYN
--PYGMWT
--PYGMWK
--PYGMWD
--PYGMVM
--PYGMVF
--PYGMVA
--PYGMTG
--PYGMQM
--PYGMMG
--PYGMLG
--PYGMIT
--PYGMIG
--PYGMHW
PKPYGM--
--PYGMYT
--PYGMYE
--PYGMTV
--PYGMTF
--PYGMRW
--PYGMNL
--PYGMMH
--PYGMLI
--PYGMHT
--PYGMHF
--PYGMGI
--PYGMFN
--PYGMET
--PYGMAH
PGPYGM--
--PYGMVS
--PYGMSE
--PYGMQY
--PYGMQR
--PYGMQL
--PYGMLV
--PYGMKM
--PYGMKI
--PYGMIN
--PYGMHH
--PYGMHG
PMPYGM--
--PYGMTL

--PYGMSD
--PYGMRV
--PYGMQT
--PYGMNQ
--PYGMMM
--PYGMKT
--PYGMIH
--PYGMID
--PYGMIA
--PYGMHQ
--PYGMFK
--PYGMFG
--PYGMEG
--PYGMDS
--PYGMWE
--PYGMQW
--PYGMNR
--PYGMNA
--PYGMKH
--PYGMII
--PYGMGL
--PYGMGG
--PYGMAS
--PYGMYS
--PYGMSF
--PYGMRC
--PYGMNY
--PYGMNV
--PYGMNM
--PYGMNE
--PYGMLK
--PYGMKE
--PYGMHA
--PYGMGH
--PYGMGF
--PYGMEV
--PYGMEI
--PYGMDA
--PYGMAQ
--PYGMWQ
--PYGMVE
--PYGMSW
--PYGMSV
--PYGMNG
--PYGMNF
--PYGMLE
--PYGMKY
--PYGMKA
--PYGMIM
--PYGMHM

--PYGMGT
--PYGMGR
--PYGMGM
--PYGMFA
--PYGMEM
--PYGMEF
--PYGMDG
--PYGMAR
PDPYGM--
--PYGMTT
--PYGMTH
--PYGMRP
--PYGMQN
--PYGMQE
--PYGMMQ
--PYGMMA
--PYGMLR
--PYGMLH
--PYGMLD
--PYGMKD
--PYGMHE
--PYGMGV
--PYGMGQ
--PYGMGK
--PYGMFT
--PYGMES
--PYGMEQ
--PYGMEN
--PYGMCN
--PYGMAM
--PYGMAK
--PYGMAE
PIPYGM--
PYPYGM--
--PYGMYH
--PYGMWR
--PYGMVV
--PYGMVN
--PYGMTW
--PYGMRE
--PYGMRD
--PYGMQV
--PYGMPK
--PYGMNN
--PYGMLQ
--PYGMIY
--PYGMIF
--PYGMIC
--PYGMHI
--PYGMER

--PYGMEH
--PYGMDT
--PYGMAW
PWPYGM--
PQPYGM--
PPPYGM--
PEPYGM--
-QPYGMR-
QRPYGM--
QGPYGM--
-QPYGMW-
-QPYGMS-
-QPYGMG-
-QPYGMT-
-QPYGMN-
QEPYGM--
-QPYGMA-
QAPYGM--
QTPYGM--
QQPYGM--
QPPYGM--
-QPYGMF-
-QPYGMV-
QLPYGM--
QKPYGM--
-QPYGMH-
QIPYGM--
QVPYGM--
-QPYGMQ-
QWPYGM--
QSPYGM--
QNPYGM--
QHPYGM--
-QPYGMI-
-RPYGMT-
RSPYGM--
-RPYGMR-
-RPYGMN-
RLPYGM--
RPPYGM--
RGPYGM--
RNPYGM--
RRPYGM--
-RPYGMY-
-RPYGMS-
-RPYGMF-
-RPYGMA-
-RPYGMM-
RIPYGM--
RDPYGM--

-RPYGMK-
-RPYGMH-
-RPYGMI-
RYPYGM--
RTPYGM--
RQPYGM--
REPYGM--
RHPYGM--
-RPYGME-
-RPYGMW-
-RPYGMG-
RAPYGM--
RCPYGM--
-RPYGMQ-
RKPYGM--
RVPYGM--
-RPYGMD-
RFPYGM--
SVPYGM--
SSPYGM--
-SPYGML-
-SPYGMR-
SKPYGM--
-SPYGMS-
-SPYGMA-
SWPYGM--
-SPYGMH-
SIPYGM--
-SPYGMV-
-SPYGMT-
SLPYGM--
-SPYGMI-
-SPYGMK-
SRPYGM--
-SPYGMQ-
SAPYGM--
SYPYGM--
STPYGM--
-SPYGMN-
SNPYGM--
SMPYGM--
SGPYGM--
SPPYGM--
SEPYGM--
-SPYGMW-
SFPYGM--
SDPYGM--
-SPYGMM-
SQPYGM--
SHPYGM--

-SPYGMY-
-SPYGME-
SCPYGM--
-TPYGMT-
-TPYGMS-
-TPYGMW-
TAPYGM--
TLPYGM--
-TPYGMR-
-TPYGML-
-TPYGMV-
TIPYGM--
TPPYGM--
TVPYGM--
-TPYGMK-
-TPYGMI-
TGPYGM--
-TPYGMY-
-TPYGMM-
TYPYGM--
-TPYGMA-
-TPYGMN-
TTPYGM--
TSPYGM--
-TPYGMQ-
-TPYGMH-
-TPYGMF-
TMPYGM--
TKPYGM--
TFPYGM--
-TPYGMG-
TDPYGM--
TRPYGM--
-TPYGME-
TEPYGM--
THPYGM--
VFPYGM--
VRPYGM--
VHPYGM--
-VPYGML-
VGPYGM--
-VPYGMS-
VWPYGM--
VSPYGM--
VTPYGM--
VAPYGM--
VQPYGM--
-VPYGMV-
-VPYGMN-
-VPYGMA-

VVPYGM--
-VPYGMT-
-VPYGMG-
-VPYGME-
VNPYGM--
VIPYGM--
VEPYGM--
VMPYGM--
-VPYGMR-
-VPYGMI-
VPPYGM--
VKPYGM--
-VPYGMF-
-VPYGMD-
-VPYGMM-
WYPYGM--
WRPYGM--
-WPYGMS-
WPPYGM--
WQPYGM--
WNPYGM--
WEPYGM--
-WPYGMA-
-WPYGMK-
WFPYGM--
-WPYGMN-
-WPYGMF-
WGPYGM--
WTPYGM--
WHPYGM--
WAPYGM--
-WPYGMV-
-WPYGMQ-
-WPYGMG-
WKPYGM--
WSPYGM--
-WPYGMM-
WMPYGM--
-YPYGMM-
-YPYGMR-
YPPYGM--
-YPYGMT-
-YPYGMQ-
YRPYGM--
YAPYGM--
-YPYGMS-
YNPYGM--
YKPYGM--
-YPYGML-
YYPYGM--

YTPYGM--
-YPYGMK-
-YPYGME-
YQPYGM--
YLPYGM--
YDPYGM--
YIPYGM--
-YPYGMH-
-YPYGMA-
YGPYGM--
YSPYGM--
YVPYGM--
-YPYGMG-
-YPYGMN-
-YPYGMF-
YMPYGM--

PMAY	223	734	49.73

--PMAYQK
AYPMAY--
-APMAYR-
AGPMAY--
-APMAYS-
AMPMAY--
-APMAYL-
-APMAYI-
ANPMAY--
AWPMAY--
-APMAYM-
AFPMAY--
ASPMAY--
ALPMAY--
ARPMAY--
AKPMAY--
-APMAYY-
-APMAYK-
-APMAYH-
-APMAYN-
-APMAYF-
AEPMAY--
-APMAYT-
ADPMAY--
-APMAYG-
-APMAYQ-
AAPMAY--
CSPMAY--
CRPMAY--
CNPMAY--
-CPMAYV-
-CPMAYR-
CCPMAY--

CPPMAY--
-CPMAYM-
-CPMAYL-
CGPMAY--
CQPMAY--
-CPMAYY-
-CPMAYK-
-CPMAYI-
-CPMAYH-
-CPMAYA-
CAPMAY--
-DPMAYT-
-DPMAYY-
-DPMAYS-
DVPMAY--
DRPMAY--
-DPMAYV-
DWPMAY--
-DPMAYM-
-DPMAYF-
DMPMAY--
DLPMAY--
DAPMAY--
DYPMAY--
DSPMAY--
DKPMAY--
-DPMAYQ-
-DPMAYK-
-DPMAYR-
-DPMAYL-
-DPMAYI-
DHPMAY--
EKPMAY--
ESPMAY--
-EPMAYS-
-EPMAYL-
-EPMAYK-
-EPMAYH-
EYPMAY--
-EPMAYR-
-EPMAYA-
EVPMAY--
-EPMAYG-
-EPMAYI-
-EPMAYF-
ENPMAY--
-EPMAYY-
EGPMAY--
EWPMAY--
EFPMAY--

-EPMAYT-
-EPMAYM-
EEPMAY--
-FPMAYK-
FVPMAY--
FIPMAY--
FHPMAY--
-FPMAYN-
FRPMAY--
FTPMAY--
-FPMAYS-
-FPMAYI-
FLPMAY--
FYPMAY--
-FPMAYW-
FGPMAY--
-FPMAYT-
-FPMAYQ-
-FPMAYV-
FSPMAY--
-FPMAYG-
FNPMAY--
FMPMAY--
FEPMAY--
FWPMAY--
-GPMAYL-
-GPMAYI-
GNPMAY--
GRPMAY--
GWPMAY--
-GPMAYT-
GSPMAY--
GVPMAY--
GIPMAY--
GGPMAY--
-GPMAYS-
-GPMAYH-
GLPMAY--
-GPMAYE-
-GPMAYN-
-GPMAYV-
-GPMAYR-
-GPMAYM-
-GPMAYQ-
-GPMAYG-
GHPMAY--
GPPMAY--
-GPMAYW-
GKPMAY--
GFPMAY--

GQPMAY--
-GPMAYY-
-GPMAYF-
GEPMAY--
GDPMAY--
HRPMAY--
HLPMAY--
-HPMAYY-
HNPMAY--
-HPMAYS-
HSPMAY--
HQPMAY--
-HPMAYW-
-HPMAYN-
-HPMAYH-
-HPMAYM-
HAPMAY--
-HPMAYR-
HIPMAY--
-HPMAYL-
HVPMAY--
-HPMAYT-
HGPMAY--
HTPMAY--
-HPMAYG-
-HPMAYA-
HMPMAY--
HYPMAY--
-HPMAYF-
-HPMAYV-
-HPMAYE-
HDPMAY--
ITPMAY--
-IPMAYL-
IEPMAY--
IRPMAY--
-IPMAYA-
ILPMAY--
IGPMAY--
-IPMAYT-
ISPMAY--
-IPMAYF-
IVPMAY--
IPPMAY--
-IPMAYR-
IKPMAY--
-IPMAYQ-
-IPMAYK-
INPMAY--
-IPMAYN-

-IPMAYW-
IAPMAY--
-IPMAYV-
-IPMAYH-
-IPMAYS-
-IPMAYG-
-IPMAYE-
-IPMAYC-
IFPMAY--
IMPMAY--
KSPMAY--
KIPMAY--
KKPMAY--
KRPMAY--
KWPMAY--
KYPMAY--
-KPMAYL-
-KPMAYG-
KTPMAY--
KQPMAY--
-KPMAYY-
KGPMAY--
-KPMAYR-
-KPMAYI-
-KPMAYH-
KDPMAY--
KVPMAY--
-KPMAYE-
KCPMAY--
-KPMAYT-
-KPMAYS-
KPPMAY--
-KPMAYN-
KAPMAY--
KHPMAY--
-KPMAYA-
-KPMAYV-
LGPMAY--
-LPMAYI-
-LPMAYS-
-LPMAYL-
LKPMAY--
LNPMAY--
LAPMAY--
-LPMAYW-
LEPMAY--
LHPMAY--
LTPMAY--
-LPMAYQ-
LSPMAY--

LIPMAY--
LPPMAY--
LQPMAY--
-LPMAYM-
LMPMAY--
-LPMAYR-
-LPMAYN-
-LPMAYK-
-LPMAYT-
-LPMAYE-
-LPMAYG-
-LPMAYD-
-LPMAYC-
-MPMAYK-
-MPMAYL-
-MPMAYR-
MLPMAY--
MSPMAY--
-MPMAYS-
MEPMAY--
-MPMAYH-
MRPMAY--
MHPMAY--
MTPMAY--
-MPMAYV-
MMPMAY--
-MPMAYA-
MGPMAY--
MAPMAY--
MPPMAY--
-MPMAYG-
-MPMAYI-
MQPMAY--
-MPMAYN-
MVPMAY--
-MPMAYW-
-MPMAYE-
MIPMAY--
MDPMAY--
NLPMAY--
NIPMAY--
NRPMAY--
NGPMAY--
-NPMAYL-
NKPMAY--
-NPMAYI-
NFPMAY--
-NPMAYT-
-NPMAYA-
NHPMAY--

NYPMAY--
NSPMAY--
NEPMAY--
NTPMAY--
-NPMAYM-
-NPMAYK-
-NPMAYF-
-NPMAYS-
NMPMAY--
NAPMAY--
NWPMAY--
NVPMAY--
-NPMAYR-
-NPMAYQ-
-NPMAYN-
-NPMAYG-
NPPMAY--
--PMAYRT
--PMAYLR
--PMAYRN
--PMAYAS
PGPMAY--
--PMAYRA
--PMAYTT
--PMAYTL
--PMAYTS
--PMAYTG
--PMAYAL
--PMAYRV
--PMAYVW
--PMAYVR
--PMAYSR
--PMAYKT
--PMAYST
--PMAYKS
--PMAYTI
--PMAYSM
--PMAYLH
--PMAYLA
--PMAYKM
--PMAYHL
--PMAYYG
--PMAYVG
--PMAYSF
--PMAYWH
--PMAYSG
--PMAYNL
--PMAYVN
--PMAYYI
--PMAYRD

--PMAYGA
--PMAYVQ
--PMAYSD
--PMAYRG
--PMAYGY
--PMAYFF
--PMAYSY
--PMAYSI
--PMAYMR
--PMAYGI
--PMAYSV
--PMAYSH
--PMAYRS
--PMAYLS
--PMAYKV
--PMAYIT
--PMAYFG
--PMAYSK
--PMAYQI
--PMAYLT
--PMAYIS
--PMAYIK
--PMAYVT
--PMAYTQ
--PMAYKA
--PMAYGS
--PMAYFT
--PMAYET
PDPMAY--
PAPMAY--
PQPMAY--
--PMAYVA
--PMAYLL
--PMAYHK
--PMAYEI
--PMAYWT
--PMAYWG
--PMAYWD
--PMAYVS
--PMAYVD
--PMAYTN
--PMAYMS
--PMAYMK
--PMAYLN
--PMAYHD
--PMAYAG
PMPMAY--
--PMAYSQ
--PMAYRM
--PMAYKK

--PMAYYS
--PMAYYR
--PMAYWS
--PMAYTY
--PMAYTA
--PMAYNM
--PMAYNH
--PMAYMH
--PMAYMF
--PMAYLE
--PMAYIV
--PMAYHR
--PMAYEM
--PMAYEA
--PMAYYH
--PMAYVE
--PMAYSW
--PMAYRY
--PMAYQL
--PMAYNY
--PMAYNI
--PMAYKF
--PMAYGW
--PMAYFS
--PMAYFR
PIPMAY--
--PMAYYK
--PMAYWW
--PMAYRQ
--PMAYMA
--PMAYHW
--PMAYHH
--PMAYHF
--PMAYEW
PWPMAY--
PNPMAY--
--PMAYSA
--PMAYRE
--PMAYQQ
--PMAYQG
--PMAYMW
--PMAYIA
--PMAYGF
--PMAYYM
--PMAYTW
--PMAYTF
--PMAYQR
--PMAYNT
--PMAYMT
--PMAYLM

--PMAYHA
--PMAYFV
--PMAYEG
--PMAYYY
--PMAYWM
--PMAYWK
--PMAYVH
--PMAYTV
--PMAYQF
--PMAYPY
--PMAYPM
--PMAYMN
--PMAYLQ
--PMAYLD
--PMAYKN
--PMAYII
--PMAYID
--PMAYHY
--PMAYHS
--PMAYGV
--PMAYFW
--PMAYFQ
--PMAYFN
--PMAYFH
--PMAYES
--PMAYEN
--PMAYDL
--PMAYDA
--PMAYAF
--PMAYAA
PKPMAY--
PYPMAY--
--PMAYYE
--PMAYWY
--PMAYWN
--PMAYVY
--PMAYQH
--PMAYPI
--PMAYPF
--PMAYNK
--PMAYMI
--PMAYLV
--PMAYLK
--PMAYGQ
--PMAYGH
--PMAYFM
--PMAYFK
--PMAYFE
--PMAYAK
PTPMAY--

--PMAYYW
--PMAYYQ
--PMAYYN
--PMAYYD
--PMAYWI
--PMAYTR
--PMAYSE
--PMAYRF
--PMAYQS
--PMAYQM
--PMAYQD
--PMAYNV
--PMAYND
--PMAYNA
--PMAYMG
--PMAYME
--PMAYKH
--PMAYKD
--PMAYHT
--PMAYGT
--PMAYEY
--PMAYER
--PMAYEL
--PMAYDT
--PMAYDS
--PMAYDG
--PMAYAT
--PMAYAR
PFPMAY--
-PPMAYT-
--PMAYYF
--PMAYWQ
--PMAYVK
--PMAYRH
--PMAYQW
--PMAYQA
--PMAYPT
--PMAYPD
--PMAYNW
--PMAYNE
--PMAYLP
--PMAYKY
--PMAYKL
--PMAYIY
--PMAYIG
--PMAYIF
--PMAYIE
--PMAYGR
--PMAYFP
--PMAYCT

--PMAYCS
--PMAYAY
PHPMAY--
PEPMAY--
-QPMAYS-
-QPMAYF-
QVPMAY--
QSPMAY--
-QPMAYQ-
QTPMAY--
-QPMAYW-
QIPMAY--
QEPMAY--
QWPMAY--
-QPMAYR-
-QPMAYH-
QRPMAY--
QPPMAY--
-QPMAYV-
-QPMAYN-
QNPMAY--
QFPMAY--
QAPMAY--
-QPMAYK-
QLPMAY--
QCPMAY--
-QPMAYL-
-QPMAYG-
-QPMAYA-
QKPMAY--
-QPMAYE-
QDPMAY--
RLPMAY--
RVPMAY--
RGPMAY--
RSPMAY--
-RPMAYL-
RMPMAY--
RAPMAY--
RPPMAY--
RTPMAY--
-RPMAYR-
-RPMAYT-
-RPMAYS-
RNPMAY--
-RPMAYM-
-RPMAYK-
REPMAY--
-RPMAYN-
RWPMAY--

-RPMAYY-
-RPMAYV-
RDPMAY--
-RPMAYH-
-RPMAYA-
-RPMAYI-
-RPMAYE-
RYPMAY--
-RPMAYQ-
RQPMAY--
RHPMAY--
RFPMAY--
SEPMAY--
SQPMAY--
SLPMAY--
SSPMAY--
-SPMAYS-
-SPMAYA-
SAPMAY--
-SPMAYF-
SGPMAY--
-SPMAYL-
-SPMAYI-
-SPMAYV-
-SPMAYN-
SPPMAY--
-SPMAYW-
STPMAY--
-SPMAYM-
SVPMAY--
-SPMAYT-
SIPMAY--
-SPMAYH-
SKPMAY--
-SPMAYG-
SDPMAY--
SYPMAY--
-SPMAYQ-
SWPMAY--
-SPMAYY-
SMPMAY--
-SPMAYE-
SNPMAY--
SCPMAY--
TSPMAY--
TVPMAY--
TKPMAY--
-TPMAYS-
-TPMAYT-
-TPMAYM-

TLPMAY--
-TPMAYI-
TEPMAY--
-TPMAYF-
TPPMAY--
TIPMAY--
-TPMAYA-
TTPMAY--
-TPMAYV-
-TPMAYR-
TYPMAY--
THPMAY--
TAPMAY--
-TPMAYH-
TWPMAY--
-TPMAYN-
-TPMAYW-
-TPMAYG-
TFPMAY--
-TPMAYK-
TCPMAY--
-VPMAYL-
VRPMAY--
-VPMAYS-
VSPMAY--
VLPMAY--
VIPMAY--
VAPMAY--
-VPMAYI-
VWPMAY--
-VPMAYG-
VHPMAY--
VNPMAY--
VYPMAY--
VMPMAY--
-VPMAYH-
-VPMAYK-
VEPMAY--
VTPMAY--
VPPMAY--
-VPMAYR-
-VPMAYN-
-VPMAYF-
VDPMAY--
-VPMAYT-
-VPMAYE-
-VPMAYQ-
VKPMAY--
VVPMAY--
-VPMAYM-

VGPMAY--
VQPMAY--
-VPMAYV-
WGPMAY--
-WPMAYI-
WVPMAY--
WRPMAY--
WQPMAY--
WCPMAY--
WSPMAY--
WLPMAY--
-WPMAYV-
-WPMAYS-
WDPMAY--
WPPMAY--
WMPMAY--
-WPMAYQ-
WAPMAY--
-WPMAYH-
-WPMAYG-
WKPMAY--
WIPMAY--
WEPMAY--
-WPMAYW-
-WPMAYT-
-WPMAYN-
-WPMAYD-
-WPMAYA-
YWPMAY--
YAPMAY--
YVPMAY--
-YPMAYV-
-YPMAYL-
-YPMAYY-
-YPMAYK-
YRPMAY--
-YPMAYA-
YIPMAY--
YGPMAY--
YSPMAY--
YKPMAY--
-YPMAYH-
YYPMAY--
YHPMAY--
YDPMAY--
YPPMAY--
-YPMAYM-
YNPMAY--
YFPMAY--
-YPMAYS-

	-YPMAYG-			
PWAM	429	648	49.72	
	-APWAML-			
	-APWAMT-			
	ARPWAM--			
	ASPWAM--			
	-APWAMI-			
	AVPWAM--			
	AMPWAM--			
	AGPWAM--			
	ATPWAM--			
	-APWAMG-			
	AWPWAM--			
	AKPWAM--			
	-APWAMY-			
	-APWAMV-			
	-APWAMA-			
	APPWAM--			
	AIPWAM--			
	AQPWAM--			
	-APWAMW-			
	-APWAMH-			
	AEPWAM--			
	ADPWAM--			
	AYPWAM--			
	AHPWAM--			
	AFPWAM--			
	CGPWAM--			
	CLPWAM--			
	-CPWAMH-			
	CYPWAM--			
	CWPWAM--			
	-CPWAMY-			
	-CPWAMS-			
	-CPWAML-			
	DRPWAM--			
	DTPWAM--			
	-DPWAMR-			
	DSPWAM--			
	DFPWAM--			
	-DPWAMI-			
	DYPWAM--			
	-DPWAMS-			
	-DPWAMK-			
	DIPWAM--			
	DVPWAM--			
	-DPWAMV-			
	-DPWAML-			
	DLPWAM--			
	DGPWAM--			

–DPWAMW–
–DPWAMT–
–DPWAMN–
–DPWAMF–
DPPWAM––
DKPWAM––
–EPWAMH–
EGPWAM––
EAPWAM––
–EPWAMT–
–EPWAMK–
–EPWAMN–
EKPWAM––
EQPWAM––
–EPWAMF–
EFPWAM––
–EPWAMV–
EMPWAM––
EWPWAM––
–EPWAMQ–
–EPWAMW–
–EPWAMA–
–EPWAMG–
–EPWAML–
ELPWAM––
EIPWAM––
–FPWAMS–
–FPWAMN–
–FPWAMK–
FRPWAM––
FFPWAM––
–FPWAMR–
–FPWAMH–
–FPWAMT–
–FPWAML–
–FPWAMA–
FHPWAM––
FGPWAM––
–FPWAMM–
FNPWAM––
–FPWAMQ–
–FPWAMG–
FIPWAM––
GTPWAM––
GVPWAM––
GDPWAM––
GHPWAM––
–GPWAMM–
–GPWAMA–
–GPWAMK–

-GPWAML-
-GPWAMR-
GQPWAM--
-GPWAMS-
GNPWAM--
-GPWAMT-
-GPWAMI-
-GPWAMW-
GWPWAM--
GSPWAM--
GRPWAM--
GKPWAM--
-GPWAMN-
GPPWAM--
GLPWAM--
GAPWAM--
GYPWAM--
-GPWAMG-
-GPWAMY-
GFPWAM--
GEPWAM--
-GPWAME-
GIPWAM--
GGPWAM--
HGPWAM--
HRPWAM--
HNPWAM--
-HPWAMI-
HSPWAM--
HIPWAM--
HTPWAM--
HAPWAM--
-HPWAMK-
-HPWAML-
-HPWAMT-
-HPWAMR-
HFPWAM--
-HPWAMS-
HLPWAM--
HHPWAM--
HDPWAM--
-HPWAMA-
HYPWAM--
-HPWAMQ-
-HPWAMG-
-HPWAME-
IRPWAM--
ISPWAM--
-IPWAMR-
INPWAM--

```
-IPWAMA-
IGPWAM--
ILPWAM--
-IPWAMV-
-IPWAMS-
IEPWAM--
IQPWAM--
-IPWAMI-
IIPWAM--
IHPWAM--
ITPWAM--
-IPWAMW-
-IPWAMQ-
IPPWAM--
IKPWAM--
-IPWAML-
-IPWAMH-
-KPWAML-
KRPWAM--
KSPWAM--
-KPWAME-
-KPWAMQ-
-KPWAMA-
-KPWAMH-
KEPWAM--
-KPWAMY-
-KPWAMN-
-KPWAMG-
KMPWAM--
KIPWAM--
KGPWAM--
KHPWAM--
KYPWAM--
-KPWAMR-
-KPWAMI-
KFPWAM--
KDPWAM--
KVPWAM--
-KPWAMS-
KPPWAM--
-KPWAMT-
-KPWAMK-
-KPWAMF-
-LPWAMM-
-LPWAMR-
LPPWAM--
LSPWAM--
LDPWAM--
LAPWAM--
-LPWAMT-
```

LMPWAM--
LLPWAM--
-LPWAMW-
-LPWAMQ-
LWPWAM--
LVPWAM--
LTPWAM--
LRPWAM--
LKPWAM--
LFPWAM--
-LPWAMV-
-LPWAMK-
-LPWAMA-
-LPWAMY-
-LPWAMH-
-LPWAMG-
-LPWAMS-
LHPWAM--
-LPWAMN-
LIPWAM--
-LPWAMI-
-MPWAMS-
-MPWAMT-
-MPWAMF-
MVPWAM--
MTPWAM--
MEPWAM--
MRPWAM--
-MPWAMR-
-MPWAMN-
MLPWAM--
MSPWAM--
-MPWAMM-
MKPWAM--
MIPWAM--
MQPWAM--
-MPWAMG-
MNPWAM--
MHPWAM--
-MPWAMY-
-MPWAMW-
-MPWAML-
MMPWAM--
-NPWAMY-
NLPWAM--
NSPWAM--
-NPWAMR-
-NPWAMT-
-NPWAMH-
NDPWAM--

NGPWAM--
NNPWAM--
NAPWAM--
NTPWAM--
-NPWAMS-
NYPWAM--
NVPWAM--
-NPWAMV-
NPPWAM--
-NPWAMN-
NKPWAM--
NWPWAM--
NRPWAM--
-NPWAMK-
NEPWAM--
-NPWAMQ-
NIPWAM--
--PWAMST
--PWAMLR
--PWAMYR
--PWAMKT
--PWAMQS
--PWAMIN
--PWAMTG
--PWAMKS
--PWAMFR
--PWAMAT
--PWAMVH
--PWAMTE
--PWAMRN
--PWAMKN
--PWAMYQ
--PWAMSS
--PWAMSN
--PWAMAS
--PWAMAL
--PWAMKG
--PWAMRT
--PWAMRF
--PWAMMR
--PWAMVI
--PWAMHG
--PWAMGA
--PWAMVG
--PWAMRV
--PWAMRK
--PWAMKY
--PWAMVV
--PWAMTS
--PWAMMT

```
--PWAMGS
--PWAMNW
--PWAMKA
--PWAMLG
--PWAMTK
--PWAMSG
--PWAMNY
--PWAMNS
--PWAMNI
--PWAMYG
--PWAMVR
--PWAMMF
--PWAMGY
PSPWAM--
--PWAMVY
--PWAMVN
--PWAMTI
--PWAMAR
--PWAMAN
--PWAMWN
--PWAMVQ
--PWAMTN
--PWAMTA
--PWAMRG
--PWAMQG
--PWAMYD
--PWAMSV
--PWAMSR
--PWAMRA
--PWAMMK
--PWAMLN
--PWAMFQ
--PWAMFG
--PWAMER
--PWAMVT
--PWAMVD
--PWAMSW
--PWAMMG
--PWAMLK
PIPWAM--
--PWAMWT
--PWAMVS
--PWAMNK
--PWAMNH
--PWAMME
--PWAMKQ
--PWAMIY
--PWAMFH
--PWAMFD
PTPWAM--
```

--PWAMVM
--PWAMSA
--PWAMRH
--PWAMHY
--PWAMHT
--PWAMFK
--PWAMEH
--PWAMAI
--PWAMWH
--PWAMQN
--PWAMMN
--PWAMLH
--PWAMKM
--PWAMKL
--PWAMHK
--PWAMGI
--PWAMYK
--PWAMVE
--PWAMTV
--PWAMTR
--PWAMSQ
--PWAMSI
--PWAMSD
--PWAMNV
--PWAMMH
--PWAMKK
--PWAMEN
--PWAMEG
--PWAMAV
--PWAMYS
--PWAMSM
--PWAMRM
--PWAMNN
--PWAMNM
--PWAMHD
--PWAMGT
--PWAMFE
--PWAMEA
--PWAMAH
PGPWAM--
PEPWAM--
--PWAMYH
--PWAMVA
--PWAMTH
--PWAMQK
--PWAMID
--PWAMHW
--PWAMHQ
--PWAMGN
--PWAMGH

--PWAMGG
--PWAMGF
--PWAMAM
--PWAMAE
PMPWAM--
PKPWAM--
--PWAMWQ
--PWAMTT
--PWAMTD
--PWAMNQ
--PWAMMY
--PWAMLQ
--PWAMIS
--PWAMIG
--PWAMHI
--PWAMEK
--PWAMAW
--PWAMAG
--PWAMAD
PVPWAM--
PNPWAM--
--PWAMYP
--PWAMYE
--PWAMWG
--PWAMTW
--PWAMTQ
--PWAMTM
--PWAMSK
--PWAMSH
--PWAMQR
--PWAMPY
--PWAMPI
--PWAMMD
--PWAMIA
--PWAMHL
--PWAMGR
--PWAMGQ
--PWAMDL
PQPWAM--
PWPWAM--
--PWAMYA
--PWAMWR
--PWAMWL
--PWAMWF
--PWAMVP
--PWAMRY
--PWAMQT
--PWAMQP
--PWAMQH
--PWAMQE

--PWAMQA
--PWAMPQ
--PWAMNT
--PWAMNA
--PWAMMP
--PWAMLD
--PWAMIT
--PWAMIK
--PWAMHV
--PWAMHF
--PWAMHC
--PWAMGV
--PWAMFW
--PWAMFN
--PWAMFM
--PWAMEV
--PWAMES
--PWAMEQ
PCPWAM--
-QPWAML-
QRPWAM--
-QPWAMV-
QSPWAM--
-QPWAMQ-
-QPWAMH-
QMPWAM--
QGPWAM--
-QPWAMN-
-QPWAMY-
-QPWAMA-
-QPWAME-
QPPWAM--
QLPWAM--
QAPWAM--
QQPWAM--
QNPWAM--
QIPWAM--
QYPWAM--
-QPWAMS-
-QPWAMG-
QEPWAM--
-QPWAMK-
-QPWAMI-
QHPWAM--
QTPWAM--
-QPWAMT-
QDPWAM--
RTPWAM--
RVPWAM--
RAPWAM--

RIPWAM--
RNPWAM--
-RPWAMV-
-RPWAMT-
-RPWAMY-
-RPWAMR-
-RPWAML-
RGPWAM--
REPWAM--
-RPWAMK-
RMPWAM--
RKPWAM--
-RPWAMA-
-RPWAME-
RSPWAM--
RQPWAM--
-RPWAMS-
RPPWAM--
-RPWAMG-
RHPWAM--
RDPWAM--
-RPWAMN-
-RPWAMI-
-RPWAMW-
-RPWAMH-
RFPWAM--
-RPWAMM-
-RPWAMF-
-RPWAMD-
-SPWAMR-
-SPWAML-
-SPWAMK-
STPWAM--
SLPWAM--
SMPWAM--
-SPWAMV-
SAPWAM--
-SPWAMW-
SSPWAM--
-SPWAMI-
SRPWAM--
SQPWAM--
SVPWAM--
-SPWAMT-
-SPWAMS-
SHPWAM--
-SPWAMG-
SPPWAM--
SKPWAM--
-SPWAMM-

SNPWAM--
SYPWAM--
-SPWAMH-
-SPWAMQ-
-SPWAMA-
SIPWAM--
SCPWAM--
SFPWAM--
-TPWAMI-
-TPWAMR-
TRPWAM--
-TPWAMV-
TPPWAM--
TSPWAM--
-TPWAMN-
-TPWAMK-
TVPWAM--
-TPWAMQ-
TMPWAM--
TIPWAM--
TKPWAM--
-TPWAMS-
-TPWAMH-
-TPWAMA-
TNPWAM--
TLPWAM--
TWPWAM--
-TPWAMT-
TQPWAM--
-TPWAMM-
-TPWAMG-
TGPWAM--
TAPWAM--
-TPWAMW-
TDPWAM--
TCPWAM--
-TPWAME-
-VPWAMR-
VVPWAM--
VSPWAM--
VRPWAM--
-VPWAMI-
VKPWAM--
-VPWAMT-
VIPWAM--
VFPWAM--
VTPWAM--
VYPWAM--
VQPWAM--
-VPWAMS-

VGPWAM--
-VPWAMN-
-VPWAMH-
-VPWAMG-
VAPWAM--
-VPWAMV-
VNPWAM--
VLPWAM--
VWPWAM--
VPPWAM--
-VPWAME-
WLPWAM--
WSPWAM--
WKPWAM--
-WPWAMR-
-WPWAMH-
WHPWAM--
WYPWAM--
WTPWAM--
WFPWAM--
WAPWAM--
-WPWAMK-
-WPWAMT-
-WPWAMA-
WIPWAM--
-WPWAMV-
-WPWAMQ-
-WPWAMM-
-WPWAME-
WEPWAM--
WCPWAM--
-YPWAMS-
YKPWAM--
YIPWAM--
YTPWAM--
YRPWAM--
-YPWAMR-
YYPWAM--
YAPWAM--
YGPWAM--
YEPWAM--
-YPWAMV-
-YPWAMK-
-YPWAMI-
-YPWAMH-
YQPWAM--
-YPWAMY-
YVPWAM--
YSPWAM--
-YPWAMA-

PLNM 41 861 49.69

--PLNMVP
--PLNMPR
-APLNMT-
ARPLNM--
-APLNML-
AAPLNM--
-APLNMW-
AKPLNM--
AVPLNM--
-APLNMF-
-APLNMG-
-APLNMR-
-APLNMS-
AEPLNM--
-APLNMI-
ANPLNM--
ATPLNM--
AQPLNM--
AHPLNM--
-APLNMA-
AIPLNM--
AFPLNM--
ALPLNM--
ACPLNM--
AWPLNM--
-APLNMY-
-APLNMV-
-APLNMQ-
APPLNM--
-APLNMN-
-APLNME-
AMPLNM--
AYPLNM--
-APLNMD-
-APLNMM-
-APLNMK-
-APLNMH-
ADPLNM--
AGPLNM--
CSPLNM--
CFPLNM--
CQPLNM--
CVPLNM--
CRPLNM--
CYPLNM--
-CPLNMI-
-CPLNMG-
CEPLNM--
CCPLNM--

CAPLNM--
-CPLNMS-
-CPLNML-
CMPLNM--
CKPLNM--
-CPLNMR-
CLPLNM--
CGPLNM--
-CPLNMV-
CNPLNM--
-DPLNML-
DHPLNM--
-DPLNMR-
DYPLNM--
DTPLNM--
-DPLNMK-
DQPLNM--
-DPLNMT-
DRPLNM--
DSPLNM--
-DPLNMS-
-DPLNMH-
DKPLNM--
-DPLNMW-
DFPLNM--
-DPLNMQ-
-DPLNMA-
-DPLNMG-
-DPLNMI-
DCPLNM--
DWPLNM--
-DPLNME-
DMPLNM--
DLPLNM--
DIPLNM--
DDPLNM--
-EPLNML-
-EPLNMR-
ELPLNM--
ERPLNM--
-EPLNMT-
-EPLNMH-
-EPLNMA-
EAPLNM--
-EPLNMY-
-EPLNMM-
-EPLNMS-
ENPLNM--
EWPLNM--
ETPLNM--

-EPLNMG-
EHPLNM--
-EPLNMK-
EYPLNM--
EQPLNM--
EMPLNM--
-EPLNMW-
-EPLNMQ-
-EPLNMD-
EEPLNM--
EPPLNM--
-EPLNMI-
-EPLNME-
EGPLNM--
EFPLNM--
FTPLNM--
FRPLNM--
FSPLNM--
-FPLNML-
-FPLNMS-
FGPLNM--
-FPLNMV-
FNPLNM--
FAPLNM--
-FPLNMM-
FKPLNM--
-FPLNMR-
-FPLNMG-
-FPLNMQ-
-FPLNMY-
-FPLNMI-
FQPLNM--
-FPLNMA-
FDPLNM--
FVPLNM--
-FPLNMN-
-FPLNME-
FFPLNM--
FMPLNM--
FLPLNM--
FPPLNM--
-FPLNMD-
FIPLNM--
-FPLNMW-
-FPLNMT-
FHPLNM--
FWPLNM--
FEPLNM--
-GPLNML-
-GPLNMW-

GTPLNM--
-GPLNMS-
-GPLNMI-
-GPLNMH-
-GPLNMR-
GAPLNM--
-GPLNMV-
GWPLNM--
GRPLNM--
-GPLNMT-
GLPLNM--
GGPLNM--
GSPLNM--
-GPLNMA-
GVPLNM--
GFPLNM--
GKPLNM--
GHPLNM--
GDPLNM--
-GPLNMK-
-GPLNMG-
GIPLNM--
GYPLNM--
GQPLNM--
-GPLNMN-
GEPLNM--
GMPLNM--
-GPLNMY-
-GPLNMQ-
GPPLNM--
-GPLNMM-
GCPLNM--
-HPLNML-
-HPLNMT-
HSPLNM--
HRPLNM--
-HPLNMY-
HGPLNM--
HVPLNM--
-HPLNMV-
-HPLNMQ-
HNPLNM--
HAPLNM--
HLPLNM--
HYPLNM--
HTPLNM--
HPPLNM--
-HPLNMN-
-HPLNMK-
-HPLNMS-

−HPLNMM−
−HPLNME−
−HPLNMW−
−HPLNMR−
HHPLNM−−
HEPLNM−−
−HPLNMH−
−HPLNMG−
HKPLNM−−
HFPLNM−−
−HPLNMF−
−HPLNMA−
HIPLNM−−
HDPLNM−−
HWPLNM−−
HQPLNM−−
−HPLNMP−
HCPLNM−−
IKPLNM−−
IVPLNM−−
−IPLNMV−
ILPLNM−−
−IPLNML−
IHPLNM−−
−IPLNMT−
IFPLNM−−
IRPLNM−−
−IPLNMS−
−IPLNMR−
IYPLNM−−
−IPLNMI−
−IPLNMW−
ITPLNM−−
INPLNM−−
IGPLNM−−
−IPLNMQ−
−IPLNMG−
−IPLNMF−
IAPLNM−−
−IPLNMA−
IPPLNM−−
−IPLNMY−
−IPLNMM−
−IPLNMK−
IIPLNM−−
IWPLNM−−
IDPLNM−−
IQPLNM−−
−IPLNMN−
IMPLNM−−

ICPLNM--
KSPLNM--
-KPLNML-
-KPLNMT-
-KPLNMV-
KMPLNM--
-KPLNMA-
KLPLNM--
KVPLNM--
-KPLNMS-
-KPLNMH-
-KPLNMG-
KNPLNM--
-KPLNMF-
-KPLNMR-
-KPLNMQ-
KTPLNM--
KQPLNM--
KAPLNM--
-KPLNMM-
-KPLNMW-
KEPLNM--
-KPLNMI-
KDPLNM--
KCPLNM--
KWPLNM--
KGPLNM--
KFPLNM--
KYPLNM--
-KPLNME-
-KPLNMY-
KIPLNM--
KHPLNM--
KPPLNM--
KKPLNM--
LSPLNM--
LEPLNM--
LKPLNM--
LHPLNM--
-LPLNMT-
-LPLNMS-
-LPLNMR-
-LPLNMQ-
LMPLNM--
LAPLNM--
LVPLNM--
LWPLNM--
LPPLNM--
LYPLNM--
-LPLNMH-

```
-LPLNML-
LIPLNM--
-LPLNMV-
-LPLNMG-
-LPLNMI-
-LPLNMA-
-LPLNMK-
LQPLNM--
-LPLNME-
LDPLNM--
-LPLNMM-
-LPLNMW-
-LPLNMN-
-LPLNMD-
LFPLNM--
-MPLNMW-
MRPLNM--
MLPLNM--
MMPLNM--
-MPLNMV-
-MPLNML-
MTPLNM--
-MPLNMI-
MQPLNM--
MKPLNM--
-MPLNME-
-MPLNMK-
MVPLNM--
-MPLNMA-
MGPLNM--
-MPLNMG-
MYPLNM--
MWPLNM--
MSPLNM--
-MPLNMT-
MNPLNM--
MPPLNM--
-MPLNMS-
MHPLNM--
MDPLNM--
-MPLNMQ-
MIPLNM--
MAPLNM--
-MPLNMF-
-MPLNMD-
-MPLNMY-
-MPLNMH-
MFPLNM--
-MPLNMC-
MEPLNM--
```

```
NLPLNM--
NEPLNM--
NMPLNM--
NQPLNM--
NGPLNM--
NRPLNM--
NTPLNM--
-NPLNMI-
NWPLNM--
NVPLNM--
NAPLNM--
-NPLNMR-
NDPLNM--
-NPLNMT-
-NPLNMS-
-NPLNMA-
-NPLNMV-
-NPLNME-
NHPLNM--
-NPLNMF-
NIPLNM--
-NPLNMH-
-NPLNMN-
-NPLNML-
-NPLNMK-
NFPLNM--
NYPLNM--
NPPLNM--
-NPLNMY-
NKPLNM--
-NPLNMQ-
-NPLNMG-
--PLNMLG
--PLNMSN
--PLNMRS
--PLNMVL
PRPLNM--
--PLNMKS
--PLNMSK
--PLNMVT
--PLNMMR
--PLNMTL
--PLNMTR
--PLNMRL
--PLNMRD
--PLNMVR
PTPLNM--
--PLNMLT
--PLNMTV
--PLNMRG
```

```
--PLNMYR
--PLNMTN
--PLNMLE
--PLNMYL
--PLNMTS
--PLNMTI
--PLNMLN
--PLNMAS
--PLNMWK
--PLNMSA
--PLNMFG
--PLNMKL
--PLNMST
--PLNMSS
--PLNMRY
--PLNMRM
--PLNMSM
--PLNMVW
--PLNMLR
--PLNMRQ
--PLNMQG
--PLNMMQ
--PLNMHA
--PLNMWG
--PLNMVS
--PLNMSL
--PLNMRK
--PLNMML
--PLNMIR
--PLNMGS
--PLNMTG
--PLNMSH
--PLNMNR
--PLNMKN
--PLNMII
--PLNMGA
--PLNMVK
--PLNMRN
--PLNMGW
--PLNMGT
--PLNMWT
--PLNMNW
--PLNMLM
--PLNMKY
--PLNMKA
--PLNMYH
--PLNMVG
--PLNMMV
--PLNMKR
--PLNMGI
```

--PLNMYK
--PLNMYA
--PLNMQW
--PLNMSI
--PLNMQQ
--PLNMMS
--PLNMLD
--PLNMKI
--PLNMKG
--PLNMAN
--PLNMYT
--PLNMVV
--PLNMVF
--PLNMTQ
--PLNMRW
--PLNMLH
--PLNMGM
--PLNMAH
--PLNMRH
--PLNMQA
--PLNMNV
--PLNMMH
--PLNMMG
--PLNMIT
--PLNMAM
PEPLNM--
--PLNMVY
--PLNMTA
--PLNMQS
--PLNMNM
--PLNMNK
--PLNMNA
--PLNMES
--PLNMAR
--PLNMAL
--PLNMRF
--PLNMQN
--PLNMNY
--PLNMNS
--PLNMMM
--PLNMIE
--PLNMGY
--PLNMGG
--PLNMDG
--PLNMAW
--PLNMAV
PGPLNM--
PNPLNM--
--PLNMYD
--PLNMWN

--PLNMVH
--PLNMTY
--PLNMTW
--PLNMSG
--PLNMQL
--PLNMNT
--PLNMIG
--PLNMHF
--PLNMEH
--PLNMEE
--PLNMAA
PHPLNM--
--PLNMYM
--PLNMQT
--PLNMID
--PLNMGL
--PLNMFD
--PLNMET
--PLNMEG
--PLNMYG
--PLNMWQ
--PLNMVI
--PLNMTT
--PLNMNQ
--PLNMKV
--PLNMKH
--PLNMGF
--PLNMFH
--PLNMER
--PLNMEK
--PLNMAT
--PLNMAI
--PLNMVE
--PLNMTF
--PLNMSQ
--PLNMSE
--PLNMFS
--PLNMFE
--PLNMDT
--PLNMWM
--PLNMVN
--PLNMVM
--PLNMTE
--PLNMQV
--PLNMQR
--PLNMMT
--PLNMLQ
--PLNMIK
--PLNMHE
--PLNMGN

--PLNMFQ
--PLNMDY
--PLNMVA
--PLNMSW
--PLNMNL
--PLNMMI
--PLNMHN
--PLNMHM
--PLNMGR
--PLNMGQ
--PLNMEA
PIPLNM--
--PLNMYN
--PLNMWD
--PLNMSD
--PLNMQY
--PLNMQH
--PLNMMK
--PLNMMD
--PLNMKT
--PLNMIA
--PLNMGK
--PLNMFN
--PLNMEM
--PLNMED
--PLNMDW
--PLNMAY
--PLNMAQ
--PLNMAG
--PLNMYE
--PLNMSV
--PLNMRP
--PLNMRE
--PLNMQE
--PLNMND
--PLNMKD
--PLNMIH
--PLNMHR
--PLNMHQ
--PLNMGE
PKPLNM--
PFPLNM--
PDPLNM--
--PLNMWE
--PLNMWA
--PLNMVQ
--PLNMTM
--PLNMTH
--PLNMNE
--PLNMMC

--PLNMKQ
--PLNMIV
--PLNMHT
--PLNMHG
--PLNMGD
--PLNMFA
--PLNMEW
--PLNMEN
--PLNMDF
--PLNMAK
PQPLNM--
-PPLNME-
--PLNMYP
--PLNMTK
--PLNMTD
--PLNMSF
--PLNMSC
--PLNMRC
--PLNMQD
--PLNMPV
--PLNMNN
--PLNMNI
--PLNMNG
--PLNMMP
--PLNMME
--PLNMKM
--PLNMHS
--PLNMHI
--PLNMEY
--PLNMEI
--PLNMEF
--PLNMDV
--PLNMDM
--PLNMDA
--PLNMCY
--PLNMCR
--PLNMCH
--PLNMAE
-QPLNMY-
-QPLNML-
QIPLNM--
QGPLNM--
QRPLNM--
QTPLNM--
-QPLNMS-
QYPLNM--
-QPLNMR-
-QPLNMH-
-QPLNMM-
-QPLNMN-

QEPLNM--
QPPLNM--
-QPLNMV-
-QPLNMG-
-QPLNMK-
QSPLNM--
QHPLNM--
QNPLNM--
QMPLNM--
QKPLNM--
QWPLNM--
-QPLNME-
-QPLNMA-
-QPLNMC-
QVPLNM--
-QPLNMQ-
QAPLNM--
RVPLNM--
-RPLNMS-
-RPLNMR-
RGPLNM--
-RPLNMM-
RLPLNM--
-RPLNMI-
-RPLNMY-
RSPLNM--
-RPLNMV-
REPLNM--
-RPLNMN-
-RPLNMH-
RKPLNM--
RFPLNM--
-RPLNMK-
RIPLNM--
-RPLNMF-
RQPLNM--
RDPLNM--
-RPLNME-
-RPLNMA-
RTPLNM--
-RPLNMQ-
RNPLNM--
RMPLNM--
RPPLNM--
RHPLNM--
RAPLNM--
RCPLNM--
-RPLNMD-
RYPLNM--
-SPLNMR-

```
-SPLNMA-
SKPLNM--
SGPLNM--
-SPLNMS-
-SPLNMI-
SWPLNM--
-SPLNMF-
SAPLNM--
-SPLNMV-
-SPLNMN-
-SPLNML-
-SPLNMG-
-SPLNMY-
-SPLNMH-
SHPLNM--
SYPLNM--
-SPLNMT-
SMPLNM--
-SPLNMQ-
SEPLNM--
SVPLNM--
SFPLNM--
STPLNM--
SQPLNM--
-SPLNMK-
-SPLNMM-
SPPLNM--
SIPLNM--
SCPLNM--
-SPLNME-
SDPLNM--
-TPLNML-
TSPLNM--
-TPLNMV-
-TPLNMG-
TVPLNM--
-TPLNMR-
-TPLNMY-
-TPLNMI-
TTPLNM--
-TPLNMA-
TWPLNM--
TFPLNM--
-TPLNMT-
TNPLNM--
TGPLNM--
THPLNM--
-TPLNMM-
TKPLNM--
TYPLNM--
```

```
-TPLNME-
TLPLNM--
TAPLNM--
TQPLNM--
-TPLNMH-
TIPLNM--
-TPLNMK-
TPPLNM--
-TPLNMQ-
TMPLNM--
TCPLNM--
TEPLNM--
TDPLNM--
-TPLNMD-
-VPLNMR-
VVPLNM--
VRPLNM--
VSPLNM--
-VPLNML-
VYPLNM--
-VPLNMI-
-VPLNMK-
-VPLNMS-
-VPLNMN-
-VPLNMV-
VEPLNM--
VAPLNM--
VLPLNM--
VTPLNM--
VQPLNM--
-VPLNMW-
-VPLNMY-
-VPLNMT-
VNPLNM--
-VPLNMF-
-VPLNMD-
VPPLNM--
VGPLNM--
VMPLNM--
-VPLNMQ-
-VPLNMH-
-VPLNMG-
-VPLNMM-
-VPLNMA-
VKPLNM--
VWPLNM--
VHPLNM--
VFPLNM--
VDPLNM--
VCPLNM--
```

WGPLNM--
WRPLNM--
WLPLNM--
-WPLNMT-
-WPLNMI-
WHPLNM--
-WPLNML-
WDPLNM--
WSPLNM--
-WPLNMR-
WQPLNM--
WKPLNM--
WTPLNM--
-WPLNMH-
WAPLNM--
-WPLNMV-
-WPLNMW-
-WPLNMN-
-WPLNMA-
-WPLNMS-
-WPLNMM-
-WPLNMF-
WNPLNM--
WFPLNM--
-WPLNMK-
-WPLNMG-
WWPLNM--
WVPLNM--
WMPLNM--
-WPLNME-
-WPLNMD-
-YPLNMK-
YSPLNM--
YYPLNM--
-YPLNMR-
-YPLNMQ-
-YPLNMN-
YKPLNM--
-YPLNMF-
YRPLNM--
YAPLNM--
YLPLNM--
YGPLNM--
-YPLNMW-
-YPLNMT-
YNPLNM--
YQPLNM--
-YPLNMV-
-YPLNMH-
YWPLNM--

	YVPLNM--		
	YFPLNM--		
	-YPLNMM-		
	-YPLNMG-		
	YMPLNM--		
	YDPLNM--		
	YHPLNM--		
	-YPLNMI-		
	YIPLNM--		
	-YPLNMD-		
	-YPLNMA-		
	YEPLNM--		
PYAY	218	737	49.62
	-GPYAYT-		
	-APYAYL-		
	AQPYAY--		
	AKPYAY--		
	AGPYAY--		
	-APYAYS-		
	ALPYAY--		
	AHPYAY--		
	AWPYAY--		
	ATPYAY--		
	-APYAYG-		
	-APYAYY-		
	ARPYAY--		
	-APYAYM-		
	AEPYAY--		
	-APYAYK-		
	-APYAYI-		
	ASPYAY--		
	-APYAYQ-		
	APPYAY--		
	ANPYAY--		
	-APYAYV-		
	AYPYAY--		
	AVPYAY--		
	ADPYAY--		
	-APYAYF-		
	-APYAYE-		
	AIPYAY--		
	AFPYAY--		
	-APYAYW-		
	-APYAYT-		
	CVPYAY--		
	-CPYAYW-		
	CSPYAY--		
	CNPYAY--		
	CHPYAY--		
	CAPYAY--		

−CPYAYL−
CMPYAY−−
CRPYAY−−
−CPYAYR−
−CPYAYF−
−CPYAYQ−
−CPYAYH−
CLPYAY−−
CKPYAY−−
CCPYAY−−
DRPYAY−−
DFPYAY−−
DIPYAY−−
DNPYAY−−
DHPYAY−−
−DPYAYF−
DKPYAY−−
−DPYAYT−
DLPYAY−−
DMPYAY−−
DAPYAY−−
DVPYAY−−
DTPYAY−−
DSPYAY−−
−DPYAYR−
−DPYAYA−
−DPYAYL−
−DPYAYI−
DDPYAY−−
DCPYAY−−
−DPYAYV−
ETPYAY−−
ERPYAY−−
−EPYAYL−
ESPYAY−−
−EPYAYT−
ELPYAY−−
EGPYAY−−
EVPYAY−−
EHPYAY−−
−EPYAYK−
ENPYAY−−
EMPYAY−−
EAPYAY−−
EWPYAY−−
−EPYAYM−
−EPYAYF−
−EPYAYE−
EDPYAY−−
−EPYAYQ−

FPPYAY--
FRPYAY--
-FPYAYA-
FSPYAY--
FNPYAY--
-FPYAYH-
FMPYAY--
FQPYAY--
-FPYAYT-
-FPYAYR-
FYPYAY--
FWPYAY--
-FPYAYW-
-FPYAYQ-
FGPYAY--
FEPYAY--
-FPYAYV-
-FPYAYM-
FIPYAY--
FVPYAY--
FKPYAY--
-FPYAYI-
-FPYAYG-
-FPYAYE-
FLPYAY--
GVPYAY--
-GPYAYS-
-GPYAYV-
-GPYAYI-
GLPYAY--
GKPYAY--
GYPYAY--
-GPYAYG-
GSPYAY--
GHPYAY--
-GPYAYA-
-GPYAYR-
GDPYAY--
GWPYAY--
GRPYAY--
GMPYAY--
GFPYAY--
GNPYAY--
GAPYAY--
-GPYAYQ-
-GPYAYH-
GGPYAY--
GQPYAY--
-GPYAYM-
-GPYAYE-

GPPYAY--
-GPYAYW-
-GPYAYN-
-GPYAYK-
GIPYAY--
HRPYAY--
HGPYAY--
-HPYAYL-
HPPYAY--
HNPYAY--
HHPYAY--
-HPYAYQ-
HSPYAY--
HWPYAY--
-HPYAYV-
HLPYAY--
-HPYAYI-
HDPYAY--
-HPYAYR-
HTPYAY--
-HPYAYS-
-HPYAYK-
-HPYAYA-
HMPYAY--
HQPYAY--
-HPYAYT-
-HPYAYF-
HFPYAY--
-HPYAYE-
HYPYAY--
-HPYAYN-
-HPYAYH-
HEPYAY--
HAPYAY--
IRPYAY--
-IPYAYS-
ISPYAY--
IKPYAY--
IAPYAY--
-IPYAYR-
INPYAY--
ITPYAY--
-IPYAYK-
IHPYAY--
IQPYAY--
IMPYAY--
IFPYAY--
-IPYAYA-
IYPYAY--
-IPYAYL-

IEPYAY--
IWPYAY--
-IPYAYM-
IVPYAY--
-IPYAYI-
ILPYAY--
IGPYAY--
-IPYAYY-
-IPYAYT-
-IPYAYN-
-IPYAYH-
-IPYAYG-
IDPYAY--
KSPYAY--
KGPYAY--
KLPYAY--
-KPYAYI-
KIPYAY--
KRPYAY--
-KPYAYF-
KTPYAY--
-KPYAYV-
-KPYAYY-
-KPYAYG-
KMPYAY--
KAPYAY--
-KPYAYT-
-KPYAYL-
KDPYAY--
KFPYAY--
-KPYAYN-
-KPYAYH-
-KPYAYS-
-KPYAYQ-
-KPYAYA-
-KPYAYE-
KVPYAY--
-KPYAYM-
KHPYAY--
KYPYAY--
KWPYAY--
-KPYAYD-
KPPYAY--
LSPYAY--
LWPYAY--
LGPYAY--
LYPYAY--
-LPYAYQ-
-LPYAYR-
LLPYAY--

LAPYAY--
LVPYAY--
LNPYAY--
LKPYAY--
-LPYAYY-
-LPYAYV-
LHPYAY--
LEPYAY--
LRPYAY--
-LPYAYK-
-LPYAYA-
LDPYAY--
-LPYAYW-
-LPYAYT-
-LPYAYS-
LFPYAY--
-LPYAYI-
LTPYAY--
LQPYAY--
LPPYAY--
LIPYAY--
-LPYAYN-
-LPYAYM-
-LPYAYE-
-MPYAYL-
MTPYAY--
MGPYAY--
MQPYAY--
MHPYAY--
MLPYAY--
MRPYAY--
-MPYAYS-
MKPYAY--
-MPYAYR-
-MPYAYF-
-MPYAYE-
MPPYAY--
MYPYAY--
-MPYAYT-
-MPYAYI-
MAPYAY--
-MPYAYA-
MMPYAY--
-MPYAYW-
MNPYAY--
MIPYAY--
MVPYAY--
-MPYAYY-
-MPYAYM-
-MPYAYH-

```
-NPYAYL-
-NPYAYR-
NSPYAY--
NVPYAY--
NGPYAY--
NAPYAY--
NPPYAY--
-NPYAYS-
NHPYAY--
-NPYAYI-
NLPYAY--
-NPYAYY-
-NPYAYW-
-NPYAYM-
NKPYAY--
-NPYAYH-
NTPYAY--
NQPYAY--
-NPYAYV-
NMPYAY--
NNPYAY--
NWPYAY--
NIPYAY--
NRPYAY--
-NPYAYQ-
-NPYAYN-
NFPYAY--
-NPYAYK-
-NPYAYF-
NDPYAY--
--PYAYRG
--PYAYRT
--PYAYAI
--PYAYRE
--PYAYMS
--PYAYRI
--PYAYYA
--PYAYSL
--PYAYRS
--PYAYSR
--PYAYFS
--PYAYFG
--PYAYTT
--PYAYTL
--PYAYSA
--PYAYLR
--PYAYKS
--PYAYKR
--PYAYAT
--PYAYSV
```

--PYAYSM
--PYAYSS
--PYAYRR
--PYAYRM
--PYAYNT
--PYAYMQ
--PYAYKG
--PYAYTK
--PYAYHL
--PYAYYS
--PYAYVR
--PYAYST
--PYAYRL
--PYAYPM
--PYAYMG
--PYAYYQ
--PYAYTS
--PYAYNF
--PYAYKV
--PYAYYT
--PYAYRA
--PYAYLQ
--PYAYFA
--PYAYVT
--PYAYSQ
--PYAYMR
--PYAYHM
--PYAYAS
--PYAYVD
--PYAYTI
--PYAYRN
--PYAYKN
--PYAYWG
--PYAYKA
--PYAYIV
--PYAYHT
--PYAYFR
--PYAYEM
--PYAYYY
--PYAYVQ
--PYAYVK
--PYAYKQ
PNPYAY--
--PYAYWL
--PYAYSI
--PYAYNL
--PYAYLH
--PYAYKL
--PYAYAG
--PYAYVH

--PYAYVA
--PYAYSD
--PYAYML
--PYAYKD
--PYAYAN
--PYAYWI
--PYAYTY
--PYAYTR
--PYAYQS
--PYAYPY
--PYAYNM
--PYAYMW
--PYAYLD
--PYAYHG
--PYAYYH
--PYAYYG
--PYAYMK
--PYAYGM
--PYAYAW
--PYAYAK
--PYAYQH
--PYAYNY
--PYAYNR
--PYAYMV
--PYAYME
--PYAYIF
--PYAYHY
--PYAYFQ
--PYAYAD
--PYAYYD
--PYAYWR
--PYAYSN
--PYAYPL
--PYAYNI
--PYAYMM
--PYAYLK
--PYAYIR
--PYAYIN
--PYAYII
--PYAYEN
--PYAYAM
--PYAYAH
PWPYAY--
--PYAYTV
--PYAYTN
--PYAYTM
--PYAYSY
--PYAYSG
--PYAYRQ
--PYAYQL

--PYAYLM
--PYAYKF
--PYAYIA
--PYAYHH
--PYAYHA
--PYAYGV
--PYAYFH
--PYAYEL
--PYAYYW
--PYAYWM
--PYAYWK
--PYAYVN
--PYAYTG
--PYAYRH
--PYAYQM
--PYAYQG
--PYAYNS
--PYAYMD
--PYAYMA
--PYAYLG
--PYAYHN
--PYAYHK
--PYAYEF
PTPYAY--
PYPYAY--
--PYAYVI
--PYAYVG
--PYAYVF
--PYAYTA
--PYAYSK
--PYAYRY
--PYAYQT
--PYAYQA
--PYAYNQ
--PYAYNN
--PYAYMT
--PYAYLT
--PYAYKH
--PYAYKE
--PYAYIY
--PYAYHS
--PYAYGT
--PYAYFE
--PYAYFD
--PYAYEG
--PYAYDS
--PYAYAR
--PYAYWT
--PYAYWS
--PYAYWD

--PYAYTH
--PYAYSH
--PYAYSF
--PYAYSE
--PYAYRF
--PYAYRD
--PYAYQD
--PYAYNH
--PYAYNA
--PYAYMI
--PYAYMF
--PYAYLE
--PYAYKK
--PYAYIQ
--PYAYGN
--PYAYGH
--PYAYGF
--PYAYEY
--PYAYET
--PYAYEA
--PYAYAV
PIPYAY--
PGPYAY--
--PYAYWW
--PYAYVV
--PYAYRW
--PYAYQQ
--PYAYQN
--PYAYQI
--PYAYNV
--PYAYLW
--PYAYIT
--PYAYHV
--PYAYGY
--PYAYGR
--PYAYFT
--PYAYEI
-PPYAYL-
PFPYAY--
--PYAYYN
--PYAYWH
--PYAYTD
--PYAYRV
--PYAYPQ
--PYAYPG
--PYAYPF
--PYAYND
--PYAYMC
--PYAYHW
--PYAYHE

--PYAYGS
--PYAYGQ
--PYAYGA
--PYAYFW
--PYAYFK
--PYAYEV
--PYAYEE
--PYAYED
PQPYAY--
-QPYAYI-
-QPYAYR-
QTPYAY--
-QPYAYT-
QSPYAY--
QAPYAY--
QRPYAY--
-QPYAYQ-
-QPYAYS-
-QPYAYN-
-QPYAYM-
QQPYAY--
-QPYAYK-
-QPYAYH-
-QPYAYE-
QFPYAY--
QEPYAY--
-QPYAYF-
QPPYAY--
QVPYAY--
QLPYAY--
QYPYAY--
-QPYAYW-
QNPYAY--
QHPYAY--
QGPYAY--
-QPYAYD-
QIPYAY--
RKPYAY--
RNPYAY--
RRPYAY--
RLPYAY--
-RPYAYA-
RFPYAY--
RDPYAY--
RSPYAY--
RYPYAY--
-RPYAYI-
-RPYAYT-
-RPYAYS-
RWPYAY--

-RPYAYM-
RQPYAY--
-RPYAYL-
REPYAY--
RAPYAY--
RPPYAY--
RIPYAY--
RHPYAY--
-RPYAYN-
-RPYAYK-
RMPYAY--
-RPYAYE-
-RPYAYQ-
-RPYAYG-
-RPYAYD-
SRPYAY--
SSPYAY--
-SPYAYR-
-SPYAYM-
SMPYAY--
SHPYAY--
-SPYAYW-
-SPYAYI-
SGPYAY--
-SPYAYL-
SWPYAY--
-SPYAYY-
-SPYAYV-
-SPYAYF-
SFPYAY--
SAPYAY--
-SPYAYS-
-SPYAYQ-
SKPYAY--
-SPYAYN-
-SPYAYA-
-SPYAYH-
SNPYAY--
-SPYAYG-
SPPYAY--
-SPYAYT-
SQPYAY--
SLPYAY--
-SPYAYE-
SIPYAY--
-SPYAYK-
TGPYAY--
-TPYAYR-
TPPYAY--
-TPYAYH-

-TPYAYQ-
TYPYAY--
TVPYAY--
TDPYAY--
-TPYAYK-
TEPYAY--
TWPYAY--
TKPYAY--
TSPYAY--
TRPYAY--
-TPYAYL-
-TPYAYI-
-TPYAYA-
TIPYAY--
TLPYAY--
TAPYAY--
-TPYAYM-
-TPYAYG-
-TPYAYS-
TNPYAY--
TFPYAY--
TTPYAY--
-TPYAYN-
-TPYAYE-
THPYAY--
TCPYAY--
VSPYAY--
VVPYAY--
VRPYAY--
-VPYAYT-
-VPYAYI-
VFPYAY--
VYPYAY--
VNPYAY--
-VPYAYR-
VTPYAY--
VIPYAY--
-VPYAYM-
-VPYAYL-
-VPYAYV-
-VPYAYQ-
VHPYAY--
-VPYAYY-
-VPYAYK-
VPPYAY--
VAPYAY--
VWPYAY--
VQPYAY--
-VPYAYS-
-VPYAYH-

-VPYAYF-
VGPYAY--
-VPYAYW-
-VPYAYN-
-VPYAYA-
VMPYAY--
VDPYAY--
-VPYAYE-
-WPYAYS-
-WPYAYL-
WNPYAY--
WGPYAY--
-WPYAYR-
WQPYAY--
-WPYAYT-
WKPYAY--
WFPYAY--
WTPYAY--
-WPYAYH-
WEPYAY--
WRPYAY--
-WPYAYI-
-WPYAYY-
-WPYAYQ-
-WPYAYG-
WHPYAY--
YRPYAY--
YKPYAY--
YFPYAY--
-YPYAYM-
YLPYAY--
-YPYAYL-
YSPYAY--
-YPYAYS-
-YPYAYR-
YDPYAY--
-YPYAYV-
-YPYAYQ-
YGPYAY--
YTPYAY--
-YPYAYY-
YYPYAY--
-YPYAYT-
YWPYAY--
-YPYAYK-
YMPYAY--
YIPYAY--
YHPYAY--
YEPYAY--
-YPYAYI-

PFPL 48 852 49.54

```
-EPFPLG-
--PFPLGR
--PFPLGK
-SPFPLG-
-YPFPLN-
-APFPLR-
ARPFPL--
ATPFPL--
-APFPLQ-
-APFPLN-
AHPFPL--
-APFPLV-
-APFPLT-
-APFPLL-
AIPFPL--
AVPFPL--
-APFPLW-
ANPFPL--
ALPFPL--
-APFPLS-
AQPFPL--
AFPFPL--
AGPFPL--
AAPFPL--
AYPFPL--
AWPFPL--
-APFPLK-
AKPFPL--
-APFPLY-
-APFPLA-
-APFPLI-
ASPFPL--
-APFPLH-
AEPFPL--
APPFPL--
ADPFPL--
CKPFPL--
-CPFPLL-
CVPFPL--
-CPFPLY-
CIPFPL--
CTPFPL--
CSPFPL--
-CPFPLK-
CYPFPL--
CRPFPL--
-CPFPLS-
-CPFPLR-
-CPFPLI-
```

-CPFPLA-
CLPFPL--
CEPFPL--
DAPFPL--
DRPFPL--
-DPFPLL-
-DPFPLM-
-DPFPLW-
DSPFPL--
-DPFPLR-
DGPFPL--
DMPFPL--
DWPFPL--
DKPFPL--
DIPFPL--
DQPFPL--
DFPFPL--
DDPFPL--
DYPFPL--
DVPFPL--
-DPFPLT-
-DPFPLQ-
-DPFPLK-
-DPFPLA-
DNPFPL--
DTPFPL--
-DPFPLY-
-DPFPLV-
-DPFPLN-
ERPFPL--
EKPFPL--
-EPFPLY-
-EPFPLF-
-EPFPLK-
-EPFPLS-
EGPFPL--
EAPFPL--
-EPFPLR-
ENPFPL--
-EPFPLI-
EMPFPL--
-EPFPLQ-
ELPFPL--
-EPFPLW-
EHPFPL--
EPPFPL--
-EPFPLT-
-EPFPLN-
-EPFPLA-
-EPFPLV-

-EPFPLH-
EYPFPL--
EWPFPL--
ETPFPL--
ESPFPL--
EFPFPL--
FGPFPL--
-FPFPLQ-
FMPFPL--
FEPFPL--
FTPFPL--
FAPFPL--
-FPFPLS-
FHPFPL--
-FPFPLW-
FLPFPL--
FRPFPL--
FNPFPL--
FDPFPL--
-FPFPLT-
-FPFPLR-
FIPFPL--
FYPFPL--
-FPFPLK-
-FPFPLG-
FVPFPL--
-FPFPLY-
-FPFPLH-
-FPFPLA-
-FPFPLV-
FKPFPL--
-FPFPLL-
FQPFPL--
FPPFPL--
-FPFPLI-
GYPFPL--
GRPFPL--
-GPFPLL-
-GPFPLR-
-GPFPLQ-
GFPFPL--
GSPFPL--
GLPFPL--
GAPFPL--
GGPFPL--
-GPFPLF-
GIPFPL--
-GPFPLM-
GTPFPL--
-GPFPLT-

GQPFPL--
GHPFPL--
GDPFPL--
GNPFPL--
-GPFPLV-
-GPFPLH-
GEPFPL--
-GPFPLI-
GMPFPL--
-GPFPLW-
-GPFPLK-
GKPFPL--
-GPFPLY-
-GPFPLG-
-GPFPLA-
GWPFPL--
GCPFPL--
-GPFPLN-
-GPFPLE-
HRPFPL--
-HPFPLR-
HMPFPL--
-HPFPLV-
HHPFPL--
-HPFPLL-
-HPFPLQ-
-HPFPLH-
HQPFPL--
-HPFPLT-
HNPFPL--
HGPFPL--
HAPFPL--
HSPFPL--
HYPFPL--
HLPFPL--
HKPFPL--
-HPFPLF-
HFPFPL--
-HPFPLI-
HCPFPL--
-HPFPLN-
HEPFPL--
-HPFPLE-
-HPFPLY-
-HPFPLK-
HWPFPL--
HVPFPL--
HPPFPL--
-HPFPLW-
-HPFPLS-

−HPFPLG−
HIPFPL−−
−IPFPLS−
−IPFPLR−
IQPFPL−−
IRPFPL−−
−IPFPLM−
IAPFPL−−
ISPFPL−−
−IPFPLL−
−IPFPLF−
−IPFPLH−
IVPFPL−−
ITPFPL−−
−IPFPLG−
−IPFPLA−
IFPFPL−−
IYPFPL−−
−IPFPLY−
−IPFPLI−
ILPFPL−−
IEPFPL−−
−IPFPLW−
−IPFPLK−
IWPFPL−−
−IPFPLV−
−IPFPLT−
−IPFPLQ−
INPFPL−−
IGPFPL−−
IDPFPL−−
−IPFPLN−
−IPFPLE−
IMPFPL−−
KSPFPL−−
−KPFPLL−
KTPFPL−−
−KPFPLR−
−KPFPLK−
KVPFPL−−
KRPFPL−−
−KPFPLS−
KPPFPL−−
KQPFPL−−
KLPFPL−−
KHPFPL−−
KYPFPL−−
−KPFPLT−
KFPFPL−−
−KPFPLI−

KMPFPL--
-KPFPLH-
KGPFPL--
-KPFPLY-
KKPFPL--
-KPFPLM-
KAPFPL--
-KPFPLW-
-KPFPLN-
-KPFPLG-
-KPFPLV-
KNPFPL--
KWPFPL--
-KPFPLQ-
-KPFPLE-
-KPFPLA-
-KPFPLF-
-LPFPLR-
LNPFPL--
LSPFPL--
LAPFPL--
LPPFPL--
LTPFPL--
LRPFPL--
LEPFPL--
-LPFPLK-
LMPFPL--
LYPFPL--
-LPFPLL-
LGPFPL--
-LPFPLV-
LVPFPL--
-LPFPLT-
-LPFPLM-
LDPFPL--
-LPFPLW-
-LPFPLA-
LFPFPL--
LQPFPL--
LIPFPL--
LWPFPL--
-LPFPLN-
-LPFPLS-
-LPFPLI-
LHPFPL--
-LPFPLQ-
-LPFPLF-
-MPFPLT-
MSPFPL--
MTPFPL--

-MPFPLR-
-MPFPLL-
MGPFPL--
MRPFPL--
-MPFPLI-
-MPFPLW-
-MPFPLK-
-MPFPLF-
-MPFPLS-
-MPFPLH-
MYPFPL--
MAPFPL--
MVPFPL--
MPPFPL--
MMPFPL--
MKPFPL--
-MPFPLG-
MWPFPL--
-MPFPLA-
MHPFPL--
MCPFPL--
-MPFPLV-
-MPFPLQ-
-MPFPLC-
MNPFPL--
MLPFPL--
MDPFPL--
NIPFPL--
-NPFPLR-
NRPFPL--
NTPFPL--
-NPFPLY-
-NPFPLS-
NVPFPL--
NGPFPL--
-NPFPLQ-
NNPFPL--
NMPFPL--
NLPFPL--
-NPFPLF-
NFPFPL--
NWPFPL--
NAPFPL--
-NPFPLV-
-NPFPLW-
-NPFPLL-
-NPFPLK-
NPPFPL--
-NPFPLI-
NKPFPL--

```
NHPFPL--
NEPFPL--
NDPFPL--
NSPFPL--
NQPFPL--
-NPFPLM-
-NPFPLA-
-NPFPLN-
--PFPLRS
--PFPLVS
--PFPLKA
--PFPLRI
--PFPLLN
--PFPLTA
--PFPLSS
--PFPLVA
PVPFPL--
--PFPLKL
--PFPLQL
--PFPLLS
--PFPLHH
PFPFPL--
--PFPLRT
--PFPLRK
--PFPLTQ
--PFPLTS
--PFPLTR
--PFPLGW
--PFPLFT
--PFPLMK
--PFPLAA
PSPFPL--
--PFPLTG
--PFPLSR
--PFPLLL
--PFPLGS
--PFPLGQ
--PFPLTT
--PFPLYS
--PFPLRA
--PFPLHS
--PFPLHM
--PFPLYW
--PFPLVQ
--PFPLRR
--PFPLRH
--PFPLFS
--PFPLWK
--PFPLVF
--PFPLTM
```

```
--PFPLSN
--PFPLFG
PAPFPL--
PTPFPL--
--PFPLSQ
--PFPLPM
--PFPLIN
--PFPLHL
--PFPLEL
--PFPLDL
PKPFPL--
--PFPLSH
--PFPLMN
--PFPLKT
--PFPLIG
--PFPLGH
PQPFPL--
PLPFPL--
--PFPLWT
--PFPLWG
--PFPLRV
--PFPLQY
--PFPLNV
--PFPLLG
--PFPLFK
--PFPLYR
--PFPLYG
--PFPLSV
--PFPLSK
--PFPLLD
--PFPLKS
--PFPLHY
--PFPLVW
--PFPLVT
--PFPLVN
--PFPLSG
--PFPLNQ
--PFPLLK
--PFPLLA
--PFPLFQ
--PFPLFA
--PFPLEA
--PFPLQH
--PFPLPS
--PFPLNL
--PFPLMG
--PFPLKW
--PFPLKR
--PFPLGF
--PFPLDM
```

--PFPLYV
--PFPLYL
--PFPLVI
--PFPLRW
--PFPLRG
--PFPLPY
--PFPLIS
--PFPLGT
--PFPLAM
--PFPLWV
--PFPLRM
--PFPLNH
--PFPLNF
--PFPLMS
--PFPLMI
--PFPLIT
--PFPLHF
--PFPLAV
PRPFPL--
--PFPLWA
--PFPLVK
--PFPLTW
--PFPLRQ
--PFPLQN
--PFPLLQ
--PFPLLH
--PFPLIM
--PFPLHW
--PFPLEQ
--PFPLAS
PWPFPL--
--PFPLYT
--PFPLWL
--PFPLVG
--PFPLTK
--PFPLPW
--PFPLNS
--PFPLNR
--PFPLMY
--PFPLIK
--PFPLHR
--PFPLGG
--PFPLFF
--PFPLES
PHPFPL--
--PFPLWR
--PFPLWN
--PFPLVH
--PFPLVD
--PFPLSY

--PFPLSA
--PFPLPF
--PFPLNW
--PFPLMV
--PFPLLW
--PFPLKQ
--PFPLKH
--PFPLHQ
--PFPLVV
--PFPLQV
--PFPLQR
--PFPLQM
--PFPLQA
--PFPLNG
--PFPLKN
--PFPLKM
--PFPLII
--PFPLHV
--PFPLHA
--PFPLGN
--PFPLEG
--PFPLAT
PYPFPL--
--PFPLYK
--PFPLTN
--PFPLSW
--PFPLSF
--PFPLQQ
--PFPLQI
--PFPLME
--PFPLKI
--PFPLKE
--PFPLKD
--PFPLIH
--PFPLHN
--PFPLGA
--PFPLDI
--PFPLAN
PDPFPL--
--PFPLWE
--PFPLRY
--PFPLRE
--PFPLQG
--PFPLPV
--PFPLMH
--PFPLKY
--PFPLID
--PFPLFM
--PFPLFD
--PFPLEW

--PFPLEV
--PFPLEN
--PFPLEI
--PFPLAQ
--PFPLAK
--PFPLAG
PMPFPL--
--PFPLRF
--PFPLPQ
--PFPLNT
--PFPLND
--PFPLMT
--PFPLMR
--PFPLMC
--PFPLIQ
--PFPLHK
--PFPLHG
--PFPLDW
--PFPLDS
--PFPLYM
--PFPLPH
--PFPLMF
--PFPLMD
--PFPLKV
--PFPLIE
--PFPLHE
--PFPLFE
--PFPLEH
--PFPLEF
--PFPLDF
--PFPLCQ
PEPFPL--
PCPFPL--
PNPFPL--
--PFPLYH
--PFPLWQ
--PFPLWH
--PFPLWD
--PFPLVE
--PFPLTH
--PFPLRP
--PFPLRC
--PFPLPT
--PFPLMW
--PFPLIV
--PFPLHT
--PFPLHI
--PFPLDV
--PFPLAR
--PFPLAH

```
--PFPLAF
-PPFPLL-
--PFPLYY
--PFPLYP
--PFPLYN
--PFPLWM
--PFPLWI
--PFPLVY
--PFPLVM
--PFPLTY
--PFPLQS
--PFPLQK
--PFPLNE
--PFPLNA
--PFPLMM
--PFPLLE
--PFPLKP
--PFPLKK
--PFPLKG
--PFPLIY
--PFPLIW
--PFPLHD
--PFPLFH
--PFPLER
--PFPLEM
--PFPLDY
--PFPLDN
--PFPLDH
--PFPLCW
-QPFPLF-
-QPFPLR-
-QPFPLT-
-QPFPLK-
QWPFPL--
QRPFPL--
QQPFPL--
-QPFPLS-
QSPFPL--
-QPFPLV-
QVPFPL--
QTPFPL--
-QPFPLL-
QMPFPL--
QKPFPL--
QLPFPL--
QFPFPL--
QPPFPL--
QNPFPL--
-QPFPLH-
-QPFPLG-
```

QAPFPL--
-QPFPLN-
QHPFPL--
QGPFPL--
-QPFPLY-
-QPFPLW-
QIPFPL--
QDPFPL--
-QPFPLA-
QYPFPL--
-QPFPLM-
-RPFPLM-
RVPFPL--
RSPFPL--
RAPFPL--
RWPFPL--
-RPFPLL-
RLPFPL--
RNPFPL--
RMPFPL--
RQPFPL--
-RPFPLS-
-RPFPLH-
-RPFPLR-
-RPFPLA-
RFPFPL--
RTPFPL--
-RPFPLK-
RDPFPL--
REPFPL--
RPPFPL--
-RPFPLF-
RIPFPL--
-RPFPLI-
RGPFPL--
-RPFPLW-
-RPFPLT-
-RPFPLN-
RHPFPL--
-RPFPLG-
RYPFPL--
RCPFPL--
-RPFPLE-
-RPFPLC-
SSPFPL--
SLPFPL--
SGPFPL--
SRPFPL--
SVPFPL--
SHPFPL--

-SPFPLY-
-SPFPLL-
SIPFPL--
STPFPL--
-SPFPLN-
-SPFPLA-
-SPFPLV-
SFPFPL--
SMPFPL--
SKPFPL--
SNPFPL--
-SPFPLT-
-SPFPLS-
-SPFPLI-
SWPFPL--
-SPFPLW-
-SPFPLQ-
-SPFPLR-
SAPFPL--
-SPFPLH-
SPPFPL--
SQPFPL--
-SPFPLM-
SEPFPL--
-SPFPLK-
SDPFPL--
-SPFPLE-
-SPFPLC-
-TPFPLK-
-TPFPLY-
TWPFPL--
-TPFPLH-
THPFPL--
TAPFPL--
TNPFPL--
TLPFPL--
TYPFPL--
-TPFPLW-
-TPFPLN-
-TPFPLQ-
TIPFPL--
TKPFPL--
TPPFPL--
TEPFPL--
TVPFPL--
-TPFPLA-
-TPFPLV-
TTPFPL--
-TPFPLM-
TGPFPL--

−TPFPLF−
TMPFPL−−
TQPFPL−−
TDPFPL−−
TCPFPL−−
VVPFPL−−
−VPFPLR−
VAPFPL−−
−VPFPLV−
VNPFPL−−
VTPFPL−−
−VPFPLY−
VRPFPL−−
−VPFPLL−
VSPFPL−−
VPPFPL−−
−VPFPLK−
−VPFPLT−
VLPFPL−−
VIPFPL−−
VYPFPL−−
−VPFPLS−
−VPFPLA−
−VPFPLM−
VMPFPL−−
VHPFPL−−
VDPFPL−−
−VPFPLQ−
−VPFPLH−
−VPFPLG−
−VPFPLW−
VEPFPL−−
VQPFPL−−
−VPFPLN−
−VPFPLE−
VCPFPL−−
VWPFPL−−
VKPFPL−−
VGPFPL−−
VFPFPL−−
−WPFPLR−
WIPFPL−−
WGPFPL−−
WTPFPL−−
−WPFPLL−
WNPFPL−−
WSPFPL−−
WAPFPL−−
WPPFPL−−
WDPFPL−−

-WPFPLY-
-WPFPLF-
WYPFPL--
-WPFPLQ-
-WPFPLK-
WLPFPL--
WEPFPL--
WVPFPL--
-WPFPLA-
-WPFPLW-
-WPFPLM-
-WPFPLI-
-WPFPLH-
-WPFPLT-
-WPFPLS-
WHPFPL--
WWPFPL--
WRPFPL--
WQPFPL--
-WPFPLV-
WMPFPL--
WFPFPL--
WCPFPL--
-YPFPLR-
YRPFPL--
YPPFPL--
YLPFPL--
YSPFPL--
YAPFPL--
-YPFPLM-
YVPFPL--
-YPFPLL-
YGPFPL--
-YPFPLA-
YYPFPL--
YQPFPL--
-YPFPLT-
YNPFPL--
-YPFPLH-
YKPFPL--
-YPFPLV-
-YPFPLI-
YMPFPL--
YHPFPL--
-YPFPLS-
YDPFPL--
-YPFPLQ-
-YPFPLF-
YEPFPL--
-YPFPLG-

YIPFPL--
-YPFPLE-
YFPFPL--

PYGI	97	808	49.52

--PYGIHP
-YPYGIL-
ASPYGI--
-APYGIN-
AKPYGI--
-APYGIL-
-APYGIH-
-APYGIS-
-APYGII-
-APYGIT-
AGPYGI--
AVPYGI--
-APYGIV-
ARPYGI--
-APYGIA-
APPYGI--
AAPYGI--
AWPYGI--
-APYGIQ-
AHPYGI--
ALPYGI--
AFPYGI--
ATPYGI--
AIPYGI--
AQPYGI--
-APYGIR-
-APYGIK-
-APYGIG-
ANPYGI--
ADPYGI--
AYPYGI--
-APYGIW-
-APYGIM-
-APYGIE-
AEPYGI--
-CPYGIK-
CKPYGI--
CQPYGI--
-CPYGIT-
CRPYGI--
-CPYGIH-
CGPYGI--
CAPYGI--
-CPYGIR-
CTPYGI--
-CPYGIQ-

-CPYGIN-
-CPYGII-
DRPYGI--
-DPYGIR-
DWPYGI--
DAPYGI--
-DPYGIF-
DQPYGI--
DSPYGI--
DLPYGI--
DVPYGI--
-DPYGIS-
DGPYGI--
-DPYGIT-
-DPYGII-
-DPYGIH-
-DPYGIA-
DEPYGI--
-DPYGIW-
-DPYGIQ-
-DPYGIP-
-DPYGIN-
-DPYGIK-
DHPYGI--
DDPYGI--
-EPYGIL-
ESPYGI--
-EPYGIS-
-EPYGIM-
ERPYGI--
ELPYGI--
EMPYGI--
EAPYGI--
-EPYGIK-
-EPYGIQ-
-EPYGIN-
EWPYGI--
-EPYGIV-
EVPYGI--
EQPYGI--
-EPYGIE-
EKPYGI--
EHPYGI--
ENPYGI--
EDPYGI--
ECPYGI--
-EPYGII-
-EPYGIC-
-EPYGIA-
EFPYGI--

EEPYGI--
FSPYGI--
FRPYGI--
-FPYGIR-
-FPYGIH-
-FPYGIL-
-FPYGIQ-
FHPYGI--
-FPYGIT-
FGPYGI--
FFPYGI--
FVPYGI--
-FPYGIV-
-FPYGIG-
FYPYGI--
-FPYGII-
FMPYGI--
FPPYGI--
FTPYGI--
-FPYGIS-
FKPYGI--
FDPYGI--
FAPYGI--
-FPYGIY-
-FPYGIK-
FCPYGI--
FWPYGI--
-FPYGIA-
FNPYGI--
-FPYGIW-
GIPYGI--
-GPYGIL-
GRPYGI--
GYPYGI--
GKPYGI--
GSPYGI--
GDPYGI--
-GPYGIV-
-GPYGIH-
GEPYGI--
GFPYGI--
-GPYGIQ-
GQPYGI--
GVPYGI--
-GPYGIS-
-GPYGII-
-GPYGIW-
-GPYGIG-
GMPYGI--
GTPYGI--

GNPYGI--
-GPYGIT-
-GPYGIN-
-GPYGIM-
-GPYGIA-
GAPYGI--
GPPYGI--
-GPYGIK-
GCPYGI--
-GPYGIE-
-GPYGID-
HRPYGI--
-HPYGIL-
HHPYGI--
-HPYGII-
-HPYGIV-
HSPYGI--
HPPYGI--
-HPYGIT-
-HPYGIQ-
HVPYGI--
HQPYGI--
-HPYGIK-
-HPYGIS-
-HPYGIH-
-HPYGIY-
-HPYGIE-
HLPYGI--
HEPYGI--
HAPYGI--
HIPYGI--
HFPYGI--
HMPYGI--
HWPYGI--
HCPYGI--
HYPYGI--
-HPYGIM-
HDPYGI--
INPYGI--
-IPYGIR-
IQPYGI--
IKPYGI--
-IPYGII-
IRPYGI--
-IPYGIK-
-IPYGIS-
-IPYGIL-
ITPYGI--
ILPYGI--
-IPYGIA-

IVPYGI--
-IPYGIE-
IIPYGI--
IGPYGI--
-IPYGIV-
-IPYGIN-
-IPYGIG-
-IPYGIW-
-IPYGIQ-
IPPYGI--
IWPYGI--
IMPYGI--
IHPYGI--
IEPYGI--
-IPYGIH-
IDPYGI--
IYPYGI--
-IPYGIT-
ICPYGI--
KSPYGI--
KLPYGI--
-KPYGII-
-KPYGIR-
KVPYGI--
KTPYGI--
KMPYGI--
-KPYGIY-
KPPYGI--
-KPYGIG-
-KPYGIF-
-KPYGIN-
KHPYGI--
KWPYGI--
-KPYGIW-
-KPYGIQ-
-KPYGIE-
KAPYGI--
KQPYGI--
-KPYGIT-
-KPYGIA-
-KPYGIV-
KYPYGI--
KGPYGI--
KNPYGI--
KDPYGI--
KKPYGI--
LRPYGI--
LPPYGI--
LWPYGI--
LTPYGI--

LAPYGI--
-LPYGIR-
LKPYGI--
-LPYGIS-
LGPYGI--
LYPYGI--
LSPYGI--
LLPYGI--
-LPYGIL-
LIPYGI--
LMPYGI--
-LPYGIT-
LFPYGI--
-LPYGIV-
-LPYGIA-
LNPYGI--
LDPYGI--
LCPYGI--
LQPYGI--
-LPYGIE-
LVPYGI--
-LPYGIN-
-LPYGII-
-LPYGIK-
-LPYGIH-
-LPYGIM-
LHPYGI--
-LPYGIG-
MLPYGI--
MNPYGI--
MPPYGI--
MRPYGI--
MGPYGI--
-MPYGIR-
MFPYGI--
MSPYGI--
MVPYGI--
MQPYGI--
-MPYGIM-
MCPYGI--
-MPYGIQ-
-MPYGIL-
-MPYGIT-
-MPYGIY-
-MPYGIA-
MHPYGI--
MYPYGI--
-MPYGIV-
MDPYGI--
MAPYGI--

```
MWPYGI--
-MPYGIK-
-MPYGIE-
MIPYGI--
MEPYGI--
MTPYGI--
-MPYGIG-
NSPYGI--
NRPYGI--
NLPYGI--
NTPYGI--
NKPYGI--
-NPYGIL-
-NPYGII-
NIPYGI--
NHPYGI--
-NPYGIT-
-NPYGIV-
NAPYGI--
-NPYGIS-
NWPYGI--
NQPYGI--
-NPYGIQ-
NNPYGI--
NYPYGI--
-NPYGIM-
NVPYGI--
-NPYGIR-
-NPYGIK-
-NPYGIA-
NFPYGI--
NEPYGI--
NDPYGI--
-NPYGIF-
NGPYGI--
NCPYGI--
-NPYGIH-
NPPYGI--
NMPYGI--
--PYGILS
--PYGIRN
--PYGITG
--PYGILT
--PYGIKS
--PYGILK
--PYGIRT
--PYGILR
--PYGIRW
--PYGISR
--PYGITR
```

--PYGIRM
--PYGIAG
--PYGISV
--PYGIRG
--PYGIVV
--PYGIST
--PYGIRF
--PYGILG
--PYGIGS
--PYGIVS
--PYGIVR
--PYGIRL
--PYGIVK
--PYGISL
--PYGIRI
--PYGIHA
--PYGIRR
--PYGIMS
--PYGIMN
--PYGISA
--PYGIKR
--PYGIIA
--PYGIYK
--PYGINR
--PYGIKY
--PYGITT
--PYGITW
--PYGISY
--PYGIHK
--PYGIFS
--PYGIYR
--PYGITH
--PYGIQA
--PYGILQ
--PYGIKG
--PYGIKA
--PYGIIS
--PYGIHG
--PYGISM
--PYGIGG
--PYGIVY
--PYGITE
--PYGIMY
--PYGIIQ
--PYGIKM
--PYGIKL
--PYGIGA
--PYGIFK
PNPYGI--
--PYGISG

--PYGIRA
--PYGIMQ
--PYGIMK
--PYGIIM
--PYGIGV
--PYGIAH
--PYGISK
--PYGIQF
--PYGIAT
--PYGIRV
--PYGIRH
--PYGIQS
--PYGILH
--PYGILD
--PYGIGW
--PYGIAW
--PYGIYN
--PYGIVQ
--PYGING
--PYGITA
--PYGINM
--PYGIIY
--PYGIHT
--PYGIAY
--PYGITY
--PYGITS
--PYGISF
--PYGINQ
--PYGIIT
--PYGIGT
--PYGIER
PVPYGI--
--PYGIYD
--PYGIVH
--PYGIVD
--PYGITM
--PYGITL
--PYGITI
--PYGILE
--PYGIKV
--PYGIKT
--PYGIKQ
--PYGIIR
--PYGIHS
--PYGIHE
--PYGIAS
--PYGIAF
--PYGIYG
--PYGIWE
--PYGIVF

--PYGIRE
--PYGIRD
--PYGIQW
--PYGIQN
--PYGIQK
--PYGIQD
--PYGIMR
--PYGIMH
--PYGIIN
--PYGIHL
--PYGIAM
--PYGIAL
PYPYGI--
--PYGIYH
--PYGIVA
--PYGITK
--PYGISQ
--PYGISD
--PYGIID
--PYGIFN
--PYGIEN
--PYGIAR
--PYGIVN
--PYGIVM
--PYGISS
--PYGIQG
--PYGINH
--PYGIKK
--PYGIHI
--PYGIDA
--PYGIAV
--PYGIAK
--PYGIWA
--PYGIVT
--PYGITF
--PYGIQV
--PYGINS
--PYGINL
--PYGINA
--PYGIMG
--PYGIIG
--PYGIGR
--PYGIES
--PYGIEA
PTPYGI--
PGPYGI--
--PYGISH
--PYGIQR
--PYGINK
--PYGINI

--PYGILW
--PYGIGM
--PYGIEY
--PYGIAQ
PQPYGI--
--PYGIWH
--PYGIRQ
--PYGIQH
--PYGIMW
--PYGIME
--PYGIKW
--PYGIIW
--PYGIIH
--PYGIHF
--PYGIGY
--PYGIGI
--PYGIAN
--PYGIAE
PKPYGI--
--PYGIWD
--PYGITV
--PYGIRY
--PYGIRP
--PYGIQM
--PYGIQI
--PYGINN
--PYGIMD
--PYGIKN
--PYGIKH
--PYGIIV
--PYGIHY
--PYGIHV
--PYGIHR
--PYGIHM
--PYGIHD
--PYGIHC
--PYGIGL
--PYGIGK
--PYGIGD
--PYGIFR
--PYGIFH
--PYGIEV
--PYGIEL
--PYGICE
--PYGIAI
--PYGIAD
PEPYGI--
PDPYGI--
PAPYGI--
--PYGIYE

--PYGIWQ
--PYGITD
--PYGISI
--PYGIRK
--PYGINW
--PYGINF
--PYGIMT
--PYGILP
--PYGIGF
--PYGIFG
--PYGIFD
--PYGIEW
--PYGIEM
--PYGIEG
--PYGIDS
--PYGICR
--PYGICM
--PYGIWK
--PYGIVE
--PYGIRC
--PYGIQP
--PYGIQE
--PYGINY
--PYGINV
--PYGINE
--PYGIND
--PYGIMM
--PYGIMC
--PYGIKP
--PYGIKI
--PYGIKE
--PYGIHQ
--PYGIHH
--PYGIFE
--PYGIET
--PYGIEQ
--PYGIEI
--PYGIEH
--PYGIDL
--PYGIDG
--PYGICQ
--PYGICL
-PPYGIQ-
PHPYGI--
QRPYGI--
QAPYGI--
-QPYGIT-
QTPYGI--
-QPYGIR-
QHPYGI--

```
-QPYGIL-
QGPYGI--
QVPYGI--
QFPYGI--
-QPYGIA-
QKPYGI--
-QPYGIM-
QMPYGI--
QSPYGI--
-QPYGIH-
QPPYGI--
-QPYGIS-
-QPYGIG-
QLPYGI--
QIPYGI--
QQPYGI--
-QPYGIK-
QWPYGI--
-QPYGIN-
QEPYGI--
QDPYGI--
-QPYGIV-
-QPYGIQ-
-RPYGIL-
-RPYGIR-
RSPYGI--
-RPYGIS-
RLPYGI--
RVPYGI--
RFPYGI--
RAPYGI--
RTPYGI--
-RPYGIA-
RWPYGI--
-RPYGIG-
-RPYGII-
RGPYGI--
RRPYGI--
-RPYGIT-
-RPYGIK-
RYPYGI--
-RPYGIV-
RNPYGI--
-RPYGIM-
REPYGI--
RHPYGI--
-RPYGIN-
RKPYGI--
RIPYGI--
RQPYGI--
```

```
-RPYGIF-
RDPYGI--
-RPYGIE-
-RPYGIH-
RPPYGI--
RMPYGI--
SVPYGI--
SRPYGI--
-SPYGIR-
-SPYGIL-
SLPYGI--
SKPYGI--
SSPYGI--
-SPYGIS-
SMPYGI--
-SPYGIM-
SNPYGI--
-SPYGIG-
-SPYGIH-
SGPYGI--
-SPYGIV-
-SPYGII-
SHPYGI--
SAPYGI--
-SPYGIQ-
-SPYGIN-
SQPYGI--
-SPYGIE-
SFPYGI--
-SPYGIK-
SEPYGI--
SYPYGI--
SIPYGI--
STPYGI--
-SPYGIT-
SPPYGI--
-SPYGIA-
SWPYGI--
-TPYGIR-
-TPYGIQ-
-TPYGIS-
TIPYGI--
TNPYGI--
-TPYGIK-
-TPYGII-
TRPYGI--
TLPYGI--
TMPYGI--
TFPYGI--
TQPYGI--
```

TSPYGI--
-TPYGIT-
TPPYGI--
TGPYGI--
TKPYGI--
-TPYGIH-
TYPYGI--
-TPYGIA-
-TPYGIE-
TTPYGI--
-TPYGIV-
-TPYGIG-
TDPYGI--
THPYGI--
TAPYGI--
TVPYGI--
-TPYGIW-
TEPYGI--
-TPYGIN-
-TPYGIP-
VCPYGI--
VQPYGI--
VAPYGI--
-VPYGII-
-VPYGIV-
-VPYGIR-
VLPYGI--
VGPYGI--
-VPYGIA-
-VPYGIS-
-VPYGIM-
VPPYGI--
VSPYGI--
-VPYGIH-
VMPYGI--
-VPYGIY-
VFPYGI--
-VPYGIQ-
-VPYGIK-
-VPYGIE-
VIPYGI--
VKPYGI--
VRPYGI--
VWPYGI--
VVPYGI--
-VPYGIT-
VYPYGI--
-VPYGIF-
VNPYGI--
-VPYGIN-

```
-VPYGIG-
-VPYGIL-
VEPYGI--
VHPYGI--
VTPYGI--
VDPYGI--
WRPYGI--
WKPYGI--
WIPYGI--
-WPYGIS-
-WPYGIK-
-WPYGIL-
WVPYGI--
WTPYGI--
WWPYGI--
WSPYGI--
WFPYGI--
WEPYGI--
-WPYGIN-
WGPYGI--
-WPYGIT-
-WPYGIE-
-WPYGIR-
-WPYGIA-
WLPYGI--
WDPYGI--
WYPYGI--
-WPYGIM-
WNPYGI--
-WPYGII-
WHPYGI--
WAPYGI--
-WPYGIG-
WPPYGI--
YGPYGI--
YTPYGI--
YLPYGI--
YAPYGI--
-YPYGIK-
YIPYGI--
-YPYGIV-
YSPYGI--
-YPYGIH-
-YPYGIT-
YHPYGI--
YEPYGI--
YPPYGI--
-YPYGIA-
YFPYGI--
YRPYGI--
```

-YPYGIY-
YMPYGI--
YKPYGI--
YVPYGI--
-YPYGIE-
YYPYGI--
YQPYGI--
-YPYGIS-
-YPYGIQ-
-YPYGIM-
-YPYGIG-
-YPYGII-

PRWM	212	742	49.52

-APRWML-
AGPRWM--
-APRWMQ-
ASPRWM--
-APRWMY-
-APRWMT-
AAPRWM--
-APRWMK-
AEPRWM--
AHPRWM--
-APRWMG-
-APRWME-
-APRWMF-
ANPRWM--
AFPRWM--
-APRWMV-
ATPRWM--
-APRWMH-
AWPRWM--
ARPRWM--
-APRWMI-
-APRWMD-
APPRWM--
-APRWMW-
AVPRWM--
AIPRWM--
-APRWMN-
AQPRWM--
AMPRWM--
CPPRWM--
-CPRWMA-
CRPRWM--
-CPRWMQ-
CAPRWM--
CSPRWM--
-CPRWMR-
-CPRWMM-

```
-DPRWMR-
DLPRWM--
DEPRWM--
DVPRWM--
DQPRWM--
-DPRWMM-
-DPRWMA-
-DPRWMS-
-DPRWMF-
-DPRWMH-
DMPRWM--
DGPRWM--
DRPRWM--
-DPRWMW-
DYPRWM--
-DPRWMQ-
-DPRWMI-
-DPRWMG-
DHPRWM--
DNPRWM--
-DPRWMY-
-DPRWMT-
DFPRWM--
DSPRWM--
-DPRWMN-
-DPRWMK-
-DPRWMD-
-DPRWMC-
ESPRWM--
EGPRWM--
-EPRWMV-
-EPRWMN-
-EPRWMW-
-EPRWMF-
-EPRWMK-
EYPRWM--
ETPRWM--
-EPRWMY-
-EPRWMI-
-EPRWMS-
EKPRWM--
EFPRWM--
-EPRWMR-
-EPRWMT-
EMPRWM--
-EPRWMA-
ERPRWM--
-EPRWML-
EIPRWM--
EWPRWM--
```

ELPRWM--
EVPRWM--
-EPRWME-
-EPRWMD-
-EPRWMM-
-EPRWMC-
EEPRWM--
ECPRWM--
-FPRWML-
FTPRWM--
FRPRWM--
-FPRWMI-
FAPRWM--
FDPRWM--
FSPRWM--
FIPRWM--
-FPRWMA-
FKPRWM--
FQPRWM--
-FPRWMN-
-FPRWMG-
-FPRWMM-
FLPRWM--
FYPRWM--
FGPRWM--
-FPRWMW-
-FPRWMQ-
FEPRWM--
FWPRWM--
-FPRWMH-
FNPRWM--
FFPRWM--
-GPRWMT-
-GPRWMM-
-GPRWML-
-GPRWMV-
-GPRWMN-
-GPRWMY-
-GPRWMK-
GTPRWM--
GSPRWM--
GKPRWM--
GNPRWM--
GHPRWM--
-GPRWMS-
GLPRWM--
GVPRWM--
GQPRWM--
GGPRWM--
-GPRWMI-

GDPRWM--
GRPRWM--
-GPRWMH-
GMPRWM--
-GPRWMF-
GIPRWM--
GFPRWM--
GPPRWM--
-GPRWMG-
GEPRWM--
-GPRWME-
-GPRWMD-
-GPRWMW-
GCPRWM--
-HPRWMT-
-HPRWMM-
-HPRWMS-
HFPRWM--
HTPRWM--
-HPRWMV-
-HPRWML-
HYPRWM--
-HPRWMI-
-HPRWMK-
HIPRWM--
-HPRWMQ-
HPPRWM--
HNPRWM--
HGPRWM--
HVPRWM--
HWPRWM--
HRPRWM--
HQPRWM--
-HPRWMN-
-HPRWME-
-HPRWMD-
-HPRWMW-
HDPRWM--
HAPRWM--
ITPRWM--
IGPRWM--
IRPRWM--
IHPRWM--
-IPRWMS-
-IPRWMI-
IKPRWM--
IIPRWM--
-IPRWMV-
-IPRWMG-
-IPRWMQ-

-IPRWML-
-IPRWMF-
IDPRWM--
-IPRWMY-
ISPRWM--
IQPRWM--
-IPRWMD-
-IPRWMA-
-IPRWMN-
-KPRWMR-
-KPRWMK-
-KPRWMS-
KPPRWM--
-KPRWMV-
-KPRWMT-
KRPRWM--
-KPRWMI-
-KPRWMH-
-KPRWMG-
-KPRWML-
KLPRWM--
KHPRWM--
-KPRWMY-
-KPRWMA-
-KPRWMN-
-KPRWMQ-
KDPRWM--
KNPRWM--
KIPRWM--
KTPRWM--
KWPRWM--
KVPRWM--
-KPRWME-
KMPRWM--
KAPRWM--
KQPRWM--
-KPRWMF-
LRPRWM--
LNPRWM--
-LPRWMV-
LSPRWM--
LQPRWM--
LTPRWM--
LGPRWM--
LAPRWM--
-LPRWMM-
-LPRWML-
LIPRWM--
-LPRWMI-
LEPRWM--

LDPRWM--
LWPRWM--
LKPRWM--
-LPRWMH-
-LPRWMT-
LYPRWM--
-LPRWMK-
LVPRWM--
-LPRWMN-
-LPRWMG-
-LPRWMF-
-LPRWMD-
LPPRWM--
-LPRWMW-
LCPRWM--
-MPRWMA-
MSPRWM--
-MPRWMN-
-MPRWMY-
-MPRWMT-
-MPRWMK-
-MPRWMH-
MGPRWM--
MWPRWM--
MPPRWM--
MMPRWM--
MVPRWM--
-MPRWMV-
-MPRWML-
MEPRWM--
-MPRWMR-
MCPRWM--
MLPRWM--
MDPRWM--
-MPRWMW-
-MPRWME-
MAPRWM--
MQPRWM--
-MPRWMS-
-MPRWMQ-
MIPRWM--
MRPRWM--
-MPRWMM-
-MPRWMD-
-NPRWMT-
-NPRWMK-
NTPRWM--
NSPRWM--
NQPRWM--
NVPRWM--

```
-NPRWMF-
NIPRWM--
NGPRWM--
-NPRWMG-
NFPRWM--
-NPRWMH-
-NPRWMY-
-NPRWMS-
-NPRWML-
-NPRWMQ-
NRPRWM--
NLPRWM--
NPPRWM--
NMPRWM--
-NPRWME-
-NPRWMM-
-NPRWMW-
-NPRWMA-
NNPRWM--
NEPRWM--
NAPRWM--
NWPRWM--
NHPRWM--
--PRWMNT
--PRWMRN
--PRWMSS
--PRWMLA
--PRWMMH
--PRWMTL
--PRWMQS
--PRWMAA
--PRWMNS
--PRWMVG
--PRWMTA
--PRWMSV
--PRWMMS
--PRWMLS
--PRWMSG
--PRWMKG
--PRWMTN
--PRWMVS
--PRWMKT
--PRWMSD
--PRWMYG
--PRWMRV
--PRWMFS
--PRWMMG
--PRWMYT
--PRWMST
--PRWMYA
```

--PRWMRT
--PRWMRD
--PRWMGT
--PRWMSY
--PRWMLT
--PRWMKD
--PRWMAD
--PRWMSH
--PRWMLH
--PRWMKY
--PRWMSE
--PRWMQQ
--PRWMHA
--PRWMQY
--PRWMQT
--PRWMMM
--PRWMKN
--PRWMRH
--PRWMNH
--PRWMLE
--PRWMII
--PRWMFD
--PRWMWS
--PRWMVT
--PRWMVD
--PRWMTT
--PRWMIT
--PRWMGM
PSPRWM--
--PRWMTE
--PRWMNM
--PRWMNA
--PRWMIA
--PRWMGQ
--PRWMGI
--PRWMFG
--PRWMVN
--PRWMVH
--PRWMSI
--PRWMNV
--PRWMIS
--PRWMHD
--PRWMYD
--PRWMVW
--PRWMTG
--PRWMSM
--PRWMQK
--PRWMLI
--PRWMIY
--PRWMHS

--PRWMES
--PRWMEN
--PRWMAQ
--PRWMTW
--PRWMQI
--PRWMQG
--PRWMIG
--PRWMDG
--PRWMAN
PGPRWM--
--PRWMVQ
--PRWMTH
--PRWMSN
--PRWMRI
--PRWMMK
--PRWMIQ
--PRWMFM
--PRWMYV
--PRWMTM
--PRWMTI
--PRWMRL
--PRWMQF
--PRWMMN
--PRWMEF
--PRWMDS
--PRWMAV
PIPRWM--
PAPRWM--
--PRWMYN
--PRWMTV
--PRWMMQ
--PRWMLQ
--PRWMKE
--PRWMHN
--PRWMGY
--PRWMEV
--PRWMAG
PNPRWM--
--PRWMYE
--PRWMRW
--PRWMQM
--PRWMEY
--PRWMAY
--PRWMWA
--PRWMVE
--PRWMLY
--PRWMKS
--PRWMHI
--PRWMHH
--PRWMGH

--PRWMFT
--PRWMDT
--PRWMAS
--PRWMYY
--PRWMSW
--PRWMQH
--PRWMML
--PRWMLM
--PRWMHW
--PRWMGW
--PRWMGN
--PRWMFK
--PRWMAH
PEPRWM--
--PRWMYK
--PRWMWT
--PRWMWN
--PRWMSQ
--PRWMSF
--PRWMRY
--PRWMNN
--PRWMKL
--PRWMIN
--PRWMIK
--PRWMIH
PHPRWM--
--PRWMWY
--PRWMWW
--PRWMQD
--PRWMGA
--PRWMEA
--PRWMAL
--PRWMYM
--PRWMTD
--PRWMQW
--PRWMQN
--PRWMKW
--PRWMKQ
--PRWMHQ
--PRWMGS
--PRWMFH
--PRWMFA
--PRWMEM
--PRWMWK
--PRWMHG
--PRWMEE
--PRWMAK
PLPRWM--
PFPRWM--
PVPRWM--

--PRWMYW
--PRWMYQ
--PRWMWR
--PRWMTF
--PRWMNI
--PRWMLD
--PRWMKF
--PRWMGK
--PRWMGD
--PRWMAF
PRPRWM--
PWPRWM--
PTPRWM--
--PRWMWI
--PRWMWH
--PRWMTQ
--PRWMQA
--PRWMNY
--PRWMNQ
--PRWMNG
--PRWMMW
--PRWMMV
--PRWMIF
--PRWMID
--PRWMHE
--PRWMFE
--PRWMEQ
--PRWMAE
PMPRWM--
PYPRWM--
--PRWMYI
--PRWMWV
--PRWMVM
--PRWMVC
--PRWMTY
--PRWMPY
--PRWMPG
--PRWMNF
--PRWMNE
--PRWMIM
--PRWMHL
--PRWMGF
--PRWMET
--PRWMER
--PRWMEH
--PRWMCT
--PRWMCR
--PRWMCH
--PRWMCF
--PRWMAM

-PPRWMS-
-PPRWMR-
-PPRWMM-
PPPRWM--
-QPRWMY-
-QPRWMR-
-QPRWMK-
-QPRWMG-
-QPRWML-
QSPRWM--
QVPRWM--
-QPRWMS-
-QPRWMI-
QGPRWM--
-QPRWMN-
QYPRWM--
-QPRWMV-
-QPRWMQ-
-QPRWMF-
-QPRWMH-
-QPRWMW-
QDPRWM--
QRPRWM--
QPPRWM--
-QPRWMT-
QAPRWM--
QLPRWM--
-QPRWMA-
QMPRWM--
QTPRWM--
-RPRWMK-
RSPRWM--
RVPRWM--
-RPRWMS-
RLPRWM--
-RPRWMN-
-RPRWMQ-
-RPRWMT-
-RPRWMI-
RYPRWM--
-RPRWMY-
-RPRWMG-
-RPRWMV-
-RPRWML-
-RPRWME-
RHPRWM--
-RPRWMF-
-RPRWMH-
RIPRWM--
-RPRWMW-

```
-RPRWMM-
RPPRWM--
REPRWM--
RWPRWM--
RRPRWM--
RKPRWM--
RDPRWM--
RQPRWM--
RCPRWM--
-SPRWMS-
-SPRWMG-
-SPRWMT-
-SPRWML-
SVPRWM--
-SPRWMI-
-SPRWMK-
SYPRWM--
STPRWM--
-SPRWMM-
-SPRWMA-
SSPRWM--
-SPRWMV-
SGPRWM--
SAPRWM--
SWPRWM--
SMPRWM--
-SPRWMD-
SDPRWM--
-SPRWMN-
-SPRWME-
SPPRWM--
SHPRWM--
SNPRWM--
-SPRWMQ-
SFPRWM--
-SPRWMW-
SKPRWM--
-SPRWMF-
SQPRWM--
SIPRWM--
-TPRWMH-
TGPRWM--
-TPRWML-
TTPRWM--
-TPRWMT-
-TPRWMG-
TRPRWM--
-TPRWMQ-
-TPRWMI-
TLPRWM--
```

TFPRWM--
TQPRWM--
TAPRWM--
TWPRWM--
-TPRWMV-
-TPRWMA-
TYPRWM--
-TPRWMN-
TPPRWM--
THPRWM--
TEPRWM--
-TPRWMK-
-TPRWMD-
TKPRWM--
-TPRWMY-
TVPRWM--
TMPRWM--
TNPRWM--
-TPRWMF-
-TPRWME-
TDPRWM--
VSPRWM--
-VPRWMK-
-VPRWMR-
VRPRWM--
VLPRWM--
VVPRWM--
-VPRWMV-
-VPRWMT-
VKPRWM--
VNPRWM--
-VPRWMN-
-VPRWMA-
VYPRWM--
VFPRWM--
-VPRWMD-
VPPRWM--
VHPRWM--
-VPRWMY-
VAPRWM--
VEPRWM--
VTPRWM--
-VPRWMQ-
VGPRWM--
VIPRWM--
VDPRWM--
-VPRWMW-
-VPRWMM-
VQPRWM--
-VPRWMG-

-VPRWME-
WSPRWM--
WEPRWM--
-WPRWML-
WRPRWM--
WVPRWM--
-WPRWMR-
WGPRWM--
WNPRWM--
WLPRWM--
WAPRWM--
WKPRWM--
WWPRWM--
-WPRWMQ-
-WPRWMD-
WFPRWM--
WYPRWM--
-WPRWMV-
-WPRWMT-
-WPRWMF-
WTPRWM--
-WPRWME-
WMPRWM--
WIPRWM--
WDPRWM--
WCPRWM--
WQPRWM--
-WPRWMH-
-WPRWMG-
WPPRWM--
YGPRWM--
-YPRWMS-
YTPRWM--
YNPRWM--
YVPRWM--
-YPRWMT-
-YPRWMV-
YAPRWM--
-YPRWML-
-YPRWMH-
YSPRWM--
YFPRWM--
YLPRWM--
-YPRWMI-
-YPRWMQ-
YMPRWM--
YEPRWM--
-YPRWMN-
-YPRWMK-
YQPRWM--

	-YPRWMM-		
	YHPRWM--		
	YRPRWM--		
	-YPRWMG-		
	-YPRWME-		
PKPY	262	717	49.46
	-KPKPYS-		
	-APKPYI-		
	ASPKPY--		
	-APKPYR-		
	AGPKPY--		
	ATPKPY--		
	ALPKPY--		
	-APKPYY-		
	AMPKPY--		
	AYPKPY--		
	-APKPYS-		
	-APKPYL-		
	-APKPYA-		
	-APKPYW-		
	-APKPYM-		
	AKPKPY--		
	ANPKPY--		
	AHPKPY--		
	-APKPYV-		
	-APKPYK-		
	-APKPYQ-		
	AWPKPY--		
	AVPKPY--		
	-APKPYG-		
	AEPKPY--		
	ACPKPY--		
	-APKPYF-		
	APPKPY--		
	ADPKPY--		
	AAPKPY--		
	CVPKPY--		
	CPPKPY--		
	CCPKPY--		
	CSPKPY--		
	-CPKPYQ-		
	-CPKPYL-		
	CDPKPY--		
	DKPKPY--		
	DYPKPY--		
	-DPKPYR-		
	-DPKPYT-		
	-DPKPYY-		
	-DPKPYK-		
	-DPKPYF-		

DTPKPY--
DIPKPY--
DVPKPY--
DSPKPY--
DMPKPY--
-DPKPYV-
-DPKPYS-
-DPKPYN-
-DPKPYH-
DHPKPY--
DGPKPY--
-EPKPYL-
-EPKPYT-
ESPKPY--
-EPKPYW-
ERPKPY--
-EPKPYI-
-EPKPYR-
-EPKPYF-
EIPKPY--
EWPKPY--
ELPKPY--
-EPKPYK-
EAPKPY--
EYPKPY--
EVPKPY--
-EPKPYY-
-EPKPYA-
EFPKPY--
-EPKPYV-
-EPKPYQ-
ETPKPY--
-EPKPYH-
-EPKPYS-
EKPKPY--
EEPKPY--
FNPKPY--
FSPKPY--
FRPKPY--
-FPKPYT-
FPPKPY--
-FPKPYY-
FYPKPY--
FGPKPY--
FAPKPY--
FHPKPY--
-FPKPYH-
FEPKPY--
FVPKPY--
FMPKPY--

FLPKPY--
-FPKPYS-
FQPKPY--
-FPKPYA-
FTPKPY--
-FPKPYV-
-FPKPYF-
FFPKPY--
FDPKPY--
-GPKPYR-
-GPKPYT-
-GPKPYL-
GLPKPY--
-GPKPYV-
GGPKPY--
GVPKPY--
GYPKPY--
GMPKPY--
GSPKPY--
-GPKPYH-
GTPKPY--
GIPKPY--
GRPKPY--
-GPKPYS-
-GPKPYM-
GFPKPY--
GDPKPY--
-GPKPYW-
GPPKPY--
-GPKPYI-
GEPKPY--
GWPKPY--
-GPKPYK-
-GPKPYF-
GQPKPY--
-GPKPYA-
GKPKPY--
GHPKPY--
GAPKPY--
HWPKPY--
HAPKPY--
HRPKPY--
HIPKPY--
-HPKPYV-
HMPKPY--
HYPKPY--
-HPKPYT-
HFPKPY--
HEPKPY--
-HPKPYS-

−HPKPYL−
HKPKPY−−
HVPKPY−−
HTPKPY−−
−HPKPYR−
−HPKPYF−
HSPKPY−−
−HPKPYK−
−HPKPYA−
HLPKPY−−
HDPKPY−−
IWPKPY−−
IVPKPY−−
IKPKPY−−
−IPKPYF−
IGPKPY−−
−IPKPYL−
INPKPY−−
ISPKPY−−
−IPKPYI−
IHPKPY−−
IIPKPY−−
IYPKPY−−
−IPKPYM−
−IPKPYR−
−IPKPYV−
IPPKPY−−
ITPKPY−−
IQPKPY−−
−IPKPYA−
IEPKPY−−
IAPKPY−−
IRPKPY−−
−IPKPYW−
−IPKPYN−
−IPKPYT−
−IPKPYS−
−IPKPYG−
−IPKPYE−
−IPKPYC−
KAPKPY−−
KYPKPY−−
KTPKPY−−
KGPKPY−−
KQPKPY−−
KPPKPY−−
KEPKPY−−
−KPKPYW−
−KPKPYL−
−KPKPYT−

```
-KPKPYM-
-KPKPYI-
KKPKPY--
KIPKPY--
KRPKPY--
-KPKPYY-
-KPKPYK-
KMPKPY--
-KPKPYV-
-KPKPYH-
KNPKPY--
KLPKPY--
KHPKPY--
LRPKPY--
-LPKPYR-
LQPKPY--
LGPKPY--
LFPKPY--
LHPKPY--
-LPKPYS-
LNPKPY--
LEPKPY--
LAPKPY--
-LPKPYL-
LTPKPY--
-LPKPYH-
LYPKPY--
LLPKPY--
LMPKPY--
-LPKPYI-
LWPKPY--
-LPKPYA-
LKPKPY--
-LPKPYF-
LIPKPY--
LSPKPY--
-LPKPYV-
-LPKPYM-
LVPKPY--
-LPKPYY-
-LPKPYT-
-LPKPYQ-
-LPKPYK-
LPPKPY--
-LPKPYW-
LCPKPY--
-LPKPYG-
LDPKPY--
MYPKPY--
-MPKPYI-
```

MWPKPY--
-MPKPYR-
MLPKPY--
MSPKPY--
-MPKPYS-
MMPKPY--
MVPKPY--
-MPKPYL-
-MPKPYF-
MEPKPY--
MNPKPY--
MPPKPY--
-MPKPYT-
MIPKPY--
MAPKPY--
MRPKPY--
MGPKPY--
-MPKPYY-
-MPKPYW-
MTPKPY--
-MPKPYM-
-MPKPYG-
MKPKPY--
MCPKPY--
NGPKPY--
NFPKPY--
NYPKPY--
NWPKPY--
NRPKPY--
NVPKPY--
NAPKPY--
-NPKPYR-
NLPKPY--
NDPKPY--
NTPKPY--
NQPKPY--
NPPKPY--
-NPKPYA-
NCPKPY--
-NPKPYV-
-NPKPYT-
-NPKPYQ-
-NPKPYF-
-NPKPYL-
-NPKPYE-
NHPKPY--
--PKPYLS
--PKPYKI
--PKPYFL
--PKPYAV

--PKPYRL
--PKPYQY
--PKPYTV
--PKPYFV
--PKPYTL
--PKPYYA
--PKPYTR
--PKPYMA
--PKPYLL
--PKPYFR
--PKPYST
--PKPYRQ
--PKPYRA
--PKPYAG
--PKPYTS
--PKPYMS
--PKPYKS
--PKPYVL
--PKPYIY
--PKPYDL
--PKPYVW
--PKPYRV
--PKPYRN
--PKPYRM
--PKPYPW
--PKPYLQ
--PKPYVR
--PKPYSH
--PKPYSF
--PKPYLT
--PKPYKG
--PKPYFS
PRPKPY--
--PKPYYS
--PKPYVT
--PKPYVH
--PKPYTT
--PKPYTQ
--PKPYRI
--PKPYRH
--PKPYMT
--PKPYLW
--PKPYFT
--PKPYVY
--PKPYVI
--PKPYTI
--PKPYSG
--PKPYLR
--PKPYFM
--PKPYFF

--PKPYAF
PGPKPY--
PVPKPY--
--PKPYYW
--PKPYMH
--PKPYLY
--PKPYAT
--PKPYYL
--PKPYPM
--PKPYPF
--PKPYLN
PLPKPY--
--PKPYWY
--PKPYSR
--PKPYSK
--PKPYPY
--PKPYPS
--PKPYNY
--PKPYNI
--PKPYMF
--PKPYLH
--PKPYEF
--PKPYAQ
--PKPYYT
--PKPYYR
--PKPYYK
--PKPYWS
--PKPYSQ
--PKPYQV
--PKPYMI
--PKPYLK
--PKPYLI
--PKPYIS
--PKPYAS
--PKPYYY
--PKPYYG
--PKPYVK
--PKPYTY
--PKPYMR
--PKPYII
--PKPYGF
--PKPYFN
--PKPYSW
--PKPYSA
--PKPYNM
--PKPYMN
--PKPYMG
--PKPYHT
--PKPYDM
--PKPYYE

--PKPYVA
--PKPYTK
--PKPYSV
--PKPYRW
--PKPYRS
--PKPYRG
--PKPYNG
--PKPYIT
--PKPYIK
--PKPYIF
--PKPYHI
--PKPYGS
--PKPYDY
--PKPYWT
--PKPYWR
--PKPYTN
--PKPYQT
--PKPYPI
--PKPYNA
--PKPYKQ
--PKPYIH
--PKPYHG
--PKPYFY
--PKPYEM
--PKPYAW
PDPKPY--
PTPKPY--
--PKPYYQ
--PKPYYH
--PKPYVQ
--PKPYVG
--PKPYTA
--PKPYSS
--PKPYQG
--PKPYIW
--PKPYHK
--PKPYHF
--PKPYGH
--PKPYFH
--PKPYEN
--PKPYEA
--PKPYDQ
--PKPYAK
PKPKPY--
PWPKPY--
--PKPYWH
--PKPYWF
--PKPYVS
--PKPYVM
--PKPYVF

--PKPYVE
--PKPYTW
--PKPYTF
--PKPYSD
--PKPYRD
--PKPYNQ
--PKPYNF
--PKPYMM
--PKPYML
--PKPYKN
--PKPYKK
--PKPYKF
--PKPYKA
--PKPYIG
--PKPYHS
--PKPYHR
--PKPYHN
--PKPYFK
--PKPYFI
--PKPYEV
--PKPYES
PSPKPY--
--PKPYRF
--PKPYQH
--PKPYPQ
--PKPYPA
--PKPYNW
--PKPYNV
--PKPYNT
--PKPYNK
--PKPYME
--PKPYLF
--PKPYKD
--PKPYIQ
--PKPYIL
--PKPYHE
--PKPYEY
--PKPYET
--PKPYER
--PKPYAM
PIPKPY--
PAPKPY--
PYPKPY--
--PKPYYM
--PKPYYD
--PKPYWM
--PKPYWG
--PKPYWA
--PKPYVP
--PKPYVD

--PKPYTH
--PKPYTD
--PKPYSN
--PKPYSE
--PKPYRY
--PKPYRE
--PKPYQS
--PKPYQA
--PKPYPN
--PKPYNR
--PKPYNP
--PKPYNH
--PKPYMK
--PKPYMD
--PKPYKY
--PKPYKV
--PKPYKT
--PKPYKH
--PKPYIM
--PKPYHM
--PKPYHH
--PKPYHA
--PKPYGW
--PKPYFG
--PKPYEQ
--PKPYDV
--PKPYCL
-QPKPYS-
QAPKPY--
QLPKPY--
QSPKPY--
QQPKPY--
QWPKPY--
-QPKPYV-
QTPKPY--
-QPKPYR-
QFPKPY--
-QPKPYL-
-QPKPYM-
QYPKPY--
QPPKPY--
QVPKPY--
-QPKPYH-
QMPKPY--
QEPKPY--
-QPKPYQ-
-QPKPYN-
-QPKPYA-
-QPKPYT-
-QPKPYI-

QKPKPY--
QCPKPY--
-RPKPYL-
RIPKPY--
RHPKPY--
RLPKPY--
-RPKPYR-
RKPKPY--
RQPKPY--
RAPKPY--
-RPKPYM-
RSPKPY--
RVPKPY--
RTPKPY--
RYPKPY--
RPPKPY--
RGPKPY--
RMPKPY--
RWPKPY--
-RPKPYV-
-RPKPYI-
RNPKPY--
RFPKPY--
-RPKPYY-
-RPKPYN-
RRPKPY--
-RPKPYA-
REPKPY--
RCPKPY--
-RPKPYT-
-RPKPYW-
-RPKPYK-
-RPKPYC-
SVPKPY--
SYPKPY--
-SPKPYR-
SRPKPY--
SWPKPY--
-SPKPYY-
SFPKPY--
-SPKPYT-
SSPKPY--
SIPKPY--
-SPKPYV-
SGPKPY--
SMPKPY--
STPKPY--
-SPKPYW-
SQPKPY--
SEPKPY--

-SPKPYQ-
-SPKPYI-
SDPKPY--
SPPKPY--
SLPKPY--
-SPKPYN-
-SPKPYA-
SKPKPY--
-SPKPYL-
-SPKPYK-
-SPKPYS-
-SPKPYM-
-SPKPYH-
SNPKPY--
SHPKPY--
TSPKPY--
TFPKPY--
TVPKPY--
TNPKPY--
-TPKPYR-
TLPKPY--
-TPKPYI-
TRPKPY--
-TPKPYH-
-TPKPYT-
TDPKPY--
TEPKPY--
TYPKPY--
TTPKPY--
-TPKPYV-
TIPKPY--
TQPKPY--
-TPKPYS-
-TPKPYM-
-TPKPYF-
-TPKPYE-
TWPKPY--
-TPKPYY-
-TPKPYG-
TGPKPY--
-VPKPYS-
VSPKPY--
-VPKPYR-
VNPKPY--
VHPKPY--
-VPKPYM-
VTPKPY--
VMPKPY--
VLPKPY--
VVPKPY--

VYPKPY--
VPPKPY--
VIPKPY--
VAPKPY--
-VPKPYA-
VGPKPY--
VQPKPY--
-VPKPYY-
VFPKPY--
-VPKPYK-
VWPKPY--
-VPKPYF-
-VPKPYI-
VKPKPY--
VRPKPY--
VDPKPY--
-VPKPYT-
-VPKPYL-
-VPKPYG-
-VPKPYE-
WGPKPY--
WSPKPY--
WRPKPY--
WQPKPY--
-WPKPYR-
-WPKPYA-
WLPKPY--
-WPKPYL-
WWPKPY--
WAPKPY--
WNPKPY--
WVPKPY--
WMPKPY--
-WPKPYY-
-WPKPYQ-
-WPKPYH-
-WPKPYE-
WKPKPY--
WCPKPY--
WYPKPY--
-WPKPYT-
-WPKPYK-
-WPKPYG-
WFPKPY--
YAPKPY--
-YPKPYR-
YWPKPY--
YLPKPY--
YGPKPY--
-YPKPYV-

	-YPKPYT-		
	YTPKPY--		
	YQPKPY--		
	-YPKPYL-		
	-YPKPYS-		
	YSPKPY--		
	YDPKPY--		
	YRPKPY--		
	-YPKPYF-		
	YYPKPY--		
	-YPKPYM-		
	-YPKPYY-		
	-YPKPYI-		
	-YPKPYQ-		
	-YPKPYK-		
	YVPKPY--		
	YMPKPY--		
	YKPKPY--		
	-YPKPYH-		
	YIPKPY--		
	-YPKPYG-		
	YHPKPY--		
	YFPKPY--		
PMWL	455	635	49.43
	ACPMWL--		
	ARPMWL--		
	AVPMWL--		
	-APMWLN-		
	ASPMWL--		
	-APMWLG-		
	AIPMWL--		
	AHPMWL--		
	-APMWLR-		
	-APMWLE-		
	-APMWLK-		
	AEPMWL--		
	AKPMWL--		
	-APMWLT-		
	-APMWLQ-		
	-APMWLA-		
	ALPMWL--		
	ADPMWL--		
	ATPMWL--		
	-APMWLI-		
	AMPMWL--		
	-APMWLS-		
	-APMWLD-		
	APPMWL--		
	AYPMWL--		
	ANPMWL--		

AQPMWL--
-APMWLV-
AFPMWL--
CTPMWL--
CQPMWL--
CPPMWL--
CGPMWL--
CSPMWL--
-CPMWLW-
CVPMWL--
-CPMWLS-
CEPMWL--
CAPMWL--
DLPMWL--
DGPMWL--
DRPMWL--
DTPMWL--
-DPMWLL-
DSPMWL--
DHPMWL--
DVPMWL--
-DPMWLV-
DAPMWL--
DYPMWL--
-DPMWLM-
-DPMWLS-
-DPMWLT-
-DPMWLG-
DMPMWL--
-DPMWLY-
-DPMWLF-
DNPMWL--
DDPMWL--
DQPMWL--
-DPMWLE-
-DPMWLA-
DKPMWL--
-DPMWLH-
-DPMWLQ-
ERPMWL--
ESPMWL--
-EPMWLH-
ETPMWL--
EGPMWL--
EKPMWL--
ELPMWL--
EIPMWL--
EWPMWL--
EAPMWL--
-EPMWLI-

EVPMWL--
EHPMWL--
-EPMWLR-
-EPMWLQ-
-EPMWLN-
-EPMWLA-
-EPMWLG-
-EPMWLE-
-EPMWLW-
-EPMWLT-
EDPMWL--
-EPMWLK-
EFPMWL--
EQPMWL--
-EPMWLS-
-EPMWLM-
ENPMWL--
EMPMWL--
FSPMWL--
FMPMWL--
FYPMWL--
-FPMWLN-
-FPMWLK-
FKPMWL--
-FPMWLT-
FHPMWL--
-FPMWLH-
-FPMWLG-
FTPMWL--
-FPMWLQ-
FWPMWL--
-FPMWLS-
-FPMWLE-
FDPMWL--
FAPMWL--
-FPMWLW-
-FPMWLM-
FNPMWL--
GKPMWL--
GAPMWL--
-GPMWLR-
GYPMWL--
GRPMWL--
GGPMWL--
GSPMWL--
GVPMWL--
GLPMWL--
GMPMWL--
GQPMWL--
-GPMWLG-

−GPMWLQ−
GFPMWL−−
GIPMWL−−
GHPMWL−−
−GPMWLK−
−GPMWLS−
−GPMWLA−
GTPMWL−−
−GPMWLM−
−GPMWLN−
−GPMWLV−
GEPMWL−−
GDPMWL−−
−GPMWLH−
GPPMWL−−
−GPMWLE−
GCPMWL−−
−GPMWLD−
−GPMWLC−
GNPMWL−−
HRPMWL−−
HSPMWL−−
−HPMWLS−
−HPMWLR−
−HPMWLG−
−HPMWLA−
HPPMWL−−
HGPMWL−−
HEPMWL−−
−HPMWLL−
HVPMWL−−
−HPMWLV−
−HPMWLT−
HWPMWL−−
−HPMWLN−
−HPMWLI−
HTPMWL−−
−HPMWLD−
−HPMWLQ−
HNPMWL−−
HKPMWL−−
−HPMWLW−
−HPMWLH−
HHPMWL−−
−IPMWLG−
−IPMWLS−
IKPMWL−−
ILPMWL−−
−IPMWLT−
IFPMWL−−

ITPMWL--
IRPMWL--
-IPMWLE-
IGPMWL--
IPPMWL--
-IPMWLR-
IEPMWL--
IDPMWL--
IYPMWL--
-IPMWLQ-
INPMWL--
-IPMWLD-
IVPMWL--
-IPMWLV-
-IPMWLN-
-IPMWLC-
IMPMWL--
ICPMWL--
KSPMWL--
KRPMWL--
-KPMWLT-
-KPMWLL-
KFPMWL--
-KPMWLG-
-KPMWLS-
-KPMWLR-
KAPMWL--
-KPMWLN-
-KPMWLH-
-KPMWLE-
KIPMWL--
KHPMWL--
KMPMWL--
KGPMWL--
KVPMWL--
-KPMWLI-
KWPMWL--
-KPMWLV-
-KPMWLA-
KNPMWL--
-KPMWLM-
KLPMWL--
-KPMWLD-
KQPMWL--
KEPMWL--
LSPMWL--
LAPMWL--
-LPMWLR-
LPPMWL--
LLPMWL--

LNPMWL--
LGPMWL--
-LPMWLK-
LKPMWL--
-LPMWLT-
LVPMWL--
LDPMWL--
LQPMWL--
LYPMWL--
LHPMWL--
LWPMWL--
-LPMWLA-
-LPMWLE-
-LPMWLD-
-LPMWLQ-
-LPMWLW-
MKPMWL--
MSPMWL--
-MPMWLK-
-MPMWLR-
MIPMWL--
-MPMWLE-
-MPMWLS-
MQPMWL--
-MPMWLI-
MAPMWL--
MYPMWL--
-MPMWLG-
MMPMWL--
-MPMWLM-
MGPMWL--
MDPMWL--
-MPMWLN-
MWPMWL--
-MPMWLT-
-MPMWLP-
-MPMWLH-
-MPMWLF-
NSPMWL--
NAPMWL--
-NPMWLT-
-NPMWLR-
NLPMWL--
NGPMWL--
NDPMWL--
NWPMWL--
-NPMWLA-
NRPMWL--
-NPMWLK-
NKPMWL--

-NPMWLG-
NHPMWL--
NFPMWL--
NEPMWL--
NPPMWL--
NMPMWL--
-NPMWLH-
NNPMWL--
-NPMWLL-
NYPMWL--
-NPMWLV-
-NPMWLI-
-NPMWLE-
--PMWLSG
--PMWLGS
--PMWLTG
--PMWLRG
--PMWLHS
--PMWLRS
--PMWLES
PGPMWL--
--PMWLSS
--PMWLGT
--PMWLSH
PMPMWL--
--PMWLTR
--PMWLYG
--PMWLRY
--PMWLNA
--PMWLTA
--PMWLNS
--PMWLLG
--PMWLST
--PMWLNT
--PMWLGN
--PMWLQH
--PMWLSN
--PMWLSA
--PMWLKG
--PMWLTN
--PMWLRN
PTPMWL--
--PMWLQT
--PMWLIN
--PMWLVG
--PMWLRW
--PMWLMS
--PMWLSE
--PMWLRD
--PMWLAT

```
--PMWLKS
PHPMWL--
--PMWLVS
--PMWLVA
--PMWLSQ
--PMWLRE
--PMWLQK
--PMWLLD
--PMWLGF
--PMWLVR
--PMWLQR
--PMWLMD
--PMWLWD
--PMWLVH
--PMWLKW
--PMWLKI
--PMWLKD
--PMWLGG
--PMWLAR
--PMWLTS
--PMWLTI
--PMWLMH
--PMWLLN
--PMWLQQ
--PMWLKV
--PMWLHE
--PMWLAE
PQPMWL--
--PMWLID
--PMWLHV
--PMWLRQ
--PMWLIT
--PMWLHM
--PMWLAS
--PMWLSF
--PMWLSD
--PMWLNV
--PMWLLH
--PMWLHL
--PMWLGR
--PMWLFG
--PMWLAH
--PMWLAG
--PMWLQS
--PMWLQG
--PMWLNR
--PMWLNG
--PMWLLM
--PMWLGK
--PMWLFQ
```

```
--PMWLET
--PMWLEM
--PMWLSV
--PMWLSR
--PMWLKA
--PMWLIH
--PMWLHR
--PMWLEH
--PMWLTK
--PMWLSM
--PMWLQI
--PMWLQD
--PMWLHG
--PMWLGY
--PMWLGW
--PMWLGI
--PMWLGA
--PMWLEY
--PMWLEV
--PMWLDS
--PMWLAM
PDPMWL--
--PMWLVK
--PMWLTE
--PMWLNW
--PMWLKY
--PMWLKL
--PMWLHY
--PMWLHW
--PMWLFF
--PMWLAQ
--PMWLAK
--PMWLAD
--PMWLYQ
--PMWLNE
--PMWLMW
--PMWLMK
--PMWLMG
--PMWLLW
--PMWLHQ
--PMWLHF
--PMWLGM
--PMWLEQ
--PMWLDA
--PMWLYV
--PMWLSW
--PMWLQN
--PMWLND
--PMWLHI
--PMWLGQ
```

--PMWLGE
--PMWLFV
--PMWLFD
--PMWLER
PKPMWL--
-PPMWLY-
-PPMWLN-
--PMWLYF
--PMWLWV
--PMWLWG
--PMWLVV
--PMWLTY
--PMWLTP
--PMWLTH
--PMWLQY
--PMWLQM
--PMWLNN
--PMWLMQ
--PMWLLP
--PMWLLI
--PMWLKQ
--PMWLKN
--PMWLKF
--PMWLHT
--PMWLHN
--PMWLGD
--PMWLEG
--PMWLEF
--PMWLDT
PEPMWL--
QLPMWL--
-QPMWLS-
QSPMWL--
QRPMWL--
-QPMWLM-
QTPMWL--
-QPMWLA-
QNPMWL--
QHPMWL--
QYPMWL--
QVPMWL--
QMPMWL--
QIPMWL--
-QPMWLI-
-QPMWLQ-
-QPMWLD-
-QPMWLV-
-QPMWLK-
QFPMWL--
QWPMWL--

-QPMWLN-
-QPMWLH-
QKPMWL--
QGPMWL--
-QPMWLR-
QDPMWL--
-QPMWLL-
-QPMWLP-
-QPMWLG-
-RPMWLS-
RGPMWL--
-RPMWLK-
-RPMWLI-
RNPMWL--
-RPMWLV-
RYPMWL--
RPPMWL--
RMPMWL--
RHPMWL--
-RPMWLA-
-RPMWLT-
RAPMWL--
-RPMWLW-
-RPMWLG-
REPMWL--
RWPMWL--
-RPMWLN-
RVPMWL--
-RPMWLE-
-RPMWLD-
RDPMWL--
RQPMWL--
RCPMWL--
-RPMWLH-
-SPMWLR-
SVPMWL--
SSPMWL--
-SPMWLS-
SNPMWL--
SLPMWL--
SRPMWL--
-SPMWLT-
SGPMWL--
-SPMWLG-
-SPMWLQ-
-SPMWLH-
SHPMWL--
SKPMWL--
SFPMWL--
SEPMWL--

−SPMWLN−
SIPMWL−−
−SPMWLA−
SPPMWL−−
−SPMWLV−
SMPMWL−−
SDPMWL−−
−SPMWLE−
SQPMWL−−
SYPMWL−−
−SPMWLD−
−SPMWLK−
SCPMWL−−
TGPMWL−−
TVPMWL−−
TPPMWL−−
−TPMWLR−
−TPMWLK−
−TPMWLN−
TYPMWL−−
−TPMWLA−
TTPMWL−−
TMPMWL−−
−TPMWLL−
TSPMWL−−
TNPMWL−−
TLPMWL−−
−TPMWLQ−
−TPMWLM−
−TPMWLV−
−TPMWLT−
TIPMWL−−
TDPMWL−−
TAPMWL−−
−TPMWLS−
TKPMWL−−
−TPMWLG−
THPMWL−−
TQPMWL−−
−TPMWLI−
−TPMWLE−
TEPMWL−−
TWPMWL−−
−TPMWLH−
VSPMWL−−
−VPMWLA−
VTPMWL−−
VHPMWL−−
−VPMWLT−
VVPMWL−−

VRPMWL--
-VPMWLG-
VNPMWL--
VKPMWL--
VLPMWL--
VAPMWL--
-VPMWLS-
-VPMWLQ-
VQPMWL--
VFPMWL--
-VPMWLK-
-VPMWLH-
VYPMWL--
VWPMWL--
VGPMWL--
-VPMWLD-
-VPMWLN-
VDPMWL--
VEPMWL--
VPPMWL--
-VPMWLM-
-VPMWLE-
-VPMWLF-
VCPMWL--
WPPMWL--
WKPMWL--
WYPMWL--
-WPMWLR-
WEPMWL--
WGPMWL--
WAPMWL--
-WPMWLI-
WNPMWL--
WHPMWL--
WFPMWL--
-WPMWLH-
-WPMWLS-
-WPMWLT-
-WPMWLN-
-WPMWLF-
YHPMWL--
-YPMWLR-
YKPMWL--
YTPMWL--
YFPMWL--
YAPMWL--
YRPMWL--
YPPMWL--
-YPMWLQ-
YGPMWL--

```
          -YPMWLH-
          YIPMWL--
          YQPMWL--
          -YPMWLV-
          YMPMWL--
          -YPMWLY-
          -YPMWLE-
          -YPMWLN-
          YSPMWL--
PYGL        10        909      49.41
          -IPYGLH-
          --PYGLPA
          --PYGLLP
          -QPYGLP-
          -APYGLR-
          ANPYGL--
          -APYGLL-
          ARPYGL--
          -APYGLQ-
          -APYGLS-
          AVPYGL--
          AGPYGL--
          -APYGLN-
          AIPYGL--
          AYPYGL--
          ATPYGL--
          -APYGLT-
          AMPYGL--
          -APYGLY-
          AQPYGL--
          AKPYGL--
          -APYGLH-
          -APYGLK-
          -APYGLE-
          -APYGLA-
          -APYGLW-
          -APYGLG-
          AWPYGL--
          ACPYGL--
          -APYGLD-
          AFPYGL--
          ADPYGL--
          -APYGLP-
          -APYGLM-
          CVPYGL--
          -CPYGLR-
          CTPYGL--
          CPPYGL--
          CSPYGL--
          -CPYGLK-
```

```
-CPYGLS-
CNPYGL--
-CPYGLQ-
-CPYGLL-
CLPYGL--
CKPYGL--
CYPYGL--
-CPYGLY-
CIPYGL--
-CPYGLG-
CEPYGL--
-CPYGLI-
CGPYGL--
CQPYGL--
-CPYGLM-
CDPYGL--
CWPYGL--
-CPYGLV-
-CPYGLT-
-CPYGLF-
CFPYGL--
DIPYGL--
DRPYGL--
-DPYGLL-
DMPYGL--
DHPYGL--
-DPYGLR-
DKPYGL--
-DPYGLW-
-DPYGLF-
DVPYGL--
-DPYGLY-
-DPYGLS-
DTPYGL--
DSPYGL--
-DPYGLT-
DWPYGL--
DLPYGL--
-DPYGLK-
-DPYGLA-
-DPYGLM-
-DPYGLH-
DNPYGL--
DDPYGL--
DAPYGL--
DQPYGL--
-DPYGLN-
DEPYGL--
-DPYGLQ-
DYPYGL--
```

−DPYGLV−
−DPYGLG−
DPPYGL−−
−EPYGLL−
EVPYGL−−
−EPYGLR−
EMPYGL−−
−EPYGLK−
ELPYGL−−
−EPYGLN−
EFPYGL−−
ESPYGL−−
−EPYGLF−
ERPYGL−−
EWPYGL−−
−EPYGLS−
−EPYGLQ−
−EPYGLH−
EEPYGL−−
EAPYGL−−
−EPYGLI−
EKPYGL−−
−EPYGLV−
−EPYGLY−
−EPYGLT−
−EPYGLM−
EHPYGL−−
EYPYGL−−
ETPYGL−−
EQPYGL−−
−EPYGLW−
EPPYGL−−
−EPYGLG−
ENPYGL−−
EIPYGL−−
EDPYGL−−
−EPYGLE−
EGPYGL−−
−EPYGLA−
FRPYGL−−
FTPYGL−−
FMPYGL−−
FSPYGL−−
FGPYGL−−
−FPYGLS−
FVPYGL−−
−FPYGLR−
−FPYGLK−
−FPYGLY−
−FPYGLN−

-FPYGLL-
FIPYGL--
-FPYGLT-
FEPYGL--
FYPYGL--
-FPYGLQ-
-FPYGLG-
-FPYGLI-
-FPYGLH-
FKPYGL--
FWPYGL--
-FPYGLV-
FHPYGL--
FAPYGL--
FFPYGL--
FPPYGL--
FQPYGL--
FDPYGL--
FNPYGL--
-FPYGLA-
-FPYGLM-
-FPYGLE-
-FPYGLF-
FCPYGL--
-GPYGLR-
-GPYGLK-
GTPYGL--
GMPYGL--
GRPYGL--
GSPYGL--
GGPYGL--
-GPYGLL-
-GPYGLV-
GYPYGL--
GVPYGL--
-GPYGLN-
-GPYGLG-
GLPYGL--
-GPYGLA-
GDPYGL--
GAPYGL--
-GPYGLH-
GIPYGL--
GWPYGL--
GNPYGL--
-GPYGLI-
GEPYGL--
-GPYGLW-
-GPYGLM-
-GPYGLY-

−GPYGLF−
−GPYGLQ−
GCPYGL−−
GHPYGL−−
GPPYGL−−
−GPYGLE−
GFPYGL−−
−HPYGLS−
−HPYGLR−
HLPYGL−−
−HPYGLN−
HWPYGL−−
HIPYGL−−
HRPYGL−−
HSPYGL−−
HPPYGL−−
−HPYGLY−
−HPYGLI−
HNPYGL−−
HYPYGL−−
−HPYGLQ−
HVPYGL−−
HAPYGL−−
HKPYGL−−
HEPYGL−−
HTPYGL−−
−HPYGLK−
−HPYGLH−
−HPYGLF−
HHPYGL−−
−HPYGLA−
HQPYGL−−
HDPYGL−−
−HPYGLT−
−HPYGLW−
−HPYGLG−
HMPYGL−−
−HPYGLM−
IRPYGL−−
−IPYGLS−
−IPYGLW−
−IPYGLA−
−IPYGLL−
−IPYGLR−
IPPYGL−−
−IPYGLY−
−IPYGLI−
IGPYGL−−
IHPYGL−−
−IPYGLQ−

ILPYGL--
-IPYGLG-
IFPYGL--
ITPYGL--
IWPYGL--
IEPYGL--
IVPYGL--
IKPYGL--
-IPYGLV-
IAPYGL--
-IPYGLK-
-IPYGLN-
IDPYGL--
IIPYGL--
IYPYGL--
-IPYGLT-
-IPYGLD-
-IPYGLM-
INPYGL--
-IPYGLE-
IQPYGL--
-IPYGLF-
KSPYGL--
KLPYGL--
KRPYGL--
KGPYGL--
-KPYGLR-
KHPYGL--
-KPYGLH-
-KPYGLV-
KTPYGL--
-KPYGLY-
-KPYGLL-
KNPYGL--
KFPYGL--
-KPYGLN-
-KPYGLM-
KWPYGL--
KAPYGL--
KYPYGL--
-KPYGLS-
-KPYGLA-
-KPYGLW-
-KPYGLQ-
KPPYGL--
-KPYGLF-
-KPYGLK-
KDPYGL--
-KPYGLE-
-KPYGLD-

KQPYGL--
KMPYGL--
KCPYGL--
LVPYGL--
LTPYGL--
LWPYGL--
-LPYGLS-
LGPYGL--
LQPYGL--
LMPYGL--
LAPYGL--
LLPYGL--
LFPYGL--
-LPYGLN-
LNPYGL--
-LPYGLL-
-LPYGLI-
-LPYGLK-
-LPYGLY-
-LPYGLF-
LYPYGL--
-LPYGLH-
-LPYGLA-
LIPYGL--
-LPYGLM-
-LPYGLE-
LHPYGL--
LPPYGL--
LEPYGL--
-LPYGLG-
-LPYGLQ-
LCPYGL--
LDPYGL--
-LPYGLC-
MRPYGL--
MKPYGL--
MVPYGL--
MTPYGL--
-MPYGLK-
MSPYGL--
-MPYGLL-
MNPYGL--
-MPYGLR-
MLPYGL--
-MPYGLI-
MGPYGL--
-MPYGLA-
-MPYGLW-
-MPYGLQ-
-MPYGLM-

MHPYGL--
-MPYGLV-
MPPYGL--
MIPYGL--
MAPYGL--
-MPYGLT-
MMPYGL--
-MPYGLN-
MFPYGL--
MEPYGL--
MDPYGL--
-MPYGLE-
-MPYGLC-
NVPYGL--
-NPYGLL-
-NPYGLR-
NSPYGL--
NLPYGL--
NGPYGL--
NKPYGL--
NRPYGL--
NAPYGL--
-NPYGLY-
NNPYGL--
-NPYGLT-
-NPYGLN-
NPPYGL--
NTPYGL--
-NPYGLI-
NEPYGL--
-NPYGLM-
NYPYGL--
NQPYGL--
NMPYGL--
NIPYGL--
-NPYGLS-
-NPYGLA-
-NPYGLK-
-NPYGLW-
-NPYGLH-
-NPYGLE-
NHPYGL--
-NPYGLV-
NCPYGL--
-NPYGLG-
NWPYGL--
NDPYGL--
NFPYGL--
-NPYGLQ-
-NPYGLD-

--PYGLQA
--PYGLLR
--PYGLTW
--PYGLGS
--PYGLTS
--PYGLRS
--PYGLRN
--PYGLKS
--PYGLSL
--PYGLSS
--PYGLVS
--PYGLRG
--PYGLTY
--PYGLST
--PYGLFR
--PYGLSN
--PYGLVR
--PYGLSG
--PYGLKR
--PYGLSR
--PYGLIR
--PYGLYS
--PYGLSV
--PYGLSH
--PYGLHL
--PYGLAL
--PYGLWR
--PYGLRT
--PYGLRK
--PYGLFN
--PYGLVG
--PYGLVA
--PYGLRD
--PYGLVN
--PYGLLG
--PYGLIA
--PYGLRQ
--PYGLTL
--PYGLTK
--PYGLKG
--PYGLLD
--PYGLTG
--PYGLNT
--PYGLMR
--PYGLHS
--PYGLYQ
--PYGLRM
--PYGLLA
--PYGLHN
--PYGLGW

--PYGLAG
--PYGLWS
--PYGLVV
--PYGLMV
--PYGLMG
--PYGLAA
--PYGLRY
--PYGLGG
--PYGLVK
--PYGLRE
--PYGLNF
--PYGLHM
--PYGLRH
--PYGLLH
--PYGLSY
--PYGLMN
--PYGLML
PVPYGL--
PQPYGL--
--PYGLSK
--PYGLNH
PIPYGL--
--PYGLQG
--PYGLLQ
--PYGLAI
PTPYGL--
--PYGLVT
--PYGLTT
--PYGLTR
--PYGLNN
--PYGLGT
--PYGLKY
--PYGLIT
--PYGLFG
--PYGLRF
--PYGLSF
--PYGLNR
--PYGLHK
--PYGLET
--PYGLSE
--PYGLSA
--PYGLQK
--PYGLKF
--PYGLHR
--PYGLAM
PRPYGL--
--PYGLYG
--PYGLTA
--PYGLNG
--PYGLYD

--PYGLQT
--PYGLTV
--PYGLMD
--PYGLLM
--PYGLGA
--PYGLFS
--PYGLAT
--PYGLAR
PNPYGL--
PHPYGL--
--PYGLYK
--PYGLWG
--PYGLMT
PMPYGL--
--PYGLKL
--PYGLIS
--PYGLEN
--PYGLAV
--PYGLES
--PYGLAN
--PYGLWT
--PYGLRW
--PYGLIH
--PYGLMQ
--PYGLHH
--PYGLGM
--PYGLGL
--PYGLYN
--PYGLNM
--PYGLKE
--PYGLHI
--PYGLGN
--PYGLYH
--PYGLQR
--PYGLIN
--PYGLHA
PKPYGL--
--PYGLTM
--PYGLQL
--PYGLMH
--PYGLKT
--PYGLIG
--PYGLFT
--PYGLEY
--PYGLWQ
--PYGLTH
--PYGLQV
--PYGLNQ
--PYGLNK
--PYGLKV

--PYGLFK
--PYGLDA
--PYGLSD
--PYGLQI
--PYGLNS
--PYGLMM
--PYGLKD
--PYGLSM
--PYGLRI
--PYGLGH
--PYGLER
--PYGLVD
--PYGLTN
--PYGLNL
--PYGLKN
--PYGLHQ
--PYGLWK
--PYGLWE
--PYGLVI
--PYGLEA
--PYGLDG
--PYGLAK
PAPYGL--
--PYGLWH
--PYGLWD
--PYGLTQ
--PYGLVW
--PYGLMW
--PYGLIV
--PYGLHT
--PYGLHG
--PYGLGF
--PYGLVE
--PYGLQE
--PYGLND
--PYGLNA
--PYGLGY
--PYGLRP
--PYGLMK
--PYGLLE
--PYGLHV
--PYGLQN
--PYGLME
--PYGLIQ
--PYGLIK
--PYGLAY
--PYGLAW
--PYGLKQ
--PYGLKH
--PYGLFQ

--PYGLFE
PFPYGL--
PEPYGL--
--PYGLYP
--PYGLQF
--PYGLID
--PYGLHE
--PYGLGI
--PYGLTD
--PYGLNI
--PYGLMA
--PYGLKM
--PYGLKA
--PYGLHF
--PYGLHD
--PYGLDS
--PYGLAH
--PYGLAF
PDPYGL--
PYPYGL--
--PYGLYW
--PYGLQQ
--PYGLQM
--PYGLPG
--PYGLNW
--PYGLNE
--PYGLIY
--PYGLGQ
--PYGLFH
--PYGLEK
--PYGLVF
--PYGLQH
--PYGLII
--PYGLGK
--PYGLEW
--PYGLED
--PYGLYE
--PYGLRC
--PYGLQY
--PYGLIW
--PYGLIM
--PYGLIE
--PYGLHW
--PYGLFD
--PYGLEV
--PYGLWN
--PYGLSC
--PYGLPS
--PYGLIC
--PYGLEH

--PYGLYC
--PYGLWM
--PYGLQD
--PYGLMP
--PYGLFC
--PYGLEQ
--PYGLEF
--PYGLEE
--PYGLDW
--PYGLWW
--PYGLVC
--PYGLPI
--PYGLNC
--PYGLKP
--PYGLKK
--PYGLKC
--PYGLIP
--PYGLHP
--PYGLGP
--PYGLGE
--PYGLDV
--PYGLDT
--PYGLDN
--PYGLDM
--PYGLCG
--PYGLCE
PWPYGL--
-PPYGLI-
-PPYGLH-
PCPYGL--
-QPYGLS-
-QPYGLR-
QGPYGL--
QLPYGL--
-QPYGLT-
QRPYGL--
-QPYGLV-
QYPYGL--
QVPYGL--
-QPYGLY-
QSPYGL--
-QPYGLK-
QPPYGL--
QMPYGL--
QTPYGL--
QFPYGL--
QHPYGL--
-QPYGLF-
-QPYGLW-
-QPYGLN-

–QPYGLA–
–QPYGLG–
–QPYGLE–
QWPYGL––
QDPYGL––
QNPYGL––
QIPYGL––
QEPYGL––
–QPYGLQ–
QCPYGL––
QAPYGL––
QQPYGL––
–QPYGLH–
QKPYGL––
–QPYGLM–
–QPYGLD–
–QPYGLC–
–RPYGLR–
RSPYGL––
RTPYGL––
REPYGL––
RRPYGL––
–RPYGLY–
–RPYGLK–
RVPYGL––
RFPYGL––
–RPYGLT–
–RPYGLG–
–RPYGLI–
RGPYGL––
–RPYGLF–
–RPYGLQ–
–RPYGLH–
RYPYGL––
RPPYGL––
–RPYGLN–
–RPYGLA–
–RPYGLM–
–RPYGLW–
–RPYGLV–
RAPYGL––
RQPYGL––
RDPYGL––
RHPYGL––
RMPYGL––
RWPYGL––
–RPYGLE–
RCPYGL––
–RPYGLD–
–RPYGLP–

-RPYGLC-
SMPYGL--
SIPYGL--
SVPYGL--
-SPYGLS-
-SPYGLV-
SSPYGL--
SGPYGL--
SWPYGL--
-SPYGLQ-
-SPYGLK-
-SPYGLH-
SYPYGL--
-SPYGLF-
-SPYGLI-
-SPYGLW-
-SPYGLA-
SKPYGL--
SEPYGL--
SNPYGL--
SDPYGL--
SFPYGL--
SAPYGL--
-SPYGLE-
SQPYGL--
-SPYGLL-
-SPYGLN-
SPPYGL--
STPYGL--
-SPYGLM-
-SPYGLG-
SCPYGL--
-SPYGLD-
TRPYGL--
TSPYGL--
-TPYGLL-
-TPYGLR-
TLPYGL--
TTPYGL--
-TPYGLK-
TWPYGL--
TNPYGL--
-TPYGLT-
-TPYGLH-
TIPYGL--
-TPYGLQ-
TMPYGL--
TVPYGL--
TPPYGL--
-TPYGLI-

TGPYGL--
-TPYGLV-
-TPYGLA-
-TPYGLY-
-TPYGLM-
TKPYGL--
THPYGL--
TEPYGL--
-TPYGLN-
TQPYGL--
TCPYGL--
-TPYGLW-
TYPYGL--
TFPYGL--
-TPYGLE-
TDPYGL--
VRPYGL--
-VPYGLR-
VSPYGL--
VTPYGL--
VLPYGL--
-VPYGLH-
-VPYGLF-
-VPYGLT-
VAPYGL--
VGPYGL--
VIPYGL--
VQPYGL--
-VPYGLQ-
VPPYGL--
-VPYGLI-
VVPYGL--
-VPYGLV-
-VPYGLA-
VMPYGL--
-VPYGLG-
-VPYGLY-
-VPYGLL-
VWPYGL--
VNPYGL--
-VPYGLE-
VEPYGL--
VKPYGL--
VYPYGL--
VDPYGL--
-VPYGLK-
-VPYGLW-
VFPYGL--
VCPYGL--
-VPYGLC-

WTPYGL--
WSPYGL--
WRPYGL--
WQPYGL--
-WPYGLR-
WAPYGL--
-WPYGLI-
-WPYGLK-
WKPYGL--
-WPYGLT-
WIPYGL--
WEPYGL--
-WPYGLN-
WFPYGL--
-WPYGLV-
WLPYGL--
WGPYGL--
WVPYGL--
WWPYGL--
-WPYGLG-
-WPYGLA-
WYPYGL--
-WPYGLS-
-WPYGLQ-
-WPYGLE-
WMPYGL--
WDPYGL--
WPPYGL--
-WPYGLH-
WCPYGL--
-WPYGLF-
WNPYGL--
-WPYGLD-
YRPYGL--
YSPYGL--
YLPYGL--
-YPYGLS-
YTPYGL--
-YPYGLT-
YQPYGL--
-YPYGLR-
YVPYGL--
-YPYGLW-
-YPYGLM-
-YPYGLF-
YPPYGL--
YGPYGL--
YWPYGL--
-YPYGLL-
-YPYGLQ-

-YPYGLA-
-YPYGLI-
-YPYGLN-
-YPYGLH-
YNPYGL--
YIPYGL--
YEPYGL--
YAPYGL--
-YPYGLK-
-YPYGLY-
YHPYGL--
YYPYGL--
-YPYGLG-
-YPYGLE-
YMPYGL--
YFPYGL--
-YPYGLV-
YDPYGL--
YCPYGL--
PYAM 309 691 49.33
--PYAMGR
-APYAMR-
ARPYAM--
-APYAML-
ASPYAM--
ALPYAM--
AHPYAM--
AEPYAM--
-APYAMF-
AMPYAM--
AQPYAM--
AWPYAM--
-APYAMM-
-APYAMK-
-APYAMS-
-APYAME-
AKPYAM--
AYPYAM--
-APYAMY-
AAPYAM--
AIPYAM--
-APYAMT-
-APYAMN-
-APYAMG-
ADPYAM--
CSPYAM--
CRPYAM--
-CPYAMR-
-CPYAMH-
-CPYAMF-

CNPYAM--
CHPYAM--
CYPYAM--
-CPYAMI-
DLPYAM--
-DPYAMH-
DRPYAM--
DHPYAM--
-DPYAMT-
-DPYAMM-
DWPYAM--
-DPYAMV-
-DPYAML-
-DPYAMI-
DVPYAM--
-DPYAMR-
-DPYAMQ-
DMPYAM--
DKPYAM--
DAPYAM--
DSPYAM--
-DPYAMN-
-DPYAMK-
DGPYAM--
ESPYAM--
-EPYAMS-
EFPYAM--
-EPYAMF-
ETPYAM--
-EPYAMH-
EYPYAM--
-EPYAMV-
-EPYAMY-
-EPYAMG-
-EPYAMA-
ENPYAM--
EVPYAM--
-EPYAML-
EMPYAM--
ERPYAM--
-EPYAMI-
-FPYAMS-
-FPYAMH-
FSPYAM--
-FPYAMQ-
-FPYAMK-
-FPYAMT-
-FPYAMA-
FIPYAM--
FAPYAM--

FRPYAM--
-FPYAMY-
-FPYAML-
FKPYAM--
FGPYAM--
FTPYAM--
FPPYAM--
FMPYAM--
FQPYAM--
-FPYAME-
FHPYAM--
-FPYAMW-
-FPYAMN-
FLPYAM--
FEPYAM--
GNPYAM--
-GPYAMN-
-GPYAMV-
GYPYAM--
GKPYAM--
GWPYAM--
GVPYAM--
GTPYAM--
-GPYAMF-
GFPYAM--
-GPYAMY-
GLPYAM--
GRPYAM--
-GPYAMT-
-GPYAML-
-GPYAMQ-
GPPYAM--
GSPYAM--
-GPYAMM-
GMPYAM--
GQPYAM--
GGPYAM--
GEPYAM--
GAPYAM--
-GPYAMW-
-GPYAMK-
-GPYAMH-
-GPYAMA-
GDPYAM--
-HPYAMR-
-HPYAMK-
HRPYAM--
-HPYAMA-
HPPYAM--
HHPYAM--

HWPYAM--
HNPYAM--
-HPYAMS-
HIPYAM--
HVPYAM--
HTPYAM--
-HPYAMQ-
-HPYAMN-
HLPYAM--
-HPYAMI-
HAPYAM--
HYPYAM--
-HPYAMW-
-HPYAMV-
-HPYAMG-
-IPYAMH-
IPPYAM--
IRPYAM--
-IPYAML-
ITPYAM--
IKPYAM--
-IPYAMK-
INPYAM--
-IPYAMT-
-IPYAMR-
IGPYAM--
-IPYAMS-
IWPYAM--
-IPYAME-
IHPYAM--
IEPYAM--
IVPYAM--
-IPYAMG-
IMPYAM--
-IPYAMV-
-IPYAMD-
IIPYAM--
IDPYAM--
ISPYAM--
-IPYAMM-
-IPYAMA-
ILPYAM--
IFPYAM--
KSPYAM--
KTPYAM--
KRPYAM--
KQPYAM--
-KPYAMQ-
KKPYAM--
-KPYAMN-

-KPYAMI-
KYPYAM--
-KPYAMR-
KNPYAM--
KFPYAM--
KVPYAM--
-KPYAMW-
KAPYAM--
-KPYAML-
KLPYAM--
-KPYAMT-
-KPYAMH-
KGPYAM--
-KPYAMV-
-KPYAMM-
-KPYAME-
KWPYAM--
-KPYAMK-
-KPYAMG-
-KPYAMA-
KDPYAM--
LSPYAM--
LLPYAM--
-LPYAMV-
LRPYAM--
LNPYAM--
-LPYAMI-
LKPYAM--
-LPYAMY-
-LPYAMA-
LEPYAM--
LTPYAM--
-LPYAMR-
LHPYAM--
LDPYAM--
LAPYAM--
LYPYAM--
LVPYAM--
-LPYAMQ-
LMPYAM--
-LPYAMK-
LQPYAM--
-LPYAMN-
-LPYAME-
LFPYAM--
-LPYAMT-
-LPYAMH-
-LPYAMF-
LCPYAM--
-LPYAMG-

LIPYAM--
-MPYAMN-
MRPYAM--
MFPYAM--
-MPYAMK-
MSPYAM--
MVPYAM--
-MPYAMH-
MNPYAM--
-MPYAMR-
-MPYAMQ-
-MPYAMG-
MTPYAM--
MDPYAM--
-MPYAMA-
MIPYAM--
MWPYAM--
MPPYAM--
MYPYAM--
-MPYAMV-
-MPYAMS-
-MPYAMI-
MGPYAM--
-NPYAMV-
NRPYAM--
NNPYAM--
-NPYAMS-
-NPYAMM-
-NPYAMF-
-NPYAMI-
-NPYAMR-
-NPYAMQ-
-NPYAMH-
NSPYAM--
-NPYAMN-
-NPYAMK-
-NPYAMY-
NEPYAM--
NPPYAM--
NLPYAM--
NFPYAM--
NGPYAM--
-NPYAMW-
-NPYAMG-
NCPYAM--
NAPYAM--
--PYAMFS
--PYAMSS
--PYAMGL
--PYAMSN

--PYAMHL
--PYAMRV
--PYAMRG
--PYAMNT
--PYAMRH
--PYAMFG
--PYAMTS
--PYAMQG
--PYAMRS
--PYAMQS
--PYAMKA
--PYAMIR
--PYAMSQ
--PYAMRE
--PYAMWA
--PYAMRT
PNPYAM--
--PYAMLS
--PYAMKG
--PYAMTW
--PYAMTA
--PYAMFN
--PYAMSR
--PYAMLK
--PYAMKR
--PYAMAF
--PYAMSW
--PYAMNR
--PYAMHR
--PYAMHM
--PYAMVS
--PYAMVD
--PYAMSG
--PYAMSA
--PYAMFA
--PYAMYT
--PYAMYR
--PYAMWG
--PYAMLH
--PYAMVN
--PYAMVI
--PYAMTG
--PYAMHA
--PYAMGA
--PYAMAS
--PYAMAQ
--PYAMVT
--PYAMSV
--PYAMST
--PYAMSL

--PYAMSI
--PYAMPL
--PYAMMR
--PYAMIT
--PYAMFH
--PYAMWS
--PYAMRD
--PYAMQR
--PYAMIA
--PYAMGV
--PYAMGG
--PYAMFT
--PYAMFR
--PYAMFQ
PWPYAM--
--PYAMWR
--PYAMWN
--PYAMVR
--PYAMVH
--PYAMSH
--PYAMNW
--PYAMLG
--PYAMES
--PYAMAH
PTPYAM--
--PYAMTH
--PYAMQT
--PYAMQD
--PYAMMM
--PYAMHS
PQPYAM--
--PYAMYD
--PYAMYA
--PYAMVK
--PYAMRI
--PYAMQL
--PYAMNF
--PYAMTN
--PYAMTL
--PYAMNY
--PYAMNI
--PYAMNH
--PYAMKK
--PYAMGT
--PYAMAK
PGPYAM--
--PYAMYP
--PYAMVY
--PYAMTK
--PYAMSF

--PYAMQH
--PYAMMT
--PYAMMS
--PYAMMQ
--PYAMLV
--PYAMLE
--PYAMLD
--PYAMGS
--PYAMNQ
--PYAMMG
--PYAMKV
--PYAMKS
--PYAMKI
--PYAMIS
--PYAMIN
--PYAMHY
--PYAMHW
--PYAMET
--PYAMAT
--PYAMYQ
--PYAMTY
--PYAMTQ
--PYAMTF
--PYAMSE
--PYAMSD
--PYAMRY
--PYAMRQ
--PYAMML
--PYAMLT
--PYAMKL
--PYAMKE
--PYAMIW
--PYAMIQ
--PYAMIE
--PYAMHK
--PYAMGW
--PYAMGF
--PYAMAV
--PYAMAR
--PYAMAL
PMPYAM--
--PYAMYN
--PYAMYE
--PYAMVV
--PYAMVM
--PYAMTT
--PYAMTM
--PYAMTD
--PYAMNN
--PYAMNL

--PYAMMY
--PYAMMV
--PYAMKY
--PYAMHI
--PYAMGN
--PYAMER
--PYAMEQ
--PYAMEM
--PYAMEL
--PYAMEI
--PYAMEH
--PYAMEG
--PYAMAE
PIPYAM--
PHPYAM--
--PYAMYS
--PYAMYH
--PYAMYG
--PYAMWQ
--PYAMWH
--PYAMWF
--PYAMVG
--PYAMVE
--PYAMTV
--PYAMTI
--PYAMSK
--PYAMQN
--PYAMQI
--PYAMQA
--PYAMMP
--PYAMMN
--PYAMMD
--PYAMKH
--PYAMII
--PYAMHG
--PYAMGE
--PYAMCV
--PYAMAN
--PYAMAG
--PYAMVP
--PYAMNV
--PYAMND
--PYAMNA
--PYAMKT
--PYAMKN
--PYAMKF
--PYAMIM
--PYAMHH
--PYAMGQ
--PYAMGK

```
--PYAMFE
--PYAMFD
--PYAMEV
--PYAMEA
--PYAMDS
--PYAMDG
--PYAMCT
--PYAMCG
--PYAMAI
-QPYAMV-
-QPYAML-
QHPYAM--
QQPYAM--
-QPYAMK-
QWPYAM--
QRPYAM--
-QPYAMS-
-QPYAMR-
QSPYAM--
-QPYAMW-
-QPYAME-
QLPYAM--
QTPYAM--
-QPYAMN-
QPPYAM--
-QPYAMY-
-QPYAMI-
QKPYAM--
QFPYAM--
QAPYAM--
-QPYAMF-
QDPYAM--
-QPYAMQ-
-QPYAMG-
-QPYAMD-
RSPYAM--
-RPYAMT-
RGPYAM--
RTPYAM--
-RPYAMI-
RQPYAM--
-RPYAMS-
-RPYAMA-
RRPYAM--
-RPYAMV-
RLPYAM--
RVPYAM--
-RPYAMW-
RKPYAM--
RNPYAM--
```

RAPYAM--
-RPYAMN-
-RPYAMY-
-RPYAML-
-RPYAMQ-
RPPYAM--
RFPYAM--
RYPYAM--
-RPYAMK-
-RPYAMG-
-RPYAME-
RHPYAM--
RDPYAM--
-RPYAMD-
RIPYAM--
-RPYAMH-
RMPYAM--
-RPYAMF-
RCPYAM--
-SPYAMS-
-SPYAML-
SRPYAM--
-SPYAMW-
-SPYAMR-
SAPYAM--
-SPYAMQ-
SSPYAM--
-SPYAMY-
SLPYAM--
-SPYAMF-
SWPYAM--
-SPYAMV-
-SPYAMT-
SKPYAM--
SEPYAM--
-SPYAMN-
-SPYAMA-
SQPYAM--
SDPYAM--
-SPYAMI-
SVPYAM--
-SPYAMM-
-SPYAMK-
-SPYAME-
SGPYAM--
SYPYAM--
SMPYAM--
SHPYAM--
SFPYAM--
-SPYAMH-

SPPYAM--
SNPYAM--
SIPYAM--
-TPYAMR-
TRPYAM--
-TPYAML-
TLPYAM--
TSPYAM--
-TPYAMS-
TNPYAM--
-TPYAMM-
-TPYAMK-
-TPYAMI-
TVPYAM--
-TPYAMN-
THPYAM--
TFPYAM--
TEPYAM--
TYPYAM--
TQPYAM--
-TPYAMG-
TTPYAM--
-TPYAMQ-
-TPYAMH-
TGPYAM--
-TPYAMW-
-TPYAMA-
TKPYAM--
TIPYAM--
TAPYAM--
TWPYAM--
-TPYAMV-
TPPYAM--
TMPYAM--
TCPYAM--
-VPYAML-
VRPYAM--
-VPYAMK-
-VPYAMA-
VLPYAM--
VKPYAM--
VGPYAM--
-VPYAMN-
-VPYAMT-
VAPYAM--
VTPYAM--
VIPYAM--
VSPYAM--
-VPYAMG-
VYPYAM--

VVPYAM--
-VPYAME-
-VPYAMS-
VNPYAM--
VWPYAM--
-VPYAMV-
-VPYAMY-
-VPYAMQ-
-VPYAMM-
VHPYAM--
VFPYAM--
-VPYAMW-
VPPYAM--
VDPYAM--
-WPYAMM-
WIPYAM--
-WPYAMS-
-WPYAMR-
-WPYAMA-
WNPYAM--
WSPYAM--
-WPYAMN-
WTPYAM--
WRPYAM--
-WPYAMI-
-WPYAML-
-WPYAMF-
WHPYAM--
-WPYAMY-
-WPYAMT-
-WPYAMQ-
-WPYAMK-
-WPYAMG-
WPPYAM--
WKPYAM--
WLPYAM--
WAPYAM--
YGPYAM--
YRPYAM--
YIPYAM--
-YPYAMR-
-YPYAMM-
-YPYAMK-
YLPYAM--
-YPYAMS-
-YPYAML-
YFPYAM--
-YPYAMQ-
-YPYAMN-
YPPYAM--

	YTPYAM--		
	YSPYAM--		
	YHPYAM--		
	YQPYAM--		
	-YPYAMV-		
	-YPYAMI-		
	-YPYAMG-		
	YCPYAM--		
	YAPYAM--		
	-YPYAMH-		
	YDPYAM--		
PMGI	80	824	49.31
	-APMGIS-		
	ASPMGI--		
	-APMGIL-		
	ANPMGI--		
	AFPMGI--		
	ADPMGI--		
	-APMGIY-		
	ALPMGI--		
	-APMGIR-		
	-APMGIH-		
	AWPMGI--		
	-APMGIF-		
	AAPMGI--		
	AKPMGI--		
	AYPMGI--		
	AVPMGI--		
	ARPMGI--		
	APPMGI--		
	-APMGIQ-		
	-APMGIN-		
	-APMGIK-		
	AQPMGI--		
	-APMGIV-		
	-APMGIG-		
	AHPMGI--		
	-APMGIM-		
	-APMGII-		
	-APMGIA-		
	AIPMGI--		
	AEPMGI--		
	ACPMGI--		
	CPPMGI--		
	-CPMGIL-		
	CIPMGI--		
	-CPMGIR-		
	CLPMGI--		
	CAPMGI--		
	-CPMGIV-		

-CPMGIS-
CVPMGI--
CSPMGI--
CRPMGI--
CMPMGI--
CDPMGI--
DKPMGI--
DRPMGI--
DFPMGI--
-DPMGIM-
DLPMGI--
-DPMGIS-
-DPMGIR-
DVPMGI--
-DPMGIL-
-DPMGIF-
-DPMGIE-
-DPMGIW-
-DPMGIK-
-DPMGII-
-DPMGIA-
DNPMGI--
DGPMGI--
DDPMGI--
-DPMGIT-
DAPMGI--
-EPMGIM-
ERPMGI--
-EPMGIS-
EYPMGI--
-EPMGIR-
-EPMGIH-
-EPMGIL-
EGPMGI--
-EPMGIQ-
-EPMGIN-
-EPMGIA-
EEPMGI--
-EPMGIV-
-EPMGIG-
-EPMGIF-
EMPMGI--
EWPMGI--
ESPMGI--
EVPMGI--
ETPMGI--
EPPMGI--
-EPMGIT-
ENPMGI--
ELPMGI--

EHPMGI--
EDPMGI--
-EPMGIW-
EKPMGI--
FLPMGI--
-FPMGIL-
-FPMGIA-
FSPMGI--
-FPMGIR-
FAPMGI--
FVPMGI--
FFPMGI--
FRPMGI--
FWPMGI--
-FPMGIH-
FKPMGI--
-FPMGIS-
FTPMGI--
-FPMGIV-
-FPMGIF-
FPPMGI--
-FPMGIQ-
-FPMGII-
-FPMGIG-
FMPMGI--
FIPMGI--
FGPMGI--
FQPMGI--
FHPMGI--
FYPMGI--
FNPMGI--
FEPMGI--
-FPMGIY-
-FPMGIT-
GRPMGI--
GMPMGI--
-GPMGIR-
GGPMGI--
GYPMGI--
GWPMGI--
-GPMGIL-
GVPMGI--
-GPMGIM-
GTPMGI--
GKPMGI--
-GPMGIV-
-GPMGIT-
-GPMGIW-
-GPMGIS-
-GPMGIK-

-GPMGIY-
GSPMGI--
GQPMGI--
-GPMGIQ-
-GPMGIF-
-GPMGIN-
-GPMGIH-
GNPMGI--
GIPMGI--
-GPMGII-
GLPMGI--
GPPMGI--
GHPMGI--
-GPMGIG-
-GPMGIE-
GAPMGI--
GFPMGI--
GDPMGI--
GCPMGI--
-HPMGIM-
-HPMGIV-
-HPMGIT-
-HPMGIL-
HLPMGI--
HIPMGI--
HYPMGI--
HSPMGI--
HKPMGI--
HTPMGI--
-HPMGIS-
-HPMGIK-
HRPMGI--
-HPMGIR-
-HPMGIA-
HGPMGI--
HMPMGI--
HVPMGI--
HHPMGI--
HWPMGI--
-HPMGIG-
-HPMGIC-
HFPMGI--
HEPMGI--
HAPMGI--
HPPMGI--
-HPMGIN-
ISPMGI--
-IPMGIR-
-IPMGIL-
-IPMGIK-

ITPMGI--
-IPMGIS-
INPMGI--
-IPMGIN-
-IPMGII-
IIPMGI--
ILPMGI--
-IPMGIV-
IKPMGI--
IQPMGI--
-IPMGIH-
-IPMGIF-
IAPMGI--
IMPMGI--
IRPMGI--
-IPMGIT-
IHPMGI--
IYPMGI--
-IPMGIY-
-IPMGIQ-
IWPMGI--
-IPMGIE-
-IPMGIA-
IEPMGI--
ICPMGI--
-IPMGIM-
IPPMGI--
-IPMGIC-
IGPMGI--
IFPMGI--
KSPMGI--
-KPMGIR-
KYPMGI--
-KPMGIT-
-KPMGII-
-KPMGIL-
KHPMGI--
KGPMGI--
KVPMGI--
-KPMGIG-
-KPMGIQ-
KQPMGI--
-KPMGIF-
KTPMGI--
KRPMGI--
KAPMGI--
KFPMGI--
KMPMGI--
KDPMGI--
-KPMGIM-

−KPMGIW−
−KPMGIK−
KIPMGI−−
−KPMGIV−
−KPMGIY−
KEPMGI−−
−KPMGIE−
−KPMGIA−
LIPMGI−−
LNPMGI−−
−LPMGII−
−LPMGIR−
LMPMGI−−
LAPMGI−−
−LPMGIL−
−LPMGIM−
LKPMGI−−
LGPMGI−−
LSPMGI−−
−LPMGIK−
−LPMGIT−
LRPMGI−−
−LPMGIF−
−LPMGIS−
LEPMGI−−
LDPMGI−−
LWPMGI−−
−LPMGIA−
LHPMGI−−
LFPMGI−−
LCPMGI−−
−LPMGIV−
−LPMGIH−
LQPMGI−−
LTPMGI−−
−LPMGIY−
LVPMGI−−
−LPMGIN−
−LPMGIG−
−LPMGIE−
LPPMGI−−
−LPMGIQ−
LYPMGI−−
−LPMGIW−
MKPMGI−−
−MPMGIR−
MRPMGI−−
MNPMGI−−
MPPMGI−−
−MPMGIV−

MIPMGI--
MSPMGI--
MLPMGI--
MTPMGI--
-MPMGIS-
-MPMGIF-
-MPMGIY-
MHPMGI--
-MPMGIK-
MYPMGI--
MGPMGI--
-MPMGIA-
MMPMGI--
-MPMGIL-
MEPMGI--
MDPMGI--
-MPMGIG-
MCPMGI--
MVPMGI--
MQPMGI--
-MPMGIW-
-MPMGIN-
-MPMGII-
MFPMGI--
MAPMGI--
-NPMGIR-
-NPMGII-
-NPMGIL-
NFPMGI--
-NPMGIT-
NYPMGI--
NQPMGI--
NLPMGI--
NNPMGI--
NKPMGI--
-NPMGIY-
NIPMGI--
NEPMGI--
-NPMGIV-
NHPMGI--
-NPMGIS-
-NPMGIF-
NTPMGI--
NWPMGI--
-NPMGIN-
-NPMGIM-
-NPMGIH-
-NPMGIA-
NVPMGI--
NMPMGI--

NGPMGI--
NPPMGI--
-NPMGIQ-
--PMGIRA
--PMGIRM
--PMGIMG
--PMGILG
--PMGILS
--PMGITM
--PMGILT
--PMGIIR
--PMGIYG
--PMGIWR
--PMGISR
--PMGISQ
--PMGIRG
--PMGIMY
--PMGISM
--PMGIRI
--PMGINS
--PMGIKW
--PMGISA
--PMGIRQ
--PMGIWQ
--PMGIRY
--PMGILY
PWPMGI--
--PMGIVA
--PMGILH
--PMGIKT
--PMGIEG
--PMGITY
--PMGIRR
--PMGIGS
--PMGIQV
PYPMGI--
--PMGISN
--PMGIHI
--PMGIYS
--PMGITL
--PMGIVH
--PMGITR
--PMGIST
--PMGISG
--PMGIRE
--PMGIQS
--PMGIHS
--PMGIHL
--PMGIAT
--PMGISH

--PMGIYR
--PMGIVY
--PMGIVG
--PMGILM
--PMGIKG
PHPMGI--
PTPMGI--
PSPMGI--
--PMGIVR
--PMGILK
--PMGIGA
--PMGIFG
--PMGIAA
--PMGITS
--PMGIRD
--PMGIMT
--PMGIFR
PAPMGI--
--PMGIYH
--PMGIVS
--PMGITV
--PMGISW
--PMGIHR
--PMGIRF
--PMGIQR
--PMGIMS
--PMGIGG
--PMGIWG
--PMGISK
--PMGIRW
--PMGIRT
--PMGIKF
--PMGIFF
--PMGIRH
--PMGIMQ
--PMGILW
--PMGILN
--PMGIIW
--PMGIIS
--PMGIII
PVPMGI--
--PMGIVT
--PMGITK
--PMGISV
--PMGINA
--PMGIHW
--PMGIHG
--PMGIAR
--PMGIAG
PIPMGI--

PMPMGI--
--PMGIKR
--PMGIKH
--PMGIHA
--PMGIEY
--PMGIAS
--PMGIAL
--PMGIYM
--PMGIWN
--PMGIVW
--PMGINR
--PMGINM
--PMGINK
--PMGIMI
--PMGIME
--PMGILV
--PMGIKI
--PMGIIY
--PMGIFQ
--PMGIFN
--PMGIFK
--PMGIEW
--PMGISL
--PMGIQF
--PMGIQA
--PMGINV
--PMGIAH
--PMGIAF
--PMGIVI
--PMGITT
--PMGISI
--PMGISE
--PMGISD
--PMGIQQ
--PMGINW
--PMGILE
--PMGIKK
--PMGIFT
--PMGIFS
--PMGIWS
--PMGIWK
--PMGIVF
--PMGIRK
--PMGIQI
--PMGIMN
--PMGIIN
--PMGIHV
--PMGIHQ
--PMGIHH
--PMGIEQ

--PMGIAQ
PGPMGI--
--PMGIVL
--PMGITG
--PMGITD
--PMGIMH
--PMGIKY
--PMGIKN
--PMGIKL
--PMGIIK
--PMGIIH
--PMGIIG
--PMGIID
--PMGIIA
--PMGIHT
--PMGIEK
--PMGIAW
--PMGIYT
--PMGIWT
--PMGIWD
--PMGIVK
--PMGIVD
--PMGITF
--PMGIQE
--PMGINT
--PMGING
--PMGILR
--PMGILQ
--PMGIKV
--PMGIIV
--PMGIHF
--PMGIEA
--PMGIWL
--PMGIWE
--PMGIVQ
--PMGISY
--PMGIRP
--PMGIQT
--PMGIMV
--PMGIMR
--PMGIKM
--PMGIHM
--PMGIHK
--PMGIGQ
--PMGIFH
--PMGIFA
--PMGIES
--PMGIDG
--PMGICS
PEPMGI--

--PMGIYK
--PMGIVN
--PMGITQ
--PMGITN
--PMGITE
--PMGITA
--PMGINQ
--PMGIMM
--PMGIMK
--PMGILD
--PMGIIF
--PMGIHD
--PMGIGL
--PMGIFD
--PMGIDS
--PMGIAY
--PMGIAN
--PMGIAK
PFPMGI--
--PMGIYN
--PMGIYF
--PMGIYE
--PMGIYA
--PMGIVM
--PMGITW
--PMGITH
--PMGIQY
--PMGINH
--PMGIKQ
--PMGIIL
--PMGIHN
--PMGIGY
--PMGIGV
--PMGIGT
--PMGIGR
--PMGIGM
--PMGIGI
--PMGIGE
--PMGIDA
--PMGIAE
--PMGIAD
--PMGIWM
--PMGIRC
--PMGIQG
--PMGINF
--PMGINC
--PMGILC
--PMGIKP
--PMGIIQ
--PMGIGP

--PMGIGK
--PMGIFW
--PMGIFE
--PMGIFC
--PMGIEV
--PMGIET
--PMGIER
--PMGIDW
--PMGIDL
--PMGIDH
--PMGICY
--PMGICN
--PMGICF
--PMGIAM
PDPMGI--
QNPMGI--
QSPMGI--
-QPMGIL-
QLPMGI--
-QPMGIN-
QGPMGI--
QVPMGI--
QRPMGI--
-QPMGIR-
-QPMGIW-
QKPMGI--
QFPMGI--
-QPMGIE-
-QPMGIT-
QTPMGI--
QYPMGI--
-QPMGIY-
-QPMGII-
QWPMGI--
-QPMGIV-
-QPMGIS-
-QPMGIF-
QMPMGI--
QDPMGI--
QQPMGI--
QIPMGI--
-QPMGIQ-
RRPMGI--
-RPMGIL-
RLPMGI--
RSPMGI--
-RPMGIT-
RMPMGI--
-RPMGIS-
-RPMGIM-

RGPMGI--
-RPMGIV-
RAPMGI--
RTPMGI--
RVPMGI--
RPPMGI--
RKPMGI--
-RPMGIN-
-RPMGIH-
-RPMGIK-
RIPMGI--
RWPMGI--
-RPMGIA-
RQPMGI--
-RPMGIG-
RFPMGI--
REPMGI--
-RPMGIY-
-RPMGIQ-
RNPMGI--
-RPMGII-
-RPMGIW-
-RPMGIE-
-RPMGIC-
RDPMGI--
SRPMGI--
-SPMGIV-
-SPMGIR-
-SPMGIL-
SYPMGI--
SLPMGI--
SSPMGI--
STPMGI--
SMPMGI--
-SPMGIT-
-SPMGIK-
SEPMGI--
SVPMGI--
SIPMGI--
SAPMGI--
-SPMGIF-
SFPMGI--
-SPMGIS-
-SPMGII-
SKPMGI--
SGPMGI--
SNPMGI--
-SPMGIA-
SHPMGI--
SDPMGI--

-SPMGIY-
-SPMGIH-
SQPMGI--
-SPMGIW-
-SPMGIM-
-SPMGIQ-
-SPMGIN-
-SPMGIG-
-SPMGIE-
-SPMGID-
TRPMGI--
TIPMGI--
-TPMGIL-
-TPMGIM-
TMPMGI--
TGPMGI--
TTPMGI--
TSPMGI--
-TPMGIH-
-TPMGIS-
TQPMGI--
TNPMGI--
-TPMGIK-
-TPMGIF-
TDPMGI--
TLPMGI--
TKPMGI--
TFPMGI--
TCPMGI--
-TPMGIY-
-TPMGIT-
TYPMGI--
-TPMGIV-
-TPMGII-
TWPMGI--
TEPMGI--
TAPMGI--
-TPMGIW-
THPMGI--
TPPMGI--
-TPMGIN-
-TPMGIG-
TVPMGI--
-TPMGIP-
-VPMGIR-
-VPMGIL-
-VPMGIF-
VKPMGI--
VQPMGI--
VMPMGI--

```
VVPMGI--
-VPMGIT-
VLPMGI--
-VPMGIS-
VRPMGI--
VGPMGI--
VSPMGI--
VHPMGI--
-VPMGII-
-VPMGIV-
-VPMGIK-
-VPMGIY-
-VPMGIA-
-VPMGIN-
VWPMGI--
VPPMGI--
VYPMGI--
VTPMGI--
VEPMGI--
VAPMGI--
-VPMGIQ-
-VPMGIW-
VFPMGI--
-VPMGIM-
VDPMGI--
-VPMGIH-
VIPMGI--
WRPMGI--
WLPMGI--
WVPMGI--
-WPMGIR-
WYPMGI--
WSPMGI--
-WPMGIL-
-WPMGIV-
WDPMGI--
WTPMGI--
-WPMGIQ-
-WPMGIH-
WHPMGI--
-WPMGIS-
WKPMGI--
WWPMGI--
-WPMGIT-
-WPMGIN-
-WPMGIM-
WNPMGI--
WMPMGI--
WPPMGI--
WEPMGI--
```

WQPMGI--
-WPMGIY-
-WPMGIE-
-WPMGIA-
WGPMGI--
YSPMGI--
-YPMGIL-
YVPMGI--
-YPMGIK-
-YPMGIH-
YNPMGI--
YKPMGI--
YIPMGI--
-YPMGIS-
YTPMGI--
YLPMGI--
YFPMGI--
YWPMGI--
YRPMGI--
-YPMGIQ-
-YPMGIE-
-YPMGIT-
YPPMGI--
-YPMGIG-
YMPMGI--
YQPMGI--
-YPMGIW-
-YPMGII-
-YPMGIR-
YDPMGI--
-YPMGIY-
YEPMGI--
YYPMGI--
-YPMGIN-
YGPMGI--
-YPMGIV-

PFNM 355 674 49.30

-SPFNMY-
-APFNMR-
AGPFNM--
AAPFNM--
ASPFNM--
-APFNMS-
AFPFNM--
-APFNMK-
ALPFNM--
ADPFNM--
-APFNMY-
-APFNMM-
AYPFNM--

AVPFNM--
AWPFNM--
ATPFNM--
ANPFNM--
-APFNMD-
-APFNMA-
-APFNMN-
-APFNMI-
ACPFNM--
CEPFNM--
CSPFNM--
CNPFNM--
CVPFNM--
-CPFNMA-
CGPFNM--
CTPFNM--
CRPFNM--
-CPFNMY-
-CPFNMV-
-CPFNMM-
-CPFNMS-
-CPFNMP-
-CPFNML-
-CPFNMF-
DVPFNM--
-DPFNMR-
DQPFNM--
DWPFNM--
-DPFNMV-
-DPFNML-
-DPFNMH-
DTPFNM--
-DPFNMY-
-DPFNMS-
DMPFNM--
DIPFNM--
DFPFNM--
-EPFNML-
EYPFNM--
-EPFNMV-
ESPFNM--
ELPFNM--
-EPFNMW-
-EPFNMY-
EHPFNM--
-EPFNMK-
-EPFNMA-
-EPFNMR-
-EPFNMM-
-EPFNMG-

EKPFNM--
EWPFNM--
ETPFNM--
-EPFNMN-
-EPFNMF-
EVPFNM--
-EPFNMQ-
-EPFNMI-
EIPFNM--
-FPFNMR-
FHPFNM--
FGPFNM--
FRPFNM--
-FPFNMK-
-FPFNMM-
-FPFNMA-
-FPFNMT-
-FPFNMN-
FDPFNM--
FNPFNM--
FPPFNM--
-FPFNML-
FFPFNM--
FSPFNM--
FAPFNM--
FVPFNM--
FTPFNM--
-FPFNMW-
-FPFNMS-
-FPFNMQ-
-FPFNME-
FMPFNM--
-FPFNMV-
-FPFNMH-
-FPFNMG-
GSPFNM--
-GPFNML-
GTPFNM--
-GPFNMK-
GLPFNM--
GEPFNM--
GAPFNM--
-GPFNMR-
GFPFNM--
GYPFNM--
-GPFNMV-
GVPFNM--
-GPFNMH-
-GPFNME-
GNPFNM--

GHPFNM--
-GPFNMQ-
-GPFNMG-
-GPFNMT-
-GPFNMC-
GMPFNM--
GRPFNM--
-GPFNMY-
-GPFNMS-
-GPFNMM-
-GPFNMI-
GGPFNM--
HGPFNM--
HRPFNM--
-HPFNMS-
HQPFNM--
HSPFNM--
-HPFNML-
-HPFNMI-
HVPFNM--
HTPFNM--
-HPFNMA-
HLPFNM--
HKPFNM--
HEPFNM--
HWPFNM--
-HPFNMW-
-HPFNMM-
HDPFNM--
-HPFNMV-
-HPFNMQ-
-HPFNMK-
HNPFNM--
HFPFNM--
HAPFNM--
HYPFNM--
HPPFNM--
-HPFNMY-
HIPFNM--
IRPFNM--
-IPFNMM-
-IPFNML-
-IPFNMK-
IKPFNM--
-IPFNMN-
-IPFNMG-
-IPFNMA-
ISPFNM--
IAPFNM--
ILPFNM--

IEPFNM--
ICPFNM--
IVPFNM--
-IPFNMS-
ITPFNM--
IHPFNM--
IGPFNM--
IFPFNM--
IYPFNM--
-IPFNMR-
-IPFNMQ-
-IPFNMH-
-IPFNME-
INPFNM--
IDPFNM--
KKPFNM--
KSPFNM--
-KPFNMV-
-KPFNMS-
-KPFNMM-
-KPFNMR-
-KPFNMH-
KMPFNM--
-KPFNMW-
KLPFNM--
-KPFNML-
KGPFNM--
KVPFNM--
-KPFNMI-
-KPFNMA-
-KPFNMK-
KRPFNM--
-KPFNMN-
KFPFNM--
-KPFNMD-
-KPFNMT-
KIPFNM--
KDPFNM--
KTPFNM--
KPPFNM--
KWPFNM--
KNPFNM--
LWPFNM--
-LPFNMT-
LVPFNM--
-LPFNMS-
LMPFNM--
-LPFNMY-
LNPFNM--
-LPFNMR-

LAPFNM--
LPPFNM--
-LPFNMV-
LYPFNM--
LTPFNM--
LRPFNM--
-LPFNME-
LFPFNM--
LEPFNM--
LKPFNM--
LIPFNM--
LDPFNM--
LSPFNM--
-LPFNML-
-LPFNMK-
-LPFNMW-
-LPFNMQ-
-LPFNMH-
-LPFNMA-
LLPFNM--
LGPFNM--
-LPFNMG-
LCPFNM--
LQPFNM--
-LPFNMN-
-LPFNMM-
-LPFNMI-
LHPFNM--
-MPFNMR-
MVPFNM--
-MPFNMS-
-MPFNMA-
-MPFNMT-
MKPFNM--
-MPFNMV-
MDPFNM--
MRPFNM--
MLPFNM--
-MPFNMY-
MFPFNM--
-MPFNMH-
MNPFNM--
-MPFNMN-
-MPFNMK-
MMPFNM--
MGPFNM--
MSPFNM--
-MPFNME-
MIPFNM--
MWPFNM--

MTPFNM--
-MPFNMQ-
-MPFNMG-
NPPFNM--
NSPFNM--
NNPFNM--
NRPFNM--
-NPFNMR-
-NPFNMW-
-NPFNMM-
NAPFNM--
NTPFNM--
NEPFNM--
NMPFNM--
-NPFNMS-
NVPFNM--
NFPFNM--
NYPFNM--
NWPFNM--
-NPFNMQ-
-NPFNMF-
-NPFNMY-
-NPFNMI-
NIPFNM--
NGPFNM--
-NPFNMT-
-NPFNMK-
--PFNMSK
--PFNMSG
--PFNMVS
--PFNMSL
--PFNMRR
--PFNMRT
--PFNMLS
--PFNMSW
--PFNMLG
--PFNMFS
--PFNMRW
--PFNMTT
--PFNMLR
--PFNMSR
--PFNMLH
--PFNMVK
--PFNMVA
--PFNMMA
--PFNMMR
--PFNMLN
--PFNMER
--PFNMGS
PMPFNM--

--PFNMRI
--PFNMTM
--PFNMRK
--PFNMNF
--PFNMRN
--PFNMEG
--PFNMTG
--PFNMSY
--PFNMSA
--PFNMGG
--PFNMFR
--PFNMFH
--PFNMYE
--PFNMRF
--PFNMQM
--PFNMQI
--PFNMFL
--PFNMFK
--PFNMAS
--PFNMYN
--PFNMVR
--PFNMVG
--PFNMTI
--PFNMSQ
--PFNMSH
--PFNMRE
--PFNMQT
--PFNMYR
--PFNMSS
--PFNMSN
--PFNMRA
--PFNMNA
--PFNMLT
--PFNMGT
--PFNMMH
--PFNMLE
--PFNMLA
--PFNMKS
--PFNMIR
--PFNMEA
PKPFNM--
--PFNMYS
--PFNMYH
--PFNMTW
--PFNMST
--PFNMMT
--PFNMHI
--PFNMHA
--PFNMEL
--PFNMAT

PYPFNM--
--PFNMYK
--PFNMWT
--PFNMTS
--PFNMTN
--PFNMSI
--PFNMML
--PFNMKT
--PFNMHS
--PFNMHM
PIPFNM--
--PFNMSE
--PFNMKI
--PFNMIS
PAPFNM--
--PFNMWI
--PFNMWE
--PFNMVY
--PFNMVI
--PFNMTF
--PFNMTD
--PFNMNR
--PFNMMS
--PFNMKR
--PFNMIN
--PFNMGW
--PFNMGM
--PFNMGK
--PFNMFF
--PFNMEQ
--PFNMAA
PQPFNM--
PGPFNM--
PFPFNM--
--PFNMWS
--PFNMTV
--PFNMTR
--PFNMTH
--PFNMSF
--PFNMRY
--PFNMRH
--PFNMRD
--PFNMNY
--PFNMNS
--PFNMNI
--PFNMND
--PFNMMK
--PFNMIT
--PFNMHT
--PFNMEN

```
--PFNMEI
--PFNMDS
--PFNMAL
--PFNMAH
--PFNMYG
--PFNMQS
--PFNMQL
--PFNMNT
--PFNMMV
--PFNMMM
--PFNMLQ
--PFNMLD
--PFNMHG
--PFNMGV
--PFNMGR
--PFNMGN
--PFNMEV
--PFNMAW
--PFNMAR
--PFNMAN
--PFNMAK
PTPFNM--
--PFNMYA
--PFNMWR
--PFNMWD
--PFNMTK
--PFNMTE
--PFNMSV
--PFNMRQ
--PFNMRM
--PFNMQG
--PFNMQF
--PFNMNW
--PFNMNL
--PFNMNE
--PFNMMQ
--PFNMMD
--PFNMLK
--PFNMKN
--PFNMIW
--PFNMHR
--PFNMHH
--PFNMGL
--PFNMGF
--PFNMFA
--PFNMCH
-PPFNMY-
-PPFNMF-
--PFNMYY
--PFNMYT
```

--PFNMWM
--PFNMWA
--PFNMVM
--PFNMTY
--PFNMTA
--PFNMQV
--PFNMQR
--PFNMPS
--PFNMNV
--PFNMNQ
--PFNMNH
--PFNMLP
--PFNMKH
--PFNMIV
--PFNMIG
--PFNMIA
--PFNMHV
--PFNMHN
--PFNMHD
--PFNMGY
--PFNMFE
--PFNMFD
--PFNMEM
--PFNMDN
--PFNMAV
--PFNMAG
QLPFNM--
QAPFNM--
-QPFNMS-
QYPFNM--
-QPFNMY-
-QPFNML-
QIPFNM--
QPPFNM--
QKPFNM--
-QPFNMT-
-QPFNMR-
-QPFNMH-
QMPFNM--
QWPFNM--
QSPFNM--
-QPFNMW-
-QPFNMG-
QNPFNM--
QCPFNM--
-QPFNMK-
-QPFNMF-
QGPFNM--
QVPFNM--
-QPFNMN-

QRPFNM--
-QPFNMM-
-QPFNME-
-RPFNMS-
-RPFNML-
RTPFNM--
RIPFNM--
-RPFNMW-
RVPFNM--
-RPFNMV-
RGPFNM--
RSPFNM--
RPPFNM--
-RPFNMK-
RRPFNM--
RKPFNM--
-RPFNMF-
RMPFNM--
RQPFNM--
RNPFNM--
REPFNM--
-RPFNMN-
RHPFNM--
RAPFNM--
-RPFNMM-
RWPFNM--
-RPFNMY-
-RPFNMG-
RYPFNM--
RDPFNM--
-SPFNMV-
SSPFNM--
SIPFNM--
SAPFNM--
SGPFNM--
SRPFNM--
SYPFNM--
SFPFNM--
-SPFNMW-
STPFNM--
-SPFNMK-
-SPFNMT-
-SPFNMS-
SVPFNM--
-SPFNMF-
SNPFNM--
-SPFNMA-
-SPFNMQ-
SHPFNM--
SQPFNM--

```
-SPFNMM-
SDPFNM--
-SPFNMG-
-SPFNMI-
SKPFNM--
-SPFNMN-
-SPFNMH-
TFPFNM--
-TPFNMS-
TNPFNM--
-TPFNMR-
TPPFNM--
-TPFNMQ-
-TPFNMM-
TLPFNM--
THPFNM--
TSPFNM--
TWPFNM--
-TPFNMN-
TVPFNM--
TRPFNM--
-TPFNMH-
-TPFNMI-
-TPFNMA-
TKPFNM--
TIPFNM--
TGPFNM--
TEPFNM--
-TPFNMF-
TYPFNM--
-TPFNMW-
-TPFNML-
TTPFNM--
-TPFNMK-
-TPFNMG-
TMPFNM--
VGPFNM--
-VPFNMR-
VTPFNM--
-VPFNML-
VYPFNM--
-VPFNMA-
VVPFNM--
-VPFNMS-
VWPFNM--
VMPFNM--
VRPFNM--
VQPFNM--
-VPFNMM-
-VPFNMQ-
```

VKPFNM--
VDPFNM--
-VPFNMN-
VEPFNM--
-VPFNMH-
VIPFNM--
-VPFNMY-
-VPFNME-
-VPFNMT-
-VPFNMG-
-VPFNMF-
VNPFNM--
VAPFNM--
VLPFNM--
-VPFNMI-
VCPFNM--
WEPFNM--
WRPFNM--
WFPFNM--
WSPFNM--
-WPFNMA-
WPPFNM--
-WPFNME-
WGPFNM--
WTPFNM--
-WPFNMV-
-WPFNMT-
WIPFNM--
WVPFNM--
WQPFNM--
-WPFNMS-
WNPFNM--
WMPFNM--
WDPFNM--
YRPFNM--
YMPFNM--
YQPFNM--
-YPFNMR-
-YPFNMW-
-YPFNMH-
-YPFNMM-
-YPFNMF-
YIPFNM--
YVPFNM--
-YPFNMQ-
-YPFNMN-
-YPFNMK-
YAPFNM--
YDPFNM--
YSPFNM--

	-YPFNMI-		
	YKPFNM--		
	YEPFNM--		
	YTPFNM--		
	-YPFNMY-		
	YNPFNM--		
	YWPFNM--		
	YPPFNM--		
	-YPFNMG-		
	-YPFNME-		
	-YPFNMA-		
PLAM	87	818	49.29
	-APLAMR-		
	ATPLAM--		
	AHPLAM--		
	AVPLAM--		
	AGPLAM--		
	-APLAMA-		
	ALPLAM--		
	-APLAMV-		
	ARPLAM--		
	-APLAMH-		
	ASPLAM--		
	-APLAML-		
	AIPLAM--		
	-APLAMW-		
	-APLAMS-		
	-APLAMN-		
	-APLAMI-		
	AQPLAM--		
	AYPLAM--		
	AKPLAM--		
	-APLAMK-		
	ANPLAM--		
	AAPLAM--		
	AMPLAM--		
	APPLAM--		
	-APLAMY-		
	-APLAMM-		
	-APLAMD-		
	-APLAMT-		
	-APLAMF-		
	-APLAMG-		
	-APLAME-		
	-APLAMQ-		
	ADPLAM--		
	-APLAMC-		
	CRPLAM--		
	CVPLAM--		
	CQPLAM--		

-CPLAMS-
CEPLAM--
CPPLAM--
CYPLAM--
-CPLAMR-
CLPLAM--
CIPLAM--
CAPLAM--
-CPLAMM-
-CPLAMA-
CGPLAM--
CDPLAM--
CTPLAM--
-CPLAMY-
-CPLAMH-
CKPLAM--
DSPLAM--
DNPLAM--
DRPLAM--
DMPLAM--
DFPLAM--
-DPLAMH-
-DPLAMY-
DLPLAM--
DWPLAM--
DVPLAM--
-DPLAML-
-DPLAMF-
-DPLAMW-
-DPLAMM-
DDPLAM--
DAPLAM--
DKPLAM--
DHPLAM--
-DPLAMN-
-DPLAMK-
-DPLAMA-
DTPLAM--
-DPLAMT-
-DPLAMQ-
DYPLAM--
-DPLAMS-
-DPLAMG-
-DPLAMI-
ERPLAM--
-EPLAML-
EVPLAM--
-EPLAMR-
ELPLAM--
EYPLAM--

```
-EPLAMI-
EAPLAM--
ETPLAM--
-EPLAMQ-
-EPLAMS-
-EPLAMY-
EGPLAM--
-EPLAMW-
ENPLAM--
EIPLAM--
-EPLAMV-
-EPLAMG-
EQPLAM--
-EPLAMH-
ESPLAM--
-EPLAMN-
EHPLAM--
EWPLAM--
-EPLAMT-
-EPLAMK-
EKPLAM--
EFPLAM--
EDPLAM--
ECPLAM--
-FPLAMR-
-FPLAMS-
FGPLAM--
-FPLAMG-
-FPLAMA-
-FPLAMK-
FKPLAM--
FVPLAM--
FEPLAM--
FYPLAM--
FSPLAM--
FPPLAM--
FHPLAM--
FFPLAM--
-FPLAMI-
-FPLAMN-
-FPLAMM-
FTPLAM--
FNPLAM--
FIPLAM--
-FPLAMQ-
FAPLAM--
-FPLAME-
FQPLAM--
-FPLAMD-
FDPLAM--
```

FWPLAM--
-FPLAMT-
GRPLAM--
-GPLAMS-
GYPLAM--
GVPLAM--
GSPLAM--
-GPLAMR-
-GPLAMV-
GIPLAM--
GHPLAM--
GWPLAM--
-GPLAMH-
GFPLAM--
-GPLAMT-
GNPLAM--
GAPLAM--
-GPLAMI-
-GPLAMA-
GGPLAM--
GKPLAM--
GTPLAM--
-GPLAMQ-
-GPLAMM-
-GPLAMK-
GLPLAM--
-GPLAMY-
GQPLAM--
-GPLAMW-
-GPLAMN-
-GPLAMF-
GEPLAM--
GMPLAM--
GDPLAM--
-GPLAMG-
-GPLAME-
-GPLAMD-
-HPLAML-
HLPLAM--
HSPLAM--
HTPLAM--
HRPLAM--
HVPLAM--
-HPLAMH-
-HPLAMK-
HNPLAM--
-HPLAMR-
HYPLAM--
-HPLAMW-
-HPLAMA-

HMPLAM--
HKPLAM--
HGPLAM--
-HPLAMT-
HFPLAM--
HEPLAM--
HAPLAM--
-HPLAMY-
-HPLAMS-
-HPLAMN-
-HPLAME-
HHPLAM--
HPPLAM--
-HPLAMV-
-HPLAMG-
-HPLAMI-
-HPLAMD-
HIPLAM--
-IPLAMT-
ITPLAM--
IIPLAM--
ILPLAM--
ISPLAM--
-IPLAMH-
-IPLAMA-
IAPLAM--
-IPLAMW-
-IPLAMK-
IQPLAM--
-IPLAMF-
IDPLAM--
IWPLAM--
-IPLAMI-
IEPLAM--
-IPLAMQ-
IMPLAM--
IFPLAM--
ICPLAM--
IVPLAM--
-IPLAMG-
-IPLAME-
INPLAM--
KSPLAM--
KVPLAM--
KGPLAM--
KAPLAM--
-KPLAMV-
-KPLAMR-
-KPLAMS-
-KPLAMH-

-KPLAMM-
KPPLAM--
-KPLAMF-
-KPLAMA-
-KPLAMK-
KYPLAM--
-KPLAMD-
-KPLAMQ-
-KPLAMN-
KWPLAM--
KMPLAM--
KLPLAM--
KIPLAM--
-KPLAMY-
-KPLAMI-
-KPLAMG-
KNPLAM--
KTPLAM--
KKPLAM--
KQPLAM--
-KPLAME-
-KPLAMW-
-LPLAMV-
LRPLAM--
LIPLAM--
-LPLAMS-
-LPLAMK-
LVPLAM--
LSPLAM--
LKPLAM--
-LPLAMG-
LHPLAM--
LFPLAM--
LAPLAM--
-LPLAMT-
-LPLAMM-
LDPLAM--
LPPLAM--
LWPLAM--
LEPLAM--
LMPLAM--
-LPLAMQ-
-LPLAMH-
LYPLAM--
-LPLAMN-
LGPLAM--
-LPLAMW-
-LPLAME-
-LPLAMC-
-LPLAMD-

−MPLAMS−
MLPLAM−−
MHPLAM−−
−MPLAMR−
MRPLAM−−
−MPLAMG−
−MPLAMY−
−MPLAMQ−
MMPLAM−−
−MPLAMI−
MIPLAM−−
MNPLAM−−
MVPLAM−−
MQPLAM−−
−MPLAME−
−MPLAMH−
−MPLAMN−
MFPLAM−−
MAPLAM−−
MDPLAM−−
−MPLAMV−
MEPLAM−−
MSPLAM−−
MKPLAM−−
MYPLAM−−
MWPLAM−−
−MPLAMC−
−MPLAMA−
MCPLAM−−
NLPLAM−−
−NPLAMR−
−NPLAMW−
−NPLAMI−
−NPLAMS−
NRPLAM−−
NVPLAM−−
−NPLAMT−
NNPLAM−−
−NPLAMF−
NIPLAM−−
NSPLAM−−
−NPLAMQ−
NYPLAM−−
−NPLAMK−
NEPLAM−−
−NPLAMH−
NAPLAM−−
−NPLAMY−
NHPLAM−−
−NPLAML−

NFPLAM--
NGPLAM--
-NPLAMV-
NMPLAM--
NWPLAM--
NPPLAM--
-NPLAMA-
-NPLAMM-
-NPLAME-
NDPLAM--
NKPLAM--
-NPLAMG-
--PLAMTE
--PLAMRN
--PLAMKT
--PLAMVS
--PLAMVR
--PLAMRT
--PLAMNR
--PLAMLG
--PLAMWR
--PLAMTW
--PLAMTL
--PLAMMK
--PLAMAL
--PLAMVA
--PLAMLD
--PLAMMT
--PLAMNA
--PLAMMR
--PLAMLA
--PLAMTG
--PLAMSK
--PLAMVG
--PLAMTI
--PLAMGL
--PLAMEG
--PLAMKS
--PLAMKL
--PLAMTH
--PLAMIG
--PLAMGG
--PLAMMG
--PLAMKQ
--PLAMVT
--PLAMTV
--PLAMTA
--PLAMRA
--PLAMQS
--PLAMST

--PLAMSG
--PLAMNV
--PLAMHG
--PLAMSA
--PLAMHL
--PLAMAR
PRPLAM--
--PLAMWN
--PLAMVV
--PLAMQL
--PLAMSI
--PLAMNY
--PLAMKG
--PLAMHV
--PLAMRI
--PLAMNL
--PLAMLH
--PLAMGR
--PLAMEK
--PLAMIT
--PLAMAS
--PLAMYQ
--PLAMRV
--PLAMRH
--PLAMMS
--PLAMFQ
--PLAMAN
--PLAMSF
--PLAMKN
--PLAMSV
--PLAMQG
--PLAMKA
--PLAMHA
--PLAMFS
--PLAMEW
--PLAMVN
--PLAMTN
--PLAMRP
--PLAMQR
--PLAMQN
--PLAMTM
--PLAMSN
--PLAMGK
--PLAMDS
--PLAMTK
--PLAMNK
--PLAMHR
--PLAMHI
--PLAMGM
--PLAMFN

--PLAMAT
--PLAMLN
--PLAMQQ
--PLAMGT
--PLAMFG
--PLAMAM
--PLAMVE
--PLAMQH
--PLAMNS
--PLAMML
--PLAMLM
--PLAMGV
--PLAMGH
--PLAMFH
PTPLAM--
--PLAMVH
--PLAMRY
--PLAMPL
--PLAMKY
--PLAMKW
--PLAMHQ
--PLAMAI
--PLAMYN
--PLAMWQ
--PLAMSD
--PLAMRQ
--PLAMRE
--PLAMRD
--PLAMQV
--PLAMNN
PGPLAM--
--PLAMWG
--PLAMVD
--PLAMTD
--PLAMSH
--PLAMQK
--PLAMQA
--PLAMNI
--PLAMLE
--PLAMHF
--PLAMGF
--PLAMER
--PLAMAY
PHPLAM--
--PLAMYS
--PLAMYG
--PLAMWF
--PLAMVM
--PLAMVI
--PLAMTY

--PLAMKF
--PLAMID
--PLAMHH
--PLAMGD
PEPLAM--
PNPLAM--
--PLAMYH
--PLAMYA
--PLAMWK
--PLAMTF
--PLAMSQ
--PLAMLQ
--PLAMKD
--PLAMFE
--PLAMFD
--PLAMEL
--PLAMEH
--PLAMAG
--PLAMVY
--PLAMSY
--PLAMSW
--PLAMQT
--PLAMNW
--PLAMMM
--PLAMMH
--PLAMKI
--PLAMIW
--PLAMGQ
--PLAMET
--PLAMAH
--PLAMYD
--PLAMWH
--PLAMSE
--PLAMQI
--PLAMQE
--PLAMQD
--PLAMMA
--PLAMKV
--PLAMHK
--PLAMGW
--PLAMGA
--PLAMDA
--PLAMAK
--PLAMYP
--PLAMPY
--PLAMKH
--PLAMIN
--PLAMIA
--PLAMHY
--PLAMHW

--PLAMYK
--PLAMQF
--PLAMPM
--PLAMNM
--PLAMHS
--PLAMHM
--PLAMEI
--PLAMDM
--PLAMCL
--PLAMYE
--PLAMVC
--PLAMRM
--PLAMRF
--PLAMIH
--PLAMEV
--PLAMDW
--PLAMAV
PDPLAM--
PCPLAM--
--PLAMVP
--PLAMPI
--PLAMNQ
--PLAMMN
--PLAMLC
--PLAMKE
--PLAMIV
--PLAMES
--PLAMEN
--PLAMDT
--PLAMCS
--PLAMAE
--PLAMAA
--PLAMNG
--PLAMMP
--PLAMKC
--PLAMHD
--PLAMCY
--PLAMAW
-PPLAMV-
-PPLAMG-
--PLAMYM
--PLAMWP
--PLAMWD
--PLAMWA
--PLAMTC
--PLAMQY
--PLAMNT
--PLAMMD
--PLAMHT
--PLAMFP

```
--PLAMEE
--PLAMED
--PLAMDY
--PLAMDV
--PLAMDG
--PLAMCT
--PLAMCK
--PLAMAQ
--PLAMAD
-QPLAMK-
-QPLAML-
QRPLAM--
QWPLAM--
QPPLAM--
-QPLAMH-
QVPLAM--
-QPLAMY-
-QPLAMR-
-QPLAMT-
-QPLAMI-
QLPLAM--
-QPLAMS-
QYPLAM--
QFPLAM--
-QPLAMW-
-QPLAMN-
-QPLAMM-
-QPLAMV-
QSPLAM--
-QPLAMG-
QTPLAM--
QNPLAM--
QIPLAM--
QHPLAM--
-QPLAMF-
QGPLAM--
QCPLAM--
-QPLAMA-
QEPLAM--
-QPLAMQ-
-QPLAME-
QDPLAM--
QAPLAM--
RSPLAM--
-RPLAMW-
RLPLAM--
RGPLAM--
-RPLAMK-
-RPLAMY-
RYPLAM--
```

-RPLAMI-
RAPLAM--
RFPLAM--
REPLAM--
RNPLAM--
RKPLAM--
RIPLAM--
RVPLAM--
RTPLAM--
-RPLAMV-
-RPLAMH-
-RPLAMG-
-RPLAME-
RMPLAM--
-RPLAMM-
RWPLAM--
-RPLAMQ-
RPPLAM--
RDPLAM--
-RPLAMD-
RCPLAM--
RQPLAM--
-RPLAMP-
-RPLAMC-
-SPLAMR-
SSPLAM--
-SPLAMA-
SVPLAM--
-SPLAMV-
-SPLAMK-
-SPLAMY-
SYPLAM--
-SPLAMW-
SWPLAM--
-SPLAMM-
SHPLAM--
SFPLAM--
-SPLAMH-
-SPLAMF-
-SPLAMN-
SPPLAM--
SGPLAM--
SNPLAM--
STPLAM--
SLPLAM--
-SPLAMI-
SDPLAM--
-SPLAME-
SIPLAM--
SQPLAM--

-SPLAMG-
SAPLAM--
-SPLAMQ-
SMPLAM--
SCPLAM--
SKPLAM--
-SPLAMD-
-SPLAMC-
SEPLAM--
-TPLAMR-
TSPLAM--
-TPLAMN-
-TPLAMI-
TLPLAM--
-TPLAMY-
-TPLAMH-
-TPLAME-
-TPLAMM-
TVPLAM--
TNPLAM--
TRPLAM--
-TPLAMV-
TAPLAM--
TTPLAM--
TPPLAM--
-TPLAMQ-
-TPLAMF-
TWPLAM--
-TPLAMS-
-TPLAMG-
TIPLAM--
TDPLAM--
TMPLAM--
-TPLAMT-
THPLAM--
TGPLAM--
TEPLAM--
TFPLAM--
TQPLAM--
-TPLAMK-
TCPLAM--
-TPLAMA-
TYPLAM--
-VPLAMS-
-VPLAML-
-VPLAMH-
VVPLAM--
VLPLAM--
VFPLAM--
VAPLAM--

-VPLAMQ-
VYPLAM--
VIPLAM--
VKPLAM--
VHPLAM--
-VPLAMG-
-VPLAMI-
VNPLAM--
-VPLAMR-
-VPLAMK-
-VPLAMN-
-VPLAMW-
VDPLAM--
VTPLAM--
VPPLAM--
VEPLAM--
-VPLAMT-
-VPLAMM-
-VPLAMV-
-VPLAMA-
VMPLAM--
VWPLAM--
VQPLAM--
-VPLAME-
-VPLAMD-
WSPLAM--
WHPLAM--
WRPLAM--
-WPLAMN-
WYPLAM--
WIPLAM--
-WPLAMK-
WAPLAM--
WKPLAM--
-WPLAMW-
WDPLAM--
WLPLAM--
-WPLAMQ-
-WPLAMI-
-WPLAMD-
-WPLAMY-
-WPLAMV-
-WPLAMM-
WGPLAM--
-WPLAMT-
-WPLAMG-
-WPLAMA-
-WPLAMS-
-YPLAML-
YLPLAM--

	-YPLAMM-			
	-YPLAMG-			
	YFPLAM--			
	YRPLAM--			
	YGPLAM--			
	-YPLAMF-			
	YVPLAM--			
	-YPLAMH-			
	YHPLAM--			
	YAPLAM--			
	-YPLAMR-			
	-YPLAMN-			
	-YPLAMV-			
	-YPLAMA-			
	YIPLAM--			
	YWPLAM--			
	YEPLAM--			
	YQPLAM--			
	-YPLAMK-			
	-YPLAMI-			
	YTPLAM--			
	-YPLAMQ-			
	YDPLAM--			
	YPPLAM--			
	-YPLAMT-			
	-YPLAME-			
	YMPLAM--			
	YYPLAM--			
	YKPLAM--			
	YCPLAM--			
PLHM	77	825	49.26	
	AMPLHM--			
	--PLHMLM			
	--PLHMGR			
	-WPLHMG-			
	ARPLHM--			
	-APLHML-			
	AKPLHM--			
	ASPLHM--			
	AYPLHM--			
	-APLHMW-			
	ALPLHM--			
	-APLHMS-			
	-APLHMN-			
	AHPLHM--			
	APPLHM--			
	-APLHMR-			
	-APLHMF-			
	ANPLHM--			
	AVPLHM--			

-APLHMY-
-APLHMI-
AIPLHM--
AGPLHM--
-APLHMT-
-APLHME-
AWPLHM--
AAPLHM--
ATPLHM--
AQPLHM--
-APLHMQ-
AFPLHM--
ADPLHM--
-APLHMC-
-APLHMA-
-APLHMM-
-APLHMK-
AEPLHM--
CRPLHM--
CGPLHM--
CYPLHM--
CVPLHM--
-CPLHMT-
-CPLHMS-
-CPLHMK-
CKPLHM--
CIPLHM--
CSPLHM--
-CPLHMY-
-CPLHMV-
-CPLHMR-
DRPLHM--
-DPLHMV-
DFPLHM--
DYPLHM--
DAPLHM--
DLPLHM--
DTPLHM--
-DPLHMK-
DSPLHM--
-DPLHML-
-DPLHMI-
DWPLHM--
-DPLHMT-
-DPLHMR-
-DPLHMA-
DMPLHM--
DEPLHM--
DDPLHM--
DVPLHM--

DQPLHM--
DKPLHM--
DGPLHM--
-DPLHMS-
-DPLHMH-
-DPLHMP-
-DPLHMG-
-DPLHMF-
EYPLHM--
ERPLHM--
-EPLHMV-
EVPLHM--
-EPLHMR-
-EPLHMS-
EMPLHM--
EAPLHM--
ETPLHM--
-EPLHMY-
-EPLHMT-
ELPLHM--
EKPLHM--
EIPLHM--
-EPLHMF-
EQPLHM--
-EPLHMQ-
-EPLHMN-
-EPLHML-
-EPLHMH-
ESPLHM--
ENPLHM--
EDPLHM--
EWPLHM--
-EPLHMW-
-EPLHMM-
-EPLHMG-
-EPLHMA-
EEPLHM--
-FPLHMS-
FQPLHM--
FRPLHM--
FGPLHM--
-FPLHML-
FPPLHM--
FFPLHM--
-FPLHME-
-FPLHMK-
FSPLHM--
FVPLHM--
-FPLHMN-
-FPLHMT-

FLPLHM--
FKPLHM--
FYPLHM--
-FPLHMQ-
FHPLHM--
-FPLHMW-
-FPLHMV-
FTPLHM--
-FPLHMY-
-FPLHMM-
FNPLHM--
FCPLHM--
-FPLHMR-
-FPLHMH-
-FPLHMF-
FWPLHM--
-FPLHMI-
-FPLHMG-
-FPLHMA-
FMPLHM--
-GPLHMT-
-GPLHMS-
GLPLHM--
GRPLHM--
-GPLHMR-
GTPLHM--
GGPLHM--
-GPLHML-
GSPLHM--
-GPLHMN-
GQPLHM--
-GPLHMY-
-GPLHMI-
GKPLHM--
GNPLHM--
GIPLHM--
GYPLHM--
-GPLHMF-
-GPLHMA-
GAPLHM--
-GPLHMG-
GDPLHM--
GWPLHM--
-GPLHMV-
GEPLHM--
GVPLHM--
-GPLHMM-
GMPLHM--
-GPLHMQ-
GHPLHM--

GPPLHM--
-GPLHME-
HRPLHM--
-HPLHMI-
-HPLHML-
-HPLHMT-
HFPLHM--
HLPLHM--
HVPLHM--
HTPLHM--
-HPLHMK-
HGPLHM--
HNPLHM--
-HPLHMM-
-HPLHMA-
HIPLHM--
HDPLHM--
-HPLHMN-
-HPLHMH-
HHPLHM--
HAPLHM--
HSPLHM--
HQPLHM--
HEPLHM--
-HPLHMG-
HMPLHM--
HCPLHM--
-HPLHMW-
-HPLHMS-
-HPLHMR-
-HPLHMQ-
HKPLHM--
-IPLHMR-
ITPLHM--
IHPLHM--
IAPLHM--
-IPLHMM-
-IPLHMY-
IDPLHM--
IRPLHM--
IEPLHM--
IWPLHM--
IPPLHM--
IVPLHM--
-IPLHML-
-IPLHMK-
ISPLHM--
IQPLHM--
-IPLHMV-
-IPLHMA-

INPLHM--
ILPLHM--
IYPLHM--
-IPLHMW-
-IPLHMT-
IMPLHM--
IFPLHM--
-IPLHMG-
IIPLHM--
-IPLHMP-
-IPLHMN-
-IPLHME-
KSPLHM--
-KPLHMM-
-KPLHMT-
KRPLHM--
KYPLHM--
-KPLHMY-
KGPLHM--
-KPLHMR-
-KPLHML-
KWPLHM--
KNPLHM--
KKPLHM--
-KPLHMI-
KQPLHM--
-KPLHME-
-KPLHMH-
-KPLHMG-
KFPLHM--
-KPLHMS-
KVPLHM--
-KPLHMV-
-KPLHMQ-
KMPLHM--
KLPLHM--
KIPLHM--
KDPLHM--
-KPLHMK-
-KPLHMW-
-KPLHMF-
KCPLHM--
KPPLHM--
-KPLHMA-
-KPLHMD-
LRPLHM--
LPPLHM--
LEPLHM--
LIPLHM--
LYPLHM--

```
-LPLHMQ-
LGPLHM--
LHPLHM--
-LPLHMS-
LLPLHM--
LDPLHM--
-LPLHML-
LTPLHM--
-LPLHMV-
LAPLHM--
LVPLHM--
LSPLHM--
LKPLHM--
LWPLHM--
-LPLHMY-
-LPLHMT-
-LPLHMH-
-LPLHMA-
-LPLHMN-
-LPLHMK-
LNPLHM--
LFPLHM--
-LPLHMM-
-LPLHMF-
LQPLHM--
-LPLHMG-
-LPLHME-
MRPLHM--
MTPLHM--
MSPLHM--
MQPLHM--
-MPLHMA-
-MPLHMW-
-MPLHMT-
-MPLHMK-
-MPLHMI-
MFPLHM--
MVPLHM--
-MPLHMR-
MNPLHM--
MHPLHM--
MPPLHM--
-MPLHMQ-
MMPLHM--
MGPLHM--
-MPLHMV-
-MPLHMM-
-MPLHML-
-MPLHME-
MYPLHM--
```

```
MWPLHM--
-MPLHMG-
-MPLHMF-
MEPLHM--
-MPLHMN-
MIPLHM--
NGPLHM--
NSPLHM--
-NPLHMY-
-NPLHMI-
NYPLHM--
NRPLHM--
-NPLHMS-
-NPLHML-
-NPLHMK-
NVPLHM--
-NPLHMF-
NKPLHM--
NIPLHM--
NAPLHM--
-NPLHMT-
-NPLHMA-
NPPLHM--
-NPLHMQ-
NFPLHM--
-NPLHMV-
-NPLHMM-
-NPLHME-
-NPLHMH-
NDPLHM--
NCPLHM--
NTPLHM--
NMPLHM--
--PLHMRG
--PLHMLS
--PLHMVS
--PLHMKA
--PLHMKG
--PLHMAA
--PLHMIT
--PLHMIS
--PLHMLT
--PLHMSS
--PLHMRN
--PLHMSV
--PLHMFR
--PLHMST
--PLHMVL
--PLHMLH
--PLHMIR
```

--PLHMGS
--PLHMVH
--PLHMHV
--PLHMTF
--PLHMSG
--PLHMLR
--PLHMHS
--PLHMYR
--PLHMWK
--PLHMRM
--PLHMFT
--PLHMYG
--PLHMSR
--PLHMQM
--PLHMHR
--PLHMAG
--PLHMTM
--PLHMVI
--PLHMRR
--PLHMLV
--PLHMAR
--PLHMTG
--PLHMRD
--PLHMQV
--PLHMMR
--PLHMLA
--PLHMHA
PTPLHM--
--PLHMTQ
--PLHMSA
--PLHMRH
--PLHMAT
PEPLHM--
--PLHMYT
--PLHMYH
--PLHMWA
--PLHMVG
--PLHMRF
--PLHMRA
--PLHMLQ
--PLHMGG
PRPLHM--
--PLHMYY
--PLHMSF
--PLHMRT
--PLHMLI
--PLHMKM
--PLHMSM
--PLHMQK
--PLHMNS

--PLHMMG
--PLHMLN
--PLHMKR
--PLHMKE
--PLHMGT
--PLHMEL
--PLHMAS
--PLHMYI
--PLHMWS
--PLHMWR
--PLHMTD
--PLHMRY
--PLHMQW
--PLHMMQ
--PLHMLK
--PLHMLE
--PLHMLD
--PLHMIV
--PLHMHG
--PLHMER
--PLHMML
--PLHMLL
--PLHMKS
--PLHMFN
--PLHMYM
--PLHMWT
--PLHMVR
--PLHMTK
--PLHMTI
--PLHMSN
--PLHMRW
--PLHMMH
--PLHMWL
--PLHMVA
--PLHMSL
--PLHMSK
--PLHMSH
--PLHMQY
--PLHMVY
--PLHMVF
--PLHMTW
--PLHMTA
--PLHMMY
--PLHMGW
--PLHMYA
--PLHMVK
--PLHMMV
--PLHMKW
--PLHMIL
--PLHMFV

--PLHMFQ
--PLHMFG
--PLHMEG
--PLHMEA
--PLHMAK
PNPLHM--
--PLHMWH
--PLHMTH
--PLHMSI
--PLHMQN
--PLHMKT
--PLHMHN
--PLHMHH
--PLHMFW
--PLHMEQ
--PLHMEN
PGPLHM--
--PLHMYL
--PLHMWF
--PLHMVV
--PLHMVT
--PLHMTL
--PLHMQT
--PLHMNL
--PLHMME
--PLHMMA
--PLHMIA
--PLHMHY
--PLHMHI
--PLHMAY
--PLHMYK
--PLHMWG
--PLHMVQ
--PLHMTV
--PLHMTE
--PLHMSW
--PLHMQR
--PLHMQQ
--PLHMQL
--PLHMQH
--PLHMNF
--PLHMLG
--PLHMKN
--PLHMKF
--PLHMIG
--PLHMHT
--PLHMHK
--PLHMET
--PLHMAV
--PLHMAH

--PLHMYD
--PLHMWW
--PLHMSQ
--PLHMQD
--PLHMNV
--PLHMNA
--PLHMMF
--PLHMKQ
--PLHMKI
--PLHMIE
--PLHMHQ
--PLHMGV
--PLHMGA
--PLHMEW
--PLHMEK
--PLHMEI
--PLHMEF
--PLHMAF
PHPLHM--
--PLHMVE
--PLHMVD
--PLHMTY
--PLHMSD
--PLHMQA
--PLHMNH
--PLHMKY
--PLHMKD
--PLHMIN
--PLHMIM
--PLHMID
--PLHMGM
--PLHMGF
--PLHMAL
--PLHMAI
--PLHMWQ
--PLHMWI
--PLHMTT
--PLHMQG
--PLHMMT
--PLHMMN
--PLHMMD
--PLHMKL
--PLHMKK
--PLHMGI
--PLHMFS
--PLHMFI
--PLHMAQ
--PLHMAP
PCPLHM--
-PPLHMQ-

--PLHMYF
--PLHMYE
--PLHMWN
--PLHMWD
--PLHMRQ
--PLHMQI
--PLHMQE
--PLHMPF
--PLHMNY
--PLHMNT
--PLHMNN
--PLHMNM
--PLHMNI
--PLHMNG
--PLHMND
--PLHMKH
--PLHMIY
--PLHMHM
--PLHMGY
--PLHMGQ
--PLHMFM
--PLHMFK
--PLHMEM
--PLHMDE
--PLHMCH
--PLHMCE
--PLHMAM
PDPLHM--
QRPLHM--
-QPLHMR-
QVPLHM--
QTPLHM--
QQPLHM--
-QPLHMA-
QKPLHM--
QYPLHM--
-QPLHMV-
QSPLHM--
-QPLHMH-
-QPLHMI-
QGPLHM--
QWPLHM--
-QPLHML-
-QPLHMQ-
-QPLHMG-
QNPLHM--
-QPLHMY-
-QPLHMN-
-QPLHMW-
-QPLHMM-

−QPLHMK−
QMPLHM−−
QPPLHM−−
−QPLHMT−
−QPLHMS−
QHPLHM−−
QFPLHM−−
QAPLHM−−
−QPLHME−
−QPLHMD−
QLPLHM−−
QIPLHM−−
RGPLHM−−
RTPLHM−−
RQPLHM−−
RAPLHM−−
−RPLHMY−
−RPLHMQ−
−RPLHML−
RIPLHM−−
−RPLHMS−
RPPLHM−−
−RPLHMN−
RMPLHM−−
RLPLHM−−
−RPLHMW−
−RPLHMI−
RNPLHM−−
RYPLHM−−
−RPLHMA−
−RPLHMK−
−RPLHMV−
RDPLHM−−
RHPLHM−−
−RPLHMG−
RKPLHM−−
−RPLHMM−
RWPLHM−−
REPLHM−−
RFPLHM−−
RVPLHM−−
−RPLHME−
RCPLHM−−
−RPLHMD−
−SPLHMR−
−SPLHML−
−SPLHMK−
SKPLHM−−
SGPLHM−−
−SPLHMV−

SAPLHM--
SSPLHM--
SHPLHM--
SVPLHM--
SRPLHM--
SIPLHM--
SFPLHM--
-SPLHMS-
-SPLHMA-
SYPLHM--
SQPLHM--
-SPLHMN-
-SPLHMF-
-SPLHMG-
SDPLHM--
SPPLHM--
-SPLHMW-
STPLHM--
-SPLHMQ-
-SPLHMM-
SMPLHM--
SWPLHM--
-SPLHMY-
-SPLHMT-
SEPLHM--
-SPLHMH-
-SPLHME-
SNPLHM--
-TPLHMF-
TRPLHM--
-TPLHML-
-TPLHMM-
TGPLHM--
TYPLHM--
TWPLHM--
TVPLHM--
-TPLHMR-
-TPLHMS-
TKPLHM--
-TPLHMY-
TAPLHM--
-TPLHMT-
TLPLHM--
-TPLHMA-
-TPLHME-
TSPLHM--
TNPLHM--
TQPLHM--
-TPLHMQ-
-TPLHMV-

```
-TPLHMN-
-TPLHMI-
TPPLHM--
TMPLHM--
TIPLHM--
TTPLHM--
TDPLHM--
-TPLHMK-
-TPLHMH-
VSPLHM--
-VPLHML-
VLPLHM--
VRPLHM--
-VPLHMH-
-VPLHMR-
VGPLHM--
-VPLHMI-
-VPLHMW-
-VPLHMK-
VHPLHM--
VYPLHM--
-VPLHMT-
VWPLHM--
-VPLHMG-
VVPLHM--
VTPLHM--
VKPLHM--
VEPLHM--
-VPLHMA-
VNPLHM--
VFPLHM--
VMPLHM--
VPPLHM--
-VPLHMM-
VAPLHM--
-VPLHMY-
VDPLHM--
-VPLHMV-
VIPLHM--
VCPLHM--
VQPLHM--
-VPLHMD-
-VPLHMC-
WSPLHM--
WRPLHM--
WVPLHM--
WGPLHM--
WFPLHM--
-WPLHMS-
-WPLHMR-
```

```
-WPLHMI-
-WPLHME-
WIPLHM--
WYPLHM--
WLPLHM--
WTPLHM--
WMPLHM--
-WPLHMT-
WAPLHM--
-WPLHMN-
-WPLHML-
-WPLHMW-
-WPLHMQ-
-WPLHMA-
WDPLHM--
WPPLHM--
-WPLHMV-
-WPLHMH-
WQPLHM--
-WPLHMM-
WNPLHM--
WKPLHM--
YSPLHM--
YFPLHM--
-YPLHMR-
-YPLHML-
YLPLHM--
-YPLHMV-
-YPLHMY-
YGPLHM--
YRPLHM--
YMPLHM--
YVPLHM--
-YPLHMF-
YHPLHM--
YWPLHM--
-YPLHMH-
-YPLHMA-
YAPLHM--
-YPLHMT-
-YPLHMQ-
YNPLHM--
-YPLHMM-
-YPLHMS-
YQPLHM--
YTPLHM--
-YPLHMN-
YPPLHM--
-YPLHME-
YKPLHM--
```

	YIPLHM--		
	YEPLHM--		
	YYPLHM--		
	-YPLHMK-		
	-YPLHMI-		
	YCPLHM--		
PWPL	83	822	49.20
	-GPWPLA-		
	--PWPLSD		
	-APWPLR-		
	ARPWPL--		
	AWPWPL--		
	-APWPLL-		
	AVPWPL--		
	ASPWPL--		
	-APWPLT-		
	AAPWPL--		
	ATPWPL--		
	AHPWPL--		
	-APWPLY-		
	-APWPLM-		
	ADPWPL--		
	AEPWPL--		
	AYPWPL--		
	-APWPLK-		
	-APWPLA-		
	ANPWPL--		
	AQPWPL--		
	AMPWPL--		
	-APWPLE-		
	ALPWPL--		
	-APWPLW-		
	AGPWPL--		
	-APWPLN-		
	-APWPLG-		
	-APWPLH-		
	AKPWPL--		
	AIPWPL--		
	-APWPLQ-		
	-APWPLI-		
	AFPWPL--		
	CLPWPL--		
	CNPWPL--		
	CKPWPL--		
	CHPWPL--		
	CGPWPL--		
	CSPWPL--		
	-CPWPLS-		
	CPPWPL--		
	CAPWPL--		

CYPWPL--
-CPWPLE-
CVPWPL--
-DPWPLY-
DAPWPL--
DGPWPL--
-DPWPLR-
-DPWPLF-
DSPWPL--
DYPWPL--
-DPWPLL-
DTPWPL--
DHPWPL--
DRPWPL--
DWPWPL--
DLPWPL--
-DPWPLW-
-DPWPLV-
-DPWPLK-
DNPWPL--
DKPWPL--
-DPWPLQ-
-DPWPLM-
DIPWPL--
DFPWPL--
DEPWPL--
DVPWPL--
DQPWPL--
-DPWPLN-
-DPWPLG-
DMPWPL--
-EPWPLR-
-EPWPLY-
EYPWPL--
EIPWPL--
EWPWPL--
EFPWPL--
-EPWPLW-
-EPWPLL-
-EPWPLH-
-EPWPLF-
ELPWPL--
ESPWPL--
-EPWPLN-
-EPWPLK-
EKPWPL--
EGPWPL--
ERPWPL--
-EPWPLE-
EVPWPL--

```
-EPWPLT-
-EPWPLA-
-EPWPLS-
EPPWPL--
EAPWPL--
ETPWPL--
-EPWPLG-
-EPWPLC-
EMPWPL--
EDPWPL--
FRPWPL--
FQPWPL--
FSPWPL--
FEPWPL--
FVPWPL--
-FPWPLR-
-FPWPLT-
-FPWPLS-
FGPWPL--
-FPWPLA-
-FPWPLH-
-FPWPLN-
FMPWPL--
FYPWPL--
FTPWPL--
FKPWPL--
-FPWPLV-
FLPWPL--
-FPWPLW-
-FPWPLE-
-FPWPLY-
-FPWPLQ-
-FPWPLK-
FIPWPL--
FFPWPL--
FAPWPL--
-FPWPLC-
FDPWPL--
FCPWPL--
-GPWPLR-
-GPWPLK-
GSPWPL--
-GPWPLN-
GRPWPL--
GWPWPL--
-GPWPLW-
-GPWPLL-
GTPWPL--
-GPWPLQ-
GMPWPL--
```

GLPWPL--
-GPWPLS-
GHPWPL--
-GPWPLT-
-GPWPLG-
GAPWPL--
-GPWPLI-
GQPWPL--
-GPWPLH-
-GPWPLV-
GPPWPL--
GNPWPL--
GGPWPL--
-GPWPLF-
-GPWPLE-
GIPWPL--
GYPWPL--
GEPWPL--
GCPWPL--
-GPWPLY-
GDPWPL--
HVPWPL--
HRPWPL--
-HPWPLR-
-HPWPLY-
-HPWPLL-
HKPWPL--
-HPWPLI-
-HPWPLS-
HLPWPL--
HNPWPL--
HTPWPL--
HPPWPL--
HGPWPL--
HEPWPL--
-HPWPLN-
-HPWPLT-
HHPWPL--
HAPWPL--
-HPWPLF-
HDPWPL--
HYPWPL--
-HPWPLK-
-HPWPLE-
HQPWPL--
-HPWPLQ-
HMPWPL--
HWPWPL--
-HPWPLG-
-HPWPLA-

HIPWPL--
ILPWPL--
-IPWPLL-
IEPWPL--
-IPWPLS-
-IPWPLQ-
IYPWPL--
IPPWPL--
INPWPL--
IFPWPL--
IGPWPL--
ISPWPL--
-IPWPLW-
IKPWPL--
-IPWPLH-
-IPWPLN-
IIPWPL--
IHPWPL--
IAPWPL--
ITPWPL--
-IPWPLR-
-IPWPLK-
-IPWPLA-
IVPWPL--
IDPWPL--
IRPWPL--
-IPWPLV-
-IPWPLT-
IMPWPL--
-IPWPLM-
-IPWPLG-
-IPWPLY-
-IPWPLI-
-IPWPLE-
KSPWPL--
KGPWPL--
-KPWPLR-
KLPWPL--
KAPWPL--
-KPWPLL-
KRPWPL--
-KPWPLK-
KNPWPL--
KQPWPL--
-KPWPLS-
-KPWPLF-
KHPWPL--
KTPWPL--
KVPWPL--
-KPWPLY-

KIPWPL--
-KPWPLQ-
-KPWPLN-
-KPWPLM-
-KPWPLA-
KFPWPL--
KWPWPL--
-KPWPLW-
KYPWPL--
-KPWPLH-
-KPWPLG-
-KPWPLE-
KPPWPL--
KMPWPL--
-KPWPLV-
-KPWPLP-
KEPWPL--
KCPWPL--
LRPWPL--
-LPWPLL-
-LPWPLR-
-LPWPLW-
-LPWPLS-
LGPWPL--
LIPWPL--
LFPWPL--
LKPWPL--
LHPWPL--
LEPWPL--
LVPWPL--
LPPWPL--
LAPWPL--
-LPWPLY-
LWPWPL--
-LPWPLK-
LQPWPL--
-LPWPLN-
LTPWPL--
LMPWPL--
LDPWPL--
-LPWPLF-
-LPWPLQ-
LCPWPL--
LYPWPL--
-LPWPLT-
-LPWPLA-
-LPWPLV-
-LPWPLH-
LNPWPL--
-LPWPLI-

```
-LPWPLM-
MSPWPL--
MYPWPL--
MLPWPL--
MNPWPL--
MWPWPL--
-MPWPLN-
MPPWPL--
MFPWPL--
-MPWPLK-
-MPWPLR-
-MPWPLQ-
MAPWPL--
-MPWPLY-
-MPWPLH-
-MPWPLI-
-MPWPLW-
-MPWPLV-
-MPWPLS-
-MPWPLL-
-MPWPLG-
MGPWPL--
MVPWPL--
MTPWPL--
MRPWPL--
MQPWPL--
-MPWPLT-
-MPWPLM-
MDPWPL--
-MPWPLA-
MKPWPL--
MHPWPL--
-MPWPLE-
MIPWPL--
-MPWPLF-
NWPWPL--
-NPWPLR-
NLPWPL--
NYPWPL--
-NPWPLS-
NIPWPL--
-NPWPLN-
NHPWPL--
NNPWPL--
NKPWPL--
-NPWPLW-
NSPWPL--
NGPWPL--
NFPWPL--
NTPWPL--
```

NQPWPL--
-NPWPLK-
NVPWPL--
NMPWPL--
-NPWPLT-
-NPWPLM-
NPPWPL--
-NPWPLV-
-NPWPLQ-
-NPWPLE-
NEPWPL--
NDPWPL--
-NPWPLY-
-NPWPLI-
-NPWPLG-
-NPWPLF-
NCPWPL--
--PWPLQL
--PWPLLR
--PWPLLG
--PWPLTL
--PWPLLS
--PWPLKG
--PWPLAL
--PWPLML
--PWPLGF
--PWPLVV
--PWPLRT
--PWPLKS
--PWPLFR
--PWPLYS
--PWPLAS
--PWPLSV
--PWPLSG
--PWPLVT
--PWPLYI
--PWPLWG
--PWPLVQ
--PWPLVR
--PWPLNL
--PWPLLK
--PWPLAG
--PWPLAF
--PWPLSS
--PWPLRS
--PWPLRG
--PWPLSR
--PWPLTH
--PWPLLV
--PWPLKY

--PWPLGS
--PWPLTR
--PWPLLT
--PWPLLM
--PWPLYR
--PWPLSA
--PWPLRW
--PWPLAA
--PWPLST
--PWPLSK
--PWPLLN
--PWPLFH
--PWPLRQ
PSPWPL--
PGPWPL--
--PWPLSQ
--PWPLDL
PVPWPL--
--PWPLTG
PLPWPL--
--PWPLVN
--PWPLIS
--PWPLHS
--PWPLHR
--PWPLGA
PTPWPL--
--PWPLTK
--PWPLMS
--PWPLSF
--PWPLKV
--PWPLIQ
--PWPLFD
--PWPLMT
--PWPLMI
--PWPLKN
--PWPLHT
PAPWPL--
--PWPLYT
--PWPLYK
--PWPLWL
--PWPLVG
--PWPLNN
--PWPLKR
--PWPLIK
--PWPLHF
--PWPLTN
--PWPLSN
--PWPLQR
--PWPLPQ
--PWPLNF

--PWPLHG
--PWPLEA
--PWPLTS
--PWPLRH
--PWPLRD
--PWPLLH
--PWPLKA
--PWPLAR
--PWPLVH
--PWPLNS
--PWPLYQ
--PWPLHV
--PWPLFA
--PWPLYA
--PWPLWY
--PWPLVY
--PWPLVI
--PWPLSY
--PWPLRE
--PWPLMQ
--PWPLIG
--PWPLGQ
--PWPLEV
--PWPLWV
--PWPLVK
--PWPLTQ
--PWPLTM
--PWPLSW
--PWPLQG
--PWPLLD
--PWPLHN
--PWPLFQ
--PWPLES
--PWPLAH
PMPWPL--
PDPWPL--
--PWPLYN
--PWPLYG
--PWPLTY
--PWPLTV
--PWPLQV
--PWPLPW
--PWPLPM
--PWPLKQ
--PWPLKK
--PWPLVS
--PWPLTT
--PWPLNV
--PWPLNH
--PWPLMY

--PWPLMV
--PWPLKW
--PWPLHH
--PWPLGN
PYPWPL--
--PWPLTD
--PWPLTA
--PWPLLA
--PWPLID
--PWPLHW
--PWPLFN
--PWPLAM
--PWPLAK
--PWPLYM
--PWPLTF
--PWPLSH
--PWPLPA
--PWPLMR
--PWPLMN
--PWPLMK
--PWPLMH
--PWPLMG
--PWPLMF
--PWPLLE
--PWPLKI
--PWPLIY
--PWPLIE
--PWPLHI
--PWPLGG
--PWPLYV
--PWPLTI
--PWPLRF
--PWPLNT
--PWPLMM
--PWPLLQ
--PWPLHK
--PWPLFG
--PWPLDM
--PWPLDI
PFPWPL--
PEPWPL--
--PWPLYH
--PWPLYD
--PWPLWT
--PWPLWS
--PWPLWD
--PWPLQK
--PWPLNW
--PWPLNA
--PWPLMA

--PWPLIN
--PWPLIA
--PWPLHY
--PWPLEH
--PWPLDS
PIPWPL--
--PWPLTW
--PWPLQY
--PWPLQN
--PWPLQI
--PWPLPS
--PWPLPN
--PWPLPF
--PWPLMW
--PWPLKT
--PWPLIT
--PWPLHE
PWPWPL--
--PWPLWH
--PWPLVD
--PWPLNK
--PWPLMD
--PWPLKH
--PWPLKE
--PWPLKD
--PWPLIH
--PWPLHD
--PWPLGT
--PWPLGH
--PWPLFK
--PWPLAW
--PWPLWN
--PWPLWE
--PWPLQQ
--PWPLQM
--PWPLQD
--PWPLNE
--PWPLFP
--PWPLEG
--PWPLDW
--PWPLDG
--PWPLAE
PQPWPL--
PKPWPL--
--PWPLYE
--PWPLWR
--PWPLWA
--PWPLVP
--PWPLVM
--PWPLSE

--PWPLQW
--PWPLQT
--PWPLQF
--PWPLPV
--PWPLKM
--PWPLEM
--PWPLDY
--PWPLDV
--PWPLDQ
--PWPLDH
--PWPLDF
--PWPLDA
--PWPLAD
-PPWPLI-
PNPWPL--
-QPWPLR-
QIPWPL--
QGPWPL--
-QPWPLY-
QYPWPL--
QRPWPL--
-QPWPLA-
QSPWPL--
-QPWPLK-
QAPWPL--
QLPWPL--
QVPWPL--
QTPWPL--
-QPWPLH-
-QPWPLI-
-QPWPLS-
-QPWPLN-
QNPWPL--
QEPWPL--
-QPWPLW-
-QPWPLQ-
-QPWPLE-
QPPWPL--
QCPWPL--
QWPWPL--
-QPWPLT-
-QPWPLM-
-QPWPLG-
QMPWPL--
QHPWPL--
-RPWPLR-
RVPWPL--
RLPWPL--
RQPWPL--
-RPWPLS-

-RPWPLQ-
RSPWPL--
RMPWPL--
-RPWPLK-
RAPWPL--
-RPWPLT-
RIPWPL--
-RPWPLI-
-RPWPLY-
REPWPL--
RWPWPL--
RHPWPL--
-RPWPLH-
RNPWPL--
RDPWPL--
-RPWPLW-
-RPWPLA-
-RPWPLV-
RFPWPL--
RGPWPL--
RKPWPL--
RCPWPL--
-RPWPLN-
RPPWPL--
-RPWPLM-
-RPWPLE-
SVPWPL--
-SPWPLK-
SRPWPL--
-SPWPLL-
SLPWPL--
-SPWPLF-
SYPWPL--
SMPWPL--
SEPWPL--
-SPWPLM-
-SPWPLW-
SNPWPL--
SFPWPL--
SIPWPL--
-SPWPLY-
-SPWPLR-
SKPWPL--
STPWPL--
-SPWPLQ-
-SPWPLH-
SHPWPL--
-SPWPLG-
SPPWPL--
SDPWPL--

-SPWPLN-
SGPWPL--
SWPWPL--
-SPWPLI-
-SPWPLA-
-SPWPLE-
SQPWPL--
SCPWPL--
TRPWPL--
-TPWPLR-
-TPWPLS-
TLPWPL--
-TPWPLK-
TPPWPL--
TIPWPL--
-TPWPLL-
-TPWPLY-
-TPWPLF-
TVPWPL--
TTPWPL--
-TPWPLI-
TNPWPL--
TEPWPL--
TMPWPL--
-TPWPLM-
-TPWPLH-
-TPWPLW-
-TPWPLT-
TWPWPL--
TFPWPL--
TQPWPL--
TYPWPL--
THPWPL--
TCPWPL--
-TPWPLQ-
-TPWPLG-
-TPWPLV-
-TPWPLE-
-TPWPLN-
-TPWPLP-
-VPWPLR-
VLPWPL--
-VPWPLK-
VHPWPL--
VSPWPL--
VVPWPL--
VGPWPL--
VWPWPL--
-VPWPLY-
-VPWPLL-

-VPWPLQ-
VMPWPL--
VKPWPL--
VRPWPL--
-VPWPLW-
VFPWPL--
-VPWPLI-
VEPWPL--
VAPWPL--
-VPWPLM-
VIPWPL--
VYPWPL--
VQPWPL--
-VPWPLS-
-VPWPLH-
VTPWPL--
-VPWPLN-
VDPWPL--
-VPWPLF-
-VPWPLA-
WMPWPL--
WEPWPL--
-WPWPLS-
WSPWPL--
WNPWPL--
WQPWPL--
-WPWPLT-
WLPWPL--
WIPWPL--
WHPWPL--
WAPWPL--
-WPWPLM-
-WPWPLV-
-WPWPLL-
WRPWPL--
-WPWPLY-
WKPWPL--
-WPWPLR-
WGPWPL--
WVPWPL--
-WPWPLN-
-WPWPLK-
-WPWPLA-
WYPWPL--
-WPWPLW-
-WPWPLG-
-WPWPLE-
WDPWPL--
YRPWPL--
YGPWPL--

	-YPWPLK-		
	-YPWPLS-		
	YHPWPL--		
	YSPWPL--		
	-YPWPLQ-		
	YLPWPL--		
	-YPWPLR-		
	YVPWPL--		
	-YPWPLT-		
	YFPWPL--		
	YNPWPL--		
	-YPWPLL-		
	YMPWPL--		
	YIPWPL--		
	-YPWPLA-		
	YEPWPL--		
	YYPWPL--		
	YDPWPL--		
	YAPWPL--		
	YTPWPL--		
	-YPWPLV-		
	YPPWPL--		
	YKPWPL--		
	YQPWPL--		
	-YPWPLE-		
	-YPWPLY-		
	-YPWPLN-		
	-YPWPLG-		
	-YPWPLF-		
PYNL	16	888	49.17
	-QPYNLV-		
	ATPYNL--		
	-APYNLR-		
	-APYNLI-		
	-APYNLT-		
	-APYNLK-		
	-APYNLS-		
	AWPYNL--		
	ASPYNL--		
	ARPYNL--		
	AQPYNL--		
	AAPYNL--		
	AMPYNL--		
	-APYNLG-		
	-APYNLW-		
	AVPYNL--		
	-APYNLN-		
	ADPYNL--		
	-APYNLV-		
	ALPYNL--		

AIPYNL--
ACPYNL--
-APYNLQ-
-APYNLH-
-APYNLE-
ANPYNL--
-APYNLM-
-APYNLF-
AEPYNL--
-APYNLA-
AHPYNL--
-APYNLY-
APPYNL--
AYPYNL--
-APYNLD-
AKPYNL--
CSPYNL--
-CPYNLL-
CCPYNL--
CAPYNL--
-CPYNLM-
CKPYNL--
CHPYNL--
CRPYNL--
-CPYNLW-
-CPYNLQ-
CGPYNL--
-CPYNLS-
-CPYNLI-
-CPYNLH-
CNPYNL--
CFPYNL--
CTPYNL--
CQPYNL--
-CPYNLN-
-CPYNLG-
CLPYNL--
CDPYNL--
DRPYNL--
DTPYNL--
-DPYNLV-
DIPYNL--
DLPYNL--
-DPYNLM-
DWPYNL--
DQPYNL--
-DPYNLF-
-DPYNLY-
DVPYNL--
-DPYNLK-

-DPYNLA-
DGPYNL--
DAPYNL--
-DPYNLN-
-DPYNLL-
DNPYNL--
DMPYNL--
-DPYNLQ-
DKPYNL--
DYPYNL--
-DPYNLW-
DSPYNL--
DEPYNL--
-DPYNLG-
DPPYNL--
DFPYNL--
-EPYNLR-
-EPYNLN-
ESPYNL--
ERPYNL--
EVPYNL--
-EPYNLL-
EWPYNL--
EKPYNL--
EGPYNL--
-EPYNLH-
-EPYNLA-
EYPYNL--
ETPYNL--
-EPYNLV-
-EPYNLS-
-EPYNLK-
-EPYNLF-
EAPYNL--
EQPYNL--
-EPYNLY-
-EPYNLW-
-EPYNLT-
-EPYNLI-
-EPYNLM-
-EPYNLG-
ELPYNL--
EIPYNL--
-EPYNLE-
EDPYNL--
ENPYNL--
EEPYNL--
-FPYNLR-
-FPYNLT-
FDPYNL--

FSPYNL--
-FPYNLA-
FGPYNL--
-FPYNLS-
-FPYNLL-
-FPYNLH-
FVPYNL--
FLPYNL--
FMPYNL--
-FPYNLG-
-FPYNLI-
-FPYNLE-
-FPYNLV-
-FPYNLW-
FAPYNL--
FKPYNL--
FIPYNL--
FFPYNL--
FTPYNL--
-FPYNLY-
FEPYNL--
-FPYNLD-
FHPYNL--
-FPYNLM-
FQPYNL--
FNPYNL--
FYPYNL--
FPPYNL--
-GPYNLT-
GAPYNL--
-GPYNLL-
GSPYNL--
GKPYNL--
GTPYNL--
-GPYNLQ-
GRPYNL--
-GPYNLR-
GGPYNL--
GWPYNL--
-GPYNLK-
-GPYNLG-
GFPYNL--
-GPYNLV-
GLPYNL--
GNPYNL--
-GPYNLW-
-GPYNLN-
-GPYNLA-
-GPYNLS-
GQPYNL--

-GPYNLE-
-GPYNLH-
GDPYNL--
GMPYNL--
GHPYNL--
-GPYNLM-
GYPYNL--
GEPYNL--
GCPYNL--
-GPYNLI-
GIPYNL--
-GPYNLD-
HRPYNL--
-HPYNLR-
HVPYNL--
-HPYNLL-
HLPYNL--
HTPYNL--
HSPYNL--
-HPYNLQ-
HWPYNL--
HYPYNL--
-HPYNLV-
HNPYNL--
HGPYNL--
HAPYNL--
-HPYNLW-
HMPYNL--
-HPYNLT-
-HPYNLS-
-HPYNLK-
HHPYNL--
-HPYNLN-
-HPYNLF-
-HPYNLI-
-HPYNLG-
-HPYNLM-
-HPYNLA-
HKPYNL--
HFPYNL--
HIPYNL--
-HPYNLD-
-HPYNLH-
-HPYNLE-
HDPYNL--
-HPYNLY-
HCPYNL--
IRPYNL--
ISPYNL--
-IPYNLM-

ITPYNL--
-IPYNLL-
IHPYNL--
IYPYNL--
-IPYNLR-
IFPYNL--
IWPYNL--
-IPYNLI-
IGPYNL--
-IPYNLV-
IIPYNL--
IEPYNL--
IQPYNL--
IPPYNL--
-IPYNLH-
INPYNL--
-IPYNLS-
-IPYNLG-
IMPYNL--
-IPYNLY-
-IPYNLN-
-IPYNLK-
ILPYNL--
IDPYNL--
-IPYNLF-
IKPYNL--
-IPYNLQ-
KSPYNL--
-KPYNLR-
-KPYNLI-
KLPYNL--
KRPYNL--
KGPYNL--
-KPYNLL-
-KPYNLS-
-KPYNLV-
-KPYNLK-
KTPYNL--
KKPYNL--
KQPYNL--
KFPYNL--
KWPYNL--
KVPYNL--
-KPYNLN-
KNPYNL--
KYPYNL--
KDPYNL--
KPPYNL--
-KPYNLG-
-KPYNLT-

-KPYNLA-
KAPYNL--
-KPYNLD-
-KPYNLE-
KIPYNL--
KEPYNL--
KMPYNL--
KHPYNL--
-KPYNLQ-
LRPYNL--
LGPYNL--
LKPYNL--
LSPYNL--
LFPYNL--
LVPYNL--
LQPYNL--
-LPYNLS-
-LPYNLL-
-LPYNLR-
LIPYNL--
LYPYNL--
-LPYNLI-
LNPYNL--
LWPYNL--
-LPYNLK-
LPPYNL--
LMPYNL--
LDPYNL--
LEPYNL--
-LPYNLT-
-LPYNLG-
-LPYNLV-
-LPYNLQ-
-LPYNLN-
-LPYNLM-
-LPYNLH-
-LPYNLY-
-LPYNLA-
LHPYNL--
-LPYNLW-
-LPYNLE-
-MPYNLR-
MSPYNL--
MGPYNL--
MRPYNL--
-MPYNLH-
MLPYNL--
-MPYNLG-
MIPYNL--
MHPYNL--

MMPYNL--
-MPYNLV-
MVPYNL--
MAPYNL--
-MPYNLS-
MTPYNL--
MFPYNL--
-MPYNLY-
-MPYNLQ-
-MPYNLN-
-MPYNLE-
-MPYNLA-
MDPYNL--
MQPYNL--
MKPYNL--
-MPYNLD-
MEPYNL--
MWPYNL--
-MPYNLI-
MPPYNL--
MCPYNL--
-MPYNLM-
-MPYNLF-
-NPYNLL-
NSPYNL--
NRPYNL--
-NPYNLR-
NLPYNL--
-NPYNLI-
NDPYNL--
NMPYNL--
NFPYNL--
NTPYNL--
NIPYNL--
NYPYNL--
NVPYNL--
NHPYNL--
NGPYNL--
-NPYNLW-
-NPYNLA-
NPPYNL--
NWPYNL--
-NPYNLK-
NQPYNL--
-NPYNLY-
-NPYNLE-
NAPYNL--
-NPYNLH-
NKPYNL--
NEPYNL--

-NPYNLT-
-NPYNLG-
-NPYNLM-
-NPYNLQ-
-NPYNLP-
-NPYNLN-
NCPYNL--
--PYNLRG
--PYNLSL
--PYNLST
--PYNLRN
--PYNLRT
--PYNLSR
--PYNLLN
--PYNLLK
--PYNLSG
--PYNLLT
--PYNLWT
--PYNLSH
--PYNLLR
--PYNLGG
--PYNLRS
--PYNLMR
PAPYNL--
--PYNLVS
--PYNLTF
--PYNLIK
--PYNLGS
--PYNLVA
--PYNLKR
--PYNLGY
--PYNLWS
--PYNLWR
--PYNLNT
--PYNLFR
--PYNLYR
--PYNLAR
--PYNLTS
--PYNLTK
--PYNLTR
--PYNLLS
--PYNLKQ
--PYNLSM
--PYNLRA
--PYNLKA
PKPYNL--
--PYNLSQ
--PYNLES
--PYNLVI
PRPYNL--

PYPYNL--
--PYNLKS
--PYNLIS
--PYNLGV
--PYNLFK
--PYNLTA
--PYNLSF
--PYNLRD
--PYNLQK
--PYNLNM
--PYNLEA
--PYNLAL
PTPYNL--
--PYNLVQ
--PYNLLH
--PYNLWN
--PYNLVG
--PYNLSV
--PYNLHG
--PYNLGT
--PYNLFH
PVPYNL--
PSPYNL--
PMPYNL--
--PYNLSY
--PYNLRK
--PYNLGL
--PYNLFT
--PYNLFS
--PYNLAA
PHPYNL--
--PYNLTI
--PYNLRF
--PYNLHK
--PYNLAT
--PYNLLE
--PYNLIH
--PYNLHA
--PYNLAW
--PYNLTD
--PYNLSW
--PYNLQY
--PYNLKN
--PYNLGW
--PYNLEL
PGPYNL--
--PYNLYH
--PYNLSD
--PYNLNG
--PYNLLQ

--PYNLLD
--PYNLIA
--PYNLGA
PNPYNL--
--PYNLQV
--PYNLMN
--PYNLGF
--PYNLFN
--PYNLAY
--PYNLTG
--PYNLRM
--PYNLQR
--PYNLMT
--PYNLKT
--PYNLHN
--PYNLHL
--PYNLGI
--PYNLAF
--PYNLWK
--PYNLWH
--PYNLVN
--PYNLMH
--PYNLIN
--PYNLDA
--PYNLVT
--PYNLNL
--PYNLNH
--PYNLKG
--PYNLII
PDPYNL--
--PYNLTT
--PYNLTE
--PYNLRY
--PYNLQG
--PYNLNA
--PYNLIW
--PYNLIG
--PYNLGM
--PYNLEG
--PYNLAV
--PYNLYQ
--PYNLMQ
--PYNLIE
--PYNLHV
--PYNLGH
--PYNLDG
PLPYNL--
PFPYNL--
--PYNLWD
--PYNLVV

--PYNLVH
--PYNLQT
--PYNLQN
--PYNLNY
--PYNLIQ
--PYNLHS
--PYNLYT
--PYNLYS
--PYNLSI
--PYNLRW
--PYNLNN
--PYNLKV
PIPYNL--
--PYNLSN
--PYNLNR
--PYNLMS
--PYNLMM
--PYNLLG
--PYNLHE
--PYNLAS
--PYNLAK
--PYNLWF
--PYNLTH
--PYNLSK
--PYNLRE
--PYNLMV
--PYNLME
--PYNLGQ
--PYNLET
--PYNLDT
PCPYNL--
--PYNLVE
--PYNLTQ
--PYNLTN
--PYNLRQ
--PYNLQW
--PYNLQS
--PYNLQF
--PYNLNI
--PYNLKH
--PYNLKD
--PYNLIM
--PYNLHD
--PYNLAQ
PEPYNL--
--PYNLWA
--PYNLVP
--PYNLQM
--PYNLNF
--PYNLNE

```
--PYNLMG
--PYNLIV
--PYNLHY
--PYNLHH
--PYNLGE
--PYNLFD
--PYNLEY
--PYNLER
--PYNLEK
--PYNLAI
--PYNLKY
--PYNLHI
--PYNLEN
--PYNLEI
--PYNLAN
--PYNLAC
--PYNLWE
--PYNLQD
--PYNLQA
--PYNLNV
--PYNLNK
--PYNLMK
--PYNLKI
PQPYNL--
--PYNLYI
--PYNLYD
--PYNLTY
--PYNLRP
--PYNLRH
--PYNLQI
--PYNLMD
--PYNLKP
--PYNLKF
--PYNLGN
--PYNLGD
--PYNLFY
--PYNLFQ
--PYNLAH
--PYNLAE
--PYNLWC
--PYNLVD
--PYNLTW
--PYNLTV
--PYNLMP
--PYNLKM
--PYNLIF
--PYNLEV
--PYNLEM
--PYNLEF
--PYNLDM
```

--PYNLCS
--PYNLCD
--PYNLAM
--PYNLAD
--PYNLYE
--PYNLTM
--PYNLQQ
--PYNLQL
--PYNLQH
--PYNLQE
--PYNLQC
--PYNLPY
--PYNLPN
--PYNLPM
--PYNLPL
--PYNLMW
--PYNLLP
--PYNLIP
--PYNLHM
--PYNLFG
--PYNLEQ
--PYNLEH
--PYNLED
--PYNLDV
--PYNLDI
--PYNLCM
PWPYNL--
-PPYNLS-
-PPYNLI-
PPPYNL--
-QPYNLL-
QGPYNL--
QRPYNL--
QTPYNL--
QHPYNL--
-QPYNLT-
QKPYNL--
-QPYNLI-
-QPYNLG-
-QPYNLS-
-QPYNLR-
QAPYNL--
QYPYNL--
QWPYNL--
QNPYNL--
QEPYNL--
QSPYNL--
-QPYNLA-
QFPYNL--
-QPYNLW-

-QPYNLF-
QPPYNL--
-QPYNLE-
QMPYNL--
-QPYNLN-
-QPYNLC-
-QPYNLM-
QIPYNL--
RVPYNL--
RLPYNL--
RDPYNL--
RGPYNL--
RSPYNL--
-RPYNLS-
RMPYNL--
-RPYNLL-
-RPYNLI-
RWPYNL--
RYPYNL--
-RPYNLK-
RTPYNL--
RRPYNL--
-RPYNLM-
RIPYNL--
RNPYNL--
-RPYNLQ-
-RPYNLN-
RQPYNL--
-RPYNLG-
RFPYNL--
-RPYNLH-
RPPYNL--
RKPYNL--
-RPYNLT-
-RPYNLF-
RAPYNL--
-RPYNLY-
RHPYNL--
-RPYNLE-
REPYNL--
-RPYNLC-
-SPYNLR-
SMPYNL--
SSPYNL--
SGPYNL--
SVPYNL--
-SPYNLS-
-SPYNLA-
-SPYNLL-
SLPYNL--

SHPYNL--
-SPYNLY-
-SPYNLT-
-SPYNLQ-
SIPYNL--
SWPYNL--
-SPYNLN-
SNPYNL--
SKPYNL--
STPYNL--
-SPYNLI-
SPPYNL--
-SPYNLK-
SFPYNL--
-SPYNLV-
SDPYNL--
SAPYNL--
-SPYNLG-
-SPYNLH-
-SPYNLF-
-SPYNLE-
SEPYNL--
-SPYNLM-
SCPYNL--
SYPYNL--
SQPYNL--
-SPYNLD-
-SPYNLW-
-TPYNLR-
TLPYNL--
TMPYNL--
TYPYNL--
TRPYNL--
-TPYNLE-
-TPYNLM-
TGPYNL--
-TPYNLV-
-TPYNLS-
-TPYNLW-
-TPYNLN-
TWPYNL--
-TPYNLA-
TKPYNL--
TIPYNL--
TAPYNL--
TSPYNL--
TTPYNL--
-TPYNLK-
-TPYNLG-
-TPYNLL-

TEPYNL--
-TPYNLT-
TVPYNL--
-TPYNLQ-
-TPYNLH-
TDPYNL--
TPPYNL--
THPYNL--
TNPYNL--
TFPYNL--
TQPYNL--
-TPYNLI-
-TPYNLD-
TCPYNL--
-TPYNLF-
VSPYNL--
-VPYNLG-
-VPYNLA-
VLPYNL--
-VPYNLT-
VRPYNL--
VTPYNL--
-VPYNLH-
VNPYNL--
VMPYNL--
VGPYNL--
-VPYNLS-
-VPYNLI-
VAPYNL--
VVPYNL--
-VPYNLL-
-VPYNLR-
-VPYNLY-
VQPYNL--
-VPYNLM-
VIPYNL--
VPPYNL--
-VPYNLQ-
VFPYNL--
-VPYNLV-
-VPYNLW-
-VPYNLE-
VDPYNL--
VCPYNL--
VYPYNL--
VWPYNL--
-VPYNLK-
-VPYNLN-
-VPYNLD-
VKPYNL--

VHPYNL--
-WPYNLR-
WSPYNL--
WWPYNL--
WRPYNL--
-WPYNLV-
-WPYNLM-
-WPYNLK-
WVPYNL--
WKPYNL--
WGPYNL--
-WPYNLA-
WMPYNL--
WTPYNL--
-WPYNLL-
WFPYNL--
-WPYNLS-
-WPYNLG-
-WPYNLN-
WEPYNL--
WQPYNL--
-WPYNLQ-
WIPYNL--
WHPYNL--
WDPYNL--
-WPYNLT-
-WPYNLI-
WPPYNL--
WAPYNL--
-WPYNLE-
WNPYNL--
-WPYNLY-
YSPYNL--
YAPYNL--
-YPYNLR-
-YPYNLL-
YKPYNL--
-YPYNLT-
-YPYNLK-
YRPYNL--
YWPYNL--
-YPYNLG-
YMPYNL--
YNPYNL--
-YPYNLI-
-YPYNLM-
-YPYNLE-
YLPYNL--
YIPYNL--
YEPYNL--

YGPYNL--
-YPYNLW-
-YPYNLH-
-YPYNLQ-
YTPYNL--
-YPYNLY-
-YPYNLV-
YHPYNL--
-YPYNLF-
YQPYNL--
-YPYNLA-
YPPYNL--
YYPYNL--
YVPYNL--
YFPYNL--
YDPYNL--
-YPYNLC-
YCPYNL--

PWAL 117 797 49.13

--PWALTP
--PWALRQ
--PWALLP
ARPWAL--
-APWALH-
-APWALG-
AMPWAL--
-APWALK-
ALPWAL--
AYPWAL--
ASPWAL--
-APWALA-
ATPWAL--
AAPWAL--
AHPWAL--
AIPWAL--
AVPWAL--
-APWALM-
AQPWAL--
-APWALV-
-APWALR-
-APWALI-
AEPWAL--
-APWALT-
ANPWAL--
AWPWAL--
-APWALQ-
AKPWAL--
AGPWAL--
-APWALE-
-APWALW-

-APWALN-
ADPWAL--
APPWAL--
CAPWAL--
CVPWAL--
CYPWAL--
CRPWAL--
-CPWALY-
CNPWAL--
CKPWAL--
CEPWAL--
CCPWAL--
-CPWALK-
CHPWAL--
CTPWAL--
CMPWAL--
CSPWAL--
-CPWALM-
-CPWALT-
-CPWALL-
DAPWAL--
DFPWAL--
-DPWALY-
DYPWAL--
DRPWAL--
DIPWAL--
-DPWALT-
DQPWAL--
-DPWALH-
DNPWAL--
DLPWAL--
DWPWAL--
DTPWAL--
DSPWAL--
-DPWALQ-
-DPWALS-
-DPWALR-
-DPWALI-
DGPWAL--
-DPWALV-
-DPWALN-
-DPWALK-
DKPWAL--
DHPWAL--
DMPWAL--
DCPWAL--
DEPWAL--
-DPWALW-
-DPWALE-
-DPWALA-

ESPWAL--
-EPWALL-
EVPWAL--
EAPWAL--
-EPWALY-
-EPWALV-
-EPWALS-
ELPWAL--
EHPWAL--
EFPWAL--
EMPWAL--
ERPWAL--
EGPWAL--
-EPWALR-
-EPWALK-
-EPWALH-
-EPWALE-
-EPWALQ-
ENPWAL--
EYPWAL--
-EPWALF-
EKPWAL--
ETPWAL--
-EPWALW-
-EPWALT-
EPPWAL--
-EPWALG-
EIPWAL--
-EPWALN-
EQPWAL--
EEPWAL--
EDPWAL--
FRPWAL--
-FPWALT-
FGPWAL--
-FPWALG-
FAPWAL--
-FPWALS-
-FPWALH-
FYPWAL--
-FPWALN-
-FPWALA-
FWPWAL--
FSPWAL--
-FPWALR-
FVPWAL--
FMPWAL--
-FPWALF-
FFPWAL--
-FPWALY-

FDPWAL--
-FPWALV-
FQPWAL--
-FPWALK-
-FPWALQ-
GVPWAL--
GRPWAL--
-GPWALS-
GTPWAL--
GLPWAL--
GSPWAL--
-GPWALQ-
GQPWAL--
-GPWALR-
-GPWALA-
-GPWALN-
-GPWALE-
GGPWAL--
GFPWAL--
GIPWAL--
GAPWAL--
GWPWAL--
-GPWALT-
-GPWALM-
-GPWALK-
GNPWAL--
GKPWAL--
GYPWAL--
GMPWAL--
-GPWALV-
-GPWALI-
GDPWAL--
GCPWAL--
GEPWAL--
-GPWALW-
-GPWALF-
GHPWAL--
-GPWALD-
HGPWAL--
-HPWALR-
HVPWAL--
HLPWAL--
HTPWAL--
-HPWALL-
HSPWAL--
-HPWALN-
-HPWALS-
-HPWALH-
HKPWAL--
-HPWALT-

HPPWAL--
HEPWAL--
HWPWAL--
HMPWAL--
HYPWAL--
HDPWAL--
HRPWAL--
-HPWALA-
-HPWALV-
-HPWALI-
-HPWALG-
-HPWALK-
HHPWAL--
HAPWAL--
-HPWALQ-
-HPWALE-
HFPWAL--
IRPWAL--
ISPWAL--
IKPWAL--
IYPWAL--
IWPWAL--
IVPWAL--
-IPWALS-
-IPWALA-
IGPWAL--
-IPWALL-
IFPWAL--
-IPWALK-
-IPWALE-
IIPWAL--
ITPWAL--
-IPWALT-
IQPWAL--
-IPWALG-
-IPWALH-
IMPWAL--
IAPWAL--
-IPWALI-
IPPWAL--
ICPWAL--
-IPWALN-
INPWAL--
IEPWAL--
IHPWAL--
-IPWALW-
-IPWALV-
-IPWALQ-
-IPWALM-
KSPWAL--

KRPWAL--
KLPWAL--
KMPWAL--
KGPWAL--
-KPWALL-
-KPWALQ-
-KPWALS-
KIPWAL--
KHPWAL--
KAPWAL--
-KPWALI-
-KPWALG-
-KPWALT-
-KPWALY-
KNPWAL--
-KPWALF-
-KPWALR-
KVPWAL--
-KPWALN-
KDPWAL--
KYPWAL--
KWPWAL--
-KPWALA-
KQPWAL--
-KPWALK-
-KPWALE-
-KPWALH-
-KPWALW-
KKPWAL--
KFPWAL--
KPPWAL--
-KPWALC-
KEPWAL--
LQPWAL--
-LPWALQ-
LHPWAL--
-LPWALT-
LIPWAL--
LGPWAL--
LTPWAL--
LNPWAL--
-LPWALI-
LVPWAL--
LAPWAL--
-LPWALK-
-LPWALN-
-LPWALA-
-LPWALW-
LLPWAL--
-LPWALH-

-LPWALG-
-LPWALV-
LWPWAL--
LDPWAL--
LKPWAL--
-LPWALM-
LMPWAL--
LCPWAL--
-MPWALT-
MVPWAL--
MKPWAL--
-MPWALS-
MGPWAL--
MSPWAL--
-MPWALH-
MEPWAL--
-MPWALK-
-MPWALI-
MMPWAL--
MTPWAL--
MDPWAL--
-MPWALG-
MPPWAL--
MNPWAL--
MIPWAL--
-MPWALR-
-MPWALE-
MWPWAL--
-MPWALV-
-MPWALA-
MFPWAL--
-MPWALQ-
MQPWAL--
MHPWAL--
MAPWAL--
-MPWALP-
NRPWAL--
NIPWAL--
NAPWAL--
NSPWAL--
NYPWAL--
NVPWAL--
-NPWALK-
NGPWAL--
-NPWALS-
-NPWALY-
NTPWAL--
NMPWAL--
NFPWAL--
NDPWAL--

```
-NPWALT-
-NPWALH-
NHPWAL--
-NPWALI-
NKPWAL--
NNPWAL--
-NPWALV-
-NPWALN-
-NPWALW-
-NPWALQ-
-NPWALG-
-NPWALA-
NPPWAL--
NQPWAL--
--PWALLD
--PWALSG
--PWALVR
--PWALNA
--PWALRT
--PWALTS
--PWALRN
--PWALTK
PRPWAL--
--PWALHL
--PWALAS
--PWALLN
--PWALKS
--PWALSY
--PWALGS
--PWALHR
--PWALRH
--PWALHS
PGPWAL--
--PWALQR
--PWALMS
--PWALGT
--PWALVS
--PWALTN
--PWALTI
--PWALAH
--PWALMG
--PWALSM
--PWALPL
--PWALAT
--PWALSV
--PWALIK
--PWALQT
--PWALVI
--PWALLT
--PWALKR
```

--PWALHK
--PWALGL
--PWALST
--PWALYR
--PWALQG
--PWALNV
--PWALNH
--PWALKT
--PWALTT
--PWALTR
PVPWAL--
--PWALNN
--PWALLQ
--PWALGG
PSPWAL--
PYPWAL--
--PWALVK
--PWALSH
--PWALQS
--PWALNT
--PWALMK
--PWALIR
--PWALGY
--PWALTA
--PWALSN
--PWALIT
--PWALAR
--PWALWR
--PWALRD
--PWALQK
--PWALNR
PKPWAL--
--PWALWS
--PWALNM
--PWALMR
--PWALIG
--PWALVY
--PWALTY
--PWALTH
--PWALLK
--PWALFD
PMPWAL--
--PWALSE
--PWALKY
--PWALHT
--PWALFG
--PWALEM
PWPWAL--
--PWALVH
--PWALHV

--PWALGV
--PWALAM
PFPWAL--
--PWALTM
--PWALNI
--PWALKQ
--PWALIS
--PWALGR
--PWALEK
--PWALAK
--PWALTD
--PWALQQ
--PWALAG
--PWALWG
--PWALRE
--PWALQN
--PWALKG
--PWALFQ
--PWALFH
--PWALWK
--PWALVQ
--PWALSQ
--PWALFV
--PWALYT
--PWALVW
--PWALRW
--PWALLH
--PWALGF
--PWALEA
--PWALTG
--PWALSI
--PWALKV
--PWALHH
--PWALHD
--PWALDS
--PWALAQ
PQPWAL--
PLPWAL--
PIPWAL--
--PWALTW
--PWALQH
--PWALNK
--PWALMT
--PWALKH
--PWALIH
--PWALHI
--PWALFR
--PWALER
--PWALAI
--PWALWH

--PWALQE
--PWALMY
--PWALKE
--PWALHM
--PWALGM
--PWALGA
--PWALAA
PTPWAL--
PDPWAL--
--PWALYG
--PWALQY
--PWALQD
--PWALKD
--PWALIQ
--PWALHE
--PWALDG
--PWALAN
--PWALAD
--PWALWD
--PWALVD
--PWALTE
--PWALSK
--PWALQA
--PWALPM
--PWALNY
--PWALMQ
--PWALGI
--PWALET
PEPWAL--
--PWALYH
--PWALYD
--PWALVE
--PWALPI
--PWALMD
--PWALKM
--PWALIN
--PWALID
--PWALFW
--PWALES
--PWALAV
PCPWAL--
--PWALYS
--PWALYN
--PWALWN
--PWALVN
--PWALMV
--PWALKF
--PWALHP
--PWALHN
--PWALGW

--PWALGK
--PWALAE
PHPWAL--
--PWALWQ
--PWALTQ
--PWALPF
--PWALNG
--PWALGQ
--PWALGH
--PWALEQ
--PWALEG
PAPWAL--
--PWALWI
--PWALQV
--PWALPY
--PWALMN
--PWALMH
--PWALHQ
--PWALFN
--PWALEE
--PWALDY
--PWALYQ
--PWALYI
--PWALQM
--PWALKN
--PWALGE
--PWALFP
--PWALEW
--PWALEN
--PWALEI
--PWALDA
-PPWALM-
PNPWAL--
--PWALYW
--PWALYC
--PWALWW
--PWALWC
--PWALSC
--PWALPQ
--PWALPH
--PWALMP
--PWALME
--PWALIW
--PWALII
--PWALHY
--PWALHA
--PWALGC
--PWALEY
--PWALEV
--PWALEH

--PWALDW
--PWALDT
--PWALDN
--PWALCY
--PWALCQ
--PWALAF
--PWALAC
-QPWALA-
QNPWAL--
QSPWAL--
-QPWALT-
-QPWALM-
QQPWAL--
-QPWALN-
QRPWAL--
QVPWAL--
QMPWAL--
QTPWAL--
QLPWAL--
QGPWAL--
-QPWALS-
QIPWAL--
QAPWAL--
-QPWALV-
-QPWALH-
-QPWALQ-
-QPWALG-
QWPWAL--
-QPWALL-
-QPWALE-
-QPWALI-
QDPWAL--
QYPWAL--
QEPWAL--
QKPWAL--
-QPWALD-
QPPWAL--
-RPWALS-
RSPWAL--
-RPWALK-
RAPWAL--
RMPWAL--
RVPWAL--
RHPWAL--
-RPWALA-
RFPWAL--
-RPWALV-
RNPWAL--
RLPWAL--
-RPWALI-

```
-RPWALM-
-RPWALF-
RYPWAL--
-RPWALE-
-RPWALY-
-RPWALN-
RPPWAL--
RQPWAL--
-RPWALW-
RWPWAL--
RDPWAL--
-RPWALH-
RIPWAL--
RCPWAL--
-RPWALD-
-RPWALC-
SRPWAL--
SGPWAL--
-SPWALR-
-SPWALL-
SYPWAL--
SLPWAL--
SAPWAL--
-SPWALV-
SNPWAL--
SHPWAL--
SQPWAL--
-SPWALN-
SIPWAL--
SSPWAL--
-SPWALH-
-SPWALI-
SVPWAL--
SWPWAL--
-SPWALE-
-SPWALG-
SMPWAL--
SEPWAL--
-SPWALY-
-SPWALQ-
-SPWALK-
-SPWALA-
-SPWALM-
SPPWAL--
SFPWAL--
-SPWALW-
-SPWALT-
SKPWAL--
TRPWAL--
-TPWALK-
```

-TPWALL-
TFPWAL--
-TPWALI-
-TPWALV-
-TPWALS-
TGPWAL--
TIPWAL--
TAPWAL--
TVPWAL--
THPWAL--
-TPWALH-
TLPWAL--
TTPWAL--
TQPWAL--
TSPWAL--
TWPWAL--
-TPWALW-
-TPWALT-
TDPWAL--
-TPWALN-
-TPWALA-
TKPWAL--
-TPWALQ-
TMPWAL--
TYPWAL--
-TPWALE-
TPPWAL--
-TPWALG-
TNPWAL--
-TPWALC-
TEPWAL--
VSPWAL--
VKPWAL--
VTPWAL--
-VPWALT-
VAPWAL--
VHPWAL--
VVPWAL--
-VPWALK-
-VPWALS-
-VPWALA-
VLPWAL--
VRPWAL--
VFPWAL--
VGPWAL--
-VPWALG-
-VPWALN-
VNPWAL--
VQPWAL--
-VPWALH-

-VPWALI-
VWPWAL--
VMPWAL--
-VPWALM-
-VPWALQ-
-VPWALE-
VPPWAL--
VIPWAL--
VCPWAL--
VEPWAL--
-VPWALP-
-VPWALC-
WSPWAL--
WKPWAL--
-WPWALT-
-WPWALK-
WGPWAL--
WAPWAL--
WLPWAL--
WYPWAL--
WHPWAL--
WEPWAL--
WFPWAL--
-WPWALR-
-WPWALN-
-WPWALE-
WVPWAL--
WQPWAL--
-WPWALV-
-WPWALA-
-WPWALQ-
WNPWAL--
-WPWALS-
-WPWALI-
WMPWAL--
-WPWALM-
-WPWALH-
YRPWAL--
-YPWALK-
YLPWAL--
YTPWAL--
-YPWALV-
-YPWALS-
YKPWAL--
-YPWALQ-
YNPWAL--
YGPWAL--
YFPWAL--
-YPWALR-
-YPWALM-

-YPWALA-
YDPWAL--
YYPWAL--
-YPWALW-
-YPWALT-
YVPWAL--
-YPWALE-
YAPWAL--
-YPWALI-
-YPWALH-
YWPWAL--
YMPWAL--
YCPWAL--
YQPWAL--
YPPWAL--
YIPWAL--

PWGI	149	777	49.09

-LPWGII-
--PWGISP
-APWGIL-
AKPWGI--
AMPWGI--
-APWGIY-
AVPWGI--
-APWGIR-
AIPWGI--
AWPWGI--
ANPWGI--
ATPWGI--
ARPWGI--
ASPWGI--
AGPWGI--
-APWGIS-
AFPWGI--
-APWGIV-
-APWGIK-
-APWGIG-
AYPWGI--
AQPWGI--
-APWGIM-
-APWGIH-
ADPWGI--
-APWGII-
AHPWGI--
AEPWGI--
ACPWGI--
AAPWGI--
-APWGIN-
-APWGIA-
ALPWGI--

CQPWGI--
CMPWGI--
CEPWGI--
CTPWGI--
-CPWGIK-
CVPWGI--
-CPWGIL-
CHPWGI--
-CPWGIQ-
CLPWGI--
CIPWGI--
DGPWGI--
DRPWGI--
-DPWGIR-
-DPWGIN-
DLPWGI--
-DPWGIV-
DFPWGI--
DWPWGI--
DHPWGI--
DVPWGI--
DTPWGI--
DSPWGI--
-DPWGIE-
-DPWGIQ-
-DPWGIA-
DIPWGI--
DEPWGI--
DYPWGI--
-DPWGIT-
-DPWGII-
-DPWGIH-
-DPWGIF-
DNPWGI--
ESPWGI--
-EPWGIR-
EQPWGI--
ERPWGI--
-EPWGIL-
EKPWGI--
-EPWGII-
-EPWGIF-
EFPWGI--
EAPWGI--
-EPWGIW-
-EPWGIE-
ELPWGI--
ENPWGI--
EGPWGI--
-EPWGIG-

EPPWGI--
EYPWGI--
-EPWGIK-
-EPWGIA-
EMPWGI--
-EPWGIH-
EIPWGI--
-EPWGIN-
-EPWGIM-
EDPWGI--
FGPWGI--
-FPWGIK-
FSPWGI--
FLPWGI--
FNPWGI--
-FPWGIT-
FDPWGI--
FRPWGI--
-FPWGIR-
-FPWGIL-
FKPWGI--
FQPWGI--
FVPWGI--
-FPWGIH-
FWPWGI--
-FPWGIM-
FPPWGI--
FIPWGI--
-FPWGIV-
-FPWGIE-
-FPWGII-
-FPWGIA-
FFPWGI--
-FPWGIS-
-FPWGIN-
FMPWGI--
FYPWGI--
-FPWGIQ-
-GPWGIR-
GRPWGI--
GVPWGI--
-GPWGIV-
-GPWGIL-
GTPWGI--
GPPWGI--
-GPWGIS-
GQPWGI--
GIPWGI--
GAPWGI--
GYPWGI--

GNPWGI--
GMPWGI--
GHPWGI--
GLPWGI--
-GPWGIN-
-GPWGIK-
-GPWGIE-
GSPWGI--
-GPWGIG-
-GPWGIA-
-GPWGIH-
GGPWGI--
GDPWGI--
-GPWGIT-
-GPWGIQ-
-GPWGIM-
GKPWGI--
GEPWGI--
HRPWGI--
-HPWGIL-
HEPWGI--
-HPWGIK-
-HPWGIR-
HNPWGI--
HVPWGI--
-HPWGIS-
-HPWGIV-
HLPWGI--
HYPWGI--
HKPWGI--
HAPWGI--
HGPWGI--
-HPWGIM-
HHPWGI--
HMPWGI--
-HPWGIN-
HPPWGI--
-HPWGIT-
-HPWGII-
HSPWGI--
HTPWGI--
-HPWGIY-
HDPWGI--
-HPWGIQ-
-HPWGIH-
-HPWGIG-
-IPWGIM-
-IPWGIV-
INPWGI--
ILPWGI--

IPPWGI--
ITPWGI--
ISPWGI--
IAPWGI--
-IPWGIR-
-IPWGIS-
-IPWGIQ-
-IPWGIN-
-IPWGII-
IKPWGI--
IIPWGI--
-IPWGIL-
IGPWGI--
-IPWGIT-
IEPWGI--
IVPWGI--
-IPWGIH-
-IPWGIK-
-IPWGIG-
-IPWGIA-
IQPWGI--
IHPWGI--
-IPWGIP-
IFPWGI--
KSPWGI--
-KPWGIL-
-KPWGIN-
-KPWGIM-
KIPWGI--
KRPWGI--
-KPWGIR-
KGPWGI--
-KPWGII-
-KPWGIS-
-KPWGIH-
KFPWGI--
KWPWGI--
-KPWGIY-
KHPWGI--
KMPWGI--
KVPWGI--
KKPWGI--
KAPWGI--
-KPWGIT-
KCPWGI--
KYPWGI--
-KPWGIV-
-KPWGIE-
-KPWGIA-
-KPWGIC-

KLPWGI--
KQPWGI--
-KPWGIK-
-KPWGIF-
KPPWGI--
KEPWGI--
KDPWGI--
LSPWGI--
LRPWGI--
LLPWGI--
LGPWGI--
-LPWGIT-
-LPWGIR-
-LPWGIH-
LKPWGI--
LTPWGI--
LPPWGI--
LMPWGI--
-LPWGIL-
-LPWGIV-
-LPWGIQ-
LQPWGI--
LHPWGI--
LYPWGI--
LNPWGI--
LWPWGI--
-LPWGIK-
LDPWGI--
LIPWGI--
-LPWGIA-
LFPWGI--
-LPWGIN-
-LPWGIM-
-LPWGIG-
-LPWGID-
MLPWGI--
MGPWGI--
-MPWGIK-
-MPWGIG-
-MPWGIH-
MNPWGI--
MKPWGI--
MHPWGI--
MYPWGI--
MSPWGI--
MRPWGI--
MMPWGI--
-MPWGIT-
MAPWGI--
-MPWGIL-

MDPWGI--
MEPWGI--
MVPWGI--
MTPWGI--
MQPWGI--
MFPWGI--
-MPWGIS-
-MPWGIN-
MIPWGI--
MWPWGI--
-MPWGIW-
-MPWGIV-
-MPWGIQ-
-MPWGIM-
NRPWGI--
-NPWGIM-
-NPWGIR-
NMPWGI--
NAPWGI--
-NPWGIS-
NIPWGI--
NLPWGI--
NVPWGI--
-NPWGIL-
-NPWGIF-
NDPWGI--
NPPWGI--
-NPWGIQ-
-NPWGII-
NKPWGI--
-NPWGIH-
NTPWGI--
NNPWGI--
-NPWGIT-
-NPWGIN-
-NPWGIV-
NWPWGI--
-NPWGIK-
NSPWGI--
-NPWGIY-
NGPWGI--
NEPWGI--
NHPWGI--
NQPWGI--
-NPWGIG-
-NPWGIA-
--PWGIRS
--PWGIVS
--PWGIRG
--PWGISS

```
--PWGIRT
--PWGIMS
--PWGILS
--PWGIRQ
--PWGIRD
--PWGIRE
--PWGIRN
--PWGIVT
--PWGISL
--PWGIRI
--PWGIVN
PGPWGI--
--PWGIKS
--PWGIKG
--PWGIHL
--PWGIAG
--PWGIVH
--PWGISM
--PWGISI
--PWGIMG
--PWGITL
--PWGIMH
--PWGILD
--PWGIHR
--PWGIVQ
--PWGITK
--PWGILN
--PWGIEA
--PWGITT
--PWGIST
--PWGIRL
--PWGINA
--PWGIGS
PRPWGI--
--PWGIVG
--PWGIQS
PSPWGI--
--PWGITW
--PWGITR
--PWGIHF
--PWGIRY
--PWGIRV
--PWGIIR
--PWGISQ
--PWGIQT
--PWGIVV
--PWGISR
--PWGIIS
--PWGIIK
--PWGITG
```

```
--PWGIRA
--PWGIKN
--PWGIWK
--PWGITQ
--PWGISH
--PWGIHA
--PWGIGG
--PWGIFS
--PWGIAR
--PWGIWG
--PWGILG
--PWGIKD
PFPWGI--
--PWGIYE
--PWGIQH
--PWGIMT
--PWGILR
--PWGIKR
--PWGIWR
--PWGITS
--PWGISV
--PWGIRK
--PWGIMY
--PWGIMA
--PWGIKV
--PWGIVF
--PWGISK
--PWGIQV
--PWGILT
--PWGIIT
--PWGIIM
--PWGIIG
PVPWGI--
PEPWGI--
PYPWGI--
PWPWGI--
--PWGIYY
--PWGISY
--PWGISA
--PWGIRM
--PWGINR
--PWGIID
--PWGIHV
PLPWGI--
--PWGIYQ
--PWGIKT
--PWGIHH
--PWGIGV
--PWGIAV
--PWGIVA
```

--PWGISE
--PWGIRW
--PWGIQG
--PWGIMN
--PWGIKI
--PWGIHT
--PWGIHG
--PWGIGA
--PWGIEV
--PWGIAS
PDPWGI--
--PWGIWE
--PWGITA
--PWGIMD
--PWGILW
--PWGIHS
--PWGIYG
--PWGITF
--PWGITE
--PWGIQF
--PWGINN
--PWGIFQ
--PWGIER
--PWGIAW
PNPWGI--
--PWGIYK
--PWGIWM
--PWGIWH
--PWGITM
--PWGIQQ
--PWGINK
--PWGIMV
--PWGIMF
--PWGILK
--PWGILH
--PWGILE
--PWGIKF
--PWGIHK
--PWGIGT
--PWGIAY
--PWGIVK
--PWGIVE
--PWGITV
--PWGITD
--PWGIQA
--PWGINM
--PWGILQ
--PWGIHE
--PWGIFR
--PWGIVR

--PWGITY
--PWGITH
--PWGISN
--PWGISD
--PWGIRP
--PWGIQY
--PWGIQE
--PWGING
--PWGIMQ
--PWGIMK
--PWGIKE
--PWGIIQ
--PWGIGI
--PWGIEY
--PWGIEQ
--PWGIEN
--PWGIAI
--PWGIYN
--PWGIVY
--PWGIQR
--PWGINQ
--PWGIKY
--PWGIKW
--PWGIIY
--PWGIIV
--PWGIIN
--PWGIIH
--PWGIHQ
--PWGIHN
--PWGIHM
--PWGIFV
--PWGIFE
--PWGIEW
--PWGIET
--PWGIED
--PWGIDS
--PWGIDG
--PWGICQ
--PWGIAA
--PWGIYM
--PWGIWD
--PWGIVD
--PWGIQL
--PWGIQI
--PWGIQD
--PWGINY
--PWGIMW
--PWGIMM
--PWGIME
--PWGIKL

--PWGIGY
--PWGIFK
--PWGIEK
--PWGIEE
--PWGIDA
--PWGIAF
PHPWGI--
--PWGIYF
--PWGIWY
--PWGIWW
--PWGIVC
--PWGITN
--PWGIPH
--PWGINI
--PWGIND
--PWGIKQ
--PWGIKM
--PWGIKK
--PWGIKH
--PWGIKA
--PWGIII
--PWGIGN
--PWGIGM
--PWGIGH
--PWGIFG
--PWGIEI
--PWGIEG
--PWGIDI
--PWGIAQ
--PWGIAM
--PWGIAK
-PPWGIY-
-PPWGIR-
-PPWGIF-
PAPWGI--
QRPWGI--
QSPWGI--
-QPWGIL-
-QPWGIQ-
QYPWGI--
QTPWGI--
QVPWGI--
QIPWGI--
QEPWGI--
-QPWGIR-
QGPWGI--
-QPWGIK-
-QPWGIG-
QFPWGI--
QCPWGI--

QDPWGI--
-QPWGIT-
QQPWGI--
-QPWGIM-
-QPWGIE-
QAPWGI--
-QPWGIV-
QNPWGI--
QLPWGI--
-QPWGIW-
-QPWGIS-
-QPWGIH-
-QPWGIN-
RSPWGI--
RTPWGI--
-RPWGIS-
-RPWGIL-
-RPWGII-
RAPWGI--
-RPWGIA-
-RPWGIR-
RFPWGI--
-RPWGIV-
-RPWGIH-
-RPWGIN-
RNPWGI--
RPPWGI--
RDPWGI--
-RPWGIG-
RMPWGI--
REPWGI--
-RPWGIK-
RLPWGI--
RGPWGI--
-RPWGIT-
-RPWGIQ-
RKPWGI--
-RPWGIM-
RRPWGI--
-RPWGIE-
RIPWGI--
RHPWGI--
RCPWGI--
RWPWGI--
RQPWGI--
-RPWGIP-
SLPWGI--
SRPWGI--
SSPWGI--
-SPWGIT-

−SPWGIK−
SKPWGI−−
−SPWGIN−
−SPWGIH−
SNPWGI−−
SVPWGI−−
−SPWGIS−
SPPWGI−−
−SPWGIM−
SIPWGI−−
−SPWGIQ−
STPWGI−−
SMPWGI−−
SYPWGI−−
−SPWGIA−
−SPWGIG−
−SPWGII−
SQPWGI−−
−SPWGIW−
SGPWGI−−
SFPWGI−−
SCPWGI−−
SHPWGI−−
SAPWGI−−
−SPWGIV−
SDPWGI−−
SWPWGI−−
TVPWGI−−
TQPWGI−−
−TPWGIK−
−TPWGIL−
TAPWGI−−
TLPWGI−−
TGPWGI−−
−TPWGII−
TEPWGI−−
TTPWGI−−
−TPWGIV−
−TPWGIN−
TSPWGI−−
TRPWGI−−
−TPWGIS−
−TPWGIR−
TDPWGI−−
−TPWGIT−
−TPWGIQ−
TFPWGI−−
TYPWGI−−
−TPWGIE−
−TPWGIM−

TKPWGI--
TIPWGI--
-TPWGIA-
TWPWGI--
TNPWGI--
-TPWGIG-
TMPWGI--
-VPWGIR-
-VPWGIK-
VRPWGI--
VYPWGI--
VHPWGI--
VLPWGI--
VVPWGI--
VSPWGI--
-VPWGII-
VWPWGI--
VFPWGI--
-VPWGIV-
-VPWGIL-
VGPWGI--
VQPWGI--
-VPWGIS-
-VPWGIM-
VKPWGI--
-VPWGIT-
VIPWGI--
VEPWGI--
VMPWGI--
VTPWGI--
-VPWGIF-
-VPWGIA-
VPPWGI--
-VPWGIQ-
-VPWGIE-
-VPWGIG-
VNPWGI--
VCPWGI--
-VPWGIN-
-VPWGIH-
WKPWGI--
WDPWGI--
-WPWGIL-
-WPWGII-
WGPWGI--
WRPWGI--
-WPWGIK-
WLPWGI--
WAPWGI--
WWPWGI--

-WPWGIV-
-WPWGIR-
-WPWGIQ-
-WPWGIH-
-WPWGIA-
WMPWGI--
WTPWGI--
WEPWGI--
WVPWGI--
WQPWGI--
-WPWGIS-
-WPWGIN-
-WPWGIM-
-WPWGIG-
WFPWGI--
YSPWGI--
YTPWGI--
YKPWGI--
YHPWGI--
YVPWGI--
YGPWGI--
YYPWGI--
-YPWGII-
YDPWGI--
YEPWGI--
YAPWGI--
YRPWGI--
-YPWGIY-
YQPWGI--
-YPWGIS-
-YPWGIF-
YNPWGI--
-YPWGIR-
-YPWGIA-
YMPWGI--
YIPWGI--
-YPWGIV-
-YPWGIN-
-YPWGIM-
-YPWGIH-
-YPWGIE-
YPPWGI--
-YPWGIT-
-YPWGIG-

PKAI 259 719 49.09

--PKAILP
-APKAIS-
AEPKAI--
-APKAIN-
AYPKAI--

-APKAIK-
AMPKAI--
ARPKAI--
ALPKAI--
AKPKAI--
AVPKAI--
-APKAIY-
-APKAIQ-
ADPKAI--
AWPKAI--
-APKAIW-
-APKAII-
-APKAIF-
-APKAIV-
-APKAIT-
-APKAIE-
-APKAIA-
AHPKAI--
ASPKAI--
-APKAIM-
-APKAIC-
-APKAIG-
ANPKAI--
AGPKAI--
CPPKAI--
CTPKAI--
CLPKAI--
-CPKAIF-
CKPKAI--
CAPKAI--
-CPKAIQ-
CRPKAI--
-CPKAIK-
CEPKAI--
CIPKAI--
-CPKAIS-
CNPKAI--
CGPKAI--
DYPKAI--
DAPKAI--
DVPKAI--
-DPKAIW-
DSPKAI--
-DPKAIL-
DQPKAI--
-DPKAIK-
-DPKAII-
-DPKAIA-
DHPKAI--
-DPKAIY-

-DPKAIS-
-DPKAIR-
-DPKAIN-
-DPKAIF-
DIPKAI--
DEPKAI--
-EPKAIL-
-EPKAII-
-EPKAIT-
EYPKAI--
ERPKAI--
EQPKAI--
-EPKAIR-
-EPKAIQ-
EVPKAI--
-EPKAIS-
-EPKAIH-
ESPKAI--
-EPKAIG-
ELPKAI--
EKPKAI--
ENPKAI--
-EPKAIV-
EIPKAI--
EDPKAI--
-EPKAIY-
ETPKAI--
-EPKAIN-
EMPKAI--
FSPKAI--
-FPKAIL-
FAPKAI--
-FPKAIM-
-FPKAIV-
FGPKAI--
FIPKAI--
FKPKAI--
FVPKAI--
-FPKAIY-
-FPKAIK-
FLPKAI--
FFPKAI--
FEPKAI--
-FPKAIH-
-FPKAII-
FPPKAI--
-FPKAIS-
-FPKAIA-
-FPKAIE-
GMPKAI--

GLPKAI--
GTPKAI--
-GPKAIQ-
-GPKAIW-
GSPKAI--
GKPKAI--
GQPKAI--
-GPKAIM-
GFPKAI--
-GPKAIV-
-GPKAIT-
GIPKAI--
-GPKAIK-
GWPKAI--
-GPKAIE-
GHPKAI--
GDPKAI--
-GPKAIY-
-GPKAIH-
GEPKAI--
-GPKAIG-
GYPKAI--
-GPKAIL-
GPPKAI--
-GPKAIN-
-GPKAII-
-GPKAIA-
GAPKAI--
HRPKAI--
HVPKAI--
HAPKAI--
HLPKAI--
HSPKAI--
-HPKAIY-
HPPKAI--
-HPKAIL-
HYPKAI--
-HPKAIF-
-HPKAIA-
-HPKAIW-
-HPKAII-
HIPKAI--
HKPKAI--
HTPKAI--
-HPKAIG-
HNPKAI--
HQPKAI--
-HPKAIH-
HCPKAI--
-IPKAIL-

INPKAI--
IIPKAI--
IKPKAI--
-IPKAIF-
ITPKAI--
-IPKAIW-
ICPKAI--
-IPKAIV-
IGPKAI--
-IPKAIG-
ISPKAI--
-IPKAIM-
IEPKAI--
-IPKAIQ-
-IPKAIK-
-IPKAII-
IFPKAI--
IWPKAI--
-IPKAIS-
IMPKAI--
IQPKAI--
-IPKAIN-
-IPKAIA-
IHPKAI--
IDPKAI--
IVPKAI--
KAPKAI--
-KPKAII-
KYPKAI--
KLPKAI--
-KPKAIW-
-KPKAIQ-
-KPKAIH-
KGPKAI--
-KPKAIF-
KHPKAI--
KEPKAI--
KTPKAI--
-KPKAIK-
KPPKAI--
-KPKAIN-
-KPKAIE-
KNPKAI--
KVPKAI--
-KPKAIS-
-KPKAIV-
KDPKAI--
-KPKAIY-
-KPKAIA-
KRPKAI--

-KPKAIG-
KIPKAI--
-KPKAIT-
-KPKAIM-
LSPKAI--
LKPKAI--
LTPKAI--
-LPKAIL-
LAPKAI--
LLPKAI--
LMPKAI--
LVPKAI--
LHPKAI--
-LPKAIF-
LNPKAI--
LWPKAI--
LRPKAI--
-LPKAIT-
LFPKAI--
LPPKAI--
LGPKAI--
-LPKAIW-
-LPKAIM-
-LPKAIH-
LEPKAI--
LDPKAI--
LQPKAI--
LIPKAI--
-LPKAIV-
LCPKAI--
-LPKAIN-
-LPKAIG-
-LPKAIC-
-MPKAIS-
MGPKAI--
MVPKAI--
-MPKAIL-
-MPKAIF-
MLPKAI--
-MPKAIN-
-MPKAIV-
MAPKAI--
MPPKAI--
MNPKAI--
-MPKAIG-
MKPKAI--
-MPKAIQ-
MDPKAI--
-MPKAIH-
MYPKAI--

```
-MPKAIW-
-MPKAIT-
-MPKAIA-
MFPKAI--
-MPKAIP-
-MPKAIE-
-MPKAIC-
NSPKAI--
NAPKAI--
NRPKAI--
NHPKAI--
NMPKAI--
NEPKAI--
-NPKAIL-
NFPKAI--
-NPKAIQ-
-NPKAIF-
NNPKAI--
NWPKAI--
NTPKAI--
-NPKAIS-
NLPKAI--
NVPKAI--
-NPKAIW-
NQPKAI--
-NPKAIR-
-NPKAIY-
NGPKAI--
NCPKAI--
-NPKAIT-
-NPKAII-
NIPKAI--
NPPKAI--
-NPKAIN-
-NPKAIA-
--PKAIRS
--PKAITS
--PKAISV
--PKAIGL
--PKAILL
--PKAIVN
--PKAIRV
--PKAIHS
--PKAIYF
--PKAILF
--PKAIMA
--PKAIWS
--PKAIYD
--PKAITY
--PKAIKL
```

--PKAIVW
--PKAILG
--PKAIYW
--PKAIYK
--PKAILS
--PKAIGS
--PKAIGI
--PKAIHQ
--PKAISM
--PKAIAS
PLPKAI--
--PKAIWV
--PKAITK
--PKAITT
--PKAIRN
--PKAIMG
--PKAIMV
--PKAIIS
--PKAIIF
--PKAIFI
PVPKAI--
--PKAIVT
--PKAILI
--PKAIYQ
--PKAISW
--PKAISL
--PKAIQS
--PKAIHL
--PKAIGG
--PKAIVV
--PKAINA
PSPKAI--
--PKAIQA
--PKAIIG
--PKAIEV
PIPKAI--
--PKAIYV
--PKAIWD
--PKAIRD
PGPKAI--
--PKAIWN
--PKAITM
--PKAIPF
--PKAIFG
--PKAIVA
--PKAIMS
--PKAILK
--PKAIKQ
--PKAISY
--PKAISA

--PKAIRM
--PKAILE
--PKAIKG
--PKAIAF
--PKAIYT
--PKAIWG
--PKAIVS
--PKAISN
--PKAIQQ
--PKAINS
--PKAILV
--PKAILT
--PKAIKA
--PKAIFF
--PKAIAW
--PKAIAL
--PKAIAG
PAPKAI--
PTPKAI--
--PKAIWK
--PKAIVI
--PKAITQ
--PKAITA
--PKAINT
--PKAINL
--PKAINH
--PKAIMH
--PKAIKN
--PKAIFQ
--PKAIFN
--PKAIAI
--PKAIYY
--PKAIWA
--PKAISI
--PKAIHK
--PKAIHH
--PKAIFY
--PKAIEL
--PKAIAV
--PKAIAH
PNPKAI--
--PKAIYS
--PKAISK
--PKAISE
--PKAIKH
--PKAIFT
--PKAIFE
--PKAIYH
--PKAIWT
--PKAIVL

--PKAISH
--PKAIRE
--PKAIQV
--PKAINY
--PKAINQ
--PKAIMK
--PKAIKD
--PKAIFM
--PKAIQG
--PKAIMD
--PKAILM
--PKAIKM
--PKAIIW
--PKAIIH
--PKAIGT
--PKAIFV
--PKAIFA
--PKAIAM
--PKAIAD
--PKAIYN
--PKAIYE
--PKAIWL
--PKAIVM
--PKAIVH
--PKAIQL
--PKAIQI
--PKAINR
--PKAIMY
--PKAIMF
--PKAIKS
--PKAIHG
--PKAIWH
--PKAITE
--PKAIRI
--PKAIQT
--PKAIQH
--PKAIPY
--PKAIPL
--PKAINW
--PKAINM
--PKAIKV
--PKAIIQ
--PKAIID
--PKAIHV
--PKAIHI
--PKAIFK
--PKAIET
--PKAIEN
--PKAIAY
--PKAIAA

--PKAIWI
--PKAIVQ
--PKAIIK
--PKAIHT
--PKAIGM
--PKAICS
PRPKAI--
--PKAIWM
--PKAITI
--PKAITH
--PKAITF
--PKAIQN
--PKAIQM
--PKAIME
--PKAIKY
--PKAIKT
--PKAIKI
--PKAIII
--PKAIGY
--PKAIFH
--PKAIFD
--PKAIEY
--PKAIEH
--PKAIDG
--PKAICM
--PKAICF
PDPKAI--
--PKAIYM
--PKAIWF
--PKAIVY
--PKAIVD
--PKAIVC
--PKAITV
--PKAISD
--PKAIRC
--PKAIQW
--PKAIPM
--PKAINN
--PKAIMN
--PKAIMM
--PKAIKW
--PKAIKF
--PKAIHW
--PKAIGW
--PKAIGQ
--PKAIGA
--PKAIEQ
--PKAIEK
--PKAIEG
--PKAICT

--PKAIAN
PHPKAI--
-PPKAIV-
-PPKAII-
-PPKAIC-
PKPKAI--
--PKAIYI
--PKAIWE
--PKAIVK
--PKAIVE
--PKAISF
--PKAIQD
--PKAIPT
--PKAIPS
--PKAINK
--PKAING
--PKAINF
--PKAIND
--PKAIKP
--PKAIKC
--PKAIHY
--PKAIHF
--PKAIHE
--PKAIGE
--PKAIFW
--PKAIDY
--PKAIDF
--PKAIDD
--PKAICR
--PKAICN
--PKAIAC
PFPKAI--
-QPKAIV-
QRPKAI--
QEPKAI--
QSPKAI--
QFPKAI--
QVPKAI--
QPPKAI--
QIPKAI--
-QPKAIN-
-QPKAIF-
QLPKAI--
QYPKAI--
-QPKAIY-
QTPKAI--
-QPKAIT-
QNPKAI--
-QPKAIW-
QGPKAI--

QWPKAI--
QDPKAI--
RGPKAI--
RIPKAI--
-RPKAIF-
-RPKAIV-
-RPKAIT-
RTPKAI--
RPPKAI--
RMPKAI--
-RPKAIQ-
-RPKAIN-
RAPKAI--
-RPKAIM-
RNPKAI--
RHPKAI--
RFPKAI--
RQPKAI--
-RPKAIW-
RDPKAI--
-RPKAIA-
-RPKAIK-
-RPKAIE-
-RPKAIY-
-RPKAIH-
-RPKAIG-
-RPKAIC-
RRPKAI--
RCPKAI--
SSPKAI--
SGPKAI--
-SPKAIT-
SRPKAI--
-SPKAII-
-SPKAIM-
-SPKAIA-
SYPKAI--
-SPKAIF-
SEPKAI--
-SPKAIS-
STPKAI--
-SPKAIN-
SKPKAI--
SDPKAI--
SPPKAI--
SIPKAI--
-SPKAIY-
-SPKAIW-
SQPKAI--
SNPKAI--

−SPKAIV−
−SPKAIG−
−SPKAIE−
SFPKAI−−
TRPKAI−−
TVPKAI−−
TQPKAI−−
−TPKAIW−
−TPKAIT−
THPKAI−−
−TPKAIH−
TKPKAI−−
−TPKAIS−
TLPKAI−−
TWPKAI−−
TEPKAI−−
TTPKAI−−
TYPKAI−−
−TPKAIV−
−TPKAIF−
TMPKAI−−
TSPKAI−−
−TPKAIQ−
−TPKAIN−
−TPKAIA−
TPPKAI−−
TNPKAI−−
−TPKAIM−
TAPKAI−−
−TPKAII−
−TPKAIE−
−VPKAIL−
VSPKAI−−
−VPKAIS−
VRPKAI−−
−VPKAIT−
−VPKAIR−
VGPKAI−−
−VPKAII−
VEPKAI−−
VPPKAI−−
−VPKAIM−
VQPKAI−−
−VPKAIW−
−VPKAIQ−
−VPKAIE−
VLPKAI−−
−VPKAIN−
VKPKAI−−
VIPKAI−−

VAPKAI--
-VPKAIY-
-VPKAIH-
VMPKAI--
VVPKAI--
VNPKAI--
-VPKAIA-
VHPKAI--
VDPKAI--
VFPKAI--
-VPKAIC-
VYPKAI--
VTPKAI--
-VPKAIG-
WRPKAI--
WYPKAI--
-WPKAIF-
WSPKAI--
-WPKAIY-
WAPKAI--
-WPKAIE-
WIPKAI--
WQPKAI--
WMPKAI--
-WPKAIW-
WPPKAI--
-WPKAIH-
WWPKAI--
WEPKAI--
WVPKAI--
-WPKAII-
WCPKAI--
WNPKAI--
-WPKAIM-
WKPKAI--
-WPKAIQ-
WGPKAI--
WFPKAI--
WDPKAI--
YGPKAI--
YSPKAI--
-YPKAIM-
-YPKAIF-
-YPKAIV-
YIPKAI--
YNPKAI--
YRPKAI--
-YPKAIS-
YQPKAI--
-YPKAII-

YWPKAI--
YVPKAI--
YYPKAI--
-YPKAIG-
YEPKAI--
YDPKAI--
-YPKAIT-
-YPKAIQ-
YCPKAI--
YTPKAI--
-YPKAIW-
YKPKAI--
YHPKAI--
-YPKAIE-

PLAY	30	869	49.04

-DPLAYK-
-KPLAYM-
--PLAYTP
-SPLAYD-
-TPLAYP-
-APLAYL-
AKPLAY--
ASPLAY--
-APLAYK-
-APLAYR-
ATPLAY--
AGPLAY--
-APLAYS-
AYPLAY--
-APLAYW-
-APLAYG-
AHPLAY--
AIPLAY--
-APLAYT-
-APLAYH-
AFPLAY--
AVPLAY--
ADPLAY--
-APLAYN-
-APLAYI-
-APLAYF-
ANPLAY--
AWPLAY--
-APLAYV-
AAPLAY--
AQPLAY--
-APLAYQ-
-APLAYE-
AMPLAY--
-APLAYM-

-APLAYY-
AEPLAY--
-APLAYA-
ACPLAY--
CQPLAY--
CSPLAY--
-CPLAYV-
CHPLAY--
CTPLAY--
CRPLAY--
-CPLAYI-
CEPLAY--
CCPLAY--
CYPLAY--
-CPLAYT-
-CPLAYM-
CAPLAY--
-CPLAYW-
-CPLAYQ-
-CPLAYE-
CKPLAY--
CVPLAY--
-CPLAYR-
-CPLAYN-
CMPLAY--
CLPLAY--
CDPLAY--
-DPLAYR-
-DPLAYS-
DSPLAY--
-DPLAYL-
-DPLAYH-
-DPLAYT-
DHPLAY--
DRPLAY--
-DPLAYA-
-DPLAYV-
DAPLAY--
DWPLAY--
DGPLAY--
DKPLAY--
-DPLAYI-
DEPLAY--
DVPLAY--
DMPLAY--
DYPLAY--
-DPLAYM-
-DPLAYF-
DLPLAY--
DFPLAY--

DQPLAY--
-DPLAYY-
-DPLAYW-
-DPLAYG-
DNPLAY--
DTPLAY--
-DPLAYC-
DDPLAY--
-EPLAYR-
-EPLAYL-
-EPLAYF-
EYPLAY--
ESPLAY--
EFPLAY--
-EPLAYV-
EVPLAY--
-EPLAYA-
EQPLAY--
ERPLAY--
-EPLAYG-
ENPLAY--
EIPLAY--
-EPLAYT-
EGPLAY--
-EPLAYQ-
EAPLAY--
EPPLAY--
-EPLAYS-
-EPLAYW-
EKPLAY--
ETPLAY--
-EPLAYY-
-EPLAYN-
-EPLAYI-
EEPLAY--
EWPLAY--
-EPLAYM-
-EPLAYC-
EMPLAY--
ECPLAY--
FTPLAY--
FGPLAY--
-FPLAYL-
-FPLAYT-
-FPLAYI-
FKPLAY--
-FPLAYR-
-FPLAYA-
-FPLAYS-
FNPLAY--

FVPLAY--
FDPLAY--
FSPLAY--
FHPLAY--
FAPLAY--
FMPLAY--
FLPLAY--
FPPLAY--
FIPLAY--
FEPLAY--
-FPLAYN-
-FPLAYW-
FFPLAY--
FQPLAY--
-FPLAYM-
-FPLAYK-
-FPLAYH-
FCPLAY--
-GPLAYS-
-GPLAYL-
-GPLAYR-
GRPLAY--
-GPLAYK-
-GPLAYI-
GLPLAY--
-GPLAYA-
GVPLAY--
GSPLAY--
GKPLAY--
-GPLAYH-
GMPLAY--
GHPLAY--
GNPLAY--
GWPLAY--
GTPLAY--
GAPLAY--
-GPLAYV-
-GPLAYF-
GFPLAY--
-GPLAYT-
-GPLAYY-
-GPLAYM-
-GPLAYN-
GDPLAY--
GGPLAY--
-GPLAYE-
GEPLAY--
-GPLAYQ-
GQPLAY--
GPPLAY--

GCPLAY--
GIPLAY--
HRPLAY--
-HPLAYV-
HVPLAY--
-HPLAYR-
HMPLAY--
HGPLAY--
HIPLAY--
HNPLAY--
-HPLAYQ-
HKPLAY--
HSPLAY--
-HPLAYS-
HAPLAY--
HWPLAY--
-HPLAYG-
HTPLAY--
-HPLAYH-
-HPLAYY-
-HPLAYE-
HDPLAY--
-HPLAYF-
HLPLAY--
HPPLAY--
-HPLAYN-
-HPLAYM-
HFPLAY--
HQPLAY--
-HPLAYT-
HCPLAY--
HEPLAY--
INPLAY--
ISPLAY--
-IPLAYS-
IMPLAY--
IYPLAY--
ILPLAY--
IKPLAY--
IQPLAY--
IAPLAY--
-IPLAYG-
IGPLAY--
IVPLAY--
-IPLAYR-
IEPLAY--
IRPLAY--
IPPLAY--
ITPLAY--
-IPLAYK-

-IPLAYE-
IIPLAY--
-IPLAYN-
-IPLAYT-
IWPLAY--
-IPLAYY-
-IPLAYM-
-IPLAYI-
IHPLAY--
-IPLAYA-
ICPLAY--
IDPLAY--
-IPLAYD-
KSPLAY--
-KPLAYR-
KVPLAY--
-KPLAYI-
-KPLAYL-
KTPLAY--
-KPLAYV-
-KPLAYA-
KWPLAY--
KGPLAY--
KLPLAY--
KDPLAY--
KEPLAY--
-KPLAYK-
KQPLAY--
-KPLAYS-
KIPLAY--
-KPLAYN-
KMPLAY--
KKPLAY--
-KPLAYY-
-KPLAYW-
-KPLAYE-
KFPLAY--
KYPLAY--
-KPLAYF-
KAPLAY--
-KPLAYT-
-KPLAYQ-
KHPLAY--
-KPLAYH-
KNPLAY--
-KPLAYG-
KPPLAY--
-KPLAYD-
KCPLAY--
-LPLAYL-

LGPLAY--
LLPLAY--
-LPLAYV-
-LPLAYI-
LEPLAY--
LSPLAY--
LNPLAY--
-LPLAYT-
LIPLAY--
-LPLAYF-
-LPLAYS-
LTPLAY--
LAPLAY--
LHPLAY--
-LPLAYM-
LMPLAY--
-LPLAYQ-
LWPLAY--
LKPLAY--
LQPLAY--
LPPLAY--
LDPLAY--
-LPLAYK-
-LPLAYH-
LCPLAY--
-LPLAYE-
-LPLAYC-
MHPLAY--
-MPLAYR-
-MPLAYN-
MRPLAY--
-MPLAYL-
MTPLAY--
MNPLAY--
MGPLAY--
-MPLAYT-
MEPLAY--
MPPLAY--
MKPLAY--
MQPLAY--
-MPLAYW-
-MPLAYK-
MIPLAY--
-MPLAYG-
MYPLAY--
-MPLAYV-
-MPLAYI-
-MPLAYA-
MAPLAY--
-MPLAYY-

```
MFPLAY--
MDPLAY--
MVPLAY--
-MPLAYQ-
-MPLAYH-
-MPLAYE-
MMPLAY--
NLPLAY--
NRPLAY--
-NPLAYR-
NIPLAY--
NSPLAY--
NGPLAY--
NVPLAY--
-NPLAYM-
NYPLAY--
NFPLAY--
-NPLAYT-
NAPLAY--
NTPLAY--
-NPLAYF-
NKPLAY--
-NPLAYK-
-NPLAYV-
NQPLAY--
-NPLAYW-
-NPLAYY-
NNPLAY--
-NPLAYN-
-NPLAYQ-
-NPLAYH-
NDPLAY--
-NPLAYA-
NMPLAY--
NWPLAY--
NPPLAY--
-NPLAYE-
-NPLAYG-
NHPLAY--
--PLAYRT
--PLAYSL
--PLAYLS
--PLAYLT
--PLAYVT
--PLAYTR
--PLAYAS
--PLAYRS
--PLAYGY
--PLAYVK
--PLAYWS
```

--PLAYVD
--PLAYSS
--PLAYMN
--PLAYGS
--PLAYMT
--PLAYHL
--PLAYSG
--PLAYSM
--PLAYLR
PRPLAY--
--PLAYVS
--PLAYTS
--PLAYRL
--PLAYLQ
--PLAYHR
--PLAYVV
--PLAYSR
--PLAYRG
--PLAYSA
--PLAYKK
--PLAYTI
--PLAYQR
--PLAYNL
--PLAYYL
--PLAYTA
--PLAYSQ
--PLAYRH
--PLAYAR
--PLAYRI
--PLAYML
--PLAYLK
--PLAYHS
--PLAYTL
--PLAYSK
--PLAYLV
--PLAYFQ
--PLAYAL
--PLAYWQ
--PLAYST
--PLAYRA
PGPLAY--
--PLAYMK
--PLAYTH
--PLAYSH
--PLAYQM
--PLAYAM
--PLAYPL
--PLAYVY
--PLAYMR
--PLAYWV

--PLAYRF
--PLAYNK
--PLAYLH
--PLAYLA
--PLAYGM
--PLAYGL
--PLAYSN
--PLAYRD
--PLAYNS
--PLAYLF
--PLAYKS
--PLAYKN
--PLAYGA
--PLAYYA
--PLAYRY
--PLAYRW
--PLAYRV
--PLAYQG
--PLAYVG
--PLAYQS
--PLAYHM
--PLAYEV
--PLAYVW
--PLAYVN
--PLAYMH
--PLAYKT
--PLAYHV
--PLAYAV
--PLAYAN
--PLAYAA
--PLAYYK
--PLAYWR
--PLAYSV
--PLAYMM
--PLAYGI
--PLAYFS
--PLAYAT
--PLAYAH
--PLAYWA
--PLAYSY
--PLAYSF
--PLAYRE
--PLAYPM
--PLAYMS
--PLAYIT
--PLAYHG
--PLAYHF
--PLAYGT
--PLAYFR
--PLAYRM

--PLAYQL
--PLAYIN
--PLAYTY
--PLAYRQ
--PLAYQT
--PLAYNW
--PLAYMA
--PLAYHT
--PLAYSD
--PLAYKY
--PLAYII
--PLAYWN
--PLAYVQ
--PLAYFH
--PLAYFD
--PLAYER
--PLAYEG
--PLAYAI
PNPLAY--
--PLAYSW
--PLAYNF
--PLAYMD
--PLAYKG
--PLAYIM
--PLAYAF
--PLAYSI
--PLAYQN
--PLAYMW
--PLAYIV
--PLAYIA
--PLAYHY
--PLAYHI
PMPLAY--
--PLAYYH
--PLAYVM
--PLAYQY
--PLAYQI
--PLAYNM
--PLAYND
--PLAYIS
--PLAYHD
--PLAYGR
--PLAYGH
--PLAYFG
PQPLAY--
--PLAYWT
--PLAYVH
--PLAYTQ
--PLAYTN
--PLAYNV

--PLAYGW
--PLAYAY
PKPLAY--
--PLAYYN
--PLAYYF
--PLAYTF
--PLAYTD
--PLAYLE
--PLAYKV
--PLAYGV
--PLAYGF
--PLAYDL
PIPLAY--
--PLAYWI
--PLAYVI
--PLAYTV
--PLAYQW
--PLAYNR
--PLAYNI
--PLAYMV
--PLAYMI
--PLAYHK
--PLAYGG
--PLAYFK
--PLAYEW
--PLAYEF
--PLAYDG
--PLAYYY
--PLAYQH
--PLAYMG
--PLAYHH
--PLAYHE
--PLAYYG
--PLAYVA
--PLAYTM
--PLAYQV
--PLAYNN
--PLAYLD
--PLAYHA
--PLAYAD
--PLAYYQ
--PLAYWK
--PLAYWG
--PLAYQK
--PLAYQA
--PLAYKI
--PLAYKH
--PLAYIK
--PLAYIG
--PLAYGQ

--PLAYGN
--PLAYEM
--PLAYDA
--PLAYAW
--PLAYAK
PHPLAY--
--PLAYYD
--PLAYWH
--PLAYRP
--PLAYPF
--PLAYNG
--PLAYMQ
--PLAYKD
--PLAYCL
--PLAYCA
PEPLAY--
--PLAYYE
--PLAYWM
--PLAYWD
--PLAYVE
--PLAYSE
--PLAYQF
--PLAYQE
--PLAYNY
--PLAYNA
--PLAYKE
--PLAYIQ
--PLAYGK
--PLAYEQ
--PLAYEI
--PLAYEH
--PLAYDT
--PLAYAQ
PFPLAY--
--PLAYTE
--PLAYPW
--PLAYKQ
--PLAYIY
--PLAYHN
--PLAYDS
--PLAYCR
--PLAYAG
-PPLAYL-
--PLAYYP
--PLAYYM
--PLAYWW
--PLAYWE
--PLAYTW
--PLAYTC
--PLAYSP

--PLAYQP
--PLAYQD
--PLAYPY
--PLAYPQ
--PLAYNQ
--PLAYNH
--PLAYMY
--PLAYMF
--PLAYLC
--PLAYKF
--PLAYKC
--PLAYIW
--PLAYIE
--PLAYID
--PLAYHC
--PLAYEY
--PLAYES
--PLAYEN
--PLAYED
--PLAYDY
--PLAYDV
--PLAYDF
--PLAYCT
--PLAYCN
--PLAYAE
QRPLAY--
-QPLAYK-
-QPLAYL-
QSPLAY--
QVPLAY--
-QPLAYS-
QAPLAY--
-QPLAYA-
-QPLAYY-
QLPLAY--
-QPLAYV-
-QPLAYR-
-QPLAYQ-
QIPLAY--
-QPLAYT-
-QPLAYN-
QMPLAY--
QWPLAY--
QTPLAY--
QHPLAY--
QPPLAY--
-QPLAYH-
QFPLAY--
QGPLAY--
QKPLAY--

QYPLAY--
QEPLAY--
-QPLAYE-
QNPLAY--
QDPLAY--
-QPLAYM-
QQPLAY--
-QPLAYG-
RQPLAY--
-RPLAYM-
RKPLAY--
RSPLAY--
-RPLAYK-
RGPLAY--
-RPLAYS-
-RPLAYI-
RTPLAY--
RLPLAY--
-RPLAYN-
-RPLAYF-
-RPLAYL-
RNPLAY--
RFPLAY--
-RPLAYQ-
-RPLAYH-
RIPLAY--
REPLAY--
RYPLAY--
RWPLAY--
-RPLAYV-
RHPLAY--
-RPLAYE-
RDPLAY--
RPPLAY--
-RPLAYC-
RCPLAY--
-SPLAYL-
STPLAY--
-SPLAYM-
-SPLAYF-
SIPLAY--
-SPLAYI-
-SPLAYN-
SQPLAY--
SNPLAY--
SGPLAY--
SKPLAY--
-SPLAYV-
SLPLAY--
SSPLAY--

-SPLAYK-
-SPLAYG-
-SPLAYY-
SMPLAY--
SHPLAY--
SWPLAY--
-SPLAYH-
SAPLAY--
-SPLAYE-
SYPLAY--
SDPLAY--
SPPLAY--
-SPLAYA-
SEPLAY--
SCPLAY--
TRPLAY--
-TPLAYL-
-TPLAYV-
-TPLAYI-
-TPLAYT-
TPPLAY--
TYPLAY--
-TPLAYS-
TVPLAY--
TGPLAY--
TWPLAY--
-TPLAYM-
TTPLAY--
THPLAY--
TAPLAY--
-TPLAYA-
-TPLAYG-
-TPLAYK-
-TPLAYQ-
TQPLAY--
-TPLAYY-
-TPLAYH-
TNPLAY--
TFPLAY--
-TPLAYN-
-TPLAYE-
-TPLAYW-
TMPLAY--
TEPLAY--
-TPLAYC-
TCPLAY--
-VPLAYQ-
-VPLAYI-
-VPLAYL-
VKPLAY--

VAPLAY--
VSPLAY--
-VPLAYS-
VTPLAY--
-VPLAYV-
VLPLAY--
VVPLAY--
-VPLAYR-
VNPLAY--
VDPLAY--
-VPLAYW-
-VPLAYM-
-VPLAYK-
-VPLAYG-
VMPLAY--
VYPLAY--
-VPLAYA-
-VPLAYY-
-VPLAYE-
-VPLAYT-
VFPLAY--
VIPLAY--
VQPLAY--
-VPLAYH-
VCPLAY--
-VPLAYD-
VHPLAY--
VEPLAY--
WYPLAY--
WEPLAY--
WTPLAY--
WSPLAY--
WNPLAY--
-WPLAYS-
WRPLAY--
WLPLAY--
-WPLAYI-
WWPLAY--
WGPLAY--
WDPLAY--
WPPLAY--
-WPLAYT-
-WPLAYH-
WMPLAY--
-WPLAYW-
-WPLAYN-
-WPLAYM-
-WPLAYA-
WVPLAY--
WQPLAY--

	-WPLAYQ-		
	-WPLAYK-		
	WFPLAY--		
	-WPLAYG-		
	WAPLAY--		
	-YPLAYL-		
	-YPLAYK-		
	YVPLAY--		
	-YPLAYM-		
	YGPLAY--		
	YSPLAY--		
	-YPLAYT-		
	-YPLAYH-		
	-YPLAYS-		
	YPPLAY--		
	YMPLAY--		
	YNPLAY--		
	-YPLAYI-		
	YIPLAY--		
	YTPLAY--		
	-YPLAYN-		
	-YPLAYF-		
	YKPLAY--		
	YAPLAY--		
	-YPLAYA-		
	YEPLAY--		
	YDPLAY--		
	YHPLAY--		
	-YPLAYE-		
	-YPLAYY-		
	-YPLAYG-		
	-YPLAYW-		
	YCPLAY--		
	-YPLAYQ-		
PLPL	3	924	49.03
	-GPLPLD-		
	-KPLPLP-		
	--PLPLPY		
	-SPLPLD-		
	-YPLPLD-		
	-APLPLR-		
	ASPLPL--		
	AIPLPL--		
	-APLPLT-		
	AYPLPL--		
	AMPLPL--		
	ALPLPL--		
	-APLPLA-		
	-APLPLV-		
	AAPLPL--		

```
-APLPLW-
AKPLPL--
-APLPLS-
AGPLPL--
ANPLPL--
-APLPLG-
AWPLPL--
AQPLPL--
-APLPLY-
-APLPLI-
APPLPL--
AFPLPL--
-APLPLM-
-APLPLN-
-APLPLQ-
-APLPLH-
AHPLPL--
AEPLPL--
ADPLPL--
-APLPLE-
-APLPLC-
CSPLPL--
CRPLPL--
-CPLPLR-
CHPLPL--
CIPLPL--
-CPLPLS-
CLPLPL--
-CPLPLT-
CVPLPL--
CQPLPL--
-CPLPLK-
CCPLPL--
-CPLPLF-
CYPLPL--
-CPLPLW-
-CPLPLL-
CGPLPL--
CAPLPL--
CTPLPL--
CMPLPL--
CWPLPL--
-CPLPLY-
-CPLPLV-
-CPLPLN-
CKPLPL--
CEPLPL--
-CPLPLQ-
-CPLPLI-
-DPLPLR-
```

DTPLPL--
DSPLPL--
DLPLPL--
DRPLPL--
-DPLPLH-
DWPLPL--
DQPLPL--
DAPLPL--
-DPLPLN-
-DPLPLL-
DKPLPL--
-DPLPLT-
DMPLPL--
DYPLPL--
-DPLPLW-
DHPLPL--
-DPLPLF-
-DPLPLA-
DIPLPL--
-DPLPLS-
DVPLPL--
-DPLPLV-
DGPLPL--
-DPLPLI-
-DPLPLQ-
-DPLPLY-
-DPLPLK-
DEPLPL--
DNPLPL--
DFPLPL--
DDPLPL--
-DPLPLG-
-DPLPLE-
EVPLPL--
-EPLPLL-
ERPLPL--
EAPLPL--
-EPLPLW-
-EPLPLR-
-EPLPLH-
EFPLPL--
-EPLPLM-
-EPLPLT-
EQPLPL--
EGPLPL--
EWPLPL--
-EPLPLV-
-EPLPLS-
-EPLPLG-
-EPLPLA-

-EPLPLQ-
EKPLPL--
EPPLPL--
-EPLPLK-
-EPLPLY-
-EPLPLF-
EHPLPL--
ETPLPL--
EMPLPL--
ENPLPL--
EYPLPL--
-EPLPLN-
-EPLPLI-
EIPLPL--
EEPLPL--
FRPLPL--
FLPLPL--
-FPLPLK-
FHPLPL--
-FPLPLL-
FNPLPL--
FYPLPL--
-FPLPLQ-
FGPLPL--
-FPLPLS-
FIPLPL--
-FPLPLI-
FPPLPL--
-FPLPLH-
-FPLPLY-
FEPLPL--
-FPLPLN-
FMPLPL--
FAPLPL--
-FPLPLV-
FFPLPL--
FSPLPL--
-FPLPLT-
FVPLPL--
-FPLPLG-
-FPLPLW-
-FPLPLF-
FQPLPL--
-FPLPLE-
FKPLPL--
FDPLPL--
FWPLPL--
-GPLPLR-
GSPLPL--
GLPLPL--

GFPLPL--
-GPLPLV-
GRPLPL--
GAPLPL--
GHPLPL--
-GPLPLF-
GGPLPL--
GKPLPL--
-GPLPLH-
-GPLPLK-
GYPLPL--
-GPLPLS-
GTPLPL--
GNPLPL--
GMPLPL--
-GPLPLN-
-GPLPLI-
-GPLPLQ-
-GPLPLT-
-GPLPLA-
GQPLPL--
GWPLPL--
-GPLPLW-
GPPLPL--
GIPLPL--
-GPLPLG-
GEPLPL--
-GPLPLY-
-GPLPLE-
GCPLPL--
GDPLPL--
HSPLPL--
HRPLPL--
HLPLPL--
HYPLPL--
-HPLPLY-
HHPLPL--
HGPLPL--
HIPLPL--
-HPLPLH-
HPPLPL--
HKPLPL--
-HPLPLM-
HNPLPL--
-HPLPLW-
-HPLPLA-
HFPLPL--
-HPLPLF-
HAPLPL--
-HPLPLR-

```
-HPLPLK-
HWPLPL--
-HPLPLN-
-HPLPLV-
-HPLPLG-
-HPLPLL-
HDPLPL--
HTPLPL--
HMPLPL--
-HPLPLT-
HEPLPL--
HQPLPL--
-HPLPLE-
-HPLPLI-
-IPLPLA-
ITPLPL--
ILPLPL--
ISPLPL--
IYPLPL--
IIPLPL--
-IPLPLL-
-IPLPLS-
IVPLPL--
INPLPL--
-IPLPLK-
-IPLPLT-
-IPLPLH-
-IPLPLF-
IEPLPL--
-IPLPLQ-
IKPLPL--
IFPLPL--
IPPLPL--
IGPLPL--
IWPLPL--
IDPLPL--
-IPLPLY-
-IPLPLN-
-IPLPLG-
IQPLPL--
-IPLPLI-
-IPLPLM-
IMPLPL--
IAPLPL--
-IPLPLW-
-IPLPLV-
-IPLPLE-
ICPLPL--
-IPLPLD-
-KPLPLR-
```

-KPLPLL-
KGPLPL--
-KPLPLK-
KIPLPL--
-KPLPLS-
-KPLPLN-
-KPLPLH-
KFPLPL--
KYPLPL--
-KPLPLY-
KLPLPL--
KMPLPL--
KAPLPL--
-KPLPLM-
-KPLPLQ-
KDPLPL--
KQPLPL--
-KPLPLT-
KNPLPL--
KWPLPL--
KTPLPL--
-KPLPLG-
-KPLPLV-
-KPLPLA-
KEPLPL--
KPPLPL--
-KPLPLW-
-KPLPLI-
-KPLPLE-
-KPLPLC-
KHPLPL--
KCPLPL--
-LPLPLK-
LGPLPL--
LTPLPL--
LYPLPL--
LVPLPL--
LQPLPL--
-LPLPLR-
LFPLPL--
LEPLPL--
LMPLPL--
LAPLPL--
LNPLPL--
-LPLPLT-
LDPLPL--
LWPLPL--
LPPLPL--
-LPLPLF-
LHPLPL--

-LPLPLN-
-LPLPLY-
-LPLPLV-
-LPLPLH-
-LPLPLQ-
LIPLPL--
-LPLPLW-
-LPLPLE-
LCPLPL--
MTPLPL--
-MPLPLL-
-MPLPLS-
MLPLPL--
MRPLPL--
-MPLPLR-
MSPLPL--
-MPLPLN-
MAPLPL--
MVPLPL--
MGPLPL--
-MPLPLG-
MYPLPL--
-MPLPLQ-
MMPLPL--
MQPLPL--
-MPLPLW-
MKPLPL--
MHPLPL--
MWPLPL--
-MPLPLI-
-MPLPLH-
MEPLPL--
-MPLPLV-
-MPLPLT-
-MPLPLY-
MNPLPL--
MIPLPL--
-MPLPLE-
MPPLPL--
MFPLPL--
MCPLPL--
-MPLPLK-
MDPLPL--
NNPLPL--
-NPLPLY-
-NPLPLR-
NRPLPL--
NYPLPL--
NAPLPL--
NLPLPL--

-NPLPLN-
NVPLPL--
-NPLPLV-
-NPLPLS-
NMPLPL--
NWPLPL--
-NPLPLT-
-NPLPLI-
-NPLPLH-
NKPLPL--
-NPLPLK-
-NPLPLF-
NQPLPL--
NTPLPL--
-NPLPLA-
NDPLPL--
NIPLPL--
-NPLPLQ-
NPPLPL--
-NPLPLM-
NCPLPL--
-NPLPLE-
-NPLPLW-
NEPLPL--
-NPLPLG-
-NPLPLC-
--PLPLVL
--PLPLRL
--PLPLSS
--PLPLTR
--PLPLSR
--PLPLLR
--PLPLLL
--PLPLSL
--PLPLSA
--PLPLLG
--PLPLGY
--PLPLRH
--PLPLVG
--PLPLAL
--PLPLVS
--PLPLML
--PLPLRG
--PLPLLT
--PLPLTL
--PLPLRA
--PLPLYL
--PLPLYR
--PLPLKT
--PLPLSK

--PLPLQT
--PLPLIS
--PLPLYQ
--PLPLRR
--PLPLRN
--PLPLWR
--PLPLLM
--PLPLKL
--PLPLGS
--PLPLLA
--PLPLAS
--PLPLSF
--PLPLRV
--PLPLVY
--PLPLKA
--PLPLRT
--PLPLIT
--PLPLHR
--PLPLTA
PRPLPL--
--PLPLYG
--PLPLVN
--PLPLFR
--PLPLRW
--PLPLGL
--PLPLRY
--PLPLNL
--PLPLYT
--PLPLVI
--PLPLTK
--PLPLII
--PLPLYS
PAPLPL--
--PLPLYY
--PLPLSV
--PLPLHG
--PLPLRF
--PLPLKI
--PLPLTY
--PLPLFS
--PLPLNA
--PLPLEM
--PLPLWG
--PLPLVA
--PLPLSY
--PLPLHI
--PLPLKG
--PLPLGW
--PLPLGI
--PLPLVW

--PLPLNW
--PLPLMK
--PLPLMH
--PLPLVQ
--PLPLTS
--PLPLRQ
--PLPLLV
--PLPLEI
PGPLPL--
--PLPLLN
--PLPLPL
--PLPLYN
--PLPLWN
--PLPLVK
--PLPLQL
PNPLPL--
--PLPLTV
--PLPLQM
--PLPLMT
--PLPLGM
--PLPLYI
--PLPLSI
--PLPLQY
--PLPLIV
--PLPLHF
--PLPLAH
--PLPLRD
--PLPLNT
--PLPLGT
--PLPLYA
--PLPLTN
--PLPLQI
--PLPLMF
--PLPLLD
--PLPLKR
--PLPLHS
--PLPLYW
--PLPLWA
--PLPLTI
--PLPLSQ
--PLPLPV
--PLPLNS
--PLPLLE
--PLPLAA
--PLPLTQ
--PLPLTF
--PLPLSM
--PLPLIG
--PLPLIF
--PLPLGF

--PLPLFN
--PLPLVV
--PLPLPF
--PLPLLH
--PLPLHN
--PLPLGQ
--PLPLFK
--PLPLYH
--PLPLLQ
--PLPLIY
--PLPLFT
--PLPLAV
--PLPLAT
--PLPLAK
--PLPLWS
--PLPLSH
--PLPLNY
--PLPLNI
--PLPLLK
--PLPLKW
PFPLPL--
--PLPLWV
--PLPLWF
--PLPLQR
--PLPLMR
--PLPLLW
--PLPLFQ
--PLPLEY
--PLPLWK
--PLPLPM
--PLPLKQ
--PLPLHV
--PLPLAY
--PLPLPI
--PLPLNH
--PLPLGV
--PLPLDL
PDPLPL--
--PLPLVT
--PLPLTT
--PLPLQK
--PLPLIW
--PLPLIH
--PLPLAG
PIPLPL--
--PLPLQW
--PLPLMN
--PLPLKN
--PLPLIM
--PLPLHW

PEPLPL--
--PLPLVH
--PLPLQV
--PLPLMV
--PLPLMI
--PLPLKM
--PLPLIQ
--PLPLIA
--PLPLAI
--PLPLAF
PQPLPL--
--PLPLTW
--PLPLTG
--PLPLSD
--PLPLQF
--PLPLNN
--PLPLHE
--PLPLES
--PLPLEF
PHPLPL--
--PLPLWH
--PLPLND
--PLPLKY
--PLPLKV
--PLPLHQ
--PLPLHA
--PLPLFG
--PLPLDY
--PLPLAN
--PLPLYK
--PLPLTE
--PLPLQA
--PLPLMW
--PLPLHT
--PLPLAM
PYPLPL--
--PLPLWI
--PLPLSW
--PLPLPS
--PLPLPQ
--PLPLMM
--PLPLME
--PLPLKK
--PLPLHY
--PLPLGR
--PLPLER
--PLPLEK
--PLPLRM
--PLPLRE
--PLPLQQ

--PLPLNR
--PLPLHK
--PLPLGK
--PLPLFH
--PLPLDM
--PLPLYM
--PLPLWQ
--PLPLWD
--PLPLVD
--PLPLSE
--PLPLQS
--PLPLQD
--PLPLNV
--PLPLNM
--PLPLNK
--PLPLNG
--PLPLIK
--PLPLID
--PLPLEG
--PLPLMY
--PLPLKH
--PLPLGH
--PLPLDS
--PLPLAD
--PLPLYP
--PLPLYE
--PLPLTD
--PLPLRP
--PLPLQN
--PLPLPW
--PLPLPH
--PLPLIE
--PLPLEN
--PLPLDQ
--PLPLDF
--PLPLAE
PMPLPL--
--PLPLWM
--PLPLNE
--PLPLMP
--PLPLKD
--PLPLFA
--PLPLEW
--PLPLEQ
--PLPLDV
--PLPLYD
--PLPLPA
--PLPLMQ
--PLPLMG
--PLPLHD

--PLPLFM
--PLPLFE
--PLPLEV
--PLPLET
--PLPLDT
--PLPLQH
--PLPLQG
--PLPLPT
--PLPLNQ
--PLPLEA
--PLPLDW
--PLPLWC
--PLPLVP
--PLPLVE
--PLPLQE
--PLPLIC
--PLPLFP
--PLPLCA
-PPLPLY-
--PLPLWW
--PLPLSC
--PLPLLP
--PLPLIP
--PLPLHH
--PLPLFD
--PLPLEC
--PLPLDG
--PLPLAP
PPPLPL--
--PLPLTP
--PLPLTC
--PLPLQP
--PLPLPK
--PLPLKP
--PLPLKC
--PLPLHM
--PLPLGE
--PLPLEH
--PLPLCV
--PLPLCS
--PLPLCG
--PLPLCF
--PLPLAQ
QLPLPL--
QSPLPL--
QGPLPL--
-QPLPLR-
QVPLPL--
QPPLPL--
QAPLPL--

QKPLPL--
QFPLPL--
-QPLPLN-
-QPLPLT-
-QPLPLI-
-QPLPLY-
-QPLPLQ-
QMPLPL--
QWPLPL--
QDPLPL--
QYPLPL--
-QPLPLF-
QTPLPL--
QQPLPL--
-QPLPLA-
QNPLPL--
QEPLPL--
QHPLPL--
-QPLPLV-
-QPLPLG-
QCPLPL--
-QPLPLM-
-QPLPLE-
QIPLPL--
-RPLPLT-
-RPLPLR-
RYPLPL--
-RPLPLS-
RGPLPL--
-RPLPLF-
-RPLPLI-
-RPLPLA-
RIPLPL--
RWPLPL--
RVPLPL--
-RPLPLN-
RAPLPL--
-RPLPLQ-
-RPLPLM-
RPPLPL--
RNPLPL--
RQPLPL--
-RPLPLH-
RHPLPL--
RDPLPL--
-RPLPLW-
-RPLPLE-
RFPLPL--
-RPLPLC-
RCPLPL--

SIPLPL--
-SPLPLS-
SVPLPL--
SHPLPL--
-SPLPLV-
SGPLPL--
-SPLPLI-
STPLPL--
SLPLPL--
SWPLPL--
SSPLPL--
-SPLPLK-
SKPLPL--
-SPLPLH-
SNPLPL--
-SPLPLA-
SYPLPL--
SAPLPL--
SEPLPL--
SQPLPL--
-SPLPLF-
-SPLPLG-
-SPLPLM-
SFPLPL--
-SPLPLW-
SMPLPL--
-SPLPLN-
SPPLPL--
-SPLPLQ-
SDPLPL--
-SPLPLE-
SCPLPL--
-SPLPLP-
TIPLPL--
TVPLPL--
TSPLPL--
-TPLPLT-
TLPLPL--
-TPLPLL-
-TPLPLR-
-TPLPLS-
TRPLPL--
TAPLPL--
-TPLPLM-
TWPLPL--
-TPLPLA-
-TPLPLN-
TFPLPL--
-TPLPLV-
TQPLPL--

-TPLPLK-
-TPLPLY-
TNPLPL--
TEPLPL--
TPPLPL--
-TPLPLI-
TGPLPL--
-TPLPLE-
TKPLPL--
THPLPL--
-TPLPLW-
-TPLPLG-
TMPLPL--
TDPLPL--
-TPLPLQ-
-TPLPLF-
VSPLPL--
VRPLPL--
-VPLPLR-
-VPLPLA-
VIPLPL--
-VPLPLK-
VKPLPL--
VAPLPL--
VLPLPL--
VVPLPL--
VWPLPL--
VMPLPL--
VYPLPL--
-VPLPLS-
VHPLPL--
-VPLPLH-
-VPLPLF-
-VPLPLV-
VDPLPL--
VFPLPL--
-VPLPLQ-
-VPLPLT-
VNPLPL--
VQPLPL--
-VPLPLG-
-VPLPLN-
-VPLPLE-
VEPLPL--
-VPLPLM-
-VPLPLC-
-WPLPLL-
WSPLPL--
-WPLPLA-
WMPLPL--

WLPLPL--
-WPLPLS-
WGPLPL--
WEPLPL--
WTPLPL--
-WPLPLK-
-WPLPLT-
-WPLPLN-
-WPLPLM-
-WPLPLV-
WAPLPL--
-WPLPLH-
WCPLPL--
WNPLPL--
-WPLPLI-
WKPLPL--
WFPLPL--
WYPLPL--
WWPLPL--
WPPLPL--
-WPLPLG-
WQPLPL--
-WPLPLQ-
-WPLPLY-
-WPLPLD-
WHPLPL--
WDPLPL--
YRPLPL--
-YPLPLL-
YQPLPL--
-YPLPLT-
YHPLPL--
YNPLPL--
YIPLPL--
YAPLPL--
YEPLPL--
YTPLPL--
YMPLPL--
-YPLPLS-
-YPLPLA-
YKPLPL--
-YPLPLY-
YDPLPL--
YGPLPL--
YPPLPL--
-YPLPLI-
YWPLPL--
-YPLPLQ-
-YPLPLK-
-YPLPLW-

	-YPLPLG-		
	-YPLPLV-		
	-YPLPLF-		
	YLPLPL--		
	-YPLPLN-		
	-YPLPLM-		
	-YPLPLH-		
	YVPLPL--		
	YCPLPL--		
PLGL	43	858	48.99
	--PLGLPL		
	--PLGLYC		
	--PLGLTP		
	--PLGLPI		
	--PLGLPE		
	--PLGLDR		
	--PLGLDK		
	--PLGLDH		
	--PLGLDD		
	--PLGLCA		
	-APLGLS-		
	-APLGLR-		
	ASPLGL--		
	ARPLGL--		
	AAPLGL--		
	-APLGLT-		
	-APLGLH-		
	-APLGLG-		
	AWPLGL--		
	AMPLGL--		
	-APLGLL-		
	ANPLGL--		
	-APLGLN-		
	AIPLGL--		
	APPLGL--		
	AHPLGL--		
	-APLGLI-		
	-APLGLV-		
	-APLGLA-		
	-APLGLQ-		
	AFPLGL--		
	-APLGLF-		
	-APLGLM-		
	AGPLGL--		
	AQPLGL--		
	-APLGLK-		
	AEPLGL--		
	ADPLGL--		
	-APLGLE-		
	-APLGLD-		

```
-APLGLC-
ACPLGL--
CRPLGL--
CSPLGL--
CGPLGL--
CPPLGL--
-CPLGLV-
-CPLGLL-
CTPLGL--
CNPLGL--
CEPLGL--
CLPLGL--
-CPLGLA-
-CPLGLS-
CMPLGL--
CCPLGL--
-CPLGLE-
-CPLGLK-
-CPLGLT-
-CPLGLQ-
CIPLGL--
-CPLGLY-
CKPLGL--
CQPLGL--
-CPLGLN-
-CPLGLI-
CHPLGL--
CDPLGL--
CAPLGL--
CVPLGL--
CFPLGL--
CYPLGL--
-CPLGLM-
-CPLGLG-
-CPLGLF-
-CPLGLW-
-CPLGLH-
DSPLGL--
-DPLGLS-
-DPLGLR-
DYPLGL--
DGPLGL--
DTPLGL--
-DPLGLL-
DQPLGL--
-DPLGLI-
-DPLGLY-
DFPLGL--
-DPLGLV-
-DPLGLT-
```

-DPLGLH-
-DPLGLN-
-DPLGLW-
-DPLGLK-
-DPLGLF-
DKPLGL--
DWPLGL--
DNPLGL--
DMPLGL--
DIPLGL--
-DPLGLQ-
-DPLGLG-
-DPLGLA-
-DPLGLE-
DHPLGL--
DCPLGL--
DDPLGL--
DPPLGL--
DAPLGL--
-DPLGLC-
ESPLGL--
EYPLGL--
-EPLGLY-
EAPLGL--
EKPLGL--
-EPLGLR-
-EPLGLT-
-EPLGLF-
-EPLGLV-
ELPLGL--
EMPLGL--
-EPLGLH-
-EPLGLA-
ERPLGL--
EFPLGL--
-EPLGLG-
EQPLGL--
EEPLGL--
EVPLGL--
EWPLGL--
-EPLGLM-
EHPLGL--
EGPLGL--
-EPLGLQ-
-EPLGLS-
-EPLGLN-
ENPLGL--
-EPLGLK-
EDPLGL--
-EPLGLE-

EPPLGL--
-EPLGLW-
-EPLGLD-
-FPLGLH-
FSPLGL--
FGPLGL--
FVPLGL--
FAPLGL--
FTPLGL--
-FPLGLN-
FLPLGL--
FNPLGL--
-FPLGLT-
FRPLGL--
-FPLGLA-
FIPLGL--
-FPLGLV-
FMPLGL--
FKPLGL--
FEPLGL--
-FPLGLG-
FQPLGL--
-FPLGLK-
FPPLGL--
-FPLGLM-
-FPLGLW-
-FPLGLQ-
-FPLGLI-
FDPLGL--
-FPLGLD-
-GPLGLS-
GSPLGL--
GVPLGL--
-GPLGLT-
GYPLGL--
GTPLGL--
GFPLGL--
GMPLGL--
-GPLGLI-
GLPLGL--
GHPLGL--
GKPLGL--
GQPLGL--
-GPLGLH-
-GPLGLN-
GIPLGL--
GGPLGL--
GAPLGL--
-GPLGLV-
-GPLGLQ-

GDPLGL--
-GPLGLE-
-GPLGLF-
GPPLGL--
-GPLGLA-
GEPLGL--
GNPLGL--
GWPLGL--
-GPLGLM-
-GPLGLC-
GCPLGL--
-HPLGLT-
HSPLGL--
-HPLGLS-
HLPLGL--
HRPLGL--
HFPLGL--
HNPLGL--
HGPLGL--
-HPLGLF-
HQPLGL--
-HPLGLA-
-HPLGLV-
HIPLGL--
-HPLGLN-
-HPLGLI-
HMPLGL--
HKPLGL--
HYPLGL--
HVPLGL--
-HPLGLH-
HAPLGL--
-HPLGLK-
-HPLGLG-
-HPLGLQ-
HWPLGL--
-HPLGLE-
HPPLGL--
-HPLGLW-
HEPLGL--
-HPLGLY-
HHPLGL--
-HPLGLM-
-HPLGLD-
HCPLGL--
ISPLGL--
-IPLGLS-
IGPLGL--
ILPLGL--
IRPLGL--

INPLGL--
-IPLGLI-
-IPLGLK-
IAPLGL--
-IPLGLL-
-IPLGLV-
-IPLGLQ-
IHPLGL--
IFPLGL--
-IPLGLA-
-IPLGLH-
IPPLGL--
-IPLGLE-
IMPLGL--
IKPLGL--
-IPLGLY-
IYPLGL--
-IPLGLF-
-IPLGLG-
IVPLGL--
-IPLGLT-
IDPLGL--
IWPLGL--
-IPLGLN-
-IPLGLM-
IEPLGL--
-IPLGLD-
-IPLGLC-
ICPLGL--
-KPLGLL-
-KPLGLR-
KTPLGL--
KLPLGL--
KGPLGL--
KVPLGL--
-KPLGLI-
-KPLGLH-
KMPLGL--
KKPLGL--
-KPLGLV-
KIPLGL--
KYPLGL--
KWPLGL--
KHPLGL--
-KPLGLW-
KQPLGL--
-KPLGLG-
-KPLGLK-
KPPLGL--
-KPLGLF-

KFPLGL--
-KPLGLQ-
-KPLGLY-
KNPLGL--
KDPLGL--
KEPLGL--
-KPLGLM-
-KPLGLE-
-KPLGLC-
-KPLGLD-
LSPLGL--
LRPLGL--
LHPLGL--
-LPLGLT-
LVPLGL--
-LPLGLK-
LFPLGL--
LAPLGL--
LYPLGL--
LQPLGL--
-LPLGLL-
LTPLGL--
LLPLGL--
LKPLGL--
LPPLGL--
-LPLGLI-
LWPLGL--
-LPLGLG-
LEPLGL--
LDPLGL--
-LPLGLN-
-LPLGLA-
-LPLGLF-
-LPLGLH-
-LPLGLQ-
-LPLGLC-
-LPLGLD-
LCPLGL--
MSPLGL--
MLPLGL--
-MPLGLR-
-MPLGLS-
-MPLGLV-
MVPLGL--
MRPLGL--
MTPLGL--
-MPLGLG-
MKPLGL--
-MPLGLY-
-MPLGLL-

MEPLGL--
MGPLGL--
MAPLGL--
-MPLGLH-
-MPLGLA-
MMPLGL--
-MPLGLF-
MPPLGL--
MNPLGL--
MHPLGL--
-MPLGLW-
-MPLGLI-
-MPLGLQ-
MFPLGL--
-MPLGLM-
MDPLGL--
MQPLGL--
-MPLGLE-
MWPLGL--
-MPLGLD-
-MPLGLC-
MCPLGL--
-NPLGLR-
NRPLGL--
NVPLGL--
-NPLGLL-
NLPLGL--
NIPLGL--
NFPLGL--
NWPLGL--
-NPLGLH-
-NPLGLI-
-NPLGLT-
NAPLGL--
-NPLGLV-
NTPLGL--
-NPLGLK-
NNPLGL--
-NPLGLY-
-NPLGLF-
-NPLGLM-
NGPLGL--
-NPLGLW-
-NPLGLA-
NQPLGL--
-NPLGLQ-
-NPLGLG-
NPPLGL--
NDPLGL--
NEPLGL--

```
-NPLGLN-
NCPLGL--
-NPLGLC-
--PLGLRS
--PLGLRT
--PLGLTS
--PLGLSS
--PLGLVS
--PLGLTR
--PLGLRI
--PLGLSI
--PLGLFS
--PLGLSA
--PLGLQR
--PLGLRA
--PLGLLV
--PLGLVR
--PLGLLN
--PLGLNR
--PLGLHR
--PLGLRV
--PLGLRH
--PLGLLK
--PLGLVG
--PLGLRD
--PLGLKR
--PLGLGS
--PLGLTG
--PLGLYR
--PLGLTI
--PLGLSQ
--PLGLGT
--PLGLFR
--PLGLTT
--PLGLLA
--PLGLST
--PLGLMR
--PLGLYT
--PLGLKT
--PLGLIS
--PLGLSN
--PLGLYH
--PLGLVK
--PLGLNS
--PLGLSM
--PLGLRK
--PLGLTN
--PLGLNV
--PLGLTH
--PLGLTL
```

PRPLGL--
--PLGLMD
--PLGLHT
PGPLGL--
--PLGLVA
--PLGLRN
--PLGLVI
--PLGLAT
--PLGLKG
--PLGLWK
--PLGLVN
--PLGLSH
--PLGLML
--PLGLLE
--PLGLAH
--PLGLNM
--PLGLKH
--PLGLAI
--PLGLRM
--PLGLLD
--PLGLGL
--PLGLWT
--PLGLTW
--PLGLQA
--PLGLIQ
--PLGLAY
--PLGLVD
--PLGLTV
--PLGLTM
--PLGLQS
--PLGLMT
--PLGLSD
--PLGLRQ
--PLGLHS
--PLGLER
PIPLGL--
--PLGLTK
--PLGLKK
--PLGLFK
--PLGLQV
--PLGLTA
--PLGLGH
--PLGLIH
--PLGLGG
--PLGLEM
--PLGLYQ
--PLGLWN
--PLGLVQ
--PLGLRP
--PLGLMA

--PLGLIM
--PLGLEK
--PLGLWS
--PLGLQG
--PLGLGW
--PLGLHF
--PLGLDS
--PLGLQT
--PLGLQL
--PLGLHI
--PLGLVY
--PLGLRE
--PLGLKA
--PLGLVH
--PLGLQW
--PLGLQN
--PLGLMV
--PLGLFQ
--PLGLGY
--PLGLAN
PNPLGL--
--PLGLVM
--PLGLNH
--PLGLNW
--PLGLAW
--PLGLNT
--PLGLKN
--PLGLIK
--PLGLGN
--PLGLMK
--PLGLKE
--PLGLIN
--PLGLWR
--PLGLEY
--PLGLAV
--PLGLID
--PLGLHV
--PLGLHQ
--PLGLNN
--PLGLHM
--PLGLHH
--PLGLHG
--PLGLAK
--PLGLTQ
--PLGLKV
--PLGLKM
--PLGLHW
--PLGLGV
--PLGLDG
--PLGLYD

--PLGLKF
--PLGLFT
--PLGLHD
--PLGLME
--PLGLLH
--PLGLFN
--PLGLTF
--PLGLIY
PEPLGL--
--PLGLYK
--PLGLMH
--PLGLGF
--PLGLEL
--PLGLDA
--PLGLAS
--PLGLAA
--PLGLFH
--PLGLVW
--PLGLTE
--PLGLSE
--PLGLQD
--PLGLSW
--PLGLIT
--PLGLIE
--PLGLWH
--PLGLQH
--PLGLEN
--PLGLEI
--PLGLNG
--PLGLEG
--PLGLWG
--PLGLWE
PWPLGL--
--PLGLQI
--PLGLIA
--PLGLAQ
--PLGLAD
--PLGLSF
--PLGLKQ
--PLGLDW
--PLGLQM
--PLGLGK
--PLGLFD
--PLGLNI
--PLGLHY
--PLGLGQ
PQPLGL--
--PLGLWQ
--PLGLIW
--PLGLEW

--PLGLED
--PLGLQY
--PLGLEF
--PLGLNQ
--PLGLMM
--PLGLHN
--PLGLNE
--PLGLWD
--PLGLFM
--PLGLAE
--PLGLYP
--PLGLQQ
--PLGLES
--PLGLND
--PLGLEH
--PLGLVE
--PLGLPA
--PLGLMQ
--PLGLMF
--PLGLGE
--PLGLYN
--PLGLQE
--PLGLLP
--PLGLHE
--PLGLPG
--PLGLYE
--PLGLMP
--PLGLKD
--PLGLGD
--PLGLDT
--PLGLDM
--PLGLCS
--PLGLNC
--PLGLKP
--PLGLPS
PHPLGL--
--PLGLWP
--PLGLHP
--PLGLEQ
--PLGLEE
--PLGLEC
--PLGLDY
--PLGLCQ
--PLGLCG
PCPLGL--
--PLGLVP
--PLGLSC
--PLGLRC
--PLGLLC
--PLGLIC

--PLGLCR
--PLGLCD
PPPLGL--
--PLGLTC
--PLGLPW
--PLGLIP
--PLGLFP
--PLGLDV
--PLGLDF
--PLGLCW
--PLGLCT
--PLGLCK
--PLGLCI
--PLGLAC
-PPLGLR-
-PPLGLQ-
-PPLGLN-
-PPLGLH-
-PPLGLG-
-PPLGLF-
--PLGLWC
--PLGLQF
--PLGLFC
--PLGLCV
--PLGLCN
--PLGLCL
PDPLGL--
-QPLGLL-
-QPLGLR-
-QPLGLY-
QSPLGL--
QFPLGL--
-QPLGLA-
QIPLGL--
-QPLGLG-
-QPLGLT-
QTPLGL--
QEPLGL--
QVPLGL--
-QPLGLS-
-QPLGLN-
-QPLGLQ-
QLPLGL--
QHPLGL--
-QPLGLH-
QGPLGL--
QPPLGL--
-QPLGLF-
-QPLGLV-
-QPLGLI-

QKPLGL--
-QPLGLM-
QQPLGL--
QDPLGL--
-QPLGLE-
QMPLGL--
QAPLGL--
-QPLGLD-
-QPLGLC-
RGPLGL--
-RPLGLK-
-RPLGLT-
-RPLGLY-
RTPLGL--
-RPLGLN-
-RPLGLA-
-RPLGLH-
RHPLGL--
RYPLGL--
REPLGL--
-RPLGLQ-
RPPLGL--
-RPLGLF-
RQPLGL--
RFPLGL--
-RPLGLE-
RDPLGL--
RCPLGL--
-RPLGLD-
-RPLGLC-
-SPLGLL-
-SPLGLS-
SSPLGL--
SNPLGL--
-SPLGLK-
SGPLGL--
SVPLGL--
-SPLGLT-
SIPLGL--
STPLGL--
-SPLGLY-
-SPLGLV-
SYPLGL--
SKPLGL--
-SPLGLN-
SWPLGL--
SQPLGL--
SMPLGL--
-SPLGLQ-
SFPLGL--

SPPLGL--
SDPLGL--
-SPLGLM-
-SPLGLI-
SHPLGL--
-SPLGLE-
SCPLGL--
TSPLGL--
-TPLGLS-
-TPLGLT-
TGPLGL--
-TPLGLH-
TKPLGL--
THPLGL--
TWPLGL--
-TPLGLK-
TAPLGL--
TVPLGL--
-TPLGLW-
-TPLGLG-
-TPLGLF-
-TPLGLN-
TIPLGL--
TTPLGL--
TDPLGL--
-TPLGLV-
-TPLGLM-
TFPLGL--
-TPLGLA-
TPPLGL--
-TPLGLQ-
TEPLGL--
TMPLGL--
TQPLGL--
TCPLGL--
-TPLGLC-
-TPLGLD-
VLPLGL--
-VPLGLL-
VRPLGL--
-VPLGLR-
VKPLGL--
-VPLGLN-
-VPLGLA-
VMPLGL--
VQPLGL--
-VPLGLV-
VVPLGL--
VNPLGL--
VIPLGL--

-VPLGLG-
VTPLGL--
VWPLGL--
VEPLGL--
-VPLGLM-
-VPLGLH-
-VPLGLE-
VHPLGL--
-VPLGLT-
-VPLGLF-
VCPLGL--
-VPLGLD-
-VPLGLC-
WSPLGL--
WKPLGL--
-WPLGLN-
-WPLGLK-
WTPLGL--
-WPLGLE-
WVPLGL--
WDPLGL--
-WPLGLT-
WYPLGL--
-WPLGLL-
-WPLGLF-
-WPLGLA-
-WPLGLV-
WEPLGL--
WFPLGL--
-WPLGLY-
WNPLGL--
WHPLGL--
WMPLGL--
-WPLGLM-
-WPLGLH-
-WPLGLG-
WWPLGL--
-WPLGLW-
-WPLGLD-
WCPLGL--
YSPLGL--
YGPLGL--
-YPLGLS-
YLPLGL--
-YPLGLN-
-YPLGLL-
-YPLGLV-
YYPLGL--
YNPLGL--
-YPLGLR-

-YPLGLA-
YTPLGL--
-YPLGLT-
-YPLGLQ-
YPPLGL--
-YPLGLE-
-YPLGLK-
YHPLGL--
YQPLGL--
YIPLGL--
-YPLGLM-
YDPLGL--
YFPLGL--
-YPLGLG-
YWPLGL--
YMPLGL--
-YPLGLW-
YCPLGL--

PFGM	229	731	48.97

--PFGMGR
-VPFGMT-
-APFGMQ-
APPFGM--
ASPFGM--
-APFGMS-
-APFGML-
-APFGMY-
AMPFGM--
ALPFGM--
-APFGMT-
AKPFGM--
AIPFGM--
AVPFGM--
-APFGMW-
AAPFGM--
-APFGMR-
-APFGMV-
-APFGMG-
ANPFGM--
AGPFGM--
AFPFGM--
AWPFGM--
-APFGMA-
ATPFGM--
AQPFGM--
-APFGME-
ADPFGM--
-APFGMI-
-APFGMF-
CRPFGM--

CEPFGM--
-CPFGMK-
CLPFGM--
CMPFGM--
CTPFGM--
-CPFGMR-
CSPFGM--
CPPFGM--
CKPFGM--
CIPFGM--
CGPFGM--
CCPFGM--
CAPFGM--
DSPFGM--
DVPFGM--
DKPFGM--
DLPFGM--
DYPFGM--
-DPFGMR-
-DPFGMM-
DRPFGM--
-DPFGMH-
DMPFGM--
DGPFGM--
DWPFGM--
DTPFGM--
-DPFGMW-
-DPFGML-
-DPFGMA-
DNPFGM--
DIPFGM--
DAPFGM--
-DPFGMS-
-DPFGMK-
DEPFGM--
-EPFGMR-
-EPFGMI-
-EPFGMF-
-EPFGML-
-EPFGMN-
-EPFGMM-
-EPFGMH-
EGPFGM--
EVPFGM--
ETPFGM--
ERPFGM--
EPPFGM--
-EPFGMK-
ENPFGM--
-EPFGMV-

EWPFGM--
EQPFGM--
EMPFGM--
ELPFGM--
EYPFGM--
-EPFGMT-
EHPFGM--
EFPFGM--
-FPFGMR-
FLPFGM--
FQPFGM--
FSPFGM--
FRPFGM--
-FPFGMS-
FAPFGM--
-FPFGML-
-FPFGMT-
FVPFGM--
-FPFGMW-
-FPFGMV-
-FPFGMM-
FTPFGM--
-FPFGMK-
FGPFGM--
-FPFGMG-
-FPFGMH-
FMPFGM--
-FPFGME-
FDPFGM--
-FPFGMQ-
-FPFGMI-
-FPFGMA-
FKPFGM--
GVPFGM--
GKPFGM--
GSPFGM--
-GPFGMI-
-GPFGMT-
GYPFGM--
GLPFGM--
-GPFGMY-
GQPFGM--
GTPFGM--
GNPFGM--
GMPFGM--
GAPFGM--
GRPFGM--
GGPFGM--
-GPFGMS-
-GPFGMM-

GIPFGM--
-GPFGMH-
-GPFGMV-
-GPFGMN-
-GPFGMG-
GDPFGM--
GHPFGM--
-GPFGMQ-
-GPFGMF-
-GPFGMK-
GFPFGM--
GEPFGM--
GWPFGM--
HLPFGM--
-HPFGMI-
-HPFGMF-
HWPFGM--
HEPFGM--
-HPFGML-
HKPFGM--
HSPFGM--
-HPFGMR-
HGPFGM--
-HPFGMK-
HYPFGM--
HIPFGM--
-HPFGMY-
-HPFGMN-
HVPFGM--
HTPFGM--
HQPFGM--
-HPFGMQ-
-HPFGMH-
-HPFGMW-
HRPFGM--
-HPFGMS-
-HPFGMA-
HFPFGM--
-HPFGME-
HCPFGM--
IRPFGM--
ISPFGM--
ILPFGM--
-IPFGMS-
-IPFGMT-
IAPFGM--
-IPFGMK-
IWPFGM--
-IPFGMM-
-IPFGML-

-IPFGMR-
IGPFGM--
-IPFGMV-
ITPFGM--
IEPFGM--
IDPFGM--
IQPFGM--
-IPFGMI-
IKPFGM--
-IPFGMG-
-IPFGMA-
INPFGM--
ICPFGM--
IYPFGM--
-IPFGMY-
-IPFGMN-
IIPFGM--
-IPFGMH-
-IPFGMF-
IFPFGM--
-IPFGMQ-
-IPFGME-
KSPFGM--
KRPFGM--
-KPFGMK-
-KPFGMT-
-KPFGMY-
-KPFGMV-
-KPFGMI-
KKPFGM--
KLPFGM--
KTPFGM--
KGPFGM--
KWPFGM--
-KPFGMS-
KHPFGM--
KAPFGM--
KQPFGM--
-KPFGMR-
KMPFGM--
-KPFGMM-
-KPFGMF-
-KPFGML-
-KPFGMG-
KYPFGM--
KIPFGM--
-KPFGMW-
KDPFGM--
LKPFGM--
-LPFGMR-

```
-LPFGMT-
LGPFGM--
LLPFGM--
LRPFGM--
LTPFGM--
-LPFGMI-
LIPFGM--
LPPFGM--
LDPFGM--
LNPFGM--
LVPFGM--
LAPFGM--
-LPFGMS-
-LPFGMY-
-LPFGMQ-
-LPFGMK-
-LPFGMV-
LWPFGM--
LHPFGM--
-LPFGMN-
LFPFGM--
-LPFGMH-
LMPFGM--
LEPFGM--
LCPFGM--
-MPFGMR-
MSPFGM--
MRPFGM--
MLPFGM--
MFPFGM--
-MPFGMA-
MKPFGM--
MVPFGM--
MMPFGM--
MAPFGM--
MNPFGM--
MYPFGM--
MQPFGM--
-MPFGMM-
MHPFGM--
MEPFGM--
MWPFGM--
-MPFGMN-
-MPFGMI-
-MPFGMG-
MTPFGM--
MPPFGM--
MCPFGM--
-MPFGMS-
-MPFGML-
```

```
-MPFGMH-
-MPFGME-
-MPFGMD-
NFPFGM--
NYPFGM--
-NPFGML-
NPPFGM--
-NPFGMH-
NRPFGM--
NQPFGM--
-NPFGMV-
-NPFGMK-
NGPFGM--
NAPFGM--
NWPFGM--
-NPFGMM-
NLPFGM--
NSPFGM--
-NPFGMI-
NEPFGM--
NVPFGM--
-NPFGMA-
-NPFGMS-
-NPFGMY-
NMPFGM--
-NPFGMT-
-NPFGMQ-
-NPFGMF-
NKPFGM--
-NPFGMR-
-NPFGMN-
NDPFGM--
--PFGMSS
--PFGMLS
--PFGMRV
--PFGMIG
--PFGMKG
--PFGMVR
--PFGMKA
--PFGMIR
--PFGMVS
--PFGMTT
--PFGMSK
--PFGMST
--PFGMFK
--PFGMRI
--PFGMKV
--PFGMRR
--PFGMRD
--PFGMVW
```

--PFGMVK
--PFGMKR
--PFGMIY
--PFGMWN
--PFGMTR
--PFGMLT
--PFGMYS
--PFGMTH
--PFGMLA
--PFGMHK
--PFGMTS
--PFGMTY
--PFGMTV
--PFGMSV
--PFGMSH
--PFGMMA
--PFGMLR
--PFGMYG
--PFGMRG
--PFGMQS
--PFGMLE
--PFGMHR
--PFGMGS
--PFGMFR
--PFGMVY
--PFGMKS
--PFGMHV
--PFGMHM
--PFGMFV
--PFGMYR
--PFGMSL
--PFGMVT
--PFGMVI
--PFGMTK
--PFGMMM
--PFGMVV
--PFGMVH
--PFGMTL
--PFGMSF
--PFGMRY
--PFGMRN
--PFGMRA
--PFGMHS
--PFGMHN
--PFGMHL
--PFGMHH
--PFGMHA
PKPFGM--
--PFGMSR
--PFGMRT

--PFGMNN
--PFGMKD
--PFGMIA
--PFGMEY
--PFGMYK
--PFGMRE
--PFGMNF
--PFGMIW
--PFGMEV
--PFGMES
PHPFGM--
--PFGMYE
--PFGMTQ
--PFGMSN
--PFGMSM
--PFGMRK
--PFGMQQ
--PFGMHD
--PFGMDG
PDPFGM--
PAPFGM--
--PFGMVN
--PFGMTN
--PFGMNS
--PFGMNM
--PFGMMV
--PFGMMK
--PFGMKQ
--PFGMHW
--PFGMAY
PIPFGM--
--PFGMWR
--PFGMTF
--PFGMSW
--PFGMSD
--PFGMQV
--PFGMMN
--PFGMIS
--PFGMFE
--PFGMAN
--PFGMYH
--PFGMYD
--PFGMQL
--PFGMMG
--PFGMLD
--PFGMIV
--PFGMIE
--PFGMHY
--PFGMGY
--PFGMWQ

--PFGMTA
--PFGMQN
--PFGMQE
--PFGMME
--PFGMKI
--PFGMIN
--PFGMIH
--PFGMFQ
--PFGMFH
--PFGMEW
--PFGMAH
PVPFGM--
PGPFGM--
--PFGMYQ
--PFGMVQ
--PFGMTI
--PFGMTG
--PFGMQT
--PFGMQD
--PFGMMS
--PFGMLH
--PFGMKK
--PFGMGV
--PFGMGT
--PFGMGM
--PFGMEN
--PFGMEF
--PFGMDA
--PFGMAK
--PFGMWG
--PFGMVG
--PFGMVD
--PFGMSY
--PFGMRM
--PFGMNY
--PFGMNR
--PFGMNK
--PFGMNI
--PFGMNH
--PFGMMH
--PFGMLG
--PFGMKW
--PFGMKT
--PFGMKN
--PFGMIT
--PFGMIM
--PFGMHT
--PFGMGW
--PFGMGG
--PFGMFI

--PFGMFG
--PFGMET
--PFGMDS
--PFGMCN
--PFGMAA
PMPFGM--
--PFGMTE
--PFGMQI
--PFGMND
--PFGMKF
--PFGMFT
--PFGMEQ
--PFGMEM
--PFGMEK
--PFGMCR
--PFGMAL
--PFGMAI
--PFGMAE
PEPFGM--
PCPFGM--
PYPFGM--
PTPFGM--
PQPFGM--
PNPFGM--
--PFGMVA
--PFGMSI
--PFGMQY
--PFGMQR
--PFGMQF
--PFGMPK
--PFGMPH
--PFGMPF
--PFGMNA
--PFGMMY
--PFGMMQ
--PFGMLQ
--PFGMKC
--PFGMII
--PFGMHQ
--PFGMHE
--PFGMGL
--PFGMGK
--PFGMGI
--PFGMGH
--PFGMGF
--PFGMFW
--PFGMFM
--PFGMEI
--PFGMCE
--PFGMAW

--PFGMAS
--PFGMAR
--PFGMAQ
--PFGMAG
--PFGMAF
-QPFGMR-
QYPFGM--
QVPFGM--
QSPFGM--
-QPFGMF-
QLPFGM--
-QPFGML-
-QPFGMH-
QIPFGM--
QRPFGM--
-QPFGMY-
QHPFGM--
-QPFGMV-
-QPFGMT-
-QPFGMI-
QMPFGM--
-QPFGMQ-
-QPFGMK-
-QPFGMW-
-QPFGMA-
QTPFGM--
-QPFGMN-
QKPFGM--
QGPFGM--
-RPFGML-
RPPFGM--
RSPFGM--
RRPFGM--
RFPFGM--
-RPFGMN-
-RPFGMV-
RTPFGM--
RMPFGM--
-RPFGMS-
-RPFGMM-
RHPFGM--
RVPFGM--
RGPFGM--
-RPFGMT-
-RPFGMI-
RAPFGM--
-RPFGMK-
-RPFGMA-
RYPFGM--
REPFGM--

-RPFGMG-
-RPFGME-
RLPFGM--
-RPFGMW-
RKPFGM--
-RPFGMH-
-RPFGMQ-
RNPFGM--
RWPFGM--
RQPFGM--
-SPFGMS-
SRPFGM--
-SPFGMY-
SFPFGM--
-SPFGMR-
-SPFGMT-
-SPFGML-
SIPFGM--
-SPFGMQ-
-SPFGMH-
SMPFGM--
SSPFGM--
-SPFGMW-
SNPFGM--
SLPFGM--
-SPFGMA-
-SPFGMK-
SHPFGM--
SKPFGM--
SDPFGM--
-SPFGMI-
SYPFGM--
SQPFGM--
SVPFGM--
SEPFGM--
SWPFGM--
SGPFGM--
-SPFGMM-
-SPFGMG-
SPPFGM--
-SPFGMN-
STPFGM--
-SPFGMC-
TWPFGM--
-TPFGMS-
-TPFGMV-
TPPFGM--
-TPFGMY-
-TPFGMK-
-TPFGMT-

−TPFGMM−
−TPFGML−
−TPFGMN−
TSPFGM−−
TMPFGM−−
TLPFGM−−
−TPFGMR−
TGPFGM−−
−TPFGMH−
TIPFGM−−
TAPFGM−−
−TPFGMG−
TFPFGM−−
TTPFGM−−
−TPFGMW−
TNPFGM−−
TYPFGM−−
TVPFGM−−
TCPFGM−−
TQPFGM−−
−TPFGMQ−
TEPFGM−−
VRPFGM−−
−VPFGML−
VTPFGM−−
VSPFGM−−
VHPFGM−−
−VPFGMS−
VYPFGM−−
VMPFGM−−
−VPFGMH−
−VPFGMI−
−VPFGMG−
VVPFGM−−
VQPFGM−−
−VPFGMK−
VNPFGM−−
VGPFGM−−
−VPFGMM−
VFPFGM−−
VEPFGM−−
−VPFGMY−
VPPFGM−−
−VPFGME−
VLPFGM−−
−VPFGMA−
−VPFGMV−
VDPFGM−−
−WPFGMK−
−WPFGMH−

WTPFGM--
-WPFGMS-
WPPFGM--
-WPFGML-
WAPFGM--
WRPFGM--
-WPFGMT-
-WPFGMN-
WNPFGM--
WFPFGM--
WYPFGM--
WSPFGM--
WMPFGM--
WLPFGM--
WHPFGM--
-WPFGMR-
-WPFGMV-
WKPFGM--
WIPFGM--
WGPFGM--
-YPFGML-
-YPFGMK-
YRPFGM--
YVPFGM--
-YPFGMT-
YYPFGM--
YSPFGM--
-YPFGMI-
YDPFGM--
-YPFGMN-
YKPFGM--
YFPFGM--
YAPFGM--
YLPFGM--
YQPFGM--
-YPFGMH-
-YPFGMR-
YGPFGM--
-YPFGMV-
YHPFGM--
YPPFGM--
YNPFGM--
YMPFGM--
YIPFGM--
YTPFGM--
-YPFGMF-
YWPFGM--
-YPFGMA-
YEPFGM--
-YPFGME-

PMNL 49 852 48.96

```
--PMNLPS
--PMNLAC
ASPMNL--
AIPMNL--
-APMNLT-
-APMNLG-
AKPMNL--
-APMNLR-
-APMNLV-
AGPMNL--
-APMNLI-
-APMNLK-
-APMNLA-
ALPMNL--
AQPMNL--
AVPMNL--
-APMNLM-
AEPMNL--
AYPMNL--
AHPMNL--
ADPMNL--
AAPMNL--
ARPMNL--
APPMNL--
AMPMNL--
AFPMNL--
-APMNLN-
-APMNLH-
ANPMNL--
-APMNLY-
-APMNLW-
AWPMNL--
-APMNLE-
-APMNLD-
CQPMNL--
-CPMNLK-
CAPMNL--
CSPMNL--
CWPMNL--
-CPMNLV-
-CPMNLL-
CMPMNL--
CKPMNL--
CGPMNL--
CCPMNL--
CVPMNL--
-CPMNLS-
-CPMNLH-
CNPMNL--
```

-CPMNLW-
-CPMNLN-
CHPMNL--
CPPMNL--
-CPMNLT-
CFPMNL--
CEPMNL--
DSPMNL--
DVPMNL--
DAPMNL--
-DPMNLL-
DRPMNL--
-DPMNLS-
DNPMNL--
DLPMNL--
-DPMNLR-
-DPMNLK-
DTPMNL--
-DPMNLY-
-DPMNLW-
-DPMNLM-
-DPMNLI-
DKPMNL--
-DPMNLV-
-DPMNLA-
DHPMNL--
-DPMNLN-
DIPMNL--
-DPMNLG-
DEPMNL--
DWPMNL--
-DPMNLQ-
-DPMNLE-
DFPMNL--
-EPMNLR-
-EPMNLL-
ERPMNL--
-EPMNLT-
-EPMNLY-
EFPMNL--
-EPMNLS-
ELPMNL--
EVPMNL--
-EPMNLK-
EAPMNL--
-EPMNLQ-
-EPMNLI-
EMPMNL--
-EPMNLM-
ESPMNL--

-EPMNLV-
EGPMNL--
-EPMNLA-
ENPMNL--
EWPMNL--
ETPMNL--
EQPMNL--
-EPMNLW-
-EPMNLG-
EHPMNL--
-FPMNLL-
FVPMNL--
FRPMNL--
-FPMNLN-
-FPMNLV-
FPPMNL--
FKPMNL--
FGPMNL--
FHPMNL--
-FPMNLS-
-FPMNLH-
-FPMNLT-
-FPMNLG-
FAPMNL--
-FPMNLF-
-FPMNLM-
FFPMNL--
-FPMNLA-
FEPMNL--
-FPMNLK-
FNPMNL--
-FPMNLY-
FQPMNL--
-FPMNLR-
-FPMNLQ-
-FPMNLE-
-FPMNLD-
FDPMNL--
GRPMNL--
-GPMNLS-
-GPMNLL-
GPPMNL--
-GPMNLQ-
GMPMNL--
-GPMNLN-
GSPMNL--
-GPMNLM-
GGPMNL--
GLPMNL--
GTPMNL--

GFPMNL--
-GPMNLK-
-GPMNLR-
-GPMNLA-
GYPMNL--
GAPMNL--
GKPMNL--
GVPMNL--
-GPMNLV-
GQPMNL--
-GPMNLG-
-GPMNLF-
GHPMNL--
-GPMNLY-
GDPMNL--
-GPMNLW-
-GPMNLD-
-GPMNLT-
-GPMNLH-
-GPMNLE-
GEPMNL--
GWPMNL--
GIPMNL--
GCPMNL--
HSPMNL--
HKPMNL--
HRPMNL--
HYPMNL--
-HPMNLV-
-HPMNLL-
-HPMNLM-
-HPMNLS-
-HPMNLA-
HMPMNL--
HGPMNL--
-HPMNLH-
HPPMNL--
-HPMNLT-
-HPMNLG-
HFPMNL--
HNPMNL--
HIPMNL--
HHPMNL--
-HPMNLR-
-HPMNLI-
HLPMNL--
-HPMNLK-
HAPMNL--
-HPMNLE-
HTPMNL--

−HPMNLY−
HEPMNL−−
HDPMNL−−
HQPMNL−−
−HPMNLN−
−HPMNLP−
−HPMNLF−
ISPMNL−−
IRPMNL−−
IYPMNL−−
−IPMNLH−
IGPMNL−−
−IPMNLR−
IVPMNL−−
−IPMNLG−
−IPMNLN−
−IPMNLL−
−IPMNLI−
−IPMNLA−
−IPMNLV−
IQPMNL−−
ILPMNL−−
IDPMNL−−
ITPMNL−−
−IPMNLT−
−IPMNLM−
IMPMNL−−
IIPMNL−−
IEPMNL−−
IWPMNL−−
INPMNL−−
−IPMNLQ−
−IPMNLW−
−IPMNLK−
−IPMNLE−
−IPMNLD−
IKPMNL−−
IHPMNL−−
IAPMNL−−
KSPMNL−−
−KPMNLY−
−KPMNLS−
KMPMNL−−
−KPMNLR−
KLPMNL−−
KGPMNL−−
KPPMNL−−
KAPMNL−−
−KPMNLV−
KVPMNL−−

KIPMNL--
KYPMNL--
-KPMNLL-
-KPMNLG-
KFPMNL--
-KPMNLI-
-KPMNLT-
-KPMNLM-
-KPMNLA-
-KPMNLW-
-KPMNLH-
-KPMNLE-
KEPMNL--
-KPMNLN-
-KPMNLF-
-KPMNLQ-
KNPMNL--
-KPMNLD-
KTPMNL--
KQPMNL--
KHPMNL--
KKPMNL--
KCPMNL--
LRPMNL--
LGPMNL--
-LPMNLR-
-LPMNLL-
LLPMNL--
LSPMNL--
-LPMNLK-
-LPMNLV-
LAPMNL--
-LPMNLH-
LKPMNL--
-LPMNLI-
LHPMNL--
-LPMNLA-
-LPMNLS-
-LPMNLT-
-LPMNLW-
-LPMNLM-
LDPMNL--
LTPMNL--
LPPMNL--
LWPMNL--
-LPMNLG-
LMPMNL--
LQPMNL--
-LPMNLN-
-LPMNLQ-

LFPMNL--
LEPMNL--
-LPMNLF-
LCPMNL--
LIPMNL--
-MPMNLN-
MWPMNL--
-MPMNLF-
-MPMNLT-
-MPMNLS-
MRPMNL--
MMPMNL--
MKPMNL--
MLPMNL--
MEPMNL--
MGPMNL--
MTPMNL--
-MPMNLA-
-MPMNLH-
MSPMNL--
-MPMNLY-
-MPMNLE-
MVPMNL--
MQPMNL--
-MPMNLV-
MHPMNL--
MDPMNL--
MAPMNL--
-MPMNLQ-
-MPMNLD-
MNPMNL--
NRPMNL--
-NPMNLR-
NFPMNL--
NSPMNL--
-NPMNLV-
NPPMNL--
NLPMNL--
-NPMNLF-
NMPMNL--
NWPMNL--
NQPMNL--
-NPMNLM-
NTPMNL--
NEPMNL--
-NPMNLT-
NGPMNL--
NIPMNL--
-NPMNLL-
-NPMNLA-

```
-NPMNLG-
NYPMNL--
-NPMNLW-
-NPMNLH-
NAPMNL--
-NPMNLY-
NHPMNL--
-NPMNLQ-
-NPMNLN-
-NPMNLE-
-NPMNLK-
-NPMNLI-
NDPMNL--
-NPMNLD-
--PMNLRR
--PMNLRL
--PMNLRW
--PMNLST
--PMNLMR
--PMNLLT
--PMNLRV
--PMNLIG
--PMNLVI
PVPMNL--
--PMNLVR
--PMNLLS
--PMNLRY
--PMNLFS
--PMNLES
--PMNLTY
--PMNLTS
--PMNLKY
--PMNLSL
--PMNLGA
--PMNLWK
--PMNLWA
--PMNLRQ
--PMNLGS
--PMNLYT
--PMNLML
--PMNLKT
PRPMNL--
--PMNLRG
--PMNLKN
--PMNLWG
--PMNLVY
--PMNLVT
--PMNLRH
--PMNLFT
--PMNLTG
```

--PMNLRN
--PMNLTK
--PMNLRD
--PMNLNS
PKPMNL--
--PMNLSV
--PMNLSI
--PMNLRF
--PMNLLE
--PMNLIA
--PMNLEA
--PMNLHR
--PMNLSK
--PMNLKA
--PMNLGN
--PMNLYH
--PMNLSQ
--PMNLRK
--PMNLRE
--PMNLGV
--PMNLYS
--PMNLLH
PLPMNL--
PGPMNL--
--PMNLSA
--PMNLSN
--PMNLRI
--PMNLMK
--PMNLHG
--PMNLMG
--PMNLAR
--PMNLRT
--PMNLMI
PWPMNL--
--PMNLVH
--PMNLSY
--PMNLKK
--PMNLIK
--PMNLGR
--PMNLAK
PIPMNL--
--PMNLWS
--PMNLKL
--PMNLFK
--PMNLEI
PEPMNL--
--PMNLYR
--PMNLYG
--PMNLSF
--PMNLQG

--PMNLNA
--PMNLFR
--PMNLAS
PHPMNL--
--PMNLVM
--PMNLMS
--PMNLMD
--PMNLGT
--PMNLWI
--PMNLSR
--PMNLSM
--PMNLQK
--PMNLNN
--PMNLLQ
--PMNLAT
PFPMNL--
PQPMNL--
--PMNLWT
--PMNLLD
--PMNLKR
--PMNLHF
--PMNLVK
--PMNLQA
--PMNLKV
--PMNLKS
--PMNLKG
--PMNLIE
--PMNLGI
--PMNLET
--PMNLAL
--PMNLVQ
--PMNLVN
--PMNLTT
--PMNLVW
--PMNLVE
--PMNLQW
--PMNLQN
--PMNLNR
--PMNLNQ
--PMNLMN
--PMNLKH
--PMNLGY
--PMNLFD
--PMNLAW
--PMNLQY
--PMNLQT
--PMNLKM
--PMNLIS
--PMNLIN
--PMNLHV

--PMNLHT
--PMNLHA
--PMNLFA
--PMNLAF
--PMNLTM
--PMNLME
--PMNLFH
--PMNLDG
--PMNLAG
--PMNLWE
--PMNLVD
--PMNLTD
--PMNLSG
--PMNLHW
--PMNLHN
--PMNLHI
--PMNLEL
PDPMNL--
PSPMNL--
--PMNLSE
--PMNLQL
--PMNLNW
--PMNLNI
--PMNLMT
--PMNLKI
--PMNLKF
--PMNLKE
--PMNLID
--PMNLHM
--PMNLGF
--PMNLFQ
--PMNLFG
--PMNLAM
PTPMNL--
PNPMNL--
--PMNLWW
--PMNLWN
--PMNLTN
--PMNLTH
--PMNLSH
--PMNLNM
--PMNLNL
--PMNLKD
--PMNLGE
--PMNLWD
--PMNLMY
--PMNLMQ
--PMNLIW
--PMNLHQ
--PMNLHK

--PMNLHD
--PMNLGW
--PMNLFW
--PMNLDT
--PMNLAN
--PMNLYQ
--PMNLYN
--PMNLYA
--PMNLWQ
--PMNLTA
--PMNLQH
--PMNLMH
--PMNLIH
--PMNLHS
--PMNLGM
--PMNLGG
--PMNLDW
--PMNLAQ
--PMNLAD
--PMNLAA
PYPMNL--
--PMNLYK
--PMNLTE
--PMNLSD
--PMNLQV
--PMNLQI
--PMNLNV
--PMNLNT
--PMNLNG
--PMNLKQ
--PMNLEV
--PMNLER
--PMNLEK
--PMNLAV
-PPMNLL-
--PMNLYE
--PMNLYD
--PMNLWM
--PMNLVP
--PMNLRP
--PMNLQS
--PMNLNK
--PMNLMA
--PMNLKW
--PMNLEN
--PMNLEG
--PMNLEF
--PMNLCS
--PMNLAY
--PMNLAH

--PMNLAE
PAPMNL--
-PPMNLG-
--PMNLYM
--PMNLWH
--PMNLWF
--PMNLNF
--PMNLNE
--PMNLMM
--PMNLHY
--PMNLGK
--PMNLGD
--PMNLEQ
--PMNLEE
--PMNLDV
--PMNLDM
--PMNLCA
--PMNLAI
PCPMNL--
--PMNLYV
--PMNLTI
--PMNLTC
--PMNLQQ
--PMNLQF
--PMNLPN
--PMNLNY
--PMNLNH
--PMNLMW
--PMNLHH
--PMNLEY
--PMNLEH
--PMNLDQ
--PMNLCR
--PMNLCG
QSPMNL--
-QPMNLY-
QWPMNL--
-QPMNLT-
-QPMNLM-
-QPMNLK-
QRPMNL--
QHPMNL--
-QPMNLS-
QPPMNL--
-QPMNLN-
-QPMNLL-
-QPMNLG-
-QPMNLR-
QGPMNL--
QVPMNL--

-QPMNLW-
-QPMNLQ-
-QPMNLF-
QFPMNL--
-QPMNLH-
-QPMNLI-
QTPMNL--
-QPMNLV-
-QPMNLA-
QNPMNL--
QAPMNL--
QQPMNL--
-QPMNLD-
QYPMNL--
-QPMNLE-
QLPMNL--
QKPMNL--
QMPMNL--
QDPMNL--
RGPMNL--
RSPMNL--
RVPMNL--
-RPMNLG-
-RPMNLR-
RHPMNL--
-RPMNLI-
-RPMNLT-
RPPMNL--
-RPMNLE-
-RPMNLH-
-RPMNLA-
-RPMNLV-
RIPMNL--
-RPMNLQ-
RNPMNL--
RAPMNL--
-RPMNLN-
RQPMNL--
-RPMNLM-
RDPMNL--
-RPMNLF-
REPMNL--
RYPMNL--
RTPMNL--
RKPMNL--
-RPMNLY-
-RPMNLD-
RCPMNL--
-RPMNLW-
SLPMNL--

SRPMNL--
SGPMNL--
-SPMNLI-
-SPMNLH-
-SPMNLS-
SMPMNL--
SEPMNL--
SWPMNL--
-SPMNLV-
-SPMNLG-
SAPMNL--
SVPMNL--
SSPMNL--
-SPMNLY-
-SPMNLN-
-SPMNLK-
-SPMNLA-
SPPMNL--
-SPMNLQ-
SKPMNL--
SDPMNL--
STPMNL--
-SPMNLF-
SIPMNL--
SHPMNL--
SYPMNL--
-SPMNLE-
-SPMNLM-
-SPMNLD-
-SPMNLW-
SQPMNL--
TWPMNL--
TLPMNL--
-TPMNLL-
TRPMNL--
TNPMNL--
-TPMNLF-
-TPMNLA-
-TPMNLK-
-TPMNLR-
TVPMNL--
TAPMNL--
TYPMNL--
-TPMNLT-
-TPMNLQ-
-TPMNLS-
TTPMNL--
TPPMNL--
-TPMNLY-
TIPMNL--

-TPMNLM-
-TPMNLN-
-TPMNLV-
TSPMNL--
THPMNL--
TEPMNL--
TMPMNL--
-TPMNLG-
-TPMNLH-
-TPMNLD-
TDPMNL--
TQPMNL--
-TPMNLI-
TKPMNL--
-TPMNLE-
TFPMNL--
VSPMNL--
VLPMNL--
-VPMNLT-
-VPMNLA-
VTPMNL--
-VPMNLN-
-VPMNLK-
-VPMNLE-
VWPMNL--
VAPMNL--
-VPMNLI-
-VPMNLG-
VVPMNL--
VPPMNL--
-VPMNLM-
VKPMNL--
VQPMNL--
VDPMNL--
VGPMNL--
-VPMNLY-
-VPMNLS-
-VPMNLH-
VIPMNL--
VHPMNL--
-VPMNLV-
VNPMNL--
VMPMNL--
VYPMNL--
-VPMNLD-
VEPMNL--
-VPMNLQ-
VFPMNL--
-VPMNLP-
WVPMNL--

WRPMNL--
WPPMNL--
-WPMNLT-
-WPMNLI-
-WPMNLS-
WKPMNL--
-WPMNLH-
WSPMNL--
-WPMNLR-
WGPMNL--
WTPMNL--
-WPMNLQ-
-WPMNLA-
WNPMNL--
WDPMNL--
-WPMNLL-
-WPMNLG-
-WPMNLV-
WEPMNL--
WQPMNL--
-WPMNLE-
WHPMNL--
-YPMNLQ-
YSPMNL--
-YPMNLM-
-YPMNLT-
YVPMNL--
YAPMNL--
YWPMNL--
-YPMNLR-
-YPMNLK-
-YPMNLS-
-YPMNLN-
YKPMNL--
-YPMNLH-
YEPMNL--
-YPMNLY-
-YPMNLV-
-YPMNLA-
YPPMNL--
YMPMNL--
YDPMNL--
YYPMNL--
YQPMNL--
-YPMNLG-
YTPMNL--
-YPMNLE-
-YPMNLI-
YNPMNL--
YHPMNL--

	YFPMNL--		
	-YPMNLD-		
	YCPMNL--		
PMAL	103	805	48.95
	-NPMALP-		
	--PMALVC		
	--PMALSP		
	ALPMAL--		
	ASPMAL--		
	AIPMAL--		
	ATPMAL--		
	AAPMAL--		
	-APMALH-		
	ANPMAL--		
	-APMALS-		
	AHPMAL--		
	AQPMAL--		
	-APMALN-		
	AVPMAL--		
	APPMAL--		
	-APMALQ-		
	-APMALI-		
	-APMALM-		
	-APMALK-		
	-APMALG-		
	-APMALT-		
	ADPMAL--		
	AKPMAL--		
	AYPMAL--		
	-APMALW-		
	-APMALA-		
	AMPMAL--		
	AEPMAL--		
	AWPMAL--		
	-APMALV-		
	AFPMAL--		
	-APMALE-		
	ACPMAL--		
	CSPMAL--		
	CVPMAL--		
	-CPMALK-		
	CNPMAL--		
	CQPMAL--		
	-CPMALR-		
	-CPMALN-		
	CAPMAL--		
	-CPMALV-		
	-CPMALL-		
	-CPMALS-		
	-CPMALA-		

CCPMAL--
-CPMALT-
-CPMALI-
-CPMALH-
CHPMAL--
CGPMAL--
CYPMAL--
-CPMALW-
-DPMALR-
-DPMALL-
DWPMAL--
DEPMAL--
DRPMAL--
DSPMAL--
-DPMALS-
DGPMAL--
DAPMAL--
DVPMAL--
-DPMALT-
DKPMAL--
-DPMALA-
DTPMAL--
DYPMAL--
DNPMAL--
-DPMALV-
-DPMALM-
-DPMALI-
DMPMAL--
DHPMAL--
-DPMALQ-
-DPMALK-
-DPMALG-
DIPMAL--
DDPMAL--
-DPMALE-
DLPMAL--
DFPMAL--
-EPMALR-
-EPMALS-
-EPMALL-
ELPMAL--
EFPMAL--
-EPMALH-
EKPMAL--
EAPMAL--
-EPMALM-
EYPMAL--
-EPMALQ-
-EPMALY-
-EPMALV-

-EPMALK-
EVPMAL--
ERPMAL--
-EPMALA-
EHPMAL--
EWPMAL--
-EPMALT-
ESPMAL--
EGPMAL--
EEPMAL--
-EPMALG-
EIPMAL--
EDPMAL--
-EPMALE-
ETPMAL--
FRPMAL--
FSPMAL--
-FPMALS-
-FPMALV-
FLPMAL--
FGPMAL--
FQPMAL--
FVPMAL--
FPPMAL--
-FPMALQ-
FAPMAL--
-FPMALT-
FIPMAL--
-FPMALM-
-FPMALA-
-FPMALN-
-FPMALG-
-FPMALK-
-FPMALH-
FHPMAL--
-FPMALW-
FMPMAL--
FEPMAL--
FDPMAL--
-FPMALE-
FFPMAL--
FCPMAL--
GRPMAL--
GSPMAL--
-GPMALL-
GAPMAL--
-GPMALS-
GNPMAL--
GKPMAL--
-GPMALR-

-GPMALK-
GLPMAL--
-GPMALV-
GTPMAL--
-GPMALM-
-GPMALQ-
-GPMALI-
GPPMAL--
-GPMALN-
-GPMALH-
GGPMAL--
GVPMAL--
-GPMALG-
-GPMALA-
GMPMAL--
GEPMAL--
GWPMAL--
GDPMAL--
-GPMALE-
GIPMAL--
GHPMAL--
GQPMAL--
HRPMAL--
-HPMALR-
HPPMAL--
HSPMAL--
-HPMALT-
-HPMALK-
HKPMAL--
-HPMALL-
HVPMAL--
-HPMALI-
HTPMAL--
-HPMALY-
-HPMALG-
-HPMALA-
HWPMAL--
-HPMALV-
HGPMAL--
-HPMALW-
HFPMAL--
HNPMAL--
HQPMAL--
-HPMALS-
-HPMALH-
HIPMAL--
HMPMAL--
-HPMALN-
-HPMALF-
HDPMAL--

-HPMALE-
HEPMAL--
-HPMALM-
-HPMALQ-
HYPMAL--
-HPMALD-
HHPMAL--
IAPMAL--
ILPMAL--
ISPMAL--
IYPMAL--
-IPMALS-
-IPMALK-
ITPMAL--
IHPMAL--
-IPMALA-
-IPMALN-
-IPMALL-
IDPMAL--
-IPMALI-
IKPMAL--
IPPMAL--
-IPMALT-
-IPMALE-
IGPMAL--
-IPMALV-
IEPMAL--
INPMAL--
-IPMALG-
IQPMAL--
IMPMAL--
IWPMAL--
-IPMALQ-
-IPMALD-
IFPMAL--
KSPMAL--
KTPMAL--
KRPMAL--
-KPMALN-
KVPMAL--
KYPMAL--
-KPMALE-
-KPMALF-
KNPMAL--
KGPMAL--
KWPMAL--
-KPMALM-
KPPMAL--
-KPMALI-
-KPMALW-

-KPMALY-
KHPMAL--
-KPMALG-
KAPMAL--
KLPMAL--
KMPMAL--
KQPMAL--
KDPMAL--
-KPMALQ-
-KPMALA-
KFPMAL--
KEPMAL--
-KPMALV-
-KPMALK-
-KPMALH-
-KPMALD-
LKPMAL--
LGPMAL--
-LPMALL-
-LPMALR-
LAPMAL--
-LPMALS-
-LPMALV-
LIPMAL--
LLPMAL--
LTPMAL--
LNPMAL--
-LPMALT-
-LPMALH-
LEPMAL--
LHPMAL--
-LPMALK-
-LPMALN-
-LPMALQ-
-LPMALM-
LPPMAL--
-LPMALA-
LYPMAL--
-LPMALG-
LCPMAL--
LDPMAL--
-MPMALN-
-MPMALL-
MPPMAL--
-MPMALR-
-MPMALA-
MLPMAL--
MGPMAL--
MAPMAL--
MWPMAL--

MTPMAL--
MKPMAL--
MHPMAL--
MIPMAL--
-MPMALG-
MDPMAL--
MYPMAL--
-MPMALS-
-MPMALQ-
-MPMALI-
MSPMAL--
-MPMALT-
-MPMALK-
-MPMALH-
MMPMAL--
MEPMAL--
-MPMALV-
-MPMALE-
MNPMAL--
-MPMALD-
MCPMAL--
NVPMAL--
-NPMALK-
NLPMAL--
NAPMAL--
-NPMALR-
-NPMALL-
NRPMAL--
NEPMAL--
NTPMAL--
-NPMALT-
-NPMALQ-
NPPMAL--
-NPMALS-
NYPMAL--
-NPMALW-
-NPMALY-
NFPMAL--
NQPMAL--
-NPMALV-
NMPMAL--
NDPMAL--
NWPMAL--
NNPMAL--
NKPMAL--
-NPMALF-
-NPMALM-
-NPMALI-
-NPMALH-
NCPMAL--

NGPMAL--
NHPMAL--
--PMALRH
--PMALMS
--PMALTR
--PMALRG
--PMALSL
--PMALKA
--PMALLR
--PMALGL
--PMALRI
--PMALLA
--PMALWR
--PMALAS
--PMALSG
--PMALTG
--PMALVR
--PMALRY
--PMALRE
--PMALNS
--PMALLG
--PMALRD
--PMALRR
--PMALRN
--PMALQR
--PMALNF
--PMALHR
--PMALPL
--PMALAL
--PMALTT
--PMALTA
PYPMAL--
--PMALWN
--PMALSY
--PMALRT
--PMALLD
--PMALHW
--PMALHL
--PMALYG
--PMALVG
--PMALTI
--PMALQV
--PMALMA
--PMALIR
--PMALWA
--PMALVQ
--PMALQS
--PMALKI
--PMALVS
--PMALAT

PVPMAL--
--PMALSS
--PMALVN
--PMALNN
--PMALMN
PIPMAL--
--PMALMG
--PMALHF
--PMALTK
--PMALKR
--PMALGS
--PMALSI
--PMALSF
--PMALRV
--PMALQT
--PMALSH
--PMALQL
--PMALLE
--PMALHM
--PMALGN
--PMALET
--PMALSN
--PMALQM
--PMALND
--PMALKM
--PMALKH
--PMALIN
--PMALSW
--PMALRK
--PMALFK
--PMALES
PAPMAL--
PTPMAL--
PNPMAL--
--PMALTN
--PMALAR
--PMALAI
--PMALSE
--PMALMI
--PMALGA
--PMALEY
--PMALAA
--PMALVK
--PMALNA
--PMALMR
--PMALLQ
--PMALKG
--PMALFD
--PMALML
--PMALKD

--PMALHG
--PMALGM
--PMALFS
--PMALEW
--PMALMV
--PMALIT
--PMALHK
--PMALAG
--PMALYF
--PMALTW
--PMALNG
--PMALKF
--PMALIQ
--PMALGT
--PMALEQ
--PMALVW
--PMALGQ
--PMALWS
--PMALVT
--PMALVH
--PMALRW
--PMALID
--PMALHD
--PMALEH
PDPMAL--
PMPMAL--
--PMALYP
--PMALSK
--PMALQK
--PMALNV
--PMALNR
--PMALMM
--PMALKK
PFPMAL--
--PMALYD
--PMALMW
--PMALKN
--PMALIG
--PMALHV
--PMALHS
--PMALHQ
--PMALGI
--PMALGH
--PMALAV
--PMALAH
PHPMAL--
--PMALVD
--PMALTQ
--PMALTH
--PMALSA

--PMALRA
--PMALNH
--PMALLH
--PMALKV
--PMALIH
--PMALGY
--PMALGV
--PMALDS
--PMALAN
--PMALLI
--PMALKT
--PMALFH
--PMALAW
--PMALWM
--PMALVE
--PMALQY
--PMALQD
--PMALPM
--PMALMY
--PMALKW
--PMALIK
--PMALHH
--PMALGK
--PMALGG
--PMALGF
PKPMAL--
PWPMAL--
--PMALWH
--PMALWF
--PMALTM
--PMALQN
--PMALPV
--PMALMH
--PMALKQ
--PMALDA
--PMALAY
--PMALAD
--PMALYH
--PMALTF
--PMALQW
--PMALMT
--PMALMQ
--PMALMD
--PMALHP
--PMALEG
--PMALED
--PMALWG
--PMALWE
--PMALRP
--PMALNI

--PMALME
--PMALKE
--PMALHE
--PMALER
--PMALSM
--PMALQG
--PMALNT
--PMALHN
--PMALEF
--PMALDY
--PMALCR
--PMALAK
--PMALYQ
--PMALVP
--PMALTV
--PMALNK
--PMALHI
--PMALEV
--PMALEK
--PMALDL
--PMALDG
--PMALCH
PEPMAL--
--PMALQQ
--PMALQI
--PMALNW
--PMALNC
--PMALLP
--PMALKY
--PMALKP
--PMALKC
--PMALIP
--PMALHY
-PPMALW-
-PPMALV-
-PPMALK-
-PPMALF-
--PMALWT
--PMALWQ
--PMALWD
--PMALTE
--PMALQA
--PMALPT
--PMALPQ
--PMALPI
--PMALPF
--PMALMC
--PMALLC
--PMALHA
--PMALGE

```
--PMALGD
--PMALFP
--PMALFC
--PMALEN
--PMALDT
--PMALDQ
--PMALDF
--PMALAM
QIPMAL--
QLPMAL--
QFPMAL--
QRPMAL--
QSPMAL--
-QPMALS-
QAPMAL--
-QPMALN-
QPPMAL--
-QPMALI-
QGPMAL--
-QPMALR-
QKPMAL--
QYPMAL--
-QPMALM-
-QPMALT-
-QPMALL-
-QPMALG-
-QPMALA-
QHPMAL--
-QPMALF-
QWPMAL--
QTPMAL--
-QPMALV-
-QPMALE-
QQPMAL--
QDPMAL--
-QPMALQ-
-QPMALD-
RSPMAL--
-RPMALR-
-RPMALK-
RTPMAL--
RGPMAL--
-RPMALL-
RRPMAL--
RQPMAL--
RPPMAL--
RAPMAL--
-RPMALV-
RWPMAL--
RIPMAL--
```

-RPMALW-
-RPMALI-
RNPMAL--
-RPMALN-
-RPMALA-
RMPMAL--
RDPMAL--
REPMAL--
-RPMALH-
-RPMALG-
-RPMALQ-
-RPMALE-
RCPMAL--
SRPMAL--
-SPMALR-
SSPMAL--
SVPMAL--
SLPMAL--
STPMAL--
-SPMALL-
-SPMALT-
-SPMALV-
SIPMAL--
-SPMALW-
SQPMAL--
SFPMAL--
SEPMAL--
SWPMAL--
SAPMAL--
-SPMALI-
SGPMAL--
-SPMALQ-
-SPMALA-
-SPMALK-
-SPMALH-
SMPMAL--
SPPMAL--
-SPMALF-
SYPMAL--
-SPMALM-
SDPMAL--
-SPMALG-
-SPMALE-
SCPMAL--
-SPMALN-
SHPMAL--
-SPMALC-
-TPMALR-
TTPMAL--
TGPMAL--

TSPMAL--
TYPMAL--
-TPMALK-
-TPMALS-
TIPMAL--
-TPMALV-
TNPMAL--
TPPMAL--
TVPMAL--
-TPMALH-
TAPMAL--
-TPMALA-
-TPMALG-
-TPMALN-
TEPMAL--
TQPMAL--
-TPMALL-
TFPMAL--
-TPMALM-
TMPMAL--
TDPMAL--
-TPMALW-
-TPMALI-
-TPMALE-
THPMAL--
TKPMAL--
TCPMAL--
VTPMAL--
-VPMALL-
VSPMAL--
VYPMAL--
VWPMAL--
VQPMAL--
-VPMALS-
-VPMALI-
VAPMAL--
VNPMAL--
VGPMAL--
-VPMALA-
VLPMAL--
-VPMALN-
-VPMALG-
VEPMAL--
-VPMALM-
VKPMAL--
VDPMAL--
VPPMAL--
-VPMALH-
VMPMAL--
-VPMALK-

VFPMAL--
-VPMALQ-
VCPMAL--
-VPMALE-
-VPMALC-
WSPMAL--
WNPMAL--
WRPMAL--
WLPMAL--
-WPMALS-
-WPMALK-
WGPMAL--
WDPMAL--
-WPMALG-
-WPMALE-
-WPMALN-
-WPMALL-
WAPMAL--
WQPMAL--
WWPMAL--
WHPMAL--
-WPMALR-
-WPMALI-
WKPMAL--
WFPMAL--
WMPMAL--
WEPMAL--
-WPMALV-
-WPMALH-
-WPMALC-
-WPMALA-
YRPMAL--
-YPMALT-
YTPMAL--
-YPMALS-
YYPMAL--
-YPMALI-
YQPMAL--
YHPMAL--
YGPMAL--
YKPMAL--
YMPMAL--
YFPMAL--
-YPMALR-
-YPMALH-
YIPMAL--
YEPMAL--
YWPMAL--
-YPMALG-
-YPMALV-

-YPMALA-
YNPMAL--
YDPMAL--
YPPMAL--
-YPMALQ-
-YPMALK-
YAPMAL--
-YPMALW-
YCPMAL--

PYGY	55	848	48.94

-HPYGYM-
TEPYGY--
ARPYGY--
ASPYGY--
-APYGYR-
ALPYGY--
AEPYGY--
AIPYGY--
-APYGYT-
-APYGYS-
-APYGYM-
-APYGYF-
AGPYGY--
-APYGYV-
-APYGYQ-
AAPYGY--
AWPYGY--
AFPYGY--
-APYGYY-
AQPYGY--
APPYGY--
ADPYGY--
-APYGYG-
ANPYGY--
ATPYGY--
-APYGYL-
-APYGYH-
-APYGYA-
AMPYGY--
AVPYGY--
AKPYGY--
-APYGYN-
-APYGYI-
-APYGYW-
-APYGYK-
CTPYGY--
-CPYGYL-
-CPYGYH-
CLPYGY--
CEPYGY--

```
-CPYGYS-
-CPYGYA-
CVPYGY--
CSPYGY--
CRPYGY--
-CPYGYR-
CKPYGY--
CYPYGY--
-CPYGYF-
CNPYGY--
-CPYGYY-
-CPYGYI-
-CPYGYG-
CAPYGY--
-DPYGYR-
DSPYGY--
DVPYGY--
-DPYGYM-
-DPYGYH-
-DPYGYT-
DLPYGY--
DRPYGY--
DQPYGY--
-DPYGYY-
-DPYGYW-
DTPYGY--
-DPYGYV-
DGPYGY--
DYPYGY--
-DPYGYF-
DNPYGY--
DFPYGY--
DCPYGY--
-DPYGYI-
DAPYGY--
-DPYGYQ-
-DPYGYN-
-DPYGYE-
DIPYGY--
DEPYGY--
-EPYGYR-
ETPYGY--
EYPYGY--
ERPYGY--
EPPYGY--
EIPYGY--
-EPYGYM-
-EPYGYS-
ESPYGY--
-EPYGYN-
```

EWPYGY--
-EPYGYL-
EQPYGY--
-EPYGYQ-
-EPYGYI-
-EPYGYF-
EVPYGY--
-EPYGYY-
ELPYGY--
ENPYGY--
EGPYGY--
EEPYGY--
ECPYGY--
EAPYGY--
-EPYGYK-
-EPYGYA-
EFPYGY--
-EPYGYV-
-EPYGYT-
EHPYGY--
EKPYGY--
FRPYGY--
FTPYGY--
FDPYGY--
-FPYGYR-
-FPYGYL-
-FPYGYV-
-FPYGYK-
-FPYGYI-
FKPYGY--
FQPYGY--
-FPYGYM-
FEPYGY--
FSPYGY--
FVPYGY--
-FPYGYQ-
-FPYGYW-
-FPYGYS-
FMPYGY--
FHPYGY--
FFPYGY--
-FPYGYY-
-FPYGYE-
FPPYGY--
FNPYGY--
FWPYGY--
-FPYGYT-
-FPYGYG-
FLPYGY--
FIPYGY--

FAPYGY--
FYPYGY--
-FPYGYF-
-FPYGYA-
-GPYGYL-
GGPYGY--
-GPYGYR-
-GPYGYY-
GKPYGY--
GLPYGY--
-GPYGYI-
GRPYGY--
GYPYGY--
-GPYGYV-
GAPYGY--
-GPYGYF-
-GPYGYK-
GFPYGY--
GIPYGY--
GTPYGY--
GQPYGY--
GWPYGY--
-GPYGYN-
-GPYGYH-
GHPYGY--
GVPYGY--
GDPYGY--
GPPYGY--
-GPYGYA-
-GPYGYW-
-GPYGYG-
GEPYGY--
-GPYGYM-
-GPYGYE-
GMPYGY--
GNPYGY--
HGPYGY--
HKPYGY--
HSPYGY--
HRPYGY--
-HPYGYL-
-HPYGYV-
-HPYGYR-
-HPYGYS-
-HPYGYK-
-HPYGYT-
HLPYGY--
HIPYGY--
HQPYGY--
-HPYGYA-

HFPYGY--
HTPYGY--
-HPYGYY-
HVPYGY--
-HPYGYF-
HMPYGY--
HAPYGY--
HHPYGY--
HPPYGY--
HNPYGY--
HWPYGY--
-HPYGYN-
-HPYGYE-
-HPYGYC-
-HPYGYQ-
-HPYGYG-
ISPYGY--
INPYGY--
-IPYGYL-
-IPYGYR-
-IPYGYH-
IYPYGY--
IGPYGY--
-IPYGYT-
-IPYGYS-
ILPYGY--
-IPYGYQ-
IRPYGY--
IVPYGY--
-IPYGYM-
IMPYGY--
IEPYGY--
-IPYGYI-
-IPYGYG-
IPPYGY--
ICPYGY--
IAPYGY--
IIPYGY--
IHPYGY--
IWPYGY--
-IPYGYP-
-IPYGYN-
-IPYGYC-
IKPYGY--
KSPYGY--
-KPYGYR-
KRPYGY--
-KPYGYS-
KLPYGY--
KIPYGY--

-KPYGYV-
KMPYGY--
KAPYGY--
KTPYGY--
KEPYGY--
KDPYGY--
KVPYGY--
KGPYGY--
-KPYGYL-
-KPYGYA-
KNPYGY--
-KPYGYN-
-KPYGYI-
-KPYGYH-
KFPYGY--
-KPYGYF-
KQPYGY--
-KPYGYQ-
KWPYGY--
-KPYGYY-
KKPYGY--
KHPYGY--
-KPYGYM-
-KPYGYG-
-KPYGYK-
-KPYGYE-
LRPYGY--
LIPYGY--
-LPYGYR-
LQPYGY--
LYPYGY--
-LPYGYT-
LKPYGY--
LGPYGY--
-LPYGYK-
LLPYGY--
-LPYGYQ-
LFPYGY--
LEPYGY--
LTPYGY--
-LPYGYL-
-LPYGYA-
-LPYGYS-
LPPYGY--
LNPYGY--
-LPYGYV-
-LPYGYI-
LDPYGY--
-LPYGYN-
-LPYGYH-

LWPYGY--
-LPYGYY-
LMPYGY--
LAPYGY--
LVPYGY--
-LPYGYM-
LCPYGY--
-LPYGYG-
-LPYGYF-
-LPYGYW-
LHPYGY--
-LPYGYE-
-MPYGYS-
MRPYGY--
-MPYGYN-
MVPYGY--
-MPYGYA-
-MPYGYW-
MGPYGY--
MWPYGY--
MFPYGY--
MYPYGY--
MSPYGY--
MPPYGY--
MMPYGY--
MLPYGY--
MAPYGY--
-MPYGYY-
-MPYGYV-
-MPYGYR-
-MPYGYM-
-MPYGYF-
MIPYGY--
MTPYGY--
MQPYGY--
MKPYGY--
-MPYGYL-
-MPYGYI-
MEPYGY--
-MPYGYG-
NVPYGY--
-NPYGYR-
-NPYGYS-
NHPYGY--
-NPYGYW-
NLPYGY--
NYPYGY--
NQPYGY--
NAPYGY--
NRPYGY--

NGPYGY--
-NPYGYT-
NPPYGY--
NKPYGY--
-NPYGYK-
-NPYGYF-
-NPYGYH-
-NPYGYL-
NIPYGY--
NMPYGY--
NDPYGY--
NSPYGY--
-NPYGYV-
-NPYGYM-
-NPYGYI-
NEPYGY--
NFPYGY--
NTPYGY--
NCPYGY--
-NPYGYY-
-NPYGYQ-
--PYGYRS
--PYGYRT
--PYGYVK
--PYGYMG
--PYGYSR
--PYGYRR
--PYGYRV
--PYGYSH
--PYGYST
--PYGYLG
--PYGYSL
--PYGYRH
--PYGYQL
--PYGYSV
--PYGYWQ
--PYGYWG
--PYGYVR
--PYGYSS
--PYGYMR
--PYGYGT
--PYGYIR
--PYGYRL
--PYGYRG
--PYGYRQ
--PYGYLD
--PYGYTV
--PYGYKA
--PYGYTL
--PYGYKV

--PYGYRI
--PYGYRE
--PYGYHV
--PYGYSQ
--PYGYSI
--PYGYRA
--PYGYVW
--PYGYTN
--PYGYRN
--PYGYKS
--PYGYHL
PSPYGY--
--PYGYTS
--PYGYSG
--PYGYSF
--PYGYQT
--PYGYNS
--PYGYRD
--PYGYKG
--PYGYHQ
--PYGYAR
--PYGYVY
--PYGYRM
--PYGYLT
--PYGYWT
--PYGYTY
--PYGYYR
--PYGYVS
--PYGYSN
--PYGYLW
--PYGYLM
PWPYGY--
--PYGYTR
--PYGYTI
--PYGYMA
--PYGYGS
--PYGYAK
--PYGYWN
--PYGYTG
--PYGYNA
--PYGYMS
--PYGYFR
--PYGYFG
--PYGYFA
--PYGYAV
--PYGYVT
--PYGYTK
--PYGYNY
--PYGYLH
--PYGYAA

--PYGYYW
--PYGYRF
--PYGYQA
--PYGYLK
--PYGYFQ
--PYGYAL
PQPYGY--
--PYGYSA
--PYGYKK
--PYGYIT
--PYGYIM
--PYGYHT
--PYGYHS
PEPYGY--
--PYGYQN
--PYGYQM
--PYGYNV
--PYGYLE
--PYGYIS
--PYGYHA
--PYGYFT
--PYGYFS
--PYGYFI
--PYGYFH
--PYGYYG
--PYGYWS
--PYGYVM
--PYGYTM
--PYGYTE
--PYGYSM
--PYGYMF
--PYGYKY
--PYGYKW
--PYGYHI
--PYGYGI
--PYGYNK
--PYGYLQ
--PYGYKL
--PYGYIW
--PYGYFD
--PYGYWR
--PYGYVV
--PYGYTT
--PYGYSK
--PYGYQS
--PYGYQI
--PYGYNR
--PYGYNL
--PYGYMD
--PYGYKE

```
--PYGYAT
--PYGYAQ
--PYGYYS
--PYGYWH
--PYGYTQ
--PYGYQY
--PYGYMT
--PYGYMI
--PYGYMH
--PYGYLI
--PYGYIQ
--PYGYII
--PYGYIG
--PYGYHR
--PYGYGM
--PYGYAS
PYPYGY--
--PYGYYN
--PYGYYE
--PYGYVE
--PYGYSY
--PYGYRY
--PYGYQK
--PYGYMM
--PYGYLN
--PYGYKH
--PYGYFN
--PYGYEY
--PYGYES
--PYGYEN
--PYGYDS
PVPYGY--
--PYGYVA
--PYGYTA
--PYGYRW
--PYGYQV
--PYGYQE
--PYGYNW
--PYGYNT
--PYGYNM
--PYGYNI
--PYGYMY
--PYGYME
--PYGYID
--PYGYHF
--PYGYFK
--PYGYEA
--PYGYAY
--PYGYAN
PKPYGY--
```

PIPYGY--
PAPYGY--
--PYGYWK
--PYGYVI
--PYGYVG
--PYGYQG
--PYGYKN
--PYGYIY
--PYGYIN
--PYGYIK
--PYGYHK
--PYGYHG
--PYGYHD
--PYGYGY
--PYGYGW
--PYGYEM
--PYGYEI
--PYGYAI
PDPYGY--
--PYGYYQ
--PYGYWE
--PYGYVQ
--PYGYVH
--PYGYTW
--PYGYTD
--PYGYSW
--PYGYSE
--PYGYSD
--PYGYNQ
--PYGYMQ
--PYGYMN
--PYGYML
--PYGYMK
--PYGYKT
--PYGYKF
--PYGYIV
--PYGYHY
--PYGYGL
--PYGYGH
--PYGYGF
--PYGYFY
--PYGYFE
--PYGYEQ
--PYGYEH
--PYGYEF
--PYGYAW
PTPYGY--
PGPYGY--
--PYGYWY
--PYGYVD

--PYGYTH
--PYGYTF
--PYGYRC
--PYGYQW
--PYGYQR
--PYGYQQ
--PYGYPA
--PYGYNN
--PYGYNE
--PYGYMP
--PYGYKI
--PYGYIH
--PYGYIE
--PYGYHN
--PYGYHM
--PYGYGV
--PYGYGR
--PYGYGG
--PYGYGD
--PYGYET
--PYGYEC
--PYGYDG
--PYGYCS
--PYGYAM
--PYGYAF
--PYGYAE
--PYGYAD
-PPYGYL-
PMPYGY--
PCPYGY--
-QPYGYR-
QYPYGY--
QVPYGY--
QGPYGY--
QFPYGY--
-QPYGYA-
QRPYGY--
-QPYGYY-
QLPYGY--
QTPYGY--
QSPYGY--
QPPYGY--
-QPYGYV-
QKPYGY--
QNPYGY--
-QPYGYQ-
-QPYGYI-
-QPYGYM-
-QPYGYK-
QEPYGY--

QHPYGY--
QIPYGY--
-QPYGYN-
-QPYGYT-
-QPYGYS-
QQPYGY--
-QPYGYW-
-QPYGYF-
QMPYGY--
QCPYGY--
QAPYGY--
RSPYGY--
RYPYGY--
-RPYGYH-
-RPYGYI-
-RPYGYR-
-RPYGYV-
RGPYGY--
-RPYGYW-
RNPYGY--
RLPYGY--
-RPYGYG-
RAPYGY--
RQPYGY--
-RPYGYS-
-RPYGYN-
RIPYGY--
REPYGY--
RTPYGY--
-RPYGYQ-
-RPYGYA-
-RPYGYF-
RMPYGY--
RRPYGY--
RKPYGY--
RVPYGY--
RPPYGY--
-RPYGYM-
-RPYGYY-
-RPYGYE-
RHPYGY--
RWPYGY--
RDPYGY--
RFPYGY--
-RPYGYT-
-SPYGYR-
SRPYGY--
SSPYGY--
-SPYGYL-
-SPYGYY-

STPYGY--
-SPYGYT-
-SPYGYM-
SVPYGY--
-SPYGYS-
SHPYGY--
SFPYGY--
-SPYGYV-
SWPYGY--
-SPYGYK-
-SPYGYW-
SEPYGY--
-SPYGYA-
SIPYGY--
SYPYGY--
SQPYGY--
SMPYGY--
SKPYGY--
-SPYGYH-
-SPYGYF-
-SPYGYN-
SCPYGY--
SPPYGY--
SAPYGY--
-SPYGYQ-
SGPYGY--
-SPYGYI-
-SPYGYG-
-SPYGYE-
TIPYGY--
TVPYGY--
-TPYGYR-
-TPYGYF-
-TPYGYL-
-TPYGYY-
-TPYGYS-
TKPYGY--
TSPYGY--
-TPYGYI-
-TPYGYG-
-TPYGYM-
-TPYGYK-
-TPYGYH-
THPYGY--
TGPYGY--
TDPYGY--
TWPYGY--
TTPYGY--
-TPYGYN-
-TPYGYT-

-TPYGYW-
-TPYGYA-
TYPYGY--
TQPYGY--
TMPYGY--
TPPYGY--
TFPYGY--
TAPYGY--
-TPYGYQ-
TNPYGY--
TLPYGY--
VGPYGY--
-VPYGYR-
VKPYGY--
VRPYGY--
VQPYGY--
VTPYGY--
VAPYGY--
-VPYGYT-
-VPYGYM-
VWPYGY--
VLPYGY--
VSPYGY--
-VPYGYI-
-VPYGYS-
-VPYGYL-
-VPYGYK-
VPPYGY--
VEPYGY--
VIPYGY--
VDPYGY--
-VPYGYQ-
-VPYGYH-
VFPYGY--
VNPYGY--
VVPYGY--
-VPYGYY-
VYPYGY--
-VPYGYF-
-VPYGYA-
-VPYGYV-
VCPYGY--
-VPYGYG-
VMPYGY--
-VPYGYW-
VHPYGY--
WLPYGY--
-WPYGYL-
-WPYGYR-
WVPYGY--

WHPYGY--
WSPYGY--
-WPYGYT-
WRPYGY--
-WPYGYM-
WQPYGY--
WKPYGY--
-WPYGYY-
-WPYGYV-
WIPYGY--
WEPYGY--
WTPYGY--
WGPYGY--
WPPYGY--
WAPYGY--
-WPYGYW-
-WPYGYI-
-WPYGYQ-
-WPYGYK-
WNPYGY--
-WPYGYN-
WMPYGY--
WDPYGY--
WYPYGY--
-WPYGYS-
-WPYGYF-
-YPYGYS-
-YPYGYR-
YRPYGY--
YPPYGY--
YWPYGY--
YYPYGY--
YVPYGY--
-YPYGYL-
YGPYGY--
YTPYGY--
-YPYGYK-
YMPYGY--
-YPYGYN-
YLPYGY--
-YPYGYW-
YSPYGY--
-YPYGYM-
YHPYGY--
-YPYGYQ-
YEPYGY--
-YPYGYT-
-YPYGYI-
-YPYGYY-
-YPYGYA-

	YKPYGY--		
	YDPYGY--		
	YAPYGY--		
	YFPYGY--		
	-YPYGYV-		
	-YPYGYF-		
	-YPYGYG-		
	YNPYGY--		
PWNI	377	667	48.92
	-RPWNII-		
	ARPWNI--		
	ASPWNI--		
	-APWNIR-		
	-APWNIL-		
	-APWNIK-		
	-APWNIY-		
	AFPWNI--		
	-APWNIG-		
	AKPWNI--		
	ANPWNI--		
	AIPWNI--		
	AVPWNI--		
	-APWNIW-		
	-APWNIS-		
	-APWNIV-		
	-APWNIA-		
	AYPWNI--		
	-APWNIQ-		
	ALPWNI--		
	AGPWNI--		
	-APWNIE-		
	ATPWNI--		
	AQPWNI--		
	APPWNI--		
	AWPWNI--		
	-APWNIN-		
	ADPWNI--		
	CRPWNI--		
	CQPWNI--		
	-CPWNIR-		
	-CPWNII-		
	CPPWNI--		
	-CPWNIS-		
	-CPWNIQ-		
	DWPWNI--		
	DRPWNI--		
	DYPWNI--		
	-DPWNIT-		
	-DPWNIR-		
	DGPWNI--		

DKPWNI--
DMPWNI--
DAPWNI--
DQPWNI--
-DPWNIS-
-DPWNIL-
-DPWNII-
-DPWNIE-
DTPWNI--
-DPWNIY-
-DPWNIW-
-DPWNIN-
DNPWNI--
DIPWNI--
DFPWNI--
-EPWNIV-
ERPWNI--
-EPWNIS-
-EPWNIT-
-EPWNIR-
EFPWNI--
EAPWNI--
EGPWNI--
EVPWNI--
-EPWNIM-
ELPWNI--
EIPWNI--
-EPWNIL-
-EPWNIY-
EWPWNI--
ESPWNI--
-EPWNIW-
-EPWNIN-
-EPWNIK-
-EPWNIH-
EKPWNI--
FDPWNI--
-FPWNIY-
FGPWNI--
FKPWNI--
FRPWNI--
FNPWNI--
FSPWNI--
-FPWNIR-
FEPWNI--
-FPWNIE-
-FPWNIS-
-FPWNIQ-
FHPWNI--
FAPWNI--

FYPWNI--
FVPWNI--
FTPWNI--
-FPWNII-
-FPWNIG-
FIPWNI--
FFPWNI--
FQPWNI--
-FPWNIV-
-FPWNIK-
-FPWNIA-
FPPWNI--
GSPWNI--
-GPWNIR-
-GPWNIM-
GEPWNI--
GWPWNI--
GAPWNI--
-GPWNIK-
GFPWNI--
-GPWNIV-
GKPWNI--
GTPWNI--
-GPWNIQ-
GGPWNI--
GPPWNI--
GIPWNI--
-GPWNIH-
-GPWNIS-
-GPWNII-
-GPWNIG-
-GPWNIA-
GVPWNI--
GRPWNI--
GNPWNI--
GLPWNI--
GMPWNI--
GHPWNI--
-GPWNIY-
-GPWNIT-
GDPWNI--
GCPWNI--
-HPWNIS-
-HPWNIY-
HSPWNI--
-HPWNIW-
HNPWNI--
HLPWNI--
-HPWNIR-
-HPWNIQ-

-HPWNIH-
-HPWNIA-
HTPWNI--
HRPWNI--
HKPWNI--
HEPWNI--
-HPWNII-
-HPWNIG-
HIPWNI--
HGPWNI--
HYPWNI--
-HPWNIM-
-HPWNIL-
HWPWNI--
-HPWNIV-
HPPWNI--
HHPWNI--
HFPWNI--
HAPWNI--
IRPWNI--
-IPWNIR-
IYPWNI--
-IPWNIM-
IMPWNI--
IAPWNI--
ISPWNI--
IQPWNI--
IPPWNI--
IFPWNI--
-IPWNIT-
-IPWNIA-
-IPWNIN-
-IPWNIS-
-IPWNIF-
IKPWNI--
-IPWNII-
ILPWNI--
-IPWNIQ-
-IPWNIE-
IIPWNI--
IEPWNI--
ICPWNI--
-IPWNIK-
-IPWNIH-
IDPWNI--
KSPWNI--
-KPWNIS-
KGPWNI--
KAPWNI--
KLPWNI--

-KPWNIT-
-KPWNIA-
KNPWNI--
-KPWNIY-
-KPWNIF-
KRPWNI--
-KPWNIR-
-KPWNIK-
-KPWNIH-
KDPWNI--
-KPWNIV-
-KPWNIG-
KFPWNI--
KVPWNI--
-KPWNIM-
KHPWNI--
KTPWNI--
KYPWNI--
-KPWNIQ-
-KPWNII-
LKPWNI--
LTPWNI--
LWPWNI--
LRPWNI--
-LPWNIR-
LNPWNI--
-LPWNIT-
LAPWNI--
-LPWNIV-
LLPWNI--
LHPWNI--
LYPWNI--
LPPWNI--
LSPWNI--
LIPWNI--
LVPWNI--
-LPWNIH-
-LPWNIY-
-LPWNIS-
-LPWNIA-
LMPWNI--
-LPWNIK-
-LPWNII-
-LPWNIE-
-LPWNIN-
LFPWNI--
LCPWNI--
-MPWNIS-
-MPWNIT-
MPPWNI--

```
MLPWNI--
MNPWNI--
MDPWNI--
MRPWNI--
-MPWNIV-
-MPWNIQ-
-MPWNIK-
-MPWNIE-
MAPWNI--
MKPWNI--
MMPWNI--
MYPWNI--
MTPWNI--
-MPWNIM-
-MPWNIH-
-MPWNIG-
MIPWNI--
NSPWNI--
-NPWNIR-
-NPWNIT-
-NPWNIW-
-NPWNIS-
NWPWNI--
NLPWNI--
-NPWNIA-
-NPWNII-
NRPWNI--
-NPWNIK-
-NPWNIH-
-NPWNIG-
NGPWNI--
-NPWNIQ-
NNPWNI--
NMPWNI--
NAPWNI--
NYPWNI--
-NPWNIL-
NEPWNI--
-NPWNIE-
NIPWNI--
NDPWNI--
NTPWNI--
-NPWNIY-
-NPWNIM-
NPPWNI--
NKPWNI--
--PWNIRS
--PWNIRR
--PWNIRN
--PWNIVR
```

--PWNILR
--PWNIIK
--PWNIST
--PWNIRG
--PWNILG
--PWNITS
--PWNIRD
--PWNISR
--PWNIRL
--PWNIQS
--PWNIWN
--PWNIVK
--PWNISN
--PWNIRI
--PWNIRK
--PWNIQT
PVPWNI--
--PWNIVQ
--PWNITR
--PWNIMS
--PWNIAV
--PWNISM
--PWNIKS
--PWNIIV
--PWNIFR
--PWNIAS
PWPWNI--
--PWNIYQ
--PWNITT
--PWNIRT
--PWNIKK
--PWNIGS
--PWNIVY
--PWNIRF
--PWNIQY
--PWNIMD
--PWNIWT
--PWNIKT
--PWNIHA
--PWNIAG
--PWNIVG
--PWNISS
--PWNIFY
--PWNIVA
--PWNISQ
--PWNISA
--PWNIQR
--PWNIQA
--PWNIIN
--PWNIHK

--PWNIGM
--PWNITA
--PWNIRQ
--PWNIRM
--PWNIMA
PEPWNI--
--PWNITD
--PWNISL
--PWNISH
--PWNINV
--PWNIKY
--PWNIIR
--PWNIES
--PWNIMY
--PWNIIS
--PWNIGA
PSPWNI--
--PWNISE
--PWNIQV
--PWNIQK
--PWNIQG
--PWNINT
--PWNIMM
--PWNILK
--PWNIHG
--PWNIAY
PYPWNI--
--PWNIYG
--PWNIWS
--PWNITI
--PWNIRA
--PWNIQD
--PWNING
--PWNILH
--PWNILF
--PWNIKR
--PWNIKA
--PWNIEI
--PWNIAR
--PWNIAD
--PWNIWH
--PWNIWD
--PWNITL
--PWNITH
--PWNIQF
--PWNIMF
--PWNILE
--PWNILD
--PWNIKI
--PWNIIG

```
--PWNIAT
PNPWNI--
PKPWNI--
--PWNIYW
--PWNIYE
--PWNIVT
--PWNITM
--PWNISY
--PWNISD
--PWNIRE
--PWNIQH
--PWNINM
--PWNIMR
--PWNIMN
--PWNIMG
--PWNILI
--PWNILA
--PWNIKV
--PWNIKN
--PWNIKL
--PWNIHM
--PWNIHI
--PWNIGV
--PWNIER
--PWNIEK
--PWNIYS
--PWNIYN
--PWNIWG
--PWNIVS
--PWNIVM
--PWNITN
--PWNISK
--PWNIRY
--PWNIQN
--PWNINA
--PWNIHS
--PWNIGW
--PWNIGN
--PWNIFD
--PWNIET
--PWNIEM
--PWNIYV
--PWNIYT
--PWNIYR
--PWNIYI
--PWNIYH
--PWNIYF
--PWNIWW
--PWNIVW
--PWNIVN
```

--PWNIVI
--PWNIRW
--PWNIRH
--PWNIMT
--PWNIMK
--PWNIKM
--PWNIKH
--PWNIIT
--PWNIIQ
--PWNIII
--PWNIHT
--PWNIHL
--PWNIHD
--PWNIGY
--PWNIGL
--PWNIEV
PTPWNI--
PAPWNI--
--PWNIWE
--PWNIVE
--PWNITG
--PWNISF
--PWNINR
--PWNINL
--PWNINK
--PWNINI
--PWNIMQ
--PWNIMH
--PWNILP
--PWNIKG
--PWNIIY
--PWNIHR
--PWNIGT
--PWNIGR
--PWNIGQ
--PWNIGK
--PWNIGG
--PWNIFV
--PWNIFN
--PWNIEE
--PWNIDS
--PWNIDA
--PWNICM
--PWNIAH
--PWNIAA
-QPWNIR-
QAPWNI--
-QPWNIT-
QIPWNI--
QRPWNI--

-QPWNIV-
-QPWNIH-
QEPWNI--
QSPWNI--
-QPWNIS-
-QPWNIW-
-QPWNIK-
-QPWNIM-
QKPWNI--
QMPWNI--
-QPWNII-
QYPWNI--
-QPWNIY-
QTPWNI--
-QPWNIN-
-QPWNIG-
QLPWNI--
RRPWNI--
RSPWNI--
-RPWNIS-
-RPWNIY-
-RPWNIV-
REPWNI--
RLPWNI--
RQPWNI--
-RPWNIH-
RIPWNI--
-RPWNIT-
-RPWNIN-
-RPWNIG-
-RPWNIW-
-RPWNIA-
-RPWNIQ-
-RPWNIK-
RMPWNI--
RWPWNI--
RVPWNI--
RYPWNI--
RFPWNI--
-RPWNIM-
RPPWNI--
RNPWNI--
RDPWNI--
-RPWNIE-
RAPWNI--
SRPWNI--
-SPWNIM-
SYPWNI--
SAPWNI--
-SPWNIR-

SNPWNI--
SLPWNI--
SPPWNI--
SKPWNI--
SQPWNI--
SHPWNI--
-SPWNIT-
SVPWNI--
-SPWNIA-
SGPWNI--
-SPWNIS-
SIPWNI--
SMPWNI--
SFPWNI--
SWPWNI--
STPWNI--
SEPWNI--
SDPWNI--
SSPWNI--
-SPWNIN-
-SPWNIG-
-SPWNIV-
-SPWNII-
-SPWNIE-
SCPWNI--
-SPWNIY-
TYPWNI--
-TPWNIR-
TPPWNI--
-TPWNIT-
TAPWNI--
-TPWNIS-
TLPWNI--
-TPWNIM-
-TPWNIE-
TIPWNI--
TSPWNI--
TRPWNI--
TDPWNI--
-TPWNIW-
-TPWNIA-
TVPWNI--
-TPWNIV-
TQPWNI--
-TPWNIG-
TMPWNI--
TKPWNI--
TFPWNI--
-TPWNIK-
TEPWNI--

TTPWNI--
-TPWNIQ-
-TPWNIN-
-TPWNIF-
-TPWNIY-
-TPWNII-
TCPWNI--
VSPWNI--
VKPWNI--
VRPWNI--
-VPWNIS-
-VPWNIR-
VEPWNI--
VAPWNI--
-VPWNIV-
-VPWNIM-
VLPWNI--
VFPWNI--
VTPWNI--
VPPWNI--
VHPWNI--
VVPWNI--
-VPWNIY-
-VPWNIH-
VGPWNI--
VMPWNI--
-VPWNIG-
-VPWNIF-
-VPWNIQ-
-VPWNIN-
VNPWNI--
-VPWNIW-
-VPWNIK-
-VPWNII-
-VPWNIA-
VDPWNI--
VYPWNI--
VQPWNI--
WVPWNI--
WIPWNI--
WRPWNI--
WSPWNI--
WGPWNI--
WTPWNI--
WYPWNI--
-WPWNIL-
WHPWNI--
-WPWNIT-
WFPWNI--
WAPWNI--

-WPWNIA-
WLPWNI--
-WPWNIV-
WNPWNI--
WMPWNI--
-WPWNIG-
WEPWNI--
YVPWNI--
-YPWNIY-
YLPWNI--
-YPWNII-
YQPWNI--
-YPWNIR-
YHPWNI--
-YPWNIV-
-YPWNIS-
YAPWNI--
YTPWNI--
YEPWNI--
-YPWNIN-
-YPWNIK-
YRPWNI--
-YPWNIQ-
-YPWNIG-
YKPWNI--
YGPWNI--
PIAY 343 678 48.92
-QPIAYG-
ASPIAY--
AYPIAY--
-APIAYR-
AAPIAY--
AWPIAY--
AVPIAY--
-APIAYV-
AFPIAY--
APPIAY--
-APIAYM-
ATPIAY--
-APIAYH-
-APIAYK-
AKPIAY--
AQPIAY--
ANPIAY--
-APIAYW-
-APIAYN-
-APIAYA-
AIPIAY--
-APIAYT-
AMPIAY--

CRPIAY--
CNPIAY--
CSPIAY--
CLPIAY--
CEPIAY--
CWPIAY--
CQPIAY--
-CPIAYM-
CVPIAY--
-CPIAYT-
-CPIAYL-
DLPIAY--
DTPIAY--
-DPIAYR-
DKPIAY--
DEPIAY--
-DPIAYI-
-DPIAYW-
DVPIAY--
DHPIAY--
DSPIAY--
-DPIAYS-
DNPIAY--
DRPIAY--
-DPIAYL-
-DPIAYF-
DAPIAY--
-DPIAYM-
DFPIAY--
-EPIAYI-
ENPIAY--
ELPIAY--
-EPIAYL-
-EPIAYY-
-EPIAYV-
EQPIAY--
-EPIAYS-
EVPIAY--
EWPIAY--
ESPIAY--
-EPIAYA-
EIPIAY--
-EPIAYW-
-EPIAYR-
-EPIAYF-
EPPIAY--
-EPIAYT-
EEPIAY--
ECPIAY--
EAPIAY--

FRPIAY--
FIPIAY--
FGPIAY--
FEPIAY--
-FPIAYI-
FKPIAY--
FLPIAY--
FQPIAY--
FDPIAY--
-FPIAYW-
-FPIAYR-
FWPIAY--
FSPIAY--
FYPIAY--
FPPIAY--
-FPIAYM-
-FPIAYK-
-FPIAYT-
-FPIAYF-
FFPIAY--
-FPIAYY-
-FPIAYE-
-FPIAYA-
-GPIAYR-
GGPIAY--
-GPIAYS-
GVPIAY--
GKPIAY--
-GPIAYM-
-GPIAYK-
GAPIAY--
GSPIAY--
GRPIAY--
-GPIAYH-
-GPIAYY-
GCPIAY--
GYPIAY--
GWPIAY--
-GPIAYV-
-GPIAYA-
GHPIAY--
GFPIAY--
GEPIAY--
-GPIAYT-
GMPIAY--
GIPIAY--
GQPIAY--
-GPIAYN-
-GPIAYI-
-GPIAYE-

HSPIAY--
HNPIAY--
HQPIAY--
-HPIAYL-
HDPIAY--
HAPIAY--
HYPIAY--
-HPIAYV-
-HPIAYF-
-HPIAYA-
-HPIAYY-
-HPIAYR-
HGPIAY--
HKPIAY--
HTPIAY--
-HPIAYS-
-HPIAYQ-
HFPIAY--
-HPIAYE-
-HPIAYT-
-HPIAYI-
-HPIAYN-
-HPIAYM-
-HPIAYK-
HHPIAY--
HPPIAY--
-HPIAYH-
HIPIAY--
IIPIAY--
IPPIAY--
-IPIAYM-
ISPIAY--
IKPIAY--
IVPIAY--
-IPIAYL-
-IPIAYQ-
IAPIAY--
-IPIAYR-
IHPIAY--
IGPIAY--
IRPIAY--
-IPIAYN-
-IPIAYV-
-IPIAYS-
-IPIAYH-
IFPIAY--
IEPIAY--
IWPIAY--
-IPIAYA-
-KPIAYL-

-KPIAYR-
-KPIAYI-
-KPIAYS-
-KPIAYV-
KTPIAY--
KNPIAY--
KHPIAY--
-KPIAYA-
-KPIAYY-
KPPIAY--
KLPIAY--
KDPIAY--
KAPIAY--
KVPIAY--
-KPIAYF-
KQPIAY--
KMPIAY--
KIPIAY--
KCPIAY--
-KPIAYE-
KGPIAY--
KWPIAY--
-KPIAYQ-
-KPIAYM-
-KPIAYG-
LSPIAY--
LDPIAY--
-LPIAYL-
LTPIAY--
LRPIAY--
LGPIAY--
-LPIAYR-
LAPIAY--
-LPIAYM-
-LPIAYS-
-LPIAYK-
LEPIAY--
-LPIAYH-
-LPIAYA-
LNPIAY--
LQPIAY--
LPPIAY--
-LPIAYN-
-LPIAYQ-
-LPIAYF-
LMPIAY--
LFPIAY--
LCPIAY--
-LPIAYE-
LHPIAY--

-MPIAYL-
MRPIAY--
MLPIAY--
MIPIAY--
MWPIAY--
-MPIAYR-
-MPIAYK-
-MPIAYH-
MHPIAY--
MVPIAY--
MEPIAY--
MSPIAY--
MNPIAY--
MQPIAY--
MPPIAY--
-MPIAYV-
-MPIAYM-
MKPIAY--
MYPIAY--
-MPIAYS-
-MPIAYI-
-MPIAYG-
-MPIAYA-
MTPIAY--
-MPIAYT-
NRPIAY--
-NPIAYR-
NQPIAY--
-NPIAYF-
NGPIAY--
-NPIAYI-
NTPIAY--
NLPIAY--
-NPIAYM-
NPPIAY--
NVPIAY--
NSPIAY--
NAPIAY--
-NPIAYY-
-NPIAYL-
NNPIAY--
NEPIAY--
NYPIAY--
-NPIAYS-
NHPIAY--
-NPIAYW-
NIPIAY--
-NPIAYV-
-NPIAYQ-
NWPIAY--

-NPIAYT-
-NPIAYN-
-NPIAYA-
NDPIAY--
--PIAYIG
--PIAYTI
--PIAYIR
--PIAYMK
--PIAYYS
--PIAYST
--PIAYRS
--PIAYRN
--PIAYRA
--PIAYLR
--PIAYES
--PIAYVT
--PIAYTK
--PIAYSS
--PIAYSL
--PIAYLS
--PIAYSI
--PIAYRT
--PIAYLA
--PIAYIS
--PIAYML
--PIAYSM
--PIAYRI
--PIAYHL
--PIAYAI
--PIAYRH
--PIAYYG
--PIAYVS
--PIAYSR
--PIAYSQ
--PIAYRG
--PIAYQS
--PIAYKS
--PIAYTS
--PIAYLM
--PIAYIT
--PIAYAL
--PIAYTV
--PIAYTL
--PIAYVA
--PIAYRV
--PIAYRE
--PIAYQR
--PIAYKT
--PIAYIH
--PIAYHT

--PIAYHS
--PIAYTR
--PIAYPY
PNPIAY--
--PIAYTG
--PIAYLT
--PIAYKA
--PIAYHQ
--PIAYWS
--PIAYWA
--PIAYVQ
--PIAYRQ
--PIAYQG
--PIAYNT
--PIAYNL
--PIAYMW
--PIAYKL
--PIAYHR
--PIAYHK
--PIAYGW
--PIAYGI
--PIAYVR
--PIAYRD
--PIAYMR
--PIAYKM
--PIAYKI
--PIAYKG
--PIAYGA
--PIAYAS
--PIAYAN
--PIAYAG
--PIAYAA
--PIAYVF
--PIAYSK
--PIAYSF
--PIAYQL
--PIAYNY
--PIAYMI
--PIAYLQ
--PIAYKN
--PIAYKH
--PIAYGS
--PIAYGM
--PIAYFH
--PIAYEV
--PIAYDL
--PIAYYQ
--PIAYVK
--PIAYTT
--PIAYTM

--PIAYTF
--PIAYSA
--PIAYNV
--PIAYMM
--PIAYLI
--PIAYHD
--PIAYGF
--PIAYAW
--PIAYAT
--PIAYYY
--PIAYWR
--PIAYVW
--PIAYSH
--PIAYSG
--PIAYRM
--PIAYQV
--PIAYQI
--PIAYPF
--PIAYLG
--PIAYKY
--PIAYKR
--PIAYHW
--PIAYFI
--PIAYEM
PFPIAY--
--PIAYYH
--PIAYVD
--PIAYTN
--PIAYTD
--PIAYQN
--PIAYLH
--PIAYIF
--PIAYID
--PIAYIA
--PIAYGQ
--PIAYFT
--PIAYEI
--PIAYAF
PEPIAY--
PKPIAY--
--PIAYYV
--PIAYYD
--PIAYWT
--PIAYWK
--PIAYVH
--PIAYQT
--PIAYNW
--PIAYNG
--PIAYMT
--PIAYLK

--PIAYIV
--PIAYHH
--PIAYHA
--PIAYGV
--PIAYGG
--PIAYFY
--PIAYEH
--PIAYEG
PDPIAY--
--PIAYYT
--PIAYWG
--PIAYVI
--PIAYSW
--PIAYSV
--PIAYSN
--PIAYRY
--PIAYPV
--PIAYPI
--PIAYNS
--PIAYNM
--PIAYNH
--PIAYMV
--PIAYMH
--PIAYMA
--PIAYIW
--PIAYIE
--PIAYHV
--PIAYHG
--PIAYFM
--PIAYEF
PYPIAY--
PTPIAY--
-PPIAYT-
PIPIAY--
--PIAYYW
--PIAYYN
--PIAYTY
--PIAYTQ
--PIAYTE
--PIAYSD
--PIAYRF
--PIAYQA
--PIAYPW
--PIAYPM
--PIAYPH
--PIAYNR
--PIAYNP
--PIAYNI
--PIAYNA
--PIAYLW

--PIAYLN
--PIAYFE
--PIAYFD
--PIAYFA
--PIAYEY
--PIAYET
--PIAYER
--PIAYDY
--PIAYDG
--PIAYAM
-QPIAYR-
QWPIAY--
QPPIAY--
-QPIAYF-
QAPIAY--
QSPIAY--
-QPIAYL-
-QPIAYI-
QLPIAY--
QYPIAY--
-QPIAYV-
-QPIAYS-
QNPIAY--
-QPIAYK-
-QPIAYY-
-QPIAYT-
-QPIAYA-
QHPIAY--
QGPIAY--
QFPIAY--
QDPIAY--
QCPIAY--
QVPIAY--
-QPIAYN-
-QPIAYE-
RSPIAY--
RAPIAY--
-RPIAYT-
RFPIAY--
RYPIAY--
-RPIAYY-
RMPIAY--
RLPIAY--
RKPIAY--
RRPIAY--
-RPIAYS-
-RPIAYH-
RWPIAY--
-RPIAYL-
RNPIAY--

```
-RPIAYM-
RPPIAY--
RHPIAY--
RTPIAY--
RQPIAY--
-RPIAYK-
-RPIAYI-
-RPIAYE-
RDPIAY--
RIPIAY--
RCPIAY--
REPIAY--
-RPIAYC-
-SPIAYT-
SKPIAY--
SGPIAY--
SYPIAY--
SQPIAY--
SRPIAY--
SVPIAY--
-SPIAYS-
-SPIAYR-
SNPIAY--
SSPIAY--
SAPIAY--
SFPIAY--
SWPIAY--
-SPIAYY-
-SPIAYL-
-SPIAYI-
-SPIAYF-
-SPIAYA-
-SPIAYV-
STPIAY--
-SPIAYM-
-SPIAYK-
SHPIAY--
-SPIAYQ-
SEPIAY--
SDPIAY--
-SPIAYW-
SIPIAY--
-SPIAYH-
-SPIAYE-
SLPIAY--
SCPIAY--
TGPIAY--
-TPIAYL-
-TPIAYR-
TQPIAY--
```

TPPIAY--
TEPIAY--
TVPIAY--
TSPIAY--
TFPIAY--
TYPIAY--
-TPIAYV-
TRPIAY--
TMPIAY--
-TPIAYI-
TKPIAY--
-TPIAYT-
-TPIAYK-
TDPIAY--
TLPIAY--
TAPIAY--
TTPIAY--
-TPIAYH-
-TPIAYA-
-TPIAYS-
TNPIAY--
-TPIAYM-
-TPIAYG-
-TPIAYF-
-TPIAYY-
THPIAY--
VSPIAY--
-VPIAYK-
-VPIAYR-
-VPIAYL-
VTPIAY--
VKPIAY--
-VPIAYM-
-VPIAYA-
VRPIAY--
-VPIAYI-
VLPIAY--
VFPIAY--
VQPIAY--
-VPIAYQ-
-VPIAYN-
VEPIAY--
VYPIAY--
VAPIAY--
-VPIAYS-
-VPIAYY-
VNPIAY--
-VPIAYT-
VDPIAY--
VWPIAY--

-VPIAYH-
VIPIAY--
VPPIAY--
-VPIAYG-
-VPIAYE-
VMPIAY--
VCPIAY--
-WPIAYT-
WKPIAY--
-WPIAYK-
WDPIAY--
WNPIAY--
WAPIAY--
-WPIAYQ-
WLPIAY--
WQPIAY--
WGPIAY--
WHPIAY--
-WPIAYV-
-WPIAYR-
-WPIAYM-
WMPIAY--
WTPIAY--
-WPIAYS-
-WPIAYA-
WEPIAY--
-WPIAYE-
-YPIAYF-
YLPIAY--
YKPIAY--
YTPIAY--
YAPIAY--
YRPIAY--
YSPIAY--
-YPIAYW-
-YPIAYV-
-YPIAYS-
YNPIAY--
YPPIAY--
-YPIAYK-
-YPIAYI-
YYPIAY--
YQPIAY--
-YPIAYR-
YHPIAY--
YVPIAY--
YGPIAY--
YDPIAY--
-YPIAYQ-
YMPIAY--

YIPIAY--
-YPIAYT-
-YPIAYN-
-YPIAYE-
YCPIAY--

PFAM	425	650	48.89

--PFAMYP
--PFAMES
ARPFAM--
-APFAMN-
AIPFAM--
ATPFAM--
-APFAMY-
APPFAM--
AYPFAM--
-APFAMH-
AFPFAM--
-APFAMV-
AGPFAM--
AQPFAM--
-APFAMI-
ANPFAM--
AEPFAM--
-APFAMT-
-APFAMA-
-APFAMM-
-APFAMG-
AVPFAM--
-APFAML-
-APFAMR-
-APFAMK-
AKPFAM--
AAPFAM--
AWPFAM--
AMPFAM--
CKPFAM--
-CPFAMT-
CNPFAM--
CLPFAM--
-CPFAMN-
-CPFAML-
CEPFAM--
-CPFAMS-
-CPFAMR-
CGPFAM--
DIPFAM--
DMPFAM--
-DPFAMK-
DRPFAM--
DWPFAM--

-DPFAMR-
DGPFAM--
-DPFAMW-
DHPFAM--
-DPFAMY-
-DPFAMH-
DQPFAM--
-DPFAMI-
DSPFAM--
-DPFAMT-
-DPFAML-
-DPFAME-
-DPFAMA-
DNPFAM--
DLPFAM--
-EPFAMT-
ESPFAM--
EKPFAM--
ERPFAM--
EAPFAM--
-EPFAMY-
-EPFAMV-
-EPFAMS-
-EPFAMI-
EWPFAM--
ETPFAM--
-EPFAMW-
EEPFAM--
-EPFAMH-
-EPFAME-
-EPFAMA-
EYPFAM--
-EPFAML-
-EPFAMF-
ELPFAM--
FFPFAM--
FPPFAM--
FSPFAM--
-FPFAMN-
FKPFAM--
FTPFAM--
-FPFAMH-
-FPFAMR-
-FPFAMQ-
FRPFAM--
-FPFAMS-
FHPFAM--
FDPFAM--
-FPFAMT-
-FPFAMM-

-FPFAMK-
FAPFAM--
FVPFAM--
-FPFAMI-
-FPFAMG-
FYPFAM--
FLPFAM--
GFPFAM--
GQPFAM--
GSPFAM--
GYPFAM--
GGPFAM--
-GPFAMV-
GKPFAM--
-GPFAMM-
GAPFAM--
-GPFAMA-
GNPFAM--
-GPFAMT-
-GPFAMK-
-GPFAMI-
GLPFAM--
GRPFAM--
GIPFAM--
-GPFAMS-
-GPFAMN-
-GPFAML-
GMPFAM--
-GPFAMH-
-GPFAMG-
GPPFAM--
-GPFAMW-
-GPFAME-
-HPFAML-
HLPFAM--
HFPFAM--
-HPFAMT-
-HPFAMR-
HTPFAM--
-HPFAMW-
HEPFAM--
HSPFAM--
-HPFAMV-
HPPFAM--
-HPFAMY-
-HPFAMS-
-HPFAMI-
-HPFAMA-
HCPFAM--
-HPFAME-

HKPFAM--
HHPFAM--
HIPFAM--
HQPFAM--
-HPFAMN-
HDPFAM--
IGPFAM--
ISPFAM--
-IPFAMV-
ITPFAM--
-IPFAML-
-IPFAMW-
INPFAM--
-IPFAMT-
-IPFAMG-
IWPFAM--
-IPFAMQ-
-IPFAMN-
-IPFAMS-
-IPFAMH-
IYPFAM--
-IPFAMR-
-IPFAMF-
IKPFAM--
-IPFAMM-
ILPFAM--
KGPFAM--
KEPFAM--
-KPFAMR-
KSPFAM--
KFPFAM--
KIPFAM--
-KPFAMN-
KNPFAM--
-KPFAMI-
-KPFAMK-
-KPFAMA-
KMPFAM--
KKPFAM--
KTPFAM--
KQPFAM--
-KPFAMH-
-KPFAMM-
-KPFAMV-
KRPFAM--
KPPFAM--
KAPFAM--
KWPFAM--
-KPFAMW-
-KPFAMG-

-KPFAME-
-KPFAMS-
-KPFAMF-
LSPFAM--
LTPFAM--
-LPFAMS-
LMPFAM--
-LPFAMK-
LNPFAM--
-LPFAME-
LDPFAM--
LLPFAM--
LIPFAM--
LHPFAM--
LVPFAM--
LGPFAM--
LKPFAM--
LPPFAM--
-LPFAMI-
-LPFAMH-
-LPFAMA-
LFPFAM--
-LPFAMM-
-LPFAMG-
LAPFAM--
LQPFAM--
-LPFAMV-
-LPFAMQ-
-LPFAMN-
LEPFAM--
MQPFAM--
-MPFAMT-
-MPFAMW-
MGPFAM--
-MPFAMS-
-MPFAMK-
-MPFAML-
-MPFAME-
-MPFAMA-
-MPFAMH-
MLPFAM--
MKPFAM--
MEPFAM--
MYPFAM--
-MPFAMN-
MRPFAM--
-MPFAMY-
MIPFAM--
-MPFAMV-
MTPFAM--

```
-MPFAMQ-
-MPFAMI-
MNPFAM--
-MPFAMM-
-MPFAMG-
-NPFAMH-
-NPFAMY-
NGPFAM--
NRPFAM--
-NPFAMI-
NYPFAM--
NKPFAM--
NSPFAM--
NIPFAM--
NTPFAM--
NHPFAM--
NNPFAM--
NLPFAM--
NAPFAM--
-NPFAMW-
NFPFAM--
-NPFAMV-
NDPFAM--
-NPFAMM-
-NPFAML-
-NPFAMS-
-NPFAMT-
--PFAMRR
--PFAMRS
--PFAMSL
--PFAMYS
PGPFAM--
--PFAMKS
--PFAMWK
--PFAMRT
--PFAMVM
--PFAMWG
--PFAMKT
--PFAMLS
--PFAMFK
--PFAMWN
--PFAMSI
--PFAMAS
--PFAMQS
--PFAMLG
--PFAMWH
--PFAMVR
--PFAMVG
--PFAMNS
--PFAMKW
```

PKPFAM--
--PFAMTG
--PFAMMT
--PFAMHR
--PFAMVH
--PFAMSG
--PFAMNW
--PFAMGF
--PFAMMA
--PFAMLR
--PFAMLN
--PFAMAT
--PFAMMR
--PFAMYG
--PFAMWD
--PFAMRG
--PFAMYR
--PFAMSV
--PFAMQE
--PFAMMS
--PFAMGY
--PFAMVY
--PFAMSK
--PFAMRV
--PFAMRN
--PFAMLK
--PFAMHG
--PFAMEM
--PFAMSF
--PFAMRQ
--PFAMMH
--PFAMGS
--PFAMYK
--PFAMTN
--PFAMRF
--PFAMRD
--PFAMPY
--PFAMIS
--PFAMVS
--PFAMTL
--PFAMMK
--PFAMLT
--PFAMLM
--PFAMGI
--PFAMGG
--PFAMTK
--PFAMSQ
--PFAMRH
--PFAMQR
--PFAMQA

--PFAMNY
--PFAMNR
--PFAMNM
--PFAMKR
--PFAMIT
--PFAMIH
--PFAMGH
--PFAMEY
--PFAMAW
--PFAMAR
--PFAMYQ
--PFAMWS
--PFAMVV
--PFAMTV
--PFAMTM
--PFAMSS
--PFAMRM
--PFAMNQ
--PFAMNL
--PFAMFS
--PFAMEG
--PFAMAF
PYPFAM--
PHPFAM--
--PFAMTT
--PFAMST
--PFAMRE
--PFAMRA
--PFAMMN
--PFAMIQ
--PFAMHN
--PFAMHI
--PFAMHD
--PFAMGT
--PFAMFW
--PFAMET
--PFAMAI
--PFAMVQ
--PFAMTE
--PFAMQM
--PFAMNA
--PFAMLQ
--PFAMHS
--PFAMGQ
--PFAMGK
--PFAMFT
--PFAMEW
--PFAMAQ
PEPFAM--
--PFAMWQ

--PFAMVN
--PFAMVA
--PFAMNV
--PFAMKI
--PFAMIR
--PFAMGV
--PFAMGM
--PFAMEQ
--PFAMAN
--PFAMSW
--PFAMSD
--PFAMSA
--PFAMQW
--PFAMQH
--PFAMNT
--PFAMLD
--PFAMLA
--PFAMKQ
--PFAMID
--PFAMHV
--PFAMFY
--PFAMER
--PFAMEL
--PFAMEH
--PFAMEE
--PFAMCT
--PFAMAM
--PFAMAE
--PFAMAA
PTPFAM--
-PPFAMS-
-PPFAMK-
PMPFAM--
--PFAMWE
--PFAMVT
--PFAMVK
--PFAMVD
--PFAMTA
--PFAMSM
--PFAMSC
--PFAMQQ
--PFAMQG
--PFAMQD
--PFAMPR
--PFAMPH
--PFAMPF
--PFAMNN
--PFAMNH
--PFAMNE
--PFAMMY

--PFAMMV
--PFAMLH
--PFAMKM
--PFAMKG
--PFAMKF
--PFAMKE
--PFAMKD
--PFAMKA
--PFAMII
--PFAMIC
--PFAMIA
--PFAMHY
--PFAMGN
--PFAMEV
--PFAMDV
--PFAMDM
--PFAMDL
--PFAMDA
--PFAMCY
-QPFAMT-
-QPFAMH-
-QPFAMR-
-QPFAMS-
-QPFAMY-
-QPFAMK-
-QPFAME-
-QPFAMW-
-QPFAMV-
-QPFAMN-
QMPFAM--
QHPFAM--
-QPFAMF-
QSPFAM--
QNPFAM--
QEPFAM--
-QPFAML-
QDPFAM--
-QPFAMG-
QFPFAM--
QYPFAM--
QVPFAM--
QTPFAM--
QRPFAM--
QIPFAM--
QCPFAM--
RSPFAM--
-RPFAMR-
RGPFAM--
-RPFAMT-
RNPFAM--

RVPFAM--
RYPFAM--
-RPFAMG-
RRPFAM--
RTPFAM--
-RPFAMQ-
-RPFAMN-
-RPFAMH-
-RPFAMY-
-RPFAMI-
-RPFAMV-
RAPFAM--
RPPFAM--
-RPFAMM-
RKPFAM--
REPFAM--
-RPFAMF-
RFPFAM--
RWPFAM--
RDPFAM--
-RPFAME-
RMPFAM--
RQPFAM--
RIPFAM--
-RPFAMA-
-SPFAMT-
SAPFAM--
-SPFAMR-
-SPFAMV-
SFPFAM--
-SPFAML-
-SPFAMS-
-SPFAMF-
-SPFAMQ-
SEPFAM--
SQPFAM--
SIPFAM--
SVPFAM--
-SPFAMH-
SHPFAM--
SNPFAM--
-SPFAMN-
-SPFAMG-
SLPFAM--
SGPFAM--
SYPFAM--
-SPFAMW-
-SPFAME-
-SPFAMA-
-SPFAMY-

SWPFAM--
SKPFAM--
STPFAM--
SPPFAM--
SMPFAM--
-TPFAMR-
TRPFAM--
-TPFAMT-
-TPFAMA-
TGPFAM--
TWPFAM--
-TPFAMV-
-TPFAMS-
-TPFAMI-
TNPFAM--
-TPFAML-
TYPFAM--
TVPFAM--
TQPFAM--
-TPFAMM-
THPFAM--
TIPFAM--
TSPFAM--
TPPFAM--
TMPFAM--
TLPFAM--
-TPFAMY-
-TPFAMN-
-TPFAMK-
TKPFAM--
-TPFAMG-
-TPFAME-
-TPFAMQ-
TAPFAM--
-TPFAMW-
VSPFAM--
VLPFAM--
VYPFAM--
-VPFAMN-
VRPFAM--
VPPFAM--
-VPFAMV-
-VPFAMA-
-VPFAMT-
VMPFAM--
-VPFAMH-
-VPFAMI-
VVPFAM--
VQPFAM--
-VPFAMR-

VIPFAM--
VEPFAM--
-VPFAML-
-VPFAMG-
VFPFAM--
VDPFAM--
-VPFAMW-
VTPFAM--
VKPFAM--
-VPFAMK-
-VPFAME-
VWPFAM--
VGPFAM--
-VPFAMS-
-VPFAMM-
-WPFAMR-
-WPFAMQ-
WKPFAM--
-WPFAMG-
WPPFAM--
WLPFAM--
WRPFAM--
WTPFAM--
WSPFAM--
-WPFAMS-
-WPFAMW-
WYPFAM--
-WPFAMT-
WAPFAM--
WDPFAM--
YHPFAM--
-YPFAMT-
-YPFAMK-
YMPFAM--
YRPFAM--
YTPFAM--
-YPFAMV-
YVPFAM--
YFPFAM--
-YPFAMN-
-YPFAMR-
-YPFAML-
YKPFAM--
-YPFAMS-
-YPFAMQ-
-YPFAMI-
YGPFAM--
YYPFAM--
-YPFAMW-
-YPFAMG-

-YPFAME-
YNPFAM--
-YPFAMM-
-YPFAMA-
YIPFAM--
YCPFAM--
PFAL 126 792 48.88
-APFALD-
--PFALVP
--PFALGE
--PFALDS
--PFALAP
ARPFAL--
AGPFAL--
-APFALS-
ALPFAL--
-APFALL-
ATPFAL--
-APFALT-
-APFALG-
AWPFAL--
-APFALK-
AYPFAL--
AHPFAL--
AAPFAL--
-APFALH-
AVPFAL--
-APFALN-
ANPFAL--
AIPFAL--
-APFALV-
-APFALI-
AMPFAL--
APPFAL--
AFPFAL--
AEPFAL--
AQPFAL--
-APFALW-
-APFALQ-
ADPFAL--
ACPFAL--
-APFALE-
CQPFAL--
CSPFAL--
CEPFAL--
CCPFAL--
CAPFAL--
CGPFAL--
CNPFAL--
CMPFAL--

CTPFAL--
CRPFAL--
-CPFALK-
CIPFAL--
CFPFAL--
CYPFAL--
CKPFAL--
CHPFAL--
CVPFAL--
-CPFALV-
-CPFALT-
-CPFALR-
-CPFALN-
-CPFALG-
-CPFALE-
CLPFAL--
CDPFAL--
DRPFAL--
DSPFAL--
-DPFALN-
DLPFAL--
DTPFAL--
DQPFAL--
-DPFALL-
DNPFAL--
DFPFAL--
DVPFAL--
-DPFALR-
-DPFALV-
-DPFALM-
-DPFALQ-
-DPFALI-
DAPFAL--
DGPFAL--
DWPFAL--
-DPFALT-
-DPFALF-
DYPFAL--
DEPFAL--
-DPFALS-
DKPFAL--
-EPFALI-
-EPFALT-
-EPFALN-
ESPFAL--
EYPFAL--
ELPFAL--
-EPFALS-
-EPFALV-
-EPFALL-

EVPFAL--
EKPFAL--
-EPFALQ-
-EPFALA-
EGPFAL--
ERPFAL--
ETPFAL--
-EPFALR-
-EPFALK-
EHPFAL--
-EPFALG-
EPPFAL--
-EPFALM-
EIPFAL--
EQPFAL--
-EPFALW-
-EPFALH-
-EPFALE-
-EPFALD-
EDPFAL--
FSPFAL--
FNPFAL--
-FPFALV-
-FPFALT-
FTPFAL--
-FPFALN-
FGPFAL--
-FPFALK-
-FPFALI-
-FPFALG-
FPPFAL--
FQPFAL--
-FPFALH-
FVPFAL--
FYPFAL--
FWPFAL--
-FPFALA-
-FPFALQ-
FEPFAL--
FLPFAL--
-FPFALF-
FAPFAL--
FKPFAL--
FDPFAL--
FCPFAL--
-FPFALE-
GVPFAL--
GIPFAL--
GQPFAL--
-GPFALR-

-GPFALN-
-GPFALI-
GLPFAL--
-GPFALS-
GFPFAL--
GAPFAL--
GSPFAL--
-GPFALL-
-GPFALT-
-GPFALH-
GNPFAL--
GGPFAL--
-GPFALM-
GKPFAL--
GHPFAL--
GWPFAL--
-GPFALV-
-GPFALK-
-GPFALG-
-GPFALQ-
GMPFAL--
GEPFAL--
GDPFAL--
-GPFALF-
-GPFALE-
-GPFALA-
GCPFAL--
GPPFAL--
HTPFAL--
HSPFAL--
HRPFAL--
HLPFAL--
-HPFALK-
HGPFAL--
-HPFALN-
HKPFAL--
HWPFAL--
-HPFALT-
-HPFALG-
HFPFAL--
-HPFALR-
-HPFALI-
HVPFAL--
HPPFAL--
HDPFAL--
-HPFALY-
-HPFALQ-
-HPFALS-
HAPFAL--
-HPFALL-

–HPFALH–
–HPFALA–
HEPFAL––
–HPFALM–
HIPFAL––
HNPFAL––
HMPFAL––
HHPFAL––
HCPFAL––
–HPFALV–
–HPFALD–
–HPFALC–
–IPFALK–
–IPFALS–
IHPFAL––
IDPFAL––
IRPFAL––
–IPFALR–
ILPFAL––
ITPFAL––
IKPFAL––
IFPFAL––
IVPFAL––
IMPFAL––
–IPFALT–
IYPFAL––
–IPFALL–
–IPFALI–
–IPFALA–
IGPFAL––
–IPFALG–
–IPFALH–
IWPFAL––
–IPFALV–
–IPFALE–
IIPFAL––
IPPFAL––
IQPFAL––
–IPFALN–
INPFAL––
–IPFALQ–
–IPFALW–
IEPFAL––
IAPFAL––
ICPFAL––
–IPFALD–
KSPFAL––
KPPFAL––
KVPFAL––
KAPFAL––

-KPFALS-
KGPFAL--
-KPFALL-
KIPFAL--
-KPFALY-
-KPFALT-
KEPFAL--
KLPFAL--
-KPFALR-
KRPFAL--
-KPFALW-
KQPFAL--
-KPFALH-
-KPFALV-
-KPFALE-
-KPFALN-
-KPFALK-
KNPFAL--
KKPFAL--
KFPFAL--
KWPFAL--
KTPFAL--
-KPFALQ-
-KPFALI-
KYPFAL--
-KPFALG-
KHPFAL--
KMPFAL--
KDPFAL--
-KPFALM-
-KPFALD-
-LPFALS-
-LPFALR-
LLPFAL--
LYPFAL--
LTPFAL--
LWPFAL--
LMPFAL--
LVPFAL--
-LPFALH-
LNPFAL--
LIPFAL--
-LPFALI-
LGPFAL--
LEPFAL--
-LPFALV-
LHPFAL--
LQPFAL--
-LPFALE-
-LPFALM-

LKPFAL--
-LPFALA-
LDPFAL--
-LPFALQ-
-LPFALG-
LCPFAL--
MLPFAL--
-MPFALR-
MSPFAL--
MEPFAL--
-MPFALI-
-MPFALT-
MVPFAL--
MNPFAL--
MRPFAL--
-MPFALE-
-MPFALA-
MTPFAL--
-MPFALS-
-MPFALH-
-MPFALY-
MMPFAL--
-MPFALV-
-MPFALN-
MKPFAL--
MWPFAL--
MIPFAL--
MHPFAL--
MAPFAL--
-MPFALK-
MPPFAL--
-MPFALW-
-MPFALG-
MFPFAL--
MYPFAL--
NIPFAL--
NRPFAL--
-NPFALR-
NLPFAL--
NKPFAL--
NEPFAL--
NSPFAL--
NHPFAL--
NTPFAL--
-NPFALT-
-NPFALV-
NPPFAL--
NGPFAL--
NYPFAL--
NFPFAL--

-NPFALH-
-NPFALS-
NVPFAL--
-NPFALN-
NNPFAL--
NAPFAL--
NQPFAL--
-NPFALM-
-NPFALK-
-NPFALW-
-NPFALG-
-NPFALA-
NDPFAL--
-NPFALE-
-NPFALD-
--PFALRS
--PFALMR
--PFALGS
--PFALRA
--PFALLT
--PFALLS
--PFALRN
--PFALRQ
--PFALSL
--PFALHR
--PFALSN
--PFALRR
--PFALRT
--PFALKR
--PFALSA
--PFALTS
--PFALTK
--PFALRM
--PFALYG
--PFALQS
--PFALVS
--PFALKG
--PFALVT
--PFALST
--PFALTM
--PFALSG
--PFALLQ
PGPFAL--
--PFALYS
--PFALTR
--PFALIN
--PFALMT
--PFALQR
--PFALKM
--PFALIH

--PFALWR
--PFALVN
--PFALLH
PNPFAL--
--PFALWS
--PFALVI
--PFALMS
--PFALGY
--PFALVG
--PFALRY
--PFALGA
--PFALAV
PKPFAL--
--PFALKH
--PFALAS
--PFALLN
--PFALHW
PSPFAL--
PAPFAL--
--PFALWD
--PFALSV
--PFALSM
--PFALLD
--PFALFN
--PFALQY
--PFALKV
--PFALKL
--PFALGT
--PFALAH
--PFALWG
--PFALVD
--PFALSW
--PFALSD
--PFALQH
--PFALNV
--PFALEV
--PFALAT
--PFALWA
--PFALVA
--PFALQG
--PFALPL
--PFALKS
--PFALIR
PQPFAL--
--PFALSY
--PFALAR
PHPFAL--
--PFALYN
--PFALWN
--PFALME

--PFALLG
--PFALEL
--PFALSI
--PFALRW
--PFALRE
--PFALRD
--PFALQT
--PFALNT
--PFALMQ
--PFALKD
--PFALHY
--PFALAA
--PFALWQ
--PFALVH
--PFALHS
--PFALHG
PIPFAL--
--PFALYQ
--PFALTG
PWPFAL--
--PFALTW
--PFALTH
--PFALQV
--PFALQI
--PFALNI
--PFALND
--PFALKI
--PFALIS
--PFALYH
--PFALYD
--PFALTT
--PFALSQ
--PFALGG
--PFALER
--PFALWK
--PFALQN
--PFALMA
--PFALHL
--PFALGN
--PFALGM
--PFALEH
PYPFAL--
--PFALTV
--PFALQD
--PFALPM
--PFALKE
--PFALHT
--PFALFG
--PFALFD
--PFALET

--PFALAM
PMPFAL--
--PFALVK
--PFALTY
--PFALQL
--PFALPF
--PFALLK
--PFALIK
--PFALHD
--PFALFH
--PFALAG
--PFALTI
--PFALRP
--PFALNE
--PFALAN
--PFALYP
--PFALKY
--PFALHQ
--PFALHM
--PFALGW
--PFALGR
--PFALES
--PFALVW
--PFALQM
--PFALNK
--PFALVQ
--PFALVE
--PFALTQ
--PFALNA
--PFALMP
--PFALHV
--PFALHN
--PFALHK
--PFALHA
--PFALFQ
PEPFAL--
--PFALSH
--PFALSE
--PFALNW
--PFALKT
--PFALKK
--PFALHF
--PFALFK
--PFALEW
--PFALEI
PDPFAL--
PFPFAL--
--PFALYK
--PFALTE
--PFALQQ

--PFALQA
--PFALPI
--PFALNN
--PFALNM
--PFALMN
--PFALKF
--PFALKA
--PFALII
--PFALHI
--PFALHH
--PFALFF
--PFALEQ
--PFALDY
--PFALDI
--PFALDG
--PFALTD
--PFALNH
--PFALKQ
--PFALEN
--PFALEM
--PFALEK
PCPFAL--
--PFALQK
--PFALMH
--PFALIV
--PFALGQ
--PFALEY
--PFALDV
--PFALDT
--PFALCH
-PPFALW-
-PPFALR-
--PFALYC
--PFALWT
--PFALTF
--PFALTC
--PFALSC
--PFALPY
--PFALPW
--PFALPV
--PFALPQ
--PFALNY
--PFALNQ
--PFALNG
--PFALNC
--PFALLP
--PFALLE
--PFALIQ
--PFALIP
--PFALID

--PFALHP
--PFALGK
--PFALGC
--PFALDM
--PFALAQ
--PFALAD
QRPFAL--
-QPFALI-
QLPFAL--
-QPFALS-
QGPFAL--
QFPFAL--
-QPFALT-
-QPFALN-
QEPFAL--
-QPFALW-
-QPFALV-
-QPFALG-
QTPFAL--
-QPFALQ-
QKPFAL--
-QPFALR-
-QPFALM-
-QPFALL-
QVPFAL--
QQPFAL--
QHPFAL--
QSPFAL--
QPPFAL--
-QPFALH-
-QPFALA-
QCPFAL--
QYPFAL--
QAPFAL--
QIPFAL--
QMPFAL--
QWPFAL--
-QPFALE-
RLPFAL--
-RPFALS-
RRPFAL--
RVPFAL--
RGPFAL--
RSPFAL--
-RPFALV-
-RPFALH-
-RPFALM-
REPFAL--
-RPFALT-
RHPFAL--

-RPFALG-
RYPFAL--
RPPFAL--
-RPFALQ-
RAPFAL--
-RPFALN-
RFPFAL--
RWPFAL--
RDPFAL--
-RPFALE-
RNPFAL--
RIPFAL--
RQPFAL--
-SPFALR-
SVPFAL--
SSPFAL--
SNPFAL--
-SPFALS-
-SPFALK-
-SPFALA-
SKPFAL--
-SPFALT-
SYPFAL--
SDPFAL--
SMPFAL--
-SPFALQ-
-SPFALV-
-SPFALW-
-SPFALM-
-SPFALI-
SHPFAL--
SGPFAL--
-SPFALH-
STPFAL--
SIPFAL--
-SPFALE-
SFPFAL--
SCPFAL--
TSPFAL--
TRPFAL--
TNPFAL--
TEPFAL--
TLPFAL--
-TPFALI-
-TPFALV-
-TPFALR-
-TPFALH-
TMPFAL--
TYPFAL--
-TPFALT-

−TPFALA−
TWPFAL−−
−TPFALS−
−TPFALQ−
−TPFALK−
−TPFALN−
TGPFAL−−
−TPFALM−
TKPFAL−−
TAPFAL−−
TTPFAL−−
−TPFALE−
TIPFAL−−
−TPFALG−
TPPFAL−−
THPFAL−−
TQPFAL−−
−TPFALC−
TCPFAL−−
−VPFALM−
VRPFAL−−
VGPFAL−−
VTPFAL−−
VNPFAL−−
−VPFALR−
−VPFALL−
VHPFAL−−
−VPFALV−
−VPFALT−
VVPFAL−−
VEPFAL−−
VKPFAL−−
VWPFAL−−
VPPFAL−−
−VPFALG−
−VPFALK−
−VPFALN−
VAPFAL−−
VFPFAL−−
VYPFAL−−
VDPFAL−−
−VPFALI−
−VPFALH−
−VPFALE−
−VPFALA−
VIPFAL−−
−VPFALC−
VMPFAL−−
−WPFALS−
WKPFAL−−

WHPFAL--
-WPFALA-
WSPFAL--
-WPFALH-
WAPFAL--
-WPFALV-
WVPFAL--
-WPFALR-
WPPFAL--
-WPFALN-
-WPFALM-
WEPFAL--
WYPFAL--
WTPFAL--
WGPFAL--
WNPFAL--
-WPFALI-
WFPFAL--
WQPFAL--
-WPFALG-
WMPFAL--
WIPFAL--
-YPFALQ-
YNPFAL--
YRPFAL--
YSPFAL--
-YPFALK-
-YPFALH-
-YPFALR-
YGPFAL--
YVPFAL--
YHPFAL--
-YPFALG-
-YPFALS-
YPPFAL--
-YPFALT-
YAPFAL--
YYPFAL--
YIPFAL--
YDPFAL--
-YPFALI-
YLPFAL--
YTPFAL--
-YPFALN-
-YPFALM-
-YPFALE-
YWPFAL--
YFPFAL--
YCPFAL--

PFNI 339 680 48.87

-APFNIA-
AQPFNI--
ADPFNI--
-APFNIV-
ATPFNI--
-APFNIT-
-APFNIR-
AHPFNI--
-APFNIS-
-APFNIG-
-APFNIQ-
AFPFNI--
AWPFNI--
ANPFNI--
ALPFNI--
AVPFNI--
AAPFNI--
ARPFNI--
ASPFNI--
APPFNI--
-APFNIN-
-APFNIH-
AIPFNI--
AYPFNI--
AGPFNI--
-APFNIY-
-APFNIM-
-APFNIE-
CVPFNI--
CRPFNI--
CMPFNI--
CSPFNI--
-CPFNIT-
-CPFNIN-
CNPFNI--
CKPFNI--
CFPFNI--
DAPFNI--
DRPFNI--
-DPFNIS-
DIPFNI--
DTPFNI--
-DPFNIR-
-DPFNII-
DMPFNI--
DKPFNI--
DSPFNI--
DQPFNI--
-DPFNIH-
DYPFNI--

DVPFNI--
-DPFNIN-
DFPFNI--
ESPFNI--
-EPFNII-
EKPFNI--
-EPFNIK-
EMPFNI--
EGPFNI--
-EPFNIY-
EAPFNI--
ERPFNI--
EFPFNI--
EYPFNI--
ETPFNI--
-EPFNIM-
-EPFNIH-
-EPFNIF-
ELPFNI--
-EPFNIS-
-EPFNIE-
-EPFNIA-
ENPFNI--
EIPFNI--
EEPFNI--
FRPFNI--
-FPFNIR-
FKPFNI--
-FPFNIS-
FMPFNI--
FLPFNI--
FSPFNI--
FQPFNI--
FPPFNI--
-FPFNIT-
-FPFNIK-
-FPFNIV-
-FPFNIE-
FAPFNI--
-FPFNIH-
FYPFNI--
-FPFNIY-
-FPFNIM-
-FPFNIA-
FGPFNI--
FVPFNI--
FNPFNI--
FDPFNI--
GLPFNI--
GRPFNI--

-GPFNIR-
-GPFNIT-
GAPFNI--
GIPFNI--
GSPFNI--
-GPFNIY-
-GPFNIN-
GKPFNI--
GFPFNI--
GGPFNI--
-GPFNIV-
-GPFNII-
GDPFNI--
GWPFNI--
GEPFNI--
-GPFNIS-
-GPFNIM-
GVPFNI--
-GPFNIK-
-GPFNIE-
GMPFNI--
GQPFNI--
-GPFNIF-
HVPFNI--
HRPFNI--
HSPFNI--
HIPFNI--
-HPFNII-
-HPFNIQ-
HTPFNI--
-HPFNIT-
HYPFNI--
HGPFNI--
-HPFNIH-
-HPFNIG-
-HPFNIV-
-HPFNIA-
HLPFNI--
HNPFNI--
HKPFNI--
HDPFNI--
HWPFNI--
HQPFNI--
HPPFNI--
HAPFNI--
-HPFNIS-
-HPFNIR-
-HPFNIE-
HHPFNI--
IRPFNI--

IHPFNI--
-IPFNIT-
IQPFNI--
-IPFNIS-
INPFNI--
-IPFNIW-
-IPFNIK-
ITPFNI--
IPPFNI--
-IPFNIY-
IMPFNI--
-IPFNIQ-
-IPFNII-
ISPFNI--
-IPFNIE-
-IPFNIR-
-IPFNIH-
IKPFNI--
IGPFNI--
IAPFNI--
IYPFNI--
-IPFNIV-
ILPFNI--
IIPFNI--
IEPFNI--
KSPFNI--
-KPFNIL-
-KPFNIV-
KAPFNI--
-KPFNIN-
KPPFNI--
-KPFNIR-
KWPFNI--
KVPFNI--
-KPFNIT-
KGPFNI--
-KPFNIM-
KTPFNI--
-KPFNIK-
KIPFNI--
KRPFNI--
-KPFNIS-
-KPFNII-
KEPFNI--
-KPFNIG-
KHPFNI--
-KPFNIQ-
-KPFNIA-
KKPFNI--
-KPFNIH-

KMPFNI--
KDPFNI--
-LPFNIR-
LRPFNI--
LTPFNI--
LAPFNI--
LGPFNI--
LLPFNI--
-LPFNIS-
LNPFNI--
LSPFNI--
-LPFNIV-
LMPFNI--
LKPFNI--
LQPFNI--
LPPFNI--
-LPFNIK-
-LPFNII-
LHPFNI--
LFPFNI--
LEPFNI--
-LPFNIY-
LYPFNI--
LWPFNI--
-LPFNIA-
-LPFNIN-
-LPFNIH-
-LPFNIE-
LDPFNI--
MSPFNI--
-MPFNIV-
MGPFNI--
-MPFNIT-
-MPFNIA-
-MPFNIM-
-MPFNIY-
-MPFNIR-
MMPFNI--
MAPFNI--
MKPFNI--
-MPFNIN-
MVPFNI--
MTPFNI--
MRPFNI--
MDPFNI--
MFPFNI--
MYPFNI--
-MPFNIH-
-MPFNIQ-
-MPFNIK-

```
-MPFNID-
MNPFNI--
MLPFNI--
MIPFNI--
MEPFNI--
NTPFNI--
NRPFNI--
-NPFNIV-
-NPFNIR-
NAPFNI--
NSPFNI--
-NPFNIQ-
NEPFNI--
NWPFNI--
-NPFNIK-
NFPFNI--
NHPFNI--
NVPFNI--
NNPFNI--
NIPFNI--
-NPFNIT-
NMPFNI--
-NPFNIA-
-NPFNIN-
NLPFNI--
NKPFNI--
NYPFNI--
-NPFNIW-
-NPFNIS-
-NPFNII-
NDPFNI--
--PFNIVR
--PFNIRR
--PFNIAS
--PFNIRS
--PFNIAG
--PFNISG
--PFNIIR
--PFNIGS
--PFNIVL
--PFNITT
--PFNIQR
--PFNIST
--PFNIVD
--PFNIMS
--PFNIVG
--PFNILN
--PFNIKI
--PFNIAR
--PFNIVS
```

--PFNIIK
--PFNIAF
--PFNIAA
PGPFNI--
--PFNIYK
--PFNIQT
--PFNIAM
--PFNIVT
--PFNILY
--PFNIEG
--PFNIAW
--PFNIYA
--PFNIVF
--PFNIQY
--PFNIMR
--PFNIGF
--PFNIVM
--PFNIVK
--PFNITV
--PFNIRD
--PFNIMG
--PFNILH
--PFNIVA
--PFNISS
--PFNISI
--PFNIRL
--PFNIRF
--PFNIMK
--PFNILQ
--PFNILE
--PFNIII
--PFNIFW
--PFNIEA
--PFNIWG
--PFNIVV
--PFNITS
--PFNIKT
--PFNIFK
--PFNIVW
--PFNITR
--PFNIRI
--PFNIRG
--PFNIKN
--PFNIIY
--PFNIIE
--PFNIGV
--PFNIWR
--PFNIWM
--PFNITH
--PFNITA

```
--PFNISN
--PFNISD
--PFNINT
--PFNINS
--PFNING
--PFNILG
--PFNIHQ
--PFNIEM
--PFNIAY
--PFNIVN
--PFNITK
--PFNIRQ
--PFNIQK
--PFNIMD
--PFNIIN
--PFNIIA
--PFNIHR
--PFNIEY
--PFNIWN
--PFNIVY
--PFNITE
--PFNIRT
--PFNIRM
--PFNIQL
--PFNINQ
--PFNINA
--PFNIMM
--PFNILW
--PFNILK
--PFNILD
--PFNIKM
--PFNIIQ
--PFNIHH
--PFNIGG
--PFNIFN
--PFNIFG
--PFNIAV
PKPFNI--
PIPFNI--
--PFNIYV
--PFNIYI
--PFNIWW
--PFNIVE
--PFNITM
--PFNITG
--PFNISW
--PFNISV
--PFNISA
--PFNIRA
--PFNIQS
```

```
--PFNIQG
--PFNIQA
--PFNINY
--PFNIMN
--PFNIMA
--PFNILM
--PFNIKV
--PFNIKA
--PFNIIV
--PFNIFR
--PFNIEL
--PFNIEI
--PFNIAT
PEPFNI--
PQPFNI--
PHPFNI--
--PFNIYE
--PFNIWK
--PFNIWE
--PFNITD
--PFNISY
--PFNISL
--PFNINF
--PFNIMT
--PFNIML
--PFNIHV
--PFNIHT
--PFNIHS
--PFNIHK
--PFNIHG
--PFNIGM
--PFNIFM
--PFNIER
--PFNIEN
--PFNIAN
--PFNIAK
--PFNIYQ
--PFNIYH
--PFNISK
--PFNISF
--PFNIRY
--PFNIRE
--PFNINH
--PFNILR
--PFNIKK
--PFNIKG
--PFNIKE
--PFNIGY
--PFNIGL
--PFNIET
```

--PFNIEF
--PFNIDS
--PFNIAQ
--PFNIAL
PCPFNI--
PWPFNI--
PTPFNI--
--PFNIYW
--PFNIWY
--PFNITQ
--PFNITN
--PFNITF
--PFNISQ
--PFNIRN
--PFNIQN
--PFNIPL
--PFNINI
--PFNIMY
--PFNIKR
--PFNIKQ
--PFNIKL
--PFNIKF
--PFNIIH
--PFNIID
--PFNIHN
--PFNIHI
--PFNIGP
--PFNIGI
--PFNIGH
--PFNIGA
--PFNIEH
--PFNICF
--PFNIAC
PDPFNI--
QRPFNI--
QKPFNI--
-QPFNIR-
-QPFNII-
-QPFNIH-
QHPFNI--
-QPFNIS-
-QPFNIN-
-QPFNIK-
QPPFNI--
-QPFNIY-
QAPFNI--
QWPFNI--
QVPFNI--
-QPFNIQ-
QLPFNI--

QNPFNI--
QGPFNI--
QYPFNI--
QTPFNI--
-QPFNIM-
-QPFNIA-
QSPFNI--
QQPFNI--
-QPFNIV-
-QPFNIF-
QIPFNI--
QEPFNI--
QDPFNI--
-RPFNIR-
RMPFNI--
RRPFNI--
REPFNI--
RAPFNI--
-RPFNIV-
-RPFNIK-
RGPFNI--
RSPFNI--
RNPFNI--
RWPFNI--
RVPFNI--
-RPFNIS-
-RPFNIQ-
-RPFNIT-
-RPFNIG-
RYPFNI--
RQPFNI--
-RPFNIF-
RHPFNI--
RFPFNI--
RKPFNI--
-RPFNII-
RIPFNI--
RPPFNI--
-RPFNIA-
-RPFNIY-
-RPFNIE-
RDPFNI--
-RPFNIH-
-RPFNIW-
-RPFNIM-
-SPFNIF-
SVPFNI--
SYPFNI--
SGPFNI--
-SPFNIS-

SSPFNI--
-SPFNIH-
SLPFNI--
SEPFNI--
-SPFNIK-
SRPFNI--
SPPFNI--
-SPFNIT-
-SPFNIM-
SIPFNI--
-SPFNIY-
-SPFNIA-
SFPFNI--
-SPFNIN-
-SPFNIG-
-SPFNIE-
SWPFNI--
STPFNI--
-SPFNIV-
-SPFNIQ-
SNPFNI--
SHPFNI--
-SPFNIC-
SMPFNI--
SAPFNI--
SDPFNI--
SCPFNI--
TRPFNI--
-TPFNIV-
TSPFNI--
-TPFNIR-
TTPFNI--
-TPFNIS-
-TPFNIM-
TLPFNI--
TFPFNI--
TPPFNI--
-TPFNIE-
TAPFNI--
-TPFNIQ-
TKPFNI--
TVPFNI--
-TPFNIT-
TMPFNI--
-TPFNIK-
TIPFNI--
TGPFNI--
-TPFNII-
TQPFNI--
-TPFNIN-

TNPFNI--
-TPFNIP-
-TPFNIH-
-TPFNIA-
-VPFNIR-
VRPFNI--
VGPFNI--
VLPFNI--
-VPFNIS-
VQPFNI--
VKPFNI--
VDPFNI--
VWPFNI--
VTPFNI--
VPPFNI--
-VPFNIN-
VYPFNI--
-VPFNIT-
VVPFNI--
-VPFNIM-
VSPFNI--
-VPFNIW-
-VPFNIV-
VNPFNI--
VAPFNI--
-VPFNIE-
-VPFNIH-
-VPFNIG-
-VPFNIF-
-VPFNIY-
-VPFNIA-
VHPFNI--
VCPFNI--
VEPFNI--
WLPFNI--
-WPFNIR-
WSPFNI--
WVPFNI--
-WPFNIS-
WEPFNI--
-WPFNIK-
WRPFNI--
WKPFNI--
WIPFNI--
WGPFNI--
WAPFNI--
WTPFNI--
WQPFNI--
-WPFNIN-
-WPFNIG-

	WDPFNI--		
	WMPFNI--		
	-YPFNIR-		
	YSPFNI--		
	-YPFNIS-		
	YHPFNI--		
	YAPFNI--		
	YWPFNI--		
	-YPFNIK-		
	YEPFNI--		
	-YPFNIM-		
	YRPFNI--		
	-YPFNIT-		
	YLPFNI--		
	-YPFNIQ-		
	-YPFNIH-		
	YKPFNI--		
	-YPFNIW-		
	YTPFNI--		
	YMPFNI--		
	YYPFNI--		
	YVPFNI--		
	YPPFNI--		
	YDPFNI--		
	-YPFNII-		
	-YPFNIF-		
	-YPFNIA-		
	YGPFNI--		
	-YPFNIN-		
	YCPFNI--		
PKGM	155	774	48.84
	--PKGMPG		
	--PKGMIP		
	--PKGMGQ		
	-APKGMS-		
	-APKGMY-		
	-APKGMF-		
	-APKGMN-		
	ARPKGM--		
	-APKGMR-		
	-APKGMT-		
	AAPKGM--		
	ASPKGM--		
	-APKGMG-		
	AMPKGM--		
	AGPKGM--		
	AVPKGM--		
	-APKGMV-		
	-APKGMM-		
	-APKGMH-		

-APKGMW-
ATPKGM--
AYPKGM--
-APKGME-
APPKGM--
AFPKGM--
AIPKGM--
-APKGMQ-
-APKGMK-
-APKGMA-
AQPKGM--
-APKGMI-
ANPKGM--
AHPKGM--
CSPKGM--
CVPKGM--
CEPKGM--
CYPKGM--
CWPKGM--
CCPKGM--
CPPKGM--
CGPKGM--
CRPKGM--
-CPKGMR-
-CPKGML-
CHPKGM--
CDPKGM--
-CPKGMV-
-CPKGMQ-
-CPKGMN-
-CPKGMG-
CMPKGM--
DWPKGM--
DNPKGM--
-DPKGMI-
-DPKGMH-
DFPKGM--
-DPKGMW-
-DPKGMR-
DMPKGM--
DSPKGM--
-DPKGMF-
-DPKGMS-
DAPKGM--
-DPKGMT-
-DPKGMQ-
-DPKGML-
DLPKGM--
DIPKGM--
-DPKGMY-

-DPKGMV-
-DPKGMA-
EPPKGM--
-EPKGMQ-
ERPKGM--
ELPKGM--
EIPKGM--
EWPKGM--
-EPKGMY-
EVPKGM--
-EPKGMW-
EEPKGM--
-EPKGMH-
EGPKGM--
-EPKGMR-
-EPKGMM-
-EPKGMF-
-EPKGML-
-EPKGME-
EFPKGM--
EDPKGM--
EYPKGM--
EQPKGM--
-EPKGMS-
-EPKGMG-
EKPKGM--
FTPKGM--
-FPKGMI-
FWPKGM--
FKPKGM--
-FPKGML-
FSPKGM--
FNPKGM--
-FPKGMV-
FEPKGM--
-FPKGMY-
FLPKGM--
FDPKGM--
FVPKGM--
FPPKGM--
-FPKGMG-
-FPKGMW-
FHPKGM--
-FPKGMS-
-FPKGMN-
FQPKGM--
-FPKGMM-
-FPKGMK-
-FPKGMF-
-FPKGMA-

FIPKGM--
-GPKGML-
GMPKGM--
-GPKGMT-
GSPKGM--
-GPKGMR-
-GPKGMW-
-GPKGMS-
GIPKGM--
GLPKGM--
-GPKGMN-
GYPKGM--
-GPKGMI-
-GPKGME-
GFPKGM--
GVPKGM--
-GPKGMF-
GKPKGM--
GWPKGM--
-GPKGMV-
GNPKGM--
-GPKGMM-
GCPKGM--
GRPKGM--
GHPKGM--
GQPKGM--
GGPKGM--
-GPKGMH-
-GPKGMG-
-GPKGMA-
GEPKGM--
-HPKGML-
HSPKGM--
HRPKGM--
HQPKGM--
-HPKGMS-
-HPKGMN-
HHPKGM--
-HPKGMF-
HLPKGM--
HTPKGM--
HKPKGM--
-HPKGMV-
-HPKGMI-
HYPKGM--
HVPKGM--
-HPKGMY-
-HPKGMT-
-HPKGMM-
-HPKGME-

HWPKGM--
HNPKGM--
HIPKGM--
-HPKGMQ-
-HPKGMK-
-HPKGMH-
-HPKGMC-
HGPKGM--
HFPKGM--
HAPKGM--
IKPKGM--
-IPKGMT-
-IPKGMV-
-IPKGML-
ISPKGM--
IAPKGM--
IRPKGM--
-IPKGMF-
-IPKGMR-
-IPKGMN-
ITPKGM--
-IPKGMQ-
ILPKGM--
IHPKGM--
IGPKGM--
-IPKGMA-
INPKGM--
IMPKGM--
IVPKGM--
IIPKGM--
-IPKGMY-
-IPKGMW-
IYPKGM--
IPPKGM--
-IPKGMH-
-IPKGME-
-IPKGMS-
-IPKGMM-
-IPKGMG-
KSPKGM--
-KPKGMR-
-KPKGML-
-KPKGMW-
-KPKGMS-
-KPKGMT-
KNPKGM--
KLPKGM--
KAPKGM--
KQPKGM--
-KPKGMF-

KGPKGM--
KDPKGM--
KCPKGM--
KYPKGM--
-KPKGMV-
-KPKGMQ-
-KPKGMM-
KEPKGM--
KTPKGM--
-KPKGMY-
-KPKGMN-
KFPKGM--
KPPKGM--
-KPKGMI-
KIPKGM--
KHPKGM--
KWPKGM--
KVPKGM--
KRPKGM--
-KPKGMH-
-KPKGMG-
-KPKGMC-
LLPKGM--
LTPKGM--
LVPKGM--
-LPKGML-
-LPKGMT-
-LPKGMS-
-LPKGMR-
-LPKGMF-
LGPKGM--
-LPKGMM-
-LPKGMH-
LHPKGM--
-LPKGMN-
LPPKGM--
-LPKGMI-
-LPKGMQ-
LSPKGM--
LCPKGM--
LMPKGM--
LKPKGM--
LYPKGM--
LIPKGM--
-LPKGMY-
-LPKGME-
LNPKGM--
LQPKGM--
-LPKGMA-
LAPKGM--

```
-LPKGMC-
-MPKGMH-
MWPKGM--
-MPKGMF-
MPPKGM--
-MPKGMV-
MYPKGM--
MTPKGM--
MSPKGM--
-MPKGMS-
MDPKGM--
-MPKGMW-
-MPKGMN-
-MPKGMY-
MNPKGM--
MEPKGM--
-MPKGMK-
-MPKGMT-
MKPKGM--
MGPKGM--
-MPKGME-
MAPKGM--
-MPKGMA-
MIPKGM--
NTPKGM--
-NPKGML-
NLPKGM--
-NPKGMW-
-NPKGMT-
-NPKGMK-
NGPKGM--
-NPKGMV-
-NPKGMQ-
NKPKGM--
NYPKGM--
NQPKGM--
-NPKGMS-
-NPKGMI-
NNPKGM--
NVPKGM--
-NPKGMF-
NHPKGM--
NFPKGM--
NDPKGM--
NPPKGM--
NEPKGM--
-NPKGMM-
-NPKGMH-
-NPKGME-
-NPKGMA-
```

NAPKGM--
-NPKGMY-
-NPKGMN-
-NPKGMG-
NMPKGM--
NCPKGM--
--PKGMVA
--PKGMLA
--PKGMLY
--PKGMSS
--PKGMFH
--PKGMTV
--PKGMST
--PKGMFG
--PKGMLT
--PKGMLF
--PKGMMT
--PKGMVV
--PKGMRT
--PKGMMV
--PKGMLL
--PKGMYM
--PKGMSA
--PKGMMW
--PKGMFI
--PKGMSR
--PKGMRG
--PKGMLN
--PKGMWS
--PKGMTS
--PKGMSY
--PKGMRQ
--PKGMYL
--PKGMWL
--PKGMRD
--PKGMRA
--PKGMLG
--PKGMGT
--PKGMII
--PKGMRI
--PKGMQS
--PKGMHA
--PKGMES
--PKGMYG
--PKGMVN
--PKGMMA
--PKGMKT
--PKGMFR
--PKGMFL
--PKGMWT

--PKGMWF
--PKGMNS
--PKGMNL
--PKGMLM
--PKGMTQ
--PKGMTK
--PKGMSK
PSPKGM--
--PKGMVT
--PKGMLQ
--PKGMHM
--PKGMFN
PLPKGM--
--PKGMYY
--PKGMVW
--PKGMVG
--PKGMTA
--PKGMIT
--PKGMIS
--PKGMHR
--PKGMSW
--PKGMQN
--PKGMLW
--PKGMIM
--PKGMHV
--PKGMHS
--PKGMAS
--PKGMTY
--PKGMTL
--PKGMRN
--PKGMRF
--PKGMLH
--PKGMKQ
--PKGMKM
--PKGMIH
--PKGMGV
--PKGMAA
--PKGMYN
--PKGMSG
--PKGMIN
--PKGMHY
--PKGMHI
--PKGMRV
--PKGMIY
--PKGMIA
--PKGMHL
--PKGMWK
--PKGMTH
--PKGMNA
--PKGMMR

--PKGMMN
--PKGMMI
--PKGMLD
--PKGMKY
--PKGMIQ
--PKGMHD
--PKGMFM
--PKGMEV
--PKGMWG
--PKGMTD
--PKGMRE
--PKGMQY
--PKGMNT
--PKGMIK
--PKGMIG
--PKGMHW
--PKGMHQ
--PKGMAI
PYPKGM--
--PKGMWE
--PKGMVQ
--PKGMVF
--PKGMTF
--PKGMSQ
--PKGMSM
--PKGMQT
--PKGMMS
--PKGMHN
--PKGMHG
--PKGMFV
--PKGMFE
--PKGMEW
PVPKGM--
--PKGMYQ
--PKGMWI
--PKGMTM
--PKGMSN
--PKGMQF
--PKGMNF
--PKGMNE
--PKGMMY
--PKGMMQ
--PKGMMK
--PKGMKL
--PKGMIF
--PKGMGR
--PKGMGG
--PKGMFA
--PKGMAW
--PKGMAL

PTPKGM--
PKPKGM--
--PKGMYV
--PKGMVH
--PKGMTI
--PKGMSH
--PKGMQL
--PKGMNY
--PKGMNQ
--PKGMMG
--PKGMLV
--PKGMKN
--PKGMKA
--PKGMGL
--PKGMGI
--PKGMGA
--PKGMFQ
--PKGMET
--PKGMEM
--PKGMAV
--PKGMAM
--PKGMYE
--PKGMWV
--PKGMWD
--PKGMVD
--PKGMTN
--PKGMQM
--PKGMNK
--PKGMNG
--PKGMIV
--PKGMAT
--PKGMAR
--PKGMAH
--PKGMAG
PIPKGM--
PRPKGM--
--PKGMYF
--PKGMYD
--PKGMVE
--PKGMTT
--PKGMSD
--PKGMRP
--PKGMRC
--PKGMQW
--PKGMQH
--PKGMNR
--PKGMMH
--PKGMHF
--PKGMGF
--PKGMFT

--PKGMFD
--PKGMAQ
--PKGMWN
--PKGMVI
--PKGMLE
--PKGMKW
--PKGMKV
--PKGMKF
--PKGMEI
--PKGMDS
--PKGMAN
--PKGMAK
PDPKGM--
PAPKGM--
--PKGMYK
--PKGMYI
--PKGMWA
--PKGMTE
--PKGMSF
--PKGMQK
--PKGMNV
--PKGMNI
--PKGMMC
--PKGMKH
--PKGMKG
--PKGMKD
--PKGMIC
--PKGMGM
--PKGMGK
--PKGMEG
--PKGMAE
-PPKGMS-
PNPKGM--
--PKGMYH
--PKGMSC
--PKGMQV
--PKGMQE
--PKGMQD
--PKGMNH
--PKGMND
--PKGMLP
--PKGMIE
--PKGMID
--PKGMGW
--PKGMGH
--PKGMFW
--PKGMER
--PKGMEK
--PKGMEH
--PKGMEA

--PKGMDH
--PKGMDG
--PKGMCY
--PKGMCM
--PKGMCH
--PKGMAP
--PKGMAF
QLPKGM--
QAPKGM--
-QPKGML-
-QPKGMS-
-QPKGMI-
QTPKGM--
-QPKGMF-
QIPKGM--
-QPKGMH-
QGPKGM--
-QPKGMR-
-QPKGMA-
QVPKGM--
-QPKGMM-
QYPKGM--
-QPKGMV-
QWPKGM--
-QPKGMK-
-QPKGMG-
-QPKGMY-
QNPKGM--
QRPKGM--
-QPKGMQ-
QEPKGM--
-QPKGMN-
QHPKGM--
-RPKGMY-
RVPKGM--
RWPKGM--
RYPKGM--
-RPKGMT-
-RPKGMI-
-RPKGMN-
RIPKGM--
-RPKGMV-
RGPKGM--
RFPKGM--
-RPKGME-
RSPKGM--
-RPKGMK-
RNPKGM--
RTPKGM--
REPKGM--

-RPKGMW-
-RPKGMQ-
-RPKGMG-
RQPKGM--
RPPKGM--
-RPKGMM-
-RPKGMF-
RMPKGM--
RDPKGM--
-SPKGMR-
-SPKGMI-
-SPKGML-
SAPKGM--
-SPKGMV-
-SPKGMS-
SYPKGM--
STPKGM--
-SPKGMW-
SKPKGM--
SHPKGM--
SLPKGM--
SSPKGM--
SIPKGM--
SGPKGM--
-SPKGMT-
-SPKGMY-
-SPKGMN-
SWPKGM--
-SPKGMG-
SNPKGM--
-SPKGME-
-SPKGMM-
-SPKGMH-
-SPKGMF-
SPPKGM--
-SPKGMA-
-SPKGMK-
-SPKGMQ-
SMPKGM--
SFPKGM--
SDPKGM--
SEPKGM--
-TPKGMW-
TLPKGM--
TSPKGM--
-TPKGMS-
TVPKGM--
-TPKGMY-
-TPKGMN-
-TPKGMR-

-TPKGMT-
-TPKGMF-
-TPKGML-
TTPKGM--
-TPKGMV-
-TPKGMI-
TRPKGM--
TQPKGM--
-TPKGMM-
-TPKGMA-
TGPKGM--
TAPKGM--
-TPKGMK-
-TPKGMH-
TIPKGM--
TEPKGM--
TCPKGM--
-TPKGMQ-
TMPKGM--
TKPKGM--
-TPKGMG-
THPKGM--
TYPKGM--
TPPKGM--
-TPKGME-
TNPKGM--
-VPKGMF-
-VPKGMS-
VHPKGM--
VSPKGM--
-VPKGMV-
VYPKGM--
VLPKGM--
-VPKGMW-
-VPKGMT-
VFPKGM--
VKPKGM--
-VPKGMN-
-VPKGMY-
-VPKGML-
VGPKGM--
VQPKGM--
VNPKGM--
VDPKGM--
VMPKGM--
-VPKGMG-
VVPKGM--
-VPKGMA-
-VPKGMH-
-VPKGME-

VTPKGM--
-VPKGMQ-
-VPKGMK-
VEPKGM--
VPPKGM--
-WPKGML-
WSPKGM--
WVPKGM--
-WPKGMY-
-WPKGMV-
WGPKGM--
-WPKGMS-
-WPKGMI-
WQPKGM--
WLPKGM--
WWPKGM--
WRPKGM--
WYPKGM--
-WPKGMM-
-WPKGMA-
WNPKGM--
WFPKGM--
-WPKGME-
WCPKGM--
-WPKGMQ-
-WPKGMH-
WIPKGM--
-WPKGMF-
WMPKGM--
WHPKGM--
WDPKGM--
-YPKGMS-
YVPKGM--
-YPKGMK-
-YPKGMT-
-YPKGMQ-
-YPKGMH-
YKPKGM--
-YPKGMR-
-YPKGMN-
YNPKGM--
-YPKGMW-
YYPKGM--
YRPKGM--
YEPKGM--
YTPKGM--
YSPKGM--
-YPKGMA-
-YPKGME-
-YPKGMF-

```
            -YPKGMV-
            -YPKGMG-
            YHPKGM--
            YCPKGM--
            YAPKGM--
PKAM         397         658      48.80
            -RPKAMP-
            -APKAMT-
            -APKAMS-
            -APKAMK-
            AKPKAM--
            -APKAMF-
            ASPKAM--
            -APKAMM-
            AVPKAM--
            -APKAML-
            ALPKAM--
            -APKAMA-
            -APKAMI-
            AQPKAM--
            AGPKAM--
            -APKAMQ-
            -APKAMY-
            -APKAMV-
            AIPKAM--
            ACPKAM--
            -APKAMR-
            -APKAMN-
            ANPKAM--
            -APKAMW-
            -APKAMG-
            AAPKAM--
            CGPKAM--
            CRPKAM--
            -CPKAMH-
            -CPKAMR-
            CHPKAM--
            CSPKAM--
            -CPKAMT-
            -CPKAMI-
            -CPKAMF-
            CMPKAM--
            CLPKAM--
            DRPKAM--
            DLPKAM--
            DWPKAM--
            DVPKAM--
            DIPKAM--
            -DPKAMK-
            DFPKAM--
```

DEPKAM--
DQPKAM--
-DPKAMN-
DKPKAM--
DHPKAM--
DAPKAM--
-DPKAMI-
DTPKAM--
DSPKAM--
-DPKAMV-
-DPKAMS-
-EPKAMY-
-EPKAML-
-EPKAMS-
ELPKAM--
-EPKAMN-
-EPKAMV-
EYPKAM--
-EPKAMI-
ENPKAM--
EIPKAM--
-EPKAME-
EEPKAM--
EVPKAM--
EQPKAM--
EKPKAM--
ESPKAM--
ERPKAM--
-EPKAMT-
-EPKAMG-
-EPKAMA-
EAPKAM--
-EPKAMQ-
-EPKAMK-
-EPKAMF-
-FPKAMY-
FFPKAM--
-FPKAMN-
FMPKAM--
FGPKAM--
FSPKAM--
FQPKAM--
-FPKAMK-
-FPKAMF-
FKPKAM--
-FPKAMT-
FLPKAM--
FPPKAM--
FDPKAM--
FIPKAM--

-FPKAME-
-GPKAMS-
GLPKAM--
GGPKAM--
-GPKAMY-
-GPKAMV-
GSPKAM--
GYPKAM--
-GPKAMF-
-GPKAMW-
-GPKAMT-
-GPKAMK-
GVPKAM--
-GPKAMQ-
-GPKAML-
-GPKAMH-
GHPKAM--
-GPKAMI-
GRPKAM--
-GPKAMG-
-GPKAMN-
-GPKAMA-
GQPKAM--
-GPKAME-
GIPKAM--
GCPKAM--
GPPKAM--
GTPKAM--
GKPKAM--
GWPKAM--
GEPKAM--
GAPKAM--
HIPKAM--
HSPKAM--
-HPKAMT-
HVPKAM--
-HPKAMF-
HAPKAM--
-HPKAMS-
HFPKAM--
HRPKAM--
-HPKAMQ-
-HPKAMI-
HGPKAM--
HQPKAM--
HPPKAM--
HMPKAM--
HKPKAM--
HDPKAM--
HWPKAM--

-HPKAMH-
-HPKAME-
ILPKAM--
-IPKAMR-
IQPKAM--
-IPKAML-
IRPKAM--
IAPKAM--
-IPKAMA-
-IPKAMN-
ISPKAM--
IVPKAM--
-IPKAMG-
-IPKAMV-
-IPKAMK-
-IPKAMH-
-IPKAMY-
IKPKAM--
KSPKAM--
KVPKAM--
-KPKAMS-
-KPKAMM-
-KPKAMI-
-KPKAMT-
-KPKAMQ-
KTPKAM--
KAPKAM--
KWPKAM--
-KPKAMK-
KQPKAM--
-KPKAMV-
-KPKAMH-
KFPKAM--
-KPKAMY-
-KPKAMN-
-KPKAMG-
KNPKAM--
KDPKAM--
-KPKAMP-
KMPKAM--
KKPKAM--
KEPKAM--
-LPKAMF-
LGPKAM--
-LPKAMM-
-LPKAMK-
-LPKAMI-
LWPKAM--
LVPKAM--
LKPKAM--

LYPKAM--
LTPKAM--
LSPKAM--
LNPKAM--
-LPKAMW-
-LPKAMQ-
-LPKAMV-
LMPKAM--
-LPKAML-
-LPKAMY-
-LPKAMS-
LHPKAM--
-LPKAME-
LEPKAM--
LQPKAM--
LPPKAM--
-LPKAMP-
-LPKAMH-
LDPKAM--
MVPKAM--
MLPKAM--
MMPKAM--
-MPKAMV-
-MPKAML-
MPPKAM--
-MPKAMQ-
MTPKAM--
-MPKAMH-
MSPKAM--
-MPKAMW-
MIPKAM--
-MPKAMY-
-MPKAMN-
MYPKAM--
MQPKAM--
-MPKAMS-
-MPKAMG-
MNPKAM--
MHPKAM--
-NPKAMR-
-NPKAMW-
NGPKAM--
NWPKAM--
NYPKAM--
NDPKAM--
NTPKAM--
NVPKAM--
-NPKAMI-
-NPKAMF-
NFPKAM--

-NPKAMN-
-NPKAMA-
NMPKAM--
NPPKAM--
-NPKAMK-
-NPKAMY-
-NPKAMT-
--PKAMYG
--PKAMAL
--PKAMRG
--PKAMNS
--PKAMSS
--PKAMSR
--PKAMLS
--PKAMVQ
--PKAMTG
--PKAMMS
--PKAMIK
--PKAMVG
--PKAMSL
--PKAMSA
--PKAMTL
--PKAMSF
--PKAMKN
--PKAMQL
--PKAMTI
--PKAMSI
--PKAMAS
--PKAMRM
--PKAMLK
--PKAMAG
--PKAMSY
--PKAMSW
--PKAMYT
--PKAMWN
--PKAMIF
--PKAMGS
--PKAMFT
--PKAMWL
--PKAMLF
PSPKAM--
--PKAMWM
--PKAMVL
--PKAMTS
--PKAMIG
--PKAMHS
--PKAMGA
--PKAMYN
--PKAMVT
--PKAMYS

--PKAMTF
--PKAMSN
--PKAMSH
--PKAMQN
--PKAMGQ
PVPKAM--
--PKAMST
--PKAMSM
--PKAMSG
--PKAMRD
--PKAMNL
--PKAMFS
--PKAMFH
--PKAMAY
--PKAMYF
--PKAMFL
--PKAMWI
--PKAMTR
--PKAMTQ
--PKAMTN
--PKAMMR
--PKAMHT
--PKAMAM
--PKAMYD
--PKAMVF
--PKAMTK
--PKAMRW
--PKAMMQ
--PKAMMK
--PKAMFN
--PKAMAI
PEPKAM--
--PKAMYY
--PKAMYV
--PKAMTV
--PKAMMA
--PKAMLI
--PKAMLG
--PKAMKV
--PKAMWV
--PKAMVM
--PKAMTH
--PKAMNR
--PKAMNF
--PKAMMF
--PKAMHN
--PKAMGW
--PKAMGI
--PKAMFM
--PKAMRI

--PKAMNI
--PKAMMI
--PKAMKW
--PKAMIA
--PKAMGV
--PKAMFY
--PKAMFG
--PKAMFF
--PKAMEM
--PKAMAV
--PKAMAF
PFPKAM--
PTPKAM--
--PKAMVI
--PKAMTY
--PKAMTA
--PKAMSQ
--PKAMRE
--PKAMQV
--PKAMLH
--PKAMIY
--PKAMEW
--PKAMAR
--PKAMAN
--PKAMAA
PLPKAM--
--PKAMYW
--PKAMVY
--PKAMVW
--PKAMVN
--PKAMQH
--PKAMNT
--PKAMMT
--PKAMMD
--PKAMLM
--PKAMKI
--PKAMIW
--PKAMIH
--PKAMFA
--PKAMET
--PKAMEA
--PKAMDL
--PKAMAT
--PKAMAQ
--PKAMYA
--PKAMWG
--PKAMVH
--PKAMQT
--PKAMQS
--PKAMMW

--PKAMLD
--PKAMKD
--PKAMHV
--PKAMGT
--PKAMFK
--PKAMVK
--PKAMSD
--PKAMQG
--PKAMPY
--PKAMMH
--PKAMKQ
--PKAMIR
--PKAMGF
--PKAMEI
PRPKAM--
--PKAMYQ
--PKAMVE
--PKAMVD
--PKAMQR
--PKAMQQ
--PKAMQA
--PKAMNM
--PKAMLE
--PKAMKM
--PKAMHF
--PKAMHA
--PKAMGG
--PKAMEH
PQPKAM--
-PPKAMR-
--PKAMWE
--PKAMVA
--PKAMTM
--PKAMTE
--PKAMSE
--PKAMRQ
--PKAMRP
--PKAMQM
--PKAMQF
--PKAMNY
--PKAMNH
--PKAMNC
--PKAMLP
--PKAMKT
--PKAMKH
--PKAMKF
--PKAMKE
--PKAMID
--PKAMGN
--PKAMFV

--PKAMFR
--PKAMES
-PPKAMW-
PNPKAM--
--PKAMYI
--PKAMYH
--PKAMYE
--PKAMWQ
--PKAMWK
--PKAMWH
--PKAMWF
--PKAMVP
--PKAMSK
--PKAMRC
--PKAMQD
--PKAMPI
--PKAMPF
--PKAMPA
--PKAMNV
--PKAMNE
--PKAMNA
--PKAMIV
--PKAMIE
--PKAMHR
--PKAMGR
--PKAMFQ
--PKAMFE
--PKAMFD
--PKAMFC
--PKAMEY
--PKAMEK
--PKAMDM
--PKAMDI
--PKAMDF
--PKAMCL
-QPKAML-
-QPKAMK-
-QPKAMT-
QGPKAM--
QRPKAM--
-QPKAMI-
-QPKAMA-
-QPKAMY-
-QPKAMS-
QVPKAM--
-QPKAMM-
QIPKAM--
-QPKAMQ-
QWPKAM--
QEPKAM--

-QPKAMF-
QMPKAM--
-QPKAMW-
-QPKAMR-
-QPKAMV-
-QPKAMG-
-QPKAME-
QDPKAM--
-RPKAMW-
-RPKAMT-
-RPKAMS-
RAPKAM--
RTPKAM--
RLPKAM--
-RPKAMI-
RGPKAM--
RYPKAM--
-RPKAMK-
-RPKAMY-
RPPKAM--
-RPKAMV-
REPKAM--
RMPKAM--
-RPKAMQ-
-RPKAMN-
RVPKAM--
-RPKAMH-
RQPKAM--
RNPKAM--
RFPKAM--
-RPKAMM-
-RPKAME-
-RPKAMC-
RRPKAM--
-RPKAMG-
RDPKAM--
-SPKAMR-
-SPKAMI-
-SPKAMV-
SMPKAM--
SGPKAM--
-SPKAMY-
SSPKAM--
SVPKAM--
-SPKAMH-
-SPKAMS-
SQPKAM--
STPKAM--
SHPKAM--
SDPKAM--

SPPKAM--
-SPKAMQ-
SAPKAM--
SNPKAM--
SYPKAM--
SKPKAM--
SEPKAM--
-SPKAMF-
-SPKAMW-
-SPKAMG-
-SPKAMN-
-SPKAME-
-TPKAMR-
TLPKAM--
-TPKAMY-
-TPKAMS-
TGPKAM--
TTPKAM--
TRPKAM--
TKPKAM--
-TPKAMT-
TSPKAM--
TIPKAM--
TFPKAM--
TVPKAM--
TWPKAM--
TAPKAM--
-TPKAMF-
-TPKAMI-
-TPKAMG-
TQPKAM--
-TPKAMW-
-TPKAMH-
-TPKAMQ-
-TPKAMA-
TMPKAM--
TYPKAM--
-TPKAMN-
-TPKAMM-
-TPKAME-
TEPKAM--
TCPKAM--
-VPKAMR-
-VPKAML-
VKPKAM--
-VPKAMK-
VVPKAM--
-VPKAMF-
VSPKAM--
VPPKAM--

```
-VPKAMW-
-VPKAMT-
VGPKAM--
VRPKAM--
VIPKAM--
-VPKAMM-
VTPKAM--
VYPKAM--
-VPKAMQ-
VAPKAM--
VEPKAM--
-VPKAMS-
-VPKAMH-
VWPKAM--
-VPKAMA-
-VPKAMN-
VDPKAM--
VNPKAM--
VHPKAM--
-WPKAML-
WRPKAM--
WLPKAM--
-WPKAMG-
WNPKAM--
-WPKAMA-
-WPKAMV-
WGPKAM--
WWPKAM--
WVPKAM--
WFPKAM--
WQPKAM--
WMPKAM--
WEPKAM--
WDPKAM--
WAPKAM--
-WPKAMQ-
-WPKAMF-
-WPKAMC-
-WPKAMN-
WIPKAM--
WTPKAM--
-WPKAMT-
-WPKAME-
-WPKAMD-
YRPKAM--
YVPKAM--
YLPKAM--
-YPKAMT-
-YPKAMS-
YFPKAM--
```

YPPKAM--
-YPKAMI-
YGPKAM--
-YPKAMQ-
-YPKAMV-
-YPKAML-
-YPKAMF-
YEPKAM--
YDPKAM--
YMPKAM--
YQPKAM--
-YPKAMA-
-YPKAMY-
YKPKAM--
PYHL 56 848 48.78
-DPYHLL-
-NPYHLG-
-SPYHLG-
-YPYHLG-
AQPYHL--
-APYHLL-
ARPYHL--
-APYHLA-
ALPYHL--
AWPYHL--
-APYHLR-
-APYHLT-
AYPYHL--
AVPYHL--
ATPYHL--
ASPYHL--
-APYHLY-
ADPYHL--
-APYHLV-
-APYHLN-
APPYHL--
-APYHLH-
-APYHLM-
-APYHLI-
AGPYHL--
-APYHLS-
-APYHLF-
ANPYHL--
-APYHLW-
AAPYHL--
-APYHLG-
AFPYHL--
-APYHLE-
AHPYHL--
AMPYHL--

AIPYHL--
-APYHLQ-
-APYHLK-
AEPYHL--
ACPYHL--
AKPYHL--
CGPYHL--
CRPYHL--
CTPYHL--
CSPYHL--
CKPYHL--
CFPYHL--
-CPYHLT-
CNPYHL--
CVPYHL--
CQPYHL--
-CPYHLN-
-CPYHLL-
CLPYHL--
-CPYHLM-
-CPYHLI-
CPPYHL--
CDPYHL--
CAPYHL--
DAPYHL--
DQPYHL--
-DPYHLR-
DWPYHL--
DRPYHL--
DLPYHL--
DIPYHL--
-DPYHLS-
DGPYHL--
-DPYHLW-
-DPYHLV-
-DPYHLT-
-DPYHLM-
DVPYHL--
DKPYHL--
DFPYHL--
DEPYHL--
-DPYHLI-
-DPYHLF-
-DPYHLY-
-DPYHLQ-
DNPYHL--
DMPYHL--
ERPYHL--
ETPYHL--
ESPYHL--

```
-EPYHLI-
-EPYHLR-
-EPYHLV-
ELPYHL--
EMPYHL--
-EPYHLL-
-EPYHLT-
EAPYHL--
-EPYHLK-
-EPYHLH-
EKPYHL--
-EPYHLS-
EGPYHL--
EFPYHL--
EEPYHL--
EVPYHL--
-EPYHLQ-
-EPYHLM-
-EPYHLA-
EHPYHL--
EWPYHL--
-EPYHLY-
-EPYHLE-
EPPYHL--
ENPYHL--
FSPYHL--
FVPYHL--
FTPYHL--
-FPYHLH-
FRPYHL--
FPPYHL--
FLPYHL--
FIPYHL--
FGPYHL--
-FPYHLR-
-FPYHLA-
FKPYHL--
-FPYHLS-
FNPYHL--
-FPYHLV-
-FPYHLI-
FEPYHL--
FYPYHL--
-FPYHLN-
FQPYHL--
-FPYHLY-
-FPYHLW-
FWPYHL--
FFPYHL--
FDPYHL--
```

-FPYHLT-
-FPYHLQ-
-FPYHLL-
-FPYHLK-
-FPYHLG-
GYPYHL--
GGPYHL--
-GPYHLI-
GRPYHL--
-GPYHLL-
GSPYHL--
-GPYHLV-
GVPYHL--
GTPYHL--
GLPYHL--
-GPYHLY-
GKPYHL--
GAPYHL--
-GPYHLR-
GIPYHL--
GFPYHL--
GNPYHL--
-GPYHLT-
-GPYHLQ-
GMPYHL--
-GPYHLW-
-GPYHLN-
-GPYHLA-
GEPYHL--
-GPYHLS-
-GPYHLE-
GDPYHL--
GQPYHL--
-GPYHLF-
GWPYHL--
GCPYHL--
-GPYHLM-
-GPYHLK-
-GPYHLG-
GPPYHL--
GHPYHL--
-GPYHLH-
-GPYHLD-
HRPYHL--
HTPYHL--
-HPYHLR-
-HPYHLV-
HFPYHL--
-HPYHLY-
-HPYHLS-

HLPYHL--
HKPYHL--
HHPYHL--
HEPYHL--
HGPYHL--
HSPYHL--
HQPYHL--
-HPYHLW-
-HPYHLT-
-HPYHLQ-
-HPYHLL-
HWPYHL--
HDPYHL--
HVPYHL--
HIPYHL--
-HPYHLK-
HYPYHL--
-HPYHLE-
-HPYHLG-
-HPYHLA-
HPPYHL--
-HPYHLN-
-HPYHLH-
-HPYHLF-
HNPYHL--
HAPYHL--
IGPYHL--
IVPYHL--
ILPYHL--
INPYHL--
-IPYHLV-
-IPYHLR-
-IPYHLA-
IDPYHL--
IPPYHL--
IFPYHL--
-IPYHLL-
-IPYHLK-
ISPYHL--
ITPYHL--
IMPYHL--
-IPYHLM-
-IPYHLH-
-IPYHLS-
-IPYHLQ-
IHPYHL--
-IPYHLE-
IAPYHL--
IYPYHL--
IRPYHL--

IQPYHL--
-IPYHLN-
-IPYHLI-
-IPYHLF-
IWPYHL--
-IPYHLY-
-IPYHLT-
IIPYHL--
IEPYHL--
KSPYHL--
KRPYHL--
KLPYHL--
-KPYHLI-
KQPYHL--
-KPYHLW-
-KPYHLL-
-KPYHLR-
-KPYHLF-
KYPYHL--
-KPYHLV-
KGPYHL--
KMPYHL--
KAPYHL--
KWPYHL--
-KPYHLS-
-KPYHLM-
-KPYHLG-
KFPYHL--
-KPYHLN-
-KPYHLA-
-KPYHLY-
KTPYHL--
-KPYHLK-
KPPYHL--
-KPYHLE-
-KPYHLQ-
KIPYHL--
KDPYHL--
-KPYHLT-
KHPYHL--
KCPYHL--
KNPYHL--
KEPYHL--
LQPYHL--
LGPYHL--
LRPYHL--
LAPYHL--
LTPYHL--
LVPYHL--
LMPYHL--

LPPYHL--
LKPYHL--
-LPYHLR-
-LPYHLT-
-LPYHLS-
LWPYHL--
-LPYHLV-
LYPYHL--
LEPYHL--
-LPYHLL-
LNPYHL--
-LPYHLQ-
-LPYHLY-
-LPYHLK-
LHPYHL--
-LPYHLM-
-LPYHLH-
-LPYHLN-
LDPYHL--
-LPYHLF-
-LPYHLG-
LCPYHL--
MSPYHL--
MRPYHL--
MGPYHL--
MLPYHL--
MVPYHL--
MYPYHL--
MTPYHL--
-MPYHLS-
MAPYHL--
MQPYHL--
-MPYHLT-
MFPYHL--
-MPYHLY-
-MPYHLL-
MKPYHL--
MDPYHL--
-MPYHLV-
MMPYHL--
MIPYHL--
-MPYHLH-
-MPYHLA-
-MPYHLW-
-MPYHLQ-
-MPYHLN-
-MPYHLM-
-MPYHLI-
MPPYHL--
MNPYHL--

```
MWPYHL--
-MPYHLF-
MCPYHL--
-MPYHLK-
-MPYHLG-
MEPYHL--
NRPYHL--
NNPYHL--
NKPYHL--
-NPYHLR-
NVPYHL--
NGPYHL--
NAPYHL--
-NPYHLS-
NSPYHL--
-NPYHLV-
NTPYHL--
NPPYHL--
-NPYHLK-
NLPYHL--
-NPYHLT-
-NPYHLY-
-NPYHLQ-
NEPYHL--
NMPYHL--
-NPYHLL-
-NPYHLE-
-NPYHLA-
-NPYHLW-
-NPYHLM-
-NPYHLF-
NYPYHL--
-NPYHLN-
NIPYHL--
NHPYHL--
NFPYHL--
NDPYHL--
--PYHLVT
--PYHLRT
--PYHLRG
--PYHLLS
--PYHLMG
--PYHLRN
--PYHLKS
--PYHLTQ
--PYHLAG
--PYHLAS
PWPYHL--
--PYHLVS
--PYHLVG
```

--PYHLSH
PTPYHL--
--PYHLSG
--PYHLLT
--PYHLTM
--PYHLRQ
--PYHLSS
--PYHLMV
--PYHLTR
--PYHLRS
--PYHLFR
--PYHLYR
--PYHLYN
--PYHLYH
--PYHLRR
--PYHLLQ
--PYHLNS
--PYHLIT
PDPYHL--
--PYHLTA
--PYHLIK
PVPYHL--
--PYHLWS
--PYHLVI
--PYHLVH
--PYHLTS
--PYHLSV
--PYHLST
--PYHLHS
--PYHLSL
--PYHLQY
--PYHLYM
--PYHLSR
--PYHLQK
--PYHLYT
--PYHLYA
--PYHLWN
--PYHLNT
--PYHLKT
--PYHLVM
--PYHLVD
--PYHLQL
--PYHLIW
PNPYHL--
--PYHLTG
--PYHLMY
PGPYHL--
--PYHLWQ
--PYHLRH
--PYHLIS

--PYHLII
--PYHLYI
--PYHLTH
--PYHLAM
--PYHLYG
--PYHLVY
--PYHLTT
--PYHLSM
--PYHLSI
--PYHLNA
--PYHLLG
--PYHLEY
--PYHLVK
--PYHLQG
--PYHLMW
--PYHLMH
--PYHLIR
--PYHLHR
--PYHLFA
PFPYHL--
--PYHLSK
--PYHLLN
--PYHLLD
--PYHLIG
--PYHLHN
--PYHLFT
--PYHLFN
PQPYHL--
--PYHLYK
--PYHLWT
--PYHLWR
--PYHLWH
--PYHLTI
--PYHLSN
--PYHLRD
--PYHLQD
--PYHLMT
--PYHLMR
--PYHLKA
--PYHLGT
--PYHLER
PMPYHL--
PHPYHL--
--PYHLVV
--PYHLVN
--PYHLSQ
--PYHLRI
--PYHLRE
--PYHLLK
--PYHLLH

--PYHLIY
--PYHLES
--PYHLAR
PYPYHL--
--PYHLWV
--PYHLVE
--PYHLTV
--PYHLRV
--PYHLQT
--PYHLLM
--PYHLKH
--PYHLHV
--PYHLEL
--PYHLAT
--PYHLWI
--PYHLWE
--PYHLTY
--PYHLTL
--PYHLTF
--PYHLTD
--PYHLRM
--PYHLQI
--PYHLMN
--PYHLKN
--PYHLKE
--PYHLIF
--PYHLEH
--PYHLED
--PYHLYS
--PYHLVQ
--PYHLTK
--PYHLQF
--PYHLIM
--PYHLHL
--PYHLFH
--PYHLFD
--PYHLAY
--PYHLAN
--PYHLAI
--PYHLWY
--PYHLQR
--PYHLMF
--PYHLKR
--PYHLKM
--PYHLKL
--PYHLGM
--PYHLGG
--PYHLEN
--PYHLAV
PEPYHL--

--PYHLVW
--PYHLRF
--PYHLQN
--PYHLIV
--PYHLGY
--PYHLGW
--PYHLFQ
--PYHLEE
--PYHLAK
--PYHLWL
--PYHLSD
--PYHLRW
--PYHLQH
--PYHLQA
--PYHLNQ
--PYHLMI
--PYHLKG
--PYHLHI
--PYHLHF
--PYHLHD
--PYHLGS
--PYHLGL
--PYHLGA
--PYHLEA
--PYHLYE
--PYHLVA
--PYHLTW
--PYHLTE
--PYHLQM
--PYHLNW
--PYHLNV
--PYHLMQ
--PYHLMM
--PYHLME
--PYHLIE
--PYHLHT
--PYHLHK
--PYHLHE
--PYHLHA
--PYHLGF
--PYHLFS
--PYHLFI
--PYHLEV
--PYHLEQ
--PYHLAQ
--PYHLAF
--PYHLAA
PKPYHL--
PIPYHL--
PCPYHL--

--PYHLYV
--PYHLYD
--PYHLWM
--PYHLWG
--PYHLSE
--PYHLQS
--PYHLNN
--PYHLKI
--PYHLHM
--PYHLHG
--PYHLAL
--PYHLAE
--PYHLAD
PAPYHL--
--PYHLYQ
--PYHLNM
--PYHLNK
--PYHLNI
--PYHLNE
--PYHLML
--PYHLMD
--PYHLMC
--PYHLMA
--PYHLLE
--PYHLKW
--PYHLKK
--PYHLKD
--PYHLHY
--PYHLFG
--PYHLET
--PYHLEI
--PYHLCN
--PYHLAH
-PPYHLS-
QRPYHL--
-QPYHLT-
-QPYHLE-
-QPYHLR-
QVPYHL--
QAPYHL--
-QPYHLV-
-QPYHLY-
QTPYHL--
-QPYHLH-
-QPYHLA-
QMPYHL--
QLPYHL--
QPPYHL--
QIPYHL--
QWPYHL--

-QPYHLL-
QHPYHL--
QKPYHL--
QYPYHL--
QNPYHL--
-QPYHLK-
-QPYHLI-
-QPYHLG-
QGPYHL--
-QPYHLF-
-QPYHLN-
QDPYHL--
RSPYHL--
-RPYHLA-
RRPYHL--
RGPYHL--
-RPYHLS-
RLPYHL--
RIPYHL--
-RPYHLR-
-RPYHLW-
RMPYHL--
-RPYHLY-
-RPYHLT-
RWPYHL--
RHPYHL--
-RPYHLQ-
-RPYHLN-
RDPYHL--
-RPYHLI-
-RPYHLV-
RYPYHL--
-RPYHLE-
-RPYHLH-
-RPYHLM-
-RPYHLG-
RPPYHL--
RAPYHL--
REPYHL--
RCPYHL--
RTPYHL--
-RPYHLF-
-RPYHLP-
SRPYHL--
SVPYHL--
-SPYHLK-
SMPYHL--
-SPYHLT-
STPYHL--
-SPYHLE-

SKPYHL--
SQPYHL--
-SPYHLR-
SYPYHL--
-SPYHLS-
SIPYHL--
-SPYHLV-
SLPYHL--
-SPYHLY-
-SPYHLI-
SFPYHL--
-SPYHLM-
SWPYHL--
-SPYHLW-
SGPYHL--
SAPYHL--
SPPYHL--
-SPYHLH-
SEPYHL--
-SPYHLQ-
-SPYHLA-
SNPYHL--
-SPYHLL-
SDPYHL--
-SPYHLN-
SHPYHL--
-SPYHLF-
SCPYHL--
TRPYHL--
TLPYHL--
TAPYHL--
-TPYHLK-
-TPYHLS-
TKPYHL--
-TPYHLR-
TNPYHL--
TVPYHL--
-TPYHLW-
-TPYHLI-
TYPYHL--
TMPYHL--
TTPYHL--
-TPYHLL-
TPPYHL--
TDPYHL--
TSPYHL--
-TPYHLY-
-TPYHLE-
TIPYHL--
TEPYHL--

-TPYHLV-
-TPYHLT-
TQPYHL--
-TPYHLF-
-TPYHLM-
-TPYHLG-
-TPYHLN-
-TPYHLH-
-TPYHLC-
-TPYHLA-
THPYHL--
TFPYHL--
-TPYHLQ-
TGPYHL--
TCPYHL--
VSPYHL--
-VPYHLS-
VQPYHL--
VGPYHL--
VYPYHL--
VWPYHL--
VTPYHL--
-VPYHLM-
-VPYHLY-
-VPYHLL-
VRPYHL--
-VPYHLR-
-VPYHLW-
-VPYHLI-
VIPYHL--
VHPYHL--
VVPYHL--
VFPYHL--
-VPYHLE-
VAPYHL--
-VPYHLV-
-VPYHLK-
-VPYHLF-
VKPYHL--
-VPYHLA-
-VPYHLH-
VNPYHL--
VEPYHL--
-VPYHLT-
-VPYHLN-
-VPYHLG-
VDPYHL--
-VPYHLQ-
-VPYHLC-
VCPYHL--

WEPYHL--
WLPYHL--
WHPYHL--
WNPYHL--
WGPYHL--
WSPYHL--
WAPYHL--
-WPYHLT-
WYPYHL--
-WPYHLH-
-WPYHLV-
-WPYHLQ-
-WPYHLI-
-WPYHLA-
WKPYHL--
WRPYHL--
WTPYHL--
WQPYHL--
WVPYHL--
-WPYHLF-
-WPYHLL-
WMPYHL--
-WPYHLK-
WWPYHL--
-WPYHLR-
-WPYHLN-
-WPYHLM-
WFPYHL--
YRPYHL--
-YPYHLS-
-YPYHLL-
YGPYHL--
-YPYHLV-
YHPYHL--
YLPYHL--
-YPYHLR-
YTPYHL--
-YPYHLI-
YNPYHL--
YYPYHL--
YVPYHL--
YIPYHL--
YKPYHL--
-YPYHLT-
YSPYHL--
YPPYHL--
-YPYHLW-
-YPYHLQ-
-YPYHLH-
-YPYHLE-

YFPYHL--
YCPYHL--
-YPYHLM-
-YPYHLA-
YWPYHL--
-YPYHLK-
YAPYHL--
-YPYHLY-
-YPYHLN-
YEPYHL--
YDPYHL--
YQPYHL--
YMPYHL--

PKGI 101 806 48.73

-DPKGIA-
MMPKGI--
ASPKGI--
-APKGIT-
-APKGIL-
-APKGIY-
-APKGII-
-APKGIW-
AMPKGI--
AWPKGI--
-APKGIS-
-APKGIF-
-APKGIA-
ATPKGI--
-APKGIH-
AGPKGI--
ARPKGI--
AQPKGI--
AKPKGI--
APPKGI--
-APKGIG-
AFPKGI--
AEPKGI--
ANPKGI--
AIPKGI--
-APKGIM-
-APKGIV-
-APKGIQ-
-APKGIE-
AVPKGI--
-APKGIC-
CQPKGI--
CLPKGI--
-CPKGIS-
-CPKGIR-
CNPKGI--

CYPKGI--
CAPKGI--
CRPKGI--
-CPKGIV-
CGPKGI--
-CPKGII-
-CPKGIF-
CKPKGI--
CFPKGI--
DKPKGI--
-DPKGIR-
-DPKGIV-
-DPKGIF-
DLPKGI--
-DPKGIL-
DHPKGI--
DYPKGI--
DTPKGI--
-DPKGII-
DPPKGI--
-DPKGIH-
DSPKGI--
DAPKGI--
DRPKGI--
-DPKGIN-
-DPKGIE-
DNPKGI--
DIPKGI--
DFPKGI--
-DPKGIW-
DMPKGI--
DGPKGI--
DVPKGI--
-DPKGIS-
ERPKGI--
-EPKGIH-
-EPKGIS-
EWPKGI--
ENPKGI--
-EPKGIR-
EHPKGI--
-EPKGIK-
-EPKGIF-
EAPKGI--
-EPKGIW-
-EPKGIY-
-EPKGIV-
-EPKGIL-
EKPKGI--
-EPKGIM-

EIPKGI--
ECPKGI--
EVPKGI--
EPPKGI--
-EPKGIT-
-EPKGIN-
EFPKGI--
EQPKGI--
ESPKGI--
EGPKGI--
ETPKGI--
ELPKGI--
FSPKGI--
FLPKGI--
FRPKGI--
-FPKGII-
FEPKGI--
-FPKGIS-
FKPKGI--
FVPKGI--
FFPKGI--
-FPKGIF-
FTPKGI--
FPPKGI--
-FPKGIV-
-FPKGIQ-
FAPKGI--
-FPKGIN-
FMPKGI--
FNPKGI--
FCPKGI--
FQPKGI--
-FPKGIH-
-FPKGIA-
-GPKGIR-
GLPKGI--
-GPKGIM-
-GPKGIL-
GRPKGI--
GSPKGI--
-GPKGII-
GMPKGI--
GPPKGI--
-GPKGIY-
GIPKGI--
-GPKGIA-
-GPKGIQ-
GGPKGI--
-GPKGIV-
GEPKGI--

GAPKGI--
GNPKGI--
-GPKGIS-
-GPKGIN-
GKPKGI--
GYPKGI--
-GPKGIH-
GHPKGI--
-GPKGIW-
-GPKGIT-
GFPKGI--
GDPKGI--
GCPKGI--
GTPKGI--
-GPKGIF-
-GPKGIE-
-GPKGIK-
-GPKGIG-
-GPKGIP-
HTPKGI--
-HPKGIR-
-HPKGIL-
HWPKGI--
-HPKGIH-
HGPKGI--
HMPKGI--
HLPKGI--
HVPKGI--
-HPKGIQ-
HYPKGI--
HSPKGI--
HDPKGI--
-HPKGIF-
-HPKGIA-
HIPKGI--
-HPKGIY-
-HPKGIV-
HKPKGI--
-HPKGIW-
HFPKGI--
HAPKGI--
-HPKGIT-
-HPKGII-
HQPKGI--
HPPKGI--
HNPKGI--
HHPKGI--
HCPKGI--
-HPKGIM-
HEPKGI--

-HPKGIN-
-HPKGIK-
ILPKGI--
INPKGI--
IPPKGI--
-IPKGIT-
IVPKGI--
ITPKGI--
-IPKGIV-
-IPKGII-
-IPKGIG-
-IPKGIY-
IWPKGI--
ISPKGI--
-IPKGIR-
-IPKGIL-
-IPKGIK-
-IPKGIE-
IHPKGI--
IAPKGI--
-IPKGIW-
-IPKGIN-
-IPKGIH-
-IPKGIA-
IFPKGI--
-IPKGIS-
-IPKGIF-
IYPKGI--
IDPKGI--
-IPKGID-
IKPKGI--
ICPKGI--
KYPKGI--
-KPKGII-
-KPKGIM-
-KPKGIS-
KDPKGI--
-KPKGIQ-
KHPKGI--
KPPKGI--
-KPKGIK-
-KPKGIH-
-KPKGIF-
KMPKGI--
KIPKGI--
KWPKGI--
KRPKGI--
KNPKGI--
KKPKGI--
-KPKGIV-

-KPKGIT-
KQPKGI--
-KPKGIW-
-KPKGIN-
-KPKGIA-
KCPKGI--
-LPKGIY-
LVPKGI--
LGPKGI--
LTPKGI--
LDPKGI--
-LPKGIR-
LAPKGI--
-LPKGIM-
LIPKGI--
-LPKGIT-
-LPKGIW-
LMPKGI--
LQPKGI--
-LPKGIS-
-LPKGIF-
-LPKGIK-
LNPKGI--
LPPKGI--
-LPKGIA-
LKPKGI--
-LPKGIN-
-LPKGIH-
-LPKGIQ-
-LPKGII-
-LPKGIE-
MFPKGI--
MLPKGI--
-MPKGIL-
MNPKGI--
MVPKGI--
-MPKGIY-
MDPKGI--
MTPKGI--
MRPKGI--
MGPKGI--
-MPKGIV-
-MPKGIK-
MWPKGI--
-MPKGIR-
-MPKGIN-
MSPKGI--
-MPKGIT-
-MPKGII-
-MPKGIW-

MKPKGI--
MEPKGI--
-MPKGIA-
-MPKGIM-
MHPKGI--
MIPKGI--
-MPKGIQ-
-MPKGIH-
-MPKGIC-
MCPKGI--
NRPKGI--
NLPKGI--
-NPKGIW-
-NPKGIY-
NFPKGI--
NHPKGI--
NGPKGI--
-NPKGII-
NTPKGI--
-NPKGIV-
-NPKGIF-
NSPKGI--
NNPKGI--
NEPKGI--
-NPKGIA-
NDPKGI--
NVPKGI--
-NPKGIT-
-NPKGIK-
NKPKGI--
NIPKGI--
NCPKGI--
NAPKGI--
-NPKGIN-
NMPKGI--
NQPKGI--
--PKGIST
--PKGIWG
--PKGISI
--PKGILH
--PKGIRM
--PKGITS
--PKGISF
--PKGILV
--PKGIRL
--PKGITT
--PKGISG
--PKGILT
--PKGIVL
--PKGISA

--PKGIYT
--PKGIIT
--PKGIVA
--PKGISS
--PKGIRG
--PKGISM
--PKGIQS
--PKGIQA
--PKGIKV
--PKGIMV
--PKGIMT
--PKGIFS
--PKGIIV
--PKGIHL
--PKGIYY
--PKGITE
PSPKGI--
--PKGITN
--PKGITA
--PKGISV
--PKGIRQ
--PKGITM
--PKGISY
PLPKGI--
--PKGINS
--PKGIMS
--PKGIKS
--PKGITY
--PKGITQ
--PKGISW
--PKGILY
--PKGIKG
--PKGIHM
--PKGIYH
--PKGIMF
--PKGILQ
--PKGIVV
--PKGINF
--PKGIMK
--PKGIMA
--PKGILN
--PKGILK
--PKGIHS
--PKGIHI
--PKGITR
--PKGILI
--PKGILF
--PKGIYR
--PKGIYI
--PKGITV

--PKGIRT
--PKGINR
--PKGINQ
--PKGIKT
--PKGIEG
--PKGIYL
--PKGIVT
--PKGISH
--PKGIMQ
--PKGIAV
--PKGIWA
--PKGIKI
--PKGIHV
--PKGIFM
--PKGIFG
--PKGIAT
PVPKGI--
--PKGIWT
--PKGIWS
--PKGIWE
--PKGIVW
--PKGIVH
--PKGITW
--PKGITL
--PKGIQR
--PKGINM
--PKGIKH
--PKGIFA
PWPKGI--
--PKGIWL
--PKGIMN
--PKGIIQ
--PKGIHW
PYPKGI--
--PKGITI
--PKGILD
--PKGIFI
--PKGIAI
--PKGIYS
--PKGIYQ
--PKGIGV
--PKGIEM
--PKGIAH
--PKGITG
--PKGIHF
--PKGIGT
--PKGIFY
--PKGIYW
--PKGIWI
--PKGISL

--PKGINV
--PKGIHT
--PKGIHR
--PKGIHQ
--PKGIFK
--PKGIWW
--PKGIVQ
--PKGIMG
--PKGILM
--PKGIIK
--PKGIII
--PKGIIA
--PKGIFT
--PKGIYV
--PKGIYN
--PKGISR
--PKGIRF
--PKGIQK
--PKGILW
--PKGIGY
PIPKGI--
PTPKGI--
PRPKGI--
--PKGIYM
--PKGIYA
--PKGIWH
--PKGIQI
--PKGIMI
--PKGIME
--PKGIGI
--PKGIFN
--PKGIEW
--PKGIAL
PQPKGI--
--PKGIYE
--PKGIWV
--PKGIVS
--PKGIVK
--PKGISE
--PKGIQV
--PKGIQN
--PKGIIF
--PKGIHK
--PKGIHG
--PKGIFQ
--PKGIEY
--PKGIAS
--PKGIAF
PGPKGI--
PAPKGI--

--PKGIVY
--PKGIRE
--PKGIRD
--PKGINI
--PKGIMY
--PKGILE
--PKGIKY
--PKGIKN
--PKGIIY
--PKGIIH
--PKGIGW
--PKGIFV
--PKGIAN
--PKGIAG
--PKGIYF
--PKGIWD
--PKGIVM
--PKGITK
--PKGISQ
--PKGIRI
--PKGIRH
--PKGIQL
--PKGIQG
--PKGINA
--PKGIMM
--PKGIMH
--PKGIGH
--PKGIES
--PKGIEH
--PKGIDA
PKPKGI--
--PKGISN
--PKGIQT
--PKGIQQ
--PKGIQF
--PKGINT
--PKGIKM
--PKGIID
--PKGIEI
--PKGIAY
--PKGIAA
--PKGIVN
--PKGIVG
--PKGIVE
--PKGITH
--PKGITF
--PKGIQM
--PKGINY
--PKGINN
--PKGILC

--PKGIKW
--PKGIKF
--PKGIKE
--PKGIKD
--PKGIGR
--PKGIGF
--PKGIGA
--PKGIFH
--PKGIFE
--PKGIEQ
--PKGIEN
--PKGIEK
--PKGIAM
--PKGIAK
PEPKGI--
PNPKGI--
--PKGIWF
--PKGIVD
--PKGINW
--PKGINK
--PKGIIM
--PKGIIE
--PKGIHY
--PKGIHA
--PKGIGQ
--PKGIGN
--PKGIGG
--PKGIFF
-PPKGIV-
-PPKGIR-
-PPKGIQ-
--PKGIYP
--PKGIYK
--PKGIYD
--PKGIWQ
--PKGISD
--PKGIQH
--PKGIQD
--PKGIPK
--PKGIPF
--PKGING
--PKGIMW
--PKGIKQ
--PKGIKK
--PKGIIP
--PKGIHH
--PKGIHD
--PKGIHC
--PKGIEE
--PKGIDS

--PKGIDG
--PKGICS
--PKGICM
--PKGICI
--PKGICD
--PKGIAQ
PFPKGI--
-QPKGIL-
QLPKGI--
-QPKGIR-
-QPKGII-
QTPKGI--
QRPKGI--
QHPKGI--
QVPKGI--
QFPKGI--
-QPKGIF-
QQPKGI--
-QPKGIW-
QYPKGI--
-QPKGIV-
-QPKGIK-
QGPKGI--
QSPKGI--
-QPKGIE-
-QPKGIA-
QIPKGI--
QDPKGI--
-QPKGIS-
QNPKGI--
QMPKGI--
QEPKGI--
QWPKGI--
QPPKGI--
-QPKGIY-
-QPKGIT-
-QPKGIG-
RAPKGI--
-RPKGIL-
RSPKGI--
-RPKGII-
-RPKGIM-
-RPKGIF-
-RPKGIA-
RLPKGI--
RTPKGI--
-RPKGIH-
RGPKGI--
-RPKGIY-
-RPKGIQ-

RCPKGI--
-RPKGIT-
RQPKGI--
RPPKGI--
-RPKGIS-
REPKGI--
-RPKGIN-
-RPKGIK-
RKPKGI--
-RPKGIV-
-RPKGIE-
-RPKGIG-
RNPKGI--
RDPKGI--
-RPKGIC-
SRPKGI--
SLPKGI--
SVPKGI--
-SPKGIL-
STPKGI--
-SPKGIS-
SWPKGI--
SMPKGI--
SYPKGI--
-SPKGIR-
-SPKGIQ-
SAPKGI--
SIPKGI--
SGPKGI--
-SPKGIK-
SSPKGI--
-SPKGIW-
-SPKGIT-
-SPKGIA-
SNPKGI--
-SPKGIH-
-SPKGIF-
SDPKGI--
-SPKGIY-
SHPKGI--
SPPKGI--
-SPKGIV-
SCPKGI--
SQPKGI--
-SPKGIN-
SEPKGI--
-SPKGIE-
-SPKGIG-
TVPKGI--
TYPKGI--

TSPKGI--
-TPKGII-
-TPKGIY-
-TPKGIS-
TGPKGI--
-TPKGIK-
TKPKGI--
-TPKGIH-
TPPKGI--
TFPKGI--
TQPKGI--
-TPKGIF-
TAPKGI--
-TPKGIV-
-TPKGIT-
-TPKGIN-
TEPKGI--
TCPKGI--
-TPKGIW-
-TPKGIE-
-TPKGIQ-
TIPKGI--
-TPKGIG-
TTPKGI--
TWPKGI--
TNPKGI--
TMPKGI--
TDPKGI--
-VPKGIT-
-VPKGIR-
VLPKGI--
VEPKGI--
-VPKGIV-
-VPKGIL-
-VPKGIW-
-VPKGII-
VFPKGI--
VYPKGI--
-VPKGIH-
VKPKGI--
-VPKGIK-
VMPKGI--
VAPKGI--
VGPKGI--
VWPKGI--
VRPKGI--
-VPKGIF-
VTPKGI--
VIPKGI--
VDPKGI--

-VPKGIN-
-VPKGIY-
-VPKGIG-
VVPKGI--
VQPKGI--
VHPKGI--
VPPKGI--
-VPKGIE-
VNPKGI--
-VPKGIA-
-VPKGIM-
-VPKGIC-
WKPKGI--
WLPKGI--
WYPKGI--
-WPKGIL-
-WPKGIT-
WAPKGI--
WQPKGI--
WRPKGI--
-WPKGIS-
WGPKGI--
-WPKGIK-
WVPKGI--
-WPKGIE-
WNPKGI--
WIPKGI--
WTPKGI--
WHPKGI--
WDPKGI--
WSPKGI--
-WPKGIW-
-WPKGIM-
-WPKGIV-
-WPKGIQ-
-WPKGIA-
-WPKGII-
-WPKGIG-
WEPKGI--
WCPKGI--
YTPKGI--
YGPKGI--
YPPKGI--
-YPKGIL-
-YPKGIT-
-YPKGIY-
-YPKGII-
YMPKGI--
YVPKGI--
YQPKGI--

YRPKGI--
-YPKGIQ-
YLPKGI--
YDPKGI--
-YPKGIM-
-YPKGIK-
-YPKGIA-
-YPKGIH-
-YPKGIF-
YEPKGI--
YHPKGI--
YWPKGI--
-YPKGIV-
YYPKGI--
-YPKGIN-
YNPKGI--
-YPKGIG-
-YPKGIE-
YIPKGI--
YCPKGI--
PWAY 344 678 48.73
ASPWAY--
-APWAYR-
-APWAYL-
-APWAYM-
APPWAY--
AKPWAY--
AGPWAY--
-APWAYT-
AAPWAY--
AVPWAY--
ARPWAY--
-APWAYA-
AQPWAY--
-APWAYG-
AMPWAY--
AYPWAY--
AWPWAY--
ALPWAY--
AHPWAY--
ATPWAY--
-APWAYN-
-APWAYK-
AIPWAY--
-APWAYS-
-APWAYQ-
AEPWAY--
-APWAYY-
-APWAYV-
-APWAYI-

-APWAYH-
CRPWAY--
CEPWAY--
-CPWAYY-
-CPWAYI-
CKPWAY--
CGPWAY--
CTPWAY--
CSPWAY--
CNPWAY--
CMPWAY--
DHPWAY--
DYPWAY--
DKPWAY--
DDPWAY--
DSPWAY--
-DPWAYI-
DFPWAY--
-DPWAYW-
-DPWAYM-
DLPWAY--
DGPWAY--
-DPWAYR-
-DPWAYL-
-DPWAYT-
-DPWAYS-
-EPWAYL-
-EPWAYI-
ESPWAY--
ERPWAY--
-EPWAYH-
EVPWAY--
EYPWAY--
ELPWAY--
EIPWAY--
-EPWAYV-
-EPWAYT-
EGPWAY--
-EPWAYS-
-EPWAYN-
EDPWAY--
EQPWAY--
-EPWAYY-
-EPWAYW-
-EPWAYR-
-EPWAYQ-
ECPWAY--
EAPWAY--
FLPWAY--
FKPWAY--

```
-FPWAYV-
FSPWAY--
FNPWAY--
FAPWAY--
FVPWAY--
FIPWAY--
FEPWAY--
FRPWAY--
-FPWAYK-
FPPWAY--
-FPWAYT-
-FPWAYW-
-FPWAYS-
-FPWAYR-
FDPWAY--
FWPWAY--
-FPWAYM-
FMPWAY--
FGPWAY--
-FPWAYH-
-FPWAYE-
GSPWAY--
GAPWAY--
-GPWAYR-
GEPWAY--
GGPWAY--
-GPWAYS-
-GPWAYI-
-GPWAYA-
-GPWAYT-
GTPWAY--
-GPWAYK-
GLPWAY--
-GPWAYH-
-GPWAYY-
GRPWAY--
-GPWAYV-
GVPWAY--
GFPWAY--
GQPWAY--
-GPWAYG-
-GPWAYF-
GMPWAY--
GHPWAY--
GDPWAY--
-GPWAYN-
-GPWAYE-
GPPWAY--
GYPWAY--
GNPWAY--
```

GIPWAY--
-GPWAYQ-
HVPWAY--
HSPWAY--
-HPWAYR-
-HPWAYL-
HRPWAY--
HGPWAY--
HLPWAY--
HTPWAY--
-HPWAYH-
-HPWAYS-
-HPWAYF-
HDPWAY--
-HPWAYY-
HEPWAY--
HAPWAY--
HIPWAY--
HCPWAY--
HQPWAY--
-HPWAYV-
-HPWAYK-
-HPWAYI-
HNPWAY--
-HPWAYQ-
-HPWAYN-
-HPWAYA-
HKPWAY--
-IPWAYT-
IRPWAY--
-IPWAYS-
ISPWAY--
IVPWAY--
-IPWAYR-
IWPWAY--
-IPWAYW-
IEPWAY--
-IPWAYI-
INPWAY--
ITPWAY--
-IPWAYV-
-IPWAYQ-
IYPWAY--
-IPWAYA-
IPPWAY--
IKPWAY--
IDPWAY--
-IPWAYG-
IGPWAY--
KNPWAY--

KMPWAY--
-KPWAYA-
KEPWAY--
KVPWAY--
-KPWAYL-
KDPWAY--
KLPWAY--
KYPWAY--
KTPWAY--
-KPWAYN-
KGPWAY--
-KPWAYM-
KHPWAY--
KKPWAY--
KAPWAY--
-KPWAYT-
-KPWAYI-
-KPWAYK-
-KPWAYH-
-KPWAYG-
-KPWAYS-
-KPWAYR-
-KPWAYY-
-KPWAYW-
KIPWAY--
KWPWAY--
KFPWAY--
LGPWAY--
-LPWAYS-
LPPWAY--
LLPWAY--
LSPWAY--
LEPWAY--
LDPWAY--
-LPWAYR-
LVPWAY--
LRPWAY--
LHPWAY--
-LPWAYH-
LAPWAY--
-LPWAYT-
-LPWAYA-
LNPWAY--
-LPWAYQ-
-LPWAYM-
LMPWAY--
LKPWAY--
LTPWAY--
LWPWAY--
-LPWAYY-

LIPWAY--
-LPWAYI-
-LPWAYE-
LQPWAY--
-LPWAYV-
LYPWAY--
-LPWAYW-
MNPWAY--
MSPWAY--
MVPWAY--
MFPWAY--
-MPWAYV-
-MPWAYK-
MHPWAY--
MTPWAY--
-MPWAYH-
-MPWAYI-
MLPWAY--
-MPWAYS-
MEPWAY--
-MPWAYT-
MGPWAY--
MAPWAY--
MMPWAY--
MKPWAY--
MDPWAY--
NRPWAY--
NYPWAY--
NDPWAY--
-NPWAYY-
NHPWAY--
NSPWAY--
NTPWAY--
NPPWAY--
NVPWAY--
-NPWAYS-
-NPWAYV-
NIPWAY--
-NPWAYI-
NFPWAY--
NAPWAY--
-NPWAYL-
NQPWAY--
-NPWAYR-
-NPWAYH-
-NPWAYA-
NGPWAY--
-NPWAYT-
-NPWAYK-
-NPWAYW-

-NPWAYG-
-NPWAYC-
--PWAYRT
--PWAYRN
--PWAYRG
--PWAYRL
--PWAYAL
--PWAYNM
--PWAYLH
--PWAYLG
PAPWAY--
--PWAYAS
--PWAYWG
PKPWAY--
--PWAYSG
PTPWAY--
--PWAYRS
--PWAYHR
--PWAYVT
--PWAYNV
--PWAYLS
--PWAYKG
--PWAYME
--PWAYIT
--PWAYFN
--PWAYSD
--PWAYRM
--PWAYQR
--PWAYGI
--PWAYKS
--PWAYYH
--PWAYRQ
--PWAYAN
--PWAYST
--PWAYAH
PEPWAY--
--PWAYVG
--PWAYRH
--PWAYQG
--PWAYML
--PWAYRD
--PWAYQT
--PWAYMA
--PWAYLR
--PWAYIR
--PWAYHK
--PWAYGS
--PWAYVH
--PWAYRA
--PWAYLN

--PWAYVS
--PWAYSQ
--PWAYIN
--PWAYTY
--PWAYHL
--PWAYGY
--PWAYGA
--PWAYAA
--PWAYTW
--PWAYTI
--PWAYSV
--PWAYQY
--PWAYQS
--PWAYQL
--PWAYNY
--PWAYLT
--PWAYAV
--PWAYYY
--PWAYYR
--PWAYSY
--PWAYRY
--PWAYNS
--PWAYIA
--PWAYAQ
--PWAYAM
--PWAYAI
--PWAYWT
--PWAYTK
--PWAYTH
--PWAYTE
--PWAYSM
--PWAYSE
--PWAYRF
--PWAYMT
--PWAYMQ
--PWAYLK
--PWAYLD
PNPWAY--
PIPWAY--
--PWAYTM
--PWAYHV
--PWAYHS
--PWAYHM
--PWAYGT
--PWAYES
--PWAYEH
PGPWAY--
PWPWAY--
--PWAYYQ
--PWAYWE

--PWAYTS
--PWAYSH
--PWAYNA
--PWAYMK
--PWAYIK
--PWAYHQ
--PWAYGG
--PWAYFS
--PWAYAF
PYPWAY--
--PWAYVV
--PWAYVN
--PWAYVM
--PWAYTN
--PWAYSK
--PWAYSI
--PWAYQW
--PWAYNI
--PWAYLW
--PWAYKW
--PWAYKN
--PWAYIQ
--PWAYIG
--PWAYGF
--PWAYAK
PHPWAY--
--PWAYYS
--PWAYWH
--PWAYVE
--PWAYVD
--PWAYTG
--PWAYQH
--PWAYMV
--PWAYKA
--PWAYFQ
--PWAYFI
--PWAYEW
--PWAYEQ
--PWAYDL
--PWAYAR
PDPWAY--
--PWAYYV
--PWAYYN
--PWAYYG
--PWAYYE
--PWAYWV
--PWAYWK
--PWAYVA
--PWAYSA
--PWAYRI

--PWAYRE
--PWAYQN
--PWAYQK
--PWAYQD
--PWAYNR
--PWAYMS
--PWAYMG
--PWAYKT
--PWAYKQ
--PWAYGH
--PWAYFW
--PWAYER
--PWAYEL
PVPWAY--
PSPWAY--
--PWAYYT
--PWAYYD
--PWAYWS
--PWAYWR
--PWAYWD
--PWAYVQ
--PWAYVK
--PWAYPY
--PWAYNN
--PWAYKV
--PWAYKF
--PWAYID
--PWAYHH
--PWAYHF
--PWAYHE
--PWAYGW
--PWAYET
--PWAYEM
--PWAYEG
PMPWAY--
--PWAYYK
--PWAYTV
--PWAYTQ
--PWAYTD
--PWAYSN
--PWAYRW
--PWAYQC
--PWAYPM
--PWAYNT
--PWAYNQ
--PWAYNE
--PWAYMW
--PWAYMM
--PWAYLE
--PWAYKL

```
--PWAYKK
--PWAYKE
--PWAYGK
--PWAYDY
--PWAYAD
PQPWAY--
-PPWAYS-
-QPWAYR-
-QPWAYL-
-QPWAYQ-
QRPWAY--
QQPWAY--
QSPWAY--
-QPWAYV-
QGPWAY--
QWPWAY--
QLPWAY--
-QPWAYT-
-QPWAYS-
-QPWAYW-
QMPWAY--
-QPWAYI-
QAPWAY--
-QPWAYY-
-QPWAYM-
QTPWAY--
-QPWAYK-
QKPWAY--
QIPWAY--
QVPWAY--
-QPWAYH-
-QPWAYF-
QPPWAY--
RSPWAY--
-RPWAYS-
-RPWAYV-
RLPWAY--
RVPWAY--
RTPWAY--
REPWAY--
-RPWAYN-
-RPWAYA-
RNPWAY--
RGPWAY--
RYPWAY--
-RPWAYQ-
-RPWAYL-
-RPWAYK-
-RPWAYW-
-RPWAYR-
```

```
-RPWAYI-
RMPWAY--
RHPWAY--
-RPWAYE-
RPPWAY--
RKPWAY--
RQPWAY--
RIPWAY--
-RPWAYG-
-RPWAYF-
RFPWAY--
RDPWAY--
-RPWAYM-
RWPWAY--
-RPWAYH-
-SPWAYR-
SRPWAY--
SVPWAY--
SKPWAY--
SSPWAY--
-SPWAYY-
-SPWAYS-
-SPWAYK-
-SPWAYQ-
STPWAY--
-SPWAYM-
SPPWAY--
-SPWAYH-
SYPWAY--
-SPWAYI-
-SPWAYW-
SLPWAY--
-SPWAYN-
-SPWAYV-
-SPWAYT-
SWPWAY--
SDPWAY--
SQPWAY--
SGPWAY--
SAPWAY--
SMPWAY--
SNPWAY--
SIPWAY--
SHPWAY--
SCPWAY--
-SPWAYA-
SFPWAY--
-SPWAYE-
SEPWAY--
-SPWAYG-
```

-TPWAYS-
TGPWAY--
TEPWAY--
-TPWAYI-
TNPWAY--
TLPWAY--
TSPWAY--
TTPWAY--
TMPWAY--
TYPWAY--
-TPWAYN-
TKPWAY--
TRPWAY--
TQPWAY--
-TPWAYQ-
-TPWAYL-
TDPWAY--
-TPWAYT-
TPPWAY--
-TPWAYA-
TFPWAY--
-TPWAYV-
-TPWAYK-
TIPWAY--
TVPWAY--
-TPWAYY-
-TPWAYM-
-TPWAYW-
-TPWAYH-
TAPWAY--
VSPWAY--
VLPWAY--
VIPWAY--
VDPWAY--
-VPWAYW-
VGPWAY--
-VPWAYS-
VKPWAY--
VFPWAY--
VTPWAY--
-VPWAYR-
-VPWAYH-
-VPWAYT-
VVPWAY--
VPPWAY--
-VPWAYI-
VRPWAY--
-VPWAYV-
-VPWAYK-
-VPWAYF-

VHPWAY--
VAPWAY--
-VPWAYN-
VMPWAY--
-VPWAYQ-
-VPWAYG-
-VPWAYE-
VWPWAY--
WEPWAY--
WRPWAY--
-WPWAYT-
WIPWAY--
WVPWAY--
WTPWAY--
WSPWAY--
-WPWAYY-
WLPWAY--
-WPWAYM-
-WPWAYI-
WDPWAY--
-WPWAYR-
-WPWAYS-
-WPWAYQ-
-WPWAYA-
WHPWAY--
WGPWAY--
-WPWAYN-
YRPWAY--
YYPWAY--
YSPWAY--
YIPWAY--
-YPWAYM-
-YPWAYS-
-YPWAYH-
YKPWAY--
YDPWAY--
YQPWAY--
-YPWAYK-
YVPWAY--
-YPWAYV-
-YPWAYW-
YTPWAY--
YMPWAY--
YGPWAY--
-YPWAYQ-
-YPWAYN-
YAPWAY--
-YPWAYG-
-YPWAYE-

PKWI 491 624 48.72

-RPKWID-
APPKWI--
AVPKWI--
ASPKWI--
-APKWIQ-
ALPKWI--
-APKWIM-
-APKWIS-
-APKWIK-
-APKWIV-
-APKWIT-
AMPKWI--
-APKWIL-
AIPKWI--
-APKWIY-
-APKWIA-
ARPKWI--
AGPKWI--
AFPKWI--
ANPKWI--
-APKWIG-
ATPKWI--
-APKWIE-
AEPKWI--
CAPKWI--
CKPKWI--
CHPKWI--
CVPKWI--
CTPKWI--
CSPKWI--
CLPKWI--
-CPKWIY-
-CPKWIV-
CMPKWI--
CGPKWI--
CFPKWI--
DRPKWI--
DEPKWI--
-DPKWIR-
DVPKWI--
DKPKWI--
DQPKWI--
-DPKWIT-
DAPKWI--
-DPKWIK-
DDPKWI--
DTPKWI--
DIPKWI--
DGPKWI--
DWPKWI--

```
-DPKWII-
-DPKWIG-
DLPKWI--
DSPKWI--
-DPKWIS-
-DPKWIL-
EAPKWI--
-EPKWIF-
-EPKWIT-
ERPKWI--
EKPKWI--
EVPKWI--
-EPKWIN-
ETPKWI--
-EPKWIL-
-EPKWIH-
ESPKWI--
-EPKWIS-
-EPKWIA-
ELPKWI--
ENPKWI--
EGPKWI--
-EPKWIR-
-EPKWIG-
-EPKWIE-
EIPKWI--
EHPKWI--
EEPKWI--
EWPKWI--
EPPKWI--
-EPKWIQ-
EFPKWI--
EDPKWI--
FSPKWI--
FLPKWI--
FGPKWI--
FIPKWI--
FQPKWI--
FTPKWI--
FAPKWI--
FNPKWI--
FMPKWI--
FHPKWI--
FPPKWI--
FDPKWI--
-FPKWIG-
-FPKWIA-
FYPKWI--
-FPKWIY-
-FPKWIK-
```

FEPKWI--
-GPKWIR-
GVPKWI--
-GPKWIG-
GYPKWI--
GEPKWI--
GSPKWI--
-GPKWIS-
GKPKWI--
-GPKWIL-
-GPKWIA-
GGPKWI--
GQPKWI--
-GPKWIK-
-GPKWII-
GLPKWI--
-GPKWIE-
GNPKWI--
GFPKWI--
-GPKWIN-
-GPKWIY-
GAPKWI--
HRPKWI--
HEPKWI--
HTPKWI--
HAPKWI--
-HPKWIS-
HNPKWI--
HMPKWI--
HIPKWI--
HSPKWI--
-HPKWIK-
HYPKWI--
-HPKWIY-
-HPKWIL-
-HPKWII-
-HPKWIA-
HLPKWI--
HWPKWI--
HQPKWI--
-HPKWIQ-
-HPKWIM-
-HPKWIG-
HPPKWI--
-HPKWIR-
HDPKWI--
IEPKWI--
IRPKWI--
ISPKWI--
IKPKWI--

-IPKWII-
IIPKWI--
IYPKWI--
IAPKWI--
IVPKWI--
-IPKWIL-
ILPKWI--
IWPKWI--
-IPKWIS-
INPKWI--
ITPKWI--
-IPKWIQ-
-IPKWIY-
-IPKWIN-
-IPKWIK-
-IPKWID-
-IPKWIA-
IHPKWI--
IFPKWI--
IDPKWI--
KLPKWI--
KEPKWI--
KMPKWI--
-KPKWIG-
-KPKWIQ-
-KPKWIF-
KIPKWI--
-KPKWIV-
-KPKWIR-
KAPKWI--
-KPKWIK-
KKPKWI--
KQPKWI--
-KPKWIY-
KNPKWI--
KYPKWI--
-KPKWIN-
-KPKWIE-
KCPKWI--
-KPKWII-
-KPKWID-
LAPKWI--
LVPKWI--
LKPKWI--
LDPKWI--
LEPKWI--
LIPKWI--
LLPKWI--
LQPKWI--
LNPKWI--

LGPKWI--
LYPKWI--
-LPKWII-
-LPKWIQ-
-LPKWIH-
-LPKWIA-
LFPKWI--
LPPKWI--
-LPKWIN-
-LPKWIM-
-LPKWIF-
-LPKWIL-
LCPKWI--
-LPKWIV-
-LPKWIT-
-LPKWIP-
MQPKWI--
MLPKWI--
MRPKWI--
MMPKWI--
MTPKWI--
MSPKWI--
MDPKWI--
MNPKWI--
MWPKWI--
MHPKWI--
-MPKWIT-
-MPKWIA-
MIPKWI--
-MPKWIQ-
MPPKWI--
-MPKWIW-
-MPKWIL-
MYPKWI--
-MPKWIF-
NWPKWI--
NGPKWI--
NPPKWI--
NSPKWI--
NQPKWI--
-NPKWIR-
NIPKWI--
-NPKWIK-
NNPKWI--
NAPKWI--
-NPKWIY-
NKPKWI--
NHPKWI--
NYPKWI--
NLPKWI--

NEPKWI--
NVPKWI--
-NPKWIT-
-NPKWIS-
-NPKWIQ-
NRPKWI--
-NPKWIV-
-NPKWII-
-NPKWIH-
NFPKWI--
--PKWIMS
--PKWILG
--PKWIVS
--PKWIRS
--PKWIVG
--PKWIMA
--PKWIGS
--PKWIKS
--PKWIAA
--PKWISQ
--PKWIMD
PVPKWI--
--PKWIRM
--PKWIGI
--PKWIMG
--PKWIFS
--PKWIAT
PLPKWI--
--PKWISN
--PKWIQG
--PKWIAS
--PKWIRV
--PKWILY
--PKWIGT
--PKWIKG
--PKWITQ
--PKWITG
--PKWITV
--PKWISA
--PKWIAK
--PKWIWN
--PKWITA
--PKWIRI
--PKWIRA
--PKWIQN
--PKWIWA
--PKWIVK
--PKWIKV
--PKWIIQ
PTPKWI--

--PKWISG
PSPKWI--
--PKWITN
--PKWIST
--PKWISE
--PKWIQK
--PKWILK
--PKWILA
--PKWIYH
--PKWIVW
--PKWITS
--PKWISK
--PKWIRQ
--PKWIKY
--PKWIKA
--PKWIAN
PFPKWI--
--PKWIYT
--PKWIYA
--PKWISV
--PKWISI
--PKWINS
--PKWIKT
--PKWIGG
--PKWIGA
--PKWIVT
--PKWIVH
--PKWITI
--PKWITH
--PKWISW
--PKWIRW
--PKWIQH
--PKWILH
--PKWIKD
--PKWIIY
--PKWIFG
--PKWITT
--PKWIMN
--PKWIMI
--PKWIKN
--PKWIIS
--PKWIIL
--PKWIHM
PIPKWI--
--PKWITY
--PKWITK
--PKWITD
--PKWIRF
--PKWIQS
--PKWILD

--PKWIIW
--PKWIIA
--PKWIHN
--PKWIGL
--PKWIFT
PQPKWI--
--PKWISM
--PKWINN
--PKWIMV
--PKWIMT
--PKWIME
--PKWIKF
--PKWIES
--PKWIEG
PHPKWI--
--PKWIYY
--PKWIYK
--PKWIWF
--PKWIVN
--PKWISL
--PKWISD
--PKWIQI
--PKWINM
--PKWILW
--PKWIKI
--PKWIHS
--PKWIEM
--PKWIYV
--PKWITM
--PKWISH
--PKWIRG
--PKWIQE
--PKWILV
--PKWILI
--PKWIKH
--PKWIIT
--PKWIIH
--PKWIHE
--PKWIGK
--PKWIAM
--PKWIVE
--PKWIVD
--PKWIRD
--PKWIQW
--PKWIQT
--PKWIQR
--PKWIQL
--PKWIQD
--PKWINY
--PKWILE

```
--PKWIKQ
--PKWIKM
--PKWIHV
--PKWIFV
--PKWIFQ
--PKWIEV
--PKWIDT
PGPKWI--
PEPKWI--
PRPKWI--
-PPKWIN-
-PPKWIA-
PNPKWI--
--PKWIYS
--PKWIYD
--PKWIWS
--PKWIVY
--PKWIVL
--PKWIVF
--PKWITF
--PKWITE
--PKWISY
--PKWIRL
--PKWIRE
--PKWIQQ
--PKWIQM
--PKWIPD
--PKWINV
--PKWINK
--PKWING
--PKWINA
--PKWIMW
--PKWILL
--PKWIKL
--PKWIIC
--PKWIHR
--PKWIHH
--PKWIHD
--PKWIHA
--PKWIGY
--PKWIGV
--PKWIGQ
--PKWIGM
--PKWIGH
--PKWIEY
--PKWIEW
--PKWIET
--PKWIAL
--PKWIAD
PKPKWI--
```

PAPKWI--
QVPKWI--
QSPKWI--
QRPKWI--
QKPKWI--
QGPKWI--
-QPKWIT-
-QPKWIK-
-QPKWIM-
-QPKWIR-
-QPKWIV-
QQPKWI--
-QPKWIN-
-QPKWIS-
QYPKWI--
-QPKWIL-
QWPKWI--
-QPKWIY-
QHPKWI--
QDPKWI--
QPPKWI--
-QPKWIQ-
QIPKWI--
-QPKWIW-
-QPKWII-
QNPKWI--
QEPKWI--
-RPKWIS-
-RPKWIT-
-RPKWIY-
REPKWI--
RLPKWI--
RQPKWI--
RPPKWI--
-RPKWIA-
RFPKWI--
RGPKWI--
-RPKWIQ-
-RPKWIL-
-RPKWII-
RKPKWI--
-RPKWIV-
-RPKWIH-
-RPKWIM-
-RPKWIW-
-RPKWIK-
RIPKWI--
RDPKWI--
RCPKWI--
SAPKWI--

SHPKWI--
STPKWI--
SKPKWI--
SLPKWI--
-SPKWIG-
SPPKWI--
SFPKWI--
SMPKWI--
-SPKWIY-
-SPKWII-
-SPKWIA-
SYPKWI--
-SPKWIW-
SDPKWI--
-SPKWIS-
SRPKWI--
-SPKWIL-
SIPKWI--
-SPKWIK-
SQPKWI--
-SPKWIT-
-SPKWIN-
SGPKWI--
SCPKWI--
-SPKWIF-
-SPKWIE-
SEPKWI--
TIPKWI--
TQPKWI--
TTPKWI--
TGPKWI--
TSPKWI--
TRPKWI--
-TPKWIR-
TMPKWI--
-TPKWIK-
-TPKWIG-
TAPKWI--
TFPKWI--
TVPKWI--
-TPKWII-
-TPKWIF-
THPKWI--
TPPKWI--
-TPKWIM-
-TPKWIE-
TKPKWI--
TDPKWI--
-TPKWIV-
TEPKWI--

```
-TPKWIT-
-TPKWIA-
TWPKWI--
TNPKWI--
VRPKWI--
VYPKWI--
VFPKWI--
VSPKWI--
VLPKWI--
VQPKWI--
VAPKWI--
VDPKWI--
VKPKWI--
VGPKWI--
VIPKWI--
VHPKWI--
VTPKWI--
-VPKWIS-
-VPKWIN-
-VPKWIM-
-VPKWIA-
-VPKWIK-
-VPKWII-
VEPKWI--
-VPKWIY-
VWPKWI--
-VPKWIG-
VNPKWI--
-VPKWIQ-
WSPKWI--
-WPKWIL-
WKPKWI--
WRPKWI--
WYPKWI--
WQPKWI--
-WPKWIV-
-WPKWIW-
-WPKWIM-
-WPKWIG-
WNPKWI--
-WPKWIQ-
WVPKWI--
WPPKWI--
-WPKWIN-
WHPKWI--
WWPKWI--
-WPKWIH-
WDPKWI--
WAPKWI--
WEPKWI--
```

	YRPKWI--		
	YQPKWI--		
	YMPKWI--		
	-YPKWIR-		
	YVPKWI--		
	YTPKWI--		
	YEPKWI--		
	YHPKWI--		
	YAPKWI--		
	YWPKWI--		
	-YPKWIV-		
	YLPKWI--		
	-YPKWIN-		
	-YPKWIM-		
	-YPKWIG-		
	YDPKWI--		
	YSPKWI--		
	-YPKWII-		
	-YPKWIH-		
	-YPKWIW-		
	-YPKWIQ-		
	-YPKWIK-		
	-YPKWIE-		
	YCPKWI--		
PQWL	303	695	48.70
	-EPQWLN-		
	--PQWLEY		
	-APQWLS-		
	ARPQWL--		
	-APQWLR-		
	-APQWLK-		
	AGPQWL--		
	APPQWL--		
	AMPQWL--		
	AIPQWL--		
	ANPQWL--		
	-APQWLL-		
	ALPQWL--		
	ATPQWL--		
	AQPQWL--		
	-APQWLG-		
	AVPQWL--		
	-APQWLT-		
	AHPQWL--		
	AFPQWL--		
	-APQWLM-		
	-APQWLY-		
	-APQWLN-		
	-APQWLD-		
	AAPQWL--		

-APQWLE-
AEPQWL--
-APQWLQ-
-APQWLI-
ASPQWL--
-APQWLA-
-APQWLV-
AKPQWL--
ADPQWL--
ACPQWL--
CQPQWL--
CRPQWL--
-CPQWLI-
CSPQWL--
CPPQWL--
CGPQWL--
-CPQWLW-
-CPQWLS-
-CPQWLR-
-CPQWLG-
CHPQWL--
CFPQWL--
DAPQWL--
DRPQWL--
-DPQWLS-
DFPQWL--
DGPQWL--
-DPQWLQ-
-DPQWLR-
DLPQWL--
DKPQWL--
DSPQWL--
-DPQWLW-
DNPQWL--
DIPQWL--
DVPQWL--
-DPQWLY-
DHPQWL--
DYPQWL--
-DPQWLT-
-DPQWLL-
-DPQWLH-
-DPQWLG-
-DPQWLA-
-EPQWLV-
-EPQWLR-
ELPQWL--
-EPQWLY-
-EPQWLT-
EKPQWL--

-EPQWLG-
ETPQWL--
-EPQWLI-
EAPQWL--
EVPQWL--
-EPQWLK-
-EPQWLS-
EGPQWL--
EQPQWL--
ENPQWL--
ERPQWL--
-EPQWLL-
EEPQWL--
EIPQWL--
FKPQWL--
-FPQWLT-
FTPQWL--
FHPQWL--
FPPQWL--
FSPQWL--
FVPQWL--
-FPQWLL-
-FPQWLA-
-FPQWLS-
FRPQWL--
-FPQWLF-
FMPQWL--
-FPQWLQ-
FFPQWL--
-FPQWLG-
-FPQWLE-
FGPQWL--
FYPQWL--
-FPQWLM-
-FPQWLK-
FNPQWL--
GSPQWL--
GRPQWL--
-GPQWLS-
-GPQWLM-
-GPQWLI-
-GPQWLL-
-GPQWLT-
GMPQWL--
GPPQWL--
GKPQWL--
-GPQWLV-
GWPQWL--
GTPQWL--
GQPQWL--

-GPQWLQ-
-GPQWLG-
-GPQWLK-
-GPQWLA-
GLPQWL--
GVPQWL--
-GPQWLH-
GAPQWL--
GYPQWL--
GEPQWL--
-GPQWLE-
-GPQWLN-
GDPQWL--
-GPQWLD-
-GPQWLC-
GFPQWL--
GCPQWL--
-HPQWLR-
HRPQWL--
-HPQWLT-
HHPQWL--
HSPQWL--
-HPQWLQ-
HDPQWL--
HGPQWL--
HFPQWL--
HQPQWL--
HLPQWL--
-HPQWLY-
-HPQWLV-
-HPQWLS-
-HPQWLI-
-HPQWLG-
-HPQWLW-
HMPQWL--
HVPQWL--
HTPQWL--
-HPQWLL-
-HPQWLK-
-HPQWLH-
HNPQWL--
ISPQWL--
IRPQWL--
-IPQWLR-
ILPQWL--
IYPQWL--
-IPQWLE-
IHPQWL--
IGPQWL--
-IPQWLT-

```
-IPQWLK-
IPPQWL--
IIPQWL--
ITPQWL--
IVPQWL--
INPQWL--
IFPQWL--
-IPQWLV-
-IPQWLA-
-IPQWLQ-
IMPQWL--
IDPQWL--
-IPQWLD-
-IPQWLN-
-IPQWLH-
IEPQWL--
ICPQWL--
KSPQWL--
KTPQWL--
KMPQWL--
KVPQWL--
-KPQWLR-
-KPQWLS-
KPPQWL--
KLPQWL--
KQPQWL--
-KPQWLN-
KNPQWL--
-KPQWLL-
-KPQWLE-
-KPQWLA-
KWPQWL--
-KPQWLT-
-KPQWLI-
-KPQWLY-
-KPQWLH-
KAPQWL--
KIPQWL--
KGPQWL--
-KPQWLM-
KHPQWL--
-KPQWLG-
KDPQWL--
-KPQWLQ-
-KPQWLD-
KCPQWL--
-LPQWLK-
LKPQWL--
LGPQWL--
LRPQWL--
```

LPPQWL--
LQPQWL--
-LPQWLR-
LSPQWL--
LMPQWL--
-LPQWLV-
LLPQWL--
LWPQWL--
LNPQWL--
LVPQWL--
LTPQWL--
-LPQWLL-
-LPQWLI-
-LPQWLH-
-LPQWLA-
LDPQWL--
LIPQWL--
-LPQWLT-
LYPQWL--
-LPQWLQ-
-LPQWLM-
-LPQWLN-
MRPQWL--
MNPQWL--
MQPQWL--
MSPQWL--
MEPQWL--
-MPQWLV-
MGPQWL--
MTPQWL--
-MPQWLQ-
MMPQWL--
MAPQWL--
-MPQWLE-
-MPQWLA-
-MPQWLT-
-MPQWLM-
-MPQWLH-
-MPQWLL-
MWPQWL--
MFPQWL--
MVPQWL--
-MPQWLG-
MLPQWL--
MHPQWL--
MKPQWL--
NKPQWL--
-NPQWLV-
NSPQWL--
NPPQWL--

```
NTPQWL--
-NPQWLK-
-NPQWLQ-
NGPQWL--
-NPQWLY-
-NPQWLN-
NIPQWL--
NMPQWL--
NHPQWL--
-NPQWLR-
-NPQWLL-
NYPQWL--
NRPQWL--
-NPQWLW-
-NPQWLT-
-NPQWLG-
NVPQWL--
-NPQWLI-
NNPQWL--
NFPQWL--
NEPQWL--
NQPQWL--
-NPQWLH-
NAPQWL--
-NPQWLE-
-NPQWLS-
NCPQWL--
--PQWLST
--PQWLTS
--PQWLRT
--PQWLSV
--PQWLRS
--PQWLSG
--PQWLLS
--PQWLKA
--PQWLTA
--PQWLVS
--PQWLLG
--PQWLGA
--PQWLAS
--PQWLVN
--PQWLRA
--PQWLIR
--PQWLMN
--PQWLIS
--PQWLVR
--PQWLGS
--PQWLRQ
--PQWLRE
--PQWLMR
```

--PQWLTG
PAPQWL--
PRPQWL--
--PQWLTK
--PQWLIG
--PQWLHG
--PQWLHA
--PQWLLN
--PQWLGV
PKPQWL--
--PQWLSA
--PQWLMI
--PQWLKT
--PQWLKK
--PQWLKH
--PQWLGT
--PQWLAG
--PQWLTM
--PQWLSN
--PQWLQT
--PQWLLV
--PQWLGL
--PQWLVG
--PQWLVA
--PQWLRV
--PQWLSD
--PQWLRN
--PQWLQR
--PQWLQG
--PQWLIT
--PQWLIH
--PQWLAR
--PQWLYN
--PQWLVD
--PQWLTQ
--PQWLSQ
--PQWLRI
--PQWLNG
--PQWLKS
--PQWLHV
--PQWLFG
PDPQWL--
--PQWLWG
--PQWLTR
--PQWLSI
--PQWLQQ
--PQWLLW
--PQWLHS
--PQWLGN
--PQWLGI

PTPQWL--
--PQWLYG
--PQWLSY
--PQWLQI
--PQWLMH
--PQWLKF
--PQWLIV
--PQWLHT
--PQWLAH
PHPQWL--
--PQWLVT
--PQWLRG
--PQWLQS
--PQWLMS
PVPQWL--
--PQWLTW
--PQWLTN
--PQWLTE
--PQWLSF
--PQWLSE
--PQWLNT
--PQWLMY
--PQWLLK
--PQWLKQ
--PQWLID
--PQWLAA
--PQWLYQ
--PQWLTD
--PQWLRW
--PQWLQV
--PQWLQN
--PQWLLD
--PQWLKI
--PQWLII
--PQWLFN
--PQWLEW
--PQWLDT
--PQWLAY
PYPQWL--
PSPQWL--
--PQWLYW
--PQWLWW
--PQWLSH
--PQWLRD
--PQWLMM
--PQWLLH
--PQWLHQ
--PQWLGY
--PQWLEG
--PQWLEA

--PQWLDS
--PQWLAN
--PQWLAM
PGPQWL--
PFPQWL--
PEPQWL--
--PQWLWS
--PQWLWR
--PQWLWH
--PQWLTV
--PQWLTH
--PQWLQH
--PQWLNA
--PQWLMA
--PQWLKW
--PQWLKV
--PQWLHH
--PQWLFM
--PQWLAQ
--PQWLAK
--PQWLAD
PNPQWL--
--PQWLYE
--PQWLIK
--PQWLIF
--PQWLHF
--PQWLGQ
--PQWLGE
--PQWLES
--PQWLER
--PQWLVH
--PQWLRH
--PQWLNV
--PQWLNQ
--PQWLNN
--PQWLND
--PQWLMW
--PQWLMK
--PQWLMF
--PQWLKG
--PQWLIW
--PQWLHM
--PQWLHD
--PQWLGR
--PQWLGH
--PQWLEV
--PQWLDG
--PQWLAE
--PQWLYH
--PQWLYD

--PQWLWL
--PQWLWI
--PQWLTT
--PQWLSW
--PQWLQE
--PQWLQC
--PQWLNE
--PQWLMD
--PQWLLE
--PQWLKM
--PQWLKD
--PQWLIP
--PQWLIN
--PQWLIE
--PQWLHI
--PQWLGM
--PQWLGF
--PQWLET
--PQWLEK
--PQWLDA
--PQWLAW
--PQWLAT
-PPQWLR-
-PPQWLM-
-QPQWLS-
QKPQWL--
-QPQWLR-
QTPQWL--
-QPQWLF-
-QPQWLK-
QRPQWL--
-QPQWLV-
QSPQWL--
-QPQWLL-
QYPQWL--
-QPQWLA-
QMPQWL--
QEPQWL--
QDPQWL--
-QPQWLT-
QLPQWL--
QIPQWL--
-QPQWLI-
-QPQWLD-
QFPQWL--
QVPQWL--
QQPQWL--
-QPQWLC-
QHPQWL--
QAPQWL--

RVPQWL--
-RPQWLV-
RSPQWL--
RTPQWL--
RRPQWL--
-RPQWLS-
RHPQWL--
RIPQWL--
-RPQWLL-
-RPQWLM-
RYPQWL--
-RPQWLN-
RGPQWL--
RMPQWL--
-RPQWLG-
RAPQWL--
-RPQWLI-
RFPQWL--
RDPQWL--
-RPQWLQ-
RNPQWL--
RWPQWL--
-RPQWLE-
-RPQWLW-
-RPQWLF-
REPQWL--
RPPQWL--
RQPQWL--
-RPQWLY-
RKPQWL--
RCPQWL--
-RPQWLD-
-SPQWLR-
-SPQWLA-
-SPQWLL-
-SPQWLS-
SGPQWL--
SSPQWL--
STPQWL--
SAPQWL--
-SPQWLG-
SWPQWL--
-SPQWLK-
-SPQWLF-
SHPQWL--
-SPQWLV-
SNPQWL--
SLPQWL--
SPPQWL--
SKPQWL--

SEPQWL--
SRPQWL--
-SPQWLM-
SYPQWL--
SFPQWL--
-SPQWLI-
-SPQWLQ-
-SPQWLE-
-SPQWLN-
-SPQWLH-
SVPQWL--
SQPQWL--
-SPQWLD-
-TPQWLR-
-TPQWLA-
-TPQWLL-
-TPQWLS-
TAPQWL--
TVPQWL--
TSPQWL--
TKPQWL--
TRPQWL--
TQPQWL--
TFPQWL--
TLPQWL--
TYPQWL--
TEPQWL--
THPQWL--
TGPQWL--
-TPQWLT-
TDPQWL--
-TPQWLG-
-TPQWLV-
TTPQWL--
-TPQWLN-
TPPQWL--
-TPQWLQ-
TWPQWL--
-TPQWLK-
-TPQWLY-
TMPQWL--
-TPQWLH-
TIPQWL--
VLPQWL--
-VPQWLT-
VVPQWL--
VGPQWL--
-VPQWLH-
-VPQWLA-
VRPQWL--

VFPQWL--
-VPQWLL-
-VPQWLG-
VTPQWL--
-VPQWLR-
-VPQWLK-
-VPQWLI-
VSPQWL--
VKPQWL--
-VPQWLE-
VAPQWL--
-VPQWLM-
VMPQWL--
VNPQWL--
VQPQWL--
VIPQWL--
VEPQWL--
VYPQWL--
-VPQWLQ-
VHPQWL--
-VPQWLD-
VWPQWL--
VPPQWL--
WSPQWL--
WFPQWL--
-WPQWLT-
-WPQWLR-
-WPQWLG-
WVPQWL--
WHPQWL--
WPPQWL--
WRPQWL--
-WPQWLA-
-WPQWLK-
WEPQWL--
-WPQWLQ-
-WPQWLH-
WNPQWL--
WYPQWL--
-WPQWLS-
-WPQWLN-
-WPQWLE-
WGPQWL--
YVPQWL--
-YPQWLA-
YGPQWL--
-YPQWLR-
-YPQWLS-
-YPQWLK-
YTPQWL--

	YYPQWL--		
	YAPQWL--		
	-YPQWLN-		
	-YPQWLH-		
	YEPQWL--		
	YQPQWL--		
	-YPQWLG-		
	YIPQWL--		
	-YPQWLT-		
	-YPQWLL-		
	YDPQWL--		
	YMPQWL--		
	YSPQWL--		
	YPPQWL--		
	YHPQWL--		
	YFPQWL--		
	-YPQWLM-		
	-YPQWLW-		
	-YPQWLI-		
	-YPQWLE-		
PYPL	22	877	48.66
	-NPYPLI-		
	--PYPLGR		
	--PYPLGP		
	ARPYPL--		
	-APYPLS-		
	-APYPLK-		
	-APYPLR-		
	-APYPLG-		
	AIPYPL--		
	AEPYPL--		
	AKPYPL--		
	AVPYPL--		
	AAPYPL--		
	-APYPLN-		
	-APYPLL-		
	-APYPLW-		
	-APYPLV-		
	-APYPLA-		
	-APYPLM-		
	-APYPLH-		
	APPYPL--		
	AQPYPL--		
	-APYPLT-		
	AHPYPL--		
	AGPYPL--		
	AYPYPL--		
	ATPYPL--		
	-APYPLY-		
	-APYPLI-		

AMPYPL--
-APYPLQ-
-APYPLF-
ALPYPL--
AWPYPL--
-APYPLE-
AFPYPL--
ADPYPL--
CLPYPL--
-CPYPLY-
-CPYPLI-
CNPYPL--
CYPYPL--
-CPYPLN-
CKPYPL--
CTPYPL--
-CPYPLK-
CRPYPL--
-CPYPLR-
-CPYPLH-
-CPYPLA-
CGPYPL--
DRPYPL--
DLPYPL--
DQPYPL--
-DPYPLL-
DMPYPL--
DFPYPL--
DTPYPL--
DAPYPL--
DHPYPL--
DVPYPL--
-DPYPLS-
DEPYPL--
-DPYPLY-
-DPYPLT-
DIPYPL--
DKPYPL--
DYPYPL--
DWPYPL--
-DPYPLW-
-DPYPLI-
-DPYPLK-
-DPYPLA-
DSPYPL--
-DPYPLV-
-DPYPLH-
EAPYPL--
ERPYPL--
-EPYPLR-

EVPYPL--
EFPYPL--
-EPYPLW-
-EPYPLI-
ETPYPL--
ELPYPL--
ESPYPL--
EKPYPL--
-EPYPLY-
-EPYPLS-
EGPYPL--
-EPYPLV-
-EPYPLA-
-EPYPLT-
-EPYPLN-
-EPYPLM-
ENPYPL--
-EPYPLF-
-EPYPLH-
EIPYPL--
-EPYPLK-
EHPYPL--
ECPYPL--
-FPYPLV-
-FPYPLM-
FGPYPL--
FSPYPL--
-FPYPLA-
-FPYPLQ-
FEPYPL--
FPPYPL--
-FPYPLT-
-FPYPLN-
FKPYPL--
FYPYPL--
FAPYPL--
FHPYPL--
FTPYPL--
FMPYPL--
FNPYPL--
FFPYPL--
FLPYPL--
FRPYPL--
-FPYPLW-
-FPYPLR-
-FPYPLG-
-FPYPLF-
-FPYPLI-
-FPYPLE-
FDPYPL--

FWPYPL--
FVPYPL--
-FPYPLY-
FIPYPL--
GRPYPL--
-GPYPLR-
GVPYPL--
GYPYPL--
GGPYPL--
-GPYPLL-
GMPYPL--
GTPYPL--
-GPYPLV-
-GPYPLS-
-GPYPLN-
-GPYPLM-
GLPYPL--
-GPYPLY-
-GPYPLW-
GWPYPL--
-GPYPLA-
-GPYPLI-
GEPYPL--
GKPYPL--
GNPYPL--
-GPYPLG-
GHPYPL--
GQPYPL--
-GPYPLK-
GPPYPL--
GIPYPL--
GFPYPL--
-GPYPLQ-
GDPYPL--
GAPYPL--
-GPYPLE-
HRPYPL--
-HPYPLR-
HVPYPL--
-HPYPLQ-
-HPYPLN-
-HPYPLT-
HSPYPL--
-HPYPLV-
HMPYPL--
-HPYPLH-
-HPYPLL-
HGPYPL--
HYPYPL--
HQPYPL--

-HPYPLM-
HPPYPL--
HLPYPL--
HAPYPL--
HTPYPL--
HNPYPL--
HHPYPL--
HEPYPL--
-HPYPLY-
-HPYPLI-
HFPYPL--
-HPYPLG-
-HPYPLF-
-HPYPLK-
-HPYPLS-
-HPYPLE-
HIPYPL--
-IPYPLS-
-IPYPLL-
-IPYPLT-
IHPYPL--
IFPYPL--
IEPYPL--
IQPYPL--
ITPYPL--
IYPYPL--
-IPYPLR-
IIPYPL--
-IPYPLV-
IAPYPL--
IVPYPL--
INPYPL--
-IPYPLY-
-IPYPLM-
-IPYPLI-
ISPYPL--
-IPYPLW-
-IPYPLE-
IWPYPL--
IPPYPL--
IMPYPL--
ILPYPL--
ICPYPL--
-IPYPLQ-
-IPYPLN-
-IPYPLA-
IDPYPL--
IGPYPL--
IRPYPL--
IKPYPL--

-IPYPLP-
-IPYPLG-
KLPYPL--
KGPYPL--
KAPYPL--
KYPYPL--
-KPYPLR-
-KPYPLL-
KRPYPL--
-KPYPLV-
KPPYPL--
KVPYPL--
-KPYPLA-
-KPYPLI-
-KPYPLH-
KIPYPL--
-KPYPLW-
-KPYPLK-
-KPYPLQ-
KKPYPL--
-KPYPLG-
KQPYPL--
KMPYPL--
KHPYPL--
-KPYPLF-
-KPYPLM-
KNPYPL--
KDPYPL--
-KPYPLY-
-KPYPLN-
-KPYPLE-
KEPYPL--
-KPYPLT-
-KPYPLS-
LLPYPL--
-LPYPLM-
LTPYPL--
-LPYPLT-
LGPYPL--
-LPYPLS-
LQPYPL--
LPPYPL--
LVPYPL--
LNPYPL--
LYPYPL--
-LPYPLQ-
LFPYPL--
LKPYPL--
LDPYPL--
-LPYPLA-

LEPYPL--
LAPYPL--
-LPYPLH-
LWPYPL--
-LPYPLI-
-LPYPLN-
LHPYPL--
-LPYPLK-
-LPYPLY-
-LPYPLV-
LIPYPL--
-LPYPLG-
-LPYPLE-
-LPYPLW-
-LPYPLF-
LCPYPL--
MRPYPL--
MGPYPL--
-MPYPLR-
-MPYPLK-
-MPYPLV-
MKPYPL--
MVPYPL--
-MPYPLS-
MMPYPL--
-MPYPLQ-
MHPYPL--
MDPYPL--
MSPYPL--
-MPYPLL-
MLPYPL--
MFPYPL--
MQPYPL--
-MPYPLI-
-MPYPLF-
MIPYPL--
-MPYPLG-
MTPYPL--
-MPYPLA-
-MPYPLH-
MYPYPL--
-MPYPLW-
MNPYPL--
MEPYPL--
MAPYPL--
-MPYPLT-
MWPYPL--
-MPYPLE-
NLPYPL--
-NPYPLF-

NSPYPL--
-NPYPLR-
-NPYPLV-
-NPYPLL-
NRPYPL--
NVPYPL--
NQPYPL--
NPPYPL--
-NPYPLH-
-NPYPLN-
-NPYPLQ-
-NPYPLM-
NNPYPL--
-NPYPLS-
NGPYPL--
NAPYPL--
-NPYPLT-
NWPYPL--
NTPYPL--
-NPYPLA-
NKPYPL--
NFPYPL--
NYPYPL--
-NPYPLY-
-NPYPLW-
NIPYPL--
NDPYPL--
NMPYPL--
-NPYPLK-
NCPYPL--
--PYPLVS
--PYPLSY
--PYPLIT
--PYPLRR
PSPYPL--
--PYPLSR
--PYPLKS
--PYPLRY
--PYPLIA
--PYPLRA
--PYPLLT
--PYPLLS
--PYPLWY
--PYPLTL
--PYPLQS
--PYPLLR
--PYPLAY
--PYPLSS
--PYPLGL
--PYPLIK

--PYPLSW
--PYPLLN
--PYPLGV
--PYPLWG
--PYPLSA
--PYPLKA
--PYPLIG
--PYPLYN
--PYPLVR
--PYPLVK
--PYPLVY
--PYPLTM
--PYPLKG
--PYPLFS
--PYPLRS
--PYPLLG
--PYPLYM
--PYPLVH
--PYPLWS
--PYPLTI
--PYPLSI
--PYPLRQ
--PYPLYG
--PYPLVI
--PYPLTS
--PYPLLA
--PYPLKR
--PYPLIS
--PYPLIN
--PYPLHR
--PYPLGS
--PYPLWL
--PYPLSH
--PYPLRE
--PYPLAW
--PYPLVM
--PYPLRI
--PYPLML
--PYPLKL
--PYPLAS
--PYPLTF
--PYPLMW
--PYPLHL
--PYPLYR
--PYPLTR
--PYPLRH
--PYPLNL
--PYPLIQ
--PYPLAG
--PYPLWK

--PYPLQL
--PYPLIR
--PYPLGF
--PYPLFG
--PYPLYL
--PYPLST
--PYPLPH
--PYPLLH
--PYPLEA
--PYPLDL
--PYPLWA
--PYPLVT
--PYPLMS
--PYPLKN
PLPYPL--
--PYPLWV
--PYPLTG
--PYPLTA
--PYPLME
--PYPLLK
PKPYPL--
PFPYPL--
--PYPLWH
--PYPLTV
--PYPLSF
--PYPLRN
--PYPLNI
--PYPLMI
--PYPLMF
--PYPLEF
PQPYPL--
--PYPLTQ
--PYPLRK
--PYPLPM
--PYPLLW
--PYPLKK
--PYPLIF
--PYPLHQ
--PYPLGN
--PYPLFT
--PYPLFK
--PYPLAF
--PYPLPS
--PYPLNM
--PYPLLM
--PYPLEI
--PYPLAT
--PYPLAQ
--PYPLYS
--PYPLYD

--PYPLWN
--PYPLVA
--PYPLPA
--PYPLMT
--PYPLLE
--PYPLKY
--PYPLFW
--PYPLYA
--PYPLSN
--PYPLQV
--PYPLIM
--PYPLIH
--PYPLHS
--PYPLGQ
--PYPLFQ
--PYPLDQ
--PYPLAI
--PYPLYI
--PYPLVV
--PYPLRM
--PYPLQA
--PYPLNT
--PYPLMV
--PYPLMQ
--PYPLGH
PAPYPL--
--PYPLWT
--PYPLVQ
--PYPLSV
--PYPLSM
--PYPLSG
--PYPLRD
--PYPLQI
--PYPLNY
--PYPLNA
--PYPLHN
--PYPLEM
--PYPLAR
--PYPLYQ
--PYPLYK
--PYPLWR
--PYPLND
--PYPLMK
--PYPLMH
--PYPLMG
--PYPLIV
--PYPLIE
--PYPLHT
--PYPLHG
--PYPLGA

--PYPLES
--PYPLAN
--PYPLAK
PGPYPL--
PYPYPL--
--PYPLYW
--PYPLVN
--PYPLVD
--PYPLTY
--PYPLSK
--PYPLRW
--PYPLQY
--PYPLPY
--PYPLIY
--PYPLHY
--PYPLGW
--PYPLGT
--PYPLFH
--PYPLDY
--PYPLAL
--PYPLYH
--PYPLTT
--PYPLTN
--PYPLTK
--PYPLTH
--PYPLTE
--PYPLQR
--PYPLQN
--PYPLHM
--PYPLET
--PYPLDM
--PYPLAA
--PYPLYT
--PYPLWE
--PYPLVF
--PYPLPT
--PYPLNW
--PYPLNS
--PYPLNG
--PYPLMY
--PYPLMN
--PYPLLD
--PYPLKM
--PYPLHK
--PYPLFF
--PYPLEW
--PYPLEQ
--PYPLEG
--PYPLDF
PIPYPL--

--PYPLYY
--PYPLWQ
--PYPLSD
--PYPLQK
--PYPLQD
--PYPLNF
--PYPLKI
--PYPLEL
--PYPLDH
--PYPLCS
PWPYPL--
PMPYPL--
--PYPLTD
--PYPLRP
--PYPLPV
--PYPLPF
--PYPLNV
--PYPLMD
--PYPLKH
--PYPLHV
--PYPLGK
--PYPLGG
--PYPLFN
--PYPLFE
PVPYPL--
PEPYPL--
--PYPLYP
--PYPLYE
--PYPLWD
--PYPLVW
--PYPLVE
--PYPLTW
--PYPLSQ
--PYPLQM
--PYPLQG
--PYPLPN
--PYPLNR
--PYPLMM
--PYPLFR
--PYPLFD
--PYPLEV
--PYPLCN
--PYPLAH
PTPYPL--
PNPYPL--
--PYPLVG
--PYPLSE
--PYPLQW
--PYPLQQ
--PYPLQH

```
--PYPLQF
--PYPLPW
--PYPLPI
--PYPLNQ
--PYPLKW
--PYPLKT
--PYPLKQ
--PYPLKE
--PYPLHF
--PYPLHE
--PYPLHA
--PYPLFP
--PYPLFC
--PYPLFA
--PYPLEY
--PYPLEN
--PYPLEH
--PYPLDV
--PYPLCW
--PYPLCV
--PYPLCH
--PYPLCA
--PYPLAV
-PPYPLL-
PHPYPL--
-QPYPLL-
QRPYPL--
-QPYPLY-
-QPYPLV-
-QPYPLT-
QFPYPL--
-QPYPLR-
-QPYPLS-
QNPYPL--
-QPYPLG-
-QPYPLN-
-QPYPLH-
-QPYPLQ-
QWPYPL--
QSPYPL--
-QPYPLW-
-QPYPLK-
QLPYPL--
QIPYPL--
QGPYPL--
QQPYPL--
-QPYPLI-
QAPYPL--
QTPYPL--
-QPYPLM-
```

```
-QPYPLA-
-QPYPLF-
-QPYPLE-
-QPYPLC-
QMPYPL--
QKPYPL--
QHPYPL--
QEPYPL--
RTPYPL--
RSPYPL--
RYPYPL--
RVPYPL--
-RPYPLW-
RFPYPL--
-RPYPLS-
-RPYPLL-
-RPYPLI-
RGPYPL--
RNPYPL--
-RPYPLK-
RMPYPL--
-RPYPLE-
-RPYPLF-
RIPYPL--
-RPYPLT-
RRPYPL--
RAPYPL--
-RPYPLV-
REPYPL--
-RPYPLH-
RQPYPL--
RPPYPL--
-RPYPLN-
-RPYPLG-
RWPYPL--
-RPYPLQ-
-RPYPLY-
RDPYPL--
RHPYPL--
RCPYPL--
STPYPL--
-SPYPLR-
-SPYPLL-
SAPYPL--
SRPYPL--
SSPYPL--
-SPYPLS-
-SPYPLF-
-SPYPLQ-
SYPYPL--
```

SVPYPL--
-SPYPLK-
SNPYPL--
SMPYPL--
SHPYPL--
SWPYPL--
-SPYPLY-
-SPYPLN-
SIPYPL--
-SPYPLA-
SKPYPL--
SGPYPL--
SFPYPL--
-SPYPLM-
SDPYPL--
-SPYPLH-
-SPYPLV-
-SPYPLE-
SPPYPL--
SEPYPL--
SQPYPL--
-SPYPLG-
SCPYPL--
-SPYPLW-
-TPYPLR-
-TPYPLV-
TAPYPL--
TSPYPL--
-TPYPLL-
THPYPL--
TVPYPL--
TEPYPL--
TRPYPL--
TTPYPL--
-TPYPLK-
-TPYPLM-
-TPYPLF-
-TPYPLY-
-TPYPLI-
TGPYPL--
TLPYPL--
TMPYPL--
TYPYPL--
-TPYPLW-
-TPYPLS-
TKPYPL--
-TPYPLA-
TFPYPL--
TQPYPL--
-TPYPLE-

TWPYPL--
-TPYPLQ-
-TPYPLH-
TIPYPL--
-TPYPLN-
-TPYPLT-
TPPYPL--
VYPYPL--
VGPYPL--
VSPYPL--
-VPYPLT-
-VPYPLS-
-VPYPLL-
VQPYPL--
VIPYPL--
VTPYPL--
-VPYPLR-
-VPYPLA-
-VPYPLV-
VPPYPL--
VVPYPL--
VFPYPL--
VEPYPL--
VAPYPL--
-VPYPLH-
VRPYPL--
-VPYPLQ-
VHPYPL--
-VPYPLI-
-VPYPLY-
-VPYPLF-
VLPYPL--
VMPYPL--
VDPYPL--
VKPYPL--
-VPYPLW-
-VPYPLG-
-VPYPLM-
-VPYPLE-
-VPYPLN-
VWPYPL--
WSPYPL--
WTPYPL--
WLPYPL--
-WPYPLT-
-WPYPLL-
WQPYPL--
WNPYPL--
WEPYPL--
WYPYPL--

-WPYPLS-
-WPYPLI-
-WPYPLA-
WAPYPL--
WKPYPL--
WRPYPL--
-WPYPLR-
-WPYPLQ-
WMPYPL--
-WPYPLW-
-WPYPLF-
WVPYPL--
-WPYPLK-
-WPYPLE-
WFPYPL--
-WPYPLH-
-WPYPLM-
WGPYPL--
YSPYPL--
YTPYPL--
YLPYPL--
YKPYPL--
-YPYPLM-
YNPYPL--
-YPYPLT-
YHPYPL--
YVPYPL--
-YPYPLV-
-YPYPLH-
-YPYPLS-
-YPYPLQ-
YAPYPL--
YYPYPL--
YMPYPL--
YEPYPL--
YPPYPL--
YIPYPL--
YFPYPL--
-YPYPLW-
YGPYPL--
YDPYPL--
YQPYPL--
-YPYPLA-
YWPYPL--
-YPYPLL-
-YPYPLI-
YCPYPL--
-YPYPLY-

PFGI 153 774 48.61

-GPFGIE-

--PFGIRP
VDPFGI--
ATPFGI--
-APFGIR-
ARPFGI--
-APFGIT-
-APFGIL-
-APFGIK-
AKPFGI--
AYPFGI--
-APFGIS-
-APFGIM-
AHPFGI--
-APFGIA-
ANPFGI--
ALPFGI--
-APFGIH-
AGPFGI--
AWPFGI--
AIPFGI--
AAPFGI--
-APFGIV-
-APFGIE-
AMPFGI--
-APFGIQ-
-APFGIG-
AFPFGI--
AVPFGI--
AQPFGI--
-APFGIC-
AEPFGI--
ACPFGI--
ASPFGI--
APPFGI--
CGPFGI--
CRPFGI--
-CPFGIT-
-CPFGIH-
CLPFGI--
CVPFGI--
CPPFGI--
-CPFGIR-
-CPFGIK-
CEPFGI--
CCPFGI--
CSPFGI--
-CPFGIW-
-CPFGIQ-
-CPFGIL-
-CPFGII-

−CPFGIF−
CNPFGI−−
CIPFGI−−
−DPFGIR−
DWPFGI−−
−DPFGIM−
DYPFGI−−
DVPFGI−−
DLPFGI−−
DFPFGI−−
DAPFGI−−
DRPFGI−−
−DPFGIT−
DGPFGI−−
−DPFGIL−
DNPFGI−−
DMPFGI−−
−DPFGII−
DKPFGI−−
DCPFGI−−
−DPFGIQ−
−DPFGIH−
DEPFGI−−
ERPFGI−−
−EPFGIR−
EHPFGI−−
ESPFGI−−
−EPFGIV−
−EPFGIL−
EKPFGI−−
EIPFGI−−
EVPFGI−−
−EPFGIT−
−EPFGIK−
−EPFGIH−
ELPFGI−−
EGPFGI−−
EYPFGI−−
−EPFGIS−
−EPFGIE−
ETPFGI−−
EQPFGI−−
EEPFGI−−
EMPFGI−−
EDPFGI−−
EAPFGI−−
FSPFGI−−
FKPFGI−−
FRPFGI−−
FLPFGI−−

FAPFGI--
-FPFGIR-
FGPFGI--
-FPFGIM-
-FPFGIA-
FTPFGI--
-FPFGIN-
FDPFGI--
-FPFGIS-
FNPFGI--
FFPFGI--
FPPFGI--
-FPFGIT-
FHPFGI--
FMPFGI--
FQPFGI--
FYPFGI--
-FPFGIY-
-FPFGIH-
-FPFGIQ-
FIPFGI--
-GPFGIL-
GRPFGI--
-GPFGIR-
GVPFGI--
GYPFGI--
-GPFGIS-
GEPFGI--
GGPFGI--
GAPFGI--
GLPFGI--
GSPFGI--
GMPFGI--
GNPFGI--
GTPFGI--
GDPFGI--
-GPFGIH-
GKPFGI--
-GPFGII-
-GPFGIA-
-GPFGIT-
-GPFGIQ-
-GPFGIK-
GQPFGI--
GIPFGI--
-GPFGIV-
GHPFGI--
GFPFGI--
GWPFGI--
-GPFGIM-

GCPFGI--
GPPFGI--
HKPFGI--
HTPFGI--
HVPFGI--
HYPFGI--
HRPFGI--
-HPFGIS-
-HPFGIA-
-HPFGIV-
HSPFGI--
-HPFGIQ-
HIPFGI--
-HPFGIT-
HWPFGI--
-HPFGIE-
HGPFGI--
HPPFGI--
HFPFGI--
-HPFGIN-
-HPFGIK-
HNPFGI--
HLPFGI--
HCPFGI--
HQPFGI--
-IPFGIV-
-IPFGIR-
IEPFGI--
IVPFGI--
ISPFGI--
-IPFGIM-
IMPFGI--
-IPFGIQ-
-IPFGIH-
-IPFGIK-
IFPFGI--
IWPFGI--
ITPFGI--
-IPFGIT-
IRPFGI--
-IPFGIS-
ILPFGI--
IYPFGI--
IKPFGI--
-IPFGIG-
ICPFGI--
-IPFGIN-
-IPFGII-
IDPFGI--
-IPFGIL-

```
-IPFGIE-
INPFGI--
IGPFGI--
IHPFGI--
IAPFGI--
KSPFGI--
-KPFGIL-
-KPFGIR-
-KPFGIV-
KPPFGI--
KQPFGI--
-KPFGIA-
-KPFGIH-
-KPFGIT-
-KPFGIS-
-KPFGIG-
KWPFGI--
KGPFGI--
KVPFGI--
KHPFGI--
KTPFGI--
KLPFGI--
KDPFGI--
KNPFGI--
KRPFGI--
-KPFGIN-
-KPFGIM-
KIPFGI--
KFPFGI--
-KPFGII-
KAPFGI--
-KPFGIF-
-KPFGIQ-
KMPFGI--
-KPFGIK-
KEPFGI--
LSPFGI--
LRPFGI--
LYPFGI--
LAPFGI--
-LPFGIR-
LKPFGI--
-LPFGIH-
-LPFGIS-
-LPFGIV-
-LPFGIM-
-LPFGII-
LNPFGI--
LHPFGI--
LTPFGI--
```

−LPFGIK−
−LPFGIQ−
−LPFGIN−
LIPFGI−−
LQPFGI−−
LFPFGI−−
LDPFGI−−
−LPFGIT−
LVPFGI−−
LPPFGI−−
LWPFGI−−
LEPFGI−−
LCPFGI−−
−LPFGIA−
−LPFGIG−
−LPFGIE−
MRPFGI−−
MNPFGI−−
MGPFGI−−
MAPFGI−−
−MPFGIL−
MVPFGI−−
MTPFGI−−
MYPFGI−−
−MPFGIR−
−MPFGIT−
−MPFGIS−
MMPFGI−−
−MPFGIV−
MIPFGI−−
MSPFGI−−
−MPFGIA−
−MPFGIM−
MWPFGI−−
−MPFGIN−
MKPFGI−−
MDPFGI−−
−MPFGIE−
MPPFGI−−
−MPFGIQ−
−MPFGII−
−MPFGIH−
MHPFGI−−
NLPFGI−−
NRPFGI−−
NSPFGI−−
NWPFGI−−
−NPFGII−
NKPFGI−−
NAPFGI−−

NFPFGI--
-NPFGIT-
NPPFGI--
-NPFGIH-
NVPFGI--
NTPFGI--
NGPFGI--
-NPFGIS-
-NPFGIR-
NEPFGI--
-NPFGIV-
-NPFGIK-
NQPFGI--
NHPFGI--
NYPFGI--
NNPFGI--
-NPFGIN-
-NPFGIQ-
-NPFGIM-
-NPFGIE-
-NPFGIA-
--PFGITG
--PFGIRA
--PFGIWG
--PFGIRS
--PFGIVR
--PFGITR
--PFGIHR
--PFGIMS
--PFGISG
--PFGIKS
--PFGILG
--PFGILH
--PFGIVT
--PFGIRT
--PFGIKR
--PFGIIR
--PFGITL
--PFGILR
--PFGIRL
--PFGISK
--PFGIST
--PFGIRV
--PFGIRG
--PFGIRF
--PFGIYN
--PFGISI
--PFGISA
--PFGIMR
--PFGIIT

```
--PFGIRN
--PFGILV
--PFGIHL
--PFGIFR
--PFGISR
--PFGIRR
--PFGIKK
--PFGIHT
--PFGIFN
--PFGIRK
--PFGIRD
--PFGIQS
--PFGILK
--PFGIKW
--PFGIIS
--PFGIRY
--PFGIMG
--PFGIYR
--PFGIAN
--PFGIAA
--PFGIVY
--PFGIHQ
--PFGITT
--PFGISM
--PFGILE
--PFGIKI
--PFGIGS
--PFGIEG
PYPFGI--
--PFGIVI
--PFGITN
--PFGISS
--PFGING
--PFGIKQ
--PFGIIV
--PFGITV
--PFGINT
--PFGIIG
--PFGITW
--PFGITQ
--PFGISL
--PFGISF
--PFGIFG
PSPFGI--
--PFGIYK
--PFGITK
--PFGITH
--PFGIMH
--PFGIFD
--PFGIAY
```

--PFGIAW
--PFGIAV
--PFGIAG
PAPFGI--
--PFGITM
--PFGIQQ
--PFGIKN
--PFGIAM
PTPFGI--
--PFGIVM
--PFGINY
--PFGINA
--PFGIHS
--PFGIVN
--PFGIRH
--PFGILN
--PFGIHG
PDPFGI--
PVPFGI--
PIPFGI--
PGPFGI--
--PFGITE
--PFGITA
--PFGIQN
--PFGIQM
--PFGIMY
--PFGIMN
--PFGIMD
--PFGIHV
--PFGIHA
--PFGIGG
--PFGIES
--PFGIEM
PRPFGI--
PQPFGI--
--PFGIVV
--PFGIVQ
--PFGIVG
--PFGIVA
--PFGITY
--PFGIRE
--PFGIMQ
--PFGILM
--PFGIIM
--PFGIER
--PFGIEI
--PFGIEF
--PFGIWD
--PFGIVK
--PFGISY

--PFGINW
--PFGILS
--PFGIIK
--PFGIII
--PFGIIE
--PFGIHY
--PFGIFE
--PFGIEY
--PFGIAS
PKPFGI--
--PFGIYH
--PFGIWE
--PFGIVH
--PFGIVD
--PFGITI
--PFGITF
--PFGIQK
--PFGIQG
--PFGINS
--PFGINN
--PFGIMW
--PFGILQ
--PFGIKE
--PFGIIY
--PFGIGT
PNPFGI--
--PFGIYT
--PFGIYD
--PFGIVW
--PFGITS
--PFGITD
--PFGISQ
--PFGIQH
--PFGINV
--PFGINR
--PFGIMK
--PFGIKV
--PFGIKM
--PFGIKL
--PFGIHN
--PFGIHK
--PFGIFQ
--PFGIEH
--PFGIAK
--PFGIAH
PEPFGI--
PHPFGI--
--PFGIWK
--PFGISW
--PFGIRQ

--PFGIQT
--PFGIQL
--PFGILD
--PFGIKT
--PFGIKH
--PFGIKG
--PFGIID
--PFGIHD
--PFGIGI
--PFGIGF
--PFGIGA
--PFGIAT
--PFGIAR
PWPFGI--
--PFGIYY
--PFGIWN
--PFGISN
--PFGIQR
--PFGIQF
--PFGIQA
--PFGINF
--PFGIMI
--PFGIIA
--PFGIHF
--PFGIGW
--PFGIGR
--PFGIGN
--PFGIGH
--PFGIFK
--PFGIFH
--PFGIEQ
--PFGIDA
-PPFGIL-
--PFGIVE
--PFGIRI
--PFGIQV
--PFGIQI
--PFGINQ
--PFGINP
--PFGINL
--PFGINK
--PFGINH
--PFGIND
--PFGIME
--PFGILC
--PFGIKF
--PFGIHM
--PFGIHI
--PFGIGV
--PFGIFW

--PFGIEW
--PFGIET
--PFGIEN
--PFGIEL
--PFGIDS
--PFGIDM
--PFGICL
--PFGIAQ
--PFGIAL
--PFGIAI
PCPFGI--
QPPFGI--
-QPFGIR-
-QPFGIV-
-QPFGIL-
QVPFGI--
-QPFGIT-
QFPFGI--
-QPFGIH-
-QPFGIS-
-QPFGIM-
QQPFGI--
-QPFGIA-
QRPFGI--
QNPFGI--
QAPFGI--
QSPFGI--
-QPFGIN-
QMPFGI--
-QPFGIK-
QWPFGI--
QLPFGI--
QHPFGI--
QYPFGI--
-QPFGIG-
QEPFGI--
QDPFGI--
QTPFGI--
-QPFGIQ-
QKPFGI--
QIPFGI--
-QPFGII-
-QPFGID-
RSPFGI--
RTPFGI--
RFPFGI--
RVPFGI--
RGPFGI--
RPPFGI--
RAPFGI--

RRPFGI--
-RPFGIS-
RLPFGI--
-RPFGII-
RMPFGI--
RKPFGI--
-RPFGIK-
-RPFGIV-
-RPFGIT-
-RPFGIM-
RNPFGI--
RIPFGI--
-RPFGIN-
RQPFGI--
RYPFGI--
RWPFGI--
-RPFGIQ-
REPFGI--
-RPFGIE-
-RPFGIG-
RHPFGI--
RDPFGI--
RCPFGI--
-SPFGIR-
SSPFGI--
SKPFGI--
SGPFGI--
-SPFGIK-
SRPFGI--
-SPFGII-
SMPFGI--
SIPFGI--
SLPFGI--
-SPFGIS-
SDPFGI--
-SPFGIN-
STPFGI--
-SPFGIT-
SNPFGI--
-SPFGIL-
-SPFGIM-
SYPFGI--
SWPFGI--
SPPFGI--
SAPFGI--
SVPFGI--
-SPFGIQ-
-SPFGIH-
SEPFGI--
SQPFGI--

SHPFGI--
-SPFGIE-
-SPFGIG-
-SPFGIA-
-SPFGIC-
SCPFGI--
TRPFGI--
-TPFGIR-
TTPFGI--
-TPFGIS-
TSPFGI--
TYPFGI--
TVPFGI--
-TPFGIT-
-TPFGIW-
TQPFGI--
-TPFGIL-
THPFGI--
-TPFGIH-
-TPFGII-
TFPFGI--
TMPFGI--
TGPFGI--
-TPFGIK-
TKPFGI--
TEPFGI--
TDPFGI--
TAPFGI--
-TPFGIA-
TNPFGI--
TWPFGI--
TPPFGI--
TIPFGI--
-TPFGIG-
-TPFGIQ-
-TPFGIE-
TCPFGI--
-VPFGIS-
-VPFGIR-
-VPFGIL-
VKPFGI--
VSPFGI--
VLPFGI--
VHPFGI--
VYPFGI--
VPPFGI--
-VPFGIV-
VIPFGI--
VVPFGI--
VTPFGI--

VRPFGI--
VFPFGI--
VAPFGI--
VWPFGI--
-VPFGIN-
-VPFGIG-
-VPFGIT-
-VPFGIH-
VNPFGI--
-VPFGIM-
-VPFGIA-
-VPFGII-
-VPFGIQ-
VCPFGI--
VQPFGI--
-VPFGIE-
-WPFGIR-
WGPFGI--
WQPFGI--
WSPFGI--
WPPFGI--
WKPFGI--
-WPFGIK-
-WPFGIH-
-WPFGIQ-
-WPFGII-
WRPFGI--
WLPFGI--
-WPFGIN-
WWPFGI--
-WPFGIS-
WYPFGI--
WIPFGI--
WVPFGI--
WNPFGI--
-WPFGIG-
-WPFGIV-
-WPFGIL-
-WPFGIF-
WDPFGI--
WMPFGI--
WCPFGI--
YMPFGI--
-YPFGIR-
YLPFGI--
YSPFGI--
YEPFGI--
YAPFGI--
-YPFGIV-
YRPFGI--

	-YPFGIS-		
	-YPFGIH-		
	-YPFGIT-		
	-YPFGIL-		
	YFPFGI--		
	YKPFGI--		
	-YPFGIA-		
	YGPFGI--		
	YDPFGI--		
	YYPFGI--		
	-YPFGIQ-		
	YNPFGI--		
	YVPFGI--		
	YQPFGI--		
	YPPFGI--		
	YTPFGI--		
	-YPFGIM-		
	-YPFGII-		
	YHPFGI--		
	YWPFGI--		
	-YPFGIW-		
	-YPFGIK-		
	-YPFGIG-		
PFGY	128	792	48.60
	--PFGYLH		
	--PFGYGR		
	--PFGYEL		
	-WPFGYS-		
	-APFGYR-		
	-APFGYM-		
	ALPFGY--		
	ADPFGY--		
	ATPFGY--		
	ARPFGY--		
	-APFGYI-		
	-APFGYH-		
	AGPFGY--		
	-APFGYN-		
	-APFGYL-		
	AMPFGY--		
	-APFGYS-		
	AWPFGY--		
	ASPFGY--		
	-APFGYK-		
	AVPFGY--		
	APPFGY--		
	-APFGYQ-		
	-APFGYT-		
	-APFGYF-		
	AQPFGY--		

ANPFGY--
AHPFGY--
ACPFGY--
-APFGYG-
-APFGYA-
AEPFGY--
AFPFGY--
AAPFGY--
CNPFGY--
CQPFGY--
CRPFGY--
CVPFGY--
CKPFGY--
CHPFGY--
CTPFGY--
CDPFGY--
CSPFGY--
-CPFGYW-
CFPFGY--
CLPFGY--
CGPFGY--
CEPFGY--
CAPFGY--
DMPFGY--
DRPFGY--
-DPFGYI-
DEPFGY--
DAPFGY--
DQPFGY--
DLPFGY--
DWPFGY--
-DPFGYY-
-DPFGYR-
DKPFGY--
DVPFGY--
-DPFGYT-
DNPFGY--
DIPFGY--
DGPFGY--
DFPFGY--
DTPFGY--
-DPFGYK-
-DPFGYW-
-DPFGYS-
-DPFGYM-
-DPFGYL-
DYPFGY--
DSPFGY--
-DPFGYF-
-EPFGYR-

-EPFGYL-
-EPFGYA-
ELPFGY--
ETPFGY--
-EPFGYS-
-EPFGYK-
EGPFGY--
EQPFGY--
EKPFGY--
-EPFGYV-
-EPFGYI-
-EPFGYF-
EMPFGY--
EYPFGY--
-EPFGYW-
ERPFGY--
-EPFGYE-
EFPFGY--
EEPFGY--
-EPFGYQ-
-EPFGYY-
-EPFGYT-
EWPFGY--
EVPFGY--
-EPFGYM-
-EPFGYH-
ENPFGY--
EAPFGY--
-FPFGYR-
FGPFGY--
FRPFGY--
FFPFGY--
FPPFGY--
FTPFGY--
FVPFGY--
FSPFGY--
FLPFGY--
-FPFGYI-
FYPFGY--
-FPFGYL-
FNPFGY--
-FPFGYA-
FEPFGY--
FDPFGY--
-FPFGYV-
-FPFGYT-
-FPFGYS-
-FPFGYH-
-FPFGYM-
FWPFGY--

-FPFGYN-
-FPFGYK-
-GPFGYT-
-GPFGYI-
GKPFGY--
-GPFGYY-
GMPFGY--
GTPFGY--
GAPFGY--
GRPFGY--
GLPFGY--
-GPFGYW-
-GPFGYR-
-GPFGYK-
GVPFGY--
GSPFGY--
-GPFGYH-
GEPFGY--
-GPFGYL-
-GPFGYA-
GYPFGY--
-GPFGYF-
GNPFGY--
GFPFGY--
-GPFGYM-
GIPFGY--
GQPFGY--
-GPFGYG-
GHPFGY--
-GPFGYV-
-GPFGYN-
-GPFGYE-
GGPFGY--
GWPFGY--
GDPFGY--
HLPFGY--
HTPFGY--
HSPFGY--
HDPFGY--
HRPFGY--
HGPFGY--
HQPFGY--
-HPFGYR-
HVPFGY--
-HPFGYN-
-HPFGYL-
-HPFGYK-
-HPFGYF-
-HPFGYS-
-HPFGYQ-

HPPFGY--
-HPFGYV-
-HPFGYH-
HAPFGY--
-HPFGYY-
-HPFGYT-
-HPFGYA-
HNPFGY--
HHPFGY--
-HPFGYW-
HIPFGY--
HFPFGY--
ITPFGY--
ILPFGY--
-IPFGYN-
IRPFGY--
INPFGY--
ISPFGY--
IVPFGY--
-IPFGYS-
-IPFGYR-
IWPFGY--
-IPFGYT-
-IPFGYM-
-IPFGYI-
IIPFGY--
-IPFGYW-
-IPFGYH-
-IPFGYA-
IHPFGY--
-IPFGYK-
-IPFGYE-
-IPFGYV-
IEPFGY--
IAPFGY--
IYPFGY--
IPPFGY--
-IPFGYG-
-IPFGYF-
IDPFGY--
KSPFGY--
-KPFGYR-
-KPFGYL-
KGPFGY--
KAPFGY--
-KPFGYQ-
KLPFGY--
-KPFGYI-
-KPFGYS-
KVPFGY--

KYPFGY--
-KPFGYN-
-KPFGYA-
KWPFGY--
KNPFGY--
KIPFGY--
KPPFGY--
-KPFGYK-
-KPFGYF-
KKPFGY--
KTPFGY--
KHPFGY--
KQPFGY--
-KPFGYM-
-KPFGYW-
-KPFGYT-
KFPFGY--
KEPFGY--
-KPFGYV-
KDPFGY--
-KPFGYE-
-KPFGYY-
-KPFGYH-
KMPFGY--
KCPFGY--
LSPFGY--
LTPFGY--
LYPFGY--
LGPFGY--
-LPFGYH-
LRPFGY--
LAPFGY--
LKPFGY--
LWPFGY--
LPPFGY--
-LPFGYY-
LQPFGY--
-LPFGYL-
LLPFGY--
LNPFGY--
LHPFGY--
-LPFGYS-
-LPFGYR-
LDPFGY--
LIPFGY--
-LPFGYW-
-LPFGYT-
-LPFGYI-
-LPFGYM-
LFPFGY--

LMPFGY--
-LPFGYV-
-LPFGYN-
-LPFGYK-
-LPFGYQ-
LEPFGY--
-LPFGYA-
MRPFGY--
-MPFGYL-
MFPFGY--
-MPFGYV-
MGPFGY--
-MPFGYY-
MLPFGY--
-MPFGYT-
-MPFGYR-
MKPFGY--
MAPFGY--
-MPFGYS-
MVPFGY--
MQPFGY--
-MPFGYF-
MMPFGY--
MTPFGY--
MDPFGY--
-MPFGYA-
MNPFGY--
MSPFGY--
-MPFGYI-
MYPFGY--
-MPFGYE-
NRPFGY--
-NPFGYR-
NLPFGY--
NWPFGY--
NVPFGY--
-NPFGYS-
NPPFGY--
NSPFGY--
-NPFGYV-
NDPFGY--
-NPFGYK-
-NPFGYN-
NYPFGY--
NGPFGY--
NTPFGY--
NMPFGY--
-NPFGYL-
NFPFGY--
NQPFGY--

-NPFGYM-
NAPFGY--
-NPFGYW-
-NPFGYT-
-NPFGYQ-
-NPFGYI-
-NPFGYH-
NNPFGY--
NKPFGY--
-NPFGYY-
-NPFGYA-
NIPFGY--
NHPFGY--
NEPFGY--
PTPFGY--
--PFGYRS
--PFGYVS
--PFGYMA
--PFGYVT
PQPFGY--
--PFGYVG
--PFGYRR
--PFGYRT
--PFGYLS
--PFGYLG
--PFGYRA
--PFGYLT
--PFGYIS
--PFGYRK
--PFGYRG
--PFGYSV
--PFGYST
--PFGYVN
PGPFGY--
--PFGYAA
--PFGYSW
--PFGYSR
--PFGYSG
--PFGYSN
--PFGYKV
--PFGYTR
--PFGYTI
--PFGYHV
--PFGYTK
--PFGYQG
--PFGYLA
--PFGYKS
--PFGYTV
--PFGYSL
--PFGYLV

--PFGYVQ
--PFGYRQ
--PFGYNS
--PFGYNR
--PFGYLM
--PFGYLK
--PFGYWH
--PFGYRV
--PFGYQA
--PFGYAT
--PFGYTH
--PFGYKT
--PFGYKL
--PFGYHL
--PFGYTA
--PFGYRY
--PFGYMH
--PFGYVF
--PFGYNV
--PFGYIQ
--PFGYYG
--PFGYTS
--PFGYRM
--PFGYHN
--PFGYAH
--PFGYSQ
--PFGYQT
--PFGYQR
--PFGYLN
--PFGYIV
--PFGYHT
--PFGYTT
--PFGYTM
--PFGYRI
--PFGYMT
--PFGYIA
--PFGYGV
--PFGYGS
--PFGYAS
--PFGYSF
--PFGYQL
--PFGYKN
--PFGYKF
--PFGYIR
--PFGYFQ
--PFGYFN
PRPFGY--
--PFGYYS
--PFGYVI
--PFGYVA

--PFGYSY
--PFGYRE
--PFGYQH
--PFGYFK
--PFGYFG
--PFGYAM
PEPFGY--
--PFGYWT
--PFGYWQ
--PFGYWN
--PFGYWG
--PFGYVH
--PFGYRN
--PFGYQF
--PFGYMK
--PFGYHS
--PFGYFH
--PFGYAL
--PFGYYN
--PFGYWK
--PFGYVY
--PFGYVR
--PFGYTQ
--PFGYTL
--PFGYNY
--PFGYNW
--PFGYMR
--PFGYMN
--PFGYKG
--PFGYKA
--PFGYGT
--PFGYEW
--PFGYEF
--PFGYAR
PVPFGY--
PMPFGY--
PHPFGY--
--PFGYYK
--PFGYVV
--PFGYSH
--PFGYSA
--PFGYRF
--PFGYMV
--PFGYIY
--PFGYGG
--PFGYFS
--PFGYES
--PFGYAV
--PFGYYH
--PFGYWL

--PFGYWD
--PFGYSM
--PFGYQS
--PFGYQI
--PFGYMY
--PFGYKI
--PFGYIM
--PFGYHK
--PFGYET
--PFGYAW
--PFGYAN
--PFGYAF
PAPFGY--
PSPFGY--
--PFGYYD
--PFGYWR
--PFGYVD
--PFGYTW
--PFGYRD
--PFGYQY
--PFGYQQ
--PFGYMS
--PFGYKY
--PFGYKW
--PFGYIG
--PFGYHY
PWPFGY--
--PFGYYR
--PFGYYQ
--PFGYVW
--PFGYTF
--PFGYTD
--PFGYNL
--PFGYNG
--PFGYLQ
--PFGYKH
--PFGYHH
--PFGYDS
--PFGYCI
--PFGYAQ
PYPFGY--
PIPFGY--
--PFGYVE
--PFGYTE
--PFGYSK
--PFGYQN
--PFGYQK
--PFGYNM
--PFGYLE
--PFGYKR

```
--PFGYIT
--PFGYIK
--PFGYGY
--PFGYGI
--PFGYCG
--PFGYAY
--PFGYAK
--PFGYAG
--PFGYWA
--PFGYVK
--PFGYTN
--PFGYSI
--PFGYQW
--PFGYQM
--PFGYML
--PFGYIW
--PFGYII
--PFGYIH
--PFGYHW
--PFGYHI
--PFGYHE
--PFGYHA
--PFGYGL
--PFGYFD
--PFGYER
--PFGYEN
--PFGYEG
--PFGYAI
PDPFGY--
PNPFGY--
--PFGYYC
--PFGYYA
--PFGYVP
--PFGYVM
--PFGYRW
--PFGYRH
--PFGYPT
--PFGYPM
--PFGYNE
--PFGYNA
--PFGYMM
--PFGYMD
--PFGYIE
--PFGYGM
--PFGYGA
--PFGYFL
--PFGYEK
--PFGYEA
--PFGYDL
--PFGYDG
```

--PFGYCS
--PFGYCN
--PFGYAE
PCPFGY--
-QPFGYI-
QAPFGY--
QTPFGY--
QKPFGY--
QQPFGY--
-QPFGYM-
-QPFGYR-
QIPFGY--
QEPFGY--
-QPFGYS-
-QPFGYV-
QVPFGY--
-QPFGYY-
QRPFGY--
-QPFGYT-
-QPFGYK-
QNPFGY--
QLPFGY--
QGPFGY--
QWPFGY--
-QPFGYQ-
-QPFGYE-
-QPFGYA-
QSPFGY--
QPPFGY--
-QPFGYW-
-QPFGYN-
-QPFGYG-
-QPFGYL-
-RPFGYR-
RLPFGY--
-RPFGYT-
RVPFGY--
-RPFGYS-
RNPFGY--
-RPFGYL-
RSPFGY--
RTPFGY--
RPPFGY--
RRPFGY--
-RPFGYY-
RMPFGY--
RKPFGY--
RGPFGY--
RHPFGY--
-RPFGYM-

−RPFGYI−
−RPFGYH−
−RPFGYV−
RIPFGY−−
RQPFGY−−
RDPFGY−−
−RPFGYK−
−RPFGYQ−
RAPFGY−−
−RPFGYG−
−RPFGYA−
RWPFGY−−
−RPFGYN−
RYPFGY−−
−RPFGYW−
−RPFGYE−
RFPFGY−−
−RPFGYF−
REPFGY−−
SVPFGY−−
−SPFGYL−
−SPFGYH−
SAPFGY−−
SSPFGY−−
SFPFGY−−
SKPFGY−−
−SPFGYI−
SLPFGY−−
−SPFGYQ−
SNPFGY−−
SIPFGY−−
SQPFGY−−
SGPFGY−−
−SPFGYS−
SMPFGY−−
STPFGY−−
−SPFGYM−
SEPFGY−−
SRPFGY−−
−SPFGYV−
−SPFGYA−
−SPFGYT−
−SPFGYN−
SPPFGY−−
SDPFGY−−
SWPFGY−−
−SPFGYK−
SCPFGY−−
−SPFGYG−
SHPFGY−−

-SPFGYC-
TKPFGY--
-TPFGYF-
-TPFGYR-
TSPFGY--
TGPFGY--
TNPFGY--
TRPFGY--
-TPFGYL-
TWPFGY--
TEPFGY--
-TPFGYA-
TLPFGY--
TFPFGY--
-TPFGYT-
-TPFGYH-
TIPFGY--
-TPFGYN-
-TPFGYI-
TAPFGY--
-TPFGYK-
TPPFGY--
TCPFGY--
-TPFGYY-
TMPFGY--
TVPFGY--
TQPFGY--
THPFGY--
TDPFGY--
-TPFGYM-
-TPFGYQ-
-TPFGYE-
VVPFGY--
-VPFGYL-
-VPFGYH-
VGPFGY--
-VPFGYA-
VQPFGY--
-VPFGYT-
VLPFGY--
VSPFGY--
-VPFGYY-
VPPFGY--
-VPFGYR-
-VPFGYK-
VMPFGY--
VKPFGY--
VFPFGY--
-VPFGYS-
VNPFGY--

VAPFGY--
VYPFGY--
-VPFGYW-
VTPFGY--
-VPFGYQ-
VWPFGY--
-VPFGYI-
VHPFGY--
-VPFGYM-
-VPFGYF-
-VPFGYG-
VIPFGY--
VEPFGY--
-VPFGYE-
-VPFGYD-
WSPFGY--
-WPFGYR-
WNPFGY--
WRPFGY--
-WPFGYM-
-WPFGYI-
-WPFGYK-
WMPFGY--
WDPFGY--
WVPFGY--
WLPFGY--
WGPFGY--
-WPFGYA-
WIPFGY--
-WPFGYH-
WKPFGY--
WHPFGY--
WAPFGY--
WWPFGY--
WQPFGY--
-WPFGYQ-
-WPFGYY-
YSPFGY--
-YPFGYR-
YGPFGY--
YMPFGY--
-YPFGYM-
YNPFGY--
YVPFGY--
-YPFGYS-
-YPFGYH-
YPPFGY--
YYPFGY--
-YPFGYY-
-YPFGYF-

```
               YDPFGY--
               YHPFGY--
               YWPFGY--
               YTPFGY--
               -YPFGYN-
               -YPFGYA-
               YKPFGY--
               YFPFGY--
               -YPFGYV-
               -YPFGYQ-
               -YPFGYK-
               YAPFGY--
               YQPFGY--
               -YPFGYW-
               YLPFGY--
               -YPFGYD-
PMGL              7         914       48.59
               -FPMGLP-
               -FPMGLD-
               -HPMGLD-
               --PMGLSP
               --PMGLRP
               --PMGLPT
               -SPMGLD-
               ARPMGL--
               ASPMGL--
               AKPMGL--
               AGPMGL--
               -APMGLV-
               AVPMGL--
               AMPMGL--
               -APMGLK-
               -APMGLR-
               -APMGLL-
               ATPMGL--
               APPMGL--
               ALPMGL--
               -APMGLW-
               AAPMGL--
               AWPMGL--
               -APMGLM-
               -APMGLE-
               AQPMGL--
               -APMGLF-
               AIPMGL--
               ADPMGL--
               -APMGLQ-
               AHPMGL--
               AFPMGL--
               -APMGLI-
```

-APMGLH-
AYPMGL--
-APMGLY-
-APMGLT-
ACPMGL--
ANPMGL--
AEPMGL--
-APMGLD-
CCPMGL--
CSPMGL--
-CPMGLS-
CLPMGL--
CRPMGL--
-CPMGLT-
-CPMGLV-
-CPMGLQ-
CNPMGL--
CAPMGL--
CVPMGL--
CTPMGL--
-CPMGLM-
-CPMGLI-
CMPMGL--
CIPMGL--
-CPMGLR-
-CPMGLH-
-CPMGLL-
CFPMGL--
-CPMGLN-
-CPMGLK-
-CPMGLA-
CKPMGL--
CGPMGL--
DWPMGL--
-DPMGLT-
DNPMGL--
-DPMGLW-
DRPMGL--
-DPMGLS-
DLPMGL--
DGPMGL--
DVPMGL--
DFPMGL--
DHPMGL--
DSPMGL--
-DPMGLQ-
DTPMGL--
-DPMGLA-
-DPMGLV-
DAPMGL--

-DPMGLH-
-DPMGLR-
-DPMGLN-
-DPMGLF-
-DPMGLY-
DMPMGL--
-DPMGLE-
DKPMGL--
DIPMGL--
DDPMGL--
DCPMGL--
-DPMGLK-
-DPMGLI-
DEPMGL--
ESPMGL--
-EPMGLL-
EQPMGL--
EGPMGL--
EVPMGL--
-EPMGLR-
-EPMGLA-
EFPMGL--
-EPMGLW-
-EPMGLK-
EAPMGL--
EEPMGL--
ETPMGL--
-EPMGLT-
-EPMGLS-
EKPMGL--
EYPMGL--
-EPMGLV-
-EPMGLF-
ENPMGL--
-EPMGLN-
-EPMGLI-
ERPMGL--
-EPMGLY-
EIPMGL--
EHPMGL--
-EPMGLQ-
-EPMGLM-
EMPMGL--
ECPMGL--
-EPMGLH-
-EPMGLG-
EWPMGL--
-EPMGLE-
-FPMGLL-
-FPMGLS-

FVPMGL--
-FPMGLR-
FFPMGL--
FEPMGL--
-FPMGLT-
FQPMGL--
FRPMGL--
-FPMGLA-
FYPMGL--
FNPMGL--
FLPMGL--
FSPMGL--
-FPMGLN-
FTPMGL--
-FPMGLI-
-FPMGLG-
FGPMGL--
-FPMGLY-
-FPMGLM-
-FPMGLK-
FIPMGL--
FDPMGL--
FPPMGL--
FCPMGL--
FMPMGL--
-FPMGLW-
FHPMGL--
-FPMGLQ-
FWPMGL--
-FPMGLF-
-FPMGLE-
-FPMGLC-
-GPMGLR-
GLPMGL--
GVPMGL--
GTPMGL--
-GPMGLS-
GYPMGL--
GSPMGL--
-GPMGLQ-
-GPMGLN-
-GPMGLK-
GNPMGL--
GGPMGL--
-GPMGLM-
-GPMGLT-
-GPMGLY-
-GPMGLG-
GKPMGL--
-GPMGLI-

GIPMGL--
GAPMGL--
GWPMGL--
GMPMGL--
GQPMGL--
GHPMGL--
-GPMGLW-
GEPMGL--
-GPMGLH-
GCPMGL--
-GPMGLE-
-GPMGLA-
-GPMGLF-
GFPMGL--
GDPMGL--
-GPMGLD-
HRPMGL--
-HPMGLS-
HVPMGL--
HYPMGL--
HGPMGL--
HIPMGL--
HFPMGL--
-HPMGLR-
-HPMGLL-
-HPMGLF-
-HPMGLW-
HAPMGL--
HSPMGL--
HHPMGL--
-HPMGLQ-
-HPMGLV-
-HPMGLI-
HEPMGL--
HPPMGL--
HNPMGL--
-HPMGLN-
-HPMGLA-
HMPMGL--
HWPMGL--
-HPMGLH-
HKPMGL--
-HPMGLY-
HDPMGL--
-HPMGLT-
HLPMGL--
HTPMGL--
-HPMGLG-
-HPMGLE-
-HPMGLK-

IRPMGL--
-IPMGLS-
ILPMGL--
-IPMGLR-
IKPMGL--
IPPMGL--
IIPMGL--
IYPMGL--
-IPMGLY-
IAPMGL--
ITPMGL--
-IPMGLM-
IDPMGL--
IGPMGL--
IVPMGL--
-IPMGLK-
-IPMGLA-
IMPMGL--
IWPMGL--
-IPMGLN-
-IPMGLH-
-IPMGLT-
-IPMGLF-
ISPMGL--
-IPMGLW-
-IPMGLG-
IHPMGL--
-IPMGLE-
IEPMGL--
IQPMGL--
IFPMGL--
-IPMGLD-
-IPMGLQ-
-KPMGLL-
KRPMGL--
KVPMGL--
KGPMGL--
KYPMGL--
KWPMGL--
KLPMGL--
KNPMGL--
-KPMGLI-
-KPMGLN-
-KPMGLY-
-KPMGLM-
-KPMGLF-
KPPMGL--
KTPMGL--
-KPMGLG-
KHPMGL--

KKPMGL--
KEPMGL--
KQPMGL--
KMPMGL--
-KPMGLQ-
KAPMGL--
KFPMGL--
-KPMGLW-
-KPMGLT-
-KPMGLH-
-KPMGLA-
KDPMGL--
-KPMGLK-
KCPMGL--
KIPMGL--
-KPMGLD-
-LPMGLR-
LLPMGL--
LVPMGL--
LRPMGL--
LEPMGL--
-LPMGLS-
LGPMGL--
-LPMGLT-
LAPMGL--
LNPMGL--
LPPMGL--
-LPMGLY-
LSPMGL--
LWPMGL--
-LPMGLI-
-LPMGLV-
-LPMGLK-
LYPMGL--
-LPMGLA-
-LPMGLQ-
-LPMGLH-
LTPMGL--
-LPMGLG-
-LPMGLW-
-LPMGLN-
LDPMGL--
LMPMGL--
LQPMGL--
LFPMGL--
-LPMGLE-
LCPMGL--
LIPMGL--
-LPMGLM-
-LPMGLC-

MRPMGL--
MSPMGL--
-MPMGLS-
MLPMGL--
MPPMGL--
-MPMGLR-
MFPMGL--
-MPMGLH-
MTPMGL--
-MPMGLV-
-MPMGLN-
MVPMGL--
-MPMGLK-
-MPMGLT-
MGPMGL--
MHPMGL--
MAPMGL--
-MPMGLI-
-MPMGLW-
MEPMGL--
MDPMGL--
MYPMGL--
-MPMGLA-
-MPMGLE-
MQPMGL--
-MPMGLG-
MMPMGL--
MKPMGL--
MWPMGL--
-MPMGLC-
MNPMGL--
-MPMGLM-
-MPMGLF-
-MPMGLD-
NLPMGL--
NRPMGL--
NMPMGL--
-NPMGLY-
-NPMGLK-
NGPMGL--
-NPMGLS-
NSPMGL--
-NPMGLT-
NYPMGL--
NEPMGL--
-NPMGLW-
NWPMGL--
-NPMGLH-
NVPMGL--
-NPMGLR-

```
-NPMGLI-
NTPMGL--
-NPMGLV-
NNPMGL--
NFPMGL--
NKPMGL--
NAPMGL--
NQPMGL--
-NPMGLM-
-NPMGLA-
-NPMGLQ-
NHPMGL--
-NPMGLN-
NCPMGL--
NIPMGL--
NDPMGL--
-NPMGLG-
-NPMGLE-
--PMGLTS
--PMGLSS
--PMGLLS
--PMGLRA
--PMGLTR
--PMGLRT
--PMGLVS
--PMGLVV
--PMGLVR
--PMGLRG
--PMGLSG
PGPMGL--
--PMGLSR
--PMGLRH
--PMGLLT
--PMGLRL
--PMGLSL
--PMGLRI
--PMGLRY
--PMGLRS
--PMGLRM
--PMGLRD
--PMGLLH
--PMGLVA
--PMGLSA
--PMGLTT
--PMGLLK
--PMGLHY
--PMGLGS
--PMGLTM
--PMGLST
--PMGLVL
```

--PMGLTA
--PMGLSN
--PMGLRV
--PMGLMR
--PMGLLQ
--PMGLIS
--PMGLGT
--PMGLVT
--PMGLKY
--PMGLFS
--PMGLKA
--PMGLHK
--PMGLKS
--PMGLER
--PMGLTL
--PMGLNS
--PMGLMS
--PMGLAK
--PMGLLA
--PMGLKL
--PMGLHL
--PMGLVW
--PMGLMT
--PMGLVQ
--PMGLQA
--PMGLKG
--PMGLSH
--PMGLAN
--PMGLYR
--PMGLWR
--PMGLRW
--PMGLHT
--PMGLTV
--PMGLRF
--PMGLQI
--PMGLDG
--PMGLWG
--PMGLVI
--PMGLGA
--PMGLRE
--PMGLLD
--PMGLKI
--PMGLTN
--PMGLTG
--PMGLHI
--PMGLYS
--PMGLVN
--PMGLAT
PLPMGL--
--PMGLNM

--PMGLGF
--PMGLEA
--PMGLVM
--PMGLSK
--PMGLNT
--PMGLIG
--PMGLFD
PRPMGL--
--PMGLWN
PNPMGL--
--PMGLKE
--PMGLTF
--PMGLSM
--PMGLNL
--PMGLNK
--PMGLMV
--PMGLKR
--PMGLFG
--PMGLYG
--PMGLNV
--PMGLHH
--PMGLEW
--PMGLAR
--PMGLMH
--PMGLKN
--PMGLIL
--PMGLIA
--PMGLHG
--PMGLEG
--PMGLAS
--PMGLAQ
--PMGLYH
--PMGLVY
--PMGLVE
--PMGLTH
--PMGLMA
--PMGLKH
--PMGLFQ
PWPMGL--
--PMGLYQ
--PMGLSF
--PMGLRQ
--PMGLQS
--PMGLQR
--PMGLNQ
--PMGLIV
--PMGLHF
--PMGLGN
PKPMGL--
PHPMGL--

PFPMGL--
--PMGLVF
--PMGLSV
--PMGLRN
--PMGLQM
--PMGLNI
--PMGLIN
--PMGLGV
--PMGLEM
--PMGLAV
PAPMGL--
--PMGLYK
--PMGLTK
--PMGLKV
--PMGLGG
--PMGLES
PSPMGL--
--PMGLSD
--PMGLNY
--PMGLMY
--PMGLMF
--PMGLHV
PMPMGL--
--PMGLVD
--PMGLTQ
--PMGLLM
--PMGLIK
--PMGLHM
--PMGLAY
--PMGLWQ
--PMGLVG
--PMGLNH
--PMGLKQ
--PMGLKD
--PMGLGW
--PMGLGI
--PMGLAI
--PMGLAH
PIPMGL--
PTPMGL--
--PMGLLE
--PMGLKF
--PMGLIY
--PMGLII
--PMGLGY
--PMGLFN
--PMGLQN
--PMGLKM
--PMGLFK
--PMGLEH

--PMGLYD
--PMGLWT
--PMGLWD
--PMGLSQ
--PMGLNR
--PMGLMQ
--PMGLMG
--PMGLHQ
--PMGLHA
--PMGLGL
--PMGLFA
--PMGLET
--PMGLAA
--PMGLYN
--PMGLMI
--PMGLID
--PMGLHW
--PMGLFH
--PMGLQL
--PMGLNA
--PMGLLW
--PMGLIH
PVPMGL--
--PMGLQW
--PMGLME
--PMGLEI
--PMGLAF
--PMGLYA
--PMGLWK
--PMGLQK
--PMGLQF
--PMGLNW
--PMGLNN
--PMGLNE
--PMGLMD
--PMGLHN
--PMGLAG
PDPMGL--
--PMGLTW
--PMGLQV
--PMGLQG
--PMGLIM
--PMGLHS
--PMGLEL
--PMGLYE
--PMGLQQ
--PMGLMK
--PMGLIQ
--PMGLGR
--PMGLGM

--PMGLGK
--PMGLAM
--PMGLWM
--PMGLND
--PMGLMW
--PMGLKW
--PMGLEY
--PMGLTD
--PMGLQE
--PMGLMN
--PMGLHE
--PMGLFF
--PMGLDT
--PMGLAE
--PMGLYP
--PMGLQD
--PMGLMM
--PMGLSW
--PMGLSC
--PMGLIE
--PMGLHD
--PMGLGQ
--PMGLEK
PYPMGL--
PQPMGL--
-PPMGLS-
--PMGLWE
--PMGLSE
--PMGLRC
--PMGLQH
--PMGLMC
--PMGLHP
--PMGLGE
--PMGLGD
--PMGLFC
--PMGLEV
--PMGLDW
--PMGLDM
--PMGLCY
--PMGLCK
PCPMGL--
-PPMGLL-
--PMGLPG
--PMGLNC
--PMGLMP
--PMGLLC
--PMGLFP
--PMGLFE
--PMGLCT
--PMGLCS

--PMGLCM
--PMGLCH
--PMGLCA
--PMGLAD
QRPMGL--
QGPMGL--
QLPMGL--
-QPMGLI-
QPPMGL--
-QPMGLL-
-QPMGLN-
QIPMGL--
QTPMGL--
-QPMGLH-
QHPMGL--
QVPMGL--
-QPMGLV-
QSPMGL--
QQPMGL--
-QPMGLK-
-QPMGLS-
-QPMGLR-
-QPMGLQ-
QNPMGL--
QFPMGL--
-QPMGLG-
-QPMGLW-
QYPMGL--
-QPMGLY-
QWPMGL--
QAPMGL--
QEPMGL--
-QPMGLT-
-QPMGLE-
QKPMGL--
-QPMGLM-
QCPMGL--
RSPMGL--
-RPMGLT-
RGPMGL--
-RPMGLL-
-RPMGLV-
RVPMGL--
RAPMGL--
RLPMGL--
-RPMGLS-
RFPMGL--
RHPMGL--
REPMGL--
RYPMGL--

RIPMGL--
-RPMGLW-
-RPMGLK-
RMPMGL--
-RPMGLH-
-RPMGLA-
-RPMGLM-
-RPMGLG-
-RPMGLF-
RPPMGL--
RQPMGL--
-RPMGLI-
RKPMGL--
RWPMGL--
-RPMGLE-
RDPMGL--
RCPMGL--
-RPMGLC-
-RPMGLD-
SVPMGL--
-SPMGLS-
SAPMGL--
SSPMGL--
-SPMGLR-
SRPMGL--
SGPMGL--
-SPMGLV-
STPMGL--
SHPMGL--
SKPMGL--
SYPMGL--
SNPMGL--
SPPMGL--
SWPMGL--
-SPMGLH-
SMPMGL--
-SPMGLG-
SQPMGL--
SIPMGL--
-SPMGLK-
SFPMGL--
-SPMGLY-
-SPMGLQ-
-SPMGLM-
-SPMGLW-
-SPMGLE-
SEPMGL--
SCPMGL--
SDPMGL--
-TPMGLS-

−TPMGLV−
−TPMGLR−
TSPMGL−−
−TPMGLL−
TTPMGL−−
TYPMGL−−
TNPMGL−−
TLPMGL−−
TKPMGL−−
TIPMGL−−
TAPMGL−−
−TPMGLG−
−TPMGLY−
TFPMGL−−
TWPMGL−−
TQPMGL−−
−TPMGLN−
−TPMGLF−
TEPMGL−−
TGPMGL−−
TCPMGL−−
−TPMGLM−
−TPMGLA−
TPPMGL−−
−TPMGLI−
−TPMGLW−
TDPMGL−−
−TPMGLE−
TMPMGL−−
−TPMGLT−
−TPMGLH−
−TPMGLK−
−TPMGLQ−
THPMGL−−
−TPMGLC−
VKPMGL−−
VLPMGL−−
VTPMGL−−
VIPMGL−−
VSPMGL−−
VMPMGL−−
VYPMGL−−
VRPMGL−−
−VPMGLE−
−VPMGLW−
−VPMGLV−
−VPMGLL−
−VPMGLQ−
−VPMGLH−
−VPMGLF−

VGPMGL--
VFPMGL--
VWPMGL--
VVPMGL--
-VPMGLK-
VQPMGL--
-VPMGLI-
-VPMGLS-
-VPMGLY-
VAPMGL--
-VPMGLG-
VHPMGL--
VCPMGL--
-VPMGLM-
VEPMGL--
-VPMGLN-
-VPMGLA-
VNPMGL--
-VPMGLD-
WIPMGL--
-WPMGLR-
WAPMGL--
-WPMGLL-
WRPMGL--
WGPMGL--
WDPMGL--
WQPMGL--
-WPMGLV-
-WPMGLE-
-WPMGLN-
WLPMGL--
WWPMGL--
WTPMGL--
-WPMGLS-
-WPMGLH-
-WPMGLM-
WFPMGL--
-WPMGLI-
WPPMGL--
-WPMGLA-
WNPMGL--
WYPMGL--
-WPMGLY-
WEPMGL--
-WPMGLG-
-WPMGLK-
WMPMGL--
WHPMGL--
-WPMGLT-
-YPMGLR-

YRPMGL--
YTPMGL--
YGPMGL--
-YPMGLS-
YVPMGL--
YKPMGL--
-YPMGLT-
-YPMGLL-
-YPMGLH-
YEPMGL--
-YPMGLV-
-YPMGLK-
YAPMGL--
YQPMGL--
YHPMGL--
-YPMGLF-
YSPMGL--
-YPMGLY-
-YPMGLQ-
-YPMGLG-
-YPMGLA-
YLPMGL--
YWPMGL--
YIPMGL--
YYPMGL--
-YPMGLM-
YNPMGL--
YDPMGL--
YMPMGL--
-YPMGLE-
-YPMGLD-

PLGI	15	897	48.56

--PLGIYP
--PLGISP
--PLGIPT
-APLGIV-
ASPLGI--
ATPLGI--
ALPLGI--
ARPLGI--
-APLGIS-
-APLGIT-
AHPLGI--
-APLGIF-
-APLGIR-
-APLGIQ-
-APLGII-
AEPLGI--
ANPLGI--
AYPLGI--

-APLGIG-
AMPLGI--
AGPLGI--
-APLGIK-
AFPLGI--
APPLGI--
-APLGIN-
AQPLGI--
AIPLGI--
AWPLGI--
-APLGIE-
-APLGIH-
ADPLGI--
-APLGID-
ACPLGI--
-APLGIC-
CRPLGI--
CKPLGI--
CLPLGI--
CTPLGI--
CSPLGI--
-CPLGIR-
CHPLGI--
CCPLGI--
CAPLGI--
CQPLGI--
-CPLGIL-
CVPLGI--
-CPLGII-
-CPLGIT-
-CPLGIS-
CEPLGI--
CPPLGI--
-CPLGIV-
-CPLGIE-
CIPLGI--
-CPLGIN-
-CPLGIM-
-CPLGIH-
-CPLGIG-
-CPLGIQ-
CGPLGI--
CFPLGI--
CDPLGI--
DLPLGI--
DMPLGI--
DAPLGI--
DHPLGI--
DIPLGI--
-DPLGIT-

-DPLGIL-
DKPLGI--
DTPLGI--
-DPLGIK-
-DPLGIA-
DWPLGI--
-DPLGIQ-
DQPLGI--
-DPLGIM-
DYPLGI--
-DPLGIS-
-DPLGII-
DSPLGI--
-DPLGIF-
DEPLGI--
-DPLGIR-
-DPLGIH-
DFPLGI--
DVPLGI--
-DPLGIN-
DGPLGI--
DDPLGI--
-DPLGIG-
-DPLGIY-
-DPLGIW-
DNPLGI--
DCPLGI--
-DPLGIV-
-EPLGIR-
EVPLGI--
ESPLGI--
EGPLGI--
-EPLGIL-
-EPLGIS-
-EPLGIK-
-EPLGIY-
-EPLGIW-
EAPLGI--
ERPLGI--
-EPLGIF-
ETPLGI--
EPPLGI--
-EPLGIN-
EYPLGI--
-EPLGIT-
-EPLGIG-
EFPLGI--
-EPLGIM-
ELPLGI--
-EPLGII-

-EPLGIE-
EHPLGI--
EDPLGI--
EWPLGI--
EMPLGI--
EIPLGI--
ENPLGI--
EEPLGI--
EKPLGI--
-EPLGIV-
-EPLGIQ-
-FPLGIH-
FKPLGI--
-FPLGIM-
FGPLGI--
FYPLGI--
-FPLGIK-
FTPLGI--
FPPLGI--
-FPLGIT-
-FPLGIQ-
FNPLGI--
FIPLGI--
FQPLGI--
-FPLGIL-
-FPLGIG-
-FPLGII-
-FPLGIN-
FFPLGI--
FAPLGI--
FWPLGI--
-FPLGIS-
FHPLGI--
FMPLGI--
-FPLGIA-
-FPLGIE-
FEPLGI--
FDPLGI--
-FPLGIC-
-GPLGIR-
GLPLGI--
GSPLGI--
-GPLGIT-
GFPLGI--
GGPLGI--
-GPLGIL-
GWPLGI--
-GPLGIS-
-GPLGII-
GAPLGI--

-GPLGIH-
-GPLGIA-
GKPLGI--
GVPLGI--
-GPLGIN-
GHPLGI--
GYPLGI--
-GPLGIW-
-GPLGIK-
-GPLGIF-
-GPLGIE-
GIPLGI--
GDPLGI--
-GPLGIQ-
GPPLGI--
-GPLGIM-
GMPLGI--
GNPLGI--
-GPLGIV-
-GPLGIG-
GQPLGI--
GCPLGI--
GEPLGI--
HRPLGI--
HKPLGI--
-HPLGIY-
-HPLGIR-
-HPLGIG-
HWPLGI--
-HPLGIS-
HGPLGI--
HNPLGI--
HHPLGI--
-HPLGIV-
-HPLGIT-
-HPLGIK-
HAPLGI--
-HPLGIF-
HFPLGI--
-HPLGIL-
-HPLGIE-
HVPLGI--
HQPLGI--
-HPLGIM-
HTPLGI--
-HPLGIH-
HPPLGI--
-HPLGIQ-
-HPLGII-
-HPLGIA-

HEPLGI--
HDPLGI--
HYPLGI--
-HPLGIW-
HIPLGI--
HCPLGI--
-IPLGIL-
-IPLGIR-
ISPLGI--
IVPLGI--
IGPLGI--
IKPLGI--
-IPLGIN-
IRPLGI--
IMPLGI--
IAPLGI--
-IPLGIM-
ITPLGI--
-IPLGIS-
IFPLGI--
-IPLGIG-
-IPLGIT-
IPPLGI--
ILPLGI--
IHPLGI--
IDPLGI--
IIPLGI--
-IPLGIH-
-IPLGIA-
IYPLGI--
-IPLGII-
-IPLGIV-
INPLGI--
IWPLGI--
IQPLGI--
IEPLGI--
-IPLGIQ-
-IPLGIK-
-IPLGIW-
-IPLGIE-
ICPLGI--
-KPLGIL-
KMPLGI--
KGPLGI--
-KPLGII-
-KPLGIS-
-KPLGIR-
-KPLGIM-
KVPLGI--
-KPLGIA-

KHPLGI--
-KPLGIY-
KAPLGI--
KYPLGI--
-KPLGIT-
KQPLGI--
KIPLGI--
KNPLGI--
KEPLGI--
-KPLGIK-
-KPLGIF-
KPPLGI--
-KPLGIW-
-KPLGIQ-
-KPLGIH-
KDPLGI--
KWPLGI--
-KPLGIE-
-KPLGIN-
LRPLGI--
-LPLGIL-
LGPLGI--
LLPLGI--
LTPLGI--
LQPLGI--
LVPLGI--
LMPLGI--
-LPLGIR-
-LPLGIS-
LHPLGI--
LAPLGI--
-LPLGIT-
LWPLGI--
LNPLGI--
-LPLGIK-
LKPLGI--
LIPLGI--
-LPLGIV-
LYPLGI--
-LPLGIM-
-LPLGIQ-
-LPLGIA-
LDPLGI--
-LPLGIN-
LEPLGI--
LFPLGI--
-LPLGII-
LPPLGI--
-LPLGIG-
-LPLGIE-

```
-LPLGIF-
LCPLGI--
-MPLGII-
-MPLGIL-
MRPLGI--
-MPLGIS-
-MPLGIH-
MTPLGI--
MSPLGI--
-MPLGIT-
-MPLGIM-
MAPLGI--
MGPLGI--
-MPLGIR-
MIPLGI--
-MPLGIY-
MFPLGI--
MNPLGI--
MVPLGI--
-MPLGIG-
MWPLGI--
-MPLGIK-
-MPLGIV-
MQPLGI--
MPPLGI--
MKPLGI--
MYPLGI--
-MPLGIA-
-MPLGIF-
MDPLGI--
-MPLGIQ-
-MPLGIN-
MHPLGI--
MEPLGI--
MCPLGI--
NRPLGI--
NKPLGI--
NLPLGI--
NVPLGI--
-NPLGIL-
NFPLGI--
NTPLGI--
NAPLGI--
NPPLGI--
NHPLGI--
-NPLGIS-
NSPLGI--
-NPLGIK-
-NPLGIM-
-NPLGII-
```

-NPLGIA-
NIPLGI--
-NPLGIH-
-NPLGIY-
NNPLGI--
NGPLGI--
NEPLGI--
NYPLGI--
-NPLGIT-
-NPLGIQ-
-NPLGIN-
NQPLGI--
NWPLGI--
-NPLGIE-
NMPLGI--
-NPLGIF-
NDPLGI--
NCPLGI--
-NPLGIC-
--PLGITR
--PLGIRL
--PLGILT
--PLGILK
--PLGIRY
--PLGIMR
--PLGIRV
--PLGIST
--PLGITL
--PLGIGS
--PLGISL
--PLGIRN
--PLGIQR
--PLGISN
--PLGIRG
--PLGILG
--PLGISS
--PLGIML
--PLGIRA
--PLGILY
--PLGIWR
--PLGIVT
--PLGIVS
--PLGIVG
--PLGIYT
--PLGIVV
--PLGIQG
--PLGIVR
--PLGILR
--PLGISG
--PLGIRM

--PLGIRW
--PLGIKG
--PLGIHL
--PLGIGT
--PLGITS
PGPLGI--
--PLGIRI
--PLGITK
--PLGILW
--PLGILV
--PLGIHR
--PLGITG
--PLGIRE
--PLGIKR
--PLGIIK
--PLGISV
--PLGIQV
--PLGIIR
--PLGITF
--PLGIMY
--PLGIFG
--PLGIYQ
--PLGITH
--PLGISA
--PLGIKT
--PLGITN
--PLGISI
--PLGIYN
--PLGIMG
--PLGIWT
--PLGISM
--PLGINV
--PLGIKY
--PLGISF
--PLGIQS
--PLGILD
--PLGIKH
--PLGIHG
--PLGIFA
--PLGILA
PTPLGI--
--PLGIQI
--PLGING
--PLGINY
--PLGINR
--PLGIIS
--PLGITY
--PLGIQA
--PLGIHS
--PLGIHI

--PLGIFN
--PLGISY
--PLGIRF
--PLGIRD
--PLGIYG
--PLGISK
PFPLGI--
--PLGIAL
--PLGIIG
--PLGIFT
--PLGISW
--PLGINS
--PLGIGG
--PLGIFH
--PLGIFS
--PLGIES
--PLGIAK
--PLGIMT
--PLGIKE
--PLGIAV
--PLGINK
--PLGIMK
--PLGIMI
--PLGIIT
--PLGIAM
--PLGIYR
--PLGIWK
--PLGIVA
--PLGIGN
--PLGIGK
--PLGIAT
--PLGIRP
--PLGIKA
--PLGINQ
--PLGINA
--PLGIIY
--PLGIEV
--PLGIVQ
--PLGIKS
--PLGIIH
--PLGIHV
--PLGIHT
--PLGIHK
--PLGIFR
--PLGIFK
PMPLGI--
--PLGIWN
--PLGIVN
--PLGITT
--PLGISD

```
--PLGINM
--PLGIII
--PLGIEY
--PLGIDS
--PLGIWG
--PLGITM
--PLGISQ
--PLGIQN
--PLGINE
--PLGIIA
--PLGIAH
--PLGIVE
--PLGIGY
--PLGIGV
--PLGIVI
--PLGITI
--PLGILQ
--PLGIIQ
PIPLGI--
PWPLGI--
--PLGIYK
--PLGIQH
--PLGINI
--PLGIME
--PLGIHM
--PLGIEI
PNPLGI--
--PLGITE
--PLGIQT
--PLGIMV
--PLGIKN
--PLGIGI
--PLGIEN
--PLGIEL
--PLGIDG
--PLGIVM
--PLGIKM
--PLGIKL
--PLGIIN
--PLGIIM
--PLGIGA
--PLGIEF
--PLGIAG
--PLGIVW
--PLGIQF
--PLGIQD
--PLGINT
--PLGIKV
--PLGIEA
--PLGIAY
```

--PLGIYH
--PLGIWQ
--PLGIVY
--PLGIVH
--PLGISH
--PLGISE
--PLGIRH
--PLGILH
--PLGIHN
--PLGIGL
--PLGIFQ
--PLGIFD
--PLGITQ
--PLGITD
--PLGIQK
--PLGIKK
--PLGIGF
--PLGIET
--PLGIAN
PHPLGI--
--PLGIWH
--PLGIQY
--PLGIMW
--PLGILE
--PLGIKQ
--PLGIID
--PLGIHD
--PLGIGW
--PLGIEW
PDPLGI--
--PLGIYD
--PLGIVD
--PLGITV
--PLGIIW
--PLGIHA
--PLGIEM
--PLGIAI
--PLGIAA
PKPLGI--
PCPLGI--
PYPLGI--
PQPLGI--
--PLGIYE
--PLGIWE
--PLGIQL
--PLGIIE
--PLGIHQ
--PLGIFE
--PLGITW
--PLGITA

--PLGINW
--PLGINN
--PLGIND
--PLGIMD
--PLGIKF
--PLGIGM
--PLGIEK
--PLGIAE
--PLGIQW
--PLGIPG
--PLGIMF
--PLGIMA
--PLGIKI
--PLGIWA
--PLGIQQ
--PLGIQM
--PLGIHF
--PLGIGE
--PLGIGD
--PLGICQ
--PLGIAD
--PLGIWD
--PLGISC
--PLGINH
--PLGIMQ
--PLGIMH
--PLGILP
--PLGIIP
--PLGIHW
--PLGIHP
--PLGIDT
--PLGIDK
--PLGIDA
--PLGICR
--PLGICL
--PLGIAQ
-PPLGIN-
-PPLGII-
-PPLGIA-
--PLGIMP
--PLGILC
--PLGIKD
--PLGIIC
--PLGIGH
--PLGIED
--PLGIEC
--PLGIDM
--PLGIDH
--PLGICM
--PLGICK

--PLGICG
--PLGIAW
PEPLGI--
-QPLGIK-
QGPLGI--
QRPLGI--
-QPLGIL-
QSPLGI--
QIPLGI--
-QPLGIV-
-QPLGIR-
QYPLGI--
QWPLGI--
QVPLGI--
-QPLGIY-
-QPLGII-
QLPLGI--
-QPLGIN-
-QPLGIH-
-QPLGIQ-
-QPLGIA-
QQPLGI--
-QPLGIM-
-QPLGIE-
QNPLGI--
QDPLGI--
QCPLGI--
-QPLGIT-
QHPLGI--
QTPLGI--
-QPLGIF-
QMPLGI--
QKPLGI--
QEPLGI--
QAPLGI--
QPPLGI--
-QPLGIG-
-RPLGIS-
RVPLGI--
-RPLGIR-
-RPLGIL-
-RPLGIT-
-RPLGIK-
RGPLGI--
RRPLGI--
RLPLGI--
RAPLGI--
RSPLGI--
RIPLGI--
-RPLGIV-

RMPLGI--
RPPLGI--
-RPLGIY-
RYPLGI--
-RPLGIN-
RKPLGI--
RTPLGI--
RWPLGI--
-RPLGIG-
RHPLGI--
RFPLGI--
-RPLGIA-
RQPLGI--
RNPLGI--
-RPLGIQ-
RDPLGI--
-RPLGIH-
REPLGI--
RCPLGI--
-RPLGIC-
-SPLGIS-
SVPLGI--
SLPLGI--
SGPLGI--
-SPLGIV-
-SPLGIQ-
-SPLGIT-
SKPLGI--
STPLGI--
-SPLGIM-
SNPLGI--
SIPLGI--
SAPLGI--
SYPLGI--
-SPLGIK-
SEPLGI--
SHPLGI--
SWPLGI--
-SPLGIH-
-SPLGIG-
-SPLGIE-
SPPLGI--
-SPLGIN-
SDPLGI--
SQPLGI--
SCPLGI--
-SPLGID-
-TPLGIR-
TSPLGI--
TGPLGI--

TTPLGI--
-TPLGIS-
TLPLGI--
TRPLGI--
-TPLGIH-
TPPLGI--
TEPLGI--
TNPLGI--
TWPLGI--
TQPLGI--
TIPLGI--
-TPLGIQ-
TKPLGI--
-TPLGIM-
-TPLGIV-
-TPLGII-
TFPLGI--
TAPLGI--
THPLGI--
-TPLGIE-
TCPLGI--
-TPLGIY-
TMPLGI--
TYPLGI--
TDPLGI--
-TPLGIT-
-TPLGIF-
-TPLGIK-
-TPLGIA-
-TPLGIG-
-TPLGID-
VRPLGI--
VSPLGI--
-VPLGIR-
VWPLGI--
-VPLGIV-
VLPLGI--
VIPLGI--
VPPLGI--
-VPLGIS-
VQPLGI--
-VPLGIG-
VNPLGI--
-VPLGIH-
VEPLGI--
VTPLGI--
-VPLGIN-
-VPLGIQ-
VGPLGI--
-VPLGII-

-VPLGIA-
VYPLGI--
-VPLGIF-
VDPLGI--
VHPLGI--
VFPLGI--
-VPLGIM-
VMPLGI--
-VPLGIE-
VAPLGI--
VCPLGI--
WSPLGI--
-WPLGIR-
WRPLGI--
WAPLGI--
-WPLGIT-
-WPLGIQ-
WYPLGI--
WFPLGI--
WIPLGI--
WGPLGI--
WQPLGI--
WLPLGI--
WVPLGI--
-WPLGIK-
-WPLGIA-
-WPLGIV-
-WPLGIG-
WDPLGI--
-WPLGIS-
WMPLGI--
WKPLGI--
-WPLGIN-
-WPLGIH-
WHPLGI--
WNPLGI--
-WPLGIM-
-WPLGII-
-WPLGIE-
WPPLGI--
-WPLGIY-
WWPLGI--
WEPLGI--
-WPLGIF-
WCPLGI--
YHPLGI--
YRPLGI--
-YPLGIS-
YSPLGI--
YKPLGI--

	-YPLGIY-		
	-YPLGIG-		
	-YPLGIH-		
	-YPLGIT-		
	-YPLGII-		
	YGPLGI--		
	YTPLGI--		
	-YPLGIR-		
	YYPLGI--		
	-YPLGIN-		
	YEPLGI--		
	YPPLGI--		
	-YPLGIA-		
	YVPLGI--		
	YLPLGI--		
	-YPLGIV-		
	-YPLGIM-		
	-YPLGIL-		
	-YPLGIK-		
	YMPLGI--		
	YNPLGI--		
	YIPLGI--		
	YFPLGI--		
	YWPLGI--		
	-YPLGIE-		
	YDPLGI--		
	-YPLGIQ-		
	YCPLGI--		
	-YPLGIC-		
PFWL	378	667	48.52
	-APFWLR-		
	AEPFWL--		
	AGPFWL--		
	-APFWLK-		
	ASPFWL--		
	-APFWLS-		
	-APFWLI-		
	ANPFWL--		
	-APFWLA-		
	ATPFWL--		
	-APFWLV-		
	-APFWLT-		
	-APFWLH-		
	AIPFWL--		
	ARPFWL--		
	AKPFWL--		
	APPFWL--		
	-APFWLG-		
	AYPFWL--		
	AQPFWL--		

AFPFWL--
AWPFWL--
AVPFWL--
-APFWLQ-
-APFWLD-
AHPFWL--
CEPFWL--
CGPFWL--
CYPFWL--
CRPFWL--
CKPFWL--
CPPFWL--
-CPFWLS-
-CPFWLI-
CMPFWL--
CLPFWL--
CAPFWL--
DVPFWL--
-DPFWLR-
DLPFWL--
-DPFWLS-
DSPFWL--
-DPFWLT-
DQPFWL--
DRPFWL--
DYPFWL--
DDPFWL--
-DPFWLI-
DGPFWL--
-DPFWLQ-
-DPFWLM-
DMPFWL--
DFPFWL--
DWPFWL--
-DPFWLE-
DIPFWL--
DHPFWL--
-DPFWLL-
-DPFWLA-
DNPFWL--
-DPFWLN-
-DPFWLK-
-DPFWLH-
-DPFWLG-
DAPFWL--
ESPFWL--
-EPFWLR-
-EPFWLG-
ERPFWL--
EKPFWL--

EQPFWL--
-EPFWLN-
ENPFWL--
ETPFWL--
ELPFWL--
-EPFWLQ-
-EPFWLW-
EWPFWL--
-EPFWLT-
EPPFWL--
-EPFWLK-
EVPFWL--
-EPFWLV-
-EPFWLI-
-EPFWLH-
-EPFWLE-
-EPFWLD-
-EPFWLS-
EGPFWL--
EFPFWL--
EEPFWL--
EIPFWL--
EDPFWL--
FSPFWL--
FKPFWL--
FAPFWL--
FQPFWL--
FPPFWL--
-FPFWLR-
-FPFWLI-
-FPFWLS-
-FPFWLH-
FNPFWL--
-FPFWLL-
-FPFWLG-
-FPFWLE-
FEPFWL--
GGPFWL--
GHPFWL--
-GPFWLR-
GLPFWL--
GTPFWL--
-GPFWLE-
GQPFWL--
-GPFWLI-
-GPFWLT-
GVPFWL--
GYPFWL--
GKPFWL--
GFPFWL--

GAPFWL--
GSPFWL--
-GPFWLA-
GDPFWL--
-GPFWLV-
-GPFWLG-
GNPFWL--
-GPFWLQ-
GIPFWL--
-GPFWLS-
-GPFWLN-
-GPFWLM-
-GPFWLH-
GWPFWL--
GPPFWL--
GRPFWL--
-GPFWLD-
GCPFWL--
-GPFWLK-
GMPFWL--
HTPFWL--
HNPFWL--
HHPFWL--
HLPFWL--
HRPFWL--
-HPFWLT-
HVPFWL--
HFPFWL--
-HPFWLA-
HPPFWL--
HMPFWL--
-HPFWLS-
-HPFWLK-
-HPFWLY-
-HPFWLH-
HGPFWL--
HSPFWL--
HQPFWL--
HKPFWL--
HAPFWL--
HYPFWL--
-HPFWLE-
-HPFWLV-
-HPFWLN-
HWPFWL--
-HPFWLD-
HEPFWL--
ISPFWL--
IRPFWL--
ITPFWL--

IQPFWL--
-IPFWLS-
IKPFWL--
IYPFWL--
-IPFWLT-
-IPFWLN-
IHPFWL--
-IPFWLH-
INPFWL--
-IPFWLG-
ILPFWL--
-IPFWLK-
IDPFWL--
IVPFWL--
-IPFWLE-
IAPFWL--
IPPFWL--
IGPFWL--
-IPFWLY-
-IPFWLV-
-IPFWLA-
KSPFWL--
-KPFWLS-
KIPFWL--
KGPFWL--
KPPFWL--
KVPFWL--
KMPFWL--
-KPFWLR-
-KPFWLH-
KAPFWL--
-KPFWLA-
KHPFWL--
-KPFWLN-
KKPFWL--
KEPFWL--
-KPFWLG-
-KPFWLQ-
-KPFWLT-
-KPFWLK-
KLPFWL--
KQPFWL--
-KPFWLE-
-KPFWLI-
-KPFWLV-
KYPFWL--
KWPFWL--
-KPFWLD-
KFPFWL--
-KPFWLL-

KNPFWL--
KDPFWL--
-KPFWLC-
LRPFWL--
LAPFWL--
LVPFWL--
-LPFWLR-
LNPFWL--
-LPFWLT-
-LPFWLS-
LQPFWL--
LHPFWL--
-LPFWLA-
LDPFWL--
-LPFWLG-
LKPFWL--
LWPFWL--
-LPFWLN-
LLPFWL--
LYPFWL--
LFPFWL--
-LPFWLM-
MVPFWL--
MPPFWL--
-MPFWLG-
MDPFWL--
MKPFWL--
MTPFWL--
MEPFWL--
MSPFWL--
-MPFWLT-
MQPFWL--
-MPFWLI-
MLPFWL--
MGPFWL--
MFPFWL--
-MPFWLH-
MMPFWL--
MHPFWL--
MCPFWL--
MNPFWL--
-MPFWLR-
MIPFWL--
-NPFWLE-
NYPFWL--
NTPFWL--
-NPFWLK-
-NPFWLQ-
NFPFWL--
NRPFWL--

NAPFWL--
NGPFWL--
NIPFWL--
NLPFWL--
-NPFWLR-
-NPFWLA-
NEPFWL--
NPPFWL--
-NPFWLF-
NMPFWL--
-NPFWLT-
-NPFWLH-
-NPFWLN-
NKPFWL--
NDPFWL--
-NPFWLY-
-NPFWLG-
NNPFWL--
--PFWLAG
--PFWLVQ
--PFWLVH
--PFWLSN
--PFWLSR
--PFWLRT
--PFWLHS
--PFWLTN
--PFWLST
--PFWLTR
--PFWLTQ
--PFWLRN
--PFWLNS
--PFWLGG
--PFWLEA
--PFWLAT
--PFWLRM
--PFWLSV
--PFWLSQ
--PFWLLQ
--PFWLQR
--PFWLEH
--PFWLYD
--PFWLRH
--PFWLYN
--PFWLKR
--PFWLKQ
--PFWLRQ
--PFWLRA
--PFWLGS
--PFWLQV
--PFWLKS

--PFWLRL
--PFWLIK
--PFWLAK
--PFWLVN
--PFWLTG
--PFWLSG
--PFWLSE
--PFWLRS
--PFWLQI
PTPFWL--
--PFWLLG
--PFWLKL
--PFWLKH
--PFWLGT
--PFWLGI
--PFWLAS
--PFWLAQ
--PFWLQM
--PFWLKT
PEPFWL--
--PFWLVS
--PFWLSD
--PFWLRK
--PFWLRG
--PFWLMQ
--PFWLKE
--PFWLIG
--PFWLHR
--PFWLAH
PYPFWL--
--PFWLSF
--PFWLQT
--PFWLLD
--PFWLKD
--PFWLIH
--PFWLHA
--PFWLGL
--PFWLDS
--PFWLAR
--PFWLTV
--PFWLTK
--PFWLTA
--PFWLQN
--PFWLQE
--PFWLNY
--PFWLNT
--PFWLLY
--PFWLKY
--PFWLKI
--PFWLHT

--PFWLGV
--PFWLEW
--PFWLES
--PFWLEG
PHPFWL--
--PFWLYH
--PFWLYA
--PFWLTS
--PFWLRY
--PFWLMN
--PFWLLK
--PFWLKM
--PFWLHH
--PFWLEI
--PFWLAN
--PFWLYG
--PFWLTD
--PFWLSW
--PFWLNA
--PFWLET
--PFWLAA
--PFWLWH
--PFWLWG
--PFWLSK
--PFWLMH
--PFWLMD
--PFWLHQ
--PFWLHK
--PFWLGW
--PFWLGR
--PFWLGQ
--PFWLAY
PGPFWL--
--PFWLYF
--PFWLWT
--PFWLTE
--PFWLQD
--PFWLMK
--PFWLMG
--PFWLGM
--PFWLGK
--PFWLFH
--PFWLFD
PDPFWL--
PWPFWL--
--PFWLWQ
--PFWLRF
--PFWLQQ
--PFWLQA
--PFWLNV

--PFWLNQ
--PFWLNE
--PFWLMS
--PFWLLM
--PFWLIQ
--PFWLHW
--PFWLGH
--PFWLEV
--PFWLEE
PFPFWL--
PMPFWL--
--PFWLYQ
--PFWLWW
--PFWLVL
--PFWLVD
--PFWLTH
--PFWLSH
--PFWLRW
--PFWLRE
--PFWLQY
--PFWLQK
--PFWLMY
--PFWLML
--PFWLLH
--PFWLKN
--PFWLKK
--PFWLID
--PFWLHV
--PFWLHL
--PFWLGA
--PFWLER
--PFWLED
-PPFWLR-
-PPFWLA-
PNPFWL--
PKPFWL--
PIPFWL--
--PFWLWV
--PFWLVM
--PFWLTM
--PFWLQW
--PFWLQS
--PFWLQH
--PFWLPP
--PFWLPG
--PFWLNK
--PFWLNF
--PFWLMI
--PFWLKW
--PFWLKV

```
--PFWLIV
--PFWLHN
--PFWLHG
--PFWLHD
--PFWLGF
--PFWLEN
--PFWLCT
--PFWLCN
--PFWLAW
--PFWLAD
-QPFWLS-
-QPFWLQ-
-QPFWLT-
QSPFWL--
-QPFWLR-
QGPFWL--
QTPFWL--
QRPFWL--
QQPFWL--
QNPFWL--
QLPFWL--
QKPFWL--
-QPFWLN-
QEPFWL--
QAPFWL--
-QPFWLM-
-QPFWLA-
QDPFWL--
QYPFWL--
QMPFWL--
-QPFWLE-
-QPFWLF-
QWPFWL--
-QPFWLK-
-QPFWLH-
QFPFWL--
RTPFWL--
RVPFWL--
RSPFWL--
RGPFWL--
-RPFWLI-
RLPFWL--
RAPFWL--
RDPFWL--
-RPFWLT-
RPPFWL--
-RPFWLR-
REPFWL--
-RPFWLS-
-RPFWLK-
```

RQPFWL--
-RPFWLH-
-RPFWLN-
RIPFWL--
RNPFWL--
RMPFWL--
RWPFWL--
-RPFWLE-
-RPFWLD-
RHPFWL--
RYPFWL--
-RPFWLQ-
SSPFWL--
SRPFWL--
SVPFWL--
SAPFWL--
-SPFWLK-
-SPFWLR-
SFPFWL--
-SPFWLS-
-SPFWLV-
SQPFWL--
-SPFWLT-
SYPFWL--
STPFWL--
-SPFWLG-
SIPFWL--
SHPFWL--
SGPFWL--
SKPFWL--
SEPFWL--
SNPFWL--
SLPFWL--
SDPFWL--
-SPFWLH-
-SPFWLA-
-SPFWLM-
-SPFWLN-
-SPFWLQ-
-SPFWLE-
-SPFWLD-
SCPFWL--
-TPFWLS-
-TPFWLR-
TAPFWL--
TLPFWL--
-TPFWLV-
-TPFWLK-
TTPFWL--
TSPFWL--

TVPFWL--
TPPFWL--
TEPFWL--
-TPFWLA-
-TPFWLG-
TMPFWL--
TKPFWL--
TFPFWL--
-TPFWLN-
TDPFWL--
TNPFWL--
TIPFWL--
-TPFWLT-
-TPFWLQ-
-TPFWLE-
-TPFWLD-
TCPFWL--
TWPFWL--
-TPFWLM-
THPFWL--
-TPFWLF-
TYPFWL--
-TPFWLI-
TQPFWL--
VSPFWL--
VNPFWL--
VRPFWL--
VPPFWL--
VTPFWL--
-VPFWLG-
-VPFWLT-
VAPFWL--
VGPFWL--
-VPFWLR-
VFPFWL--
-VPFWLS-
VKPFWL--
-VPFWLH-
VLPFWL--
-VPFWLQ-
-VPFWLI-
VVPFWL--
-VPFWLK-
VIPFWL--
VDPFWL--
VWPFWL--
-VPFWLN-
-VPFWLF-
-VPFWLA-
-VPFWLD-

	WEPFWL--		
	-WPFWLG-		
	-WPFWLR-		
	WNPFWL--		
	WTPFWL--		
	WRPFWL--		
	-WPFWLS-		
	-WPFWLK-		
	WDPFWL--		
	WLPFWL--		
	WKPFWL--		
	WSPFWL--		
	-WPFWLH-		
	-WPFWLE-		
	WFPFWL--		
	-WPFWLN-		
	-WPFWLF-		
	WHPFWL--		
	-YPFWLR-		
	YKPFWL--		
	-YPFWLA-		
	YLPFWL--		
	-YPFWLQ-		
	-YPFWLM-		
	YGPFWL--		
	-YPFWLV-		
	-YPFWLT-		
	-YPFWLS-		
	YPPFWL--		
	-YPFWLH-		
	YDPFWL--		
	YHPFWL--		
	YTPFWL--		
	YWPFWL--		
	YVPFWL--		
	-YPFWLN-		
	-YPFWLG-		
	-YPFWLE-		
	YFPFWL--		
	-YPFWLF-		
	YQPFWL--		
	-YPFWLY-		
	-YPFWLD-		
	YMPFWL--		
	YIPFWL--		
	YEPFWL--		
	YAPFWL--		
PKAY	288	703	48.50
	-KPKAYE-		
	-MPKAYG-		

ATPKAY--
AAPKAY--
AFPKAY--
AQPKAY--
-APKAYA-
-APKAYY-
-APKAYK-
ALPKAY--
AEPKAY--
-APKAYM-
AIPKAY--
-APKAYV-
AKPKAY--
ADPKAY--
-APKAYG-
AVPKAY--
-APKAYN-
AYPKAY--
ANPKAY--
AGPKAY--
-APKAYQ-
-APKAYE-
APPKAY--
-APKAYT-
CRPKAY--
CQPKAY--
-CPKAYR-
CKPKAY--
CHPKAY--
CGPKAY--
-CPKAYT-
-CPKAYI-
CVPKAY--
CTPKAY--
-CPKAYL-
CLPKAY--
CFPKAY--
CYPKAY--
-CPKAYW-
DWPKAY--
DTPKAY--
DLPKAY--
DRPKAY--
DEPKAY--
DQPKAY--
-DPKAYT-
DSPKAY--
-DPKAYR-
-DPKAYL-
-DPKAYF-

DYPKAY--
DVPKAY--
-DPKAYY-
-DPKAYM-
DGPKAY--
-DPKAYV-
-DPKAYS-
-DPKAYK-
-DPKAYA-
DIPKAY--
-DPKAYI-
DKPKAY--
DDPKAY--
ESPKAY--
-EPKAYK-
EWPKAY--
-EPKAYT-
-EPKAYR-
EMPKAY--
ELPKAY--
-EPKAYA-
EGPKAY--
EYPKAY--
EQPKAY--
-EPKAYQ-
EDPKAY--
EAPKAY--
-EPKAYI-
-EPKAYV-
-EPKAYS-
-EPKAYL-
-EPKAYF-
EKPKAY--
EFPKAY--
EVPKAY--
EPPKAY--
-EPKAYH-
-EPKAYW-
-EPKAYN-
FRPKAY--
FSPKAY--
-FPKAYS-
FNPKAY--
FDPKAY--
FTPKAY--
-FPKAYW-
FGPKAY--
FVPKAY--
FIPKAY--
FFPKAY--

FPPKAY--
-FPKAYY-
-FPKAYI-
FEPKAY--
-FPKAYM-
FLPKAY--
FQPKAY--
-FPKAYG-
FMPKAY--
FAPKAY--
-FPKAYF-
FKPKAY--
-GPKAYS-
-GPKAYQ-
-GPKAYL-
GTPKAY--
-GPKAYI-
GSPKAY--
GYPKAY--
-GPKAYK-
GMPKAY--
GPPKAY--
-GPKAYF-
GNPKAY--
-GPKAYH-
GWPKAY--
-GPKAYM-
-GPKAYY-
-GPKAYN-
GLPKAY--
GDPKAY--
GQPKAY--
-GPKAYW-
-GPKAYT-
-GPKAYA-
GAPKAY--
-GPKAYG-
GKPKAY--
GIPKAY--
-HPKAYR-
-HPKAYS-
-HPKAYL-
HLPKAY--
HYPKAY--
HGPKAY--
HMPKAY--
HKPKAY--
-HPKAYH-
-HPKAYK-
HAPKAY--

-HPKAYT-
HHPKAY--
-HPKAYA-
HRPKAY--
HEPKAY--
HPPKAY--
-HPKAYW-
-HPKAYQ-
-HPKAYP-
-HPKAYF-
HIPKAY--
IVPKAY--
IRPKAY--
ITPKAY--
-IPKAYH-
IGPKAY--
-IPKAYQ-
-IPKAYI-
-IPKAYV-
ISPKAY--
IEPKAY--
-IPKAYY-
-IPKAYK-
IPPKAY--
-IPKAYW-
-IPKAYG-
-IPKAYF-
-IPKAYE-
IIPKAY--
IMPKAY--
-IPKAYS-
IHPKAY--
-IPKAYM-
IAPKAY--
KSPKAY--
KAPKAY--
-KPKAYL-
KWPKAY--
-KPKAYY-
KYPKAY--
-KPKAYI-
KIPKAY--
KVPKAY--
-KPKAYA-
KTPKAY--
KGPKAY--
KEPKAY--
-KPKAYV-
KLPKAY--
KRPKAY--

KQPKAY--
-KPKAYN-
KNPKAY--
-KPKAYQ-
-KPKAYF-
KDPKAY--
-KPKAYK-
-KPKAYG-
KMPKAY--
-KPKAYM-
-KPKAYH-
KHPKAY--
LVPKAY--
LSPKAY--
LAPKAY--
LGPKAY--
-LPKAYK-
LDPKAY--
LTPKAY--
-LPKAYY-
LMPKAY--
LQPKAY--
-LPKAYV-
LLPKAY--
-LPKAYS-
-LPKAYF-
LEPKAY--
LKPKAY--
LHPKAY--
LIPKAY--
LPPKAY--
-LPKAYM-
-LPKAYI-
LYPKAY--
-LPKAYH-
-LPKAYA-
-LPKAYQ-
-LPKAYL-
-LPKAYN-
LCPKAY--
MKPKAY--
MLPKAY--
-MPKAYR-
-MPKAYH-
MWPKAY--
-MPKAYN-
-MPKAYL-
MRPKAY--
-MPKAYV-
MSPKAY--

MVPKAY--
MDPKAY--
-MPKAYI-
MTPKAY--
-MPKAYS-
MGPKAY--
-MPKAYA-
MAPKAY--
MQPKAY--
-MPKAYM-
MNPKAY--
MMPKAY--
MFPKAY--
-MPKAYW-
-MPKAYF-
-MPKAYE-
-NPKAYY-
NKPKAY--
-NPKAYL-
NIPKAY--
NRPKAY--
NNPKAY--
NHPKAY--
-NPKAYR-
NLPKAY--
NTPKAY--
NPPKAY--
-NPKAYH-
NDPKAY--
NQPKAY--
-NPKAYS-
NAPKAY--
-NPKAYF-
NEPKAY--
NCPKAY--
-NPKAYI-
-NPKAYA-
-NPKAYM-
-NPKAYW-
-NPKAYT-
-NPKAYN-
NVPKAY--
-NPKAYQ-
NWPKAY--
NFPKAY--
--PKAYVN
--PKAYGY
--PKAYAW
--PKAYLT
--PKAYMT

--PKAYMI
--PKAYLG
--PKAYSG
--PKAYSI
--PKAYLN
--PKAYHS
--PKAYSH
--PKAYWT
--PKAYSN
--PKAYSF
--PKAYVS
--PKAYLW
--PKAYKY
--PKAYIK
--PKAYWS
--PKAYVH
--PKAYMG
--PKAYIM
--PKAYHL
--PKAYFT
--PKAYTW
--PKAYMR
--PKAYHR
PSPKAY--
--PKAYLA
--PKAYIS
--PKAYYT
--PKAYSW
--PKAYMN
--PKAYYN
--PKAYSY
--PKAYML
--PKAYFS
--PKAYAL
--PKAYYI
--PKAYLI
--PKAYIA
--PKAYGF
--PKAYFY
--PKAYFV
--PKAYES
--PKAYTA
--PKAYRD
--PKAYGM
--PKAYYL
--PKAYMQ
--PKAYKG
--PKAYIN
--PKAYHT
PVPKAY--

--PKAYWI
--PKAYTV
--PKAYTG
--PKAYLQ
--PKAYLF
--PKAYGI
--PKAYTT
--PKAYNT
--PKAYMY
--PKAYAH
--PKAYWY
--PKAYSM
--PKAYRE
--PKAYQS
--PKAYQR
--PKAYNY
--PKAYNA
PKPKAY--
--PKAYFN
--PKAYFI
--PKAYYS
--PKAYYM
--PKAYVQ
--PKAYVE
--PKAYIV
--PKAYIQ
--PKAYIF
--PKAYFH
--PKAYFG
--PKAYVV
--PKAYVG
--PKAYTQ
--PKAYSE
--PKAYRF
--PKAYNS
--PKAYKH
--PKAYKF
--PKAYGV
--PKAYFR
--PKAYEW
--PKAYEH
PTPKAY--
--PKAYYF
--PKAYWG
--PKAYWF
--PKAYVT
--PKAYTN
--PKAYTF
--PKAYQW
--PKAYQT

--PKAYPV
--PKAYLE
--PKAYGN
PFPKAY--
PQPKAY--
--PKAYYY
--PKAYVA
--PKAYSD
--PKAYNN
--PKAYLH
--PKAYKI
--PKAYIL
--PKAYFD
--PKAYDL
--PKAYYA
--PKAYWK
--PKAYVK
--PKAYTI
--PKAYRV
--PKAYQV
--PKAYPI
--PKAYPF
--PKAYNM
--PKAYLD
--PKAYKT
--PKAYKN
--PKAYIG
--PKAYGS
--PKAYGG
--PKAYEV
--PKAYAM
PRPKAY--
--PKAYYK
--PKAYWQ
--PKAYWN
--PKAYWH
--PKAYVW
--PKAYQQ
--PKAYQA
--PKAYMW
--PKAYGW
--PKAYAV
--PKAYAQ
--PKAYAI
PGPKAY--
--PKAYST
--PKAYSA
--PKAYRM
--PKAYQG
--PKAYMK

--PKAYLM
--PKAYHV
--PKAYHQ
--PKAYHN
--PKAYET
PHPKAY--
-PPKAYL-
--PKAYYQ
--PKAYVM
--PKAYVF
--PKAYQY
--PKAYQD
--PKAYPM
--PKAYLY
--PKAYHY
--PKAYHF
--PKAYHE
--PKAYGT
--PKAYEM
--PKAYDI
--PKAYWM
--PKAYVY
--PKAYVI
--PKAYTY
--PKAYQK
--PKAYQF
--PKAYNH
--PKAYNE
--PKAYMV
--PKAYMH
--PKAYHM
--PKAYGQ
--PKAYFF
--PKAYEI
--PKAYEG
PEPKAY--
--PKAYYC
--PKAYTS
--PKAYTE
--PKAYNQ
--PKAYNI
--PKAYKD
--PKAYIY
--PKAYGH
--PKAYEF
--PKAYDS
--PKAYAY
-PPKAYV-
PNPKAY--
--PKAYYV

--PKAYYG
--PKAYYE
--PKAYWL
--PKAYTM
--PKAYSQ
--PKAYSC
--PKAYNG
--PKAYME
--PKAYMD
--PKAYID
--PKAYHI
--PKAYHH
--PKAYGK
--PKAYFM
--PKAYDV
--PKAYCY
--PKAYCN
--PKAYCH
--PKAYAK
-QPKAYS-
-QPKAYL-
-QPKAYM-
QLPKAY--
QGPKAY--
QYPKAY--
QPPKAY--
QHPKAY--
-QPKAYN-
-QPKAYT-
QNPKAY--
-QPKAYF-
QRPKAY--
-QPKAYK-
-QPKAYI-
QKPKAY--
QWPKAY--
QVPKAY--
QTPKAY--
-QPKAYG-
-QPKAYY-
-QPKAYQ-
-QPKAYE-
QMPKAY--
QIPKAY--
QEPKAY--
RVPKAY--
RSPKAY--
RPPKAY--
RGPKAY--
-RPKAYT-

-RPKAYV-
RWPKAY--
-RPKAYA-
-RPKAYN-
-RPKAYH-
-RPKAYM-
RHPKAY--
RRPKAY--
RDPKAY--
RCPKAY--
-RPKAYE-
RKPKAY--
-RPKAYG-
SFPKAY--
-SPKAYL-
-SPKAYT-
SWPKAY--
SNPKAY--
SVPKAY--
SSPKAY--
SRPKAY--
SQPKAY--
SIPKAY--
-SPKAYV-
-SPKAYS-
SHPKAY--
-SPKAYN-
SAPKAY--
STPKAY--
-SPKAYW-
-SPKAYF-
SMPKAY--
-SPKAYY-
-SPKAYQ-
SDPKAY--
-SPKAYA-
-SPKAYG-
SEPKAY--
SPPKAY--
-SPKAYK-
-SPKAYH-
SKPKAY--
-SPKAYE-
-SPKAYC-
TLPKAY--
TRPKAY--
-TPKAYL-
-TPKAYT-
TGPKAY--
TVPKAY--

−TPKAYW−
TMPKAY−−
−TPKAYA−
−TPKAYF−
−TPKAYS−
−TPKAYR−
TWPKAY−−
−TPKAYK−
TEPKAY−−
TPPKAY−−
−TPKAYH−
TIPKAY−−
TAPKAY−−
TQPKAY−−
−TPKAYQ−
−TPKAYM−
TNPKAY−−
TKPKAY−−
−TPKAYG−
−TPKAYN−
THPKAY−−
−TPKAYE−
−VPKAYV−
VRPKAY−−
VAPKAY−−
−VPKAYL−
VWPKAY−−
VVPKAY−−
VGPKAY−−
VTPKAY−−
VIPKAY−−
−VPKAYY−
−VPKAYS−
VYPKAY−−
VDPKAY−−
−VPKAYN−
VPPKAY−−
−VPKAYT−
VHPKAY−−
−VPKAYI−
VNPKAY−−
VSPKAY−−
VEPKAY−−
VQPKAY−−
−VPKAYQ−
−VPKAYH−
−VPKAYG−
−VPKAYE−
VFPKAY−−
−VPKAYC−

-VPKAYM-
-WPKAYL-
-WPKAYI-
WTPKAY--
WRPKAY--
WGPKAY--
WSPKAY--
-WPKAYY-
-WPKAYE-
WPPKAY--
-WPKAYF-
WMPKAY--
-WPKAYQ-
WLPKAY--
-WPKAYN-
WDPKAY--
WCPKAY--
-WPKAYW-
-WPKAYV-
WFPKAY--
-WPKAYC-
-WPKAYM-
WYPKAY--
-WPKAYT-
WAPKAY--
YYPKAY--
YSPKAY--
YRPKAY--
-YPKAYW-
YVPKAY--
YKPKAY--
-YPKAYS-
YTPKAY--
-YPKAYQ-
YLPKAY--
YNPKAY--
YGPKAY--
YAPKAY--
-YPKAYR-
YIPKAY--
YPPKAY--
-YPKAYT-
-YPKAYM-
-YPKAYF-
-YPKAYE-
-YPKAYH-
YDPKAY--
-YPKAYY-
-YPKAYI-
-YPKAYG-

	YMPKAY--		
PQAL	17	886	48.46
	-MPQALH-		
	--PQALPK		
	--PQALGR		
	-RPQALP-		
	APPQAL--		
	ARPQAL--		
	-APQALR-		
	AMPQAL--		
	-APQALA-		
	-APQALS-		
	AVPQAL--		
	-APQALV-		
	ALPQAL--		
	AAPQAL--		
	-APQALF-		
	-APQALK-		
	ATPQAL--		
	AIPQAL--		
	-APQALQ-		
	AQPQAL--		
	AEPQAL--		
	-APQALM-		
	AGPQAL--		
	AYPQAL--		
	-APQALT-		
	AKPQAL--		
	ASPQAL--		
	-APQALY-		
	AWPQAL--		
	-APQALH-		
	-APQALI-		
	ACPQAL--		
	ADPQAL--		
	ANPQAL--		
	AFPQAL--		
	-APQALC-		
	-APQALE-		
	CSPQAL--		
	CTPQAL--		
	-CPQALM-		
	-CPQALT-		
	CYPQAL--		
	-CPQALR-		
	-CPQALH-		
	-CPQALA-		
	CKPQAL--		
	CQPQAL--		
	-CPQALY-		

-CPQALK-
-CPQALF-
CLPQAL--
CDPQAL--
CWPQAL--
-CPQALV-
-CPQALS-
CMPQAL--
CHPQAL--
CEPQAL--
DSPQAL--
-DPQALI-
DTPQAL--
-DPQALS-
DGPQAL--
DVPQAL--
DLPQAL--
DNPQAL--
DKPQAL--
DMPQAL--
DYPQAL--
DAPQAL--
DRPQAL--
-DPQALL-
-DPQALW-
-DPQALR-
DHPQAL--
-DPQALA-
DQPQAL--
-DPQALN-
-DPQALK-
DIPQAL--
DFPQAL--
DWPQAL--
-DPQALT-
-DPQALQ-
-EPQALT-
-EPQALR-
-EPQALK-
ELPQAL--
EVPQAL--
ERPQAL--
-EPQALV-
-EPQALS-
-EPQALL-
-EPQALQ-
EEPQAL--
EWPQAL--
EIPQAL--
EAPQAL--

EKPQAL--
ETPQAL--
-EPQALI-
-EPQALH-
EYPQAL--
ESPQAL--
EQPQAL--
-EPQALY-
-EPQALM-
-EPQALG-
EMPQAL--
-EPQALN-
-EPQALE-
EGPQAL--
EFPQAL--
-FPQALI-
FRPQAL--
FFPQAL--
FAPQAL--
-FPQALF-
FVPQAL--
FTPQAL--
-FPQALS-
-FPQALQ-
FNPQAL--
FPPQAL--
FGPQAL--
FQPQAL--
-FPQALV-
-FPQALR-
-FPQALL-
-FPQALK-
-FPQALY-
FIPQAL--
FLPQAL--
-FPQALN-
-FPQALH-
FKPQAL--
FDPQAL--
FMPQAL--
FHPQAL--
-FPQALT-
-FPQALM-
-FPQALW-
FCPQAL--
-FPQALE-
-FPQALA-
FEPQAL--
GRPQAL--
GSPQAL--

GFPQAL--
GYPQAL--
-GPQALT-
-GPQALK-
-GPQALH-
GAPQAL--
-GPQALS-
-GPQALR-
-GPQALV-
-GPQALM-
GTPQAL--
GPPQAL--
GGPQAL--
-GPQALF-
-GPQALA-
-GPQALQ-
GNPQAL--
GKPQAL--
GIPQAL--
GHPQAL--
GVPQAL--
GMPQAL--
-GPQALN-
-GPQALI-
-GPQALW-
-GPQALG-
-GPQALE-
-GPQALY-
GDPQAL--
-HPQALS-
HRPQAL--
-HPQALI-
HIPQAL--
HSPQAL--
HGPQAL--
HVPQAL--
-HPQALR-
-HPQALT-
-HPQALL-
HKPQAL--
HNPQAL--
HFPQAL--
HAPQAL--
-HPQALN-
HLPQAL--
HEPQAL--
-HPQALV-
-HPQALK-
-HPQALF-
HTPQAL--

HYPQAL--
HWPQAL--
-HPQALH-
HMPQAL--
-HPQALG-
-HPQALA-
HQPQAL--
-HPQALQ-
-HPQALM-
-HPQALE-
HDPQAL--
-IPQALR-
-IPQALS-
ILPQAL--
IVPQAL--
IMPQAL--
IHPQAL--
-IPQALT-
ISPQAL--
IPPQAL--
IWPQAL--
-IPQALW-
-IPQALN-
IKPQAL--
-IPQALH-
IRPQAL--
IGPQAL--
IFPQAL--
-IPQALI-
-IPQALG-
IAPQAL--
-IPQALA-
INPQAL--
-IPQALL-
IYPQAL--
-IPQALQ-
-IPQALF-
-IPQALV-
IEPQAL--
IDPQAL--
IIPQAL--
-IPQALY-
-IPQALK-
-IPQALD-
-KPQALI-
KIPQAL--
KLPQAL--
KGPQAL--
-KPQALT-
KQPQAL--

-KPQALA-
KPPQAL--
KRPQAL--
KVPQAL--
-KPQALQ-
-KPQALV-
KYPQAL--
-KPQALS-
KWPQAL--
-KPQALN-
-KPQALF-
KMPQAL--
KFPQAL--
KEPQAL--
-KPQALM-
KAPQAL--
KTPQAL--
-KPQALY-
-KPQALK-
-KPQALH-
-KPQALG-
KDPQAL--
-KPQALE-
KNPQAL--
KHPQAL--
KCPQAL--
LRPQAL--
LLPQAL--
LTPQAL--
LYPQAL--
-LPQALW-
-LPQALR-
LMPQAL--
LGPQAL--
LAPQAL--
LDPQAL--
-LPQALV-
LEPQAL--
-LPQALT-
LNPQAL--
LKPQAL--
-LPQALH-
LHPQAL--
LPPQAL--
-LPQALS-
-LPQALQ-
-LPQALF-
LQPQAL--
-LPQALK-
-LPQALI-

LIPQAL--
-LPQALA-
LFPQAL--
-LPQALN-
-LPQALM-
-LPQALG-
LCPQAL--
-LPQALE-
-MPQALS-
-MPQALL-
-MPQALI-
-MPQALV-
MGPQAL--
MLPQAL--
-MPQALR-
MRPQAL--
MFPQAL--
MIPQAL--
MDPQAL--
MYPQAL--
MWPQAL--
-MPQALY-
MVPQAL--
-MPQALT-
-MPQALF-
MPPQAL--
MKPQAL--
MHPQAL--
-MPQALG-
MNPQAL--
-MPQALW-
-MPQALN-
MEPQAL--
MSPQAL--
MQPQAL--
-MPQALQ-
MMPQAL--
MTPQAL--
-MPQALA-
-MPQALM-
-MPQALE-
MAPQAL--
NRPQAL--
NYPQAL--
-NPQALS-
-NPQALT-
NHPQAL--
-NPQALI-
NTPQAL--
NMPQAL--

NGPQAL--
NWPQAL--
NVPQAL--
NSPQAL--
NLPQAL--
NPPQAL--
NAPQAL--
-NPQALY-
-NPQALH-
NKPQAL--
NNPQAL--
-NPQALV-
-NPQALK-
-NPQALL-
NEPQAL--
-NPQALQ-
-NPQALN-
NQPQAL--
-NPQALW-
-NPQALM-
-NPQALF-
-NPQALA-
NIPQAL--
--PQALSS
--PQALST
--PQALLT
--PQALLK
--PQALSL
--PQALRF
--PQALRT
--PQALRL
--PQALAL
--PQALRS
PTPQAL--
--PQALTL
--PQALLR
--PQALIS
--PQALVR
--PQALLA
--PQALHL
--PQALTS
--PQALSW
--PQALMR
--PQALYI
--PQALVQ
--PQALWK
--PQALTT
--PQALHR
--PQALFM
--PQALWT

--PQALHS
--PQALSA
--PQALRA
--PQALLS
--PQALVL
--PQALLL
--PQALPL
--PQALIA
--PQALWL
--PQALKV
--PQALRN
--PQALMS
--PQALWR
--PQALVH
--PQALSI
--PQALYG
--PQALVA
--PQALQL
--PQALIW
--PQALAS
PWPQAL--
--PQALWS
--PQALWA
--PQALRW
--PQALRV
--PQALSY
--PQALSG
--PQALMI
--PQALMA
--PQALHM
--PQALGI
--PQALES
--PQALYE
--PQALNL
--PQALGS
--PQALFT
--PQALAF
PIPQAL--
--PQALRH
--PQALYL
--PQALVV
--PQALVN
--PQALTR
--PQALTA
--PQALRG
--PQALLH
PVPQAL--
--PQALVI
--PQALTF
--PQALNS

--PQALML
--PQALKL
--PQALHF
--PQALEY
--PQALSK
--PQALSF
--PQALNV
--PQALNM
--PQALFS
--PQALFR
--PQALYA
--PQALSR
--PQALMV
--PQALAT
--PQALYQ
--PQALRK
--PQALHY
--PQALEV
--PQALRY
--PQALQN
--PQALQK
--PQALNI
--PQALNA
--PQALLQ
--PQALKR
--PQALIV
--PQALYF
--PQALWM
--PQALVW
--PQALTH
--PQALIL
--PQALGL
PLPQAL--
PKPQAL--
--PQALWH
--PQALVK
--PQALTW
--PQALTQ
--PQALNF
--PQALIN
--PQALGF
--PQALAI
--PQALYS
--PQALYH
--PQALVS
--PQALQF
--PQALLD
--PQALHA
PFPQAL--
PQPQAL--

--PQALVF
--PQALVD
--PQALQM
--PQALMM
--PQALLI
--PQALLE
--PQALKG
--PQALIT
--PQALHN
--PQALFH
--PQALEW
--PQALAQ
PGPQAL--
--PQALWY
--PQALTG
--PQALSN
--PQALQD
--PQALNT
--PQALIG
--PQALGT
PMPQAL--
--PQALYN
--PQALTI
--PQALSH
--PQALKY
--PQALKK
--PQALKA
--PQALFG
--PQALEL
--PQALAW
--PQALAH
--PQALYT
--PQALWE
--PQALSQ
--PQALNR
--PQALKN
--PQALHV
--PQALHQ
PRPQAL--
--PQALWQ
--PQALRQ
--PQALQT
--PQALQA
--PQALMQ
--PQALMH
--PQALME
--PQALKI
--PQALIH
--PQALGY
--PQALFE

--PQALER
--PQALEG
--PQALYK
--PQALWW
--PQALID
--PQALGM
--PQALGH
--PQALGA
--PQALAR
PYPQAL--
--PQALYW
--PQALWG
--PQALSM
--PQALLN
--PQALKW
--PQALKE
--PQALII
--PQALHH
--PQALGQ
PEPQAL--
PAPQAL--
--PQALTY
--PQALSV
--PQALQS
--PQALQR
--PQALNQ
--PQALMW
--PQALMP
--PQALMD
--PQALLW
--PQALAV
PNPQAL--
PSPQAL--
--PQALTV
--PQALTM
--PQALSD
--PQALRP
--PQALRE
--PQALQW
--PQALQH
--PQALPF
--PQALMK
--PQALKQ
--PQALIF
--PQALGN
--PQALEH
--PQALDS
--PQALAY
--PQALAK
--PQALAA

PHPQAL--
PDPQAL--
--PQALVE
--PQALTE
--PQALQV
--PQALQE
--PQALNN
--PQALNE
--PQALHI
--PQALHD
--PQALGV
--PQALGG
--PQALFK
--PQALFA
--PQALET
--PQALEA
--PQALDL
--PQALCI
--PQALYP
--PQALYD
--PQALSE
--PQALQQ
--PQALPM
--PQALNK
--PQALNG
--PQALND
--PQALMF
--PQALHT
--PQALFQ
--PQALEN
--PQALEM
--PQALEK
--PQALDY
--PQALDM
--PQALCG
--PQALAD
--PQALVM
--PQALVC
--PQALRC
--PQALQY
--PQALQI
--PQALQG
--PQALPY
--PQALPG
--PQALMT
--PQALKT
--PQALKM
--PQALKF
--PQALIQ
--PQALEI

--PQALDR
--PQALDN
--PQALDF
--PQALDD
--PQALCY
--PQALCQ
--PQALAE
-PPQALR-
-PPQALM-
PPPQAL--
QSPQAL--
QIPQAL--
QVPQAL--
-QPQALI-
QRPQAL--
-QPQALN-
QYPQAL--
-QPQALY-
-QPQALH-
QQPQAL--
QTPQAL--
-QPQALL-
-QPQALK-
QKPQAL--
QDPQAL--
-QPQALT-
-QPQALQ-
QFPQAL--
-QPQALS-
-QPQALR-
-QPQALW-
QGPQAL--
QWPQAL--
QLPQAL--
-QPQALA-
QAPQAL--
-QPQALG-
-QPQALV-
QMPQAL--
-QPQALF-
-QPQALD-
QPPQAL--
QNPQAL--
QEPQAL--
RTPQAL--
RSPQAL--
-RPQALL-
-RPQALY-
-RPQALR-
RGPQAL--

RVPQAL--
RAPQAL--
RIPQAL--
RLPQAL--
-RPQALN-
RQPQAL--
-RPQALW-
-RPQALI-
-RPQALT-
RPPQAL--
RMPQAL--
RWPQAL--
-RPQALH-
RHPQAL--
REPQAL--
-RPQALM-
-RPQALK-
RYPQAL--
RKPQAL--
-RPQALV-
-RPQALE-
-RPQALA-
RNPQAL--
RDPQAL--
-RPQALF-
RCPQAL--
-RPQALQ-
RFPQAL--
-RPQALC-
-SPQALR-
SRPQAL--
SHPQAL--
-SPQALL-
SSPQAL--
SGPQAL--
SYPQAL--
STPQAL--
SLPQAL--
-SPQALK-
SNPQAL--
-SPQALT-
SPPQAL--
-SPQALM-
SKPQAL--
SMPQAL--
SWPQAL--
-SPQALQ-
-SPQALN-
-SPQALA-
-SPQALV-

SIPQAL--
SEPQAL--
-SPQALS-
SQPQAL--
SDPQAL--
-SPQALY-
SFPQAL--
-SPQALH-
-SPQALW-
-SPQALI-
-SPQALF-
SCPQAL--
-SPQALG-
-SPQALE-
TTPQAL--
-TPQALR-
TRPQAL--
TFPQAL--
TGPQAL--
-TPQALV-
TPPQAL--
-TPQALL-
-TPQALW-
TIPQAL--
TKPQAL--
TWPQAL--
-TPQALS-
-TPQALI-
TSPQAL--
TDPQAL--
THPQAL--
TNPQAL--
-TPQALY-
TQPQAL--
-TPQALH-
TMPQAL--
-TPQALA-
-TPQALG-
TAPQAL--
TYPQAL--
TVPQAL--
-TPQALF-
-TPQALK-
-TPQALN-
-TPQALE-
TCPQAL--
-VPQALR-
VRPQAL--
-VPQALL-
VSPQAL--

-VPQALT-
-VPQALS-
-VPQALW-
-VPQALK-
VMPQAL--
-VPQALM-
VLPQAL--
VIPQAL--
VGPQAL--
VYPQAL--
VWPQAL--
VPPQAL--
VKPQAL--
VCPQAL--
-VPQALG-
-VPQALA-
VHPQAL--
-VPQALH-
VFPQAL--
VNPQAL--
-VPQALE-
-VPQALN-
VEPQAL--
WVPQAL--
WSPQAL--
WLPQAL--
WQPQAL--
-WPQALS-
-WPQALW-
-WPQALN-
WRPQAL--
WTPQAL--
WYPQAL--
-WPQALM-
WFPQAL--
-WPQALV-
WAPQAL--
-WPQALH-
-WPQALK-
WMPQAL--
-WPQALQ-
WNPQAL--
WKPQAL--
WHPQAL--
-WPQALL-
-WPQALI-
-WPQALT-
-WPQALG-
-WPQALF-
-WPQALA-

	WDPQAL--		
	-WPQALC-		
	WCPQAL--		
	YSPQAL--		
	YMPQAL--		
	YRPQAL--		
	-YPQALK-		
	YQPQAL--		
	-YPQALH-		
	YHPQAL--		
	-YPQALY-		
	-YPQALR-		
	-YPQALL-		
	-YPQALI-		
	YVPQAL--		
	YPPQAL--		
	YKPQAL--		
	YTPQAL--		
	-YPQALN-		
	YGPQAL--		
	-YPQALS-		
	YLPQAL--		
	YAPQAL--		
	YNPQAL--		
	YIPQAL--		
	-YPQALW-		
	-YPQALA-		
	YDPQAL--		
	YWPQAL--		
	YCPQAL--		
	YYPQAL--		
	-YPQALG-		
	-YPQALT-		
	YEPQAL--		
	-YPQALQ-		
	-YPQALM-		
	-YPQALE-		
	-YPQALD-		
PKGL	88	818	48.41
	-APKGLD-		
	-CPKGLK-		
	-DPKGLG-		
	--PKGLPL		
	--PKGLPS		
	--PKGLPA		
	--PKGLIP		
	-TPKGLC-		
	-WPKGLD-		
	-APKGLL-		
	ALPKGL--		

AVPKGL--
-APKGLY-
-APKGLR-
ASPKGL--
-APKGLV-
AIPKGL--
-APKGLS-
AYPKGL--
AAPKGL--
-APKGLT-
AHPKGL--
ATPKGL--
-APKGLW-
AGPKGL--
-APKGLK-
-APKGLI-
APPKGL--
ANPKGL--
-APKGLH-
AMPKGL--
AKPKGL--
AQPKGL--
AEPKGL--
-APKGLM-
-APKGLG-
-APKGLF-
-APKGLA-
-APKGLE-
ADPKGL--
-APKGLN-
ACPKGL--
-APKGLC-
CHPKGL--
CRPKGL--
CSPKGL--
CLPKGL--
CPPKGL--
CGPKGL--
CVPKGL--
CIPKGL--
CTPKGL--
-CPKGLF-
-CPKGLS-
-CPKGLN-
-CPKGLL-
CAPKGL--
CYPKGL--
CWPKGL--
-CPKGLV-
-CPKGLQ-

CKPKGL--
-CPKGLY-
CMPKGL--
-CPKGLT-
-CPKGLI-
CNPKGL--
CFPKGL--
-CPKGLW-
-CPKGLH-
-CPKGLG-
-CPKGLE-
-CPKGLA-
DSPKGL--
DAPKGL--
-DPKGLV-
DIPKGL--
DFPKGL--
-DPKGLL-
DLPKGL--
DYPKGL--
-DPKGLY-
-DPKGLF-
-DPKGLN-
-DPKGLA-
DKPKGL--
-DPKGLT-
-DPKGLW-
-DPKGLR-
DNPKGL--
DQPKGL--
-DPKGLI-
-DPKGLK-
DHPKGL--
DGPKGL--
-DPKGLM-
-DPKGLH-
DMPKGL--
-DPKGLQ-
DWPKGL--
-DPKGLE-
-DPKGLS-
DEPKGL--
DDPKGL--
DCPKGL--
-EPKGLR-
EMPKGL--
EVPKGL--
EIPKGL--
ESPKGL--
EGPKGL--

EFPKGL--
ERPKGL--
-EPKGLV-
-EPKGLL-
-EPKGLQ-
-EPKGLH-
-EPKGLY-
-EPKGLS-
ETPKGL--
-EPKGLM-
-EPKGLK-
EWPKGL--
-EPKGLF-
EHPKGL--
EYPKGL--
ELPKGL--
EPPKGL--
EAPKGL--
EQPKGL--
EKPKGL--
-EPKGLA-
-EPKGLN-
-EPKGLI-
-EPKGLE-
ECPKGL--
-EPKGLW-
EEPKGL--
-EPKGLG-
ENPKGL--
EDPKGL--
FVPKGL--
-FPKGLY-
FNPKGL--
FTPKGL--
FLPKGL--
-FPKGLT-
FGPKGL--
FDPKGL--
-FPKGLK-
-FPKGLN-
FKPKGL--
FPPKGL--
-FPKGLW-
FMPKGL--
FWPKGL--
FQPKGL--
-FPKGLM-
-FPKGLI-
-FPKGLF-
FIPKGL--

-FPKGLD-
GAPKGL--
-GPKGLT-
-GPKGLL-
GYPKGL--
GSPKGL--
-GPKGLG-
-GPKGLV-
-GPKGLM-
GIPKGL--
-GPKGLY-
GVPKGL--
GPPKGL--
-GPKGLS-
GLPKGL--
-GPKGLA-
-GPKGLN-
GEPKGL--
-GPKGLK-
GQPKGL--
-GPKGLW-
-GPKGLE-
GNPKGL--
GGPKGL--
-GPKGLQ-
-GPKGLF-
GWPKGL--
GHPKGL--
GDPKGL--
GTPKGL--
-GPKGLC-
GCPKGL--
-HPKGLL-
-HPKGLT-
HSPKGL--
-HPKGLS-
HVPKGL--
-HPKGLQ-
HTPKGL--
-HPKGLA-
HPPKGL--
-HPKGLN-
HYPKGL--
-HPKGLY-
HAPKGL--
HMPKGL--
HIPKGL--
HFPKGL--
HQPKGL--
HGPKGL--

HHPKGL--
-HPKGLH-
-HPKGLM-
-HPKGLF-
-HPKGLG-
HEPKGL--
HCPKGL--
-HPKGLE-
HNPKGL--
-HPKGLK-
-IPKGLL-
IYPKGL--
IIPKGL--
IRPKGL--
ISPKGL--
-IPKGLR-
IGPKGL--
ILPKGL--
-IPKGLT-
IAPKGL--
-IPKGLF-
IQPKGL--
-IPKGLS-
-IPKGLN-
-IPKGLK-
-IPKGLM-
-IPKGLA-
IMPKGL--
-IPKGLY-
-IPKGLI-
ICPKGL--
-IPKGLH-
IHPKGL--
-IPKGLE-
IEPKGL--
INPKGL--
IDPKGL--
-IPKGLG-
-IPKGLW-
-IPKGLC-
-IPKGLD-
IKPKGL--
-IPKGLP-
KSPKGL--
-KPKGLL-
KGPKGL--
-KPKGLT-
-KPKGLS-
-KPKGLA-
KIPKGL--

−KPKGLY−
KVPKGL−−
KLPKGL−−
−KPKGLI−
KTPKGL−−
−KPKGLQ−
KAPKGL−−
KNPKGL−−
KPPKGL−−
−KPKGLW−
KKPKGL−−
KHPKGL−−
KEPKGL−−
−KPKGLN−
−KPKGLM−
−KPKGLF−
KQPKGL−−
−KPKGLH−
−KPKGLG−
KCPKGL−−
−KPKGLE−
−KPKGLP−
LVPKGL−−
−LPKGLR−
LSPKGL−−
LYPKGL−−
LLPKGL−−
−LPKGLT−
−LPKGLF−
−LPKGLH−
LNPKGL−−
LHPKGL−−
LMPKGL−−
−LPKGLS−
−LPKGLY−
−LPKGLV−
LGPKGL−−
−LPKGLN−
LDPKGL−−
LTPKGL−−
LEPKGL−−
−LPKGLA−
LPPKGL−−
−LPKGLQ−
LCPKGL−−
−LPKGLG−
−LPKGLE−
−LPKGLC−
−MPKGLL−
MSPKGL−−

MQPKGL--
-MPKGLQ-
MYPKGL--
MMPKGL--
MKPKGL--
MAPKGL--
-MPKGLT-
MIPKGL--
-MPKGLV-
-MPKGLR-
-MPKGLS-
-MPKGLG-
-MPKGLK-
-MPKGLY-
-MPKGLH-
-MPKGLA-
MNPKGL--
-MPKGLN-
MHPKGL--
MDPKGL--
-MPKGLM-
MPPKGL--
MEPKGL--
MGPKGL--
MCPKGL--
-MPKGLF-
-MPKGLE-
-MPKGLC-
NIPKGL--
NGPKGL--
-NPKGLL-
NYPKGL--
NVPKGL--
NSPKGL--
-NPKGLT-
NAPKGL--
-NPKGLI-
-NPKGLH-
NWPKGL--
-NPKGLM-
-NPKGLS-
-NPKGLK-
NTPKGL--
NQPKGL--
-NPKGLW-
-NPKGLV-
-NPKGLF-
NDPKGL--
-NPKGLQ-
-NPKGLN-

NPPKGL--
NFPKGL--
NEPKGL--
-NPKGLY-
NNPKGL--
-NPKGLE-
-NPKGLA-
NCPKGL--
-NPKGLG-
-NPKGLD-
--PKGLVG
--PKGLYL
--PKGLLF
--PKGLTS
--PKGLVL
--PKGLYK
--PKGLSF
--PKGLKS
--PKGLAS
--PKGLYR
--PKGLSW
--PKGLRG
--PKGLIL
--PKGLYH
--PKGLVN
--PKGLSI
--PKGLLG
--PKGLRT
--PKGLHT
--PKGLSY
--PKGLVV
--PKGLWA
--PKGLWS
--PKGLWG
--PKGLVT
--PKGLSG
PVPKGL--
--PKGLVF
--PKGLTG
--PKGLLH
--PKGLNW
--PKGLWD
--PKGLHH
--PKGLFL
--PKGLSA
--PKGLTT
--PKGLSL
--PKGLLE
--PKGLKG
--PKGLFV

--PKGLAV
--PKGLVI
--PKGLGV
--PKGLNM
--PKGLMA
--PKGLGT
PYPKGL--
--PKGLVW
--PKGLNV
--PKGLIS
--PKGLTF
--PKGLWY
--PKGLSM
--PKGLMI
--PKGLLY
--PKGLFK
--PKGLAG
--PKGLRE
--PKGLYV
--PKGLMH
--PKGLMG
--PKGLII
--PKGLIH
PSPKGL--
--PKGLYT
PLPKGL--
--PKGLYS
--PKGLGA
--PKGLYI
--PKGLRD
--PKGLQS
--PKGLLD
PGPKGL--
--PKGLRQ
--PKGLNT
--PKGLLK
--PKGLAY
--PKGLWV
--PKGLTV
--PKGLLQ
--PKGLKN
--PKGLIK
--PKGLSV
PHPKGL--
PRPKGL--
--PKGLTY
--PKGLSN
--PKGLNS
--PKGLNI
--PKGLSK

--PKGLIA
--PKGLHN
--PKGLGS
--PKGLNF
PQPKGL--
--PKGLVA
--PKGLIV
PMPKGL--
--PKGLYF
--PKGLYA
--PKGLNY
--PKGLFM
--PKGLAI
PEPKGL--
--PKGLTM
--PKGLWE
--PKGLIG
--PKGLYN
--PKGLYM
--PKGLQL
--PKGLQA
--PKGLML
--PKGLFI
--PKGLHS
--PKGLHG
--PKGLGI
--PKGLFQ
--PKGLFN
--PKGLMD
--PKGLIM
PTPKGL--
--PKGLQW
--PKGLMT
--PKGLFY
--PKGLQI
--PKGLFH
--PKGLAA
--PKGLTH
--PKGLIT
--PKGLGG
--PKGLES
--PKGLAK
--PKGLKM
--PKGLHV
--PKGLDS
PCPKGL--
--PKGLWQ
--PKGLNK
--PKGLEM
--PKGLTN

--PKGLTI
--PKGLQG
--PKGLKT
--PKGLKQ
--PKGLIN
--PKGLAT
--PKGLYY
--PKGLWT
--PKGLVD
--PKGLSQ
--PKGLKH
--PKGLIF
--PKGLEF
--PKGLNR
--PKGLID
--PKGLHE
--PKGLFS
--PKGLTQ
--PKGLTK
--PKGLIW
--PKGLFF
--PKGLAQ
--PKGLQK
--PKGLHW
--PKGLEG
PDPKGL--
--PKGLQT
--PKGLMN
--PKGLIQ
--PKGLHY
--PKGLHF
--PKGLAN
--PKGLQV
--PKGLNN
--PKGLKF
--PKGLIE
--PKGLFD
--PKGLEW
--PKGLMW
--PKGLKE
--PKGLEY
--PKGLEV
--PKGLWN
--PKGLVQ
--PKGLSE
--PKGLNH
--PKGLNG
--PKGLND
--PKGLMK
--PKGLER

--PKGLYQ
--PKGLQM
--PKGLMQ
--PKGLMF
--PKGLGL
--PKGLAW
PKPKGL--
--PKGLWW
--PKGLVE
--PKGLTW
--PKGLNA
--PKGLEN
--PKGLDG
--PKGLQF
--PKGLNE
--PKGLGH
PNPKGL--
--PKGLTD
--PKGLGQ
--PKGLEH
--PKGLGK
--PKGLWM
--PKGLQE
--PKGLMM
--PKGLEA
--PKGLCN
--PKGLAF
--PKGLQD
--PKGLDA
--PKGLYC
--PKGLQQ
--PKGLKK
--PKGLYD
--PKGLTE
--PKGLME
--PKGLET
--PKGLEQ
--PKGLAD
--PKGLKC
--PKGLGW
--PKGLEL
--PKGLEI
--PKGLNC
--PKGLHM
--PKGLEE
--PKGLDY
--PKGLCL
--PKGLCI
-PPKGLY-
-PPKGLS-

-PPKGLR-
-PPKGLM-
-PPKGLL-
--PKGLRC
--PKGLPN
--PKGLPH
--PKGLHQ
--PKGLHK
--PKGLHI
--PKGLHD
--PKGLGE
--PKGLEK
--PKGLED
--PKGLEC
--PKGLDW
--PKGLCW
--PKGLCV
--PKGLCR
--PKGLCQ
--PKGLCM
--PKGLCH
--PKGLCF
--PKGLAE
--PKGLAC
PAPKGL--
QLPKGL--
QRPKGL--
QTPKGL--
-QPKGLL-
-QPKGLY-
-QPKGLK-
QSPKGL--
QPPKGL--
QHPKGL--
QVPKGL--
-QPKGLT-
-QPKGLN-
QIPKGL--
QNPKGL--
-QPKGLF-
-QPKGLI-
QKPKGL--
-QPKGLW-
QYPKGL--
-QPKGLS-
-QPKGLH-
QGPKGL--
QEPKGL--
QQPKGL--
-QPKGLA-

-QPKGLQ-
-QPKGLE-
-QPKGLG-
QAPKGL--
-QPKGLM-
QDPKGL--
QMPKGL--
QCPKGL--
-RPKGLL-
RMPKGL--
RTPKGL--
RIPKGL--
-RPKGLV-
REPKGL--
-RPKGLF-
-RPKGLW-
RDPKGL--
RNPKGL--
RYPKGL--
-RPKGLH-
-RPKGLI-
RGPKGL--
RPPKGL--
-RPKGLE-
-RPKGLN-
RQPKGL--
-RPKGLA-
RCPKGL--
-RPKGLQ-
-SPKGLL-
SLPKGL--
-SPKGLT-
SMPKGL--
-SPKGLV-
SHPKGL--
-SPKGLY-
SNPKGL--
-SPKGLH-
-SPKGLW-
-SPKGLN-
-SPKGLM-
SGPKGL--
SDPKGL--
-SPKGLQ-
-SPKGLG-
-SPKGLE-
SQPKGL--
SCPKGL--
SKPKGL--
SEPKGL--

-SPKGLC-
-SPKGLD-
-TPKGLI-
TGPKGL--
-TPKGLY-
TSPKGL--
-TPKGLS-
TEPKGL--
TYPKGL--
TLPKGL--
TTPKGL--
TPPKGL--
TQPKGL--
-TPKGLA-
-TPKGLV-
TAPKGL--
-TPKGLF-
TKPKGL--
TWPKGL--
-TPKGLW-
-TPKGLQ-
-TPKGLM-
-TPKGLN-
-TPKGLE-
-TPKGLG-
TMPKGL--
TIPKGL--
-TPKGLK-
TDPKGL--
TCPKGL--
-VPKGLL-
-VPKGLT-
VVPKGL--
VPPKGL--
-VPKGLR-
-VPKGLH-
VIPKGL--
VAPKGL--
VTPKGL--
-VPKGLK-
-VPKGLF-
-VPKGLN-
-VPKGLA-
VQPKGL--
-VPKGLM-
VMPKGL--
-VPKGLV-
VGPKGL--
VHPKGL--
VEPKGL--

-VPKGLI-
-VPKGLE-
-VPKGLY-
VDPKGL--
-VPKGLW-
VCPKGL--
WVPKGL--
WSPKGL--
-WPKGLY-
-WPKGLL-
WKPKGL--
WGPKGL--
-WPKGLV-
-WPKGLG-
WQPKGL--
WTPKGL--
-WPKGLT-
WMPKGL--
WWPKGL--
-WPKGLE-
-WPKGLS-
-WPKGLM-
WFPKGL--
WYPKGL--
-WPKGLA-
WDPKGL--
WHPKGL--
WEPKGL--
-WPKGLQ-
-WPKGLN-
WPPKGL--
-WPKGLC-
-YPKGLL-
YLPKGL--
YWPKGL--
-YPKGLY-
-YPKGLM-
YSPKGL--
-YPKGLS-
-YPKGLF-
YIPKGL--
YYPKGL--
-YPKGLV-
-YPKGLA-
-YPKGLQ-
YMPKGL--
YGPKGL--
YAPKGL--
YEPKGL--
YVPKGL--

```
           -YPKGLW-
           YDPKGL--
           -YPKGLE-
           -YPKGLN-
           -YPKGLG-
           YPPKGL--
           YNPKGL--
PRNM        150         776      48.40
           --PRNMNP
           --PRNMNG
           --PRNMDS
           -VPRNMP-
           -YPRNMD-
           -APRNML-
           -APRNMS-
           -APRNMK-
           -APRNMY-
           -APRNMH-
           AVPRNM--
           -APRNMI-
           -APRNMM-
           ARPRNM--
           ASPRNM--
           AHPRNM--
           AAPRNM--
           -APRNMG-
           AKPRNM--
           AIPRNM--
           -APRNMW-
           ATPRNM--
           AGPRNM--
           -APRNMT-
           ANPRNM--
           -APRNMF-
           -APRNMA-
           AQPRNM--
           -APRNME-
           AEPRNM--
           ADPRNM--
           -APRNMN-
           AMPRNM--
           -APRNMQ-
           -APRNMC-
           APPRNM--
           ACPRNM--
           CRPRNM--
           -CPRNML-
           CGPRNM--
           CCPRNM--
           CVPRNM--
```

CMPRNM--
-CPRNMV-
-CPRNMT-
CKPRNM--
CFPRNM--
CDPRNM--
CSPRNM--
-CPRNMI-
-CPRNMG-
CNPRNM--
-CPRNMS-
CPPRNM--
CLPRNM--
CHPRNM--
DVPRNM--
DMPRNM--
-DPRNMR-
DYPRNM--
-DPRNMS-
DRPRNM--
DLPRNM--
DKPRNM--
DTPRNM--
-DPRNMG-
DEPRNM--
-DPRNMM-
-DPRNML-
DSPRNM--
-DPRNMA-
DDPRNM--
DAPRNM--
-DPRNMK-
-DPRNMF-
DIPRNM--
DWPRNM--
-DPRNMT-
-DPRNMI-
-DPRNMH-
DHPRNM--
DGPRNM--
DFPRNM--
ESPRNM--
EVPRNM--
-EPRNMR-
EQPRNM--
EHPRNM--
-EPRNMY-
-EPRNMQ-
EYPRNM--
EFPRNM--

-EPRNMA-
EKPRNM--
-EPRNMT-
EIPRNM--
EGPRNM--
EEPRNM--
ETPRNM--
-EPRNMM-
-EPRNML-
ERPRNM--
-EPRNMH-
-EPRNMF-
-EPRNMS-
-EPRNMK-
-EPRNMG-
-EPRNMV-
-EPRNMN-
-EPRNME-
ENPRNM--
EDPRNM--
EAPRNM--
-EPRNMI-
FRPRNM--
-FPRNMG-
FSPRNM--
-FPRNML-
-FPRNMS-
FTPRNM--
FNPRNM--
FMPRNM--
-FPRNMY-
-FPRNMQ-
FAPRNM--
-FPRNMV-
-FPRNMH-
FLPRNM--
-FPRNME-
FIPRNM--
-FPRNMN-
-FPRNMK-
FVPRNM--
-FPRNMD-
FHPRNM--
FEPRNM--
FDPRNM--
FCPRNM--
-GPRNMM-
-GPRNMW-
GRPRNM--
-GPRNMT-

GYPRNM--
GVPRNM--
GSPRNM--
-GPRNMR-
-GPRNMV-
GTPRNM--
GLPRNM--
GIPRNM--
GAPRNM--
-GPRNMQ-
-GPRNME-
-GPRNMG-
-GPRNMS-
-GPRNMI-
GWPRNM--
GKPRNM--
-GPRNMA-
-GPRNMH-
-GPRNMK-
-GPRNMY-
GQPRNM--
GNPRNM--
-GPRNMN-
GEPRNM--
-GPRNMF-
GHPRNM--
-HPRNMY-
HSPRNM--
-HPRNMS-
-HPRNMT-
-HPRNML-
HVPRNM--
HTPRNM--
HLPRNM--
-HPRNMR-
HQPRNM--
HYPRNM--
-HPRNMV-
-HPRNMG-
-HPRNMA-
HGPRNM--
HRPRNM--
-HPRNMM-
HHPRNM--
HEPRNM--
-HPRNMW-
-HPRNMN-
-HPRNMH-
HCPRNM--
-HPRNMI-

HMPRNM--
-HPRNMF-
HPPRNM--
HKPRNM--
HDPRNM--
ISPRNM--
-IPRNMR-
IGPRNM--
IIPRNM--
-IPRNMM-
ILPRNM--
-IPRNMW-
-IPRNMG-
-IPRNMH-
INPRNM--
-IPRNMS-
-IPRNMV-
IEPRNM--
-IPRNMK-
-IPRNMF-
-IPRNMN-
-IPRNMQ-
IQPRNM--
-IPRNMT-
IPPRNM--
-IPRNMI-
-IPRNME-
IHPRNM--
IDPRNM--
ICPRNM--
IAPRNM--
IFPRNM--
ITPRNM--
IKPRNM--
-KPRNMS-
-KPRNML-
KLPRNM--
-KPRNMW-
KVPRNM--
-KPRNMY-
KGPRNM--
KQPRNM--
KYPRNM--
KWPRNM--
-KPRNMV-
-KPRNMM-
-KPRNMG-
KDPRNM--
KMPRNM--
-KPRNMH-

KRPRNM--
-KPRNMK-
-KPRNMI-
KPPRNM--
KTPRNM--
-KPRNME-
KNPRNM--
KKPRNM--
-KPRNMD-
LLPRNM--
-LPRNMY-
-LPRNMT-
-LPRNMH-
LGPRNM--
LVPRNM--
LIPRNM--
LNPRNM--
LYPRNM--
LTPRNM--
-LPRNMN-
LPPRNM--
-LPRNMA-
LMPRNM--
-LPRNMW-
-LPRNMG-
-LPRNMQ-
-LPRNMM-
LKPRNM--
LCPRNM--
LQPRNM--
-LPRNMF-
-LPRNMV-
-LPRNME-
LDPRNM--
LEPRNM--
-LPRNMC-
-MPRNML-
MGPRNM--
-MPRNMI-
MVPRNM--
-MPRNMK-
-MPRNMS-
-MPRNMA-
-MPRNMF-
MPPRNM--
MAPRNM--
MTPRNM--
-MPRNMM-
-MPRNMG-
-MPRNMQ-

MDPRNM--
MYPRNM--
-MPRNMV-
MMPRNM--
-MPRNMH-
MHPRNM--
-MPRNMY-
-MPRNMT-
-MPRNME-
MNPRNM--
-MPRNMN-
MIPRNM--
NLPRNM--
-NPRNML-
NGPRNM--
-NPRNMW-
-NPRNMT-
-NPRNMA-
NRPRNM--
NVPRNM--
NSPRNM--
-NPRNMV-
-NPRNMR-
-NPRNMY-
NKPRNM--
NNPRNM--
-NPRNMF-
NTPRNM--
NAPRNM--
NYPRNM--
NPPRNM--
NMPRNM--
-NPRNMQ-
-NPRNMN-
-NPRNMI-
-NPRNME-
-NPRNMG-
NHPRNM--
NEPRNM--
-NPRNMM-
--PRNMTS
--PRNMSS
--PRNMRV
--PRNMYL
--PRNMIS
--PRNMFL
--PRNMSV
--PRNMIT
--PRNMTL
--PRNMSG

--PRNMFT
--PRNMSY
--PRNMIG
--PRNMLM
--PRNMYR
--PRNMGI
--PRNMYS
--PRNMSQ
--PRNMVS
--PRNMWV
--PRNMMS
--PRNMNS
--PRNMIM
--PRNMGS
--PRNMYI
--PRNMFA
--PRNMSK
--PRNMYG
--PRNMVI
--PRNMLH
--PRNMES
--PRNMRD
--PRNMFG
--PRNMVN
--PRNMVM
--PRNMTT
--PRNMTF
--PRNMRT
--PRNMRM
--PRNMQL
--PRNMLT
--PRNMIR
--PRNMAL
--PRNMFS
--PRNMAK
PYPRNM--
--PRNMWW
--PRNMRW
--PRNMNY
--PRNMLF
--PRNMLE
--PRNMRI
--PRNMLI
--PRNMWY
--PRNMWH
--PRNMVW
--PRNMLY
--PRNMVT
--PRNMRA
--PRNMNI

--PRNMHW
--PRNMAW
PGPRNM--
--PRNMWS
--PRNMSF
--PRNMGL
--PRNMEL
--PRNMAS
--PRNMVF
--PRNMST
--PRNMMV
--PRNMMN
PVPRNM--
--PRNMGK
--PRNMAT
--PRNMTW
--PRNMLG
--PRNMIY
--PRNMYT
--PRNMNV
--PRNMNR
--PRNMMR
--PRNMLQ
--PRNMIE
--PRNMHR
--PRNMFQ
--PRNMFD
--PRNMVA
--PRNMTM
--PRNMSD
--PRNMSA
--PRNMQY
--PRNMKL
--PRNMFH
--PRNMEI
--PRNMAD
PSPRNM--
--PRNMYY
--PRNMYK
--PRNMYH
--PRNMRY
--PRNMLN
--PRNMKT
--PRNMIV
--PRNMHS
--PRNMAF
--PRNMWD
--PRNMTA
--PRNMRE
--PRNMQA

--PRNMLD
--PRNMFR
--PRNMFF
--PRNMEY
--PRNMAY
--PRNMAN
--PRNMVE
--PRNMTH
--PRNMTD
--PRNMNW
--PRNMNN
--PRNMNF
--PRNMMY
--PRNMGG
--PRNMER
PKPRNM--
--PRNMYD
--PRNMWE
--PRNMTN
--PRNMTI
--PRNMQK
--PRNMKS
--PRNMHN
--PRNMGQ
--PRNMGA
--PRNMFW
PIPRNM--
PAPRNM--
--PRNMYV
--PRNMWQ
--PRNMQW
--PRNMQV
--PRNMNM
--PRNMMH
--PRNMMA
--PRNMKV
--PRNMIW
--PRNMGT
--PRNMAQ
PHPRNM--
--PRNMYM
--PRNMYE
--PRNMVD
--PRNMRQ
--PRNMQT
--PRNMNL
--PRNMMI
--PRNMKF
--PRNMHE
--PRNMGW

--PRNMGV
--PRNMFM
--PRNMFE
--PRNMED
--PRNMAV
--PRNMAM
--PRNMAG
--PRNMAA
PFPRNM--
PWPRNM--
--PRNMYN
--PRNMWF
--PRNMMD
--PRNMKY
--PRNMHV
--PRNMHF
--PRNMAI
PEPRNM--
PRPRNM--
--PRNMQG
--PRNMQE
--PRNMNA
--PRNMKM
--PRNMHH
--PRNMGR
--PRNMGM
--PRNMGF
--PRNMFK
--PRNMEW
--PRNMEM
--PRNMTV
--PRNMTQ
--PRNMRP
--PRNMLW
--PRNMKW
--PRNMKA
--PRNMIA
--PRNMHM
--PRNMHK
--PRNMHI
--PRNMEV
--PRNMET
--PRNMTE
--PRNMSE
--PRNMQM
--PRNMME
--PRNMKQ
--PRNMKD
--PRNMHT
--PRNMHQ

--PRNMHG
--PRNMHA
--PRNMFY
--PRNMFI
--PRNMEG
--PRNMEF
--PRNMDA
PMPRNM--
--PRNMYF
--PRNMTY
--PRNMNK
--PRNMKN
--PRNMKG
--PRNMGH
--PRNMGE
--PRNMGD
--PRNMEN
--PRNMDW
--PRNMDV
--PRNMDT
--PRNMDM
--PRNMCS
PNPRNM--
PTPRNM--
--PRNMYA
--PRNMVP
--PRNMTC
--PRNMQS
--PRNMQQ
--PRNMQD
--PRNMPL
--PRNMMM
--PRNMKE
--PRNMID
--PRNMHY
--PRNMHD
--PRNMEA
--PRNMDN
--PRNMAE
-PPRNMR-
-PPRNMP-
PDPRNM--
QYPRNM--
-QPRNMR-
-QPRNMW-
-QPRNMS-
-QPRNML-
-QPRNMT-
QPPRNM--
-QPRNMV-

QFPRNM--
QWPRNM--
-QPRNMN-
-QPRNMY-
-QPRNMQ-
-QPRNMH-
-QPRNMM-
QAPRNM--
QSPRNM--
QQPRNM--
-QPRNMA-
QMPRNM--
QIPRNM--
QGPRNM--
QTPRNM--
QNPRNM--
QVPRNM--
-QPRNMG-
-QPRNMF-
-QPRNMI-
-QPRNMC-
QDPRNM--
QCPRNM--
-RPRNML-
-RPRNMV-
-RPRNMS-
-RPRNMY-
RVPRNM--
-RPRNMF-
REPRNM--
-RPRNMN-
RHPRNM--
-RPRNMA-
-RPRNMG-
-RPRNMH-
RPPRNM--
RQPRNM--
-RPRNMI-
-RPRNMW-
-RPRNME-
RTPRNM--
RAPRNM--
RDPRNM--
-RPRNMM-
RCPRNM--
-RPRNMC-
RNPRNM--
-SPRNML-
-SPRNMV-
SRPRNM--

SNPRNM--
SKPRNM--
SLPRNM--
-SPRNMI-
-SPRNMH-
-SPRNMM-
-SPRNMW-
SQPRNM--
-SPRNMN-
SGPRNM--
STPRNM--
-SPRNMA-
SEPRNM--
-SPRNMG-
-SPRNMY-
-SPRNMF-
-SPRNMK-
SHPRNM--
SCPRNM--
-SPRNME-
SDPRNM--
-SPRNMQ-
SPPRNM--
SIPRNM--
-TPRNML-
TLPRNM--
-TPRNMR-
-TPRNMH-
-TPRNMK-
TTPRNM--
-TPRNMI-
-TPRNMW-
TGPRNM--
TKPRNM--
TAPRNM--
TSPRNM--
TVPRNM--
-TPRNMV-
TEPRNM--
-TPRNMG-
TWPRNM--
-TPRNMF-
THPRNM--
-TPRNMY-
-TPRNMN-
-TPRNMA-
TMPRNM--
TPPRNM--
TYPRNM--
TQPRNM--

-TPRNMS-
TNPRNM--
-TPRNMM-
TDPRNM--
TIPRNM--
TCPRNM--
-VPRNMA-
VSPRNM--
-VPRNML-
-VPRNMS-
VVPRNM--
-VPRNMY-
VLPRNM--
-VPRNMI-
VQPRNM--
VGPRNM--
VHPRNM--
VMPRNM--
-VPRNMG-
VNPRNM--
-VPRNMW-
-VPRNMH-
VEPRNM--
-VPRNMK-
-VPRNMM-
-VPRNMT-
-VPRNMN-
VTPRNM--
-VPRNMQ-
VDPRNM--
VPPRNM--
-VPRNMD-
VKPRNM--
VFPRNM--
VAPRNM--
WVPRNM--
WQPRNM--
WGPRNM--
-WPRNMS-
WAPRNM--
-WPRNMV-
WKPRNM--
WTPRNM--
WPPRNM--
-WPRNML-
WLPRNM--
WHPRNM--
WNPRNM--
WFPRNM--
-WPRNMN-

	-WPRNMH-		
	WYPRNM--		
	WRPRNM--		
	WWPRNM--		
	WIPRNM--		
	-WPRNMG-		
	-YPRNMK-		
	-YPRNMA-		
	YAPRNM--		
	-YPRNMT-		
	YYPRNM--		
	-YPRNMQ-		
	-YPRNMN-		
	YLPRNM--		
	YRPRNM--		
	-YPRNMS-		
	YSPRNM--		
	YKPRNM--		
	YVPRNM--		
	-YPRNMV-		
	-YPRNMH-		
	-YPRNMI-		
	YTPRNM--		
	YHPRNM--		
	YMPRNM--		
	-YPRNMG-		
	-YPRNME-		
	-YPRNMM-		
	YPPRNM--		
	YEPRNM--		
	YDPRNM--		
	-YPRNMW-		
	-YPRNMF-		
	YCPRNM--		
PFNL	47	854	48.39
	-LPFNLP-		
	--PFNLIT		
	-SPFNLP-		
	-SPFNLD-		
	AKPFNL--		
	ANPFNL--		
	ASPFNL--		
	AVPFNL--		
	AQPFNL--		
	-APFNLT-		
	-APFNLR-		
	-APFNLL-		
	ALPFNL--		
	ARPFNL--		
	-APFNLI-		

AYPFNL--
-APFNLQ-
-APFNLE-
AWPFNL--
AMPFNL--
-APFNLH-
-APFNLY-
-APFNLN-
AIPFNL--
ATPFNL--
-APFNLK-
AFPFNL--
-APFNLV-
-APFNLG-
-APFNLF-
AEPFNL--
AGPFNL--
AHPFNL--
ADPFNL--
AAPFNL--
-APFNLM-
APPFNL--
-APFNLA-
ACPFNL--
CLPFNL--
CHPFNL--
CGPFNL--
CQPFNL--
CFPFNL--
-CPFNLH-
CNPFNL--
CIPFNL--
CAPFNL--
CVPFNL--
CTPFNL--
-CPFNLY-
CCPFNL--
CEPFNL--
CRPFNL--
-CPFNLM-
-CPFNLA-
CKPFNL--
DVPFNL--
-DPFNLR-
DAPFNL--
DIPFNL--
DRPFNL--
DTPFNL--
DWPFNL--
DGPFNL--

-DPFNLM-
-DPFNLV-
DLPFNL--
-DPFNLY-
-DPFNLK-
DMPFNL--
-DPFNLS-
-DPFNLL-
DEPFNL--
DNPFNL--
DYPFNL--
-DPFNLW-
-DPFNLA-
DQPFNL--
DFPFNL--
DKPFNL--
-DPFNLQ-
-DPFNLH-
-DPFNLN-
-DPFNLI-
-DPFNLE-
DHPFNL--
-DPFNLT-
-DPFNLG-
-DPFNLC-
DDPFNL--
ERPFNL--
-EPFNLS-
EQPFNL--
ESPFNL--
EVPFNL--
-EPFNLQ-
-EPFNLA-
ETPFNL--
-EPFNLG-
-EPFNLR-
-EPFNLF-
EYPFNL--
EFPFNL--
EEPFNL--
-EPFNLT-
-EPFNLM-
-EPFNLK-
EPPFNL--
-EPFNLW-
-EPFNLV-
EKPFNL--
EGPFNL--
-EPFNLI-
EIPFNL--

EWPFNL--
-EPFNLY-
EDPFNL--
EMPFNL--
-EPFNLN-
-EPFNLH-
ENPFNL--
EHPFNL--
-EPFNLD-
ELPFNL--
FVPFNL--
-FPFNLR-
FIPFNL--
-FPFNLQ-
FQPFNL--
-FPFNLA-
-FPFNLS-
-FPFNLK-
FTPFNL--
-FPFNLT-
FNPFNL--
FWPFNL--
FRPFNL--
-FPFNLM-
FKPFNL--
FGPFNL--
FPPFNL--
-FPFNLH-
FMPFNL--
FHPFNL--
-FPFNLN-
-FPFNLD-
-FPFNLG-
-FPFNLV-
FEPFNL--
FDPFNL--
-FPFNLE-
-FPFNLI-
FFPFNL--
-FPFNLY-
-FPFNLF-
-GPFNLK-
-GPFNLS-
-GPFNLR-
GYPFNL--
GSPFNL--
-GPFNLN-
GKPFNL--
GFPFNL--
-GPFNLQ-

-GPFNLY-
-GPFNLA-
-GPFNLG-
GNPFNL--
-GPFNLV-
-GPFNLE-
GIPFNL--
GTPFNL--
-GPFNLT-
-GPFNLF-
-GPFNLH-
GLPFNL--
GWPFNL--
GVPFNL--
GGPFNL--
GEPFNL--
GDPFNL--
GQPFNL--
-GPFNLM-
-GPFNLI-
GHPFNL--
GCPFNL--
GPPFNL--
-GPFNLW-
-GPFNLC-
HVPFNL--
HRPFNL--
HFPFNL--
HKPFNL--
HYPFNL--
HQPFNL--
-HPFNLF-
HSPFNL--
-HPFNLL-
HGPFNL--
HTPFNL--
HWPFNL--
HHPFNL--
HAPFNL--
HPPFNL--
-HPFNLW-
-HPFNLV-
-HPFNLR-
-HPFNLT-
-HPFNLA-
-HPFNLG-
-HPFNLY-
-HPFNLQ-
HDPFNL--
HNPFNL--

```
-HPFNLM-
HIPFNL--
-HPFNLS-
-HPFNLN-
HEPFNL--
-HPFNLP-
-HPFNLI-
-IPFNLR-
IVPFNL--
IKPFNL--
-IPFNLS-
INPFNL--
IIPFNL--
ITPFNL--
ISPFNL--
-IPFNLF-
-IPFNLQ-
-IPFNLA-
IGPFNL--
IEPFNL--
-IPFNLG-
-IPFNLT-
IYPFNL--
IMPFNL--
IFPFNL--
-IPFNLN-
IPPFNL--
-IPFNLH-
IWPFNL--
IHPFNL--
-IPFNLM-
-IPFNLE-
ICPFNL--
-IPFNLV-
-IPFNLK-
IQPFNL--
-IPFNLY-
IDPFNL--
-IPFNLD-
IAPFNL--
KLPFNL--
KGPFNL--
KVPFNL--
KPPFNL--
KTPFNL--
-KPFNLS-
-KPFNLL-
KRPFNL--
KAPFNL--
-KPFNLY-
```

–KPFNLW–
–KPFNLN–
–KPFNLK–
–KPFNLA–
KNPFNL––
–KPFNLV–
KKPFNL––
KEPFNL––
–KPFNLH–
KYPFNL––
KDPFNL––
–KPFNLI–
–KPFNLE–
KWPFNL––
KFPFNL––
–KPFNLF–
KIPFNL––
KMPFNL––
–KPFNLD–
KCPFNL––
–KPFNLM–
LRPFNL––
–LPFNLR–
LGPFNL––
LLPFNL––
LPPFNL––
–LPFNLS–
LKPFNL––
–LPFNLT–
LNPFNL––
–LPFNLH–
LEPFNL––
LIPFNL––
LDPFNL––
–LPFNLK–
–LPFNLE–
LAPFNL––
–LPFNLV–
LSPFNL––
–LPFNLA–
LTPFNL––
–LPFNLM–
LFPFNL––
LMPFNL––
–LPFNLQ–
LQPFNL––
–LPFNLI–
LYPFNL––
LWPFNL––
–LPFNLW–

```
-LPFNLN-
-LPFNLY-
-LPFNLG-
LHPFNL--
-LPFNLC-
MRPFNL--
-MPFNLS-
MAPFNL--
-MPFNLT-
-MPFNLR-
MMPFNL--
MQPFNL--
-MPFNLQ-
-MPFNLA-
-MPFNLN-
MYPFNL--
MNPFNL--
MPPFNL--
-MPFNLV-
-MPFNLG-
MFPFNL--
MWPFNL--
MSPFNL--
MTPFNL--
MKPFNL--
-MPFNLW-
MVPFNL--
MHPFNL--
-MPFNLH-
-MPFNLD-
-MPFNLK-
-MPFNLF-
MDPFNL--
-MPFNLE-
MEPFNL--
MCPFNL--
-NPFNLR-
NRPFNL--
NPPFNL--
NYPFNL--
NIPFNL--
NSPFNL--
-NPFNLV-
-NPFNLL-
NWPFNL--
NVPFNL--
NTPFNL--
-NPFNLA-
NKPFNL--
-NPFNLT-
```

NLPFNL--
-NPFNLQ-
NEPFNL--
-NPFNLS-
NNPFNL--
NAPFNL--
NQPFNL--
NFPFNL--
NHPFNL--
-NPFNLI-
NGPFNL--
-NPFNLH-
-NPFNLE-
-NPFNLK-
-NPFNLG-
-NPFNLM-
-NPFNLF-
-NPFNLY-
NDPFNL--
-NPFNLW-
--PFNLVS
--PFNLTR
--PFNLVR
PRPFNL--
--PFNLST
--PFNLVG
--PFNLRN
PVPFNL--
--PFNLKT
--PFNLES
--PFNLSM
--PFNLNW
--PFNLGT
--PFNLYR
--PFNLSR
--PFNLSQ
--PFNLRT
--PFNLVK
--PFNLSL
--PFNLKG
--PFNLAS
--PFNLTI
--PFNLRK
--PFNLGS
PNPFNL--
--PFNLMR
PGPFNL--
--PFNLSK
--PFNLWS
--PFNLVW

--PFNLRV
--PFNLRQ
--PFNLNA
--PFNLLK
PWPFNL--
--PFNLTA
--PFNLRH
--PFNLRA
--PFNLIR
--PFNLRR
--PFNLLT
--PFNLIG
--PFNLVV
--PFNLMS
--PFNLFE
PEPFNL--
PAPFNL--
--PFNLTT
--PFNLTL
--PFNLSV
--PFNLSH
--PFNLQR
--PFNLMA
--PFNLHS
--PFNLYA
--PFNLMT
--PFNLTN
--PFNLNS
--PFNLAA
--PFNLMG
--PFNLLN
--PFNLSN
--PFNLMM
--PFNLLG
--PFNLFT
--PFNLSS
--PFNLSD
--PFNLSA
--PFNLRF
--PFNLKL
--PFNLHL
--PFNLHK
PTPFNL--
--PFNLVH
--PFNLLE
--PFNLKQ
--PFNLGN
PIPFNL--
--PFNLKE
--PFNLGM

--PFNLAN
--PFNLAK
--PFNLQV
--PFNLKR
--PFNLGV
--PFNLGA
--PFNLAT
--PFNLWR
--PFNLVT
--PFNLTM
--PFNLTH
--PFNLSI
--PFNLSG
--PFNLKA
--PFNLHQ
--PFNLFG
--PFNLSE
--PFNLNR
PFPFNL--
--PFNLVY
--PFNLVA
--PFNLTV
--PFNLQN
--PFNLQK
--PFNLLH
--PFNLKN
--PFNLHG
--PFNLFS
--PFNLET
--PFNLWG
--PFNLVN
--PFNLTE
--PFNLSY
--PFNLRE
--PFNLIS
--PFNLFK
--PFNLAV
--PFNLYT
--PFNLIK
--PFNLHT
--PFNLHD
--PFNLGG
--PFNLYG
--PFNLWV
--PFNLRD
--PFNLNT
--PFNLIY
--PFNLHR
--PFNLHH
--PFNLFQ

--PFNLFN
--PFNLNY
--PFNLIV
--PFNLFH
--PFNLEI
PKPFNL--
--PFNLWM
--PFNLQA
--PFNLHN
--PFNLGY
--PFNLAQ
PYPFNL--
--PFNLYK
--PFNLTQ
--PFNLQQ
--PFNLMD
--PFNLEA
--PFNLAG
PLPFNL--
--PFNLWQ
--PFNLQW
--PFNLIH
--PFNLGR
--PFNLGL
--PFNLGK
--PFNLYN
--PFNLVM
--PFNLVD
--PFNLNK
--PFNLHM
--PFNLEV
--PFNLEN
--PFNLTY
--PFNLTD
--PFNLSF
--PFNLLD
--PFNLHY
--PFNLGW
--PFNLGF
--PFNLEM
--PFNLAE
--PFNLQS
--PFNLQI
--PFNLQF
--PFNLNV
--PFNLKK
--PFNLFD
--PFNLER
--PFNLYM
--PFNLTF

--PFNLQY
--PFNLNH
--PFNLMH
--PFNLHA
--PFNLEQ
--PFNLEL
--PFNLDS
--PFNLDG
--PFNLAF
PMPFNL--
--PFNLYD
--PFNLWD
--PFNLTK
--PFNLRW
--PFNLNL
--PFNLMP
--PFNLMK
--PFNLLQ
--PFNLKV
--PFNLKH
--PFNLIE
--PFNLHE
--PFNLAH
--PFNLNN
--PFNLNI
--PFNLMN
--PFNLLM
--PFNLHW
--PFNLHF
--PFNLFW
--PFNLEE
PDPFNL--
PSPFNL--
--PFNLYQ
--PFNLWK
--PFNLVI
--PFNLVE
--PFNLTW
--PFNLQT
--PFNLQL
--PFNLQG
--PFNLNQ
--PFNLMQ
--PFNLGH
--PFNLGD
--PFNLEH
--PFNLDH
--PFNLDA
--PFNLCW
--PFNLCS

--PFNLCQ
-PPFNLT-
-PPFNLS-
-PPFNLN-
-PPFNLL-
--PFNLYE
--PFNLWN
--PFNLWE
--PFNLVP
--PFNLQM
--PFNLQH
--PFNLKY
--PFNLKW
--PFNLKM
--PFNLKD
--PFNLKC
--PFNLEW
--PFNLDV
--PFNLDM
--PFNLCT
--PFNLCL
--PFNLCD
--PFNLAD
-QPFNLL-
QRPFNL--
-QPFNLR-
QVPFNL--
-QPFNLV-
-QPFNLK-
-QPFNLI-
-QPFNLA-
-QPFNLS-
QLPFNL--
QGPFNL--
QYPFNL--
QTPFNL--
QKPFNL--
-QPFNLM-
QHPFNL--
-QPFNLW-
-QPFNLE-
QAPFNL--
-QPFNLT-
-QPFNLG-
QEPFNL--
-QPFNLN-
QNPFNL--
QFPFNL--
QPPFNL--
QMPFNL--

-QPFNLC-
QSPFNL--
-QPFNLY-
QIPFNL--
QDPFNL--
-RPFNLR-
RLPFNL--
RVPFNL--
RNPFNL--
-RPFNLL-
RTPFNL--
-RPFNLV-
RGPFNL--
RHPFNL--
REPFNL--
-RPFNLY-
RYPFNL--
RWPFNL--
RFPFNL--
RMPFNL--
RIPFNL--
-RPFNLM-
-RPFNLF-
RAPFNL--
-RPFNLK-
-RPFNLI-
-RPFNLE-
-RPFNLH-
-RPFNLN-
RDPFNL--
-RPFNLW-
-RPFNLD-
RQPFNL--
RCPFNL--
-SPFNLR-
SGPFNL--
SSPFNL--
-SPFNLL-
SVPFNL--
-SPFNLT-
SYPFNL--
-SPFNLS-
SLPFNL--
-SPFNLA-
-SPFNLI-
STPFNL--
SAPFNL--
-SPFNLN-
SHPFNL--
-SPFNLK-

SMPFNL--
SNPFNL--
-SPFNLQ-
-SPFNLG-
SPPFNL--
-SPFNLY-
SEPFNL--
SDPFNL--
-SPFNLM-
SWPFNL--
SIPFNL--
-SPFNLH-
-SPFNLE-
SCPFNL--
TSPFNL--
TLPFNL--
-TPFNLR-
TRPFNL--
TWPFNL--
-TPFNLS-
-TPFNLE-
-TPFNLL-
-TPFNLK-
TAPFNL--
-TPFNLW-
TFPFNL--
-TPFNLT-
TYPFNL--
-TPFNLN-
TQPFNL--
TTPFNL--
TPPFNL--
-TPFNLQ-
TNPFNL--
TVPFNL--
-TPFNLV-
THPFNL--
TMPFNL--
-TPFNLA-
TIPFNL--
-TPFNLH-
-TPFNLM-
-TPFNLG-
TGPFNL--
TCPFNL--
TEPFNL--
TDPFNL--
-TPFNLY-
-TPFNLI-
VKPFNL--

VSPFNL--
-VPFNLS-
VRPFNL--
-VPFNLL-
VLPFNL--
VMPFNL--
VYPFNL--
-VPFNLV-
VTPFNL--
VGPFNL--
-VPFNLM-
-VPFNLR-
-VPFNLW-
-VPFNLA-
-VPFNLN-
VHPFNL--
-VPFNLK-
-VPFNLG-
-VPFNLY-
-VPFNLE-
VWPFNL--
-VPFNLI-
VNPFNL--
-VPFNLF-
VVPFNL--
-VPFNLT-
VFPFNL--
VAPFNL--
-VPFNLQ-
VDPFNL--
-VPFNLH-
VEPFNL--
VPPFNL--
-VPFNLD-
VIPFNL--
WKPFNL--
WSPFNL--
-WPFNLA-
-WPFNLH-
WRPFNL--
-WPFNLQ-
WTPFNL--
-WPFNLR-
WVPFNL--
WNPFNL--
WIPFNL--
WEPFNL--
WDPFNL--
WAPFNL--
WQPFNL--

WHPFNL--
WGPFNL--
WYPFNL--
-WPFNLI-
-WPFNLT-
-WPFNLN-
WPPFNL--
-WPFNLM-
-WPFNLK-
-WPFNLG-
-WPFNLF-
YKPFNL--
YGPFNL--
YTPFNL--
YRPFNL--
-YPFNLM-
YHPFNL--
-YPFNLL-
YAPFNL--
-YPFNLK-
-YPFNLV-
YSPFNL--
-YPFNLS-
-YPFNLE-
YFPFNL--
-YPFNLT-
YEPFNL--
YYPFNL--
YDPFNL--
-YPFNLQ-
YLPFNL--
YMPFNL--
-YPFNLN-
YWPFNL--
YIPFNL--
-YPFNLG-
-YPFNLH-
-YPFNLI-
-YPFNLY-

PLAL 193 753 48.38

--PLALPP
--PLALDN
-RPLALP-
-YPLALP-
-YPLALD-
-APLALT-
ALPLAL--
ASPLAL--
AIPLAL--
-APLALS-

```
-APLALE-
-APLALH-
-APLALV-
AYPLAL--
AQPLAL--
ATPLAL--
AMPLAL--
-APLALG-
AKPLAL--
-APLALI-
AFPLAL--
AVPLAL--
-APLALA-
APPLAL--
-APLALN-
AEPLAL--
-APLALM-
AHPLAL--
AWPLAL--
-APLALK-
-APLALQ-
AGPLAL--
ADPLAL--
ACPLAL--
-APLALD-
-APLALC-
CTPLAL--
CRPLAL--
CMPLAL--
CKPLAL--
CVPLAL--
CSPLAL--
-CPLALR-
CIPLAL--
-CPLALL-
CNPLAL--
CAPLAL--
-CPLALK-
CGPLAL--
CEPLAL--
-CPLALV-
-CPLALT-
CQPLAL--
-CPLALS-
CPPLAL--
CFPLAL--
-CPLALW-
-CPLALG-
-CPLALN-
-CPLALA-
```

CCPLAL--
-CPLALH-
-CPLALF-
-CPLALE-
CLPLAL--
CDPLAL--
-DPLALL-
DMPLAL--
DLPLAL--
DVPLAL--
DSPLAL--
DAPLAL--
-DPLALT-
-DPLALN-
DTPLAL--
DKPLAL--
DNPLAL--
DQPLAL--
DGPLAL--
-DPLALI-
-DPLALV-
DWPLAL--
DHPLAL--
DIPLAL--
-DPLALH-
DEPLAL--
DYPLAL--
DFPLAL--
-DPLALM-
-DPLALQ-
-DPLALK-
-DPLALA-
-DPLALG-
DDPLAL--
DCPLAL--
ESPLAL--
EKPLAL--
-EPLALR-
ERPLAL--
ELPLAL--
EGPLAL--
-EPLALI-
-EPLALV-
ETPLAL--
-EPLALS-
-EPLALG-
-EPLALK-
EVPLAL--
EPPLAL--
EHPLAL--

EAPLAL--
EWPLAL--
-EPLALN-
EFPLAL--
-EPLALQ-
-EPLALH-
-EPLALA-
EQPLAL--
EEPLAL--
EDPLAL--
EMPLAL--
-EPLALE-
ENPLAL--
ECPLAL--
-FPLALN-
FSPLAL--
-FPLALS-
-FPLALQ-
FLPLAL--
-FPLALT-
FNPLAL--
FAPLAL--
FQPLAL--
-FPLALM-
FEPLAL--
FGPLAL--
FDPLAL--
-FPLALH-
-FPLALA-
-FPLALE-
-FPLALK-
-FPLALI-
-FPLALG-
FYPLAL--
FMPLAL--
-FPLALD-
FCPLAL--
-GPLALR-
GRPLAL--
GKPLAL--
GLPLAL--
-GPLALS-
GAPLAL--
GWPLAL--
-GPLALT-
GYPLAL--
GEPLAL--
-GPLALM-
GNPLAL--
GPPLAL--

GGPLAL--
GQPLAL--
-GPLALK-
-GPLALG-
-GPLALN-
GFPLAL--
-GPLALV-
-GPLALQ-
-GPLALA-
-GPLALE-
-GPLALC-
GCPLAL--
-GPLALD-
HGPLAL--
HLPLAL--
HSPLAL--
HRPLAL--
HKPLAL--
-HPLALN-
HVPLAL--
HYPLAL--
-HPLALH-
-HPLALV-
-HPLALR-
HIPLAL--
-HPLALL-
HNPLAL--
-HPLALI-
HFPLAL--
HTPLAL--
-HPLALS-
HMPLAL--
-HPLALA-
-HPLALT-
-HPLALM-
HQPLAL--
HWPLAL--
HEPLAL--
-HPLALK-
HHPLAL--
HDPLAL--
-HPLALE-
-HPLALD-
-HPLALC-
-IPLALS-
ISPLAL--
IAPLAL--
-IPLALL-
-IPLALA-
-IPLALH-

IKPLAL--
-IPLALN-
IHPLAL--
IVPLAL--
IIPLAL--
ITPLAL--
-IPLALM-
IMPLAL--
INPLAL--
ILPLAL--
IQPLAL--
IFPLAL--
-IPLALG-
-IPLALQ-
-IPLALK-
IPPLAL--
IEPLAL--
IYPLAL--
IDPLAL--
IWPLAL--
-IPLALE-
ICPLAL--
-IPLALD-
-IPLALC-
KWPLAL--
-KPLALS-
KVPLAL--
-KPLALA-
-KPLALN-
-KPLALG-
KLPLAL--
-KPLALH-
KTPLAL--
-KPLALQ-
KHPLAL--
KYPLAL--
-KPLALK-
-KPLALV-
KAPLAL--
KKPLAL--
-KPLALE-
KPPLAL--
KIPLAL--
KEPLAL--
KMPLAL--
KDPLAL--
KFPLAL--
-KPLALM-
KQPLAL--
-KPLALC-

LQPLAL--
LTPLAL--
LRPLAL--
-LPLALS-
-LPLALK-
LMPLAL--
LPPLAL--
-LPLALN-
-LPLALT-
-LPLALV-
LKPLAL--
-LPLALH-
LNPLAL--
LIPLAL--
-LPLALM-
LHPLAL--
LFPLAL--
LDPLAL--
LWPLAL--
-LPLALQ-
-LPLALA-
LCPLAL--
MLPLAL--
-MPLALL-
MTPLAL--
MGPLAL--
-MPLALS-
MRPLAL--
-MPLALV-
-MPLALK-
-MPLALA-
MIPLAL--
MAPLAL--
MVPLAL--
MNPLAL--
MQPLAL--
-MPLALH-
MHPLAL--
-MPLALQ-
-MPLALN-
MKPLAL--
-MPLALG-
-MPLALE-
MFPLAL--
MEPLAL--
MMPLAL--
MWPLAL--
MCPLAL--
-NPLALR-
-NPLALT-

NSPLAL--
NRPLAL--
-NPLALL-
NVPLAL--
-NPLALS-
NGPLAL--
NIPLAL--
NPPLAL--
-NPLALK-
-NPLALQ-
NYPLAL--
NWPLAL--
-NPLALW-
-NPLALI-
NAPLAL--
-NPLALN-
NQPLAL--
NNPLAL--
NTPLAL--
NEPLAL--
-NPLALF-
-NPLALA-
NFPLAL--
-NPLALV-
-NPLALM-
NMPLAL--
-NPLALE-
-NPLALG-
-NPLALH-
NKPLAL--
NCPLAL--
NHPLAL--
NDPLAL--
-NPLALC-
--PLALRR
--PLALTS
--PLALVR
--PLALRI
--PLALAL
--PLALNS
--PLALRG
--PLALRA
--PLALMS
--PLALYG
--PLALST
--PLALHR
--PLALSV
--PLALRN
--PLALIN
--PLALGR

--PLALLK
--PLALSN
--PLALSS
--PLALLN
PGPLAL--
--PLALRK
--PLALWS
--PLALKL
--PLALNR
--PLALTK
--PLALSI
--PLALGG
--PLALRP
--PLALNL
--PLALSH
--PLALRW
--PLALMN
--PLALQR
--PLALKT
--PLALKK
--PLALSQ
--PLALRD
--PLALAS
--PLALAH
--PLALSK
--PLALPL
--PLALER
--PLALGY
--PLALKM
--PLALGA
--PLALKG
--PLALHT
--PLALES
PEPLAL--
--PLALSM
--PLALTA
--PLALHG
--PLALWN
PTPLAL--
--PLALFG
--PLALNH
--PLALIR
--PLALGT
PDPLAL--
--PLALSY
--PLALQS
--PLALSE
--PLALKN
--PLALAI
--PLALYN

--PLALTM
--PLALSF
--PLALKS
--PLALHL
--PLALGN
--PLALAN
--PLALNV
--PLALLD
--PLALHF
--PLALGI
--PLALSD
--PLALVK
--PLALTV
--PLALTI
--PLALRQ
--PLALNA
--PLALYH
--PLALRE
--PLALMG
--PLALHN
--PLALHM
--PLALAK
--PLALAA
--PLALNQ
--PLALNI
--PLALKV
--PLALET
--PLALQA
--PLALMK
--PLALKQ
--PLALVG
--PLALHA
--PLALQK
--PLALNY
--PLALNN
--PLALTD
--PLALKY
--PLALFH
--PLALTH
--PLALTF
--PLALSW
--PLALQG
--PLALNM
--PLALMP
--PLALID
--PLALEL
--PLALEA
--PLALTE
--PLALQM
--PLALKW

--PLALGQ
--PLALVE
--PLALQY
--PLALQF
--PLALNT
--PLALHH
--PLALEN
--PLALAW
--PLALHS
--PLALGW
--PLALGV
--PLALKE
--PLALTN
--PLALMT
--PLALEK
--PLALNW
--PLALKD
--PLALHY
--PLALGF
--PLALTQ
--PLALNE
--PLALLH
--PLALHV
--PLALDS
--PLALQT
--PLALNG
--PLALAG
--PLALYD
--PLALDL
--PLALQV
--PLALQN
--PLALLP
--PLALTW
--PLALQL
--PLALPY
--PLALKH
--PLALHP
--PLALQH
--PLALHI
--PLALEW
--PLALEM
--PLALVP
--PLALPI
--PLALPF
--PLALMQ
--PLALKF
--PLALAD
PNPLAL--
--PLALNK
--PLALHW

--PLALHD
--PLALDY
--PLALDA
--PLALQD
--PLALNF
--PLALIP
--PLALGM
--PLALFP
--PLALEY
--PLALND
--PLALHK
--PLALFN
--PLALQQ
--PLALGD
--PLALTP
--PLALEG
--PLALEE
--PLALAE
--PLALWD
--PLALTC
--PLALLQ
--PLALEQ
--PLALDV
--PLALCS
--PLALYP
--PLALSC
--PLALQE
--PLALIH
--PLALGE
--PLALEI
--PLALCG
--PLALAQ
-PPLALT-
--PLALSP
--PLALQW
--PLALPM
--PLALHE
--PLALDG
--PLALCL
--PLALCA
--PLALKP
--PLALDT
--PLALDM
--PLALDF
--PLALAC
-PPLALL-
--PLALQP
--PLALED
--PLALEC
--PLALDI

--PLALCT
-PPLALV-
-PPLALS-
--PLALWC
--PLALNC
--PLALLC
--PLALFC
--PLALDQ
--PLALDH
--PLALCK
--PLALCF
-QPLALS-
-QPLALT-
-QPLALR-
QRPLAL--
QTPLAL--
QSPLAL--
QPPLAL--
QQPLAL--
QWPLAL--
-QPLALG-
-QPLALH-
QLPLAL--
QEPLAL--
QYPLAL--
QVPLAL--
-QPLALL-
-QPLALM-
-QPLALK-
-QPLALA-
-QPLALE-
QMPLAL--
QAPLAL--
QNPLAL--
-QPLALQ-
QKPLAL--
QIPLAL--
QDPLAL--
QFPLAL--
-QPLALD-
QCPLAL--
RSPLAL--
RIPLAL--
RGPLAL--
RAPLAL--
-RPLALV-
RNPLAL--
RDPLAL--
-RPLALK-
RFPLAL--

RQPLAL--
-RPLALG-
RYPLAL--
RHPLAL--
-RPLALE-
REPLAL--
RMPLAL--
-RPLALH-
RWPLAL--
-RPLALM-
-RPLALQ-
-RPLALD-
RCPLAL--
-SPLALR-
SGPLAL--
SSPLAL--
-SPLALS-
SKPLAL--
-SPLALK-
-SPLALA-
SMPLAL--
SFPLAL--
STPLAL--
-SPLALQ-
SAPLAL--
-SPLALV-
SYPLAL--
SPPLAL--
-SPLALI-
SEPLAL--
SQPLAL--
-SPLALG-
SHPLAL--
-SPLALM-
SDPLAL--
SCPLAL--
-TPLALL-
TVPLAL--
TSPLAL--
-TPLALT-
TAPLAL--
-TPLALS-
-TPLALG-
TTPLAL--
THPLAL--
TGPLAL--
-TPLALH-
TQPLAL--
-TPLALN-
-TPLALQ-

-TPLALM-
TWPLAL--
-TPLALE-
TNPLAL--
TIPLAL--
-TPLALK-
TMPLAL--
TFPLAL--
TDPLAL--
TEPLAL--
TCPLAL--
-TPLALD-
VSPLAL--
-VPLALT-
-VPLALR-
VRPLAL--
-VPLALL-
VIPLAL--
-VPLALS-
VKPLAL--
VYPLAL--
VEPLAL--
VAPLAL--
VHPLAL--
VTPLAL--
-VPLALN-
VMPLAL--
VNPLAL--
VWPLAL--
-VPLALH-
VPPLAL--
-VPLALE-
-VPLALA-
-VPLALQ-
-VPLALI-
VQPLAL--
VDPLAL--
-VPLALD-
-VPLALC-
VCPLAL--
WGPLAL--
WMPLAL--
-WPLALR-
WPPLAL--
-WPLALT-
-WPLALA-
-WPLALV-
-WPLALK-
WEPLAL--
-WPLALH-

WYPLAL--
-WPLALM-
WNPLAL--
-WPLALG-
WFPLAL--
-WPLALQ-
WAPLAL--
WVPLAL--
-WPLALE-
WHPLAL--
WDPLAL--
-WPLALD-
WCPLAL--
WIPLAL--
-YPLALV-
-YPLALR-
YSPLAL--
YRPLAL--
-YPLALN-
-YPLALH-
-YPLALS-
YTPLAL--
YAPLAL--
YYPLAL--
-YPLALT-
YMPLAL--
-YPLALK-
YWPLAL--
YFPLAL--
YLPLAL--
-YPLALA-
-YPLALQ-
-YPLALG-
YIPLAL--
YQPLAL--
YEPLAL--
-YPLALE-
-YPLALM-
-YPLALI-
YHPLAL--
YDPLAL--
YCPLAL--
-YPLALC-

PRAL	714	557	48.37

-FPRALP-
-FPRALD-
-HPRALD-
-KPRALP-
--PRALQP
--PRALPT

--PRALPS
--PRALPQ
--PRALNP
--PRALDK
-RPRALD-
-RPRALP-
-SPRALC-
-YPRALD-
ASPRAL--
-APRALN-
ATPRAL--
AGPRAL--
AYPRAL--
AVPRAL--
AQPRAL--
-APRALA-
-APRALV-
AAPRAL--
-APRALQ-
-APRALH-
-APRALE-
AEPRAL--
ADPRAL--
-APRALM-
ACPRAL--
-APRALD-
-APRALC-
CLPRAL--
-CPRALK-
CCPRAL--
CVPRAL--
CNPRAL--
CSPRAL--
CKPRAL--
CRPRAL--
CMPRAL--
CHPRAL--
-CPRALQ-
-CPRALF-
-CPRALM-
-CPRALS-
-CPRALL-
-CPRALH-
-CPRALY-
CPPRAL--
CGPRAL--
-CPRALV-
-CPRALT-
CEPRAL--
-CPRALI-

-CPRALG-
-CPRALW-
CFPRAL--
CYPRAL--
DSPRAL--
-DPRALR-
-DPRALA-
DWPRAL--
DFPRAL--
DYPRAL--
-DPRALS-
DGPRAL--
DIPRAL--
-DPRALV-
-DPRALT-
DVPRAL--
DNPRAL--
DKPRAL--
-DPRALH-
-DPRALY-
DEPRAL--
DQPRAL--
-DPRALM-
-DPRALK-
-DPRALQ-
-DPRALN-
-DPRALW-
-DPRALF-
DDPRAL--
DMPRAL--
-DPRALG-
DCPRAL--
DHPRAL--
ERPRAL--
-EPRALR-
ETPRAL--
ESPRAL--
-EPRALT-
EAPRAL--
EKPRAL--
-EPRALS-
EWPRAL--
EVPRAL--
EIPRAL--
EGPRAL--
-EPRALI-
-EPRALV-
-EPRALF-
-EPRALN-
EFPRAL--

-EPRALY-
-EPRALW-
-EPRALH-
-EPRALK-
-EPRALA-
EMPRAL--
EHPRAL--
-EPRALE-
EPPRAL--
-EPRALQ-
EDPRAL--
-EPRALM-
-EPRALG-
ECPRAL--
-EPRALC-
-EPRALP-
FTPRAL--
FGPRAL--
-FPRALN-
-FPRALY-
FQPRAL--
-FPRALW-
FEPRAL--
-FPRALE-
FDPRAL--
FIPRAL--
FCPRAL--
-FPRALC-
GIPRAL--
-GPRALL-
GLPRAL--
-GPRALK-
-GPRALN-
-GPRALA-
-GPRALY-
-GPRALG-
GHPRAL--
GQPRAL--
-GPRALM-
-GPRALT-
-GPRALI-
GKPRAL--
-GPRALF-
GPPRAL--
-GPRALW-
-GPRALQ-
-GPRALV-
-GPRALH-
GCPRAL--
-GPRALE-

-GPRALD-
GDPRAL--
-GPRALC-
HIPRAL--
HTPRAL--
HSPRAL--
-HPRALG-
HAPRAL--
-HPRALT-
HPPRAL--
-HPRALV-
-HPRALM-
HNPRAL--
HEPRAL--
-HPRALN-
HQPRAL--
-HPRALW-
-HPRALE-
HFPRAL--
-HPRALQ-
HDPRAL--
IIPRAL--
-IPRALA-
-IPRALV-
-IPRALN-
IMPRAL--
INPRAL--
IQPRAL--
-IPRALH-
IPPRAL--
IEPRAL--
IGPRAL--
-IPRALQ-
IHPRAL--
-IPRALE-
-IPRALM-
-IPRALW-
IDPRAL--
-IPRALI-
ICPRAL--
-IPRALC-
KGPRAL--
-KPRALY-
KHPRAL--
-KPRALF-
KTPRAL--
-KPRALA-
KKPRAL--
-KPRALN-
-KPRALG-

-KPRALW-
-KPRALK-
KPPRAL--
KEPRAL--
KIPRAL--
-KPRALV-
-KPRALH-
KQPRAL--
KNPRAL--
KDPRAL--
-KPRALM-
-KPRALE-
KCPRAL--
-KPRALD-
-KPRALC-
LSPRAL--
LVPRAL--
-LPRALI-
-LPRALT-
LDPRAL--
-LPRALY-
-LPRALN-
LMPRAL--
LQPRAL--
LEPRAL--
-LPRALE-
LCPRAL--
-LPRALC-
-MPRALT-
MSPRAL--
-MPRALH-
MKPRAL--
-MPRALQ-
MTPRAL--
-MPRALV-
-MPRALG-
-MPRALN-
MMPRAL--
MEPRAL--
MDPRAL--
-MPRALK-
MCPRAL--
MWPRAL--
-MPRALM-
-MPRALE-
-MPRALD-
-MPRALP-
NSPRAL--
-NPRALK-
-NPRALW-

-NPRALS-
-NPRALV-
-NPRALI-
-NPRALY-
NQPRAL--
NGPRAL--
NDPRAL--
NAPRAL--
NMPRAL--
-NPRALQ-
NHPRAL--
-NPRALN-
-NPRALM-
-NPRALH-
NKPRAL--
-NPRALG-
NCPRAL--
-NPRALA-
-NPRALC-
--PRALWG
--PRALVI
--PRALWS
--PRALLV
--PRALIS
--PRALVV
--PRALGT
--PRALSG
PSPRAL--
PLPRAL--
--PRALHS
--PRALSI
--PRALYA
--PRALSN
--PRALTH
--PRALTT
--PRALVQ
--PRALHL
--PRALFG
--PRALIW
--PRALGG
--PRALGA
--PRALYT
--PRALNT
--PRALSK
--PRALNL
--PRALKS
--PRALWA
--PRALTK
--PRALLH
--PRALFK

--PRALII
--PRALID
--PRALFA
--PRALNR
--PRALYW
--PRALMK
--PRALVM
--PRALVD
--PRALIN
--PRALMD
--PRALFN
--PRALTV
--PRALNA
--PRALFQ
--PRALYK
--PRALIV
--PRALQN
--PRALNK
--PRALLM
--PRALFM
--PRALES
--PRALYQ
--PRALYD
PIPRAL--
--PRALMG
--PRALGN
--PRALTM
--PRALQF
--PRALWL
--PRALLE
--PRALHK
--PRALAH
--PRALSQ
--PRALGV
PMPRAL--
--PRALYG
--PRALWK
--PRALNG
--PRALIM
--PRALHF
--PRALYN
--PRALYE
--PRALQH
--PRALGF
--PRALEL
--PRALAV
--PRALMN
--PRALFV
--PRALYY
--PRALYH

--PRALTN
--PRALQW
--PRALQT
--PRALER
--PRALTD
--PRALNE
--PRALSD
--PRALWE
--PRALSE
--PRALNN
--PRALMP
--PRALVW
--PRALSM
--PRALHY
--PRALTQ
--PRALEG
PQPRAL--
PKPRAL--
--PRALQG
--PRALFD
--PRALET
--PRALAQ
--PRALNH
--PRALIQ
--PRALHN
--PRALTE
--PRALEN
--PRALDL
--PRALNV
--PRALIF
--PRALHI
--PRALHG
--PRALDS
PHPRAL--
--PRALIK
--PRALWQ
--PRALKN
--PRALQD
--PRALMQ
--PRALFE
--PRALEY
--PRALME
PEPRAL--
--PRALNQ
--PRALMF
--PRALHA
--PRALEI
--PRALVE
--PRALLC
--PRALGE

--PRALWM
--PRALPV
--PRALAW
--PRALAE
--PRALDI
--PRALDG
PAPRAL--
--PRALHH
--PRALEW
--PRALEV
--PRALEF
--PRALIE
--PRALKP
--PRALHD
--PRALFP
--PRALDY
--PRALDT
--PRALWW
--PRALSC
--PRALQE
--PRALIP
--PRALHW
--PRALEE
--PRALDW
--PRALCL
--PRALCH
--PRALPH
--PRALND
--PRALEH
--PRALDR
--PRALCG
--PRALCF
PCPRAL--
--PRALTC
--PRALGD
--PRALDF
--PRALCT
--PRALCQ
-PPRALG-
--PRALYC
--PRALVC
--PRALNC
--PRALDQ
--PRALCV
--PRALCN
--PRALCK
--PRALCA
--PRALAC
-PPRALV-
-PPRALS-

-PPRALL-
-PPRALH-
-PPRALE-
PPPRAL--
-QPRALL-
-QPRALY-
QVPRAL--
QAPRAL--
-QPRALV-
-QPRALQ-
QTPRAL--
-QPRALK-
-QPRALH-
-QPRALI-
QPPRAL--
-QPRALT-
-QPRALA-
-QPRALS-
QMPRAL--
QEPRAL--
-QPRALF-
QDPRAL--
QYPRAL--
-QPRALM-
QCPRAL--
-QPRALE-
-QPRALC-
RSPRAL--
RGPRAL--
RPPRAL--
-RPRALW-
-RPRALH-
-RPRALF-
RQPRAL--
-RPRALQ-
REPRAL--
-RPRALE-
RDPRAL--
RCPRAL--
-RPRALC-
SAPRAL--
-SPRALN-
-SPRALY-
-SPRALQ-
-SPRALE-
SEPRAL--
SCPRAL--
-TPRALN-
-TPRALI-
-TPRALY-

TMPRAL--
TKPRAL--
-TPRALG-
TNPRAL--
TQPRAL--
TEPRAL--
THPRAL--
TDPRAL--
-TPRALA-
-TPRALM-
-TPRALD-
TCPRAL--
-TPRALC-
-VPRALL-
VSPRAL--
VKPRAL--
VPPRAL--
VGPRAL--
VNPRAL--
VVPRAL--
VQPRAL--
VEPRAL--
-VPRALI-
-VPRALH-
-VPRALE-
-VPRALW-
VCPRAL--
-VPRALD-
WQPRAL--
WNPRAL--
-WPRALY-
WFPRAL--
-WPRALH-
WAPRAL--
WDPRAL--
-WPRALE-
-WPRALN-
WCPRAL--
-WPRALD-
YTPRAL--
-YPRALL-
-YPRALY-
-YPRALN-
-YPRALW-
-YPRALE-
YMPRAL--
-YPRALI-
YQPRAL--
YIPRAL--
YDPRAL--

	YKPRAL--			
PKAL	216	740	48.36	
	--PKALVP			
	--PKALPS			
	-APKALS-			
	ATPKAL--			
	-APKALN-			
	ALPKAL--			
	-APKALK-			
	-APKALR-			
	-APKALG-			
	-APKALY-			
	ANPKAL--			
	AEPKAL--			
	-APKALV-			
	-APKALQ-			
	AYPKAL--			
	-APKALT-			
	AMPKAL--			
	AIPKAL--			
	-APKALM-			
	AVPKAL--			
	AQPKAL--			
	AKPKAL--			
	-APKALF-			
	AAPKAL--			
	-APKALE-			
	-APKALA-			
	APPKAL--			
	-APKALW-			
	-APKALL-			
	-APKALH-			
	AGPKAL--			
	ADPKAL--			
	-APKALC-			
	CKPKAL--			
	-CPKALH-			
	CSPKAL--			
	CCPKAL--			
	CRPKAL--			
	CPPKAL--			
	CAPKAL--			
	CTPKAL--			
	CGPKAL--			
	CYPKAL--			
	-CPKALS-			
	CLPKAL--			
	CIPKAL--			
	-CPKALV-			
	-CPKALL-			

-CPKALF-
-CPKALK-
CHPKAL--
CFPKAL--
CDPKAL--
CQPKAL--
-CPKALN-
-CPKALA-
CEPKAL--
CWPKAL--
-CPKALQ-
-CPKALG-
CMPKAL--
DLPKAL--
-DPKALR-
DIPKAL--
DYPKAL--
DSPKAL--
-DPKALS-
DQPKAL--
DVPKAL--
DTPKAL--
DPPKAL--
DMPKAL--
-DPKALI-
-DPKALV-
DFPKAL--
-DPKALT-
-DPKALW-
-DPKALH-
DNPKAL--
DWPKAL--
-DPKALM-
DAPKAL--
-DPKALF-
-DPKALA-
-DPKALN-
-DPKALY-
DDPKAL--
DCPKAL--
-EPKALL-
-EPKALT-
-EPKALY-
ESPKAL--
-EPKALR-
ELPKAL--
EVPKAL--
-EPKALK-
-EPKALS-
EGPKAL--

ETPKAL--
-EPKALM-
-EPKALF-
EMPKAL--
-EPKALV-
-EPKALI-
-EPKALA-
EHPKAL--
EFPKAL--
EAPKAL--
EWPKAL--
EEPKAL--
EYPKAL--
EQPKAL--
EPPKAL--
-EPKALQ-
-EPKALG-
-EPKALN-
EDPKAL--
-EPKALW-
-EPKALC-
EIPKAL--
FLPKAL--
FRPKAL--
FMPKAL--
-FPKALA-
FFPKAL--
FYPKAL--
-FPKALM-
FSPKAL--
FNPKAL--
-FPKALE-
-FPKALY-
FEPKAL--
FVPKAL--
-FPKALQ-
-FPKALG-
FAPKAL--
-FPKALV-
FHPKAL--
FCPKAL--
-FPKALW-
FPPKAL--
GLPKAL--
-GPKALT-
-GPKALM-
-GPKALR-
GMPKAL--
GRPKAL--
-GPKALV-

GSPKAL--
-GPKALA-
-GPKALK-
-GPKALW-
-GPKALF-
-GPKALS-
GYPKAL--
GAPKAL--
-GPKALN-
GPPKAL--
GEPKAL--
-GPKALH-
-GPKALQ-
GNPKAL--
GKPKAL--
-GPKALI-
GHPKAL--
-GPKALG-
GWPKAL--
-GPKALE-
GTPKAL--
GCPKAL--
HLPKAL--
HVPKAL--
-HPKALV-
HRPKAL--
HIPKAL--
HMPKAL--
HPPKAL--
HSPKAL--
HNPKAL--
HWPKAL--
-HPKALS-
-HPKALK-
-HPKALY-
HYPKAL--
-HPKALL-
HGPKAL--
-HPKALH-
-HPKALE-
-HPKALW-
HKPKAL--
HQPKAL--
-HPKALA-
HDPKAL--
-HPKALM-
-HPKALQ-
-HPKALG-
-HPKALT-
HEPKAL--

IAPKAL--
ISPKAL--
-IPKALS-
IVPKAL--
INPKAL--
IHPKAL--
-IPKALT-
ITPKAL--
-IPKALY-
-IPKALI-
IQPKAL--
IYPKAL--
-IPKALH-
IDPKAL--
IMPKAL--
-IPKALV-
-IPKALE-
IGPKAL--
-IPKALN-
-IPKALG-
-IPKALA-
ICPKAL--
-IPKALQ-
-IPKALM-
-KPKALV-
-KPKALL-
-KPKALI-
-KPKALS-
KGPKAL--
KVPKAL--
KTPKAL--
-KPKALK-
KQPKAL--
KHPKAL--
KAPKAL--
-KPKALQ-
-KPKALH-
-KPKALG-
KNPKAL--
KMPKAL--
-KPKALA-
-KPKALW-
-KPKALM-
-KPKALN-
KDPKAL--
KCPKAL--
KEPKAL--
LGPKAL--
-LPKALL-
LMPKAL--

LKPKAL--
LYPKAL--
-LPKALM-
-LPKALT-
-LPKALI-
LLPKAL--
-LPKALV-
LPPKAL--
-LPKALF-
LQPKAL--
LDPKAL--
-LPKALH-
-LPKALY-
LEPKAL--
-LPKALW-
-LPKALA-
-LPKALG-
-LPKALE-
LCPKAL--
-MPKALS-
-MPKALV-
MLPKAL--
MSPKAL--
MGPKAL--
-MPKALW-
MIPKAL--
-MPKALK-
-MPKALI-
MMPKAL--
-MPKALA-
MVPKAL--
-MPKALT-
MTPKAL--
-MPKALH-
MKPKAL--
MAPKAL--
MWPKAL--
-MPKALQ-
-MPKALN-
MDPKAL--
-MPKALE-
-MPKALG-
MEPKAL--
MNPKAL--
-MPKALM-
MFPKAL--
MCPKAL--
NVPKAL--
-NPKALM-
-NPKALF-

-NPKALV-
NMPKAL--
-NPKALW-
-NPKALN-
NEPKAL--
NQPKAL--
NHPKAL--
NDPKAL--
NNPKAL--
-NPKALA-
NSPKAL--
NGPKAL--
-NPKALG-
-NPKALQ-
-NPKALH-
NCPKAL--
--PKALNT
--PKALNL
--PKALLL
--PKALSY
--PKALSS
--PKALTA
PKPKAL--
--PKALLK
--PKALVT
--PKALRV
--PKALYH
--PKALLQ
--PKALTW
--PKALFS
--PKALVI
--PKALSF
--PKALLA
--PKALLN
PRPKAL--
--PKALYG
--PKALML
--PKALFL
PLPKAL--
--PKALWN
--PKALST
--PKALMT
--PKALYK
--PKALWG
--PKALSK
--PKALFT
--PKALYV
--PKALAS
--PKALYN
--PKALWL

--PKALAL
--PKALTG
--PKALTY
--PKALKS
--PKALGL
--PKALAY
--PKALVM
--PKALII
--PKALRE
--PKALMG
--PKALIL
--PKALSG
--PKALIN
--PKALHS
--PKALTT
--PKALTK
--PKALQQ
--PKALIH
--PKALAA
--PKALGY
--PKALGA
PFPKAL--
PSPKAL--
--PKALWS
--PKALMI
--PKALIF
--PKALHG
--PKALWH
--PKALTI
--PKALFN
--PKALFI
--PKALAT
--PKALWY
--PKALHN
--PKALYT
--PKALWM
--PKALSD
--PKALNW
--PKALNK
--PKALMY
--PKALQS
--PKALNY
--PKALIK
--PKALVA
--PKALME
--PKALHV
--PKALGV
--PKALFH
--PKALTQ
--PKALNV

--PKALKD
--PKALGF
--PKALAG
--PKALYD
--PKALQV
--PKALGT
--PKALWQ
--PKALNF
--PKALNA
--PKALHI
--PKALHF
--PKALGI
--PKALLV
--PKALFK
--PKALEV
--PKALAR
--PKALAI
PMPKAL--
--PKALWV
--PKALMK
--PKALAV
--PKALYI
--PKALVG
--PKALSW
--PKALSN
--PKALPL
--PKALER
PVPKAL--
--PKALYF
--PKALKT
--PKALGG
PHPKAL--
PNPKAL--
--PKALYQ
--PKALYP
--PKALWA
--PKALVE
--PKALTH
--PKALRQ
--PKALNE
--PKALLD
--PKALKQ
--PKALHK
--PKALFG
--PKALDS
--PKALAF
--PKALQT
--PKALMV
--PKALIM
--PKALFV

```
--PKALFM
--PKALES
--PKALEN
--PKALNN
--PKALKF
--PKALHW
--PKALYE
--PKALMW
--PKALHA
PQPKAL--
--PKALVD
--PKALQK
--PKALQE
--PKALMP
--PKALIG
--PKALHD
--PKALGQ
--PKALEH
--PKALAM
--PKALSH
--PKALMN
--PKALMM
--PKALYW
--PKALTE
--PKALRC
--PKALQY
--PKALQW
--PKALNQ
--PKALKM
--PKALIW
--PKALIV
--PKALIT
--PKALCL
PGPKAL--
PAPKAL--
--PKALSE
--PKALQN
--PKALND
--PKALHH
--PKALFW
--PKALAK
PDPKAL--
--PKALWT
--PKALTF
--PKALSQ
--PKALQL
--PKALPY
--PKALMF
--PKALMA
--PKALIE
```

--PKALIA
--PKALGK
--PKALGH
PEPKAL--
--PKALYA
--PKALWE
--PKALTN
--PKALTM
--PKALQF
--PKALNH
--PKALDM
--PKALCF
--PKALAD
--PKALWK
--PKALQG
--PKALPM
--PKALMQ
--PKALKK
--PKALGR
--PKALED
--PKALEA
--PKALDI
--PKALCI
--PKALWD
--PKALPI
--PKALPF
--PKALIQ
--PKALGW
--PKALGM
--PKALET
--PKALEK
--PKALEF
--PKALCS
--PKALAH
--PKALYM
--PKALLP
--PKALKW
--PKALHE
--PKALCN
--PKALAE
--PKALYC
--PKALPV
--PKALPQ
--PKALNM
--PKALMC
--PKALHY
--PKALHQ
--PKALEY
--PKALEI
--PKALDY

--PKALCT
PIPKAL--
PYPKAL--
PTPKAL--
-PPKALI-
-PPKALG-
--PKALWW
--PKALWP
--PKALWC
--PKALVC
--PKALQD
--PKALQC
--PKALPH
--PKALPG
--PKALPE
--PKALMD
--PKALLC
--PKALKC
--PKALHM
--PKALHC
--PKALGD
--PKALGC
--PKALFD
--PKALDW
--PKALDK
--PKALDG
--PKALCY
--PKALCV
--PKALCR
--PKALCG
--PKALCD
QSPKAL--
QRPKAL--
-QPKALR-
-QPKALT-
-QPKALH-
-QPKALK-
QLPKAL--
QPPKAL--
-QPKALV-
-QPKALW-
-QPKALL-
QVPKAL--
QAPKAL--
QNPKAL--
-QPKALM-
QYPKAL--
-QPKALA-
QTPKAL--
-QPKALF-

QQPKAL--
-QPKALQ-
QHPKAL--
QEPKAL--
QWPKAL--
QGPKAL--
-QPKALI-
-QPKALE-
QIPKAL--
-QPKALG-
QDPKAL--
-QPKALN-
QCPKAL--
RIPKAL--
RTPKAL--
-RPKALA-
-RPKALM-
RVPKAL--
-RPKALV-
-RPKALG-
REPKAL--
-RPKALH-
-RPKALN-
RHPKAL--
-RPKALE-
RKPKAL--
SLPKAL--
-SPKALT-
STPKAL--
-SPKALS-
SKPKAL--
-SPKALL-
-SPKALF-
-SPKALH-
-SPKALA-
SVPKAL--
SIPKAL--
-SPKALW-
-SPKALV-
SEPKAL--
SPPKAL--
-SPKALI-
SQPKAL--
SGPKAL--
-SPKALE-
SNPKAL--
-SPKALQ-
SDPKAL--
-SPKALM-
SCPKAL--

TKPKAL--
TVPKAL--
-TPKALM-
-TPKALK-
-TPKALA-
TTPKAL--
TYPKAL--
TGPKAL--
TPPKAL--
TLPKAL--
-TPKALV-
TAPKAL--
-TPKALN-
-TPKALQ-
-TPKALS-
TFPKAL--
-TPKALY-
-TPKALW-
TMPKAL--
TNPKAL--
-TPKALE-
TDPKAL--
THPKAL--
TEPKAL--
TWPKAL--
TCPKAL--
-VPKALV-
-VPKALN-
VVPKAL--
-VPKALA-
VSPKAL--
-VPKALM-
VLPKAL--
-VPKALW-
VTPKAL--
-VPKALL-
VMPKAL--
VHPKAL--
VGPKAL--
VEPKAL--
VQPKAL--
VNPKAL--
-VPKALI-
VDPKAL--
-VPKALE-
-VPKALQ-
VCPKAL--
-VPKALF-
-VPKALC-
WLPKAL--

```
            WKPKAL--
            WPPKAL--
            -WPKALT-
            -WPKALN-
            WYPKAL--
            WQPKAL--
            WMPKAL--
            WNPKAL--
            WFPKAL--
            -WPKALA-
            WDPKAL--
            WEPKAL--
            WWPKAL--
            -WPKALW-
            -WPKALF-
            -WPKALM-
            -WPKALH-
            -WPKALE-
            -WPKALD-
            -WPKALC-
            WCPKAL--
            -YPKALS-
            YTPKAL--
            YKPKAL--
            YGPKAL--
            YSPKAL--
            YIPKAL--
            YAPKAL--
            -YPKALH-
            YEPKAL--
            -YPKALL-
            YYPKAL--
            YPPKAL--
            -YPKALQ-
            YNPKAL--
            -YPKALI-
            YMPKAL--
            YDPKAL--
            -YPKALE-
            YCPKAL--
            -YPKALC-
            -YPKALP-
PLPI          59         847     48.36
            -LPLPIN-
            -NPLPII-
            --PLPIPG
            --PLPIPA
            -APLPIT-
            AKPLPI--
            -APLPIH-
```

APPLPI--
-APLPIR-
AIPLPI--
-APLPIF-
ALPLPI--
-APLPIS-
-APLPIL-
AGPLPI--
-APLPIW-
-APLPIN-
ASPLPI--
-APLPII-
AVPLPI--
ARPLPI--
AEPLPI--
AWPLPI--
ATPLPI--
-APLPIY-
-APLPIA-
ANPLPI--
AHPLPI--
-APLPIM-
-APLPIG-
ADPLPI--
AYPLPI--
AQPLPI--
ACPLPI--
-APLPIV-
-APLPIE-
AMPLPI--
AFPLPI--
AAPLPI--
CMPLPI--
-CPLPIY-
CSPLPI--
-CPLPIL-
CNPLPI--
-CPLPIQ-
CDPLPI--
CQPLPI--
-CPLPII-
CGPLPI--
CTPLPI--
CRPLPI--
-CPLPIK-
-CPLPIC-
CLPLPI--
CCPLPI--
CAPLPI--
-DPLPIR-

DWPLPI--
DLPLPI--
DMPLPI--
-DPLPIW-
DGPLPI--
DAPLPI--
DTPLPI--
-DPLPIQ-
-DPLPIL-
-DPLPII-
DYPLPI--
DRPLPI--
-DPLPIY-
-DPLPIS-
-DPLPIN-
-DPLPIA-
DFPLPI--
DQPLPI--
-DPLPIV-
DVPLPI--
-DPLPIM-
DHPLPI--
ERPLPI--
-EPLPIY-
EVPLPI--
-EPLPIW-
-EPLPIR-
-EPLPIS-
-EPLPIL-
-EPLPIV-
ELPLPI--
EGPLPI--
EWPLPI--
EKPLPI--
EIPLPI--
EHPLPI--
EFPLPI--
EAPLPI--
EYPLPI--
-EPLPIT-
-EPLPIQ-
-EPLPIK-
-EPLPII-
-EPLPIA-
ESPLPI--
-EPLPIM-
-EPLPIE-
ENPLPI--
EMPLPI--
EDPLPI--

-FPLPIR-
-FPLPIT-
FSPLPI--
-FPLPIL-
FRPLPI--
-FPLPIS-
FNPLPI--
FYPLPI--
-FPLPIK-
FMPLPI--
FGPLPI--
FEPLPI--
-FPLPIV-
FFPLPI--
-FPLPIG-
FHPLPI--
FWPLPI--
FDPLPI--
FTPLPI--
-FPLPIW-
FIPLPI--
-FPLPII-
-FPLPIH-
FQPLPI--
FPPLPI--
-FPLPIA-
-FPLPIQ-
-FPLPIN-
-FPLPIM-
FVPLPI--
-FPLPIP-
-FPLPIF-
GRPLPI--
-GPLPIR-
-GPLPIW-
GVPLPI--
GGPLPI--
-GPLPIL-
GFPLPI--
GKPLPI--
-GPLPIS-
GYPLPI--
-GPLPIM-
GSPLPI--
GAPLPI--
-GPLPIV-
GIPLPI--
-GPLPIT-
-GPLPIK-
GMPLPI--

-GPLPIH-
-GPLPIG-
GTPLPI--
GDPLPI--
GQPLPI--
GPPLPI--
-GPLPIE-
-GPLPIQ-
-GPLPIN-
-GPLPII-
-GPLPIF-
-GPLPIA-
GWPLPI--
GEPLPI--
GCPLPI--
HRPLPI--
HFPLPI--
HTPLPI--
-HPLPIS-
-HPLPIA-
HPPLPI--
-HPLPIR-
-HPLPII-
HWPLPI--
-HPLPIM-
-HPLPIL-
-HPLPIF-
HLPLPI--
HSPLPI--
HKPLPI--
HIPLPI--
HHPLPI--
-HPLPIY-
-HPLPIT-
HQPLPI--
HNPLPI--
HAPLPI--
HVPLPI--
-HPLPIW-
-HPLPIG-
-HPLPIN-
HDPLPI--
-HPLPIQ-
-HPLPIP-
-HPLPIH-
HMPLPI--
ISPLPI--
IRPLPI--
-IPLPIQ-
IVPLPI--

-IPLPIA-
ILPLPI--
-IPLPIT-
IFPLPI--
-IPLPIL-
IYPLPI--
IGPLPI--
-IPLPIH-
-IPLPIG-
IWPLPI--
ITPLPI--
-IPLPIS-
-IPLPIK-
INPLPI--
-IPLPIF-
IIPLPI--
-IPLPII-
IDPLPI--
IKPLPI--
IPPLPI--
-IPLPIV-
-IPLPIN-
IEPLPI--
-IPLPIY-
-IPLPIM-
-IPLPIE-
IMPLPI--
ICPLPI--
-KPLPIR-
KGPLPI--
KTPLPI--
-KPLPIT-
KKPLPI--
-KPLPIQ-
KVPLPI--
KMPLPI--
-KPLPIS-
-KPLPIM-
KAPLPI--
KWPLPI--
KYPLPI--
-KPLPII-
KDPLPI--
-KPLPIK-
KLPLPI--
-KPLPIV-
-KPLPIG-
-KPLPIA-
-KPLPIN-
-KPLPIY-

-KPLPIH-
KNPLPI--
KCPLPI--
KPPLPI--
KIPLPI--
-KPLPIE-
KFPLPI--
KEPLPI--
KQPLPI--
LAPLPI--
LLPLPI--
LGPLPI--
LYPLPI--
LVPLPI--
LIPLPI--
LNPLPI--
LKPLPI--
LTPLPI--
LQPLPI--
-LPLPIL-
LWPLPI--
-LPLPIY-
LHPLPI--
LSPLPI--
-LPLPIF-
-LPLPIS-
-LPLPIT-
-LPLPIM-
-LPLPIV-
LMPLPI--
LPPLPI--
-LPLPIK-
-LPLPII-
-LPLPIH-
LEPLPI--
LDPLPI--
-LPLPIA-
-LPLPIQ-
LFPLPI--
-LPLPIG-
-LPLPIC-
-MPLPIL-
MSPLPI--
MHPLPI--
-MPLPIY-
MRPLPI--
MQPLPI--
MKPLPI--
-MPLPIT-
-MPLPII-

MFPLPI--
MEPLPI--
-MPLPIS-
-MPLPIH-
MMPLPI--
-MPLPIV-
MIPLPI--
MPPLPI--
-MPLPIK-
MAPLPI--
-MPLPIW-
MLPLPI--
-MPLPIE-
-MPLPIM-
-MPLPIG-
MTPLPI--
MDPLPI--
MYPLPI--
-MPLPIA-
MCPLPI--
-MPLPIR-
-MPLPIN-
MNPLPI--
NGPLPI--
NSPLPI--
-NPLPIT-
-NPLPIK-
NKPLPI--
NIPLPI--
-NPLPIR-
NNPLPI--
NVPLPI--
NTPLPI--
NPPLPI--
NWPLPI--
-NPLPIS-
NRPLPI--
-NPLPIY-
NMPLPI--
-NPLPIV-
-NPLPIQ-
NFPLPI--
-NPLPIF-
NEPLPI--
NYPLPI--
NQPLPI--
-NPLPIN-
-NPLPIE-
-NPLPIA-
NCPLPI--

--PLPILA
--PLPIRS
--PLPISR
--PLPIRT
--PLPIVG
--PLPIVL
--PLPIRV
--PLPIMT
--PLPILV
--PLPILS
--PLPIVR
--PLPILH
--PLPIFR
--PLPIRK
--PLPIRI
--PLPIPL
--PLPINL
--PLPISI
--PLPIAL
--PLPILW
PRPLPI--
--PLPISS
--PLPISA
--PLPISQ
--PLPIRN
--PLPIAY
PKPLPI--
--PLPIYL
--PLPIVS
--PLPITL
--PLPIHS
--PLPISF
--PLPIHR
--PLPITR
--PLPIQG
--PLPITA
--PLPIRH
--PLPILN
--PLPILG
--PLPITY
--PLPIST
--PLPIMA
--PLPILK
--PLPIFF
PTPLPI--
--PLPIYH
--PLPITS
--PLPIRY
--PLPIQR
--PLPIAI

--PLPIWT
--PLPIWG
--PLPIKV
--PLPIIR
--PLPIFT
--PLPIVH
--PLPITT
--PLPIWR
--PLPIVV
--PLPIQA
--PLPIGG
PGPLPI--
--PLPIRA
--PLPIIG
--PLPIFH
--PLPISV
--PLPIGS
--PLPIFG
--PLPIFA
--PLPITG
--PLPIKS
--PLPIIL
--PLPIVT
--PLPITQ
--PLPINY
--PLPIGY
--PLPIAR
--PLPITW
--PLPISM
--PLPIMR
--PLPIGF
--PLPIAS
--PLPIYN
--PLPIYE
--PLPIVW
--PLPIVK
--PLPINA
--PLPIMG
--PLPIDY
PWPLPI--
--PLPIWL
--PLPIIV
--PLPIIQ
--PLPIHH
--PLPIHG
--PLPIAA
--PLPIYS
--PLPIYM
--PLPIWS
--PLPISW

--PLPIRD
--PLPIPY
--PLPIKK
--PLPIKI
--PLPIAV
--PLPIYK
--PLPIVF
--PLPISY
--PLPIRQ
--PLPIQT
--PLPIQQ
--PLPINS
--PLPIKM
--PLPIHN
--PLPIHM
--PLPIFK
--PLPIAT
--PLPITK
--PLPISD
--PLPIMN
--PLPIMH
--PLPIIS
--PLPIAK
--PLPIYI
--PLPIYG
--PLPIYD
--PLPIWW
--PLPIWE
--PLPISN
--PLPIRG
--PLPINI
--PLPIKN
--PLPIIT
--PLPIGA
--PLPIFS
--PLPIAG
PQPLPI--
--PLPIYY
--PLPIVD
--PLPIQI
--PLPINK
--PLPILY
--PLPIGW
--PLPIWF
--PLPIVN
--PLPIRW
--PLPIRE
--PLPIQV
--PLPIPW
--PLPIMQ

--PLPILM
--PLPIKA
--PLPIIK
--PLPIIA
--PLPIHA
--PLPIGK
--PLPIGI
--PLPIGH
--PLPIFN
--PLPIEI
--PLPIEA
--PLPITV
--PLPITN
--PLPITI
--PLPIQN
--PLPIQK
--PLPIPV
--PLPIPM
--PLPIKH
--PLPIHT
--PLPIHL
--PLPIHI
--PLPIFV
--PLPIEL
--PLPIAF
PEPLPI--
--PLPIWH
--PLPITH
--PLPISH
--PLPISE
--PLPIPF
--PLPINQ
--PLPIMW
--PLPIMP
--PLPIMF
--PLPILI
--PLPIKT
--PLPIFD
--PLPIEK
--PLPIAW
--PLPIAN
--PLPIAH
PNPLPI--
--PLPIWQ
--PLPIVI
--PLPITM
--PLPITF
--PLPIQS
--PLPINM
--PLPIMV

--PLPIMK
--PLPILE
--PLPIIY
--PLPIIW
--PLPIIN
--PLPIGV
--PLPIGN
--PLPIGM
--PLPIEV
--PLPIDL
--PLPIYQ
--PLPIWI
--PLPIWD
--PLPIVY
--PLPITD
--PLPIQY
--PLPIQW
--PLPIQM
--PLPIQD
--PLPIKY
--PLPIKQ
--PLPIID
--PLPIHQ
--PLPIHK
--PLPIGT
--PLPIFE
--PLPIET
--PLPIER
--PLPIEG
--PLPIDM
PDPLPI--
PPPLPI--
--PLPIWY
--PLPIWV
--PLPIWN
--PLPIWA
--PLPIVE
--PLPIPH
--PLPINW
--PLPINV
--PLPIKG
--PLPIKD
--PLPIIH
--PLPIHE
--PLPIFY
--PLPIFM
--PLPIEW
--PLPIAD
PCPLPI--
--PLPIYV

--PLPITE
--PLPIRP
--PLPIRF
--PLPINF
--PLPIND
--PLPIKE
--PLPIHY
--PLPIHV
--PLPIHC
--PLPIFW
--PLPIES
--PLPIEQ
--PLPIEH
--PLPIDI
--PLPIDA
--PLPICY
--PLPICS
--PLPICG
--PLPICC
--PLPIAM
PIPLPI--
PHPLPI--
-QPLPIK-
-QPLPIR-
-QPLPIL-
QRPLPI--
QAPLPI--
-QPLPIW-
-QPLPII-
QSPLPI--
QMPLPI--
QVPLPI--
-QPLPIY-
QYPLPI--
QGPLPI--
QLPLPI--
QHPLPI--
QTPLPI--
-QPLPIV-
-QPLPIA-
QIPLPI--
QWPLPI--
QPPLPI--
-QPLPIN-
-QPLPIM-
QNPLPI--
QQPLPI--
-QPLPIS-
-QPLPIQ-
QDPLPI--

QFPLPI--
QCPLPI--
RSPLPI--
RGPLPI--
RLPLPI--
-RPLPIV-
-RPLPIK-
RFPLPI--
-RPLPII-
RIPLPI--
RVPLPI--
-RPLPIM-
-RPLPIS-
RQPLPI--
-RPLPIQ-
RHPLPI--
RWPLPI--
-RPLPIA-
RAPLPI--
RYPLPI--
-RPLPIN-
RDPLPI--
RTPLPI--
RPPLPI--
RNPLPI--
-RPLPIH-
REPLPI--
-RPLPIG-
-RPLPIE-
RCPLPI--
SRPLPI--
SVPLPI--
SSPLPI--
-SPLPIS-
-SPLPIR-
SWPLPI--
-SPLPII-
SKPLPI--
SYPLPI--
SGPLPI--
-SPLPIK-
-SPLPIY-
SLPLPI--
SIPLPI--
-SPLPIN-
-SPLPIW-
SEPLPI--
STPLPI--
SAPLPI--
SQPLPI--

-SPLPIT-
-SPLPIA-
SFPLPI--
SDPLPI--
SCPLPI--
-SPLPIM-
-SPLPIG-
SHPLPI--
-SPLPIV-
-SPLPIQ-
-SPLPIF-
SNPLPI--
-SPLPIE-
-TPLPIL-
TSPLPI--
-TPLPIF-
-TPLPIW-
-TPLPIR-
-TPLPIT-
TIPLPI--
TRPLPI--
TTPLPI--
-TPLPIK-
-TPLPIH-
-TPLPIA-
TYPLPI--
TEPLPI--
TFPLPI--
TWPLPI--
-TPLPII-
TPPLPI--
TGPLPI--
TMPLPI--
-TPLPIG-
-TPLPIQ-
-TPLPIY-
TNPLPI--
TKPLPI--
TCPLPI--
-TPLPIE-
TQPLPI--
THPLPI--
TAPLPI--
-TPLPIM-
TDPLPI--
-VPLPIL-
-VPLPIR-
VMPLPI--
VLPLPI--
VDPLPI--

VRPLPI--
VVPLPI--
VPPLPI--
-VPLPIV-
VNPLPI--
VTPLPI--
VGPLPI--
VQPLPI--
VSPLPI--
-VPLPIA-
VIPLPI--
VWPLPI--
-VPLPII-
VKPLPI--
VEPLPI--
VYPLPI--
VFPLPI--
-VPLPIS-
VHPLPI--
-VPLPIH-
-VPLPIY-
-VPLPIT-
-VPLPIK-
-VPLPIG-
-VPLPIW-
-VPLPIQ-
VAPLPI--
-VPLPIM-
-VPLPIF-
-VPLPIE-
VCPLPI--
-VPLPIN-
WAPLPI--
WRPLPI--
-WPLPIS-
WLPLPI--
WDPLPI--
WTPLPI--
WHPLPI--
WEPLPI--
WSPLPI--
WQPLPI--
-WPLPIY-
-WPLPIK-
WMPLPI--
WFPLPI--
-WPLPIT-
-WPLPII-
WNPLPI--
WKPLPI--

	WVPLPI--		
	-WPLPIQ-		
	-WPLPIH-		
	WIPLPI--		
	WGPLPI--		
	-WPLPIN-		
	-WPLPIM-		
	-WPLPIL-		
	-WPLPIF-		
	-WPLPIE-		
	-WPLPIA-		
	WWPLPI--		
	WPPLPI--		
	-YPLPIF-		
	YLPLPI--		
	YGPLPI--		
	YSPLPI--		
	-YPLPIR-		
	YRPLPI--		
	-YPLPIY-		
	-YPLPIL-		
	YIPLPI--		
	YYPLPI--		
	-YPLPIK-		
	YWPLPI--		
	YHPLPI--		
	YAPLPI--		
	YKPLPI--		
	-YPLPIT-		
	-YPLPIH-		
	-YPLPIS-		
	YDPLPI--		
	YVPLPI--		
	-YPLPIW-		
	-YPLPIV-		
	-YPLPIQ-		
	YPPLPI--		
	-YPLPIG-		
	YEPLPI--		
	YNPLPI--		
PMHM	632	577	48.30
	APPMHM--		
	AYPMHM--		
	AIPMHM--		
	AAPMHM--		
	-APMHMW-		
	-APMHMV-		
	ATPMHM--		
	-APMHMQ-		
	-APMHME-		

-APMHMS-
ANPMHM--
AFPMHM--
ASPMHM--
-APMHMM-
-APMHML-
AGPMHM--
-APMHMN-
-APMHMK-
-APMHMA-
AQPMHM--
-APMHMG-
AKPMHM--
CVPMHM--
-CPMHMT-
DSPMHM--
DVPMHM--
DIPMHM--
DRPMHM--
DAPMHM--
DWPMHM--
-DPMHMT-
-DPMHMQ-
DFPMHM--
DYPMHM--
-DPMHMY-
-DPMHMW-
DLPMHM--
-DPMHMV-
-DPMHMS-
DKPMHM--
DGPMHM--
-EPMHMY-
ESPMHM--
-EPMHMK-
EYPMHM--
EKPMHM--
-EPMHMV-
-EPMHMH-
ETPMHM--
ERPMHM--
-EPMHMI-
-EPMHMA-
ELPMHM--
EIPMHM--
EFPMHM--
-FPMHMT-
FLPMHM--
-FPMHMK-
-FPMHMI-

-FPMHMS-
FRPMHM--
FGPMHM--
-FPMHMV-
-FPMHMH-
-FPMHMF-
-FPMHML-
FIPMHM--
FPPMHM--
-FPMHMN-
FMPMHM--
FHPMHM--
-FPMHME-
-FPMHMW-
-FPMHMA-
FFPMHM--
GVPMHM--
GNPMHM--
GEPMHM--
-GPMHMS-
GAPMHM--
-GPMHMM-
GRPMHM--
-GPMHMA-
GLPMHM--
GYPMHM--
GMPMHM--
GFPMHM--
-GPMHMY-
GDPMHM--
-GPMHMR-
GWPMHM--
GTPMHM--
-GPMHMV-
GKPMHM--
GSPMHM--
GPPMHM--
-GPMHML-
-GPMHMW-
-GPMHMN-
-GPMHMI-
-GPMHMH-
-GPMHME-
GIPMHM--
GGPMHM--
-HPMHML-
-HPMHMI-
-HPMHMT-
HLPMHM--
-HPMHMS-

-HPMHMF-
HKPMHM--
HAPMHM--
HGPMHM--
-HPMHMR-
-HPMHMH-
HYPMHM--
HPPMHM--
-HPMHMW-
HRPMHM--
-HPMHMM-
HFPMHM--
HEPMHM--
ISPMHM--
-IPMHMV-
-IPMHMK-
IVPMHM--
IRPMHM--
-IPMHMS-
-IPMHMR-
-IPMHMI-
-IPMHML-
IYPMHM--
-IPMHMT-
IHPMHM--
-IPMHMY-
-IPMHMW-
-IPMHMH-
IMPMHM--
ILPMHM--
IAPMHM--
ITPMHM--
IQPMHM--
-IPMHME-
IDPMHM--
KSPMHM--
KLPMHM--
-KPMHMS-
-KPMHMF-
KWPMHM--
KHPMHM--
KPPMHM--
KEPMHM--
KGPMHM--
KTPMHM--
KRPMHM--
-KPMHML-
-KPMHMA-
KFPMHM--
-KPMHMY-

-KPMHMV-
-KPMHMR-
-KPMHMK-
KDPMHM--
KAPMHM--
-KPMHMT-
LAPMHM--
LSPMHM--
LFPMHM--
-LPMHML-
LLPMHM--
LNPMHM--
LTPMHM--
LRPMHM--
-LPMHMT-
-LPMHMR-
LYPMHM--
-LPMHMQ-
-LPMHMK-
LEPMHM--
-LPMHMV-
LVPMHM--
LHPMHM--
-LPMHMW-
-LPMHMM-
-LPMHMA-
-LPMHME-
LMPMHM--
-LPMHMH-
MGPMHM--
MNPMHM--
-MPMHMR-
MSPMHM--
-MPMHMI-
-MPMHMM-
-MPMHML-
MMPMHM--
MFPMHM--
MPPMHM--
MEPMHM--
MWPMHM--
-MPMHMW-
-MPMHME-
MRPMHM--
MQPMHM--
MLPMHM--
MIPMHM--
NRPMHM--
NDPMHM--
NNPMHM--

NTPMHM--
-NPMHMF-
NSPMHM--
-NPMHMS-
-NPMHMR-
NKPMHM--
-NPMHMW-
-NPMHMV-
NMPMHM--
NFPMHM--
NAPMHM--
NVPMHM--
NPPMHM--
-NPMHMY-
-NPMHMT-
-NPMHMM-
-NPMHML-
NQPMHM--
-NPMHMK-
NIPMHM--
--PMHMWA
--PMHMVN
--PMHMRN
--PMHMLA
--PMHMST
--PMHMTR
--PMHMSY
--PMHMSA
--PMHMTT
--PMHMRR
--PMHMVA
--PMHMLG
--PMHMRS
--PMHMQR
--PMHMLW
PTPMHM--
--PMHMTS
--PMHMSS
--PMHMSH
--PMHMRA
--PMHMQS
--PMHMMM
--PMHMMK
--PMHMIR
--PMHMFK
--PMHMSQ
--PMHMKS
--PMHMYK
--PMHMWT
--PMHMTG

--PMHMSR
--PMHMML
--PMHMLN
--PMHMLM
--PMHMLK
--PMHMIV
--PMHMAM
--PMHMRL
--PMHMKH
--PMHMFR
--PMHMWI
--PMHMVT
--PMHMSG
--PMHMRW
--PMHMRV
--PMHMNS
--PMHMKA
--PMHMHW
--PMHMGA
--PMHMTF
--PMHMSV
--PMHMIY
--PMHMIL
--PMHMFI
--PMHMWR
--PMHMVW
--PMHMVQ
--PMHMTW
--PMHMTN
--PMHMTH
--PMHMMY
--PMHMMA
--PMHMLT
--PMHMEF
--PMHMAY
--PMHMAA
PNPMHM--
--PMHMYG
--PMHMYD
--PMHMVK
--PMHMTL
--PMHMTA
--PMHMRD
--PMHMME
--PMHMLY
--PMHMLV
--PMHMKN
--PMHMIF
--PMHMHF
--PMHMAG

PHPMHM--
--PMHMYW
--PMHMYF
--PMHMVI
--PMHMVG
--PMHMTY
--PMHMTK
--PMHMSN
--PMHMSM
--PMHMRH
--PMHMRG
--PMHMQD
--PMHMIE
--PMHMER
--PMHMYY
--PMHMYN
--PMHMYM
--PMHMYA
--PMHMWV
--PMHMTV
--PMHMTE
--PMHMSF
--PMHMQL
--PMHMQG
--PMHMMS
--PMHMKY
--PMHMKQ
--PMHMKM
--PMHMKG
--PMHMIT
--PMHMIA
--PMHMHV
--PMHMHS
--PMHMFH
--PMHMDG
--PMHMYS
--PMHMYL
--PMHMYH
--PMHMWS
--PMHMWF
--PMHMVR
--PMHMVM
--PMHMVL
--PMHMVD
--PMHMTQ
--PMHMTI
--PMHMSL
--PMHMSI
--PMHMRM
--PMHMQK

--PMHMQI
--PMHMQF
--PMHMNT
--PMHMNA
--PMHMMV
--PMHMMH
--PMHMLS
--PMHMLR
--PMHMLQ
--PMHMLL
--PMHMHR
--PMHMHK
--PMHMGS
--PMHMGM
--PMHMFW
--PMHMFT
--PMHMFM
--PMHMEL
--PMHMAS
--PMHMAR
--PMHMAH
--PMHMAF
-PPMHMS-
--PMHMWH
--PMHMVY
--PMHMVV
--PMHMVS
--PMHMVH
--PMHMTM
--PMHMTD
--PMHMSW
--PMHMSK
--PMHMRY
--PMHMRI
--PMHMQA
--PMHMPP
--PMHMNN
--PMHMLI
--PMHMIS
--PMHMIN
--PMHMGV
--PMHMGT
--PMHMGL
--PMHMFL
--PMHMFE
--PMHMFC
--PMHMEY
--PMHMEN
--PMHMEG
--PMHMDF

--PMHMAT
QNPMHM--
QMPMHM--
QFPMHM--
-QPMHMQ-
QTPMHM--
QRPMHM--
QVPMHM--
QPPMHM--
-QPMHMY-
-QPMHMI-
-QPMHMH-
QEPMHM--
QIPMHM--
QDPMHM--
QSPMHM--
-QPMHMW-
-QPMHMN-
-QPMHMF-
QLPMHM--
QGPMHM--
QWPMHM--
-QPMHML-
RNPMHM--
RWPMHM--
-RPMHMR-
RLPMHM--
RQPMHM--
-RPMHMS-
RSPMHM--
-RPMHMI-
-RPMHMT-
-RPMHMA-
-RPMHMQ-
-RPMHMV-
RHPMHM--
RAPMHM--
RTPMHM--
RGPMHM--
RFPMHM--
RMPMHM--
RKPMHM--
RYPMHM--
-RPMHMH-
-RPMHMM-
RPPMHM--
-RPMHMK-
REPMHM--
-RPMHMY-
RIPMHM--

RDPMHM--
-RPMHMN-
-RPMHML-
-RPMHME-
RCPMHM--
-SPMHMR-
-SPMHMK-
SNPMHM--
-SPMHMI-
SVPMHM--
SIPMHM--
SLPMHM--
-SPMHML-
-SPMHMY-
SGPMHM--
SYPMHM--
-SPMHMQ-
-SPMHMV-
-SPMHMS-
SMPMHM--
-SPMHMT-
SFPMHM--
SWPMHM--
-SPMHMM-
STPMHM--
SRPMHM--
-SPMHMW-
-SPMHMN-
SHPMHM--
SPPMHM--
SAPMHM--
SDPMHM--
SCPMHM--
TVPMHM--
-TPMHMK-
TRPMHM--
TLPMHM--
TQPMHM--
-TPMHMV-
TMPMHM--
TDPMHM--
TTPMHM--
-TPMHMR-
TKPMHM--
THPMHM--
-TPMHME-
-TPMHMW-
-TPMHMM-
-TPMHMH-
TGPMHM--

TSPMHM--
-TPMHML-
TIPMHM--
-TPMHMS-
TFPMHM--
TAPMHM--
VRPMHM--
VVPMHM--
-VPMHMW-
VLPMHM--
-VPMHMR-
VKPMHM--
VAPMHM--
-VPMHMV-
-VPMHMA-
VSPMHM--
-VPMHML-
VMPMHM--
VEPMHM--
-VPMHMI-
VIPMHM--
VDPMHM--
-VPMHMG-
-VPMHME-
VHPMHM--
VWPMHM--
-VPMHMQ-
-VPMHMF-
VGPMHM--
VTPMHM--
WNPMHM--
WGPMHM--
WRPMHM--
WPPMHM--
WIPMHM--
WYPMHM--
WAPMHM--
-WPMHMR-
-WPMHMY-
-WPMHML-
-WPMHMH-
-WPMHMF-
-WPMHMW-
-WPMHMT-
-WPMHMI-
-WPMHMA-
WDPMHM--
-WPMHMN-
-WPMHMG-
WLPMHM--

WEPMHM--
-YPMHMS-
YSPMHM--
-YPMHMT-
YYPMHM--
-YPMHMY-
-YPMHMF-
-YPMHMA-
YLPMHM--
YVPMHM--
YAPMHM--
-YPMHMI-
YDPMHM--
YIPMHM--
YRPMHM--
-YPMHML-
YFPMHM--
YKPMHM--

PYNI	294	700	48.29

--PYNIGL
ARPYNI--
AVPYNI--
ASPYNI--
-APYNIS-
-APYNIV-
AKPYNI--
AAPYNI--
-APYNIR-
AMPYNI--
ALPYNI--
AHPYNI--
-APYNIL-
APPYNI--
AFPYNI--
ANPYNI--
AWPYNI--
ATPYNI--
-APYNIG-
-APYNIQ-
-APYNIN-
-APYNIE-
AEPYNI--
AQPYNI--
-APYNII-
AYPYNI--
-APYNIH-
ADPYNI--
CRPYNI--
CKPYNI--
CCPYNI--

CYPYNI--
CSPYNI--
-CPYNIT-
-CPYNIR-
CEPYNI--
CWPYNI--
CTPYNI--
-CPYNIK-
CLPYNI--
CAPYNI--
DRPYNI--
DLPYNI--
-DPYNIR-
DGPYNI--
DQPYNI--
-DPYNIY-
-DPYNIS-
DNPYNI--
DYPYNI--
-DPYNIN-
-DPYNIK-
-DPYNIA-
DMPYNI--
DFPYNI--
DEPYNI--
EYPYNI--
ETPYNI--
-EPYNIR-
-EPYNIL-
-EPYNIF-
EAPYNI--
ERPYNI--
ESPYNI--
-EPYNIE-
ENPYNI--
ELPYNI--
EGPYNI--
EWPYNI--
EVPYNI--
EQPYNI--
-EPYNIN-
-EPYNIM-
-EPYNII-
EMPYNI--
EIPYNI--
EEPYNI--
FSPYNI--
FQPYNI--
FAPYNI--
FYPYNI--

FLPYNI--
-FPYNIA-
-FPYNIL-
FMPYNI--
FTPYNI--
FIPYNI--
FVPYNI--
-FPYNIT-
-FPYNIR-
FFPYNI--
FRPYNI--
-FPYNIQ-
-FPYNIM-
-FPYNIV-
-FPYNIG-
FGPYNI--
-FPYNIS-
FNPYNI--
-FPYNIK-
-FPYNIH-
FPPYNI--
FDPYNI--
-FPYNIY-
-FPYNIN-
FEPYNI--
-GPYNIR-
GKPYNI--
-GPYNIL-
-GPYNIS-
GRPYNI--
GSPYNI--
-GPYNII-
GVPYNI--
-GPYNIY-
-GPYNIV-
-GPYNIQ-
-GPYNIF-
GHPYNI--
GQPYNI--
GAPYNI--
-GPYNIK-
GTPYNI--
-GPYNIH-
GGPYNI--
-GPYNIT-
GMPYNI--
GFPYNI--
GWPYNI--
-GPYNIE-
GIPYNI--

-HPYNIR-
-HPYNIF-
HTPYNI--
HSPYNI--
-HPYNIL-
HRPYNI--
HFPYNI--
-HPYNIY-
HHPYNI--
HEPYNI--
HKPYNI--
HDPYNI--
HYPYNI--
-HPYNIS-
-HPYNIV-
HNPYNI--
HQPYNI--
-HPYNIT-
-HPYNIK-
-HPYNII-
HIPYNI--
-HPYNIH-
HCPYNI--
-HPYNIN-
-HPYNIM-
-HPYNIG-
ITPYNI--
-IPYNIT-
IHPYNI--
IGPYNI--
ISPYNI--
-IPYNIS-
-IPYNIL-
IKPYNI--
-IPYNIR-
ILPYNI--
IRPYNI--
-IPYNIY-
IVPYNI--
IYPYNI--
INPYNI--
-IPYNIG-
-IPYNIF-
-IPYNIV-
-IPYNIK-
-IPYNIE-
IPPYNI--
IFPYNI--
KSPYNI--
-KPYNIR-

-KPYNIL-
KVPYNI--
-KPYNIV-
KWPYNI--
KGPYNI--
KQPYNI--
-KPYNIY-
KMPYNI--
KLPYNI--
KEPYNI--
-KPYNIS-
-KPYNIK-
-KPYNIG-
-KPYNIM-
-KPYNII-
-KPYNIW-
-KPYNIN-
KAPYNI--
KYPYNI--
KNPYNI--
KKPYNI--
KIPYNI--
LLPYNI--
LSPYNI--
LYPYNI--
LTPYNI--
-LPYNIT-
LKPYNI--
LVPYNI--
-LPYNIV-
LIPYNI--
LRPYNI--
LEPYNI--
-LPYNIS-
-LPYNIA-
-LPYNIW-
LGPYNI--
-LPYNIY-
LNPYNI--
-LPYNIE-
LMPYNI--
-LPYNIG-
LFPYNI--
-LPYNIQ-
-LPYNIN-
LPPYNI--
-LPYNIR-
-LPYNIH-
LDPYNI--
LHPYNI--

LWPYNI--
LQPYNI--
-LPYNIK-
MGPYNI--
-MPYNIL-
MSPYNI--
-MPYNIS-
-MPYNIA-
MNPYNI--
MLPYNI--
MTPYNI--
-MPYNIK-
MPPYNI--
-MPYNIR-
MIPYNI--
MRPYNI--
MKPYNI--
-MPYNIY-
-MPYNII-
-MPYNIQ-
-MPYNIW-
-MPYNIE-
MHPYNI--
MVPYNI--
MQPYNI--
-MPYNIV-
-MPYNIN-
-MPYNIG-
-MPYNIM-
-MPYNIH-
MDPYNI--
MAPYNI--
NRPYNI--
NSPYNI--
-NPYNIS-
NKPYNI--
NLPYNI--
NAPYNI--
-NPYNIR-
NEPYNI--
NVPYNI--
NGPYNI--
-NPYNIW-
-NPYNIT-
-NPYNIA-
NYPYNI--
NTPYNI--
-NPYNIF-
NNPYNI--
NFPYNI--

-NPYNIY-
-NPYNIM-
-NPYNII-
-NPYNIH-
NDPYNI--
NWPYNI--
-NPYNIQ-
NPPYNI--
NMPYNI--
NIPYNI--
-NPYNIN-
--PYNILR
--PYNILG
--PYNIRS
--PYNIIR
--PYNIRR
--PYNIRA
--PYNISR
--PYNIRT
--PYNIMR
--PYNIVK
--PYNILS
--PYNIVG
--PYNIRG
--PYNILN
--PYNITA
--PYNIWA
--PYNIST
--PYNIVN
--PYNIVS
--PYNIVL
--PYNITS
--PYNIEV
--PYNISA
--PYNIMK
--PYNIRV
--PYNIKI
--PYNIYM
--PYNIWT
--PYNISW
--PYNILK
--PYNIKS
--PYNIWG
--PYNITT
--PYNIRY
--PYNIRK
--PYNIHS
--PYNIFR
--PYNISL
--PYNIRQ

--PYNILQ
--PYNIAS
--PYNIWN
--PYNIVT
--PYNIVA
--PYNITK
--PYNISN
--PYNIQA
--PYNIMS
--PYNILW
--PYNIAT
--PYNIAI
--PYNISQ
--PYNIRL
--PYNIRI
--PYNIKV
--PYNIIA
--PYNIFK
--PYNIYE
--PYNISD
--PYNIKG
--PYNIYN
--PYNIVR
--PYNITR
--PYNITG
--PYNINS
--PYNIKL
--PYNIHH
--PYNIFF
--PYNIYT
--PYNITI
--PYNISG
--PYNIRH
--PYNIIV
--PYNIIH
--PYNIHQ
--PYNIHA
--PYNIGY
--PYNIEQ
--PYNIEA
--PYNIAQ
--PYNIYQ
--PYNIWM
--PYNIVY
--PYNIVI
--PYNITY
--PYNIRW
--PYNIRN
--PYNIQW
--PYNIQT

--PYNIQR
--PYNINQ
--PYNIMG
--PYNIKN
--PYNIKM
--PYNIID
--PYNIHT
--PYNIHK
PQPYNI--
--PYNIYS
--PYNIWF
--PYNIVV
--PYNIVD
--PYNITE
--PYNINR
--PYNILD
--PYNIKK
--PYNIIN
--PYNIHY
--PYNIHL
--PYNIGA
--PYNIER
--PYNIAG
PTPYNI--
PNPYNI--
PGPYNI--
--PYNIYY
--PYNIYR
--PYNIYK
--PYNIVW
--PYNIVM
--PYNITV
--PYNITM
--PYNISM
--PYNIRM
--PYNIQV
--PYNIMI
--PYNIIW
--PYNIGI
--PYNIES
--PYNIAR
--PYNIAL
--PYNIAF
--PYNIAE
--PYNIAA
PFPYNI--
--PYNIYG
--PYNIYD
--PYNIWH
--PYNIVQ

--PYNISY
--PYNIRD
--PYNIQM
--PYNIMN
--PYNIME
--PYNIMD
--PYNIGW
--PYNIGN
--PYNIFS
--PYNIFN
--PYNIEN
--PYNIEG
--PYNIAY
--PYNIWR
--PYNITH
--PYNINN
--PYNINM
--PYNILI
--PYNIKW
--PYNIKR
--PYNIKF
--PYNIKE
--PYNIKA
--PYNIIS
--PYNIII
--PYNIIE
--PYNIGT
--PYNIGR
--PYNIGH
--PYNIGG
--PYNIFY
--PYNIFE
--PYNIDS
PDPYNI--
--PYNIYW
--PYNIYV
--PYNIVP
--PYNIVE
--PYNITD
--PYNISV
--PYNISH
--PYNISC
--PYNIQY
--PYNIQQ
--PYNIQH
--PYNIQG
--PYNIQE
--PYNINV
--PYNINP
--PYNINI

--PYNINH
--PYNIMM
--PYNILP
--PYNIIT
--PYNIHW
--PYNIHR
--PYNIHF
--PYNIHC
--PYNIGS
--PYNIGK
--PYNIFT
--PYNIFI
--PYNIFG
--PYNIFD
--PYNIEI
--PYNICK
--PYNICE
--PYNIAN
--PYNIAH
-PPYNIT-
PHPYNI--
-QPYNIS-
QGPYNI--
QSPYNI--
QEPYNI--
QRPYNI--
-QPYNIT-
-QPYNIM-
-QPYNIY-
QKPYNI--
QVPYNI--
-QPYNIL-
-QPYNIV-
-QPYNIR-
-QPYNII-
QNPYNI--
QIPYNI--
-QPYNIK-
-QPYNIQ-
-QPYNIH-
QPPYNI--
QLPYNI--
QQPYNI--
QMPYNI--
-RPYNIL-
RSPYNI--
RTPYNI--
RRPYNI--
-RPYNIR-
-RPYNIT-

RMPYNI--
-RPYNIM-
-RPYNIY-
RGPYNI--
-RPYNIS-
REPYNI--
-RPYNIA-
-RPYNIQ-
RYPYNI--
RNPYNI--
-RPYNIH-
-RPYNIF-
RFPYNI--
RAPYNI--
-RPYNIV-
RVPYNI--
RLPYNI--
RQPYNI--
RKPYNI--
RDPYNI--
-RPYNIN-
-RPYNIK-
-RPYNIW-
RIPYNI--
RHPYNI--
-RPYNII-
-RPYNIE-
-RPYNIG-
RPPYNI--
RCPYNI--
SKPYNI--
SLPYNI--
-SPYNIR-
SSPYNI--
-SPYNIT-
SMPYNI--
-SPYNIH-
-SPYNIS-
STPYNI--
SRPYNI--
-SPYNIV-
-SPYNII-
SVPYNI--
SIPYNI--
SGPYNI--
SEPYNI--
-SPYNIA-
SHPYNI--
-SPYNIQ-
-SPYNIK-

```
-SPYNIN-
SAPYNI--
-SPYNIM-
SDPYNI--
SPPYNI--
SWPYNI--
-SPYNIE-
SNPYNI--
SFPYNI--
SQPYNI--
-SPYNIY-
-SPYNIG-
-SPYNIP-
-TPYNIH-
TLPYNI--
TSPYNI--
TGPYNI--
-TPYNIR-
TTPYNI--
TYPYNI--
-TPYNIS-
TEPYNI--
-TPYNIG-
TAPYNI--
-TPYNIK-
TNPYNI--
TVPYNI--
-TPYNIW-
-TPYNIV-
-TPYNIQ-
TRPYNI--
-TPYNIY-
-TPYNIT-
-TPYNIL-
TPPYNI--
TIPYNI--
TQPYNI--
-TPYNIN-
-TPYNIE-
TMPYNI--
TFPYNI--
TCPYNI--
-VPYNIR-
VLPYNI--
VTPYNI--
-VPYNIY-
VGPYNI--
-VPYNIS-
VYPYNI--
VIPYNI--
```

VEPYNI--
VVPYNI--
-VPYNIM-
-VPYNIQ-
VAPYNI--
VRPYNI--
-VPYNII-
-VPYNIE-
VMPYNI--
VHPYNI--
VQPYNI--
VNPYNI--
-VPYNIT-
VPPYNI--
VSPYNI--
-VPYNIW-
-VPYNIK-
VKPYNI--
VDPYNI--
-VPYNIV-
-VPYNIG-
-VPYNIA-
VFPYNI--
-WPYNIR-
-WPYNIY-
WRPYNI--
-WPYNIK-
WGPYNI--
WLPYNI--
WPPYNI--
WQPYNI--
-WPYNIQ-
-WPYNIW-
-WPYNIT-
-WPYNIH-
WKPYNI--
WAPYNI--
WYPYNI--
-WPYNIN-
WWPYNI--
WVPYNI--
WHPYNI--
YLPYNI--
YTPYNI--
YRPYNI--
YEPYNI--
YYPYNI--
YSPYNI--
YHPYNI--
YVPYNI--

-YPYNIT-
-YPYNIV-
-YPYNIS-
-YPYNII-
YAPYNI--
YQPYNI--
-YPYNIM-
-YPYNIG-
YPPYNI--
-YPYNIW-
-YPYNIQ-
YNPYNI--
-YPYNIY-
YIPYNI--
YGPYNI--
-YPYNIN-
-YPYNIK-
YMPYNI--
YFPYNI--

PRNL	346	678	48.27

-EPRNLD-
-MPRNLD-
--PRNLYP
--PRNLWW
--PRNLPW
--PRNLPL
--PRNLHP
--PRNLDR
ASPRNL--
-APRNLS-
-APRNLY-
AHPRNL--
-APRNLL-
-APRNLV-
AAPRNL--
-APRNLW-
AGPRNL--
-APRNLF-
ANPRNL--
-APRNLE-
-APRNLA-
AKPRNL--
-APRNLN-
AEPRNL--
ADPRNL--
-APRNLH-
AQPRNL--
-APRNLK-
ACPRNL--
-APRNLD-

CAPRNL--
-CPRNLL-
CCPRNL--
-CPRNLT-
CGPRNL--
CTPRNL--
CIPRNL--
CVPRNL--
CSPRNL--
CPPRNL--
CHPRNL--
CQPRNL--
-CPRNLW-
CLPRNL--
CKPRNL--
CRPRNL--
-CPRNLV-
-CPRNLK-
-CPRNLH-
-CPRNLE-
-CPRNLY-
-CPRNLI-
CFPRNL--
CWPRNL--
-CPRNLR-
CMPRNL--
-CPRNLQ-
-CPRNLN-
CNPRNL--
CYPRNL--
-CPRNLM-
-CPRNLF-
-CPRNLD-
-CPRNLA-
-DPRNLL-
DRPRNL--
-DPRNLI-
DTPRNL--
DLPRNL--
DIPRNL--
DVPRNL--
-DPRNLK-
-DPRNLR-
DSPRNL--
-DPRNLT-
DMPRNL--
DHPRNL--
DYPRNL--
-DPRNLS-
-DPRNLY-

DAPRNL--
DQPRNL--
DWPRNL--
-DPRNLW-
-DPRNLA-
-DPRNLF-
DNPRNL--
DKPRNL--
-DPRNLV-
-DPRNLE-
DGPRNL--
-DPRNLQ-
-DPRNLN-
-DPRNLG-
-DPRNLM-
DDPRNL--
DCPRNL--
ELPRNL--
EWPRNL--
-EPRNLS-
-EPRNLL-
-EPRNLK-
EVPRNL--
-EPRNLI-
EGPRNL--
-EPRNLY-
EQPRNL--
EMPRNL--
EYPRNL--
-EPRNLT-
-EPRNLV-
-EPRNLF-
-EPRNLH-
ENPRNL--
-EPRNLQ-
-EPRNLN-
EKPRNL--
EIPRNL--
-EPRNLM-
-EPRNLE-
EHPRNL--
EAPRNL--
ETPRNL--
EPPRNL--
EEPRNL--
FVPRNL--
-FPRNLL-
-FPRNLW-
FSPRNL--
-FPRNLY-

FGPRNL--
FMPRNL--
FDPRNL--
-FPRNLE-
-FPRNLF-
FNPRNL--
FQPRNL--
-FPRNLQ-
FHPRNL--
-FPRNLM-
FEPRNL--
FPPRNL--
FCPRNL--
-FPRNLD-
-GPRNLL-
-GPRNLS-
-GPRNLY-
GLPRNL--
GNPRNL--
-GPRNLM-
-GPRNLG-
GGPRNL--
-GPRNLH-
GQPRNL--
GVPRNL--
GEPRNL--
-GPRNLN-
GMPRNL--
GHPRNL--
-GPRNLV-
-GPRNLQ-
-GPRNLA-
-GPRNLW-
GAPRNL--
-GPRNLE-
GDPRNL--
-GPRNLD-
GPPRNL--
-HPRNLT-
-HPRNLW-
HGPRNL--
-HPRNLL-
-HPRNLS-
HTPRNL--
HIPRNL--
-HPRNLQ-
HFPRNL--
HHPRNL--
HQPRNL--
-HPRNLY-

−HPRNLF−
HYPRNL−−
−HPRNLG−
HMPRNL−−
HKPRNL−−
−HPRNLV−
−HPRNLI−
HPPRNL−−
HNPRNL−−
HDPRNL−−
−HPRNLH−
−HPRNLE−
−HPRNLM−
−HPRNLD−
HEPRNL−−
−HPRNLN−
HCPRNL−−
−IPRNLW−
ISPRNL−−
−IPRNLT−
−IPRNLH−
IHPRNL−−
−IPRNLM−
IPPRNL−−
−IPRNLG−
−IPRNLS−
−IPRNLN−
−IPRNLK−
IGPRNL−−
IDPRNL−−
IKPRNL−−
−IPRNLE−
IIPRNL−−
IQPRNL−−
IEPRNL−−
ICPRNL−−
−IPRNLD−
−KPRNLL−
KVPRNL−−
−KPRNLN−
−KPRNLG−
−KPRNLY−
−KPRNLW−
KHPRNL−−
−KPRNLV−
−KPRNLI−
KGPRNL−−
−KPRNLT−
KEPRNL−−
−KPRNLK−

KNPRNL--
KQPRNL--
-KPRNLE-
-KPRNLH-
KDPRNL--
KYPRNL--
-KPRNLQ-
KCPRNL--
-KPRNLD-
LSPRNL--
LKPRNL--
LGPRNL--
-LPRNLI-
LIPRNL--
-LPRNLV-
-LPRNLQ-
-LPRNLN-
LEPRNL--
LHPRNL--
-LPRNLW-
LDPRNL--
-LPRNLE-
LQPRNL--
-LPRNLM-
LCPRNL--
-LPRNLD-
MSPRNL--
-MPRNLY-
MAPRNL--
MGPRNL--
-MPRNLK-
-MPRNLS-
-MPRNLA-
MNPRNL--
MHPRNL--
-MPRNLH-
MTPRNL--
MQPRNL--
-MPRNLV-
-MPRNLQ-
-MPRNLM-
-MPRNLG-
MEPRNL--
-MPRNLE-
MPPRNL--
-MPRNLN-
MCPRNL--
MFPRNL--
MDPRNL--
-NPRNLL-

-NPRNLY-
-NPRNLQ-
NGPRNL--
-NPRNLV-
NPPRNL--
-NPRNLH-
-NPRNLK-
NQPRNL--
-NPRNLA-
NNPRNL--
-NPRNLG-
NEPRNL--
NKPRNL--
-NPRNLM-
-NPRNLW-
NDPRNL--
-NPRNLF-
-NPRNLE-
NCPRNL--
-NPRNLC-
--PRNLRT
--PRNLIE
--PRNLFK
--PRNLFT
--PRNLVS
--PRNLLY
--PRNLFN
--PRNLRW
--PRNLYS
--PRNLGV
--PRNLFI
--PRNLMK
--PRNLWK
--PRNLIN
--PRNLSN
--PRNLFL
--PRNLYQ
--PRNLWG
--PRNLYL
--PRNLTV
--PRNLLQ
--PRNLMY
--PRNLVY
--PRNLRI
--PRNLFS
--PRNLYG
--PRNLTI
--PRNLYI
--PRNLRA
--PRNLKT

--PRNLFV
--PRNLIA
--PRNLAF
--PRNLYT
--PRNLWN
--PRNLKG
--PRNLFM
--PRNLAH
--PRNLTY
--PRNLTG
--PRNLGW
--PRNLTN
--PRNLMT
--PRNLAS
--PRNLTA
--PRNLST
--PRNLQS
--PRNLFH
--PRNLSY
--PRNLGL
--PRNLIV
PVPRNL--
--PRNLWT
--PRNLWA
--PRNLNL
--PRNLAY
--PRNLWR
--PRNLMD
--PRNLLE
--PRNLYM
--PRNLTF
--PRNLSW
--PRNLLD
--PRNLYR
--PRNLQT
--PRNLNI
--PRNLMM
--PRNLYV
--PRNLRQ
--PRNLKV
--PRNLET
--PRNLAW
--PRNLVV
--PRNLGY
--PRNLSM
--PRNLGG
--PRNLAN
--PRNLQI
--PRNLNS
PMPRNL--

--PRNLRE
--PRNLVQ
--PRNLII
--PRNLHV
PQPRNL--
--PRNLSE
PAPRNL--
--PRNLHT
--PRNLNG
--PRNLHG
--PRNLAA
--PRNLVM
--PRNLTT
--PRNLNQ
--PRNLHI
--PRNLFY
PTPRNL--
--PRNLYW
--PRNLTE
--PRNLGH
--PRNLGA
--PRNLTQ
--PRNLDG
--PRNLDA
--PRNLWY
--PRNLWE
--PRNLKH
--PRNLHF
--PRNLVE
--PRNLSK
--PRNLLM
--PRNLIF
--PRNLYE
--PRNLYD
--PRNLWM
--PRNLHM
--PRNLQL
--PRNLQG
--PRNLFD
--PRNLWH
--PRNLQY
--PRNLNR
--PRNLGN
--PRNLFQ
--PRNLEV
--PRNLAM
--PRNLNV
--PRNLME
--PRNLKQ
--PRNLKN

--PRNLKE
--PRNLIM
--PRNLHA
--PRNLGM
--PRNLAD
--PRNLQK
--PRNLFA
--PRNLEW
--PRNLEL
--PRNLYY
--PRNLTM
--PRNLID
--PRNLEN
--PRNLVP
--PRNLVD
--PRNLQV
--PRNLNY
PNPRNL--
--PRNLTD
--PRNLMF
--PRNLIQ
--PRNLDT
--PRNLDS
--PRNLMI
--PRNLMQ
--PRNLEY
--PRNLQQ
--PRNLNK
--PRNLEI
--PRNLWD
--PRNLSD
--PRNLNH
--PRNLMW
--PRNLHD
--PRNLGQ
--PRNLEH
--PRNLQN
--PRNLNT
--PRNLNE
--PRNLHY
--PRNLQF
--PRNLQD
--PRNLNW
--PRNLKD
--PRNLFE
--PRNLGE
--PRNLEQ
--PRNLDV
--PRNLAE
PDPRNL--

--PRNLHK
--PRNLEM
--PRNLED
PKPRNL--
--PRNLQM
--PRNLMH
--PRNLEA
--PRNLHQ
--PRNLHH
--PRNLHE
--PRNLQW
--PRNLMP
--PRNLLC
--PRNLGD
--PRNLFC
--PRNLCF
PHPRNL--
PEPRNL--
--PRNLQH
--PRNLEK
--PRNLEE
--PRNLVC
--PRNLTC
--PRNLRC
--PRNLNC
--PRNLDM
--PRNLDI
--PRNLCY
--PRNLCV
--PRNLCS
--PRNLCI
--PRNLCH
--PRNLCA
-PPRNLY-
-PPRNLI-
--PRNLWC
--PRNLSC
--PRNLQP
--PRNLND
--PRNLKC
--PRNLIC
--PRNLGC
--PRNLDN
--PRNLDH
--PRNLCW
--PRNLCG
--PRNLCE
-PPRNLS-
-PPRNLR-
PPPRNL--

QSPRNL--
-QPRNLY-
-QPRNLS-
-QPRNLK-
QPPRNL--
QQPRNL--
-QPRNLL-
-QPRNLA-
QTPRNL--
-QPRNLI-
-QPRNLF-
-QPRNLH-
-QPRNLT-
-QPRNLM-
-QPRNLG-
QHPRNL--
QAPRNL--
-QPRNLV-
-QPRNLQ-
-QPRNLW-
QIPRNL--
QEPRNL--
QGPRNL--
QNPRNL--
-QPRNLE-
QDPRNL--
-QPRNLD-
-RPRNLQ-
-RPRNLI-
-RPRNLW-
-RPRNLM-
RPPRNL--
-RPRNLV-
-RPRNLE-
RNPRNL--
-RPRNLD-
RDPRNL--
-RPRNLC-
-SPRNLR-
SSPRNL--
-SPRNLY-
-SPRNLL-
-SPRNLV-
-SPRNLG-
-SPRNLA-
-SPRNLK-
STPRNL--
-SPRNLW-
-SPRNLM-
-SPRNLI-

-SPRNLF-
SQPRNL--
-SPRNLQ-
SAPRNL--
SHPRNL--
SNPRNL--
SEPRNL--
SPPRNL--
SMPRNL--
SCPRNL--
-SPRNLD-
-SPRNLC-
TSPRNL--
TLPRNL--
TIPRNL--
TAPRNL--
-TPRNLV-
-TPRNLA-
-TPRNLG-
-TPRNLM-
-TPRNLY-
TQPRNL--
-TPRNLS-
TGPRNL--
-TPRNLT-
TNPRNL--
-TPRNLQ-
-TPRNLH-
-TPRNLN-
-TPRNLW-
TPPRNL--
THPRNL--
TKPRNL--
TEPRNL--
TDPRNL--
TCPRNL--
-TPRNLE-
-TPRNLD-
-VPRNLL-
VKPRNL--
-VPRNLA-
VGPRNL--
-VPRNLI-
-VPRNLN-
-VPRNLG-
-VPRNLV-
-VPRNLK-
VAPRNL--
-VPRNLW-
VPPRNL--

VNPRNL--
-VPRNLM-
-VPRNLE-
-VPRNLF-
-VPRNLQ-
VIPRNL--
-VPRNLD-
VEPRNL--
VDPRNL--
VCPRNL--
-VPRNLC-
-WPRNLS-
WLPRNL--
WVPRNL--
WPPRNL--
WFPRNL--
-WPRNLY-
WEPRNL--
WMPRNL--
WAPRNL--
WTPRNL--
-WPRNLL-
WGPRNL--
-WPRNLM-
WQPRNL--
-WPRNLE-
WDPRNL--
-WPRNLD-
WCPRNL--
YLPRNL--
-YPRNLW-
YAPRNL--
YVPRNL--
-YPRNLA-
YSPRNL--
YTPRNL--
YQPRNL--
YKPRNL--
YNPRNL--
YMPRNL--
-YPRNLY-
YPPRNL--
-YPRNLE-
YEPRNL--
-YPRNLD-
YDPRNL--
-YPRNLQ-
YCPRNL--

PYWL	323	687	48.27

ASPYWL--

-APYWLR-
-APYWLI-
AGPYWL--
AFPYWL--
ATPYWL--
-APYWLH-
-APYWLA-
-APYWLT-
AHPYWL--
-APYWLG-
AKPYWL--
-APYWLM-
AIPYWL--
AVPYWL--
AWPYWL--
-APYWLS-
-APYWLE-
AEPYWL--
-APYWLD-
-APYWLQ-
ADPYWL--
-APYWLK-
ANPYWL--
AYPYWL--
-APYWLV-
APPYWL--
ACPYWL--
AAPYWL--
CNPYWL--
CKPYWL--
CGPYWL--
CRPYWL--
-CPYWLR-
-CPYWLK-
CSPYWL--
-CPYWLN-
-CPYWLL-
-CPYWLG-
CMPYWL--
CLPYWL--
CHPYWL--
CDPYWL--
CCPYWL--
DRPYWL--
-DPYWLT-
DGPYWL--
-DPYWLS-
DTPYWL--
-DPYWLA-
-DPYWLI-

DLPYWL--
-DPYWLV-
-DPYWLL-
DVPYWL--
DIPYWL--
DFPYWL--
DYPYWL--
-DPYWLM-
-DPYWLG-
DMPYWL--
-DPYWLK-
-DPYWLY-
-DPYWLQ-
DKPYWL--
-EPYWLH-
-EPYWLR-
-EPYWLV-
-EPYWLF-
ERPYWL--
-EPYWLK-
EKPYWL--
EVPYWL--
-EPYWLA-
-EPYWLL-
EIPYWL--
EWPYWL--
-EPYWLI-
-EPYWLE-
EQPYWL--
-EPYWLN-
ETPYWL--
-EPYWLS-
ELPYWL--
EAPYWL--
ESPYWL--
-EPYWLQ-
EHPYWL--
EGPYWL--
EFPYWL--
EYPYWL--
-EPYWLT-
-EPYWLD-
EMPYWL--
EEPYWL--
FDPYWL--
-FPYWLT-
FKPYWL--
FTPYWL--
FSPYWL--
-FPYWLG-

-FPYWLK-
-FPYWLL-
-FPYWLA-
-FPYWLH-
FNPYWL--
FWPYWL--
-FPYWLF-
-FPYWLV-
-FPYWLW-
-FPYWLP-
FPPYWL--
FMPYWL--
GRPYWL--
-GPYWLR-
GVPYWL--
GSPYWL--
-GPYWLS-
-GPYWLH-
GAPYWL--
GKPYWL--
-GPYWLL-
-GPYWLT-
GNPYWL--
-GPYWLV-
-GPYWLM-
-GPYWLI-
GWPYWL--
GEPYWL--
GGPYWL--
GQPYWL--
-GPYWLE-
GTPYWL--
GMPYWL--
GLPYWL--
-GPYWLA-
GPPYWL--
GYPYWL--
-GPYWLQ-
-GPYWLK-
-GPYWLG-
GFPYWL--
GDPYWL--
-GPYWLN-
GIPYWL--
GCPYWL--
HSPYWL--
-HPYWLR-
HVPYWL--
-HPYWLK-
HRPYWL--

-HPYWLV-
HKPYWL--
HWPYWL--
HGPYWL--
-HPYWLQ-
-HPYWLG-
HLPYWL--
HYPYWL--
HFPYWL--
-HPYWLN-
-HPYWLE-
HIPYWL--
-HPYWLS-
-HPYWLD-
HMPYWL--
HQPYWL--
-HPYWLY-
-HPYWLH-
HPPYWL--
-HPYWLL-
-HPYWLA-
HEPYWL--
-HPYWLT-
-HPYWLI-
HDPYWL--
IKPYWL--
IGPYWL--
IRPYWL--
IMPYWL--
-IPYWLN-
IEPYWL--
IYPYWL--
IVPYWL--
IQPYWL--
-IPYWLR-
-IPYWLA-
INPYWL--
IIPYWL--
-IPYWLL-
IDPYWL--
-IPYWLG-
IPPYWL--
-IPYWLK-
ILPYWL--
IWPYWL--
-IPYWLV-
-IPYWLH-
-IPYWLD-
-IPYWLE-
-IPYWLS-

-IPYWLQ-
IAPYWL--
KSPYWL--
KMPYWL--
KRPYWL--
KTPYWL--
KAPYWL--
-KPYWLL-
KLPYWL--
KGPYWL--
-KPYWLA-
-KPYWLK-
-KPYWLT-
-KPYWLI-
KWPYWL--
KPPYWL--
KHPYWL--
KFPYWL--
-KPYWLS-
-KPYWLQ-
KDPYWL--
-KPYWLN-
-KPYWLV-
-KPYWLH-
KKPYWL--
KEPYWL--
KYPYWL--
-KPYWLG-
KIPYWL--
-KPYWLM-
LSPYWL--
LRPYWL--
LKPYWL--
-LPYWLT-
LNPYWL--
LQPYWL--
-LPYWLS-
-LPYWLR-
-LPYWLM-
LGPYWL--
-LPYWLA-
-LPYWLL-
LPPYWL--
LAPYWL--
LIPYWL--
LFPYWL--
LWPYWL--
LMPYWL--
LYPYWL--
LEPYWL--

LTPYWL--
LLPYWL--
LDPYWL--
-LPYWLK-
-LPYWLH-
-LPYWLG-
-LPYWLQ-
LHPYWL--
-LPYWLV-
-LPYWLN-
LCPYWL--
-MPYWLS-
MNPYWL--
-MPYWLR-
-MPYWLK-
MSPYWL--
MGPYWL--
MLPYWL--
MKPYWL--
MPPYWL--
-MPYWLV-
-MPYWLN-
-MPYWLH-
MEPYWL--
MDPYWL--
MTPYWL--
MRPYWL--
-MPYWLY-
-MPYWLT-
-MPYWLG-
MMPYWL--
-MPYWLM-
-MPYWLI-
-MPYWLA-
MAPYWL--
-MPYWLF-
MIPYWL--
MHPYWL--
MFPYWL--
NAPYWL--
NGPYWL--
NVPYWL--
-NPYWLG-
NLPYWL--
-NPYWLR-
-NPYWLA-
NTPYWL--
-NPYWLK-
NQPYWL--
NFPYWL--

NDPYWL--
-NPYWLT-
-NPYWLN-
-NPYWLM-
NNPYWL--
NEPYWL--
-NPYWLV-
NMPYWL--
NRPYWL--
-NPYWLH-
NYPYWL--
-NPYWLY-
-NPYWLQ-
NKPYWL--
NHPYWL--
NWPYWL--
-NPYWLS-
-NPYWLP-
-NPYWLI-
-NPYWLE-
NPPYWL--
-NPYWLF-
--PYWLNN
--PYWLSG
--PYWLSS
--PYWLRS
--PYWLGS
--PYWLRT
--PYWLRI
--PYWLVS
--PYWLRN
--PYWLKN
--PYWLTT
--PYWLRV
--PYWLDS
--PYWLGA
--PYWLQS
--PYWLAS
--PYWLSW
--PYWLSH
--PYWLKA
--PYWLSQ
--PYWLAD
--PYWLQT
--PYWLQN
--PYWLKS
--PYWLRH
--PYWLSA
--PYWLNA
--PYWLVY

PWPYWL--
--PYWLYG
--PYWLTS
--PYWLRM
--PYWLQH
--PYWLQG
--PYWLHS
--PYWLTV
--PYWLSR
--PYWLNS
--PYWLKI
--PYWLSN
--PYWLRG
--PYWLQR
--PYWLRY
--PYWLVT
--PYWLES
--PYWLTY
--PYWLSI
--PYWLSE
--PYWLGR
--PYWLDA
--PYWLAH
--PYWLTG
--PYWLKM
--PYWLSK
--PYWLKV
PGPYWL--
--PYWLVK
--PYWLKY
--PYWLKE
--PYWLAK
--PYWLVD
--PYWLSD
--PYWLAG
PIPYWL--
--PYWLYD
--PYWLMK
--PYWLKT
--PYWLKG
--PYWLGY
--PYWLER
--PYWLVH
--PYWLTQ
--PYWLRD
--PYWLQV
--PYWLIH
--PYWLEH
--PYWLVN
--PYWLST

--PYWLNT
--PYWLLR
PNPYWL--
--PYWLYY
--PYWLVG
--PYWLRQ
--PYWLNG
--PYWLMQ
--PYWLMD
--PYWLKK
--PYWLHY
--PYWLHA
--PYWLAR
PTPYWL--
--PYWLTI
--PYWLID
--PYWLHQ
--PYWLHG
--PYWLGK
--PYWLAW
--PYWLAT
--PYWLTE
--PYWLQE
--PYWLNV
--PYWLKF
--PYWLHR
--PYWLGN
--PYWLGF
--PYWLGE
--PYWLET
--PYWLEG
--PYWLAM
--PYWLQF
--PYWLML
--PYWLIN
--PYWLGV
--PYWLGQ
--PYWLFV
--PYWLEA
--PYWLAV
PFPYWL--
--PYWLWT
--PYWLRW
--PYWLNQ
--PYWLME
--PYWLLE
--PYWLLD
--PYWLIM
--PYWLIG
--PYWLAN

--PYWLTN
--PYWLTH
--PYWLRE
--PYWLMG
--PYWLHV
--PYWLHK
--PYWLHH
--PYWLGT
--PYWLGD
--PYWLEW
--PYWLWG
--PYWLTA
--PYWLSM
--PYWLQY
--PYWLPQ
--PYWLNR
--PYWLNE
--PYWLLM
--PYWLIQ
--PYWLHN
--PYWLGM
--PYWLGI
--PYWLGH
--PYWLVW
--PYWLVF
--PYWLTK
--PYWLQD
--PYWLPY
--PYWLND
--PYWLKW
--PYWLHW
--PYWLHT
--PYWLHM
--PYWLHE
--PYWLFQ
--PYWLFN
--PYWLEK
--PYWLED
--PYWLCA
--PYWLAQ
--PYWLAE
-PPYWLM-
PHPYWL--
QSPYWL--
-QPYWLR-
-QPYWLS-
-QPYWLT-
QLPYWL--
QKPYWL--
QTPYWL--

-QPYWLN-
QNPYWL--
QHPYWL--
-QPYWLQ-
-QPYWLM-
QVPYWL--
-QPYWLA-
QMPYWL--
QQPYWL--
-QPYWLK-
-QPYWLH-
QEPYWL--
QPPYWL--
QGPYWL--
QFPYWL--
QRPYWL--
-QPYWLV-
-QPYWLD-
-RPYWLS-
-RPYWLG-
-RPYWLM-
RYPYWL--
RSPYWL--
-RPYWLI-
RLPYWL--
RTPYWL--
-RPYWLK-
-RPYWLH-
RHPYWL--
RGPYWL--
-RPYWLR-
RNPYWL--
-RPYWLL-
RVPYWL--
-RPYWLQ-
RRPYWL--
-RPYWLN-
-RPYWLV-
-RPYWLA-
RMPYWL--
RKPYWL--
-RPYWLE-
RAPYWL--
RFPYWL--
RDPYWL--
RWPYWL--
REPYWL--
RPPYWL--
RQPYWL--
-SPYWLA-

-SPYWLN-
SNPYWL--
SSPYWL--
STPYWL--
SAPYWL--
SRPYWL--
SKPYWL--
SGPYWL--
SQPYWL--
-SPYWLK-
SYPYWL--
SEPYWL--
-SPYWLM-
SWPYWL--
SHPYWL--
-SPYWLS-
SVPYWL--
-SPYWLT-
-SPYWLH-
-SPYWLG-
-SPYWLE-
-SPYWLW-
SMPYWL--
SDPYWL--
-SPYWLQ-
SPPYWL--
SCPYWL--
-SPYWLD-
TLPYWL--
-TPYWLS-
TTPYWL--
TVPYWL--
TEPYWL--
-TPYWLL-
-TPYWLA-
TPPYWL--
TGPYWL--
TDPYWL--
TYPYWL--
TFPYWL--
-TPYWLH-
TNPYWL--
TKPYWL--
-TPYWLT-
-TPYWLM-
TAPYWL--
-TPYWLV-
-TPYWLK-
-TPYWLI-
-TPYWLW-

```
THPYWL--
TWPYWL--
-TPYWLN-
TQPYWL--
-TPYWLE-
-TPYWLG-
-TPYWLD-
TCPYWL--
VGPYWL--
VNPYWL--
VRPYWL--
VSPYWL--
-VPYWLT-
VLPYWL--
-VPYWLR-
VHPYWL--
VYPYWL--
-VPYWLV-
VMPYWL--
-VPYWLN-
VAPYWL--
-VPYWLS-
-VPYWLD-
VVPYWL--
-VPYWLM-
VQPYWL--
-VPYWLA-
VFPYWL--
-VPYWLH-
VPPYWL--
VEPYWL--
VTPYWL--
-VPYWLL-
-VPYWLK-
-VPYWLF-
VKPYWL--
VDPYWL--
-VPYWLG-
-VPYWLI-
VWPYWL--
WEPYWL--
WRPYWL--
-WPYWLR-
-WPYWLS-
WFPYWL--
WVPYWL--
-WPYWLL-
WWPYWL--
WTPYWL--
-WPYWLT-
```

	WLPYWL--		
	-WPYWLV-		
	-WPYWLI-		
	-WPYWLH-		
	WKPYWL--		
	WHPYWL--		
	-WPYWLG-		
	-WPYWLN-		
	-WPYWLK-		
	-YPYWLL-		
	YRPYWL--		
	YQPYWL--		
	-YPYWLS-		
	YNPYWL--		
	YKPYWL--		
	YLPYWL--		
	-YPYWLK-		
	YGPYWL--		
	YTPYWL--		
	-YPYWLQ-		
	YPPYWL--		
	YEPYWL--		
	-YPYWLN-		
	YDPYWL--		
	-YPYWLI-		
	-YPYWLG-		
	-YPYWLM-		
	YVPYWL--		
	YHPYWL--		
	-YPYWLV-		
	-YPYWLF-		
	-YPYWLA-		
	YMPYWL--		
	-YPYWLT-		
	-YPYWLR-		
	-YPYWLD-		
PKPL	73	829	48.26
	AAPKPL--		
	-APKPLQ-		
	AIPKPL--		
	-APKPLH-		
	-APKPLF-		
	-APKPLV-		
	-APKPLA-		
	-APKPLW-		
	-APKPLR-		
	ALPKPL--		
	AHPKPL--		
	-APKPLK-		
	AKPKPL--		

AQPKPL--
APPKPL--
-APKPLS-
AVPKPL--
-APKPLM-
ADPKPL--
AMPKPL--
AWPKPL--
ANPKPL--
-APKPLE-
AYPKPL--
AGPKPL--
AEPKPL--
-APKPLP-
ACPKPL--
CTPKPL--
CYPKPL--
-CPKPLT-
CLPKPL--
CFPKPL--
CGPKPL--
-CPKPLV-
CVPKPL--
CRPKPL--
-CPKPLW-
-CPKPLF-
CKPKPL--
CIPKPL--
-CPKPLY-
-CPKPLR-
-CPKPLI-
-CPKPLH-
CCPKPL--
CAPKPL--
CWPKPL--
CSPKPL--
-CPKPLN-
-CPKPLL-
-CPKPLD-
DTPKPL--
DLPKPL--
-DPKPLF-
DVPKPL--
-DPKPLR-
-DPKPLT-
-DPKPLL-
-DPKPLK-
DEPKPL--
DKPKPL--
DIPKPL--

DAPKPL--
DRPKPL--
-DPKPLS-
DFPKPL--
DYPKPL--
-DPKPLI-
-DPKPLN-
DQPKPL--
-DPKPLY-
DMPKPL--
DHPKPL--
DSPKPL--
-DPKPLM-
DNPKPL--
-EPKPLL-
-EPKPLT-
-EPKPLR-
-EPKPLW-
ERPKPL--
-EPKPLF-
EVPKPL--
EQPKPL--
-EPKPLA-
EAPKPL--
EWPKPL--
ESPKPL--
-EPKPLV-
EGPKPL--
-EPKPLK-
EMPKPL--
ETPKPL--
-EPKPLQ-
EEPKPL--
-EPKPLS-
-EPKPLH-
ENPKPL--
-EPKPLN-
-EPKPLE-
EKPKPL--
EPPKPL--
-EPKPLI-
EFPKPL--
ECPKPL--
EDPKPL--
-FPKPLL-
-FPKPLY-
FRPKPL--
FLPKPL--
FMPKPL--
-FPKPLF-

FAPKPL--
FTPKPL--
-FPKPLS-
FNPKPL--
FHPKPL--
FYPKPL--
FWPKPL--
FIPKPL--
FKPKPL--
-FPKPLR-
-FPKPLI-
FVPKPL--
-FPKPLN-
FFPKPL--
FEPKPL--
-FPKPLK-
FPPKPL--
-FPKPLV-
-FPKPLH-
FGPKPL--
FQPKPL--
-FPKPLQ-
GAPKPL--
-GPKPLS-
-GPKPLI-
GTPKPL--
-GPKPLK-
-GPKPLV-
-GPKPLR-
-GPKPLM-
-GPKPLF-
GQPKPL--
GRPKPL--
-GPKPLA-
GIPKPL--
GPPKPL--
-GPKPLN-
-GPKPLW-
-GPKPLQ-
-GPKPLL-
GYPKPL--
GVPKPL--
GFPKPL--
GSPKPL--
GLPKPL--
GCPKPL--
-GPKPLT-
GNPKPL--
GKPKPL--
GWPKPL--

-GPKPLH-
GMPKPL--
GGPKPL--
-GPKPLY-
-GPKPLG-
GEPKPL--
GHPKPL--
-HPKPLL-
HSPKPL--
HGPKPL--
-HPKPLR-
HLPKPL--
HAPKPL--
HYPKPL--
-HPKPLK-
-HPKPLS-
HRPKPL--
-HPKPLA-
-HPKPLT-
-HPKPLH-
HWPKPL--
HFPKPL--
HEPKPL--
HDPKPL--
-HPKPLY-
-HPKPLW-
-HPKPLN-
HKPKPL--
-HPKPLM-
HNPKPL--
HPPKPL--
-HPKPLE-
HQPKPL--
-HPKPLV-
-HPKPLG-
-HPKPLC-
IVPKPL--
-IPKPLI-
IWPKPL--
ILPKPL--
ISPKPL--
-IPKPLH-
IRPKPL--
IAPKPL--
-IPKPLY-
IQPKPL--
-IPKPLR-
INPKPL--
-IPKPLM-
-IPKPLT-

-IPKPLL-
-IPKPLV-
-IPKPLK-
IFPKPL--
ITPKPL--
IYPKPL--
-IPKPLQ-
-IPKPLN-
-IPKPLF-
-IPKPLS-
IKPKPL--
-IPKPLG-
IIPKPL--
IDPKPL--
-IPKPLW-
IMPKPL--
KSPKPL--
-KPKPLL-
KGPKPL--
KVPKPL--
-KPKPLS-
KWPKPL--
KTPKPL--
KEPKPL--
KHPKPL--
-KPKPLF-
-KPKPLI-
-KPKPLH-
KIPKPL--
-KPKPLY-
-KPKPLW-
-KPKPLN-
-KPKPLK-
-KPKPLQ-
KQPKPL--
KPPKPL--
-KPKPLA-
-KPKPLM-
-KPKPLG-
KDPKPL--
KCPKPL--
-KPKPLC-
-LPKPLL-
LLPKPL--
LHPKPL--
LAPKPL--
-LPKPLI-
-LPKPLK-
-LPKPLW-
-LPKPLS-

-LPKPLR-
LTPKPL--
LFPKPL--
LVPKPL--
-LPKPLV-
LPPKPL--
-LPKPLT-
-LPKPLM-
-LPKPLF-
-LPKPLA-
LNPKPL--
LWPKPL--
LKPKPL--
LYPKPL--
LDPKPL--
-LPKPLN-
-LPKPLG-
LQPKPL--
-LPKPLC-
-MPKPLI-
MRPKPL--
-MPKPLF-
MMPKPL--
MIPKPL--
MLPKPL--
MYPKPL--
-MPKPLS-
-MPKPLW-
MAPKPL--
MTPKPL--
-MPKPLR-
-MPKPLK-
MVPKPL--
-MPKPLQ-
MWPKPL--
MPPKPL--
-MPKPLA-
MDPKPL--
MKPKPL--
-MPKPLM-
-MPKPLG-
MNPKPL--
-MPKPLV-
MHPKPL--
MEPKPL--
MQPKPL--
-MPKPLN-
-MPKPLY-
-MPKPLH-
-MPKPLE-

MCPKPL--
-NPKPLL-
NLPKPL--
NSPKPL--
NTPKPL--
-NPKPLH-
-NPKPLR-
NIPKPL--
NWPKPL--
-NPKPLV-
NAPKPL--
NVPKPL--
-NPKPLY-
-NPKPLS-
-NPKPLG-
-NPKPLF-
NCPKPL--
-NPKPLA-
NRPKPL--
-NPKPLI-
NNPKPL--
NMPKPL--
NPPKPL--
-NPKPLM-
-NPKPLT-
-NPKPLQ-
NHPKPL--
NDPKPL--
-NPKPLP-
-NPKPLN-
--PKPLLS
--PKPLSS
--PKPLFW
--PKPLSL
--PKPLMS
--PKPLVN
--PKPLSW
--PKPLLY
--PKPLIF
--PKPLYG
--PKPLMR
--PKPLRA
--PKPLYL
--PKPLVA
--PKPLSA
--PKPLFT
--PKPLQL
--PKPLLV
--PKPLAV
--PKPLHQ

--PKPLWA
--PKPLWG
--PKPLHF
--PKPLFY
--PKPLTF
--PKPLRV
--PKPLVG
--PKPLTY
--PKPLHS
--PKPLTI
--PKPLST
--PKPLLQ
--PKPLNV
--PKPLVQ
--PKPLRT
--PKPLNQ
--PKPLFR
--PKPLAA
--PKPLTG
--PKPLYS
--PKPLVI
--PKPLRY
--PKPLRI
--PKPLYF
--PKPLWS
--PKPLVL
--PKPLFH
--PKPLTT
--PKPLSF
--PKPLRH
--PKPLMY
--PKPLLW
--PKPLIN
--PKPLVS
--PKPLII
--PKPLGT
--PKPLVT
--PKPLRD
--PKPLIG
--PKPLYT
--PKPLYI
--PKPLWY
--PKPLPW
--PKPLIV
--PKPLHM
--PKPLEQ
--PKPLAL
--PKPLVF
--PKPLHA
--PKPLYQ

--PKPLVY
--PKPLRQ
--PKPLNS
--PKPLKN
--PKPLGF
--PKPLAG
--PKPLWN
--PKPLTA
--PKPLQI
--PKPLMG
--PKPLLH
--PKPLMA
--PKPLLE
--PKPLKT
--PKPLFL
--PKPLEY
--PKPLAH
PEPKPL--
PVPKPL--
PQPKPL--
--PKPLYK
--PKPLVW
--PKPLTW
--PKPLPQ
--PKPLMM
--PKPLLD
--PKPLAN
PWPKPL--
PSPKPL--
--PKPLWL
--PKPLWK
--PKPLSV
--PKPLQT
--PKPLLM
--PKPLIH
--PKPLGH
--PKPLFS
--PKPLES
--PKPLWH
--PKPLVK
--PKPLTR
--PKPLSY
--PKPLSH
--PKPLSG
--PKPLRN
--PKPLRK
--PKPLQW
--PKPLMW
--PKPLLF
--PKPLKK

--PKPLKI
--PKPLIK
--PKPLGG
--PKPLFA
PGPKPL--
--PKPLYW
--PKPLWF
--PKPLTV
--PKPLTH
--PKPLRW
--PKPLQK
--PKPLNW
--PKPLNH
--PKPLMV
--PKPLMN
--PKPLKL
--PKPLIY
--PKPLGR
--PKPLGA
--PKPLAT
PRPKPL--
--PKPLYN
--PKPLTN
--PKPLQS
--PKPLNT
--PKPLNA
--PKPLKV
--PKPLIT
--PKPLIA
--PKPLFG
--PKPLEI
--PKPLAQ
--PKPLYY
--PKPLWT
--PKPLSK
--PKPLRM
--PKPLNG
--PKPLLI
--PKPLHG
--PKPLGW
--PKPLGK
--PKPLFV
--PKPLDL
--PKPLDF
PKPKPL--
PMPKPL--
--PKPLWI
--PKPLWD
--PKPLQA
--PKPLNL

--PKPLMK
--PKPLKF
--PKPLIQ
--PKPLHW
--PKPLHH
--PKPLFK
--PKPLFI
--PKPLEW
--PKPLAM
PFPKPL--
--PKPLTQ
--PKPLPF
--PKPLPA
--PKPLME
--PKPLKA
--PKPLIL
--PKPLEL
--PKPLEG
--PKPLMT
--PKPLHY
--PKPLEF
--PKPLAR
--PKPLYM
--PKPLWV
--PKPLVV
--PKPLQN
--PKPLQG
--PKPLMH
--PKPLLK
--PKPLKM
--PKPLHT
--PKPLGS
--PKPLFF
--PKPLET
--PKPLDV
PAPKPL--
--PKPLYV
--PKPLQV
--PKPLQH
--PKPLQE
--PKPLIW
--PKPLHE
--PKPLFN
--PKPLFE
--PKPLEV
--PKPLEH
--PKPLDA
PHPKPL--
PCPKPL--
--PKPLYD

```
--PKPLRP
--PKPLPN
--PKPLMQ
--PKPLMF
--PKPLIE
--PKPLHR
--PKPLHK
--PKPLHD
--PKPLGN
--PKPLFQ
--PKPLEA
--PKPLAW
--PKPLAK
PIPKPL--
--PKPLYE
--PKPLTK
--PKPLSE
--PKPLSD
--PKPLQQ
--PKPLNN
--PKPLNK
--PKPLMI
--PKPLMD
--PKPLLC
--PKPLID
--PKPLGQ
--PKPLFM
--PKPLDY
--PKPLDS
--PKPLCV
PPPKPL--
-PPKPLT-
--PKPLWQ
--PKPLVP
--PKPLTM
--PKPLRE
--PKPLQF
--PKPLQD
--PKPLPP
--PKPLNP
--PKPLKY
--PKPLKW
--PKPLKR
--PKPLKQ
--PKPLKG
--PKPLKD
--PKPLFC
--PKPLEM
--PKPLDQ
--PKPLDI
```

--PKPLCR
--PKPLCL
--PKPLCH
--PKPLCF
--PKPLAF
PDPKPL--
-QPKPLK-
-QPKPLL-
-QPKPLF-
QVPKPL--
QRPKPL--
-QPKPLS-
QSPKPL--
-QPKPLT-
-QPKPLA-
-QPKPLM-
-QPKPLH-
QLPKPL--
QIPKPL--
QEPKPL--
-QPKPLW-
QQPKPL--
-QPKPLV-
QMPKPL--
QYPKPL--
QGPKPL--
QDPKPL--
-QPKPLN-
-QPKPLC-
QNPKPL--
QFPKPL--
QWPKPL--
-QPKPLQ-
-QPKPLI-
-QPKPLG-
QTPKPL--
-QPKPLY-
QKPKPL--
QCPKPL--
RSPKPL--
-RPKPLK-
RVPKPL--
RLPKPL--
RMPKPL--
-RPKPLR-
-RPKPLT-
-RPKPLN-
-RPKPLA-
-RPKPLY-
-RPKPLS-

RAPKPL--
RFPKPL--
RHPKPL--
RIPKPL--
RTPKPL--
RKPKPL--
RWPKPL--
-RPKPLM-
RDPKPL--
RPPKPL--
RYPKPL--
-RPKPLI-
RQPKPL--
-RPKPLG-
-RPKPLE-
RCPKPL--
-RPKPLC-
-SPKPLY-
SSPKPL--
-SPKPLT-
SLPKPL--
SRPKPL--
-SPKPLK-
SYPKPL--
-SPKPLV-
SVPKPL--
-SPKPLW-
-SPKPLS-
-SPKPLI-
-SPKPLN-
SWPKPL--
SQPKPL--
STPKPL--
SHPKPL--
SIPKPL--
SAPKPL--
SKPKPL--
-SPKPLA-
SDPKPL--
-SPKPLQ-
-SPKPLF-
SMPKPL--
SEPKPL--
SPPKPL--
-SPKPLM-
-SPKPLE-
-SPKPLH-
-SPKPLC-
-TPKPLR-
TFPKPL--

TRPKPL--
TLPKPL--
-TPKPLL-
-TPKPLH-
TSPKPL--
-TPKPLY-
-TPKPLV-
-TPKPLS-
-TPKPLI-
TGPKPL--
TAPKPL--
-TPKPLW-
-TPKPLK-
TVPKPL--
TPPKPL--
-TPKPLA-
TMPKPL--
TIPKPL--
THPKPL--
TQPKPL--
TNPKPL--
-TPKPLQ-
-TPKPLM-
TKPKPL--
TDPKPL--
-TPKPLN-
TEPKPL--
TYPKPL--
-TPKPLG-
-VPKPLR-
VVPKPL--
-VPKPLL-
VYPKPL--
VQPKPL--
VSPKPL--
VRPKPL--
VHPKPL--
VLPKPL--
VKPKPL--
-VPKPLA-
-VPKPLM-
-VPKPLV-
-VPKPLN-
VTPKPL--
-VPKPLS-
VNPKPL--
-VPKPLT-
VAPKPL--
-VPKPLQ-
VDPKPL--

VGPKPL--
-VPKPLF-
VCPKPL--
-VPKPLY-
-VPKPLH-
-VPKPLG-
VWPKPL--
VEPKPL--
-VPKPLI-
-WPKPLR-
WRPKPL--
WDPKPL--
-WPKPLK-
WVPKPL--
-WPKPLL-
WLPKPL--
-WPKPLS-
WTPKPL--
WKPKPL--
WSPKPL--
-WPKPLI-
WPPKPL--
WQPKPL--
WGPKPL--
-WPKPLV-
WMPKPL--
WHPKPL--
-WPKPLT-
WIPKPL--
WAPKPL--
WYPKPL--
WEPKPL--
-WPKPLN-
-WPKPLE-
-WPKPLA-
WWPKPL--
-WPKPLQ-
WNPKPL--
YVPKPL--
YLPKPL--
YHPKPL--
YAPKPL--
YFPKPL--
YPPKPL--
YRPKPL--
-YPKPLS-
YNPKPL--
YGPKPL--
-YPKPLI-
YYPKPL--

	-YPKPLM-		
	YDPKPL--		
	-YPKPLH-		
	YMPKPL--		
	YTPKPL--		
	YQPKPL--		
	-YPKPLY-		
	YEPKPL--		
	YIPKPL--		
	-YPKPLE-		
	YKPKPL--		
	-YPKPLV-		
	-YPKPLF-		
	-YPKPLK-		
	YCPKPL--		
	-YPKPLC-		
PLAI	67	837	48.25
	--PLAIPR		
	--PLAIIP		
	-RPLAID-		
	-SPLAID-		
	-TPLAIP-		
	ARPLAI--		
	ASPLAI--		
	AVPLAI--		
	AWPLAI--		
	-APLAIK-		
	-APLAIT-		
	-APLAIV-		
	AIPLAI--		
	AAPLAI--		
	AKPLAI--		
	AFPLAI--		
	AYPLAI--		
	ALPLAI--		
	-APLAIM-		
	AQPLAI--		
	ANPLAI--		
	-APLAIH-		
	-APLAIW-		
	-APLAIS-		
	APPLAI--		
	-APLAIG-		
	AHPLAI--		
	ATPLAI--		
	AGPLAI--		
	AEPLAI--		
	-APLAIA-		
	ADPLAI--		
	-APLAII-		

```
-APLAIE-
-APLAIF-
-APLAIY-
-APLAIN-
CIPLAI--
CSPLAI--
CNPLAI--
CRPLAI--
-CPLAIT-
CVPLAI--
CTPLAI--
CWPLAI--
-CPLAIM-
-CPLAIH-
-CPLAII-
-CPLAIS-
-CPLAIL-
-CPLAIF-
CKPLAI--
CEPLAI--
CDPLAI--
CYPLAI--
-CPLAIY-
-CPLAIW-
-CPLAIV-
-CPLAIR-
CLPLAI--
CHPLAI--
DVPLAI--
-DPLAIQ-
DHPLAI--
DSPLAI--
-DPLAIL-
-DPLAIK-
DRPLAI--
-DPLAIR-
DWPLAI--
-DPLAIS-
DAPLAI--
DIPLAI--
-DPLAIH-
DFPLAI--
DQPLAI--
-DPLAIT-
DYPLAI--
DMPLAI--
DTPLAI--
-DPLAIV-
-DPLAII-
DNPLAI--
```

-DPLAIW-
-DPLAIN-
-DPLAIA-
-DPLAIF-
DKPLAI--
-DPLAIM-
-DPLAIE-
DLPLAI--
ERPLAI--
-EPLAIL-
EAPLAI--
EWPLAI--
ETPLAI--
-EPLAIH-
EYPLAI--
-EPLAIR-
EKPLAI--
-EPLAIQ-
ESPLAI--
-EPLAIS-
-EPLAIT-
-EPLAIF-
EMPLAI--
EVPLAI--
ELPLAI--
EFPLAI--
EQPLAI--
-EPLAIN-
-EPLAIM-
-EPLAIK-
-EPLAIG-
EGPLAI--
-EPLAIW-
-EPLAIV-
-EPLAIA-
ENPLAI--
EDPLAI--
EPPLAI--
-EPLAII-
-EPLAIE-
EIPLAI--
EEPLAI--
FNPLAI--
FVPLAI--
FSPLAI--
FGPLAI--
-FPLAIN-
FKPLAI--
-FPLAIK-
-FPLAIS-

-FPLAIG-
-FPLAIE-
FQPLAI--
FEPLAI--
FPPLAI--
FHPLAI--
FDPLAI--
FWPLAI--
-FPLAIW-
FMPLAI--
-FPLAIT-
-FPLAIH-
FFPLAI--
FCPLAI--
FAPLAI--
GSPLAI--
-GPLAIS-
-GPLAIL-
GFPLAI--
GWPLAI--
GHPLAI--
-GPLAIT-
GGPLAI--
-GPLAIR-
GVPLAI--
-GPLAIA-
-GPLAIV-
-GPLAII-
GQPLAI--
-GPLAIM-
-GPLAIG-
GAPLAI--
GNPLAI--
-GPLAIE-
GKPLAI--
-GPLAIH-
GEPLAI--
GYPLAI--
GMPLAI--
-GPLAIQ-
-GPLAIN-
-GPLAIK-
-GPLAIW-
GIPLAI--
GPPLAI--
-GPLAIY-
HRPLAI--
HVPLAI--
-HPLAIL-
-HPLAIR-

HNPLAI--
-HPLAIT-
-HPLAIY-
-HPLAIV-
HLPLAI--
-HPLAIQ-
-HPLAIN-
HHPLAI--
HQPLAI--
HMPLAI--
HTPLAI--
HAPLAI--
HSPLAI--
-HPLAIS-
-HPLAIH-
-HPLAIG-
HPPLAI--
-HPLAIA-
HEPLAI--
-HPLAIW-
-HPLAII-
HKPLAI--
HGPLAI--
-HPLAIF-
HYPLAI--
HWPLAI--
-HPLAIM-
HCPLAI--
-HPLAIK-
IGPLAI--
ISPLAI--
IVPLAI--
-IPLAIS-
IRPLAI--
-IPLAIH-
IPPLAI--
-IPLAIL-
IMPLAI--
IHPLAI--
INPLAI--
ILPLAI--
-IPLAII-
IAPLAI--
-IPLAIT-
IIPLAI--
IEPLAI--
IQPLAI--
-IPLAIQ-
IKPLAI--
-IPLAIV-

ITPLAI--
-IPLAIW-
-IPLAIA-
IYPLAI--
-IPLAIG-
-IPLAIM-
IDPLAI--
IWPLAI--
-IPLAIK-
ICPLAI--
KSPLAI--
-KPLAIS-
-KPLAIM-
KIPLAI--
-KPLAIR-
-KPLAII-
KAPLAI--
KVPLAI--
KLPLAI--
-KPLAIT-
-KPLAIK-
KHPLAI--
-KPLAIH-
KGPLAI--
-KPLAIQ-
-KPLAIF-
KEPLAI--
KYPLAI--
-KPLAIY-
KKPLAI--
-KPLAIG-
KFPLAI--
KQPLAI--
-KPLAIV-
-KPLAIL-
KWPLAI--
KPPLAI--
-KPLAIN-
-KPLAIE-
KMPLAI--
KNPLAI--
-KPLAIW-
KCPLAI--
KDPLAI--
-LPLAIR-
-LPLAIM-
LRPLAI--
LQPLAI--
-LPLAIT-
-LPLAIL-

LGPLAI--
-LPLAIV-
-LPLAIS-
LLPLAI--
LIPLAI--
LPPLAI--
LYPLAI--
-LPLAIN-
LKPLAI--
LEPLAI--
LWPLAI--
-LPLAIH-
-LPLAIF-
LNPLAI--
LAPLAI--
-LPLAIQ-
-LPLAII-
LMPLAI--
-LPLAIW-
LCPLAI--
-LPLAIA-
-LPLAIE-
MQPLAI--
-MPLAIL-
MRPLAI--
-MPLAIR-
MAPLAI--
-MPLAIS-
MIPLAI--
MGPLAI--
MKPLAI--
MSPLAI--
-MPLAIG-
MEPLAI--
-MPLAIV-
-MPLAIA-
MFPLAI--
MNPLAI--
-MPLAIK-
MWPLAI--
MVPLAI--
MTPLAI--
MYPLAI--
-MPLAIH-
-MPLAIN-
MMPLAI--
-MPLAIT-
-MPLAIM-
MLPLAI--
-MPLAIQ-

```
MDPLAI--
MPPLAI--
-MPLAII-
MCPLAI--
NVPLAI--
NKPLAI--
-NPLAIR-
NLPLAI--
NQPLAI--
NRPLAI--
-NPLAIY-
-NPLAIS-
NSPLAI--
NNPLAI--
NPPLAI--
NFPLAI--
-NPLAIH-
-NPLAIQ-
NAPLAI--
NTPLAI--
-NPLAIK-
-NPLAIM-
-NPLAIA-
NYPLAI--
-NPLAIF-
NWPLAI--
-NPLAIN-
NHPLAI--
NEPLAI--
-NPLAIV-
-NPLAIE-
NIPLAI--
NMPLAI--
-NPLAII-
NCPLAI--
--PLAIRS
--PLAILT
--PLAILS
--PLAIRR
--PLAISG
--PLAIHM
--PLAIMR
--PLAILR
--PLAIHA
--PLAIIA
--PLAIVA
--PLAISR
--PLAIYR
--PLAINR
--PLAIVN
```

```
--PLAIGG
--PLAITL
--PLAISN
--PLAIAS
--PLAIYS
--PLAIFS
--PLAIRQ
--PLAIRE
--PLAIMV
--PLAIVS
--PLAIKY
--PLAIKT
PGPLAI--
--PLAITQ
--PLAIRT
--PLAIKS
--PLAIWT
--PLAIST
--PLAISK
--PLAIRF
--PLAIRH
--PLAILQ
--PLAIWH
--PLAIAG
--PLAIIQ
--PLAIGS
--PLAIRI
--PLAIGV
--PLAILN
--PLAIKL
--PLAIRM
--PLAIQA
--PLAISM
--PLAIIS
--PLAINQ
--PLAIYY
--PLAITT
--PLAIRW
--PLAIMS
--PLAIMK
--PLAIFK
--PLAIVY
--PLAIVK
--PLAIRA
--PLAIMG
--PLAIIR
--PLAIGA
--PLAIFR
--PLAIFG
--PLAIYD
```

--PLAITN
--PLAIQR
--PLAIKN
--PLAIKA
--PLAITG
--PLAIPL
--PLAIHG
--PLAIGF
--PLAIHV
--PLAIGT
--PLAIAA
--PLAIYT
--PLAISS
--PLAIRP
--PLAIRN
--PLAIHT
--PLAIEH
--PLAIWS
--PLAIWN
--PLAITA
--PLAIRD
--PLAINW
--PLAINT
--PLAIMQ
--PLAIKK
--PLAIKG
--PLAIID
--PLAIEM
--PLAIEG
--PLAIAL
--PLAIWK
--PLAISQ
PHPLAI--
--PLAITM
--PLAIQY
--PLAIQT
--PLAILH
--PLAIHQ
--PLAIET
--PLAIAQ
--PLAIVV
--PLAIVR
--PLAIVG
--PLAISV
--PLAIMW
--PLAIGM
--PLAIYK
--PLAIYG
--PLAISH
--PLAIQI

--PLAINL
--PLAILD
--PLAIHK
--PLAIFH
--PLAIES
--PLAIAH
--PLAIVT
--PLAIVQ
--PLAITV
--PLAIMA
--PLAILM
--PLAIKV
--PLAIHE
--PLAIFT
PTPLAI--
PNPLAI--
--PLAIYM
--PLAISW
--PLAINY
--PLAIIV
--PLAIHL
--PLAIFN
--PLAIEK
--PLAIAV
PWPLAI--
--PLAIWG
--PLAITD
--PLAIMM
--PLAIHY
--PLAIHN
--PLAITK
--PLAIQN
--PLAIMY
--PLAIMI
--PLAILW
--PLAIHR
--PLAIER
--PLAIYI
--PLAIWY
--PLAIVD
--PLAIQV
--PLAIQG
--PLAINE
--PLAIMH
--PLAIIN
--PLAIGN
--PLAIDG
--PLAIYH
--PLAIPM
--PLAINV

```
--PLAINH
--PLAILE
--PLAIGK
--PLAISY
--PLAIQH
--PLAINK
--PLAIKI
--PLAIIY
--PLAIII
--PLAIHS
--PLAIVH
--PLAISF
--PLAIQS
--PLAIKH
--PLAIIW
--PLAIHW
--PLAIAK
--PLAIYQ
--PLAIYN
--PLAIVE
--PLAITI
--PLAITE
--PLAINS
--PLAINA
--PLAIKR
--PLAIIT
--PLAIEA
PDPLAI--
--PLAIWD
--PLAIVP
--PLAISE
--PLAIQK
--PLAIKE
--PLAIKD
--PLAIIK
--PLAIGI
--PLAIFD
--PLAIEF
--PLAIDS
--PLAIAN
--PLAIYP
--PLAIWE
--PLAISC
--PLAIRY
--PLAINI
--PLAING
--PLAIMD
--PLAIKM
--PLAIIH
--PLAIHI
```

--PLAIGH
--PLAIFW
--PLAIEN
--PLAIEL
--PLAICK
PEPLAI--
--PLAIVW
--PLAITH
--PLAISD
--PLAIRC
--PLAIQD
--PLAILP
--PLAIKP
--PLAIHP
--PLAIHH
--PLAIHF
--PLAIEE
--PLAIAT
PCPLAI--
-PPLAIV-
-PPLAIP-
-PPLAIG-
--PLAIYC
--PLAIWV
--PLAIWQ
--PLAIWP
--PLAITC
--PLAIQP
--PLAIPS
--PLAIPI
--PLAINN
--PLAINF
--PLAIND
--PLAIKQ
--PLAIHD
--PLAIHC
--PLAIGC
--PLAIFM
--PLAIEW
--PLAIEV
--PLAIEQ
--PLAIEI
--PLAIED
--PLAIEC
--PLAIDA
--PLAICV
--PLAIAY
--PLAIAW
--PLAIAP
-QPLAIR-

QRPLAI--
-QPLAIS-
QYPLAI--
QTPLAI--
QLPLAI--
QGPLAI--
QVPLAI--
QPPLAI--
-QPLAIL-
QAPLAI--
QSPLAI--
-QPLAIK-
QKPLAI--
QMPLAI--
-QPLAIN-
-QPLAIV-
-QPLAIH-
QQPLAI--
QHPLAI--
QWPLAI--
QIPLAI--
-QPLAIQ-
-QPLAIW-
-QPLAIE-
-QPLAIT-
-QPLAIF-
QNPLAI--
-QPLAIA-
QFPLAI--
QEPLAI--
QDPLAI--
RSPLAI--
-RPLAIK-
RGPLAI--
RTPLAI--
RVPLAI--
-RPLAIM-
RNPLAI--
-RPLAIG-
-RPLAIH-
-RPLAII-
REPLAI--
-RPLAIF-
RHPLAI--
RDPLAI--
RIPLAI--
-RPLAIA-
RAPLAI--
-RPLAIV-
-RPLAIN-

RWPLAI--
RMPLAI--
RQPLAI--
-RPLAIE-
RFPLAI--
-RPLAIQ-
RPPLAI--
RYPLAI--
-RPLAIC-
SLPLAI--
-SPLAIR-
STPLAI--
SAPLAI--
-SPLAIH-
SSPLAI--
SNPLAI--
-SPLAIA-
-SPLAIN-
-SPLAII-
-SPLAIF-
SHPLAI--
-SPLAIQ-
SKPLAI--
SYPLAI--
-SPLAIK-
SQPLAI--
-SPLAIT-
-SPLAIV-
-SPLAIG-
SFPLAI--
SMPLAI--
SEPLAI--
-SPLAIW-
-SPLAIM-
SPPLAI--
SCPLAI--
-SPLAIE-
TGPLAI--
-TPLAIS-
-TPLAIR-
TSPLAI--
TAPLAI--
TLPLAI--
TVPLAI--
TTPLAI--
TRPLAI--
TNPLAI--
TFPLAI--
-TPLAIT-
TKPLAI--

TDPLAI--
-TPLAIY-
-TPLAIN-
-TPLAIF-
TYPLAI--
TQPLAI--
TPPLAI--
-TPLAIH-
TMPLAI--
TWPLAI--
-TPLAIE-
TIPLAI--
-TPLAIG-
-TPLAIQ-
TEPLAI--
-TPLAIA-
-TPLAIV-
THPLAI--
-TPLAIW-
-TPLAIK-
-VPLAIR-
VRPLAI--
VSPLAI--
VGPLAI--
-VPLAIK-
VAPLAI--
VVPLAI--
VFPLAI--
-VPLAIV-
VTPLAI--
VEPLAI--
VDPLAI--
VWPLAI--
-VPLAIH-
VNPLAI--
VQPLAI--
-VPLAIT-
-VPLAIN-
VHPLAI--
-VPLAII-
-VPLAIQ-
-VPLAIE-
-VPLAIY-
-VPLAIA-
-VPLAIG-
VYPLAI--
VPPLAI--
-VPLAIC-
WTPLAI--
-WPLAIS-

WEPLAI--
WYPLAI--
WVPLAI--
WPPLAI--
-WPLAIG-
WMPLAI--
-WPLAII-
WAPLAI--
-WPLAIQ-
-WPLAIR-
-WPLAIN-
WHPLAI--
WFPLAI--
-WPLAIV-
WDPLAI--
WWPLAI--
WIPLAI--
WNPLAI--
WCPLAI--
-WPLAIH-
-WPLAIE-
WQPLAI--
-WPLAIW-
-WPLAIM-
-WPLAIK-
WLPLAI--
YSPLAI--
YLPLAI--
YEPLAI--
-YPLAIS-
YHPLAI--
YGPLAI--
YRPLAI--
-YPLAIR-
YYPLAI--
YVPLAI--
YTPLAI--
YQPLAI--
YPPLAI--
-YPLAIE-
YNPLAI--
YDPLAI--
YAPLAI--
YWPLAI--
-YPLAII-
-YPLAIV-
YIPLAI--
-YPLAIW-
-YPLAIN-
-YPLAIA-

	YFPLAI--		
	-YPLAIT-		
	-YPLAIM-		
	-YPLAIK-		
PRPM	70	833	48.23
	-GPRPMG-		
	-QPRPMP-		
	-APRPMR-		
	ASPRPM--		
	-APRPMY-		
	ATPRPM--		
	-APRPMA-		
	AVPRPM--		
	-APRPMS-		
	AGPRPM--		
	AAPRPM--		
	ARPRPM--		
	-APRPMI-		
	ANPRPM--		
	AIPRPM--		
	AFPRPM--		
	-APRPMM-		
	AMPRPM--		
	AYPRPM--		
	AHPRPM--		
	-APRPMN-		
	-APRPME-		
	AKPRPM--		
	AQPRPM--		
	-APRPMV-		
	-APRPMW-		
	-APRPMT-		
	-APRPMQ-		
	-APRPMG-		
	AEPRPM--		
	-CPRPMT-		
	CGPRPM--		
	-CPRPMH-		
	CCPRPM--		
	CWPRPM--		
	-CPRPMS-		
	CQPRPM--		
	-CPRPMY-		
	-CPRPMA-		
	CEPRPM--		
	CAPRPM--		
	CVPRPM--		
	-CPRPMW-		
	-CPRPMK-		
	-CPRPMF-		

CIPRPM--
-DPRPMK-
-DPRPMI-
DTPRPM--
DLPRPM--
DRPRPM--
-DPRPMY-
-DPRPMS-
DSPRPM--
DFPRPM--
DAPRPM--
-DPRPMM-
-DPRPMR-
DEPRPM--
DVPRPM--
-DPRPMA-
DWPRPM--
-DPRPMQ-
DNPRPM--
DKPRPM--
DYPRPM--
DQPRPM--
-DPRPMN-
-DPRPMH-
-DPRPMG-
DDPRPM--
EVPRPM--
-EPRPML-
ERPRPM--
EGPRPM--
-EPRPMK-
EIPRPM--
EMPRPM--
EFPRPM--
ETPRPM--
ELPRPM--
EWPRPM--
ESPRPM--
-EPRPMY-
-EPRPMQ-
ENPRPM--
-EPRPMM-
-EPRPMI-
EQPRPM--
-EPRPMT-
EKPRPM--
-EPRPMH-
-EPRPME-
EEPRPM--
ECPRPM--

EAPRPM--
FRPRPM--
-FPRPMV-
-FPRPMF-
FTPRPM--
FIPRPM--
FLPRPM--
-FPRPMT-
FAPRPM--
-FPRPMS-
-FPRPMY-
FYPRPM--
-FPRPMI-
FFPRPM--
FQPRPM--
FMPRPM--
FWPRPM--
-FPRPMM-
-FPRPMW-
FDPRPM--
FHPRPM--
-FPRPMH-
FVPRPM--
-FPRPME-
FGPRPM--
FSPRPM--
-FPRPMG-
-FPRPMA-
FPPRPM--
GLPRPM--
GVPRPM--
-GPRPMT-
GAPRPM--
GRPRPM--
-GPRPMS-
GYPRPM--
-GPRPMV-
GSPRPM--
-GPRPMR-
GIPRPM--
GFPRPM--
GWPRPM--
-GPRPMA-
-GPRPMY-
-GPRPMI-
GHPRPM--
GKPRPM--
GTPRPM--
-GPRPMK-
GMPRPM--

GEPRPM--
-GPRPMF-
GNPRPM--
GQPRPM--
-GPRPMN-
-GPRPME-
-GPRPMD-
GDPRPM--
-HPRPML-
HRPRPM--
-HPRPMR-
-HPRPMW-
HVPRPM--
HNPRPM--
-HPRPMK-
HSPRPM--
HAPRPM--
-HPRPMT-
HGPRPM--
-HPRPMI-
-HPRPMH-
HTPRPM--
-HPRPMN-
-HPRPMV-
-HPRPME-
-HPRPMS-
HWPRPM--
HQPRPM--
-HPRPMG-
-HPRPMC-
HLPRPM--
-HPRPMY-
-HPRPMM-
HFPRPM--
HYPRPM--
HPPRPM--
-IPRPML-
-IPRPMH-
-IPRPMY-
ISPRPM--
IQPRPM--
ILPRPM--
ITPRPM--
IVPRPM--
-IPRPMW-
-IPRPMN-
IPPRPM--
-IPRPMI-
-IPRPMR-
IEPRPM--

IDPRPM--
IAPRPM--
IRPRPM--
-IPRPMV-
-IPRPMT-
IYPRPM--
IWPRPM--
-IPRPMF-
IMPRPM--
IIPRPM--
IHPRPM--
IGPRPM--
IFPRPM--
-IPRPMQ-
-IPRPMK-
INPRPM--
KSPRPM--
-KPRPMF-
-KPRPMT-
-KPRPML-
-KPRPMS-
-KPRPMK-
-KPRPMI-
KEPRPM--
KLPRPM--
KRPRPM--
KGPRPM--
KIPRPM--
KAPRPM--
-KPRPMY-
-KPRPMM-
-KPRPMA-
-KPRPMN-
-KPRPMH-
KYPRPM--
KPPRPM--
KTPRPM--
KFPRPM--
KVPRPM--
KHPRPM--
KDPRPM--
-KPRPMW-
KWPRPM--
-KPRPMQ-
-KPRPMG-
-KPRPME-
-KPRPMC-
LSPRPM--
LRPRPM--
-LPRPML-

```
-LPRPMI-
-LPRPMQ-
LLPRPM--
-LPRPMY-
LTPRPM--
LVPRPM--
LAPRPM--
-LPRPMS-
LMPRPM--
LFPRPM--
LNPRPM--
LKPRPM--
LYPRPM--
LPPRPM--
-LPRPMM-
LEPRPM--
LGPRPM--
-LPRPMG-
-LPRPMA-
-LPRPMW-
LIPRPM--
-LPRPMH-
LHPRPM--
-LPRPMN-
LDPRPM--
LQPRPM--
-LPRPME-
LCPRPM--
-MPRPMQ-
-MPRPMF-
-MPRPMY-
MAPRPM--
MTPRPM--
MVPRPM--
MLPRPM--
-MPRPML-
MDPRPM--
MNPRPM--
MYPRPM--
MEPRPM--
-MPRPMN-
MSPRPM--
-MPRPMT-
-MPRPMI-
-MPRPMH-
-MPRPMA-
-MPRPMV-
-MPRPMR-
MGPRPM--
MFPRPM--
```

-MPRPMG-
MPPRPM--
MKPRPM--
-MPRPMS-
MMPRPM--
MIPRPM--
-MPRPMM-
-MPRPMK-
-MPRPMC-
NWPRPM--
-NPRPMA-
-NPRPML-
NFPRPM--
NRPRPM--
-NPRPMR-
NYPRPM--
-NPRPMS-
-NPRPMI-
NHPRPM--
-NPRPMF-
-NPRPMV-
-NPRPMH-
NLPRPM--
NSPRPM--
-NPRPMN-
NPPRPM--
-NPRPMQ-
NNPRPM--
NIPRPM--
NVPRPM--
NGPRPM--
-NPRPMW-
-NPRPMT-
-NPRPMM-
-NPRPMK-
NDPRPM--
-NPRPMY-
-NPRPMG-
-NPRPME-
--PRPMNW
--PRPMLL
--PRPMRS
--PRPMLG
--PRPMFS
--PRPMLS
--PRPMSY
--PRPMTI
--PRPMRM
--PRPMRF
--PRPMIT

--PRPMLH
--PRPMYT
--PRPMYL
--PRPMSW
--PRPMLV
--PRPMHL
--PRPMLK
--PRPMTL
--PRPMMI
--PRPMIY
--PRPMVN
--PRPMVL
--PRPMLF
--PRPMVT
--PRPMPF
--PRPMNS
--PRPMIV
--PRPMYS
--PRPMQM
--PRPMFT
--PRPMRN
--PRPMMT
--PRPMFV
--PRPMFM
--PRPMAG
--PRPMWN
--PRPMVV
--PRPMSS
--PRPMRI
--PRPMLR
--PRPMIG
--PRPMGF
--PRPMAS
--PRPMVA
--PRPMIL
--PRPMVR
--PRPMTW
--PRPMQL
--PRPMGI
--PRPMVY
--PRPMVF
--PRPMTT
--PRPMSF
--PRPMNI
--PRPMIA
--PRPMAV
--PRPMVG
--PRPMTH
--PRPMIQ
--PRPMFG

--PRPMST
--PRPMQG
--PRPMIR
--PRPMES
--PRPMDL
PSPRPM--
--PRPMTF
--PRPMSG
--PRPMRD
--PRPMKS
--PRPMFY
--PRPMAT
--PRPMWS
--PRPMVS
--PRPMSI
--PRPMPY
--PRPMHR
--PRPMFF
--PRPMEL
--PRPMAW
--PRPMYV
--PRPMWT
--PRPMRR
--PRPMII
--PRPMEY
--PRPMYG
--PRPMTS
--PRPMTN
--PRPMSQ
--PRPMQN
--PRPMMV
--PRPMGT
--PRPMWQ
--PRPMWI
--PRPMQA
--PRPMLM
--PRPMKF
--PRPMGA
--PRPMFQ
--PRPMEF
--PRPMDV
--PRPMAI
--PRPMYM
--PRPMWV
--PRPMWF
--PRPMSR
--PRPMSK
--PRPMSH
--PRPMQI
--PRPMMS

--PRPMKV
--PRPMIW
--PRPMIH
--PRPMAH
--PRPMNN
--PRPMLE
--PRPMKL
--PRPMKH
--PRPMKG
--PRPMGQ
--PRPMYN
--PRPMTY
--PRPMSA
--PRPMGY
--PRPMWK
--PRPMRH
--PRPMFA
--PRPMTQ
--PRPMNF
--PRPMMH
--PRPMKT
--PRPMHM
--PRPMGW
--PRPMFH
--PRPMAQ
--PRPMAN
PGPRPM--
--PRPMYA
--PRPMWH
--PRPMWG
--PRPMMG
--PRPMIM
--PRPMIF
--PRPMGV
--PRPMFN
--PRPMFK
--PRPMAF
--PRPMRQ
--PRPMPM
--PRPMPI
--PRPMNA
--PRPMKI
--PRPMHY
--PRPMHV
--PRPMTD
--PRPMTA
--PRPMSD
--PRPMRW
--PRPMKY
--PRPMKW

--PRPMHW
--PRPMEI
--PRPMYY
--PRPMYH
--PRPMWW
--PRPMTV
--PRPMTR
--PRPMQS
--PRPMPS
--PRPMNV
--PRPMKN
--PRPMYQ
--PRPMYF
--PRPMYD
--PRPMRA
--PRPMLD
--PRPMHQ
--PRPMGG
--PRPMFI
--PRPMEN
--PRPMEG
--PRPMDY
PFPRPM--
PWPRPM--
--PRPMWM
--PRPMVQ
--PRPMTK
--PRPMQF
--PRPMNT
--PRPMMR
--PRPMHT
--PRPMGK
--PRPMDS
--PRPMRK
--PRPMRE
--PRPMPW
--PRPMNQ
--PRPMMN
--PRPMME
--PRPMKA
--PRPMHS
--PRPMHG
--PRPMHA
--PRPMFD
--PRPMEA
--PRPMDI
--PRPMDF
--PRPMCT
--PRPMAR
--PRPMYK

--PRPMQT
--PRPMPA
--PRPMNH
--PRPMNG
--PRPMMY
--PRPMMQ
--PRPMMF
--PRPMMA
--PRPMLQ
--PRPMKK
--PRPMIN
--PRPMID
--PRPMGR
--PRPMGM
--PRPMFE
--PRPMEW
--PRPMDW
--PRPMDQ
--PRPMAY
--PRPMAK
PLPRPM--
PYPRPM--
PVPRPM--
PTPRPM--
--PRPMYW
--PRPMYI
--PRPMWE
--PRPMWA
--PRPMVD
--PRPMSE
--PRPMQR
--PRPMQH
--PRPMQE
--PRPMQD
--PRPMNY
--PRPMNR
--PRPMNE
--PRPMMC
--PRPMKQ
--PRPMKM
--PRPMIE
--PRPMHE
--PRPMAA
PEPRPM--
PRPRPM--
--PRPMVE
--PRPMTM
--PRPMPQ
--PRPMPN
--PRPMNK

--PRPMMW
--PRPMMD
--PRPMKD
--PRPMHF
--PRPMEM
--PRPMCG
--PRPMAM
PDPRPM--
--PRPMWR
--PRPMVP
--PRPMRC
--PRPMQQ
--PRPMQK
--PRPMPV
--PRPMPT
--PRPMNM
--PRPMND
--PRPMLC
--PRPMKP
--PRPMIP
--PRPMIK
--PRPMHI
--PRPMGN
--PRPMEQ
--PRPMDN
--PRPMDM
--PRPMCS
--PRPMCI
-PPRPMT-
-PPRPMM-
-PPRPML-
-PPRPMF-
PNPRPM--
PAPRPM--
-QPRPMT-
-QPRPML-
-QPRPMR-
-QPRPMW-
QYPRPM--
QPPRPM--
QHPRPM--
-QPRPMM-
-QPRPMK-
QVPRPM--
-QPRPMI-
QTPRPM--
-QPRPMF-
QMPRPM--
-QPRPMV-
QEPRPM--

−QPRPMQ−
QAPRPM−−
−QPRPMA−
QWPRPM−−
−QPRPMH−
QNPRPM−−
QLPRPM−−
QDPRPM−−
−QPRPMN−
−QPRPMG−
QKPRPM−−
QGPRPM−−
QFPRPM−−
−RPRPML−
RSPRPM−−
RVPRPM−−
RLPRPM−−
−RPRPMW−
RYPRPM−−
RFPRPM−−
RAPRPM−−
−RPRPMI−
−RPRPMF−
−RPRPMS−
RQPRPM−−
−RPRPMN−
−RPRPMT−
−RPRPMY−
−RPRPMM−
RIPRPM−−
RWPRPM−−
−RPRPMG−
−RPRPMA−
−RPRPMH−
RRPRPM−−
RMPRPM−−
RHPRPM−−
RCPRPM−−
REPRPM−−
−RPRPME−
RDPRPM−−
−RPRPMV−
−RPRPMK−
−RPRPMQ−
RPPRPM−−
−RPRPMC−
SVPRPM−−
SSPRPM−−
SRPRPM−−
−SPRPML−

-SPRPMV-
-SPRPMY-
SLPRPM--
-SPRPMR-
-SPRPMT-
SQPRPM--
-SPRPMI-
SFPRPM--
STPRPM--
-SPRPMS-
-SPRPMH-
-SPRPMF-
SYPRPM--
SMPRPM--
SKPRPM--
SAPRPM--
-SPRPMW-
-SPRPMK-
SGPRPM--
-SPRPMQ-
-SPRPMA-
-SPRPMM-
-SPRPMG-
-SPRPMC-
SNPRPM--
-TPRPML-
-TPRPMS-
-TPRPMR-
-TPRPMI-
-TPRPMA-
TSPRPM--
TVPRPM--
TTPRPM--
-TPRPMQ-
-TPRPMN-
TPPRPM--
TYPRPM--
-TPRPMT-
TIPRPM--
TWPRPM--
TLPRPM--
TGPRPM--
TFPRPM--
TQPRPM--
-TPRPMF-
TAPRPM--
TNPRPM--
-TPRPMY-
TKPRPM--
-TPRPME-

-TPRPMH-
-TPRPMG-
-TPRPMM-
VRPRPM--
-VPRPMQ-
VSPRPM--
-VPRPMS-
-VPRPMR-
-VPRPMH-
-VPRPMA-
-VPRPMV-
-VPRPMN-
VLPRPM--
VIPRPM--
VPPRPM--
-VPRPML-
-VPRPMG-
-VPRPMI-
VMPRPM--
-VPRPMM-
VWPRPM--
VFPRPM--
VVPRPM--
VTPRPM--
-VPRPMY-
VHPRPM--
VDPRPM--
VCPRPM--
VNPRPM--
-VPRPMC-
-VPRPME-
VGPRPM--
VAPRPM--
-WPRPMK-
WTPRPM--
WLPRPM--
WVPRPM--
WRPRPM--
WNPRPM--
-WPRPMF-
WGPRPM--
WSPRPM--
-WPRPMT-
WQPRPM--
-WPRPMM-
WKPRPM--
-WPRPMS-
-WPRPMG-
WWPRPM--
-WPRPMV-

-WPRPMN-
WFPRPM--
WEPRPM--
-WPRPME-
WPPRPM--
WDPRPM--
-WPRPMH-
WIPRPM--
WHPRPM--
-YPRPMT-
-YPRPMS-
YNPRPM--
-YPRPMF-
YKPRPM--
YRPRPM--
-YPRPMY-
YSPRPM--
YAPRPM--
-YPRPMN-
-YPRPMV-
YLPRPM--
YTPRPM--
YPPRPM--
YEPRPM--
YVPRPM--
-YPRPMA-
-YPRPMR-
YCPRPM--
-YPRPMQ-
-YPRPMI-
YIPRPM--
YWPRPM--
-YPRPMG-
YDPRPM--
YGPRPM--
-YPRPMW-
-YPRPMM-
YFPRPM--
PAWM 417 653 48.23
-NPAWML-
--PAWMTL
-RPAWMV-
-APAWMR-
AQPAWM--
ARPAWM--
ASPAWM--
-APAWMQ-
AVPAWM--
-APAWML-
-APAWMY-

```
-APAWMT-
ALPAWM--
-APAWMA-
ANPAWM--
AYPAWM--
-APAWMS-
-APAWMH-
-APAWMG-
-APAWMW-
-APAWMK-
APPAWM--
-APAWMV-
-APAWMI-
-APAWMN-
AEPAWM--
ATPAWM--
-APAWMD-
AKPAWM--
AHPAWM--
AFPAWM--
-APAWMF-
-APAWME-
AGPAWM--
AAPAWM--
CPPAWM--
-CPAWMA-
CIPAWM--
CTPAWM--
CSPAWM--
CNPAWM--
DGPAWM--
DRPAWM--
DVPAWM--
DSPAWM--
DHPAWM--
-DPAWML-
-DPAWMT-
DYPAWM--
-DPAWMY-
-DPAWMR-
-DPAWMK-
-DPAWMF-
DDPAWM--
-EPAWMR-
-EPAWMI-
EYPAWM--
-EPAWMK-
ERPAWM--
-EPAWMQ-
ESPAWM--
```

-EPAWMS-
-EPAWMF-
-EPAWMA-
-EPAWMT-
-EPAWMM-
EVPAWM--
-EPAWML-
ELPAWM--
EEPAWM--
ETPAWM--
-EPAWMH-
EMPAWM--
EHPAWM--
EGPAWM--
EAPAWM--
FRPAWM--
FVPAWM--
FGPAWM--
FQPAWM--
FIPAWM--
FYPAWM--
FSPAWM--
-FPAWMI-
-FPAWML-
-FPAWMG-
FLPAWM--
FDPAWM--
-FPAWMV-
-FPAWMM-
FMPAWM--
-FPAWMK-
FKPAWM--
-GPAWMR-
GFPAWM--
-GPAWMA-
-GPAWMY-
GKPAWM--
GRPAWM--
-GPAWMN-
-GPAWMH-
-GPAWMG-
-GPAWME-
GTPAWM--
-GPAWMK-
GNPAWM--
GSPAWM--
-GPAWMW-
-GPAWMV-
-GPAWMF-
-GPAWML-

GIPAWM--
GGPAWM--
GAPAWM--
-GPAWMS-
GLPAWM--
GQPAWM--
GPPAWM--
GYPAWM--
-GPAWMT-
-GPAWMD-
-GPAWMM-
GMPAWM--
GEPAWM--
HTPAWM--
-HPAWMR-
-HPAWMS-
HNPAWM--
HRPAWM--
-HPAWMY-
-HPAWMQ-
HIPAWM--
HYPAWM--
-HPAWMG-
HVPAWM--
-HPAWMK-
-HPAWMI-
-HPAWMF-
HLPAWM--
HKPAWM--
HHPAWM--
HFPAWM--
-IPAWMA-
IRPAWM--
-IPAWMS-
IFPAWM--
-IPAWMR-
-IPAWMV-
-IPAWMG-
-IPAWMK-
IGPAWM--
IQPAWM--
IHPAWM--
IAPAWM--
-IPAWMQ-
ISPAWM--
IDPAWM--
IVPAWM--
-IPAWMH-
ILPAWM--
-IPAWMN-

```
-IPAWMF-
-IPAWMD-
INPAWM--
KSPAWM--
-KPAWMS-
-KPAWMI-
KYPAWM--
KGPAWM--
KQPAWM--
KRPAWM--
-KPAWMT-
-KPAWMA-
KMPAWM--
KAPAWM--
KTPAWM--
-KPAWMF-
-KPAWMM-
KNPAWM--
KIPAWM--
KVPAWM--
-KPAWMN-
-KPAWMY-
KLPAWM--
-KPAWMG-
-KPAWME-
-KPAWMD-
KWPAWM--
KPPAWM--
-KPAWMV-
KKPAWM--
KFPAWM--
KDPAWM--
LQPAWM--
LPPAWM--
LEPAWM--
-LPAWMR-
LYPAWM--
-LPAWMS-
-LPAWMK-
LRPAWM--
-LPAWMV-
-LPAWMG-
LLPAWM--
-LPAWMT-
-LPAWML-
LMPAWM--
LGPAWM--
LVPAWM--
-LPAWMQ-
-LPAWMM-
```

LFPAWM--
LSPAWM--
-LPAWMY-
LNPAWM--
LDPAWM--
-LPAWMF-
-LPAWMN-
LWPAWM--
LHPAWM--
LAPAWM--
MAPAWM--
MWPAWM--
MSPAWM--
MRPAWM--
-MPAWMA-
MVPAWM--
MEPAWM--
-MPAWMV-
-MPAWMH-
MNPAWM--
MMPAWM--
MIPAWM--
MPPAWM--
-MPAWMY-
-MPAWMT-
-MPAWMG-
MYPAWM--
MTPAWM--
-MPAWMQ-
-MPAWMK-
MHPAWM--
MQPAWM--
-MPAWML-
MDPAWM--
-NPAWMN-
NSPAWM--
-NPAWMT-
-NPAWMI-
NLPAWM--
NGPAWM--
NDPAWM--
-NPAWMG-
NVPAWM--
-NPAWMS-
NMPAWM--
NWPAWM--
NRPAWM--
-NPAWMK-
NKPAWM--
NFPAWM--

-NPAWMF-
-NPAWMA-
NTPAWM--
NPPAWM--
-NPAWMY-
-NPAWMQ-
-NPAWMH-
NIPAWM--
--PAWMLA
--PAWMGT
--PAWMSG
--PAWMSA
--PAWMRV
--PAWMTV
--PAWMRH
--PAWMFN
--PAWMFS
--PAWMRS
--PAWMMS
--PAWMLT
--PAWMFG
--PAWMMT
--PAWMAA
--PAWMYH
--PAWMRG
--PAWMIH
--PAWMLG
--PAWMMH
--PAWMSW
--PAWMIA
--PAWMGL
--PAWMQA
--PAWMLR
--PAWMVN
--PAWMVH
--PAWMQT
--PAWMIR
--PAWMGA
--PAWMSY
--PAWMKA
--PAWMWS
--PAWMRN
--PAWMKR
--PAWMGS
--PAWMAS
--PAWMTR
--PAWMST
--PAWMSM
--PAWMLS
--PAWMIN

PVPAWM--
PSPAWM--
--PAWMVG
--PAWMSL
--PAWMSH
--PAWMLD
--PAWMGM
--PAWMTI
--PAWMSV
--PAWMSN
--PAWMMA
--PAWMIS
--PAWMES
--PAWMAV
--PAWMYI
--PAWMYA
--PAWMWV
--PAWMVT
--PAWMVA
--PAWMQG
--PAWMKG
--PAWMIK
--PAWMIE
--PAWMFQ
PAPAWM--
PWPAWM--
PQPAWM--
--PAWMTH
--PAWMTG
--PAWMSS
--PAWMSD
--PAWMLV
--PAWMLK
--PAWMGF
--PAWMYG
--PAWMVE
--PAWMVD
--PAWMTK
--PAWMSI
--PAWMRQ
--PAWMLQ
--PAWMKT
--PAWMHG
--PAWMGV
--PAWMFT
--PAWMFM
PGPAWM--
PFPAWM--
--PAWMWG
--PAWMVV

--PAWMVR
--PAWMTD
--PAWMRY
--PAWMQM
--PAWMQI
--PAWMQH
--PAWMLN
--PAWMLE
--PAWMKY
--PAWMIT
--PAWMHS
--PAWMHN
--PAWMGR
--PAWMGI
--PAWMGH
--PAWMAM
PTPAWM--
PNPAWM--
--PAWMWY
--PAWMWF
--PAWMSQ
--PAWMRE
--PAWMQS
--PAWMNS
--PAWMIV
--PAWMID
--PAWMFV
--PAWMDS
PYPAWM--
--PAWMWN
--PAWMVW
--PAWMTN
--PAWMRW
--PAWMQV
--PAWMQK
--PAWMNW
--PAWMMR
--PAWMMK
--PAWMLI
--PAWMLF
--PAWMKV
--PAWMKM
--PAWMKD
--PAWMIQ
--PAWMIG
--PAWMHW
--PAWMHI
--PAWMGY
--PAWMGW
--PAWMGK

--PAWMFR
--PAWMEN
--PAWMYT
--PAWMYR
--PAWMVK
--PAWMSF
--PAWMSE
--PAWMRM
--PAWMRL
--PAWMQE
--PAWMNY
--PAWMNQ
--PAWMKS
--PAWMKL
--PAWMIW
--PAWMHQ
--PAWMGQ
--PAWMGN
--PAWMGG
--PAWMFE
--PAWMEW
--PAWMET
--PAWMAR
--PAWMAQ
PIPAWM--
--PAWMYW
--PAWMYL
--PAWMVS
--PAWMTE
--PAWMSK
--PAWMRD
--PAWMQN
--PAWMPG
--PAWMNA
--PAWMMV
--PAWMMD
--PAWMLM
--PAWMLH
--PAWMKI
--PAWMKF
--PAWMKE
--PAWMIY
--PAWMHM
--PAWMHA
--PAWMFK
--PAWMFA
--PAWMEE
--PAWMEA
--PAWMDA
--PAWMAN

--PAWMAL
--PAWMAH
--PAWMAG
--PAWMAD
QGPAWM--
-QPAWMR-
QVPAWM--
-QPAWMY-
-QPAWMS-
QTPAWM--
QRPAWM--
-QPAWMI-
QNPAWM--
-QPAWMV-
-QPAWML-
-QPAWMH-
-QPAWMK-
QKPAWM--
QDPAWM--
-QPAWMQ-
-QPAWMA-
QLPAWM--
QAPAWM--
-QPAWMW-
QMPAWM--
QHPAWM--
QFPAWM--
QEPAWM--
-RPAWMS-
RSPAWM--
-RPAWMR-
RLPAWM--
RGPAWM--
RRPAWM--
-RPAWMG-
RFPAWM--
RTPAWM--
-RPAWMY-
-RPAWMT-
RIPAWM--
RNPAWM--
RVPAWM--
-RPAWMK-
-RPAWMA-
RWPAWM--
-RPAWMH-
RKPAWM--
RDPAWM--
-RPAWMQ-
-RPAWMN-

RYPAWM--
-RPAWME-
RAPAWM--
RQPAWM--
-RPAWMW-
-RPAWMI-
REPAWM--
RHPAWM--
SRPAWM--
-SPAWMR-
SFPAWM--
SVPAWM--
-SPAWML-
STPAWM--
-SPAWMT-
SSPAWM--
SKPAWM--
SPPAWM--
-SPAWMV-
SHPAWM--
SQPAWM--
-SPAWMA-
-SPAWMS-
-SPAWMK-
-SPAWMG-
SYPAWM--
SMPAWM--
SLPAWM--
-SPAWMH-
SIPAWM--
SAPAWM--
-SPAWMM-
SWPAWM--
-SPAWMY-
-SPAWMQ-
-SPAWMF-
-SPAWMN-
SNPAWM--
SEPAWM--
SGPAWM--
SCPAWM--
TQPAWM--
TRPAWM--
TMPAWM--
-TPAWMI-
TLPAWM--
-TPAWMS-
-TPAWMH-
TAPAWM--
-TPAWMR-

-TPAWMY-
THPAWM--
TTPAWM--
TSPAWM--
-TPAWMQ-
-TPAWMF-
TWPAWM--
TPPAWM--
TNPAWM--
-TPAWMW-
-TPAWMV-
-TPAWMM-
-TPAWMA-
TFPAWM--
TVPAWM--
TCPAWM--
-TPAWME-
-TPAWMT-
-TPAWML-
-TPAWMK-
TGPAWM--
-VPAWMR-
VKPAWM--
-VPAWMS-
VTPAWM--
VVPAWM--
VQPAWM--
-VPAWMA-
VNPAWM--
-VPAWMH-
VIPAWM--
-VPAWML-
-VPAWMI-
VYPAWM--
-VPAWMT-
VAPAWM--
VSPAWM--
-VPAWMY-
VGPAWM--
VPPAWM--
-VPAWMK-
-VPAWMF-
VRPAWM--
-VPAWMV-
VDPAWM--
-VPAWMQ-
-VPAWMG-
-VPAWME-
VFPAWM--
VEPAWM--

	WRPAWM--		
	-WPAWMM-		
	WIPAWM--		
	-WPAWMK-		
	WQPAWM--		
	-WPAWMY-		
	-WPAWMA-		
	WAPAWM--		
	WSPAWM--		
	-WPAWMF-		
	WLPAWM--		
	WVPAWM--		
	WNPAWM--		
	-WPAWMV-		
	-WPAWMS-		
	-WPAWMH-		
	-WPAWMG-		
	-YPAWMR-		
	YGPAWM--		
	-YPAWMS-		
	-YPAWMK-		
	-YPAWMT-		
	YTPAWM--		
	YSPAWM--		
	YPPAWM--		
	YLPAWM--		
	YKPAWM--		
	YYPAWM--		
	YQPAWM--		
	YIPAWM--		
	-YPAWMV-		
	-YPAWMG-		
	YMPAWM--		
	-YPAWMM-		
	-YPAWMH-		
	-YPAWMA-		
	YNPAWM--		
	YRPAWM--		
	-YPAWMW-		
	-YPAWMI-		
	-YPAWMF-		
	YEPAWM--		
PLSL	387	664	48.17
	-APLSLP-		
	-FPLSLP-		
	-IPLSLP-		
	-LPLSLD-		
	-NPLSLP-		
	-NPLSLC-		
	--PLSLVP		

--PLSLPL
--PLSLAP
--PLSLHP
--PLSLTC
--PLSLPR
--PLSLPH
--PLSLLP
--PLSLIP
--PLSLHC
--PLSLGP
--PLSLGE
--PLSLGC
--PLSLEP
--PLSLDW
--PLSLDR
--PLSLDM
--PLSLDL
--PLSLDE
-QPLSLP-
-SPLSLP-
-SPLSLD-
-TPLSLP-
-YPLSLD-
-APLSLL-
-APLSLS-
AKPLSL--
-APLSLA-
-APLSLN-
-APLSLQ-
AWPLSL--
ATPLSL--
-APLSLH-
-APLSLG-
-APLSLK-
AHPLSL--
-APLSLI-
APPLSL--
AQPLSL--
-APLSLM-
-APLSLE-
AEPLSL--
ADPLSL--
ACPLSL--
-APLSLD-
-APLSLC-
CLPLSL--
CRPLSL--
CCPLSL--
-CPLSLR-
CKPLSL--

-CPLSLT-
CSPLSL--
CGPLSL--
CIPLSL--
CAPLSL--
CQPLSL--
CTPLSL--
-CPLSLK-
CMPLSL--
CEPLSL--
CDPLSL--
CYPLSL--
-CPLSLS-
-CPLSLI-
CVPLSL--
-CPLSLQ-
-CPLSLM-
CNPLSL--
CWPLSL--
-CPLSLY-
-CPLSLV-
-CPLSLA-
-CPLSLN-
-CPLSLL-
CFPLSL--
-CPLSLP-
-CPLSLE-
DRPLSL--
DSPLSL--
-DPLSLR-
DKPLSL--
DFPLSL--
DTPLSL--
DVPLSL--
DMPLSL--
DAPLSL--
-DPLSLL-
-DPLSLI-
-DPLSLT-
-DPLSLF-
-DPLSLK-
DGPLSL--
DIPLSL--
DWPLSL--
DHPLSL--
DLPLSL--
-DPLSLV-
DYPLSL--
DQPLSL--
-DPLSLH-

-DPLSLA-
-DPLSLQ-
DEPLSL--
-DPLSLE-
-DPLSLN-
DCPLSL--
DPPLSL--
DDPLSL--
-DPLSLC-
ESPLSL--
ERPLSL--
-EPLSLS-
-EPLSLK-
EVPLSL--
-EPLSLI-
ELPLSL--
EMPLSL--
-EPLSLH-
EGPLSL--
EYPLSL--
ETPLSL--
EKPLSL--
EAPLSL--
-EPLSLV-
-EPLSLA-
-EPLSLM-
EHPLSL--
ENPLSL--
-EPLSLF-
EIPLSL--
-EPLSLQ-
-EPLSLN-
EFPLSL--
EQPLSL--
-EPLSLG-
-EPLSLE-
EPPLSL--
EDPLSL--
ECPLSL--
-EPLSLC-
FSPLSL--
FKPLSL--
-FPLSLS-
FGPLSL--
-FPLSLT-
FNPLSL--
FHPLSL--
-FPLSLN-
FIPLSL--
-FPLSLQ-

-FPLSLG-
-FPLSLA-
FPPLSL--
-FPLSLE-
-FPLSLI-
FQPLSL--
FEPLSL--
-FPLSLM-
FDPLSL--
FCPLSL--
-FPLSLC-
GTPLSL--
GYPLSL--
GRPLSL--
-GPLSLT-
-GPLSLA-
-GPLSLK-
GAPLSL--
GWPLSL--
GKPLSL--
GGPLSL--
GMPLSL--
GFPLSL--
GPPLSL--
-GPLSLH-
GEPLSL--
GIPLSL--
-GPLSLI-
-GPLSLN-
-GPLSLQ-
GNPLSL--
-GPLSLE-
GHPLSL--
GDPLSL--
GCPLSL--
-GPLSLC-
HSPLSL--
-HPLSLT-
-HPLSLY-
HTPLSL--
-HPLSLL-
HPPLSL--
-HPLSLN-
-HPLSLS-
HFPLSL--
-HPLSLV-
HGPLSL--
-HPLSLK-
HIPLSL--
HHPLSL--

–HPLSLI–
–HPLSLH–
HDPLSL––
–HPLSLQ–
–HPLSLA–
HQPLSL––
HNPLSL––
–HPLSLW–
–HPLSLG–
–HPLSLF–
HMPLSL––
–HPLSLE–
–HPLSLC–
–IPLSLR–
IQPLSL––
–IPLSLN–
IAPLSL––
–IPLSLG–
IFPLSL––
IPPLSL––
IKPLSL––
–IPLSLA–
IMPLSL––
INPLSL––
IYPLSL––
IEPLSL––
IWPLSL––
–IPLSLM–
–IPLSLE–
–IPLSLC–
ICPLSL––
KGPLSL––
–KPLSLK–
KNPLSL––
KTPLSL––
–KPLSLA–
KAPLSL––
–KPLSLI–
KFPLSL––
–KPLSLR–
KYPLSL––
–KPLSLN–
KDPLSL––
KEPLSL––
KHPLSL––
KQPLSL––
–KPLSLE–
KCPLSL––
–KPLSLD–
–KPLSLC–

LSPLSL--
-LPLSLS-
-LPLSLN-
LLPLSL--
-LPLSLL-
LEPLSL--
LAPLSL--
LYPLSL--
LHPLSL--
LDPLSL--
LNPLSL--
-LPLSLV-
-LPLSLM-
-LPLSLK-
-LPLSLQ-
-LPLSLA-
LQPLSL--
-LPLSLE-
LCPLSL--
-LPLSLC-
MSPLSL--
-MPLSLL-
-MPLSLS-
MTPLSL--
-MPLSLM-
MWPLSL--
MYPLSL--
MQPLSL--
MNPLSL--
MIPLSL--
-MPLSLH-
-MPLSLN-
-MPLSLI-
-MPLSLG-
-MPLSLV-
-MPLSLQ-
MEPLSL--
MHPLSL--
MMPLSL--
-MPLSLE-
-MPLSLP-
-MPLSLD-
-MPLSLC-
MCPLSL--
NLPLSL--
NVPLSL--
NPPLSL--
NAPLSL--
-NPLSLS-
NNPLSL--

-NPLSLI-
NIPLSL--
-NPLSLT-
NFPLSL--
NHPLSL--
-NPLSLQ-
-NPLSLK-
-NPLSLH-
-NPLSLA-
NEPLSL--
NYPLSL--
-NPLSLN-
NMPLSL--
NWPLSL--
-NPLSLF-
-NPLSLE-
-NPLSLG-
NDPLSL--
NCPLSL--
--PLSLGV
--PLSLRG
--PLSLLG
--PLSLSR
--PLSLSS
--PLSLSG
--PLSLLK
--PLSLIR
--PLSLRN
--PLSLGS
--PLSLRV
--PLSLTT
--PLSLTS
--PLSLSK
--PLSLRA
--PLSLNS
--PLSLTR
--PLSLHR
--PLSLVA
--PLSLMG
--PLSLSV
--PLSLVG
--PLSLFG
--PLSLGA
--PLSLTG
--PLSLLH
--PLSLAA
--PLSLWS
--PLSLST
--PLSLYG
--PLSLQS

--PLSLMR
--PLSLKS
--PLSLRD
--PLSLHS
--PLSLHG
--PLSLAR
--PLSLNR
--PLSLGG
--PLSLLD
--PLSLHT
--PLSLSQ
--PLSLSN
--PLSLRW
--PLSLVT
--PLSLGT
--PLSLTV
--PLSLVD
--PLSLEW
--PLSLWG
--PLSLKN
--PLSLKV
--PLSLQA
--PLSLNT
--PLSLSW
--PLSLIG
--PLSLMT
--PLSLKA
--PLSLKT
--PLSLSE
--PLSLRQ
--PLSLYN
--PLSLNV
--PLSLMM
--PLSLKM
--PLSLAW
--PLSLTM
--PLSLLQ
--PLSLTN
--PLSLTH
--PLSLKD
--PLSLTI
--PLSLQT
--PLSLHQ
--PLSLMN
--PLSLHL
--PLSLQW
--PLSLHK
--PLSLKI
--PLSLEA
--PLSLTW

--PLSLIQ
--PLSLHW
--PLSLTQ
--PLSLNK
--PLSLHE
--PLSLER
--PLSLQN
--PLSLHF
--PLSLET
--PLSLEH
--PLSLAH
--PLSLAT
--PLSLAM
--PLSLVE
--PLSLNM
--PLSLTF
--PLSLNW
--PLSLES
--PLSLKK
--PLSLHV
--PLSLGY
--PLSLKE
--PLSLYD
--PLSLNN
--PLSLHM
--PLSLQI
--PLSLKY
--PLSLIH
--PLSLGM
--PLSLGI
--PLSLWH
--PLSLKF
--PLSLGH
--PLSLDS
--PLSLAK
PEPLSL--
--PLSLTE
--PLSLHN
--PLSLDG
--PLSLYQ
PDPLSL--
--PLSLME
--PLSLEL
--PLSLQM
--PLSLEN
--PLSLEI
--PLSLWD
--PLSLQF
--PLSLTD
--PLSLHD

--PLSLEY
--PLSLFH
--PLSLAQ
--PLSLAE
--PLSLHH
--PLSLNH
--PLSLHY
--PLSLQH
--PLSLID
--PLSLQY
--PLSLEQ
--PLSLNG
--PLSLNE
--PLSLEK
--PLSLCS
--PLSLQE
--PLSLEF
--PLSLAD
PCPLSL--
--PLSLWF
--PLSLEE
--PLSLDT
--PLSLSC
--PLSLNQ
--PLSLCV
--PLSLDA
--PLSLRC
--PLSLMP
--PLSLCY
--PLSLCK
--PLSLWC
--PLSLCG
-PPLSLR-
--PLSLKP
--PLSLFP
--PLSLAC
--PLSLVC
--PLSLPY
--PLSLMC
--PLSLKC
--PLSLIC
--PLSLED
--PLSLDI
--PLSLCW
-PPLSLT-
-PPLSLL-
-PPLSLK-
-PPLSLI-
-PPLSLH-
-PPLSLF-

-PPLSLE-
-PPLSLA-
--PLSLYC
--PLSLPC
--PLSLNC
--PLSLFC
--PLSLEC
--PLSLCQ
--PLSLCN
QSPLSL--
-QPLSLR-
-QPLSLS-
-QPLSLA-
QFPLSL--
QGPLSL--
QKPLSL--
QAPLSL--
QLPLSL--
QVPLSL--
-QPLSLN-
-QPLSLG-
QMPLSL--
QYPLSL--
-QPLSLI-
QHPLSL--
QQPLSL--
QIPLSL--
QWPLSL--
-QPLSLV-
QNPLSL--
QDPLSL--
-QPLSLH-
QCPLSL--
-QPLSLC-
-RPLSLT-
RTPLSL--
-RPLSLN-
-RPLSLG-
-RPLSLA-
RNPLSL--
-RPLSLM-
-RPLSLQ-
REPLSL--
-RPLSLE-
RCPLSL--
-RPLSLC-
-RPLSLD-
SSPLSL--
SVPLSL--
SLPLSL--

SNPLSL--
-SPLSLT-
-SPLSLK-
SAPLSL--
SFPLSL--
-SPLSLV-
-SPLSLH-
SIPLSL--
-SPLSLQ-
-SPLSLI-
SWPLSL--
-SPLSLF-
-SPLSLA-
-SPLSLN-
SQPLSL--
SHPLSL--
-SPLSLG-
SEPLSL--
SDPLSL--
SPPLSL--
SCPLSL--
-SPLSLC-
-TPLSLR-
-TPLSLT-
TLPLSL--
TVPLSL--
TKPLSL--
TTPLSL--
-TPLSLS-
TIPLSL--
-TPLSLK-
-TPLSLI-
-TPLSLA-
TYPLSL--
TQPLSL--
THPLSL--
-TPLSLQ-
-TPLSLH-
TEPLSL--
TDPLSL--
-TPLSLE-
TCPLSL--
-TPLSLC-
-VPLSLS-
-VPLSLR-
-VPLSLK-
VKPLSL--
VLPLSL--
VPPLSL--
-VPLSLN-

VYPLSL--
VIPLSL--
VGPLSL--
VEPLSL--
VHPLSL--
VNPLSL--
VQPLSL--
-VPLSLV-
-VPLSLH-
VAPLSL--
-VPLSLE-
-VPLSLQ-
-VPLSLG-
-VPLSLM-
VCPLSL--
-VPLSLD-
-VPLSLC-
WKPLSL--
WSPLSL--
WPPLSL--
WQPLSL--
WFPLSL--
WNPLSL--
WMPLSL--
-WPLSLN-
-WPLSLV-
WEPLSL--
WAPLSL--
-WPLSLG-
-WPLSLH-
-WPLSLE-
WWPLSL--
WCPLSL--
-WPLSLM-
-YPLSLS-
YLPLSL--
-YPLSLL-
YTPLSL--
-YPLSLI-
YKPLSL--
-YPLSLV-
YAPLSL--
YIPLSL--
-YPLSLT-
-YPLSLQ-
YNPLSL--
YFPLSL--
YQPLSL--
-YPLSLH-
-YPLSLG-

	-YPLSLM-		
	YHPLSL--		
	-YPLSLN-		
	YDPLSL--		
	YPPLSL--		
	-YPLSLE-		
	YCPLSL--		
PAAM	192	754	48.15
	-HPAAMG-		
	-NPAAMT-		
	--PAAMNG		
	--PAAMER		
	-APAAMK-		
	-APAAMS-		
	-APAAMH-		
	ALPAAM--		
	ASPAAM--		
	-APAAMT-		
	-APAAMA-		
	AIPAAM--		
	-APAAMM-		
	-APAAMI-		
	AYPAAM--		
	ANPAAM--		
	ACPAAM--		
	-APAAML-		
	-APAAMG-		
	AWPAAM--		
	-APAAMQ-		
	ATPAAM--		
	ARPAAM--		
	APPAAM--		
	-APAAMF-		
	AGPAAM--		
	-APAAME-		
	AMPAAM--		
	AFPAAM--		
	-APAAMW-		
	-APAAMV-		
	-APAAMN-		
	AHPAAM--		
	ADPAAM--		
	CQPAAM--		
	CSPAAM--		
	-CPAAMT-		
	-CPAAMQ-		
	-CPAAML-		
	CPPAAM--		
	CMPAAM--		
	CAPAAM--		

DRPAAM--
DFPAAM--
DYPAAM--
DSPAAM--
-DPAAML-
-DPAAMK-
DTPAAM--
-DPAAMY-
-DPAAMW-
-DPAAMR-
DLPAAM--
DAPAAM--
DEPAAM--
DVPAAM--
-DPAAMT-
-DPAAMA-
DMPAAM--
DHPAAM--
ERPAAM--
-EPAAMR-
ESPAAM--
-EPAAML-
-EPAAMV-
EIPAAM--
ELPAAM--
EHPAAM--
-EPAAMF-
-EPAAMA-
-EPAAMY-
-EPAAMW-
EKPAAM--
ETPAAM--
-EPAAMI-
EWPAAM--
-EPAAMH-
ENPAAM--
EFPAAM--
EYPAAM--
-EPAAMS-
-EPAAMK-
ECPAAM--
FSPAAM--
FFPAAM--
-FPAAMR-
-FPAAMF-
-FPAAMV-
FEPAAM--
FDPAAM--
FRPAAM--
-FPAAMQ-

FMPAAM--
-FPAAMT-
-FPAAMY-
-FPAAMS-
FHPAAM--
-FPAAML-
-FPAAMN-
FYPAAM--
FWPAAM--
-FPAAMH-
-FPAAMA-
FKPAAM--
FCPAAM--
FAPAAM--
-GPAAMS-
-GPAAML-
GTPAAM--
GRPAAM--
GSPAAM--
-GPAAMK-
GVPAAM--
GEPAAM--
-GPAAMR-
GGPAAM--
-GPAAMT-
-GPAAMF-
GLPAAM--
GFPAAM--
-GPAAMA-
GKPAAM--
-GPAAMN-
GAPAAM--
-GPAAMG-
GIPAAM--
GQPAAM--
GNPAAM--
GYPAAM--
-GPAAMQ-
-GPAAMW-
-GPAAMM-
-GPAAMI-
-GPAAMH-
GMPAAM--
-GPAAMD-
-GPAAMC-
GDPAAM--
-HPAAML-
-HPAAMS-
-HPAAMR-
-HPAAMF-

HHPAAM--
-HPAAMY-
-HPAAMT-
-HPAAMI-
HGPAAM--
HIPAAM--
HSPAAM--
HRPAAM--
HTPAAM--
-HPAAMA-
HCPAAM--
HWPAAM--
-HPAAMV-
-HPAAMK-
HLPAAM--
HDPAAM--
HAPAAM--
-IPAAML-
-IPAAMR-
-IPAAMS-
-IPAAMM-
-IPAAMF-
ISPAAM--
-IPAAMY-
-IPAAMW-
-IPAAMK-
IGPAAM--
IWPAAM--
INPAAM--
IFPAAM--
IYPAAM--
IVPAAM--
-IPAAMT-
-IPAAMQ-
-IPAAMH-
ILPAAM--
IKPAAM--
IIPAAM--
ITPAAM--
-IPAAMA-
IHPAAM--
-IPAAMG-
-KPAAMS-
KSPAAM--
-KPAAMT-
KGPAAM--
-KPAAMR-
-KPAAMQ-
KRPAAM--
KLPAAM--

-KPAAMF-
KYPAAM--
-KPAAMW-
-KPAAML-
-KPAAMI-
KTPAAM--
-KPAAMH-
-KPAAMA-
KQPAAM--
-KPAAMK-
KAPAAM--
-KPAAMV-
KNPAAM--
-KPAAMY-
KKPAAM--
KFPAAM--
KDPAAM--
KVPAAM--
KMPAAM--
KEPAAM--
-LPAAMR-
LNPAAM--
-LPAAMS-
LTPAAM--
LRPAAM--
-LPAAMW-
-LPAAML-
LGPAAM--
LAPAAM--
LIPAAM--
-LPAAMY-
-LPAAMK-
LSPAAM--
LLPAAM--
LHPAAM--
-LPAAMI-
LVPAAM--
-LPAAMT-
LMPAAM--
LYPAAM--
-LPAAMH-
LKPAAM--
-LPAAMF-
LPPAAM--
-LPAAMQ-
-LPAAMA-
-LPAAMN-
LEPAAM--
LDPAAM--
LCPAAM--

```
-LPAAMG-
LFPAAM--
-MPAAML-
-MPAAMV-
MQPAAM--
-MPAAMR-
MFPAAM--
MRPAAM--
MHPAAM--
MTPAAM--
-MPAAMY-
-MPAAMM-
MAPAAM--
-MPAAMS-
-MPAAMT-
-MPAAMH-
MNPAAM--
MDPAAM--
MVPAAM--
MIPAAM--
MSPAAM--
-MPAAMQ-
-MPAAMW-
-MPAAMA-
MGPAAM--
-MPAAMI-
-MPAAMF-
MLPAAM--
MKPAAM--
-NPAAMV-
NAPAAM--
-NPAAMY-
-NPAAML-
NYPAAM--
NGPAAM--
-NPAAMH-
NMPAAM--
-NPAAMW-
-NPAAMR-
NNPAAM--
NIPAAM--
NWPAAM--
NVPAAM--
NKPAAM--
NHPAAM--
-NPAAMM-
-NPAAMI-
NCPAAM--
NTPAAM--
NSPAAM--
```

NRPAAM--
-NPAAMS-
NDPAAM--
--PAAMRN
--PAAMVR
--PAAMRS
--PAAMTR
--PAAMRG
--PAAMLR
--PAAMTV
--PAAMAW
--PAAMHL
--PAAMVG
--PAAMLQ
--PAAMKM
--PAAMYS
--PAAMYK
--PAAMWS
--PAAMWR
--PAAMYH
--PAAMVT
--PAAMSL
--PAAMPL
--PAAMRR
--PAAMKS
--PAAMRM
--PAAMLK
--PAAMFL
--PAAMRD
--PAAMFH
--PAAMVN
--PAAMPY
--PAAMNT
--PAAMIA
--PAAMAS
--PAAMWE
--PAAMRT
--PAAMRK
--PAAMLV
--PAAMLL
--PAAMLH
--PAAMWL
--PAAMRY
--PAAMRA
--PAAMIS
--PAAMAL
--PAAMSA
--PAAMMG
--PAAMIR
--PAAMIG

--PAAMRV
--PAAMQQ
--PAAMLG
--PAAMSH
--PAAMKR
--PAAMHN
--PAAMFD
--PAAMEG
--PAAMVS
--PAAMIM
--PAAMFS
PRPAAM--
--PAAMYQ
--PAAMWQ
--PAAMVQ
--PAAMFR
--PAAMYN
--PAAMVW
--PAAMTW
--PAAMWI
--PAAMVE
--PAAMTH
--PAAMSG
--PAAMRI
--PAAMMS
--PAAMLN
--PAAMHS
--PAAMGL
--PAAMAA
PHPAAM--
--PAAMTF
--PAAMSQ
--PAAMNL
--PAAMKN
--PAAMYL
--PAAMWN
--PAAMVV
--PAAMSR
--PAAMRW
--PAAMRH
--PAAMQG
--PAAMLS
--PAAMGY
--PAAMGA
PFPAAM--
--PAAMST
--PAAMRF
--PAAMKG
--PAAMIN
--PAAMHR

--PAAMAT
PNPAAM--
--PAAMMV
--PAAMLA
--PAAMIL
--PAAMHY
--PAAMFG
--PAAMAI
--PAAMAF
PLPAAM--
--PAAMYT
--PAAMYR
--PAAMYG
--PAAMVA
--PAAMQW
--PAAMQS
--PAAMKI
--PAAMGS
--PAAMGF
--PAAMFM
--PAAMTS
--PAAMTK
--PAAMTG
--PAAMSY
--PAAMQT
--PAAMFT
PEPAAM--
--PAAMWM
--PAAMVM
--PAAMVI
--PAAMNY
--PAAMMF
--PAAMMA
--PAAMKY
--PAAMKQ
--PAAMKL
--PAAMIF
--PAAMGI
--PAAMFQ
PIPAAM--
--PAAMVD
--PAAMTT
--PAAMSI
--PAAMQL
--PAAMPM
--PAAMLM
--PAAMLE
--PAAMHA
--PAAMYV
--PAAMYA

--PAAMRE
--PAAMQK
--PAAMPI
--PAAMNS
--PAAMLD
--PAAMKW
--PAAMKA
--PAAMID
--PAAMAY
--PAAMAH
PCPAAM--
--PAAMYM
--PAAMTY
--PAAMSW
--PAAMRQ
--PAAMQH
--PAAMPF
--PAAMMN
--PAAMMI
--PAAMMH
--PAAMKV
--PAAMKT
--PAAMIY
--PAAMHW
--PAAMGV
--PAAMGM
--PAAMFW
--PAAMEW
--PAAMEA
--PAAMAN
PWPAAM--
PAPAAM--
--PAAMYW
--PAAMYD
--PAAMWT
--PAAMWA
--PAAMTL
--PAAMTI
--PAAMSN
--PAAMSK
--PAAMSF
--PAAMSD
--PAAMQV
--PAAMQR
--PAAMQI
--PAAMNM
--PAAMMW
--PAAMMT
--PAAMMR
--PAAMMQ

--PAAMLI
--PAAMKH
--PAAMIQ
--PAAMHF
--PAAMGH
--PAAMFY
--PAAMFV
--PAAMFK
PVPAAM--
PQPAAM--
PGPAAM--
--PAAMYI
--PAAMWV
--PAAMWD
--PAAMVH
--PAAMTM
--PAAMTE
--PAAMTD
--PAAMSV
--PAAMSE
--PAAMRC
--PAAMQF
--PAAMQE
--PAAMNN
--PAAMNI
--PAAMLC
--PAAMIT
--PAAMIH
--PAAMIC
--PAAMHM
--PAAMHI
--PAAMGT
--PAAMGG
--PAAMET
--PAAMEL
--PAAMEK
--PAAMDL
--PAAMAV
-PPAAMR-
PKPAAM--
PDPAAM--
--PAAMYE
--PAAMWW
--PAAMSC
--PAAMNV
--PAAMNR
--PAAMNP
--PAAMKD
--PAAMIW
--PAAMIV

--PAAMHV
--PAAMFP
--PAAMEV
--PAAMEN
--PAAMEI
--PAAMDR
--PAAMCL
--PAAMCI
--PAAMAQ
--PAAMAM
QRPAAM--
-QPAAMT-
-QPAAMI-
-QPAAML-
-QPAAMY-
-QPAAMS-
-QPAAMK-
QTPAAM--
-QPAAMV-
QAPAAM--
-QPAAMH-
-QPAAMR-
-QPAAMQ-
-QPAAMN-
QIPAAM--
QFPAAM--
-QPAAMF-
QHPAAM--
QGPAAM--
QYPAAM--
QSPAAM--
QNPAAM--
QMPAAM--
QLPAAM--
QEPAAM--
-RPAAML-
-RPAAMT-
-RPAAMV-
RTPAAM--
RSPAAM--
-RPAAMR-
-RPAAMS-
-RPAAMW-
RKPAAM--
RGPAAM--
RIPAAM--
RRPAAM--
RYPAAM--
-RPAAMG-
RMPAAM--

RLPAAM--
-RPAAMI-
RVPAAM--
-RPAAMK-
RWPAAM--
-RPAAMQ-
RNPAAM--
RQPAAM--
RCPAAM--
RHPAAM--
-RPAAMA-
-RPAAMN-
RAPAAM--
RPPAAM--
REPAAM--
-RPAAMM-
-RPAAMH-
RFPAAM--
-RPAAMF-
-RPAAME-
-RPAAMC-
RDPAAM--
SSPAAM--
-SPAAMK-
SLPAAM--
SVPAAM--
-SPAAMR-
-SPAAMV-
-SPAAMS-
-SPAAMF-
STPAAM--
-SPAAMH-
SKPAAM--
SRPAAM--
-SPAAMW-
-SPAAMI-
SGPAAM--
SNPAAM--
-SPAAMT-
-SPAAMN-
-SPAAML-
-SPAAMA-
SWPAAM--
-SPAAMQ-
SYPAAM--
SPPAAM--
-SPAAMM-
SFPAAM--
SCPAAM--
-SPAAMY-

SAPAAM--
SMPAAM--
SHPAAM--
SEPAAM--
-SPAAMG-
SIPAAM--
-TPAAML-
-TPAAMR-
TKPAAM--
TYPAAM--
TRPAAM--
TWPAAM--
-TPAAMW-
THPAAM--
TTPAAM--
TLPAAM--
-TPAAMT-
-TPAAMA-
TAPAAM--
TVPAAM--
-TPAAMS-
-TPAAMQ-
-TPAAMY-
-TPAAMV-
TSPAAM--
-TPAAMN-
-TPAAMI-
TGPAAM--
-TPAAME-
TNPAAM--
TEPAAM--
-VPAAML-
-VPAAMK-
VRPAAM--
-VPAAMY-
-VPAAMA-
VGPAAM--
-VPAAMW-
VTPAAM--
VFPAAM--
-VPAAMR-
-VPAAMI-
VLPAAM--
VKPAAM--
-VPAAMV-
-VPAAMF-
VNPAAM--
-VPAAMS-
-VPAAMT-
-VPAAMH-

VIPAAM--
VYPAAM--
VAPAAM--
VPPAAM--
VMPAAM--
VHPAAM--
VDPAAM--
VVPAAM--
-VPAAMG-
-VPAAME-
VSPAAM--
-VPAAMQ-
-VPAAMN-
VCPAAM--
-WPAAML-
-WPAAMY-
-WPAAMM-
-WPAAMS-
-WPAAMR-
WRPAAM--
-WPAAMH-
-WPAAMN-
WVPAAM--
-WPAAMF-
-WPAAMQ-
WPPAAM--
WNPAAM--
WKPAAM--
-WPAAMW-
-WPAAMG-
WGPAAM--
WYPAAM--
WWPAAM--
-WPAAMI-
-WPAAMA-
WMPAAM--
-YPAAML-
YVPAAM--
-YPAAMV-
-YPAAMI-
YSPAAM--
YAPAAM--
YNPAAM--
YLPAAM--
YHPAAM--
-YPAAMR-
-YPAAMS-
YFPAAM--
-YPAAMM-
-YPAAMY-

	-YPAAMN-		
	YKPAAM--		
	YPPAAM--		
	-YPAAMQ-		
	-YPAAMK-		
	-YPAAMH-		
	-YPAAMC-		
	YYPAAM--		
	YTPAAM--		
	YQPAAM--		
	-YPAAMT-		
	YEPAAM--		
PHAY	316	688	48.15
	--PHAYIE		
	-RPHAYG-		
	AKPHAY--		
	ASPHAY--		
	-APHAYS-		
	AAPHAY--		
	-APHAYR-		
	ALPHAY--		
	AFPHAY--		
	-APHAYI-		
	AVPHAY--		
	AQPHAY--		
	-APHAYH-		
	-APHAYA-		
	-APHAYL-		
	AMPHAY--		
	AWPHAY--		
	-APHAYV-		
	AIPHAY--		
	-APHAYF-		
	AEPHAY--		
	ARPHAY--		
	ADPHAY--		
	ATPHAY--		
	-APHAYK-		
	ANPHAY--		
	-APHAYT-		
	CAPHAY--		
	CTPHAY--		
	CWPHAY--		
	CGPHAY--		
	CRPHAY--		
	-CPHAYT-		
	-CPHAYL-		
	CMPHAY--		
	CHPHAY--		
	CDPHAY--		

-CPHAYS-
-CPHAYR-
CIPHAY--
DSPHAY--
DIPHAY--
DFPHAY--
-DPHAYR-
DTPHAY--
DAPHAY--
DQPHAY--
-DPHAYV-
-DPHAYK-
DNPHAY--
DKPHAY--
DGPHAY--
DVPHAY--
-DPHAYT-
-DPHAYS-
-DPHAYM-
DLPHAY--
-DPHAYH-
DEPHAY--
DDPHAY--
ESPHAY--
-EPHAYF-
EGPHAY--
EAPHAY--
EVPHAY--
ERPHAY--
EQPHAY--
EKPHAY--
-EPHAYS-
-EPHAYL-
EEPHAY--
-EPHAYR-
ENPHAY--
EWPHAY--
-EPHAYQ-
FTPHAY--
-FPHAYM-
FGPHAY--
FHPHAY--
FRPHAY--
-FPHAYL-
-FPHAYY-
FSPHAY--
FQPHAY--
FPPHAY--
FNPHAY--
-FPHAYV-

FIPHAY--
-FPHAYW-
-FPHAYA-
FWPHAY--
-FPHAYK-
-FPHAYT-
-FPHAYR-
-FPHAYN-
FCPHAY--
GYPHAY--
-GPHAYL-
GVPHAY--
GRPHAY--
GSPHAY--
-GPHAYV-
-GPHAYR-
GWPHAY--
GTPHAY--
-GPHAYS-
GAPHAY--
GQPHAY--
-GPHAYF-
GLPHAY--
GKPHAY--
-GPHAYM-
-GPHAYQ-
-GPHAYN-
-GPHAYH-
GPPHAY--
-GPHAYY-
-GPHAYA-
GGPHAY--
GEPHAY--
-GPHAYI-
GNPHAY--
GDPHAY--
-GPHAYT-
HSPHAY--
HLPHAY--
-HPHAYW-
-HPHAYM-
-HPHAYT-
-HPHAYL-
HHPHAY--
HAPHAY--
HRPHAY--
HYPHAY--
HVPHAY--
-HPHAYS-
HGPHAY--

HFPHAY--
HQPHAY--
-HPHAYY-
-HPHAYV-
HWPHAY--
-HPHAYR-
-HPHAYK-
HNPHAY--
HMPHAY--
HTPHAY--
-HPHAYI-
-HPHAYH-
-HPHAYC-
-HPHAYA-
IVPHAY--
ISPHAY--
-IPHAYT-
-IPHAYS-
IAPHAY--
ILPHAY--
IKPHAY--
-IPHAYR-
-IPHAYW-
-IPHAYY-
IHPHAY--
IGPHAY--
IMPHAY--
IQPHAY--
-IPHAYI-
IFPHAY--
-IPHAYN-
-IPHAYL-
ITPHAY--
-IPHAYK-
-IPHAYE-
-IPHAYG-
-IPHAYA-
KSPHAY--
-KPHAYL-
KAPHAY--
-KPHAYS-
KLPHAY--
KMPHAY--
KRPHAY--
-KPHAYV-
-KPHAYN-
-KPHAYA-
-KPHAYI-
-KPHAYT-
-KPHAYM-

-KPHAYH-
KHPHAY--
KQPHAY--
KEPHAY--
KYPHAY--
-KPHAYY-
-KPHAYW-
-KPHAYR-
LGPHAY--
LRPHAY--
-LPHAYL-
LVPHAY--
LKPHAY--
LQPHAY--
LNPHAY--
LLPHAY--
LAPHAY--
-LPHAYR-
LMPHAY--
-LPHAYT-
-LPHAYS-
LYPHAY--
LIPHAY--
-LPHAYM-
LHPHAY--
LPPHAY--
LWPHAY--
-LPHAYQ-
-LPHAYF-
LDPHAY--
LTPHAY--
-LPHAYY-
-LPHAYV-
-LPHAYK-
-LPHAYH-
LCPHAY--
MRPHAY--
-MPHAYR-
MKPHAY--
-MPHAYH-
MSPHAY--
-MPHAYS-
MTPHAY--
-MPHAYF-
-MPHAYQ-
MDPHAY--
MAPHAY--
MYPHAY--
MVPHAY--
MLPHAY--

-MPHAYY-
-MPHAYI-
MPPHAY--
-MPHAYT-
-MPHAYA-
MQPHAY--
-MPHAYV-
-MPHAYN-
-MPHAYM-
-MPHAYL-
-MPHAYK-
MGPHAY--
NWPHAY--
NRPHAY--
NGPHAY--
NSPHAY--
-NPHAYT-
NLPHAY--
NTPHAY--
-NPHAYL-
NMPHAY--
NVPHAY--
-NPHAYM-
-NPHAYR-
NHPHAY--
-NPHAYK-
NKPHAY--
NDPHAY--
-NPHAYS-
NAPHAY--
-NPHAYV-
-NPHAYF-
-NPHAYY-
NNPHAY--
NIPHAY--
-NPHAYI-
--PHAYHS
--PHAYFL
--PHAYRL
--PHAYHT
--PHAYLT
--PHAYSG
--PHAYRS
--PHAYTA
--PHAYSR
--PHAYRM
--PHAYFT
--PHAYTS
--PHAYRG
--PHAYYL

--PHAYSY
--PHAYKA
--PHAYYK
--PHAYSS
--PHAYSN
--PHAYMS
--PHAYLS
--PHAYLN
--PHAYAL
PLPHAY--
--PHAYSK
--PHAYRH
--PHAYRD
--PHAYMT
--PHAYLK
--PHAYLL
--PHAYIS
--PHAYTK
--PHAYTG
--PHAYST
--PHAYRT
--PHAYHK
--PHAYRQ
--PHAYRI
--PHAYKT
--PHAYVG
--PHAYTY
--PHAYTT
--PHAYFH
--PHAYAT
--PHAYWN
--PHAYVS
--PHAYVI
--PHAYSI
--PHAYMV
--PHAYGI
--PHAYAV
--PHAYTL
--PHAYNT
--PHAYKY
--PHAYKQ
--PHAYGF
--PHAYFG
--PHAYFD
PFPHAY--
PMPHAY--
PKPHAY--
--PHAYVL
--PHAYTR
--PHAYQS

--PHAYYW
--PHAYWS
--PHAYVA
--PHAYSM
--PHAYLV
--PHAYKW
--PHAYKL
--PHAYIT
--PHAYII
PWPHAY--
--PHAYYV
--PHAYWL
--PHAYVV
--PHAYVT
--PHAYVR
--PHAYTI
--PHAYTF
--PHAYRA
--PHAYPY
--PHAYNL
--PHAYLY
--PHAYLF
--PHAYLA
--PHAYKV
--PHAYIV
--PHAYIF
--PHAYIA
--PHAYHQ
--PHAYFF
PVPHAY--
--PHAYYI
--PHAYWV
--PHAYWQ
--PHAYWH
--PHAYVQ
--PHAYRN
--PHAYNV
--PHAYNG
--PHAYMW
--PHAYLM
--PHAYGS
--PHAYGA
--PHAYAI
--PHAYAG
--PHAYAF
PRPHAY--
--PHAYYS
--PHAYYN
--PHAYWT
--PHAYTN

--PHAYSV
--PHAYSH
--PHAYQL
--PHAYKS
--PHAYKD
--PHAYIM
--PHAYHD
--PHAYEL
--PHAYEA
--PHAYWG
--PHAYWF
--PHAYVK
--PHAYRW
--PHAYRV
--PHAYQR
--PHAYQM
--PHAYQA
--PHAYPM
--PHAYPI
--PHAYNF
--PHAYNA
--PHAYMN
--PHAYMM
--PHAYMD
--PHAYHR
--PHAYGQ
--PHAYFY
--PHAYFR
--PHAYFN
--PHAYES
--PHAYEG
--PHAYAH
PGPHAY--
PYPHAY--
PTPHAY--
--PHAYYH
--PHAYYF
--PHAYWK
--PHAYVN
--PHAYTE
--PHAYRE
--PHAYQQ
--PHAYQN
--PHAYNI
--PHAYMI
--PHAYLI
--PHAYLD
--PHAYKG
--PHAYIW
--PHAYIR

--PHAYHL
--PHAYGG
--PHAYFV
--PHAYDL
--PHAYAS
--PHAYAQ
PNPHAY--
--PHAYYY
--PHAYYM
--PHAYYA
--PHAYWI
--PHAYVC
--PHAYSD
--PHAYNS
--PHAYNM
--PHAYMH
--PHAYKI
--PHAYIL
--PHAYGY
--PHAYFW
--PHAYFQ
--PHAYEW
--PHAYDI
--PHAYAW
--PHAYAE
PSPHAY--
PPPHAY--
--PHAYYT
--PHAYYG
--PHAYWR
--PHAYWA
--PHAYVF
--PHAYTV
--PHAYTH
--PHAYSQ
--PHAYQW
--PHAYQI
--PHAYQH
--PHAYPF
--PHAYNK
--PHAYMY
--PHAYMA
--PHAYLW
--PHAYLP
--PHAYLH
--PHAYKH
--PHAYIQ
--PHAYIK
--PHAYIG
--PHAYHP

--PHAYHN
--PHAYHM
--PHAYHE
--PHAYHA
--PHAYGN
--PHAYGH
--PHAYFS
--PHAYFM
--PHAYAK
QSPHAY--
QRPHAY--
-QPHAYK-
-QPHAYW-
-QPHAYR-
-QPHAYQ-
-QPHAYV-
QWPHAY--
QVPHAY--
QNPHAY--
QAPHAY--
-QPHAYI-
-QPHAYY-
-QPHAYL-
QIPHAY--
QFPHAY--
-QPHAYS-
QMPHAY--
QLPHAY--
QGPHAY--
QYPHAY--
QPPHAY--
-QPHAYN-
-QPHAYH-
QEPHAY--
-RPHAYR-
RGPHAY--
-RPHAYS-
RSPHAY--
-RPHAYL-
-RPHAYV-
RVPHAY--
-RPHAYM-
RLPHAY--
RWPHAY--
-RPHAYY-
-RPHAYW-
RPPHAY--
RMPHAY--
RFPHAY--
RAPHAY--

-RPHAYQ-
-RPHAYT-
-RPHAYI-
RIPHAY--
-RPHAYH-
-RPHAYA-
RHPHAY--
REPHAY--
RNPHAY--
RKPHAY--
RDPHAY--
RRPHAY--
RQPHAY--
-RPHAYN-
RYPHAY--
-RPHAYF-
-SPHAYR-
SKPHAY--
-SPHAYS-
SLPHAY--
SQPHAY--
SSPHAY--
SVPHAY--
-SPHAYA-
STPHAY--
SHPHAY--
SAPHAY--
-SPHAYL-
-SPHAYM-
-SPHAYF-
SYPHAY--
-SPHAYT-
SPPHAY--
-SPHAYH-
SIPHAY--
SMPHAY--
SGPHAY--
SFPHAY--
SDPHAY--
SCPHAY--
SWPHAY--
-SPHAYY-
-SPHAYV-
-SPHAYN-
-SPHAYE-
SNPHAY--
SEPHAY--
-SPHAYC-
TGPHAY--
TLPHAY--

TRPHAY--
TEPHAY--
TTPHAY--
TWPHAY--
-TPHAYY-
-TPHAYK-
TVPHAY--
TKPHAY--
TMPHAY--
TAPHAY--
TPPHAY--
-TPHAYV-
-TPHAYR-
-TPHAYQ-
-TPHAYT-
-TPHAYI-
THPHAY--
TIPHAY--
TFPHAY--
-TPHAYA-
-TPHAYS-
-TPHAYM-
TNPHAY--
TDPHAY--
VIPHAY--
VPPHAY--
-VPHAYT-
VMPHAY--
VLPHAY--
VKPHAY--
VEPHAY--
VVPHAY--
-VPHAYY-
-VPHAYS-
-VPHAYK-
VSPHAY--
VRPHAY--
VQPHAY--
-VPHAYR-
-VPHAYA-
-VPHAYI-
VFPHAY--
-VPHAYV-
VNPHAY--
VAPHAY--
-VPHAYL-
-VPHAYF-
VTPHAY--
-VPHAYW-
-VPHAYH-

-VPHAYN-
-VPHAYM-
VCPHAY--
-WPHAYS-
WSPHAY--
WAPHAY--
-WPHAYR-
WTPHAY--
WHPHAY--
WPPHAY--
WGPHAY--
-WPHAYM-
-WPHAYV-
WVPHAY--
-WPHAYL-
-WPHAYW-
WKPHAY--
WFPHAY--
-WPHAYK-
WMPHAY--
WWPHAY--
-WPHAYY-
-WPHAYT-
-WPHAYQ-
WNPHAY--
WEPHAY--
-YPHAYS-
-YPHAYY-
YIPHAY--
-YPHAYN-
-YPHAYH-
-YPHAYR-
-YPHAYL-
-YPHAYI-
YNPHAY--
YEPHAY--
-YPHAYM-
-YPHAYF-
-YPHAYA-
YKPHAY--
YWPHAY--
YLPHAY--
YVPHAY--
YSPHAY--
-YPHAYQ-
-YPHAYK-
YMPHAY--
YTPHAY--
YFPHAY--
YRPHAY--

	-YPHAYE-		
PLNI	25	873	48.14
	--PLNITM		
	--PLNISP		
	-APLNIR-		
	APPLNI--		
	-APLNIT-		
	AMPLNI--		
	AVPLNI--		
	-APLNIG-		
	-APLNIF-		
	ASPLNI--		
	-APLNIM-		
	AHPLNI--		
	-APLNIS-		
	AGPLNI--		
	ANPLNI--		
	ATPLNI--		
	-APLNIH-		
	AKPLNI--		
	AFPLNI--		
	AIPLNI--		
	-APLNIN-		
	AAPLNI--		
	-APLNIK-		
	-APLNII-		
	-APLNIW-		
	-APLNIQ-		
	AQPLNI--		
	AEPLNI--		
	ACPLNI--		
	-APLNIY-		
	-CPLNIL-		
	CYPLNI--		
	CRPLNI--		
	CAPLNI--		
	CKPLNI--		
	-CPLNIR-		
	CLPLNI--		
	CVPLNI--		
	CQPLNI--		
	-CPLNIT-		
	-CPLNIS-		
	CFPLNI--		
	CWPLNI--		
	CSPLNI--		
	-CPLNIM-		
	CMPLNI--		
	CTPLNI--		
	CPPLNI--		

-CPLNIY-
-CPLNIQ-
-CPLNIH-
CIPLNI--
CEPLNI--
DVPLNI--
DSPLNI--
DWPLNI--
-DPLNIT-
DFPLNI--
-DPLNIL-
-DPLNIF-
DMPLNI--
DNPLNI--
DRPLNI--
DLPLNI--
DYPLNI--
-DPLNIW-
-DPLNIS-
-DPLNIR-
DIPLNI--
-DPLNIH-
DAPLNI--
-DPLNIY-
DTPLNI--
-DPLNII-
DGPLNI--
DQPLNI--
-DPLNIV-
-DPLNIG-
DEPLNI--
-DPLNIM-
-DPLNIK-
-DPLNIE-
-DPLNIC-
-DPLNIA-
DHPLNI--
DDPLNI--
-EPLNIR-
-EPLNII-
ESPLNI--
EWPLNI--
-EPLNIK-
-EPLNIF-
-EPLNIS-
-EPLNIM-
-EPLNIL-
EYPLNI--
-EPLNIV-
ETPLNI--

ERPLNI--
EGPLNI--
EFPLNI--
EKPLNI--
-EPLNIY-
ENPLNI--
EDPLNI--
EVPLNI--
EQPLNI--
-EPLNIT-
-EPLNIH-
EMPLNI--
EEPLNI--
EAPLNI--
-EPLNIN-
-EPLNIA-
EIPLNI--
FTPLNI--
FRPLNI--
-FPLNIS-
FVPLNI--
-FPLNIR-
-FPLNIQ-
FLPLNI--
-FPLNIK-
FSPLNI--
-FPLNIM-
-FPLNII-
FGPLNI--
-FPLNIV-
-FPLNIA-
FNPLNI--
FKPLNI--
FHPLNI--
-FPLNIH-
FAPLNI--
-FPLNIY-
FFPLNI--
FPPLNI--
-FPLNIL-
-FPLNIE-
FIPLNI--
FWPLNI--
FEPLNI--
FYPLNI--
FMPLNI--
-FPLNIW-
-GPLNIR-
-GPLNIL-
GSPLNI--

GVPLNI--
GTPLNI--
-GPLNIG-
GHPLNI--
-GPLNIH-
-GPLNIT-
GKPLNI--
-GPLNIS-
-GPLNIV-
GGPLNI--
GWPLNI--
GMPLNI--
-GPLNIM-
GEPLNI--
GFPLNI--
-GPLNIY-
-GPLNIW-
-GPLNIF-
GAPLNI--
GQPLNI--
GYPLNI--
-GPLNIN-
GIPLNI--
-GPLNIQ-
-GPLNIA-
GLPLNI--
-GPLNIK-
GNPLNI--
-GPLNIE-
-GPLNII-
-GPLNID-
GDPLNI--
-HPLNIW-
HSPLNI--
-HPLNIL-
HRPLNI--
-HPLNIV-
HVPLNI--
HLPLNI--
-HPLNIQ-
-HPLNIR-
HAPLNI--
-HPLNIA-
HIPLNI--
HNPLNI--
HGPLNI--
HFPLNI--
HPPLNI--
-HPLNII-
HYPLNI--

HQPLNI--
-HPLNIS-
HDPLNI--
HKPLNI--
-HPLNIY-
-HPLNIN-
-HPLNIE-
HMPLNI--
HHPLNI--
HTPLNI--
-HPLNIM-
-HPLNIK-
-HPLNIC-
HEPLNI--
-HPLNIF-
ILPLNI--
-IPLNIL-
IKPLNI--
IRPLNI--
IGPLNI--
-IPLNII-
-IPLNIR-
-IPLNIS-
-IPLNIM-
IAPLNI--
-IPLNIG-
IVPLNI--
IPPLNI--
IEPLNI--
-IPLNIY-
-IPLNIQ-
-IPLNIV-
-IPLNIE-
IHPLNI--
ITPLNI--
-IPLNIT-
INPLNI--
-IPLNIW-
IFPLNI--
-IPLNIA-
IIPLNI--
IDPLNI--
IYPLNI--
-IPLNIH-
IQPLNI--
-IPLNIN-
-IPLNIK-
KSPLNI--
KGPLNI--
-KPLNIL-

-KPLNIV-
-KPLNIR-
KRPLNI--
-KPLNIA-
KLPLNI--
-KPLNIF-
KNPLNI--
KTPLNI--
KVPLNI--
-KPLNIT-
-KPLNIM-
KYPLNI--
KMPLNI--
-KPLNIH-
KPPLNI--
-KPLNIG-
-KPLNIQ-
-KPLNIN-
KFPLNI--
KWPLNI--
-KPLNIK-
-KPLNIE-
-KPLNIY-
KDPLNI--
-KPLNIW-
KKPLNI--
KIPLNI--
KHPLNI--
KQPLNI--
-KPLNIC-
LRPLNI--
LSPLNI--
LYPLNI--
LGPLNI--
LWPLNI--
-LPLNIH-
LAPLNI--
-LPLNIS-
-LPLNIL-
-LPLNIA-
LMPLNI--
-LPLNIY-
LDPLNI--
-LPLNII-
LFPLNI--
LEPLNI--
-LPLNIV-
LNPLNI--
LQPLNI--
-LPLNIQ-

```
-LPLNIE-
-LPLNIT-
-LPLNIK-
LHPLNI--
-LPLNIN-
LCPLNI--
-LPLNIW-
-LPLNIG-
-LPLNIC-
-MPLNIS-
MSPLNI--
MKPLNI--
-MPLNIL-
MMPLNI--
MQPLNI--
-MPLNIV-
MGPLNI--
-MPLNII-
-MPLNIT-
MIPLNI--
MPPLNI--
MLPLNI--
-MPLNIY-
-MPLNIK-
MTPLNI--
MDPLNI--
MWPLNI--
MAPLNI--
-MPLNIA-
MFPLNI--
MYPLNI--
-MPLNIN-
-MPLNIH-
-MPLNIE-
-MPLNIR-
-MPLNIW-
-MPLNID-
MNPLNI--
MHPLNI--
MCPLNI--
NPPLNI--
-NPLNIT-
NSPLNI--
NIPLNI--
NQPLNI--
-NPLNIR-
-NPLNIV-
-NPLNIK-
NGPLNI--
-NPLNIL-
```

NLPLNI--
NVPLNI--
NYPLNI--
-NPLNIY-
NNPLNI--
NKPLNI--
NFPLNI--
-NPLNIQ-
-NPLNIG-
-NPLNIS-
-NPLNIA-
NWPLNI--
-NPLNIM-
-NPLNIW-
-NPLNII-
NMPLNI--
NDPLNI--
NCPLNI--
--PLNILT
--PLNIRL
--PLNIVS
--PLNIVG
--PLNIVR
--PLNISL
--PLNIVA
--PLNILR
--PLNIGS
--PLNISR
--PLNIYR
--PLNIST
--PLNIRG
--PLNIRI
--PLNISS
--PLNIVY
--PLNIVT
--PLNIRA
--PLNIRR
--PLNIKH
--PLNIRV
--PLNISN
--PLNILN
--PLNITL
--PLNIRY
--PLNIRK
--PLNIIS
--PLNIAS
--PLNILH
--PLNISK
--PLNIWK
--PLNISI

--PLNIFS
--PLNIMS
--PLNILK
--PLNIRM
--PLNIGG
PGPLNI--
--PLNIWS
--PLNIKW
--PLNIHV
--PLNIFK
--PLNIFR
--PLNITS
--PLNIKV
--PLNIKA
--PLNIAN
PRPLNI--
--PLNITR
--PLNISV
--PLNIMR
--PLNIGW
--PLNISA
--PLNIHN
--PLNIEA
--PLNIQR
--PLNIQG
--PLNIKR
--PLNIIK
--PLNITQ
--PLNITK
--PLNIMG
--PLNILG
--PLNIGA
--PLNIAF
PWPLNI--
--PLNIYS
--PLNIIR
--PLNIHR
--PLNIYV
--PLNIYA
--PLNIWH
--PLNIWF
--PLNITT
--PLNISH
--PLNISG
--PLNILQ
PKPLNI--
--PLNIWN
--PLNIRF
--PLNIWR
--PLNIKS

--PLNIWG
--PLNIVW
--PLNIRP
--PLNIMA
--PLNILE
--PLNIGY
--PLNIGT
--PLNIET
--PLNIES
--PLNIVH
--PLNITH
--PLNITG
--PLNISY
--PLNIAT
--PLNIAL
--PLNIYF
--PLNIRQ
--PLNIQV
--PLNIML
--PLNIKY
--PLNIKM
--PLNIAM
--PLNIAG
--PLNIAA
--PLNIYG
--PLNIWQ
--PLNIVV
--PLNIVQ
--PLNIRH
--PLNIQY
--PLNIMW
--PLNIMN
--PLNILD
--PLNIIV
--PLNIIN
--PLNIFN
--PLNIWT
--PLNIVE
--PLNIMV
--PLNIMQ
--PLNIMI
--PLNIMH
--PLNIHT
--PLNIYW
--PLNIVM
--PLNITV
--PLNITN
--PLNISD
--PLNIQH
--PLNIKK

--PLNIIT
--PLNIIQ
--PLNIHK
--PLNIHA
--PLNIGV
--PLNIER
--PLNIEK
--PLNIEI
--PLNIAV
PFPLNI--
--PLNIVI
--PLNITA
--PLNIQT
--PLNIQQ
--PLNIMT
--PLNIMK
--PLNILW
--PLNIGL
--PLNIFY
--PLNIFT
--PLNIFE
--PLNIYH
--PLNINS
--PLNIKT
--PLNIKD
--PLNIAI
PTPLNI--
PNPLNI--
PMPLNI--
--PLNISM
--PLNINK
--PLNING
--PLNIMD
--PLNIKQ
--PLNIIP
--PLNIID
--PLNIHW
--PLNIHD
--PLNIGN
PDPLNI--
--PLNIYN
--PLNIYD
--PLNIVK
--PLNIRD
--PLNINI
--PLNIIY
--PLNIHS
--PLNIGR
--PLNIAK
--PLNIWY

--PLNIVP
--PLNITD
--PLNISW
--PLNINT
--PLNIMY
--PLNIIH
--PLNIIG
--PLNIHQ
--PLNIHH
--PLNIGF
--PLNIDT
--PLNIDG
--PLNIAW
--PLNIYM
--PLNIYK
--PLNIVD
--PLNITY
--PLNIQS
--PLNINV
--PLNINQ
--PLNINA
--PLNIIA
--PLNIFW
--PLNIFF
--PLNIEW
--PLNIEM
--PLNIEG
PEPLNI--
PQPLNI--
--PLNIYY
--PLNIYT
--PLNIYQ
--PLNIYI
--PLNIWD
--PLNISQ
--PLNIQA
--PLNINY
--PLNINW
--PLNINM
--PLNIKI
--PLNIHG
--PLNIGK
--PLNIEQ
--PLNIEL
--PLNIAR
--PLNITC
--PLNIQE
--PLNINN
--PLNINL
--PLNIMP

--PLNILC
--PLNIKN
--PLNIHY
--PLNIHI
--PLNIHE
--PLNIFH
--PLNIAQ
PIPLNI--
-PPLNIT-
--PLNIWE
--PLNITW
--PLNITP
--PLNITI
--PLNITE
--PLNIQW
--PLNIPT
--PLNIPN
--PLNIPM
--PLNIPL
--PLNINE
--PLNIMM
--PLNIME
--PLNIGQ
--PLNIGH
--PLNIGC
--PLNIFQ
--PLNIFC
--PLNIEY
--PLNIEN
--PLNIDW
--PLNIDR
--PLNIDA
--PLNIAE
--PLNIAD
PCPLNI--
QGPLNI--
-QPLNII-
QAPLNI--
-QPLNIK-
-QPLNIS-
-QPLNIR-
QRPLNI--
QSPLNI--
QQPLNI--
-QPLNIW-
-QPLNIM-
-QPLNIH-
-QPLNIQ-
QTPLNI--
QMPLNI--

QYPLNI--
-QPLNIV-
-QPLNIA-
QNPLNI--
QKPLNI--
-QPLNIT-
-QPLNIF-
QLPLNI--
QHPLNI--
QEPLNI--
QWPLNI--
QVPLNI--
QPPLNI--
-QPLNIE-
-QPLNIL-
QIPLNI--
-QPLNIY-
-QPLNIG-
-QPLNIC-
QDPLNI--
-RPLNIR-
-RPLNIS-
-RPLNIL-
-RPLNIT-
RVPLNI--
-RPLNII-
-RPLNIG-
RLPLNI--
RIPLNI--
RSPLNI--
RPPLNI--
RRPLNI--
-RPLNIV-
-RPLNIY-
RTPLNI--
-RPLNIF-
RKPLNI--
RWPLNI--
-RPLNIN-
RAPLNI--
-RPLNIW-
-RPLNIH-
REPLNI--
-RPLNIA-
RGPLNI--
RDPLNI--
RYPLNI--
RQPLNI--
-RPLNIK-
RHPLNI--

RMPLNI--
-RPLNIQ-
-RPLNIM-
-RPLNID-
-RPLNIC-
-RPLNIE-
RCPLNI--
-SPLNIL-
SKPLNI--
SVPLNI--
SRPLNI--
-SPLNIS-
SIPLNI--
-SPLNIV-
-SPLNII-
SSPLNI--
-SPLNIF-
SHPLNI--
SLPLNI--
-SPLNIK-
-SPLNIY-
SNPLNI--
-SPLNIM-
-SPLNIQ-
SMPLNI--
SFPLNI--
-SPLNIR-
SQPLNI--
-SPLNIT-
-SPLNIG-
SAPLNI--
SPPLNI--
SYPLNI--
SEPLNI--
-SPLNIN-
-SPLNIA-
SGPLNI--
SWPLNI--
-SPLNIE-
-SPLNIH-
SDPLNI--
-SPLNIW-
SCPLNI--
-SPLNID-
TSPLNI--
-TPLNIL-
TRPLNI--
-TPLNIR-
-TPLNIT-
-TPLNIF-

-TPLNIA-
TKPLNI--
-TPLNIS-
-TPLNII-
-TPLNIY-
-TPLNIH-
TIPLNI--
TYPLNI--
TMPLNI--
TGPLNI--
-TPLNIM-
-TPLNIK-
-TPLNIG-
TLPLNI--
TAPLNI--
TFPLNI--
TPPLNI--
TQPLNI--
TTPLNI--
THPLNI--
TEPLNI--
TWPLNI--
TNPLNI--
-TPLNIN-
-TPLNIE-
TDPLNI--
-TPLNIQ-
-TPLNIC-
TCPLNI--
-VPLNIR-
VLPLNI--
-VPLNIS-
-VPLNIW-
VRPLNI--
VGPLNI--
VNPLNI--
VMPLNI--
VAPLNI--
-VPLNIM-
VSPLNI--
-VPLNIY-
-VPLNII-
-VPLNIA-
-VPLNIK-
VWPLNI--
-VPLNIQ-
VIPLNI--
-VPLNIV-
-VPLNIG-
-VPLNIT-

VVPLNI--
VPPLNI--
VEPLNI--
VYPLNI--
-VPLNIN-
-VPLNIH-
VTPLNI--
VFPLNI--
VDPLNI--
VQPLNI--
-VPLNIF-
-VPLNIE-
WKPLNI--
WRPLNI--
-WPLNIS-
WGPLNI--
WMPLNI--
-WPLNIK-
-WPLNIR-
WNPLNI--
-WPLNIQ-
WHPLNI--
-WPLNIE-
-WPLNIH-
WLPLNI--
WPPLNI--
WDPLNI--
WAPLNI--
WSPLNI--
-WPLNIT-
-WPLNIM-
-WPLNIY-
WTPLNI--
-WPLNIV-
-WPLNIF-
WFPLNI--
WEPLNI--
-WPLNII-
-WPLNIA-
WIPLNI--
-WPLNIN-
-WPLNIP-
-WPLNIG-
-YPLNIS-
YFPLNI--
-YPLNIL-
YGPLNI--
-YPLNIR-
YSPLNI--
-YPLNIH-

	YNPLNI--		
	YKPLNI--		
	YHPLNI--		
	YVPLNI--		
	YTPLNI--		
	-YPLNIT-		
	YAPLNI--		
	-YPLNIE-		
	YLPLNI--		
	YYPLNI--		
	-YPLNIG-		
	-YPLNIM-		
	-YPLNII-		
	YPPLNI--		
	-YPLNIQ-		
	YIPLNI--		
	YQPLNI--		
	-YPLNIK-		
	-YPLNIA-		
	-YPLNIW-		
	-YPLNIN-		
	YWPLNI--		
	-YPLNID-		
	YEPLNI--		
	YCPLNI--		
PQAI	252	722	48.13
	-APQAIS-		
	--PQAIAR		
	AWPQAI--		
	AAPQAI--		
	ARPQAI--		
	AHPQAI--		
	-APQAIM-		
	-APQAIR-		
	ALPQAI--		
	ATPQAI--		
	ASPQAI--		
	-APQAIL-		
	-APQAIF-		
	AYPQAI--		
	AVPQAI--		
	AQPQAI--		
	-APQAIY-		
	AFPQAI--		
	-APQAIH-		
	AGPQAI--		
	-APQAIA-		
	AIPQAI--		
	-APQAII-		
	-APQAIG-		

ACPQAI--
-APQAIV-
-APQAIQ-
CQPQAI--
-CPQAIL-
CKPQAI--
CGPQAI--
CRPQAI--
-CPQAIY-
-CPQAIT-
-CPQAIM-
-CPQAIF-
CPPQAI--
CMPQAI--
CLPQAI--
DRPQAI--
-DPQAIR-
DLPQAI--
-DPQAIL-
DGPQAI--
DWPQAI--
DQPQAI--
DNPQAI--
DMPQAI--
DHPQAI--
DAPQAI--
DTPQAI--
-DPQAIY-
-DPQAIW-
-DPQAIQ-
DIPQAI--
DVPQAI--
-DPQAIH-
DKPQAI--
ERPQAI--
EVPQAI--
-EPQAIY-
-EPQAIL-
EYPQAI--
-EPQAIF-
-EPQAIM-
-EPQAIW-
-EPQAIS-
-EPQAIR-
EMPQAI--
ESPQAI--
-EPQAII-
ENPQAI--
ELPQAI--
-EPQAIK-

EIPQAI--
EEPQAI--
FIPQAI--
FKPQAI--
FFPQAI--
FRPQAI--
FGPQAI--
-FPQAIF-
FSPQAI--
FVPQAI--
-FPQAIM-
FLPQAI--
FEPQAI--
-FPQAII-
-FPQAIH-
-FPQAIG-
FNPQAI--
FMPQAI--
FAPQAI--
FTPQAI--
FPPQAI--
FDPQAI--
-FPQAIY-
-FPQAIQ-
-FPQAIE-
-FPQAIA-
GKPQAI--
GRPQAI--
GLPQAI--
GIPQAI--
GSPQAI--
-GPQAIS-
-GPQAIL-
GVPQAI--
-GPQAIY-
-GPQAIQ-
-GPQAIG-
-GPQAIF-
-GPQAIV-
GEPQAI--
GGPQAI--
GWPQAI--
GHPQAI--
GFPQAI--
GTPQAI--
GQPQAI--
-GPQAIM-
GPPQAI--
GNPQAI--
GDPQAI--

-GPQAIR-
GYPQAI--
-GPQAIT-
-GPQAIH-
-GPQAIN-
-GPQAII-
HGPQAI--
HMPQAI--
-HPQAIH-
HTPQAI--
HWPQAI--
HRPQAI--
-HPQAII-
HNPQAI--
-HPQAIV-
-HPQAIT-
-HPQAIN-
-HPQAIM-
HHPQAI--
HVPQAI--
-HPQAIL-
HIPQAI--
HFPQAI--
-HPQAIS-
-HPQAIR-
-HPQAIK-
-HPQAIA-
HLPQAI--
-IPQAIR-
-IPQAIL-
IVPQAI--
IIPQAI--
IKPQAI--
INPQAI--
IAPQAI--
IRPQAI--
IDPQAI--
ISPQAI--
-IPQAIV-
-IPQAIF-
IQPQAI--
-IPQAIS-
IEPQAI--
-IPQAIQ-
ILPQAI--
-IPQAIT-
-IPQAIN-
IGPQAI--
ITPQAI--
-IPQAIA-

-IPQAIY-
-IPQAIM-
-IPQAII-
IMPQAI--
-IPQAIK-
-IPQAIH-
-IPQAIG-
IFPQAI--
KSPQAI--
KVPQAI--
KLPQAI--
-KPQAIT-
KNPQAI--
-KPQAIQ-
KGPQAI--
-KPQAIK-
KIPQAI--
-KPQAIR-
KHPQAI--
-KPQAIY-
KKPQAI--
-KPQAIF-
KYPQAI--
-KPQAII-
-KPQAIH-
KWPQAI--
KPPQAI--
-KPQAIS-
-KPQAIL-
-KPQAIG-
-KPQAIE-
-KPQAIA-
KMPQAI--
KAPQAI--
KQPQAI--
-KPQAIN-
KRPQAI--
-KPQAIV-
-KPQAIM-
-KPQAIC-
KDPQAI--
LRPQAI--
LSPQAI--
-LPQAIR-
LVPQAI--
-LPQAIY-
-LPQAIM-
LTPQAI--
LQPQAI--
-LPQAIF-

LGPQAI--
-LPQAII-
LLPQAI--
LYPQAI--
LIPQAI--
LHPQAI--
-LPQAIL-
-LPQAIT-
-LPQAIK-
LPPQAI--
LWPQAI--
-LPQAIA-
-LPQAIW-
-LPQAIQ-
LAPQAI--
-LPQAIV-
LNPQAI--
LEPQAI--
-LPQAIS-
-LPQAIN-
LMPQAI--
-LPQAIH-
-LPQAIE-
MAPQAI--
-MPQAIL-
-MPQAIK-
MLPQAI--
-MPQAIT-
MWPQAI--
MMPQAI--
MVPQAI--
MSPQAI--
-MPQAIH-
MPPQAI--
MKPQAI--
MYPQAI--
MTPQAI--
-MPQAIR-
MQPQAI--
-MPQAIY-
-MPQAIV-
MGPQAI--
-MPQAIF-
MDPQAI--
-MPQAIM-
-MPQAIA-
NRPQAI--
NVPQAI--
NIPQAI--
NSPQAI--

-NPQAIF-
NAPQAI--
NTPQAI--
-NPQAIW-
-NPQAIT-
-NPQAIS-
-NPQAIM-
-NPQAIL-
NLPQAI--
NKPQAI--
-NPQAIK-
NPPQAI--
NMPQAI--
NEPQAI--
NNPQAI--
NWPQAI--
-NPQAIN-
NFPQAI--
NCPQAI--
--PQAILS
--PQAIVR
--PQAIRK
--PQAIAL
--PQAIRS
--PQAILA
--PQAIWR
--PQAIKR
--PQAIRQ
--PQAILT
--PQAIRI
--PQAIFS
--PQAIFH
--PQAIRN
--PQAILR
--PQAIRA
--PQAINA
--PQAITG
--PQAITT
--PQAISV
--PQAIST
--PQAILM
--PQAILG
--PQAISF
PMPQAI--
PSPQAI--
--PQAIWS
--PQAIVT
--PQAIRV
--PQAIHS
--PQAIFY

--PQAIYT
--PQAIVL
--PQAISS
--PQAIVQ
--PQAISW
--PQAIRG
--PQAIKL
--PQAIHA
--PQAIAA
--PQAIYS
--PQAIYH
--PQAISY
--PQAISG
--PQAILV
--PQAIKY
--PQAIIL
PLPQAI--
--PQAIRY
--PQAIQM
--PQAIIG
--PQAIGS
--PQAIWL
--PQAIWF
--PQAIVG
--PQAISL
--PQAINS
--PQAIMR
--PQAIMD
--PQAIHW
--PQAIFA
--PQAISI
--PQAISH
--PQAIRR
--PQAIII
PAPQAI--
--PQAIYL
--PQAIYI
--PQAIWG
--PQAIWA
--PQAIVV
--PQAIVM
--PQAITA
--PQAILY
--PQAIKT
--PQAIGL
--PQAIGI
--PQAIFW
--PQAIFT
PKPQAI--
PVPQAI--

--PQAIYM
--PQAIWV
--PQAIRW
--PQAIRF
--PQAIML
--PQAIKN
--PQAIKM
--PQAIFQ
PRPQAI--
--PQAITY
--PQAITW
--PQAIQA
--PQAIIR
--PQAIHI
--PQAIGG
--PQAIAS
--PQAIAG
--PQAIWI
--PQAIVN
--PQAISK
--PQAIRM
--PQAIPL
--PQAILQ
--PQAILD
--PQAIHL
--PQAIHG
--PQAIGV
--PQAIFI
PGPQAI--
--PQAITQ
--PQAITM
--PQAIRE
--PQAING
--PQAINF
--PQAIMQ
--PQAIMK
--PQAILI
--PQAIHT
--PQAIFN
--PQAIEV
--PQAIVF
--PQAITK
--PQAITH
--PQAIQR
--PQAINN
--PQAIMN
--PQAIMA
--PQAILN
--PQAILL
--PQAIKH

--PQAIGM
--PQAIEY
PTPQAI--
--PQAIYV
--PQAIYK
--PQAIYG
--PQAITN
--PQAITF
--PQAITD
--PQAISN
--PQAISA
--PQAIRT
--PQAIRH
--PQAIMH
--PQAIKF
--PQAIIT
--PQAIHY
--PQAIHR
--PQAIFM
--PQAIFL
PHPQAI--
PWPQAI--
--PQAIYY
--PQAITL
--PQAIRP
--PQAIRL
--PQAIRD
--PQAIQV
--PQAIQS
--PQAIQN
--PQAIQL
--PQAINL
--PQAIME
--PQAILK
--PQAILH
--PQAIKI
--PQAIIY
--PQAIID
--PQAIHK
--PQAIHF
--PQAIFK
--PQAIAW
--PQAIAT
--PQAIYA
--PQAIWM
--PQAIVW
--PQAIVS
--PQAIVH
--PQAIVD
--PQAITS

--PQAITI
--PQAITC
--PQAISM
--PQAIQT
--PQAIQI
--PQAIQH
--PQAIQF
--PQAIPF
--PQAINY
--PQAINR
--PQAIMG
--PQAILW
--PQAIKV
--PQAIKQ
--PQAIIV
--PQAIIK
--PQAIHQ
--PQAIHH
--PQAIGW
--PQAIFR
--PQAIET
--PQAIES
--PQAIEK
--PQAIAI
--PQAIAH
PIPQAI--
--PQAIYR
--PQAIWT
--PQAIWK
--PQAIVY
--PQAISQ
--PQAISE
--PQAIQY
--PQAIQE
--PQAIPY
--PQAIPI
--PQAIPH
--PQAINT
--PQAINK
--PQAINI
--PQAIMS
--PQAIKS
--PQAIKE
--PQAIIS
--PQAIHV
--PQAIGY
--PQAIGF
--PQAIGA
--PQAIFV
--PQAIER

--PQAIEM
--PQAIEH
--PQAIAK
-PPQAIR-
PNPQAI--
PEPQAI--
QRPQAI--
-QPQAIR-
QYPQAI--
QGPQAI--
QLPQAI--
QWPQAI--
QVPQAI--
QSPQAI--
-QPQAIL-
-QPQAII-
-QPQAIA-
-QPQAIY-
-QPQAIT-
-QPQAIN-
QKPQAI--
-QPQAIH-
QIPQAI--
-QPQAIM-
-QPQAIE-
QDPQAI--
-QPQAIC-
QNPQAI--
QAPQAI--
-RPQAIF-
-RPQAIL-
-RPQAII-
RGPQAI--
RYPQAI--
-RPQAIH-
RQPQAI--
-RPQAIR-
RIPQAI--
-RPQAIS-
RHPQAI--
REPQAI--
RVPQAI--
-RPQAIW-
RFPQAI--
-RPQAIA-
RKPQAI--
-RPQAIM-
RWPQAI--
RRPQAI--
-RPQAIK-

-RPQAIG-
RNPQAI--
RAPQAI--
-RPQAIQ-
-RPQAIN-
-RPQAIV-
RMPQAI--
RDPQAI--
-RPQAIE-
STPQAI--
-SPQAIS-
SIPQAI--
SRPQAI--
SFPQAI--
-SPQAIM-
SQPQAI--
-SPQAIV-
SSPQAI--
SKPQAI--
SWPQAI--
-SPQAIT-
-SPQAIR-
SAPQAI--
-SPQAIK-
SEPQAI--
SYPQAI--
SVPQAI--
-SPQAIL-
SNPQAI--
SHPQAI--
-SPQAIW-
-SPQAIF-
-SPQAIA-
SDPQAI--
SGPQAI--
-SPQAII-
SPPQAI--
SLPQAI--
-SPQAIN-
-SPQAIQ-
-SPQAIH-
SMPQAI--
TRPQAI--
-TPQAIT-
TNPQAI--
-TPQAIR-
TSPQAI--
TVPQAI--
TGPQAI--
-TPQAIL-

-TPQAII-
TEPQAI--
-TPQAIV-
TPPQAI--
TLPQAI--
TQPQAI--
-TPQAIY-
TKPQAI--
TAPQAI--
-TPQAIS-
-TPQAIH-
TIPQAI--
THPQAI--
TFPQAI--
TWPQAI--
-TPQAIW-
-TPQAIG-
-TPQAIE-
TCPQAI--
-TPQAIQ-
-TPQAIF-
-VPQAIR-
VMPQAI--
VWPQAI--
VVPQAI--
VSPQAI--
VLPQAI--
VAPQAI--
-VPQAIS-
VRPQAI--
VIPQAI--
-VPQAIW-
-VPQAIN-
-VPQAIF-
VKPQAI--
-VPQAIY-
VGPQAI--
VQPQAI--
-VPQAIA-
VDPQAI--
VYPQAI--
VTPQAI--
VEPQAI--
-VPQAIV-
-VPQAIK-
-VPQAIQ-
-VPQAIL-
-VPQAII-
-VPQAIH-
VNPQAI--

```
               VFPQAI--
               WWPQAI--
               WLPQAI--
               WYPQAI--
               WGPQAI--
               WRPQAI--
               -WPQAIQ-
               -WPQAIW-
               WNPQAI--
               WTPQAI--
               WDPQAI--
               WQPQAI--
               -WPQAIV-
               -WPQAIS-
               WVPQAI--
               -WPQAIR-
               -WPQAIM-
               -WPQAIG-
               -WPQAIA-
               -WPQAIT-
               -WPQAIK-
               WFPQAI--
               WPPQAI--
               YGPQAI--
               YTPQAI--
               -YPQAIM-
               YVPQAI--
               YQPQAI--
               -YPQAIT-
               -YPQAIR-
               -YPQAIF-
               -YPQAIH-
               YHPQAI--
               YMPQAI--
               -YPQAIS-
               -YPQAIL-
               YFPQAI--
               -YPQAIV-
               -YPQAII-
               -YPQAIA-
               YAPQAI--
               YYPQAI--
               YRPQAI--
               YLPQAI--
               YIPQAI--
               YSPQAI--
               -YPQAIQ-
               -YPQAIN-
PLNL            112        800     48.09
               -GPLNLP-
```

```
--PLNLYP
--PLNLGP
--PLNLDI
-RPLNLP-
-SPLNLC-
-APLNLT-
ASPLNL--
-APLNLS-
AMPLNL--
-APLNLH-
-APLNLN-
-APLNLR-
-APLNLV-
ALPLNL--
AKPLNL--
AVPLNL--
-APLNLG-
APPLNL--
ANPLNL--
AGPLNL--
AWPLNL--
AFPLNL--
-APLNLQ-
-APLNLI-
AQPLNL--
AHPLNL--
-APLNLM-
-APLNLK-
AAPLNL--
AYPLNL--
-APLNLE-
ADPLNL--
-APLNLA-
AEPLNL--
-APLNLD-
-APLNLC-
CTPLNL--
CCPLNL--
CIPLNL--
-CPLNLR-
CAPLNL--
CWPLNL--
-CPLNLS-
CLPLNL--
CKPLNL--
CSPLNL--
-CPLNLL-
-CPLNLK-
CGPLNL--
CPPLNL--
```

```
-CPLNLT-
CEPLNL--
CRPLNL--
CQPLNL--
CNPLNL--
-CPLNLY-
-CPLNLN-
-CPLNLI-
CHPLNL--
CDPLNL--
CYPLNL--
-CPLNLH-
-CPLNLD-
CFPLNL--
DTPLNL--
-DPLNLR-
DRPLNL--
DHPLNL--
DVPLNL--
DIPLNL--
DWPLNL--
DSPLNL--
DKPLNL--
-DPLNLW-
-DPLNLV-
-DPLNLL-
-DPLNLI-
-DPLNLF-
-DPLNLQ-
-DPLNLT-
-DPLNLH-
DYPLNL--
-DPLNLM-
-DPLNLK-
DFPLNL--
-DPLNLE-
DMPLNL--
-DPLNLN-
-DPLNLA-
DNPLNL--
-DPLNLS-
DDPLNL--
-DPLNLG-
-DPLNLC-
-EPLNLS-
EGPLNL--
-EPLNLL-
ERPLNL--
-EPLNLR-
ESPLNL--
```

```
-EPLNLT-
-EPLNLV-
EVPLNL--
-EPLNLG-
-EPLNLY-
-EPLNLA-
EYPLNL--
-EPLNLQ-
-EPLNLI-
ENPLNL--
-EPLNLH-
EHPLNL--
EFPLNL--
ETPLNL--
-EPLNLK-
EMPLNL--
EPPLNL--
-EPLNLM-
EKPLNL--
-EPLNLW-
EQPLNL--
ELPLNL--
EWPLNL--
-EPLNLF-
-EPLNLN-
-EPLNLE-
EDPLNL--
ECPLNL--
FSPLNL--
-FPLNLR-
-FPLNLV-
FRPLNL--
FTPLNL--
-FPLNLQ-
-FPLNLA-
-FPLNLH-
-FPLNLG-
-FPLNLS-
-FPLNLN-
FPPLNL--
-FPLNLK-
FNPLNL--
-FPLNLT-
FQPLNL--
FHPLNL--
FEPLNL--
FVPLNL--
-FPLNLE-
FKPLNL--
-FPLNLM-
```

FCPLNL--
-FPLNLD-
GFPLNL--
GRPLNL--
GGPLNL--
GIPLNL--
GTPLNL--
GLPLNL--
-GPLNLS-
-GPLNLM-
-GPLNLL-
-GPLNLI-
GYPLNL--
-GPLNLG-
-GPLNLT-
-GPLNLK-
GWPLNL--
-GPLNLH-
GAPLNL--
GNPLNL--
GEPLNL--
GQPLNL--
-GPLNLV-
GDPLNL--
-GPLNLN-
-GPLNLA-
-GPLNLQ-
GMPLNL--
-GPLNLD-
-GPLNLE-
GCPLNL--
-GPLNLC-
HRPLNL--
HLPLNL--
HSPLNL--
-HPLNLV-
HTPLNL--
-HPLNLS-
-HPLNLA-
HEPLNL--
-HPLNLL-
-HPLNLH-
-HPLNLG-
-HPLNLT-
HQPLNL--
HGPLNL--
HAPLNL--
HFPLNL--
HWPLNL--
HKPLNL--

HIPLNL--
-HPLNLM-
-HPLNLF-
HYPLNL--
HMPLNL--
-HPLNLE-
-HPLNLK-
HPPLNL--
-HPLNLY-
-HPLNLQ-
-HPLNLW-
HNPLNL--
HDPLNL--
-HPLNLD-
-HPLNLP-
-HPLNLC-
-IPLNLK-
-IPLNLL-
-IPLNLR-
IGPLNL--
-IPLNLT-
IVPLNL--
ITPLNL--
-IPLNLS-
-IPLNLN-
IIPLNL--
IAPLNL--
IMPLNL--
IPPLNL--
-IPLNLE-
IYPLNL--
-IPLNLM-
IQPLNL--
-IPLNLH-
-IPLNLA-
IKPLNL--
-IPLNLQ-
IEPLNL--
-IPLNLG-
-IPLNLD-
IHPLNL--
INPLNL--
IDPLNL--
ICPLNL--
KSPLNL--
-KPLNLR-
-KPLNLL-
-KPLNLT-
-KPLNLI-
-KPLNLV-

KAPLNL--
-KPLNLW-
KHPLNL--
KGPLNL--
KYPLNL--
KIPLNL--
KEPLNL--
-KPLNLA-
KTPLNL--
-KPLNLY-
-KPLNLN-
KPPLNL--
-KPLNLH-
-KPLNLE-
-KPLNLQ-
KMPLNL--
KDPLNL--
KQPLNL--
KNPLNL--
-KPLNLD-
KCPLNL--
LSPLNL--
-LPLNLR-
-LPLNLT-
LTPLNL--
LWPLNL--
-LPLNLQ-
LAPLNL--
LPPLNL--
LEPLNL--
LQPLNL--
-LPLNLM-
-LPLNLI-
LDPLNL--
-LPLNLV-
-LPLNLH-
LHPLNL--
-LPLNLN-
-LPLNLG-
LYPLNL--
-LPLNLA-
-LPLNLE-
-LPLNLK-
LIPLNL--
LKPLNL--
LCPLNL--
MSPLNL--
MVPLNL--
MGPLNL--
-MPLNLR-

```
-MPLNLS-
-MPLNLV-
MAPLNL--
MRPLNL--
-MPLNLT-
-MPLNLG-
-MPLNLK-
MMPLNL--
-MPLNLN-
-MPLNLM-
MNPLNL--
MFPLNL--
MYPLNL--
MIPLNL--
-MPLNLI-
MHPLNL--
MQPLNL--
MEPLNL--
-MPLNLQ-
-MPLNLH-
-MPLNLE-
MKPLNL--
MCPLNL--
MDPLNL--
-MPLNLD-
-MPLNLP-
-MPLNLC-
NRPLNL--
NVPLNL--
NSPLNL--
-NPLNLV-
NLPLNL--
NAPLNL--
-NPLNLS-
NGPLNL--
NMPLNL--
NWPLNL--
NQPLNL--
-NPLNLM-
NPPLNL--
-NPLNLY-
NTPLNL--
NFPLNL--
-NPLNLT-
-NPLNLQ-
-NPLNLK-
NIPLNL--
NHPLNL--
NNPLNL--
NKPLNL--
```

-NPLNLE-
-NPLNLA-
NEPLNL--
NDPLNL--
-NPLNLH-
-NPLNLN-
-NPLNLD-
NCPLNL--
--PLNLLG
--PLNLSR
--PLNLRR
--PLNLGS
--PLNLRT
--PLNLST
--PLNLSS
--PLNLTI
--PLNLLS
--PLNLTR
--PLNLRA
--PLNLLH
--PLNLAR
--PLNLTL
--PLNLTS
--PLNLKS
--PLNLLK
--PLNLIS
--PLNLSG
--PLNLRQ
--PLNLWN
--PLNLTV
--PLNLSY
--PLNLIR
PRPLNL--
--PLNLNS
--PLNLAI
--PLNLAM
--PLNLSV
--PLNLLN
--PLNLRH
--PLNLKR
--PLNLTT
--PLNLSK
--PLNLTM
--PLNLQT
--PLNLHN
--PLNLAW
--PLNLGL
--PLNLAS
--PLNLYS
--PLNLGI

--PLNLAT
--PLNLGA
PGPLNL--
--PLNLTA
--PLNLMK
--PLNLGG
--PLNLAL
--PLNLTG
--PLNLSH
--PLNLQL
PTPLNL--
--PLNLVT
--PLNLVN
--PLNLLD
--PLNLGV
--PLNLSN
--PLNLYG
--PLNLSI
--PLNLMH
--PLNLKQ
--PLNLES
--PLNLKT
--PLNLHT
--PLNLEL
--PLNLSQ
--PLNLQS
--PLNLQR
--PLNLSM
--PLNLIN
--PLNLMW
--PLNLHS
--PLNLEV
--PLNLQN
--PLNLAH
--PLNLTH
--PLNLMS
--PLNLMR
--PLNLFG
--PLNLNM
--PLNLKK
--PLNLKG
--PLNLET
--PLNLAG
--PLNLWQ
--PLNLTN
--PLNLNT
--PLNLGT
--PLNLYN
--PLNLQI
--PLNLAA

--PLNLRE
--PLNLMA
--PLNLLE
--PLNLKM
--PLNLGN
--PLNLYH
--PLNLTQ
--PLNLNV
--PLNLKL
--PLNLGK
--PLNLDS
--PLNLVK
--PLNLNR
--PLNLKY
--PLNLHK
--PLNLHI
--PLNLHH
--PLNLHL
--PLNLHG
--PLNLTW
--PLNLMT
--PLNLKV
--PLNLNL
--PLNLNA
--PLNLLQ
--PLNLKN
--PLNLGF
--PLNLTY
--PLNLSE
--PLNLHW
--PLNLQQ
--PLNLAN
--PLNLQG
--PLNLID
--PLNLHA
--PLNLQK
--PLNLER
--PLNLYA
--PLNLTD
--PLNLML
--PLNLIH
--PLNLGM
--PLNLGH
--PLNLFN
--PLNLEI
--PLNLWH
--PLNLSW
--PLNLQH
--PLNLMY
--PLNLHR

--PLNLEG
--PLNLRP
--PLNLRD
--PLNLQF
--PLNLMV
--PLNLKI
--PLNLWD
--PLNLVP
--PLNLSD
--PLNLNQ
--PLNLKF
--PLNLFD
--PLNLAK
--PLNLWG
--PLNLVE
--PLNLMG
--PLNLHF
--PLNLAQ
--PLNLWK
--PLNLVD
--PLNLTE
--PLNLNK
--PLNLME
--PLNLKD
--PLNLHM
--PLNLEY
--PLNLEQ
--PLNLYD
--PLNLNH
--PLNLMD
--PLNLED
PEPLNL--
--PLNLMQ
--PLNLKA
--PLNLEW
--PLNLDT
--PLNLVW
--PLNLIP
--PLNLHQ
PNPLNL--
--PLNLLP
--PLNLHY
--PLNLDW
--PLNLWA
--PLNLQW
--PLNLQM
--PLNLNY
--PLNLMP
--PLNLGY
--PLNLFQ

--PLNLEK
--PLNLQV
--PLNLNW
--PLNLNN
--PLNLKW
--PLNLKH
--PLNLIE
--PLNLHE
--PLNLHD
--PLNLDV
--PLNLDM
--PLNLDG
--PLNLQY
--PLNLQE
--PLNLNE
--PLNLND
--PLNLEE
--PLNLMN
--PLNLGD
--PLNLEF
--PLNLGW
--PLNLDF
--PLNLCS
--PLNLAE
--PLNLAD
--PLNLRC
--PLNLQD
--PLNLKC
--PLNLEH
--PLNLDY
--PLNLDN
--PLNLDA
--PLNLCL
PDPLNL--
PPPLNL--
--PLNLSC
--PLNLPR
--PLNLNG
--PLNLKP
--PLNLKE
--PLNLEN
--PLNLCW
--PLNLCG
--PLNLAC
-PPLNLV-
-PPLNLS-
-PPLNLR-
-PPLNLL-
--PLNLVC
--PLNLPI

--PLNLNC
--PLNLLC
--PLNLDH
--PLNLCY
--PLNLCT
--PLNLCK
--PLNLCA
-QPLNLL-
QRPLNL--
-QPLNLS-
QLPLNL--
-QPLNLR-
-QPLNLG-
QTPLNL--
-QPLNLT-
-QPLNLA-
-QPLNLV-
-QPLNLI-
QAPLNL--
-QPLNLW-
QFPLNL--
QVPLNL--
QKPLNL--
QGPLNL--
-QPLNLQ-
-QPLNLM-
QMPLNL--
QEPLNL--
QWPLNL--
QPPLNL--
-QPLNLH-
-QPLNLF-
QNPLNL--
-QPLNLN-
QQPLNL--
-QPLNLE-
-QPLNLY-
-QPLNLK-
QDPLNL--
-QPLNLD-
QHPLNL--
-RPLNLT-
RVPLNL--
-RPLNLS-
RLPLNL--
RGPLNL--
RTPLNL--
RMPLNL--
RIPLNL--
RWPLNL--

-RPLNLA-
-RPLNLQ-
REPLNL--
-RPLNLI-
RHPLNL--
-RPLNLM-
-RPLNLW-
-RPLNLE-
RQPLNL--
-RPLNLD-
RDPLNL--
SRPLNL--
SGPLNL--
-SPLNLL-
-SPLNLF-
-SPLNLR-
SSPLNL--
-SPLNLK-
SKPLNL--
SAPLNL--
-SPLNLV-
-SPLNLI-
STPLNL--
-SPLNLN-
SHPLNL--
-SPLNLM-
SWPLNL--
-SPLNLT-
SNPLNL--
SMPLNL--
SFPLNL--
SYPLNL--
SQPLNL--
SPPLNL--
-SPLNLQ-
-SPLNLA-
-SPLNLH-
SIPLNL--
SEPLNL--
SDPLNL--
-SPLNLE-
SCPLNL--
-SPLNLD-
TSPLNL--
TLPLNL--
-TPLNLS-
-TPLNLH-
TGPLNL--
TVPLNL--
TWPLNL--

TAPLNL--
-TPLNLN-
TKPLNL--
TTPLNL--
TFPLNL--
-TPLNLK-
-TPLNLM-
-TPLNLA-
TYPLNL--
-TPLNLI-
TPPLNL--
-TPLNLQ-
TNPLNL--
-TPLNLE-
TDPLNL--
THPLNL--
TEPLNL--
-TPLNLD-
-VPLNLR-
VSPLNL--
VKPLNL--
-VPLNLL-
VRPLNL--
-VPLNLS-
-VPLNLN-
VMPLNL--
VLPLNL--
-VPLNLV-
VAPLNL--
-VPLNLQ-
VPPLNL--
-VPLNLK-
-VPLNLH-
-VPLNLM-
-VPLNLI-
-VPLNLT-
-VPLNLA-
VEPLNL--
VGPLNL--
-VPLNLG-
VIPLNL--
-VPLNLE-
-VPLNLD-
VFPLNL--
VNPLNL--
VHPLNL--
VDPLNL--
VCPLNL--
-WPLNLT-
-WPLNLS-

WPPLNL--
WVPLNL--
WEPLNL--
-WPLNLG-
WGPLNL--
-WPLNLQ-
-WPLNLL-
WTPLNL--
-WPLNLK-
WQPLNL--
WKPLNL--
-WPLNLV-
WDPLNL--
-WPLNLN-
-WPLNLM-
-WPLNLD-
WNPLNL--
-WPLNLH-
-YPLNLS-
YVPLNL--
YSPLNL--
YKPLNL--
-YPLNLR-
YLPLNL--
-YPLNLL-
YEPLNL--
-YPLNLK-
YAPLNL--
YTPLNL--
-YPLNLH-
-YPLNLV-
YPPLNL--
-YPLNLM-
YIPLNL--
-YPLNLI-
YNPLNL--
YQPLNL--
-YPLNLE-
-YPLNLA-
-YPLNLQ-
YMPLNL--
-YPLNLT-
-YPLNLN-
-YPLNLF-
-YPLNLG-
-YPLNLD-
YHPLNL--
YDPLNL--
YCPLNL--

PRWI 394 660 48.08

```
-APRWIG-
-APRWIL-
AVPRWI--
-APRWIY-
ASPRWI--
ALPRWI--
AYPRWI--
-APRWIR-
ANPRWI--
AHPRWI--
-APRWIN-
AWPRWI--
-APRWIK-
-APRWIF-
-APRWII-
ADPRWI--
AQPRWI--
AKPRWI--
-APRWID-
AEPRWI--
-APRWIV-
-APRWIE-
AFPRWI--
-APRWIW-
CSPRWI--
CNPRWI--
CMPRWI--
-CPRWIS-
CLPRWI--
CPPRWI--
-CPRWIR-
-CPRWIK-
CFPRWI--
CWPRWI--
CRPRWI--
-CPRWIQ-
CHPRWI--
-CPRWIL-
-CPRWIH-
CKPRWI--
CIPRWI--
CGPRWI--
CDPRWI--
DRPRWI--
DTPRWI--
-DPRWIS-
-DPRWIR-
DHPRWI--
DWPRWI--
DVPRWI--
```

DNPRWI--
DFPRWI--
DSPRWI--
-DPRWIW-
DAPRWI--
DMPRWI--
DLPRWI--
DKPRWI--
-DPRWIH-
DIPRWI--
DGPRWI--
-DPRWIT-
-DPRWIQ-
-DPRWIK-
DQPRWI--
-DPRWIY-
-DPRWIV-
-DPRWIG-
ESPRWI--
ERPRWI--
EGPRWI--
-EPRWIR-
EKPRWI--
EAPRWI--
EWPRWI--
-EPRWIA-
ENPRWI--
EVPRWI--
ETPRWI--
-EPRWIW-
-EPRWIT-
-EPRWIY-
ELPRWI--
-EPRWIM-
-EPRWII-
-EPRWIH-
-EPRWIS-
EHPRWI--
-EPRWIG-
-EPRWIN-
EEPRWI--
-EPRWIQ-
EQPRWI--
EIPRWI--
EDPRWI--
FEPRWI--
FSPRWI--
FTPRWI--
FHPRWI--
-FPRWII-

FGPRWI--
-FPRWIQ-
FKPRWI--
FFPRWI--
-FPRWIN-
FMPRWI--
FYPRWI--
-FPRWIK-
-FPRWIV-
FPPRWI--
FIPRWI--
FNPRWI--
FDPRWI--
FVPRWI--
-FPRWIM-
-GPRWIS-
-GPRWIL-
GKPRWI--
GVPRWI--
GGPRWI--
GTPRWI--
-GPRWIR-
GNPRWI--
-GPRWIM-
GRPRWI--
-GPRWIQ-
-GPRWIG-
-GPRWIK-
GHPRWI--
-GPRWIV-
-GPRWIN-
GEPRWI--
GIPRWI--
-GPRWIW-
-GPRWIY-
-GPRWIT-
-GPRWIH-
-GPRWIE-
GSPRWI--
GQPRWI--
GDPRWI--
GCPRWI--
-GPRWIF-
GPPRWI--
HNPRWI--
HTPRWI--
HGPRWI--
-HPRWIQ-
-HPRWIL-
HSPRWI--

-HPRWIH-
HEPRWI--
-HPRWIS-
HDPRWI--
-HPRWII-
-HPRWIV-
HKPRWI--
-HPRWIY-
-HPRWIT-
HQPRWI--
-HPRWIW-
-HPRWIK-
ISPRWI--
-IPRWIR-
-IPRWIT-
ILPRWI--
INPRWI--
IPPRWI--
-IPRWIV-
-IPRWIL-
IQPRWI--
IGPRWI--
IWPRWI--
-IPRWIS-
IVPRWI--
-IPRWIM-
-IPRWIY-
-IPRWIG-
ITPRWI--
-IPRWIK-
IMPRWI--
-IPRWIW-
-IPRWIF-
-IPRWIN-
-IPRWIA-
IFPRWI--
IYPRWI--
-IPRWIQ-
-IPRWIE-
IIPRWI--
IHPRWI--
KQPRWI--
KVPRWI--
-KPRWIY-
-KPRWIG-
-KPRWIS-
KPPRWI--
KIPRWI--
-KPRWIT-
-KPRWII-

-KPRWIF-
-KPRWIV-
-KPRWIQ-
-KPRWIM-
KWPRWI--
-KPRWIN-
KDPRWI--
-KPRWIH-
-KPRWIE-
KEPRWI--
KCPRWI--
-KPRWIW-
-KPRWIR-
LEPRWI--
-LPRWIG-
-LPRWIT-
-LPRWIS-
LNPRWI--
LGPRWI--
-LPRWIL-
-LPRWIK-
-LPRWIN-
-LPRWIH-
-LPRWII-
LDPRWI--
LYPRWI--
-LPRWIF-
LPPRWI--
LIPRWI--
-LPRWIV-
-LPRWIP-
MSPRWI--
MKPRWI--
MRPRWI--
MVPRWI--
MGPRWI--
-MPRWIA-
MLPRWI--
MDPRWI--
-MPRWIN-
-MPRWIG-
-MPRWIV-
-MPRWIK-
MQPRWI--
MPPRWI--
-MPRWIH-
MYPRWI--
-MPRWIY-
MAPRWI--
MHPRWI--

MTPRWI--
MIPRWI--
-MPRWIQ-
MEPRWI--
MWPRWI--
-MPRWIE-
NPPRWI--
NVPRWI--
NHPRWI--
NMPRWI--
NTPRWI--
NRPRWI--
-NPRWIR-
NQPRWI--
-NPRWIS-
NIPRWI--
-NPRWIA-
-NPRWIT-
-NPRWII-
-NPRWIF-
NGPRWI--
NNPRWI--
-NPRWIY-
-NPRWIL-
NKPRWI--
-NPRWIN-
-NPRWIH-
NYPRWI--
-NPRWIM-
-NPRWIG-
NEPRWI--
-NPRWIV-
-NPRWIQ-
-NPRWIK-
-NPRWID-
--PRWITG
PSPRWI--
--PRWITS
--PRWISN
--PRWIHG
PRPRWI--
PTPRWI--
--PRWITT
--PRWIAG
PWPRWI--
--PRWISV
--PRWINS
--PRWIFS
--PRWISK
--PRWIRI

--PRWISH
--PRWIRQ
--PRWIKA
--PRWIGA
--PRWIWS
--PRWITA
--PRWIQS
--PRWIRN
--PRWIKM
--PRWIID
--PRWIMV
--PRWIYK
--PRWIAY
--PRWISQ
--PRWILQ
--PRWIAI
--PRWIWG
--PRWISG
--PRWISA
PEPRWI--
--PRWIYH
--PRWIVD
--PRWIRY
--PRWIRA
--PRWIGL
PLPRWI--
PYPRWI--
--PRWIIK
--PRWISY
--PRWIMD
--PRWILY
--PRWILN
--PRWIFG
--PRWIAL
--PRWIAA
--PRWIYV
--PRWIMT
--PRWIKG
--PRWIGQ
--PRWIYN
--PRWIWH
--PRWITD
--PRWIME
--PRWIKN
--PRWIWT
--PRWIWK
--PRWITN
--PRWIFA
--PRWIAV
PVPRWI--

--PRWIYD
--PRWIVQ
--PRWIRW
--PRWIQT
--PRWIAN
--PRWINT
--PRWIKQ
--PRWIIS
--PRWIHS
PAPRWI--
--PRWIVE
--PRWITQ
--PRWIRD
--PRWIEY
--PRWIVT
--PRWIQG
--PRWIMH
--PRWIHM
--PRWIDS
--PRWIAT
--PRWIVW
--PRWIKD
PQPRWI--
--PRWIYY
--PRWIQV
--PRWIQK
--PRWIFD
--PRWITV
--PRWITH
--PRWIQW
--PRWINV
--PRWIHW
--PRWIHI
--PRWIEA
--PRWIAQ
PMPRWI--
--PRWIYS
--PRWIWV
--PRWISW
--PRWISE
--PRWINY
--PRWIKH
--PRWIHQ
--PRWIGF
--PRWIFW
--PRWIVY
--PRWIVV
--PRWITW
--PRWITE
--PRWISM

--PRWIQE
--PRWINA
--PRWILD
--PRWIGN
--PRWIFT
--PRWIEV
--PRWIET
--PRWIWQ
--PRWIQQ
--PRWING
--PRWILE
--PRWIIT
--PRWIII
--PRWIGY
--PRWIEK
--PRWIAH
--PRWIVF
--PRWIRF
--PRWIQN
--PRWIQA
--PRWIKW
--PRWIIY
--PRWIHK
--PRWINH
--PRWIIV
--PRWIHH
--PRWIER
PHPRWI--
PDPRWI--
--PRWIYE
--PRWIWN
--PRWIWL
--PRWIQY
--PRWIQI
--PRWIMW
--PRWIMN
--PRWIHT
--PRWIHD
--PRWIFE
--PRWIEW
--PRWIEN
--PRWIEM
--PRWIEG
PIPRWI--
--PRWIYQ
--PRWIWD
--PRWIVS
--PRWIVM
--PRWIQL
--PRWIQF

--PRWIQD
--PRWILW
--PRWILC
--PRWIKL
--PRWIKE
--PRWIIM
--PRWIIE
--PRWIHE
--PRWIGM
--PRWIGH
--PRWIEQ
--PRWIEF
--PRWIED
--PRWIDA
--PRWIYA
--PRWITC
--PRWISC
--PRWIPH
--PRWINQ
--PRWINK
--PRWINF
--PRWIKC
--PRWIIW
--PRWIIH
--PRWIHP
--PRWIGW
--PRWIGE
--PRWIFV
--PRWIFN
--PRWIFH
--PRWIDT
--PRWIDK
--PRWICK
-PPRWIQ-
-PPRWIF-
QRPRWI--
-QPRWIR-
-QPRWIM-
QMPRWI--
QEPRWI--
-QPRWIV-
QLPRWI--
-QPRWIQ-
-QPRWIH-
QPPRWI--
QGPRWI--
QTPRWI--
QQPRWI--
QIPRWI--
-QPRWIA-

QVPRWI--
QFPRWI--
-QPRWIK-
QYPRWI--
-QPRWIS-
-QPRWIG-
QKPRWI--
-QPRWIT-
QNPRWI--
-QPRWIF-
-QPRWIN-
-QPRWIE-
-QPRWIY-
-QPRWII-
QCPRWI--
RNPRWI--
-RPRWIM-
RIPRWI--
REPRWI--
RQPRWI--
-RPRWIW-
-RPRWIQ-
-RPRWIK-
RDPRWI--
-RPRWIY-
-RPRWIG-
RPPRWI--
-RPRWIN-
-RPRWII-
-RPRWIH-
-RPRWIE-
RRPRWI--
-SPRWIS-
-SPRWIR-
-SPRWIM-
SPPRWI--
STPRWI--
-SPRWIN-
-SPRWIG-
-SPRWIE-
SYPRWI--
SDPRWI--
-SPRWIH-
-SPRWIY-
SAPRWI--
-SPRWIQ-
-SPRWIF-
SKPRWI--
-SPRWIV-
SIPRWI--

-SPRWII-
-SPRWIW-
-SPRWID-
TRPRWI--
TGPRWI--
TQPRWI--
TAPRWI--
TTPRWI--
-TPRWIF-
-TPRWIA-
-TPRWIQ-
TYPRWI--
-TPRWIR-
TKPRWI--
TEPRWI--
-TPRWIN-
-TPRWIM-
-TPRWIG-
TPPRWI--
TIPRWI--
-TPRWII-
-TPRWIH-
-TPRWIT-
-TPRWIS-
-TPRWIK-
-TPRWIE-
TFPRWI--
TVPRWI--
-TPRWID-
-TPRWIV-
TNPRWI--
-TPRWIW-
TDPRWI--
TCPRWI--
VRPRWI--
-VPRWIT-
-VPRWIA-
VSPRWI--
VNPRWI--
-VPRWIR-
VLPRWI--
VEPRWI--
VGPRWI--
VQPRWI--
VDPRWI--
-VPRWIQ-
VFPRWI--
-VPRWIH-
-VPRWIE-
-VPRWIG-

VVPRWI--
-VPRWIF-
-VPRWIM-
-VPRWII-
VPPRWI--
VHPRWI--
VYPRWI--
-VPRWIN-
-VPRWIW-
WTPRWI--
WSPRWI--
WNPRWI--
WVPRWI--
WGPRWI--
WEPRWI--
WHPRWI--
WAPRWI--
WQPRWI--
-WPRWIH-
WDPRWI--
-WPRWIL-
WWPRWI--
-WPRWIQ-
-WPRWII-
WLPRWI--
WKPRWI--
WIPRWI--
YRPRWI--
YKPRWI--
YSPRWI--
-YPRWIK-
YVPRWI--
YIPRWI--
-YPRWIS-
YGPRWI--
-YPRWIR-
-YPRWIG-
-YPRWIM-
YLPRWI--
YHPRWI--
YYPRWI--
YWPRWI--
-YPRWIQ-
-YPRWIT-
YTPRWI--
YEPRWI--
-YPRWIY-
-YPRWIF-
YAPRWI--
-YPRWIW-

				-YPRWII-
				-YPRWIE-
				YMPRWI--
				YDPRWI--
				-YPRWIV-
				-YPRWIH-
				YPPRWI--
				YNPRWI--
				YFPRWI--
				YQPRWI--
PMNM	321	687	48.08	
				--PMNMSS
				ALPMNM--
				-APMNMS-
				-APMNMF-
				AIPMNM--
				AVPMNM--
				-APMNML-
				AGPMNM--
				AFPMNM--
				-APMNMY-
				-APMNMR-
				ARPMNM--
				-APMNMN-
				AKPMNM--
				ANPMNM--
				-APMNMG-
				AYPMNM--
				ATPMNM--
				AQPMNM--
				-APMNMW-
				-APMNMV-
				AMPMNM--
				ADPMNM--
				-APMNMT-
				-APMNMK-
				-APMNMA-
				AHPMNM--
				AEPMNM--
				APPMNM--
				-APMNME-
				-CPMNML-
				CTPMNM--
				-CPMNMY-
				-CPMNMT-
				CCPMNM--
				-CPMNMI-
				CKPMNM--
				CAPMNM--
				CVPMNM--

CSPMNM--
CNPMNM--
-DPMNML-
DNPMNM--
DTPMNM--
-DPMNMR-
DMPMNM--
DYPMNM--
-DPMNMQ-
-DPMNMF-
-DPMNMS-
-DPMNMA-
DLPMNM--
DKPMNM--
DEPMNM--
-DPMNMT-
-DPMNMN-
DIPMNM--
DGPMNM--
DCPMNM--
DAPMNM--
DRPMNM--
-DPMNMH-
-EPMNMV-
-EPMNMS-
-EPMNMT-
-EPMNMH-
-EPMNMF-
EVPMNM--
EYPMNM--
EPPMNM--
-EPMNMM-
ENPMNM--
ELPMNM--
-EPMNMK-
EGPMNM--
-EPMNMQ-
-EPMNMN-
-EPMNMA-
ECPMNM--
FSPMNM--
FTPMNM--
-FPMNMR-
FRPMNM--
-FPMNMY-
FNPMNM--
-FPMNMH-
-FPMNMW-
-FPMNML-
FYPMNM--

-FPMNMN-
-FPMNMA-
-FPMNMV-
-FPMNMQ-
-FPMNMT-
FKPMNM--
-FPMNMS-
FGPMNM--
FAPMNM--
FWPMNM--
-FPMNMI-
-FPMNMF-
FCPMNM--
-GPMNML-
-GPMNMA-
GWPMNM--
-GPMNMG-
GLPMNM--
GEPMNM--
-GPMNMR-
-GPMNMS-
-GPMNMV-
GGPMNM--
GSPMNM--
-GPMNMN-
GRPMNM--
GFPMNM--
-GPMNMH-
GDPMNM--
-GPMNMT-
GNPMNM--
GKPMNM--
GMPMNM--
GAPMNM--
GTPMNM--
-GPMNMM-
-GPMNMK-
GHPMNM--
-HPMNML-
HKPMNM--
HSPMNM--
HRPMNM--
HAPMNM--
-HPMNME-
-HPMNMV-
HWPMNM--
HFPMNM--
-HPMNMS-
-HPMNMT-
-HPMNMI-

-HPMNMQ-
-HPMNMN-
HYPMNM--
HMPMNM--
-HPMNMH-
-HPMNMG-
HLPMNM--
HTPMNM--
-HPMNMY-
-HPMNMR-
-HPMNMF-
HNPMNM--
HPPMNM--
-HPMNMA-
HGPMNM--
INPMNM--
IRPMNM--
-IPMNMV-
-IPMNMR-
IKPMNM--
-IPMNML-
-IPMNMY-
ISPMNM--
-IPMNMT-
IMPMNM--
IEPMNM--
IVPMNM--
IDPMNM--
IWPMNM--
ITPMNM--
-IPMNMA-
ILPMNM--
-IPMNMS-
-IPMNMI-
-IPMNMN-
-IPMNMH-
-IPMNMG-
IGPMNM--
KSPMNM--
-KPMNMR-
KLPMNM--
KGPMNM--
-KPMNMN-
KVPMNM--
KYPMNM--
KTPMNM--
-KPMNMW-
-KPMNMV-
KDPMNM--
KAPMNM--

KRPMNM--
KQPMNM--
-KPMNMY-
-KPMNMH-
-KPMNMG-
-KPMNMM-
-KPMNMA-
-KPMNMT-
-KPMNML-
KIPMNM--
KHPMNM--
-LPMNMS-
-LPMNMR-
LGPMNM--
LRPMNM--
LFPMNM--
LKPMNM--
LHPMNM--
LSPMNM--
-LPMNML-
LLPMNM--
-LPMNMM-
LTPMNM--
LNPMNM--
LEPMNM--
-LPMNMA-
LQPMNM--
-LPMNMT-
-LPMNMQ-
-LPMNMW-
-LPMNMV-
-LPMNMK-
-LPMNMF-
LAPMNM--
LVPMNM--
LIPMNM--
-LPMNMN-
-LPMNMG-
LYPMNM--
-LPMNMH-
LWPMNM--
-LPMNMD-
LDPMNM--
-MPMNMR-
MVPMNM--
MYPMNM--
MQPMNM--
MPPMNM--
MNPMNM--
MFPMNM--

MHPMNM--
-MPMNMS-
-MPMNME-
MAPMNM--
-MPMNML-
MWPMNM--
-MPMNMN-
MLPMNM--
MGPMNM--
-MPMNMM-
-MPMNMA-
MSPMNM--
MRPMNM--
-MPMNMH-
MEPMNM--
-NPMNML-
NLPMNM--
NGPMNM--
NSPMNM--
NHPMNM--
NPPMNM--
-NPMNMY-
-NPMNMQ-
NTPMNM--
-NPMNMS-
NYPMNM--
NQPMNM--
NMPMNM--
-NPMNMG-
NDPMNM--
-NPMNMN-
-NPMNMA-
NFPMNM--
-NPMNMH-
-NPMNMF-
-NPMNME-
NNPMNM--
NEPMNM--
--PMNMRL
--PMNMVR
--PMNMRI
--PMNMYR
--PMNMLT
--PMNMAY
--PMNMGS
--PMNMLR
--PMNMLG
--PMNMVW
--PMNMWT
--PMNMSG

--PMNMRY
--PMNMSL
--PMNMYA
--PMNMVG
--PMNMLN
--PMNMSH
--PMNMMT
--PMNMTH
--PMNMRE
--PMNMIQ
--PMNMWV
--PMNMQN
--PMNMVQ
--PMNMKT
--PMNMYS
--PMNMTT
--PMNMTI
--PMNMTA
--PMNMMS
--PMNMLH
--PMNMAS
--PMNMTR
--PMNMTD
--PMNMSF
--PMNMRW
--PMNMGV
--PMNMFH
--PMNMYV
--PMNMYM
--PMNMYK
--PMNMTL
--PMNMSV
--PMNMLD
--PMNMIR
--PMNMRN
--PMNMNM
--PMNMVL
--PMNMIW
--PMNMIA
--PMNMHL
--PMNMVD
--PMNMSA
--PMNMQY
--PMNMLQ
--PMNMKN
--PMNMIY
--PMNMHR
--PMNMAI
PWPMNM--
--PMNMYE

--PMNMVA
--PMNMTS
--PMNMSY
--PMNMSM
--PMNMMR
--PMNMKM
--PMNMES
PIPMNM--
--PMNMWL
--PMNMRG
--PMNMRD
--PMNMLM
--PMNMKF
--PMNMGM
--PMNMGI
--PMNMWK
--PMNMTN
--PMNMSK
--PMNMSI
--PMNMRQ
--PMNMQS
--PMNMQA
--PMNMMF
--PMNMLV
--PMNMLL
--PMNMKW
--PMNMKV
--PMNMKH
--PMNMHK
--PMNMGR
--PMNMFT
--PMNMFR
--PMNMAL
--PMNMWI
--PMNMWH
--PMNMVF
--PMNMTM
--PMNMSN
--PMNMRT
--PMNMRR
--PMNMNT
--PMNMMW
--PMNMKI
--PMNMIS
--PMNMGT
--PMNMGL
--PMNMFS
--PMNMEG
--PMNMYD
--PMNMWG

--PMNMVV
--PMNMVT
--PMNMVK
--PMNMLK
--PMNMKQ
--PMNMKL
--PMNMIV
--PMNMGK
--PMNMGF
--PMNMEQ
--PMNMAR
--PMNMAH
--PMNMAF
--PMNMAA
--PMNMYH
--PMNMYG
--PMNMQH
--PMNMNS
--PMNMNN
--PMNMNH
--PMNMMD
--PMNMHS
--PMNMGY
--PMNMGN
--PMNMGG
--PMNMFG
--PMNMEL
--PMNMDS
--PMNMAK
PDPMNM--
PQPMNM--
--PMNMVS
--PMNMTK
--PMNMTF
--PMNMSQ
--PMNMQR
--PMNMNW
--PMNMNL
--PMNMMV
--PMNMLW
--PMNMIM
--PMNMIG
--PMNMHF
--PMNMFY
--PMNMAQ
PTPMNM--
-PPMNMR-
--PMNMYQ
--PMNMWW
--PMNMWR

--PMNMWF
--PMNMVH
--PMNMTV
--PMNMSD
--PMNMRM
--PMNMQT
--PMNMQG
--PMNMPR
--PMNMNR
--PMNMNQ
--PMNMMI
--PMNMLY
--PMNMKS
--PMNMKG
--PMNMIK
--PMNMHY
--PMNMHV
--PMNMGW
--PMNMGQ
--PMNMGH
--PMNMFN
--PMNMFD
--PMNMEY
--PMNMEM
--PMNMDA
--PMNMCL
--PMNMAW
--PMNMAV
--PMNMAN
PYPMNM--
--PMNMYY
--PMNMYW
--PMNMWD
--PMNMVP
--PMNMVM
--PMNMTG
--PMNMST
--PMNMRS
--PMNMRP
--PMNMRH
--PMNMQV
--PMNMQL
--PMNMQF
--PMNMPV
--PMNMNK
--PMNMMY
--PMNMMM
--PMNMLE
--PMNMKK
--PMNMKD

--PMNMIF
--PMNMHT
--PMNMGD
--PMNMGC
--PMNMEV
--PMNMDL
--PMNMCT
--PMNMAT
--PMNMAG
PGPMNM--
QAPMNM--
-QPMNMS-
QNPMNM--
-QPMNMR-
-QPMNMH-
QIPMNM--
QLPMNM--
-QPMNML-
-QPMNMT-
-QPMNMF-
QYPMNM--
QVPMNM--
-QPMNMG-
-QPMNMM-
QHPMNM--
QDPMNM--
-QPMNMV-
QGPMNM--
QFPMNM--
QWPMNM--
-QPMNMN-
-QPMNMI-
-RPMNMR-
RHPMNM--
RLPMNM--
RSPMNM--
-RPMNMS-
-RPMNMM-
-RPMNML-
-RPMNMY-
-RPMNMV-
RGPMNM--
-RPMNMK-
-RPMNMW-
-RPMNMI-
-RPMNMG-
-RPMNMF-
RWPMNM--
-RPMNMT-
RFPMNM--

RAPMNM--
RPPMNM--
-RPMNME-
-RPMNMA-
RRPMNM--
RYPMNM--
-RPMNMH-
-RPMNMQ-
REPMNM--
RVPMNM--
RIPMNM--
-RPMNMN-
RQPMNM--
-RPMNMD-
-SPMNMV-
-SPMNMH-
SSPMNM--
-SPMNMW-
-SPMNMS-
SMPMNM--
-SPMNML-
SLPMNM--
SEPMNM--
SHPMNM--
SWPMNM--
-SPMNMM-
SQPMNM--
-SPMNMY-
-SPMNMR-
-SPMNMQ-
SRPMNM--
-SPMNMA-
SVPMNM--
STPMNM--
-SPMNME-
SNPMNM--
SFPMNM--
SYPMNM--
-SPMNMK-
SGPMNM--
SPPMNM--
SCPMNM--
-TPMNML-
-TPMNMR-
TYPMNM--
TGPMNM--
-TPMNMT-
-TPMNMQ-
-TPMNMK-
TTPMNM--

-TPMNMY-
TEPMNM--
TVPMNM--
-TPMNMV-
-TPMNMH-
TNPMNM--
TMPMNM--
TLPMNM--
-TPMNME-
-TPMNMW-
-TPMNMS-
-TPMNMG-
TKPMNM--
-TPMNMI-
-TPMNMM-
-TPMNMD-
TIPMNM--
THPMNM--
TDPMNM--
-VPMNMM-
VSPMNM--
-VPMNMR-
VVPMNM--
VKPMNM--
VNPMNM--
VDPMNM--
VLPMNM--
-VPMNMW-
VGPMNM--
-VPMNMA-
-VPMNMY-
-VPMNMV-
-VPMNMI-
-VPMNMQ-
VIPMNM--
VRPMNM--
VHPMNM--
-VPMNMT-
VWPMNM--
-VPMNMN-
-VPMNMG-
VAPMNM--
-VPMNML-
VQPMNM--
-VPMNMS-
-VPMNMD-
-VPMNMH-
VMPMNM--
VFPMNM--
-WPMNMR-

-WPMNML-
-WPMNMS-
WDPMNM--
WPPMNM--
WGPMNM--
WLPMNM--
WSPMNM--
WRPMNM--
-WPMNMQ-
-WPMNMI-
WQPMNM--
-WPMNMM-
-WPMNMF-
WMPMNM--
WTPMNM--
WNPMNM--
WHPMNM--
-WPMNME-
WKPMNM--
-WPMNMG-
-WPMNMV-
WAPMNM--
YRPMNM--
-YPMNMA-
-YPMNMN-
-YPMNMI-
YLPMNM--
YAPMNM--
YGPMNM--
YQPMNM--
-YPMNMT-
-YPMNMS-
YKPMNM--
-YPMNMQ-
YIPMNM--
-YPMNMW-
-YPMNMR-
-YPMNMH-
YNPMNM--
YHPMNM--
YFPMNM--
YVPMNM--
YPPMNM--
-YPMNMF-
YYPMNM--
YTPMNM--
-YPMNML-
-YPMNMC-

PWNY 376 668 48.07

-GPWNYS-

ASPWNY--
ARPWNY--
AVPWNY--
AQPWNY--
AGPWNY--
APPWNY--
-APWNYM-
AAPWNY--
-APWNYE-
-APWNYQ-
-APWNYT-
ALPWNY--
-APWNYS-
-APWNYI-
AFPWNY--
-APWNYY-
AMPWNY--
-APWNYW-
-APWNYF-
AIPWNY--
AHPWNY--
AEPWNY--
AWPWNY--
-APWNYH-
AKPWNY--
AYPWNY--
-APWNYR-
ATPWNY--
-APWNYK-
-APWNYG-
-APWNYD-
-APWNYA-
ANPWNY--
ADPWNY--
CSPWNY--
-CPWNYL-
CGPWNY--
CRPWNY--
DHPWNY--
DAPWNY--
-DPWNYI-
DRPWNY--
DEPWNY--
-DPWNYS-
DFPWNY--
-DPWNYW-
-DPWNYY-
-DPWNYL-
-DPWNYV-
DLPWNY--

DVPWNY--
-DPWNYF-
DWPWNY--
DTPWNY--
-DPWNYK-
-DPWNYH-
DNPWNY--
DIPWNY--
ESPWNY--
EVPWNY--
-EPWNYY-
-EPWNYV-
EGPWNY--
-EPWNYW-
-EPWNYL-
ERPWNY--
-EPWNYF-
EAPWNY--
EQPWNY--
-EPWNYR-
-EPWNYH-
EIPWNY--
EEPWNY--
-EPWNYA-
-EPWNYS-
-EPWNYQ-
-EPWNYG-
EYPWNY--
-EPWNYN-
-EPWNYK-
EDPWNY--
FNPWNY--
-FPWNYR-
FQPWNY--
-FPWNYY-
-FPWNYK-
-FPWNYV-
FRPWNY--
-FPWNYS-
-FPWNYH-
FSPWNY--
FHPWNY--
FGPWNY--
FVPWNY--
-FPWNYM-
FAPWNY--
-FPWNYQ-
-FPWNYG-
-FPWNYF-
-FPWNYE-

-GPWNYL-
-GPWNYR-
GSPWNY--
GLPWNY--
GEPWNY--
GRPWNY--
-GPWNYH-
-GPWNYA-
-GPWNYV-
GFPWNY--
-GPWNYK-
GVPWNY--
-GPWNYT-
-GPWNYI-
GHPWNY--
GGPWNY--
-GPWNYM-
GMPWNY--
-GPWNYY-
-GPWNYW-
GKPWNY--
GYPWNY--
GTPWNY--
-GPWNYG-
GNPWNY--
GWPWNY--
-GPWNYN-
-GPWNYF-
GAPWNY--
HRPWNY--
HGPWNY--
HSPWNY--
HNPWNY--
-HPWNYT-
-HPWNYL-
-HPWNYY-
HPPWNY--
HKPWNY--
HIPWNY--
-HPWNYQ-
-HPWNYV-
-HPWNYK-
-HPWNYI-
HEPWNY--
HTPWNY--
-HPWNYS-
HMPWNY--
HYPWNY--
-HPWNYR-
-HPWNYM-

HVPWNY--
HQPWNY--
HDPWNY--
-HPWNYG-
HLPWNY--
HWPWNY--
HFPWNY--
IPPWNY--
ITPWNY--
IQPWNY--
ISPWNY--
-IPWNYI-
INPWNY--
IWPWNY--
IVPWNY--
-IPWNYS-
IEPWNY--
IAPWNY--
-IPWNYR-
-IPWNYL-
IKPWNY--
-IPWNYQ-
-IPWNYG-
-IPWNYD-
IGPWNY--
IDPWNY--
-IPWNYK-
-IPWNYT-
-IPWNYH-
IMPWNY--
IHPWNY--
IFPWNY--
KSPWNY--
KGPWNY--
-KPWNYL-
-KPWNYT-
KQPWNY--
KNPWNY--
-KPWNYV-
KLPWNY--
-KPWNYI-
KRPWNY--
-KPWNYN-
KFPWNY--
KYPWNY--
-KPWNYR-
KVPWNY--
-KPWNYS-
KDPWNY--
KAPWNY--

-KPWNYY-
-KPWNYQ-
-KPWNYW-
-KPWNYK-
-KPWNYH-
KEPWNY--
KKPWNY--
-KPWNYM-
-KPWNYG-
-KPWNYD-
-KPWNYA-
KMPWNY--
KHPWNY--
-LPWNYV-
-LPWNYS-
LLPWNY--
LDPWNY--
LAPWNY--
-LPWNYI-
LFPWNY--
-LPWNYL-
LPPWNY--
LWPWNY--
LSPWNY--
LGPWNY--
LTPWNY--
LRPWNY--
-LPWNYA-
LMPWNY--
-LPWNYT-
LVPWNY--
-LPWNYM-
-LPWNYK-
LYPWNY--
-LPWNYW-
-LPWNYQ-
-LPWNYF-
-LPWNYE-
LNPWNY--
LCPWNY--
MKPWNY--
MHPWNY--
MQPWNY--
MGPWNY--
-MPWNYR-
MWPWNY--
MVPWNY--
MNPWNY--
MEPWNY--
MAPWNY--

MMPWNY--
MLPWNY--
-MPWNYA-
MIPWNY--
-MPWNYY-
-MPWNYT-
-MPWNYN-
MTPWNY--
MSPWNY--
MRPWNY--
-MPWNYS-
-MPWNYM-
MFPWNY--
-NPWNYM-
NLPWNY--
NGPWNY--
NTPWNY--
-NPWNYR-
-NPWNYI-
NRPWNY--
NPPWNY--
-NPWNYS-
NSPWNY--
NYPWNY--
NQPWNY--
-NPWNYA-
NHPWNY--
-NPWNYT-
NNPWNY--
NKPWNY--
NVPWNY--
-NPWNYF-
NMPWNY--
-NPWNYL-
-NPWNYW-
-NPWNYH-
NAPWNY--
NWPWNY--
-NPWNYG-
-NPWNYE-
-NPWNYC-
NEPWNY--
--PWNYLS
PAPWNY--
--PWNYSS
--PWNYLG
--PWNYTL
--PWNYES
--PWNYTS
--PWNYKG

--PWNYTR
--PWNYRA
--PWNYLH
--PWNYLQ
PSPWNY--
--PWNYYR
--PWNYTG
--PWNYTH
--PWNYMR
--PWNYME
--PWNYYQ
--PWNYRG
--PWNYFR
--PWNYVM
--PWNYFT
--PWNYRF
--PWNYQY
PQPWNY--
--PWNYVR
--PWNYRD
--PWNYIN
--PWNYAG
--PWNYTQ
--PWNYGV
--PWNYGS
PGPWNY--
--PWNYST
--PWNYSF
--PWNYRS
--PWNYRL
--PWNYKV
--PWNYAV
--PWNYVS
--PWNYNS
--PWNYKR
--PWNYAA
--PWNYHA
--PWNYGT
--PWNYEV
--PWNYAL
PKPWNY--
--PWNYSQ
--PWNYRT
--PWNYRI
--PWNYQA
--PWNYMA
--PWNYLK
--PWNYLI
PEPWNY--
--PWNYYT

--PWNYYK
--PWNYTV
--PWNYSM
--PWNYQV
--PWNYQR
--PWNYQL
--PWNYQF
--PWNYNG
--PWNYGA
PNPWNY--
--PWNYYY
--PWNYWT
--PWNYVQ
--PWNYVH
--PWNYTK
--PWNYTD
--PWNYSL
--PWNYQK
--PWNYLT
--PWNYKT
--PWNYIM
--PWNYHQ
PTPWNY--
--PWNYYM
--PWNYWH
--PWNYTT
--PWNYTI
--PWNYSN
--PWNYSD
--PWNYMS
--PWNYKN
--PWNYKL
--PWNYIA
--PWNYHS
--PWNYGG
--PWNYFS
--PWNYFN
--PWNYFM
--PWNYFD
PYPWNY--
--PWNYTF
--PWNYRV
--PWNYRN
--PWNYMD
--PWNYIH
--PWNYHT
--PWNYHI
--PWNYHF
--PWNYDA
--PWNYAS

PHPWNY--
--PWNYYN
--PWNYVN
--PWNYVK
--PWNYVG
--PWNYVA
--PWNYRM
--PWNYRH
--PWNYNM
--PWNYMT
--PWNYML
--PWNYLE
--PWNYIS
--PWNYEA
--PWNYAT
--PWNYAM
--PWNYAI
--PWNYWL
--PWNYWD
--PWNYVW
--PWNYVV
--PWNYSR
--PWNYSH
--PWNYSE
--PWNYRY
--PWNYQS
--PWNYQN
--PWNYMV
--PWNYKK
--PWNYKI
--PWNYIR
--PWNYHV
--PWNYFG
--PWNYEL
--PWNYEG
--PWNYAK
--PWNYYV
--PWNYYG
--PWNYYD
--PWNYYA
--PWNYWR
--PWNYWK
--PWNYTM
--PWNYSY
--PWNYSG
--PWNYSA
--PWNYQT
--PWNYNV
--PWNYNH
--PWNYKS

--PWNYIT
--PWNYHY
--PWNYGR
--PWNYGN
--PWNYEQ
--PWNYAW
PIPWNY--
--PWNYYW
--PWNYYS
--PWNYWG
--PWNYVT
--PWNYVI
--PWNYVE
--PWNYTY
--PWNYSW
--PWNYRK
--PWNYNY
--PWNYNR
--PWNYNQ
--PWNYMQ
--PWNYLY
--PWNYKQ
--PWNYIK
--PWNYIG
--PWNYHM
--PWNYHK
--PWNYER
--PWNYEF
--PWNYDT
--PWNYAQ
PVPWNY--
PMPWNY--
PFPWNY--
PDPWNY--
-QPWNYQ-
-QPWNYY-
-QPWNYS-
-QPWNYI-
QDPWNY--
QWPWNY--
QKPWNY--
QLPWNY--
-QPWNYV-
-QPWNYE-
QMPWNY--
QYPWNY--
QRPWNY--
-QPWNYM-
-QPWNYG-
-QPWNYF-

QSPWNY--
QGPWNY--
-QPWNYT-
-QPWNYR-
-QPWNYL-
-QPWNYH-
QHPWNY--
RSPWNY--
RGPWNY--
-RPWNYS-
RYPWNY--
-RPWNYI-
-RPWNYV-
-RPWNYR-
RVPWNY--
RQPWNY--
-RPWNYW-
-RPWNYG-
RLPWNY--
-RPWNYY-
RAPWNY--
RKPWNY--
-RPWNYM-
RTPWNY--
RWPWNY--
-RPWNYT-
RNPWNY--
RMPWNY--
-RPWNYQ-
-RPWNYL-
RDPWNY--
REPWNY--
-RPWNYF-
RHPWNY--
-RPWNYK-
RIPWNY--
-RPWNYH-
-RPWNYA-
RPPWNY--
RRPWNY--
-RPWNYN-
-SPWNYS-
SRPWNY--
-SPWNYR-
SLPWNY--
SVPWNY--
-SPWNYM-
-SPWNYA-
SGPWNY--
SKPWNY--

SSPWNY--
-SPWNYI-
SMPWNY--
-SPWNYQ-
SHPWNY--
-SPWNYW-
-SPWNYL-
-SPWNYE-
SFPWNY--
SYPWNY--
-SPWNYF-
SIPWNY--
STPWNY--
SNPWNY--
SAPWNY--
SQPWNY--
-SPWNYK-
-SPWNYT-
SEPWNY--
-SPWNYV-
-SPWNYN-
SPPWNY--
SDPWNY--
-TPWNYM-
TFPWNY--
TNPWNY--
-TPWNYT-
-TPWNYL-
TRPWNY--
-TPWNYR-
-TPWNYS-
TGPWNY--
-TPWNYW-
TEPWNY--
THPWNY--
TQPWNY--
TKPWNY--
TSPWNY--
TAPWNY--
TTPWNY--
-TPWNYN-
-TPWNYH-
TVPWNY--
-TPWNYQ-
TPPWNY--
TLPWNY--
-TPWNYA-
TCPWNY--
TYPWNY--
TWPWNY--

-TPWNYG-
-TPWNYE-
TDPWNY--
VMPWNY--
VRPWNY--
VEPWNY--
VSPWNY--
VTPWNY--
-VPWNYS-
VPPWNY--
VGPWNY--
-VPWNYM-
-VPWNYA-
-VPWNYY-
-VPWNYQ-
VVPWNY--
-VPWNYV-
VWPWNY--
-VPWNYI-
VAPWNY--
VQPWNY--
-VPWNYH-
VFPWNY--
VYPWNY--
-VPWNYR-
-VPWNYN-
-VPWNYG-
-VPWNYF-
VNPWNY--
-VPWNYT-
VKPWNY--
VCPWNY--
WSPWNY--
-WPWNYY-
-WPWNYI-
-WPWNYA-
WMPWNY--
WRPWNY--
WFPWNY--
-WPWNYQ-
-WPWNYK-
WIPWNY--
WEPWNY--
-WPWNYM-
-WPWNYR-
-WPWNYG-
YKPWNY--
-YPWNYR-
YSPWNY--
-YPWNYT-

YGPWNY--
YYPWNY--
YRPWNY--
-YPWNYM-
YNPWNY--
-YPWNYS-
YAPWNY--
YLPWNY--
YTPWNY--
YQPWNY--
-YPWNYQ-
-YPWNYL-
-YPWNYK-
YDPWNY--
YVPWNY--
-YPWNYG-
YHPWNY--

PFGL	31	868	48.06

AHPFGL--
-DPFGLK-
-GPFGLP-
KMPFGL--
--PFGLPS
--PFGLPL
--PFGLHP
--PFGLGK
-RPFGLD-
-APFGLR-
ASPFGL--
-APFGLS-
-APFGLA-
AVPFGL--
ATPFGL--
-APFGLL-
ARPFGL--
-APFGLK-
AKPFGL--
AGPFGL--
AFPFGL--
AQPFGL--
ALPFGL--
-APFGLN-
AAPFGL--
-APFGLE-
-APFGLV-
ANPFGL--
AEPFGL--
-APFGLY-
AYPFGL--
-APFGLT-

-APFGLI-
-APFGLG-
-APFGLW-
-APFGLH-
AIPFGL--
AMPFGL--
AWPFGL--
-APFGLQ-
APPFGL--
ADPFGL--
-APFGLF-
-APFGLM-
ACPFGL--
-APFGLD-
-APFGLC-
-CPFGLS-
CNPFGL--
CTPFGL--
-CPFGLM-
CAPFGL--
CKPFGL--
CVPFGL--
-CPFGLL-
CSPFGL--
-CPFGLQ-
-CPFGLV-
-CPFGLK-
-CPFGLT-
-CPFGLR-
CCPFGL--
CRPFGL--
-CPFGLA-
CGPFGL--
CYPFGL--
-CPFGLW-
CEPFGL--
CQPFGL--
-CPFGLN-
-CPFGLI-
CMPFGL--
CLPFGL--
-DPFGLT-
DSPFGL--
-DPFGLR-
DKPFGL--
DMPFGL--
DRPFGL--
DHPFGL--
DAPFGL--
DTPFGL--

```
-DPFGLH-
DLPFGL--
-DPFGLY-
DFPFGL--
-DPFGLI-
DEPFGL--
-DPFGLS-
DNPFGL--
DIPFGL--
-DPFGLW-
DGPFGL--
DYPFGL--
-DPFGLQ-
-DPFGLN-
-DPFGLA-
DQPFGL--
DWPFGL--
-DPFGLM-
DDPFGL--
-EPFGLR-
-EPFGLV-
EGPFGL--
ERPFGL--
-EPFGLS-
-EPFGLL-
-EPFGLT-
EMPFGL--
-EPFGLI-
-EPFGLM-
-EPFGLK-
-EPFGLW-
-EPFGLH-
-EPFGLA-
-EPFGLF-
EFPFGL--
-EPFGLG-
-EPFGLE-
EKPFGL--
ETPFGL--
EHPFGL--
EEPFGL--
EAPFGL--
EQPFGL--
-EPFGLQ-
ENPFGL--
-EPFGLN-
EWPFGL--
ELPFGL--
EIPFGL--
ECPFGL--
```

FRPFGL--
-FPFGLS-
FVPFGL--
FTPFGL--
-FPFGLL-
FKPFGL--
FSPFGL--
FQPFGL--
-FPFGLQ-
FLPFGL--
-FPFGLR-
FHPFGL--
FFPFGL--
FPPFGL--
-FPFGLV-
-FPFGLH-
FGPFGL--
-FPFGLK-
-FPFGLE-
FEPFGL--
-FPFGLY-
FWPFGL--
-FPFGLN-
-FPFGLG-
-FPFGLA-
FDPFGL--
FNPFGL--
FIPFGL--
-FPFGLI-
-FPFGLM-
FMPFGL--
GRPFGL--
-GPFGLR-
-GPFGLS-
GIPFGL--
GTPFGL--
GHPFGL--
GFPFGL--
GVPFGL--
GKPFGL--
-GPFGLG-
GSPFGL--
-GPFGLI-
GDPFGL--
-GPFGLA-
GLPFGL--
GAPFGL--
-GPFGLM-
GMPFGL--
-GPFGLT-

-GPFGLQ-
-GPFGLF-
-GPFGLE-
GWPFGL--
GYPFGL--
GQPFGL--
-GPFGLH-
-GPFGLW-
-GPFGLN-
-GPFGLV-
-GPFGLK-
GEPFGL--
GNPFGL--
GCPFGL--
-GPFGLC-
-HPFGLL-
-HPFGLQ-
-HPFGLS-
HGPFGL--
HSPFGL--
HFPFGL--
-HPFGLK-
HTPFGL--
-HPFGLG-
HMPFGL--
-HPFGLH-
HRPFGL--
HLPFGL--
HKPFGL--
HWPFGL--
HIPFGL--
-HPFGLF-
-HPFGLV-
-HPFGLN-
-HPFGLI-
HVPFGL--
-HPFGLW-
-HPFGLR-
HPPFGL--
HYPFGL--
-HPFGLE-
HNPFGL--
-HPFGLY-
HHPFGL--
HAPFGL--
HEPFGL--
HCPFGL--
HQPFGL--
-HPFGLA-
HDPFGL--

ITPFGL--
-IPFGLS-
IHPFGL--
ISPFGL--
-IPFGLR-
-IPFGLV-
IKPFGL--
IMPFGL--
IPPFGL--
IAPFGL--
ILPFGL--
IRPFGL--
-IPFGLK-
IFPFGL--
-IPFGLN-
-IPFGLL-
-IPFGLM-
-IPFGLF-
-IPFGLG-
IWPFGL--
IGPFGL--
-IPFGLQ-
IQPFGL--
INPFGL--
IVPFGL--
-IPFGLE-
IIPFGL--
IYPFGL--
-IPFGLT-
-IPFGLI-
IDPFGL--
-IPFGLH-
IEPFGL--
ICPFGL--
KSPFGL--
KPPFGL--
-KPFGLS-
-KPFGLV-
-KPFGLR-
KLPFGL--
-KPFGLQ-
-KPFGLT-
KGPFGL--
KEPFGL--
KAPFGL--
KIPFGL--
KYPFGL--
KTPFGL--
-KPFGLL-
-KPFGLN-

```
-KPFGLI-
-KPFGLH-
-KPFGLY-
KNPFGL--
KFPFGL--
-KPFGLE-
KWPFGL--
KHPFGL--
KDPFGL--
-KPFGLM-
KKPFGL--
-KPFGLK-
-KPFGLF-
KCPFGL--
LRPFGL--
LVPFGL--
LLPFGL--
-LPFGLL-
LKPFGL--
-LPFGLK-
-LPFGLS-
LTPFGL--
LYPFGL--
-LPFGLH-
LFPFGL--
LDPFGL--
LAPFGL--
LMPFGL--
-LPFGLN-
LHPFGL--
LEPFGL--
-LPFGLQ-
-LPFGLV-
-LPFGLA-
LWPFGL--
LIPFGL--
-LPFGLT-
-LPFGLM-
-LPFGLF-
-LPFGLE-
MRPFGL--
-MPFGLR-
MKPFGL--
MSPFGL--
MAPFGL--
-MPFGLL-
MIPFGL--
-MPFGLY-
MFPFGL--
MYPFGL--
```

MNPFGL--
MGPFGL--
-MPFGLE-
-MPFGLQ-
MPPFGL--
-MPFGLN-
-MPFGLV-
-MPFGLA-
MLPFGL--
-MPFGLM-
-MPFGLK-
MMPFGL--
-MPFGLS-
MWPFGL--
MTPFGL--
-MPFGLG-
-MPFGLF-
MEPFGL--
-MPFGLH-
MCPFGL--
MQPFGL--
MHPFGL--
MDPFGL--
NRPFGL--
NPPFGL--
NTPFGL--
NLPFGL--
NSPFGL--
-NPFGLR-
-NPFGLL-
NQPFGL--
-NPFGLT-
NWPFGL--
NHPFGL--
-NPFGLH-
-NPFGLQ-
NAPFGL--
-NPFGLA-
-NPFGLV-
-NPFGLI-
NKPFGL--
NNPFGL--
-NPFGLN-
NGPFGL--
NDPFGL--
-NPFGLF-
-NPFGLE-
NMPFGL--
NVPFGL--
NCPFGL--

NIPFGL--
--PFGLSS
--PFGLSR
--PFGLRR
PRPFGL--
--PFGLRH
--PFGLSV
--PFGLTR
--PFGLVR
--PFGLLR
--PFGLVS
--PFGLAS
--PFGLST
--PFGLLG
--PFGLTS
--PFGLGS
--PFGLFR
--PFGLRV
--PFGLRL
--PFGLRT
--PFGLAY
--PFGLSK
--PFGLHT
--PFGLAT
--PFGLRG
--PFGLLT
--PFGLYR
--PFGLHR
--PFGLTK
--PFGLTG
PYPFGL--
--PFGLNS
--PFGLIQ
--PFGLTL
--PFGLSI
--PFGLIR
--PFGLAG
PAPFGL--
--PFGLRF
--PFGLLA
--PFGLRQ
--PFGLNT
--PFGLII
--PFGLKV
--PFGLIS
--PFGLEV
PLPFGL--
--PFGLYS
--PFGLTN
PTPFGL--

--PFGLTH
--PFGLKG
--PFGLTA
--PFGLMS
--PFGLMK
--PFGLKN
--PFGLKH
--PFGLSH
--PFGLRD
--PFGLKK
--PFGLIG
--PFGLRK
--PFGLQF
--PFGLLN
--PFGLGT
--PFGLFS
--PFGLWG
--PFGLVD
--PFGLVQ
--PFGLTV
--PFGLHH
--PFGLVV
--PFGLVI
--PFGLSN
--PFGLSG
--PFGLQS
--PFGLLQ
--PFGLKT
PFPFGL--
PMPFGL--
--PFGLYT
--PFGLYG
--PFGLWQ
--PFGLAV
--PFGLQR
--PFGLNA
--PFGLAL
--PFGLVM
--PFGLKQ
--PFGLVH
--PFGLIT
--PFGLWE
--PFGLRY
--PFGLRW
--PFGLMQ
--PFGLMM
--PFGLHG
--PFGLGA
--PFGLFQ
--PFGLFE

--PFGLVA
--PFGLRN
--PFGLNG
--PFGLMH
--PFGLKF
--PFGLIW
--PFGLIK
--PFGLGV
--PFGLGG
PIPFGL--
--PFGLTI
--PFGLNR
--PFGLLH
--PFGLKS
--PFGLKA
--PFGLFK
--PFGLWY
--PFGLSE
--PFGLMT
--PFGLHY
--PFGLAN
--PFGLRM
--PFGLMN
--PFGLHS
--PFGLES
--PFGLTQ
--PFGLQL
--PFGLIA
--PFGLAK
--PFGLAA
PSPFGL--
--PFGLYQ
--PFGLWK
--PFGLSA
--PFGLNV
--PFGLKI
--PFGLKE
--PFGLKD
--PFGLAM
--PFGLNN
--PFGLNF
--PFGLID
--PFGLHF
PDPFGL--
PVPFGL--
--PFGLYK
--PFGLVK
--PFGLTT
--PFGLSF
--PFGLQK

--PFGLMD
--PFGLKY
--PFGLGI
--PFGLEA
--PFGLAI
--PFGLNW
--PFGLWV
--PFGLWR
--PFGLTF
--PFGLSQ
--PFGLSD
--PFGLLE
--PFGLIY
--PFGLHM
--PFGLHK
--PFGLGN
--PFGLAR
PNPFGL--
--PFGLYN
--PFGLSW
--PFGLQN
--PFGLQA
--PFGLNH
--PFGLMA
--PFGLHD
--PFGLET
--PFGLAH
--PFGLTM
--PFGLQG
--PFGLLD
--PFGLKM
--PFGLVN
--PFGLQI
--PFGLQE
--PFGLNK
--PFGLHA
--PFGLRE
--PFGLIE
--PFGLGM
--PFGLFT
--PFGLER
--PFGLVE
--PFGLTE
--PFGLQD
--PFGLNY
--PFGLNQ
--PFGLMG
--PFGLEK
PQPFGL--
PKPFGL--

--PFGLVW
--PFGLNI
--PFGLHL
--PFGLGW
--PFGLGF
--PFGLFH
--PFGLDG
--PFGLAD
--PFGLGR
--PFGLGH
--PFGLDS
--PFGLDA
PGPFGL--
--PFGLYE
--PFGLWH
--PFGLWD
--PFGLRP
--PFGLIN
--PFGLIM
--PFGLGY
--PFGLFN
--PFGLEI
--PFGLEG
PEPFGL--
PHPFGL--
--PFGLYH
--PFGLTW
--PFGLTD
--PFGLQH
--PFGLMW
--PFGLME
--PFGLAQ
--PFGLWN
--PFGLQY
--PFGLKW
--PFGLGQ
--PFGLFD
--PFGLEF
--PFGLQV
--PFGLHQ
--PFGLHN
--PFGLEQ
--PFGLEM
--PFGLMV
--PFGLHV
--PFGLHI
--PFGLEN
--PFGLEL
PWPFGL--
--PFGLYD

--PFGLVC
--PFGLQW
--PFGLQM
--PFGLPG
--PFGLPA
--PFGLMY
--PFGLFM
--PFGLAC
-PPFGLL-
--PFGLNE
--PFGLGP
--PFGLGC
--PFGLFW
--PFGLEY
--PFGLEE
--PFGLED
--PFGLDW
--PFGLCR
--PFGLCI
--PFGLCF
PCPFGL--
QRPFGL--
QPPFGL--
-QPFGLS-
-QPFGLI-
QQPFGL--
-QPFGLV-
QGPFGL--
-QPFGLN-
QLPFGL--
-QPFGLY-
-QPFGLT-
QSPFGL--
QKPFGL--
QTPFGL--
-QPFGLM-
-QPFGLL-
-QPFGLK-
-QPFGLH-
QWPFGL--
QVPFGL--
QMPFGL--
QIPFGL--
QYPFGL--
-QPFGLG-
QEPFGL--
-QPFGLQ-
QDPFGL--
-QPFGLE-
QAPFGL--

−QPFGLW−
−QPFGLA−
QNPFGL−−
QHPFGL−−
−RPFGLR−
RVPFGL−−
−RPFGLS−
RLPFGL−−
−RPFGLN−
RGPFGL−−
RHPFGL−−
RIPFGL−−
−RPFGLH−
−RPFGLA−
RNPFGL−−
RTPFGL−−
RMPFGL−−
−RPFGLI−
RAPFGL−−
RYPFGL−−
−RPFGLM−
RWPFGL−−
RQPFGL−−
RDPFGL−−
RKPFGL−−
RFPFGL−−
−RPFGLQ−
−RPFGLV−
−RPFGLG−
−RPFGLE−
−RPFGLF−
RPPFGL−−
REPFGL−−
RCPFGL−−
−SPFGLL−
SLPFGL−−
−SPFGLK−
SFPFGL−−
SMPFGL−−
SAPFGL−−
−SPFGLH−
−SPFGLS−
SYPFGL−−
SVPFGL−−
SNPFGL−−
STPFGL−−
−SPFGLN−
SIPFGL−−
−SPFGLR−
SHPFGL−−

-SPFGLQ-
-SPFGLA-
-SPFGLV-
-SPFGLF-
-SPFGLM-
SPPFGL--
SEPFGL--
SDPFGL--
SWPFGL--
SKPFGL--
SQPFGL--
-SPFGLE-
SCPFGL--
TFPFGL--
TVPFGL--
TSPFGL--
TRPFGL--
TYPFGL--
-TPFGLT-
-TPFGLG-
TLPFGL--
TGPFGL--
TPPFGL--
TDPFGL--
-TPFGLN-
-TPFGLK-
-TPFGLH-
TKPFGL--
-TPFGLS-
TNPFGL--
TTPFGL--
-TPFGLA-
-TPFGLQ-
THPFGL--
-TPFGLE-
-TPFGLM-
TWPFGL--
TQPFGL--
-TPFGLF-
TMPFGL--
TCPFGL--
TAPFGL--
-TPFGLI-
TIPFGL--
-TPFGLD-
-VPFGLA-
VRPFGL--
-VPFGLS-
VNPFGL--
VFPFGL--

-VPFGLT-
-VPFGLY-
VIPFGL--
-VPFGLK-
VSPFGL--
VPPFGL--
VGPFGL--
-VPFGLH-
VDPFGL--
-VPFGLQ-
-VPFGLN-
-VPFGLM-
VTPFGL--
-VPFGLG-
VHPFGL--
VMPFGL--
VKPFGL--
-VPFGLV-
-VPFGLI-
VQPFGL--
VWPFGL--
VYPFGL--
VAPFGL--
-VPFGLE-
VEPFGL--
VCPFGL--
-VPFGLD-
WSPFGL--
WRPFGL--
-WPFGLS-
WTPFGL--
-WPFGLR-
-WPFGLQ-
-WPFGLV-
-WPFGLT-
WEPFGL--
-WPFGLM-
WIPFGL--
WGPFGL--
WQPFGL--
WPPFGL--
WAPFGL--
WKPFGL--
WHPFGL--
WNPFGL--
-WPFGLH-
-WPFGLN-
WFPFGL--
-WPFGLK-
-WPFGLA-

	WMPFGL--		
	-WPFGLY-		
	-WPFGLG-		
	-WPFGLE-		
	WYPFGL--		
	WWPFGL--		
	YVPFGL--		
	YRPFGL--		
	-YPFGLS-		
	YSPFGL--		
	-YPFGLR-		
	YGPFGL--		
	YPPFGL--		
	YIPFGL--		
	-YPFGLK-		
	-YPFGLN-		
	YKPFGL--		
	YQPFGL--		
	YHPFGL--		
	-YPFGLL-		
	-YPFGLT-		
	-YPFGLQ-		
	-YPFGLA-		
	YFPFGL--		
	-YPFGLE-		
	-YPFGLI-		
	-YPFGLF-		
	YNPFGL--		
	YMPFGL--		
	YDPFGL--		
	YYPFGL--		
	YWPFGL--		
	-YPFGLH-		
	-YPFGLG-		
	YLPFGL--		
	YCPFGL--		
PFAY	330	682	48.04
	ARPFAY--		
	AAPFAY--		
	-APFAYQ-		
	-APFAYA-		
	ALPFAY--		
	AGPFAY--		
	AHPFAY--		
	AQPFAY--		
	-APFAYI-		
	-APFAYV-		
	-APFAYS-		
	-APFAYK-		
	ASPFAY--		

-APFAYY-
AIPFAY--
ATPFAY--
-APFAYT-
-APFAYH-
AMPFAY--
AKPFAY--
AEPFAY--
-APFAYN-
-APFAYG-
ANPFAY--
ADPFAY--
AWPFAY--
AVPFAY--
CLPFAY--
CVPFAY--
-CPFAYI-
CRPFAY--
CDPFAY--
CSPFAY--
-CPFAYS-
CMPFAY--
CKPFAY--
DIPFAY--
DRPFAY--
-DPFAYS-
DSPFAY--
-DPFAYH-
DMPFAY--
DKPFAY--
DAPFAY--
-DPFAYL-
DGPFAY--
-DPFAYT-
-DPFAYR-
DHPFAY--
DQPFAY--
-DPFAYW-
-DPFAYV-
DCPFAY--
-DPFAYY-
EGPFAY--
-EPFAYN-
-EPFAYS-
-EPFAYK-
EAPFAY--
EYPFAY--
ERPFAY--
-EPFAYR-
EQPFAY--

EWPFAY--
-EPFAYH-
EKPFAY--
-EPFAYI-
EVPFAY--
-EPFAYE-
EFPFAY--
EDPFAY--
-EPFAYT-
-EPFAYM-
-EPFAYG-
-EPFAYA-
ETPFAY--
ESPFAY--
-EPFAYF-
FGPFAY--
FRPFAY--
FSPFAY--
FDPFAY--
-FPFAYS-
-FPFAYQ-
-FPFAYH-
FNPFAY--
FHPFAY--
FAPFAY--
-FPFAYT-
FTPFAY--
-FPFAYK-
FVPFAY--
FMPFAY--
FFPFAY--
FQPFAY--
-FPFAYI-
-FPFAYM-
-FPFAYE-
GYPFAY--
GNPFAY--
-GPFAYL-
GVPFAY--
GTPFAY--
GFPFAY--
GKPFAY--
-GPFAYS-
-GPFAYN-
GWPFAY--
-GPFAYH-
-GPFAYF-
GLPFAY--
-GPFAYR-
-GPFAYV-

GSPFAY--
-GPFAYA-
GMPFAY--
GAPFAY--
GGPFAY--
-GPFAYW-
GDPFAY--
GEPFAY--
GQPFAY--
-GPFAYM-
GPPFAY--
-GPFAYY-
-GPFAYK-
-GPFAYG-
-GPFAYE-
GIPFAY--
GHPFAY--
-HPFAYR-
HGPFAY--
-HPFAYI-
-HPFAYF-
-HPFAYH-
HVPFAY--
HTPFAY--
HSPFAY--
HYPFAY--
HEPFAY--
HMPFAY--
-HPFAYW-
HNPFAY--
-HPFAYT-
-HPFAYA-
HIPFAY--
HWPFAY--
HPPFAY--
-HPFAYL-
HFPFAY--
-HPFAYS-
HHPFAY--
-IPFAYS-
IRPFAY--
IVPFAY--
IKPFAY--
IYPFAY--
-IPFAYK-
IPPFAY--
ITPFAY--
IAPFAY--
ISPFAY--
-IPFAYV-

-IPFAYF-
-IPFAYA-
-IPFAYI-
-IPFAYT-
-IPFAYQ-
IQPFAY--
-IPFAYN-
IIPFAY--
INPFAY--
KSPFAY--
KLPFAY--
KTPFAY--
KMPFAY--
-KPFAYR-
KRPFAY--
-KPFAYL-
KNPFAY--
KGPFAY--
KKPFAY--
KAPFAY--
-KPFAYS-
-KPFAYA-
-KPFAYY-
KIPFAY--
KVPFAY--
-KPFAYG-
KYPFAY--
-KPFAYN-
KHPFAY--
-KPFAYT-
KDPFAY--
KQPFAY--
-KPFAYV-
-KPFAYK-
-KPFAYE-
LRPFAY--
LGPFAY--
-LPFAYR-
LVPFAY--
LHPFAY--
-LPFAYS-
LDPFAY--
LSPFAY--
LQPFAY--
-LPFAYI-
-LPFAYT-
LTPFAY--
-LPFAYW-
LIPFAY--
LNPFAY--

-LPFAYM-
-LPFAYA-
LAPFAY--
-LPFAYV-
LWPFAY--
LPPFAY--
-LPFAYH-
-LPFAYN-
LKPFAY--
LEPFAY--
-MPFAYT-
MSPFAY--
MMPFAY--
MRPFAY--
-MPFAYI-
MDPFAY--
-MPFAYA-
MGPFAY--
-MPFAYL-
MVPFAY--
-MPFAYR-
MLPFAY--
MAPFAY--
MYPFAY--
MFPFAY--
MEPFAY--
-MPFAYV-
MTPFAY--
MIPFAY--
MQPFAY--
MHPFAY--
MPPFAY--
-MPFAYS-
-MPFAYQ-
NSPFAY--
NGPFAY--
-NPFAYR-
-NPFAYM-
-NPFAYL-
NRPFAY--
NNPFAY--
NEPFAY--
NIPFAY--
NTPFAY--
-NPFAYI-
NFPFAY--
-NPFAYA-
NLPFAY--
NAPFAY--
NYPFAY--

NMPFAY--
NVPFAY--
-NPFAYS-
-NPFAYF-
NKPFAY--
NQPFAY--
-NPFAYV-
-NPFAYQ-
-NPFAYN-
--PFAYRL
--PFAYQR
--PFAYRG
--PFAYLS
--PFAYWQ
--PFAYSL
--PFAYMS
--PFAYMR
--PFAYHL
--PFAYGS
--PFAYSR
--PFAYSG
--PFAYRT
--PFAYMT
PYPFAY--
--PFAYRA
--PFAYMA
--PFAYIG
--PFAYWN
--PFAYHS
--PFAYFT
--PFAYWT
--PFAYVA
--PFAYAS
--PFAYQT
--PFAYNS
--PFAYKT
--PFAYYG
--PFAYVL
--PFAYTR
--PFAYTH
--PFAYRD
--PFAYLR
--PFAYLA
--PFAYKS
--PFAYSM
--PFAYRN
--PFAYIN
--PFAYWK
--PFAYVT
PGPFAY--

--PFAYYR
--PFAYSA
--PFAYRK
--PFAYNT
--PFAYFR
--PFAYAL
--PFAYAG
--PFAYYK
--PFAYRH
--PFAYQS
--PFAYIT
--PFAYIR
--PFAYHA
PHPFAY--
--PFAYYT
--PFAYTN
--PFAYTA
--PFAYSV
--PFAYRM
--PFAYQF
--PFAYNL
--PFAYNA
--PFAYKG
--PFAYAA
--PFAYYH
--PFAYVR
--PFAYTG
--PFAYRF
--PFAYNQ
--PFAYLY
--PFAYLK
--PFAYFN
PTPFAY--
--PFAYTM
--PFAYSS
--PFAYRE
--PFAYQY
--PFAYLH
--PFAYKF
--PFAYFK
PAPFAY--
--PFAYWS
--PFAYGY
--PFAYAK
--PFAYWA
--PFAYVS
--PFAYSN
--PFAYFQ
--PFAYEA
PQPFAY--

--PFAYWV
--PFAYVK
--PFAYVG
--PFAYTT
--PFAYTQ
--PFAYTK
--PFAYTD
--PFAYQW
--PFAYIK
--PFAYGA
--PFAYEY
--PFAYYY
--PFAYWM
--PFAYVN
--PFAYVI
--PFAYVH
--PFAYRY
--PFAYQL
--PFAYQG
--PFAYNW
--PFAYMN
--PFAYMH
--PFAYME
--PFAYLE
--PFAYIS
--PFAYHQ
--PFAYGW
--PFAYEL
--PFAYAN
--PFAYYS
--PFAYWD
--PFAYVE
--PFAYSH
--PFAYRW
--PFAYRI
--PFAYQK
--PFAYNV
--PFAYNM
--PFAYNK
--PFAYMV
--PFAYMD
--PFAYIY
--PFAYHH
--PFAYGV
--PFAYFG
--PFAYFD
--PFAYEW
PKPFAY--
--PFAYYQ
--PFAYVQ

--PFAYTI
--PFAYSW
--PFAYST
--PFAYPM
--PFAYMG
--PFAYLG
--PFAYKA
--PFAYIQ
--PFAYIE
--PFAYHE
--PFAYGF
--PFAYFY
--PFAYER
--PFAYEI
--PFAYVV
--PFAYSY
--PFAYSQ
--PFAYPI
--PFAYNG
--PFAYMQ
--PFAYKL
--PFAYKE
--PFAYIM
--PFAYHV
--PFAYHK
--PFAYHF
--PFAYGT
--PFAYGQ
--PFAYGH
--PFAYGG
--PFAYAY
--PFAYAR
PEPFAY--
--PFAYYD
--PFAYWG
--PFAYVD
--PFAYSF
--PFAYSE
--PFAYLN
--PFAYLD
--PFAYKW
--PFAYKV
--PFAYKH
--PFAYKD
--PFAYIH
--PFAYHR
--PFAYAV
--PFAYAT
--PFAYAC
PNPFAY--

--PFAYYV
--PFAYYN
--PFAYTY
--PFAYTE
--PFAYSK
--PFAYSD
--PFAYQQ
--PFAYQI
--PFAYQD
--PFAYPQ
--PFAYNR
--PFAYNI
--PFAYMF
--PFAYIV
--PFAYII
--PFAYIA
--PFAYGN
--PFAYGK
--PFAYET
--PFAYES
--PFAYDG
--PFAYDA
--PFAYCR
--PFAYCM
--PFAYCI
PDPFAY--
QMPFAY--
-QPFAYY-
-QPFAYL-
QKPFAY--
QQPFAY--
QNPFAY--
-QPFAYG-
QSPFAY--
QRPFAY--
QGPFAY--
QPPFAY--
-QPFAYA-
QEPFAY--
QDPFAY--
-QPFAYV-
-QPFAYT-
-QPFAYM-
-QPFAYH-
QIPFAY--
QFPFAY--
QAPFAY--
QWPFAY--
QVPFAY--
QTPFAY--

-QPFAYS-
QLPFAY--
-RPFAYL-
RVPFAY--
RGPFAY--
-RPFAYI-
RLPFAY--
-RPFAYR-
RNPFAY--
RTPFAY--
RRPFAY--
RPPFAY--
RWPFAY--
RKPFAY--
RYPFAY--
-RPFAYV-
-RPFAYK-
RMPFAY--
-RPFAYM-
-RPFAYW-
RIPFAY--
RDPFAY--
-RPFAYT-
-RPFAYA-
-RPFAYF-
RHPFAY--
REPFAY--
-RPFAYH-
-RPFAYE-
RQPFAY--
RFPFAY--
-RPFAYG-
-RPFAYP-
SSPFAY--
-SPFAYR-
SRPFAY--
SKPFAY--
-SPFAYM-
SMPFAY--
-SPFAYI-
SGPFAY--
STPFAY--
-SPFAYL-
-SPFAYK-
SVPFAY--
SLPFAY--
-SPFAYS-
SAPFAY--
-SPFAYT-
SWPFAY--

SHPFAY--
-SPFAYY-
-SPFAYW-
-SPFAYQ-
-SPFAYA-
SYPFAY--
SNPFAY--
SDPFAY--
-SPFAYH-
-SPFAYV-
-SPFAYN-
-SPFAYE-
SEPFAY--
-SPFAYG-
-SPFAYF-
SIPFAY--
SCPFAY--
TRPFAY--
TLPFAY--
TTPFAY--
-TPFAYI-
-TPFAYS-
-TPFAYR-
TKPFAY--
-TPFAYV-
TNPFAY--
-TPFAYH-
THPFAY--
-TPFAYT-
-TPFAYN-
-TPFAYM-
-TPFAYK-
TYPFAY--
TSPFAY--
TIPFAY--
TQPFAY--
TWPFAY--
-TPFAYG-
-TPFAYY-
TMPFAY--
TEPFAY--
TAPFAY--
TVPFAY--
-TPFAYE-
VSPFAY--
VRPFAY--
VHPFAY--
-VPFAYA-
VKPFAY--
VPPFAY--

```
-VPFAYY-
-VPFAYR-
-VPFAYV-
-VPFAYN-
-VPFAYT-
-VPFAYG-
-VPFAYS-
VMPFAY--
VAPFAY--
VVPFAY--
VGPFAY--
VQPFAY--
VYPFAY--
VLPFAY--
VIPFAY--
-VPFAYI-
VTPFAY--
-VPFAYQ-
-VPFAYF-
-VPFAYE-
VNPFAY--
WKPFAY--
-WPFAYV-
WTPFAY--
WRPFAY--
-WPFAYR-
WHPFAY--
WVPFAY--
WNPFAY--
WMPFAY--
-WPFAYT-
WGPFAY--
WDPFAY--
WAPFAY--
WYPFAY--
-WPFAYH-
-WPFAYG-
WWPFAY--
-WPFAYQ-
-WPFAYM-
-WPFAYI-
WLPFAY--
WIPFAY--
YKPFAY--
-YPFAYS-
-YPFAYI-
YHPFAY--
-YPFAYK-
YSPFAY--
-YPFAYT-
```

YFPFAY--
YRPFAY--
YPPFAY--
YAPFAY--
-YPFAYY-
YLPFAY--
YIPFAY--
-YPFAYW-
YWPFAY--
-YPFAYQ-
YYPFAY--
-YPFAYV-
YGPFAY--
YEPFAY--
YTPFAY--
-YPFAYR-
YMPFAY--
YDPFAY--
YCPFAY--
PYPY 298 698 47.96
-VPYPYG-
ASPYPY--
-APYPYR-
-APYPYI-
AIPYPY--
-APYPYS-
-APYPYY-
ARPYPY--
AGPYPY--
-APYPYL-
AKPYPY--
AQPYPY--
ANPYPY--
AVPYPY--
-APYPYT-
AHPYPY--
AMPYPY--
-APYPYG-
-APYPYA-
-APYPYK-
-APYPYH-
AEPYPY--
-APYPYQ-
-APYPYM-
ALPYPY--
AWPYPY--
-APYPYV-
-APYPYN-
-APYPYF-
AAPYPY--

-CPYPYQ-
CTPYPY--
CSPYPY--
CKPYPY--
CAPYPY--
-CPYPYL-
DTPYPY--
DMPYPY--
-DPYPYR-
DRPYPY--
DYPYPY--
-DPYPYW-
-DPYPYM-
DDPYPY--
DSPYPY--
-DPYPYQ-
-DPYPYV-
-DPYPYL-
-DPYPYS-
-DPYPYN-
-DPYPYK-
-DPYPYF-
-DPYPYA-
DLPYPY--
DKPYPY--
DEPYPY--
EVPYPY--
ERPYPY--
EGPYPY--
EIPYPY--
EYPYPY--
ETPYPY--
-EPYPYR-
-EPYPYF-
ESPYPY--
-EPYPYA-
ENPYPY--
-EPYPYT-
-EPYPYI-
-EPYPYH-
ELPYPY--
EKPYPY--
FRPYPY--
-FPYPYS-
FEPYPY--
FIPYPY--
FSPYPY--
FGPYPY--
-FPYPYK-
-FPYPYH-

FAPYPY--
-FPYPYR-
FHPYPY--
-FPYPYW-
FKPYPY--
FVPYPY--
FMPYPY--
FWPYPY--
-FPYPYL-
-FPYPYF-
-FPYPYA-
FPPYPY--
FLPYPY--
FFPYPY--
GRPYPY--
GSPYPY--
GIPYPY--
GFPYPY--
GMPYPY--
GTPYPY--
GYPYPY--
-GPYPYH-
GPPYPY--
-GPYPYV-
GVPYPY--
GQPYPY--
-GPYPYY-
GLPYPY--
GAPYPY--
-GPYPYK-
-GPYPYI-
-GPYPYF-
-GPYPYE-
GHPYPY--
-GPYPYW-
-GPYPYR-
-GPYPYQ-
-GPYPYN-
GWPYPY--
-GPYPYM-
GKPYPY--
GGPYPY--
GDPYPY--
-GPYPYT-
-GPYPYA-
GCPYPY--
HRPYPY--
-HPYPYR-
-HPYPYA-
HKPYPY--

HSPYPY--
HQPYPY--
-HPYPYQ-
HNPYPY--
HAPYPY--
HLPYPY--
-HPYPYF-
HGPYPY--
HEPYPY--
HVPYPY--
-HPYPYT-
-HPYPYL-
-HPYPYK-
HTPYPY--
-HPYPYV-
-HPYPYM-
-HPYPYG-
-HPYPYE-
-HPYPYY-
HHPYPY--
-IPYPYQ-
-IPYPYL-
IHPYPY--
-IPYPYR-
ISPYPY--
ILPYPY--
-IPYPYS-
IPPYPY--
IIPYPY--
-IPYPYF-
-IPYPYT-
IWPYPY--
IRPYPY--
-IPYPYA-
IEPYPY--
IAPYPY--
-IPYPYW-
IKPYPY--
IQPYPY--
-IPYPYV-
-IPYPYH-
IMPYPY--
IVPYPY--
-IPYPYI-
IGPYPY--
KSPYPY--
-KPYPYL-
KTPYPY--
KWPYPY--
KVPYPY--

-KPYPYM-
-KPYPYR-
-KPYPYF-
-KPYPYS-
KPPYPY--
-KPYPYQ-
KLPYPY--
KRPYPY--
-KPYPYN-
-KPYPYW-
-KPYPYT-
KAPYPY--
-KPYPYI-
KCPYPY--
-KPYPYV-
-KPYPYH-
-KPYPYA-
KMPYPY--
KHPYPY--
KNPYPY--
KGPYPY--
KIPYPY--
KEPYPY--
KYPYPY--
KQPYPY--
LSPYPY--
-LPYPYR-
LGPYPY--
LRPYPY--
-LPYPYT-
-LPYPYM-
LKPYPY--
LAPYPY--
LWPYPY--
LNPYPY--
-LPYPYF-
LPPYPY--
LYPYPY--
LTPYPY--
-LPYPYS-
LMPYPY--
-LPYPYL-
LVPYPY--
-LPYPYI-
-LPYPYH-
LEPYPY--
LDPYPY--
LIPYPY--
LQPYPY--
-LPYPYV-

-LPYPYK-
-LPYPYQ-
-LPYPYA-
-LPYPYY-
LFPYPY--
-MPYPYS-
-MPYPYL-
-MPYPYR-
MAPYPY--
-MPYPYY-
MLPYPY--
MGPYPY--
-MPYPYA-
-MPYPYV-
MSPYPY--
-MPYPYF-
MRPYPY--
-MPYPYN-
-MPYPYH-
-MPYPYQ-
-MPYPYG-
MNPYPY--
MTPYPY--
-MPYPYK-
MMPYPY--
MHPYPY--
MDPYPY--
-NPYPYR-
NSPYPY--
-NPYPYW-
-NPYPYV-
-NPYPYS-
NGPYPY--
NPPYPY--
NWPYPY--
-NPYPYM-
NQPYPY--
NTPYPY--
-NPYPYQ-
-NPYPYA-
NHPYPY--
NVPYPY--
NAPYPY--
-NPYPYT-
NKPYPY--
NFPYPY--
NDPYPY--
-NPYPYL-
NMPYPY--
NLPYPY--

-NPYPYK-
-NPYPYI-
-NPYPYF-
NNPYPY--
NIPYPY--
NCPYPY--
--PYPYRL
--PYPYLG
--PYPYRS
--PYPYVT
--PYPYRA
--PYPYSG
--PYPYSD
--PYPYRH
--PYPYHA
--PYPYRG
--PYPYLR
--PYPYAS
--PYPYVA
--PYPYFS
--PYPYYF
--PYPYHM
--PYPYYG
--PYPYTT
--PYPYSH
--PYPYML
--PYPYLW
--PYPYKI
--PYPYTL
--PYPYTA
--PYPYSS
--PYPYMS
--PYPYHR
--PYPYWL
--PYPYVS
--PYPYTH
--PYPYSN
--PYPYRN
PGPYPY--
--PYPYVI
--PYPYRQ
--PYPYRI
--PYPYMT
--PYPYGS
--PYPYFT
--PYPYYT
--PYPYLS
--PYPYLL
PHPYPY--
--PYPYYW

--PYPYRY
--PYPYRT
--PYPYHS
--PYPYGF
--PYPYQG
--PYPYLN
--PYPYKK
PNPYPY--
--PYPYVH
--PYPYTY
--PYPYTK
--PYPYSR
--PYPYSA
--PYPYKQ
--PYPYHG
--PYPYGQ
--PYPYFW
PIPYPY--
--PYPYWR
--PYPYVF
--PYPYQA
--PYPYNV
--PYPYMA
--PYPYLI
--PYPYLA
--PYPYES
--PYPYEQ
--PYPYVV
--PYPYSF
--PYPYRW
--PYPYRK
--PYPYQY
--PYPYMN
--PYPYMK
--PYPYLM
--PYPYLH
--PYPYKS
--PYPYKH
--PYPYIR
--PYPYYR
--PYPYYL
--PYPYWG
--PYPYNF
--PYPYLQ
--PYPYAW
--PYPYYK
--PYPYYI
--PYPYWV
--PYPYWQ
--PYPYST

--PYPYNY
--PYPYMI
--PYPYMG
--PYPYKA
--PYPYFQ
--PYPYFH
--PYPYWF
--PYPYVR
--PYPYVQ
--PYPYTM
--PYPYTG
--PYPYSK
--PYPYQI
--PYPYLT
--PYPYKV
--PYPYFV
--PYPYFI
PQPYPY--
--PYPYYS
--PYPYYN
--PYPYYA
--PYPYTS
--PYPYTR
--PYPYTI
--PYPYQF
--PYPYPY
--PYPYNK
--PYPYMF
--PYPYLD
--PYPYKY
--PYPYIL
--PYPYIH
--PYPYIG
--PYPYIF
--PYPYHF
--PYPYGK
--PYPYDM
--PYPYDI
PDPYPY--
--PYPYYY
--PYPYYQ
--PYPYWY
--PYPYVG
--PYPYTQ
--PYPYRF
--PYPYQW
--PYPYPW
--PYPYNT
--PYPYNQ
--PYPYMY

--PYPYMV
--PYPYMR
--PYPYMH
--PYPYLK
--PYPYKT
--PYPYII
--PYPYHT
--PYPYGW
--PYPYGG
--PYPYGA
--PYPYFF
--PYPYEV
--PYPYYV
--PYPYYH
--PYPYWD
--PYPYVN
--PYPYVD
--PYPYSE
--PYPYPS
--PYPYPI
--PYPYNN
--PYPYNA
--PYPYMM
--PYPYKM
--PYPYIY
--PYPYIS
--PYPYIN
--PYPYIE
--PYPYIA
--PYPYHY
--PYPYHQ
--PYPYHN
--PYPYHI
--PYPYHE
--PYPYFR
--PYPYEY
--PYPYET
--PYPYEA
--PYPYDV
--PYPYDF
--PYPYAR
--PYPYAK
--PYPYAF
PMPYPY--
PEPYPY--
PCPYPY--
--PYPYYM
--PYPYYD
--PYPYWT
--PYPYWE

--PYPYVW
--PYPYVK
--PYPYVE
--PYPYTV
--PYPYSW
--PYPYRM
--PYPYRE
--PYPYQV
--PYPYPV
--PYPYPQ
--PYPYPH
--PYPYNR
--PYPYNM
--PYPYLE
--PYPYKR
--PYPYID
--PYPYHH
--PYPYGH
--PYPYFN
--PYPYEE
--PYPYDS
--PYPYCL
--PYPYAN
PTPYPY--
PFPYPY--
QAPYPY--
QRPYPY--
-QPYPYW-
QNPYPY--
-QPYPYA-
QVPYPY--
QKPYPY--
QSPYPY--
-QPYPYT-
-QPYPYI-
-QPYPYH-
-QPYPYV-
QQPYPY--
-QPYPYQ-
QIPYPY--
QHPYPY--
-QPYPYL-
QLPYPY--
QEPYPY--
QYPYPY--
-QPYPYY-
-QPYPYR-
-QPYPYG-
QGPYPY--
-QPYPYF-

QPPYPY--
-RPYPYL-
RLPYPY--
RSPYPY--
RAPYPY--
RPPYPY--
REPYPY--
-RPYPYR-
-RPYPYW-
-RPYPYI-
RGPYPY--
RFPYPY--
-RPYPYY-
RYPYPY--
RVPYPY--
RTPYPY--
-RPYPYV-
RIPYPY--
-RPYPYA-
RDPYPY--
-RPYPYN-
RMPYPY--
RHPYPY--
-RPYPYM-
-RPYPYG-
RNPYPY--
RWPYPY--
-RPYPYS-
-RPYPYF-
-RPYPYT-
-RPYPYE-
-RPYPYC-
RKPYPY--
RCPYPY--
SRPYPY--
STPYPY--
SSPYPY--
-SPYPYR-
-SPYPYL-
-SPYPYV-
SIPYPY--
SAPYPY--
SPPYPY--
-SPYPYS-
SYPYPY--
SGPYPY--
SVPYPY--
SLPYPY--
-SPYPYM-
SKPYPY--

SFPYPY--
-SPYPYH-
SNPYPY--
-SPYPYF-
SMPYPY--
SEPYPY--
-SPYPYT-
-SPYPYN-
-SPYPYA-
SQPYPY--
-SPYPYQ-
-SPYPYI-
-SPYPYE-
SHPYPY--
TAPYPY--
TVPYPY--
TLPYPY--
TRPYPY--
TMPYPY--
-TPYPYY-
-TPYPYI-
TWPYPY--
TSPYPY--
TKPYPY--
TYPYPY--
-TPYPYV-
-TPYPYS-
TEPYPY--
TNPYPY--
TGPYPY--
TPPYPY--
TIPYPY--
TQPYPY--
-TPYPYL-
-TPYPYF-
-TPYPYQ-
TTPYPY--
TFPYPY--
-TPYPYH-
-TPYPYE-
-TPYPYA-
-TPYPYP-
THPYPY--
-VPYPYT-
-VPYPYL-
VTPYPY--
-VPYPYS-
VHPYPY--
VKPYPY--
-VPYPYR-

VAPYPY--
VSPYPY--
VRPYPY--
VMPYPY--
VFPYPY--
-VPYPYV-
VNPYPY--
-VPYPYA-
VPPYPY--
VGPYPY--
VYPYPY--
-VPYPYM-
VDPYPY--
VVPYPY--
-VPYPYE-
-VPYPYW-
-VPYPYK-
-VPYPYH-
VCPYPY--
WSPYPY--
WEPYPY--
WPPYPY--
WNPYPY--
WHPYPY--
-WPYPYR-
WDPYPY--
WLPYPY--
WIPYPY--
WGPYPY--
-WPYPYA-
WVPYPY--
WTPYPY--
-WPYPYN-
-WPYPYL-
WKPYPY--
-WPYPYV-
WMPYPY--
WFPYPY--
WQPYPY--
-WPYPYY-
-WPYPYK-
YSPYPY--
YLPYPY--
YIPYPY--
YVPYPY--
-YPYPYL-
YYPYPY--
YKPYPY--
-YPYPYS-
-YPYPYA-

	YRPYPY--		
	-YPYPYK-		
	YEPYPY--		
	-YPYPYV-		
	-YPYPYR-		
	YMPYPY--		
	YGPYPY--		
	-YPYPYT-		
	-YPYPYI-		
	YDPYPY--		
	YTPYPY--		
	-YPYPYQ-		
	-YPYPYN-		
	-YPYPYM-		
	-YPYPYF-		
	YWPYPY--		
	-YPYPYE-		
	YFPYPY--		
PFWM	789	539	47.90
	-RPFWMD-		
	-APFWMR-		
	AYPFWM--		
	ALPFWM--		
	AAPFWM--		
	-APFWMQ-		
	ASPFWM--		
	AGPFWM--		
	-APFWMN-		
	AEPFWM--		
	-APFWMA-		
	ARPFWM--		
	-APFWMI-		
	-APFWMT-		
	ANPFWM--		
	AMPFWM--		
	-APFWML-		
	-APFWME-		
	ACPFWM--		
	AWPFWM--		
	-APFWMH-		
	AVPFWM--		
	-APFWMK-		
	CRPFWM--		
	CNPFWM--		
	CSPFWM--		
	CGPFWM--		
	-DPFWMR-		
	DSPFWM--		
	-DPFWMT-		
	DKPFWM--		

DVPFWM--
DAPFWM--
-DPFWMY-
DGPFWM--
DLPFWM--
DTPFWM--
DQPFWM--
-DPFWMS-
-DPFWMK-
-DPFWMF-
DNPFWM--
DCPFWM--
DWPFWM--
-DPFWMV-
-DPFWMQ-
-DPFWMH-
-EPFWMS-
ESPFWM--
-EPFWMQ-
EYPFWM--
-EPFWMA-
-EPFWMV-
-EPFWMR-
-EPFWMG-
EWPFWM--
ETPFWM--
EMPFWM--
EIPFWM--
EDPFWM--
-EPFWMP-
-EPFWMM-
-EPFWML-
-EPFWMH-
-EPFWMF-
EEPFWM--
-FPFWMT-
FNPFWM--
FDPFWM--
FVPFWM--
FQPFWM--
FTPFWM--
-FPFWMR-
-FPFWMV-
-FPFWMM-
FKPFWM--
FPPFWM--
-FPFWML-
FRPFWM--
-FPFWMS-
-FPFWMQ-

FFPFWM--
FAPFWM--
FSPFWM--
-FPFWMW-
-FPFWME-
GHPFWM--
-GPFWMM-
-GPFWMG-
-GPFWMT-
GGPFWM--
GVPFWM--
GTPFWM--
-GPFWML-
GRPFWM--
-GPFWMQ-
GYPFWM--
-GPFWMA-
GAPFWM--
-GPFWMS-
-GPFWMI-
-GPFWME-
GMPFWM--
GKPFWM--
GIPFWM--
GFPFWM--
-GPFWMY-
GNPFWM--
GCPFWM--
GWPFWM--
-GPFWMK-
HSPFWM--
-HPFWMK-
-HPFWMS-
-HPFWMM-
HTPFWM--
-HPFWMV-
-HPFWMH-
HIPFWM--
-HPFWMQ-
HWPFWM--
HGPFWM--
HFPFWM--
-HPFWMR-
-HPFWMT-
-HPFWMG-
-HPFWMA-
-HPFWML-
HEPFWM--
INPFWM--
ISPFWM--

IMPFWM--
-IPFWMR-
-IPFWML-
-IPFWMS-
IAPFWM--
-IPFWMV-
IGPFWM--
IIPFWM--
-IPFWMM-
-IPFWMK-
IVPFWM--
-IPFWMD-
ILPFWM--
-KPFWMR-
KTPFWM--
KRPFWM--
KEPFWM--
-KPFWMN-
-KPFWMV-
-KPFWML-
-KPFWMA-
KYPFWM--
KNPFWM--
-KPFWMI-
KHPFWM--
KVPFWM--
-KPFWMY-
-KPFWMS-
-KPFWMF-
KIPFWM--
KAPFWM--
KGPFWM--
-KPFWMW-
-KPFWMQ-
-KPFWMG-
LVPFWM--
LHPFWM--
LQPFWM--
-LPFWMT-
LKPFWM--
LPPFWM--
-LPFWMS-
LGPFWM--
LTPFWM--
LAPFWM--
-LPFWMY-
LYPFWM--
-LPFWMV-
-LPFWMR-
-LPFWMM-

LMPFWM--
-LPFWMQ-
LSPFWM--
LLPFWM--
LDPFWM--
-LPFWMN-
-LPFWMA-
-MPFWMT-
MEPFWM--
MLPFWM--
-MPFWMS-
MHPFWM--
MQPFWM--
-MPFWMK-
MMPFWM--
MAPFWM--
-MPFWMG-
MDPFWM--
MVPFWM--
MIPFWM--
MWPFWM--
-MPFWMQ-
MKPFWM--
MRPFWM--
-MPFWMR-
-MPFWME-
-MPFWMA-
-MPFWMV-
MFPFWM--
NSPFWM--
-NPFWMR-
NLPFWM--
NMPFWM--
NRPFWM--
-NPFWMS-
-NPFWMT-
-NPFWMK-
NYPFWM--
-NPFWMA-
NAPFWM--
NTPFWM--
-NPFWMV-
NWPFWM--
NQPFWM--
-NPFWMG-
-NPFWMF-
-NPFWME-
NNPFWM--
NPPFWM--
-NPFWMH-

--PFWMSG
--PFWMKS
--PFWMSR
--PFWMSV
--PFWMHA
--PFWMSH
--PFWMMV
--PFWMHS
--PFWMYN
--PFWMVT
--PFWMVA
--PFWMRQ
--PFWMAT
--PFWMTT
--PFWMKY
--PFWMGS
--PFWMTS
--PFWMRG
--PFWMLG
--PFWMRV
--PFWMRA
--PFWMLN
--PFWMIN
--PFWMVQ
--PFWMRT
--PFWMSN
--PFWMSI
--PFWMAV
--PFWMSS
--PFWMQM
--PFWMQH
--PFWMKV
PHPFWM--
--PFWMYT
--PFWMYQ
--PFWMYG
--PFWMRY
--PFWMNS
--PFWMIR
--PFWMHG
--PFWMGI
--PFWMGG
--PFWMTD
--PFWMMN
--PFWMLV
--PFWMLT
--PFWMVD
--PFWMSQ
--PFWMRH
--PFWMQT

--PFWMNQ
--PFWMLQ
--PFWMLE
--PFWMVR
--PFWMTI
--PFWMTE
--PFWMSM
--PFWMSK
--PFWMRD
--PFWMLR
--PFWMKH
--PFWMKE
--PFWMGR
--PFWMGA
--PFWMFA
--PFWMYK
--PFWMWK
--PFWMVN
--PFWMTA
--PFWMSA
--PFWMMD
--PFWMMA
--PFWMKK
--PFWMGQ
--PFWMGK
--PFWMAH
PEPFWM--
--PFWMYS
--PFWMYM
--PFWMYD
--PFWMWY
--PFWMWR
--PFWMTN
--PFWMQA
--PFWMNV
--PFWMMY
--PFWMMR
--PFWMLH
--PFWMHR
--PFWMHI
--PFWMFG
--PFWMYR
--PFWMYI
--PFWMWG
--PFWMVH
--PFWMTK
--PFWMSY
--PFWMST
--PFWMSE
--PFWMRE

--PFWMQD
--PFWMNL
--PFWMMG
--PFWMKT
--PFWMIM
--PFWMIH
--PFWMHV
--PFWMHN
--PFWMGT
--PFWMES
--PFWMEG
--PFWMDG
--PFWMAS
--PFWMAD
-PPFWMK-
PGPFWM--
--PFWMWV
--PFWMWS
--PFWMVK
--PFWMTW
--PFWMTG
--PFWMSD
--PFWMQY
--PFWMQW
--PFWMQR
--PFWMQI
--PFWMPH
--PFWMNW
--PFWMNH
--PFWMNG
--PFWMMH
--PFWMKQ
--PFWMKL
--PFWMIQ
--PFWMIL
--PFWMIA
--PFWMHH
--PFWMGL
--PFWMGF
--PFWMGE
--PFWMFS
--PFWMEN
--PFWMEH
--PFWMAE
-QPFWMQ-
QLPFWM--
-QPFWMT-
QHPFWM--
-QPFWMK-
-QPFWMM-

```
-QPFWMA-
QSPFWM--
QRPFWM--
QAPFWM--
QVPFWM--
-QPFWMV-
-QPFWMH-
-QPFWMI-
-QPFWMG-
QEPFWM--
QWPFWM--
-QPFWMS-
-QPFWMN-
-QPFWMF-
-QPFWMD-
QGPFWM--
RSPFWM--
-RPFWMT-
-RPFWMN-
RLPFWM--
RIPFWM--
-RPFWMG-
RAPFWM--
RQPFWM--
-RPFWMS-
-RPFWMV-
RMPFWM--
RTPFWM--
-RPFWME-
-RPFWMY-
-RPFWMA-
RRPFWM--
-RPFWML-
-RPFWMH-
-RPFWMF-
-RPFWMQ-
RHPFWM--
RGPFWM--
REPFWM--
RPPFWM--
-RPFWMM-
-RPFWMI-
-RPFWMW-
-RPFWMC-
RCPFWM--
-SPFWMR-
-SPFWMS-
SSPFWM--
-SPFWMA-
-SPFWMK-
```

SRPFWM--
-SPFWMI-
SLPFWM--
SWPFWM--
STPFWM--
SNPFWM--
SQPFWM--
SAPFWM--
-SPFWMQ-
SKPFWM--
SGPFWM--
-SPFWMH-
SFPFWM--
-SPFWMN-
-SPFWMD-
SMPFWM--
SDPFWM--
SPPFWM--
-SPFWMT-
-SPFWME-
SYPFWM--
-SPFWMV-
-TPFWMT-
-TPFWMS-
TRPFWM--
TWPFWM--
-TPFWMF-
TEPFWM--
-TPFWMR-
TGPFWM--
-TPFWMY-
-TPFWMG-
TSPFWM--
-TPFWMM-
-TPFWMV-
-TPFWMI-
TMPFWM--
TFPFWM--
TVPFWM--
-TPFWMW-
-TPFWMN-
-TPFWME-
TIPFWM--
TDPFWM--
TTPFWM--
TPPFWM--
-TPFWMK-
-TPFWMA-
TNPFWM--
THPFWM--

-VPFWMT-
-VPFWMV-
VMPFWM--
-VPFWMR-
-VPFWMK-
-VPFWMH-
-VPFWME-
-VPFWML-
VSPFWM--
VNPFWM--
-VPFWMG-
VIPFWM--
-VPFWMY-
-VPFWMA-
-VPFWMQ-
VTPFWM--
VQPFWM--
-VPFWMN-
-VPFWMI-
VLPFWM--
VDPFWM--
VAPFWM--
VPPFWM--
-VPFWMP-
-VPFWMM-
VKPFWM--
VGPFWM--
WSPFWM--
WRPFWM--
-WPFWMG-
WYPFWM--
-WPFWMY-
WVPFWM--
WAPFWM--
-WPFWMS-
WMPFWM--
WCPFWM--
-WPFWMN-
-YPFWMR-
YSPFWM--
YPPFWM--
YTPFWM--
YEPFWM--
-YPFWMT-
-YPFWMK-
YGPFWM--
YAPFWM--
-YPFWMV-
-YPFWMM-
-YPFWMS-

	YVPFWM--		
	-YPFWMY-		
	-YPFWMI-		
	-YPFWMG-		
	YNPFWM--		
	YRPFWM--		
	-YPFWMW-		
	-YPFWME-		
PKNL	140	785	47.89
	--PKNLRP		
	--PKNLLP		
	-SPKNLC-		
	-APKNLI-		
	-APKNLL-		
	AAPKNL--		
	ASPKNL--		
	-APKNLR-		
	-APKNLV-		
	-APKNLW-		
	AMPKNL--		
	AGPKNL--		
	-APKNLY-		
	ATPKNL--		
	AKPKNL--		
	APPKNL--		
	-APKNLK-		
	AHPKNL--		
	AQPKNL--		
	AYPKNL--		
	-APKNLS-		
	-APKNLG-		
	-APKNLE-		
	ADPKNL--		
	AVPKNL--		
	-APKNLA-		
	-APKNLM-		
	-APKNLH-		
	AIPKNL--		
	-APKNLQ-		
	AEPKNL--		
	-APKNLD-		
	CRPKNL--		
	CQPKNL--		
	CYPKNL--		
	CSPKNL--		
	CTPKNL--		
	CPPKNL--		
	-CPKNLQ-		
	CHPKNL--		
	-CPKNLL-		

-CPKNLA-
CGPKNL--
CVPKNL--
-CPKNLV-
-CPKNLT-
CLPKNL--
CIPKNL--
-CPKNLS-
-CPKNLR-
-CPKNLM-
-CPKNLG-
-CPKNLF-
-CPKNLE-
CMPKNL--
CFPKNL--
CAPKNL--
DWPKNL--
DTPKNL--
-DPKNLL-
-DPKNLR-
DFPKNL--
-DPKNLK-
DMPKNL--
DKPKNL--
DGPKNL--
-DPKNLV-
-DPKNLQ-
DHPKNL--
DAPKNL--
DSPKNL--
DRPKNL--
-DPKNLY-
-DPKNLT-
-DPKNLG-
DLPKNL--
DYPKNL--
DVPKNL--
-DPKNLS-
-DPKNLM-
-DPKNLA-
DNPKNL--
DQPKNL--
-DPKNLI-
DEPKNL--
-EPKNLR-
EVPKNL--
-EPKNLI-
EGPKNL--
-EPKNLQ-
ELPKNL--

ESPKNL--
-EPKNLH-
-EPKNLY-
EYPKNL--
-EPKNLT-
-EPKNLL-
-EPKNLK-
EFPKNL--
-EPKNLF-
-EPKNLW-
-EPKNLV-
-EPKNLS-
-EPKNLG-
EIPKNL--
EHPKNL--
EQPKNL--
-EPKNLA-
EAPKNL--
ERPKNL--
EPPKNL--
ENPKNL--
EMPKNL--
EKPKNL--
FSPKNL--
FAPKNL--
-FPKNLL-
-FPKNLV-
FTPKNL--
FLPKNL--
FNPKNL--
FGPKNL--
FWPKNL--
-FPKNLM-
FMPKNL--
FHPKNL--
-FPKNLW-
FPPKNL--
FVPKNL--
-FPKNLH-
FQPKNL--
-FPKNLE-
-FPKNLQ-
-FPKNLF-
FKPKNL--
FEPKNL--
-FPKNLC-
FCPKNL--
GLPKNL--
-GPKNLQ-
-GPKNLL-

GFPKNL--
-GPKNLK-
-GPKNLM-
-GPKNLR-
-GPKNLW-
-GPKNLA-
GRPKNL--
-GPKNLV-
-GPKNLI-
GEPKNL--
-GPKNLY-
-GPKNLN-
GIPKNL--
GAPKNL--
GYPKNL--
-GPKNLT-
-GPKNLH-
GDPKNL--
GVPKNL--
GGPKNL--
-GPKNLG-
GQPKNL--
GPPKNL--
-GPKNLE-
GNPKNL--
GMPKNL--
-GPKNLD-
GCPKNL--
-GPKNLF-
GKPKNL--
HRPKNL--
-HPKNLS-
-HPKNLL-
HWPKNL--
HVPKNL--
HHPKNL--
HYPKNL--
-HPKNLR-
HQPKNL--
HPPKNL--
HSPKNL--
HAPKNL--
-HPKNLV-
-HPKNLM-
-HPKNLW-
-HPKNLI-
HIPKNL--
-HPKNLG-
-HPKNLE-
HGPKNL--

HTPKNL--
-HPKNLK-
HNPKNL--
-HPKNLA-
HEPKNL--
-HPKNLF-
-HPKNLY-
HMPKNL--
HKPKNL--
-HPKNLH-
-IPKNLV-
-IPKNLS-
-IPKNLG-
IIPKNL--
IVPKNL--
IGPKNL--
-IPKNLY-
IEPKNL--
-IPKNLH-
INPKNL--
ISPKNL--
IMPKNL--
IWPKNL--
-IPKNLL-
-IPKNLK-
IDPKNL--
-IPKNLW-
IPPKNL--
-IPKNLI-
IHPKNL--
-IPKNLE-
IFPKNL--
-IPKNLF-
IKPKNL--
IAPKNL--
ICPKNL--
-IPKNLD-
-IPKNLA-
KTPKNL--
KVPKNL--
-KPKNLT-
-KPKNLS-
-KPKNLG-
-KPKNLY-
KYPKNL--
KRPKNL--
-KPKNLH-
-KPKNLA-
KPPKNL--
KMPKNL--

-KPKNLE-
KGPKNL--
KWPKNL--
-KPKNLF-
-KPKNLW-
-KPKNLV-
-KPKNLM-
KNPKNL--
KIPKNL--
KHPKNL--
KDPKNL--
-KPKNLC-
KQPKNL--
KKPKNL--
LSPKNL--
LLPKNL--
LVPKNL--
LAPKNL--
LGPKNL--
LTPKNL--
-LPKNLQ-
-LPKNLW-
-LPKNLT-
LKPKNL--
LEPKNL--
LMPKNL--
LHPKNL--
-LPKNLH-
LFPKNL--
-LPKNLY-
-LPKNLS-
-LPKNLA-
LDPKNL--
-LPKNLI-
-LPKNLV-
LWPKNL--
-LPKNLN-
LYPKNL--
-LPKNLC-
MTPKNL--
-MPKNLY-
-MPKNLS-
-MPKNLL-
-MPKNLA-
MMPKNL--
-MPKNLN-
MVPKNL--
MAPKNL--
-MPKNLQ-
-MPKNLI-

MLPKNL--
MSPKNL--
-MPKNLM-
-MPKNLH-
MDPKNL--
-MPKNLV-
-MPKNLT-
MGPKNL--
MKPKNL--
MIPKNL--
MHPKNL--
MEPKNL--
MPPKNL--
MFPKNL--
-MPKNLK-
MCPKNL--
NVPKNL--
-NPKNLR-
NSPKNL--
NYPKNL--
-NPKNLL-
-NPKNLA-
NAPKNL--
NPPKNL--
-NPKNLV-
NWPKNL--
-NPKNLY-
NIPKNL--
NGPKNL--
-NPKNLM-
-NPKNLK-
NHPKNL--
NTPKNL--
NNPKNL--
NQPKNL--
-NPKNLT-
NDPKNL--
NCPKNL--
-NPKNLS-
-NPKNLH-
-NPKNLF-
-NPKNLG-
-NPKNLE-
NKPKNL--
--PKNLYL
--PKNLSS
--PKNLVL
--PKNLFG
--PKNLSL
--PKNLSV

--PKNLMF
--PKNLFR
--PKNLYV
--PKNLLG
--PKNLGT
--PKNLWA
--PKNLLN
--PKNLYA
--PKNLVT
--PKNLYS
--PKNLAS
--PKNLAA
PMPKNL--
--PKNLTL
--PKNLWY
--PKNLWV
--PKNLVA
--PKNLNS
--PKNLSW
--PKNLRM
--PKNLLV
--PKNLVV
--PKNLYN
--PKNLSH
--PKNLSF
--PKNLLE
--PKNLTS
--PKNLTQ
--PKNLKS
--PKNLFI
PLPKNL--
--PKNLVH
--PKNLSA
--PKNLFT
--PKNLST
--PKNLLM
--PKNLSK
--PKNLIA
--PKNLTT
--PKNLTA
--PKNLIN
--PKNLAV
--PKNLYR
--PKNLTF
--PKNLRV
--PKNLLW
--PKNLYH
PFPKNL--
--PKNLRA
--PKNLIS

--PKNLWM
--PKNLLY
--PKNLYG
--PKNLTI
--PKNLSN
--PKNLLH
--PKNLFN
--PKNLFL
--PKNLSI
--PKNLSG
--PKNLKF
--PKNLIG
--PKNLGG
--PKNLAI
--PKNLVQ
--PKNLTM
--PKNLTK
--PKNLTG
--PKNLFS
--PKNLAW
--PKNLAK
--PKNLRQ
--PKNLMT
--PKNLMH
PQPKNL--
--PKNLVS
--PKNLVK
--PKNLQR
--PKNLNM
--PKNLLT
--PKNLGF
--PKNLGA
--PKNLWT
--PKNLWI
--PKNLVN
--PKNLRD
--PKNLQG
--PKNLKW
--PKNLWQ
--PKNLVE
--PKNLTD
--PKNLMK
--PKNLHY
--PKNLGH
--PKNLFM
--PKNLES
--PKNLYY
--PKNLNA
--PKNLHS
--PKNLGW

--PKNLGV
--PKNLGR
PVPKNL--
--PKNLYM
--PKNLWE
--PKNLVY
--PKNLRI
--PKNLHL
--PKNLHI
--PKNLFK
--PKNLFA
--PKNLER
--PKNLEM
--PKNLAM
--PKNLAH
--PKNLYE
--PKNLVW
--PKNLMI
--PKNLLQ
--PKNLHW
--PKNLGN
--PKNLYW
--PKNLYT
--PKNLQT
--PKNLHV
--PKNLHF
--PKNLFQ
--PKNLAN
PSPKNL--
--PKNLWG
--PKNLVM
--PKNLQS
--PKNLQL
--PKNLNT
--PKNLLF
--PKNLLD
--PKNLHN
--PKNLFV
--PKNLEW
--PKNLAL
PHPKNL--
PNPKNL--
--PKNLWN
--PKNLTV
--PKNLSE
--PKNLQW
--PKNLNN
--PKNLGY
--PKNLFY
--PKNLFH

--PKNLFD
--PKNLEH
--PKNLAQ
--PKNLAF
PAPKNL--
--PKNLWF
--PKNLTH
--PKNLSM
--PKNLQV
--PKNLQH
--PKNLQE
--PKNLNW
--PKNLKD
--PKNLEF
--PKNLAT
--PKNLYF
--PKNLTN
--PKNLQD
--PKNLNF
--PKNLKM
--PKNLIM
PEPKNL--
--PKNLYI
--PKNLYD
--PKNLQF
--PKNLNR
--PKNLNK
--PKNLME
--PKNLKN
--PKNLIL
--PKNLIE
--PKNLET
--PKNLAE
PKPKNL--
--PKNLTY
--PKNLQQ
--PKNLNY
--PKNLMQ
--PKNLMM
--PKNLIV
--PKNLGQ
--PKNLGI
--PKNLFF
--PKNLEK
--PKNLEG
--PKNLEA
--PKNLTE
--PKNLNV
--PKNLKY
--PKNLIP

--PKNLHE
--PKNLFE
--PKNLDA
--PKNLCT
--PKNLCN
PIPKNL--
PGPKNL--
--PKNLYQ
--PKNLWS
--PKNLTW
--PKNLTC
--PKNLQY
--PKNLQM
--PKNLNC
--PKNLKT
--PKNLIQ
--PKNLHH
--PKNLHG
--PKNLGE
--PKNLEY
--PKNLEV
--PKNLDT
-PPKNLW-
--PKNLWW
--PKNLWD
--PKNLSC
--PKNLRE
--PKNLPP
--PKNLPA
--PKNLNI
--PKNLNG
--PKNLNE
--PKNLND
--PKNLMD
--PKNLKI
--PKNLKE
--PKNLKC
--PKNLHQ
--PKNLHD
--PKNLGD
--PKNLEN
--PKNLEE
--PKNLDW
--PKNLDG
--PKNLDE
--PKNLCV
--PKNLCS
--PKNLCL
--PKNLCG
--PKNLCA

QLPKNL--
QSPKNL--
-QPKNLR-
QGPKNL--
-QPKNLH-
-QPKNLY-
-QPKNLN-
QFPKNL--
QAPKNL--
-QPKNLL-
QDPKNL--
-QPKNLA-
-QPKNLS-
-QPKNLT-
QYPKNL--
-QPKNLV-
-QPKNLQ-
QMPKNL--
QVPKNL--
-QPKNLG-
QEPKNL--
-QPKNLM-
QIPKNL--
QPPKNL--
QWPKNL--
-QPKNLF-
QNPKNL--
QHPKNL--
QCPKNL--
QTPKNL--
-QPKNLK-
-QPKNLC-
QKPKNL--
-RPKNLQ-
RQPKNL--
-RPKNLS-
RTPKNL--
RAPKNL--
RPPKNL--
REPKNL--
RYPKNL--
-RPKNLW-
-RPKNLM-
RGPKNL--
-RPKNLN-
-RPKNLG-
RMPKNL--
-RPKNLH-
RCPKNL--
RNPKNL--

RKPKNL--
-SPKNLY-
SAPKNL--
SMPKNL--
-SPKNLL-
-SPKNLT-
-SPKNLS-
SVPKNL--
-SPKNLA-
SQPKNL--
SPPKNL--
STPKNL--
SYPKNL--
-SPKNLV-
-SPKNLH-
-SPKNLN-
-SPKNLQ-
SEPKNL--
-SPKNLW-
SKPKNL--
-SPKNLM-
-SPKNLK-
SDPKNL--
-SPKNLF-
-SPKNLE-
SGPKNL--
SCPKNL--
TAPKNL--
TDPKNL--
TVPKNL--
TWPKNL--
-TPKNLS-
-TPKNLR-
TMPKNL--
TKPKNL--
TQPKNL--
TSPKNL--
TNPKNL--
TRPKNL--
-TPKNLI-
TIPKNL--
-TPKNLT-
TTPKNL--
-TPKNLF-
TLPKNL--
-TPKNLW-
TEPKNL--
TYPKNL--
-TPKNLK-
-TPKNLV-

-TPKNLM-
-TPKNLE-
THPKNL--
TPPKNL--
-TPKNLQ-
-TPKNLN-
TGPKNL--
-TPKNLH-
-TPKNLA-
VSPKNL--
VRPKNL--
VLPKNL--
-VPKNLS-
-VPKNLY-
-VPKNLQ-
VTPKNL--
-VPKNLI-
-VPKNLA-
-VPKNLR-
VWPKNL--
VVPKNL--
-VPKNLV-
-VPKNLM-
-VPKNLF-
-VPKNLG-
-VPKNLE-
VYPKNL--
VPPKNL--
VNPKNL--
-VPKNLW-
VDPKNL--
-VPKNLN-
VQPKNL--
VCPKNL--
-VPKNLH-
VEPKNL--
-VPKNLD-
WRPKNL--
WKPKNL--
WVPKNL--
WMPKNL--
-WPKNLL-
WLPKNL--
WHPKNL--
WPPKNL--
-WPKNLN-
-WPKNLM-
-WPKNLG-
WNPKNL--
-WPKNLW-

WDPKNL--
WIPKNL--
WGPKNL--
WQPKNL--
-WPKNLH-
WEPKNL--
-WPKNLT-
-WPKNLQ-
WTPKNL--
-WPKNLF-
WYPKNL--
-WPKNLA-
-WPKNLE-
YVPKNL--
YSPKNL--
YLPKNL--
-YPKNLL-
-YPKNLQ-
-YPKNLI-
YFPKNL--
-YPKNLT-
YHPKNL--
YAPKNL--
YQPKNL--
YRPKNL--
YKPKNL--
YGPKNL--
-YPKNLK-
-YPKNLG-
YTPKNL--
YIPKNL--
-YPKNLF-
YEPKNL--
YNPKNL--
YDPKNL--
YYPKNL--
YPPKNL--
-YPKNLY-
-YPKNLE-
-YPKNLN-
-YPKNLM-
-YPKNLV-
YMPKNL--
-YPKNLH-

PYHI	454	635	47.89

--PYHIYL
-APYHIT-
-APYHIR-
ALPYHI--
AVPYHI--

AEPYHI--
-APYHII-
AKPYHI--
-APYHIV-
-APYHIS-
AYPYHI--
AMPYHI--
-APYHIG-
-APYHIY-
-APYHIL-
-APYHIF-
-APYHIA-
ANPYHI--
AIPYHI--
AFPYHI--
AGPYHI--
AAPYHI--
AWPYHI--
-APYHIK-
ADPYHI--
ATPYHI--
-APYHIN-
-APYHIH-
-APYHIE-
AHPYHI--
ACPYHI--
CLPYHI--
CAPYHI--
CMPYHI--
-CPYHIR-
-CPYHIW-
-CPYHIF-
CFPYHI--
CDPYHI--
DWPYHI--
-DPYHIR-
DMPYHI--
DAPYHI--
DRPYHI--
-DPYHIY-
DVPYHI--
DQPYHI--
-DPYHIK-
DLPYHI--
DHPYHI--
DSPYHI--
-DPYHIA-
DKPYHI--
DEPYHI--
DDPYHI--

EQPYHI--
ETPYHI--
-EPYHIL-
-EPYHIV-
-EPYHIT-
EYPYHI--
-EPYHIW-
-EPYHIR-
-EPYHII-
ELPYHI--
ESPYHI--
-EPYHIS-
-EPYHIA-
EKPYHI--
EHPYHI--
EFPYHI--
EMPYHI--
EAPYHI--
FRPYHI--
FNPYHI--
FVPYHI--
FEPYHI--
FSPYHI--
-FPYHIR-
-FPYHIM-
FAPYHI--
FMPYHI--
FLPYHI--
-FPYHIA-
FKPYHI--
-FPYHIK-
FHPYHI--
FDPYHI--
FQPYHI--
FGPYHI--
-FPYHIQ-
-FPYHIH-
FIPYHI--
FTPYHI--
-FPYHIT-
-FPYHIF-
GTPYHI--
-GPYHIR-
-GPYHII-
GRPYHI--
-GPYHIM-
GIPYHI--
GGPYHI--
GMPYHI--
GAPYHI--

-GPYHIS-
GFPYHI--
GSPYHI--
GLPYHI--
-GPYHIL-
GNPYHI--
-GPYHIA-
GQPYHI--
-GPYHIW-
-GPYHIT-
-GPYHIQ-
GHPYHI--
GVPYHI--
-GPYHIY-
-GPYHIN-
-GPYHIH-
-GPYHIE-
GPPYHI--
GKPYHI--
-GPYHIK-
-GPYHIG-
-GPYHIF-
GEPYHI--
GDPYHI--
-HPYHIL-
HRPYHI--
HVPYHI--
HTPYHI--
HLPYHI--
-HPYHIR-
HMPYHI--
-HPYHIV-
-HPYHIT-
HAPYHI--
-HPYHIH-
-HPYHIE-
HWPYHI--
-HPYHIM-
IRPYHI--
ILPYHI--
IWPYHI--
IGPYHI--
-IPYHIS-
IEPYHI--
-IPYHIG-
ITPYHI--
IQPYHI--
IKPYHI--
IAPYHI--
-IPYHIL-

-IPYHII-
INPYHI--
ISPYHI--
-IPYHIR-
IYPYHI--
-IPYHIV-
IDPYHI--
-IPYHIY-
IPPYHI--
IHPYHI--
IVPYHI--
-IPYHIT-
-IPYHIQ-
-IPYHIE-
ICPYHI--
KSPYHI--
KPPYHI--
KLPYHI--
-KPYHIL-
-KPYHIQ-
KAPYHI--
KGPYHI--
-KPYHIT-
-KPYHIH-
-KPYHIS-
-KPYHIG-
KYPYHI--
KRPYHI--
KWPYHI--
KEPYHI--
KVPYHI--
-KPYHIW-
-KPYHIE-
-KPYHIA-
KNPYHI--
KCPYHI--
LTPYHI--
LIPYHI--
LLPYHI--
-LPYHIR-
LWPYHI--
LVPYHI--
LAPYHI--
LYPYHI--
LFPYHI--
LSPYHI--
LKPYHI--
LPPYHI--
LNPYHI--
LGPYHI--

-LPYHIH-
-LPYHIW-
LQPYHI--
-LPYHIY-
-LPYHIK-
-LPYHIE-
-LPYHIT-
-LPYHIS-
-LPYHIL-
LHPYHI--
LEPYHI--
MAPYHI--
MMPYHI--
MVPYHI--
-MPYHIF-
MYPYHI--
-MPYHIS-
-MPYHIM-
MTPYHI--
MSPYHI--
-MPYHIV-
MNPYHI--
MGPYHI--
-MPYHIT-
-MPYHII-
-MPYHIH-
-MPYHIE-
MCPYHI--
-MPYHIR-
-MPYHIA-
NYPYHI--
NVPYHI--
NTPYHI--
NLPYHI--
NQPYHI--
NSPYHI--
NIPYHI--
-NPYHIF-
NKPYHI--
-NPYHIV-
-NPYHIR-
NPPYHI--
NWPYHI--
NRPYHI--
-NPYHII-
NHPYHI--
NAPYHI--
-NPYHIT-
-NPYHIM-
NNPYHI--

NGPYHI--
-NPYHIY-
-NPYHIS-
NMPYHI--
NFPYHI--
NDPYHI--
--PYHILS
--PYHIRS
--PYHIRG
--PYHIYS
--PYHITG
--PYHIRT
--PYHIRN
--PYHITY
--PYHIMT
--PYHITS
--PYHIRI
--PYHIST
--PYHIRA
--PYHIMQ
--PYHIVG
--PYHIQQ
--PYHINS
--PYHIMK
--PYHIKS
--PYHIFY
--PYHIWS
--PYHISN
--PYHIRY
--PYHIMG
--PYHILR
--PYHIIS
--PYHIGS
--PYHIWA
--PYHIVQ
--PYHIVA
--PYHISS
--PYHIKM
--PYHIHV
--PYHIFK
--PYHIAG
--PYHIYW
--PYHIYA
--PYHIVR
--PYHITT
--PYHITR
--PYHIMS
--PYHIMN
--PYHIIG
--PYHIHF

--PYHIET
--PYHIAS
--PYHIYV
--PYHIVM
--PYHIRW
--PYHIQA
--PYHIML
--PYHIME
--PYHIKT
--PYHIAA
PQPYHI--
--PYHIYY
--PYHIWH
--PYHIVY
--PYHIVW
--PYHIVV
--PYHITV
--PYHITA
--PYHIRL
--PYHILG
--PYHIGG
--PYHIGA
--PYHIFR
--PYHIEY
--PYHIAR
--PYHIAF
--PYHIYM
--PYHITH
--PYHIRH
--PYHIMM
--PYHILT
--PYHILK
--PYHIHH
--PYHIFG
--PYHIWY
--PYHIWF
--PYHIVL
--PYHIVI
--PYHITF
--PYHISI
--PYHIRV
--PYHIRM
--PYHIQW
--PYHINT
--PYHINA
--PYHIMA
--PYHILQ
--PYHILA
--PYHIKW
--PYHIIT

--PYHIIK
--PYHIIH
--PYHIHG
--PYHIFT
--PYHIYK
--PYHIYF
--PYHIWN
--PYHIWE
--PYHIVN
--PYHIVK
--PYHIVD
--PYHITN
--PYHITD
--PYHISL
--PYHISH
--PYHISF
--PYHIQL
--PYHINW
--PYHINV
--PYHINN
--PYHILC
--PYHIKG
--PYHIKA
--PYHIIQ
--PYHIIL
--PYHIID
--PYHIFQ
--PYHIFM
--PYHIFI
--PYHIEA
--PYHIAH
PHPYHI--
--PYHIWR
--PYHITK
--PYHITE
--PYHISR
--PYHIRE
--PYHIQY
--PYHIMR
--PYHIMD
--PYHILW
--PYHILH
--PYHILF
--PYHIKK
--PYHIIR
--PYHIIA
--PYHIHK
--PYHIHA
--PYHIGT
--PYHIES

--PYHIER
--PYHIEF
--PYHIAQ
PEPYHI--
PCPYHI--
--PYHIYR
--PYHIWV
--PYHIVS
--PYHIVP
--PYHITM
--PYHITL
--PYHITI
--PYHISY
--PYHISA
--PYHING
--PYHIMH
--PYHILE
--PYHIKY
--PYHIKR
--PYHIKL
--PYHIIM
--PYHIHY
--PYHIHQ
--PYHIHN
--PYHIHM
--PYHIHE
--PYHIGV
--PYHIGI
--PYHIFW
--PYHIFH
--PYHIFE
--PYHIEM
--PYHICV
--PYHIAY
-PPYHIT-
-QPYHIR-
-QPYHIT-
QRPYHI--
QLPYHI--
QGPYHI--
QWPYHI--
QVPYHI--
QKPYHI--
-QPYHIL-
QSPYHI--
-QPYHII-
QNPYHI--
-QPYHIF-
QIPYHI--
-QPYHIK-

QPPYHI--
QDPYHI--
-QPYHIW-
-QPYHIQ-
-QPYHIH-
QMPYHI--
RLPYHI--
RGPYHI--
-RPYHIV-
RYPYHI--
RQPYHI--
-RPYHIS-
-RPYHIK-
RRPYHI--
RVPYHI--
RAPYHI--
-RPYHIL-
RTPYHI--
RSPYHI--
RPPYHI--
RHPYHI--
RMPYHI--
RKPYHI--
REPYHI--
-RPYHIR-
-RPYHII-
RWPYHI--
-RPYHIY-
-RPYHIQ-
-RPYHIF-
RFPYHI--
RDPYHI--
-RPYHIT-
-RPYHIH-
-RPYHIN-
-RPYHIM-
RIPYHI--
-RPYHIE-
-RPYHIG-
-RPYHIW-
RCPYHI--
SRPYHI--
-SPYHIT-
SNPYHI--
SSPYHI--
SIPYHI--
-SPYHIV-
SKPYHI--
-SPYHIM-
-SPYHIL-

STPYHI--
SVPYHI--
-SPYHIS-
SLPYHI--
SFPYHI--
SYPYHI--
-SPYHIW-
SEPYHI--
SQPYHI--
-SPYHIR-
SMPYHI--
SAPYHI--
SHPYHI--
SGPYHI--
SDPYHI--
SWPYHI--
-SPYHIH-
-SPYHIE-
-SPYHIA-
-SPYHIQ-
-SPYHIF-
-SPYHIC-
TRPYHI--
TSPYHI--
-TPYHIR-
TGPYHI--
TAPYHI--
TKPYHI--
TYPYHI--
TVPYHI--
-TPYHIL-
TNPYHI--
THPYHI--
-TPYHIS-
-TPYHIM-
TPPYHI--
TLPYHI--
TEPYHI--
-TPYHII-
TDPYHI--
TWPYHI--
TQPYHI--
-TPYHIY-
-TPYHIQ-
-TPYHIH-
TIPYHI--
-TPYHIA-
TMPYHI--
TTPYHI--
-TPYHIT-

-TPYHIF-
VGPYHI--
VSPYHI--
-VPYHII-
VAPYHI--
VKPYHI--
VYPYHI--
VTPYHI--
VNPYHI--
-VPYHIY-
VPPYHI--
VRPYHI--
-VPYHIM-
-VPYHIL-
VMPYHI--
VQPYHI--
-VPYHIH-
VIPYHI--
-VPYHIT-
-VPYHIF-
VEPYHI--
VDPYHI--
VWPYHI--
-VPYHIQ-
-VPYHIG-
-VPYHIA-
VFPYHI--
-VPYHIW-
-VPYHIV-
-VPYHIR-
-VPYHIE-
-WPYHIG-
-WPYHIR-
WGPYHI--
WIPYHI--
WQPYHI--
-WPYHII-
WAPYHI--
WPPYHI--
WSPYHI--
-WPYHIK-
-WPYHIV-
-WPYHIA-
WLPYHI--
WDPYHI--
WWPYHI--
WVPYHI--
-WPYHIM-
WKPYHI--
WYPYHI--

	-WPYHIT-		
	-WPYHIQ-		
	-WPYHIL-		
	WNPYHI--		
	WHPYHI--		
	YVPYHI--		
	YTPYHI--		
	-YPYHIL-		
	YPPYHI--		
	-YPYHIR-		
	YYPYHI--		
	YRPYHI--		
	YFPYHI--		
	YSPYHI--		
	-YPYHIA-		
	-YPYHIK-		
	YKPYHI--		
	YEPYHI--		
	YQPYHI--		
	-YPYHIQ-		
	YLPYHI--		
	YGPYHI--		
	-YPYHIS-		
	YDPYHI--		
	-YPYHIW-		
	-YPYHIV-		
	-YPYHII-		
	YIPYHI--		
	YHPYHI--		
	YAPYHI--		
PQGL	4	920	47.86
	IYPQGL--		
	--PQGLLP		
	--PQGLEG		
	ARPQGL--		
	AGPQGL--		
	AIPQGL--		
	-APQGLL-		
	ASPQGL--		
	AVPQGL--		
	ATPQGL--		
	-APQGLN-		
	-APQGLR-		
	-APQGLW-		
	AYPQGL--		
	-APQGLS-		
	AKPQGL--		
	-APQGLA-		
	AQPQGL--		
	-APQGLT-		

-APQGLI-
AFPQGL--
AWPQGL--
-APQGLG-
-APQGLV-
-APQGLY-
-APQGLH-
ADPQGL--
-APQGLQ-
ANPQGL--
-APQGLE-
APPQGL--
AAPQGL--
-APQGLM-
AMPQGL--
-APQGLK-
-APQGLF-
AHPQGL--
AEPQGL--
CKPQGL--
CTPQGL--
CLPQGL--
CSPQGL--
CQPQGL--
CYPQGL--
CRPQGL--
-CPQGLR-
CCPQGL--
-CPQGLW-
CAPQGL--
CVPQGL--
-CPQGLS-
CIPQGL--
-CPQGLT-
-CPQGLK-
-CPQGLH-
-CPQGLL-
CHPQGL--
-CPQGLY-
-CPQGLV-
-CPQGLN-
-CPQGLM-
-CPQGLG-
-CPQGLF-
CNPQGL--
-DPQGLR-
-DPQGLW-
-DPQGLY-
DFPQGL--
DVPQGL--

DWPQGL--
-DPQGLH-
DLPQGL--
DKPQGL--
-DPQGLF-
DAPQGL--
DSPQGL--
-DPQGLQ-
DNPQGL--
DYPQGL--
DIPQGL--
DGPQGL--
DTPQGL--
-DPQGLT-
-DPQGLS-
DHPQGL--
-DPQGLV-
-DPQGLM-
-DPQGLI-
-DPQGLL-
DMPQGL--
DEPQGL--
DQPQGL--
-DPQGLN-
-DPQGLK-
-EPQGLV-
-EPQGLS-
ESPQGL--
ETPQGL--
ELPQGL--
-EPQGLK-
-EPQGLY-
-EPQGLI-
EVPQGL--
ENPQGL--
EWPQGL--
-EPQGLL-
-EPQGLW-
-EPQGLR-
EAPQGL--
EYPQGL--
ERPQGL--
EQPQGL--
-EPQGLM-
EGPQGL--
-EPQGLT-
-EPQGLG-
EKPQGL--
EEPQGL--
-EPQGLA-

EMPQGL--
EFPQGL--
-EPQGLH-
EDPQGL--
-FPQGLR-
FKPQGL--
FSPQGL--
FLPQGL--
-FPQGLK-
FMPQGL--
FVPQGL--
-FPQGLL-
FIPQGL--
-FPQGLT-
FRPQGL--
FFPQGL--
FTPQGL--
-FPQGLF-
FAPQGL--
FGPQGL--
-FPQGLH-
FDPQGL--
-FPQGLW-
-FPQGLQ-
FQPQGL--
FWPQGL--
-FPQGLM-
FNPQGL--
-FPQGLS-
-FPQGLA-
FPPQGL--
-FPQGLI-
-FPQGLG-
FHPQGL--
-FPQGLY-
-FPQGLN-
-GPQGLR-
-GPQGLV-
GFPQGL--
GRPQGL--
GKPQGL--
-GPQGLW-
-GPQGLL-
GMPQGL--
-GPQGLT-
-GPQGLQ-
GLPQGL--
GVPQGL--
GQPQGL--
-GPQGLF-

GTPQGL--
GPPQGL--
-GPQGLI-
-GPQGLM-
-GPQGLN-
GNPQGL--
GDPQGL--
GHPQGL--
GGPQGL--
GAPQGL--
GYPQGL--
GEPQGL--
GIPQGL--
GWPQGL--
-GPQGLY-
-GPQGLK-
-GPQGLG-
GCPQGL--
-GPQGLE-
-GPQGLA-
-GPQGLH-
-HPQGLR-
HLPQGL--
HVPQGL--
HQPQGL--
HGPQGL--
-HPQGLF-
HRPQGL--
-HPQGLS-
HSPQGL--
HFPQGL--
HHPQGL--
HTPQGL--
-HPQGLM-
-HPQGLQ-
HAPQGL--
-HPQGLN-
HNPQGL--
-HPQGLY-
-HPQGLI-
-HPQGLV-
-HPQGLH-
HIPQGL--
HWPQGL--
-HPQGLT-
-HPQGLL-
HKPQGL--
HDPQGL--
-HPQGLW-
-HPQGLK-

HMPQGL--
HCPQGL--
-HPQGLG-
-HPQGLE-
-IPQGLR-
-IPQGLV-
-IPQGLH-
-IPQGLK-
ISPQGL--
-IPQGLS-
IRPQGL--
-IPQGLI-
-IPQGLT-
INPQGL--
-IPQGLN-
IFPQGL--
-IPQGLG-
-IPQGLA-
-IPQGLF-
IVPQGL--
IQPQGL--
ILPQGL--
IKPQGL--
IPPQGL--
IGPQGL--
IAPQGL--
IMPQGL--
-IPQGLQ-
-IPQGLM-
IEPQGL--
-IPQGLE-
-IPQGLY-
-IPQGLW-
IIPQGL--
IHPQGL--
IDPQGL--
ICPQGL--
-KPQGLK-
KGPQGL--
KNPQGL--
-KPQGLS-
KYPQGL--
-KPQGLF-
KKPQGL--
-KPQGLI-
-KPQGLN-
KVPQGL--
-KPQGLY-
-KPQGLR-
KTPQGL--

-KPQGLA-
KFPQGL--
KEPQGL--
KHPQGL--
KLPQGL--
-KPQGLH-
KIPQGL--
-KPQGLV-
-KPQGLQ-
KRPQGL--
KQPQGL--
-KPQGLM-
KWPQGL--
-KPQGLG-
KPPQGL--
KMPQGL--
KAPQGL--
-KPQGLE-
KDPQGL--
-LPQGLR-
LGPQGL--
LTPQGL--
-LPQGLH-
-LPQGLS-
LMPQGL--
LLPQGL--
-LPQGLT-
LKPQGL--
-LPQGLQ-
LPPQGL--
LNPQGL--
LAPQGL--
-LPQGLW-
LYPQGL--
LWPQGL--
LVPQGL--
-LPQGLI-
LIPQGL--
-LPQGLF-
LEPQGL--
-LPQGLV-
LFPQGL--
-LPQGLY-
-LPQGLN-
-LPQGLK-
-LPQGLA-
LHPQGL--
LDPQGL--
-LPQGLM-
-LPQGLG-

LQPQGL--
-LPQGLE-
MEPQGL--
-MPQGLT-
-MPQGLL-
-MPQGLR-
MSPQGL--
MRPQGL--
MGPQGL--
MVPQGL--
MLPQGL--
MYPQGL--
-MPQGLN-
-MPQGLH-
-MPQGLF-
MMPQGL--
-MPQGLG-
-MPQGLA-
MFPQGL--
-MPQGLS-
-MPQGLI-
-MPQGLK-
-MPQGLW-
MKPQGL--
MWPQGL--
-MPQGLY-
-MPQGLV-
MIPQGL--
MTPQGL--
MPPQGL--
MNPQGL--
-MPQGLM-
-MPQGLE-
MDPQGL--
MQPQGL--
NSPQGL--
NMPQGL--
-NPQGLR-
NAPQGL--
-NPQGLY-
NRPQGL--
-NPQGLT-
-NPQGLS-
NYPQGL--
-NPQGLI-
NHPQGL--
NVPQGL--
NTPQGL--
-NPQGLW-
-NPQGLL-

-NPQGLF-
NPPQGL--
NCPQGL--
NWPQGL--
-NPQGLN-
-NPQGLK-
-NPQGLA-
NFPQGL--
-NPQGLV-
NEPQGL--
-NPQGLQ-
-NPQGLM-
NDPQGL--
NQPQGL--
-NPQGLG-
NNPQGL--
NKPQGL--
--PQGLRS
--PQGLLR
--PQGLSG
--PQGLRA
--PQGLLT
--PQGLST
--PQGLFR
--PQGLMS
--PQGLVR
--PQGLRT
--PQGLLY
--PQGLEM
--PQGLRY
--PQGLKG
--PQGLLG
--PQGLSS
--PQGLLK
--PQGLHL
--PQGLLW
--PQGLRR
--PQGLRH
--PQGLKR
--PQGLTR
--PQGLRL
--PQGLIA
--PQGLVS
--PQGLHS
--PQGLWS
--PQGLMT
--PQGLTG
--PQGLSR
--PQGLLQ
--PQGLSF

--PQGLRI
--PQGLMA
--PQGLLS
--PQGLAS
--PQGLLM
PVPQGL--
--PQGLYR
--PQGLVV
--PQGLSV
--PQGLLV
--PQGLLH
PLPQGL--
--PQGLVA
--PQGLAG
PHPQGL--
--PQGLSL
--PQGLFL
--PQGLNF
--PQGLWR
--PQGLRK
--PQGLFS
--PQGLFN
--PQGLTL
--PQGLIF
--PQGLVL
--PQGLNS
--PQGLNA
--PQGLMV
PNPQGL--
PSPQGL--
--PQGLYS
--PQGLWG
--PQGLVT
--PQGLNG
--PQGLGV
--PQGLAT
--PQGLYV
--PQGLVG
--PQGLTF
--PQGLTA
--PQGLSN
--PQGLNM
--PQGLKS
--PQGLTS
--PQGLTQ
--PQGLKK
--PQGLTM
--PQGLMN
--PQGLHD
PRPQGL--

--PQGLTV
--PQGLTH
--PQGLMG
--PQGLHR
--PQGLFI
--PQGLFD
--PQGLWV
--PQGLRM
--PQGLFT
--PQGLVK
--PQGLNY
--PQGLMK
--PQGLIN
--PQGLHT
--PQGLGS
--PQGLFA
--PQGLYA
--PQGLWT
--PQGLRN
--PQGLKT
--PQGLFK
--PQGLYQ
--PQGLYK
--PQGLWK
--PQGLVI
--PQGLTK
--PQGLTI
--PQGLRF
--PQGLGA
--PQGLTW
--PQGLLF
--PQGLIR
--PQGLIL
--PQGLII
--PQGLHM
--PQGLFG
--PQGLAW
--PQGLAF
--PQGLYN
--PQGLSW
--PQGLQN
--PQGLKL
--PQGLIY
--PQGLHN
--PQGLFY
--PQGLWH
--PQGLQS
--PQGLKE
--PQGLHH
PIPQGL--

PEPQGL--
PWPQGL--
--PQGLWN
--PQGLWM
--PQGLVD
--PQGLSY
--PQGLSQ
--PQGLQL
--PQGLMF
--PQGLKI
--PQGLHI
--PQGLYF
--PQGLSD
--PQGLNT
--PQGLMQ
--PQGLIK
--PQGLIG
--PQGLIE
--PQGLEW
--PQGLWL
--PQGLVN
--PQGLQR
--PQGLNV
--PQGLKW
--PQGLKV
--PQGLIQ
--PQGLAA
--PQGLYG
--PQGLWI
--PQGLVY
--PQGLVF
--PQGLVE
--PQGLTD
--PQGLRQ
--PQGLMI
--PQGLIV
PYPQGL--
--PQGLYW
--PQGLYI
--PQGLIS
PKPQGL--
PGPQGL--
--PQGLSM
--PQGLSI
--PQGLNL
--PQGLHF
--PQGLGI
--PQGLAM
PMPQGL--
--PQGLYM

--PQGLVW
--PQGLVH
--PQGLQI
--PQGLMH
--PQGLMD
--PQGLIH
--PQGLGY
--PQGLYY
--PQGLLA
--PQGLKA
--PQGLHV
--PQGLHK
--PQGLGF
--PQGLAN
--PQGLAL
--PQGLAH
PDPQGL--
PAPQGL--
--PQGLRE
--PQGLRD
--PQGLLD
--PQGLKN
--PQGLFW
--PQGLYH
--PQGLYD
--PQGLQV
--PQGLQQ
--PQGLFM
--PQGLFH
--PQGLET
--PQGLAY
--PQGLAI
--PQGLYT
--PQGLSH
--PQGLKM
--PQGLKH
--PQGLHY
--PQGLDG
--PQGLAV
--PQGLIT
--PQGLIM
--PQGLHW
--PQGLHA
--PQGLFQ
--PQGLEY
--PQGLES
--PQGLEF
--PQGLEA
--PQGLDA
--PQGLWW

--PQGLQA
--PQGLNK
--PQGLND
--PQGLLE
--PQGLKD
--PQGLHG
--PQGLHE
--PQGLGL
--PQGLEN
--PQGLEK
PFPQGL--
PTPQGL--
--PQGLYE
--PQGLWD
--PQGLWC
--PQGLWA
--PQGLVQ
--PQGLSC
--PQGLQM
--PQGLQH
--PQGLNQ
--PQGLNN
--PQGLNI
--PQGLNH
--PQGLME
--PQGLGM
--PQGLER
--PQGLCS
--PQGLCQ
--PQGLAE
--PQGLWE
--PQGLVM
--PQGLQT
--PQGLQF
--PQGLNE
--PQGLGW
--PQGLFC
--PQGLEV
--PQGLDS
--PQGLCE
--PQGLCA
--PQGLAQ
--PQGLRP
--PQGLQE
--PQGLKF
--PQGLHQ
--PQGLGQ
--PQGLFE
--PQGLEL
--PQGLEH

--PQGLEC
--PQGLDR
--PQGLCY
--PQGLCW
--PQGLCG
--PQGLAD
-PPQGLI-
-PPQGLF-
-QPQGLR-
-QPQGLS-
QVPQGL--
QCPQGL--
QKPQGL--
-QPQGLT-
-QPQGLN-
-QPQGLY-
QAPQGL--
-QPQGLL-
-QPQGLF-
QYPQGL--
-QPQGLQ-
-QPQGLH-
QLPQGL--
QTPQGL--
QHPQGL--
-QPQGLA-
QEPQGL--
QWPQGL--
QQPQGL--
QPPQGL--
QSPQGL--
QDPQGL--
QIPQGL--
QGPQGL--
-QPQGLK-
QMPQGL--
-QPQGLG-
-QPQGLM-
-QPQGLI-
-QPQGLE-
QFPQGL--
-QPQGLW-
-QPQGLC-
RGPQGL--
-RPQGLA-
-RPQGLS-
-RPQGLR-
RSPQGL--
-RPQGLL-
RMPQGL--

RRPQGL--
-RPQGLM-
RWPQGL--
-RPQGLT-
-RPQGLY-
RFPQGL--
RAPQGL--
-RPQGLN-
RPPQGL--
RIPQGL--
-RPQGLK-
RYPQGL--
-RPQGLV-
-RPQGLI-
-RPQGLG-
RHPQGL--
RNPQGL--
RDPQGL--
-RPQGLW-
-RPQGLF-
-RPQGLQ-
REPQGL--
-RPQGLH-
RQPQGL--
-RPQGLE-
RCPQGL--
RKPQGL--
-SPQGLL-
SYPQGL--
SLPQGL--
SIPQGL--
-SPQGLR-
-SPQGLT-
-SPQGLW-
SEPQGL--
-SPQGLY-
-SPQGLS-
SHPQGL--
STPQGL--
-SPQGLI-
SGPQGL--
SKPQGL--
SNPQGL--
SSPQGL--
SMPQGL--
SQPQGL--
-SPQGLV-
SAPQGL--
-SPQGLM-
-SPQGLA-

SRPQGL--
-SPQGLE-
-SPQGLK-
-SPQGLH-
-SPQGLQ-
-SPQGLF-
-SPQGLG-
SFPQGL--
SDPQGL--
-SPQGLN-
SWPQGL--
SPPQGL--
SCPQGL--
TSPQGL--
-TPQGLS-
TLPQGL--
-TPQGLH-
-TPQGLL-
TQPQGL--
-TPQGLT-
TRPQGL--
-TPQGLI-
TGPQGL--
TVPQGL--
TTPQGL--
TYPQGL--
-TPQGLV-
TMPQGL--
TAPQGL--
TFPQGL--
TKPQGL--
TIPQGL--
-TPQGLQ-
-TPQGLW-
TEPQGL--
TNPQGL--
THPQGL--
TWPQGL--
-TPQGLE-
TDPQGL--
-TPQGLK-
-TPQGLF-
TPPQGL--
-TPQGLG-
-TPQGLA-
-TPQGLN-
-TPQGLM-
TCPQGL--
VLPQGL--
-VPQGLS-

VVPQGL--
VRPQGL--
-VPQGLR-
-VPQGLL-
-VPQGLI-
-VPQGLA-
VAPQGL--
VWPQGL--
-VPQGLN-
VNPQGL--
VMPQGL--
VGPQGL--
VHPQGL--
VQPQGL--
-VPQGLY-
-VPQGLW-
-VPQGLM-
VIPQGL--
VSPQGL--
-VPQGLT-
VFPQGL--
-VPQGLV-
VYPQGL--
-VPQGLK-
VTPQGL--
-VPQGLQ-
-VPQGLH-
-VPQGLG-
-VPQGLF-
VEPQGL--
-VPQGLE-
VCPQGL--
VDPQGL--
-WPQGLR-
-WPQGLL-
WSPQGL--
WAPQGL--
WDPQGL--
WTPQGL--
-WPQGLK-
WEPQGL--
-WPQGLH-
WMPQGL--
WGPQGL--
-WPQGLQ-
WNPQGL--
-WPQGLI-
WKPQGL--
-WPQGLM-
WPPQGL--

-WPQGLT-
-WPQGLW-
WYPQGL--
-WPQGLY-
WRPQGL--
WFPQGL--
-WPQGLV-
-WPQGLF-
WVPQGL--
WIPQGL--
-WPQGLN-
-WPQGLG-
WWPQGL--
WQPQGL--
-WPQGLS-
YQPQGL--
-YPQGLR-
YVPQGL--
YSPQGL--
YIPQGL--
YKPQGL--
-YPQGLK-
-YPQGLN-
-YPQGLV-
-YPQGLS-
-YPQGLL-
YLPQGL--
YWPQGL--
YNPQGL--
YMPQGL--
YGPQGL--
-YPQGLT-
-YPQGLQ-
-YPQGLM-
-YPQGLH-
-YPQGLA-
YTPQGL--
-YPQGLF-
-YPQGLY-
YFPQGL--
YAPQGL--
YDPQGL--
-YPQGLW-
YEPQGL--
YHPQGL--
-YPQGLI-
-YPQGLG-
-YPQGLE-
-YPQGLD-
-YPQGLC-

YCPQGL--
PWNM 435 646 47.84
-APWNML-
ALPWNM--
-APWNMK-
AGPWNM--
-APWNMR-
ANPWNM--
-APWNMS-
-APWNMH-
AWPWNM--
ATPWNM--
ASPWNM--
-APWNMF-
AEPWNM--
AYPWNM--
ARPWNM--
-APWNMV-
AVPWNM--
AQPWNM--
-APWNMY-
-APWNMT-
-APWNMM-
-APWNMI-
AMPWNM--
-APWNMQ-
-APWNMG-
AKPWNM--
-APWNMA-
CCPWNM--
CLPWNM--
CVPWNM--
CRPWNM--
-CPWNMY-
-CPWNMA-
CDPWNM--
DLPWNM--
DSPWNM--
DRPWNM--
-DPWNMI-
DVPWNM--
-DPWNMS-
DYPWNM--
-DPWNMR-
-DPWNMA-
-DPWNMH-
-DPWNMN-
DTPWNM--
-DPWNMT-
DIPWNM--

DHPWNM--
DDPWNM--
EKPWNM--
ESPWNM--
ERPWNM--
-EPWNMM-
EAPWNM--
-EPWNMS-
-EPWNMR-
ETPWNM--
EMPWNM--
-EPWNMV-
-EPWNMI-
ELPWNM--
-EPWNML-
EVPWNM--
-EPWNMH-
-EPWNMA-
ENPWNM--
EQPWNM--
-EPWNMQ-
EFPWNM--
-FPWNMM-
-FPWNMK-
FDPWNM--
FAPWNM--
-FPWNMT-
-FPWNMA-
FNPWNM--
FKPWNM--
-FPWNMR-
FLPWNM--
-FPWNMS-
-FPWNMG-
-FPWNMF-
FGPWNM--
FWPWNM--
-FPWNMQ-
-FPWNMN-
-FPWNMI-
-FPWNMH-
FRPWNM--
FHPWNM--
FEPWNM--
GTPWNM--
GAPWNM--
GVPWNM--
-GPWNME-
GLPWNM--
-GPWNMN-

-GPWNMR-
-GPWNMM-
GFPWNM--
-GPWNML-
GGPWNM--
-GPWNMS-
-GPWNMI-
-GPWNMH-
-GPWNMT-
GSPWNM--
-GPWNMW-
-GPWNMV-
-GPWNMK-
-GPWNMF-
-GPWNMA-
GKPWNM--
GRPWNM--
-GPWNMG-
GPPWNM--
GMPWNM--
GWPWNM--
GQPWNM--
GYPWNM--
GHPWNM--
HIPWNM--
HYPWNM--
HNPWNM--
-HPWNMR-
-HPWNMT-
HTPWNM--
HSPWNM--
-HPWNMK-
HKPWNM--
-HPWNMN-
HHPWNM--
-HPWNML-
HEPWNM--
HRPWNM--
-HPWNMS-
-HPWNMG-
-HPWNMF-
HWPWNM--
-HPWNMA-
-HPWNME-
HPPWNM--
HLPWNM--
HGPWNM--
-HPWNMH-
HAPWNM--
HQPWNM--

HDPWNM--
-IPWNMA-
IRPWNM--
IGPWNM--
ISPWNM--
ITPWNM--
-IPWNMI-
IKPWNM--
IFPWNM--
-IPWNMW-
-IPWNMN-
IQPWNM--
-IPWNMV-
-IPWNMT-
INPWNM--
-IPWNME-
IMPWNM--
IHPWNM--
-IPWNMS-
-IPWNMG-
IAPWNM--
-IPWNMP-
-IPWNMM-
KSPWNM--
-KPWNML-
-KPWNMT-
-KPWNMW-
KDPWNM--
KGPWNM--
-KPWNMY-
KIPWNM--
KRPWNM--
KFPWNM--
KTPWNM--
-KPWNMR-
-KPWNMV-
-KPWNMH-
-KPWNMD-
-KPWNMA-
KNPWNM--
KCPWNM--
-KPWNMS-
KWPWNM--
-KPWNMK-
-KPWNMI-
KKPWNM--
KEPWNM--
KHPWNM--
KYPWNM--
KQPWNM--

KPPWNM--
-LPWNML-
LMPWNM--
LHPWNM--
LGPWNM--
-LPWNMT-
LSPWNM--
-LPWNMF-
LQPWNM--
-LPWNMI-
LTPWNM--
-LPWNMV-
LLPWNM--
-LPWNMS-
LAPWNM--
-LPWNMR-
-LPWNMQ-
LIPWNM--
LYPWNM--
LWPWNM--
LVPWNM--
-LPWNMH-
-LPWNMA-
-LPWNME-
LKPWNM--
LFPWNM--
LCPWNM--
MSPWNM--
-MPWNMY-
-MPWNMS-
-MPWNMT-
MVPWNM--
-MPWNMA-
MGPWNM--
-MPWNMN-
MRPWNM--
MNPWNM--
MTPWNM--
MQPWNM--
-MPWNMV-
-MPWNMI-
-MPWNMG-
MHPWNM--
-MPWNML-
-MPWNMK-
MPPWNM--
-MPWNMW-
-MPWNMR-
-MPWNMM-
MMPWNM--

MKPWNM--
NWPWNM--
-NPWNMR-
NLPWNM--
-NPWNMH-
NTPWNM--
-NPWNMS-
-NPWNMA-
-NPWNMQ-
NDPWNM--
-NPWNMV-
-NPWNME-
NAPWNM--
-NPWNMK-
NIPWNM--
NYPWNM--
NRPWNM--
-NPWNMN-
-NPWNMM-
NPPWNM--
NHPWNM--
NGPWNM--
-NPWNMF-
--PWNMYA
--PWNMRS
--PWNMLQ
--PWNMKR
--PWNMAS
--PWNMYG
--PWNMSM
--PWNMVS
--PWNMMR
--PWNMSG
--PWNMKG
--PWNMWT
--PWNMRG
--PWNMHS
--PWNMVR
--PWNMSK
--PWNMQS
--PWNMLG
--PWNMVA
--PWNMMG
--PWNMKN
--PWNMTR
--PWNMRA
--PWNMHA
--PWNMVN
--PWNMTS
--PWNMQT

--PWNMQV
--PWNMLA
--PWNMIR
--PWNMGS
--PWNMWH
--PWNMSQ
--PWNMIG
--PWNMTF
--PWNMQL
--PWNMQF
--PWNMHT
--PWNMAG
--PWNMYV
--PWNMVY
--PWNMTY
--PWNMTT
--PWNMQN
--PWNMLT
--PWNMLD
--PWNMHM
--PWNMGV
--PWNMYS
--PWNMSI
--PWNMQG
--PWNMNV
--PWNMMH
--PWNMAR
PDPWNM--
PYPWNM--
--PWNMYT
--PWNMST
--PWNMMK
--PWNMKA
--PWNMGG
--PWNMAT
--PWNMYN
--PWNMWG
--PWNMTN
--PWNMTL
--PWNMRK
--PWNMRI
--PWNMLS
--PWNMKQ
--PWNMKH
--PWNMIT
--PWNMIA
--PWNMEG
PMPWNM--
--PWNMYM
--PWNMWR

--PWNMVK
--PWNMVH
--PWNMNR
--PWNMMS
--PWNMLW
--PWNMLK
--PWNMFV
--PWNMFR
--PWNMEY
--PWNMYQ
--PWNMWV
--PWNMVW
--PWNMVI
--PWNMVD
--PWNMTI
--PWNMSW
--PWNMSN
--PWNMSF
--PWNMSA
--PWNMRT
--PWNMRM
--PWNMQR
--PWNMNS
--PWNMMT
--PWNMLH
--PWNMFN
PSPWNM--
PNPWNM--
--PWNMTM
--PWNMTG
--PWNMSS
--PWNMNN
--PWNMME
--PWNMLN
--PWNMKY
--PWNMKV
--PWNMIS
--PWNMGT
--PWNMGL
--PWNMFT
--PWNMEL
PTPWNM--
PKPWNM--
PWPWNM--
--PWNMVQ
--PWNMSD
--PWNMNA
--PWNMML
--PWNMKT
--PWNMFQ

--PWNMFK
--PWNMFD
--PWNMET
--PWNMER
--PWNMEH
PVPWNM--
PGPWNM--
--PWNMYH
--PWNMWS
--PWNMVM
--PWNMVG
--PWNMTV
--PWNMSV
--PWNMRE
--PWNMRD
--PWNMQW
--PWNMQM
--PWNMQE
--PWNMMN
--PWNMLE
--PWNMKS
--PWNMKE
--PWNMIY
--PWNMHH
--PWNMGY
--PWNMGR
--PWNMGH
--PWNMGA
--PWNMFS
--PWNMEV
--PWNMEK
--PWNMCS
--PWNMAV
--PWNMAN
--PWNMAL
--PWNMAD
PIPWNM--
--PWNMYR
--PWNMYK
--PWNMYF
--PWNMYD
--PWNMWQ
--PWNMWE
--PWNMWA
--PWNMVV
--PWNMVE
--PWNMTQ
--PWNMSY
--PWNMQK
--PWNMNG

--PWNMMI
--PWNMIN
--PWNMHW
--PWNMHF
--PWNMHE
--PWNMGK
--PWNMGI
--PWNMGE
--PWNMDA
--PWNMCM
-PPWNMG-
QVPWNM--
QSPWNM--
-QPWNMY-
-QPWNMR-
-QPWNMI-
QHPWNM--
-QPWNMT-
QNPWNM--
QLPWNM--
QAPWNM--
QPPWNM--
QRPWNM--
-QPWNML-
QIPWNM--
-QPWNMS-
-QPWNMK-
QWPWNM--
-QPWNMG-
QFPWNM--
-QPWNMQ-
-QPWNMN-
-QPWNMM-
QYPWNM--
QTPWNM--
-QPWNMW-
-RPWNMS-
-RPWNMF-
RSPWNM--
RNPWNM--
RKPWNM--
-RPWNMI-
RPPWNM--
RTPWNM--
RRPWNM--
RGPWNM--
-RPWNMK-
RLPWNM--
RIPWNM--
-RPWNML-

-RPWNMM-
-RPWNME-
-RPWNMA-
RHPWNM--
-RPWNMV-
-RPWNMQ-
REPWNM--
RVPWNM--
-RPWNMT-
-RPWNMN-
-RPWNMG-
RAPWNM--
RFPWNM--
-RPWNMY-
-RPWNMR-
RDPWNM--
-RPWNMW-
RWPWNM--
-RPWNMD-
RMPWNM--
RQPWNM--
-RPWNMH-
RYPWNM--
SRPWNM--
-SPWNML-
SNPWNM--
SLPWNM--
SMPWNM--
-SPWNMH-
-SPWNMT-
-SPWNMK-
-SPWNMI-
-SPWNMY-
STPWNM--
-SPWNMV-
-SPWNMS-
SSPWNM--
SAPWNM--
-SPWNMN-
SIPWNM--
SVPWNM--
SPPWNM--
SGPWNM--
-SPWNMG-
-SPWNMM-
SEPWNM--
SYPWNM--
SWPWNM--
TSPWNM--
-TPWNMH-

TTPWNM--
TAPWNM--
TLPWNM--
TVPWNM--
TNPWNM--
TGPWNM--
-TPWNMY-
-TPWNMV-
-TPWNMM-
-TPWNMW-
-TPWNMK-
-TPWNMA-
-TPWNMR-
TDPWNM--
TQPWNM--
-TPWNMT-
-TPWNMN-
TIPWNM--
TYPWNM--
-TPWNMI-
TRPWNM--
-TPWNMF-
-TPWNME-
TPPWNM--
TWPWNM--
-TPWNMQ-
TFPWNM--
VSPWNM--
-VPWNMR-
-VPWNML-
-VPWNMT-
VHPWNM--
-VPWNMN-
VMPWNM--
-VPWNMY-
-VPWNMS-
-VPWNME-
VRPWNM--
-VPWNMH-
-VPWNMK-
VGPWNM--
VQPWNM--
-VPWNMQ-
-VPWNMI-
VPPWNM--
VEPWNM--
-VPWNMM-
-VPWNMG-
VYPWNM--
VCPWNM--

VTPWNM--
VFPWNM--
-VPWNMV-
VNPWNM--
VLPWNM--
VIPWNM--
VDPWNM--
WHPWNM--
WMPWNM--
-WPWNMT-
WVPWNM--
WSPWNM--
-WPWNMS-
WKPWNM--
WIPWNM--
-WPWNMG-
WLPWNM--
WDPWNM--
WWPWNM--
-WPWNMR-
-WPWNMQ-
-WPWNMN-
-WPWNMH-
WGPWNM--
WAPWNM--
YRPWNM--
YNPWNM--
YGPWNM--
-YPWNMV-
-YPWNMT-
-YPWNMK-
-YPWNMG-
-YPWNMF-
YSPWNM--
YVPWNM--
YMPWNM--
YLPWNM--
YQPWNM--
-YPWNMR-
-YPWNMQ-
-YPWNMA-
YPPWNM--
YWPWNM--
YFPWNM--
YDPWNM--
-YPWNMI-
-YPWNME-
YHPWNM--

PRAI	365	672	47.84

--PRAISP

--PRAIPP
--PRAINP
-RPRAIP-
ALPRAI--
ASPRAI--
-APRAIL-
AYPRAI--
ANPRAI--
ARPRAI--
AFPRAI--
APPRAI--
AMPRAI--
-APRAIW-
-APRAIK-
AGPRAI--
-APRAIV-
-APRAIN-
AHPRAI--
-APRAIQ-
-APRAIA-
AVPRAI--
ACPRAI--
AEPRAI--
-APRAIM-
ADPRAI--
-CPRAIR-
CVPRAI--
CTPRAI--
CLPRAI--
CAPRAI--
-CPRAIL-
CGPRAI--
CCPRAI--
-CPRAIS-
-CPRAIK-
CHPRAI--
CYPRAI--
CRPRAI--
-CPRAIH-
CSPRAI--
-CPRAIQ-
-CPRAIM-
CIPRAI--
CFPRAI--
CEPRAI--
CWPRAI--
-CPRAII-
-CPRAIF-
-CPRAIA-
CPPRAI--

CQPRAI--
-CPRAIN-
-CPRAIE-
DRPRAI--
DKPRAI--
-DPRAIR-
-DPRAIL-
DVPRAI--
-DPRAIK-
DHPRAI--
-DPRAIT-
-DPRAIV-
DNPRAI--
DFPRAI--
DSPRAI--
-DPRAIS-
-DPRAIM-
DMPRAI--
DLPRAI--
DTPRAI--
-DPRAIH-
-DPRAII-
-DPRAIW-
DIPRAI--
DYPRAI--
DQPRAI--
-DPRAIF-
-DPRAIQ-
-DPRAIA-
DGPRAI--
DEPRAI--
DAPRAI--
-DPRAIE-
DDPRAI--
ERPRAI--
ESPRAI--
-EPRAIL-
EYPRAI--
ELPRAI--
-EPRAII-
-EPRAIS-
ETPRAI--
-EPRAIT-
-EPRAIM-
EMPRAI--
EVPRAI--
-EPRAIG-
-EPRAIW-
EKPRAI--
EQPRAI--

-EPRAIN-
-EPRAIK-
EIPRAI--
-EPRAIH-
ENPRAI--
EFPRAI--
EAPRAI--
-EPRAIV-
EPPRAI--
EHPRAI--
-EPRAIA-
EEPRAI--
-EPRAIE-
FIPRAI--
FLPRAI--
FHPRAI--
FGPRAI--
FVPRAI--
FTPRAI--
-FPRAIN-
-FPRAIV-
FEPRAI--
FAPRAI--
FPPRAI--
FDPRAI--
-FPRAIQ-
FNPRAI--
-FPRAIG-
-FPRAIE-
FCPRAI--
-GPRAIL-
GQPRAI--
GMPRAI--
-GPRAIS-
GVPRAI--
-GPRAIT-
GIPRAI--
-GPRAIV-
GTPRAI--
GKPRAI--
GSPRAI--
-GPRAII-
-GPRAIK-
GNPRAI--
-GPRAIY-
-GPRAIN-
GYPRAI--
GAPRAI--
GDPRAI--
-GPRAIM-

-GPRAIH-
GPPRAI--
-GPRAIF-
GEPRAI--
-GPRAIG-
-GPRAIE-
GCPRAI--
-HPRAIL-
HFPRAI--
-HPRAIK-
HGPRAI--
-HPRAIT-
HKPRAI--
HDPRAI--
HPPRAI--
HYPRAI--
-HPRAIM-
HLPRAI--
HMPRAI--
HEPRAI--
-HPRAIQ-
-HPRAII-
HVPRAI--
HQPRAI--
-HPRAIY-
HCPRAI--
-HPRAIE-
IPPRAI--
-IPRAIH-
IAPRAI--
ISPRAI--
IRPRAI--
-IPRAIR-
IYPRAI--
-IPRAIS-
IEPRAI--
IWPRAI--
-IPRAII-
-IPRAIW-
-IPRAIQ-
-IPRAIK-
-IPRAIV-
IGPRAI--
INPRAI--
IQPRAI--
IDPRAI--
-IPRAIY-
-IPRAIG-
-IPRAIT-
-IPRAIE-

ICPRAI--
KVPRAI--
-KPRAII-
KLPRAI--
-KPRAIV-
KPPRAI--
KKPRAI--
-KPRAIY-
KIPRAI--
KDPRAI--
-KPRAIH-
-KPRAIQ-
-KPRAIG-
-KPRAIE-
LKPRAI--
LPPRAI--
LVPRAI--
LQPRAI--
LGPRAI--
-LPRAIV-
-LPRAIH-
-LPRAII-
-LPRAIY-
-LPRAIN-
-LPRAIQ-
LDPRAI--
LNPRAI--
LEPRAI--
-LPRAIG-
-LPRAIM-
-LPRAIW-
-LPRAIE-
-LPRAIC-
MTPRAI--
-MPRAIT-
MSPRAI--
-MPRAIH-
MGPRAI--
MEPRAI--
-MPRAIY-
MFPRAI--
MAPRAI--
-MPRAIQ-
-MPRAII-
-MPRAIF-
MNPRAI--
-MPRAIA-
MPPRAI--
-MPRAIN-
MIPRAI--

```
-MPRAIV-
-MPRAIM-
-MPRAIE-
MDPRAI--
-MPRAIS-
MYPRAI--
MQPRAI--
MHPRAI--
MCPRAI--
NLPRAI--
-NPRAIS-
-NPRAIF-
-NPRAIT-
NYPRAI--
NVPRAI--
-NPRAIL-
NEPRAI--
-NPRAIK-
NHPRAI--
NIPRAI--
-NPRAIE-
NQPRAI--
-NPRAIY-
NNPRAI--
-NPRAIV-
-NPRAIQ-
NDPRAI--
-NPRAIW-
-NPRAIA-
NKPRAI--
-NPRAIH-
-NPRAIM-
NCPRAI--
--PRAIVS
--PRAILT
--PRAISA
--PRAITL
--PRAISG
--PRAILK
--PRAILG
--PRAIYG
--PRAIAG
--PRAIVL
--PRAIGL
--PRAIWV
--PRAIMS
--PRAILV
--PRAIMG
--PRAIYS
--PRAITW
```

--PRAIVT
--PRAITG
--PRAINL
--PRAISY
--PRAIKG
--PRAIHT
--PRAIVG
--PRAISH
--PRAIWS
--PRAIIA
--PRAIGF
--PRAISQ
PGPRAI--
--PRAIHS
--PRAIML
--PRAITQ
--PRAIIN
--PRAIFG
--PRAIAI
--PRAIRV
--PRAIMN
--PRAIAF
--PRAIWT
--PRAIVF
--PRAITT
--PRAIQS
--PRAIHR
--PRAIHI
--PRAIGM
--PRAIHG
--PRAIST
--PRAISM
--PRAILI
--PRAILE
--PRAIYT
--PRAIWK
--PRAIGS
PLPRAI--
--PRAIWW
--PRAIIT
--PRAIQI
--PRAIFA
--PRAIVK
--PRAIME
--PRAIIY
--PRAIGA
--PRAIES
--PRAIEA
--PRAIAH
--PRAIQG

--PRAIFN
PMPRAI--
--PRAISN
--PRAIHV
--PRAIGY
--PRAIFT
--PRAIKV
--PRAIIQ
--PRAIFI
--PRAILN
--PRAIYK
--PRAIQV
--PRAILD
--PRAIFD
--PRAISK
--PRAIYN
--PRAIQT
--PRAIKH
--PRAIHN
--PRAIEY
--PRAIAQ
--PRAIQD
--PRAIIH
--PRAIYW
--PRAIYA
--PRAIWY
--PRAIVQ
--PRAISD
--PRAIHQ
PSPRAI--
--PRAIVH
--PRAITE
--PRAIQA
--PRAIKF
--PRAIKA
--PRAIHM
--PRAIGG
PDPRAI--
--PRAITK
--PRAINT
--PRAIGI
--PRAIYF
--PRAIWQ
--PRAITV
--PRAIRW
--PRAIPL
--PRAIPI
--PRAINS
--PRAING
--PRAIFE

PQPRAI--
--PRAIKQ
--PRAIII
--PRAIHK
--PRAIGQ
--PRAIFH
--PRAIAY
PNPRAI--
--PRAIVY
--PRAIVA
--PRAIIW
--PRAIIF
--PRAIHY
--PRAIHH
--PRAIGK
--PRAIEN
--PRAIEL
--PRAIYD
--PRAIWG
--PRAINA
--PRAIMY
--PRAIMQ
--PRAIHA
--PRAIFW
--PRAIWF
--PRAIWD
--PRAIVE
--PRAIQN
--PRAIPY
--PRAIMW
--PRAIMT
--PRAIHD
--PRAIAV
--PRAIAT
--PRAIWI
--PRAIVM
--PRAIVI
--PRAITH
--PRAIQW
--PRAINW
--PRAIKE
--PRAIIK
--PRAIET
--PRAIWE
--PRAIVV
--PRAINY
--PRAIIE
--PRAIYE
--PRAISW
--PRAIMF

--PRAIGH
--PRAIEQ
--PRAIEF
--PRAIQM
--PRAINE
--PRAIKM
--PRAIHE
--PRAIAN
--PRAIAA
PFPRAI--
--PRAIYQ
--PRAIWH
--PRAIVN
--PRAINQ
--PRAINN
--PRAINF
--PRAIMD
--PRAITM
--PRAITD
--PRAIPF
--PRAIIS
--PRAIEK
PAPRAI--
--PRAISE
--PRAIQF
--PRAIPV
--PRAINV
--PRAINK
--PRAIKD
--PRAIAM
--PRAIWM
--PRAIRC
--PRAIQE
--PRAIQC
--PRAIPW
--PRAINM
--PRAINH
--PRAILC
--PRAIEW
--PRAIEH
--PRAICI
PVPRAI--
--PRAIYC
--PRAIRP
--PRAIQY
--PRAIQH
--PRAIMP
--PRAIFP
--PRAIFC
--PRAIEM

--PRAIEI
--PRAIEG
--PRAICS
--PRAIAE
--PRAIVC
--PRAITC
--PRAIKW
--PRAIIP
--PRAIHW
--PRAIGE
--PRAIGC
--PRAIEE
--PRAIDY
--PRAIDW
--PRAIDM
--PRAIDI
--PRAIDA
--PRAICT
--PRAICR
--PRAICM
--PRAICG
-PPRAIV-
-PPRAIQ-
-PPRAIM-
QGPRAI--
-QPRAIS-
-QPRAIL-
-QPRAIF-
QLPRAI--
QMPRAI--
-QPRAIA-
QIPRAI--
-QPRAIK-
QAPRAI--
QKPRAI--
QNPRAI--
-QPRAII-
QFPRAI--
QEPRAI--
-QPRAIW-
QQPRAI--
-QPRAIQ-
-QPRAIM-
QSPRAI--
-QPRAIG-
QYPRAI--
QTPRAI--
QWPRAI--
-QPRAIN-
-QPRAIE-

-QPRAIY-
-QPRAIV-
QHPRAI--
QDPRAI--
QPPRAI--
-QPRAIC-
-RPRAIS-
-RPRAIT-
RQPRAI--
RNPRAI--
REPRAI--
-RPRAIW-
-RPRAIY-
RDPRAI--
-RPRAIN-
-RPRAIM-
RPPRAI--
-RPRAIE-
RKPRAI--
RCPRAI--
-RPRAIC-
SSPRAI--
-SPRAIS-
STPRAI--
SGPRAI--
-SPRAIK-
-SPRAIY-
-SPRAII-
SNPRAI--
SQPRAI--
SPPRAI--
SEPRAI--
-SPRAIV-
-SPRAIF-
-SPRAIM-
SHPRAI--
-SPRAIN-
-SPRAIE-
SCPRAI--
-TPRAII-
TSPRAI--
TLPRAI--
-TPRAIS-
TVPRAI--
TIPRAI--
-TPRAIR-
-TPRAIQ-
-TPRAIF-
TKPRAI--
-TPRAIH-

-TPRAIK-
-TPRAIA-
TWPRAI--
TDPRAI--
-TPRAIV-
THPRAI--
-TPRAIN-
-TPRAIG-
TEPRAI--
TPPRAI--
-TPRAIE-
VLPRAI--
VKPRAI--
-VPRAIS-
-VPRAIK-
VNPRAI--
VPPRAI--
VGPRAI--
VQPRAI--
VVPRAI--
-VPRAIA-
VAPRAI--
-VPRAII-
-VPRAIW-
-VPRAIV-
VIPRAI--
VEPRAI--
-VPRAIY-
VYPRAI--
-VPRAIM-
-VPRAIE-
-VPRAIG-
VDPRAI--
VCPRAI--
WGPRAI--
WKPRAI--
WTPRAI--
WMPRAI--
WEPRAI--
-WPRAIL-
-WPRAIM-
WLPRAI--
WDPRAI--
-WPRAIV-
-WPRAIQ-
-WPRAIH-
WPPRAI--
-WPRAIF-
WNPRAI--
WHPRAI--

```
            WYPRAI--
            -WPRAID-
            -YPRAIH-
            YNPRAI--
            YVPRAI--
            YAPRAI--
            YTPRAI--
            -YPRAIV-
            YPPRAI--
            YDPRAI--
            YRPRAI--
            -YPRAII-
            -YPRAIQ-
            -YPRAIW-
            YQPRAI--
            YEPRAI--
            -YPRAIY-
            YMPRAI--
            YYPRAI--
            YKPRAI--
            -YPRAIC-
PMNY         350        675      47.80
            ARPMNY--
            -APMNYI-
            -APMNYR-
            -APMNYT-
            -APMNYL-
            AFPMNY--
            -APMNYG-
            ALPMNY--
            AMPMNY--
            -APMNYW-
            -APMNYV-
            AVPMNY--
            -APMNYA-
            AIPMNY--
            -APMNYK-
            AGPMNY--
            -APMNYY-
            -APMNYF-
            AKPMNY--
            AAPMNY--
            -APMNYM-
            AYPMNY--
            ASPMNY--
            AHPMNY--
            ADPMNY--
            AEPMNY--
            APPMNY--
            -APMNYS-
```

-APMNYN-
-APMNYD-
CCPMNY--
CTPMNY--
CYPMNY--
CSPMNY--
CVPMNY--
-CPMNYW-
CNPMNY--
CMPMNY--
CFPMNY--
DNPMNY--
DWPMNY--
-DPMNYS-
DVPMNY--
-DPMNYF-
DKPMNY--
DAPMNY--
DYPMNY--
DSPMNY--
-DPMNYT-
-DPMNYR-
DHPMNY--
DFPMNY--
DCPMNY--
EGPMNY--
-EPMNYL-
ESPMNY--
ETPMNY--
ELPMNY--
-EPMNYE-
EKPMNY--
EFPMNY--
EDPMNY--
EWPMNY--
-EPMNYA-
EEPMNY--
-EPMNYR-
-EPMNYG-
ENPMNY--
FSPMNY--
-FPMNYL-
FTPMNY--
-FPMNYK-
FWPMNY--
FYPMNY--
FGPMNY--
FMPMNY--
-FPMNYS-
-FPMNYE-

FRPMNY--
FHPMNY--
FNPMNY--
FLPMNY--
FIPMNY--
-FPMNYY-
FQPMNY--
-FPMNYG-
FEPMNY--
FVPMNY--
-FPMNYV-
FDPMNY--
-FPMNYA-
GNPMNY--
-GPMNYL-
-GPMNYM-
GIPMNY--
GEPMNY--
GRPMNY--
-GPMNYS-
GKPMNY--
GPPMNY--
-GPMNYV-
GFPMNY--
-GPMNYH-
GDPMNY--
GTPMNY--
GSPMNY--
-GPMNYN-
-GPMNYR-
GAPMNY--
-GPMNYY-
-GPMNYF-
-GPMNYT-
-GPMNYQ-
-GPMNYC-
-GPMNYA-
GYPMNY--
GWPMNY--
-GPMNYI-
-GPMNYP-
GLPMNY--
-HPMNYR-
-HPMNYL-
HFPMNY--
-HPMNYT-
-HPMNYS-
HNPMNY--
HGPMNY--
-HPMNYF-

HTPMNY--
-HPMNYV-
-HPMNYI-
HLPMNY--
HSPMNY--
HCPMNY--
HRPMNY--
HEPMNY--
HYPMNY--
HVPMNY--
HPPMNY--
HIPMNY--
-IPMNYI-
IVPMNY--
IQPMNY--
-IPMNYR-
IKPMNY--
-IPMNYW-
IFPMNY--
IMPMNY--
-IPMNYS-
IYPMNY--
-IPMNYV-
IGPMNY--
IAPMNY--
-IPMNYA-
IHPMNY--
ICPMNY--
-IPMNYN-
IEPMNY--
IDPMNY--
ITPMNY--
-IPMNYQ-
INPMNY--
KSPMNY--
-KPMNYS-
-KPMNYW-
KLPMNY--
KTPMNY--
-KPMNYL-
-KPMNYY-
-KPMNYM-
KGPMNY--
KDPMNY--
KRPMNY--
-KPMNYR-
-KPMNYF-
KVPMNY--
-KPMNYT-
KHPMNY--

-KPMNYV-
-KPMNYA-
-KPMNYG-
KKPMNY--
KYPMNY--
KNPMNY--
KIPMNY--
KEPMNY--
LGPMNY--
LPPMNY--
-LPMNYI-
-LPMNYL-
LLPMNY--
LWPMNY--
-LPMNYV-
-LPMNYM-
LMPMNY--
LKPMNY--
LEPMNY--
LHPMNY--
LYPMNY--
LQPMNY--
-LPMNYS-
-LPMNYA-
LNPMNY--
-LPMNYF-
LFPMNY--
LDPMNY--
-LPMNYW-
LRPMNY--
LAPMNY--
LTPMNY--
-LPMNYH-
-LPMNYY-
-LPMNYR-
-LPMNYP-
-LPMNYN-
-LPMNYK-
-LPMNYG-
-LPMNYE-
LIPMNY--
LCPMNY--
-MPMNYL-
MKPMNY--
-MPMNYI-
-MPMNYT-
MTPMNY--
MLPMNY--
MRPMNY--
-MPMNYH-

MYPMNY--
-MPMNYS-
-MPMNYN-
MHPMNY--
-MPMNYV-
MVPMNY--
-MPMNYR-
-MPMNYG-
-MPMNYA-
MGPMNY--
MPPMNY--
-MPMNYY-
-NPMNYT-
NSPMNY--
-NPMNYL-
NQPMNY--
NVPMNY--
-NPMNYR-
NLPMNY--
NEPMNY--
-NPMNYV-
NFPMNY--
-NPMNYM-
-NPMNYY-
NTPMNY--
-NPMNYS-
-NPMNYF-
NAPMNY--
NYPMNY--
NRPMNY--
NPPMNY--
-NPMNYQ-
-NPMNYK-
-NPMNYH-
-NPMNYG-
-NPMNYA-
NNPMNY--
NMPMNY--
NGPMNY--
--PMNYLG
--PMNYLT
--PMNYRS
--PMNYRT
--PMNYLV
--PMNYAS
--PMNYHS
--PMNYWQ
--PMNYGS
--PMNYQA
--PMNYMS

--PMNYAL
--PMNYIS
--PMNYFG
--PMNYFS
--PMNYWA
--PMNYRL
--PMNYVS
--PMNYSS
--PMNYKT
--PMNYTS
--PMNYSY
--PMNYMG
--PMNYSL
--PMNYFN
--PMNYEA
--PMNYSA
PWPMNY--
--PMNYYG
--PMNYRH
--PMNYLQ
--PMNYLK
--PMNYAI
--PMNYVY
--PMNYTK
--PMNYTE
--PMNYST
--PMNYRD
--PMNYNG
--PMNYLS
--PMNYLR
--PMNYID
--PMNYFK
--PMNYFA
--PMNYEV
--PMNYVT
--PMNYQV
--PMNYLI
--PMNYSV
--PMNYRM
--PMNYMA
--PMNYFW
--PMNYAY
--PMNYWH
--PMNYTR
--PMNYTI
--PMNYTG
--PMNYSG
--PMNYSF
--PMNYRW
--PMNYFQ

PGPMNY--
--PMNYYL
--PMNYWL
--PMNYRQ
--PMNYLL
--PMNYLF
--PMNYKS
--PMNYYY
--PMNYVD
--PMNYRY
--PMNYQG
--PMNYNT
--PMNYMN
--PMNYLH
--PMNYLD
--PMNYIG
--PMNYGV
--PMNYAA
PYPMNY--
PTPMNY--
--PMNYWV
--PMNYVA
--PMNYTL
--PMNYSR
--PMNYSK
--PMNYRI
--PMNYQM
--PMNYIQ
--PMNYGT
--PMNYGG
--PMNYVW
--PMNYVR
--PMNYVI
--PMNYRV
--PMNYRG
--PMNYNH
--PMNYGN
--PMNYGL
--PMNYGA
--PMNYFI
--PMNYEY
--PMNYAK
--PMNYAE
--PMNYVH
--PMNYTV
--PMNYSM
--PMNYSE
--PMNYRN
--PMNYQI
--PMNYKW

--PMNYKQ
--PMNYIM
--PMNYIH
--PMNYFT
--PMNYAH
--PMNYYW
--PMNYYM
--PMNYWG
--PMNYWD
--PMNYVK
--PMNYTW
--PMNYTT
--PMNYQT
--PMNYNS
--PMNYKV
--PMNYKL
--PMNYIA
--PMNYHW
--PMNYHM
--PMNYHH
--PMNYES
--PMNYEN
--PMNYEL
--PMNYYT
--PMNYYS
--PMNYYI
--PMNYYA
--PMNYWS
--PMNYTN
--PMNYTH
--PMNYSW
--PMNYSN
--PMNYSH
--PMNYSD
--PMNYQL
--PMNYNV
--PMNYNF
--PMNYME
--PMNYLN
--PMNYLA
--PMNYKN
--PMNYKM
--PMNYKK
--PMNYKD
--PMNYHQ
--PMNYHL
--PMNYHI
--PMNYHF
--PMNYGW
--PMNYGM

--PMNYFM
--PMNYER
--PMNYAG
--PMNYAF
PKPMNY--
PDPMNY--
--PMNYTC
--PMNYRF
--PMNYRE
--PMNYQS
--PMNYQF
--PMNYNR
--PMNYNL
--PMNYKR
--PMNYKA
--PMNYIY
--PMNYIE
--PMNYHV
--PMNYHT
--PMNYGY
--PMNYGF
--PMNYEE
--PMNYED
--PMNYDG
--PMNYAT
PIPMNY--
-PPMNYD-
--PMNYWI
--PMNYWF
--PMNYVV
--PMNYVG
--PMNYTA
--PMNYSP
--PMNYSI
--PMNYQQ
--PMNYQK
--PMNYQE
--PMNYPA
--PMNYNW
--PMNYMY
--PMNYMV
--PMNYMT
--PMNYMD
--PMNYLM
--PMNYLE
--PMNYIK
--PMNYHY
--PMNYHG
--PMNYGH
--PMNYFE

--PMNYET
--PMNYCG
--PMNYAD
PHPMNY--
PCPMNY--
QRPMNY--
-QPMNYI-
QNPMNY--
QKPMNY--
-QPMNYT-
QLPMNY--
QWPMNY--
-QPMNYR-
-QPMNYW-
-QPMNYF-
QEPMNY--
QGPMNY--
-QPMNYS-
-QPMNYN-
-QPMNYG-
-QPMNYE-
-QPMNYK-
QHPMNY--
QIPMNY--
QTPMNY--
-QPMNYV-
QFPMNY--
QYPMNY--
-QPMNYM-
-QPMNYL-
-QPMNYH-
-QPMNYC-
QMPMNY--
RVPMNY--
-RPMNYA-
RGPMNY--
RSPMNY--
-RPMNYR-
-RPMNYV-
-RPMNYF-
-RPMNYL-
-RPMNYQ-
-RPMNYI-
-RPMNYS-
-RPMNYM-
RLPMNY--
RMPMNY--
RFPMNY--
-RPMNYT-
RNPMNY--

RDPMNY--
RIPMNY--
RWPMNY--
RPPMNY--
-RPMNYH-
-RPMNYG-
REPMNY--
RYPMNY--
RTPMNY--
-RPMNYN-
-RPMNYE-
-RPMNYK-
RAPMNY--
-RPMNYW-
RQPMNY--
RKPMNY--
-SPMNYW-
-SPMNYM-
-SPMNYF-
SSPMNY--
SHPMNY--
-SPMNYS-
SRPMNY--
SNPMNY--
-SPMNYQ-
SFPMNY--
STPMNY--
SGPMNY--
SPPMNY--
-SPMNYY-
-SPMNYN-
-SPMNYA-
SKPMNY--
SAPMNY--
-SPMNYR-
-SPMNYG-
SWPMNY--
-SPMNYL-
SVPMNY--
SQPMNY--
-SPMNYK-
-SPMNYC-
-SPMNYH-
SMPMNY--
SIPMNY--
SDPMNY--
-TPMNYV-
TGPMNY--
TLPMNY--
-TPMNYI-

TVPMNY--
-TPMNYS-
-TPMNYQ-
TFPMNY--
-TPMNYT-
TMPMNY--
TRPMNY--
TPPMNY--
-TPMNYR-
TKPMNY--
-TPMNYY-
-TPMNYK-
TIPMNY--
TTPMNY--
-TPMNYN-
TYPMNY--
-TPMNYW-
THPMNY--
TSPMNY--
TAPMNY--
-TPMNYA-
TDPMNY--
VVPMNY--
-VPMNYY-
-VPMNYL-
-VPMNYT-
VIPMNY--
VGPMNY--
VKPMNY--
-VPMNYW-
VPPMNY--
-VPMNYS-
VSPMNY--
-VPMNYI-
VHPMNY--
-VPMNYV-
VTPMNY--
-VPMNYQ-
-VPMNYE-
-VPMNYN-
VAPMNY--
VYPMNY--
-VPMNYG-
VNPMNY--
VMPMNY--
VLPMNY--
VRPMNY--
VFPMNY--
VDPMNY--
VQPMNY--

-VPMNYM-
WTPMNY--
-WPMNYR-
WRPMNY--
WNPMNY--
-WPMNYM-
WPPMNY--
WQPMNY--
-WPMNYS-
-WPMNYT-
WGPMNY--
WDPMNY--
-WPMNYN-
WSPMNY--
-WPMNYH-
WLPMNY--
WKPMNY--
WIPMNY--
WHPMNY--
-WPMNYL-
-WPMNYC-
YRPMNY--
-YPMNYR-
-YPMNYH-
YNPMNY--
-YPMNYF-
YQPMNY--
YKPMNY--
-YPMNYT-
-YPMNYS-
YTPMNY--
-YPMNYA-
YVPMNY--
-YPMNYL-
-YPMNYW-
YMPMNY--
YGPMNY--
-YPMNYM-
YLPMNY--
-YPMNYV-
-YPMNYG-
YFPMNY--
-YPMNYY-
-YPMNYQ-
YAPMNY--
YSPMNY--
-YPMNYN-

PLNY 27 870 47.78

-SPLNYP-
-WPLNYG-

-APLNYR-
-APLNYM-
-APLNYL-
-APLNYE-
ALPLNY--
-APLNYV-
AVPLNY--
AYPLNY--
ASPLNY--
ARPLNY--
ADPLNY--
AKPLNY--
-APLNYH-
-APLNYS-
ATPLNY--
-APLNYA-
ANPLNY--
-APLNYQ-
AGPLNY--
AAPLNY--
-APLNYF-
AWPLNY--
AQPLNY--
-APLNYW-
-APLNYK-
AHPLNY--
APPLNY--
-APLNYI-
-APLNYY-
-APLNYN-
AEPLNY--
-APLNYT-
-APLNYD-
AFPLNY--
-APLNYG-
ACPLNY--
-CPLNYM-
CSPLNY--
CNPLNY--
CEPLNY--
-CPLNYT-
-CPLNYI-
CMPLNY--
-CPLNYW-
-CPLNYL-
CPPLNY--
-CPLNYR-
CIPLNY--
CVPLNY--
CRPLNY--

CQPLNY--
CKPLNY--
CDPLNY--
CAPLNY--
DRPLNY--
DKPLNY--
-DPLNYR-
DVPLNY--
-DPLNYV-
DLPLNY--
DTPLNY--
-DPLNYS-
DSPLNY--
DMPLNY--
-DPLNYI-
DFPLNY--
-DPLNYT-
-DPLNYQ-
-DPLNYH-
DWPLNY--
-DPLNYY-
-DPLNYK-
DNPLNY--
DIPLNY--
DAPLNY--
-DPLNYN-
-DPLNYM-
-DPLNYL-
DGPLNY--
DCPLNY--
DYPLNY--
-DPLNYW-
-DPLNYF-
-DPLNYE-
DEPLNY--
ESPLNY--
ERPLNY--
ETPLNY--
-EPLNYL-
EFPLNY--
-EPLNYW-
-EPLNYI-
ELPLNY--
EQPLNY--
-EPLNYK-
-EPLNYH-
EGPLNY--
-EPLNYY-
-EPLNYR-
-EPLNYM-

EVPLNY--
EKPLNY--
EHPLNY--
EMPLNY--
EAPLNY--
-EPLNYT-
-EPLNYA-
EIPLNY--
EEPLNY--
EYPLNY--
-EPLNYV-
-EPLNYS-
-EPLNYP-
-EPLNYF-
-EPLNYD-
ENPLNY--
-FPLNYT-
-FPLNYS-
FSPLNY--
FLPLNY--
-FPLNYR-
FMPLNY--
FAPLNY--
-FPLNYV-
FVPLNY--
FWPLNY--
FGPLNY--
FTPLNY--
-FPLNYA-
FQPLNY--
FEPLNY--
-FPLNYN-
-FPLNYQ-
-FPLNYW-
-FPLNYK-
-FPLNYH-
-FPLNYY-
FPPLNY--
FIPLNY--
FDPLNY--
-FPLNYG-
-FPLNYF-
FNPLNY--
FFPLNY--
-FPLNYD-
FKPLNY--
FCPLNY--
-GPLNYL-
GSPLNY--
-GPLNYW-

-GPLNYM-
GGPLNY--
-GPLNYR-
-GPLNYT-
GLPLNY--
-GPLNYK-
-GPLNYV-
GIPLNY--
GAPLNY--
-GPLNYS-
GQPLNY--
-GPLNYE-
GFPLNY--
-GPLNYF-
GHPLNY--
-GPLNYH-
GMPLNY--
GYPLNY--
-GPLNYG-
-GPLNYY-
-GPLNYN-
GDPLNY--
GVPLNY--
-GPLNYQ-
-GPLNYA-
GTPLNY--
GEPLNY--
GPPLNY--
GKPLNY--
-GPLNYI-
-GPLNYD-
HRPLNY--
HSPLNY--
-HPLNYL-
HQPLNY--
HVPLNY--
-HPLNYE-
HTPLNY--
-HPLNYW-
-HPLNYR-
-HPLNYI-
-HPLNYM-
HYPLNY--
HNPLNY--
-HPLNYT-
-HPLNYY-
HMPLNY--
HFPLNY--
-HPLNYG-
-HPLNYF-

HGPLNY--
HAPLNY--
HKPLNY--
-HPLNYQ-
HLPLNY--
HDPLNY--
HWPLNY--
-HPLNYN-
-HPLNYK-
-HPLNYV-
-HPLNYH-
HIPLNY--
HHPLNY--
HEPLNY--
HCPLNY--
-IPLNYS-
-IPLNYR-
-IPLNYL-
IRPLNY--
-IPLNYV-
IGPLNY--
ILPLNY--
-IPLNYK-
-IPLNYM-
IIPLNY--
IKPLNY--
IPPLNY--
IDPLNY--
IYPLNY--
-IPLNYA-
IQPLNY--
-IPLNYF-
IWPLNY--
-IPLNYW-
-IPLNYE-
IFPLNY--
-IPLNYY-
-IPLNYG-
INPLNY--
ITPLNY--
-IPLNYD-
IHPLNY--
ICPLNY--
-IPLNYQ-
IEPLNY--
IVPLNY--
-IPLNYT-
-IPLNYH-
KSPLNY--
-KPLNYS-

-KPLNYM-
-KPLNYL-
KMPLNY--
KNPLNY--
-KPLNYI-
-KPLNYV-
-KPLNYA-
KTPLNY--
KVPLNY--
-KPLNYY-
-KPLNYH-
-KPLNYG-
-KPLNYK-
-KPLNYF-
-KPLNYW-
-KPLNYQ-
KFPLNY--
KAPLNY--
KRPLNY--
KEPLNY--
KWPLNY--
KQPLNY--
KIPLNY--
KDPLNY--
-KPLNYE-
KLPLNY--
KYPLNY--
-KPLNYN-
KCPLNY--
LRPLNY--
LSPLNY--
-LPLNYF-
LTPLNY--
LHPLNY--
-LPLNYS-
LYPLNY--
LLPLNY--
-LPLNYT-
LAPLNY--
LQPLNY--
LFPLNY--
-LPLNYM-
LMPLNY--
LDPLNY--
-LPLNYW-
-LPLNYI-
LKPLNY--
LPPLNY--
-LPLNYA-
LNPLNY--

LIPLNY--
-LPLNYN-
-LPLNYE-
-LPLNYY-
-LPLNYH-
-LPLNYG-
LEPLNY--
-LPLNYK-
MSPLNY--
MRPLNY--
-MPLNYL-
-MPLNYF-
-MPLNYM-
-MPLNYS-
-MPLNYT-
MGPLNY--
-MPLNYR-
-MPLNYV-
MYPLNY--
-MPLNYY-
-MPLNYE-
-MPLNYG-
MIPLNY--
MLPLNY--
MQPLNY--
MAPLNY--
MPPLNY--
-MPLNYA-
MNPLNY--
MKPLNY--
MEPLNY--
MHPLNY--
MFPLNY--
MWPLNY--
-MPLNYH-
MDPLNY--
MCPLNY--
MVPLNY--
-MPLNYP-
-MPLNYK-
NRPLNY--
-NPLNYK-
-NPLNYT-
-NPLNYY-
NVPLNY--
-NPLNYL-
NLPLNY--
NSPLNY--
-NPLNYA-
-NPLNYS-

NAPLNY--
-NPLNYV-
-NPLNYQ-
NFPLNY--
-NPLNYR-
NGPLNY--
NQPLNY--
NPPLNY--
-NPLNYW-
NTPLNY--
NNPLNY--
NIPLNY--
-NPLNYH-
-NPLNYE-
NCPLNY--
NYPLNY--
-NPLNYN-
-NPLNYM-
NMPLNY--
NHPLNY--
--PLNYLR
--PLNYFS
--PLNYST
--PLNYTH
--PLNYRS
--PLNYRL
--PLNYLT
--PLNYRH
--PLNYTG
--PLNYTA
--PLNYRT
--PLNYMS
--PLNYYI
--PLNYQR
--PLNYKA
--PLNYMR
--PLNYLN
--PLNYLH
--PLNYKN
--PLNYHS
--PLNYTR
--PLNYLV
--PLNYVS
--PLNYRN
--PLNYRE
--PLNYWS
--PLNYSH
--PLNYKM
--PLNYVR
--PLNYLG

--PLNYYM
--PLNYYL
--PLNYSR
--PLNYSQ
--PLNYSG
--PLNYYG
--PLNYVW
--PLNYVA
--PLNYTT
--PLNYMT
--PLNYFL
--PLNYFG
PRPLNY--
--PLNYTN
--PLNYAS
--PLNYVT
--PLNYTL
--PLNYSA
--PLNYTI
--PLNYNS
--PLNYKG
--PLNYVV
--PLNYTS
--PLNYRQ
--PLNYLI
--PLNYIR
--PLNYIK
--PLNYFA
--PLNYWR
--PLNYIG
--PLNYGG
--PLNYAG
--PLNYAA
--PLNYVG
--PLNYKT
--PLNYHM
--PLNYER
PWPLNY--
--PLNYLM
--PLNYIT
--PLNYFR
--PLNYYS
--PLNYWG
--PLNYVK
--PLNYVI
--PLNYSD
--PLNYQF
--PLNYGV
--PLNYGA
--PLNYET

--PLNYQA
--PLNYMQ
--PLNYHR
--PLNYEI
--PLNYAL
PTPLNY--
--PLNYYQ
--PLNYRY
--PLNYIE
--PLNYHT
PMPLNY--
--PLNYVN
--PLNYSK
--PLNYQG
--PLNYMG
--PLNYMD
--PLNYIY
--PLNYIS
--PLNYAN
--PLNYWI
--PLNYTV
--PLNYKI
--PLNYIH
--PLNYGI
--PLNYAR
--PLNYYN
--PLNYTK
--PLNYTE
--PLNYRF
--PLNYKW
--PLNYKL
--PLNYKF
--PLNYIW
--PLNYYW
--PLNYVD
--PLNYNF
--PLNYMK
--PLNYIV
--PLNYAW
--PLNYSY
--PLNYRM
--PLNYQI
--PLNYLK
--PLNYLD
--PLNYKS
--PLNYHH
--PLNYFK
--PLNYEK
PKPLNY--
--PLNYWT

--PLNYWD
--PLNYSN
--PLNYSM
--PLNYQY
--PLNYNI
--PLNYMV
--PLNYML
--PLNYLQ
--PLNYHI
--PLNYHG
--PLNYGY
--PLNYEG
--PLNYEA
--PLNYAY
PIPLNY--
PGPLNY--
--PLNYWM
--PLNYWE
--PLNYRI
--PLNYQL
--PLNYNA
--PLNYIF
--PLNYHL
--PLNYGR
--PLNYDA
--PLNYAQ
--PLNYTW
--PLNYSE
--PLNYNL
--PLNYNH
--PLNYMN
--PLNYMH
--PLNYKK
--PLNYIM
--PLNYFT
PQPLNY--
--PLNYWK
--PLNYTM
--PLNYRD
--PLNYQW
--PLNYMI
PFPLNY--
--PLNYYA
--PLNYVM
--PLNYNY
--PLNYNQ
--PLNYKY
--PLNYIQ
--PLNYHY
--PLNYHV

--PLNYHK
--PLNYGL
--PLNYGH
--PLNYFN
--PLNYEV
--PLNYEQ
--PLNYEL
--PLNYDT
--PLNYAV
--PLNYAF
PDPLNY--
--PLNYYY
--PLNYWW
--PLNYVQ
--PLNYTY
--PLNYRV
--PLNYQQ
--PLNYNT
--PLNYNR
--PLNYKH
--PLNYKD
--PLNYID
--PLNYHW
--PLNYFM
--PLNYEY
--PLNYAH
--PLNYAE
PHPLNY--
--PLNYYV
--PLNYYK
--PLNYVE
--PLNYTQ
--PLNYTD
--PLNYQM
--PLNYQE
--PLNYNW
--PLNYMY
--PLNYMM
--PLNYMF
--PLNYLE
--PLNYKV
--PLNYKE
--PLNYHE
--PLNYDS
--PLNYAK
--PLNYVH
--PLNYQS
--PLNYQN
--PLNYNN
--PLNYND

--PLNYME
--PLNYHN
--PLNYHA
--PLNYGW
--PLNYGM
--PLNYFE
--PLNYFD
--PLNYEW
--PLNYDG
--PLNYAD
PPPLNY--
-PPLNYT-
-PPLNYQ-
--PLNYWH
--PLNYVC
--PLNYRP
--PLNYQV
--PLNYQT
--PLNYQH
--PLNYNE
--PLNYLC
--PLNYIN
--PLNYIA
--PLNYHD
--PLNYGK
--PLNYES
--PLNYEM
--PLNYEH
--PLNYDW
--PLNYDC
--PLNYCT
--PLNYCR
--PLNYCM
--PLNYCI
--PLNYCG
--PLNYCA
--PLNYAI
PEPLNY--
-QPLNYL-
QGPLNY--
QSPLNY--
QEPLNY--
QMPLNY--
QAPLNY--
-QPLNYF-
QVPLNY--
-QPLNYV-
QRPLNY--
QFPLNY--
-QPLNYS-

-QPLNYR-
-QPLNYI-
-QPLNYT-
-QPLNYG-
QQPLNY--
-QPLNYY-
-QPLNYQ-
QTPLNY--
QPPLNY--
QWPLNY--
-QPLNYE-
QNPLNY--
QIPLNY--
-QPLNYN-
-QPLNYK-
-QPLNYH-
QLPLNY--
QDPLNY--
QKPLNY--
-QPLNYA-
-QPLNYW-
-RPLNYS-
-RPLNYI-
RGPLNY--
-RPLNYV-
RSPLNY--
-RPLNYY-
RLPLNY--
-RPLNYR-
-RPLNYW-
-RPLNYT-
RWPLNY--
RMPLNY--
RVPLNY--
-RPLNYH-
RKPLNY--
RQPLNY--
-RPLNYF-
-RPLNYQ-
RFPLNY--
-RPLNYA-
-RPLNYK-
RNPLNY--
RPPLNY--
-RPLNYN-
-RPLNYG-
REPLNY--
-RPLNYM-
RAPLNY--
RHPLNY--

RIPLNY--
-RPLNYE-
RDPLNY--
RCPLNY--
-RPLNYD-
-SPLNYL-
-SPLNYR-
-SPLNYV-
SSPLNY--
SGPLNY--
-SPLNYS-
SAPLNY--
-SPLNYQ-
-SPLNYA-
-SPLNYF-
SKPLNY--
SVPLNY--
SMPLNY--
-SPLNYI-
SIPLNY--
-SPLNYM-
SHPLNY--
-SPLNYK-
SNPLNY--
SFPLNY--
-SPLNYW-
SYPLNY--
SEPLNY--
SPPLNY--
-SPLNYN-
-SPLNYY-
SQPLNY--
-SPLNYH-
-SPLNYE-
SDPLNY--
-SPLNYG-
-TPLNYR-
-TPLNYL-
-TPLNYS-
-TPLNYK-
TRPLNY--
-TPLNYA-
TGPLNY--
-TPLNYM-
-TPLNYH-
TSPLNY--
TPPLNY--
-TPLNYV-
-TPLNYT-
TTPLNY--

TAPLNY--
TLPLNY--
TDPLNY--
TYPLNY--
TMPLNY--
TVPLNY--
-TPLNYF-
TWPLNY--
-TPLNYY-
-TPLNYQ-
TKPLNY--
TQPLNY--
THPLNY--
-TPLNYN-
-TPLNYI-
TNPLNY--
TEPLNY--
-TPLNYG-
-TPLNYD-
-TPLNYE-
TCPLNY--
TFPLNY--
VSPLNY--
-VPLNYL-
VLPLNY--
VWPLNY--
-VPLNYT-
VKPLNY--
VMPLNY--
-VPLNYY-
VAPLNY--
VGPLNY--
VNPLNY--
VTPLNY--
-VPLNYA-
-VPLNYS-
-VPLNYR-
VDPLNY--
VFPLNY--
VQPLNY--
-VPLNYW-
-VPLNYV-
VPPLNY--
-VPLNYH-
-VPLNYF-
-VPLNYK-
VIPLNY--
-VPLNYG-
VHPLNY--
-VPLNYQ-

-VPLNYI-
-VPLNYE-
VYPLNY--
-VPLNYM-
-VPLNYN-
VEPLNY--
VCPLNY--
WGPLNY--
-WPLNYS-
WTPLNY--
WSPLNY--
-WPLNYL-
WFPLNY--
WDPLNY--
WPPLNY--
-WPLNYA-
WNPLNY--
-WPLNYK-
-WPLNYF-
-WPLNYR-
WAPLNY--
WWPLNY--
WVPLNY--
-WPLNYT-
WLPLNY--
-WPLNYN-
-WPLNYE-
WMPLNY--
-WPLNYM-
-WPLNYH-
-WPLNYI-
WKPLNY--
WEPLNY--
YRPLNY--
-YPLNYR-
YVPLNY--
-YPLNYT-
YPPLNY--
-YPLNYI-
YSPLNY--
YYPLNY--
-YPLNYL-
YAPLNY--
-YPLNYK-
YNPLNY--
-YPLNYS-
YTPLNY--
YQPLNY--
-YPLNYF-
YHPLNY--

-YPLNYW-
YMPLNY--
YLPLNY--
YKPLNY--
YEPLNY--
-YPLNYM-
-YPLNYH-
-YPLNYE-
YGPLNY--
YFPLNY--
-YPLNYG-
-YPLNYA-
YIPLNY--
-YPLNYY-
YDPLNY--
-YPLNYQ-
-YPLNYN-
YCPLNY--

PRWL	392	663	47.78

--PRWLDY
-RPRWLP-
ANPRWL--
AGPRWL--
ARPRWL--
-APRWLT-
-APRWLS-
AMPRWL--
-APRWLH-
AAPRWL--
ASPRWL--
-APRWLA-
AEPRWL--
APPRWL--
AHPRWL--
ADPRWL--
ATPRWL--
-APRWLW-
AKPRWL--
CAPRWL--
CMPRWL--
CTPRWL--
-CPRWLA-
-CPRWLS-
CLPRWL--
CGPRWL--
CCPRWL--
CQPRWL--
-CPRWLT-
CRPRWL--
CPPRWL--

CIPRWL--
-CPRWLW-
-CPRWLR-
-CPRWLQ-
-CPRWLE-
CNPRWL--
CFPRWL--
CEPRWL--
-DPRWLR-
-DPRWLI-
DWPRWL--
DSPRWL--
DRPRWL--
-DPRWLV-
DIPRWL--
DAPRWL--
-DPRWLS-
-DPRWLA-
DYPRWL--
DVPRWL--
DKPRWL--
DQPRWL--
DEPRWL--
DLPRWL--
DGPRWL--
DMPRWL--
DTPRWL--
DHPRWL--
-DPRWLG-
-DPRWLY-
-DPRWLT-
-DPRWLQ-
-DPRWLH-
-DPRWLN-
-DPRWLM-
DFPRWL--
DDPRWL--
-EPRWLL-
EYPRWL--
ESPRWL--
-EPRWLW-
-EPRWLS-
-EPRWLK-
EMPRWL--
ETPRWL--
ERPRWL--
-EPRWLT-
-EPRWLF-
-EPRWLN-
EFPRWL--

-EPRWLA-
-EPRWLQ-
EKPRWL--
-EPRWLI-
EIPRWL--
EVPRWL--
-EPRWLG-
EEPRWL--
-EPRWLY-
EQPRWL--
-EPRWLM-
ELPRWL--
-EPRWLV-
-EPRWLH-
-EPRWLE-
EAPRWL--
EHPRWL--
EDPRWL--
-EPRWLD-
EWPRWL--
FSPRWL--
FEPRWL--
FIPRWL--
FNPRWL--
FKPRWL--
FLPRWL--
FTPRWL--
-FPRWLV-
-FPRWLQ-
FQPRWL--
-FPRWLN-
-FPRWLM-
-FPRWLE-
FMPRWL--
FDPRWL--
-FPRWLY-
-FPRWLH-
FYPRWL--
-FPRWLA-
-FPRWLI-
-FPRWLF-
FPPRWL--
FCPRWL--
GSPRWL--
-GPRWLR-
-GPRWLM-
GVPRWL--
GTPRWL--
-GPRWLG-
-GPRWLA-

GYPRWL--
-GPRWLV-
GKPRWL--
GQPRWL--
GIPRWL--
-GPRWLH-
GNPRWL--
GGPRWL--
GRPRWL--
-GPRWLN-
GWPRWL--
-GPRWLI-
-GPRWLE-
GEPRWL--
GDPRWL--
-GPRWLD-
-GPRWLY-
GHPRWL--
-GPRWLC-
HGPRWL--
-HPRWLS-
-HPRWLL-
HVPRWL--
HSPRWL--
HRPRWL--
HTPRWL--
-HPRWLH-
-HPRWLM-
HAPRWL--
-HPRWLA-
HPPRWL--
-HPRWLV-
-HPRWLN-
-HPRWLK-
HDPRWL--
-HPRWLQ-
-HPRWLI-
HNPRWL--
-HPRWLF-
-HPRWLT-
-HPRWLE-
HYPRWL--
-HPRWLD-
-HPRWLC-
-IPRWLN-
-IPRWLI-
-IPRWLK-
IEPRWL--
ITPRWL--
IKPRWL--

```
-IPRWLR-
-IPRWLY-
IVPRWL--
IRPRWL--
-IPRWLL-
IDPRWL--
IMPRWL--
-IPRWLM-
-IPRWLG-
IPPRWL--
-IPRWLF-
IYPRWL--
-IPRWLV-
-IPRWLH-
-IPRWLD-
ICPRWL--
IFPRWL--
-KPRWLS-
KYPRWL--
-KPRWLH-
KLPRWL--
-KPRWLA-
-KPRWLG-
-KPRWLQ-
-KPRWLN-
KEPRWL--
-KPRWLV-
-KPRWLI-
KRPRWL--
KQPRWL--
-KPRWLK-
KHPRWL--
KGPRWL--
KIPRWL--
-KPRWLD-
KNPRWL--
KWPRWL--
-KPRWLE-
-KPRWLT-
-KPRWLM-
LVPRWL--
-LPRWLS-
-LPRWLV-
LHPRWL--
-LPRWLG-
LQPRWL--
LAPRWL--
-LPRWLQ-
LKPRWL--
-LPRWLW-
```

LFPRWL--
-LPRWLE-
LCPRWL--
MTPRWL--
MVPRWL--
MHPRWL--
MPPRWL--
MFPRWL--
-MPRWLK-
-MPRWLH-
-MPRWLL-
-MPRWLN-
-MPRWLA-
MEPRWL--
MQPRWL--
MNPRWL--
-MPRWLM-
-MPRWLI-
-MPRWLQ-
MMPRWL--
MYPRWL--
MDPRWL--
-MPRWLY-
MAPRWL--
-MPRWLE-
-MPRWLD-
NVPRWL--
NRPRWL--
-NPRWLV-
NLPRWL--
-NPRWLS-
NAPRWL--
NSPRWL--
NTPRWL--
NFPRWL--
NYPRWL--
-NPRWLN-
NHPRWL--
-NPRWLK-
NNPRWL--
NPPRWL--
NQPRWL--
NGPRWL--
-NPRWLH-
-NPRWLI-
-NPRWLG-
NMPRWL--
NDPRWL--
-NPRWLY-
-NPRWLQ-

```
-NPRWLA-
-NPRWLE-
-NPRWLD-
NCPRWL--
-NPRWLC-
--PRWLLS
--PRWLRT
PSPRWL--
--PRWLGL
--PRWLLN
PKPRWL--
--PRWLSV
PRPRWL--
--PRWLLA
--PRWLSN
--PRWLLT
--PRWLKT
--PRWLAN
--PRWLES
--PRWLIG
--PRWLSA
--PRWLGA
--PRWLNT
--PRWLMS
--PRWLYG
--PRWLVK
--PRWLTI
--PRWLKG
--PRWLHN
--PRWLHA
--PRWLHV
--PRWLYK
--PRWLVT
--PRWLGG
PAPRWL--
--PRWLRD
--PRWLQS
--PRWLIA
--PRWLRY
--PRWLHS
--PRWLYS
--PRWLRM
--PRWLMK
--PRWLIS
--PRWLKE
--PRWLTA
--PRWLSW
--PRWLRE
--PRWLLQ
--PRWLHG
```

--PRWLVM
--PRWLTY
PTPRWL--
--PRWLSH
--PRWLAD
PEPRWL--
--PRWLYN
--PRWLKA
--PRWLAH
--PRWLYT
--PRWLSQ
--PRWLQG
--PRWLIH
--PRWLAQ
--PRWLMH
--PRWLVN
--PRWLTW
--PRWLNA
--PRWLLK
--PRWLEA
--PRWLYH
--PRWLSM
--PRWLSD
--PRWLNV
--PRWLWV
--PRWLWG
--PRWLKQ
--PRWLGW
--PRWLGR
PYPRWL--
--PRWLTV
--PRWLTD
--PRWLNW
--PRWLLM
--PRWLKY
--PRWLHH
--PRWLSF
--PRWLQD
--PRWLME
--PRWLIQ
--PRWLEN
--PRWLAA
--PRWLLD
--PRWLGH
--PRWLFT
PGPRWL--
--PRWLGM
--PRWLIW
--PRWLHK
--PRWLQA

--PRWLLI
--PRWLEM
PIPRWL--
--PRWLYM
--PRWLMY
--PRWLLV
--PRWLFN
--PRWLKF
PVPRWL--
--PRWLVD
--PRWLTQ
--PRWLHW
--PRWLFQ
PMPRWL--
PFPRWL--
--PRWLYV
--PRWLYD
--PRWLET
PDPRWL--
--PRWLTM
--PRWLTF
--PRWLRI
--PRWLQY
--PRWLQN
--PRWLYI
--PRWLWT
--PRWLTE
--PRWLMD
--PRWLLW
--PRWLIV
--PRWLAM
--PRWLQT
--PRWLKH
--PRWLEH
--PRWLEG
--PRWLIN
--PRWLHQ
--PRWLGN
--PRWLFD
--PRWLEV
--PRWLEI
--PRWLNR
--PRWLNN
--PRWLWH
--PRWLVY
--PRWLVW
--PRWLQV
--PRWLNY
--PRWLNQ
--PRWLNM

--PRWLKW
--PRWLDG
--PRWLAY
PHPRWL--
--PRWLTH
--PRWLKD
--PRWLHF
--PRWLER
--PRWLEE
--PRWLQQ
--PRWLNI
--PRWLND
--PRWLMM
--PRWLFY
--PRWLEK
--PRWLWQ
--PRWLQW
--PRWLQI
PWPRWL--
--PRWLWD
--PRWLGD
--PRWLQE
--PRWLNK
--PRWLNH
--PRWLIE
--PRWLHD
--PRWLGK
--PRWLFK
--PRWLWN
--PRWLWA
--PRWLQK
--PRWLQH
--PRWLPL
--PRWLHT
--PRWLEL
--PRWLWW
--PRWLLC
--PRWLII
--PRWLYY
--PRWLWM
--PRWLWE
--PRWLNE
--PRWLKM
--PRWLFE
--PRWLEW
--PRWLCS
--PRWLYE
--PRWLWI
--PRWLQM
--PRWLPY

--PRWLPR
--PRWLPF
--PRWLHM
--PRWLHE
--PRWLHC
--PRWLED
--PRWLDI
--PRWLCT
--PRWLCN
-PPRWLT-
-PPRWLS-
-PPRWLR-
-PPRWLK-
-PPRWLG-
PCPRWL--
-QPRWLS-
-QPRWLL-
QEPRWL--
QGPRWL--
QSPRWL--
-QPRWLA-
QLPRWL--
QVPRWL--
QTPRWL--
-QPRWLT-
-QPRWLH-
-QPRWLV-
QQPRWL--
QYPRWL--
-QPRWLF-
-QPRWLN-
-QPRWLG-
QNPRWL--
-QPRWLE-
QDPRWL--
-QPRWLQ-
QKPRWL--
QHPRWL--
QFPRWL--
-QPRWLI-
-QPRWLM-
-QPRWLC-
QIPRWL--
-RPRWLT-
-RPRWLL-
-RPRWLS-
-RPRWLY-
RPPRWL--
RHPRWL--
-RPRWLK-

-RPRWLQ-
-RPRWLG-
REPRWL--
RDPRWL--
-RPRWLE-
-RPRWLN-
RKPRWL--
RQPRWL--
-RPRWLI-
-RPRWLW-
-RPRWLF-
-RPRWLD-
RCPRWL--
SGPRWL--
SRPRWL--
-SPRWLV-
-SPRWLA-
-SPRWLK-
SAPRWL--
SVPRWL--
-SPRWLH-
SSPRWL--
SQPRWL--
SEPRWL--
-SPRWLQ-
SPPRWL--
STPRWL--
-SPRWLN-
SFPRWL--
-SPRWLE-
SNPRWL--
-SPRWLI-
-SPRWLF-
-SPRWLD-
-SPRWLM-
SHPRWL--
-TPRWLL-
TVPRWL--
TKPRWL--
TGPRWL--
TPPRWL--
THPRWL--
-TPRWLA-
TRPRWL--
-TPRWLY-
TLPRWL--
-TPRWLS-
-TPRWLF-
-TPRWLT-
-TPRWLI-

TYPRWL--
-TPRWLQ-
TAPRWL--
TWPRWL--
-TPRWLW-
-TPRWLH-
TTPRWL--
-TPRWLE-
TQPRWL--
-TPRWLD-
-TPRWLG-
TCPRWL--
-VPRWLL-
VEPRWL--
VTPRWL--
-VPRWLE-
-VPRWLN-
-VPRWLM-
VIPRWL--
-VPRWLV-
VYPRWL--
VAPRWL--
-VPRWLW-
-VPRWLH-
VVPRWL--
VKPRWL--
-VPRWLD-
-VPRWLF-
VQPRWL--
VCPRWL--
WNPRWL--
WTPRWL--
WRPRWL--
-WPRWLS-
WYPRWL--
WVPRWL--
WKPRWL--
-WPRWLG-
WGPRWL--
WQPRWL--
-WPRWLQ-
WPPRWL--
-WPRWLA-
WLPRWL--
WIPRWL--
WEPRWL--
WWPRWL--
-WPRWLH-
WHPRWL--
-WPRWLI-

```
            -WPRWLE-
            WFPRWL--
            -WPRWLD-
            -WPRWLN-
            YTPRWL--
            YKPRWL--
            YYPRWL--
            -YPRWLQ-
            -YPRWLS-
            -YPRWLH-
            YPPRWL--
            YEPRWL--
            YDPRWL--
            YAPRWL--
            -YPRWLA-
            YQPRWL--
            -YPRWLK-
            YWPRWL--
            -YPRWLG-
            -YPRWLT-
            -YPRWLR-
            YNPRWL--
            -YPRWLD-
            -YPRWLV-
            -YPRWLN-
            -YPRWLF-
            -YPRWLE-
            -YPRWLM-
            -YPRWLW-
            YIPRWL--
            -YPRWLY-
            YCPRWL--
PMHL          69        834     47.77
            --PMHLGP
            -APMHLR-
            AWPMHL--
            -APMHLS-
            ASPMHL--
            -APMHLH-
            -APMHLW-
            AGPMHL--
            -APMHLV-
            ANPMHL--
            AIPMHL--
            AEPMHL--
            -APMHLK-
            APPMHL--
            -APMHLM-
            -APMHLL-
            AQPMHL--
```

−APMHLI−
−APMHLE−
AVPMHL−−
ARPMHL−−
AFPMHL−−
ADPMHL−−
AMPMHL−−
−APMHLT−
ACPMHL−−
AAPMHL−−
−APMHLN−
−APMHLG−
−APMHLA−
AKPMHL−−
AHPMHL−−
AYPMHL−−
−APMHLF−
−APMHLY−
−APMHLQ−
−APMHLD−
ALPMHL−−
CNPMHL−−
CYPMHL−−
CRPMHL−−
CGPMHL−−
CSPMHL−−
−CPMHLL−
CVPMHL−−
CTPMHL−−
−CPMHLW−
−CPMHLT−
−CPMHLK−
−CPMHLE−
−CPMHLA−
CLPMHL−−
CKPMHL−−
DIPMHL−−
DYPMHL−−
DRPMHL−−
DWPMHL−−
DVPMHL−−
−DPMHLK−
DHPMHL−−
−DPMHLT−
DNPMHL−−
−DPMHLI−
DMPMHL−−
DTPMHL−−
−DPMHLH−
DSPMHL−−

-DPMHLA-
DLPMHL--
-DPMHLL-
DGPMHL--
-DPMHLV-
-DPMHLM-
-DPMHLF-
DKPMHL--
DFPMHL--
-DPMHLY-
-DPMHLE-
DEPMHL--
ERPMHL--
ESPMHL--
ELPMHL--
-EPMHLI-
EAPMHL--
-EPMHLW-
-EPMHLS-
-EPMHLR-
EFPMHL--
EYPMHL--
-EPMHLV-
EIPMHL--
EGPMHL--
EPPMHL--
-EPMHLM-
-EPMHLL-
EEPMHL--
EKPMHL--
-EPMHLH-
-EPMHLG-
ETPMHL--
-EPMHLT-
-EPMHLF-
-EPMHLE-
ENPMHL--
EMPMHL--
EWPMHL--
EQPMHL--
-EPMHLN-
-EPMHLA-
EDPMHL--
FRPMHL--
FWPMHL--
-FPMHLL-
-FPMHLQ-
FDPMHL--
FAPMHL--
FEPMHL--

FYPMHL--
-FPMHLY-
-FPMHLT-
FHPMHL--
-FPMHLS-
-FPMHLH-
FSPMHL--
FGPMHL--
FFPMHL--
-FPMHLW-
FKPMHL--
-FPMHLN-
-FPMHLV-
-FPMHLE-
FNPMHL--
-FPMHLK-
GYPMHL--
GHPMHL--
GGPMHL--
GVPMHL--
-GPMHLR-
GAPMHL--
-GPMHLL-
GIPMHL--
GPPMHL--
GRPMHL--
-GPMHLA-
GQPMHL--
-GPMHLS-
-GPMHLE-
GEPMHL--
-GPMHLV-
-GPMHLK-
-GPMHLI-
GSPMHL--
-GPMHLY-
-GPMHLQ-
-GPMHLG-
-GPMHLW-
-GPMHLF-
GKPMHL--
GTPMHL--
GFPMHL--
GLPMHL--
GWPMHL--
GMPMHL--
-GPMHLT-
-GPMHLN-
-GPMHLH-
GCPMHL--

-GPMHLM-
GDPMHL--
-HPMHLR-
HRPMHL--
HPPMHL--
-HPMHLS-
HAPMHL--
HMPMHL--
HHPMHL--
HIPMHL--
HFPMHL--
HSPMHL--
-HPMHLQ-
HEPMHL--
HDPMHL--
-HPMHLV-
HTPMHL--
-HPMHLY-
-HPMHLT-
-HPMHLH-
HVPMHL--
-HPMHLL-
HNPMHL--
HKPMHL--
HQPMHL--
-HPMHLE-
-HPMHLM-
-HPMHLF-
-HPMHLA-
-HPMHLI-
-HPMHLN-
-IPMHLT-
ITPMHL--
-IPMHLV-
-IPMHLY-
-IPMHLR-
IRPMHL--
IVPMHL--
-IPMHLQ-
INPMHL--
-IPMHLM-
IQPMHL--
ILPMHL--
IGPMHL--
IPPMHL--
IIPMHL--
IDPMHL--
ISPMHL--
-IPMHLE-
-IPMHLL-

IAPMHL--
-IPMHLS-
-IPMHLK-
IFPMHL--
IHPMHL--
-IPMHLW-
-IPMHLI-
-IPMHLH-
-IPMHLN-
-IPMHLG-
IWPMHL--
-IPMHLF-
-IPMHLA-
IMPMHL--
IEPMHL--
KSPMHL--
KAPMHL--
KNPMHL--
KVPMHL--
KQPMHL--
-KPMHLW-
-KPMHLR-
-KPMHLI-
KGPMHL--
-KPMHLE-
KWPMHL--
-KPMHLV-
-KPMHLQ-
-KPMHLM-
KTPMHL--
-KPMHLS-
KIPMHL--
-KPMHLT-
KDPMHL--
KFPMHL--
-KPMHLN-
-KPMHLK-
-KPMHLA-
KMPMHL--
KHPMHL--
KYPMHL--
KKPMHL--
-KPMHLY-
KEPMHL--
-KPMHLL-
-KPMHLH-
-KPMHLG-
LRPMHL--
LVPMHL--
LGPMHL--

-LPMHLK-
LMPMHL--
-LPMHLS-
-LPMHLM-
LYPMHL--
LSPMHL--
LEPMHL--
LPPMHL--
-LPMHLV-
-LPMHLL-
LFPMHL--
-LPMHLY-
LLPMHL--
LTPMHL--
-LPMHLF-
LAPMHL--
-LPMHLH-
LDPMHL--
LIPMHL--
LHPMHL--
LQPMHL--
-LPMHLE-
LNPMHL--
LKPMHL--
-LPMHLQ-
-LPMHLA-
-LPMHLI-
-LPMHLN-
-MPMHLY-
MLPMHL--
MRPMHL--
MVPMHL--
MEPMHL--
MNPMHL--
-MPMHLT-
MGPMHL--
-MPMHLL-
MTPMHL--
-MPMHLM-
MFPMHL--
MAPMHL--
MQPMHL--
MDPMHL--
-MPMHLE-
MSPMHL--
-MPMHLW-
-MPMHLV-
MWPMHL--
MPPMHL--
-MPMHLN-

```
-MPMHLH-
-MPMHLG-
-MPMHLS-
-MPMHLR-
-MPMHLK-
-MPMHLQ-
MYPMHL--
-MPMHLI-
MHPMHL--
-MPMHLF-
-MPMHLA-
NSPMHL--
NVPMHL--
NPPMHL--
NRPMHL--
NMPMHL--
NLPMHL--
-NPMHLL-
NNPMHL--
NHPMHL--
NIPMHL--
NTPMHL--
-NPMHLY-
-NPMHLT-
-NPMHLE-
-NPMHLR-
NYPMHL--
-NPMHLI-
-NPMHLF-
-NPMHLA-
NQPMHL--
-NPMHLV-
-NPMHLQ-
-NPMHLM-
-NPMHLH-
NFPMHL--
-NPMHLW-
-NPMHLS-
NDPMHL--
--PMHLVN
--PMHLLS
--PMHLAA
--PMHLSS
--PMHLGS
--PMHLRN
--PMHLSM
--PMHLTF
--PMHLRT
--PMHLVS
--PMHLMK
```

--PMHLML
--PMHLTS
--PMHLRM
--PMHLKA
--PMHLIK
--PMHLVA
--PMHLIS
--PMHLYG
--PMHLLQ
--PMHLIG
--PMHLQG
--PMHLVR
--PMHLVF
--PMHLNT
--PMHLLM
--PMHLYY
--PMHLSI
PGPMHL--
PFPMHL--
PTPMHL--
--PMHLYT
--PMHLKH
--PMHLVV
--PMHLRG
--PMHLMG
--PMHLHQ
--PMHLTT
--PMHLSK
--PMHLRD
--PMHLMQ
--PMHLYS
--PMHLWV
--PMHLWT
--PMHLKS
--PMHLQW
--PMHLHV
--PMHLYK
--PMHLKW
--PMHLRA
--PMHLFY
PEPMHL--
--PMHLNW
--PMHLLV
--PMHLLA
--PMHLVG
--PMHLTL
--PMHLRV
--PMHLQL
--PMHLMF
PLPMHL--

PQPMHL--
--PMHLYW
--PMHLYV
--PMHLSV
--PMHLST
--PMHLRE
--PMHLIV
--PMHLIM
--PMHLHT
--PMHLEA
PIPMHL--
PSPMHL--
--PMHLWS
--PMHLVT
--PMHLQT
--PMHLMM
--PMHLKR
--PMHLKG
--PMHLEV
PVPMHL--
--PMHLWF
--PMHLSN
--PMHLSD
--PMHLQA
--PMHLLW
--PMHLLL
--PMHLLD
--PMHLIH
--PMHLEI
--PMHLVD
--PMHLTR
--PMHLTE
--PMHLSA
--PMHLKL
--PMHLHW
--PMHLHI
--PMHLHA
--PMHLGG
--PMHLAW
PAPMHL--
PNPMHL--
--PMHLYI
--PMHLVM
--PMHLTV
--PMHLRQ
--PMHLQK
--PMHLNY
--PMHLLE
--PMHLHK
--PMHLGV

--PMHLER
--PMHLDS
PMPMHL--
--PMHLYA
--PMHLQV
--PMHLNG
--PMHLMA
--PMHLKT
--PMHLHL
--PMHLAH
--PMHLYL
--PMHLYF
--PMHLWR
--PMHLVK
--PMHLTK
--PMHLNS
--PMHLMR
--PMHLME
--PMHLID
--PMHLFV
--PMHLFS
--PMHLAV
--PMHLAT
--PMHLYQ
--PMHLYM
--PMHLWL
--PMHLSQ
--PMHLRI
--PMHLMY
--PMHLMS
--PMHLLK
--PMHLHM
--PMHLGM
--PMHLFK
--PMHLEF
PHPMHL--
PYPMHL--
PWPMHL--
--PMHLWY
--PMHLWD
--PMHLWA
--PMHLVW
--PMHLSF
--PMHLQI
--PMHLNH
--PMHLNF
--PMHLNA
--PMHLKV
--PMHLFQ
--PMHLAQ

--PMHLYE
--PMHLVE
--PMHLSH
--PMHLSG
--PMHLRH
--PMHLMH
--PMHLIT
--PMHLHY
--PMHLHS
--PMHLHH
--PMHLFT
--PMHLEQ
--PMHLEN
--PMHLYD
--PMHLWQ
--PMHLVI
--PMHLTM
--PMHLTA
--PMHLSW
--PMHLQQ
--PMHLQF
--PMHLNV
--PMHLMT
--PMHLMN
--PMHLMI
--PMHLMD
--PMHLLN
--PMHLKK
--PMHLIA
--PMHLHG
--PMHLFR
--PMHLFM
--PMHLFH
--PMHLAY
PDPMHL--
--PMHLYR
--PMHLYN
--PMHLWI
--PMHLVY
--PMHLTW
--PMHLSY
--PMHLSE
--PMHLRF
--PMHLQN
--PMHLQM
--PMHLIR
--PMHLHE
--PMHLFW
--PMHLEW
--PMHLES

--PMHLAL
--PMHLAF
PCPMHL--
--PMHLWN
--PMHLWK
--PMHLVQ
--PMHLTY
--PMHLTI
--PMHLTH
--PMHLQY
--PMHLQS
--PMHLQH
--PMHLNR
--PMHLKQ
--PMHLKN
--PMHLHF
--PMHLHC
--PMHLGW
--PMHLGL
--PMHLGA
--PMHLFN
--PMHLFG
--PMHLEY
--PMHLEH
--PMHLDG
--PMHLAI
-PPMHLY-
-PPMHLN-
-PPMHLK-
--PMHLWH
--PMHLWE
--PMHLVH
--PMHLTC
--PMHLQD
--PMHLNM
--PMHLNL
--PMHLNK
--PMHLKI
--PMHLIY
--PMHLIW
--PMHLIF
--PMHLIE
--PMHLHN
--PMHLGT
--PMHLGH
--PMHLFD
--PMHLET
--PMHLDF
--PMHLDE
--PMHLCS

--PMHLAM
--PMHLAK
PKPMHL--
QAPMHL--
-QPMHLS-
QVPMHL--
QIPMHL--
-QPMHLE-
QRPMHL--
QTPMHL--
-QPMHLA-
-QPMHLK-
-QPMHLM-
-QPMHLR-
QMPMHL--
-QPMHLH-
QLPMHL--
QPPMHL--
-QPMHLY-
-QPMHLN-
QKPMHL--
-QPMHLQ-
QFPMHL--
QSPMHL--
-QPMHLV-
QHPMHL--
QDPMHL--
QYPMHL--
QQPMHL--
-QPMHLG-
-QPMHLT-
-QPMHLF-
-QPMHLC-
QNPMHL--
RAPMHL--
RLPMHL--
RSPMHL--
-RPMHLS-
-RPMHLR-
REPMHL--
-RPMHLA-
RVPMHL--
-RPMHLM-
-RPMHLL-
RFPMHL--
RGPMHL--
-RPMHLW-
-RPMHLF-
-RPMHLK-
RIPMHL--

RMPMHL--
RQPMHL--
-RPMHLT-
RWPMHL--
RPPMHL--
-RPMHLY-
RCPMHL--
RYPMHL--
-RPMHLV-
-RPMHLH-
-RPMHLG-
-RPMHLN-
RHPMHL--
RDPMHL--
-RPMHLQ-
-RPMHLE-
-SPMHLR-
SLPMHL--
SSPMHL--
SYPMHL--
-SPMHLK-
-SPMHLY-
SAPMHL--
STPMHL--
-SPMHLI-
-SPMHLS-
SKPMHL--
-SPMHLV-
SDPMHL--
SIPMHL--
-SPMHLL-
SMPMHL--
SQPMHL--
SNPMHL--
-SPMHLM-
SHPMHL--
-SPMHLH-
-SPMHLW-
SGPMHL--
SFPMHL--
-SPMHLN-
-SPMHLA-
-SPMHLT-
SCPMHL--
-SPMHLQ-
-SPMHLE-
-SPMHLG-
SEPMHL--
TIPMHL--
TVPMHL--

TTPMHL--
-TPMHLR-
TNPMHL--
TLPMHL--
-TPMHLS-
-TPMHLY-
TWPMHL--
TFPMHL--
TYPMHL--
TSPMHL--
-TPMHLL-
TKPMHL--
TEPMHL--
-TPMHLT-
-TPMHLI-
THPMHL--
TGPMHL--
TAPMHL--
-TPMHLK-
TMPMHL--
TPPMHL--
-TPMHLF-
-TPMHLA-
-TPMHLM-
-TPMHLQ-
TDPMHL--
-TPMHLN-
TQPMHL--
-TPMHLW-
-TPMHLG-
VSPMHL--
VRPMHL--
VGPMHL--
-VPMHLL-
VLPMHL--
-VPMHLS-
-VPMHLR-
VNPMHL--
VIPMHL--
VVPMHL--
VEPMHL--
-VPMHLT-
-VPMHLW-
-VPMHLV-
-VPMHLM-
-VPMHLK-
VMPMHL--
VPPMHL--
-VPMHLQ-
-VPMHLI-

VFPMHL--
-VPMHLN-
VQPMHL--
-VPMHLH-
-VPMHLA-
-VPMHLE-
VYPMHL--
-VPMHLF-
VHPMHL--
-VPMHLY-
-VPMHLG-
WSPMHL--
WYPMHL--
WEPMHL--
-WPMHLS-
WKPMHL--
WLPMHL--
WTPMHL--
-WPMHLM-
-WPMHLT-
-WPMHLR-
-WPMHLN-
WQPMHL--
WNPMHL--
WMPMHL--
-WPMHLV-
-WPMHLG-
WGPMHL--
-WPMHLL-
WIPMHL--
-WPMHLW-
-WPMHLY-
-WPMHLE-
-WPMHLQ-
WFPMHL--
WPPMHL--
-WPMHLK-
YLPMHL--
-YPMHLS-
YKPMHL--
YGPMHL--
YYPMHL--
YEPMHL--
-YPMHLR-
YHPMHL--
YTPMHL--
-YPMHLT-
-YPMHLE-
YSPMHL--
-YPMHLI-

	YDPMHL--		
	-YPMHLA-		
	-YPMHLY-		
	-YPMHLL-		
	YNPMHL--		
	YIPMHL--		
	YPPMHL--		
	-YPMHLV-		
	-YPMHLM-		
	-YPMHLK-		
	-YPMHLF-		
	YMPMHL--		
	-YPMHLN-		
	-YPMHLG-		
	YAPMHL--		
	YWPMHL--		
	-YPMHLH-		
PIGL	24	874	47.70
	-APIGLD-		
	-HPIGLG-		
	-KPIGLP-		
	--PIGLTP		
	--PIGLSP		
	--PIGLGL		
	--PIGLGE		
	-RPIGLP-		
	-RPIGLD-		
	-SPIGLP-		
	-APIGLS-		
	APPIGL--		
	AGPIGL--		
	ASPIGL--		
	ARPIGL--		
	-APIGLR-		
	-APIGLL-		
	AYPIGL--		
	-APIGLT-		
	-APIGLG-		
	AVPIGL--		
	AAPIGL--		
	AIPIGL--		
	-APIGLH-		
	AQPIGL--		
	-APIGLM-		
	-APIGLI-		
	AHPIGL--		
	-APIGLA-		
	AMPIGL--		
	AFPIGL--		
	-APIGLN-		

ALPIGL--
-APIGLY-
-APIGLV-
ATPIGL--
-APIGLK-
ANPIGL--
AWPIGL--
-APIGLE-
ADPIGL--
AEPIGL--
ACPIGL--
-APIGLQ-
-APIGLF-
CSPIGL--
CKPIGL--
CCPIGL--
CWPIGL--
-CPIGLR-
CFPIGL--
CPPIGL--
CAPIGL--
CTPIGL--
-CPIGLV-
-CPIGLT-
-CPIGLL-
CGPIGL--
CRPIGL--
-CPIGLY-
-CPIGLI-
CNPIGL--
CHPIGL--
CVPIGL--
CQPIGL--
CIPIGL--
-CPIGLQ-
CLPIGL--
CYPIGL--
-CPIGLH-
-CPIGLA-
CEPIGL--
-DPIGLR-
DTPIGL--
-DPIGLL-
DMPIGL--
-DPIGLY-
DNPIGL--
DVPIGL--
DEPIGL--
-DPIGLI-
DSPIGL--

-DPIGLH-
DLPIGL--
DYPIGL--
DWPIGL--
DKPIGL--
-DPIGLW-
-DPIGLT-
-DPIGLM-
-DPIGLK-
DFPIGL--
DAPIGL--
-DPIGLN-
-DPIGLF-
DCPIGL--
DQPIGL--
-DPIGLQ-
-DPIGLA-
DHPIGL--
DGPIGL--
ETPIGL--
ERPIGL--
ESPIGL--
-EPIGLV-
EYPIGL--
-EPIGLT-
-EPIGLM-
-EPIGLL-
-EPIGLF-
-EPIGLN-
-EPIGLY-
EVPIGL--
-EPIGLI-
EMPIGL--
EGPIGL--
-EPIGLS-
EAPIGL--
EIPIGL--
EWPIGL--
-EPIGLQ-
-EPIGLW-
ELPIGL--
-EPIGLA-
-EPIGLG-
-EPIGLE-
EFPIGL--
ENPIGL--
EDPIGL--
-EPIGLK-
EKPIGL--
-EPIGLH-

EQPIGL--
EHPIGL--
ECPIGL--
FEPIGL--
FTPIGL--
-FPIGLT-
FGPIGL--
-FPIGLF-
-FPIGLS-
-FPIGLY-
FSPIGL--
-FPIGLK-
FWPIGL--
FIPIGL--
-FPIGLR-
FHPIGL--
FYPIGL--
-FPIGLV-
FDPIGL--
-FPIGLA-
-FPIGLG-
FLPIGL--
FMPIGL--
-FPIGLW-
FQPIGL--
-FPIGLH-
-FPIGLE-
FPPIGL--
-FPIGLM-
-FPIGLN-
-FPIGLQ-
-FPIGLI-
GRPIGL--
-GPIGLL-
GTPIGL--
-GPIGLK-
GSPIGL--
GWPIGL--
GLPIGL--
GVPIGL--
-GPIGLQ-
-GPIGLI-
-GPIGLN-
-GPIGLS-
GIPIGL--
GFPIGL--
GKPIGL--
-GPIGLF-
-GPIGLT-
GQPIGL--

GMPIGL--
GAPIGL--
-GPIGLA-
GGPIGL--
GEPIGL--
GNPIGL--
GHPIGL--
GPPIGL--
-GPIGLH-
-GPIGLE-
GDPIGL--
-GPIGLM-
-GPIGLG-
GCPIGL--
-HPIGLL-
HSPIGL--
-HPIGLR-
HRPIGL--
HKPIGL--
-HPIGLK-
-HPIGLV-
-HPIGLM-
HMPIGL--
HQPIGL--
-HPIGLS-
HTPIGL--
HVPIGL--
HLPIGL--
HEPIGL--
-HPIGLT-
-HPIGLW-
HIPIGL--
-HPIGLA-
HWPIGL--
HPPIGL--
HFPIGL--
HAPIGL--
-HPIGLF-
HYPIGL--
-HPIGLQ-
HGPIGL--
-HPIGLN-
HDPIGL--
-HPIGLY-
HHPIGL--
HNPIGL--
ILPIGL--
ITPIGL--
-IPIGLT-
-IPIGLS-

-IPIGLR-
IDPIGL--
IRPIGL--
-IPIGLQ-
IFPIGL--
ISPIGL--
INPIGL--
IPPIGL--
IAPIGL--
-IPIGLI-
IEPIGL--
-IPIGLW-
-IPIGLN-
IHPIGL--
-IPIGLH-
IQPIGL--
-IPIGLE-
IKPIGL--
-IPIGLM-
IVPIGL--
-IPIGLV-
IYPIGL--
-IPIGLA-
IGPIGL--
-IPIGLK-
IWPIGL--
ICPIGL--
-IPIGLG-
IIPIGL--
KSPIGL--
KVPIGL--
KRPIGL--
KGPIGL--
-KPIGLT-
KFPIGL--
KPPIGL--
KNPIGL--
-KPIGLY-
KQPIGL--
KHPIGL--
-KPIGLH-
KLPIGL--
-KPIGLQ-
KTPIGL--
-KPIGLN-
-KPIGLF-
KDPIGL--
KAPIGL--
KYPIGL--
-KPIGLV-

−KPIGLK−
KWPIGL−−
KEPIGL−−
KIPIGL−−
−KPIGLI−
−KPIGLA−
KCPIGL−−
KMPIGL−−
−KPIGLM−
−KPIGLG−
−KPIGLE−
−LPIGLN−
LSPIGL−−
LMPIGL−−
LLPIGL−−
−LPIGLS−
−LPIGLR−
−LPIGLT−
−LPIGLL−
LTPIGL−−
−LPIGLI−
LFPIGL−−
LGPIGL−−
LQPIGL−−
LWPIGL−−
LNPIGL−−
LDPIGL−−
LYPIGL−−
−LPIGLV−
LVPIGL−−
−LPIGLM−
LHPIGL−−
−LPIGLH−
−LPIGLA−
−LPIGLQ−
LEPIGL−−
LPPIGL−−
−LPIGLE−
−LPIGLC−
MRPIGL−−
−MPIGLT−
MLPIGL−−
MTPIGL−−
−MPIGLY−
MSPIGL−−
MNPIGL−−
−MPIGLS−
MKPIGL−−
MGPIGL−−
−MPIGLV−

-MPIGLK-
MHPIGL--
MAPIGL--
-MPIGLA-
MPPIGL--
-MPIGLR-
-MPIGLL-
-MPIGLI-
MWPIGL--
-MPIGLH-
MEPIGL--
MFPIGL--
-MPIGLW-
MVPIGL--
-MPIGLF-
-MPIGLQ-
-MPIGLN-
-MPIGLG-
-MPIGLE-
-MPIGLM-
MYPIGL--
MIPIGL--
MMPIGL--
MDPIGL--
MCPIGL--
-NPIGLL-
-NPIGLI-
NSPIGL--
NHPIGL--
NAPIGL--
NVPIGL--
NNPIGL--
NLPIGL--
NTPIGL--
-NPIGLR-
NEPIGL--
-NPIGLK-
NYPIGL--
NKPIGL--
-NPIGLT-
NGPIGL--
-NPIGLQ-
NDPIGL--
-NPIGLH-
-NPIGLM-
-NPIGLN-
NIPIGL--
NWPIGL--
NQPIGL--
-NPIGLV-

-NPIGLF-
NFPIGL--
NPPIGL--
-NPIGLG-
NCPIGL--
-NPIGLE-
--PIGLVR
--PIGLRA
--PIGLTS
--PIGLST
--PIGLRN
--PIGLRG
--PIGLMR
--PIGLVS
--PIGLRS
--PIGLSS
--PIGLLG
--PIGLWS
--PIGLRY
--PIGLRV
--PIGLRL
--PIGLGT
--PIGLYS
--PIGLHR
--PIGLFT
--PIGLVA
--PIGLGS
--PIGLLR
--PIGLRI
--PIGLMS
--PIGLSN
--PIGLLT
--PIGLKG
--PIGLQS
--PIGLRK
--PIGLVM
--PIGLVQ
--PIGLTK
--PIGLSG
--PIGLRM
--PIGLMY
--PIGLYT
--PIGLRH
--PIGLVT
--PIGLVL
--PIGLTI
--PIGLYG
--PIGLSW
PSPIGL--
--PIGLKR

--PIGLYQ
--PIGLSF
--PIGLSA
--PIGLRW
--PIGLMG
--PIGLFS
--PIGLTA
--PIGLSI
--PIGLRD
--PIGLIN
--PIGLLQ
--PIGLIS
--PIGLAA
--PIGLVY
--PIGLRF
--PIGLIA
--PIGLHS
--PIGLES
--PIGLML
--PIGLKS
--PIGLNY
--PIGLHL
--PIGLHA
--PIGLTL
--PIGLRQ
--PIGLYD
--PIGLWQ
--PIGLSQ
--PIGLSK
--PIGLQV
--PIGLWD
--PIGLVE
--PIGLKT
--PIGLFH
--PIGLEA
--PIGLQT
--PIGLAN
--PIGLAH
PLPIGL--
--PIGLMA
--PIGLKL
--PIGLHY
--PIGLAG
PHPIGL--
--PIGLHV
--PIGLAK
--PIGLKA
--PIGLVN
--PIGLNW
--PIGLKY

--PIGLKH
PWPIGL--
--PIGLWG
--PIGLVV
--PIGLRE
--PIGLNL
--PIGLNI
--PIGLNA
--PIGLLN
--PIGLLE
--PIGLKQ
PFPIGL--
PKPIGL--
--PIGLWH
--PIGLTT
--PIGLTN
--PIGLKF
--PIGLHG
PTPIGL--
PQPIGL--
--PIGLTW
--PIGLTG
--PIGLNR
--PIGLMI
--PIGLMH
--PIGLKN
--PIGLHI
PAPIGL--
--PIGLME
--PIGLHE
--PIGLFD
--PIGLAV
--PIGLVH
--PIGLVF
--PIGLTM
--PIGLQA
--PIGLKD
PYPIGL--
--PIGLYN
--PIGLYK
--PIGLHM
--PIGLYH
--PIGLQG
--PIGLNG
--PIGLIH
--PIGLFQ
PVPIGL--
--PIGLMF
--PIGLIK
--PIGLGA

--PIGLAY
--PIGLVI
--PIGLTE
--PIGLNV
--PIGLII
--PIGLHF
--PIGLGG
--PIGLVK
--PIGLNM
--PIGLMW
--PIGLKI
--PIGLIW
PNPIGL--
--PIGLTY
--PIGLSV
--PIGLQR
--PIGLQM
--PIGLTH
--PIGLQY
--PIGLMN
--PIGLMK
--PIGLIT
--PIGLHT
--PIGLHN
--PIGLHK
--PIGLGW
--PIGLQI
--PIGLNT
--PIGLLD
--PIGLHW
--PIGLTQ
--PIGLLK
--PIGLID
--PIGLAM
--PIGLAL
PEPIGL--
--PIGLHD
--PIGLQF
--PIGLNK
--PIGLNE
--PIGLMT
--PIGLIG
--PIGLFA
--PIGLEH
--PIGLDG
--PIGLAF
--PIGLYE
--PIGLVD
--PIGLQN
--PIGLQK

--PIGLNN
--PIGLGH
--PIGLQL
--PIGLGY
--PIGLEV
--PIGLAR
--PIGLTD
--PIGLSM
--PIGLMM
--PIGLMD
--PIGLKM
--PIGLGR
--PIGLGF
--PIGLEM
--PIGLEK
--PIGLCT
--PIGLNF
--PIGLKV
--PIGLHQ
--PIGLER
--PIGLEL
--PIGLAI
--PIGLWN
--PIGLSD
--PIGLNH
--PIGLMV
--PIGLET
--PIGLAQ
PDPIGL--
--PIGLGK
--PIGLGI
--PIGLEW
--PIGLEG
--PIGLDS
-PPIGLS-
--PIGLWE
--PIGLTC
--PIGLQD
--PIGLPY
--PIGLPL
--PIGLPG
--PIGLNQ
--PIGLMQ
--PIGLKW
--PIGLIQ
--PIGLHH
--PIGLGV
--PIGLGN
--PIGLEN
--PIGLEI

--PIGLCR
PPPIGL--
-PPIGLM-
-PPIGLI-
PMPIGL--
--PIGLQQ
--PIGLKE
--PIGLKC
--PIGLIE
--PIGLIC
--PIGLGQ
--PIGLEQ
--PIGLEF
--PIGLDV
--PIGLDT
--PIGLCY
--PIGLCQ
--PIGLCF
--PIGLCA
QRPIGL--
-QPIGLS-
QLPIGL--
-QPIGLY-
QIPIGL--
QSPIGL--
QKPIGL--
QPPIGL--
-QPIGLL-
-QPIGLI-
-QPIGLF-
QTPIGL--
QQPIGL--
-QPIGLW-
-QPIGLN-
-QPIGLR-
QFPIGL--
-QPIGLT-
-QPIGLM-
QNPIGL--
QMPIGL--
QYPIGL--
QWPIGL--
QDPIGL--
QGPIGL--
-QPIGLH-
-QPIGLG-
QHPIGL--
-QPIGLK-
QAPIGL--
QVPIGL--

-QPIGLA-
-QPIGLQ-
-QPIGLC-
-QPIGLE-
RLPIGL--
RIPIGL--
RGPIGL--
RAPIGL--
RRPIGL--
RWPIGL--
RVPIGL--
-RPIGLT-
REPIGL--
RSPIGL--
-RPIGLV-
-RPIGLH-
RDPIGL--
-RPIGLN-
-RPIGLK-
-RPIGLA-
RMPIGL--
RHPIGL--
RNPIGL--
RFPIGL--
-RPIGLW-
-RPIGLE-
RQPIGL--
-RPIGLI-
RPPIGL--
RCPIGL--
-RPIGLC-
SSPIGL--
-SPIGLR-
SNPIGL--
-SPIGLS-
-SPIGLY-
SAPIGL--
SVPIGL--
SLPIGL--
SGPIGL--
SMPIGL--
SQPIGL--
SWPIGL--
STPIGL--
SFPIGL--
-SPIGLW-
-SPIGLT-
-SPIGLV-
SDPIGL--
-SPIGLH-

-SPIGLF-
-SPIGLQ-
-SPIGLN-
-SPIGLK-
SEPIGL--
SHPIGL--
SKPIGL--
-SPIGLA-
SIPIGL--
SYPIGL--
SPPIGL--
-SPIGLG-
-SPIGLI-
-SPIGLE-
-SPIGLC-
SCPIGL--
-TPIGLI-
TVPIGL--
TSPIGL--
TRPIGL--
-TPIGLR-
TGPIGL--
-TPIGLL-
-TPIGLY-
TTPIGL--
TPPIGL--
TFPIGL--
-TPIGLT-
TKPIGL--
THPIGL--
-TPIGLS-
TYPIGL--
TWPIGL--
-TPIGLF-
-TPIGLV-
-TPIGLN-
TMPIGL--
-TPIGLH-
-TPIGLM-
-TPIGLA-
-TPIGLW-
TEPIGL--
TQPIGL--
-TPIGLK-
-TPIGLG-
-TPIGLE-
-TPIGLQ-
TIPIGL--
TDPIGL--
TCPIGL--

-VPIGLR-
-VPIGLT-
VRPIGL--
VTPIGL--
VHPIGL--
VSPIGL--
VVPIGL--
-VPIGLA-
VKPIGL--
-VPIGLS-
-VPIGLN-
VDPIGL--
VLPIGL--
-VPIGLH-
-VPIGLV-
VAPIGL--
-VPIGLK-
VWPIGL--
VFPIGL--
VEPIGL--
VIPIGL--
VPPIGL--
VMPIGL--
-VPIGLQ-
-VPIGLG-
-VPIGLE-
VNPIGL--
VYPIGL--
-VPIGLI-
VCPIGL--
VQPIGL--
-WPIGLR-
WSPIGL--
WPPIGL--
-WPIGLT-
WAPIGL--
-WPIGLL-
WVPIGL--
WLPIGL--
WTPIGL--
-WPIGLV-
WGPIGL--
-WPIGLN-
-WPIGLI-
-WPIGLM-
WFPIGL--
-WPIGLY-
WIPIGL--
-WPIGLS-
-WPIGLF-

-WPIGLE-
WNPIGL--
WEPIGL--
-WPIGLK-
-WPIGLH-
WWPIGL--
WHPIGL--
-WPIGLQ-
-WPIGLA-
WCPIGL--
-WPIGLD-
WDPIGL--
YLPIGL--
YSPIGL--
-YPIGLR-
-YPIGLQ-
-YPIGLK-
-YPIGLV-
-YPIGLH-
YMPIGL--
YTPIGL--
-YPIGLS-
YGPIGL--
YFPIGL--
-YPIGLW-
YVPIGL--
YNPIGL--
YEPIGL--
YYPIGL--
YQPIGL--
-YPIGLA-
-YPIGLI-
YKPIGL--
-YPIGLF-
-YPIGLM-
-YPIGLT-
-YPIGLN-
-YPIGLE-
YHPIGL--
-YPIGLG-
YDPIGL--
YWPIGL--
PRGM 84 822 47.70
-IPRGMP-
-NPRGMP-
--PRGMPY
--PRGMMP
--PRGMLP
--PRGMET
--PRGMER

--PRGMDL
AGPRGM--
-APRGMI-
-APRGMN-
-APRGMA-
-APRGMY-
ALPRGM--
ATPRGM--
-APRGML-
AFPRGM--
ARPRGM--
-APRGMK-
APPRGM--
AVPRGM--
-APRGMS-
-APRGMM-
-APRGMT-
AIPRGM--
ADPRGM--
AHPRGM--
AAPRGM--
-APRGMH-
-APRGMQ-
-APRGMG-
-APRGME-
AKPRGM--
AQPRGM--
-APRGMV-
ANPRGM--
AYPRGM--
AMPRGM--
-APRGMF-
-APRGMD-
-APRGMC-
CRPRGM--
CCPRGM--
CAPRGM--
-CPRGMH-
-CPRGMV-
CVPRGM--
CSPRGM--
CKPRGM--
-CPRGMF-
-CPRGML-
-CPRGMR-
-CPRGMA-
CGPRGM--
CDPRGM--
-CPRGMT-
-CPRGMN-

CTPRGM--
-CPRGMY-
-CPRGMG-
CPPRGM--
-CPRGMW-
CNPRGM--
CEPRGM--
DLPRGM--
DTPRGM--
DWPRGM--
DQPRGM--
-DPRGMF-
-DPRGMT-
-DPRGMW-
-DPRGMK-
DAPRGM--
-DPRGMR-
-DPRGMS-
-DPRGMA-
DRPRGM--
-DPRGMI-
-DPRGMH-
DKPRGM--
-DPRGMV-
-DPRGMQ-
DSPRGM--
-DPRGMM-
DNPRGM--
DGPRGM--
DFPRGM--
DVPRGM--
-DPRGMY-
-DPRGMN-
-DPRGMG-
-EPRGMM-
-EPRGMS-
EKPRGM--
ERPRGM--
ELPRGM--
-EPRGML-
EVPRGM--
-EPRGMY-
-EPRGMW-
-EPRGMI-
EYPRGM--
EMPRGM--
ESPRGM--
ETPRGM--
-EPRGMT-
-EPRGME-

-EPRGMH-
-EPRGMN-
-EPRGMG-
ENPRGM--
EQPRGM--
EAPRGM--
EHPRGM--
EWPRGM--
-EPRGMV-
EGPRGM--
-EPRGMQ-
EEPRGM--
-EPRGMA-
EPPRGM--
EDPRGM--
FHPRGM--
-FPRGMN-
FFPRGM--
-FPRGMS-
FSPRGM--
-FPRGMQ-
-FPRGMG-
-FPRGMV-
FGPRGM--
FYPRGM--
FEPRGM--
FVPRGM--
FTPRGM--
FIPRGM--
FQPRGM--
-FPRGMW-
-FPRGMM-
FPPRGM--
-FPRGMT-
FNPRGM--
FDPRGM--
FKPRGM--
FMPRGM--
-FPRGME-
-FPRGMA-
FCPRGM--
-GPRGMS-
-GPRGMR-
GTPRGM--
GNPRGM--
GSPRGM--
-GPRGMV-
-GPRGMT-
GHPRGM--
-GPRGMM-

-GPRGML-
GAPRGM--
-GPRGMG-
-GPRGMA-
-GPRGMQ-
GRPRGM--
-GPRGMF-
-GPRGME-
GQPRGM--
GGPRGM--
-GPRGMH-
GKPRGM--
GDPRGM--
GVPRGM--
GYPRGM--
-GPRGMK-
GMPRGM--
-GPRGMI-
GIPRGM--
-GPRGMW-
GPPRGM--
GWPRGM--
GEPRGM--
GCPRGM--
-GPRGMN-
-GPRGMD-
-HPRGMT-
-HPRGML-
HSPRGM--
-HPRGMF-
HFPRGM--
HAPRGM--
HMPRGM--
-HPRGMY-
-HPRGMH-
HVPRGM--
-HPRGMN-
-HPRGMA-
HRPRGM--
HQPRGM--
HKPRGM--
HGPRGM--
HIPRGM--
-HPRGMG-
-HPRGMV-
HHPRGM--
-HPRGMS-
-HPRGMI-
-HPRGMK-
HEPRGM--

HYPRGM--
HTPRGM--
-HPRGMW-
-HPRGMM-
IAPRGM--
-IPRGML-
ITPRGM--
-IPRGMY-
IKPRGM--
IRPRGM--
-IPRGMI-
-IPRGMS-
IHPRGM--
-IPRGMK-
IYPRGM--
-IPRGMT-
-IPRGMM-
-IPRGMW-
IMPRGM--
IGPRGM--
-IPRGMA-
IEPRGM--
IVPRGM--
-IPRGMG-
IIPRGM--
ISPRGM--
ICPRGM--
-IPRGMH-
IDPRGM--
IQPRGM--
-IPRGME-
INPRGM--
IPPRGM--
-IPRGMQ-
-IPRGMN-
-IPRGMC-
-KPRGMI-
-KPRGML-
-KPRGMT-
-KPRGMV-
-KPRGMM-
KGPRGM--
-KPRGMF-
-KPRGMS-
-KPRGMW-
-KPRGMH-
KVPRGM--
-KPRGMA-
KLPRGM--
-KPRGMG-

KDPRGM--
-KPRGME-
KFPRGM--
-KPRGMK-
-KPRGMN-
KTPRGM--
KQPRGM--
KEPRGM--
KYPRGM--
KMPRGM--
KNPRGM--
KKPRGM--
KIPRGM--
KCPRGM--
KPPRGM--
-KPRGMQ-
LLPRGM--
LRPRGM--
LYPRGM--
-LPRGMS-
-LPRGMF-
LIPRGM--
LFPRGM--
-LPRGMW-
-LPRGMQ-
-LPRGMV-
LTPRGM--
LNPRGM--
-LPRGMA-
LEPRGM--
-LPRGMH-
LPPRGM--
-LPRGMI-
LAPRGM--
LKPRGM--
-LPRGMY-
-LPRGMM-
LQPRGM--
-LPRGME-
-LPRGMG-
LCPRGM--
-LPRGMC-
-MPRGMS-
-MPRGMF-
-MPRGML-
-MPRGMW-
-MPRGMR-
-MPRGMQ-
MAPRGM--
MVPRGM--

MIPRGM--
-MPRGMV-
-MPRGMH-
-MPRGMK-
MQPRGM--
-MPRGMN-
-MPRGMI-
MHPRGM--
-MPRGMM-
-MPRGMA-
-MPRGMT-
MSPRGM--
MTPRGM--
-MPRGMY-
-MPRGMG-
MPPRGM--
MEPRGM--
MDPRGM--
MKPRGM--
MMPRGM--
MGPRGM--
MNPRGM--
-MPRGME-
MCPRGM--
NSPRGM--
-NPRGMY-
NYPRGM--
-NPRGML-
NEPRGM--
-NPRGMI-
-NPRGMF-
-NPRGMR-
NFPRGM--
-NPRGMV-
NWPRGM--
-NPRGMS-
NGPRGM--
-NPRGMH-
NMPRGM--
NTPRGM--
NRPRGM--
NNPRGM--
NIPRGM--
-NPRGMK-
NPPRGM--
-NPRGMQ-
-NPRGMN-
-NPRGME-
NVPRGM--
-NPRGMW-

```
-NPRGMM-
NDPRGM--
-NPRGMG-
NQPRGM--
NKPRGM--
NCPRGM--
--PRGMGA
--PRGMFS
--PRGMLT
--PRGMLS
--PRGMYV
--PRGMVK
--PRGMST
--PRGMVL
--PRGMSS
--PRGMTS
--PRGMYR
--PRGMMF
--PRGMWS
--PRGMTG
--PRGMTL
--PRGMYS
--PRGMVT
--PRGMSV
--PRGMSL
--PRGMSA
--PRGMFK
--PRGMIN
--PRGMTF
--PRGMWL
--PRGMMN
--PRGMNS
--PRGMLI
--PRGMYL
--PRGMVM
--PRGMNM
--PRGMRQ
--PRGMRE
--PRGMTY
--PRGMTN
--PRGMSN
--PRGMLY
--PRGMLQ
--PRGMFQ
--PRGMMH
--PRGMSY
--PRGMIL
--PRGMFH
--PRGMVS
--PRGMLH
```

--PRGMLE
--PRGMKY
--PRGMFT
--PRGMIA
--PRGMKW
--PRGMSQ
--PRGMSK
--PRGMQR
--PRGMLM
--PRGMYT
--PRGMLF
--PRGMAN
PSPRGM--
--PRGMWN
--PRGMKA
--PRGMTK
--PRGMTI
--PRGMSF
--PRGMMV
--PRGMMK
--PRGMMA
--PRGMGS
--PRGMTT
--PRGMWT
--PRGMHW
--PRGMHR
--PRGMSI
--PRGMMG
--PRGMHG
PWPRGM--
--PRGMYH
--PRGMSW
--PRGMQS
--PRGMMI
--PRGMHL
--PRGMFL
--PRGMAG
--PRGMWI
--PRGMQG
--PRGMKN
--PRGMIK
--PRGMES
--PRGMNR
--PRGMKG
--PRGMYD
--PRGMNN
PRPRGM--
--PRGMYW
--PRGMYK
--PRGMVV

--PRGMQT
--PRGMMW
--PRGMIY
--PRGMYE
--PRGMMM
--PRGMHM
--PRGMHK
--PRGMHF
--PRGMWG
--PRGMKH
--PRGMFM
--PRGMAY
--PRGMYQ
--PRGMMY
--PRGMLD
--PRGMIQ
--PRGMID
--PRGMHS
PAPRGM--
--PRGMYY
PGPRGM--
--PRGMTM
--PRGMSM
--PRGMSD
--PRGMNF
--PRGMKT
--PRGMFN
--PRGMFD
--PRGMYI
--PRGMYA
--PRGMWF
--PRGMWA
--PRGMVH
--PRGMSE
--PRGMIF
--PRGMFR
--PRGMYM
--PRGMQQ
--PRGMQF
--PRGMNW
--PRGMNG
--PRGMGF
--PRGMFA
--PRGMAK
--PRGMWV
--PRGMWK
--PRGMVQ
--PRGMMT
--PRGMII
--PRGMHV

--PRGMGT
PFPRGM--
--PRGMWH
--PRGMWE
--PRGMQI
--PRGMQA
--PRGMHT
--PRGMGV
--PRGMFG
--PRGMAR
--PRGMAM
--PRGMAF
--PRGMWY
--PRGMQN
--PRGMIV
--PRGMEN
PVPRGM--
--PRGMVF
--PRGMVE
--PRGMTQ
--PRGMRW
--PRGMQK
--PRGMNV
--PRGMHQ
--PRGMGY
--PRGMGM
--PRGMGI
PEPRGM--
PTPRGM--
--PRGMQY
--PRGMQW
--PRGMNQ
--PRGMMQ
--PRGMHI
--PRGMHH
--PRGMHA
PYPRGM--
--PRGMQE
--PRGMNI
--PRGMNH
--PRGMKD
--PRGMGW
--PRGMEW
--PRGMDS
PDPRGM--
--PRGMTE
--PRGMQM
--PRGMQD
--PRGMGG
--PRGMEG

--PRGMAH
PKPRGM--
--PRGMWM
--PRGMNY
--PRGMNK
--PRGMEI
PIPRGM--
PHPRGM--
--PRGMWW
--PRGMTD
--PRGMKS
--PRGMEF
--PRGMDA
--PRGMCS
--PRGMNT
--PRGMMD
--PRGMKE
--PRGMIE
--PRGMGQ
--PRGMGK
--PRGMFY
--PRGMFE
--PRGMAE
PMPRGM--
--PRGMTH
--PRGMRP
--PRGMQH
--PRGMME
--PRGMGD
--PRGMEK
--PRGMEH
--PRGMDG
--PRGMCN
PNPRGM--
PCPRGM--
--PRGMNA
--PRGMKF
--PRGMHY
--PRGMHD
--PRGMGN
--PRGMFI
--PRGMEM
PQPRGM--
--PRGMVD
--PRGMIP
--PRGMEQ
--PRGMCV
--PRGMCQ
--PRGMAI
--PRGMAD

--PRGMSC
--PRGMNE
--PRGMGC
--PRGMFP
--PRGMDV
--PRGMCY
--PRGMCH
--PRGMCA
--PRGMAQ
--PRGMAC
-PPRGMY-
-PPRGMT-
-PPRGMG-
-PPRGMA-
QVPRGM--
-QPRGMS-
-QPRGMT-
-QPRGMI-
-QPRGMR-
-QPRGMF-
QSPRGM--
QLPRGM--
QEPRGM--
QGPRGM--
QPPRGM--
-QPRGMN-
QMPRGM--
-QPRGMV-
-QPRGMH-
-QPRGME-
QNPRGM--
QAPRGM--
-QPRGMM-
-QPRGMG-
-QPRGMA-
QTPRGM--
-QPRGMW-
QQPRGM--
-QPRGMQ-
-QPRGMK-
QCPRGM--
-RPRGMY-
-RPRGMI-
RLPRGM--
-RPRGMW-
-RPRGMQ-
RDPRGM--
RGPRGM--
-RPRGML-
RMPRGM--

-RPRGMS-
-RPRGMV-
-RPRGMH-
RSPRGM--
RAPRGM--
-RPRGMK-
-RPRGMG-
RIPRGM--
-RPRGMM-
-RPRGMF-
RPPRGM--
RQPRGM--
REPRGM--
-RPRGMA-
-RPRGMT-
-RPRGMN-
RHPRGM--
-RPRGME-
RKPRGM--
-RPRGMC-
-SPRGMS-
SLPRGM--
STPRGM--
-SPRGMY-
-SPRGMI-
-SPRGMV-
SAPRGM--
SRPRGM--
-SPRGMT-
-SPRGMF-
SGPRGM--
-SPRGMA-
SMPRGM--
-SPRGMM-
SSPRGM--
-SPRGMK-
SQPRGM--
SKPRGM--
SHPRGM--
SPPRGM--
-SPRGMH-
SDPRGM--
SNPRGM--
-SPRGMQ-
-SPRGME-
-SPRGMG-
SEPRGM--
-SPRGMW-
-SPRGMD-
SCPRGM--

-TPRGMV-
TSPRGM--
-TPRGMH-
TGPRGM--
-TPRGMY-
-TPRGML-
TLPRGM--
-TPRGMF-
-TPRGMT-
TEPRGM--
TPPRGM--
-TPRGMS-
TKPRGM--
-TPRGMA-
-TPRGMW-
-TPRGMM-
TDPRGM--
-TPRGMN-
TNPRGM--
TYPRGM--
-TPRGMI-
TVPRGM--
TIPRGM--
-TPRGMG-
TTPRGM--
TQPRGM--
-TPRGMQ-
-TPRGME-
TAPRGM--
THPRGM--
-TPRGMK-
-TPRGMC-
-VPRGMY-
-VPRGML-
-VPRGMS-
-VPRGMV-
-VPRGMI-
VTPRGM--
VAPRGM--
-VPRGMK-
-VPRGMH-
VRPRGM--
VYPRGM--
VKPRGM--
-VPRGMN-
-VPRGMF-
VIPRGM--
-VPRGMM-
VHPRGM--
VEPRGM--

-VPRGMG-
-VPRGMA-
VFPRGM--
VGPRGM--
-VPRGME-
VNPRGM--
VDPRGM--
VQPRGM--
VPPRGM--
VCPRGM--
VMPRGM--
-VPRGMQ-
WDPRGM--
-WPRGMH-
-WPRGMA-
WNPRGM--
WSPRGM--
-WPRGML-
WEPRGM--
WAPRGM--
-WPRGMF-
-WPRGMN-
WMPRGM--
WIPRGM--
-WPRGMQ-
WKPRGM--
WQPRGM--
WGPRGM--
-WPRGMW-
-WPRGMG-
-WPRGME-
WHPRGM--
-WPRGMY-
-WPRGMK-
WCPRGM--
YYPRGM--
YLPRGM--
YSPRGM--
YRPRGM--
-YPRGMN-
-YPRGML-
YGPRGM--
-YPRGMV-
-YPRGMI-
-YPRGMK-
-YPRGMF-
YHPRGM--
YMPRGM--
YVPRGM--
-YPRGMA-

	-YPRGMY-		
	-YPRGMM-		
	-YPRGMG-		
	YEPRGM--		
	YKPRGM--		
	-YPRGMW-		
	YDPRGM--		
	YPPRGM--		
	YAPRGM--		
	-YPRGME-		
	-YPRGMD-		
	-YPRGMC-		
	YNPRGM--		
	YFPRGM--		
	YCPRGM--		
PWHL	137	788	47.70
	-HPWHLG-		
	--PWHLNL		
	-RPWHLP-		
	AKPWHL--		
	ATPWHL--		
	AGPWHL--		
	-APWHLR-		
	ASPWHL--		
	AMPWHL--		
	ALPWHL--		
	-APWHLI-		
	-APWHLF-		
	-APWHLK-		
	AWPWHL--		
	-APWHLA-		
	ADPWHL--		
	AYPWHL--		
	-APWHLN-		
	AVPWHL--		
	ANPWHL--		
	AHPWHL--		
	-APWHLT-		
	APPWHL--		
	-APWHLM-		
	-APWHLY-		
	AFPWHL--		
	AEPWHL--		
	-APWHLE-		
	-APWHLQ-		
	AIPWHL--		
	ACPWHL--		
	AQPWHL--		
	-APWHLC-		
	CSPWHL--		

CIPWHL--
CGPWHL--
CKPWHL--
CAPWHL--
CCPWHL--
CRPWHL--
CVPWHL--
CMPWHL--
CTPWHL--
-CPWHLH-
CHPWHL--
DRPWHL--
DKPWHL--
DYPWHL--
-DPWHLL-
DLPWHL--
DGPWHL--
-DPWHLE-
DSPWHL--
-DPWHLV-
-DPWHLF-
DHPWHL--
DAPWHL--
DTPWHL--
-DPWHLS-
DIPWHL--
-DPWHLK-
-DPWHLH-
DMPWHL--
-DPWHLR-
DNPWHL--
DQPWHL--
-DPWHLW-
-DPWHLT-
-DPWHLM-
DDPWHL--
-EPWHLM-
-EPWHLR-
EKPWHL--
EGPWHL--
-EPWHLK-
-EPWHLI-
ERPWHL--
EIPWHL--
-EPWHLS-
ETPWHL--
-EPWHLW-
-EPWHLL-
EQPWHL--
ESPWHL--

-EPWHLV-
EVPWHL--
-EPWHLY-
-EPWHLA-
EHPWHL--
EFPWHL--
EWPWHL--
-EPWHLT-
-EPWHLN-
ELPWHL--
EEPWHL--
EDPWHL--
-EPWHLQ-
FHPWHL--
FGPWHL--
FKPWHL--
FSPWHL--
-FPWHLK-
FQPWHL--
FTPWHL--
-FPWHLV-
FIPWHL--
-FPWHLT-
FPPWHL--
FYPWHL--
FWPWHL--
-FPWHLY-
-FPWHLQ-
FFPWHL--
FEPWHL--
FMPWHL--
FAPWHL--
-FPWHLS-
-FPWHLI-
FDPWHL--
-FPWHLL-
-FPWHLE-
FNPWHL--
-GPWHLL-
GFPWHL--
-GPWHLR-
GMPWHL--
GSPWHL--
GLPWHL--
GRPWHL--
GAPWHL--
GIPWHL--
GVPWHL--
-GPWHLS-
GWPWHL--

-GPWHLF-
-GPWHLK-
-GPWHLI-
GEPWHL--
GNPWHL--
GQPWHL--
GKPWHL--
GGPWHL--
-GPWHLT-
-GPWHLH-
GYPWHL--
GTPWHL--
-GPWHLY-
-GPWHLA-
GHPWHL--
GCPWHL--
-GPWHLW-
-GPWHLN-
-GPWHLM-
-GPWHLG-
-GPWHLE-
GPPWHL--
GDPWHL--
-GPWHLV-
HLPWHL--
HKPWHL--
HTPWHL--
HHPWHL--
-HPWHLL-
-HPWHLH-
-HPWHLI-
HGPWHL--
HFPWHL--
HAPWHL--
HRPWHL--
HPPWHL--
HWPWHL--
-HPWHLS-
HVPWHL--
-HPWHLK-
HNPWHL--
HSPWHL--
-HPWHLY-
-HPWHLV-
-HPWHLR-
-HPWHLM-
HEPWHL--
-HPWHLQ-
-HPWHLF-
HMPWHL--

HIPWHL--
-HPWHLN-
HDPWHL--
HYPWHL--
HQPWHL--
-HPWHLW-
-HPWHLA-
IKPWHL--
INPWHL--
IRPWHL--
IVPWHL--
ISPWHL--
-IPWHLK-
IQPWHL--
ILPWHL--
IGPWHL--
ITPWHL--
-IPWHLI-
IPPWHL--
IHPWHL--
-IPWHLE-
IYPWHL--
-IPWHLS-
-IPWHLN-
-IPWHLQ-
-IPWHLM-
IMPWHL--
IEPWHL--
IDPWHL--
-IPWHLT-
-IPWHLH-
-IPWHLY-
-IPWHLW-
-IPWHLV-
-IPWHLR-
-IPWHLL-
-IPWHLG-
-IPWHLF-
-IPWHLD-
IFPWHL--
IAPWHL--
KSPWHL--
KLPWHL--
KVPWHL--
KIPWHL--
-KPWHLT-
KKPWHL--
KGPWHL--
KNPWHL--
-KPWHLR-

KHPWHL--
KMPWHL--
-KPWHLI-
KTPWHL--
-KPWHLY-
-KPWHLK-
-KPWHLS-
-KPWHLN-
KFPWHL--
-KPWHLA-
-KPWHLE-
KAPWHL--
-KPWHLF-
KDPWHL--
-KPWHLW-
-KPWHLL-
-KPWHLV-
-KPWHLH-
-KPWHLG-
LSPWHL--
LRPWHL--
LGPWHL--
LVPWHL--
-LPWHLR-
LHPWHL--
LKPWHL--
LAPWHL--
-LPWHLT-
LLPWHL--
LWPWHL--
LPPWHL--
LMPWHL--
-LPWHLA-
-LPWHLL-
-LPWHLK-
-LPWHLQ-
-LPWHLH-
LFPWHL--
-LPWHLV-
LDPWHL--
LNPWHL--
-LPWHLW-
-LPWHLI-
LQPWHL--
LIPWHL--
LEPWHL--
LYPWHL--
-LPWHLM-
-LPWHLE-
-LPWHLN-

-LPWHLG-
-LPWHLF-
MRPWHL--
MGPWHL--
MEPWHL--
-MPWHLR-
MHPWHL--
-MPWHLL-
MLPWHL--
MSPWHL--
MPPWHL--
MAPWHL--
MKPWHL--
MQPWHL--
-MPWHLH-
MDPWHL--
-MPWHLS-
MMPWHL--
MTPWHL--
MYPWHL--
MVPWHL--
-MPWHLW-
-MPWHLI-
-MPWHLY-
-MPWHLF-
-MPWHLM-
-MPWHLG-
MNPWHL--
MIPWHL--
-MPWHLQ-
MFPWHL--
NLPWHL--
-NPWHLR-
-NPWHLV-
NAPWHL--
NDPWHL--
NRPWHL--
NIPWHL--
NEPWHL--
NYPWHL--
-NPWHLY-
-NPWHLS-
NNPWHL--
-NPWHLL-
NTPWHL--
-NPWHLT-
NVPWHL--
-NPWHLM-
-NPWHLI-
NWPWHL--

NFPWHL--
-NPWHLW-
NKPWHL--
NHPWHL--
NGPWHL--
-NPWHLN-
NQPWHL--
-NPWHLH-
-NPWHLF-
-NPWHLA-
--PWHLVR
--PWHLSS
PRPWHL--
PSPWHL--
--PWHLRS
--PWHLHT
--PWHLRN
--PWHLLS
--PWHLSR
--PWHLVS
--PWHLTL
--PWHLSG
--PWHLIS
PTPWHL--
--PWHLSN
--PWHLTV
--PWHLST
--PWHLSQ
--PWHLKG
--PWHLSV
--PWHLRG
--PWHLLT
--PWHLFH
--PWHLNS
--PWHLLQ
--PWHLSA
--PWHLRR
--PWHLEA
--PWHLTA
--PWHLLH
--PWHLHS
--PWHLHG
--PWHLGA
--PWHLAG
--PWHLYT
--PWHLVY
--PWHLTG
--PWHLNA
--PWHLGS
--PWHLWS

--PWHLTR
--PWHLMK
--PWHLFN
--PWHLYA
--PWHLSI
--PWHLMY
--PWHLKV
--PWHLEG
--PWHLRT
--PWHLLV
--PWHLIN
--PWHLEK
--PWHLAV
--PWHLAA
PLPWHL--
--PWHLYL
--PWHLVD
--PWHLTS
--PWHLRY
--PWHLQR
--PWHLQG
--PWHLHH
--PWHLFK
--PWHLYM
--PWHLVW
--PWHLVK
--PWHLVG
--PWHLTM
--PWHLSH
--PWHLRH
--PWHLQT
--PWHLQN
--PWHLLG
--PWHLLA
--PWHLIM
PAPWHL--
--PWHLYS
--PWHLWR
--PWHLIY
--PWHLIK
--PWHLHR
--PWHLFY
--PWHLFS
--PWHLFA
PHPWHL--
PYPWHL--
--PWHLYH
--PWHLVE
--PWHLSY
--PWHLSF

--PWHLQS
--PWHLMT
--PWHLMR
--PWHLML
--PWHLMD
--PWHLKT
--PWHLGG
--PWHLFT
--PWHLEY
PEPWHL--
--PWHLWK
--PWHLTY
--PWHLRK
--PWHLMS
--PWHLIH
--PWHLIG
--PWHLHE
--PWHLGV
--PWHLGT
--PWHLAS
--PWHLAQ
--PWHLVI
--PWHLTN
--PWHLTH
--PWHLLN
--PWHLKL
--PWHLHV
PNPWHL--
PMPWHL--
--PWHLYV
--PWHLVT
--PWHLVQ
--PWHLVH
--PWHLTT
--PWHLTQ
--PWHLSD
--PWHLRQ
--PWHLMI
--PWHLLR
--PWHLLK
--PWHLKW
--PWHLIR
--PWHLAI
--PWHLTW
--PWHLTF
--PWHLSK
--PWHLNF
--PWHLKR
--PWHLHL
--PWHLFR

--PWHLAT
--PWHLAR
PKPWHL--
--PWHLWN
--PWHLWI
--PWHLSM
--PWHLRM
--PWHLQH
--PWHLQE
--PWHLNW
--PWHLNV
--PWHLLI
--PWHLLE
--PWHLFE
--PWHLET
--PWHLES
--PWHLAH
PVPWHL--
PGPWHL--
--PWHLYI
--PWHLYD
--PWHLWG
--PWHLTD
--PWHLNT
--PWHLKS
--PWHLHN
--PWHLHI
--PWHLFD
--PWHLER
PFPWHL--
--PWHLWT
--PWHLQV
--PWHLNQ
--PWHLNN
--PWHLMQ
--PWHLLW
--PWHLLD
--PWHLKN
--PWHLKM
--PWHLIV
--PWHLIQ
--PWHLIA
--PWHLHK
--PWHLHD
--PWHLHA
--PWHLGL
--PWHLDS
--PWHLYY
--PWHLYK
--PWHLYF

--PWHLWY
--PWHLWA
--PWHLVN
--PWHLVF
--PWHLQL
--PWHLQF
--PWHLQA
--PWHLNG
--PWHLMG
--PWHLME
--PWHLLM
--PWHLKF
--PWHLKD
--PWHLKA
--PWHLHW
--PWHLFQ
--PWHLEH
--PWHLEE
--PWHLDG
--PWHLAN
--PWHLAL
--PWHLAF
--PWHLAE
--PWHLYQ
--PWHLWW
--PWHLWV
--PWHLTC
--PWHLSC
--PWHLNI
--PWHLIE
--PWHLHM
--PWHLGY
--PWHLFM
--PWHLEM
--PWHLED
--PWHLAW
--PWHLAK
--PWHLAD
-PPWHLI-
PPPWHL--
QRPWHL--
QTPWHL--
QSPWHL--
QNPWHL--
-QPWHLW-
-QPWHLY-
-QPWHLR-
QLPWHL--
QYPWHL--
-QPWHLF-

QFPWHL--
QHPWHL--
-QPWHLQ-
QPPWHL--
-QPWHLK-
QMPWHL--
QGPWHL--
QWPWHL--
-QPWHLL-
-QPWHLE-
QKPWHL--
-QPWHLT-
-QPWHLI-
QEPWHL--
QVPWHL--
-QPWHLH-
QIPWHL--
-RPWHLS-
RSPWHL--
RVPWHL--
-RPWHLY-
REPWHL--
RYPWHL--
RHPWHL--
RDPWHL--
RTPWHL--
-RPWHLL-
RLPWHL--
RKPWHL--
RMPWHL--
RPPWHL--
-RPWHLH-
-RPWHLW-
-RPWHLF-
-RPWHLM-
-RPWHLA-
RAPWHL--
-RPWHLN-
-RPWHLQ-
-RPWHLT-
-RPWHLR-
RNPWHL--
RIPWHL--
RFPWHL--
-RPWHLI-
RWPWHL--
-RPWHLK-
-RPWHLG-
-RPWHLE-
-RPWHLD-

STPWHL--
SRPWHL--
SLPWHL--
SSPWHL--
-SPWHLR-
-SPWHLL-
SWPWHL--
SGPWHL--
SDPWHL--
-SPWHLQ-
SKPWHL--
SNPWHL--
SPPWHL--
-SPWHLT-
-SPWHLK-
SAPWHL--
SQPWHL--
-SPWHLA-
SVPWHL--
-SPWHLH-
-SPWHLN-
-SPWHLW-
-SPWHLV-
-SPWHLY-
SEPWHL--
SHPWHL--
SYPWHL--
-SPWHLE-
-SPWHLM-
-SPWHLF-
SMPWHL--
SFPWHL--
SCPWHL--
TLPWHL--
TEPWHL--
TVPWHL--
TNPWHL--
-TPWHLS-
TSPWHL--
TRPWHL--
-TPWHLV-
-TPWHLL-
-TPWHLR-
-TPWHLA-
-TPWHLT-
TTPWHL--
TQPWHL--
TIPWHL--
-TPWHLY-
-TPWHLQ-

TKPWHL--
-TPWHLM-
-TPWHLW-
TFPWHL--
TWPWHL--
TAPWHL--
TYPWHL--
-TPWHLN-
TDPWHL--
-TPWHLI-
-TPWHLK-
-TPWHLG-
-TPWHLF-
-TPWHLE-
-TPWHLC-
VLPWHL--
VRPWHL--
-VPWHLL-
VNPWHL--
VSPWHL--
VPPWHL--
VTPWHL--
-VPWHLT-
-VPWHLY-
-VPWHLR-
-VPWHLQ-
-VPWHLN-
VDPWHL--
-VPWHLH-
-VPWHLS-
-VPWHLI-
-VPWHLK-
VKPWHL--
VGPWHL--
VVPWHL--
-VPWHLA-
VHPWHL--
VQPWHL--
VAPWHL--
VYPWHL--
-VPWHLM-
-VPWHLE-
VIPWHL--
VEPWHL--
VWPWHL--
-VPWHLV-
-VPWHLW-
-VPWHLF-
-VPWHLC-
WGPWHL--

WNPWHL--
WIPWHL--
WSPWHL--
WMPWHL--
WRPWHL--
WLPWHL--
WKPWHL--
-WPWHLH-
WTPWHL--
-WPWHLM-
-WPWHLV-
-WPWHLS-
-WPWHLR-
-WPWHLA-
WEPWHL--
-WPWHLT-
-WPWHLG-
WAPWHL--
WYPWHL--
WVPWHL--
-WPWHLY-
-WPWHLI-
WWPWHL--
-WPWHLN-
WHPWHL--
WFPWHL--
YAPWHL--
YRPWHL--
YSPWHL--
-YPWHLM-
YMPWHL--
YYPWHL--
-YPWHLT-
-YPWHLR-
YIPWHL--
YQPWHL--
-YPWHLE-
-YPWHLA-
YDPWHL--
YVPWHL--
YTPWHL--
-YPWHLS-
YGPWHL--
-YPWHLL-
-YPWHLK-
YNPWHL--
YKPWHL--
-YPWHLY-
-YPWHLG-
-YPWHLW-

YPPWHL--
YHPWHL--
-YPWHLH-
YFPWHL--

PIAI	255	720	47.69

--PIAIVH
--PIAIGT
--PIAIGR
-SPIAIN-
AKPIAI--
-APIAIL-
-APIAIK-
-APIAIQ-
AAPIAI--
AVPIAI--
-APIAIY-
-APIAIV-
-APIAIA-
AWPIAI--
ARPIAI--
AMPIAI--
AEPIAI--
-APIAIR-
AGPIAI--
-APIAIT-
-APIAIS-
-APIAIH-
AFPIAI--
AYPIAI--
ASPIAI--
APPIAI--
-APIAIM-
-APIAII-
-APIAIG-
ANPIAI--
ACPIAI--
-APIAIN-
-APIAIE-
ATPIAI--
AHPIAI--
ADPIAI--
CSPIAI--
CRPIAI--
CVPIAI--
CNPIAI--
CDPIAI--
CQPIAI--
-CPIAIY-
-CPIAIV-
-CPIAIR-

CLPIAI--
CEPIAI--
DRPIAI--
DHPIAI--
DNPIAI--
DAPIAI--
-DPIAIR-
DSPIAI--
-DPIAIY-
-DPIAIV-
DMPIAI--
DVPIAI--
-DPIAIK-
-DPIAII-
-DPIAIS-
-DPIAIQ-
-DPIAIF-
DEPIAI--
DYPIAI--
-DPIAIM-
-DPIAIL-
DLPIAI--
DKPIAI--
EIPIAI--
-EPIAIL-
ETPIAI--
EFPIAI--
-EPIAIQ-
ESPIAI--
EPPIAI--
-EPIAIY-
-EPIAII-
EAPIAI--
EWPIAI--
EVPIAI--
-EPIAIR-
EMPIAI--
ELPIAI--
EYPIAI--
-EPIAIV-
-EPIAIT-
-EPIAIH-
-EPIAIC-
-EPIAIA-
EKPIAI--
FFPIAI--
FAPIAI--
-FPIAIR-
FSPIAI--
FEPIAI--

FKPIAI--
FDPIAI--
-FPIAIL-
FHPIAI--
-FPIAIQ-
-FPIAIS-
-FPIAIN-
FWPIAI--
-FPIAIW-
FNPIAI--
FIPIAI--
-FPIAIT-
-FPIAIK-
FVPIAI--
FTPIAI--
-FPIAIP-
-FPIAIH-
GGPIAI--
-GPIAIL-
-GPIAIR-
-GPIAIK-
GSPIAI--
GQPIAI--
-GPIAIV-
-GPIAIM-
-GPIAIH-
GVPIAI--
-GPIAIS-
GFPIAI--
-GPIAIY-
-GPIAIT-
GKPIAI--
GHPIAI--
GRPIAI--
GLPIAI--
GTPIAI--
-GPIAII-
GAPIAI--
GNPIAI--
GDPIAI--
-GPIAIQ-
GIPIAI--
GEPIAI--
GWPIAI--
-GPIAIF-
GPPIAI--
-GPIAIW-
HRPIAI--
HVPIAI--
HMPIAI--

HGPIAI--
-HPIAIL-
HNPIAI--
HFPIAI--
HHPIAI--
HLPIAI--
-HPIAIV-
HSPIAI--
-HPIAIT-
-HPIAIR-
-HPIAIK-
HWPIAI--
-HPIAIA-
HEPIAI--
-HPIAIY-
-HPIAIQ-
-HPIAIH-
HDPIAI--
-HPIAIN-
-HPIAIF-
-HPIAIS-
-HPIAIG-
ILPIAI--
IGPIAI--
INPIAI--
IQPIAI--
IVPIAI--
-IPIAIY-
IEPIAI--
-IPIAIW-
-IPIAIT-
-IPIAII-
IWPIAI--
-IPIAIV-
-IPIAIR-
-IPIAIS-
-IPIAIQ-
-IPIAIL-
ICPIAI--
ITPIAI--
IYPIAI--
IRPIAI--
IPPIAI--
-IPIAIN-
-IPIAIK-
IMPIAI--
IKPIAI--
IIPIAI--
KSPIAI--
-KPIAIW-

KQPIAI--
KWPIAI--
KPPIAI--
-KPIAIF-
KRPIAI--
KTPIAI--
-KPIAIH-
KYPIAI--
KIPIAI--
KNPIAI--
-KPIAIV-
KKPIAI--
-KPIAIQ-
KGPIAI--
-KPIAIR-
KLPIAI--
-KPIAIT-
-KPIAIM-
KVPIAI--
KHPIAI--
KEPIAI--
-KPIAIK-
-KPIAIL-
-KPIAIA-
KAPIAI--
-KPIAIN-
KMPIAI--
KFPIAI--
-KPIAIP-
-KPIAIC-
LRPIAI--
LYPIAI--
LKPIAI--
-LPIAIR-
LSPIAI--
LEPIAI--
LIPIAI--
LNPIAI--
LPPIAI--
LTPIAI--
-LPIAIV-
-LPIAIS-
LMPIAI--
LQPIAI--
-LPIAIY-
-LPIAIL-
-LPIAII-
-LPIAIT-
-LPIAIQ-
LLPIAI--

LAPIAI--
-LPIAIK-
-LPIAIH-
-LPIAIM-
LDPIAI--
-LPIAIA-
LHPIAI--
-LPIAIN-
-LPIAIF-
-LPIAIC-
LCPIAI--
MMPIAI--
MKPIAI--
-MPIAIR-
MNPIAI--
MIPIAI--
MLPIAI--
-MPIAIM-
MTPIAI--
-MPIAIV-
-MPIAIH-
MSPIAI--
-MPIAIS-
-MPIAIW-
-MPIAIN-
MRPIAI--
-MPIAIY-
-MPIAIQ-
-MPIAIF-
MDPIAI--
MYPIAI--
MWPIAI--
MPPIAI--
-MPIAIK-
-MPIAIA-
MHPIAI--
MVPIAI--
-MPIAII-
-MPIAIT-
-MPIAIE-
MGPIAI--
NVPIAI--
NMPIAI--
NLPIAI--
-NPIAIR-
NNPIAI--
-NPIAIV-
-NPIAIL-
-NPIAIH-
NHPIAI--

NPPIAI--
-NPIAIT-
NKPIAI--
NTPIAI--
NAPIAI--
NGPIAI--
NFPIAI--
NYPIAI--
NIPIAI--
NDPIAI--
NWPIAI--
-NPIAIW-
-NPIAII-
-NPIAIG-
-NPIAIF-
-NPIAIA-
--PIAIRS
--PIAIRM
--PIAILT
--PIAIRL
--PIAIRI
--PIAIYR
--PIAISS
--PIAILG
--PIAIKT
--PIAISV
--PIAITA
--PIAISL
--PIAIFK
--PIAILS
--PIAIIN
--PIAITS
--PIAIMG
--PIAIYK
--PIAIVT
--PIAIKR
--PIAITV
--PIAIRG
--PIAIEG
--PIAIVS
--PIAITR
--PIAIKS
--PIAIKI
--PIAIWQ
--PIAIPL
--PIAINS
--PIAIKL
--PIAIYA
--PIAIWH
--PIAISA

--PIAIRV
--PIAILH
--PIAIKG
--PIAIRQ
--PIAIRH
--PIAIHG
--PIAIYT
--PIAIYG
--PIAIMK
--PIAIHA
--PIAIFS
--PIAIWR
--PIAIKK
--PIAIHL
--PIAIGV
--PIAIGA
--PIAIFT
--PIAIAS
PHPIAI--
--PIAIWW
--PIAIRT
--PIAIRN
--PIAIQR
--PIAIPI
--PIAINT
--PIAIMS
--PIAIMA
--PIAIHR
--PIAIGL
--PIAIAA
--PIAIWG
--PIAITT
--PIAISG
--PIAIRR
--PIAIQV
--PIAIQM
--PIAILN
--PIAIIR
--PIAIAY
PEPIAI--
PAPIAI--
--PIAISW
--PIAIRA
--PIAINM
--PIAIIG
--PIAIGF
--PIAIFQ
--PIAIES
PYPIAI--
--PIAIYS

--PIAITH
--PIAISQ
--PIAIQY
--PIAIQS
--PIAINA
--PIAILR
--PIAIEL
PFPIAI--
--PIAIYF
--PIAIWD
--PIAITW
--PIAITG
--PIAINY
--PIAILE
--PIAIWL
--PIAIWK
--PIAIVQ
--PIAIST
--PIAISM
--PIAIRY
--PIAIPF
--PIAIKN
--PIAIKA
--PIAIIT
--PIAIGY
--PIAIFG
--PIAIEY
--PIAIAF
PWPIAI--
PTPIAI--
--PIAIVR
--PIAITL
--PIAITK
--PIAISI
--PIAINV
--PIAINN
--PIAIMD
--PIAIKY
--PIAIHN
--PIAIHE
--PIAIHD
--PIAIFN
PKPIAI--
--PIAIYV
--PIAIYN
--PIAIWV
--PIAIVG
--PIAITQ
--PIAITM
--PIAIQK

--PIAIQH
--PIAIQE
--PIAIPY
--PIAIPV
--PIAINF
--PIAIMH
--PIAILM
--PIAILD
--PIAIGG
--PIAIAR
--PIAIAM
--PIAIAI
PDPIAI--
PNPIAI--
--PIAIWF
--PIAIWE
--PIAIVA
--PIAITN
--PIAITF
--PIAISN
--PIAISK
--PIAIQL
--PIAINK
--PIAILQ
--PIAILK
--PIAIKW
--PIAIKD
--PIAIIA
--PIAIHH
--PIAIHF
--PIAIEW
--PIAIYY
--PIAIYQ
--PIAIYL
--PIAIYI
--PIAIYH
--PIAIVI
--PIAITE
--PIAISY
--PIAISH
--PIAIQA
--PIAINL
--PIAINI
--PIAINC
--PIAIIK
--PIAIHY
--PIAIHW
--PIAIHV
--PIAIGS
--PIAIGM

--PIAIGH
--PIAIFA
--PIAIER
--PIAIEF
--PIAIAW
--PIAIAN
--PIAIAL
--PIAIAK
--PIAIYW
--PIAIWY
--PIAIWT
--PIAIVW
--PIAITI
--PIAISF
--PIAISE
--PIAIQW
--PIAIQG
--PIAINR
--PIAIMP
--PIAIMN
--PIAIMM
--PIAILW
--PIAIKE
--PIAIIH
--PIAIID
--PIAIHT
--PIAIHM
--PIAIGW
--PIAIGI
--PIAIFI
--PIAIEV
--PIAIET
--PIAIEK
--PIAIDM
--PIAIDG
--PIAICT
--PIAICS
--PIAIAQ
PGPIAI--
QRPIAI--
QYPIAI--
QAPIAI--
QKPIAI--
QVPIAI--
-QPIAIR-
QQPIAI--
-QPIAII-
QSPIAI--
QLPIAI--
-QPIAIV-

QGPIAI--
QEPIAI--
QTPIAI--
-QPIAIT-
-QPIAIQ-
QWPIAI--
QPPIAI--
-QPIAIM-
-QPIAIH-
-QPIAIA-
-QPIAIY-
-QPIAIL-
-QPIAIK-
-QPIAIF-
QDPIAI--
-RPIAIA-
RLPIAI--
RGPIAI--
RSPIAI--
RTPIAI--
-RPIAIR-
-RPIAIS-
-RPIAIQ-
RKPIAI--
RMPIAI--
-RPIAIT-
RYPIAI--
RQPIAI--
RWPIAI--
-RPIAIK-
-RPIAIW-
RPPIAI--
RVPIAI--
RHPIAI--
RCPIAI--
-RPIAIV-
-RPIAIM-
-RPIAIH-
-RPIAIF-
-RPIAIN-
-RPIAII-
-RPIAIG-
-RPIAIP-
RFPIAI--
-RPIAID-
SRPIAI--
SLPIAI--
-SPIAIR-
-SPIAIL-
-SPIAIH-

-SPIAII-
-SPIAIM-
SFPIAI--
SVPIAI--
SPPIAI--
-SPIAIS-
SMPIAI--
SAPIAI--
-SPIAIY-
-SPIAIV-
SIPIAI--
-SPIAIK-
SHPIAI--
STPIAI--
-SPIAIT-
-SPIAIA-
SGPIAI--
SYPIAI--
SQPIAI--
-SPIAIW-
SNPIAI--
SSPIAI--
-SPIAIF-
SEPIAI--
TAPIAI--
TFPIAI--
-TPIAIW-
TMPIAI--
TRPIAI--
-TPIAIR-
-TPIAIF-
TVPIAI--
-TPIAIK-
TYPIAI--
TTPIAI--
TSPIAI--
TQPIAI--
-TPIAIL-
TKPIAI--
-TPIAIS-
TIPIAI--
-TPIAIQ-
TEPIAI--
-TPIAIV-
-TPIAIA-
-TPIAIY-
TLPIAI--
-TPIAIH-
TGPIAI--
TDPIAI--

```
-TPIAIN-
-TPIAII-
THPIAI--
VRPIAI--
-VPIAIL-
-VPIAIS-
VMPIAI--
VFPIAI--
-VPIAIK-
VSPIAI--
VTPIAI--
VLPIAI--
-VPIAIQ-
VNPIAI--
-VPIAIA-
VIPIAI--
-VPIAIT-
-VPIAIY-
VVPIAI--
VDPIAI--
VYPIAI--
VKPIAI--
VGPIAI--
VEPIAI--
VWPIAI--
VQPIAI--
VAPIAI--
VPPIAI--
-VPIAIW-
-VPIAIV-
-VPIAIN-
-VPIAIG-
WRPIAI--
-WPIAIR-
WVPIAI--
WIPIAI--
WSPIAI--
WNPIAI--
WDPIAI--
-WPIAIS-
-WPIAIK-
WAPIAI--
WKPIAI--
-WPIAIT-
-WPIAIH-
WTPIAI--
-WPIAIQ-
-WPIAIL-
-WPIAII-
WFPIAI--
```

```
              -YPIAIR-
              YNPIAI--
              YYPIAI--
              YTPIAI--
              YSPIAI--
              YKPIAI--
              YVPIAI--
              -YPIAIT-
              YGPIAI--
              YFPIAI--
              -YPIAIS-
              -YPIAIN-
              YLPIAI--
              YDPIAI--
              -YPIAIM-
              -YPIAIL-
              -YPIAIK-
              -YPIAII-
              YMPIAI--
              YHPIAI--
              -YPIAIA-
              YPPIAI--
              -YPIAIQ-
              -YPIAIH-
              YCPIAI--
PRPY            26         871      47.69
              -HPRPYG-
              -IPRPYW-
              --PRPYPT
              -RPRPYC-
              -APRPYV-
              ASPRPY--
              AVPRPY--
              -APRPYT-
              -APRPYK-
              -APRPYL-
              -APRPYI-
              -APRPYQ-
              AGPRPY--
              ARPRPY--
              APPRPY--
              AIPRPY--
              AAPRPY--
              -APRPYR-
              -APRPYS-
              -APRPYN-
              AWPRPY--
              -APRPYY-
              -APRPYM-
              -APRPYH-
```

AFPRPY--
ANPRPY--
AMPRPY--
-APRPYW-
-APRPYG-
AEPRPY--
-APRPYF-
-APRPYE-
AQPRPY--
AKPRPY--
AHPRPY--
ACPRPY--
CAPRPY--
-CPRPYR-
CVPRPY--
-CPRPYS-
-CPRPYY-
CMPRPY--
CHPRPY--
CEPRPY--
CWPRPY--
CTPRPY--
CQPRPY--
-CPRPYW-
-CPRPYA-
CLPRPY--
-CPRPYV-
-CPRPYM-
-CPRPYF-
CPPRPY--
CNPRPY--
CYPRPY--
CSPRPY--
CRPRPY--
-CPRPYL-
CKPRPY--
CGPRPY--
CFPRPY--
DWPRPY--
-DPRPYI-
DFPRPY--
-DPRPYL-
-DPRPYY-
DYPRPY--
-DPRPYR-
DLPRPY--
DIPRPY--
DSPRPY--
DEPRPY--
DTPRPY--

DQPRPY--
DMPRPY--
DNPRPY--
DVPRPY--
DAPRPY--
-DPRPYW-
-DPRPYF-
DHPRPY--
DDPRPY--
DRPRPY--
-DPRPYM-
-DPRPYT-
-DPRPYK-
-DPRPYV-
-DPRPYQ-
DKPRPY--
-DPRPYE-
-EPRPYL-
ETPRPY--
EGPRPY--
EIPRPY--
-EPRPYI-
-EPRPYR-
EVPRPY--
-EPRPYW-
EMPRPY--
ELPRPY--
EFPRPY--
EYPRPY--
-EPRPYY-
-EPRPYH-
-EPRPYA-
EKPRPY--
EEPRPY--
EAPRPY--
EQPRPY--
-EPRPYK-
-EPRPYF-
ENPRPY--
ESPRPY--
EHPRPY--
-EPRPYT-
-EPRPYM-
EPPRPY--
EWPRPY--
ERPRPY--
-EPRPYN-
-EPRPYE-
-EPRPYC-
EDPRPY--

−FPRPYF−
FIPRPY−−
FVPRPY−−
−FPRPYH−
−FPRPYI−
FTPRPY−−
FSPRPY−−
FMPRPY−−
FLPRPY−−
−FPRPYL−
−FPRPYS−
−FPRPYR−
FKPRPY−−
FYPRPY−−
−FPRPYN−
−FPRPYA−
FAPRPY−−
−FPRPYK−
FQPRPY−−
−FPRPYW−
FNPRPY−−
−FPRPYV−
FEPRPY−−
FHPRPY−−
FGPRPY−−
FFPRPY−−
FDPRPY−−
−FPRPYY−
GVPRPY−−
−GPRPYY−
GLPRPY−−
−GPRPYL−
−GPRPYM−
−GPRPYK−
GSPRPY−−
−GPRPYV−
−GPRPYS−
GYPRPY−−
GRPRPY−−
−GPRPYW−
GMPRPY−−
GGPRPY−−
GTPRPY−−
GKPRPY−−
GIPRPY−−
−GPRPYA−
GNPRPY−−
−GPRPYR−
GFPRPY−−
−GPRPYF−

GEPRPY--
GQPRPY--
-GPRPYI-
GCPRPY--
GAPRPY--
GWPRPY--
-GPRPYN-
-GPRPYG-
-GPRPYH-
GHPRPY--
-HPRPYR-
HSPRPY--
HHPRPY--
HAPRPY--
HYPRPY--
-HPRPYL-
HVPRPY--
-HPRPYT-
-HPRPYM-
HKPRPY--
HRPRPY--
HTPRPY--
HLPRPY--
HWPRPY--
HQPRPY--
-HPRPYS-
-HPRPYI-
HPPRPY--
-HPRPYQ-
HMPRPY--
-HPRPYA-
HDPRPY--
-HPRPYW-
-HPRPYF-
-HPRPYV-
-HPRPYH-
-HPRPYC-
HGPRPY--
-HPRPYE-
HEPRPY--
-IPRPYL-
ISPRPY--
IRPRPY--
-IPRPYK-
IYPRPY--
-IPRPYI-
-IPRPYS-
ILPRPY--
INPRPY--
-IPRPYQ-

IVPRPY--
-IPRPYR-
IGPRPY--
ITPRPY--
IIPRPY--
-IPRPYT-
-IPRPYN-
-IPRPYH-
-IPRPYG-
IPPRPY--
-IPRPYF-
IFPRPY--
IHPRPY--
IDPRPY--
-IPRPYM-
IEPRPY--
IKPRPY--
-IPRPYA-
IAPRPY--
ICPRPY--
-IPRPYV-
IMPRPY--
-KPRPYV-
-KPRPYL-
KLPRPY--
KIPRPY--
-KPRPYY-
-KPRPYR-
KNPRPY--
KFPRPY--
KTPRPY--
KVPRPY--
KHPRPY--
-KPRPYM-
KEPRPY--
-KPRPYQ-
KYPRPY--
-KPRPYW-
KMPRPY--
KGPRPY--
KAPRPY--
-KPRPYA-
KKPRPY--
KQPRPY--
-KPRPYN-
-KPRPYK-
-KPRPYF-
-KPRPYE-
LVPRPY--
LTPRPY--

LGPRPY--
-LPRPYL-
LIPRPY--
LAPRPY--
-LPRPYY-
-LPRPYR-
LRPRPY--
-LPRPYS-
-LPRPYV-
LLPRPY--
LPPRPY--
LYPRPY--
-LPRPYT-
LHPRPY--
-LPRPYW-
LQPRPY--
-LPRPYM-
-LPRPYK-
-LPRPYQ-
LEPRPY--
-LPRPYI-
LMPRPY--
LWPRPY--
-LPRPYH-
-LPRPYA-
LDPRPY--
LKPRPY--
-LPRPYN-
-LPRPYF-
-LPRPYG-
LCPRPY--
-MPRPYL-
MNPRPY--
MKPRPY--
MYPRPY--
-MPRPYQ-
-MPRPYK-
-MPRPYI-
MRPRPY--
-MPRPYF-
MTPRPY--
MSPRPY--
-MPRPYV-
MIPRPY--
MFPRPY--
-MPRPYT-
-MPRPYA-
-MPRPYH-
MVPRPY--
-MPRPYN-

MMPRPY--
MWPRPY--
-MPRPYW-
-MPRPYM-
MDPRPY--
-MPRPYY-
MGPRPY--
-MPRPYR-
-MPRPYG-
MPPRPY--
MEPRPY--
MAPRPY--
MQPRPY--
-MPRPYE-
NGPRPY--
-NPRPYR-
NVPRPY--
NFPRPY--
NTPRPY--
NNPRPY--
NHPRPY--
NAPRPY--
-NPRPYV-
NYPRPY--
NSPRPY--
-NPRPYT-
-NPRPYF-
-NPRPYY-
-NPRPYW-
NKPRPY--
NMPRPY--
NEPRPY--
-NPRPYM-
NPPRPY--
-NPRPYI-
NIPRPY--
-NPRPYA-
NLPRPY--
NDPRPY--
-NPRPYK-
NQPRPY--
-NPRPYH-
--PRPYRL
--PRPYSL
--PRPYTL
--PRPYHL
--PRPYSI
--PRPYLT
--PRPYRT
--PRPYMS

--PRPYLS
--PRPYLA
--PRPYVG
--PRPYTF
--PRPYFQ
--PRPYFV
--PRPYST
--PRPYFL
--PRPYVS
--PRPYLQ
--PRPYDV
--PRPYTT
--PRPYSS
--PRPYSG
--PRPYML
--PRPYVR
--PRPYVL
--PRPYTG
--PRPYSR
--PRPYLN
--PRPYWA
--PRPYWI
--PRPYVW
--PRPYKL
--PRPYAQ
PVPRPY--
--PRPYVV
--PRPYLM
--PRPYSA
--PRPYRS
--PRPYAY
--PRPYVY
--PRPYVT
--PRPYIS
--PRPYSV
--PRPYRM
--PRPYRI
--PRPYKT
--PRPYGV
--PRPYYG
--PRPYSQ
--PRPYRV
--PRPYRG
--PRPYHY
--PRPYWN
--PRPYTV
--PRPYNS
--PRPYKG
--PRPYGM
--PRPYWT

PFPRPY--
--PRPYMK
--PRPYMH
--PRPYKI
PSPRPY--
--PRPYGK
--PRPYIT
--PRPYFA
--PRPYWQ
--PRPYVH
--PRPYTN
--PRPYSN
--PRPYRN
--PRPYMA
--PRPYLF
--PRPYIF
--PRPYGY
--PRPYFT
--PRPYVM
--PRPYNV
--PRPYLK
--PRPYIL
--PRPYFM
--PRPYYS
--PRPYYA
--PRPYTA
--PRPYKV
--PRPYIA
--PRPYAS
--PRPYIV
--PRPYIG
--PRPYGT
--PRPYEF
--PRPYAL
--PRPYWE
--PRPYTM
--PRPYGQ
--PRPYEL
--PRPYRA
--PRPYIH
--PRPYGF
--PRPYFF
--PRPYAT
--PRPYAI
--PRPYTW
--PRPYRY
--PRPYRQ
--PRPYQI
--PRPYHQ
--PRPYSF

--PRPYLI
--PRPYFG
--PRPYAR
--PRPYAG
--PRPYWS
--PRPYVQ
--PRPYVD
--PRPYRD
--PRPYLW
--PRPYKS
--PRPYIY
--PRPYGI
--PRPYGH
--PRPYGA
--PRPYEN
--PRPYSY
--PRPYPM
--PRPYPF
--PRPYDW
--PRPYAV
PAPRPY--
PYPRPY--
--PRPYTS
--PRPYSW
--PRPYMM
--PRPYIK
--PRPYHT
--PRPYAA
PGPRPY--
--PRPYYV
--PRPYWV
--PRPYNK
--PRPYMT
--PRPYEY
--PRPYDL
PQPRPY--
--PRPYVN
--PRPYVA
--PRPYRF
--PRPYQL
--PRPYMI
--PRPYHV
--PRPYEI
--PRPYAK
PRPRPY--
--PRPYWF
--PRPYTY
--PRPYRH
--PRPYPY
--PRPYAN

--PRPYWR
--PRPYWG
--PRPYRE
--PRPYMQ
--PRPYKW
--PRPYAH
--PRPYAF
--PRPYYI
--PRPYSK
--PRPYQG
--PRPYPW
--PRPYPH
--PRPYLH
PLPRPY--
--PRPYWL
--PRPYVF
--PRPYTH
--PRPYMN
--PRPYMG
--PRPYHM
--PRPYHG
--PRPYGS
--PRPYGN
--PRPYFS
--PRPYYT
--PRPYVK
--PRPYPS
--PRPYPA
--PRPYMY
--PRPYMV
--PRPYKY
--PRPYHR
--PRPYGG
--PRPYFD
--PRPYYF
--PRPYQW
--PRPYQT
--PRPYNY
--PRPYNT
--PRPYNH
--PRPYFK
PTPRPY--
--PRPYIE
--PRPYID
--PRPYEM
--PRPYTQ
--PRPYQH
--PRPYNQ
--PRPYMF
--PRPYME

--PRPYFY
--PRPYDI
--PRPYCM
PNPRPY--
--PRPYYW
--PRPYWW
--PRPYSE
--PRPYQV
--PRPYNA
--PRPYLD
--PRPYKQ
--PRPYHI
--PRPYEG
--PRPYEA
--PRPYDF
--PRPYWM
--PRPYWK
--PRPYVE
--PRPYQY
--PRPYPQ
--PRPYNN
--PRPYKN
--PRPYKH
--PRPYIN
--PRPYGW
--PRPYDS
--PRPYAM
--PRPYYY
--PRPYYM
--PRPYYH
--PRPYTK
--PRPYQQ
--PRPYQM
--PRPYNF
--PRPYII
--PRPYHS
--PRPYEH
--PRPYDM
--PRPYCL
--PRPYAD
PKPRPY--
PEPRPY--
--PRPYWY
--PRPYTD
--PRPYQS
--PRPYQN
--PRPYQK
--PRPYPV
--PRPYPR
--PRPYPN

--PRPYMD
--PRPYKF
--PRPYKE
--PRPYHN
--PRPYHK
--PRPYHD
--PRPYGR
--PRPYFI
--PRPYFH
--PRPYFE
--PRPYET
--PRPYDQ
--PRPYCR
-PPRPYS-
PIPRPY--
PHPRPY--
PDPRPY--
--PRPYTE
--PRPYSC
--PRPYRC
--PRPYQR
--PRPYPI
--PRPYNR
--PRPYNC
--PRPYMW
--PRPYEV
--PRPYCT
--PRPYCK
--PRPYCI
--PRPYCA
--PRPYAE
-PPRPYH-
PMPRPY--
PCPRPY--
-QPRPYR-
QSPRPY--
QLPRPY--
QHPRPY--
-QPRPYI-
QAPRPY--
-QPRPYT-
-QPRPYM-
-QPRPYY-
-QPRPYS-
QKPRPY--
QWPRPY--
QVPRPY--
QPPRPY--
-QPRPYH-
-QPRPYK-

QIPRPY--
QYPRPY--
-QPRPYV-
QQPRPY--
QNPRPY--
QFPRPY--
QRPRPY--
-QPRPYL-
-QPRPYF-
QEPRPY--
QTPRPY--
-QPRPYA-
-QPRPYE-
QCPRPY--
-QPRPYW-
-QPRPYQ-
-QPRPYN-
RSPRPY--
-RPRPYL-
RGPRPY--
RYPRPY--
-RPRPYV-
RVPRPY--
-RPRPYF-
-RPRPYM-
RTPRPY--
-RPRPYY-
RIPRPY--
-RPRPYA-
RAPRPY--
RFPRPY--
RMPRPY--
RWPRPY--
-RPRPYH-
RNPRPY--
REPRPY--
RQPRPY--
-RPRPYT-
-RPRPYS-
RPPRPY--
-RPRPYQ-
-RPRPYK-
RDPRPY--
-RPRPYG-
-RPRPYE-
RRPRPY--
RCPRPY--
-SPRPYL-
SLPRPY--
SRPRPY--

-SPRPYI-
SNPRPY--
SGPRPY--
-SPRPYA-
SVPRPY--
-SPRPYV-
-SPRPYR-
SMPRPY--
SQPRPY--
SHPRPY--
SAPRPY--
SSPRPY--
-SPRPYY-
SDPRPY--
STPRPY--
-SPRPYM-
SKPRPY--
SFPRPY--
-SPRPYF-
-SPRPYW-
SPPRPY--
-SPRPYQ-
-SPRPYH-
SEPRPY--
SIPRPY--
-SPRPYN-
SCPRPY--
-SPRPYE-
-SPRPYC-
TGPRPY--
-TPRPYR-
TYPRPY--
-TPRPYS-
-TPRPYI-
TFPRPY--
TLPRPY--
-TPRPYL-
TMPRPY--
TAPRPY--
TIPRPY--
-TPRPYV-
TEPRPY--
-TPRPYW-
THPRPY--
-TPRPYY-
-TPRPYA-
TKPRPY--
-TPRPYT-
-TPRPYQ-
-TPRPYH-

TPPRPY--
-TPRPYF-
TVPRPY--
TCPRPY--
TQPRPY--
-TPRPYE-
-TPRPYN-
-TPRPYM-
-TPRPYG-
TDPRPY--
VIPRPY--
-VPRPYL-
VSPRPY--
-VPRPYV-
-VPRPYM-
VRPRPY--
-VPRPYH-
VYPRPY--
-VPRPYA-
-VPRPYS-
-VPRPYT-
VPPRPY--
-VPRPYI-
-VPRPYR-
VGPRPY--
VVPRPY--
VTPRPY--
VQPRPY--
VDPRPY--
VAPRPY--
-VPRPYW-
VHPRPY--
-VPRPYQ-
-VPRPYN-
VEPRPY--
VMPRPY--
-VPRPYF-
-VPRPYG-
VWPRPY--
WVPRPY--
WGPRPY--
WFPRPY--
-WPRPYW-
WAPRPY--
-WPRPYG-
-WPRPYA-
WMPRPY--
WIPRPY--
WTPRPY--
-WPRPYK-

	-WPRPYV-		
	WEPRPY--		
	-WPRPYI-		
	-WPRPYF-		
	WLPRPY--		
	-WPRPYS-		
	WHPRPY--		
	WYPRPY--		
	WWPRPY--		
	WRPRPY--		
	WQPRPY--		
	-WPRPYM-		
	-WPRPYT-		
	-WPRPYH-		
	-WPRPYD-		
	WDPRPY--		
	YRPRPY--		
	-YPRPYV-		
	-YPRPYR-		
	-YPRPYA-		
	YMPRPY--		
	YTPRPY--		
	-YPRPYN-		
	YIPRPY--		
	-YPRPYT-		
	YNPRPY--		
	-YPRPYH-		
	YYPRPY--		
	-YPRPYW-		
	YPPRPY--		
	YWPRPY--		
	-YPRPYS-		
	-YPRPYF-		
	YHPRPY--		
	-YPRPYY-		
	YLPRPY--		
	YAPRPY--		
	YQPRPY--		
	YKPRPY--		
	YDPRPY--		
	YGPRPY--		
	YVPRPY--		
	-YPRPYM-		
	YEPRPY--		
	-YPRPYQ-		
	-YPRPYP-		
	YFPRPY--		
PAAL	8	911	47.63
	-APAALE-		
	-KPAALD-		

```
-LPAALG-
-NPAALE-
--PAALRP
--PAALPQ
--PAALNP
--PAALMP
--PAALDI
-TPAALP-
AIPAAL--
-APAALL-
ASPAAL--
AGPAAL--
-APAALS-
-APAALF-
AMPAAL--
-APAALT-
-APAALK-
APPAAL--
AWPAAL--
AHPAAL--
-APAALW-
-APAALH-
-APAALY-
ALPAAL--
AYPAAL--
-APAALI-
-APAALN-
-APAALA-
AKPAAL--
ANPAAL--
-APAALG-
AFPAAL--
-APAALQ-
AAPAAL--
AQPAAL--
-APAALM-
-APAALC-
AEPAAL--
CQPAAL--
CLPAAL--
CCPAAL--
CMPAAL--
CIPAAL--
CSPAAL--
CFPAAL--
CVPAAL--
CTPAAL--
-CPAALH-
-CPAALS-
-CPAALK-
```

−CPAALN−
−CPAALI−
−CPAALF−
CNPAAL−−
CGPAAL−−
CYPAAL−−
CHPAAL−−
CRPAAL−−
−CPAALT−
−CPAALL−
CWPAAL−−
−CPAALW−
−CPAALR−
CKPAAL−−
CDPAAL−−
CAPAAL−−
DRPAAL−−
DTPAAL−−
DVPAAL−−
DIPAAL−−
−DPAALR−
DKPAAL−−
DAPAAL−−
DSPAAL−−
DMPAAL−−
−DPAALM−
DWPAAL−−
DYPAAL−−
DLPAAL−−
−DPAALH−
DHPAAL−−
DGPAAL−−
−DPAALY−
−DPAALL−
−DPAALW−
−DPAALK−
−DPAALQ−
DEPAAL−−
DQPAAL−−
−DPAALT−
−DPAALS−
DNPAAL−−
−DPAALV−
−DPAALA−
−DPAALF−
−EPAALR−
ERPAAL−−
−EPAALL−
−EPAALK−
ELPAAL−−

-EPAALS-
-EPAALH-
-EPAALW-
-EPAALV-
EGPAAL--
-EPAALM-
EVPAAL--
-EPAALT-
-EPAALN-
-EPAALQ-
EAPAAL--
-EPAALF-
ETPAAL--
EEPAAL--
ENPAAL--
EIPAAL--
-EPAALI-
EFPAAL--
EQPAAL--
-EPAALG-
-EPAALE-
-EPAALA-
FRPAAL--
FSPAAL--
FGPAAL--
FYPAAL--
FTPAAL--
-FPAALV-
-FPAALR-
-FPAALS-
FWPAAL--
FFPAAL--
FAPAAL--
-FPAALI-
-FPAALL-
FNPAAL--
-FPAALQ-
FLPAAL--
-FPAALT-
FHPAAL--
-FPAALH-
FDPAAL--
-FPAALN-
-FPAALM-
-FPAALF-
FVPAAL--
-FPAALA-
-FPAALK-
FMPAAL--
FQPAAL--

FPPAAL--
-FPAALC-
-GPAALI-
-GPAALL-
GLPAAL--
-GPAALK-
GVPAAL--
GQPAAL--
GWPAAL--
-GPAALR-
-GPAALT-
GFPAAL--
GTPAAL--
GRPAAL--
-GPAALY-
GAPAAL--
GGPAAL--
-GPAALS-
GMPAAL--
-GPAALW-
-GPAALH-
-GPAALN-
GNPAAL--
GPPAAL--
GKPAAL--
GHPAAL--
-GPAALA-
-GPAALV-
-GPAALQ-
-GPAALE-
GYPAAL--
GDPAAL--
GIPAAL--
-GPAALG-
GCPAAL--
-GPAALC-
-GPAALP-
-GPAALM-
HRPAAL--
HVPAAL--
HGPAAL--
HAPAAL--
-HPAALL-
-HPAALR-
HEPAAL--
-HPAALT-
HLPAAL--
-HPAALS-
-HPAALY-
-HPAALQ-

HFPAAL--
HNPAAL--
-HPAALV-
-HPAALN-
-HPAALK-
HYPAAL--
HQPAAL--
-HPAALG-
HCPAAL--
-HPAALH-
-HPAALE-
HTPAAL--
-HPAALA-
HKPAAL--
HDPAAL--
-HPAALI-
HPPAAL--
HWPAAL--
-HPAALW-
-HPAALC-
HMPAAL--
-IPAALR-
ILPAAL--
IGPAAL--
-IPAALL-
-IPAALT-
IRPAAL--
-IPAALV-
IVPAAL--
INPAAL--
-IPAALS-
-IPAALI-
IYPAAL--
IWPAAL--
ISPAAL--
IQPAAL--
-IPAALN-
IKPAAL--
-IPAALW-
IAPAAL--
IPPAAL--
IEPAAL--
IDPAAL--
IMPAAL--
ITPAAL--
-IPAALY-
-IPAALM-
-IPAALG-
IIPAAL--
-IPAALK-

-IPAALA-
IHPAAL--
-IPAALH-
-IPAALE-
-IPAALQ-
IFPAAL--
ICPAAL--
KSPAAL--
-KPAALR-
KTPAAL--
-KPAALY-
KLPAAL--
KHPAAL--
KGPAAL--
KMPAAL--
KKPAAL--
KPPAAL--
KIPAAL--
-KPAALW-
KQPAAL--
-KPAALH-
KYPAAL--
-KPAALT-
-KPAALS-
KAPAAL--
-KPAALV-
-KPAALI-
KNPAAL--
KWPAAL--
-KPAALQ-
KEPAAL--
-KPAALE-
-KPAALM-
KDPAAL--
-KPAALF-
KCPAAL--
LRPAAL--
LGPAAL--
LSPAAL--
-LPAALL-
LLPAAL--
LPPAAL--
-LPAALY-
-LPAALT-
-LPAALI-
LAPAAL--
-LPAALK-
LYPAAL--
LVPAAL--
LNPAAL--

-LPAALV-
LTPAAL--
LHPAAL--
-LPAALH-
-LPAALS-
LMPAAL--
-LPAALR-
LKPAAL--
-LPAALF-
LFPAAL--
LWPAAL--
-LPAALN-
-LPAALW-
-LPAALA-
LDPAAL--
-LPAALM-
-LPAALE-
LCPAAL--
MSPAAL--
-MPAALA-
MIPAAL--
-MPAALM-
MQPAAL--
-MPAALT-
-MPAALL-
MNPAAL--
MDPAAL--
MTPAAL--
-MPAALV-
MPPAAL--
MLPAAL--
MKPAAL--
-MPAALS-
MVPAAL--
MMPAAL--
MHPAAL--
-MPAALH-
-MPAALN-
MGPAAL--
MWPAAL--
MAPAAL--
MFPAAL--
MYPAAL--
-MPAALQ-
-MPAALF-
-MPAALY-
-MPAALW-
NLPAAL--
NGPAAL--
NVPAAL--

NTPAAL--
-NPAALR-
NSPAAL--
-NPAALY-
NWPAAL--
NQPAAL--
NYPAAL--
NIPAAL--
-NPAALT-
-NPAALK-
NNPAAL--
NHPAAL--
NEPAAL--
-NPAALS-
NPPAAL--
-NPAALW-
NAPAAL--
-NPAALF-
NKPAAL--
-NPAALV-
-NPAALH-
-NPAALA-
NCPAAL--
-NPAALN-
-NPAALM-
-NPAALC-
NDPAAL--
--PAALYL
--PAALRR
--PAALST
--PAALRK
--PAALRF
PRPAAL--
--PAALLK
--PAALTR
--PAALWL
--PAALFR
--PAALRI
--PAALRH
--PAALVR
--PAALVS
--PAALSL
--PAALTL
--PAALSR
--PAALRS
--PAALLS
--PAALLI
--PAALYK
--PAALFT
--PAALKL

--PAALSY
--PAALSG
--PAALSV
--PAALLW
--PAALGS
--PAALLR
--PAALLG
--PAALMS
--PAALLQ
--PAALRT
--PAALSH
PNPAAL--
--PAALGL
--PAALWS
--PAALTK
--PAALPY
--PAALQS
--PAALFQ
--PAALVY
--PAALTS
--PAALSA
--PAALNL
--PAALFK
--PAALVK
--PAALQR
PLPAAL--
--PAALVW
--PAALRQ
--PAALKQ
--PAALYS
--PAALMY
--PAALRW
--PAALSK
--PAALMG
--PAALHK
--PAALYN
--PAALVT
--PAALRD
--PAALVG
--PAALRY
--PAALRA
--PAALFS
--PAALMM
--PAALKN
--PAALII
PWPAAL--
PFPAAL--
--PAALYE
--PAALVA
--PAALMV

--PAALLV
--PAALKS
--PAALAY
--PAALTT
--PAALSQ
--PAALQV
--PAALMA
--PAALHM
--PAALHH
PGPAAL--
--PAALML
--PAALKY
--PAALRG
--PAALQA
--PAALNS
--PAALMK
PTPAAL--
PIPAAL--
--PAALTI
--PAALRE
--PAALLT
--PAALKG
--PAALIF
--PAALHS
--PAALVN
--PAALVM
--PAALQN
--PAALMD
--PAALLD
--PAALLA
--PAALKT
--PAALHR
--PAALAM
--PAALVI
--PAALRM
--PAALAL
--PAALAA
PQPAAL--
--PAALYF
--PAALQH
--PAALNY
--PAALMF
--PAALIL
--PAALWK
--PAALTH
--PAALKI
--PAALIW
PMPAAL--
--PAALTG
--PAALSM

--PAALIV
--PAALAW
--PAALTN
--PAALLN
--PAALHG
--PAALYW
--PAALYV
--PAALTA
--PAALRV
--PAALQK
--PAALGY
--PAALGF
--PAALET
--PAALAQ
PSPAAL--
PKPAAL--
--PAALWI
--PAALQL
--PAALNT
--PAALMN
--PAALLH
--PAALHT
--PAALHI
--PAALFD
--PAALPL
--PAALKA
--PAALHA
--PAALEL
--PAALAV
--PAALWN
--PAALSN
--PAALNV
--PAALNR
--PAALNI
--PAALKV
--PAALKM
--PAALEA
--PAALSI
--PAALIK
--PAALAF
--PAALVF
--PAALTM
--PAALNW
--PAALIH
--PAALFI
--PAALAT
--PAALYM
--PAALYA
--PAALWV
--PAALWG

--PAALWF
--PAALVE
--PAALTQ
--PAALTE
--PAALQT
--PAALMQ
PEPAAL--
--PAALQY
--PAALMI
--PAALMH
--PAALKW
--PAALHY
--PAALGM
--PAALGG
--PAALFH
--PAALES
PYPAAL--
--PAALYT
--PAALTF
--PAALNF
PVPAAL--
--PAALWD
--PAALSW
--PAALMT
--PAALIN
--PAALIG
--PAALFW
--PAALEG
--PAALAR
--PAALAK
--PAALYQ
--PAALYH
--PAALQI
--PAALHQ
--PAALHF
--PAALGQ
PHPAAL--
--PAALQF
--PAALNK
--PAALIY
--PAALIM
--PAALIA
--PAALAS
--PAALWT
--PAALVH
--PAALTD
--PAALQG
--PAALNN
--PAALMW
--PAALIQ

--PAALGV
--PAALGA
--PAALEI
--PAALAH
--PAALPI
--PAALNQ
--PAALNA
--PAALMR
--PAALHV
--PAALHN
--PAALGN
--PAALEN
--PAALEF
--PAALDG
--PAALYG
--PAALWQ
--PAALWE
--PAALSE
--PAALSC
--PAALQD
--PAALKF
--PAALKE
--PAALHE
--PAALFG
--PAALEQ
--PAALDY
--PAALAN
PAPAAL--
--PAALTW
--PAALRC
--PAALNM
--PAALNE
--PAALGW
--PAALGH
--PAALEY
--PAALEV
--PAALDS
--PAALYD
--PAALWH
--PAALSD
--PAALNG
--PAALND
--PAALKH
--PAALIE
--PAALHW
--PAALFE
PCPAAL--
--PAALYP
--PAALWM
--PAALQQ

--PAALPF
--PAALNH
--PAALHD
--PAALGK
--PAALER
--PAALDM
--PAALVC
--PAALQW
--PAALKD
--PAALFC
--PAALEK
--PAALDL
--PAALCN
--PAALCD
PPPAAL--
-PPAALR-
--PAALVP
--PAALQM
--PAALQE
--PAALQC
--PAALPW
--PAALPM
--PAALNC
--PAALMC
--PAALLC
--PAALID
--PAALHC
--PAALGE
--PAALEW
--PAALCW
--PAALCV
--PAALCS
--PAALCL
--PAALCI
--PAALAC
-QPAALL-
-QPAALR-
QGPAAL--
QRPAAL--
-QPAALS-
QAPAAL--
-QPAALW-
QKPAAL--
-QPAALK-
QSPAAL--
-QPAALH-
QVPAAL--
QTPAAL--
QHPAAL--
QYPAAL--

QEPAAL--
QWPAAL--
-QPAALV-
QNPAAL--
QQPAAL--
-QPAALY-
-QPAALN-
-QPAALA-
-QPAALT-
-QPAALG-
-QPAALF-
QFPAAL--
-QPAALQ-
QMPAAL--
QPPAAL--
-QPAALM-
-QPAALI-
-QPAALE-
REPAAL--
RSPAAL--
-RPAALT-
RLPAAL--
-RPAALL-
RRPAAL--
RVPAAL--
-RPAALS-
RHPAAL--
RAPAAL--
RFPAAL--
RNPAAL--
RWPAAL--
-RPAALQ-
RYPAAL--
RTPAAL--
-RPAALN-
RIPAAL--
RPPAAL--
-RPAALK-
RMPAAL--
-RPAALG-
-RPAALF-
RQPAAL--
-RPAALV-
-RPAALM-
-RPAALA-
RDPAAL--
-RPAALE-
RCPAAL--
-RPAALC-
SRPAAL--

-SPAALR-
SIPAAL--
SVPAAL--
-SPAALY-
-SPAALT-
SYPAAL--
-SPAALM-
-SPAALS-
SQPAAL--
-SPAALF-
-SPAALN-
SWPAAL--
SPPAAL--
SHPAAL--
STPAAL--
-SPAALV-
-SPAALQ-
SKPAAL--
SFPAAL--
SAPAAL--
-SPAALW-
-SPAALI-
SGPAAL--
-SPAALH-
SMPAAL--
SDPAAL--
-SPAALA-
-SPAALK-
SEPAAL--
-SPAALG-
-SPAALE-
SCPAAL--
TRPAAL--
-TPAALY-
TFPAAL--
TLPAAL--
TVPAAL--
-TPAALQ-
-TPAALS-
-TPAALH-
TMPAAL--
-TPAALW-
-TPAALN-
-TPAALT-
TAPAAL--
TYPAAL--
THPAAL--
TGPAAL--
TTPAAL--
TIPAAL--

TSPAAL--
-TPAALV-
-TPAALK-
-TPAALA-
TKPAAL--
-TPAALM-
-TPAALI-
TEPAAL--
-TPAALG-
TNPAAL--
TDPAAL--
TCPAAL--
TPPAAL--
TWPAAL--
-TPAALE-
-TPAALC-
-VPAALR-
-VPAALK-
VNPAAL--
VFPAAL--
-VPAALL-
VSPAAL--
VRPAAL--
VLPAAL--
VHPAAL--
VVPAAL--
-VPAALV-
-VPAALM-
-VPAALW-
VYPAAL--
-VPAALS-
VKPAAL--
-VPAALT-
-VPAALN-
-VPAALH-
VAPAAL--
VTPAAL--
-VPAALY-
VPPAAL--
-VPAALQ-
-VPAALF-
VDPAAL--
VGPAAL--
VIPAAL--
VEPAAL--
-VPAALG-
-VPAALA-
-VPAALE-
VCPAAL--
-VPAALC-

-WPAALR-
WGPAAL--
WLPAAL--
-WPAALT-
WRPAAL--
-WPAALM-
-WPAALS-
WWPAAL--
-WPAALH-
WPPAAL--
-WPAALN-
WHPAAL--
-WPAALI-
WFPAAL--
WQPAAL--
WVPAAL--
WNPAAL--
-WPAALE-
WCPAAL--
WTPAAL--
-WPAALF-
WAPAAL--
WEPAAL--
WYPAAL--
-WPAALQ-
-WPAALK-
-WPAALC-
YRPAAL--
YLPAAL--
-YPAALR-
YVPAAL--
YSPAAL--
-YPAALY-
YGPAAL--
-YPAALS-
-YPAALT-
YAPAAL--
-YPAALL-
YTPAAL--
YPPAAL--
-YPAALK-
YNPAAL--
-YPAALH-
-YPAALQ-
YIPAAL--
YMPAAL--
YFPAAL--
YEPAAL--
-YPAALV-
-YPAALI-

	-YPAALG-		
	-YPAALF-		
	-YPAALE-		
	YYPAAL--		
	YWPAAL--		
	-YPAALM-		
	YQPAAL--		
	-YPAALA-		
	YHPAAL--		
PLPM	86	819	47.63
	AEPLPM--		
	-APLPMR-		
	-APLPMS-		
	AIPLPM--		
	-APLPML-		
	AFPLPM--		
	AYPLPM--		
	ASPLPM--		
	ATPLPM--		
	ARPLPM--		
	-APLPMN-		
	AWPLPM--		
	AKPLPM--		
	-APLPMI-		
	-APLPMY-		
	AVPLPM--		
	AMPLPM--		
	AAPLPM--		
	-APLPMH-		
	ADPLPM--		
	-APLPMM-		
	ALPLPM--		
	-APLPMW-		
	-APLPMT-		
	-APLPMG-		
	AHPLPM--		
	AQPLPM--		
	APPLPM--		
	-APLPMV-		
	-APLPMF-		
	-APLPMQ-		
	ANPLPM--		
	ACPLPM--		
	CVPLPM--		
	CKPLPM--		
	-CPLPMY-		
	CMPLPM--		
	CSPLPM--		
	-CPLPMV-		
	-CPLPMM-		

CGPLPM--
CAPLPM--
CWPLPM--
CRPLPM--
CPPLPM--
-CPLPMT-
-CPLPMN-
-CPLPME-
CIPLPM--
CEPLPM--
-DPLPML-
-DPLPMS-
DTPLPM--
DRPLPM--
-DPLPMQ-
DLPLPM--
-DPLPMT-
DNPLPM--
-DPLPMY-
DQPLPM--
-DPLPMK-
DKPLPM--
DVPLPM--
DIPLPM--
DGPLPM--
-DPLPMW-
-DPLPMN-
-DPLPMM-
DYPLPM--
-DPLPMI-
-DPLPMA-
-DPLPMV-
-DPLPMH-
-DPLPMG-
DEPLPM--
-EPLPMM-
-EPLPMR-
EVPLPM--
ETPLPM--
-EPLPML-
EIPLPM--
EFPLPM--
ERPLPM--
EHPLPM--
-EPLPMT-
-EPLPMS-
ESPLPM--
EPPLPM--
EWPLPM--
-EPLPMW-

-EPLPMH-
EKPLPM--
EYPLPM--
-EPLPMK-
ELPLPM--
-EPLPME-
ENPLPM--
EGPLPM--
-EPLPMA-
EAPLPM--
FRPLPM--
-FPLPMS-
-FPLPMR-
FFPLPM--
-FPLPMV-
FKPLPM--
-FPLPML-
-FPLPMT-
FSPLPM--
FMPLPM--
FLPLPM--
-FPLPMH-
FNPLPM--
FEPLPM--
FVPLPM--
-FPLPMI-
FYPLPM--
FWPLPM--
FPPLPM--
-FPLPMK-
FIPLPM--
FHPLPM--
FAPLPM--
-FPLPMY-
-FPLPMQ-
-FPLPMF-
-FPLPME-
FTPLPM--
-FPLPMG-
GLPLPM--
GTPLPM--
-GPLPMR-
GVPLPM--
GKPLPM--
-GPLPMT-
-GPLPMQ-
-GPLPMN-
GMPLPM--
GRPLPM--
GSPLPM--

-GPLPMW-
GYPLPM--
GGPLPM--
-GPLPMK-
-GPLPMA-
GAPLPM--
-GPLPMG-
GFPLPM--
-GPLPMS-
-GPLPMI-
-GPLPMH-
GCPLPM--
GNPLPM--
-GPLPME-
GIPLPM--
GQPLPM--
GPPLPM--
-GPLPMV-
-GPLPMP-
GHPLPM--
GEPLPM--
HLPLPM--
HRPLPM--
-HPLPMW-
-HPLPML-
HSPLPM--
-HPLPMA-
HKPLPM--
-HPLPMH-
-HPLPMR-
HWPLPM--
HVPLPM--
HPPLPM--
HMPLPM--
HAPLPM--
-HPLPMN-
-HPLPMI-
-HPLPMF-
HGPLPM--
HFPLPM--
-HPLPMT-
HIPLPM--
-HPLPMY-
-HPLPMV-
-HPLPMQ-
-HPLPMK-
HEPLPM--
HDPLPM--
-HPLPMM-
-HPLPME-

-HPLPMG-
HHPLPM--
IRPLPM--
ILPLPM--
-IPLPMT-
-IPLPMY-
IFPLPM--
-IPLPMK-
IKPLPM--
IAPLPM--
ISPLPM--
IYPLPM--
-IPLPMS-
-IPLPMA-
INPLPM--
IEPLPM--
-IPLPMR-
-IPLPMH-
IQPLPM--
IPPLPM--
-IPLPMF-
IVPLPM--
-IPLPMW-
-IPLPMI-
-IPLPMN-
IDPLPM--
IMPLPM--
IGPLPM--
-IPLPMQ-
-IPLPME-
IIPLPM--
ICPLPM--
KSPLPM--
-KPLPML-
KWPLPM--
KGPLPM--
-KPLPMF-
-KPLPMM-
KLPLPM--
-KPLPMY-
KEPLPM--
KRPLPM--
KVPLPM--
KTPLPM--
KNPLPM--
-KPLPMH-
-KPLPMS-
-KPLPMV-
-KPLPMW-
-KPLPMQ-

-KPLPMN-
-KPLPMI-
-KPLPMA-
KAPLPM--
KYPLPM--
-KPLPMG-
KHPLPM--
-KPLPMT-
KPPLPM--
-KPLPME-
KDPLPM--
-LPLPMR-
LRPLPM--
LSPLPM--
-LPLPMW-
-LPLPMK-
-LPLPMV-
-LPLPMS-
LHPLPM--
-LPLPML-
-LPLPMT-
-LPLPMH-
LMPLPM--
LNPLPM--
LQPLPM--
LIPLPM--
LGPLPM--
LAPLPM--
LTPLPM--
-LPLPMY-
LYPLPM--
LFPLPM--
-LPLPMN-
LLPLPM--
LEPLPM--
-LPLPMM-
LWPLPM--
LVPLPM--
-LPLPMQ-
LPPLPM--
-LPLPME-
-LPLPMD-
LDPLPM--
MRPLPM--
MLPLPM--
-MPLPML-
-MPLPMR-
MNPLPM--
MYPLPM--
-MPLPMF-

-MPLPMS-
-MPLPMK-
MWPLPM--
MMPLPM--
-MPLPMT-
MTPLPM--
-MPLPMI-
MPPLPM--
MAPLPM--
-MPLPMY-
-MPLPMQ-
-MPLPMG-
-MPLPMA-
MKPLPM--
-MPLPMV-
MHPLPM--
MVPLPM--
-MPLPME-
MIPLPM--
MEPLPM--
-NPLPML-
-NPLPMY-
-NPLPMR-
NMPLPM--
NYPLPM--
-NPLPMV-
-NPLPMT-
NLPLPM--
NVPLPM--
-NPLPMN-
-NPLPMQ-
NRPLPM--
NKPLPM--
-NPLPMF-
NNPLPM--
NEPLPM--
NQPLPM--
NSPLPM--
-NPLPMH-
-NPLPMA-
NHPLPM--
NTPLPM--
-NPLPMI-
-NPLPMS-
NIPLPM--
NGPLPM--
NFPLPM--
NAPLPM--
NPPLPM--
-NPLPMM-

```
-NPLPMK-
-NPLPMG-
NDPLPM--
--PLPMVS
--PLPMSI
--PLPMTD
--PLPMYI
--PLPMMS
--PLPMTL
--PLPMIR
--PLPMSL
--PLPMVL
--PLPMTR
--PLPMFR
--PLPMRT
--PLPMLM
--PLPMRL
--PLPMST
--PLPMVN
--PLPMLV
--PLPMAT
--PLPMSK
--PLPMFT
--PLPMAS
--PLPMWL
--PLPMVR
--PLPMGQ
--PLPMGI
--PLPMVM
--PLPMYG
--PLPMSN
--PLPMAG
--PLPMYA
--PLPMRW
--PLPMQN
--PLPMIL
--PLPMAY
--PLPMRY
--PLPMRS
--PLPMQM
--PLPMRF
--PLPMLH
--PLPMKV
--PLPMGV
--PLPMAR
--PLPMLR
--PLPMII
--PLPMET
--PLPMAL
--PLPMWG
```

--PLPMVF
--PLPMTS
--PLPMLA
--PLPMKK
--PLPMVT
--PLPMQS
--PLPMNT
--PLPMGS
--PLPMTG
--PLPMRK
--PLPMFQ
--PLPMYH
--PLPMPY
--PLPMNI
--PLPMLT
--PLPMLK
--PLPMLG
--PLPMAF
--PLPMAA
--PLPMAI
--PLPMVH
--PLPMVG
--PLPMMY
--PLPMIW
--PLPMFS
--PLPMEV
--PLPMAM
--PLPMSH
--PLPMKM
PRPLPM--
--PLPMYE
--PLPMWR
--PLPMVY
--PLPMNS
--PLPMIM
PGPLPM--
--PLPMYV
--PLPMWH
--PLPMVK
--PLPMVD
--PLPMTQ
--PLPMTA
--PLPMIS
--PLPMIA
--PLPMHS
--PLPMGM
--PLPMEY
--PLPMEI
--PLPMEH
--PLPMWT

--PLPMTH
--PLPMMN
--PLPMLQ
--PLPMKE
--PLPMGN
--PLPMTE
--PLPMRV
--PLPMPH
--PLPMID
--PLPMGA
--PLPMYQ
--PLPMWQ
--PLPMSS
--PLPMRQ
--PLPMRG
--PLPMRD
--PLPMQT
--PLPMNY
--PLPMNV
--PLPMKL
--PLPMGH
--PLPMYK
--PLPMQV
--PLPMQR
--PLPMQF
--PLPMPW
--PLPMNE
--PLPMMR
--PLPMME
--PLPMKG
--PLPMIH
--PLPMIE
--PLPMGT
--PLPMGG
--PLPMDF
--PLPMAK
--PLPMYD
--PLPMWV
--PLPMTT
--PLPMTK
--PLPMTF
--PLPMRN
--PLPMLE
--PLPMIK
--PLPMFV
--PLPMFK
--PLPMYS
--PLPMWD
--PLPMVA
--PLPMSQ

--PLPMSA
--PLPMQL
--PLPMQH
--PLPMQA
--PLPMPV
--PLPMPM
--PLPMNH
--PLPMML
--PLPMKT
--PLPMHA
--PLPMEW
--PLPMYW
--PLPMWA
--PLPMSW
--PLPMSM
--PLPMQY
--PLPMPF
--PLPMLD
--PLPMKN
--PLPMIY
--PLPMIF
--PLPMHW
--PLPMGR
--PLPMDL
--PLPMAV
--PLPMYR
--PLPMYM
--PLPMWN
--PLPMWF
--PLPMVV
--PLPMVQ
--PLPMTI
--PLPMSD
--PLPMNQ
--PLPMNG
--PLPMHQ
--PLPMGK
--PLPMYN
--PLPMWK
--PLPMTW
--PLPMTV
--PLPMRA
--PLPMQE
--PLPMPS
--PLPMMW
--PLPMMG
--PLPMKQ
--PLPMKA
--PLPMIG
--PLPMHG

--PLPMGY
--PLPMFN
--PLPMFM
PEPLPM--
PTPLPM--
--PLPMWW
--PLPMWI
--PLPMVW
--PLPMVI
--PLPMSE
--PLPMNR
--PLPMNM
--PLPMNF
--PLPMNA
--PLPMIV
--PLPMHV
--PLPMHN
--PLPMHK
--PLPMHI
--PLPMHD
--PLPMES
--PLPMEK
--PLPMDH
--PLPMAW
--PLPMSV
--PLPMPI
--PLPMKR
--PLPMKD
--PLPMHR
--PLPMHE
--PLPMFI
--PLPMFD
--PLPMFC
--PLPMEF
--PLPMDS
--PLPMDM
--PLPMDI
-PPLPMH-
--PLPMWY
--PLPMWE
--PLPMVP
--PLPMTY
--PLPMTM
--PLPMSY
--PLPMRP
--PLPMQI
--PLPMQG
--PLPMQD
--PLPMNW
--PLPMNN

--PLPMND
--PLPMMV
--PLPMMK
--PLPMMD
--PLPMMA
--PLPMKF
--PLPMIQ
--PLPMHY
--PLPMHH
--PLPMFE
--PLPMEQ
--PLPMEN
--PLPMEA
--PLPMDY
--PLPMDV
--PLPMDG
--PLPMCM
--PLPMCI
QVPLPM--
-QPLPMF-
QAPLPM--
QYPLPM--
QFPLPM--
QRPLPM--
-QPLPMW-
QPPLPM--
QIPLPM--
-QPLPMH-
QTPLPM--
-QPLPMS-
-QPLPMR-
-QPLPMK-
-QPLPMI-
QWPLPM--
QSPLPM--
-QPLPMV-
-QPLPMT-
-QPLPMG-
QMPLPM--
-QPLPMQ-
QQPLPM--
-QPLPMN-
QHPLPM--
QEPLPM--
QDPLPM--
-QPLPMM-
-QPLPME-
QGPLPM--
QCPLPM--
RNPLPM--

RTPLPM--
-RPLPMI-
RHPLPM--
RSPLPM--
RYPLPM--
RLPLPM--
-RPLPMN-
-RPLPMG-
RVPLPM--
RDPLPM--
-RPLPMK-
-RPLPMY-
RKPLPM--
RIPLPM--
RAPLPM--
RGPLPM--
-RPLPMV-
-RPLPMT-
RMPLPM--
RFPLPM--
-RPLPMS-
-RPLPMW-
REPLPM--
-RPLPMF-
-RPLPME-
RWPLPM--
RPPLPM--
-RPLPMQ-
-RPLPMC-
RCPLPM--
SRPLPM--
-SPLPMW-
-SPLPMH-
SKPLPM--
-SPLPMY-
STPLPM--
-SPLPMR-
-SPLPML-
SGPLPM--
-SPLPMF-
SVPLPM--
-SPLPMA-
-SPLPMK-
SNPLPM--
SLPLPM--
SAPLPM--
SYPLPM--
SQPLPM--
SSPLPM--
SWPLPM--

-SPLPMV-
-SPLPMI-
SEPLPM--
SMPLPM--
SHPLPM--
-SPLPMQ-
-SPLPMM-
SDPLPM--
-SPLPMN-
-SPLPMG-
SIPLPM--
SPPLPM--
-SPLPME-
SFPLPM--
-TPLPMR-
-TPLPML-
-TPLPMI-
TDPLPM--
TTPLPM--
-TPLPMQ-
TIPLPM--
TLPLPM--
-TPLPMA-
TSPLPM--
TRPLPM--
TVPLPM--
-TPLPMV-
TGPLPM--
-TPLPMY-
-TPLPMN-
-TPLPMM-
-TPLPMS-
TPPLPM--
TFPLPM--
TAPLPM--
-TPLPMW-
-TPLPMG-
-TPLPMT-
TNPLPM--
-TPLPMH-
-TPLPME-
-VPLPMS-
-VPLPMR-
-VPLPMI-
-VPLPMA-
VLPLPM--
VAPLPM--
VHPLPM--
VVPLPM--
-VPLPMK-

VSPLPM--
VGPLPM--
VYPLPM--
VQPLPM--
-VPLPMQ-
VKPLPM--
VTPLPM--
VRPLPM--
-VPLPMV-
VIPLPM--
VDPLPM--
VNPLPM--
-VPLPMW-
VMPLPM--
-VPLPMT-
-VPLPMG-
VEPLPM--
VCPLPM--
-VPLPMH-
VPPLPM--
VFPLPM--
-VPLPMN-
-VPLPMM-
-WPLPMS-
WKPLPM--
-WPLPML-
WAPLPM--
-WPLPMV-
-WPLPMR-
WGPLPM--
WPPLPM--
-WPLPMY-
-WPLPMT-
WMPLPM--
WSPLPM--
WQPLPM--
-WPLPMQ-
-WPLPMG-
WIPLPM--
WTPLPM--
-WPLPMK-
WHPLPM--
WVPLPM--
-WPLPMH-
WNPLPM--
WLPLPM--
WEPLPM--
-WPLPMW-
-WPLPMI-
-WPLPMF-

```
          -YPLPMR-
          YHPLPM--
          YLPLPM--
          YTPLPM--
          YSPLPM--
          YYPLPM--
          -YPLPMT-
          -YPLPMI-
          -YPLPMF-
          -YPLPMQ-
          YRPLPM--
          -YPLPMW-
          -YPLPME-
          YWPLPM--
          -YPLPMA-
          YGPLPM--
          YVPLPM--
          YPPLPM--
          -YPLPMK-
          -YPLPMN-
          YKPLPM--
          -YPLPMY-
          YEPLPM--
          -YPLPMV-
          -YPLPMS-
          -YPLPMH-
          -YPLPMG-
          YAPLPM--
          YNPLPM--
PSAL        14        900     47.61
          -FPSALP-
          -FPSALE-
          -NPSALP-
          --PSALVP
          --PSALDL
          --PSALPQ
          --PSALKC
          --PSALDI
          QDPSAL--
          -RPSALP-
          -RPSALD-
          -SPSALD-
          ALPSAL--
          ASPSAL--
          -APSALM-
          -APSALR-
          AVPSAL--
          APPSAL--
          ATPSAL--
          -APSALY-
```

−APSALS−
−APSALK−
AYPSAL−−
AWPSAL−−
AGPSAL−−
AKPSAL−−
AIPSAL−−
−APSALH−
−APSALT−
−APSALL−
−APSALF−
−APSALA−
−APSALI−
AEPSAL−−
AAPSAL−−
−APSALV−
ADPSAL−−
AMPSAL−−
ANPSAL−−
AQPSAL−−
−APSALW−
−APSALE−
−APSALQ−
−APSALG−
AHPSAL−−
−APSALN−
AFPSAL−−
ACPSAL−−
−APSALC−
CSPSAL−−
CMPSAL−−
CVPSAL−−
CIPSAL−−
CRPSAL−−
CGPSAL−−
CAPSAL−−
CNPSAL−−
CEPSAL−−
CTPSAL−−
−CPSALR−
−CPSALL−
CKPSAL−−
−CPSALY−
CFPSAL−−
−CPSALK−
CLPSAL−−
−CPSALV−
CPPSAL−−
−CPSALW−
−CPSALQ−

-CPSALM-
-CPSALI-
CDPSAL--
CCPSAL--
CQPSAL--
-CPSALS-
CHPSAL--
-CPSALN-
DSPSAL--
DRPSAL--
DIPSAL--
-DPSALL-
DFPSAL--
DYPSAL--
DQPSAL--
DKPSAL--
DAPSAL--
DTPSAL--
DMPSAL--
DGPSAL--
DLPSAL--
DWPSAL--
-DPSALR-
DVPSAL--
-DPSALF-
-DPSALV-
-DPSALY-
-DPSALW-
-DPSALI-
-DPSALH-
-DPSALS-
-DPSALT-
DEPSAL--
DHPSAL--
-DPSALN-
DNPSAL--
-DPSALM-
-DPSALQ-
-EPSALR-
EAPSAL--
ERPSAL--
-EPSALT-
ELPSAL--
-EPSALL-
ESPSAL--
EKPSAL--
-EPSALI-
EMPSAL--
EVPSAL--
-EPSALV-

-EPSALM-
-EPSALH-
-EPSALS-
EGPSAL--
EHPSAL--
EEPSAL--
EQPSAL--
EIPSAL--
-EPSALW-
-EPSALA-
EYPSAL--
-EPSALK-
EFPSAL--
-EPSALN-
-EPSALF-
EWPSAL--
-EPSALQ-
-EPSALP-
-EPSALG-
-EPSALE-
-FPSALT-
FRPSAL--
-FPSALR-
FGPSAL--
-FPSALL-
FLPSAL--
FMPSAL--
FPPSAL--
FSPSAL--
-FPSALW-
FAPSAL--
-FPSALS-
FTPSAL--
FEPSAL--
FVPSAL--
FKPSAL--
-FPSALV-
FNPSAL--
FHPSAL--
FYPSAL--
FIPSAL--
FFPSAL--
-FPSALY-
-FPSALI-
-FPSALH-
FWPSAL--
-FPSALK-
-FPSALF-
FDPSAL--
-FPSALN-

-FPSALA-
FQPSAL--
-FPSALM-
-FPSALC-
-GPSALM-
-GPSALR-
-GPSALL-
GRPSAL--
GWPSAL--
GGPSAL--
GVPSAL--
-GPSALF-
-GPSALS-
GMPSAL--
-GPSALT-
GQPSAL--
-GPSALW-
-GPSALQ-
GHPSAL--
-GPSALK-
-GPSALV-
-GPSALA-
-GPSALY-
-GPSALN-
GKPSAL--
GAPSAL--
GFPSAL--
GEPSAL--
-GPSALH-
GIPSAL--
-GPSALG-
GPPSAL--
-GPSALE-
GNPSAL--
-GPSALI-
GDPSAL--
GCPSAL--
HRPSAL--
HVPSAL--
HLPSAL--
HSPSAL--
-HPSALR-
-HPSALS-
-HPSALT-
HGPSAL--
HTPSAL--
-HPSALL-
HFPSAL--
-HPSALV-
HAPSAL--

-HPSALI-
HKPSAL--
-HPSALN-
HWPSAL--
-HPSALY-
-HPSALK-
-HPSALH-
HMPSAL--
-HPSALW-
HPPSAL--
HNPSAL--
HYPSAL--
-HPSALF-
HQPSAL--
-HPSALQ-
-HPSALA-
HDPSAL--
-HPSALG-
HEPSAL--
-IPSALR-
IRPSAL--
ISPSAL--
-IPSALQ-
IVPSAL--
-IPSALV-
-IPSALS-
-IPSALW-
ITPSAL--
-IPSALL-
-IPSALY-
-IPSALF-
-IPSALH-
IAPSAL--
IMPSAL--
IHPSAL--
IGPSAL--
-IPSALK-
IFPSAL--
IIPSAL--
IYPSAL--
-IPSALN-
INPSAL--
IKPSAL--
-IPSALE-
IQPSAL--
IWPSAL--
IDPSAL--
IPPSAL--
IEPSAL--
-IPSALM-

-IPSALG-
-IPSALA-
ICPSAL--
KRPSAL--
KFPSAL--
-KPSALR-
KWPSAL--
-KPSALL-
-KPSALV-
KNPSAL--
KGPSAL--
KYPSAL--
KPPSAL--
KAPSAL--
KIPSAL--
-KPSALT-
-KPSALI-
KTPSAL--
-KPSALN-
-KPSALK-
-KPSALY-
KMPSAL--
-KPSALA-
-KPSALQ-
KEPSAL--
-KPSALW-
-KPSALG-
KHPSAL--
-KPSALM-
-KPSALH-
KQPSAL--
KDPSAL--
-KPSALE-
KCPSAL--
LRPSAL--
LSPSAL--
-LPSALL-
LAPSAL--
LWPSAL--
-LPSALK-
LQPSAL--
LIPSAL--
-LPSALS-
-LPSALF-
LMPSAL--
LPPSAL--
LYPSAL--
LVPSAL--
-LPSALW-
-LPSALR-

```
-LPSALI-
LTPSAL--
LFPSAL--
LNPSAL--
-LPSALQ-
LDPSAL--
LKPSAL--
-LPSALH-
-LPSALY-
-LPSALM-
-LPSALN-
-LPSALV-
-LPSALA-
LHPSAL--
LCPSAL--
-LPSALC-
-MPSALL-
MVPSAL--
MRPSAL--
MSPSAL--
MKPSAL--
MTPSAL--
MFPSAL--
-MPSALT-
-MPSALS-
MGPSAL--
-MPSALI-
-MPSALR-
-MPSALF-
MPPSAL--
MMPSAL--
MYPSAL--
MWPSAL--
-MPSALY-
-MPSALW-
-MPSALH-
MAPSAL--
MQPSAL--
MHPSAL--
-MPSALV-
-MPSALK-
MNPSAL--
-MPSALN-
MDPSAL--
-MPSALA-
-MPSALG-
-MPSALC-
-MPSALM-
-MPSALE-
MCPSAL--
```

NRPSAL--
-NPSALR-
NKPSAL--
NSPSAL--
NLPSAL--
NTPSAL--
-NPSALL-
NEPSAL--
NAPSAL--
NYPSAL--
-NPSALS-
-NPSALV-
NHPSAL--
NGPSAL--
-NPSALH-
-NPSALT-
NFPSAL--
-NPSALQ-
NNPSAL--
-NPSALN-
NDPSAL--
NQPSAL--
-NPSALI-
-NPSALM-
NMPSAL--
NPPSAL--
-NPSALW-
-NPSALF-
-NPSALK-
NCPSAL--
-NPSALC-
--PSALLS
--PSALRV
--PSALLL
--PSALST
--PSALRG
--PSALVS
--PSALLG
--PSALAL
--PSALRF
--PSALIR
--PSALVR
--PSALLV
--PSALLT
--PSALHR
--PSALLN
PSPSAL--
--PSALTR
--PSALQA
--PSALYA

--PSALSM
--PSALIN
--PSALAS
--PSALTS
--PSALWS
--PSALKS
--PSALGL
--PSALSR
--PSALSG
--PSALMS
--PSALHL
--PSALSS
--PSALLY
--PSALKL
PAPSAL--
--PSALRT
--PSALKG
--PSALRQ
--PSALYW
--PSALRE
PTPSAL--
--PSALVG
--PSALTG
--PSALSN
--PSALSV
--PSALWG
--PSALRH
--PSALMV
--PSALYS
--PSALRD
--PSALVK
--PSALAT
--PSALHG
--PSALWQ
--PSALVH
--PSALMA
--PSALGA
--PSALTV
--PSALLH
--PSALLD
--PSALGM
PVPSAL--
--PSALYR
--PSALVT
--PSALTM
--PSALTA
--PSALSH
--PSALFT
--PSALYQ
--PSALVA

--PSALAY
--PSALNS
--PSALKQ
--PSALFE
--PSALSW
--PSALMW
--PSALHS
--PSALLA
--PSALKR
--PSALKN
--PSALKI
--PSALHK
--PSALAG
--PSALMK
--PSALVF
--PSALTT
--PSALQT
--PSALPL
--PSALFN
--PSALVN
--PSALLM
--PSALKA
--PSALGT
--PSALFG
PWPSAL--
--PSALVV
--PSALQG
--PSALSA
--PSALMN
--PSALIV
--PSALIL
--PSALFQ
--PSALQW
--PSALNY
--PSALMF
--PSALHT
--PSALWI
--PSALQV
--PSALAV
--PSALTQ
--PSALTN
--PSALNM
--PSALMG
--PSALFA
PIPSAL--
--PSALSK
--PSALLQ
--PSALGG
--PSALFI
--PSALAI

--PSALSQ
--PSALYI
--PSALAF
PHPSAL--
--PSALTY
--PSALTW
--PSALIG
PMPSAL--
--PSALWK
--PSALTH
--PSALQL
--PSALIH
--PSALHQ
--PSALWV
--PSALQH
--PSALKW
--PSALKV
--PSALIQ
--PSALHA
--PSALES
PLPSAL--
--PSALYM
--PSALSI
--PSALMD
--PSALEA
PKPSAL--
--PSALVI
--PSALNV
--PSALMT
--PSALWN
--PSALNA
--PSALHI
--PSALGV
--PSALQR
--PSALQM
--PSALMM
--PSALHV
PQPSAL--
PNPSAL--
--PSALYN
--PSALYG
--PSALKH
--PSALAM
--PSALAA
PFPSAL--
--PSALNR
--PSALMH
--PSALAN
--PSALYE
--PSALQY

PEPSAL--
--PSALYY
--PSALWT
--PSALQN
--PSALKM
--PSALHE
--PSALWH
--PSALMQ
--PSALGF
--PSALFM
--PSALFH
--PSALVM
--PSALVD
--PSALNI
--PSALHN
--PSALGY
--PSALAW
--PSALQK
--PSALIT
--PSALIF
--PSALWM
--PSALNQ
--PSALII
--PSALHY
--PSALLE
--PSALFD
--PSALEV
--PSALEG
--PSALWY
--PSALPY
--PSALNT
--PSALNH
--PSALMI
--PSALHH
--PSALGH
--PSALEY
--PSALAR
--PSALAH
--PSALQS
--PSALKE
--PSALWW
--PSALWD
--PSALPI
--PSALKY
--PSALID
--PSALHD
--PSALAK
--PSALYD
--PSALQI
--PSALQE

--PSALIY
--PSALVQ
--PSALTE
--PSALGW
--PSALGQ
--PSALEQ
PYPSAL--
--PSALYP
--PSALVE
--PSALSE
--PSALSD
--PSALRP
--PSALNK
--PSALHF
--PSALPF
--PSALNE
--PSALKF
--PSALIE
--PSALHM
PCPSAL--
--PSALWF
--PSALNG
--PSALND
--PSALET
--PSALEL
--PSALEW
--PSALER
--PSALAQ
--PSALQQ
--PSALPM
--PSALEK
--PSALEI
--PSALWC
--PSALFC
--PSALEH
--PSALEF
--PSALDS
--PSALDM
--PSALCV
--PSALRC
--PSALQF
--PSALQD
--PSALMP
--PSALLC
--PSALHP
--PSALGE
--PSALFP
--PSALDA
--PSALCS
--PSALCN

--PSALCL
--PSALCE
--PSALCA
--PSALVC
--PSALSC
--PSALPD
--PSALIC
--PSALEN
--PSALDG
--PSALCM
--PSALCG
--PSALAD
--PSALAC
-PPSALQ-
-PPSALM-
-PPSALL-
-PPSALK-
-PPSALI-
-PPSALF-
PDPSAL--
-QPSALL-
QRPSAL--
QLPSAL--
QWPSAL--
-QPSALK-
QVPSAL--
QIPSAL--
QGPSAL--
-QPSALI-
-QPSALM-
QTPSAL--
QNPSAL--
QKPSAL--
-QPSALA-
QPPSAL--
QAPSAL--
QQPSAL--
-QPSALR-
QFPSAL--
QEPSAL--
-QPSALV-
QMPSAL--
-QPSALW-
-QPSALQ-
-QPSALF-
-QPSALS-
QHPSAL--
-QPSALH-
-QPSALE-
-QPSALP-

-RPSALS-
RVPSAL--
RIPSAL--
-RPSALT-
-RPSALK-
RGPSAL--
-RPSALN-
-RPSALA-
RTPSAL--
-RPSALG-
RPPSAL--
-RPSALH-
-RPSALF-
-RPSALV-
RQPSAL--
-RPSALY-
-RPSALM-
REPSAL--
RMPSAL--
RYPSAL--
-RPSALW-
RFPSAL--
-RPSALQ-
RDPSAL--
-RPSALE-
RCPSAL--
-RPSALC-
SMPSAL--
-SPSALR-
SKPSAL--
SVPSAL--
SLPSAL--
STPSAL--
SAPSAL--
SFPSAL--
SHPSAL--
-SPSALV-
-SPSALH-
-SPSALA-
SNPSAL--
-SPSALM-
SQPSAL--
-SPSALN-
-SPSALF-
SEPSAL--
SYPSAL--
-SPSALI-
-SPSALK-
-SPSALQ-
-SPSALE-

SCPSAL--
-SPSALG-
TRPSAL--
TLPSAL--
TVPSAL--
-TPSALL-
TFPSAL--
TSPSAL--
-TPSALY-
TIPSAL--
TTPSAL--
-TPSALF-
-TPSALT-
TYPSAL--
TNPSAL--
-TPSALK-
TPPSAL--
TKPSAL--
TAPSAL--
-TPSALN-
-TPSALW-
-TPSALV-
TGPSAL--
TEPSAL--
-TPSALH-
TDPSAL--
-TPSALM-
TWPSAL--
-TPSALQ-
-TPSALA-
TCPSAL--
TQPSAL--
THPSAL--
-TPSALG-
VLPSAL--
VSPSAL--
-VPSALR-
VPPSAL--
-VPSALL-
VRPSAL--
VAPSAL--
-VPSALS-
-VPSALT-
VTPSAL--
VNPSAL--
-VPSALI-
VWPSAL--
-VPSALY-
VMPSAL--
-VPSALW-

-VPSALQ-
-VPSALV-
VGPSAL--
-VPSALN-
-VPSALM-
-VPSALH-
-VPSALA-
VIPSAL--
VHPSAL--
-VPSALK-
VYPSAL--
VQPSAL--
VEPSAL--
VFPSAL--
VCPSAL--
-VPSALF-
VDPSAL--
WLPSAL--
WGPSAL--
-WPSALR-
WIPSAL--
WVPSAL--
WNPSAL--
WFPSAL--
WAPSAL--
WKPSAL--
WEPSAL--
WHPSAL--
-WPSALY-
-WPSALT-
-WPSALM-
WWPSAL--
WTPSAL--
-WPSALA-
-WPSALV-
WDPSAL--
-WPSALH-
WMPSAL--
-WPSALI-
WPPSAL--
-WPSALQ-
-WPSALF-
-WPSALG-
-WPSALN-
-WPSALE-
WCPSAL--
-YPSALL-
-YPSALR-
YSPSAL--
YTPSAL--

	YIPSAL--		
	YNPSAL--		
	YYPSAL--		
	-YPSALH-		
	-YPSALT-		
	YLPSAL--		
	YHPSAL--		
	YVPSAL--		
	-YPSALS-		
	YKPSAL--		
	-YPSALI-		
	-YPSALN-		
	YAPSAL--		
	-YPSALY-		
	-YPSALM-		
	YQPSAL--		
	YPPSAL--		
	-YPSALK-		
	YMPSAL--		
	YGPSAL--		
	-YPSALF-		
	YDPSAL--		
	-YPSALA-		
	-YPSALV-		
	-YPSALQ-		
	-YPSALE-		
	YEPSAL--		
	-YPSALG-		
	YCPSAL--		
	-YPSALC-		
PYSL	63	840	47.60
	-DPYSLD-		
	-LPYSLP-		
	--PYSLTP		
	--PYSLPY		
	--PYSLPS		
	--PYSLGE		
	--PYSLGD		
	-RPYSLP-		
	-SPYSLD-		
	-YPYSLG-		
	ASPYSL--		
	-APYSLS-		
	ATPYSL--		
	ARPYSL--		
	-APYSLL-		
	AAPYSL--		
	ALPYSL--		
	AQPYSL--		
	-APYSLV-		

AVPYSL--
AFPYSL--
APPYSL--
AHPYSL--
-APYSLN-
-APYSLT-
-APYSLH-
AKPYSL--
-APYSLK-
AWPYSL--
ADPYSL--
-APYSLQ-
-APYSLG-
ACPYSL--
-APYSLA-
AYPYSL--
-APYSLM-
ANPYSL--
-APYSLW-
-APYSLE-
-APYSLD-
-APYSLC-
-APYSLP-
CSPYSL--
CTPYSL--
CCPYSL--
CWPYSL--
CVPYSL--
-CPYSLT-
CLPYSL--
-CPYSLK-
CGPYSL--
-CPYSLR-
CDPYSL--
CQPYSL--
-CPYSLS-
-CPYSLN-
CMPYSL--
CHPYSL--
CEPYSL--
CYPYSL--
-CPYSLV-
CAPYSL--
-CPYSLY-
-CPYSLL-
-CPYSLG-
-CPYSLA-
CKPYSL--
DLPYSL--
DTPYSL--

DRPYSL--
DSPYSL--
-DPYSLR-
DKPYSL--
DIPYSL--
DFPYSL--
-DPYSLV-
DQPYSL--
-DPYSLM-
-DPYSLI-
DWPYSL--
-DPYSLQ-
DNPYSL--
DMPYSL--
-DPYSLH-
DAPYSL--
DYPYSL--
-DPYSLT-
-DPYSLK-
DHPYSL--
-DPYSLW-
DGPYSL--
-DPYSLS-
-DPYSLN-
DPPYSL--
-DPYSLC-
DEPYSL--
DCPYSL--
-EPYSLS-
ERPYSL--
ESPYSL--
ELPYSL--
-EPYSLR-
-EPYSLL-
EYPYSL--
EVPYSL--
-EPYSLK-
-EPYSLQ-
EFPYSL--
-EPYSLH-
-EPYSLV-
ENPYSL--
-EPYSLY-
-EPYSLW-
EEPYSL--
EKPYSL--
-EPYSLN-
EAPYSL--
ETPYSL--
-EPYSLM-

EHPYSL--
EDPYSL--
EPPYSL--
-EPYSLG-
EQPYSL--
ECPYSL--
-FPYSLR-
-FPYSLK-
FSPYSL--
-FPYSLT-
-FPYSLL-
FNPYSL--
FHPYSL--
FGPYSL--
FTPYSL--
-FPYSLS-
FAPYSL--
-FPYSLV-
FEPYSL--
-FPYSLH-
-FPYSLI-
-FPYSLQ-
FIPYSL--
-FPYSLN-
-FPYSLG-
FDPYSL--
FMPYSL--
FPPYSL--
FYPYSL--
-FPYSLE-
FQPYSL--
FCPYSL--
-GPYSLS-
GRPYSL--
GSPYSL--
GGPYSL--
-GPYSLK-
GKPYSL--
-GPYSLT-
-GPYSLI-
GFPYSL--
GVPYSL--
GYPYSL--
GWPYSL--
GHPYSL--
GTPYSL--
-GPYSLN-
-GPYSLA-
GMPYSL--
-GPYSLH-

−GPYSLY−
−GPYSLM−
GNPYSL−−
−GPYSLF−
GEPYSL−−
−GPYSLW−
−GPYSLE−
GDPYSL−−
−GPYSLQ−
−GPYSLG−
GIPYSL−−
GCPYSL−−
GPPYSL−−
GQPYSL−−
HSPYSL−−
HGPYSL−−
HQPYSL−−
HVPYSL−−
HKPYSL−−
HIPYSL−−
−HPYSLQ−
−HPYSLL−
−HPYSLS−
HEPYSL−−
HWPYSL−−
−HPYSLK−
−HPYSLV−
−HPYSLW−
−HPYSLI−
HAPYSL−−
−HPYSLN−
HNPYSL−−
HFPYSL−−
HHPYSL−−
HTPYSL−−
−HPYSLA−
HPPYSL−−
−HPYSLG−
−HPYSLF−
HDPYSL−−
HYPYSL−−
−HPYSLM−
−HPYSLH−
HCPYSL−−
−IPYSLS−
ITPYSL−−
IVPYSL−−
−IPYSLR−
IGPYSL−−
IQPYSL−−

-IPYSLH-
IKPYSL--
IHPYSL--
IMPYSL--
IWPYSL--
IEPYSL--
IPPYSL--
-IPYSLA-
-IPYSLQ-
-IPYSLK-
-IPYSLT-
INPYSL--
IFPYSL--
IDPYSL--
-IPYSLG-
IAPYSL--
-IPYSLV-
-IPYSLM-
-IPYSLI-
IIPYSL--
-IPYSLE-
-IPYSLN-
KSPYSL--
-KPYSLR-
KLPYSL--
-KPYSLS-
-KPYSLT-
-KPYSLL-
KNPYSL--
KTPYSL--
KGPYSL--
KVPYSL--
KHPYSL--
KWPYSL--
KAPYSL--
KYPYSL--
-KPYSLQ-
KFPYSL--
KEPYSL--
-KPYSLA-
KKPYSL--
-KPYSLN-
KMPYSL--
KQPYSL--
-KPYSLY-
KIPYSL--
-KPYSLG-
-KPYSLH-
KPPYSL--
KDPYSL--

-KPYSLE-
-KPYSLK-
-KPYSLV-
-KPYSLD-
-KPYSLC-
KCPYSL--
LSPYSL--
LVPYSL--
-LPYSLT-
LTPYSL--
LYPYSL--
LGPYSL--
LPPYSL--
LKPYSL--
LNPYSL--
LAPYSL--
LWPYSL--
-LPYSLS-
LHPYSL--
-LPYSLH-
-LPYSLY-
-LPYSLV-
LMPYSL--
-LPYSLK-
-LPYSLN-
LQPYSL--
LEPYSL--
-LPYSLQ-
LDPYSL--
-LPYSLI-
-LPYSLM-
-LPYSLA-
-LPYSLE-
-LPYSLG-
MHPYSL--
MVPYSL--
MRPYSL--
-MPYSLL-
-MPYSLN-
MTPYSL--
-MPYSLR-
MAPYSL--
MLPYSL--
-MPYSLT-
MGPYSL--
MFPYSL--
MIPYSL--
MMPYSL--
-MPYSLS-
MNPYSL--

-MPYSLI-
-MPYSLA-
MSPYSL--
-MPYSLV-
-MPYSLK-
-MPYSLF-
-MPYSLE-
-MPYSLH-
MYPYSL--
MWPYSL--
MPPYSL--
-MPYSLQ-
-MPYSLM-
MEPYSL--
-MPYSLW-
MDPYSL--
-MPYSLG-
MKPYSL--
MQPYSL--
MCPYSL--
-MPYSLC-
-MPYSLP-
NRPYSL--
-NPYSLR-
NKPYSL--
NVPYSL--
NGPYSL--
-NPYSLT-
-NPYSLH-
NHPYSL--
-NPYSLV-
-NPYSLF-
NFPYSL--
NWPYSL--
NYPYSL--
-NPYSLY-
NEPYSL--
NAPYSL--
-NPYSLL-
NTPYSL--
NQPYSL--
NNPYSL--
NIPYSL--
-NPYSLI-
-NPYSLK-
NDPYSL--
-NPYSLQ-
-NPYSLN-
-NPYSLE-
NPPYSL--

NMPYSL--
NCPYSL--
-NPYSLD-
--PYSLNT
--PYSLRT
--PYSLTT
--PYSLRD
--PYSLRG
--PYSLLR
--PYSLTR
--PYSLTS
--PYSLLT
--PYSLSV
--PYSLSR
--PYSLRA
--PYSLRK
--PYSLLS
--PYSLAS
--PYSLYG
--PYSLWK
--PYSLSS
--PYSLGG
--PYSLSY
--PYSLWG
--PYSLLK
--PYSLSM
--PYSLSL
--PYSLFR
--PYSLKT
--PYSLNS
--PYSLMN
--PYSLMR
--PYSLLH
--PYSLYS
--PYSLVA
--PYSLQR
PGPYSL--
--PYSLEA
--PYSLIG
--PYSLTM
--PYSLMV
--PYSLKS
--PYSLIR
--PYSLAG
PKPYSL--
--PYSLSK
--PYSLMK
--PYSLMG
--PYSLVN
--PYSLTA

--PYSLKG
--PYSLIH
--PYSLHS
PTPYSL--
--PYSLRE
--PYSLLE
--PYSLVV
--PYSLKR
--PYSLVT
--PYSLVK
--PYSLSH
--PYSLRH
--PYSLGS
PYPYSL--
--PYSLRF
--PYSLFH
--PYSLAI
--PYSLTH
--PYSLSF
--PYSLWR
--PYSLTG
--PYSLSG
--PYSLAL
--PYSLWN
--PYSLVY
--PYSLQS
--PYSLLN
--PYSLGT
--PYSLVH
--PYSLQW
--PYSLMS
--PYSLRN
--PYSLKA
--PYSLFG
PMPYSL--
--PYSLTV
--PYSLYN
--PYSLTN
--PYSLTL
--PYSLSN
--PYSLTW
PEPYSL--
--PYSLWA
--PYSLVM
--PYSLEN
--PYSLAT
PAPYSL--
--PYSLYD
--PYSLWS
--PYSLVD

--PYSLTK
--PYSLRW
--PYSLQN
--PYSLIW
--PYSLHY
--PYSLHN
--PYSLRQ
--PYSLQV
--PYSLNL
--PYSLIQ
--PYSLHA
--PYSLYH
--PYSLWH
--PYSLVG
--PYSLNF
--PYSLMH
--PYSLKM
--PYSLIT
PNPYSL--
--PYSLVE
--PYSLEV
--PYSLEG
--PYSLVQ
--PYSLNM
--PYSLLQ
--PYSLKY
--PYSLFE
--PYSLAQ
PWPYSL--
PIPYSL--
--PYSLSI
--PYSLQY
--PYSLMW
--PYSLKF
--PYSLYT
--PYSLVI
--PYSLID
--PYSLHR
--PYSLFQ
--PYSLAA
--PYSLTI
--PYSLPL
--PYSLNV
--PYSLKW
--PYSLHH
--PYSLHE
--PYSLSE
--PYSLNA
--PYSLHM
--PYSLQD

--PYSLNY
--PYSLGM
--PYSLGL
--PYSLTQ
--PYSLMI
--PYSLHI
--PYSLGW
--PYSLYE
--PYSLTY
--PYSLSD
--PYSLNQ
--PYSLNH
--PYSLKH
--PYSLHW
--PYSLES
--PYSLEM
--PYSLAV
--PYSLQA
--PYSLKD
--PYSLHG
--PYSLGA
--PYSLFN
--PYSLMD
--PYSLIE
--PYSLAR
--PYSLWM
--PYSLTF
--PYSLFD
--PYSLEQ
--PYSLDS
--PYSLDG
--PYSLAM
--PYSLAE
--PYSLQK
--PYSLNG
--PYSLKQ
--PYSLGH
--PYSLAW
--PYSLAN
--PYSLWQ
--PYSLVW
--PYSLQH
--PYSLNK
--PYSLNI
--PYSLII
--PYSLFK
--PYSLET
--PYSLEH
--PYSLQI
--PYSLNN

--PYSLMQ
--PYSLGN
--PYSLTE
--PYSLQT
--PYSLQF
--PYSLNW
--PYSLKE
--PYSLAK
--PYSLAH
--PYSLYQ
--PYSLQM
--PYSLND
--PYSLMM
--PYSLHL
--PYSLHD
--PYSLWW
--PYSLWE
--PYSLNE
--PYSLIN
--PYSLHF
--PYSLGY
--PYSLFI
--PYSLEY
--PYSLER
--PYSLEF
--PYSLHQ
--PYSLGI
--PYSLEK
PFPYSL--
--PYSLHV
--PYSLGK
--PYSLGF
--PYSLEI
--PYSLCA
PHPYSL--
--PYSLWD
--PYSLVC
--PYSLKI
--PYSLEL
--PYSLDT
--PYSLCS
--PYSLAD
PDPYSL--
--PYSLYP
--PYSLQE
--PYSLDA
--PYSLCG
-PPYSLY-
--PYSLYC
--PYSLMC

--PYSLLC
--PYSLEW
--PYSLEE
--PYSLCY
--PYSLCV
--PYSLCQ
--PYSLCK
--PYSLCH
--PYSLAC
-PPYSLS-
-PPYSLN-
-QPYSLR-
QGPYSL--
QSPYSL--
QLPYSL--
QVPYSL--
-QPYSLS-
QAPYSL--
-QPYSLL-
QFPYSL--
QYPYSL--
QTPYSL--
-QPYSLK-
-QPYSLW-
-QPYSLA-
-QPYSLT-
QWPYSL--
QMPYSL--
-QPYSLI-
-QPYSLN-
QEPYSL--
QIPYSL--
-QPYSLH-
QKPYSL--
QNPYSL--
QDPYSL--
-QPYSLM-
-QPYSLE-
QHPYSL--
QPPYSL--
QCPYSL--
QQPYSL--
RIPYSL--
-RPYSLR-
RGPYSL--
RLPYSL--
-RPYSLK-
RTPYSL--
-RPYSLS-
-RPYSLA-

-RPYSLY-
-RPYSLT-
RQPYSL--
-RPYSLH-
RAPYSL--
RWPYSL--
-RPYSLG-
-RPYSLW-
REPYSL--
RYPYSL--
-RPYSLM-
-RPYSLF-
RDPYSL--
-RPYSLQ-
RHPYSL--
-RPYSLE-
-RPYSLD-
-RPYSLC-
SRPYSL--
SSPYSL--
-SPYSLL-
SNPYSL--
-SPYSLT-
SLPYSL--
SHPYSL--
SAPYSL--
-SPYSLH-
SIPYSL--
SGPYSL--
SFPYSL--
SMPYSL--
-SPYSLK-
-SPYSLA-
SQPYSL--
SKPYSL--
-SPYSLW-
-SPYSLI-
-SPYSLQ-
-SPYSLV-
-SPYSLM-
STPYSL--
SYPYSL--
-SPYSLN-
SEPYSL--
SDPYSL--
SPPYSL--
-SPYSLG-
-SPYSLE-
SCPYSL--
TSPYSL--

TVPYSL--
-TPYSLL-
TLPYSL--
TGPYSL--
-TPYSLV-
-TPYSLY-
-TPYSLS-
TMPYSL--
TAPYSL--
THPYSL--
-TPYSLH-
TYPYSL--
-TPYSLT-
TKPYSL--
TNPYSL--
TIPYSL--
-TPYSLK-
TTPYSL--
-TPYSLW-
TPPYSL--
-TPYSLQ-
TWPYSL--
-TPYSLN-
-TPYSLG-
-TPYSLA-
TFPYSL--
TEPYSL--
TQPYSL--
-TPYSLM-
-TPYSLI-
TDPYSL--
TCPYSL--
-TPYSLE-
-VPYSLR-
VSPYSL--
VLPYSL--
-VPYSLL-
VYPYSL--
VTPYSL--
VKPYSL--
-VPYSLV-
VQPYSL--
VVPYSL--
VGPYSL--
-VPYSLT-
-VPYSLH-
-VPYSLF-
-VPYSLQ-
-VPYSLN-
-VPYSLW-

VNPYSL--
VPPYSL--
VIPYSL--
VHPYSL--
-VPYSLK-
VAPYSL--
-VPYSLM-
VWPYSL--
VFPYSL--
VEPYSL--
VDPYSL--
-VPYSLE-
VCPYSL--
-WPYSLR-
WLPYSL--
-WPYSLT-
WGPYSL--
WKPYSL--
WPPYSL--
WTPYSL--
WIPYSL--
-WPYSLS-
-WPYSLK-
WAPYSL--
WSPYSL--
WEPYSL--
WMPYSL--
WDPYSL--
-WPYSLV-
WQPYSL--
-WPYSLM-
-WPYSLN-
WWPYSL--
-WPYSLH-
-WPYSLA-
WHPYSL--
-WPYSLF-
-WPYSLI-
-WPYSLG-
-WPYSLE-
YGPYSL--
-YPYSLR-
YRPYSL--
YLPYSL--
YSPYSL--
YTPYSL--
-YPYSLH-
YKPYSL--
YAPYSL--
YMPYSL--

-YPYSLL-
-YPYSLV-
YNPYSL--
YYPYSL--
YFPYSL--
YIPYSL--
YVPYSL--
YWPYSL--
YHPYSL--
-YPYSLI-
YEPYSL--
-YPYSLN-
YQPYSL--
YPPYSL--
-YPYSLK-
-YPYSLA-
-YPYSLM-
-YPYSLE-
YDPYSL--
-YPYSLY-
-YPYSLW-
PIGM 185 756 47.60
--PIGMLL
--PIGMTW
AYPIGM--
-APIGMR-
ARPIGM--
AFPIGM--
-APIGMS-
AVPIGM--
AWPIGM--
ALPIGM--
-APIGML-
AEPIGM--
-APIGMT-
-APIGMQ-
AMPIGM--
-APIGMK-
-APIGMY-
-APIGMN-
ASPIGM--
AIPIGM--
-APIGMM-
-APIGMW-
-APIGMI-
AGPIGM--
ATPIGM--
AAPIGM--
-APIGMV-
-APIGMG-

-APIGME-
-APIGMA-
APPIGM--
-APIGMH-
AHPIGM--
ADPIGM--
CCPIGM--
CNPIGM--
-CPIGMT-
CIPIGM--
CVPIGM--
-CPIGMR-
CGPIGM--
CTPIGM--
-CPIGMY-
-CPIGMM-
-CPIGMN-
-DPIGMR-
DRPIGM--
-DPIGMK-
DSPIGM--
DMPIGM--
-DPIGMI-
DYPIGM--
DTPIGM--
DKPIGM--
DIPIGM--
DVPIGM--
-DPIGMS-
-DPIGMM-
DWPIGM--
-DPIGMV-
-DPIGML-
-DPIGMH-
DLPIGM--
DHPIGM--
DGPIGM--
DFPIGM--
-DPIGMW-
DDPIGM--
DAPIGM--
-EPIGMR-
-EPIGMK-
-EPIGMT-
ETPIGM--
-EPIGMS-
ERPIGM--
-EPIGMH-
ESPIGM--
EGPIGM--

ENPIGM--
EIPIGM--
-EPIGMN-
-EPIGMM-
-EPIGMF-
EKPIGM--
EYPIGM--
EQPIGM--
-EPIGMG-
-FPIGMT-
FQPIGM--
-FPIGMR-
-FPIGMI-
FRPIGM--
-FPIGMK-
FNPIGM--
FPPIGM--
-FPIGMS-
FHPIGM--
-FPIGMV-
-FPIGMQ-
-FPIGML-
FMPIGM--
FAPIGM--
FWPIGM--
FTPIGM--
-FPIGMY-
-FPIGMF-
FGPIGM--
FDPIGM--
FYPIGM--
-FPIGME-
-FPIGMH-
FFPIGM--
-FPIGMW-
-FPIGMN-
-FPIGMA-
FLPIGM--
FEPIGM--
-GPIGML-
GLPIGM--
-GPIGMR-
GRPIGM--
GGPIGM--
-GPIGMS-
-GPIGMA-
GAPIGM--
-GPIGMT-
GQPIGM--
-GPIGMK-

-GPIGMF-
GTPIGM--
GVPIGM--
-GPIGMI-
-GPIGMN-
GHPIGM--
-GPIGMY-
-GPIGMM-
-GPIGME-
GMPIGM--
GFPIGM--
GNPIGM--
GYPIGM--
-GPIGMV-
GEPIGM--
GDPIGM--
GCPIGM--
GIPIGM--
-GPIGMG-
-HPIGML-
HMPIGM--
-HPIGMR-
-HPIGMI-
HWPIGM--
-HPIGMN-
HVPIGM--
-HPIGMT-
HRPIGM--
HGPIGM--
HYPIGM--
-HPIGMV-
-HPIGMH-
-HPIGMS-
-HPIGMQ-
HLPIGM--
HAPIGM--
HPPIGM--
-HPIGME-
-HPIGMY-
-HPIGMG-
-HPIGMD-
HNPIGM--
HIPIGM--
HHPIGM--
ISPIGM--
-IPIGML-
IRPIGM--
IVPIGM--
-IPIGMS-
-IPIGMY-

-IPIGMW-
ILPIGM--
-IPIGMR-
-IPIGMT-
-IPIGMK-
-IPIGMQ-
ITPIGM--
IMPIGM--
INPIGM--
IFPIGM--
-IPIGMF-
IDPIGM--
-IPIGMV-
-IPIGMN-
-IPIGMI-
-IPIGME-
IGPIGM--
KSPIGM--
-KPIGMI-
-KPIGML-
-KPIGMK-
KPPIGM--
KAPIGM--
KVPIGM--
-KPIGMN-
-KPIGMG-
KGPIGM--
KMPIGM--
KIPIGM--
KYPIGM--
-KPIGMH-
-KPIGMQ-
-KPIGMF-
KNPIGM--
KLPIGM--
-KPIGMY-
-KPIGMT-
-KPIGMM-
-KPIGME-
KQPIGM--
KCPIGM--
-KPIGMW-
KEPIGM--
KDPIGM--
LGPIGM--
-LPIGMW-
LSPIGM--
LMPIGM--
LIPIGM--
LRPIGM--

-LPIGMY-
-LPIGML-
-LPIGMT-
LAPIGM--
-LPIGMV-
LLPIGM--
LKPIGM--
LFPIGM--
-LPIGMM-
LYPIGM--
LDPIGM--
-LPIGMI-
-LPIGMS-
LNPIGM--
LVPIGM--
LTPIGM--
-LPIGMK-
LQPIGM--
-LPIGMF-
LHPIGM--
LPPIGM--
-LPIGMN-
-LPIGMA-
-LPIGMQ-
LWPIGM--
-LPIGMG-
-MPIGMF-
-MPIGML-
MIPIGM--
MDPIGM--
MGPIGM--
MFPIGM--
MRPIGM--
-MPIGMV-
MEPIGM--
-MPIGMT-
-MPIGMR-
MWPIGM--
-MPIGMN-
MYPIGM--
MVPIGM--
MSPIGM--
-MPIGMQ-
-MPIGMI-
MQPIGM--
-MPIGMK-
-MPIGMH-
-MPIGME-
MNPIGM--
NRPIGM--

-NPIGMR-
NLPIGM--
NVPIGM--
NGPIGM--
NEPIGM--
-NPIGMY-
NAPIGM--
NSPIGM--
NQPIGM--
NPPIGM--
NIPIGM--
NFPIGM--
-NPIGMM-
-NPIGML-
NYPIGM--
-NPIGMS-
-NPIGMF-
-NPIGMH-
NNPIGM--
-NPIGMV-
-NPIGMT-
-NPIGMQ-
NHPIGM--
NTPIGM--
-NPIGMW-
-NPIGMN-
NKPIGM--
-NPIGMK-
--PIGMGI
--PIGMTD
--PIGMRI
--PIGMTG
--PIGMIV
--PIGMRT
--PIGMST
--PIGMSM
--PIGMHS
--PIGMTS
--PIGMFS
--PIGMFQ
--PIGMVN
--PIGMRV
--PIGMVI
--PIGMRR
--PIGMLT
--PIGMLA
--PIGMSL
--PIGMMA
--PIGMVR
--PIGMSA

--PIGMSS
--PIGMWG
--PIGMTA
--PIGMRF
--PIGMLS
--PIGMYS
--PIGMVS
--PIGMTL
--PIGMRK
--PIGMLG
--PIGMFG
--PIGMYH
--PIGMRN
--PIGMRD
--PIGMKS
--PIGMIM
--PIGMVA
--PIGMLR
--PIGMFH
--PIGMVL
--PIGMTH
--PIGMNW
--PIGMMS
--PIGMMG
--PIGMIY
--PIGMIA
--PIGMHF
--PIGMAV
--PIGMVY
--PIGMSW
--PIGMSR
--PIGMNR
--PIGMMR
--PIGMME
--PIGMLH
--PIGMWS
--PIGMTV
--PIGMSN
--PIGMRQ
--PIGMQY
--PIGMQS
--PIGMMN
--PIGMLK
--PIGMLD
--PIGMKG
--PIGMIT
--PIGMGT
PGPIGM--
PDPIGM--
PTPIGM--

PIPIGM--
--PIGMWT
--PIGMVV
--PIGMTY
--PIGMSY
--PIGMKI
--PIGMIS
--PIGMFT
--PIGMAS
--PIGMTM
--PIGMSQ
--PIGMLV
--PIGMIR
--PIGMHL
PMPIGM--
--PIGMYG
--PIGMRE
--PIGMQN
--PIGMQA
--PIGMNS
--PIGMNM
--PIGMMY
--PIGMLN
--PIGMDG
--PIGMAG
--PIGMSI
--PIGMSH
--PIGMRA
--PIGMMV
--PIGMMT
--PIGMKY
--PIGMKA
--PIGMHY
--PIGMFA
--PIGMAA
--PIGMWR
--PIGMWK
--PIGMTI
--PIGMQW
--PIGMQV
--PIGMQR
--PIGMQI
--PIGMMK
--PIGMHK
--PIGMFR
--PIGMET
--PIGMYY
--PIGMVW
--PIGMVM
--PIGMTQ

--PIGMSV
--PIGMRG
--PIGMQQ
--PIGMLQ
--PIGMLE
--PIGMIK
--PIGMER
--PIGMAI
--PIGMYI
--PIGMYD
--PIGMSK
--PIGMNN
--PIGMNK
--PIGMNH
--PIGMNF
--PIGMKN
--PIGMKK
--PIGMKE
--PIGMGA
--PIGMAW
PQPIGM--
--PIGMYN
--PIGMWY
--PIGMTN
--PIGMRW
--PIGMNT
--PIGMNQ
--PIGMML
--PIGMKT
--PIGMKR
--PIGMKL
--PIGMIN
--PIGMIG
--PIGMIE
--PIGMHV
--PIGMHG
--PIGMHD
--PIGMGN
--PIGMGM
--PIGMFK
--PIGMEY
--PIGMEF
--PIGMDS
--PIGMAQ
--PIGMAM
PWPIGM--
--PIGMYT
--PIGMYR
--PIGMYE
--PIGMWV

--PIGMWD
--PIGMWC
--PIGMVK
--PIGMVD
--PIGMRL
--PIGMQG
--PIGMNI
--PIGMND
--PIGMMI
--PIGMMH
--PIGMKQ
--PIGMKF
--PIGMIQ
--PIGMIL
--PIGMHR
--PIGMHE
--PIGMHA
--PIGMGG
--PIGMFN
--PIGMFE
--PIGMEG
--PIGMAH
PYPIGM--
--PIGMWH
--PIGMWE
--PIGMVT
--PIGMVG
--PIGMSG
--PIGMSE
--PIGMQT
--PIGMQF
--PIGMNY
--PIGMNV
--PIGMNL
--PIGMLI
--PIGMKW
--PIGMKH
--PIGMIW
--PIGMIH
--PIGMIF
--PIGMHQ
--PIGMHI
--PIGMGV
--PIGMEV
--PIGMES
--PIGMAN
--PIGMAK
PHPIGM--
-PPIGMY-
--PIGMYQ

--PIGMWN
--PIGMVH
--PIGMTT
--PIGMTR
--PIGMSD
--PIGMQH
--PIGMQD
--PIGMKV
--PIGMKD
--PIGMII
--PIGMGY
--PIGMGR
--PIGMGF
--PIGMEW
--PIGMEL
--PIGMEA
--PIGMDW
--PIGMDI
--PIGMDC
--PIGMCN
--PIGMCI
--PIGMAL
PFPIGM--
PAPIGM--
-QPIGMS-
QRPIGM--
QKPIGM--
-QPIGMV-
QTPIGM--
-QPIGMR-
QVPIGM--
QFPIGM--
-QPIGMT-
-QPIGMH-
-QPIGMF-
QGPIGM--
QWPIGM--
QNPIGM--
QMPIGM--
QEPIGM--
QYPIGM--
QLPIGM--
QHPIGM--
QSPIGM--
QQPIGM--
-QPIGML-
QDPIGM--
-QPIGMI-
-QPIGMG-
-QPIGMA-

RSPIGM--
RVPIGM--
-RPIGMR-
-RPIGMS-
-RPIGMY-
-RPIGMV-
RFPIGM--
RMPIGM--
RQPIGM--
-RPIGMA-
RKPIGM--
RLPIGM--
-RPIGMQ-
RWPIGM--
-RPIGMT-
-RPIGML-
RAPIGM--
-RPIGMI-
RNPIGM--
-RPIGMF-
RTPIGM--
-RPIGMH-
-RPIGMG-
RGPIGM--
REPIGM--
RDPIGM--
RIPIGM--
RHPIGM--
RYPIGM--
-RPIGMK-
RPPIGM--
-RPIGMW-
-RPIGMN-
-SPIGMR-
-SPIGMI-
SSPIGM--
SVPIGM--
-SPIGML-
-SPIGMT-
-SPIGMA-
SRPIGM--
-SPIGMF-
-SPIGMS-
SFPIGM--
-SPIGMH-
SIPIGM--
-SPIGMQ-
SKPIGM--
SAPIGM--
SNPIGM--

```
-SPIGMM-
SYPIGM--
SQPIGM--
-SPIGMY-
-SPIGMK-
SMPIGM--
STPIGM--
-SPIGMN-
SHPIGM--
SDPIGM--
SCPIGM--
-SPIGMG-
-SPIGME-
TRPIGM--
-TPIGML-
TVPIGM--
TYPIGM--
-TPIGMR-
TFPIGM--
-TPIGMK-
-TPIGMF-
TPPIGM--
TTPIGM--
TSPIGM--
-TPIGMY-
-TPIGMS-
TCPIGM--
-TPIGMN-
-TPIGMI-
TMPIGM--
THPIGM--
TGPIGM--
-TPIGMV-
-TPIGMM-
TLPIGM--
TWPIGM--
-TPIGMQ-
-TPIGMH-
-TPIGMG-
-TPIGMA-
TIPIGM--
TEPIGM--
TDPIGM--
VVPIGM--
-VPIGMR-
VSPIGM--
VFPIGM--
VTPIGM--
VRPIGM--
-VPIGML-
```

```
-VPIGMS-
VLPIGM--
VHPIGM--
VGPIGM--
VKPIGM--
VYPIGM--
VIPIGM--
VDPIGM--
VPPIGM--
VNPIGM--
VAPIGM--
VWPIGM--
-VPIGMY-
VQPIGM--
-VPIGMQ-
-VPIGMT-
-VPIGMM-
-VPIGMI-
-VPIGMH-
-VPIGME-
-VPIGMA-
VEPIGM--
-VPIGMW-
-VPIGMN-
-VPIGMK-
-WPIGML-
WVPIGM--
WNPIGM--
WYPIGM--
-WPIGMR-
WHPIGM--
WRPIGM--
-WPIGMT-
-WPIGMF-
-WPIGMH-
WCPIGM--
WSPIGM--
WQPIGM--
-WPIGMY-
-WPIGMW-
WDPIGM--
WIPIGM--
WPPIGM--
-WPIGMS-
-WPIGMQ-
-WPIGMK-
-WPIGMG-
-WPIGMA-
WMPIGM--
-WPIGMN-
```

YLPIGM--
-YPIGMR-
YQPIGM--
YRPIGM--
-YPIGMM-
-YPIGML-
YFPIGM--
YTPIGM--
-YPIGMK-
-YPIGMT-
-YPIGMV-
YDPIGM--
YVPIGM--
-YPIGMN-
YSPIGM--
-YPIGMY-
-YPIGMI-
-YPIGMH-
YEPIGM--
YAPIGM--
YYPIGM--
-YPIGMQ-
-YPIGMF-
-YPIGMA-
YNPIGM--
YWPIGM--
-YPIGMG-
YIPIGM--

PYSM	89	814	47.59

-DPYSMI-
-FPYSMG-
-APYSMR-
ARPYSM--
-APYSML-
ASPYSM--
-APYSMF-
AVPYSM--
-APYSMM-
AWPYSM--
-APYSMA-
ATPYSM--
-APYSMS-
-APYSMY-
ALPYSM--
AHPYSM--
-APYSMG-
-APYSMK-
AMPYSM--
AIPYSM--
AFPYSM--

AEPYSM--
-APYSMT-
-APYSMH-
AKPYSM--
-APYSMV-
AYPYSM--
AAPYSM--
-APYSMN-
APPYSM--
AGPYSM--
-APYSMQ-
-APYSMI-
AQPYSM--
-APYSMW-
ADPYSM--
CHPYSM--
CEPYSM--
-CPYSMT-
CGPYSM--
CQPYSM--
-CPYSMY-
CLPYSM--
CVPYSM--
CIPYSM--
-CPYSMR-
-CPYSML-
-CPYSMH-
CKPYSM--
CFPYSM--
CCPYSM--
DRPYSM--
DWPYSM--
-DPYSMR-
-DPYSMW-
-DPYSMH-
DYPYSM--
DVPYSM--
DLPYSM--
DHPYSM--
DTPYSM--
DMPYSM--
DKPYSM--
-DPYSMY-
-DPYSMV-
-DPYSMM-
DAPYSM--
-DPYSMS-
DQPYSM--
-DPYSMQ-
DGPYSM--

DFPYSM--
DSPYSM--
-DPYSMT-
-DPYSML-
DNPYSM--
DDPYSM--
-EPYSMR-
-EPYSML-
-EPYSMW-
EHPYSM--
ETPYSM--
-EPYSMS-
-EPYSMH-
ERPYSM--
EGPYSM--
EWPYSM--
ESPYSM--
-EPYSMN-
-EPYSMA-
EAPYSM--
EVPYSM--
EQPYSM--
-EPYSMY-
-EPYSMI-
-EPYSMG-
ELPYSM--
EIPYSM--
-EPYSMV-
-EPYSMP-
FGPYSM--
-FPYSMS-
-FPYSMR-
FYPYSM--
-FPYSML-
FRPYSM--
FHPYSM--
FTPYSM--
FFPYSM--
FLPYSM--
-FPYSMM-
FQPYSM--
-FPYSMV-
-FPYSMI-
-FPYSMY-
-FPYSMT-
FMPYSM--
-FPYSMF-
-FPYSMA-
-FPYSMW-
-FPYSMQ-

-FPYSMK-
FWPYSM--
FSPYSM--
FNPYSM--
FDPYSM--
FVPYSM--
-FPYSME-
GGPYSM--
-GPYSMR-
GVPYSM--
GIPYSM--
GYPYSM--
GKPYSM--
-GPYSMK-
-GPYSMF-
GTPYSM--
GSPYSM--
GRPYSM--
GLPYSM--
-GPYSMV-
-GPYSMS-
-GPYSML-
GPPYSM--
-GPYSMQ-
-GPYSMI-
GHPYSM--
GWPYSM--
-GPYSMY-
-GPYSMH-
-GPYSMA-
GMPYSM--
GEPYSM--
GNPYSM--
-GPYSMN-
GFPYSM--
GCPYSM--
GDPYSM--
-GPYSMG-
-GPYSME-
-GPYSMC-
HTPYSM--
-HPYSMR-
HSPYSM--
-HPYSML-
HRPYSM--
-HPYSMS-
HHPYSM--
HNPYSM--
HLPYSM--
HAPYSM--

HEPYSM--
-HPYSMV-
-HPYSMF-
HMPYSM--
HYPYSM--
HQPYSM--
-HPYSMA-
-HPYSMI-
HIPYSM--
HGPYSM--
HDPYSM--
-HPYSMY-
-HPYSMT-
-HPYSMK-
-HPYSMG-
HVPYSM--
-HPYSMQ-
-HPYSMH-
-HPYSMW-
-HPYSMM-
-HPYSME-
HPPYSM--
HKPYSM--
ILPYSM--
-IPYSMR-
-IPYSML-
-IPYSMK-
ISPYSM--
IGPYSM--
-IPYSMG-
-IPYSMS-
IVPYSM--
IWPYSM--
-IPYSMN-
IFPYSM--
-IPYSMY-
INPYSM--
-IPYSMW-
-IPYSMI-
IAPYSM--
IMPYSM--
IEPYSM--
-IPYSMT-
IKPYSM--
IIPYSM--
IYPYSM--
ITPYSM--
-IPYSMF-
IPPYSM--
-IPYSMQ-

-IPYSMH-
IHPYSM--
IDPYSM--
ICPYSM--
KSPYSM--
-KPYSMR-
KVPYSM--
KPPYSM--
KRPYSM--
-KPYSMV-
-KPYSMI-
-KPYSMF-
-KPYSMA-
KTPYSM--
KEPYSM--
-KPYSML-
KLPYSM--
KAPYSM--
KYPYSM--
KWPYSM--
-KPYSMY-
-KPYSMH-
KHPYSM--
KMPYSM--
-KPYSMT-
-KPYSMN-
KGPYSM--
-KPYSMS-
-KPYSMM-
KNPYSM--
-KPYSMW-
-KPYSMQ-
-KPYSMG-
-KPYSME-
KFPYSM--
KQPYSM--
-KPYSMP-
KCPYSM--
LRPYSM--
LGPYSM--
LLPYSM--
-LPYSMR-
-LPYSML-
LKPYSM--
-LPYSMY-
LSPYSM--
-LPYSMS-
-LPYSMA-
LIPYSM--
LYPYSM--

-LPYSMM-
LQPYSM--
-LPYSMF-
LAPYSM--
LTPYSM--
-LPYSMT-
LNPYSM--
LHPYSM--
-LPYSMI-
-LPYSMH-
LMPYSM--
LWPYSM--
-LPYSMQ-
LEPYSM--
LCPYSM--
LDPYSM--
-MPYSMV-
-MPYSMT-
-MPYSMI-
MRPYSM--
-MPYSML-
-MPYSMW-
-MPYSMM-
MSPYSM--
-MPYSMR-
MGPYSM--
MYPYSM--
-MPYSMN-
MTPYSM--
-MPYSMH-
MNPYSM--
MPPYSM--
MLPYSM--
-MPYSMY-
-MPYSMA-
-MPYSMQ-
-MPYSMK-
MWPYSM--
-MPYSMG-
-MPYSMF-
MQPYSM--
MIPYSM--
MFPYSM--
MKPYSM--
MEPYSM--
MCPYSM--
NRPYSM--
-NPYSMW-
NAPYSM--
NVPYSM--

-NPYSMR-
NDPYSM--
-NPYSMT-
NLPYSM--
NYPYSM--
NSPYSM--
NKPYSM--
NPPYSM--
-NPYSMF-
-NPYSML-
-NPYSMI-
-NPYSMS-
-NPYSMQ-
NIPYSM--
NGPYSM--
NQPYSM--
-NPYSMV-
NNPYSM--
NMPYSM--
NWPYSM--
-NPYSMY-
-NPYSMM-
-NPYSMK-
-NPYSMA-
NHPYSM--
-NPYSMH-
NEPYSM--
--PYSMRT
--PYSMYR
--PYSMRI
--PYSMLS
--PYSMIS
--PYSMVR
--PYSMRH
--PYSMKS
--PYSMSG
--PYSMRN
--PYSMTT
--PYSMVS
PAPYSM--
--PYSMTL
--PYSMSV
--PYSMLR
--PYSMFR
--PYSMLM
--PYSMIV
--PYSMGA
--PYSMTA
--PYSMSN
--PYSMNT

--PYSMSK
--PYSMRV
--PYSMRF
--PYSMFS
--PYSMAA
--PYSMSL
--PYSMLA
--PYSMKK
--PYSMIE
--PYSMES
--PYSMST
--PYSMGS
--PYSMVT
--PYSMME
--PYSMEW
--PYSMSW
--PYSMRY
--PYSMKR
--PYSMWG
--PYSMVA
--PYSMTV
--PYSMRA
--PYSMMG
--PYSMHG
--PYSMGV
--PYSMYT
--PYSMTR
--PYSMRD
--PYSMWN
--PYSMTN
--PYSMSA
--PYSMNL
--PYSMMR
--PYSMMK
--PYSMWV
--PYSMWD
--PYSMVV
--PYSMRE
--PYSMKY
--PYSMVG
--PYSMQA
--PYSMMS
--PYSMLV
--PYSMLH
--PYSMLE
--PYSMIK
--PYSMEY
--PYSMLK
--PYSMLD
--PYSMII

--PYSMGG
--PYSMYV
--PYSMVH
--PYSMTM
--PYSMRG
--PYSMMN
--PYSMLQ
--PYSMIR
--PYSMFA
--PYSMEA
PVPYSM--
--PYSMTW
--PYSMTS
--PYSMQN
--PYSMKE
--PYSMIG
--PYSMHD
--PYSMGT
--PYSMFG
PNPYSM--
--PYSMYK
--PYSMVM
--PYSMSQ
--PYSMNS
--PYSMMY
--PYSMKM
--PYSMIT
--PYSMFQ
--PYSMAL
PTPYSM--
--PYSMYS
--PYSMYG
--PYSMWT
--PYSMVQ
--PYSMSH
--PYSMRM
--PYSMQT
--PYSMMW
--PYSMLN
--PYSMHR
--PYSMHM
--PYSMFF
--PYSMAW
--PYSMYA
--PYSMVI
--PYSMVE
--PYSMVD
--PYSMSM
--PYSMRQ
--PYSMNV

--PYSMNN
--PYSMNI
--PYSMNH
--PYSMNG
--PYSMMQ
--PYSMKN
--PYSMFN
--PYSMEG
--PYSMAM
PQPYSM--
PFPYSM--
--PYSMYN
--PYSMTQ
--PYSMTK
--PYSMSF
--PYSMQS
--PYSMQI
--PYSMLI
--PYSMKW
--PYSMKV
--PYSMKQ
--PYSMIH
--PYSMHS
--PYSMHL
PHPYSM--
--PYSMWR
--PYSMVN
--PYSMTI
--PYSMTG
--PYSMSI
--PYSMQK
--PYSMNR
--PYSMNQ
--PYSMMT
--PYSMKG
--PYSMIY
--PYSMIA
--PYSMHV
--PYSMGL
--PYSMFH
--PYSMAT
PKPYSM--
--PYSMWK
--PYSMSY
--PYSMQR
--PYSMNW
--PYSMMM
--PYSMMH
--PYSMMA
--PYSMIN

--PYSMIM
--PYSMHK
--PYSMHI
--PYSMEV
--PYSMET
--PYSMEF
--PYSMAN
--PYSMAG
PDPYSM--
--PYSMQY
--PYSMQF
--PYSMNM
--PYSMND
--PYSMKI
--PYSMIW
--PYSMIQ
--PYSMHA
--PYSMGM
--PYSMGF
--PYSMEM
--PYSMEH
PIPYSM--
--PYSMVC
--PYSMTH
--PYSMRW
--PYSMRC
--PYSMQD
--PYSMPL
--PYSMNK
--PYSMMD
--PYSMGR
--PYSMEQ
--PYSMAV
--PYSMAR
--PYSMAK
PWPYSM--
--PYSMYH
--PYSMWH
--PYSMWA
--PYSMVK
--PYSMTE
--PYSMSD
--PYSMQM
--PYSMQL
--PYSMQG
--PYSMNF
--PYSMNA
--PYSMIC
--PYSMHY
--PYSMHH

--PYSMGW
--PYSMGI
--PYSMER
--PYSMEN
--PYSMEL
--PYSMEK
--PYSMDS
--PYSMDG
--PYSMCM
--PYSMCH
--PYSMAQ
--PYSMAD
PYPYSM--
-PPYSMY-
-PPYSMG-
PMPYSM--
PGPYSM--
QRPYSM--
QGPYSM--
QSPYSM--
-QPYSMS-
-QPYSMT-
QTPYSM--
-QPYSMF-
-QPYSMN-
QEPYSM--
-QPYSML-
-QPYSMK-
-QPYSMI-
QHPYSM--
-QPYSMW-
QVPYSM--
QPPYSM--
QNPYSM--
QLPYSM--
QWPYSM--
QIPYSM--
QFPYSM--
-QPYSMV-
-QPYSMY-
-QPYSMH-
QKPYSM--
-QPYSMM-
-QPYSME-
-QPYSMR-
-QPYSMG-
QCPYSM--
-RPYSMS-
RLPYSM--
-RPYSMR-

-RPYSML-
RSPYSM--
-RPYSMT-
-RPYSMQ-
-RPYSMM-
RWPYSM--
-RPYSMW-
-RPYSMK-
-RPYSMH-
-RPYSMV-
RTPYSM--
-RPYSMI-
RMPYSM--
RVPYSM--
RPPYSM--
-RPYSMN-
RKPYSM--
RNPYSM--
RIPYSM--
-RPYSMG-
RFPYSM--
-RPYSMY-
-RPYSMF-
RAPYSM--
RHPYSM--
RDPYSM--
-RPYSMA-
-RPYSME-
RGPYSM--
REPYSM--
RYPYSM--
RCPYSM--
-SPYSMR-
SLPYSM--
SVPYSM--
STPYSM--
SFPYSM--
SKPYSM--
SNPYSM--
SSPYSM--
SYPYSM--
-SPYSMS-
SGPYSM--
SQPYSM--
SWPYSM--
-SPYSMM-
-SPYSMI-
SAPYSM--
-SPYSMF-
-SPYSML-

SIPYSM--
-SPYSMV-
-SPYSMK-
-SPYSMT-
SMPYSM--
-SPYSMY-
-SPYSMA-
-SPYSMW-
SHPYSM--
SPPYSM--
-SPYSMQ-
-SPYSMH-
-SPYSMC-
SDPYSM--
-SPYSMN-
-SPYSMG-
-SPYSME-
-TPYSMR-
-TPYSMK-
TLPYSM--
TRPYSM--
-TPYSMV-
-TPYSMT-
-TPYSMI-
TGPYSM--
TVPYSM--
-TPYSMY-
TSPYSM--
-TPYSMM-
-TPYSMF-
-TPYSMH-
-TPYSMS-
TYPYSM--
TKPYSM--
TFPYSM--
TMPYSM--
-TPYSMA-
TTPYSM--
TAPYSM--
TIPYSM--
TNPYSM--
-TPYSMG-
TPPYSM--
THPYSM--
-TPYSMN-
-TPYSMW-
-TPYSMQ-
TEPYSM--
TDPYSM--
TCPYSM--

VKPYSM--
VVPYSM--
VSPYSM--
VRPYSM--
-VPYSMV-
VLPYSM--
-VPYSMS-
-VPYSMT-
VNPYSM--
VMPYSM--
-VPYSMR-
VYPYSM--
VQPYSM--
VWPYSM--
-VPYSMA-
VGPYSM--
VEPYSM--
VTPYSM--
-VPYSML-
VHPYSM--
-VPYSMQ-
-VPYSMK-
VIPYSM--
-VPYSMN-
-VPYSMG-
-VPYSMI-
-VPYSMH-
VAPYSM--
-VPYSMW-
-VPYSMM-
VPPYSM--
VDPYSM--
VFPYSM--
VCPYSM--
WVPYSM--
WSPYSM--
-WPYSMK-
WAPYSM--
WRPYSM--
WGPYSM--
WIPYSM--
-WPYSMT-
WKPYSM--
-WPYSMI-
WTPYSM--
-WPYSMY-
-WPYSMR-
-WPYSMF-
WPPYSM--
-WPYSMV-

-WPYSME-
-WPYSMQ-
WQPYSM--
-WPYSMW-
-WPYSMG-
WLPYSM--
-WPYSMN-
-WPYSMM-
-WPYSMA-
WNPYSM--
WDPYSM--
YVPYSM--
YRPYSM--
-YPYSMK-
YTPYSM--
-YPYSMH-
-YPYSML-
-YPYSMV-
YAPYSM--
-YPYSMR-
-YPYSMY-
-YPYSMS-
-YPYSMQ-
-YPYSMI-
YLPYSM--
YGPYSM--
YQPYSM--
-YPYSMG-
YDPYSM--
YWPYSM--
-YPYSMA-
YPPYSM--
-YPYSMT-
YKPYSM--
YHPYSM--
YYPYSM--
-YPYSMM-
YSPYSM--
-YPYSMW-
-YPYSMF-
YMPYSM--
YIPYSM--
YEPYSM--
PHAI 266 714 47.59
PGPHAI--
-RPHAIR-
-APHAIK-
-APHAIR-
-APHAIL-
-APHAIV-

AYPHAI--
ARPHAI--
-APHAIN-
AKPHAI--
-APHAIF-
AVPHAI--
-APHAIH-
-APHAIT-
-APHAIQ-
AGPHAI--
AFPHAI--
AQPHAI--
ANPHAI--
ATPHAI--
-APHAIY-
AMPHAI--
ALPHAI--
AHPHAI--
ASPHAI--
-APHAIE-
AIPHAI--
AEPHAI--
ADPHAI--
-APHAII-
CKPHAI--
CAPHAI--
CSPHAI--
CMPHAI--
CIPHAI--
CRPHAI--
-CPHAIS-
-CPHAIL-
-CPHAIH-
CNPHAI--
DSPHAI--
DRPHAI--
DQPHAI--
DYPHAI--
DGPHAI--
DVPHAI--
-DPHAIL-
DEPHAI--
DWPHAI--
DLPHAI--
EVPHAI--
-EPHAIY-
EGPHAI--
-EPHAIV-
EIPHAI--
ETPHAI--

ESPHAI--
EQPHAI--
-EPHAIF-
ELPHAI--
EWPHAI--
ERPHAI--
-EPHAIK-
ENPHAI--
EAPHAI--
EYPHAI--
-EPHAIR-
-EPHAIQ-
-EPHAIM-
EDPHAI--
-FPHAIR-
-FPHAIT-
FVPHAI--
FIPHAI--
FMPHAI--
FRPHAI--
-FPHAIV-
-FPHAIK-
-FPHAIH-
FKPHAI--
FDPHAI--
FLPHAI--
-FPHAIL-
-FPHAIF-
FGPHAI--
FWPHAI--
-FPHAIS-
FQPHAI--
FEPHAI--
FPPHAI--
-FPHAIN-
FFPHAI--
FYPHAI--
-FPHAIW-
-FPHAIQ-
-FPHAII-
GGPHAI--
-GPHAIW-
-GPHAIL-
GYPHAI--
GIPHAI--
-GPHAIY-
-GPHAIS-
GWPHAI--
GVPHAI--
GTPHAI--

```
-GPHAIV-
GSPHAI--
-GPHAIR-
-GPHAIM-
GKPHAI--
-GPHAIT-
GAPHAI--
GRPHAI--
GEPHAI--
-GPHAIG-
-GPHAIA-
GNPHAI--
GLPHAI--
GQPHAI--
-GPHAIK-
-GPHAIQ-
HRPHAI--
HKPHAI--
HNPHAI--
HTPHAI--
HMPHAI--
HYPHAI--
-HPHAIS-
HQPHAI--
-HPHAIQ-
HWPHAI--
-HPHAIW-
HLPHAI--
HGPHAI--
-HPHAIY-
-HPHAIV-
-HPHAIK-
HPPHAI--
HIPHAI--
HHPHAI--
HCPHAI--
HVPHAI--
HSPHAI--
-HPHAIP-
-HPHAIM-
-HPHAII-
-HPHAIF-
HEPHAI--
IVPHAI--
ISPHAI--
-IPHAIR-
ITPHAI--
-IPHAIF-
IMPHAI--
ILPHAI--
```

INPHAI--
IRPHAI--
-IPHAIS-
IYPHAI--
IIPHAI--
IAPHAI--
-IPHAIY-
-IPHAIH-
IQPHAI--
IPPHAI--
-IPHAIM-
-IPHAIA-
-IPHAIV-
-IPHAIL-
IHPHAI--
IDPHAI--
IWPHAI--
-IPHAIN-
-IPHAII-
IKPHAI--
IGPHAI--
-IPHAID-
KSPHAI--
-KPHAIL-
-KPHAIR-
KRPHAI--
KTPHAI--
KGPHAI--
KWPHAI--
KLPHAI--
KIPHAI--
KVPHAI--
-KPHAIY-
-KPHAII-
-KPHAIT-
KFPHAI--
-KPHAIS-
-KPHAIA-
-KPHAIV-
-KPHAIK-
KPPHAI--
-KPHAIW-
-KPHAIM-
-KPHAIH-
-KPHAIG-
KNPHAI--
KQPHAI--
-KPHAIF-
-KPHAIE-
KHPHAI--

KCPHAI--
KAPHAI--
-LPHAIR-
LLPHAI--
LHPHAI--
LPPHAI--
LRPHAI--
LQPHAI--
LKPHAI--
LTPHAI--
LAPHAI--
-LPHAIT-
-LPHAIL-
LYPHAI--
LIPHAI--
-LPHAIS-
-LPHAIW-
LEPHAI--
LVPHAI--
-LPHAIK-
LGPHAI--
-LPHAIH-
LFPHAI--
-LPHAIY-
-LPHAIV-
-LPHAIN-
-LPHAII-
LNPHAI--
-LPHAIM-
-LPHAIF-
LDPHAI--
MVPHAI--
MYPHAI--
-MPHAIR-
-MPHAIM-
MWPHAI--
MLPHAI--
MSPHAI--
MPPHAI--
MRPHAI--
MFPHAI--
-MPHAIL-
MMPHAI--
MKPHAI--
MTPHAI--
-MPHAIW-
-MPHAIF-
-MPHAIS-
MGPHAI--
-MPHAIV-

```
-MPHAIT-
-MPHAII-
MNPHAI--
MAPHAI--
-MPHAIK-
-MPHAIA-
MIPHAI--
MHPHAI--
-MPHAIY-
MEPHAI--
NMPHAI--
NYPHAI--
NSPHAI--
-NPHAIY-
NRPHAI--
-NPHAIT-
NGPHAI--
NFPHAI--
NAPHAI--
NVPHAI--
NTPHAI--
NNPHAI--
NKPHAI--
-NPHAIV-
-NPHAIF-
-NPHAIW-
-NPHAIR-
NLPHAI--
NIPHAI--
NWPHAI--
-NPHAII-
--PHAIIS
--PHAILS
--PHAIRR
--PHAIKH
--PHAISL
--PHAIYL
--PHAIYA
--PHAIFR
--PHAIYQ
--PHAIVS
--PHAILT
--PHAIRF
--PHAIRD
--PHAIWL
--PHAISS
--PHAIRS
PRPHAI--
--PHAIFY
--PHAIRA
```

--PHAIFA
--PHAISA
--PHAINL
--PHAISK
--PHAILG
--PHAIYH
--PHAITT
--PHAIKS
--PHAIKG
--PHAIWV
--PHAITR
--PHAIRI
--PHAIFV
--PHAITS
--PHAIRH
--PHAIST
--PHAISR
--PHAIRQ
--PHAIFN
--PHAIYM
--PHAIYG
--PHAIYF
--PHAISN
--PHAIPL
--PHAIMY
--PHAILF
--PHAIIR
--PHAIFD
--PHAIVA
--PHAIFH
--PHAIYY
--PHAISV
--PHAIRK
--PHAINV
--PHAIHS
--PHAIGL
--PHAIFS
PFPHAI--
PVPHAI--
--PHAIVM
--PHAITM
--PHAISW
--PHAIQG
--PHAINM
--PHAILK
--PHAIGA
--PHAIYK
--PHAITI
--PHAISI
--PHAILR

--PHAILN
--PHAIIY
--PHAIAY
--PHAITK
--PHAIMT
--PHAIMG
PKPHAI--
--PHAIYE
--PHAIVY
--PHAIVL
--PHAISY
--PHAIQA
--PHAILA
--PHAIKR
--PHAIGI
--PHAIFL
--PHAIWW
--PHAIVH
--PHAISF
--PHAIRT
--PHAILW
--PHAIKT
--PHAIIT
--PHAIIA
--PHAIHT
--PHAIHR
--PHAIHL
--PHAIFQ
--PHAIAL
PWPHAI--
--PHAIYV
--PHAIYT
--PHAIYS
--PHAIYI
--PHAIWY
--PHAIWT
--PHAIWH
--PHAIVK
--PHAITF
--PHAIRG
--PHAIQI
--PHAILE
--PHAIHG
--PHAIFW
--PHAIFT
--PHAIFF
--PHAIAS
--PHAIYR
--PHAIWS
--PHAIWK

--PHAITY
--PHAITA
--PHAISM
--PHAIRW
--PHAIRN
--PHAIQM
--PHAIMI
--PHAILQ
--PHAIKD
--PHAIIM
--PHAIEG
--PHAIAR
--PHAIYN
--PHAIWQ
--PHAIVR
--PHAIVN
--PHAIVI
--PHAIVE
--PHAITV
--PHAITN
--PHAINF
--PHAIMK
--PHAIMF
--PHAIKV
--PHAIKL
--PHAIIK
--PHAIIG
--PHAIGY
--PHAIAA
--PHAIWM
--PHAIWI
--PHAIVW
--PHAIVQ
--PHAIVG
--PHAITL
--PHAITG
--PHAISG
--PHAIQS
--PHAIQH
--PHAIPY
--PHAIPI
--PHAIPF
--PHAINY
--PHAIMV
--PHAIMR
--PHAIML
--PHAILY
--PHAILM
--PHAIII
--PHAIIF

--PHAIHK
--PHAIGS
--PHAIFI
--PHAIEW
--PHAIES
--PHAIAG
--PHAIAF
PMPHAI--
PIPHAI--
--PHAIYW
--PHAIWA
--PHAIVT
--PHAITQ
--PHAISH
--PHAIRE
--PHAIQT
--PHAIQQ
--PHAIQK
--PHAINN
--PHAING
--PHAIMQ
--PHAIMN
--PHAILV
--PHAILI
--PHAIKK
--PHAIIV
--PHAIIQ
--PHAIIL
--PHAIGW
--PHAIGT
--PHAIGG
--PHAIGF
--PHAIEC
PEPHAI--
PAPHAI--
PQPHAI--
-PPHAIY-
-PPHAIS-
-PPHAIP-
PNPHAI--
PLPHAI--
PHPHAI--
--PHAIWN
--PHAIWE
--PHAIVD
--PHAITE
--PHAISD
--PHAIQV
--PHAIQF
--PHAIQE

--PHAIPM
--PHAINI
--PHAIMW
--PHAILH
--PHAILD
--PHAIKY
--PHAIHN
--PHAIHD
--PHAIGV
--PHAIFK
--PHAIEY
--PHAIAW
--PHAIAV
--PHAIAM
--PHAIAK
PDPHAI--
PCPHAI--
QRPHAI--
QGPHAI--
-QPHAIY-
QLPHAI--
-QPHAIT-
QTPHAI--
-QPHAIW-
-QPHAIR-
QNPHAI--
-QPHAIS-
-QPHAIL-
QSPHAI--
QIPHAI--
QEPHAI--
QPPHAI--
-QPHAIV-
-QPHAIQ-
-QPHAIM-
-QPHAIK-
QDPHAI--
QAPHAI--
RGPHAI--
-RPHAIL-
RRPHAI--
-RPHAIY-
RVPHAI--
RSPHAI--
-RPHAIT-
RIPHAI--
-RPHAIS-
-RPHAII-
RLPHAI--
RTPHAI--

-RPHAIM-
-RPHAIQ-
RPPHAI--
RKPHAI--
REPHAI--
-RPHAIK-
-RPHAIH-
RFPHAI--
RQPHAI--
RYPHAI--
RWPHAI--
-RPHAIN-
-RPHAIF-
-RPHAIW-
-RPHAIA-
RNPHAI--
-SPHAIL-
SGPHAI--
SVPHAI--
-SPHAIY-
SLPHAI--
-SPHAIR-
SIPHAI--
-SPHAIS-
-SPHAIM-
SKPHAI--
-SPHAIQ-
SEPHAI--
STPHAI--
-SPHAIW-
-SPHAII-
-SPHAIT-
SYPHAI--
SMPHAI--
SWPHAI--
SHPHAI--
-SPHAIH-
SPPHAI--
SAPHAI--
-SPHAIF-
SFPHAI--
SSPHAI--
-SPHAIV-
-SPHAIK-
-SPHAIA-
SNPHAI--
SDPHAI--
SQPHAI--
-SPHAIE-
-TPHAIR-

TRPHAI--
TPPHAI--
TAPHAI--
TGPHAI--
-TPHAIS-
TTPHAI--
-TPHAIY-
-TPHAIQ-
TYPHAI--
-TPHAIL-
TVPHAI--
-TPHAIW-
TNPHAI--
TLPHAI--
TSPHAI--
-TPHAIV-
TMPHAI--
TQPHAI--
-TPHAIT-
TFPHAI--
TWPHAI--
-TPHAIK-
-TPHAII-
-TPHAIF-
TKPHAI--
TDPHAI--
VGPHAI--
VNPHAI--
VKPHAI--
-VPHAIR-
-VPHAIM-
-VPHAIK-
VSPHAI--
-VPHAIY-
VYPHAI--
VIPHAI--
VLPHAI--
-VPHAIV-
VWPHAI--
-VPHAIW-
VMPHAI--
VVPHAI--
VPPHAI--
-VPHAII-
-VPHAIA-
VTPHAI--
-VPHAIF-
VRPHAI--
VFPHAI--
VQPHAI--

-VPHAIL-
-VPHAIS-
VAPHAI--
-VPHAIT-
-VPHAIQ-
-VPHAIN-
VHPHAI--
-VPHAIE-
-VPHAID-
-VPHAIC-
VDPHAI--
VCPHAI--
WRPHAI--
WAPHAI--
-WPHAIL-
WQPHAI--
-WPHAIQ-
WNPHAI--
WGPHAI--
WEPHAI--
-WPHAIR-
WTPHAI--
WIPHAI--
WHPHAI--
WDPHAI--
-WPHAIY-
WLPHAI--
WFPHAI--
-WPHAIT-
-WPHAIE-
-WPHAIV-
-WPHAIS-
-WPHAIA-
WKPHAI--
YTPHAI--
YGPHAI--
-YPHAIL-
YAPHAI--
YFPHAI--
-YPHAIY-
-YPHAIM-
YLPHAI--
YWPHAI--
YNPHAI--
YYPHAI--
YSPHAI--
-YPHAIH-
YIPHAI--
-YPHAIW-
-YPHAIS-

	-YPHAII-		
	YQPHAI--		
	-YPHAIN-		
	YRPHAI--		
	-YPHAIF-		
	YVPHAI--		
	-YPHAIR-		
	YHPHAI--		
PLGY	19	881	47.55
	--PLGYPS		
	--PLGYIP		
	-APLGYS-		
	-APLGYI-		
	-APLGYG-		
	AQPLGY--		
	ALPLGY--		
	AIPLGY--		
	ASPLGY--		
	-APLGYR-		
	-APLGYK-		
	AHPLGY--		
	-APLGYL-		
	-APLGYA-		
	AFPLGY--		
	-APLGYF-		
	AVPLGY--		
	-APLGYV-		
	-APLGYM-		
	AAPLGY--		
	AWPLGY--		
	-APLGYQ-		
	-APLGYE-		
	ATPLGY--		
	-APLGYH-		
	-APLGYT-		
	AGPLGY--		
	APPLGY--		
	-APLGYN-		
	AYPLGY--		
	ANPLGY--		
	AEPLGY--		
	ACPLGY--		
	AKPLGY--		
	AMPLGY--		
	ADPLGY--		
	-APLGYD-		
	CGPLGY--		
	CSPLGY--		
	CKPLGY--		
	CHPLGY--		

CRPLGY--
CAPLGY--
CWPLGY--
CTPLGY--
CCPLGY--
-CPLGYT-
-CPLGYK-
CVPLGY--
-CPLGYV-
-CPLGYS-
CDPLGY--
CYPLGY--
-CPLGYQ-
-CPLGYN-
-CPLGYE-
-CPLGYA-
CMPLGY--
CLPLGY--
-DPLGYT-
DLPLGY--
-DPLGYL-
DWPLGY--
-DPLGYM-
DRPLGY--
DQPLGY--
-DPLGYI-
DTPLGY--
-DPLGYF-
-DPLGYS-
DGPLGY--
DSPLGY--
-DPLGYR-
DAPLGY--
-DPLGYK-
DMPLGY--
-DPLGYH-
-DPLGYA-
DKPLGY--
DIPLGY--
DFPLGY--
DVPLGY--
-DPLGYV-
DNPLGY--
DYPLGY--
-DPLGYY-
DDPLGY--
DCPLGY--
-DPLGYQ-
-DPLGYW-
-DPLGYG-

DHPLGY--
DEPLGY--
ERPLGY--
-EPLGYI-
EVPLGY--
ETPLGY--
-EPLGYY-
EAPLGY--
EMPLGY--
EYPLGY--
-EPLGYS-
-EPLGYF-
EKPLGY--
ESPLGY--
-EPLGYR-
EDPLGY--
-EPLGYW-
-EPLGYQ-
EIPLGY--
EWPLGY--
-EPLGYG-
EEPLGY--
ENPLGY--
ELPLGY--
-EPLGYK-
-EPLGYV-
EFPLGY--
-EPLGYN-
-EPLGYE-
-EPLGYA-
EQPLGY--
EGPLGY--
EHPLGY--
FSPLGY--
-FPLGYK-
-FPLGYS-
FEPLGY--
FQPLGY--
-FPLGYR-
FMPLGY--
FVPLGY--
-FPLGYT-
-FPLGYV-
-FPLGYN-
-FPLGYY-
-FPLGYM-
-FPLGYA-
FPPLGY--
-FPLGYG-
FTPLGY--

FDPLGY--
FGPLGY--
FYPLGY--
-FPLGYQ-
FFPLGY--
-FPLGYW-
FAPLGY--
-FPLGYH-
FNPLGY--
-FPLGYI-
FHPLGY--
FWPLGY--
FIPLGY--
-FPLGYE-
-GPLGYS-
GSPLGY--
-GPLGYL-
-GPLGYR-
-GPLGYH-
GRPLGY--
GTPLGY--
GGPLGY--
-GPLGYI-
GVPLGY--
-GPLGYA-
-GPLGYN-
-GPLGYV-
GFPLGY--
GKPLGY--
GAPLGY--
-GPLGYF-
GQPLGY--
GYPLGY--
-GPLGYE-
GNPLGY--
-GPLGYY-
GIPLGY--
-GPLGYK-
-GPLGYG-
-GPLGYW-
-GPLGYT-
GMPLGY--
GDPLGY--
-GPLGYM-
GPPLGY--
-GPLGYQ-
GEPLGY--
-GPLGYC-
-GPLGYD-
GHPLGY--

-HPLGYS-
-HPLGYN-
-HPLGYL-
HAPLGY--
-HPLGYA-
HFPLGY--
HIPLGY--
HSPLGY--
-HPLGYV-
-HPLGYK-
HTPLGY--
-HPLGYR-
HWPLGY--
-HPLGYW-
HMPLGY--
HNPLGY--
HLPLGY--
-HPLGYY-
-HPLGYT-
HHPLGY--
HGPLGY--
HPPLGY--
HVPLGY--
-HPLGYM-
HYPLGY--
HKPLGY--
-HPLGYE-
HQPLGY--
-HPLGYI-
HEPLGY--
HDPLGY--
-HPLGYQ-
-HPLGYG-
-HPLGYH-
ITPLGY--
IGPLGY--
IRPLGY--
IAPLGY--
-IPLGYA-
ISPLGY--
-IPLGYK-
-IPLGYY-
IQPLGY--
-IPLGYM-
-IPLGYR-
-IPLGYE-
-IPLGYH-
IDPLGY--
-IPLGYI-
INPLGY--

IMPLGY--
IHPLGY--
IEPLGY--
-IPLGYG-
-IPLGYS-
-IPLGYN-
-IPLGYW-
-IPLGYF-
IWPLGY--
IIPLGY--
-IPLGYQ-
IKPLGY--
IYPLGY--
ICPLGY--
KSPLGY--
-KPLGYR-
-KPLGYS-
KGPLGY--
KLPLGY--
KYPLGY--
-KPLGYF-
-KPLGYK-
-KPLGYI-
-KPLGYT-
KAPLGY--
-KPLGYA-
KWPLGY--
-KPLGYH-
KPPLGY--
KNPLGY--
-KPLGYG-
KHPLGY--
KTPLGY--
KVPLGY--
KIPLGY--
-KPLGYM-
KFPLGY--
-KPLGYN-
-KPLGYY-
-KPLGYE-
KEPLGY--
-KPLGYW-
KMPLGY--
KQPLGY--
-LPLGYW-
-LPLGYL-
LGPLGY--
LSPLGY--
-LPLGYS-
-LPLGYR-

LAPLGY--
-LPLGYH-
LTPLGY--
LIPLGY--
LEPLGY--
LVPLGY--
LPPLGY--
LDPLGY--
LNPLGY--
-LPLGYQ-
-LPLGYT-
LFPLGY--
-LPLGYK-
-LPLGYI-
-LPLGYN-
-LPLGYA-
LQPLGY--
LMPLGY--
LWPLGY--
-LPLGYG-
-LPLGYF-
LHPLGY--
-LPLGYE-
MSPLGY--
MGPLGY--
MIPLGY--
MFPLGY--
-MPLGYS-
-MPLGYL-
-MPLGYG-
MTPLGY--
MLPLGY--
-MPLGYM-
-MPLGYH-
MNPLGY--
-MPLGYT-
MVPLGY--
MKPLGY--
-MPLGYV-
-MPLGYQ-
-MPLGYW-
MQPLGY--
-MPLGYI-
MAPLGY--
-MPLGYN-
MYPLGY--
-MPLGYA-
MHPLGY--
MDPLGY--
-MPLGYY-

MMPLGY--
MEPLGY--
-MPLGYK-
MCPLGY--
MPPLGY--
-NPLGYR-
-NPLGYS-
NSPLGY--
-NPLGYI-
NAPLGY--
NQPLGY--
NKPLGY--
NVPLGY--
-NPLGYL-
NTPLGY--
NWPLGY--
-NPLGYY-
-NPLGYH-
NFPLGY--
NLPLGY--
NGPLGY--
NPPLGY--
NIPLGY--
-NPLGYN-
-NPLGYK-
NNPLGY--
-NPLGYV-
-NPLGYA-
NMPLGY--
NYPLGY--
NEPLGY--
-NPLGYF-
NCPLGY--
NHPLGY--
NDPLGY--
-NPLGYQ-
-NPLGYW-
-NPLGYG-
--PLGYWN
--PLGYLR
--PLGYRL
--PLGYRV
--PLGYRY
--PLGYVS
--PLGYRD
--PLGYSH
--PLGYLG
--PLGYST
--PLGYLK
--PLGYSA

--PLGYTR
--PLGYMI
--PLGYSS
--PLGYKG
--PLGYYK
--PLGYSK
--PLGYVH
--PLGYIR
--PLGYRT
--PLGYMR
--PLGYWR
--PLGYSI
--PLGYLA
--PLGYLT
--PLGYSV
--PLGYVG
--PLGYLQ
--PLGYSN
--PLGYMH
--PLGYLH
--PLGYQR
--PLGYTN
--PLGYKY
--PLGYYT
--PLGYMK
--PLGYKI
--PLGYFR
PTPLGY--
--PLGYNS
--PLGYAS
--PLGYTT
--PLGYSQ
--PLGYVI
--PLGYTH
--PLGYRN
--PLGYTS
--PLGYML
--PLGYLI
--PLGYVY
--PLGYQA
--PLGYFK
--PLGYTD
--PLGYHG
--PLGYAI
--PLGYLN
--PLGYKQ
--PLGYGW
--PLGYAY
--PLGYYM
--PLGYWQ

```
--PLGYSD
--PLGYNK
--PLGYVT
--PLGYRK
--PLGYLM
--PLGYKS
--PLGYFS
--PLGYNT
PFPLGY--
PRPLGY--
--PLGYNR
--PLGYYS
--PLGYRM
--PLGYQK
--PLGYAW
--PLGYAA
PKPLGY--
--PLGYRE
--PLGYIS
--PLGYSG
--PLGYRA
--PLGYQV
--PLGYKR
--PLGYKN
--PLGYGL
--PLGYVM
--PLGYRH
--PLGYQG
--PLGYIT
--PLGYGS
--PLGYFM
--PLGYSF
--PLGYKL
--PLGYIN
--PLGYHH
--PLGYGG
--PLGYAR
--PLGYQN
--PLGYQM
--PLGYNV
--PLGYMD
--PLGYVN
--PLGYTA
--PLGYNW
--PLGYMG
--PLGYIM
--PLGYIG
--PLGYFT
--PLGYTY
--PLGYQY
```

--PLGYNL
--PLGYMS
--PLGYKF
--PLGYHV
--PLGYHR
--PLGYTG
--PLGYSE
--PLGYLD
--PLGYII
--PLGYHT
--PLGYHS
--PLGYGI
--PLGYTV
--PLGYFG
--PLGYAK
--PLGYIQ
--PLGYHF
--PLGYVQ
--PLGYTI
--PLGYRQ
--PLGYGF
--PLGYGA
--PLGYAV
--PLGYSM
--PLGYMT
PQPLGY--
--PLGYQS
--PLGYKT
--PLGYAN
PNPLGY--
--PLGYYG
--PLGYVW
--PLGYVD
--PLGYTM
--PLGYSY
--PLGYQW
--PLGYNQ
PEPLGY--
--PLGYWE
--PLGYVA
--PLGYTK
--PLGYMN
--PLGYIA
--PLGYGV
--PLGYVK
--PLGYNA
--PLGYFH
--PLGYAL
--PLGYTE
--PLGYNY

--PLGYNI
--PLGYIK
--PLGYGR
--PLGYEY
--PLGYYH
--PLGYWV
--PLGYVF
--PLGYNN
--PLGYNM
--PLGYKD
--PLGYGM
--PLGYER
--PLGYEG
PGPLGY--
--PLGYWD
--PLGYNH
--PLGYNF
--PLGYEA
--PLGYAT
PIPLGY--
--PLGYWK
--PLGYMW
--PLGYIY
--PLGYIE
--PLGYHM
--PLGYHE
--PLGYHD
--PLGYGY
--PLGYGN
--PLGYFN
--PLGYEN
--PLGYDG
--PLGYAF
--PLGYYD
--PLGYQQ
--PLGYNG
--PLGYKE
--PLGYHA
--PLGYGK
--PLGYEV
--PLGYEI
--PLGYDA
--PLGYAH
--PLGYYQ
--PLGYWG
--PLGYVE
--PLGYQH
--PLGYMA
--PLGYKM
--PLGYKH

--PLGYEH
--PLGYAE
--PLGYAD
--PLGYWW
--PLGYWT
--PLGYWH
--PLGYWA
--PLGYQI
--PLGYLE
--PLGYIH
--PLGYHW
--PLGYFQ
--PLGYFD
--PLGYEW
--PLGYEL
--PLGYTF
--PLGYQF
--PLGYME
--PLGYGQ
--PLGYES
--PLGYDS
PYPLGY--
PWPLGY--
PMPLGY--
--PLGYYN
--PLGYMQ
--PLGYGH
--PLGYCL
--PLGYAM
PHPLGY--
--PLGYRP
--PLGYIW
--PLGYID
--PLGYHI
--PLGYET
--PLGYDT
--PLGYCN
PPPLGY--
--PLGYNC
--PLGYGD
--PLGYFE
PCPLGY--
-PPLGYV-
-PPLGYN-
--PLGYQE
--PLGYPV
--PLGYPR
--PLGYNE
--PLGYND
--PLGYIC

--PLGYHK
--PLGYHC
--PLGYGE
--PLGYDW
--PLGYDM
--PLGYCR
--PLGYAC
-QPLGYR-
QLPLGY--
-QPLGYY-
-QPLGYL-
QGPLGY--
QSPLGY--
QQPLGY--
-QPLGYS-
-QPLGYK-
-QPLGYG-
QHPLGY--
QWPLGY--
-QPLGYQ-
QVPLGY--
-QPLGYV-
-QPLGYT-
-QPLGYI-
-QPLGYA-
QNPLGY--
QIPLGY--
-QPLGYW-
-QPLGYM-
QDPLGY--
QAPLGY--
QTPLGY--
QPPLGY--
QMPLGY--
-QPLGYF-
QFPLGY--
QYPLGY--
-QPLGYH-
-QPLGYN-
QEPLGY--
-QPLGYE-
-QPLGYD-
-RPLGYL-
-RPLGYM-
RVPLGY--
RLPLGY--
-RPLGYS-
-RPLGYT-
RPPLGY--
-RPLGYW-

-RPLGYK-
-RPLGYG-
RTPLGY--
REPLGY--
-RPLGYF-
-RPLGYY-
RKPLGY--
RDPLGY--
-RPLGYQ-
RGPLGY--
RAPLGY--
RIPLGY--
RMPLGY--
RYPLGY--
-RPLGYH-
-RPLGYE-
RHPLGY--
RQPLGY--
-RPLGYN-
RNPLGY--
RCPLGY--
-RPLGYD-
-RPLGYC-
SSPLGY--
-SPLGYR-
SAPLGY--
SGPLGY--
SLPLGY--
SKPLGY--
-SPLGYS-
-SPLGYV-
STPLGY--
SFPLGY--
-SPLGYI-
-SPLGYG-
SHPLGY--
-SPLGYN-
-SPLGYQ-
-SPLGYH-
SYPLGY--
SVPLGY--
-SPLGYA-
SMPLGY--
-SPLGYY-
SNPLGY--
SWPLGY--
SPPLGY--
-SPLGYW-
-SPLGYE-
SIPLGY--

```
-SPLGYM-
SDPLGY--
SEPLGY--
SQPLGY--
SCPLGY--
-TPLGYN-
-TPLGYS-
TVPLGY--
-TPLGYL-
-TPLGYH-
-TPLGYI-
TAPLGY--
TMPLGY--
-TPLGYK-
TGPLGY--
THPLGY--
-TPLGYV-
-TPLGYY-
-TPLGYM-
TTPLGY--
-TPLGYG-
TKPLGY--
TWPLGY--
TPPLGY--
TFPLGY--
-TPLGYT-
-TPLGYW-
TIPLGY--
TQPLGY--
-TPLGYQ-
-TPLGYA-
TYPLGY--
-TPLGYF-
TEPLGY--
TDPLGY--
TNPLGY--
TCPLGY--
-TPLGYD-
-VPLGYS-
VSPLGY--
-VPLGYL-
-VPLGYR-
VGPLGY--
VKPLGY--
-VPLGYT-
-VPLGYI-
VMPLGY--
VYPLGY--
-VPLGYG-
VPPLGY--
```

-VPLGYK-
-VPLGYV-
-VPLGYY-
-VPLGYM-
-VPLGYA-
VVPLGY--
VHPLGY--
-VPLGYN-
VFPLGY--
-VPLGYH-
VWPLGY--
VEPLGY--
-VPLGYE-
-VPLGYQ-
-VPLGYF-
VDPLGY--
VQPLGY--
-VPLGYW-
VIPLGY--
VAPLGY--
-VPLGYC-
VCPLGY--
-VPLGYD-
WFPLGY--
WAPLGY--
-WPLGYR-
-WPLGYL-
WKPLGY--
WQPLGY--
-WPLGYK-
WNPLGY--
WMPLGY--
WLPLGY--
-WPLGYT-
WGPLGY--
-WPLGYG-
-WPLGYS-
-WPLGYI-
-WPLGYQ-
WHPLGY--
WEPLGY--
WVPLGY--
WPPLGY--
-WPLGYN-
-WPLGYH-
-WPLGYA-
WDPLGY--
WCPLGY--
WWPLGY--
-WPLGYV-

	-WPLGYF-		
	-WPLGYM-		
	WYPLGY--		
	WTPLGY--		
	-WPLGYE-		
	-WPLGYY-		
	WIPLGY--		
	-YPLGYR-		
	-YPLGYH-		
	YVPLGY--		
	YSPLGY--		
	-YPLGYN-		
	YAPLGY--		
	YKPLGY--		
	-YPLGYL-		
	-YPLGYA-		
	-YPLGYF-		
	-YPLGYM-		
	-YPLGYT-		
	YDPLGY--		
	YFPLGY--		
	YTPLGY--		
	-YPLGYY-		
	YGPLGY--		
	-YPLGYG-		
	YNPLGY--		
	YMPLGY--		
	-YPLGYS-		
	YHPLGY--		
	YPPLGY--		
	YYPLGY--		
	-YPLGYQ-		
	-YPLGYI-		
	YWPLGY--		
	-YPLGYW-		
	YIPLGY--		
	YEPLGY--		
	-YPLGYK-		
	YQPLGY--		
	YCPLGY--		
PMQL	284	705	47.53
	ASPMQL--		
	-APMQLV-		
	AMPMQL--		
	APPMQL--		
	ARPMQL--		
	-APMQLR-		
	-APMQLS-		
	ALPMQL--		
	-APMQLN-		

AVPMQL--
AIPMQL--
AGPMQL--
-APMQLK-
AWPMQL--
ATPMQL--
AKPMQL--
-APMQLT-
-APMQLA-
-APMQLI-
AAPMQL--
-APMQLM-
-APMQLG-
AFPMQL--
AQPMQL--
-APMQLH-
ANPMQL--
AEPMQL--
CSPMQL--
CYPMQL--
CFPMQL--
CCPMQL--
CTPMQL--
CRPMQL--
CQPMQL--
-CPMQLR-
CMPMQL--
CIPMQL--
CVPMQL--
CDPMQL--
CWPMQL--
-CPMQLS-
CNPMQL--
CAPMQL--
DHPMQL--
DNPMQL--
DYPMQL--
DWPMQL--
-DPMQLR-
DTPMQL--
DFPMQL--
DAPMQL--
-DPMQLF-
-DPMQLL-
DVPMQL--
DSPMQL--
-DPMQLS-
DIPMQL--
-DPMQLV-
-DPMQLK-

-DPMQLW-
-DPMQLI-
DRPMQL--
-DPMQLN-
DKPMQL--
DGPMQL--
-DPMQLT-
-DPMQLQ-
DMPMQL--
DLPMQL--
-EPMQLN-
ERPMQL--
ESPMQL--
EYPMQL--
-EPMQLS-
EGPMQL--
EAPMQL--
EQPMQL--
EMPMQL--
-EPMQLR-
-EPMQLV-
EIPMQL--
EEPMQL--
EWPMQL--
-EPMQLQ-
ELPMQL--
-EPMQLT-
EHPMQL--
ETPMQL--
-EPMQLL-
-EPMQLK-
-EPMQLI-
-FPMQLI-
FRPMQL--
-FPMQLV-
-FPMQLR-
FSPMQL--
FDPMQL--
-FPMQLS-
-FPMQLN-
FMPMQL--
FGPMQL--
FTPMQL--
-FPMQLK-
FKPMQL--
FQPMQL--
-FPMQLA-
FPPMQL--
-FPMQLM-
FIPMQL--

FAPMQL--
FHPMQL--
FYPMQL--
FNPMQL--
-FPMQLQ-
-FPMQLH-
GFPMQL--
GAPMQL--
GRPMQL--
-GPMQLK-
-GPMQLS-
GSPMQL--
-GPMQLM-
GQPMQL--
-GPMQLR-
GKPMQL--
GIPMQL--
GTPMQL--
-GPMQLQ-
GLPMQL--
-GPMQLT-
GGPMQL--
-GPMQLN-
GYPMQL--
-GPMQLI-
-GPMQLH-
-GPMQLW-
GMPMQL--
GNPMQL--
GPPMQL--
GDPMQL--
-GPMQLL-
GWPMQL--
GHPMQL--
GEPMQL--
-GPMQLV-
-GPMQLG-
GCPMQL--
HQPMQL--
-HPMQLR-
HSPMQL--
HFPMQL--
-HPMQLL-
HAPMQL--
HLPMQL--
HKPMQL--
HGPMQL--
-HPMQLN-
-HPMQLK-
HRPMQL--

-HPMQLT-
HTPMQL--
HPPMQL--
HIPMQL--
-HPMQLV-
HVPMQL--
-HPMQLW-
-HPMQLH-
HNPMQL--
HEPMQL--
HDPMQL--
HCPMQL--
-IPMQLS-
ILPMQL--
ISPMQL--
-IPMQLR-
IVPMQL--
IRPMQL--
IYPMQL--
-IPMQLA-
INPMQL--
IEPMQL--
ITPMQL--
IAPMQL--
-IPMQLK-
-IPMQLT-
-IPMQLG-
-IPMQLI-
-IPMQLF-
IGPMQL--
IKPMQL--
IWPMQL--
-IPMQLM-
IFPMQL--
IDPMQL--
-IPMQLH-
KSPMQL--
-KPMQLR-
KYPMQL--
-KPMQLL-
KLPMQL--
KFPMQL--
KGPMQL--
-KPMQLS-
-KPMQLV-
KVPMQL--
-KPMQLW-
KIPMQL--
KHPMQL--
KTPMQL--

KMPMQL--
KPPMQL--
KQPMQL--
-KPMQLT-
-KPMQLN-
-KPMQLM-
KAPMQL--
-KPMQLY-
-KPMQLH-
-KPMQLG-
KKPMQL--
KWPMQL--
-KPMQLQ-
-KPMQLK-
-KPMQLI-
LSPMQL--
LKPMQL--
LYPMQL--
-LPMQLT-
LRPMQL--
-LPMQLL-
LAPMQL--
-LPMQLR-
LVPMQL--
-LPMQLV-
LIPMQL--
LPPMQL--
-LPMQLN-
LLPMQL--
LGPMQL--
LTPMQL--
LNPMQL--
-LPMQLM-
LMPMQL--
LWPMQL--
-LPMQLS-
-LPMQLK-
LQPMQL--
LFPMQL--
-LPMQLI-
-LPMQLQ-
-LPMQLA-
LDPMQL--
LEPMQL--
-LPMQLG-
-LPMQLH-
-MPMQLR-
MGPMQL--
-MPMQLL-
MRPMQL--

MVPMQL--
-MPMQLM-
-MPMQLN-
-MPMQLS-
MLPMQL--
MWPMQL--
MTPMQL--
MPPMQL--
-MPMQLT-
MSPMQL--
-MPMQLI-
MKPMQL--
-MPMQLV-
-MPMQLH-
-MPMQLG-
MFPMQL--
MYPMQL--
-MPMQLW-
MMPMQL--
-MPMQLQ-
-MPMQLC-
-MPMQLF-
-NPMQLR-
-NPMQLW-
NKPMQL--
NRPMQL--
NWPMQL--
NIPMQL--
NMPMQL--
NTPMQL--
-NPMQLV-
NNPMQL--
NGPMQL--
NSPMQL--
NPPMQL--
-NPMQLY-
-NPMQLN-
NFPMQL--
NVPMQL--
NYPMQL--
-NPMQLS-
-NPMQLK-
NEPMQL--
NDPMQL--
-NPMQLL-
NLPMQL--
-NPMQLH-
NHPMQL--
NAPMQL--
-NPMQLI-

-NPMQLC-
--PMQLRR
--PMQLRT
--PMQLVK
--PMQLRV
--PMQLTT
--PMQLSS
--PMQLAG
--PMQLTS
--PMQLFG
--PMQLLT
--PMQLGS
--PMQLLS
--PMQLTN
--PMQLNF
--PMQLWS
--PMQLVR
--PMQLRY
--PMQLRF
--PMQLIT
--PMQLML
--PMQLVS
--PMQLSW
--PMQLRQ
--PMQLNG
--PMQLMG
--PMQLLK
--PMQLVY
--PMQLKY
--PMQLKV
--PMQLRL
--PMQLQV
--PMQLTG
--PMQLTA
--PMQLSH
--PMQLSG
--PMQLIS
--PMQLRD
--PMQLHT
--PMQLVL
--PMQLST
--PMQLWQ
--PMQLVN
--PMQLRW
--PMQLIW
--PMQLFY
--PMQLAT
--PMQLTW
--PMQLTF
--PMQLRN

--PMQLQK
--PMQLMH
--PMQLII
--PMQLIG
--PMQLGA
PNPMQL--
--PMQLTD
--PMQLSR
--PMQLSA
--PMQLNQ
--PMQLIK
--PMQLAS
--PMQLQL
--PMQLES
--PMQLAA
PMPMQL--
PFPMQL--
--PMQLVT
--PMQLMR
--PMQLMA
--PMQLIA
--PMQLHW
--PMQLHS
--PMQLWG
--PMQLVE
--PMQLLG
--PMQLKL
--PMQLKA
PIPMQL--
--PMQLTK
--PMQLSM
--PMQLNW
--PMQLMT
--PMQLLA
--PMQLKM
--PMQLYS
--PMQLYN
--PMQLWR
--PMQLVH
--PMQLTV
--PMQLSN
--PMQLRH
--PMQLNS
--PMQLNN
--PMQLNE
--PMQLMS
--PMQLHR
PKPMQL--
PAPMQL--
--PMQLVQ

--PMQLSF
--PMQLRM
--PMQLRE
--PMQLNR
--PMQLLQ
--PMQLKQ
--PMQLKH
--PMQLHI
PGPMQL--
--PMQLYH
--PMQLWN
--PMQLTR
--PMQLTL
--PMQLTH
--PMQLSV
--PMQLQG
--PMQLMY
--PMQLLH
--PMQLIN
--PMQLHV
--PMQLEW
PTPMQL--
--PMQLSQ
--PMQLQA
--PMQLNI
--PMQLMD
--PMQLKR
--PMQLIL
--PMQLGT
--PMQLAW
--PMQLAV
--PMQLYT
--PMQLQR
--PMQLQM
--PMQLMN
--PMQLLD
--PMQLKD
--PMQLIH
--PMQLVM
--PMQLVG
--PMQLVA
--PMQLSK
--PMQLSI
--PMQLQN
--PMQLNT
--PMQLND
--PMQLMW
--PMQLMV
--PMQLME
--PMQLKT

--PMQLHN
--PMQLHL
--PMQLHG
--PMQLEA
--PMQLAY
--PMQLAL
--PMQLWD
--PMQLTI
--PMQLSL
--PMQLQD
--PMQLNV
--PMQLKI
--PMQLHY
--PMQLFE
--PMQLAN
PEPMQL--
PQPMQL--
--PMQLYW
--PMQLYK
--PMQLVW
--PMQLVD
--PMQLTE
--PMQLSC
--PMQLQI
--PMQLQE
--PMQLNK
--PMQLMK
--PMQLLE
--PMQLID
--PMQLHD
--PMQLHC
--PMQLGI
--PMQLEY
--PMQLCT
--PMQLAM
-PPMQLN-
--PMQLYQ
--PMQLYC
--PMQLWK
--PMQLTQ
--PMQLTM
--PMQLSP
--PMQLSD
--PMQLQY
--PMQLQT
--PMQLQS
--PMQLKN
--PMQLHE
--PMQLGY
--PMQLGV

--PMQLGM
--PMQLGG
--PMQLEH
--PMQLDS
--PMQLCV
--PMQLAK
--PMQLAH
PCPMQL--
QSPMQL--
QRPMQL--
QHPMQL--
-QPMQLT-
-QPMQLI-
-QPMQLS-
-QPMQLR-
QLPMQL--
QKPMQL--
QEPMQL--
QMPMQL--
QTPMQL--
-QPMQLH-
QNPMQL--
QGPMQL--
QAPMQL--
QPPMQL--
-QPMQLE-
-QPMQLV-
-QPMQLQ-
QFPMQL--
QYPMQL--
-QPMQLN-
-QPMQLL-
-QPMQLA-
QVPMQL--
QQPMQL--
-QPMQLG-
QIPMQL--
QDPMQL--
REPMQL--
RSPMQL--
RLPMQL--
RGPMQL--
-RPMQLS-
-RPMQLK-
-RPMQLT-
RQPMQL--
RHPMQL--
-RPMQLY-
-RPMQLQ-
-RPMQLI-

-RPMQLH-
RYPMQL--
RIPMQL--
RVPMQL--
RKPMQL--
-RPMQLM-
RNPMQL--
RAPMQL--
-RPMQLN-
-RPMQLA-
RDPMQL--
-RPMQLG-
-RPMQLV-
-RPMQLE-
-RPMQLW-
-SPMQLT-
-SPMQLL-
SLPMQL--
SVPMQL--
SFPMQL--
SSPMQL--
SIPMQL--
-SPMQLN-
SRPMQL--
-SPMQLR-
SMPMQL--
-SPMQLI-
SWPMQL--
-SPMQLK-
-SPMQLS-
SAPMQL--
SGPMQL--
SPPMQL--
SKPMQL--
SDPMQL--
SEPMQL--
SHPMQL--
SYPMQL--
-SPMQLH-
-SPMQLG-
-SPMQLA-
SQPMQL--
-SPMQLV-
-SPMQLQ-
-SPMQLM-
SNPMQL--
TRPMQL--
-TPMQLR-
-TPMQLL-
-TPMQLS-

TAPMQL--
-TPMQLH-
TLPMQL--
TTPMQL--
-TPMQLK-
THPMQL--
TSPMQL--
-TPMQLT-
TIPMQL--
-TPMQLV-
-TPMQLA-
-TPMQLN-
TGPMQL--
TNPMQL--
-TPMQLG-
-TPMQLW-
-TPMQLI-
TDPMQL--
TQPMQL--
TPPMQL--
TFPMQL--
TEPMQL--
TVPMQL--
TCPMQL--
-TPMQLM-
-TPMQLE-
TMPMQL--
VRPMQL--
-VPMQLR-
VLPMQL--
VMPMQL--
-VPMQLL-
VAPMQL--
VIPMQL--
-VPMQLT-
-VPMQLS-
-VPMQLN-
VWPMQL--
VPPMQL--
VEPMQL--
VNPMQL--
VTPMQL--
VHPMQL--
VGPMQL--
-VPMQLM-
VDPMQL--
VSPMQL--
-VPMQLH-
-VPMQLG-
VQPMQL--

-VPMQLE-
-VPMQLK-
VCPMQL--
-WPMQLS-
-WPMQLR-
WSPMQL--
WAPMQL--
-WPMQLQ-
WTPMQL--
WKPMQL--
-WPMQLM-
-WPMQLK-
-WPMQLH-
WNPMQL--
WDPMQL--
-WPMQLT-
WRPMQL--
-WPMQLN-
-WPMQLE-
-WPMQLV-
-WPMQLG-
-WPMQLA-
WEPMQL--
-YPMQLV-
-YPMQLK-
YLPMQL--
-YPMQLQ-
YGPMQL--
YAPMQL--
YSPMQL--
-YPMQLI-
YNPMQL--
YIPMQL--
-YPMQLS-
-YPMQLR-
YKPMQL--
-YPMQLL-
YYPMQL--
YTPMQL--
YDPMQL--
-YPMQLA-
YEPMQL--
-YPMQLG-
-YPMQLE-
-YPMQLH-
YFPMQL--
PWHM 774 541 47.53
--PWHMAR
-APWHMR-
-APWHML-

AVPWHM--
ANPWHM--
ATPWHM--
-APWHMM-
AHPWHM--
AFPWHM--
-APWHMV-
-APWHMK-
-APWHMQ-
-APWHMY-
-APWHMI-
ARPWHM--
AQPWHM--
ALPWHM--
AYPWHM--
-APWHMW-
AIPWHM--
CSPWHM--
-CPWHML-
DRPWHM--
DLPWHM--
DQPWHM--
DFPWHM--
-DPWHMR-
DMPWHM--
DIPWHM--
DWPWHM--
DVPWHM--
-DPWHMV-
-DPWHME-
DGPWHM--
DSPWHM--
-DPWHMQ-
-DPWHMF-
ESPWHM--
-EPWHMR-
ERPWHM--
-EPWHME-
EWPWHM--
EYPWHM--
-EPWHMF-
-EPWHMT-
-EPWHMS-
ELPWHM--
EFPWHM--
-EPWHMW-
-FPWHMR-
FRPWHM--
FLPWHM--
FAPWHM--

FGPWHM--
FEPWHM--
-FPWHMS-
-FPWHMA-
FKPWHM--
FYPWHM--
-FPWHMI-
FFPWHM--
FWPWHM--
-FPWHMK-
GKPWHM--
GVPWHM--
-GPWHML-
GQPWHM--
GTPWHM--
-GPWHMR-
GYPWHM--
GWPWHM--
-GPWHMK-
GSPWHM--
-GPWHMH-
GRPWHM--
-GPWHMT-
GLPWHM--
GAPWHM--
-GPWHMY-
-GPWHME-
-GPWHMV-
GNPWHM--
-GPWHMS-
-GPWHMA-
GHPWHM--
-GPWHMQ-
-GPWHMF-
GGPWHM--
GFPWHM--
GDPWHM--
GCPWHM--
HYPWHM--
HNPWHM--
-HPWHMR-
-HPWHMY-
HPPWHM--
HLPWHM--
HSPWHM--
-HPWHML-
HVPWHM--
-HPWHMA-
HRPWHM--
-HPWHMI-

-HPWHMV-
-HPWHMQ-
HGPWHM--
-HPWHMF-
-HPWHME-
HQPWHM--
-HPWHMT-
HTPWHM--
-HPWHMS-
HIPWHM--
HCPWHM--
IRPWHM--
ISPWHM--
-IPWHML-
IVPWHM--
IQPWHM--
IDPWHM--
ITPWHM--
-IPWHMM-
-IPWHMI-
INPWHM--
ILPWHM--
IWPWHM--
-IPWHMN-
IHPWHM--
-IPWHMS-
-IPWHMQ-
IMPWHM--
IYPWHM--
-IPWHMW-
-IPWHMK-
KSPWHM--
KTPWHM--
-KPWHMI-
-KPWHML-
-KPWHMF-
-KPWHMR-
-KPWHMH-
KLPWHM--
-KPWHMV-
KIPWHM--
KQPWHM--
-KPWHMS-
KCPWHM--
-KPWHMW-
KKPWHM--
KAPWHM--
KRPWHM--
-KPWHMY-
-KPWHMK-

-KPWHMA-
KHPWHM--
KGPWHM--
LRPWHM--
LAPWHM--
LLPWHM--
LGPWHM--
LPPWHM--
-LPWHMV-
-LPWHML-
LFPWHM--
LKPWHM--
-LPWHMS-
-LPWHMT-
LHPWHM--
-LPWHMR-
LMPWHM--
LYPWHM--
LTPWHM--
-LPWHMY-
LIPWHM--
LWPWHM--
-LPWHMW-
-LPWHMM-
-LPWHMK-
-LPWHMA-
-LPWHMH-
MSPWHM--
-MPWHMI-
MYPWHM--
MRPWHM--
MVPWHM--
MTPWHM--
-MPWHMT-
-MPWHMS-
-MPWHMQ-
MGPWHM--
-MPWHML-
-MPWHMK-
-MPWHMF-
MLPWHM--
MAPWHM--
MMPWHM--
MFPWHM--
NGPWHM--
NSPWHM--
NLPWHM--
NRPWHM--
-NPWHMI-
NAPWHM--

-NPWHMS-
NMPWHM--
-NPWHMQ-
NKPWHM--
NFPWHM--
NPPWHM--
NEPWHM--
-NPWHMV-
NWPWHM--
-NPWHMT-
-NPWHME-
-NPWHMR-
NDPWHM--
--PWHMSG
--PWHMLG
--PWHMIA
--PWHMRL
--PWHMTR
--PWHMTT
--PWHMVQ
--PWHMRT
--PWHMIG
--PWHMKT
PMPWHM--
--PWHMMT
--PWHMLS
--PWHMTA
--PWHMRK
--PWHMAS
--PWHMRH
--PWHMRA
--PWHMFS
PFPWHM--
--PWHMST
--PWHMRG
--PWHMMN
--PWHMKI
--PWHMYH
--PWHMVD
--PWHMMR
--PWHMIM
PIPWHM--
--PWHMSS
--PWHMQT
--PWHMVA
--PWHMTS
--PWHMSA
--PWHMLE
--PWHMAA
PKPWHM--

PEPWHM--
PYPWHM--
--PWHMWG
--PWHMVK
--PWHMTW
--PWHMTQ
--PWHMSM
--PWHMNS
--PWHMKR
--PWHMGG
--PWHMWN
--PWHMSN
--PWHMSI
--PWHMRW
--PWHMRS
--PWHMRI
--PWHMQG
--PWHMML
--PWHMLR
--PWHMLQ
--PWHMLA
--PWHMKV
--PWHMIS
--PWHMIQ
--PWHMEV
PTPWHM--
--PWHMYV
--PWHMSW
--PWHMSV
--PWHMSF
--PWHMQV
--PWHMNA
--PWHMMV
--PWHMLD
--PWHMIT
--PWHMIN
--PWHMAY
--PWHMAT
PNPWHM--
--PWHMTF
--PWHMRM
--PWHMRE
--PWHMQY
--PWHMLH
--PWHMKQ
--PWHMIK
--PWHMHG
--PWHMGS
--PWHMEM
--PWHMAM

--PWHMYG
--PWHMWY
--PWHMWI
--PWHMWA
--PWHMTK
--PWHMSY
--PWHMSL
--PWHMSK
--PWHMSH
--PWHMSD
--PWHMMM
--PWHMLI
--PWHMKM
--PWHMHL
--PWHMHH
--PWHMFW
--PWHMFT
--PWHMYR
--PWHMVY
--PWHMVT
--PWHMVS
--PWHMTM
--PWHMTI
--PWHMTE
--PWHMRD
--PWHMNT
--PWHMNN
--PWHMMS
--PWHMME
--PWHMLT
--PWHMLM
--PWHMKW
--PWHMKF
--PWHMKE
--PWHMIY
--PWHMIR
--PWHMIL
--PWHMII
--PWHMIH
--PWHMIF
--PWHMHK
--PWHMGV
--PWHMER
--PWHMDS
--PWHMAV
--PWHMAN
--PWHMAL
--PWHMAK
--PWHMAH
PAPWHM--

PWPWHM--
--PWHMYI
--PWHMTC
--PWHMSR
--PWHMRV
--PWHMRQ
--PWHMQQ
--PWHMQN
--PWHMNW
--PWHMNR
--PWHMNL
--PWHMMI
--PWHMLK
--PWHMLF
--PWHMKY
--PWHMKS
--PWHMKH
--PWHMKG
--PWHMKD
--PWHMHY
--PWHMHR
--PWHMHN
--PWHMFM
--PWHMFG
--PWHMFD
--PWHMAG
-QPWHMR-
QGPWHM--
-QPWHMH-
-QPWHMS-
QRPWHM--
QLPWHM--
-QPWHMV-
-QPWHME-
QPPWHM--
QEPWHM--
QVPWHM--
-QPWHML-
-QPWHMK-
-QPWHMN-
QCPWHM--
QSPWHM--
-QPWHMY-
-QPWHMF-
-QPWHMD-
-RPWHMR-
-RPWHMK-
-RPWHMW-
RTPWHM--
RAPWHM--

RIPWHM--
-RPWHMS-
RPPWHM--
RRPWHM--
REPWHM--
RWPWHM--
-RPWHMQ-
RVPWHM--
-RPWHME-
-RPWHMA-
-RPWHMT-
-RPWHMN-
RNPWHM--
RKPWHM--
RGPWHM--
-RPWHML-
-RPWHMG-
-RPWHMF-
RYPWHM--
RSPWHM--
-RPWHMM-
RHPWHM--
-RPWHMV-
-RPWHMH-
RLPWHM--
RFPWHM--
-RPWHMY-
RDPWHM--
RCPWHM--
SQPWHM--
SSPWHM--
-SPWHMW-
SIPWHM--
SGPWHM--
SRPWHM--
-SPWHMY-
STPWHM--
-SPWHMS-
-SPWHML-
-SPWHMQ-
-SPWHMF-
-SPWHME-
SNPWHM--
SAPWHM--
-SPWHMT-
SLPWHM--
SYPWHM--
SVPWHM--
-SPWHMR-
-SPWHMK-

```
-SPWHMH-
-SPWHMI-
SFPWHM--
-SPWHMV-
SPPWHM--
SMPWHM--
SEPWHM--
SKPWHM--
SHPWHM--
TSPWHM--
-TPWHMT-
TMPWHM--
-TPWHMI-
-TPWHMM-
-TPWHMR-
-TPWHMH-
TRPWHM--
-TPWHMV-
-TPWHML-
-TPWHMF-
TYPWHM--
TNPWHM--
TGPWHM--
TWPWHM--
-TPWHMY-
-TPWHMS-
-TPWHMQ-
-TPWHMN-
TLPWHM--
VKPWHM--
VRPWHM--
VMPWHM--
VLPWHM--
VFPWHM--
-VPWHMV-
-VPWHMR-
-VPWHMI-
-VPWHMQ-
VTPWHM--
VVPWHM--
VNPWHM--
VIPWHM--
VSPWHM--
-VPWHMG-
-VPWHMF-
-VPWHMA-
VGPWHM--
VCPWHM--
VYPWHM--
-VPWHMT-
```

	-VPWHMS-		
	-VPWHML-		
	-VPWHMH-		
	VEPWHM--		
	WKPWHM--		
	WSPWHM--		
	WEPWHM--		
	WQPWHM--		
	WYPWHM--		
	-WPWHMT-		
	-WPWHMQ-		
	WMPWHM--		
	WDPWHM--		
	WAPWHM--		
	WRPWHM--		
	-WPWHME-		
	WHPWHM--		
	-WPWHMY-		
	-WPWHMW-		
	-WPWHMV-		
	-WPWHMG-		
	WLPWHM--		
	WFPWHM--		
	YIPWHM--		
	-YPWHMR-		
	-YPWHMI-		
	YEPWHM--		
	YVPWHM--		
	YFPWHM--		
	YSPWHM--		
	YQPWHM--		
	-YPWHMT-		
	YMPWHM--		
	YTPWHM--		
	-YPWHML-		
	YLPWHM--		
	YHPWHM--		
	YYPWHM--		
PKWL	227	732	47.49
	-SPKWLE-		
	-APKWLR-		
	ALPKWL--		
	-APKWLA-		
	-APKWLL-		
	-APKWLI-		
	AIPKWL--		
	ARPKWL--		
	-APKWLG-		
	-APKWLV-		
	-APKWLS-		

AAPKWL--
AVPKWL--
-APKWLQ-
AQPKWL--
ATPKWL--
-APKWLN-
AEPKWL--
AFPKWL--
AWPKWL--
-APKWLE-
ANPKWL--
AHPKWL--
APPKWL--
AKPKWL--
AYPKWL--
ASPKWL--
-APKWLM-
-APKWLT-
AGPKWL--
-APKWLK-
-APKWLF-
ADPKWL--
CYPKWL--
-CPKWLS-
CAPKWL--
CGPKWL--
CSPKWL--
CRPKWL--
-CPKWLT-
-CPKWLR-
-CPKWLQ-
-CPKWLI-
-CPKWLA-
-DPKWLK-
-DPKWLS-
-DPKWLV-
-DPKWLF-
DRPKWL--
-DPKWLN-
-DPKWLG-
DVPKWL--
-DPKWLR-
DGPKWL--
DMPKWL--
-DPKWLT-
DHPKWL--
-DPKWLI-
-DPKWLA-
DTPKWL--
DQPKWL--

-DPKWLL-
-DPKWLE-
DLPKWL--
DKPKWL--
DEPKWL--
DWPKWL--
-DPKWLY-
-DPKWLH-
DIPKWL--
DAPKWL--
DYPKWL--
DSPKWL--
-DPKWLQ-
-EPKWLS-
-EPKWLI-
-EPKWLL-
EVPKWL--
EIPKWL--
EGPKWL--
EAPKWL--
-EPKWLQ-
ERPKWL--
-EPKWLR-
-EPKWLH-
ESPKWL--
EYPKWL--
-EPKWLT-
EMPKWL--
EPPKWL--
ENPKWL--
-EPKWLE-
EHPKWL--
EFPKWL--
ETPKWL--
-EPKWLF-
EKPKWL--
-EPKWLV-
EWPKWL--
EQPKWL--
-EPKWLN-
-EPKWLM-
-FPKWLT-
FLPKWL--
FHPKWL--
FTPKWL--
FQPKWL--
FIPKWL--
FVPKWL--
FSPKWL--
-FPKWLI-

FEPKWL--
FDPKWL--
-FPKWLG-
-FPKWLK-
FAPKWL--
-FPKWLV-
-FPKWLN-
-FPKWLL-
FYPKWL--
FPPKWL--
-FPKWLF-
GLPKWL--
GVPKWL--
-GPKWLG-
GAPKWL--
GWPKWL--
-GPKWLM-
GEPKWL--
GTPKWL--
GGPKWL--
-GPKWLV-
-GPKWLL-
GPPKWL--
GQPKWL--
-GPKWLW-
-GPKWLA-
GRPKWL--
-GPKWLQ-
GNPKWL--
GIPKWL--
GSPKWL--
-GPKWLY-
GKPKWL--
GFPKWL--
-GPKWLI-
-GPKWLD-
GYPKWL--
-GPKWLF-
GHPKWL--
HRPKWL--
HGPKWL--
-HPKWLI-
-HPKWLA-
HKPKWL--
HNPKWL--
HSPKWL--
-HPKWLT-
-HPKWLS-
HVPKWL--
-HPKWLR-

-HPKWLQ-
HLPKWL--
-HPKWLV-
-HPKWLK-
HWPKWL--
-HPKWLL-
HAPKWL--
HTPKWL--
-HPKWLN-
-HPKWLG-
HFPKWL--
HPPKWL--
-HPKWLY-
-HPKWLM-
HQPKWL--
HIPKWL--
HYPKWL--
-HPKWLW-
-HPKWLE-
ITPKWL--
-IPKWLS-
IGPKWL--
IVPKWL--
IAPKWL--
-IPKWLT-
-IPKWLH-
ISPKWL--
IQPKWL--
-IPKWLN-
-IPKWLG-
-IPKWLY-
-IPKWLW-
IKPKWL--
-IPKWLI-
INPKWL--
IEPKWL--
IYPKWL--
-IPKWLE-
IMPKWL--
IWPKWL--
-IPKWLQ-
-IPKWLM-
-IPKWLK-
-IPKWLF-
IFPKWL--
KMPKWL--
-KPKWLL-
KRPKWL--
-KPKWLV-
-KPKWLI-

KHPKWL--
KVPKWL--
KGPKWL--
-KPKWLN-
-KPKWLS-
-KPKWLY-
-KPKWLG-
KYPKWL--
KLPKWL--
KTPKWL--
-KPKWLK-
-KPKWLD-
KEPKWL--
KDPKWL--
KPPKWL--
-KPKWLF-
KNPKWL--
-KPKWLW-
-KPKWLT-
-KPKWLM-
-KPKWLH-
-KPKWLE-
LVPKWL--
LNPKWL--
LIPKWL--
-LPKWLK-
LAPKWL--
LWPKWL--
-LPKWLA-
-LPKWLS-
LGPKWL--
-LPKWLI-
LDPKWL--
-LPKWLV-
-LPKWLQ-
-LPKWLM-
LHPKWL--
LMPKWL--
LCPKWL--
LQPKWL--
-LPKWLH-
-LPKWLF-
-LPKWLE-
-LPKWLD-
MTPKWL--
-MPKWLL-
MNPKWL--
MSPKWL--
MRPKWL--
-MPKWLI-

MVPKWL--
-MPKWLK-
-MPKWLV-
-MPKWLA-
-MPKWLW-
-MPKWLN-
MGPKWL--
MFPKWL--
-MPKWLG-
-MPKWLR-
-MPKWLQ-
MPPKWL--
-MPKWLY-
MEPKWL--
MYPKWL--
MQPKWL--
-MPKWLS-
-MPKWLT-
MMPKWL--
MDPKWL--
MAPKWL--
MIPKWL--
MHPKWL--
MCPKWL--
-MPKWLE-
NDPKWL--
NVPKWL--
-NPKWLV-
-NPKWLK-
-NPKWLL-
NRPKWL--
-NPKWLI-
-NPKWLR-
NGPKWL--
NKPKWL--
NEPKWL--
NPPKWL--
NNPKWL--
-NPKWLG-
NWPKWL--
-NPKWLA-
NLPKWL--
NYPKWL--
-NPKWLY-
-NPKWLQ-
NQPKWL--
-NPKWLF-
-NPKWLE-
-NPKWLT-
NTPKWL--

-NPKWLH-
NCPKWL--
NHPKWL--
NFPKWL--
--PKWLSG
--PKWLTT
--PKWLVS
--PKWLLS
--PKWLST
--PKWLRV
--PKWLLG
--PKWLSN
--PKWLLK
--PKWLRG
--PKWLKG
--PKWLGA
--PKWLTN
--PKWLSV
--PKWLAT
--PKWLGS
--PKWLVT
--PKWLVI
--PKWLIK
--PKWLRM
--PKWLIS
--PKWLWT
--PKWLVV
--PKWLSQ
--PKWLRH
--PKWLQG
--PKWLIH
--PKWLLA
--PKWLAQ
--PKWLYN
--PKWLTK
--PKWLGG
--PKWLNS
--PKWLGT
--PKWLFI
--PKWLES
--PKWLEA
--PKWLVN
--PKWLAY
--PKWLVE
--PKWLFG
--PKWLQS
--PKWLLH
--PKWLKT
--PKWLKS
PSPKWL--

--PKWLVA
--PKWLFA
--PKWLET
--PKWLQD
--PKWLQA
--PKWLFS
--PKWLTV
--PKWLRL
--PKWLKK
--PKWLFT
--PKWLAN
PHPKWL--
--PKWLTM
--PKWLNT
--PKWLIV
--PKWLHM
--PKWLFH
--PKWLYY
--PKWLSH
--PKWLDS
--PKWLAG
--PKWLQH
--PKWLGL
--PKWLEG
PTPKWL--
--PKWLYM
--PKWLWY
--PKWLTY
--PKWLHY
--PKWLYE
--PKWLVG
--PKWLKM
--PKWLIT
--PKWLIN
PGPKWL--
PRPKWL--
--PKWLWA
--PKWLTA
--PKWLRF
--PKWLLQ
--PKWLHV
--PKWLFY
--PKWLYF
--PKWLSM
--PKWLIM
--PKWLYI
--PKWLYH
--PKWLWG
--PKWLSI
--PKWLNA

--PKWLKF
--PKWLIG
--PKWLWV
--PKWLVM
--PKWLKY
--PKWLHG
--PKWLWH
--PKWLVW
--PKWLTE
--PKWLMD
--PKWLIA
--PKWLHI
--PKWLHA
--PKWLGR
--PKWLFQ
--PKWLWS
--PKWLTD
--PKWLRD
--PKWLQE
--PKWLNM
--PKWLNH
--PKWLMW
--PKWLKE
--PKWLGK
--PKWLEV
--PKWLYS
--PKWLYG
--PKWLVQ
--PKWLSA
--PKWLQT
--PKWLMV
--PKWLGF
--PKWLAD
PEPKWL--
--PKWLWN
--PKWLTF
--PKWLQI
--PKWLNQ
--PKWLKV
--PKWLHF
--PKWLHE
--PKWLDT
--PKWLDA
--PKWLAA
PAPKWL--
PYPKWL--
--PKWLTW
--PKWLTQ
--PKWLTI
--PKWLSE

--PKWLQM
--PKWLNN
--PKWLMK
--PKWLKQ
--PKWLKD
--PKWLHT
--PKWLGI
--PKWLEY
--PKWLEN
PFPKWL--
PDPKWL--
--PKWLYW
--PKWLVY
--PKWLMQ
--PKWLKH
--PKWLGY
--PKWLGM
--PKWLEH
--PKWLAK
--PKWLSY
--PKWLQY
--PKWLQN
--PKWLNY
--PKWLNG
--PKWLMH
--PKWLHS
--PKWLGN
--PKWLFE
--PKWLEQ
--PKWLEM
--PKWLAW
--PKWLAV
--PKWLAE
-PPKWLS-
PNPKWL--
--PKWLYK
--PKWLQQ
--PKWLNF
--PKWLMA
--PKWLLM
--PKWLKW
--PKWLIW
--PKWLHW
--PKWLHN
--PKWLHK
--PKWLDG
PQPKWL--
--PKWLWI
--PKWLSW
--PKWLNI

--PKWLND
--PKWLLW
--PKWLIY
--PKWLHH
--PKWLHD
--PKWLGQ
--PKWLEK
--PKWLEE
--PKWLCK
--PKWLAM
--PKWLAH
PCPKWL--
-PPKWLH-
--PKWLWM
--PKWLRW
--PKWLQW
--PKWLPM
--PKWLMM
--PKWLMF
--PKWLIQ
--PKWLHL
--PKWLGH
--PKWLDV
PKPKWL--
QMPKWL--
-QPKWLS-
-QPKWLL-
QTPKWL--
QPPKWL--
-QPKWLI-
-QPKWLG-
QRPKWL--
-QPKWLA-
-QPKWLN-
QNPKWL--
-QPKWLK-
QSPKWL--
-QPKWLQ-
-QPKWLT-
QLPKWL--
QHPKWL--
QYPKWL--
-QPKWLW-
-QPKWLM-
QIPKWL--
QKPKWL--
QWPKWL--
QDPKWL--
QQPKWL--
-QPKWLH-

-QPKWLD-
QCPKWL--
-RPKWLT-
RVPKWL--
RFPKWL--
RLPKWL--
RQPKWL--
-RPKWLI-
RYPKWL--
-RPKWLL-
RGPKWL--
RPPKWL--
-RPKWLN-
-RPKWLM-
-RPKWLV-
-RPKWLG-
REPKWL--
-RPKWLK-
-RPKWLE-
RDPKWL--
-RPKWLW-
RHPKWL--
-RPKWLQ-
RRPKWL--
-RPKWLF-
-RPKWLD-
RNPKWL--
SSPKWL--
STPKWL--
-SPKWLY-
SRPKWL--
-SPKWLT-
-SPKWLH-
-SPKWLM-
SFPKWL--
SKPKWL--
SIPKWL--
-SPKWLW-
SPPKWL--
SNPKWL--
-SPKWLQ-
-SPKWLA-
SEPKWL--
-SPKWLG-
-SPKWLK-
-SPKWLV-
SDPKWL--
SWPKWL--
-SPKWLN-
-SPKWLF-

SHPKWL--
SCPKWL--
SYPKWL--
-SPKWLD-
TRPKWL--
TSPKWL--
TAPKWL--
TPPKWL--
TWPKWL--
-TPKWLN-
TEPKWL--
-TPKWLV-
-TPKWLL-
TGPKWL--
-TPKWLT-
TVPKWL--
TTPKWL--
-TPKWLR-
TNPKWL--
TMPKWL--
TQPKWL--
-TPKWLQ-
-TPKWLK-
TIPKWL--
-TPKWLM-
-TPKWLI-
-TPKWLA-
TKPKWL--
-TPKWLG-
-TPKWLY-
TYPKWL--
-TPKWLH-
-TPKWLF-
TCPKWL--
THPKWL--
TDPKWL--
-TPKWLE-
-TPKWLW-
-TPKWLD-
VTPKWL--
VKPKWL--
VVPKWL--
-VPKWLK-
VSPKWL--
VHPKWL--
-VPKWLY-
-VPKWLL-
-VPKWLT-
VLPKWL--
VAPKWL--

VQPKWL--
-VPKWLV-
-VPKWLN-
VIPKWL--
-VPKWLA-
VNPKWL--
-VPKWLM-
VDPKWL--
-VPKWLW-
VCPKWL--
VWPKWL--
VFPKWL--
WSPKWL--
WRPKWL--
WNPKWL--
WKPKWL--
WAPKWL--
WEPKWL--
WPPKWL--
WQPKWL--
-WPKWLS-
-WPKWLL-
WIPKWL--
WFPKWL--
WTPKWL--
-WPKWLN-
-WPKWLY-
WYPKWL--
WHPKWL--
WGPKWL--
-WPKWLV-
-WPKWLT-
WLPKWL--
WDPKWL--
WWPKWL--
-WPKWLD-
-WPKWLF-
-WPKWLE-
WMPKWL--
YTPKWL--
YRPKWL--
YEPKWL--
YVPKWL--
YQPKWL--
-YPKWLT-
-YPKWLM-
YLPKWL--
YIPKWL--
YYPKWL--
YKPKWL--

YWPKWL--
-YPKWLL-
-YPKWLG-
-YPKWLK-
YSPKWL--
-YPKWLV-
-YPKWLH-
YFPKWL--
-YPKWLQ-
-YPKWLI-
-YPKWLF-
YAPKWL--
YPPKWL--
-YPKWLS-
-YPKWLN-
YMPKWL--
YNPKWL--
YHPKWL--
-YPKWLW-
YGPKWL--
YDPKWL--

PSNL 2 925 47.47

-IPSNLD-
-NPSNLG-
--PSNLPS
--PSNLGR
--PSNLPL
--PSNLNI
--PSNLFP
-RPSNLP-
-TPSNLD-
-APSNLR-
ALPSNL--
AAPSNL--
AKPSNL--
-APSNLS-
ASPSNL--
-APSNLL-
-APSNLI-
-APSNLA-
AGPSNL--
-APSNLV-
-APSNLT-
ATPSNL--
-APSNLM-
AIPSNL--
AWPSNL--
-APSNLQ-
AQPSNL--
AMPSNL--

-APSNLG-
AYPSNL--
AVPSNL--
-APSNLY-
-APSNLH-
AFPSNL--
ANPSNL--
APPSNL--
-APSNLF-
-APSNLE-
AEPSNL--
AHPSNL--
ADPSNL--
-APSNLN-
-APSNLD-
ACPSNL--
CIPSNL--
CYPSNL--
-CPSNLR-
CSPSNL--
CLPSNL--
CAPSNL--
CKPSNL--
CTPSNL--
CQPSNL--
-CPSNLQ-
-CPSNLL-
CHPSNL--
-CPSNLY-
-CPSNLV-
-CPSNLS-
-CPSNLM-
-CPSNLK-
-CPSNLF-
CMPSNL--
CVPSNL--
-CPSNLH-
-CPSNLA-
CNPSNL--
CFPSNL--
CCPSNL--
CPPSNL--
DAPSNL--
DVPSNL--
DKPSNL--
-DPSNLS-
DIPSNL--
-DPSNLR-
DTPSNL--
DRPSNL--

-DPSNLL-
DSPSNL--
DMPSNL--
DLPSNL--
-DPSNLV-
-DPSNLI-
-DPSNLM-
DGPSNL--
DYPSNL--
DWPSNL--
-DPSNLT-
DQPSNL--
DFPSNL--
-DPSNLY-
-DPSNLK-
-DPSNLW-
-DPSNLQ-
DNPSNL--
DDPSNL--
-DPSNLF-
DEPSNL--
-EPSNLR-
ELPSNL--
ESPSNL--
EKPSNL--
ERPSNL--
ETPSNL--
-EPSNLV-
-EPSNLK-
EVPSNL--
EYPSNL--
-EPSNLY-
-EPSNLF-
-EPSNLW-
EIPSNL--
EFPSNL--
ENPSNL--
EHPSNL--
-EPSNLS-
-EPSNLA-
EMPSNL--
EQPSNL--
-EPSNLM-
EWPSNL--
-EPSNLH-
EAPSNL--
-EPSNLT-
-EPSNLQ-
-EPSNLN-
-EPSNLI-

EEPSNL--
EGPSNL--
EPPSNL--
EDPSNL--
-FPSNLL-
FLPSNL--
-FPSNLR-
-FPSNLV-
FEPSNL--
FRPSNL--
FVPSNL--
FAPSNL--
FIPSNL--
-FPSNLI-
FFPSNL--
FGPSNL--
FTPSNL--
-FPSNLK-
-FPSNLN-
-FPSNLY-
FKPSNL--
-FPSNLT-
FWPSNL--
FDPSNL--
-FPSNLE-
-FPSNLW-
FNPSNL--
-FPSNLA-
-FPSNLF-
-FPSNLQ-
-FPSNLH-
FMPSNL--
-FPSNLM-
FHPSNL--
FQPSNL--
-FPSNLG-
FCPSNL--
-GPSNLL-
-GPSNLR-
GRPSNL--
GTPSNL--
GLPSNL--
-GPSNLA-
-GPSNLV-
-GPSNLW-
-GPSNLH-
GGPSNL--
GWPSNL--
-GPSNLQ-
GSPSNL--

-GPSNLS-
GYPSNL--
GVPSNL--
-GPSNLY-
GEPSNL--
-GPSNLI-
GMPSNL--
GIPSNL--
GHPSNL--
-GPSNLK-
GQPSNL--
-GPSNLM-
GKPSNL--
GPPSNL--
GAPSNL--
-GPSNLF-
-GPSNLG-
GNPSNL--
GDPSNL--
-GPSNLN-
-GPSNLE-
GCPSNL--
-GPSNLC-
-GPSNLD-
HLPSNL--
HRPSNL--
-HPSNLL-
HGPSNL--
HAPSNL--
HVPSNL--
-HPSNLY-
HWPSNL--
HQPSNL--
HHPSNL--
HYPSNL--
-HPSNLK-
-HPSNLT-
-HPSNLH-
HEPSNL--
HIPSNL--
-HPSNLA-
-HPSNLR-
HFPSNL--
-HPSNLV-
HPPSNL--
-HPSNLE-
-HPSNLI-
HSPSNL--
-HPSNLM-
HMPSNL--

HKPSNL--
-HPSNLG-
HNPSNL--
-HPSNLW-
-HPSNLQ-
-HPSNLF-
HCPSNL--
HDPSNL--
-IPSNLS-
ILPSNL--
ISPSNL--
IIPSNL--
-IPSNLL-
IRPSNL--
IYPSNL--
-IPSNLK-
ITPSNL--
-IPSNLW-
IVPSNL--
-IPSNLQ-
IWPSNL--
-IPSNLY-
IMPSNL--
IQPSNL--
-IPSNLT-
-IPSNLF-
IHPSNL--
IAPSNL--
-IPSNLR-
-IPSNLI-
-IPSNLA-
IPPSNL--
IEPSNL--
INPSNL--
IDPSNL--
-IPSNLV-
-IPSNLN-
-IPSNLM-
-IPSNLH-
-IPSNLG-
-IPSNLE-
ICPSNL--
KSPSNL--
-KPSNLL-
-KPSNLR-
KLPSNL--
KGPSNL--
-KPSNLY-
-KPSNLS-
KWPSNL--

KFPSNL--
-KPSNLH-
KMPSNL--
-KPSNLF-
KAPSNL--
KQPSNL--
-KPSNLK-
-KPSNLW-
-KPSNLQ-
KTPSNL--
-KPSNLG-
KYPSNL--
-KPSNLI-
-KPSNLN-
-KPSNLA-
KIPSNL--
-KPSNLT-
KPPSNL--
KEPSNL--
KKPSNL--
KHPSNL--
KNPSNL--
-KPSNLE-
KDPSNL--
-KPSNLM-
KCPSNL--
LSPSNL--
LLPSNL--
-LPSNLR-
-LPSNLT-
-LPSNLY-
LAPSNL--
LMPSNL--
-LPSNLV-
-LPSNLH-
LFPSNL--
LVPSNL--
LGPSNL--
LTPSNL--
LQPSNL--
LYPSNL--
LHPSNL--
LDPSNL--
-LPSNLS-
LIPSNL--
LKPSNL--
-LPSNLI-
-LPSNLW-
-LPSNLA-
-LPSNLM-

LEPSNL--
-LPSNLK-
-LPSNLE-
-LPSNLN-
-LPSNLQ-
-LPSNLG-
LCPSNL--
-LPSNLP-
-LPSNLC-
-MPSNLL-
MRPSNL--
MIPSNL--
MLPSNL--
MVPSNL--
MSPSNL--
-MPSNLV-
-MPSNLK-
-MPSNLT-
-MPSNLS-
-MPSNLA-
MGPSNL--
MQPSNL--
MEPSNL--
MMPSNL--
MKPSNL--
MWPSNL--
MNPSNL--
-MPSNLY-
MPPSNL--
-MPSNLW-
-MPSNLQ-
-MPSNLM-
MAPSNL--
-MPSNLN-
-MPSNLI-
-MPSNLH-
-MPSNLF-
MFPSNL--
MYPSNL--
-MPSNLG-
MHPSNL--
-MPSNLE-
MDPSNL--
MCPSNL--
-NPSNLR-
NAPSNL--
NIPSNL--
NWPSNL--
NSPSNL--
NHPSNL--

NVPSNL--
-NPSNLI-
NLPSNL--
-NPSNLV-
-NPSNLT-
NGPSNL--
-NPSNLY-
NPPSNL--
NTPSNL--
-NPSNLK-
NNPSNL--
NKPSNL--
NQPSNL--
NYPSNL--
-NPSNLH-
NEPSNL--
-NPSNLQ-
-NPSNLW-
-NPSNLF-
-NPSNLN-
-NPSNLE-
-NPSNLM-
-NPSNLA-
NDPSNL--
NMPSNL--
--PSNLRS
--PSNLRL
--PSNLRT
--PSNLRA
--PSNLLT
--PSNLWS
--PSNLVS
--PSNLSS
--PSNLLS
--PSNLST
--PSNLFS
--PSNLVR
--PSNLTL
--PSNLSV
--PSNLRV
--PSNLRY
--PSNLRR
--PSNLRQ
--PSNLLR
--PSNLWR
--PSNLLH
--PSNLTS
--PSNLFR
--PSNLVG
--PSNLRG

--PSNLTR
--PSNLRI
--PSNLKS
--PSNLRK
--PSNLLL
--PSNLVI
--PSNLSR
--PSNLSM
--PSNLLN
--PSNLYS
--PSNLSA
--PSNLSG
--PSNLSL
--PSNLLQ
--PSNLVW
--PSNLAG
--PSNLWQ
--PSNLYN
--PSNLFV
--PSNLSW
--PSNLAS
--PSNLTH
--PSNLRM
--PSNLFT
--PSNLLW
--PSNLVK
--PSNLMR
--PSNLLI
--PSNLFI
--PSNLWT
--PSNLTV
--PSNLTI
--PSNLKY
PVPSNL--
PSPSNL--
--PSNLQS
--PSNLES
--PSNLTT
--PSNLGS
--PSNLTM
--PSNLVV
--PSNLSK
--PSNLSH
--PSNLQL
--PSNLVM
--PSNLHS
--PSNLEV
--PSNLRW
--PSNLIT
--PSNLAT

--PSNLVY
--PSNLQG
--PSNLKG
--PSNLYA
--PSNLWN
--PSNLMS
--PSNLLK
--PSNLLA
PYPSNL--
--PSNLYR
--PSNLYL
--PSNLVF
--PSNLTG
--PSNLTA
--PSNLMA
--PSNLMF
--PSNLHT
--PSNLFG
--PSNLYT
--PSNLYG
--PSNLTD
--PSNLQR
--PSNLIN
--PSNLHL
--PSNLGT
--PSNLWF
--PSNLQA
--PSNLIS
--PSNLAA
PAPSNL--
--PSNLTY
--PSNLTQ
--PSNLMD
--PSNLHM
--PSNLFL
PGPSNL--
--PSNLWK
--PSNLVN
--PSNLSQ
--PSNLMN
--PSNLFN
--PSNLWL
--PSNLKN
--PSNLFM
--PSNLAW
--PSNLAF
--PSNLWV
--PSNLSY
--PSNLFW
--PSNLYI

--PSNLRD
--PSNLQN
--PSNLQH
--PSNLMK
--PSNLKK
PHPSNL--
--PSNLWM
--PSNLRF
--PSNLHA
--PSNLAV
--PSNLAI
--PSNLWI
--PSNLMQ
--PSNLKT
--PSNLIV
--PSNLFQ
--PSNLEA
PQPSNL--
PLPSNL--
--PSNLVQ
--PSNLTF
--PSNLSD
--PSNLQI
--PSNLIQ
--PSNLHV
--PSNLFY
--PSNLRE
--PSNLNT
--PSNLGF
--PSNLEW
--PSNLTK
--PSNLMT
--PSNLIW
--PSNLGG
--PSNLFK
--PSNLKH
--PSNLIH
--PSNLHQ
PKPSNL--
--PSNLYF
--PSNLSN
--PSNLIA
PIPSNL--
PFPSNL--
--PSNLTW
--PSNLNM
--PSNLMH
--PSNLKV
--PSNLGV
--PSNLFE

--PSNLYK
--PSNLWD
--PSNLVD
--PSNLTN
--PSNLQW
--PSNLIY
--PSNLIK
--PSNLHI
--PSNLET
--PSNLEL
--PSNLEF
--PSNLYW
--PSNLVH
--PSNLII
--PSNLHR
--PSNLFH
--PSNLER
--PSNLEH
--PSNLDG
--PSNLAH
PTPSNL--
--PSNLWW
--PSNLWA
--PSNLVE
--PSNLLD
--PSNLKW
--PSNLKQ
--PSNLFD
--PSNLAQ
PWPSNL--
--PSNLYY
--PSNLWH
--PSNLWE
--PSNLSE
--PSNLQY
--PSNLNV
--PSNLNR
--PSNLIM
--PSNLHG
PNPSNL--
--PSNLWG
--PSNLQE
--PSNLMV
--PSNLMI
--PSNLLM
--PSNLKM
--PSNLHH
--PSNLHF
--PSNLGM
--PSNLEQ

--PSNLEI
--PSNLEG
--PSNLYD
--PSNLNW
--PSNLNH
--PSNLNG
--PSNLID
--PSNLHD
--PSNLGI
--PSNLAK
--PSNLYE
--PSNLHK
--PSNLAY
--PSNLAN
--PSNLAM
--PSNLQM
--PSNLQD
--PSNLKI
--PSNLKD
--PSNLIG
--PSNLHW
--PSNLHN
--PSNLHE
--PSNLDS
--PSNLNL
--PSNLNF
--PSNLEM
PEPSNL--
PDPSNL--
--PSNLQV
--PSNLNN
--PSNLHY
--PSNLGY
--PSNLCE
--PSNLCA
--PSNLND
--PSNLMP
--PSNLKE
--PSNLIE
--PSNLED
--PSNLAD
--PSNLVC
--PSNLTC
--PSNLNQ
--PSNLMG
--PSNLGN
--PSNLGH
--PSNLEN
--PSNLEE
--PSNLDW

--PSNLDA
--PSNLCV
--PSNLCS
--PSNLCF
--PSNLYC
--PSNLTP
--PSNLQF
--PSNLPF
--PSNLNK
--PSNLMY
--PSNLGQ
--PSNLGK
--PSNLDI
--PSNLCY
--PSNLCG
-PPSNLY-
-PPSNLL-
-PPSNLF-
PCPSNL--
-QPSNLR-
QRPSNL--
QVPSNL--
QLPSNL--
-QPSNLS-
-QPSNLL-
QPPSNL--
-QPSNLW-
-QPSNLT-
-QPSNLK-
-QPSNLF-
QNPSNL--
QAPSNL--
-QPSNLY-
QKPSNL--
QFPSNL--
QWPSNL--
QMPSNL--
QYPSNL--
-QPSNLM-
-QPSNLG-
QQPSNL--
-QPSNLQ-
-QPSNLH-
QHPSNL--
-QPSNLA-
-QPSNLN-
QDPSNL--
QEPSNL--
QIPSNL--
-QPSNLI-

−QPSNLV−
QCPSNL−−
RVPSNL−−
−RPSNLL−
−RPSNLS−
RIPSNL−−
RSPSNL−−
−RPSNLI−
RGPSNL−−
RRPSNL−−
−RPSNLW−
−RPSNLK−
RMPSNL−−
RWPSNL−−
RTPSNL−−
−RPSNLV−
RFPSNL−−
RKPSNL−−
−RPSNLY−
RAPSNL−−
RNPSNL−−
RYPSNL−−
RPPSNL−−
RHPSNL−−
−RPSNLE−
−RPSNLA−
−RPSNLN−
−RPSNLH−
−RPSNLM−
RQPSNL−−
RDPSNL−−
−RPSNLQ−
RCPSNL−−
−RPSNLD−
−RPSNLC−
−SPSNLR−
−SPSNLL−
SSPSNL−−
−SPSNLS−
SRPSNL−−
−SPSNLI−
SKPSNL−−
−SPSNLV−
SMPSNL−−
SWPSNL−−
−SPSNLM−
SNPSNL−−
STPSNL−−
SGPSNL−−
SQPSNL−−

-SPSNLW-
-SPSNLQ-
SYPSNL--
-SPSNLN-
-SPSNLK-
-SPSNLH-
-SPSNLF-
-SPSNLA-
SFPSNL--
-SPSNLY-
SAPSNL--
SEPSNL--
SPPSNL--
-SPSNLG-
-SPSNLE-
SDPSNL--
-SPSNLC-
SCPSNL--
TVPSNL--
-TPSNLR-
TLPSNL--
TTPSNL--
TRPSNL--
TNPSNL--
-TPSNLL-
TIPSNL--
-TPSNLF-
-TPSNLS-
TPPSNL--
TGPSNL--
-TPSNLH-
TAPSNL--
-TPSNLW-
THPSNL--
-TPSNLV-
TQPSNL--
-TPSNLM-
-TPSNLE-
TKPSNL--
-TPSNLA-
-TPSNLI-
-TPSNLK-
-TPSNLQ-
TMPSNL--
TWPSNL--
-TPSNLN-
-TPSNLY-
-TPSNLG-
TDPSNL--
-TPSNLC-

VLPSNL--
-VPSNLT-
-VPSNLL-
VRPSNL--
VSPSNL--
VTPSNL--
-VPSNLA-
-VPSNLM-
-VPSNLV-
VHPSNL--
VGPSNL--
VMPSNL--
VAPSNL--
VYPSNL--
VFPSNL--
VNPSNL--
VEPSNL--
-VPSNLN-
VPPSNL--
-VPSNLK-
-VPSNLI-
-VPSNLY-
-VPSNLH-
-VPSNLF-
VWPSNL--
-VPSNLG-
VQPSNL--
-VPSNLQ-
-VPSNLE-
VDPSNL--
-VPSNLD-
-VPSNLC-
-WPSNLA-
-WPSNLL-
WSPSNL--
-WPSNLR-
WQPSNL--
WVPSNL--
-WPSNLT-
WKPSNL--
-WPSNLK-
-WPSNLY-
-WPSNLS-
WMPSNL--
WGPSNL--
WFPSNL--
WDPSNL--
-WPSNLQ-
-WPSNLV-
WPPSNL--

-WPSNLM-
-WPSNLW-
-WPSNLH-
WWPSNL--
WHPSNL--
-WPSNLG-
WIPSNL--
WAPSNL--
-WPSNLN-
-WPSNLI-
WNPSNL--
WYPSNL--
-WPSNLE-
WEPSNL--
-WPSNLF-
YYPSNL--
-YPSNLR-
YSPSNL--
YGPSNL--
-YPSNLV-
YTPSNL--
YHPSNL--
YVPSNL--
-YPSNLA-
YFPSNL--
YLPSNL--
-YPSNLS-
-YPSNLT-
-YPSNLQ-
-YPSNLH-
YKPSNL--
-YPSNLI-
YMPSNL--
YPPSNL--
YIPSNL--
YNPSNL--
YQPSNL--
-YPSNLW-
YAPSNL--
-YPSNLK-
YEPSNL--
-YPSNLM-
-YPSNLF-
-YPSNLG-
-YPSNLN-
YWPSNL--
YDPSNL--

PYPM 508 616 47.47

--PYPMGR
-SPYPMS-

SGPYPM--
-APYPMR-
ASPYPM--
-APYPML-
-APYPMV-
-APYPMT-
-APYPMK-
ATPYPM--
-APYPMY-
-APYPMG-
AYPYPM--
-APYPMF-
ANPYPM--
AVPYPM--
AQPYPM--
-APYPMH-
-APYPMA-
AKPYPM--
AHPYPM--
AGPYPM--
-APYPMS-
-APYPME-
APPYPM--
AFPYPM--
AAPYPM--
CHPYPM--
CFPYPM--
-CPYPMK-
CGPYPM--
-CPYPMH-
CIPYPM--
CDPYPM--
-DPYPMR-
-DPYPMK-
DKPYPM--
-DPYPMT-
DFPYPM--
DRPYPM--
-DPYPMV-
-DPYPMI-
-DPYPMF-
DYPYPM--
DTPYPM--
DSPYPM--
-DPYPMS-
-DPYPMN-
DIPYPM--
-EPYPMT-
EYPYPM--
ETPYPM--

-EPYPMS-
ERPYPM--
-EPYPML-
EVPYPM--
-EPYPMK-
EMPYPM--
ELPYPM--
EQPYPM--
-EPYPMW-
-EPYPMA-
EAPYPM--
-EPYPMF-
EHPYPM--
EFPYPM--
EEPYPM--
-FPYPMY-
FSPYPM--
-FPYPMS-
FDPYPM--
-FPYPMM-
-FPYPMF-
-FPYPMA-
FMPYPM--
-FPYPMK-
FWPYPM--
-FPYPMR-
FKPYPM--
FYPYPM--
FTPYPM--
FQPYPM--
-FPYPMN-
FVPYPM--
-FPYPMW-
-FPYPMI-
FNPYPM--
FGPYPM--
GRPYPM--
-GPYPMI-
-GPYPMY-
-GPYPMK-
GVPYPM--
GTPYPM--
GSPYPM--
-GPYPMV-
GEPYPM--
-GPYPMM-
GAPYPM--
GQPYPM--
-GPYPMT-
-GPYPMN-

GMPYPM--
GKPYPM--
GWPYPM--
-GPYPMW-
-GPYPMS-
GPPYPM--
GIPYPM--
GHPYPM--
GGPYPM--
GDPYPM--
-GPYPMQ-
-GPYPMH-
-GPYPMA-
GYPYPM--
-GPYPMF-
-GPYPME-
-GPYPMC-
GLPYPM--
GFPYPM--
-HPYPMR-
HMPYPM--
HGPYPM--
HRPYPM--
-HPYPMK-
-HPYPMF-
HYPYPM--
HLPYPM--
HTPYPM--
-HPYPMT-
-HPYPMS-
HEPYPM--
HVPYPM--
-HPYPMW-
-HPYPMV-
HKPYPM--
-HPYPMN-
-HPYPME-
HHPYPM--
HQPYPM--
-HPYPMQ-
-HPYPMM-
-HPYPMG-
HPPYPM--
HAPYPM--
IRPYPM--
-IPYPMK-
INPYPM--
-IPYPMV-
IAPYPM--
-IPYPMR-

```
-IPYPML-
IKPYPM--
IDPYPM--
-IPYPMQ-
-IPYPMM-
ISPYPM--
IIPYPM--
IGPYPM--
IYPYPM--
IVPYPM--
IMPYPM--
-IPYPMS-
-IPYPMI-
-IPYPMH-
-IPYPMT-
ILPYPM--
KSPYPM--
-KPYPMT-
-KPYPML-
-KPYPMH-
-KPYPMY-
KLPYPM--
KFPYPM--
KRPYPM--
KPPYPM--
KHPYPM--
KVPYPM--
KTPYPM--
-KPYPMR-
-KPYPMI-
KGPYPM--
-KPYPMA-
-KPYPMS-
KIPYPM--
KAPYPM--
-KPYPMW-
-KPYPMV-
-KPYPMQ-
-KPYPMG-
KQPYPM--
-KPYPMP-
-KPYPMN-
-KPYPMF-
-KPYPME-
KEPYPM--
LLPYPM--
LNPYPM--
-LPYPMF-
-LPYPMK-
-LPYPML-
```

LKPYPM--
LRPYPM--
-LPYPMM-
LTPYPM--
LIPYPM--
-LPYPMY-
LGPYPM--
-LPYPMH-
LVPYPM--
-LPYPMS-
-LPYPMT-
LFPYPM--
LSPYPM--
LHPYPM--
LYPYPM--
-LPYPMV-
-LPYPMA-
LDPYPM--
LAPYPM--
LWPYPM--
-MPYPMF-
MRPYPM--
MVPYPM--
-MPYPMR-
-MPYPMN-
-MPYPMI-
MIPYPM--
-MPYPMQ-
-MPYPML-
MFPYPM--
-MPYPMS-
MTPYPM--
MMPYPM--
MSPYPM--
MNPYPM--
MLPYPM--
MKPYPM--
MGPYPM--
-MPYPMV-
-MPYPMT-
NIPYPM--
NLPYPM--
NTPYPM--
-NPYPML-
NAPYPM--
NQPYPM--
-NPYPMH-
NPPYPM--
NSPYPM--
NRPYPM--

-NPYPMY-
NFPYPM--
-NPYPMW-
NVPYPM--
-NPYPMT-
NYPYPM--
-NPYPMN-
-NPYPMF-
-NPYPMA-
-NPYPMM-
NMPYPM--
NGPYPM--
--PYPMRS
--PYPMML
--PYPMLS
--PYPMRT
--PYPMSS
--PYPMLV
--PYPMRL
--PYPMQI
--PYPMLN
--PYPMIL
--PYPMKT
--PYPMVA
--PYPMRY
--PYPMVL
--PYPMIA
--PYPMMT
--PYPMYK
--PYPMQR
--PYPMLL
--PYPMLG
--PYPMGS
--PYPMYV
--PYPMRI
--PYPMRF
--PYPMHS
--PYPMQS
--PYPMFM
--PYPMWS
--PYPMWI
--PYPMVH
--PYPMSR
--PYPMSN
--PYPMQK
--PYPMMW
--PYPMIR
--PYPMTA
--PYPMRN
--PYPMLQ

--PYPMLF
--PYPMHG
--PYPMYD
--PYPMTT
--PYPMRH
--PYPMMY
--PYPMLM
--PYPMIE
--PYPMWG
--PYPMTI
--PYPMLH
--PYPMKN
--PYPMIT
--PYPMIG
--PYPMHR
--PYPMFG
--PYPMVN
--PYPMSH
--PYPMRM
--PYPMRG
--PYPMNA
--PYPMGQ
--PYPMGH
--PYPMAG
--PYPMWK
--PYPMVT
--PYPMVM
--PYPMST
--PYPMRQ
--PYPMRE
--PYPMRD
--PYPMMR
--PYPMHA
--PYPMFD
--PYPMYT
--PYPMYN
--PYPMYL
--PYPMWH
--PYPMTW
--PYPMRW
--PYPMMN
--PYPMKR
--PYPMKQ
--PYPMKM
--PYPMKD
--PYPMIN
--PYPMAN
--PYPMYA
--PYPMVW
--PYPMTY

--PYPMTR
--PYPMMS
--PYPMKV
--PYPMKF
--PYPMKA
--PYPMIY
--PYPMHD
--PYPMES
--PYPMYM
--PYPMWE
--PYPMVK
--PYPMTG
--PYPMRV
--PYPMQA
--PYPMNS
--PYPMHI
--PYPMGN
--PYPMFS
--PYPMEQ
--PYPMAR
--PYPMYS
--PYPMYI
--PYPMWW
--PYPMVQ
--PYPMTQ
--PYPMSG
--PYPMQW
--PYPMPV
--PYPMNT
--PYPMME
--PYPMLK
--PYPMKY
--PYPMIH
--PYPMHY
--PYPMFK
--PYPMFA
--PYPMET
--PYPMER
--PYPMAF
--PYPMWR
--PYPMWM
--PYPMVR
--PYPMVG
--PYPMTV
--PYPMTS
--PYPMPS
--PYPMND
--PYPMMK
--PYPMMH
--PYPMHF

--PYPMGA
--PYPMFE
--PYPMEH
--PYPMEA
--PYPMAT
--PYPMYH
--PYPMVS
--PYPMTN
--PYPMTH
--PYPMRK
--PYPMNE
--PYPMIV
--PYPMFT
--PYPMEY
--PYPMEW
--PYPMEK
--PYPMEI
--PYPMEF
--PYPMDS
--PYPMYG
--PYPMWV
--PYPMWN
--PYPMWL
--PYPMVV
--PYPMTD
--PYPMSD
--PYPMQM
--PYPMQG
--PYPMQD
--PYPMNQ
--PYPMNK
--PYPMNH
--PYPMMD
--PYPMLE
--PYPMKE
--PYPMIW
--PYPMIK
--PYPMGT
--PYPMGG
--PYPMFN
--PYPMFH
--PYPMDW
--PYPMAW
--PYPMAS
QRPYPM--
QVPYPM--
-QPYPMS-
QYPYPM--
-QPYPMK-
-QPYPMV-

QWPYPM--
-QPYPMT-
-QPYPML-
-QPYPMQ-
-QPYPMI-
QPPYPM--
QKPYPM--
-QPYPMN-
QEPYPM--
-QPYPMR-
-QPYPMG-
-QPYPMF-
-QPYPMA-
QGPYPM--
QFPYPM--
RIPYPM--
-RPYPMT-
RAPYPM--
RSPYPM--
RNPYPM--
-RPYPMV-
RRPYPM--
RLPYPM--
RVPYPM--
-RPYPMG-
-RPYPMA-
RGPYPM--
RTPYPM--
-RPYPMR-
RMPYPM--
-RPYPMF-
RYPYPM--
RPPYPM--
-RPYPMH-
-RPYPMS-
-RPYPMY-
-RPYPMN-
-RPYPMI-
RKPYPM--
REPYPM--
-RPYPMM-
-RPYPMK-
RDPYPM--
-RPYPMP-
-RPYPME-
SSPYPM--
SIPYPM--
-SPYPMR-
SRPYPM--
SHPYPM--

STPYPM--
SYPYPM--
SMPYPM--
-SPYPMY-
-SPYPMV-
SLPYPM--
-SPYPMF-
SAPYPM--
SPPYPM--
SNPYPM--
-SPYPMK-
SKPYPM--
-SPYPMG-
-SPYPMN-
-SPYPMA-
-TPYPMR-
TSPYPM--
-TPYPMS-
-TPYPMQ-
-TPYPMM-
-TPYPML-
TDPYPM--
-TPYPMI-
TRPYPM--
-TPYPMK-
TNPYPM--
TLPYPM--
TAPYPM--
TVPYPM--
TGPYPM--
-TPYPMT-
TMPYPM--
-TPYPMF-
TYPYPM--
-TPYPMV-
TKPYPM--
TTPYPM--
-TPYPMA-
TPPYPM--
-TPYPMH-
-TPYPMG-
-VPYPMT-
VFPYPM--
-VPYPMQ-
VRPYPM--
-VPYPMR-
VSPYPM--
-VPYPMS-
-VPYPMI-
-VPYPML-

VIPYPM--
VHPYPM--
VDPYPM--
VKPYPM--
-VPYPMM-
-VPYPMK-
-VPYPMG-
-VPYPMF-
VLPYPM--
-VPYPMV-
-VPYPMC-
VGPYPM--
VAPYPM--
VYPYPM--
VVPYPM--
-VPYPME-
-VPYPMA-
VPPYPM--
VMPYPM--
WKPYPM--
WQPYPM--
WPPYPM--
-WPYPMW-
WSPYPM--
-WPYPMH-
-WPYPMY-
WEPYPM--
-WPYPMK-
WIPYPM--
-WPYPMA-
-WPYPMT-
-WPYPMS-
-WPYPML-
-WPYPMN-
-WPYPMM-
YMPYPM--
YFPYPM--
YVPYPM--
YSPYPM--
-YPYPMR-
-YPYPMT-
-YPYPMA-
YYPYPM--
-YPYPMY-
YRPYPM--
YWPYPM--
-YPYPML-
YAPYPM--
YTPYPM--
-YPYPMS-

	YLPYPM--		
	YGPYPM--		
	YDPYPM--		
	-YPYPMV-		
	-YPYPMH-		
	-YPYPMF-		
	YQPYPM--		
	-YPYPMM-		
	-YPYPMI-		
	-YPYPMW-		
	-YPYPMP-		
	-YPYPMK-		
	-YPYPMG-		
	YKPYPM--		
PMGY	93	810	47.46
	-APMGYF-		
	AFPMGY--		
	-APMGYS-		
	-APMGYI-		
	AYPMGY--		
	AWPMGY--		
	-APMGYT-		
	AMPMGY--		
	-APMGYK-		
	AKPMGY--		
	ARPMGY--		
	ASPMGY--		
	-APMGYG-		
	-APMGYY-		
	-APMGYM-		
	APPMGY--		
	-APMGYR-		
	ANPMGY--		
	-APMGYN-		
	AQPMGY--		
	ATPMGY--		
	-APMGYW-		
	-APMGYL-		
	-APMGYA-		
	-APMGYV-		
	AEPMGY--		
	AAPMGY--		
	AVPMGY--		
	-APMGYH-		
	AHPMGY--		
	AGPMGY--		
	-APMGYQ-		
	-APMGYE-		
	ADPMGY--		
	-CPMGYS-		

CMPMGY--
CPPMGY--
CVPMGY--
CTPMGY--
CSPMGY--
CRPMGY--
CQPMGY--
-CPMGYA-
CHPMGY--
CGPMGY--
-CPMGYY-
CKPMGY--
CCPMGY--
-CPMGYR-
CNPMGY--
CIPMGY--
DMPMGY--
-DPMGYI-
DVPMGY--
DQPMGY--
DNPMGY--
-DPMGYY-
DSPMGY--
-DPMGYR-
DKPMGY--
-DPMGYK-
DLPMGY--
DTPMGY--
-DPMGYS-
DHPMGY--
-DPMGYL-
-DPMGYA-
DIPMGY--
DDPMGY--
-DPMGYF-
DGPMGY--
DAPMGY--
-DPMGYG-
-DPMGYE-
-EPMGYV-
-EPMGYF-
EGPMGY--
-EPMGYL-
ETPMGY--
-EPMGYM-
ESPMGY--
-EPMGYI-
-EPMGYR-
EMPMGY--
ERPMGY--

-EPMGYK-
EEPMGY--
EVPMGY--
-EPMGYT-
ELPMGY--
-EPMGYA-
EIPMGY--
EYPMGY--
-EPMGYG-
EPPMGY--
-EPMGYQ-
-EPMGYH-
EHPMGY--
EAPMGY--
-EPMGYE-
ENPMGY--
-FPMGYT-
FVPMGY--
-FPMGYV-
FGPMGY--
FQPMGY--
-FPMGYN-
FFPMGY--
-FPMGYS-
-FPMGYL-
-FPMGYA-
FKPMGY--
FSPMGY--
-FPMGYK-
-FPMGYF-
-FPMGYW-
FAPMGY--
-FPMGYY-
FEPMGY--
FRPMGY--
FTPMGY--
-FPMGYQ-
-FPMGYM-
-FPMGYH-
FLPMGY--
FNPMGY--
FHPMGY--
FYPMGY--
FDPMGY--
-FPMGYG-
GTPMGY--
-GPMGYS-
-GPMGYV-
-GPMGYY-
-GPMGYR-

-GPMGYL-
GNPMGY--
GRPMGY--
-GPMGYT-
-GPMGYG-
GMPMGY--
GKPMGY--
GSPMGY--
-GPMGYM-
GWPMGY--
GAPMGY--
GVPMGY--
-GPMGYQ-
-GPMGYI-
-GPMGYH-
GLPMGY--
GCPMGY--
-GPMGYK-
GYPMGY--
-GPMGYW-
-GPMGYE-
GDPMGY--
-GPMGYN-
-GPMGYF-
-GPMGYA-
GIPMGY--
GEPMGY--
-HPMGYL-
HSPMGY--
HTPMGY--
-HPMGYT-
HGPMGY--
-HPMGYR-
HKPMGY--
HYPMGY--
HQPMGY--
-HPMGYH-
-HPMGYS-
HRPMGY--
HVPMGY--
-HPMGYA-
HLPMGY--
HAPMGY--
-HPMGYK-
-HPMGYW-
-HPMGYF-
HNPMGY--
HWPMGY--
-HPMGYQ-
-HPMGYI-

-HPMGYG-
-HPMGYY-
HHPMGY--
HDPMGY--
ISPMGY--
-IPMGYA-
-IPMGYK-
IDPMGY--
IRPMGY--
ILPMGY--
INPMGY--
-IPMGYQ-
-IPMGYF-
IPPMGY--
-IPMGYV-
IQPMGY--
-IPMGYL-
-IPMGYY-
-IPMGYT-
IMPMGY--
IEPMGY--
-IPMGYS-
-IPMGYM-
IAPMGY--
IWPMGY--
ITPMGY--
-IPMGYW-
-IPMGYR-
IYPMGY--
-IPMGYN-
-IPMGYH-
-IPMGYE-
IIPMGY--
IFPMGY--
KSPMGY--
-KPMGYL-
KWPMGY--
KAPMGY--
KKPMGY--
KFPMGY--
-KPMGYT-
-KPMGYK-
KTPMGY--
-KPMGYN-
-KPMGYY-
-KPMGYV-
-KPMGYA-
KPPMGY--
-KPMGYW-
-KPMGYR-

-KPMGYM-
-KPMGYI-
KNPMGY--
KLPMGY--
-KPMGYH-
-KPMGYF-
KRPMGY--
KQPMGY--
-KPMGYS-
KGPMGY--
KVPMGY--
-KPMGYG-
KYPMGY--
KDPMGY--
KEPMGY--
LRPMGY--
LMPMGY--
-LPMGYR-
-LPMGYM-
LVPMGY--
LSPMGY--
LTPMGY--
LGPMGY--
LHPMGY--
LKPMGY--
LPPMGY--
LIPMGY--
LLPMGY--
LAPMGY--
-LPMGYT-
LFPMGY--
-LPMGYV-
LWPMGY--
-LPMGYI-
LNPMGY--
LYPMGY--
-LPMGYH-
LDPMGY--
-LPMGYK-
-LPMGYF-
LEPMGY--
-LPMGYS-
LQPMGY--
-LPMGYQ-
-LPMGYN-
-LPMGYY-
-LPMGYA-
MSPMGY--
-MPMGYS-
MKPMGY--

-MPMGYV-
MRPMGY--
-MPMGYR-
MWPMGY--
MTPMGY--
-MPMGYM-
-MPMGYL-
MEPMGY--
MFPMGY--
MPPMGY--
MHPMGY--
MGPMGY--
-MPMGYK-
MAPMGY--
-MPMGYQ-
MMPMGY--
MIPMGY--
MYPMGY--
-MPMGYN-
MDPMGY--
-MPMGYY-
-MPMGYH-
-MPMGYT-
-MPMGYE-
-MPMGYI-
MCPMGY--
NSPMGY--
NYPMGY--
NVPMGY--
NAPMGY--
-NPMGYT-
NRPMGY--
NLPMGY--
NDPMGY--
-NPMGYY-
-NPMGYS-
NNPMGY--
NMPMGY--
NTPMGY--
NGPMGY--
-NPMGYN-
-NPMGYK-
-NPMGYM-
-NPMGYI-
-NPMGYL-
-NPMGYF-
NQPMGY--
NHPMGY--
NWPMGY--
-NPMGYW-

-NPMGYR-
-NPMGYQ-
-NPMGYH-
-NPMGYV-
-NPMGYA-
NIPMGY--
--PMGYRS
--PMGYRG
--PMGYTS
--PMGYVT
--PMGYTL
--PMGYSH
--PMGYWR
--PMGYTG
--PMGYSR
--PMGYLR
--PMGYLN
--PMGYVR
--PMGYVA
--PMGYWN
--PMGYMS
--PMGYAG
--PMGYYR
--PMGYTT
--PMGYRT
--PMGYLH
--PMGYWT
--PMGYVH
--PMGYSK
--PMGYLA
--PMGYIV
--PMGYRH
--PMGYLS
--PMGYQT
--PMGYVV
--PMGYVS
--PMGYKS
--PMGYYM
--PMGYYI
--PMGYMW
--PMGYMR
--PMGYRA
--PMGYNR
--PMGYLT
--PMGYKN
--PMGYIT
--PMGYFQ
--PMGYRN
--PMGYLQ
--PMGYRV

--PMGYQL
--PMGYMG
--PMGYIS
PVPMGY--
--PMGYSW
--PMGYRY
--PMGYRD
--PMGYHN
--PMGYYV
--PMGYTR
--PMGYST
--PMGYRQ
--PMGYQR
--PMGYNY
--PMGYKV
--PMGYEG
--PMGYYN
--PMGYYG
--PMGYWL
--PMGYQG
--PMGYNS
--PMGYLV
--PMGYHW
--PMGYWG
--PMGYTD
--PMGYSI
--PMGYRM
--PMGYMN
--PMGYMA
--PMGYKW
--PMGYKI
--PMGYHV
--PMGYHR
--PMGYFG
--PMGYWE
--PMGYSQ
--PMGYRW
--PMGYQS
--PMGYNT
--PMGYLI
--PMGYKY
--PMGYKR
--PMGYHD
--PMGYFT
--PMGYFK
PEPMGY--
PYPMGY--
PMPMGY--
--PMGYYS
--PMGYRF

--PMGYQK
--PMGYML
--PMGYLK
--PMGYKT
--PMGYII
--PMGYFM
PWPMGY--
--PMGYYT
--PMGYVK
--PMGYTN
--PMGYTA
--PMGYSM
--PMGYQH
--PMGYQA
--PMGYKH
--PMGYGS
--PMGYGA
--PMGYAA
PQPMGY--
--PMGYVY
--PMGYTY
--PMGYSY
--PMGYNA
--PMGYKL
--PMGYHT
PIPMGY--
--PMGYYA
--PMGYWH
--PMGYVM
--PMGYSV
--PMGYSN
--PMGYMF
--PMGYFF
--PMGYFA
--PMGYAQ
--PMGYAF
--PMGYTE
--PMGYQV
--PMGYNQ
--PMGYNL
--PMGYNG
--PMGYMQ
--PMGYLM
--PMGYIR
--PMGYIN
--PMGYIK
--PMGYIA
--PMGYER
--PMGYAV
PTPMGY--

--PMGYYQ
--PMGYYH
--PMGYWS
--PMGYVE
--PMGYVD
--PMGYSG
--PMGYNH
--PMGYKF
--PMGYKD
--PMGYAM
PGPMGY--
--PMGYWW
--PMGYTK
--PMGYRE
--PMGYNW
--PMGYNF
--PMGYMI
--PMGYKA
--PMGYGY
--PMGYGF
--PMGYAY
--PMGYAI
PHPMGY--
--PMGYVI
--PMGYVG
--PMGYSF
--PMGYNN
--PMGYNM
--PMGYKK
--PMGYIH
--PMGYGV
--PMGYGT
--PMGYGM
--PMGYGI
--PMGYEM
--PMGYYE
--PMGYTI
--PMGYTF
--PMGYQQ
--PMGYQN
--PMGYME
--PMGYMD
--PMGYHG
--PMGYGW
--PMGYGL
--PMGYGG
--PMGYFR
--PMGYEY
--PMGYES
PAPMGY--

--PMGYWA
--PMGYNI
--PMGYMY
--PMGYLC
--PMGYKG
--PMGYIM
--PMGYIG
--PMGYET
--PMGYEQ
--PMGYEH
--PMGYDG
--PMGYAT
--PMGYAH
--PMGYYK
--PMGYTV
--PMGYTQ
--PMGYSE
--PMGYQI
--PMGYNK
--PMGYNE
--PMGYMV
--PMGYKM
--PMGYIQ
--PMGYGK
--PMGYFH
--PMGYEK
--PMGYCA
--PMGYAK
-PPMGYA-
PNPMGY--
--PMGYWV
--PMGYWD
--PMGYVQ
--PMGYTW
--PMGYQD
--PMGYPL
--PMGYPK
--PMGYPF
--PMGYNV
--PMGYMK
--PMGYMH
--PMGYLD
--PMGYHY
--PMGYHI
--PMGYHH
--PMGYHF
--PMGYGH
--PMGYEV
--PMGYEL
--PMGYDS

--PMGYCE
QKPMGY--
QAPMGY--
QSPMGY--
-QPMGYI-
-QPMGYA-
-QPMGYM-
-QPMGYH-
QRPMGY--
-QPMGYL-
QFPMGY--
QIPMGY--
-QPMGYS-
QTPMGY--
QMPMGY--
QLPMGY--
QQPMGY--
-QPMGYF-
QYPMGY--
-QPMGYQ-
QVPMGY--
-QPMGYN-
QEPMGY--
-QPMGYR-
QDPMGY--
QPPMGY--
-QPMGYK-
RRPMGY--
-RPMGYL-
-RPMGYS-
-RPMGYT-
RMPMGY--
RVPMGY--
RFPMGY--
-RPMGYR-
RGPMGY--
RTPMGY--
RNPMGY--
-RPMGYI-
RSPMGY--
RPPMGY--
-RPMGYA-
-RPMGYN-
RDPMGY--
RHPMGY--
RAPMGY--
RLPMGY--
REPMGY--
-RPMGYY-
-RPMGYQ-

-RPMGYW-
-RPMGYK-
-RPMGYV-
-RPMGYH-
-RPMGYF-
RQPMGY--
-RPMGYE-
-RPMGYG-
-RPMGYM-
RWPMGY--
RYPMGY--
-RPMGYC-
RCPMGY--
-SPMGYS-
STPMGY--
-SPMGYR-
SRPMGY--
SSPMGY--
SYPMGY--
SLPMGY--
-SPMGYV-
-SPMGYI-
-SPMGYK-
SDPMGY--
SGPMGY--
-SPMGYF-
SIPMGY--
-SPMGYT-
SEPMGY--
SHPMGY--
SPPMGY--
-SPMGYQ-
-SPMGYG-
SNPMGY--
SFPMGY--
SAPMGY--
SMPMGY--
-SPMGYA-
-SPMGYH-
SWPMGY--
-SPMGYW-
-SPMGYM-
-SPMGYY-
-SPMGYN-
SQPMGY--
-TPMGYS-
TGPMGY--
-TPMGYV-
-TPMGYR-
-TPMGYL-

TAPMGY--
TWPMGY--
TRPMGY--
TLPMGY--
-TPMGYY-
TNPMGY--
-TPMGYI-
TTPMGY--
-TPMGYW-
-TPMGYT-
TVPMGY--
-TPMGYF-
-TPMGYH-
TKPMGY--
TQPMGY--
TSPMGY--
TIPMGY--
-TPMGYN-
TDPMGY--
TCPMGY--
-TPMGYQ-
THPMGY--
TPPMGY--
TEPMGY--
TMPMGY--
TYPMGY--
-TPMGYG-
-TPMGYP-
-TPMGYE-
VSPMGY--
-VPMGYH-
-VPMGYV-
VKPMGY--
VIPMGY--
-VPMGYT-
VRPMGY--
-VPMGYK-
VGPMGY--
-VPMGYF-
-VPMGYY-
-VPMGYA-
VTPMGY--
VDPMGY--
-VPMGYN-
-VPMGYG-
VHPMGY--
VPPMGY--
VAPMGY--
-VPMGYS-
VLPMGY--

-VPMGYM-
VFPMGY--
VQPMGY--
-VPMGYI-
-VPMGYE-
VNPMGY--
VMPMGY--
VEPMGY--
VWPMGY--
-VPMGYQ-
-VPMGYD-
-VPMGYC-
WSPMGY--
-WPMGYL-
-WPMGYS-
WKPMGY--
WMPMGY--
WHPMGY--
-WPMGYI-
WRPMGY--
-WPMGYQ-
-WPMGYG-
-WPMGYF-
-WPMGYT-
-WPMGYM-
WLPMGY--
-WPMGYV-
WTPMGY--
WQPMGY--
-WPMGYK-
-WPMGYA-
WGPMGY--
-WPMGYR-
-WPMGYE-
WFPMGY--
WEPMGY--
WIPMGY--
-WPMGYN-
-WPMGYH-
WYPMGY--
WWPMGY--
WVPMGY--
WPPMGY--
-WPMGYW-
WAPMGY--
WDPMGY--
-YPMGYR-
-YPMGYL-
YRPMGY--
-YPMGYS-

	YSPMGY--		
	YGPMGY--		
	YTPMGY--		
	-YPMGYV-		
	YYPMGY--		
	YVPMGY--		
	YMPMGY--		
	YWPMGY--		
	YLPMGY--		
	YEPMGY--		
	-YPMGYY-		
	-YPMGYA-		
	YAPMGY--		
	-YPMGYQ-		
	-YPMGYN-		
	-YPMGYM-		
	-YPMGYH-		
	-YPMGYF-		
	YDPMGY--		
	-YPMGYK-		
	YPPMGY--		
	-YPMGYT-		
	YCPMGY--		
	-YPMGYG-		
	YIPMGY--		
PYAF	386	664	47.46
	-FPYAFA-		
	-GPYAFH-		
	-SPYAFN-		
	-TPYAFN-		
	-TPYAFE-		
	ATPYAF--		
	-APYAFR-		
	-APYAFM-		
	ASPYAF--		
	AVPYAF--		
	-APYAFT-		
	-APYAFW-		
	ANPYAF--		
	AIPYAF--		
	AGPYAF--		
	AWPYAF--		
	AKPYAF--		
	AMPYAF--		
	AEPYAF--		
	ADPYAF--		
	-APYAFN-		
	-APYAFF-		
	AFPYAF--		
	AAPYAF--		

-APYAFY-
-APYAFI-
APPYAF--
AQPYAF--
-APYAFH-
AHPYAF--
-APYAFP-
-CPYAFT-
-CPYAFR-
CTPYAF--
CGPYAF--
CAPYAF--
CQPYAF--
-CPYAFL-
CNPYAF--
CMPYAF--
CLPYAF--
CEPYAF--
CDPYAF--
DSPYAF--
DQPYAF--
DGPYAF--
DYPYAF--
DFPYAF--
DRPYAF--
DMPYAF--
DIPYAF--
-DPYAFS-
DTPYAF--
-DPYAFY-
-DPYAFT-
-DPYAFH-
DNPYAF--
DVPYAF--
-DPYAFW-
-DPYAFM-
-DPYAFI-
DLPYAF--
DEPYAF--
DAPYAF--
ESPYAF--
ERPYAF--
EEPYAF--
EKPYAF--
EQPYAF--
-EPYAFR-
EIPYAF--
-EPYAFL-
-EPYAFV-
-EPYAFK-

-EPYAFM-
EVPYAF--
ETPYAF--
-EPYAFT-
ENPYAF--
ELPYAF--
EGPYAF--
EDPYAF--
EAPYAF--
FQPYAF--
FGPYAF--
FNPYAF--
FKPYAF--
-FPYAFM-
FSPYAF--
-FPYAFT-
-FPYAFH-
FAPYAF--
FVPYAF--
FTPYAF--
FRPYAF--
-FPYAFQ-
FMPYAF--
FWPYAF--
-FPYAFS-
-FPYAFK-
FIPYAF--
FDPYAF--
-FPYAFN-
-FPYAFP-
FLPYAF--
GRPYAF--
GSPYAF--
GAPYAF--
-GPYAFS-
GVPYAF--
GWPYAF--
GIPYAF--
GKPYAF--
GLPYAF--
GGPYAF--
GTPYAF--
-GPYAFV-
-GPYAFI-
-GPYAFW-
GNPYAF--
GFPYAF--
-GPYAFK-
-GPYAFN-
-GPYAFQ-

GEPYAF--
-GPYAFL-
GHPYAF--
-GPYAFR-
-GPYAFM-
-GPYAFF-
GMPYAF--
GDPYAF--
-HPYAFR-
HRPYAF--
-HPYAFS-
HQPYAF--
HSPYAF--
HLPYAF--
HVPYAF--
-HPYAFT-
-HPYAFW-
-HPYAFA-
-HPYAFL-
HKPYAF--
HTPYAF--
-HPYAFM-
-HPYAFN-
-HPYAFK-
HNPYAF--
HEPYAF--
HAPYAF--
-IPYAFL-
ISPYAF--
IRPYAF--
IPPYAF--
-IPYAFM-
-IPYAFS-
IWPYAF--
IGPYAF--
INPYAF--
IYPYAF--
-IPYAFQ-
IEPYAF--
-IPYAFK-
IVPYAF--
IKPYAF--
-IPYAFV-
-IPYAFI-
-IPYAFH-
-IPYAFA-
ILPYAF--
IIPYAF--
KSPYAF--
KTPYAF--

-KPYAFL-
KYPYAF--
KKPYAF--
KGPYAF--
KPPYAF--
-KPYAFT-
-KPYAFS-
-KPYAFR-
-KPYAFM-
-KPYAFA-
KRPYAF--
KVPYAF--
KMPYAF--
-KPYAFK-
-KPYAFY-
KEPYAF--
-KPYAFQ-
KLPYAF--
-KPYAFF-
KHPYAF--
-KPYAFI-
-KPYAFV-
-KPYAFN-
KFPYAF--
-KPYAFE-
KNPYAF--
KAPYAF--
-KPYAFW-
-KPYAFC-
LRPYAF--
LGPYAF--
LQPYAF--
LTPYAF--
LKPYAF--
LYPYAF--
LLPYAF--
LHPYAF--
LAPYAF--
LSPYAF--
-LPYAFH-
-LPYAFS-
-LPYAFK-
LDPYAF--
-LPYAFI-
LEPYAF--
-LPYAFQ-
LNPYAF--
LVPYAF--
-LPYAFM-
LWPYAF--

−LPYAFW−
MRPYAF−−
MNPYAF−−
MYPYAF−−
MGPYAF−−
MQPYAF−−
−MPYAFR−
MIPYAF−−
MTPYAF−−
−MPYAFV−
−MPYAFA−
−MPYAFK−
MVPYAF−−
−MPYAFN−
−MPYAFS−
−MPYAFM−
−MPYAFL−
MKPYAF−−
MAPYAF−−
−MPYAFH−
MPPYAF−−
MHPYAF−−
MDPYAF−−
MCPYAF−−
NAPYAF−−
NNPYAF−−
NEPYAF−−
−NPYAFR−
NLPYAF−−
−NPYAFW−
NTPYAF−−
NVPYAF−−
−NPYAFK−
−NPYAFH−
−NPYAFS−
−NPYAFV−
−NPYAFL−
NSPYAF−−
−NPYAFT−
−NPYAFM−
NQPYAF−−
−NPYAFI−
NYPYAF−−
NMPYAF−−
NKPYAF−−
NGPYAF−−
NFPYAF−−
NDPYAF−−
NWPYAF−−
NRPYAF−−

-NPYAFA-
NIPYAF--
NHPYAF--
--PYAFSM
--PYAFRI
--PYAFRA
--PYAFRS
--PYAFAS
--PYAFTY
--PYAFRL
--PYAFSG
--PYAFAG
--PYAFNL
--PYAFSS
--PYAFRT
--PYAFSF
--PYAFRQ
--PYAFVS
--PYAFSL
--PYAFMN
--PYAFLG
--PYAFAA
--PYAFRG
--PYAFQT
--PYAFNS
--PYAFPL
--PYAFTL
--PYAFST
--PYAFSA
--PYAFKS
--PYAFHY
--PYAFGY
PGPYAF--
--PYAFVR
--PYAFVQ
--PYAFLS
--PYAFYW
--PYAFRW
--PYAFNA
--PYAFHA
--PYAFVT
--PYAFTG
--PYAFSQ
--PYAFRN
--PYAFRH
--PYAFQY
--PYAFHL
--PYAFAL
--PYAFKT
--PYAFNT

--PYAFNF
--PYAFMS
--PYAFKR
--PYAFIE
--PYAFHQ
--PYAFQV
--PYAFKM
--PYAFVH
--PYAFTQ
--PYAFSY
--PYAFQS
--PYAFKI
--PYAFIS
--PYAFHS
--PYAFHN
--PYAFHM
--PYAFHF
--PYAFGL
--PYAFAM
--PYAFWL
--PYAFQR
--PYAFKG
--PYAFKF
--PYAFAQ
--PYAFTK
--PYAFTA
--PYAFSR
--PYAFNM
--PYAFLN
--PYAFKK
--PYAFIG
--PYAFTN
--PYAFSD
--PYAFRD
--PYAFQM
--PYAFQG
--PYAFPY
--PYAFNN
--PYAFLA
--PYAFSH
--PYAFQW
--PYAFQL
--PYAFMA
--PYAFLK
--PYAFHT
--PYAFHG
--PYAFGS
--PYAFFQ
--PYAFYQ
--PYAFPM

--PYAFMT
--PYAFMQ
--PYAFKN
--PYAFKE
--PYAFIK
--PYAFHV
--PYAFHR
--PYAFGV
--PYAFGI
--PYAFFI
--PYAFFG
--PYAFFD
--PYAFWT
--PYAFWR
--PYAFWK
--PYAFVK
--PYAFVF
--PYAFTR
--PYAFSN
--PYAFSK
--PYAFRE
--PYAFNI
--PYAFME
--PYAFKY
--PYAFHI
--PYAFHH
--PYAFFN
--PYAFEG
PTPYAF--
PQPYAF--
--PYAFYG
--PYAFWG
--PYAFVN
--PYAFVG
--PYAFTT
--PYAFTH
--PYAFSW
--PYAFQK
--PYAFQF
--PYAFNR
--PYAFLR
--PYAFLH
--PYAFKV
--PYAFIQ
--PYAFGG
--PYAFFY
--PYAFEW
--PYAFAY
--PYAFAF
PIPYAF--

PDPYAF--
--PYAFWS
--PYAFVM
--PYAFVI
--PYAFTM
--PYAFTI
--PYAFQI
--PYAFQH
--PYAFPF
--PYAFNW
--PYAFNG
--PYAFLI
--PYAFKQ
--PYAFKH
--PYAFKD
--PYAFIM
--PYAFIH
--PYAFGQ
--PYAFGF
--PYAFFF
--PYAFES
--PYAFEI
--PYAFEF
--PYAFAN
PWPYAF--
-PPYAFL-
PHPYAF--
--PYAFYC
--PYAFWI
--PYAFWF
--PYAFWD
--PYAFVD
--PYAFTD
--PYAFQQ
--PYAFNK
--PYAFNH
--PYAFMV
--PYAFMG
--PYAFMF
--PYAFLE
--PYAFII
--PYAFHK
--PYAFFC
--PYAFET
--PYAFEN
--PYAFEM
--PYAFDF
--PYAFAT
--PYAFAK
PMPYAF--

PEPYAF--
PCPYAF--
QRPYAF--
QLPYAF--
QIPYAF--
-QPYAFR-
-QPYAFL-
-QPYAFM-
QPPYAF--
-QPYAFS-
QAPYAF--
QSPYAF--
-QPYAFH-
QYPYAF--
QFPYAF--
QQPYAF--
-QPYAFK-
QMPYAF--
-QPYAFW-
QKPYAF--
QGPYAF--
-QPYAFV-
QEPYAF--
QVPYAF--
-QPYAFP-
QNPYAF--
RLPYAF--
RMPYAF--
RYPYAF--
RAPYAF--
-RPYAFL-
-RPYAFS-
-RPYAFT-
-RPYAFI-
RFPYAF--
RTPYAF--
-RPYAFH-
-RPYAFR-
-RPYAFQ-
RGPYAF--
RSPYAF--
RRPYAF--
-RPYAFN-
-RPYAFW-
RWPYAF--
-RPYAFK-
REPYAF--
-RPYAFM-
RKPYAF--
RCPYAF--

RPPYAF--
RNPYAF--
RQPYAF--
-RPYAFE-
RDPYAF--
-RPYAFG-
-RPYAFA-
-RPYAFV-
RHPYAF--
SRPYAF--
SNPYAF--
-SPYAFH-
STPYAF--
-SPYAFF-
SYPYAF--
SSPYAF--
-SPYAFK-
SLPYAF--
SWPYAF--
-SPYAFW-
-SPYAFM-
-SPYAFV-
-SPYAFS-
SVPYAF--
SIPYAF--
-SPYAFQ-
SKPYAF--
SMPYAF--
-SPYAFI-
SAPYAF--
SHPYAF--
SQPYAF--
SPPYAF--
SFPYAF--
SGPYAF--
-SPYAFT-
-SPYAFA-
SDPYAF--
SEPYAF--
TRPYAF--
-TPYAFR-
TSPYAF--
TTPYAF--
TGPYAF--
TYPYAF--
THPYAF--
TAPYAF--
TDPYAF--
TPPYAF--
TNPYAF--

TVPYAF--
TIPYAF--
-TPYAFI-
TKPYAF--
TLPYAF--
-TPYAFY-
-TPYAFW-
TQPYAF--
-TPYAFQ-
-TPYAFM-
-TPYAFH-
-TPYAFA-
TMPYAF--
TWPYAF--
-TPYAFS-
-TPYAFK-
TFPYAF--
-TPYAFG-
-TPYAFC-
VLPYAF--
VKPYAF--
-VPYAFR-
-VPYAFL-
-VPYAFT-
VVPYAF--
VFPYAF--
VMPYAF--
VDPYAF--
VNPYAF--
VEPYAF--
VRPYAF--
VSPYAF--
VQPYAF--
-VPYAFV-
-VPYAFI-
-VPYAFH-
VTPYAF--
VPPYAF--
VGPYAF--
-VPYAFS-
-VPYAFK-
VIPYAF--
VWPYAF--
VAPYAF--
VYPYAF--
VHPYAF--
-VPYAFN-
-VPYAFE-
-WPYAFS-
WSPYAF--

-WPYAFR-
WEPYAF--
WTPYAF--
WYPYAF--
WVPYAF--
WGPYAF--
WRPYAF--
WIPYAF--
-WPYAFT-
-WPYAFK-
WDPYAF--
WLPYAF--
WHPYAF--
WQPYAF--
-WPYAFY-
-WPYAFV-
-WPYAFM-
-WPYAFA-
WPPYAF--
-YPYAFR-
YTPYAF--
YPPYAF--
-YPYAFM-
YWPYAF--
YKPYAF--
YHPYAF--
YSPYAF--
YEPYAF--
YDPYAF--
-YPYAFK-
YQPYAF--
YMPYAF--
YYPYAF--
-YPYAFS-
-YPYAFN-
YNPYAF--
YVPYAF--
-YPYAFW-
YLPYAF--
-YPYAFC-
PWGL 57 848 47.45
--PWGLVP
-APWGLR-
ARPWGL--
AVPWGL--
ASPWGL--
-APWGLA-
-APWGLL-
-APWGLT-
-APWGLK-

AEPWGL--
AKPWGL--
-APWGLG-
ANPWGL--
ALPWGL--
-APWGLI-
APPWGL--
AHPWGL--
-APWGLH-
AGPWGL--
AFPWGL--
-APWGLN-
-APWGLF-
AAPWGL--
-APWGLQ-
ACPWGL--
AMPWGL--
-APWGLE-
-APWGLS-
AIPWGL--
ADPWGL--
AWPWGL--
-APWGLM-
CTPWGL--
CEPWGL--
CRPWGL--
-CPWGLS-
CFPWGL--
CKPWGL--
CCPWGL--
-CPWGLM-
-CPWGLK-
CVPWGL--
-CPWGLT-
-CPWGLN-
-CPWGLA-
CSPWGL--
-CPWGLV-
-CPWGLE-
-CPWGLQ-
-CPWGLL-
CIPWGL--
CHPWGL--
DLPWGL--
DSPWGL--
DVPWGL--
DRPWGL--
-DPWGLV-
-DPWGLR-
DYPWGL--

DWPWGL--
DMPWGL--
-DPWGLT-
-DPWGLN-
-DPWGLI-
DFPWGL--
-DPWGLS-
-DPWGLQ-
DQPWGL--
-DPWGLY-
DTPWGL--
-DPWGLL-
DNPWGL--
DDPWGL--
-DPWGLH-
-DPWGLE-
DEPWGL--
-DPWGLM-
-DPWGLK-
DKPWGL--
DGPWGL--
DPPWGL--
-DPWGLF-
DCPWGL--
-EPWGLR-
EVPWGL--
-EPWGLN-
-EPWGLT-
ETPWGL--
-EPWGLK-
ELPWGL--
-EPWGLI-
-EPWGLF-
ESPWGL--
-EPWGLM-
ENPWGL--
-EPWGLQ-
EHPWGL--
-EPWGLG-
-EPWGLL-
-EPWGLH-
EWPWGL--
EQPWGL--
-EPWGLA-
EFPWGL--
-EPWGLS-
EMPWGL--
EDPWGL--
ECPWGL--
-EPWGLW-

EEPWGL--
EAPWGL--
-EPWGLE-
EKPWGL--
EIPWGL--
FVPWGL--
FGPWGL--
FTPWGL--
FWPWGL--
-FPWGLN-
FLPWGL--
-FPWGLS-
FIPWGL--
FNPWGL--
-FPWGLY-
FRPWGL--
-FPWGLL-
FKPWGL--
FYPWGL--
FSPWGL--
-FPWGLR-
-FPWGLK-
-FPWGLA-
FEPWGL--
FDPWGL--
FMPWGL--
-FPWGLM-
FQPWGL--
-FPWGLI-
-FPWGLH-
-FPWGLE-
-FPWGLT-
-FPWGLQ-
FCPWGL--
-FPWGLV-
FPPWGL--
FHPWGL--
FFPWGL--
FAPWGL--
-FPWGLG-
GRPWGL--
GQPWGL--
GSPWGL--
GGPWGL--
-GPWGLK-
GNPWGL--
GKPWGL--
-GPWGLT-
GVPWGL--
GLPWGL--

-GPWGLF-
GTPWGL--
GMPWGL--
GWPWGL--
-GPWGLA-
-GPWGLI-
GAPWGL--
-GPWGLM-
-GPWGLG-
GHPWGL--
-GPWGLV-
GPPWGL--
-GPWGLN-
GFPWGL--
GIPWGL--
-GPWGLW-
-GPWGLH-
-GPWGLY-
-GPWGLE-
GYPWGL--
GCPWGL--
-GPWGLQ-
GEPWGL--
GDPWGL--
-HPWGLN-
HVPWGL--
HLPWGL--
-HPWGLI-
-HPWGLL-
-HPWGLS-
HHPWGL--
-HPWGLA-
HTPWGL--
HAPWGL--
-HPWGLY-
HQPWGL--
-HPWGLF-
-HPWGLR-
HSPWGL--
-HPWGLG-
HYPWGL--
HKPWGL--
-HPWGLT-
-HPWGLM-
HEPWGL--
-HPWGLK-
HIPWGL--
HFPWGL--
-HPWGLE-
-HPWGLH-

HGPWGL--
HDPWGL--
HCPWGL--
-HPWGLQ-
-HPWGLV-
HWPWGL--
-HPWGLD-
HPPWGL--
IRPWGL--
-IPWGLE-
IVPWGL--
-IPWGLT-
-IPWGLL-
INPWGL--
IMPWGL--
-IPWGLK-
-IPWGLA-
IGPWGL--
IQPWGL--
IEPWGL--
-IPWGLI-
-IPWGLR-
IDPWGL--
-IPWGLM-
IPPWGL--
-IPWGLN-
-IPWGLF-
IYPWGL--
-IPWGLY-
IFPWGL--
-IPWGLH-
IWPWGL--
-IPWGLV-
IAPWGL--
IKPWGL--
IIPWGL--
ITPWGL--
IHPWGL--
-IPWGLW-
-IPWGLG-
-IPWGLC-
KSPWGL--
-KPWGLI-
-KPWGLS-
-KPWGLH-
KIPWGL--
KYPWGL--
KVPWGL--
KHPWGL--
-KPWGLT-

-KPWGLV-
KGPWGL--
-KPWGLW-
-KPWGLK-
KWPWGL--
-KPWGLL-
KFPWGL--
-KPWGLY-
-KPWGLR-
-KPWGLG-
-KPWGLN-
KNPWGL--
KMPWGL--
KEPWGL--
KKPWGL--
KDPWGL--
-KPWGLF-
KPPWGL--
KQPWGL--
-KPWGLA-
-KPWGLQ-
-KPWGLM-
-KPWGLE-
KCPWGL--
LSPWGL--
LNPWGL--
LPPWGL--
LTPWGL--
-LPWGLL-
LAPWGL--
LYPWGL--
LMPWGL--
LVPWGL--
-LPWGLK-
LQPWGL--
LKPWGL--
-LPWGLN-
LGPWGL--
-LPWGLV-
-LPWGLI-
-LPWGLM-
-LPWGLA-
-LPWGLH-
LWPWGL--
LCPWGL--
-LPWGLQ-
LEPWGL--
-LPWGLG-
LHPWGL--
-LPWGLE-

MRPWGL--
MGPWGL--
MLPWGL--
-MPWGLS-
MTPWGL--
-MPWGLN-
-MPWGLA-
-MPWGLL-
-MPWGLV-
MNPWGL--
MVPWGL--
-MPWGLT-
-MPWGLK-
-MPWGLI-
MIPWGL--
MKPWGL--
MYPWGL--
MQPWGL--
MDPWGL--
-MPWGLG-
MSPWGL--
MPPWGL--
-MPWGLE-
-MPWGLH-
MWPWGL--
MMPWGL--
MAPWGL--
-MPWGLF-
-MPWGLW-
MCPWGL--
NRPWGL--
-NPWGLS-
NAPWGL--
NGPWGL--
NVPWGL--
NMPWGL--
NNPWGL--
-NPWGLT-
-NPWGLA-
NIPWGL--
NEPWGL--
NQPWGL--
-NPWGLY-
-NPWGLN-
-NPWGLL-
-NPWGLK-
-NPWGLV-
NPPWGL--
NKPWGL--
NYPWGL--

-NPWGLE-
NFPWGL--
NDPWGL--
-NPWGLI-
-NPWGLM-
-NPWGLG-
-NPWGLF-
NHPWGL--
NWPWGL--
-NPWGLD-
--PWGLRN
--PWGLRS
--PWGLRT
--PWGLVA
--PWGLLT
--PWGLSS
--PWGLRH
--PWGLTE
PRPWGL--
--PWGLMR
--PWGLTS
--PWGLSN
--PWGLMH
--PWGLAS
--PWGLSR
--PWGLYH
--PWGLST
--PWGLRR
--PWGLTL
--PWGLLN
--PWGLGT
--PWGLYG
--PWGLQS
--PWGLGS
PSPWGL--
--PWGLVR
--PWGLTG
--PWGLKT
--PWGLTR
--PWGLTT
--PWGLSK
--PWGLTV
--PWGLRA
--PWGLLA
--PWGLVV
--PWGLVK
--PWGLVI
--PWGLNT
PNPWGL--
--PWGLKR

--PWGLIS
--PWGLHV
--PWGLVN
--PWGLIG
--PWGLSI
--PWGLTF
--PWGLSY
--PWGLNM
--PWGLVG
--PWGLTN
--PWGLTK
--PWGLSH
PMPWGL--
--PWGLML
--PWGLLQ
--PWGLLH
--PWGLKG
--PWGLFR
PLPWGL--
--PWGLYR
--PWGLNV
--PWGLKY
--PWGLTA
--PWGLSA
--PWGLNA
--PWGLHI
--PWGLSW
--PWGLHS
--PWGLYQ
--PWGLVT
--PWGLVM
--PWGLSF
--PWGLQG
--PWGLKS
--PWGLFD
--PWGLEG
PIPWGL--
--PWGLVH
--PWGLQR
--PWGLFH
--PWGLES
PQPWGL--
PKPWGL--
--PWGLTD
--PWGLQT
--PWGLKV
--PWGLGG
--PWGLFG
--PWGLAW
--PWGLYT

--PWGLTY
--PWGLIT
--PWGLAG
PEPWGL--
PYPWGL--
--PWGLHK
--PWGLYS
--PWGLYN
--PWGLHR
--PWGLER
--PWGLWN
--PWGLSV
--PWGLLM
--PWGLKI
--PWGLKA
--PWGLFK
--PWGLAA
--PWGLWR
--PWGLWK
--PWGLRE
--PWGLRD
--PWGLME
--PWGLIW
--PWGLFQ
--PWGLVY
--PWGLVE
--PWGLMQ
--PWGLHN
--PWGLDS
--PWGLYK
--PWGLWT
--PWGLRQ
--PWGLQF
--PWGLHF
--PWGLDA
PGPWGL--
PWPWGL--
--PWGLYF
--PWGLTW
--PWGLSQ
--PWGLNH
--PWGLII
--PWGLAV
--PWGLSD
--PWGLNL
--PWGLMD
--PWGLMA
--PWGLIN
--PWGLIK
--PWGLGA

--PWGLEM
PAPWGL--
--PWGLWH
--PWGLRM
--PWGLIY
--PWGLID
--PWGLHQ
--PWGLHH
--PWGLGW
--PWGLGL
--PWGLAT
PVPWGL--
PTPWGL--
PDPWGL--
--PWGLYD
--PWGLQK
--PWGLNY
--PWGLNE
--PWGLND
--PWGLLE
--PWGLKN
--PWGLEQ
--PWGLEN
--PWGLVQ
--PWGLQH
--PWGLKK
--PWGLIA
PHPWGL--
--PWGLTI
--PWGLNK
--PWGLNG
--PWGLMV
--PWGLMI
--PWGLLD
--PWGLKW
--PWGLKD
--PWGLIM
--PWGLHL
--PWGLFS
--PWGLEV
--PWGLWQ
--PWGLQI
--PWGLMG
--PWGLIV
--PWGLHA
--PWGLAR
--PWGLAK
--PWGLTQ
--PWGLQQ
--PWGLQN

--PWGLNQ
--PWGLGM
--PWGLGI
--PWGLFN
--PWGLEY
--PWGLEW
--PWGLEI
--PWGLNI
--PWGLHW
--PWGLHG
--PWGLHE
--PWGLHD
--PWGLEL
--PWGLEA
--PWGLWE
--PWGLTH
--PWGLNW
--PWGLNS
--PWGLGQ
--PWGLGN
--PWGLFT
--PWGLAI
--PWGLRW
--PWGLQL
--PWGLNR
--PWGLGY
--PWGLGR
--PWGLEF
--PWGLVD
--PWGLMM
--PWGLKM
--PWGLIH
--PWGLED
--PWGLAH
--PWGLSE
--PWGLQY
--PWGLQE
--PWGLDT
--PWGLCV
--PWGLKQ
--PWGLKF
--PWGLHT
--PWGLET
--PWGLAN
--PWGLAE
--PWGLAD
--PWGLSC
--PWGLQD
--PWGLQA
--PWGLMF

--PWGLKH
--PWGLKE
--PWGLHM
--PWGLGK
--PWGLGH
--PWGLGF
--PWGLEH
--PWGLYP
--PWGLTM
--PWGLQW
--PWGLQV
--PWGLQP
--PWGLPG
--PWGLNC
--PWGLMP
--PWGLLC
--PWGLEC
--PWGLDV
--PWGLDP
--PWGLDM
--PWGLCH
-PPWGLA-
QRPWGL--
QAPWGL--
QTPWGL--
-QPWGLY-
-QPWGLQ-
QVPWGL--
-QPWGLH-
QSPWGL--
-QPWGLT-
-QPWGLV-
QLPWGL--
QFPWGL--
-QPWGLK-
-QPWGLA-
QHPWGL--
-QPWGLR-
-QPWGLN-
-QPWGLF-
QQPWGL--
QKPWGL--
QIPWGL--
QNPWGL--
QPPWGL--
-QPWGLM-
-QPWGLE-
QGPWGL--
-QPWGLG-
-QPWGLC-

QYPWGL--
QMPWGL--
QEPWGL--
-RPWGLL-
-RPWGLA-
RSPWGL--
RLPWGL--
-RPWGLV-
-RPWGLF-
-RPWGLY-
RIPWGL--
-RPWGLS-
RAPWGL--
RFPWGL--
-RPWGLR-
-RPWGLG-
RTPWGL--
RGPWGL--
RWPWGL--
-RPWGLK-
-RPWGLI-
-RPWGLH-
-RPWGLN-
RQPWGL--
REPWGL--
RDPWGL--
-RPWGLQ-
RHPWGL--
RKPWGL--
-RPWGLE-
RMPWGL--
RYPWGL--
-RPWGLM-
-RPWGLD-
RCPWGL--
-RPWGLP-
SLPWGL--
-SPWGLS-
SSPWGL--
-SPWGLR-
SRPWGL--
SHPWGL--
SKPWGL--
-SPWGLL-
-SPWGLV-
SYPWGL--
STPWGL--
-SPWGLK-
SIPWGL--
-SPWGLA-

SVPWGL--
-SPWGLN-
SEPWGL--
-SPWGLI-
SAPWGL--
SMPWGL--
-SPWGLH-
SWPWGL--
-SPWGLQ-
-SPWGLM-
-SPWGLE-
-SPWGLW-
-SPWGLG-
SQPWGL--
SCPWGL--
-TPWGLR-
-TPWGLL-
-TPWGLT-
TSPWGL--
TAPWGL--
TYPWGL--
-TPWGLN-
TIPWGL--
TLPWGL--
-TPWGLY-
TRPWGL--
TTPWGL--
-TPWGLK-
TGPWGL--
-TPWGLV-
TFPWGL--
-TPWGLS-
TNPWGL--
-TPWGLQ-
TMPWGL--
-TPWGLM-
-TPWGLE-
TEPWGL--
TQPWGL--
-TPWGLA-
THPWGL--
-TPWGLG-
-TPWGLH-
TPPWGL--
TDPWGL--
-TPWGLI-
TWPWGL--
TKPWGL--
-VPWGLL-
VVPWGL--

-VPWGLR-
VSPWGL--
VRPWGL--
VHPWGL--
VGPWGL--
-VPWGLT-
VLPWGL--
VAPWGL--
-VPWGLM-
VPPWGL--
VKPWGL--
-VPWGLV-
-VPWGLA-
VIPWGL--
VYPWGL--
-VPWGLK-
VFPWGL--
VTPWGL--
-VPWGLG-
VNPWGL--
VMPWGL--
-VPWGLI-
-VPWGLE-
-VPWGLF-
VDPWGL--
VQPWGL--
-VPWGLH-
-VPWGLQ-
VEPWGL--
-VPWGLD-
WSPWGL--
WGPWGL--
-WPWGLR-
WTPWGL--
WRPWGL--
-WPWGLT-
-WPWGLV-
-WPWGLA-
-WPWGLN-
WAPWGL--
-WPWGLE-
WVPWGL--
-WPWGLS-
-WPWGLH-
WKPWGL--
-WPWGLM-
WHPWGL--
-WPWGLL-
-WPWGLG-
WDPWGL--

-WPWGLK-
WQPWGL--
WFPWGL--
WYPWGL--
-WPWGLQ-
-WPWGLI-
-WPWGLF-
-WPWGLW-
WCPWGL--
-YPWGLR-
YRPWGL--
YKPWGL--
YSPWGL--
-YPWGLL-
-YPWGLI-
YNPWGL--
YMPWGL--
YQPWGL--
-YPWGLT-
YAPWGL--
-YPWGLN-
YIPWGL--
-YPWGLA-
YTPWGL--
YHPWGL--
YVPWGL--
-YPWGLK-
-YPWGLV-
YGPWGL--
YEPWGL--
-YPWGLH-
YDPWGL--
YWPWGL--
-YPWGLQ-
YPPWGL--
YYPWGL--
-YPWGLG-
-YPWGLM-
-YPWGLE-

PKNY 313 689 47.41

GFPKNY--
ALPKNY--
-APKNYL-
-APKNYS-
ATPKNY--
AHPKNY--
ASPKNY--
-APKNYK-
-APKNYA-
APPKNY--

-APKNYV-
AMPKNY--
AKPKNY--
AIPKNY--
AGPKNY--
AVPKNY--
ARPKNY--
-APKNYW-
-APKNYI-
AAPKNY--
-APKNYY-
-APKNYT-
AFPKNY--
-APKNYM-
ANPKNY--
AEPKNY--
ADPKNY--
ACPKNY--
-APKNYN-
-APKNYG-
-APKNYC-
AYPKNY--
AQPKNY--
-APKNYE-
CTPKNY--
CAPKNY--
CQPKNY--
-CPKNYW-
CRPKNY--
-CPKNYR-
CIPKNY--
-CPKNYK-
-CPKNYS-
CKPKNY--
CVPKNY--
CSPKNY--
-CPKNYH-
CMPKNY--
CGPKNY--
-DPKNYV-
-DPKNYS-
-DPKNYI-
-DPKNYT-
-DPKNYK-
DMPKNY--
DGPKNY--
-DPKNYR-
-DPKNYH-
DKPKNY--
DFPKNY--

DWPKNY--
DRPKNY--
-DPKNYL-
DLPKNY--
DHPKNY--
DVPKNY--
-DPKNYY-
-DPKNYQ-
DIPKNY--
DAPKNY--
-EPKNYR-
-EPKNYK-
EHPKNY--
EGPKNY--
ELPKNY--
ESPKNY--
-EPKNYQ-
EQPKNY--
-EPKNYI-
EVPKNY--
-EPKNYY-
-EPKNYS-
-EPKNYL-
EMPKNY--
-EPKNYW-
EIPKNY--
EYPKNY--
-EPKNYG-
EWPKNY--
-EPKNYT-
-EPKNYM-
FAPKNY--
FTPKNY--
-FPKNYI-
FFPKNY--
FNPKNY--
FWPKNY--
FLPKNY--
FIPKNY--
FHPKNY--
FDPKNY--
FYPKNY--
-FPKNYH-
FVPKNY--
-FPKNYW-
-FPKNYV-
FEPKNY--
FSPKNY--
-FPKNYT-
FRPKNY--

-FPKNYK-
FPPKNY--
-FPKNYM-
-FPKNYF-
-FPKNYE-
-GPKNYV-
GLPKNY--
-GPKNYI-
-GPKNYH-
GRPKNY--
-GPKNYS-
-GPKNYL-
-GPKNYA-
-GPKNYR-
GSPKNY--
GQPKNY--
GIPKNY--
GGPKNY--
GTPKNY--
-GPKNYM-
-GPKNYG-
GMPKNY--
GWPKNY--
-GPKNYW-
GEPKNY--
GYPKNY--
-GPKNYY-
GAPKNY--
-GPKNYE-
GVPKNY--
-GPKNYQ-
-GPKNYK-
GKPKNY--
GHPKNY--
GPPKNY--
GNPKNY--
GCPKNY--
HVPKNY--
-HPKNYI-
-HPKNYM-
HEPKNY--
-HPKNYS-
HWPKNY--
HSPKNY--
-HPKNYT-
HHPKNY--
HAPKNY--
-HPKNYV-
-HPKNYK-
-HPKNYF-

HLPKNY--
HRPKNY--
HTPKNY--
HYPKNY--
-HPKNYY-
-HPKNYW-
-HPKNYN-
HDPKNY--
ISPKNY--
-IPKNYR-
IRPKNY--
-IPKNYV-
-IPKNYL-
-IPKNYM-
IIPKNY--
-IPKNYW-
-IPKNYK-
-IPKNYT-
ITPKNY--
-IPKNYY-
-IPKNYG-
-IPKNYE-
IDPKNY--
IPPKNY--
-IPKNYF-
IMPKNY--
-IPKNYQ-
IFPKNY--
IAPKNY--
IYPKNY--
-IPKNYS-
INPKNY--
IQPKNY--
-IPKNYI-
-IPKNYC-
ILPKNY--
KSPKNY--
KGPKNY--
-KPKNYE-
KLPKNY--
KNPKNY--
KHPKNY--
KTPKNY--
KVPKNY--
KWPKNY--
-KPKNYS-
-KPKNYK-
-KPKNYI-
-KPKNYF-
KQPKNY--

KPPKNY--
-KPKNYQ-
KIPKNY--
KYPKNY--
KDPKNY--
-KPKNYY-
-KPKNYV-
KFPKNY--
KCPKNY--
LGPKNY--
LTPKNY--
LSPKNY--
-LPKNYM-
LNPKNY--
LLPKNY--
LIPKNY--
LVPKNY--
LHPKNY--
-LPKNYV-
LWPKNY--
LFPKNY--
LPPKNY--
-LPKNYH-
LRPKNY--
-LPKNYY-
-LPKNYW-
LMPKNY--
LEPKNY--
LQPKNY--
-LPKNYT-
-LPKNYQ-
-LPKNYI-
-LPKNYF-
LDPKNY--
-LPKNYC-
-MPKNYI-
-MPKNYW-
MIPKNY--
MRPKNY--
MHPKNY--
MFPKNY--
MLPKNY--
MMPKNY--
MYPKNY--
MNPKNY--
MTPKNY--
-MPKNYQ-
-MPKNYA-
MVPKNY--
MSPKNY--

-MPKNYV-
-MPKNYT-
-MPKNYM-
-MPKNYG-
-MPKNYC-
MKPKNY--
MGPKNY--
MQPKNY--
-MPKNYR-
-MPKNYP-
-MPKNYN-
-MPKNYH-
-MPKNYF-
MAPKNY--
NFPKNY--
NGPKNY--
-NPKNYW-
-NPKNYM-
NSPKNY--
-NPKNYS-
NYPKNY--
NVPKNY--
-NPKNYR-
-NPKNYI-
NIPKNY--
NRPKNY--
NLPKNY--
-NPKNYQ-
-NPKNYF-
NNPKNY--
-NPKNYY-
NHPKNY--
NQPKNY--
-NPKNYK-
NTPKNY--
NPPKNY--
-NPKNYL-
-NPKNYV-
-NPKNYT-
--PKNYNS
--PKNYST
--PKNYSG
--PKNYLA
--PKNYVA
--PKNYAH
--PKNYVG
--PKNYIR
--PKNYRF
--PKNYLS
--PKNYIT

--PKNYTR
--PKNYNV
--PKNYML
--PKNYVS
--PKNYRA
--PKNYLT
--PKNYLQ
--PKNYAA
--PKNYVK
--PKNYTS
--PKNYFT
PMPKNY--
--PKNYWT
--PKNYIG
--PKNYVL
--PKNYIW
--PKNYWR
--PKNYSR
--PKNYRY
--PKNYIL
--PKNYFG
--PKNYTV
--PKNYLW
--PKNYLV
--PKNYLL
--PKNYYS
--PKNYTG
--PKNYSS
--PKNYSQ
--PKNYVF
--PKNYTT
--PKNYWY
--PKNYVN
--PKNYQS
--PKNYIK
--PKNYAL
PRPKNY--
--PKNYQT
--PKNYYL
--PKNYMQ
--PKNYHG
--PKNYGA
--PKNYYI
--PKNYWA
--PKNYTI
--PKNYSN
--PKNYSA
--PKNYIN
--PKNYYA
--PKNYHQ

--PKNYGG
--PKNYAF
PGPKNY--
PYPKNY--
--PKNYWS
--PKNYWN
--PKNYIF
--PKNYWQ
--PKNYQK
--PKNYKD
--PKNYFL
--PKNYAM
--PKNYYK
--PKNYWK
--PKNYWI
--PKNYVY
--PKNYTQ
--PKNYSY
--PKNYSV
--PKNYLG
--PKNYIS
--PKNYHA
--PKNYGV
--PKNYGM
--PKNYGF
--PKNYFI
--PKNYFH
--PKNYSW
--PKNYMI
--PKNYMF
--PKNYME
--PKNYKH
--PKNYIM
--PKNYFK
--PKNYEV
--PKNYEH
--PKNYEA
--PKNYAS
PLPKNY--
--PKNYVT
--PKNYVE
--PKNYTL
--PKNYTH
--PKNYRM
--PKNYQN
--PKNYNW
--PKNYMM
--PKNYKW
--PKNYIY
--PKNYAI

PFPKNY--
--PKNYYT
--PKNYYH
--PKNYWW
--PKNYWF
--PKNYVW
--PKNYTE
--PKNYSH
--PKNYRI
--PKNYQR
--PKNYQQ
--PKNYLD
--PKNYIA
--PKNYHS
--PKNYHM
--PKNYFW
--PKNYFS
--PKNYFR
--PKNYFM
--PKNYEN
--PKNYEI
--PKNYDS
--PKNYAY
--PKNYAK
--PKNYAG
PVPKNY--
--PKNYWD
--PKNYTK
--PKNYTA
--PKNYSK
--PKNYSE
--PKNYRH
--PKNYRG
--PKNYQA
--PKNYNY
--PKNYNA
--PKNYLH
--PKNYLF
--PKNYKY
--PKNYKA
--PKNYII
--PKNYIE
--PKNYHW
--PKNYGW
--PKNYEK
--PKNYCT
PQPKNY--
--PKNYSI
--PKNYSF
--PKNYMS

--PKNYMN
--PKNYMG
--PKNYMD
--PKNYKM
--PKNYKI
--PKNYIQ
--PKNYHY
--PKNYHV
--PKNYHR
--PKNYHN
--PKNYHI
--PKNYGR
--PKNYGI
--PKNYFN
--PKNYFD
--PKNYEY
--PKNYEM
--PKNYEG
PDPKNY--
PPPKNY--
--PKNYYY
--PKNYYN
--PKNYWG
--PKNYVD
--PKNYTM
--PKNYTF
--PKNYTC
--PKNYQM
--PKNYQG
--PKNYQE
--PKNYPE
--PKNYMY
--PKNYMV
--PKNYMA
--PKNYKS
--PKNYIP
--PKNYHT
--PKNYHK
--PKNYGY
--PKNYGT
--PKNYGN
--PKNYFV
--PKNYFQ
--PKNYEW
--PKNYES
--PKNYER
--PKNYAW
--PKNYAV
--PKNYAT
PHPKNY--

PEPKNY--
QVPKNY--
QSPKNY--
-QPKNYY-
QGPKNY--
-QPKNYS-
-QPKNYV-
QEPKNY--
QLPKNY--
-QPKNYM-
-QPKNYK-
-QPKNYA-
QYPKNY--
QMPKNY--
-QPKNYQ-
QQPKNY--
-QPKNYN-
-QPKNYG-
-QPKNYE-
QNPKNY--
QHPKNY--
QDPKNY--
QAPKNY--
RMPKNY--
RSPKNY--
RWPKNY--
REPKNY--
RLPKNY--
-RPKNYL-
RGPKNY--
RQPKNY--
-RPKNYF-
RAPKNY--
-RPKNYS-
-RPKNYI-
-RPKNYG-
RIPKNY--
RYPKNY--
RVPKNY--
RPPKNY--
-RPKNYH-
-RPKNYY-
RNPKNY--
RKPKNY--
-RPKNYC-
-RPKNYA-
RDPKNY--
-RPKNYE-
-RPKNYD-
RCPKNY--

-SPKNYR-
SVPKNY--
-SPKNYS-
-SPKNYV-
-SPKNYL-
SSPKNY--
-SPKNYT-
-SPKNYY-
SLPKNY--
STPKNY--
-SPKNYI-
-SPKNYG-
SNPKNY--
SGPKNY--
SPPKNY--
-SPKNYW-
-SPKNYQ-
-SPKNYF-
-SPKNYE-
SIPKNY--
-SPKNYN-
-SPKNYK-
-SPKNYH-
SMPKNY--
SEPKNY--
SFPKNY--
SDPKNY--
SCPKNY--
SWPKNY--
SYPKNY--
-TPKNYY-
TVPKNY--
-TPKNYL-
TIPKNY--
TSPKNY--
TLPKNY--
-TPKNYW-
-TPKNYS-
-TPKNYT-
-TPKNYV-
-TPKNYE-
-TPKNYM-
TRPKNY--
-TPKNYA-
-TPKNYG-
-TPKNYF-
TQPKNY--
TNPKNY--
TMPKNY--
TTPKNY--

TPPKNY--
TFPKNY--
TCPKNY--
-TPKNYQ-
TKPKNY--
TWPKNY--
-TPKNYN-
TEPKNY--
VRPKNY--
VWPKNY--
-VPKNYR-
VSPKNY--
-VPKNYS-
VVPKNY--
-VPKNYW-
VQPKNY--
-VPKNYI-
VMPKNY--
VHPKNY--
VPPKNY--
-VPKNYY-
-VPKNYT-
-VPKNYK-
VNPKNY--
-VPKNYL-
-VPKNYM-
-VPKNYF-
VLPKNY--
VFPKNY--
VDPKNY--
VTPKNY--
-VPKNYQ-
VEPKNY--
VAPKNY--
-VPKNYV-
-VPKNYN-
-VPKNYH-
-VPKNYE-
-VPKNYA-
WTPKNY--
WWPKNY--
-WPKNYV-
WMPKNY--
WSPKNY--
-WPKNYF-
WDPKNY--
WIPKNY--
WAPKNY--
-WPKNYR-
WGPKNY--

-WPKNYY-
-WPKNYG-
WNPKNY--
WKPKNY--
WVPKNY--
WRPKNY--
WQPKNY--
-WPKNYM-
YSPKNY--
YYPKNY--
YGPKNY--
-YPKNYL-
YLPKNY--
-YPKNYF-
YIPKNY--
-YPKNYM-
YAPKNY--
YRPKNY--
YWPKNY--
YVPKNY--
YPPKNY--
-YPKNYK-
YNPKNY--
YKPKNY--
YQPKNY--
-YPKNYT-
-YPKNYI-
-YPKNYE-
YCPKNY--
PIGY 151 775 47.39
-DPIGYL-
--PIGYLL
-TPIGYG-
-APIGYL-
ASPIGY--
-APIGYS-
-APIGYR-
AVPIGY--
-APIGYT-
AMPIGY--
ARPIGY--
-APIGYI-
-APIGYA-
-APIGYM-
ALPIGY--
-APIGYY-
AWPIGY--
-APIGYW-
-APIGYG-
AEPIGY--

AQPIGY--
AFPIGY--
-APIGYV-
-APIGYQ-
-APIGYK-
AAPIGY--
APPIGY--
-APIGYE-
AIPIGY--
AYPIGY--
-APIGYH-
-APIGYN-
AHPIGY--
ADPIGY--
-APIGYC-
AGPIGY--
CYPIGY--
-CPIGYY-
CMPIGY--
CKPIGY--
CTPIGY--
-CPIGYR-
CAPIGY--
CWPIGY--
CLPIGY--
CSPIGY--
CNPIGY--
CCPIGY--
-DPIGYR-
DSPIGY--
-DPIGYI-
DYPIGY--
DRPIGY--
DAPIGY--
DTPIGY--
-DPIGYT-
DMPIGY--
DQPIGY--
-DPIGYM-
DLPIGY--
-DPIGYS-
DEPIGY--
-DPIGYW-
-DPIGYQ-
DDPIGY--
DVPIGY--
DIPIGY--
DFPIGY--
-EPIGYR-
-EPIGYT-

ESPIGY--
-EPIGYL-
-EPIGYW-
-EPIGYM-
ERPIGY--
-EPIGYS-
-EPIGYI-
-EPIGYQ-
EMPIGY--
EWPIGY--
-EPIGYY-
EKPIGY--
EGPIGY--
EAPIGY--
EVPIGY--
EPPIGY--
-EPIGYV-
EIPIGY--
EHPIGY--
EEPIGY--
ETPIGY--
-EPIGYE-
-EPIGYA-
ELPIGY--
-FPIGYR-
FRPIGY--
FKPIGY--
FPPIGY--
FTPIGY--
-FPIGYM-
FNPIGY--
FHPIGY--
FGPIGY--
-FPIGYI-
FYPIGY--
-FPIGYT-
-FPIGYS-
-FPIGYL-
-FPIGYK-
FEPIGY--
FWPIGY--
FMPIGY--
FSPIGY--
FAPIGY--
-FPIGYN-
-FPIGYA-
FQPIGY--
-FPIGYW-
-FPIGYF-
FDPIGY--

-FPIGYV-
-FPIGYP-
-FPIGYH-
-FPIGYG-
FLPIGY--
FIPIGY--
GRPIGY--
GSPIGY--
-GPIGYL-
-GPIGYM-
-GPIGYV-
-GPIGYR-
GTPIGY--
GLPIGY--
GHPIGY--
GGPIGY--
-GPIGYK-
-GPIGYI-
-GPIGYH-
-GPIGYA-
-GPIGYS-
GMPIGY--
-GPIGYW-
-GPIGYT-
GWPIGY--
-GPIGYN-
-GPIGYF-
GYPIGY--
-GPIGYY-
GVPIGY--
GQPIGY--
GIPIGY--
-GPIGYQ-
-GPIGYG-
-GPIGYE-
GFPIGY--
GEPIGY--
GDPIGY--
HGPIGY--
-HPIGYR-
-HPIGYL-
HRPIGY--
-HPIGYT-
HQPIGY--
HSPIGY--
HKPIGY--
HWPIGY--
-HPIGYW-
HHPIGY--
HAPIGY--

-HPIGYS-
HLPIGY--
HYPIGY--
HVPIGY--
-HPIGYN-
HFPIGY--
HTPIGY--
-HPIGYM-
-HPIGYF-
-HPIGYY-
-HPIGYV-
-HPIGYH-
-HPIGYE-
-HPIGYA-
HMPIGY--
HEPIGY--
HCPIGY--
-HPIGYG-
-IPIGYL-
ISPIGY--
ITPIGY--
IKPIGY--
IVPIGY--
-IPIGYI-
-IPIGYT-
IRPIGY--
IFPIGY--
-IPIGYK-
-IPIGYY-
-IPIGYR-
INPIGY--
IMPIGY--
IAPIGY--
IPPIGY--
ILPIGY--
IIPIGY--
-IPIGYS-
-IPIGYV-
IHPIGY--
IEPIGY--
IDPIGY--
-IPIGYN-
IGPIGY--
-IPIGYM-
-IPIGYF-
IYPIGY--
IQPIGY--
-IPIGYE-
-IPIGYH-
-IPIGYD-

ICPIGY--
-KPIGYT-
-KPIGYR-
-KPIGYS-
KTPIGY--
-KPIGYV-
-KPIGYF-
KIPIGY--
KKPIGY--
KFPIGY--
KPPIGY--
KVPIGY--
KMPIGY--
-KPIGYM-
-KPIGYL-
KRPIGY--
KEPIGY--
-KPIGYQ-
-KPIGYW-
-KPIGYH-
KNPIGY--
KGPIGY--
KQPIGY--
-KPIGYN-
KWPIGY--
-KPIGYI-
KHPIGY--
KDPIGY--
KAPIGY--
-KPIGYY-
LLPIGY--
LRPIGY--
LSPIGY--
LMPIGY--
LGPIGY--
LTPIGY--
LNPIGY--
-LPIGYV-
-LPIGYR-
LAPIGY--
LYPIGY--
-LPIGYY-
-LPIGYI-
-LPIGYS-
LVPIGY--
LPPIGY--
LIPIGY--
-LPIGYH-
LWPIGY--
LKPIGY--

LHPIGY--
-LPIGYT-
-LPIGYN-
LQPIGY--
-LPIGYK-
LEPIGY--
LCPIGY--
-LPIGYQ-
-LPIGYF-
LDPIGY--
MSPIGY--
MKPIGY--
MLPIGY--
MYPIGY--
-MPIGYR-
MVPIGY--
MRPIGY--
-MPIGYA-
MAPIGY--
-MPIGYW-
MGPIGY--
MDPIGY--
-MPIGYL-
-MPIGYH-
-MPIGYM-
MIPIGY--
-MPIGYI-
-MPIGYF-
MPPIGY--
MMPIGY--
-MPIGYY-
MWPIGY--
-MPIGYV-
-MPIGYN-
-MPIGYG-
-MPIGYC-
NKPIGY--
NAPIGY--
NLPIGY--
NSPIGY--
NGPIGY--
NHPIGY--
-NPIGYR-
NTPIGY--
NEPIGY--
NPPIGY--
-NPIGYM-
-NPIGYS-
-NPIGYK-
NYPIGY--

NVPIGY--
NQPIGY--
NNPIGY--
NCPIGY--
NRPIGY--
-NPIGYI-
-NPIGYH-
NFPIGY--
-NPIGYW-
-NPIGYT-
-NPIGYE-
NMPIGY--
NDPIGY--
--PIGYLS
--PIGYGS
--PIGYLT
--PIGYST
--PIGYSG
--PIGYRL
--PIGYSR
--PIGYSV
--PIGYWS
--PIGYTR
--PIGYVS
--PIGYVG
--PIGYTS
--PIGYRS
--PIGYRA
--PIGYSA
--PIGYRT
--PIGYAL
--PIGYTF
--PIGYRD
--PIGYRV
--PIGYLV
--PIGYSY
--PIGYLG
--PIGYIA
--PIGYSH
--PIGYSS
--PIGYRH
--PIGYHN
PFPIGY--
--PIGYWG
--PIGYTK
--PIGYLR
--PIGYKS
--PIGYYR
--PIGYMK
--PIGYRQ

--PIGYTQ
PKPIGY--
--PIGYTY
--PIGYFG
PAPIGY--
--PIGYTV
--PIGYIM
--PIGYHA
--PIGYMS
--PIGYIK
--PIGYTM
--PIGYQG
--PIGYLA
--PIGYYF
--PIGYTH
--PIGYTG
--PIGYTA
--PIGYRW
--PIGYRN
--PIGYQA
--PIGYNI
--PIGYLM
--PIGYIT
--PIGYHV
--PIGYFM
--PIGYAS
--PIGYWN
--PIGYRI
--PIGYNG
--PIGYLQ
--PIGYLK
--PIGYKT
--PIGYGA
--PIGYFR
PDPIGY--
--PIGYWW
--PIGYRF
--PIGYQS
--PIGYMV
--PIGYHW
--PIGYHT
--PIGYVI
--PIGYTI
--PIGYNT
--PIGYNL
--PIGYMG
--PIGYLN
--PIGYHK
--PIGYFS
--PIGYAK

PGPIGY--
--PIGYTT
--PIGYNQ
--PIGYMQ
--PIGYLI
--PIGYKN
--PIGYKK
--PIGYII
--PIGYHL
--PIGYEW
--PIGYEG
--PIGYAI
--PIGYRG
--PIGYIR
--PIGYFV
--PIGYFT
--PIGYES
--PIGYAY
--PIGYAV
PTPIGY--
--PIGYVW
--PIGYSW
--PIGYRM
--PIGYKY
--PIGYGM
--PIGYAM
--PIGYYG
--PIGYVK
--PIGYVH
--PIGYTL
--PIGYTE
--PIGYSN
--PIGYRY
--PIGYQV
--PIGYQT
--PIGYNV
--PIGYNS
--PIGYMI
--PIGYMA
--PIGYKL
--PIGYIH
--PIGYIG
--PIGYGL
--PIGYFA
PIPIGY--
--PIGYYT
--PIGYYK
--PIGYYA
--PIGYWD
--PIGYTW

--PIGYNW
--PIGYMF
--PIGYLF
--PIGYKA
--PIGYIY
--PIGYIS
--PIGYFN
--PIGYAT
--PIGYAA
PQPIGY--
--PIGYYN
--PIGYYH
--PIGYVY
--PIGYVM
--PIGYSF
--PIGYSD
--PIGYQL
--PIGYNY
--PIGYNA
--PIGYMT
--PIGYIN
--PIGYHH
--PIGYHF
--PIGYGI
--PIGYFK
--PIGYEA
--PIGYAG
PHPIGY--
--PIGYWK
--PIGYVT
--PIGYVA
--PIGYQR
--PIGYQN
--PIGYQM
--PIGYPL
--PIGYNM
--PIGYNH
--PIGYND
--PIGYKQ
--PIGYKF
--PIGYIW
--PIGYID
--PIGYHI
--PIGYHE
--PIGYGG
--PIGYFF
--PIGYAH
--PIGYAE
--PIGYWR
--PIGYVQ

--PIGYVE
--PIGYSQ
--PIGYSI
--PIGYSE
--PIGYSC
--PIGYQK
--PIGYKW
--PIGYKV
--PIGYKI
--PIGYKG
--PIGYHR
--PIGYFQ
--PIGYEY
--PIGYER
PEPIGY--
PMPIGY--
--PIGYYS
--PIGYYQ
--PIGYYE
--PIGYWE
--PIGYVN
--PIGYTN
--PIGYRC
--PIGYQQ
--PIGYNN
--PIGYNK
--PIGYKD
--PIGYKC
--PIGYIQ
--PIGYIF
--PIGYIE
--PIGYHQ
--PIGYHG
--PIGYGY
--PIGYGT
--PIGYGF
--PIGYGD
--PIGYFD
--PIGYFC
--PIGYEN
--PIGYEI
--PIGYEF
--PIGYCG
QTPIGY--
-QPIGYS-
QRPIGY--
-QPIGYV-
QLPIGY--
QFPIGY--
QSPIGY--

QVPIGY--
-QPIGYR-
-QPIGYH-
QGPIGY--
-QPIGYY-
-QPIGYL-
-QPIGYI-
QKPIGY--
-QPIGYT-
-QPIGYF-
QDPIGY--
-QPIGYN-
-QPIGYK-
QHPIGY--
-QPIGYE-
-QPIGYA-
QMPIGY--
QIPIGY--
QWPIGY--
QPPIGY--
-QPIGYM-
QNPIGY--
QCPIGY--
RVPIGY--
-RPIGYL-
-RPIGYH-
RSPIGY--
RGPIGY--
-RPIGYY-
-RPIGYR-
-RPIGYF-
RDPIGY--
RNPIGY--
RRPIGY--
-RPIGYT-
RFPIGY--
-RPIGYI-
-RPIGYQ-
RKPIGY--
RTPIGY--
-RPIGYK-
RYPIGY--
RIPIGY--
-RPIGYM-
-RPIGYW-
RMPIGY--
RQPIGY--
-RPIGYE-
-RPIGYN-
RAPIGY--

-RPIGYA-
REPIGY--
RPPIGY--
-RPIGYG-
RHPIGY--
RCPIGY--
SRPIGY--
-SPIGYT-
-SPIGYL-
SMPIGY--
SSPIGY--
SEPIGY--
SNPIGY--
-SPIGYR-
SAPIGY--
SKPIGY--
SGPIGY--
-SPIGYH-
SYPIGY--
SFPIGY--
-SPIGYS-
SQPIGY--
SLPIGY--
SIPIGY--
SHPIGY--
-SPIGYN-
-SPIGYW-
SWPIGY--
-SPIGYA-
SVPIGY--
-SPIGYF-
SPPIGY--
-SPIGYY-
SCPIGY--
-SPIGYK-
-SPIGYI-
-SPIGYV-
SDPIGY--
STPIGY--
-SPIGYQ-
TKPIGY--
-TPIGYR-
-TPIGYL-
TRPIGY--
TPPIGY--
-TPIGYV-
TNPIGY--
TYPIGY--
TGPIGY--
TAPIGY--

TLPIGY--
TSPIGY--
TTPIGY--
TVPIGY--
TQPIGY--
-TPIGYW-
TMPIGY--
-TPIGYQ-
-TPIGYS-
TWPIGY--
-TPIGYF-
-TPIGYA-
TEPIGY--
TFPIGY--
-TPIGYI-
-TPIGYH-
-TPIGYM-
-TPIGYE-
TDPIGY--
-TPIGYN-
TIPIGY--
THPIGY--
VLPIGY--
-VPIGYR-
VKPIGY--
VVPIGY--
-VPIGYV-
VWPIGY--
-VPIGYS-
VAPIGY--
VNPIGY--
VDPIGY--
-VPIGYL-
VQPIGY--
VGPIGY--
VSPIGY--
VMPIGY--
-VPIGYW-
VYPIGY--
VTPIGY--
-VPIGYT-
-VPIGYA-
-VPIGYY-
-VPIGYN-
-VPIGYI-
-VPIGYM-
VPPIGY--
-VPIGYG-
VEPIGY--
VFPIGY--

VHPIGY--
-VPIGYQ-
-VPIGYE-
-VPIGYH-
-WPIGYR-
WRPIGY--
WSPIGY--
WPPIGY--
-WPIGYA-
WEPIGY--
-WPIGYN-
WTPIGY--
WLPIGY--
WFPIGY--
-WPIGYS-
-WPIGYM-
-WPIGYL-
-WPIGYF-
-WPIGYI-
WQPIGY--
-WPIGYQ-
-WPIGYK-
WMPIGY--
WGPIGY--
WDPIGY--
WAPIGY--
-WPIGYV-
-WPIGYT-
WHPIGY--
YYPIGY--
YLPIGY--
YTPIGY--
YRPIGY--
-YPIGYI-
-YPIGYM-
YIPIGY--
-YPIGYS-
YAPIGY--
YQPIGY--
YKPIGY--
YDPIGY--
-YPIGYL-
YHPIGY--
YFPIGY--
-YPIGYH-
YNPIGY--
YMPIGY--
-YPIGYV-
-YPIGYR-
YPPIGY--

-YPIGYQ-
-YPIGYF-
-YPIGYG-
-YPIGYA-
YEPIGY--

PSGL	11	902	47.39

-DPSGLG-
-DPSGLA-
-FPSGLG-
-FPSGLC-
-NPSGLG-
--PSGLGL
--PSGLGK
--PSGLVP
--PSGLPI
--PSGLPA
--PSGLLC
--PSGLEN
--PSGLEE
--PSGLAE
-TPSGLP-
-TPSGLD-
-APSGLV-
ALPSGL--
ASPSGL--
-APSGLR-
ATPSGL--
ARPSGL--
AFPSGL--
-APSGLL-
-APSGLT-
AGPSGL--
-APSGLW-
-APSGLM-
AQPSGL--
AYPSGL--
AVPSGL--
-APSGLI-
AIPSGL--
-APSGLN-
-APSGLQ-
ANPSGL--
AAPSGL--
-APSGLH-
AMPSGL--
AWPSGL--
-APSGLK-
AKPSGL--
-APSGLF-
AHPSGL--

-APSGLG-
-APSGLY-
-APSGLC-
-APSGLE-
ADPSGL--
ACPSGL--
CGPSGL--
CVPSGL--
CTPSGL--
CKPSGL--
CRPSGL--
CWPSGL--
CLPSGL--
-CPSGLH-
CSPSGL--
-CPSGLT-
-CPSGLY-
-CPSGLV-
CMPSGL--
-CPSGLR-
-CPSGLK-
CYPSGL--
-CPSGLQ-
CPPSGL--
CAPSGL--
CFPSGL--
-CPSGLL-
CEPSGL--
CDPSGL--
-CPSGLW-
-CPSGLF-
CNPSGL--
CIPSGL--
CHPSGL--
CQPSGL--
-CPSGLN-
CCPSGL--
-CPSGLM-
-CPSGLI-
DVPSGL--
DKPSGL--
-DPSGLR-
DGPSGL--
-DPSGLL-
DRPSGL--
DLPSGL--
DMPSGL--
DQPSGL--
DWPSGL--
-DPSGLM-

DHPSGL--
DYPSGL--
DIPSGL--
DSPSGL--
DFPSGL--
DTPSGL--
-DPSGLS-
DAPSGL--
-DPSGLW-
-DPSGLK-
-DPSGLF-
DNPSGL--
-DPSGLV-
-DPSGLH-
-DPSGLT-
DEPSGL--
-DPSGLI-
-DPSGLN-
-EPSGLR-
ERPSGL--
EMPSGL--
ESPSGL--
-EPSGLL-
-EPSGLI-
-EPSGLY-
EVPSGL--
-EPSGLS-
-EPSGLK-
ELPSGL--
-EPSGLW-
EQPSGL--
EAPSGL--
-EPSGLH-
-EPSGLN-
ETPSGL--
-EPSGLV-
EIPSGL--
EYPSGL--
-EPSGLM-
EFPSGL--
EWPSGL--
-EPSGLQ-
-EPSGLF-
EHPSGL--
EDPSGL--
-EPSGLT-
-EPSGLA-
EPPSGL--
-EPSGLE-
EEPSGL--

−EPSGLG−
−FPSGLR−
FRPSGL−−
FLPSGL−−
FAPSGL−−
FGPSGL−−
FYPSGL−−
−FPSGLS−
−FPSGLL−
FHPSGL−−
FTPSGL−−
FSPSGL−−
−FPSGLK−
FWPSGL−−
FFPSGL−−
FVPSGL−−
−FPSGLY−
FIPSGL−−
FKPSGL−−
−FPSGLW−
FQPSGL−−
−FPSGLI−
−FPSGLM−
FNPSGL−−
−FPSGLN−
FEPSGL−−
−FPSGLT−
−FPSGLF−
FCPSGL−−
−FPSGLV−
FMPSGL−−
FPPSGL−−
FDPSGL−−
−FPSGLA−
−FPSGLE−
−GPSGLL−
−GPSGLR−
−GPSGLS−
−GPSGLT−
GKPSGL−−
GTPSGL−−
GRPSGL−−
−GPSGLM−
−GPSGLW−
GSPSGL−−
GFPSGL−−
−GPSGLI−
−GPSGLF−
GVPSGL−−
−GPSGLK−

-GPSGLY-
GIPSGL--
-GPSGLN-
GHPSGL--
GYPSGL--
-GPSGLV-
GQPSGL--
GNPSGL--
GMPSGL--
-GPSGLQ-
GDPSGL--
GEPSGL--
GPPSGL--
-GPSGLG-
-GPSGLE-
GCPSGL--
-GPSGLC-
HRPSGL--
-HPSGLL-
HIPSGL--
-HPSGLR-
HSPSGL--
HLPSGL--
HGPSGL--
HKPSGL--
-HPSGLS-
HTPSGL--
-HPSGLT-
HYPSGL--
-HPSGLM-
HQPSGL--
HNPSGL--
-HPSGLY-
HVPSGL--
-HPSGLI-
HPPSGL--
HMPSGL--
-HPSGLN-
HAPSGL--
HWPSGL--
HEPSGL--
HFPSGL--
HDPSGL--
-HPSGLK-
-HPSGLA-
-HPSGLF-
-HPSGLH-
-HPSGLG-
HCPSGL--
HHPSGL--

−HPSGLW−
−HPSGLE−
−HPSGLC−
−IPSGLS−
ISPSGL−−
IRPSGL−−
ILPSGL−−
−IPSGLL−
−IPSGLY−
IIPSGL−−
−IPSGLV−
IFPSGL−−
IVPSGL−−
IYPSGL−−
−IPSGLW−
−IPSGLR−
−IPSGLM−
−IPSGLF−
IGPSGL−−
ITPSGL−−
IMPSGL−−
−IPSGLK−
INPSGL−−
−IPSGLT−
IAPSGL−−
IWPSGL−−
IHPSGL−−
−IPSGLI−
−IPSGLQ−
−IPSGLN−
IKPSGL−−
IPPSGL−−
−IPSGLE−
IQPSGL−−
−IPSGLA−
−IPSGLH−
IEPSGL−−
IDPSGL−−
ICPSGL−−
KSPSGL−−
KRPSGL−−
−KPSGLL−
KLPSGL−−
KWPSGL−−
KGPSGL−−
−KPSGLF−
−KPSGLI−
KFPSGL−−
KYPSGL−−
−KPSGLR−

KIPSGL--
-KPSGLT-
KAPSGL--
-KPSGLY-
-KPSGLV-
-KPSGLN-
KTPSGL--
-KPSGLS-
KMPSGL--
KNPSGL--
-KPSGLA-
-KPSGLW-
KKPSGL--
KQPSGL--
KPPSGL--
-KPSGLG-
-KPSGLM-
-KPSGLE-
KHPSGL--
-KPSGLK-
-KPSGLQ-
KDPSGL--
-KPSGLH-
KCPSGL--
-KPSGLD-
LRPSGL--
LLPSGL--
LTPSGL--
LSPSGL--
LVPSGL--
LKPSGL--
LGPSGL--
LYPSGL--
LNPSGL--
LAPSGL--
-LPSGLL-
LPPSGL--
-LPSGLW-
-LPSGLY-
LMPSGL--
-LPSGLH-
LIPSGL--
-LPSGLV-
-LPSGLM-
LHPSGL--
-LPSGLI-
LDPSGL--
-LPSGLF-
-LPSGLN-
LCPSGL--

-MPSGLR-
MTPSGL--
MRPSGL--
MGPSGL--
-MPSGLT-
MAPSGL--
MSPSGL--
-MPSGLY-
MDPSGL--
MKPSGL--
-MPSGLS-
-MPSGLN-
-MPSGLI-
MLPSGL--
-MPSGLK-
MMPSGL--
MHPSGL--
MYPSGL--
MIPSGL--
-MPSGLH-
MEPSGL--
-MPSGLM-
-MPSGLA-
MQPSGL--
-MPSGLF-
-MPSGLQ-
-MPSGLE-
-MPSGLG-
MCPSGL--
NVPSGL--
NLPSGL--
NRPSGL--
-NPSGLR-
-NPSGLW-
NSPSGL--
NAPSGL--
-NPSGLY-
NTPSGL--
NYPSGL--
NKPSGL--
NGPSGL--
-NPSGLH-
-NPSGLL-
-NPSGLS-
NMPSGL--
-NPSGLK-
-NPSGLQ-
NWPSGL--
-NPSGLT-
NPPSGL--

NQPSGL--
-NPSGLI-
NEPSGL--
-NPSGLV-
NNPSGL--
-NPSGLM-
NHPSGL--
NDPSGL--
-NPSGLN-
-NPSGLA-
NFPSGL--
NCPSGL--
-NPSGLC-
--PSGLRS
--PSGLRL
--PSGLSS
--PSGLFS
--PSGLRG
--PSGLLT
--PSGLSG
--PSGLRT
--PSGLRN
--PSGLRE
--PSGLTG
--PSGLMS
--PSGLHR
--PSGLLV
--PSGLSR
--PSGLRA
PLPSGL--
PWPSGL--
--PSGLQA
--PSGLRV
--PSGLLQ
--PSGLQS
--PSGLYL
--PSGLVS
--PSGLYG
--PSGLFT
--PSGLST
--PSGLRH
--PSGLSN
--PSGLTT
--PSGLLM
--PSGLFG
--PSGLTR
--PSGLSF
--PSGLTS
--PSGLLK
--PSGLVF

--PSGLTH
--PSGLYR
--PSGLAG
--PSGLNV
--PSGLWK
--PSGLIS
--PSGLYT
--PSGLTL
--PSGLMR
--PSGLLA
--PSGLWS
--PSGLLW
--PSGLRF
--PSGLMA
--PSGLKS
--PSGLFN
--PSGLSL
--PSGLFE
--PSGLYS
--PSGLGV
--PSGLYA
--PSGLIR
--PSGLFM
--PSGLTN
--PSGLKW
PIPSGL--
--PSGLYI
--PSGLWR
--PSGLNS
--PSGLMW
--PSGLGS
--PSGLVY
--PSGLTF
--PSGLNA
--PSGLMK
PEPSGL--
PYPSGL--
--PSGLVW
--PSGLSH
PAPSGL--
PSPSGL--
--PSGLMT
--PSGLHL
--PSGLFL
--PSGLEA
--PSGLFA
--PSGLAS
PVPSGL--
--PSGLIM
--PSGLTI

--PSGLVM
--PSGLNT
--PSGLKN
--PSGLIL
--PSGLVN
--PSGLVG
--PSGLSY
--PSGLIT
--PSGLAA
--PSGLYY
--PSGLQG
--PSGLHY
--PSGLWA
--PSGLKH
--PSGLIA
--PSGLES
--PSGLAF
--PSGLYM
--PSGLSW
--PSGLGT
--PSGLYH
--PSGLVT
--PSGLKG
--PSGLQN
--PSGLMQ
--PSGLLY
PTPSGL--
--PSGLIF
--PSGLWN
--PSGLSK
--PSGLIY
--PSGLHG
--PSGLVV
--PSGLLH
--PSGLKA
--PSGLHI
--PSGLAT
--PSGLWG
--PSGLNF
PKPSGL--
--PSGLYV
--PSGLQY
--PSGLKT
--PSGLHN
--PSGLSM
--PSGLMY
--PSGLHK
--PSGLGA
PMPSGL--
--PSGLYK

--PSGLML
--PSGLKV
--PSGLIV
--PSGLHA
--PSGLFQ
--PSGLTA
--PSGLSQ
--PSGLII
--PSGLHV
--PSGLAR
--PSGLVQ
--PSGLHF
--PSGLQT
--PSGLWM
--PSGLQR
--PSGLMI
--PSGLHM
--PSGLFI
--PSGLYQ
--PSGLNM
--PSGLKK
--PSGLIQ
--PSGLIG
--PSGLAV
--PSGLYE
--PSGLMN
--PSGLMD
--PSGLIN
--PSGLQF
--PSGLNR
--PSGLNH
--PSGLMM
--PSGLIH
--PSGLFW
--PSGLAN
PHPSGL--
--PSGLNW
--PSGLMV
--PSGLLE
--PSGLRM
--PSGLEG
--PSGLAM
PFPSGL--
--PSGLYD
--PSGLLD
--PSGLKI
--PSGLHH
--PSGLEF
--PSGLQQ
--PSGLMH

--PSGLIK
--PSGLKQ
--PSGLHW
--PSGLAK
--PSGLVK
--PSGLKF
--PSGLHT
--PSGLFD
--PSGLWQ
--PSGLQI
--PSGLQE
--PSGLNN
--PSGLTQ
--PSGLEV
--PSGLAY
PDPSGL--
--PSGLTD
--PSGLWH
--PSGLVD
--PSGLSD
--PSGLQM
--PSGLNI
--PSGLMF
--PSGLEY
--PSGLET
--PSGLTE
--PSGLQH
--PSGLAH
--PSGLVE
--PSGLHQ
--PSGLTK
--PSGLQV
--PSGLQL
--PSGLQK
--PSGLGY
--PSGLFH
--PSGLEW
--PSGLEH
--PSGLVI
--PSGLER
--PSGLEM
--PSGLAQ
--PSGLWE
--PSGLSE
--PSGLQW
--PSGLNK
--PSGLME
--PSGLGF
--PSGLEK
--PSGLTW

--PSGLKD
--PSGLGI
PQPSGL--
--PSGLWW
--PSGLDA
--PSGLND
--PSGLKE
--PSGLHE
PCPSGL--
--PSGLIE
--PSGLGW
--PSGLEI
--PSGLDG
--PSGLCT
--PSGLCG
--PSGLDS
--PSGLCV
--PSGLNG
--PSGLMC
--PSGLGM
--PSGLGH
--PSGLCD
--PSGLCA
--PSGLPM
--PSGLIC
--PSGLEL
--PSGLCR
-PPSGLT-
-PPSGLR-
--PSGLQD
--PSGLQC
--PSGLNE
--PSGLNC
--PSGLHC
--PSGLDM
--PSGLCM
--PSGLCK
--PSGLCE
-PPSGLK-
-PPSGLH-
PPPSGL--
QVPSGL--
QRPSGL--
-QPSGLT-
-QPSGLW-
-QPSGLS-
QLPSGL--
QSPSGL--
QWPSGL--
-QPSGLY-

-QPSGLK-
QGPSGL--
QTPSGL--
QAPSGL--
-QPSGLV-
QQPSGL--
-QPSGLM-
-QPSGLF-
QPPSGL--
QFPSGL--
QNPSGL--
-QPSGLQ-
-QPSGLA-
QHPSGL--
-QPSGLI-
-QPSGLG-
QIPSGL--
QEPSGL--
-QPSGLH-
QDPSGL--
QYPSGL--
-QPSGLE-
QMPSGL--
-QPSGLC-
QCPSGL--
-RPSGLS-
RSPSGL--
RLPSGL--
-RPSGLN-
-RPSGLY-
RYPSGL--
RNPSGL--
-RPSGLF-
-RPSGLK-
RAPSGL--
-RPSGLV-
RIPSGL--
RQPSGL--
-RPSGLA-
RHPSGL--
-RPSGLQ-
RPPSGL--
RWPSGL--
RDPSGL--
REPSGL--
-RPSGLE-
RKPSGL--
-RPSGLG-
RCPSGL--
SRPSGL--

-SPSGLR-
-SPSGLL-
STPSGL--
SLPSGL--
SSPSGL--
SKPSGL--
SVPSGL--
SAPSGL--
SFPSGL--
-SPSGLV-
-SPSGLS-
-SPSGLF-
SNPSGL--
-SPSGLY-
SHPSGL--
-SPSGLW-
-SPSGLH-
-SPSGLQ-
-SPSGLI-
-SPSGLN-
SQPSGL--
SDPSGL--
SEPSGL--
-SPSGLA-
-SPSGLE-
SCPSGL--
-SPSGLC-
TPPSGL--
-TPSGLR-
TVPSGL--
TLPSGL--
-TPSGLL-
TGPSGL--
TFPSGL--
-TPSGLI-
-TPSGLY-
TTPSGL--
TRPSGL--
TMPSGL--
-TPSGLT-
-TPSGLK-
TKPSGL--
-TPSGLF-
-TPSGLV-
TSPSGL--
-TPSGLQ-
TWPSGL--
TAPSGL--
-TPSGLH-
THPSGL--

TQPSGL--
-TPSGLW-
TDPSGL--
TYPSGL--
-TPSGLN-
-TPSGLM-
TEPSGL--
-TPSGLG-
-TPSGLE-
TCPSGL--
-TPSGLC-
VRPSGL--
VNPSGL--
VSPSGL--
-VPSGLF-
VVPSGL--
-VPSGLW-
-VPSGLL-
VMPSGL--
VFPSGL--
VTPSGL--
VAPSGL--
-VPSGLV-
-VPSGLS-
VGPSGL--
VPPSGL--
-VPSGLM-
-VPSGLQ-
-VPSGLH-
-VPSGLY-
-VPSGLN-
-VPSGLG-
VKPSGL--
VHPSGL--
VEPSGL--
VDPSGL--
-VPSGLK-
-VPSGLE-
VCPSGL--
-VPSGLC-
-WPSGLT-
WAPSGL--
WSPSGL--
WRPSGL--
WLPSGL--
-WPSGLR-
WVPSGL--
WTPSGL--
WIPSGL--
WWPSGL--

−WPSGLK−
WFPSGL−−
−WPSGLY−
−WPSGLL−
−WPSGLS−
−WPSGLI−
−WPSGLV−
WKPSGL−−
WNPSGL−−
−WPSGLN−
−WPSGLH−
WPPSGL−−
−WPSGLQ−
WHPSGL−−
WDPSGL−−
WMPSGL−−
−WPSGLW−
WGPSGL−−
−WPSGLM−
−WPSGLA−
WEPSGL−−
WQPSGL−−
−WPSGLF−
−WPSGLG−
−WPSGLE−
WCPSGL−−
−YPSGLR−
YGPSGL−−
−YPSGLT−
YLPSGL−−
−YPSGLS−
−YPSGLL−
YEPSGL−−
YTPSGL−−
YVPSGL−−
−YPSGLM−
YYPSGL−−
−YPSGLY−
YMPSGL−−
YFPSGL−−
−YPSGLV−
−YPSGLI−
YHPSGL−−
YKPSGL−−
−YPSGLN−
−YPSGLH−
YAPSGL−−
−YPSGLW−
−YPSGLQ−
−YPSGLK−

	-YPSGLF-		
	YQPSGL--		
	-YPSGLA-		
	YNPSGL--		
	YDPSGL--		
	YCPSGL--		
	-YPSGLE-		
	-YPSGLD-		
PRGL	517	614	47.38
	-GPRGLC-		
	-LPRGLC-		
	-MPRGLP-		
	-NPRGLD-		
	--PRGLDR		
	--PRGLDL		
	--PRGLVP		
	--PRGLPV		
	--PRGLPS		
	--PRGLPD		
	--PRGLLC		
	--PRGLAP		
	-QPRGLD-		
	-RPRGLD-		
	-TPRGLD-		
	-VPRGLP-		
	-YPRGLP-		
	ASPRGL--		
	AVPRGL--		
	-APRGLS-		
	-APRGLI-		
	AGPRGL--		
	-APRGLV-		
	-APRGLT-		
	-APRGLG-		
	-APRGLN-		
	-APRGLE-		
	-APRGLY-		
	-APRGLA-		
	-APRGLM-		
	APPRGL--		
	ATPRGL--		
	AQPRGL--		
	AEPRGL--		
	-APRGLF-		
	-APRGLH-		
	-APRGLW-		
	ACPRGL--		
	ADPRGL--		
	-APRGLD-		
	-APRGLC-		

-APRGLP-
CLPRGL--
CKPRGL--
-CPRGLL-
-CPRGLR-
CFPRGL--
CSPRGL--
CGPRGL--
CCPRGL--
-CPRGLH-
CVPRGL--
-CPRGLT-
CDPRGL--
CRPRGL--
-CPRGLW-
-CPRGLK-
CIPRGL--
CAPRGL--
-CPRGLI-
-CPRGLY-
CMPRGL--
CNPRGL--
CWPRGL--
-CPRGLA-
CHPRGL--
CEPRGL--
CQPRGL--
-CPRGLQ-
-CPRGLF-
-DPRGLL-
DTPRGL--
DSPRGL--
-DPRGLV-
-DPRGLR-
-DPRGLI-
DIPRGL--
-DPRGLT-
-DPRGLE-
DQPRGL--
DLPRGL--
DVPRGL--
-DPRGLY-
-DPRGLK-
DYPRGL--
-DPRGLH-
-DPRGLA-
DMPRGL--
DGPRGL--
-DPRGLF-
DNPRGL--

DEPRGL--
-DPRGLG-
DFPRGL--
-DPRGLQ-
DHPRGL--
-DPRGLN-
DDPRGL--
DCPRGL--
-DPRGLC-
ESPRGL--
-EPRGLT-
-EPRGLL-
ERPRGL--
ELPRGL--
-EPRGLS-
-EPRGLW-
EMPRGL--
ETPRGL--
EFPRGL--
EKPRGL--
-EPRGLY-
-EPRGLV-
EGPRGL--
-EPRGLQ-
EAPRGL--
-EPRGLE-
-EPRGLK-
-EPRGLI-
-EPRGLH-
EHPRGL--
EEPRGL--
EQPRGL--
-EPRGLM-
EIPRGL--
ENPRGL--
-EPRGLF-
EDPRGL--
EPPRGL--
ECPRGL--
-EPRGLD-
FLPRGL--
FAPRGL--
FDPRGL--
-FPRGLH-
FVPRGL--
-FPRGLQ-
FYPRGL--
-FPRGLY-
FEPRGL--
FMPRGL--

FPPRGL--
FKPRGL--
-FPRGLE-
-FPRGLC-
-FPRGLD-
GLPRGL--
GSPRGL--
GYPRGL--
-GPRGLR-
-GPRGLN-
-GPRGLT-
GNPRGL--
-GPRGLW-
GKPRGL--
-GPRGLM-
-GPRGLA-
-GPRGLV-
-GPRGLQ-
-GPRGLH-
-GPRGLI-
-GPRGLY-
-GPRGLF-
GPPRGL--
GDPRGL--
-GPRGLE-
GCPRGL--
-HPRGLS-
HGPRGL--
-HPRGLT-
HAPRGL--
HYPRGL--
HEPRGL--
-HPRGLM-
HKPRGL--
HVPRGL--
-HPRGLN-
-HPRGLY-
-HPRGLG-
HPPRGL--
HDPRGL--
-HPRGLF-
-HPRGLE-
HFPRGL--
-HPRGLK-
HQPRGL--
HHPRGL--
HNPRGL--
HCPRGL--
-HPRGLQ-
-IPRGLT-

−IPRGLS−
ITPRGL−−
−IPRGLH−
ISPRGL−−
−IPRGLY−
IGPRGL−−
−IPRGLE−
−IPRGLQ−
IDPRGL−−
IKPRGL−−
INPRGL−−
IEPRGL−−
−IPRGLN−
IHPRGL−−
−IPRGLA−
−IPRGLM−
−IPRGLG−
−IPRGLD−
−IPRGLC−
−KPRGLT−
−KPRGLL−
−KPRGLH−
−KPRGLK−
KGPRGL−−
−KPRGLI−
−KPRGLF−
KTPRGL−−
−KPRGLN−
KIPRGL−−
−KPRGLM−
KQPRGL−−
−KPRGLV−
−KPRGLA−
−KPRGLQ−
−KPRGLW−
KPPRGL−−
KNPRGL−−
KCPRGL−−
−KPRGLE−
KDPRGL−−
−KPRGLD−
−KPRGLC−
−LPRGLK−
−LPRGLS−
−LPRGLH−
LHPRGL−−
−LPRGLN−
−LPRGLY−
−LPRGLQ−
−LPRGLF−

-LPRGLE-
-MPRGLL-
-MPRGLS-
MGPRGL--
-MPRGLQ-
-MPRGLN-
-MPRGLI-
-MPRGLG-
MMPRGL--
MNPRGL--
-MPRGLV-
-MPRGLW-
-MPRGLY-
MEPRGL--
MDPRGL--
MAPRGL--
MCPRGL--
MQPRGL--
-MPRGLC-
NRPRGL--
-NPRGLT-
NYPRGL--
-NPRGLV-
-NPRGLY-
-NPRGLQ-
-NPRGLL-
-NPRGLS-
-NPRGLW-
-NPRGLK-
-NPRGLN-
NKPRGL--
NAPRGL--
NEPRGL--
-NPRGLI-
-NPRGLF-
-NPRGLH-
-NPRGLA-
-NPRGLM-
NPPRGL--
-NPRGLG-
-NPRGLE-
NCPRGL--
--PRGLTL
--PRGLSS
--PRGLFL
--PRGLST
--PRGLAT
--PRGLSV
--PRGLIQ
--PRGLTT

--PRGLSK
PLPRGL--
--PRGLYS
--PRGLRF
--PRGLTF
--PRGLTW
--PRGLLM
--PRGLSH
--PRGLWT
--PRGLVK
--PRGLLW
--PRGLFS
--PRGLAL
--PRGLWG
--PRGLAR
--PRGLYV
--PRGLMI
--PRGLGG
--PRGLNM
--PRGLWS
--PRGLNR
--PRGLMG
--PRGLTK
--PRGLTH
--PRGLNL
--PRGLYD
--PRGLAG
--PRGLME
--PRGLAV
--PRGLAH
--PRGLYN
--PRGLSF
--PRGLAS
--PRGLYI
--PRGLFH
--PRGLFN
--PRGLLD
--PRGLGV
--PRGLWI
--PRGLSQ
--PRGLQG
--PRGLRD
--PRGLIM
--PRGLWR
--PRGLAW
PSPRGL--
--PRGLNF
--PRGLHF
--PRGLWV
--PRGLES

--PRGLEN
--PRGLAI
--PRGLNI
--PRGLMA
--PRGLFQ
--PRGLQK
--PRGLSD
--PRGLQQ
--PRGLIY
--PRGLIG
--PRGLYA
--PRGLVV
--PRGLMT
--PRGLYQ
--PRGLWA
--PRGLAY
--PRGLQT
--PRGLIE
--PRGLVE
--PRGLMD
--PRGLEW
--PRGLTQ
--PRGLMN
--PRGLKN
--PRGLYY
--PRGLTY
--PRGLNY
--PRGLIN
--PRGLIH
--PRGLYT
--PRGLNA
--PRGLHA
--PRGLFG
--PRGLHT
--PRGLMK
--PRGLSE
--PRGLAM
--PRGLWQ
--PRGLRE
--PRGLKQ
--PRGLVW
--PRGLTD
--PRGLQH
PIPRGL--
PEPRGL--
--PRGLMW
--PRGLII
--PRGLET
--PRGLHK
--PRGLDG

PAPRGL--
--PRGLNN
--PRGLEY
PWPRGL--
--PRGLEK
--PRGLKF
--PRGLEF
--PRGLAN
--PRGLMM
--PRGLNQ
--PRGLEV
--PRGLQY
--PRGLFW
PMPRGL--
PKPRGL--
--PRGLNH
--PRGLID
--PRGLKI
--PRGLFE
--PRGLDS
--PRGLQN
PVPRGL--
--PRGLNW
--PRGLHV
--PRGLNK
--PRGLGQ
--PRGLGM
--PRGLFK
PNPRGL--
--PRGLWW
--PRGLWD
--PRGLND
--PRGLEL
--PRGLMQ
PFPRGL--
--PRGLYP
--PRGLYE
--PRGLKE
--PRGLKD
--PRGLFA
--PRGLAD
--PRGLWE
--PRGLNG
--PRGLEH
PDPRGL--
PCPRGL--
--PRGLTE
--PRGLPG
--PRGLHQ
--PRGLEG

--PRGLQF
--PRGLFD
--PRGLWM
--PRGLQV
--PRGLQM
--PRGLQI
--PRGLEI
PQPRGL--
--PRGLTM
--PRGLHD
--PRGLQE
--PRGLHP
--PRGLEA
--PRGLDT
--PRGLAQ
--PRGLEQ
--PRGLCS
--PRGLWH
--PRGLMP
--PRGLDW
--PRGLCL
--PRGLVC
--PRGLHE
--PRGLQW
--PRGLCR
--PRGLSC
--PRGLIP
--PRGLED
--PRGLCV
--PRGLCT
--PRGLCN
--PRGLRC
--PRGLHC
--PRGLGE
--PRGLDY
-PPRGLG-
--PRGLPM
--PRGLFP
--PRGLDN
--PRGLDE
--PRGLCI
--PRGLCH
--PRGLCA
--PRGLYC
--PRGLEE
--PRGLDM
--PRGLCY
--PRGLCW
--PRGLCQ
--PRGLCD

--PRGLAC
-PPRGLY-
-PPRGLM-
-QPRGLS-
-QPRGLL-
QSPRGL--
-QPRGLT-
QRPRGL--
-QPRGLN-
-QPRGLV-
QNPRGL--
QTPRGL--
QPPRGL--
-QPRGLK-
QQPRGL--
QIPRGL--
QYPRGL--
QMPRGL--
-QPRGLW-
QGPRGL--
-QPRGLH-
-QPRGLQ-
-QPRGLA-
-QPRGLG-
-QPRGLY-
QFPRGL--
QEPRGL--
QAPRGL--
-QPRGLM-
QDPRGL--
QKPRGL--
RSPRGL--
RIPRGL--
REPRGL--
-RPRGLM-
-RPRGLG-
-RPRGLE-
-RPRGLH-
RPPRGL--
-RPRGLN-
RDPRGL--
RKPRGL--
RCPRGL--
-RPRGLC-
-SPRGLS-
STPRGL--
SAPRGL--
-SPRGLY-
-SPRGLA-
-SPRGLQ-

SPPRGL--
-SPRGLH-
-SPRGLM-
SNPRGL--
-SPRGLE-
-SPRGLF-
SEPRGL--
SCPRGL--
-SPRGLD-
TLPRGL--
-TPRGLY-
-TPRGLK-
-TPRGLT-
-TPRGLW-
TNPRGL--
-TPRGLN-
TIPRGL--
-TPRGLA-
-TPRGLV-
TQPRGL--
-TPRGLQ-
-TPRGLE-
TDPRGL--
TCPRGL--
-TPRGLC-
-VPRGLG-
-VPRGLY-
-VPRGLQ-
-VPRGLF-
VPPRGL--
-VPRGLV-
-VPRGLN-
-VPRGLA-
-VPRGLE-
-VPRGLH-
VEPRGL--
VAPRGL--
-VPRGLW-
-VPRGLM-
VKPRGL--
VDPRGL--
VCPRGL--
WSPRGL--
WRPRGL--
WVPRGL--
WFPRGL--
-WPRGLF-
WMPRGL--
-WPRGLN-
-WPRGLI-

WGPRGL--
WNPRGL--
WPPRGL--
-WPRGLH-
-WPRGLE-
WDPRGL--
-WPRGLQ-
-WPRGLM-
WCPRGL--
YSPRGL--
-YPRGLI-
YIPRGL--
-YPRGLG-
-YPRGLN-
YDPRGL--
YKPRGL--
YFPRGL--
YEPRGL--
-YPRGLF-
-YPRGLE-
-YPRGLM-
YCPRGL--
-YPRGLC-

PMHI	515	614	47.38

ALPMHI--
-APMHIR-
ATPMHI--
AAPMHI--
-APMHIW-
-APMHIM-
AKPMHI--
AVPMHI--
-APMHIV-
-APMHIS-
ADPMHI--
AQPMHI--
AYPMHI--
-APMHIE-
AIPMHI--
AMPMHI--
AHPMHI--
AGPMHI--
AFPMHI--
CRPMHI--
CSPMHI--
-CPMHIF-
-CPMHIR-
CTPMHI--
-CPMHIM-
-CPMHIL-

CNPMHI--
CLPMHI--
CIPMHI--
DWPMHI--
DSPMHI--
-DPMHIW-
-DPMHIR-
DVPMHI--
DTPMHI--
-DPMHIT-
-DPMHIQ-
DFPMHI--
DRPMHI--
-DPMHIL-
DNPMHI--
DMPMHI--
DLPMHI--
DIPMHI--
DGPMHI--
DEPMHI--
-EPMHIR-
-EPMHIL-
ESPMHI--
-EPMHIA-
ELPMHI--
EVPMHI--
EGPMHI--
ERPMHI--
-EPMHIT-
EYPMHI--
-EPMHIH-
EDPMHI--
-EPMHIM-
-EPMHIE-
EAPMHI--
FSPMHI--
-FPMHIK-
-FPMHIT-
-FPMHIS-
-FPMHIA-
FAPMHI--
-FPMHIY-
-FPMHIV-
-FPMHII-
FNPMHI--
FHPMHI--
FGPMHI--
FFPMHI--
FRPMHI--
-FPMHIH-

FEPMHI--
FWPMHI--
FKPMHI--
FIPMHI--
FYPMHI--
-FPMHIR-
-FPMHIN-
-FPMHIM-
-FPMHIG-
-FPMHIF-
FMPMHI--
GKPMHI--
GRPMHI--
-GPMHIK-
-GPMHIT-
-GPMHIA-
GTPMHI--
GHPMHI--
GNPMHI--
GMPMHI--
-GPMHIW-
-GPMHIM-
-GPMHII-
-GPMHIL-
-GPMHIE-
GVPMHI--
GPPMHI--
-GPMHIY-
-GPMHIR-
-GPMHIN-
GGPMHI--
GEPMHI--
GAPMHI--
GYPMHI--
-GPMHIS-
GLPMHI--
GIPMHI--
GDPMHI--
HSPMHI--
HRPMHI--
HKPMHI--
HIPMHI--
HPPMHI--
-HPMHIK-
-HPMHIY-
HLPMHI--
-HPMHIL-
-HPMHIS-
HYPMHI--
HEPMHI--

HDPMHI--
HVPMHI--
-HPMHIM-
HCPMHI--
HAPMHI--
IRPMHI--
-IPMHIT-
-IPMHIR-
IEPMHI--
IQPMHI--
-IPMHIK-
IDPMHI--
-IPMHIS-
ITPMHI--
ISPMHI--
IMPMHI--
ILPMHI--
-IPMHIM-
-IPMHIL-
IIPMHI--
IHPMHI--
IPPMHI--
-IPMHIV-
-IPMHIF-
IKPMHI--
-IPMHIH-
IFPMHI--
KVPMHI--
KSPMHI--
KFPMHI--
KPPMHI--
-KPMHIQ-
KGPMHI--
KAPMHI--
-KPMHIW-
-KPMHIA-
KWPMHI--
KRPMHI--
-KPMHIY-
-KPMHIR-
-KPMHIL-
-KPMHIE-
KLPMHI--
KEPMHI--
-KPMHIS-
KYPMHI--
-KPMHIV-
-KPMHIF-
KDPMHI--
LKPMHI--

LRPMHI--
LVPMHI--
LGPMHI--
LWPMHI--
-LPMHIR-
LYPMHI--
LTPMHI--
LIPMHI--
-LPMHIM-
-LPMHIK-
-LPMHIS-
LEPMHI--
LDPMHI--
-LPMHIY-
-LPMHIH-
LHPMHI--
-LPMHIT-
-LPMHIL-
LFPMHI--
LNPMHI--
LLPMHI--
LPPMHI--
-LPMHII-
-LPMHIF-
LMPMHI--
LCPMHI--
LAPMHI--
MQPMHI--
-MPMHIL-
MVPMHI--
MSPMHI--
MWPMHI--
MFPMHI--
-MPMHIS-
MEPMHI--
-MPMHIV-
MLPMHI--
-MPMHIR-
MRPMHI--
MGPMHI--
-MPMHIF-
-MPMHIE-
MKPMHI--
MPPMHI--
MHPMHI--
MAPMHI--
MTPMHI--
-MPMHIT-
-MPMHIQ-
-MPMHIM-

-MPMHII-
-MPMHIH-
MMPMHI--
MIPMHI--
NMPMHI--
NLPMHI--
NTPMHI--
NSPMHI--
NRPMHI--
-NPMHIR-
NHPMHI--
NGPMHI--
NFPMHI--
NKPMHI--
NYPMHI--
NVPMHI--
NEPMHI--
NPPMHI--
-NPMHIY-
-NPMHIV-
-NPMHIT-
-NPMHIF-
-NPMHIM-
-NPMHII-
--PMHISG
--PMHIKA
--PMHIRS
PGPMHI--
--PMHINA
--PMHIYQ
PMPMHI--
--PMHILS
--PMHIGA
--PMHIRA
--PMHIHG
--PMHIAF
--PMHIRR
PHPMHI--
--PMHIVR
--PMHISQ
--PMHISK
--PMHIRH
--PMHIFW
PQPMHI--
--PMHIVM
--PMHIQG
--PMHIKF
--PMHIVA
--PMHISV
--PMHIMS

--PMHIAN
--PMHIYS
--PMHIVN
--PMHISS
--PMHISA
--PMHIIW
--PMHIFV
--PMHIAA
--PMHIYN
--PMHISR
--PMHIRI
--PMHIFR
--PMHITF
--PMHIRL
--PMHIRK
--PMHILK
--PMHILG
--PMHIKR
--PMHIIN
--PMHIFI
PCPMHI--
--PMHITD
--PMHISY
--PMHIQV
--PMHILN
--PMHILM
--PMHIKV
--PMHIIV
--PMHIGS
--PMHIAT
--PMHIYH
--PMHIWT
--PMHIWS
--PMHIVT
--PMHITW
--PMHITQ
--PMHITG
--PMHISI
--PMHIRT
--PMHIRF
--PMHIRD
--PMHIMW
--PMHIIR
--PMHIIL
--PMHIII
--PMHIEF
--PMHIYK
--PMHIYG
--PMHIWV
--PMHIVL

--PMHITN
--PMHIST
--PMHIRN
--PMHIQL
--PMHILT
--PMHILA
--PMHIKH
--PMHIFY
--PMHIFT
--PMHIYV
--PMHIYR
--PMHIWW
--PMHIWN
--PMHIWK
--PMHITT
--PMHITR
--PMHIRV
--PMHIQW
--PMHIQR
--PMHINW
--PMHIMV
--PMHIMM
--PMHIKW
--PMHIHS
--PMHIHI
--PMHIFH
--PMHIFE
--PMHIAG
PDPMHI--
PTPMHI--
--PMHIWE
--PMHIVH
--PMHISW
--PMHISH
--PMHIRQ
--PMHIRM
--PMHIQI
--PMHIML
--PMHIKK
--PMHIKD
--PMHIIT
--PMHIHV
--PMHIAY
--PMHIYW
--PMHIYI
--PMHIYF
--PMHIYE
--PMHIVY
--PMHIVI
--PMHIVD

--PMHITM
--PMHISM
--PMHISF
--PMHISE
--PMHIRG
--PMHIQY
--PMHIQN
--PMHIQK
--PMHIMR
--PMHIME
--PMHILW
--PMHILQ
--PMHIKS
--PMHIKI
--PMHIKG
--PMHIIS
--PMHIIK
--PMHIIH
--PMHIID
--PMHIHR
--PMHIHL
--PMHIHH
--PMHIGG
--PMHIFN
--PMHIFK
PWPMHI--
--PMHIYM
--PMHIWL
--PMHIVQ
--PMHIVF
--PMHITL
--PMHITI
--PMHISP
--PMHISN
--PMHISL
--PMHISD
--PMHIQS
--PMHIPQ
--PMHIPL
--PMHINY
--PMHINV
--PMHINT
--PMHINN
--PMHING
--PMHIMF
--PMHILY
--PMHIKT
--PMHIKQ
--PMHIIF
--PMHIIA

--PMHIHD
--PMHIGW
--PMHIFS
--PMHIFD
--PMHIFC
--PMHIEV
--PMHIES
--PMHIEL
--PMHIEI
--PMHIEG
--PMHIAM
--PMHIAL
--PMHIAK
--PMHIAI
QGPMHI--
QPPMHI--
-QPMHIV-
QLPMHI--
QQPMHI--
-QPMHIT-
QKPMHI--
QRPMHI--
QSPMHI--
-QPMHIL-
QMPMHI--
-QPMHIH-
QYPMHI--
-QPMHIS-
-QPMHIM-
-QPMHIF-
QTPMHI--
-QPMHIQ-
QAPMHI--
QVPMHI--
-QPMHIK-
-QPMHII-
QHPMHI--
QEPMHI--
RVPMHI--
-RPMHIR-
RIPMHI--
RLPMHI--
RSPMHI--
-RPMHIK-
-RPMHIS-
RRPMHI--
-RPMHIH-
RAPMHI--
-RPMHII-
-RPMHIL-

RGPMHI--
RYPMHI--
RPPMHI--
RHPMHI--
-RPMHIF-
RNPMHI--
-RPMHIM-
-RPMHIE-
-RPMHIV-
-RPMHIT-
-RPMHIY-
RDPMHI--
-RPMHIG-
RKPMHI--
RFPMHI--
RQPMHI--
-RPMHIN-
REPMHI--
SVPMHI--
SAPMHI--
SSPMHI--
SLPMHI--
-SPMHIR-
SRPMHI--
-SPMHIY-
-SPMHIM-
SFPMHI--
SYPMHI--
SKPMHI--
-SPMHIK-
SIPMHI--
-SPMHIF-
STPMHI--
-SPMHIL-
SNPMHI--
SHPMHI--
SQPMHI--
-SPMHIS-
SEPMHI--
SPPMHI--
-SPMHIQ-
SMPMHI--
-SPMHIV-
-SPMHIT-
SCPMHI--
TSPMHI--
-TPMHIR-
TGPMHI--
TYPMHI--
-TPMHIQ-

TVPMHI--
-TPMHIY-
-TPMHIT-
-TPMHIH-
TNPMHI--
TLPMHI--
TKPMHI--
TIPMHI--
-TPMHIV-
TEPMHI--
TAPMHI--
TWPMHI--
TPPMHI--
-TPMHIW-
-TPMHIM-
TDPMHI--
-TPMHIS-
-TPMHIF-
VTPMHI--
-VPMHIR-
-VPMHIS-
-VPMHIL-
VMPMHI--
VIPMHI--
VFPMHI--
-VPMHIV-
-VPMHIW-
VKPMHI--
VPPMHI--
VLPMHI--
VEPMHI--
-VPMHIY-
VHPMHI--
-VPMHII-
-VPMHIG-
-VPMHIE-
VGPMHI--
-VPMHIA-
VRPMHI--
-VPMHIQ-
-VPMHIK-
-VPMHIF-
-WPMHIM-
WPPMHI--
WMPMHI--
WQPMHI--
WGPMHI--
-WPMHIA-
WRPMHI--
-WPMHIS-

	WLPMHI--		
	-WPMHIT-		
	-WPMHIK-		
	-WPMHII-		
	-WPMHIH-		
	WKPMHI--		
	WYPMHI--		
	WAPMHI--		
	WSPMHI--		
	-WPMHIL-		
	WFPMHI--		
	WDPMHI--		
	YIPMHI--		
	YNPMHI--		
	YGPMHI--		
	YQPMHI--		
	-YPMHIL-		
	-YPMHIF-		
	YFPMHI--		
	YSPMHI--		
	-YPMHII-		
	YKPMHI--		
	YHPMHI--		
	-YPMHIR-		
	-YPMHIT-		
	YLPMHI--		
	YAPMHI--		
	YTPMHI--		
	YPPMHI--		
	-YPMHIM-		
	-YPMHIE-		
	-YPMHIA-		
	YVPMHI--		
	-YPMHIV-		
	-YPMHIN-		
	-YPMHIK-		
	YMPMHI--		
	YCPMHI--		
PLSM	35	863	47.34
	-LPLSMG-		
	--PLSMPS		
	--PLSMPN		
	--PLSMPL		
	--PLSMNE		
	-RPLSMP-		
	-VPLSMP-		
	ARPLSM--		
	AAPLSM--		
	-APLSMN-		
	-APLSMG-		

AVPLSM--
-APLSML-
AKPLSM--
AMPLSM--
-APLSMT-
AQPLSM--
AHPLSM--
ALPLSM--
AFPLSM--
AYPLSM--
-APLSMY-
-APLSMK-
AIPLSM--
-APLSMA-
-APLSMI-
APPLSM--
AWPLSM--
-APLSMF-
AEPLSM--
-APLSMQ-
-APLSME-
ANPLSM--
ADPLSM--
-APLSMM-
-APLSMH-
ACPLSM--
-APLSMW-
-APLSMD-
CKPLSM--
CCPLSM--
-CPLSMR-
CVPLSM--
CSPLSM--
-CPLSMS-
CNPLSM--
CHPLSM--
CFPLSM--
CYPLSM--
-CPLSMV-
CRPLSM--
-CPLSML-
-CPLSMA-
CIPLSM--
CLPLSM--
CEPLSM--
CPPLSM--
-CPLSMW-
-CPLSMI-
-CPLSMF-
CDPLSM--

CAPLSM--
DRPLSM--
-DPLSMW-
DLPLSM--
DVPLSM--
-DPLSMF-
-DPLSMI-
DMPLSM--
DFPLSM--
-DPLSMR-
DHPLSM--
DAPLSM--
DTPLSM--
DSPLSM--
DKPLSM--
-DPLSMT-
-DPLSMK-
-DPLSMY-
-DPLSML-
DWPLSM--
-DPLSMH-
DIPLSM--
-DPLSMQ-
DNPLSM--
DGPLSM--
-DPLSMM-
DYPLSM--
-DPLSMV-
-DPLSMA-
DQPLSM--
-DPLSMN-
-DPLSME-
DCPLSM--
-DPLSMS-
DDPLSM--
-EPLSML-
-EPLSMR-
-EPLSMW-
-EPLSMI-
ELPLSM--
EGPLSM--
ESPLSM--
ERPLSM--
EVPLSM--
ENPLSM--
EWPLSM--
-EPLSMN-
-EPLSMG-
EQPLSM--
-EPLSMT-

```
-EPLSMQ-
EIPLSM--
-EPLSMY-
EFPLSM--
ETPLSM--
EPPLSM--
-EPLSMV-
-EPLSMM-
EEPLSM--
EHPLSM--
-EPLSMF-
-EPLSME-
-EPLSMA-
EKPLSM--
EDPLSM--
-EPLSMK-
ECPLSM--
-FPLSML-
FSPLSM--
-FPLSMM-
FQPLSM--
-FPLSMS-
FLPLSM--
FIPLSM--
-FPLSMK-
FYPLSM--
FTPLSM--
-FPLSMV-
-FPLSMI-
FPPLSM--
FKPLSM--
-FPLSMA-
FWPLSM--
FHPLSM--
FDPLSM--
FNPLSM--
-FPLSMQ-
-FPLSMW-
FAPLSM--
-FPLSMY-
FFPLSM--
-FPLSMT-
FEPLSM--
-FPLSMF-
-FPLSMH-
-FPLSMG-
FCPLSM--
-FPLSME-
-GPLSMS-
GNPLSM--
```

GLPLSM--
GRPLSM--
GVPLSM--
-GPLSMI-
GAPLSM--
-GPLSMY-
GGPLSM--
GSPLSM--
-GPLSMA-
-GPLSMN-
-GPLSMK-
GIPLSM--
-GPLSMF-
GMPLSM--
GEPLSM--
-GPLSMT-
-GPLSMG-
-GPLSMV-
-GPLSMH-
GKPLSM--
GYPLSM--
GTPLSM--
-GPLSMM-
-GPLSME-
-GPLSMW-
GHPLSM--
GQPLSM--
GPPLSM--
GWPLSM--
GDPLSM--
-GPLSMQ-
GFPLSM--
GCPLSM--
HSPLSM--
-HPLSML-
-HPLSMI-
HLPLSM--
HYPLSM--
HRPLSM--
-HPLSMT-
HAPLSM--
-HPLSMS-
HNPLSM--
HVPLSM--
-HPLSMW-
-HPLSMF-
-HPLSMR-
HGPLSM--
-HPLSMV-
-HPLSMA-

HKPLSM--
HHPLSM--
HTPLSM--
-HPLSMY-
HPPLSM--
-HPLSMK-
HFPLSM--
HWPLSM--
-HPLSMG-
HIPLSM--
HEPLSM--
HMPLSM--
HQPLSM--
-HPLSMN-
-HPLSMM-
-IPLSMS-
IVPLSM--
ISPLSM--
-IPLSMR-
ITPLSM--
IYPLSM--
-IPLSMH-
-IPLSML-
IAPLSM--
IRPLSM--
IKPLSM--
IIPLSM--
IPPLSM--
-IPLSMI-
-IPLSMA-
IGPLSM--
IFPLSM--
-IPLSMV-
-IPLSMT-
IQPLSM--
-IPLSMY-
IHPLSM--
-IPLSMN-
-IPLSMF-
-IPLSME-
IDPLSM--
-IPLSMG-
IWPLSM--
IMPLSM--
ILPLSM--
INPLSM--
-IPLSMQ-
IEPLSM--
ICPLSM--
-IPLSMK-

-IPLSMM-
-KPLSML-
-KPLSMS-
-KPLSMK-
KVPLSM--
KRPLSM--
-KPLSMT-
-KPLSMV-
-KPLSMH-
-KPLSMM-
KLPLSM--
-KPLSMW-
KYPLSM--
KPPLSM--
-KPLSMN-
-KPLSMF-
KAPLSM--
KGPLSM--
-KPLSMI-
KFPLSM--
-KPLSMY-
KIPLSM--
-KPLSMG-
KNPLSM--
-KPLSMA-
KHPLSM--
-KPLSMQ-
KDPLSM--
KQPLSM--
KWPLSM--
KTPLSM--
KMPLSM--
KEPLSM--
-KPLSME-
-KPLSMC-
KCPLSM--
LRPLSM--
LSPLSM--
LVPLSM--
LTPLSM--
LWPLSM--
LEPLSM--
-LPLSMS-
-LPLSMH-
LKPLSM--
-LPLSMT-
LYPLSM--
LAPLSM--
LQPLSM--
-LPLSMY-

-LPLSMV-
LPPLSM--
LLPLSM--
-LPLSMI-
LFPLSM--
LHPLSM--
-LPLSMN-
-LPLSMM-
-LPLSMQ-
LIPLSM--
-LPLSMW-
-LPLSMA-
LMPLSM--
-MPLSML-
MRPLSM--
-MPLSMV-
-MPLSMR-
-MPLSMK-
MNPLSM--
MTPLSM--
-MPLSMT-
-MPLSMS-
-MPLSMF-
-MPLSMQ-
-MPLSMH-
MFPLSM--
MVPLSM--
MLPLSM--
MPPLSM--
MHPLSM--
MKPLSM--
-MPLSMN-
MSPLSM--
MAPLSM--
MMPLSM--
MYPLSM--
MDPLSM--
-MPLSMI-
MGPLSM--
MEPLSM--
-MPLSMY-
MWPLSM--
-MPLSME-
-MPLSMA-
-MPLSMG-
-MPLSMM-
MCPLSM--
-NPLSMR-
NSPLSM--
NYPLSM--

-NPLSMI-
NEPLSM--
-NPLSMV-
NHPLSM--
-NPLSML-
NVPLSM--
NKPLSM--
-NPLSMK-
-NPLSMS-
-NPLSMM-
NMPLSM--
-NPLSMY-
-NPLSMT-
NNPLSM--
-NPLSMW-
NFPLSM--
NAPLSM--
NPPLSM--
NGPLSM--
NIPLSM--
-NPLSMQ-
-NPLSMA-
NWPLSM--
NQPLSM--
-NPLSME-
NDPLSM--
-NPLSMF-
NCPLSM--
--PLSMMN
--PLSMSS
--PLSMRG
--PLSMRS
--PLSMTS
--PLSMRA
--PLSMFG
--PLSMTL
--PLSMST
--PLSMTR
--PLSMKR
--PLSMLT
--PLSMSR
--PLSMYT
--PLSMIG
--PLSMIS
--PLSMMS
--PLSMRV
--PLSMFS
--PLSMSL
--PLSMMR
--PLSMGA

--PLSMAG
--PLSMYS
--PLSMLG
--PLSMVY
--PLSMKT
--PLSMVT
--PLSMLV
--PLSMIN
--PLSMGS
--PLSMFK
--PLSMME
--PLSMSM
--PLSMHR
--PLSMLN
PRPLSM--
--PLSMVI
--PLSMNS
--PLSMLQ
--PLSMLD
PTPLSM--
--PLSMTN
--PLSMNY
--PLSMWT
--PLSMGT
--PLSMKH
--PLSMAS
--PLSMSK
--PLSMRQ
--PLSMTT
--PLSMTF
--PLSMSY
--PLSMSN
--PLSMHA
--PLSMAV
--PLSMWS
--PLSMRN
--PLSMQL
--PLSMIT
PGPLSM--
--PLSMVQ
--PLSMSV
--PLSMSG
--PLSMIK
--PLSMHV
--PLSMAL
--PLSMVK
--PLSMSI
PNPLSM--
--PLSMSH
--PLSMNT

--PLSMMK
--PLSMHF
--PLSMVM
--PLSMSA
--PLSMRE
--PLSMNR
--PLSMHY
--PLSMAI
--PLSMWA
PQPLSM--
--PLSMKA
--PLSMYN
--PLSMTQ
--PLSMWG
--PLSMTG
--PLSMQV
--PLSMQS
--PLSMHL
--PLSMGG
--PLSMEW
--PLSMWM
--PLSMQR
--PLSMQQ
--PLSMQA
--PLSMMI
--PLSMHS
--PLSMEA
--PLSMSE
--PLSMTV
--PLSMSW
--PLSMMA
--PLSMLM
--PLSMFE
--PLSMTD
--PLSMQN
--PLSMKV
--PLSMHG
--PLSMFN
--PLSMYA
--PLSMWK
--PLSMVW
--PLSMHN
--PLSMVA
--PLSMNF
--PLSMML
--PLSMHH
--PLSMGM
--PLSMET
--PLSMYQ
--PLSMVD

--PLSMKL
--PLSMAT
PKPLSM--
--PLSMTY
--PLSMTM
--PLSMTH
--PLSMQG
--PLSMNA
--PLSMEI
--PLSMNV
--PLSMEV
--PLSMAY
--PLSMAW
--PLSMTK
--PLSMTI
--PLSMNM
--PLSMKI
--PLSMKG
--PLSMHD
--PLSMFD
--PLSMES
--PLSMYD
--PLSMQW
--PLSMNW
--PLSMNN
PFPLSM--
PWPLSM--
--PLSMRW
--PLSMNQ
--PLSMMV
--PLSMHM
--PLSMHK
--PLSMEY
--PLSMAN
--PLSMMD
--PLSMGR
PIPLSM--
--PLSMSQ
--PLSMMT
--PLSMKQ
--PLSMKD
--PLSMGY
--PLSMEL
--PLSMAQ
--PLSMAA
--PLSMWD
--PLSMVH
--PLSMKN
--PLSMYG
--PLSMTW

```
--PLSMNI
--PLSMMQ
--PLSMKW
--PLSMII
--PLSMHI
--PLSMER
--PLSMID
--PLSMGQ
--PLSMAM
--PLSMYE
--PLSMWN
--PLSMVN
--PLSMSD
--PLSMQT
--PLSMMM
--PLSMDG
--PLSMDA
--PLSMYH
--PLSMIQ
--PLSMHW
--PLSMGF
--PLSMEM
PHPLSM--
PEPLSM--
PDPLSM--
--PLSMTE
--PLSMRM
--PLSMQD
--PLSMNH
--PLSMKM
--PLSMIV
--PLSMGN
--PLSMFY
--PLSMFH
--PLSMEQ
--PLSMEH
--PLSMQI
--PLSMKK
--PLSMIE
--PLSMEK
--PLSMDS
--PLSMAH
--PLSMQY
--PLSMQE
--PLSMNK
--PLSMKE
--PLSMEG
--PLSMPI
--PLSMHQ
--PLSMEN
```

--PLSMCR
--PLSMCD
-PPLSMR-
-PPLSMK-
--PLSMSC
--PLSMQK
--PLSMQF
--PLSMPR
--PLSMHE
--PLSMEF
--PLSMCK
--PLSMAK
--PLSMAE
-PPLSMP-
-PPLSMF-
--PLSMYC
--PLSMWH
--PLSMQM
--PLSMQC
--PLSMIH
--PLSMGK
--PLSMGH
--PLSMGE
--PLSMFP
--PLSMDE
--PLSMCY
--PLSMCW
--PLSMCT
--PLSMCN
PCPLSM--
QRPLSM--
QAPLSM--
QFPLSM--
-QPLSMR-
-QPLSML-
-QPLSMN-
QKPLSM--
QNPLSM--
QSPLSM--
-QPLSMW-
-QPLSMS-
-QPLSMK-
QYPLSM--
QHPLSM--
QGPLSM--
QTPLSM--
-QPLSMV-
-QPLSMA-
-QPLSMF-
QPPLSM--

```
-QPLSMY-
-QPLSMH-
QWPLSM--
-QPLSMT-
QLPLSM--
QMPLSM--
-QPLSMM-
QEPLSM--
-QPLSMG-
-QPLSMI-
QCPLSM--
QDPLSM--
RVPLSM--
RLPLSM--
-RPLSML-
-RPLSMT-
RGPLSM--
RRPLSM--
RNPLSM--
RAPLSM--
-RPLSMK-
RYPLSM--
-RPLSMA-
RTPLSM--
-RPLSMY-
RKPLSM--
-RPLSMQ-
RFPLSM--
-RPLSMH-
-RPLSMM-
REPLSM--
RPPLSM--
-RPLSMG-
RMPLSM--
RQPLSM--
-RPLSME-
-RPLSMV-
RHPLSM--
-RPLSMW-
-RPLSMF-
RDPLSM--
RCPLSM--
-RPLSMD-
SVPLSM--
SSPLSM--
SLPLSM--
STPLSM--
-SPLSMK-
-SPLSML-
-SPLSMS-
```

SRPLSM--
-SPLSMT-
SFPLSM--
SQPLSM--
-SPLSMV-
-SPLSMY-
-SPLSMA-
SKPLSM--
SIPLSM--
SAPLSM--
SWPLSM--
-SPLSMF-
SGPLSM--
SNPLSM--
SHPLSM--
-SPLSMH-
SMPLSM--
-SPLSME-
SCPLSM--
SPPLSM--
SYPLSM--
SEPLSM--
SDPLSM--
-TPLSMR-
TRPLSM--
TSPLSM--
-TPLSMS-
TGPLSM--
-TPLSMV-
-TPLSMH-
THPLSM--
TVPLSM--
TIPLSM--
TNPLSM--
TAPLSM--
-TPLSMK-
TPPLSM--
-TPLSMF-
-TPLSMM-
TFPLSM--
TTPLSM--
TLPLSM--
-TPLSMY-
-TPLSMW-
-TPLSMT-
TYPLSM--
TMPLSM--
-TPLSMA-
-TPLSMG-
TEPLSM--

TKPLSM--
-TPLSMI-
TWPLSM--
TQPLSM--
-TPLSME-
-TPLSMQ-
TDPLSM--
TCPLSM--
-TPLSMD-
-VPLSML-
VSPLSM--
VGPLSM--
VQPLSM--
VFPLSM--
-VPLSMS-
VVPLSM--
-VPLSMA-
VLPLSM--
VTPLSM--
-VPLSMV-
-VPLSMI-
VKPLSM--
VAPLSM--
-VPLSMN-
-VPLSMK-
VMPLSM--
VNPLSM--
VPPLSM--
VIPLSM--
VDPLSM--
-VPLSMQ-
-VPLSMW-
VYPLSM--
VWPLSM--
-VPLSME-
VEPLSM--
VCPLSM--
-VPLSMM-
WSPLSM--
WRPLSM--
-WPLSMH-
WAPLSM--
WHPLSM--
WDPLSM--
-WPLSMR-
WVPLSM--
-WPLSMT-
-WPLSMM-
WIPLSM--
WEPLSM--

-WPLSMS-
WPPLSM--
-WPLSMN-
WNPLSM--
-WPLSMK-
WKPLSM--
-WPLSMQ-
WLPLSM--
WMPLSM--
WWPLSM--
-WPLSMG-
WFPLSM--
WYPLSM--
-WPLSMV-
-WPLSMW-
WQPLSM--
WGPLSM--
-WPLSME-
-WPLSMP-
-WPLSMI-
YRPLSM--
-YPLSMR-
YVPLSM--
YAPLSM--
-YPLSMI-
-YPLSMY-
YFPLSM--
-YPLSMS-
YPPLSM--
-YPLSMW-
-YPLSMA-
-YPLSMV-
-YPLSMT-
-YPLSMK-
-YPLSMQ-
-YPLSMH-
YKPLSM--
-YPLSMF-
YNPLSM--
-YPLSME-
-YPLSMM-
YQPLSM--
-YPLSMG-
YEPLSM--
YIPLSM--
YDPLSM--
-YPLSMN-
YHPLSM--
YGPLSM--
YYPLSM--

	YMPLSM--		
	-YPLSMP-		
PSPL	5	917	47.34
	-APSPLP-		
	DNPSPL--		
	-IPSPLG-		
	-KPSPLP-		
	-KPSPLG-		
	-KPSPLE-		
	-NPSPLG-		
	-NPSPLA-		
	--PSPLGR		
	--PSPLVW		
	--PSPLTP		
	--PSPLPR		
	--PSPLEN		
	-SPSPLE-		
	-TPSPLG-		
	-YPSPLG-		
	-APSPLR-		
	-APSPLY-		
	-APSPLL-		
	AVPSPL--		
	ARPSPL--		
	AYPSPL--		
	APPSPL--		
	ALPSPL--		
	ATPSPL--		
	-APSPLS-		
	AEPSPL--		
	-APSPLK-		
	AFPSPL--		
	AWPSPL--		
	ASPSPL--		
	AKPSPL--		
	AAPSPL--		
	-APSPLT-		
	-APSPLH-		
	AGPSPL--		
	AIPSPL--		
	-APSPLV-		
	AHPSPL--		
	AMPSPL--		
	-APSPLW-		
	-APSPLF-		
	AQPSPL--		
	-APSPLM-		
	ADPSPL--		
	-APSPLG-		
	-APSPLA-		

−APSPLE−
−APSPLC−
ACPSPL−−
−APSPLQ−
CWPSPL−−
CMPSPL−−
CTPSPL−−
−CPSPLI−
CEPSPL−−
−CPSPLM−
CPPSPL−−
CGPSPL−−
CKPSPL−−
CVPSPL−−
CQPSPL−−
−CPSPLL−
CCPSPL−−
CAPSPL−−
CSPSPL−−
CNPSPL−−
−CPSPLK−
−CPSPLA−
CIPSPL−−
CFPSPL−−
CYPSPL−−
−CPSPLT−
−CPSPLS−
−CPSPLR−
−CPSPLQ−
−CPSPLH−
CDPSPL−−
−DPSPLR−
DRPSPL−−
DVPSPL−−
−DPSPLY−
DIPSPL−−
DFPSPL−−
DSPSPL−−
DTPSPL−−
−DPSPLW−
−DPSPLF−
DAPSPL−−
DWPSPL−−
DQPSPL−−
DYPSPL−−
−DPSPLK−
−DPSPLI−
−DPSPLT−
−DPSPLS−
−DPSPLN−

-DPSPLM-
DGPSPL--
-DPSPLQ-
DMPSPL--
-DPSPLH-
-DPSPLC-
-EPSPLL-
ESPSPL--
-EPSPLY-
ERPSPL--
-EPSPLI-
-EPSPLF-
-EPSPLK-
-EPSPLW-
-EPSPLT-
EKPSPL--
-EPSPLM-
-EPSPLV-
-EPSPLH-
EWPSPL--
EYPSPL--
EFPSPL--
-EPSPLQ-
ENPSPL--
EVPSPL--
EIPSPL--
-EPSPLA-
EHPSPL--
EGPSPL--
EQPSPL--
-EPSPLN-
ETPSPL--
-EPSPLC-
-EPSPLG-
EAPSPL--
FLPSPL--
-FPSPLR-
FEPSPL--
FRPSPL--
FTPSPL--
FQPSPL--
FIPSPL--
-FPSPLH-
FGPSPL--
FAPSPL--
FNPSPL--
FHPSPL--
FVPSPL--
FYPSPL--
FSPSPL--

-FPSPLW-
FWPSPL--
FFPSPL--
-FPSPLS-
-FPSPLY-
FPPSPL--
FKPSPL--
-FPSPLM-
FMPSPL--
FDPSPL--
-FPSPLK-
-FPSPLF-
-FPSPLV-
-FPSPLN-
-FPSPLI-
-FPSPLG-
-FPSPLA-
-FPSPLQ-
FCPSPL--
GVPSPL--
GAPSPL--
-GPSPLY-
-GPSPLL-
GSPSPL--
GYPSPL--
GFPSPL--
-GPSPLV-
GKPSPL--
GTPSPL--
GMPSPL--
-GPSPLM-
-GPSPLH-
-GPSPLK-
-GPSPLF-
GLPSPL--
GDPSPL--
-GPSPLW-
-GPSPLI-
GQPSPL--
-GPSPLT-
-GPSPLA-
GNPSPL--
-GPSPLN-
GPPSPL--
-GPSPLQ-
GIPSPL--
GHPSPL--
HSPSPL--
HGPSPL--
-HPSPLR-

−HPSPLK−
HRPSPL−−
HLPSPL−−
HAPSPL−−
HMPSPL−−
−HPSPLY−
HHPSPL−−
−HPSPLS−
HFPSPL−−
HVPSPL−−
HKPSPL−−
−HPSPLW−
−HPSPLL−
−HPSPLQ−
−HPSPLM−
HYPSPL−−
HQPSPL−−
−HPSPLF−
−HPSPLH−
−HPSPLI−
HNPSPL−−
−HPSPLA−
HPPSPL−−
HWPSPL−−
−HPSPLV−
−HPSPLN−
−HPSPLT−
HDPSPL−−
−HPSPLC−
−HPSPLG−
IRPSPL−−
ITPSPL−−
IFPSPL−−
ISPSPL−−
−IPSPLL−
IAPSPL−−
−IPSPLR−
IPPSPL−−
IKPSPL−−
−IPSPLM−
IVPSPL−−
−IPSPLS−
IGPSPL−−
−IPSPLN−
−IPSPLA−
−IPSPLF−
IIPSPL−−
IEPSPL−−
−IPSPLY−
IYPSPL−−

IWPSPL--
-IPSPLH-
-IPSPLQ-
IHPSPL--
-IPSPLV-
IMPSPL--
-IPSPLW-
-IPSPLK-
-IPSPLE-
IQPSPL--
IDPSPL--
-IPSPLI-
INPSPL--
KSPSPL--
KWPSPL--
-KPSPLR-
KLPSPL--
KIPSPL--
KTPSPL--
-KPSPLM-
-KPSPLF-
-KPSPLY-
-KPSPLS-
KMPSPL--
-KPSPLI-
-KPSPLV-
-KPSPLL-
-KPSPLK-
-KPSPLH-
-KPSPLT-
-KPSPLQ-
-KPSPLN-
KEPSPL--
KYPSPL--
KPPSPL--
KNPSPL--
KVPSPL--
-KPSPLA-
KQPSPL--
KFPSPL--
KKPSPL--
KDPSPL--
KHPSPL--
KCPSPL--
LHPSPL--
LLPSPL--
-LPSPLR-
LQPSPL--
LIPSPL--
LGPSPL--

LKPSPL--
LNPSPL--
LPPSPL--
LTPSPL--
-LPSPLL-
-LPSPLT-
LVPSPL--
-LPSPLM-
LYPSPL--
-LPSPLK-
LMPSPL--
LEPSPL--
LWPSPL--
-LPSPLY-
-LPSPLV-
-LPSPLH-
-LPSPLW-
LDPSPL--
-LPSPLQ-
LCPSPL--
-LPSPLN-
-LPSPLA-
MSPSPL--
-MPSPLK-
-MPSPLI-
MYPSPL--
MLPSPL--
MRPSPL--
-MPSPLV-
-MPSPLH-
-MPSPLL-
MKPSPL--
-MPSPLQ-
-MPSPLY-
MGPSPL--
MTPSPL--
MQPSPL--
MNPSPL--
-MPSPLW-
MHPSPL--
MVPSPL--
MPPSPL--
MWPSPL--
MDPSPL--
-MPSPLT-
MAPSPL--
-MPSPLM-
-MPSPLA-
MMPSPL--
MFPSPL--

MIPSPL--
MEPSPL--
-MPSPLN-
-MPSPLF-
-MPSPLE-
MCPSPL--
-NPSPLR-
-NPSPLL-
NTPSPL--
NLPSPL--
-NPSPLY-
NVPSPL--
NSPSPL--
NAPSPL--
-NPSPLV-
NPPSPL--
-NPSPLI-
-NPSPLF-
NEPSPL--
NKPSPL--
-NPSPLS-
-NPSPLK-
NMPSPL--
-NPSPLH-
-NPSPLW-
NGPSPL--
NNPSPL--
NFPSPL--
-NPSPLT-
-NPSPLQ-
NYPSPL--
-NPSPLM-
NHPSPL--
NDPSPL--
-NPSPLN-
NCPSPL--
--PSPLRS
--PSPLSV
--PSPLLG
--PSPLGL
--PSPLSG
--PSPLWS
--PSPLVS
--PSPLRG
--PSPLYI
--PSPLRY
--PSPLLY
--PSPLKA
--PSPLLN
--PSPLFS

PRPSPL--
--PSPLQL
--PSPLMT
--PSPLAL
--PSPLWL
--PSPLTL
--PSPLTI
--PSPLIR
--PSPLGF
--PSPLSI
--PSPLRR
--PSPLKG
PVPSPL--
--PSPLIN
--PSPLIG
--PSPLQS
--PSPLWI
--PSPLTY
--PSPLST
--PSPLFY
PSPSPL--
--PSPLLF
--PSPLKY
--PSPLYS
--PSPLVY
--PSPLDL
--PSPLWN
--PSPLTS
--PSPLLW
--PSPLLK
--PSPLGV
--PSPLKT
--PSPLKS
--PSPLFV
--PSPLFW
--PSPLFG
--PSPLTG
--PSPLWV
--PSPLVR
--PSPLRT
--PSPLGY
--PSPLGS
--PSPLAS
--PSPLWM
--PSPLTA
PAPSPL--
--PSPLRA
--PSPLNF
--PSPLIH
PMPSPL--

--PSPLVM
--PSPLRF
--PSPLML
--PSPLKL
PLPSPL--
PTPSPL--
--PSPLYT
--PSPLIT
PGPSPL--
--PSPLWR
--PSPLSN
--PSPLRI
--PSPLYY
--PSPLFL
--PSPLYK
--PSPLPM
--PSPLMS
--PSPLMR
--PSPLHI
--PSPLAW
--PSPLVL
--PSPLTM
--PSPLMY
--PSPLKW
--PSPLHR
--PSPLAT
--PSPLWQ
--PSPLRV
--PSPLNS
--PSPLGH
--PSPLVH
--PSPLHL
--PSPLVV
--PSPLTR
PIPSPL--
--PSPLLR
--PSPLLQ
--PSPLKI
--PSPLKF
--PSPLYA
--PSPLVG
--PSPLIM
--PSPLGI
--PSPLSR
--PSPLHG
--PSPLSW
--PSPLRH
--PSPLIA
--PSPLDM
--PSPLGQ

--PSPLQG
--PSPLKK
--PSPLIS
--PSPLWK
--PSPLTK
--PSPLSH
--PSPLPV
--PSPLHS
--PSPLAF
--PSPLYG
--PSPLVN
--PSPLQM
--PSPLHN
--PSPLVI
--PSPLMV
--PSPLMK
--PSPLHA
--PSPLEG
--PSPLAH
--PSPLYN
--PSPLTW
--PSPLTF
--PSPLSM
--PSPLRQ
--PSPLFT
--PSPLAG
--PSPLVK
--PSPLKV
--PSPLAI
--PSPLQT
--PSPLLI
--PSPLIW
--PSPLHF
--PSPLYV
--PSPLYD
--PSPLNY
--PSPLEL
--PSPLDY
--PSPLYE
--PSPLWE
--PSPLSQ
--PSPLSK
--PSPLRM
--PSPLPI
--PSPLMA
--PSPLKH
--PSPLGW
PEPSPL--
--PSPLQW
--PSPLNH

--PSPLMN
--PSPLIK
PFPSPL--
--PSPLWD
--PSPLRD
--PSPLNN
--PSPLNA
--PSPLKR
--PSPLII
--PSPLFI
--PSPLAV
--PSPLMW
--PSPLME
--PSPLKM
--PSPLWG
--PSPLTQ
--PSPLQR
--PSPLMI
--PSPLKN
--PSPLIQ
--PSPLGG
--PSPLFQ
--PSPLAY
--PSPLWH
--PSPLVQ
--PSPLRW
--PSPLPW
--PSPLLD
--PSPLIF
--PSPLHH
PWPSPL--
--PSPLYM
--PSPLYF
--PSPLVD
--PSPLQQ
--PSPLPF
--PSPLNG
--PSPLIL
--PSPLGN
--PSPLGA
--PSPLQA
--PSPLPY
--PSPLKQ
--PSPLHY
--PSPLHW
--PSPLGM
--PSPLEW
--PSPLES
--PSPLAN
--PSPLAM

--PSPLPS
--PSPLPH
--PSPLNV
--PSPLNT
--PSPLNM
--PSPLMH
--PSPLMF
--PSPLHT
--PSPLHM
--PSPLHD
--PSPLFN
--PSPLFH
--PSPLAA
--PSPLYQ
--PSPLNI
--PSPLMG
PYPSPL--
--PSPLQY
--PSPLQF
--PSPLNR
--PSPLHK
--PSPLAK
PKPSPL--
--PSPLWY
--PSPLWW
--PSPLWT
--PSPLVE
--PSPLTN
--PSPLTD
--PSPLQV
--PSPLMM
--PSPLFD
--PSPLEV
PQPSPL--
--PSPLQN
--PSPLHQ
--PSPLEY
--PSPLEA
PDPSPL--
--PSPLQI
--PSPLQH
--PSPLPN
--PSPLPA
--PSPLNQ
--PSPLKD
--PSPLDV
--PSPLDA
--PSPLWA
--PSPLTE
--PSPLSD

--PSPLPQ
--PSPLNK
--PSPLHV
--PSPLGT
--PSPLFE
--PSPLFA
--PSPLEM
--PSPLDS
--PSPLDH
--PSPLYW
--PSPLQK
--PSPLMD
--PSPLKE
--PSPLEI
PNPSPL--
PHPSPL--
--PSPLQE
--PSPLPT
--PSPLLE
--PSPLDW
--PSPLCV
--PSPLCI
-PPSPLW-
-PPSPLN-
--PSPLRC
--PSPLQC
--PSPLNP
--PSPLND
--PSPLIE
--PSPLID
--PSPLHE
--PSPLGK
--PSPLGD
--PSPLFM
--PSPLFC
--PSPLET
--PSPLEQ
--PSPLEF
--PSPLEC
--PSPLDI
--PSPLDD
--PSPLCL
--PSPLCG
--PSPLAP
--PSPLAC
-PPSPLY-
-PPSPLV-
-PPSPLI-
-QPSPLR-
QWPSPL--

```
-QPSPLT-
QTPSPL--
QYPSPL--
QPPSPL--
QIPSPL--
-QPSPLW-
-QPSPLS-
QRPSPL--
-QPSPLY-
-QPSPLV-
QGPSPL--
QAPSPL--
QKPSPL--
QNPSPL--
-QPSPLN-
QEPSPL--
QQPSPL--
QFPSPL--
-QPSPLK-
-QPSPLA-
QHPSPL--
-QPSPLM-
-QPSPLI-
-QPSPLH-
-QPSPLF-
QLPSPL--
-QPSPLQ-
QMPSPL--
QDPSPL--
-QPSPLE-
QCPSPL--
RSPSPL--
-RPSPLS-
RIPSPL--
RMPSPL--
RVPSPL--
RAPSPL--
RTPSPL--
RRPSPL--
REPSPL--
-RPSPLI-
-RPSPLY-
RWPSPL--
-RPSPLF-
RPPSPL--
RNPSPL--
RYPSPL--
RFPSPL--
RKPSPL--
RQPSPL--
```

−RPSPLW−
−RPSPLN−
−RPSPLA−
−RPSPLH−
RHPSPL−−
−RPSPLQ−
−RPSPLT−
RDPSPL−−
−RPSPLG−
−RPSPLE−
RCPSPL−−
−SPSPLR−
SSPSPL−−
−SPSPLY−
SVPSPL−−
SLPSPL−−
−SPSPLL−
SMPSPL−−
SIPSPL−−
SAPSPL−−
−SPSPLH−
SKPSPL−−
−SPSPLI−
SWPSPL−−
STPSPL−−
SHPSPL−−
SQPSPL−−
−SPSPLS−
SPPSPL−−
−SPSPLT−
SFPSPL−−
−SPSPLW−
−SPSPLQ−
−SPSPLM−
SNPSPL−−
SDPSPL−−
−SPSPLV−
−SPSPLK−
−SPSPLA−
SCPSPL−−
−TPSPLR−
TRPSPL−−
TAPSPL−−
TLPSPL−−
TNPSPL−−
TVPSPL−−
−TPSPLL−
TQPSPL−−
TMPSPL−−
−TPSPLY−

-TPSPLI-
TEPSPL--
TIPSPL--
TTPSPL--
-TPSPLV-
THPSPL--
TSPSPL--
-TPSPLS-
-TPSPLT-
-TPSPLF-
-TPSPLK-
-TPSPLH-
-TPSPLW-
TFPSPL--
TWPSPL--
-TPSPLQ-
-TPSPLM-
-TPSPLN-
-TPSPLA-
VVPSPL--
-VPSPLR-
VLPSPL--
VRPSPL--
-VPSPLK-
VIPSPL--
-VPSPLN-
VMPSPL--
-VPSPLL-
-VPSPLT-
-VPSPLS-
VYPSPL--
-VPSPLY-
-VPSPLV-
VKPSPL--
VPPSPL--
VGPSPL--
VEPSPL--
-VPSPLI-
-VPSPLQ-
VWPSPL--
VQPSPL--
VFPSPL--
-VPSPLW-
-VPSPLM-
-VPSPLH-
VHPSPL--
-VPSPLA-
-VPSPLG-
-VPSPLF-
VDPSPL--

-VPSPLE-
VCPSPL--
-VPSPLC-
WAPSPL--
WSPSPL--
-WPSPLR-
-WPSPLL-
WTPSPL--
WKPSPL--
WLPSPL--
WGPSPL--
WVPSPL--
WEPSPL--
-WPSPLM-
WPPSPL--
-WPSPLH-
WYPSPL--
-WPSPLI-
WIPSPL--
-WPSPLK-
-WPSPLV-
-WPSPLF-
WHPSPL--
-WPSPLS-
WQPSPL--
WWPSPL--
-WPSPLT-
-WPSPLA-
WDPSPL--
WCPSPL--
-WPSPLN-
WNPSPL--
-WPSPLG-
-WPSPLD-
-WPSPLW-
-WPSPLQ-
-WPSPLY-
-WPSPLE-
YRPSPL--
YAPSPL--
YVPSPL--
-YPSPLS-
-YPSPLR-
YSPSPL--
YDPSPL--
YTPSPL--
YMPSPL--
-YPSPLM-
YQPSPL--
YGPSPL--

	YYPSPL--		
	-YPSPLV-		
	YIPSPL--		
	-YPSPLN-		
	-YPSPLL-		
	-YPSPLK-		
	YEPSPL--		
	YHPSPL--		
	YWPSPL--		
	-YPSPLT-		
	-YPSPLH-		
	-YPSPLW-		
	YPPSPL--		
	-YPSPLI-		
	-YPSPLQ-		
	YKPSPL--		
	-YPSPLA-		
	-YPSPLE-		
	YCPSPL--		
	-YPSPLC-		
PVAY	152	775	47.28
	-GPVAYG-		
	-RPVAYP-		
	-SPVAYT-		
	-APVAYR-		
	AHPVAY--		
	-APVAYL-		
	AAPVAY--		
	ATPVAY--		
	AKPVAY--		
	-APVAYS-		
	-APVAYA-		
	AVPVAY--		
	AYPVAY--		
	ASPVAY--		
	-APVAYI-		
	-APVAYM-		
	-APVAYK-		
	APPVAY--		
	ALPVAY--		
	AWPVAY--		
	-APVAYT-		
	ACPVAY--		
	AQPVAY--		
	-APVAYV-		
	-APVAYN-		
	AIPVAY--		
	ANPVAY--		
	-APVAYQ-		
	AFPVAY--		

AMPVAY--
-APVAYH-
-APVAYG-
AEPVAY--
CLPVAY--
CTPVAY--
CSPVAY--
CGPVAY--
CYPVAY--
CKPVAY--
-CPVAYT-
CPPVAY--
CAPVAY--
CQPVAY--
CRPVAY--
-CPVAYV-
-CPVAYS-
CFPVAY--
CDPVAY--
-CPVAYY-
-CPVAYH-
CIPVAY--
DRPVAY--
-DPVAYR-
-DPVAYY-
DSPVAY--
DVPVAY--
-DPVAYK-
-DPVAYT-
DTPVAY--
DNPVAY--
-DPVAYW-
-DPVAYI-
DAPVAY--
DYPVAY--
-DPVAYS-
-DPVAYM-
-DPVAYV-
-DPVAYL-
DKPVAY--
DIPVAY--
DFPVAY--
DLPVAY--
-EPVAYT-
EVPVAY--
ERPVAY--
ESPVAY--
-EPVAYV-
ELPVAY--
-EPVAYR-

ETPVAY--
-EPVAYL-
EPPVAY--
EMPVAY--
EGPVAY--
-EPVAYF-
-EPVAYY-
EHPVAY--
-EPVAYS-
-EPVAYM-
-EPVAYI-
ENPVAY--
EIPVAY--
-EPVAYW-
-EPVAYK-
EYPVAY--
-EPVAYG-
EFPVAY--
EDPVAY--
ECPVAY--
EAPVAY--
FSPVAY--
FNPVAY--
FKPVAY--
FMPVAY--
FEPVAY--
FGPVAY--
FYPVAY--
-FPVAYT-
-FPVAYR-
FAPVAY--
-FPVAYV-
-FPVAYF-
FQPVAY--
-FPVAYK-
FIPVAY--
FWPVAY--
-FPVAYL-
FHPVAY--
-FPVAYH-
FVPVAY--
-FPVAYY-
-FPVAYQ-
-FPVAYA-
-FPVAYW-
-FPVAYS-
-FPVAYN-
-FPVAYC-
FDPVAY--
GAPVAY--

GRPVAY--
-GPVAYS-
-GPVAYV-
-GPVAYT-
GVPVAY--
GIPVAY--
-GPVAYY-
-GPVAYR-
-GPVAYL-
GGPVAY--
-GPVAYM-
GHPVAY--
GSPVAY--
GNPVAY--
GKPVAY--
GLPVAY--
GTPVAY--
GDPVAY--
GWPVAY--
GMPVAY--
GYPVAY--
GEPVAY--
-GPVAYH-
GFPVAY--
-GPVAYQ-
GQPVAY--
-GPVAYK-
-GPVAYN-
-GPVAYE-
GPPVAY--
GCPVAY--
HSPVAY--
HLPVAY--
HHPVAY--
-HPVAYR-
-HPVAYS-
HMPVAY--
HGPVAY--
HRPVAY--
-HPVAYY-
HVPVAY--
-HPVAYK-
HTPVAY--
HIPVAY--
HYPVAY--
-HPVAYT-
-HPVAYV-
-HPVAYQ-
-HPVAYA-
HPPVAY--

HCPVAY--
-HPVAYL-
-HPVAYH-
HKPVAY--
HQPVAY--
-HPVAYN-
HNPVAY--
HDPVAY--
-HPVAYF-
HAPVAY--
-HPVAYC-
-IPVAYL-
IQPVAY--
IGPVAY--
-IPVAYQ-
ISPVAY--
-IPVAYV-
-IPVAYF-
ITPVAY--
IRPVAY--
-IPVAYN-
INPVAY--
IHPVAY--
IMPVAY--
-IPVAYA-
IPPVAY--
IDPVAY--
-IPVAYT-
-IPVAYS-
-IPVAYK-
IVPVAY--
IWPVAY--
-IPVAYY-
-IPVAYM-
IEPVAY--
IKPVAY--
-IPVAYH-
-IPVAYE-
IFPVAY--
IAPVAY--
IYPVAY--
-IPVAYP-
KSPVAY--
-KPVAYR-
-KPVAYS-
-KPVAYT-
-KPVAYQ-
KGPVAY--
-KPVAYM-
-KPVAYL-

KVPVAY--
KLPVAY--
-KPVAYI-
-KPVAYY-
KFPVAY--
KDPVAY--
KEPVAY--
KTPVAY--
KYPVAY--
-KPVAYK-
KIPVAY--
KWPVAY--
-KPVAYW-
-KPVAYH-
-KPVAYG-
-KPVAYF-
-KPVAYE-
KNPVAY--
KMPVAY--
-KPVAYN-
-KPVAYA-
KCPVAY--
KQPVAY--
LSPVAY--
-LPVAYL-
LMPVAY--
-LPVAYR-
LFPVAY--
LVPVAY--
LTPVAY--
LAPVAY--
-LPVAYV-
-LPVAYS-
LNPVAY--
LYPVAY--
-LPVAYW-
LHPVAY--
-LPVAYT-
-LPVAYA-
-LPVAYQ-
LPPVAY--
LWPVAY--
LQPVAY--
-LPVAYH-
-LPVAYE-
-LPVAYN-
-LPVAYC-
LEPVAY--
LDPVAY--
LCPVAY--

MSPVAY--
MRPVAY--
-MPVAYR-
MLPVAY--
-MPVAYL-
-MPVAYH-
MGPVAY--
-MPVAYS-
-MPVAYQ-
MPPVAY--
MNPVAY--
MIPVAY--
MTPVAY--
MAPVAY--
-MPVAYY-
MVPVAY--
-MPVAYT-
MKPVAY--
MYPVAY--
MQPVAY--
-MPVAYV-
MHPVAY--
-MPVAYA-
MMPVAY--
-MPVAYW-
-MPVAYF-
MWPVAY--
-MPVAYG-
MEPVAY--
MCPVAY--
NSPVAY--
-NPVAYR-
NNPVAY--
-NPVAYS-
NGPVAY--
-NPVAYM-
NLPVAY--
NVPVAY--
-NPVAYL-
NPPVAY--
-NPVAYV-
NMPVAY--
NFPVAY--
NKPVAY--
-NPVAYK-
NIPVAY--
NEPVAY--
NYPVAY--
-NPVAYW-
-NPVAYF-

-NPVAYY-
NWPVAY--
NHPVAY--
NDPVAY--
NTPVAY--
-NPVAYI-
-NPVAYE-
-NPVAYA-
--PVAYRS
--PVAYSL
--PVAYLG
--PVAYVK
--PVAYRN
--PVAYLS
--PVAYRY
--PVAYTS
--PVAYGL
--PVAYVT
--PVAYTR
--PVAYMR
--PVAYKL
--PVAYSR
--PVAYRA
--PVAYMG
--PVAYLT
--PVAYKS
--PVAYIS
--PVAYVS
--PVAYSS
--PVAYRH
--PVAYNL
--PVAYLN
--PVAYKT
--PVAYKG
--PVAYSI
--PVAYRV
--PVAYRL
--PVAYRI
--PVAYIG
--PVAYLM
--PVAYAL
--PVAYHT
--PVAYLA
--PVAYAS
--PVAYRT
--PVAYQL
--PVAYAA
--PVAYRF
--PVAYMI
--PVAYFG

--PVAYYR
--PVAYTH
--PVAYMQ
--PVAYWS
--PVAYRQ
--PVAYLR
--PVAYKQ
--PVAYSH
--PVAYSV
--PVAYSA
--PVAYWN
--PVAYSG
--PVAYRG
--PVAYQV
--PVAYIA
--PVAYEL
--PVAYWH
--PVAYTV
--PVAYSN
--PVAYNF
--PVAYML
--PVAYVN
--PVAYPF
--PVAYMN
--PVAYGY
--PVAYGA
--PVAYTD
--PVAYSK
--PVAYFN
--PVAYTN
--PVAYRD
PRPVAY--
--PVAYRM
--PVAYNT
--PVAYMK
--PVAYKW
--PVAYIR
--PVAYHQ
--PVAYHM
--PVAYGI
--PVAYYH
--PVAYVQ
--PVAYTT
--PVAYSW
--PVAYQR
--PVAYFK
PQPVAY--
PHPVAY--
PYPVAY--
--PVAYQS

--PVAYPM
--PVAYPI
--PVAYLQ
PNPVAY--
PWPVAY--
--PVAYWA
--PVAYQY
--PVAYQM
--PVAYQF
--PVAYKN
PSPVAY--
PKPVAY--
--PVAYWR
--PVAYVA
--PVAYNV
--PVAYMF
--PVAYHH
--PVAYFQ
--PVAYYM
--PVAYST
--PVAYSM
--PVAYQG
--PVAYLH
--PVAYIN
--PVAYES
--PVAYEA
--PVAYDL
--PVAYAY
--PVAYAT
PTPVAY--
PMPVAY--
--PVAYYQ
--PVAYVM
--PVAYTI
--PVAYPY
--PVAYNS
--PVAYNA
--PVAYKA
--PVAYHV
--PVAYHG
--PVAYHF
--PVAYAI
--PVAYTG
--PVAYSY
--PVAYQK
--PVAYII
--PVAYGM
--PVAYEG
--PVAYAM
--PVAYWE

--PVAYTF
--PVAYQQ
--PVAYGW
--PVAYAN
--PVAYAK
--PVAYAG
--PVAYAF
--PVAYYW
--PVAYWG
--PVAYVV
--PVAYVG
--PVAYRW
--PVAYLD
--PVAYKM
--PVAYHL
--PVAYGV
--PVAYET
--PVAYEI
--PVAYAR
PAPVAY--
--PVAYYG
--PVAYWT
--PVAYWQ
--PVAYTE
--PVAYSD
--PVAYRE
--PVAYPQ
--PVAYNW
--PVAYNQ
--PVAYKY
--PVAYKV
--PVAYHI
--PVAYGG
PGPVAY--
PEPVAY--
--PVAYYK
--PVAYWM
--PVAYVD
--PVAYQN
--PVAYNG
--PVAYMM
--PVAYME
--PVAYKF
--PVAYKD
--PVAYHE
PCPVAY--
--PVAYWK
--PVAYTQ
--PVAYSQ
--PVAYPV

--PVAYMW
--PVAYMH
--PVAYLE
--PVAYIY
--PVAYIH
--PVAYHR
--PVAYHK
--PVAYHA
--PVAYGT
--PVAYGS
--PVAYFC
--PVAYFA
--PVAYEY
--PVAYDM
--PVAYAV
--PVAYAH
--PVAYYV
--PVAYYD
--PVAYVE
--PVAYTY
--PVAYTM
--PVAYQH
--PVAYQE
--PVAYPW
--PVAYNK
--PVAYNI
--PVAYND
--PVAYMA
--PVAYLC
--PVAYKP
--PVAYIQ
--PVAYID
--PVAYHD
--PVAYGQ
--PVAYGD
--PVAYFH
--PVAYEW
--PVAYER
--PVAYEN
--PVAYDT
--PVAYDI
--PVAYCW
--PVAYCR
--PVAYCA
--PVAYAW
--PVAYAQ
-PPVAYS-
-PPVAYL-
PDPVAY--
QGPVAY--

QSPVAY--
QKPVAY--
QNPVAY--
-QPVAYV-
QEPVAY--
-QPVAYK-
QLPVAY--
QWPVAY--
QRPVAY--
-QPVAYT-
-QPVAYM-
-QPVAYL-
QTPVAY--
-QPVAYS-
-QPVAYR-
-QPVAYF-
QAPVAY--
-QPVAYH-
QPPVAY--
-QPVAYY-
QVPVAY--
QQPVAY--
-QPVAYW-
-QPVAYQ-
QMPVAY--
QYPVAY--
-QPVAYA-
-QPVAYD-
QFPVAY--
QDPVAY--
RSPVAY--
-RPVAYM-
RAPVAY--
-RPVAYL-
RIPVAY--
RLPVAY--
-RPVAYT-
RMPVAY--
RYPVAY--
-RPVAYK-
RTPVAY--
-RPVAYV-
-RPVAYH-
-RPVAYN-
RCPVAY--
-RPVAYA-
RFPVAY--
-RPVAYF-
-RPVAYE-
RNPVAY--

RQPVAY--
REPVAY--
-RPVAYQ-
RDPVAY--
RPPVAY--
RHPVAY--
-RPVAYG-
-SPVAYR-
SSPVAY--
SLPVAY--
SFPVAY--
-SPVAYL-
SVPVAY--
STPVAY--
-SPVAYS-
SNPVAY--
-SPVAYV-
-SPVAYY-
SWPVAY--
-SPVAYI-
SIPVAY--
SEPVAY--
-SPVAYF-
-SPVAYW-
-SPVAYM-
SGPVAY--
-SPVAYA-
-SPVAYK-
SPPVAY--
-SPVAYQ-
SYPVAY--
SHPVAY--
SDPVAY--
SCPVAY--
SAPVAY--
SMPVAY--
-SPVAYN-
-SPVAYG-
-TPVAYY-
TKPVAY--
TYPVAY--
-TPVAYS-
-TPVAYH-
-TPVAYV-
TTPVAY--
TVPVAY--
TNPVAY--
-TPVAYL-
-TPVAYR-
TMPVAY--

TIPVAY--
TWPVAY--
THPVAY--
TEPVAY--
TQPVAY--
-TPVAYK-
-TPVAYF-
TGPVAY--
-TPVAYQ-
TPPVAY--
TFPVAY--
-TPVAYT-
-TPVAYN-
-TPVAYM-
TDPVAY--
-TPVAYI-
-TPVAYA-
TLPVAY--
TCPVAY--
-TPVAYE-
-VPVAYR-
VSPVAY--
VTPVAY--
VLPVAY--
-VPVAYT-
VAPVAY--
VVPVAY--
-VPVAYA-
-VPVAYI-
VKPVAY--
VNPVAY--
-VPVAYH-
-VPVAYL-
-VPVAYQ-
-VPVAYM-
-VPVAYK-
VDPVAY--
VMPVAY--
VWPVAY--
VHPVAY--
VGPVAY--
-VPVAYW-
-VPVAYN-
VPPVAY--
VIPVAY--
-VPVAYE-
VQPVAY--
VCPVAY--
VEPVAY--
WNPVAY--

```
            WRPVAY--
            -WPVAYM-
            -WPVAYR-
            WTPVAY--
            WVPVAY--
            WLPVAY--
            WHPVAY--
            -WPVAYK-
            WIPVAY--
            WFPVAY--
            WGPVAY--
            WDPVAY--
            -WPVAYV-
            WQPVAY--
            -WPVAYN-
            -WPVAYH-
            WAPVAY--
            -WPVAYA-
            WSPVAY--
            -WPVAYS-
            -WPVAYQ-
            -YPVAYR-
            YSPVAY--
            YLPVAY--
            YGPVAY--
            YAPVAY--
            YIPVAY--
            YDPVAY--
            -YPVAYS-
            YHPVAY--
            -YPVAYL-
            YKPVAY--
            YYPVAY--
            YQPVAY--
            -YPVAYH-
            -YPVAYA-
            -YPVAYW-
            -YPVAYM-
            YVPVAY--
            YTPVAY--
            -YPVAYV-
            -YPVAYQ-
            -YPVAYN-
            YPPVAY--
            -YPVAYG-
            -YPVAYE-
PANL           1        925     47.25
            -NPANLN-
            --PANLIP
            -APANLT-
```

-APANLL-
AFPANL--
-APANLR-
-APANLS-
-APANLF-
ALPANL--
-APANLN-
-APANLK-
AAPANL--
AGPANL--
ARPANL--
-APANLM-
-APANLG-
ANPANL--
ATPANL--
-APANLW-
-APANLV-
AIPANL--
AVPANL--
-APANLH-
-APANLE-
ASPANL--
-APANLI-
APPANL--
AMPANL--
AKPANL--
AQPANL--
-APANLA-
AHPANL--
ACPANL--
-APANLQ-
-APANLD-
AYPANL--
ADPANL--
AEPANL--
CVPANL--
-CPANLI-
CCPANL--
CGPANL--
CIPANL--
CAPANL--
CSPANL--
-CPANLL-
CMPANL--
CKPANL--
CYPANL--
CWPANL--
-CPANLF-
-CPANLK-
CLPANL--

−CPANLV−
−CPANLS−
−CPANLR−
−CPANLG−
CNPANL−−
CFPANL−−
CTPANL−−
CPPANL−−
−CPANLY−
−CPANLQ−
−CPANLM−
−CPANLH−
DTPANL−−
DRPANL−−
DHPANL−−
−DPANLM−
DSPANL−−
−DPANLR−
DWPANL−−
−DPANLY−
DVPANL−−
−DPANLK−
DIPANL−−
−DPANLA−
DMPANL−−
DYPANL−−
DLPANL−−
DKPANL−−
DFPANL−−
−DPANLT−
−DPANLL−
DAPANL−−
−DPANLI−
−DPANLW−
−DPANLH−
−DPANLV−
DNPANL−−
DEPANL−−
DDPANL−−
−DPANLS−
−EPANLL−
−EPANLR−
EIPANL−−
ESPANL−−
−EPANLT−
−EPANLI−
ERPANL−−
−EPANLM−
−EPANLK−
ELPANL−−

−EPANLQ−
EQPANL−−
EVPANL−−
−EPANLS−
−EPANLW−
EGPANL−−
EAPANL−−
EMPANL−−
−EPANLY−
−EPANLG−
EEPANL−−
EWPANL−−
−EPANLH−
ENPANL−−
EYPANL−−
−EPANLV−
−EPANLA−
EHPANL−−
−EPANLN−
−EPANLF−
−EPANLC−
FSPANL−−
FGPANL−−
FAPANL−−
−FPANLR−
−FPANLM−
FVPANL−−
FFPANL−−
FIPANL−−
FPPANL−−
−FPANLV−
FLPANL−−
FKPANL−−
−FPANLT−
−FPANLL−
FNPANL−−
FQPANL−−
−FPANLY−
−FPANLQ−
−FPANLI−
−FPANLH−
FYPANL−−
FEPANL−−
−FPANLG−
FHPANL−−
−FPANLE−
FDPANL−−
FTPANL−−
−FPANLN−
−FPANLF−

-FPANLA-
FMPANL--
-FPANLK-
FWPANL--
-FPANLW-
FCPANL--
GVPANL--
-GPANLR-
GLPANL--
-GPANLL-
-GPANLS-
GMPANL--
GGPANL--
GSPANL--
GKPANL--
-GPANLY-
GIPANL--
-GPANLF-
-GPANLV-
-GPANLT-
GWPANL--
GYPANL--
-GPANLW-
-GPANLA-
GTPANL--
GQPANL--
-GPANLH-
-GPANLE-
-GPANLG-
-GPANLK-
GEPANL--
-GPANLN-
-GPANLI-
GAPANL--
GRPANL--
GHPANL--
GPPANL--
GDPANL--
GCPANL--
-GPANLQ-
GNPANL--
-HPANLY-
-HPANLR-
HLPANL--
HRPANL--
HEPANL--
HKPANL--
HSPANL--
HIPANL--
HGPANL--

HWPANL--
-HPANLL-
-HPANLS-
HYPANL--
-HPANLQ-
HHPANL--
HQPANL--
HFPANL--
HAPANL--
-HPANLI-
HMPANL--
HNPANL--
HVPANL--
-HPANLV-
-HPANLE-
-HPANLW-
-HPANLA-
-HPANLN-
-HPANLK-
-HPANLG-
-HPANLT-
HTPANL--
HPPANL--
-HPANLF-
-HPANLM-
HCPANL--
-IPANLS-
ITPANL--
-IPANLI-
-IPANLL-
IRPANL--
IVPANL--
-IPANLR-
IAPANL--
ISPANL--
IQPANL--
IMPANL--
IYPANL--
-IPANLT-
-IPANLM-
IGPANL--
IFPANL--
IHPANL--
IWPANL--
-IPANLK-
-IPANLW-
-IPANLY-
IEPANL--
-IPANLV-
IKPANL--

-IPANLH-
-IPANLE-
-IPANLN-
-IPANLQ-
-IPANLF-
-IPANLG-
ILPANL--
ICPANL--
INPANL--
IDPANL--
-KPANLL-
KTPANL--
-KPANLR-
KVPANL--
KLPANL--
-KPANLT-
KAPANL--
KPPANL--
KIPANL--
-KPANLF-
KEPANL--
KMPANL--
-KPANLW-
KNPANL--
KYPANL--
-KPANLS-
-KPANLQ-
KQPANL--
-KPANLV-
-KPANLG-
-KPANLY-
-KPANLE-
-KPANLA-
-KPANLI-
-KPANLK-
-KPANLN-
KGPANL--
KWPANL--
-KPANLM-
KDPANL--
KHPANL--
KCPANL--
-LPANLV-
LLPANL--
LAPANL--
LGPANL--
LQPANL--
LMPANL--
LTPANL--
-LPANLI-

−LPANLW−
−LPANLT−
LFPANL−−
−LPANLK−
LWPANL−−
−LPANLM−
LNPANL−−
LVPANL−−
LKPANL−−
LPPANL−−
LIPANL−−
−LPANLY−
−LPANLE−
−LPANLA−
−LPANLH−
−LPANLF−
LEPANL−−
−LPANLQ−
−LPANLN−
−LPANLG−
LHPANL−−
MTPANL−−
−MPANLY−
MLPANL−−
−MPANLR−
MSPANL−−
−MPANLS−
MRPANL−−
−MPANLT−
MHPANL−−
MAPANL−−
MNPANL−−
−MPANLH−
−MPANLN−
MVPANL−−
−MPANLG−
−MPANLL−
MFPANL−−
MQPANL−−
MKPANL−−
−MPANLI−
−MPANLV−
−MPANLE−
MWPANL−−
MEPANL−−
−MPANLQ−
MIPANL−−
MYPANL−−
−MPANLM−
MMPANL−−

MCPANL--
MPPANL--
-MPANLW-
-MPANLF-
NSPANL--
NRPANL--
NFPANL--
NNPANL--
NLPANL--
NEPANL--
NYPANL--
NVPANL--
-NPANLV-
-NPANLI-
NGPANL--
-NPANLR-
-NPANLY-
-NPANLS-
-NPANLT-
-NPANLF-
NTPANL--
-NPANLA-
-NPANLL-
NKPANL--
NIPANL--
NWPANL--
NAPANL--
-NPANLK-
NMPANL--
NQPANL--
NCPANL--
NPPANL--
-NPANLH-
-NPANLM-
-NPANLG-
-NPANLE-
-NPANLQ-
NDPANL--
--PANLRS
--PANLRL
--PANLVL
--PANLRT
--PANLRG
--PANLRA
--PANLLT
--PANLVR
--PANLSL
--PANLSR
--PANLTR
--PANLRI

--PANLLR
--PANLSS
--PANLRM
--PANLYR
--PANLMR
--PANLLH
--PANLYS
--PANLIS
PSPANL--
--PANLNW
--PANLLW
--PANLYL
--PANLVS
--PANLRN
--PANLLL
--PANLWS
--PANLST
--PANLSA
--PANLRQ
--PANLTL
--PANLLS
--PANLWH
--PANLTS
--PANLVV
--PANLTG
--PANLQV
--PANLRV
--PANLLA
--PANLAG
--PANLSV
--PANLMG
--PANLLK
--PANLKV
--PANLVQ
--PANLFN
PWPANL--
PGPANL--
--PANLWR
--PANLWN
--PANLTV
--PANLSI
--PANLKH
--PANLVT
--PANLTT
--PANLLD
--PANLAS
--PANLYI
--PANLSY
--PANLLM
PLPANL--

```
--PANLLV
--PANLAF
--PANLVI
--PANLVH
--PANLTA
--PANLSH
--PANLRK
--PANLLG
--PANLFH
PRPANL--
--PANLVA
--PANLSW
--PANLKS
--PANLGS
--PANLTK
--PANLSN
--PANLSK
--PANLKA
--PANLIY
--PANLYA
--PANLTI
--PANLQI
--PANLLQ
--PANLKT
--PANLIF
--PANLFA
--PANLAT
--PANLVG
--PANLFQ
--PANLFK
--PANLAY
--PANLYG
--PANLRD
--PANLMT
--PANLKI
--PANLEN
--PANLAL
--PANLVF
--PANLTY
--PANLRH
--PANLHT
--PANLYN
--PANLMA
--PANLLE
--PANLFT
--PANLWG
--PANLVY
--PANLSM
--PANLQR
--PANLGT
```

PYPANL--
--PANLYT
--PANLWT
--PANLVN
--PANLSF
--PANLRW
--PANLIT
--PANLFS
PAPANL--
--PANLVD
--PANLKR
--PANLIK
--PANLFL
--PANLEA
--PANLVW
--PANLVM
--PANLMY
--PANLLN
--PANLIR
--PANLIH
--PANLIA
--PANLHN
--PANLFG
--PANLYY
--PANLYH
--PANLSD
--PANLRF
--PANLRE
--PANLQW
--PANLNT
--PANLHG
--PANLEG
--PANLAI
--PANLYD
--PANLWV
--PANLVK
--PANLTM
--PANLTF
--PANLTE
--PANLSQ
--PANLSG
--PANLQY
--PANLMS
--PANLHR
--PANLFY
--PANLAK
--PANLYF
--PANLTN
--PANLSE
--PANLQL

--PANLIQ
--PANLHW
--PANLAH
PTPANL--
--PANLYV
--PANLQM
--PANLQH
--PANLMV
--PANLMI
--PANLMF
--PANLII
--PANLHQ
PMPANL--
PIPANL--
PFPANL--
--PANLYQ
--PANLVE
--PANLQS
--PANLQN
--PANLNA
--PANLKG
--PANLHL
--PANLGA
--PANLFD
--PANLNG
--PANLMK
--PANLKE
--PANLIV
--PANLGI
--PANLEV
--PANLAQ
PHPANL--
--PANLTD
--PANLNS
--PANLNR
--PANLMH
--PANLKN
--PANLKL
--PANLHS
--PANLHM
--PANLGG
--PANLER
--PANLEF
PVPANL--
PKPANL--
--PANLYM
--PANLYE
--PANLWQ
--PANLWF
--PANLQF

--PANLNM
--PANLNI
--PANLIN
--PANLIG
--PANLHF
--PANLES
PNPANL--
--PANLWI
--PANLQG
--PANLNV
--PANLKM
--PANLIW
--PANLID
--PANLFM
--PANLEH
--PANLYW
--PANLTH
--PANLKY
--PANLKQ
--PANLKF
--PANLKD
--PANLHE
--PANLAA
PEPANL--
--PANLTQ
--PANLNY
--PANLNH
--PANLME
--PANLKW
--PANLHI
--PANLHD
--PANLHA
--PANLGK
--PANLGD
--PANLEW
--PANLEM
--PANLEE
--PANLDT
--PANLDS
--PANLWL
--PANLGY
--PANLGR
--PANLGM
--PANLEI
--PANLDG
--PANLAW
--PANLYK
--PANLYC
--PANLWM
--PANLWE

--PANLWD
--PANLQQ
--PANLNF
--PANLMQ
--PANLGW
--PANLGN
--PANLGH
--PANLFE
--PANLEY
--PANLEL
--PANLED
--PANLCA
--PANLAN
PDPANL--
--PANLVC
--PANLTW
--PANLRC
--PANLQK
--PANLNN
--PANLNL
--PANLNK
--PANLND
--PANLGL
--PANLEQ
--PANLCN
--PANLCG
--PANLAR
--PANLAD
--PANLWK
--PANLQE
--PANLPS
--PANLNQ
--PANLKK
--PANLGQ
--PANLGF
--PANLFC
--PANLEK
--PANLDV
--PANLDK
--PANLDA
--PANLCK
--PANLCF
--PANLAE
QLPANL--
QYPANL--
-QPANLL-
QWPANL--
QVPANL--
-QPANLS-
-QPANLI-

-QPANLF-
-QPANLR-
QAPANL--
QRPANL--
QGPANL--
QSPANL--
-QPANLA-
-QPANLY-
QPPANL--
QHPANL--
QQPANL--
-QPANLW-
QTPANL--
-QPANLV-
-QPANLN-
-QPANLH-
-QPANLE-
-QPANLM-
QKPANL--
-QPANLT-
-QPANLQ-
QFPANL--
-QPANLK-
QNPANL--
QMPANL--
QDPANL--
QIPANL--
RVPANL--
-RPANLS-
-RPANLL-
-RPANLV-
RLPANL--
RSPANL--
-RPANLT-
RIPANL--
RGPANL--
-RPANLR-
RFPANL--
RAPANL--
-RPANLI-
-RPANLA-
RPPANL--
-RPANLK-
-RPANLQ-
RMPANL--
-RPANLF-
RTPANL--
-RPANLW-
RNPANL--
-RPANLG-

RKPANL--
-RPANLN-
RYPANL--
REPANL--
-RPANLM-
RHPANL--
-RPANLE-
RDPANL--
-RPANLC-
-RPANLD-
RCPANL--
SRPANL--
SGPANL--
SAPANL--
-SPANLR-
-SPANLS-
SVPANL--
SSPANL--
-SPANLT-
SWPANL--
-SPANLF-
SEPANL--
-SPANLK-
STPANL--
SIPANL--
SHPANL--
-SPANLV-
SPPANL--
-SPANLI-
-SPANLA-
-SPANLY-
SYPANL--
SKPANL--
-SPANLW-
-SPANLQ-
-SPANLM-
-SPANLH-
SNPANL--
SQPANL--
SMPANL--
SFPANL--
SDPANL--
-SPANLG-
-SPANLE-
SCPANL--
TRPANL--
-TPANLL-
TSPANL--
TPPANL--
-TPANLS-

```
-TPANLR-
TIPANL--
TYPANL--
TAPANL--
-TPANLH-
TKPANL--
TFPANL--
-TPANLA-
-TPANLQ-
-TPANLW-
TLPANL--
TMPANL--
TTPANL--
THPANL--
TEPANL--
-TPANLN-
-TPANLF-
TWPANL--
-TPANLY-
TNPANL--
TCPANL--
-TPANLV-
-TPANLK-
-TPANLG-
TQPANL--
-TPANLM-
-TPANLE-
-VPANLL-
VVPANL--
VLPANL--
-VPANLV-
-VPANLK-
VSPANL--
VIPANL--
VYPANL--
VMPANL--
VHPANL--
VFPANL--
-VPANLT-
VGPANL--
VNPANL--
VKPANL--
VWPANL--
-VPANLW-
VAPANL--
VQPANL--
-VPANLA-
-VPANLE-
VPPANL--
-VPANLN-
```

-VPANLI-
-VPANLG-
-VPANLF-
VCPANL--
-VPANLM-
VDPANL--
-VPANLQ-
VEPANL--
-VPANLC-
WRPANL--
WAPANL--
-WPANLR-
WKPANL--
-WPANLL-
WPPANL--
WGPANL--
WLPANL--
-WPANLF-
WHPANL--
-WPANLY-
-WPANLA-
-WPANLH-
WQPANL--
WEPANL--
-WPANLS-
-WPANLV-
WMPANL--
WSPANL--
-WPANLW-
-WPANLK-
WDPANL--
-WPANLM-
-WPANLI-
-WPANLE-
WFPANL--
-WPANLQ-
WWPANL--
-WPANLG-
-WPANLC-
YSPANL--
YRPANL--
YAPANL--
-YPANLT-
YFPANL--
YGPANL--
-YPANLR-
-YPANLY-
YLPANL--
YIPANL--
-YPANLV-

```
            -YPANLK-
            YNPANL--
            -YPANLL-
            YYPANL--
            -YPANLH-
            -YPANLI-
            YVPANL--
            YEPANL--
            -YPANLM-
            YMPANL--
            YTPANL--
            -YPANLS-
            YHPANL--
            YQPANL--
            YPPANL--
            -YPANLN-
            YDPANL--
            -YPANLG-
            -YPANLA-
            -YPANLW-
            YKPANL--
            -YPANLE-
PVPL          18        884      47.23
            -DPVPLN-
            --PVPLLP
            -WPVPLA-
            -APVPLR-
            -APVPLL-
            ARPVPL--
            -APVPLS-
            ASPVPL--
            AAPVPL--
            -APVPLT-
            -APVPLH-
            AYPVPL--
            AKPVPL--
            ALPVPL--
            -APVPLK-
            AVPVPL--
            -APVPLI-
            ATPVPL--
            -APVPLY-
            -APVPLV-
            AFPVPL--
            AHPVPL--
            -APVPLA-
            -APVPLF-
            AGPVPL--
            -APVPLQ-
            APPVPL--
```

ANPVPL--
AMPVPL--
-APVPLG-
AQPVPL--
-APVPLM-
AEPVPL--
ADPVPL--
AWPVPL--
-APVPLE-
AIPVPL--
CRPVPL--
CSPVPL--
CNPVPL--
CHPVPL--
CVPVPL--
CFPVPL--
CCPVPL--
-CPVPLT-
-CPVPLR-
-CPVPLL-
CPPVPL--
CMPVPL--
CIPVPL--
-CPVPLM-
CLPVPL--
CAPVPL--
-DPVPLR-
DRPVPL--
DLPVPL--
DKPVPL--
DWPVPL--
-DPVPLW-
DGPVPL--
DIPVPL--
DSPVPL--
-DPVPLV-
-DPVPLA-
-DPVPLT-
DHPVPL--
DFPVPL--
DAPVPL--
-DPVPLQ-
DEPVPL--
DDPVPL--
DTPVPL--
DQPVPL--
DYPVPL--
-DPVPLM-
-DPVPLL-
DMPVPL--

-EPVPLT-
ESPVPL--
-EPVPLR-
-EPVPLW-
-EPVPLK-
EGPVPL--
-EPVPLL-
EWPVPL--
-EPVPLS-
EKPVPL--
ERPVPL--
EVPVPL--
EAPVPL--
ELPVPL--
-EPVPLM-
EQPVPL--
-EPVPLY-
-EPVPLQ-
-EPVPLH-
ETPVPL--
ENPVPL--
EIPVPL--
-EPVPLA-
-EPVPLN-
-EPVPLF-
EEPVPL--
-EPVPLI-
EYPVPL--
-EPVPLV-
FRPVPL--
FVPVPL--
FSPVPL--
FEPVPL--
FYPVPL--
FGPVPL--
-FPVPLR-
-FPVPLY-
-FPVPLM-
FKPVPL--
-FPVPLV-
FDPVPL--
-FPVPLK-
-FPVPLS-
-FPVPLT-
-FPVPLH-
FIPVPL--
FAPVPL--
FTPVPL--
FQPVPL--
-FPVPLI-

-FPVPLN-
FPPVPL--
FNPVPL--
FCPVPL--
-FPVPLQ-
-FPVPLF-
FFPVPL--
-FPVPLA-
FMPVPL--
-FPVPLG-
-FPVPLW-
GTPVPL--
GSPVPL--
-GPVPLW-
-GPVPLK-
GLPVPL--
GRPVPL--
-GPVPLV-
GNPVPL--
-GPVPLH-
-GPVPLF-
-GPVPLS-
GVPVPL--
GWPVPL--
-GPVPLQ-
GFPVPL--
GYPVPL--
GKPVPL--
GDPVPL--
-GPVPLT-
GIPVPL--
-GPVPLM-
GMPVPL--
-GPVPLI-
GHPVPL--
-GPVPLA-
-GPVPLN-
GEPVPL--
GAPVPL--
GQPVPL--
GPPVPL--
-GPVPLG-
-GPVPLE-
HSPVPL--
HFPVPL--
HRPVPL--
HNPVPL--
-HPVPLW-
-HPVPLL-
HHPVPL--

-HPVPLS-
HGPVPL--
HAPVPL--
HKPVPL--
HIPVPL--
HWPVPL--
-HPVPLA-
HLPVPL--
HMPVPL--
HYPVPL--
-HPVPLM-
-HPVPLK-
-HPVPLY-
HEPVPL--
-HPVPLT-
HPPVPL--
HDPVPL--
-HPVPLH-
-HPVPLG-
-HPVPLI-
-HPVPLV-
HTPVPL--
HQPVPL--
-HPVPLQ-
-HPVPLN-
ISPVPL--
IGPVPL--
IFPVPL--
-IPVPLR-
IRPVPL--
-IPVPLK-
-IPVPLI-
-IPVPLT-
ILPVPL--
ITPVPL--
-IPVPLL-
IAPVPL--
IKPVPL--
-IPVPLF-
-IPVPLW-
-IPVPLH-
IPPVPL--
IYPVPL--
-IPVPLA-
INPVPL--
IHPVPL--
IIPVPL--
-IPVPLS-
-IPVPLM-
IDPVPL--

-IPVPLV-
IEPVPL--
IWPVPL--
-IPVPLQ-
-IPVPLN-
-IPVPLE-
KYPVPL--
KMPVPL--
-KPVPLR-
-KPVPLS-
-KPVPLT-
KEPVPL--
KQPVPL--
-KPVPLH-
KGPVPL--
-KPVPLK-
KRPVPL--
KWPVPL--
KKPVPL--
-KPVPLM-
-KPVPLI-
KNPVPL--
-KPVPLN-
-KPVPLW-
-KPVPLV-
-KPVPLQ-
KIPVPL--
KAPVPL--
-KPVPLG-
KPPVPL--
-KPVPLE-
-KPVPLA-
KCPVPL--
LRPVPL--
LTPVPL--
LSPVPL--
LGPVPL--
LHPVPL--
LYPVPL--
LLPVPL--
LKPVPL--
LVPVPL--
LAPVPL--
-LPVPLT-
LWPVPL--
-LPVPLS-
-LPVPLM-
-LPVPLR-
-LPVPLQ-
-LPVPLV-

-LPVPLK-
LIPVPL--
LFPVPL--
-LPVPLN-
-LPVPLP-
LNPVPL--
LDPVPL--
-LPVPLG-
LQPVPL--
LMPVPL--
-LPVPLH-
-LPVPLA-
-LPVPLE-
LCPVPL--
MIPVPL--
-MPVPLR-
-MPVPLT-
-MPVPLW-
-MPVPLI-
MWPVPL--
MPPVPL--
-MPVPLK-
MKPVPL--
MFPVPL--
MGPVPL--
MTPVPL--
MSPVPL--
-MPVPLL-
MYPVPL--
MVPVPL--
-MPVPLV-
-MPVPLF-
MNPVPL--
-MPVPLS-
-MPVPLQ-
-MPVPLN-
MDPVPL--
MQPVPL--
-MPVPLM-
-MPVPLA-
MMPVPL--
MHPVPL--
MCPVPL--
MAPVPL--
NSPVPL--
NKPVPL--
NRPVPL--
NTPVPL--
NFPVPL--
NIPVPL--

-NPVPLR-
-NPVPLF-
NPPVPL--
NGPVPL--
NWPVPL--
NQPVPL--
NNPVPL--
-NPVPLT-
NYPVPL--
NLPVPL--
NHPVPL--
NEPVPL--
NAPVPL--
NVPVPL--
-NPVPLY-
-NPVPLS-
-NPVPLQ-
-NPVPLK-
NMPVPL--
-NPVPLW-
-NPVPLH-
-NPVPLP-
-NPVPLN-
NCPVPL--
-NPVPLI-
NDPVPL--
-NPVPLA-
--PVPLRL
--PVPLRA
--PVPLRT
--PVPLYS
--PVPLWL
--PVPLKL
--PVPLFR
--PVPLVS
--PVPLLH
--PVPLLY
--PVPLAS
--PVPLRG
--PVPLWS
--PVPLSY
--PVPLHL
--PVPLSL
--PVPLRY
--PVPLRN
--PVPLMR
--PVPLGL
--PVPLSF
--PVPLKI
--PVPLRR

--PVPLSV
--PVPLST
--PVPLMG
--PVPLWW
--PVPLRH
--PVPLFL
--PVPLLV
--PVPLYV
--PVPLLG
--PVPLWV
--PVPLWI
PSPVPL--
--PVPLTK
--PVPLTA
--PVPLLR
--PVPLVA
--PVPLLI
--PVPLIQ
--PVPLGV
--PVPLVY
--PVPLHA
--PVPLVF
--PVPLSA
--PVPLVT
--PVPLLN
--PVPLIR
--PVPLNM
--PVPLMK
--PVPLLM
--PVPLVR
--PVPLNL
--PVPLFG
--PVPLSS
--PVPLIL
--PVPLGS
--PVPLWA
--PVPLSM
--PVPLSH
--PVPLLW
--PVPLEL
--PVPLYN
--PVPLYM
--PVPLTS
--PVPLSG
--PVPLRM
--PVPLPY
--PVPLIG
--PVPLFS
--PVPLEV
--PVPLLA

--PVPLMQ
--PVPLGH
PRPVPL--
--PVPLTG
--PVPLMY
--PVPLLT
--PVPLII
PMPVPL--
--PVPLTT
--PVPLNQ
--PVPLKK
--PVPLGF
--PVPLMA
--PVPLKV
--PVPLKT
--PVPLKR
--PVPLKF
--PVPLHI
--PVPLAF
PFPVPL--
PVPVPL--
--PVPLYI
--PVPLYE
--PVPLQI
--PVPLNF
--PVPLLQ
--PVPLAL
PAPVPL--
PYPVPL--
--PVPLYY
--PVPLRQ
--PVPLQS
--PVPLQA
--PVPLPI
--PVPLFT
--PVPLYR
--PVPLVN
--PVPLVI
--PVPLIV
--PVPLHV
--PVPLFY
--PVPLAY
--PVPLRF
--PVPLRD
--PVPLPS
--PVPLTM
--PVPLNW
--PVPLIN
--PVPLGT
--PVPLAQ

--PVPLWY
--PVPLTY
--PVPLTH
--PVPLRI
--PVPLKQ
--PVPLKN
--PVPLKG
--PVPLKA
--PVPLHT
--PVPLFA
PLPVPL--
--PVPLWT
--PVPLWG
--PVPLVH
--PVPLTW
--PVPLLF
--PVPLIT
PTPVPL--
--PVPLTN
--PVPLLK
--PVPLIK
--PVPLIH
--PVPLDL
--PVPLAK
--PVPLAG
PIPVPL--
PHPVPL--
--PVPLYG
--PVPLRK
--PVPLQY
--PVPLQW
--PVPLPQ
--PVPLNI
--PVPLHR
--PVPLWE
--PVPLTQ
--PVPLNT
--PVPLEG
--PVPLDF
--PVPLYT
--PVPLYF
--PVPLVM
--PVPLTV
--PVPLTI
--PVPLRE
--PVPLPF
--PVPLNG
--PVPLHF
--PVPLFQ
--PVPLFH

--PVPLEA
--PVPLYQ
--PVPLYA
--PVPLWK
--PVPLVD
--PVPLNS
--PVPLNH
--PVPLIS
--PVPLHK
--PVPLFK
--PVPLAV
--PVPLWH
--PVPLSI
--PVPLQV
--PVPLHM
--PVPLFW
--PVPLES
--PVPLEF
--PVPLAI
PNPVPL--
--PVPLYD
--PVPLTF
--PVPLQN
--PVPLMN
--PVPLKM
--PVPLHN
--PVPLHG
--PVPLGQ
--PVPLGN
--PVPLFN
PGPVPL--
--PVPLSW
--PVPLSN
--PVPLNV
--PVPLIW
--PVPLHD
--PVPLEW
--PVPLQM
--PVPLQG
--PVPLQF
--PVPLLD
--PVPLID
--PVPLIA
--PVPLDQ
--PVPLAW
--PVPLAM
--PVPLSK
--PVPLQH
--PVPLMW
--PVPLMV

--PVPLKD
--PVPLHH
--PVPLEY
--PVPLEI
PQPVPL--
--PVPLPN
--PVPLPH
--PVPLLE
--PVPLKW
--PVPLIE
--PVPLHW
--PVPLDS
--PVPLAN
--PVPLAA
PWPVPL--
--PVPLWQ
--PVPLWF
--PVPLQR
--PVPLPV
--PVPLPM
--PVPLNR
--PVPLMF
--PVPLMD
--PVPLDY
--PVPLDW
--PVPLDH
--PVPLAT
PEPVPL--
--PVPLYW
--PVPLYH
--PVPLWM
--PVPLVE
--PVPLQK
--PVPLPA
--PVPLMI
--PVPLKY
--PVPLGR
--PVPLGG
--PVPLDM
--PVPLAH
PKPVPL--
--PVPLPW
--PVPLNY
--PVPLNN
--PVPLKH
--PVPLGW
--PVPLFF
--PVPLFE
--PVPLTD
--PVPLQE

--PVPLPT
--PVPLMM
--PVPLHQ
--PVPLHE
--PVPLEH
--PVPLDV
--PVPLDI
--PVPLWD
--PVPLTE
--PVPLSE
--PVPLSD
--PVPLNE
--PVPLNA
--PVPLME
--PVPLGP
--PVPLFD
--PVPLEQ
--PVPLEM
--PVPLCV
--PVPLCL
--PVPLAE
--PVPLQD
--PVPLPP
--PVPLPG
--PVPLNC
--PVPLMT
--PVPLGE
--PVPLGC
--PVPLFP
--PVPLET
--PVPLEN
--PVPLCW
--PVPLCR
--PVPLCN
--PVPLCK
--PVPLCE
--PVPLCD
-PPVPLT-
-PPVPLH-
-PPVPLD-
PDPVPL--
-QPVPLR-
QHPVPL--
-QPVPLL-
QVPVPL--
-QPVPLV-
-QPVPLQ-
QRPVPL--
-QPVPLS-
-QPVPLT-

QLPVPL--
QTPVPL--
QSPVPL--
QEPVPL--
QNPVPL--
-QPVPLA-
QQPVPL--
QKPVPL--
-QPVPLN-
QPPVPL--
QIPVPL--
-QPVPLM-
-QPVPLK-
QMPVPL--
QWPVPL--
-QPVPLW-
QFPVPL--
QAPVPL--
-QPVPLY-
-QPVPLH-
-QPVPLI-
-QPVPLG-
QDPVPL--
QYPVPL--
-RPVPLR-
RLPVPL--
-RPVPLY-
-RPVPLS-
-RPVPLT-
RVPVPL--
-RPVPLK-
RNPVPL--
-RPVPLV-
-RPVPLM-
RRPVPL--
RIPVPL--
RDPVPL--
RAPVPL--
-RPVPLI-
RPPVPL--
REPVPL--
RHPVPL--
-RPVPLQ-
-RPVPLA-
RQPVPL--
-RPVPLN-
RCPVPL--
-RPVPLE-
-RPVPLC-
SRPVPL--

-SPVPLR-
SKPVPL--
SVPVPL--
SSPVPL--
STPVPL--
SGPVPL--
SEPVPL--
SWPVPL--
-SPVPLV-
-SPVPLS-
-SPVPLI-
-SPVPLT-
SNPVPL--
-SPVPLH-
SQPVPL--
-SPVPLW-
SFPVPL--
SHPVPL--
-SPVPLY-
-SPVPLN-
-SPVPLM-
-SPVPLQ-
SPPVPL--
SDPVPL--
SCPVPL--
-SPVPLP-
-TPVPLK-
-TPVPLL-
TLPVPL--
-TPVPLT-
TTPVPL--
TSPVPL--
-TPVPLS-
TFPVPL--
TAPVPL--
-TPVPLW-
TGPVPL--
TRPVPL--
-TPVPLV-
TVPVPL--
-TPVPLY-
-TPVPLM-
TEPVPL--
-TPVPLH-
-TPVPLF-
TPPVPL--
-TPVPLI-
-TPVPLA-
TKPVPL--
TYPVPL--

-TPVPLN-
TMPVPL--
TNPVPL--
THPVPL--
TQPVPL--
-TPVPLG-
-TPVPLE-
TDPVPL--
TWPVPL--
TCPVPL--
-VPVPLT-
VLPVPL--
VVPVPL--
VSPVPL--
VYPVPL--
-VPVPLL-
-VPVPLW-
VQPVPL--
-VPVPLQ-
-VPVPLI-
VKPVPL--
VTPVPL--
VMPVPL--
-VPVPLS-
VNPVPL--
VEPVPL--
VGPVPL--
-VPVPLK-
-VPVPLH-
VPPVPL--
-VPVPLV-
-VPVPLN-
-VPVPLY-
-VPVPLF-
VIPVPL--
VDPVPL--
VHPVPL--
-VPVPLM-
VFPVPL--
-VPVPLG-
-VPVPLE-
-VPVPLA-
VCPVPL--
WSPVPL--
WRPVPL--
WKPVPL--
WGPVPL--
WVPVPL--
WMPVPL--
WIPVPL--

-WPVPLW-
-WPVPLR-
WAPVPL--
WNPVPL--
WDPVPL--
WTPVPL--
-WPVPLS-
-WPVPLL-
WLPVPL--
WQPVPL--
-WPVPLK-
-WPVPLN-
-WPVPLI-
-WPVPLM-
-WPVPLY-
-WPVPLH-
WYPVPL--
-WPVPLV-
-WPVPLG-
WCPVPL--
WHPVPL--
WEPVPL--
WFPVPL--
YLPVPL--
-YPVPLN-
YSPVPL--
YIPVPL--
YTPVPL--
-YPVPLS-
YPPVPL--
YAPVPL--
YWPVPL--
-YPVPLR-
-YPVPLV-
-YPVPLT-
YMPVPL--
-YPVPLF-
YYPVPL--
-YPVPLY-
YKPVPL--
YGPVPL--
YFPVPL--
-YPVPLQ-
-YPVPLM-
YEPVPL--
-YPVPLL-
-YPVPLK-
YDPVPL--
YNPVPL--
-YPVPLA-

	YQPVPL--		
	-YPVPLW-		
	YVPVPL--		
	-YPVPLI-		
	YHPVPL--		
	-YPVPLG-		
	YCPVPL--		
PAGL	6	914	47.23
	-APAGLP-		
	-LPAGLG-		
	--PAGLRP		
	--PAGLVP		
	--PAGLPL		
	--PAGLPK		
	--PAGLHP		
	--PAGLEL		
	--PAGLDR		
	--PAGLCS		
	--PAGLAD		
	-RPAGLD-		
	-RPAGLP-		
	-SPAGLE-		
	-YPAGLG-		
	ARPAGL--		
	AVPAGL--		
	ASPAGL--		
	-APAGLR-		
	ALPAGL--		
	-APAGLA-		
	-APAGLT-		
	-APAGLS-		
	-APAGLL-		
	AFPAGL--		
	-APAGLF-		
	-APAGLH-		
	-APAGLG-		
	AYPAGL--		
	ATPAGL--		
	AHPAGL--		
	AMPAGL--		
	-APAGLV-		
	-APAGLQ-		
	-APAGLM-		
	APPAGL--		
	-APAGLK-		
	AWPAGL--		
	-APAGLW-		
	-APAGLN-		
	AEPAGL--		
	AQPAGL--		

-APAGLE-
ADPAGL--
ACPAGL--
AAPAGL--
ANPAGL--
CRPAGL--
CSPAGL--
CQPAGL--
CEPAGL--
-CPAGLL-
-CPAGLH-
-CPAGLK-
-CPAGLM-
CPPAGL--
-CPAGLV-
-CPAGLR-
-CPAGLQ-
CMPAGL--
CKPAGL--
CFPAGL--
CTPAGL--
CLPAGL--
CCPAGL--
CAPAGL--
-CPAGLY-
-CPAGLW-
-CPAGLS-
-CPAGLF-
CNPAGL--
CIPAGL--
CGPAGL--
DRPAGL--
DYPAGL--
DVPAGL--
DGPAGL--
-DPAGLR-
-DPAGLT-
DKPAGL--
-DPAGLY-
DLPAGL--
DIPAGL--
DNPAGL--
DTPAGL--
-DPAGLM-
-DPAGLL-
DQPAGL--
-DPAGLS-
-DPAGLK-
-DPAGLF-
DEPAGL--

DSPAGL--
DWPAGL--
-DPAGLV-
DMPAGL--
DHPAGL--
DCPAGL--
-DPAGLW-
-DPAGLQ-
-DPAGLN-
DFPAGL--
DAPAGL--
-DPAGLP-
DDPAGL--
ERPAGL--
-EPAGLL-
ELPAGL--
EFPAGL--
ESPAGL--
-EPAGLR-
-EPAGLK-
ETPAGL--
-EPAGLS-
EAPAGL--
-EPAGLW-
EKPAGL--
EGPAGL--
-EPAGLH-
EIPAGL--
-EPAGLV-
-EPAGLT-
EQPAGL--
EMPAGL--
EYPAGL--
-EPAGLM-
-EPAGLY-
EEPAGL--
EWPAGL--
EVPAGL--
-EPAGLI-
-EPAGLE-
-EPAGLA-
ENPAGL--
EDPAGL--
EHPAGL--
-EPAGLN-
-EPAGLQ-
-EPAGLF-
ECPAGL--
FSPAGL--
FYPAGL--

-FPAGLY-
-FPAGLS-
FVPAGL--
-FPAGLL-
FLPAGL--
FHPAGL--
-FPAGLR-
FQPAGL--
FTPAGL--
FAPAGL--
-FPAGLN-
-FPAGLV-
-FPAGLT-
-FPAGLK-
-FPAGLM-
FDPAGL--
FNPAGL--
FMPAGL--
FEPAGL--
FWPAGL--
-FPAGLW-
FFPAGL--
-FPAGLQ-
-FPAGLH-
-FPAGLA-
-FPAGLI-
FIPAGL--
FPPAGL--
FCPAGL--
-FPAGLF-
-FPAGLC-
-GPAGLK-
GLPAGL--
-GPAGLH-
-GPAGLY-
-GPAGLS-
GYPAGL--
-GPAGLM-
GAPAGL--
GSPAGL--
GVPAGL--
GIPAGL--
-GPAGLL-
-GPAGLV-
GNPAGL--
GTPAGL--
GPPAGL--
-GPAGLN-
GMPAGL--
-GPAGLI-

−GPAGLF−
−GPAGLT−
GEPAGL−−
GQPAGL−−
−GPAGLQ−
GKPAGL−−
−GPAGLA−
GHPAGL−−
GWPAGL−−
−GPAGLW−
−GPAGLG−
GDPAGL−−
−GPAGLE−
−GPAGLC−
GCPAGL−−
HTPAGL−−
−HPAGLR−
HRPAGL−−
HEPAGL−−
HMPAGL−−
HIPAGL−−
−HPAGLL−
−HPAGLH−
−HPAGLN−
HLPAGL−−
HVPAGL−−
−HPAGLF−
HGPAGL−−
HHPAGL−−
−HPAGLI−
HKPAGL−−
HFPAGL−−
HYPAGL−−
−HPAGLY−
−HPAGLS−
−HPAGLQ−
HAPAGL−−
−HPAGLM−
HPPAGL−−
−HPAGLW−
−HPAGLK−
HDPAGL−−
HQPAGL−−
−HPAGLV−
−HPAGLT−
HNPAGL−−
HCPAGL−−
−HPAGLE−
−HPAGLD−
−HPAGLA−

−IPAGLL−
IKPAGL−−
−IPAGLR−
IRPAGL−−
−IPAGLY−
−IPAGLS−
IAPAGL−−
IWPAGL−−
ITPAGL−−
−IPAGLT−
−IPAGLA−
IHPAGL−−
−IPAGLV−
IIPAGL−−
IYPAGL−−
−IPAGLF−
INPAGL−−
IDPAGL−−
−IPAGLM−
IFPAGL−−
ISPAGL−−
−IPAGLN−
−IPAGLI−
−IPAGLQ−
−IPAGLH−
IVPAGL−−
IMPAGL−−
−IPAGLK−
−IPAGLE−
IEPAGL−−
ICPAGL−−
IPPAGL−−
−IPAGLG−
IQPAGL−−
−IPAGLP−
KSPAGL−−
KRPAGL−−
KTPAGL−−
KAPAGL−−
KPPAGL−−
−KPAGLL−
−KPAGLT−
KLPAGL−−
−KPAGLK−
KGPAGL−−
KVPAGL−−
KIPAGL−−
KFPAGL−−
−KPAGLA−
KYPAGL−−

-KPAGLV-
KKPAGL--
-KPAGLY-
KWPAGL--
-KPAGLW-
KEPAGL--
KQPAGL--
-KPAGLI-
KDPAGL--
-KPAGLH-
KHPAGL--
-KPAGLM-
-KPAGLE-
KNPAGL--
KMPAGL--
KCPAGL--
LRPAGL--
LLPAGL--
LQPAGL--
LSPAGL--
LAPAGL--
LVPAGL--
LKPAGL--
LHPAGL--
-LPAGLT-
LGPAGL--
-LPAGLK-
-LPAGLW-
LPPAGL--
LMPAGL--
LYPAGL--
LNPAGL--
LDPAGL--
-LPAGLI-
-LPAGLH-
LFPAGL--
-LPAGLQ-
-LPAGLA-
LEPAGL--
-LPAGLN-
-LPAGLF-
-LPAGLM-
LCPAGL--
-LPAGLE-
-LPAGLC-
-MPAGLR-
MLPAGL--
-MPAGLS-
-MPAGLH-
MRPAGL--

MYPAGL--
MSPAGL--
MIPAGL--
-MPAGLV-
-MPAGLI-
MPPAGL--
-MPAGLN-
MNPAGL--
MGPAGL--
-MPAGLY-
-MPAGLW-
-MPAGLA-
MWPAGL--
MMPAGL--
MEPAGL--
-MPAGLM-
MKPAGL--
-MPAGLK-
MVPAGL--
-MPAGLQ-
MAPAGL--
-MPAGLG-
-MPAGLE-
MFPAGL--
MQPAGL--
MHPAGL--
MTPAGL--
-MPAGLF-
MDPAGL--
NSPAGL--
NGPAGL--
NYPAGL--
NWPAGL--
NTPAGL--
NRPAGL--
-NPAGLS-
-NPAGLL-
NHPAGL--
NAPAGL--
-NPAGLV-
NQPAGL--
NMPAGL--
-NPAGLR-
NNPAGL--
NVPAGL--
-NPAGLW-
-NPAGLF-
-NPAGLM-
-NPAGLI-
NEPAGL--

NIPAGL--
NPPAGL--
NLPAGL--
NFPAGL--
-NPAGLA-
NDPAGL--
-NPAGLN-
-NPAGLK-
-NPAGLQ-
NCPAGL--
-NPAGLG-
-NPAGLC-
--PAGLVG
--PAGLRS
--PAGLLS
--PAGLRV
--PAGLRG
--PAGLTS
--PAGLRR
--PAGLSR
--PAGLLG
--PAGLMR
--PAGLWT
--PAGLRI
--PAGLHL
--PAGLYR
--PAGLRN
--PAGLRF
--PAGLSG
--PAGLLT
--PAGLLR
--PAGLTT
--PAGLTG
--PAGLTL
--PAGLKA
--PAGLRT
--PAGLFR
--PAGLKS
--PAGLIS
--PAGLTR
--PAGLIR
--PAGLWK
--PAGLVT
--PAGLRY
--PAGLLQ
--PAGLLH
PVPAGL--
PSPAGL--
--PAGLRA
--PAGLFS

--PAGLYT
--PAGLKW
--PAGLFH
--PAGLVR
--PAGLVL
--PAGLSV
--PAGLVA
--PAGLSL
--PAGLKT
--PAGLVH
--PAGLIF
--PAGLRQ
--PAGLRH
--PAGLMM
--PAGLMS
--PAGLLL
--PAGLVS
--PAGLKL
--PAGLTY
--PAGLHS
--PAGLWH
--PAGLML
--PAGLWS
--PAGLSI
--PAGLFI
--PAGLAV
--PAGLVV
--PAGLIK
PQPAGL--
--PAGLVY
--PAGLST
--PAGLKG
--PAGLAG
--PAGLYK
--PAGLTA
--PAGLRW
--PAGLLW
--PAGLTI
--PAGLTN
--PAGLSM
--PAGLSF
--PAGLIN
--PAGLVF
PTPAGL--
--PAGLSN
--PAGLNW
--PAGLWM
--PAGLNT
--PAGLLK
--PAGLHY

PWPAGL--
PHPAGL--
--PAGLWN
--PAGLHR
--PAGLHK
--PAGLMK
--PAGLHV
--PAGLAS
--PAGLAF
PYPAGL--
--PAGLLM
--PAGLIY
--PAGLTK
--PAGLMW
--PAGLMG
PGPAGL--
--PAGLYV
--PAGLWG
--PAGLVN
--PAGLVM
--PAGLQS
--PAGLMV
--PAGLIW
--PAGLIL
PMPAGL--
--PAGLMF
--PAGLKN
--PAGLVK
--PAGLQY
--PAGLQA
--PAGLMH
--PAGLHG
--PAGLGS
--PAGLEW
--PAGLEG
--PAGLWR
--PAGLRD
--PAGLYI
--PAGLVD
--PAGLFN
--PAGLAR
--PAGLKM
--PAGLIH
--PAGLGT
--PAGLFK
--PAGLYN
--PAGLQG
--PAGLNN
--PAGLKR
--PAGLTQ

--PAGLRE
--PAGLMA
--PAGLGG
--PAGLFM
--PAGLYE
--PAGLWQ
--PAGLVW
--PAGLHW
--PAGLFT
PFPAGL--
--PAGLKV
--PAGLHD
PLPAGL--
--PAGLQN
--PAGLQF
--PAGLNV
--PAGLHI
--PAGLAY
--PAGLYG
--PAGLYA
--PAGLTM
--PAGLSQ
--PAGLNL
--PAGLMT
--PAGLVI
--PAGLNA
--PAGLME
--PAGLIG
--PAGLHM
--PAGLGY
--PAGLET
--PAGLAH
--PAGLNY
--PAGLNR
--PAGLMQ
--PAGLKY
--PAGLHT
PNPAGL--
PKPAGL--
--PAGLTF
--PAGLSD
--PAGLMY
--PAGLHF
--PAGLFV
--PAGLTW
--PAGLQW
--PAGLQV
--PAGLQR
--PAGLSH
--PAGLQL

--PAGLQH
--PAGLNQ
--PAGLNK
--PAGLAK
--PAGLYM
--PAGLWW
--PAGLVE
--PAGLSE
--PAGLQT
--PAGLNF
--PAGLGM
--PAGLGL
--PAGLGA
--PAGLFQ
--PAGLYH
--PAGLWE
--PAGLGF
--PAGLFG
--PAGLEN
PAPAGL--
--PAGLYW
--PAGLYQ
--PAGLTV
--PAGLQK
--PAGLQI
--PAGLMD
--PAGLKF
--PAGLIV
--PAGLYD
--PAGLWD
--PAGLMI
--PAGLEF
--PAGLEA
--PAGLDG
--PAGLQM
--PAGLLD
--PAGLKQ
--PAGLIM
--PAGLHH
--PAGLFA
--PAGLAN
--PAGLKK
--PAGLHN
--PAGLGV
--PAGLEY
--PAGLTD
--PAGLND
--PAGLFW
--PAGLAQ
--PAGLQQ

--PAGLQD
--PAGLLE
--PAGLIQ
--PAGLGW
--PAGLFD
--PAGLER
--PAGLDA
PIPAGL--
PDPAGL--
--PAGLKD
--PAGLDS
--PAGLAA
PEPAGL--
--PAGLWA
--PAGLTH
--PAGLNH
--PAGLNE
--PAGLMC
--PAGLIC
--PAGLEH
PCPAGL--
--PAGLVC
--PAGLPI
--PAGLIE
--PAGLID
--PAGLHE
--PAGLGQ
--PAGLGN
--PAGLEI
--PAGLDM
--PAGLAM
--PAGLAE
--PAGLAC
-PPAGLW-
-PPAGLM-
-PPAGLL-
--PAGLWC
--PAGLTE
--PAGLLC
--PAGLKC
--PAGLEV
--PAGLEK
--PAGLCW
--PAGLCL
--PAGLCI
QRPAGL--
-QPAGLL-
-QPAGLT-
-QPAGLY-
-QPAGLR-

QTPAGL--
QVPAGL--
-QPAGLS-
QAPAGL--
-QPAGLF-
-QPAGLW-
QLPAGL--
-QPAGLV-
QSPAGL--
QGPAGL--
-QPAGLA-
QNPAGL--
QWPAGL--
QPPAGL--
-QPAGLI-
QKPAGL--
QIPAGL--
QYPAGL--
-QPAGLN-
QFPAGL--
QEPAGL--
-QPAGLH-
-QPAGLG-
QMPAGL--
-QPAGLE-
QHPAGL--
QDPAGL--
QQPAGL--
RSPAGL--
RVPAGL--
RGPAGL--
RLPAGL--
-RPAGLR-
RYPAGL--
-RPAGLS-
-RPAGLY-
-RPAGLT-
RTPAGL--
RKPAGL--
-RPAGLF-
RPPAGL--
-RPAGLW-
-RPAGLK-
-RPAGLH-
RIPAGL--
-RPAGLQ-
-RPAGLN-
RNPAGL--
-RPAGLI-
REPAGL--

RWPAGL--
RHPAGL--
RMPAGL--
-RPAGLE-
-RPAGLA-
RDPAGL--
-RPAGLG-
RCPAGL--
SSPAGL--
-SPAGLR-
SRPAGL--
SLPAGL--
-SPAGLL-
SWPAGL--
STPAGL--
-SPAGLH-
SGPAGL--
-SPAGLK-
-SPAGLF-
SHPAGL--
SAPAGL--
-SPAGLI-
SMPAGL--
SVPAGL--
-SPAGLN-
-SPAGLV-
SNPAGL--
SYPAGL--
SKPAGL--
-SPAGLM-
SEPAGL--
-SPAGLY-
-SPAGLW-
-SPAGLA-
SFPAGL--
SQPAGL--
-SPAGLQ-
-SPAGLG-
SDPAGL--
SCPAGL--
-SPAGLC-
-SPAGLD-
-TPAGLL-
TVPAGL--
TRPAGL--
TSPAGL--
-TPAGLR-
TLPAGL--
TGPAGL--
-TPAGLK-

-TPAGLY-
-TPAGLH-
-TPAGLT-
-TPAGLI-
TNPAGL--
-TPAGLV-
TMPAGL--
THPAGL--
TFPAGL--
TQPAGL--
TIPAGL--
-TPAGLF-
TKPAGL--
TDPAGL--
TWPAGL--
TYPAGL--
TEPAGL--
-TPAGLQ-
TPPAGL--
-TPAGLA-
-TPAGLN-
-TPAGLW-
TAPAGL--
-TPAGLG-
-TPAGLM-
-TPAGLE-
TCPAGL--
-TPAGLC-
-VPAGLR-
VLPAGL--
VVPAGL--
-VPAGLV-
VSPAGL--
-VPAGLW-
-VPAGLL-
-VPAGLK-
-VPAGLH-
-VPAGLT-
VTPAGL--
VIPAGL--
VEPAGL--
-VPAGLN-
VAPAGL--
-VPAGLY-
VNPAGL--
-VPAGLM-
-VPAGLG-
VPPAGL--
VYPAGL--
VGPAGL--

-VPAGLE-
VCPAGL--
VMPAGL--
VWPAGL--
-VPAGLA-
-VPAGLI-
VFPAGL--
-VPAGLQ-
-VPAGLP-
VDPAGL--
WLPAGL--
WTPAGL--
-WPAGLR-
-WPAGLL-
WSPAGL--
WIPAGL--
WVPAGL--
WKPAGL--
-WPAGLW-
-WPAGLH-
WAPAGL--
-WPAGLS-
-WPAGLN-
WMPAGL--
-WPAGLA-
-WPAGLV-
WFPAGL--
WHPAGL--
-WPAGLK-
-WPAGLQ-
WGPAGL--
-WPAGLG-
-WPAGLY-
WEPAGL--
WPPAGL--
WYPAGL--
WDPAGL--
WCPAGL--
-WPAGLF-
-WPAGLM-
-WPAGLE-
YQPAGL--
YNPAGL--
-YPAGLR-
YVPAGL--
YTPAGL--
YMPAGL--
YLPAGL--
-YPAGLI-
YSPAGL--

	-YPAGLY-		
	-YPAGLT-		
	YGPAGL--		
	-YPAGLF-		
	-YPAGLK-		
	YAPAGL--		
	-YPAGLW-		
	-YPAGLS-		
	YEPAGL--		
	-YPAGLL-		
	YDPAGL--		
	-YPAGLN-		
	-YPAGLA-		
	-YPAGLQ-		
	YIPAGL--		
	YHPAGL--		
	YWPAGL--		
	-YPAGLV-		
	YYPAGL--		
	-YPAGLE-		
	YFPAGL--		
	-YPAGLP-		
PYWY	720	556	47.21
	-GPYWYG-		
	-APYWYV-		
	-APYWYS-		
	ATPYWY--		
	ASPYWY--		
	AIPYWY--		
	-APYWYK-		
	-APYWYA-		
	AKPYWY--		
	ANPYWY--		
	-APYWYI-		
	AFPYWY--		
	AAPYWY--		
	AMPYWY--		
	AHPYWY--		
	AQPYWY--		
	-APYWYY-		
	-APYWYW-		
	-APYWYR-		
	-APYWYN-		
	-APYWYM-		
	ADPYWY--		
	AWPYWY--		
	-APYWYT-		
	-APYWYL-		
	CLPYWY--		
	CHPYWY--		

DKPYWY--
DEPYWY--
DRPYWY--
DAPYWY--
DSPYWY--
-DPYWYM-
DWPYWY--
-DPYWYK-
-DPYWYA-
DFPYWY--
-DPYWYV-
-DPYWYR-
DMPYWY--
DHPYWY--
-DPYWYT-
-DPYWYQ-
DLPYWY--
DTPYWY--
-DPYWYS-
-DPYWYL-
EKPYWY--
ERPYWY--
-EPYWYS-
-EPYWYA-
EEPYWY--
ELPYWY--
-EPYWYQ-
ENPYWY--
EMPYWY--
EGPYWY--
-EPYWYF-
EQPYWY--
EAPYWY--
ETPYWY--
-EPYWYN-
-EPYWYL-
EHPYWY--
-EPYWYI-
-EPYWYH-
-EPYWYE-
FNPYWY--
FGPYWY--
FRPYWY--
-FPYWYR-
FTPYWY--
-FPYWYA-
FLPYWY--
FQPYWY--
-FPYWYK-
FMPYWY--

FDPYWY--
FAPYWY--
-FPYWYT-
FKPYWY--
-FPYWYS-
-FPYWYQ-
FEPYWY--
FSPYWY--
-FPYWYH-
FPPYWY--
GRPYWY--
-GPYWYR-
GYPYWY--
GAPYWY--
GIPYWY--
-GPYWYK-
GPPYWY--
GLPYWY--
GFPYWY--
GQPYWY--
-GPYWYQ-
GSPYWY--
GKPYWY--
GTPYWY--
-GPYWYM-
-GPYWYW-
GMPYWY--
GEPYWY--
GWPYWY--
-GPYWYV-
-GPYWYS-
-GPYWYI-
-GPYWYH-
-GPYWYF-
GDPYWY--
-GPYWYT-
-GPYWYL-
HVPYWY--
-HPYWYT-
HRPYWY--
-HPYWYL-
HSPYWY--
-HPYWYA-
HWPYWY--
-HPYWYR-
HTPYWY--
HKPYWY--
-HPYWYK-
HMPYWY--
HDPYWY--

-HPYWYG-
HNPYWY--
HLPYWY--
HGPYWY--
HEPYWY--
-HPYWYW-
HPPYWY--
-IPYWYR-
IRPYWY--
INPYWY--
IGPYWY--
IYPYWY--
-IPYWYV-
IHPYWY--
IWPYWY--
IIPYWY--
ISPYWY--
-IPYWYA-
IKPYWY--
IMPYWY--
IQPYWY--
-IPYWYS-
-IPYWYL-
-IPYWYH-
ITPYWY--
-IPYWYW-
-IPYWYM-
-IPYWYK-
KSPYWY--
KRPYWY--
KEPYWY--
KGPYWY--
-KPYWYT-
KAPYWY--
-KPYWYM-
-KPYWYL-
KYPYWY--
KVPYWY--
KKPYWY--
-KPYWYS-
KPPYWY--
KQPYWY--
-KPYWYF-
-KPYWYR-
KIPYWY--
-KPYWYV-
-KPYWYN-
-KPYWYA-
KMPYWY--
-KPYWYY-

KNPYWY--
LKPYWY--
LPPYWY--
LRPYWY--
LNPYWY--
LGPYWY--
LDPYWY--
-LPYWYV-
LVPYWY--
-LPYWYR-
LMPYWY--
-LPYWYS-
-LPYWYI-
LIPYWY--
LEPYWY--
LWPYWY--
LFPYWY--
-LPYWYQ-
LYPYWY--
-LPYWYL-
-LPYWYK-
-LPYWYF-
MRPYWY--
MGPYWY--
MQPYWY--
MVPYWY--
MWPYWY--
MNPYWY--
MKPYWY--
MSPYWY--
-MPYWYL-
MFPYWY--
MHPYWY--
-MPYWYI-
-MPYWYG-
MDPYWY--
-MPYWYY-
-MPYWYT-
MLPYWY--
NRPYWY--
NGPYWY--
NWPYWY--
-NPYWYS-
NSPYWY--
NHPYWY--
NAPYWY--
NTPYWY--
NKPYWY--
NVPYWY--
NIPYWY--

NPPYWY--
-NPYWYV-
-NPYWYT-
-NPYWYF-
NEPYWY--
-NPYWYQ-
-NPYWYG-
NFPYWY--
NDPYWY--
NCPYWY--
--PYWYRS
--PYWYRG
--PYWYKT
--PYWYTQ
--PYWYSS
--PYWYSG
--PYWYRA
--PYWYFS
--PYWYVT
--PYWYTT
--PYWYIH
--PYWYVG
--PYWYLG
--PYWYRQ
--PYWYGY
--PYWYAT
--PYWYAL
PNPYWY--
--PYWYWV
--PYWYTS
--PYWYST
--PYWYRV
--PYWYRL
--PYWYHV
--PYWYAS
--PYWYGS
--PYWYFT
--PYWYES
--PYWYYS
--PYWYTV
--PYWYSN
--PYWYSD
--PYWYQN
--PYWYHA
PEPYWY--
--PYWYSV
--PYWYRH
--PYWYQM
--PYWYKG
--PYWYKA

--PYWYIA
--PYWYHG
--PYWYDA
--PYWYVY
--PYWYTG
--PYWYSH
--PYWYMT
--PYWYGA
--PYWYYQ
--PYWYYA
--PYWYWS
--PYWYTI
--PYWYRT
--PYWYRI
--PYWYRE
--PYWYQR
--PYWYMI
--PYWYKH
--PYWYIW
--PYWYHS
--PYWYYK
--PYWYVS
--PYWYVN
--PYWYNT
--PYWYLN
--PYWYIT
--PYWYIG
--PYWYGT
--PYWYGI
--PYWYAR
--PYWYAH
--PYWYAA
--PYWYYT
--PYWYYR
--PYWYSL
--PYWYSI
--PYWYQV
--PYWYQT
--PYWYMM
--PYWYMK
--PYWYLH
--PYWYKV
--PYWYKF
--PYWYKD
--PYWYIV
--PYWYID
--PYWYGG
--PYWYFA
--PYWYAI
--PYWYWA

--PYWYVR
--PYWYTL
--PYWYSM
--PYWYSA
--PYWYRD
--PYWYNA
--PYWYLT
--PYWYKN
--PYWYIQ
--PYWYFN
--PYWYFM
--PYWYFI
--PYWYEG
--PYWYDS
--PYWYAQ
PGPYWY--
--PYWYYL
--PYWYYH
--PYWYWT
--PYWYWN
--PYWYWI
--PYWYTM
--PYWYTH
--PYWYTF
--PYWYSR
--PYWYQK
--PYWYMH
--PYWYLR
--PYWYLF
--PYWYIR
--PYWYHN
--PYWYGN
--PYWYGL
--PYWYGH
--PYWYFR
--PYWYAF
--PYWYAE
--PYWYYM
--PYWYYG
--PYWYWG
--PYWYVQ
--PYWYVE
--PYWYVD
--PYWYTR
--PYWYTK
--PYWYSW
--PYWYQG
--PYWYNQ
--PYWYNN
--PYWYNM

--PYWYMN
--PYWYMD
--PYWYLW
--PYWYLQ
--PYWYLM
--PYWYLK
--PYWYLI
--PYWYKM
--PYWYKI
--PYWYIY
--PYWYIP
--PYWYIN
--PYWYIK
--PYWYHY
--PYWYFY
--PYWYFQ
--PYWYFG
--PYWYEE
--PYWYAY
--PYWYAV
--PYWYAN
PDPYWY--
QSPYWY--
QMPYWY--
-QPYWYV-
QFPYWY--
-QPYWYR-
QYPYWY--
QKPYWY--
QRPYWY--
QHPYWY--
QGPYWY--
QWPYWY--
-QPYWYY-
QLPYWY--
-QPYWYT-
QNPYWY--
-QPYWYS-
-QPYWYW-
-RPYWYT-
-RPYWYQ-
RSPYWY--
RTPYWY--
-RPYWYL-
-RPYWYM-
-RPYWYF-
-RPYWYR-
RHPYWY--
RDPYWY--
RNPYWY--

REPYWY--
RYPYWY--
RWPYWY--
-RPYWYV-
-RPYWYS-
RQPYWY--
-RPYWYY-
-RPYWYK-
-RPYWYA-
RAPYWY--
RVPYWY--
-RPYWYN-
RRPYWY--
-RPYWYI-
-RPYWYH-
-RPYWYG-
RPPYWY--
RIPYWY--
SRPYWY--
SSPYWY--
SQPYWY--
SGPYWY--
SIPYWY--
SKPYWY--
SYPYWY--
SVPYWY--
SLPYWY--
-SPYWYA-
SNPYWY--
-SPYWYV-
-SPYWYM-
SPPYWY--
SFPYWY--
-SPYWYL-
-SPYWYG-
SWPYWY--
-SPYWYY-
-SPYWYW-
SHPYWY--
SEPYWY--
SAPYWY--
-SPYWYI-
SDPYWY--
-SPYWYN-
-SPYWYF-
TSPYWY--
TLPYWY--
-TPYWYT-
-TPYWYF-
TPPYWY--

TAPYWY--
-TPYWYQ-
TGPYWY--
TDPYWY--
TRPYWY--
-TPYWYM-
TMPYWY--
TYPYWY--
TTPYWY--
-TPYWYW-
TIPYWY--
TFPYWY--
-TPYWYS-
TKPYWY--
-TPYWYK-
-TPYWYR-
TWPYWY--
-TPYWYY-
-TPYWYV-
-TPYWYH-
-TPYWYA-
TNPYWY--
VSPYWY--
-VPYWYM-
VMPYWY--
VNPYWY--
VAPYWY--
-VPYWYH-
VKPYWY--
-VPYWYK-
-VPYWYL-
-VPYWYS-
VIPYWY--
-VPYWYQ-
-VPYWYA-
VPPYWY--
VGPYWY--
-VPYWYN-
VTPYWY--
-VPYWYI-
-VPYWYG-
VFPYWY--
VYPYWY--
-VPYWYY-
-WPYWYS-
-WPYWYA-
WVPYWY--
-WPYWYQ-
WSPYWY--
WRPYWY--

WKPYWY--
WAPYWY--
WTPYWY--
-WPYWYM-
WLPYWY--
WEPYWY--
-WPYWYK-
YRPYWY--
YKPYWY--
-YPYWYS-
YVPYWY--
YPPYWY--
YSPYWY--
YDPYWY--
YTPYWY--
-YPYWYQ-
YLPYWY--
YHPYWY--
YQPYWY--
-YPYWYV-
YIPYWY--
-YPYWYT-
-YPYWYN-
-YPYWYK-
-YPYWYI-
-YPYWYH-
YNPYWY--
YMPYWY--
YFPYWY--

PYQL	198	749	47.21

-IPYQLG-
--PYQLRP
ALPYQL--
ATPYQL--
AQPYQL--
ARPYQL--
-APYQLR-
ASPYQL--
AVPYQL--
AEPYQL--
-APYQLK-
AGPYQL--
-APYQLS-
APPYQL--
-APYQLT-
-APYQLN-
-APYQLI-
AWPYQL--
-APYQLV-
-APYQLH-

AAPYQL--
ANPYQL--
AKPYQL--
-APYQLG-
AHPYQL--
AFPYQL--
AMPYQL--
-APYQLE-
AYPYQL--
-APYQLF-
ACPYQL--
-APYQLQ-
-APYQLM-
CVPYQL--
-CPYQLK-
CYPYQL--
-CPYQLN-
CGPYQL--
CRPYQL--
-CPYQLL-
CLPYQL--
DVPYQL--
DLPYQL--
DRPYQL--
-DPYQLN-
-DPYQLR-
-DPYQLK-
DQPYQL--
DMPYQL--
DWPYQL--
-DPYQLL-
DHPYQL--
DYPYQL--
DTPYQL--
DKPYQL--
DSPYQL--
-DPYQLW-
DIPYQL--
DGPYQL--
DEPYQL--
-DPYQLY-
-DPYQLS-
-DPYQLQ-
-DPYQLM-
-DPYQLH-
-DPYQLV-
-DPYQLI-
DNPYQL--
DAPYQL--
-EPYQLK-

EAPYQL--
EKPYQL--
ELPYQL--
-EPYQLN-
EQPYQL--
EVPYQL--
-EPYQLT-
-EPYQLS-
EWPYQL--
-EPYQLV-
EGPYQL--
EEPYQL--
-EPYQLR-
-EPYQLA-
-EPYQLH-
-EPYQLG-
EPPYQL--
EYPYQL--
ESPYQL--
ERPYQL--
-EPYQLW-
EDPYQL--
ETPYQL--
-EPYQLQ-
ENPYQL--
EHPYQL--
-EPYQLC-
FSPYQL--
FLPYQL--
FAPYQL--
FKPYQL--
-FPYQLA-
FTPYQL--
-FPYQLQ-
-FPYQLR-
FCPYQL--
FPPYQL--
FMPYQL--
FGPYQL--
FQPYQL--
-FPYQLV-
-FPYQLF-
-FPYQLN-
FVPYQL--
-FPYQLT-
-FPYQLS-
-FPYQLI-
FDPYQL--
-FPYQLH-
FNPYQL--

GQPYQL--
-GPYQLI-
-GPYQLK-
-GPYQLM-
-GPYQLS-
-GPYQLA-
GIPYQL--
GVPYQL--
GRPYQL--
GYPYQL--
GSPYQL--
-GPYQLR-
GNPYQL--
GMPYQL--
-GPYQLH-
-GPYQLG-
-GPYQLN-
-GPYQLL-
GTPYQL--
GWPYQL--
GGPYQL--
GEPYQL--
GDPYQL--
-GPYQLT-
-GPYQLQ-
GFPYQL--
-GPYQLV-
GLPYQL--
GKPYQL--
GHPYQL--
HRPYQL--
-HPYQLL-
-HPYQLT-
HIPYQL--
-HPYQLS-
-HPYQLN-
HVPYQL--
HMPYQL--
HWPYQL--
HTPYQL--
HGPYQL--
HYPYQL--
-HPYQLK-
HHPYQL--
-HPYQLM-
-HPYQLH-
HLPYQL--
HEPYQL--
-HPYQLY-
-HPYQLR-

-HPYQLA-
HPPYQL--
HSPYQL--
HQPYQL--
-HPYQLG-
-HPYQLP-
-HPYQLI-
HFPYQL--
IRPYQL--
-IPYQLR-
ISPYQL--
IVPYQL--
-IPYQLV-
-IPYQLN-
-IPYQLT-
-IPYQLS-
-IPYQLI-
ITPYQL--
IIPYQL--
IGPYQL--
-IPYQLH-
ILPYQL--
-IPYQLL-
IEPYQL--
IAPYQL--
-IPYQLQ-
IMPYQL--
IHPYQL--
-IPYQLM-
-IPYQLA-
INPYQL--
IFPYQL--
KSPYQL--
-KPYQLN-
-KPYQLL-
KMPYQL--
-KPYQLT-
-KPYQLA-
KRPYQL--
-KPYQLR-
KWPYQL--
KLPYQL--
KYPYQL--
-KPYQLS-
-KPYQLQ-
KGPYQL--
-KPYQLV-
-KPYQLY-
-KPYQLK-
-KPYQLI-

KTPYQL--
-KPYQLW-
KNPYQL--
KEPYQL--
-KPYQLH-
-KPYQLG-
KIPYQL--
KAPYQL--
KPPYQL--
KFPYQL--
-KPYQLC-
KCPYQL--
-KPYQLE-
KKPYQL--
KDPYQL--
LSPYQL--
LGPYQL--
-LPYQLR-
LEPYQL--
LKPYQL--
LVPYQL--
LMPYQL--
-LPYQLS-
-LPYQLK-
LYPYQL--
LTPYQL--
-LPYQLN-
LAPYQL--
-LPYQLI-
LIPYQL--
LLPYQL--
LFPYQL--
-LPYQLH-
LWPYQL--
-LPYQLQ-
-LPYQLV-
LNPYQL--
-LPYQLT-
LPPYQL--
LQPYQL--
-LPYQLA-
LDPYQL--
-LPYQLW-
LCPYQL--
LHPYQL--
MKPYQL--
-MPYQLL-
-MPYQLA-
-MPYQLK-
-MPYQLS-

MTPYQL--
-MPYQLR-
MVPYQL--
MEPYQL--
MRPYQL--
MGPYQL--
-MPYQLV-
-MPYQLT-
-MPYQLN-
MFPYQL--
-MPYQLI-
MLPYQL--
-MPYQLQ-
MMPYQL--
MIPYQL--
MHPYQL--
MWPYQL--
MYPYQL--
-MPYQLH-
NRPYQL--
-NPYQLR-
NFPYQL--
NSPYQL--
-NPYQLL-
NAPYQL--
NIPYQL--
NMPYQL--
NLPYQL--
NEPYQL--
NNPYQL--
-NPYQLT-
NWPYQL--
-NPYQLI-
-NPYQLY-
NPPYQL--
NVPYQL--
NTPYQL--
NKPYQL--
NGPYQL--
NDPYQL--
-NPYQLW-
-NPYQLS-
NCPYQL--
NQPYQL--
-NPYQLV-
NYPYQL--
-NPYQLA-
NHPYQL--
-NPYQLQ-
-NPYQLN-

-NPYQLK-
-NPYQLH-
--PYQLRS
--PYQLTS
--PYQLWH
--PYQLWS
--PYQLIS
--PYQLRY
PQPYQL--
--PYQLRT
--PYQLAS
--PYQLST
--PYQLSH
--PYQLRA
PIPYQL--
--PYQLRL
--PYQLSG
--PYQLMS
--PYQLKT
--PYQLSN
--PYQLLN
--PYQLLG
--PYQLRR
--PYQLMR
PEPYQL--
--PYQLWK
--PYQLVQ
--PYQLVS
--PYQLRI
--PYQLMA
--PYQLFT
--PYQLQN
--PYQLKR
--PYQLYS
--PYQLWG
--PYQLVA
--PYQLNH
--PYQLVN
--PYQLLR
--PYQLWN
--PYQLFG
--PYQLWQ
--PYQLTG
--PYQLRE
--PYQLGA
PWPYQL--
--PYQLQR
--PYQLNT
--PYQLLS
--PYQLIN

--PYQLIH
--PYQLGT
--PYQLVY
--PYQLVK
--PYQLSI
--PYQLRD
--PYQLKL
--PYQLHV
--PYQLHT
--PYQLVT
--PYQLVI
--PYQLKV
--PYQLQT
--PYQLLK
--PYQLHG
PNPYQL--
--PYQLRV
--PYQLRG
--PYQLNN
--PYQLLT
--PYQLKN
--PYQLGS
PKPYQL--
--PYQLTW
--PYQLRQ
--PYQLNV
--PYQLIG
--PYQLVG
--PYQLTR
--PYQLTQ
--PYQLSK
--PYQLNM
--PYQLIK
--PYQLME
--PYQLKQ
--PYQLVD
--PYQLTF
--PYQLFM
--PYQLAT
--PYQLTL
--PYQLSW
--PYQLQV
--PYQLQG
--PYQLLH
--PYQLKS
--PYQLKM
--PYQLKD
--PYQLHR
--PYQLTA
--PYQLSQ

--PYQLNK
--PYQLNA
--PYQLMM
--PYQLEW
--PYQLAG
--PYQLYN
--PYQLYG
--PYQLTK
--PYQLSM
--PYQLQM
--PYQLNL
--PYQLII
--PYQLHW
--PYQLGV
--PYQLGG
--PYQLEA
--PYQLAM
--PYQLAI
PTPYQL--
PMPYQL--
--PYQLTN
--PYQLSY
--PYQLQA
--PYQLNY
--PYQLIA
--PYQLHN
--PYQLHE
--PYQLVM
--PYQLVH
--PYQLTM
--PYQLRF
--PYQLQQ
--PYQLQI
--PYQLNR
--PYQLMW
--PYQLMH
--PYQLFK
--PYQLAR
PYPYQL--
PHPYQL--
--PYQLYQ
--PYQLYH
--PYQLWT
--PYQLTV
--PYQLTT
--PYQLRW
--PYQLQS
--PYQLMG
--PYQLLD
--PYQLKE

--PYQLIR
--PYQLIM
--PYQLHS
--PYQLFH
--PYQLDS
--PYQLAV
PGPYQL--
--PYQLWE
--PYQLVE
--PYQLTE
--PYQLTD
--PYQLSD
--PYQLRM
--PYQLQL
--PYQLQK
--PYQLQF
--PYQLNI
--PYQLMD
--PYQLKW
--PYQLKF
--PYQLHF
--PYQLHA
--PYQLFY
--PYQLEY
--PYQLEV
--PYQLEG
--PYQLAW
PFPYQL--
--PYQLTY
--PYQLTI
--PYQLRN
--PYQLQH
--PYQLNS
--PYQLNG
--PYQLMQ
--PYQLMK
--PYQLLQ
--PYQLKH
--PYQLKG
--PYQLKA
--PYQLHY
--PYQLHH
--PYQLHD
--PYQLGM
--PYQLGH
--PYQLFN
--PYQLET
--PYQLAN
PCPYQL--
--PYQLYD

--PYQLVW
--PYQLTH
--PYQLSE
--PYQLRH
--PYQLRC
--PYQLQY
--PYQLQW
--PYQLPS
--PYQLNW
--PYQLNQ
--PYQLND
--PYQLMF
--PYQLLE
--PYQLKY
--PYQLKI
--PYQLIV
--PYQLID
--PYQLHQ
--PYQLGL
--PYQLGF
--PYQLFD
--PYQLEM
--PYQLEF
--PYQLCW
--PYQLAH
-PPYQLR-
-PPYQLG-
PDPYQL--
-QPYQLR-
QGPYQL--
QRPYQL--
QSPYQL--
QTPYQL--
-QPYQLM-
QYPYQL--
QIPYQL--
-QPYQLF-
QVPYQL--
-QPYQLT-
QLPYQL--
-QPYQLW-
-QPYQLS-
-QPYQLN-
QDPYQL--
-QPYQLV-
QHPYQL--
-QPYQLQ-
QFPYQL--
-QPYQLG-
QMPYQL--

QKPYQL--
-QPYQLY-
-QPYQLK-
-QPYQLA-
QNPYQL--
QEPYQL--
RRPYQL--
RAPYQL--
-RPYQLR-
-RPYQLT-
RGPYQL--
RLPYQL--
RFPYQL--
RVPYQL--
-RPYQLI-
-RPYQLV-
-RPYQLQ-
RPPYQL--
RTPYQL--
-RPYQLW-
RNPYQL--
RQPYQL--
-RPYQLN-
RHPYQL--
-RPYQLG-
-RPYQLH-
RIPYQL--
-RPYQLK-
RWPYQL--
RCPYQL--
RYPYQL--
-RPYQLA-
-RPYQLF-
-RPYQLM-
-RPYQLE-
SIPYQL--
SSPYQL--
-SPYQLR-
-SPYQLV-
SLPYQL--
SFPYQL--
SRPYQL--
SGPYQL--
SWPYQL--
STPYQL--
SYPYQL--
-SPYQLS-
SAPYQL--
-SPYQLT-
-SPYQLM-

SHPYQL--
-SPYQLK-
SMPYQL--
-SPYQLH-
SKPYQL--
SEPYQL--
-SPYQLA-
-SPYQLI-
SPPYQL--
SNPYQL--
-SPYQLW-
SQPYQL--
SDPYQL--
-SPYQLG-
-SPYQLE-
TGPYQL--
-TPYQLR-
-TPYQLI-
-TPYQLH-
-TPYQLF-
TEPYQL--
TVPYQL--
-TPYQLK-
TRPYQL--
-TPYQLT-
-TPYQLS-
-TPYQLN-
TTPYQL--
TNPYQL--
TSPYQL--
TMPYQL--
TLPYQL--
TAPYQL--
TKPYQL--
TIPYQL--
-TPYQLV-
TYPYQL--
TWPYQL--
-TPYQLQ-
-TPYQLC-
TPPYQL--
THPYQL--
TCPYQL--
-VPYQLT-
VRPYQL--
-VPYQLR-
-VPYQLL-
VVPYQL--
VTPYQL--
-VPYQLK-

VYPYQL--
-VPYQLS-
VWPYQL--
VKPYQL--
-VPYQLI-
VMPYQL--
-VPYQLQ-
VLPYQL--
VGPYQL--
-VPYQLW-
-VPYQLN-
VPPYQL--
VQPYQL--
-VPYQLM-
-VPYQLA-
-VPYQLV-
VFPYQL--
VNPYQL--
VIPYQL--
VHPYQL--
VEPYQL--
VDPYQL--
-VPYQLG-
VCPYQL--
VAPYQL--
-VPYQLH-
-VPYQLE-
WTPYQL--
WRPYQL--
WQPYQL--
WVPYQL--
-WPYQLT-
-WPYQLY-
WSPYQL--
-WPYQLS-
WFPYQL--
-WPYQLM-
WMPYQL--
-WPYQLK-
WLPYQL--
WEPYQL--
-WPYQLH-
WAPYQL--
-WPYQLN-
-WPYQLI-
WGPYQL--
-WPYQLR-
-WPYQLQ-
-WPYQLE-
WPPYQL--

WKPYQL--
YRPYQL--
YLPYQL--
YGPYQL--
YVPYQL--
-YPYQLT-
YSPYQL--
YKPYQL--
-YPYQLN-
-YPYQLS-
YYPYQL--
-YPYQLK-
-YPYQLH-
YIPYQL--
YFPYQL--
-YPYQLV-
YPPYQL--
YAPYQL--
YTPYQL--
YQPYQL--
-YPYQLQ-
-YPYQLL-
-YPYQLI-
-YPYQLM-
-YPYQLA-
YNPYQL--
YDPYQL--
-YPYQLF-
YMPYQL--

PRHM	249	725	47.20

-HPRHMG-
-LPRHMN-
LEPRHM--
--PRHMGL
AGPRHM--
-APRHMA-
-APRHMI-
-APRHMV-
ASPRHM--
ALPRHM--
AQPRHM--
AVPRHM--
AMPRHM--
-APRHML-
-APRHMW-
-APRHMT-
ATPRHM--
-APRHMH-
-APRHME-
AAPRHM--

-APRHMN-
-APRHMG-
AEPRHM--
-APRHMS-
-APRHMM-
APPRHM--
-APRHMY-
-APRHMK-
AIPRHM--
-APRHMQ-
ADPRHM--
-APRHMF-
-APRHMD-
ANPRHM--
CLPRHM--
CMPRHM--
CTPRHM--
-CPRHMQ-
CCPRHM--
CGPRHM--
-CPRHMW-
-CPRHMV-
-CPRHMR-
CKPRHM--
CVPRHM--
CSPRHM--
CQPRHM--
-CPRHMY-
-CPRHMT-
-DPRHMR-
DSPRHM--
DVPRHM--
DGPRHM--
DKPRHM--
DFPRHM--
DQPRHM--
-DPRHML-
DTPRHM--
DLPRHM--
DWPRHM--
-DPRHMV-
DYPRHM--
-DPRHMI-
DAPRHM--
-DPRHMT-
DNPRHM--
DDPRHM--
DEPRHM--
-DPRHMY-
-DPRHMS-

-DPRHMK-
-DPRHMA-
DMPRHM--
DIPRHM--
ETPRHM--
-EPRHMW-
EHPRHM--
-EPRHML-
-EPRHMM-
EGPRHM--
-EPRHMK-
-EPRHMI-
ELPRHM--
ESPRHM--
EKPRHM--
ERPRHM--
EMPRHM--
EFPRHM--
EWPRHM--
-EPRHMY-
-EPRHMR-
-EPRHMT-
-EPRHME-
-EPRHMV-
EDPRHM--
-EPRHMS-
-EPRHMF-
ENPRHM--
EEPRHM--
EVPRHM--
-EPRHMN-
EAPRHM--
-FPRHMI-
FSPRHM--
-FPRHMS-
FQPRHM--
FGPRHM--
FIPRHM--
FTPRHM--
-FPRHMY-
-FPRHMK-
-FPRHMM-
FNPRHM--
FMPRHM--
FDPRHM--
FVPRHM--
-FPRHMV-
-FPRHMH-
-FPRHMF-
FHPRHM--

FKPRHM--
-FPRHMN-
FYPRHM--
-FPRHME-
-FPRHMA-
-GPRHMR-
GLPRHM--
GVPRHM--
-GPRHMS-
GYPRHM--
GIPRHM--
-GPRHML-
GGPRHM--
GTPRHM--
-GPRHMT-
-GPRHMH-
-GPRHMW-
GQPRHM--
-GPRHMI-
-GPRHMN-
-GPRHMF-
GPPRHM--
GNPRHM--
GHPRHM--
-GPRHMA-
GSPRHM--
GMPRHM--
GRPRHM--
GEPRHM--
-GPRHMV-
-GPRHME-
-GPRHMK-
-GPRHMD-
GDPRHM--
GCPRHM--
HHPRHM--
HMPRHM--
HSPRHM--
-HPRHMV-
HTPRHM--
-HPRHMY-
-HPRHMK-
HLPRHM--
-HPRHMT-
-HPRHMM-
-HPRHMN-
-HPRHMF-
HEPRHM--
HVPRHM--
HNPRHM--

HKPRHM--
HQPRHM--
-HPRHMS-
-HPRHMR-
-HPRHMQ-
-HPRHMI-
HAPRHM--
-HPRHMW-
HPPRHM--
HIPRHM--
ISPRHM--
IYPRHM--
-IPRHML-
-IPRHMR-
IGPRHM--
-IPRHMM-
ITPRHM--
-IPRHMI-
IAPRHM--
-IPRHMH-
-IPRHME-
IVPRHM--
-IPRHMV-
-IPRHMT-
IKPRHM--
-IPRHMN-
IIPRHM--
IHPRHM--
-IPRHMG-
-IPRHMA-
-IPRHMY-
-IPRHMF-
INPRHM--
IQPRHM--
-IPRHMQ-
-IPRHMK-
IEPRHM--
IDPRHM--
IPPRHM--
KVPRHM--
-KPRHMS-
-KPRHML-
KTPRHM--
-KPRHMY-
-KPRHMA-
-KPRHMT-
KMPRHM--
-KPRHMW-
-KPRHMR-
-KPRHMI-

KEPRHM--
-KPRHMQ-
KYPRHM--
-KPRHMN-
-KPRHMH-
KQPRHM--
-KPRHMM-
-KPRHMG-
-KPRHME-
-LPRHML-
LLPRHM--
LVPRHM--
LGPRHM--
LMPRHM--
LTPRHM--
LPPRHM--
LYPRHM--
-LPRHMQ-
-LPRHMV-
-LPRHMS-
-LPRHMA-
LQPRHM--
LAPRHM--
-LPRHMH-
-LPRHMY-
LNPRHM--
-LPRHMT-
LDPRHM--
-LPRHMM-
-LPRHMK-
-LPRHMF-
-LPRHMW-
LHPRHM--
-LPRHME-
-MPRHMT-
MDPRHM--
-MPRHMI-
MNPRHM--
MSPRHM--
MYPRHM--
-MPRHMR-
MVPRHM--
MAPRHM--
-MPRHMS-
-MPRHMG-
-MPRHMA-
MEPRHM--
-MPRHMY-
-MPRHMM-
-MPRHMK-

MPPRHM--
MRPRHM--
-MPRHMN-
MTPRHM--
MHPRHM--
MQPRHM--
-MPRHMW-
-MPRHMQ-
MLPRHM--
-MPRHMH-
-MPRHME-
-MPRHMC-
NPPRHM--
NSPRHM--
NNPRHM--
NTPRHM--
-NPRHMR-
NEPRHM--
-NPRHML-
NQPRHM--
NDPRHM--
NAPRHM--
-NPRHMW-
-NPRHMK-
-NPRHMF-
NVPRHM--
-NPRHMV-
NRPRHM--
-NPRHMM-
-NPRHMS-
NGPRHM--
-NPRHMQ-
-NPRHMH-
NMPRHM--
NHPRHM--
-NPRHMY-
-NPRHMA-
--PRHMLS
--PRHMIR
--PRHMFG
--PRHMRT
--PRHMKS
--PRHMWS
PSPRHM--
--PRHMVI
--PRHMRI
--PRHMYS
--PRHMFW
--PRHMGG
--PRHMVK

--PRHMSS
PAPRHM--
--PRHMVT
--PRHMLN
--PRHMTL
--PRHMFS
--PRHMVM
--PRHMLF
--PRHMIF
--PRHMYN
--PRHMVV
--PRHMLW
--PRHMLA
--PRHMYQ
--PRHMRG
--PRHMHT
--PRHMFH
--PRHMAS
PLPRHM--
PVPRHM--
--PRHMWI
--PRHMTY
--PRHMSV
--PRHMQA
--PRHMMY
--PRHMHS
PGPRHM--
--PRHMYR
--PRHMHA
--PRHMAG
--PRHMVQ
--PRHMVD
--PRHMSG
--PRHMMW
--PRHMLT
--PRHMIN
--PRHMTV
--PRHMQT
--PRHMGS
PMPRHM--
PYPRHM--
--PRHMWY
--PRHMWN
--PRHMVY
--PRHMRH
--PRHMMT
--PRHMMS
--PRHMMI
--PRHMKY
--PRHMYG

--PRHMWM
--PRHMTG
--PRHMST
PKPRHM--
--PRHMYT
--PRHMYM
--PRHMQW
--PRHMTH
--PRHMSA
--PRHMRD
--PRHMNS
--PRHMMH
--PRHMLV
--PRHMES
--PRHMAT
--PRHMVS
--PRHMVF
--PRHMVA
--PRHMTW
--PRHMMV
--PRHMIT
--PRHMFQ
--PRHMVW
--PRHMQM
--PRHMLQ
--PRHMLE
--PRHMIS
--PRHMFR
--PRHMFK
--PRHMAY
--PRHMYW
--PRHMYE
--PRHMWA
--PRHMTT
--PRHMMN
--PRHMKH
--PRHMGA
--PRHMFI
--PRHMTI
--PRHMSY
--PRHMSQ
--PRHMQH
--PRHMNV
--PRHMIL
--PRHMAH
--PRHMSK
--PRHMRM
--PRHMKN
--PRHMID
--PRHMHN

--PRHMFY
--PRHMFM
PRPRHM--
--PRHMTF
--PRHMSD
--PRHMQS
--PRHMKL
--PRHMKI
--PRHMII
--PRHMGW
--PRHMFA
--PRHMEW
--PRHMEV
--PRHMYV
--PRHMSM
--PRHMKV
--PRHMKG
--PRHMET
--PRHMEG
--PRHMAA
--PRHMYY
--PRHMVN
--PRHMLM
--PRHMLI
--PRHMLH
--PRHMHL
--PRHMGT
--PRHMNW
--PRHMMD
--PRHMKW
--PRHMKT
--PRHMKE
--PRHMKD
--PRHMIM
--PRHMHV
--PRHMHG
--PRHMFE
--PRHMAW
--PRHMWF
--PRHMSI
--PRHMQY
--PRHMNM
--PRHMMA
--PRHMFV
--PRHMFF
--PRHMFD
--PRHMEY
--PRHMEI
PIPRHM--
--PRHMVE

--PRHMTQ
--PRHMTD
--PRHMQV
--PRHMQL
--PRHMQD
--PRHMHQ
--PRHMHM
--PRHMGI
--PRHMFT
--PRHMEQ
--PRHMEN
--PRHMAL
--PRHMAI
--PRHMYA
--PRHMWV
--PRHMTK
--PRHMRE
--PRHMQN
--PRHMMQ
--PRHMIY
--PRHMIW
--PRHMIV
--PRHMIE
--PRHMGF
--PRHMFL
--PRHMER
--PRHMDT
--PRHMCS
--PRHMCN
PNPRHM--
PCPRHM--
--PRHMWL
--PRHMSF
--PRHMQF
--PRHMNY
--PRHMNT
--PRHMNF
--PRHMKM
--PRHMKF
--PRHMIG
--PRHMHI
--PRHMGV
--PRHMEH
--PRHMDS
--PRHMAV
PHPRHM--
--PRHMYH
--PRHMYD
--PRHMWQ
--PRHMTE

--PRHMQI
--PRHMQE
--PRHMPT
--PRHMNR
--PRHMNK
--PRHMNI
--PRHMMG
--PRHMLP
--PRHMIP
--PRHMGN
--PRHMGM
--PRHMGK
--PRHMEL
--PRHMDM
--PRHMDG
--PRHMCQ
--PRHMAQ
--PRHMAM
--PRHMAK
PEPRHM--
-QPRHMR-
QGPRHM--
-QPRHMY-
-QPRHMS-
-QPRHMI-
-QPRHMT-
-QPRHMQ-
QSPRHM--
-QPRHML-
QYPRHM--
QAPRHM--
QVPRHM--
QIPRHM--
-QPRHMM-
-QPRHMH-
QKPRHM--
-QPRHMA-
QHPRHM--
QRPRHM--
QTPRHM--
-QPRHMG-
QMPRHM--
QEPRHM--
-QPRHMN-
-QPRHMK-
QPPRHM--
QCPRHM--
QQPRHM--
-QPRHMV-
QDPRHM--

RVPRHM--
-RPRHMV-
RNPRHM--
-RPRHMT-
-RPRHMW-
-RPRHMQ-
-RPRHMM-
-RPRHMK-
-RPRHMF-
-RPRHMY-
REPRHM--
RDPRHM--
RPPRHM--
RAPRHM--
-RPRHMS-
-RPRHME-
-RPRHMA-
-RPRHMG-
-RPRHMI-
SLPRHM--
STPRHM--
SEPRHM--
SSPRHM--
-SPRHMR-
-SPRHMT-
SAPRHM--
SNPRHM--
-SPRHMV-
-SPRHMY-
-SPRHMS-
-SPRHMM-
SPPRHM--
-SPRHMA-
-SPRHMK-
-SPRHMW-
-SPRHML-
-SPRHMI-
-SPRHMF-
SVPRHM--
-SPRHMH-
SQPRHM--
-SPRHMQ-
-SPRHMN-
SYPRHM--
SMPRHM--
-SPRHMG-
-SPRHME-
SKPRHM--
TEPRHM--
TAPRHM--

```
-TPRHMT-
-TPRHMF-
TNPRHM--
TGPRHM--
TSPRHM--
TQPRHM--
TVPRHM--
TTPRHM--
-TPRHMH-
-TPRHMV-
TIPRHM--
TYPRHM--
TLPRHM--
-TPRHMS-
-TPRHML-
TRPRHM--
-TPRHMW-
-TPRHMQ-
-TPRHMM-
THPRHM--
-TPRHMY-
-TPRHMN-
-TPRHMG-
-TPRHMA-
TPPRHM--
-TPRHMI-
-TPRHME-
TMPRHM--
-VPRHMM-
VYPRHM--
VAPRHM--
-VPRHMS-
VEPRHM--
VLPRHM--
VNPRHM--
VVPRHM--
-VPRHMY-
VQPRHM--
-VPRHMR-
VDPRHM--
-VPRHMI-
VGPRHM--
VTPRHM--
-VPRHMA-
-VPRHMT-
-VPRHML-
-VPRHMF-
-VPRHMV-
-VPRHMG-
-VPRHME-
```

VIPRHM--
VCPRHM--
-VPRHMQ-
-VPRHMN-
-VPRHMK-
-VPRHMW-
-VPRHMH-
VPPRHM--
VMPRHM--
WSPRHM--
WNPRHM--
-WPRHMQ-
WRPRHM--
-WPRHMS-
-WPRHMI-
WYPRHM--
WLPRHM--
WVPRHM--
WMPRHM--
-WPRHMT-
-WPRHMM-
-WPRHMY-
WIPRHM--
WEPRHM--
-WPRHMN-
WPPRHM--
WKPRHM--
-WPRHMV-
-WPRHMF-
WGPRHM--
YSPRHM--
YGPRHM--
-YPRHMT-
YYPRHM--
-YPRHMV-
YHPRHM--
YTPRHM--
YRPRHM--
-YPRHMM-
-YPRHML-
YAPRHM--
-YPRHMS-
-YPRHMK-
YPPRHM--
-YPRHMF-
-YPRHMQ-
YNPRHM--
YKPRHM--
-YPRHMY-
YEPRHM--

	YDPRHM--		
	YIPRHM--		
	-YPRHMP-		
	-YPRHMH-		
	-YPRHMG-		
PVGL	37	863	47.20
	-APVGLP-		
	-EPVGLG-		
	-KPVGLD-		
	-LPVGLG-		
	-MPVGLP-		
	--PVGLSP		
	--PVGLRP		
	--PVGLVP		
	--PVGLMP		
	--PVGLIK		
	--PVGLGK		
	--PVGLDT		
	--PVGLDR		
	-VPVGLD-		
	-APVGLS-		
	-APVGLR-		
	ASPVGL--		
	-APVGLL-		
	ATPVGL--		
	AGPVGL--		
	AYPVGL--		
	-APVGLY-		
	AVPVGL--		
	AWPVGL--		
	-APVGLV-		
	-APVGLT-		
	-APVGLK-		
	AMPVGL--		
	AFPVGL--		
	AAPVGL--		
	-APVGLQ-		
	ALPVGL--		
	-APVGLM-		
	-APVGLN-		
	-APVGLF-		
	APPVGL--		
	-APVGLH-		
	AEPVGL--		
	-APVGLW-		
	-APVGLG-		
	AKPVGL--		
	AQPVGL--		
	ANPVGL--		
	-APVGLE-		

AHPVGL--
ADPVGL--
ACPVGL--
CVPVGL--
CRPVGL--
CSPVGL--
CAPVGL--
CPPVGL--
-CPVGLW-
-CPVGLV-
CLPVGL--
CKPVGL--
CFPVGL--
-CPVGLS-
CWPVGL--
-CPVGLR-
-CPVGLL-
CDPVGL--
CCPVGL--
-CPVGLI-
CNPVGL--
-CPVGLN-
-CPVGLA-
CMPVGL--
CEPVGL--
-CPVGLQ-
-CPVGLK-
-CPVGLH-
CHPVGL--
CGPVGL--
-DPVGLT-
DYPVGL--
DQPVGL--
-DPVGLR-
-DPVGLH-
DNPVGL--
DTPVGL--
DSPVGL--
DRPVGL--
DMPVGL--
-DPVGLL-
DGPVGL--
-DPVGLS-
DAPVGL--
-DPVGLK-
DLPVGL--
-DPVGLW-
-DPVGLV-
DWPVGL--
DFPVGL--

-DPVGLN-
-DPVGLM-
-DPVGLI-
DKPVGL--
DEPVGL--
DIPVGL--
DDPVGL--
DVPVGL--
-DPVGLQ-
-DPVGLE-
-EPVGLR-
ESPVGL--
-EPVGLL-
-EPVGLY-
-EPVGLM-
ERPVGL--
EWPVGL--
EVPVGL--
-EPVGLK-
EGPVGL--
ELPVGL--
-EPVGLI-
-EPVGLH-
-EPVGLW-
EYPVGL--
-EPVGLT-
-EPVGLS-
EAPVGL--
EPPVGL--
ETPVGL--
-EPVGLF-
EFPVGL--
EEPVGL--
EKPVGL--
EQPVGL--
EHPVGL--
-EPVGLQ-
EMPVGL--
-EPVGLN-
-EPVGLE-
EIPVGL--
ECPVGL--
FSPVGL--
-FPVGLF-
-FPVGLV-
FNPVGL--
-FPVGLS-
FGPVGL--
-FPVGLT-
FYPVGL--

FTPVGL--
-FPVGLL-
FKPVGL--
FFPVGL--
-FPVGLK-
FWPVGL--
FQPVGL--
-FPVGLI-
FDPVGL--
-FPVGLM-
FPPVGL--
FEPVGL--
-FPVGLA-
-FPVGLH-
-FPVGLG-
FIPVGL--
-FPVGLQ-
-FPVGLE-
-FPVGLC-
FCPVGL--
-GPVGLS-
GLPVGL--
GTPVGL--
-GPVGLR-
-GPVGLV-
-GPVGLT-
GNPVGL--
GKPVGL--
GGPVGL--
-GPVGLW-
GYPVGL--
-GPVGLL-
-GPVGLK-
GSPVGL--
GIPVGL--
GFPVGL--
GMPVGL--
-GPVGLI-
GVPVGL--
-GPVGLH-
GEPVGL--
GAPVGL--
GQPVGL--
GWPVGL--
-GPVGLM-
-GPVGLG-
-GPVGLE-
-GPVGLA-
GHPVGL--
-GPVGLQ-

GPPVGL--
GDPVGL--
GCPVGL--
-GPVGLC-
-GPVGLD-
HLPVGL--
HKPVGL--
-HPVGLW-
HMPVGL--
HVPVGL--
-HPVGLH-
HSPVGL--
-HPVGLM-
HWPVGL--
-HPVGLN-
HIPVGL--
HQPVGL--
-HPVGLY-
HNPVGL--
-HPVGLF-
HPPVGL--
-HPVGLK-
HEPVGL--
HFPVGL--
-HPVGLV-
HAPVGL--
-HPVGLS-
HYPVGL--
-HPVGLQ-
-HPVGLG-
-HPVGLA-
HHPVGL--
HCPVGL--
-HPVGLE-
IRPVGL--
-IPVGLL-
ISPVGL--
ILPVGL--
IWPVGL--
-IPVGLT-
-IPVGLN-
-IPVGLA-
IPPVGL--
-IPVGLY-
-IPVGLH-
ITPVGL--
IKPVGL--
-IPVGLS-
IMPVGL--
IGPVGL--

IYPVGL--
-IPVGLK-
IQPVGL--
-IPVGLQ-
IEPVGL--
IIPVGL--
IFPVGL--
IDPVGL--
INPVGL--
-IPVGLV-
-IPVGLE-
IHPVGL--
IAPVGL--
ICPVGL--
-IPVGLM-
-IPVGLD-
-IPVGLP-
-IPVGLC-
-KPVGLR-
KVPVGL--
-KPVGLV-
-KPVGLW-
KGPVGL--
KRPVGL--
KLPVGL--
KAPVGL--
KKPVGL--
-KPVGLT-
-KPVGLA-
KYPVGL--
KFPVGL--
KHPVGL--
KNPVGL--
-KPVGLN-
-KPVGLM-
KEPVGL--
KWPVGL--
KDPVGL--
KCPVGL--
KPPVGL--
-KPVGLE-
LFPVGL--
LRPVGL--
-LPVGLR-
LLPVGL--
LNPVGL--
-LPVGLT-
LWPVGL--
LIPVGL--
LKPVGL--

-LPVGLW-
LAPVGL--
-LPVGLH-
LYPVGL--
LGPVGL--
LTPVGL--
-LPVGLV-
-LPVGLY-
-LPVGLM-
LHPVGL--
-LPVGLI-
-LPVGLK-
LPPVGL--
-LPVGLQ-
-LPVGLN-
-LPVGLF-
LEPVGL--
-LPVGLA-
-LPVGLE-
LCPVGL--
MTPVGL--
-MPVGLS-
-MPVGLR-
MGPVGL--
MRPVGL--
-MPVGLV-
-MPVGLT-
MYPVGL--
-MPVGLL-
MSPVGL--
MPPVGL--
MLPVGL--
-MPVGLK-
MMPVGL--
MHPVGL--
MFPVGL--
-MPVGLN-
-MPVGLA-
MAPVGL--
-MPVGLM-
-MPVGLF-
-MPVGLI-
MWPVGL--
MKPVGL--
-MPVGLY-
MIPVGL--
MQPVGL--
-MPVGLG-
MDPVGL--
-MPVGLW-

```
-MPVGLE-
-MPVGLQ-
-NPVGLR-
NHPVGL--
NLPVGL--
NWPVGL--
-NPVGLW-
NIPVGL--
-NPVGLK-
NSPVGL--
NGPVGL--
NVPVGL--
NAPVGL--
NKPVGL--
NTPVGL--
NFPVGL--
-NPVGLV-
-NPVGLT-
NYPVGL--
-NPVGLH-
NEPVGL--
-NPVGLS-
NPPVGL--
-NPVGLI-
NDPVGL--
-NPVGLF-
NQPVGL--
-NPVGLY-
NMPVGL--
-NPVGLM-
NNPVGL--
-NPVGLA-
NCPVGL--
-NPVGLQ-
-NPVGLN-
-NPVGLG-
-NPVGLE-
--PVGLLS
--PVGLLR
--PVGLRT
--PVGLVR
--PVGLSS
--PVGLRS
--PVGLRG
--PVGLLG
--PVGLLA
--PVGLRR
--PVGLHS
--PVGLVA
--PVGLGS
```

--PVGLRW
--PVGLST
--PVGLIS
--PVGLSV
--PVGLRA
--PVGLKV
--PVGLNS
--PVGLSL
--PVGLMT
--PVGLTR
--PVGLSY
--PVGLKT
--PVGLSG
--PVGLRN
--PVGLRY
--PVGLTS
--PVGLRQ
--PVGLRK
--PVGLYS
--PVGLLN
--PVGLVS
--PVGLTG
--PVGLYA
--PVGLMG
--PVGLFG
--PVGLQS
--PVGLHR
--PVGLKS
--PVGLYH
--PVGLRM
--PVGLRF
--PVGLGA
--PVGLRD
--PVGLAS
--PVGLFQ
PGPVGL--
--PVGLIT
--PVGLWN
--PVGLVT
PVPVGL--
--PVGLMK
PWPVGL--
--PVGLTM
--PVGLML
--PVGLTT
--PVGLLV
--PVGLWG
--PVGLSI
--PVGLMS
--PVGLLI

--PVGLVN
--PVGLRH
--PVGLNM
--PVGLMR
--PVGLKA
--PVGLIA
--PVGLRE
--PVGLNR
--PVGLKY
--PVGLIR
--PVGLTN
--PVGLTF
--PVGLSM
--PVGLGG
--PVGLHW
--PVGLTV
--PVGLSA
--PVGLQR
--PVGLLQ
--PVGLYQ
--PVGLTK
--PVGLKF
--PVGLHL
--PVGLGT
--PVGLTA
--PVGLMM
--PVGLKR
--PVGLWK
--PVGLNV
--PVGLHA
--PVGLKW
--PVGLSW
--PVGLKG
--PVGLIN
--PVGLFH
--PVGLAG
--PVGLQA
--PVGLFT
--PVGLAW
PLPVGL--
PHPVGL--
--PVGLWF
--PVGLLE
PKPVGL--
--PVGLTY
--PVGLEV
--PVGLLK
--PVGLKI
--PVGLHH
--PVGLAK

PYPVGL--
--PVGLGV
--PVGLNT
--PVGLNL
--PVGLMA
--PVGLHI
--PVGLAA
PAPVGL--
--PVGLQY
--PVGLQT
--PVGLNH
--PVGLIG
--PVGLFN
--PVGLAL
--PVGLYN
--PVGLTI
--PVGLQK
--PVGLKK
--PVGLHD
--PVGLVQ
--PVGLVI
--PVGLAY
--PVGLWM
--PVGLKH
--PVGLAT
--PVGLMN
--PVGLMI
--PVGLHG
--PVGLEA
--PVGLSD
--PVGLQW
PIPVGL--
--PVGLIQ
--PVGLHN
--PVGLYD
--PVGLVH
--PVGLVE
--PVGLSQ
--PVGLNY
--PVGLNN
--PVGLMY
--PVGLMH
--PVGLGW
PSPVGL--
--PVGLTH
--PVGLNI
--PVGLYK
--PVGLVD
--PVGLQH
--PVGLNA

--PVGLHT
--PVGLHK
--PVGLNQ
--PVGLMV
--PVGLHV
--PVGLWD
--PVGLVV
--PVGLGL
--PVGLEW
--PVGLAR
--PVGLAM
--PVGLVW
--PVGLQG
--PVGLAH
--PVGLVM
--PVGLQL
--PVGLMW
--PVGLKE
--PVGLGY
--PVGLFD
PFPVGL--
--PVGLQV
--PVGLAI
--PVGLEY
PTPVGL--
--PVGLQN
--PVGLAN
--PVGLWH
--PVGLTQ
--PVGLKN
--PVGLHQ
--PVGLHF
--PVGLWW
--PVGLQE
--PVGLQD
--PVGLAQ
PCPVGL--
--PVGLNF
--PVGLME
--PVGLLD
--PVGLEG
--PVGLEF
--PVGLWQ
--PVGLSE
--PVGLKQ
--PVGLKD
--PVGLGI
--PVGLMD
--PVGLHE
--PVGLGN

--PVGLFA
--PVGLAV
--PVGLWE
--PVGLQM
--PVGLQI
--PVGLNW
--PVGLHY
--PVGLHM
--PVGLEH
--PVGLSC
--PVGLAD
PQPVGL--
PDPVGL--
--PVGLYE
--PVGLRC
--PVGLQQ
--PVGLMQ
--PVGLEQ
--PVGLMF
--PVGLII
--PVGLEK
--PVGLEI
PNPVGL--
--PVGLTD
--PVGLNG
--PVGLKC
--PVGLIE
--PVGLWA
--PVGLQF
--PVGLER
--PVGLCL
--PVGLCF
--PVGLND
--PVGLGM
--PVGLEN
--PVGLEL
--PVGLCR
--PVGLCQ
--PVGLCK
--PVGLCH
-PPVGLR-
-PPVGLK-
-PPVGLG-
PEPVGL--
--PVGLWC
--PVGLVC
--PVGLPY
--PVGLNE
--PVGLMC
--PVGLEM

```
--PVGLEC
--PVGLDV
--PVGLDD
--PVGLCV
--PVGLCT
--PVGLCN
--PVGLCI
--PVGLCG
-PPVGLV-
-PPVGLT-
-QPVGLR-
QSPVGL--
QRPVGL--
-QPVGLY-
QGPVGL--
-QPVGLS-
-QPVGLI-
QLPVGL--
QVPVGL--
QTPVGL--
QHPVGL--
-QPVGLW-
-QPVGLL-
-QPVGLT-
-QPVGLV-
QKPVGL--
QEPVGL--
-QPVGLF-
QNPVGL--
-QPVGLK-
-QPVGLQ-
QPPVGL--
QYPVGL--
-QPVGLH-
QAPVGL--
QWPVGL--
-QPVGLN-
-QPVGLA-
QMPVGL--
QIPVGL--
-QPVGLE-
-QPVGLM-
-QPVGLG-
QDPVGL--
QQPVGL--
-QPVGLC-
QCPVGL--
RYPVGL--
RAPVGL--
RWPVGL--
```

-RPVGLK-
RIPVGL--
RTPVGL--
-RPVGLN-
-RPVGLA-
RGPVGL--
-RPVGLH-
REPVGL--
-RPVGLM-
RDPVGL--
RHPVGL--
-RPVGLG-
RQPVGL--
RCPVGL--
-RPVGLC-
SLPVGL--
-SPVGLV-
SYPVGL--
SGPVGL--
STPVGL--
SKPVGL--
-SPVGLI-
SNPVGL--
SFPVGL--
SVPVGL--
-SPVGLA-
SWPVGL--
-SPVGLT-
-SPVGLM-
SIPVGL--
SAPVGL--
-SPVGLQ-
-SPVGLK-
SHPVGL--
-SPVGLF-
-SPVGLN-
-SPVGLW-
SPPVGL--
SQPVGL--
-SPVGLH-
SDPVGL--
SEPVGL--
-SPVGLE-
-SPVGLG-
SCPVGL--
-SPVGLC-
-TPVGLL-
TRPVGL--
TSPVGL--
TLPVGL--

TGPVGL--
-TPVGLS-
TYPVGL--
TMPVGL--
-TPVGLR-
TKPVGL--
TPPVGL--
-TPVGLK-
-TPVGLT-
TNPVGL--
TAPVGL--
-TPVGLF-
-TPVGLM-
TFPVGL--
-TPVGLY-
-TPVGLV-
-TPVGLN-
TWPVGL--
-TPVGLH-
TIPVGL--
-TPVGLA-
-TPVGLI-
TEPVGL--
TDPVGL--
THPVGL--
-TPVGLQ-
-TPVGLE-
TQPVGL--
-TPVGLW-
-TPVGLG-
-VPVGLR-
VVPVGL--
-VPVGLK-
-VPVGLL-
VSPVGL--
VTPVGL--
-VPVGLA-
VMPVGL--
VQPVGL--
VGPVGL--
-VPVGLY-
-VPVGLH-
VEPVGL--
-VPVGLM-
-VPVGLT-
VPPVGL--
-VPVGLN-
-VPVGLI-
VKPVGL--
-VPVGLV-

VAPVGL--
VCPVGL--
VYPVGL--
VIPVGL--
-VPVGLG-
VHPVGL--
VDPVGL--
VWPVGL--
VFPVGL--
-VPVGLE-
-VPVGLC-
WLPVGL--
WSPVGL--
WNPVGL--
-WPVGLL-
-WPVGLR-
WKPVGL--
-WPVGLY-
-WPVGLS-
-WPVGLI-
-WPVGLT-
-WPVGLN-
WYPVGL--
WFPVGL--
WEPVGL--
WDPVGL--
-WPVGLQ-
-WPVGLA-
WPPVGL--
WVPVGL--
-WPVGLH-
-WPVGLM-
WGPVGL--
-WPVGLK-
-WPVGLF-
-WPVGLV-
-WPVGLE-
WQPVGL--
-YPVGLR-
YAPVGL--
YQPVGL--
YLPVGL--
YGPVGL--
-YPVGLK-
-YPVGLH-
YEPVGL--
YRPVGL--
YTPVGL--
-YPVGLS-
YMPVGL--

YNPVGL--
-YPVGLL-
-YPVGLI-
YDPVGL--
-YPVGLT-
YFPVGL--
-YPVGLA-
-YPVGLV-
-YPVGLM-
YHPVGL--
YKPVGL--
-YPVGLN-
-YPVGLF-
YPPVGL--
YYPVGL--
-YPVGLQ-
-YPVGLE-
YWPVGL--
YCPVGL--

PRGI 202 747 47.20

-DPRGID-
-LPRGIP-
--PRGIPY
--PRGIDV
-RPRGIP-
-SPRGIP-
-VPRGID-
AGPRGI--
AVPRGI--
-APRGIV-
-APRGIR-
-APRGIL-
AKPRGI--
-APRGIY-
AQPRGI--
-APRGIM-
AMPRGI--
-APRGIH-
-APRGIK-
-APRGIQ-
-APRGIA-
AAPRGI--
AIPRGI--
-APRGIT-
-APRGIW-
ADPRGI--
AHPRGI--
-APRGIN-
AEPRGI--
-APRGIG-

-APRGIF-
ACPRGI--
AFPRGI--
-APRGIE-
APPRGI--
CTPRGI--
CQPRGI--
CMPRGI--
CHPRGI--
-CPRGIY-
CCPRGI--
-CPRGIL-
CLPRGI--
CSPRGI--
-CPRGIS-
CGPRGI--
-CPRGIA-
CIPRGI--
CAPRGI--
-CPRGIV-
-CPRGIT-
-CPRGIM-
-CPRGIF-
CWPRGI--
-CPRGIW-
CYPRGI--
-CPRGIK-
-CPRGIE-
CNPRGI--
CKPRGI--
CPPRGI--
CFPRGI--
DSPRGI--
-DPRGIL-
DLPRGI--
-DPRGIS-
-DPRGIR-
-DPRGII-
DTPRGI--
-DPRGIT-
DYPRGI--
DGPRGI--
DAPRGI--
DQPRGI--
-DPRGIV-
-DPRGIH-
DFPRGI--
DRPRGI--
-DPRGIF-
DMPRGI--

DHPRGI--
-DPRGIM-
-DPRGIY-
-DPRGIW-
DKPRGI--
-DPRGIA-
DDPRGI--
-DPRGIQ-
-DPRGIK-
-DPRGIG-
-DPRGIE-
DNPRGI--
-EPRGIH-
ESPRGI--
-EPRGIL-
-EPRGIY-
EVPRGI--
-EPRGIR-
-EPRGIV-
ELPRGI--
EGPRGI--
EWPRGI--
ERPRGI--
-EPRGIW-
-EPRGII-
EIPRGI--
EKPRGI--
-EPRGIS-
EHPRGI--
EMPRGI--
-EPRGIK-
-EPRGIG-
EAPRGI--
ETPRGI--
-EPRGIM-
EEPRGI--
EQPRGI--
-EPRGIT-
EYPRGI--
ENPRGI--
EDPRGI--
EPPRGI--
-FPRGII-
-FPRGIH-
FKPRGI--
FRPRGI--
FQPRGI--
-FPRGIS-
FGPRGI--
-FPRGIF-

FVPRGI--
-FPRGIN-
FAPRGI--
FMPRGI--
FEPRGI--
FDPRGI--
-FPRGIV-
FYPRGI--
FNPRGI--
FIPRGI--
-FPRGIM-
FPPRGI--
-FPRGIK-
-FPRGIE-
-GPRGIS-
-GPRGIL-
GNPRGI--
GRPRGI--
GKPRGI--
-GPRGIF-
-GPRGIT-
-GPRGIV-
-GPRGIY-
GEPRGI--
-GPRGIM-
GQPRGI--
-GPRGIE-
GAPRGI--
-GPRGIQ-
-GPRGIK-
GDPRGI--
-GPRGIN-
-GPRGII-
GIPRGI--
GHPRGI--
GWPRGI--
-GPRGIA-
-GPRGIW-
-GPRGIC-
GCPRGI--
HSPRGI--
-HPRGIS-
-HPRGIA-
HKPRGI--
-HPRGIL-
-HPRGIT-
-HPRGIV-
HNPRGI--
HLPRGI--
-HPRGIK-

HHPRGI--
HYPRGI--
HVPRGI--
-HPRGIR-
HMPRGI--
HTPRGI--
HGPRGI--
-HPRGIN-
HPPRGI--
-HPRGIW-
HQPRGI--
-HPRGIH-
HIPRGI--
-HPRGIY-
-HPRGIM-
-HPRGIQ-
HEPRGI--
HDPRGI--
-HPRGIE-
HCPRGI--
HWPRGI--
-IPRGII-
IIPRGI--
IAPRGI--
-IPRGIN-
IWPRGI--
IRPRGI--
-IPRGIA-
IVPRGI--
-IPRGIH-
-IPRGIY-
ILPRGI--
-IPRGIT-
IKPRGI--
ISPRGI--
-IPRGIG-
-IPRGIM-
IHPRGI--
-IPRGIK-
IGPRGI--
-IPRGIQ-
-IPRGIE-
IDPRGI--
IMPRGI--
IPPRGI--
-IPRGIW-
IQPRGI--
IEPRGI--
KVPRGI--
KPPRGI--

-KPRGIW-
-KPRGIV-
-KPRGIQ-
KEPRGI--
-KPRGIG-
-KPRGIM-
KYPRGI--
-KPRGIA-
KNPRGI--
-KPRGIN-
KQPRGI--
-KPRGIF-
KLPRGI--
KCPRGI--
KTPRGI--
KHPRGI--
-KPRGIE-
-KPRGIP-
-KPRGID-
-LPRGIS-
LNPRGI--
LQPRGI--
LRPRGI--
-LPRGIF-
LHPRGI--
-LPRGIY-
LAPRGI--
LKPRGI--
-LPRGIH-
LEPRGI--
-LPRGIM-
-LPRGIN-
-LPRGIQ-
LYPRGI--
-LPRGIV-
LIPRGI--
-LPRGIW-
-LPRGII-
-LPRGIE-
LCPRGI--
-LPRGIG-
LPPRGI--
-LPRGIC-
-MPRGIS-
MAPRGI--
MVPRGI--
-MPRGIN-
-MPRGIT-
MGPRGI--
MHPRGI--

-MPRGIQ-
MMPRGI--
MYPRGI--
MIPRGI--
MTPRGI--
MQPRGI--
-MPRGIK-
-MPRGII-
-MPRGIH-
-MPRGIG-
-MPRGIE-
-MPRGIW-
MPPRGI--
MDPRGI--
MCPRGI--
-MPRGID-
-NPRGIF-
-NPRGIY-
-NPRGIS-
NFPRGI--
-NPRGIT-
NRPRGI--
NWPRGI--
-NPRGIN-
-NPRGIW-
-NPRGIV-
NDPRGI--
NEPRGI--
NQPRGI--
-NPRGII-
-NPRGIH-
-NPRGIQ-
NKPRGI--
NAPRGI--
-NPRGIA-
NCPRGI--
-NPRGIM-
NTPRGI--
NGPRGI--
-NPRGIE-
NMPRGI--
NHPRGI--
--PRGIRS
--PRGISS
--PRGISI
--PRGIYS
--PRGITG
--PRGIVS
--PRGISG
--PRGIVL

--PRGIRT
--PRGIYV
--PRGIKI
--PRGILH
--PRGIKA
--PRGITV
--PRGIMT
--PRGIYK
--PRGILM
--PRGIAS
--PRGIIN
--PRGIVG
--PRGIGG
--PRGISM
--PRGIIV
--PRGIHL
--PRGIHH
--PRGIWK
--PRGIYG
--PRGITY
--PRGIFV
--PRGISH
--PRGIML
PSPRGI--
--PRGISF
--PRGIMY
--PRGIYE
--PRGILQ
--PRGIFE
--PRGIVI
--PRGITI
--PRGIIK
--PRGIIL
--PRGIWT
--PRGIVA
--PRGIHS
PLPRGI--
--PRGITS
--PRGIKS
--PRGIFQ
PRPRGI--
--PRGIHK
--PRGINT
--PRGIAV
--PRGIYM
--PRGIWD
--PRGISN
--PRGINA
--PRGIGV
--PRGIYT

--PRGIYN
--PRGIVV
--PRGIVT
--PRGIHT
--PRGIFN
--PRGIFG
--PRGIFD
--PRGIRH
--PRGIIA
--PRGIRQ
--PRGIQS
--PRGIAT
--PRGILE
--PRGIKV
PVPRGI--
--PRGISK
--PRGIFH
--PRGIQV
--PRGIIM
--PRGIAH
--PRGIAG
--PRGIHA
--PRGIKK
--PRGIDG
--PRGIAY
--PRGIIW
--PRGIHW
--PRGIAK
--PRGIMG
--PRGILA
--PRGIKN
--PRGIKG
--PRGIHR
--PRGIET
--PRGIYW
--PRGIYH
--PRGIWA
--PRGITK
--PRGIVY
--PRGINY
--PRGIFF
--PRGIYD
--PRGIMD
--PRGITM
--PRGILD
--PRGIHF
--PRGIFM
--PRGIAQ
--PRGIVQ
--PRGISW

--PRGISD
--PRGIRE
--PRGIGT
--PRGIAL
--PRGIYI
--PRGINK
--PRGIFW
--PRGIVK
--PRGISE
--PRGINM
--PRGIHN
PAPRGI--
PYPRGI--
--PRGIKD
--PRGIFA
--PRGIEH
--PRGIAR
--PRGIWV
--PRGIVH
--PRGITQ
--PRGIKE
--PRGIHI
PGPRGI--
--PRGIWW
--PRGIVD
--PRGITW
--PRGIRM
--PRGINH
--PRGIMK
--PRGIAA
--PRGIQT
--PRGIQK
--PRGIHQ
PWPRGI--
--PRGIWE
--PRGITA
--PRGIQA
--PRGIMH
--PRGIKY
--PRGIIQ
--PRGIGF
--PRGIWQ
--PRGIWN
--PRGIQI
--PRGIID
--PRGIEY
--PRGIVW
--PRGITD
--PRGIQD
--PRGIMN

--PRGIFT
--PRGIEA
--PRGIAI
--PRGIMI
--PRGIES
PIPRGI--
--PRGIWF
--PRGIKW
--PRGIKF
--PRGIGY
--PRGIGN
--PRGIEG
--PRGIDA
--PRGIAF
--PRGIYQ
--PRGIQH
--PRGIQG
--PRGIQE
PKPRGI--
PHPRGI--
PEPRGI--
--PRGIVM
--PRGIQF
--PRGINR
--PRGIHG
--PRGIHD
--PRGIGW
--PRGIGI
PNPRGI--
PTPRGI--
--PRGINI
--PRGIKM
--PRGIWM
--PRGINQ
--PRGIMW
--PRGIKQ
--PRGIHY
--PRGIGM
--PRGIEV
--PRGIEN
--PRGIAE
--PRGITF
--PRGIMA
--PRGIEK
--PRGIDS
--PRGIYP
--PRGIIH
--PRGIGH
--PRGICT
--PRGIAN

--PRGIAD
PQPRGI--
--PRGIQW
--PRGIND
--PRGIHE
--PRGICR
--PRGIVE
--PRGINC
--PRGILC
--PRGIIE
--PRGIHC
--PRGIGQ
--PRGIEW
--PRGIEM
--PRGIEF
PCPRGI--
--PRGIYY
--PRGITC
--PRGISC
--PRGIRC
--PRGING
--PRGIMQ
--PRGIMM
--PRGIPQ
--PRGIPH
--PRGIME
--PRGIMC
--PRGIDW
--PRGIDM
--PRGICY
--PRGICW
--PRGICV
--PRGICI
--PRGICA
--PRGIAW
-PPRGIS-
-PPRGIM-
-PPRGIA-
QLPRGI--
-QPRGIL-
-QPRGIS-
-QPRGIT-
QSPRGI--
-QPRGIV-
QFPRGI--
QGPRGI--
QAPRGI--
QTPRGI--
-QPRGIN-
-QPRGIH-

-QPRGIW-
QYPRGI--
QVPRGI--
-QPRGII-
QIPRGI--
QRPRGI--
QDPRGI--
-QPRGIM-
QQPRGI--
-QPRGIQ-
-QPRGIY-
-QPRGIF-
QHPRGI--
-QPRGIR-
-QPRGIA-
-QPRGID-
QPPRGI--
QNPRGI--
QEPRGI--
QCPRGI--
-RPRGIL-
RGPRGI--
RSPRGI--
RPPRGI--
-RPRGIY-
RDPRGI--
REPRGI--
-RPRGIF-
RMPRGI--
-RPRGIV-
-RPRGIQ-
-RPRGIH-
-RPRGIE-
RRPRGI--
RHPRGI--
-RPRGIW-
RNPRGI--
RKPRGI--
RQPRGI--
RCPRGI--
-RPRGIC-
-SPRGIS-
-SPRGIW-
-SPRGIL-
STPRGI--
-SPRGIE-
SFPRGI--
-SPRGIY-
-SPRGIH-
SAPRGI--

-SPRGIF-
SYPRGI--
-SPRGIA-
SIPRGI--
SMPRGI--
-SPRGIQ-
-SPRGIN-
-SPRGIK-
SEPRGI--
-SPRGIM-
-SPRGIV-
SPPRGI--
-SPRGIG-
SCPRGI--
SDPRGI--
-SPRGIC-
-TPRGIR-
TAPRGI--
-TPRGIV-
TRPRGI--
-TPRGIS-
-TPRGIY-
TKPRGI--
TTPRGI--
-TPRGIW-
-TPRGII-
TIPRGI--
TEPRGI--
TYPRGI--
-TPRGIN-
-TPRGIA-
-TPRGIG-
-TPRGIK-
TGPRGI--
-TPRGIM-
TDPRGI--
TQPRGI--
-TPRGIE-
-TPRGIQ-
TCPRGI--
VSPRGI--
VLPRGI--
-VPRGIY-
-VPRGIS-
-VPRGIR-
-VPRGIL-
-VPRGIN-
-VPRGIT-
VTPRGI--
VMPRGI--

−VPRGIK−
VNPRGI−−
VPPRGI−−
−VPRGIM−
VVPRGI−−
−VPRGII−
VWPRGI−−
−VPRGIW−
VDPRGI−−
VQPRGI−−
VIPRGI−−
VEPRGI−−
VYPRGI−−
−VPRGIA−
−VPRGIE−
−VPRGIQ−
VCPRGI−−
WSPRGI−−
WPPRGI−−
WLPRGI−−
WGPRGI−−
WTPRGI−−
WQPRGI−−
−WPRGIL−
WDPRGI−−
WEPRGI−−
−WPRGIY−
−WPRGIM−
−WPRGIN−
−WPRGII−
−WPRGIH−
WMPRGI−−
−WPRGIT−
−WPRGIS−
WIPRGI−−
WKPRGI−−
−WPRGIE−
WCPRGI−−
−WPRGIF−
−YPRGIL−
−YPRGIS−
YLPRGI−−
YIPRGI−−
YTPRGI−−
YPPRGI−−
−YPRGIT−
−YPRGIQ−
−YPRGIF−
−YPRGIM−
YVPRGI−−

	-YPRGIH-		
	YMPRGI--		
	YFPRGI--		
	YAPRGI--		
	YYPRGI--		
	YNPRGI--		
	-YPRGIN-		
	YEPRGI--		
	-YPRGII-		
	YDPRGI--		
	YKPRGI--		
	-YPRGIG-		
	YQPRGI--		
	-YPRGIA-		
	-YPRGIW-		
	-YPRGIC-		
	YCPRGI--		
PFWI	842	523	47.19
	-SPFWIN-		
	-APFWIT-		
	AMPFWI--		
	AAPFWI--		
	AQPFWI--		
	AKPFWI--		
	AGPFWI--		
	-APFWIR-		
	ATPFWI--		
	-APFWIG-		
	AYPFWI--		
	ASPFWI--		
	-APFWIQ-		
	ALPFWI--		
	AVPFWI--		
	AEPFWI--		
	-APFWIK-		
	-APFWII-		
	-APFWIV-		
	-APFWID-		
	-APFWIC-		
	CQPFWI--		
	DRPFWI--		
	DSPFWI--		
	DAPFWI--		
	-DPFWIK-		
	DLPFWI--		
	DTPFWI--		
	-DPFWIR-		
	-DPFWIF-		
	DNPFWI--		
	-DPFWIT-		

DMPFWI--
DFPFWI--
DHPFWI--
DDPFWI--
DQPFWI--
-DPFWIS-
-DPFWIV-
-DPFWIL-
-DPFWIH-
DGPFWI--
EAPFWI--
ETPFWI--
-EPFWIR-
ERPFWI--
-EPFWIT-
-EPFWIH-
EKPFWI--
-EPFWIG-
EMPFWI--
EGPFWI--
EVPFWI--
-EPFWIY-
-EPFWIV-
-EPFWIL-
EHPFWI--
EWPFWI--
-EPFWIS-
-EPFWIA-
EFPFWI--
ESPFWI--
-EPFWIC-
ELPFWI--
EIPFWI--
-FPFWIK-
FGPFWI--
FVPFWI--
FIPFWI--
FEPFWI--
FRPFWI--
FFPFWI--
-FPFWIR-
-FPFWIF-
FLPFWI--
FHPFWI--
-FPFWIS-
-FPFWIH-
FCPFWI--
FAPFWI--
GKPFWI--
GSPFWI--

GEPFWI--
GTPFWI--
GGPFWI--
GRPFWI--
GQPFWI--
-GPFWIH-
-GPFWIV-
GLPFWI--
GYPFWI--
-GPFWIQ-
GMPFWI--
GVPFWI--
GAPFWI--
-GPFWIT-
-GPFWIM-
GIPFWI--
GDPFWI--
-GPFWIR-
GHPFWI--
-GPFWIS-
-GPFWIE-
GPPFWI--
-GPFWII-
HSPFWI--
-HPFWIY-
HKPFWI--
-HPFWIA-
-HPFWIR-
HNPFWI--
-HPFWIV-
HIPFWI--
-HPFWIK-
-HPFWII-
HMPFWI--
HLPFWI--
-HPFWIQ-
-HPFWIE-
HVPFWI--
HQPFWI--
-HPFWIS-
-HPFWIH-
HRPFWI--
HEPFWI--
INPFWI--
IKPFWI--
IRPFWI--
IPPFWI--
-IPFWIA-
IHPFWI--
IVPFWI--

IDPFWI--
ITPFWI--
-IPFWIN-
-IPFWIG-
-IPFWIY-
IIPFWI--
IAPFWI--
-IPFWIS-
-IPFWIR-
-IPFWIL-
-IPFWID-
ILPFWI--
ISPFWI--
IQPFWI--
-IPFWIQ-
IFPFWI--
KSPFWI--
KLPFWI--
KKPFWI--
KQPFWI--
KYPFWI--
KRPFWI--
KPPFWI--
KVPFWI--
KNPFWI--
KHPFWI--
KIPFWI--
-KPFWIH-
KFPFWI--
KTPFWI--
KGPFWI--
KDPFWI--
-KPFWIS-
-KPFWIY-
-KPFWII-
-KPFWIF-
-KPFWIA-
LEPFWI--
LAPFWI--
LGPFWI--
LVPFWI--
LRPFWI--
LHPFWI--
LDPFWI--
LYPFWI--
-LPFWIS-
LKPFWI--
-LPFWIT-
-LPFWIG-
LPPFWI--

-LPFWIK-
-LPFWII-
-LPFWIH-
LIPFWI--
LQPFWI--
LLPFWI--
-LPFWIW-
-LPFWIQ-
-LPFWIF-
-LPFWIE-
LMPFWI--
MRPFWI--
MVPFWI--
MLPFWI--
MGPFWI--
MIPFWI--
MNPFWI--
-MPFWIT-
MQPFWI--
-MPFWIW-
-MPFWIF-
MYPFWI--
-MPFWIS-
MWPFWI--
-MPFWIN-
MHPFWI--
-NPFWIR-
NLPFWI--
-NPFWIT-
-NPFWIK-
NEPFWI--
NRPFWI--
-NPFWIV-
NNPFWI--
NWPFWI--
NVPFWI--
NSPFWI--
NHPFWI--
NPPFWI--
-NPFWIH-
NMPFWI--
NIPFWI--
NTPFWI--
-NPFWIG-
NKPFWI--
NDPFWI--
PGPFWI--
--PFWIVK
--PFWIRD
--PFWIRV

```
--PFWISS
--PFWIRT
--PFWIIQ
--PFWIGA
--PFWIHS
PEPFWI--
--PFWIRQ
--PFWIRN
--PFWILK
--PFWIIR
--PFWIMA
--PFWIVS
--PFWIST
--PFWIRE
--PFWIIG
--PFWIVG
--PFWISK
--PFWIQK
PHPFWI--
--PFWIRA
--PFWIMS
--PFWIAR
--PFWIVE
--PFWITQ
--PFWINS
--PFWIMY
PDPFWI--
--PFWIYR
--PFWITR
--PFWITG
--PFWISH
--PFWIRG
--PFWIQG
--PFWINN
--PFWIKM
--PFWIGW
--PFWIGL
--PFWIGG
--PFWIET
--PFWIES
--PFWITH
--PFWIMR
--PFWIKY
--PFWIIT
--PFWIIS
--PFWIHE
--PFWIVA
--PFWISR
--PFWISD
--PFWIKA
```

PNPFWI--
--PFWIVR
--PFWISI
--PFWISA
--PFWIMH
--PFWIKT
--PFWIKL
--PFWIGR
--PFWIGQ
--PFWIAH
--PFWIAG
--PFWIYN
--PFWIVN
--PFWITV
--PFWINT
--PFWINM
--PFWINA
--PFWILR
--PFWIKR
--PFWIKF
--PFWIHM
--PFWIHG
--PFWIHA
--PFWIGH
--PFWIFQ
--PFWIAQ
--PFWIWS
--PFWIWH
--PFWIWA
--PFWITM
--PFWITK
--PFWISE
--PFWIRY
--PFWIQS
--PFWIQD
--PFWIMK
--PFWIMD
--PFWILH
--PFWIKG
--PFWIIN
--PFWIID
--PFWIGT
--PFWIGS
--PFWIGD
--PFWIFN
--PFWIFK
--PFWIEV
--PFWIEK
--PFWIDT
--PFWIDG

-PPFWIN-
-PPFWIG-
--PFWIYL
--PFWIVH
--PFWIVD
--PFWITT
--PFWISW
--PFWISN
--PFWISG
--PFWIRL
--PFWIQY
--PFWIPS
--PFWINY
--PFWINV
--PFWINQ
--PFWINK
--PFWINH
--PFWILY
--PFWIKS
--PFWIHW
--PFWIHQ
--PFWIHK
--PFWIHH
--PFWIGY
--PFWIFT
--PFWIFS
--PFWIEY
--PFWIER
--PFWIAT
QVPFWI--
QMPFWI--
QAPFWI--
-QPFWIV-
QDPFWI--
QKPFWI--
QIPFWI--
QHPFWI--
QLPFWI--
-QPFWIK-
-QPFWIR-
-QPFWIA-
QEPFWI--
QRPFWI--
QQPFWI--
-QPFWIG-
QGPFWI--
QWPFWI--
QSPFWI--
-QPFWIW-
-QPFWIT-

-QPFWIH-
RNPFWI--
RVPFWI--
RSPFWI--
-RPFWIK-
RRPFWI--
-RPFWIS-
RLPFWI--
RTPFWI--
-RPFWIG-
RMPFWI--
-RPFWIR-
RHPFWI--
-RPFWIM-
-RPFWIA-
REPFWI--
RAPFWI--
RDPFWI--
-RPFWII-
RGPFWI--
RWPFWI--
RQPFWI--
-RPFWIN-
-RPFWIE-
RYPFWI--
-RPFWIV-
-RPFWIT-
RFPFWI--
-RPFWIH-
-SPFWIR-
SRPFWI--
SSPFWI--
SVPFWI--
SGPFWI--
STPFWI--
SFPFWI--
SYPFWI--
-SPFWIT-
SNPFWI--
-SPFWIV-
SMPFWI--
-SPFWIH-
SAPFWI--
-SPFWIY-
-SPFWIQ-
-SPFWIF-
-SPFWIE-
SDPFWI--
SIPFWI--
SQPFWI--

SPPFWI--
-SPFWII-
TMPFWI--
TSPFWI--
TTPFWI--
-TPFWIK-
TRPFWI--
THPFWI--
TKPFWI--
TFPFWI--
TVPFWI--
-TPFWIS-
TIPFWI--
TEPFWI--
TYPFWI--
TQPFWI--
-TPFWIV-
-TPFWIG-
-TPFWIA-
TAPFWI--
-TPFWIY-
-TPFWIT-
-TPFWIR-
-TPFWIQ-
TLPFWI--
-TPFWIF-
TGPFWI--
TDPFWI--
-TPFWIW-
-TPFWIH-
-TPFWID-
-VPFWIR-
VSPFWI--
VLPFWI--
VEPFWI--
VTPFWI--
VMPFWI--
VKPFWI--
VHPFWI--
-VPFWIQ-
VAPFWI--
VVPFWI--
-VPFWIT-
-VPFWIA-
-VPFWIS-
-VPFWIG-
VRPFWI--
VPPFWI--
-VPFWIM-
VYPFWI--

-VPFWIY-
VGPFWI--
-VPFWIL-
-VPFWII-
VWPFWI--
VNPFWI--
-VPFWIN-
VIPFWI--
VFPFWI--
WRPFWI--
WHPFWI--
WTPFWI--
WSPFWI--
-WPFWIS-
-WPFWIR-
WEPFWI--
-WPFWIL-
WQPFWI--
-WPFWIN-
WMPFWI--
WLPFWI--
WKPFWI--
WAPFWI--
-YPFWIR-
YKPFWI--
-YPFWIS-
-YPFWIM-
YIPFWI--
YSPFWI--
YEPFWI--
YTPFWI--
YGPFWI--
YNPFWI--
YDPFWI--
YVPFWI--
YPPFWI--
YRPFWI--
-YPFWIW-
-YPFWIA-
YLPFWI--
YAPFWI--
PIAL 217 737 47.18
GEPIAL--
-LPIALG-
--PIALRP
--PIALGR
--PIALGN
--PIALPS
--PIALLP
-YPIALG-

AGPIAL--
ANPIAL--
-APIALL-
ASPIAL--
ALPIAL--
ARPIAL--
AFPIAL--
AYPIAL--
-APIALT-
AMPIAL--
AHPIAL--
AKPIAL--
-APIALM-
AAPIAL--
-APIALS-
-APIALV-
AQPIAL--
-APIALH-
-APIALG-
-APIALK-
AEPIAL--
AIPIAL--
ADPIAL--
-APIALA-
-APIALN-
AWPIAL--
APPIAL--
ATPIAL--
-APIALQ-
ACPIAL--
-APIALE-
CLPIAL--
CQPIAL--
CVPIAL--
CTPIAL--
CRPIAL--
-CPIALL-
CSPIAL--
-CPIALW-
-CPIALY-
-CPIALN-
CNPIAL--
CMPIAL--
CGPIAL--
CEPIAL--
DSPIAL--
DKPIAL--
DMPIAL--
DLPIAL--
DTPIAL--

DIPIAL--
DDPIAL--
-DPIALH-
DYPIAL--
DVPIAL--
-DPIALS-
DAPIAL--
-DPIALI-
DRPIAL--
-DPIALL-
DGPIAL--
DFPIAL--
DEPIAL--
-DPIALV-
-DPIALK-
-DPIALT-
-DPIALQ-
-DPIALM-
DNPIAL--
-DPIALN-
-EPIALV-
-EPIALR-
EYPIAL--
EAPIAL--
ESPIAL--
EQPIAL--
-EPIALT-
-EPIALN-
EGPIAL--
-EPIALY-
-EPIALL-
ENPIAL--
EFPIAL--
EVPIAL--
-EPIALM-
EMPIAL--
EWPIAL--
-EPIALW-
EKPIAL--
ETPIAL--
EEPIAL--
-EPIALQ-
-EPIALI-
ELPIAL--
ERPIAL--
-EPIALK-
-EPIALH-
-EPIALG-
-EPIALA-
-EPIALS-

-EPIALE-
EIPIAL--
EHPIAL--
FSPIAL--
-FPIALT-
-FPIALR-
-FPIALK-
FEPIAL--
FGPIAL--
FTPIAL--
FQPIAL--
FAPIAL--
-FPIALA-
FNPIAL--
FIPIAL--
-FPIALQ-
-FPIALM-
FMPIAL--
FHPIAL--
-FPIALH-
-FPIALG-
-FPIALN-
FKPIAL--
-FPIALE-
-GPIALL-
-GPIALR-
GYPIAL--
GWPIAL--
GKPIAL--
GNPIAL--
-GPIALT-
-GPIALS-
GSPIAL--
GLPIAL--
-GPIALA-
GDPIAL--
GAPIAL--
GTPIAL--
GQPIAL--
GHPIAL--
-GPIALK-
GGPIAL--
GIPIAL--
-GPIALM-
-GPIALV-
-GPIALE-
-GPIALG-
-GPIALQ-
-GPIALH-
-GPIALN-

GPPIAL--
GCPIAL--
HVPIAL--
-HPIALT-
HMPIAL--
HLPIAL--
HAPIAL--
HGPIAL--
-HPIALR-
HIPIAL--
HTPIAL--
HKPIAL--
HEPIAL--
-HPIALL-
HWPIAL--
-HPIALW-
-HPIALS-
-HPIALK-
-HPIALQ-
-HPIALI-
-HPIALE-
HPPIAL--
-HPIALM-
-HPIALH-
-HPIALA-
HDPIAL--
-HPIALV-
-HPIALN-
-HPIALG-
HNPIAL--
HHPIAL--
HYPIAL--
HQPIAL--
-IPIALR-
-IPIALT-
ISPIAL--
ILPIAL--
IVPIAL--
-IPIALS-
IGPIAL--
-IPIALA-
-IPIALQ-
IAPIAL--
IKPIAL--
IQPIAL--
-IPIALK-
IEPIAL--
-IPIALH-
-IPIALV-
IYPIAL--

```
-IPIALG-
IIPIAL--
IPPIAL--
-IPIALN-
IFPIAL--
-IPIALW-
-IPIALL-
-IPIALE-
IMPIAL--
KSPIAL--
-KPIALS-
KGPIAL--
KLPIAL--
KRPIAL--
-KPIALV-
KFPIAL--
KYPIAL--
KTPIAL--
-KPIALM-
-KPIALK-
KKPIAL--
KVPIAL--
-KPIALN-
KNPIAL--
-KPIALR-
KIPIAL--
-KPIALW-
KWPIAL--
KPPIAL--
KEPIAL--
KCPIAL--
KHPIAL--
-KPIALG-
KMPIAL--
-KPIALT-
-KPIALH-
-KPIALQ-
KDPIAL--
KQPIAL--
-KPIALE-
LNPIAL--
LLPIAL--
LVPIAL--
LGPIAL--
-LPIALT-
LYPIAL--
-LPIALR-
LHPIAL--
LPPIAL--
LKPIAL--
```

-LPIALL-
LEPIAL--
LAPIAL--
-LPIALH-
-LPIALI-
-LPIALM-
LDPIAL--
-LPIALN-
-LPIALA-
-LPIALS-
-LPIALE-
-LPIALQ-
LCPIAL--
MRPIAL--
-MPIALR-
-MPIALH-
MHPIAL--
MGPIAL--
MSPIAL--
-MPIALL-
MTPIAL--
MMPIAL--
MVPIAL--
-MPIALT-
-MPIALN-
MYPIAL--
-MPIALK-
MKPIAL--
MFPIAL--
-MPIALS-
-MPIALM-
MIPIAL--
-MPIALA-
MAPIAL--
MQPIAL--
-MPIALG-
MEPIAL--
MDPIAL--
MCPIAL--
-MPIALQ-
-MPIALE-
MNPIAL--
NRPIAL--
NIPIAL--
NAPIAL--
NHPIAL--
NTPIAL--
-NPIALH-
NLPIAL--
NKPIAL--

NPPIAL--
NSPIAL--
NYPIAL--
NEPIAL--
-NPIALL-
-NPIALT-
-NPIALK-
NVPIAL--
NGPIAL--
NDPIAL--
-NPIALW-
NQPIAL--
-NPIALR-
-NPIALF-
NWPIAL--
-NPIALV-
NNPIAL--
-NPIALM-
-NPIALA-
-NPIALQ-
-NPIALS-
-NPIALN-
-NPIALE-
NMPIAL--
--PIALRG
--PIALTR
--PIALGL
--PIALGM
--PIALGS
--PIALRY
--PIALRK
--PIALRH
--PIALKG
--PIALKR
--PIALRN
--PIALLN
PFPIAL--
--PIALST
--PIALRD
--PIALNV
--PIALSV
--PIALTS
--PIALSA
--PIALSQ
--PIALIG
--PIALHS
--PIALGI
--PIALTA
--PIALRT
--PIALRE

--PIALNL
--PIALQG
--PIALSG
--PIALAL
PWPIAL--
--PIALTY
--PIALSS
--PIALSK
--PIALMH
--PIALKF
--PIALVQ
--PIALTV
--PIALQM
PQPIAL--
--PIALQS
--PIALKL
--PIALKH
--PIALHL
--PIALSM
PGPIAL--
PAPIAL--
--PIALWN
--PIALVG
--PIALTN
--PIALTK
--PIALLD
--PIALIN
--PIALHW
--PIALHQ
--PIALGT
PEPIAL--
PIPIAL--
--PIALNM
PHPIAL--
--PIALRM
--PIALQV
--PIALGV
--PIALTG
--PIALSF
--PIALKK
--PIALTQ
--PIALMS
--PIALVN
--PIALNR
--PIALME
--PIALHG
--PIALPY
--PIALNG
--PIALMA
--PIALHN

--PIALGA
--PIALFG
--PIALAS
--PIALTH
--PIALSW
--PIALES
--PIALWH
--PIALMR
--PIALHK
--PIALVM
--PIALIH
--PIALSH
--PIALMD
--PIALHF
PTPIAL--
--PIALYA
--PIALWD
--PIALVH
--PIALQL
--PIALPI
--PIALNE
--PIALLG
--PIALHV
--PIALGH
--PIALET
--PIALEL
--PIALAN
--PIALAG
--PIALTI
--PIALTD
--PIALQT
--PIALQN
--PIALPL
--PIALKE
--PIALIF
--PIALIA
--PIALAT
PNPIAL--
--PIALTM
--PIALTF
--PIALQI
--PIALNT
--PIALMT
--PIALMI
--PIALMG
--PIALKT
--PIALHI
--PIALGF
--PIALAH
--PIALSE

--PIALRW
--PIALQA
--PIALPF
--PIALHM
--PIALGQ
--PIALGG
--PIALAW
--PIALTT
--PIALQE
--PIALMQ
--PIALKN
--PIALHA
--PIALQY
--PIALQW
--PIALMN
--PIALMK
--PIALKY
--PIALHR
--PIALGY
--PIALEN
--PIALEA
--PIALDY
PDPIAL--
--PIALSI
--PIALRQ
--PIALQH
--PIALPM
--PIALNW
--PIALNN
--PIALNK
--PIALMF
--PIALKQ
--PIALHH
--PIALFD
--PIALDS
--PIALCA
--PIALNI
--PIALNA
--PIALLH
--PIALHY
--PIALFW
--PIALER
--PIALEH
PMPIAL--
--PIALRC
--PIALQK
--PIALQD
--PIALLE
--PIALKV
--PIALKM

--PIALHT
--PIALHE
--PIALEM
--PIALEG
--PIALDM
--PIALAD
--PIALYP
--PIALSD
--PIALND
--PIALGK
--PIALEY
--PIALDL
--PIALCI
--PIALCD
--PIALVD
--PIALTC
--PIALQQ
--PIALQP
--PIALQF
--PIALPQ
--PIALNQ
--PIALKI
--PIALKD
--PIALKC
--PIALHD
--PIALFI
--PIALEV
--PIALEK
--PIALEF
--PIALDQ
--PIALDG
--PIALDA
--PIALCT
--PIALAK
--PIALAC
-QPIALR-
QGPIAL--
QVPIAL--
QAPIAL--
QLPIAL--
QTPIAL--
-QPIALL-
QMPIAL--
-QPIALN-
-QPIALH-
QWPIAL--
QSPIAL--
-QPIALM-
-QPIALS-
QKPIAL--

-QPIALE-
QEPIAL--
QQPIAL--
QPPIAL--
-QPIALI-
-QPIALA-
QHPIAL--
-QPIALT-
-QPIALQ-
-QPIALG-
QDPIAL--
QYPIAL--
-QPIALC-
QFPIAL--
RLPIAL--
RGPIAL--
RTPIAL--
-RPIALR-
RSPIAL--
-RPIALS-
RKPIAL--
RPPIAL--
RIPIAL--
RVPIAL--
RFPIAL--
RAPIAL--
-RPIALM-
RMPIAL--
RDPIAL--
RNPIAL--
RYPIAL--
-RPIALN-
-RPIALQ-
-RPIALI-
REPIAL--
-RPIALV-
RHPIAL--
-RPIALA-
-RPIALG-
RWPIAL--
-RPIALE-
RCPIAL--
SLPIAL--
SVPIAL--
SNPIAL--
SGPIAL--
-SPIALR-
-SPIALL-
-SPIALT-
-SPIALS-

-SPIALV-
-SPIALQ-
SFPIAL--
SAPIAL--
-SPIALK-
SEPIAL--
STPIAL--
SKPIAL--
SSPIAL--
-SPIALI-
SHPIAL--
-SPIALM-
SMPIAL--
-SPIALH-
SWPIAL--
SIPIAL--
SQPIAL--
-SPIALN-
SPPIAL--
-SPIALE-
SDPIAL--
-SPIALG-
-SPIALC-
SCPIAL--
-TPIALR-
TRPIAL--
-TPIALL-
TAPIAL--
TSPIAL--
TYPIAL--
TPPIAL--
-TPIALS-
TFPIAL--
TLPIAL--
-TPIALA-
TWPIAL--
TVPIAL--
-TPIALI-
-TPIALQ-
TGPIAL--
TNPIAL--
-TPIALK-
-TPIALE-
TKPIAL--
TEPIAL--
TTPIAL--
-TPIALN-
THPIAL--
-TPIALM-
TQPIAL--

TDPIAL--
-TPIALG-
VKPIAL--
VLPIAL--
-VPIALL-
VSPIAL--
-VPIALT-
-VPIALR-
VTPIAL--
-VPIALS-
VNPIAL--
-VPIALM-
-VPIALH-
-VPIALK-
VPPIAL--
VQPIAL--
VDPIAL--
VWPIAL--
VFPIAL--
VMPIAL--
VGPIAL--
VYPIAL--
-VPIALN-
-VPIALI-
VAPIAL--
VHPIAL--
VCPIAL--
-VPIALV-
-VPIALQ-
-VPIALG-
-VPIALE-
-VPIALA-
WLPIAL--
-WPIALL-
WAPIAL--
-WPIALR-
WGPIAL--
WFPIAL--
WHPIAL--
WPPIAL--
WQPIAL--
-WPIALK-
-WPIALQ-
WDPIAL--
-WPIALN-
-WPIALM-
-WPIALH-
-WPIALA-
WEPIAL--
-WPIALS-

	-WPIALG-		
	-WPIALE-		
	YRPIAL--		
	YSPIAL--		
	-YPIALH-		
	-YPIALR-		
	YFPIAL--		
	YTPIAL--		
	-YPIALT-		
	YHPIAL--		
	YYPIAL--		
	YPPIAL--		
	YLPIAL--		
	YKPIAL--		
	YVPIAL--		
	YNPIAL--		
	YDPIAL--		
	-YPIALA-		
	YAPIAL--		
	YMPIAL--		
	-YPIALV-		
	YEPIAL--		
	-YPIALK-		
	YIPIAL--		
	YGPIAL--		
	YQPIAL--		
	-YPIALW-		
	-YPIALN-		
	-YPIALE-		
PLEM	270	712	47.16
	-IPLEMT-		
	-LPLEMS-		
	-APLEMH-		
	-APLEMN-		
	-APLEMW-		
	-APLEMT-		
	-APLEML-		
	ALPLEM--		
	AHPLEM--		
	-APLEMV-		
	ASPLEM--		
	AFPLEM--		
	ARPLEM--		
	-APLEMS-		
	-APLEMK-		
	-APLEMF-		
	ATPLEM--		
	AGPLEM--		
	ANPLEM--		
	AKPLEM--		

AVPLEM--
-APLEMA-
-APLEMG-
-APLEMM-
AIPLEM--
AEPLEM--
-APLEME-
ACPLEM--
APPLEM--
-APLEMR-
ADPLEM--
CRPLEM--
CLPLEM--
CTPLEM--
CSPLEM--
-CPLEMV-
-CPLEMN-
CGPLEM--
CPPLEM--
-CPLEMH-
CVPLEM--
CFPLEM--
CCPLEM--
-DPLEMR-
-DPLEMW-
DRPLEM--
-DPLEMI-
DSPLEM--
-DPLEMS-
DHPLEM--
DGPLEM--
DTPLEM--
DVPLEM--
-DPLEMT-
DMPLEM--
DAPLEM--
-DPLEMY-
-DPLEMN-
-DPLEMF-
DNPLEM--
DLPLEM--
DIPLEM--
DDPLEM--
ESPLEM--
ERPLEM--
-EPLEMR-
-EPLEMN-
-EPLEML-
-EPLEMW-
-EPLEMK-

EGPLEM--
EPPLEM--
-EPLEMV-
-EPLEMQ-
-EPLEMM-
-EPLEMI-
EKPLEM--
EAPLEM--
-EPLEMA-
ETPLEM--
-EPLEMT-
-EPLEMS-
-EPLEMC-
ENPLEM--
EMPLEM--
ELPLEM--
FGPLEM--
-FPLEMH-
-FPLEMR-
-FPLEMS-
FAPLEM--
FRPLEM--
FNPLEM--
FQPLEM--
-FPLEMW-
FIPLEM--
FPPLEM--
FFPLEM--
-FPLEMM-
FSPLEM--
-FPLEMV-
FVPLEM--
FTPLEM--
-FPLEML-
-FPLEMI-
FKPLEM--
-FPLEMQ-
-FPLEMF-
FDPLEM--
-FPLEMN-
-FPLEMG-
FWPLEM--
-FPLEMK-
-FPLEMA-
FLPLEM--
FHPLEM--
FCPLEM--
GRPLEM--
-GPLEMR-
GQPLEM--

GVPLEM--
-GPLEMT-
-GPLEMH-
GMPLEM--
GAPLEM--
-GPLEMI-
-GPLEMW-
GTPLEM--
-GPLEMS-
-GPLEMA-
GKPLEM--
GIPLEM--
GGPLEM--
GYPLEM--
-GPLEMY-
-GPLEMV-
GLPLEM--
GSPLEM--
-GPLEMG-
GDPLEM--
GFPLEM--
-GPLEMN-
-GPLEML-
-GPLEMQ-
GPPLEM--
GNPLEM--
GEPLEM--
HVPLEM--
-HPLEMH-
-HPLEMN-
HTPLEM--
-HPLEMY-
-HPLEMK-
-HPLEMG-
-HPLEMQ-
HNPLEM--
HGPLEM--
HYPLEM--
-HPLEMR-
-HPLEMT-
HWPLEM--
-HPLEMW-
-HPLEMS-
-HPLEMA-
HLPLEM--
HEPLEM--
-HPLEMV-
-HPLEMC-
HIPLEM--
HPPLEM--

-HPLEML-
IRPLEM--
IVPLEM--
-IPLEMR-
-IPLEMV-
-IPLEMM-
IQPLEM--
-IPLEMF-
-IPLEMK-
-IPLEMW-
IWPLEM--
-IPLEMQ-
ITPLEM--
ILPLEM--
-IPLEMN-
-IPLEML-
-IPLEMI-
INPLEM--
IKPLEM--
IGPLEM--
IAPLEM--
IPPLEM--
-IPLEMG-
-IPLEMA-
IEPLEM--
IYPLEM--
ISPLEM--
-IPLEMS-
-IPLEME-
IMPLEM--
IIPLEM--
IFPLEM--
-KPLEML-
-KPLEMS-
-KPLEMY-
-KPLEMR-
-KPLEMH-
-KPLEMF-
KVPLEM--
KRPLEM--
-KPLEMV-
-KPLEMW-
KKPLEM--
KIPLEM--
-KPLEMG-
KGPLEM--
KTPLEM--
-KPLEMQ-
-KPLEMN-
KPPLEM--

-KPLEMK-
KMPLEM--
-KPLEMI-
-KPLEMA-
-KPLEMT-
KFPLEM--
KNPLEM--
KQPLEM--
KLPLEM--
LGPLEM--
-LPLEMR-
LQPLEM--
LLPLEM--
LFPLEM--
-LPLEMT-
-LPLEMN-
-LPLEML-
LKPLEM--
LVPLEM--
LIPLEM--
-LPLEMH-
LMPLEM--
LPPLEM--
-LPLEMK-
LNPLEM--
LWPLEM--
LTPLEM--
-LPLEMQ-
LHPLEM--
-LPLEMV-
-LPLEMA-
LSPLEM--
-LPLEMM-
LEPLEM--
LCPLEM--
LAPLEM--
MRPLEM--
-MPLEML-
-MPLEMR-
MKPLEM--
-MPLEMS-
-MPLEMN-
MNPLEM--
MAPLEM--
-MPLEMK-
-MPLEMF-
MVPLEM--
MSPLEM--
MYPLEM--
-MPLEMQ-

-MPLEMH-
MGPLEM--
-MPLEMT-
-MPLEMG-
MLPLEM--
MWPLEM--
MQPLEM--
-MPLEMV-
-MPLEMM-
-MPLEMI-
NRPLEM--
-NPLEMR-
-NPLEMI-
NKPLEM--
NSPLEM--
-NPLEMY-
-NPLEML-
NHPLEM--
NTPLEM--
-NPLEMS-
NFPLEM--
NYPLEM--
-NPLEMV-
NGPLEM--
NNPLEM--
NIPLEM--
NEPLEM--
-NPLEMN-
-NPLEMK-
-NPLEMH-
NLPLEM--
NAPLEM--
NWPLEM--
NVPLEM--
-NPLEMT-
-NPLEMQ-
NPPLEM--
-NPLEMG-
--PLEMRL
--PLEMRR
--PLEMVS
--PLEMVW
--PLEMLT
--PLEMSS
--PLEMRY
--PLEMLS
--PLEMWS
--PLEMSR
--PLEMRW
--PLEMWA

--PLEMSQ
--PLEMSG
--PLEMLR
--PLEMLG
--PLEMKM
--PLEMRG
--PLEMSA
--PLEMKS
--PLEMAT
--PLEMSM
--PLEMRA
--PLEMVK
--PLEMTK
--PLEMIS
--PLEMFS
--PLEMMS
--PLEMKL
--PLEMYS
--PLEMWG
--PLEMSK
--PLEMQT
--PLEMNA
--PLEMKR
--PLEMKG
--PLEMIN
--PLEMIG
--PLEMWR
--PLEMVN
--PLEMVG
--PLEMSL
--PLEMSH
--PLEMRT
--PLEMNR
--PLEMMQ
--PLEMML
--PLEMMH
--PLEMLH
--PLEMKY
--PLEMGA
--PLEMVQ
--PLEMTW
--PLEMQK
--PLEMMA
--PLEMKV
--PLEMII
--PLEMGS
--PLEMWE
--PLEMRD
--PLEMLQ
--PLEMAV

--PLEMYA
--PLEMVR
--PLEMSW
--PLEMRV
--PLEMLN
--PLEMKA
--PLEMGV
--PLEMAW
--PLEMTM
--PLEMSF
--PLEMQR
--PLEMNL
--PLEMNG
--PLEMMD
--PLEMLK
--PLEMIW
--PLEMHS
--PLEMTL
--PLEMTG
--PLEMNW
--PLEMNV
--PLEMKW
--PLEMIA
--PLEMHF
--PLEMYN
--PLEMVV
--PLEMTV
--PLEMSV
--PLEMQW
--PLEMQQ
--PLEMMN
--PLEMIV
--PLEMIQ
--PLEMYQ
--PLEMWF
--PLEMTT
--PLEMTQ
--PLEMTN
--PLEMSN
--PLEMRN
--PLEMRE
--PLEMQV
--PLEMQS
--PLEMQL
--PLEMQH
--PLEMKE
--PLEMIT
--PLEMIK
--PLEMHR
--PLEMHG

--PLEMEW
--PLEMES
--PLEMWN
--PLEMWD
--PLEMNH
--PLEMMI
--PLEMKF
--PLEMHI
--PLEMGI
--PLEMGG
--PLEMFY
--PLEMFF
--PLEMCS
--PLEMAS
--PLEMAG
--PLEMYY
--PLEMYK
--PLEMWI
--PLEMTY
--PLEMTR
--PLEMRK
--PLEMNT
--PLEMNS
--PLEMNE
--PLEMND
--PLEMLE
--PLEMKH
--PLEMHY
--PLEMHV
--PLEMGT
--PLEMGN
--PLEMGM
--PLEMFG
--PLEMAQ
PGPLEM--
--PLEMYR
--PLEMWQ
--PLEMVH
--PLEMVD
--PLEMSI
--PLEMSE
--PLEMRQ
--PLEMRM
--PLEMRI
--PLEMRC
--PLEMQE
--PLEMNY
--PLEMMV
--PLEMMK
--PLEMMG

--PLEMKK
--PLEMIE
--PLEMID
--PLEMHN
--PLEMFH
--PLEMAR
--PLEMAN
--PLEMAH
PCPLEM--
-PPLEMN-
--PLEMWV
--PLEMTE
--PLEMTD
--PLEMST
--PLEMSD
--PLEMSC
--PLEMRH
--PLEMQI
--PLEMNQ
--PLEMNI
--PLEMMT
--PLEMKQ
--PLEMKN
--PLEMHQ
--PLEMHH
--PLEMHA
--PLEMGR
--PLEMGH
--PLEMGF
--PLEMGD
--PLEMET
--PLEMEG
--PLEMEA
--PLEMCT
--PLEMAF
--PLEMAA
PDPLEM--
QRPLEM--
QVPLEM--
-QPLEMV-
-QPLEMR-
QNPLEM--
QYPLEM--
QTPLEM--
QWPLEM--
-QPLEMS-
-QPLEML-
-QPLEMH-
-QPLEMW-
-QPLEMT-

-QPLEMA-
QLPLEM--
-QPLEMK-
-QPLEMI-
-QPLEMG-
QIPLEM--
-QPLEMY-
QHPLEM--
QPPLEM--
QMPLEM--
QGPLEM--
RLPLEM--
-RPLEML-
-RPLEMQ-
RRPLEM--
-RPLEMW-
RVPLEM--
-RPLEMT-
-RPLEMR-
RSPLEM--
-RPLEMK-
-RPLEMH-
RGPLEM--
RPPLEM--
-RPLEMS-
-RPLEMG-
REPLEM--
RIPLEM--
-RPLEMN-
-RPLEMI-
RFPLEM--
RYPLEM--
-RPLEMM-
RQPLEM--
-RPLEMF-
-RPLEMA-
RTPLEM--
-RPLEMV-
RWPLEM--
RKPLEM--
-RPLEME-
RHPLEM--
RDPLEM--
RAPLEM--
RCPLEM--
-RPLEMD-
-RPLEMC-
RNPLEM--
-SPLEMI-
-SPLEMR-

-SPLEMS-
-SPLEML-
SRPLEM--
-SPLEMV-
SMPLEM--
SIPLEM--
SLPLEM--
SSPLEM--
-SPLEMW-
SAPLEM--
SVPLEM--
-SPLEMH-
SKPLEM--
SPPLEM--
SFPLEM--
-SPLEMN-
SDPLEM--
SHPLEM--
-SPLEMM-
-SPLEMT-
-SPLEMK-
SNPLEM--
SWPLEM--
SQPLEM--
STPLEM--
-SPLEMF-
-SPLEMA-
SGPLEM--
TVPLEM--
TGPLEM--
-TPLEMV-
TSPLEM--
TRPLEM--
-TPLEMR-
-TPLEMF-
TWPLEM--
-TPLEMS-
-TPLEMW-
-TPLEMT-
-TPLEMN-
-TPLEMM-
TAPLEM--
TKPLEM--
TFPLEM--
-TPLEMK-
TNPLEM--
-TPLEML-
TMPLEM--
THPLEM--
TYPLEM--

TEPLEM--
TPPLEM--
-TPLEMA-
TDPLEM--
TQPLEM--
-TPLEMY-
-TPLEMQ-
-TPLEMH-
-TPLEME-
-VPLEMH-
-VPLEMR-
VQPLEM--
VTPLEM--
-VPLEMV-
-VPLEMT-
-VPLEMS-
VVPLEM--
-VPLEMN-
-VPLEML-
-VPLEMM-
VLPLEM--
VKPLEM--
VYPLEM--
-VPLEMY-
VSPLEM--
-VPLEMA-
VIPLEM--
-VPLEMK-
VNPLEM--
VMPLEM--
VFPLEM--
VAPLEM--
VRPLEM--
VHPLEM--
VDPLEM--
-VPLEMW-
-VPLEMG-
VPPLEM--
-VPLEME-
-VPLEMD-
VEPLEM--
-WPLEMR-
WRPLEM--
WYPLEM--
-WPLEMH-
WKPLEM--
-WPLEMM-
-WPLEMV-
WMPLEM--
WIPLEM--

	WHPLEM--		
	WSPLEM--		
	WAPLEM--		
	WPPLEM--		
	-WPLEMT-		
	-WPLEMS-		
	WVPLEM--		
	-WPLEMQ-		
	-WPLEMG-		
	-WPLEMA-		
	WNPLEM--		
	WGPLEM--		
	-WPLEMN-		
	-YPLEMN-		
	-YPLEMS-		
	-YPLEMR-		
	YQPLEM--		
	YSPLEM--		
	YTPLEM--		
	YRPLEM--		
	YKPLEM--		
	-YPLEMV-		
	-YPLEMA-		
	YGPLEM--		
	YIPLEM--		
	YEPLEM--		
	YYPLEM--		
	-YPLEMW-		
	-YPLEML-		
	YVPLEM--		
	YLPLEM--		
	YHPLEM--		
	YNPLEM--		
	YWPLEM--		
	-YPLEMT-		
	-YPLEMM-		
	-YPLEMG-		
	YMPLEM--		
	-YPLEMQ-		
	-YPLEMH-		
	YAPLEM--		
	YFPLEM--		
PWQM	1148	466	47.15
	-HPWQMG-		
	-APWQMT-		
	-APWQMR-		
	-APWQMI-		
	ADPWQM--		
	ATPWQM--		
	-APWQMW-		

ARPWQM--
-APWQMY-
ASPWQM--
-APWQMS-
ALPWQM--
AKPWQM--
AAPWQM--
ANPWQM--
AVPWQM--
-APWQMK-
AIPWQM--
-APWQMQ-
-APWQMN-
-APWQMA-
CHPWQM--
-CPWQMT-
-CPWQMS-
CPPWQM--
CNPWQM--
DTPWQM--
DRPWQM--
-DPWQMF-
DGPWQM--
DEPWQM--
DFPWQM--
DSPWQM--
-DPWQMR-
-DPWQMN-
-DPWQMQ-
DYPWQM--
DHPWQM--
-EPWQMR-
-EPWQMI-
ESPWQM--
-EPWQML-
-EPWQMT-
EAPWQM--
EKPWQM--
EHPWQM--
-EPWQMM-
-EPWQMW-
-EPWQMK-
-EPWQMF-
-FPWQMR-
-FPWQMV-
FYPWQM--
FAPWQM--
FSPWQM--
-FPWQMS-
FNPWQM--

FLPWQM--
FFPWQM--
-FPWQMW-
-FPWQMK-
FGPWQM--
-FPWQMT-
FHPWQM--
GSPWQM--
-GPWQMS-
-GPWQMR-
-GPWQMN-
GRPWQM--
-GPWQMK-
GNPWQM--
GHPWQM--
-GPWQMF-
GYPWQM--
-GPWQMI-
GIPWQM--
GFPWQM--
GWPWQM--
-GPWQML-
-GPWQMH-
GAPWQM--
-GPWQMA-
-GPWQMW-
-GPWQMV-
GTPWQM--
-GPWQMQ-
GLPWQM--
GDPWQM--
GCPWQM--
HVPWQM--
HRPWQM--
-HPWQMN-
-HPWQMV-
-HPWQML-
HWPWQM--
-HPWQMS-
HSPWQM--
HNPWQM--
HFPWQM--
-HPWQMM-
HTPWQM--
-HPWQMY-
HAPWQM--
IIPWQM--
-IPWQMM-
IAPWQM--
IRPWQM--

-IPWQMQ-
-IPWQML-
-IPWQMS-
IYPWQM--
IGPWQM--
-IPWQMA-
INPWQM--
IQPWQM--
-IPWQMY-
-IPWQMT-
IDPWQM--
KSPWQM--
-KPWQMM-
-KPWQML-
KGPWQM--
KQPWQM--
-KPWQMK-
KVPWQM--
-KPWQMR-
-KPWQMA-
KNPWQM--
KTPWQM--
KYPWQM--
KWPWQM--
KIPWQM--
-KPWQMI-
-KPWQMF-
KLPWQM--
KHPWQM--
-KPWQMH-
-KPWQME-
LKPWQM--
LFPWQM--
-LPWQMI-
LQPWQM--
-LPWQMV-
-LPWQMT-
-LPWQML-
LPPWQM--
LHPWQM--
LTPWQM--
-LPWQMR-
-LPWQMQ-
LIPWQM--
-LPWQMS-
LYPWQM--
LRPWQM--
LEPWQM--
LSPWQM--
-LPWQMH-

-LPWQMF-
LDPWQM--
-MPWQMR-
-MPWQMV-
-MPWQMK-
MSPWQM--
MIPWQM--
MTPWQM--
MVPWQM--
MEPWQM--
MRPWQM--
-MPWQMW-
-MPWQMA-
MPPWQM--
-MPWQMY-
-MPWQMS-
MAPWQM--
NLPWQM--
-NPWQMM-
NRPWQM--
-NPWQMF-
NTPWQM--
-NPWQMR-
NGPWQM--
-NPWQMV-
-NPWQMS-
NWPWQM--
NMPWQM--
NEPWQM--
NDPWQM--
-NPWQMW-
NIPWQM--
--PWQMWS
--PWQMRT
--PWQMRR
--PWQMVS
--PWQMRS
--PWQMRM
--PWQMRG
--PWQMSA
--PWQMRH
--PWQMRF
--PWQMMH
--PWQMQN
--PWQMFS
--PWQMRA
--PWQMKT
--PWQMKN
--PWQMHH
--PWQMYS

--PWQMVN
--PWQMSS
--PWQMRK
--PWQMKA
--PWQMIT
--PWQMWG
--PWQMRN
--PWQMRD
--PWQMQT
--PWQMKS
--PWQMIS
--PWQMYA
--PWQMWR
--PWQMNY
--PWQMNA
--PWQMIW
--PWQMHG
--PWQMFQ
--PWQMYN
--PWQMYM
--PWQMVA
--PWQMSM
--PWQMLK
--PWQMLF
--PWQMIQ
--PWQMGS
PMPWQM--
--PWQMVQ
--PWQMVF
--PWQMTF
--PWQMTE
--PWQMTA
--PWQMST
--PWQMNT
--PWQMMM
--PWQMMF
--PWQMLS
--PWQMIY
--PWQMAT
--PWQMAG
--PWQMTV
--PWQMTH
--PWQMSR
--PWQMRV
--PWQMRE
--PWQMQR
--PWQMNS
--PWQMNF
--PWQMMA
--PWQMID

--PWQMHK
--PWQMHA
--PWQMFG
--PWQMFF
--PWQMFE
--PWQMEG
--PWQMEA
--PWQMAY
PTPWQM--
--PWQMYW
--PWQMYR
--PWQMYH
--PWQMWW
--PWQMVW
--PWQMVK
--PWQMTW
--PWQMTR
--PWQMTK
--PWQMSH
--PWQMSG
--PWQMNR
--PWQMNM
--PWQMNK
--PWQMMV
--PWQMLR
--PWQMGV
--PWQMGG
--PWQMFT
--PWQMET
--PWQMES
--PWQMAV
--PWQMAM
--PWQMWL
--PWQMWE
--PWQMWD
--PWQMVT
--PWQMTS
--PWQMTQ
--PWQMTI
--PWQMTD
--PWQMSW
--PWQMSV
--PWQMSQ
--PWQMSN
--PWQMSL
--PWQMSI
--PWQMSE
--PWQMRQ
--PWQMQS
--PWQMQH

--PWQMNI
--PWQMMK
--PWQMLQ
--PWQMLA
--PWQMKV
--PWQMKM
--PWQMKI
--PWQMKH
--PWQMIR
--PWQMIG
--PWQMIE
--PWQMHS
--PWQMHE
--PWQMFN
--PWQMEW
--PWQMCY
-PPWQMG-
QRPWQM--
QSPWQM--
-QPWQMY-
QWPWQM--
-QPWQMV-
-QPWQMS-
-QPWQMW-
-QPWQMH-
-QPWQMT-
-QPWQMR-
QLPWQM--
-QPWQML-
-QPWQMK-
QTPWQM--
-QPWQMN-
-QPWQMF-
QMPWQM--
QIPWQM--
QGPWQM--
-RPWQMM-
RIPWQM--
-RPWQMS-
-RPWQMY-
RAPWQM--
RQPWQM--
-RPWQMT-
RPPWQM--
RSPWQM--
RNPWQM--
-RPWQMK-
RTPWQM--
-RPWQMI-
-RPWQMR-

REPWQM--
-RPWQMV-
-RPWQMQ-
-RPWQMF-
-RPWQMA-
RGPWQM--
-RPWQMW-
-RPWQMN-
-RPWQME-
RVPWQM--
RKPWQM--
RHPWQM--
RDPWQM--
RCPWQM--
SMPWQM--
SSPWQM--
-SPWQMS-
SHPWQM--
SVPWQM--
SGPWQM--
-SPWQMR-
-SPWQMH-
-SPWQMF-
-SPWQMN-
-SPWQMA-
SAPWQM--
SRPWQM--
SPPWQM--
STPWQM--
SLPWQM--
-SPWQMK-
SNPWQM--
SIPWQM--
-SPWQMW-
SWPWQM--
-SPWQMV-
-SPWQMT-
SDPWQM--
-SPWQMY-
-TPWQMR-
-TPWQMK-
TTPWQM--
-TPWQMQ-
TPPWQM--
TSPWQM--
TVPWQM--
-TPWQMS-
-TPWQMN-
TLPWQM--
TWPWQM--

TRPWQM--
-TPWQMM-
TFPWQM--
TDPWQM--
TAPWQM--
-TPWQMY-
-TPWQMT-
-TPWQMI-
-TPWQMH-
-TPWQMF-
-VPWQMR-
-VPWQMV-
VLPWQM--
VTPWQM--
VVPWQM--
-VPWQMT-
VHPWQM--
-VPWQMW-
VFPWQM--
-VPWQMY-
-VPWQMS-
-VPWQMI-
VPPWQM--
VAPWQM--
-VPWQMQ-
-VPWQMH-
VRPWQM--
-VPWQMN-
VKPWQM--
-VPWQMF-
WGPWQM--
-WPWQMR-
-WPWQMK-
WYPWQM--
WSPWQM--
WLPWQM--
WRPWQM--
-WPWQMT-
WAPWQM--
-WPWQMG-
-WPWQME-
WKPWQM--
WDPWQM--
-YPWQMW-
-YPWQMV-
-YPWQMY-
-YPWQMF-
YAPWQM--
-YPWQMN-
YMPWQM--

	YKPWQM--		
	YYPWQM--		
	YTPWQM--		
	-YPWQMT-		
	YPPWQM--		
	YSPWQM--		
	YRPWQM--		
	-YPWQMH-		
	YNPWQM--		
PQPL	66	837	47.13
	--PQPLRP		
	-APQPLT-		
	-APQPLW-		
	ATPQPL--		
	-APQPLV-		
	-APQPLA-		
	AAPQPL--		
	-APQPLM-		
	ALPQPL--		
	-APQPLL-		
	AIPQPL--		
	ARPQPL--		
	-APQPLK-		
	-APQPLN-		
	AWPQPL--		
	-APQPLE-		
	ASPQPL--		
	AFPQPL--		
	AKPQPL--		
	AVPQPL--		
	-APQPLH-		
	-APQPLR-		
	ANPQPL--		
	AGPQPL--		
	-APQPLF-		
	AMPQPL--		
	ADPQPL--		
	-APQPLS-		
	-APQPLQ-		
	-APQPLI-		
	-APQPLC-		
	CCPQPL--		
	CAPQPL--		
	CTPQPL--		
	-CPQPLL-		
	CLPQPL--		
	CSPQPL--		
	CEPQPL--		
	CWPQPL--		
	CRPQPL--		

−CPQPLV−
−CPQPLR−
CMPQPL−−
CIPQPL−−
−CPQPLT−
−CPQPLH−
CKPQPL−−
CHPQPL−−
CFPQPL−−
−DPQPLR−
−DPQPLT−
DLPQPL−−
DGPQPL−−
DSPQPL−−
−DPQPLL−
DIPQPL−−
−DPQPLY−
DEPQPL−−
DVPQPL−−
−DPQPLF−
DFPQPL−−
DWPQPL−−
DRPQPL−−
−DPQPLS−
−DPQPLI−
DHPQPL−−
DYPQPL−−
−DPQPLW−
−EPQPLK−
EGPQPL−−
−EPQPLH−
ELPQPL−−
ETPQPL−−
ESPQPL−−
EFPQPL−−
EVPQPL−−
−EPQPLM−
−EPQPLL−
EYPQPL−−
−EPQPLT−
−EPQPLS−
ERPQPL−−
−EPQPLA−
−EPQPLN−
EEPQPL−−
EAPQPL−−
−EPQPLW−
−EPQPLV−
−EPQPLR−
EKPQPL−−

-FPQPLI-
FKPQPL--
FVPQPL--
-FPQPLV-
FSPQPL--
FEPQPL--
FHPQPL--
FTPQPL--
FAPQPL--
-FPQPLY-
-FPQPLW-
FPPQPL--
-FPQPLT-
-FPQPLM-
-FPQPLF-
FWPQPL--
-FPQPLS-
FGPQPL--
FFPQPL--
-FPQPLR-
-FPQPLH-
-FPQPLA-
FNPQPL--
FYPQPL--
-FPQPLG-
-GPQPLL-
GRPQPL--
GVPQPL--
GIPQPL--
GFPQPL--
GYPQPL--
-GPQPLN-
GLPQPL--
GQPQPL--
GWPQPL--
GGPQPL--
GNPQPL--
GAPQPL--
GTPQPL--
-GPQPLT-
-GPQPLK-
GMPQPL--
GHPQPL--
-GPQPLW-
GEPQPL--
GSPQPL--
-GPQPLF-
-GPQPLM-
-GPQPLI-
-GPQPLR-

−GPQPLH−
−GPQPLE−
−GPQPLA−
−GPQPLG−
−GPQPLC−
−GPQPLQ−
−GPQPLV−
GCPQPL−−
HTPQPL−−
HPPQPL−−
HWPQPL−−
−HPQPLR−
HNPQPL−−
−HPQPLW−
HLPQPL−−
HKPQPL−−
−HPQPLT−
−HPQPLL−
−HPQPLI−
−HPQPLK−
HSPQPL−−
HIPQPL−−
HEPQPL−−
HCPQPL−−
HRPQPL−−
−HPQPLN−
HGPQPL−−
−HPQPLS−
−HPQPLG−
HHPQPL−−
−HPQPLV−
−HPQPLF−
HDPQPL−−
ITPQPL−−
IYPQPL−−
IMPQPL−−
IGPQPL−−
IVPQPL−−
−IPQPLI−
−IPQPLH−
IQPQPL−−
−IPQPLK−
INPQPL−−
−IPQPLS−
−IPQPLQ−
−IPQPLL−
IDPQPL−−
IRPQPL−−
−IPQPLT−
−IPQPLY−

-IPQPLA-
-IPQPLR-
ILPQPL--
ISPQPL--
IPPQPL--
-IPQPLN-
-IPQPLE-
IWPQPL--
-IPQPLV-
-IPQPLF-
IKPQPL--
IEPQPL--
-IPQPLP-
KSPQPL--
-KPQPLR-
KDPQPL--
KTPQPL--
-KPQPLY-
-KPQPLL-
-KPQPLF-
KVPQPL--
-KPQPLT-
KKPQPL--
-KPQPLM-
KHPQPL--
-KPQPLS-
KRPQPL--
KMPQPL--
KIPQPL--
-KPQPLV-
KNPQPL--
KEPQPL--
-KPQPLK-
-KPQPLQ-
-KPQPLN-
-KPQPLH-
-KPQPLA-
KAPQPL--
KWPQPL--
KQPQPL--
KPPQPL--
KGPQPL--
KYPQPL--
-KPQPLE-
KLPQPL--
KFPQPL--
-LPQPLR-
LGPQPL--
LRPQPL--
LVPQPL--

-LPQPLL-
LTPQPL--
LWPQPL--
LQPQPL--
-LPQPLW-
LLPQPL--
-LPQPLK-
LPPQPL--
LSPQPL--
LIPQPL--
LHPQPL--
LMPQPL--
-LPQPLM-
-LPQPLS-
-LPQPLI-
LNPQPL--
-LPQPLY-
-LPQPLF-
LAPQPL--
-LPQPLV-
-LPQPLH-
-LPQPLA-
LDPQPL--
LFPQPL--
LYPQPL--
-LPQPLN-
-LPQPLC-
LEPQPL--
-LPQPLG-
-LPQPLT-
-LPQPLE-
LCPQPL--
MKPQPL--
-MPQPLL-
-MPQPLR-
MGPQPL--
MTPQPL--
MRPQPL--
MSPQPL--
-MPQPLM-
MVPQPL--
-MPQPLV-
-MPQPLS-
MFPQPL--
-MPQPLY-
-MPQPLW-
MWPQPL--
MCPQPL--
-MPQPLA-
MMPQPL--

MLPQPL--
MEPQPL--
-MPQPLN-
MYPQPL--
MQPQPL--
-MPQPLT-
-MPQPLG-
MPPQPL--
MDPQPL--
-MPQPLQ-
-MPQPLE-
-MPQPLC-
MHPQPL--
NIPQPL--
NAPQPL--
NLPQPL--
-NPQPLL-
NGPQPL--
-NPQPLR-
NYPQPL--
NRPQPL--
-NPQPLI-
NNPQPL--
NKPQPL--
NHPQPL--
NSPQPL--
-NPQPLK-
-NPQPLY-
-NPQPLS-
NDPQPL--
-NPQPLW-
-NPQPLT-
-NPQPLM-
NPPQPL--
-NPQPLN-
-NPQPLF-
NEPQPL--
-NPQPLV-
-NPQPLH-
NFPQPL--
NVPQPL--
NTPQPL--
NMPQPL--
NCPQPL--
--PQPLTA
--PQPLRG
--PQPLRL
--PQPLYS
--PQPLNL
--PQPLLT

--PQPLLS
--PQPLST
--PQPLSL
--PQPLWS
--PQPLRT
--PQPLMR
--PQPLIS
--PQPLRS
PRPQPL--
--PQPLRF
--PQPLTL
--PQPLLV
--PQPLLL
--PQPLVY
--PQPLRA
--PQPLWL
--PQPLYG
--PQPLTS
--PQPLSV
--PQPLRN
--PQPLMS
--PQPLLF
--PQPLTF
--PQPLSW
--PQPLGF
--PQPLRR
--PQPLNS
--PQPLFL
--PQPLWT
--PQPLVW
--PQPLRI
--PQPLLA
--PQPLRV
--PQPLGN
--PQPLTI
--PQPLRY
--PQPLAM
PFPQPL--
--PQPLSF
--PQPLMT
--PQPLLR
--PQPLKM
--PQPLHS
--PQPLFM
--PQPLAL
--PQPLYR
--PQPLYI
--PQPLWN
--PQPLSN
--PQPLSG

--PQPLRH
--PQPLQV
--PQPLFI
--PQPLNH
--PQPLKS
--PQPLGT
PGPQPL--
PSPQPL--
--PQPLVT
--PQPLTR
--PQPLSS
--PQPLQA
--PQPLNA
--PQPLKH
--PQPLIF
--PQPLGW
PAPQPL--
--PQPLVM
--PQPLKI
--PQPLHV
--PQPLFW
--PQPLFN
--PQPLAF
--PQPLAA
PIPQPL--
PTPQPL--
--PQPLYQ
--PQPLVS
--PQPLVA
--PQPLSA
--PQPLNQ
--PQPLHT
--PQPLGQ
--PQPLAI
--PQPLWA
--PQPLVR
--PQPLVG
--PQPLTQ
--PQPLQL
--PQPLNV
--PQPLKR
--PQPLES
--PQPLYW
--PQPLRM
--PQPLKT
--PQPLFV
PLPQPL--
--PQPLTH
--PQPLGS
--PQPLYT

--PQPLPW
--PQPLNT
--PQPLMK
--PQPLMA
--PQPLLW
--PQPLLI
--PQPLIY
--PQPLIV
--PQPLIR
--PQPLHR
--PQPLHQ
--PQPLHI
--PQPLAG
--PQPLYN
--PQPLWG
--PQPLVK
--PQPLTG
--PQPLSH
--PQPLRD
--PQPLQS
--PQPLMG
--PQPLLH
--PQPLHY
--PQPLFE
--PQPLVN
--PQPLRW
--PQPLQY
--PQPLQQ
--PQPLQI
--PQPLPS
--PQPLLK
--PQPLIT
--PQPLIN
--PQPLHH
--PQPLEQ
--PQPLDQ
--PQPLWQ
--PQPLVI
--PQPLTT
--PQPLTN
--PQPLNF
--PQPLKK
--PQPLHL
--PQPLHA
--PQPLEM
--PQPLWR
--PQPLWK
--PQPLWD
--PQPLVQ
--PQPLSY

--PQPLNN
--PQPLMI
--PQPLLQ
--PQPLLN
--PQPLLM
--PQPLIW
--PQPLIG
--PQPLFK
--PQPLFG
--PQPLAT
--PQPLAH
--PQPLYY
--PQPLYA
--PQPLWM
--PQPLVF
--PQPLVD
--PQPLTW
--PQPLRK
--PQPLMY
--PQPLMW
--PQPLMV
--PQPLMN
--PQPLMF
--PQPLKW
--PQPLKG
--PQPLKD
--PQPLIK
--PQPLIH
--PQPLHK
--PQPLGA
--PQPLEL
--PQPLEG
--PQPLDY
--PQPLDV
--PQPLAV
PMPQPL--
PYPQPL--
PQPQPL--
--PQPLYD
--PQPLWE
--PQPLTK
--PQPLSQ
--PQPLQT
--PQPLQM
--PQPLPV
--PQPLPQ
--PQPLNW
--PQPLNR
--PQPLKQ
--PQPLIM

--PQPLID
--PQPLIA
--PQPLHN
--PQPLGK
--PQPLET
--PQPLEI
--PQPLDS
--PQPLYF
--PQPLWV
--PQPLTM
--PQPLRE
--PQPLQW
--PQPLPF
--PQPLPA
--PQPLNG
--PQPLMQ
--PQPLLE
--PQPLKL
--PQPLKE
--PQPLIQ
--PQPLIL
--PQPLHF
--PQPLGH
--PQPLFY
--PQPLFT
--PQPLFF
--PQPLFA
--PQPLEN
--PQPLCN
--PQPLCD
--PQPLAS
PKPQPL--
PEPQPL--
PVPQPL--
--PQPLYV
--PQPLYP
--PQPLYM
--PQPLYK
--PQPLYH
--PQPLYE
--PQPLVH
--PQPLTV
--PQPLTE
--PQPLTD
--PQPLSK
--PQPLQF
--PQPLPT
--PQPLPN
--PQPLPH
--PQPLKA

--PQPLIC
--PQPLHW
--PQPLHM
--PQPLHG
--PQPLHE
--PQPLEY
--PQPLEV
--PQPLDR
--PQPLDL
--PQPLDG
--PQPLCT
--PQPLCS
--PQPLAW
--PQPLAN
--PQPLAK
--PQPLAE
-PPQPLR-
-PPQPLL-
-PPQPLH-
PNPQPL--
-QPQPLR-
QRPQPL--
-QPQPLS-
-QPQPLF-
QGPQPL--
-QPQPLK-
QTPQPL--
-QPQPLL-
QLPQPL--
QQPQPL--
-QPQPLY-
-QPQPLT-
-QPQPLH-
QSPQPL--
QYPQPL--
-QPQPLV-
-QPQPLI-
QWPQPL--
QHPQPL--
QFPQPL--
QVPQPL--
-QPQPLA-
QPPQPL--
QDPQPL--
-QPQPLW-
-QPQPLG-
QMPQPL--
QAPQPL--
RRPQPL--
RTPQPL--

RGPQPL--
-RPQPLR-
-RPQPLS-
RLPQPL--
-RPQPLT-
-RPQPLL-
RYPQPL--
RSPQPL--
RPPQPL--
RMPQPL--
-RPQPLN-
-RPQPLK-
RAPQPL--
RFPQPL--
RDPQPL--
-RPQPLF-
-RPQPLM-
-RPQPLI-
-RPQPLQ-
RWPQPL--
RKPQPL--
RIPQPL--
RVPQPL--
RNPQPL--
-RPQPLA-
-RPQPLV-
RHPQPL--
RCPQPL--
-RPQPLW-
-RPQPLG-
REPQPL--
-RPQPLY-
-RPQPLH-
-SPQPLR-
SLPQPL--
-SPQPLK-
SAPQPL--
STPQPL--
SSPQPL--
SRPQPL--
-SPQPLV-
-SPQPLI-
SVPQPL--
-SPQPLF-
SIPQPL--
SGPQPL--
SYPQPL--
SHPQPL--
-SPQPLH-
-SPQPLT-

-SPQPLY-
-SPQPLW-
-SPQPLS-
-SPQPLN-
SPPQPL--
SKPQPL--
SFPQPL--
SWPQPL--
SQPQPL--
SMPQPL--
-SPQPLQ-
-SPQPLA-
SEPQPL--
-SPQPLM-
-SPQPLG-
SNPQPL--
SDPQPL--
TAPQPL--
-TPQPLR-
-TPQPLY-
TGPQPL--
TPPQPL--
-TPQPLK-
TRPQPL--
-TPQPLH-
TLPQPL--
-TPQPLI-
-TPQPLL-
-TPQPLS-
THPQPL--
TKPQPL--
TWPQPL--
-TPQPLW-
-TPQPLT-
TSPQPL--
-TPQPLV-
-TPQPLA-
TNPQPL--
TTPQPL--
-TPQPLN-
-TPQPLM-
-TPQPLQ-
TDPQPL--
-TPQPLF-
TMPQPL--
TYPQPL--
-TPQPLG-
TFPQPL--
TVPQPL--
VRPQPL--

```
-VPQPLS-
VTPQPL--
-VPQPLL-
VLPQPL--
VSPQPL--
VAPQPL--
-VPQPLH-
VHPQPL--
VFPQPL--
VWPQPL--
-VPQPLA-
VVPQPL--
-VPQPLY-
-VPQPLM-
-VPQPLI-
VGPQPL--
-VPQPLN-
-VPQPLT-
-VPQPLW-
VIPQPL--
-VPQPLR-
-VPQPLF-
VMPQPL--
-VPQPLK-
-VPQPLG-
VPPQPL--
VYPQPL--
VQPQPL--
-VPQPLV-
VKPQPL--
-VPQPLQ-
VNPQPL--
WRPQPL--
WIPQPL--
-WPQPLF-
WYPQPL--
-WPQPLR-
-WPQPLK-
WSPQPL--
-WPQPLT-
WTPQPL--
-WPQPLY-
WNPQPL--
-WPQPLN-
-WPQPLM-
-WPQPLS-
WKPQPL--
WVPQPL--
WGPQPL--
WCPQPL--
```

-WPQPLI-
WMPQPL--
WEPQPL--
WQPQPL--
WDPQPL--
-WPQPLH-
WLPQPL--
-WPQPLV-
WAPQPL--
WWPQPL--
-WPQPLQ-
-WPQPLL-
WHPQPL--
WFPQPL--
YVPQPL--
YFPQPL--
YRPQPL--
YEPQPL--
-YPQPLR-
YSPQPL--
YPPQPL--
YMPQPL--
-YPQPLL-
-YPQPLS-
YHPQPL--
YQPQPL--
YYPQPL--
-YPQPLW-
YNPQPL--
YLPQPL--
YIPQPL--
-YPQPLT-
-YPQPLY-
-YPQPLQ-
-YPQPLF-
YKPQPL--
YTPQPL--
-YPQPLK-
-YPQPLG-
YGPQPL--
YWPQPL--
-YPQPLN-
-YPQPLA-
YAPQPL--
-YPQPLM-
YDPQPL--
YCPQPL--

PMSL	129	791	47.13

-LPMSLD-
-NPMSLG-

--PMSLYP
--PMSLSP
--PMSLPS
--PMSLPR
--PMSLGK
-VPMSLP-
ASPMSL--
AVPMSL--
AKPMSL--
-APMSLT-
AAPMSL--
-APMSLS-
AGPMSL--
-APMSLR-
AIPMSL--
-APMSLA-
-APMSLN-
AHPMSL--
AYPMSL--
AMPMSL--
AFPMSL--
ATPMSL--
-APMSLI-
-APMSLM-
ANPMSL--
-APMSLK-
AEPMSL--
-APMSLV-
AQPMSL--
-APMSLH-
APPMSL--
-APMSLW-
ACPMSL--
-APMSLE-
ADPMSL--
-APMSLC-
CTPMSL--
CGPMSL--
CEPMSL--
CSPMSL--
-CPMSLK-
CPPMSL--
CYPMSL--
CVPMSL--
CQPMSL--
-CPMSLT-
CHPMSL--
-CPMSLV-
-CPMSLI-
CMPMSL--

CLPMSL--
CCPMSL--
-CPMSLY-
-CPMSLN-
-CPMSLL-
-CPMSLF-
CNPMSL--
CWPMSL--
-CPMSLS-
CIPMSL--
CFPMSL--
CDPMSL--
CAPMSL--
-DPMSLR-
DRPMSL--
DGPMSL--
DWPMSL--
DSPMSL--
-DPMSLW-
-DPMSLL-
DHPMSL--
DFPMSL--
DTPMSL--
DQPMSL--
DKPMSL--
DAPMSL--
-DPMSLM-
DMPMSL--
-DPMSLV-
DVPMSL--
DIPMSL--
DYPMSL--
DNPMSL--
-DPMSLS-
-DPMSLT-
-DPMSLQ-
DEPMSL--
-DPMSLI-
-DPMSLN-
-DPMSLH-
-DPMSLE-
-DPMSLA-
-DPMSLK-
DDPMSL--
DPPMSL--
DCPMSL--
-EPMSLR-
-EPMSLT-
ERPMSL--
-EPMSLL-

-EPMSLN-
EAPMSL--
-EPMSLQ-
EKPMSL--
-EPMSLY-
EGPMSL--
-EPMSLK-
EYPMSL--
-EPMSLV-
ETPMSL--
-EPMSLW-
-EPMSLA-
EFPMSL--
-EPMSLI-
EWPMSL--
-EPMSLM-
-EPMSLE-
EQPMSL--
EIPMSL--
-EPMSLG-
ENPMSL--
EEPMSL--
-EPMSLH-
EDPMSL--
FGPMSL--
-FPMSLT-
-FPMSLR-
FFPMSL--
FKPMSL--
-FPMSLL-
-FPMSLS-
FNPMSL--
-FPMSLV-
-FPMSLM-
FPPMSL--
-FPMSLQ-
-FPMSLK-
FHPMSL--
-FPMSLN-
-FPMSLA-
FQPMSL--
-FPMSLH-
FEPMSL--
FDPMSL--
-FPMSLG-
-FPMSLE-
-FPMSLI-
FCPMSL--
-GPMSLR-
GSPMSL--

-GPMSLL-
-GPMSLQ-
GGPMSL--
GKPMSL--
GQPMSL--
-GPMSLS-
GHPMSL--
GAPMSL--
-GPMSLV-
-GPMSLM-
-GPMSLK-
GLPMSL--
-GPMSLW-
GWPMSL--
GIPMSL--
GFPMSL--
GYPMSL--
-GPMSLT-
GMPMSL--
-GPMSLN-
-GPMSLI-
-GPMSLG-
-GPMSLE-
-GPMSLA-
GDPMSL--
-GPMSLH-
-GPMSLC-
HLPMSL--
HGPMSL--
HNPMSL--
HHPMSL--
-HPMSLT-
HKPMSL--
HIPMSL--
-HPMSLQ-
-HPMSLE-
HWPMSL--
HVPMSL--
HSPMSL--
HFPMSL--
-HPMSLL-
HYPMSL--
HEPMSL--
-HPMSLH-
HQPMSL--
-HPMSLN-
-HPMSLS-
-HPMSLI-
HMPMSL--
-HPMSLV-

HTPMSL--
HDPMSL--
HPPMSL--
HAPMSL--
-HPMSLM-
-HPMSLF-
-HPMSLY-
-HPMSLG-
-HPMSLC-
-HPMSLW-
-HPMSLK-
-HPMSLA-
ILPMSL--
IRPMSL--
IFPMSL--
IVPMSL--
IGPMSL--
-IPMSLR-
IIPMSL--
IYPMSL--
-IPMSLS-
-IPMSLL-
IDPMSL--
ISPMSL--
IKPMSL--
IWPMSL--
-IPMSLV-
-IPMSLT-
INPMSL--
IPPMSL--
-IPMSLN-
-IPMSLK-
IQPMSL--
-IPMSLQ-
IHPMSL--
IEPMSL--
-IPMSLI-
-IPMSLM-
-IPMSLA-
-IPMSLG-
IMPMSL--
ICPMSL--
-KPMSLS-
KGPMSL--
-KPMSLR-
KTPMSL--
-KPMSLL-
KVPMSL--
KQPMSL--
KRPMSL--

-KPMSLW-
-KPMSLH-
KPPMSL--
KEPMSL--
-KPMSLT-
KKPMSL--
-KPMSLM-
KYPMSL--
-KPMSLF-
-KPMSLV-
-KPMSLI-
-KPMSLN-
KAPMSL--
KIPMSL--
-KPMSLE-
KHPMSL--
-KPMSLQ-
KNPMSL--
-KPMSLY-
KDPMSL--
KFPMSL--
-KPMSLG-
KMPMSL--
LLPMSL--
-LPMSLR-
-LPMSLT-
LFPMSL--
-LPMSLS-
-LPMSLL-
LHPMSL--
LTPMSL--
LYPMSL--
-LPMSLV-
LPPMSL--
LNPMSL--
-LPMSLK-
-LPMSLI-
LAPMSL--
-LPMSLN-
LDPMSL--
-LPMSLH-
LEPMSL--
-LPMSLQ-
LMPMSL--
-LPMSLM-
-LPMSLA-
LQPMSL--
-LPMSLE-
LCPMSL--
MLPMSL--

-MPMSLS-
MRPMSL--
MTPMSL--
MSPMSL--
-MPMSLQ-
-MPMSLN-
-MPMSLI-
MWPMSL--
MQPMSL--
-MPMSLV-
-MPMSLT-
-MPMSLM-
MAPMSL--
MHPMSL--
MIPMSL--
MVPMSL--
MMPMSL--
-MPMSLA-
-MPMSLK-
-MPMSLH-
MYPMSL--
-MPMSLE-
-MPMSLG-
MDPMSL--
NSPMSL--
-NPMSLR-
NRPMSL--
NFPMSL--
NIPMSL--
NVPMSL--
-NPMSLT-
NTPMSL--
NMPMSL--
-NPMSLL-
-NPMSLI-
NPPMSL--
NYPMSL--
NGPMSL--
-NPMSLV-
-NPMSLF-
-NPMSLW-
NEPMSL--
-NPMSLM-
-NPMSLS-
-NPMSLH-
NWPMSL--
-NPMSLN-
NQPMSL--
-NPMSLQ-
NKPMSL--

-NPMSLK-
-NPMSLE-
-NPMSLA-
NCPMSL--
--PMSLEV
--PMSLLG
--PMSLRN
--PMSLSS
--PMSLSG
--PMSLLR
--PMSLRG
--PMSLWR
--PMSLHR
--PMSLLS
--PMSLYG
--PMSLTR
--PMSLMT
--PMSLLA
--PMSLSA
--PMSLKR
--PMSLMR
--PMSLSR
--PMSLVS
--PMSLGS
--PMSLTA
PAPMSL--
--PMSLKG
--PMSLVW
--PMSLKV
--PMSLRI
--PMSLMS
--PMSLTK
--PMSLYT
--PMSLMQ
--PMSLTV
--PMSLIT
--PMSLIK
--PMSLHS
--PMSLTN
--PMSLTG
--PMSLSM
--PMSLIR
PWPMSL--
--PMSLAS
--PMSLNT
--PMSLGT
--PMSLVN
--PMSLWG
--PMSLRQ
--PMSLTH

--PMSLVK
--PMSLVH
--PMSLKL
--PMSLHG
--PMSLFS
--PMSLTM
--PMSLNS
--PMSLKQ
--PMSLAR
--PMSLSY
--PMSLST
--PMSLLN
--PMSLFN
--PMSLRD
--PMSLHL
--PMSLFH
--PMSLAG
PVPMSL--
--PMSLVT
--PMSLQT
--PMSLMG
--PMSLIA
--PMSLNA
--PMSLMK
--PMSLKT
PEPMSL--
--PMSLHF
PNPMSL--
--PMSLMV
--PMSLTY
--PMSLNY
--PMSLMW
--PMSLIH
--PMSLGG
--PMSLWN
--PMSLVM
--PMSLSK
--PMSLQS
--PMSLKS
--PMSLTI
--PMSLQG
--PMSLHY
--PMSLAN
--PMSLWQ
--PMSLTQ
--PMSLTL
--PMSLNK
--PMSLLH
--PMSLAY
PTPMSL--

--PMSLWT
--PMSLNL
--PMSLKN
--PMSLSQ
--PMSLQI
--PMSLHM
--PMSLEH
--PMSLSW
--PMSLSF
--PMSLMA
--PMSLIG
--PMSLHV
--PMSLAH
PMPMSL--
--PMSLNQ
--PMSLHQ
--PMSLGW
--PMSLAV
--PMSLVG
--PMSLQQ
--PMSLKA
--PMSLII
--PMSLHW
--PMSLFD
--PMSLWD
--PMSLTE
--PMSLNV
--PMSLNM
--PMSLMH
--PMSLAA
--PMSLYW
--PMSLYH
--PMSLSE
--PMSLQR
--PMSLNR
--PMSLVQ
--PMSLNW
--PMSLID
--PMSLHA
--PMSLGY
--PMSLGV
--PMSLEW
--PMSLYE
--PMSLQN
--PMSLHK
--PMSLEK
--PMSLAM
--PMSLMY
--PMSLMM
--PMSLDS

PIPMSL--
PFPMSL--
--PMSLQV
--PMSLKK
--PMSLKI
--PMSLIN
--PMSLGI
--PMSLAI
--PMSLGF
--PMSLVD
--PMSLTW
--PMSLKM
--PMSLHN
--PMSLGM
--PMSLEG
PHPMSL--
--PMSLWE
--PMSLKH
--PMSLIQ
--PMSLAK
--PMSLYD
--PMSLWW
--PMSLTD
PDPMSL--
--PMSLQK
--PMSLND
--PMSLMD
--PMSLKF
--PMSLEL
PQPMSL--
--PMSLVE
--PMSLQH
--PMSLNN
--PMSLGL
--PMSLEI
PKPMSL--
--PMSLYN
--PMSLWH
--PMSLSD
--PMSLHH
--PMSLER
--PMSLEQ
-PPMSLV-
--PMSLYQ
--PMSLYC
--PMSLKW
--PMSLGQ
--PMSLMI
--PMSLHD
--PMSLGN

--PMSLGH
--PMSLEF
--PMSLEA
--PMSLRP
--PMSLQM
--PMSLQF
--PMSLQE
--PMSLNI
--PMSLNG
--PMSLMF
--PMSLKE
--PMSLEY
--PMSLCM
--PMSLAD
--PMSLQY
--PMSLPL
--PMSLHE
--PMSLEN
--PMSLEM
--PMSLED
--PMSLCK
PCPMSL--
PPPMSL--
--PMSLTC
--PMSLSC
--PMSLQW
--PMSLQD
--PMSLDV
--PMSLCV
--PMSLCN
-PPMSLT-
-PPMSLL-
--PMSLPM
--PMSLNE
--PMSLNC
--PMSLGE
--PMSLFC
--PMSLDG
--PMSLDA
--PMSLCS
--PMSLCL
--PMSLCE
QSPMSL--
QVPMSL--
-QPMSLS-
QTPMSL--
QFPMSL--
-QPMSLV-
QLPMSL--
QIPMSL--

QHPMSL--
QKPMSL--
QAPMSL--
-QPMSLL-
QNPMSL--
-QPMSLF-
QGPMSL--
-QPMSLH-
-QPMSLM-
-QPMSLK-
QEPMSL--
-QPMSLR-
-QPMSLQ-
-QPMSLN-
QQPMSL--
-QPMSLA-
-QPMSLT-
QYPMSL--
QWPMSL--
-QPMSLY-
-QPMSLE-
QMPMSL--
-QPMSLW-
QDPMSL--
-QPMSLG-
QCPMSL--
QPPMSL--
RTPMSL--
-RPMSLT-
RSPMSL--
RFPMSL--
RYPMSL--
-RPMSLV-
RHPMSL--
RAPMSL--
REPMSL--
RPPMSL--
-RPMSLM-
-RPMSLI-
-RPMSLA-
RQPMSL--
RNPMSL--
-RPMSLG-
-RPMSLW-
RDPMSL--
-RPMSLE-
-RPMSLQ-
RCPMSL--
-RPMSLC-
SAPMSL--

-SPMSLL-
-SPMSLW-
SVPMSL--
-SPMSLS-
-SPMSLT-
SNPMSL--
-SPMSLI-
-SPMSLV-
SMPMSL--
SPPMSL--
SWPMSL--
STPMSL--
-SPMSLK-
SKPMSL--
-SPMSLN-
-SPMSLA-
SIPMSL--
SQPMSL--
SHPMSL--
-SPMSLY-
SFPMSL--
SEPMSL--
SDPMSL--
-SPMSLE-
-SPMSLG-
-SPMSLM-
SCPMSL--
-SPMSLC-
TRPMSL--
-TPMSLL-
TGPMSL--
TLPMSL--
TIPMSL--
THPMSL--
TKPMSL--
-TPMSLV-
-TPMSLT-
-TPMSLW-
-TPMSLK-
TYPMSL--
-TPMSLH-
TNPMSL--
TMPMSL--
TAPMSL--
-TPMSLA-
-TPMSLS-
-TPMSLN-
-TPMSLM-
TQPMSL--
-TPMSLQ-

TDPMSL--
TFPMSL--
TEPMSL--
-TPMSLG-
-TPMSLF-
-TPMSLE-
VMPMSL--
VSPMSL--
VVPMSL--
VPPMSL--
VNPMSL--
VKPMSL--
VTPMSL--
-VPMSLT-
VIPMSL--
VGPMSL--
VAPMSL--
VYPMSL--
-VPMSLR-
-VPMSLK-
VDPMSL--
-VPMSLV-
VQPMSL--
VFPMSL--
-VPMSLA-
VEPMSL--
-VPMSLH-
-VPMSLN-
-VPMSLM-
-VPMSLG-
VHPMSL--
-VPMSLQ-
VCPMSL--
-VPMSLE-
WRPMSL--
WNPMSL--
WLPMSL--
-WPMSLK-
-WPMSLM-
-WPMSLS-
-WPMSLR-
WSPMSL--
WMPMSL--
WGPMSL--
-WPMSLN-
WQPMSL--
WKPMSL--
-WPMSLT-
-WPMSLH-
-WPMSLG-

	WHPMSL--		
	WFPMSL--		
	WWPMSL--		
	-WPMSLQ-		
	-WPMSLV-		
	WDPMSL--		
	-WPMSLA-		
	-WPMSLE-		
	YRPMSL--		
	YVPMSL--		
	-YPMSLT-		
	-YPMSLR-		
	YLPMSL--		
	YKPMSL--		
	YGPMSL--		
	YSPMSL--		
	-YPMSLL-		
	YDPMSL--		
	YTPMSL--		
	-YPMSLQ-		
	-YPMSLS-		
	-YPMSLN-		
	-YPMSLV-		
	YWPMSL--		
	YPPMSL--		
	YIPMSL--		
	-YPMSLK-		
	YEPMSL--		
	YNPMSL--		
	YAPMSL--		
	YYPMSL--		
	YHPMSL--		
	YQPMSL--		
	-YPMSLM-		
	-YPMSLG-		
	YMPMSL--		
	-YPMSLA-		
	-YPMSLC-		
	-YPMSLE-		
PQAM	418	652	47.11
	ARPQAM--		
	-APQAMR-		
	AGPQAM--		
	AIPQAM--		
	AFPQAM--		
	-APQAMY-		
	-APQAMS-		
	AAPQAM--		
	-APQAMW-		
	AQPQAM--		

-APQAMA-
-APQAMH-
ALPQAM--
-APQAMQ-
-APQAMT-
-APQAMK-
AMPQAM--
AHPQAM--
-APQAMG-
ANPQAM--
AKPQAM--
-APQAMM-
AWPQAM--
AVPQAM--
ATPQAM--
-APQAMV-
-APQAML-
-APQAMF-
CIPQAM--
CRPQAM--
CHPQAM--
CGPQAM--
CCPQAM--
-CPQAMI-
DRPQAM--
-DPQAMR-
DAPQAM--
DVPQAM--
DFPQAM--
-DPQAMF-
DSPQAM--
-DPQAMK-
DLPQAM--
-DPQAMQ-
-DPQAML-
-DPQAMH-
DNPQAM--
DGPQAM--
EKPQAM--
ERPQAM--
-EPQAMI-
EQPQAM--
-EPQAMM-
EYPQAM--
ETPQAM--
-EPQAMH-
EPPQAM--
-EPQAMS-
-EPQAMG-
EAPQAM--

EVPQAM--
-EPQAML-
-EPQAMF-
ECPQAM--
FKPQAM--
FSPQAM--
FVPQAM--
-FPQAMR-
FTPQAM--
-FPQAMQ-
-FPQAML-
FWPQAM--
FQPQAM--
FGPQAM--
-FPQAMY-
-FPQAMS-
FRPQAM--
FLPQAM--
FEPQAM--
-FPQAMN-
FYPQAM--
-FPQAMG-
-FPQAMA-
FHPQAM--
GFPQAM--
GWPQAM--
GSPQAM--
GVPQAM--
-GPQAMT-
GMPQAM--
-GPQAML-
GTPQAM--
-GPQAMV-
GPPQAM--
-GPQAMY-
-GPQAMS-
-GPQAMR-
-GPQAMH-
-GPQAMI-
GYPQAM--
GKPQAM--
GHPQAM--
GGPQAM--
GQPQAM--
GLPQAM--
GRPQAM--
-GPQAMM-
GIPQAM--
GAPQAM--
-GPQAME-

GEPQAM--
GDPQAM--
-GPQAMG-
HQPQAM--
-HPQAML-
-HPQAMS-
HFPQAM--
-HPQAMR-
-HPQAMM-
-HPQAMF-
HWPQAM--
HSPQAM--
-HPQAMI-
HGPQAM--
-HPQAMK-
HLPQAM--
HKPQAM--
-HPQAMY-
-HPQAMQ-
-HPQAMH-
HIPQAM--
HHPQAM--
-IPQAML-
IPPQAM--
-IPQAMK-
-IPQAMF-
-IPQAMS-
IVPQAM--
IRPQAM--
-IPQAMA-
IMPQAM--
-IPQAMY-
-IPQAMR-
ISPQAM--
IKPQAM--
IWPQAM--
-IPQAMQ-
INPQAM--
IIPQAM--
IYPQAM--
ITPQAM--
-IPQAMW-
-IPQAMT-
ILPQAM--
IAPQAM--
-IPQAMV-
KSPQAM--
-KPQAMY-
-KPQAMS-
KTPQAM--

KEPQAM--
-KPQAML-
KVPQAM--
-KPQAMT-
-KPQAMI-
-KPQAMH-
KMPQAM--
KFPQAM--
-KPQAMN-
KRPQAM--
-KPQAMA-
KAPQAM--
KYPQAM--
-KPQAMV-
-KPQAMR-
-KPQAMQ-
-KPQAMK-
KCPQAM--
KWPQAM--
-KPQAMP-
-KPQAMG-
KNPQAM--
KIPQAM--
LRPQAM--
LLPQAM--
LTPQAM--
LHPQAM--
-LPQAMK-
-LPQAMR-
LSPQAM--
-LPQAML-
LMPQAM--
LKPQAM--
-LPQAMQ-
-LPQAMI-
-LPQAMA-
LNPQAM--
LAPQAM--
-LPQAMS-
-LPQAMW-
LEPQAM--
LWPQAM--
-LPQAMY-
-LPQAMV-
-LPQAMT-
-LPQAMH-
LPPQAM--
LDPQAM--
LCPQAM--
-LPQAMN-

-LPQAMG-
LGPQAM--
-MPQAMY-
-MPQAMN-
MEPQAM--
-MPQAME-
-MPQAML-
MSPQAM--
MRPQAM--
-MPQAMH-
-MPQAMF-
-MPQAMT-
MTPQAM--
MPPQAM--
MNPQAM--
MIPQAM--
MVPQAM--
MKPQAM--
MDPQAM--
MWPQAM--
-MPQAMW-
-MPQAMI-
-MPQAMS-
-MPQAMA-
MYPQAM--
MGPQAM--
NVPQAM--
NTPQAM--
-NPQAMM-
-NPQAMK-
-NPQAMH-
-NPQAML-
NGPQAM--
NSPQAM--
NRPQAM--
-NPQAMY-
-NPQAMT-
NNPQAM--
NMPQAM--
-NPQAMR-
-NPQAMI-
NWPQAM--
-NPQAMV-
-NPQAMF-
-NPQAMS-
-NPQAMW-
NLPQAM--
NKPQAM--
NEPQAM--
NAPQAM--

--PQAMFG
--PQAMSQ
--PQAMTV
--PQAMRY
--PQAMSL
--PQAMLR
--PQAMRL
--PQAMWG
--PQAMRN
--PQAMLS
--PQAMQA
--PQAMVK
--PQAMRS
--PQAMSF
--PQAMVT
--PQAMKL
--PQAMLA
--PQAMGS
--PQAMSK
--PQAMRT
--PQAMIQ
--PQAMHS
--PQAMVG
--PQAMTA
--PQAMIW
--PQAMFT
PTPQAM--
--PQAMYA
--PQAMTS
--PQAMTR
--PQAMKT
--PQAMIA
--PQAMHQ
--PQAMEA
--PQAMAY
--PQAMAG
--PQAMAA
--PQAMSV
--PQAMFK
PSPQAM--
--PQAMWW
--PQAMSG
--PQAMRW
--PQAMRK
--PQAMMS
--PQAMLY
--PQAMVY
--PQAMVR
--PQAMTW
--PQAMHL

--PQAMGF
--PQAMFV
--PQAMYT
--PQAMYI
--PQAMSN
--PQAMRH
--PQAMLN
--PQAMLD
--PQAMQN
--PQAMMR
--PQAMLG
--PQAMKF
--PQAMHH
--PQAMVL
--PQAMTG
--PQAMTF
--PQAMRQ
--PQAMMG
--PQAMLL
--PQAMKN
--PQAMVW
--PQAMTK
--PQAMSY
--PQAMSS
--PQAMRR
--PQAMRF
--PQAMRE
--PQAMNF
--PQAMKW
--PQAMIS
--PQAMIR
--PQAMID
--PQAMHT
--PQAMGY
--PQAMFI
--PQAMAF
PWPQAM--
--PQAMWR
--PQAMVN
--PQAMSI
--PQAMRV
--PQAMPI
--PQAMLQ
--PQAMIM
--PQAMGW
--PQAMFE
--PQAMEV
PFPQAM--
--PQAMYD
--PQAMWH

--PQAMRI
--PQAMRG
--PQAMQY
--PQAMQL
--PQAMMH
--PQAMLW
--PQAMLK
--PQAMKS
--PQAMKA
--PQAMAS
--PQAMAN
--PQAMAL
--PQAMWV
--PQAMWT
--PQAMWA
--PQAMVV
--PQAMSA
--PQAMQV
--PQAMML
--PQAMLM
--PQAMLH
--PQAMLE
--PQAMKM
--PQAMGG
--PQAMFW
--PQAMFQ
--PQAMFA
--PQAMET
--PQAMAM
--PQAMAI
--PQAMWQ
--PQAMTN
--PQAMTL
--PQAMNN
--PQAMMY
--PQAMIG
--PQAMHV
--PQAMGV
--PQAMAV
PIPQAM--
--PQAMYY
--PQAMYL
--PQAMVH
--PQAMVF
--PQAMVA
--PQAMTD
--PQAMQK
--PQAMQG
--PQAMPF
--PQAMNW

--PQAMNS
--PQAMMK
--PQAMKQ
--PQAMHM
--PQAMFL
--PQAMAT
--PQAMAK
PEPQAM--
PYPQAM--
--PQAMYN
--PQAMWM
--PQAMWE
--PQAMVM
--PQAMTM
--PQAMRM
--PQAMRD
--PQAMNY
--PQAMNQ
--PQAMNI
--PQAMNG
--PQAMMV
--PQAMMT
--PQAMMN
--PQAMMM
--PQAMLT
--PQAMKK
--PQAMKG
--PQAMIL
--PQAMFY
--PQAMFS
--PQAMFR
--PQAMFN
--PQAMFH
--PQAMEL
--PQAMEK
--PQAMEI
--PQAMEF
--PQAMDY
--PQAMAW
--PQAMAR
PAPQAM--
--PQAMYS
--PQAMYG
--PQAMWS
--PQAMWN
--PQAMVI
--PQAMVD
--PQAMTI
--PQAMTC
--PQAMSW

--PQAMRA
--PQAMPY
--PQAMNR
--PQAMNM
--PQAMNA
--PQAMLI
--PQAMKE
--PQAMIY
--PQAMIV
--PQAMIN
--PQAMHR
--PQAMHA
--PQAMGQ
--PQAMGI
--PQAMGE
--PQAMFM
--PQAMFF
--PQAMER
-PPQAMY-
-PPQAML-
PDPQAM--
-QPQAMA-
QIPQAM--
QTPQAM--
-QPQAMF-
QKPQAM--
QSPQAM--
-QPQAMT-
-QPQAML-
QDPQAM--
QEPQAM--
-QPQAMY-
-QPQAMR-
-QPQAMK-
QRPQAM--
-QPQAMS-
-QPQAME-
QAPQAM--
QQPQAM--
-QPQAMN-
-QPQAMI-
-QPQAMG-
-RPQAMQ-
-RPQAMT-
RTPQAM--
RSPQAM--
-RPQAMN-
-RPQAML-
RIPQAM--
RYPQAM--

-RPQAMF-
RGPQAM--
-RPQAMI-
-RPQAMY-
-RPQAMM-
-RPQAMS-
-RPQAMR-
RRPQAM--
RHPQAM--
-RPQAMH-
-RPQAMA-
RLPQAM--
-RPQAMV-
RFPQAM--
-RPQAME-
RPPQAM--
-RPQAMG-
RNPQAM--
RMPQAM--
RAPQAM--
RVPQAM--
RQPQAM--
-SPQAMV-
-SPQAMR-
SLPQAM--
-SPQAMK-
SVPQAM--
SNPQAM--
SMPQAM--
SIPQAM--
SKPQAM--
-SPQAMW-
SGPQAM--
STPQAM--
-SPQAMS-
SEPQAM--
SFPQAM--
SYPQAM--
-SPQAMY-
-SPQAMQ-
-SPQAMA-
SWPQAM--
SSPQAM--
-SPQAMT-
-SPQAMF-
-SPQAMG-
SDPQAM--
-SPQAMN-
-SPQAMM-
-SPQAME-

```
-SPQAMC-
-TPQAML-
TRPQAM--
-TPQAMM-
TSPQAM--
-TPQAMS-
TLPQAM--
TIPQAM--
TQPQAM--
-TPQAMY-
TAPQAM--
-TPQAMK-
-TPQAMQ-
-TPQAMN-
-TPQAMI-
TKPQAM--
TEPQAM--
-TPQAMV-
-TPQAMR-
TGPQAM--
TTPQAM--
TPPQAM--
TWPQAM--
TVPQAM--
-TPQAMA-
TNPQAM--
TMPQAM--
THPQAM--
VRPQAM--
VWPQAM--
-VPQAMF-
VNPQAM--
VSPQAM--
VLPQAM--
VFPQAM--
VQPQAM--
-VPQAML-
-VPQAMH-
-VPQAMW-
-VPQAMI-
VIPQAM--
VVPQAM--
-VPQAME-
-VPQAMV-
-VPQAMS-
-VPQAMK-
-VPQAMR-
VKPQAM--
VTPQAM--
-VPQAMY-
```

	VPPQAM--		
	VGPQAM--		
	-VPQAMG-		
	VMPQAM--		
	-WPQAMV-		
	WRPQAM--		
	-WPQAML-		
	-WPQAMK-		
	WDPQAM--		
	WAPQAM--		
	WTPQAM--		
	-WPQAMT-		
	-WPQAMS-		
	WHPQAM--		
	WYPQAM--		
	WGPQAM--		
	-WPQAMR-		
	-WPQAMH-		
	-WPQAMW-		
	WVPQAM--		
	-WPQAMY-		
	-WPQAMN-		
	YIPQAM--		
	YSPQAM--		
	-YPQAMY-		
	-YPQAMI-		
	-YPQAMH-		
	-YPQAMT-		
	YLPQAM--		
	YVPQAM--		
	-YPQAMW-		
	-YPQAMQ-		
	-YPQAMK-		
	-YPQAMN-		
	YPPQAM--		
	YTPQAM--		
	-YPQAMV-		
	-YPQAML-		
	-YPQAMS-		
	-YPQAMR-		
	-YPQAMG-		
	-YPQAMA-		
PMGM	148	777	47.08
	-LPMGMN-		
	--PMGMGL		
	-APMGMR-		
	-APMGMS-		
	-APMGMA-		
	ALPMGM--		
	AVPMGM--		

−APMGMY−
−APMGMK−
−APMGMI−
AFPMGM−−
AIPMGM−−
−APMGMV−
AHPMGM−−
−APMGMG−
ATPMGM−−
ARPMGM−−
−APMGML−
AEPMGM−−
APPMGM−−
−APMGMN−
AAPMGM−−
−APMGMT−
−APMGME−
ASPMGM−−
−APMGMQ−
ANPMGM−−
AKPMGM−−
−APMGMW−
AMPMGM−−
AWPMGM−−
−APMGMF−
AGPMGM−−
CRPMGM−−
−CPMGMK−
CDPMGM−−
−CPMGMT−
CHPMGM−−
CYPMGM−−
CTPMGM−−
CPPMGM−−
−CPMGMQ−
CKPMGM−−
CEPMGM−−
−CPMGMR−
−CPMGMH−
−CPMGMA−
CFPMGM−−
DVPMGM−−
DKPMGM−−
−DPMGML−
−DPMGMK−
−DPMGMY−
−DPMGMV−
DNPMGM−−
DRPMGM−−
−DPMGMW−

-DPMGMR-
-DPMGMI-
-DPMGMF-
DFPMGM--
DYPMGM--
-DPMGMT-
-DPMGMQ-
DWPMGM--
DTPMGM--
DMPMGM--
DAPMGM--
-DPMGMS-
DLPMGM--
DEPMGM--
-EPMGMY-
EGPMGM--
-EPMGMM-
-EPMGMA-
EVPMGM--
ERPMGM--
EKPMGM--
ESPMGM--
ETPMGM--
-EPMGMW-
-EPMGML-
-EPMGMI-
-EPMGMV-
ELPMGM--
EYPMGM--
EWPMGM--
-EPMGMD-
-EPMGMR-
-EPMGMK-
-EPMGMG-
-EPMGME-
EMPMGM--
EFPMGM--
-FPMGMS-
-FPMGMH-
-FPMGML-
FVPMGM--
-FPMGMV-
-FPMGMN-
FHPMGM--
-FPMGMR-
FMPMGM--
FLPMGM--
FKPMGM--
FRPMGM--
-FPMGMT-

FAPMGM--
-FPMGMI-
FDPMGM--
FPPMGM--
-FPMGMK-
-FPMGMF-
FGPMGM--
FWPMGM--
-FPMGMY-
-FPMGMW-
-FPMGMA-
FSPMGM--
FIPMGM--
-FPMGMG-
-FPMGMQ-
FNPMGM--
FYPMGM--
-FPMGMM-
GMPMGM--
GYPMGM--
GSPMGM--
-GPMGMW-
GTPMGM--
-GPMGML-
-GPMGMK-
GRPMGM--
GVPMGM--
-GPMGMR-
GAPMGM--
-GPMGMS-
GLPMGM--
-GPMGMM-
GDPMGM--
-GPMGMY-
-GPMGMT-
-GPMGMQ-
GFPMGM--
GNPMGM--
GGPMGM--
-GPMGMI-
-GPMGMH-
-GPMGMF-
-GPMGMA-
GHPMGM--
GPPMGM--
-GPMGMV-
-GPMGMD-
HRPMGM--
-HPMGMS-
HGPMGM--

HVPMGM--
HWPMGM--
-HPMGMY-
-HPMGMV-
-HPMGMM-
HYPMGM--
-HPMGMW-
-HPMGMN-
-HPMGML-
-HPMGMK-
HPPMGM--
HIPMGM--
HFPMGM--
-HPMGMR-
-HPMGMH-
HHPMGM--
HQPMGM--
-HPMGMT-
-HPMGMI-
HLPMGM--
HDPMGM--
-HPMGME-
-HPMGMA-
HKPMGM--
-IPMGMS-
-IPMGMR-
IGPMGM--
IAPMGM--
IYPMGM--
IVPMGM--
ILPMGM--
-IPMGMG-
ISPMGM--
IEPMGM--
-IPMGMN-
-IPMGML-
IWPMGM--
IRPMGM--
-IPMGMK-
-IPMGMA-
INPMGM--
IKPMGM--
IIPMGM--
-IPMGMT-
ITPMGM--
IQPMGM--
IHPMGM--
-IPMGMY-
-IPMGMI-
IFPMGM--

-IPMGMM-
-IPMGMH-
-IPMGMF-
ICPMGM--
KSPMGM--
KGPMGM--
-KPMGMS-
KAPMGM--
-KPMGMA-
-KPMGMN-
-KPMGMF-
KWPMGM--
KLPMGM--
KYPMGM--
-KPMGML-
KKPMGM--
-KPMGMG-
KRPMGM--
KPPMGM--
-KPMGMV-
KHPMGM--
KQPMGM--
-KPMGMT-
-KPMGMK-
-KPMGMY-
-KPMGMR-
KNPMGM--
KTPMGM--
-KPMGMM-
KEPMGM--
-KPMGMI-
KDPMGM--
-KPMGMH-
-KPMGMD-
KIPMGM--
KFPMGM--
-LPMGMK-
LRPMGM--
LNPMGM--
LVPMGM--
-LPMGMS-
-LPMGMR-
LGPMGM--
-LPMGMH-
-LPMGMY-
-LPMGMV-
-LPMGMQ-
LDPMGM--
LMPMGM--
LFPMGM--

LSPMGM--
-LPMGMW-
-LPMGMI-
LTPMGM--
LAPMGM--
LQPMGM--
-LPMGMA-
LHPMGM--
LYPMGM--
-LPMGML-
-LPMGME-
-LPMGMM-
-LPMGMT-
-LPMGMG-
LIPMGM--
LPPMGM--
LEPMGM--
LCPMGM--
MRPMGM--
MAPMGM--
-MPMGMS-
MFPMGM--
MQPMGM--
MNPMGM--
MLPMGM--
MSPMGM--
MWPMGM--
-MPMGMV-
-MPMGMK-
-MPMGMA-
-MPMGMI-
MTPMGM--
-MPMGMW-
-MPMGML-
-MPMGMM-
-MPMGMF-
-MPMGME-
MKPMGM--
MEPMGM--
MVPMGM--
-MPMGMR-
-MPMGMN-
MMPMGM--
-MPMGMH-
MIPMGM--
MGPMGM--
-MPMGMY-
-MPMGMG-
MHPMGM--
-NPMGMR-

NSPMGM--
NLPMGM--
NWPMGM--
-NPMGMK-
-NPMGMN-
NGPMGM--
-NPMGMY-
NIPMGM--
-NPMGMS-
-NPMGML-
NHPMGM--
-NPMGMG-
NRPMGM--
-NPMGMI-
-NPMGMH-
-NPMGMA-
NNPMGM--
NMPMGM--
NEPMGM--
-NPMGMV-
-NPMGMM-
NVPMGM--
--PMGMKT
--PMGMSY
--PMGMSG
--PMGMRH
--PMGMLK
--PMGMRS
--PMGMLV
--PMGMWR
--PMGMTT
--PMGMVR
--PMGMYA
--PMGMRG
--PMGMVS
--PMGMLR
--PMGMLS
--PMGMLQ
--PMGMKS
--PMGMYQ
--PMGMTR
--PMGMYT
--PMGMHR
--PMGMSW
--PMGMRR
--PMGMRQ
--PMGMKV
--PMGMKN
--PMGMYR
--PMGMSR

--PMGMQR
--PMGMMH
--PMGMAS
--PMGMRI
--PMGMQS
--PMGMSM
--PMGMRA
--PMGMMG
--PMGMFR
--PMGMMT
--PMGMFM
--PMGMWH
--PMGMVG
--PMGMRV
--PMGMTH
--PMGMST
--PMGMSI
--PMGMSD
--PMGMKM
--PMGMFN
--PMGMFF
--PMGMYH
--PMGMVY
--PMGMVD
--PMGMTF
--PMGMRF
--PMGMNG
PMPMGM--
--PMGMVI
--PMGMTV
--PMGMRT
--PMGMIV
--PMGMWS
--PMGMWN
--PMGMRM
--PMGMRK
--PMGMNM
--PMGMAI
--PMGMTA
--PMGMSL
--PMGMSA
--PMGMNS
--PMGMIT
--PMGMAL
--PMGMTI
--PMGMSH
--PMGMMN
--PMGMMD
PIPMGM--
--PMGMYK

--PMGMYG
--PMGMWE
--PMGMVL
--PMGMTN
--PMGMTD
--PMGMRW
--PMGMIF
--PMGMDG
PYPMGM--
--PMGMVN
--PMGMTM
--PMGMSV
--PMGMNT
--PMGMMW
--PMGMMV
--PMGMMI
--PMGMGT
--PMGMFD
--PMGMES
--PMGMAV
--PMGMVT
--PMGMTY
--PMGMNR
--PMGMML
--PMGMLI
--PMGMLH
--PMGMLD
--PMGMIH
--PMGMHL
--PMGMHE
--PMGMGS
--PMGMGR
--PMGMGA
--PMGMEA
--PMGMAH
--PMGMAG
PAPMGM--
--PMGMSQ
--PMGMRE
--PMGMRD
--PMGMMR
--PMGMKF
--PMGMIK
--PMGMHM
--PMGMGG
--PMGMFT
--PMGMEW
--PMGMEG
PKPMGM--
PWPMGM--

--PMGMWA
--PMGMVM
--PMGMVE
--PMGMVA
--PMGMTK
--PMGMTG
--PMGMQG
--PMGMQE
--PMGMLT
--PMGMLA
--PMGMKA
--PMGMIS
--PMGMIA
--PMGMHQ
--PMGMFH
--PMGMFG
--PMGMFA
--PMGMAA
--PMGMWM
--PMGMRN
--PMGMQA
--PMGMMQ
--PMGMLM
--PMGMKD
--PMGMID
--PMGMGW
--PMGMYE
--PMGMSN
--PMGMSE
--PMGMQN
--PMGMQI
--PMGMNN
--PMGMNA
--PMGMKY
--PMGMIY
--PMGMIG
--PMGMHK
--PMGMGV
--PMGMGN
--PMGMFK
--PMGMED
--PMGMDA
PGPMGM--
PVPMGM--
--PMGMYY
--PMGMYS
--PMGMWG
--PMGMVK
--PMGMQY
--PMGMQQ

--PMGMQK
--PMGMQH
--PMGMNL
--PMGMMK
--PMGMMF
--PMGMMA
--PMGMKG
--PMGMII
--PMGMGY
--PMGMGE
--PMGMFV
--PMGMEY
--PMGMET
--PMGMAN
PNPMGM--
--PMGMWK
--PMGMVH
--PMGMTE
--PMGMRP
--PMGMQL
--PMGMND
--PMGMMS
--PMGMLE
--PMGMKQ
--PMGMKE
--PMGMIQ
--PMGMIE
--PMGMHV
--PMGMHN
--PMGMHG
--PMGMFL
--PMGMEI
--PMGMEF
--PMGMAR
-PPMGMF-
--PMGMVV
--PMGMVQ
--PMGMVF
--PMGMQM
--PMGMNQ
--PMGMNF
--PMGMNE
--PMGMLN
--PMGMIR
--PMGMHY
--PMGMHI
--PMGMHA
--PMGMGQ
--PMGMFS
--PMGMER

```
--PMGMEK
--PMGMDM
--PMGMCK
--PMGMCE
--PMGMAK
--PMGMAD
-QPMGMR-
QRPMGM--
-QPMGMK-
QSPMGM--
-QPMGML-
-QPMGMI-
QIPMGM--
QLPMGM--
QVPMGM--
-QPMGMM-
QKPMGM--
QYPMGM--
-QPMGME-
-QPMGMW-
-QPMGMN-
QTPMGM--
-QPMGMY-
-QPMGMQ-
-QPMGMA-
QAPMGM--
QQPMGM--
-QPMGMT-
RSPMGM--
-RPMGMR-
RLPMGM--
-RPMGMF-
-RPMGML-
RGPMGM--
RHPMGM--
RFPMGM--
RRPMGM--
-RPMGMK-
RYPMGM--
-RPMGMH-
-RPMGMI-
RPPMGM--
-RPMGMV-
-RPMGMY-
RKPMGM--
RAPMGM--
-RPMGMT-
RTPMGM--
-RPMGMW-
-RPMGMA-
```

RWPMGM--
-RPMGMG-
-RPMGMN-
RMPMGM--
-RPMGMM-
RVPMGM--
RDPMGM--
-RPMGME-
-RPMGMQ-
RCPMGM--
RIPMGM--
REPMGM--
RQPMGM--
RNPMGM--
-SPMGMS-
-SPMGML-
-SPMGMY-
-SPMGMQ-
-SPMGMI-
SNPMGM--
SHPMGM--
SFPMGM--
-SPMGMH-
SVPMGM--
STPMGM--
-SPMGMV-
-SPMGMF-
-SPMGMG-
-SPMGMK-
-SPMGMW-
-SPMGMT-
-SPMGMM-
SSPMGM--
-SPMGMA-
SLPMGM--
SYPMGM--
SPPMGM--
SAPMGM--
SKPMGM--
SIPMGM--
SQPMGM--
SDPMGM--
SWPMGM--
-SPMGME-
SGPMGM--
-SPMGMN-
TRPMGM--
TWPMGM--
-TPMGMW-
-TPMGMI-

TLPMGM--
-TPMGML-
TAPMGM--
TVPMGM--
-TPMGMK-
-TPMGMA-
TGPMGM--
-TPMGMM-
TNPMGM--
TSPMGM--
-TPMGMS-
TDPMGM--
TYPMGM--
TTPMGM--
-TPMGMT-
-TPMGMH-
TPPMGM--
-TPMGMR-
TFPMGM--
TQPMGM--
-TPMGMN-
TMPMGM--
TKPMGM--
-TPMGMY-
-TPMGMV-
-TPMGMQ-
-TPMGME-
TIPMGM--
TEPMGM--
THPMGM--
-VPMGML-
VLPMGM--
VRPMGM--
-VPMGMY-
-VPMGMH-
VKPMGM--
VTPMGM--
VHPMGM--
VSPMGM--
-VPMGMF-
-VPMGMS-
VIPMGM--
VAPMGM--
-VPMGMA-
-VPMGMG-
VVPMGM--
-VPMGMV-
VGPMGM--
VPPMGM--
-VPMGMW-

-VPMGMI-
VNPMGM--
VFPMGM--
-VPMGMT-
-VPMGMK-
-VPMGME-
VYPMGM--
VWPMGM--
VQPMGM--
-VPMGMQ-
-VPMGMM-
VDPMGM--
-VPMGMN-
-VPMGMD-
-VPMGMC-
-WPMGMS-
WKPMGM--
-WPMGMF-
WVPMGM--
WLPMGM--
-WPMGMK-
-WPMGMA-
-WPMGMH-
WWPMGM--
-WPMGMT-
WDPMGM--
WSPMGM--
WPPMGM--
WMPMGM--
-WPMGMV-
-WPMGMN-
-WPMGMG-
WTPMGM--
-WPMGMW-
-WPMGMR-
-WPMGML-
WIPMGM--
WEPMGM--
-WPMGMI-
WGPMGM--
-YPMGMR-
-YPMGML-
-YPMGMS-
YKPMGM--
YFPMGM--
-YPMGMT-
-YPMGMN-
-YPMGMH-
-YPMGMF-
YVPMGM--

YYPMGM--
-YPMGMG-
YTPMGM--
-YPMGMK-
-YPMGME-
YDPMGM--
YSPMGM--
YLPMGM--
YGPMGM--
YWPMGM--
-YPMGMY-
YIPMGM--
-YPMGMV-
-YPMGMM-
-YPMGMI-
-YPMGMA-
YHPMGM--
-YPMGMQ-
-YPMGMP-
YAPMGM--

PLSI	39	862	47.05

-DPLSIA-
-LPLSIE-
-NPLSIG-
-NPLSIE-
--PLSIRP
--PLSIPW
--PLSIPR
--PLSIDK
--PLSIDA
--PLSIAP
ARPLSI--
-APLSIR-
AQPLSI--
-APLSIM-
AVPLSI--
-APLSIY-
-APLSIT-
-APLSIL-
-APLSIK-
AAPLSI--
AGPLSI--
AIPLSI--
APPLSI--
-APLSIV-
-APLSIA-
-APLSIN-
-APLSII-
ANPLSI--
ATPLSI--

-APLSIF-
AWPLSI--
-APLSIW-
-APLSIG-
AFPLSI--
-APLSIH-
AMPLSI--
AEPLSI--
ADPLSI--
-APLSIE-
-APLSIP-
-APLSID-
CPPLSI--
CSPLSI--
CFPLSI--
CTPLSI--
CLPLSI--
CGPLSI--
CCPLSI--
CEPLSI--
-CPLSIR-
CAPLSI--
CVPLSI--
-CPLSIM-
CNPLSI--
CQPLSI--
-CPLSIF-
CKPLSI--
CWPLSI--
-CPLSIV-
-CPLSIT-
-CPLSIS-
-CPLSIK-
-CPLSIA-
CIPLSI--
CYPLSI--
-CPLSIY-
-CPLSIW-
-CPLSII-
CMPLSI--
CDPLSI--
DVPLSI--
DRPLSI--
-DPLSIR-
-DPLSIL-
DMPLSI--
DKPLSI--
-DPLSIF-
DWPLSI--
DFPLSI--

DIPLSI--
DAPLSI--
-DPLSIT-
DGPLSI--
DYPLSI--
DNPLSI--
-DPLSIV-
DLPLSI--
DQPLSI--
DTPLSI--
-DPLSIM-
-DPLSIK-
-DPLSIQ-
-DPLSII-
DHPLSI--
-DPLSIS-
-DPLSIW-
DCPLSI--
-EPLSIL-
ERPLSI--
EVPLSI--
ELPLSI--
EGPLSI--
-EPLSIV-
EQPLSI--
-EPLSIS-
-EPLSIK-
-EPLSIH-
EAPLSI--
-EPLSII-
-EPLSIT-
-EPLSIR-
-EPLSIA-
-EPLSIY-
ETPLSI--
ENPLSI--
EIPLSI--
EPPLSI--
-EPLSIW-
EFPLSI--
-EPLSIN-
EKPLSI--
EMPLSI--
-EPLSIQ-
-EPLSIG-
EWPLSI--
EEPLSI--
EYPLSI--
-EPLSIM-
-FPLSIL-

FRPLSI--
FQPLSI--
-FPLSIR-
-FPLSIT-
-FPLSIH-
FLPLSI--
FGPLSI--
FAPLSI--
FTPLSI--
FSPLSI--
FYPLSI--
-FPLSIQ-
FHPLSI--
FVPLSI--
FNPLSI--
-FPLSIY-
FEPLSI--
-FPLSIK-
FFPLSI--
-FPLSII-
-FPLSIN-
-FPLSIG-
FIPLSI--
-FPLSIW-
-FPLSIE-
-FPLSIA-
FDPLSI--
FPPLSI--
FCPLSI--
FMPLSI--
-GPLSIR-
GGPLSI--
GKPLSI--
GVPLSI--
GLPLSI--
-GPLSIL-
GTPLSI--
GRPLSI--
-GPLSIT-
GYPLSI--
-GPLSIS-
GIPLSI--
GDPLSI--
-GPLSIW-
GAPLSI--
-GPLSIA-
-GPLSIN-
GWPLSI--
GNPLSI--
GMPLSI--

GHPLSI--
-GPLSIH-
GFPLSI--
-GPLSIY-
-GPLSII-
GEPLSI--
-GPLSIM-
-GPLSIK-
-GPLSIG-
GPPLSI--
-GPLSIQ-
-GPLSIE-
-GPLSIP-
-GPLSIC-
GCPLSI--
-HPLSIR-
HAPLSI--
-HPLSIL-
HSPLSI--
HLPLSI--
HEPLSI--
-HPLSIS-
HVPLSI--
-HPLSII-
-HPLSIV-
HYPLSI--
HGPLSI--
HPPLSI--
HTPLSI--
HMPLSI--
HWPLSI--
-HPLSIK-
HKPLSI--
-HPLSIH-
-HPLSIA-
-HPLSIY-
HIPLSI--
-HPLSIQ-
HFPLSI--
-HPLSIE-
HHPLSI--
HQPLSI--
-HPLSIM-
-HPLSIG-
-HPLSIF-
HDPLSI--
IRPLSI--
ILPLSI--
-IPLSIR-
IKPLSI--

IVPLSI--
IGPLSI--
-IPLSIM-
ISPLSI--
-IPLSIV-
IPPLSI--
-IPLSIL-
-IPLSIT-
IMPLSI--
IAPLSI--
-IPLSIA-
IDPLSI--
IYPLSI--
-IPLSIN-
ITPLSI--
-IPLSIQ-
-IPLSII-
IQPLSI--
-IPLSIK-
INPLSI--
IHPLSI--
IEPLSI--
-IPLSIW-
IIPLSI--
-IPLSIG-
-IPLSIE-
-IPLSIC-
ICPLSI--
KSPLSI--
KVPLSI--
-KPLSIL-
KGPLSI--
KAPLSI--
-KPLSIY-
KRPLSI--
-KPLSIS-
KNPLSI--
KEPLSI--
-KPLSIT-
KIPLSI--
-KPLSII-
-KPLSIF-
KMPLSI--
KHPLSI--
-KPLSIK-
KKPLSI--
-KPLSIG-
KFPLSI--
KYPLSI--
-KPLSIQ-

-KPLSIA-
-KPLSIM-
-KPLSIW-
KPPLSI--
-KPLSIE-
KWPLSI--
KDPLSI--
-KPLSIN-
KQPLSI--
-KPLSIC-
KCPLSI--
-LPLSIR-
LRPLSI--
LSPLSI--
LTPLSI--
LKPLSI--
LGPLSI--
-LPLSII-
LNPLSI--
-LPLSIY-
LEPLSI--
LPPLSI--
LWPLSI--
LQPLSI--
-LPLSIT-
-LPLSIM-
LMPLSI--
LFPLSI--
-LPLSIV-
-LPLSIW-
-LPLSIH-
LDPLSI--
-LPLSIQ-
-LPLSIK-
LYPLSI--
-LPLSIA-
LCPLSI--
MRPLSI--
-MPLSIL-
MSPLSI--
MKPLSI--
-MPLSIS-
MGPLSI--
-MPLSIR-
MIPLSI--
MWPLSI--
MTPLSI--
MPPLSI--
-MPLSIK-
MNPLSI--

MAPLSI--
-MPLSIY-
-MPLSII-
-MPLSIW-
-MPLSIA-
MDPLSI--
-MPLSIT-
-MPLSIN-
-MPLSIH-
-MPLSIM-
MEPLSI--
-MPLSIV-
-MPLSIF-
-MPLSIG-
-MPLSIQ-
MQPLSI--
-MPLSIE-
MMPLSI--
MHPLSI--
MCPLSI--
NVPLSI--
-NPLSIR-
NTPLSI--
NKPLSI--
NLPLSI--
NSPLSI--
-NPLSII-
NQPLSI--
-NPLSIY-
NAPLSI--
NMPLSI--
NYPLSI--
NPPLSI--
-NPLSIL-
NHPLSI--
-NPLSIK-
-NPLSIS-
-NPLSIH-
-NPLSIW-
-NPLSIV-
-NPLSIT-
-NPLSIM-
-NPLSIF-
-NPLSIQ-
NEPLSI--
-NPLSIN-
NIPLSI--
NFPLSI--
NDPLSI--
NWPLSI--

```
-NPLSIA-
NCPLSI--
--PLSISI
--PLSILS
--PLSIRL
PRPLSI--
--PLSISS
--PLSIWR
--PLSIRH
--PLSILT
--PLSIRA
--PLSIRK
--PLSIRI
--PLSISG
--PLSIGS
--PLSIYR
--PLSIRG
--PLSIMR
--PLSITR
--PLSILG
--PLSIWS
--PLSIAS
PGPLSI--
--PLSIST
--PLSILN
--PLSIAA
--PLSIVS
--PLSIVK
--PLSINS
--PLSIHG
--PLSIVT
--PLSIHR
--PLSIAR
--PLSIRV
--PLSIFR
--PLSIKN
--PLSIVY
--PLSISM
--PLSIRF
--PLSIIS
--PLSINT
--PLSIYS
--PLSIRD
--PLSIQR
--PLSIYT
--PLSIRE
--PLSIKF
--PLSIIT
--PLSIRQ
--PLSIMS
```

--PLSILQ
--PLSIKS
--PLSIAG
--PLSIYV
--PLSIQA
--PLSIIA
--PLSIYK
--PLSISH
--PLSIHL
--PLSIAV
--PLSIYD
--PLSIMN
--PLSIMG
--PLSIHT
--PLSIHN
--PLSIHM
--PLSITS
--PLSIFK
--PLSIQS
--PLSIMT
--PLSIKG
--PLSITW
--PLSINM
--PLSIML
--PLSIHW
--PLSIHS
--PLSIYQ
--PLSISW
--PLSIIN
--PLSIFT
--PLSIFG
--PLSIYM
--PLSITM
--PLSIKM
--PLSIKL
--PLSIFS
--PLSIVI
--PLSIVA
--PLSIHQ
--PLSIWY
--PLSIVD
--PLSITN
--PLSISK
--PLSIQT
--PLSILW
--PLSILD
--PLSIET
--PLSIAN
PNPLSI--
--PLSITT

--PLSITQ
--PLSIWK
--PLSIWH
--PLSITA
--PLSIRY
--PLSIQN
--PLSILE
--PLSINA
--PLSIKT
--PLSIIG
--PLSIIE
--PLSIGV
PEPLSI--
--PLSINR
--PLSIMD
--PLSIES
--PLSIAK
--PLSIQK
--PLSIKV
--PLSIYG
--PLSISQ
--PLSIMV
--PLSIEG
--PLSIYY
--PLSIWN
--PLSIVV
--PLSITG
--PLSIQQ
--PLSIHA
--PLSIAW
--PLSIWT
--PLSIIY
--PLSIFD
PDPLSI--
--PLSIKK
PTPLSI--
--PLSIVQ
--PLSIVN
--PLSISY
--PLSING
--PLSIMQ
--PLSIMK
--PLSIFE
PWPLSI--
--PLSIYN
--PLSIYI
--PLSINY
--PLSINW
--PLSIKW
--PLSIGW

--PLSISE
--PLSISD
--PLSIQM
--PLSIHK
--PLSIAT
PKPLSI--
--PLSIQG
--PLSIKA
--PLSIHF
--PLSIFQ
--PLSIER
--PLSIEA
PHPLSI--
--PLSISN
--PLSIQV
--PLSIWG
--PLSITF
--PLSIGM
--PLSIWQ
--PLSIQE
--PLSIMW
--PLSIMH
--PLSIIH
--PLSIGY
--PLSIGA
--PLSIEI
--PLSIAQ
--PLSIWV
--PLSIVW
--PLSITY
--PLSIQI
--PLSIKD
--PLSIQY
--PLSIQW
--PLSINV
--PLSIKQ
--PLSIIW
--PLSIHD
--PLSIGN
--PLSIEF
--PLSIAH
--PLSIVE
--PLSIQL
--PLSIQF
--PLSINN
--PLSIMM
--PLSIIV
--PLSIFN
PQPLSI--
--PLSIYW

```
--PLSIME
--PLSIGR
--PLSIEN
--PLSIYH
--PLSIWM
--PLSIVC
--PLSITE
--PLSIQH
--PLSINL
--PLSINH
--PLSIMI
--PLSIKI
--PLSIKE
--PLSIID
--PLSIHV
--PLSIGI
--PLSIGH
--PLSIGF
--PLSIEW
--PLSIEM
--PLSIEL
--PLSIYE
--PLSINF
--PLSIEY
--PLSIEV
--PLSIAM
--PLSIAI
PCPLSI--
--PLSIWW
--PLSIWD
--PLSITD
--PLSISC
--PLSINQ
--PLSIIM
--PLSIHY
--PLSIGK
--PLSIDS
--PLSIAE
--PLSIYC
--PLSINE
--PLSIND
--PLSICR
--PLSIAD
PFPLSI--
-PPLSIV-
-PPLSIT-
--PLSIQD
--PLSINI
--PLSIIQ
--PLSIHI
```

--PLSIGQ
--PLSIFC
--PLSIEQ
--PLSIEK
--PLSIEH
--PLSIDG
--PLSICT
--PLSIAC
-PPLSIY-
-PPLSIS-
-PPLSIM-
-PPLSIG-
--PLSIPT
--PLSINP
--PLSIMP
--PLSIMC
--PLSIKH
--PLSIHE
--PLSIGC
--PLSIFH
--PLSIDW
--PLSICY
--PLSICN
--PLSICK
--PLSICI
-QPLSIM-
QTPLSI--
-QPLSIT-
QLPLSI--
-QPLSIR-
-QPLSIL-
QSPLSI--
-QPLSIY-
-QPLSIS-
-QPLSIW-
-QPLSIA-
QAPLSI--
QQPLSI--
QGPLSI--
QVPLSI--
-QPLSIH-
QWPLSI--
QPPLSI--
QYPLSI--
-QPLSIK-
-QPLSIV-
-QPLSIG-
-QPLSIF-
QNPLSI--
QDPLSI--

QKPLSI--
QIPLSI--
-QPLSIN-
QEPLSI--
-QPLSIE-
QHPLSI--
-QPLSIQ-
QCPLSI--
RSPLSI--
-RPLSIL-
RGPLSI--
-RPLSIS-
RMPLSI--
RVPLSI--
-RPLSIR-
RAPLSI--
RIPLSI--
RWPLSI--
-RPLSIM-
RTPLSI--
-RPLSIK-
-RPLSIV-
-RPLSIY-
REPLSI--
-RPLSIT-
-RPLSIA-
-RPLSIQ-
-RPLSIH-
-RPLSIG-
RFPLSI--
RNPLSI--
-RPLSIW-
RQPLSI--
-RPLSII-
-RPLSIF-
RDPLSI--
-RPLSIE-
RCPLSI--
SRPLSI--
SLPLSI--
-SPLSIS-
SVPLSI--
-SPLSII-
SSPLSI--
SAPLSI--
SFPLSI--
SNPLSI--
SMPLSI--
STPLSI--
-SPLSIV-

-SPLSIL-
SKPLSI--
-SPLSIY-
-SPLSIF-
SIPLSI--
SHPLSI--
-SPLSIH-
-SPLSIK-
SEPLSI--
-SPLSIW-
-SPLSIA-
-SPLSIN-
SWPLSI--
-SPLSIM-
SDPLSI--
SQPLSI--
SPPLSI--
-SPLSIG-
-SPLSIE-
SCPLSI--
-SPLSIC-
TRPLSI--
-TPLSIR-
TTPLSI--
TVPLSI--
-TPLSIL-
TGPLSI--
TLPLSI--
-TPLSIS-
-TPLSIT-
TWPLSI--
-TPLSIY-
-TPLSIK-
TPPLSI--
TAPLSI--
TFPLSI--
-TPLSIM-
TNPLSI--
TEPLSI--
TDPLSI--
TQPLSI--
-TPLSIA-
-TPLSIN-
TYPLSI--
THPLSI--
-TPLSIE-
-VPLSIL-
VRPLSI--
VTPLSI--
VKPLSI--

VLPLSI--
VPPLSI--
-VPLSIS-
-VPLSIQ-
VYPLSI--
-VPLSIV-
-VPLSII-
VAPLSI--
VMPLSI--
VDPLSI--
-VPLSIK-
VEPLSI--
-VPLSIW-
VFPLSI--
-VPLSIH-
-VPLSIN-
-VPLSIF-
-VPLSIA-
VWPLSI--
VIPLSI--
-VPLSIE-
VCPLSI--
WSPLSI--
WHPLSI--
WRPLSI--
WNPLSI--
WGPLSI--
WPPLSI--
-WPLSIT-
-WPLSIR-
WQPLSI--
WVPLSI--
-WPLSIV-
-WPLSIS-
-WPLSIQ-
-WPLSIK-
-WPLSIY-
-WPLSIW-
WDPLSI--
-WPLSIL-
-WPLSIA-
WYPLSI--
-WPLSIM-
WAPLSI--
-WPLSII-
WIPLSI--
WFPLSI--
-WPLSIG-
-WPLSIE-
YRPLSI--

	YSPLSI--		
	YLPLSI--		
	-YPLSIR-		
	YTPLSI--		
	YKPLSI--		
	-YPLSIT-		
	-YPLSIS-		
	YMPLSI--		
	YAPLSI--		
	YFPLSI--		
	YVPLSI--		
	-YPLSIV-		
	-YPLSIL-		
	YGPLSI--		
	-YPLSIQ-		
	YWPLSI--		
	-YPLSIN-		
	YPPLSI--		
	YNPLSI--		
	-YPLSII-		
	-YPLSIY-		
	YDPLSI--		
	YYPLSI--		
	-YPLSIK-		
	-YPLSIH-		
	YEPLSI--		
	-YPLSIA-		
	YHPLSI--		
	YQPLSI--		
	-YPLSIW-		
	-YPLSIM-		
	YCPLSI--		
	-YPLSIE-		
PLPY	50	850	47.02
	FTPLPY--		
	-LPLPYN-		
	-APLPYT-		
	ASPLPY--		
	AKPLPY--		
	-APLPYK-		
	-APLPYS-		
	AMPLPY--		
	AVPLPY--		
	ALPLPY--		
	-APLPYM-		
	-APLPYI-		
	AYPLPY--		
	ARPLPY--		
	-APLPYL-		
	-APLPYY-		

AWPLPY--
ATPLPY--
-APLPYW-
-APLPYR-
-APLPYH-
AAPLPY--
AQPLPY--
-APLPYF-
ANPLPY--
AGPLPY--
AHPLPY--
-APLPYE-
AEPLPY--
-APLPYN-
AIPLPY--
-APLPYV-
-APLPYC-
CAPLPY--
CVPLPY--
CSPLPY--
CRPLPY--
CEPLPY--
CTPLPY--
-CPLPYL-
-CPLPYI-
CNPLPY--
CHPLPY--
CFPLPY--
-CPLPYV-
-CPLPYK-
CMPLPY--
DQPLPY--
-DPLPYR-
DVPLPY--
-DPLPYL-
DTPLPY--
DSPLPY--
-DPLPYS-
DLPLPY--
-DPLPYI-
DYPLPY--
DRPLPY--
-DPLPYT-
DAPLPY--
-DPLPYY-
DHPLPY--
DGPLPY--
DFPLPY--
-DPLPYV-
-DPLPYH-

−DPLPYF−
DNPLPY−−
−DPLPYN−
DWPLPY−−
DKPLPY−−
DIPLPY−−
DEPLPY−−
ELPLPY−−
EVPLPY−−
−EPLPYK−
−EPLPYR−
ETPLPY−−
EKPLPY−−
−EPLPYL−
−EPLPYS−
EWPLPY−−
ERPLPY−−
−EPLPYQ−
−EPLPYI−
ENPLPY−−
ESPLPY−−
EIPLPY−−
−EPLPYM−
−EPLPYA−
−EPLPYH−
EFPLPY−−
EQPLPY−−
−EPLPYV−
−EPLPYG−
−EPLPYE−
EHPLPY−−
EAPLPY−−
−EPLPYF−
−EPLPYC−
EDPLPY−−
FRPLPY−−
FSPLPY−−
−FPLPYR−
−FPLPYK−
FVPLPY−−
−FPLPYI−
−FPLPYQ−
FHPLPY−−
−FPLPYW−
−FPLPYT−
FAPLPY−−
−FPLPYM−
−FPLPYF−
FGPLPY−−
−FPLPYS−

FEPLPY--
-FPLPYY-
-FPLPYA-
FLPLPY--
-FPLPYG-
FNPLPY--
FPPLPY--
-FPLPYV-
-FPLPYH-
FMPLPY--
FWPLPY--
FKPLPY--
FFPLPY--
FYPLPY--
FQPLPY--
GRPLPY--
-GPLPYR-
GTPLPY--
GKPLPY--
-GPLPYL-
-GPLPYS-
GIPLPY--
GVPLPY--
-GPLPYQ-
GYPLPY--
-GPLPYF-
GFPLPY--
-GPLPYH-
GNPLPY--
-GPLPYM-
-GPLPYT-
GAPLPY--
-GPLPYK-
GSPLPY--
-GPLPYW-
GDPLPY--
GQPLPY--
-GPLPYA-
GHPLPY--
GWPLPY--
GMPLPY--
-GPLPYV-
-GPLPYG-
GEPLPY--
-GPLPYN-
HMPLPY--
HRPLPY--
HVPLPY--
-HPLPYI-
HQPLPY--

HSPLPY--
HIPLPY--
HTPLPY--
-HPLPYS-
HDPLPY--
-HPLPYL-
-HPLPYK-
HWPLPY--
-HPLPYF-
HLPLPY--
HFPLPY--
HAPLPY--
-HPLPYW-
HPPLPY--
-HPLPYH-
HNPLPY--
-HPLPYT-
-HPLPYA-
HYPLPY--
HKPLPY--
HGPLPY--
-HPLPYQ-
-HPLPYY-
HHPLPY--
HEPLPY--
-HPLPYV-
-HPLPYM-
ITPLPY--
ILPLPY--
-IPLPYR-
-IPLPYT-
IQPLPY--
-IPLPYF-
-IPLPYV-
-IPLPYL-
INPLPY--
-IPLPYK-
ISPLPY--
-IPLPYW-
IAPLPY--
IHPLPY--
IKPLPY--
-IPLPYI-
-IPLPYS-
-IPLPYN-
IMPLPY--
IYPLPY--
IVPLPY--
-IPLPYG-
-IPLPYQ-

IGPLPY--
-IPLPYY-
IPPLPY--
-IPLPYM-
IDPLPY--
IFPLPY--
-IPLPYA-
-IPLPYE-
ICPLPY--
KTPLPY--
KKPLPY--
KRPLPY--
-KPLPYS-
KMPLPY--
KVPLPY--
KYPLPY--
KLPLPY--
-KPLPYK-
-KPLPYY-
-KPLPYI-
-KPLPYA-
KIPLPY--
KFPLPY--
KQPLPY--
-KPLPYE-
KEPLPY--
KAPLPY--
-KPLPYN-
-KPLPYG-
KNPLPY--
-KPLPYW-
-KPLPYQ-
LRPLPY--
LLPLPY--
LGPLPY--
LKPLPY--
-LPLPYT-
-LPLPYR-
LSPLPY--
LAPLPY--
LYPLPY--
-LPLPYL-
LPPLPY--
LFPLPY--
-LPLPYS-
-LPLPYV-
LEPLPY--
-LPLPYM-
-LPLPYA-
LIPLPY--

–LPLPYF–
LNPLPY––
LQPLPY––
LWPLPY––
–LPLPYW–
–LPLPYQ–
–LPLPYK–
–LPLPYI–
LCPLPY––
–LPLPYH–
LDPLPY––
–LPLPYG–
MRPLPY––
MSPLPY––
–MPLPYH–
MLPLPY––
–MPLPYR–
MQPLPY––
MFPLPY––
–MPLPYT–
MAPLPY––
MNPLPY––
MYPLPY––
MTPLPY––
MPPLPY––
–MPLPYI–
MIPLPY––
MMPLPY––
MHPLPY––
–MPLPYS–
–MPLPYF–
–MPLPYY–
–MPLPYN–
–MPLPYA–
–MPLPYW–
MEPLPY––
MKPLPY––
MDPLPY––
MGPLPY––
–MPLPYQ–
MVPLPY––
–MPLPYK–
–MPLPYG–
MCPLPY––
–MPLPYE–
–NPLPYL–
NTPLPY––
–NPLPYR–
NVPLPY––
–NPLPYV–

NRPLPY--
NKPLPY--
-NPLPYS-
NLPLPY--
-NPLPYT-
NEPLPY--
NAPLPY--
-NPLPYW-
-NPLPYH-
NYPLPY--
NMPLPY--
NGPLPY--
-NPLPYY-
-NPLPYK-
-NPLPYI-
NWPLPY--
NPPLPY--
NHPLPY--
-NPLPYM-
-NPLPYF-
-NPLPYA-
-NPLPYN-
NQPLPY--
NFPLPY--
--PLPYLS
--PLPYLT
--PLPYRL
--PLPYSR
--PLPYYL
--PLPYYA
--PLPYVV
--PLPYGS
--PLPYSS
--PLPYVS
--PLPYLG
--PLPYAS
--PLPYMR
--PLPYRS
--PLPYFV
--PLPYLA
--PLPYGT
--PLPYTS
--PLPYAL
--PLPYST
--PLPYRG
--PLPYLQ
PGPLPY--
--PLPYLM
--PLPYSY
--PLPYRN

--PLPYYT
--PLPYLK
--PLPYVH
--PLPYTL
--PLPYLN
--PLPYLL
--PLPYIM
--PLPYAR
PRPLPY--
--PLPYGA
--PLPYYQ
--PLPYVA
--PLPYIT
--PLPYHV
--PLPYRA
--PLPYLY
--PLPYYG
--PLPYRW
--PLPYAF
--PLPYYS
--PLPYRM
--PLPYQI
--PLPYQG
--PLPYIA
--PLPYVQ
--PLPYSF
--PLPYRI
--PLPYIL
--PLPYMK
--PLPYIW
--PLPYVR
--PLPYTR
--PLPYSH
--PLPYSG
--PLPYKR
--PLPYGM
--PLPYFN
--PLPYTT
--PLPYTM
--PLPYRY
--PLPYRV
--PLPYLF
--PLPYII
--PLPYWS
--PLPYVL
--PLPYQK
--PLPYKT
--PLPYIH
--PLPYAY
--PLPYTG

--PLPYSK
--PLPYSA
--PLPYNA
--PLPYMN
--PLPYML
--PLPYMA
--PLPYLH
PIPLPY--
--PLPYWY
--PLPYTI
--PLPYQT
--PLPYNS
--PLPYMV
--PLPYLW
--PLPYKI
--PLPYHL
--PLPYAV
--PLPYYV
--PLPYMT
--PLPYGV
--PLPYWR
--PLPYVW
--PLPYVF
--PLPYTV
--PLPYLV
--PLPYKN
--PLPYKA
--PLPYFK
--PLPYES
--PLPYYH
--PLPYWI
--PLPYTY
--PLPYRE
--PLPYQV
--PLPYKQ
--PLPYIR
--PLPYFY
--PLPYFS
--PLPYFL
--PLPYAQ
--PLPYAA
--PLPYYN
--PLPYVG
--PLPYTK
--PLPYTH
--PLPYSN
--PLPYQS
--PLPYQN
--PLPYNI
--PLPYMW

--PLPYKV
--PLPYKL
--PLPYHQ
--PLPYFG
--PLPYFA
--PLPYEW
--PLPYAG
--PLPYYI
--PLPYMS
--PLPYMG
--PLPYKS
--PLPYKH
--PLPYIS
--PLPYGG
--PLPYAW
PHPLPY--
PTPLPY--
--PLPYSD
--PLPYMM
--PLPYLD
--PLPYKW
--PLPYHW
--PLPYFM
--PLPYTN
--PLPYQY
--PLPYMQ
--PLPYLE
--PLPYEN
--PLPYYY
--PLPYYD
--PLPYWL
--PLPYNN
--PLPYKF
--PLPYHR
--PLPYGY
--PLPYFE
--PLPYFD
--PLPYEY
--PLPYDL
--PLPYAN
PEPLPY--
--PLPYYW
--PLPYYM
--PLPYWN
--PLPYVI
--PLPYSQ
--PLPYNF
--PLPYHS
--PLPYHH
--PLPYGK

--PLPYFH
--PLPYEI
PWPLPY--
--PLPYWK
--PLPYWE
--PLPYWD
--PLPYVM
--PLPYSV
--PLPYQL
--PLPYQH
--PLPYPV
--PLPYKY
--PLPYEL
PKPLPY--
PNPLPY--
--PLPYYK
--PLPYWT
--PLPYTD
--PLPYPQ
--PLPYME
--PLPYIY
--PLPYIV
--PLPYIG
--PLPYIF
--PLPYGQ
--PLPYER
--PLPYDM
--PLPYWH
--PLPYTQ
--PLPYSW
--PLPYSI
--PLPYPM
--PLPYNR
--PLPYNM
--PLPYNH
--PLPYMI
--PLPYGH
--PLPYFT
--PLPYET
--PLPYCM
--PLPYYR
--PLPYYE
--PLPYWA
--PLPYTF
--PLPYTE
--PLPYPW
--PLPYNT
--PLPYNK
--PLPYKM
--PLPYKG

--PLPYKE
--PLPYKD
--PLPYIE
--PLPYHI
--PLPYHA
--PLPYGW
--PLPYDS
--PLPYDF
--PLPYCI
--PLPYCG
--PLPYAM
--PLPYAD
--PLPYWV
--PLPYVD
--PLPYTA
--PLPYSM
--PLPYSE
--PLPYQF
--PLPYPI
--PLPYNW
--PLPYNQ
--PLPYLC
--PLPYIN
--PLPYIK
--PLPYHN
--PLPYHM
--PLPYHG
--PLPYGN
--PLPYGF
--PLPYFQ
--PLPYEV
--PLPYEK
--PLPYDV
--PLPYDI
--PLPYCS
PDPLPY--
-PPLPYR-
--PLPYYF
--PLPYWW
--PLPYVY
--PLPYRH
--PLPYQM
--PLPYQE
--PLPYQA
--PLPYPY
--PLPYPF
--PLPYNV
--PLPYNG
--PLPYMY
--PLPYMD

--PLPYIQ
--PLPYIP
--PLPYHK
--PLPYEM
--PLPYEG
--PLPYEF
--PLPYDG
--PLPYCW
--PLPYCT
--PLPYCR
--PLPYCF
--PLPYAK
--PLPYAI
--PLPYAH
--PLPYAE
QRPLPY--
-QPLPYI-
-QPLPYR-
-QPLPYL-
QSPLPY--
QLPLPY--
-QPLPYY-
QKPLPY--
-QPLPYK-
QVPLPY--
QHPLPY--
QYPLPY--
QGPLPY--
QQPLPY--
QEPLPY--
QTPLPY--
-QPLPYH-
-QPLPYM-
-QPLPYF-
-QPLPYG-
QFPLPY--
-QPLPYW-
-QPLPYS-
-QPLPYQ-
QNPLPY--
QAPLPY--
-QPLPYV-
-QPLPYN-
-QPLPYA-
QMPLPY--
QIPLPY--
RSPLPY--
RAPLPY--
RLPLPY--
-RPLPYS-

RGPLPY--
RNPLPY--
RYPLPY--
-RPLPYK-
RQPLPY--
-RPLPYN-
-RPLPYV-
-RPLPYR-
-RPLPYT-
-RPLPYF-
RMPLPY--
RTPLPY--
-RPLPYY-
RFPLPY--
RVPLPY--
RIPLPY--
RWPLPY--
-RPLPYA-
-RPLPYE-
-RPLPYG-
RDPLPY--
RPPLPY--
-RPLPYW-
-RPLPYM-
RHPLPY--
REPLPY--
-RPLPYQ-
-RPLPYP-
-SPLPYL-
SLPLPY--
SQPLPY--
SMPLPY--
SAPLPY--
-SPLPYR-
SSPLPY--
SFPLPY--
SVPLPY--
-SPLPYY-
SRPLPY--
-SPLPYK-
-SPLPYS-
-SPLPYI-
SKPLPY--
STPLPY--
-SPLPYW-
SGPLPY--
SHPLPY--
SYPLPY--
SDPLPY--
-SPLPYM-

-SPLPYH-
SPPLPY--
-SPLPYV-
SNPLPY--
-SPLPYA-
SCPLPY--
-SPLPYQ-
-SPLPYT-
-SPLPYG-
-SPLPYE-
-SPLPYN-
SEPLPY--
TLPLPY--
TMPLPY--
-TPLPYR-
-TPLPYT-
TFPLPY--
TTPLPY--
TSPLPY--
-TPLPYS-
-TPLPYI-
TIPLPY--
-TPLPYL-
-TPLPYA-
TEPLPY--
TQPLPY--
TPPLPY--
-TPLPYH-
TAPLPY--
TVPLPY--
-TPLPYM-
TYPLPY--
TWPLPY--
-TPLPYQ-
THPLPY--
TGPLPY--
-TPLPYK-
-TPLPYW-
-TPLPYY-
TNPLPY--
TDPLPY--
TCPLPY--
-TPLPYV-
-TPLPYN-
-TPLPYE-
VSPLPY--
VLPLPY--
VRPLPY--
VQPLPY--
VTPLPY--

-VPLPYR-
-VPLPYF-
-VPLPYS-
VIPLPY--
-VPLPYL-
-VPLPYV-
VGPLPY--
VVPLPY--
-VPLPYY-
-VPLPYT-
-VPLPYW-
VPPLPY--
-VPLPYN-
VFPLPY--
VAPLPY--
VNPLPY--
VHPLPY--
VYPLPY--
-VPLPYI-
-VPLPYG-
VMPLPY--
VEPLPY--
VDPLPY--
-VPLPYQ-
-VPLPYE-
-VPLPYH-
VWPLPY--
-VPLPYK-
WEPLPY--
-WPLPYQ-
WSPLPY--
WAPLPY--
WKPLPY--
WGPLPY--
WRPLPY--
WVPLPY--
-WPLPYR-
WIPLPY--
WHPLPY--
WLPLPY--
WDPLPY--
WFPLPY--
WQPLPY--
-WPLPYS-
WMPLPY--
-WPLPYV-
-WPLPYT-
-WPLPYG-
-WPLPYN-
-WPLPYL-

	-WPLPYK-		
	-WPLPYI-		
	-WPLPYY-		
	-WPLPYF-		
	-WPLPYA-		
	YFPLPY--		
	YSPLPY--		
	YGPLPY--		
	-YPLPYS-		
	YRPLPY--		
	YPPLPY--		
	-YPLPYT-		
	-YPLPYL-		
	-YPLPYV-		
	-YPLPYR-		
	YIPLPY--		
	YVPLPY--		
	YLPLPY--		
	YAPLPY--		
	YNPLPY--		
	YMPLPY--		
	YTPLPY--		
	YKPLPY--		
	-YPLPYI-		
	YQPLPY--		
	-YPLPYN-		
	-YPLPYG-		
	-YPLPYM-		
	-YPLPYW-		
	YHPLPY--		
	-YPLPYH-		
	YEPLPY--		
	YCPLPY--		
	-YPLPYA-		
PLWM	367	671	47.02
	-APLWMR-		
	AAPLWM--		
	-APLWMH-		
	ANPLWM--		
	AIPLWM--		
	ARPLWM--		
	AKPLWM--		
	ASPLWM--		
	-APLWML-		
	-APLWMM-		
	-APLWMQ-		
	-APLWMK-		
	ATPLWM--		
	AMPLWM--		
	AGPLWM--		

AHPLWM--
-APLWMW-
-APLWMT-
-APLWMS-
-APLWMD-
-APLWMV-
AFPLWM--
ADPLWM--
-APLWME-
-APLWMA-
ACPLWM--
AQPLWM--
-APLWMN-
CVPLWM--
CGPLWM--
CSPLWM--
CRPLWM--
CLPLWM--
CDPLWM--
-CPLWML-
-CPLWMA-
CCPLWM--
-DPLWMR-
-DPLWMG-
DGPLWM--
-DPLWMK-
-DPLWMV-
-DPLWML-
DRPLWM--
-DPLWMM-
DTPLWM--
-DPLWMQ-
DYPLWM--
-DPLWMA-
-DPLWMF-
DNPLWM--
DKPLWM--
DFPLWM--
DAPLWM--
-DPLWMT-
-DPLWMS-
-DPLWMN-
-DPLWMH-
DIPLWM--
DWPLWM--
DSPLWM--
-DPLWMI-
DLPLWM--
ESPLWM--
-EPLWML-

-EPLWMT-
ERPLWM--
-EPLWMR-
EEPLWM--
-EPLWMN-
EKPLWM--
EAPLWM--
-EPLWMA-
-EPLWMV-
-EPLWMI-
EDPLWM--
EYPLWM--
-EPLWMM-
EWPLWM--
-EPLWMQ-
EQPLWM--
-EPLWMW-
EVPLWM--
ETPLWM--
-EPLWMS-
-EPLWME-
ENPLWM--
ELPLWM--
EIPLWM--
FGPLWM--
-FPLWML-
-FPLWMS-
-FPLWMT-
-FPLWMR-
FVPLWM--
-FPLWMH-
FRPLWM--
-FPLWMA-
FDPLWM--
FQPLWM--
FTPLWM--
-FPLWMN-
FNPLWM--
FPPLWM--
FEPLWM--
-FPLWMY-
-FPLWMQ-
-FPLWMM-
-FPLWMK-
-FPLWMD-
FMPLWM--
-FPLWME-
GGPLWM--
GWPLWM--
GVPLWM--

-GPLWMS-
GFPLWM--
GLPLWM--
-GPLWMY-
-GPLWMN-
-GPLWMK-
-GPLWMT-
-GPLWML-
GKPLWM--
GHPLWM--
GTPLWM--
GRPLWM--
-GPLWME-
-GPLWMH-
GNPLWM--
-GPLWMA-
GYPLWM--
-GPLWMQ-
-GPLWMI-
GEPLWM--
GPPLWM--
-GPLWMG-
GIPLWM--
-GPLWMD-
-GPLWMM-
GMPLWM--
-GPLWMV-
-GPLWMP-
GDPLWM--
-HPLWML-
-HPLWMT-
HKPLWM--
HGPLWM--
-HPLWMM-
HSPLWM--
-HPLWMG-
HEPLWM--
HHPLWM--
HVPLWM--
HFPLWM--
HTPLWM--
-HPLWMY-
-HPLWMR-
-HPLWMD-
-HPLWMS-
HMPLWM--
-HPLWMQ-
-HPLWMN-
HIPLWM--
HRPLWM--

```
-HPLWMV-
-HPLWMK-
-HPLWMF-
-HPLWME-
HLPLWM--
HDPLWM--
HAPLWM--
-HPLWMH-
-HPLWMA-
HCPLWM--
IYPLWM--
IAPLWM--
-IPLWMG-
ISPLWM--
-IPLWMR-
ITPLWM--
-IPLWMA-
IGPLWM--
-IPLWMT-
-IPLWML-
INPLWM--
IHPLWM--
IIPLWM--
IRPLWM--
IKPLWM--
IQPLWM--
-IPLWMQ-
-IPLWMI-
IPPLWM--
IMPLWM--
-IPLWMV-
-IPLWMF-
IVPLWM--
-IPLWMM-
-IPLWMH-
IFPLWM--
KPPLWM--
-KPLWMG-
KLPLWM--
-KPLWMV-
-KPLWMH-
-KPLWMA-
KAPLWM--
-KPLWMS-
-KPLWMM-
-KPLWMT-
KEPLWM--
-KPLWMI-
KYPLWM--
-KPLWML-
```

KVPLWM--
-KPLWMW-
-KPLWMD-
KMPLWM--
KTPLWM--
-KPLWMK-
KKPLWM--
KFPLWM--
KDPLWM--
KGPLWM--
-KPLWMN-
LSPLWM--
LVPLWM--
LGPLWM--
-LPLWMR-
-LPLWMS-
LLPLWM--
LMPLWM--
LIPLWM--
LQPLWM--
-LPLWMH-
-LPLWME-
-LPLWMV-
LKPLWM--
-LPLWMN-
-LPLWMY-
-LPLWMK-
LAPLWM--
LYPLWM--
LTPLWM--
LPPLWM--
-LPLWMD-
LCPLWM--
-LPLWMM-
LEPLWM--
LDPLWM--
-MPLWMM-
MRPLWM--
-MPLWMR-
MSPLWM--
-MPLWMS-
MAPLWM--
-MPLWMG-
MGPLWM--
-MPLWMV-
MLPLWM--
MVPLWM--
-MPLWMH-
MIPLWM--
-MPLWMK-

-MPLWMI-
-MPLWMA-
MFPLWM--
MPPLWM--
-MPLWMQ-
-MPLWMN-
-MPLWML-
MTPLWM--
MEPLWM--
MYPLWM--
MWPLWM--
MQPLWM--
-MPLWME-
-MPLWMT-
MNPLWM--
MKPLWM--
MHPLWM--
NRPLWM--
-NPLWMA-
NGPLWM--
NMPLWM--
NSPLWM--
-NPLWMK-
-NPLWMS-
-NPLWMQ-
-NPLWMG-
-NPLWMR-
NNPLWM--
NWPLWM--
NAPLWM--
-NPLWMI-
NQPLWM--
-NPLWMN-
NLPLWM--
NEPLWM--
NDPLWM--
NTPLWM--
NPPLWM--
-NPLWMT-
-NPLWME-
NIPLWM--
NYPLWM--
NVPLWM--
--PLWMGS
--PLWMTS
--PLWMVS
--PLWMRG
--PLWMSR
--PLWMSA
--PLWMIR

--PLWMAG
--PLWMKG
--PLWMQT
--PLWMQS
--PLWMTT
--PLWMQV
--PLWMNS
--PLWMGR
--PLWMSD
--PLWMRQ
--PLWMRS
--PLWMHT
--PLWMSH
--PLWMGQ
--PLWMSG
--PLWMAR
--PLWMWH
--PLWMVK
--PLWMST
--PLWMKT
--PLWMGI
--PLWMEV
--PLWMTN
--PLWMSK
--PLWMRY
--PLWMAA
--PLWMSW
--PLWMSE
--PLWMRE
--PLWMNV
PEPLWM--
--PLWMQR
--PLWMKH
--PLWMAN
--PLWMYD
--PLWMVA
--PLWMRH
--PLWMRA
--PLWMIT
--PLWMHA
--PLWMTM
--PLWMNN
--PLWMMN
--PLWMGV
--PLWMDS
--PLWMRV
--PLWMKV
--PLWMKE
--PLWMGE
--PLWMEG

```
PDPLWM--
--PLWMYG
--PLWMTH
--PLWMSN
--PLWMNG
--PLWMLE
--PLWMKY
--PLWMGN
--PLWMVT
--PLWMSI
--PLWMQN
--PLWMQA
--PLWMMK
--PLWMLH
--PLWMKQ
--PLWMIG
--PLWMHG
--PLWMGA
--PLWMEH
--PLWMVE
--PLWMTW
--PLWMML
--PLWMKL
--PLWMKA
--PLWMGT
--PLWMGL
--PLWMAS
--PLWMWG
--PLWMTE
--PLWMSQ
--PLWMRD
--PLWMNA
--PLWMMG
--PLWMIM
--PLWMIE
--PLWMGH
--PLWMFL
--PLWMEQ
--PLWMTI
--PLWMNT
--PLWMMQ
--PLWMMI
--PLWMLD
--PLWMHI
--PLWMAK
--PLWMAH
--PLWMYN
--PLWMYI
--PLWMYH
--PLWMYE
```

--PLWMTK
--PLWMSM
--PLWMRM
--PLWMQK
--PLWMME
--PLWMHE
--PLWMGK
--PLWMGG
--PLWMES
--PLWMDA
--PLWMAT
--PLWMWN
--PLWMTR
--PLWMTD
--PLWMQE
--PLWMNK
--PLWMNI
--PLWMKK
--PLWMHS
--PLWMHR
--PLWMHN
--PLWMEK
--PLWMDG
--PLWMAM
--PLWMYT
--PLWMWQ
--PLWMTQ
--PLWMTA
--PLWMQQ
--PLWMQF
--PLWMMH
--PLWMLW
--PLWMKM
--PLWMID
--PLWMHY
--PLWMER
--PLWMAI
PCPLWM--
--PLWMYM
--PLWMTV
--PLWMTG
--PLWMQG
--PLWMMT
--PLWMFG
--PLWMEW
--PLWMEN
--PLWMEA
--PLWMDT
--PLWMAD
-PPLWMS-

-PPLWMR-
--PLWMWE
--PLWMRN
--PLWMNY
--PLWMNR
--PLWMNE
--PLWMMD
--PLWMLQ
--PLWMKD
--PLWMIN
--PLWMHL
--PLWMHD
--PLWMED
--PLWMDY
-QPLWMS-
-QPLWMR-
-QPLWML-
-QPLWMK-
QSPLWM--
QGPLWM--
QRPLWM--
-QPLWMV-
-QPLWMH-
-QPLWMG-
QKPLWM--
-QPLWMT-
-QPLWME-
QVPLWM--
-QPLWMM-
QAPLWM--
-QPLWMY-
QIPLWM--
-QPLWMA-
QHPLWM--
-QPLWMW-
-QPLWMN-
-QPLWMI-
QDPLWM--
RAPLWM--
-RPLWMG-
RNPLWM--
-RPLWMA-
-RPLWMK-
-RPLWMT-
-RPLWMS-
RIPLWM--
RVPLWM--
-RPLWMQ-
-RPLWME-
RGPLWM--

```
-RPLWMW-
RLPLWM--
RTPLWM--
RDPLWM--
RWPLWM--
-RPLWML-
-RPLWMH-
RQPLWM--
REPLWM--
-RPLWMN-
RYPLWM--
-RPLWMY-
-RPLWMD-
RCPLWM--
-RPLWMV-
RPPLWM--
-RPLWMM-
-SPLWMS-
-SPLWMR-
SKPLWM--
SRPLWM--
-SPLWMI-
SLPLWM--
SSPLWM--
-SPLWMT-
SMPLWM--
SQPLWM--
SAPLWM--
STPLWM--
SFPLWM--
-SPLWMQ-
SDPLWM--
-SPLWMW-
-SPLWMA-
SPPLWM--
-SPLWMM-
SNPLWM--
SEPLWM--
SWPLWM--
-SPLWMK-
SYPLWM--
-SPLWMG-
-SPLWMV-
-SPLWMH-
SIPLWM--
SHPLWM--
-SPLWMN-
-SPLWME-
SGPLWM--
-TPLWMS-
```

TRPLWM--
TTPLWM--
-TPLWMA-
TIPLWM--
-TPLWMK-
TSPLWM--
-TPLWMI-
TNPLWM--
-TPLWMT-
-TPLWMV-
-TPLWMN-
TPPLWM--
TLPLWM--
TAPLWM--
-TPLWML-
TQPLWM--
-TPLWMW-
TGPLWM--
TVPLWM--
-TPLWMY-
TMPLWM--
TKPLWM--
-TPLWMG-
TFPLWM--
TYPLWM--
-TPLWMQ-
-TPLWMD-
THPLWM--
TEPLWM--
-VPLWMR-
VPPLWM--
VRPLWM--
-VPLWMK-
VSPLWM--
-VPLWMT-
-VPLWMI-
VHPLWM--
VGPLWM--
VAPLWM--
VTPLWM--
-VPLWME-
VLPLWM--
VKPLWM--
VYPLWM--
-VPLWMH-
VQPLWM--
-VPLWML-
-VPLWMS-
-VPLWMA-
VEPLWM--

VNPLWM--
VFPLWM--
-VPLWMD-
VMPLWM--
VDPLWM--
-VPLWMQ-
-VPLWMG-
-VPLWMN-
-VPLWMV-
-VPLWMM-
VIPLWM--
VVPLWM--
WHPLWM--
WQPLWM--
WGPLWM--
WPPLWM--
-WPLWMS-
WAPLWM--
WVPLWM--
WTPLWM--
-WPLWMR-
-WPLWMI-
WKPLWM--
-WPLWMQ-
-WPLWMT-
-WPLWMP-
-WPLWML-
-WPLWME-
WNPLWM--
WMPLWM--
YKPLWM--
-YPLWMS-
YVPLWM--
-YPLWMR-
YSPLWM--
YDPLWM--
YNPLWM--
YIPLWM--
YMPLWM--
YHPLWM--
YQPLWM--
YRPLWM--
-YPLWMM-
YGPLWM--
-YPLWMT-
-YPLWMV-
-YPLWMD-
YPPLWM--
-YPLWMN-
-YPLWMH-

YYPLWM--
-YPLWML-
-YPLWMI-
-YPLWMA-
YAPLWM--
YTPLWM--
PHAM 460 633 47.01
-RPHAMA-
-SPHAMG-
AVPHAM--
-APHAML-
-APHAMT-
ANPHAM--
-APHAMR-
ASPHAM--
AMPHAM--
AIPHAM--
-APHAMK-
AHPHAM--
-APHAMS-
-APHAMN-
-APHAMI-
AAPHAM--
APPHAM--
-APHAMQ-
ARPHAM--
-APHAMY-
-APHAME-
ATPHAM--
-APHAMW-
AKPHAM--
AYPHAM--
-APHAMC-
AGPHAM--
CAPHAM--
CRPHAM--
CCPHAM--
-CPHAMV-
-CPHAMK-
CTPHAM--
CQPHAM--
-CPHAMT-
-CPHAMH-
DTPHAM--
-DPHAMI-
DWPHAM--
-DPHAMS-
DKPHAM--
DRPHAM--
DAPHAM--

DSPHAM--
-DPHAMV-
DVPHAM--
DQPHAM--
DDPHAM--
-EPHAML-
-EPHAMH-
ELPHAM--
-EPHAMT-
EWPHAM--
ESPHAM--
ERPHAM--
ENPHAM--
EGPHAM--
-FPHAMR-
FIPHAM--
-FPHAMV-
FVPHAM--
FRPHAM--
-FPHAML-
-FPHAMF-
FWPHAM--
-FPHAMK-
FNPHAM--
FYPHAM--
-FPHAMT-
FSPHAM--
FLPHAM--
-FPHAMS-
-FPHAMQ-
GRPHAM--
GLPHAM--
-GPHAML-
-GPHAMI-
GMPHAM--
GKPHAM--
-GPHAMV-
-GPHAMN-
GWPHAM--
GHPHAM--
GSPHAM--
-GPHAMA-
GQPHAM--
GDPHAM--
GYPHAM--
GAPHAM--
-GPHAMS-
-GPHAMQ-
-GPHAMT-
-GPHAMG-

-GPHAMF-
GIPHAM--
-GPHAMY-
-GPHAMR-
GFPHAM--
-GPHAMM-
-GPHAMK-
GPPHAM--
-GPHAMW-
-GPHAME-
GNPHAM--
HSPHAM--
-HPHAMI-
HRPHAM--
-HPHAMY-
HEPHAM--
-HPHAMV-
-HPHAMF-
-HPHAMK-
HKPHAM--
HYPHAM--
-HPHAMS-
HLPHAM--
-HPHAML-
HGPHAM--
HQPHAM--
-HPHAMT-
-HPHAMR-
IRPHAM--
ITPHAM--
-IPHAMI-
-IPHAMS-
IMPHAM--
-IPHAMW-
-IPHAMR-
IVPHAM--
-IPHAMF-
IAPHAM--
ISPHAM--
-IPHAMY-
-IPHAMK-
IIPHAM--
IYPHAM--
IWPHAM--
IQPHAM--
IPPHAM--
INPHAM--
-IPHAMN-
-IPHAMH-
IGPHAM--

KSPHAM--
-KPHAMR-
-KPHAMH-
-KPHAML-
-KPHAMW-
-KPHAMN-
KLPHAM--
-KPHAMI-
-KPHAMF-
KKPHAM--
KVPHAM--
-KPHAMM-
-KPHAMA-
KAPHAM--
KRPHAM--
-KPHAMS-
-KPHAMK-
-KPHAME-
KNPHAM--
KGPHAM--
KDPHAM--
KCPHAM--
-LPHAMR-
LSPHAM--
-LPHAMH-
LRPHAM--
LTPHAM--
LNPHAM--
LGPHAM--
LYPHAM--
-LPHAML-
-LPHAMI-
LEPHAM--
-LPHAMV-
LLPHAM--
LKPHAM--
LQPHAM--
-LPHAMS-
LMPHAM--
LIPHAM--
LPPHAM--
-LPHAMK-
LHPHAM--
LDPHAM--
LCPHAM--
LWPHAM--
LVPHAM--
-LPHAMQ-
-LPHAMP-
-LPHAMN-

MVPHAM--
MLPHAM--
MGPHAM--
-MPHAMV-
-MPHAMT-
MRPHAM--
-MPHAMR-
-MPHAMF-
MTPHAM--
-MPHAMS-
-MPHAMQ-
MEPHAM--
MAPHAM--
MQPHAM--
-MPHAML-
MMPHAM--
MPPHAM--
-MPHAMY-
MKPHAM--
MYPHAM--
NGPHAM--
-NPHAMR-
NPPHAM--
NNPHAM--
NHPHAM--
NRPHAM--
-NPHAMI-
-NPHAMT-
-NPHAMH-
-NPHAMF-
-NPHAMW-
-NPHAMM-
NYPHAM--
NSPHAM--
NMPHAM--
NLPHAM--
NFPHAM--
NTPHAM--
-NPHAMY-
-NPHAMS-
-NPHAMQ-
-NPHAML-
-NPHAMA-
NDPHAM--
--PHAMRL
--PHAMKG
--PHAMRT
--PHAMVY
--PHAMVT
--PHAMLS

--PHAMTS
--PHAMRD
--PHAMFS
--PHAMVK
--PHAMRS
--PHAMYA
--PHAMYS
--PHAMTV
--PHAMSS
--PHAMVI
--PHAMQV
--PHAMYI
--PHAMWW
--PHAMSA
--PHAMRN
PMPHAM--
--PHAMWF
--PHAMVR
--PHAMTG
--PHAMRH
--PHAMLV
--PHAMIS
--PHAMWS
--PHAMWR
--PHAMTQ
--PHAMSR
--PHAMRV
PSPHAM--
--PHAMVA
--PHAMTK
--PHAMTI
--PHAMFN
PRPHAM--
--PHAMYK
--PHAMTM
--PHAMRQ
--PHAMLR
--PHAMLN
--PHAMLL
--PHAMLG
--PHAMKT
--PHAMKL
--PHAMHR
--PHAMGY
--PHAMFY
PWPHAM--
--PHAMVV
--PHAMTL
--PHAMSK
--PHAMRG

--PHAMLY
--PHAMIK
--PHAMFR
PVPHAM--
--PHAMVG
--PHAMTT
--PHAMMY
--PHAMIM
--PHAMHY
--PHAMGI
--PHAMVF
--PHAMRR
--PHAMRI
--PHAMRE
--PHAMQY
--PHAMQS
--PHAMLT
--PHAMLH
--PHAMKI
--PHAMKA
--PHAMHV
--PHAMHG
--PHAMGG
PIPHAM--
--PHAMWT
--PHAMTW
--PHAMTF
--PHAMSY
--PHAMST
--PHAMSN
--PHAMSH
--PHAMNF
--PHAMKS
--PHAMIQ
--PHAMIH
--PHAMHL
--PHAMGF
--PHAMEL
--PHAMAL
--PHAMWY
--PHAMVM
--PHAMSM
--PHAMNL
--PHAMMM
--PHAMLQ
--PHAMLK
--PHAMLA
--PHAMII
--PHAMHM
--PHAMFM

PKPHAM--
--PHAMWA
--PHAMVN
--PHAMVH
--PHAMTD
--PHAMRA
--PHAMNT
--PHAMNS
--PHAMML
--PHAMMK
--PHAMIN
--PHAMHA
--PHAMFK
--PHAMFG
--PHAMEW
--PHAMYG
--PHAMWG
--PHAMSG
--PHAMSF
--PHAMNR
--PHAMMT
--PHAMKV
--PHAMHH
--PHAMFF
--PHAMAH
--PHAMVL
--PHAMQT
--PHAMPM
--PHAMNG
--PHAMLM
--PHAMKY
--PHAMKF
--PHAMKD
--PHAMIV
--PHAMIT
--PHAMHQ
--PHAMHF
--PHAMFQ
--PHAMFD
--PHAMAW
--PHAMAT
--PHAMAM
--PHAMWV
--PHAMWE
--PHAMVW
--PHAMVQ
--PHAMTY
--PHAMTR
--PHAMSQ
--PHAMRY

--PHAMQW
--PHAMQM
--PHAMNY
--PHAMNW
--PHAMKK
--PHAMKH
--PHAMHT
--PHAMHD
--PHAMGT
--PHAMGM
--PHAMGA
--PHAMFT
--PHAMFE
--PHAMET
--PHAMEG
--PHAMDL
PGPHAM--
--PHAMYV
--PHAMYN
--PHAMYD
--PHAMWI
--PHAMVE
--PHAMSV
--PHAMQF
--PHAMNQ
--PHAMNI
--PHAMMV
--PHAMMR
--PHAMLF
--PHAMLE
--PHAMKN
--PHAMIG
--PHAMIF
--PHAMGW
--PHAMGS
--PHAMES
--PHAMEI
--PHAMCS
--PHAMAS
PEPHAM--
--PHAMYR
--PHAMYH
--PHAMYE
--PHAMWN
--PHAMTN
--PHAMSW
--PHAMSI
--PHAMSD
--PHAMQI
--PHAMQG

--PHAMPV
--PHAMPT
--PHAMNN
--PHAMNH
--PHAMMS
--PHAMMI
--PHAMMG
--PHAMMF
--PHAMLI
--PHAMLD
--PHAMKW
--PHAMKC
--PHAMHW
--PHAMGV
--PHAMFV
--PHAMFL
--PHAMEH
--PHAMDS
--PHAMAR
PCPHAM--
QIPHAM--
QLPHAM--
-QPHAMK-
QTPHAM--
-QPHAML-
-QPHAMH-
QYPHAM--
QQPHAM--
-QPHAMI-
QAPHAM--
-QPHAMN-
QVPHAM--
-QPHAMR-
QHPHAM--
QRPHAM--
-QPHAMT-
-QPHAMS-
-QPHAMQ-
-QPHAMF-
-QPHAME-
RIPHAM--
-RPHAMI-
RVPHAM--
-RPHAML-
-RPHAMM-
RGPHAM--
RNPHAM--
-RPHAMF-
RRPHAM--
-RPHAMK-

RYPHAM--
RSPHAM--
RQPHAM--
-RPHAMR-
RPPHAM--
-RPHAMW-
-RPHAMY-
-RPHAMV-
REPHAM--
RWPHAM--
-RPHAMT-
-RPHAMN-
-RPHAMQ-
-RPHAMH-
RAPHAM--
RTPHAM--
-RPHAMS-
RMPHAM--
-RPHAME-
RFPHAM--
-RPHAMD-
RLPHAM--
RDPHAM--
-SPHAML-
-SPHAMR-
SVPHAM--
SWPHAM--
-SPHAMS-
SLPHAM--
SAPHAM--
-SPHAMY-
SSPHAM--
SIPHAM--
SFPHAM--
SEPHAM--
-SPHAMW-
-SPHAMV-
-SPHAMM-
-SPHAMH-
-SPHAMQ-
SMPHAM--
SHPHAM--
SQPHAM--
-SPHAMT-
SPPHAM--
-SPHAMN-
-SPHAMK-
SKPHAM--
-SPHAMI-
-SPHAMF-

-SPHAME-
SRPHAM--
SDPHAM--
SYPHAM--
-SPHAMA-
-TPHAML-
-TPHAMR-
TVPHAM--
-TPHAMV-
TRPHAM--
-TPHAMQ-
TMPHAM--
TQPHAM--
-TPHAMK-
-TPHAMI-
TGPHAM--
TAPHAM--
-TPHAMS-
TYPHAM--
TPPHAM--
-TPHAMA-
TIPHAM--
TFPHAM--
-TPHAMW-
-TPHAMY-
-TPHAMN-
-TPHAMM-
TKPHAM--
TDPHAM--
TLPHAM--
TEPHAM--
-VPHAMS-
-VPHAML-
VVPHAM--
-VPHAMR-
-VPHAMV-
VAPHAM--
-VPHAMY-
-VPHAMW-
VEPHAM--
VWPHAM--
VTPHAM--
-VPHAMH-
-VPHAMA-
VPPHAM--
-VPHAMQ-
-VPHAMI-
-VPHAMF-
VKPHAM--
-VPHAMT-

-VPHAMG-
-VPHAME-
VYPHAM--
VLPHAM--
VHPHAM--
VGPHAM--
WVPHAM--
WKPHAM--
-WPHAMT-
WLPHAM--
-WPHAMV-
-WPHAML-
WWPHAM--
-WPHAMH-
WRPHAM--
-WPHAMW-
-WPHAMR-
WFPHAM--
-WPHAMS-
-WPHAMQ-
WPPHAM--
-WPHAMG-
-YPHAMT-
YEPHAM--
-YPHAMV-
YSPHAM--
-YPHAMI-
-YPHAMF-
YAPHAM--
-YPHAML-
YNPHAM--
YFPHAM--
YYPHAM--
-YPHAMQ-
YLPHAM--
YWPHAM--
YTPHAM--
-YPHAMH-
YMPHAM--
YIPHAM--

PRNY 157 772 46.99

-DPRNYG-
-FPRNYP-
--PRNYYP
-RPRNYD-
-WPRNYI-
AYPRNY--
AVPRNY--
AAPRNY--
-APRNYL-

ASPRNY--
-APRNYM-
AFPRNY--
AMPRNY--
ATPRNY--
-APRNYW-
AIPRNY--
AGPRNY--
-APRNYV-
-APRNYN-
-APRNYF-
-APRNYT-
-APRNYK-
-APRNYY-
-APRNYQ-
-APRNYA-
AQPRNY--
-APRNYH-
AKPRNY--
AHPRNY--
APPRNY--
-APRNYG-
AEPRNY--
ADPRNY--
-APRNYE-
ANPRNY--
-APRNYD-
-APRNYC-
ACPRNY--
CRPRNY--
CSPRNY--
CNPRNY--
-CPRNYI-
CIPRNY--
CCPRNY--
CVPRNY--
CTPRNY--
CQPRNY--
CMPRNY--
-CPRNYK-
CPPRNY--
-CPRNYM-
CKPRNY--
-CPRNYY-
-CPRNYN-
-CPRNYG-
-CPRNYF-
-CPRNYA-
CEPRNY--
CDPRNY--

-DPRNYS-
-DPRNYL-
-DPRNYM-
DAPRNY--
-DPRNYR-
DQPRNY--
DSPRNY--
DLPRNY--
DIPRNY--
DVPRNY--
DRPRNY--
-DPRNYW-
DDPRNY--
DTPRNY--
-DPRNYY-
-DPRNYT-
DGPRNY--
-DPRNYH-
-DPRNYA-
DYPRNY--
-DPRNYI-
-DPRNYE-
DMPRNY--
DEPRNY--
-DPRNYF-
-DPRNYK-
DKPRNY--
DFPRNY--
-EPRNYL-
-EPRNYV-
-EPRNYS-
ESPRNY--
ELPRNY--
EYPRNY--
EGPRNY--
ETPRNY--
-EPRNYR-
EMPRNY--
EKPRNY--
EIPRNY--
ERPRNY--
-EPRNYK-
EEPRNY--
EVPRNY--
-EPRNYG-
EAPRNY--
-EPRNYH-
-EPRNYW-
-EPRNYI-
EWPRNY--

EHPRNY--
EFPRNY--
-EPRNYY-
-EPRNYF-
-EPRNYE-
-EPRNYD-
-FPRNYL-
FGPRNY--
FRPRNY--
FIPRNY--
FKPRNY--
FDPRNY--
-FPRNYN-
FVPRNY--
FSPRNY--
FQPRNY--
FFPRNY--
-FPRNYW-
FEPRNY--
-FPRNYI-
-FPRNYG-
FWPRNY--
FMPRNY--
FAPRNY--
-FPRNYE-
FCPRNY--
FYPRNY--
FNPRNY--
FHPRNY--
-GPRNYL-
-GPRNYI-
-GPRNYK-
GIPRNY--
-GPRNYV-
GLPRNY--
GQPRNY--
-GPRNYT-
-GPRNYA-
GAPRNY--
GTPRNY--
GRPRNY--
-GPRNYY-
-GPRNYQ-
GGPRNY--
GYPRNY--
GVPRNY--
-GPRNYN-
-GPRNYG-
-GPRNYW-
GKPRNY--

```
-GPRNYM-
-GPRNYH-
GNPRNY--
GMPRNY--
GWPRNY--
-GPRNYE-
GDPRNY--
HTPRNY--
HSPRNY--
-HPRNYL-
HGPRNY--
HVPRNY--
HMPRNY--
HPPRNY--
-HPRNYV-
-HPRNYY-
HEPRNY--
-HPRNYW-
HLPRNY--
HKPRNY--
-HPRNYT-
-HPRNYK-
HNPRNY--
HIPRNY--
HQPRNY--
HAPRNY--
-HPRNYI-
-HPRNYQ-
-HPRNYH-
-HPRNYN-
-HPRNYM-
HDPRNY--
HYPRNY--
-HPRNYF-
HCPRNY--
ICPRNY--
ISPRNY--
ILPRNY--
IVPRNY--
IKPRNY--
-IPRNYS-
IFPRNY--
-IPRNYG-
-IPRNYH-
INPRNY--
IEPRNY--
IYPRNY--
-IPRNYY-
IIPRNY--
ITPRNY--
```

IPPRNY--
IQPRNY--
-IPRNYW-
-IPRNYI-
-IPRNYA-
IDPRNY--
-IPRNYL-
-IPRNYE-
IMPRNY--
-IPRNYV-
-IPRNYT-
IGPRNY--
IAPRNY--
-IPRNYD-
KSPRNY--
-KPRNYL-
-KPRNYV-
-KPRNYT-
-KPRNYI-
KTPRNY--
-KPRNYF-
-KPRNYS-
KLPRNY--
KGPRNY--
KVPRNY--
-KPRNYA-
KRPRNY--
-KPRNYG-
-KPRNYW-
-KPRNYE-
-KPRNYQ-
-KPRNYM-
KPPRNY--
-KPRNYN-
-KPRNYH-
KHPRNY--
KNPRNY--
KAPRNY--
KQPRNY--
KMPRNY--
KEPRNY--
KCPRNY--
KKPRNY--
-KPRNYY-
-KPRNYC-
KIPRNY--
-LPRNYV-
LNPRNY--
LTPRNY--
LAPRNY--

LSPRNY--
LGPRNY--
-LPRNYM-
LHPRNY--
LVPRNY--
-LPRNYE-
-LPRNYS-
-LPRNYQ-
LPPRNY--
LIPRNY--
-LPRNYH-
LKPRNY--
LEPRNY--
-LPRNYN-
LQPRNY--
-LPRNYG-
-LPRNYI-
-LPRNYK-
LDPRNY--
LCPRNY--
-MPRNYL-
MSPRNY--
-MPRNYV-
-MPRNYQ-
MAPRNY--
MGPRNY--
MPPRNY--
MLPRNY--
-MPRNYK-
MMPRNY--
MYPRNY--
MNPRNY--
MDPRNY--
MKPRNY--
-MPRNYM-
-MPRNYE-
-MPRNYF-
-MPRNYI-
-MPRNYH-
MHPRNY--
-MPRNYN-
MEPRNY--
-MPRNYG-
MCPRNY--
-NPRNYS-
NVPRNY--
NSPRNY--
-NPRNYV-
-NPRNYF-
NIPRNY--

-NPRNYI-
NGPRNY--
-NPRNYY-
-NPRNYW-
NEPRNY--
NRPRNY--
NTPRNY--
-NPRNYT-
NLPRNY--
NYPRNY--
-NPRNYL-
-NPRNYH-
NMPRNY--
-NPRNYQ-
-NPRNYN-
-NPRNYM-
NNPRNY--
NFPRNY--
NQPRNY--
NDPRNY--
NAPRNY--
-NPRNYK-
-NPRNYE-
NCPRNY--
--PRNYSQ
--PRNYRL
--PRNYSL
--PRNYTL
--PRNYST
--PRNYFS
--PRNYML
--PRNYLN
--PRNYIL
--PRNYLY
--PRNYVQ
--PRNYFA
--PRNYHS
--PRNYIT
--PRNYSG
--PRNYMR
--PRNYGS
--PRNYMG
--PRNYWI
--PRNYTI
--PRNYGV
--PRNYYS
--PRNYRH
--PRNYNA
--PRNYSS
--PRNYSI

--PRNYFH
--PRNYSR
--PRNYSM
--PRNYVT
--PRNYVG
--PRNYVF
--PRNYRY
--PRNYSV
--PRNYGA
--PRNYVA
--PRNYYA
--PRNYTA
--PRNYMS
--PRNYLH
--PRNYVV
--PRNYWV
--PRNYVK
--PRNYLV
--PRNYIG
--PRNYYI
--PRNYAS
--PRNYWR
--PRNYHR
--PRNYHL
--PRNYVW
--PRNYVD
--PRNYTG
--PRNYRQ
--PRNYRE
--PRNYLF
--PRNYIS
--PRNYVI
PWPRNY--
--PRNYSF
--PRNYLW
--PRNYLE
--PRNYFV
--PRNYYR
--PRNYWS
--PRNYWK
--PRNYWA
--PRNYHA
--PRNYMI
--PRNYGW
--PRNYEG
--PRNYYM
--PRNYVY
--PRNYSY
--PRNYAI
PRPRNY--

--PRNYNS
--PRNYIA
--PRNYFW
--PRNYFG
--PRNYSN
--PRNYSA
--PRNYHT
--PRNYFI
--PRNYAT
--PRNYYV
--PRNYYT
--PRNYQS
--PRNYHH
--PRNYHG
--PRNYEF
--PRNYAK
--PRNYYD
--PRNYWE
--PRNYGT
PSPRNY--
--PRNYQV
--PRNYNV
--PRNYNF
--PRNYGM
--PRNYGI
--PRNYYK
--PRNYVH
--PRNYSK
--PRNYKL
--PRNYIQ
--PRNYIH
--PRNYHY
--PRNYFT
--PRNYFN
--PRNYAL
--PRNYAD
PTPRNY--
--PRNYSW
--PRNYQI
--PRNYQG
--PRNYMV
--PRNYLM
--PRNYII
--PRNYWQ
--PRNYTD
--PRNYQM
--PRNYMH
--PRNYKT
--PRNYGF
--PRNYAA

--PRNYWT
--PRNYTV
--PRNYTN
--PRNYTM
--PRNYMF
--PRNYME
--PRNYMA
--PRNYKF
--PRNYGH
--PRNYFM
--PRNYEM
--PRNYAG
--PRNYQQ
--PRNYNM
--PRNYIF
--PRNYEW
--PRNYAV
--PRNYAR
PFPRNY--
--PRNYYE
--PRNYWH
--PRNYWD
--PRNYVM
--PRNYRD
--PRNYQN
--PRNYQH
--PRNYMT
--PRNYLD
--PRNYKI
--PRNYID
--PRNYGN
--PRNYGG
--PRNYES
--PRNYAW
--PRNYAQ
--PRNYYQ
--PRNYQT
--PRNYNY
--PRNYNI
--PRNYNH
--PRNYNG
--PRNYKG
--PRNYHQ
--PRNYFK
--PRNYET
--PRNYAN
PQPRNY--
--PRNYYH
--PRNYSD
--PRNYQL

--PRNYNL
--PRNYEV
--PRNYED
--PRNYYN
--PRNYVE
--PRNYTY
--PRNYTE
--PRNYMN
--PRNYMK
--PRNYKY
--PRNYKD
--PRNYGY
--PRNYFY
--PRNYFD
--PRNYER
--PRNYAY
--PRNYTQ
--PRNYTF
--PRNYQY
--PRNYMQ
--PRNYIV
--PRNYAH
PKPRNY--
PHPRNY--
PAPRNY--
PVPRNY--
--PRNYVN
--PRNYTW
--PRNYRW
--PRNYQD
--PRNYNT
--PRNYIY
--PRNYIW
--PRNYIK
--PRNYIE
--PRNYHK
--PRNYFQ
--PRNYFE
--PRNYEH
--PRNYDG
--PRNYWY
--PRNYTK
--PRNYKE
--PRNYGQ
--PRNYFF
--PRNYEQ
--PRNYAM
--PRNYAF
PNPRNY--
PEPRNY--

--PRNYWW
--PRNYNQ
--PRNYKQ
--PRNYKM
--PRNYHF
--PRNYEL
--PRNYDT
--PRNYDS
--PRNYCV
PMPRNY--
PIPRNY--
PGPRNY--
PCPRNY--
PYPRNY--
--PRNYYW
--PRNYTH
--PRNYSE
--PRNYQE
--PRNYPN
--PRNYHW
--PRNYHI
--PRNYHE
--PRNYEE
--PRNYDV
--PRNYAE
-PPRNYS-
-PPRNYL-
-QPRNYL-
QVPRNY--
-QPRNYG-
-QPRNYV-
-QPRNYI-
QSPRNY--
-QPRNYM-
-QPRNYH-
-QPRNYA-
QTPRNY--
-QPRNYS-
-QPRNYT-
-QPRNYF-
QQPRNY--
-QPRNYY-
QHPRNY--
QIPRNY--
QNPRNY--
QFPRNY--
-QPRNYN-
QPPRNY--
QAPRNY--
-QPRNYW-

QKPRNY--
QYPRNY--
-QPRNYE-
QEPRNY--
QCPRNY--
-RPRNYL-
-RPRNYS-
RGPRNY--
REPRNY--
-RPRNYV-
RTPRNY--
RMPRNY--
-RPRNYI-
RHPRNY--
-RPRNYG-
-RPRNYH-
-RPRNYM-
-RPRNYY-
RAPRNY--
RQPRNY--
-RPRNYE-
RCPRNY--
RPPRNY--
RNPRNY--
-RPRNYQ-
RKPRNY--
-RPRNYF-
RIPRNY--
-SPRNYL-
SGPRNY--
-SPRNYV-
SYPRNY--
-SPRNYF-
SMPRNY--
-SPRNYQ-
-SPRNYA-
SLPRNY--
SIPRNY--
-SPRNYY-
-SPRNYT-
SKPRNY--
-SPRNYH-
STPRNY--
-SPRNYW-
-SPRNYM-
-SPRNYI-
SPPRNY--
-SPRNYG-
-SPRNYE-
SQPRNY--

−SPRNYD−
−SPRNYC−
TKPRNY−−
−TPRNYS−
TTPRNY−−
TSPRNY−−
−TPRNYW−
−TPRNYL−
−TPRNYT−
TPPRNY−−
TEPRNY−−
TAPRNY−−
−TPRNYR−
−TPRNYG−
−TPRNYF−
TYPRNY−−
−TPRNYV−
TQPRNY−−
TRPRNY−−
TWPRNY−−
−TPRNYY−
TMPRNY−−
TNPRNY−−
−TPRNYA−
−TPRNYN−
TGPRNY−−
−TPRNYE−
−TPRNYH−
TFPRNY−−
−TPRNYM−
TIPRNY−−
TCPRNY−−
THPRNY−−
−VPRNYL−
VVPRNY−−
−VPRNYV−
−VPRNYS−
VLPRNY−−
VSPRNY−−
VQPRNY−−
VNPRNY−−
−VPRNYG−
−VPRNYQ−
VYPRNY−−
−VPRNYT−
−VPRNYN−
VGPRNY−−
−VPRNYY−
VAPRNY−−
VDPRNY−−

-VPRNYE-
VPPRNY--
-VPRNYW-
VHPRNY--
-VPRNYK-
-VPRNYI-
VFPRNY--
VEPRNY--
-VPRNYH-
-VPRNYA-
-VPRNYD-
VCPRNY--
-WPRNYM-
WVPRNY--
WSPRNY--
WGPRNY--
-WPRNYV-
WLPRNY--
WYPRNY--
WIPRNY--
WFPRNY--
WMPRNY--
WRPRNY--
-WPRNYW-
WKPRNY--
WTPRNY--
WDPRNY--
-WPRNYF-
-WPRNYY-
WNPRNY--
WAPRNY--
WQPRNY--
WEPRNY--
YSPRNY--
-YPRNYL-
YLPRNY--
YGPRNY--
-YPRNYT-
YAPRNY--
YRPRNY--
YPPRNY--
-YPRNYM-
-YPRNYY-
YMPRNY--
YFPRNY--
-YPRNYI-
YQPRNY--
YVPRNY--
YTPRNY--
-YPRNYQ-

-YPRNYG-
YIPRNY--
YNPRNY--
YEPRNY--
-YPRNYE-
YHPRNY--
-YPRNYF-
YYPRNY--
-YPRNYK-
YDPRNY--
YCPRNY--
-YPRNYW-
-YPRNYC-

PHAL	45	856	46.96

-APHALM-
-IPHALA-
-LPHALE-
--PHALRP
-RPHALP-
-SPHALD-
ALPHAL--
AAPHAL--
-APHALL-
-APHALR-
-APHALY-
AYPHAL--
AWPHAL--
-APHALK-
AIPHAL--
-APHALS-
ARPHAL--
APPHAL--
-APHALI-
AMPHAL--
AKPHAL--
ATPHAL--
-APHALN-
-APHALT-
ANPHAL--
AHPHAL--
-APHALQ-
AVPHAL--
-APHALV-
-APHALE-
-APHALH-
AGPHAL--
AQPHAL--
-APHALA-
AFPHAL--
ADPHAL--

CLPHAL--
CVPHAL--
-CPHALL-
CTPHAL--
CKPHAL--
CAPHAL--
CEPHAL--
CNPHAL--
CCPHAL--
CWPHAL--
-CPHALS-
CGPHAL--
CSPHAL--
CQPHAL--
CPPHAL--
CIPHAL--
DSPHAL--
DRPHAL--
DWPHAL--
DFPHAL--
DYPHAL--
DQPHAL--
-DPHALI-
DAPHAL--
DVPHAL--
DMPHAL--
DEPHAL--
-DPHALW-
-DPHALK-
DNPHAL--
DGPHAL--
DDPHAL--
-DPHALY-
-DPHALR-
-DPHALT-
-DPHALS-
-DPHALQ-
-DPHALM-
-DPHALF-
DKPHAL--
DCPHAL--
-EPHALR-
ERPHAL--
ELPHAL--
EPPHAL--
EKPHAL--
EVPHAL--
EYPHAL--
ESPHAL--
-EPHALL-

ETPHAL--
-EPHALY-
EWPHAL--
EFPHAL--
-EPHALI-
-EPHALK-
-EPHALE-
EQPHAL--
-EPHALW-
-EPHALH-
EMPHAL--
EHPHAL--
EAPHAL--
-EPHALN-
-EPHALG-
-EPHALA-
EIPHAL--
EGPHAL--
EDPHAL--
FRPHAL--
-FPHALR-
FNPHAL--
FKPHAL--
FFPHAL--
FVPHAL--
FYPHAL--
FQPHAL--
FPPHAL--
-FPHALW-
-FPHALV-
FGPHAL--
-FPHALF-
-FPHALT-
-FPHALL-
FIPHAL--
FLPHAL--
FTPHAL--
-FPHALY-
FMPHAL--
FEPHAL--
FWPHAL--
-FPHALK-
-FPHALN-
-FPHALM-
-FPHALA-
-FPHALQ-
-FPHALI-
FDPHAL--
FHPHAL--
GRPHAL--

-GPHALV-
GVPHAL--
-GPHALL-
GSPHAL--
-GPHALH-
-GPHALS-
GNPHAL--
GGPHAL--
GLPHAL--
-GPHALR-
-GPHALA-
-GPHALK-
-GPHALT-
GFPHAL--
GKPHAL--
GIPHAL--
GMPHAL--
-GPHALI-
-GPHALN-
GHPHAL--
-GPHALQ-
GEPHAL--
GTPHAL--
GQPHAL--
GPPHAL--
-GPHALW-
-GPHALY-
GAPHAL--
-GPHALE-
GDPHAL--
GCPHAL--
GYPHAL--
-GPHALF-
-GPHALM-
HRPHAL--
-HPHALL-
HVPHAL--
HTPHAL--
HPPHAL--
-HPHALY-
-HPHALV-
HIPHAL--
HWPHAL--
-HPHALN-
HLPHAL--
HGPHAL--
-HPHALR-
HMPHAL--
HAPHAL--
HSPHAL--

```
-HPHALT-
HQPHAL--
HKPHAL--
HHPHAL--
HYPHAL--
-HPHALA-
HDPHAL--
-HPHALK-
-HPHALI-
-HPHALG-
HNPHAL--
-HPHALS-
-HPHALQ-
-HPHALE-
IKPHAL--
ISPHAL--
IRPHAL--
IGPHAL--
-IPHALT-
ILPHAL--
IPPHAL--
-IPHALL-
IYPHAL--
-IPHALK-
IVPHAL--
-IPHALR-
-IPHALS-
IHPHAL--
INPHAL--
IIPHAL--
ITPHAL--
IDPHAL--
IFPHAL--
IEPHAL--
IWPHAL--
-IPHALY-
-IPHALQ-
-IPHALM-
-IPHALG-
IQPHAL--
-IPHALW-
-IPHALI-
IAPHAL--
-KPHALL-
-KPHALR-
-KPHALW-
-KPHALQ-
KVPHAL--
KFPHAL--
-KPHALS-
```

KAPHAL--
KYPHAL--
-KPHALT-
-KPHALF-
KPPHAL--
KGPHAL--
-KPHALA-
KIPHAL--
KWPHAL--
KLPHAL--
-KPHALV-
KNPHAL--
KMPHAL--
-KPHALI-
-KPHALY-
-KPHALM-
-KPHALG-
KDPHAL--
KKPHAL--
-KPHALH-
KTPHAL--
KQPHAL--
KHPHAL--
KEPHAL--
KRPHAL--
-KPHALP-
-KPHALN-
KCPHAL--
LTPHAL--
LLPHAL--
LMPHAL--
LKPHAL--
LPPHAL--
-LPHALI-
LSPHAL--
-LPHALK-
LAPHAL--
-LPHALS-
LNPHAL--
LFPHAL--
LHPHAL--
LQPHAL--
-LPHALR-
-LPHALL-
LIPHAL--
LWPHAL--
-LPHALW-
-LPHALA-
-LPHALT-
-LPHALN-

```
-LPHALM-
LDPHAL--
-LPHALV-
LYPHAL--
-LPHALQ-
-LPHALF-
-LPHALG-
LCPHAL--
MSPHAL--
-MPHALS-
-MPHALN-
MRPHAL--
-MPHALI-
-MPHALL-
MQPHAL--
-MPHALR-
-MPHALF-
MIPHAL--
MYPHAL--
MMPHAL--
MKPHAL--
MHPHAL--
-MPHALY-
-MPHALT-
MLPHAL--
MWPHAL--
MTPHAL--
MPPHAL--
-MPHALV-
-MPHALM-
MFPHAL--
-MPHALK-
-MPHALE-
MVPHAL--
-MPHALH-
MAPHAL--
-MPHALA-
MGPHAL--
-MPHALG-
MEPHAL--
-MPHALQ-
MDPHAL--
MCPHAL--
NVPHAL--
NKPHAL--
NHPHAL--
NYPHAL--
-NPHALY-
NAPHAL--
-NPHALL-
```

NGPHAL--
NEPHAL--
-NPHALR-
NLPHAL--
NIPHAL--
NWPHAL--
NQPHAL--
-NPHALI-
NDPHAL--
-NPHALH-
NFPHAL--
NPPHAL--
-NPHALT-
NTPHAL--
-NPHALM-
-NPHALV-
-NPHALQ-
-NPHALW-
-NPHALS-
NMPHAL--
-NPHALN-
PYPHAL--
--PHALYR
--PHALRT
--PHALVS
--PHALLG
--PHALRV
--PHALYS
--PHALVA
--PHALSS
--PHALRS
--PHALTL
--PHALIR
--PHALTG
--PHALMG
--PHALSI
--PHALTS
--PHALRF
--PHALRD
--PHALSG
--PHALQG
--PHALSR
--PHALGS
--PHALSL
--PHALRY
--PHALFS
PMPHAL--
--PHALVV
--PHALSA
--PHALAI

--PHALRN
--PHALRI
--PHALWR
--PHALLT
--PHALYT
--PHALSM
--PHALLR
PTPHAL--
--PHALYW
--PHALVT
--PHALRG
--PHALRA
--PHALKT
--PHALMR
--PHALLA
--PHALKA
--PHALWG
--PHALVY
--PHALYA
--PHALVM
--PHALSV
--PHALKV
--PHALKG
--PHALHY
--PHALHV
--PHALAL
--PHALRQ
--PHALQI
--PHALKS
--PHALKF
--PHALGG
--PHALWQ
--PHALGL
--PHALTT
--PHALTI
--PHALRE
--PHALKI
--PHALIS
--PHALPL
--PHALMV
--PHALLF
--PHALIQ
--PHALHS
--PHALFL
--PHALST
--PHALNL
--PHALMQ
--PHALGM
--PHALFQ
--PHALVG

--PHALTV
--PHALTQ
--PHALTF
--PHALNA
--PHALHL
--PHALES
--PHALVR
--PHALVI
--PHALVH
--PHALTH
--PHALLQ
--PHALAA
--PHALSH
--PHALQS
--PHALMY
--PHALIL
--PHALHN
--PHALAW
--PHALWT
--PHALWN
--PHALNW
--PHALLV
--PHALHR
--PHALHG
--PHALFY
--PHALFA
--PHALYL
--PHALVF
--PHALTN
--PHALQL
--PHALLK
--PHALLH
--PHALKM
--PHALGY
--PHALAG
PIPHAL--
--PHALYF
--PHALVN
--PHALMK
--PHALLE
--PHALKN
--PHALFE
--PHALAM
--PHALVL
--PHALTA
--PHALSQ
--PHALSK
--PHALNR
--PHALNM
--PHALNH

--PHALKK
--PHALGV
--PHALFT
--PHALEG
PLPHAL--
--PHALWY
--PHALMS
--PHALIT
--PHALIG
--PHALHT
--PHALHI
--PHALAY
--PHALAN
--PHALAF
PKPHAL--
--PHALRM
--PHALNV
--PHALLM
--PHALVE
--PHALSN
--PHALMT
--PHALLN
--PHALKY
--PHALIW
--PHALIK
--PHALHW
--PHALFK
PAPHAL--
--PHALVD
--PHALTK
--PHALSY
--PHALQH
--PHALML
--PHALIA
--PHALHF
--PHALAV
--PHALAT
--PHALAK
--PHALYN
--PHALYK
--PHALSF
--PHALQT
--PHALNT
--PHALNK
--PHALLD
--PHALEV
--PHALYQ
--PHALVK
--PHALSW
--PHALRH

```
--PHALPY
--PHALNY
--PHALMN
--PHALHE
--PHALFV
--PHALFD
--PHALEQ
--PHALAR
--PHALAH
--PHALWL
--PHALVQ
--PHALRW
--PHALNQ
--PHALME
--PHALMA
--PHALKD
--PHALHM
--PHALGF
--PHALFH
--PHALEI
PQPHAL--
PHPHAL--
--PHALWK
--PHALWD
--PHALWA
--PHALIM
--PHALIH
--PHALHK
--PHALFN
--PHALFF
--PHALAQ
PFPHAL--
--PHALWW
--PHALWF
--PHALQQ
--PHALND
--PHALKH
--PHALIY
--PHALII
--PHALHQ
--PHALHA
--PHALGA
--PHALET
--PHALEA
PGPHAL--
PWPHAL--
PSPHAL--
--PHALYY
--PHALYV
--PHALYM
```

--PHALYG
--PHALWI
--PHALSD
--PHALQY
--PHALQF
--PHALPI
--PHALNF
--PHALLC
--PHALEW
--PHALEL
--PHALYH
--PHALWS
--PHALTD
--PHALQW
--PHALPM
--PHALIE
--PHALHH
--PHALGW
--PHALFI
--PHALFG
--PHALEN
--PHALEK
--PHALEH
--PHALCD
PDPHAL--
-PPHALV-
-PPHALQ-
--PHALWE
--PHALVC
--PHALQV
--PHALQE
--PHALQA
--PHALPS
--PHALLP
--PHALKQ
--PHALIV
--PHALIP
--PHALHD
--PHALGQ
--PHALGN
--PHALGK
--PHALGH
--PHALGD
--PHALFW
--PHALEY
--PHALDY
--PHALDM
--PHALDA
--PHALCI
PCPHAL--

QGPHAL--
QRPHAL--
QFPHAL--
-QPHALR-
-QPHALS-
QHPHAL--
QAPHAL--
QSPHAL--
-QPHALT-
QIPHAL--
-QPHALL-
QVPHAL--
QTPHAL--
-QPHALV-
QLPHAL--
-QPHALH-
-QPHALF-
QWPHAL--
-QPHALQ-
-QPHALG-
QKPHAL--
-QPHALN-
QEPHAL--
-QPHALA-
-QPHALK-
-QPHALI-
QNPHAL--
QMPHAL--
-QPHALY-
-QPHALW-
-QPHALC-
-RPHALL-
RSPHAL--
-RPHALI-
RMPHAL--
RKPHAL--
-RPHALT-
RPPHAL--
-RPHALS-
RLPHAL--
RRPHAL--
-RPHALR-
RAPHAL--
RYPHAL--
RVPHAL--
-RPHALW-
-RPHALY-
-RPHALV-
-RPHALM-
RHPHAL--

RTPHAL--
RGPHAL--
-RPHALH-
REPHAL--
-RPHALE-
-RPHALN-
-RPHALG-
RNPHAL--
-RPHALQ-
-RPHALF-
RCPHAL--
RIPHAL--
SRPHAL--
-SPHALL-
SHPHAL--
-SPHALY-
SLPHAL--
SAPHAL--
-SPHALI-
SQPHAL--
-SPHALH-
-SPHALK-
-SPHALW-
SEPHAL--
SVPHAL--
-SPHALV-
SYPHAL--
-SPHALS-
SMPHAL--
SWPHAL--
SIPHAL--
-SPHALQ-
-SPHALN-
SSPHAL--
SGPHAL--
SFPHAL--
SPPHAL--
-SPHALT-
-SPHALM-
STPHAL--
SNPHAL--
-SPHALA-
SCPHAL--
-SPHALG-
SDPHAL--
-SPHALE-
-TPHALR-
TVPHAL--
-TPHALT-
-TPHALL-

-TPHALF-
-TPHALI-
TKPHAL--
-TPHALW-
TSPHAL--
TQPHAL--
-TPHALS-
TMPHAL--
TGPHAL--
TIPHAL--
TAPHAL--
-TPHALH-
TNPHAL--
-TPHALY-
-TPHALV-
TFPHAL--
TEPHAL--
TPPHAL--
TYPHAL--
TTPHAL--
TWPHAL--
-TPHALK-
THPHAL--
-TPHALE-
-TPHALG-
-TPHALA-
TCPHAL--
-TPHALQ-
VLPHAL--
-VPHALT-
VMPHAL--
-VPHALY-
VAPHAL--
VRPHAL--
VHPHAL--
-VPHALL-
VIPHAL--
-VPHALQ-
VQPHAL--
VPPHAL--
VKPHAL--
-VPHALI-
-VPHALF-
VYPHAL--
VVPHAL--
-VPHALK-
-VPHALH-
VGPHAL--
VNPHAL--
-VPHALW-

VWPHAL--
-VPHALM-
-VPHALA-
VEPHAL--
-VPHALV-
-VPHALN-
VDPHAL--
VCPHAL--
-VPHALG-
-VPHALE-
VFPHAL--
VTPHAL--
WVPHAL--
WGPHAL--
-WPHALT-
-WPHALI-
WLPHAL--
-WPHALF-
WFPHAL--
WYPHAL--
WKPHAL--
WSPHAL--
-WPHALA-
WAPHAL--
WWPHAL--
-WPHALK-
WEPHAL--
WTPHAL--
WMPHAL--
WIPHAL--
WCPHAL--
WQPHAL--
-WPHALG-
WHPHAL--
-WPHALV-
-WPHALM-
-WPHALH-
-WPHALE-
WNPHAL--
-YPHALY-
-YPHALH-
YMPHAL--
YSPHAL--
YRPHAL--
-YPHALL-
-YPHALF-
YIPHAL--
YTPHAL--
YAPHAL--
YPPHAL--

YGPHAL--
-YPHALK-
-YPHALS-
-YPHALE-
-YPHALV-
-YPHALA-
YLPHAL--
YHPHAL--
-YPHALT-
-YPHALI-
YFPHAL--
YWPHAL--
-YPHALN-
YVPHAL--
YCPHAL--
YEPHAL--
-YPHALQ-
-YPHALM-
-YPHALD-
YDPHAL--

PIAM	523	612	46.96

--PIAMKL
ASPIAM--
ARPIAM--
AMPIAM--
AFPIAM--
-APIAMT-
-APIAMM-
-APIAML-
AYPIAM--
-APIAMQ-
-APIAMG-
ANPIAM--
AWPIAM--
AGPIAM--
ATPIAM--
-APIAMI-
-APIAME-
APPIAM--
-APIAMK-
AKPIAM--
-APIAMW-
-APIAMN-
-APIAMH-
ADPIAM--
AAPIAM--
CCPIAM--
-CPIAMV-
CIPIAM--
-CPIAML-

CLPIAM--
CHPIAM--
CFPIAM--
DRPIAM--
-DPIAMY-
DFPIAM--
DTPIAM--
-DPIAMT-
-DPIAMR-
-DPIAML-
-DPIAMH-
DWPIAM--
-DPIAMV-
DKPIAM--
DCPIAM--
DSPIAM--
-DPIAMW-
-DPIAMK-
-DPIAMF-
DIPIAM--
-EPIAMV-
-EPIAMR-
ESPIAM--
-EPIAMH-
-EPIAMI-
EWPIAM--
-EPIAML-
-EPIAMW-
-EPIAMT-
EVPIAM--
ERPIAM--
ENPIAM--
EPPIAM--
-EPIAMQ-
EGPIAM--
-EPIAME-
EMPIAM--
EHPIAM--
EAPIAM--
-FPIAMI-
FIPIAM--
FHPIAM--
FRPIAM--
FQPIAM--
-FPIAMK-
-FPIAMS-
FAPIAM--
FDPIAM--
FFPIAM--
FWPIAM--

-FPIAML-
FLPIAM--
FYPIAM--
FSPIAM--
-FPIAMH-
-FPIAMR-
FGPIAM--
FEPIAM--
-FPIAMA-
FCPIAM--
-GPIAMR-
GLPIAM--
GGPIAM--
GKPIAM--
GVPIAM--
-GPIAML-
-GPIAMI-
GFPIAM--
GRPIAM--
-GPIAMT-
-GPIAMS-
GSPIAM--
-GPIAMV-
-GPIAMN-
-GPIAMK-
GNPIAM--
GIPIAM--
-GPIAMM-
-GPIAMG-
GAPIAM--
GYPIAM--
GWPIAM--
-GPIAMH-
-GPIAMY-
-GPIAMQ-
-GPIAME-
-GPIAMA-
HGPIAM--
HDPIAM--
HFPIAM--
-HPIAML-
-HPIAMW-
HRPIAM--
HHPIAM--
HQPIAM--
HKPIAM--
HWPIAM--
HMPIAM--
HTPIAM--
HPPIAM--

-HPIAMT-
-HPIAMM-
HIPIAM--
-HPIAMY-
-HPIAMA-
HAPIAM--
HLPIAM--
HYPIAM--
-HPIAMQ-
HCPIAM--
HSPIAM--
-HPIAMI-
HEPIAM--
IYPIAM--
-IPIAMI-
ILPIAM--
IQPIAM--
-IPIAMT-
IWPIAM--
-IPIAMS-
IRPIAM--
ISPIAM--
-IPIAMM-
ITPIAM--
IFPIAM--
INPIAM--
-IPIAMK-
-IPIAMH-
IGPIAM--
-IPIAML-
KSPIAM--
-KPIAMR-
KMPIAM--
KWPIAM--
-KPIAMQ-
-KPIAMI-
KFPIAM--
-KPIAML-
KQPIAM--
-KPIAMK-
KYPIAM--
-KPIAMH-
-KPIAMG-
-KPIAMV-
KGPIAM--
-KPIAMW-
KHPIAM--
-LPIAMR-
LSPIAM--
LMPIAM--

LGPIAM--
LEPIAM--
LAPIAM--
-LPIAML-
LIPIAM--
-LPIAMM-
LFPIAM--
LHPIAM--
LPPIAM--
LNPIAM--
LLPIAM--
LQPIAM--
LKPIAM--
-LPIAMS-
-LPIAMT-
LWPIAM--
-LPIAMQ-
-LPIAMK-
LDPIAM--
-MPIAMK-
-MPIAMT-
-MPIAMV-
MLPIAM--
MHPIAM--
MRPIAM--
-MPIAMS-
MFPIAM--
MAPIAM--
MPPIAM--
-MPIAMM-
-MPIAMI-
-MPIAML-
MYPIAM--
MSPIAM--
-MPIAMN-
-MPIAMH-
-MPIAMF-
-MPIAMA-
MGPIAM--
-MPIAME-
MNPIAM--
MIPIAM--
NFPIAM--
NRPIAM--
-NPIAMR-
NVPIAM--
NTPIAM--
NSPIAM--
-NPIAMW-
-NPIAMT-

-NPIAML-
-NPIAMV-
-NPIAMI-
-NPIAMF-
NQPIAM--
NGPIAM--
-NPIAMN-
-NPIAMM-
NHPIAM--
-NPIAMQ-
NNPIAM--
NPPIAM--
-NPIAMS-
-NPIAMK-
-NPIAMG-
-NPIAMA-
--PIAMIG
--PIAMRL
--PIAMRR
--PIAMHR
--PIAMLH
--PIAMHL
--PIAMTM
--PIAMSS
--PIAMTG
--PIAMRG
--PIAMLT
--PIAMLS
--PIAMHG
--PIAMRT
--PIAMRM
--PIAMGY
--PIAMKA
--PIAMTL
--PIAMSR
--PIAMHV
--PIAMYG
--PIAMNL
--PIAMMN
--PIAMGS
--PIAMTT
--PIAMTA
--PIAMNT
--PIAMHF
--PIAMHA
--PIAMGA
--PIAMTV
--PIAMIV
--PIAMGW
--PIAMTR

--PIAMRA
--PIAMLG
--PIAMIN
--PIAMTN
--PIAMQS
--PIAMHM
--PIAMFG
--PIAMAL
PQPIAM--
--PIAMST
--PIAMSI
--PIAMKS
--PIAMKR
--PIAMHI
--PIAMFS
--PIAMYH
--PIAMYF
--PIAMWQ
--PIAMVQ
--PIAMTI
--PIAMRY
--PIAMGV
--PIAMES
PDPIAM--
--PIAMRE
--PIAMNQ
--PIAMNM
--PIAMHH
--PIAMGG
--PIAMVT
--PIAMSF
--PIAMKT
--PIAMKN
--PIAMAA
--PIAMYS
--PIAMWL
--PIAMWG
--PIAMVN
--PIAMVD
--PIAMTK
--PIAMRI
--PIAMPI
--PIAMMQ
--PIAMMK
--PIAMKV
--PIAMIH
--PIAMIF
--PIAMGF
--PIAMAF
PTPIAM--

--PIAMVG
--PIAMSV
--PIAMSG
--PIAMSA
--PIAMRV
--PIAMQQ
--PIAMQM
--PIAMQI
--PIAMQG
--PIAMMS
--PIAMLY
--PIAMGM
--PIAMEA
--PIAMDL
--PIAMYE
--PIAMWE
--PIAMSM
--PIAMSH
--PIAMNW
--PIAMKG
--PIAMFT
--PIAMET
--PIAMEL
--PIAMEH
--PIAMAS
--PIAMYN
--PIAMSW
--PIAMRD
--PIAMQY
--PIAMQN
--PIAMQA
--PIAMPW
--PIAMNY
--PIAMKW
--PIAMIQ
--PIAMGI
--PIAMFD
--PIAMEG
PGPIAM--
--PIAMYD
--PIAMVY
--PIAMVH
--PIAMVF
--PIAMSD
--PIAMRQ
--PIAMRN
--PIAMRK
--PIAMQW
--PIAMQH
--PIAMQE

--PIAMNK
--PIAMMA
--PIAMLN
--PIAMLK
--PIAMKQ
--PIAMHQ
--PIAMEM
--PIAMDS
--PIAMAY
--PIAMAV
--PIAMAQ
--PIAMYP
--PIAMWN
--PIAMWC
--PIAMTF
--PIAMTD
--PIAMSK
--PIAMQR
--PIAMQK
--PIAMPY
--PIAMPV
--PIAMPQ
--PIAMNV
--PIAMMH
--PIAMLD
--PIAMKY
--PIAMKM
--PIAMKK
--PIAMKH
--PIAMKC
--PIAMIC
--PIAMHW
--PIAMHN
--PIAMFR
--PIAMFH
--PIAMEY
--PIAMEQ
--PIAMEI
--PIAMDM
--PIAMDA
--PIAMCS
--PIAMCR
--PIAMCN
--PIAMCK
--PIAMCG
--PIAMAW
--PIAMAT
--PIAMAH
QSPIAM--
-QPIAMQ-

-QPIAMR-
QIPIAM--
-QPIAMT-
-QPIAMY-
QLPIAM--
QYPIAM--
QQPIAM--
-QPIAMV-
QMPIAM--
QNPIAM--
QFPIAM--
QRPIAM--
-QPIAMK-
-QPIAMG-
QTPIAM--
QHPIAM--
-QPIAMS-
QAPIAM--
RSPIAM--
RRPIAM--
RGPIAM--
REPIAM--
-RPIAMT-
-RPIAML-
-RPIAMR-
-RPIAMW-
-RPIAMA-
-RPIAMV-
-RPIAMQ-
RTPIAM--
RMPIAM--
RKPIAM--
RNPIAM--
RWPIAM--
-RPIAMI-
-RPIAMN-
-RPIAMK-
-RPIAMM-
RHPIAM--
-RPIAMY-
RIPIAM--
RDPIAM--
RQPIAM--
-RPIAME-
RPPIAM--
RVPIAM--
-RPIAMG-
RYPIAM--
RLPIAM--
-RPIAMD-

-SPIAMK-
-SPIAMT-
STPIAM--
SDPIAM--
SSPIAM--
SRPIAM--
-SPIAML-
SWPIAM--
SVPIAM--
SGPIAM--
SIPIAM--
SLPIAM--
-SPIAMH-
SKPIAM--
SEPIAM--
SAPIAM--
SYPIAM--
-SPIAMF-
SCPIAM--
-SPIAMA-
SFPIAM--
-SPIAMW-
-SPIAMQ-
-SPIAMN-
SMPIAM--
-SPIAMV-
-SPIAMS-
-SPIAMI-
SHPIAM--
SPPIAM--
-SPIAMM-
-SPIAME-
-SPIAMD-
-SPIAMC-
TSPIAM--
-TPIAMR-
TVPIAM--
TGPIAM--
THPIAM--
-TPIAMS-
TLPIAM--
-TPIAMT-
TIPIAM--
TEPIAM--
-TPIAMH-
TRPIAM--
-TPIAMY-
TWPIAM--
-TPIAMV-
-TPIAMQ-

-TPIAMM-
-TPIAML-
TTPIAM--
TNPIAM--
-TPIAMA-
TYPIAM--
TPPIAM--
-TPIAMK-
TAPIAM--
-TPIAMI-
-TPIAMG-
TFPIAM--
VSPIAM--
-VPIAMS-
VYPIAM--
-VPIAML-
VFPIAM--
-VPIAMI-
-VPIAMQ-
VHPIAM--
VAPIAM--
VVPIAM--
VTPIAM--
-VPIAME-
VKPIAM--
VDPIAM--
-VPIAMN-
-VPIAMM-
VEPIAM--
-VPIAMR-
VWPIAM--
VRPIAM--
VPPIAM--
-VPIAMT-
-VPIAMG-
-VPIAMV-
-VPIAMK-
-VPIAMA-
VMPIAM--
VLPIAM--
WLPIAM--
-WPIAMM-
WVPIAM--
WTPIAM--
WKPIAM--
WFPIAM--
-WPIAMS-
-WPIAMQ-
-WPIAMI-
WNPIAM--

-WPIAMK-
WYPIAM--
-WPIAMV-
-WPIAMR-
-WPIAMN-
-WPIAML-
WDPIAM--
WAPIAM--
YHPIAM--
-YPIAMR-
-YPIAMI-
YVPIAM--
-YPIAMS-
-YPIAML-
YMPIAM--
YAPIAM--
-YPIAMN-
YKPIAM--
YSPIAM--
-YPIAMV-
YRPIAM--
YNPIAM--
-YPIAMM-
-YPIAMT-
-YPIAMQ-
-YPIAMK-
YPPIAM--
-YPIAMA-
-YPIAMW-
-YPIAMH-
YIPIAM--
YEPIAM--
YCPIAM--

PLHY 166 766 46.95

-APLHYR-
ASPLHY--
-APLHYV-
-APLHYS-
-APLHYY-
ALPLHY--
ARPLHY--
-APLHYM-
-APLHYK-
AYPLHY--
AVPLHY--
-APLHYI-
-APLHYH-
AQPLHY--
AFPLHY--
-APLHYA-

ANPLHY--
ADPLHY--
AIPLHY--
-APLHYQ-
-APLHYW-
-APLHYF-
-APLHYT-
AEPLHY--
-APLHYE-
ATPLHY--
-APLHYN-
-APLHYG-
AKPLHY--
CCPLHY--
CVPLHY--
CKPLHY--
CLPLHY--
CSPLHY--
-CPLHYK-
CNPLHY--
CDPLHY--
CPPLHY--
-CPLHYT-
-CPLHYS-
CRPLHY--
-CPLHYL-
-CPLHYI-
CEPLHY--
DRPLHY--
DYPLHY--
DKPLHY--
DTPLHY--
DSPLHY--
DLPLHY--
DVPLHY--
DFPLHY--
DAPLHY--
-DPLHYM-
-DPLHYK-
DCPLHY--
-DPLHYE-
DNPLHY--
DQPLHY--
-DPLHYS-
-DPLHYR-
DIPLHY--
DDPLHY--
DWPLHY--
DEPLHY--
-EPLHYT-

EVPLHY--
ELPLHY--
EKPLHY--
-EPLHYS-
-EPLHYY-
-EPLHYV-
ESPLHY--
EQPLHY--
-EPLHYM-
-EPLHYA-
EMPLHY--
EEPLHY--
EWPLHY--
-EPLHYK-
-EPLHYI-
EGPLHY--
EFPLHY--
EAPLHY--
-EPLHYR-
-EPLHYL-
EIPLHY--
ETPLHY--
EDPLHY--
-FPLHYV-
-FPLHYI-
FVPLHY--
FSPLHY--
FRPLHY--
FTPLHY--
FDPLHY--
FLPLHY--
FHPLHY--
FNPLHY--
-FPLHYM-
-FPLHYT-
-FPLHYH-
-FPLHYR-
-FPLHYF-
FGPLHY--
FEPLHY--
FAPLHY--
FMPLHY--
FFPLHY--
FYPLHY--
FPPLHY--
-FPLHYY-
-FPLHYS-
-FPLHYQ-
-FPLHYK-
FKPLHY--

GSPLHY--
GLPLHY--
-GPLHYI-
-GPLHYL-
GRPLHY--
-GPLHYT-
-GPLHYA-
GYPLHY--
GFPLHY--
GWPLHY--
-GPLHYV-
-GPLHYM-
-GPLHYR-
-GPLHYY-
GVPLHY--
GPPLHY--
-GPLHYK-
GIPLHY--
GAPLHY--
GQPLHY--
-GPLHYN-
-GPLHYW-
GKPLHY--
GNPLHY--
GMPLHY--
-GPLHYE-
-GPLHYG-
-GPLHYF-
GGPLHY--
-HPLHYL-
HAPLHY--
HGPLHY--
HRPLHY--
HWPLHY--
HSPLHY--
-HPLHYS-
-HPLHYI-
HYPLHY--
HLPLHY--
HNPLHY--
HIPLHY--
-HPLHYM-
HTPLHY--
HEPLHY--
HQPLHY--
-HPLHYY-
-HPLHYV-
-HPLHYK-
HPPLHY--
-HPLHYE-

-HPLHYT-
-HPLHYA-
HMPLHY--
HHPLHY--
-HPLHYQ-
IKPLHY--
IRPLHY--
IWPLHY--
-IPLHYM-
IDPLHY--
IHPLHY--
IQPLHY--
ITPLHY--
-IPLHYT-
-IPLHYL-
-IPLHYI-
ISPLHY--
-IPLHYE-
ILPLHY--
IIPLHY--
IYPLHY--
IPPLHY--
-IPLHYR-
IGPLHY--
IFPLHY--
-IPLHYN-
-IPLHYK-
-IPLHYC-
-IPLHYQ-
-IPLHYF-
-IPLHYA-
INPLHY--
IAPLHY--
-IPLHYH-
KSPLHY--
-KPLHYF-
-KPLHYQ-
KTPLHY--
-KPLHYS-
KRPLHY--
KVPLHY--
-KPLHYL-
-KPLHYR-
KWPLHY--
KYPLHY--
-KPLHYV-
-KPLHYM-
-KPLHYH-
KFPLHY--
-KPLHYE-

KNPLHY--
KIPLHY--
KAPLHY--
-KPLHYY-
-KPLHYN-
-KPLHYW-
KGPLHY--
KDPLHY--
-KPLHYG-
-KPLHYA-
KLPLHY--
KHPLHY--
KEPLHY--
KQPLHY--
KPPLHY--
-KPLHYK-
KCPLHY--
LRPLHY--
-LPLHYT-
LEPLHY--
LTPLHY--
LWPLHY--
LLPLHY--
LMPLHY--
LHPLHY--
LPPLHY--
-LPLHYL-
-LPLHYS-
-LPLHYQ-
LVPLHY--
-LPLHYK-
-LPLHYI-
LNPLHY--
LIPLHY--
-LPLHYY-
-LPLHYW-
-LPLHYH-
-LPLHYF-
-LPLHYG-
-LPLHYA-
-LPLHYV-
-LPLHYE-
MNPLHY--
-MPLHYK-
MRPLHY--
-MPLHYS-
MGPLHY--
MPPLHY--
MLPLHY--
MKPLHY--

MSPLHY--
MWPLHY--
MFPLHY--
MVPLHY--
MMPLHY--
MIPLHY--
-MPLHYF-
-MPLHYV-
-MPLHYE-
MAPLHY--
MTPLHY--
-MPLHYM-
MEPLHY--
-MPLHYT-
-MPLHYG-
-MPLHYR-
-MPLHYA-
MCPLHY--
NRPLHY--
-NPLHYR-
NVPLHY--
NLPLHY--
NSPLHY--
NPPLHY--
-NPLHYM-
NGPLHY--
-NPLHYI-
-NPLHYQ-
NYPLHY--
NTPLHY--
-NPLHYS-
NFPLHY--
-NPLHYY-
-NPLHYA-
NHPLHY--
NEPLHY--
NAPLHY--
-NPLHYV-
NNPLHY--
-NPLHYL-
NIPLHY--
NWPLHY--
NQPLHY--
-NPLHYW-
-NPLHYT-
NMPLHY--
NKPLHY--
NCPLHY--
-NPLHYK-
NDPLHY--

-NPLHYF-
--PLHYSS
--PLHYRG
--PLHYTS
--PLHYRT
--PLHYIA
--PLHYIG
--PLHYRA
--PLHYLS
--PLHYRL
--PLHYYT
--PLHYRN
--PLHYAY
--PLHYLA
--PLHYNS
--PLHYVR
--PLHYTA
--PLHYSG
--PLHYIK
--PLHYFR
--PLHYMA
--PLHYST
--PLHYEV
--PLHYLV
--PLHYMM
--PLHYHS
PGPLHY--
--PLHYLG
--PLHYFS
--PLHYLW
--PLHYHT
--PLHYTT
--PLHYQR
--PLHYMR
--PLHYLN
--PLHYFG
--PLHYVT
--PLHYRS
--PLHYII
--PLHYAS
--PLHYYL
--PLHYFM
--PLHYVV
--PLHYTQ
--PLHYTN
--PLHYSM
--PLHYMV
--PLHYMN
--PLHYIR
--PLHYET

--PLHYAV
--PLHYVF
--PLHYSK
--PLHYNA
--PLHYMT
--PLHYMK
--PLHYGV
--PLHYYS
--PLHYVS
--PLHYTL
--PLHYSR
--PLHYSF
--PLHYQQ
--PLHYQI
--PLHYLK
--PLHYKG
PNPLHY--
--PLHYSW
--PLHYMG
--PLHYKM
--PLHYAQ
--PLHYSV
--PLHYRM
--PLHYRH
--PLHYQY
--PLHYNF
--PLHYKI
--PLHYIS
--PLHYWR
--PLHYVY
--PLHYVA
--PLHYTM
--PLHYQG
--PLHYKK
--PLHYGS
--PLHYAA
--PLHYRY
--PLHYQA
--PLHYLT
--PLHYKL
--PLHYFH
--PLHYEW
--PLHYAW
--PLHYWY
--PLHYTH
--PLHYTG
--PLHYRQ
--PLHYNG
--PLHYLF
--PLHYIW

--PLHYIM
--PLHYVI
--PLHYTF
--PLHYRW
--PLHYRD
--PLHYMY
--PLHYMD
--PLHYKS
--PLHYHA
--PLHYWT
--PLHYWM
--PLHYTW
--PLHYTV
--PLHYSI
--PLHYSH
--PLHYQS
--PLHYQN
--PLHYNL
--PLHYKF
--PLHYIN
--PLHYIE
--PLHYHI
--PLHYER
--PLHYAM
--PLHYAI
--PLHYAH
--PLHYYA
--PLHYVN
--PLHYVH
--PLHYVG
--PLHYTE
--PLHYSY
--PLHYSN
--PLHYQT
--PLHYHQ
--PLHYHN
--PLHYHG
--PLHYEM
--PLHYAT
--PLHYYW
--PLHYYH
--PLHYWI
--PLHYWF
--PLHYVW
--PLHYVQ
--PLHYVK
--PLHYNM
--PLHYHY
--PLHYHF
--PLHYGG

--PLHYFW
--PLHYAR
--PLHYAL
PEPLHY--
PDPLHY--
PCPLHY--
PTPLHY--
--PLHYYG
--PLHYTD
--PLHYSE
--PLHYRF
--PLHYRE
--PLHYQH
--PLHYNT
--PLHYHL
--PLHYGM
--PLHYGL
--PLHYGA
--PLHYFQ
--PLHYAN
--PLHYAE
-PPLHYQ-
--PLHYYF
--PLHYWW
--PLHYTI
--PLHYSA
--PLHYQM
--PLHYQF
--PLHYMI
--PLHYME
--PLHYLM
--PLHYKT
--PLHYKQ
--PLHYKN
--PLHYKA
--PLHYIV
--PLHYIQ
--PLHYIL
--PLHYHR
--PLHYGY
--PLHYGT
--PLHYFL
--PLHYEK
--PLHYWV
--PLHYWQ
--PLHYWG
--PLHYRV
--PLHYNW
--PLHYML
--PLHYLY

--PLHYLE
--PLHYKD
--PLHYIT
--PLHYID
--PLHYHV
--PLHYGI
--PLHYEH
--PLHYEG
--PLHYDS
--PLHYDA
--PLHYAK
--PLHYAG
PPPLHY--
--PLHYYQ
--PLHYYM
--PLHYVM
--PLHYVE
--PLHYTK
--PLHYSQ
--PLHYQV
--PLHYQL
--PLHYQE
--PLHYPF
--PLHYNI
--PLHYMH
--PLHYLI
--PLHYKW
--PLHYIF
--PLHYHH
--PLHYGW
--PLHYGQ
--PLHYGF
--PLHYFN
--PLHYEY
--PLHYEQ
--PLHYDR
--PLHYAF
QYPLHY--
QKPLHY--
QAPLHY--
QRPLHY--
QGPLHY--
-QPLHYS-
QSPLHY--
QPPLHY--
-QPLHYA-
QFPLHY--
QEPLHY--
QVPLHY--
-QPLHYY-

QNPLHY--
-QPLHYL-
QTPLHY--
-QPLHYM-
-QPLHYT-
-QPLHYI-
-QPLHYW-
-QPLHYV-
QIPLHY--
-QPLHYN-
-QPLHYH-
-QPLHYE-
QLPLHY--
QWPLHY--
-QPLHYQ-
QMPLHY--
QHPLHY--
RSPLHY--
RGPLHY--
-RPLHYL-
-RPLHYS-
-RPLHYT-
-RPLHYI-
RPPLHY--
-RPLHYA-
RAPLHY--
-RPLHYY-
RVPLHY--
RYPLHY--
-RPLHYQ-
-RPLHYH-
RIPLHY--
RDPLHY--
RQPLHY--
-RPLHYM-
-RPLHYW-
REPLHY--
-RPLHYK-
RKPLHY--
-RPLHYF-
RHPLHY--
RFPLHY--
RWPLHY--
-RPLHYN-
RCPLHY--
-RPLHYE-
-SPLHYS-
SKPLHY--
-SPLHYR-
-SPLHYT-

-SPLHYV-
-SPLHYM-
SRPLHY--
-SPLHYL-
SWPLHY--
SPPLHY--
SSPLHY--
SGPLHY--
SYPLHY--
-SPLHYA-
-SPLHYI-
SDPLHY--
SQPLHY--
SAPLHY--
SMPLHY--
SIPLHY--
SEPLHY--
SHPLHY--
-SPLHYY-
-SPLHYF-
SVPLHY--
-SPLHYW-
-SPLHYH-
-SPLHYE-
-SPLHYQ-
SFPLHY--
-SPLHYN-
SCPLHY--
-SPLHYK-
-SPLHYG-
-TPLHYL-
TSPLHY--
TAPLHY--
TRPLHY--
TEPLHY--
-TPLHYI-
TVPLHY--
TTPLHY--
-TPLHYS-
TLPLHY--
THPLHY--
TGPLHY--
-TPLHYT-
TIPLHY--
-TPLHYR-
-TPLHYM-
TMPLHY--
-TPLHYF-
-TPLHYY-
-TPLHYQ-

TDPLHY--
TQPLHY--
-TPLHYV-
TNPLHY--
TYPLHY--
TPPLHY--
-TPLHYW-
-TPLHYA-
-TPLHYH-
TKPLHY--
TWPLHY--
-TPLHYK-
-TPLHYE-
-VPLHYR-
VVPLHY--
VRPLHY--
VSPLHY--
VMPLHY--
VYPLHY--
-VPLHYS-
VIPLHY--
VGPLHY--
-VPLHYL-
-VPLHYI-
-VPLHYM-
VFPLHY--
-VPLHYF-
-VPLHYT-
-VPLHYA-
VWPLHY--
VQPLHY--
VAPLHY--
-VPLHYK-
VEPLHY--
-VPLHYV-
-VPLHYQ-
-VPLHYH-
-VPLHYY-
-VPLHYN-
VDPLHY--
VTPLHY--
VPPLHY--
VNPLHY--
-VPLHYW-
-VPLHYG-
-VPLHYC-
VCPLHY--
-WPLHYT-
WNPLHY--
WMPLHY--

WHPLHY--
WLPLHY--
WAPLHY--
WQPLHY--
-WPLHYS-
-WPLHYM-
WGPLHY--
-WPLHYQ-
WKPLHY--
WRPLHY--
-WPLHYI-
WSPLHY--
WPPLHY--
WFPLHY--
-WPLHYV-
-WPLHYL-
WYPLHY--
-WPLHYA-
WEPLHY--
WCPLHY--
-WPLHYY-
-WPLHYG-
WDPLHY--
YRPLHY--
-YPLHYK-
YPPLHY--
-YPLHYQ-
-YPLHYI-
YVPLHY--
-YPLHYT-
-YPLHYR-
YNPLHY--
YIPLHY--
YMPLHY--
YGPLHY--
YSPLHY--
YLPLHY--
YKPLHY--
YEPLHY--
-YPLHYM-
YQPLHY--
YFPLHY--
YYPLHY--
-YPLHYY-
YAPLHY--
YTPLHY--
-YPLHYF-
-YPLHYV-
-YPLHYH-
-YPLHYE-

PRPI 142 783 46.91

```
--PRPIEF
--PRPICR
-RPRPIG-
-APRPIR-
ALPRPI--
ASPRPI--
-APRPIH-
AWPRPI--
AGPRPI--
-APRPIY-
-APRPIQ-
-APRPIT-
APPRPI--
AYPRPI--
AQPRPI--
-APRPIV-
-APRPIM-
ATPRPI--
ARPRPI--
AAPRPI--
-APRPIA-
-APRPIW-
-APRPIN-
-APRPII-
-APRPIS-
-APRPIF-
AEPRPI--
-APRPIK-
AHPRPI--
ADPRPI--
ANPRPI--
-APRPIG-
-APRPIC-
ACPRPI--
CLPRPI--
CWPRPI--
CQPRPI--
CSPRPI--
CVPRPI--
-CPRPIW-
CFPRPI--
CMPRPI--
CRPRPI--
CPPRPI--
CHPRPI--
CGPRPI--
-CPRPIY-
-CPRPIS-
-CPRPIQ-
```

-CPRPIH-
-CPRPIF-
CAPRPI--
-DPRPIK-
-DPRPIL-
-DPRPIY-
-DPRPIT-
DYPRPI--
DLPRPI--
DSPRPI--
-DPRPIR-
-DPRPII-
DGPRPI--
-DPRPIV-
DVPRPI--
DHPRPI--
-DPRPIW-
-DPRPIF-
DWPRPI--
DIPRPI--
DMPRPI--
-DPRPIM-
DNPRPI--
DKPRPI--
DEPRPI--
DDPRPI--
DCPRPI--
DAPRPI--
ESPRPI--
-EPRPIR-
-EPRPIL-
EGPRPI--
-EPRPIV-
EVPRPI--
-EPRPIN-
ETPRPI--
EMPRPI--
EFPRPI--
-EPRPIS-
-EPRPIK-
-EPRPII-
ELPRPI--
ERPRPI--
-EPRPIT-
-EPRPIH-
EHPRPI--
EAPRPI--
EWPRPI--
EKPRPI--
-EPRPIY-

-EPRPIF-
EQPRPI--
-EPRPIW-
-EPRPIQ-
-EPRPIM-
-EPRPIA-
EIPRPI--
FSPRPI--
FIPRPI--
FKPRPI--
FPPRPI--
FTPRPI--
-FPRPIR-
FEPRPI--
FQPRPI--
FLPRPI--
-FPRPIH-
FDPRPI--
FVPRPI--
-FPRPIK-
-FPRPIF-
-FPRPIM-
-FPRPIQ-
FMPRPI--
FFPRPI--
FGPRPI--
-FPRPIN-
FYPRPI--
FAPRPI--
FWPRPI--
FHPRPI--
-FPRPIW-
-GPRPIR-
-GPRPIS-
GLPRPI--
GVPRPI--
GYPRPI--
GFPRPI--
GSPRPI--
-GPRPIH-
GNPRPI--
GGPRPI--
GHPRPI--
GKPRPI--
GDPRPI--
-GPRPIT-
-GPRPIM-
-GPRPIK-
-GPRPIY-
-GPRPIN-

-GPRPII-
GIPRPI--
GWPRPI--
GAPRPI--
GQPRPI--
-GPRPIV-
GMPRPI--
-GPRPIE-
-GPRPIQ-
-GPRPIA-
GPPRPI--
-GPRPIF-
HAPRPI--
HSPRPI--
HVPRPI--
-HPRPIY-
HRPRPI--
HIPRPI--
-HPRPII-
-HPRPIM-
-HPRPIL-
HTPRPI--
HLPRPI--
HGPRPI--
HFPRPI--
HWPRPI--
HNPRPI--
HDPRPI--
-HPRPIV-
-HPRPIK-
-HPRPIW-
-HPRPIT-
-HPRPIS-
-HPRPIF-
HMPRPI--
-HPRPIN-
-HPRPIH-
-HPRPIE-
-HPRPIA-
HEPRPI--
HHPRPI--
-HPRPIG-
IVPRPI--
ILPRPI--
-IPRPIY-
-IPRPIR-
IGPRPI--
-IPRPIG-
IMPRPI--
IAPRPI--

-IPRPIM-
INPRPI--
IPPRPI--
IKPRPI--
IYPRPI--
ITPRPI--
-IPRPIT-
IDPRPI--
IIPRPI--
IFPRPI--
-IPRPIS-
-IPRPIH-
IEPRPI--
IWPRPI--
-IPRPIN-
-IPRPIK-
-IPRPIA-
-IPRPIW-
-IPRPIF-
-IPRPII-
-IPRPIE-
KSPRPI--
-KPRPIF-
KVPRPI--
KGPRPI--
KAPRPI--
KFPRPI--
-KPRPIR-
-KPRPIY-
-KPRPIH-
KPPRPI--
KLPRPI--
-KPRPII-
KIPRPI--
-KPRPIA-
-KPRPIS-
-KPRPIM-
KQPRPI--
KDPRPI--
KWPRPI--
-KPRPIV-
KKPRPI--
KNPRPI--
-KPRPIQ-
-KPRPIK-
-KPRPIW-
KYPRPI--
LGPRPI--
LYPRPI--
LLPRPI--

LKPRPI--
LQPRPI--
LIPRPI--
LSPRPI--
LRPRPI--
-LPRPIY-
-LPRPIV-
LEPRPI--
LMPRPI--
LPPRPI--
LWPRPI--
LAPRPI--
LVPRPI--
LFPRPI--
LTPRPI--
-LPRPIA-
-LPRPIM-
-LPRPIW-
-LPRPIH-
-LPRPIN-
-LPRPIK-
LDPRPI--
MLPRPI--
MTPRPI--
MVPRPI--
-MPRPIL-
-MPRPIR-
-MPRPIY-
MWPRPI--
MAPRPI--
MYPRPI--
MRPRPI--
MPPRPI--
MQPRPI--
-MPRPIW-
-MPRPIV-
MSPRPI--
MHPRPI--
-MPRPIT-
-MPRPIK-
MDPRPI--
MIPRPI--
MKPRPI--
MEPRPI--
MFPRPI--
-MPRPIF-
MMPRPI--
-MPRPIN-
-MPRPIQ-
-MPRPII-

-MPRPIG-
-MPRPIE-
NDPRPI--
NGPRPI--
NLPRPI--
NTPRPI--
NQPRPI--
-NPRPII-
-NPRPIH-
NKPRPI--
NYPRPI--
-NPRPIW-
NNPRPI--
-NPRPIY-
-NPRPIM-
-NPRPIL-
NVPRPI--
-NPRPIV-
-NPRPIS-
NHPRPI--
NRPRPI--
-NPRPIF-
NMPRPI--
NEPRPI--
NCPRPI--
--PRPISL
--PRPIPY
--PRPIRL
--PRPILT
--PRPINL
--PRPIFL
--PRPITA
--PRPIVG
--PRPIHY
--PRPIST
--PRPIKS
--PRPISV
--PRPISI
--PRPIIS
--PRPIWT
--PRPITY
--PRPISF
--PRPIWI
--PRPIFM
--PRPIYS
--PRPILV
--PRPISN
--PRPILK
--PRPIIT
--PRPIHG

--PRPIFS
--PRPIEL
--PRPIRN
--PRPISW
--PRPIRA
--PRPIGV
--PRPIYL
--PRPIFH
--PRPITR
--PRPISY
--PRPIRV
--PRPIQR
--PRPINS
--PRPIHR
--PRPIFI
PGPRPI--
--PRPIYY
--PRPITS
--PRPIVM
--PRPIML
--PRPIHA
--PRPIQG
--PRPIGS
--PRPIAS
--PRPIYI
--PRPIMY
--PRPIHL
--PRPIGQ
--PRPIWY
--PRPILF
--PRPISM
PSPRPI--
--PRPINV
--PRPIKH
--PRPIAL
--PRPIVR
--PRPIQL
--PRPIFQ
--PRPIFA
--PRPIVS
--PRPITN
--PRPIMT
--PRPIGH
--PRPIAF
--PRPISG
--PRPIKG
--PRPIKA
PKPRPI--
--PRPIYA
--PRPIRF

--PRPINW
--PRPIAT
--PRPIWW
--PRPIWM
--PRPITV
--PRPISA
--PRPIRM
--PRPIRE
--PRPIPF
--PRPIKV
--PRPIGF
--PRPIFW
PEPRPI--
--PRPIWQ
--PRPIWG
--PRPISS
--PRPIRH
--PRPIMF
--PRPIIG
--PRPIAI
--PRPIAA
--PRPIYT
--PRPIYN
--PRPIWA
--PRPIPM
--PRPINF
--PRPIFE
--PRPIYK
--PRPIVV
--PRPIVD
--PRPIVA
--PRPISR
--PRPIMS
--PRPIMM
--PRPIKT
--PRPIIR
--PRPIIF
--PRPIHK
--PRPIHI
--PRPIGY
--PRPIDI
--PRPIDF
--PRPIAV
PHPRPI--
--PRPIYF
--PRPIWV
--PRPIRR
--PRPIQI
--PRPIIW
--PRPIII

--PRPIAY
--PRPIRY
--PRPIRW
--PRPINH
--PRPIHT
--PRPIGA
PYPRPI--
--PRPIRQ
--PRPIQV
--PRPIPS
--PRPINY
--PRPINT
--PRPIHH
--PRPIYV
--PRPIWF
--PRPIVY
--PRPITK
--PRPITG
--PRPITF
--PRPISH
--PRPIQT
--PRPIHV
--PRPIHQ
--PRPIGN
--PRPIYH
--PRPIWK
--PRPIMD
--PRPIIK
--PRPIGW
--PRPIGM
--PRPIDY
--PRPIVN
--PRPIRD
--PRPIKR
--PRPIGT
PDPRPI--
--PRPIVT
--PRPIQH
--PRPIPW
--PRPINM
--PRPINI
--PRPIMQ
--PRPIMA
--PRPIIH
--PRPIGG
--PRPIFF
--PRPISQ
--PRPIQS
--PRPIQN
--PRPING

--PRPIMI
--PRPIKK
--PRPIIA
--PRPIHW
--PRPIHS
--PRPIAK
PQPRPI--
--PRPIWH
--PRPIVW
--PRPIQK
--PRPIPI
--PRPIPH
--PRPIMN
--PRPILM
--PRPIKY
--PRPIGK
--PRPIFY
--PRPICL
--PRPIAQ
--PRPIAM
PLPRPI--
--PRPIYM
--PRPIYG
--PRPIWN
--PRPIQM
--PRPIPV
--PRPIPA
--PRPINQ
--PRPINA
--PRPIMV
--PRPIKM
--PRPIKI
--PRPIFD
--PRPIES
PIPRPI--
--PRPIVK
--PRPITD
--PRPIQY
--PRPIKF
--PRPIIY
--PRPIAN
PNPRPI--
PMPRPI--
--PRPIYD
--PRPIQQ
--PRPIQA
--PRPIPN
--PRPIMH
--PRPILE
--PRPIIV

--PRPIHE
--PRPIFC
--PRPIYW
--PRPIWE
--PRPIVE
--PRPISD
--PRPINK
--PRPIMW
--PRPIKL
--PRPIHM
--PRPIEV
--PRPIET
--PRPIEG
--PRPIAG
--PRPITH
--PRPIPQ
--PRPIME
--PRPIKN
--PRPIFV
--PRPIEW
--PRPIEN
--PRPIEA
--PRPIDQ
--PRPICH
PAPRPI--
PWPRPI--
--PRPITW
--PRPITE
--PRPISE
--PRPISC
--PRPIRC
--PRPIQE
--PRPIQD
--PRPINN
--PRPINE
--PRPIMK
--PRPIIP
--PRPIIC
--PRPIHN
--PRPIFP
--PRPIER
--PRPIEK
--PRPIEI
--PRPIDS
--PRPIDR
--PRPIDM
--PRPICM
--PRPICK
-PPRPIR-
-PPRPIL-

QRPRPI--
QTPRPI--
-QPRPIS-
-QPRPII-
QLPRPI--
QVPRPI--
QGPRPI--
QSPRPI--
-QPRPIL-
-QPRPIM-
QAPRPI--
-QPRPIT-
QIPRPI--
QWPRPI--
QMPRPI--
-QPRPIQ-
-QPRPIY-
-QPRPIH-
-QPRPIN-
QPPRPI--
QDPRPI--
QCPRPI--
-QPRPIW-
-QPRPIG-
-QPRPIF-
-QPRPIK-
QKPRPI--
QFPRPI--
RFPRPI--
RVPRPI--
RLPRPI--
-RPRPIL-
-RPRPII-
RGPRPI--
RHPRPI--
RTPRPI--
-RPRPIT-
-RPRPIY-
RDPRPI--
-RPRPIF-
-RPRPIS-
RQPRPI--
RYPRPI--
RNPRPI--
RMPRPI--
RPPRPI--
-RPRPIH-
-RPRPIQ-
REPRPI--
-RPRPIV-

-RPRPIN-
RKPRPI--
RCPRPI--
RRPRPI--
-RPRPIE-
-RPRPIC-
SVPRPI--
SSPRPI--
-SPRPIR-
SMPRPI--
SLPRPI--
-SPRPIF-
STPRPI--
-SPRPIT-
-SPRPIM-
SQPRPI--
SIPRPI--
SNPRPI--
SKPRPI--
SEPRPI--
-SPRPIQ-
SWPRPI--
SGPRPI--
-SPRPIH-
-SPRPIA-
SPPRPI--
SCPRPI--
-SPRPIN-
TVPRPI--
-TPRPIR-
-TPRPIL-
TGPRPI--
THPRPI--
-TPRPIV-
TQPRPI--
-TPRPIY-
-TPRPIF-
-TPRPIM-
-TPRPIS-
TMPRPI--
TEPRPI--
-TPRPIK-
TDPRPI--
-TPRPIH-
TAPRPI--
-TPRPIN-
-TPRPIQ-
-TPRPIA-
-TPRPIG-
TFPRPI--

TWPRPI--
-TPRPIE-
-TPRPIC-
TCPRPI--
VPPRPI--
VQPRPI--
VTPRPI--
-VPRPII-
-VPRPIL-
VKPRPI--
VRPRPI--
VMPRPI--
VLPRPI--
VSPRPI--
-VPRPIM-
VWPRPI--
-VPRPIT-
VNPRPI--
VYPRPI--
VVPRPI--
-VPRPIK-
VGPRPI--
-VPRPIQ-
-VPRPIV-
VEPRPI--
VDPRPI--
VHPRPI--
-VPRPIF-
-VPRPIG-
VFPRPI--
-VPRPIE-
-VPRPIA-
WSPRPI--
WFPRPI--
WEPRPI--
WMPRPI--
WNPRPI--
WIPRPI--
WTPRPI--
WGPRPI--
WVPRPI--
WHPRPI--
-WPRPIT-
WPPRPI--
-WPRPIA-
-WPRPIF-
-WPRPIK-
-WPRPIY-
WYPRPI--
WWPRPI--

	WQPRPI--		
	-WPRPIN-		
	WAPRPI--		
	-WPRPIM-		
	-WPRPIH-		
	-WPRPIE-		
	WRPRPI--		
	WKPRPI--		
	WCPRPI--		
	YVPRPI--		
	-YPRPIY-		
	-YPRPIH-		
	YTPRPI--		
	-YPRPIT-		
	-YPRPII-		
	YIPRPI--		
	YFPRPI--		
	YHPRPI--		
	YAPRPI--		
	YGPRPI--		
	-YPRPIS-		
	-YPRPIQ-		
	-YPRPIK-		
	YDPRPI--		
	-YPRPIA-		
	-YPRPIV-		
	YPPRPI--		
	-YPRPIN-		
	-YPRPIE-		
	YYPRPI--		
	YWPRPI--		
	YKPRPI--		
	-YPRPIW-		
	YEPRPI--		
PWGY	194	753	46.91
	-QPWGYG-		
	ALPWGY--		
	-APWGYV-		
	-APWGYT-		
	-APWGYM-		
	AYPWGY--		
	ATPWGY--		
	-APWGYL-		
	ARPWGY--		
	-APWGYR-		
	AQPWGY--		
	-APWGYS-		
	AWPWGY--		
	ASPWGY--		
	-APWGYA-		

AHPWGY--
ANPWGY--
AAPWGY--
AVPWGY--
-APWGYK-
-APWGYG-
-APWGYE-
AEPWGY--
ADPWGY--
-APWGYI-
-APWGYQ-
AKPWGY--
-APWGYF-
-APWGYW-
-APWGYH-
AMPWGY--
AIPWGY--
-APWGYN-
CSPWGY--
CNPWGY--
CVPWGY--
CLPWGY--
CTPWGY--
-CPWGYK-
CKPWGY--
CHPWGY--
CGPWGY--
-CPWGYS-
-CPWGYR-
-CPWGYM-
CMPWGY--
CAPWGY--
DSPWGY--
DAPWGY--
-DPWGYI-
DTPWGY--
-DPWGYR-
DRPWGY--
-DPWGYM-
-DPWGYL-
-DPWGYF-
DGPWGY--
DYPWGY--
-DPWGYT-
DMPWGY--
-DPWGYY-
-DPWGYK-
DIPWGY--
DWPWGY--
-DPWGYS-

-DPWGYQ-
DNPWGY--
DLPWGY--
DHPWGY--
EVPWGY--
EFPWGY--
-EPWGYM-
-EPWGYR-
EKPWGY--
ETPWGY--
-EPWGYS-
ESPWGY--
-EPWGYI-
EIPWGY--
EDPWGY--
EWPWGY--
EHPWGY--
EAPWGY--
-EPWGYK-
ERPWGY--
-EPWGYW-
ENPWGY--
-EPWGYV-
-EPWGYL-
EGPWGY--
-EPWGYT-
-EPWGYQ-
-EPWGYG-
-EPWGYF-
EMPWGY--
FSPWGY--
FKPWGY--
FRPWGY--
FVPWGY--
FPPWGY--
FLPWGY--
FAPWGY--
FIPWGY--
FYPWGY--
FTPWGY--
FNPWGY--
-FPWGYH-
FDPWGY--
FQPWGY--
-FPWGYT-
-FPWGYS-
-FPWGYL-
-FPWGYA-
-FPWGYV-
-FPWGYR-

-FPWGYQ-
FFPWGY--
FWPWGY--
-FPWGYN-
FGPWGY--
FEPWGY--
GRPWGY--
-GPWGYR-
-GPWGYS-
GVPWGY--
GGPWGY--
GKPWGY--
GSPWGY--
-GPWGYY-
-GPWGYV-
-GPWGYA-
GEPWGY--
GTPWGY--
-GPWGYQ-
GPPWGY--
GQPWGY--
GLPWGY--
-GPWGYK-
-GPWGYE-
-GPWGYI-
GMPWGY--
-GPWGYT-
GYPWGY--
-GPWGYN-
GDPWGY--
-GPWGYF-
GFPWGY--
-GPWGYM-
-GPWGYH-
GHPWGY--
GCPWGY--
GWPWGY--
GAPWGY--
HDPWGY--
-HPWGYR-
HRPWGY--
-HPWGYY-
HGPWGY--
HVPWGY--
-HPWGYM-
HNPWGY--
-HPWGYT-
HIPWGY--
HSPWGY--
HPPWGY--

HQPWGY--
HEPWGY--
-HPWGYI-
HFPWGY--
HTPWGY--
-HPWGYF-
HKPWGY--
-HPWGYL-
-HPWGYA-
-HPWGYQ-
HAPWGY--
HYPWGY--
HLPWGY--
IRPWGY--
IKPWGY--
-IPWGYS-
ISPWGY--
-IPWGYL-
IMPWGY--
-IPWGYQ-
-IPWGYK-
IAPWGY--
-IPWGYM-
IEPWGY--
-IPWGYN-
IGPWGY--
IVPWGY--
-IPWGYA-
IIPWGY--
-IPWGYW-
IYPWGY--
-IPWGYT-
-IPWGYI-
IDPWGY--
IFPWGY--
-IPWGYV-
-IPWGYR-
-IPWGYE-
-IPWGYD-
-IPWGYH-
-IPWGYG-
IPPWGY--
KSPWGY--
KKPWGY--
-KPWGYR-
KAPWGY--
KTPWGY--
KGPWGY--
KRPWGY--
-KPWGYW-

KNPWGY--
KMPWGY--
KHPWGY--
-KPWGYQ-
KVPWGY--
-KPWGYI-
-KPWGYL-
KLPWGY--
-KPWGYT-
-KPWGYA-
-KPWGYS-
KFPWGY--
KYPWGY--
-KPWGYY-
-KPWGYG-
-KPWGYH-
KIPWGY--
-KPWGYM-
KPPWGY--
KEPWGY--
-KPWGYN-
-KPWGYK-
KDPWGY--
LSPWGY--
LEPWGY--
LDPWGY--
LVPWGY--
-LPWGYS-
LHPWGY--
LGPWGY--
LAPWGY--
LLPWGY--
-LPWGYT-
-LPWGYY-
LNPWGY--
-LPWGYM-
LKPWGY--
LTPWGY--
LFPWGY--
LYPWGY--
LMPWGY--
LRPWGY--
-LPWGYV-
-LPWGYA-
LPPWGY--
LQPWGY--
-LPWGYI-
-LPWGYN-
-LPWGYQ-
-LPWGYW-

LWPWGY--
-LPWGYE-
-LPWGYK-
LIPWGY--
MSPWGY--
-MPWGYR-
MKPWGY--
MVPWGY--
-MPWGYS-
-MPWGYL-
MTPWGY--
MIPWGY--
MYPWGY--
-MPWGYA-
MFPWGY--
-MPWGYN-
MGPWGY--
-MPWGYM-
-MPWGYF-
MMPWGY--
-MPWGYV-
MLPWGY--
MWPWGY--
-MPWGYH-
MNPWGY--
MEPWGY--
-MPWGYI-
-MPWGYT-
MQPWGY--
-MPWGYQ-
-MPWGYP-
-MPWGYE-
MHPWGY--
MDPWGY--
NSPWGY--
-NPWGYR-
NHPWGY--
NRPWGY--
NKPWGY--
-NPWGYS-
NQPWGY--
NFPWGY--
NPPWGY--
NAPWGY--
NTPWGY--
NVPWGY--
NMPWGY--
-NPWGYY-
-NPWGYW-
-NPWGYI-

NNPWGY--
NGPWGY--
NIPWGY--
NYPWGY--
-NPWGYL-
NEPWGY--
-NPWGYQ-
-NPWGYN-
-NPWGYT-
-NPWGYH-
-NPWGYV-
-NPWGYF-
-NPWGYE-
NDPWGY--
-NPWGYM-
-NPWGYK-
--PWGYQA
--PWGYTR
--PWGYSS
--PWGYRT
--PWGYLG
--PWGYRS
--PWGYVT
--PWGYIT
--PWGYSV
--PWGYLS
--PWGYHN
--PWGYVG
--PWGYSN
--PWGYRI
--PWGYRG
--PWGYRH
--PWGYSI
--PWGYSG
--PWGYKT
--PWGYYG
--PWGYWR
--PWGYTS
--PWGYSY
--PWGYSQ
--PWGYTI
--PWGYLT
--PWGYFG
--PWGYMA
--PWGYMY
--PWGYAS
--PWGYFK
--PWGYAG
--PWGYVS
--PWGYMN

--PWGYVN
--PWGYTV
--PWGYQS
--PWGYLA
--PWGYGS
--PWGYTF
--PWGYSK
--PWGYIV
--PWGYIS
PRPWGY--
--PWGYYQ
--PWGYWN
--PWGYSA
--PWGYNS
--PWGYET
--PWGYTG
--PWGYRV
--PWGYRQ
--PWGYQR
--PWGYKG
--PWGYAY
--PWGYTK
--PWGYSH
--PWGYQT
--PWGYLN
--PWGYKQ
--PWGYHT
--PWGYTW
--PWGYTL
--PWGYSR
--PWGYRE
--PWGYQK
--PWGYHL
PWPWGY--
--PWGYYT
--PWGYTE
--PWGYRA
--PWGYNT
--PWGYMT
--PWGYKH
--PWGYAH
PSPWGY--
PDPWGY--
--PWGYYR
--PWGYTQ
--PWGYQY
--PWGYMV
--PWGYLK
--PWGYLH
--PWGYKV

--PWGYKA
--PWGYGG
--PWGYYK
--PWGYTA
--PWGYNA
--PWGYMQ
--PWGYLR
--PWGYKS
--PWGYIQ
--PWGYHW
--PWGYFT
--PWGYES
PHPWGY--
--PWGYYS
--PWGYYH
--PWGYVV
--PWGYTT
--PWGYTN
--PWGYRY
--PWGYQG
--PWGYMR
--PWGYIF
--PWGYHK
--PWGYEG
--PWGYAT
--PWGYVR
--PWGYVK
--PWGYRF
--PWGYQI
--PWGYNN
--PWGYML
--PWGYKN
--PWGYKL
--PWGYKF
--PWGYIR
--PWGYIM
--PWGYHV
--PWGYHI
--PWGYGV
--PWGYGT
--PWGYWS
--PWGYVH
--PWGYVA
--PWGYRN
--PWGYRD
--PWGYNR
--PWGYNQ
--PWGYNL
--PWGYIA
--PWGYGL

--PWGYFA
--PWGYAV
--PWGYAR
--PWGYAN
--PWGYAF
PAPWGY--
PYPWGY--
--PWGYYD
--PWGYWM
--PWGYWL
--PWGYWE
--PWGYTM
--PWGYSW
--PWGYSM
--PWGYSD
--PWGYNK
--PWGYNF
--PWGYNE
--PWGYMS
--PWGYMM
--PWGYKI
--PWGYID
--PWGYHS
--PWGYHF
--PWGYHD
--PWGYGA
--PWGYFS
--PWGYFR
--PWGYAL
PVPWGY--
PQPWGY--
PNPWGY--
--PWGYYM
--PWGYWY
--PWGYWD
--PWGYVY
--PWGYVQ
--PWGYTD
--PWGYQH
--PWGYNY
--PWGYNH
--PWGYNG
--PWGYND
--PWGYMD
--PWGYLQ
--PWGYKM
--PWGYFQ
--PWGYFN
--PWGYFD
--PWGYEA

--PWGYCR
--PWGYAK
--PWGYWP
--PWGYWG
--PWGYVD
--PWGYTH
--PWGYSF
--PWGYQL
--PWGYQE
--PWGYPI
--PWGYNV
--PWGYMW
--PWGYMG
--PWGYMF
--PWGYME
--PWGYKD
--PWGYII
--PWGYIG
--PWGYIE
--PWGYHG
--PWGYHE
--PWGYGW
--PWGYGN
--PWGYGF
--PWGYEV
--PWGYEQ
--PWGYDG
--PWGYAM
--PWGYAI
PTPWGY--
PMPWGY--
PFPWGY--
QNPWGY--
-QPWGYR-
QSPWGY--
-QPWGYT-
QRPWGY--
QTPWGY--
QMPWGY--
QGPWGY--
QQPWGY--
QEPWGY--
-QPWGYS-
QPPWGY--
QYPWGY--
-QPWGYL-
-QPWGYM-
-QPWGYK-
QLPWGY--
-QPWGYE-

QKPWGY--
QWPWGY--
-QPWGYY-
-QPWGYF-
QDPWGY--
-QPWGYV-
QHPWGY--
QCPWGY--
-QPWGYW-
-RPWGYT-
RLPWGY--
RAPWGY--
RSPWGY--
RNPWGY--
-RPWGYM-
-RPWGYI-
RDPWGY--
RYPWGY--
-RPWGYQ-
RVPWGY--
RMPWGY--
-RPWGYY-
RPPWGY--
RHPWGY--
-RPWGYS-
-RPWGYR-
-RPWGYH-
-RPWGYA-
RKPWGY--
RFPWGY--
-RPWGYV-
RQPWGY--
-RPWGYK-
RWPWGY--
RIPWGY--
-RPWGYW-
-RPWGYF-
-RPWGYL-
-RPWGYE-
REPWGY--
-RPWGYN-
-RPWGYG-
SLPWGY--
SRPWGY--
SEPWGY--
SGPWGY--
SSPWGY--
-SPWGYR-
STPWGY--
SKPWGY--

SFPWGY--
SAPWGY--
-SPWGYT-
SNPWGY--
SVPWGY--
SMPWGY--
SDPWGY--
SWPWGY--
-SPWGYK-
SQPWGY--
SPPWGY--
SYPWGY--
-SPWGYW-
-SPWGYM-
-SPWGYV-
-SPWGYS-
SIPWGY--
-SPWGYN-
-SPWGYE-
-SPWGYA-
-SPWGYI-
-SPWGYH-
SHPWGY--
-SPWGYG-
TRPWGY--
-TPWGYI-
TLPWGY--
-TPWGYL-
TGPWGY--
TFPWGY--
-TPWGYR-
TSPWGY--
-TPWGYS-
TMPWGY--
TKPWGY--
-TPWGYM-
-TPWGYT-
TEPWGY--
-TPWGYH-
-TPWGYF-
TAPWGY--
THPWGY--
TVPWGY--
TWPWGY--
TIPWGY--
TYPWGY--
-TPWGYA-
TPPWGY--
TTPWGY--
-TPWGYQ-

TDPWGY--
TCPWGY--
-TPWGYN-
-TPWGYK-
-TPWGYG-
-TPWGYV-
TQPWGY--
-TPWGYE-
VSPWGY--
VRPWGY--
-VPWGYL-
VAPWGY--
VFPWGY--
VVPWGY--
-VPWGYT-
VLPWGY--
VIPWGY--
VYPWGY--
VMPWGY--
VTPWGY--
-VPWGYV-
VNPWGY--
VQPWGY--
VKPWGY--
-VPWGYM-
VEPWGY--
VGPWGY--
-VPWGYH-
-VPWGYA-
-VPWGYQ-
-VPWGYK-
-VPWGYS-
VPPWGY--
-VPWGYN-
VHPWGY--
-VPWGYY-
-VPWGYF-
-VPWGYG-
-VPWGYE-
WIPWGY--
WKPWGY--
WGPWGY--
WNPWGY--
WVPWGY--
-WPWGYL-
-WPWGYR-
WLPWGY--
WSPWGY--
WRPWGY--
WMPWGY--

WEPWGY--
-WPWGYS-
WTPWGY--
-WPWGYT-
-WPWGYA-
-WPWGYK-
-WPWGYV-
-WPWGYM-
WHPWGY--
YAPWGY--
YGPWGY--
-YPWGYR-
YRPWGY--
YFPWGY--
YTPWGY--
-YPWGYN-
YIPWGY--
-YPWGYL-
YSPWGY--
YMPWGY--
-YPWGYH-
-YPWGYV-
-YPWGYM-
YNPWGY--
-YPWGYK-
-YPWGYY-
-YPWGYT-
-YPWGYA-
YPPWGY--
YLPWGY--
YVPWGY--
-YPWGYS-
-YPWGYQ-
-YPWGYI-
YKPWGY--
YDPWGY--
-YPWGYE-
YEPWGY--

PVAL	160	769	46.86	

--PVALRP
--PVALGN
--PVALGK
--PVALYP
--PVALIP
--PVALAC
-TPVALP-
-APVALR-
AKPVAL--
AWPVAL--
ALPVAL--

ATPVAL--
ASPVAL--
AVPVAL--
-APVALS-
AMPVAL--
APPVAL--
-APVALV-
AYPVAL--
AGPVAL--
-APVALK-
AIPVAL--
AQPVAL--
-APVALH-
AAPVAL--
-APVALN-
AEPVAL--
-APVALQ-
-APVALA-
AFPVAL--
AHPVAL--
ADPVAL--
ACPVAL--
-APVALG-
-APVALE-
-APVALP-
CRPVAL--
CGPVAL--
CSPVAL--
CHPVAL--
CAPVAL--
CLPVAL--
CKPVAL--
CVPVAL--
CIPVAL--
-CPVALT-
-CPVALR-
CTPVAL--
CQPVAL--
CPPVAL--
CNPVAL--
CFPVAL--
CYPVAL--
CWPVAL--
-CPVALS-
-CPVALN-
-CPVALI-
CEPVAL--
-CPVALG-
DRPVAL--
-DPVALR-

-DPVALV-
-DPVALY-
DAPVAL--
DKPVAL--
DTPVAL--
DMPVAL--
DYPVAL--
DVPVAL--
DGPVAL--
DFPVAL--
DEPVAL--
-DPVALQ-
DWPVAL--
-DPVALM-
DHPVAL--
-DPVALF-
-DPVALH-
-DPVALW-
-DPVALT-
-DPVALS-
-DPVALL-
DLPVAL--
DNPVAL--
DIPVAL--
DQPVAL--
-DPVALI-
-DPVALA-
DDPVAL--
-DPVALN-
-DPVALK-
DPPVAL--
DCPVAL--
-EPVALR-
-EPVALV-
-EPVALL-
EVPVAL--
ERPVAL--
-EPVALH-
EWPVAL--
-EPVALQ-
EKPVAL--
-EPVALW-
ELPVAL--
-EPVALN-
ETPVAL--
EMPVAL--
EAPVAL--
-EPVALI-
EFPVAL--
-EPVALS-

ESPVAL--
EYPVAL--
EQPVAL--
-EPVALK-
EGPVAL--
EEPVAL--
ENPVAL--
EIPVAL--
-EPVALT-
-EPVALG-
EPPVAL--
EHPVAL--
-EPVALE-
-EPVALC-
-EPVALA-
-FPVALR-
FYPVAL--
FGPVAL--
FNPVAL--
FLPVAL--
FHPVAL--
FPPVAL--
-FPVALH-
FEPVAL--
-FPVALN-
-FPVALV-
-FPVALM-
-FPVALT-
-FPVALK-
FQPVAL--
FAPVAL--
-FPVALG-
FDPVAL--
-FPVALA-
FCPVAL--
-GPVALR-
GSPVAL--
GYPVAL--
GKPVAL--
GAPVAL--
-GPVALS-
GLPVAL--
-GPVALT-
GTPVAL--
GIPVAL--
GNPVAL--
GVPVAL--
GQPVAL--
-GPVALI-
-GPVALM-

-GPVALK-
-GPVALH-
GDPVAL--
GGPVAL--
-GPVALN-
-GPVALL-
GFPVAL--
GHPVAL--
-GPVALQ-
-GPVALA-
-GPVALG-
GCPVAL--
GEPVAL--
GPPVAL--
HRPVAL--
HQPVAL--
HLPVAL--
HAPVAL--
HSPVAL--
HGPVAL--
HVPVAL--
HTPVAL--
-HPVALI-
-HPVALT-
HIPVAL--
HEPVAL--
-HPVALV-
HMPVAL--
HPPVAL--
HNPVAL--
HFPVAL--
-HPVALF-
HYPVAL--
HWPVAL--
-HPVALY-
-HPVALN-
-HPVALA-
-HPVALW-
HDPVAL--
-HPVALM-
-HPVALK-
-HPVALQ-
-HPVALH-
-HPVALP-
-HPVALG-
HCPVAL--
-IPVALR-
ILPVAL--
ISPVAL--
-IPVALL-

IGPVAL--
IAPVAL--
INPVAL--
IKPVAL--
IYPVAL--
IPPVAL--
-IPVALA-
-IPVALQ-
-IPVALH-
IQPVAL--
-IPVALS-
IDPVAL--
IWPVAL--
IEPVAL--
ITPVAL--
IMPVAL--
IHPVAL--
-IPVALI-
-IPVALT-
-IPVALV-
-IPVALN-
-IPVALK-
-IPVALG-
-IPVALM-
-IPVALE-
-IPVALC-
ICPVAL--
KSPVAL--
-KPVALT-
KGPVAL--
-KPVALQ-
KAPVAL--
-KPVALK-
KVPVAL--
-KPVALS-
-KPVALR-
KHPVAL--
-KPVALY-
KTPVAL--
KFPVAL--
KNPVAL--
-KPVALI-
-KPVALV-
KMPVAL--
-KPVALF-
KDPVAL--
KQPVAL--
KEPVAL--
-KPVALM-
-KPVALN-

KWPVAL--
KIPVAL--
KCPVAL--
-KPVALG-
KPPVAL--
-LPVALR-
LGPVAL--
LYPVAL--
-LPVALK-
LHPVAL--
LPPVAL--
-LPVALS-
LQPVAL--
LNPVAL--
-LPVALT-
LFPVAL--
LDPVAL--
-LPVALH-
-LPVALQ-
-LPVALM-
-LPVALV-
-LPVALI-
-LPVALN-
-LPVALA-
LCPVAL--
-MPVALR-
MKPVAL--
MPPVAL--
MIPVAL--
-MPVALF-
-MPVALT-
MTPVAL--
MNPVAL--
MGPVAL--
MWPVAL--
MAPVAL--
-MPVALA-
-MPVALM-
-MPVALH-
MYPVAL--
-MPVALV-
-MPVALK-
MFPVAL--
MHPVAL--
MQPVAL--
-MPVALQ-
-MPVALN-
MDPVAL--
-MPVALI-
-MPVALE-

```
MEPVAL--
-MPVALG-
NRPVAL--
NFPVAL--
NSPVAL--
NNPVAL--
NVPVAL--
NIPVAL--
NHPVAL--
NGPVAL--
NTPVAL--
-NPVALI-
NLPVAL--
-NPVALR-
NAPVAL--
NWPVAL--
-NPVALQ-
-NPVALT-
-NPVALL-
-NPVALS-
-NPVALV-
NQPVAL--
-NPVALN-
-NPVALK-
NEPVAL--
NMPVAL--
-NPVALM-
-NPVALH-
NKPVAL--
NDPVAL--
NCPVAL--
-NPVALA-
--PVALRS
--PVALRG
--PVALVR
PRPVAL--
--PVALRY
--PVALRN
--PVALRQ
--PVALTS
--PVALVG
--PVALLT
--PVALRR
--PVALGS
--PVALST
--PVALTR
--PVALSA
--PVALTI
--PVALRA
--PVALTL
```

--PVALTT
--PVALMS
--PVALSF
--PVALGL
--PVALLK
--PVALMR
--PVALSG
--PVALSV
--PVALYG
--PVALKR
--PVALRK
--PVALSI
--PVALRT
--PVALKT
--PVALAS
--PVALLN
--PVALSN
--PVALTN
--PVALNY
--PVALHT
PVPVAL--
--PVALNR
--PVALAI
--PVALMA
--PVALKL
--PVALTM
--PVALAA
PTPVAL--
--PVALLH
--PVALKA
--PVALQA
PIPVAL--
--PVALSY
--PVALQN
--PVALGT
--PVALSW
--PVALQR
--PVALHR
--PVALNS
--PVALLG
--PVALKG
--PVALRH
--PVALQV
--PVALSM
--PVALHK
--PVALVN
--PVALPI
--PVALNA
--PVALMH
--PVALTY

--PVALKN
--PVALHG
--PVALGY
--PVALTH
--PVALNT
--PVALGI
--PVALGA
--PVALAN
PHPVAL--
PGPVAL--
--PVALTV
--PVALTK
--PVALNG
--PVALTA
PSPVAL--
--PVALVK
--PVALQS
--PVALNN
--PVALNF
PWPVAL--
--PVALYH
--PVALSQ
--PVALTE
--PVALNQ
--PVALKH
--PVALIN
--PVALGH
--PVALNK
--PVALMT
--PVALES
--PVALTW
--PVALTF
--PVALPF
--PVALKY
--PVALHQ
--PVALHN
--PVALQL
--PVALAT
--PVALAM
PDPVAL--
--PVALRW
--PVALDL
--PVALAR
--PVALVD
--PVALQT
--PVALHH
--PVALAG
--PVALQF
--PVALNM
--PVALHM

--PVALHF
--PVALSH
--PVALQY
--PVALHD
--PVALGV
PAPVAL--
--PVALSD
--PVALQI
--PVALHA
--PVALGQ
--PVALFH
--PVALFG
--PVALWD
--PVALGG
--PVALYD
--PVALRD
--PVALQM
--PVALNV
--PVALLQ
--PVALIG
--PVALTD
--PVALQG
--PVALNW
--PVALKI
--PVALKE
--PVALQH
--PVALKF
--PVALEL
--PVALWN
--PVALNI
--PVALMW
--PVALMG
--PVALGF
--PVALFN
--PVALEV
--PVALKW
--PVALDY
PNPVAL--
--PVALQW
--PVALHY
--PVALET
--PVALEQ
--PVALAH
PYPVAL--
--PVALTQ
--PVALEK
--PVALKM
--PVALKK
--PVALGM
--PVALEG

--PVALEA
--PVALPL
--PVALKD
--PVALID
--PVALHV
--PVALEN
PQPVAL--
PMPVAL--
PFPVAL--
--PVALQE
--PVALNH
--PVALND
--PVALME
--PVALMD
PEPVAL--
--PVALSE
--PVALPW
--PVALPQ
--PVALPM
--PVALMQ
--PVALMN
--PVALHW
--PVALEY
--PVALEI
--PVALAQ
--PVALWH
--PVALQK
--PVALPV
--PVALKQ
--PVALHE
--PVALEH
PCPVAL--
--PVALQD
--PVALNE
--PVALER
--PVALEF
--PVALDS
--PVALAE
-PPVALT-
--PVALVC
--PVALRC
--PVALPH
--PVALEM
--PVALDM
--PVALCS
--PVALAD
--PVALWP
--PVALQQ
--PVALPT
--PVALPC

--PVALLC
--PVALHP
--PVALFD
--PVALFC
--PVALEW
--PVALDW
--PVALDQ
--PVALCT
--PVALCG
--PVALCF
--PVALCD
-PPVALS-
-PPVALN-
-PPVALH-
PPPVAL--
QSPVAL--
QLPVAL--
-QPVALL-
QQPVAL--
QVPVAL--
-QPVALI-
-QPVALS-
QAPVAL--
-QPVALN-
-QPVALK-
-QPVALT-
QFPVAL--
-QPVALV-
-QPVALM-
QTPVAL--
QGPVAL--
QKPVAL--
QEPVAL--
QPPVAL--
QNPVAL--
QWPVAL--
-QPVALE-
QDPVAL--
-QPVALG-
QMPVAL--
-QPVALH-
-QPVALA-
QIPVAL--
QYPVAL--
RMPVAL--
RGPVAL--
RVPVAL--
RNPVAL--
RQPVAL--
-RPVALW-

RAPVAL--
-RPVALH-
RKPVAL--
REPVAL--
-RPVALT-
RTPVAL--
RHPVAL--
-RPVALI-
-RPVALN-
RPPVAL--
RIPVAL--
-RPVALV-
RDPVAL--
-RPVALG-
RCPVAL--
-RPVALM-
-RPVALE-
-RPVALC-
-SPVALL-
-SPVALR-
SGPVAL--
-SPVALI-
SLPVAL--
-SPVALT-
-SPVALS-
SHPVAL--
SAPVAL--
SPPVAL--
SVPVAL--
SIPVAL--
SSPVAL--
SFPVAL--
SEPVAL--
SWPVAL--
-SPVALK-
-SPVALH-
SMPVAL--
SQPVAL--
SYPVAL--
-SPVALN-
-SPVALQ-
-SPVALV-
-SPVALA-
SDPVAL--
-SPVALG-
-SPVALE-
SCPVAL--
-TPVALL-
-TPVALK-
-TPVALR-

TRPVAL--
TVPVAL--
TSPVAL--
TLPVAL--
TTPVAL--
TAPVAL--
TKPVAL--
TNPVAL--
-TPVALS-
-TPVALV-
TGPVAL--
TWPVAL--
TIPVAL--
TDPVAL--
-TPVALN-
-TPVALH-
TFPVAL--
TYPVAL--
THPVAL--
-TPVALQ-
TPPVAL--
TEPVAL--
-TPVALT-
TQPVAL--
-TPVALA-
-TPVALE-
TCPVAL--
-TPVALG-
-TPVALD-
-VPVALR-
-VPVALK-
VSPVAL--
VHPVAL--
-VPVALN-
-VPVALS-
VPPVAL--
VTPVAL--
-VPVALL-
VGPVAL--
-VPVALT-
VVPVAL--
-VPVALA-
VIPVAL--
VMPVAL--
VEPVAL--
VQPVAL--
VNPVAL--
VYPVAL--
-VPVALV-
-VPVALH-

−VPVALM−
VWPVAL−−
−VPVALQ−
VDPVAL−−
−VPVALE−
−WPVALR−
WKPVAL−−
WAPVAL−−
−WPVALS−
WMPVAL−−
WSPVAL−−
−WPVALT−
−WPVALL−
−WPVALK−
−WPVALV−
WYPVAL−−
−WPVALN−
WNPVAL−−
WWPVAL−−
WTPVAL−−
−WPVALQ−
−WPVALA−
WEPVAL−−
WDPVAL−−
WQPVAL−−
−WPVALG−
WPPVAL−−
−WPVALE−
WCPVAL−−
YRPVAL−−
−YPVALR−
−YPVALH−
YTPVAL−−
−YPVALS−
YQPVAL−−
YKPVAL−−
YVPVAL−−
YGPVAL−−
−YPVALK−
YFPVAL−−
−YPVALT−
YYPVAL−−
−YPVALL−
YLPVAL−−
YIPVAL−−
YEPVAL−−
YNPVAL−−
YAPVAL−−
YWPVAL−−
−YPVALN−

				-YPVALG-
				-YPVALQ-
				-YPVALM-
				-YPVALA-
				-YPVALE-
				YPPVAL--
				YDPVAL--
				-YPVALP-
PQNL	58	848	46.86	
				-WPQNLG-
				ASPQNL--
				AYPQNL--
				-APQNLL-
				AVPQNL--
				-APQNLR-
				-APQNLV-
				-APQNLQ-
				-APQNLT-
				AGPQNL--
				AKPQNL--
				AIPQNL--
				AHPQNL--
				ALPQNL--
				-APQNLS-
				APPQNL--
				AFPQNL--
				ARPQNL--
				-APQNLH-
				-APQNLG-
				-APQNLA-
				-APQNLN-
				AEPQNL--
				ATPQNL--
				ADPQNL--
				AWPQNL--
				-APQNLF-
				-APQNLK-
				-APQNLY-
				-APQNLW-
				-APQNLI-
				-APQNLE-
				ANPQNL--
				AQPQNL--
				-APQNLC-
				CWPQNL--
				CSPQNL--
				-CPQNLR-
				CCPQNL--
				-CPQNLY-
				-CPQNLI-

CEPQNL--
-CPQNLS-
CHPQNL--
CFPQNL--
CVPQNL--
CTPQNL--
-CPQNLV-
-CPQNLT-
-CPQNLL-
CNPQNL--
CMPQNL--
CLPQNL--
DFPQNL--
DWPQNL--
DVPQNL--
-DPQNLK-
DLPQNL--
DSPQNL--
-DPQNLW-
-DPQNLR-
DAPQNL--
DYPQNL--
DRPQNL--
-DPQNLM-
-DPQNLI-
-DPQNLF-
DGPQNL--
DIPQNL--
-DPQNLY-
-DPQNLT-
-DPQNLS-
-DPQNLC-
-DPQNLV-
-DPQNLQ-
ERPQNL--
EGPQNL--
EVPQNL--
-EPQNLL-
ELPQNL--
EPPQNL--
ESPQNL--
-EPQNLS-
-EPQNLQ-
-EPQNLH-
EHPQNL--
-EPQNLV-
-EPQNLI-
EKPQNL--
EDPQNL--
-EPQNLY-

-EPQNLT-
EIPQNL--
EEPQNL--
EQPQNL--
-EPQNLK-
-EPQNLG-
ENPQNL--
-FPQNLR-
FLPQNL--
FMPQNL--
FGPQNL--
FVPQNL--
FSPQNL--
FRPQNL--
FAPQNL--
FYPQNL--
FHPQNL--
-FPQNLI-
FKPQNL--
FTPQNL--
-FPQNLF-
-FPQNLM-
-FPQNLL-
FFPQNL--
-FPQNLA-
FQPQNL--
-FPQNLG-
FPPQNL--
-FPQNLT-
-FPQNLH-
FDPQNL--
-FPQNLY-
FWPQNL--
-FPQNLQ-
-FPQNLE-
-FPQNLV-
FCPQNL--
GRPQNL--
GLPQNL--
GVPQNL--
-GPQNLN-
-GPQNLI-
GSPQNL--
GIPQNL--
GWPQNL--
-GPQNLL-
-GPQNLS-
-GPQNLT-
GKPQNL--
-GPQNLQ-

GTPQNL--
GAPQNL--
-GPQNLM-
GHPQNL--
GYPQNL--
-GPQNLV-
-GPQNLY-
-GPQNLR-
GGPQNL--
-GPQNLK-
GMPQNL--
GQPQNL--
-GPQNLF-
GFPQNL--
-GPQNLE-
GPPQNL--
-GPQNLW-
-GPQNLG-
GNPQNL--
GDPQNL--
-HPQNLR-
HRPQNL--
HTPQNL--
-HPQNLY-
-HPQNLL-
HNPQNL--
-HPQNLF-
HYPQNL--
HWPQNL--
-HPQNLW-
-HPQNLT-
HQPQNL--
HVPQNL--
HPPQNL--
-HPQNLV-
HFPQNL--
HIPQNL--
-HPQNLK-
-HPQNLA-
HSPQNL--
HMPQNL--
HGPQNL--
-HPQNLH-
HKPQNL--
-HPQNLS-
-HPQNLI-
-HPQNLE-
HDPQNL--
IRPQNL--
ITPQNL--

ILPQNL--
IVPQNL--
-IPQNLH-
-IPQNLQ-
-IPQNLM-
-IPQNLR-
-IPQNLL-
ISPQNL--
-IPQNLV-
IIPQNL--
-IPQNLS-
-IPQNLK-
IGPQNL--
IMPQNL--
IKPQNL--
IDPQNL--
-IPQNLW-
IAPQNL--
-IPQNLI-
-IPQNLG-
-IPQNLN-
IPPQNL--
-IPQNLY-
-IPQNLT-
-IPQNLA-
INPQNL--
IHPQNL--
-IPQNLF-
IFPQNL--
IEPQNL--
IWPQNL--
-IPQNLE-
ICPQNL--
KSPQNL--
-KPQNLR-
-KPQNLY-
-KPQNLV-
KLPQNL--
KAPQNL--
-KPQNLL-
KPPQNL--
-KPQNLA-
-KPQNLE-
KIPQNL--
KQPQNL--
-KPQNLG-
KYPQNL--
KRPQNL--
-KPQNLM-
KFPQNL--

KEPQNL--
KNPQNL--
KWPQNL--
-KPQNLW-
KHPQNL--
-KPQNLQ-
KKPQNL--
-KPQNLT-
-KPQNLN-
-KPQNLH-
KGPQNL--
-KPQNLK-
-KPQNLF-
KDPQNL--
KCPQNL--
-KPQNLI-
LRPQNL--
-LPQNLR-
LGPQNL--
LLPQNL--
LTPQNL--
-LPQNLS-
LWPQNL--
-LPQNLI-
LKPQNL--
LPPQNL--
LHPQNL--
-LPQNLV-
-LPQNLT-
LIPQNL--
-LPQNLW-
LYPQNL--
LVPQNL--
-LPQNLL-
-LPQNLA-
-LPQNLY-
LNPQNL--
LDPQNL--
-LPQNLK-
LMPQNL--
LEPQNL--
LAPQNL--
LQPQNL--
-LPQNLQ-
-LPQNLF-
LFPQNL--
-LPQNLH-
-LPQNLG-
MSPQNL--
MTPQNL--

MVPQNL--
MFPQNL--
MLPQNL--
-MPQNLY-
-MPQNLN-
-MPQNLA-
MKPQNL--
MHPQNL--
-MPQNLV-
MRPQNL--
-MPQNLS-
-MPQNLT-
-MPQNLL-
-MPQNLQ-
-MPQNLK-
MNPQNL--
MYPQNL--
-MPQNLE-
MWPQNL--
MAPQNL--
MQPQNL--
-MPQNLW-
MIPQNL--
MCPQNL--
-MPQNLR-
-NPQNLR-
NSPQNL--
NPPQNL--
-NPQNLY-
NKPQNL--
-NPQNLS-
-NPQNLL-
NRPQNL--
-NPQNLW-
-NPQNLV-
-NPQNLQ-
-NPQNLA-
NHPQNL--
NTPQNL--
NGPQNL--
NLPQNL--
NWPQNL--
-NPQNLT-
-NPQNLI-
-NPQNLM-
-NPQNLK-
-NPQNLH-
NEPQNL--
NYPQNL--
NVPQNL--

-NPQNLG-
-NPQNLN-
-NPQNLF-
NIPQNL--
NFPQNL--
NQPQNL--
--PQNLRA
--PQNLRT
--PQNLRL
--PQNLLG
--PQNLLS
--PQNLRV
--PQNLLA
--PQNLTR
PSPQNL--
--PQNLLR
--PQNLVR
--PQNLRN
--PQNLLW
--PQNLKS
--PQNLLK
--PQNLSL
--PQNLRM
--PQNLFM
--PQNLTT
--PQNLIT
--PQNLGS
--PQNLVT
--PQNLTN
--PQNLLT
--PQNLKL
--PQNLTL
--PQNLIR
--PQNLFK
PRPQNL--
--PQNLVY
--PQNLTS
--PQNLLQ
--PQNLVS
--PQNLTG
--PQNLSH
--PQNLTA
--PQNLRW
--PQNLQL
--PQNLLV
--PQNLLL
--PQNLFT
PLPQNL--
--PQNLYM
--PQNLYH

--PQNLVV
--PQNLVH
--PQNLRI
--PQNLIK
--PQNLHW
--PQNLFW
--PQNLIS
--PQNLFR
--PQNLSV
--PQNLWA
--PQNLVI
--PQNLRY
--PQNLMW
--PQNLKY
PFPQNL--
--PQNLVW
--PQNLSS
--PQNLQS
--PQNLMT
--PQNLWS
--PQNLSA
--PQNLRH
--PQNLLN
--PQNLHS
--PQNLAG
--PQNLWL
--PQNLWI
--PQNLTQ
--PQNLSY
--PQNLRS
--PQNLAT
PHPQNL--
--PQNLTI
--PQNLQN
--PQNLMA
--PQNLLI
--PQNLLD
--PQNLHN
--PQNLGT
--PQNLGG
--PQNLAL
--PQNLYT
--PQNLWN
--PQNLSI
--PQNLQT
--PQNLMN
--PQNLLM
--PQNLHR
--PQNLAN
--PQNLWR

--PQNLWH
--PQNLVG
--PQNLVA
--PQNLQR
--PQNLNS
--PQNLMQ
--PQNLMG
--PQNLKR
--PQNLIW
--PQNLHK
PAPQNL--
--PQNLWV
--PQNLVL
--PQNLST
--PQNLSQ
--PQNLQG
--PQNLNY
--PQNLMH
--PQNLIY
--PQNLIM
--PQNLAI
PNPQNL--
PGPQNL--
--PQNLYS
--PQNLWT
--PQNLWK
--PQNLSN
--PQNLRE
--PQNLQD
--PQNLNT
--PQNLNM
--PQNLKK
--PQNLKG
--PQNLHT
--PQNLEW
--PQNLEG
PQPQNL--
--PQNLYG
--PQNLWQ
--PQNLVM
--PQNLNV
--PQNLMK
--PQNLKQ
--PQNLHA
--PQNLFG
PVPQNL--
--PQNLYN
--PQNLTY
--PQNLSF
--PQNLQY

--PQNLNF
--PQNLNA
--PQNLLF
--PQNLII
PIPQNL--
--PQNLVK
--PQNLTV
--PQNLMI
--PQNLKA
--PQNLIA
--PQNLHI
--PQNLFH
--PQNLAV
--PQNLAF
--PQNLYL
--PQNLYF
--PQNLSW
--PQNLSD
--PQNLRF
--PQNLQA
--PQNLFV
--PQNLFF
--PQNLEM
--PQNLAH
--PQNLAA
--PQNLVF
--PQNLSM
--PQNLQV
--PQNLQI
--PQNLNW
--PQNLNK
--PQNLIV
--PQNLGV
--PQNLFI
--PQNLFA
--PQNLEL
--PQNLAY
PWPQNL--
--PQNLWE
--PQNLVN
--PQNLTH
--PQNLTF
--PQNLMY
--PQNLKW
--PQNLKN
--PQNLKM
--PQNLIF
--PQNLHH
--PQNLGF
--PQNLGA

--PQNLFS
--PQNLES
--PQNLEI
--PQNLWG
--PQNLNG
--PQNLMS
--PQNLMF
--PQNLKI
--PQNLHY
--PQNLGM
--PQNLEV
--PQNLAD
PYPQNL--
--PQNLYE
--PQNLWW
--PQNLSK
--PQNLKH
--PQNLKF
--PQNLKD
--PQNLIQ
--PQNLIN
--PQNLGY
--PQNLER
--PQNLAW
--PQNLAS
--PQNLAK
PKPQNL--
PTPQNL--
--PQNLYI
--PQNLVD
--PQNLTM
--PQNLSE
--PQNLQK
--PQNLNR
--PQNLMR
--PQNLMP
--PQNLML
--PQNLKV
--PQNLGW
--PQNLGQ
--PQNLFQ
--PQNLEN
--PQNLEF
--PQNLCA
--PQNLAM
--PQNLAE
PMPQNL--
--PQNLYQ
--PQNLYP
--PQNLYK

--PQNLYD
--PQNLVQ
--PQNLVE
--PQNLTK
--PQNLNN
--PQNLNL
--PQNLNH
--PQNLMV
--PQNLMD
--PQNLIG
--PQNLID
--PQNLHE
--PQNLHD
--PQNLGK
--PQNLGI
--PQNLFY
--PQNLFN
--PQNLFE
--PQNLFD
--PQNLEY
--PQNLEQ
--PQNLEK
--PQNLDS
--PQNLDG
--PQNLDA
--PQNLCY
--PQNLCR
--PQNLCH
--PQNLCG
-PPQNLF-
-QPQNLR-
QLPQNL--
QSPQNL--
QVPQNL--
-QPQNLF-
-QPQNLI-
QRPQNL--
QMPQNL--
QWPQNL--
-QPQNLY-
-QPQNLS-
-QPQNLH-
QIPQNL--
QTPQNL--
-QPQNLV-
-QPQNLA-
QYPQNL--
-QPQNLW-
-QPQNLQ-
-QPQNLM-

−QPQNLK−
−QPQNLG−
QEPQNL−−
−QPQNLT−
QHPQNL−−
−QPQNLN−
QPPQNL−−
QKPQNL−−
QFPQNL−−
QGPQNL−−
QDPQNL−−
QQPQNL−−
−QPQNLE−
−RPQNLR−
RGPQNL−−
RSPQNL−−
RVPQNL−−
RLPQNL−−
−RPQNLS−
−RPQNLI−
RMPQNL−−
−RPQNLF−
−RPQNLT−
RIPQNL−−
RYPQNL−−
RTPQNL−−
−RPQNLL−
−RPQNLG−
−RPQNLM−
RQPQNL−−
−RPQNLY−
RRPQNL−−
−RPQNLQ−
−RPQNLA−
−RPQNLV−
RPPQNL−−
RFPQNL−−
−RPQNLN−
−RPQNLW−
RDPQNL−−
−RPQNLK−
REPQNL−−
RHPQNL−−
RAPQNL−−
−RPQNLE−
−RPQNLH−
RCPQNL−−
−SPQNLR−
SGPQNL−−
−SPQNLL−

-SPQNLI-
-SPQNLS-
SHPQNL--
-SPQNLT-
SLPQNL--
SYPQNL--
SSPQNL--
SRPQNL--
SMPQNL--
SNPQNL--
-SPQNLV-
-SPQNLA-
SWPQNL--
SFPQNL--
SKPQNL--
SAPQNL--
SQPQNL--
-SPQNLM-
SDPQNL--
-SPQNLN-
-SPQNLH-
-SPQNLE-
-SPQNLW-
-SPQNLG-
SPPQNL--
SIPQNL--
-SPQNLY-
SEPQNL--
-SPQNLK-
-SPQNLF-
-SPQNLQ-
-SPQNLD-
TFPQNL--
TRPQNL--
TSPQNL--
-TPQNLS-
TPPQNL--
-TPQNLR-
-TPQNLW-
TMPQNL--
TLPQNL--
-TPQNLT-
TYPQNL--
TAPQNL--
TTPQNL--
TVPQNL--
-TPQNLQ-
-TPQNLV-
-TPQNLL-
-TPQNLA-

TKPQNL--
TGPQNL--
-TPQNLN-
-TPQNLM-
-TPQNLY-
-TPQNLE-
-TPQNLF-
TIPQNL--
-TPQNLK-
-TPQNLG-
TDPQNL--
TWPQNL--
-TPQNLH-
TCPQNL--
TNPQNL--
-VPQNLL-
-VPQNLR-
VRPQNL--
-VPQNLS-
VYPQNL--
-VPQNLI-
VLPQNL--
VWPQNL--
-VPQNLM-
VSPQNL--
-VPQNLA-
VVPQNL--
VGPQNL--
-VPQNLT-
VPPQNL--
VQPQNL--
VNPQNL--
VIPQNL--
VHPQNL--
VTPQNL--
-VPQNLH-
-VPQNLW-
-VPQNLG-
VAPQNL--
-VPQNLN-
-VPQNLV-
VDPQNL--
-VPQNLQ-
-VPQNLE-
VKPQNL--
VEPQNL--
-VPQNLY-
-VPQNLK-
-VPQNLF-
VMPQNL--

VFPQNL--
WSPQNL--
-WPQNLL-
WRPQNL--
WEPQNL--
-WPQNLK-
WVPQNL--
-WPQNLI-
-WPQNLR-
WNPQNL--
-WPQNLS-
-WPQNLW-
WFPQNL--
-WPQNLY-
-WPQNLT-
WIPQNL--
WGPQNL--
WTPQNL--
WPPQNL--
WCPQNL--
WQPQNL--
-WPQNLF-
-WPQNLA-
WAPQNL--
-WPQNLE-
WWPQNL--
-WPQNLQ-
-WPQNLH-
WDPQNL--
-WPQNLV-
-YPQNLR-
YYPQNL--
YRPQNL--
YAPQNL--
-YPQNLV-
YQPQNL--
-YPQNLT-
YTPQNL--
-YPQNLN-
-YPQNLK-
YKPQNL--
YPPQNL--
YMPQNL--
YHPQNL--
YGPQNL--
YSPQNL--
-YPQNLI-
YFPQNL--
YNPQNL--
-YPQNLH-

YDPQNL--
-YPQNLY-
-YPQNLG-
YEPQNL--
-YPQNLE-
-YPQNLW-
-YPQNLM-

PKGY	143	782	46.85

--PKGYLL
--PKGYPA
-SPKGYG-
-APKGYL-
ANPKGY--
AAPKGY--
AIPKGY--
-APKGYS-
-APKGYQ-
AHPKGY--
AVPKGY--
ARPKGY--
ALPKGY--
ATPKGY--
-APKGYH-
-APKGYT-
-APKGYG-
ADPKGY--
AGPKGY--
AWPKGY--
AYPKGY--
ASPKGY--
-APKGYN-
-APKGYA-
AFPKGY--
-APKGYV-
-APKGYM-
-APKGYE-
AKPKGY--
-APKGYI-
ACPKGY--
-APKGYF-
-APKGYW-
-APKGYK-
CIPKGY--
-CPKGYL-
CGPKGY--
CSPKGY--
CCPKGY--
-CPKGYY-
CNPKGY--
CYPKGY--

CWPKGY--
-CPKGYR-
CLPKGY--
CFPKGY--
CEPKGY--
CTPKGY--
-CPKGYV-
CAPKGY--
DLPKGY--
DSPKGY--
-DPKGYR-
DRPKGY--
-DPKGYW-
DAPKGY--
DMPKGY--
DQPKGY--
DHPKGY--
DFPKGY--
-DPKGYY-
-DPKGYS-
-DPKGYL-
DYPKGY--
-DPKGYF-
DNPKGY--
DEPKGY--
-DPKGYV-
-DPKGYT-
DWPKGY--
DTPKGY--
-DPKGYP-
-DPKGYI-
ESPKGY--
ETPKGY--
-EPKGYK-
EGPKGY--
ERPKGY--
EWPKGY--
-EPKGYS-
-EPKGYI-
-EPKGYL-
ENPKGY--
ELPKGY--
EKPKGY--
-EPKGYR-
-EPKGYM-
EAPKGY--
EVPKGY--
EMPKGY--
EYPKGY--
EHPKGY--

-EPKGYW-
-EPKGYA-
EFPKGY--
EQPKGY--
-EPKGYH-
-EPKGYF-
-EPKGYV-
-EPKGYN-
EDPKGY--
-FPKGYV-
FRPKGY--
FNPKGY--
FGPKGY--
-FPKGYY-
FKPKGY--
FSPKGY--
FTPKGY--
-FPKGYF-
FFPKGY--
FWPKGY--
FIPKGY--
FPPKGY--
FAPKGY--
-FPKGYW-
-FPKGYQ-
-FPKGYI-
-FPKGYT-
FYPKGY--
FLPKGY--
FDPKGY--
-FPKGYE-
-FPKGYC-
FHPKGY--
FEPKGY--
GWPKGY--
GSPKGY--
GRPKGY--
GAPKGY--
-GPKGYW-
-GPKGYL-
GMPKGY--
-GPKGYS-
-GPKGYI-
GYPKGY--
GNPKGY--
GGPKGY--
-GPKGYV-
GVPKGY--
GHPKGY--
-GPKGYQ-

-GPKGYH-
-GPKGYK-
-GPKGYN-
GLPKGY--
-GPKGYY-
-GPKGYT-
-GPKGYG-
GQPKGY--
GDPKGY--
GEPKGY--
-GPKGYF-
GTPKGY--
-GPKGYE-
GCPKGY--
HSPKGY--
-HPKGYH-
HAPKGY--
HWPKGY--
-HPKGYL-
HFPKGY--
-HPKGYG-
HTPKGY--
-HPKGYS-
-HPKGYI-
HNPKGY--
HKPKGY--
HEPKGY--
-HPKGYV-
HQPKGY--
-HPKGYP-
-HPKGYN-
HMPKGY--
HIPKGY--
HGPKGY--
HCPKGY--
HPPKGY--
HDPKGY--
-HPKGYT-
-HPKGYQ-
-HPKGYK-
-HPKGYF-
-HPKGYC-
-IPKGYL-
IRPKGY--
INPKGY--
IGPKGY--
-IPKGYF-
ISPKGY--
-IPKGYT-
IMPKGY--

IVPKGY--
-IPKGYM-
-IPKGYK-
IKPKGY--
IPPKGY--
-IPKGYV-
IYPKGY--
-IPKGYY-
IEPKGY--
ILPKGY--
ITPKGY--
IWPKGY--
-IPKGYA-
IIPKGY--
IAPKGY--
IQPKGY--
-IPKGYQ-
-IPKGYG-
-IPKGYP-
-IPKGYN-
KSPKGY--
-KPKGYS-
-KPKGYL-
KGPKGY--
-KPKGYV-
KVPKGY--
KAPKGY--
KLPKGY--
-KPKGYM-
KMPKGY--
KYPKGY--
KRPKGY--
KQPKGY--
KDPKGY--
KWPKGY--
-KPKGYY-
KEPKGY--
KCPKGY--
KPPKGY--
-KPKGYH-
-KPKGYN-
-KPKGYK-
-KPKGYF-
-KPKGYA-
KTPKGY--
-KPKGYT-
-KPKGYE-
LGPKGY--
-LPKGYA-
LSPKGY--

LNPKGY--
LVPKGY--
LEPKGY--
-LPKGYH-
-LPKGYY-
LLPKGY--
LIPKGY--
LQPKGY--
LMPKGY--
LTPKGY--
LHPKGY--
-LPKGYW-
LAPKGY--
-LPKGYV-
LFPKGY--
-LPKGYS-
-LPKGYK-
LDPKGY--
-LPKGYF-
LYPKGY--
LPPKGY--
-LPKGYQ-
LCPKGY--
-LPKGYN-
MTPKGY--
MAPKGY--
-MPKGYT-
MGPKGY--
-MPKGYN-
-MPKGYL-
MYPKGY--
MHPKGY--
MEPKGY--
-MPKGYK-
MRPKGY--
-MPKGYV-
-MPKGYM-
-MPKGYH-
-MPKGYF-
-MPKGYS-
MIPKGY--
-MPKGYQ-
MSPKGY--
MQPKGY--
-MPKGYY-
MWPKGY--
MVPKGY--
-MPKGYG-
MMPKGY--
MKPKGY--

MPPKGY--
-MPKGYE-
MDPKGY--
MCPKGY--
NSPKGY--
-NPKGYL-
NTPKGY--
-NPKGYR-
-NPKGYS-
NHPKGY--
NAPKGY--
-NPKGYT-
-NPKGYM-
NNPKGY--
NMPKGY--
NLPKGY--
NRPKGY--
-NPKGYF-
NEPKGY--
NYPKGY--
NWPKGY--
-NPKGYV-
-NPKGYH-
NFPKGY--
NVPKGY--
-NPKGYY-
-NPKGYN-
-NPKGYK-
-NPKGYA-
NGPKGY--
NQPKGY--
-NPKGYQ-
NIPKGY--
--PKGYNT
--PKGYVV
--PKGYSL
--PKGYRI
--PKGYSS
--PKGYLS
--PKGYMS
--PKGYTL
--PKGYVF
--PKGYLV
--PKGYYA
--PKGYRN
--PKGYSR
--PKGYSA
--PKGYWS
--PKGYIS
--PKGYYS

--PKGYIT
--PKGYTS
--PKGYSM
--PKGYWR
--PKGYTK
--PKGYLA
--PKGYYT
--PKGYVT
--PKGYHI
--PKGYAI
--PKGYHG
--PKGYYR
--PKGYSW
--PKGYMG
--PKGYMV
--PKGYAG
--PKGYAF
--PKGYTA
--PKGYSF
--PKGYYH
--PKGYKY
--PKGYWY
--PKGYHS
--PKGYSY
--PKGYHL
--PKGYGA
--PKGYWN
--PKGYSV
--PKGYQY
--PKGYLN
--PKGYAA
PVPKGY--
--PKGYYL
--PKGYTY
--PKGYSN
--PKGYMT
--PKGYLI
--PKGYFL
--PKGYYF
--PKGYTG
--PKGYQG
--PKGYML
--PKGYLY
--PKGYAL
--PKGYVG
--PKGYNW
--PKGYNL
--PKGYLQ
--PKGYLK
--PKGYFK

PSPKGY--
--PKGYVY
--PKGYKS
--PKGYHK
--PKGYHE
--PKGYGS
--PKGYWG
--PKGYTN
--PKGYQA
--PKGYMW
--PKGYIR
--PKGYGT
--PKGYEY
--PKGYVW
--PKGYKT
--PKGYIQ
--PKGYWH
--PKGYTQ
--PKGYTF
--PKGYNF
--PKGYLF
--PKGYHV
--PKGYAN
--PKGYYY
--PKGYYK
--PKGYRQ
--PKGYNS
--PKGYMH
--PKGYLD
--PKGYAY
--PKGYWW
--PKGYVN
--PKGYSK
--PKGYRE
--PKGYIY
--PKGYHF
--PKGYES
PRPKGY--
--PKGYYN
--PKGYVK
--PKGYSG
--PKGYHH
--PKGYGW
--PKGYAM
PGPKGY--
--PKGYWL
--PKGYVQ
--PKGYTM
--PKGYSI
--PKGYFS

PDPKGY--
--PKGYSQ
--PKGYLH
--PKGYIL
--PKGYIK
--PKGYER
PYPKGY--
-PPKGYR-
--PKGYVM
--PKGYTR
--PKGYTH
--PKGYLW
--PKGYKL
--PKGYFQ
--PKGYFA
--PKGYAR
PMPKGY--
--PKGYYM
--PKGYVL
--PKGYRD
--PKGYQL
--PKGYQI
--PKGYNM
--PKGYIV
--PKGYST
--PKGYNV
--PKGYMF
--PKGYKW
--PKGYKV
--PKGYKF
--PKGYFY
--PKGYFN
--PKGYFG
--PKGYEG
PWPKGY--
--PKGYVI
--PKGYSH
--PKGYQN
--PKGYHR
--PKGYGY
--PKGYFI
--PKGYWF
--PKGYVH
--PKGYRF
--PKGYQT
--PKGYQF
--PKGYNA
--PKGYME
--PKGYAV
--PKGYAT

--PKGYWM
--PKGYTT
--PKGYQS
--PKGYNI
--PKGYLE
--PKGYIM
--PKGYHW
--PKGYHT
--PKGYHQ
--PKGYGG
--PKGYFM
--PKGYFD
PHPKGY--
PLPKGY--
--PKGYYV
--PKGYYG
--PKGYYD
--PKGYQK
--PKGYQH
--PKGYNN
--PKGYMI
--PKGYMD
--PKGYII
--PKGYFH
--PKGYAK
--PKGYWQ
--PKGYWK
--PKGYWD
--PKGYTD
--PKGYSD
--PKGYNY
--PKGYNH
--PKGYMN
--PKGYMM
--PKGYKD
--PKGYHM
--PKGYHA
--PKGYGH
--PKGYGF
--PKGYEW
--PKGYEQ
--PKGYEK
--PKGYEH
PAPKGY--
PTPKGY--
PQPKGY--
--PKGYYI
--PKGYYC
--PKGYWT
--PKGYWA

--PKGYVC
--PKGYTI
--PKGYQQ
--PKGYQM
--PKGYQD
--PKGYMQ
--PKGYMK
--PKGYKM
--PKGYIW
--PKGYIG
--PKGYIE
--PKGYIA
--PKGYHN
--PKGYHD
--PKGYGV
--PKGYGI
--PKGYEF
PFPKGY--
PEPKGY--
PNPKGY--
--PKGYYQ
--PKGYWI
--PKGYVP
--PKGYVD
--PKGYTV
--PKGYTE
--PKGYRC
--PKGYID
--PKGYGM
--PKGYET
--PKGYDS
--PKGYDK
--PKGYDA
--PKGYCY
--PKGYCL
--PKGYAH
PIPKGY--
-QPKGYS-
-QPKGYH-
QFPKGY--
QLPKGY--
-QPKGYW-
QRPKGY--
QWPKGY--
QSPKGY--
-QPKGYM-
-QPKGYY-
QAPKGY--
QVPKGY--
-QPKGYT-

QYPKGY--
-QPKGYF-
QEPKGY--
-QPKGYA-
QGPKGY--
QNPKGY--
QIPKGY--
QDPKGY--
QTPKGY--
QQPKGY--
-QPKGYQ-
QHPKGY--
-QPKGYK-
-QPKGYE-
QMPKGY--
QKPKGY--
-RPKGYL-
RMPKGY--
-RPKGYS-
RSPKGY--
-RPKGYF-
RIPKGY--
-RPKGYY-
-RPKGYH-
RGPKGY--
RYPKGY--
RVPKGY--
-RPKGYV-
REPKGY--
RTPKGY--
RDPKGY--
RNPKGY--
RWPKGY--
-RPKGYM-
-RPKGYI-
-RPKGYT-
-RPKGYQ-
-RPKGYN-
RHPKGY--
RPPKGY--
-RPKGYA-
RAPKGY--
RQPKGY--
-RPKGYW-
RCPKGY--
-RPKGYP-
-RPKGYG-
-RPKGYE-
-SPKGYI-
SLPKGY--

SSPKGY--
SIPKGY--
-SPKGYV-
-SPKGYL-
SKPKGY--
SMPKGY--
SRPKGY--
SAPKGY--
-SPKGYF-
SVPKGY--
SYPKGY--
-SPKGYY-
SNPKGY--
SQPKGY--
-SPKGYW-
SHPKGY--
-SPKGYN-
-SPKGYM-
-SPKGYA-
-SPKGYQ-
SCPKGY--
TAPKGY--
TSPKGY--
TLPKGY--
-TPKGYY-
TTPKGY--
-TPKGYS-
TFPKGY--
TRPKGY--
-TPKGYV-
TMPKGY--
TVPKGY--
TIPKGY--
-TPKGYT-
TWPKGY--
-TPKGYI-
-TPKGYK-
-TPKGYM-
TNPKGY--
TYPKGY--
-TPKGYW-
THPKGY--
-TPKGYQ-
-TPKGYH-
-TPKGYA-
TEPKGY--
TGPKGY--
-TPKGYE-
TPPKGY--
-TPKGYG-

VNPKGY--
VLPKGY--
-VPKGYR-
VSPKGY--
VVPKGY--
-VPKGYS-
VAPKGY--
VQPKGY--
VTPKGY--
VIPKGY--
VYPKGY--
-VPKGYL-
-VPKGYK-
VWPKGY--
-VPKGYV-
VHPKGY--
VGPKGY--
-VPKGYT-
-VPKGYM-
VKPKGY--
-VPKGYY-
VMPKGY--
-VPKGYH-
-VPKGYE-
VPPKGY--
-VPKGYQ-
-VPKGYF-
-VPKGYN-
VEPKGY--
-VPKGYA-
-VPKGYW-
-VPKGYG-
-VPKGYI-
VDPKGY--
WLPKGY--
WTPKGY--
-WPKGYT-
WRPKGY--
WYPKGY--
-WPKGYH-
WQPKGY--
-WPKGYV-
-WPKGYL-
WSPKGY--
-WPKGYS-
WVPKGY--
WIPKGY--
-WPKGYM-
WMPKGY--
WGPKGY--

-WPKGYW-
-WPKGYI-
WPPKGY--
-WPKGYY-
WNPKGY--
WAPKGY--
-WPKGYE-
WWPKGY--
-WPKGYN-
WEPKGY--
WDPKGY--
WCPKGY--
-WPKGYF-
WFPKGY--
YGPKGY--
YMPKGY--
-YPKGYT-
-YPKGYL-
YQPKGY--
YSPKGY--
-YPKGYY-
YLPKGY--
YVPKGY--
-YPKGYQ-
YYPKGY--
YPPKGY--
YAPKGY--
-YPKGYA-
YFPKGY--
YWPKGY--
-YPKGYW-
-YPKGYM-
YIPKGY--
YTPKGY--
YRPKGY--
-YPKGYH-
-YPKGYN-
YEPKGY--
-YPKGYV-
YCPKGY--

PYNY 342 678 46.83

--PYNYGR
-APYNYS-
-APYNYL-
-APYNYQ-
-APYNYR-
-APYNYE-
AKPYNY--
AIPYNY--
ALPYNY--

AEPYNY--
AAPYNY--
ASPYNY--
-APYNYA-
AGPYNY--
AFPYNY--
-APYNYT-
-APYNYG-
AYPYNY--
AWPYNY--
AQPYNY--
-APYNYM-
-APYNYH-
AMPYNY--
-APYNYY-
ANPYNY--
-APYNYI-
ADPYNY--
ATPYNY--
-APYNYW-
AHPYNY--
CTPYNY--
CKPYNY--
CEPYNY--
CLPYNY--
DKPYNY--
-DPYNYV-
-DPYNYL-
DGPYNY--
DQPYNY--
DAPYNY--
-DPYNYE-
DLPYNY--
DRPYNY--
DSPYNY--
-DPYNYT-
-DPYNYS-
-DPYNYI-
-DPYNYA-
DTPYNY--
-DPYNYY-
-DPYNYG-
DFPYNY--
-EPYNYS-
EYPYNY--
EWPYNY--
-EPYNYI-
-EPYNYF-
-EPYNYL-
-EPYNYK-

ELPYNY--
-EPYNYY-
EQPYNY--
-EPYNYT-
ESPYNY--
EHPYNY--
EVPYNY--
-EPYNYW-
-EPYNYR-
-EPYNYM-
ENPYNY--
ERPYNY--
-EPYNYV-
EFPYNY--
EEPYNY--
FRPYNY--
FTPYNY--
FSPYNY--
-FPYNYW-
FNPYNY--
FWPYNY--
-FPYNYL-
-FPYNYT-
-FPYNYR-
-FPYNYK-
FLPYNY--
FYPYNY--
-FPYNYQ-
FEPYNY--
-FPYNYI-
FDPYNY--
-FPYNYN-
-FPYNYM-
-FPYNYC-
FIPYNY--
-GPYNYV-
-GPYNYI-
GKPYNY--
GVPYNY--
GTPYNY--
-GPYNYL-
-GPYNYH-
GIPYNY--
GRPYNY--
GWPYNY--
-GPYNYK-
GAPYNY--
GEPYNY--
-GPYNYS-
-GPYNYQ-

GMPYNY--
-GPYNYG-
GQPYNY--
GYPYNY--
-GPYNYM-
GGPYNY--
GDPYNY--
GSPYNY--
-GPYNYR-
-GPYNYA-
-GPYNYT-
-GPYNYD-
GNPYNY--
GLPYNY--
HRPYNY--
HSPYNY--
HFPYNY--
HYPYNY--
-HPYNYR-
HKPYNY--
HVPYNY--
HMPYNY--
HTPYNY--
-HPYNYF-
HPPYNY--
HEPYNY--
-HPYNYY-
-HPYNYG-
-HPYNYT-
-HPYNYS-
-HPYNYL-
-HPYNYQ-
-HPYNYI-
HNPYNY--
HIPYNY--
-HPYNYM-
HAPYNY--
IRPYNY--
IGPYNY--
-IPYNYR-
-IPYNYI-
IIPYNY--
IKPYNY--
-IPYNYT-
-IPYNYA-
IYPYNY--
INPYNY--
-IPYNYS-
-IPYNYQ-
ISPYNY--

IQPYNY--
-IPYNYL-
IFPYNY--
ITPYNY--
-IPYNYW-
IVPYNY--
IPPYNY--
IMPYNY--
IDPYNY--
KSPYNY--
-KPYNYR-
KKPYNY--
KGPYNY--
KWPYNY--
-KPYNYW-
KLPYNY--
KYPYNY--
KRPYNY--
KAPYNY--
KMPYNY--
KVPYNY--
KTPYNY--
-KPYNYV-
-KPYNYN-
-KPYNYI-
-KPYNYF-
-KPYNYA-
-KPYNYM-
KIPYNY--
KFPYNY--
KEPYNY--
KDPYNY--
-KPYNYK-
-KPYNYG-
KPPYNY--
KNPYNY--
-KPYNYH-
-LPYNYL-
LKPYNY--
-LPYNYS-
LRPYNY--
LYPYNY--
-LPYNYV-
-LPYNYR-
-LPYNYE-
LGPYNY--
LAPYNY--
LPPYNY--
LSPYNY--
LTPYNY--

-LPYNYW-
LWPYNY--
LVPYNY--
-LPYNYY-
-LPYNYT-
-LPYNYK-
LQPYNY--
-LPYNYM-
-LPYNYI-
LFPYNY--
LDPYNY--
-LPYNYA-
LNPYNY--
-LPYNYP-
-LPYNYH-
-LPYNYG-
-LPYNYF-
LMPYNY--
LEPYNY--
LCPYNY--
-MPYNYI-
-MPYNYR-
MGPYNY--
MRPYNY--
-MPYNYL-
-MPYNYV-
MKPYNY--
MAPYNY--
MEPYNY--
-MPYNYG-
MWPYNY--
-MPYNYA-
MYPYNY--
-MPYNYH-
MIPYNY--
MVPYNY--
MSPYNY--
-MPYNYY-
-MPYNYN-
MMPYNY--
MTPYNY--
-MPYNYM-
MCPYNY--
-MPYNYW-
-MPYNYS-
MNPYNY--
-NPYNYI-
NLPYNY--
-NPYNYS-
NWPYNY--

NRPYNY--
-NPYNYA-
NIPYNY--
NSPYNY--
-NPYNYM-
NVPYNY--
NTPYNY--
-NPYNYW-
NMPYNY--
NQPYNY--
-NPYNYV-
-NPYNYK-
-NPYNYF-
NKPYNY--
-NPYNYR-
-NPYNYQ-
NPPYNY--
NNPYNY--
NGPYNY--
-NPYNYY-
-NPYNYT-
-NPYNYE-
NAPYNY--
--PYNYLA
--PYNYVS
--PYNYLT
--PYNYLG
--PYNYTS
--PYNYFS
--PYNYLR
--PYNYLK
--PYNYSM
--PYNYRT
--PYNYRA
--PYNYLS
--PYNYRG
--PYNYVT
--PYNYVG
--PYNYTN
--PYNYRV
--PYNYMR
--PYNYYL
--PYNYEI
--PYNYWK
--PYNYRS
--PYNYGS
--PYNYWA
--PYNYFA
--PYNYTW
--PYNYSS

--PYNYSV
--PYNYRI
--PYNYQH
--PYNYKG
--PYNYTV
--PYNYTG
--PYNYTA
--PYNYSY
--PYNYSG
--PYNYRE
--PYNYQA
--PYNYFR
--PYNYAF
--PYNYEK
--PYNYEG
--PYNYAV
--PYNYAL
--PYNYSW
--PYNYRW
--PYNYMA
--PYNYLN
--PYNYWH
--PYNYLQ
--PYNYKS
--PYNYEA
--PYNYAA
--PYNYYV
--PYNYVH
--PYNYSK
--PYNYRN
--PYNYMQ
--PYNYLH
--PYNYKL
--PYNYKA
--PYNYHA
--PYNYEF
--PYNYAG
--PYNYWS
--PYNYVI
--PYNYTL
--PYNYST
--PYNYSH
--PYNYRL
--PYNYQL
--PYNYKH
--PYNYIN
--PYNYGV
--PYNYGG
--PYNYAR
PEPYNY--

--PYNYSL
--PYNYRF
--PYNYHH
--PYNYGA
--PYNYVW
--PYNYVD
--PYNYTR
--PYNYTF
--PYNYSN
--PYNYQG
--PYNYNG
--PYNYLF
--PYNYHF
--PYNYFK
--PYNYYN
--PYNYVQ
--PYNYTK
--PYNYSA
--PYNYQT
--PYNYMV
--PYNYMN
--PYNYMM
--PYNYLV
--PYNYKQ
--PYNYIR
--PYNYIL
--PYNYIH
--PYNYGT
--PYNYFN
--PYNYFH
--PYNYAT
--PYNYAS
PHPYNY--
--PYNYYS
--PYNYWW
--PYNYWM
--PYNYVM
--PYNYVL
--PYNYSQ
--PYNYSD
--PYNYRH
--PYNYNS
--PYNYKF
--PYNYIM
--PYNYGY
--PYNYGN
--PYNYGF
--PYNYFV
--PYNYET
--PYNYEL

PTPYNY--
--PYNYYH
--PYNYYA
--PYNYWT
--PYNYVN
--PYNYVA
--PYNYTQ
--PYNYTH
--PYNYSI
--PYNYSF
--PYNYQS
--PYNYQN
--PYNYML
--PYNYMH
--PYNYLD
--PYNYIT
--PYNYHW
--PYNYHN
--PYNYAH
PMPYNY--
PGPYNY--
--PYNYWN
--PYNYWG
--PYNYVK
--PYNYTT
--PYNYSE
--PYNYQK
--PYNYQE
--PYNYNY
--PYNYNA
--PYNYMF
--PYNYIQ
--PYNYIK
--PYNYIG
--PYNYID
--PYNYIA
--PYNYHR
--PYNYHL
--PYNYHE
--PYNYGL
--PYNYGI
--PYNYGH
--PYNYFT
--PYNYEV
--PYNYES
--PYNYER
--PYNYCG
--PYNYAM
--PYNYAD
PWPYNY--

--PYNYYR
--PYNYYI
--PYNYWR
--PYNYTD
--PYNYRY
--PYNYRQ
--PYNYQW
--PYNYQQ
--PYNYQD
--PYNYNV
--PYNYNN
--PYNYNL
--PYNYNK
--PYNYNH
--PYNYNF
--PYNYLC
--PYNYKY
--PYNYKR
--PYNYIW
--PYNYHS
--PYNYHG
--PYNYGP
--PYNYEQ
--PYNYEH
--PYNYDK
--PYNYCT
PQPYNY--
-PPYNYL-
-QPYNYS-
-QPYNYV-
QGPYNY--
QRPYNY--
-QPYNYI-
-QPYNYR-
QTPYNY--
QHPYNY--
QSPYNY--
-QPYNYT-
-QPYNYG-
QPPYNY--
QYPYNY--
-QPYNYW-
-QPYNYL-
QNPYNY--
-QPYNYY-
-QPYNYH-
-QPYNYK-
-QPYNYF-
QWPYNY--
QVPYNY--

QQPYNY--
QLPYNY--
-RPYNYK-
RVPYNY--
-RPYNYR-
-RPYNYL-
RYPYNY--
-RPYNYY-
-RPYNYS-
-RPYNYI-
RRPYNY--
RNPYNY--
RAPYNY--
RSPYNY--
RLPYNY--
RQPYNY--
-RPYNYC-
RFPYNY--
-RPYNYF-
-RPYNYE-
-RPYNYA-
-RPYNYT-
RKPYNY--
RHPYNY--
-RPYNYW-
REPYNY--
-RPYNYG-
RMPYNY--
RIPYNY--
-RPYNYN-
-RPYNYH-
-RPYNYD-
RTPYNY--
RPPYNY--
-SPYNYR-
SSPYNY--
SRPYNY--
SIPYNY--
-SPYNYV-
SLPYNY--
-SPYNYL-
SVPYNY--
SKPYNY--
-SPYNYS-
-SPYNYH-
SAPYNY--
SYPYNY--
-SPYNYK-
-SPYNYG-
-SPYNYI-

SFPYNY--
STPYNY--
SPPYNY--
-SPYNYQ-
SMPYNY--
SCPYNY--
SHPYNY--
SDPYNY--
-SPYNYA-
SGPYNY--
SEPYNY--
-SPYNYW-
-SPYNYT-
-SPYNYE-
TRPYNY--
-TPYNYS-
TSPYNY--
TAPYNY--
TWPYNY--
-TPYNYV-
TKPYNY--
TGPYNY--
-TPYNYT-
-TPYNYR-
TLPYNY--
TYPYNY--
-TPYNYM-
-TPYNYF-
-TPYNYH-
-TPYNYE-
-TPYNYY-
-TPYNYK-
-TPYNYI-
TFPYNY--
TEPYNY--
TDPYNY--
-TPYNYN-
-TPYNYG-
TNPYNY--
TIPYNY--
VTPYNY--
VNPYNY--
VRPYNY--
-VPYNYS-
-VPYNYM-
VHPYNY--
VSPYNY--
VKPYNY--
VVPYNY--
-VPYNYL-

VDPYNY--
VFPYNY--
-VPYNYG-
-VPYNYQ-
-VPYNYE-
VWPYNY--
-VPYNYV-
-VPYNYK-
VAPYNY--
VQPYNY--
-VPYNYR-
-VPYNYW-
-VPYNYA-
VGPYNY--
VYPYNY--
-VPYNYI-
VEPYNY--
-VPYNYN-
VLPYNY--
-VPYNYY-
-VPYNYF-
VIPYNY--
WSPYNY--
WDPYNY--
WVPYNY--
WLPYNY--
WKPYNY--
-WPYNYR-
WNPYNY--
WGPYNY--
WYPYNY--
WEPYNY--
-WPYNYL-
WMPYNY--
WTPYNY--
-WPYNYS-
-WPYNYN-
-WPYNYH-
-WPYNYA-
WAPYNY--
YRPYNY--
-YPYNYI-
YTPYNY--
YSPYNY--
YKPYNY--
-YPYNYV-
YVPYNY--
-YPYNYK-
-YPYNYE-
YYPYNY--

-YPYNYT-
YNPYNY--
YIPYNY--
YGPYNY--
-YPYNYL-
YWPYNY--
YLPYNY--
-YPYNYW-
-YPYNYR-
-YPYNYA-
YPPYNY--
YHPYNY--
-YPYNYY-
YAPYNY--
-YPYNYS-
-YPYNYH-
-YPYNYG-
YMPYNY--
YDPYNY--

PQWI	781	540	46.82

-APQWIS-
-APQWIR-
ASPQWI--
AEPQWI--
-APQWIV-
-APQWII-
AYPQWI--
-APQWIM-
AGPQWI--
-APQWIQ-
-APQWIL-
-APQWIK-
-APQWIH-
ALPQWI--
AWPQWI--
ATPQWI--
-APQWIE-
-APQWID-
AMPQWI--
AFPQWI--
CPPQWI--
CSPQWI--
-CPQWIF-
CVPQWI--
-CPQWIS-
-CPQWIR-
CHPQWI--
CAPQWI--
-DPQWIR-
DGPQWI--

DIPQWI--
DFPQWI--
DTPQWI--
-DPQWIK-
DEPQWI--
-DPQWIT-
-DPQWIQ-
DLPQWI--
DWPQWI--
DSPQWI--
-DPQWIS-
DKPQWI--
ETPQWI--
-EPQWIW-
EGPQWI--
ERPQWI--
ESPQWI--
-EPQWIY-
EQPQWI--
EVPQWI--
ELPQWI--
-EPQWIS-
-EPQWIG-
EMPQWI--
EWPQWI--
EAPQWI--
-EPQWIL-
ENPQWI--
EKPQWI--
EDPQWI--
-EPQWIR-
-EPQWIQ-
EFPQWI--
FTPQWI--
FSPQWI--
FLPQWI--
FMPQWI--
-FPQWIT-
-FPQWIR-
FCPQWI--
FRPQWI--
-FPQWIA-
FAPQWI--
FYPQWI--
-FPQWIL-
-FPQWII-
FHPQWI--
FDPQWI--
GTPQWI--
-GPQWIV-

GFPQWI--
GQPQWI--
GEPQWI--
GSPQWI--
-GPQWIG-
-GPQWIR-
GPPQWI--
-GPQWII-
GNPQWI--
GKPQWI--
GMPQWI--
GRPQWI--
-GPQWIM-
-GPQWIK-
GWPQWI--
GVPQWI--
GDPQWI--
-GPQWIT-
-GPQWIL-
GIPQWI--
GHPQWI--
GAPQWI--
HGPQWI--
HLPQWI--
-HPQWIV-
-HPQWIK-
HRPQWI--
HTPQWI--
HSPQWI--
HIPQWI--
HHPQWI--
-HPQWIS-
HVPQWI--
-HPQWIG-
HKPQWI--
HEPQWI--
HAPQWI--
ISPQWI--
IYPQWI--
ILPQWI--
-IPQWIK-
IQPQWI--
IWPQWI--
-IPQWIS-
IDPQWI--
IPPQWI--
IIPQWI--
IFPQWI--
-IPQWIV-
-IPQWIR-

-IPQWIM-
-IPQWIL-
-IPQWIG-
IKPQWI--
KSPQWI--
KLPQWI--
-KPQWIL-
-KPQWII-
KWPQWI--
KYPQWI--
KHPQWI--
KIPQWI--
-KPQWIS-
KVPQWI--
-KPQWIG-
KQPQWI--
-KPQWIT-
KNPQWI--
-KPQWIY-
-KPQWIV-
-KPQWIK-
KEPQWI--
KRPQWI--
-KPQWIQ-
-KPQWIN-
-KPQWIH-
-KPQWIA-
KPPQWI--
KTPQWI--
-KPQWIR-
-KPQWIF-
-KPQWIE-
KFPQWI--
LSPQWI--
LAPQWI--
LTPQWI--
LIPQWI--
LHPQWI--
LQPQWI--
-LPQWIK-
-LPQWIG-
LLPQWI--
LGPQWI--
LRPQWI--
-LPQWIM-
LNPQWI--
LDPQWI--
-LPQWIR-
-LPQWIA-
LKPQWI--

LWPQWI--
-LPQWIS-
LPPQWI--
LFPQWI--
-LPQWIY-
-LPQWIV-
-LPQWIT-
-LPQWIL-
-LPQWII-
-LPQWIF-
LEPQWI--
-LPQWIQ-
-LPQWIN-
MYPQWI--
MVPQWI--
MTPQWI--
MFPQWI--
-MPQWIT-
-MPQWIS-
MGPQWI--
MSPQWI--
MRPQWI--
MLPQWI--
-MPQWIV-
-MPQWIG-
-MPQWIA-
-NPQWIR-
NAPQWI--
NQPQWI--
NRPQWI--
NGPQWI--
-NPQWIA-
NKPQWI--
NYPQWI--
-NPQWIS-
NVPQWI--
-NPQWIQ-
-NPQWIK-
NLPQWI--
-NPQWIT-
-NPQWII-
--PQWIRK
--PQWIRS
--PQWILG
--PQWIRV
--PQWITS
--PQWIRH
--PQWILY
--PQWIRG
--PQWITG

--PQWISG
--PQWILT
--PQWIVQ
PAPQWI--
--PQWIYN
--PQWIVV
--PQWIFG
--PQWIVH
--PQWIQS
--PQWIMT
PSPQWI--
--PQWITH
--PQWISS
PDPQWI--
--PQWIWA
--PQWISN
--PQWIIN
--PQWIFV
--PQWIFH
--PQWIAA
--PQWIRT
--PQWIYT
--PQWITR
--PQWITQ
--PQWIST
--PQWIKY
--PQWIKS
--PQWIYS
--PQWIWH
--PQWISQ
--PQWIRW
--PQWIQN
--PQWINS
--PQWIMG
--PQWIKT
--PQWIKG
--PQWIHS
--PQWIHN
--PQWIAS
PNPQWI--
--PQWIYK
--PQWILA
--PQWIKW
--PQWIAR
--PQWIAG
--PQWIYR
--PQWIWM
--PQWITY
--PQWISV
--PQWIRQ

--PQWIQY
--PQWIQR
--PQWING
--PQWIFS
--PQWIFK
--PQWIVT
--PQWIVS
--PQWIVG
--PQWITN
--PQWISH
--PQWIRN
--PQWIRI
--PQWIQF
--PQWIMH
--PQWIMD
--PQWILN
--PQWIKD
--PQWIHV
--PQWIGY
--PQWIGS
--PQWIGK
--PQWIGG
--PQWITV
--PQWITK
--PQWISW
--PQWIRY
--PQWIRD
--PQWIQQ
--PQWIQI
--PQWIMY
--PQWIMI
--PQWIKA
--PQWIIM
--PQWIIH
--PQWIGL
--PQWIEA
--PQWIAV
--PQWIAM
--PQWIAE
PKPQWI--
PFPQWI--
--PQWIYD
--PQWIRL
--PQWIQD
--PQWINT
--PQWIKV
--PQWIKL
--PQWIIW
--PQWIIT
--PQWIHT

--PQWIHG
--PQWIGA
--PQWIFN
--PQWIAH
PIPQWI--
--PQWIYW
--PQWIWS
--PQWIWI
--PQWIVY
--PQWITM
--PQWITA
--PQWISR
--PQWIQH
--PQWIQG
--PQWIMS
--PQWILF
--PQWIKF
--PQWIIG
--PQWIIE
--PQWIGV
--PQWIGN
--PQWIEQ
--PQWIAT
PEPQWI--
PVPQWI--
PTPQWI--
--PQWIVR
--PQWITD
--PQWISK
--PQWISD
--PQWISA
--PQWIQW
--PQWIQE
--PQWIMR
--PQWILR
--PQWILH
--PQWIKI
--PQWIIS
--PQWIGQ
--PQWIGM
--PQWIFW
--PQWIFE
--PQWIEM
--PQWIEG
--PQWIDV
--PQWICQ
--PQWIAQ
PQPQWI--
QGPQWI--
QVPQWI--

-QPQWIR-
QMPQWI--
QDPQWI--
QPPQWI--
-QPQWIM-
QNPQWI--
QIPQWI--
-QPQWIY-
-QPQWIS-
-QPQWIQ-
-QPQWIK-
QKPQWI--
QTPQWI--
QRPQWI--
-QPQWIT-
QLPQWI--
QAPQWI--
QWPQWI--
-QPQWIV-
QHPQWI--
QSPQWI--
-QPQWIN-
-QPQWIL-
-QPQWIH-
-RPQWIR-
RTPQWI--
RSPQWI--
-RPQWIK-
RLPQWI--
RNPQWI--
-RPQWIG-
RRPQWI--
RGPQWI--
RIPQWI--
RKPQWI--
RWPQWI--
RDPQWI--
-RPQWIL-
-RPQWIH-
-RPQWIF-
RAPQWI--
RVPQWI--
-RPQWIT-
-RPQWIM-
-RPQWII-
-RPQWIA-
REPQWI--
RYPQWI--
RQPQWI--
-RPQWIE-

RPPQWI--
RMPQWI--
-RPQWIY-
-RPQWIQ-
RFPQWI--
RCPQWI--
SSPQWI--
SLPQWI--
SVPQWI--
STPQWI--
SMPQWI--
-SPQWIR-
SFPQWI--
SRPQWI--
-SPQWIQ-
SKPQWI--
-SPQWIT-
SEPQWI--
SAPQWI--
-SPQWII-
-SPQWIH-
SPPQWI--
-SPQWIS-
-SPQWIK-
-SPQWIA-
SIPQWI--
SQPQWI--
SWPQWI--
SNPQWI--
-SPQWIY-
-SPQWIV-
TSPQWI--
TRPQWI--
-TPQWIT-
TAPQWI--
-TPQWIV-
-TPQWIH-
THPQWI--
-TPQWIS-
-TPQWIR-
-TPQWII-
TPPQWI--
-TPQWIA-
TGPQWI--
-TPQWIY-
TYPQWI--
TQPQWI--
-TPQWIQ-
TIPQWI--
TFPQWI--

TNPQWI--
TKPQWI--
VRPQWI--
VEPQWI--
VPPQWI--
VVPQWI--
-VPQWIL-
VLPQWI--
-VPQWII-
VSPQWI--
VHPQWI--
-VPQWIY-
-VPQWIS-
-VPQWIR-
-VPQWIE-
VKPQWI--
VYPQWI--
-VPQWIQ-
-VPQWIV-
VGPQWI--
VCPQWI--
-VPQWIW-
-VPQWIT-
-VPQWIK-
VTPQWI--
-VPQWIF-
-VPQWIA-
VNPQWI--
VIPQWI--
WVPQWI--
WSPQWI--
-WPQWIR-
WRPQWI--
-WPQWIS-
WTPQWI--
WKPQWI--
WGPQWI--
-WPQWIA-
WHPQWI--
WAPQWI--
WFPQWI--
WIPQWI--
YRPQWI--
YSPQWI--
-YPQWIL-
YNPQWI--
YEPQWI--
YLPQWI--
-YPQWIR-
YTPQWI--

YAPQWI--
-YPQWII-
YHPQWI--
YGPQWI--
YDPQWI--
-YPQWIS-
YMPQWI--
YIPQWI--
-YPQWIK-
-YPQWIA-

PKPI	416	653	46.80

-NPKPIL-
--PKPIGR
ALPKPI--
-APKPIV-
ASPKPI--
-APKPIW-
-APKPIL-
-APKPIH-
AKPKPI--
AVPKPI--
ARPKPI--
-APKPIT-
ATPKPI--
-APKPIN-
-APKPII-
-APKPIF-
AFPKPI--
AAPKPI--
-APKPIS-
AYPKPI--
AQPKPI--
-APKPIR-
-APKPIQ-
AMPKPI--
AEPKPI--
-APKPIM-
-APKPIE-
ANPKPI--
ADPKPI--
AWPKPI--
APPKPI--
-APKPIY-
-APKPIK-
CMPKPI--
CFPKPI--
CRPKPI--
CYPKPI--
CTPKPI--
CIPKPI--

CVPKPI--
CQPKPI--
CNPKPI--
CKPKPI--
CLPKPI--
DAPKPI--
-DPKPII-
DQPKPI--
-DPKPIQ-
-DPKPIK-
DGPKPI--
DTPKPI--
DRPKPI--
-DPKPIV-
-DPKPIT-
DMPKPI--
DIPKPI--
DWPKPI--
DVPKPI--
-DPKPIY-
-DPKPIW-
-DPKPIM-
-DPKPIA-
DLPKPI--
-EPKPIK-
-EPKPII-
-EPKPIT-
-EPKPIN-
-EPKPIL-
EGPKPI--
EVPKPI--
EPPKPI--
-EPKPIW-
EIPKPI--
ESPKPI--
-EPKPIS-
-EPKPIF-
EEPKPI--
EAPKPI--
ETPKPI--
EQPKPI--
-EPKPIA-
EFPKPI--
FKPKPI--
FSPKPI--
FIPKPI--
-FPKPIH-
FLPKPI--
FRPKPI--
-FPKPIV-

FTPKPI--
FPPKPI--
FNPKPI--
FFPKPI--
-FPKPIL-
-FPKPIR-
FHPKPI--
-FPKPII-
-FPKPIT-
-FPKPIM-
FEPKPI--
-FPKPIA-
FVPKPI--
-FPKPIS-
-FPKPIQ-
-FPKPIK-
-FPKPIC-
FAPKPI--
-GPKPIL-
GRPKPI--
GKPKPI--
GFPKPI--
GEPKPI--
GSPKPI--
-GPKPIR-
GAPKPI--
GPPKPI--
GMPKPI--
-GPKPIK-
GWPKPI--
GQPKPI--
-GPKPIF-
-GPKPIT-
-GPKPIQ-
-GPKPIA-
GNPKPI--
GGPKPI--
GVPKPI--
-GPKPIY-
-GPKPIS-
GHPKPI--
GYPKPI--
-GPKPIW-
-GPKPIM-
GLPKPI--
GIPKPI--
-HPKPIL-
HRPKPI--
HWPKPI--
-HPKPIR-

HVPKPI--
-HPKPII-
HSPKPI--
HNPKPI--
HEPKPI--
HIPKPI--
HAPKPI--
HQPKPI--
-HPKPIM-
HGPKPI--
-HPKPIQ-
-HPKPIH-
HMPKPI--
HLPKPI--
HKPKPI--
HDPKPI--
HTPKPI--
-HPKPIS-
-HPKPIN-
-HPKPIF-
-IPKPIR-
IVPKPI--
-IPKPII-
IQPKPI--
ISPKPI--
-IPKPIF-
IMPKPI--
IGPKPI--
IEPKPI--
ITPKPI--
-IPKPIH-
IFPKPI--
-IPKPIK-
INPKPI--
IRPKPI--
IKPKPI--
-IPKPIW-
-IPKPIM-
IYPKPI--
-IPKPIG-
IDPKPI--
ICPKPI--
KSPKPI--
KGPKPI--
-KPKPIV-
-KPKPIL-
KYPKPI--
-KPKPIS-
-KPKPIR-
-KPKPII-

KFPKPI--
KQPKPI--
-KPKPIY-
KPPKPI--
-KPKPIW-
-KPKPIH-
-KPKPIA-
KLPKPI--
KTPKPI--
KRPKPI--
KIPKPI--
KHPKPI--
KEPKPI--
KAPKPI--
LSPKPI--
LAPKPI--
-LPKPIR-
-LPKPIY-
LWPKPI--
LMPKPI--
LLPKPI--
LGPKPI--
LEPKPI--
LRPKPI--
LQPKPI--
-LPKPIF-
LTPKPI--
LFPKPI--
LYPKPI--
-LPKPIM-
LPPKPI--
LHPKPI--
LVPKPI--
-LPKPIQ-
-LPKPIH-
LDPKPI--
-LPKPIT-
-LPKPIS-
-LPKPIK-
-LPKPII-
-LPKPIC-
-LPKPIV-
-LPKPIL-
LNPKPI--
-MPKPII-
MWPKPI--
-MPKPIR-
MEPKPI--
MLPKPI--
MIPKPI--

MAPKPI--
MTPKPI--
-MPKPIY-
-MPKPIS-
MMPKPI--
MPPKPI--
-MPKPIK-
-MPKPIL-
MFPKPI--
-MPKPIV-
-MPKPIF-
MVPKPI--
MQPKPI--
-MPKPIN-
-MPKPIM-
MNPKPI--
MSPKPI--
MRPKPI--
-MPKPIQ-
MDPKPI--
-NPKPIR-
NRPKPI--
NMPKPI--
NAPKPI--
NFPKPI--
NVPKPI--
NSPKPI--
NLPKPI--
-NPKPII-
NGPKPI--
NEPKPI--
-NPKPIV-
-NPKPIT-
NNPKPI--
NKPKPI--
NDPKPI--
NCPKPI--
--PKPISW
--PKPIWI
--PKPIMI
--PKPIRV
--PKPIMT
--PKPIVS
--PKPIRY
--PKPIKV
--PKPILT
--PKPIRS
--PKPIRW
--PKPIRL
--PKPISY

--PKPIFK
--PKPIRA
PVPKPI--
--PKPIYN
--PKPIWY
--PKPIWV
--PKPISV
--PKPIMS
--PKPIKS
--PKPIHV
--PKPIFA
--PKPIYS
--PKPIWR
--PKPILS
--PKPILL
--PKPIKY
--PKPIIW
--PKPIFT
--PKPIVG
--PKPILM
--PKPIGV
--PKPIYL
--PKPIVY
--PKPIVV
--PKPITL
--PKPIIY
--PKPIFY
--PKPIFW
--PKPIFR
--PKPIWS
--PKPISH
--PKPIPY
--PKPILK
--PKPIIG
--PKPIES
--PKPIMD
--PKPIDM
--PKPIYR
--PKPISF
--PKPIQV
--PKPIMN
--PKPIMA
--PKPILV
PFPKPI--
--PKPIYT
--PKPIYQ
--PKPITR
--PKPISA
--PKPIQT
--PKPIMV

--PKPIKQ
--PKPIIK
--PKPIIA
--PKPIFI
--PKPIAG
PAPKPI--
--PKPITI
--PKPITA
--PKPIRT
--PKPIRM
--PKPILI
--PKPIIN
--PKPIFM
--PKPIAF
--PKPIWL
--PKPIVH
--PKPIQS
--PKPIPF
--PKPILW
--PKPIGS
--PKPIFS
PHPKPI--
--PKPIWD
--PKPIVM
--PKPIVA
--PKPITV
--PKPITH
--PKPISK
--PKPIRQ
--PKPILY
--PKPILQ
--PKPIKR
--PKPIGF
--PKPIEV
--PKPIDF
PWPKPI--
--PKPIVQ
--PKPIVN
--PKPIQG
--PKPIQA
--PKPIMR
--PKPIMG
--PKPIKF
--PKPIIR
--PKPIHN
--PKPIFF
--PKPIEL
--PKPIAA
PNPKPI--
--PKPIYV

--PKPIWG
--PKPITG
--PKPITF
--PKPISG
--PKPIRN
--PKPIRH
--PKPILN
--PKPIKH
--PKPIIT
--PKPIEI
--PKPIEF
--PKPIAT
--PKPIAQ
--PKPIYW
--PKPIYK
--PKPIYH
--PKPIYA
--PKPITQ
--PKPIRF
--PKPIQL
--PKPIQF
--PKPIPH
--PKPINS
--PKPINA
--PKPIMH
--PKPIKT
--PKPIKK
--PKPIKG
--PKPIIM
--PKPIIF
--PKPIGG
--PKPIFQ
--PKPIFN
PGPKPI--
--PKPIVR
--PKPIVF
--PKPITK
--PKPITD
--PKPIRK
--PKPIPQ
--PKPINT
--PKPIIQ
--PKPIHF
--PKPIFG
--PKPIEQ
--PKPICG
--PKPIAV
--PKPIAS
--PKPIWA
--PKPITM

--PKPIQW
--PKPIPW
--PKPINY
--PKPINV
--PKPINH
--PKPIML
--PKPILF
--PKPIKI
--PKPIII
--PKPIHY
--PKPIHA
--PKPIGA
--PKPIEY
PTPKPI--
--PKPIYM
--PKPIYE
--PKPIWW
--PKPIWT
--PKPIWP
--PKPIVE
--PKPIVC
--PKPITY
--PKPITW
--PKPITT
--PKPITE
--PKPISD
--PKPIRE
--PKPIRD
--PKPIQY
--PKPIQR
--PKPIQN
--PKPIQI
--PKPINQ
--PKPINK
--PKPINI
--PKPIMM
--PKPILH
--PKPIKD
--PKPIHS
--PKPIHI
--PKPIHG
--PKPIFL
--PKPIEW
PCPKPI--
QYPKPI--
QTPKPI--
QEPKPI--
QSPKPI--
-QPKPIL-
QLPKPI--

-QPKPIR-
QMPKPI--
QGPKPI--
QVPKPI--
-QPKPIV-
-QPKPIT-
-QPKPIH-
QIPKPI--
-QPKPIY-
-QPKPIS-
-QPKPIQ-
QHPKPI--
QWPKPI--
QQPKPI--
-QPKPIF-
RFPKPI--
RVPKPI--
-RPKPIF-
-RPKPIV-
RPPKPI--
-RPKPIR-
RGPKPI--
-RPKPII-
REPKPI--
RWPKPI--
RMPKPI--
RIPKPI--
-RPKPIY-
-RPKPIK-
-RPKPIH-
-RPKPIA-
-RPKPIQ-
-RPKPIM-
RCPKPI--
RRPKPI--
-RPKPIG-
-RPKPID-
RHPKPI--
RAPKPI--
SLPKPI--
SYPKPI--
SRPKPI--
SEPKPI--
-SPKPIL-
SGPKPI--
-SPKPIF-
SIPKPI--
STPKPI--
-SPKPII-
SPPKPI--

-SPKPIR-
SMPKPI--
SAPKPI--
SQPKPI--
-SPKPIK-
-SPKPIM-
-SPKPIH-
SKPKPI--
-SPKPIY-
-SPKPIV-
-SPKPIA-
SNPKPI--
SDPKPI--
-SPKPIW-
-SPKPIT-
-SPKPIQ-
-SPKPIS-
-SPKPIC-
TVPKPI--
TSPKPI--
TWPKPI--
-TPKPIY-
TEPKPI--
TLPKPI--
-TPKPIR-
TNPKPI--
TAPKPI--
TMPKPI--
THPKPI--
-TPKPIS-
-TPKPIN-
TGPKPI--
-TPKPIM-
-TPKPIL-
TDPKPI--
-TPKPIK-
-TPKPIH-
-TPKPIG-
TRPKPI--
TQPKPI--
-TPKPIV-
-TPKPII-
-TPKPIF-
TKPKPI--
VRPKPI--
VAPKPI--
VYPKPI--
VQPKPI--
-VPKPIF-
VLPKPI--

VVPKPI--
-VPKPIL-
VTPKPI--
-VPKPIW-
VKPKPI--
VWPKPI--
VFPKPI--
-VPKPIH-
VPPKPI--
-VPKPIM-
VMPKPI--
VIPKPI--
VEPKPI--
-VPKPIY-
-VPKPII-
VDPKPI--
-VPKPIT-
-VPKPIS-
-VPKPIC-
WSPKPI--
WMPKPI--
WTPKPI--
-WPKPIQ-
WEPKPI--
WPPKPI--
-WPKPIF-
-WPKPIR-
-WPKPIN-
WLPKPI--
WKPKPI--
WGPKPI--
WAPKPI--
-WPKPIY-
-WPKPIW-
-WPKPIT-
-WPKPIS-
-WPKPIL-
WVPKPI--
-WPKPIH-
WNPKPI--
YLPKPI--
YRPKPI--
-YPKPIL-
YQPKPI--
YGPKPI--
YSPKPI--
YAPKPI--
-YPKPIT-
-YPKPIR-
YVPKPI--

YTPKPI--
-YPKPIW-
-YPKPIF-
-YPKPIS-
YYPKPI--
YPPKPI--
YWPKPI--
YIPKPI--
YFPKPI--
-YPKPIP-
-YPKPIK-
-YPKPIA-
YMPKPI--
YHPKPI--
PFHL 81 824 46.79
--PFHLYP
-TPFHLG-
-WPFHLG-
AVPFHL--
ARPFHL--
ASPFHL--
AYPFHL--
ANPFHL--
ATPFHL--
-APFHLR-
-APFHLY-
-APFHLE-
AGPFHL--
-APFHLT-
AWPFHL--
-APFHLQ-
-APFHLH-
-APFHLA-
-APFHLS-
-APFHLK-
AFPFHL--
ALPFHL--
APPFHL--
AQPFHL--
AAPFHL--
-APFHLN-
AMPFHL--
-APFHLW-
-APFHLF-
AEPFHL--
-APFHLV-
CTPFHL--
CRPFHL--
CNPFHL--
CKPFHL--

CDPFHL--
-CPFHLV-
-CPFHLH-
CWPFHL--
CVPFHL--
CEPFHL--
CAPFHL--
-CPFHLS-
-CPFHLR-
-CPFHLQ-
CLPFHL--
DIPFHL--
DSPFHL--
DKPFHL--
DHPFHL--
DVPFHL--
-DPFHLR-
-DPFHLK-
DGPFHL--
DAPFHL--
DLPFHL--
DFPFHL--
DTPFHL--
DYPFHL--
-DPFHLT-
-DPFHLF-
DEPFHL--
DQPFHL--
-DPFHLQ-
-DPFHLM-
-DPFHLI-
DNPFHL--
-DPFHLY-
-DPFHLL-
-DPFHLH-
-DPFHLA-
-EPFHLR-
ERPFHL--
-EPFHLW-
-EPFHLL-
EGPFHL--
EVPFHL--
ESPFHL--
EYPFHL--
ETPFHL--
EKPFHL--
-EPFHLT-
ELPFHL--
-EPFHLF-
-EPFHLY-

EFPFHL--
EPPFHL--
-EPFHLV-
EMPFHL--
-EPFHLS-
ENPFHL--
EHPFHL--
EEPFHL--
-EPFHLQ-
-EPFHLK-
-EPFHLI-
-EPFHLA-
EQPFHL--
-EPFHLN-
-EPFHLM-
-EPFHLG-
FGPFHL--
FSPFHL--
FHPFHL--
FRPFHL--
FDPFHL--
FEPFHL--
-FPFHLT-
FMPFHL--
-FPFHLK-
-FPFHLS-
FWPFHL--
FTPFHL--
FKPFHL--
-FPFHLR-
FAPFHL--
-FPFHLH-
-FPFHLE-
-FPFHLA-
FNPFHL--
FYPFHL--
-FPFHLL-
-FPFHLQ-
FPPFHL--
-FPFHLI-
-FPFHLG-
FQPFHL--
-FPFHLY-
-FPFHLN-
GRPFHL--
-GPFHLR-
GKPFHL--
-GPFHLS-
GLPFHL--
GTPFHL--

−GPFHLK−
GSPFHL−−
−GPFHLL−
−GPFHLT−
GGPFHL−−
GFPFHL−−
GMPFHL−−
−GPFHLV−
−GPFHLQ−
GIPFHL−−
GAPFHL−−
−GPFHLE−
GHPFHL−−
GEPFHL−−
GYPFHL−−
GNPFHL−−
GVPFHL−−
−GPFHLN−
GWPFHL−−
−GPFHLW−
−GPFHLI−
GCPFHL−−
−GPFHLM−
−GPFHLH−
GQPFHL−−
−GPFHLG−
−GPFHLF−
−GPFHLA−
−GPFHLC−
GDPFHL−−
HSPFHL−−
HTPFHL−−
HRPFHL−−
HIPFHL−−
HKPFHL−−
HNPFHL−−
−HPFHLI−
HPPFHL−−
−HPFHLV−
−HPFHLR−
HYPFHL−−
−HPFHLW−
HWPFHL−−
HEPFHL−−
HMPFHL−−
HLPFHL−−
−HPFHLM−
−HPFHLE−
−HPFHLL−
−HPFHLK−

-HPFHLA-
HQPFHL--
-HPFHLY-
-HPFHLS-
-HPFHLF-
HGPFHL--
HAPFHL--
-HPFHLN-
HCPFHL--
IKPFHL--
ITPFHL--
IGPFHL--
ILPFHL--
IQPFHL--
-IPFHLH-
IRPFHL--
IDPFHL--
-IPFHLR-
IYPFHL--
IHPFHL--
IPPFHL--
-IPFHLY-
-IPFHLT-
-IPFHLS-
IEPFHL--
IVPFHL--
-IPFHLL-
-IPFHLI-
-IPFHLQ-
-IPFHLE-
-IPFHLW-
IAPFHL--
-IPFHLV-
-IPFHLF-
IMPFHL--
-IPFHLM-
-IPFHLG-
INPFHL--
IIPFHL--
IWPFHL--
-IPFHLN-
KSPFHL--
-KPFHLS-
-KPFHLT-
KLPFHL--
KVPFHL--
KTPFHL--
KNPFHL--
-KPFHLR-
KGPFHL--

-KPFHLW-
-KPFHLE-
KPPFHL--
-KPFHLL-
KEPFHL--
-KPFHLV-
-KPFHLI-
-KPFHLA-
KWPFHL--
KDPFHL--
-KPFHLK-
-KPFHLY-
-KPFHLH-
-KPFHLQ-
KQPFHL--
-KPFHLN-
KHPFHL--
KFPFHL--
KIPFHL--
-KPFHLM-
KKPFHL--
KMPFHL--
KCPFHL--
LRPFHL--
LMPFHL--
LPPFHL--
LTPFHL--
LYPFHL--
-LPFHLR-
-LPFHLV-
LFPFHL--
LQPFHL--
-LPFHLT-
LAPFHL--
LLPFHL--
LDPFHL--
LKPFHL--
LHPFHL--
LGPFHL--
-LPFHLH-
-LPFHLL-
-LPFHLF-
-LPFHLA-
-LPFHLN-
LEPFHL--
-LPFHLE-
LCPFHL--
-LPFHLM-
-MPFHLT-
-MPFHLS-

MGPFHL--
MSPFHL--
MTPFHL--
MQPFHL--
MIPFHL--
MFPFHL--
-MPFHLR-
-MPFHLV-
MEPFHL--
-MPFHLF-
MHPFHL--
-MPFHLL-
MKPFHL--
MMPFHL--
MYPFHL--
-MPFHLY-
-MPFHLM-
MDPFHL--
MLPFHL--
MNPFHL--
-MPFHLN-
-MPFHLA-
MPPFHL--
-MPFHLG-
-MPFHLE-
-MPFHLK-
MAPFHL--
NSPFHL--
NEPFHL--
-NPFHLR-
NMPFHL--
NRPFHL--
-NPFHLT-
NLPFHL--
NWPFHL--
NVPFHL--
-NPFHLL-
NTPFHL--
-NPFHLH-
-NPFHLS-
NFPFHL--
NPPFHL--
-NPFHLY-
-NPFHLF-
NYPFHL--
-NPFHLI-
NNPFHL--
NIPFHL--
-NPFHLM-
NKPFHL--

NDPFHL--
-NPFHLV-
NAPFHL--
NQPFHL--
-NPFHLW-
NGPFHL--
-NPFHLQ-
-NPFHLK-
-NPFHLE-
-NPFHLA-
--PFHLRS
--PFHLLS
--PFHLQT
--PFHLRG
--PFHLSS
--PFHLLA
--PFHLFT
PSPFHL--
PRPFHL--
--PFHLSR
--PFHLLR
--PFHLSG
--PFHLRR
--PFHLAG
--PFHLLH
--PFHLAS
--PFHLVS
--PFHLEA
--PFHLRM
--PFHLLT
PTPFHL--
PVPFHL--
--PFHLLK
--PFHLKT
--PFHLVA
PKPFHL--
--PFHLWT
--PFHLLQ
--PFHLAA
--PFHLST
--PFHLVG
--PFHLQN
PFPFHL--
--PFHLTT
--PFHLRF
--PFHLRE
--PFHLFS
--PFHLIR
--PFHLIK
--PFHLYA

--PFHLTG
--PFHLRK
--PFHLVR
--PFHLTS
--PFHLTR
--PFHLRL
--PFHLFA
PAPFHL--
PYPFHL--
PWPFHL--
--PFHLRV
--PFHLQS
--PFHLLN
--PFHLWV
--PFHLRQ
--PFHLRN
--PFHLRH
--PFHLQA
--PFHLLG
--PFHLIS
--PFHLIM
--PFHLET
PNPFHL--
--PFHLTY
--PFHLTH
--PFHLML
--PFHLGS
--PFHLEY
PLPFHL--
--PFHLWS
--PFHLVT
--PFHLSN
--PFHLKM
--PFHLEV
--PFHLYN
--PFHLYH
--PFHLVM
--PFHLNS
--PFHLKA
--PFHLIY
--PFHLYT
--PFHLTW
--PFHLTI
--PFHLSI
--PFHLRY
--PFHLMG
--PFHLKR
--PFHLHA
--PFHLAR
PEPFHL--

--PFHLWA
--PFHLMD
--PFHLKW
--PFHLKK
PQPFHL--
PGPFHL--
--PFHLYV
--PFHLVN
--PFHLVH
--PFHLSH
--PFHLLI
--PFHLID
--PFHLHK
--PFHLEM
--PFHLAT
--PFHLTK
--PFHLSV
--PFHLSL
--PFHLIN
--PFHLFK
--PFHLYQ
--PFHLYK
--PFHLTN
--PFHLKG
--PFHLIT
--PFHLEN
--PFHLTV
--PFHLSE
--PFHLRD
--PFHLNA
--PFHLMH
--PFHLLV
--PFHLKN
--PFHLKI
--PFHLHE
--PFHLFY
--PFHLFM
--PFHLAL
--PFHLYI
--PFHLVW
--PFHLRW
--PFHLNT
--PFHLKQ
--PFHLIG
--PFHLHI
--PFHLHH
--PFHLHD
--PFHLFN
--PFHLER
--PFHLAV

PIPFHL--
--PFHLYG
--PFHLWG
--PFHLWE
--PFHLVV
--PFHLVK
--PFHLVE
--PFHLTQ
--PFHLSM
--PFHLSK
--PFHLQH
--PFHLMA
--PFHLIQ
--PFHLHW
--PFHLHT
--PFHLHQ
--PFHLFW
--PFHLAY
--PFHLVF
--PFHLTA
--PFHLSA
--PFHLNV
--PFHLNQ
--PFHLHR
--PFHLES
--PFHLEG
PHPFHL--
--PFHLYW
--PFHLYM
--PFHLYL
--PFHLWQ
--PFHLWD
--PFHLVI
--PFHLTL
--PFHLSW
--PFHLSQ
--PFHLSD
--PFHLQI
--PFHLQE
--PFHLMY
--PFHLKS
--PFHLIV
--PFHLGT
--PFHLGM
--PFHLGA
--PFHLFH
--PFHLFF
--PFHLEF
--PFHLDS
--PFHLYY

--PFHLYE
--PFHLVY
--PFHLVQ
--PFHLTF
--PFHLTE
--PFHLTD
--PFHLSF
--PFHLQK
--PFHLNM
--PFHLMM
--PFHLLD
--PFHLKF
--PFHLHN
--PFHLGG
--PFHLAQ
--PFHLAF
PDPFHL--
--PFHLQQ
--PFHLQD
--PFHLNF
--PFHLMR
--PFHLMQ
--PFHLKV
--PFHLKE
--PFHLIF
--PFHLHV
--PFHLHL
--PFHLFG
--PFHLEW
--PFHLEH
--PFHLAW
--PFHLAN
PMPFHL--
--PFHLYR
--PFHLWM
--PFHLWL
--PFHLWK
--PFHLWF
--PFHLPS
--PFHLMI
--PFHLLE
--PFHLIW
--PFHLGY
--PFHLGL
--PFHLFV
--PFHLEQ
--PFHLEL
--PFHLEI
--PFHLAM
--PFHLAK

-PPFHLI-
--PFHLVC
--PFHLRC
--PFHLQF
--PFHLPN
--PFHLNW
--PFHLNN
--PFHLNL
--PFHLNE
--PFHLMC
--PFHLKY
--PFHLKL
--PFHLKH
--PFHLIH
--PFHLHS
--PFHLGW
--PFHLFQ
--PFHLFD
--PFHLEK
--PFHLAH
QRPFHL--
-QPFHLS-
QGPFHL--
-QPFHLT-
QVPFHL--
QPPFHL--
QQPFHL--
-QPFHLI-
-QPFHLR-
QYPFHL--
QNPFHL--
QLPFHL--
QAPFHL--
QKPFHL--
QSPFHL--
-QPFHLW-
-QPFHLQ-
QHPFHL--
-QPFHLY-
-QPFHLE-
-QPFHLA-
QMPFHL--
-QPFHLV-
-QPFHLF-
-QPFHLL-
-QPFHLK-
QTPFHL--
-QPFHLM-
QIPFHL--
-QPFHLD-

RSPFHL--
RGPFHL--
-RPFHLR-
-RPFHLH-
RMPFHL--
RYPFHL--
RDPFHL--
-RPFHLQ-
-RPFHLM-
RKPFHL--
-RPFHLL-
-RPFHLK-
RPPFHL--
RQPFHL--
-RPFHLT-
RLPFHL--
-RPFHLS-
RNPFHL--
RAPFHL--
-RPFHLE-
RFPFHL--
REPFHL--
RWPFHL--
-RPFHLV-
-RPFHLF-
-RPFHLA-
RHPFHL--
RIPFHL--
-RPFHLW-
-RPFHLN-
-RPFHLI-
-RPFHLY-
-RPFHLG-
RCPFHL--
SRPFHL--
SSPFHL--
-SPFHLR-
SGPFHL--
SKPFHL--
SWPFHL--
-SPFHLS-
STPFHL--
-SPFHLT-
SMPFHL--
SYPFHL--
-SPFHLK-
-SPFHLL-
SIPFHL--
SPPFHL--
SAPFHL--

-SPFHLV-
SDPFHL--
-SPFHLI-
SEPFHL--
-SPFHLH-
-SPFHLF-
-SPFHLW-
SHPFHL--
-SPFHLY-
-SPFHLQ-
SQPFHL--
-SPFHLA-
SFPFHL--
-SPFHLG-
SCPFHL--
-SPFHLN-
-TPFHLR-
TRPFHL--
TVPFHL--
TGPFHL--
TWPFHL--
-TPFHLK-
TMPFHL--
TDPFHL--
TLPFHL--
-TPFHLW-
-TPFHLF-
TIPFHL--
TTPFHL--
TSPFHL--
-TPFHLL-
TEPFHL--
-TPFHLS-
-TPFHLE-
TYPFHL--
TKPFHL--
-TPFHLV-
-TPFHLI-
-TPFHLY-
-TPFHLQ-
TNPFHL--
THPFHL--
TPPFHL--
TFPFHL--
-TPFHLM-
TAPFHL--
-TPFHLH-
TQPFHL--
-TPFHLA-
TCPFHL--

VGPFHL--
VYPFHL--
VAPFHL--
-VPFHLY-
VLPFHL--
-VPFHLL-
-VPFHLK-
VEPFHL--
VIPFHL--
VQPFHL--
VSPFHL--
VPPFHL--
VMPFHL--
-VPFHLR-
-VPFHLH-
-VPFHLE-
VTPFHL--
-VPFHLM-
-VPFHLS-
VDPFHL--
VFPFHL--
-VPFHLQ-
-VPFHLA-
-VPFHLI-
-VPFHLF-
VHPFHL--
-VPFHLT-
-VPFHLW-
-VPFHLV-
VCPFHL--
VKPFHL--
-VPFHLG-
WSPFHL--
WGPFHL--
WRPFHL--
WMPFHL--
WAPFHL--
WKPFHL--
WNPFHL--
WHPFHL--
WLPFHL--
WYPFHL--
WVPFHL--
-WPFHLS-
WIPFHL--
-WPFHLV-
-WPFHLA-
-WPFHLI-
-WPFHLH-
WFPFHL--

	WWPFHL--		
	-WPFHLK-		
	-WPFHLT-		
	-WPFHLR-		
	-WPFHLN-		
	-WPFHLM-		
	-WPFHLL-		
	-WPFHLE-		
	YQPFHL--		
	YYPFHL--		
	YRPFHL--		
	YSPFHL--		
	YLPFHL--		
	YVPFHL--		
	YHPFHL--		
	YTPFHL--		
	-YPFHLV-		
	-YPFHLS-		
	YNPFHL--		
	-YPFHLT-		
	YIPFHL--		
	YEPFHL--		
	YDPFHL--		
	-YPFHLK-		
	YFPFHL--		
	YPPFHL--		
	-YPFHLR-		
	-YPFHLM-		
	-YPFHLF-		
	-YPFHLA-		
	YWPFHL--		
	-YPFHLQ-		
	-YPFHLN-		
	-YPFHLE-		
	YAPFHL--		
	YKPFHL--		
	YGPFHL--		
	-YPFHLY-		
	-YPFHLH-		
PRGY	120	795	46.79
	-DPRGYE-		
	GPPRGY--		
	-IPRGYD-		
	--PRGYEI		
	--PRGYCR		
	--PRGYAK		
	-RPRGYP-		
	-YPRGYD-		
	-APRGYS-		
	-APRGYR-		

-APRGYV-
-APRGYI-
-APRGYT-
AVPRGY--
AMPRGY--
ANPRGY--
ASPRGY--
ATPRGY--
-APRGYQ-
-APRGYN-
AAPRGY--
-APRGYW-
-APRGYA-
-APRGYM-
AGPRGY--
-APRGYY-
-APRGYG-
AHPRGY--
-APRGYK-
APPRGY--
AKPRGY--
-APRGYE-
-APRGYF-
-APRGYH-
ADPRGY--
CGPRGY--
CTPRGY--
CCPRGY--
CLPRGY--
CPPRGY--
-CPRGYR-
CAPRGY--
CNPRGY--
CKPRGY--
CQPRGY--
-CPRGYA-
CDPRGY--
-CPRGYY-
-CPRGYN-
-CPRGYL-
CEPRGY--
-CPRGYT-
-CPRGYK-
CYPRGY--
CWPRGY--
-CPRGYW-
-CPRGYQ-
-CPRGYM-
DRPRGY--
DLPRGY--

-DPRGYR-
-DPRGYF-
DMPRGY--
DYPRGY--
-DPRGYT-
DGPRGY--
-DPRGYL-
DSPRGY--
-DPRGYQ-
-DPRGYS-
DNPRGY--
DKPRGY--
-DPRGYM-
-DPRGYA-
-DPRGYK-
-DPRGYI-
DAPRGY--
DHPRGY--
DWPRGY--
-DPRGYW-
DEPRGY--
-DPRGYN-
-DPRGYH-
DTPRGY--
-DPRGYV-
DCPRGY--
ESPRGY--
ELPRGY--
EQPRGY--
-EPRGYI-
ERPRGY--
-EPRGYL-
-EPRGYW-
-EPRGYS-
-EPRGYR-
-EPRGYF-
EFPRGY--
ETPRGY--
EYPRGY--
EAPRGY--
-EPRGYT-
-EPRGYH-
-EPRGYV-
-EPRGYN-
EHPRGY--
EEPRGY--
-EPRGYA-
EGPRGY--
-EPRGYE-
ENPRGY--

EDPRGY--
EKPRGY--
EVPRGY--
-EPRGYY-
EMPRGY--
-EPRGYQ-
-EPRGYK-
-EPRGYG-
FAPRGY--
-FPRGYT-
-FPRGYS-
FSPRGY--
-FPRGYW-
FRPRGY--
FTPRGY--
FGPRGY--
-FPRGYF-
FEPRGY--
FYPRGY--
-FPRGYV-
FIPRGY--
FHPRGY--
-FPRGYQ-
FDPRGY--
FPPRGY--
-FPRGYH-
FMPRGY--
FKPRGY--
-FPRGYE-
-FPRGYN-
FVPRGY--
FCPRGY--
GVPRGY--
GMPRGY--
GGPRGY--
-GPRGYM-
-GPRGYS-
GLPRGY--
GKPRGY--
GEPRGY--
GQPRGY--
-GPRGYF-
GTPRGY--
-GPRGYT-
-GPRGYV-
-GPRGYR-
-GPRGYI-
-GPRGYQ-
GNPRGY--
-GPRGYH-

GIPRGY--
-GPRGYN-
GHPRGY--
GFPRGY--
-GPRGYG-
GRPRGY--
-GPRGYE-
-GPRGYA-
GAPRGY--
GDPRGY--
-GPRGYY-
GWPRGY--
-GPRGYW-
-GPRGYC-
GCPRGY--
-HPRGYA-
-HPRGYH-
HVPRGY--
-HPRGYV-
-HPRGYT-
HAPRGY--
HSPRGY--
-HPRGYL-
-HPRGYI-
HYPRGY--
-HPRGYY-
HPPRGY--
HGPRGY--
HRPRGY--
-HPRGYK-
HLPRGY--
-HPRGYS-
HTPRGY--
-HPRGYM-
HDPRGY--
HEPRGY--
HNPRGY--
-HPRGYG-
-HPRGYQ-
-HPRGYF-
-HPRGYE-
HKPRGY--
HHPRGY--
-HPRGYW-
-HPRGYN-
-IPRGYS-
-IPRGYL-
-IPRGYK-
ISPRGY--
-IPRGYV-

-IPRGYR-
-IPRGYM-
ILPRGY--
IGPRGY--
-IPRGYY-
IYPRGY--
-IPRGYT-
IAPRGY--
ITPRGY--
IWPRGY--
IDPRGY--
IHPRGY--
IQPRGY--
IKPRGY--
IMPRGY--
IVPRGY--
-IPRGYW-
-IPRGYG-
IIPRGY--
IFPRGY--
-IPRGYN-
IPPRGY--
INPRGY--
-IPRGYE-
IEPRGY--
-KPRGYL-
KLPRGY--
KGPRGY--
-KPRGYI-
-KPRGYT-
KVPRGY--
KNPRGY--
KFPRGY--
KYPRGY--
-KPRGYS-
-KPRGYV-
KWPRGY--
KTPRGY--
KAPRGY--
KMPRGY--
KKPRGY--
-KPRGYF-
KPPRGY--
KQPRGY--
-KPRGYG-
-KPRGYW-
-KPRGYH-
KDPRGY--
KHPRGY--
-KPRGYE-

-KPRGYA-
-KPRGYN-
KCPRGY--
KIPRGY--
LSPRGY--
-LPRGYL-
LGPRGY--
LKPRGY--
LVPRGY--
LNPRGY--
LPPRGY--
-LPRGYI-
-LPRGYF-
LAPRGY--
LYPRGY--
-LPRGYQ-
-LPRGYN-
-LPRGYA-
-LPRGYM-
LHPRGY--
-LPRGYY-
-LPRGYW-
LEPRGY--
LDPRGY--
LCPRGY--
-LPRGYE-
MSPRGY--
-MPRGYS-
-MPRGYL-
MGPRGY--
-MPRGYV-
MNPRGY--
MYPRGY--
-MPRGYH-
-MPRGYM-
MPPRGY--
-MPRGYT-
MHPRGY--
MMPRGY--
-MPRGYW-
-MPRGYK-
MAPRGY--
MKPRGY--
-MPRGYI-
MDPRGY--
-MPRGYE-
-MPRGYY-
-MPRGYF-
MCPRGY--
-MPRGYG-

-MPRGYN-
MIPRGY--
-MPRGYQ-
-NPRGYS-
NGPRGY--
NSPRGY--
-NPRGYT-
NVPRGY--
-NPRGYL-
NMPRGY--
-NPRGYA-
NAPRGY--
-NPRGYY-
-NPRGYV-
NYPRGY--
NTPRGY--
NIPRGY--
NNPRGY--
NRPRGY--
NKPRGY--
NCPRGY--
-NPRGYN-
-NPRGYM-
-NPRGYK-
-NPRGYI-
NPPRGY--
-NPRGYQ-
-NPRGYW-
NDPRGY--
-NPRGYC-
NQPRGY--
-NPRGYG-
-NPRGYE-
--PRGYSS
--PRGYSL
--PRGYGS
--PRGYFS
--PRGYLF
--PRGYSI
--PRGYWY
--PRGYRQ
--PRGYRI
--PRGYLW
--PRGYVT
--PRGYSQ
--PRGYVS
--PRGYFT
--PRGYTQ
--PRGYIW
--PRGYTS

--PRGYVV
--PRGYNR
--PRGYST
--PRGYHR
--PRGYTA
--PRGYSV
--PRGYYR
--PRGYMF
--PRGYGG
--PRGYTF
--PRGYFN
--PRGYAS
--PRGYTY
--PRGYTL
--PRGYSM
--PRGYWK
--PRGYWL
--PRGYIA
PGPRGY--
--PRGYVK
--PRGYMT
--PRGYIV
--PRGYWS
PSPRGY--
--PRGYWN
--PRGYES
--PRGYMR
--PRGYHS
--PRGYHI
--PRGYSF
--PRGYRD
--PRGYGT
--PRGYQS
--PRGYMA
--PRGYLK
--PRGYHG
--PRGYYT
--PRGYVH
--PRGYRV
--PRGYGA
--PRGYNL
--PRGYHN
--PRGYFK
--PRGYYS
--PRGYWT
--PRGYRH
--PRGYIL
--PRGYGV
--PRGYVQ
--PRGYRF

--PRGYNQ
--PRGYFY
--PRGYVA
--PRGYLM
--PRGYAG
--PRGYRN
--PRGYKF
--PRGYAY
--PRGYWG
--PRGYVF
--PRGYSK
--PRGYSH
--PRGYKG
--PRGYIT
--PRGYHA
--PRGYFA
--PRGYMI
--PRGYIH
--PRGYIG
--PRGYAA
--PRGYNV
--PRGYFF
--PRGYTH
--PRGYMN
--PRGYIK
--PRGYAT
--PRGYAF
--PRGYWH
--PRGYTN
--PRGYQT
--PRGYKM
--PRGYSY
--PRGYSN
--PRGYQM
--PRGYNA
--PRGYMV
--PRGYMK
--PRGYMD
--PRGYKY
--PRGYAW
--PRGYAV
--PRGYTI
--PRGYQL
--PRGYNN
--PRGYKI
--PRGYII
--PRGYIF
--PRGYHM
--PRGYFR
--PRGYFI

PKPRGY--
PRPRGY--
--PRGYYG
PTPRGY--
--PRGYTD
--PRGYNM
--PRGYGM
--PRGYYW
--PRGYYA
--PRGYMH
--PRGYET
PEPRGY--
--PRGYYM
--PRGYWI
--PRGYSW
--PRGYSD
--PRGYGI
--PRGYEA
--PRGYVI
--PRGYKH
--PRGYTM
--PRGYMG
--PRGYLQ
--PRGYIM
--PRGYAN
--PRGYWW
--PRGYNH
PHPRGY--
--PRGYYQ
--PRGYWM
--PRGYRW
--PRGYNW
--PRGYNT
--PRGYEH
--PRGYEG
--PRGYWD
--PRGYTW
--PRGYQI
--PRGYLE
--PRGYAM
--PRGYYK
--PRGYWE
--PRGYQH
--PRGYKW
--PRGYGW
--PRGYFH
--PRGYAH
PNPRGY--
--PRGYYV
--PRGYWF

--PRGYQN
--PRGYQA
--PRGYME
--PRGYHQ
PVPRGY--
--PRGYWV
--PRGYTE
--PRGYEN
--PRGYRE
--PRGYQQ
--PRGYQG
--PRGYGY
--PRGYEY
--PRGYYY
--PRGYSE
--PRGYQV
--PRGYIQ
--PRGYHT
--PRGYHF
--PRGYDS
--PRGYQK
--PRGYHD
--PRGYFM
--PRGYFG
--PRGYAQ
--PRGYAI
--PRGYYN
--PRGYQW
--PRGYQF
--PRGYQD
--PRGYHW
--PRGYHH
--PRGYHE
--PRGYEW
--PRGYEV
--PRGYAD
--PRGYVE
--PRGYVD
--PRGYNF
--PRGYLD
--PRGYIE
--PRGYGN
--PRGYGF
--PRGYFV
--PRGYYE
--PRGYYD
--PRGYWQ
--PRGYND
--PRGYMQ
PQPRGY--

PDPRGY--
--PRGYQY
--PRGYPL
--PRGYNG
--PRGYMY
--PRGYKE
--PRGYGK
--PRGYEL
--PRGYQE
--PRGYHV
--PRGYFD
--PRGYEM
--PRGYEK
--PRGYDA
--PRGYAE
PCPRGY--
--PRGYKN
--PRGYKD
--PRGYFE
--PRGYEQ
--PRGYCG
-PPRGYS-
--PRGYYF
--PRGYWC
--PRGYVC
--PRGYPQ
--PRGYNP
--PRGYNE
--PRGYMC
--PRGYKC
--PRGYED
--PRGYDY
--PRGYCN
--PRGYCL
--PRGYCK
--PRGYCI
--PRGYCH
--PRGYAC
PPPRGY--
-QPRGYL-
QSPRGY--
QFPRGY--
-QPRGYI-
-QPRGYF-
QTPRGY--
QVPRGY--
-QPRGYN-
QMPRGY--
QLPRGY--
QEPRGY--

-QPRGYS-
-QPRGYT-
QGPRGY--
QKPRGY--
QRPRGY--
-QPRGYW-
-QPRGYQ-
QAPRGY--
QNPRGY--
QDPRGY--
-QPRGYV-
-QPRGYM-
-QPRGYK-
-QPRGYH-
-QPRGYA-
QQPRGY--
QHPRGY--
-QPRGYG-
QIPRGY--
QPPRGY--
-RPRGYL-
-RPRGYS-
-RPRGYI-
RGPRGY--
RAPRGY--
RVPRGY--
-RPRGYM-
RTPRGY--
-RPRGYV-
-RPRGYF-
RNPRGY--
REPRGY--
RHPRGY--
RQPRGY--
-RPRGYN-
RDPRGY--
-RPRGYW-
RPPRGY--
RKPRGY--
-RPRGYY-
-RPRGYE-
RCPRGY--
SLPRGY--
-SPRGYN-
-SPRGYM-
STPRGY--
SYPRGY--
SSPRGY--
-SPRGYY-
-SPRGYR-

-SPRGYV-
-SPRGYH-
-SPRGYW-
-SPRGYF-
SGPRGY--
SIPRGY--
-SPRGYK-
-SPRGYG-
-SPRGYA-
SEPRGY--
SHPRGY--
SDPRGY--
SAPRGY--
SFPRGY--
SQPRGY--
SPPRGY--
SCPRGY--
-SPRGYQ-
-SPRGYE-
-TPRGYS-
TSPRGY--
-TPRGYV-
-TPRGYA-
TQPRGY--
-TPRGYL-
TAPRGY--
-TPRGYT-
TVPRGY--
TNPRGY--
TIPRGY--
-TPRGYY-
TKPRGY--
TWPRGY--
-TPRGYW-
-TPRGYK-
THPRGY--
TPPRGY--
TFPRGY--
TMPRGY--
-TPRGYM-
TTPRGY--
-TPRGYI-
-TPRGYF-
-TPRGYN-
TYPRGY--
-TPRGYH-
-TPRGYE-
-TPRGYG-
TCPRGY--
TEPRGY--

-TPRGYQ-
TDPRGY--
-TPRGYC-
VRPRGY--
VSPRGY--
VFPRGY--
-VPRGYS-
-VPRGYR-
VTPRGY--
VIPRGY--
VQPRGY--
VKPRGY--
-VPRGYH-
-VPRGYN-
VNPRGY--
-VPRGYT-
VVPRGY--
-VPRGYY-
-VPRGYG-
VGPRGY--
VHPRGY--
VAPRGY--
VYPRGY--
VMPRGY--
VEPRGY--
VPPRGY--
-VPRGYW-
VDPRGY--
-VPRGYQ-
-VPRGYI-
-VPRGYF-
-VPRGYE-
VCPRGY--
WRPRGY--
-WPRGYM-
WNPRGY--
WHPRGY--
-WPRGYY-
WTPRGY--
WGPRGY--
WSPRGY--
-WPRGYN-
WKPRGY--
WWPRGY--
-WPRGYW-
WPPRGY--
-WPRGYF-
WEPRGY--
-WPRGYQ-
WAPRGY--

WDPRGY--
WFPRGY--
WMPRGY--
WYPRGY--
-WPRGYE-
WQPRGY--
-WPRGYG-
WCPRGY--
-YPRGYM-
YSPRGY--
-YPRGYH-
-YPRGYV-
YLPRGY--
YGPRGY--
YMPRGY--
YEPRGY--
-YPRGYF-
-YPRGYY-
-YPRGYN-
YDPRGY--
-YPRGYQ-
-YPRGYG-
-YPRGYS-
-YPRGYW-
YKPRGY--
YPPRGY--
-YPRGYA-
YQPRGY--
-YPRGYT-
YNPRGY--
YVPRGY--
-YPRGYE-
YAPRGY--
YYPRGY--
YCPRGY--

PRPL	165	766	46.79

-FPRPLD-
-GPRPLP-
-MPRPLC-
--PRPLNG
--PRPLLP
--PRPLGE
--PRPLDP
--PRPLAP
-APRPLR-
-APRPLS-
AVPRPL--
AWPRPL--
AGPRPL--
ARPRPL--

AHPRPL--
AQPRPL--
AKPRPL--
-APRPLQ-
AAPRPL--
APPRPL--
-APRPLK-
-APRPLI-
-APRPLG-
-APRPLF-
-APRPLY-
-APRPLA-
AYPRPL--
-APRPLH-
-APRPLN-
AFPRPL--
-APRPLE-
-APRPLC-
ACPRPL--
CCPRPL--
CSPRPL--
-CPRPLL-
-CPRPLS-
CMPRPL--
CKPRPL--
CFPRPL--
-CPRPLY-
CPPRPL--
CVPRPL--
-CPRPLV-
CEPRPL--
CYPRPL--
-CPRPLI-
-CPRPLR-
-CPRPLN-
-CPRPLT-
-CPRPLM-
CNPRPL--
-CPRPLW-
-CPRPLQ-
CGPRPL--
CAPRPL--
CTPRPL--
-CPRPLG-
-CPRPLF-
-CPRPLE-
CIPRPL--
-DPRPLR-
DMPRPL--
DSPRPL--

-DPRPLL-
DVPRPL--
-DPRPLA-
DHPRPL--
DAPRPL--
DWPRPL--
DGPRPL--
-DPRPLW-
DEPRPL--
DIPRPL--
DQPRPL--
-DPRPLT-
DFPRPL--
-DPRPLY-
-DPRPLQ-
-DPRPLH-
-DPRPLF-
DKPRPL--
-DPRPLS-
DYPRPL--
-DPRPLV-
-DPRPLK-
-DPRPLE-
DNPRPL--
DDPRPL--
DCPRPL--
-DPRPLM-
-DPRPLG-
ELPRPL--
ESPRPL--
ERPRPL--
-EPRPLR-
-EPRPLQ-
EWPRPL--
EVPRPL--
-EPRPLW-
-EPRPLK-
EPPRPL--
EFPRPL--
-EPRPLL-
-EPRPLM-
EEPRPL--
EAPRPL--
ETPRPL--
-EPRPLI-
-EPRPLF-
EIPRPL--
-EPRPLY-
-EPRPLG-
EYPRPL--

-EPRPLT-
-EPRPLH-
ENPRPL--
-EPRPLA-
EDPRPL--
EQPRPL--
-EPRPLN-
-EPRPLE-
-EPRPLC-
EHPRPL--
EMPRPL--
FRPRPL--
FHPRPL--
FYPRPL--
FAPRPL--
-FPRPLW-
-FPRPLS-
FLPRPL--
-FPRPLF-
FGPRPL--
-FPRPLH-
FMPRPL--
-FPRPLN-
FFPRPL--
FDPRPL--
FQPRPL--
-FPRPLL-
FWPRPL--
-FPRPLE-
-FPRPLG-
FCPRPL--
-GPRPLL-
GTPRPL--
GRPRPL--
-GPRPLY-
GYPRPL--
GWPRPL--
-GPRPLI-
GIPRPL--
GFPRPL--
-GPRPLV-
-GPRPLT-
GEPRPL--
-GPRPLF-
-GPRPLW-
-GPRPLQ-
GMPRPL--
GDPRPL--
GQPRPL--
GHPRPL--

-GPRPLH-
-GPRPLM-
-GPRPLN-
-GPRPLG-
-GPRPLE-
GPPRPL--
-GPRPLD-
-HPRPLR-
HIPRPL--
HVPRPL--
HSPRPL--
-HPRPLH-
-HPRPLY-
-HPRPLL-
HMPRPL--
-HPRPLS-
HRPRPL--
-HPRPLK-
-HPRPLI-
HWPRPL--
-HPRPLN-
HFPRPL--
HEPRPL--
HGPRPL--
HYPRPL--
-HPRPLW-
HQPRPL--
-HPRPLV-
-HPRPLQ-
-HPRPLG-
HDPRPL--
-HPRPLM-
HPPRPL--
HAPRPL--
HHPRPL--
-HPRPLF-
-HPRPLP-
-HPRPLC-
INPRPL--
ILPRPL--
IRPRPL--
IGPRPL--
-IPRPLH-
IVPRPL--
-IPRPLS-
IYPRPL--
-IPRPLY-
ITPRPL--
-IPRPLA-
IEPRPL--

IAPRPL--
-IPRPLV-
IMPRPL--
-IPRPLG-
-IPRPLI-
IQPRPL--
IIPRPL--
-IPRPLW-
-IPRPLT-
-IPRPLM-
-IPRPLF-
-IPRPLN-
IPPRPL--
IFPRPL--
-IPRPLQ-
ICPRPL--
-IPRPLE-
-IPRPLC-
KGPRPL--
-KPRPLF-
-KPRPLR-
KYPRPL--
-KPRPLY-
KIPRPL--
KFPRPL--
-KPRPLS-
KEPRPL--
-KPRPLM-
-KPRPLI-
-KPRPLH-
-KPRPLA-
-KPRPLT-
-KPRPLV-
-KPRPLK-
KHPRPL--
-KPRPLN-
-KPRPLG-
KNPRPL--
-KPRPLW-
KCPRPL--
KAPRPL--
KDPRPL--
-KPRPLE-
LGPRPL--
LAPRPL--
LTPRPL--
-LPRPLI-
LIPRPL--
LNPRPL--
LDPRPL--

LHPRPL--
LFPRPL--
LQPRPL--
-LPRPLK-
-LPRPLW-
LKPRPL--
-LPRPLH-
-LPRPLM-
-LPRPLF-
-LPRPLG-
-LPRPLE-
-LPRPLP-
LCPRPL--
MTPRPL--
MRPRPL--
-MPRPLS-
MIPRPL--
MGPRPL--
MAPRPL--
-MPRPLK-
-MPRPLQ-
-MPRPLW-
-MPRPLF-
MVPRPL--
MQPRPL--
-MPRPLI-
MLPRPL--
MHPRPL--
MDPRPL--
-MPRPLV-
-MPRPLN-
MWPRPL--
-MPRPLM-
MNPRPL--
-MPRPLG-
MPPRPL--
MCPRPL--
MEPRPL--
-MPRPLY-
-MPRPLE-
NSPRPL--
-NPRPLL-
NHPRPL--
NRPRPL--
NVPRPL--
NLPRPL--
NTPRPL--
NMPRPL--
-NPRPLY-
NWPRPL--

```
-NPRPLW-
-NPRPLQ-
-NPRPLS-
-NPRPLM-
NIPRPL--
-NPRPLH-
-NPRPLI-
-NPRPLT-
NNPRPL--
-NPRPLN-
NAPRPL--
NKPRPL--
NGPRPL--
-NPRPLA-
-NPRPLK-
NEPRPL--
-NPRPLE-
NDPRPL--
-NPRPLD-
--PRPLVL
--PRPLYL
--PRPLAL
--PRPLWT
--PRPLIS
--PRPLFS
--PRPLFI
--PRPLYS
--PRPLFL
--PRPLSK
--PRPLVR
--PRPLLN
--PRPLKL
--PRPLFR
--PRPLVT
--PRPLSR
--PRPLRG
--PRPLTV
--PRPLFG
--PRPLAT
--PRPLLK
--PRPLTA
--PRPLII
--PRPLAW
--PRPLMS
--PRPLVH
--PRPLTI
--PRPLQL
--PRPLVA
--PRPLRH
--PRPLNF
```

--PRPLWL
--PRPLIT
--PRPLEL
--PRPLYG
--PRPLYA
--PRPLWA
--PRPLKT
--PRPLGN
--PRPLYK
--PRPLWS
--PRPLSN
--PRPLHY
--PRPLWV
--PRPLSV
--PRPLNS
--PRPLKH
--PRPLVK
--PRPLSA
--PRPLMG
--PRPLFQ
--PRPLFM
PRPRPL--
--PRPLYV
--PRPLLI
--PRPLVY
--PRPLVQ
--PRPLQN
--PRPLAF
--PRPLNN
--PRPLQS
--PRPLAG
--PRPLFY
--PRPLAN
--PRPLQT
--PRPLNA
--PRPLLQ
--PRPLTN
--PRPLSH
--PRPLVG
--PRPLRQ
--PRPLIV
--PRPLHT
--PRPLYH
--PRPLNT
--PRPLNH
--PRPLRM
--PRPLLD
--PRPLVD
--PRPLTG
--PRPLSG

--PRPLMV
--PRPLGH
--PRPLWG
--PRPLEV
--PRPLRE
--PRPLYY
--PRPLVE
--PRPLIG
--PRPLKW
--PRPLYI
--PRPLYF
--PRPLMY
--PRPLHA
--PRPLFK
--PRPLFH
--PRPLIM
--PRPLHH
PIPRPL--
--PRPLTK
--PRPLLH
--PRPLVW
--PRPLRY
--PRPLMK
--PRPLKY
--PRPLHS
--PRPLQV
--PRPLVM
--PRPLID
--PRPLEW
--PRPLYM
--PRPLYD
--PRPLRF
--PRPLKF
--PRPLHG
--PRPLGG
--PRPLWI
--PRPLTT
--PRPLTH
--PRPLSW
--PRPLLE
--PRPLKR
--PRPLIY
--PRPLHV
--PRPLMF
--PRPLKM
--PRPLAK
--PRPLWN
--PRPLQY
--PRPLQA
--PRPLIN

--PRPLGK
--PRPLDI
--PRPLAA
--PRPLWD
--PRPLMM
--PRPLHQ
--PRPLGA
--PRPLEM
--PRPLTQ
--PRPLMW
PGPRPL--
--PRPLWK
--PRPLNR
--PRPLNQ
--PRPLMN
--PRPLKI
--PRPLIE
--PRPLDF
--PRPLYE
--PRPLWQ
--PRPLQW
--PRPLPA
--PRPLKN
--PRPLHN
--PRPLFD
--PRPLEY
--PRPLET
PNPRPL--
PWPRPL--
--PRPLWM
--PRPLTF
--PRPLQK
--PRPLFN
--PRPLAH
--PRPLPW
--PRPLHF
--PRPLDS
--PRPLQG
--PRPLPH
--PRPLFW
--PRPLES
--PRPLEH
--PRPLDY
--PRPLQF
--PRPLPV
--PRPLPQ
--PRPLME
--PRPLMD
--PRPLIQ
--PRPLKA

--PRPLEQ
--PRPLSE
--PRPLEN
--PRPLEI
--PRPLEG
PEPRPL--
PDPRPL--
--PRPLRW
PMPRPL--
--PRPLYN
--PRPLTD
--PRPLDM
--PRPLSD
--PRPLKD
--PRPLWE
--PRPLTM
--PRPLDW
--PRPLWW
--PRPLRD
--PRPLPN
--PRPLTE
--PRPLQQ
--PRPLCL
PKPRPL--
--PRPLPT
--PRPLMQ
--PRPLIK
--PRPLHK
--PRPLER
--PRPLCT
--PRPLYP
--PRPLWY
--PRPLQI
--PRPLEK
--PRPLAE
--PRPLQE
--PRPLKK
--PRPLHD
--PRPLDA
--PRPLCS
--PRPLCG
--PRPLVC
--PRPLRC
--PRPLKE
--PRPLFP
--PRPLEA
--PRPLDH
--PRPLDG
--PRPLCY
PHPRPL--

--PRPLQH
--PRPLNE
--PRPLND
--PRPLMC
--PRPLCH
--PRPLCF
--PRPLWP
--PRPLWF
--PRPLQD
--PRPLQC
--PRPLKQ
--PRPLIC
--PRPLEP
--PRPLED
--PRPLDV
--PRPLDT
--PRPLDQ
--PRPLDN
--PRPLCQ
--PRPLCM
--PRPLCI
--PRPLCE
PQPRPL--
-PPRPLY-
-PPRPLN-
-PPRPLL-
-QPRPLT-
-QPRPLL-
QFPRPL--
-QPRPLR-
QGPRPL--
QSPRPL--
-QPRPLY-
-QPRPLI-
QWPRPL--
-QPRPLF-
-QPRPLN-
QRPRPL--
-QPRPLA-
-QPRPLQ-
QAPRPL--
QNPRPL--
QIPRPL--
-QPRPLW-
QDPRPL--
QCPRPL--
QYPRPL--
QMPRPL--
-QPRPLK-
-QPRPLH-

−QPRPLG−
QEPRPL−−
QHPRPL−−
−RPRPLT−
−RPRPLR−
RAPRPL−−
RVPRPL−−
RYPRPL−−
RHPRPL−−
−RPRPLS−
RGPRPL−−
REPRPL−−
RQPRPL−−
−RPRPLV−
−RPRPLN−
RNPRPL−−
RDPRPL−−
−RPRPLI−
−RPRPLH−
−RPRPLM−
−RPRPLK−
−RPRPLW−
−RPRPLG−
RCPRPL−−
−RPRPLE−
−RPRPLC−
−RPRPLD−
SAPRPL−−
−SPRPLK−
SNPRPL−−
−SPRPLW−
STPRPL−−
SMPRPL−−
SRPRPL−−
SIPRPL−−
SHPRPL−−
SKPRPL−−
−SPRPLT−
−SPRPLA−
SQPRPL−−
−SPRPLQ−
−SPRPLN−
SEPRPL−−
−SPRPLH−
SDPRPL−−
−SPRPLG−
−SPRPLM−
−SPRPLE−
SCPRPL−−
−TPRPLL−

−TPRPLR−
−TPRPLW−
TFPRPL−−
TGPRPL−−
TIPRPL−−
−TPRPLN−
TPPRPL−−
−TPRPLT−
TQPRPL−−
−TPRPLS−
TEPRPL−−
−TPRPLY−
TYPRPL−−
−TPRPLK−
−TPRPLV−
TMPRPL−−
−TPRPLI−
−TPRPLH−
−TPRPLA−
−TPRPLF−
TAPRPL−−
−TPRPLM−
−TPRPLG−
TCPRPL−−
−TPRPLE−
TDPRPL−−
−TPRPLD−
−VPRPLL−
VRPRPL−−
−VPRPLV−
VFPRPL−−
−VPRPLG−
−VPRPLK−
VYPRPL−−
−VPRPLA−
VMPRPL−−
−VPRPLW−
VAPRPL−−
VHPRPL−−
VQPRPL−−
−VPRPLT−
−VPRPLQ−
−VPRPLH−
VEPRPL−−
VDPRPL−−
−VPRPLE−
VCPRPL−−
VGPRPL−−
−VPRPLD−
−WPRPLL−

WKPRPL--
WGPRPL--
-WPRPLV-
-WPRPLS-
WIPRPL--
WVPRPL--
WQPRPL--
WDPRPL--
-WPRPLK-
WMPRPL--
WLPRPL--
-WPRPLT-
-WPRPLY-
WHPRPL--
WAPRPL--
-WPRPLQ-
WTPRPL--
WWPRPL--
-WPRPLH-
-WPRPLA-
WEPRPL--
-WPRPLM-
-WPRPLF-
-WPRPLG-
-WPRPLE-
-WPRPLI-
WPPRPL--
WCPRPL--
-WPRPLW-
-WPRPLD-
-WPRPLC-
YTPRPL--
YLPRPL--
-YPRPLL-
-YPRPLI-
YIPRPL--
YAPRPL--
-YPRPLA-
YHPRPL--
YFPRPL--
-YPRPLY-
YNPRPL--
-YPRPLQ-
-YPRPLF-
YMPRPL--
-YPRPLN-
-YPRPLM-
-YPRPLG-
-YPRPLE-
-YPRPLH-

	YEPRPL--		
	YCPRPL--		
PLHL	61	842	46.77
	ACPLHL--		
	-HPLHLD-		
	-KPLHLD-		
	--PLHLRP		
	--PLHLPP		
	--PLHLPL		
	--PLHLLP		
	--PLHLGD		
	--PLHLDW		
	--PLHLAP		
	-RPLHLP-		
	ARPLHL--		
	-APLHLR-		
	ALPLHL--		
	ATPLHL--		
	AGPLHL--		
	AVPLHL--		
	-APLHLT-		
	-APLHLQ-		
	-APLHLK-		
	-APLHLL-		
	-APLHLV-		
	AKPLHL--		
	AYPLHL--		
	-APLHLS-		
	ANPLHL--		
	-APLHLM-		
	AWPLHL--		
	-APLHLH-		
	ADPLHL--		
	AQPLHL--		
	-APLHLW-		
	-APLHLN-		
	AHPLHL--		
	AEPLHL--		
	AAPLHL--		
	-APLHLI-		
	ASPLHL--		
	APPLHL--		
	-APLHLY-		
	-APLHLE-		
	-APLHLF-		
	-APLHLD-		
	-APLHLC-		
	CLPLHL--		
	CAPLHL--		
	CCPLHL--		

CRPLHL--
CHPLHL--
CGPLHL--
CPPLHL--
CNPLHL--
CIPLHL--
CSPLHL--
CMPLHL--
CEPLHL--
CVPLHL--
-CPLHLW-
-CPLHLR-
-CPLHLK-
-CPLHLE-
CYPLHL--
CTPLHL--
-CPLHLY-
-CPLHLV-
-CPLHLM-
-CPLHLI-
CDPLHL--
DRPLHL--
DWPLHL--
DVPLHL--
-DPLHLR-
DSPLHL--
DHPLHL--
DGPLHL--
DNPLHL--
DYPLHL--
DIPLHL--
-DPLHLS-
-DPLHLQ-
DTPLHL--
-DPLHLM-
-DPLHLK-
DLPLHL--
-DPLHLY-
-DPLHLL-
DMPLHL--
-DPLHLF-
DQPLHL--
-DPLHLV-
DAPLHL--
-DPLHLE-
-DPLHLW-
DKPLHL--
DFPLHL--
DEPLHL--
-DPLHLT-

–DPLHLI–
DDPLHL––
ESPLHL––
–EPLHLS–
EYPLHL––
EGPLHL––
–EPLHLY–
–EPLHLR–
EVPLHL––
–EPLHLV–
ELPLHL––
EIPLHL––
ERPLHL––
–EPLHLW–
EWPLHL––
ETPLHL––
EFPLHL––
EMPLHL––
–EPLHLF–
–EPLHLM–
ENPLHL––
EKPLHL––
–EPLHLN–
–EPLHLK–
EEPLHL––
–EPLHLQ–
–EPLHLT–
–EPLHLA–
–EPLHLI–
EHPLHL––
EDPLHL––
EQPLHL––
–EPLHLE–
–EPLHLC–
ECPLHL––
–FPLHLS–
FLPLHL––
–FPLHLL–
FEPLHL––
FIPLHL––
FGPLHL––
FRPLHL––
FKPLHL––
–FPLHLR–
FTPLHL––
–FPLHLN–
–FPLHLY–
–FPLHLW–
FQPLHL––
–FPLHLH–

FSPLHL--
-FPLHLI-
FAPLHL--
FDPLHL--
-FPLHLG-
FPPLHL--
FHPLHL--
-FPLHLV-
FMPLHL--
-FPLHLM-
FYPLHL--
FWPLHL--
-FPLHLF-
-FPLHLQ-
FNPLHL--
GRPLHL--
-GPLHLL-
-GPLHLR-
GFPLHL--
GSPLHL--
GAPLHL--
-GPLHLT-
GLPLHL--
GTPLHL--
GKPLHL--
GNPLHL--
-GPLHLH-
GYPLHL--
-GPLHLA-
GPPLHL--
GMPLHL--
GQPLHL--
-GPLHLQ-
-GPLHLV-
-GPLHLY-
-GPLHLM-
-GPLHLE-
GDPLHL--
-GPLHLN-
GWPLHL--
-GPLHLG-
-GPLHLW-
GHPLHL--
GCPLHL--
-GPLHLF-
-HPLHLR-
HWPLHL--
HLPLHL--
-HPLHLV-
HGPLHL--

HSPLHL--
HPPLHL--
HVPLHL--
HAPLHL--
HTPLHL--
HKPLHL--
-HPLHLM-
-HPLHLT-
-HPLHLY-
HFPLHL--
-HPLHLW-
-HPLHLH-
-HPLHLL-
-HPLHLS-
-HPLHLG-
-HPLHLI-
-HPLHLF-
-HPLHLA-
HIPLHL--
HHPLHL--
-HPLHLN-
HNPLHL--
-HPLHLE-
HMPLHL--
-HPLHLQ-
HCPLHL--
-IPLHLM-
IRPLHL--
ITPLHL--
IKPLHL--
IGPLHL--
ISPLHL--
IVPLHL--
IHPLHL--
-IPLHLH-
IMPLHL--
-IPLHLS-
IYPLHL--
ILPLHL--
-IPLHLR-
IEPLHL--
-IPLHLK-
-IPLHLY-
-IPLHLL-
-IPLHLV-
-IPLHLQ-
IIPLHL--
IFPLHL--
IQPLHL--
IDPLHL--

−IPLHLG−
−IPLHLF−
INPLHL−−
−IPLHLA−
IAPLHL−−
−IPLHLE−
IWPLHL−−
ICPLHL−−
−IPLHLW−
−IPLHLC−
KRPLHL−−
−KPLHLR−
−KPLHLL−
KAPLHL−−
−KPLHLV−
KIPLHL−−
KLPLHL−−
KYPLHL−−
KNPLHL−−
−KPLHLY−
KGPLHL−−
−KPLHLM−
KWPLHL−−
−KPLHLS−
−KPLHLH−
KPPLHL−−
−KPLHLQ−
−KPLHLK−
−KPLHLN−
−KPLHLA−
−KPLHLT−
−KPLHLE−
−KPLHLI−
KMPLHL−−
−KPLHLG−
−KPLHLF−
KFPLHL−−
KQPLHL−−
KEPLHL−−
KHPLHL−−
KDPLHL−−
−KPLHLC−
KCPLHL−−
LSPLHL−−
LGPLHL−−
LVPLHL−−
LYPLHL−−
−LPLHLL−
−LPLHLR−
−LPLHLT−

LPPLHL--
LLPLHL--
LNPLHL--
-LPLHLV-
-LPLHLI-
LQPLHL--
LFPLHL--
LEPLHL--
LDPLHL--
-LPLHLS-
-LPLHLH-
LIPLHL--
-LPLHLK-
-LPLHLA-
-LPLHLN-
-LPLHLQ-
-LPLHLM-
-LPLHLF-
-LPLHLW-
-LPLHLG-
LCPLHL--
MTPLHL--
-MPLHLH-
-MPLHLS-
MAPLHL--
-MPLHLQ-
MSPLHL--
MWPLHL--
MGPLHL--
MMPLHL--
MIPLHL--
-MPLHLY-
-MPLHLL-
-MPLHLI-
MQPLHL--
-MPLHLT-
-MPLHLA-
MNPLHL--
MPPLHL--
MHPLHL--
-MPLHLW-
-MPLHLN-
MEPLHL--
MDPLHL--
-MPLHLM-
-MPLHLG-
-MPLHLE-
MYPLHL--
-MPLHLF-
-MPLHLK-

NNPLHL--
NVPLHL--
NSPLHL--
NHPLHL--
NRPLHL--
NQPLHL--
NGPLHL--
NTPLHL--
-NPLHLI-
-NPLHLT-
NIPLHL--
NWPLHL--
NAPLHL--
-NPLHLR-
-NPLHLM-
NKPLHL--
-NPLHLS-
-NPLHLW-
NMPLHL--
NYPLHL--
-NPLHLQ-
NDPLHL--
-NPLHLK-
NEPLHL--
NPPLHL--
-NPLHLH-
-NPLHLF-
-NPLHLE-
NCPLHL--
-NPLHLV-
-NPLHLN-
-NPLHLA-
--PLHLMS
--PLHLVR
--PLHLTR
--PLHLRN
--PLHLKA
PRPLHL--
--PLHLYR
--PLHLRS
--PLHLRH
--PLHLVT
--PLHLNS
--PLHLTG
--PLHLRT
--PLHLSI
--PLHLAS
--PLHLTM
--PLHLSV
--PLHLQR

--PLHLLG
--PLHLTT
--PLHLLS
--PLHLFS
--PLHLST
--PLHLSK
--PLHLSG
--PLHLYD
--PLHLTS
--PLHLTL
--PLHLGS
--PLHLYG
--PLHLVW
--PLHLMN
--PLHLIV
--PLHLIT
--PLHLWS
--PLHLRD
--PLHLWG
--PLHLIY
--PLHLGG
--PLHLGA
--PLHLER
--PLHLSQ
--PLHLSM
--PLHLRM
--PLHLRE
--PLHLLA
--PLHLYY
--PLHLYK
--PLHLKH
--PLHLIS
--PLHLYA
PIPLHL--
--PLHLYW
--PLHLHK
--PLHLAR
--PLHLSN
--PLHLHS
--PLHLET
--PLHLEL
PKPLHL--
--PLHLKV
--PLHLHG
--PLHLFK
--PLHLSH
--PLHLMT
--PLHLLH
--PLHLAH
--PLHLAA

--PLHLYN
--PLHLVY
--PLHLMV
--PLHLKT
--PLHLIM
--PLHLES
--PLHLVH
--PLHLVG
--PLHLMR
--PLHLKS
--PLHLKR
--PLHLWT
--PLHLQG
--PLHLGT
--PLHLAV
--PLHLAT
PTPLHL--
--PLHLLM
--PLHLLL
--PLHLQF
--PLHLQA
--PLHLLI
--PLHLTV
--PLHLQL
--PLHLML
--PLHLMA
--PLHLIQ
--PLHLAN
--PLHLQM
--PLHLFN
--PLHLYH
--PLHLVD
--PLHLLN
--PLHLAW
PWPLHL--
--PLHLWH
--PLHLMI
--PLHLKI
--PLHLHV
--PLHLYV
--PLHLWA
--PLHLVQ
--PLHLVK
--PLHLMD
--PLHLKN
--PLHLKG
--PLHLIW
--PLHLHR
--PLHLGV
--PLHLFR

```
--PLHLAG
--PLHLLE
--PLHLAQ
--PLHLYQ
--PLHLYI
--PLHLVI
--PLHLTH
--PLHLSD
--PLHLMG
--PLHLIR
--PLHLIH
--PLHLYT
--PLHLYF
--PLHLWN
--PLHLSA
--PLHLQE
--PLHLNY
--PLHLTN
--PLHLQV
--PLHLNV
--PLHLHQ
--PLHLHL
PDPLHL--
--PLHLQT
--PLHLNH
--PLHLMK
--PLHLKK
--PLHLIA
--PLHLHT
--PLHLFD
--PLHLAI
--PLHLWQ
--PLHLTF
--PLHLRG
--PLHLMY
--PLHLMQ
--PLHLKQ
--PLHLHN
--PLHLGM
--PLHLFW
--PLHLEY
--PLHLEK
--PLHLWV
--PLHLQI
--PLHLMM
--PLHLKY
--PLHLKW
--PLHLFL
--PLHLEQ
--PLHLVF
```

--PLHLSE
--PLHLNW
--PLHLNR
--PLHLNN
--PLHLHD
--PLHLGW
--PLHLEV
--PLHLWI
--PLHLWD
--PLHLVV
--PLHLVM
--PLHLVE
--PLHLTI
--PLHLQY
--PLHLIE
--PLHLHA
--PLHLGI
--PLHLEH
--PLHLAE
--PLHLAD
--PLHLTY
--PLHLQH
--PLHLNM
--PLHLNK
--PLHLKD
--PLHLFH
--PLHLED
--PLHLTD
--PLHLNF
--PLHLIK
--PLHLHY
--PLHLEG
--PLHLDG
--PLHLQW
--PLHLNQ
--PLHLKE
--PLHLGQ
--PLHLGH
--PLHLDA
--PLHLWE
--PLHLTW
--PLHLNG
--PLHLKF
--PLHLHM
--PLHLGN
--PLHLEM
--PLHLQD
--PLHLMW
--PLHLME
--PLHLII

--PLHLHH
--PLHLGK
--PLHLEA
--PLHLDS
--PLHLAK
PNPLHL--
--PLHLYE
--PLHLQN
--PLHLNI
--PLHLIN
--PLHLID
--PLHLHF
--PLHLGL
--PLHLFQ
--PLHLAF
PEPLHL--
PCPLHL--
-PPLHLN-
--PLHLTE
--PLHLGY
--PLHLFE
--PLHLEW
--PLHLEN
--PLHLEE
--PLHLCG
--PLHLWW
--PLHLSC
--PLHLQK
--PLHLMF
--PLHLKM
--PLHLHW
--PLHLHI
--PLHLDT
--PLHLCM
--PLHLCF
-PPLHLS-
--PLHLPE
--PLHLNE
--PLHLMP
--PLHLLC
--PLHLGE
--PLHLCY
--PLHLCV
--PLHLCR
--PLHLCA
QRPLHL--
-QPLHLT-
-QPLHLL-
-QPLHLR-
-QPLHLS-

QMPLHL--
QSPLHL--
QLPLHL--
QTPLHL--
QVPLHL--
-QPLHLV-
QAPLHL--
QPPLHL--
QIPLHL--
-QPLHLQ-
-QPLHLM-
QWPLHL--
-QPLHLF-
-QPLHLE-
QKPLHL--
QYPLHL--
QNPLHL--
QFPLHL--
-QPLHLN-
-QPLHLK-
-QPLHLG-
-QPLHLW-
QEPLHL--
QDPLHL--
QQPLHL--
QHPLHL--
RPPLHL--
-RPLHLT-
-RPLHLS-
-RPLHLV-
RAPLHL--
-RPLHLL-
RNPLHL--
RIPLHL--
-RPLHLK-
RMPLHL--
RQPLHL--
RHPLHL--
RWPLHL--
-RPLHLA-
-RPLHLM-
RYPLHL--
-RPLHLH-
RDPLHL--
-RPLHLE-
-RPLHLY-
-RPLHLQ-
RCPLHL--
SGPLHL--
-SPLHLR-

SSPLHL--
-SPLHLT-
SPPLHL--
-SPLHLW-
SAPLHL--
SVPLHL--
SQPLHL--
SLPLHL--
SYPLHL--
-SPLHLA-
-SPLHLK-
-SPLHLV-
SNPLHL--
SHPLHL--
-SPLHLH-
SFPLHL--
SWPLHL--
-SPLHLE-
-SPLHLF-
SEPLHL--
-SPLHLN-
-SPLHLM-
SCPLHL--
-SPLHLG-
SDPLHL--
-TPLHLS-
TSPLHL--
TGPLHL--
TIPLHL--
-TPLHLQ-
-TPLHLR-
TLPLHL--
-TPLHLN-
TPPLHL--
TTPLHL--
-TPLHLA-
-TPLHLY-
TMPLHL--
-TPLHLT-
TVPLHL--
-TPLHLL-
TWPLHL--
TAPLHL--
-TPLHLK-
TKPLHL--
-TPLHLI-
TNPLHL--
-TPLHLM-
TYPLHL--
TQPLHL--

TDPLHL--
-TPLHLG-
TEPLHL--
-TPLHLH-
TFPLHL--
-TPLHLW-
TCPLHL--
VSPLHL--
VAPLHL--
VRPLHL--
-VPLHLY-
VVPLHL--
VYPLHL--
-VPLHLS-
VIPLHL--
VTPLHL--
-VPLHLL-
-VPLHLA-
VGPLHL--
VFPLHL--
-VPLHLV-
-VPLHLK-
-VPLHLF-
-VPLHLI-
-VPLHLW-
-VPLHLM-
-VPLHLQ-
VWPLHL--
-VPLHLH-
VQPLHL--
VHPLHL--
VPPLHL--
VEPLHL--
VDPLHL--
-VPLHLE-
-VPLHLD-
VCPLHL--
WGPLHL--
WFPLHL--
WSPLHL--
-WPLHLA-
WLPLHL--
WKPLHL--
WDPLHL--
WRPLHL--
WHPLHL--
-WPLHLG-
WVPLHL--
WIPLHL--
-WPLHLT-

	-WPLHLR-		
	WNPLHL--		
	WQPLHL--		
	WPPLHL--		
	-WPLHLK-		
	-WPLHLS-		
	-WPLHLE-		
	WWPLHL--		
	-WPLHLW-		
	-WPLHLV-		
	-WPLHLI-		
	WYPLHL--		
	WEPLHL--		
	-WPLHLQ-		
	-WPLHLF-		
	-WPLHLM-		
	YGPLHL--		
	-YPLHLR-		
	YTPLHL--		
	-YPLHLT-		
	YNPLHL--		
	YYPLHL--		
	-YPLHLS-		
	YMPLHL--		
	-YPLHLV-		
	-YPLHLM-		
	-YPLHLY-		
	YHPLHL--		
	YEPLHL--		
	YWPLHL--		
	-YPLHLI-		
	-YPLHLH-		
	YPPLHL--		
	-YPLHLA-		
	YIPLHL--		
	YAPLHL--		
	-YPLHLK-		
	YQPLHL--		
	-YPLHLL-		
	-YPLHLE-		
	-YPLHLN-		
	-YPLHLG-		
	YKPLHL--		
	YDPLHL--		
	YCPLHL--		
PFSL	135	788	46.74
	-NPFSLP-		
	--PFSLPI		
	--PFSLPF		
	--PFSLGR		

--PFSLGQ
--PFSLGK
-SPFSLD-
-TPFSLE-
-WPFSLG-
-APFSLL-
-APFSLK-
-APFSLS-
AVPFSL--
AMPFSL--
AGPFSL--
ASPFSL--
ALPFSL--
ANPFSL--
AQPFSL--
ATPFSL--
-APFSLI-
-APFSLT-
APPFSL--
-APFSLH-
-APFSLG-
AHPFSL--
-APFSLV-
AAPFSL--
AEPFSL--
AKPFSL--
AWPFSL--
ADPFSL--
-APFSLN-
-APFSLQ-
AYPFSL--
-APFSLW-
-APFSLM-
AFPFSL--
-APFSLA-
ACPFSL--
-APFSLE-
CGPFSL--
CKPFSL--
CSPFSL--
CPPFSL--
-CPFSLR-
CLPFSL--
CIPFSL--
CCPFSL--
CAPFSL--
CQPFSL--
CYPFSL--
-CPFSLL-
CMPFSL--

CNPFSL--
CVPFSL--
CRPFSL--
-CPFSLK-
-CPFSLH-
-CPFSLY-
-CPFSLV-
-CPFSLT-
-CPFSLQ-
-CPFSLM-
CFPFSL--
CEPFSL--
CDPFSL--
-DPFSLR-
DVPFSL--
DRPFSL--
-DPFSLI-
DLPFSL--
DYPFSL--
DEPFSL--
DSPFSL--
DMPFSL--
DTPFSL--
DQPFSL--
DKPFSL--
DWPFSL--
-DPFSLT-
-DPFSLF-
DFPFSL--
-DPFSLV-
-DPFSLY-
-DPFSLK-
DGPFSL--
DNPFSL--
-DPFSLQ-
-DPFSLM-
-DPFSLH-
DDPFSL--
DCPFSL--
DAPFSL--
EVPFSL--
-EPFSLR-
-EPFSLS-
ERPFSL--
ESPFSL--
-EPFSLV-
-EPFSLL-
EMPFSL--
EKPFSL--
ETPFSL--

−EPFSLK−
−EPFSLI−
EAPFSL−−
−EPFSLT−
−EPFSLF−
−EPFSLH−
EYPFSL−−
EQPFSL−−
ELPFSL−−
EWPFSL−−
−EPFSLA−
EIPFSL−−
EGPFSL−−
EHPFSL−−
−EPFSLW−
ENPFSL−−
EPPFSL−−
−EPFSLQ−
−EPFSLN−
EFPFSL−−
EEPFSL−−
−EPFSLE−
ECPFSL−−
−FPFSLS−
−FPFSLK−
−FPFSLH−
−FPFSLR−
FNPFSL−−
FPPFSL−−
FQPFSL−−
−FPFSLI−
−FPFSLM−
FKPFSL−−
−FPFSLV−
FEPFSL−−
FWPFSL−−
FFPFSL−−
FHPFSL−−
FMPFSL−−
FIPFSL−−
FDPFSL−−
−FPFSLN−
−FPFSLW−
−FPFSLQ−
−FPFSLA−
FYPFSL−−
−FPFSLE−
−FPFSLF−
−GPFSLR−
GVPFSL−−

GTPFSL--
GRPFSL--
GGPFSL--
GKPFSL--
GAPFSL--
GLPFSL--
-GPFSLV-
-GPFSLY-
-GPFSLT-
GEPFSL--
GYPFSL--
-GPFSLQ-
-GPFSLL-
-GPFSLK-
GIPFSL--
GQPFSL--
-GPFSLM-
-GPFSLN-
-GPFSLI-
GWPFSL--
-GPFSLW-
-GPFSLG-
-GPFSLF-
GDPFSL--
-GPFSLH-
GPPFSL--
-GPFSLA-
GHPFSL--
-GPFSLC-
GCPFSL--
-GPFSLE-
HRPFSL--
HSPFSL--
-HPFSLL-
-HPFSLR-
HGPFSL--
HAPFSL--
HVPFSL--
HTPFSL--
HLPFSL--
HHPFSL--
HQPFSL--
-HPFSLK-
HEPFSL--
-HPFSLV-
-HPFSLT-
-HPFSLN-
-HPFSLS-
-HPFSLH-
-HPFSLY-

HFPPFSL--
-HPFSLF-
HWPFSL--
HNPFSL--
HDPFSL--
HYPFSL--
HIPFSL--
HKPFSL--
-HPFSLW-
HMPFSL--
-HPFSLM-
-HPFSLA-
HPPFSL--
-HPFSLE-
HCPFSL--
-HPFSLQ-
-HPFSLC-
IGPFSL--
ILPFSL--
IVPFSL--
ITPFSL--
-IPFSLR-
ISPFSL--
-IPFSLH-
-IPFSLK-
-IPFSLV-
IKPFSL--
IAPFSL--
IFPFSL--
-IPFSLS-
-IPFSLA-
IPPFSL--
-IPFSLN-
IDPFSL--
IIPFSL--
IWPFSL--
-IPFSLG-
IMPFSL--
-IPFSLI-
-IPFSLW-
-IPFSLE-
IYPFSL--
IEPFSL--
-IPFSLC-
ICPFSL--
KSPFSL--
-KPFSLL-
KVPFSL--
KTPFSL--
-KPFSLS-

KMPFSL--
KEPFSL--
-KPFSLT-
-KPFSLV-
KFPFSL--
KWPFSL--
-KPFSLI-
KAPFSL--
KKPFSL--
KYPFSL--
-KPFSLG-
KQPFSL--
-KPFSLH-
KHPFSL--
-KPFSLY-
-KPFSLF-
-KPFSLN-
KNPFSL--
KPPFSL--
-KPFSLE-
KGPFSL--
KIPFSL--
-KPFSLQ-
KDPFSL--
LSPFSL--
LGPFSL--
-LPFSLT-
LLPFSL--
LQPFSL--
LAPFSL--
LVPFSL--
LWPFSL--
-LPFSLS-
-LPFSLV-
LFPFSL--
LPPFSL--
LMPFSL--
-LPFSLM-
LNPFSL--
-LPFSLI-
-LPFSLQ-
LHPFSL--
LEPFSL--
-LPFSLK-
-LPFSLA-
LDPFSL--
-LPFSLN-
-LPFSLE-
LCPFSL--
MYPFSL--

MRPFSL--
MMPFSL--
MPPFSL--
MVPFSL--
-MPFSLR-
MLPFSL--
MKPFSL--
MGPFSL--
-MPFSLK-
MAPFSL--
MFPFSL--
MNPFSL--
-MPFSLA-
MIPFSL--
-MPFSLV-
-MPFSLN-
-MPFSLS-
-MPFSLG-
-MPFSLT-
MSPFSL--
MHPFSL--
-MPFSLQ-
MEPFSL--
-MPFSLY-
-MPFSLH-
MDPFSL--
MQPFSL--
MWPFSL--
-MPFSLE-
-MPFSLC-
MCPFSL--
NRPFSL--
NGPFSL--
-NPFSLR-
NVPFSL--
NWPFSL--
-NPFSLM-
NKPFSL--
NAPFSL--
-NPFSLI-
NFPFSL--
NPPFSL--
-NPFSLS-
NMPFSL--
NIPFSL--
NNPFSL--
-NPFSLL-
-NPFSLT-
NEPFSL--
-NPFSLK-

-NPFSLW-
-NPFSLN-
-NPFSLQ-
-NPFSLA-
NHPFSL--
-NPFSLH-
NDPFSL--
-NPFSLV-
NQPFSL--
-NPFSLE-
-NPFSLC-
--PFSLSS
--PFSLRS
--PFSLVS
--PFSLRG
--PFSLSA
--PFSLTR
--PFSLTA
PRPFSL--
--PFSLVT
--PFSLIR
--PFSLLQ
--PFSLSV
--PFSLYN
--PFSLLR
--PFSLST
--PFSLMG
--PFSLKM
--PFSLTG
--PFSLRQ
--PFSLKA
--PFSLGS
--PFSLRN
--PFSLVQ
--PFSLYH
PSPFSL--
--PFSLMS
--PFSLAG
--PFSLLK
--PFSLLG
--PFSLRH
--PFSLAL
--PFSLYT
--PFSLYS
--PFSLTI
--PFSLSR
--PFSLKT
--PFSLAS
--PFSLTT
--PFSLKG

--PFSLVV
--PFSLLN
--PFSLHT
--PFSLSN
--PFSLNA
--PFSLKQ
--PFSLNV
--PFSLNS
--PFSLIG
--PFSLFG
PIPFSL--
--PFSLTW
--PFSLMN
--PFSLMR
--PFSLFH
--PFSLRE
--PFSLTH
--PFSLKL
PTPFSL--
--PFSLTN
--PFSLNM
--PFSLMH
--PFSLHQ
--PFSLFN
--PFSLTY
--PFSLTM
--PFSLTK
--PFSLSQ
--PFSLIS
--PFSLEW
--PFSLAR
--PFSLSH
--PFSLIA
PHPFSL--
--PFSLGG
--PFSLAT
--PFSLRM
--PFSLHH
PAPFSL--
--PFSLGV
--PFSLGA
--PFSLYG
--PFSLTV
PVPFSL--
--PFSLVK
--PFSLVG
--PFSLNR
--PFSLNG
--PFSLKN
--PFSLRT

--PFSLQR
--PFSLQG
--PFSLMV
--PFSLMT
--PFSLMK
--PFSLGL
--PFSLWT
--PFSLMA
--PFSLKI
--PFSLHS
--PFSLAA
--PFSLWA
--PFSLRF
--PFSLQT
--PFSLQH
--PFSLWH
--PFSLPL
--PFSLIH
--PFSLHG
--PFSLYD
--PFSLVN
--PFSLQN
--PFSLKD
PDPFSL--
--PFSLEN
--PFSLQS
--PFSLHE
--PFSLFQ
PKPFSL--
--PFSLQM
--PFSLKE
PYPFSL--
--PFSLNF
--PFSLMM
--PFSLHF
--PFSLGT
--PFSLWK
--PFSLQY
--PFSLKK
--PFSLHY
--PFSLYE
--PFSLVH
--PFSLVD
--PFSLTD
--PFSLNY
--PFSLQW
--PFSLQD
--PFSLIV
--PFSLIN
--PFSLHA

--PFSLEG
PCPFSL--
PMPFSL--
--PFSLYQ
--PFSLTQ
--PFSLNW
--PFSLNT
--PFSLIQ
--PFSLIE
--PFSLFR
--PFSLFE
--PFSLEM
--PFSLAK
PFPFSL--
--PFSLWN
--PFSLSW
--PFSLME
--PFSLHN
--PFSLGY
--PFSLDS
--PFSLAV
--PFSLQI
--PFSLQA
--PFSLNH
--PFSLES
--PFSLWR
--PFSLSI
--PFSLQL
--PFSLNK
--PFSLKY
--PFSLIK
--PFSLGM
--PFSLGI
--PFSLYW
--PFSLMQ
--PFSLHK
--PFSLGW
--PFSLAQ
--PFSLSE
--PFSLQV
--PFSLQQ
--PFSLQF
--PFSLHW
--PFSLEF
--PFSLTE
--PFSLNN
--PFSLGH
--PFSLAN
--PFSLWE
--PFSLPY

--PFSLPM
--PFSLNI
--PFSLHV
--PFSLFD
-PPFSLT-
--PFSLWQ
--PFSLVE
--PFSLNQ
--PFSLNE
--PFSLLE
--PFSLEY
--PFSLEV
--PFSLEH
PEPFSL--
--PFSLYC
--PFSLHM
--PFSLGN
--PFSLGF
--PFSLET
--PFSLEQ
--PFSLAH
PQPFSL--
PPPFSL--
--PFSLQE
--PFSLND
--PFSLMD
--PFSLEK
-PPFSLR-
-PPFSLQ-
-PPFSLN-
-PPFSLL-
--PFSLTC
--PFSLIP
--PFSLID
--PFSLHI
--PFSLEP
--PFSLEC
--PFSLCR
--PFSLCN
--PFSLCL
--PFSLAE
--PFSLAD
-QPFSLR-
-QPFSLN-
QVPFSL--
-QPFSLI-
-QPFSLK-
-QPFSLT-
QAPFSL--
QHPFSL--

−QPFSLS−
−QPFSLM−
QMPFSL−−
QKPFSL−−
QWPFSL−−
−QPFSLL−
QPPFSL−−
QGPFSL−−
−QPFSLA−
−QPFSLW−
−QPFSLQ−
QTPFSL−−
QFPFSL−−
QEPFSL−−
QQPFSL−−
QNPFSL−−
−QPFSLH−
QYPFSL−−
−QPFSLG−
−QPFSLE−
QCPFSL−−
−QPFSLP−
QIPFSL−−
RSPFSL−−
−RPFSLR−
RHPFSL−−
RGPFSL−−
RLPFSL−−
−RPFSLV−
RTPFSL−−
RPPFSL−−
−RPFSLW−
REPFSL−−
RYPFSL−−
RMPFSL−−
RFPFSL−−
RNPFSL−−
RAPFSL−−
−RPFSLH−
RWPFSL−−
−RPFSLM−
−RPFSLN−
−RPFSLI−
−RPFSLQ−
RDPFSL−−
RQPFSL−−
−RPFSLG−
−RPFSLE−
−RPFSLD−
RCPFSL−−

-RPFSLC-
SRPFSL--
SSPFSL--
-SPFSLK-
SKPFSL--
STPFSL--
SAPFSL--
SQPFSL--
-SPFSLV-
SVPFSL--
-SPFSLI-
SGPFSL--
-SPFSLH-
-SPFSLT-
SNPFSL--
SPPFSL--
SYPFSL--
-SPFSLQ-
SFPFSL--
-SPFSLW-
-SPFSLM-
SHPFSL--
SEPFSL--
-SPFSLA-
-SPFSLN-
-SPFSLE-
SDPFSL--
SCPFSL--
-SPFSLC-
-TPFSLR-
TVPFSL--
TLPFSL--
TSPFSL--
TGPFSL--
TMPFSL--
-TPFSLS-
TQPFSL--
-TPFSLV-
TYPFSL--
-TPFSLI-
THPFSL--
-TPFSLM-
-TPFSLW-
TNPFSL--
-TPFSLH-
TAPFSL--
-TPFSLK-
TDPFSL--
TKPFSL--
TIPFSL--

TPPFSL--
TEPFSL--
-TPFSLA-
TFPFSL--
-TPFSLG-
-TPFSLC-
VSPFSL--
-VPFSLS-
-VPFSLR-
-VPFSLT-
VMPFSL--
-VPFSLK-
VTPFSL--
VKPFSL--
VAPFSL--
VHPFSL--
-VPFSLI-
VVPFSL--
-VPFSLV-
VPPFSL--
-VPFSLH-
VQPFSL--
-VPFSLN-
-VPFSLA-
VEPFSL--
-VPFSLG-
VFPFSL--
VDPFSL--
VWPFSL--
-VPFSLE-
-VPFSLQ-
WRPFSL--
WAPFSL--
WVPFSL--
WLPFSL--
WQPFSL--
WTPFSL--
-WPFSLS-
WHPFSL--
WEPFSL--
WMPFSL--
WPPFSL--
WGPFSL--
WKPFSL--
-WPFSLV-
-WPFSLM-
WNPFSL--
WWPFSL--
-WPFSLT-
-WPFSLQ-

	-WPFSLK-		
	WDPFSL--		
	-WPFSLN-		
	-WPFSLH-		
	-WPFSLE-		
	WFPFSL--		
	-WPFSLA-		
	YVPFSL--		
	YSPFSL--		
	-YPFSLR-		
	YIPFSL--		
	YTPFSL--		
	YKPFSL--		
	YAPFSL--		
	-YPFSLT-		
	YEPFSL--		
	-YPFSLV-		
	YMPFSL--		
	YYPFSL--		
	YQPFSL--		
	-YPFSLS-		
	-YPFSLM-		
	-YPFSLQ-		
	-YPFSLN-		
	-YPFSLA-		
	YGPFSL--		
	YWPFSL--		
	YNPFSL--		
	YFPFSL--		
	-YPFSLH-		
	YDPFSL--		
	-YPFSLE-		
	-YPFSLG-		
	YHPFSL--		
PHNM	595	588	46.73
	ALPHNM--		
	AIPHNM--		
	AHPHNM--		
	AEPHNM--		
	AMPHNM--		
	AYPHNM--		
	ASPHNM--		
	ANPHNM--		
	AWPHNM--		
	AVPHNM--		
	ATPHNM--		
	AGPHNM--		
	-APHNMF-		
	AAPHNM--		
	-APHNMV-		

-APHNML-
-APHNMI-
-APHNMH-
-APHNMG-
-APHNMA-
APPHNM--
-APHNMW-
-APHNMT-
AKPHNM--
ARPHNM--
-APHNMY-
-APHNMR-
-APHNMQ-
-APHNMK-
AFPHNM--
-APHNME-
CCPHNM--
CGPHNM--
CNPHNM--
CKPHNM--
CHPHNM--
CPPHNM--
-CPHNMS-
-CPHNML-
-CPHNMK-
-CPHNMI-
CAPHNM--
DKPHNM--
DVPHNM--
-DPHNMS-
-DPHNMT-
-DPHNMK-
-DPHNMI-
-DPHNMF-
DWPHNM--
DTPHNM--
DSPHNM--
DRPHNM--
-DPHNMV-
-DPHNML-
-DPHNMH-
-DPHNMC-
DLPHNM--
DCPHNM--
-EPHNMS-
ESPHNM--
-EPHNML-
-EPHNMK-
-EPHNMA-
-EPHNMR-

-EPHNMF-
ELPHNM--
EAPHNM--
EYPHNM--
EWPHNM--
-EPHNMG-
EIPHNM--
EHPHNM--
FVPHNM--
FTPHNM--
FRPHNM--
-FPHNMY-
-FPHNML-
-FPHNMF-
FGPHNM--
FAPHNM--
FLPHNM--
FFPHNM--
-FPHNMM-
FSPHNM--
-FPHNMN-
FIPHNM--
FHPHNM--
FEPHNM--
FPPHNM--
-FPHNMW-
-FPHNMV-
-FPHNMS-
-FPHNMI-
-FPHNMG-
-FPHNME-
FKPHNM--
-GPHNMV-
-GPHNMR-
GGPHNM--
GSPHNM--
GWPHNM--
-GPHNMI-
-GPHNMA-
GLPHNM--
GYPHNM--
-GPHNMQ-
-GPHNMM-
-GPHNML-
GNPHNM--
GEPHNM--
GRPHNM--
-GPHNMY-
-GPHNMK-
GFPHNM--

GTPHNM--
-GPHNMS-
GIPHNM--
GPPHNM--
GCPHNM--
HRPHNM--
-HPHNMY-
HSPHNM--
-HPHNMS-
-HPHNMR-
HEPHNM--
HVPHNM--
-HPHNMW-
-HPHNMK-
-HPHNMV-
HLPHNM--
-HPHNMT-
-HPHNMF-
HMPHNM--
HDPHNM--
ITPHNM--
-IPHNMR-
-IPHNMY-
IYPHNM--
IGPHNM--
IMPHNM--
ISPHNM--
INPHNM--
-IPHNMV-
-IPHNMT-
-IPHNML-
IHPHNM--
IFPHNM--
IQPHNM--
-IPHNMI-
-IPHNMN-
-IPHNMK-
-IPHNMH-
-IPHNME-
-KPHNMV-
KVPHNM--
KSPHNM--
KAPHNM--
-KPHNMS-
KMPHNM--
-KPHNMT-
KYPHNM--
KWPHNM--
KQPHNM--
-KPHNMR-

-KPHNMA-
-KPHNMN-
-KPHNMM-
-KPHNMH-
KLPHNM--
KPPHNM--
-KPHNML-
-KPHNMK-
-KPHNMI-
KGPHNM--
KFPHNM--
KDPHNM--
LSPHNM--
LHPHNM--
LRPHNM--
LMPHNM--
-LPHNMW-
LFPHNM--
LVPHNM--
LKPHNM--
LGPHNM--
LTPHNM--
-LPHNMS-
-LPHNMR-
LNPHNM--
LQPHNM--
-LPHNMT-
LDPHNM--
LAPHNM--
LPPHNM--
-LPHNML-
-LPHNMI-
-LPHNME-
-LPHNMV-
-LPHNMQ-
-LPHNMM-
LLPHNM--
-LPHNMN-
LEPHNM--
MAPHNM--
MMPHNM--
MHPHNM--
MPPHNM--
-MPHNMS-
MYPHNM--
-MPHNMV-
MLPHNM--
-MPHNMT-
-MPHNMF-
-MPHNMG-

NSPHNM--
-NPHNMI-
-NPHNMR-
NRPHNM--
NPPHNM--
-NPHNMW-
-NPHNMA-
-NPHNMS-
-NPHNMY-
-NPHNMK-
NVPHNM--
-NPHNMV-
-NPHNMN-
NKPHNM--
--PHNMSV
--PHNMTG
--PHNMLS
--PHNMML
--PHNMWS
--PHNMTS
--PHNMRT
--PHNMYR
--PHNMSS
--PHNMKS
--PHNMFR
--PHNMYH
--PHNMAA
--PHNMVY
--PHNMVH
PLPHNM--
--PHNMIT
--PHNMLT
--PHNMIS
--PHNMYS
--PHNMWV
--PHNMVV
--PHNMRL
--PHNMRH
--PHNMYG
--PHNMVN
--PHNMTF
--PHNMQW
--PHNMKA
PGPHNM--
--PHNMVF
--PHNMIL
--PHNMES
PSPHNM--
--PHNMRY
--PHNMRS

--PHNMRK
--PHNMLD
--PHNMKQ
--PHNMAS
PWPHNM--
PKPHNM--
--PHNMYL
--PHNMYK
--PHNMWK
--PHNMVT
--PHNMTR
--PHNMTN
--PHNMNG
--PHNMLY
--PHNMLV
--PHNMLN
--PHNMLA
--PHNMGT
--PHNMFL
--PHNMEV
--PHNMEG
--PHNMAV
--PHNMWN
--PHNMSR
--PHNMSQ
--PHNMSL
--PHNMSH
--PHNMNS
--PHNMMN
--PHNMMG
--PHNMLR
--PHNMIF
--PHNMGS
--PHNMET
--PHNMYN
--PHNMYM
--PHNMVR
--PHNMVI
--PHNMTA
--PHNMSG
--PHNMMR
--PHNMLK
--PHNMLE
--PHNMFM
--PHNMEA
--PHNMYV
--PHNMWH
--PHNMVS
--PHNMVM
--PHNMTV

--PHNMSY
--PHNMSN
--PHNMSM
--PHNMRQ
--PHNMRN
--PHNMMW
--PHNMMQ
--PHNMMH
--PHNMLH
--PHNMIG
--PHNMFV
--PHNMAH
PQPHNM--
PIPHNM--
--PHNMWY
--PHNMVK
--PHNMVG
--PHNMTK
--PHNMRW
--PHNMRG
--PHNMQM
--PHNMNM
--PHNMKT
--PHNMIY
--PHNMFQ
PFPHNM--
PNPHNM--
--PHNMYY
--PHNMYA
--PHNMTT
--PHNMRF
--PHNMRE
--PHNMRD
--PHNMRA
--PHNMQT
--PHNMQR
--PHNMQN
--PHNMQH
--PHNMNV
--PHNMNA
--PHNMLW
--PHNMLL
--PHNMLG
--PHNMIV
--PHNMHR
--PHNMHG
--PHNMGM
--PHNMGG
--PHNMFY
--PHNMFG

--PHNMEI
--PHNMEH
--PHNMAW
--PHNMAI
PAPHNM--
PRPHNM--
-PPHNMR-
--PHNMYP
--PHNMYI
--PHNMYE
--PHNMWT
--PHNMWI
--PHNMWG
--PHNMWD
--PHNMVW
--PHNMVD
--PHNMTL
--PHNMTH
--PHNMTE
--PHNMSF
--PHNMRV
--PHNMQY
--PHNMQQ
--PHNMQL
--PHNMQK
--PHNMQI
--PHNMPH
--PHNMME
--PHNMLQ
--PHNMLM
--PHNMLI
--PHNMKW
--PHNMKN
--PHNMKK
--PHNMKH
--PHNMIM
--PHNMII
--PHNMID
--PHNMHL
--PHNMHK
--PHNMHE
--PHNMGV
--PHNMGI
--PHNMFS
--PHNMFK
--PHNMFE
--PHNMFD
--PHNMEQ
--PHNMEN
--PHNMEM

--PHNMDG
--PHNMAL
QSPHNM--
-QPHNMW-
QMPHNM--
QRPHNM--
-QPHNMV-
QWPHNM--
QNPHNM--
-QPHNMS-
-QPHNMH-
QHPHNM--
QYPHNM--
-QPHNMR-
-QPHNMA-
QVPHNM--
QTPHNM--
-QPHNML-
QKPHNM--
RSPHNM--
-RPHNML-
-RPHNMT-
-RPHNMS-
RAPHNM--
-RPHNMR-
RQPHNM--
RVPHNM--
-RPHNMQ-
-RPHNME-
RIPHNM--
RMPHNM--
RHPHNM--
-RPHNMA-
-RPHNMN-
RDPHNM--
-RPHNMK-
RLPHNM--
REPHNM--
RYPHNM--
RWPHNM--
-RPHNMY-
-RPHNMF-
RFPHNM--
RGPHNM--
RTPHNM--
-RPHNMC-
RNPHNM--
RCPHNM--
SVPHNM--
-SPHNMR-

SRPHNM--
-SPHNMK-
-SPHNMM-
SMPHNM--
-SPHNMY-
-SPHNMS-
SLPHNM--
-SPHNML-
SHPHNM--
-SPHNMA-
SSPHNM--
-SPHNMW-
-SPHNMG-
SIPHNM--
-SPHNMV-
-SPHNMF-
SGPHNM--
SYPHNM--
STPHNM--
SQPHNM--
-SPHNMI-
SFPHNM--
SDPHNM--
SKPHNM--
-SPHNMN-
SAPHNM--
-TPHNMT-
TLPHNM--
TKPHNM--
-TPHNMV-
-TPHNMW-
TRPHNM--
-TPHNMM-
TNPHNM--
TEPHNM--
TYPHNM--
TVPHNM--
TTPHNM--
TSPHNM--
-TPHNMN-
TAPHNM--
-TPHNMI-
-TPHNMA-
TIPHNM--
TQPHNM--
-TPHNMS-
-TPHNMK-
TFPHNM--
TDPHNM--
TCPHNM--

TWPHNM--
-TPHNML-
-TPHNMH-
-TPHNMG-
TGPHNM--
-VPHNML-
-VPHNMY-
-VPHNMR-
VAPHNM--
VFPHNM--
VTPHNM--
VRPHNM--
-VPHNMA-
-VPHNMT-
VSPHNM--
VLPHNM--
-VPHNMW-
-VPHNMI-
-VPHNMG-
VKPHNM--
VVPHNM--
VQPHNM--
-VPHNMV-
-VPHNMS-
-VPHNMQ-
-VPHNMN-
-VPHNMK-
VIPHNM--
VEPHNM--
VYPHNM--
-VPHNMF-
VNPHNM--
VMPHNM--
VWPHNM--
-VPHNMM-
-VPHNME-
WQPHNM--
-WPHNMK-
WIPHNM--
WSPHNM--
WFPHNM--
-WPHNML-
WNPHNM--
-WPHNMY-
-WPHNMI-
WVPHNM--
-WPHNMV-
WMPHNM--
-WPHNMT-
WGPHNM--

WEPHNM--
WDPHNM--
WRPHNM--
-WPHNMN-
-WPHNMH-
WLPHNM--
YRPHNM--
-YPHNMS-
YLPHNM--
YSPHNM--
-YPHNMQ-
YGPHNM--
-YPHNMV-
-YPHNMW-
-YPHNMA-
YYPHNM--
YVPHNM--
-YPHNMY-
-YPHNMH-
-YPHNME-
YAPHNM--
-YPHNMT-
YFPHNM--

PAAY 127 792 46.72

ASPAAY--
-APAAYR-
-APAAYL-
AGPAAY--
-APAAYV-
ARPAAY--
ALPAAY--
AMPAAY--
ANPAAY--
-APAAYH-
AYPAAY--
AVPAAY--
-APAAYK-
-APAAYM-
-APAAYF-
-APAAYI-
-APAAYG-
-APAAYE-
APPAAY--
-APAAYT-
AIPAAY--
-APAAYW-
-APAAYS-
-APAAYQ-
-APAAYA-
AKPAAY--

AFPAAY--
-APAAYY-
AWPAAY--
ATPAAY--
-APAAYD-
ACPAAY--
-APAAYN-
AAPAAY--
CMPAAY--
CLPAAY--
CIPAAY--
-CPAAYK-
-CPAAYH-
CFPAAY--
CYPAAY--
CSPAAY--
CNPAAY--
CGPAAY--
CDPAAY--
-DPAAYR-
DFPAAY--
DGPAAY--
DQPAAY--
DVPAAY--
DYPAAY--
DTPAAY--
-DPAAYK-
DWPAAY--
-DPAAYS-
DRPAAY--
-DPAAYV-
-DPAAYL-
-DPAAYH-
DLPAAY--
DKPAAY--
DAPAAY--
-DPAAYY-
-DPAAYI-
DEPAAY--
-DPAAYM-
EAPAAY--
-EPAAYR-
ESPAAY--
EMPAAY--
EGPAAY--
-EPAAYS-
ENPAAY--
EYPAAY--
-EPAAYL-
EVPAAY--

EWPAAY--
ERPAAY--
EQPAAY--
ELPAAY--
EEPAAY--
-EPAAYV-
EFPAAY--
-EPAAYY-
-EPAAYW-
-EPAAYQ-
-EPAAYF-
EHPAAY--
-EPAAYI-
-EPAAYG-
EKPAAY--
EDPAAY--
-EPAAYT-
-FPAAYR-
FSPAAY--
FWPAAY--
FYPAAY--
-FPAAYS-
FQPAAY--
-FPAAYL-
-FPAAYI-
FKPAAY--
-FPAAYN-
FVPAAY--
-FPAAYK-
FNPAAY--
FLPAAY--
-FPAAYV-
-FPAAYQ-
FTPAAY--
FPPAAY--
-FPAAYT-
FEPAAY--
-FPAAYM-
-FPAAYC-
-FPAAYA-
FHPAAY--
FDPAAY--
-GPAAYR-
GWPAAY--
-GPAAYL-
GRPAAY--
-GPAAYS-
GGPAAY--
GTPAAY--
GKPAAY--

GLPAAY--
GVPAAY--
-GPAAYK-
GAPAAY--
GYPAAY--
GSPAAY--
GFPAAY--
-GPAAYF-
GIPAAY--
GHPAAY--
GEPAAY--
GDPAAY--
-GPAAYH-
GNPAAY--
-GPAAYT-
-GPAAYN-
GPPAAY--
-GPAAYA-
GQPAAY--
-GPAAYM-
-GPAAYV-
-GPAAYG-
-GPAAYE-
GMPAAY--
-GPAAYY-
-GPAAYI-
-GPAAYW-
HSPAAY--
-HPAAYR-
HLPAAY--
-HPAAYH-
HFPAAY--
-HPAAYL-
HNPAAY--
-HPAAYM-
-HPAAYK-
HKPAAY--
HRPAAY--
-HPAAYS-
HYPAAY--
-HPAAYT-
-HPAAYI-
HIPAAY--
HGPAAY--
HPPAAY--
HTPAAY--
-HPAAYA-
-HPAAYV-
HHPAAY--
HEPAAY--

HAPAAY--
ITPAAY--
-IPAAYT-
IVPAAY--
-IPAAYR-
IYPAAY--
INPAAY--
-IPAAYL-
-IPAAYW-
-IPAAYA-
IGPAAY--
ISPAAY--
-IPAAYI-
IPPAAY--
-IPAAYM-
IDPAAY--
-IPAAYY-
IHPAAY--
IAPAAY--
-IPAAYV-
ICPAAY--
IKPAAY--
IFPAAY--
IEPAAY--
IQPAAY--
-IPAAYN-
-IPAAYK-
ILPAAY--
-IPAAYS-
-IPAAYE-
KSPAAY--
-KPAAYS-
KAPAAY--
-KPAAYV-
-KPAAYT-
-KPAAYR-
KYPAAY--
KTPAAY--
-KPAAYI-
-KPAAYW-
-KPAAYM-
KIPAAY--
KMPAAY--
-KPAAYN-
KPPAAY--
-KPAAYQ-
KLPAAY--
KKPAAY--
KNPAAY--
KHPAAY--

KVPAAY--
KGPAAY--
-KPAAYK-
-KPAAYA-
KWPAAY--
-KPAAYE-
KEPAAY--
KQPAAY--
KCPAAY--
LRPAAY--
-LPAAYR-
LAPAAY--
LWPAAY--
LKPAAY--
-LPAAYI-
-LPAAYH-
LMPAAY--
LLPAAY--
-LPAAYS-
-LPAAYL-
LNPAAY--
LIPAAY--
LGPAAY--
LSPAAY--
LQPAAY--
-LPAAYW-
LYPAAY--
LTPAAY--
LPPAAY--
-LPAAYT-
LHPAAY--
LCPAAY--
-LPAAYF-
LEPAAY--
-LPAAYV-
LFPAAY--
LVPAAY--
LDPAAY--
-LPAAYN-
-LPAAYM-
-LPAAYK-
-MPAAYM-
-MPAAYS-
-MPAAYT-
MSPAAY--
MTPAAY--
MGPAAY--
MKPAAY--
-MPAAYL-
MAPAAY--

```
-MPAAYY-
MRPAAY--
MVPAAY--
MMPAAY--
-MPAAYI-
MLPAAY--
MHPAAY--
MYPAAY--
MWPAAY--
MNPAAY--
-MPAAYV-
-MPAAYN-
MDPAAY--
-MPAAYF-
MPPAAY--
-MPAAYH-
-MPAAYW-
-MPAAYQ-
-MPAAYK-
-MPAAYG-
-NPAAYR-
NGPAAY--
NVPAAY--
-NPAAYL-
NRPAAY--
-NPAAYT-
NMPAAY--
NLPAAY--
NPPAAY--
NQPAAY--
NEPAAY--
NSPAAY--
NWPAAY--
-NPAAYH-
-NPAAYF-
-NPAAYI-
NTPAAY--
-NPAAYA-
-NPAAYY-
-NPAAYV-
-NPAAYS-
-NPAAYQ-
-NPAAYM-
-NPAAYK-
NFPAAY--
NDPAAY--
--PAAYRS
--PAAYRL
--PAAYLS
--PAAYAL
```

--PAAYGL
--PAAYLT
--PAAYVT
--PAAYSK
--PAAYAG
PLPAAY--
--PAAYRH
--PAAYYS
--PAAYSS
--PAAYNL
--PAAYRN
--PAAYRG
--PAAYMK
--PAAYTK
--PAAYTS
--PAAYPY
--PAAYVV
--PAAYTG
--PAAYHA
--PAAYFR
--PAAYSF
--PAAYHT
--PAAYST
--PAAYSL
--PAAYSI
--PAAYTL
--PAAYWS
--PAAYVS
--PAAYVR
--PAAYRY
--PAAYMA
--PAAYLG
--PAAYWR
--PAAYTA
--PAAYSA
--PAAYRD
--PAAYMR
--PAAYRI
--PAAYLN
--PAAYIV
--PAAYSG
--PAAYMN
--PAAYKN
--PAAYVH
--PAAYNM
--PAAYMH
--PAAYLI
--PAAYKV
--PAAYSY
--PAAYSV

--PAAYRM
--PAAYAM
--PAAYYT
--PAAYRE
--PAAYLV
--PAAYLM
--PAAYGF
PYPAAY--
--PAAYYD
--PAAYRV
--PAAYQR
--PAAYNV
--PAAYML
--PAAYHV
--PAAYGI
--PAAYWA
--PAAYVN
--PAAYRF
--PAAYRA
--PAAYNW
--PAAYLR
--PAAYHR
--PAAYVK
--PAAYGS
--PAAYES
PVPAAY--
--PAAYSM
--PAAYRQ
--PAAYQT
--PAAYPL
--PAAYNS
--PAAYMW
--PAAYMI
--PAAYII
--PAAYAV
--PAAYWI
--PAAYVW
--PAAYTH
--PAAYQY
--PAAYQN
--PAAYMT
--PAAYKI
--PAAYIT
--PAAYAK
PWPAAY--
PFPAAY--
--PAAYYI
--PAAYQL
--PAAYNY
--PAAYLL

--PAAYKL
--PAAYIW
--PAAYGY
--PAAYEV
PRPAAY--
PKPAAY--
PGPAAY--
--PAAYNG
--PAAYMV
--PAAYLD
--PAAYIY
--PAAYIG
--PAAYHW
--PAAYHG
--PAAYGM
--PAAYFI
PEPAAY--
--PAAYTN
--PAAYQD
--PAAYMQ
--PAAYMD
--PAAYKT
--PAAYIS
--PAAYIQ
--PAAYHQ
--PAAYFE
--PAAYFA
PSPAAY--
--PAAYYH
--PAAYYG
--PAAYWT
--PAAYSW
--PAAYSN
--PAAYMM
--PAAYKH
--PAAYHE
--PAAYFT
--PAAYAY
--PAAYAS
PHPAAY--
--PAAYYY
--PAAYYF
--PAAYVL
--PAAYVG
--PAAYTQ
--PAAYTE
--PAAYSD
--PAAYNR
--PAAYMF
--PAAYLH

--PAAYKS
--PAAYKR
--PAAYKG
--PAAYHY
--PAAYGT
--PAAYGA
--PAAYFQ
--PAAYFK
--PAAYFG
--PAAYEA
PMPAAY--
PDPAAY--
PAPAAY--
--PAAYWV
--PAAYWN
--PAAYWE
--PAAYVM
--PAAYTV
--PAAYQI
--PAAYKY
--PAAYIA
--PAAYHH
--PAAYHF
--PAAYFY
--PAAYAW
--PAAYAH
--PAAYAA
--PAAYYM
--PAAYVY
--PAAYSH
--PAAYQW
--PAAYQH
--PAAYNI
--PAAYKW
--PAAYGV
--PAAYGG
--PAAYFN
--PAAYFD
--PAAYEI
--PAAYYN
--PAAYWH
--PAAYVE
--PAAYQE
--PAAYKM
--PAAYIH
--PAAYHK
PNPAAY--
PCPAAY--
--PAAYYQ
--PAAYYK

--PAAYWL
--PAAYVA
--PAAYTM
--PAAYSQ
--PAAYRW
--PAAYNN
--PAAYLQ
--PAAYKA
--PAAYIN
--PAAYHS
--PAAYHD
--PAAYFS
--PAAYFM
--PAAYFH
--PAAYEY
--PAAYEL
--PAAYDM
--PAAYAF
PPPAAY--
-PPAAYY-
-PPAAYN-
-PPAAYK-
PIPAAY--
--PAAYYR
--PAAYYA
--PAAYWF
--PAAYWD
--PAAYTI
--PAAYTF
--PAAYQS
--PAAYQG
--PAAYPW
--PAAYPI
--PAAYPF
--PAAYNT
--PAAYNK
--PAAYNH
--PAAYNA
--PAAYMY
--PAAYMP
--PAAYMG
--PAAYLE
--PAAYLC
--PAAYKF
--PAAYKE
--PAAYIK
--PAAYHI
--PAAYDY
--PAAYDL
--PAAYDI

--PAAYAT
--PAAYAE
-QPAAYW-
QYPAAY--
-QPAAYL-
QRPAAY--
-QPAAYM-
QSPAAY--
QQPAAY--
-QPAAYI-
-QPAAYQ-
-QPAAYY-
QMPAAY--
QGPAAY--
-QPAAYR-
QPPAAY--
-QPAAYT-
-QPAAYK-
QKPAAY--
QEPAAY--
QWPAAY--
-QPAAYA-
QNPAAY--
QHPAAY--
QFPAAY--
QAPAAY--
QVPAAY--
-QPAAYV-
-QPAAYS-
-QPAAYG-
-QPAAYE-
QIPAAY--
-QPAAYF-
QLPAAY--
RRPAAY--
RVPAAY--
-RPAAYY-
-RPAAYR-
RLPAAY--
-RPAAYS-
RGPAAY--
RSPAAY--
RIPAAY--
-RPAAYV-
-RPAAYI-
-RPAAYQ-
RKPAAY--
RYPAAY--
REPAAY--
RAPAAY--

RPPAAY--
RFPAAY--
-RPAAYT-
RMPAAY--
-RPAAYK-
-RPAAYM-
RTPAAY--
RQPAAY--
RHPAAY--
-RPAAYN-
-RPAAYW-
-RPAAYH-
-RPAAYA-
-RPAAYE-
-RPAAYG-
RDPAAY--
-SPAAYS-
-SPAAYR-
SRPAAY--
-SPAAYV-
SGPAAY--
-SPAAYL-
-SPAAYF-
SFPAAY--
SNPAAY--
SLPAAY--
SYPAAY--
SSPAAY--
STPAAY--
SQPAAY--
-SPAAYY-
-SPAAYH-
-SPAAYT-
-SPAAYM-
SKPAAY--
SAPAAY--
SWPAAY--
SVPAAY--
SEPAAY--
-SPAAYW-
SPPAAY--
-SPAAYK-
SHPAAY--
SMPAAY--
-SPAAYE-
SCPAAY--
-SPAAYA-
-SPAAYP-
SDPAAY--
TRPAAY--

TMPAAY--
-TPAAYL-
-TPAAYI-
-TPAAYS-
TQPAAY--
-TPAAYV-
TWPAAY--
-TPAAYR-
-TPAAYM-
TAPAAY--
TVPAAY--
-TPAAYT-
TIPAAY--
-TPAAYK-
TGPAAY--
THPAAY--
TSPAAY--
-TPAAYW-
TNPAAY--
-TPAAYQ-
-TPAAYF-
TYPAAY--
TPPAAY--
-TPAAYY-
-TPAAYG-
-TPAAYE-
TTPAAY--
-TPAAYA-
TKPAAY--
TFPAAY--
TEPAAY--
TCPAAY--
-VPAAYR-
-VPAAYS-
-VPAAYL-
VKPAAY--
VGPAAY--
VSPAAY--
VRPAAY--
VPPAAY--
VMPAAY--
VLPAAY--
-VPAAYT-
VIPAAY--
-VPAAYW-
VQPAAY--
VFPAAY--
-VPAAYI-
-VPAAYQ-
VYPAAY--

-VPAAYV-
-VPAAYG-
VNPAAY--
VAPAAY--
VTPAAY--
-VPAAYN-
-VPAAYK-
-VPAAYH-
VCPAAY--
VWPAAY--
VVPAAY--
VEPAAY--
-VPAAYM-
-VPAAYF-
VHPAAY--
-VPAAYY-
WTPAAY--
WSPAAY--
WLPAAY--
-WPAAYW-
WQPAAY--
-WPAAYV-
WAPAAY--
WIPAAY--
WNPAAY--
WYPAAY--
WWPAAY--
-WPAAYM-
WEPAAY--
-WPAAYQ-
-WPAAYN-
-WPAAYE-
WFPAAY--
WDPAAY--
WPPAAY--
-WPAAYT-
-WPAAYH-
WMPAAY--
YLPAAY--
YWPAAY--
YVPAAY--
YSPAAY--
-YPAAYS-
-YPAAYQ-
YMPAAY--
YKPAAY--
YHPAAY--
-YPAAYL-
-YPAAYK-
YRPAAY--

YNPAAY--
YYPAAY--
-YPAAYT-
-YPAAYM-
YFPAAY--
YTPAAY--
YEPAAY--
YPPAAY--
-YPAAYW-
YDPAAY--
YQPAAY--
-YPAAYY-
-YPAAYN-
-YPAAYG-
-YPAAYA-
YIPAAY--

PLWI 390 663 46.70

-QPLWIY-
AGPLWI--
AKPLWI--
-APLWIS-
-APLWIH-
-APLWIT-
ASPLWI--
-APLWIF-
-APLWIQ-
-APLWIV-
-APLWIG-
-APLWIA-
-APLWIY-
-APLWIR-
AAPLWI--
ADPLWI--
AEPLWI--
APPLWI--
-APLWIN-
AFPLWI--
-APLWIM-
-APLWIW-
ANPLWI--
AHPLWI--
ACPLWI--
AWPLWI--
-APLWID-
CKPLWI--
CAPLWI--
CSPLWI--
-CPLWIK-
CGPLWI--
CYPLWI--

CNPLWI--
CMPLWI--
CLPLWI--
CDPLWI--
-DPLWIK-
DKPLWI--
DTPLWI--
DRPLWI--
DLPLWI--
-DPLWIT-
DEPLWI--
DSPLWI--
DQPLWI--
-DPLWIS-
DNPLWI--
-DPLWIG-
-DPLWIF-
DHPLWI--
DIPLWI--
-DPLWIH-
-DPLWIW-
-DPLWIQ-
-DPLWIM-
DGPLWI--
DDPLWI--
-DPLWIY-
-DPLWIN-
DMPLWI--
DFPLWI--
ESPLWI--
ELPLWI--
-EPLWIK-
EEPLWI--
EYPLWI--
EQPLWI--
ETPLWI--
EMPLWI--
EKPLWI--
EGPLWI--
ERPLWI--
-EPLWIM-
EAPLWI--
-EPLWIG-
ENPLWI--
-EPLWIV-
-EPLWIS-
-EPLWIR-
-EPLWIE-
-EPLWIW-
-EPLWIQ-

```
-EPLWIT-
-EPLWII-
EIPLWI--
EDPLWI--
EVPLWI--
-EPLWIN-
-EPLWID-
FEPLWI--
FTPLWI--
FPPLWI--
-FPLWIR-
-FPLWIQ-
FNPLWI--
FMPLWI--
FAPLWI--
FVPLWI--
FKPLWI--
-FPLWIS-
FRPLWI--
FSPLWI--
FHPLWI--
FYPLWI--
FGPLWI--
-FPLWIK-
-FPLWIG-
-FPLWIA-
-FPLWIW-
-FPLWIT-
FDPLWI--
FLPLWI--
FIPLWI--
-GPLWIT-
GEPLWI--
GVPLWI--
-GPLWIR-
GLPLWI--
GTPLWI--
-GPLWIQ-
-GPLWII-
GPPLWI--
-GPLWIS-
GQPLWI--
GAPLWI--
-GPLWIY-
GGPLWI--
-GPLWIE-
-GPLWIG-
GMPLWI--
GKPLWI--
GDPLWI--
```

-GPLWIV-
-GPLWIA-
-GPLWIN-
GIPLWI--
GWPLWI--
-GPLWIK-
-GPLWID-
GFPLWI--
-GPLWIH-
-GPLWIW-
GNPLWI--
GSPLWI--
-GPLWIP-
HSPLWI--
HAPLWI--
-HPLWIN-
HPPLWI--
HLPLWI--
HKPLWI--
HHPLWI--
HGPLWI--
HRPLWI--
HYPLWI--
HTPLWI--
-HPLWIR-
-HPLWII-
HMPLWI--
-HPLWIG-
HVPLWI--
-HPLWIM-
-HPLWIW-
-HPLWIK-
HEPLWI--
HNPLWI--
HFPLWI--
-HPLWIV-
-HPLWIS-
-HPLWIH-
-HPLWIE-
-HPLWID-
IQPLWI--
-IPLWIG-
-IPLWIK-
ITPLWI--
IGPLWI--
IAPLWI--
-IPLWIR-
INPLWI--
-IPLWIH-
ILPLWI--

IHPLWI--
-IPLWIV-
IFPLWI--
IVPLWI--
ISPLWI--
-IPLWIE-
IPPLWI--
-IPLWIN-
-IPLWID-
IEPLWI--
IYPLWI--
-IPLWIA-
IWPLWI--
IKPLWI--
ICPLWI--
KSPLWI--
-KPLWIR-
-KPLWIS-
KAPLWI--
-KPLWIT-
KHPLWI--
-KPLWII-
KKPLWI--
-KPLWIM-
KEPLWI--
KLPLWI--
-KPLWIQ-
KVPLWI--
-KPLWIA-
KDPLWI--
-KPLWIN-
-KPLWIG-
KQPLWI--
KYPLWI--
-KPLWIY-
-KPLWIV-
-KPLWIK-
-KPLWIL-
-KPLWIE-
KPPLWI--
KTPLWI--
KMPLWI--
KGPLWI--
-KPLWID-
KFPLWI--
-KPLWIW-
KNPLWI--
LGPLWI--
LTPLWI--
LNPLWI--

LLPLWI--
LAPLWI--
LPPLWI--
LKPLWI--
LDPLWI--
LEPLWI--
LMPLWI--
LVPLWI--
-LPLWIQ-
LHPLWI--
-LPLWIA-
LQPLWI--
-LPLWIV-
-LPLWIN-
-LPLWIH-
-MPLWIT-
MSPLWI--
MNPLWI--
MLPLWI--
-MPLWIS-
MVPLWI--
MEPLWI--
MTPLWI--
MAPLWI--
-MPLWIK-
MIPLWI--
MGPLWI--
-MPLWIN-
MQPLWI--
-MPLWIH-
-MPLWIY-
MYPLWI--
MPPLWI--
MHPLWI--
MDPLWI--
-MPLWIG-
MKPLWI--
-MPLWIE-
NGPLWI--
NVPLWI--
-NPLWIS-
NEPLWI--
NDPLWI--
-NPLWII-
-NPLWIR-
-NPLWIA-
NKPLWI--
-NPLWIM-
-NPLWIG-
NLPLWI--

NHPLWI--
NYPLWI--
NTPLWI--
-NPLWIT-
-NPLWIQ-
-NPLWIK-
NSPLWI--
NWPLWI--
-NPLWIY-
-NPLWIV-
-NPLWIH-
-NPLWIE-
NFPLWI--
NPPLWI--
NAPLWI--
NNPLWI--
NQPLWI--
NMPLWI--
NIPLWI--
-NPLWIW-
-NPLWIN-
--PLWILG
--PLWIKS
--PLWIRS
--PLWISS
--PLWIRT
--PLWISK
--PLWIGS
--PLWISV
--PLWIHG
--PLWITR
--PLWIKK
--PLWITA
--PLWINA
--PLWIAA
--PLWISR
--PLWIKG
--PLWIST
--PLWIQS
--PLWIKY
--PLWIRQ
--PLWIQR
--PLWITN
--PLWISN
--PLWISG
--PLWIRY
--PLWIMS
--PLWIYG
--PLWIVS
--PLWIVN

--PLWIES
--PLWISA
--PLWIRN
--PLWIMR
--PLWIGI
--PLWIGA
--PLWIRH
--PLWIIR
--PLWIWQ
--PLWILH
--PLWIFR
--PLWIYD
--PLWITT
--PLWITQ
--PLWIQG
--PLWIMQ
--PLWIGY
--PLWIGW
--PLWITS
--PLWISD
--PLWIRL
--PLWIGM
--PLWIYN
--PLWIYK
--PLWIYH
--PLWITK
--PLWISQ
--PLWIRE
--PLWILK
--PLWIHS
--PLWIHA
--PLWIGN
--PLWIFS
--PLWIVK
--PLWIRD
--PLWIQN
--PLWINS
--PLWIMD
--PLWIHR
--PLWIHH
--PLWIQK
--PLWIIN
--PLWIHT
--PLWIGR
--PLWIEG
PEPLWI--
--PLWIVW
--PLWITW
--PLWISH
--PLWILE

--PLWILD
--PLWIKN
--PLWIAE
--PLWITG
--PLWITD
--PLWIQH
--PLWINV
--PLWILQ
--PLWIIS
--PLWIGT
--PLWIDA
--PLWIWH
--PLWIRW
--PLWINW
--PLWIAN
--PLWIAG
--PLWIWG
--PLWIQD
--PLWIQA
--PLWING
--PLWIMN
--PLWIKD
--PLWIHE
--PLWIGK
--PLWIGF
--PLWIFD
--PLWIWN
--PLWINN
--PLWIKV
--PLWIIH
--PLWIGG
--PLWIFA
--PLWIEA
--PLWIDT
--PLWIDS
--PLWIAM
--PLWIWS
--PLWITI
--PLWISW
--PLWIKW
--PLWIKA
--PLWIIY
--PLWIIK
--PLWIFE
--PLWIYS
--PLWIYA
--PLWIWK
--PLWIQV
--PLWINH
--PLWIME

--PLWIIG
--PLWIHI
--PLWIGV
--PLWIGH
--PLWIFN
--PLWIFM
--PLWIFK
--PLWIFH
--PLWIFG
--PLWIEN
--PLWIEE
--PLWIAV
--PLWIAH
-PPLWIR-
--PLWIYM
--PLWIVY
--PLWITV
--PLWIQQ
--PLWIMW
--PLWIKT
--PLWIKQ
--PLWIIQ
--PLWIHM
--PLWIGQ
--PLWIGD
--PLWIEF
PPPLWI--
-PPLWIS-
-PPLWIM-
--PLWIYE
--PLWIWF
--PLWIWE
--PLWIVV
--PLWISI
--PLWISC
--PLWIQM
--PLWINL
--PLWIMM
--PLWILI
--PLWIIL
--PLWIHK
--PLWIGL
--PLWIGE
--PLWIFY
--PLWIEV
--PLWIEK
--PLWICR
-QPLWIR-
-QPLWIT-
QRPLWI--

-QPLWIQ-
QNPLWI--
QGPLWI--
QHPLWI--
QAPLWI--
-QPLWII-
-QPLWIG-
QTPLWI--
-QPLWIK-
-QPLWIN-
QPPLWI--
QYPLWI--
QEPLWI--
QVPLWI--
QDPLWI--
QLPLWI--
QKPLWI--
QIPLWI--
-QPLWIH-
QFPLWI--
QWPLWI--
-QPLWIW-
-QPLWIE-
RRPLWI--
-RPLWIS-
RLPLWI--
RGPLWI--
RSPLWI--
RDPLWI--
RHPLWI--
RVPLWI--
-RPLWIQ-
-RPLWIT-
RNPLWI--
RPPLWI--
RYPLWI--
-RPLWIA-
RAPLWI--
RMPLWI--
RIPLWI--
RQPLWI--
REPLWI--
-RPLWIW-
-RPLWIH-
-RPLWIF-
-RPLWIE-
RWPLWI--
-RPLWIK-
-RPLWIM-
-RPLWID-

-RPLWIP-
-RPLWIC-
RCPLWI--
SRPLWI--
SKPLWI--
SSPLWI--
SGPLWI--
-SPLWIS-
STPLWI--
-SPLWIT-
-SPLWIK-
-SPLWIQ-
-SPLWIG-
SQPLWI--
SAPLWI--
-SPLWII-
SEPLWI--
SYPLWI--
-SPLWIN-
SHPLWI--
-SPLWIW-
SFPLWI--
SPPLWI--
SIPLWI--
SMPLWI--
SNPLWI--
-SPLWIY-
-SPLWIV-
-SPLWIE-
-SPLWIA-
SDPLWI--
-SPLWID-
SCPLWI--
SWPLWI--
-SPLWIH-
TGPLWI--
TAPLWI--
TSPLWI--
-TPLWIG-
-TPLWIT-
TLPLWI--
TYPLWI--
-TPLWIS-
TNPLWI--
TIPLWI--
-TPLWIH-
-TPLWII-
-TPLWIA-
TQPLWI--
TVPLWI--

TEPLWI--
TDPLWI--
-TPLWIV-
-TPLWIN-
TKPLWI--
TMPLWI--
-TPLWIE-
THPLWI--
-TPLWID-
TCPLWI--
VSPLWI--
-VPLWIR-
VKPLWI--
VRPLWI--
VGPLWI--
VPPLWI--
VNPLWI--
-VPLWIH-
-VPLWIM-
-VPLWIG-
VMPLWI--
VDPLWI--
VTPLWI--
VYPLWI--
-VPLWIS-
VLPLWI--
VVPLWI--
-VPLWIN-
-VPLWIA-
VWPLWI--
VEPLWI--
VAPLWI--
-VPLWIW-
-VPLWIV-
-VPLWIE-
VHPLWI--
-VPLWIK-
WSPLWI--
WQPLWI--
WRPLWI--
WNPLWI--
WLPLWI--
WTPLWI--
WVPLWI--
-WPLWIS-
-WPLWIK-
WMPLWI--
WDPLWI--
WPPLWI--
-WPLWIT-

	-WPLWIR-		
	WKPLWI--		
	WEPLWI--		
	WAPLWI--		
	WYPLWI--		
	WIPLWI--		
	WFPLWI--		
	YTPLWI--		
	YKPLWI--		
	YSPLWI--		
	-YPLWIG-		
	YQPLWI--		
	-YPLWIK-		
	YEPLWI--		
	YNPLWI--		
	YLPLWI--		
	YVPLWI--		
	-YPLWIS-		
	YDPLWI--		
	YPPLWI--		
	-YPLWIN-		
	YRPLWI--		
	-YPLWIT-		
	-YPLWIQ-		
	-YPLWIV-		
	YMPLWI--		
	-YPLWIW-		
	YAPLWI--		
	YIPLWI--		
	YFPLWI--		
PSWL	171	761	46.70
	-LPSWLE-		
	--PSWLSF		
	--PSWLPL		
	--PSWLLP		
	--PSWLGD		
	-SPSWLP-		
	-WPSWLK-		
	ASPSWL--		
	-APSWLL-		
	-APSWLR-		
	AFPSWL--		
	ANPSWL--		
	ALPSWL--		
	-APSWLK-		
	AAPSWL--		
	ATPSWL--		
	AVPSWL--		
	AIPSWL--		
	AHPSWL--		

AMPSWL--
AKPSWL--
-APSWLI-
AQPSWL--
APPSWL--
-APSWLY-
-APSWLT-
-APSWLQ-
-APSWLV-
AGPSWL--
AEPSWL--
AYPSWL--
AWPSWL--
-APSWLM-
ADPSWL--
-APSWLE-
-APSWLN-
-APSWLH-
-APSWLD-
ACPSWL--
CRPSWL--
CKPSWL--
CLPSWL--
CVPSWL--
CHPSWL--
CAPSWL--
CSPSWL--
CQPSWL--
CNPSWL--
-CPSWLK-
CTPSWL--
-CPSWLS-
CMPSWL--
CGPSWL--
-CPSWLR-
CIPSWL--
CEPSWL--
-CPSWLQ-
CPPSWL--
CYPSWL--
-CPSWLW-
-CPSWLT-
-CPSWLN-
-CPSWLI-
-CPSWLF-
-CPSWLA-
CCPSWL--
DSPSWL--
DGPSWL--
DVPSWL--

DRPSWL--
-DPSWLR-
-DPSWLS-
DYPSWL--
DTPSWL--
DKPSWL--
DAPSWL--
DIPSWL--
-DPSWLY-
-DPSWLL-
-DPSWLI-
-DPSWLF-
DMPSWL--
DQPSWL--
-DPSWLM-
DFPSWL--
DEPSWL--
DDPSWL--
DWPSWL--
-DPSWLV-
DHPSWL--
-DPSWLH-
-DPSWLG-
-DPSWLA-
-DPSWLT-
-DPSWLQ-
-DPSWLN-
-DPSWLK-
DNPSWL--
-EPSWLR-
ERPSWL--
ESPSWL--
-EPSWLT-
ETPSWL--
-EPSWLS-
-EPSWLI-
EWPSWL--
EVPSWL--
-EPSWLH-
-EPSWLK-
ELPSWL--
-EPSWLQ-
-EPSWLM-
EHPSWL--
EMPSWL--
-EPSWLV-
-EPSWLY-
EIPSWL--
-EPSWLN-
EGPSWL--

EPPSWL--
EKPSWL--
EYPSWL--
EFPSWL--
-EPSWLA-
EAPSWL--
EDPSWL--
-EPSWLG-
-EPSWLF-
-EPSWLE-
EQPSWL--
ENPSWL--
ECPSWL--
FTPSWL--
FIPSWL--
FHPSWL--
FGPSWL--
FLPSWL--
FAPSWL--
FQPSWL--
FRPSWL--
-FPSWLS-
FKPSWL--
-FPSWLQ-
FMPSWL--
-FPSWLA-
FEPSWL--
-FPSWLK-
FNPSWL--
FYPSWL--
-FPSWLH-
-FPSWLI-
FPPSWL--
-FPSWLN-
-FPSWLG-
FVPSWL--
-FPSWLV-
FDPSWL--
FCPSWL--
-GPSWLL-
GRPSWL--
GLPSWL--
GYPSWL--
GVPSWL--
-GPSWLG-
GQPSWL--
GTPSWL--
GSPSWL--
-GPSWLT-
-GPSWLK-

GEPSWL--
-GPSWLH-
GHPSWL--
-GPSWLN-
GGPSWL--
GIPSWL--
GNPSWL--
-GPSWLA-
-GPSWLS-
-GPSWLI-
GWPSWL--
-GPSWLQ-
GKPSWL--
-GPSWLY-
GCPSWL--
GAPSWL--
-GPSWLE-
GDPSWL--
HVPSWL--
HRPSWL--
HSPSWL--
HTPSWL--
HPPSWL--
-HPSWLR-
-HPSWLS-
HQPSWL--
-HPSWLA-
-HPSWLL-
HNPSWL--
HHPSWL--
HIPSWL--
HYPSWL--
-HPSWLM-
-HPSWLI-
-HPSWLT-
-HPSWLH-
-HPSWLY-
HAPSWL--
-HPSWLQ-
-HPSWLN-
-HPSWLK-
HKPSWL--
-HPSWLW-
-HPSWLG-
HEPSWL--
-HPSWLE-
HFPSWL--
HDPSWL--
IRPSWL--
IGPSWL--

INPSWL--
-IPSWLS-
IKPSWL--
-IPSWLL-
IVPSWL--
ILPSWL--
IQPSWL--
ITPSWL--
IHPSWL--
-IPSWLG-
-IPSWLI-
IIPSWL--
-IPSWLT-
IPPSWL--
IMPSWL--
IYPSWL--
IEPSWL--
-IPSWLA-
-IPSWLQ-
-IPSWLV-
IFPSWL--
IDPSWL--
-IPSWLH-
-IPSWLM-
IAPSWL--
IWPSWL--
-IPSWLN-
ICPSWL--
-IPSWLK-
KSPSWL--
KLPSWL--
-KPSWLT-
KVPSWL--
-KPSWLQ-
KTPSWL--
-KPSWLS-
-KPSWLA-
KAPSWL--
-KPSWLK-
KYPSWL--
-KPSWLI-
KFPSWL--
KMPSWL--
KKPSWL--
KHPSWL--
-KPSWLG-
-KPSWLF-
KPPSWL--
KEPSWL--
KDPSWL--

```
-KPSWLV-
-KPSWLH-
KNPSWL--
-KPSWLY-
-KPSWLM-
-KPSWLW-
KWPSWL--
KCPSWL--
KIPSWL--
-KPSWLE-
LHPSWL--
LTPSWL--
LEPSWL--
LIPSWL--
LKPSWL--
LQPSWL--
LAPSWL--
-LPSWLK-
LDPSWL--
-LPSWLH-
LWPSWL--
LYPSWL--
LFPSWL--
-LPSWLL-
-LPSWLN-
-LPSWLM-
LCPSWL--
MSPSWL--
MNPSWL--
MGPSWL--
-MPSWLK-
MKPSWL--
MEPSWL--
MIPSWL--
-MPSWLG-
MFPSWL--
MWPSWL--
-MPSWLT-
-MPSWLM-
-MPSWLV-
-MPSWLI-
MPPSWL--
MHPSWL--
MAPSWL--
-MPSWLN-
-MPSWLH-
-MPSWLQ-
MDPSWL--
-MPSWLE-
MQPSWL--
```

MCPSWL--
NRPSWL--
NSPSWL--
NTPSWL--
-NPSWLT-
NYPSWL--
-NPSWLR-
NKPSWL--
-NPSWLY-
NHPSWL--
-NPSWLS-
NVPSWL--
NGPSWL--
NMPSWL--
NFPSWL--
-NPSWLA-
NPPSWL--
-NPSWLV-
-NPSWLG-
NWPSWL--
-NPSWLH-
NNPSWL--
NEPSWL--
-NPSWLM-
NQPSWL--
NDPSWL--
-NPSWLQ-
-NPSWLE-
-NPSWLK-
-NPSWLW-
-NPSWLN-
-NPSWLF-
NCPSWL--
--PSWLTS
--PSWLRN
--PSWLVS
--PSWLGS
--PSWLVA
PLPSWL--
--PSWLRH
--PSWLSN
--PSWLRG
--PSWLVG
--PSWLSG
--PSWLNS
--PSWLSA
--PSWLMS
--PSWLMR
--PSWLLT
PRPSWL--

--PSWLTR
PSPSWL--
--PSWLLN
--PSWLKS
--PSWLRI
--PSWLVN
--PSWLKG
--PSWLKA
--PSWLIH
--PSWLSV
--PSWLGG
--PSWLAG
--PSWLMA
--PSWLGV
--PSWLIQ
--PSWLHG
--PSWLGT
PAPSWL--
--PSWLLQ
--PSWLIS
--PSWLHS
--PSWLAV
PTPSWL--
--PSWLTT
--PSWLSI
--PSWLNA
--PSWLTI
--PSWLNG
--PSWLTQ
--PSWLIT
--PSWLAY
--PSWLLH
PVPSWL--
--PSWLSY
--PSWLQR
--PSWLGA
--PSWLSM
--PSWLQG
--PSWLIR
--PSWLGY
--PSWLEG
--PSWLTY
--PSWLSQ
--PSWLQV
--PSWLMK
--PSWLKV
--PSWLIV
--PSWLVH
--PSWLTN
--PSWLTG

--PSWLSW
--PSWLSD
--PSWLQS
--PSWLNT
--PSWLMG
PHPSWL--
--PSWLFG
--PSWLAT
--PSWLAA
--PSWLVK
--PSWLQY
--PSWLQN
--PSWLVV
--PSWLDS
PGPSWL--
--PSWLYE
--PSWLTA
--PSWLKH
PQPSWL--
PKPSWL--
--PSWLWG
--PSWLRF
--PSWLMH
--PSWLTW
--PSWLNN
--PSWLEA
PYPSWL--
--PSWLYG
--PSWLTL
--PSWLHT
--PSWLSH
--PSWLNW
--PSWLLK
--PSWLKK
--PSWLIG
--PSWLGN
--PSWLFN
--PSWLSK
--PSWLRD
--PSWLKN
PIPSWL--
--PSWLVQ
--PSWLVE
--PSWLRW
--PSWLNV
--PSWLKT
--PSWLKD
--PSWLIE
--PSWLFE
--PSWLAK

--PSWLYQ
--PSWLTH
--PSWLQF
--PSWLMD
--PSWLES
--PSWLAR
--PSWLWN
--PSWLWH
--PSWLMN
--PSWLMI
--PSWLLD
--PSWLAN
--PSWLAH
--PSWLAF
PEPSWL--
--PSWLTE
--PSWLQW
--PSWLQH
--PSWLMW
--PSWLID
--PSWLHV
--PSWLHD
--PSWLQA
--PSWLME
--PSWLKQ
--PSWLHM
--PSWLHA
--PSWLKI
--PSWLHN
--PSWLEW
--PSWLAQ
--PSWLAE
PNPSWL--
--PSWLVD
--PSWLHH
PDPSWL--
--PSWLYK
--PSWLSE
--PSWLNQ
--PSWLHW
--PSWLFT
--PSWLAI
--PSWLWK
--PSWLWF
--PSWLTD
--PSWLMM
--PSWLIK
--PSWLEV
--PSWLMT
--PSWLLW

--PSWLFH
--PSWLYD
--PSWLQD
--PSWLII
--PSWLHF
--PSWLEN
--PSWLDT
--PSWLCA
--PSWLYW
--PSWLQE
--PSWLNM
--PSWLNI
--PSWLKE
--PSWLHY
--PSWLHQ
--PSWLHE
--PSWLGH
--PSWLFD
--PSWLER
--PSWLEE
--PSWLED
-PPSWLS-
PFPSWL--
--PSWLWR
--PSWLWQ
--PSWLWE
--PSWLWD
--PSWLPE
--PSWLNH
--PSWLMP
--PSWLET
--PSWLEQ
--PSWLEI
--PSWLEH
--PSWLDA
--PSWLCV
--PSWLCS
--PSWLCN
--PSWLAP
--PSWLAM
-PPSWLV-
-PPSWLT-
PPPSWL--
PCPSWL--
QRPSWL--
-QPSWLL-
QLPSWL--
-QPSWLA-
QVPSWL--
QGPSWL--

QSPSWL--
-QPSWLS-
QTPSWL--
-QPSWLT-
QYPSWL--
-QPSWLI-
QMPSWL--
QHPSWL--
QFPSWL--
QPPSWL--
-QPSWLN-
QDPSWL--
-QPSWLK-
-QPSWLG-
QWPSWL--
-QPSWLH-
QIPSWL--
-QPSWLQ-
QQPSWL--
-QPSWLY-
-QPSWLM-
QNPSWL--
-QPSWLV-
QKPSWL--
QEPSWL--
RRPSWL--
RTPSWL--
-RPSWLA-
RSPSWL--
-RPSWLT-
-RPSWLV-
-RPSWLG-
-RPSWLK-
RYPSWL--
-RPSWLI-
RNPSWL--
-RPSWLL-
RQPSWL--
-RPSWLH-
-RPSWLW-
-RPSWLY-
-RPSWLF-
RHPSWL--
-RPSWLM-
-RPSWLN-
RPPSWL--
REPSWL--
-RPSWLE-
RCPSWL--
-RPSWLD-

SRPSWL--
SVPSWL--
STPSWL--
SAPSWL--
SGPSWL--
-SPSWLR-
SKPSWL--
SYPSWL--
-SPSWLT-
-SPSWLV-
-SPSWLK-
-SPSWLG-
SNPSWL--
-SPSWLY-
-SPSWLL-
SQPSWL--
-SPSWLA-
-SPSWLM-
-SPSWLH-
SPPSWL--
SFPSWL--
SMPSWL--
SEPSWL--
-SPSWLN-
SDPSWL--
-SPSWLE-
SHPSWL--
-SPSWLQ-
SCPSWL--
TRPSWL--
TSPSWL--
-TPSWLS-
-TPSWLR-
TKPSWL--
TLPSWL--
TTPSWL--
TPPSWL--
-TPSWLL-
TGPSWL--
TAPSWL--
THPSWL--
-TPSWLA-
TEPSWL--
-TPSWLT-
-TPSWLG-
TYPSWL--
-TPSWLH-
-TPSWLV-
TIPSWL--
-TPSWLI-

```
-TPSWLM-
-TPSWLQ-
TQPSWL--
-TPSWLK-
TFPSWL--
TWPSWL--
TDPSWL--
-TPSWLN-
TCPSWL--
-TPSWLE-
-TPSWLD-
VRPSWL--
VSPSWL--
-VPSWLL-
-VPSWLT-
VYPSWL--
-VPSWLH-
VVPSWL--
-VPSWLA-
-VPSWLM-
VNPSWL--
VEPSWL--
-VPSWLV-
VHPSWL--
-VPSWLQ-
-VPSWLN-
VDPSWL--
VWPSWL--
VQPSWL--
-VPSWLD-
VCPSWL--
-VPSWLP-
-VPSWLE-
-VPSWLC-
WRPSWL--
WTPSWL--
WAPSWL--
-WPSWLS-
WEPSWL--
WFPSWL--
WWPSWL--
WQPSWL--
WPPSWL--
WKPSWL--
WHPSWL--
WDPSWL--
-WPSWLT-
WMPSWL--
WVPSWL--
-WPSWLG-
```

	-WPSWLA-		
	-WPSWLQ-		
	WIPSWL--		
	-WPSWLM-		
	-WPSWLH-		
	WCPSWL--		
	-WPSWLE-		
	-WPSWLD-		
	WNPSWL--		
	YLPSWL--		
	YSPSWL--		
	-YPSWLR-		
	YRPSWL--		
	YVPSWL--		
	YNPSWL--		
	-YPSWLQ-		
	YTPSWL--		
	YPPSWL--		
	-YPSWLS-		
	YQPSWL--		
	-YPSWLT-		
	-YPSWLK-		
	YKPSWL--		
	YHPSWL--		
	YEPSWL--		
	YYPSWL--		
	-YPSWLL-		
	-YPSWLF-		
	YMPSWL--		
	YDPSWL--		
	-YPSWLY-		
	YFPSWL--		
	-YPSWLG-		
	-YPSWLA-		
	YGPSWL--		
	-YPSWLI-		
	-YPSWLV-		
	-YPSWLN-		
	-YPSWLM-		
	-YPSWLE-		
	-YPSWLH-		
	YCPSWL--		
PIPL	72	831	46.69
	-EPIPLI-		
	-LPIPLA-		
	--PIPLWY		
	--PIPLSP		
	--PIPLIP		
	-APIPLA-		
	AKPIPL--		

AAPIPL--
-APIPLQ-
-APIPLM-
-APIPLT-
-APIPLL-
AIPIPL--
AYPIPL--
AHPIPL--
ADPIPL--
-APIPLS-
AQPIPL--
AVPIPL--
AWPIPL--
-APIPLK-
-APIPLV-
-APIPLN-
-APIPLH-
AMPIPL--
-APIPLG-
-APIPLE-
ANPIPL--
ASPIPL--
-APIPLI-
-APIPLF-
AGPIPL--
AEPIPL--
-APIPLW-
APPIPL--
AFPIPL--
-APIPLD-
ACPIPL--
CCPIPL--
CFPIPL--
CYPIPL--
CRPIPL--
CAPIPL--
CVPIPL--
-CPIPLL-
CHPIPL--
CSPIPL--
CLPIPL--
-CPIPLV-
DSPIPL--
-DPIPLT-
DYPIPL--
DRPIPL--
-DPIPLM-
DAPIPL--
DKPIPL--
DLPIPL--

DWPIPL--
-DPIPLY-
DQPIPL--
-DPIPLL-
-DPIPLR-
-DPIPLK-
-DPIPLI-
DFPIPL--
DVPIPL--
DHPIPL--
-DPIPLV-
DNPIPL--
DMPIPL--
DDPIPL--
DTPIPL--
-DPIPLS-
-DPIPLQ-
-DPIPLF-
DIPIPL--
-EPIPLL-
-EPIPLA-
-EPIPLR-
EAPIPL--
ENPIPL--
EIPIPL--
EYPIPL--
ERPIPL--
EEPIPL--
EFPIPL--
EWPIPL--
-EPIPLG-
EVPIPL--
-EPIPLV-
-EPIPLH-
EMPIPL--
EHPIPL--
ETPIPL--
-EPIPLY-
-EPIPLN-
-EPIPLM-
ELPIPL--
EGPIPL--
EQPIPL--
-EPIPLS-
-EPIPLQ-
-EPIPLK-
FQPIPL--
FYPIPL--
-FPIPLY-
FGPIPL--

FSPIPL--
FFPIPL--
FIPIPL--
FWPIPL--
FTPIPL--
-FPIPLR-
-FPIPLV-
FHPIPL--
-FPIPLN-
-FPIPLT-
-FPIPLA-
FDPIPL--
FEPIPL--
FNPIPL--
FMPIPL--
-FPIPLS-
FAPIPL--
-FPIPLI-
-FPIPLH-
-FPIPLE-
FKPIPL--
FVPIPL--
-FPIPLG-
-FPIPLF-
FPPIPL--
GVPIPL--
GSPIPL--
GYPIPL--
GMPIPL--
GKPIPL--
-GPIPLK-
-GPIPLT-
GRPIPL--
GNPIPL--
GGPIPL--
-GPIPLR-
GEPIPL--
-GPIPLI-
-GPIPLQ-
GAPIPL--
GTPIPL--
GFPIPL--
GHPIPL--
-GPIPLW-
GQPIPL--
GIPIPL--
-GPIPLS-
-GPIPLM-
-GPIPLV-
GLPIPL--

-GPIPLF-
-GPIPLN-
GDPIPL--
GWPIPL--
-GPIPLH-
GPPIPL--
GCPIPL--
-GPIPLY-
-GPIPLG-
-GPIPLC-
HAPIPL--
-HPIPLR-
HRPIPL--
HTPIPL--
HVPIPL--
HMPIPL--
-HPIPLT-
-HPIPLH-
HWPIPL--
-HPIPLF-
HQPIPL--
-HPIPLL-
HNPIPL--
-HPIPLK-
HLPIPL--
HSPIPL--
HGPIPL--
-HPIPLA-
HFPIPL--
-HPIPLY-
-HPIPLM-
-HPIPLQ-
-HPIPLN-
HIPIPL--
HHPIPL--
HEPIPL--
HDPIPL--
-HPIPLG-
HKPIPL--
HPPIPL--
-HPIPLS-
-HPIPLI-
-HPIPLE-
IRPIPL--
IVPIPL--
ISPIPL--
IPPIPL--
-IPIPLR-
IDPIPL--
IYPIPL--

```
-IPIPLS-
IHPIPL--
IEPIPL--
IIPIPL--
IGPIPL--
-IPIPLY-
-IPIPLL-
IKPIPL--
-IPIPLV-
INPIPL--
-IPIPLG-
-IPIPLA-
IAPIPL--
IWPIPL--
IFPIPL--
ILPIPL--
IQPIPL--
-IPIPLQ-
-IPIPLH-
ICPIPL--
-IPIPLT-
-IPIPLF-
-IPIPLW-
KSPIPL--
KGPIPL--
-KPIPLL-
-KPIPLA-
-KPIPLT-
KAPIPL--
-KPIPLH-
-KPIPLQ-
-KPIPLI-
KMPIPL--
-KPIPLY-
KYPIPL--
KTPIPL--
KFPIPL--
KWPIPL--
-KPIPLG-
KVPIPL--
-KPIPLS-
KKPIPL--
KQPIPL--
KEPIPL--
-KPIPLN-
KHPIPL--
-KPIPLV-
-KPIPLF-
KPPIPL--
KLPIPL--
```

-KPIPLW-
-KPIPLK-
-KPIPLE-
-KPIPLC-
LRPIPL--
LSPIPL--
LAPIPL--
LVPIPL--
LIPIPL--
-LPIPLL-
LHPIPL--
LLPIPL--
-LPIPLY-
LNPIPL--
-LPIPLH-
-LPIPLR-
LDPIPL--
-LPIPLT-
LFPIPL--
LYPIPL--
LKPIPL--
-LPIPLV-
LQPIPL--
LPPIPL--
-LPIPLS-
-LPIPLM-
LTPIPL--
-LPIPLQ-
LEPIPL--
-LPIPLK-
-LPIPLG-
-LPIPLI-
-LPIPLF-
LCPIPL--
-LPIPLN-
-MPIPLR-
MGPIPL--
MPPIPL--
MQPIPL--
MLPIPL--
-MPIPLY-
-MPIPLI-
MWPIPL--
-MPIPLV-
MIPIPL--
-MPIPLT-
MKPIPL--
MVPIPL--
-MPIPLF-
-MPIPLS-

MHPIPL--
-MPIPLN-
MNPIPL--
-MPIPLW-
MAPIPL--
-MPIPLL-
-MPIPLH-
MFPIPL--
-MPIPLQ-
MYPIPL--
-MPIPLM-
MMPIPL--
MEPIPL--
-NPIPLR-
-NPIPLY-
NSPIPL--
NGPIPL--
NFPIPL--
NMPIPL--
-NPIPLV-
NYPIPL--
NIPIPL--
NTPIPL--
NWPIPL--
-NPIPLI-
NVPIPL--
NNPIPL--
-NPIPLW-
-NPIPLH-
NQPIPL--
NAPIPL--
-NPIPLS-
-NPIPLF-
-NPIPLA-
-NPIPLT-
-NPIPLN-
-NPIPLL-
-NPIPLK-
-NPIPLE-
NHPIPL--
-NPIPLG-
NEPIPL--
--PIPLSL
--PIPLWL
--PIPLAL
--PIPLVR
--PIPLRS
--PIPLLL
--PIPLRN
--PIPLKL

--PIPLYR
--PIPLLG
--PIPLTL
--PIPLVA
--PIPLKY
--PIPLHG
--PIPLTT
--PIPLST
--PIPLSA
--PIPLRR
--PIPLRI
--PIPLDL
--PIPLSW
--PIPLNQ
--PIPLVG
--PIPLTK
--PIPLFI
--PIPLTR
--PIPLSG
--PIPLFG
--PIPLLI
--PIPLSS
--PIPLRA
--PIPLNL
--PIPLML
--PIPLLN
--PIPLRT
PTPIPL--
--PIPLYS
--PIPLYN
--PIPLQS
--PIPLLR
--PIPLWR
--PIPLRH
--PIPLLH
--PIPLRF
--PIPLLT
PSPIPL--
--PIPLTI
--PIPLRM
--PIPLKN
--PIPLGF
--PIPLAS
--PIPLYG
--PIPLLS
--PIPLIT
--PIPLHI
--PIPLFM
--PIPLAI
--PIPLNW

--PIPLKS
--PIPLHS
--PIPLKR
--PIPLHF
--PIPLYT
--PIPLVV
--PIPLVI
--PIPLSK
--PIPLQR
--PIPLMA
--PIPLGQ
--PIPLSV
--PIPLLV
--PIPLLA
PGPIPL--
--PIPLWT
--PIPLWS
--PIPLWM
--PIPLSN
--PIPLRW
--PIPLRK
--PIPLQL
--PIPLLM
--PIPLIR
PAPIPL--
--PIPLIK
--PIPLGS
--PIPLAF
--PIPLRY
--PIPLLK
--PIPLES
PWPIPL--
--PIPLNS
--PIPLHT
--PIPLHA
--PIPLGA
--PIPLFA
--PIPLYH
--PIPLWW
--PIPLRQ
--PIPLQT
--PIPLQG
--PIPLFS
--PIPLFN
PVPIPL--
--PIPLYV
--PIPLVH
--PIPLTW
--PIPLSY
--PIPLSF

--PIPLNA
--PIPLMG
--PIPLAT
--PIPLYW
--PIPLVW
--PIPLVM
--PIPLTN
--PIPLQV
--PIPLQQ
--PIPLNF
--PIPLLD
--PIPLGN
PDPIPL--
PMPIPL--
--PIPLYI
--PIPLTY
--PIPLMS
--PIPLLE
--PIPLKV
--PIPLKQ
--PIPLKG
--PIPLHM
--PIPLFK
--PIPLYM
--PIPLWV
--PIPLVQ
--PIPLTS
--PIPLPF
--PIPLHN
--PIPLFL
--PIPLDV
--PIPLAM
PHPIPL--
--PIPLWD
--PIPLVT
--PIPLQA
--PIPLIY
--PIPLGH
--PIPLFY
--PIPLYY
--PIPLVE
--PIPLTG
--PIPLQY
--PIPLMR
--PIPLMN
--PIPLKM
--PIPLIS
--PIPLFW
--PIPLAV
--PIPLAN

--PIPLAG
--PIPLWQ
--PIPLTM
--PIPLTH
--PIPLTA
--PIPLMI
--PIPLKT
--PIPLHR
--PIPLGW
--PIPLDF
--PIPLAR
--PIPLWK
--PIPLWA
--PIPLSQ
--PIPLSH
--PIPLQI
--PIPLQH
--PIPLIV
--PIPLIQ
--PIPLIN
--PIPLGG
PFPIPL--
--PIPLWH
--PIPLTQ
--PIPLPW
--PIPLNV
--PIPLNH
--PIPLMV
--PIPLLQ
--PIPLKE
--PIPLIM
--PIPLHV
--PIPLGK
--PIPLFH
--PIPLAK
--PIPLRD
--PIPLQK
--PIPLQD
--PIPLPA
--PIPLLW
--PIPLHK
--PIPLEY
--PIPLEH
--PIPLEA
--PIPLAQ
--PIPLAA
PNPIPL--
--PIPLYA
--PIPLWE
--PIPLPV

--PIPLPQ
--PIPLMW
--PIPLMK
--PIPLMF
--PIPLME
--PIPLKK
--PIPLEN
--PIPLSD
--PIPLQE
--PIPLNR
--PIPLMM
--PIPLHW
--PIPLHQ
--PIPLGT
--PIPLGR
--PIPLFD
--PIPLEQ
--PIPLEG
--PIPLDS
--PIPLDI
--PIPLDA
--PIPLAH
PEPIPL--
--PIPLYK
--PIPLTE
--PIPLSE
--PIPLQN
--PIPLPS
--PIPLNE
--PIPLKH
--PIPLKA
--PIPLFV
--PIPLFE
--PIPLEW
--PIPLEV
--PIPLER
--PIPLEM
PYPIPL--
-PPIPLS-
-PPIPLQ-
--PIPLYQ
--PIPLYD
--PIPLWN
--PIPLWI
--PIPLTF
--PIPLTC
--PIPLQF
--PIPLPT
--PIPLNN
--PIPLNG

--PIPLKF
--PIPLIG
--PIPLID
--PIPLHY
--PIPLHH
--PIPLFQ
--PIPLET
--PIPLDR
--PIPLCQ
--PIPLCL
QYPIPL--
QNPIPL--
QIPIPL--
QSPIPL--
-QPIPLI-
QTPIPL--
-QPIPLR-
-QPIPLK-
QMPIPL--
-QPIPLT-
-QPIPLL-
-QPIPLY-
-QPIPLS-
QHPIPL--
QWPIPL--
QQPIPL--
-QPIPLF-
QRPIPL--
QPPIPL--
-QPIPLN-
-QPIPLH-
QLPIPL--
QFPIPL--
-QPIPLQ-
-QPIPLA-
QKPIPL--
QEPIPL--
QAPIPL--
QGPIPL--
QDPIPL--
-QPIPLE-
-RPIPLV-
RAPIPL--
-RPIPLK-
-RPIPLS-
RTPIPL--
-RPIPLR-
-RPIPLH-
RWPIPL--
RVPIPL--

RNPIPL--
RMPIPL--
RQPIPL--
-RPIPLW-
-RPIPLQ-
-RPIPLI-
RHPIPL--
-RPIPLT-
-RPIPLN-
RIPIPL--
-RPIPLA-
RPPIPL--
REPIPL--
RDPIPL--
RYPIPL--
-RPIPLM-
-RPIPLG-
-RPIPLE-
RCPIPL--
SGPIPL--
SRPIPL--
SWPIPL--
-SPIPLR-
SLPIPL--
SVPIPL--
-SPIPLV-
SSPIPL--
-SPIPLI-
-SPIPLW-
-SPIPLT-
SAPIPL--
SYPIPL--
-SPIPLH-
-SPIPLF-
SIPIPL--
SPPIPL--
SQPIPL--
-SPIPLY-
SHPIPL--
SKPIPL--
STPIPL--
SMPIPL--
SFPIPL--
SEPIPL--
-SPIPLQ-
-SPIPLK-
-SPIPLN-
-SPIPLG-
SDPIPL--
-SPIPLA-

-SPIPLE-
-SPIPLM-
TGPIPL--
-TPIPLR-
-TPIPLK-
-TPIPLL-
TVPIPL--
-TPIPLT-
TSPIPL--
-TPIPLI-
TIPIPL--
TKPIPL--
-TPIPLS-
TYPIPL--
TPPIPL--
TMPIPL--
TWPIPL--
TLPIPL--
-TPIPLY-
-TPIPLW-
TQPIPL--
TNPIPL--
-TPIPLH-
-TPIPLN-
TFPIPL--
-TPIPLV-
THPIPL--
-TPIPLQ-
-TPIPLG-
TDPIPL--
TAPIPL--
-TPIPLE-
TEPIPL--
-TPIPLA-
-VPIPLR-
VSPIPL--
-VPIPLT-
-VPIPLY-
VIPIPL--
VAPIPL--
-VPIPLL-
VLPIPL--
VDPIPL--
VKPIPL--
-VPIPLV-
-VPIPLS-
-VPIPLA-
-VPIPLM-
VFPIPL--
VVPIPL--

VMPIPL--
VPPIPL--
-VPIPLW-
-VPIPLF-
VYPIPL--
VTPIPL--
-VPIPLK-
VHPIPL--
VNPIPL--
-VPIPLN-
VGPIPL--
-VPIPLQ-
-VPIPLG-
VCPIPL--
VWPIPL--
-VPIPLH-
-VPIPLE-
WSPIPL--
WAPIPL--
-WPIPLS-
WLPIPL--
WGPIPL--
WIPIPL--
WRPIPL--
WPPIPL--
WYPIPL--
-WPIPLT-
-WPIPLV-
WNPIPL--
WFPIPL--
WTPIPL--
-WPIPLQ-
-WPIPLK-
-WPIPLG-
WDPIPL--
WKPIPL--
-WPIPLR-
-WPIPLL-
WQPIPL--
-WPIPLF-
-WPIPLA-
-WPIPLN-
-WPIPLC-
YIPIPL--
YLPIPL--
YEPIPL--
-YPIPLY-
YAPIPL--
-YPIPLR-
YFPIPL--

YGPIPL--
YSPIPL--
-YPIPLI-
YVPIPL--
YQPIPL--
YDPIPL--
YPPIPL--
YKPIPL--
-YPIPLV-
YWPIPL--
-YPIPLL-
-YPIPLK-
-YPIPLM-
-YPIPLN-
YHPIPL--
-YPIPLT-
-YPIPLE-
YMPIPL--
YNPIPL--
-YPIPLW-
-YPIPLG-
-YPIPLQ-
-YPIPLH-
-YPIPLA-
PKNI 443 640 46.69
-APKNIV-
-APKNIA-
-APKNIR-
-APKNIL-
AVPKNI--
AWPKNI--
ARPKNI--
-APKNIY-
AIPKNI--
-APKNIG-
AEPKNI--
-APKNIS-
-APKNIM-
-APKNII-
-APKNIH-
AGPKNI--
-APKNIK-
AHPKNI--
-APKNIT-
-APKNIN-
AMPKNI--
CRPKNI--
CKPKNI--
CTPKNI--
CSPKNI--

CEPKNI--
-CPKNIK-
-CPKNIA-
CYPKNI--
CWPKNI--
CPPKNI--
-CPKNIR-
-CPKNIF-
CNPKNI--
CHPKNI--
CGPKNI--
-DPKNIT-
-DPKNIR-
-DPKNII-
DLPKNI--
DRPKNI--
-DPKNIW-
DMPKNI--
-DPKNIV-
-DPKNIS-
-DPKNIL-
-DPKNIE-
DHPKNI--
DGPKNI--
DFPKNI--
DVPKNI--
-DPKNIQ-
-DPKNIN-
-EPKNIY-
-EPKNIL-
-EPKNIW-
EYPKNI--
EQPKNI--
-EPKNIN-
EMPKNI--
-EPKNIK-
-EPKNIA-
EAPKNI--
-EPKNIR-
-EPKNII-
-EPKNIG-
ERPKNI--
-EPKNIT-
EKPKNI--
EEPKNI--
ECPKNI--
FMPKNI--
FAPKNI--
FKPKNI--
FRPKNI--

−FPKNII−
FSPKNI−−
−FPKNIA−
FVPKNI−−
−FPKNIV−
−FPKNIH−
FYPKNI−−
FNPKNI−−
FLPKNI−−
FHPKNI−−
−FPKNIM−
FWPKNI−−
FTPKNI−−
FFPKNI−−
FEPKNI−−
−FPKNIW−
FGPKNI−−
FDPKNI−−
FQPKNI−−
FPPKNI−−
−FPKNIY−
−FPKNIF−
FIPKNI−−
−GPKNIS−
GVPKNI−−
−GPKNIR−
−GPKNIF−
−GPKNII−
−GPKNIV−
GRPKNI−−
GLPKNI−−
GGPKNI−−
−GPKNIT−
−GPKNIN−
GEPKNI−−
−GPKNIQ−
GAPKNI−−
−GPKNIH−
GMPKNI−−
GWPKNI−−
GTPKNI−−
−GPKNIY−
−GPKNIL−
−GPKNIK−
−GPKNIG−
GKPKNI−−
GIPKNI−−
GHPKNI−−
−GPKNIE−
GCPKNI−−

-HPKNIL-
-HPKNIS-
-HPKNIF-
HRPKNI--
HGPKNI--
-HPKNIM-
-HPKNIT-
-HPKNII-
HMPKNI--
HHPKNI--
HVPKNI--
-HPKNIV-
HAPKNI--
HQPKNI--
-HPKNIW-
HLPKNI--
HEPKNI--
HSPKNI--
-HPKNIA-
HNPKNI--
IRPKNI--
-IPKNIR-
-IPKNIL-
-IPKNIT-
-IPKNII-
IVPKNI--
-IPKNIH-
IIPKNI--
IYPKNI--
-IPKNIS-
-IPKNIM-
IAPKNI--
ITPKNI--
-IPKNIK-
IKPKNI--
IPPKNI--
-IPKNIW-
-IPKNIV-
-IPKNIF-
IMPKNI--
IWPKNI--
-IPKNIQ-
IDPKNI--
IGPKNI--
-IPKNIN-
-IPKNIA-
IFPKNI--
KEPKNI--
-KPKNIS-
KVPKNI--

KPPKNI--
-KPKNIR-
-KPKNII-
-KPKNIF-
KYPKNI--
-KPKNIY-
KMPKNI--
-KPKNIQ-
-KPKNIL-
-KPKNIK-
KNPKNI--
-KPKNIV-
KLPKNI--
-KPKNIM-
-KPKNIG-
KAPKNI--
KQPKNI--
-KPKNIE-
KIPKNI--
KFPKNI--
-KPKNIN-
-KPKNIH-
KKPKNI--
LVPKNI--
LKPKNI--
LTPKNI--
LSPKNI--
LRPKNI--
-LPKNIV-
-LPKNIM-
LFPKNI--
LGPKNI--
LIPKNI--
LHPKNI--
LMPKNI--
-LPKNIQ-
LAPKNI--
-LPKNIL-
-LPKNIF-
LQPKNI--
-LPKNIT-
LPPKNI--
LNPKNI--
LDPKNI--
-LPKNIS-
-LPKNIK-
LEPKNI--
LWPKNI--
-LPKNII-
-MPKNIS-

MTPKNI--
MLPKNI--
MRPKNI--
-MPKNIV-
-MPKNIR-
-MPKNIL-
MNPKNI--
-MPKNIT-
-MPKNIQ-
MVPKNI--
MPPKNI--
-MPKNII-
MMPKNI--
-MPKNIF-
MDPKNI--
MYPKNI--
MWPKNI--
MQPKNI--
-MPKNIY-
-MPKNIG-
MAPKNI--
MSPKNI--
-MPKNIH-
NWPKNI--
NQPKNI--
-NPKNIM-
-NPKNIF-
NLPKNI--
NFPKNI--
-NPKNIW-
-NPKNIT-
NPPKNI--
-NPKNIL-
NEPKNI--
NAPKNI--
NTPKNI--
NRPKNI--
-NPKNIY-
-NPKNIC-
NNPKNI--
NKPKNI--
--PKNILT
--PKNIVG
--PKNIRS
--PKNIRH
--PKNIMS
--PKNILL
--PKNILS
--PKNIVS
--PKNIVN

--PKNISR
--PKNIVL
--PKNILG
PSPKNI--
--PKNIVR
--PKNISN
--PKNILA
--PKNIWY
--PKNIVQ
--PKNISV
--PKNIRY
--PKNIRF
PRPKNI--
--PKNIWT
--PKNIWL
--PKNIKL
--PKNIRG
--PKNIRA
--PKNIYL
--PKNITA
--PKNISG
--PKNIIR
--PKNIFW
--PKNIFI
--PKNIGS
--PKNIFA
--PKNIAV
--PKNIWS
--PKNIVI
--PKNIVH
--PKNITK
--PKNIHF
--PKNIFY
--PKNIFK
--PKNILV
--PKNIKA
--PKNITT
--PKNIRN
--PKNIML
--PKNIIV
--PKNIIS
--PKNIGV
--PKNIFV
--PKNIAT
PTPKNI--
--PKNIWW
--PKNIVY
--PKNINW
--PKNIHG
--PKNIFS

--PKNIAI
PFPKNI--
--PKNIYV
--PKNITM
--PKNITF
--PKNISW
--PKNIME
--PKNIHS
--PKNIWN
--PKNIQG
--PKNIIY
--PKNIGW
--PKNIAR
--PKNIAG
PWPKNI--
--PKNIYN
--PKNIWQ
--PKNIQA
--PKNINM
--PKNIMV
--PKNILN
--PKNIKI
--PKNIFT
--PKNIEG
--PKNIYI
--PKNISY
--PKNISS
--PKNISE
--PKNIQT
--PKNIMW
--PKNILM
--PKNIIF
--PKNIID
--PKNIHV
--PKNIER
--PKNIYW
--PKNIYH
--PKNIYG
--PKNIWA
--PKNIVT
--PKNINY
--PKNING
--PKNIMT
--PKNIKF
--PKNIHT
--PKNIHL
--PKNIGT
--PKNIGI
--PKNIGG
--PKNIFL

--PKNIES
--PKNIAA
PIPKNI--
PAPKNI--
--PKNIWD
--PKNIVM
--PKNIVA
--PKNITV
--PKNISM
--PKNISI
--PKNIQW
--PKNINV
--PKNIMA
--PKNIKV
--PKNIKT
--PKNIKG
--PKNIIN
--PKNIHR
--PKNIEW
--PKNIEN
--PKNIEK
--PKNIAS
PHPKNI--
PMPKNI--
--PKNIYA
--PKNIVV
--PKNIVK
--PKNIVE
--PKNITY
--PKNITW
--PKNIQL
--PKNINT
--PKNINI
--PKNINF
--PKNILI
--PKNILC
--PKNIIG
--PKNIHI
--PKNIGY
--PKNIFG
--PKNIEM
--PKNIEF
--PKNIAK
PGPKNI--
PVPKNI--
PQPKNI--
-PPKNIS-
PLPKNI--
--PKNIYY
--PKNIYT

--PKNIYQ
--PKNIWG
--PKNIVW
--PKNIVC
--PKNITN
--PKNITE
--PKNISD
--PKNIQV
--PKNIQN
--PKNIPS
--PKNIPL
--PKNINQ
--PKNINN
--PKNIMN
--PKNIMM
--PKNILH
--PKNILD
--PKNIKD
--PKNIIW
--PKNIHK
--PKNIHE
--PKNIGR
--PKNIGN
--PKNIGF
--PKNIGE
--PKNIFM
--PKNIEY
--PKNIEQ
--PKNIEL
--PKNIAN
--PKNIAL
PDPKNI--
-QPKNIS-
-QPKNIL-
QEPKNI--
QFPKNI--
QSPKNI--
-QPKNIY-
-QPKNIT-
-QPKNIR-
-QPKNIF-
QIPKNI--
QPPKNI--
-QPKNIA-
QLPKNI--
QKPKNI--
-QPKNIV-
-QPKNIK-
-QPKNIH-
QMPKNI--

−QPKNIE−
−RPKNIS−
−RPKNIW−
−RPKNIL−
RIPKNI−−
−RPKNIF−
RYPKNI−−
−RPKNII−
RSPKNI−−
−RPKNIY−
−RPKNIT−
−RPKNIH−
−RPKNIG−
RNPKNI−−
−RPKNIQ−
−RPKNIK−
−RPKNIA−
RMPKNI−−
RKPKNI−−
−RPKNIN−
RDPKNI−−
RCPKNI−−
RRPKNI−−
RQPKNI−−
−SPKNIL−
STPKNI−−
SPPKNI−−
SLPKNI−−
SSPKNI−−
−SPKNIV−
−SPKNIF−
SHPKNI−−
−SPKNIY−
SFPKNI−−
SKPKNI−−
SAPKNI−−
SEPKNI−−
−SPKNIS−
SGPKNI−−
−SPKNIM−
−SPKNII−
−SPKNIH−
−SPKNIG−
−SPKNIW−
SYPKNI−−
−SPKNIA−
−SPKNIK−
−SPKNIE−
SIPKNI−−
−SPKNIQ−

-SPKNIN-
SNPKNI--
-SPKNIC-
SDPKNI--
SCPKNI--
-TPKNIL-
TIPKNI--
TSPKNI--
-TPKNIM-
TLPKNI--
TRPKNI--
-TPKNIF-
TKPKNI--
-TPKNIA-
TAPKNI--
-TPKNIW-
-TPKNIS-
TFPKNI--
-TPKNII-
-TPKNIY-
-TPKNIR-
TGPKNI--
TEPKNI--
-TPKNIK-
TPPKNI--
TNPKNI--
TTPKNI--
-TPKNIV-
-TPKNIN-
-TPKNIH-
-TPKNIG-
-TPKNIQ-
-TPKNIP-
-TPKNID-
THPKNI--
TDPKNI--
VMPKNI--
-VPKNIW-
-VPKNIV-
-VPKNIL-
VPPKNI--
VVPKNI--
-VPKNIY-
VYPKNI--
VWPKNI--
VSPKNI--
VRPKNI--
VFPKNI--
VHPKNI--
VIPKNI--

VTPKNI--
VQPKNI--
VGPKNI--
-VPKNIM-
VKPKNI--
VEPKNI--
VNPKNI--
-VPKNIG-
-VPKNIQ-
-VPKNIF-
VDPKNI--
-VPKNIN-
-WPKNIL-
WKPKNI--
-WPKNIE-
-WPKNIA-
-WPKNIF-
WSPKNI--
WQPKNI--
WPPKNI--
WYPKNI--
WLPKNI--
-WPKNIK-
WTPKNI--
WEPKNI--
WDPKNI--
WVPKNI--
-WPKNIN-
-WPKNII-
WHPKNI--
WAPKNI--
-WPKNIT-
-WPKNIS-
-WPKNIQ-
WGPKNI--
WRPKNI--
WIPKNI--
YLPKNI--
-YPKNIY-
YRPKNI--
YVPKNI--
YSPKNI--
-YPKNIV-
YEPKNI--
-YPKNIF-
YTPKNI--
-YPKNIK-
YWPKNI--
-YPKNIW-
-YPKNIS-

-YPKNIE-
YIPKNI--
YHPKNI--
YQPKNI--
-YPKNIH-
YKPKNI--
-YPKNIA-
YMPKNI--
YGPKNI--
YAPKNI--
YPPKNI--
-YPKNIT-
-YPKNII-
YFPKNI--
YCPKNI--

PIPM	441	640	46.69

ARPIPM--
-APIPMI-
-APIPML-
AYPIPM--
AAPIPM--
ASPIPM--
-APIPMS-
-APIPMA-
-APIPMN-
AKPIPM--
AHPIPM--
ATPIPM--
-APIPMR-
AMPIPM--
APPIPM--
-APIPMW-
ADPIPM--
-APIPMF-
ALPIPM--
AGPIPM--
AFPIPM--
-APIPMV-
ANPIPM--
AIPIPM--
AWPIPM--
AVPIPM--
-APIPMY-
-APIPMT-
-APIPMM-
CGPIPM--
CKPIPM--
CIPIPM--
CTPIPM--
CLPIPM--

CAPIPM--
CRPIPM--
DTPIPM--
DEPIPM--
-DPIPMR-
DVPIPM--
DSPIPM--
-DPIPMM-
-DPIPMH-
DIPIPM--
DYPIPM--
-DPIPMY-
-DPIPMS-
-DPIPML-
DMPIPM--
DLPIPM--
ERPIPM--
-EPIPMR-
-EPIPMV-
EAPIPM--
EWPIPM--
ETPIPM--
-EPIPME-
ELPIPM--
EVPIPM--
ESPIPM--
EPPIPM--
-EPIPML-
EHPIPM--
FHPIPM--
FRPIPM--
FGPIPM--
FYPIPM--
FLPIPM--
FMPIPM--
FVPIPM--
-FPIPMS-
-FPIPMF-
-FPIPMR-
FSPIPM--
-FPIPMK-
-FPIPMH-
FIPIPM--
FFPIPM--
FAPIPM--
FPPIPM--
-FPIPML-
-FPIPMC-
FTPIPM--
-FPIPMQ-

-FPIPMN-
-FPIPMI-
-FPIPMA-
FDPIPM--
GSPIPM--
-GPIPMM-
-GPIPMR-
GRPIPM--
-GPIPML-
-GPIPMQ-
-GPIPMV-
-GPIPMA-
GDPIPM--
-GPIPMH-
GMPIPM--
GVPIPM--
GGPIPM--
-GPIPMT-
GWPIPM--
GNPIPM--
GHPIPM--
GTPIPM--
-GPIPMY-
GCPIPM--
-GPIPMF-
-GPIPME-
GLPIPM--
-GPIPMS-
-GPIPMN-
GKPIPM--
GFPIPM--
GEPIPM--
HRPIPM--
HMPIPM--
-HPIPMF-
-HPIPMR-
HGPIPM--
-HPIPMK-
-HPIPMT-
-HPIPML-
HWPIPM--
HHPIPM--
HTPIPM--
HSPIPM--
-HPIPMS-
HNPIPM--
HKPIPM--
HIPIPM--
-HPIPMW-
-HPIPMQ-

-HPIPMH-
-HPIPMA-
IGPIPM--
IAPIPM--
-IPIPMT-
-IPIPMR-
ILPIPM--
IFPIPM--
IHPIPM--
-IPIPMA-
IWPIPM--
IRPIPM--
IIPIPM--
-IPIPMN-
IEPIPM--
-IPIPMS-
INPIPM--
ICPIPM--
IQPIPM--
-IPIPMI-
IVPIPM--
ITPIPM--
-IPIPMY-
ISPIPM--
IPPIPM--
-IPIPMK-
-IPIPMH-
-IPIPMG-
IMPIPM--
KSPIPM--
-KPIPMW-
KMPIPM--
KAPIPM--
KGPIPM--
KPPIPM--
KVPIPM--
-KPIPMR-
-KPIPMT-
-KPIPMY-
-KPIPMH-
-KPIPMF-
KCPIPM--
KTPIPM--
KIPIPM--
KQPIPM--
-KPIPMV-
-KPIPMQ-
-KPIPMA-
KFPIPM--
KYPIPM--

−KPIPMN−
−KPIPMG−
−KPIPMC−
LRPIPM−−
LGPIPM−−
−LPIPMR−
LPPIPM−−
LVPIPM−−
−LPIPMT−
−LPIPMK−
−LPIPMY−
−LPIPML−
LSPIPM−−
LAPIPM−−
−LPIPMH−
LLPIPM−−
LYPIPM−−
LDPIPM−−
LWPIPM−−
LIPIPM−−
−LPIPMQ−
−LPIPMN−
LNPIPM−−
LQPIPM−−
LMPIPM−−
MSPIPM−−
−MPIPMS−
MKPIPM−−
MNPIPM−−
MLPIPM−−
−MPIPML−
MGPIPM−−
MYPIPM−−
MRPIPM−−
MAPIPM−−
MVPIPM−−
−MPIPMV−
MWPIPM−−
−MPIPMY−
−MPIPMM−
MPPIPM−−
−MPIPMW−
MFPIPM−−
MQPIPM−−
−MPIPME−
MDPIPM−−
NVPIPM−−
NFPIPM−−
NTPIPM−−
−NPIPMF−

NYPIPM--
NRPIPM--
NIPIPM--
NSPIPM--
NQPIPM--
-NPIPML-
NLPIPM--
-NPIPMY-
NGPIPM--
NWPIPM--
NPPIPM--
-NPIPMV-
-NPIPMN-
-NPIPMM-
-NPIPMK-
-NPIPMI-
-NPIPMQ-
-NPIPMC-
NNPIPM--
NMPIPM--
NKPIPM--
NDPIPM--
--PIPMLM
--PIPMRH
--PIPMHY
--PIPMRV
--PIPMIK
--PIPMVS
--PIPMLS
--PIPMGS
--PIPMSS
--PIPMRR
--PIPMTM
--PIPMRA
--PIPMFQ
--PIPMQG
--PIPMIT
--PIPMNV
--PIPMIR
--PIPMFH
--PIPMTS
--PIPMYY
--PIPMWS
--PIPMLL
--PIPMRT
--PIPMRN
--PIPMMA
--PIPMLK
--PIPMFW
--PIPMVW

--PIPMRK
--PIPMKV
--PIPMFI
--PIPMVI
--PIPMQS
--PIPMLH
--PIPMYR
--PIPMRS
--PIPMRD
--PIPMLA
--PIPMFS
--PIPMVN
--PIPMTV
--PIPMRW
--PIPMLF
--PIPMIW
--PIPMYK
--PIPMVQ
--PIPMVM
--PIPMVL
--PIPMTT
--PIPMLR
--PIPMIV
--PIPMHR
--PIPMVD
--PIPMNA
--PIPMHT
--PIPMHA
--PIPMWR
--PIPMVV
--PIPMSH
--PIPMLQ
--PIPMGA
--PIPMFV
--PIPMFR
--PIPMWA
--PIPMVA
--PIPMTG
--PIPMMR
--PIPMKA
--PIPMIM
--PIPMIA
--PIPMGH
--PIPMFE
--PIPMAN
PEPIPM--
--PIPMYW
--PIPMYN
--PIPMYL
--PIPMNF

--PIPMIN
--PIPMEG
--PIPMDI
PGPIPM--
PNPIPM--
--PIPMVY
--PIPMTN
--PIPMRM
--PIPMPA
--PIPMNS
--PIPMMV
--PIPMLN
--PIPMLG
--PIPMKT
--PIPMKS
--PIPMKQ
--PIPMKG
--PIPMHM
--PIPMGN
--PIPMFK
--PIPMAT
PFPIPM--
PMPIPM--
--PIPMYT
--PIPMWV
--PIPMTR
--PIPMTI
--PIPMRF
--PIPMQY
--PIPMLT
--PIPMKR
--PIPMKM
--PIPMIL
--PIPMIH
--PIPMID
--PIPMHQ
--PIPMES
PTPIPM--
--PIPMWT
--PIPMWK
--PIPMVR
--PIPMTD
--PIPMTA
--PIPMST
--PIPMSR
--PIPMSF
--PIPMSD
--PIPMQW
--PIPMPY
--PIPMPS

--PIPMNN
--PIPMLY
--PIPMKI
--PIPMIY
--PIPMIQ
--PIPMIE
--PIPMFM
--PIPMAS
--PIPMYG
--PIPMWN
--PIPMWE
--PIPMVT
--PIPMSK
--PIPMSA
--PIPMRG
--PIPMQM
--PIPMNQ
--PIPMKY
--PIPMKE
--PIPMII
--PIPMHS
--PIPMGK
--PIPMGG
--PIPMAK
--PIPMVG
--PIPMVE
--PIPMTK
--PIPMTH
--PIPMSQ
--PIPMRE
--PIPMQF
--PIPMPM
--PIPMMT
--PIPMMG
--PIPMMD
--PIPMIS
--PIPMHG
--PIPMDS
--PIPMCF
--PIPMAQ
PKPIPM--
--PIPMYI
--PIPMYH
--PIPMYE
--PIPMYA
--PIPMWW
--PIPMVH
--PIPMTY
--PIPMTF
--PIPMSE

--PIPMRQ
--PIPMRI
--PIPMQV
--PIPMQR
--PIPMPQ
--PIPMPI
--PIPMNT
--PIPMNR
--PIPMNH
--PIPMNG
--PIPMMN
--PIPMMI
--PIPMLW
--PIPMLE
--PIPMKW
--PIPMIG
--PIPMHV
--PIPMHH
--PIPMGW
--PIPMGT
--PIPMGR
--PIPMGQ
--PIPMFP
--PIPMFF
--PIPMFC
--PIPMEP
--PIPMAW
--PIPMAR
QGPIPM--
QSPIPM--
QRPIPM--
-QPIPMV-
-QPIPML-
-QPIPMI-
QNPIPM--
QMPIPM--
QLPIPM--
QPPIPM--
-QPIPMR-
QWPIPM--
-QPIPMS-
-QPIPMA-
QKPIPM--
QDPIPM--
QAPIPM--
-QPIPMG-
-QPIPMT-
-QPIPMN-
QIPIPM--
RLPIPM--

RRPIPM--
RSPIPM--
-RPIPMI-
RFPIPM--
-RPIPMN-
-RPIPML-
RNPIPM--
-RPIPMH-
RGPIPM--
-RPIPMT-
RTPIPM--
RMPIPM--
RVPIPM--
RHPIPM--
-RPIPMS-
RQPIPM--
-RPIPMK-
-RPIPMY-
RAPIPM--
-RPIPMA-
RYPIPM--
-RPIPMR-
-RPIPMF-
REPIPM--
RWPIPM--
RPPIPM--
-RPIPMM-
-RPIPMG-
-RPIPME-
-RPIPMW-
-RPIPMD-
SSPIPM--
SIPIPM--
-SPIPMI-
SNPIPM--
SRPIPM--
-SPIPMT-
-SPIPMS-
SVPIPM--
SMPIPM--
-SPIPML-
-SPIPMY-
-SPIPMW-
SAPIPM--
SYPIPM--
-SPIPMM-
-SPIPMH-
-SPIPMA-
STPIPM--
-SPIPMR-

-SPIPMQ-
SHPIPM--
SGPIPM--
SFPIPM--
SEPIPM--
SDPIPM--
SCPIPM--
-SPIPMN-
-SPIPMK-
-SPIPMG-
SKPIPM--
-TPIPMR-
TLPIPM--
-TPIPML-
TSPIPM--
-TPIPMV-
-TPIPMS-
TAPIPM--
-TPIPMY-
TDPIPM--
TWPIPM--
TFPIPM--
TYPIPM--
-TPIPMF-
TIPIPM--
TVPIPM--
-TPIPMW-
-TPIPMQ-
-TPIPMM-
TRPIPM--
-TPIPMA-
-TPIPMT-
-TPIPMK-
TGPIPM--
TKPIPM--
THPIPM--
-TPIPMG-
-TPIPMN-
-TPIPME-
TNPIPM--
TMPIPM--
VQPIPM--
VYPIPM--
VRPIPM--
VGPIPM--
VFPIPM--
VKPIPM--
VHPIPM--
-VPIPMF-
-VPIPMY-

-VPIPML-
VNPIPM--
VLPIPM--
-VPIPMQ-
-VPIPMS-
-VPIPMN-
-VPIPMH-
VAPIPM--
VIPIPM--
-VPIPMW-
-VPIPMV-
-VPIPMA-
VTPIPM--
-VPIPMT-
VVPIPM--
-VPIPMM-
-VPIPMG-
-VPIPME-
VMPIPM--
WRPIPM--
WVPIPM--
WNPIPM--
WQPIPM--
WIPIPM--
-WPIPMS-
-WPIPMK-
-WPIPMA-
-WPIPML-
WAPIPM--
WSPIPM--
-WPIPMY-
-WPIPMT-
-WPIPMR-
WFPIPM--
-YPIPMH-
YLPIPM--
-YPIPMM-
-YPIPMS-
-YPIPMV-
-YPIPMA-
YPPIPM--
YYPIPM--
YTPIPM--
-YPIPMI-
-YPIPMY-
-YPIPMK-
YGPIPM--
YWPIPM--
-YPIPMT-
YDPIPM--

	-YPIPMW-		
	-YPIPMN-		
	-YPIPML-		
	-YPIPMD-		
	YMPIPM--		
	YEPIPM--		
PMPM	732	554	46.67
	-APMPMR-		
	AFPMPM--		
	-APMPMN-		
	-APMPMM-		
	-APMPMW-		
	ATPMPM--		
	-APMPMT-		
	-APMPMK-		
	-APMPMH-		
	AGPMPM--		
	-APMPMF-		
	AMPMPM--		
	AAPMPM--		
	-APMPMI-		
	-APMPMG-		
	AHPMPM--		
	AWPMPM--		
	AVPMPM--		
	ASPMPM--		
	APPMPM--		
	-APMPMY-		
	ANPMPM--		
	-CPMPMS-		
	-CPMPMW-		
	-CPMPMR-		
	CWPMPM--		
	CSPMPM--		
	CKPMPM--		
	-DPMPMT-		
	-DPMPMF-		
	DIPMPM--		
	DRPMPM--		
	-DPMPMH-		
	-DPMPMS-		
	-DPMPMN-		
	-DPMPML-		
	-DPMPMV-		
	-DPMPMI-		
	DNPMPM--		
	DLPMPM--		
	DYPMPM--		
	-DPMPMY-		
	-DPMPMM-		

DDPMPM--
DAPMPM--
-EPMPMY-
ETPMPM--
EWPMPM--
-EPMPMV-
-EPMPMR-
ELPMPM--
-EPMPMM-
EYPMPM--
EVPMPM--
-EPMPMN-
EQPMPM--
-EPMPMI-
EIPMPM--
EFPMPM--
-FPMPMW-
FTPMPM--
-FPMPMF-
FYPMPM--
FSPMPM--
-FPMPMS-
FNPMPM--
-FPMPMI-
FDPMPM--
-FPMPMY-
-FPMPMR-
-FPMPMN-
FFPMPM--
-FPMPMM-
FCPMPM--
FLPMPM--
FWPMPM--
-FPMPMK-
-FPMPMA-
FVPMPM--
FRPMPM--
-FPMPMV-
-FPMPME-
-FPMPMC-
FKPMPM--
-GPMPML-
-GPMPMY-
-GPMPMR-
GSPMPM--
GYPMPM--
GLPMPM--
GMPMPM--
GRPMPM--
-GPMPMW-

-GPMPMF-
-GPMPMS-
GWPMPM--
GVPMPM--
-GPMPMN-
GGPMPM--
GTPMPM--
GHPMPM--
-GPMPMV-
GKPMPM--
HTPMPM--
HIPMPM--
-HPMPMS-
HWPMPM--
HQPMPM--
-HPMPMQ-
-HPMPML-
-HPMPMA-
HGPMPM--
HFPMPM--
HEPMPM--
-HPMPMK-
HKPMPM--
HVPMPM--
-HPMPMR-
-HPMPMH-
HNPMPM--
-IPMPML-
IGPMPM--
-IPMPMF-
IFPMPM--
IWPMPM--
-IPMPMQ-
-IPMPMK-
ILPMPM--
ISPMPM--
-IPMPMW-
IDPMPM--
-IPMPMR-
-IPMPMI-
IPPMPM--
-IPMPMY-
-IPMPME-
IEPMPM--
IAPMPM--
KSPMPM--
KPPMPM--
-KPMPMR-
-KPMPMK-
KRPMPM--

KGPMPM--
-KPMPML-
-KPMPMS-
KWPMPM--
KVPMPM--
KLPMPM--
-KPMPMH-
KQPMPM--
-KPMPMA-
-KPMPMV-
-KPMPMW-
-KPMPMI-
KTPMPM--
-KPMPMT-
-KPMPMQ-
-KPMPMN-
-KPMPMM-
-LPMPMK-
-LPMPMV-
-LPMPMA-
LYPMPM--
-LPMPMT-
LMPMPM--
LGPMPM--
LVPMPM--
LTPMPM--
-LPMPMW-
-LPMPMR-
-LPMPMY-
LAPMPM--
LSPMPM--
LQPMPM--
LNPMPM--
LWPMPM--
LPPMPM--
-LPMPMM-
-LPMPML-
-LPMPMN-
LLPMPM--
LFPMPM--
-LPMPMF-
MRPMPM--
-MPMPML-
-MPMPMF-
MGPMPM--
MSPMPM--
-MPMPMR-
-MPMPMH-
MMPMPM--
MQPMPM--

MNPMPM--
MAPMPM--
-MPMPMS-
-MPMPMK-
MYPMPM--
-MPMPMW-
-MPMPMT-
MLPMPM--
MHPMPM--
MVPMPM--
-MPMPMI-
-MPMPMM-
MIPMPM--
MEPMPM--
-MPMPMV-
MFPMPM--
-NPMPMY-
NNPMPM--
-NPMPMK-
-NPMPMW-
NIPMPM--
NLPMPM--
-NPMPML-
NGPMPM--
NRPMPM--
NPPMPM--
-NPMPMI-
NEPMPM--
NSPMPM--
NVPMPM--
-NPMPMV-
-NPMPMR-
NFPMPM--
NDPMPM--
NTPMPM--
-NPMPMT-
-NPMPMS-
NKPMPM--
--PMPMLS
--PMPMHS
--PMPMRK
--PMPMFM
--PMPMRG
--PMPMVI
--PMPMTA
--PMPMMG
--PMPMIG
--PMPMYL
--PMPMVS
--PMPMVF

--PMPMIT
--PMPMTS
--PMPMGS
--PMPMWT
--PMPMVY
--PMPMVL
--PMPMSS
--PMPMRV
--PMPMLK
--PMPMYQ
--PMPMSN
--PMPMSA
--PMPMQG
--PMPMST
--PMPMRR
--PMPMYG
--PMPMVR
--PMPMFG
--PMPMAH
--PMPMVN
--PMPMSH
--PMPMRS
--PMPMQY
--PMPMLH
--PMPMKT
--PMPMWR
--PMPMVK
--PMPMVH
--PMPMTI
--PMPMNT
--PMPMLT
--PMPMYK
--PMPMYA
--PMPMVV
--PMPMTK
--PMPMRY
--PMPMRQ
--PMPMPM
--PMPMLN
--PMPMLL
--PMPMLG
--PMPMKE
--PMPMIR
--PMPMIA
--PMPMYS
--PMPMVA
--PMPMIY
--PMPMIL
--PMPMHT
--PMPMAS

--PMPMAR
--PMPMAA
--PMPMYT
--PMPMYD
--PMPMWV
--PMPMWG
--PMPMLY
--PMPMAT
PQPMPM--
--PMPMWI
--PMPMVG
--PMPMTW
--PMPMTV
--PMPMRA
--PMPMPW
--PMPMKM
--PMPMHQ
PWPMPM--
--PMPMWA
--PMPMQR
--PMPMQM
--PMPMNR
--PMPMMV
--PMPMMF
--PMPMLR
--PMPMLQ
--PMPMLE
--PMPMLA
--PMPMIH
--PMPMHH
--PMPMFS
--PMPMEA
--PMPMAN
PNPMPM--
--PMPMYY
--PMPMYE
--PMPMWH
--PMPMTG
--PMPMSD
--PMPMRF
--PMPMRD
--PMPMQT
--PMPMKQ
--PMPMKH
--PMPMFN
--PMPMYR
--PMPMWM
--PMPMPA
--PMPMMH
--PMPMKR

--PMPMEI
--PMPMAW
--PMPMTR
--PMPMRN
--PMPMRE
--PMPMQW
--PMPMQE
--PMPMPQ
--PMPMNS
--PMPMNA
--PMPMMQ
--PMPMIS
--PMPMHV
--PMPMHR
--PMPMHN
--PMPMGN
--PMPMEV
--PMPMEN
--PMPMDF
--PMPMYN
--PMPMWN
--PMPMVD
--PMPMSG
--PMPMQK
--PMPMQA
--PMPMNW
--PMPMNG
--PMPMMR
--PMPMLV
--PMPMLI
--PMPMLD
--PMPMKV
--PMPMKK
--PMPMGG
--PMPMEF
--PMPMDY
--PMPMDQ
--PMPMCT
PGPMPM--
PEPMPM--
PTPMPM--
--PMPMYW
--PMPMWK
--PMPMVQ
--PMPMTQ
--PMPMSW
--PMPMSP
--PMPMQN
--PMPMPE
--PMPMNP

--PMPMMT
--PMPMMM
--PMPMME
--PMPMLC
--PMPMKY
--PMPMID
--PMPMHA
--PMPMGE
--PMPMFV
--PMPMFQ
--PMPMFC
--PMPMEY
--PMPMES
--PMPMCR
--PMPMCG
--PMPMAQ
--PMPMAK
--PMPMAE
-QPMPMW-
QMPMPM--
-QPMPMH-
-QPMPMR-
QNPMPM--
-QPMPMI-
QFPMPM--
QYPMPM--
QVPMPM--
-QPMPMT-
-QPMPMS-
-QPMPMQ-
-QPMPMY-
-QPMPMN-
RVPMPM--
RGPMPM--
RRPMPM--
RTPMPM--
RNPMPM--
RSPMPM--
RPPMPM--
-RPMPMV-
-RPMPMK-
RMPMPM--
RIPMPM--
RWPMPM--
RQPMPM--
-RPMPMQ-
-RPMPMN-
REPMPM--
-RPMPMR-
RYPMPM--

-RPMPMM-
RFPMPM--
RAPMPM--
-RPMPMY-
-RPMPMH-
RHPMPM--
RDPMPM--
-RPMPMI-
-RPMPMG-
-RPMPMA-
-SPMPMH-
-SPMPMV-
-SPMPML-
-SPMPMT-
SRPMPM--
STPMPM--
-SPMPMQ-
-SPMPMK-
SEPMPM--
SSPMPM--
SQPMPM--
-SPMPMW-
SVPMPM--
-SPMPMM-
SFPMPM--
SLPMPM--
SPPMPM--
SAPMPM--
SYPMPM--
-SPMPMF-
SHPMPM--
-SPMPMN-
-SPMPMG-
-SPMPME-
-SPMPMA-
SDPMPM--
-TPMPMR-
TTPMPM--
-TPMPMV-
-TPMPMW-
TYPMPM--
-TPMPMY-
-TPMPMA-
TSPMPM--
TRPMPM--
-TPMPMN-
TNPMPM--
THPMPM--
TPPMPM--
-TPMPMS-

TKPMPM--
TVPMPM--
-TPMPMT-
-TPMPML-
TGPMPM--
VVPMPM--
-VPMPMW-
VRPMPM--
VHPMPM--
VWPMPM--
-VPMPMS-
VTPMPM--
-VPMPMI-
-VPMPMT-
-VPMPMQ-
-VPMPML-
-VPMPMH-
VLPMPM--
VSPMPM--
VKPMPM--
VAPMPM--
-VPMPMN-
-VPMPME-
VQPMPM--
-VPMPMV-
-VPMPMR-
VNPMPM--
VFPMPM--
VPPMPM--
WPPMPM--
WSPMPM--
WVPMPM--
WGPMPM--
WRPMPM--
-WPMPMT-
-WPMPMH-
-WPMPMN-
WAPMPM--
WYPMPM--
-WPMPMV-
-WPMPMS-
WNPMPM--
-WPMPMY-
YTPMPM--
-YPMPMI-
-YPMPMK-
-YPMPMQ-
YFPMPM--
YWPMPM--
YRPMPM--

```
            -YPMPMR-
            -YPMPMH-
            YLPMPM--
            YVPMPM--
            YMPMPM--
            -YPMPMY-
            YEPMPM--
            -YPMPMS-
            -YPMPMN-
            -YPMPMG-
            -YPMPME-
PSAY          20         881      46.65
            -APSAYP-
            -NPSAYN-
            --PSAYGR
            --PSAYPR
            --PSAYIP
            --PSAYHD
            -YPSAYE-
            -APSAYL-
            -APSAYR-
            -APSAYM-
            AMPSAY--
            AIPSAY--
            ASPSAY--
            -APSAYV-
            AYPSAY--
            -APSAYI-
            ARPSAY--
            -APSAYY-
            -APSAYT-
            AQPSAY--
            -APSAYA-
            -APSAYK-
            AKPSAY--
            AGPSAY--
            -APSAYF-
            AWPSAY--
            AFPSAY--
            AAPSAY--
            -APSAYQ-
            ALPSAY--
            ACPSAY--
            AVPSAY--
            ADPSAY--
            AEPSAY--
            APPSAY--
            -APSAYH-
            AHPSAY--
            -APSAYN-
```

-APSAYG-
ATPSAY--
-APSAYE-
CTPSAY--
CGPSAY--
CRPSAY--
CVPSAY--
CKPSAY--
CEPSAY--
CSPSAY--
CAPSAY--
CQPSAY--
-CPSAYT-
CHPSAY--
CFPSAY--
-CPSAYR-
-CPSAYI-
-CPSAYV-
-CPSAYQ-
CLPSAY--
CYPSAY--
-CPSAYY-
-CPSAYS-
-CPSAYK-
CPPSAY--
DTPSAY--
DGPSAY--
DWPSAY--
DQPSAY--
DKPSAY--
DVPSAY--
DRPSAY--
-DPSAYY-
DEPSAY--
DLPSAY--
DAPSAY--
DIPSAY--
-DPSAYS-
-DPSAYQ-
-DPSAYL-
-DPSAYI-
DHPSAY--
-DPSAYT-
-DPSAYK-
-DPSAYF-
-DPSAYD-
DNPSAY--
DMPSAY--
DFPSAY--
ERPSAY--

-EPSAYR-
EYPSAY--
EKPSAY--
EWPSAY--
ESPSAY--
ELPSAY--
-EPSAYS-
-EPSAYQ-
EVPSAY--
EGPSAY--
EFPSAY--
-EPSAYL-
ETPSAY--
-EPSAYH-
EAPSAY--
EMPSAY--
EHPSAY--
-EPSAYM-
-EPSAYI-
EEPSAY--
-EPSAYT-
-EPSAYW-
ENPSAY--
EDPSAY--
-EPSAYY-
EIPSAY--
EQPSAY--
-EPSAYV-
-EPSAYN-
-EPSAYA-
EPPSAY--
ECPSAY--
-FPSAYR-
FSPSAY--
FTPSAY--
FIPSAY--
FLPSAY--
FYPSAY--
FQPSAY--
-FPSAYV-
-FPSAYM-
FHPSAY--
FFPSAY--
-FPSAYL-
FAPSAY--
-FPSAYT-
FMPSAY--
-FPSAYI-
FNPSAY--
FKPSAY--

-FPSAYY-
-FPSAYS-
-FPSAYF-
FWPSAY--
FDPSAY--
-FPSAYQ-
-FPSAYH-
FGPSAY--
FEPSAY--
FVPSAY--
FPPSAY--
-FPSAYW-
-FPSAYN-
-FPSAYK-
FCPSAY--
GRPSAY--
-GPSAYL-
GGPSAY--
GYPSAY--
GMPSAY--
-GPSAYM-
-GPSAYR-
GKPSAY--
GWPSAY--
-GPSAYY-
GAPSAY--
GQPSAY--
GLPSAY--
-GPSAYT-
-GPSAYK-
GVPSAY--
GSPSAY--
-GPSAYF-
GTPSAY--
GNPSAY--
GEPSAY--
-GPSAYV-
GIPSAY--
-GPSAYQ-
-GPSAYS-
GFPSAY--
-GPSAYH-
GDPSAY--
-GPSAYW-
-GPSAYI-
-GPSAYG-
-GPSAYE-
GPPSAY--
GCPSAY--
GHPSAY--

HTPSAY--
-HPSAYR-
HRPSAY--
-HPSAYT-
HAPSAY--
-HPSAYV-
HNPSAY--
-HPSAYY-
-HPSAYL-
-HPSAYS-
HLPSAY--
HKPSAY--
-HPSAYK-
HGPSAY--
-HPSAYH-
HYPSAY--
-HPSAYA-
-HPSAYQ-
-HPSAYI-
HFPSAY--
HSPSAY--
-HPSAYM-
HQPSAY--
-HPSAYW-
HMPSAY--
HHPSAY--
HWPSAY--
HVPSAY--
HEPSAY--
-HPSAYF-
HPPSAY--
HIPSAY--
HCPSAY--
-IPSAYR-
-IPSAYL-
IAPSAY--
ILPSAY--
IIPSAY--
IVPSAY--
-IPSAYI-
IEPSAY--
-IPSAYK-
-IPSAYH-
IGPSAY--
-IPSAYS-
IYPSAY--
-IPSAYW-
-IPSAYT-
-IPSAYM-
-IPSAYV-

```
-IPSAYY-
IPPSAY--
ISPSAY--
ITPSAY--
IRPSAY--
-IPSAYQ-
INPSAY--
-IPSAYN-
-IPSAYE-
IMPSAY--
-IPSAYF-
IFPSAY--
-IPSAYG-
-IPSAYA-
IDPSAY--
IWPSAY--
ICPSAY--
KSPSAY--
-KPSAYL-
-KPSAYI-
KGPSAY--
KRPSAY--
KVPSAY--
-KPSAYT-
KKPSAY--
KFPSAY--
-KPSAYS-
KNPSAY--
KQPSAY--
KLPSAY--
KAPSAY--
-KPSAYK-
-KPSAYM-
-KPSAYQ-
-KPSAYY-
-KPSAYF-
KWPSAY--
-KPSAYA-
KYPSAY--
-KPSAYV-
KEPSAY--
-KPSAYH-
-KPSAYW-
KCPSAY--
KPPSAY--
KHPSAY--
-KPSAYN-
-KPSAYE-
-KPSAYC-
KMPSAY--
```

LRPSAY--
LTPSAY--
-LPSAYT-
LGPSAY--
LLPSAY--
LKPSAY--
LVPSAY--
LNPSAY--
LWPSAY--
LEPSAY--
-LPSAYV-
LIPSAY--
LQPSAY--
LYPSAY--
LHPSAY--
-LPSAYY-
-LPSAYR-
-LPSAYM-
LMPSAY--
-LPSAYW-
-LPSAYI-
LCPSAY--
-LPSAYH-
LPPSAY--
-LPSAYS-
-LPSAYK-
LDPSAY--
MRPSAY--
MTPSAY--
MYPSAY--
MAPSAY--
MWPSAY--
MVPSAY--
-MPSAYA-
-MPSAYL-
MMPSAY--
MHPSAY--
MGPSAY--
-MPSAYK-
-MPSAYM-
MKPSAY--
-MPSAYS-
-MPSAYI-
MNPSAY--
MEPSAY--
-MPSAYV-
-MPSAYT-
MFPSAY--
MQPSAY--
-MPSAYQ-

-MPSAYH-
MPPSAY--
MDPSAY--
-MPSAYW-
-MPSAYE-
-MPSAYF-
MLPSAY--
MCPSAY--
NSPSAY--
NRPSAY--
NFPSAY--
-NPSAYR-
NWPSAY--
NMPSAY--
-NPSAYK-
NKPSAY--
NYPSAY--
-NPSAYM-
NNPSAY--
NLPSAY--
NTPSAY--
-NPSAYV-
NAPSAY--
-NPSAYY-
-NPSAYT-
-NPSAYI-
NGPSAY--
NQPSAY--
-NPSAYS-
NIPSAY--
-NPSAYW-
-NPSAYQ-
-NPSAYL-
-NPSAYH-
-NPSAYF-
NPPSAY--
NVPSAY--
NEPSAY--
NDPSAY--
-NPSAYC-
NHPSAY--
--PSAYFS
--PSAYRG
--PSAYFR
--PSAYLT
--PSAYGL
--PSAYSL
--PSAYHS
--PSAYSA
--PSAYRH

--PSAYSS
--PSAYMK
--PSAYRQ
--PSAYTR
--PSAYLS
--PSAYYG
--PSAYWA
--PSAYMR
--PSAYVM
--PSAYRI
PRPSAY--
--PSAYSY
--PSAYVF
--PSAYHT
--PSAYSG
--PSAYAS
PNPSAY--
--PSAYRA
--PSAYLV
PGPSAY--
--PSAYMG
--PSAYLY
--PSAYIA
--PSAYFA
--PSAYSV
--PSAYTL
--PSAYSW
--PSAYRY
--PSAYGM
PYPSAY--
--PSAYVS
--PSAYMW
--PSAYVG
--PSAYMS
--PSAYKV
--PSAYIR
--PSAYYS
--PSAYWL
--PSAYMV
--PSAYLH
--PSAYTV
--PSAYLN
PSPSAY--
--PSAYWT
--PSAYTH
--PSAYRF
--PSAYQV
--PSAYLM
--PSAYVN
--PSAYTW

--PSAYSH
--PSAYKR
--PSAYIS
--PSAYFK
--PSAYAG
--PSAYWH
--PSAYVK
--PSAYFN
--PSAYAY
--PSAYYH
--PSAYVY
--PSAYSM
--PSAYRE
--PSAYNS
--PSAYLG
--PSAYSQ
--PSAYRT
--PSAYHR
--PSAYST
--PSAYMT
--PSAYKS
--PSAYGS
--PSAYFG
PKPSAY--
--PSAYSI
--PSAYQY
--PSAYNY
--PSAYIG
--PSAYES
--PSAYAI
PHPSAY--
PEPSAY--
--PSAYTK
--PSAYTI
--PSAYPL
--PSAYMA
--PSAYII
--PSAYHN
--PSAYLW
--PSAYKT
--PSAYGY
--PSAYFM
PLPSAY--
--PSAYYK
--PSAYRV
--PSAYRD
--PSAYQH
--PSAYQA
--PSAYIY
--PSAYHH

--PSAYEG
--PSAYYM
--PSAYWR
--PSAYVT
--PSAYVA
--PSAYTT
--PSAYTN
--PSAYQL
--PSAYIW
--PSAYHK
--PSAYAL
--PSAYWG
--PSAYVI
--PSAYSN
--PSAYNF
--PSAYLK
--PSAYIK
--PSAYYR
--PSAYVW
--PSAYVQ
--PSAYRW
--PSAYIM
--PSAYHY
--PSAYHM
--PSAYHL
--PSAYAM
PAPSAY--
PTPSAY--
--PSAYYN
--PSAYTY
--PSAYRM
--PSAYKI
--PSAYKG
--PSAYGA
--PSAYKQ
--PSAYAA
PVPSAY--
--PSAYNA
--PSAYKM
--PSAYKL
--PSAYKD
--PSAYIV
--PSAYIH
--PSAYHA
--PSAYGW
--PSAYGF
PFPSAY--
--PSAYVV
--PSAYTA
--PSAYKH

--PSAYHG
--PSAYHE
--PSAYGT
--PSAYFH
--PSAYFF
--PSAYWW
--PSAYTM
--PSAYMN
--PSAYKK
--PSAYKF
--PSAYGQ
--PSAYEY
--PSAYEA
PMPSAY--
--PSAYYW
--PSAYYE
--PSAYYD
--PSAYWN
--PSAYNV
--PSAYNQ
--PSAYNL
--PSAYMH
--PSAYHQ
--PSAYGV
--PSAYWS
--PSAYSF
--PSAYQS
--PSAYQM
--PSAYNT
--PSAYLQ
--PSAYIQ
--PSAYHF
--PSAYFT
--PSAYDL
--PSAYAT
PCPSAY--
--PSAYYA
--PSAYWE
--PSAYVH
--PSAYPI
--PSAYNW
--PSAYMM
--PSAYMF
--PSAYHV
--PSAYHI
--PSAYAV
--PSAYAF
--PSAYWM
--PSAYQR
--PSAYNN

--PSAYNG
--PSAYML
--PSAYME
--PSAYLE
--PSAYKY
--PSAYKA
--PSAYIT
--PSAYIL
--PSAYFD
--PSAYET
--PSAYAR
--PSAYAN
--PSAYAH
PQPSAY--
--PSAYWK
--PSAYQN
--PSAYQF
--PSAYNI
--PSAYMD
--PSAYLF
--PSAYID
--PSAYFQ
--PSAYEQ
--PSAYCS
--PSAYWQ
--PSAYWD
--PSAYVE
--PSAYVD
--PSAYTQ
--PSAYTF
--PSAYTE
--PSAYSD
--PSAYQQ
--PSAYQK
--PSAYPY
--PSAYNR
--PSAYNH
--PSAYMI
--PSAYKW
--PSAYIN
--PSAYIE
--PSAYEM
--PSAYAW
--PSAYAQ
PIPSAY--
PWPSAY--
--PSAYTD
--PSAYQE
--PSAYQD
--PSAYPM

--PSAYNM
--PSAYNC
--PSAYMY
--PSAYKN
--PSAYGI
--PSAYFE
--PSAYEW
--PSAYEV
--PSAYEN
--PSAYEL
--PSAYEI
--PSAYDY
--PSAYCN
--PSAYCM
--PSAYCL
--PSAYAK
-PPSAYS-
QSPSAY--
QLPSAY--
QRPSAY--
-QPSAYS-
QDPSAY--
QGPSAY--
-QPSAYL-
QHPSAY--
QAPSAY--
QKPSAY--
QVPSAY--
-QPSAYK-
QIPSAY--
QTPSAY--
-QPSAYY-
QPPSAY--
-QPSAYI-
QWPSAY--
-QPSAYR-
-QPSAYF-
QMPSAY--
QQPSAY--
QYPSAY--
-QPSAYW-
-QPSAYN-
-QPSAYM-
-QPSAYQ-
-QPSAYH-
-QPSAYA-
RSPSAY--
-RPSAYS-
RVPSAY--
RNPSAY--

RGPSAY--
-RPSAYI-
RRPSAY--
-RPSAYW-
RAPSAY--
-RPSAYF-
RFPSAY--
-RPSAYV-
-RPSAYQ-
RQPSAY--
RIPSAY--
REPSAY--
RKPSAY--
RYPSAY--
RHPSAY--
RWPSAY--
-RPSAYN-
-RPSAYM-
-RPSAYT-
RDPSAY--
-RPSAYY-
-RPSAYH-
RPPSAY--
RCPSAY--
-RPSAYG-
-RPSAYE-
-RPSAYC-
SRPSAY--
STPSAY--
-SPSAYL-
SSPSAY--
SLPSAY--
SGPSAY--
-SPSAYT-
SIPSAY--
SHPSAY--
-SPSAYS-
-SPSAYK-
SPPSAY--
SAPSAY--
-SPSAYI-
SKPSAY--
SNPSAY--
SQPSAY--
-SPSAYY-
SDPSAY--
SYPSAY--
SFPSAY--
SWPSAY--
SMPSAY--

-SPSAYA-
SEPSAY--
-SPSAYF-
-SPSAYH-
-SPSAYM-
-SPSAYW-
-SPSAYE-
SCPSAY--
-SPSAYG-
-SPSAYQ-
TLPSAY--
TVPSAY--
TSPSAY--
-TPSAYT-
TNPSAY--
TAPSAY--
TMPSAY--
TYPSAY--
TGPSAY--
-TPSAYK-
-TPSAYL-
-TPSAYS-
TTPSAY--
TFPSAY--
TKPSAY--
-TPSAYH-
-TPSAYY-
TQPSAY--
TPPSAY--
TDPSAY--
-TPSAYV-
-TPSAYA-
-TPSAYF-
-TPSAYM-
-TPSAYI-
TEPSAY--
-TPSAYW-
-TPSAYN-
TIPSAY--
-TPSAYQ-
TCPSAY--
THPSAY--
TWPSAY--
-TPSAYG-
-TPSAYE-
-TPSAYC-
-VPSAYS-
VLPSAY--
VSPSAY--
VRPSAY--

VGPSAY--
-VPSAYH-
VYPSAY--
-VPSAYM-
VKPSAY--
VVPSAY--
VTPSAY--
-VPSAYI-
-VPSAYQ-
VNPSAY--
VAPSAY--
VWPSAY--
-VPSAYW-
-VPSAYK-
VIPSAY--
VDPSAY--
VFPSAY--
-VPSAYF-
VMPSAY--
VCPSAY--
-VPSAYT-
-VPSAYE-
VHPSAY--
VEPSAY--
VPPSAY--
-VPSAYN-
-VPSAYA-
VQPSAY--
WNPSAY--
WGPSAY--
WRPSAY--
WYPSAY--
WKPSAY--
WAPSAY--
WSPSAY--
WTPSAY--
WQPSAY--
-WPSAYF-
WPPSAY--
WHPSAY--
-WPSAYV-
-WPSAYS-
WMPSAY--
-WPSAYA-
WLPSAY--
-WPSAYY-
-WPSAYM-
WIPSAY--
-WPSAYH-
-WPSAYQ-

-WPSAYW-
WWPSAY--
WCPSAY--
-WPSAYT-
-WPSAYI-
-WPSAYK-
WFPSAY--
YRPSAY--
-YPSAYT-
-YPSAYL-
YDPSAY--
-YPSAYR-
YAPSAY--
YHPSAY--
YTPSAY--
YSPSAY--
-YPSAYS-
YYPSAY--
-YPSAYM-
YPPSAY--
YFPSAY--
-YPSAYA-
YVPSAY--
-YPSAYW-
YMPSAY--
YNPSAY--
YIPSAY--
YQPSAY--
-YPSAYK-
YLPSAY--
YKPSAY--
-YPSAYF-
YEPSAY--
-YPSAYI-
YGPSAY--
-YPSAYY-
-YPSAYQ-
-YPSAYN-
-YPSAYH-
YWPSAY--
-YPSAYV-
YCPSAY--
-YPSAYC-
PWPY 476 628 46.64
-LPWPYG-
AAPWPY--
ASPWPY--
ALPWPY--
ARPWPY--
AKPWPY--

-APWPYS-
-APWPYL-
-APWPYI-
AGPWPY--
APPWPY--
AMPWPY--
AYPWPY--
-APWPYQ-
AWPWPY--
ANPWPY--
-APWPYR-
-APWPYK-
AIPWPY--
ATPWPY--
-APWPYN-
-APWPYY-
AQPWPY--
-APWPYM-
-APWPYG-
ADPWPY--
-APWPYV-
-APWPYH-
-APWPYF-
-APWPYA-
CYPWPY--
CRPWPY--
CLPWPY--
CFPWPY--
CVPWPY--
CTPWPY--
-CPWPYK-
-CPWPYF-
DHPWPY--
-DPWPYF-
DAPWPY--
DRPWPY--
-DPWPYR-
-DPWPYM-
DVPWPY--
DTPWPY--
-DPWPYI-
-DPWPYA-
DLPWPY--
-DPWPYT-
-DPWPYH-
-EPWPYR-
EVPWPY--
ESPWPY--
-EPWPYH-
EHPWPY--

-EPWPYT-
EIPWPY--
-EPWPYL-
-EPWPYF-
-EPWPYI-
ERPWPY--
-EPWPYS-
EPPWPY--
EMPWPY--
FNPWPY--
-FPWPYR-
FHPWPY--
-FPWPYL-
FTPWPY--
FGPWPY--
FPPWPY--
-FPWPYI-
FEPWPY--
-FPWPYM-
-FPWPYN-
-FPWPYK-
FLPWPY--
FRPWPY--
-FPWPYT-
-FPWPYW-
-FPWPYF-
FFPWPY--
FCPWPY--
FAPWPY--
GGPWPY--
GMPWPY--
GRPWPY--
GAPWPY--
-GPWPYT-
GTPWPY--
-GPWPYL-
-GPWPYH-
-GPWPYS-
GFPWPY--
GSPWPY--
GNPWPY--
GDPWPY--
GQPWPY--
-GPWPYW-
-GPWPYM-
-GPWPYA-
GPPWPY--
GLPWPY--
GHPWPY--
-GPWPYN-

-GPWPYV-
GIPWPY--
-GPWPYY-
-GPWPYK-
-GPWPYE-
GYPWPY--
GVPWPY--
GKPWPY--
-HPWPYI-
HSPWPY--
HKPWPY--
HPPWPY--
HHPWPY--
HRPWPY--
HTPWPY--
-HPWPYY-
-HPWPYR-
HNPWPY--
-HPWPYW-
HLPWPY--
-HPWPYQ-
-HPWPYF-
HMPWPY--
HDPWPY--
HGPWPY--
-HPWPYL-
HIPWPY--
HYPWPY--
HQPWPY--
-HPWPYV-
HAPWPY--
IRPWPY--
-IPWPYR-
ILPWPY--
IFPWPY--
IQPWPY--
IAPWPY--
IYPWPY--
-IPWPYA-
-IPWPYN-
-IPWPYK-
IIPWPY--
ISPWPY--
-IPWPYH-
IGPWPY--
INPWPY--
-IPWPYT-
-IPWPYG-
IKPWPY--
IDPWPY--

-IPWPYV-
-IPWPYS-
-IPWPYI-
-IPWPYY-
IPPWPY--
KSPWPY--
-KPWPYS-
KTPWPY--
KRPWPY--
-KPWPYQ-
-KPWPYF-
KLPWPY--
KAPWPY--
KGPWPY--
KFPWPY--
KVPWPY--
-KPWPYH-
KQPWPY--
KKPWPY--
KWPWPY--
-KPWPYA-
-KPWPYT-
-KPWPYR-
-KPWPYE-
KEPWPY--
KYPWPY--
-KPWPYI-
KIPWPY--
LRPWPY--
LGPWPY--
LLPWPY--
LFPWPY--
LNPWPY--
LSPWPY--
LAPWPY--
-LPWPYV-
-LPWPYI-
-LPWPYS-
-LPWPYL-
LIPWPY--
LQPWPY--
LKPWPY--
LEPWPY--
LTPWPY--
-LPWPYT-
-LPWPYH-
LHPWPY--
LWPWPY--
-LPWPYW-
LDPWPY--

LYPWPY--
-LPWPYQ-
-LPWPYK-
-LPWPYF-
-LPWPYA-
LPPWPY--
-MPWPYR-
MHPWPY--
-MPWPYL-
MPPWPY--
MRPWPY--
MLPWPY--
MMPWPY--
MAPWPY--
-MPWPYI-
MGPWPY--
MFPWPY--
MSPWPY--
-MPWPYT-
MKPWPY--
MQPWPY--
-MPWPYV-
-MPWPYF-
-MPWPYA-
MIPWPY--
MVPWPY--
MEPWPY--
-MPWPYG-
NRPWPY--
NHPWPY--
NSPWPY--
NLPWPY--
NFPWPY--
NWPWPY--
NGPWPY--
NMPWPY--
-NPWPYS-
-NPWPYI-
NQPWPY--
-NPWPYM-
NVPWPY--
NTPWPY--
-NPWPYV-
-NPWPYF-
NAPWPY--
NNPWPY--
NIPWPY--
-NPWPYA-
NKPWPY--
NEPWPY--

--PWPYLL
--PWPYLS
--PWPYSS
--PWPYRT
--PWPYYL
--PWPYLG
--PWPYVS
--PWPYVF
--PWPYRV
--PWPYFL
--PWPYSH
--PWPYLT
--PWPYTN
--PWPYFS
--PWPYSK
--PWPYHI
--PWPYDY
--PWPYMS
--PWPYMG
--PWPYQS
--PWPYHG
--PWPYRI
--PWPYRD
--PWPYVW
--PWPYVV
--PWPYRQ
--PWPYPW
--PWPYFV
--PWPYFG
PSPWPY--
--PWPYVN
--PWPYQY
--PWPYQN
--PWPYMH
--PWPYVQ
--PWPYTG
--PWPYSF
--PWPYLV
--PWPYLR
--PWPYHK
--PWPYVT
--PWPYTA
--PWPYSR
--PWPYNF
--PWPYIN
--PWPYGQ
--PWPYTI
--PWPYKV
--PWPYHY
PVPWPY--

--PWPYQI
--PWPYQH
--PWPYLY
--PWPYKR
--PWPYAH
PIPWPY--
PAPWPY--
--PWPYYR
--PWPYWS
--PWPYVI
--PWPYTW
--PWPYRK
--PWPYRE
--PWPYMQ
--PWPYKS
PNPWPY--
PGPWPY--
--PWPYYT
--PWPYYK
--PWPYVY
--PWPYVG
--PWPYTS
--PWPYTR
--PWPYSA
--PWPYRY
--PWPYQT
--PWPYNW
--PWPYLH
--PWPYKI
--PWPYIV
--PWPYIL
--PWPYHF
--PWPYFK
--PWPYFA
PMPWPY--
--PWPYYM
--PWPYYA
--PWPYWA
--PWPYVR
--PWPYVA
--PWPYPM
--PWPYMT
--PWPYMR
--PWPYMI
--PWPYMF
--PWPYLW
--PWPYKY
--PWPYIR
--PWPYHT
--PWPYHS

--PWPYET
--PWPYAA
--PWPYYG
--PWPYWY
--PWPYWK
--PWPYWG
--PWPYTT
--PWPYTH
--PWPYTF
--PWPYSG
--PWPYSD
--PWPYPS
--PWPYNS
--PWPYNH
--PWPYLD
--PWPYLA
--PWPYIH
--PWPYHH
--PWPYFH
--PWPYEV
--PWPYAS
PKPWPY--
PEPWPY--
PDPWPY--
--PWPYYY
--PWPYYS
--PWPYYN
--PWPYYH
--PWPYWD
--PWPYVD
--PWPYTM
--PWPYTK
--PWPYSQ
--PWPYSN
--PWPYRN
--PWPYRG
--PWPYQW
--PWPYQR
--PWPYQG
--PWPYQA
--PWPYMY
--PWPYME
--PWPYKA
--PWPYGW
--PWPYDF
--PWPYAN
--PWPYYI
--PWPYYD
--PWPYWV
--PWPYST

--PWPYRW
--PWPYRS
--PWPYRF
--PWPYRA
--PWPYQK
--PWPYPH
--PWPYNQ
--PWPYNK
--PWPYMM
--PWPYLK
--PWPYKK
--PWPYKG
--PWPYIY
--PWPYIM
--PWPYGS
--PWPYGG
--PWPYFR
--PWPYFN
--PWPYES
--PWPYDQ
--PWPYAG
--PWPYYW
--PWPYYV
--PWPYYQ
--PWPYYF
--PWPYVH
--PWPYTQ
--PWPYQV
--PWPYQD
--PWPYNT
--PWPYLN
--PWPYLI
--PWPYKM
--PWPYKE
--PWPYIW
--PWPYIS
--PWPYIQ
--PWPYIA
--PWPYHN
--PWPYHE
--PWPYHD
--PWPYEN
--PWPYEI
--PWPYCL
--PWPYAR
PWPWPY--
PTPWPY--
-PPWPYV-
QGPWPY--
-QPWPYL-

QSPWPY--
QHPWPY--
-QPWPYH-
-QPWPYI-
QRPWPY--
QQPWPY--
-QPWPYY-
-QPWPYS-
QAPWPY--
-QPWPYM-
-QPWPYW-
QNPWPY--
QLPWPY--
QIPWPY--
QWPWPY--
QPPWPY--
-QPWPYN-
-QPWPYG-
QMPWPY--
RSPWPY--
RKPWPY--
-RPWPYS-
-RPWPYL-
RRPWPY--
-RPWPYV-
RNPWPY--
-RPWPYT-
-RPWPYK-
RGPWPY--
-RPWPYR-
RQPWPY--
-RPWPYH-
-RPWPYF-
RPPWPY--
RWPWPY--
RVPWPY--
RLPWPY--
RIPWPY--
RFPWPY--
REPWPY--
-RPWPYY-
-RPWPYW-
RMPWPY--
RTPWPY--
-RPWPYI-
RYPWPY--
-RPWPYQ-
-RPWPYN-
-RPWPYM-
-RPWPYA-

RHPWPY--
SRPWPY--
SLPWPY--
STPWPY--
-SPWPYY-
SVPWPY--
SSPWPY--
-SPWPYM-
SFPWPY--
SKPWPY--
SYPWPY--
-SPWPYS-
-SPWPYF-
SMPWPY--
-SPWPYT-
-SPWPYW-
-SPWPYA-
SIPWPY--
SQPWPY--
SHPWPY--
SGPWPY--
-SPWPYV-
SWPWPY--
-SPWPYI-
-SPWPYR-
-SPWPYK-
SNPWPY--
SAPWPY--
-SPWPYE-
SPPWPY--
SEPWPY--
SDPWPY--
TSPWPY--
TLPWPY--
TRPWPY--
-TPWPYR-
-TPWPYS-
TQPWPY--
TPPWPY--
TFPWPY--
TEPWPY--
TTPWPY--
TNPWPY--
TGPWPY--
TAPWPY--
-TPWPYI-
TVPWPY--
-TPWPYT-
-TPWPYH-
-TPWPYW-

-TPWPYL-
-TPWPYF-
TWPWPY--
-TPWPYV-
THPWPY--
-TPWPYK-
-TPWPYA-
TCPWPY--
-TPWPYN-
TKPWPY--
VTPWPY--
VSPWPY--
VEPWPY--
VNPWPY--
-VPWPYW-
VVPWPY--
VGPWPY--
VLPWPY--
-VPWPYI-
-VPWPYY-
-VPWPYS-
VYPWPY--
-VPWPYN-
-VPWPYL-
VPPWPY--
VMPWPY--
VIPWPY--
VWPWPY--
VRPWPY--
-VPWPYK-
-VPWPYF-
-VPWPYT-
-VPWPYM-
-VPWPYG-
VHPWPY--
-VPWPYA-
VCPWPY--
WKPWPY--
WLPWPY--
-WPWPYR-
WGPWPY--
WSPWPY--
WYPWPY--
-WPWPYV-
-WPWPYL-
WMPWPY--
WRPWPY--
-WPWPYS-
WEPWPY--
WDPWPY--

-WPWPYT-
WFPWPY--
WNPWPY--
YLPWPY--
YIPWPY--
YGPWPY--
YTPWPY--
-YPWPYK-
-YPWPYT-
YKPWPY--
-YPWPYR-
-YPWPYI-
YHPWPY--
YSPWPY--
-YPWPYM-
YFPWPY--
YQPWPY--
-YPWPYV-
-YPWPYQ-
-YPWPYY-
-YPWPYH-
YAPWPY--

PFNY	349	676	46.63

--PFNYGV
--PFNYGL
-APFNYK-
ARPFNY--
AVPFNY--
ASPFNY--
-APFNYY-
AAPFNY--
-APFNYL-
AKPFNY--
-APFNYR-
-APFNYV-
-APFNYS-
-APFNYA-
-APFNYW-
-APFNYF-
AYPFNY--
ALPFNY--
AHPFNY--
AIPFNY--
ATPFNY--
-APFNYN-
AMPFNY--
AGPFNY--
-APFNYG-
-APFNYE-
AFPFNY--

ACPFNY--
-APFNYT-
-APFNYM-
ANPFNY--
CRPFNY--
-CPFNYR-
CHPFNY--
CCPFNY--
CNPFNY--
CGPFNY--
CVPFNY--
CPPFNY--
-CPFNYY-
-CPFNYS-
DHPFNY--
DVPFNY--
DSPFNY--
DRPFNY--
DGPFNY--
-DPFNYR-
-DPFNYQ-
DLPFNY--
DTPFNY--
-DPFNYV-
-DPFNYS-
DQPFNY--
-DPFNYY-
-DPFNYL-
DNPFNY--
DMPFNY--
-EPFNYL-
-EPFNYW-
ERPFNY--
-EPFNYK-
-EPFNYH-
ESPFNY--
-EPFNYV-
ELPFNY--
-EPFNYT-
-EPFNYR-
EVPFNY--
ETPFNY--
EHPFNY--
-EPFNYM-
-EPFNYA-
EGPFNY--
EFPFNY--
-EPFNYS-
-EPFNYN-
-EPFNYF-

-FPFNYR-
-FPFNYT-
-FPFNYS-
-FPFNYQ-
FSPFNY--
FVPFNY--
-FPFNYM-
FKPFNY--
FTPFNY--
-FPFNYK-
-FPFNYA-
FWPFNY--
-FPFNYN-
FLPFNY--
-FPFNYY-
-FPFNYW-
FHPFNY--
FEPFNY--
FDPFNY--
FAPFNY--
FQPFNY--
-FPFNYV-
FGPFNY--
GRPFNY--
GQPFNY--
GMPFNY--
GIPFNY--
-GPFNYS-
-GPFNYR-
GWPFNY--
-GPFNYL-
GSPFNY--
-GPFNYV-
GVPFNY--
GNPFNY--
GLPFNY--
GKPFNY--
GAPFNY--
GDPFNY--
GTPFNY--
GYPFNY--
-GPFNYF-
-GPFNYT-
-GPFNYA-
GPPFNY--
-GPFNYQ-
-GPFNYN-
-GPFNYI-
-GPFNYG-
GFPFNY--

GCPFNY--
-HPFNYL-
HMPFNY--
HRPFNY--
HYPFNY--
HQPFNY--
-HPFNYS-
HNPFNY--
-HPFNYV-
HWPFNY--
HKPFNY--
-HPFNYT-
-HPFNYA-
HLPFNY--
HFPFNY--
HAPFNY--
HTPFNY--
-HPFNYQ-
HIPFNY--
HHPFNY--
HSPFNY--
-HPFNYG-
HPPFNY--
-HPFNYR-
-HPFNYK-
-HPFNYF-
HDPFNY--
-IPFNYS-
IWPFNY--
ITPFNY--
IGPFNY--
IEPFNY--
-IPFNYR-
-IPFNYL-
-IPFNYF-
ISPFNY--
IRPFNY--
IKPFNY--
-IPFNYN-
-IPFNYA-
-IPFNYG-
INPFNY--
IQPFNY--
-IPFNYM-
-IPFNYK-
-IPFNYI-
IIPFNY--
IDPFNY--
KSPFNY--
-KPFNYF-

KTPFNY--
-KPFNYL-
-KPFNYR-
KIPFNY--
KEPFNY--
KYPFNY--
KAPFNY--
KLPFNY--
KNPFNY--
-KPFNYT-
-KPFNYQ-
-KPFNYS-
-KPFNYW-
KMPFNY--
-KPFNYN-
-KPFNYK-
-KPFNYI-
-KPFNYH-
-KPFNYG-
KWPFNY--
KQPFNY--
-KPFNYY-
-KPFNYV-
-KPFNYD-
-KPFNYA-
KKPFNY--
KGPFNY--
KFPFNY--
KDPFNY--
LRPFNY--
LQPFNY--
-LPFNYL-
-LPFNYR-
-LPFNYV-
LTPFNY--
LGPFNY--
-LPFNYY-
LYPFNY--
-LPFNYS-
-LPFNYA-
LHPFNY--
-LPFNYF-
-LPFNYT-
LSPFNY--
LVPFNY--
-LPFNYM-
LEPFNY--
LWPFNY--
LLPFNY--
LIPFNY--

LPPFNY--
-LPFNYK-
-LPFNYI-
LNPFNY--
LMPFNY--
LDPFNY--
-LPFNYW-
-LPFNYH-
-LPFNYN-
MRPFNY--
-MPFNYV-
-MPFNYR-
-MPFNYA-
MTPFNY--
-MPFNYM-
MWPFNY--
-MPFNYS-
-MPFNYL-
-MPFNYI-
-MPFNYY-
-MPFNYT-
MKPFNY--
MMPFNY--
MGPFNY--
MFPFNY--
MSPFNY--
MDPFNY--
MPPFNY--
-MPFNYE-
MCPFNY--
-MPFNYK-
MVPFNY--
-MPFNYN-
MNPFNY--
NRPFNY--
-NPFNYR-
-NPFNYV-
NGPFNY--
NVPFNY--
NSPFNY--
-NPFNYT-
-NPFNYE-
NAPFNY--
NTPFNY--
-NPFNYM-
-NPFNYH-
NWPFNY--
-NPFNYQ-
-NPFNYI-
NNPFNY--

NFPFNY--
NMPFNY--
-NPFNYY-
-NPFNYA-
NLPFNY--
NPPFNY--
NIPFNY--
--PFNYSS
--PFNYMS
--PFNYYR
--PFNYAT
PIPFNY--
--PFNYLA
--PFNYSL
--PFNYRF
--PFNYRL
--PFNYKS
--PFNYLR
--PFNYFK
--PFNYTS
--PFNYTR
--PFNYKW
PEPFNY--
PAPFNY--
--PFNYFH
--PFNYRN
--PFNYLN
--PFNYVQ
--PFNYGS
--PFNYVV
--PFNYRM
--PFNYKA
--PFNYTM
--PFNYRR
--PFNYWS
--PFNYVM
--PFNYII
--PFNYHG
PYPFNY--
PGPFNY--
--PFNYNS
--PFNYMR
--PFNYLT
--PFNYLH
--PFNYIA
--PFNYFS
PKPFNY--
--PFNYVI
--PFNYTV
--PFNYTT

--PFNYRS
--PFNYRQ
--PFNYID
--PFNYAR
PMPFNY--
--PFNYWR
--PFNYVW
--PFNYVT
--PFNYSD
--PFNYRG
--PFNYQH
--PFNYKH
--PFNYHL
PTPFNY--
--PFNYYQ
--PFNYTA
--PFNYSW
--PFNYST
--PFNYSH
--PFNYGT
--PFNYAV
--PFNYVR
--PFNYSR
--PFNYSK
--PFNYNA
--PFNYML
--PFNYIY
--PFNYHV
--PFNYFT
--PFNYAW
--PFNYAS
--PFNYYG
--PFNYWV
--PFNYVH
--PFNYVD
--PFNYTG
--PFNYSM
--PFNYRA
--PFNYQG
--PFNYLG
--PFNYKM
--PFNYHS
--PFNYAG
--PFNYVK
--PFNYTD
--PFNYSN
--PFNYSG
--PFNYMW
--PFNYMK
--PFNYMA

--PFNYHA
--PFNYGY
--PFNYGG
--PFNYFR
--PFNYYT
--PFNYYE
--PFNYVN
--PFNYTY
--PFNYTQ
--PFNYTE
--PFNYSY
--PFNYSA
--PFNYQA
--PFNYMQ
--PFNYKY
--PFNYIW
--PFNYIS
--PFNYHM
--PFNYFQ
--PFNYFN
--PFNYAM
PNPFNY--
--PFNYYS
--PFNYYA
--PFNYWF
--PFNYTN
--PFNYTK
--PFNYQR
--PFNYLE
--PFNYLD
--PFNYIV
--PFNYIK
--PFNYHR
--PFNYES
--PFNYEK
--PFNYAL
--PFNYAE
--PFNYYY
--PFNYYV
--PFNYWN
--PFNYVG
--PFNYVA
--PFNYTL
--PFNYTH
--PFNYNW
--PFNYMV
--PFNYKI
--PFNYKF
--PFNYHQ
--PFNYEW

--PFNYER
PQPFNY--
--PFNYWT
--PFNYTF
--PFNYSQ
--PFNYSI
--PFNYRY
--PFNYRV
--PFNYRI
--PFNYQT
--PFNYQQ
--PFNYQM
--PFNYQF
--PFNYKL
--PFNYIT
--PFNYIG
--PFNYHN
--PFNYGW
--PFNYFY
--PFNYFF
--PFNYEQ
--PFNYEN
--PFNYEG
--PFNYDS
--PFNYAI
PDPFNY--
PWPFNY--
PFPFNY--
--PFNYYD
--PFNYWE
--PFNYVP
--PFNYVE
--PFNYSV
--PFNYSE
--PFNYRW
--PFNYRT
--PFNYRD
--PFNYQW
--PFNYNV
--PFNYNN
--PFNYND
--PFNYMI
--PFNYLK
--PFNYLC
--PFNYKR
--PFNYKC
--PFNYIR
--PFNYIN
--PFNYIH
--PFNYIF

--PFNYIC
--PFNYHY
--PFNYHD
--PFNYGM
--PFNYGI
--PFNYGF
--PFNYFI
--PFNYFG
--PFNYFC
--PFNYEM
--PFNYEL
--PFNYDH
--PFNYCK
--PFNYAN
--PFNYAD
-QPFNYR-
-QPFNYS-
QRPFNY--
-QPFNYK-
-QPFNYL-
QKPFNY--
-QPFNYY-
QSPFNY--
-QPFNYG-
QIPFNY--
QGPFNY--
QMPFNY--
QVPFNY--
-QPFNYV-
-QPFNYE-
QPPFNY--
QNPFNY--
QAPFNY--
QWPFNY--
-QPFNYT-
-QPFNYQ-
-QPFNYN-
-QPFNYH-
-QPFNYF-
QDPFNY--
-RPFNYL-
-RPFNYS-
-RPFNYI-
-RPFNYQ-
-RPFNYM-
RIPFNY--
RQPFNY--
-RPFNYK-
-RPFNYH-
RKPFNY--

RNPFNY--
RSPFNY--
-RPFNYT-
RLPFNY--
REPFNY--
RYPFNY--
RVPFNY--
-RPFNYA-
-RPFNYF-
RHPFNY--
RMPFNY--
RFPFNY--
-RPFNYV-
-RPFNYN-
-RPFNYG-
RDPFNY--
RTPFNY--
-RPFNYY-
-RPFNYE-
RGPFNY--
-SPFNYV-
SSPFNY--
-SPFNYR-
SRPFNY--
SIPFNY--
SKPFNY--
-SPFNYS-
SVPFNY--
-SPFNYY-
-SPFNYQ-
SHPFNY--
SLPFNY--
SYPFNY--
-SPFNYW-
SQPFNY--
SGPFNY--
-SPFNYT-
-SPFNYG-
SMPFNY--
SPPFNY--
-SPFNYM-
-SPFNYI-
-SPFNYA-
-SPFNYE-
SWPFNY--
-SPFNYN-
-SPFNYH-
SCPFNY--
SAPFNY--
STPFNY--

SNPFNY--
-TPFNYW-
-TPFNYV-
-TPFNYS-
-TPFNYL-
TLPFNY--
-TPFNYY-
-TPFNYT-
-TPFNYM-
TVPFNY--
-TPFNYI-
TGPFNY--
TDPFNY--
-TPFNYR-
-TPFNYK-
TPPFNY--
TNPFNY--
TAPFNY--
TWPFNY--
TSPFNY--
THPFNY--
TRPFNY--
-TPFNYH-
-TPFNYF-
TIPFNY--
-TPFNYD-
TEPFNY--
VPPFNY--
VRPFNY--
VMPFNY--
-VPFNYS-
-VPFNYI-
-VPFNYL-
VTPFNY--
-VPFNYA-
-VPFNYM-
-VPFNYW-
-VPFNYR-
VKPFNY--
VLPFNY--
VQPFNY--
-VPFNYQ-
VYPFNY--
-VPFNYG-
VGPFNY--
-VPFNYV-
-VPFNYN-
VNPFNY--
-VPFNYF-
VIPFNY--

VHPFNY--
VEPFNY--
WPPFNY--
WTPFNY--
-WPFNYS-
WVPFNY--
WRPFNY--
-WPFNYI-
WGPFNY--
-WPFNYW-
-WPFNYA-
WNPFNY--
-WPFNYR-
-WPFNYK-
WYPFNY--
-WPFNYH-
-WPFNYV-
-WPFNYT-
-WPFNYG-
WLPFNY--
WDPFNY--
WAPFNY--
YPPFNY--
-YPFNYY-
-YPFNYM-
YRPFNY--
YVPFNY--
YTPFNY--
-YPFNYK-
-YPFNYS-
YLPFNY--
YAPFNY--
YQPFNY--
-YPFNYI-
YMPFNY--
YKPFNY--
YSPFNY--
-YPFNYH-
YNPFNY--
YEPFNY--
-YPFNYG-
-YPFNYE-
-YPFNYV-
-YPFNYR-
-YPFNYQ-
-YPFNYF-
YYPFNY--
-YPFNYC-
YIPFNY--

PLPF 98 807 46.62

-LPLPFA-
--PLPFET
AKPLPF--
ARPLPF--
AVPLPF--
AWPLPF--
ALPLPF--
-APLPFS-
ANPLPF--
AMPLPF--
AAPLPF--
ASPLPF--
-APLPFY-
AGPLPF--
ATPLPF--
-APLPFT-
-APLPFN-
AFPLPF--
APPLPF--
AIPLPF--
AEPLPF--
AYPLPF--
-APLPFV-
-APLPFA-
-APLPFQ-
-APLPFF-
-APLPFM-
-APLPFE-
AQPLPF--
-APLPFW-
-APLPFH-
ADPLPF--
CLPLPF--
CCPLPF--
CTPLPF--
CPPLPF--
CVPLPF--
-CPLPFL-
CHPLPF--
CAPLPF--
-CPLPFR-
-CPLPFQ-
CNPLPF--
CSPLPF--
-CPLPFY-
-CPLPFK-
CKPLPF--
DRPLPF--
DWPLPF--
DVPLPF--

DTPLPF--
DSPLPF--
-DPLPFF-
DIPLPF--
DLPLPF--
-DPLPFR-
-DPLPFL-
DAPLPF--
DYPLPF--
DHPLPF--
DQPLPF--
DPPLPF--
DGPLPF--
-DPLPFM-
-DPLPFK-
-DPLPFH-
DFPLPF--
-DPLPFV-
-DPLPFT-
-DPLPFQ-
-DPLPFI-
-DPLPFW-
-DPLPFG-
DKPLPF--
EAPLPF--
-EPLPFM-
ESPLPF--
EMPLPF--
-EPLPFV-
EYPLPF--
-EPLPFH-
EVPLPF--
ERPLPF--
EIPLPF--
EGPLPF--
-EPLPFF-
ENPLPF--
ELPLPF--
EEPLPF--
ETPLPF--
-EPLPFQ-
EQPLPF--
-EPLPFY-
-EPLPFR-
-EPLPFA-
EKPLPF--
EPPLPF--
-EPLPFS-
-EPLPFN-
-EPLPFL-

EFPLPF--
FAPLPF--
-FPLPFR-
-FPLPFV-
-FPLPFL-
-FPLPFT-
FGPLPF--
FMPLPF--
FQPLPF--
FNPLPF--
FKPLPF--
FSPLPF--
FPPLPF--
FYPLPF--
FVPLPF--
FHPLPF--
-FPLPFY-
FLPLPF--
FTPLPF--
-FPLPFS-
-FPLPFK-
-FPLPFH-
-FPLPFQ-
-FPLPFW-
-FPLPFN-
-FPLPFM-
-FPLPFF-
FFPLPF--
FEPLPF--
-FPLPFA-
FWPLPF--
-GPLPFM-
-GPLPFS-
-GPLPFY-
GIPLPF--
GHPLPF--
-GPLPFR-
GTPLPF--
GSPLPF--
GLPLPF--
GGPLPF--
GVPLPF--
-GPLPFW-
-GPLPFL-
GFPLPF--
-GPLPFF-
-GPLPFV-
GYPLPF--
GPPLPF--
GKPLPF--

GQPLPF--
-GPLPFN-
-GPLPFK-
GMPLPF--
GAPLPF--
-GPLPFA-
-GPLPFT-
-GPLPFH-
-GPLPFQ-
GEPLPF--
GCPLPF--
HRPLPF--
-HPLPFR-
HVPLPF--
-HPLPFT-
HGPLPF--
HTPLPF--
HQPLPF--
-HPLPFS-
HMPLPF--
HIPLPF--
-HPLPFQ-
HLPLPF--
HSPLPF--
-HPLPFW-
HPPLPF--
-HPLPFL-
HNPLPF--
HEPLPF--
HKPLPF--
-HPLPFI-
HFPLPF--
HAPLPF--
HWPLPF--
-HPLPFY-
-HPLPFM-
-HPLPFA-
-HPLPFH-
HDPLPF--
HYPLPF--
-HPLPFN-
-HPLPFK-
HHPLPF--
HCPLPF--
-IPLPFR-
ILPLPF--
ISPLPF--
-IPLPFS-
IWPLPF--
IVPLPF--

IGPLPF--
-IPLPFN-
IIPLPF--
-IPLPFQ-
-IPLPFL-
IHPLPF--
-IPLPFT-
IAPLPF--
IPPLPF--
-IPLPFV-
-IPLPFA-
-IPLPFI-
-IPLPFF-
-IPLPFY-
-IPLPFK-
IMPLPF--
IKPLPF--
-IPLPFH-
IYPLPF--
-IPLPFW-
-IPLPFP-
-IPLPFE-
IFPLPF--
IDPLPF--
KSPLPF--
-KPLPFR-
KWPLPF--
KAPLPF--
KHPLPF--
KQPLPF--
-KPLPFS-
-KPLPFT-
KIPLPF--
KGPLPF--
KTPLPF--
-KPLPFL-
KLPLPF--
-KPLPFM-
-KPLPFF-
KMPLPF--
-KPLPFW-
KNPLPF--
KPPLPF--
KYPLPF--
-KPLPFN-
-KPLPFH-
KVPLPF--
-KPLPFV-
-KPLPFQ-
-KPLPFA-

KEPLPF--
KKPLPF--
KFPLPF--
KCPLPF--
LSPLPF--
LYPLPF--
LTPLPF--
LGPLPF--
LVPLPF--
LHPLPF--
LDPLPF--
LQPLPF--
-LPLPFY-
LFPLPF--
LAPLPF--
LLPLPF--
LPPLPF--
-LPLPFI-
LWPLPF--
-LPLPFR-
-LPLPFM-
-LPLPFK-
-LPLPFS-
LMPLPF--
LKPLPF--
-LPLPFQ-
-LPLPFH-
-LPLPFF-
-LPLPFV-
-LPLPFC-
-LPLPFN-
LCPLPF--
MKPLPF--
MSPLPF--
MEPLPF--
-MPLPFV-
-MPLPFR-
MVPLPF--
-MPLPFS-
MHPLPF--
MYPLPF--
MRPLPF--
-MPLPFL-
-MPLPFA-
-MPLPFY-
MDPLPF--
-MPLPFI-
-MPLPFH-
MGPLPF--
MNPLPF--

MPPLPF--
-MPLPFT-
-MPLPFN-
-MPLPFM-
MIPLPF--
MCPLPF--
MAPLPF--
MQPLPF--
-MPLPFQ-
-MPLPFK-
MMPLPF--
NRPLPF--
NFPLPF--
NYPLPF--
NAPLPF--
NSPLPF--
NPPLPF--
NQPLPF--
-NPLPFT-
-NPLPFR-
NVPLPF--
-NPLPFI-
NNPLPF--
NLPLPF--
NKPLPF--
NGPLPF--
NWPLPF--
-NPLPFF-
NMPLPF--
-NPLPFK-
NHPLPF--
NEPLPF--
NTPLPF--
-NPLPFV-
-NPLPFS-
-NPLPFH-
NIPLPF--
NDPLPF--
-NPLPFW-
-NPLPFQ-
-NPLPFM-
-NPLPFA-
--PLPFSL
--PLPFKL
--PLPFRS
--PLPFRV
--PLPFKS
--PLPFYS
--PLPFRT
--PLPFTL

--PLPFLT
PRPLPF--
--PLPFTT
--PLPFSR
--PLPFSQ
--PLPFLH
PGPLPF--
--PLPFAG
--PLPFTG
--PLPFTA
--PLPFQL
--PLPFFG
--PLPFST
--PLPFSS
--PLPFRG
PTPLPF--
--PLPFVS
--PLPFTI
--PLPFVV
--PLPFRY
--PLPFNY
--PLPFYA
--PLPFWA
--PLPFML
--PLPFNS
--PLPFEG
--PLPFTF
--PLPFSF
--PLPFSA
--PLPFMG
--PLPFIG
--PLPFSI
--PLPFRH
--PLPFPM
--PLPFLV
--PLPFTS
--PLPFQT
--PLPFMS
--PLPFLA
--PLPFNI
--PLPFNA
--PLPFKR
--PLPFHT
--PLPFYY
--PLPFVW
--PLPFTN
--PLPFIS
--PLPFGI
--PLPFSV
--PLPFFV

--PLPFYV
--PLPFVT
--PLPFFK
--PLPFDI
--PLPFMQ
--PLPFGH
--PLPFVN
--PLPFTQ
--PLPFQA
--PLPFKT
--PLPFAL
--PLPFYN
--PLPFVR
--PLPFRF
--PLPFLQ
--PLPFLN
--PLPFLK
--PLPFIT
--PLPFWI
--PLPFVH
--PLPFSY
--PLPFRN
--PLPFAS
PEPLPF--
--PLPFWM
--PLPFVA
--PLPFTR
--PLPFSM
--PLPFRI
--PLPFMW
--PLPFLW
--PLPFKQ
--PLPFIR
--PLPFIN
--PLPFHF
--PLPFWV
--PLPFVG
--PLPFRE
--PLPFQY
--PLPFMH
--PLPFHY
--PLPFGV
--PLPFGQ
--PLPFES
--PLPFTH
--PLPFRW
--PLPFQM
--PLPFKF
--PLPFIQ
--PLPFHI

--PLPFHG
--PLPFEY
--PLPFAM
--PLPFYL
--PLPFYG
--PLPFTW
--PLPFSW
--PLPFSK
--PLPFRA
--PLPFPQ
--PLPFPF
--PLPFMT
--PLPFKM
--PLPFKH
--PLPFGM
--PLPFEF
--PLPFAI
--PLPFYW
--PLPFYI
--PLPFTY
--PLPFRD
--PLPFQV
--PLPFQS
--PLPFNM
--PLPFHV
--PLPFGA
--PLPFFA
--PLPFAQ
--PLPFYK
--PLPFWS
--PLPFMA
--PLPFKV
--PLPFKK
--PLPFHS
--PLPFFN
--PLPFEI
--PLPFAY
--PLPFAH
--PLPFAA
PNPLPF--
--PLPFYT
--PLPFVE
--PLPFQG
--PLPFMN
--PLPFIF
--PLPFGG
--PLPFAV
--PLPFAT
--PLPFAF
--PLPFWT

--PLPFVI
--PLPFTV
--PLPFTM
--PLPFTK
--PLPFQW
--PLPFNW
--PLPFNF
--PLPFMI
--PLPFKI
--PLPFIK
--PLPFHW
--PLPFHN
--PLPFGT
--PLPFEL
--PLPFEA
--PLPFDY
--PLPFDL
PCPLPF--
--PLPFYQ
--PLPFYH
--PLPFVQ
--PLPFVL
--PLPFVF
--PLPFPY
--PLPFPV
--PLPFPI
--PLPFMV
--PLPFMM
--PLPFIW
--PLPFFH
--PLPFEW
PDPLPF--
--PLPFYD
--PLPFWN
--PLPFQR
--PLPFQQ
--PLPFQK
--PLPFNV
--PLPFNN
--PLPFKY
--PLPFKN
--PLPFIV
--PLPFGS
--PLPFGF
--PLPFFT
--PLPFEQ
--PLPFEM
--PLPFCL
--PLPFWW
--PLPFSD

--PLPFRQ
--PLPFQN
--PLPFQH
--PLPFPT
--PLPFPH
--PLPFNT
--PLPFNH
--PLPFMD
--PLPFLM
--PLPFHD
--PLPFFI
--PLPFFE
--PLPFFD
--PLPFEN
--PLPFDQ
--PLPFCG
--PLPFCA
--PLPFAR
--PLPFYE
--PLPFVK
--PLPFVD
--PLPFVC
--PLPFTD
--PLPFSE
--PLPFQD
--PLPFPS
--PLPFNR
--PLPFNK
--PLPFNG
--PLPFMP
--PLPFLD
--PLPFKW
--PLPFKE
--PLPFKD
--PLPFIP
--PLPFIH
--PLPFID
--PLPFHQ
--PLPFHK
--PLPFHE
--PLPFHA
--PLPFEV
--PLPFEH
--PLPFDV
--PLPFCS
--PLPFAW
--PLPFAN
--PLPFAK
--PLPFAE
QRPLPF--

```
-QPLPFR-
QHPLPF--
QTPLPF--
QVPLPF--
-QPLPFY-
-QPLPFK-
QAPLPF--
-QPLPFT-
QYPLPF--
QFPLPF--
-QPLPFW-
QWPLPF--
QPPLPF--
-QPLPFL-
QSPLPF--
QEPLPF--
-QPLPFS-
-QPLPFV-
-QPLPFI-
QGPLPF--
-QPLPFH-
QNPLPF--
QKPLPF--
QCPLPF--
-QPLPFQ-
-QPLPFN-
QLPLPF--
-RPLPFR-
RWPLPF--
RIPLPF--
RGPLPF--
-RPLPFI-
RAPLPF--
-RPLPFS-
-RPLPFM-
-RPLPFT-
-RPLPFH-
RPPLPF--
RVPLPF--
-RPLPFK-
RHPLPF--
RTPLPF--
RMPLPF--
RYPLPF--
RNPLPF--
-RPLPFA-
REPLPF--
-RPLPFV-
-RPLPFQ-
-RPLPFW-
```

-RPLPFN-
RKPLPF--
RQPLPF--
RDPLPF--
-RPLPFG-
-RPLPFE-
RCPLPF--
SLPLPF--
SSPLPF--
SGPLPF--
STPLPF--
SWPLPF--
SAPLPF--
SRPLPF--
SNPLPF--
-SPLPFM-
SIPLPF--
SVPLPF--
SFPLPF--
SEPLPF--
-SPLPFK-
-SPLPFR-
SQPLPF--
-SPLPFW-
-SPLPFS-
SKPLPF--
SMPLPF--
SYPLPF--
-SPLPFT-
SHPLPF--
SDPLPF--
-SPLPFI-
-SPLPFA-
SCPLPF--
SPPLPF--
-SPLPFH-
-SPLPFY-
-SPLPFV-
-SPLPFN-
TRPLPF--
TSPLPF--
-TPLPFK-
TFPLPF--
-TPLPFL-
TKPLPF--
-TPLPFR-
TPPLPF--
TMPLPF--
TVPLPF--
TGPLPF--

TLPLPF--
-TPLPFI-
-TPLPFS-
-TPLPFA-
TAPLPF--
TWPLPF--
-TPLPFV-
-TPLPFT-
TNPLPF--
TDPLPF--
TYPLPF--
-TPLPFM-
-TPLPFH-
TEPLPF--
-TPLPFY-
THPLPF--
-TPLPFP-
TIPLPF--
-TPLPFW-
-TPLPFN-
TQPLPF--
TCPLPF--
-VPLPFQ-
VIPLPF--
VQPLPF--
VSPLPF--
VHPLPF--
-VPLPFY-
VNPLPF--
VFPLPF--
-VPLPFM-
-VPLPFI-
-VPLPFR-
-VPLPFT-
-VPLPFL-
VDPLPF--
-VPLPFV-
VMPLPF--
VPPLPF--
-VPLPFS-
-VPLPFH-
VGPLPF--
VWPLPF--
VLPLPF--
VAPLPF--
VVPLPF--
-VPLPFW-
VEPLPF--
-VPLPFN-
-VPLPFE-

VCPLPF--
VYPLPF--
-VPLPFG-
WRPLPF--
WKPLPF--
WYPLPF--
-WPLPFS-
-WPLPFT-
WGPLPF--
WSPLPF--
WDPLPF--
WTPLPF--
WAPLPF--
-WPLPFA-
-WPLPFV-
-WPLPFQ-
-WPLPFH-
WPPLPF--
-WPLPFR-
-WPLPFL-
WVPLPF--
-WPLPFM-
-WPLPFF-
WWPLPF--
-WPLPFY-
-WPLPFW-
WLPLPF--
-WPLPFG-
-WPLPFE-
YSPLPF--
-YPLPFR-
YIPLPF--
YVPLPF--
YTPLPF--
YGPLPF--
YMPLPF--
-YPLPFY-
YAPLPF--
YWPLPF--
-YPLPFT-
YKPLPF--
YPPLPF--
-YPLPFN-
-YPLPFK-
YEPLPF--
YFPLPF--
YLPLPF--
YHPLPF--
YDPLPF--
YQPLPF--

	-YPLPFV-		
	-YPLPFM-		
	-YPLPFF-		
	YNPLPF--		
	-YPLPFW-		
	-YPLPFH-		
	-YPLPFP-		
PQNY	514	614	46.57
	-APQNYL-		
	AKPQNY--		
	ASPQNY--		
	-APQNYV-		
	AVPQNY--		
	AAPQNY--		
	AYPQNY--		
	-APQNYN-		
	-APQNYM-		
	ATPQNY--		
	-APQNYR-		
	AIPQNY--		
	-APQNYS-		
	-APQNYF-		
	-APQNYA-		
	AQPQNY--		
	-APQNYW-		
	ANPQNY--		
	AFPQNY--		
	-APQNYY-		
	ACPQNY--		
	CGPQNY--		
	CRPQNY--		
	-CPQNYM-		
	CYPQNY--		
	-CPQNYY-		
	-CPQNYS-		
	-CPQNYL-		
	-CPQNYI-		
	CMPQNY--		
	CIPQNY--		
	DGPQNY--		
	DIPQNY--		
	DYPQNY--		
	DRPQNY--		
	-DPQNYL-		
	-DPQNYS-		
	DAPQNY--		
	-DPQNYR-		
	-DPQNYM-		
	-DPQNYW-		
	-DPQNYT-		

−DPQNYI−
DHPQNY−−
DDPQNY−−
−EPQNYL−
ESPQNY−−
EQPQNY−−
−EPQNYR−
EFPQNY−−
ERPQNY−−
−EPQNYV−
−EPQNYK−
EKPQNY−−
−EPQNYI−
−EPQNYH−
FRPQNY−−
FPPQNY−−
−FPQNYL−
FHPQNY−−
FDPQNY−−
−FPQNYS−
−FPQNYR−
−FPQNYM−
−FPQNYI−
−FPQNYW−
FGPQNY−−
−FPQNYV−
−FPQNYA−
−FPQNYT−
FLPQNY−−
−FPQNYG−
GGPQNY−−
GVPQNY−−
−GPQNYR−
−GPQNYL−
GLPQNY−−
GYPQNY−−
GSPQNY−−
GIPQNY−−
GRPQNY−−
GQPQNY−−
−GPQNYW−
−GPQNYV−
GFPQNY−−
−GPQNYF−
GEPQNY−−
−GPQNYS−
−GPQNYQ−
GPPQNY−−
−GPQNYI−
−GPQNYH−

GMPQNY--
GKPQNY--
GDPQNY--
HWPQNY--
-HPQNYL-
HKPQNY--
-HPQNYM-
HTPQNY--
HPPQNY--
HRPQNY--
-HPQNYV-
-HPQNYS-
HFPQNY--
-HPQNYW-
-HPQNYT-
-HPQNYY-
-HPQNYN-
-HPQNYK-
-HPQNYI-
HMPQNY--
HLPQNY--
HAPQNY--
-IPQNYR-
-IPQNYT-
IWPQNY--
ITPQNY--
IKPQNY--
IFPQNY--
ISPQNY--
-IPQNYN-
-IPQNYI-
ILPQNY--
IRPQNY--
-IPQNYL-
IIPQNY--
IYPQNY--
-IPQNYM-
-IPQNYG-
-IPQNYE-
IGPQNY--
IVPQNY--
-IPQNYS-
-IPQNYH-
-IPQNYA-
KSPQNY--
-KPQNYV-
-KPQNYS-
-KPQNYR-
-KPQNYL-
-KPQNYH-

KRPQNY--
KTPQNY--
KGPQNY--
KPPQNY--
-KPQNYQ-
KAPQNY--
KMPQNY--
KLPQNY--
KVPQNY--
KQPQNY--
-KPQNYN-
-KPQNYI-
-KPQNYF-
-KPQNYA-
KFPQNY--
LRPQNY--
LNPQNY--
LSPQNY--
LWPQNY--
-LPQNYS-
LKPQNY--
LTPQNY--
LQPQNY--
-LPQNYM-
LFPQNY--
-LPQNYR-
-LPQNYL-
-LPQNYY-
LMPQNY--
-LPQNYV-
-LPQNYQ-
LLPQNY--
LEPQNY--
LVPQNY--
-LPQNYE-
LHPQNY--
LDPQNY--
-LPQNYT-
-LPQNYK-
-LPQNYI-
-LPQNYH-
-LPQNYG-
-LPQNYA-
LPPQNY--
LAPQNY--
-MPQNYT-
MSPQNY--
MRPQNY--
-MPQNYM-
MGPQNY--

MLPQNY--
-MPQNYF-
-MPQNYA-
-MPQNYW-
-MPQNYE-
MPPQNY--
MKPQNY--
MMPQNY--
MFPQNY--
MDPQNY--
MYPQNY--
MWPQNY--
MIPQNY--
MTPQNY--
-MPQNYS-
-MPQNYQ-
-MPQNYN-
-MPQNYL-
-MPQNYK-
NSPQNY--
NRPQNY--
-NPQNYS-
-NPQNYR-
-NPQNYL-
NMPQNY--
-NPQNYV-
NPPQNY--
NAPQNY--
NYPQNY--
NQPQNY--
NIPQNY--
NHPQNY--
NLPQNY--
NKPQNY--
-NPQNYK-
NVPQNY--
NTPQNY--
NNPQNY--
NFPQNY--
--PQNYSH
--PQNYLV
--PQNYLK
--PQNYFA
--PQNYVS
--PQNYRS
--PQNYRT
--PQNYKM
--PQNYTR
--PQNYSV
--PQNYRV

--PQNYLS
--PQNYYS
--PQNYTL
--PQNYIM
--PQNYAG
--PQNYYR
--PQNYIG
--PQNYVV
--PQNYSG
--PQNYWS
--PQNYMF
--PQNYLM
--PQNYLA
--PQNYFN
--PQNYAT
--PQNYYY
--PQNYYF
--PQNYVH
--PQNYLG
--PQNYFR
--PQNYTM
--PQNYSN
--PQNYSF
--PQNYFH
--PQNYAY
--PQNYAS
--PQNYTG
--PQNYNS
--PQNYLN
--PQNYIS
--PQNYHV
--PQNYTF
--PQNYST
--PQNYRQ
--PQNYIA
--PQNYGS
--PQNYFW
--PQNYWK
--PQNYVI
--PQNYSY
--PQNYSL
--PQNYLI
--PQNYIR
--PQNYIF
--PQNYAL
--PQNYWV
--PQNYVQ
--PQNYVL
--PQNYSR
--PQNYRY

--PQNYQF
--PQNYMY
--PQNYLQ
--PQNYYV
--PQNYVR
--PQNYTY
--PQNYTD
--PQNYQG
--PQNYNG
--PQNYMS
--PQNYMM
--PQNYKV
--PQNYIQ
--PQNYIL
--PQNYHR
--PQNYEG
--PQNYYT
--PQNYYL
--PQNYYG
--PQNYVM
--PQNYTV
--PQNYMN
--PQNYLL
--PQNYIV
--PQNYIE
--PQNYGT
--PQNYFQ
--PQNYFG
PWPQNY--
PSPQNY--
--PQNYYM
--PQNYWT
--PQNYWG
--PQNYWD
--PQNYVK
--PQNYVG
--PQNYTK
--PQNYTH
--PQNYSS
--PQNYSK
--PQNYRM
--PQNYRK
--PQNYRG
--PQNYQT
--PQNYQR
--PQNYNW
--PQNYMG
--PQNYME
--PQNYLW
--PQNYLT

--PQNYLH
--PQNYFD
--PQNYES
--PQNYYI
--PQNYWA
--PQNYVW
--PQNYVE
--PQNYTT
--PQNYRN
--PQNYRF
--PQNYRD
--PQNYNT
--PQNYNN
--PQNYMW
--PQNYMH
--PQNYKF
--PQNYHQ
--PQNYFT
--PQNYEH
PGPQNY--
PTPQNY--
--PQNYYW
--PQNYYH
--PQNYYA
--PQNYWN
--PQNYWM
--PQNYVT
--PQNYTI
--PQNYTE
--PQNYSQ
--PQNYRI
--PQNYRH
--PQNYRA
--PQNYMV
--PQNYMK
--PQNYLD
--PQNYKR
--PQNYKN
--PQNYKA
--PQNYGV
--PQNYGI
--PQNYGF
--PQNYEM
--PQNYAH
PAPQNY--
--PQNYYN
--PQNYWY
--PQNYWR
--PQNYWH
--PQNYWE

--PQNYVN
--PQNYVA
--PQNYTC
--PQNYSM
--PQNYSI
--PQNYSA
--PQNYRE
--PQNYQK
--PQNYQA
--PQNYPT
--PQNYPL
--PQNYNM
--PQNYNL
--PQNYNI
--PQNYML
--PQNYLE
--PQNYKY
--PQNYKT
--PQNYKS
--PQNYKG
--PQNYII
--PQNYHS
--PQNYHN
--PQNYHM
--PQNYHL
--PQNYHK
--PQNYHH
--PQNYHF
--PQNYHE
--PQNYGW
--PQNYGN
--PQNYGK
--PQNYFS
--PQNYFE
--PQNYEY
--PQNYER
--PQNYDR
--PQNYCK
--PQNYCG
--PQNYAF
PPPQNY--
QSPQNY--
QLPQNY--
QAPQNY--
QVPQNY--
-QPQNYI-
-QPQNYE-
-QPQNYT-
QRPQNY--
-QPQNYY-

```
-QPQNYS-
QWPQNY--
-QPQNYH-
QGPQNY--
-QPQNYM-
QIPQNY--
QTPQNY--
QQPQNY--
-QPQNYQ-
-QPQNYF-
QDPQNY--
RLPQNY--
RAPQNY--
-RPQNYS-
-RPQNYV-
RVPQNY--
-RPQNYY-
RGPQNY--
RSPQNY--
-RPQNYT-
RPPQNY--
-RPQNYM-
-RPQNYR-
-RPQNYA-
-RPQNYQ-
RYPQNY--
RWPQNY--
RTPQNY--
-RPQNYF-
RHPQNY--
RIPQNY--
RDPQNY--
RNPQNY--
-RPQNYK-
-RPQNYW-
RRPQNY--
-RPQNYG-
-RPQNYE-
-RPQNYN-
-RPQNYH-
RKPQNY--
RCPQNY--
-SPQNYL-
-SPQNYR-
SWPQNY--
-SPQNYT-
-SPQNYM-
SFPQNY--
SSPQNY--
SGPQNY--
```

SAPQNY--
-SPQNYH-
SVPQNY--
SRPQNY--
SLPQNY--
-SPQNYY-
STPQNY--
-SPQNYV-
-SPQNYQ-
-SPQNYF-
-SPQNYA-
SMPQNY--
SYPQNY--
-SPQNYI-
SNPQNY--
SIPQNY--
SHPQNY--
-SPQNYW-
SQPQNY--
-SPQNYG-
SPPQNY--
SDPQNY--
-SPQNYN-
TPPQNY--
TVPQNY--
-TPQNYL-
TGPQNY--
TTPQNY--
TSPQNY--
TQPQNY--
-TPQNYI-
TKPQNY--
TEPQNY--
-TPQNYT-
-TPQNYR-
TAPQNY--
-TPQNYW-
-TPQNYM-
TMPQNY--
TDPQNY--
-TPQNYQ-
-TPQNYK-
-TPQNYE-
-TPQNYA-
TIPQNY--
TYPQNY--
-TPQNYF-
TLPQNY--
-TPQNYS-
-TPQNYV-

```
-TPQNYH-
-TPQNYG-
TNPQNY--
-VPQNYV-
-VPQNYM-
-VPQNYR-
VHPQNY--
-VPQNYS-
VIPQNY--
VSPQNY--
-VPQNYE-
VEPQNY--
VWPQNY--
-VPQNYA-
VVPQNY--
VRPQNY--
VLPQNY--
VAPQNY--
-VPQNYY-
-VPQNYK-
VTPQNY--
VQPQNY--
-VPQNYW-
-VPQNYI-
VMPQNY--
-VPQNYT-
-VPQNYQ-
-VPQNYL-
-VPQNYG-
-VPQNYN-
VGPQNY--
VFPQNY--
WRPQNY--
WEPQNY--
-WPQNYR-
WGPQNY--
WNPQNY--
WKPQNY--
-WPQNYK-
WVPQNY--
WIPQNY--
WQPQNY--
-WPQNYM-
WPPQNY--
-WPQNYV-
-WPQNYF-
WLPQNY--
-WPQNYY-
YPPQNY--
YKPQNY--
```

```
            -YPQNYT-
            -YPQNYL-
            -YPQNYK-
            YSPQNY--
            -YPQNYS-
            YVPQNY--
            YNPQNY--
            YLPQNY--
            YGPQNY--
            YFPQNY--
            -YPQNYI-
            -YPQNYF-
            YMPQNY--
            YWPQNY--
            YRPQNY--
            -YPQNYR-
            -YPQNYQ-
            -YPQNYA-
            -YPQNYY-
            -YPQNYH-
            -YPQNYE-
            YEPQNY--
PRNI         275         710      46.57
            -NPRNIP-
            --PRNIRP
            --PRNIDW
            -APRNIL-
            AVPRNI--
            AFPRNI--
            AYPRNI--
            -APRNIA-
            ALPRNI--
            AKPRNI--
            -APRNIT-
            AQPRNI--
            -APRNIR-
            AHPRNI--
            -APRNIW-
            -APRNIH-
            APPRNI--
            AGPRNI--
            AAPRNI--
            -APRNIE-
            ATPRNI--
            -APRNIY-
            ANPRNI--
            ADPRNI--
            ACPRNI--
            -APRNIF-
            -APRNIQ-
```

```
-APRNIP-
AEPRNI--
CRPRNI--
CMPRNI--
CCPRNI--
CTPRNI--
CSPRNI--
CHPRNI--
CAPRNI--
CYPRNI--
-CPRNIL-
-CPRNIA-
CQPRNI--
-CPRNIR-
CNPRNI--
CEPRNI--
-CPRNIT-
CLPRNI--
CGPRNI--
CVPRNI--
-CPRNIW-
-CPRNIQ-
-CPRNII-
CPPRNI--
-DPRNIR-
DVPRNI--
-DPRNIL-
DRPRNI--
-DPRNIW-
-DPRNIH-
DWPRNI--
DQPRNI--
-DPRNIV-
DTPRNI--
DFPRNI--
-DPRNIY-
-DPRNIT-
-DPRNIS-
DLPRNI--
DKPRNI--
DIPRNI--
DAPRNI--
DYPRNI--
-DPRNIM-
-DPRNIA-
DGPRNI--
-DPRNIQ-
-DPRNIN-
-DPRNIK-
-DPRNIF-
```

DHPRNI--
DSPRNI--
-DPRNII-
DPPRNI--
DEPRNI--
ESPRNI--
-EPRNIL-
-EPRNIR-
-EPRNIF-
-EPRNIS-
-EPRNIV-
ERPRNI--
ELPRNI--
EVPRNI--
EGPRNI--
EAPRNI--
EHPRNI--
-EPRNIM-
EIPRNI--
EQPRNI--
-EPRNIA-
EMPRNI--
EYPRNI--
EEPRNI--
ETPRNI--
EKPRNI--
EFPRNI--
-EPRNIT-
-EPRNIE-
-EPRNIH-
-EPRNIY-
ENPRNI--
FSPRNI--
FAPRNI--
FGPRNI--
FTPRNI--
FVPRNI--
-FPRNIV-
FIPRNI--
-FPRNIW-
-FPRNIL-
-FPRNIQ-
FQPRNI--
-FPRNIA-
FPPRNI--
FKPRNI--
-FPRNIY-
FEPRNI--
-FPRNIE-
-FPRNIN-

FNPRNI--
FDPRNI--
-GPRNIL-
-GPRNIT-
-GPRNIR-
-GPRNIA-
-GPRNII-
-GPRNIS-
GRPRNI--
GIPRNI--
GMPRNI--
-GPRNIM-
GTPRNI--
GSPRNI--
-GPRNIN-
GGPRNI--
-GPRNIV-
-GPRNIF-
GLPRNI--
-GPRNIY-
GNPRNI--
GAPRNI--
GYPRNI--
GWPRNI--
-GPRNIW-
-GPRNIK-
-GPRNIQ-
-GPRNIG-
GDPRNI--
-GPRNIE-
GPPRNI--
GHPRNI--
GEPRNI--
GVPRNI--
GQPRNI--
GKPRNI--
GCPRNI--
-GPRNIC-
HKPRNI--
HAPRNI--
-HPRNIW-
HVPRNI--
HSPRNI--
-HPRNII-
HLPRNI--
HGPRNI--
HTPRNI--
-HPRNIV-
-HPRNIG-
HQPRNI--

-HPRNIQ-
-HPRNIS-
HIPRNI--
HYPRNI--
-HPRNIN-
-HPRNIM-
-HPRNIE-
-HPRNIK-
HDPRNI--
-HPRNIY-
-HPRNIH-
INPRNI--
IVPRNI--
-IPRNII-
IAPRNI--
-IPRNIV-
IQPRNI--
-IPRNIG-
IDPRNI--
IIPRNI--
-IPRNIW-
-IPRNIT-
IGPRNI--
-IPRNIM-
-IPRNIF-
-IPRNIE-
IHPRNI--
-IPRNIY-
IPPRNI--
IMPRNI--
-IPRNIH-
-IPRNID-
IEPRNI--
-KPRNIL-
KDPRNI--
-KPRNIM-
KGPRNI--
KQPRNI--
-KPRNIV-
KPPRNI--
-KPRNIA-
-KPRNIF-
KIPRNI--
-KPRNIK-
-KPRNIH-
-KPRNII-
KTPRNI--
-KPRNIY-
KHPRNI--
-KPRNIW-

-KPRNIN-
KKPRNI--
-KPRNIQ-
-KPRNIG-
-KPRNIE-
-LPRNIT-
LQPRNI--
-LPRNIL-
LHPRNI--
-LPRNIW-
-LPRNIN-
LDPRNI--
-LPRNIA-
LEPRNI--
LPPRNI--
LNPRNI--
-LPRNIM-
-LPRNIH-
LYPRNI--
-LPRNIF-
-LPRNII-
-LPRNIY-
-LPRNIQ-
-LPRNIP-
-LPRNIE-
LKPRNI--
MVPRNI--
-MPRNIQ-
-MPRNIL-
-MPRNIY-
MSPRNI--
MRPRNI--
MIPRNI--
-MPRNIN-
-MPRNIS-
MMPRNI--
-MPRNIH-
MTPRNI--
-MPRNII-
MNPRNI--
MLPRNI--
-MPRNIK-
MYPRNI--
-MPRNIW-
-MPRNIV-
MFPRNI--
MEPRNI--
-MPRNIF-
-MPRNIE-
MGPRNI--

MDPRNI--
MAPRNI--
-MPRNIA-
-MPRNIG-
MQPRNI--
-MPRNIT-
MPPRNI--
MHPRNI--
NVPRNI--
NRPRNI--
-NPRNII-
-NPRNIM-
-NPRNIL-
-NPRNIR-
-NPRNIA-
NSPRNI--
NLPRNI--
-NPRNIV-
NYPRNI--
NGPRNI--
-NPRNIY-
NEPRNI--
NDPRNI--
NQPRNI--
-NPRNIQ-
NNPRNI--
NIPRNI--
-NPRNIF-
NPPRNI--
-NPRNIH-
-NPRNIE-
-NPRNIN-
-NPRNIG-
--PRNISL
--PRNISS
--PRNILY
--PRNIKT
--PRNISQ
--PRNIST
--PRNIFV
--PRNIRA
--PRNISF
--PRNIFM
--PRNIYL
--PRNITG
--PRNISV
--PRNIYF
--PRNIWS
--PRNIWM
--PRNIVN

--PRNIRY
--PRNIVM
--PRNIMA
--PRNIGG
--PRNILM
--PRNITW
--PRNISI
--PRNIWK
--PRNITT
--PRNISG
--PRNIYT
PAPRNI--
--PRNITI
--PRNIYS
--PRNISA
--PRNINA
--PRNIKS
--PRNIVF
--PRNIIH
--PRNIGI
--PRNIIS
--PRNIIK
--PRNITE
--PRNIAA
--PRNITA
--PRNIQS
--PRNIGY
--PRNIGQ
PTPRNI--
--PRNILE
--PRNIIM
--PRNIYM
PMPRNI--
--PRNIVS
--PRNIVQ
--PRNISW
--PRNIMV
--PRNILV
--PRNILD
--PRNIHW
--PRNIHG
--PRNIVI
--PRNIIY
--PRNIVE
--PRNIMI
--PRNIKI
--PRNIIN
--PRNIGT
PGPRNI--
--PRNIHQ

PKPRNI--
--PRNIVV
--PRNIVD
--PRNIRW
--PRNIQY
--PRNIQL
--PRNIFY
--PRNIEV
--PRNIES
--PRNINT
--PRNIHF
--PRNIAV
--PRNIAT
--PRNIYG
--PRNIKA
--PRNIGW
--PRNIAN
--PRNIAG
PNPRNI--
PVPRNI--
--PRNIYY
--PRNITD
--PRNIRF
--PRNIQK
--PRNIQD
--PRNINY
--PRNIMN
--PRNIAR
--PRNIWE
--PRNIRD
--PRNIQA
--PRNINW
--PRNIGV
--PRNIAW
--PRNIRE
--PRNIKM
--PRNIIQ
--PRNIIA
--PRNIHL
--PRNIGH
--PRNIFG
--PRNIFA
--PRNIEL
--PRNIVW
--PRNITY
--PRNITN
--PRNIQG
--PRNINR
--PRNINF
--PRNIMM

--PRNIIT
--PRNIGM
--PRNIDS
--PRNIAK
PQPRNI--
--PRNIQQ
--PRNIQF
--PRNIFK
--PRNIFF
--PRNIAD
PEPRNI--
--PRNIYI
--PRNIQW
--PRNIQV
--PRNIQT
--PRNIIF
--PRNIHM
--PRNIHK
--PRNIHH
--PRNIGN
--PRNIFE
--PRNIEI
PHPRNI--
PSPRNI--
--PRNIYD
--PRNIYA
--PRNISD
--PRNIQN
--PRNIQM
--PRNIKW
--PRNIKE
--PRNIII
--PRNIHY
--PRNIEA
--PRNIWV
--PRNIWN
--PRNISE
--PRNIQH
--PRNINM
--PRNING
--PRNIKQ
--PRNIID
--PRNIER
--PRNIEQ
--PRNIAL
--PRNIAF
--PRNIYN
--PRNIYE
--PRNIWY
--PRNITQ

--PRNITF
--PRNINS
--PRNIMQ
--PRNIME
--PRNIIE
--PRNIHN
--PRNIFD
--PRNIEM
--PRNIEF
--PRNIAM
--PRNIAH
--PRNIND
--PRNIKY
--PRNIHV
--PRNIHE
--PRNIEH
--PRNIDG
--PRNIAY
--PRNIAI
PCPRNI--
--PRNIYK
--PRNIQI
--PRNINI
--PRNIHA
--PRNIFC
--PRNIEK
--PRNIDT
--PRNICT
--PRNICL
--PRNICA
--PRNIAC
PDPRNI--
--PRNIYQ
--PRNIVP
--PRNITH
--PRNISC
--PRNIPY
--PRNIPT
--PRNIPL
--PRNINP
--PRNIMW
--PRNIEW
--PRNIEN
--PRNIEE
--PRNIEC
--PRNICS
--PRNICI
--PRNIAE
-PPRNIV-
-PPRNIS-

-PPRNIL-
-PPRNIA-
PPPRNI--
QSPRNI--
-QPRNIS-
-QPRNIR-
-QPRNIF-
QRPRNI--
-QPRNIQ-
-QPRNIV-
QYPRNI--
QEPRNI--
QFPRNI--
-QPRNII-
QVPRNI--
-QPRNIM-
-QPRNIT-
QHPRNI--
QGPRNI--
-QPRNIG-
QTPRNI--
QIPRNI--
-QPRNIA-
QPPRNI--
QNPRNI--
QKPRNI--
-QPRNIN-
-QPRNIE-
QDPRNI--
QQPRNI--
-QPRNIW-
-QPRNID-
-RPRNIL-
-RPRNIT-
-RPRNII-
-RPRNIV-
REPRNI--
-RPRNIS-
-RPRNIK-
RPPRNI--
-RPRNIQ-
-RPRNIW-
-RPRNIG-
-RPRNIA-
-RPRNIN-
-RPRNIE-
-RPRNIY-
RNPRNI--
RHPRNI--
SVPRNI--

STPRNI--
SAPRNI--
SIPRNI--
-SPRNIW-
-SPRNII-
-SPRNIT-
-SPRNIS-
SGPRNI--
-SPRNIY-
SEPRNI--
SYPRNI--
-SPRNIQ-
-SPRNIH-
-SPRNIG-
SMPRNI--
-SPRNIA-
SHPRNI--
-SPRNIN-
SKPRNI--
SDPRNI--
-SPRNIV-
-SPRNIK-
-SPRNIE-
SPPRNI--
-TPRNIL-
TRPRNI--
TSPRNI--
-TPRNIV-
-TPRNIF-
-TPRNIR-
-TPRNIK-
TGPRNI--
-TPRNII-
TPPRNI--
-TPRNIS-
-TPRNIQ-
-TPRNIT-
-TPRNIM-
TLPRNI--
TVPRNI--
-TPRNIN-
TEPRNI--
-TPRNIH-
TTPRNI--
-TPRNIG-
TYPRNI--
-TPRNIY-
TFPRNI--
TNPRNI--
TQPRNI--

THPRNI--
TCPRNI--
-TPRNIE-
TDPRNI--
-VPRNIR-
VSPRNI--
-VPRNIS-
-VPRNII-
-VPRNIW-
VLPRNI--
-VPRNIG-
-VPRNIF-
VYPRNI--
VMPRNI--
-VPRNIV-
-VPRNIT-
VVPRNI--
-VPRNIH-
-VPRNIE-
-VPRNIA-
VNPRNI--
VGPRNI--
VEPRNI--
-VPRNIQ-
VPPRNI--
-VPRNIK-
VIPRNI--
VFPRNI--
-VPRNIN-
-VPRNID-
VDPRNI--
-VPRNIM-
VCPRNI--
VHPRNI--
WPPRNI--
WLPRNI--
WKPRNI--
-WPRNIF-
-WPRNII-
WQPRNI--
-WPRNIM-
WNPRNI--
WEPRNI--
WSPRNI--
WDPRNI--
WTPRNI--
WVPRNI--
-WPRNIH-
WYPRNI--
WMPRNI--

	WGPRNI--		
	-WPRNIY-		
	WFPRNI--		
	WWPRNI--		
	WHPRNI--		
	-WPRNIW-		
	-WPRNIE-		
	WAPRNI--		
	YTPRNI--		
	-YPRNIM-		
	YVPRNI--		
	-YPRNIY-		
	YSPRNI--		
	-YPRNIG-		
	YLPRNI--		
	YHPRNI--		
	-YPRNIK-		
	YGPRNI--		
	-YPRNIL-		
	YIPRNI--		
	-YPRNIW-		
	YAPRNI--		
	YKPRNI--		
	-YPRNIA-		
	-YPRNIH-		
	YNPRNI--		
	-YPRNIT-		
	YPPRNI--		
	YMPRNI--		
	YDPRNI--		
	YYPRNI--		
	-YPRNIN-		
	YCPRNI--		
PKAF	607	585	46.56
	-DPKAFL-		
	-EPKAFL-		
	-APKAFI-		
	ATPKAF--		
	-APKAFV-		
	-APKAFS-		
	AKPKAF--		
	AMPKAF--		
	AGPKAF--		
	-APKAFY-		
	-APKAFL-		
	-APKAFH-		
	AIPKAF--		
	AQPKAF--		
	ARPKAF--		
	-APKAFW-		

-APKAFA-
-APKAFN-
AYPKAF--
-APKAFQ-
ANPKAF--
-APKAFF-
AHPKAF--
APPKAF--
-APKAFK-
-APKAFG-
-APKAFE-
CGPKAF--
CTPKAF--
CLPKAF--
CQPKAF--
CPPKAF--
CMPKAF--
CYPKAF--
CVPKAF--
CRPKAF--
-CPKAFW-
-CPKAFH-
CHPKAF--
CCPKAF--
DIPKAF--
DVPKAF--
DTPKAF--
DGPKAF--
DRPKAF--
-DPKAFR-
DLPKAF--
DWPKAF--
DMPKAF--
DYPKAF--
-DPKAFI-
DKPKAF--
-DPKAFW-
-DPKAFV-
-DPKAFT-
-DPKAFK-
DFPKAF--
DEPKAF--
DAPKAF--
ESPKAF--
EGPKAF--
-EPKAFR-
EMPKAF--
ETPKAF--
-EPKAFT-
-EPKAFA-

EYPKAF--
EWPKAF--
-EPKAFF-
ELPKAF--
EFPKAF--
-EPKAFS-
EVPKAF--
-EPKAFY-
-EPKAFI-
-EPKAFH-
EKPKAF--
EIPKAF--
EHPKAF--
-EPKAFW-
-EPKAFV-
ERPKAF--
ENPKAF--
EEPKAF--
FSPKAF--
FVPKAF--
FRPKAF--
FTPKAF--
FAPKAF--
FDPKAF--
-FPKAFI-
FIPKAF--
FNPKAF--
FMPKAF--
-FPKAFH-
FHPKAF--
FWPKAF--
FPPKAF--
FGPKAF--
FEPKAF--
-FPKAFV-
-FPKAFP-
-FPKAFN-
-FPKAFA-
-GPKAFL-
GGPKAF--
-GPKAFS-
GKPKAF--
-GPKAFK-
-GPKAFF-
GSPKAF--
-GPKAFT-
-GPKAFM-
-GPKAFI-
GHPKAF--
-GPKAFY-

GNPKAF--
GAPKAF--
GMPKAF--
-GPKAFA-
GQPKAF--
GLPKAF--
GRPKAF--
-GPKAFN-
-GPKAFQ-
GDPKAF--
GPPKAF--
GCPKAF--
GTPKAF--
-GPKAFG-
-GPKAFE-
GIPKAF--
HWPKAF--
-HPKAFS-
HGPKAF--
HVPKAF--
HYPKAF--
HLPKAF--
HRPKAF--
-HPKAFR-
HEPKAF--
HPPKAF--
HDPKAF--
-HPKAFY-
HAPKAF--
-HPKAFT-
-HPKAFK-
HIPKAF--
-HPKAFQ-
-HPKAFM-
-HPKAFH-
IRPKAF--
IAPKAF--
INPKAF--
IGPKAF--
-IPKAFM-
-IPKAFY-
IMPKAF--
IEPKAF--
-IPKAFF-
ILPKAF--
-IPKAFI-
-IPKAFA-
IIPKAF--
-IPKAFN-
IHPKAF--

ICPKAF--
KSPKAF--
-KPKAFL-
KQPKAF--
-KPKAFV-
KIPKAF--
-KPKAFS-
KEPKAF--
-KPKAFM-
KGPKAF--
-KPKAFR-
KRPKAF--
KWPKAF--
-KPKAFA-
KMPKAF--
KHPKAF--
KPPKAF--
-KPKAFY-
-KPKAFF-
KFPKAF--
KVPKAF--
KKPKAF--
KDPKAF--
KCPKAF--
KTPKAF--
KAPKAF--
-KPKAFQ-
-KPKAFI-
-KPKAFH-
KNPKAF--
LRPKAF--
-LPKAFY-
LVPKAF--
LPPKAF--
LNPKAF--
LGPKAF--
LIPKAF--
-LPKAFI-
LYPKAF--
LHPKAF--
LMPKAF--
-LPKAFV-
LEPKAF--
LCPKAF--
-LPKAFH-
-LPKAFT-
LDPKAF--
LKPKAF--
-LPKAFN-
-LPKAFL-

-LPKAFQ-
-LPKAFA-
MGPKAF--
MSPKAF--
-MPKAFS-
MLPKAF--
MIPKAF--
MPPKAF--
MAPKAF--
-MPKAFL-
-MPKAFM-
MQPKAF--
-MPKAFA-
-MPKAFV-
-MPKAFH-
MEPKAF--
MWPKAF--
-MPKAFW-
-MPKAFQ-
-MPKAFI-
MVPKAF--
MRPKAF--
MYPKAF--
MTPKAF--
NFPKAF--
NYPKAF--
NLPKAF--
NGPKAF--
NVPKAF--
NTPKAF--
NAPKAF--
NPPKAF--
NNPKAF--
NSPKAF--
NQPKAF--
-NPKAFH-
NMPKAF--
-NPKAFF-
NIPKAF--
NHPKAF--
-NPKAFV-
-NPKAFT-
-NPKAFS-
-NPKAFQ-
-NPKAFK-
NKPKAF--
NEPKAF--
--PKAFTG
--PKAFMY
--PKAFYS

--PKAFVS
--PKAFVN
--PKAFLA
--PKAFFS
--PKAFYY
--PKAFYL
--PKAFVA
--PKAFPM
PQPKAF--
--PKAFSI
PTPKAF--
--PKAFWG
--PKAFSW
--PKAFHW
--PKAFLM
--PKAFKS
--PKAFMK
--PKAFVM
--PKAFMA
--PKAFFT
--PKAFYV
PSPKAF--
--PKAFTY
--PKAFVT
--PKAFTA
--PKAFHY
--PKAFAS
--PKAFQI
--PKAFNA
--PKAFMG
--PKAFKA
--PKAFAI
PRPKAF--
--PKAFYQ
--PKAFTI
--PKAFQL
--PKAFLY
--PKAFHA
--PKAFFG
--PKAFEY
--PKAFWR
--PKAFVV
--PKAFTV
--PKAFTQ
--PKAFQV
--PKAFAF
--PKAFYT
--PKAFNV
--PKAFNL
--PKAFMM

--PKAFLK
--PKAFLF
--PKAFIV
--PKAFGI
--PKAFVW
--PKAFTK
--PKAFQA
--PKAFGM
--PKAFGF
--PKAFYN
--PKAFYD
--PKAFNY
--PKAFLT
--PKAFGA
--PKAFFQ
--PKAFFI
--PKAFDL
--PKAFYG
--PKAFWV
--PKAFVQ
--PKAFTW
--PKAFSY
--PKAFSM
--PKAFMF
--PKAFLN
--PKAFLG
--PKAFKF
--PKAFIY
--PKAFIH
--PKAFIA
--PKAFES
--PKAFAH
--PKAFYH
--PKAFYE
--PKAFWK
--PKAFVY
--PKAFSN
--PKAFQK
--PKAFNT
--PKAFLV
--PKAFKW
--PKAFIN
--PKAFIK
--PKAFFY
--PKAFFA
--PKAFSQ
--PKAFQF
--PKAFHM
--PKAFHH
--PKAFGS

--PKAFFM
--PKAFDM
--PKAFWN
--PKAFWH
--PKAFVF
--PKAFQQ
--PKAFME
--PKAFHV
--PKAFFW
--PKAFFR
--PKAFAT
--PKAFAQ
--PKAFAA
PMPKAF--
--PKAFYW
--PKAFYI
--PKAFWD
--PKAFRE
--PKAFQT
--PKAFMI
--PKAFID
--PKAFGQ
--PKAFFH
--PKAFFD
--PKAFEG
PHPKAF--
PNPKAF--
--PKAFWY
--PKAFVH
--PKAFTE
--PKAFRW
--PKAFNQ
--PKAFNM
--PKAFNG
--PKAFMV
--PKAFLE
--PKAFKE
--PKAFGW
--PKAFGG
--PKAFFV
--PKAFEV
--PKAFDI
--PKAFCA
--PKAFAK
PYPKAF--
PVPKAF--
PKPKAF--
--PKAFYA
--PKAFWI
--PKAFWF

--PKAFVG
--PKAFVD
--PKAFSC
--PKAFRC
--PKAFQH
--PKAFQG
--PKAFPY
--PKAFPI
--PKAFPC
--PKAFNR
--PKAFNF
--PKAFMQ
--PKAFMD
--PKAFIQ
--PKAFII
--PKAFIE
--PKAFHS
--PKAFGT
--PKAFGK
--PKAFFE
--PKAFET
--PKAFEM
--PKAFCV
--PKAFAM
-QPKAFY-
-QPKAFI-
-QPKAFV-
-QPKAFS-
QIPKAF--
QNPKAF--
QWPKAF--
-QPKAFM-
QPPKAF--
QSPKAF--
QGPKAF--
QRPKAF--
-QPKAFW-
-QPKAFR-
-QPKAFN-
QEPKAF--
QMPKAF--
-QPKAFF-
-QPKAFL-
QHPKAF--
QAPKAF--
QYPKAF--
QVPKAF--
QTPKAF--
QQPKAF--
-QPKAFQ-

QDPKAF--
RGPKAF--
RAPKAF--
-RPKAFM-
-RPKAFT-
RFPKAF--
RNPKAF--
RTPKAF--
-RPKAFF-
RSPKAF--
RDPKAF--
RPPKAF--
-RPKAFQ-
REPKAF--
-RPKAFA-
RHPKAF--
SLPKAF--
-SPKAFL-
SIPKAF--
SVPKAF--
-SPKAFF-
-SPKAFT-
SPPKAF--
-SPKAFS-
-SPKAFY-
SAPKAF--
STPKAF--
-SPKAFW-
-SPKAFK-
SKPKAF--
-SPKAFM-
SCPKAF--
-SPKAFN-
-SPKAFA-
SEPKAF--
SDPKAF--
SQPKAF--
TPPKAF--
TLPKAF--
TTPKAF--
TIPKAF--
TEPKAF--
TVPKAF--
-TPKAFS-
-TPKAFF-
TGPKAF--
TWPKAF--
TQPKAF--
-TPKAFW-
-TPKAFT-

-TPKAFQ-
TAPKAF--
TSPKAF--
-TPKAFI-
TCPKAF--
-TPKAFV-
-TPKAFH-
-TPKAFY-
-TPKAFC-
TFPKAF--
VRPKAF--
VGPKAF--
VSPKAF--
-VPKAFM-
-VPKAFI-
-VPKAFY-
VIPKAF--
VYPKAF--
VQPKAF--
VTPKAF--
VKPKAF--
VHPKAF--
-VPKAFL-
-VPKAFA-
VEPKAF--
VAPKAF--
-VPKAFW-
VNPKAF--
-VPKAFT-
-VPKAFV-
-VPKAFN-
VCPKAF--
-VPKAFF-
-VPKAFK-
WRPKAF--
-WPKAFT-
WVPKAF--
-WPKAFY-
-WPKAFL-
WLPKAF--
-WPKAFF-
WNPKAF--
WAPKAF--
-WPKAFS-
WIPKAF--
WHPKAF--
-WPKAFQ-
WSPKAF--
WMPKAF--
WKPKAF--

```
            WYPKAF--
            WTPKAF--
            -WPKAFP-
            YLPKAF--
            YHPKAF--
            -YPKAFT-
            YSPKAF--
            YMPKAF--
            YKPKAF--
            YTPKAF--
            YPPKAF--
            -YPKAFV-
            -YPKAFQ-
            YEPKAF--
            YQPKAF--
            YDPKAF--
            -YPKAFW-
            -YPKAFF-
            -YPKAFA-
PKSM         234        730      46.51
            --PKSMGT
            --PKSMGN
            --PKSMEL
            -APKSML-
            -APKSMI-
            -APKSMS-
            -APKSMV-
            -APKSMT-
            AVPKSM--
            -APKSMY-
            AYPKSM--
            -APKSMQ-
            ASPKSM--
            APPKSM--
            AFPKSM--
            AEPKSM--
            AQPKSM--
            -APKSMN-
            -APKSMH-
            -APKSME-
            -APKSMA-
            AHPKSM--
            AIPKSM--
            -APKSMG-
            ACPKSM--
            ADPKSM--
            AMPKSM--
            AKPKSM--
            AGPKSM--
            AWPKSM--
```

CSPKSM--
CIPKSM--
CRPKSM--
-CPKSMY-
CCPKSM--
CAPKSM--
CPPKSM--
-CPKSMV-
-CPKSMR-
-CPKSMF-
-CPKSMS-
-CPKSMA-
CKPKSM--
CGPKSM--
CFPKSM--
CWPKSM--
CVPKSM--
-CPKSMW-
-CPKSMM-
-CPKSML-
-CPKSMH-
CMPKSM--
CLPKSM--
CEPKSM--
CDPKSM--
DMPKSM--
DSPKSM--
-DPKSMR-
DRPKSM--
-DPKSMY-
DVPKSM--
-DPKSMI-
DYPKSM--
DKPKSM--
-DPKSMW-
-DPKSMH-
DWPKSM--
DQPKSM--
-DPKSMT-
-DPKSMS-
-DPKSML-
DLPKSM--
DFPKSM--
DDPKSM--
DTPKSM--
-DPKSMQ-
-DPKSMC-
DNPKSM--
DCPKSM--
EHPKSM--

-EPKSML-
EAPKSM--
-EPKSMS-
-EPKSMV-
-EPKSMI-
ERPKSM--
EIPKSM--
-EPKSMF-
EVPKSM--
-EPKSMA-
EMPKSM--
EGPKSM--
-EPKSMM-
-EPKSMH-
EWPKSM--
ENPKSM--
EKPKSM--
EYPKSM--
ESPKSM--
EQPKSM--
-EPKSMY-
ETPKSM--
-EPKSMN-
-EPKSMQ-
EFPKSM--
EEPKSM--
EPPKSM--
-EPKSMW-
-EPKSMK-
-FPKSMY-
FLPKSM--
FYPKSM--
FRPKSM--
FDPKSM--
FQPKSM--
-FPKSMI-
FAPKSM--
-FPKSMW-
FIPKSM--
FHPKSM--
-FPKSMN-
FEPKSM--
FNPKSM--
FSPKSM--
FVPKSM--
-FPKSMV-
FMPKSM--
FCPKSM--
FKPKSM--
-FPKSMM-

-FPKSMD-
GAPKSM--
-GPKSML-
-GPKSMW-
GSPKSM--
GLPKSM--
GFPKSM--
-GPKSMV-
-GPKSMA-
GWPKSM--
-GPKSMS-
GQPKSM--
GTPKSM--
GIPKSM--
GKPKSM--
GMPKSM--
GHPKSM--
GEPKSM--
-GPKSMT-
-GPKSMK-
-GPKSMH-
-GPKSMF-
GVPKSM--
GGPKSM--
-GPKSMQ-
GRPKSM--
GDPKSM--
GYPKSM--
GNPKSM--
GCPKSM--
-GPKSMN-
-GPKSME-
-GPKSMM-
-GPKSMG-
GPPKSM--
-GPKSMC-
HSPKSM--
HYPKSM--
HGPKSM--
HLPKSM--
-HPKSMV-
HWPKSM--
HRPKSM--
-HPKSML-
-HPKSMS-
HFPKSM--
-HPKSMY-
HNPKSM--
HDPKSM--
-HPKSMK-

HVPKSM--
HQPKSM--
-HPKSMH-
HHPKSM--
HPPKSM--
-HPKSMF-
-HPKSMA-
-HPKSMN-
-HPKSMM-
-HPKSMI-
HIPKSM--
ISPKSM--
-IPKSMK-
IRPKSM--
IEPKSM--
IAPKSM--
IMPKSM--
-IPKSMV-
-IPKSMM-
-IPKSML-
-IPKSMT-
-IPKSMS-
-IPKSMY-
IYPKSM--
-IPKSMA-
IKPKSM--
-IPKSMH-
-IPKSMF-
IWPKSM--
IVPKSM--
-IPKSMN-
IQPKSM--
-IPKSMI-
-IPKSMG-
INPKSM--
IGPKSM--
-IPKSMQ-
-IPKSME-
-IPKSMC-
IHPKSM--
KSPKSM--
KYPKSM--
KMPKSM--
-KPKSMT-
-KPKSMW-
-KPKSMM-
KLPKSM--
-KPKSMA-
KQPKSM--
KHPKSM--

-KPKSMG-
KVPKSM--
KKPKSM--
KIPKSM--
KCPKSM--
KTPKSM--
KPPKSM--
-KPKSMV-
-KPKSMN-
-LPKSML-
LRPKSM--
-LPKSMV-
LSPKSM--
LYPKSM--
-LPKSMY-
LAPKSM--
LWPKSM--
-LPKSMT-
-LPKSMW-
LTPKSM--
LMPKSM--
LKPKSM--
LHPKSM--
LDPKSM--
-LPKSMS-
LQPKSM--
-LPKSMH-
LPPKSM--
LGPKSM--
-LPKSMM-
LNPKSM--
LCPKSM--
-LPKSMI-
-LPKSMF-
-LPKSMN-
-LPKSMQ-
MLPKSM--
MSPKSM--
-MPKSMF-
MTPKSM--
MGPKSM--
-MPKSML-
-MPKSMR-
MVPKSM--
MPPKSM--
MAPKSM--
-MPKSMH-
MYPKSM--
-MPKSMT-
-MPKSMM-

MMPKSM--
MEPKSM--
-MPKSMI-
MFPKSM--
-MPKSMY-
-MPKSMA-
-MPKSME-
MNPKSM--
-MPKSMV-
-MPKSMN-
MHPKSM--
-MPKSMQ-
MDPKSM--
MCPKSM--
NGPKSM--
NSPKSM--
NRPKSM--
-NPKSMT-
-NPKSMF-
NVPKSM--
NNPKSM--
NHPKSM--
NTPKSM--
-NPKSMV-
-NPKSML-
-NPKSMS-
-NPKSMK-
-NPKSMH-
NEPKSM--
NPPKSM--
-NPKSMY-
NAPKSM--
NWPKSM--
-NPKSMW-
NKPKSM--
NYPKSM--
NQPKSM--
NLPKSM--
NIPKSM--
--PKSMMG
--PKSMWT
--PKSMLS
--PKSMIM
--PKSMVF
--PKSMST
--PKSMYT
PLPKSM--
--PKSMAG
--PKSMSS
--PKSMMS

--PKSMVS
--PKSMTT
--PKSMSG
--PKSMML
--PKSMSA
--PKSMMI
--PKSMVY
--PKSMLW
--PKSMKS
--PKSMSV
--PKSMVV
--PKSMFR
--PKSMAV
--PKSMIS
--PKSMNW
--PKSMIN
--PKSMLN
--PKSMAL
PSPKSM--
--PKSMYG
--PKSMWV
--PKSMWS
--PKSMMA
--PKSMTG
--PKSMMQ
--PKSMIL
--PKSMFV
--PKSMFT
--PKSMVG
--PKSMIG
--PKSMHL
--PKSMFS
--PKSMSL
--PKSMNL
--PKSMYA
--PKSMHG
--PKSMYI
--PKSMTN
--PKSMTA
--PKSMLK
--PKSMFY
--PKSMYL
--PKSMYE
--PKSMMK
--PKSMIA
--PKSMFM
--PKSMYY
--PKSMVT
--PKSMVK
--PKSMHA

PAPKSM--
--PKSMTF
--PKSMQT
--PKSMMH
--PKSMGL
--PKSMVN
--PKSMLF
--PKSMKV
--PKSMHT
--PKSMAA
--PKSMVH
--PKSMSH
--PKSMSF
PGPKSM--
--PKSMWA
--PKSMTQ
--PKSMNY
--PKSMNM
--PKSMNA
--PKSMIT
PNPKSM--
--PKSMYS
--PKSMWI
--PKSMVI
--PKSMTH
--PKSMLL
--PKSMWW
--PKSMRD
--PKSMQM
--PKSMES
--PKSMAS
PIPKSM--
--PKSMYD
--PKSMWQ
--PKSMTV
--PKSMTI
--PKSMRQ
--PKSMQL
--PKSMNS
--PKSMNF
--PKSMIV
--PKSMFQ
--PKSMFI
--PKSMAT
--PKSMWF
--PKSMTM
--PKSMSN
--PKSMKM
--PKSMHY
--PKSMHE

--PKSMFA
PVPKSM--
--PKSMYN
--PKSMYF
--PKSMSW
--PKSMSQ
--PKSMMW
--PKSMKW
--PKSMKG
PEPKSM--
PWPKSM--
PRPKSM--
--PKSMYK
--PKSMVQ
--PKSMQF
--PKSMNQ
--PKSMHM
--PKSMHF
--PKSMGY
--PKSMVM
--PKSMTE
--PKSMQS
--PKSMNG
--PKSMLD
--PKSMKN
--PKSMHD
--PKSMAR
PHPKSM--
--PKSMTD
--PKSMSE
--PKSMQY
--PKSMQN
--PKSMMN
--PKSMAH
--PKSMYQ
--PKSMYH
--PKSMWL
--PKSMVE
--PKSMQK
--PKSMQG
--PKSMLH
--PKSMLE
--PKSMKI
--PKSMHQ
--PKSMGG
--PKSMFW
--PKSMEG
--PKSMAN
--PKSMAI
PDPKSM--

--PKSMWM
--PKSMWK
--PKSMWE
--PKSMRE
--PKSMNT
--PKSMMY
--PKSMIE
--PKSMEI
--PKSMCR
--PKSMAM
PFPKSM--
PYPKSM--
PMPKSM--
--PKSMWN
--PKSMQA
--PKSMPL
--PKSMNN
--PKSMNK
--PKSMKH
--PKSMKE
--PKSMIH
--PKSMHW
--PKSMHN
--PKSMGW
--PKSMGF
--PKSMET
--PKSMEK
--PKSMCA
--PKSMAQ
PCPKSM--
PTPKSM--
PQPKSM--
--PKSMYM
--PKSMWC
--PKSMTK
--PKSMQI
--PKSMQH
--PKSMNI
--PKSMND
--PKSMKY
--PKSMKD
--PKSMIQ
--PKSMHS
--PKSMHK
--PKSMHH
--PKSMGM
--PKSMEY
--PKSMEV
--PKSMER
--PKSMEQ

--PKSMEH
--PKSMEA
--PKSMDT
--PKSMDS
--PKSMDG
--PKSMCV
--PKSMCM
--PKSMCK
-QPKSMR-
QVPKSM--
-QPKSMI-
QLPKSM--
-QPKSML-
-QPKSMK-
-QPKSMH-
-QPKSMM-
QAPKSM--
QGPKSM--
QTPKSM--
QIPKSM--
QFPKSM--
-QPKSMT-
-QPKSMS-
-QPKSMY-
QKPKSM--
QEPKSM--
QHPKSM--
QYPKSM--
QPPKSM--
-QPKSMQ-
-QPKSMG-
-QPKSMA-
QNPKSM--
QMPKSM--
QSPKSM--
QRPKSM--
QQPKSM--
-QPKSME-
QWPKSM--
-QPKSMV-
RLPKSM--
-RPKSMY-
RVPKSM--
RAPKSM--
RNPKSM--
RWPKSM--
-RPKSMF-
-RPKSMV-
RMPKSM--
-RPKSMH-

RGPKSM--
RDPKSM--
RIPKSM--
RQPKSM--
RPPKSM--
RTPKSM--
-RPKSMI-
-RPKSMQ-
REPKSM--
RCPKSM--
-RPKSMG-
-RPKSME-
-RPKSMT-
-SPKSMS-
SRPKSM--
-SPKSMI-
SVPKSM--
-SPKSMT-
SSPKSM--
SPPKSM--
-SPKSML-
SGPKSM--
SEPKSM--
-SPKSMV-
SAPKSM--
-SPKSMF-
SFPKSM--
-SPKSMH-
SKPKSM--
SHPKSM--
SYPKSM--
SMPKSM--
-SPKSMY-
-SPKSMN-
SNPKSM--
-SPKSMK-
-SPKSMA-
SQPKSM--
-SPKSMQ-
SCPKSM--
-SPKSMG-
SDPKSM--
-SPKSMM-
TSPKSM--
-TPKSML-
-TPKSMM-
TNPKSM--
TVPKSM--
-TPKSMS-
-TPKSMK-

TAPKSM--
TFPKSM--
TDPKSM--
TYPKSM--
TEPKSM--
TTPKSM--
TQPKSM--
-TPKSMV-
TGPKSM--
TWPKSM--
-TPKSMI-
-TPKSMH-
-TPKSMY-
-TPKSMT-
TPPKSM--
-TPKSMF-
-TPKSMW-
-TPKSMN-
TIPKSM--
THPKSM--
TCPKSM--
-TPKSME-
-VPKSMW-
-VPKSMY-
VHPKSM--
VYPKSM--
VTPKSM--
VMPKSM--
-VPKSMV-
-VPKSML-
VIPKSM--
-VPKSMQ-
VQPKSM--
VGPKSM--
VFPKSM--
-VPKSMS-
VKPKSM--
-VPKSMM-
VEPKSM--
VAPKSM--
VPPKSM--
-VPKSMA-
-VPKSMI-
-VPKSME-
-VPKSMH-
-VPKSMF-
-VPKSMG-
-VPKSMN-
VDPKSM--
WSPKSM--

-WPKSMY-
-WPKSMV-
WLPKSM--
WVPKSM--
WHPKSM--
WQPKSM--
-WPKSMW-
WIPKSM--
WAPKSM--
-WPKSMH-
WKPKSM--
WTPKSM--
WRPKSM--
-WPKSME-
WYPKSM--
-WPKSMI-
-WPKSMG-
WMPKSM--
-WPKSMQ-
WNPKSM--
WGPKSM--
WFPKSM--
WDPKSM--
WPPKSM--
WEPKSM--
WCPKSM--
YHPKSM--
YVPKSM--
-YPKSMS-
YMPKSM--
YDPKSM--
YQPKSM--
YNPKSM--
-YPKSMH-
-YPKSMF-
YYPKSM--
YIPKSM--
YTPKSM--
-YPKSMW-
-YPKSMT-
YKPKSM--
-YPKSMI-
YRPKSM--
YPPKSM--
YEPKSM--
-YPKSMQ-
-YPKSMY-
YGPKSM--
YCPKSM--
YAPKSM--

PKHL 311 689 46.51

--PKHLGF
-QPKHLG-
-QPKHLE-
AYPKHL--
ALPKHL--
APPKHL--
-APKHLT-
-APKHLR-
-APKHLL-
-APKHLY-
-APKHLV-
AIPKHL--
-APKHLH-
ASPKHL--
AGPKHL--
AKPKHL--
-APKHLW-
AMPKHL--
AEPKHL--
AQPKHL--
-APKHLI-
AHPKHL--
ADPKHL--
AAPKHL--
-APKHLA-
-APKHLG-
ANPKHL--
-APKHLM-
-APKHLS-
ACPKHL--
-APKHLK-
AVPKHL--
-APKHLN-
CGPKHL--
CAPKHL--
CMPKHL--
CKPKHL--
CIPKHL--
CRPKHL--
CCPKHL--
CVPKHL--
CPPKHL--
CHPKHL--
-CPKHLK-
-CPKHLH-
CFPKHL--
-CPKHLR-
-CPKHLQ-
-CPKHLA-

CLPKHL--
DGPKHL--
DTPKHL--
DRPKHL--
DSPKHL--
DWPKHL--
DMPKHL--
-DPKHLL-
DAPKHL--
DYPKHL--
-DPKHLI-
DIPKHL--
-DPKHLT-
DVPKHL--
-DPKHLS-
-DPKHLM-
-DPKHLK-
-DPKHLR-
-DPKHLA-
DNPKHL--
DQPKHL--
DHPKHL--
DFPKHL--
DEPKHL--
-EPKHLI-
-EPKHLK-
-EPKHLM-
-EPKHLQ-
EVPKHL--
-EPKHLF-
EIPKHL--
ETPKHL--
-EPKHLH-
EYPKHL--
-EPKHLY-
-EPKHLW-
-EPKHLV-
-EPKHLS-
EGPKHL--
EKPKHL--
-EPKHLL-
-EPKHLA-
EMPKHL--
EQPKHL--
-EPKHLT-
EHPKHL--
FLPKHL--
FWPKHL--
FTPKHL--
FYPKHL--

FVPKHL--
-FPKHLI-
FIPKHL--
FSPKHL--
FQPKHL--
FGPKHL--
FDPKHL--
FEPKHL--
-FPKHLL-
FPPKHL--
-FPKHLW-
FNPKHL--
FAPKHL--
-FPKHLQ-
-FPKHLF-
-FPKHLE-
GTPKHL--
-GPKHLS-
GVPKHL--
-GPKHLQ-
GYPKHL--
GSPKHL--
GGPKHL--
GMPKHL--
GLPKHL--
-GPKHLV-
-GPKHLL-
GWPKHL--
-GPKHLW-
-GPKHLM-
-GPKHLE-
GAPKHL--
-GPKHLT-
-GPKHLH-
-GPKHLR-
-GPKHLI-
GIPKHL--
-GPKHLK-
GNPKHL--
GEPKHL--
-GPKHLN-
GDPKHL--
GHPKHL--
-GPKHLG-
-GPKHLF-
-GPKHLA-
GQPKHL--
HTPKHL--
-HPKHLL-
-HPKHLT-

HVPKHL--
-HPKHLW-
HPPKHL--
HMPKHL--
HFPKHL--
-HPKHLR-
HNPKHL--
HHPKHL--
-HPKHLV-
-HPKHLI-
HEPKHL--
HQPKHL--
HIPKHL--
HYPKHL--
-HPKHLY-
HGPKHL--
-HPKHLH-
HDPKHL--
-HPKHLF-
HLPKHL--
HSPKHL--
-HPKHLM-
IAPKHL--
-IPKHLR-
IFPKHL--
IDPKHL--
-IPKHLL-
IIPKHL--
ITPKHL--
IQPKHL--
-IPKHLE-
IGPKHL--
IVPKHL--
-IPKHLS-
-IPKHLT-
-IPKHLV-
-IPKHLA-
-IPKHLW-
INPKHL--
-IPKHLN-
-IPKHLF-
IEPKHL--
IPPKHL--
-IPKHLY-
-IPKHLM-
-IPKHLK-
-IPKHLG-
-IPKHLQ-
KPPKHL--
-KPKHLM-

-KPKHLI-
KNPKHL--
KIPKHL--
KMPKHL--
KTPKHL--
KGPKHL--
KRPKHL--
-KPKHLT-
-KPKHLY-
-KPKHLS-
KKPKHL--
KEPKHL--
-KPKHLV-
-KPKHLL-
-KPKHLW-
-KPKHLQ-
-KPKHLN-
-KPKHLE-
-KPKHLA-
KDPKHL--
KCPKHL--
LNPKHL--
-LPKHLR-
LVPKHL--
-LPKHLT-
-LPKHLM-
LEPKHL--
-LPKHLS-
LMPKHL--
LGPKHL--
LIPKHL--
LDPKHL--
-LPKHLF-
-LPKHLV-
-LPKHLE-
-LPKHLQ-
-LPKHLI-
-LPKHLH-
-LPKHLA-
-LPKHLW-
-LPKHLN-
MVPKHL--
MGPKHL--
MAPKHL--
-MPKHLF-
-MPKHLR-
MYPKHL--
MEPKHL--
MQPKHL--
MTPKHL--

-MPKHLI-
-MPKHLS-
-MPKHLL-
MNPKHL--
-MPKHLH-
-MPKHLA-
MIPKHL--
-MPKHLE-
MPPKHL--
-MPKHLN-
-MPKHLW-
MCPKHL--
MWPKHL--
-MPKHLY-
-MPKHLM-
-MPKHLG-
NGPKHL--
NPPKHL--
-NPKHLR-
NSPKHL--
NAPKHL--
-NPKHLV-
-NPKHLT-
-NPKHLI-
NMPKHL--
NNPKHL--
-NPKHLM-
NEPKHL--
-NPKHLL-
NIPKHL--
NWPKHL--
-NPKHLY-
-NPKHLK-
-NPKHLH-
-NPKHLF-
NQPKHL--
-NPKHLS-
-NPKHLQ-
-NPKHLN-
-NPKHLE-
-NPKHLC-
NDPKHL--
--PKHLSA
--PKHLKT
--PKHLLS
--PKHLST
PSPKHL--
--PKHLIF
--PKHLYI
--PKHLTM

--PKHLRI
--PKHLIK
--PKHLIA
--PKHLSI
--PKHLQA
--PKHLSW
--PKHLIN
--PKHLVN
--PKHLRQ
--PKHLQS
--PKHLTL
--PKHLNS
--PKHLLT
PMPKHL--
--PKHLSN
--PKHLVH
--PKHLFV
PHPKHL--
PTPKHL--
--PKHLWT
--PKHLLA
--PKHLKS
--PKHLAT
PLPKHL--
--PKHLVK
--PKHLET
--PKHLVY
--PKHLVW
--PKHLVS
--PKHLEW
--PKHLTG
--PKHLSQ
--PKHLMT
--PKHLMV
--PKHLYY
--PKHLQL
--PKHLNW
--PKHLKN
--PKHLIG
PGPKHL--
--PKHLVA
--PKHLTW
--PKHLMA
--PKHLVV
--PKHLVL
--PKHLTY
--PKHLTA
--PKHLSG
--PKHLHH
--PKHLYV

--PKHLYQ
--PKHLWI
--PKHLSV
--PKHLSM
--PKHLQG
--PKHLFS
--PKHLFA
PAPKHL--
--PKHLQM
--PKHLMN
--PKHLMM
--PKHLKA
--PKHLAG
--PKHLVQ
--PKHLQQ
--PKHLQF
--PKHLLV
--PKHLLM
--PKHLKF
--PKHLWG
--PKHLVI
--PKHLTI
--PKHLSH
--PKHLRE
--PKHLMG
--PKHLIV
--PKHLFD
--PKHLEV
--PKHLAK
--PKHLYK
--PKHLWV
--PKHLTQ
--PKHLMY
--PKHLLH
--PKHLAF
--PKHLVD
--PKHLTV
--PKHLSK
--PKHLQH
--PKHLLE
--PKHLES
PYPKHL--
--PKHLYF
--PKHLYD
--PKHLWS
--PKHLVM
--PKHLSL
--PKHLRM
--PKHLHA
PNPKHL--

```
--PKHLYS
--PKHLWR
--PKHLWD
--PKHLTF
--PKHLLF
--PKHLLD
--PKHLIL
--PKHLID
--PKHLGG
--PKHLGA
--PKHLFQ
--PKHLFK
--PKHLEY
--PKHLEG
--PKHLAW
--PKHLYN
--PKHLYL
--PKHLWF
--PKHLVF
--PKHLRW
--PKHLNY
--PKHLNT
--PKHLIT
--PKHLIR
--PKHLIM
--PKHLIH
--PKHLFN
--PKHLFI
--PKHLAI
PEPKHL--
--PKHLYH
--PKHLWK
--PKHLWA
--PKHLVE
--PKHLTE
--PKHLQN
--PKHLQD
--PKHLNV
--PKHLNQ
--PKHLMQ
--PKHLKM
--PKHLHW
--PKHLHV
--PKHLHS
--PKHLHG
--PKHLHD
--PKHLAL
--PKHLWL
--PKHLTD
--PKHLQK
```

--PKHLNM
--PKHLMF
--PKHLKH
--PKHLHR
--PKHLHN
--PKHLHL
--PKHLHE
--PKHLGT
--PKHLEL
--PKHLDS
--PKHLAQ
--PKHLAM
--PKHLYW
--PKHLWQ
--PKHLWN
--PKHLSY
--PKHLQT
--PKHLNN
--PKHLNL
--PKHLNA
--PKHLMI
--PKHLKV
--PKHLKD
--PKHLIY
--PKHLIQ
--PKHLHI
--PKHLGS
--PKHLFT
--PKHLFM
--PKHLFH
--PKHLFG
--PKHLFE
--PKHLER
--PKHLEM
--PKHLCN
--PKHLAV
--PKHLAR
-PPKHLR-
-PPKHLE-
--PKHLYG
--PKHLYE
--PKHLWY
--PKHLWW
--PKHLWM
--PKHLWE
--PKHLQI
--PKHLNK
--PKHLNF
--PKHLME
--PKHLKW

--PKHLIP
--PKHLHF
--PKHLGV
--PKHLGQ
--PKHLGM
--PKHLGI
--PKHLFW
--PKHLEQ
--PKHLEN
--PKHLEH
--PKHLCV
--PKHLCR
--PKHLCG
PIPKHL--
PDPKHL--
QMPKHL--
-QPKHLR-
-QPKHLL-
QFPKHL--
QGPKHL--
QSPKHL--
QEPKHL--
QPPKHL--
-QPKHLH-
-QPKHLT-
QHPKHL--
-QPKHLM-
QKPKHL--
-QPKHLI-
QCPKHL--
QVPKHL--
-QPKHLS-
QDPKHL--
QQPKHL--
QIPKHL--
QTPKHL--
QNPKHL--
-QPKHLY-
-QPKHLQ-
-QPKHLK-
QYPKHL--
-QPKHLV-
RQPKHL--
RPPKHL--
RNPKHL--
-RPKHLI-
-RPKHLS-
-RPKHLH-
-RPKHLV-
RDPKHL--

−RPKHLL−
−RPKHLF−
REPKHL−−
−RPKHLN−
−RPKHLE−
−RPKHLK−
−RPKHLA−
RCPKHL−−
−RPKHLY−
−RPKHLT−
−RPKHLQ−
RKPKHL−−
−RPKHLP−
SVPKHL−−
SEPKHL−−
SRPKHL−−
−SPKHLS−
−SPKHLI−
−SPKHLV−
SGPKHL−−
SIPKHL−−
−SPKHLH−
SPPKHL−−
−SPKHLL−
−SPKHLT−
−SPKHLF−
−SPKHLK−
SKPKHL−−
−SPKHLY−
SDPKHL−−
−SPKHLW−
−SPKHLN−
−SPKHLE−
−SPKHLA−
−SPKHLG−
SCPKHL−−
TSPKHL−−
TLPKHL−−
TKPKHL−−
−TPKHLT−
TWPKHL−−
TTPKHL−−
TRPKHL−−
−TPKHLY−
−TPKHLL−
TGPKHL−−
−TPKHLF−
TIPKHL−−
TPPKHL−−
−TPKHLV−

-TPKHLI-
TEPKHL--
TQPKHL--
-TPKHLS-
-TPKHLA-
TYPKHL--
TNPKHL--
-TPKHLE-
TMPKHL--
-TPKHLH-
THPKHL--
-TPKHLQ-
TDPKHL--
-TPKHLN-
-TPKHLW-
-TPKHLG-
-TPKHLM-
-VPKHLS-
-VPKHLR-
VLPKHL--
VHPKHL--
-VPKHLI-
-VPKHLT-
VTPKHL--
-VPKHLH-
VQPKHL--
VMPKHL--
-VPKHLN-
VPPKHL--
-VPKHLF-
-VPKHLA-
-VPKHLY-
-VPKHLK-
-VPKHLW-
-VPKHLE-
-VPKHLL-
VEPKHL--
VNPKHL--
-VPKHLG-
VWPKHL--
VDPKHL--
-VPKHLQ-
-VPKHLM-
VCPKHL--
WLPKHL--
WTPKHL--
-WPKHLY-
WRPKHL--
-WPKHLE-
WYPKHL--

WGPKHL--
-WPKHLK-
-WPKHLV-
-WPKHLN-
WMPKHL--
WQPKHL--
-WPKHLL-
WDPKHL--
-WPKHLQ-
WNPKHL--
WEPKHL--
-WPKHLI-
-WPKHLM-
-WPKHLD-
YVPKHL--
-YPKHLL-
YRPKHL--
YQPKHL--
-YPKHLR-
YMPKHL--
-YPKHLT-
-YPKHLK-
YSPKHL--
-YPKHLV-
YIPKHL--
YDPKHL--
-YPKHLS-
-YPKHLF-
YKPKHL--
YTPKHL--
-YPKHLM-
YNPKHL--
-YPKHLY-
-YPKHLI-
YHPKHL--
-YPKHLH-
-YPKHLQ-
-YPKHLG-
YEPKHL--
YCPKHL--
PMSM 106 804 46.50
-DPMSMV-
--PMSMEI
ARPMSM--
ASPMSM--
-APMSMT-
AKPMSM--
ATPMSM--
ALPMSM--
-APMSMR-

AAPMSM--
AGPMSM--
-APMSMI-
-APMSMA-
-APMSMW-
-APMSMV-
-APMSMS-
-APMSMK-
-APMSMH-
AVPMSM--
AMPMSM--
AHPMSM--
ANPMSM--
-APMSMY-
-APMSMN-
AQPMSM--
APPMSM--
-APMSMM-
-APMSML-
AEPMSM--
AYPMSM--
-APMSMQ-
AWPMSM--
-APMSMF-
-APMSMG-
AIPMSM--
ADPMSM--
-APMSMD-
CTPMSM--
CVPMSM--
CSPMSM--
CRPMSM--
CQPMSM--
-CPMSMR-
-CPMSMI-
CMPMSM--
CGPMSM--
-CPMSMY-
-CPMSMT-
-CPMSMG-
-CPMSMF-
CNPMSM--
CLPMSM--
CIPMSM--
CCPMSM--
-DPMSMI-
-DPMSMR-
DYPMSM--
DQPMSM--
DGPMSM--

DEPMSM--
DNPMSM--
-DPMSMF-
DHPMSM--
DTPMSM--
-DPMSMM-
DSPMSM--
DRPMSM--
-DPMSMY-
-DPMSMK-
DLPMSM--
DAPMSM--
DVPMSM--
-DPMSMT-
DDPMSM--
DWPMSM--
-DPMSMH-
DIPMSM--
EMPMSM--
-EPMSMI-
-EPMSML-
-EPMSMT-
ETPMSM--
EGPMSM--
ESPMSM--
-EPMSMS-
ENPMSM--
ELPMSM--
-EPMSMR-
EQPMSM--
-EPMSMF-
EIPMSM--
ERPMSM--
-EPMSMQ-
-EPMSMM-
-EPMSMA-
EAPMSM--
EKPMSM--
EVPMSM--
-EPMSMW-
-EPMSMN-
-EPMSMY-
-EPMSMH-
EWPMSM--
EPPMSM--
-EPMSMV-
-EPMSMK-
-EPMSMG-
-EPMSME-
FVPMSM--

-FPMSMM-
-FPMSMK-
-FPMSMS-
FSPMSM--
FNPMSM--
FLPMSM--
FYPMSM--
-FPMSMV-
FTPMSM--
-FPMSMT-
FDPMSM--
FPPMSM--
FFPMSM--
-FPMSMR-
-FPMSMY-
FKPMSM--
FIPMSM--
FAPMSM--
FCPMSM--
-FPMSMQ-
-FPMSMI-
FMPMSM--
-FPMSMP-
-FPMSMH-
FHPMSM--
FGPMSM--
GEPMSM--
-GPMSML-
-GPMSMW-
-GPMSMS-
-GPMSMK-
GQPMSM--
GPPMSM--
GLPMSM--
-GPMSMV-
-GPMSMN-
GRPMSM--
-GPMSMI-
GNPMSM--
GKPMSM--
-GPMSMR-
GTPMSM--
-GPMSMA-
GYPMSM--
-GPMSMH-
-GPMSMT-
GHPMSM--
GDPMSM--
GGPMSM--
-GPMSMY-

GFPMSM--
GSPMSM--
-GPMSMF-
GIPMSM--
GCPMSM--
-GPMSME-
GAPMSM--
-GPMSMM-
-GPMSMG-
-HPMSMW-
-HPMSMS-
-HPMSMR-
HGPMSM--
HVPMSM--
-HPMSMV-
-HPMSML-
HSPMSM--
HMPMSM--
HEPMSM--
HTPMSM--
HIPMSM--
HFPMSM--
HKPMSM--
HRPMSM--
-HPMSMM-
HYPMSM--
-HPMSMT-
-HPMSMN-
-HPMSMK-
-HPMSMH-
HLPMSM--
HDPMSM--
HAPMSM--
-HPMSMA-
HNPMSM--
HWPMSM--
-HPMSMG-
-IPMSMR-
-IPMSMM-
-IPMSML-
ISPMSM--
IFPMSM--
ITPMSM--
-IPMSMT-
IEPMSM--
ILPMSM--
IIPMSM--
IPPMSM--
IVPMSM--
-IPMSMI-

IGPMSM--
IDPMSM--
-IPMSMS-
INPMSM--
IAPMSM--
IQPMSM--
IKPMSM--
IRPMSM--
-IPMSMQ-
-IPMSMV-
-IPMSMG-
IMPMSM--
IYPMSM--
-IPMSMW-
IHPMSM--
IWPMSM--
-IPMSMH-
KSPMSM--
-KPMSML-
-KPMSMH-
KLPMSM--
-KPMSMR-
KYPMSM--
-KPMSMS-
-KPMSMV-
-KPMSMF-
KIPMSM--
KTPMSM--
-KPMSMA-
-KPMSMI-
KFPMSM--
KPPMSM--
-KPMSMG-
KWPMSM--
KVPMSM--
KRPMSM--
-KPMSMN-
-KPMSMW-
KAPMSM--
KNPMSM--
KGPMSM--
KDPMSM--
-KPMSMY-
-KPMSMK-
KEPMSM--
LIPMSM--
-LPMSMR-
LSPMSM--
-LPMSML-
-LPMSMK-

LNPMSM--
LHPMSM--
LGPMSM--
LLPMSM--
LMPMSM--
LFPMSM--
-LPMSMT-
LPPMSM--
-LPMSMS-
-LPMSMV-
-LPMSMY-
-LPMSMN-
LEPMSM--
-LPMSMF-
LQPMSM--
-LPMSMM-
-LPMSMA-
LAPMSM--
-LPMSMQ-
LKPMSM--
-LPMSMG-
-MPMSMI-
-MPMSMS-
MVPMSM--
-MPMSMV-
MKPMSM--
MGPMSM--
-MPMSML-
MHPMSM--
MFPMSM--
-MPMSMR-
MEPMSM--
MSPMSM--
MAPMSM--
-MPMSMK-
-MPMSMF-
MWPMSM--
-MPMSMY-
-MPMSMW-
-MPMSMG-
MLPMSM--
MPPMSM--
-MPMSMN-
MIPMSM--
-MPMSMT-
MTPMSM--
MNPMSM--
-MPMSMM-
-MPMSMH-
-MPMSME-

-MPMSMA-
MDPMSM--
-NPMSMR-
NLPMSM--
NRPMSM--
-NPMSML-
NYPMSM--
-NPMSMV-
-NPMSMT-
NPPMSM--
-NPMSMK-
NGPMSM--
NSPMSM--
-NPMSMH-
NHPMSM--
NKPMSM--
NIPMSM--
NEPMSM--
-NPMSMY-
-NPMSMS-
NNPMSM--
NFPMSM--
NWPMSM--
-NPMSMQ-
NVPMSM--
NTPMSM--
-NPMSME-
NCPMSM--
-NPMSMI-
-NPMSMF-
NAPMSM--
--PMSMVL
--PMSMLS
--PMSMRA
--PMSMRS
--PMSMQS
--PMSMTS
--PMSMSA
--PMSMRK
--PMSMVG
--PMSMKS
--PMSMRW
--PMSMLV
--PMSMSM
--PMSMSS
--PMSMVA
--PMSMTL
--PMSMYG
--PMSMTR
--PMSMRN

--PMSMLA
--PMSMKR
PFPMSM--
--PMSMIS
--PMSMFR
--PMSMFG
--PMSMAG
--PMSMTQ
--PMSMTK
--PMSMRT
--PMSMKV
--PMSMLH
--PMSMKT
--PMSMHS
--PMSMES
--PMSMVR
--PMSMTI
--PMSMST
--PMSMLM
--PMSMAS
--PMSMYM
--PMSMTG
--PMSMSR
--PMSMQV
--PMSMYR
--PMSMAM
PEPMSM--
--PMSMYS
--PMSMWT
--PMSMLN
--PMSMTT
--PMSMSL
PHPMSM--
--PMSMMY
--PMSMKM
--PMSMHV
--PMSMHL
--PMSMSY
--PMSMMS
--PMSMMR
--PMSMKH
--PMSMHG
--PMSMFK
--PMSMEG
--PMSMYA
--PMSMVS
--PMSMSF
--PMSMRY
--PMSMLT
--PMSMLK

--PMSMII
--PMSMFA
--PMSMYK
PNPMSM--
--PMSMTM
--PMSMRV
--PMSMRH
--PMSMIT
PWPMSM--
--PMSMVM
--PMSMSI
--PMSMNA
--PMSMMT
--PMSMMG
--PMSMKL
--PMSMFM
PAPMSM--
--PMSMVT
--PMSMTV
--PMSMSN
--PMSMQY
--PMSMMW
--PMSMKG
--PMSMAR
--PMSMYT
--PMSMWK
--PMSMVN
--PMSMTH
--PMSMSK
--PMSMKQ
--PMSMFH
--PMSMAA
PIPMSM--
--PMSMNN
--PMSMMN
--PMSMIV
--PMSMHI
--PMSMGS
--PMSMAW
--PMSMAT
--PMSMAH
PYPMSM--
--PMSMYI
--PMSMYE
--PMSMWS
--PMSMWE
--PMSMVD
--PMSMSW
--PMSMSE
--PMSMNW

--PMSMIY
--PMSMFV
PCPMSM--
--PMSMVI
--PMSMRM
--PMSMQN
--PMSMIW
--PMSMGG
--PMSMAQ
PMPMSM--
--PMSMYH
--PMSMVW
--PMSMVV
--PMSMSV
--PMSMML
--PMSMIN
--PMSMIG
--PMSMHT
--PMSMHQ
--PMSMFN
PQPMSM--
--PMSMVQ
--PMSMVK
--PMSMMK
--PMSMIA
--PMSMHH
--PMSMFQ
--PMSMEY
PKPMSM--
--PMSMWV
--PMSMWN
--PMSMSG
--PMSMQT
--PMSMNG
--PMSMKE
--PMSMHE
--PMSMGV
--PMSMGA
--PMSMFD
--PMSMAN
--PMSMAF
--PMSMWH
--PMSMWG
--PMSMQR
--PMSMQL
--PMSMNR
--PMSMNQ
--PMSMKD
--PMSMHR
--PMSMHF

--PMSMHA
--PMSMEN
--PMSMEF
PDPMSM--
--PMSMYV
--PMSMYN
--PMSMWR
--PMSMWQ
--PMSMRE
--PMSMQG
--PMSMQF
--PMSMNF
--PMSMMQ
--PMSMME
--PMSMMA
--PMSMLD
--PMSMKY
--PMSMKN
--PMSMKI
--PMSMKA
--PMSMIQ
--PMSMIK
--PMSMGT
--PMSMFI
--PMSMFE
--PMSMAY
--PMSMAV
PVPMSM--
--PMSMYD
--PMSMVH
--PMSMTY
--PMSMSQ
--PMSMQK
--PMSMNV
--PMSMNT
--PMSMNL
--PMSMLE
--PMSMKW
--PMSMKF
--PMSMIR
--PMSMIH
--PMSMHY
--PMSMHM
--PMSMHK
--PMSMGW
--PMSMGM
--PMSMFS
--PMSMEW
--PMSMDS
PTPMSM--

-PPMSMW-
--PMSMVE
--PMSMTE
--PMSMRQ
--PMSMRD
--PMSMQM
--PMSMQH
--PMSMPL
--PMSMND
--PMSMMH
--PMSMKK
--PMSMIP
--PMSMHN
--PMSMGP
--PMSMET
--PMSMEM
--PMSMEL
--PMSMEK
--PMSMEA
--PMSMCV
--PMSMCL
--PMSMCG
--PMSMAI
QSPMSM--
QNPMSM--
QKPMSM--
-QPMSMR-
-QPMSMY-
-QPMSMW-
-QPMSMK-
QLPMSM--
QGPMSM--
-QPMSMS-
-QPMSMH-
QIPMSM--
-QPMSMN-
-QPMSML-
-QPMSMA-
QVPMSM--
-QPMSMI-
-QPMSMV-
QPPMSM--
-QPMSMT-
-QPMSMF-
-QPMSMM-
QHPMSM--
QDPMSM--
QYPMSM--
QTPMSM--
-QPMSMG-

-QPMSME-
QCPMSM--
QAPMSM--
QMPMSM--
QFPMSM--
-RPMSML-
RLPMSM--
RRPMSM--
-RPMSMS-
-RPMSMV-
RGPMSM--
-RPMSMY-
RSPMSM--
-RPMSMI-
RVPMSM--
-RPMSMM-
RPPMSM--
RNPMSM--
RMPMSM--
-RPMSMN-
-RPMSMA-
RFPMSM--
-RPMSMT-
-RPMSMH-
RHPMSM--
-RPMSMW-
RTPMSM--
RKPMSM--
RQPMSM--
-RPMSMK-
-RPMSMF-
RWPMSM--
-RPMSMQ-
-RPMSMG-
-RPMSME-
RDPMSM--
-RPMSMC-
RCPMSM--
-RPMSMD-
-SPMSML-
-SPMSMR-
SIPMSM--
SGPMSM--
SKPMSM--
SFPMSM--
SSPMSM--
STPMSM--
-SPMSMI-
-SPMSMM-
-SPMSMK-

SAPMSM--
-SPMSMY-
-SPMSMT-
-SPMSMN-
SYPMSM--
-SPMSMF-
-SPMSMA-
-SPMSMV-
SWPMSM--
SHPMSM--
SPPMSM--
SMPMSM--
-SPMSMQ-
-SPMSMH-
-SPMSMW-
SVPMSM--
SEPMSM--
SDPMSM--
-SPMSMG-
SNPMSM--
SQPMSM--
-SPMSME-
SCPMSM--
TSPMSM--
TRPMSM--
-TPMSMR-
-TPMSML-
TFPMSM--
-TPMSMV-
TPPMSM--
-TPMSMY-
TNPMSM--
TKPMSM--
TMPMSM--
-TPMSMI-
TVPMSM--
-TPMSMW-
TYPMSM--
TIPMSM--
TLPMSM--
TQPMSM--
-TPMSMA-
TTPMSM--
-TPMSMG-
TEPMSM--
TDPMSM--
-TPMSMK-
-TPMSMF-
TCPMSM--
TWPMSM--

-TPMSME-
THPMSM--
-TPMSMQ-
-TPMSMN-
-TPMSMH-
VRPMSM--
-VPMSMR-
-VPMSMS-
-VPMSMI-
VPPMSM--
VKPMSM--
VLPMSM--
-VPMSMY-
-VPMSMA-
VDPMSM--
VIPMSM--
VVPMSM--
-VPMSMH-
-VPMSMT-
VMPMSM--
VTPMSM--
-VPMSMG-
VWPMSM--
-VPMSMW-
-VPMSMK-
VGPMSM--
-VPMSMM-
-VPMSMF-
VHPMSM--
-VPMSME-
VFPMSM--
VAPMSM--
VQPMSM--
VYPMSM--
-VPMSMV-
-VPMSMQ-
VNPMSM--
VCPMSM--
-WPMSML-
WHPMSM--
WDPMSM--
WEPMSM--
WLPMSM--
WMPMSM--
WPPMSM--
-WPMSMN-
-WPMSMF-
-WPMSMS-
-WPMSMR-
-WPMSMQ-

	WFPMSM--		
	WYPMSM--		
	WWPMSM--		
	WQPMSM--		
	-WPMSMV-		
	-WPMSMM-		
	WGPMSM--		
	-WPMSMY-		
	-WPMSMH-		
	WVPMSM--		
	-WPMSMI-		
	WIPMSM--		
	YSPMSM--		
	-YPMSML-		
	-YPMSMR-		
	-YPMSMI-		
	YRPMSM--		
	YQPMSM--		
	-YPMSMT-		
	-YPMSMS-		
	-YPMSMY-		
	YGPMSM--		
	YPPMSM--		
	-YPMSMN-		
	YDPMSM--		
	YWPMSM--		
	-YPMSMW-		
	-YPMSMH-		
	-YPMSMF-		
	YLPMSM--		
	YIPMSM--		
	-YPMSMV-		
	-YPMSMQ-		
	YYPMSM--		
	-YPMSME-		
	YNPMSM--		
	YEPMSM--		
	YAPMSM--		
	-YPMSMM-		
	-YPMSMG-		
	-YPMSMA-		
	YFPMSM--		
	YVPMSM--		
	YTPMSM--		
	-YPMSMK-		
PSAM	52	850	46.49
	GDPSAM--		
	-LPSAMG-		
	--PSAMPV		
	--PSAMGN		

--PSAMDS
-SPSAMG-
-SPSAME-
ALPSAM--
-APSAML-
AVPSAM--
AIPSAM--
-APSAMS-
AAPSAM--
-APSAMY-
-APSAMV-
-APSAMF-
AFPSAM--
ATPSAM--
-APSAMA-
-APSAMN-
AKPSAM--
AEPSAM--
-APSAMW-
-APSAMI-
AHPSAM--
ARPSAM--
-APSAMM-
AGPSAM--
APPSAM--
-APSAMR-
AQPSAM--
AMPSAM--
ASPSAM--
-APSAMK-
ACPSAM--
AWPSAM--
-APSAMP-
-APSAMG-
-APSAMD-
ADPSAM--
CRPSAM--
CSPSAM--
-CPSAMI-
CVPSAM--
CLPSAM--
CCPSAM--
CTPSAM--
-CPSAMT-
-CPSAMF-
CGPSAM--
-CPSAMY-
CMPSAM--
CYPSAM--
CWPSAM--

−CPSAMQ−
−CPSAMN−
−CPSAMH−
CHPSAM−−
CEPSAM−−
DRPSAM−−
DVPSAM−−
DGPSAM−−
DAPSAM−−
DSPSAM−−
DTPSAM−−
DLPSAM−−
−DPSAMY−
DKPSAM−−
DFPSAM−−
−DPSAMR−
DMPSAM−−
−DPSAMS−
−DPSAML−
−DPSAMI−
−DPSAMQ−
−DPSAMK−
DQPSAM−−
−DPSAMV−
−DPSAMT−
−DPSAMM−
−DPSAMF−
DNPSAM−−
DIPSAM−−
−EPSAMR−
ESPSAM−−
−EPSAMW−
−EPSAMN−
ERPSAM−−
EQPSAM−−
−EPSAMI−
−EPSAML−
−EPSAMK−
EIPSAM−−
EVPSAM−−
−EPSAMS−
−EPSAMQ−
−EPSAMH−
EHPSAM−−
−EPSAMF−
−EPSAMT−
−EPSAMM−
ENPSAM−−
EFPSAM−−
−EPSAMY−

-EPSAMV-
EMPSAM--
ELPSAM--
EKPSAM--
-EPSAMG-
EGPSAM--
EDPSAM--
-FPSAMR-
FRPSAM--
-FPSAML-
-FPSAMS-
-FPSAMH-
FVPSAM--
-FPSAMT-
FNPSAM--
-FPSAMK-
FSPSAM--
-FPSAMV-
FLPSAM--
FKPSAM--
-FPSAMY-
-FPSAMF-
-FPSAMA-
-FPSAMW-
FYPSAM--
-FPSAMM-
FHPSAM--
FGPSAM--
FTPSAM--
-FPSAMQ-
FMPSAM--
FWPSAM--
-FPSAMN-
FPPSAM--
FIPSAM--
FFPSAM--
FDPSAM--
-FPSAMG-
-FPSAME-
FAPSAM--
-GPSAMR-
GLPSAM--
-GPSAMH-
GRPSAM--
GSPSAM--
GQPSAM--
GIPSAM--
-GPSAML-
GNPSAM--
-GPSAMV-

GGPSAM--
-GPSAMN-
-GPSAMK-
GWPSAM--
-GPSAMA-
GVPSAM--
-GPSAMT-
-GPSAMI-
-GPSAMS-
-GPSAMG-
GYPSAM--
GFPSAM--
GAPSAM--
-GPSAMY-
GTPSAM--
-GPSAMM-
-GPSAMW-
GMPSAM--
GKPSAM--
-GPSAMQ-
-GPSAME-
GPPSAM--
-GPSAMF-
-HPSAMR-
HGPSAM--
HWPSAM--
HMPSAM--
-HPSAMT-
HSPSAM--
HRPSAM--
HTPSAM--
-HPSAMS-
-HPSAMI-
HAPSAM--
-HPSAMW-
-HPSAMQ-
-HPSAMF-
HNPSAM--
HKPSAM--
HQPSAM--
-HPSAMV-
HHPSAM--
HVPSAM--
-HPSAMN-
HEPSAM--
-HPSAMM-
HIPSAM--
-HPSAMK-
HDPSAM--
-HPSAMH-

-HPSAMC-
-HPSAMA-
HFPSAM--
HCPSAM--
-IPSAMS-
-IPSAML-
IRPSAM--
IYPSAM--
-IPSAMF-
IFPSAM--
ISPSAM--
-IPSAMY-
-IPSAMV-
-IPSAMM-
-IPSAMI-
IPPSAM--
IQPSAM--
IHPSAM--
IMPSAM--
ILPSAM--
IAPSAM--
-IPSAMW-
-IPSAMT-
-IPSAMR-
-IPSAMN-
ITPSAM--
-IPSAMK-
-IPSAMA-
IGPSAM--
INPSAM--
IVPSAM--
-IPSAME-
IWPSAM--
-IPSAMG-
-IPSAMD-
KSPSAM--
-KPSAML-
KLPSAM--
KNPSAM--
KRPSAM--
-KPSAMH-
KVPSAM--
-KPSAMS-
-KPSAMR-
KGPSAM--
KYPSAM--
-KPSAMA-
KFPSAM--
-KPSAMW-
-KPSAMI-

-KPSAMT-
-KPSAMQ-
KTPSAM--
-KPSAMF-
-KPSAMY-
-KPSAMN-
-KPSAMG-
-KPSAME-
KAPSAM--
KPPSAM--
-KPSAMV-
KKPSAM--
-KPSAMM-
LRPSAM--
-LPSAMR-
LLPSAM--
-LPSAML-
LAPSAM--
LDPSAM--
LSPSAM--
-LPSAMT-
-LPSAMH-
-LPSAMS-
LKPSAM--
LGPSAM--
LPPSAM--
LIPSAM--
-LPSAMY-
LYPSAM--
-LPSAMF-
-LPSAMI-
LHPSAM--
-LPSAMW-
-LPSAMV-
-LPSAMK-
LTPSAM--
LNPSAM--
-LPSAMM-
LMPSAM--
LWPSAM--
LQPSAM--
LEPSAM--
LFPSAM--
LVPSAM--
-LPSAMQ-
-LPSAMC-
-LPSAMA-
MRPSAM--
MTPSAM--
-MPSAMY-

MSPSAM--
-MPSAMI-
-MPSAML-
MLPSAM--
MAPSAM--
MVPSAM--
-MPSAMH-
-MPSAMQ-
MIPSAM--
-MPSAMV-
-MPSAMM-
-MPSAMT-
MMPSAM--
MPPSAM--
-MPSAMS-
-MPSAMN-
-MPSAMK-
MWPSAM--
-MPSAMG-
MCPSAM--
MYPSAM--
-MPSAMF-
MHPSAM--
MGPSAM--
-MPSAMA-
NLPSAM--
NRPSAM--
NFPSAM--
NTPSAM--
NVPSAM--
NSPSAM--
-NPSAMS-
NAPSAM--
-NPSAML-
NHPSAM--
-NPSAMW-
-NPSAMV-
-NPSAMT-
NGPSAM--
NYPSAM--
NIPSAM--
NWPSAM--
NKPSAM--
-NPSAMK-
NNPSAM--
NQPSAM--
-NPSAMQ-
NPPSAM--
-NPSAMA-
NMPSAM--

--PSAMRG
--PSAMLS
--PSAMSL
--PSAMSS
--PSAMIS
--PSAMRI
--PSAMYG
PRPSAM--
--PSAMLT
--PSAMRR
--PSAMST
--PSAMLH
--PSAMRH
--PSAMRN
--PSAMRL
--PSAMHW
--PSAMTG
--PSAMTA
--PSAMKR
--PSAMHR
--PSAMYL
--PSAMVR
--PSAMQG
--PSAMYR
--PSAMWL
--PSAMVG
--PSAMSV
--PSAMRM
--PSAMFS
PTPSAM--
--PSAMWA
--PSAMSG
--PSAMQH
--PSAMML
--PSAMVV
--PSAMVS
--PSAMVF
--PSAMTW
--PSAMFT
PVPSAM--
--PSAMVA
--PSAMLN
--PSAMHS
--PSAMPY
--PSAMKA
--PSAMHT
--PSAMMN
--PSAMFL
--PSAMYT
--PSAMVM

--PSAMLR
--PSAMKG
--PSAMWG
--PSAMSW
--PSAMNL
--PSAMIM
--PSAMGL
--PSAMSR
--PSAMRW
--PSAMRF
--PSAMNS
--PSAMHA
--PSAMWR
--PSAMVK
--PSAMTF
--PSAMND
--PSAMMV
--PSAMGS
--PSAMYV
--PSAMWS
--PSAMQR
PGPSAM--
--PSAMYI
--PSAMVY
--PSAMTY
--PSAMTT
--PSAMTL
--PSAMLW
--PSAMLG
--PSAMLD
--PSAMHD
--PSAMFA
--PSAMAA
--PSAMSN
--PSAMSH
--PSAMLY
--PSAMFQ
--PSAMAN
--PSAMYN
--PSAMTI
--PSAMNF
--PSAMLV
--PSAMIN
--PSAMFK
--PSAMFE
--PSAMAG
PQPSAM--
PAPSAM--
--PSAMNG
--PSAMMI

--PSAMFG
--PSAMWD
--PSAMVL
--PSAMLK
--PSAMYW
--PSAMWI
--PSAMTV
--PSAMSI
--PSAMRQ
--PSAMMA
--PSAMLE
--PSAMKY
--PSAMIL
--PSAMHF
--PSAMFN
--PSAMAL
PWPSAM--
--PSAMYM
--PSAMYK
--PSAMYA
--PSAMVI
--PSAMSY
--PSAMHL
--PSAMFM
--PSAMAF
PNPSAM--
PKPSAM--
--PSAMWT
--PSAMKH
--PSAMIG
--PSAMIA
--PSAMGY
--PSAMGA
--PSAMFW
PFPSAM--
--PSAMWQ
--PSAMTK
--PSAMIQ
--PSAMHK
--PSAMHI
--PSAMAY
--PSAMAV
PEPSAM--
--PSAMVE
--PSAMTS
--PSAMSF
--PSAMNW
--PSAMKK
--PSAMIT
--PSAMHV

--PSAMHM
--PSAMHG
--PSAMGV
--PSAMGI
--PSAMAW
--PSAMAS
--PSAMAM
--PSAMWW
--PSAMSQ
--PSAMQA
--PSAMPL
--PSAMMF
--PSAMKI
--PSAMIR
--PSAMYD
--PSAMTH
--PSAMQV
--PSAMQN
--PSAMKT
--PSAMKS
--PSAMFD
PLPSAM--
PSPSAM--
--PSAMYH
--PSAMVQ
--PSAMSD
--PSAMRY
--PSAMRE
--PSAMNN
--PSAMNA
--PSAMMT
--PSAMMG
--PSAMIH
--PSAMHQ
--PSAMHH
--PSAMFV
--PSAMES
--PSAMYE
--PSAMWF
--PSAMPM
--PSAMNV
--PSAMMQ
--PSAMME
--PSAMMD
--PSAMKV
--PSAMKL
--PSAMHE
--PSAMAH
PHPSAM--
--PSAMVW

--PSAMVT
--PSAMTD
--PSAMSM
--PSAMSK
--PSAMQI
--PSAMNY
--PSAMNK
--PSAMLM
--PSAMKE
--PSAMII
--PSAMIF
--PSAMHN
--PSAMFR
--PSAMEW
--PSAMAQ
PYPSAM--
--PSAMYQ
--PSAMWM
--PSAMWE
--PSAMVH
--PSAMTM
--PSAMQT
--PSAMQQ
--PSAMQL
--PSAMQF
--PSAMNR
--PSAMNM
--PSAMNI
--PSAMNH
--PSAMMK
--PSAMKN
--PSAMGT
--PSAMGM
--PSAMFI
--PSAMFH
--PSAMEQ
--PSAMEN
--PSAMEF
--PSAMDG
--PSAMYY
--PSAMTE
--PSAMSE
--PSAMQK
--PSAMPI
--PSAMPF
--PSAMNT
--PSAMMM
--PSAMLQ
--PSAMLF
--PSAMKC

--PSAMIK
--PSAMGF
--PSAMEL
--PSAMCF
--PSAMCC
--PSAMAK
PIPSAM--
PDPSAM--
--PSAMWH
--PSAMTQ
--PSAMSC
--PSAMRC
--PSAMQY
--PSAMQD
--PSAMPW
--PSAMMW
--PSAMMR
--PSAMMH
--PSAMLC
--PSAMKM
--PSAMKD
--PSAMIV
--PSAMID
--PSAMGW
--PSAMDY
--PSAMDN
--PSAMCS
--PSAMCR
--PSAMCD
--PSAMCA
--PSAMAI
--PSAMAD
-QPSAMR-
QRPSAM--
QGPSAM--
QTPSAM--
-QPSAMT-
-QPSAMK-
-QPSAMW-
QVPSAM--
-QPSAMV-
-QPSAMF-
QLPSAM--
QWPSAM--
-QPSAMS-
QYPSAM--
QSPSAM--
QPPSAM--
QKPSAM--
-QPSAMI-

−QPSAMH−
QIPSAM−−
QHPSAM−−
QQPSAM−−
−QPSAMQ−
−QPSAMA−
QMPSAM−−
QEPSAM−−
−QPSAMN−
QFPSAM−−
−QPSAME−
QAPSAM−−
−QPSAMY−
−QPSAML−
−RPSAMS−
RGPSAM−−
RNPSAM−−
RTPSAM−−
RSPSAM−−
RVPSAM−−
−RPSAMY−
RYPSAM−−
RKPSAM−−
−RPSAMI−
−RPSAMN−
−RPSAMF−
−RPSAMV−
−RPSAMK−
−RPSAMA−
RFPSAM−−
RMPSAM−−
−RPSAMQ−
−RPSAMM−
RAPSAM−−
RPPSAM−−
RIPSAM−−
RWPSAM−−
RHPSAM−−
RQPSAM−−
REPSAM−−
−RPSAME−
RDPSAM−−
SLPSAM−−
STPSAM−−
−SPSAML−
−SPSAMR−
SIPSAM−−
SFPSAM−−
−SPSAMV−
SMPSAM−−

-SPSAMH-
-SPSAMF-
SAPSAM--
SYPSAM--
SVPSAM--
SHPSAM--
SGPSAM--
-SPSAMT-
-SPSAMI-
SPPSAM--
SQPSAM--
-SPSAMQ-
-SPSAMY-
-SPSAMW-
SNPSAM--
-SPSAMN-
SKPSAM--
-SPSAMM-
SEPSAM--
SDPSAM--
-SPSAMC-
SCPSAM--
-TPSAMR-
-TPSAMT-
-TPSAMY-
-TPSAMH-
-TPSAMS-
TRPSAM--
TLPSAM--
-TPSAML-
TVPSAM--
TIPSAM--
-TPSAMM-
TSPSAM--
-TPSAMV-
-TPSAMI-
-TPSAMF-
TPPSAM--
TMPSAM--
TGPSAM--
TDPSAM--
-TPSAMN-
TTPSAM--
THPSAM--
TYPSAM--
TKPSAM--
-TPSAMQ-
-TPSAMW-
TWPSAM--
TNPSAM--

TFPSAM--
TEPSAM--
TQPSAM--
-TPSAMA-
TCPSAM--
-TPSAMK-
-VPSAMS-
VRPSAM--
VSPSAM--
-VPSAMH-
VWPSAM--
-VPSAML-
VIPSAM--
VDPSAM--
VLPSAM--
VKPSAM--
-VPSAMW-
-VPSAMM-
-VPSAMK-
VTPSAM--
VVPSAM--
VNPSAM--
VMPSAM--
VGPSAM--
VEPSAM--
-VPSAMT-
-VPSAMA-
VYPSAM--
-VPSAMV-
-VPSAMQ-
VPPSAM--
VFPSAM--
VQPSAM--
-VPSAMG-
-VPSAMN-
-VPSAME-
-VPSAMI-
-VPSAMF-
VAPSAM--
-WPSAMR-
WFPSAM--
WVPSAM--
WGPSAM--
WIPSAM--
WDPSAM--
-WPSAMF-
-WPSAML-
WSPSAM--
WMPSAM--
-WPSAMT-

-WPSAMN-
WLPSAM--
-WPSAMK-
-WPSAMS-
WHPSAM--
-WPSAMH-
-WPSAMA-
WNPSAM--
WTPSAM--
-WPSAMV-
-WPSAMM-
-WPSAME-
WKPSAM--
-WPSAMY-
-WPSAMI-
-YPSAMR-
YSPSAM--
YVPSAM--
YTPSAM--
-YPSAMS-
YLPSAM--
-YPSAMH-
-YPSAMK-
-YPSAMM-
-YPSAML-
YHPSAM--
YGPSAM--
YFPSAM--
-YPSAMF-
YMPSAM--
YAPSAM--
YQPSAM--
-YPSAMW-
YEPSAM--
-YPSAMY-
-YPSAMV-
-YPSAMI-
YKPSAM--
-YPSAMN-
-YPSAME-
-YPSAMQ-
YNPSAM--
YIPSAM--
YDPSAM--
-YPSAMP-
YWPSAM--

PHNL	40	862	46.48

DDPHNL--
-NPHNLN-
-NPHNLG-

AVPHNL--
-APHNLR-
ASPHNL--
ATPHNL--
-APHNLA-
-APHNLL-
ALPHNL--
-APHNLW-
-APHNLG-
AGPHNL--
AKPHNL--
ARPHNL--
-APHNLT-
AAPHNL--
AWPHNL--
APPHNL--
-APHNLK-
AIPHNL--
AFPHNL--
ADPHNL--
-APHNLH-
AHPHNL--
-APHNLY-
-APHNLS-
-APHNLE-
AMPHNL--
AQPHNL--
-APHNLM-
ANPHNL--
AYPHNL--
-APHNLI-
AEPHNL--
-APHNLN-
-APHNLF-
-APHNLV-
-APHNLQ-
-APHNLP-
CRPHNL--
CPPHNL--
-CPHNLI-
CAPHNL--
CLPHNL--
-CPHNLL-
CMPHNL--
CKPHNL--
-CPHNLM-
CDPHNL--
CYPHNL--
CWPHNL--
CQPHNL--

CIPHNL--
CGPHNL--
CEPHNL--
DRPHNL--
DVPHNL--
DSPHNL--
-DPHNLS-
-DPHNLL-
DMPHNL--
DWPHNL--
DTPHNL--
-DPHNLT-
DLPHNL--
DYPHNL--
DQPHNL--
-DPHNLR-
-DPHNLM-
-DPHNLI-
DNPHNL--
DIPHNL--
DFPHNL--
DEPHNL--
DAPHNL--
-EPHNLR-
-EPHNLM-
ESPHNL--
ETPHNL--
-EPHNLS-
-EPHNLY-
EGPHNL--
EAPHNL--
EKPHNL--
EFPHNL--
EVPHNL--
-EPHNLH-
-EPHNLL-
ERPHNL--
EQPHNL--
ELPHNL--
EIPHNL--
EWPHNL--
-EPHNLV-
-EPHNLT-
-EPHNLF-
EMPHNL--
-FPHNLS-
FLPHNL--
FQPHNL--
FAPHNL--
-FPHNLH-

FSPHNL--
FWPHNL--
-FPHNLL-
FTPHNL--
-FPHNLY-
-FPHNLR-
FMPHNL--
FIPHNL--
FFPHNL--
FEPHNL--
FRPHNL--
-FPHNLN-
FPPHNL--
-FPHNLT-
-FPHNLG-
-FPHNLE-
FYPHNL--
-FPHNLF-
-FPHNLV-
-FPHNLA-
-FPHNLQ-
-FPHNLK-
FKPHNL--
FGPHNL--
FNPHNL--
-FPHNLC-
-GPHNLR-
GKPHNL--
GRPHNL--
GMPHNL--
GVPHNL--
GTPHNL--
GLPHNL--
GGPHNL--
-GPHNLL-
GPPHNL--
-GPHNLW-
GHPHNL--
GSPHNL--
GFPHNL--
GNPHNL--
GAPHNL--
GYPHNL--
-GPHNLT-
-GPHNLY-
-GPHNLV-
-GPHNLS-
-GPHNLQ-
-GPHNLH-
GIPHNL--

-GPHNLN-
-GPHNLK-
-GPHNLE-
GWPHNL--
-GPHNLG-
GQPHNL--
-GPHNLI-
-GPHNLF-
-GPHNLM-
HRPHNL--
-HPHNLW-
HAPHNL--
-HPHNLI-
HFPHNL--
HQPHNL--
HPPHNL--
HGPHNL--
-HPHNLL-
HLPHNL--
HSPHNL--
HVPHNL--
-HPHNLM-
-HPHNLH-
-HPHNLV-
-HPHNLS-
HWPHNL--
-HPHNLR-
-HPHNLG-
HMPHNL--
-HPHNLK-
-HPHNLF-
HNPHNL--
HEPHNL--
-HPHNLQ-
HDPHNL--
IRPHNL--
ISPHNL--
IVPHNL--
IGPHNL--
-IPHNLK-
IFPHNL--
-IPHNLL-
-IPHNLR-
ILPHNL--
IIPHNL--
-IPHNLW-
INPHNL--
IAPHNL--
-IPHNLS-
-IPHNLF-

ITPHNL--
IPPHNL--
-IPHNLI-
IMPHNL--
IKPHNL--
IEPHNL--
IQPHNL--
IHPHNL--
IDPHNL--
-IPHNLT-
IYPHNL--
-IPHNLQ-
-IPHNLH-
-IPHNLY-
-IPHNLV-
-IPHNLG-
-IPHNLM-
-KPHNLT-
-KPHNLY-
-KPHNLS-
-KPHNLR-
-KPHNLL-
KGPHNL--
KWPHNL--
KRPHNL--
KYPHNL--
KVPHNL--
KPPHNL--
-KPHNLK-
KIPHNL--
-KPHNLW-
KHPHNL--
KQPHNL--
-KPHNLQ-
KFPHNL--
KAPHNL--
-KPHNLG-
-KPHNLF-
-KPHNLV-
-KPHNLI-
-KPHNLE-
KTPHNL--
-KPHNLM-
KNPHNL--
KLPHNL--
KKPHNL--
-KPHNLH-
-KPHNLC-
-KPHNLA-
LRPHNL--

LSPHNL--
LLPHNL--
LVPHNL--
LKPHNL--
LGPHNL--
LWPHNL--
-LPHNLY-
LYPHNL--
-LPHNLV-
LDPHNL--
LQPHNL--
-LPHNLM-
-LPHNLR-
LNPHNL--
-LPHNLS-
LEPHNL--
LFPHNL--
LAPHNL--
LIPHNL--
-LPHNLQ-
-LPHNLL-
-LPHNLT-
-LPHNLI-
-LPHNLF-
LTPHNL--
LHPHNL--
-LPHNLW-
-LPHNLA-
LMPHNL--
LPPHNL--
-LPHNLH-
-LPHNLK-
LCPHNL--
-LPHNLN-
-LPHNLE-
-LPHNLP-
-LPHNLC-
MSPHNL--
MGPHNL--
-MPHNLR-
MVPHNL--
MKPHNL--
-MPHNLY-
-MPHNLS-
MAPHNL--
MLPHNL--
-MPHNLK-
-MPHNLI-
MIPHNL--
MTPHNL--

-MPHNLQ-
MMPHNL--
MRPHNL--
MQPHNL--
MYPHNL--
-MPHNLN-
MDPHNL--
-MPHNLM-
MHPHNL--
MEPHNL--
-MPHNLW-
-MPHNLH-
MCPHNL--
MWPHNL--
-MPHNLT-
-MPHNLD-
-MPHNLA-
NVPHNL--
NWPHNL--
NRPHNL--
-NPHNLR-
NQPHNL--
-NPHNLL-
NLPHNL--
-NPHNLW-
NGPHNL--
-NPHNLK-
NFPHNL--
-NPHNLT-
NMPHNL--
NIPHNL--
NHPHNL--
-NPHNLI-
NTPHNL--
NPPHNL--
-NPHNLM-
-NPHNLA-
NKPHNL--
NAPHNL--
-NPHNLH-
-NPHNLY-
-NPHNLV-
-NPHNLS-
-NPHNLF-
-NPHNLE-
NNPHNL--
NYPHNL--
-NPHNLQ-
-NPHNLC-
NDPHNL--

--PHNLSS
--PHNLLT
--PHNLLQ
--PHNLYA
--PHNLLH
--PHNLSA
--PHNLLG
--PHNLGS
--PHNLTL
--PHNLYS
--PHNLRT
--PHNLLS
PVPHNL--
--PHNLYQ
--PHNLWH
--PHNLIG
--PHNLRK
--PHNLKA
PLPHNL--
--PHNLRN
--PHNLWS
--PHNLVL
--PHNLAV
--PHNLTT
--PHNLVR
--PHNLSI
--PHNLRH
--PHNLQT
--PHNLFY
--PHNLVK
--PHNLRI
--PHNLRG
--PHNLMH
--PHNLAA
--PHNLYH
--PHNLYF
--PHNLSM
--PHNLRA
--PHNLLA
--PHNLWT
--PHNLVM
--PHNLTH
--PHNLSY
--PHNLLR
--PHNLGT
--PHNLGG
--PHNLVY
--PHNLSG
--PHNLYR
--PHNLYL

--PHNLWF
--PHNLTI
--PHNLWD
--PHNLMV
--PHNLIS
--PHNLFW
--PHNLFR
--PHNLFI
--PHNLWN
--PHNLVS
--PHNLTG
--PHNLLD
--PHNLKY
PRPHNL--
--PHNLYT
--PHNLTR
--PHNLQG
--PHNLMR
--PHNLKL
--PHNLIR
--PHNLES
--PHNLWI
--PHNLRF
--PHNLIT
--PHNLEI
--PHNLAY
--PHNLWG
--PHNLMD
--PHNLIQ
--PHNLHN
--PHNLLY
--PHNLHS
--PHNLFQ
--PHNLAK
--PHNLAG
PYPHNL--
--PHNLTF
--PHNLSN
--PHNLRW
--PHNLRE
--PHNLMT
--PHNLMN
--PHNLMA
--PHNLLL
--PHNLKF
--PHNLIK
--PHNLGA
--PHNLQS
--PHNLQI
--PHNLNN

--PHNLKV
--PHNLIF
PWPHNL--
--PHNLWL
--PHNLVD
--PHNLSF
--PHNLQY
--PHNLLN
--PHNLLI
--PHNLKG
--PHNLIL
--PHNLII
--PHNLHG
--PHNLFL
PMPHNL--
--PHNLYW
--PHNLYN
--PHNLVT
--PHNLVN
--PHNLVI
--PHNLVH
--PHNLSK
--PHNLSH
--PHNLSE
--PHNLQA
--PHNLMK
--PHNLKT
--PHNLFA
--PHNLET
--PHNLAS
--PHNLAM
--PHNLYK
--PHNLYG
--PHNLQN
--PHNLML
--PHNLLV
--PHNLLM
--PHNLGW
--PHNLFG
--PHNLAI
PIPHNL--
PHPHNL--
--PHNLWR
--PHNLVQ
--PHNLTM
--PHNLST
--PHNLSL
--PHNLRQ
--PHNLQF
--PHNLNV

--PHNLNT
--PHNLNM
--PHNLNA
--PHNLMS
--PHNLMG
--PHNLKH
--PHNLIA
--PHNLHQ
--PHNLAL
PTPHNL--
PSPHNL--
--PHNLYD
--PHNLWQ
--PHNLVA
--PHNLSQ
--PHNLQW
--PHNLQL
--PHNLNW
--PHNLNS
--PHNLIV
--PHNLIN
--PHNLFS
--PHNLAW
PKPHNL--
--PHNLYY
--PHNLYM
--PHNLVE
--PHNLTV
--PHNLTD
--PHNLSV
--PHNLQV
--PHNLNQ
--PHNLMW
--PHNLMI
--PHNLHW
--PHNLHV
--PHNLEK
--PHNLAT
--PHNLAF
PNPHNL--
--PHNLWV
--PHNLWM
--PHNLVW
--PHNLVG
--PHNLTN
--PHNLTK
--PHNLSW
--PHNLQR
--PHNLNL
--PHNLNI

--PHNLNH
--PHNLMY
--PHNLME
--PHNLLE
--PHNLKM
--PHNLKE
--PHNLIY
--PHNLIH
--PHNLHR
--PHNLHH
--PHNLHD
--PHNLGM
--PHNLFT
--PHNLFN
--PHNLFH
--PHNLFE
--PHNLEH
--PHNLDA
--PHNLAR
PQPHNL--
--PHNLYE
--PHNLWW
--PHNLVP
--PHNLTE
--PHNLTA
--PHNLSD
--PHNLRD
--PHNLQD
--PHNLPI
--PHNLNY
--PHNLNF
--PHNLMQ
--PHNLMM
--PHNLMF
--PHNLLW
--PHNLKN
--PHNLHT
--PHNLHM
--PHNLHE
--PHNLER
--PHNLEN
--PHNLDS
--PHNLCI
--PHNLAE
--PHNLAD
-PPHNLM-
--PHNLYC
--PHNLWK
--PHNLWE
--PHNLSP

--PHNLSC
--PHNLPK
--PHNLND
--PHNLKW
--PHNLIM
--PHNLID
--PHNLHL
--PHNLGV
--PHNLGN
--PHNLGL
--PHNLGK
--PHNLGI
--PHNLGH
--PHNLGF
--PHNLGE
--PHNLFV
--PHNLFM
--PHNLFF
--PHNLFD
--PHNLEW
--PHNLEQ
--PHNLEM
--PHNLEF
--PHNLEC
--PHNLEA
--PHNLDG
--PHNLDC
--PHNLCQ
--PHNLAP
--PHNLAN
PEPHNL--
QVPHNL--
QAPHNL--
QPPHNL--
-QPHNLF-
QSPHNL--
-QPHNLR-
QRPHNL--
-QPHNLL-
-QPHNLS-
QYPHNL--
QWPHNL--
QTPHNL--
-QPHNLI-
-QPHNLW-
-QPHNLH-
QKPHNL--
QHPHNL--
QIPHNL--
-QPHNLK-

–QPHNLA–
–QPHNLN–
–QPHNLG–
QQPHNL––
–QPHNLV–
–QPHNLT–
QLPHNL––
–QPHNLQ–
QNPHNL––
–QPHNLC–
RYPHNL––
RGPHNL––
RTPHNL––
RVPHNL––
–RPHNLF–
RMPHNL––
–RPHNLL–
RNPHNL––
–RPHNLS–
RIPHNL––
RRPHNL––
–RPHNLV–
–RPHNLM–
–RPHNLW–
–RPHNLI–
RAPHNL––
–RPHNLT–
RFPHNL––
–RPHNLG–
–RPHNLE–
–RPHNLY–
–RPHNLN–
RPPHNL––
RWPHNL––
RHPHNL––
REPHNL––
–RPHNLA–
–RPHNLQ–
–RPHNLH–
RQPHNL––
RCPHNL––
RDPHNL––
RKPHNL––
–RPHNLP–
SRPHNL––
SMPHNL––
SIPHNL––
SGPHNL––
SFPHNL––
–SPHNLS–

SKPHNL--
-SPHNLL-
SVPHNL--
SSPHNL--
-SPHNLV-
SLPHNL--
-SPHNLR-
-SPHNLM-
SQPHNL--
SWPHNL--
STPHNL--
-SPHNLA-
SYPHNL--
SHPHNL--
-SPHNLQ-
-SPHNLN-
-SPHNLH-
SNPHNL--
-SPHNLT-
-SPHNLY-
-SPHNLE-
-SPHNLF-
SAPHNL--
-SPHNLG-
SEPHNL--
-SPHNLW-
SDPHNL--
-SPHNLI-
SCPHNL--
-TPHNLR-
TRPHNL--
-TPHNLL-
-TPHNLV-
-TPHNLT-
TSPHNL--
TIPHNL--
TVPHNL--
TFPHNL--
TKPHNL--
-TPHNLW-
TGPHNL--
TTPHNL--
-TPHNLF-
TQPHNL--
-TPHNLQ-
-TPHNLA-
-TPHNLS-
TYPHNL--
-TPHNLI-
TMPHNL--

TLPHNL--
-TPHNLM-
TNPHNL--
-TPHNLY-
TDPHNL--
TEPHNL--
TWPHNL--
-TPHNLN-
-TPHNLE-
TPPHNL--
-TPHNLG-
THPHNL--
TCPHNL--
-VPHNLL-
-VPHNLS-
VSPHNL--
-VPHNLI-
-VPHNLR-
-VPHNLA-
VTPHNL--
VRPHNL--
VAPHNL--
VYPHNL--
VNPHNL--
VVPHNL--
-VPHNLV-
VHPHNL--
VMPHNL--
VGPHNL--
VQPHNL--
-VPHNLH-
-VPHNLM-
-VPHNLG-
VPPHNL--
-VPHNLQ-
-VPHNLT-
-VPHNLN-
VLPHNL--
-VPHNLY-
VFPHNL--
VWPHNL--
-VPHNLK-
-VPHNLF-
VKPHNL--
VDPHNL--
VIPHNL--
VCPHNL--
-VPHNLE-
WSPHNL--
WRPHNL--

```
-WPHNLT-
-WPHNLL-
WIPHNL--
WAPHNL--
WVPHNL--
WKPHNL--
WQPHNL--
WPPHNL--
-WPHNLR-
WLPHNL--
-WPHNLK-
-WPHNLA-
-WPHNLI-
-WPHNLY-
-WPHNLQ-
-WPHNLS-
WGPHNL--
WWPHNL--
WCPHNL--
WNPHNL--
WEPHNL--
-WPHNLV-
WMPHNL--
WFPHNL--
-WPHNLN-
-WPHNLH-
-WPHNLF-
-WPHNLE-
WHPHNL--
YRPHNL--
YVPHNL--
-YPHNLL-
YMPHNL--
YGPHNL--
-YPHNLF-
-YPHNLM-
YIPHNL--
-YPHNLS-
YAPHNL--
YHPHNL--
YQPHNL--
YTPHNL--
YYPHNL--
YKPHNL--
YSPHNL--
-YPHNLA-
-YPHNLW-
-YPHNLV-
-YPHNLY-
YWPHNL--
```

-YPHNLH-
YNPHNL--
YLPHNL--
YEPHNL--
YPPHNL--
-YPHNLN-
-YPHNLG-
-YPHNLI-
YDPHNL--

PWSL	123	793	46.42

-EPWSLG-
--PWSLPS
--PWSLPL
--PWSLGP
-RPWSLD-
-APWSLR-
-APWSLY-
AMPWSL--
AYPWSL--
ALPWSL--
AAPWSL--
-APWSLI-
AIPWSL--
AHPWSL--
-APWSLW-
-APWSLA-
AKPWSL--
-APWSLV-
AVPWSL--
AQPWSL--
AGPWSL--
ATPWSL--
-APWSLM-
ANPWSL--
AFPWSL--
-APWSLF-
-APWSLT-
AEPWSL--
-APWSLE-
APPWSL--
ACPWSL--
-APWSLQ-
-APWSLN-
AWPWSL--
-APWSLC-
CIPWSL--
CVPWSL--
CAPWSL--
CRPWSL--
CLPWSL--

CCPWSL--
CTPWSL--
CSPWSL--
CKPWSL--
CDPWSL--
-CPWSLK-
CMPWSL--
CGPWSL--
CEPWSL--
CQPWSL--
-CPWSLV-
-CPWSLL-
-CPWSLI-
CNPWSL--
CWPWSL--
-CPWSLT-
-CPWSLS-
-CPWSLQ-
DRPWSL--
DIPWSL--
DSPWSL--
DKPWSL--
-DPWSLR-
-DPWSLL-
DHPWSL--
DAPWSL--
DVPWSL--
-DPWSLY-
-DPWSLK-
DMPWSL--
DTPWSL--
DLPWSL--
DYPWSL--
-DPWSLM-
DQPWSL--
-DPWSLH-
-DPWSLT-
-DPWSLW-
-DPWSLI-
-DPWSLQ-
DNPWSL--
DGPWSL--
DFPWSL--
-DPWSLS-
-DPWSLN-
DWPWSL--
-DPWSLF-
-DPWSLA-
-DPWSLV-
DCPWSL--

```
-DPWSLE-
ERPWSL--
-EPWSLR-
ESPWSL--
-EPWSLS-
-EPWSLI-
EIPWSL--
-EPWSLT-
EFPWSL--
-EPWSLY-
EMPWSL--
EGPWSL--
-EPWSLN-
-EPWSLK-
EVPWSL--
EKPWSL--
EYPWSL--
ETPWSL--
-EPWSLQ-
-EPWSLW-
-EPWSLM-
-EPWSLA-
-EPWSLH-
EHPWSL--
EWPWSL--
ELPWSL--
EQPWSL--
ENPWSL--
EEPWSL--
EPPWSL--
-EPWSLF-
-EPWSLE-
EDPWSL--
EAPWSL--
FSPWSL--
-FPWSLH-
FAPWSL--
FLPWSL--
FIPWSL--
FHPWSL--
FNPWSL--
FGPWSL--
-FPWSLT-
-FPWSLN-
-FPWSLR-
-FPWSLA-
FMPWSL--
FEPWSL--
-FPWSLK-
FPPWSL--
```

-FPWSLQ-
FTPWSL--
-FPWSLS-
-FPWSLM-
FWPWSL--
-FPWSLV-
FDPWSL--
FFPWSL--
-FPWSLG-
-FPWSLE-
-GPWSLS-
GVPWSL--
GLPWSL--
-GPWSLL-
-GPWSLR-
-GPWSLH-
GAPWSL--
-GPWSLN-
-GPWSLA-
-GPWSLV-
GIPWSL--
-GPWSLT-
GKPWSL--
-GPWSLY-
GYPWSL--
GNPWSL--
GWPWSL--
-GPWSLM-
-GPWSLK-
GHPWSL--
GTPWSL--
GQPWSL--
GFPWSL--
GEPWSL--
GPPWSL--
GMPWSL--
-GPWSLQ-
GDPWSL--
-GPWSLG-
-GPWSLI-
-GPWSLW-
-GPWSLE-
GCPWSL--
-GPWSLF-
-HPWSLR-
HRPWSL--
HTPWSL--
-HPWSLS-
HNPWSL--
HLPWSL--

HKPWSL--
-HPWSLT-
-HPWSLH-
HGPWSL--
-HPWSLY-
-HPWSLV-
-HPWSLL-
HDPWSL--
HFPWSL--
HAPWSL--
HIPWSL--
-HPWSLM-
HYPWSL--
-HPWSLN-
-HPWSLI-
-HPWSLA-
HVPWSL--
-HPWSLG-
HPPWSL--
-HPWSLK-
-HPWSLF-
HHPWSL--
HMPWSL--
HQPWSL--
-HPWSLW-
-HPWSLQ-
HEPWSL--
-HPWSLE-
HWPWSL--
IRPWSL--
-IPWSLR-
IAPWSL--
IGPWSL--
ITPWSL--
-IPWSLK-
-IPWSLS-
-IPWSLM-
-IPWSLE-
-IPWSLY-
IQPWSL--
-IPWSLN-
IEPWSL--
-IPWSLL-
-IPWSLI-
IMPWSL--
IDPWSL--
IIPWSL--
-IPWSLV-
INPWSL--
IKPWSL--

IYPWSL--
ILPWSL--
IHPWSL--
IFPWSL--
-IPWSLW-
-IPWSLA-
-IPWSLC-
-IPWSLQ-
-IPWSLH-
ICPWSL--
KGPWSL--
-KPWSLR-
-KPWSLT-
KVPWSL--
KFPWSL--
KAPWSL--
KTPWSL--
-KPWSLQ-
-KPWSLL-
-KPWSLI-
-KPWSLA-
-KPWSLS-
KHPWSL--
KQPWSL--
-KPWSLG-
KYPWSL--
-KPWSLH-
-KPWSLN-
-KPWSLK-
KMPWSL--
KWPWSL--
KIPWSL--
-KPWSLW-
KKPWSL--
KEPWSL--
KNPWSL--
-KPWSLM-
-KPWSLE-
KDPWSL--
-KPWSLY-
-KPWSLC-
KPPWSL--
KCPWSL--
LSPWSL--
LGPWSL--
-LPWSLS-
LMPWSL--
LAPWSL--
LNPWSL--
-LPWSLK-

LHPWSL--
LTPWSL--
LPPWSL--
-LPWSLT-
-LPWSLA-
-LPWSLI-
LLPWSL--
LQPWSL--
LEPWSL--
LWPWSL--
-LPWSLV-
-LPWSLN-
-LPWSLM-
LDPWSL--
-LPWSLQ-
-LPWSLW-
LCPWSL--
-LPWSLG-
-LPWSLE-
MSPWSL--
MTPWSL--
-MPWSLR-
MRPWSL--
MVPWSL--
MLPWSL--
MGPWSL--
MNPWSL--
MIPWSL--
-MPWSLS-
-MPWSLT-
-MPWSLM-
-MPWSLN-
MFPWSL--
-MPWSLH-
MEPWSL--
MYPWSL--
-MPWSLQ-
-MPWSLY-
-MPWSLI-
MQPWSL--
-MPWSLK-
-MPWSLG-
-MPWSLV-
-MPWSLA-
-MPWSLW-
MMPWSL--
MCPWSL--
MKPWSL--
-MPWSLE-
NSPWSL--

-NPWSLR-
NKPWSL--
NQPWSL--
NAPWSL--
-NPWSLI-
NLPWSL--
NNPWSL--
NGPWSL--
-NPWSLH-
NHPWSL--
NVPWSL--
-NPWSLT-
-NPWSLM-
-NPWSLK-
NTPWSL--
-NPWSLV-
NWPWSL--
-NPWSLA-
NPPWSL--
NDPWSL--
-NPWSLN-
NIPWSL--
NFPWSL--
-NPWSLY-
-NPWSLQ-
-NPWSLE-
NYPWSL--
NCPWSL--
-NPWSLD-
--PWSLSS
--PWSLTS
--PWSLVS
--PWSLLR
PLPWSL--
--PWSLRT
--PWSLLS
--PWSLYG
--PWSLRQ
--PWSLKS
--PWSLYS
--PWSLLN
--PWSLKT
--PWSLSA
--PWSLRN
--PWSLSK
--PWSLRE
PSPWSL--
--PWSLLG
--PWSLIS
--PWSLSV

--PWSLRY
--PWSLNS
PTPWSL--
--PWSLKR
--PWSLST
--PWSLVN
--PWSLSN
--PWSLRH
--PWSLVA
--PWSLSM
PKPWSL--
PQPWSL--
--PWSLVK
--PWSLKG
--PWSLFQ
--PWSLTT
--PWSLMT
--PWSLGA
--PWSLVQ
--PWSLNV
--PWSLAV
--PWSLYN
--PWSLWS
--PWSLLH
--PWSLVV
--PWSLSY
--PWSLMS
--PWSLWG
--PWSLSI
--PWSLHV
--PWSLTG
--PWSLIG
--PWSLHS
PAPWSL--
--PWSLSR
--PWSLAG
PRPWSL--
--PWSLNR
--PWSLYT
--PWSLSG
--PWSLQT
--PWSLNM
--PWSLHT
--PWSLEM
--PWSLSQ
--PWSLFR
--PWSLYK
--PWSLTN
--PWSLMN
--PWSLKV

PWPWSL--
--PWSLIN
--PWSLKL
--PWSLES
PMPWSL--
--PWSLVT
--PWSLRW
--PWSLQY
--PWSLNL
--PWSLIA
--PWSLHR
--PWSLHI
PDPWSL--
--PWSLLK
--PWSLVD
--PWSLTF
--PWSLTQ
--PWSLHF
--PWSLTI
--PWSLSH
--PWSLRM
--PWSLQG
--PWSLMD
--PWSLEN
--PWSLSW
--PWSLHM
--PWSLLE
--PWSLHL
--PWSLHG
--PWSLGS
--PWSLYE
--PWSLWR
--PWSLTK
--PWSLTA
--PWSLNT
--PWSLFN
--PWSLAT
--PWSLAA
PHPWSL--
PEPWSL--
--PWSLQS
--PWSLNN
--PWSLKW
--PWSLFT
PNPWSL--
--PWSLKQ
--PWSLIE
--PWSLHD
--PWSLWN
--PWSLTD

--PWSLQI
--PWSLQA
--PWSLNF
--PWSLMH
--PWSLKF
--PWSLAN
--PWSLVI
--PWSLSD
--PWSLEY
PIPWSL--
--PWSLYD
--PWSLQN
--PWSLMQ
--PWSLKD
--PWSLHH
--PWSLHA
--PWSLWQ
--PWSLTH
--PWSLQM
--PWSLQE
--PWSLLD
--PWSLHK
--PWSLFD
--PWSLAQ
PVPWSL--
--PWSLTW
--PWSLQR
--PWSLQK
--PWSLKK
--PWSLHN
--PWSLGL
--PWSLAR
--PWSLWD
--PWSLTE
--PWSLNQ
--PWSLME
--PWSLKN
--PWSLEV
--PWSLWE
--PWSLID
--PWSLEA
--PWSLAK
--PWSLWA
--PWSLQH
--PWSLNI
--PWSLNE
--PWSLIK
--PWSLII
--PWSLHQ
--PWSLER

--PWSLEG
--PWSLMK
--PWSLIT
--PWSLQD
--PWSLEH
--PWSLDG
--PWSLAH
--PWSLTC
--PWSLQV
--PWSLQF
--PWSLNW
--PWSLNG
--PWSLGN
--PWSLEW
--PWSLDS
--PWSLSE
--PWSLRP
--PWSLKE
--PWSLIF
--PWSLGY
--PWSLGQ
--PWSLGM
--PWSLFH
--PWSLWK
--PWSLWH
--PWSLWF
--PWSLMC
--PWSLKY
--PWSLHW
--PWSLEL
--PWSLDW
--PWSLCG
-PPWSLT-
-PPWSLN-
--PWSLTM
--PWSLQW
--PWSLND
--PWSLHE
--PWSLEQ
--PWSLEF
--PWSLDA
--PWSLCT
--PWSLCN
--PWSLCL
-PPWSLK-
-PPWSLA-
PPPWSL--
PCPWSL--
QDPWSL--
QRPWSL--

```
-QPWSLL-
QSPWSL--
QVPWSL--
QGPWSL--
-QPWSLK-
QLPWSL--
-QPWSLS-
-QPWSLI-
QTPWSL--
-QPWSLV-
-QPWSLT-
QAPWSL--
QMPWSL--
QKPWSL--
-QPWSLN-
QNPWSL--
QIPWSL--
-QPWSLW-
QQPWSL--
QHPWSL--
-QPWSLH-
-QPWSLE-
QWPWSL--
-QPWSLG-
QCPWSL--
-QPWSLQ-
QPPWSL--
-QPWSLM-
-QPWSLA-
-RPWSLR-
RSPWSL--
RIPWSL--
RGPWSL--
-RPWSLT-
-RPWSLH-
RNPWSL--
-RPWSLK-
RAPWSL--
-RPWSLV-
-RPWSLA-
-RPWSLN-
-RPWSLI-
RFPWSL--
-RPWSLM-
-RPWSLQ-
-RPWSLY-
-RPWSLG-
RPPWSL--
RHPWSL--
-RPWSLW-
```

-RPWSLF-
RQPWSL--
-RPWSLC-
RCPWSL--
-SPWSLR-
SLPWSL--
-SPWSLS-
SYPWSL--
-SPWSLL-
SVPWSL--
SMPWSL--
-SPWSLT-
SGPWSL--
-SPWSLY-
SFPWSL--
SQPWSL--
SAPWSL--
STPWSL--
-SPWSLH-
SNPWSL--
-SPWSLM-
SPPWSL--
SWPWSL--
-SPWSLI-
SEPWSL--
SHPWSL--
-SPWSLQ-
-SPWSLF-
-SPWSLV-
-SPWSLE-
-SPWSLA-
-SPWSLW-
-SPWSLG-
SCPWSL--
-TPWSLR-
TRPWSL--
TSPWSL--
TVPWSL--
THPWSL--
TTPWSL--
-TPWSLS-
-TPWSLT-
TEPWSL--
TNPWSL--
TAPWSL--
TFPWSL--
-TPWSLK-
TQPWSL--
-TPWSLL-
TMPWSL--

TKPWSL--
-TPWSLW-
-TPWSLI-
-TPWSLY-
-TPWSLQ-
TYPWSL--
TGPWSL--
-TPWSLV-
-TPWSLH-
TDPWSL--
-TPWSLF-
-TPWSLG-
-TPWSLE-
TCPWSL--
TWPWSL--
-TPWSLN-
VLPWSL--
VKPWSL--
VTPWSL--
VHPWSL--
VEPWSL--
-VPWSLK-
VAPWSL--
VGPWSL--
VIPWSL--
VNPWSL--
-VPWSLQ-
VYPWSL--
-VPWSLG-
VMPWSL--
-VPWSLS-
-VPWSLH-
VPPWSL--
VWPWSL--
-VPWSLY-
-VPWSLI-
-VPWSLF-
-VPWSLM-
-VPWSLA-
VQPWSL--
-VPWSLE-
VCPWSL--
-VPWSLW-
WLPWSL--
WVPWSL--
WTPWSL--
WGPWSL--
WEPWSL--
-WPWSLS-
-WPWSLL-

```
-WPWSLV-
WAPWSL--
WSPWSL--
-WPWSLR-
-WPWSLK-
WKPWSL--
-WPWSLT-
-WPWSLQ-
-WPWSLI-
-WPWSLF-
WNPWSL--
WQPWSL--
-WPWSLH-
-WPWSLG-
-WPWSLA-
WYPWSL--
WHPWSL--
WWPWSL--
-WPWSLP-
-WPWSLM-
-WPWSLN-
WCPWSL--
YRPWSL--
YKPWSL--
-YPWSLL-
-YPWSLR-
YWPWSL--
-YPWSLH-
YGPWSL--
YTPWSL--
-YPWSLV-
-YPWSLS-
YYPWSL--
YVPWSL--
-YPWSLT-
YIPWSL--
YMPWSL--
YHPWSL--
YFPWSL--
YPPWSL--
YNPWSL--
YQPWSL--
YEPWSL--
YDPWSL--
-YPWSLA-
YAPWSL--
-YPWSLQ-
-YPWSLK-
-YPWSLM-
-YPWSLW-
```

	-YPWSLE-		
	-YPWSLI-		
	-YPWSLG-		
PRWY	308	692	46.42
	--PRWYLP		
	-APRWYI-		
	AVPRWY--		
	AKPRWY--		
	-APRWYT-		
	AAPRWY--		
	ALPRWY--		
	AFPRWY--		
	AMPRWY--		
	AIPRWY--		
	AHPRWY--		
	-APRWYG-		
	-APRWYQ-		
	-APRWYA-		
	-APRWYN-		
	-APRWYF-		
	ADPRWY--		
	-APRWYH-		
	-APRWYE-		
	AEPRWY--		
	AYPRWY--		
	ATPRWY--		
	ACPRWY--		
	-APRWYV-		
	AWPRWY--		
	APPRWY--		
	ANPRWY--		
	AGPRWY--		
	CTPRWY--		
	CSPRWY--		
	CMPRWY--		
	CVPRWY--		
	CAPRWY--		
	-CPRWYH-		
	-CPRWYF-		
	CRPRWY--		
	-CPRWYV-		
	CFPRWY--		
	CDPRWY--		
	-CPRWYR-		
	CGPRWY--		
	DVPRWY--		
	DAPRWY--		
	DMPRWY--		
	DSPRWY--		
	DKPRWY--		

DRPRWY--
DIPRWY--
DTPRWY--
-DPRWYT-
DQPRWY--
-DPRWYK-
-DPRWYI-
DLPRWY--
DWPRWY--
-DPRWYM-
DEPRWY--
DYPRWY--
-DPRWYV-
-DPRWYQ-
-DPRWYN-
-DPRWYL-
-DPRWYG-
DNPRWY--
DFPRWY--
DDPRWY--
-EPRWYL-
ESPRWY--
ELPRWY--
-EPRWYY-
ETPRWY--
EYPRWY--
-EPRWYV-
ERPRWY--
-EPRWYF-
-EPRWYT-
-EPRWYI-
EFPRWY--
EWPRWY--
-EPRWYA-
EGPRWY--
EAPRWY--
-EPRWYS-
EEPRWY--
-EPRWYH-
-EPRWYW-
EVPRWY--
EQPRWY--
EPPRWY--
-EPRWYG-
EIPRWY--
-EPRWYM-
-EPRWYK-
EMPRWY--
EKPRWY--
EDPRWY--

-EPRWYQ-
ENPRWY--
ECPRWY--
FQPRWY--
FGPRWY--
FRPRWY--
FKPRWY--
FYPRWY--
FHPRWY--
FVPRWY--
FSPRWY--
-FPRWYI-
FNPRWY--
-FPRWYT-
FTPRWY--
-FPRWYH-
FLPRWY--
FCPRWY--
FWPRWY--
-FPRWYM-
-FPRWYG-
FEPRWY--
-FPRWYN-
-FPRWYE-
FIPRWY--
FDPRWY--
-FPRWYY-
-FPRWYV-
-GPRWYL-
GEPRWY--
-GPRWYS-
GVPRWY--
GSPRWY--
GWPRWY--
GLPRWY--
GIPRWY--
GFPRWY--
GQPRWY--
-GPRWYM-
-GPRWYT-
-GPRWYH-
-GPRWYW-
GNPRWY--
-GPRWYQ-
GYPRWY--
-GPRWYF-
GTPRWY--
GKPRWY--
-GPRWYV-
-GPRWYI-

GPPRWY--
GGPRWY--
-GPRWYY-
-GPRWYN-
-GPRWYG-
GAPRWY--
GDPRWY--
-GPRWYE-
HMPRWY--
HLPRWY--
-HPRWYV-
HSPRWY--
HFPRWY--
HGPRWY--
-HPRWYT-
HQPRWY--
-HPRWYI-
HTPRWY--
HEPRWY--
-HPRWYH-
-HPRWYM-
HAPRWY--
-HPRWYA-
-HPRWYL-
HHPRWY--
HDPRWY--
HKPRWY--
HYPRWY--
HWPRWY--
-HPRWYQ-
-HPRWYG-
HPPRWY--
HVPRWY--
IQPRWY--
INPRWY--
IKPRWY--
ISPRWY--
IHPRWY--
IYPRWY--
IRPRWY--
-IPRWYL-
-IPRWYQ-
IPPRWY--
-IPRWYS-
IGPRWY--
-IPRWYM-
-IPRWYA-
-IPRWYH-
-IPRWYE-
IMPRWY--

```
-IPRWYY-
-IPRWYT-
-IPRWYI-
ITPRWY--
-IPRWYN-
-IPRWYF-
-IPRWYC-
IEPRWY--
KSPRWY--
-KPRWYS-
KGPRWY--
KLPRWY--
KYPRWY--
KVPRWY--
KEPRWY--
KAPRWY--
-KPRWYI-
KMPRWY--
KTPRWY--
-KPRWYA-
KWPRWY--
-KPRWYG-
-KPRWYN-
KRPRWY--
-KPRWYW-
-KPRWYK-
-KPRWYY-
KKPRWY--
KDPRWY--
-KPRWYM-
KIPRWY--
KCPRWY--
LAPRWY--
LRPRWY--
LGPRWY--
-LPRWYS-
LKPRWY--
LIPRWY--
LTPRWY--
LPPRWY--
LWPRWY--
LNPRWY--
LVPRWY--
LYPRWY--
LQPRWY--
LDPRWY--
-LPRWYL-
-LPRWYQ-
-LPRWYT-
-LPRWYM-
```

-LPRWYF-
-LPRWYY-
LHPRWY--
-LPRWYG-
-LPRWYA-
-LPRWYN-
-LPRWYI-
-LPRWYW-
-LPRWYE-
LEPRWY--
MRPRWY--
MSPRWY--
MVPRWY--
MLPRWY--
MEPRWY--
MFPRWY--
MYPRWY--
-MPRWYG-
-MPRWYQ-
MHPRWY--
MDPRWY--
MTPRWY--
MQPRWY--
-MPRWYA-
-MPRWYW-
MGPRWY--
MMPRWY--
-MPRWYF-
-MPRWYY-
MPPRWY--
MCPRWY--
-MPRWYL-
-MPRWYD-
MNPRWY--
NAPRWY--
NVPRWY--
NHPRWY--
NRPRWY--
-NPRWYT-
NGPRWY--
NMPRWY--
NSPRWY--
-NPRWYQ-
NWPRWY--
-NPRWYS-
NYPRWY--
-NPRWYA-
NNPRWY--
-NPRWYI-
-NPRWYH-

-NPRWYV-
NPPRWY--
NQPRWY--
-NPRWYN-
-NPRWYM-
-NPRWYG-
-NPRWYF-
NEPRWY--
--PRWYEG
--PRWYRT
--PRWYVS
--PRWYTS
--PRWYSG
--PRWYQS
--PRWYST
--PRWYSS
--PRWYYA
PMPRWY--
--PRWYLG
--PRWYAS
--PRWYLN
--PRWYSV
--PRWYVN
PSPRWY--
--PRWYLT
--PRWYTA
--PRWYSD
--PRWYWT
--PRWYTG
--PRWYFR
--PRWYEA
--PRWYIS
--PRWYGT
--PRWYMG
--PRWYIT
--PRWYQG
--PRWYNS
--PRWYAT
--PRWYAA
--PRWYVK
--PRWYRV
--PRWYIK
--PRWYGS
--PRWYAV
--PRWYKT
PIPRWY--
--PRWYTW
--PRWYMA
--PRWYLV
--PRWYLM

--PRWYKG
--PRWYVR
--PRWYTT
--PRWYNG
--PRWYKD
--PRWYIG
--PRWYTI
--PRWYSH
--PRWYQT
--PRWYLR
--PRWYKF
--PRWYFS
--PRWYSF
--PRWYQA
--PRWYTH
--PRWYSY
--PRWYSE
--PRWYRH
--PRWYQL
--PRWYLK
--PRWYGG
--PRWYGA
--PRWYSA
--PRWYMS
--PRWYLW
--PRWYWG
--PRWYSQ
--PRWYNT
--PRWYKV
--PRWYIA
--PRWYGM
--PRWYYN
--PRWYYG
--PRWYWS
--PRWYVT
--PRWYSW
--PRWYQM
--PRWYMY
--PRWYFG
--PRWYAY
--PRWYAG
--PRWYYT
--PRWYTM
--PRWYSI
--PRWYQI
--PRWYNA
--PRWYMK
--PRWYKY
PQPRWY--
PTPRWY--

--PRWYYI
--PRWYWQ
--PRWYRN
--PRWYMH
--PRWYHV
--PRWYHS
--PRWYGY
PFPRWY--
--PRWYWI
--PRWYQN
--PRWYQD
--PRWYIQ
--PRWYHF
--PRWYFA
--PRWYQK
--PRWYLQ
--PRWYKM
--PRWYKH
--PRWYAQ
--PRWYAM
--PRWYKL
--PRWYKI
--PRWYHY
--PRWYAN
PLPRWY--
--PRWYYW
--PRWYYS
--PRWYWV
--PRWYVQ
--PRWYTY
--PRWYTN
--PRWYQV
--PRWYMT
--PRWYLE
--PRWYHA
--PRWYGI
--PRWYGH
--PRWYET
PAPRWY--
--PRWYYY
--PRWYYF
--PRWYWN
--PRWYTE
--PRWYMD
--PRWYLH
--PRWYLD
--PRWYIF
--PRWYHM
--PRWYGV
--PRWYGQ

--PRWYFN
--PRWYFM
PHPRWY--
--PRWYWK
--PRWYQQ
--PRWYNV
--PRWYNL
--PRWYKW
--PRWYIH
--PRWYID
--PRWYHW
--PRWYHQ
--PRWYHN
--PRWYGN
--PRWYDS
--PRWYDA
--PRWYAH
PGPRWY--
PDPRWY--
PCPRWY--
--PRWYNM
--PRWYMQ
--PRWYHT
--PRWYFQ
--PRWYFE
--PRWYEY
--PRWYEK
--PRWYAD
--PRWYYQ
--PRWYWY
--PRWYVD
--PRWYRW
--PRWYNN
--PRWYMV
--PRWYMN
--PRWYME
--PRWYIN
--PRWYHH
--PRWYFF
--PRWYEQ
--PRWYEN
--PRWYAE
PEPRWY--
--PRWYYK
--PRWYYE
--PRWYTQ
--PRWYQH
--PRWYNI
--PRWYNH
--PRWYND

--PRWYKC
--PRWYIE
--PRWYHI
--PRWYHE
--PRWYGW
--PRWYFK
--PRWYFD
--PRWYER
--PRWYDM
--PRWYCT
--PRWYAF
PNPRWY--
-QPRWYV-
QGPRWY--
QDPRWY--
QEPRWY--
-QPRWYM-
-QPRWYL-
-QPRWYG-
QMPRWY--
QLPRWY--
QAPRWY--
QYPRWY--
-QPRWYS-
QVPRWY--
QTPRWY--
-QPRWYH-
QNPRWY--
-QPRWYK-
QPPRWY--
-QPRWYN-
-QPRWYY-
QQPRWY--
QHPRWY--
-QPRWYT-
-QPRWYQ-
QKPRWY--
-QPRWYD-
QWPRWY--
-QPRWYW-
-QPRWYI-
-QPRWYF-
QCPRWY--
RAPRWY--
-RPRWYT-
RVPRWY--
RYPRWY--
RQPRWY--
RMPRWY--
RLPRWY--

RSPRWY--
-RPRWYL-
-RPRWYI-
RDPRWY--
-RPRWYH-
-RPRWYM-
-RPRWYV-
-RPRWYF-
RNPRWY--
RHPRWY--
RIPRWY--
RPPRWY--
-RPRWYG-
-RPRWYQ-
-RPRWYN-
RKPRWY--
REPRWY--
RFPRWY--
-RPRWYW-
RCPRWY--
-RPRWYA-
SQPRWY--
SAPRWY--
SGPRWY--
SSPRWY--
-SPRWYY-
SYPRWY--
-SPRWYA-
-SPRWYV-
SVPRWY--
SIPRWY--
-SPRWYM-
SRPRWY--
-SPRWYS-
SLPRWY--
SWPRWY--
-SPRWYL-
SDPRWY--
SMPRWY--
-SPRWYI-
-SPRWYW-
-SPRWYT-
SHPRWY--
SNPRWY--
SEPRWY--
STPRWY--
-SPRWYK-
-SPRWYG-
SPPRWY--
SCPRWY--

−SPRWYN−
−SPRWYH−
SFPRWY−−
−SPRWYE−
−TPRWYM−
TSPRWY−−
TAPRWY−−
TVPRWY−−
−TPRWYT−
TGPRWY−−
TQPRWY−−
TTPRWY−−
−TPRWYS−
TRPRWY−−
TMPRWY−−
−TPRWYQ−
−TPRWYI−
TIPRWY−−
TFPRWY−−
TYPRWY−−
TWPRWY−−
THPRWY−−
−TPRWYW−
−TPRWYN−
−TPRWYA−
−TPRWYY−
−TPRWYF−
TPPRWY−−
TEPRWY−−
−TPRWYV−
VAPRWY−−
−VPRWYA−
VKPRWY−−
−VPRWYS−
−VPRWYL−
VPPRWY−−
−VPRWYN−
−VPRWYF−
−VPRWYM−
−VPRWYV−
−VPRWYH−
−VPRWYT−
−VPRWYI−
VYPRWY−−
VNPRWY−−
−VPRWYQ−
−VPRWYY−
−VPRWYD−
VEPRWY−−
VWPRWY−−

VQPRWY--
-VPRWYE-
VHPRWY--
-VPRWYW-
WQPRWY--
WKPRWY--
WNPRWY--
WSPRWY--
-WPRWYT-
WTPRWY--
WRPRWY--
WGPRWY--
WYPRWY--
-WPRWYS-
WMPRWY--
-WPRWYN-
WIPRWY--
WFPRWY--
-WPRWYM-
-WPRWYH-
-WPRWYE-
WAPRWY--
YNPRWY--
YGPRWY--
-YPRWYS-
YTPRWY--
YRPRWY--
-YPRWYL-
YFPRWY--
YVPRWY--
YPPRWY--
YKPRWY--
YDPRWY--
YQPRWY--
YAPRWY--
-YPRWYV-
YMPRWY--
-YPRWYG-
YEPRWY--
-YPRWYN-
-YPRWYK-
-YPRWYI-
-YPRWYM-
-YPRWYW-
-YPRWYF-
YCPRWY--

PVPM 169 765 46.36

-TPVPMN-
-APVPMR-
-APVPMW-

-APVPMI-
AYPVPM--
AVPVPM--
-APVPMT-
ARPVPM--
-APVPMH-
AWPVPM--
-APVPMS-
-APVPMV-
AGPVPM--
-APVPML-
ASPVPM--
APPVPM--
AAPVPM--
ATPVPM--
-APVPMN-
-APVPMA-
ALPVPM--
-APVPMY-
-APVPMQ-
-APVPMK-
ANPVPM--
AIPVPM--
-APVPMG-
AMPVPM--
ADPVPM--
AKPVPM--
AHPVPM--
CVPVPM--
CKPVPM--
-CPVPMW-
CNPVPM--
CMPVPM--
CTPVPM--
CSPVPM--
-CPVPMS-
-CPVPMR-
-CPVPMK-
-CPVPMI-
CPPVPM--
CIPVPM--
CHPVPM--
DRPVPM--
-DPVPMR-
DSPVPM--
DIPVPM--
-DPVPMV-
DFPVPM--
-DPVPMW-
DYPVPM--

-DPVPMS-
-DPVPML-
-DPVPMH-
-DPVPMT-
-DPVPMI-
DPPVPM--
DHPVPM--
EMPVPM--
-EPVPMQ-
-EPVPMR-
-EPVPMI-
-EPVPMH-
-EPVPMF-
EEPVPM--
-EPVPMY-
EWPVPM--
ESPVPM--
ERPVPM--
-EPVPMK-
-EPVPMV-
EFPVPM--
EVPVPM--
-EPVPMW-
-EPVPMT-
-EPVPMS-
-EPVPML-
ENPVPM--
EIPVPM--
EAPVPM--
FSPVPM--
FRPVPM--
FNPVPM--
-FPVPMR-
-FPVPMT-
FAPVPM--
FEPVPM--
-FPVPMM-
-FPVPMW-
-FPVPML-
FKPVPM--
FLPVPM--
-FPVPMS-
-FPVPMI-
FTPVPM--
FWPVPM--
FQPVPM--
FYPVPM--
-FPVPMH-
FPPVPM--
FCPVPM--

FVPVPM--
-FPVPMY-
-FPVPMG-
-FPVPME-
FMPVPM--
-GPVPMR-
-GPVPMS-
GVPVPM--
GIPVPM--
GTPVPM--
GNPVPM--
GHPVPM--
-GPVPMW-
-GPVPML-
GMPVPM--
-GPVPMK-
GRPVPM--
GAPVPM--
-GPVPMT-
GLPVPM--
GSPVPM--
-GPVPMV-
GEPVPM--
-GPVPMA-
GGPVPM--
-GPVPMI-
GKPVPM--
GWPVPM--
-GPVPMM-
-GPVPMF-
-GPVPMQ-
-GPVPMH-
GDPVPM--
-HPVPMR-
HNPVPM--
HTPVPM--
-HPVPMK-
HAPVPM--
HWPVPM--
HSPVPM--
HRPVPM--
HIPVPM--
HGPVPM--
-HPVPML-
HQPVPM--
-HPVPMV-
-HPVPMS-
-HPVPMQ-
HKPVPM--
-HPVPMT-

-HPVPMY-
-HPVPMI-
HMPVPM--
HLPVPM--
-HPVPMH-
-HPVPMA-
HPPVPM--
-IPVPMR-
-IPVPMT-
IIPVPM--
-IPVPMK-
-IPVPMI-
IQPVPM--
IYPVPM--
IMPVPM--
-IPVPMM-
-IPVPMW-
IHPVPM--
IVPVPM--
-IPVPMN-
-IPVPMG-
-IPVPMS-
-IPVPMA-
ILPVPM--
ISPVPM--
IGPVPM--
IWPVPM--
-IPVPMH-
INPVPM--
IFPVPM--
ITPVPM--
-IPVPMV-
-IPVPML-
IPPVPM--
IKPVPM--
IDPVPM--
ICPVPM--
-IPVPMY-
-IPVPMF-
KSPVPM--
-KPVPMR-
-KPVPMS-
-KPVPMK-
-KPVPMT-
-KPVPMM-
-KPVPMW-
KVPVPM--
-KPVPMH-
KYPVPM--
KFPVPM--

KWPVPM--
KQPVPM--
-KPVPMV-
KNPVPM--
KMPVPM--
KRPVPM--
KHPVPM--
KGPVPM--
KDPVPM--
-KPVPMQ-
-KPVPMN-
-KPVPMG-
KPPVPM--
-KPVPMI-
-KPVPMF-
-KPVPMA-
KLPVPM--
KAPVPM--
LGPVPM--
LYPVPM--
LWPVPM--
LAPVPM--
-LPVPMI-
LIPVPM--
-LPVPMY-
-LPVPMH-
-LPVPMW-
-LPVPMT-
LKPVPM--
LHPVPM--
LSPVPM--
LFPVPM--
-LPVPMV-
LPPVPM--
LLPVPM--
LQPVPM--
-LPVPML-
LMPVPM--
LDPVPM--
LEPVPM--
-LPVPMM-
-LPVPMK-
-LPVPMQ-
-LPVPMN-
LNPVPM--
-MPVPMR-
-MPVPML-
MSPVPM--
MRPVPM--
-MPVPMT-

MGPVPM--
MVPVPM--
MMPVPM--
MNPVPM--
MHPVPM--
MAPVPM--
MTPVPM--
MLPVPM--
MKPVPM--
-MPVPMY-
-MPVPMS-
-MPVPMK-
MEPVPM--
-MPVPMQ-
MIPVPM--
-MPVPMV-
MPPVPM--
MWPVPM--
-MPVPMM-
-MPVPMI-
MFPVPM--
NSPVPM--
NIPVPM--
NGPVPM--
NAPVPM--
NVPVPM--
-NPVPMR-
NYPVPM--
NQPVPM--
-NPVPML-
-NPVPMT-
-NPVPMM-
-NPVPMH-
NLPVPM--
-NPVPMW-
NKPVPM--
NTPVPM--
NRPVPM--
-NPVPMY-
NFPVPM--
-NPVPMI-
NPPVPM--
NNPVPM--
NHPVPM--
-NPVPMV-
-NPVPMS-
-NPVPMN-
-NPVPMF-
--PVPMLY
--PVPMHA

--PVPMYS
--PVPMML
--PVPMSS
--PVPMLS
--PVPMTL
--PVPMTS
--PVPMRR
--PVPMYG
--PVPMRF
--PVPMRT
--PVPMSA
--PVPMTI
--PVPMGQ
--PVPMQG
PGPVPM--
--PVPMGS
--PVPMYR
--PVPMYF
--PVPMLT
--PVPMVT
--PVPMST
--PVPMRA
--PVPMLG
--PVPMFL
--PVPMVN
--PVPMNS
--PVPMFV
--PVPMVR
--PVPMRQ
--PVPMEL
--PVPMVG
--PVPMSV
--PVPMPW
--PVPMFI
--PVPMAS
--PVPMWY
--PVPMWL
--PVPMVV
--PVPMTR
--PVPMSY
--PVPMRI
--PVPMLL
--PVPMKS
--PVPMFS
--PVPMTT
--PVPMKW
--PVPMIN
--PVPMIM
--PVPMIG
--PVPMHT

--PVPMFA
--PVPMAY
--PVPMPS
--PVPMKG
PEPVPM--
PAPVPM--
--PVPMWG
--PVPMVY
--PVPMVL
--PVPMSH
--PVPMRY
--PVPMQY
--PVPMLK
--PVPMKF
--PVPMWM
--PVPMTV
--PVPMRM
--PVPMPF
--PVPMIY
--PVPMHG
--PVPMAQ
--PVPMQS
--PVPMLN
--PVPMFY
--PVPMFN
--PVPMYY
--PVPMVW
--PVPMRH
--PVPMLR
--PVPMKA
--PVPMIA
--PVPMFH
--PVPMVI
--PVPMTY
--PVPMTQ
--PVPMQM
--PVPMPA
--PVPMLI
--PVPMYT
--PVPMYE
--PVPMWW
--PVPMWT
--PVPMVS
--PVPMSQ
--PVPMRN
--PVPMRK
--PVPMNW
--PVPMNV
--PVPMNT
--PVPMLV

--PVPMLM
--PVPMKI
--PVPMGV
--PVPMDF
PVPVPM--
--PVPMYW
--PVPMWN
--PVPMVF
--PVPMTW
--PVPMPY
--PVPMMN
--PVPMMF
--PVPMIV
--PVPMHY
--PVPMGG
--PVPMFT
PKPVPM--
--PVPMYV
--PVPMTG
--PVPMTA
--PVPMSN
--PVPMNM
--PVPMKH
--PVPMID
--PVPMHR
--PVPMGA
--PVPMFK
PWPVPM--
--PVPMYH
--PVPMTF
--PVPMSW
--PVPMSG
--PVPMRE
--PVPMQW
--PVPMNI
--PVPMMR
--PVPMMA
--PVPMLF
--PVPMLA
--PVPMHQ
--PVPMFR
--PVPMFQ
--PVPMES
--PVPMAT
PRPVPM--
--PVPMWS
--PVPMWQ
--PVPMRW
--PVPMNY
--PVPMMM

--PVPMKQ
--PVPMII
--PVPMHM
--PVPMGN
--PVPMDY
--PVPMDL
--PVPMAR
--PVPMAH
--PVPMAF
PSPVPM--
--PVPMYN
--PVPMYA
--PVPMWH
--PVPMVM
--PVPMVA
--PVPMTM
--PVPMTH
--PVPMPI
--PVPMMV
--PVPMMT
--PVPMLD
--PVPMKR
--PVPMKK
--PVPMIS
--PVPMGH
--PVPMFG
--PVPMAN
--PVPMSK
--PVPMLQ
--PVPMKN
--PVPMKM
--PVPMIT
--PVPMIL
--PVPMHW
--PVPMFW
--PVPMEH
--PVPMEA
--PVPMDI
--PVPMAV
PQPVPM--
--PVPMYD
--PVPMWI
--PVPMVD
--PVPMQK
--PVPMPV
--PVPMMI
--PVPMLE
--PVPMKT
--PVPMKE
--PVPMIK

--PVPMIH
--PVPMIF
--PVPMHS
--PVPMGW
--PVPMFD
--PVPMEQ
--PVPMAK
--PVPMAI
--PVPMYK
--PVPMWR
--PVPMTD
--PVPMSR
--PVPMSF
--PVPMQV
--PVPMQN
--PVPMPM
--PVPMNF
--PVPMMG
--PVPMLC
--PVPMGT
--PVPMEI
--PVPMEF
--PVPMDQ
--PVPMDM
--PVPMCY
--PVPMAW
--PVPMAM
PMPVPM--
--PVPMWV
--PVPMVH
--PVPMVC
--PVPMTN
--PVPMQT
--PVPMQD
--PVPMPH
--PVPMNR
--PVPMNQ
--PVPMNH
--PVPMMS
--PVPMKV
--PVPMHK
--PVPMHI
--PVPMHC
--PVPMGK
--PVPMFE
--PVPMEW
--PVPMEV
--PVPMEN
--PVPMDS
--PVPMDG

--PVPMCQ
--PVPMCL
--PVPMCF
--PVPMAE
--PVPMAD
-PPVPMY-
-PPVPMR-
-QPVPMR-
QWPVPM--
-QPVPMF-
QRPVPM--
-QPVPML-
-QPVPMV-
-QPVPMS-
QVPVPM--
-QPVPMI-
QIPVPM--
-QPVPMH-
-QPVPMY-
-QPVPMT-
-QPVPMQ-
QEPVPM--
QDPVPM--
-QPVPMN-
QMPVPM--
QFPVPM--
QAPVPM--
QTPVPM--
-QPVPMK-
QLPVPM--
QGPVPM--
QYPVPM--
QQPVPM--
-QPVPMM-
-QPVPMG-
QKPVPM--
-RPVPMI-
-RPVPMR-
RGPVPM--
RSPVPM--
RVPVPM--
-RPVPMY-
-RPVPMQ-
RKPVPM--
-RPVPMT-
RPPVPM--
-RPVPMH-
-RPVPMW-
RLPVPM--
-RPVPMM-

RQPVPM--
-RPVPMF-
RTPVPM--
RMPVPM--
RYPVPM--
-RPVPMV-
-RPVPMK-
RDPVPM--
RFPVPM--
REPVPM--
RIPVPM--
RHPVPM--
RAPVPM--
RWPVPM--
-RPVPMG-
RNPVPM--
-RPVPME-
-RPVPMD-
-RPVPMA-
-SPVPMR-
SRPVPM--
-SPVPMW-
STPVPM--
-SPVPMS-
-SPVPML-
-SPVPMV-
SVPVPM--
SKPVPM--
-SPVPMY-
SLPVPM--
-SPVPMK-
SYPVPM--
SMPVPM--
SGPVPM--
-SPVPMH-
SWPVPM--
-SPVPMT-
SQPVPM--
SAPVPM--
SEPVPM--
-SPVPMM-
SHPVPM--
SSPVPM--
-SPVPMA-
-SPVPMQ-
SFPVPM--
SDPVPM--
-SPVPMN-
-SPVPMI-
-SPVPMF-

SIPVPM--
-SPVPMP-
-TPVPMR-
TRPVPM--
TWPVPM--
-TPVPMS-
TVPVPM--
TSPVPM--
-TPVPMK-
-TPVPMA-
-TPVPMF-
TNPVPM--
TIPVPM--
TYPVPM--
TTPVPM--
-TPVPMQ-
-TPVPMH-
TPPVPM--
TMPVPM--
-TPVPMV-
TLPVPM--
TFPVPM--
TGPVPM--
TDPVPM--
TEPVPM--
TAPVPM--
-TPVPMT-
-TPVPMG-
-TPVPMY-
-TPVPMW-
-TPVPMI-
-VPVPMR-
-VPVPMQ-
-VPVPML-
-VPVPMF-
VLPVPM--
VSPVPM--
-VPVPMW-
VMPVPM--
VRPVPM--
VKPVPM--
VTPVPM--
VAPVPM--
-VPVPMT-
-VPVPMK-
VFPVPM--
-VPVPMM-
-VPVPMY-
VIPVPM--
VVPVPM--

-VPVPMV-
VCPVPM--
VWPVPM--
-VPVPMG-
-VPVPMA-
VGPVPM--
-VPVPMS-
-VPVPMN-
VYPVPM--
VQPVPM--
-VPVPMH-
VHPVPM--
VEPVPM--
WPPVPM--
WSPVPM--
WNPVPM--
-WPVPMT-
WGPVPM--
-WPVPMR-
-WPVPMY-
WLPVPM--
WKPVPM--
-WPVPMA-
WYPVPM--
WRPVPM--
-WPVPMQ-
WDPVPM--
-WPVPMS-
-WPVPMN-
-WPVPMW-
-WPVPML-
-WPVPMK-
WVPVPM--
WTPVPM--
-WPVPMI-
WAPVPM--
WMPVPM--
YRPVPM--
YIPVPM--
-YPVPMR-
YSPVPM--
YHPVPM--
-YPVPMT-
YAPVPM--
-YPVPMW-
-YPVPMV-
-YPVPMS-
YNPVPM--
-YPVPMF-
YDPVPM--

-YPVPML-
-YPVPMI-
YYPVPM--
YEPVPM--
-YPVPMM-
YMPVPM--
-YPVPMA-
-YPVPMY-
-YPVPMQ-
-YPVPMK-
YKPVPM--
YGPVPM--
PSPM 95 810 46.34
-HPSPMN-
--PSPMRP
--PSPMHK
ASPSPM--
-APSPMR-
-APSPML-
AWPSPM--
AMPSPM--
-APSPMF-
AQPSPM--
-APSPMV-
-APSPMW-
ARPSPM--
-APSPMT-
-APSPMS-
AIPSPM--
-APSPMM-
AHPSPM--
-APSPMY-
ANPSPM--
-APSPMK-
-APSPMI-
-APSPMH-
ALPSPM--
AKPSPM--
ATPSPM--
AGPSPM--
AYPSPM--
AEPSPM--
AVPSPM--
-APSPMA-
APPSPM--
ADPSPM--
AFPSPM--
-APSPMG-
-APSPMN-
-APSPMC-

-APSPMQ-
-APSPME-
CKPSPM--
-CPSPMW-
CMPSPM--
CRPSPM--
-CPSPML-
CIPSPM--
CTPSPM--
CSPSPM--
-CPSPMR-
-CPSPMM-
CPPSPM--
CCPSPM--
DVPSPM--
-DPSPMR-
DTPSPM--
-DPSPMF-
DNPSPM--
-DPSPMV-
-DPSPMT-
-DPSPMW-
DGPSPM--
DSPSPM--
DRPSPM--
DLPSPM--
DYPSPM--
-DPSPML-
-DPSPMH-
DIPSPM--
DDPSPM--
DAPSPM--
DQPSPM--
-DPSPMS-
-DPSPMN-
DMPSPM--
DHPSPM--
-EPSPMM-
-EPSPMR-
ERPSPM--
-EPSPMQ-
EIPSPM--
-EPSPMF-
-EPSPMY-
-EPSPML-
EFPSPM--
-EPSPMS-
ESPSPM--
EQPSPM--
-EPSPMW-

ELPSPM--
-EPSPMV-
EYPSPM--
EVPSPM--
ETPSPM--
EHPSPM--
EAPSPM--
-EPSPMK-
-EPSPMI-
-EPSPMH-
-EPSPMA-
EPPSPM--
FHPSPM--
FTPSPM--
-FPSPMR-
FAPSPM--
FNPSPM--
FFPSPM--
FRPSPM--
FLPSPM--
-FPSPMY-
FIPSPM--
-FPSPMF-
FMPSPM--
FGPSPM--
-FPSPMW-
-FPSPMV-
-FPSPMQ-
FVPSPM--
FSPSPM--
-FPSPMT-
-FPSPMS-
-FPSPMI-
FKPSPM--
-FPSPMN-
-FPSPMM-
-FPSPMC-
-FPSPMA-
-GPSPMR-
GRPSPM--
GYPSPM--
GFPSPM--
-GPSPMM-
GNPSPM--
-GPSPMV-
-GPSPMI-
GAPSPM--
GSPSPM--
-GPSPMT-
GLPSPM--

-GPSPMY-
-GPSPMH-
GTPSPM--
-GPSPMK-
GKPSPM--
GVPSPM--
GQPSPM--
-GPSPMW-
-GPSPMQ-
-GPSPMF-
GWPSPM--
-GPSPML-
GPPSPM--
GMPSPM--
GHPSPM--
GDPSPM--
-GPSPMA-
GCPSPM--
-HPSPMF-
HLPSPM--
HSPSPM--
-HPSPMY-
HMPSPM--
HWPSPM--
HTPSPM--
-HPSPMV-
HNPSPM--
HIPSPM--
HDPSPM--
-HPSPMT-
-HPSPMR-
-HPSPMI-
-HPSPMH-
HYPSPM--
HVPSPM--
HRPSPM--
-HPSPMW-
-HPSPMS-
HQPSPM--
HKPSPM--
HEPSPM--
HAPSPM--
-HPSPML-
HGPSPM--
ISPSPM--
IVPSPM--
IGPSPM--
-IPSPMY-
IRPSPM--
INPSPM--

-IPSPMR-
IYPSPM--
-IPSPMH-
-IPSPMI-
-IPSPMV-
-IPSPMN-
-IPSPMA-
ITPSPM--
-IPSPMS-
IIPSPM--
IFPSPM--
IHPSPM--
IPPSPM--
IQPSPM--
-IPSPMW-
-IPSPMK-
-IPSPML-
-IPSPMC-
KSPSPM--
KTPSPM--
-KPSPMM-
-KPSPML-
-KPSPMT-
KGPSPM--
-KPSPMS-
KVPSPM--
-KPSPMK-
KPPSPM--
-KPSPMY-
KAPSPM--
-KPSPMR-
-KPSPMV-
-KPSPMI-
KLPSPM--
KIPSPM--
KRPSPM--
-KPSPMQ-
KHPSPM--
-KPSPMN-
KQPSPM--
KYPSPM--
-KPSPMH-
-KPSPMF-
KDPSPM--
-KPSPMW-
-KPSPMC-
KKPSPM--
KEPSPM--
-LPSPMR-
LYPSPM--

LRPSPM--
LLPSPM--
LSPSPM--
LVPSPM--
LIPSPM--
LGPSPM--
LFPSPM--
LTPSPM--
-LPSPMY-
-LPSPMK-
LWPSPM--
-LPSPMI-
LEPSPM--
LPPSPM--
-LPSPMT-
LKPSPM--
-LPSPMS-
LNPSPM--
-LPSPMH-
-LPSPMV-
-LPSPMW-
-LPSPMM-
LDPSPM--
LMPSPM--
-LPSPMQ-
LHPSPM--
LAPSPM--
-MPSPMR-
-MPSPML-
MRPSPM--
MIPSPM--
-MPSPMS-
MWPSPM--
-MPSPMV-
MSPSPM--
MYPSPM--
-MPSPMY-
-MPSPMI-
MGPSPM--
MAPSPM--
-MPSPMN-
-MPSPMH-
MFPSPM--
MVPSPM--
MTPSPM--
-MPSPMW-
-MPSPMK-
-MPSPMT-
MNPSPM--
MMPSPM--

MKPSPM--
MEPSPM--
NYPSPM--
NSPSPM--
-NPSPMY-
NTPSPM--
-NPSPMF-
NWPSPM--
-NPSPMV-
NGPSPM--
NEPSPM--
-NPSPML-
NLPSPM--
-NPSPMH-
NQPSPM--
-NPSPMT-
-NPSPMS-
NAPSPM--
NRPSPM--
-NPSPMW-
-NPSPMM-
NFPSPM--
-NPSPMK-
-NPSPMI-
NKPSPM--
-NPSPMQ-
NNPSPM--
NMPSPM--
-NPSPMR-
-NPSPMN-
-NPSPMA-
NIPSPM--
NHPSPM--
--PSPMAS
--PSPMQV
--PSPMLG
--PSPMLY
--PSPMSL
--PSPMLL
--PSPMTL
--PSPMFS
--PSPMLS
--PSPMWI
--PSPMTS
--PSPMSS
--PSPMML
--PSPMVG
--PSPMIS
--PSPMKL
--PSPMYL

--PSPMIT
--PSPMRT
--PSPMAW
--PSPMRS
--PSPMLQ
--PSPMWA
--PSPMRM
--PSPMRH
--PSPMNA
--PSPMYS
--PSPMYR
--PSPMFN
--PSPMAL
--PSPMVT
--PSPMSY
--PSPMSV
--PSPMGF
--PSPMAH
--PSPMVY
--PSPMSF
--PSPMKS
--PSPMIW
PGPSPM--
--PSPMWL
--PSPMFI
PRPSPM--
--PSPMHS
--PSPMWT
--PSPMAF
--PSPMMA
--PSPMHM
--PSPMYI
--PSPMWS
--PSPMVS
--PSPMVA
--PSPMTI
--PSPMSI
--PSPMRG
--PSPMLA
--PSPMIY
--PSPMYA
--PSPMSG
--PSPMSA
--PSPMRN
--PSPMGS
--PSPMFH
--PSPMTG
--PSPMNF
--PSPMGV
--PSPMVH

--PSPMRD
--PSPMFL
--PSPMEI
--PSPMVR
--PSPMLT
--PSPMIV
--PSPMYN
--PSPMST
--PSPMQS
--PSPMMN
--PSPMKI
--PSPMHF
--PSPMFG
--PSPMWQ
--PSPMWH
--PSPMRW
--PSPMNY
--PSPMKA
--PSPMFM
PLPSPM--
--PSPMYV
--PSPMVW
--PSPMSM
--PSPMLW
--PSPMHV
--PSPMGY
--PSPMFR
PYPSPM--
PVPSPM--
--PSPMRA
--PSPMNH
--PSPMLV
--PSPMIF
--PSPMFF
--PSPMTW
--PSPMTA
--PSPMRQ
--PSPMQG
--PSPMMT
--PSPMMK
--PSPMKW
--PSPMFT
--PSPMEL
--PSPMDY
--PSPMAT
--PSPMAA
--PSPMYH
--PSPMTR
--PSPMTM
--PSPMQL

--PSPMNQ
--PSPMMV
--PSPMMM
--PSPMMI
--PSPMLH
--PSPMKN
--PSPMKG
--PSPMIA
--PSPMHA
PWPSPM--
--PSPMVV
--PSPMVQ
--PSPMRR
--PSPMMH
--PSPMLI
--PSPMKY
--PSPMKT
--PSPMKQ
--PSPMHY
--PSPMGH
--PSPMFW
--PSPMWM
--PSPMTT
--PSPMPI
--PSPMNW
--PSPMMR
--PSPMGN
PEPSPM--
--PSPMYG
--PSPMVL
--PSPMTN
--PSPMTK
--PSPMTF
--PSPMSQ
--PSPMRK
--PSPMNS
--PSPMMW
--PSPMMF
--PSPMME
--PSPMKD
--PSPMGM
--PSPMFQ
--PSPMEA
--PSPMAG
PMPSPM--
PKPSPM--
PAPSPM--
PTPSPM--
--PSPMVN
--PSPMTV

--PSPMTH
--PSPMRI
--PSPMMG
--PSPMKR
--PSPMIQ
--PSPMIK
--PSPMGT
--PSPMFD
--PSPMEM
--PSPMDL
--PSPMYY
--PSPMYK
--PSPMWE
--PSPMWD
--PSPMVM
--PSPMTY
--PSPMSN
--PSPMQM
--PSPMNG
--PSPMLM
--PSPMKK
--PSPMHI
--PSPMFK
--PSPMCM
--PSPMWR
--PSPMWN
--PSPMWK
--PSPMSR
--PSPMSH
--PSPMRE
--PSPMQR
--PSPMQK
--PSPMQA
--PSPMPY
--PSPMPS
--PSPMPH
--PSPMPF
--PSPMNV
--PSPMNR
--PSPMNI
--PSPMIR
--PSPMIN
--PSPMIM
--PSPMHG
--PSPMGG
--PSPMEW
--PSPMEF
--PSPMDM
--PSPMAI
PNPSPM--

--PSPMYT
--PSPMYM
--PSPMWF
--PSPMVD
--PSPMRV
--PSPMQT
--PSPMQH
--PSPMNN
--PSPMMY
--PSPMMQ
--PSPMLK
--PSPMKH
--PSPMKF
--PSPMIL
--PSPMHT
--PSPMHQ
--PSPMHN
--PSPMHH
--PSPMFA
--PSPMES
--PSPMDS
--PSPMDI
--PSPMAV
--PSPMAC
--PSPMYW
--PSPMYD
--PSPMYC
--PSPMVP
--PSPMVI
--PSPMVF
--PSPMTE
--PSPMTD
--PSPMTC
--PSPMSW
--PSPMSK
--PSPMRC
--PSPMQN
--PSPMQI
--PSPMPV
--PSPMPA
--PSPMNT
--PSPMMS
--PSPMIG
--PSPMIE
--PSPMHD
--PSPMGW
--PSPMGA
--PSPMFE
--PSPMET
--PSPMEG

--PSPMDF
--PSPMCK
--PSPMAY
--PSPMAR
-PPSPMR-
PCPSPM--
-QPSPMR-
QRPSPM--
QLPSPM--
QSPSPM--
-QPSPMV-
QKPSPM--
-QPSPMS-
-QPSPMI-
QHPSPM--
QVPSPM--
-QPSPMT-
QIPSPM--
QFPSPM--
-QPSPMF-
QQPSPM--
QPPSPM--
QYPSPM--
QTPSPM--
-QPSPMW-
QNPSPM--
QGPSPM--
-QPSPMM-
-QPSPMK-
QAPSPM--
-QPSPML-
QEPSPM--
QWPSPM--
-QPSPMY-
-QPSPMG-
-QPSPMA-
QMPSPM--
-RPSPMR-
RRPSPM--
RSPSPM--
RMPSPM--
RFPSPM--
RYPSPM--
-RPSPMY-
RHPSPM--
RGPSPM--
-RPSPMH-
-RPSPMS-
-RPSPMQ-
-RPSPMV-

-RPSPMT-
RNPSPM--
-RPSPMI-
RPPSPM--
RIPSPM--
-RPSPMM-
REPSPM--
-RPSPMN-
-RPSPMF-
RWPSPM--
RQPSPM--
RCPSPM--
-RPSPMG-
-RPSPMA-
RDPSPM--
-RPSPME-
SSPSPM--
-SPSPMR-
-SPSPML-
SKPSPM--
SAPSPM--
SFPSPM--
SRPSPM--
-SPSPMS-
SWPSPM--
SNPSPM--
SIPSPM--
SQPSPM--
SYPSPM--
-SPSPMV-
-SPSPMH-
SLPSPM--
-SPSPMT-
STPSPM--
-SPSPMY-
SPPSPM--
-SPSPMQ-
-SPSPMM-
-SPSPMK-
SMPSPM--
-SPSPMF-
SHPSPM--
-SPSPMA-
SCPSPM--
SGPSPM--
SVPSPM--
-SPSPMN-
-SPSPME-
SEPSPM--
-TPSPMR-

TSPSPM--
TKPSPM--
-TPSPMW-
TYPSPM--
TVPSPM--
-TPSPMK-
TLPSPM--
TRPSPM--
-TPSPMI-
TFPSPM--
TPPSPM--
TMPSPM--
TQPSPM--
-TPSPMM-
-TPSPMS-
-TPSPMA-
-TPSPMY-
THPSPM--
TWPSPM--
TTPSPM--
-TPSPMH-
-TPSPMF-
TNPSPM--
-TPSPMV-
-TPSPMT-
-TPSPMG-
-TPSPMQ-
TIPSPM--
TGPSPM--
TAPSPM--
-TPSPMN-
TDPSPM--
VDPSPM--
-VPSPMR-
VYPSPM--
VRPSPM--
-VPSPML-
VKPSPM--
VLPSPM--
-VPSPMS-
VSPSPM--
VGPSPM--
-VPSPMM-
-VPSPMQ-
VHPSPM--
VEPSPM--
-VPSPMY-
VAPSPM--
-VPSPMH-
-VPSPMT-

```
-VPSPMV-
-VPSPMA-
VFPSPM--
-VPSPMI-
-VPSPMF-
VPPSPM--
VMPSPM--
VIPSPM--
VWPSPM--
VTPSPM--
VQPSPM--
VNPSPM--
-VPSPMW-
VVPSPM--
-VPSPMK-
-VPSPMN-
-WPSPMV-
WRPSPM--
WGPSPM--
WYPSPM--
WTPSPM--
-WPSPML-
-WPSPMT-
WVPSPM--
-WPSPMK-
WPPSPM--
-WPSPMR-
-WPSPMM-
WWPSPM--
WQPSPM--
WNPSPM--
WHPSPM--
WAPSPM--
WSPSPM--
-WPSPMW-
WIPSPM--
-WPSPMN-
-WPSPMA-
-WPSPMH-
WMPSPM--
WFPSPM--
WEPSPM--
WLPSPM--
WKPSPM--
WDPSPM--
YRPSPM--
YAPSPM--
-YPSPML-
YIPSPM--
-YPSPMR-
```

-YPSPMH-
YPPSPM--
YLPSPM--
-YPSPMS-
-YPSPMM-
YVPSPM--
-YPSPMF-
YMPSPM--
YYPSPM--
YTPSPM--
-YPSPMW-
YNPSPM--
-YPSPMV-
-YPSPMI-
-YPSPMA-
YDPSPM--
YWPSPM--
-YPSPMK-
YKPSPM--
YCPSPM--
-YPSPMT-
-YPSPMN-
YHPSPM--

PQNM 589 590 46.34

-APQNMK-
-APQNML-
-APQNMF-
ATPQNM--
AQPQNM--
-APQNMS-
ANPQNM--
ALPQNM--
AIPQNM--
ARPQNM--
AFPQNM--
-APQNMR-
-APQNMH-
ASPQNM--
-APQNMN-
AKPQNM--
AGPQNM--
AAPQNM--
-APQNMV-
-APQNMI-
-APQNMG-
ADPQNM--
-APQNMM-
AEPQNM--
CYPQNM--
CVPQNM--

−CPQNMS−
CGPQNM−−
−CPQNMF−
DWPQNM−−
−DPQNML−
DGPQNM−−
DSPQNM−−
−DPQNMR−
−DPQNMA−
−DPQNMV−
DLPQNM−−
DKPQNM−−
DFPQNM−−
DVPQNM−−
DDPQNM−−
DCPQNM−−
EQPQNM−−
−EPQNMQ−
−EPQNMR−
−EPQNMK−
ESPQNM−−
EFPQNM−−
EVPQNM−−
−EPQNMV−
ETPQNM−−
ENPQNM−−
ELPQNM−−
EGPQNM−−
EAPQNM−−
−FPQNMR−
FVPQNM−−
−FPQNMI−
FYPQNM−−
FGPQNM−−
FSPQNM−−
−FPQNMK−
FLPQNM−−
−FPQNMT−
FHPQNM−−
FMPQNM−−
FIPQNM−−
−FPQNML−
FNPQNM−−
FAPQNM−−
−FPQNMQ−
FFPQNM−−
FTPQNM−−
−FPQNMF−
GQPQNM−−
−GPQNMS−

-GPQNMR-
GRPQNM--
GSPQNM--
-GPQNMK-
-GPQNMQ-
GLPQNM--
GKPQNM--
-GPQNMT-
GIPQNM--
GTPQNM--
-GPQNMY-
GVPQNM--
-GPQNMW-
GYPQNM--
GWPQNM--
-GPQNMN-
GMPQNM--
-GPQNMI-
-GPQNME-
GPPQNM--
GNPQNM--
GDPQNM--
HRPQNM--
-HPQNML-
-HPQNMS-
HWPQNM--
-HPQNMW-
HSPQNM--
-HPQNMA-
HFPQNM--
HMPQNM--
-HPQNMV-
-HPQNMH-
HPPQNM--
HNPQNM--
HIPQNM--
-HPQNME-
HLPQNM--
HHPQNM--
HGPQNM--
HTPQNM--
-HPQNMT-
-HPQNMK-
HAPQNM--
ILPQNM--
IGPQNM--
IRPQNM--
-IPQNML-
IKPQNM--
IVPQNM--

ISPQNM--
-IPQNMV-
-IPQNMR-
INPQNM--
-IPQNMS-
-IPQNMG-
-IPQNMY-
-IPQNMH-
IFPQNM--
IAPQNM--
ITPQNM--
-IPQNMN-
KVPQNM--
-KPQNMS-
-KPQNMH-
KRPQNM--
-KPQNMR-
-KPQNMI-
-KPQNMW-
-KPQNML-
-KPQNMK-
-KPQNMV-
-KPQNMA-
-KPQNMT-
KLPQNM--
KMPQNM--
-KPQNMQ-
-KPQNMG-
KIPQNM--
KCPQNM--
KWPQNM--
KSPQNM--
KNPQNM--
KGPQNM--
KFPQNM--
KAPQNM--
LSPQNM--
-LPQNML-
LFPQNM--
-LPQNMI-
-LPQNMS-
-LPQNMY-
LYPQNM--
LIPQNM--
LWPQNM--
LMPQNM--
-LPQNMK-
LLPQNM--
LPPQNM--
-LPQNMF-

LDPQNM--
-LPQNMV-
-LPQNMT-
LGPQNM--
LTPQNM--
LVPQNM--
LRPQNM--
LHPQNM--
-LPQNMR-
-LPQNMQ-
-LPQNMH-
-LPQNMG-
-MPQNML-
-MPQNMY-
-MPQNMV-
-MPQNMT-
MMPQNM--
MRPQNM--
MLPQNM--
MTPQNM--
MSPQNM--
MAPQNM--
MWPQNM--
-MPQNMM-
-MPQNMA-
MNPQNM--
-MPQNMS-
-MPQNMR-
MDPQNM--
MVPQNM--
-MPQNMI-
-MPQNMH-
-MPQNMC-
MGPQNM--
MCPQNM--
-NPQNMR-
-NPQNML-
-NPQNMV-
-NPQNMF-
NNPQNM--
NSPQNM--
-NPQNMM-
NHPQNM--
NFPQNM--
-NPQNMS-
NKPQNM--
NIPQNM--
NGPQNM--
NVPQNM--
NTPQNM--

-NPQNMH-
NDPQNM--
-NPQNMQ-
-NPQNMK-
-NPQNMI-
NLPQNM--
--PQNMRS
--PQNMVT
--PQNMIG
--PQNMRG
--PQNMST
--PQNMTT
--PQNMWR
--PQNMRT
--PQNMRR
--PQNMRN
--PQNMHN
--PQNMYR
--PQNMFA
--PQNMQG
--PQNMVW
--PQNMSS
--PQNMRA
--PQNMKI
--PQNMIH
--PQNMLG
--PQNMFR
--PQNMWN
--PQNMSV
--PQNMMI
--PQNMMG
--PQNMSG
PAPQNM--
--PQNMTG
--PQNMLY
--PQNMWA
--PQNMTA
--PQNMSM
--PQNMRV
--PQNMKA
--PQNMEW
--PQNMEG
PWPQNM--
--PQNMYE
--PQNMVR
--PQNMVK
--PQNMSF
--PQNMLN
--PQNMKW
--PQNMHV

--PQNMHI
--PQNMVS
--PQNMVN
--PQNMQN
--PQNMLW
--PQNMLT
--PQNMLR
--PQNMIV
--PQNMES
--PQNMYF
--PQNMTF
--PQNMSR
--PQNMMW
--PQNMLQ
--PQNMLL
--PQNMLH
--PQNMIS
--PQNMIF
--PQNMFM
PKPQNM--
--PQNMSK
--PQNMRL
--PQNMML
--PQNMLS
--PQNMIT
--PQNMIR
--PQNMIQ
--PQNMIK
--PQNMGS
--PQNMAW
--PQNMYI
--PQNMYG
--PQNMWT
--PQNMWE
--PQNMVL
--PQNMVD
--PQNMTR
--PQNMTL
--PQNMSH
--PQNMRE
--PQNMMR
--PQNMMD
--PQNMMA
--PQNMKV
--PQNMKS
--PQNMKR
--PQNMGW
--PQNMTS
--PQNMTN
--PQNMTK

--PQNMTI
--PQNMSY
--PQNMSW
--PQNMSL
--PQNMLF
--PQNMKQ
--PQNMII
--PQNMHH
--PQNMGG
--PQNMYV
--PQNMYN
--PQNMYK
--PQNMYD
--PQNMWS
--PQNMWD
--PQNMVF
--PQNMSI
--PQNMSD
--PQNMSA
--PQNMQS
--PQNMNT
--PQNMMY
--PQNMMF
--PQNMLM
--PQNMLI
--PQNMKD
--PQNMIY
--PQNMIW
--PQNMID
--PQNMIA
--PQNMHQ
--PQNMHK
--PQNMHG
--PQNMHA
--PQNMGV
--PQNMFS
--PQNMFQ
--PQNMEY
--PQNMAQ
--PQNMAA
PIPQNM--
PTPQNM--
--PQNMWQ
--PQNMVI
--PQNMVG
--PQNMTV
--PQNMTE
--PQNMQM
--PQNMQI
--PQNMNG

--PQNMNF
--PQNMND
--PQNMKM
--PQNMKK
--PQNMHD
--PQNMAL
--PQNMAK
--PQNMAG
PQPQNM--
--PQNMYT
--PQNMYQ
--PQNMWV
--PQNMWG
--PQNMVQ
--PQNMVH
--PQNMVE
--PQNMSQ
--PQNMRW
--PQNMRF
--PQNMRD
--PQNMQW
--PQNMQQ
--PQNMQK
--PQNMQH
--PQNMNV
--PQNMNL
--PQNMNK
--PQNMMT
--PQNMLV
--PQNMHR
--PQNMHL
--PQNMGQ
--PQNMGM
--PQNMGI
--PQNMGH
--PQNMFL
--PQNMFH
--PQNMFG
--PQNMET
--PQNMEL
--PQNMDG
--PQNMAD
-PPQNMF-
PFPQNM--
QRPQNM--
QSPQNM--
QVPQNM--
-QPQNMA-
QGPQNM--
QFPQNM--

-QPQNMS-
-QPQNMH-
QKPQNM--
-QPQNMQ-
QPPQNM--
-QPQNMT-
QLPQNM--
-QPQNMV-
-QPQNMK-
-QPQNMF-
-RPQNMI-
RVPQNM--
-RPQNMR-
-RPQNML-
RQPQNM--
RLPQNM--
-RPQNMN-
-RPQNMM-
RMPQNM--
-RPQNMA-
RWPQNM--
RGPQNM--
-RPQNMV-
RYPQNM--
RSPQNM--
-RPQNMF-
RRPQNM--
-RPQNMW-
-RPQNMT-
-RPQNMS-
-RPQNMK-
RKPQNM--
RAPQNM--
RTPQNM--
REPQNM--
-RPQNMG-
RHPQNM--
-RPQNME-
RPPQNM--
RFPQNM--
-RPQNMQ-
RNPQNM--
-SPQNMY-
-SPQNMT-
-SPQNML-
SRPQNM--
SSPQNM--
-SPQNMF-
-SPQNMS-
SMPQNM--

SQPQNM--
SVPQNM--
-SPQNMI-
-SPQNMG-
-SPQNMR-
-SPQNMQ-
-SPQNMM-
-SPQNMA-
SLPQNM--
-SPQNMK-
-SPQNME-
SEPQNM--
SKPQNM--
SDPQNM--
SIPQNM--
-SPQNMW-
SGPQNM--
SAPQNM--
SYPQNM--
SWPQNM--
-SPQNMN-
SNPQNM--
-TPQNMR-
TSPQNM--
TRPQNM--
-TPQNMS-
-TPQNMM-
TTPQNM--
-TPQNMK-
TQPQNM--
-TPQNMW-
-TPQNML-
-TPQNMY-
-TPQNMF-
-TPQNMA-
TMPQNM--
TAPQNM--
-TPQNMT-
-TPQNMH-
TPPQNM--
TIPQNM--
TGPQNM--
-TPQNMV-
-TPQNMN-
-TPQNMI-
-TPQNMC-
TKPQNM--
THPQNM--
TCPQNM--
-VPQNMR-

```
-VPQNML-
VVPQNM--
VSPQNM--
-VPQNMT-
VGPQNM--
-VPQNMS-
VKPQNM--
VAPQNM--
-VPQNMK-
VRPQNM--
VNPQNM--
VQPQNM--
-VPQNMI-
-VPQNMF-
VPPQNM--
VTPQNM--
-VPQNMY-
-VPQNMH-
VIPQNM--
VYPQNM--
-VPQNMV-
-VPQNMN-
-VPQNMG-
VMPQNM--
VHPQNM--
VCPQNM--
-WPQNMI-
WGPQNM--
-WPQNML-
WEPQNM--
WIPQNM--
-WPQNMS-
WLPQNM--
WMPQNM--
WSPQNM--
-WPQNMW-
-WPQNMT-
WNPQNM--
-WPQNMV-
-WPQNMR-
-WPQNMN-
-WPQNMA-
YRPQNM--
-YPQNML-
YFPQNM--
YYPQNM--
YGPQNM--
YTPQNM--
-YPQNMW-
-YPQNMS-
```

-YPQNMF-
-YPQNMT-
-YPQNMR-
YPPQNM--
-YPQNMN-
-YPQNMK-
-YPQNMA-
YWPQNM--
YVPQNM--
YSPQNM--
-YPQNMV-
-YPQNME-
YMPQNM--
YDPQNM--

PANI 176 760 46.32

-LPANIK-
TTPANI--
-APANIR-
AQPANI--
ASPANI--
AMPANI--
-APANIW-
ANPANI--
ARPANI--
APPANI--
-APANIH-
AVPANI--
-APANIM-
AKPANI--
-APANIK-
AGPANI--
-APANIS-
-APANIN-
-APANIL-
AFPANI--
AAPANI--
ALPANI--
AYPANI--
ATPANI--
-APANIV-
AWPANI--
-APANII-
-APANIQ-
-APANIA-
AEPANI--
-APANIY-
-APANIF-
-APANIE-
AIPANI--
AHPANI--

−APANIP−
ADPANI−−
CSPANI−−
CKPANI−−
CEPANI−−
CYPANI−−
−CPANIR−
CIPANI−−
CVPANI−−
−CPANIV−
−CPANIS−
−CPANIQ−
−CPANIL−
−CPANIF−
CAPANI−−
DRPANI−−
−DPANIR−
−DPANII−
DLPANI−−
−DPANIV−
DTPANI−−
DSPANI−−
DYPANI−−
DGPANI−−
DEPANI−−
DVPANI−−
DAPANI−−
DNPANI−−
DMPANI−−
DKPANI−−
DWPANI−−
DQPANI−−
−DPANIT−
−DPANIL−
−EPANIR−
−EPANIV−
−EPANIM−
−EPANIW−
−EPANIL−
−EPANII−
−EPANIY−
EKPANI−−
EWPANI−−
EPPANI−−
−EPANIE−
ETPANI−−
ESPANI−−
EIPANI−−
EFPANI−−
EVPANI−−

EGPANI--
EEPANI--
-EPANIS-
-EPANIK-
ENPANI--
EMPANI--
EDPANI--
-FPANIR-
FNPANI--
FAPANI--
FMPANI--
FKPANI--
FGPANI--
-FPANIM-
FWPANI--
-FPANIL-
-FPANII-
FSPANI--
FHPANI--
FRPANI--
-FPANIV-
FQPANI--
FIPANI--
FFPANI--
-FPANIQ-
-FPANIT-
-FPANIH-
-FPANIA-
-FPANIS-
-FPANIN-
FLPANI--
FDPANI--
GTPANI--
-GPANIL-
-GPANIM-
GYPANI--
GMPANI--
GGPANI--
GQPANI--
-GPANIW-
-GPANIS-
-GPANIR-
GHPANI--
-GPANIY-
-GPANIT-
GAPANI--
GRPANI--
-GPANIH-
GLPANI--
GFPANI--

GVPANI--
GKPANI--
GIPANI--
GSPANI--
-GPANIG-
-GPANIF-
-GPANIV-
-GPANIK-
GEPANI--
GWPANI--
-GPANIQ-
-GPANIP-
-GPANID-
-GPANIA-
HRPANI--
-HPANIM-
HVPANI--
HHPANI--
HAPANI--
-HPANIR-
HYPANI--
HLPANI--
HKPANI--
HSPANI--
-HPANIL-
HGPANI--
-HPANIS-
HFPANI--
HIPANI--
-HPANIW-
HTPANI--
-HPANIE-
-HPANIY-
-HPANIH-
-HPANIF-
HMPANI--
ISPANI--
IVPANI--
IRPANI--
-IPANIL-
-IPANIR-
-IPANIY-
-IPANIV-
IGPANI--
IQPANI--
-IPANIS-
IFPANI--
-IPANIQ-
-IPANIG-
ILPANI--

IKPANI--
IHPANI--
IAPANI--
-IPANIM-
INPANI--
-IPANIT-
IYPANI--
-IPANIN-
IIPANI--
IEPANI--
-IPANIE-
IDPANI--
KSPANI--
KAPANI--
KRPANI--
-KPANIL-
-KPANIQ-
KPPANI--
-KPANIK-
KNPANI--
-KPANIV-
-KPANIF-
KQPANI--
-KPANII-
KYPANI--
-KPANIY-
KLPANI--
-KPANIW-
KTPANI--
-KPANIM-
KWPANI--
-KPANIS-
-KPANIE-
KKPANI--
KHPANI--
KCPANI--
KVPANI--
-KPANIH-
-KPANIA-
KIPANI--
KFPANI--
KEPANI--
KDPANI--
LKPANI--
-LPANIR-
LGPANI--
LRPANI--
-LPANIV-
LMPANI--
LLPANI--

LWPANI--
-LPANIM-
LTPANI--
LPPANI--
LNPANI--
LVPANI--
LQPANI--
-LPANIT-
-LPANIS-
LSPANI--
-LPANIL-
-LPANIF-
LFPANI--
-LPANIQ-
-LPANIA-
LEPANI--
LYPANI--
-LPANIY-
-LPANIN-
LIPANI--
-LPANIH-
LHPANI--
LDPANI--
MLPANI--
-MPANIW-
MMPANI--
MVPANI--
MNPANI--
MPPANI--
-MPANIL-
MYPANI--
MWPANI--
MSPANI--
-MPANIT-
-MPANIR-
-MPANIA-
-MPANIY-
-MPANIM-
MTPANI--
-MPANIQ-
-MPANIF-
MKPANI--
MHPANI--
MEPANI--
-MPANII-
-MPANIH-
-MPANIG-
MQPANI--
-MPANIV-
-MPANIS-

MGPANI--
MFPANI--
NLPANI--
NRPANI--
NKPANI--
-NPANIT-
-NPANIL-
NMPANI--
-NPANIR-
NSPANI--
NVPANI--
NAPANI--
-NPANIM-
NGPANI--
NQPANI--
-NPANII-
NWPANI--
-NPANIV-
-NPANIS-
NNPANI--
NIPANI--
NFPANI--
NEPANI--
-NPANIW-
-NPANIA-
NTPANI--
NPPANI--
-NPANIY-
-NPANIH-
--PANIRV
--PANIRR
--PANIST
--PANIYK
--PANIRL
--PANIAS
--PANIRI
--PANILS
--PANIVR
--PANIRA
--PANIHS
--PANIMS
--PANILR
--PANIFV
--PANIRS
--PANIRG
--PANILG
--PANIWV
--PANIVG
--PANISS
--PANIMR

--PANISG
--PANIRY
--PANIRM
--PANIAG
--PANIVK
--PANISA
--PANIYH
--PANIWS
PFPANI--
PAPANI--
--PANIYV
--PANIVT
--PANISF
--PANIHA
--PANIFH
PQPANI--
--PANIWM
--PANIIR
--PANIFS
--PANISN
--PANIYL
--PANIWN
--PANIWK
--PANILH
--PANIFA
--PANIYF
--PANIML
--PANIKS
--PANIHT
--PANIHG
--PANIFG
PRPANI--
--PANIYT
--PANIVV
--PANISQ
--PANIQS
--PANIMH
--PANILM
--PANIFW
--PANIYS
--PANIYA
--PANIVW
--PANITR
--PANITK
--PANISW
--PANISI
--PANIRH
--PANIQF
--PANILY
--PANIIG

--PANIHR
--PANIHH
--PANIYW
--PANIWD
--PANIVQ
--PANIRD
--PANINS
--PANIMM
--PANIMG
--PANILK
--PANIKW
--PANIKK
--PANIGG
--PANIFN
--PANIWR
--PANITM
--PANISV
--PANIRN
--PANIQY
--PANIQH
--PANIQG
--PANINA
--PANIMD
--PANIIY
--PANIHY
--PANIHI
--PANIEG
PWPANI--
--PANIVS
--PANITS
--PANITL
--PANITH
--PANIRF
--PANIQV
--PANINF
--PANIMT
--PANIME
--PANILI
--PANIFY
--PANIFR
--PANIFM
--PANIEY
--PANIAA
PKPANI--
PGPANI--
--PANIWE
--PANITQ
--PANITI
--PANISR
--PANISK

--PANIRW
--PANIQA
--PANIMK
--PANIMA
--PANILL
--PANILA
--PANIIH
--PANIGV
--PANIGS
--PANIGA
--PANIFQ
--PANIEV
PMPANI--
--PANIYY
--PANIVL
--PANIVI
--PANITW
--PANIRE
--PANIQK
--PANILT
--PANILQ
--PANIKY
--PANIKA
--PANIIE
--PANIIA
--PANIFT
--PANIES
--PANIAW
PNPANI--
PIPANI--
PHPANI--
PEPANI--
--PANIYN
--PANIWT
--PANIWH
--PANIVM
--PANIVF
--PANIVD
--PANITF
--PANIQN
--PANING
--PANIMV
--PANILV
--PANILE
--PANIKT
--PANIKH
--PANIIT
--PANIHL
--PANIGT
--PANIGM

--PANIER
--PANIEK
--PANIAV
--PANIAF
PVPANI--
PSPANI--
PLPANI--
--PANIYG
--PANIYD
--PANIWY
--PANIWA
--PANITN
--PANITG
--PANITE
--PANISM
--PANISE
--PANIRK
--PANIQT
--PANIQQ
--PANIQI
--PANINT
--PANILF
--PANIKL
--PANIKG
--PANIIF
--PANIHV
--PANIGW
--PANIAM
-PPANIY-
-PPANIR-
-PPANIL-
--PANIYE
--PANIWF
--PANIVE
--PANIVA
--PANITY
--PANITD
--PANITA
--PANISY
--PANIQD
--PANIPT
--PANIPR
--PANINK
--PANINI
--PANIMY
--PANILW
--PANIKR
--PANIKF
--PANIKD
--PANIIV

--PANIIQ
--PANIGY
--PANIFP
--PANIET
--PANIEN
--PANIEI
--PANIEH
--PANIED
--PANICV
--PANICS
--PANIAY
--PANIAT
--PANIAL
--PANIAI
-QPANII-
QGPANI--
QRPANI--
QLPANI--
QQPANI--
-QPANIY-
-QPANIM-
-QPANIR-
-QPANIS-
QSPANI--
-QPANIW-
-QPANIK-
QIPANI--
QKPANI--
QPPANI--
QVPANI--
QTPANI--
-QPANIL-
-QPANIV-
-QPANIA-
QHPANI--
QYPANI--
QFPANI--
QEPANI--
QWPANI--
-QPANIN-
RSPANI--
-RPANIY-
RVPANI--
-RPANIS-
-RPANIL-
RGPANI--
RKPANI--
-RPANIA-
RRPANI--
RMPANI--

-RPANIV-
-RPANIR-
RFPANI--
-RPANII-
-RPANIM-
RLPANI--
-RPANIT-
RHPANI--
RWPANI--
RTPANI--
-RPANIN-
RQPANI--
RIPANI--
REPANI--
RYPANI--
RNPANI--
RAPANI--
-RPANIH-
RPPANI--
-RPANIK-
-RPANIE-
-RPANIQ-
-RPANIF-
RDPANI--
-RPANIW-
RCPANI--
-SPANIR-
SQPANI--
SRPANI--
-SPANIS-
SKPANI--
-SPANIT-
SGPANI--
SLPANI--
SVPANI--
SNPANI--
SAPANI--
SYPANI--
-SPANIV-
STPANI--
SMPANI--
-SPANII-
-SPANIL-
-SPANIY-
-SPANIF-
-SPANIW-
SSPANI--
SIPANI--
SHPANI--
SPPANI--

−SPANIQ−
−SPANIK−
SWPANI−−
−SPANIN−
−SPANIH−
−SPANIE−
SEPANI−−
SCPANI−−
−SPANIM−
SDPANI−−
TRPANI−−
TGPANI−−
−TPANIL−
TVPANI−−
TWPANI−−
−TPANIW−
TPPANI−−
−TPANIT−
−TPANIV−
−TPANII−
TYPANI−−
−TPANIF−
−TPANIK−
−TPANIY−
−TPANIR−
TLPANI−−
−TPANIQ−
−TPANIH−
TMPANI−−
TKPANI−−
TFPANI−−
−TPANIA−
THPANI−−
TEPANI−−
TCPANI−−
−TPANIM−
−TPANIG−
−TPANIE−
TIPANI−−
VGPANI−−
VRPANI−−
−VPANIL−
VQPANI−−
VTPANI−−
−VPANIY−
−VPANIS−
−VPANIT−
VLPANI−−
VSPANI−−
−VPANIV−

VIPANI--
VFPANI--
VVPANI--
VPPANI--
VDPANI--
VNPANI--
-VPANIK-
-VPANIN-
-VPANIH-
-VPANIA-
VEPANI--
-VPANII-
VHPANI--
-VPANIG-
-VPANIF-
VMPANI--
VKPANI--
-VPANIW-
VAPANI--
WKPANI--
-WPANIR-
-WPANIL-
-WPANIT-
WYPANI--
WTPANI--
WEPANI--
WMPANI--
-WPANIQ-
-WPANIY-
-WPANIS-
WSPANI--
WHPANI--
-WPANIV-
WLPANI--
WGPANI--
WRPANI--
-WPANIH-
WQPANI--
WPPANI--
WIPANI--
WWPANI--
-WPANIM-
-WPANIK-
-WPANII-
WNPANI--
WDPANI--
YKPANI--
YRPANI--
YAPANI--
YTPANI--

-YPANIR-
YWPANI--
YLPANI--
YQPANI--
YSPANI--
-YPANIT-
-YPANIS-
-YPANIM-
-YPANIQ-
YVPANI--
-YPANIF-
-YPANIV-
-YPANIH-
YIPANI--
YHPANI--
YFPANI--
-YPANII-
YEPANI--
YMPANI--
YDPANI--
YYPANI--
YGPANI--
YCPANI--
YPPANI--
-YPANIW-
PMEL 283 706 46.31
-APMELS-
-APMELR-
AMPMEL--
-APMELV-
-APMELM-
AAPMEL--
ALPMEL--
ATPMEL--
ARPMEL--
-APMELT-
ANPMEL--
AWPMEL--
AHPMEL--
-APMELK-
-APMELH-
-APMELA-
AIPMEL--
AEPMEL--
ACPMEL--
AYPMEL--
-APMELL-
-APMELQ-
APPMEL--
-APMELG-

AGPMEL--
AFPMEL--
-APMELY-
-APMELI-
ADPMEL--
AVPMEL--
ASPMEL--
AKPMEL--
CGPMEL--
CPPMEL--
CMPMEL--
CSPMEL--
CCPMEL--
CYPMEL--
CVPMEL--
-CPMELF-
CAPMEL--
CTPMEL--
CLPMEL--
CHPMEL--
-DPMELR-
DVPMEL--
DRPMEL--
DSPMEL--
DTPMEL--
DNPMEL--
DLPMEL--
-DPMELT-
DMPMEL--
DKPMEL--
DIPMEL--
DHPMEL--
-DPMELV-
DEPMEL--
DAPMEL--
-DPMELS-
-DPMELI-
DQPMEL--
EVPMEL--
-EPMELR-
ESPMEL--
-EPMELL-
ELPMEL--
-EPMELT-
EWPMEL--
ETPMEL--
EYPMEL--
-EPMELM-
-EPMELW-
-EPMELV-

-EPMELQ-
-EPMELH-
-EPMELG-
EEPMEL--
-EPMELS-
-EPMELI-
-EPMELA-
EGPMEL--
ERPMEL--
ENPMEL--
EKPMEL--
-EPMELK-
-EPMELF-
EDPMEL--
-FPMELR-
-FPMELN-
FVPMEL--
FSPMEL--
FPPMEL--
-FPMELK-
-FPMELS-
FKPMEL--
-FPMELT-
-FPMELH-
FNPMEL--
FLPMEL--
FGPMEL--
FQPMEL--
FAPMEL--
-FPMELW-
-FPMELA-
FWPMEL--
FTPMEL--
-FPMELV-
-FPMELE-
-FPMELD-
-GPMELR-
-GPMELV-
GIPMEL--
GWPMEL--
GLPMEL--
-GPMELH-
GGPMEL--
GTPMEL--
GRPMEL--
GVPMEL--
-GPMELL-
GSPMEL--
GAPMEL--
-GPMELN-

-GPMELM-
GHPMEL--
GPPMEL--
GYPMEL--
GNPMEL--
-GPMELT-
-GPMELG-
GMPMEL--
GQPMEL--
-GPMELK-
GFPMEL--
GEPMEL--
-GPMELQ-
-GPMELI-
GKPMEL--
GDPMEL--
-GPMELE-
-GPMELC-
-GPMELA-
GCPMEL--
HLPMEL--
HTPMEL--
HRPMEL--
HAPMEL--
HIPMEL--
HVPMEL--
-HPMELY-
HGPMEL--
-HPMELL-
-HPMELK-
-HPMELR-
-HPMELH-
-HPMELS-
-HPMELT-
-HPMELA-
-HPMELW-
HMPMEL--
HKPMEL--
-HPMELI-
-HPMELF-
HHPMEL--
HFPMEL--
HEPMEL--
HNPMEL--
-HPMELN-
HSPMEL--
HQPMEL--
-HPMELP-
HCPMEL--
-IPMELR-

IRPMEL--
IIPMEL--
IHPMEL--
ILPMEL--
-IPMELH-
IKPMEL--
-IPMELN-
IQPMEL--
IVPMEL--
-IPMELL-
IGPMEL--
-IPMELQ-
ISPMEL--
IPPMEL--
-IPMELS-
-IPMELK-
-IPMELG-
IFPMEL--
IAPMEL--
ITPMEL--
-IPMELT-
IMPMEL--
IEPMEL--
INPMEL--
-IPMELA-
IDPMEL--
KSPMEL--
KRPMEL--
KAPMEL--
-KPMELR-
-KPMELH-
-KPMELS-
KHPMEL--
-KPMELF-
KTPMEL--
-KPMELT-
-KPMELL-
KFPMEL--
KVPMEL--
-KPMELI-
-KPMELA-
KQPMEL--
-KPMELV-
KMPMEL--
-KPMELN-
KKPMEL--
-KPMELG-
KIPMEL--
KYPMEL--
-KPMELY-

-KPMELQ-
-KPMELK-
KLPMEL--
KGPMEL--
-KPMELM-
-LPMELR-
LRPMEL--
LGPMEL--
LSPMEL--
LIPMEL--
LAPMEL--
LQPMEL--
LHPMEL--
LKPMEL--
-LPMELK-
LTPMEL--
LEPMEL--
-LPMELV-
-LPMELN-
LNPMEL--
-LPMELI-
-LPMELA-
LMPMEL--
LYPMEL--
LDPMEL--
LFPMEL--
-LPMELS-
-LPMELM-
-LPMELH-
-LPMELT-
-LPMELG-
-MPMELS-
-MPMELR-
MYPMEL--
MVPMEL--
MRPMEL--
MTPMEL--
MGPMEL--
MAPMEL--
MHPMEL--
MWPMEL--
-MPMELT-
-MPMELN-
MMPMEL--
MQPMEL--
MIPMEL--
MLPMEL--
-MPMELK-
MKPMEL--
MPPMEL--

MSPMEL--
-MPMELL-
-MPMELI-
-MPMELH-
-MPMELG-
-MPMELF-
MFPMEL--
-MPMELA-
MNPMEL--
-MPMELV-
MDPMEL--
-NPMELR-
NRPMEL--
-NPMELN-
NLPMEL--
NTPMEL--
NSPMEL--
NGPMEL--
NMPMEL--
NHPMEL--
NAPMEL--
NPPMEL--
-NPMELW-
NNPMEL--
NKPMEL--
NWPMEL--
NIPMEL--
-NPMELH-
NYPMEL--
-NPMELK-
NVPMEL--
NFPMEL--
NQPMEL--
-NPMELI-
NEPMEL--
NDPMEL--
-NPMELM-
-NPMELL-
-NPMELQ-
-NPMELG-
--PMELRS
--PMELRR
--PMELTV
--PMELKL
--PMELTS
--PMELRA
--PMELHS
--PMELRV
--PMELGS
--PMELRH

--PMELLS
--PMELQR
--PMELRT
--PMELLG
--PMELSA
--PMELRG
--PMELKS
--PMELHM
--PMELWS
--PMELVK
--PMELKA
--PMELRM
--PMELRI
--PMELYG
--PMELSS
--PMELFN
--PMELKN
--PMELAS
--PMELTA
--PMELNF
--PMELMW
--PMELKG
--PMELFG
--PMELRW
--PMELQS
--PMELNK
--PMELLH
--PMELVT
--PMELSV
--PMELQA
--PMELMT
--PMELWD
--PMELTN
--PMELTG
--PMELSR
--PMELLK
--PMELIG
--PMELYR
--PMELWR
--PMELTY
--PMELSG
--PMELRY
--PMELNV
--PMELNL
--PMELAG
--PMELAA
PEPMEL--
--PMELVG
--PMELSY
--PMELSN

--PMELQT
--PMELMS
--PMELMN
--PMELLD
--PMELKT
--PMELKK
--PMELFH
--PMELSM
--PMELIR
--PMELGA
PWPMEL--
PTPMEL--
--PMELRQ
--PMELKI
--PMELFY
PAPMEL--
--PMELSK
--PMELKW
--PMELHY
--PMELGW
--PMELGG
--PMELWG
--PMELRK
--PMELMG
--PMELKY
--PMELKH
--PMELKE
--PMELID
--PMELHV
--PMELAT
PYPMEL--
--PMELYD
--PMELWN
--PMELVA
--PMELTM
--PMELRF
--PMELQQ
--PMELNN
--PMELND
--PMELMH
--PMELLR
--PMELLQ
--PMELHA
--PMELVE
--PMELVD
--PMELTR
--PMELTK
--PMELRE
--PMELNH
--PMELHR

--PMELHL
--PMELGT
--PMELEG
PNPMEL--
--PMELWW
--PMELST
--PMELSL
--PMELSI
--PMELQW
--PMELQK
--PMELMY
--PMELLN
--PMELHN
PQPMEL--
--PMELVH
--PMELTQ
--PMELTL
--PMELTH
--PMELQF
--PMELMR
--PMELME
--PMELLE
--PMELKF
--PMELIN
--PMELIK
PIPMEL--
PHPMEL--
PGPMEL--
PMPMEL--
--PMELYN
--PMELWQ
--PMELVQ
--PMELTW
--PMELSW
--PMELSF
--PMELQL
--PMELNR
--PMELNE
--PMELIH
--PMELHT
--PMELGV
--PMELGL
--PMELGF
--PMELFD
--PMELAK
--PMELYS
--PMELWH
--PMELVN
--PMELTI
--PMELSQ

--PMELQD
--PMELNT
--PMELNG
--PMELNA
--PMELMM
--PMELMK
--PMELHH
--PMELFF
--PMELES
--PMELEF
--PMELCY
--PMELAM
--PMELAL
-PPMELS-
--PMELYK
--PMELYH
--PMELWK
--PMELVC
--PMELQV
--PMELQN
--PMELQG
--PMELMD
--PMELKV
--PMELKM
--PMELHE
--PMELEY
--PMELDS
--PMELCT
--PMELCS
--PMELCM
--PMELCL
--PMELAV
--PMELAR
--PMELAN
--PMELAI
PKPMEL--
QRPMEL--
QGPMEL--
QAPMEL--
QWPMEL--
-QPMELR-
-QPMELS-
QSPMEL--
QNPMEL--
-QPMELA-
QVPMEL--
-QPMELG-
-QPMELH-
QMPMEL--
QIPMEL--

QEPMEL--
-QPMELL-
QLPMEL--
QPPMEL--
-QPMELK-
QTPMEL--
-QPMELQ-
QKPMEL--
QYPMEL--
-QPMELN-
-QPMELM-
QFPMEL--
RRPMEL--
RLPMEL--
-RPMELV-
RVPMEL--
-RPMELQ-
RIPMEL--
RSPMEL--
-RPMELS-
-RPMELL-
REPMEL--
-RPMELH-
-RPMELT-
-RPMELK-
RNPMEL--
RYPMEL--
RTPMEL--
RPPMEL--
RMPMEL--
RWPMEL--
-RPMELM-
-RPMELG-
-RPMELI-
-RPMELN-
-RPMELA-
RFPMEL--
-RPMELC-
RHPMEL--
SRPMEL--
-SPMELR-
-SPMELL-
STPMEL--
SYPMEL--
SVPMEL--
SKPMEL--
-SPMELI-
-SPMELK-
SLPMEL--
-SPMELV-

-SPMELN-
-SPMELT-
SHPMEL--
SPPMEL--
SAPMEL--
SSPMEL--
-SPMELA-
-SPMELS-
-SPMELM-
-SPMELF-
SMPMEL--
SWPMEL--
SNPMEL--
SFPMEL--
-SPMELH-
SEPMEL--
-SPMELQ-
SIPMEL--
-TPMELR-
TRPMEL--
TVPMEL--
TPPMEL--
-TPMELI-
-TPMELL-
-TPMELS-
-TPMELQ-
TLPMEL--
TSPMEL--
-TPMELV-
-TPMELF-
TGPMEL--
TTPMEL--
-TPMELM-
-TPMELH-
TQPMEL--
TMPMEL--
-TPMELN-
TWPMEL--
TYPMEL--
TKPMEL--
-TPMELK-
-TPMELG-
-TPMELT-
THPMEL--
TAPMEL--
TFPMEL--
TEPMEL--
TIPMEL--
-TPMELC-
TNPMEL--

-TPMELA-
TCPMEL--
VNPMEL--
VSPMEL--
-VPMELK-
-VPMELL-
-VPMELN-
-VPMELQ-
VVPMEL--
-VPMELH-
-VPMELT-
-VPMELA-
-VPMELV-
-VPMELS-
VLPMEL--
VGPMEL--
-VPMELI-
VKPMEL--
-VPMELG-
VMPMEL--
VAPMEL--
VPPMEL--
VIPMEL--
VFPMEL--
VQPMEL--
VYPMEL--
-VPMELM-
VHPMEL--
-VPMELE-
VCPMEL--
VWPMEL--
-VPMELD-
WSPMEL--
WLPMEL--
WKPMEL--
WNPMEL--
WVPMEL--
WGPMEL--
WTPMEL--
WYPMEL--
WRPMEL--
-WPMELT-
-WPMELQ-
WHPMEL--
-WPMELS-
-WPMELH-
-WPMELG-
WDPMEL--
-WPMELN-
WQPMEL--

-WPMELM-
-WPMELK-
-WPMELD-
-WPMELA-
YGPMEL--
YPPMEL--
YLPMEL--
-YPMELT-
YWPMEL--
YTPMEL--
-YPMELN-
-YPMELL-
YFPMEL--
YSPMEL--
YEPMEL--
-YPMELV-
YRPMEL--
YKPMEL--
YVPMEL--
YYPMEL--
-YPMELR-
-YPMELH-
-YPMELS-
YAPMEL--
-YPMELK-
-YPMELI-
-YPMELA-
-YPMELG-
-YPMELW-
YCPMEL--
YQPMEL--
-YPMELM-

PASL 51 850 46.30

-DPASLA-
-GPASLC-
-KPASLC-
-LPASLA-
-LPASLP-
-LPASLE-
-MPASLG-
--PASLNR
--PASLPS
--PASLSP
--PASLPT
--PASLPM
--PASLNA
--PASLGN
--PASLER
--PASLDL
--PASLAD

-SPASLE-
-SPASLD-
-TPASLG-
-TPASLE-
-YPASLP-
-APASLR-
-APASLL-
AIPASL--
ATPASL--
ALPASL--
ASPASL--
-APASLS-
ARPASL--
AVPASL--
AKPASL--
AWPASL--
-APASLV-
-APASLK-
-APASLF-
-APASLW-
AGPASL--
ANPASL--
AYPASL--
-APASLM-
APPASL--
-APASLI-
AMPASL--
AQPASL--
-APASLH-
AHPASL--
-APASLQ-
AAPASL--
-APASLG-
-APASLA-
AEPASL--
-APASLE-
-APASLC-
CRPASL--
CKPASL--
CTPASL--
-CPASLR-
CHPASL--
CSPASL--
CAPASL--
CEPASL--
CWPASL--
-CPASLK-
CIPASL--
-CPASLV-
CNPASL--

CCPASL--
CQPASL--
-CPASLS-
-CPASLM-
-CPASLL-
CFPASL--
CVPASL--
CPPASL--
-CPASLT-
-CPASLF-
-CPASLY-
DRPASL--
DVPASL--
DYPASL--
DKPASL--
DIPASL--
DTPASL--
DSPASL--
-DPASLR-
DLPASL--
DNPASL--
DQPASL--
-DPASLW-
DFPASL--
DGPASL--
-DPASLV-
DMPASL--
DHPASL--
-DPASLH-
-DPASLM-
-DPASLL-
-DPASLI-
DEPASL--
DWPASL--
-DPASLT-
-DPASLK-
-DPASLY-
-DPASLF-
DAPASL--
DPPASL--
-DPASLS-
DCPASL--
-DPASLQ-
-DPASLN-
DDPASL--
-EPASLK-
-EPASLL-
-EPASLR-
ELPASL--
EKPASL--

EGPASL--
ESPASL--
-EPASLH-
ERPASL--
EIPASL--
EHPASL--
-EPASLV-
EQPASL--
-EPASLS-
EYPASL--
EVPASL--
-EPASLF-
EAPASL--
ETPASL--
-EPASLW-
EMPASL--
-EPASLY-
-EPASLQ-
-EPASLA-
EEPASL--
EFPASL--
-EPASLN-
ENPASL--
-EPASLG-
-EPASLE-
EWPASL--
EPPASL--
-EPASLM-
EDPASL--
ECPASL--
FAPASL--
FSPASL--
-FPASLR-
FQPASL--
FYPASL--
FPPASL--
FMPASL--
FGPASL--
FWPASL--
-FPASLV-
-FPASLL-
FHPASL--
FEPASL--
-FPASLY-
-FPASLN-
-FPASLT-
-FPASLH-
FKPASL--
-FPASLK-
-FPASLW-

-FPASLS-
-FPASLQ-
-FPASLM-
FDPASL--
-FPASLF-
-FPASLE-
FCPASL--
-FPASLC-
-GPASLR-
GSPASL--
-GPASLK-
GRPASL--
GVPASL--
-GPASLL-
GMPASL--
-GPASLI-
-GPASLH-
-GPASLW-
GTPASL--
-GPASLS-
GEPASL--
GYPASL--
GFPASL--
GPPASL--
GAPASL--
GHPASL--
-GPASLV-
-GPASLY-
GQPASL--
GIPASL--
GDPASL--
-GPASLN-
-GPASLE-
GCPASL--
HLPASL--
HRPASL--
HGPASL--
HFPASL--
HVPASL--
HHPASL--
-HPASLY-
-HPASLL-
HAPASL--
-HPASLM-
-HPASLH-
-HPASLV-
HIPASL--
HYPASL--
HTPASL--
HPPASL--

HWPASL--
-HPASLQ-
-HPASLF-
HNPASL--
HEPASL--
-HPASLI-
-HPASLK-
HDPASL--
-HPASLN-
HQPASL--
-HPASLE-
-HPASLA-
-HPASLC-
-HPASLD-
IRPASL--
-IPASLR-
IGPASL--
ILPASL--
IVPASL--
-IPASLL-
IYPASL--
-IPASLT-
-IPASLS-
ISPASL--
IFPASL--
-IPASLV-
-IPASLY-
-IPASLK-
INPASL--
IMPASL--
IAPASL--
IIPASL--
-IPASLH-
-IPASLM-
IEPASL--
IPPASL--
IKPASL--
-IPASLN-
-IPASLF-
IHPASL--
IWPASL--
-IPASLW-
-IPASLQ-
ICPASL--
IQPASL--
-IPASLG-
-IPASLE-
IDPASL--
-IPASLC-
KSPASL--

KGPASL--
-KPASLW-
-KPASLS-
KLPASL--
-KPASLF-
KPPASL--
-KPASLT-
KAPASL--
KMPASL--
-KPASLM-
KFPASL--
-KPASLI-
KYPASL--
-KPASLV-
-KPASLY-
KIPASL--
KNPASL--
-KPASLK-
KKPASL--
KEPASL--
-KPASLA-
-KPASLQ-
KQPASL--
KWPASL--
KDPASL--
KCPASL--
-KPASLE-
KHPASL--
-KPASLG-
LSPASL--
LLPASL--
LNPASL--
LTPASL--
-LPASLI-
-LPASLK-
LQPASL--
LYPASL--
LMPASL--
LEPASL--
LWPASL--
-LPASLM-
-LPASLS-
LFPASL--
-LPASLH-
-LPASLV-
LHPASL--
LDPASL--
-LPASLY-
-LPASLF-
-LPASLW-

–LPASLN–
LCPASL––
MRPASL––
MTPASL––
MVPASL––
MSPASL––
–MPASLR–
–MPASLL–
MGPASL––
–MPASLS–
MPPASL––
MMPASL––
–MPASLY–
–MPASLK–
MEPASL––
–MPASLW–
–MPASLF–
MNPASL––
–MPASLI–
MAPASL––
MYPASL––
–MPASLQ–
MFPASL––
–MPASLV–
MHPASL––
–MPASLT–
–MPASLH–
–MPASLN–
–MPASLM–
MIPASL––
MDPASL––
–MPASLA–
–MPASLE–
NRPASL––
–NPASLR–
NAPASL––
NSPASL––
NGPASL––
NTPASL––
NIPASL––
NMPASL––
NFPASL––
NQPASL––
NHPASL––
–NPASLH–
–NPASLM–
–NPASLK–
NPPASL––
NWPASL––
–NPASLW–

NEPASL--
NYPASL--
NKPASL--
-NPASLI-
-NPASLT-
-NPASLV-
NDPASL--
-NPASLS-
-NPASLF-
-NPASLQ-
-NPASLA-
NNPASL--
-NPASLN-
NCPASL--
--PASLRS
--PASLRR
--PASLLS
--PASLLT
--PASLSS
--PASLLR
--PASLVR
--PASLSG
--PASLLG
PTPASL--
--PASLWA
--PASLRA
--PASLRT
--PASLIS
--PASLAG
--PASLYS
--PASLFR
--PASLRN
--PASLFT
--PASLHS
--PASLLV
--PASLIG
--PASLVF
--PASLRK
--PASLKT
--PASLMS
--PASLTT
--PASLNS
--PASLLN
--PASLIY
--PASLRV
--PASLRH
PRPASL--
PSPASL--
--PASLTH
--PASLTG

--PASLSA
--PASLAS
--PASLKG
--PASLST
--PASLRY
--PASLFH
--PASLHW
PFPASL--
--PASLQG
PKPASL--
--PASLWR
--PASLTW
--PASLFG
--PASLWG
--PASLVL
--PASLRQ
--PASLMI
--PASLIT
--PASLHR
--PASLWS
--PASLKY
--PASLHY
PLPASL--
--PASLVT
--PASLMR
--PASLGS
--PASLYW
--PASLIA
--PASLHI
--PASLYD
--PASLQS
--PASLYV
--PASLWT
--PASLTN
--PASLLH
--PASLRD
--PASLMH
--PASLTV
--PASLMG
--PASLEA
--PASLKR
--PASLKI
--PASLRM
--PASLLQ
--PASLFS
PAPASL--
PIPASL--
--PASLYL
--PASLRW
--PASLHV

--PASLSY
--PASLSV
--PASLNL
--PASLYQ
--PASLTI
--PASLTM
--PASLES
--PASLVH
--PASLTY
--PASLSQ
--PASLRE
--PASLFV
--PASLFN
--PASLWQ
--PASLTQ
--PASLKN
--PASLIQ
--PASLIL
--PASLWV
--PASLMN
PYPASL--
--PASLQR
--PASLMY
PGPASL--
--PASLYK
--PASLWW
--PASLSM
--PASLKL
--PASLYY
--PASLYI
--PASLHF
--PASLQA
--PASLME
--PASLMA
--PASLFK
--PASLWK
--PASLTK
--PASLNG
--PASLIN
--PASLIM
--PASLFA
--PASLWH
--PASLVN
--PASLVM
--PASLTL
--PASLSI
--PASLQN
--PASLYH
--PASLWL
--PASLSK

--PASLLD
--PASLHQ
PMPASL--
--PASLYN
--PASLYE
--PASLQT
--PASLKM
--PASLKA
--PASLAN
--PASLQY
--PASLHN
--PASLHL
--PASLAW
--PASLMW
--PASLKH
--PASLAT
--PASLAI
--PASLVI
--PASLGL
PNPASL--
--PASLYA
--PASLVK
--PASLVE
--PASLQM
--PASLIW
--PASLHD
--PASLHA
PWPASL--
--PASLTA
--PASLNF
--PASLHT
--PASLHM
--PASLEW
--PASLAF
--PASLWE
--PASLNT
PQPASL--
PEPASL--
--PASLYG
--PASLNH
--PASLMV
--PASLMK
--PASLFI
PDPASL--
--PASLWI
--PASLQV
--PASLNY
--PASLNM
--PASLMQ
--PASLKV

--PASLKF
--PASLHH
--PASLGY
PCPASL--
--PASLTF
--PASLQW
--PASLQL
--PASLMD
--PASLWN
--PASLWD
--PASLVD
--PASLQK
--PASLNI
--PASLLE
--PASLHE
--PASLGT
--PASLAR
--PASLQI
--PASLNK
--PASLIE
--PASLGV
--PASLET
--PASLMM
--PASLEV
--PASLEG
--PASLAM
--PASLNW
--PASLID
--PASLFQ
--PASLTD
--PASLQF
--PASLKE
--PASLKD
--PASLFW
--PASLFE
--PASLEQ
--PASLEN
--PASLEM
--PASLNN
--PASLNE
--PASLKQ
--PASLGW
--PASLGM
--PASLGA
--PASLAQ
--PASLWM
--PASLQQ
--PASLPL
--PASLNQ
--PASLML

--PASLHK
--PASLGI
--PASLFD
--PASLCA
--PASLAV
--PASLAH
--PASLQH
PHPASL--
--PASLSD
--PASLMF
--PASLEY
--PASLCG
--PASLTP
--PASLKW
--PASLDM
--PASLCS
--PASLCN
--PASLAK
PPPASL--
-PPASLS-
-PPASLR-
-PPASLL-
-PPASLI-
--PASLTE
--PASLQD
--PASLHP
--PASLEL
--PASLEH
--PASLDW
--PASLCV
--PASLCR
--PASLCQ
--PASLCK
--PASLCH
--PASLCF
-QPASLT-
-QPASLL-
-QPASLR-
QFPASL--
QVPASL--
-QPASLM-
-QPASLH-
QTPASL--
-QPASLF-
QYPASL--
QSPASL--
-QPASLK-
-QPASLI-
-QPASLW-
-QPASLV-

-QPASLN-
QIPASL--
QAPASL--
QQPASL--
QPPASL--
QMPASL--
QKPASL--
QWPASL--
-QPASLY-
QEPASL--
-QPASLQ-
-QPASLA-
QNPASL--
-QPASLE-
QHPASL--
QCPASL--
-QPASLC-
QDPASL--
RVPASL--
RSPASL--
-RPASLS-
RIPASL--
RLPASL--
RYPASL--
RGPASL--
RFPASL--
RAPASL--
RWPASL--
-RPASLK-
-RPASLY-
-RPASLM-
-RPASLF-
RNPASL--
-RPASLT-
-RPASLQ-
RPPASL--
-RPASLH-
-RPASLI-
RTPASL--
-RPASLA-
RHPASL--
-RPASLE-
RCPASL--
-SPASLM-
SVPASL--
SLPASL--
SGPASL--
SIPASL--
SKPASL--
-SPASLT-

SHPASL--
SAPASL--
SMPASL--
SYPASL--
-SPASLF-
SPPASL--
-SPASLW-
SFPASL--
SQPASL--
-SPASLH-
-SPASLK-
-SPASLA-
SNPASL--
-SPASLN-
SEPASL--
SDPASL--
-SPASLC-
SCPASL--
TRPASL--
TWPASL--
-TPASLY-
-TPASLK-
TLPASL--
-TPASLT-
TSPASL--
TVPASL--
-TPASLS-
TIPASL--
TMPASL--
-TPASLM-
TFPASL--
TPPASL--
TAPASL--
-TPASLI-
-TPASLF-
TQPASL--
-TPASLV-
TNPASL--
-TPASLQ-
-TPASLH-
TYPASL--
THPASL--
-TPASLN-
-TPASLW-
-TPASLA-
TEPASL--
TDPASL--
TCPASL--
VLPASL--
VKPASL--

VRPASL--
VSPASL--
VGPASL--
VTPASL--
-VPASLK-
VFPASL--
VPPASL--
-VPASLT-
-VPASLH-
-VPASLY-
VNPASL--
VAPASL--
-VPASLV-
VEPASL--
-VPASLW-
-VPASLA-
-VPASLN-
VWPASL--
-VPASLQ-
VYPASL--
-VPASLF-
VHPASL--
VQPASL--
VDPASL--
-VPASLG-
-VPASLM-
VCPASL--
-VPASLE-
-VPASLD-
-WPASLR-
WWPASL--
-WPASLS-
WGPASL--
WFPASL--
WEPASL--
WPPASL--
-WPASLT-
-WPASLL-
WNPASL--
WTPASL--
-WPASLI-
-WPASLY-
-WPASLV-
WDPASL--
WQPASL--
-WPASLN-
-WPASLM-
-WPASLW-
-WPASLG-
-WPASLK-

```
              -WPASLH-
              WCPASL--
              -WPASLE-
              YEPASL--
              -YPASLI-
              -YPASLR-
              YVPASL--
              YSPASL--
              -YPASLK-
              YAPASL--
              -YPASLY-
              YYPASL--
              YQPASL--
              YKPASL--
              YFPASL--
              -YPASLL-
              -YPASLW-
              -YPASLS-
              YGPASL--
              YHPASL--
              YIPASL--
              -YPASLH-
              -YPASLQ-
              -YPASLN-
              -YPASLF-
              YNPASL--
              -YPASLM-
              YDPASL--
              -YPASLV-
              -YPASLA-
              YWPASL--
              -YPASLE-
              YCPASL--
PHGL            13        900      46.28
              --PHGLVP
              --PHGLPF
              --PHGLFP
              -RPHGLP-
              -APHGLL-
              ASPHGL--
              -APHGLS-
              -APHGLA-
              ARPHGL--
              AKPHGL--
              AAPHGL--
              -APHGLQ-
              APPHGL--
              ATPHGL--
              -APHGLH-
              AGPHGL--
```

AYPHGL--
AWPHGL--
-APHGLK-
-APHGLF-
AIPHGL--
AQPHGL--
AFPHGL--
-APHGLW-
-APHGLN-
AEPHGL--
-APHGLT-
-APHGLV-
AVPHGL--
-APHGLY-
-APHGLG-
ANPHGL--
ADPHGL--
ALPHGL--
AHPHGL--
ACPHGL--
-APHGLI-
-APHGLC-
AMPHGL--
-CPHGLY-
CCPHGL--
CGPHGL--
CLPHGL--
-CPHGLI-
CRPHGL--
-CPHGLL-
-CPHGLR-
CTPHGL--
CHPHGL--
CYPHGL--
CSPHGL--
-CPHGLS-
-CPHGLF-
CFPHGL--
CVPHGL--
CQPHGL--
-CPHGLN-
CNPHGL--
DRPHGL--
DWPHGL--
DIPHGL--
-DPHGLF-
-DPHGLR-
DVPHGL--
DSPHGL--
DHPHGL--

−DPHGLK−
−DPHGLY−
DQPHGL−−
−DPHGLH−
DNPHGL−−
DFPHGL−−
DTPHGL−−
−DPHGLN−
DMPHGL−−
DEPHGL−−
−DPHGLT−
−DPHGLM−
DLPHGL−−
DGPHGL−−
DAPHGL−−
−DPHGLS−
−DPHGLI−
DKPHGL−−
ERPHGL−−
−EPHGLT−
−EPHGLK−
−EPHGLW−
EQPHGL−−
EMPHGL−−
ETPHGL−−
ELPHGL−−
−EPHGLL−
−EPHGLR−
−EPHGLM−
−EPHGLI−
EVPHGL−−
−EPHGLS−
−EPHGLN−
−EPHGLA−
EAPHGL−−
EYPHGL−−
EWPHGL−−
ENPHGL−−
EKPHGL−−
−EPHGLY−
−EPHGLV−
−EPHGLF−
EHPHGL−−
EEPHGL−−
−FPHGLR−
−FPHGLT−
FAPHGL−−
FPPHGL−−
FSPHGL−−
−FPHGLS−

−FPHGLL−
FGPHGL−−
FLPHGL−−
FTPHGL−−
FEPHGL−−
−FPHGLK−
−FPHGLM−
−FPHGLI−
FVPHGL−−
−FPHGLV−
−FPHGLH−
FIPHGL−−
−FPHGLN−
FFPHGL−−
FQPHGL−−
FHPHGL−−
FDPHGL−−
−FPHGLF−
−FPHGLA−
−FPHGLY−
FMPHGL−−
FKPHGL−−
FNPHGL−−
FCPHGL−−
GRPHGL−−
GFPHGL−−
GVPHGL−−
−GPHGLY−
GKPHGL−−
−GPHGLS−
−GPHGLK−
−GPHGLW−
−GPHGLL−
GEPHGL−−
GAPHGL−−
−GPHGLH−
−GPHGLF−
GMPHGL−−
−GPHGLQ−
GQPHGL−−
−GPHGLT−
GTPHGL−−
GHPHGL−−
GLPHGL−−
GYPHGL−−
GPPHGL−−
GWPHGL−−
−GPHGLG−
−GPHGLM−
−GPHGLE−

GIPHGL--
GCPHGL--
GDPHGL--
-GPHGLN-
-GPHGLA-
HRPHGL--
-HPHGLI-
HGPHGL--
-HPHGLR-
-HPHGLL-
HVPHGL--
HLPHGL--
HKPHGL--
-HPHGLV-
-HPHGLY-
HFPHGL--
HMPHGL--
HWPHGL--
-HPHGLK-
HTPHGL--
HHPHGL--
HAPHGL--
HSPHGL--
-HPHGLN-
HNPHGL--
HYPHGL--
-HPHGLH-
HQPHGL--
-HPHGLF-
-HPHGLA-
HCPHGL--
HIPHGL--
-HPHGLS-
-HPHGLG-
-HPHGLM-
HEPHGL--
-HPHGLW-
-HPHGLQ-
HDPHGL--
ITPHGL--
-IPHGLR-
-IPHGLT-
IRPHGL--
-IPHGLL-
IFPHGL--
IVPHGL--
-IPHGLI-
IIPHGL--
-IPHGLA-
-IPHGLW-

IKPHGL--
IGPHGL--
ISPHGL--
IEPHGL--
-IPHGLF-
-IPHGLN-
IQPHGL--
IAPHGL--
IMPHGL--
IWPHGL--
-IPHGLY-
-IPHGLG-
ICPHGL--
-IPHGLH-
IYPHGL--
-IPHGLV-
-IPHGLE-
IHPHGL--
INPHGL--
IDPHGL--
-IPHGLC-
-KPHGLR-
-KPHGLT-
KGPHGL--
-KPHGLY-
KLPHGL--
KVPHGL--
-KPHGLK-
KFPHGL--
KAPHGL--
-KPHGLL-
-KPHGLI-
-KPHGLV-
KQPHGL--
KPPHGL--
-KPHGLS-
KNPHGL--
KIPHGL--
KKPHGL--
KHPHGL--
-KPHGLF-
-KPHGLH-
-KPHGLM-
-KPHGLA-
KTPHGL--
-KPHGLN-
-KPHGLE-
KCPHGL--
-KPHGLW-
-KPHGLG-

KEPHGL--
-KPHGLC-
KMPHGL--
KDPHGL--
LRPHGL--
-LPHGLR-
LSPHGL--
LNPHGL--
LTPHGL--
LYPHGL--
LVPHGL--
LPPHGL--
LWPHGL--
LLPHGL--
LKPHGL--
LMPHGL--
LGPHGL--
-LPHGLM-
LFPHGL--
LEPHGL--
LAPHGL--
LIPHGL--
LHPHGL--
-LPHGLK-
-LPHGLS-
-LPHGLV-
-LPHGLI-
LQPHGL--
LDPHGL--
-LPHGLL-
-LPHGLF-
-LPHGLT-
-LPHGLY-
-LPHGLQ-
-LPHGLW-
-LPHGLN-
-LPHGLA-
-LPHGLH-
-LPHGLE-
LCPHGL--
-LPHGLD-
-LPHGLC-
MRPHGL--
-MPHGLT-
MVPHGL--
-MPHGLR-
-MPHGLY-
MSPHGL--
-MPHGLS-
-MPHGLL-

MFPHGL--
MYPHGL--
MPPHGL--
-MPHGLH-
-MPHGLV-
MEPHGL--
MGPHGL--
MAPHGL--
-MPHGLW-
MKPHGL--
-MPHGLK-
MNPHGL--
MLPHGL--
-MPHGLI-
MDPHGL--
-MPHGLM-
MIPHGL--
-MPHGLF-
-MPHGLE-
-MPHGLN-
-MPHGLQ-
-MPHGLA-
MTPHGL--
MQPHGL--
-MPHGLP-
-MPHGLG-
NMPHGL--
NVPHGL--
NRPHGL--
NWPHGL--
NAPHGL--
NLPHGL--
-NPHGLS-
-NPHGLL-
-NPHGLI-
NIPHGL--
NFPHGL--
-NPHGLV-
NGPHGL--
NSPHGL--
-NPHGLW-
NNPHGL--
NHPHGL--
NTPHGL--
-NPHGLY-
-NPHGLK-
NEPHGL--
NYPHGL--
-NPHGLR-
NKPHGL--

NPPHGL--
-NPHGLT-
-NPHGLA-
-NPHGLQ-
-NPHGLH-
-NPHGLN-
-NPHGLM-
-NPHGLF-
NQPHGL--
--PHGLFS
--PHGLLR
--PHGLVS
--PHGLTG
--PHGLRS
--PHGLLT
--PHGLRR
--PHGLWT
--PHGLRN
--PHGLVG
--PHGLLG
--PHGLSM
--PHGLTT
--PHGLRK
--PHGLMS
--PHGLWS
--PHGLSN
--PHGLRG
--PHGLLS
--PHGLYA
--PHGLSA
PAPHGL--
--PHGLSS
--PHGLRE
--PHGLKG
--PHGLYL
--PHGLST
PLPHGL--
--PHGLTY
--PHGLSV
--PHGLLN
--PHGLLV
--PHGLRA
--PHGLAS
--PHGLTR
--PHGLNM
--PHGLIS
--PHGLSG
--PHGLYT
--PHGLRY
--PHGLFT

--PHGLNA
--PHGLLQ
--PHGLLM
PRPHGL--
--PHGLMY
--PHGLSY
--PHGLRF
--PHGLFR
--PHGLRL
--PHGLVK
--PHGLTV
--PHGLSL
--PHGLRW
--PHGLML
--PHGLKR
--PHGLVN
--PHGLTS
--PHGLKT
--PHGLVR
--PHGLSI
--PHGLMV
--PHGLFY
--PHGLFN
--PHGLAW
PYPHGL--
--PHGLLL
--PHGLIA
--PHGLHR
--PHGLAY
--PHGLQS
--PHGLNR
--PHGLMT
--PHGLLF
--PHGLVL
--PHGLNG
--PHGLKS
--PHGLHS
--PHGLMM
--PHGLIN
--PHGLHE
--PHGLFH
--PHGLYH
--PHGLVM
--PHGLTL
--PHGLTK
--PHGLLI
--PHGLKY
PNPHGL--
--PHGLTI
--PHGLQG

--PHGLLK
--PHGLIV
--PHGLIM
--PHGLHI
--PHGLAF
PFPHGL--
PVPHGL--
--PHGLVV
--PHGLTN
--PHGLWA
--PHGLVQ
--PHGLVA
--PHGLRM
--PHGLMG
--PHGLMF
--PHGLKV
--PHGLKD
--PHGLHL
--PHGLFI
--PHGLYG
--PHGLWD
--PHGLRD
--PHGLQR
--PHGLMW
--PHGLMK
--PHGLKW
--PHGLHG
--PHGLFW
--PHGLYS
--PHGLYK
--PHGLWM
--PHGLWI
--PHGLVI
--PHGLIF
--PHGLGA
--PHGLFQ
PGPHGL--
--PHGLYV
--PHGLYR
--PHGLTQ
--PHGLTH
--PHGLNS
--PHGLKH
--PHGLKA
--PHGLIK
--PHGLGG
--PHGLFV
--PHGLWN
--PHGLVF
--PHGLSH

--PHGLIT
--PHGLES
--PHGLYI
--PHGLWR
--PHGLWG
--PHGLVY
--PHGLTM
--PHGLNV
--PHGLKF
--PHGLHY
--PHGLHV
--PHGLHF
--PHGLGV
--PHGLFF
--PHGLAI
--PHGLVT
--PHGLSK
--PHGLQA
--PHGLKI
--PHGLHM
--PHGLAV
PTPHGL--
--PHGLWH
--PHGLSD
--PHGLMN
--PHGLGS
--PHGLFL
--PHGLFK
PMPHGL--
--PHGLQY
--PHGLNI
--PHGLEG
--PHGLAM
--PHGLYM
--PHGLWK
--PHGLVW
--PHGLVD
--PHGLQL
--PHGLQK
--PHGLNT
--PHGLNF
--PHGLMQ
--PHGLME
--PHGLLW
--PHGLKN
--PHGLGT
PSPHGL--
--PHGLYE
--PHGLWY
--PHGLWW

--PHGLWV
--PHGLWQ
--PHGLRQ
--PHGLQT
--PHGLNY
--PHGLNL
--PHGLIY
--PHGLHT
--PHGLEV
--PHGLEN
--PHGLEM
--PHGLAQ
--PHGLAN
--PHGLAL
PIPHGL--
--PHGLYQ
--PHGLYN
--PHGLYF
--PHGLNH
--PHGLLD
--PHGLKM
--PHGLKL
--PHGLKK
--PHGLAT
PHPHGL--
--PHGLTD
--PHGLSQ
--PHGLQM
--PHGLQF
--PHGLMA
--PHGLIL
--PHGLGF
--PHGLFA
--PHGLAR
--PHGLYW
--PHGLTW
--PHGLQW
--PHGLQV
--PHGLQQ
--PHGLNN
--PHGLNK
--PHGLKE
--PHGLII
--PHGLIH
--PHGLID
--PHGLHN
--PHGLFM
--PHGLFG
--PHGLEY
--PHGLET

--PHGLAH
PWPHGL--
--PHGLTE
--PHGLSW
--PHGLQH
--PHGLMI
--PHGLMH
--PHGLLE
--PHGLHW
--PHGLHQ
--PHGLHA
--PHGLGL
--PHGLER
--PHGLNE
--PHGLIQ
--PHGLGR
--PHGLFD
--PHGLEK
--PHGLDS
--PHGLDA
--PHGLCY
--PHGLCW
--PHGLAA
--PHGLYD
--PHGLSE
--PHGLQE
--PHGLNQ
--PHGLND
--PHGLMC
--PHGLIW
--PHGLIC
--PHGLGY
--PHGLGM
--PHGLDG
--PHGLAK
PCPHGL--
PPPHGL--
-PPHGLS-
-PPHGLR-
-PPHGLI-
--PHGLYY
--PHGLVH
--PHGLTC
--PHGLQI
--PHGLQD
--PHGLPP
--PHGLPK
--PHGLPH
--PHGLNW
--PHGLKQ

--PHGLKC
--PHGLIE
--PHGLHH
--PHGLGI
--PHGLEQ
--PHGLCS
PEPHGL--
QRPHGL--
QVPHGL--
QGPHGL--
QKPHGL--
-QPHGLV-
QNPHGL--
QEPHGL--
-QPHGLY-
QAPHGL--
QWPHGL--
-QPHGLQ-
-QPHGLL-
QIPHGL--
QYPHGL--
-QPHGLH-
QFPHGL--
-QPHGLN-
-QPHGLM-
-QPHGLT-
-QPHGLS-
QDPHGL--
-QPHGLK-
-QPHGLA-
-QPHGLW-
-QPHGLG-
QTPHGL--
QPPHGL--
-QPHGLI-
-QPHGLD-
QMPHGL--
QCPHGL--
-RPHGLY-
-RPHGLT-
RWPHGL--
RPPHGL--
-RPHGLQ-
-RPHGLH-
-RPHGLS-
RLPHGL--
RAPHGL--
-RPHGLV-
RVPHGL--
-RPHGLN-

-RPHGLL-
RMPHGL--
-RPHGLR-
RGPHGL--
-RPHGLK-
RHPHGL--
RIPHGL--
-RPHGLF-
RQPHGL--
RDPHGL--
-RPHGLW-
-RPHGLA-
-RPHGLM-
RFPHGL--
-RPHGLG-
-RPHGLE-
REPHGL--
RNPHGL--
RCPHGL--
-RPHGLC-
RKPHGL--
SRPHGL--
SSPHGL--
SGPHGL--
SYPHGL--
-SPHGLH-
-SPHGLL-
-SPHGLR-
SFPHGL--
SVPHGL--
SAPHGL--
-SPHGLM-
SNPHGL--
SLPHGL--
STPHGL--
SQPHGL--
SIPHGL--
-SPHGLA-
-SPHGLS-
-SPHGLY-
-SPHGLQ-
-SPHGLV-
SKPHGL--
-SPHGLN-
-SPHGLK-
-SPHGLF-
SPPHGL--
-SPHGLW-
SWPHGL--
-SPHGLT-

-SPHGLI-
-SPHGLE-
SCPHGL--
SDPHGL--
SHPHGL--
TVPHGL--
-TPHGLR-
TRPHGL--
-TPHGLT-
TFPHGL--
TWPHGL--
-TPHGLV-
-TPHGLS-
TAPHGL--
-TPHGLL-
TGPHGL--
-TPHGLY-
TKPHGL--
TIPHGL--
-TPHGLA-
TSPHGL--
TLPHGL--
TTPHGL--
-TPHGLW-
-TPHGLK-
-TPHGLI-
-TPHGLH-
TQPHGL--
TDPHGL--
-TPHGLQ-
-TPHGLF-
TMPHGL--
TYPHGL--
TPPHGL--
-TPHGLN-
-TPHGLG-
TEPHGL--
THPHGL--
-TPHGLE-
-VPHGLR-
-VPHGLS-
VKPHGL--
VGPHGL--
VRPHGL--
-VPHGLY-
VLPHGL--
-VPHGLT-
VTPHGL--
VEPHGL--
VVPHGL--

-VPHGLV-
VSPHGL--
-VPHGLN-
-VPHGLF-
VPPHGL--
VMPHGL--
-VPHGLM-
-VPHGLK-
-VPHGLI-
VFPHGL--
VAPHGL--
VNPHGL--
-VPHGLH-
-VPHGLE-
VDPHGL--
VIPHGL--
VWPHGL--
VHPHGL--
VQPHGL--
-VPHGLQ-
WAPHGL--
WVPHGL--
WNPHGL--
WTPHGL--
-WPHGLY-
-WPHGLF-
WFPHGL--
WGPHGL--
WWPHGL--
-WPHGLV-
-WPHGLN-
WRPHGL--
WKPHGL--
WIPHGL--
WDPHGL--
-WPHGLK-
WPPHGL--
-WPHGLS-
WSPHGL--
-WPHGLA-
WMPHGL--
WQPHGL--
WHPHGL--
WEPHGL--
-WPHGLT-
-WPHGLE-
WLPHGL--
-WPHGLL-
-WPHGLW-
-WPHGLQ-

	-YPHGLL-		
	YGPHGL--		
	YSPHGL--		
	YLPHGL--		
	-YPHGLK-		
	YHPHGL--		
	YAPHGL--		
	YIPHGL--		
	YYPHGL--		
	-YPHGLH-		
	YQPHGL--		
	YFPHGL--		
	YWPHGL--		
	YVPHGL--		
	YPPHGL--		
	-YPHGLQ-		
	-YPHGLT-		
	-YPHGLS-		
	-YPHGLN-		
	YMPHGL--		
	-YPHGLY-		
	-YPHGLV-		
	-YPHGLI-		
	-YPHGLF-		
	-YPHGLA-		
	YNPHGL--		
	YKPHGL--		
	-YPHGLW-		
	YEPHGL--		
	-YPHGLM-		
	-YPHGLE-		
	YCPHGL--		
	YTPHGL--		
	YDPHGL--		
PSSL	78	825	46.26
	-EPSSLN-		
	EDPSSL--		
	-FPSSLG-		
	-GPSSLP-		
	-IPSSLD-		
	-MPSSLG-		
	-MPSSLP-		
	--PSSLGL		
	--PSSLNR		
	--PSSLAR		
	--PSSLFP		
	--PSSLDS		
	--PSSLAK		
	--PSSLSP		
	--PSSLSC		

--PSSLPT
--PSSLPS
--PSSLPP
--PSSLPL
--PSSLKP
--PSSLIP
--PSSLHP
--PSSLGN
--PSSLGD
--PSSLEI
--PSSLDT
--PSSLDL
--PSSLCC
--PSSLAE
-RPSSLC-
-TPSSLG-
-VPSSLD-
-WPSSLG-
-YPSSLG-
-APSSLR-
-APSSLL-
AVPSSL--
AKPSSL--
-APSSLS-
-APSSLT-
AIPSSL--
APPSSL--
-APSSLW-
AGPSSL--
AMPSSL--
AYPSSL--
AFPSSL--
-APSSLI-
-APSSLK-
-APSSLF-
AQPSSL--
AHPSSL--
AEPSSL--
-APSSLV-
AWPSSL--
-APSSLQ-
-APSSLM-
-APSSLE-
-APSSLA-
ADPSSL--
ACPSSL--
-APSSLG-
-APSSLP-
-APSSLC-
CVPSSL--

CLPSSL--
CCPSSL--
CRPSSL--
CSPSSL--
CKPSSL--
CTPSSL--
CQPSSL--
CPPSSL--
CIPSSL--
-CPSSLR-
-CPSSLI-
CAPSSL--
CMPSSL--
CEPSSL--
-CPSSLM-
-CPSSLL-
-CPSSLY-
-CPSSLW-
CFPSSL--
CYPSSL--
-CPSSLK-
-CPSSLV-
-CPSSLS-
-CPSSLF-
-CPSSLT-
CHPSSL--
-CPSSLH-
CDPSSL--
DLPSSL--
DMPSSL--
DVPSSL--
DSPSSL--
DIPSSL--
DKPSSL--
DYPSSL--
DHPSSL--
DTPSSL--
DGPSSL--
DAPSSL--
DWPSSL--
DFPSSL--
DQPSSL--
-DPSSLF-
-DPSSLW-
-DPSSLI-
DNPSSL--
-DPSSLK-
-DPSSLY-
-DPSSLV-
-DPSSLM-

−DPSSLT−
−DPSSLL−
−DPSSLH−
−DPSSLS−
−DPSSLQ−
−DPSSLN−
ESPSSL−−
EAPSSL−−
ERPSSL−−
EVPSSL−−
−EPSSLL−
−EPSSLS−
−EPSSLR−
EMPSSL−−
EGPSSL−−
−EPSSLK−
EKPSSL−−
ETPSSL−−
EIPSSL−−
−EPSSLT−
−EPSSLV−
−EPSSLY−
−EPSSLI−
−EPSSLW−
−EPSSLQ−
EFPSSL−−
−EPSSLH−
EEPSSL−−
EYPSSL−−
−EPSSLM−
EHPSSL−−
ENPSSL−−
−EPSSLA−
EPPSSL−−
−EPSSLC−
ECPSSL−−
FLPSSL−−
−FPSSLR−
−FPSSLI−
FTPSSL−−
FVPSSL−−
FKPSSL−−
−FPSSLT−
FNPSSL−−
FHPSSL−−
FWPSSL−−
FAPSSL−−
FFPSSL−−
FQPSSL−−
FMPSSL−−

FYPSSL--
FEPSSL--
-FPSSLY-
-FPSSLV-
-FPSSLK-
FIPSSL--
-FPSSLM-
-FPSSLQ-
-FPSSLW-
FPPSSL--
FDPSSL--
FCPSSL--
-FPSSLH-
-FPSSLN-
-FPSSLE-
-GPSSLR-
GVPSSL--
GLPSSL--
GSPSSL--
GRPSSL--
GTPSSL--
GYPSSL--
GWPSSL--
-GPSSLL-
GMPSSL--
GQPSSL--
-GPSSLY-
-GPSSLS-
GGPSSL--
GFPSSL--
-GPSSLM-
-GPSSLK-
GNPSSL--
GAPSSL--
-GPSSLF-
GPPSSL--
-GPSSLW-
-GPSSLV-
GHPSSL--
-GPSSLH-
GEPSSL--
-GPSSLQ-
-GPSSLA-
-GPSSLN-
-GPSSLE-
GCPSSL--
GDPSSL--
-GPSSLC-
HVPSSL--
HSPSSL--

HYPSSL--
HQPSSL--
HKPSSL--
HFPSSL--
-HPSSLI-
-HPSSLL-
-HPSSLK-
HAPSSL--
HNPSSL--
HHPSSL--
-HPSSLR-
-HPSSLV-
-HPSSLS-
-HPSSLY-
HEPSSL--
-HPSSLH-
HWPSSL--
-HPSSLW-
HMPSSL--
-HPSSLM-
HPPSSL--
HDPSSL--
HCPSSL--
-HPSSLQ-
-HPSSLE-
-HPSSLA-
ITPSSL--
-IPSSLR-
ILPSSL--
ISPSSL--
-IPSSLT-
IKPSSL--
IHPSSL--
-IPSSLL-
-IPSSLV-
IGPSSL--
IQPSSL--
IWPSSL--
IYPSSL--
-IPSSLK-
IAPSSL--
-IPSSLQ-
-IPSSLW-
-IPSSLM-
-IPSSLF-
IFPSSL--
INPSSL--
IPPSSL--
-IPSSLY-
-IPSSLN-

IMPSSL--
-IPSSLH-
-IPSSLI-
IIPSSL--
IEPSSL--
-IPSSLE-
IDPSSL--
ICPSSL--
-IPSSLC-
-IPSSLG-
KSPSSL--
KVPSSL--
-KPSSLR-
KLPSSL--
KRPSSL--
-KPSSLI-
KAPSSL--
KMPSSL--
KWPSSL--
KHPSSL--
-KPSSLS-
KTPSSL--
-KPSSLK-
KKPSSL--
-KPSSLT-
-KPSSLY-
-KPSSLF-
-KPSSLV-
-KPSSLQ-
KPPSSL--
KEPSSL--
-KPSSLA-
-KPSSLN-
KNPSSL--
-KPSSLH-
KDPSSL--
-KPSSLW-
-KPSSLE-
KCPSSL--
LRPSSL--
LLPSSL--
LMPSSL--
LVPSSL--
LSPSSL--
LKPSSL--
LTPSSL--
LIPSSL--
LYPSSL--
LNPSSL--
LHPSSL--

LPPSSL--
LFPSSL--
-LPSSLY-
-LPSSLM-
-LPSSLH-
-LPSSLK-
LEPSSL--
-LPSSLI-
-LPSSLT-
-LPSSLW-
LDPSSL--
-LPSSLQ-
-LPSSLA-
LCPSSL--
-LPSSLE-
MSPSSL--
MVPSSL--
MGPSSL--
MAPSSL--
-MPSSLR-
MIPSSL--
-MPSSLT-
MMPSSL--
MPPSSL--
MNPSSL--
-MPSSLW-
-MPSSLL-
-MPSSLY-
MKPSSL--
MEPSSL--
-MPSSLI-
-MPSSLS-
MFPSSL--
-MPSSLK-
-MPSSLH-
MQPSSL--
MWPSSL--
-MPSSLQ-
-MPSSLV-
-MPSSLF-
-MPSSLM-
-MPSSLA-
MDPSSL--
MCPSSL--
-MPSSLN-
-MPSSLE-
-MPSSLD-
-MPSSLC-
NFPSSL--
-NPSSLR-

NVPSSL--
NTPSSL--
NGPSSL--
NWPSSL--
-NPSSLL-
NIPSSL--
NQPSSL--
NAPSSL--
NMPSSL--
NYPSSL--
NHPSSL--
NNPSSL--
-NPSSLV-
-NPSSLY-
-NPSSLT-
NPPSSL--
NEPSSL--
-NPSSLH-
-NPSSLW-
-NPSSLS-
NDPSSL--
NCPSSL--
-NPSSLI-
-NPSSLA-
--PSSLRS
PSPSSL--
--PSSLRG
--PSSLIS
--PSSLRN
--PSSLVR
--PSSLRT
--PSSLSG
--PSSLVS
--PSSLTA
PVPSSL--
PRPSSL--
--PSSLLT
--PSSLHS
--PSSLLR
--PSSLVG
--PSSLSA
--PSSLKG
PLPSSL--
--PSSLWS
--PSSLMA
--PSSLYT
--PSSLNS
--PSSLRF
--PSSLTR
PTPSSL--

--PSSLRI
--PSSLMS
--PSSLKA
PGPSSL--
--PSSLIR
--PSSLFS
--PSSLMT
--PSSLTT
--PSSLRH
--PSSLHG
--PSSLVT
--PSSLVK
--PSSLWA
--PSSLWG
--PSSLTG
PQPSSL--
--PSSLLH
--PSSLWH
--PSSLNA
--PSSLRY
--PSSLIV
--PSSLLQ
--PSSLYN
--PSSLVW
--PSSLWV
--PSSLVM
--PSSLMI
--PSSLLK
--PSSLAG
--PSSLST
--PSSLSN
--PSSLKT
PHPSSL--
--PSSLTV
--PSSLQT
--PSSLIF
--PSSLHM
--PSSLSF
--PSSLQS
--PSSLYG
--PSSLFA
PIPSSL--
--PSSLVY
--PSSLHF
--PSSLFT
--PSSLVN
--PSSLQA
--PSSLNV
PNPSSL--
--PSSLSY

--PSSLSR
--PSSLSM
--PSSLES
--PSSLTN
--PSSLTF
--PSSLGA
--PSSLYW
PAPSSL--
--PSSLVH
--PSSLMK
--PSSLYM
--PSSLGS
--PSSLFG
--PSSLTY
--PSSLTL
--PSSLWT
--PSSLWR
--PSSLLY
--PSSLTW
--PSSLFN
--PSSLKR
--PSSLMN
--PSSLFY
--PSSLFH
--PSSLVQ
--PSSLYQ
--PSSLYF
--PSSLWN
--PSSLMF
--PSSLYE
--PSSLTH
PFPSSL--
--PSSLTQ
--PSSLRE
--PSSLQV
--PSSLWW
--PSSLWD
--PSSLEG
PEPSSL--
PWPSSL--
--PSSLWQ
--PSSLIM
--PSSLFW
PMPSSL--
--PSSLYI
--PSSLFK
--PSSLAW
--PSSLSH
--PSSLHH
--PSSLHA

--PSSLTM
--PSSLTK
--PSSLMH
--PSSLLD
--PSSLYK
--PSSLKK
--PSSLMY
--PSSLFD
--PSSLIK
--PSSLHY
--PSSLHQ
--PSSLQH
--PSSLIY
--PSSLMW
--PSSLLI
--PSSLHT
--PSSLKI
--PSSLYH
--PSSLTD
--PSSLMQ
--PSSLKF
--PSSLAT
--PSSLSQ
--PSSLQG
--PSSLQY
--PSSLNM
--PSSLME
--PSSLNW
--PSSLMM
--PSSLHV
--PSSLAM
--PSSLAI
--PSSLQN
--PSSLKM
--PSSLIH
--PSSLGG
--PSSLEV
--PSSLAV
--PSSLYD
--PSSLQK
--PSSLWK
--PSSLTI
--PSSLKQ
--PSSLHK
--PSSLEW
--PSSLVE
--PSSLSK
--PSSLNG
--PSSLHN
--PSSLHE

--PSSLHD
--PSSLQR
--PSSLIE
--PSSLID
--PSSLVI
--PSSLVD
--PSSLQQ
--PSSLNT
--PSSLET
--PSSLSI
--PSSLQL
--PSSLMD
--PSSLSD
--PSSLIQ
--PSSLFE
--PSSLAH
--PSSLWI
--PSSLEF
--PSSLHL
--PSSLGM
--PSSLQF
--PSSLKE
--PSSLAQ
PYPSSL--
--PSSLSE
--PSSLQM
PCPSSL--
--PSSLTE
--PSSLGY
--PSSLFQ
--PSSLNH
--PSSLEY
--PSSLAF
--PSSLWE
--PSSLNN
--PSSLCE
--PSSLNK
--PSSLND
--PSSLLC
--PSSLKD
--PSSLGW
--PSSLEH
--PSSLYC
--PSSLNQ
--PSSLNI
--PSSLNE
--PSSLKC
--PSSLEM
--PSSLDA
--PSSLCY

--PSSLCG
--PSSLCD
--PSSLCA
-PPSSLL-
--PSSLVC
--PSSLQP
--PSSLQI
--PSSLQE
--PSSLPM
--PSSLIC
--PSSLFC
--PSSLCK
-PPSSLQ-
-PPSSLM-
-PPSSLF-
-PPSSLA-
-QPSSLR-
QVPSSL--
QSPSSL--
QAPSSL--
-QPSSLS-
QFPSSL--
QHPSSL--
-QPSSLH-
-QPSSLV-
QQPSSL--
QEPSSL--
QNPSSL--
-QPSSLF-
QIPSSL--
-QPSSLY-
-QPSSLM-
-QPSSLK-
-QPSSLT-
QPPSSL--
-QPSSLW-
QMPSSL--
QDPSSL--
-QPSSLN-
-QPSSLQ-
-QPSSLA-
-QPSSLG-
-QPSSLC-
QCPSSL--
RSPSSL--
RAPSSL--
RGPSSL--
RMPSSL--
RTPSSL--
RFPSSL--

−RPSSLS−
−RPSSLY−
−RPSSLI−
−RPSSLV−
RPPSSL−−
RWPSSL−−
−RPSSLT−
RYPSSL−−
RIPSSL−−
−RPSSLH−
−RPSSLK−
−RPSSLF−
REPSSL−−
RQPSSL−−
−RPSSLA−
RDPSSL−−
−RPSSLQ−
−RPSSLE−
RCPSSL−−
−RPSSLG−
SRPSSL−−
−SPSSLL−
SSPSSL−−
SFPSSL−−
−SPSSLY−
SIPSSL−−
SKPSSL−−
−SPSSLV−
SAPSSL−−
SYPSSL−−
SHPSSL−−
SMPSSL−−
−SPSSLI−
−SPSSLF−
−SPSSLH−
−SPSSLK−
−SPSSLM−
SPPSSL−−
SEPSSL−−
−SPSSLQ−
SQPSSL−−
SDPSSL−−
−SPSSLN−
−SPSSLA−
−SPSSLE−
SCPSSL−−
−SPSSLC−
TRPSSL−−
TSPSSL−−
−TPSSLR−

TLPSSL--
TTPSSL--
TAPSSL--
TGPSSL--
TFPSSL--
TMPSSL--
-TPSSLL-
-TPSSLW-
-TPSSLT-
-TPSSLK-
-TPSSLY-
TWPSSL--
-TPSSLM-
-TPSSLI-
TQPSSL--
-TPSSLV-
TKPSSL--
-TPSSLS-
-TPSSLF-
TYPSSL--
TNPSSL--
THPSSL--
-TPSSLN-
TEPSSL--
TCPSSL--
-TPSSLA-
TDPSSL--
-TPSSLE-
VSPSSL--
VTPSSL--
VYPSSL--
VNPSSL--
VPPSSL--
-VPSSLK-
-VPSSLY-
VIPSSL--
-VPSSLI-
VFPSSL--
VAPSSL--
-VPSSLH-
-VPSSLM-
-VPSSLV-
-VPSSLQ-
VWPSSL--
-VPSSLF-
VMPSSL--
-VPSSLW-
VEPSSL--
VHPSSL--
VQPSSL--

-VPSSLN-
VDPSSL--
-VPSSLE-
-VPSSLG-
VCPSSL--
WGPSSL--
WSPSSL--
WTPSSL--
-WPSSLR-
-WPSSLH-
-WPSSLM-
WEPSSL--
-WPSSLV-
WAPSSL--
-WPSSLT-
WFPSSL--
-WPSSLS-
-WPSSLK-
WDPSSL--
-WPSSLI-
WQPSSL--
-WPSSLW-
WHPSSL--
WYPSSL--
WNPSSL--
WPPSSL--
-WPSSLQ-
-WPSSLE-
-WPSSLA-
WCPSSL--
YLPSSL--
YGPSSL--
YRPSSL--
YVPSSL--
YYPSSL--
-YPSSLR-
YIPSSL--
YFPSSL--
-YPSSLK-
YPPSSL--
YNPSSL--
YKPSSL--
-YPSSLI-
-YPSSLT-
YAPSSL--
-YPSSLF-
YMPSSL--
-YPSSLY-
-YPSSLS-
-YPSSLW-

	YDPSSL--		
	-YPSSLQ-		
	-YPSSLH-		
	YQPSSL--		
	YHPSSL--		
	YWPSSL--		
	YEPSSL--		
	-YPSSLA-		
	YCPSSL--		
	-YPSSLE-		
PMQM	934	504	46.26
	--PMQMSD		
	-APMQMR-		
	-APMQMN-		
	AKPMQM--		
	AIPMQM--		
	-APMQMW-		
	AVPMQM--		
	-APMQMV-		
	AMPMQM--		
	APPMQM--		
	-APMQMS-		
	-APMQMT-		
	AEPMQM--		
	ADPMQM--		
	-APMQML-		
	-APMQMK-		
	-APMQMG-		
	-APMQMA-		
	AQPMQM--		
	-APMQMQ-		
	-APMQME-		
	AGPMQM--		
	AFPMQM--		
	CGPMQM--		
	CLPMQM--		
	CRPMQM--		
	CWPMQM--		
	-CPMQMI-		
	-CPMQMS-		
	CHPMQM--		
	CCPMQM--		
	CAPMQM--		
	DHPMQM--		
	DVPMQM--		
	DAPMQM--		
	DFPMQM--		
	DYPMQM--		
	DTPMQM--		
	-DPMQMW-		

-DPMQMS-
-DPMQML-
DMPMQM--
-EPMQMV-
-EPMQMK-
EWPMQM--
-EPMQMY-
-EPMQMS-
-EPMQML-
-EPMQMI-
EIPMQM--
ERPMQM--
EKPMQM--
EGPMQM--
EAPMQM--
EMPMQM--
ELPMQM--
-FPMQML-
FWPMQM--
-FPMQMY-
FKPMQM--
FRPMQM--
-FPMQMR-
-FPMQMA-
FFPMQM--
FEPMQM--
FSPMQM--
-FPMQMM-
FVPMQM--
-FPMQMH-
-FPMQMV-
-FPMQMS-
-FPMQMF-
FIPMQM--
FHPMQM--
-GPMQMR-
GSPMQM--
GIPMQM--
GTPMQM--
-GPMQMT-
GMPMQM--
GLPMQM--
GYPMQM--
-GPMQMM-
-GPMQML-
-GPMQMK-
GKPMQM--
-GPMQMA-
GVPMQM--
-GPMQMS-

GRPMQM--
-GPMQMV-
-GPMQMH-
GWPMQM--
-GPMQMF-
GHPMQM--
-HPMQMY-
HGPMQM--
HFPMQM--
-HPMQMF-
-HPMQMH-
-HPMQMR-
-HPMQMN-
-HPMQML-
HLPMQM--
HIPMQM--
HYPMQM--
HSPMQM--
-HPMQMI-
HEPMQM--
HRPMQM--
-IPMQMI-
IAPMQM--
-IPMQMV-
-IPMQMN-
-IPMQMK-
IFPMQM--
ITPMQM--
ICPMQM--
-IPMQMW-
IMPMQM--
-IPMQMY-
-IPMQML-
IWPMQM--
-KPMQMR-
-KPMQMS-
KAPMQM--
KTPMQM--
KHPMQM--
KWPMQM--
KRPMQM--
-KPMQMT-
-KPMQMG-
KLPMQM--
KPPMQM--
-KPMQMY-
-KPMQML-
-KPMQMF-
KNPMQM--
KIPMQM--

KVPMQM--
KSPMQM--
KDPMQM--
-LPMQML-
-LPMQMR-
LAPMQM--
LRPMQM--
LGPMQM--
LLPMQM--
LMPMQM--
LPPMQM--
LYPMQM--
-LPMQMT-
-LPMQMS-
LIPMQM--
LEPMQM--
LVPMQM--
LSPMQM--
-LPMQMV-
-LPMQMN-
-LPMQMH-
-LPMQMF-
-LPMQMW-
-LPMQMI-
LNPMQM--
LDPMQM--
LCPMQM--
-LPMQMY-
-LPMQMG-
-LPMQMA-
LKPMQM--
-MPMQMV-
MAPMQM--
MFPMQM--
-MPMQMR-
-MPMQMN-
MPPMQM--
-MPMQMI-
MKPMQM--
-MPMQMT-
MVPMQM--
-MPMQMS-
-MPMQMQ-
-MPMQMG-
MLPMQM--
-NPMQML-
NFPMQM--
-NPMQMT-
NWPMQM--
NTPMQM--

NLPMQM--
-NPMQMV-
-NPMQMI-
NNPMQM--
NRPMQM--
-NPMQMN-
NGPMQM--
NVPMQM--
NSPMQM--
-NPMQMM-
-NPMQMH-
NEPMQM--
NAPMQM--
--PMQMRR
--PMQMFT
--PMQMNQ
--PMQMTS
--PMQMYM
--PMQMEA
--PMQMSG
--PMQMLG
--PMQMYT
--PMQMVV
--PMQMYS
--PMQMWS
--PMQMVG
--PMQMSV
--PMQMMA
--PMQMYR
--PMQMMT
--PMQMQA
--PMQMLH
--PMQMIY
--PMQMIH
--PMQMWN
--PMQMRL
--PMQMKF
--PMQMVL
--PMQMVW
--PMQMRN
--PMQMRA
--PMQMNS
--PMQMIS
--PMQMIQ
--PMQMYN
--PMQMRT
--PMQMRH
--PMQMKW
--PMQMIK
--PMQMFH

--PMQMVY
--PMQMTG
--PMQMRY
--PMQMRF
--PMQMMV
--PMQMTH
--PMQMSH
--PMQMNV
--PMQMLK
--PMQMIG
--PMQMHV
--PMQMFQ
--PMQMEG
--PMQMVA
--PMQMSS
--PMQMNT
--PMQMKN
--PMQMIR
--PMQMHS
--PMQMYE
--PMQMWK
--PMQMWD
--PMQMVQ
--PMQMTY
--PMQMSW
--PMQMRV
--PMQMRK
--PMQMME
--PMQMKV
--PMQMKI
--PMQMKA
--PMQMIW
--PMQMHH
--PMQMGS
--PMQMFG
--PMQMAS
--PMQMAG
--PMQMWM
--PMQMTA
--PMQMSN
--PMQMSE
--PMQMRM
--PMQMLS
--PMQMKR
--PMQMIV
--PMQMHN
--PMQMHA
--PMQMFV
--PMQMAA
--PMQMYQ

--PMQMWV
--PMQMVS
--PMQMSM
--PMQMRI
--PMQMRE
--PMQMQI
--PMQMQH
--PMQMQF
--PMQMQD
--PMQMLT
--PMQMKL
--PMQMKD
--PMQMGA
--PMQMFS
--PMQMAV
--PMQMYV
--PMQMWQ
--PMQMWG
--PMQMVK
--PMQMVI
--PMQMSR
--PMQMSL
--PMQMSI
--PMQMSF
--PMQMRG
--PMQMNI
--PMQMMW
--PMQMML
--PMQMLR
--PMQMKG
--PMQMIN
--PMQMIA
--PMQMHT
--PMQMGV
--PMQMGT
--PMQMFN
--PMQMFE
--PMQMES
-PPMQMI-
--PMQMYI
--PMQMYD
--PMQMVR
--PMQMVM
--PMQMTW
--PMQMTR
--PMQMTD
--PMQMSY
--PMQMQV
--PMQMQM
--PMQMQK

--PMQMNY
--PMQMNR
--PMQMNL
--PMQMMR
--PMQMLQ
--PMQMLF
--PMQMLD
--PMQMKQ
--PMQMKK
--PMQMKE
--PMQMHW
--PMQMHQ
--PMQMHM
--PMQMHE
--PMQMFW
--PMQMFA
--PMQMEW
--PMQMAN
-QPMQMV-
-QPMQML-
-QPMQMR-
QWPMQM--
QFPMQM--
QQPMQM--
-QPMQMS-
-QPMQMM-
-QPMQMA-
QLPMQM--
QAPMQM--
QMPMQM--
-QPMQMN-
-QPMQMI-
-QPMQMH-
-RPMQML-
RAPMQM--
RGPMQM--
RWPMQM--
RSPMQM--
-RPMQMR-
-RPMQMY-
RTPMQM--
-RPMQMA-
-RPMQMS-
RNPMQM--
-RPMQMK-
-RPMQMG-
RLPMQM--
RVPMQM--
-RPMQMH-
RFPMQM--

-RPMQMM-
-RPMQMI-
-RPMQME-
RDPMQM--
RPPMQM--
RMPMQM--
RHPMQM--
REPMQM--
RYPMQM--
RQPMQM--
-RPMQMT-
-RPMQMF-
RKPMQM--
SRPMQM--
-SPMQMI-
-SPMQMY-
SHPMQM--
SAPMQM--
SGPMQM--
SVPMQM--
-SPMQMS-
-SPMQML-
SSPMQM--
-SPMQMR-
-SPMQMA-
SQPMQM--
SIPMQM--
STPMQM--
-SPMQMV-
-SPMQMT-
SPPMQM--
-SPMQMF-
-SPMQMH-
-SPMQMN-
-SPMQMK-
SMPMQM--
SEPMQM--
-SPMQMG-
SFPMQM--
-TPMQMR-
-TPMQMK-
-TPMQMI-
TRPMQM--
-TPMQMF-
-TPMQML-
-TPMQMS-
TSPMQM--
TQPMQM--
-TPMQMW-
TTPMQM--

-TPMQMH-
TFPMQM--
TAPMQM--
TPPMQM--
-TPMQMN-
-TPMQMA-
TLPMQM--
TIPMQM--
TVPMQM--
-TPMQMY-
TYPMQM--
-TPMQME-
TCPMQM--
-VPMQMR-
VTPMQM--
-VPMQML-
-VPMQMW-
VSPMQM--
-VPMQMY-
-VPMQMS-
VFPMQM--
-VPMQMK-
-VPMQMG-
-VPMQMM-
VLPMQM--
VKPMQM--
VGPMQM--
VYPMQM--
VWPMQM--
VQPMQM--
VMPMQM--
VEPMQM--
VAPMQM--
-VPMQMV-
-VPMQMA-
VCPMQM--
WFPMQM--
-WPMQMA-
WPPMQM--
-WPMQMS-
-WPMQMH-
WMPMQM--
-WPMQMM-
WYPMQM--
WQPMQM--
-WPMQMN-
-WPMQMT-
-WPMQMR-
-WPMQMG-
-YPMQML-

```
          -YPMQMN-
          -YPMQMI-
          -YPMQMT-
          -YPMQMV-
          -YPMQMM-
          YKPMQM--
          YTPMQM--
          -YPMQMY-
          -YPMQMK-
          YRPMQM--
          YMPMQM--
          YYPMQM--
          YVPMQM--
          -YPMQMQ-
          YAPMQM--
PAPL        12        900      46.24
          --PAPLPR
          -RPAPLP-
          -VPAPLP-
          -APAPLK-
          AVPAPL--
          ASPAPL--
          -APAPLL-
          -APAPLA-
          AIPAPL--
          AFPAPL--
          ALPAPL--
          AAPAPL--
          AWPAPL--
          -APAPLY-
          -APAPLS-
          -APAPLR-
          APPAPL--
          -APAPLM-
          -APAPLF-
          AMPAPL--
          AYPAPL--
          -APAPLV-
          ANPAPL--
          ARPAPL--
          -APAPLQ-
          AQPAPL--
          -APAPLI-
          -APAPLG-
          ATPAPL--
          -APAPLN-
          -APAPLT-
          AEPAPL--
          ACPAPL--
          AHPAPL--
```

AGPAPL--
-APAPLH-
-APAPLE-
CRPAPL--
CSPAPL--
-CPAPLR-
CGPAPL--
CWPAPL--
-CPAPLS-
CAPAPL--
-CPAPLY-
-CPAPLL-
-CPAPLH-
CMPAPL--
CYPAPL--
CVPAPL--
CTPAPL--
CPPAPL--
-CPAPLW-
-CPAPLV-
-CPAPLQ-
-CPAPLK-
CKPAPL--
CCPAPL--
DSPAPL--
-DPAPLF-
DWPAPL--
-DPAPLL-
DFPAPL--
DRPAPL--
DQPAPL--
-DPAPLY-
-DPAPLR-
DKPAPL--
-DPAPLW-
DVPAPL--
DTPAPL--
-DPAPLM-
-DPAPLH-
DNPAPL--
-DPAPLS-
-DPAPLK-
DMPAPL--
DLPAPL--
-DPAPLT-
-DPAPLI-
DIPAPL--
DGPAPL--
DEPAPL--
DAPAPL--

-EPAPLL-
-EPAPLT-
EGPAPL--
-EPAPLW-
-EPAPLH-
ESPAPL--
-EPAPLY-
-EPAPLI-
-EPAPLK-
EMPAPL--
-EPAPLM-
EVPAPL--
-EPAPLR-
-EPAPLS-
ELPAPL--
EWPAPL--
EYPAPL--
EEPAPL--
ETPAPL--
EQPAPL--
-EPAPLQ-
EIPAPL--
EAPAPL--
ERPAPL--
-EPAPLN-
EFPAPL--
EPPAPL--
-EPAPLG-
-EPAPLE-
FRPAPL--
FGPAPL--
FNPAPL--
-FPAPLY-
-FPAPLA-
FSPAPL--
FQPAPL--
FPPAPL--
FIPAPL--
FLPAPL--
-FPAPLR-
FFPAPL--
-FPAPLS-
-FPAPLN-
-FPAPLK-
FHPAPL--
-FPAPLT-
-FPAPLL-
FTPAPL--
-FPAPLW-
-FPAPLF-

FYPAPL--
FVPAPL--
-FPAPLV-
-FPAPLM-
-FPAPLI-
-FPAPLQ-
-FPAPLE-
GKPAPL--
-GPAPLR-
GTPAPL--
-GPAPLS-
-GPAPLL-
GGPAPL--
-GPAPLI-
GYPAPL--
GHPAPL--
-GPAPLM-
GQPAPL--
GRPAPL--
-GPAPLV-
GFPAPL--
-GPAPLF-
GLPAPL--
-GPAPLY-
-GPAPLK-
-GPAPLA-
-GPAPLT-
-GPAPLH-
GNPAPL--
GMPAPL--
GEPAPL--
-GPAPLQ-
GVPAPL--
GDPAPL--
-GPAPLN-
-GPAPLW-
GIPAPL--
HIPAPL--
HGPAPL--
HRPAPL--
-HPAPLK-
HKPAPL--
-HPAPLY-
HLPAPL--
-HPAPLF-
-HPAPLW-
HNPAPL--
HVPAPL--
-HPAPLL-
HFPAPL--

HAPAPL--
-HPAPLS-
HYPAPL--
HWPAPL--
HQPAPL--
HMPAPL--
HTPAPL--
-HPAPLV-
-HPAPLT-
-HPAPLQ-
-HPAPLA-
-HPAPLN-
HHPAPL--
HEPAPL--
HDPAPL--
HCPAPL--
-HPAPLI-
-HPAPLH-
-IPAPLR-
IGPAPL--
ISPAPL--
IVPAPL--
ITPAPL--
IEPAPL--
IRPAPL--
ILPAPL--
-IPAPLK-
IKPAPL--
IYPAPL--
IPPAPL--
-IPAPLS-
-IPAPLH-
IMPAPL--
IIPAPL--
-IPAPLN-
-IPAPLI-
INPAPL--
-IPAPLL-
-IPAPLA-
-IPAPLY-
-IPAPLT-
IHPAPL--
-IPAPLQ-
-IPAPLV-
-IPAPLF-
IAPAPL--
-IPAPLE-
IDPAPL--
-IPAPLW-
-IPAPLG-

-IPAPLM-
-KPAPLR-
-KPAPLV-
KVPAPL--
KAPAPL--
KIPAPL--
-KPAPLT-
KFPAPL--
-KPAPLY-
-KPAPLQ-
-KPAPLA-
KLPAPL--
KTPAPL--
-KPAPLK-
KHPAPL--
KYPAPL--
KRPAPL--
KGPAPL--
KEPAPL--
KPPAPL--
-KPAPLN-
-KPAPLI-
KWPAPL--
KKPAPL--
KDPAPL--
KQPAPL--
-KPAPLM-
KNPAPL--
KMPAPL--
LRPAPL--
-LPAPLR-
LVPAPL--
LKPAPL--
LGPAPL--
-LPAPLM-
LYPAPL--
-LPAPLY-
LTPAPL--
LSPAPL--
LEPAPL--
LQPAPL--
LPPAPL--
-LPAPLK-
-LPAPLH-
-LPAPLA-
-LPAPLS-
-LPAPLL-
LMPAPL--
LNPAPL--
LFPAPL--

LWPAPL--
LLPAPL--
LHPAPL--
LDPAPL--
-LPAPLV-
-LPAPLT-
LIPAPL--
-LPAPLI-
-LPAPLQ-
-LPAPLN-
-LPAPLF-
-LPAPLG-
-LPAPLP-
-LPAPLD-
MYPAPL--
-MPAPLR-
MRPAPL--
MLPAPL--
MTPAPL--
-MPAPLT-
MGPAPL--
MPPAPL--
-MPAPLW-
-MPAPLS-
-MPAPLK-
-MPAPLQ-
MVPAPL--
-MPAPLV-
-MPAPLH-
MIPAPL--
MFPAPL--
MWPAPL--
-MPAPLI-
MNPAPL--
-MPAPLA-
MHPAPL--
MDPAPL--
-MPAPLY-
MMPAPL--
MKPAPL--
MAPAPL--
-MPAPLG-
-MPAPLF-
-MPAPLM-
-MPAPLL-
NYPAPL--
NWPAPL--
NRPAPL--
NTPAPL--
NIPAPL--

NSPAPL--
NKPAPL--
-NPAPLY-
NLPAPL--
-NPAPLF-
-NPAPLR-
NNPAPL--
NAPAPL--
-NPAPLL-
-NPAPLS-
NGPAPL--
NEPAPL--
NVPAPL--
-NPAPLH-
NMPAPL--
-NPAPLK-
NPPAPL--
-NPAPLV-
-NPAPLI-
NHPAPL--
NQPAPL--
-NPAPLN-
-NPAPLM-
NDPAPL--
-NPAPLQ-
--PAPLVK
--PAPLHH
--PAPLRG
--PAPLAL
--PAPLHS
--PAPLVL
--PAPLRS
--PAPLSS
--PAPLEL
--PAPLAA
--PAPLRI
--PAPLLT
--PAPLLN
--PAPLYL
--PAPLNL
--PAPLFR
--PAPLLG
--PAPLVS
--PAPLRT
--PAPLFS
--PAPLTM
--PAPLSV
--PAPLSG
--PAPLQA
--PAPLIR

--PAPLYS
--PAPLTI
--PAPLTG
--PAPLVY
--PAPLFN
--PAPLLV
--PAPLFL
--PAPLWQ
--PAPLST
--PAPLGS
--PAPLMS
--PAPLIM
--PAPLGH
--PAPLML
--PAPLYI
--PAPLWG
--PAPLRQ
PVPAPL--
--PAPLYA
--PAPLWY
--PAPLWH
--PAPLNF
--PAPLLW
--PAPLHL
--PAPLGI
--PAPLYT
--PAPLSY
--PAPLSM
--PAPLQL
--PAPLNV
--PAPLAV
--PAPLVR
--PAPLVG
--PAPLSF
--PAPLRN
PSPAPL--
--PAPLYV
--PAPLTL
--PAPLRA
--PAPLMT
--PAPLIL
--PAPLLR
--PAPLKL
--PAPLFG
--PAPLGF
--PAPLRM
--PAPLLA
--PAPLYK
--PAPLWV
--PAPLKG

--PAPLHF
--PAPLDL
PRPAPL--
PLPAPL--
--PAPLYW
--PAPLVV
--PAPLQS
--PAPLTS
--PAPLTR
--PAPLSQ
--PAPLSA
--PAPLRY
--PAPLRK
--PAPLFV
--PAPLLS
--PAPLFT
--PAPLDI
--PAPLWN
--PAPLRW
--PAPLPV
--PAPLLK
--PAPLKR
--PAPLGY
--PAPLGA
--PAPLEF
--PAPLSN
--PAPLQG
--PAPLLQ
--PAPLKA
--PAPLIT
--PAPLAN
--PAPLYY
--PAPLVM
--PAPLSH
--PAPLMH
--PAPLKF
--PAPLHR
--PAPLNH
--PAPLMV
PKPAPL--
--PAPLVN
--PAPLRD
--PAPLMA
--PAPLKV
--PAPLIK
--PAPLGN
--PAPLDY
--PAPLWR
--PAPLMR
--PAPLLI

--PAPLIW
--PAPLIN
--PAPLFH
--PAPLVT
--PAPLTV
--PAPLTT
--PAPLSR
--PAPLQT
--PAPLQQ
--PAPLPS
--PAPLNY
--PAPLKY
--PAPLKT
PGPAPL--
--PAPLTA
--PAPLRH
--PAPLLF
--PAPLSW
--PAPLNN
--PAPLKM
--PAPLIY
--PAPLIS
--PAPLIF
--PAPLHV
--PAPLHK
--PAPLHI
--PAPLET
--PAPLWD
--PAPLVI
--PAPLVF
--PAPLTY
--PAPLTN
--PAPLTK
--PAPLPF
--PAPLNA
--PAPLMQ
--PAPLMG
--PAPLKW
--PAPLKS
--PAPLIH
--PAPLIG
--PAPLHT
--PAPLHM
--PAPLFQ
--PAPLYQ
--PAPLVW
--PAPLVE
--PAPLPI
--PAPLKI
--PAPLIV

--PAPLAM
--PAPLYG
--PAPLWM
--PAPLVD
--PAPLQK
--PAPLHG
--PAPLGG
--PAPLDM
--PAPLAW
--PAPLQM
--PAPLNT
--PAPLNG
--PAPLLD
--PAPLDV
PIPAPL--
PFPAPL--
--PAPLWK
--PAPLSK
--PAPLRE
--PAPLPY
--PAPLME
--PAPLIQ
--PAPLGQ
--PAPLES
PMPAPL--
--PAPLWF
--PAPLTD
--PAPLQW
--PAPLQH
--PAPLHY
--PAPLGW
--PAPLGV
--PAPLDW
--PAPLDF
--PAPLAI
PQPAPL--
--PAPLYN
--PAPLWT
--PAPLTQ
--PAPLLM
--PAPLKQ
--PAPLIA
--PAPLEQ
--PAPLAH
PHPAPL--
--PAPLYD
--PAPLVH
--PAPLTW
--PAPLQY
--PAPLPW

--PAPLNI
--PAPLMN
--PAPLKH
--PAPLII
--PAPLHN
--PAPLGT
--PAPLGK
--PAPLEI
--PAPLAT
--PAPLAK
--PAPLAG
PYPAPL--
--PAPLYF
--PAPLQI
--PAPLPM
--PAPLPH
--PAPLNQ
--PAPLLH
--PAPLHQ
--PAPLFW
--PAPLFA
--PAPLEK
--PAPLCK
--PAPLAF
PWPAPL--
--PAPLYH
--PAPLPT
--PAPLNE
--PAPLMK
--PAPLFM
--PAPLDH
PEPAPL--
PDPAPL--
--PAPLWI
--PAPLWE
--PAPLWA
--PAPLVQ
--PAPLQN
--PAPLQF
--PAPLMM
--PAPLLE
--PAPLKD
--PAPLHA
--PAPLGE
--PAPLFK
--PAPLFD
--PAPLEH
--PAPLDS
--PAPLCV
--PAPLCR

--PAPLAQ
--PAPLAE
-PPAPLY-
-PPAPLV-
-PPAPLS-
-PPAPLQ-
-PPAPLP-
-PPAPLL-
-PPAPLH-
-PPAPLG-
PNPAPL--
--PAPLYM
--PAPLWW
--PAPLWC
--PAPLTF
--PAPLTE
--PAPLSD
--PAPLSC
--PAPLPN
--PAPLPA
--PAPLNW
--PAPLNK
--PAPLND
--PAPLMI
--PAPLKE
--PAPLKC
--PAPLIE
--PAPLID
--PAPLHD
--PAPLEW
--PAPLDT
--PAPLDK
--PAPLDA
--PAPLCT
--PAPLCN
--PAPLCF
--PAPLAC
-QPAPLR-
QWPAPL--
-QPAPLI-
-QPAPLY-
-QPAPLW-
QYPAPL--
QAPAPL--
QIPAPL--
-QPAPLS-
-QPAPLV-
QRPAPL--
QKPAPL--
QTPAPL--

QSPAPL--
-QPAPLL-
QVPAPL--
-QPAPLK-
-QPAPLF-
QNPAPL--
QMPAPL--
QLPAPL--
QQPAPL--
-QPAPLT-
-QPAPLM-
-QPAPLH-
-QPAPLA-
QGPAPL--
-QPAPLN-
QEPAPL--
QPPAPL--
QHPAPL--
QDPAPL--
-RPAPLT-
-RPAPLR-
RLPAPL--
RSPAPL--
RVPAPL--
-RPAPLH-
-RPAPLS-
-RPAPLQ-
RNPAPL--
RIPAPL--
REPAPL--
RYPAPL--
-RPAPLK-
RAPAPL--
RPPAPL--
-RPAPLI-
RTPAPL--
-RPAPLV-
RDPAPL--
-RPAPLN-
-RPAPLW-
-RPAPLA-
RFPAPL--
RHPAPL--
-RPAPLM-
RQPAPL--
-RPAPLG-
RCPAPL--
RMPAPL--
-RPAPLE-
-RPAPLC-

-SPAPLR-
-SPAPLW-
SVPAPL--
-SPAPLF-
SAPAPL--
SEPAPL--
SMPAPL--
SSPAPL--
SRPAPL--
SLPAPL--
STPAPL--
SPPAPL--
-SPAPLK-
SYPAPL--
SFPAPL--
SIPAPL--
-SPAPLS-
-SPAPLT-
SNPAPL--
SGPAPL--
SWPAPL--
-SPAPLV-
SQPAPL--
-SPAPLA-
-SPAPLI-
-SPAPLY-
-SPAPLN-
-SPAPLH-
SKPAPL--
-SPAPLM-
-SPAPLG-
SDPAPL--
-SPAPLQ-
-SPAPLE-
-SPAPLC-
SCPAPL--
TGPAPL--
-TPAPLR-
-TPAPLY-
-TPAPLF-
-TPAPLL-
-TPAPLH-
-TPAPLT-
TVPAPL--
TSPAPL--
TEPAPL--
TRPAPL--
-TPAPLV-
-TPAPLM-
TKPAPL--

TYPAPL--
TTPAPL--
-TPAPLK-
TNPAPL--
TPPAPL--
-TPAPLS-
-TPAPLN-
-TPAPLI-
TDPAPL--
THPAPL--
TAPAPL--
-TPAPLW-
TWPAPL--
TQPAPL--
-TPAPLA-
TMPAPL--
-TPAPLG-
-TPAPLQ-
VVPAPL--
-VPAPLW-
-VPAPLR-
VFPAPL--
VRPAPL--
VKPAPL--
-VPAPLL-
-VPAPLT-
VLPAPL--
VQPAPL--
VGPAPL--
-VPAPLF-
VTPAPL--
VIPAPL--
-VPAPLY-
-VPAPLI-
-VPAPLK-
-VPAPLQ-
VNPAPL--
VYPAPL--
-VPAPLM-
-VPAPLA-
VPPAPL--
-VPAPLS-
-VPAPLV-
-VPAPLH-
VWPAPL--
-VPAPLN-
-VPAPLE-
VEPAPL--
VAPAPL--
-VPAPLG-

WIPAPL--
WGPAPL--
WRPAPL--
WNPAPL--
WTPAPL--
WVPAPL--
-WPAPLY-
-WPAPLF-
WSPAPL--
-WPAPLK-
-WPAPLW-
-WPAPLS-
-WPAPLR-
WKPAPL--
WHPAPL--
WFPAPL--
WWPAPL--
-WPAPLA-
-WPAPLT-
-WPAPLN-
-WPAPLL-
-WPAPLV-
WQPAPL--
WDPAPL--
WAPAPL--
-WPAPLG-
-WPAPLQ-
-WPAPLI-
WMPAPL--
WEPAPL--
WPPAPL--
WCPAPL--
-YPAPLR-
YSPAPL--
-YPAPLI-
YIPAPL--
YRPAPL--
YAPAPL--
YTPAPL--
YQPAPL--
-YPAPLT-
YNPAPL--
-YPAPLS-
YLPAPL--
-YPAPLA-
YVPAPL--
YHPAPL--
YFPAPL--
YYPAPL--
-YPAPLY-

-YPAPLH-
-YPAPLK-
-YPAPLF-
YWPAPL--
-YPAPLV-
-YPAPLN-
YDPAPL--
-YPAPLW-
-YPAPLQ-
-YPAPLM-
YMPAPL--
YPPAPL--
-YPAPLG-
-YPAPLE-
YKPAPL--
YGPAPL--

PRAY	348	677	46.22

--PRAYFP
-APRAYL-
AAPRAY--
AFPRAY--
ANPRAY--
AGPRAY--
-APRAYM-
AIPRAY--
-APRAYF-
AVPRAY--
ATPRAY--
-APRAYY-
-APRAYH-
AQPRAY--
-APRAYQ-
-APRAYG-
-APRAYI-
-APRAYV-
ADPRAY--
-APRAYE-
AMPRAY--
-APRAYN-
-APRAYW-
AYPRAY--
AEPRAY--
APPRAY--
AHPRAY--
-APRAYC-
CRPRAY--
-CPRAYT-
CHPRAY--
CEPRAY--
CKPRAY--

-CPRAYS-
CVPRAY--
CWPRAY--
CNPRAY--
CSPRAY--
-CPRAYL-
CCPRAY--
CAPRAY--
-CPRAYR-
-CPRAYI-
-CPRAYK-
CLPRAY--
CTPRAY--
CQPRAY--
-CPRAYM-
CPPRAY--
CYPRAY--
-CPRAYV-
CIPRAY--
CGPRAY--
CFPRAY--
DYPRAY--
DSPRAY--
DAPRAY--
-DPRAYK-
-DPRAYS-
DRPRAY--
DQPRAY--
DKPRAY--
-DPRAYH-
DFPRAY--
-DPRAYI-
DDPRAY--
DMPRAY--
DLPRAY--
DTPRAY--
-DPRAYA-
-DPRAYL-
-DPRAYY-
-DPRAYN-
-DPRAYF-
-DPRAYT-
DNPRAY--
DVPRAY--
-DPRAYV-
DGPRAY--
DHPRAY--
-DPRAYQ-
-DPRAYM-
-DPRAYE-

DPPRAY--
-EPRAYS-
ESPRAY--
ELPRAY--
-EPRAYA-
-EPRAYL-
EVPRAY--
-EPRAYF-
-EPRAYR-
ETPRAY--
EKPRAY--
EYPRAY--
EQPRAY--
EMPRAY--
-EPRAYG-
ERPRAY--
EIPRAY--
-EPRAYT-
-EPRAYK-
-EPRAYI-
EAPRAY--
-EPRAYW-
-EPRAYQ-
-EPRAYM-
EHPRAY--
EFPRAY--
-EPRAYN-
-EPRAYH-
ENPRAY--
EGPRAY--
-EPRAYV-
-EPRAYE-
EDPRAY--
FVPRAY--
FGPRAY--
FRPRAY--
FDPRAY--
FYPRAY--
FQPRAY--
-FPRAYN-
FKPRAY--
FIPRAY--
FPPRAY--
-FPRAYE-
FNPRAY--
FEPRAY--
GGPRAY--
GIPRAY--
-GPRAYW-
GRPRAY--

-GPRAYM-
GLPRAY--
GAPRAY--
GNPRAY--
GMPRAY--
GYPRAY--
GHPRAY--
-GPRAYL-
-GPRAYQ-
GSPRAY--
GFPRAY--
-GPRAYA-
GTPRAY--
-GPRAYT-
GPPRAY--
-GPRAYV-
-GPRAYN-
GDPRAY--
-GPRAYH-
-GPRAYG-
-GPRAYY-
GQPRAY--
-GPRAYS-
-GPRAYI-
-GPRAYE-
GEPRAY--
HRPRAY--
HSPRAY--
HPPRAY--
-HPRAYI-
-HPRAYL-
HMPRAY--
HAPRAY--
-HPRAYH-
HYPRAY--
HIPRAY--
-HPRAYN-
HGPRAY--
HEPRAY--
HQPRAY--
HHPRAY--
HVPRAY--
HNPRAY--
-HPRAYG-
-HPRAYE-
HFPRAY--
-HPRAYW-
-IPRAYS-
ISPRAY--
IPPRAY--

-IPRAYV-
IIPRAY--
IEPRAY--
-IPRAYQ-
-IPRAYH-
IDPRAY--
IGPRAY--
IMPRAY--
-IPRAYT-
IVPRAY--
IHPRAY--
INPRAY--
-IPRAYG-
IAPRAY--
IQPRAY--
-IPRAYC-
-KPRAYL-
KNPRAY--
-KPRAYV-
KMPRAY--
-KPRAYI-
-KPRAYN-
KGPRAY--
KRPRAY--
-KPRAYA-
-KPRAYM-
-KPRAYF-
KQPRAY--
-KPRAYK-
-KPRAYY-
KHPRAY--
-KPRAYG-
KEPRAY--
-KPRAYE-
KPPRAY--
KDPRAY--
-KPRAYD-
-LPRAYS-
-LPRAYT-
-LPRAYV-
LGPRAY--
-LPRAYQ-
LPPRAY--
LAPRAY--
-LPRAYM-
-LPRAYW-
-LPRAYI-
-LPRAYF-
LMPRAY--
-LPRAYY-

-LPRAYN-
LYPRAY--
LDPRAY--
LQPRAY--
LHPRAY--
-LPRAYE-
LEPRAY--
LCPRAY--
MSPRAY--
-MPRAYT-
-MPRAYF-
MTPRAY--
-MPRAYI-
MMPRAY--
-MPRAYS-
MVPRAY--
MGPRAY--
MFPRAY--
MQPRAY--
-MPRAYA-
MPPRAY--
MHPRAY--
MAPRAY--
MDPRAY--
-MPRAYN-
-MPRAYY-
MKPRAY--
MIPRAY--
-MPRAYM-
MEPRAY--
-MPRAYE-
-MPRAYK-
MCPRAY--
-MPRAYQ-
-NPRAYS-
NTPRAY--
NVPRAY--
NWPRAY--
NGPRAY--
-NPRAYV-
NIPRAY--
NQPRAY--
NHPRAY--
-NPRAYY-
NPPRAY--
-NPRAYF-
NNPRAY--
-NPRAYH-
-NPRAYM-
NEPRAY--

-NPRAYQ-
-NPRAYI-
NFPRAY--
-NPRAYA-
NCPRAY--
--PRAYMV
--PRAYFL
--PRAYVV
--PRAYTL
--PRAYWA
--PRAYTS
--PRAYVY
--PRAYAG
--PRAYSG
--PRAYHS
--PRAYIT
--PRAYGA
--PRAYKA
--PRAYIS
--PRAYYV
--PRAYSF
PVPRAY--
PGPRAY--
--PRAYQL
--PRAYHG
--PRAYYM
--PRAYYR
--PRAYYI
--PRAYTN
--PRAYLM
--PRAYKT
--PRAYLT
--PRAYSW
--PRAYLW
--PRAYKN
--PRAYYN
PKPRAY--
--PRAYLH
PAPRAY--
PRPRAY--
--PRAYSK
PLPRAY--
--PRAYNL
--PRAYYS
--PRAYGV
--PRAYVN
--PRAYVK
--PRAYTF
--PRAYNT
--PRAYVH

--PRAYSQ
--PRAYAN
--PRAYVI
--PRAYTI
--PRAYGT
--PRAYAQ
--PRAYYL
--PRAYYA
--PRAYVW
--PRAYYG
--PRAYSD
--PRAYKW
--PRAYIG
--PRAYGM
PMPRAY--
PYPRAY--
--PRAYMA
--PRAYIL
--PRAYGQ
--PRAYSN
--PRAYPF
--PRAYEL
--PRAYRE
--PRAYLI
--PRAYIM
--PRAYFN
--PRAYAS
--PRAYAA
--PRAYWF
--PRAYRI
--PRAYMY
--PRAYLQ
--PRAYAK
--PRAYQV
--PRAYQT
--PRAYMN
--PRAYLN
--PRAYWI
--PRAYIV
--PRAYAT
--PRAYYD
--PRAYWG
--PRAYVE
--PRAYTQ
--PRAYTE
--PRAYRM
--PRAYQY
--PRAYGI
--PRAYTV
--PRAYNY

--PRAYNV
--PRAYKM
--PRAYET
PQPRAY--
PTPRAY--
--PRAYYQ
--PRAYLY
--PRAYKH
--PRAYGW
--PRAYFE
--PRAYFA
--PRAYWM
--PRAYNA
--PRAYKV
--PRAYFQ
--PRAYDL
PDPRAY--
--PRAYWT
--PRAYTH
--PRAYLD
--PRAYIY
PNPRAY--
--PRAYTK
--PRAYMI
--PRAYIF
--PRAYID
--PRAYAM
--PRAYYY
--PRAYWE
--PRAYEA
--PRAYTD
--PRAYNH
--PRAYHE
--PRAYPM
--PRAYIQ
--PRAYHQ
--PRAYFH
--PRAYAY
PIPRAY--
PHPRAY--
--PRAYWV
--PRAYQE
--PRAYNK
--PRAYME
--PRAYMD
--PRAYHD
--PRAYGF
--PRAYFM
--PRAYYW
--PRAYQG

--PRAYNF
--PRAYAH
--PRAYVM
--PRAYQQ
--PRAYNW
--PRAYIH
--PRAYGN
--PRAYEH
--PRAYEF
--PRAYYH
--PRAYYF
--PRAYSE
--PRAYQD
--PRAYPV
--PRAYMF
--PRAYII
--PRAYIE
--PRAYIA
--PRAYHW
--PRAYHN
--PRAYHI
--PRAYGK
--PRAYFI
--PRAYEG
--PRAYAI
--PRAYYE
--PRAYQK
--PRAYQF
--PRAYKE
--PRAYGR
--PRAYGG
--PRAYQM
--PRAYNM
--PRAYNI
--PRAYHY
--PRAYHH
--PRAYDM
--PRAYMQ
--PRAYKF
--PRAYHM
--PRAYFW
--PRAYEV
--PRAYEI
--PRAYDI
--PRAYWD
--PRAYTY
--PRAYIW
--PRAYHF
--PRAYHA
--PRAYER

--PRAYWW
--PRAYWQ
--PRAYVF
--PRAYPY
--PRAYPA
--PRAYNG
--PRAYND
--PRAYMH
--PRAYKP
--PRAYES
--PRAYEQ
--PRAYEN
--PRAYDG
--PRAYDA
--PRAYCN
--PRAYCA
--PRAYAD
--PRAYSC
--PRAYQC
--PRAYPT
--PRAYNN
--PRAYLC
--PRAYFD
--PRAYEY
--PRAYDS
--PRAYCK
--PRAYCG
--PRAYCF
--PRAYCD
PPPRAY--
PEPRAY--
QAPRAY--
QYPRAY--
-QPRAYS-
-QPRAYT-
-QPRAYA-
QGPRAY--
QNPRAY--
QHPRAY--
-QPRAYY-
QMPRAY--
-QPRAYV-
QVPRAY--
-QPRAYH-
QPPRAY--
-QPRAYI-
-QPRAYK-
QLPRAY--
QDPRAY--
QTPRAY--

-QPRAYN-
QEPRAY--
-QPRAYQ-
-QPRAYF-
-QPRAYE-
QKPRAY--
-QPRAYG-
QIPRAY--
-QPRAYW-
QCPRAY--
-RPRAYV-
-RPRAYS-
-RPRAYT-
-RPRAYY-
RGPRAY--
-RPRAYM-
RDPRAY--
RPPRAY--
RTPRAY--
-RPRAYA-
RIPRAY--
-RPRAYN-
RNPRAY--
-RPRAYW-
RHPRAY--
RAPRAY--
-RPRAYG-
-RPRAYH-
-RPRAYE-
RCPRAY--
-RPRAYC-
RKPRAY--
-RPRAYD-
-SPRAYS-
SVPRAY--
-SPRAYL-
SSPRAY--
SHPRAY--
-SPRAYT-
SAPRAY--
STPRAY--
SKPRAY--
-SPRAYW-
-SPRAYH-
SMPRAY--
-SPRAYA-
-SPRAYQ-
-SPRAYY-
SFPRAY--
-SPRAYV-

−SPRAYG−
SDPRAY−−
−SPRAYE−
−SPRAYN−
SEPRAY−−
SPPRAY−−
−SPRAYM−
SCPRAY−−
TLPRAY−−
TVPRAY−−
TSPRAY−−
−TPRAYS−
−TPRAYN−
TKPRAY−−
TIPRAY−−
TGPRAY−−
−TPRAYT−
TNPRAY−−
TMPRAY−−
THPRAY−−
−TPRAYV−
TPPRAY−−
TEPRAY−−
−TPRAYH−
−TPRAYQ−
TDPRAY−−
−TPRAYG−
TQPRAY−−
−TPRAYM−
TYPRAY−−
TAPRAY−−
−TPRAYE−
TCPRAY−−
VTPRAY−−
VSPRAY−−
−VPRAYV−
VGPRAY−−
−VPRAYS−
−VPRAYK−
VPPRAY−−
−VPRAYA−
VYPRAY−−
−VPRAYI−
−VPRAYN−
VLPRAY−−
−VPRAYM−
−VPRAYY−
−VPRAYH−
VEPRAY−−
−VPRAYQ−

-VPRAYG-
VHPRAY--
VQPRAY--
VDPRAY--
-VPRAYF-
-VPRAYE-
-VPRAYD-
WTPRAY--
WRPRAY--
WKPRAY--
WAPRAY--
-WPRAYV-
WGPRAY--
WEPRAY--
WPPRAY--
WMPRAY--
WYPRAY--
WQPRAY--
WLPRAY--
-WPRAYN-
WHPRAY--
WFPRAY--
WDPRAY--
-WPRAYE-
YGPRAY--
YTPRAY--
-YPRAYI-
YAPRAY--
-YPRAYW-
-YPRAYM-
-YPRAYA-
-YPRAYV-
-YPRAYF-
YQPRAY--
-YPRAYN-
YYPRAY--
YKPRAY--
YNPRAY--
YPPRAY--
-YPRAYH-
YHPRAY--
YDPRAY--
-YPRAYE-
YVPRAY--

PAWL 190 755 46.22

--PAWLGP
--PAWLEK
-APAWLT-
ASPAWL--
-APAWLY-

AVPAWL--
AFPAWL--
-APAWLS-
AIPAWL--
AEPAWL--
AAPAWL--
AKPAWL--
AWPAWL--
ANPAWL--
ATPAWL--
-APAWLM-
-APAWLH-
-APAWLA-
AYPAWL--
-APAWLE-
-APAWLG-
-APAWLN-
AGPAWL--
AQPAWL--
-APAWLV-
ALPAWL--
-APAWLF-
-APAWLD-
AHPAWL--
-APAWLK-
-APAWLI-
APPAWL--
-APAWLQ-
ACPAWL--
AMPAWL--
CRPAWL--
-CPAWLR-
CPPAWL--
CEPAWL--
-CPAWLS-
CQPAWL--
CNPAWL--
CIPAWL--
CSPAWL--
-CPAWLT-
CHPAWL--
CAPAWL--
-CPAWLY-
-CPAWLK-
CLPAWL--
DLPAWL--
DQPAWL--
DHPAWL--
DGPAWL--
DMPAWL--

DVPAWL--
DAPAWL--
DTPAWL--
DIPAWL--
-DPAWLS-
-DPAWLI-
-DPAWLY-
-DPAWLL-
-DPAWLG-
DNPAWL--
DWPAWL--
DYPAWL--
DRPAWL--
-DPAWLR-
-DPAWLK-
-DPAWLH-
DSPAWL--
-DPAWLF-
-DPAWLW-
-DPAWLV-
-DPAWLT-
DPPAWL--
-DPAWLN-
-DPAWLA-
DKPAWL--
EAPAWL--
ESPAWL--
-EPAWLA-
-EPAWLR-
-EPAWLS-
-EPAWLH-
-EPAWLL-
EVPAWL--
-EPAWLK-
EKPAWL--
ELPAWL--
EHPAWL--
EYPAWL--
ENPAWL--
ETPAWL--
EGPAWL--
-EPAWLG-
ERPAWL--
EPPAWL--
-EPAWLQ-
-EPAWLN-
-EPAWLF-
EQPAWL--
-EPAWLT-
-EPAWLI-

EIPAWL--
-EPAWLM-
EMPAWL--
EFPAWL--
EWPAWL--
-EPAWLW-
-EPAWLV-
-EPAWLE-
-FPAWLK-
FTPAWL--
FSPAWL--
-FPAWLR-
FDPAWL--
-FPAWLA-
FAPAWL--
FKPAWL--
FVPAWL--
FQPAWL--
-FPAWLI-
FPPAWL--
-FPAWLT-
-FPAWLE-
FLPAWL--
FWPAWL--
-FPAWLH-
-FPAWLG-
-FPAWLM-
FHPAWL--
FYPAWL--
-FPAWLV-
-FPAWLL-
FNPAWL--
-GPAWLS-
GVPAWL--
GRPAWL--
-GPAWLR-
-GPAWLT-
GGPAWL--
GLPAWL--
GTPAWL--
GSPAWL--
-GPAWLL-
-GPAWLA-
-GPAWLV-
-GPAWLG-
GMPAWL--
GIPAWL--
GAPAWL--
GWPAWL--
-GPAWLK-

GHPAWL--
-GPAWLQ-
GDPAWL--
-GPAWLH-
GKPAWL--
GYPAWL--
-GPAWLI-
-GPAWLF-
GPPAWL--
-GPAWLM-
GNPAWL--
GEPAWL--
GQPAWL--
-GPAWLN-
-GPAWLE-
GCPAWL--
-GPAWLD-
HSPAWL--
HEPAWL--
-HPAWLL-
HAPAWL--
-HPAWLK-
HQPAWL--
-HPAWLV-
-HPAWLI-
-HPAWLT-
HTPAWL--
HGPAWL--
HDPAWL--
HPPAWL--
-HPAWLR-
HWPAWL--
HLPAWL--
HYPAWL--
-HPAWLQ-
-HPAWLN-
HNPAWL--
HRPAWL--
-HPAWLY-
-HPAWLF-
-HPAWLW-
-HPAWLG-
-HPAWLA-
HFPAWL--
-HPAWLH-
-IPAWLR-
IGPAWL--
IKPAWL--
-IPAWLT-
-IPAWLS-

IFPAWL--
IVPAWL--
IIPAWL--
ITPAWL--
-IPAWLI-
-IPAWLG-
IHPAWL--
-IPAWLA-
IMPAWL--
ILPAWL--
IYPAWL--
ISPAWL--
-IPAWLL-
-IPAWLN-
INPAWL--
-IPAWLH-
IPPAWL--
-IPAWLM-
IEPAWL--
IQPAWL--
-IPAWLW-
IWPAWL--
-IPAWLK-
IAPAWL--
-IPAWLQ-
-IPAWLE-
IDPAWL--
KSPAWL--
-KPAWLS-
KIPAWL--
KVPAWL--
KLPAWL--
KPPAWL--
-KPAWLA-
KNPAWL--
-KPAWLH-
KAPAWL--
KYPAWL--
KRPAWL--
-KPAWLI-
-KPAWLG-
-KPAWLY-
KDPAWL--
-KPAWLQ-
-KPAWLK-
KQPAWL--
-KPAWLW-
-KPAWLF-
KGPAWL--
-KPAWLT-

-KPAWLN-
KWPAWL--
KEPAWL--
KTPAWL--
KKPAWL--
KHPAWL--
LRPAWL--
LPPAWL--
LVPAWL--
-LPAWLS-
LEPAWL--
-LPAWLT-
LNPAWL--
LQPAWL--
-LPAWLI-
LIPAWL--
LYPAWL--
LLPAWL--
LDPAWL--
LFPAWL--
LCPAWL--
LWPAWL--
-LPAWLV-
-LPAWLG-
-LPAWLQ-
-LPAWLA-
MNPAWL--
MWPAWL--
-MPAWLI-
MVPAWL--
-MPAWLR-
MSPAWL--
-MPAWLT-
MQPAWL--
MKPAWL--
MAPAWL--
MGPAWL--
MHPAWL--
-MPAWLV-
-MPAWLL-
MLPAWL--
-MPAWLA-
-MPAWLS-
MFPAWL--
MPPAWL--
-MPAWLY-
MTPAWL--
-MPAWLQ-
MEPAWL--
-MPAWLN-

```
-MPAWLM-
-MPAWLG-
-MPAWLF-
-MPAWLK-
-MPAWLH-
-MPAWLD-
MMPAWL--
MDPAWL--
NRPAWL--
NTPAWL--
NKPAWL--
-NPAWLS-
NSPAWL--
NWPAWL--
NPPAWL--
NMPAWL--
-NPAWLR-
NHPAWL--
NLPAWL--
-NPAWLG-
NIPAWL--
-NPAWLT-
NFPAWL--
NVPAWL--
NYPAWL--
-NPAWLQ-
-NPAWLI-
-NPAWLH-
NGPAWL--
NAPAWL--
NQPAWL--
NEPAWL--
-NPAWLY-
-NPAWLW-
-NPAWLK-
-NPAWLA-
-NPAWLN-
-NPAWLL-
NNPAWL--
-NPAWLM-
-NPAWLF-
--PAWLGS
--PAWLRN
--PAWLYG
--PAWLAA
--PAWLLN
--PAWLVG
--PAWLTS
--PAWLRG
--PAWLQS
```

--PAWLVS
--PAWLRH
PMPAWL--
--PAWLTK
--PAWLTG
--PAWLFS
PSPAWL--
--PAWLMH
--PAWLRF
--PAWLKR
--PAWLTT
--PAWLSK
--PAWLES
--PAWLTW
--PAWLIG
--PAWLGT
--PAWLMS
--PAWLKS
--PAWLGG
--PAWLTN
--PAWLSM
--PAWLMG
--PAWLRD
--PAWLIS
--PAWLHT
--PAWLVT
PVPAWL--
--PAWLYS
--PAWLLG
--PAWLSQ
--PAWLVA
--PAWLDS
--PAWLMT
--PAWLMK
--PAWLGV
--PAWLRV
--PAWLKY
--PAWLIN
--PAWLAN
--PAWLAS
PKPAWL--
--PAWLWS
--PAWLVR
--PAWLTE
--PAWLST
--PAWLSN
--PAWLKK
--PAWLGY
--PAWLAY
--PAWLRA

--PAWLNG
--PAWLKL
PEPAWL--
--PAWLTQ
--PAWLLK
--PAWLKE
--PAWLIK
PAPAWL--
--PAWLKI
--PAWLKH
--PAWLIQ
--PAWLIA
--PAWLTI
--PAWLLW
--PAWLIR
--PAWLIH
--PAWLGA
--PAWLEN
--PAWLWG
--PAWLVQ
--PAWLSI
--PAWLRY
--PAWLQA
--PAWLHG
--PAWLGI
PIPAWL--
--PAWLYT
--PAWLQH
--PAWLNT
--PAWLNA
--PAWLIV
--PAWLGN
PFPAWL--
--PAWLVW
--PAWLVI
--PAWLVD
--PAWLSY
--PAWLSW
--PAWLRI
--PAWLQN
--PAWLNY
--PAWLME
--PAWLMA
--PAWLKQ
--PAWLHR
--PAWLAM
PDPAWL--
--PAWLYH
--PAWLVK
--PAWLTV

--PAWLTD
--PAWLSE
--PAWLSD
--PAWLQW
--PAWLMD
--PAWLLH
--PAWLHS
--PAWLEH
--PAWLAF
--PAWLRM
--PAWLQT
--PAWLQR
--PAWLQQ
--PAWLNM
--PAWLMQ
--PAWLMN
--PAWLHA
--PAWLGK
--PAWLEA
--PAWLYN
--PAWLTM
--PAWLQY
--PAWLQI
--PAWLMI
--PAWLKT
--PAWLYK
--PAWLYD
--PAWLTH
--PAWLTA
--PAWLSH
--PAWLQG
--PAWLNW
--PAWLMW
--PAWLHI
--PAWLFN
--PAWLWQ
--PAWLVH
--PAWLNQ
--PAWLLE
--PAWLKD
--PAWLIM
--PAWLHN
--PAWLGQ
--PAWLFH
--PAWLAK
--PAWLAD
PYPAWL--
PGPAWL--
--PAWLWN
--PAWLQV

--PAWLDT
--PAWLWH
--PAWLTY
--PAWLLI
--PAWLKW
--PAWLKM
--PAWLIE
--PAWLHV
--PAWLHH
--PAWLHD
--PAWLGF
--PAWLFE
--PAWLEY
--PAWLET
--PAWLEM
--PAWLEG
PQPAWL--
PNPAWL--
PHPAWL--
--PAWLWK
--PAWLTF
--PAWLNV
--PAWLNN
--PAWLLM
--PAWLHK
--PAWLGW
--PAWLGR
--PAWLGH
--PAWLFD
-PPAWLT-
--PAWLYW
--PAWLYM
--PAWLWE
--PAWLQF
--PAWLPA
--PAWLNK
--PAWLNH
--PAWLKG
--PAWLIY
--PAWLID
--PAWLHL
--PAWLHF
--PAWLEW
--PAWLEV
--PAWLEQ
--PAWLDG
--PAWLDA
--PAWLAE
QTPAWL--
QIPAWL--

-QPAWLS-
-QPAWLR-
QRPAWL--
-QPAWLV-
-QPAWLQ-
QGPAWL--
QPPAWL--
-QPAWLA-
-QPAWLL-
QMPAWL--
-QPAWLG-
QSPAWL--
-QPAWLW-
QVPAWL--
-QPAWLT-
-QPAWLI-
QYPAWL--
-QPAWLK-
QNPAWL--
QHPAWL--
-QPAWLH-
QFPAWL--
QQPAWL--
-QPAWLY-
-QPAWLN-
QAPAWL--
-QPAWLF-
-QPAWLE-
QLPAWL--
QKPAWL--
-QPAWLM-
-QPAWLD-
QDPAWL--
QCPAWL--
RSPAWL--
RGPAWL--
-RPAWLT-
RHPAWL--
REPAWL--
-RPAWLK-
-RPAWLS-
RTPAWL--
-RPAWLY-
-RPAWLI-
-RPAWLH-
RIPAWL--
-RPAWLV-
-RPAWLN-
-RPAWLM-
RLPAWL--

RDPAWL--
-RPAWLG-
RNPAWL--
RQPAWL--
RMPAWL--
RAPAWL--
-RPAWLA-
-RPAWLE-
RYPAWL--
RWPAWL--
RCPAWL--
SVPAWL--
-SPAWLR-
SSPAWL--
SKPAWL--
SRPAWL--
-SPAWLG-
SAPAWL--
SMPAWL--
-SPAWLI-
-SPAWLY-
SYPAWL--
-SPAWLS-
STPAWL--
-SPAWLV-
SLPAWL--
SIPAWL--
-SPAWLL-
-SPAWLT-
-SPAWLK-
SHPAWL--
-SPAWLQ-
SQPAWL--
-SPAWLM-
-SPAWLA-
SDPAWL--
-SPAWLH-
SPPAWL--
-SPAWLN-
SNPAWL--
-SPAWLD-
-SPAWLE-
SEPAWL--
TAPAWL--
TPPAWL--
-TPAWLL-
TNPAWL--
-TPAWLA-
-TPAWLQ-
-TPAWLS-

```
-TPAWLM-
THPAWL--
TVPAWL--
-TPAWLN-
-TPAWLT-
TTPAWL--
TWPAWL--
-TPAWLY-
-TPAWLH-
TIPAWL--
-TPAWLK-
-TPAWLG-
TQPAWL--
-TPAWLV-
-TPAWLI-
TEPAWL--
TKPAWL--
-TPAWLE-
-TPAWLF-
TDPAWL--
-TPAWLW-
TCPAWL--
-TPAWLD-
VSPAWL--
-VPAWLS-
-VPAWLR-
VNPAWL--
-VPAWLI-
VQPAWL--
-VPAWLK-
VTPAWL--
VAPAWL--
VHPAWL--
VFPAWL--
-VPAWLQ-
-VPAWLG-
-VPAWLV-
VLPAWL--
VEPAWL--
VDPAWL--
-VPAWLL-
VMPAWL--
-VPAWLM-
-VPAWLH-
VGPAWL--
-VPAWLE-
-VPAWLA-
VWPAWL--
VIPAWL--
-VPAWLD-
```

VPPAWL--
WPPAWL--
WQPAWL--
WTPAWL--
-WPAWLQ-
WAPAWL--
WSPAWL--
-WPAWLT-
WVPAWL--
-WPAWLI-
WHPAWL--
WEPAWL--
WWPAWL--
-WPAWLH-
WLPAWL--
WNPAWL--
-WPAWLS-
-WPAWLR-
WDPAWL--
-WPAWLM-
-WPAWLD-
-WPAWLW-
-WPAWLV-
-WPAWLA-
YEPAWL--
-YPAWLK-
YFPAWL--
-YPAWLG-
YHPAWL--
YMPAWL--
YTPAWL--
-YPAWLQ-
-YPAWLS-
-YPAWLH-
YNPAWL--
YVPAWL--
YGPAWL--
-YPAWLY-
-YPAWLN-
-YPAWLV-
-YPAWLA-
YAPAWL--
-YPAWLW-
-YPAWLT-
YCPAWL--
YQPAWL--
YWPAWL--
-YPAWLL-
-YPAWLI-
YDPAWL--

PKEL 99 807 46.22

```
--PKELLL
--PKELGN
--PKELAR
-RPKELE-
STPKEL--
ARPKEL--
ASPKEL--
AGPKEL--
AVPKEL--
-APKELV-
-APKELY-
-APKELR-
-APKELQ-
-APKELG-
-APKELF-
-APKELA-
APPKEL--
-APKELW-
AIPKEL--
-APKELL-
AFPKEL--
AKPKEL--
AHPKEL--
-APKELM-
-APKELS-
AEPKEL--
-APKELN-
ATPKEL--
-APKELT-
AAPKEL--
-APKELK-
ACPKEL--
-APKELI-
ALPKEL--
AYPKEL--
AMPKEL--
ADPKEL--
CTPKEL--
CGPKEL--
CVPKEL--
CKPKEL--
CIPKEL--
CHPKEL--
CEPKEL--
CSPKEL--
CRPKEL--
CPPKEL--
-CPKELR-
-CPKELM-
```

CLPKEL--
-DPKELK-
DKPKEL--
DWPKEL--
DSPKEL--
-DPKELW-
-DPKELR-
DYPKEL--
DFPKEL--
-DPKELF-
DTPKEL--
DQPKEL--
DVPKEL--
-DPKELS-
-DPKELI-
DNPKEL--
DMPKEL--
DHPKEL--
DGPKEL--
DRPKEL--
DPPKEL--
-DPKELL-
-DPKELH-
DIPKEL--
DAPKEL--
ESPKEL--
-EPKELR-
EYPKEL--
-EPKELS-
EVPKEL--
-EPKELL-
EKPKEL--
EGPKEL--
-EPKELT-
ETPKEL--
EFPKEL--
EAPKEL--
ERPKEL--
-EPKELN-
EQPKEL--
-EPKELK-
EHPKEL--
-EPKELY-
-EPKELI-
EMPKEL--
EWPKEL--
-EPKELV-
-EPKELQ-
-EPKELH-
EDPKEL--

FRPKEL--
FVPKEL--
FSPKEL--
FAPKEL--
-FPKELN-
-FPKELL-
FWPKEL--
FFPKEL--
FNPKEL--
FMPKEL--
FQPKEL--
-FPKELS-
-FPKELM-
-FPKELK-
FGPKEL--
-FPKELI-
-FPKELF-
FLPKEL--
-FPKELY-
-FPKELQ-
FYPKEL--
-FPKELT-
FDPKEL--
FCPKEL--
-FPKELP-
-FPKELH-
-FPKELG-
-GPKELR-
-GPKELL-
GFPKEL--
-GPKELT-
GWPKEL--
GTPKEL--
-GPKELH-
-GPKELI-
-GPKELY-
GGPKEL--
GRPKEL--
GNPKEL--
GLPKEL--
GPPKEL--
GSPKEL--
-GPKELS-
-GPKELM-
GHPKEL--
GYPKEL--
-GPKELQ-
GIPKEL--
GQPKEL--
-GPKELV-

-GPKELN-
-GPKELF-
GAPKEL--
-GPKELA-
GDPKEL--
-GPKELW-
-GPKELG-
-GPKELK-
-GPKELD-
GEPKEL--
HRPKEL--
-HPKELK-
HYPKEL--
HWPKEL--
HLPKEL--
-HPKELQ-
-HPKELL-
-HPKELT-
-HPKELI-
HVPKEL--
HSPKEL--
-HPKELS-
HNPKEL--
HAPKEL--
HHPKEL--
-HPKELV-
-HPKELH-
HTPKEL--
HPPKEL--
HQPKEL--
-HPKELN-
HIPKEL--
HGPKEL--
-HPKELY-
-HPKELG-
-HPKELA-
HFPKEL--
HEPKEL--
IHPKEL--
IRPKEL--
-IPKELK-
ITPKEL--
-IPKELF-
ILPKEL--
ISPKEL--
IWPKEL--
-IPKELR-
-IPKELW-
IGPKEL--
-IPKELV-

-IPKELN-
IIPKEL--
IFPKEL--
IAPKEL--
-IPKELT-
IMPKEL--
IEPKEL--
-IPKELS-
IQPKEL--
-IPKELL-
IDPKEL--
-IPKELI-
IKPKEL--
-IPKELA-
-IPKELH-
IYPKEL--
-IPKELM-
IPPKEL--
-IPKELY-
-IPKELG-
KSPKEL--
KTPKEL--
-KPKELW-
KRPKEL--
KLPKEL--
KAPKEL--
-KPKELF-
KPPKEL--
KGPKEL--
KYPKEL--
-KPKELV-
KVPKEL--
KHPKEL--
-KPKELL-
-KPKELA-
-KPKELR-
-KPKELY-
-KPKELH-
-KPKELG-
-KPKELN-
KMPKEL--
-KPKELS-
-KPKELK-
-KPKELI-
-KPKELT-
-KPKELQ-
-KPKELM-
KKPKEL--
KFPKEL--
LAPKEL--

LVPKEL--
-LPKELR-
LSPKEL--
LGPKEL--
LFPKEL--
LTPKEL--
-LPKELK-
LYPKEL--
LIPKEL--
LLPKEL--
LQPKEL--
LNPKEL--
LKPKEL--
-LPKELS-
LPPKEL--
-LPKELY-
-LPKELL-
-LPKELT-
-LPKELF-
-LPKELM-
-LPKELI-
LMPKEL--
-LPKELW-
-LPKELV-
-LPKELN-
LHPKEL--
LEPKEL--
-LPKELQ-
LDPKEL--
-LPKELH-
MSPKEL--
-MPKELN-
-MPKELK-
MIPKEL--
MWPKEL--
-MPKELR-
MNPKEL--
MYPKEL--
MVPKEL--
-MPKELV-
MAPKEL--
-MPKELW-
-MPKELL-
MRPKEL--
MPPKEL--
-MPKELY-
-MPKELG-
MMPKEL--
-MPKELS-
MQPKEL--

```
-MPKELT-
-MPKELI-
MTPKEL--
MLPKEL--
MHPKEL--
MFPKEL--
MDPKEL--
-MPKELM-
MGPKEL--
-MPKELH-
-MPKELA-
NLPKEL--
NRPKEL--
NYPKEL--
NGPKEL--
-NPKELW-
NSPKEL--
-NPKELR-
-NPKELK-
NAPKEL--
NKPKEL--
NEPKEL--
-NPKELL-
NTPKEL--
-NPKELV-
-NPKELS-
-NPKELN-
NNPKEL--
NHPKEL--
NFPKEL--
-NPKELY-
-NPKELH-
NMPKEL--
NIPKEL--
NVPKEL--
NQPKEL--
-NPKELM-
-NPKELG-
-NPKELA-
NDPKEL--
NCPKEL--
--PKELIT
--PKELRN
--PKELRR
--PKELRI
--PKELLS
--PKELKS
--PKELVD
--PKELRA
--PKELRY
```

--PKELNM
--PKELLG
--PKELVW
--PKELSW
--PKELRM
--PKELLY
--PKELIS
--PKELAS
--PKELRW
--PKELMA
--PKELVR
--PKELRF
--PKELNL
PRPKEL--
--PKELVQ
--PKELTA
--PKELRQ
--PKELVM
--PKELRV
--PKELRG
--PKELLT
--PKELKL
--PKELIR
PVPKEL--
--PKELWV
--PKELTS
--PKELST
--PKELYQ
--PKELVN
--PKELSI
--PKELKV
PSPKEL--
--PKELTT
--PKELSV
--PKELWS
--PKELSY
--PKELNY
--PKELKF
--PKELIG
--PKELIA
--PKELAA
--PKELWF
--PKELVY
--PKELVA
--PKELLW
--PKELLV
--PKELFT
--PKELVL
--PKELTN
--PKELIY

--PKELHH
--PKELTY
--PKELTW
--PKELRK
--PKELLH
--PKELKG
--PKELKA
PIPKEL--
PYPKEL--
--PKELWA
--PKELVG
--PKELTV
--PKELSQ
--PKELRE
--PKELQT
--PKELQS
--PKELMW
--PKELMV
PMPKEL--
--PKELYL
--PKELQK
--PKELFS
--PKELFR
--PKELFA
PAPKEL--
PLPKEL--
--PKELYV
--PKELYK
--PKELYG
--PKELWY
--PKELWQ
--PKELVF
--PKELVE
--PKELSR
--PKELSL
--PKELMR
--PKELMG
--PKELLI
--PKELKR
--PKELKN
--PKELHW
--PKELFL
--PKELYF
--PKELVT
--PKELVI
--PKELSN
--PKELQL
--PKELNT
--PKELNS
--PKELHN

--PKELGS
--PKELFF
PTPKEL--
--PKELRD
--PKELMS
--PKELME
--PKELIL
--PKELFY
--PKELFW
--PKELFV
PNPKEL--
--PKELYS
--PKELWL
--PKELVV
--PKELTK
--PKELTG
--PKELSH
--PKELQA
--PKELNV
--PKELML
--PKELLR
--PKELKW
PGPKEL--
PWPKEL--
--PKELYN
--PKELYE
--PKELWR
--PKELTI
--PKELSF
--PKELNW
--PKELNH
--PKELMK
--PKELKH
--PKELIW
--PKELEA
--PKELAF
PKPKEL--
--PKELYA
--PKELQV
--PKELQR
--PKELQG
--PKELMM
--PKELLQ
--PKELHS
--PKELHI
--PKELHF
--PKELFK
--PKELAW
--PKELAI
--PKELYI

--PKELWW
--PKELWK
--PKELWH
--PKELVH
--PKELTR
--PKELTM
--PKELQF
--PKELMN
--PKELMH
--PKELKE
--PKELIN
--PKELIK
--PKELHL
--PKELFI
--PKELEG
--PKELAV
--PKELAH
--PKELTQ
--PKELTL
--PKELTF
--PKELSK
--PKELQY
--PKELQN
--PKELNK
--PKELNA
--PKELMQ
--PKELMF
--PKELKK
--PKELIM
--PKELIE
--PKELHG
--PKELHD
--PKELGW
--PKELGV
--PKELGH
--PKELGA
--PKELFG
--PKELAQ
--PKELAN
--PKELYD
--PKELWE
--PKELWC
--PKELTH
--PKELTE
--PKELSS
--PKELSM
--PKELRP
--PKELRH
--PKELQW
--PKELQI

--PKELNG
--PKELNE
--PKELND
--PKELLE
--PKELLC
--PKELKY
--PKELKT
--PKELII
--PKELDQ
--PKELCT
--PKELCA
--PKELAY
PHPKEL--
PQPKEL--
--PKELWT
--PKELVK
--PKELTD
--PKELSD
--PKELQH
--PKELNI
--PKELMT
--PKELLM
--PKELLD
--PKELKI
--PKELIH
--PKELHM
--PKELHE
--PKELHA
--PKELGT
--PKELFQ
--PKELFN
--PKELFM
--PKELFH
--PKELFE
--PKELFC
--PKELEY
--PKELES
--PKELEH
PEPKEL--
QAPKEL--
QRPKEL--
-QPKELR-
QGPKEL--
QVPKEL--
QNPKEL--
-QPKELS-
-QPKELN-
QTPKEL--
QSPKEL--
-QPKELL-

QLPKEL--
QFPKEL--
QPPKEL--
-QPKELW-
-QPKELV-
QKPKEL--
QWPKEL--
QHPKEL--
QQPKEL--
-QPKELM-
QYPKEL--
-QPKELT-
-QPKELQ-
-QPKELI-
-QPKELH-
QMPKEL--
QCPKEL--
-QPKELY-
-QPKELK-
QIPKEL--
RLPKEL--
RWPKEL--
RVPKEL--
RMPKEL--
RFPKEL--
RAPKEL--
-RPKELY-
-RPKELT-
-RPKELR-
RSPKEL--
RQPKEL--
-RPKELK-
-RPKELW-
RPPKEL--
RHPKEL--
-RPKELG-
-RPKELA-
RRPKEL--
-RPKELS-
-RPKELQ-
-RPKELI-
-RPKELV-
-RPKELH-
-RPKELF-
RNPKEL--
-RPKELN-
-RPKELM-
RCPKEL--
RGPKEL--
RDPKEL--

REPKEL--
SVPKEL--
-SPKELW-
-SPKELN-
-SPKELR-
SPPKEL--
SRPKEL--
-SPKELL-
-SPKELT-
SGPKEL--
SSPKEL--
-SPKELS-
SMPKEL--
SKPKEL--
-SPKELF-
-SPKELK-
SHPKEL--
SEPKEL--
SWPKEL--
SFPKEL--
-SPKELI-
-SPKELY-
-SPKELQ-
-SPKELH-
-SPKELM-
-SPKELG-
SNPKEL--
-SPKELA-
SIPKEL--
SQPKEL--
SDPKEL--
SCPKEL--
TAPKEL--
TPPKEL--
TLPKEL--
-TPKELY-
-TPKELV-
TMPKEL--
-TPKELS-
-TPKELK-
-TPKELA-
-TPKELQ-
TGPKEL--
TRPKEL--
TQPKEL--
-TPKELL-
TWPKEL--
TKPKEL--
TIPKEL--
TSPKEL--

-TPKELH-
TNPKEL--
TDPKEL--
-TPKELT-
-TPKELN-
-TPKELI-
THPKEL--
TTPKEL--
-TPKELG-
TYPKEL--
-TPKELM-
TCPKEL--
-VPKELR-
-VPKELN-
VLPKEL--
VAPKEL--
VPPKEL--
VHPKEL--
VSPKEL--
-VPKELS-
VTPKEL--
VWPKEL--
-VPKELV-
-VPKELI-
VYPKEL--
VKPKEL--
VRPKEL--
-VPKELK-
-VPKELW-
VNPKEL--
VVPKEL--
VEPKEL--
-VPKELT-
-VPKELF-
-VPKELQ-
-VPKELM-
VMPKEL--
VQPKEL--
VIPKEL--
-VPKELY-
-VPKELH-
-VPKELA-
VGPKEL--
VFPKEL--
VCPKEL--
-VPKELG-
WSPKEL--
-WPKELS-
WVPKEL--
WDPKEL--

WGPKEL--
WWPKEL--
WIPKEL--
WTPKEL--
WLPKEL--
-WPKELH-
WHPKEL--
-WPKELA-
-WPKELQ-
-WPKELL-
WAPKEL--
WQPKEL--
-WPKELV-
-WPKELN-
WNPKEL--
WKPKEL--
WEPKEL--
WPPKEL--
-WPKELI-
-WPKELW-
-WPKELM-
-WPKELK-
WFPKEL--
-WPKELY-
-WPKELT-
-WPKELF-
-WPKELE-
YTPKEL--
YLPKEL--
YVPKEL--
YRPKEL--
YEPKEL--
YNPKEL--
-YPKELK-
YMPKEL--
YPPKEL--
YAPKEL--
-YPKELR-
-YPKELS-
YKPKEL--
YYPKEL--
YSPKEL--
-YPKELN-
-YPKELY-
YIPKEL--
YFPKEL--
-YPKELT-
-YPKELI-
-YPKELH-
-YPKELF-

	-YPKELA-		
	YWPKEL--		
	-YPKELL-		
	-YPKELW-		
	YGPKEL--		
	-YPKELQ-		
	-YPKELG-		
	YDPKEL--		
PQAY	291	702	46.21
	AGPQAY--		
	-APQAYV-		
	-APQAYR-		
	AVPQAY--		
	ARPQAY--		
	AMPQAY--		
	ALPQAY--		
	ASPQAY--		
	-APQAYQ-		
	-APQAYI-		
	ATPQAY--		
	-APQAYW-		
	-APQAYH-		
	-APQAYA-		
	AKPQAY--		
	-APQAYY-		
	AWPQAY--		
	-APQAYG-		
	APPQAY--		
	AFPQAY--		
	ACPQAY--		
	AYPQAY--		
	AQPQAY--		
	ANPQAY--		
	AAPQAY--		
	-APQAYN-		
	-APQAYM-		
	-APQAYE-		
	AEPQAY--		
	ADPQAY--		
	CSPQAY--		
	CLPQAY--		
	-CPQAYH-		
	CPPQAY--		
	CGPQAY--		
	CFPQAY--		
	-CPQAYL-		
	-CPQAYF-		
	CMPQAY--		
	CEPQAY--		
	-CPQAYA-		

DLPQAY--
DRPQAY--
-DPQAYR-
-DPQAYY-
-DPQAYT-
DSPQAY--
DWPQAY--
DVPQAY--
DTPQAY--
DQPQAY--
-DPQAYH-
-DPQAYS-
-DPQAYM-
-DPQAYK-
-DPQAYI-
-DPQAYA-
DIPQAY--
DEPQAY--
-DPQAYV-
ERPQAY--
EEPQAY--
-EPQAYR-
-EPQAYV-
-EPQAYT-
-EPQAYL-
EFPQAY--
-EPQAYQ-
EWPQAY--
ESPQAY--
-EPQAYY-
-EPQAYN-
-EPQAYK-
-EPQAYH-
EIPQAY--
-EPQAYA-
EPPQAY--
EQPQAY--
-EPQAYI-
-EPQAYD-
ELPQAY--
EKPQAY--
EAPQAY--
FRPQAY--
-FPQAYY-
FVPQAY--
-FPQAYV-
-FPQAYS-
FLPQAY--
FTPQAY--
-FPQAYN-

FNPQAY--
FSPQAY--
-FPQAYT-
-FPQAYK-
-FPQAYI-
-FPQAYR-
FEPQAY--
-FPQAYM-
FKPQAY--
FGPQAY--
FWPQAY--
FHPQAY--
FFPQAY--
FDPQAY--
FPPQAY--
FMPQAY--
FAPQAY--
GRPQAY--
-GPQAYH-
-GPQAYL-
-GPQAYS-
GNPQAY--
GIPQAY--
-GPQAYK-
GMPQAY--
GSPQAY--
-GPQAYA-
GLPQAY--
GYPQAY--
-GPQAYT-
-GPQAYN-
GWPQAY--
GVPQAY--
GQPQAY--
-GPQAYR-
GFPQAY--
GKPQAY--
GTPQAY--
-GPQAYW-
-GPQAYI-
-GPQAYV-
-GPQAYM-
GHPQAY--
HRPQAY--
HGPQAY--
-HPQAYI-
HAPQAY--
HYPQAY--
HFPQAY--
-HPQAYY-

−HPQAYL−
HTPQAY−−
−HPQAYR−
HNPQAY−−
HLPQAY−−
−HPQAYF−
HSPQAY−−
−HPQAYH−
−HPQAYV−
−HPQAYS−
HPPQAY−−
HKPQAY−−
−HPQAYN−
−HPQAYG−
HIPQAY−−
IGPQAY−−
−IPQAYL−
ISPQAY−−
−IPQAYS−
−IPQAYH−
IPPQAY−−
IHPQAY−−
−IPQAYT−
IWPQAY−−
IRPQAY−−
−IPQAYF−
IQPQAY−−
−IPQAYY−
IEPQAY−−
−IPQAYR−
−IPQAYV−
ILPQAY−−
IKPQAY−−
IDPQAY−−
IMPQAY−−
IAPQAY−−
−IPQAYQ−
−IPQAYE−
−IPQAYA−
IFPQAY−−
−IPQAYN−
−IPQAYG−
KSPQAY−−
KYPQAY−−
KGPQAY−−
KRPQAY−−
−KPQAYS−
−KPQAYW−
KTPQAY−−
KWPQAY−−

-KPQAYV-
KVPQAY--
KQPQAY--
-KPQAYT-
-KPQAYI-
-KPQAYL-
KLPQAY--
-KPQAYH-
-KPQAYK-
KPPQAY--
KFPQAY--
-KPQAYE-
KMPQAY--
-KPQAYG-
KNPQAY--
KEPQAY--
KCPQAY--
-KPQAYY-
-KPQAYQ-
-KPQAYF-
KIPQAY--
KDPQAY--
KAPQAY--
LSPQAY--
LRPQAY--
LKPQAY--
-LPQAYR-
LAPQAY--
-LPQAYV-
LLPQAY--
LPPQAY--
LMPQAY--
LGPQAY--
LYPQAY--
LEPQAY--
-LPQAYL-
LIPQAY--
-LPQAYK-
LVPQAY--
-LPQAYY-
-LPQAYM-
LTPQAY--
LQPQAY--
-LPQAYH-
LHPQAY--
-LPQAYI-
-LPQAYA-
LNPQAY--
-LPQAYT-
LCPQAY--

```
-MPQAYT-
MMPQAY--
MTPQAY--
-MPQAYY-
MQPQAY--
MSPQAY--
-MPQAYR-
-MPQAYF-
MIPQAY--
-MPQAYL-
MWPQAY--
-MPQAYV-
-MPQAYS-
-MPQAYE-
-MPQAYM-
MKPQAY--
MGPQAY--
-MPQAYH-
-MPQAYA-
MPPQAY--
-MPQAYW-
-MPQAYK-
MCPQAY--
NSPQAY--
-NPQAYR-
NGPQAY--
NKPQAY--
NWPQAY--
-NPQAYL-
NYPQAY--
NVPQAY--
NDPQAY--
NQPQAY--
-NPQAYF-
NLPQAY--
NPPQAY--
-NPQAYI-
NRPQAY--
-NPQAYM-
NFPQAY--
NTPQAY--
-NPQAYS-
-NPQAYK-
-NPQAYN-
NNPQAY--
NHPQAY--
--PQAYVK
--PQAYLS
--PQAYSL
--PQAYRL
```

--PQAYNS
--PQAYRT
--PQAYRG
--PQAYKS
--PQAYYL
PRPQAY--
--PQAYRA
PTPQAY--
--PQAYRS
--PQAYRK
--PQAYMK
--PQAYVM
--PQAYTR
--PQAYTQ
--PQAYSA
--PQAYAS
--PQAYSS
--PQAYSR
--PQAYRM
--PQAYMG
--PQAYFH
--PQAYYA
--PQAYLW
--PQAYLT
--PQAYYG
--PQAYWD
--PQAYQS
--PQAYSY
--PQAYQR
--PQAYIM
PGPQAY--
--PQAYQL
--PQAYLV
--PQAYLG
--PQAYHL
--PQAYAI
PSPQAY--
--PQAYYW
--PQAYVI
--PQAYTF
--PQAYRV
--PQAYYY
--PQAYYR
--PQAYYQ
--PQAYKT
--PQAYIS
--PQAYFK
--PQAYAT
--PQAYWW
--PQAYTY

--PQAYTL
--PQAYRH
--PQAYIN
--PQAYVL
--PQAYQV
--PQAYKG
--PQAYIH
--PQAYHW
--PQAYDY
--PQAYDL
PVPQAY--
--PQAYWT
--PQAYTS
--PQAYTA
--PQAYMR
--PQAYLY
--PQAYLK
--PQAYIR
--PQAYHV
--PQAYHM
--PQAYFR
--PQAYAG
--PQAYYM
--PQAYST
--PQAYRE
--PQAYNW
--PQAYMI
--PQAYLL
--PQAYLH
--PQAYKR
--PQAYKL
--PQAYIF
--PQAYFG
--PQAYEL
--PQAYAL
--PQAYYS
--PQAYYF
--PQAYWH
--PQAYVS
--PQAYSK
--PQAYSI
--PQAYRI
--PQAYQW
--PQAYNI
--PQAYMS
--PQAYMH
--PQAYIL
--PQAYGW
--PQAYGV
--PQAYGS

PAPQAY--
PWPQAY--
--PQAYWR
--PQAYVR
--PQAYTH
--PQAYSF
--PQAYRF
--PQAYQQ
--PQAYPM
--PQAYNM
--PQAYNF
--PQAYMQ
--PQAYML
--PQAYIV
--PQAYGI
--PQAYFY
--PQAYET
--PQAYAY
--PQAYYT
--PQAYWL
--PQAYVV
--PQAYVQ
--PQAYVN
--PQAYSG
--PQAYMD
--PQAYKN
--PQAYKD
--PQAYIA
--PQAYHR
--PQAYGY
--PQAYGG
--PQAYGA
--PQAYYN
--PQAYYI
--PQAYWS
--PQAYWK
--PQAYRW
--PQAYRQ
--PQAYRN
--PQAYQM
--PQAYQK
--PQAYPI
--PQAYNT
--PQAYMA
--PQAYLI
--PQAYKW
--PQAYIT
--PQAYGT
--PQAYGH
--PQAYFN

--PQAYFD
--PQAYFA
--PQAYAR
--PQAYAQ
--PQAYAH
--PQAYAF
--PQAYWV
--PQAYWM
--PQAYWG
--PQAYSQ
--PQAYRY
--PQAYNL
--PQAYMW
--PQAYKI
--PQAYKA
--PQAYIW
--PQAYIK
--PQAYHH
--PQAYYK
--PQAYWE
--PQAYTN
--PQAYTK
--PQAYTG
--PQAYTE
--PQAYSH
--PQAYQY
--PQAYQN
--PQAYPY
--PQAYPV
--PQAYNQ
--PQAYMF
--PQAYLN
--PQAYIQ
--PQAYHS
--PQAYGM
--PQAYFL
--PQAYAW
PKPQAY--
PQPQAY--
--PQAYYD
--PQAYWY
--PQAYWP
--PQAYWA
--PQAYVG
--PQAYSN
--PQAYPQ
--PQAYNN
--PQAYNH
--PQAYMY
--PQAYMV

--PQAYMN
--PQAYLE
--PQAYLA
--PQAYKH
--PQAYII
--PQAYIG
--PQAYHT
--PQAYHE
--PQAYGN
--PQAYGF
--PQAYFV
--PQAYES
--PQAYEN
--PQAYDK
PIPQAY--
QSPQAY--
QRPQAY--
QWPQAY--
-QPQAYR-
-QPQAYV-
-QPQAYL-
QQPQAY--
-QPQAYK-
-QPQAYH-
QFPQAY--
-QPQAYS-
-QPQAYI-
QGPQAY--
QAPQAY--
-QPQAYN-
QNPQAY--
-QPQAYY-
-QPQAYW-
QPPQAY--
RSPQAY--
-RPQAYR-
-RPQAYL-
RMPQAY--
-RPQAYK-
REPQAY--
RGPQAY--
-RPQAYY-
-RPQAYF-
-RPQAYI-
RVPQAY--
-RPQAYQ-
RTPQAY--
RIPQAY--
-RPQAYA-
RYPQAY--

```
-RPQAYV-
RRPQAY--
RAPQAY--
RQPQAY--
RPPQAY--
RHPQAY--
-RPQAYS-
RKPQAY--
RDPQAY--
RWPQAY--
-RPQAYG-
-RPQAYN-
-RPQAYH-
-RPQAYE-
RNPQAY--
-RPQAYW-
RCPQAY--
SAPQAY--
SSPQAY--
-SPQAYT-
SMPQAY--
SVPQAY--
-SPQAYR-
-SPQAYV-
-SPQAYL-
SRPQAY--
-SPQAYM-
SNPQAY--
SLPQAY--
SQPQAY--
-SPQAYA-
-SPQAYI-
SEPQAY--
STPQAY--
-SPQAYQ-
SDPQAY--
SYPQAY--
SKPQAY--
SFPQAY--
-SPQAYW-
-SPQAYH-
SCPQAY--
SPPQAY--
SGPQAY--
-SPQAYY-
SIPQAY--
-SPQAYN-
-SPQAYF-
-SPQAYC-
SHPQAY--
```

-TPQAYR-
TAPQAY--
TRPQAY--
TVPQAY--
TMPQAY--
TYPQAY--
-TPQAYF-
-TPQAYS-
TLPQAY--
TGPQAY--
TQPQAY--
-TPQAYA-
TKPQAY--
TFPQAY--
TTPQAY--
-TPQAYV-
TSPQAY--
-TPQAYM-
-TPQAYW-
-TPQAYQ-
-TPQAYN-
-TPQAYH-
TNPQAY--
TDPQAY--
TWPQAY--
-TPQAYK-
-TPQAYI-
-TPQAYY-
TIPQAY--
-TPQAYG-
-TPQAYT-
TPPQAY--
THPQAY--
-VPQAYR-
VRPQAY--
-VPQAYM-
VVPQAY--
VTPQAY--
-VPQAYW-
-VPQAYV-
VLPQAY--
VWPQAY--
VQPQAY--
-VPQAYK-
-VPQAYA-
VFPQAY--
VYPQAY--
-VPQAYF-
VIPQAY--
-VPQAYS-

VCPQAY--
-VPQAYH-
VPPQAY--
VMPQAY--
-VPQAYY-
-VPQAYL-
VNPQAY--
VKPQAY--
VAPQAY--
VDPQAY--
VSPQAY--
-VPQAYG-
WLPQAY--
WQPQAY--
-WPQAYV-
-WPQAYS-
WGPQAY--
WWPQAY--
WNPQAY--
WMPQAY--
WEPQAY--
-WPQAYR-
WPPQAY--
WKPQAY--
WHPQAY--
WFPQAY--
WAPQAY--
-WPQAYW-
WVPQAY--
WRPQAY--
-WPQAYN-
-WPQAYA-
-WPQAYL-
-WPQAYQ-
-WPQAYG-
YRPQAY--
-YPQAYR-
YHPQAY--
YMPQAY--
YIPQAY--
YDPQAY--
YLPQAY--
-YPQAYQ-
YGPQAY--
YFPQAY--
-YPQAYV-
-YPQAYF-
YWPQAY--
YTPQAY--
-YPQAYY-

	-YPQAYM-		
	-YPQAYK-		
	YNPQAY--		
	YAPQAY--		
	YSPQAY--		
	-YPQAYW-		
	YVPQAY--		
	-YPQAYT-		
	-YPQAYN-		
	-YPQAYC-		
	-YPQAYA-		
PKQM	1017	487	46.21
	ARPKQM--		
	AMPKQM--		
	AVPKQM--		
	AQPKQM--		
	-APKQML-		
	AGPKQM--		
	-APKQMQ-		
	AWPKQM--		
	APPKQM--		
	-APKQMM-		
	-APKQMF-		
	-APKQME-		
	-APKQMS-		
	AYPKQM--		
	-APKQMY-		
	-APKQMG-		
	ANPKQM--		
	AHPKQM--		
	AFPKQM--		
	CSPKQM--		
	CVPKQM--		
	CCPKQM--		
	-CPKQMT-		
	-DPKQMK-		
	-DPKQMI-		
	DMPKQM--		
	DRPKQM--		
	-DPKQMY-		
	DHPKQM--		
	DFPKQM--		
	DEPKQM--		
	DVPKQM--		
	DSPKQM--		
	-DPKQMH-		
	-DPKQMN-		
	-DPKQML-		
	DGPKQM--		
	DAPKQM--		

-EPKQMK-
-EPKQML-
-EPKQMF-
EMPKQM--
-EPKQMV-
-EPKQMI-
ELPKQM--
EGPKQM--
ETPKQM--
-EPKQMT-
-EPKQMS-
-EPKQMN-
EIPKQM--
FHPKQM--
-FPKQML-
-FPKQMT-
FYPKQM--
FIPKQM--
FSPKQM--
-FPKQMF-
FTPKQM--
FQPKQM--
-FPKQMN-
FRPKQM--
-FPKQMA-
FFPKQM--
-GPKQMR-
-GPKQMQ-
-GPKQML-
GEPKQM--
GYPKQM--
-GPKQMV-
-GPKQMT-
GIPKQM--
GAPKQM--
-GPKQMM-
-GPKQMN-
GNPKQM--
GRPKQM--
-GPKQMK-
GMPKQM--
-GPKQMF-
GLPKQM--
GKPKQM--
GGPKQM--
GPPKQM--
-GPKQMY-
-GPKQMW-
-GPKQMH-
HFPKQM--

HAPKQM--
HGPKQM--
HHPKQM--
HIPKQM--
-HPKQMR-
-HPKQML-
HVPKQM--
HQPKQM--
-HPKQMY-
-HPKQMS-
IFPKQM--
IYPKQM--
-IPKQMS-
ILPKQM--
IIPKQM--
IVPKQM--
-IPKQMK-
-IPKQMH-
IDPKQM--
IQPKQM--
-IPKQMN-
IPPKQM--
IKPKQM--
IGPKQM--
IAPKQM--
ISPKQM--
-IPKQMT-
-IPKQMG-
IHPKQM--
-KPKQMK-
KTPKQM--
KSPKQM--
-KPKQMI-
-KPKQMV-
-KPKQML-
-KPKQMS-
KDPKQM--
KWPKQM--
-KPKQMT-
KIPKQM--
KAPKQM--
KRPKQM--
-KPKQMY-
KGPKQM--
-LPKQML-
LIPKQM--
-LPKQMR-
-LPKQMK-
LAPKQM--
LTPKQM--

LNPKQM--
LVPKQM--
LMPKQM--
LLPKQM--
LDPKQM--
LWPKQM--
-LPKQMS-
LSPKQM--
-LPKQMI-
-LPKQMA-
LKPKQM--
LFPKQM--
LCPKQM--
-LPKQMV-
LGPKQM--
-MPKQMS-
-MPKQMR-
-MPKQMH-
-MPKQML-
-MPKQMF-
-MPKQMT-
-MPKQMI-
-MPKQMK-
MEPKQM--
MHPKQM--
MWPKQM--
MLPKQM--
MAPKQM--
MYPKQM--
-MPKQMN-
-MPKQMA-
MMPKQM--
NSPKQM--
NPPKQM--
-NPKQMT-
-NPKQMN-
NKPKQM--
-NPKQMS-
-NPKQMR-
-NPKQMQ-
-NPKQMI-
NIPKQM--
--PKQMRL
--PKQMNS
--PKQMRG
--PKQMIS
--PKQMAG
--PKQMNT
--PKQMVS
--PKQMQS

--PKQMLT
--PKQMIT
--PKQMST
--PKQMRF
--PKQMLG
PRPKQM--
--PKQMLW
--PKQMAV
--PKQMNH
--PKQMIM
--PKQMSG
--PKQMLY
PVPKQM--
--PKQMTV
--PKQMHA
--PKQMFS
--PKQMSN
--PKQMRA
--PKQMMN
--PKQMQN
--PKQMLS
--PKQMLQ
--PKQMYG
--PKQMHT
--PKQMFV
PTPKQM--
--PKQMYM
--PKQMWY
--PKQMWS
--PKQMRQ
--PKQMQT
--PKQMHV
PSPKQM--
--PKQMYR
--PKQMWN
--PKQMVW
--PKQMVH
--PKQMVA
--PKQMSA
--PKQMQW
--PKQMNN
--PKQMMQ
--PKQMIV
--PKQMIN
--PKQMHQ
--PKQMAS
--PKQMYW
--PKQMYE
--PKQMVT
--PKQMRN

--PKQMNL
--PKQMMS
--PKQMMH
--PKQMMG
--PKQMLV
--PKQMKN
--PKQMIF
--PKQMHW
PIPKQM--
PLPKQM--
--PKQMWT
--PKQMWQ
--PKQMTN
--PKQMRY
--PKQMMW
--PKQMIH
--PKQMFR
--PKQMFK
--PKQMYD
--PKQMWH
--PKQMVR
--PKQMVN
--PKQMVM
--PKQMVI
--PKQMTF
--PKQMSM
--PKQMRI
--PKQMRD
--PKQMNW
--PKQMLL
--PKQMLH
--PKQMLF
--PKQMKG
--PKQMFY
--PKQMAW
--PKQMYS
--PKQMYN
--PKQMVY
--PKQMVQ
--PKQMRK
--PKQMQM
--PKQMQK
--PKQMNR
--PKQMMV
--PKQMLR
--PKQMKH
--PKQMIW
--PKQMIK
--PKQMHN
--PKQMGG

--PKQMFE
--PKQMET
--PKQMYK
--PKQMYI
--PKQMWI
--PKQMWE
--PKQMVV
--PKQMVD
--PKQMTM
--PKQMTL
--PKQMTK
--PKQMSY
--PKQMSV
--PKQMSQ
--PKQMRM
--PKQMMI
--PKQMMF
--PKQMLN
--PKQMLD
--PKQMKD
--PKQMIL
--PKQMIE
--PKQMGS
PNPKQM--
--PKQMYY
--PKQMYV
--PKQMYL
--PKQMYC
--PKQMWW
--PKQMWL
--PKQMWK
--PKQMTT
--PKQMTS
--PKQMTQ
--PKQMTE
--PKQMSL
--PKQMSF
--PKQMSD
--PKQMRE
--PKQMQV
--PKQMQG
--PKQMQD
--PKQMNQ
--PKQMNF
--PKQMND
--PKQMNA
--PKQMMK
--PKQMLK
--PKQMLI
--PKQMKS

--PKQMKQ
--PKQMKL
--PKQMKF
--PKQMIY
--PKQMIQ
--PKQMIA
--PKQMHL
--PKQMGY
--PKQMGA
--PKQMFW
--PKQMFN
--PKQMFM
--PKQMFA
--PKQMEA
--PKQMDW
--PKQMAN
--PKQMAM
PAPKQM--
-QPKQML-
QVPKQM--
-QPKQMS-
QSPKQM--
QIPKQM--
QEPKQM--
QGPKQM--
QTPKQM--
-QPKQMW-
QHPKQM--
QDPKQM--
-QPKQMT-
-RPKQML-
RMPKQM--
-RPKQMM-
RTPKQM--
-RPKQMW-
RPPKQM--
RLPKQM--
-RPKQMK-
RAPKQM--
RWPKQM--
-RPKQMS-
RHPKQM--
-RPKQMV-
-RPKQMQ-
RGPKQM--
RDPKQM--
-RPKQMH-
-RPKQMF-
-RPKQMY-
RNPKQM--

RKPKQM--
SHPKQM--
SVPKQM--
SAPKQM--
SSPKQM--
-SPKQMY-
-SPKQMV-
SKPKQM--
-SPKQMM-
-SPKQMF-
SIPKQM--
SYPKQM--
SPPKQM--
SNPKQM--
-SPKQMW-
-SPKQMS-
-SPKQMN-
SMPKQM--
STPKQM--
SRPKQM--
SQPKQM--
-SPKQMK-
-SPKQMI-
SEPKQM--
SLPKQM--
-SPKQMQ-
-TPKQMR-
TEPKQM--
TAPKQM--
TFPKQM--
-TPKQMT-
-TPKQML-
TNPKQM--
TYPKQM--
-TPKQMS-
TLPKQM--
TWPKQM--
TSPKQM--
-TPKQMY-
-TPKQMV-
-TPKQMI-
-TPKQMF-
-TPKQMW-
-TPKQMK-
TGPKQM--
VSPKQM--
VWPKQM--
VVPKQM--
VQPKQM--
-VPKQMM-

-VPKQML-
VMPKQM--
-VPKQMT-
VKPKQM--
VYPKQM--
VRPKQM--
-VPKQMY-
-VPKQMH-
VTPKQM--
-VPKQMK-
-VPKQMI-
VLPKQM--
VHPKQM--
-VPKQMR-
-VPKQME-
VNPKQM--
VGPKQM--
-WPKQMT-
WLPKQM--
-WPKQMF-
-WPKQMS-
-WPKQML-
-WPKQMI-
WIPKQM--
WTPKQM--
-WPKQMM-
-WPKQMN-
-WPKQMA-
WEPKQM--
WRPKQM--
WSPKQM--
-YPKQMW-
YRPKQM--
-YPKQMR-
YWPKQM--
-YPKQMT-
-YPKQML-
YFPKQM--
YNPKQM--
-YPKQMS-
-YPKQMQ-
-YPKQMN-
-YPKQMM-
-YPKQMH-
-YPKQMF-
YQPKQM--
-YPKQMK-
-YPKQMA-
YIPKQM--

PLHI 125 792 46.21

-LPLHIN-
--PLHIGL
-SPLHIP-
-SPLHIA-
ASPLHI--
-APLHIR-
-APLHIL-
AVPLHI--
ANPLHI--
AMPLHI--
AQPLHI--
ALPLHI--
-APLHIF-
ATPLHI--
AGPLHI--
-APLHIS-
-APLHIH-
-APLHIQ-
-APLHIG-
AKPLHI--
-APLHIY-
-APLHII-
AYPLHI--
-APLHIN-
ARPLHI--
-APLHIA-
AEPLHI--
APPLHI--
-APLHIW-
-APLHIV-
AIPLHI--
CSPLHI--
CQPLHI--
CPPLHI--
CHPLHI--
CTPLHI--
CRPLHI--
-CPLHIS-
-CPLHIN-
-CPLHIK-
-CPLHIE-
CFPLHI--
CAPLHI--
CYPLHI--
-CPLHIY-
-CPLHIL-
-CPLHIA-
CCPLHI--
DSPLHI--
DRPLHI--

DQPLHI--
-DPLHIW-
-DPLHIF-
DWPLHI--
-DPLHIH-
DIPLHI--
DAPLHI--
DVPLHI--
DYPLHI--
-DPLHIA-
DKPLHI--
DHPLHI--
-DPLHIL-
DEPLHI--
DDPLHI--
-DPLHIT-
-DPLHIS-
-DPLHIR-
-DPLHIK-
DMPLHI--
DLPLHI--
DGPLHI--
ETPLHI--
ERPLHI--
EKPLHI--
-EPLHIR-
ELPLHI--
EGPLHI--
-EPLHIK-
EAPLHI--
ESPLHI--
-EPLHIV-
ENPLHI--
EVPLHI--
-EPLHIT-
EIPLHI--
EYPLHI--
EQPLHI--
-EPLHII-
-EPLHIF-
EMPLHI--
-EPLHIY-
-EPLHIW-
-EPLHIN-
-EPLHIM-
FSPLHI--
FLPLHI--
FRPLHI--
-FPLHIR-
-FPLHIT-

FTPLHI--
FDPLHI--
FPPLHI--
FQPLHI--
FYPLHI--
FMPLHI--
FKPLHI--
FWPLHI--
FVPLHI--
-FPLHIV-
-FPLHII-
-FPLHIW-
-FPLHIF-
-FPLHIA-
FHPLHI--
-FPLHIN-
FGPLHI--
-FPLHIY-
-FPLHIS-
FNPLHI--
-FPLHIM-
FIPLHI--
FAPLHI--
-FPLHIK-
FEPLHI--
-FPLHIH-
GRPLHI--
GGPLHI--
GIPLHI--
-GPLHIR-
GAPLHI--
-GPLHIF-
-GPLHII-
GYPLHI--
-GPLHIQ-
GKPLHI--
GPPLHI--
-GPLHIS-
-GPLHIL-
GNPLHI--
GVPLHI--
-GPLHIV-
-GPLHIH-
GHPLHI--
GWPLHI--
-GPLHIW-
-GPLHIM-
-GPLHIA-
GFPLHI--
GDPLHI--

GLPLHI--
GQPLHI--
-GPLHIG-
GMPLHI--
-GPLHIY-
-GPLHIN-
-GPLHIK-
HRPLHI--
HGPLHI--
HVPLHI--
-HPLHIR-
HSPLHI--
-HPLHIL-
-HPLHIY-
HYPLHI--
-HPLHIQ-
-HPLHIF-
HHPLHI--
HFPLHI--
HWPLHI--
HPPLHI--
-HPLHIS-
HMPLHI--
HKPLHI--
HDPLHI--
-HPLHIW-
-HPLHIT-
-HPLHIG-
-HPLHIA-
-HPLHII-
-HPLHIH-
HEPLHI--
-HPLHIK-
HLPLHI--
HIPLHI--
IRPLHI--
IIPLHI--
IPPLHI--
ILPLHI--
IVPLHI--
-IPLHIK-
IAPLHI--
IEPLHI--
-IPLHIT-
-IPLHIM-
-IPLHIR-
IGPLHI--
-IPLHIA-
-IPLHIF-
IMPLHI--

IKPLHI--
IFPLHI--
-IPLHIS-
INPLHI--
-IPLHIV-
-IPLHIN-
IQPLHI--
-IPLHIY-
-IPLHIQ-
-IPLHII-
-IPLHIE-
ITPLHI--
ISPLHI--
-IPLHIH-
-IPLHIG-
IHPLHI--
KSPLHI--
KRPLHI--
-KPLHIY-
KAPLHI--
-KPLHIM-
-KPLHIS-
-KPLHIH-
KGPLHI--
-KPLHIK-
-KPLHII-
KIPLHI--
KYPLHI--
-KPLHIW-
KLPLHI--
KNPLHI--
KTPLHI--
KEPLHI--
-KPLHIA-
KVPLHI--
-KPLHIQ-
-KPLHIL-
-KPLHIG-
KFPLHI--
-KPLHIN-
-KPLHIE-
KPPLHI--
-KPLHIF-
-KPLHIV-
-KPLHIT-
KMPLHI--
KHPLHI--
KDPLHI--
KKPLHI--
LGPLHI--

-LPLHIR-
LKPLHI--
LHPLHI--
LEPLHI--
LVPLHI--
-LPLHIT-
LYPLHI--
-LPLHIS-
LPPLHI--
LIPLHI--
LTPLHI--
-LPLHIL-
-LPLHIV-
LQPLHI--
LWPLHI--
LMPLHI--
-LPLHII-
LFPLHI--
LDPLHI--
-LPLHIF-
-LPLHIA-
-LPLHIH-
LCPLHI--
-LPLHIY-
-LPLHIK-
LAPLHI--
-LPLHIG-
MSPLHI--
MGPLHI--
-MPLHIR-
MRPLHI--
-MPLHIS-
MLPLHI--
MVPLHI--
MQPLHI--
MHPLHI--
MNPLHI--
MFPLHI--
MYPLHI--
-MPLHII-
-MPLHIT-
-MPLHIL-
MAPLHI--
MTPLHI--
-MPLHIW-
-MPLHIM-
MIPLHI--
-MPLHIG-
-MPLHIA-
MKPLHI--

MPPLHI--
MMPLHI--
MWPLHI--
-MPLHIY-
-MPLHIN-
MEPLHI--
MCPLHI--
NGPLHI--
NWPLHI--
NSPLHI--
NRPLHI--
NFPLHI--
NHPLHI--
NKPLHI--
NLPLHI--
NTPLHI--
NIPLHI--
NEPLHI--
NMPLHI--
NPPLHI--
-NPLHIT-
-NPLHIR-
-NPLHIM-
-NPLHIW-
-NPLHIV-
-NPLHIQ-
NNPLHI--
NAPLHI--
-NPLHIY-
-NPLHIS-
-NPLHII-
-NPLHIH-
NDPLHI--
-NPLHIK-
-NPLHIA-
NQPLHI--
-NPLHIE-
-NPLHIF-
NCPLHI--
--PLHIRS
--PLHISS
--PLHIRT
--PLHIFS
--PLHIRA
--PLHILS
--PLHIFV
--PLHIVK
--PLHITR
PGPLHI--
--PLHIVS

--PLHIRF
--PLHIQR
--PLHIYH
--PLHIVR
--PLHISV
--PLHISG
--PLHIRV
--PLHIMS
--PLHIIG
--PLHITL
--PLHITG
--PLHIIH
--PLHIHG
--PLHIST
--PLHIYS
--PLHISY
--PLHISR
--PLHILA
--PLHIWH
--PLHIRG
--PLHIFT
--PLHIAS
PRPLHI--
--PLHISF
--PLHIRD
--PLHIIT
--PLHIGS
--PLHIAR
--PLHIQS
--PLHIKM
--PLHIFG
--PLHIWS
--PLHIVV
--PLHIVG
--PLHIVA
--PLHITS
--PLHISL
--PLHISK
--PLHITH
--PLHISH
--PLHIKK
--PLHIKG
--PLHIYA
--PLHIVT
--PLHIVL
--PLHITN
--PLHISM
--PLHIQM
--PLHIML
--PLHILM

--PLHIHW
--PLHIFN
PNPLHI--
--PLHITV
--PLHILT
--PLHIAV
--PLHIAA
--PLHIMT
--PLHIIA
--PLHIFM
--PLHIVN
--PLHITK
--PLHITA
--PLHISA
--PLHIRY
--PLHIIR
--PLHIHQ
--PLHIHM
PEPLHI--
--PLHITY
--PLHIRM
--PLHIMG
--PLHIME
--PLHIKA
--PLHIGG
--PLHIYT
--PLHIWG
--PLHIVM
--PLHISW
--PLHINV
--PLHIKV
--PLHIIL
--PLHIGV
--PLHIAM
--PLHIAG
--PLHIKS
--PLHIGA
--PLHIAK
--PLHIYV
--PLHIYR
--PLHIYK
--PLHIVQ
--PLHISI
--PLHINT
--PLHINA
--PLHIKQ
--PLHIGW
--PLHIFF
--PLHIYG
--PLHIWY

--PLHIWR
--PLHIWN
--PLHIVD
--PLHIRH
--PLHIMM
--PLHIMF
--PLHILY
--PLHIKL
--PLHIII
--PLHIIF
--PLHIAT
--PLHIYQ
--PLHIWA
--PLHIVH
--PLHITW
--PLHITI
--PLHIRN
--PLHIRE
--PLHIQI
--PLHILN
--PLHILD
--PLHIKY
--PLHIKR
--PLHIIQ
--PLHIHN
--PLHIFW
--PLHIEH
--PLHIEF
--PLHIAY
--PLHIAN
--PLHIAL
PTPLHI--
--PLHIYY
--PLHIWM
--PLHISN
--PLHIQT
--PLHINN
--PLHIMA
--PLHILL
--PLHILH
--PLHIIV
--PLHIIN
--PLHIID
--PLHIHT
--PLHIHA
--PLHIEV
--PLHIEL
--PLHIEG
--PLHIDS
--PLHIWI

--PLHITQ
--PLHINY
--PLHINW
--PLHINS
--PLHIMN
--PLHIKN
--PLHIKD
--PLHIIY
--PLHIAQ
--PLHIAF
--PLHIWV
--PLHIWQ
--PLHIWF
--PLHIVI
--PLHIQG
--PLHINQ
--PLHING
--PLHIMV
--PLHIMQ
--PLHIKI
--PLHIIK
--PLHIHL
--PLHIHH
--PLHIGM
--PLHIFQ
--PLHIFK
--PLHIFA
--PLHIER
--PLHIAI
--PLHIVY
--PLHIVE
--PLHITF
--PLHISE
--PLHIQK
--PLHIQH
--PLHIQE
--PLHIQD
--PLHINR
--PLHINM
--PLHIMY
--PLHIMR
--PLHIMK
--PLHILK
--PLHIKW
--PLHIHV
--PLHIHR
--PLHIGY
--PLHIEA
--PLHIAW
--PLHIAH

-PPLHIL-
-PPLHIF-
--PLHIYI
--PLHIWL
--PLHIWK
--PLHIVW
--PLHISD
--PLHIQN
--PLHIQL
--PLHIPL
--PLHIMD
--PLHILQ
--PLHILC
--PLHIKT
--PLHIKH
--PLHIKF
--PLHIKE
--PLHIIE
--PLHIHS
--PLHIHE
--PLHIGT
--PLHIGR
--PLHIGI
--PLHIGH
--PLHIGF
--PLHIFD
--PLHIED
--PLHIDT
--PLHIDG
--PLHICS
PDPLHI--
QSPLHI--
QGPLHI--
QIPLHI--
-QPLHIV-
-QPLHII-
-QPLHIS-
QAPLHI--
QHPLHI--
QRPLHI--
-QPLHIW-
-QPLHIK-
QQPLHI--
-QPLHIF-
-QPLHIT-
QVPLHI--
QPPLHI--
QMPLHI--
QLPLHI--
-QPLHIL-

QEPLHI--
QYPLHI--
QWPLHI--
-QPLHIH-
QDPLHI--
QCPLHI--
-QPLHIN-
QTPLHI--
-QPLHIP-
QKPLHI--
QFPLHI--
-RPLHIR-
RYPLHI--
RPPLHI--
RFPLHI--
RVPLHI--
-RPLHIL-
RWPLHI--
RMPLHI--
RNPLHI--
-RPLHIS-
-RPLHIK-
RDPLHI--
-RPLHIV-
RQPLHI--
RKPLHI--
RHPLHI--
-RPLHIW-
-RPLHIM-
RIPLHI--
-RPLHIF-
-RPLHIA-
-RPLHII-
RTPLHI--
-RPLHIY-
-RPLHIE-
-RPLHIQ-
-RPLHIG-
REPLHI--
-RPLHIH-
-RPLHIN-
-RPLHIC-
-SPLHIL-
SVPLHI--
-SPLHIR-
SGPLHI--
SKPLHI--
SRPLHI--
SAPLHI--
SQPLHI--

-SPLHIY-
SMPLHI--
SWPLHI--
SSPLHI--
SFPLHI--
-SPLHIV-
-SPLHII-
SPPLHI--
-SPLHIT-
-SPLHIW-
-SPLHIF-
-SPLHIS-
-SPLHIQ-
-SPLHIM-
-SPLHIK-
SEPLHI--
STPLHI--
-SPLHIE-
SHPLHI--
SYPLHI--
-SPLHIG-
SCPLHI--
SDPLHI--
TGPLHI--
TRPLHI--
-TPLHIL-
-TPLHIR-
TVPLHI--
-TPLHIW-
TTPLHI--
TQPLHI--
TPPLHI--
-TPLHIF-
TYPLHI--
TSPLHI--
-TPLHIY-
TAPLHI--
TLPLHI--
TIPLHI--
-TPLHIK-
-TPLHIM-
TEPLHI--
TWPLHI--
-TPLHIV-
-TPLHIS-
-TPLHIA-
-TPLHIE-
TKPLHI--
-TPLHIQ-
THPLHI--

TDPLHI--
TFPLHI--
-TPLHIN-
-TPLHIP-
-TPLHII-
VRPLHI--
VKPLHI--
VQPLHI--
VVPLHI--
VAPLHI--
VLPLHI--
-VPLHIL-
-VPLHIQ-
VSPLHI--
-VPLHIF-
-VPLHIR-
VYPLHI--
VTPLHI--
-VPLHIM-
-VPLHIY-
VHPLHI--
VFPLHI--
VIPLHI--
VGPLHI--
-VPLHIT-
-VPLHIA-
-VPLHIG-
-VPLHIE-
VWPLHI--
VPPLHI--
-VPLHIN-
-VPLHIH-
-VPLHIW-
-VPLHIV-
-VPLHIK-
-VPLHII-
VMPLHI--
VEPLHI--
VDPLHI--
VCPLHI--
WMPLHI--
WAPLHI--
WSPLHI--
-WPLHIK-
WEPLHI--
WHPLHI--
WQPLHI--
WVPLHI--
-WPLHIY-
WLPLHI--

	-WPLHIS-		
	WGPLHI--		
	-WPLHIR-		
	WDPLHI--		
	-WPLHIW-		
	-WPLHIT-		
	-WPLHIV-		
	WNPLHI--		
	WWPLHI--		
	-WPLHIN-		
	-WPLHIL-		
	-WPLHIE-		
	WIPLHI--		
	YIPLHI--		
	YRPLHI--		
	YGPLHI--		
	YSPLHI--		
	YNPLHI--		
	YQPLHI--		
	YVPLHI--		
	-YPLHIL-		
	-YPLHIR-		
	YEPLHI--		
	-YPLHIS-		
	YTPLHI--		
	-YPLHIA-		
	-YPLHIV-		
	-YPLHIY-		
	YYPLHI--		
	-YPLHIQ-		
	YHPLHI--		
	YAPLHI--		
	YMPLHI--		
	YFPLHI--		
	YDPLHI--		
	-YPLHIT-		
	-YPLHIK-		
	-YPLHII-		
	YWPLHI--		
	YPPLHI--		
	-YPLHIN-		
	-YPLHIM-		
PWEL	351	675	46.21
	--PWELER		
	AHPWEL--		
	ARPWEL--		
	-APWELQ-		
	-APWELT-		
	-APWELR-		
	AVPWEL--		

-APWELS-
AKPWEL--
AEPWEL--
-APWELV-
-APWELM-
-APWELG-
AAPWEL--
AYPWEL--
ATPWEL--
AIPWEL--
ANPWEL--
ADPWEL--
-APWELN-
ALPWEL--
AQPWEL--
-APWELK-
-APWELI-
AWPWEL--
CRPWEL--
CKPWEL--
-CPWELQ-
CTPWEL--
CGPWEL--
CAPWEL--
CSPWEL--
-CPWELR-
-CPWELI-
CDPWEL--
DVPWEL--
DKPWEL--
DHPWEL--
DSPWEL--
-DPWELR-
-DPWELN-
DWPWEL--
DMPWEL--
DFPWEL--
-DPWELT-
DGPWEL--
DYPWEL--
DTPWEL--
-DPWELM-
-DPWELG-
DIPWEL--
-DPWELV-
-DPWELS-
DNPWEL--
DAPWEL--
DQPWEL--
-DPWELK-

-DPWELI-
-DPWELA-
DEPWEL--
ERPWEL--
-EPWELV-
ESPWEL--
-EPWELI-
-EPWELS-
-EPWELA-
EAPWEL--
-EPWELQ-
-EPWELK-
EGPWEL--
-EPWELT-
ELPWEL--
-EPWELW-
EEPWEL--
EWPWEL--
-EPWELN-
-EPWELE-
-EPWELY-
-EPWELH-
-EPWELG-
FVPWEL--
FYPWEL--
FRPWEL--
-FPWELT-
-FPWELH-
FAPWEL--
FLPWEL--
FNPWEL--
FSPWEL--
FEPWEL--
FKPWEL--
-FPWELN-
FWPWEL--
-FPWELK-
FTPWEL--
-FPWELR-
-FPWELQ-
-FPWELM-
-FPWELA-
FIPWEL--
FFPWEL--
-FPWELV-
GRPWEL--
-GPWELT-
GTPWEL--
-GPWELR-
-GPWELV-

-GPWELA-
-GPWELK-
GGPWEL--
GNPWEL--
-GPWELS-
GSPWEL--
GIPWEL--
-GPWELH-
GEPWEL--
GLPWEL--
-GPWELW-
GQPWEL--
GFPWEL--
GCPWEL--
GMPWEL--
GHPWEL--
-GPWELN-
GWPWEL--
-GPWELM-
-GPWELI-
GKPWEL--
GAPWEL--
-GPWELQ-
-GPWELG-
-GPWELE-
-GPWELC-
GPPWEL--
HRPWEL--
-HPWELR-
-HPWELK-
-HPWELW-
-HPWELN-
-HPWELV-
HLPWEL--
-HPWELS-
HQPWEL--
-HPWELQ-
HKPWEL--
-HPWELL-
-HPWELH-
HWPWEL--
-HPWELI-
HGPWEL--
HFPWEL--
HNPWEL--
HHPWEL--
HSPWEL--
-HPWELT-
-HPWELA-
HTPWEL--

HAPWEL--
HIPWEL--
HDPWEL--
-HPWELM-
HPPWEL--
HMPWEL--
IRPWEL--
ISPWEL--
IAPWEL--
-IPWELS-
-IPWELR-
IQPWEL--
IVPWEL--
IIPWEL--
-IPWELV-
IKPWEL--
-IPWELT-
-IPWELG-
INPWEL--
ILPWEL--
IHPWEL--
IGPWEL--
ITPWEL--
-IPWELL-
IFPWEL--
IEPWEL--
IYPWEL--
-IPWELQ-
-IPWELI-
IPPWEL--
IMPWEL--
IDPWEL--
-IPWELK-
-IPWELH-
IWPWEL--
-IPWELA-
KSPWEL--
KRPWEL--
-KPWELI-
-KPWELR-
-KPWELV-
KGPWEL--
-KPWELA-
KTPWEL--
-KPWELN-
KFPWEL--
-KPWELS-
KEPWEL--
-KPWELT-
-KPWELQ-

-KPWELH-
KYPWEL--
KQPWEL--
KAPWEL--
KVPWEL--
KNPWEL--
KIPWEL--
KWPWEL--
KKPWEL--
KMPWEL--
-KPWELK-
KHPWEL--
-KPWELW-
-KPWELM-
KLPWEL--
KCPWEL--
LTPWEL--
LSPWEL--
LVPWEL--
LKPWEL--
LPPWEL--
-LPWELR-
LLPWEL--
LIPWEL--
-LPWELK-
LNPWEL--
-LPWELN-
LEPWEL--
LHPWEL--
LAPWEL--
-LPWELS-
-LPWELV-
LMPWEL--
LFPWEL--
-LPWELM-
-LPWELH-
-LPWELI-
-LPWELA-
-LPWELW-
-LPWELT-
-LPWELD-
LCPWEL--
MSPWEL--
MRPWEL--
-MPWELR-
-MPWELH-
MIPWEL--
MDPWEL--
-MPWELI-
MNPWEL--

MVPWEL--
MGPWEL--
MTPWEL--
-MPWELM-
MEPWEL--
MLPWEL--
-MPWELV-
MPPWEL--
MYPWEL--
MQPWEL--
-MPWELY-
-MPWELN-
MWPWEL--
MKPWEL--
NRPWEL--
-NPWELQ-
NTPWEL--
-NPWELV-
NLPWEL--
-NPWELK-
-NPWELN-
NKPWEL--
-NPWELW-
NFPWEL--
NYPWEL--
NNPWEL--
NIPWEL--
NHPWEL--
-NPWELY-
NGPWEL--
NVPWEL--
-NPWELI-
NMPWEL--
-NPWELH-
NEPWEL--
NCPWEL--
-NPWELS-
NAPWEL--
--PWELRS
--PWELHM
--PWELHT
--PWELLG
--PWELSS
--PWELKT
--PWELTA
--PWELKL
--PWELSR
--PWELRD
--PWELRF
--PWELSG

--PWELNA
--PWELTH
--PWELSM
--PWELKK
--PWELKG
--PWELGG
--PWELWG
--PWELRT
--PWELNH
--PWELVS
--PWELRY
--PWELRI
--PWELIS
--PWELHK
--PWELTT
--PWELRG
--PWELRE
--PWELTR
--PWELNV
--PWELKI
--PWELKH
--PWELHS
--PWELFG
--PWELTQ
--PWELSQ
--PWELNT
--PWELKW
--PWELKE
PNPWEL--
--PWELTG
--PWELQH
--PWELKN
--PWELIA
--PWELAA
PGPWEL--
--PWELYD
--PWELWN
--PWELSY
--PWELNR
--PWELKS
--PWELYN
--PWELYG
--PWELVW
--PWELVR
--PWELRQ
--PWELHR
--PWELVN
--PWELSW
--PWELSK
--PWELSD

--PWELHA
--PWELSN
--PWELNM
--PWELNF
--PWELND
--PWELKV
--PWELIY
--PWELHV
--PWELGS
--PWELAN
PAPWEL--
--PWELWR
--PWELVD
--PWELTV
--PWELSF
--PWELSA
--PWELIT
--PWELIH
--PWELTK
--PWELNK
--PWELMA
--PWELLH
--PWELHD
PVPWEL--
PIPWEL--
--PWELYT
--PWELVA
--PWELRN
--PWELYS
--PWELTS
--PWELSI
--PWELQV
--PWELNW
--PWELMS
--PWELMK
--PWELLQ
--PWELLK
--PWELKM
--PWELIG
--PWELFN
--PWELAL
PFPWEL--
--PWELVE
--PWELNN
--PWELNE
--PWELLM
--PWELKR
--PWELIR
--PWELGT
--PWELAH

PTPWEL--
PDPWEL--
--PWELYR
--PWELVI
--PWELTI
--PWELRH
--PWELQK
--PWELMY
--PWELMT
--PWELMI
--PWELIN
--PWELIK
--PWELHQ
--PWELHG
--PWELHF
--PWELGW
--PWELGF
--PWELFW
--PWELEG
--PWELAS
--PWELAQ
PSPWEL--
PKPWEL--
--PWELRW
--PWELQR
--PWELNS
--PWELNG
--PWELMR
--PWELMM
--PWELLE
--PWELIQ
--PWELHE
--PWELAG
--PWELAE
--PWELYK
--PWELYH
--PWELWY
--PWELWS
--PWELWD
--PWELSL
--PWELMD
--PWELLF
--PWELLD
--PWELHL
--PWELGA
--PWELES
--PWELAD
--PWELYQ
--PWELWM
--PWELWK

--PWELVT
--PWELVK
--PWELTN
--PWELTE
--PWELSV
--PWELSE
--PWELQQ
--PWELNP
--PWELMH
--PWELKD
--PWELHI
--PWELGY
--PWELGL
--PWELGI
--PWELFH
--PWELEY
--PWELDS
--PWELCM
--PWELAY
--PWELAT
--PWELAI
-PPWELT-
PMPWEL--
PEPWEL--
-QPWELR-
-QPWELN-
QYPWEL--
-QPWELK-
QAPWEL--
QSPWEL--
QGPWEL--
QTPWEL--
-QPWELT-
QKPWEL--
QIPWEL--
QWPWEL--
QVPWEL--
QRPWEL--
-QPWELS-
-QPWELH-
QQPWEL--
QLPWEL--
-QPWELA-
QMPWEL--
QEPWEL--
-QPWELV-
-QPWELI-
QNPWEL--
RVPWEL--
-RPWELV-

RGPWEL--
RRPWEL--
-RPWELY-
RDPWEL--
-RPWELA-
RNPWEL--
-RPWELN-
RHPWEL--
RFPWEL--
RSPWEL--
-RPWELT-
-RPWELS-
-RPWELQ-
RPPWEL--
RIPWEL--
RTPWEL--
REPWEL--
RMPWEL--
-RPWELM-
-RPWELK-
-RPWELH-
RKPWEL--
RQPWEL--
-RPWELI-
RYPWEL--
RWPWEL--
-RPWELG-
-RPWELE-
SRPWEL--
STPWEL--
SKPWEL--
SSPWEL--
-SPWELN-
-SPWELI-
-SPWELA-
SVPWEL--
SYPWEL--
-SPWELW-
-SPWELK-
SIPWEL--
SLPWEL--
-SPWELY-
SHPWEL--
-SPWELT-
-SPWELR-
SGPWEL--
SMPWEL--
SEPWEL--
SAPWEL--
-SPWELM-

SPPWEL--
SNPWEL--
-SPWELQ-
SFPWEL--
SDPWEL--
-SPWELP-
-SPWELG-
-TPWELR-
TSPWEL--
-TPWELS-
THPWEL--
TVPWEL--
TAPWEL--
TRPWEL--
TPPWEL--
-TPWELN-
-TPWELI-
-TPWELA-
TGPWEL--
-TPWELT-
TKPWEL--
-TPWELM-
-TPWELK-
TMPWEL--
TIPWEL--
-TPWELV-
TYPWEL--
-TPWELG-
-TPWELH-
TFPWEL--
TQPWEL--
TNPWEL--
TEPWEL--
TDPWEL--
-VPWELR-
VLPWEL--
VVPWEL--
VRPWEL--
VYPWEL--
VTPWEL--
-VPWELN-
-VPWELK-
-VPWELG-
VGPWEL--
-VPWELV-
-VPWELS-
-VPWELT-
VNPWEL--
VFPWEL--
VQPWEL--

VDPWEL--
-VPWELI-
-VPWELA-
VKPWEL--
VAPWEL--
VSPWEL--
VWPWEL--
-VPWELQ-
VMPWEL--
-VPWELW-
-VPWELM-
VCPWEL--
-VPWELY-
-VPWELP-
VIPWEL--
VHPWEL--
WVPWEL--
WGPWEL--
-WPWELS-
-WPWELN-
WAPWEL--
WRPWEL--
-WPWELR-
-WPWELT-
-WPWELH-
WFPWEL--
WEPWEL--
-WPWELW-
WLPWEL--
WIPWEL--
WWPWEL--
-WPWELG-
WMPWEL--
WYPWEL--
WTPWEL--
-WPWELM-
-WPWELI-
WDPWEL--
WHPWEL--
-YPWELR-
YSPWEL--
YIPWEL--
YTPWEL--
-YPWELS-
YKPWEL--
-YPWELK-
YLPWEL--
YGPWEL--
-YPWELM-
YAPWEL--

YRPWEL--
-YPWELT-
-YPWELI-
YQPWEL--
-YPWELV-
YEPWEL--
YDPWEL--
-YPWELW-
YNPWEL--
YHPWEL--
YWPWEL--
YFPWEL--
YYPWEL--
-YPWELQ-
-YPWELH-
-YPWELG-
-YPWELA-
YCPWEL--

PAAI	122	793	46.16

--PAAIRI
-APAAIL-
-APAAIS-
AQPAAI--
APPAAI--
AVPAAI--
-APAAIW-
-APAAIR-
-APAAIM-
-APAAIV-
ANPAAI--
ALPAAI--
ASPAAI--
-APAAIT-
ATPAAI--
-APAAII-
AWPAAI--
AHPAAI--
ADPAAI--
-APAAIH-
AFPAAI--
AEPAAI--
AAPAAI--
-APAAIN-
-APAAIK-
-APAAIE-
-APAAIA-
-APAAIQ-
-APAAIG-
AMPAAI--
CRPAAI--

CMPAAI--
CLPAAI--
-CPAAIR-
CKPAAI--
CIPAAI--
CTPAAI--
CQPAAI--
CSPAAI--
CPPAAI--
-CPAAIH-
-CPAAIK-
-CPAAIF-
CGPAAI--
-DPAAIR-
DNPAAI--
DKPAAI--
DVPAAI--
DIPAAI--
DWPAAI--
DMPAAI--
DSPAAI--
-DPAAIV-
-DPAAIT-
DAPAAI--
DTPAAI--
DPPAAI--
DLPAAI--
DFPAAI--
DRPAAI--
-DPAAIW-
-DPAAII-
-DPAAIG-
ERPAAI--
-EPAAIL-
ESPAAI--
-EPAAIM-
-EPAAIR-
EAPAAI--
-EPAAIT-
-EPAAIQ-
EYPAAI--
ELPAAI--
-EPAAIH-
EIPAAI--
EQPAAI--
EEPAAI--
EWPAAI--
EVPAAI--
EMPAAI--
EDPAAI--

-EPAAIS-
-EPAAIN-
EKPAAI--
EGPAAI--
-EPAAIY-
-EPAAIV-
-EPAAIK-
-EPAAII-
ENPAAI--
EFPAAI--
FAPAAI--
FVPAAI--
FRPAAI--
FLPAAI--
-FPAAIM-
FGPAAI--
-FPAAIW-
FMPAAI--
FHPAAI--
-FPAAII-
FSPAAI--
FEPAAI--
FDPAAI--
-FPAAIS-
FNPAAI--
-FPAAIT-
FIPAAI--
-FPAAIL-
FYPAAI--
FTPAAI--
FPPAAI--
-FPAAIA-
FWPAAI--
-FPAAIF-
-FPAAIC-
-FPAAIE-
-GPAAIK-
-GPAAIL-
GVPAAI--
-GPAAIT-
-GPAAIV-
GRPAAI--
-GPAAIW-
GIPAAI--
GGPAAI--
GFPAAI--
-GPAAIY-
GYPAAI--
GTPAAI--
GLPAAI--

-GPAAIR-
-GPAAIH-
GEPAAI--
GAPAAI--
-GPAAIA-
GNPAAI--
-GPAAII-
-GPAAIG-
GDPAAI--
GCPAAI--
GQPAAI--
GMPAAI--
-GPAAIN-
-GPAAIM-
-GPAAIF-
-GPAAIE-
HAPAAI--
HPPAAI--
-HPAAIL-
-HPAAIR-
HLPAAI--
-HPAAIW-
HKPAAI--
HSPAAI--
HRPAAI--
HGPAAI--
-HPAAIK-
HNPAAI--
HFPAAI--
HWPAAI--
-HPAAIT-
-HPAAIM-
HQPAAI--
-HPAAIF-
HYPAAI--
HVPAAI--
-HPAAIV-
HTPAAI--
-HPAAIS-
-HPAAIQ-
-HPAAII-
-HPAAIE-
HHPAAI--
HEPAAI--
-IPAAIR-
IFPAAI--
IEPAAI--
-IPAAIL-
IRPAAI--
-IPAAIT-

IYPAAI--
ILPAAI--
IGPAAI--
-IPAAIY-
ISPAAI--
-IPAAIV-
IAPAAI--
-IPAAII-
IIPAAI--
-IPAAIS-
-IPAAIM-
IMPAAI--
IVPAAI--
-IPAAIN-
IHPAAI--
-IPAAIW-
-IPAAIH-
IKPAAI--
ITPAAI--
INPAAI--
-IPAAIE-
-IPAAIA-
IWPAAI--
IPPAAI--
-IPAAIQ-
IDPAAI--
ICPAAI--
KSPAAI--
KVPAAI--
KAPAAI--
KQPAAI--
KIPAAI--
-KPAAIS-
-KPAAIL-
-KPAAII-
-KPAAIF-
-KPAAIY-
KTPAAI--
KWPAAI--
-KPAAIH-
KFPAAI--
KLPAAI--
KGPAAI--
KMPAAI--
KKPAAI--
KRPAAI--
KPPAAI--
-KPAAIT-
-KPAAIN-
KEPAAI--

-KPAAIV-
-KPAAIM-
-KPAAIK-
-KPAAIE-
-KPAAID-
-KPAAIA-
KDPAAI--
LGPAAI--
LLPAAI--
LVPAAI--
LAPAAI--
LSPAAI--
LQPAAI--
-LPAAIK-
-LPAAIF-
LIPAAI--
LTPAAI--
-LPAAIL-
-LPAAIY-
LEPAAI--
LKPAAI--
LPPAAI--
-LPAAIR-
LYPAAI--
LMPAAI--
LFPAAI--
-LPAAII-
-LPAAIT-
-LPAAIQ-
-LPAAIM-
LHPAAI--
-LPAAIS-
-LPAAIV-
-LPAAIH-
LWPAAI--
-LPAAIW-
-LPAAIN-
LNPAAI--
-LPAAIA-
MSPAAI--
MHPAAI--
-MPAAIW-
MPPAAI--
-MPAAIR-
MQPAAI--
MNPAAI--
-MPAAII-
MMPAAI--
MWPAAI--
-MPAAIF-

-MPAAIN-
-MPAAIH-
MFPAAI--
-MPAAIQ-
MKPAAI--
MYPAAI--
-MPAAIV-
MEPAAI--
-MPAAIY-
-MPAAIT-
MDPAAI--
MAPAAI--
MVPAAI--
-MPAAIC-
MTPAAI--
MIPAAI--
MGPAAI--
NVPAAI--
NQPAAI--
NRPAAI--
-NPAAIR-
-NPAAIY-
-NPAAIM-
NLPAAI--
-NPAAIL-
NMPAAI--
-NPAAIS-
NPPAAI--
NGPAAI--
NYPAAI--
-NPAAII-
NIPAAI--
NWPAAI--
-NPAAIK-
NHPAAI--
NSPAAI--
-NPAAIV-
NKPAAI--
NAPAAI--
-NPAAIT-
-NPAAIH-
NEPAAI--
NTPAAI--
-NPAAIG-
NFPAAI--
NCPAAI--
--PAAIQE
--PAAITG
--PAAIRG
--PAAILG

--PAAILS
--PAAITR
--PAAILY
--PAAISL
--PAAIRT
--PAAIRK
--PAAISS
--PAAIVK
PWPAAI--
--PAAILT
--PAAIFS
--PAAIFK
--PAAIYK
--PAAITK
--PAAIKG
--PAAIVS
--PAAIST
--PAAITV
--PAAITS
--PAAIRS
--PAAIAL
--PAAIRY
--PAAIKA
PLPAAI--
--PAAIWR
--PAAIVT
--PAAIMI
--PAAITL
--PAAISG
--PAAIGL
PFPAAI--
--PAAITT
--PAAISR
--PAAILR
--PAAIIA
--PAAIAG
--PAAISA
--PAAIMA
--PAAIGG
--PAAIRV
PAPAAI--
--PAAIYR
--PAAIVL
--PAAISV
--PAAIQS
--PAAILQ
--PAAIFN
--PAAIRD
--PAAIMY
--PAAIAT

--PAAIGI
PGPAAI--
--PAAIYF
--PAAIVI
--PAAITN
--PAAIRA
--PAAILM
--PAAIFH
--PAAIYY
--PAAIYT
--PAAIWS
--PAAIIG
--PAAIHW
--PAAIVM
--PAAITA
--PAAIQT
--PAAIPY
--PAAILK
--PAAIKH
--PAAIIR
--PAAIIL
--PAAIFE
--PAAIAH
--PAAIAA
--PAAIVY
--PAAITQ
--PAAIPL
--PAAINM
--PAAIIS
--PAAIGS
--PAAIGA
--PAAIYS
--PAAIWF
--PAAISW
--PAAIRF
--PAAILN
--PAAIKY
--PAAIKV
--PAAIFR
--PAAITW
--PAAISY
--PAAIQR
--PAAIQN
--PAAIQH
--PAAIKT
--PAAIHL
PSPAAI--
--PAAISQ
--PAAISN
--PAAIKR

--PAAIKL
--PAAIKE
--PAAIHV
--PAAIFM
--PAAIEN
--PAAIAN
--PAAIYN
--PAAIWT
--PAAIWI
--PAAITH
--PAAIRN
--PAAIRH
--PAAIQK
--PAAINS
--PAAIMM
--PAAIML
--PAAIMG
--PAAILH
--PAAILF
--PAAILD
--PAAIKN
--PAAIHG
--PAAIHE
--PAAIFQ
--PAAIEA
PRPAAI--
--PAAIYM
--PAAIYL
--PAAIWL
--PAAIVH
--PAAIVA
--PAAIQQ
--PAAINV
--PAAINR
--PAAIMR
--PAAILW
--PAAIKS
--PAAIHT
--PAAIHN
--PAAIHD
--PAAIEL
PDPAAI--
--PAAIYD
--PAAIWD
--PAAIVV
--PAAIVQ
--PAAITM
--PAAISH
--PAAIRW
--PAAIQL

--PAAING
--PAAIMV
--PAAIKW
--PAAIKF
--PAAIIV
--PAAIEW
--PAAIAF
PVPAAI--
PIPAAI--
--PAAIWV
--PAAIVG
--PAAIRQ
--PAAIRM
--PAAIQC
--PAAILL
--PAAIKD
--PAAIIQ
--PAAIIM
--PAAIIK
--PAAIHM
--PAAIHI
--PAAIGM
--PAAIEG
--PAAIAV
PYPAAI--
--PAAIYQ
--PAAIYH
--PAAISC
--PAAIQW
--PAAINY
--PAAINL
--PAAIMD
--PAAIIN
--PAAIIH
--PAAIHR
--PAAIGY
--PAAIGH
--PAAIFY
--PAAIFD
--PAAICN
--PAAICI
PNPAAI--
--PAAIYW
--PAAIYA
--PAAIWY
--PAAIWA
--PAAITI
--PAAISK
--PAAISE
--PAAIRE

--PAAIQV
--PAAIQG
--PAAIQA
--PAAINN
--PAAINA
--PAAIME
--PAAILI
--PAAIHY
--PAAIHQ
--PAAIHC
--PAAIGV
--PAAIGF
--PAAIFT
--PAAIEV
--PAAIAW
PQPAAI--
-PPAAIW-
-PPAAIR-
PMPAAI--
PKPAAI--
--PAAIVR
--PAAIVF
--PAAIVD
--PAAITY
--PAAITE
--PAAITC
--PAAISM
--PAAISD
--PAAIQY
--PAAIQD
--PAAINW
--PAAINT
--PAAINQ
--PAAINF
--PAAIMS
--PAAIMK
--PAAILV
--PAAILC
--PAAILA
--PAAIKP
--PAAIKM
--PAAIIY
--PAAIIF
--PAAIHA
--PAAIGW
--PAAIGT
--PAAIFG
--PAAIEY
--PAAIET
--PAAIES

--PAAIER
--PAAICS
--PAAICF
QGPAAI--
-QPAAIR-
QPPAAI--
QRPAAI--
QNPAAI--
-QPAAIW-
-QPAAIS-
QIPAAI--
QAPAAI--
QLPAAI--
QKPAAI--
QWPAAI--
QMPAAI--
QFPAAI--
QTPAAI--
-QPAAIN-
-QPAAII-
-QPAAIH-
QHPAAI--
-QPAAIQ-
-QPAAIA-
QEPAAI--
QQPAAI--
-QPAAIT-
RLPAAI--
RSPAAI--
RRPAAI--
-RPAAIL-
-RPAAIY-
-RPAAIH-
-RPAAIR-
-RPAAIS-
RYPAAI--
RHPAAI--
RNPAAI--
-RPAAIT-
RFPAAI--
-RPAAIF-
RAPAAI--
RDPAAI--
RTPAAI--
-RPAAIV-
-RPAAIK-
RVPAAI--
RMPAAI--
RQPAAI--
RPPAAI--

```
-RPAAIW-
RKPAAI--
RWPAAI--
REPAAI--
-RPAAIN-
-RPAAIM-
-RPAAIQ-
RCPAAI--
-RPAAIG-
-RPAAIE-
-RPAAIC-
SSPAAI--
SNPAAI--
SRPAAI--
-SPAAIY-
-SPAAIL-
-SPAAIR-
-SPAAIV-
SIPAAI--
SVPAAI--
SKPAAI--
-SPAAIT-
-SPAAIM-
-SPAAIF-
SMPAAI--
STPAAI--
SLPAAI--
-SPAAII-
SEPAAI--
SQPAAI--
SGPAAI--
SAPAAI--
SYPAAI--
-SPAAIN-
SPPAAI--
SHPAAI--
-SPAAIW-
-SPAAIK-
SFPAAI--
SWPAAI--
-SPAAIQ-
-SPAAIA-
-SPAAIG-
SDPAAI--
-SPAAIS-
-SPAAIH-
SCPAAI--
-TPAAIV-
TVPAAI--
TLPAAI--
```

TSPAAI--
TAPAAI--
TNPAAI--
TMPAAI--
THPAAI--
-TPAAIM-
-TPAAIY-
TQPAAI--
-TPAAIH-
TYPAAI--
-TPAAIW-
-TPAAIL-
TFPAAI--
TGPAAI--
TCPAAI--
-TPAAIT-
TKPAAI--
TDPAAI--
-TPAAIK-
TWPAAI--
TIPAAI--
-TPAAII-
-TPAAIE-
-TPAAIA-
-VPAAIV-
VSPAAI--
VMPAAI--
-VPAAIL-
VRPAAI--
VPPAAI--
VLPAAI--
VVPAAI--
-VPAAIY-
-VPAAII-
VGPAAI--
VEPAAI--
VKPAAI--
-VPAAIT-
VWPAAI--
-VPAAIM-
-VPAAIA-
-VPAAIW-
VIPAAI--
VHPAAI--
VTPAAI--
-VPAAIS-
VQPAAI--
-VPAAIK-
VNPAAI--
VAPAAI--

-VPAAIH-
-VPAAIQ-
-VPAAIF-
-VPAAIG-
VFPAAI--
WVPAAI--
WKPAAI--
WDPAAI--
-WPAAIW-
-WPAAIR-
-WPAAIL-
-WPAAIT-
WTPAAI--
WHPAAI--
-WPAAIS-
WMPAAI--
WSPAAI--
WAPAAI--
WQPAAI--
WGPAAI--
WYPAAI--
WPPAAI--
-WPAAII-
-WPAAIN-
-WPAAIV-
WLPAAI--
-WPAAIK-
WEPAAI--
WCPAAI--
-WPAAIM-
-WPAAIH-
-WPAAIG-
-WPAAIA-
YRPAAI--
YLPAAI--
-YPAAIS-
YVPAAI--
YSPAAI--
YIPAAI--
YEPAAI--
YNPAAI--
YPPAAI--
-YPAAIW-
YKPAAI--
YTPAAI--
-YPAAIK-
YMPAAI--
YAPAAI--
-YPAAIL-
YWPAAI--

	-YPAAIY-		
	YGPAAI--		
	YYPAAI--		
	-YPAAIV-		
	YDPAAI--		
	YQPAAI--		
	-YPAAIR-		
	-YPAAIQ-		
	-YPAAIN-		
	-YPAAIF-		
	-YPAAIT-		
	-YPAAIG-		
PQGM	258	719	46.14
	-GPQGMG-		
	AKPQGM--		
	ASPQGM--		
	-APQGMS-		
	AWPQGM--		
	ATPQGM--		
	AMPQGM--		
	AIPQGM--		
	AGPQGM--		
	AVPQGM--		
	-APQGMY-		
	-APQGMH-		
	-APQGMF-		
	ARPQGM--		
	-APQGMT-		
	-APQGMA-		
	AQPQGM--		
	-APQGML-		
	ALPQGM--		
	AAPQGM--		
	AYPQGM--		
	-APQGMN-		
	-APQGMW-		
	-APQGMR-		
	-APQGMK-		
	-APQGME-		
	AHPQGM--		
	AEPQGM--		
	-APQGMQ-		
	-APQGMM-		
	-APQGMI-		
	-APQGMG-		
	-APQGMD-		
	APPQGM--		
	CNPQGM--		
	CMPQGM--		
	CIPQGM--		

-CPQGMI-
CVPQGM--
CQPQGM--
-CPQGMV-
-CPQGMT-
-CPQGMR-
-CPQGML-
-CPQGMH-
CLPQGM--
CFPQGM--
CEPQGM--
CCPQGM--
CAPQGM--
-DPQGMR-
-DPQGMT-
DIPQGM--
DAPQGM--
-DPQGMI-
DYPQGM--
DVPQGM--
-DPQGMH-
DMPQGM--
DWPQGM--
DTPQGM--
-DPQGMY-
-DPQGMW-
-DPQGMM-
-DPQGML-
-DPQGMK-
DNPQGM--
DHPQGM--
DCPQGM--
-EPQGMF-
ESPQGM--
-EPQGMH-
-EPQGMS-
-EPQGML-
EQPQGM--
EMPQGM--
-EPQGMY-
EGPQGM--
EWPQGM--
EAPQGM--
-EPQGMM-
EIPQGM--
EFPQGM--
FWPQGM--
FVPQGM--
FSPQGM--
-FPQGMR-

FQPQGM--
FLPQGM--
-FPQGMY-
FHPQGM--
-FPQGMH-
FRPQGM--
-FPQGML-
FIPQGM--
FFPQGM--
-FPQGMT-
-FPQGMF-
-FPQGMV-
FNPQGM--
FYPQGM--
-FPQGMN-
-FPQGMI-
FKPQGM--
FGPQGM--
-GPQGMS-
-GPQGML-
-GPQGMR-
GQPQGM--
GLPQGM--
-GPQGMM-
-GPQGMY-
-GPQGMI-
-GPQGMW-
GVPQGM--
GFPQGM--
-GPQGMQ-
GRPQGM--
GNPQGM--
GKPQGM--
GSPQGM--
GMPQGM--
-GPQGMF-
GHPQGM--
GAPQGM--
GPPQGM--
-GPQGMT-
-GPQGME-
GYPQGM--
GWPQGM--
-GPQGMV-
-GPQGMN-
-GPQGMH-
-GPQGMA-
GIPQGM--
GGPQGM--
HGPQGM--

-HPQGMS-
-HPQGMM-
-HPQGMR-
HFPQGM--
HAPQGM--
HRPQGM--
-HPQGMA-
HMPQGM--
HIPQGM--
HEPQGM--
HPPQGM--
HWPQGM--
HVPQGM--
-HPQGMY-
-HPQGML-
-HPQGMI-
-HPQGME-
-HPQGMW-
-HPQGMQ-
-HPQGMG-
HNPQGM--
HLPQGM--
HHPQGM--
ISPQGM--
-IPQGMF-
IVPQGM--
IFPQGM--
ITPQGM--
IYPQGM--
-IPQGMW-
IGPQGM--
-IPQGMH-
-IPQGMV-
IEPQGM--
-IPQGMS-
IHPQGM--
-IPQGMK-
IAPQGM--
IRPQGM--
IQPQGM--
-IPQGMI-
-IPQGME-
IWPQGM--
-IPQGMT-
-IPQGMR-
-IPQGMG-
IIPQGM--
IDPQGM--
-IPQGMM-
-IPQGML-

-IPQGMA-
IPPQGM--
ICPQGM--
KSPQGM--
-KPQGMI-
-KPQGMS-
-KPQGML-
KVPQGM--
KRPQGM--
-KPQGMK-
KGPQGM--
KDPQGM--
-KPQGMT-
-KPQGMF-
-KPQGMY-
KMPQGM--
KAPQGM--
-KPQGMA-
KNPQGM--
-KPQGMG-
KHPQGM--
-KPQGMR-
-KPQGMQ-
-KPQGMM-
KCPQGM--
-KPQGMV-
-KPQGME-
KKPQGM--
KFPQGM--
LVPQGM--
-LPQGMR-
-LPQGML-
LRPQGM--
LSPQGM--
LWPQGM--
LLPQGM--
LTPQGM--
-LPQGMT-
LHPQGM--
LIPQGM--
LAPQGM--
LPPQGM--
-LPQGMY-
LMPQGM--
-LPQGMN-
-LPQGMK-
LNPQGM--
LKPQGM--
LFPQGM--
LYPQGM--

-LPQGMV-
-LPQGMI-
-LPQGMF-
-LPQGMM-
LEPQGM--
LQPQGM--
-LPQGMW-
-LPQGME-
-LPQGMC-
LDPQGM--
-MPQGMR-
-MPQGMV-
-MPQGMT-
-MPQGMA-
MPPQGM--
-MPQGMM-
MSPQGM--
-MPQGMS-
-MPQGML-
MLPQGM--
MAPQGM--
MFPQGM--
MKPQGM--
-MPQGMK-
-MPQGMF-
-MPQGME-
MVPQGM--
MTPQGM--
MRPQGM--
-MPQGMN-
MNPQGM--
-MPQGMW-
-MPQGMQ-
-MPQGMH-
NLPQGM--
NRPQGM--
NYPQGM--
-NPQGMR-
-NPQGMV-
NVPQGM--
NGPQGM--
-NPQGMW-
NSPQGM--
NQPQGM--
-NPQGMT-
-NPQGMS-
NTPQGM--
NKPQGM--
NIPQGM--
NWPQGM--

-NPQGMK-
NHPQGM--
-NPQGML-
-NPQGMI-
NFPQGM--
NAPQGM--
-NPQGMQ-
NNPQGM--
NMPQGM--
--PQGMWK
--PQGMMG
--PQGMRS
--PQGMVA
--PQGMIS
--PQGMSI
--PQGMWT
--PQGMLT
--PQGMLS
--PQGMRT
--PQGMHF
--PQGMLQ
--PQGMTS
--PQGMVS
--PQGMFT
--PQGMST
--PQGMSG
--PQGMAA
--PQGMLG
--PQGMIF
--PQGMFS
--PQGMVY
--PQGMVF
--PQGMRG
--PQGMLH
--PQGMRN
--PQGMWR
--PQGMSA
--PQGMIR
--PQGMGA
--PQGMYT
--PQGMYN
--PQGMYG
--PQGMSQ
--PQGMRL
--PQGMKS
--PQGMHR
--PQGMYS
--PQGMWA
--PQGMVN
--PQGMSR

--PQGMSM
--PQGMRY
--PQGMLV
--PQGMFQ
--PQGMYA
--PQGMTK
--PQGMTI
--PQGMTG
--PQGMQH
--PQGMNQ
--PQGMTT
--PQGMRQ
--PQGMQY
--PQGMLA
--PQGMFG
--PQGMVM
--PQGMTA
--PQGMMT
--PQGMKV
PLPQGM--
--PQGMVR
--PQGMVL
--PQGMSF
--PQGMRI
--PQGMNT
--PQGMLY
--PQGMLM
--PQGMHS
--PQGMHA
--PQGMVT
--PQGMSL
--PQGMKW
--PQGMHL
--PQGMGT
--PQGMGG
--PQGMFV
--PQGMFK
PVPQGM--
PSPQGM--
--PQGMWH
--PQGMTN
--PQGMTH
--PQGMSY
--PQGMSW
--PQGMSH
--PQGMRH
--PQGMRE
--PQGMMI
--PQGMKT
--PQGMIV

--PQGMII
--PQGMHQ
--PQGMHK
--PQGMYL
--PQGMWN
--PQGMVQ
--PQGMTM
--PQGMLN
--PQGMKY
--PQGMIG
--PQGMHT
--PQGMAY
PRPQGM--
--PQGMYK
--PQGMTR
--PQGMTL
--PQGMSV
--PQGMRM
--PQGMNV
--PQGMNS
--PQGMMK
--PQGMKG
--PQGMGS
--PQGMFL
--PQGMEV
--PQGMAG
PKPQGM--
--PQGMWL
--PQGMWG
--PQGMVV
--PQGMVK
--PQGMVG
--PQGMTV
--PQGMTQ
--PQGMSS
--PQGMSN
--PQGMRV
--PQGMQG
--PQGMNI
--PQGMNA
--PQGMLE
--PQGMKM
--PQGMIT
--PQGMIK
--PQGMFI
--PQGMFD
--PQGMCA
--PQGMYV
--PQGMVW
--PQGMVH

--PQGMRK
--PQGMMA
--PQGMKN
--PQGMKK
--PQGMIY
--PQGMIH
--PQGMHV
--PQGMHN
--PQGMGW
--PQGMFM
--PQGMAI
--PQGMAF
PHPQGM--
PEPQGM--
PQPQGM--
--PQGMTY
--PQGMRW
--PQGMRD
--PQGMRA
--PQGMQI
--PQGMQD
--PQGMQA
--PQGMNY
--PQGMMV
--PQGMLK
--PQGMLI
--PQGMKI
--PQGMKF
--PQGMKD
--PQGMKA
--PQGMIQ
--PQGMIL
--PQGMHM
--PQGMGI
--PQGMFN
--PQGMES
PTPQGM--
--PQGMYD
--PQGMWI
--PQGMWE
--PQGMVI
--PQGMVE
--PQGMQL
--PQGMNW
--PQGMNM
--PQGMMR
--PQGMML
--PQGMMD
--PQGMLL
--PQGMLD

--PQGMGV
--PQGMFR
--PQGMFE
--PQGMEY
--PQGMEG
--PQGMCV
--PQGMCT
--PQGMAT
--PQGMAN
PFPQGM--
--PQGMYM
--PQGMWV
--PQGMWF
--PQGMVD
--PQGMSK
--PQGMSE
--PQGMNR
--PQGMNN
--PQGMNK
--PQGMNG
--PQGMNF
--PQGMND
--PQGMMS
--PQGMKR
--PQGMKQ
--PQGMKL
--PQGMKE
--PQGMIN
--PQGMIE
--PQGMIA
--PQGMHC
--PQGMGH
--PQGMFH
--PQGMEN
--PQGMEI
--PQGMDT
--PQGMDS
--PQGMDG
--PQGMCK
--PQGMAR
--PQGMAP
--PQGMAD
PCPQGM--
QRPQGM--
-QPQGML-
QWPQGM--
-QPQGMQ-
QLPQGM--
-QPQGMM-
QMPQGM--

-QPQGMT-
-QPQGMI-
-QPQGMF-
QNPQGM--
QFPQGM--
QSPQGM--
-QPQGMA-
-QPQGMY-
-QPQGMW-
-QPQGMN-
-QPQGMS-
QIPQGM--
-QPQGMK-
QDPQGM--
QAPQGM--
-QPQGMV-
-QPQGMG-
QEPQGM--
-RPQGMV-
RLPQGM--
-RPQGMR-
RGPQGM--
RTPQGM--
-RPQGMS-
RRPQGM--
RYPQGM--
-RPQGMK-
RAPQGM--
RVPQGM--
RWPQGM--
-RPQGMT-
RIPQGM--
RSPQGM--
-RPQGMW-
-RPQGMI-
RNPQGM--
RPPQGM--
-RPQGMF-
-RPQGMN-
-RPQGML-
-RPQGMG-
RQPQGM--
-RPQGMH-
-RPQGME-
RMPQGM--
-RPQGMM-
RHPQGM--
REPQGM--
-RPQGMY-
RKPQGM--

-RPQGMA-
-RPQGMQ-
RCPQGM--
-SPQGMR-
SVPQGM--
SRPQGM--
SSPQGM--
SFPQGM--
-SPQGMK-
SHPQGM--
SAPQGM--
SQPQGM--
-SPQGMW-
-SPQGMS-
-SPQGMI-
SYPQGM--
SLPQGM--
SCPQGM--
-SPQGMF-
SKPQGM--
SIPQGM--
-SPQGMT-
-SPQGME-
SEPQGM--
-SPQGMV-
-SPQGML-
-SPQGMA-
SPPQGM--
STPQGM--
SNPQGM--
SMPQGM--
-TPQGMY-
-TPQGMV-
TEPQGM--
-TPQGMA-
TAPQGM--
TTPQGM--
TRPQGM--
-TPQGMS-
-TPQGML-
TPPQGM--
TFPQGM--
-TPQGMI-
TVPQGM--
-TPQGMR-
-TPQGMN-
TLPQGM--
TKPQGM--
TGPQGM--
TMPQGM--

TSPQGM--
THPQGM--
TQPQGM--
-TPQGMQ-
-TPQGMM-
-TPQGMF-
-TPQGME-
TNPQGM--
-TPQGMK-
-TPQGMC-
TCPQGM--
VWPQGM--
VPPQGM--
VLPQGM--
VRPQGM--
-VPQGMV-
VFPQGM--
-VPQGMR-
-VPQGMN-
VYPQGM--
VSPQGM--
VDPQGM--
VVPQGM--
VNPQGM--
VIPQGM--
-VPQGMQ-
VQPQGM--
-VPQGMH-
VAPQGM--
VTPQGM--
-VPQGMK-
-VPQGMA-
VMPQGM--
VGPQGM--
-VPQGME-
VEPQGM--
-VPQGMW-
-VPQGMS-
-VPQGML-
-VPQGMI-
VHPQGM--
-WPQGMR-
WRPQGM--
-WPQGML-
-WPQGMK-
WYPQGM--
WQPQGM--
-WPQGMS-
WTPQGM--
WVPQGM--

-WPQGMY-
WWPQGM--
WSPQGM--
WLPQGM--
WGPQGM--
-WPQGME-
WNPQGM--
WDPQGM--
-WPQGMF-
WHPQGM--
-YPQGMS-
YRPQGM--
YFPQGM--
YAPQGM--
YSPQGM--
-YPQGMF-
YLPQGM--
-YPQGMY-
YVPQGM--
YTPQGM--
-YPQGMK-
YDPQGM--
YYPQGM--
-YPQGMV-
-YPQGMA-
YMPQGM--
YWPQGM--
-YPQGMW-
-YPQGMT-
-YPQGMI-
-YPQGME-
YKPQGM--

PFPM 572 595 46.12

-NPFPMT-
-APFPMT-
ASPFPM--
ARPFPM--
-APFPMS-
-APFPMW-
AMPFPM--
AAPFPM--
AGPFPM--
-APFPMR-
-APFPMQ-
-APFPML-
-APFPME-
-APFPMY-
-APFPMH-
-APFPMF-
ANPFPM--

AIPFPM--
AVPFPM--
-APFPMN-
-APFPMI-
-APFPMG-
-APFPMA-
ALPFPM--
AFPFPM--
AEPFPM--
ACPFPM--
AYPFPM--
AWPFPM--
AQPFPM--
CYPFPM--
CTPFPM--
CMPFPM--
-CPFPMK-
CLPFPM--
-CPFPMR-
-DPFPMW-
-DPFPML-
DSPFPM--
DHPFPM--
DVPFPM--
DGPFPM--
-DPFPMI-
DNPFPM--
DTPFPM--
-DPFPMN-
DIPFPM--
ELPFPM--
EYPFPM--
-EPFPMY-
-EPFPMT-
-EPFPMF-
EIPFPM--
ESPFPM--
EQPFPM--
-EPFPMQ-
EVPFPM--
ERPFPM--
-EPFPMM-
EMPFPM--
-EPFPMK-
-EPFPMD-
-FPFPMI-
FDPFPM--
-FPFPMW-
FGPFPM--
FSPFPM--

FRPFPM--
-FPFPMS-
-FPFPMY-
FYPFPM--
FVPFPM--
FLPFPM--
-FPFPMV-
-FPFPMQ-
FMPFPM--
FKPFPM--
FIPFPM--
FAPFPM--
-FPFPMM-
-FPFPMA-
-FPFPMH-
FTPFPM--
-GPFPMR-
GKPFPM--
GSPFPM--
GEPFPM--
GTPFPM--
GRPFPM--
-GPFPMM-
-GPFPMK-
GFPFPM--
-GPFPMT-
GMPFPM--
GGPFPM--
GWPFPM--
-GPFPMN-
-GPFPML-
GNPFPM--
GQPFPM--
-GPFPMS-
GVPFPM--
GPPFPM--
-GPFPMQ-
-GPFPMH-
-GPFPMA-
GDPFPM--
-GPFPMG-
-GPFPME-
GIPFPM--
HTPFPM--
HRPFPM--
HAPFPM--
HYPFPM--
-HPFPMH-
HMPFPM--
-HPFPMY-

-HPFPMV-
-HPFPML-
HFPFPM--
HQPFPM--
-HPFPMT-
-HPFPMQ-
HKPFPM--
HVPFPM--
HSPFPM--
-HPFPMR-
IRPFPM--
IHPFPM--
-IPFPMT-
-IPFPMR-
-IPFPMS-
IDPFPM--
ITPFPM--
IFPFPM--
-IPFPMY-
IEPFPM--
IVPFPM--
ISPFPM--
IWPFPM--
IYPFPM--
IPPFPM--
-IPFPML-
-IPFPMK-
-IPFPMF-
-IPFPMA-
IQPFPM--
-IPFPMG-
IIPFPM--
KSPFPM--
-KPFPML-
-KPFPMT-
-KPFPMI-
KRPFPM--
KNPFPM--
KVPFPM--
KTPFPM--
-KPFPMQ-
-KPFPMS-
-KPFPMR-
KDPFPM--
KYPFPM--
-KPFPME-
KLPFPM--
-KPFPMN-
-KPFPMA-
KHPFPM--

KAPFPM--
-KPFPMY-
-KPFPMG-
KFPFPM--
KEPFPM--
LPPFPM--
LKPFPM--
LIPFPM--
LGPFPM--
LDPFPM--
LTPFPM--
LRPFPM--
LLPFPM--
-LPFPMR-
LNPFPM--
LQPFPM--
LAPFPM--
-LPFPMT-
-LPFPMF-
-LPFPML-
-LPFPMA-
LHPFPM--
LSPFPM--
-LPFPMY-
-LPFPMK-
-LPFPMH-
-LPFPMV-
LWPFPM--
-LPFPMS-
-LPFPMM-
LCPFPM--
LVPFPM--
-LPFPMW-
LMPFPM--
-MPFPMK-
MLPFPM--
-MPFPML-
MMPFPM--
MRPFPM--
MAPFPM--
MPPFPM--
-MPFPMY-
-MPFPMI-
MYPFPM--
MTPFPM--
-MPFPMV-
MFPFPM--
MKPFPM--
MVPFPM--
-MPFPMW-

```
-MPFPMS-
-MPFPMR-
MIPFPM--
MGPFPM--
MQPFPM--
-MPFPMH-
-NPFPMR-
NIPFPM--
NAPFPM--
NMPFPM--
NYPFPM--
NRPFPM--
-NPFPMW-
-NPFPMY-
-NPFPML-
-NPFPMK-
-NPFPMI-
-NPFPMV-
-NPFPMF-
NFPFPM--
-NPFPMM-
NNPFPM--
NVPFPM--
NTPFPM--
-NPFPMD-
-NPFPMS-
NLPFPM--
NHPFPM--
NPPFPM--
--PFPMRN
--PFPMRL
--PFPMVG
--PFPMVF
--PFPMEV
--PFPMTI
--PFPMNF
--PFPMLT
--PFPMRS
--PFPMRK
--PFPMKY
--PFPMYT
--PFPMSG
--PFPMKS
--PFPMYG
--PFPMVM
--PFPMIW
--PFPMHR
PGPFPM--
--PFPMVD
--PFPMKG
```

--PFPMHG
--PFPMFE
--PFPMYH
--PFPMWI
--PFPMST
--PFPMRV
--PFPMRI
--PFPMMY
--PFPMLS
--PFPMTM
--PFPMGS
--PFPMAG
PNPFPM--
--PFPMWM
--PFPMTT
--PFPMMA
--PFPMHS
--PFPMHM
--PFPMAS
--PFPMAF
--PFPMYV
--PFPMVT
--PFPMTY
--PFPMRY
--PFPMRQ
--PFPMDM
PTPFPM--
--PFPMSR
--PFPMRG
--PFPMQY
--PFPMMS
--PFPMLN
--PFPMKR
--PFPMTR
--PFPMTA
--PFPMQH
--PFPMLG
--PFPMII
--PFPMGQ
--PFPMGA
--PFPMFN
--PFPMEW
--PFPMEG
--PFPMYR
--PFPMYL
--PFPMVR
--PFPMSW
--PFPMSA
--PFPMRW
--PFPMRT

--PFPMMF
--PFPMKV
--PFPMIT
--PFPMIR
--PFPMIM
--PFPMWN
--PFPMTV
--PFPMTS
--PFPMRH
--PFPMRF
--PFPMRD
--PFPMQA
--PFPMPV
--PFPMPM
--PFPMNS
--PFPMMV
--PFPMLA
--PFPMKT
--PFPMIQ
--PFPMFF
--PFPMES
--PFPMAH
PEPFPM--
--PFPMWS
--PFPMVE
--PFPMTG
--PFPMTF
--PFPMSN
--PFPMSE
--PFPMRA
--PFPMQM
--PFPMNW
--PFPMMQ
--PFPMMN
--PFPMLE
--PFPMIV
--PFPMIS
--PFPMIH
--PFPMIE
--PFPMFT
--PFPMEI
PYPFPM--
--PFPMYQ
--PFPMWY
--PFPMWA
--PFPMVW
--PFPMVV
--PFPMSF
--PFPMQW
--PFPMQT

--PFPMQQ
--PFPMNN
--PFPMME
--PFPMLM
--PFPMKA
--PFPMIK
--PFPMHW
--PFPMHV
--PFPMHI
--PFPMHA
--PFPMGW
--PFPMGH
--PFPMFS
--PFPMFH
PIPFPM--
PHPFPM--
--PFPMWR
--PFPMWH
--PFPMVA
--PFPMTN
--PFPMRE
--PFPMQV
--PFPMNT
--PFPMIN
--PFPMHF
--PFPMGT
--PFPMFK
PQPFPM--
--PFPMYN
--PFPMWT
--PFPMVS
--PFPMVI
--PFPMTK
--PFPMTD
--PFPMSK
--PFPMSH
--PFPMQR
--PFPMPW
--PFPMPH
--PFPMNQ
--PFPMLQ
--PFPMLD
--PFPMLC
--PFPMKM
--PFPMKF
--PFPMKD
--PFPMHN
--PFPMHE
--PFPMGN
--PFPMGK

--PFPMFR
--PFPMEM
--PFPMDS
--PFPMDR
--PFPMCQ
--PFPMCL
--PFPMAT
--PFPMAR
--PFPMAN
--PFPMAK
QYPFPM--
QRPFPM--
-QPFPMR-
-QPFPMT-
-QPFPMQ-
QAPFPM--
-QPFPMY-
-QPFPMH-
QLPFPM--
-QPFPMM-
-QPFPMK-
QNPFPM--
-QPFPML-
-QPFPMF-
QTPFPM--
QSPFPM--
-QPFPMW-
-QPFPME-
QGPFPM--
QVPFPM--
-QPFPMS-
QDPFPM--
RPPFPM--
RWPFPM--
RLPFPM--
RIPFPM--
RSPFPM--
RHPFPM--
RYPFPM--
RFPFPM--
-RPFPMF-
-RPFPMA-
-RPFPMS-
RVPFPM--
-RPFPMT-
RTPFPM--
-RPFPMM-
RKPFPM--
-RPFPML-
-RPFPMK-

-RPFPMV-
RDPFPM--
-RPFPMN-
RAPFPM--
-RPFPMI-
-RPFPMH-
-RPFPME-
-SPFPMM-
SRPFPM--
SIPFPM--
SVPFPM--
-SPFPMK-
SAPFPM--
-SPFPMR-
SMPFPM--
SLPFPM--
-SPFPMS-
SSPFPM--
-SPFPML-
SPPFPM--
SYPFPM--
STPFPM--
SQPFPM--
-SPFPMQ-
-SPFPMH-
-SPFPMF-
SGPFPM--
SDPFPM--
SHPFPM--
-SPFPMN-
SNPFPM--
SFPFPM--
-SPFPMY-
-SPFPMV-
SWPFPM--
-SPFPMD-
-SPFPMA-
TRPFPM--
TSPFPM--
-TPFPMY-
TFPFPM--
-TPFPMI-
TVPFPM--
-TPFPMR-
TNPFPM--
-TPFPMK-
TEPFPM--
TTPFPM--
-TPFPML-
TIPFPM--

-TPFPMW-
-TPFPMM-
TLPFPM--
-TPFPMA-
-TPFPMF-
TQPFPM--
-TPFPMN-
TDPFPM--
TPPFPM--
THPFPM--
-TPFPMS-
-TPFPMG-
TGPFPM--
VRPFPM--
-VPFPMR-
-VPFPML-
VFPFPM--
-VPFPMK-
VAPFPM--
VVPFPM--
-VPFPMA-
VKPFPM--
-VPFPMH-
VTPFPM--
VSPFPM--
VGPFPM--
-VPFPMS-
VHPFPM--
VEPFPM--
-VPFPMN-
-VPFPMF-
-VPFPMY-
VPPFPM--
-VPFPMT-
-VPFPMI-
VNPFPM--
VLPFPM--
VQPFPM--
-VPFPMQ-
VWPFPM--
-VPFPMG-
VMPFPM--
-WPFPML-
-WPFPMK-
WSPFPM--
-WPFPMV-
-WPFPMF-
WKPFPM--
WWPFPM--
WVPFPM--

WPPFPM--
WIPFPM--
-WPFPMS-
WCPFPM--
WYPFPM--
-WPFPMY-
-YPFPMW-
YPPFPM--
-YPFPMR-
-YPFPMT-
-YPFPMK-
YRPFPM--
YLPFPM--
YVPFPM--
YHPFPM--
YAPFPM--
YYPFPM--
YTPFPM--
-YPFPMV-
-YPFPMN-
-YPFPMA-
YFPFPM--
YSPFPM--
-YPFPMS-
-YPFPMH-
YNPFPM--
YCPFPM--
-YPFPMQ-

PHPL	104	805	46.11

-RPHPLG-
ALPHPL--
ARPHPL--
-APHPLL-
AYPHPL--
ATPHPL--
ASPHPL--
-APHPLK-
-APHPLT-
AAPHPL--
-APHPLR-
APPHPL--
-APHPLY-
-APHPLS-
-APHPLN-
-APHPLF-
AMPHPL--
AGPHPL--
ANPHPL--
AFPHPL--
AWPHPL--

AQPHPL--
-APHPLE-
AIPHPL--
-APHPLV-
-APHPLG-
AKPHPL--
AHPHPL--
ADPHPL--
-APHPLA-
CRPHPL--
CKPHPL--
CSPHPL--
-CPHPLV-
-CPHPLR-
-CPHPLN-
-CPHPLI-
CMPHPL--
CHPHPL--
CAPHPL--
-CPHPLY-
-CPHPLW-
-CPHPLL-
CFPHPL--
DRPHPL--
DLPHPL--
-DPHPLL-
-DPHPLV-
-DPHPLQ-
DSPHPL--
-DPHPLR-
-DPHPLK-
-DPHPLH-
DWPHPL--
DVPHPL--
DAPHPL--
DYPHPL--
-DPHPLW-
DKPHPL--
DIPHPL--
DGPHPL--
DFPHPL--
DEPHPL--
-EPHPLR-
-EPHPLY-
ERPHPL--
-EPHPLL-
EAPHPL--
EIPHPL--
ETPHPL--
ESPHPL--

-EPHPLQ-
-EPHPLI-
EVPHPL--
-EPHPLS-
-EPHPLH-
-EPHPLF-
EFPHPL--
EQPHPL--
-EPHPLV-
EMPHPL--
EKPHPL--
EWPHPL--
-EPHPLT-
-EPHPLN-
EEPHPL--
FVPHPL--
FIPHPL--
FSPHPL--
-FPHPLL-
FQPHPL--
FTPHPL--
FRPHPL--
-FPHPLY-
FGPHPL--
FHPHPL--
-FPHPLR-
FNPHPL--
FMPHPL--
FEPHPL--
-FPHPLK-
FAPHPL--
FWPHPL--
-FPHPLA-
-FPHPLS-
-FPHPLN-
-FPHPLI-
-FPHPLH-
-FPHPLG-
-FPHPLV-
FKPHPL--
FFPHPL--
-FPHPLQ-
-FPHPLF-
FCPHPL--
-FPHPLM-
GLPHPL--
-GPHPLR-
GMPHPL--
GQPHPL--
-GPHPLL-

GYPHPL--
GSPHPL--
GVPHPL--
GFPHPL--
-GPHPLI-
-GPHPLH-
GKPHPL--
-GPHPLK-
-GPHPLV-
GIPHPL--
-GPHPLF-
GGPHPL--
GTPHPL--
-GPHPLM-
GNPHPL--
GEPHPL--
GWPHPL--
-GPHPLT-
-GPHPLY-
-GPHPLS-
-GPHPLN-
GHPHPL--
GDPHPL--
GCPHPL--
HIPHPL--
HRPHPL--
HSPHPL--
-HPHPLS-
-HPHPLR-
HGPHPL--
HYPHPL--
-HPHPLY-
HQPHPL--
HWPHPL--
-HPHPLM-
HKPHPL--
-HPHPLQ-
HLPHPL--
HAPHPL--
HVPHPL--
-HPHPLV-
-HPHPLI-
-HPHPLF-
HPPHPL--
-HPHPLH-
HNPHPL--
-HPHPLT-
HFPHPL--
HCPHPL--
-HPHPLL-

-HPHPLG-
HEPHPL--
-HPHPLN-
HHPHPL--
HTPHPL--
-IPHPLR-
IRPHPL--
-IPHPLY-
-IPHPLK-
ILPHPL--
IVPHPL--
IGPHPL--
-IPHPLS-
IHPHPL--
-IPHPLL-
IYPHPL--
-IPHPLT-
INPHPL--
ITPHPL--
ISPHPL--
IDPHPL--
IQPHPL--
-IPHPLW-
-IPHPLV-
-IPHPLN-
-IPHPLA-
IKPHPL--
IIPHPL--
IFPHPL--
IEPHPL--
ICPHPL--
IAPHPL--
IWPHPL--
-IPHPLC-
-IPHPLI-
KSPHPL--
KAPHPL--
KVPHPL--
-KPHPLT-
-KPHPLR-
-KPHPLL-
KMPHPL--
KHPHPL--
KRPHPL--
-KPHPLS-
KLPHPL--
KTPHPL--
KKPHPL--
-KPHPLV-
-KPHPLM-

-KPHPLF-
KIPHPL--
-KPHPLY-
-KPHPLN-
-KPHPLK-
KNPHPL--
KGPHPL--
KWPHPL--
-KPHPLI-
-KPHPLH-
-KPHPLA-
KPPHPL--
-KPHPLG-
KYPHPL--
KCPHPL--
LAPHPL--
LRPHPL--
LMPHPL--
-LPHPLR-
-LPHPLW-
LNPHPL--
LSPHPL--
LYPHPL--
LGPHPL--
LHPHPL--
-LPHPLT-
LIPHPL--
LQPHPL--
LLPHPL--
LKPHPL--
-LPHPLY-
-LPHPLK-
LWPHPL--
LTPHPL--
-LPHPLS-
-LPHPLL-
-LPHPLI-
-LPHPLA-
-LPHPLH-
-LPHPLF-
LPPHPL--
LFPHPL--
LEPHPL--
LDPHPL--
-LPHPLV-
-LPHPLN-
MSPHPL--
MFPHPL--
MNPHPL--
MIPHPL--

-MPHPLR-
-MPHPLL-
MRPHPL--
MPPHPL--
MVPHPL--
-MPHPLS-
MLPHPL--
MHPHPL--
MTPHPL--
-MPHPLA-
MKPHPL--
MQPHPL--
MYPHPL--
-MPHPLF-
-MPHPLQ-
MMPHPL--
MWPHPL--
-MPHPLT-
-MPHPLI-
-MPHPLY-
-MPHPLH-
MDPHPL--
MAPHPL--
-MPHPLM-
MCPHPL--
-MPHPLG-
-MPHPLE-
MEPHPL--
NSPHPL--
-NPHPLI-
NGPHPL--
-NPHPLL-
NNPHPL--
NYPHPL--
-NPHPLR-
NQPHPL--
NLPHPL--
NTPHPL--
-NPHPLT-
NKPHPL--
NEPHPL--
-NPHPLK-
NRPHPL--
NIPHPL--
NVPHPL--
NPPHPL--
NFPHPL--
-NPHPLV-
-NPHPLS-
-NPHPLH-

-NPHPLF-
-NPHPLQ-
-NPHPLG-
-NPHPLA-
NDPHPL--
--PHPLLT
--PHPLTS
--PHPLLR
--PHPLRI
--PHPLFL
--PHPLMT
--PHPLLS
--PHPLRM
--PHPLAL
--PHPLIS
--PHPLLL
--PHPLRS
--PHPLLM
--PHPLLG
--PHPLAS
--PHPLRQ
--PHPLKL
--PHPLGF
--PHPLTL
--PHPLST
PRPHPL--
--PHPLFS
--PHPLYM
--PHPLVA
--PHPLLF
--PHPLVR
--PHPLVG
--PHPLIR
--PHPLHS
--PHPLRR
--PHPLMS
--PHPLLN
--PHPLVV
--PHPLMY
--PHPLKS
PHPHPL--
--PHPLWR
--PHPLRY
--PHPLNL
--PHPLGT
--PHPLWL
--PHPLRK
--PHPLVL
--PHPLNS
--PHPLLA

--PHPLGA
--PHPLWS
--PHPLVT
--PHPLTG
--PHPLRT
--PHPLQH
--PHPLFV
--PHPLAH
--PHPLAF
PVPHPL--
--PHPLYV
--PHPLYF
--PHPLVF
--PHPLTW
--PHPLTT
--PHPLTN
--PHPLMV
--PHPLKV
--PHPLHF
PMPHPL--
--PHPLYR
--PHPLTR
--PHPLSY
--PHPLSS
--PHPLQL
--PHPLLQ
--PHPLIY
--PHPLYL
--PHPLTI
--PHPLRG
--PHPLQQ
--PHPLMN
--PHPLMG
--PHPLKG
--PHPLIM
--PHPLFR
--PHPLEW
PIPHPL--
--PHPLYQ
--PHPLSA
--PHPLNW
--PHPLLK
--PHPLLI
--PHPLFQ
--PHPLYE
--PHPLWH
--PHPLVS
--PHPLVM
--PHPLSQ
--PHPLNV

--PHPLMQ
--PHPLKW
--PHPLIV
--PHPLGS
PWPHPL--
--PHPLYI
--PHPLWF
--PHPLVH
--PHPLSV
--PHPLSN
--PHPLRW
--PHPLRF
--PHPLPS
--PHPLKM
--PHPLIF
PGPHPL--
--PHPLQV
--PHPLMM
--PHPLKT
--PHPLIG
--PHPLFN
--PHPLFK
--PHPLVY
--PHPLVI
--PHPLTH
--PHPLQS
--PHPLQI
--PHPLMI
--PHPLFT
--PHPLEA
PSPHPL--
--PHPLYY
--PHPLWW
--PHPLWG
--PHPLTM
--PHPLTA
--PHPLSF
--PHPLQM
--PHPLPF
--PHPLLW
--PHPLKQ
--PHPLKI
--PHPLIT
--PHPLHV
--PHPLHI
--PHPLFH
--PHPLEQ
--PHPLDF
--PHPLWK
--PHPLVK

--PHPLRN
--PHPLRA
--PHPLQT
--PHPLPA
--PHPLNF
--PHPLKN
--PHPLID
--PHPLHN
--PHPLES
PTPHPL--
--PHPLWQ
--PHPLTV
--PHPLTQ
--PHPLRH
--PHPLRE
--PHPLMW
--PHPLIL
--PHPLHW
--PHPLHK
--PHPLGG
--PHPLFA
--PHPLEF
--PHPLAT
PAPHPL--
--PHPLWV
--PHPLTY
--PHPLQN
--PHPLNQ
--PHPLMF
--PHPLKR
--PHPLIQ
--PHPLFM
--PHPLFF
--PHPLFD
PNPHPL--
--PHPLYT
--PHPLYK
--PHPLYH
--PHPLWN
--PHPLVN
--PHPLTF
--PHPLSR
--PHPLQG
--PHPLPW
--PHPLNT
--PHPLNH
--PHPLKK
--PHPLFE
--PHPLEL
--PHPLAN

PFPHPL--
PLPHPL--
PKPHPL--
--PHPLYW
--PHPLWI
--PHPLWD
--PHPLQA
--PHPLPH
--PHPLMR
--PHPLME
--PHPLIW
--PHPLIH
--PHPLHT
--PHPLHQ
--PHPLFI
--PHPLEI
--PHPLDM
--PHPLDL
--PHPLDI
--PHPLAW
--PHPLAQ
--PHPLAG
PEPHPL--
--PHPLWA
--PHPLVD
--PHPLTK
--PHPLSW
--PHPLQR
--PHPLPV
--PHPLNG
--PHPLMH
--PHPLMA
--PHPLLY
--PHPLLD
--PHPLKY
--PHPLII
--PHPLHY
--PHPLHA
--PHPLGH
--PHPLDW
--PHPLDV
--PHPLDS
--PHPLAA
--PHPLYS
--PHPLYN
--PHPLYA
--PHPLWT
--PHPLTE
--PHPLSK
--PHPLSH

--PHPLSG
--PHPLSD
--PHPLRV
--PHPLQY
--PHPLQK
--PHPLQF
--PHPLNR
--PHPLNN
--PHPLND
--PHPLLH
--PHPLLE
--PHPLKD
--PHPLIN
--PHPLHR
--PHPLHE
--PHPLHD
--PHPLFY
--PHPLDY
--PHPLDQ
--PHPLCV
--PHPLCR
--PHPLCQ
--PHPLAK
PCPHPL--
-QPHPLL-
QFPHPL--
QSPHPL--
-QPHPLV-
-QPHPLT-
QGPHPL--
QNPHPL--
-QPHPLI-
-QPHPLR-
-QPHPLF-
-QPHPLW-
QLPHPL--
QWPHPL--
QTPHPL--
QRPHPL--
-QPHPLS-
-QPHPLN-
QAPHPL--
-QPHPLY-
QHPHPL--
QEPHPL--
QDPHPL--
QYPHPL--
QVPHPL--
QQPHPL--
RVPHPL--

```
-RPHPLR-
RLPHPL--
RQPHPL--
RYPHPL--
RMPHPL--
-RPHPLH-
RNPHPL--
RRPHPL--
-RPHPLK-
RIPHPL--
-RPHPLV-
-RPHPLI-
-RPHPLT-
RFPHPL--
-RPHPLS-
-RPHPLM-
RTPHPL--
-RPHPLW-
-RPHPLF-
RWPHPL--
RHPHPL--
RGPHPL--
RPPHPL--
REPHPL--
-RPHPLE-
-RPHPLY-
-RPHPLQ-
-RPHPLA-
RCPHPL--
RDPHPL--
SAPHPL--
SIPHPL--
SVPHPL--
-SPHPLR-
SMPHPL--
-SPHPLM-
SKPHPL--
-SPHPLK-
SYPHPL--
SGPHPL--
SFPHPL--
-SPHPLT-
-SPHPLY-
-SPHPLI-
-SPHPLF-
SWPHPL--
SRPHPL--
-SPHPLV-
SQPHPL--
-SPHPLQ-
```

-SPHPLL-
SPPHPL--
SDPHPL--
SNPHPL--
-SPHPLW-
-SPHPLS-
SHPHPL--
-SPHPLH-
-SPHPLN-
-SPHPLG-
SEPHPL--
SCPHPL--
-TPHPLI-
TLPHPL--
TKPHPL--
TPPHPL--
TVPHPL--
-TPHPLM-
-TPHPLY-
-TPHPLR-
-TPHPLF-
TYPHPL--
-TPHPLL-
TDPHPL--
-TPHPLT-
TSPHPL--
TRPHPL--
-TPHPLW-
-TPHPLS-
-TPHPLH-
TFPHPL--
TTPHPL--
THPHPL--
TGPHPL--
TWPHPL--
TQPHPL--
TNPHPL--
-TPHPLQ-
-TPHPLK-
-TPHPLG-
-TPHPLA-
TMPHPL--
-TPHPLV-
TCPHPL--
VLPHPL--
VPPHPL--
VFPHPL--
VRPHPL--
-VPHPLL-
VTPHPL--

-VPHPLR-
VIPHPL--
VGPHPL--
VKPHPL--
VVPHPL--
-VPHPLV-
VDPHPL--
-VPHPLW-
-VPHPLS-
-VPHPLP-
-VPHPLA-
VMPHPL--
VSPHPL--
-VPHPLQ-
VWPHPL--
-VPHPLI-
VQPHPL--
-VPHPLY-
-VPHPLH-
-VPHPLG-
VEPHPL--
VAPHPL--
-VPHPLM-
-VPHPLN-
-VPHPLE-
VNPHPL--
-VPHPLT-
-VPHPLF-
VHPHPL--
-VPHPLK-
WPPHPL--
WGPHPL--
WLPHPL--
WNPHPL--
WYPHPL--
WRPHPL--
-WPHPLW-
-WPHPLR-
WQPHPL--
WAPHPL--
-WPHPLL-
-WPHPLK-
-WPHPLV-
WKPHPL--
-WPHPLS-
-WPHPLN-
-WPHPLM-
-WPHPLA-
WFPHPL--
WWPHPL--

	-WPHPLQ-		
	-WPHPLH-		
	WTPHPL--		
	WHPHPL--		
	WMPHPL--		
	YHPHPL--		
	YPPHPL--		
	YGPHPL--		
	-YPHPLR-		
	YTPHPL--		
	YAPHPL--		
	YMPHPL--		
	YLPHPL--		
	YKPHPL--		
	-YPHPLV-		
	YRPHPL--		
	-YPHPLK-		
	-YPHPLS-		
	YFPHPL--		
	-YPHPLY-		
	-YPHPLT-		
	-YPHPLL-		
	YIPHPL--		
	YSPHPL--		
	YQPHPL--		
	YEPHPL--		
	YDPHPL--		
	YYPHPL--		
	YVPHPL--		
	-YPHPLI-		
	-YPHPLF-		
	-YPHPLW-		
	-YPHPLA-		
	-YPHPLN-		
PIPI	421	651	46.10
	AIPIPI--		
	-APIPIS-		
	-APIPIV-		
	ASPIPI--		
	AWPIPI--		
	-APIPIR-		
	-APIPIL-		
	ARPIPI--		
	-APIPIA-		
	AAPIPI--		
	ATPIPI--		
	AKPIPI--		
	-APIPII-		
	AGPIPI--		
	-APIPIM-		

-APIPIF-
AEPIPI--
APPIPI--
AYPIPI--
AHPIPI--
ADPIPI--
-APIPIE-
AMPIPI--
CAPIPI--
CWPIPI--
CRPIPI--
CSPIPI--
CDPIPI--
CTPIPI--
CQPIPI--
CKPIPI--
CCPIPI--
DRPIPI--
DQPIPI--
DKPIPI--
-DPIPIY-
-DPIPIF-
-DPIPII-
DVPIPI--
DTPIPI--
-DPIPIW-
-DPIPIH-
-DPIPIK-
DMPIPI--
DGPIPI--
DFPIPI--
DYPIPI--
-DPIPIS-
DLPIPI--
DIPIPI--
DAPIPI--
-EPIPIK-
-EPIPIR-
EGPIPI--
-EPIPIT-
EIPIPI--
-EPIPIL-
ERPIPI--
-EPIPIY-
-EPIPIV-
-EPIPII-
ESPIPI--
EQPIPI--
ETPIPI--
-EPIPIS-

EMPIPI--
EDPIPI--
FVPIPI--
-FPIPIK-
-FPIPIR-
FIPIPI--
FPPIPI--
FDPIPI--
-FPIPIA-
FSPIPI--
FRPIPI--
FNPIPI--
FGPIPI--
FFPIPI--
-FPIPIL-
-FPIPII-
-FPIPIS-
FQPIPI--
-FPIPIG-
FHPIPI--
FWPIPI--
FTPIPI--
-FPIPIT-
-FPIPIQ-
-FPIPIM-
FMPIPI--
FKPIPI--
-GPIPIT-
GSPIPI--
GTPIPI--
GIPIPI--
-GPIPIR-
GFPIPI--
GAPIPI--
GRPIPI--
GEPIPI--
-GPIPIY-
-GPIPIA-
GVPIPI--
-GPIPIL-
-GPIPII-
GLPIPI--
-GPIPIV-
GHPIPI--
GQPIPI--
-GPIPIF-
-GPIPIS-
-GPIPIM-
-GPIPIH-
-GPIPIG-

−HPIPIR−
−HPIPIL−
HRPIPI−−
HPPIPI−−
HSPIPI−−
HQPIPI−−
−HPIPIY−
HVPIPI−−
−HPIPIT−
HKPIPI−−
HYPIPI−−
−HPIPIH−
HIPIPI−−
HGPIPI−−
HLPIPI−−
HWPIPI−−
−HPIPIF−
−HPIPIA−
HTPIPI−−
−HPIPIS−
ILPIPI−−
−IPIPIW−
INPIPI−−
IGPIPI−−
IKPIPI−−
−IPIPIR−
−IPIPIH−
−IPIPIN−
−IPIPIV−
−IPIPIT−
−IPIPIS−
−IPIPIM−
−IPIPIA−
IDPIPI−−
−IPIPIL−
IMPIPI−−
IRPIPI−−
IQPIPI−−
−IPIPIY−
KSPIPI−−
KGPIPI−−
−KPIPIR−
KTPIPI−−
−KPIPIS−
−KPIPIM−
−KPIPIL−
−KPIPIH−
KMPIPI−−
KHPIPI−−
−KPIPIK−

KFPIPI--
KNPIPI--
KWPIPI--
-KPIPII-
KVPIPI--
KRPIPI--
KPPIPI--
-KPIPIV-
-KPIPIT-
-KPIPIG-
-KPIPIE-
KQPIPI--
-KPIPIA-
KKPIPI--
KIPIPI--
KCPIPI--
LGPIPI--
LYPIPI--
LNPIPI--
LSPIPI--
LLPIPI--
LTPIPI--
-LPIPIR-
LMPIPI--
LWPIPI--
LQPIPI--
LKPIPI--
LVPIPI--
-LPIPIY-
LHPIPI--
-LPIPIV-
-LPIPIT-
-LPIPIM-
LIPIPI--
-LPIPIL-
-LPIPIA-
LEPIPI--
LAPIPI--
-LPIPIH-
-LPIPII-
LDPIPI--
LPPIPI--
-LPIPIQ-
-LPIPIK-
MRPIPI--
MSPIPI--
MAPIPI--
MVPIPI--
MPPIPI--
-MPIPIY-

MLPIPI--
MWPIPI--
MTPIPI--
MQPIPI--
-MPIPIS-
-MPIPIK-
-MPIPIH-
MKPIPI--
MIPIPI--
-MPIPIR-
MFPIPI--
MYPIPI--
-MPIPIG-
MNPIPI--
MGPIPI--
NSPIPI--
-NPIPIH-
NRPIPI--
NVPIPI--
NTPIPI--
NKPIPI--
NGPIPI--
NPPIPI--
-NPIPII-
NYPIPI--
-NPIPIS-
-NPIPIM-
-NPIPIL-
NMPIPI--
NEPIPI--
-NPIPIT-
-NPIPIR-
-NPIPIF-
NLPIPI--
NIPIPI--
NDPIPI--
NAPIPI--
NWPIPI--
NQPIPI--
-NPIPIQ-
NNPIPI--
--PIPIRL
--PIPISL
--PIPIQL
--PIPILS
--PIPILA
PHPIPI--
--PIPILY
--PIPIMS
--PIPIMG

--PIPIYG
--PIPITW
--PIPIRV
--PIPIRS
--PIPIRY
--PIPINS
--PIPITR
--PIPITL
--PIPILT
--PIPILG
--PIPIKI
--PIPIIY
--PIPIFG
--PIPIVM
--PIPIVG
--PIPIKG
--PIPIFI
--PIPIYV
--PIPITT
--PIPISA
--PIPIRN
--PIPIIH
--PIPIVD
--PIPIRT
--PIPIRH
--PIPIMW
--PIPIMV
--PIPIFH
--PIPILN
PFPIPI--
--PIPIYH
--PIPIVI
--PIPIVH
--PIPIRQ
--PIPIRA
--PIPILQ
--PIPIIS
--PIPIGV
--PIPIAS
--PIPIYI
--PIPIWT
--PIPIWA
--PIPITS
--PIPINI
--PIPILV
--PIPIGQ
--PIPIGH
--PIPIYR
--PIPIVV
--PIPITV

--PIPIPW
--PIPIML
--PIPIKM
--PIPIGF
--PIPIDL
--PIPIAK
--PIPIAH
PWPIPI--
PQPIPI--
--PIPIWL
--PIPIVA
--PIPITH
--PIPIRM
--PIPIRK
--PIPIKT
--PIPIHS
--PIPIHG
--PIPIGS
--PIPIEG
--PIPIYL
--PIPISN
--PIPISG
--PIPIRG
--PIPIQW
--PIPIQR
--PIPIKS
--PIPIIL
PEPIPI--
--PIPIWN
--PIPIVL
--PIPITG
--PIPISS
--PIPIQY
--PIPIQM
--PIPIQG
--PIPIMR
--PIPIMF
--PIPIKH
--PIPIKA
--PIPIIK
--PIPIFT
--PIPIFA
--PIPIAT
--PIPIAG
--PIPIRW
--PIPINW
--PIPINQ
--PIPING
--PIPIMY
--PIPIMQ

--PIPIMM
--PIPIMH
--PIPILW
--PIPIFS
--PIPIAV
--PIPIWW
--PIPIVE
--PIPITQ
--PIPISV
--PIPISR
--PIPIRI
--PIPINM
--PIPIMT
--PIPIKN
--PIPIKF
--PIPIIN
--PIPIHW
--PIPIHR
--PIPIHM
--PIPIHF
--PIPIGW
--PIPIFR
--PIPIFQ
--PIPIEW
--PIPIDI
PGPIPI--
PTPIPI--
PNPIPI--
--PIPIYD
--PIPIWI
--PIPIVT
--PIPIVQ
--PIPITM
--PIPITK
--PIPISK
--PIPIQT
--PIPIQQ
--PIPILH
--PIPIKV
--PIPIKR
--PIPIKE
--PIPIIA
--PIPIHH
--PIPIGT
--PIPIGA
--PIPIFF
--PIPIFE
--PIPIEM
--PIPIEI
--PIPIDY

--PIPIWY
--PIPIWG
--PIPIWF
--PIPIVY
--PIPIVS
--PIPITA
--PIPIQN
--PIPIPF
--PIPINY
--PIPINH
--PIPINA
--PIPIKW
--PIPIKK
--PIPIIT
--PIPIHD
--PIPIHA
--PIPIGK
PKPIPI--
--PIPIYY
--PIPIYT
--PIPIWH
--PIPIWE
--PIPIWD
--PIPIVW
--PIPIVR
--PIPITI
--PIPITF
--PIPISY
--PIPISW
--PIPIST
--PIPISQ
--PIPISF
--PIPISE
--PIPIRD
--PIPIQI
--PIPIQF
--PIPIPY
--PIPIPS
--PIPIPI
--PIPIME
--PIPIMD
--PIPIMA
--PIPILE
--PIPILD
--PIPIKD
--PIPIIM
--PIPIIF
--PIPIGE
--PIPIFV
--PIPIEY

--PIPIEA
--PIPIDQ
--PIPIAN
--PIPIAM
PDPIPI--
QYPIPI--
QKPIPI--
QRPIPI--
-QPIPIL-
-QPIPII-
QLPIPI--
QHPIPI--
QGPIPI--
QSPIPI--
-QPIPIK-
-QPIPIH-
-QPIPIA-
-QPIPIQ-
-QPIPIF-
QTPIPI--
QPPIPI--
QAPIPI--
QWPIPI--
-QPIPIT-
QIPIPI--
RFPIPI--
-RPIPIY-
-RPIPIR-
RVPIPI--
RRPIPI--
RIPIPI--
-RPIPIF-
RLPIPI--
RGPIPI--
-RPIPIM-
RTPIPI--
-RPIPIV-
RYPIPI--
-RPIPIH-
RNPIPI--
RDPIPI--
-RPIPIK-
RKPIPI--
-RPIPIT-
-RPIPIQ-
-RPIPIN-
RMPIPI--
RWPIPI--
-RPIPIS-
RQPIPI--

-RPIPIG-
-RPIPIE-
-RPIPIA-
RHPIPI--
SRPIPI--
SSPIPI--
SVPIPI--
SGPIPI--
SYPIPI--
-SPIPIL-
SPPIPI--
STPIPI--
SHPIPI--
SIPIPI--
SQPIPI--
-SPIPIM-
-SPIPIK-
-SPIPIS-
SMPIPI--
-SPIPIW-
-SPIPIQ-
SEPIPI--
SAPIPI--
-SPIPIR-
-SPIPII-
-SPIPIF-
-SPIPIV-
SNPIPI--
SKPIPI--
-SPIPIH-
SDPIPI--
-SPIPIT-
-SPIPIG-
TVPIPI--
TRPIPI--
TSPIPI--
TLPIPI--
-TPIPIS-
-TPIPIQ-
-TPIPIL-
-TPIPIT-
TNPIPI--
-TPIPIV-
-TPIPIM-
TMPIPI--
TTPIPI--
-TPIPIR-
-TPIPII-
TKPIPI--
TEPIPI--

THPIPI--
TGPIPI--
TPPIPI--
-TPIPIK-
TIPIPI--
TFPIPI--
-TPIPIY-
-TPIPIN-
-TPIPIH-
-TPIPIF-
TAPIPI--
-VPIPIQ-
-VPIPIL-
-VPIPIF-
VTPIPI--
VIPIPI--
VGPIPI--
VRPIPI--
VNPIPI--
VHPIPI--
-VPIPII-
VMPIPI--
-VPIPIS-
VFPIPI--
-VPIPIW-
-VPIPIY-
-VPIPIK-
VAPIPI--
VLPIPI--
VSPIPI--
-VPIPIH-
VKPIPI--
-VPIPIG-
-VPIPIA-
VYPIPI--
-VPIPIV-
-VPIPIT-
-VPIPIE-
-VPIPIC-
WYPIPI--
WTPIPI--
WQPIPI--
-WPIPIL-
-WPIPIR-
WSPIPI--
-WPIPIS-
WMPIPI--
WKPIPI--
WGPIPI--
WFPIPI--

WIPIPI--
WAPIPI--
WRPIPI--
-WPIPIK-
WLPIPI--
WHPIPI--
-WPIPIY-
WVPIPI--
-WPIPIH-
-WPIPIA-
-YPIPIT-
YGPIPI--
YDPIPI--
YVPIPI--
YHPIPI--
YRPIPI--
YEPIPI--
YWPIPI--
YSPIPI--
-YPIPII-
-YPIPIH-
-YPIPIL-
-YPIPIF-
YNPIPI--
YAPIPI--
-YPIPIR-
-YPIPIW-
-YPIPIA-
YLPIPI--
YYPIPI--
-YPIPIY-
-YPIPIV-
-YPIPIQ-
-YPIPIK-
YKPIPI--
YIPIPI--

PLSY 94 810 46.10

-KPLSYG-
-LPLSYG-
--PLSYEL
--PLSYWP
--PLSYRP
--PLSYPV
--PLSYPI
-VPLSYC-
ARPLSY--
AVPLSY--
-APLSYM-
-APLSYL-
ALPLSY--

ATPLSY--
ASPLSY--
AYPLSY--
AHPLSY--
APPLSY--
ANPLSY--
AIPLSY--
AMPLSY--
-APLSYI-
-APLSYH-
AGPLSY--
-APLSYS-
AAPLSY--
ADPLSY--
-APLSYQ-
AWPLSY--
AFPLSY--
AKPLSY--
-APLSYA-
-APLSYF-
-APLSYK-
-APLSYN-
-APLSYG-
-APLSYY-
-APLSYW-
-APLSYE-
ACPLSY--
CRPLSY--
CVPLSY--
CAPLSY--
CCPLSY--
CSPLSY--
CMPLSY--
CIPLSY--
CDPLSY--
-CPLSYV-
-CPLSYQ-
-CPLSYL-
CKPLSY--
CGPLSY--
CTPLSY--
-CPLSYW-
-CPLSYT-
CLPLSY--
CWPLSY--
CQPLSY--
-CPLSYR-
-CPLSYK-
-CPLSYG-
-CPLSYF-

-CPLSYA-
CHPLSY--
DRPLSY--
DLPLSY--
DKPLSY--
DYPLSY--
DQPLSY--
DAPLSY--
DSPLSY--
-DPLSYR-
DVPLSY--
DHPLSY--
-DPLSYL-
-DPLSYK-
DDPLSY--
-DPLSYM-
-DPLSYS-
DFPLSY--
DWPLSY--
-DPLSYY-
-DPLSYQ-
DTPLSY--
-DPLSYT-
-DPLSYF-
DGPLSY--
DEPLSY--
DMPLSY--
DIPLSY--
-DPLSYI-
-DPLSYW-
-DPLSYV-
-DPLSYA-
-DPLSYE-
DCPLSY--
-EPLSYL-
EFPLSY--
ESPLSY--
-EPLSYH-
EMPLSY--
-EPLSYR-
ERPLSY--
-EPLSYV-
ETPLSY--
EKPLSY--
-EPLSYM-
EIPLSY--
-EPLSYI-
EYPLSY--
ELPLSY--
EGPLSY--

-EPLSYY-
EEPLSY--
EVPLSY--
-EPLSYQ-
EQPLSY--
-EPLSYW-
-EPLSYS-
-EPLSYK-
EHPLSY--
EAPLSY--
EWPLSY--
-EPLSYF-
-EPLSYA-
ENPLSY--
ECPLSY--
-EPLSYE-
FAPLSY--
FRPLSY--
FTPLSY--
-FPLSYT-
-FPLSYV-
FIPLSY--
FPPLSY--
FVPLSY--
-FPLSYH-
FSPLSY--
FLPLSY--
-FPLSYS-
-FPLSYA-
FNPLSY--
FFPLSY--
FEPLSY--
-FPLSYQ-
-FPLSYK-
FWPLSY--
-FPLSYM-
FKPLSY--
-FPLSYN-
-FPLSYF-
-FPLSYY-
FDPLSY--
-FPLSYE-
-FPLSYC-
FCPLSY--
-GPLSYR-
-GPLSYL-
GTPLSY--
GSPLSY--
GKPLSY--
GRPLSY--

-GPLSYT-
GYPLSY--
-GPLSYS-
GGPLSY--
-GPLSYW-
-GPLSYQ-
-GPLSYV-
GVPLSY--
-GPLSYM-
GFPLSY--
-GPLSYK-
-GPLSYA-
GNPLSY--
GEPLSY--
GHPLSY--
GWPLSY--
-GPLSYN-
GMPLSY--
GAPLSY--
GIPLSY--
GPPLSY--
GDPLSY--
GQPLSY--
-GPLSYY-
-GPLSYG-
-GPLSYE-
-HPLSYR-
HQPLSY--
HVPLSY--
HSPLSY--
-HPLSYS-
HPPLSY--
HTPLSY--
-HPLSYL-
HKPLSY--
-HPLSYT-
-HPLSYQ-
HGPLSY--
HYPLSY--
HNPLSY--
HIPLSY--
-HPLSYV-
-HPLSYK-
-HPLSYY-
HAPLSY--
HFPLSY--
-HPLSYH-
-HPLSYA-
HMPLSY--
HEPLSY--

-HPLSYM-
HCPLSY--
-HPLSYC-
HWPLSY--
-HPLSYN-
-HPLSYI-
HHPLSY--
-IPLSYR-
ITPLSY--
ISPLSY--
IRPLSY--
-IPLSYS-
-IPLSYM-
INPLSY--
IGPLSY--
-IPLSYA-
ILPLSY--
IKPLSY--
-IPLSYK-
-IPLSYV-
-IPLSYN-
IIPLSY--
IEPLSY--
IYPLSY--
IVPLSY--
IHPLSY--
-IPLSYT-
-IPLSYH-
IMPLSY--
IFPLSY--
IWPLSY--
-IPLSYF-
-IPLSYG-
IQPLSY--
-IPLSYQ-
IPPLSY--
-IPLSYW-
-IPLSYI-
KSPLSY--
KGPLSY--
KTPLSY--
KNPLSY--
KAPLSY--
KFPLSY--
-KPLSYA-
-KPLSYF-
KLPLSY--
KYPLSY--
-KPLSYY-
-KPLSYI-

KEPLSY--
-KPLSYT-
KWPLSY--
KMPLSY--
KHPLSY--
-KPLSYN-
KIPLSY--
KKPLSY--
KVPLSY--
-KPLSYE-
-KPLSYQ-
KDPLSY--
KQPLSY--
-KPLSYW-
KCPLSY--
-LPLSYT-
LGPLSY--
LKPLSY--
LNPLSY--
LPPLSY--
LAPLSY--
-LPLSYR-
LVPLSY--
LYPLSY--
-LPLSYK-
LHPLSY--
-LPLSYI-
-LPLSYV-
LQPLSY--
LMPLSY--
LIPLSY--
-LPLSYF-
LDPLSY--
-LPLSYY-
-LPLSYW-
-LPLSYQ-
-LPLSYN-
-LPLSYH-
LCPLSY--
-LPLSYP-
-MPLSYL-
-MPLSYR-
MKPLSY--
MLPLSY--
MNPLSY--
MGPLSY--
MAPLSY--
MVPLSY--
-MPLSYV-
MTPLSY--

MHPLSY--
MPPLSY--
-MPLSYQ-
MWPLSY--
-MPLSYK-
-MPLSYM-
-MPLSYH-
MQPLSY--
-MPLSYN-
-MPLSYA-
MDPLSY--
-MPLSYE-
MEPLSY--
MMPLSY--
-MPLSYI-
-MPLSYF-
-MPLSYG-
MFPLSY--
-MPLSYC-
MCPLSY--
NFPLSY--
NRPLSY--
NSPLSY--
-NPLSYR-
-NPLSYS-
NGPLSY--
NAPLSY--
-NPLSYT-
NPPLSY--
-NPLSYL-
NVPLSY--
NQPLSY--
NWPLSY--
NKPLSY--
NYPLSY--
NTPLSY--
NLPLSY--
-NPLSYY-
-NPLSYI-
-NPLSYQ-
NMPLSY--
NDPLSY--
NNPLSY--
NHPLSY--
-NPLSYM-
-NPLSYA-
NEPLSY--
NCPLSY--
-NPLSYF-
-NPLSYE-

--PLSYLG
PRPLSY--
--PLSYTR
--PLSYST
--PLSYKT
--PLSYRA
--PLSYSG
--PLSYHS
--PLSYSN
--PLSYRS
--PLSYMR
--PLSYWN
--PLSYVT
--PLSYMG
--PLSYES
--PLSYTM
--PLSYTL
--PLSYVR
--PLSYRM
--PLSYRE
--PLSYTS
--PLSYAT
--PLSYRQ
--PLSYIR
--PLSYNS
--PLSYLK
--PLSYIA
--PLSYIS
--PLSYIT
--PLSYVG
--PLSYTG
--PLSYMS
--PLSYVS
--PLSYRH
--PLSYVK
--PLSYYT
--PLSYSV
--PLSYFS
--PLSYRN
--PLSYRG
--PLSYGS
--PLSYVH
--PLSYWS
--PLSYLN
--PLSYHG
--PLSYQT
--PLSYYG
--PLSYTV
--PLSYSK
--PLSYYA

--PLSYRV
--PLSYKH
--PLSYVN
--PLSYTN
--PLSYTF
--PLSYWL
--PLSYNA
--PLSYMK
--PLSYMV
--PLSYKS
--PLSYTW
--PLSYGA
--PLSYYK
--PLSYRY
--PLSYKN
--PLSYKL
--PLSYVV
--PLSYKV
--PLSYHA
--PLSYQK
--PLSYIQ
PGPLSY--
--PLSYQN
--PLSYMQ
--PLSYKQ
--PLSYIM
--PLSYYQ
--PLSYTK
--PLSYMT
--PLSYMM
--PLSYGL
--PLSYYS
--PLSYTI
--PLSYIH
--PLSYTT
--PLSYNW
--PLSYGY
--PLSYFN
--PLSYSM
--PLSYHR
--PLSYAL
--PLSYQR
--PLSYMY
--PLSYGG
--PLSYAV
--PLSYVD
--PLSYSF
--PLSYRF
--PLSYIN
--PLSYGT

PNPLSY--
--PLSYYM
--PLSYQA
--PLSYMN
--PLSYAN
--PLSYAG
--PLSYQV
--PLSYQS
--PLSYNF
--PLSYHV
--PLSYHT
--PLSYEW
--PLSYWQ
--PLSYWA
--PLSYTD
--PLSYLE
--PLSYHI
--PLSYGV
--PLSYFM
--PLSYEA
--PLSYWD
--PLSYVA
--PLSYNM
--PLSYHM
--PLSYFG
--PLSYTE
--PLSYKW
--PLSYWV
--PLSYRW
--PLSYQW
--PLSYQG
--PLSYMD
--PLSYLD
--PLSYGM
--PLSYEH
--PLSYWM
--PLSYWK
--PLSYQQ
--PLSYQF
--PLSYNY
--PLSYKI
--PLSYHF
--PLSYAI
--PLSYVQ
--PLSYTQ
--PLSYET
--PLSYAH
--PLSYYD
--PLSYQY
--PLSYNH

--PLSYMI
--PLSYME
--PLSYKD
--PLSYHL
--PLSYFQ
--PLSYER
--PLSYAQ
--PLSYTH
--PLSYSQ
--PLSYHY
--PLSYFE
--PLSYAR
--PLSYYE
--PLSYWG
--PLSYVW
--PLSYNV
--PLSYDS
PEPLSY--
--PLSYVI
--PLSYSE
--PLSYMA
--PLSYKY
--PLSYII
--PLSYGI
--PLSYCG
--PLSYWE
--PLSYND
--PLSYMW
--PLSYFD
--PLSYEY
--PLSYEI
--PLSYWH
--PLSYTY
--PLSYNQ
--PLSYNN
--PLSYNI
--PLSYHQ
--PLSYGF
PDPLSY--
--PLSYYH
--PLSYQI
--PLSYKF
--PLSYID
--PLSYHH
--PLSYEN
--PLSYAD
--PLSYQH
--PLSYQE
--PLSYNR
--PLSYMH

--PLSYHN
--PLSYGW
--PLSYEM
--PLSYVE
--PLSYSP
--PLSYQL
--PLSYQD
--PLSYPY
--PLSYIP
--PLSYIE
--PLSYHK
--PLSYHD
--PLSYGN
--PLSYEV
--PLSYEQ
-PPLSYT-
-PPLSYM-
-PPLSYL-
-PPLSYI-
--PLSYPN
--PLSYNK
--PLSYNC
--PLSYMC
--PLSYKC
--PLSYFC
--PLSYEK
--PLSYDQ
--PLSYDK
--PLSYDG
--PLSYDA
--PLSYCR
--PLSYCN
--PLSYCM
--PLSYCK
QTPLSY--
QSPLSY--
-QPLSYR-
-QPLSYL-
QLPLSY--
QKPLSY--
QMPLSY--
-QPLSYF-
QRPLSY--
QGPLSY--
-QPLSYS-
-QPLSYV-
QPPLSY--
-QPLSYI-
QAPLSY--
QIPLSY--

QVPLSY--
QWPLSY--
QHPLSY--
-QPLSYT-
QYPLSY--
-QPLSYY-
-QPLSYW-
-QPLSYQ-
QQPLSY--
-QPLSYM-
-QPLSYH-
-QPLSYA-
QFPLSY--
QNPLSY--
QDPLSY--
-QPLSYK-
-QPLSYN-
-QPLSYG-
-QPLSYE-
-QPLSYD-
QEPLSY--
RLPLSY--
-RPLSYS-
RGPLSY--
-RPLSYA-
RPPLSY--
-RPLSYM-
RNPLSY--
RFPLSY--
RYPLSY--
-RPLSYK-
RTPLSY--
RAPLSY--
RHPLSY--
-RPLSYH-
RWPLSY--
-RPLSYW-
-RPLSYQ-
REPLSY--
-RPLSYY-
-RPLSYF-
-RPLSYN-
-RPLSYG-
RDPLSY--
RCPLSY--
-RPLSYC-
SLPLSY--
SSPLSY--
SGPLSY--
SKPLSY--

SAPLSY--
-SPLSYT-
-SPLSYI-
STPLSY--
-SPLSYM-
SVPLSY--
SIPLSY--
-SPLSYS-
-SPLSYA-
SDPLSY--
SEPLSY--
SNPLSY--
SQPLSY--
SHPLSY--
-SPLSYY-
SWPLSY--
-SPLSYN-
SMPLSY--
-SPLSYH-
-SPLSYW-
-SPLSYF-
-SPLSYG-
SPPLSY--
SCPLSY--
-SPLSYE-
TRPLSY--
-TPLSYV-
TSPLSY--
-TPLSYL-
-TPLSYI-
TTPLSY--
TVPLSY--
TAPLSY--
TLPLSY--
-TPLSYT-
-TPLSYM-
-TPLSYS-
TNPLSY--
TPPLSY--
TIPLSY--
TGPLSY--
TMPLSY--
TKPLSY--
TEPLSY--
-TPLSYA-
TQPLSY--
TYPLSY--
TWPLSY--
TFPLSY--
THPLSY--

-TPLSYQ-
-TPLSYN-
-TPLSYK-
-TPLSYH-
TDPLSY--
-TPLSYG-
TCPLSY--
-TPLSYE-
VRPLSY--
-VPLSYL-
VLPLSY--
-VPLSYI-
-VPLSYT-
VTPLSY--
-VPLSYS-
-VPLSYV-
-VPLSYR-
VGPLSY--
VVPLSY--
VDPLSY--
VFPLSY--
VEPLSY--
VAPLSY--
-VPLSYM-
-VPLSYN-
VQPLSY--
VMPLSY--
-VPLSYA-
VIPLSY--
-VPLSYK-
VPPLSY--
VNPLSY--
-VPLSYH-
VHPLSY--
VWPLSY--
-VPLSYW-
-VPLSYQ-
-VPLSYG-
-VPLSYE-
WKPLSY--
WSPLSY--
WNPLSY--
-WPLSYQ-
-WPLSYT-
-WPLSYK-
-WPLSYI-
-WPLSYV-
WEPLSY--
WIPLSY--
-WPLSYA-

WAPLSY--
-WPLSYM-
WYPLSY--
WFPLSY--
WDPLSY--
-WPLSYN-
-WPLSYY-
WQPLSY--
-WPLSYL-
WHPLSY--
-WPLSYG-
WCPLSY--
-WPLSYE-
-YPLSYR-
YRPLSY--
YLPLSY--
-YPLSYY-
YNPLSY--
YGPLSY--
YHPLSY--
YSPLSY--
YTPLSY--
YQPLSY--
-YPLSYT-
YAPLSY--
YVPLSY--
-YPLSYS-
-YPLSYH-
YIPLSY--
-YPLSYM-
-YPLSYK-
YDPLSY--
YYPLSY--
-YPLSYF-
YMPLSY--
YPPLSY--
YWPLSY--
-YPLSYW-
YKPLSY--
YEPLSY--
-YPLSYN-
-YPLSYQ-
-YPLSYE-
-YPLSYV-
-YPLSYA-
-YPLSYG-
YCPLSY--

PWPI 539 605 46.09

--PWPINK
-APWPIY-

-APWPIW-
-APWPIS-
-APWPIR-
AMPWPI--
AHPWPI--
AWPWPI--
ASPWPI--
-APWPIK-
APPWPI--
AGPWPI--
ARPWPI--
AQPWPI--
AEPWPI--
AAPWPI--
-APWPIA-
AIPWPI--
AVPWPI--
ATPWPI--
ANPWPI--
AFPWPI--
-APWPII-
ALPWPI--
AKPWPI--
AYPWPI--
-APWPIG-
-APWPIQ-
ACPWPI--
-CPWPIH-
CIPWPI--
CCPWPI--
CSPWPI--
CRPWPI--
-CPWPIL-
DRPWPI--
-DPWPIH-
DKPWPI--
DAPWPI--
-DPWPIS-
-DPWPIL-
DFPWPI--
DNPWPI--
-DPWPIY-
DLPWPI--
EVPWPI--
-EPWPIR-
-EPWPIF-
EMPWPI--
EAPWPI--
-EPWPIL-
-EPWPIK-

EYPWPI--
ESPWPI--
EQPWPI--
-EPWPIM-
EFPWPI--
-EPWPIT-
-EPWPIN-
-EPWPII-
-EPWPIG-
ELPWPI--
FMPWPI--
-FPWPIS-
FRPWPI--
FGPWPI--
-FPWPIK-
FVPWPI--
FLPWPI--
-FPWPIW-
FNPWPI--
FWPWPI--
-FPWPIR-
-FPWPIT-
FYPWPI--
-FPWPIY-
-FPWPIV-
-FPWPIF-
FIPWPI--
FTPWPI--
FQPWPI--
-FPWPIN-
FKPWPI--
FAPWPI--
GVPWPI--
GKPWPI--
-GPWPIW-
-GPWPII-
GRPWPI--
GQPWPI--
-GPWPIL-
-GPWPIY-
GMPWPI--
-GPWPIF-
GLPWPI--
-GPWPIA-
GIPWPI--
GDPWPI--
GTPWPI--
-GPWPIQ-
GHPWPI--
-GPWPIV-

-GPWPIT-
-GPWPIP-
GNPWPI--
GFPWPI--
HMPWPI--
-HPWPIR-
-HPWPIH-
HGPWPI--
-HPWPIF-
HSPWPI--
HRPWPI--
-HPWPIA-
-HPWPIL-
HTPWPI--
-HPWPIM-
-HPWPIK-
HHPWPI--
-HPWPIT-
-HPWPIQ-
HLPWPI--
-HPWPIW-
-HPWPIS-
HNPWPI--
HVPWPI--
HQPWPI--
-HPWPII-
-HPWPIE-
HIPWPI--
HDPWPI--
ISPWPI--
-IPWPIR-
-IPWPIF-
ITPWPI--
ILPWPI--
IIPWPI--
-IPWPIQ-
IRPWPI--
-IPWPIH-
-IPWPIL-
IHPWPI--
-IPWPIN-
INPWPI--
IGPWPI--
IWPWPI--
IQPWPI--
-IPWPIT-
IPPWPI--
IKPWPI--
IDPWPI--
KSPWPI--

-KPWPIY-
KAPWPI--
-KPWPIR-
KLPWPI--
-KPWPIT-
-KPWPIK-
KVPWPI--
-KPWPIH-
KFPWPI--
-KPWPIS-
-KPWPIN-
KRPWPI--
-KPWPIQ-
KEPWPI--
KTPWPI--
KDPWPI--
KWPWPI--
-KPWPIM-
-KPWPIL-
-KPWPII-
KGPWPI--
LAPWPI--
LGPWPI--
LSPWPI--
LRPWPI--
LVPWPI--
LDPWPI--
LMPWPI--
-LPWPIK-
-LPWPIR-
LKPWPI--
-LPWPIM-
-LPWPIF-
LPPWPI--
LHPWPI--
LQPWPI--
-LPWPIT-
-LPWPII-
-LPWPIH-
LLPWPI--
LYPWPI--
-LPWPIW-
-LPWPIQ-
-LPWPIL-
LFPWPI--
LWPWPI--
-LPWPIN-
LIPWPI--
-MPWPIR-
MSPWPI--

MMPWPI--
MKPWPI--
MGPWPI--
MDPWPI--
MAPWPI--
MVPWPI--
-MPWPIW-
MRPWPI--
MFPWPI--
MNPWPI--
MWPWPI--
-MPWPIS-
-MPWPIQ-
-MPWPIH-
-MPWPIA-
MYPWPI--
-MPWPIK-
MLPWPI--
MEPWPI--
NRPWPI--
NLPWPI--
-NPWPIL-
NGPWPI--
NIPWPI--
NSPWPI--
-NPWPIR-
NWPWPI--
-NPWPIW-
-NPWPII-
-NPWPIF-
NMPWPI--
NNPWPI--
NEPWPI--
-NPWPIY-
-NPWPIK-
-NPWPIH-
NPPWPI--
NDPWPI--
NTPWPI--
-NPWPIQ-
NFPWPI--
--PWPINS
--PWPIRS
--PWPILG
--PWPIHS
--PWPISS
--PWPIKS
--PWPIVT
--PWPITT
--PWPIRV

--PWPINA
--PWPIMM
--PWPIWS
--PWPIRG
--PWPIQS
--PWPIIG
--PWPIMS
--PWPIWM
--PWPILT
--PWPITS
--PWPIVG
--PWPIVA
--PWPINM
--PWPIAT
--PWPIRL
--PWPILS
--PWPIIR
--PWPIYT
--PWPIWR
--PWPIRN
--PWPIQG
--PWPIAS
--PWPITR
--PWPIRT
--PWPIFD
PIPWPI--
--PWPIYR
--PWPIQR
PMPWPI--
--PWPIVV
--PWPITY
--PWPISG
--PWPIPW
--PWPIFH
--PWPIAN
PFPWPI--
PYPWPI--
--PWPIYV
--PWPIYK
--PWPISQ
--PWPIRM
--PWPIHV
--PWPIFT
--PWPIFI
PSPWPI--
--PWPIMT
--PWPIHG
--PWPIGS
PAPWPI--
--PWPIVN

--PWPISR
--PWPIRQ
--PWPIQN
--PWPIMY
--PWPIMK
--PWPIKL
--PWPIFS
--PWPIAG
--PWPIST
--PWPIRY
--PWPIRR
--PWPIPV
--PWPIKW
--PWPIFN
PVPWPI--
--PWPIYF
--PWPITK
--PWPITI
--PWPIRI
--PWPIQL
--PWPIPF
--PWPINT
--PWPILQ
--PWPILK
--PWPIKA
--PWPIHY
--PWPIHN
--PWPIFE
--PWPIYG
--PWPIWV
--PWPIWD
--PWPIVM
--PWPITN
--PWPITH
--PWPISK
--PWPILW
--PWPIIT
--PWPIIN
--PWPIIA
--PWPIES
--PWPIAV
--PWPIAA
PTPWPI--
--PWPIWQ
--PWPIWE
--PWPIVI
--PWPISN
--PWPISD
--PWPIRF
--PWPIRE

--PWPIRD
--PWPIQY
--PWPIQK
--PWPIPH
--PWPINR
--PWPINF
--PWPIMQ
--PWPILI
--PWPIKQ
--PWPIKM
--PWPIKK
--PWPIKI
--PWPIIY
--PWPIGW
--PWPIGT
--PWPIEL
--PWPIEG
--PWPIAH
PQPWPI--
PGPWPI--
--PWPIYS
--PWPIYQ
--PWPIYN
--PWPIYH
--PWPIWT
--PWPIWK
--PWPIVY
--PWPIVW
--PWPIVF
--PWPISW
--PWPIRA
--PWPIQE
--PWPIQD
--PWPIMV
--PWPIMI
--PWPILE
--PWPIKY
--PWPIIV
--PWPIHI
--PWPIGK
--PWPIGG
--PWPIFK
--PWPIEY
--PWPIEW
--PWPIEV
--PWPIAR
--PWPIAK
PKPWPI--
--PWPIYY
--PWPIYA

--PWPIWN
--PWPIWL
--PWPIWG
--PWPIVE
--PWPIVD
--PWPITW
--PWPITE
--PWPISH
--PWPISA
--PWPIRW
--PWPIQV
--PWPIQT
--PWPINW
--PWPINV
--PWPIMG
--PWPIME
--PWPILN
--PWPILH
--PWPILC
--PWPIKV
--PWPIKT
--PWPIIS
--PWPIGF
--PWPIFG
--PWPIEK
--PWPIEI
--PWPIEA
--PWPIDS
--PWPIDN
-PPWPIF-
QIPWPI--
QVPWPI--
QPPWPI--
QYPWPI--
-QPWPIR-
QHPWPI--
-QPWPIL-
QMPWPI--
QLPWPI--
-QPWPIY-
QRPWPI--
-QPWPIN-
QTPWPI--
QQPWPI--
-QPWPIS-
QKPWPI--
-QPWPIV-
QDPWPI--
QSPWPI--
-QPWPIQ-

QAPWPI--
QWPWPI--
-QPWPIT-
QGPWPI--
-RPWPIL-
RSPWPI--
RGPWPI--
RMPWPI--
RFPWPI--
RAPWPI--
-RPWPIR-
RLPWPI--
RTPWPI--
RIPWPI--
-RPWPIH-
RVPWPI--
RYPWPI--
-RPWPIA-
-RPWPIY-
-RPWPIT-
RKPWPI--
RHPWPI--
-RPWPIK-
RQPWPI--
-RPWPIV-
-RPWPIS-
RWPWPI--
-RPWPIW-
-RPWPIE-
RRPWPI--
-RPWPIQ-
-RPWPIG-
REPWPI--
RCPWPI--
SRPWPI--
-SPWPIY-
SMPWPI--
SQPWPI--
-SPWPIW-
SYPWPI--
SVPWPI--
SAPWPI--
STPWPI--
SNPWPI--
-SPWPIT-
-SPWPIQ-
-SPWPIF-
SFPWPI--
SHPWPI--
-SPWPIR-

-SPWPIK-
-SPWPIA-
SLPWPI--
SSPWPI--
-SPWPIS-
SPPWPI--
-SPWPIV-
-SPWPIM-
-SPWPII-
SKPWPI--
SEPWPI--
SGPWPI--
-SPWPIN-
SDPWPI--
-SPWPIL-
-SPWPIH-
TRPWPI--
TIPWPI--
TAPWPI--
TLPWPI--
TVPWPI--
-TPWPIT-
TKPWPI--
-TPWPIY-
-TPWPIW-
TGPWPI--
TSPWPI--
-TPWPIQ-
TQPWPI--
-TPWPIR-
-TPWPIF-
TPPWPI--
TWPWPI--
-TPWPIV-
-TPWPIH-
TEPWPI--
-TPWPII-
THPWPI--
TCPWPI--
TYPWPI--
-TPWPIS-
-TPWPIL-
VAPWPI--
VRPWPI--
VSPWPI--
VWPWPI--
-VPWPIS-
VLPWPI--
VTPWPI--
-VPWPIT-

-VPWPIL-
VPPWPI--
VYPWPI--
VVPWPI--
-VPWPIV-
-VPWPIM-
-VPWPIK-
-VPWPIE-
VHPWPI--
VQPWPI--
-VPWPII-
VMPWPI--
VGPWPI--
-VPWPIR-
-VPWPIG-
VNPWPI--
VEPWPI--
WSPWPI--
WVPWPI--
-WPWPIF-
WMPWPI--
WTPWPI--
-WPWPIT-
WIPWPI--
WEPWPI--
WAPWPI--
WRPWPI--
WQPWPI--
-WPWPIR-
-WPWPIQ-
-WPWPIK-
WGPWPI--
YAPWPI--
YSPWPI--
-YPWPIL-
-YPWPIK-
YEPWPI--
YVPWPI--
-YPWPIR-
YKPWPI--
YTPWPI--
-YPWPIT-
-YPWPIH-
-YPWPIQ-
YMPWPI--
YRPWPI--
YLPWPI--
YIPWPI--
YQPWPI--
-YPWPIF-

YDPWPI--
-YPWPII-
YCPWPI--

PVNL	124	793	46.06

-EPVNLG-
EDPVNL--
-GPVNLG-
-NPVNLG-
-NPVNLN-
--PVNLGL
--PVNLGR
--PVNLVP
--PVNLPP
--PVNLHL
--PVNLGI
--PVNLDV
--PVNLDN
-VPVNLP-
-APVNLL-
ASPVNL--
-APVNLS-
AVPVNL--
-APVNLI-
ALPVNL--
-APVNLH-
-APVNLR-
AIPVNL--
AKPVNL--
AHPVNL--
-APVNLQ-
-APVNLM-
AYPVNL--
ANPVNL--
AMPVNL--
AFPVNL--
APPVNL--
AEPVNL--
-APVNLT-
-APVNLN-
-APVNLA-
AQPVNL--
AWPVNL--
-APVNLG-
-APVNLK-
AGPVNL--
-APVNLV-
ACPVNL--
-APVNLE-
-APVNLD-
-APVNLC-

ADPVNL--
CSPVNL--
CGPVNL--
CRPVNL--
CLPVNL--
CVPVNL--
CQPVNL--
CEPVNL--
-CPVNLT-
CMPVNL--
CCPVNL--
CHPVNL--
-CPVNLS-
-CPVNLR-
-CPVNLL-
-CPVNLK-
-CPVNLI-
CNPVNL--
CIPVNL--
CDPVNL--
CYPVNL--
CWPVNL--
CTPVNL--
-CPVNLY-
-CPVNLW-
-CPVNLF-
CFPVNL--
CAPVNL--
DKPVNL--
DGPVNL--
DRPVNL--
-DPVNLL-
DLPVNL--
DHPVNL--
DTPVNL--
-DPVNLR-
DAPVNL--
DVPVNL--
-DPVNLY-
DYPVNL--
DWPVNL--
DSPVNL--
DMPVNL--
-DPVNLT-
-DPVNLF-
DIPVNL--
-DPVNLV-
-DPVNLS-
-DPVNLE-
DFPVNL--

−DPVNLI−
DNPVNL−−
−DPVNLH−
DCPVNL−−
EVPVNL−−
−EPVNLR−
ERPVNL−−
−EPVNLL−
−EPVNLT−
ESPVNL−−
−EPVNLW−
EKPVNL−−
ELPVNL−−
−EPVNLY−
−EPVNLK−
−EPVNLS−
EQPVNL−−
EEPVNL−−
EYPVNL−−
EMPVNL−−
−EPVNLQ−
−EPVNLF−
−EPVNLA−
EAPVNL−−
−EPVNLH−
EGPVNL−−
EFPVNL−−
ETPVNL−−
−EPVNLV−
−EPVNLN−
EWPVNL−−
−EPVNLM−
−EPVNLI−
ENPVNL−−
EIPVNL−−
−EPVNLE−
EPPVNL−−
EHPVNL−−
FSPVNL−−
FLPVNL−−
−FPVNLA−
FRPVNL−−
−FPVNLR−
−FPVNLS−
FAPVNL−−
FHPVNL−−
FMPVNL−−
FGPVNL−−
FNPVNL−−
FQPVNL−−

-FPVNLE-
-FPVNLH-
-FPVNLV-
-FPVNLK-
FYPVNL--
-FPVNLQ-
-FPVNLL-
FKPVNL--
-FPVNLT-
FEPVNL--
FDPVNL--
-FPVNLN-
FPPVNL--
-FPVNLM-
FCPVNL--
GRPVNL--
GSPVNL--
-GPVNLR-
-GPVNLL-
GLPVNL--
GTPVNL--
-GPVNLV-
GMPVNL--
-GPVNLW-
GKPVNL--
-GPVNLT-
-GPVNLS-
GHPVNL--
GWPVNL--
GYPVNL--
GPPVNL--
GGPVNL--
GVPVNL--
-GPVNLI-
-GPVNLM-
-GPVNLA-
GNPVNL--
-GPVNLK-
GEPVNL--
-GPVNLQ-
-GPVNLH-
-GPVNLE-
GQPVNL--
GDPVNL--
-GPVNLN-
GAPVNL--
GCPVNL--
HLPVNL--
HSPVNL--
-HPVNLR-

HMPVNL--
-HPVNLS-
HGPVNL--
HNPVNL--
HVPVNL--
HAPVNL--
-HPVNLL-
HKPVNL--
-HPVNLI-
-HPVNLY-
-HPVNLT-
-HPVNLM-
HYPVNL--
-HPVNLF-
HIPVNL--
HHPVNL--
-HPVNLW-
HDPVNL--
-HPVNLV-
HTPVNL--
HFPVNL--
-HPVNLH-
-HPVNLE-
-HPVNLN-
-HPVNLK-
HCPVNL--
-HPVNLQ-
HWPVNL--
-HPVNLA-
HPPVNL--
ITPVNL--
IMPVNL--
IVPVNL--
-IPVNLT-
-IPVNLM-
-IPVNLL-
INPVNL--
-IPVNLK-
IHPVNL--
IAPVNL--
-IPVNLV-
-IPVNLQ-
ISPVNL--
IQPVNL--
-IPVNLG-
IGPVNL--
-IPVNLA-
-IPVNLN-
IKPVNL--
IEPVNL--

```
-IPVNLE-
IFPVNL--
IDPVNL--
-IPVNLH-
IYPVNL--
-IPVNLI-
-IPVNLD-
KSPVNL--
-KPVNLR-
KAPVNL--
KPPVNL--
KVPVNL--
KGPVNL--
KLPVNL--
-KPVNLV-
-KPVNLL-
-KPVNLM-
-KPVNLA-
KMPVNL--
-KPVNLW-
-KPVNLI-
KIPVNL--
KFPVNL--
KEPVNL--
KTPVNL--
KHPVNL--
-KPVNLH-
-KPVNLF-
KYPVNL--
-KPVNLN-
KQPVNL--
KNPVNL--
-KPVNLQ-
KDPVNL--
-KPVNLG-
KCPVNL--
-KPVNLC-
LLPVNL--
LGPVNL--
LAPVNL--
LMPVNL--
LSPVNL--
-LPVNLL-
LFPVNL--
-LPVNLV-
-LPVNLM-
LQPVNL--
LNPVNL--
LDPVNL--
-LPVNLQ-
```

LPPVNL--
LWPVNL--
-LPVNLI-
LKPVNL--
-LPVNLK-
LEPVNL--
-LPVNLS-
-LPVNLH-
-LPVNLA-
-LPVNLE-
-LPVNLN-
LCPVNL--
MRPVNL--
-MPVNLK-
-MPVNLL-
MSPVNL--
MAPVNL--
MFPVNL--
MTPVNL--
MDPVNL--
-MPVNLT-
MPPVNL--
MNPVNL--
-MPVNLS-
-MPVNLQ-
MIPVNL--
-MPVNLE-
MEPVNL--
-MPVNLV-
MGPVNL--
MCPVNL--
MYPVNL--
-MPVNLI-
-MPVNLM-
MQPVNL--
-MPVNLN-
-MPVNLA-
MWPVNL--
-MPVNLG-
-MPVNLF-
MHPVNL--
NSPVNL--
NVPVNL--
NLPVNL--
-NPVNLR-
NWPVNL--
NTPVNL--
-NPVNLL-
NAPVNL--
NHPVNL--

-NPVNLK-
NQPVNL--
-NPVNLM-
-NPVNLI-
NGPVNL--
-NPVNLQ-
-NPVNLV-
NNPVNL--
-NPVNLS-
-NPVNLW-
NKPVNL--
NIPVNL--
NMPVNL--
NDPVNL--
NYPVNL--
-NPVNLT-
-NPVNLA-
-NPVNLY-
NEPVNL--
-NPVNLH-
NPPVNL--
-NPVNLE-
-NPVNLF-
NCPVNL--
--PVNLLG
--PVNLSG
--PVNLRS
--PVNLRG
--PVNLVR
--PVNLRT
--PVNLVG
--PVNLRH
--PVNLRV
--PVNLRR
--PVNLST
--PVNLSS
PRPVNL--
--PVNLLN
--PVNLFH
--PVNLVS
--PVNLTS
--PVNLRA
--PVNLTT
--PVNLMR
--PVNLRM
--PVNLLR
--PVNLVK
--PVNLSV
--PVNLMN
--PVNLKN

--PVNLKT
--PVNLGS
--PVNLTR
--PVNLRN
--PVNLRY
--PVNLTA
--PVNLRK
--PVNLTH
--PVNLSN
--PVNLIG
PNPVNL--
--PVNLFN
PSPVNL--
--PVNLTG
--PVNLLK
--PVNLSM
--PVNLSF
--PVNLLH
--PVNLVA
--PVNLRW
--PVNLQW
--PVNLHA
--PVNLSI
--PVNLTQ
--PVNLTN
--PVNLQG
--PVNLKG
--PVNLEG
--PVNLVN
--PVNLRF
--PVNLNA
--PVNLRD
--PVNLKR
--PVNLKQ
--PVNLYG
--PVNLSY
--PVNLMG
--PVNLYN
--PVNLTV
--PVNLSR
--PVNLAS
--PVNLMY
--PVNLEV
--PVNLMA
--PVNLKL
--PVNLKI
--PVNLWG
--PVNLKM
--PVNLAG
--PVNLYQ

--PVNLWH
--PVNLHG
--PVNLFG
--PVNLES
PVPVNL--
--PVNLYK
--PVNLTI
--PVNLHM
--PVNLVQ
--PVNLSK
--PVNLQS
--PVNLNG
--PVNLSA
--PVNLMK
--PVNLVT
--PVNLNS
--PVNLMH
--PVNLAH
PTPVNL--
--PVNLQT
--PVNLHT
PKPVNL--
--PVNLTW
--PVNLTM
--PVNLIN
--PVNLAT
PAPVNL--
--PVNLVH
--PVNLVD
--PVNLKY
--PVNLGA
PQPVNL--
PLPVNL--
--PVNLYH
--PVNLHQ
--PVNLAA
--PVNLSQ
--PVNLMI
--PVNLFQ
--PVNLAI
--PVNLTK
--PVNLQQ
--PVNLQK
--PVNLMD
--PVNLLD
--PVNLHF
--PVNLEM
PHPVNL--
PGPVNL--
--PVNLVE

--PVNLTL
--PVNLNM
--PVNLLM
--PVNLET
--PVNLEI
--PVNLEF
PEPVNL--
--PVNLWQ
--PVNLWF
--PVNLSW
--PVNLNT
--PVNLMW
--PVNLME
--PVNLIK
--PVNLAR
--PVNLQY
--PVNLKW
--PVNLHY
--PVNLQR
--PVNLNW
--PVNLKF
--PVNLID
--PVNLGG
--PVNLER
--PVNLEL
--PVNLSH
--PVNLQL
--PVNLKH
--PVNLHR
--PVNLHH
--PVNLGV
PCPVNL--
PYPVNL--
--PVNLWD
--PVNLKD
--PVNLEH
--PVNLAM
PDPVNL--
--PVNLTD
--PVNLMQ
--PVNLKV
--PVNLGM
--PVNLAQ
PIPVNL--
--PVNLYD
--PVNLTY
--PVNLSD
--PVNLQH
--PVNLNH
--PVNLHN

--PVNLEQ
--PVNLTE
--PVNLQV
--PVNLNN
--PVNLNI
--PVNLHK
--PVNLQM
--PVNLQE
--PVNLNV
--PVNLNR
--PVNLIM
--PVNLIH
--PVNLHE
--PVNLGW
--PVNLEN
--PVNLSE
--PVNLQI
--PVNLQF
--PVNLNF
--PVNLMF
--PVNLEW
--PVNLEK
--PVNLEA
--PVNLDS
PWPVNL--
--PVNLYE
--PVNLWK
--PVNLWE
--PVNLKE
--PVNLHD
--PVNLGN
--PVNLAN
--PVNLAE
--PVNLAD
--PVNLVC
--PVNLTC
--PVNLRC
--PVNLQD
--PVNLNQ
--PVNLNK
--PVNLKC
--PVNLGE
--PVNLEY
--PVNLCT
--PVNLCN
--PVNLCI
--PVNLCD
-PPVNLW-
-PPVNLL-
PPPVNL--

−QPVNLR−
QVPVNL−−
QRPVNL−−
QLPVNL−−
−QPVNLL−
−QPVNLA−
−QPVNLV−
QSPVNL−−
QWPVNL−−
−QPVNLS−
−QPVNLT−
QGPVNL−−
−QPVNLH−
QPPVNL−−
QMPVNL−−
QFPVNL−−
−QPVNLM−
QQPVNL−−
QTPVNL−−
QDPVNL−−
−QPVNLY−
−QPVNLK−
QIPVNL−−
QEPVNL−−
QNPVNL−−
−QPVNLQ−
−QPVNLE−
QHPVNL−−
−QPVNLN−
QKPVNL−−
−QPVNLG−
−QPVNLF−
QYPVNL−−
RVPVNL−−
−RPVNLL−
−RPVNLV−
RPPVNL−−
RMPVNL−−
−RPVNLK−
RYPVNL−−
RGPVNL−−
−RPVNLI−
RIPVNL−−
RQPVNL−−
−RPVNLG−
RDPVNL−−
RTPVNL−−
−RPVNLF−
−RPVNLH−
−RPVNLW−

−RPVNLM−
REPVNL−−
−RPVNLN−
RNPVNL−−
−RPVNLE−
RHPVNL−−
RCPVNL−−
−RPVNLC−
−SPVNLR−
SLPVNL−−
SFPVNL−−
−SPVNLY−
SGPVNL−−
SSPVNL−−
STPVNL−−
SHPVNL−−
−SPVNLL−
−SPVNLS−
SWPVNL−−
SAPVNL−−
−SPVNLK−
−SPVNLI−
SMPVNL−−
SVPVNL−−
SEPVNL−−
SNPVNL−−
−SPVNLF−
−SPVNLE−
−SPVNLW−
SPPVNL−−
SDPVNL−−
SQPVNL−−
−SPVNLH−
SCPVNL−−
−SPVNLN−
−SPVNLA−
−SPVNLC−
−TPVNLR−
TLPVNL−−
TGPVNL−−
TSPVNL−−
−TPVNLL−
TPPVNL−−
TEPVNL−−
−TPVNLY−
−TPVNLK−
TVPVNL−−
TAPVNL−−
−TPVNLV−
TQPVNL−−

-TPVNLS-
TIPVNL--
-TPVNLF-
-TPVNLT-
THPVNL--
TMPVNL--
-TPVNLH-
TNPVNL--
TFPVNL--
-TPVNLM-
TDPVNL--
-TPVNLW-
-TPVNLG-
-TPVNLQ-
-TPVNLE-
TCPVNL--
VSPVNL--
-VPVNLR-
VRPVNL--
VNPVNL--
VVPVNL--
-VPVNLH-
VAPVNL--
VMPVNL--
VYPVNL--
-VPVNLK-
VLPVNL--
VPPVNL--
VGPVNL--
VFPVNL--
VWPVNL--
VHPVNL--
VEPVNL--
VTPVNL--
-VPVNLA-
-VPVNLT-
-VPVNLQ-
-VPVNLN-
-VPVNLG-
VIPVNL--
-VPVNLE-
VDPVNL--
VKPVNL--
-VPVNLM-
WEPVNL--
WSPVNL--
WMPVNL--
WYPVNL--
WTPVNL--
WNPVNL--

```
          WGPVNL--
          WVPVNL--
          -WPVNLR-
          -WPVNLS-
          -WPVNLM-
          -WPVNLT-
          -WPVNLL-
          WQPVNL--
          -WPVNLA-
          WAPVNL--
          -WPVNLK-
          WPPVNL--
          WHPVNL--
          -WPVNLN-
          -WPVNLI-
          -WPVNLH-
          -WPVNLG-
          WCPVNL--
          -WPVNLY-
          -WPVNLQ-
          YKPVNL--
          YSPVNL--
          -YPVNLR-
          -YPVNLS-
          YEPVNL--
          YRPVNL--
          -YPVNLT-
          -YPVNLM-
          -YPVNLL-
          YFPVNL--
          -YPVNLY-
          YPPVNL--
          YYPVNL--
          -YPVNLK-
          YGPVNL--
          YAPVNL--
          -YPVNLI-
          YMPVNL--
          -YPVNLA-
          YNPVNL--
          -YPVNLQ-
          -YPVNLN-
          -YPVNLE-
          YHPVNL--
          YDPVNL--
          YCPVNL--
          -YPVNLG-
PIGI       138       786     46.06
          --PIGIGR
          --PIGIGL
```

--PIGIER
--PIGIAL
ARPIGI--
ALPIGI--
AAPIGI--
-APIGIS-
ASPIGI--
AHPIGI--
-APIGIT-
-APIGIA-
AKPIGI--
AYPIGI--
AWPIGI--
-APIGIL-
AFPIGI--
-APIGIN-
ANPIGI--
AEPIGI--
APPIGI--
ATPIGI--
-APIGIV-
AMPIGI--
AIPIGI--
-APIGIW-
-APIGIR-
-APIGIK-
AGPIGI--
AVPIGI--
ADPIGI--
-APIGIM-
-APIGIY-
-APIGII-
-APIGIH-
-APIGIF-
-APIGIG-
ACPIGI--
AQPIGI--
-APIGIC-
CVPIGI--
CSPIGI--
CIPIGI--
CCPIGI--
CQPIGI--
CPPIGI--
CKPIGI--
CTPIGI--
-CPIGIT-
CAPIGI--
CMPIGI--
-CPIGIR-

-CPIGIF-
-DPIGII-
DLPIGI--
DFPIGI--
-DPIGIK-
DNPIGI--
DVPIGI--
DRPIGI--
DQPIGI--
-DPIGIF-
DYPIGI--
-DPIGIV-
DEPIGI--
DSPIGI--
-DPIGIL-
DAPIGI--
DWPIGI--
DTPIGI--
-DPIGIT-
-DPIGIS-
-DPIGIR-
-DPIGIH-
DKPIGI--
DIPIGI--
DHPIGI--
-EPIGIL-
-EPIGIR-
ERPIGI--
EGPIGI--
-EPIGIK-
ESPIGI--
-EPIGIS-
-EPIGIQ-
ENPIGI--
-EPIGIM-
ELPIGI--
EIPIGI--
EAPIGI--
-EPIGIV-
EYPIGI--
-EPIGII-
EHPIGI--
-EPIGIW-
EEPIGI--
EPPIGI--
-EPIGIT-
FLPIGI--
FVPIGI--
FTPIGI--
FYPIGI--

FHPIGI--
FIPIGI--
FKPIGI--
FRPIGI--
FPPIGI--
-FPIGIV-
FWPIGI--
-FPIGIN-
FGPIGI--
-FPIGIH-
FSPIGI--
-FPIGIT-
FMPIGI--
-FPIGIM-
FQPIGI--
FNPIGI--
FAPIGI--
-FPIGIA-
FEPIGI--
FDPIGI--
-FPIGII-
-FPIGIG-
GSPIGI--
-GPIGIR-
-GPIGIY-
-GPIGIL-
GYPIGI--
GHPIGI--
GIPIGI--
-GPIGIF-
-GPIGIS-
GDPIGI--
GTPIGI--
-GPIGIW-
GLPIGI--
GKPIGI--
GMPIGI--
GGPIGI--
GWPIGI--
GPPIGI--
GAPIGI--
GQPIGI--
-GPIGIQ-
-GPIGIN-
GNPIGI--
GRPIGI--
-GPIGIM-
-GPIGIE-
GVPIGI--
-GPIGIV-

-GPIGIA-
GFPIGI--
-GPIGII-
GEPIGI--
-GPIGIT-
-GPIGIH-
GCPIGI--
HRPIGI--
-HPIGIL-
-HPIGIF-
HYPIGI--
HGPIGI--
HNPIGI--
HKPIGI--
HVPIGI--
HEPIGI--
-HPIGIM-
HDPIGI--
HWPIGI--
HLPIGI--
HAPIGI--
HQPIGI--
-HPIGIW-
-HPIGIK-
-HPIGII-
HCPIGI--
-HPIGIT-
-HPIGIV-
-HPIGIH-
HPPIGI--
-HPIGIE-
-HPIGIA-
HFPIGI--
HSPIGI--
-HPIGIS-
-HPIGIG-
-HPIGID-
HMPIGI--
-IPIGIL-
IPPIGI--
IVPIGI--
IRPIGI--
ILPIGI--
-IPIGIR-
IAPIGI--
IWPIGI--
-IPIGIM-
-IPIGIK-
-IPIGIS-
-IPIGIH-

-IPIGIF-
IMPIGI--
IKPIGI--
IIPIGI--
IEPIGI--
INPIGI--
IYPIGI--
-IPIGIY-
-IPIGIQ-
IGPIGI--
IDPIGI--
IQPIGI--
-IPIGIE-
-IPIGIA-
ISPIGI--
-IPIGIV-
-IPIGIP-
-IPIGID-
IHPIGI--
ICPIGI--
KSPIGI--
KGPIGI--
-KPIGIT-
-KPIGII-
KRPIGI--
-KPIGIS-
-KPIGIF-
-KPIGIM-
-KPIGIQ-
-KPIGIY-
KFPIGI--
-KPIGIW-
KDPIGI--
KPPIGI--
-KPIGIH-
KVPIGI--
KMPIGI--
-KPIGIN-
KIPIGI--
KHPIGI--
KWPIGI--
KQPIGI--
-KPIGIV-
-KPIGIG-
-KPIGIA-
KAPIGI--
KEPIGI--
KCPIGI--
-LPIGIR-
LPPIGI--

LDPIGI--
-LPIGIY-
-LPIGIV-
LIPIGI--
LRPIGI--
LGPIGI--
LMPIGI--
LHPIGI--
LFPIGI--
LYPIGI--
LAPIGI--
LTPIGI--
LSPIGI--
LKPIGI--
-LPIGIF-
LVPIGI--
LNPIGI--
LQPIGI--
-LPIGIS-
-LPIGIM-
-LPIGIN-
-LPIGIK-
-LPIGIA-
LWPIGI--
-LPIGIT-
-LPIGIH-
LCPIGI--
-LPIGIQ-
LEPIGI--
MSPIGI--
MRPIGI--
-MPIGIL-
MGPIGI--
-MPIGIQ-
MFPIGI--
MKPIGI--
MLPIGI--
-MPIGIT-
-MPIGII-
MTPIGI--
-MPIGIR-
MDPIGI--
MAPIGI--
MYPIGI--
-MPIGIN-
MNPIGI--
MVPIGI--
-MPIGIM-
-MPIGIH-
-MPIGIS-

-MPIGIE-
-MPIGIA-
MCPIGI--
-MPIGIG-
-MPIGIF-
NTPIGI--
NRPIGI--
NGPIGI--
-NPIGIR-
NKPIGI--
NYPIGI--
-NPIGIK-
NSPIGI--
-NPIGIS-
NVPIGI--
-NPIGIM-
NPPIGI--
-NPIGIV-
-NPIGIH-
NQPIGI--
-NPIGIY-
-NPIGIW-
NMPIGI--
NNPIGI--
-NPIGIL-
NFPIGI--
-NPIGII-
NLPIGI--
NIPIGI--
-NPIGIQ-
-NPIGIN-
-NPIGIC-
NHPIGI--
NEPIGI--
--PIGISS
--PIGIRL
--PIGILS
--PIGITT
--PIGISR
PVPIGI--
--PIGITG
--PIGITM
--PIGIMG
--PIGILV
--PIGIRG
--PIGIKS
--PIGIFS
--PIGIRS
--PIGILG
PWPIGI--

--PIGILF
--PIGIIG
--PIGIST
--PIGIRY
--PIGIRM
--PIGIHG
--PIGILA
--PIGIHV
--PIGIHL
--PIGITA
--PIGIQA
PRPIGI--
--PIGIYT
--PIGIRK
--PIGILT
--PIGITL
--PIGISM
--PIGIFM
--PIGIVR
--PIGING
PYPIGI--
--PIGIVK
--PIGISG
--PIGIIA
--PIGIHN
--PIGIYE
--PIGIRQ
--PIGIYR
--PIGIVS
--PIGISY
--PIGISV
--PIGISL
--PIGILY
--PIGIKY
--PIGIIT
PGPIGI--
--PIGIYQ
--PIGIMT
--PIGIMQ
--PIGIII
PAPIGI--
PTPIGI--
PNPIGI--
--PIGIYS
--PIGITS
--PIGILR
--PIGILH
--PIGIIW
--PIGISI
--PIGIKK

--PIGIHW
--PIGIWW
--PIGITR
--PIGIRT
--PIGILQ
--PIGIHR
--PIGIML
--PIGIMF
--PIGIIS
--PIGIGV
PHPIGI--
--PIGIYG
--PIGIVM
--PIGIVG
--PIGIFT
--PIGIFG
--PIGIAS
--PIGIYH
--PIGIVV
--PIGITK
--PIGIRR
--PIGIRN
--PIGIRF
--PIGIQH
--PIGIMY
--PIGIMI
--PIGIKV
--PIGIHA
--PIGIFI
--PIGIFH
--PIGIEA
--PIGIAK
PFPIGI--
--PIGINF
--PIGIHK
--PIGIFW
--PIGIFR
--PIGIWG
--PIGIWD
--PIGIRW
--PIGIRV
--PIGIMR
--PIGIMD
--PIGIIM
--PIGIWR
--PIGIVN
--PIGITY
--PIGITV
--PIGINN
--PIGILK

--PIGILE
--PIGIKR
--PIGIIK
--PIGIIH
--PIGIYK
--PIGIVF
--PIGIRE
--PIGIRA
--PIGIKA
--PIGIFQ
PCPIGI--
PQPIGI--
--PIGIVT
--PIGIVQ
--PIGIVH
--PIGISQ
--PIGIQQ
--PIGINV
--PIGINK
--PIGILD
--PIGIKI
--PIGIIR
--PIGIHY
--PIGIFK
--PIGIES
--PIGIWT
--PIGIVE
--PIGITF
--PIGISN
--PIGINW
--PIGILN
--PIGIKT
--PIGIKN
--PIGIKG
--PIGIGT
--PIGIEN
--PIGIAR
--PIGIYY
--PIGITQ
--PIGIQW
--PIGINS
--PIGIMW
--PIGIMN
--PIGIMA
--PIGIKW
--PIGIHE
--PIGIET
--PIGIEG
--PIGIAW
--PIGIAV

PMPIGI--
PKPIGI--
--PIGITD
--PIGISF
--PIGISD
--PIGIQT
--PIGIID
--PIGIIC
--PIGIHM
--PIGIHH
--PIGIHF
--PIGIGG
--PIGIFD
--PIGIAG
--PIGIAA
PEPIGI--
--PIGIWH
--PIGIVW
--PIGIVI
--PIGIVA
--PIGIQR
--PIGINH
--PIGILW
--PIGILM
--PIGIIQ
--PIGIIN
--PIGIHS
--PIGICF
--PIGIAT
--PIGIAM
PIPIGI--
--PIGIWN
--PIGIWK
--PIGITC
--PIGISE
--PIGINT
--PIGINI
--PIGIMV
--PIGIKC
--PIGIIV
--PIGIIF
--PIGIHD
--PIGIGY
--PIGIGW
--PIGIDG
--PIGIAI
-PPIGIN-
--PIGIYN
--PIGIWQ
--PIGITW

--PIGISH
--PIGISA
--PIGIRH
--PIGIQS
--PIGIQN
--PIGIQL
--PIGIQK
--PIGIQG
--PIGIPT
--PIGIPK
--PIGIPH
--PIGINR
--PIGINE
--PIGINC
--PIGINA
--PIGIMM
--PIGIMK
--PIGIKL
--PIGIHC
--PIGIGM
--PIGIEW
--PIGIEV
--PIGIEM
--PIGIEI
--PIGIEF
--PIGIDN
--PIGICQ
-QPIGIW-
QVPIGI--
-QPIGIN-
-QPIGIL-
QIPIGI--
QYPIGI--
QSPIGI--
-QPIGIR-
-QPIGIF-
QLPIGI--
QHPIGI--
QTPIGI--
QNPIGI--
QGPIGI--
QPPIGI--
QKPIGI--
-QPIGIS-
QDPIGI--
-QPIGIT-
QEPIGI--
-QPIGIV-
-QPIGIA-
QWPIGI--

```
-QPIGIM-
QMPIGI--
-QPIGIK-
QCPIGI--
RGPIGI--
RSPIGI--
RTPIGI--
RVPIGI--
-RPIGIY-
RLPIGI--
-RPIGIT-
-RPIGIS-
RMPIGI--
RRPIGI--
-RPIGIV-
-RPIGIH-
-RPIGIR-
RAPIGI--
REPIGI--
RNPIGI--
RPPIGI--
-RPIGIQ-
-RPIGIK-
RHPIGI--
RFPIGI--
RIPIGI--
RYPIGI--
-RPIGIM-
RDPIGI--
-RPIGIN-
-RPIGIW-
-RPIGIG-
RWPIGI--
-RPIGIC-
-RPIGIA-
SSPIGI--
-SPIGIR-
SYPIGI--
SGPIGI--
-SPIGIW-
SFPIGI--
-SPIGIM-
SIPIGI--
STPIGI--
-SPIGIS-
SAPIGI--
-SPIGIK-
SKPIGI--
-SPIGIH-
SLPIGI--
```

-SPIGIL-
SEPIGI--
-SPIGIN-
SNPIGI--
-SPIGIQ-
SVPIGI--
-SPIGIA-
SMPIGI--
SCPIGI--
-SPIGIT-
-SPIGIV-
SDPIGI--
-SPIGIE-
SPPIGI--
-SPIGII-
TGPIGI--
TLPIGI--
-TPIGIR-
TRPIGI--
-TPIGIL-
TAPIGI--
-TPIGIS-
TTPIGI--
TMPIGI--
TKPIGI--
TWPIGI--
TFPIGI--
TPPIGI--
TIPIGI--
TYPIGI--
-TPIGIH-
THPIGI--
TQPIGI--
-TPIGIW-
-TPIGIV-
-TPIGIT-
-TPIGII-
-TPIGIY-
-TPIGIF-
TNPIGI--
-TPIGIM-
-TPIGIK-
-TPIGIE-
VRPIGI--
VSPIGI--
-VPIGIR-
VGPIGI--
-VPIGIL-
VKPIGI--
VNPIGI--

-VPIGIM-
-VPIGIV-
VLPIGI--
VHPIGI--
VVPIGI--
VPPIGI--
-VPIGIT-
-VPIGIS-
-VPIGIN-
VIPIGI--
VQPIGI--
-VPIGIH-
VFPIGI--
VYPIGI--
VDPIGI--
VAPIGI--
VTPIGI--
-VPIGIY-
-VPIGIW-
-VPIGIK-
VMPIGI--
VEPIGI--
-VPIGIE-
-VPIGIA-
VCPIGI--
-VPIGIG-
-VPIGIF-
WPPIGI--
WFPIGI--
WRPIGI--
WEPIGI--
WAPIGI--
WVPIGI--
-WPIGIV-
WLPIGI--
WSPIGI--
WKPIGI--
-WPIGIL-
WMPIGI--
WDPIGI--
-WPIGIY-
WYPIGI--
-WPIGIT-
-WPIGIQ-
-WPIGIM-
WIPIGI--
WGPIGI--
WTPIGI--
-WPIGIN-
-WPIGIK-

	-WPIGIH-		
	-WPIGIA-		
	WQPIGI--		
	-WPIGII-		
	-YPIGIR-		
	YRPIGI--		
	YSPIGI--		
	YLPIGI--		
	YGPIGI--		
	YFPIGI--		
	-YPIGIS-		
	-YPIGIV-		
	YKPIGI--		
	-YPIGIK-		
	YTPIGI--		
	YHPIGI--		
	YEPIGI--		
	-YPIGIY-		
	-YPIGIT-		
	-YPIGIL-		
	-YPIGII-		
	YIPIGI--		
	-YPIGIA-		
	YWPIGI--		
	YVPIGI--		
	-YPIGIW-		
	YMPIGI--		
	-YPIGIN-		
	-YPIGIF-		
	YDPIGI--		
	YYPIGI--		
	YPPIGI--		
	-YPIGIM-		
	-YPIGIH-		
PVGI	32	866	46.04
	-GPVGIG-		
	--PVGIGY		
	--PVGIGV		
	--PVGIGL		
	--PVGIDS		
	--PVGIAR		
	-TPVGIG-		
	-VPVGIG-		
	-APVGIR-		
	ARPVGI--		
	-APVGIY-		
	AIPVGI--		
	AYPVGI--		
	AWPVGI--		
	ASPVGI--		

AKPVGI--
-APVGII-
ATPVGI--
-APVGIT-
AVPVGI--
-APVGIS-
-APVGIL-
AAPVGI--
-APVGIW-
-APVGIA-
AEPVGI--
-APVGIV-
-APVGIF-
AMPVGI--
-APVGIH-
-APVGIQ-
AQPVGI--
-APVGIK-
AHPVGI--
-APVGIG-
ANPVGI--
AFPVGI--
-APVGIM-
APPVGI--
ACPVGI--
-APVGIE-
-APVGIN-
CRPVGI--
CKPVGI--
CSPVGI--
CQPVGI--
CTPVGI--
CMPVGI--
-CPVGIR-
CLPVGI--
CGPVGI--
CYPVGI--
CVPVGI--
CPPVGI--
CNPVGI--
CAPVGI--
-CPVGII-
CHPVGI--
CDPVGI--
-CPVGIV-
-CPVGIT-
-CPVGIS-
-CPVGIQ-
-CPVGIF-
DQPVGI--

DVPVGI--
DRPVGI--
DGPVGI--
DTPVGI--
DYPVGI--
DSPVGI--
-DPVGIY-
-DPVGIL-
DHPVGI--
DNPVGI--
DKPVGI--
DWPVGI--
-DPVGIT-
-DPVGIH-
-DPVGIS-
DLPVGI--
-DPVGIV-
-DPVGIM-
-DPVGIW-
-DPVGIK-
DIPVGI--
-EPVGIR-
ESPVGI--
-EPVGIV-
-EPVGIL-
EVPVGI--
-EPVGIW-
EFPVGI--
-EPVGIS-
EGPVGI--
-EPVGIT-
EIPVGI--
EWPVGI--
-EPVGIH-
ERPVGI--
-EPVGIM-
-EPVGII-
EMPVGI--
EEPVGI--
-EPVGIY-
-EPVGIQ-
ENPVGI--
ELPVGI--
EAPVGI--
-EPVGIN-
ETPVGI--
EQPVGI--
-EPVGIG-
-EPVGIF-
EPPVGI--

EKPVGI--
ECPVGI--
FLPVGI--
-FPVGIL-
-FPVGIS-
-FPVGIY-
FRPVGI--
-FPVGIT-
FDPVGI--
FGPVGI--
FTPVGI--
-FPVGIV-
FFPVGI--
FSPVGI--
FKPVGI--
FQPVGI--
-FPVGIM-
FHPVGI--
FVPVGI--
-FPVGIN-
-FPVGIQ-
-FPVGIK-
FIPVGI--
-FPVGIR-
FNPVGI--
FEPVGI--
FYPVGI--
-FPVGII-
-FPVGIA-
FMPVGI--
-FPVGIH-
-FPVGIE-
-GPVGIH-
-GPVGIR-
GGPVGI--
GRPVGI--
-GPVGIL-
GVPVGI--
GIPVGI--
-GPVGIS-
GLPVGI--
-GPVGIW-
-GPVGIQ-
GSPVGI--
-GPVGII-
-GPVGIT-
GYPVGI--
GTPVGI--
GQPVGI--
-GPVGIK-

GNPVGI--
GWPVGI--
GHPVGI--
GEPVGI--
-GPVGIM-
GPPVGI--
GAPVGI--
GFPVGI--
-GPVGIN-
-GPVGIV-
GMPVGI--
GDPVGI--
-GPVGIA-
GCPVGI--
-GPVGIE-
HLPVGI--
-HPVGIR-
HGPVGI--
HWPVGI--
HTPVGI--
-HPVGIL-
HVPVGI--
HQPVGI--
HYPVGI--
HIPVGI--
HAPVGI--
-HPVGIV-
-HPVGIK-
-HPVGIH-
HFPVGI--
HHPVGI--
-HPVGIT-
-HPVGIY-
HSPVGI--
-HPVGIF-
HNPVGI--
HMPVGI--
-HPVGIM-
HPPVGI--
-HPVGIW-
-HPVGII-
-HPVGIG-
-HPVGIA-
HEPVGI--
IRPVGI--
IGPVGI--
-IPVGIL-
IPPVGI--
ILPVGI--
IYPVGI--

IVPVGI--
-IPVGIM-
-IPVGIR-
-IPVGII-
-IPVGIK-
-IPVGIY-
-IPVGIF-
IMPVGI--
-IPVGIN-
-IPVGIT-
-IPVGIS-
-IPVGIV-
IWPVGI--
IQPVGI--
-IPVGIW-
-IPVGIE-
IHPVGI--
-IPVGIH-
ICPVGI--
IKPVGI--
ITPVGI--
INPVGI--
IIPVGI--
-IPVGIG-
-IPVGIA-
IFPVGI--
IDPVGI--
IAPVGI--
-IPVGIQ-
KSPVGI--
-KPVGIR-
KLPVGI--
-KPVGIL-
-KPVGIK-
KGPVGI--
KVPVGI--
KRPVGI--
KNPVGI--
KIPVGI--
-KPVGIM-
-KPVGIV-
-KPVGIS-
-KPVGIF-
KYPVGI--
KAPVGI--
-KPVGIY-
KEPVGI--
-KPVGII-
KHPVGI--
-KPVGIQ-

KTPVGI--
KQPVGI--
-KPVGIH-
KDPVGI--
KPPVGI--
-KPVGIT-
-KPVGIN-
-KPVGIG-
-KPVGIA-
KMPVGI--
KCPVGI--
-KPVGIE-
LRPVGI--
LTPVGI--
LSPVGI--
LNPVGI--
-LPVGIR-
LIPVGI--
LLPVGI--
LMPVGI--
LAPVGI--
LGPVGI--
LKPVGI--
LYPVGI--
LWPVGI--
-LPVGIM-
LQPVGI--
LFPVGI--
LHPVGI--
-LPVGIH-
-LPVGII-
LEPVGI--
LPPVGI--
-LPVGIY-
-LPVGIK-
-LPVGIW-
-LPVGIN-
-LPVGIQ-
-LPVGIA-
LDPVGI--
LVPVGI--
-LPVGIV-
MGPVGI--
MQPVGI--
MSPVGI--
-MPVGIR-
-MPVGIF-
MPPVGI--
-MPVGIV-
-MPVGIH-

-MPVGIW-
-MPVGIT-
-MPVGIK-
MLPVGI--
MTPVGI--
MDPVGI--
MWPVGI--
-MPVGIM-
MVPVGI--
-MPVGIS-
-MPVGII-
MYPVGI--
-MPVGIY-
MNPVGI--
-MPVGIL-
MFPVGI--
MIPVGI--
-MPVGIG-
-MPVGID-
MEPVGI--
NRPVGI--
NTPVGI--
NLPVGI--
NKPVGI--
NAPVGI--
NNPVGI--
-NPVGIL-
-NPVGIR-
NGPVGI--
NHPVGI--
-NPVGIK-
NSPVGI--
-NPVGIM-
NWPVGI--
-NPVGIT-
-NPVGIF-
NQPVGI--
-NPVGIH-
-NPVGII-
NYPVGI--
NIPVGI--
-NPVGIS-
-NPVGIN-
NCPVGI--
-NPVGIW-
NFPVGI--
-NPVGIV-
NEPVGI--
-NPVGIE-
NPPVGI--

NMPVGI--
NDPVGI--
--PVGIRM
--PVGIRS
--PVGIRN
--PVGIRV
--PVGIRK
--PVGIRL
--PVGILS
--PVGISG
--PVGIFN
--PVGIYK
--PVGIRT
--PVGIVS
--PVGISR
--PVGIWG
--PVGIRG
--PVGIVA
--PVGIHS
--PVGIYT
PAPVGI--
--PVGIYR
PLPVGI--
--PVGISA
--PVGITT
--PVGIRF
--PVGILR
PMPVGI--
--PVGITK
--PVGISW
--PVGISV
--PVGILW
--PVGIAG
--PVGITR
--PVGIIV
--PVGIRH
--PVGITV
--PVGILG
--PVGIHY
--PVGIFQ
PRPVGI--
--PVGITS
--PVGIRA
--PVGIQS
--PVGIHL
PYPVGI--
--PVGIWS
--PVGIVK
--PVGITG
--PVGIKK

--PVGIGS
--PVGIVR
--PVGITY
--PVGILH
--PVGIKI
--PVGIKG
--PVGIFS
--PVGIFG
--PVGIWK
--PVGIVV
--PVGIVT
--PVGITA
--PVGIRY
--PVGIMQ
--PVGISS
--PVGIMK
--PVGIFR
--PVGIYS
--PVGIWN
--PVGIVM
--PVGITN
--PVGIQG
--PVGIAA
--PVGIRW
--PVGIMS
--PVGILK
--PVGILA
--PVGIAT
PTPVGI--
PKPVGI--
--PVGIVQ
--PVGIST
--PVGING
--PVGIML
--PVGIMG
--PVGILF
--PVGISM
--PVGIIK
--PVGIFH
--PVGIVW
--PVGILY
--PVGILT
--PVGIHT
--PVGIYN
--PVGIWH
--PVGISY
--PVGIIW
--PVGIHW
--PVGIHR
PSPVGI--

PVPVGI--
--PVGIQR
--PVGIHG
--PVGITM
--PVGIHV
--PVGIAS
--PVGIVH
--PVGIVG
--PVGITL
--PVGIRQ
--PVGIMN
--PVGILV
--PVGIKV
--PVGIFY
--PVGIWW
--PVGISI
--PVGILN
--PVGIIR
--PVGIES
--PVGIVN
--PVGIRI
--PVGIRD
--PVGILM
--PVGIKT
--PVGIGG
--PVGIYH
--PVGISF
--PVGIMY
PNPVGI--
--PVGIYY
--PVGIWR
--PVGITW
--PVGITF
--PVGISQ
--PVGISN
--PVGIQW
--PVGINR
--PVGIHK
PFPVGI--
PGPVGI--
--PVGIYW
--PVGIWE
--PVGITI
--PVGINS
--PVGIMH
--PVGIKS
--PVGIFE
PHPVGI--
--PVGIVF
--PVGISL

--PVGINA
--PVGIMT
--PVGIKA
--PVGIGA
PIPVGI--
PWPVGI--
--PVGIYG
--PVGIYF
--PVGIWD
--PVGINF
--PVGIMW
--PVGIMM
--PVGIMF
--PVGIKR
--PVGIKL
--PVGIII
--PVGIHN
--PVGIHI
--PVGIHH
--PVGIFT
--PVGIVD
--PVGIQA
--PVGINM
--PVGILQ
--PVGIKF
--PVGIIA
--PVGIAY
--PVGIAV
PQPVGI--
--PVGISK
--PVGIRE
--PVGIQH
--PVGIEY
PDPVGI--
--PVGIVI
--PVGITQ
--PVGINV
--PVGIMV
--PVGIIT
--PVGIIH
--PVGIIG
--PVGIID
--PVGIGN
--PVGIEV
--PVGIEF
--PVGIAN
--PVGIAM
PCPVGI--
--PVGIYA
--PVGISD

--PVGIQL
--PVGIMD
--PVGILL
--PVGIKD
--PVGIIE
--PVGIHF
--PVGIAI
--PVGIYQ
--PVGITD
--PVGIQT
--PVGINK
--PVGIMA
--PVGILE
--PVGIKW
--PVGIKH
--PVGIIQ
--PVGIIF
--PVGIGF
--PVGIEW
--PVGIEN
--PVGIAW
--PVGIAQ
--PVGISC
--PVGIQY
--PVGIQN
--PVGIQI
--PVGIQF
--PVGINY
--PVGINT
--PVGINQ
--PVGINN
--PVGINL
--PVGINI
--PVGINH
--PVGIKY
--PVGIKQ
--PVGIIS
--PVGIHM
--PVGIHE
--PVGIGT
--PVGIFK
--PVGIFC
--PVGIET
--PVGIEK
--PVGIEA
--PVGIDA
--PVGICF
--PVGIAL
-PPVGIV-
--PVGIWQ

```
--PVGIVE
--PVGITE
--PVGITC
--PVGISE
--PVGIRC
--PVGIQQ
--PVGIPS
--PVGIPK
--PVGIPG
--PVGIPF
--PVGINW
--PVGILC
--PVGIKM
--PVGIHQ
--PVGIGM
--PVGIER
--PVGIEH
--PVGIEG
--PVGIDM
--PVGIDH
--PVGIDG
--PVGICT
--PVGICQ
--PVGIAH
--PVGIAF
-PPVGIR-
-PPVGIK-
-PPVGIH-
PPPVGI--
PEPVGI--
-QPVGIR-
QSPVGI--
-QPVGIK-
QRPVGI--
QPPVGI--
-QPVGIW-
-QPVGIV-
-QPVGIL-
-QPVGII-
-QPVGIT-
QTPVGI--
-QPVGIY-
-QPVGIS-
-QPVGIH-
QWPVGI--
QVPVGI--
QHPVGI--
QFPVGI--
-QPVGIA-
QLPVGI--
```

QGPVGI--
-QPVGIQ-
QMPVGI--
QYPVGI--
QQPVGI--
-QPVGIN-
-QPVGIM-
-QPVGIF-
QIPVGI--
QCPVGI--
QKPVGI--
QNPVGI--
QDPVGI--
-RPVGIR-
RLPVGI--
RGPVGI--
-RPVGIY-
RWPVGI--
-RPVGIF-
RTPVGI--
RHPVGI--
-RPVGIS-
-RPVGIK-
RIPVGI--
-RPVGIM-
-RPVGIN-
-RPVGIQ-
-RPVGII-
RNPVGI--
RYPVGI--
RVPVGI--
-RPVGIV-
-RPVGIH-
-RPVGIW-
RFPVGI--
REPVGI--
RQPVGI--
RPPVGI--
-RPVGIT-
RDPVGI--
RAPVGI--
-RPVGIG-
-RPVGIA-
-RPVGIE-
RCPVGI--
-SPVGIR-
SSPVGI--
-SPVGIY-
SAPVGI--
SLPVGI--

SGPVGI--
SYPVGI--
SRPVGI--
SHPVGI--
-SPVGIH-
-SPVGIS-
-SPVGIV-
-SPVGIQ-
SWPVGI--
SMPVGI--
SIPVGI--
SPPVGI--
-SPVGIT-
SFPVGI--
-SPVGIF-
-SPVGIN-
-SPVGII-
SNPVGI--
-SPVGIW-
SEPVGI--
-SPVGIA-
SQPVGI--
SDPVGI--
-SPVGIE-
SCPVGI--
-SPVGIG-
-TPVGIR-
TIPVGI--
-TPVGIL-
TVPVGI--
TTPVGI--
-TPVGIS-
TAPVGI--
-TPVGIY-
TSPVGI--
TNPVGI--
TGPVGI--
-TPVGIF-
TQPVGI--
-TPVGIW-
-TPVGIQ-
-TPVGIK-
TWPVGI--
-TPVGIT-
TMPVGI--
TFPVGI--
-TPVGIH-
TPPVGI--
-TPVGIA-
-TPVGIN-

TYPVGI--
-TPVGIM-
TDPVGI--
-TPVGIE-
THPVGI--
TEPVGI--
TCPVGI--
-TPVGII-
-VPVGIL-
VYPVGI--
VKPVGI--
VRPVGI--
-VPVGIF-
VGPVGI--
VSPVGI--
VQPVGI--
-VPVGIR-
-VPVGIY-
-VPVGIT-
VHPVGI--
-VPVGIS-
-VPVGIK-
VAPVGI--
VWPVGI--
VFPVGI--
VPPVGI--
VIPVGI--
VTPVGI--
-VPVGIV-
VVPVGI--
VLPVGI--
VMPVGI--
VNPVGI--
-VPVGIN-
VDPVGI--
-VPVGIH-
VEPVGI--
-VPVGIQ-
-VPVGIM-
-VPVGIE-
-VPVGII-
-VPVGID-
WDPVGI--
WLPVGI--
-WPVGIH-
WPPVGI--
WSPVGI--
WVPVGI--
-WPVGIR-
WMPVGI--

WNPVGI--
WKPVGI--
WGPVGI--
WFPVGI--
WYPVGI--
WQPVGI--
-WPVGIS-
WAPVGI--
WTPVGI--
WEPVGI--
-WPVGIT-
-WPVGIL-
-WPVGIK-
-WPVGIV-
WIPVGI--
-WPVGIQ-
-WPVGIN-
-WPVGIM-
-WPVGIA-
WHPVGI--
-YPVGIK-
YVPVGI--
YKPVGI--
YSPVGI--
YTPVGI--
-YPVGIR-
YLPVGI--
YIPVGI--
YRPVGI--
-YPVGIL-
YQPVGI--
-YPVGIS-
YAPVGI--
-YPVGIW-
YEPVGI--
-YPVGIT-
YMPVGI--
-YPVGIY-
YGPVGI--
-YPVGII-
YHPVGI--
-YPVGIV-
-YPVGIN-
YCPVGI--
YDPVGI--
-YPVGIQ-
-YPVGIM-
-YPVGIF-
-YPVGIA-
YPPVGI--

	-YPVGIH-		
PFSM	159	771	46.04
	--PFSMPS		
	--PFSMGR		
	-TPFSMW-		
	ANPFSM--		
	-APFSMT-		
	-APFSMR-		
	ARPFSM--		
	-APFSMY-		
	ASPFSM--		
	AGPFSM--		
	-APFSMS-		
	-APFSML-		
	AIPFSM--		
	AWPFSM--		
	-APFSMK-		
	ALPFSM--		
	ATPFSM--		
	-APFSMF-		
	AFPFSM--		
	-APFSMA-		
	-APFSME-		
	AMPFSM--		
	AKPFSM--		
	AAPFSM--		
	AQPFSM--		
	ADPFSM--		
	AVPFSM--		
	-APFSMQ-		
	-APFSMN-		
	AYPFSM--		
	-APFSMV-		
	-APFSMM-		
	-APFSMI-		
	-APFSMH-		
	AHPFSM--		
	ACPFSM--		
	CKPFSM--		
	CFPFSM--		
	CMPFSM--		
	CHPFSM--		
	CCPFSM--		
	CIPFSM--		
	CAPFSM--		
	-CPFSML-		
	-CPFSMA-		
	CVPFSM--		
	CSPFSM--		
	CRPFSM--		

-CPFSMV-
-CPFSMY-
-CPFSMM-
DVPFSM--
-DPFSMR-
DSPFSM--
DTPFSM--
DRPFSM--
DGPFSM--
-DPFSMV-
-DPFSMT-
-DPFSMF-
DNPFSM--
DMPFSM--
-DPFSMY-
-DPFSMK-
DIPFSM--
DHPFSM--
DFPFSM--
-DPFSMM-
DLPFSM--
-DPFSMW-
-DPFSML-
ERPFSM--
-EPFSMR-
EVPFSM--
-EPFSMV-
-EPFSMS-
EQPFSM--
EIPFSM--
EAPFSM--
EWPFSM--
-EPFSMT-
-EPFSMI-
ENPFSM--
EYPFSM--
EPPFSM--
-EPFSMF-
EMPFSM--
EHPFSM--
-EPFSMY-
-EPFSMW-
ESPFSM--
-EPFSME-
-EPFSMC-
ELPFSM--
EGPFSM--
FLPFSM--
-FPFSMV-
FIPFSM--

FRPFSM--
-FPFSMS-
FMPFSM--
FKPFSM--
-FPFSMH-
FNPFSM--
FWPFSM--
FSPFSM--
-FPFSMT-
FQPFSM--
FFPFSM--
FTPFSM--
FAPFSM--
FGPFSM--
FYPFSM--
FPPFSM--
-FPFSMY-
FDPFSM--
-FPFSMQ-
-FPFSMR-
-FPFSME-
-FPFSMA-
-FPFSMW-
FEPFSM--
-FPFSMN-
-FPFSMK-
GRPFSM--
-GPFSMS-
GQPFSM--
GVPFSM--
-GPFSMV-
GTPFSM--
GGPFSM--
-GPFSMY-
GLPFSM--
GIPFSM--
-GPFSMR-
-GPFSMT-
GWPFSM--
GYPFSM--
-GPFSMI-
GNPFSM--
GMPFSM--
-GPFSMF-
GSPFSM--
GFPFSM--
-GPFSMM-
-GPFSME-
-GPFSMN-
-GPFSML-

GDPFSM--
-GPFSMK-
GAPFSM--
-GPFSMH-
-GPFSMQ-
GHPFSM--
GEPFSM--
GPPFSM--
-GPFSMG-
-GPFSMA-
-HPFSMR-
HSPFSM--
HVPFSM--
HAPFSM--
-HPFSMT-
-HPFSMH-
HGPFSM--
HLPFSM--
-HPFSMS-
-HPFSMN-
HKPFSM--
-HPFSMV-
-HPFSMK-
-HPFSMI-
-HPFSMA-
HYPFSM--
HWPFSM--
HQPFSM--
HPPFSM--
HTPFSM--
-HPFSMY-
HMPFSM--
HHPFSM--
-HPFSMQ-
-HPFSML-
HNPFSM--
HEPFSM--
HDPFSM--
IRPFSM--
IMPFSM--
ILPFSM--
IVPFSM--
-IPFSMT-
IGPFSM--
IIPFSM--
IHPFSM--
-IPFSMS-
-IPFSMR-
ISPFSM--
IPPFSM--

ITPFSM--
-IPFSMI-
INPFSM--
-IPFSMW-
IYPFSM--
IWPFSM--
IAPFSM--
-IPFSMM-
-IPFSMK-
-IPFSMV-
IDPFSM--
-IPFSMN-
-IPFSMG-
-IPFSME-
IFPFSM--
-KPFSMS-
KVPFSM--
-KPFSMY-
KRPFSM--
KPPFSM--
KTPFSM--
KSPFSM--
-KPFSML-
-KPFSMM-
-KPFSMH-
-KPFSMQ-
-KPFSMW-
-KPFSMA-
-KPFSMT-
KMPFSM--
KAPFSM--
KFPFSM--
KIPFSM--
KGPFSM--
KQPFSM--
-KPFSMK-
KDPFSM--
KKPFSM--
-KPFSMG-
KHPFSM--
KEPFSM--
KWPFSM--
-KPFSMI-
KCPFSM--
LTPFSM--
LVPFSM--
LRPFSM--
LSPFSM--
LLPFSM--
LGPFSM--

-LPFSMV-
-LPFSMR-
LKPFSM--
-LPFSMI-
-LPFSMY-
-LPFSML-
LMPFSM--
LEPFSM--
LWPFSM--
LPPFSM--
-LPFSMF-
LYPFSM--
-LPFSMS-
LDPFSM--
LAPFSM--
-LPFSMT-
-LPFSMQ-
-LPFSMK-
LFPFSM--
-LPFSMW-
LHPFSM--
LQPFSM--
-LPFSMN-
-LPFSMH-
LCPFSM--
LNPFSM--
-LPFSMM-
MSPFSM--
MLPFSM--
MRPFSM--
-MPFSMI-
MVPFSM--
-MPFSML-
MTPFSM--
MAPFSM--
MKPFSM--
MGPFSM--
-MPFSMS-
-MPFSMF-
-MPFSMA-
MNPFSM--
MEPFSM--
MFPFSM--
MQPFSM--
-MPFSMK-
-MPFSMN-
MIPFSM--
-MPFSMH-
-MPFSME-
MWPFSM--

-MPFSMR-
MMPFSM--
MDPFSM--
MPPFSM--
-MPFSMT-
-MPFSMQ-
-MPFSMG-
MHPFSM--
NRPFSM--
NGPFSM--
NIPFSM--
NNPFSM--
NYPFSM--
NAPFSM--
NLPFSM--
NKPFSM--
-NPFSMR-
-NPFSMV-
-NPFSMS-
-NPFSML-
NHPFSM--
-NPFSMY-
NVPFSM--
NSPFSM--
-NPFSMH-
NEPFSM--
NQPFSM--
NFPFSM--
NPPFSM--
-NPFSMK-
NMPFSM--
NTPFSM--
-NPFSMI-
-NPFSMF-
NWPFSM--
-NPFSMT-
NDPFSM--
-NPFSMN-
-NPFSMM-
-NPFSMA-
--PFSMRS
--PFSMRG
--PFSMLS
--PFSMLT
--PFSMLG
--PFSMVS
--PFSMRR
--PFSMMS
--PFSMSS
--PFSMVR

--PFSMTS
--PFSMVA
--PFSMRN
--PFSMRY
PRPFSM--
--PFSMYS
--PFSMSG
--PFSMMM
--PFSMFS
--PFSMVK
--PFSMST
--PFSMFR
PWPFSM--
--PFSMTQ
--PFSMNS
--PFSMKS
--PFSMIS
--PFSMVI
--PFSMTA
--PFSMIT
--PFSMTL
--PFSMSN
PSPFSM--
--PFSMYR
--PFSMTI
--PFSMHG
--PFSMSV
--PFSMSQ
--PFSMHN
--PFSMWS
--PFSMTK
--PFSMTH
--PFSMQG
--PFSMKA
--PFSMFQ
--PFSMTT
--PFSMSH
--PFSMRH
--PFSMLR
--PFSMGS
--PFSMEG
--PFSMAM
PYPFSM--
--PFSMYN
--PFSMVM
--PFSMMT
--PFSMMN
--PFSMKG
--PFSMTN
--PFSMIR

--PFSMGG
--PFSMGA
--PFSMFH
--PFSMAG
--PFSMTR
--PFSMIK
--PFSMHH
PAPFSM--
--PFSMVT
--PFSMSY
--PFSMLH
--PFSMLD
--PFSMIN
--PFSMHL
PVPFSM--
PTPFSM--
PHPFSM--
--PFSMYA
--PFSMWT
--PFSMVN
--PFSMVG
--PFSMRA
--PFSMQT
--PFSMNA
--PFSMLQ
--PFSMLE
--PFSMHQ
--PFSMYH
--PFSMWA
--PFSMVD
--PFSMTM
--PFSMQK
--PFSMMQ
--PFSMKR
--PFSMIA
--PFSMHS
--PFSMHE
--PFSMFT
--PFSMAS
--PFSMWK
--PFSMNY
--PFSMMH
--PFSMHI
--PFSMGV
--PFSMYQ
--PFSMYM
--PFSMWN
--PFSMVY
--PFSMVH
--PFSMTG

--PFSMSF
--PFSMNL
--PFSMMR
--PFSMKQ
--PFSMIQ
--PFSMFD
--PFSMET
--PFSMTV
--PFSMSW
--PFSMSM
--PFSMRD
--PFSMQY
--PFSMNM
--PFSMKV
--PFSMGT
--PFSMFK
--PFSMAL
PNPFSM--
PGPFSM--
--PFSMRT
--PFSMQR
--PFSMQM
--PFSMNT
--PFSMMK
--PFSMLK
--PFSMHY
--PFSMHF
--PFSMHA
--PFSMAT
--PFSMYK
--PFSMSK
--PFSMKT
--PFSMKE
--PFSMHM
--PFSMES
--PFSMAQ
--PFSMAH
PDPFSM--
--PFSMYD
--PFSMWR
--PFSMVE
--PFSMQQ
--PFSMQI
--PFSMNI
--PFSMIV
--PFSMHR
--PFSMFV
--PFSMFN
--PFSMFE
--PFSMAV

PMPFSM--
--PFSMYG
--PFSMTD
--PFSMPL
--PFSMMA
--PFSMKL
--PFSMKH
--PFSMII
--PFSMIH
--PFSMIE
--PFSMHV
--PFSMEM
--PFSMEA
PFPFSM--
PQPFSM--
--PFSMYV
--PFSMYT
--PFSMTY
--PFSMSE
--PFSMSD
--PFSMQL
--PFSMQH
--PFSMQC
--PFSMNR
--PFSMNQ
--PFSMNK
--PFSMNF
--PFSMMG
--PFSMMD
--PFSMLN
--PFSMKW
--PFSMHT
--PFSMGF
--PFSMFG
--PFSMEW
--PFSMEQ
--PFSMEH
--PFSMDS
--PFSMCS
-PPFSMQ-
--PFSMYE
--PFSMWW
--PFSMWH
--PFSMWG
--PFSMWD
--PFSMVQ
--PFSMTE
--PFSMPY
--PFSMNG
--PFSMKF

--PFSMHK
--PFSMHC
--PFSMGW
--PFSMEV
--PFSMEN
--PFSMEI
--PFSMDL
--PFSMDE
--PFSMDA
--PFSMCW
--PFSMAR
--PFSMAE
-QPFSML-
QRPFSM--
QSPFSM--
QPPFSM--
QNPFSM--
QFPFSM--
-QPFSMA-
QVPFSM--
QGPFSM--
-QPFSMH-
QWPFSM--
-QPFSMS-
-QPFSMI-
-QPFSMR-
-QPFSMQ-
QEPFSM--
QTPFSM--
QQPFSM--
QKPFSM--
-QPFSMT-
QDPFSM--
QYPFSM--
-QPFSMF-
-QPFSMV-
-QPFSMN-
-QPFSMM-
-RPFSMR-
-RPFSMT-
RSPFSM--
-RPFSMI-
-RPFSMH-
RNPFSM--
-RPFSMY-
RMPFSM--
-RPFSMW-
RGPFSM--
RAPFSM--
RVPFSM--

-RPFSMK-
REPFSM--
RPPFSM--
RRPFSM--
RQPFSM--
RFPFSM--
RYPFSM--
-RPFSMA-
RIPFSM--
-RPFSMN-
-RPFSMF-
-RPFSMG-
RHPFSM--
-RPFSMQ-
-RPFSMM-
RDPFSM--
-RPFSMD-
-RPFSME-
RCPFSM--
SRPFSM--
-SPFSMR-
SLPFSM--
SSPFSM--
-SPFSML-
SVPFSM--
SAPFSM--
-SPFSMT-
SGPFSM--
SFPFSM--
-SPFSMS-
-SPFSMK-
SNPFSM--
-SPFSMI-
SHPFSM--
STPFSM--
-SPFSMV-
-SPFSMH-
SMPFSM--
-SPFSMA-
-SPFSMY-
SIPFSM--
SEPFSM--
SYPFSM--
SCPFSM--
SQPFSM--
-SPFSMW-
-SPFSMF-
SPPFSM--
SWPFSM--
-SPFSMN-

-SPFSMQ-
-SPFSMG-
-SPFSMM-
-SPFSME-
TRPFSM--
TSPFSM--
-TPFSML-
-TPFSMK-
TIPFSM--
TMPFSM--
-TPFSMV-
TNPFSM--
TLPFSM--
TYPFSM--
THPFSM--
-TPFSMS-
TGPFSM--
TVPFSM--
TPPFSM--
-TPFSMY-
TAPFSM--
-TPFSMN-
-TPFSMH-
TWPFSM--
TKPFSM--
TQPFSM--
-TPFSMT-
-TPFSMM-
-TPFSMG-
TEPFSM--
-TPFSME-
TFPFSM--
TDPFSM--
-TPFSMQ-
-TPFSMA-
VNPFSM--
VLPFSM--
-VPFSMR-
VIPFSM--
VTPFSM--
VVPFSM--
VFPFSM--
VGPFSM--
VMPFSM--
VKPFSM--
VSPFSM--
-VPFSMQ-
VPPFSM--
-VPFSMT-
-VPFSMF-

VYPFSM--
VAPFSM--
VDPFSM--
-VPFSMH-
VQPFSM--
-VPFSMY-
-VPFSMM-
-VPFSMW-
VHPFSM--
-VPFSMV-
-VPFSMN-
-VPFSME-
VCPFSM--
VWPFSM--
-VPFSMK-
VEPFSM--
-VPFSMI-
-VPFSMG-
WSPFSM--
-WPFSMR-
-WPFSMM-
WLPFSM--
WTPFSM--
WPPFSM--
WMPFSM--
-WPFSMA-
-WPFSMI-
WAPFSM--
WIPFSM--
WFPFSM--
-WPFSMY-
-WPFSMV-
-WPFSMN-
WGPFSM--
-WPFSMS-
WDPFSM--
WYPFSM--
-WPFSMW-
-WPFSMH-
WNPFSM--
WHPFSM--
WEPFSM--
-YPFSMR-
YRPFSM--
YVPFSM--
-YPFSML-
YSPFSM--
-YPFSMS-
-YPFSMK-
YAPFSM--

YFPFSM--
YEPFSM--
-YPFSMH-
YDPFSM--
YTPFSM--
YLPFSM--
YHPFSM--
YGPFSM--
YYPFSM--
-YPFSMQ-
YMPFSM--
YIPFSM--
YWPFSM--
-YPFSMT-
-YPFSMI-
-YPFSMM-
-YPFSMA-
YKPFSM--
YCPFSM--
-YPFSMV-
YPPFSM--
-YPFSMY-
-YPFSMG-

PSNY 42 859 45.99

-DPSNYL-
--PSNYSI
--PSNYRP
--PSNYKV
--PSNYKI
-RPSNYD-
-APSNYL-
AVPSNY--
-APSNYS-
ARPSNY--
-APSNYR-
ATPSNY--
ALPSNY--
AKPSNY--
-APSNYT-
-APSNYI-
-APSNYW-
AAPSNY--
-APSNYY-
AGPSNY--
-APSNYM-
-APSNYV-
-APSNYF-
APPSNY--
AWPSNY--
ASPSNY--

-APSNYG-
-APSNYE-
AMPSNY--
AEPSNY--
-APSNYN-
ANPSNY--
AIPSNY--
AHPSNY--
AQPSNY--
-APSNYQ-
AYPSNY--
-APSNYK-
-APSNYA-
AFPSNY--
-APSNYH-
ADPSNY--
ACPSNY--
CFPSNY--
CSPSNY--
CAPSNY--
CRPSNY--
-CPSNYT-
CLPSNY--
CEPSNY--
-CPSNYL-
-CPSNYF-
CNPSNY--
CHPSNY--
CGPSNY--
-CPSNYV-
-CPSNYA-
CPPSNY--
CDPSNY--
CCPSNY--
DRPSNY--
-DPSNYS-
DKPSNY--
DQPSNY--
DMPSNY--
DGPSNY--
DVPSNY--
DSPSNY--
-DPSNYR-
DWPSNY--
DFPSNY--
DAPSNY--
-DPSNYY-
-DPSNYF-
-DPSNYA-
DNPSNY--

DHPSNY--
DEPSNY--
-EPSNYL-
-EPSNYR-
-EPSNYQ-
ELPSNY--
ERPSNY--
-EPSNYT-
EWPSNY--
-EPSNYM-
EVPSNY--
ESPSNY--
-EPSNYY-
EMPSNY--
EGPSNY--
ENPSNY--
EAPSNY--
EQPSNY--
-EPSNYS-
-EPSNYI-
EFPSNY--
-EPSNYV-
-EPSNYE-
EIPSNY--
ECPSNY--
-EPSNYK-
-EPSNYH-
-EPSNYG-
-EPSNYA-
EPPSNY--
FLPSNY--
-FPSNYL-
FSPSNY--
FGPSNY--
FMPSNY--
FIPSNY--
-FPSNYV-
FQPSNY--
-FPSNYS-
-FPSNYW-
FYPSNY--
FNPSNY--
FEPSNY--
FVPSNY--
FDPSNY--
FAPSNY--
-FPSNYK-
-FPSNYI-
FKPSNY--
FRPSNY--

-FPSNYT-
-FPSNYH-
-FPSNYY-
-FPSNYQ-
-FPSNYA-
FPPSNY--
FHPSNY--
FFPSNY--
FTPSNY--
-FPSNYE-
FWPSNY--
-FPSNYG-
GRPSNY--
GLPSNY--
-GPSNYR-
-GPSNYV-
GIPSNY--
-GPSNYL-
-GPSNYY-
GTPSNY--
GSPSNY--
GYPSNY--
GMPSNY--
GHPSNY--
GWPSNY--
-GPSNYS-
GKPSNY--
GGPSNY--
GVPSNY--
-GPSNYW-
-GPSNYK-
GAPSNY--
-GPSNYQ-
-GPSNYN-
-GPSNYH-
-GPSNYA-
-GPSNYM-
GNPSNY--
GFPSNY--
GQPSNY--
GEPSNY--
-GPSNYF-
-GPSNYT-
-GPSNYI-
-GPSNYE-
GDPSNY--
GCPSNY--
HKPSNY--
HEPSNY--
HSPSNY--

-HPSNYR-
HRPSNY--
-HPSNYS-
HDPSNY--
HGPSNY--
HVPSNY--
HYPSNY--
HTPSNY--
-HPSNYI-
HLPSNY--
HFPSNY--
-HPSNYF-
HWPSNY--
HQPSNY--
-HPSNYT-
HIPSNY--
HAPSNY--
HHPSNY--
-HPSNYM-
-HPSNYW-
-HPSNYV-
-HPSNYK-
HMPSNY--
-HPSNYY-
-HPSNYH-
HPPSNY--
-IPSNYV-
-IPSNYL-
IRPSNY--
-IPSNYR-
IDPSNY--
IWPSNY--
IPPSNY--
-IPSNYA-
IAPSNY--
IFPSNY--
-IPSNYM-
ILPSNY--
IVPSNY--
-IPSNYS-
-IPSNYW-
ITPSNY--
IGPSNY--
-IPSNYT-
IEPSNY--
-IPSNYQ-
-IPSNYI-
-IPSNYH-
-IPSNYF-
IHPSNY--

ISPSNY--
-IPSNYK-
IIPSNY--
IQPSNY--
-IPSNYG-
-IPSNYE-
-IPSNYC-
IKPSNY--
KSPSNY--
KVPSNY--
KGPSNY--
-KPSNYL-
-KPSNYI-
-KPSNYT-
-KPSNYR-
-KPSNYA-
-KPSNYV-
-KPSNYS-
KNPSNY--
KWPSNY--
KTPSNY--
KRPSNY--
-KPSNYQ-
KLPSNY--
KQPSNY--
KPPSNY--
KYPSNY--
KIPSNY--
KCPSNY--
-KPSNYY-
-KPSNYH-
KAPSNY--
-KPSNYK-
-KPSNYG-
-KPSNYF-
KEPSNY--
-KPSNYN-
KKPSNY--
KHPSNY--
KFPSNY--
KDPSNY--
LKPSNY--
LRPSNY--
LVPSNY--
LSPSNY--
LTPSNY--
LWPSNY--
-LPSNYL-
LFPSNY--
LAPSNY--

LLPSNY--
LNPSNY--
LGPSNY--
LPPSNY--
LMPSNY--
-LPSNYR-
-LPSNYM-
-LPSNYI-
LEPSNY--
-LPSNYV-
-LPSNYT-
-LPSNYS-
LHPSNY--
LQPSNY--
-LPSNYQ-
-LPSNYA-
LYPSNY--
-LPSNYK-
-LPSNYH-
-LPSNYF-
-LPSNYE-
LIPSNY--
LDPSNY--
-LPSNYY-
-LPSNYW-
LCPSNY--
-MPSNYT-
MRPSNY--
MMPSNY--
MGPSNY--
MYPSNY--
MSPSNY--
-MPSNYR-
-MPSNYL-
MWPSNY--
MVPSNY--
-MPSNYF-
MLPSNY--
-MPSNYV-
-MPSNYH-
-MPSNYK-
-MPSNYQ-
MQPSNY--
-MPSNYW-
MKPSNY--
MHPSNY--
-MPSNYM-
MPPSNY--
MIPSNY--
-MPSNYN-

```
-MPSNYG-
MDPSNY--
MTPSNY--
-MPSNYA-
MNPSNY--
MFPSNY--
MAPSNY--
NLPSNY--
NRPSNY--
NQPSNY--
-NPSNYR-
NYPSNY--
-NPSNYV-
-NPSNYL-
NVPSNY--
-NPSNYS-
NHPSNY--
NGPSNY--
-NPSNYF-
NMPSNY--
NFPSNY--
NTPSNY--
-NPSNYM-
NSPSNY--
NNPSNY--
-NPSNYI-
-NPSNYY-
NEPSNY--
NAPSNY--
-NPSNYW-
NCPSNY--
-NPSNYH-
-NPSNYA-
NIPSNY--
-NPSNYK-
NDPSNY--
NWPSNY--
-NPSNYT-
-NPSNYN-
-NPSNYE-
--PSNYQT
--PSNYRS
--PSNYLG
--PSNYRA
--PSNYSS
--PSNYTS
--PSNYLW
--PSNYFA
--PSNYFV
--PSNYRT
```

--PSNYGS
--PSNYAS
--PSNYVL
--PSNYTY
--PSNYSV
--PSNYRV
PYPSNY--
--PSNYYG
--PSNYRN
--PSNYLH
--PSNYVW
--PSNYLF
--PSNYSW
--PSNYVS
--PSNYWH
--PSNYWA
--PSNYRW
--PSNYMT
PVPSNY--
--PSNYSG
--PSNYRM
--PSNYVG
--PSNYSL
--PSNYNS
--PSNYIH
--PSNYFL
--PSNYVH
--PSNYSR
--PSNYSH
--PSNYWR
--PSNYVT
--PSNYVR
--PSNYTL
--PSNYQR
--PSNYHG
--PSNYFG
--PSNYWS
--PSNYVY
--PSNYTR
--PSNYTG
--PSNYLY
--PSNYLV
--PSNYLS
--PSNYRL
--PSNYHT
--PSNYWT
--PSNYVV
--PSNYST
--PSNYSN
--PSNYSM

--PSNYMQ
--PSNYAL
PRPSNY--
--PSNYYT
--PSNYYS
--PSNYVK
--PSNYQN
--PSNYMG
--PSNYMA
--PSNYAR
--PSNYTV
--PSNYMS
--PSNYLA
--PSNYIT
--PSNYIA
--PSNYHW
--PSNYHS
PWPSNY--
--PSNYVI
--PSNYMN
--PSNYIF
--PSNYFT
--PSNYTA
--PSNYRI
--PSNYKN
--PSNYKG
--PSNYGG
--PSNYRD
--PSNYMI
--PSNYYR
--PSNYYH
--PSNYVF
--PSNYVA
--PSNYTQ
--PSNYRF
--PSNYMR
--PSNYMM
--PSNYML
--PSNYLQ
--PSNYFI
--PSNYYI
--PSNYWN
--PSNYWL
--PSNYWG
--PSNYTN
--PSNYSF
--PSNYRG
--PSNYKT
--PSNYKA
--PSNYET

PMPSNY--
PIPSNY--
--PSNYQW
--PSNYNT
--PSNYHV
--PSNYFR
--PSNYVQ
--PSNYVN
--PSNYTF
--PSNYRY
--PSNYLD
--PSNYIS
--PSNYII
--PSNYIG
--PSNYEH
--PSNYAT
PNPSNY--
PEPSNY--
PTPSNY--
--PSNYYK
--PSNYWY
--PSNYSK
--PSNYQS
--PSNYKS
--PSNYIQ
--PSNYEW
--PSNYAN
--PSNYYL
--PSNYYA
--PSNYWQ
--PSNYQG
--PSNYME
--PSNYLK
--PSNYKF
--PSNYIV
--PSNYHA
--PSNYFK
--PSNYFH
--PSNYES
--PSNYAV
PLPSNY--
PKPSNY--
PGPSNY--
--PSNYWK
--PSNYWD
--PSNYTI
--PSNYMF
--PSNYKL
--PSNYIR
--PSNYIL

--PSNYFM
--PSNYER
--PSNYEA
--PSNYAW
--PSNYAF
--PSNYAA
PHPSNY--
--PSNYWI
--PSNYWF
--PSNYSD
--PSNYQI
--PSNYMW
--PSNYLE
--PSNYKQ
--PSNYIY
--PSNYIE
--PSNYID
--PSNYFW
--PSNYFN
--PSNYEF
--PSNYAQ
PAPSNY--
--PSNYYW
--PSNYYQ
--PSNYWV
--PSNYVM
--PSNYVD
--PSNYSE
--PSNYSA
--PSNYNV
--PSNYNM
--PSNYNG
--PSNYMD
--PSNYLI
--PSNYHY
--PSNYHQ
--PSNYHF
--PSNYGM
--PSNYGF
--PSNYGA
--PSNYFY
--PSNYFE
--PSNYFD
--PSNYEQ
--PSNYAK
PFPSNY--
--PSNYYY
--PSNYYN
--PSNYYF
--PSNYYE

--PSNYYD
--PSNYTK
--PSNYQC
--PSNYQA
--PSNYNQ
--PSNYNH
--PSNYLN
--PSNYIK
--PSNYHL
--PSNYGY
--PSNYAD
PQPSNY--
PDPSNY--
--PSNYWW
--PSNYTT
--PSNYQK
--PSNYNY
--PSNYNF
--PSNYKD
--PSNYHN
--PSNYGI
--PSNYCV
--PSNYAM
--PSNYYV
--PSNYWE
--PSNYTH
--PSNYTE
--PSNYRQ
--PSNYQV
--PSNYQL
--PSNYQF
--PSNYQE
--PSNYQD
--PSNYPH
--PSNYNW
--PSNYNN
--PSNYKY
--PSNYKH
--PSNYIM
--PSNYHR
--PSNYHM
--PSNYHH
--PSNYGV
--PSNYGT
--PSNYEN
--PSNYEI
--PSNYEG
--PSNYDF
--PSNYCY
--PSNYCT

--PSNYCS
--PSNYCN
-PPSNYI-
PPPSNY--
QRPSNY--
QWPSNY--
QLPSNY--
QGPSNY--
-QPSNYR-
QVPSNY--
-QPSNYV-
-QPSNYS-
-QPSNYI-
QKPSNY--
QSPSNY--
-QPSNYK-
QIPSNY--
QYPSNY--
-QPSNYM-
QPPSNY--
QDPSNY--
QAPSNY--
-QPSNYA-
QNPSNY--
QQPSNY--
-QPSNYN-
QEPSNY--
QTPSNY--
-QPSNYW-
-QPSNYQ-
QFPSNY--
-QPSNYG-
-QPSNYF-
-QPSNYL-
-RPSNYR-
RVPSNY--
RAPSNY--
RGPSNY--
RSPSNY--
-RPSNYV-
REPSNY--
-RPSNYI-
RTPSNY--
RLPSNY--
-RPSNYQ-
-RPSNYS-
RNPSNY--
RQPSNY--
-RPSNYT-
RPPSNY--

-RPSNYW-
RFPSNY--
RWPSNY--
-RPSNYF-
RMPSNY--
RKPSNY--
-RPSNYK-
-RPSNYM-
RYPSNY--
-RPSNYY-
-RPSNYH-
RDPSNY--
-RPSNYE-
RHPSNY--
-RPSNYG-
RIPSNY--
-RPSNYN-
-SPSNYR-
SRPSNY--
SIPSNY--
-SPSNYL-
SVPSNY--
SSPSNY--
-SPSNYS-
SMPSNY--
SAPSNY--
STPSNY--
SGPSNY--
SKPSNY--
-SPSNYV-
-SPSNYA-
-SPSNYW-
SYPSNY--
-SPSNYM-
SNPSNY--
-SPSNYT-
SQPSNY--
-SPSNYY-
-SPSNYK-
-SPSNYQ-
-SPSNYH-
SPPSNY--
SFPSNY--
SWPSNY--
-SPSNYE-
SCPSNY--
SDPSNY--
-SPSNYC-
SEPSNY--
-SPSNYF-

SHPSNY--
-SPSNYI-
-SPSNYD-
TSPSNY--
TQPSNY--
-TPSNYW-
-TPSNYT-
TRPSNY--
TNPSNY--
TLPSNY--
TPPSNY--
TTPSNY--
-TPSNYS-
-TPSNYL-
TVPSNY--
-TPSNYV-
-TPSNYF-
TMPSNY--
TFPSNY--
TAPSNY--
THPSNY--
TGPSNY--
-TPSNYM-
-TPSNYK-
-TPSNYI-
-TPSNYE-
-TPSNYH-
TYPSNY--
-TPSNYQ-
TIPSNY--
TCPSNY--
TEPSNY--
-TPSNYR-
-TPSNYG-
TWPSNY--
-TPSNYY-
-TPSNYN-
-TPSNYA-
TKPSNY--
-VPSNYR-
VTPSNY--
VKPSNY--
VLPSNY--
VEPSNY--
-VPSNYY-
-VPSNYI-
-VPSNYT-
-VPSNYF-
VSPSNY--
VAPSNY--

VRPSNY--
-VPSNYM-
VIPSNY--
VGPSNY--
VVPSNY--
-VPSNYL-
-VPSNYV-
VFPSNY--
-VPSNYS-
-VPSNYH-
VNPSNY--
VMPSNY--
VQPSNY--
VHPSNY--
-VPSNYW-
-VPSNYA-
VPPSNY--
VYPSNY--
-VPSNYN-
VWPSNY--
-VPSNYE-
-VPSNYG-
-VPSNYQ-
-WPSNYR-
WTPSNY--
WLPSNY--
WVPSNY--
-WPSNYV-
WQPSNY--
WKPSNY--
WAPSNY--
WIPSNY--
WFPSNY--
-WPSNYL-
WPPSNY--
-WPSNYQ-
WGPSNY--
-WPSNYS-
WWPSNY--
-WPSNYH-
WNPSNY--
-WPSNYF-
-WPSNYA-
WMPSNY--
WEPSNY--
-WPSNYK-
WHPSNY--
-WPSNYT-
-WPSNYM-
-WPSNYE-

WCPSNY--
-YPSNYV-
-YPSNYL-
-YPSNYR-
YGPSNY--
YYPSNY--
YVPSNY--
YNPSNY--
-YPSNYS-
YKPSNY--
YFPSNY--
YRPSNY--
-YPSNYQ-
-YPSNYI-
-YPSNYW-
-YPSNYM-
YHPSNY--
YSPSNY--
YQPSNY--
-YPSNYA-
YPPSNY--
YAPSNY--
YWPSNY--
-YPSNYE-
YTPSNY--
-YPSNYK-
-YPSNYY-
YMPSNY--
-YPSNYH-
YIPSNY--
-YPSNYG-
YEPSNY--
YCPSNY--

PYPI	402	657	45.98

-APYPIT-
-APYPIS-
ASPYPI--
AGPYPI--
AFPYPI--
AHPYPI--
-APYPIL-
ALPYPI--
AMPYPI--
-APYPIR-
AVPYPI--
-APYPIY-
ANPYPI--
AIPYPI--
-APYPIQ-
-APYPII-

-APYPIA-
AAPYPI--
-APYPIN-
ACPYPI--
AWPYPI--
ATPYPI--
-APYPIF-
AQPYPI--
-APYPIV-
APPYPI--
ADPYPI--
CVPYPI--
CKPYPI--
CAPYPI--
CSPYPI--
CHPYPI--
CCPYPI--
-CPYPIT-
-CPYPIL-
-CPYPIK-
-CPYPIF-
CEPYPI--
DRPYPI--
-DPYPIW-
-DPYPII-
DKPYPI--
DSPYPI--
-DPYPIY-
-DPYPIL-
DMPYPI--
DAPYPI--
-DPYPIS-
DHPYPI--
EVPYPI--
-EPYPIR-
ESPYPI--
EMPYPI--
EYPYPI--
EQPYPI--
-EPYPIW-
-EPYPIV-
EKPYPI--
EDPYPI--
-EPYPIN-
-EPYPIL-
-EPYPIF-
ENPYPI--
EHPYPI--
EFPYPI--
FVPYPI--

FLPYPI--
FAPYPI--
-FPYPIH-
-FPYPIF-
FNPYPI--
FEPYPI--
FSPYPI--
-FPYPIV-
-FPYPIR-
FRPYPI--
FIPYPI--
FYPYPI--
-FPYPIL-
FMPYPI--
FKPYPI--
-FPYPIY-
-FPYPII-
-FPYPIQ-
-FPYPIG-
FGPYPI--
FFPYPI--
-FPYPIW-
-FPYPIN-
FDPYPI--
GRPYPI--
GSPYPI--
GYPYPI--
GLPYPI--
GVPYPI--
GWPYPI--
GTPYPI--
GIPYPI--
GQPYPI--
GFPYPI--
-GPYPIS-
-GPYPII-
-GPYPIA-
-GPYPIW-
-GPYPIK-
GKPYPI--
GGPYPI--
-GPYPIL-
-GPYPIQ-
-GPYPIH-
-GPYPIE-
GNPYPI--
GMPYPI--
GHPYPI--
-HPYPIR-
HGPYPI--

HPPYPI--
-HPYPIL-
HVPYPI--
HMPYPI--
HLPYPI--
-HPYPIS-
HSPYPI--
-HPYPIM-
-HPYPIG-
-HPYPIF-
HAPYPI--
HWPYPI--
HKPYPI--
HTPYPI--
HNPYPI--
IGPYPI--
IAPYPI--
-IPYPIR-
-IPYPIW-
ITPYPI--
ISPYPI--
IRPYPI--
-IPYPIA-
ILPYPI--
IQPYPI--
-IPYPIS-
IKPYPI--
IFPYPI--
-IPYPIT-
-IPYPIN-
-IPYPIE-
IMPYPI--
INPYPI--
IVPYPI--
-IPYPIY-
-IPYPIQ-
IIPYPI--
IEPYPI--
-IPYPIK-
IYPYPI--
-IPYPIV-
-IPYPIF-
IDPYPI--
KSPYPI--
-KPYPIF-
-KPYPII-
-KPYPIM-
KMPYPI--
-KPYPIT-
KRPYPI--

KGPYPI--
KVPYPI--
-KPYPIL-
KKPYPI--
KNPYPI--
KAPYPI--
-KPYPIN-
KFPYPI--
KDPYPI--
-KPYPIW-
-KPYPIQ-
-KPYPIS-
KIPYPI--
-KPYPIA-
KLPYPI--
-KPYPIV-
-KPYPIR-
-KPYPIH-
-KPYPIG-
KYPYPI--
-KPYPIE-
KPPYPI--
LRPYPI--
LSPYPI--
LPPYPI--
-LPYPIW-
-LPYPIH-
LKPYPI--
LTPYPI--
-LPYPIR-
-LPYPII-
-LPYPIL-
-LPYPIF-
LGPYPI--
LDPYPI--
LMPYPI--
LAPYPI--
LYPYPI--
-LPYPIS-
LLPYPI--
-LPYPIT-
LFPYPI--
-LPYPIN-
-LPYPIK-
LWPYPI--
-LPYPIV-
-LPYPIQ-
LHPYPI--
LQPYPI--
-LPYPIM-

LNPYPI--
-MPYPIR-
MWPYPI--
MRPYPI--
-MPYPIH-
-MPYPIM-
MMPYPI--
MFPYPI--
MYPYPI--
-MPYPIS-
-MPYPIL-
-MPYPIF-
MSPYPI--
MIPYPI--
MTPYPI--
-MPYPII-
MLPYPI--
-MPYPIT-
-MPYPIQ-
-MPYPIN-
-MPYPIG-
MPPYPI--
MCPYPI--
NRPYPI--
NSPYPI--
NHPYPI--
-NPYPIR-
NKPYPI--
NAPYPI--
NYPYPI--
-NPYPIK-
NVPYPI--
-NPYPIL-
-NPYPIV-
-NPYPIS-
NGPYPI--
NFPYPI--
-NPYPIY-
-NPYPIM-
-NPYPIF-
NLPYPI--
-NPYPII-
NTPYPI--
-NPYPIT-
NQPYPI--
-NPYPIN-
-NPYPIH-
--PYPILG
--PYPIYR
--PYPIFR

--PYPIFH
--PYPISA
--PYPIHS
--PYPIFK
--PYPIRL
--PYPIHN
--PYPIST
--PYPIRS
--PYPIRK
--PYPILN
--PYPIAF
--PYPISV
--PYPILQ
--PYPITV
--PYPISG
--PYPIKH
--PYPIVS
--PYPIVF
--PYPIRR
--PYPIQV
--PYPILY
--PYPILS
--PYPIKT
--PYPIAQ
--PYPIVL
--PYPIVG
--PYPISL
--PYPIRM
--PYPILH
--PYPIYL
--PYPIWS
PMPYPI--
--PYPIRD
--PYPIMS
--PYPIGQ
--PYPIWT
--PYPILW
--PYPIKL
--PYPIYG
--PYPIWR
--PYPIVH
--PYPIRA
--PYPILT
--PYPIHA
--PYPIYW
--PYPITW
--PYPIRI
--PYPIQW
--PYPIKD
--PYPIIV

--PYPIGV
--PYPIRW
--PYPIQS
--PYPINQ
--PYPINM
--PYPIKS
--PYPIIN
--PYPIIK
--PYPIGK
--PYPIFS
--PYPIYN
--PYPIYK
--PYPIVI
--PYPITR
--PYPISR
--PYPISH
--PYPIQL
--PYPIQI
--PYPIPW
--PYPILA
--PYPIIS
--PYPIIR
--PYPIWH
--PYPIWE
--PYPIVT
--PYPIVR
--PYPITH
--PYPIRV
--PYPING
--PYPIMQ
--PYPIMF
--PYPIKM
--PYPIII
--PYPIIA
--PYPIGG
--PYPIFY
--PYPIAS
--PYPIWA
--PYPITS
--PYPIRN
--PYPIRF
--PYPIQN
--PYPINS
--PYPIMT
--PYPIIL
--PYPIGS
--PYPIFM
--PYPIFE
--PYPIEY
--PYPIYS

--PYPIYQ
--PYPIYA
--PYPIWY
--PYPIWW
--PYPIWQ
--PYPIVY
--PYPISY
--PYPIRT
--PYPIQF
--PYPIMM
--PYPIML
--PYPIID
--PYPIFF
--PYPIAV
--PYPIAA
--PYPIWN
--PYPIVW
--PYPITA
--PYPISE
--PYPIQM
--PYPIPY
--PYPIMN
--PYPIGA
--PYPIDY
--PYPIAN
--PYPIAG
PTPYPI--
--PYPIYY
--PYPIWG
--PYPIWF
--PYPIVQ
--PYPIVN
--PYPIVA
--PYPITM
--PYPISN
--PYPIRC
--PYPIQY
--PYPIQQ
--PYPIPI
--PYPINY
--PYPILR
--PYPILI
--PYPIKY
--PYPIKF
--PYPIIG
--PYPIHT
--PYPIHR
--PYPIHI
--PYPIHF
--PYPIFT

--PYPIEL
--PYPIEH
--PYPIAW
PHPYPI--
PGPYPI--
--PYPIYV
--PYPIVD
--PYPITQ
--PYPISQ
--PYPISK
--PYPISF
--PYPIQR
--PYPIPM
--PYPINA
--PYPIMG
--PYPIME
--PYPILE
--PYPIKR
--PYPIKN
--PYPIKG
--PYPIIT
--PYPIHG
--PYPIGH
--PYPIFI
--PYPIEM
--PYPIEA
--PYPIDL
PNPYPI--
--PYPIYE
--PYPITE
--PYPITD
--PYPISW
--PYPISS
--PYPIRG
--PYPIQK
--PYPIQG
--PYPIQA
--PYPIPH
--PYPIPG
--PYPINV
--PYPINR
--PYPIMR
--PYPIKV
--PYPIKK
--PYPIKE
--PYPIIY
--PYPIIM
--PYPIIH
--PYPIHM
--PYPIHE

--PYPIHD
--PYPIGW
--PYPIEW
--PYPIEG
--PYPIDN
--PYPIDM
--PYPIDI
--PYPICS
--PYPICH
--PYPIAT
PQPYPI--
-PPYPII-
QTPYPI--
QRPYPI--
-QPYPIL-
-QPYPIR-
-QPYPIT-
QYPYPI--
-QPYPIH-
QQPYPI--
-QPYPIM-
-QPYPIS-
QNPYPI--
QFPYPI--
QAPYPI--
QVPYPI--
-QPYPII-
QLPYPI--
QHPYPI--
QWPYPI--
QSPYPI--
-QPYPIK-
QGPYPI--
-QPYPIW-
-QPYPIV-
-QPYPIE-
RSPYPI--
RAPYPI--
-RPYPIL-
RLPYPI--
RIPYPI--
RVPYPI--
-RPYPIT-
RYPYPI--
-RPYPIR-
-RPYPIH-
RKPYPI--
-RPYPIS-
-RPYPIA-
-RPYPIQ-

-RPYPIW-
-RPYPIN-
-RPYPIF-
RFPYPI--
RTPYPI--
-RPYPII-
-RPYPIG-
RPPYPI--
RHPYPI--
-RPYPIM-
RMPYPI--
REPYPI--
RWPYPI--
-RPYPIY-
RQPYPI--
-RPYPIK-
-SPYPIY-
SRPYPI--
-SPYPIR-
-SPYPIL-
SLPYPI--
STPYPI--
SSPYPI--
SMPYPI--
SYPYPI--
-SPYPIV-
-SPYPIS-
-SPYPIT-
SIPYPI--
SGPYPI--
-SPYPIA-
SHPYPI--
SVPYPI--
-SPYPIM-
SAPYPI--
SPPYPI--
SKPYPI--
-SPYPIW-
-SPYPII-
-SPYPIQ-
SNPYPI--
SFPYPI--
SWPYPI--
SQPYPI--
SEPYPI--
-SPYPIN-
-SPYPIK-
TGPYPI--
-TPYPIR-
TTPYPI--

TSPYPI--
TKPYPI--
TWPYPI--
TVPYPI--
-TPYPIF-
TAPYPI--
-TPYPIW-
-TPYPIL-
TYPYPI--
TQPYPI--
-TPYPIV-
-TPYPIA-
TPPYPI--
TMPYPI--
-TPYPIQ-
-TPYPIG-
TLPYPI--
TIPYPI--
THPYPI--
TEPYPI--
-TPYPIM-
-TPYPIK-
-TPYPII-
-TPYPIN-
TFPYPI--
-VPYPIS-
VPPYPI--
-VPYPIT-
-VPYPIR-
VVPYPI--
-VPYPIY-
-VPYPIK-
VMPYPI--
VLPYPI--
-VPYPIL-
VTPYPI--
-VPYPIV-
-VPYPII-
VGPYPI--
-VPYPIM-
-VPYPIH-
-VPYPIW-
-VPYPIF-
VKPYPI--
VWPYPI--
VRPYPI--
-VPYPIQ-
-VPYPIE-
-VPYPIA-
VEPYPI--

VAPYPI--
VYPYPI--
VSPYPI--
-VPYPIG-
VIPYPI--
VDPYPI--
WAPYPI--
WRPYPI--
WIPYPI--
WGPYPI--
WMPYPI--
-WPYPIH-
-WPYPIT-
-WPYPIK-
WPPYPI--
WWPYPI--
-WPYPIY-
WFPYPI--
WTPYPI--
WQPYPI--
WSPYPI--
-WPYPIR-
-YPYPIY-
-YPYPIM-
YDPYPI--
YGPYPI--
-YPYPIS-
YLPYPI--
YEPYPI--
YTPYPI--
-YPYPIR-
YIPYPI--
-YPYPIH-
-YPYPIA-
-YPYPIT-
YNPYPI--
YMPYPI--
YKPYPI--
YFPYPI--
YSPYPI--
-YPYPIQ-

PSAI	23	877	45.94

-FPSAIG-
-HPSAIN-
-LPSAIA-
--PSAIRP
--PSAIHT
--PSAIGN
-QPSAIE-
AGPSAI--

ALPSAI--
AWPSAI--
-APSAIR-
-APSAIL-
-APSAIT-
AMPSAI--
-APSAIF-
AYPSAI--
AVPSAI--
-APSAIS-
AQPSAI--
ASPSAI--
-APSAIH-
ANPSAI--
ATPSAI--
-APSAIA-
-APSAIQ-
-APSAIV-
AAPSAI--
-APSAII-
AIPSAI--
ADPSAI--
AKPSAI--
AEPSAI--
APPSAI--
-APSAIN-
AFPSAI--
-APSAIM-
-APSAIK-
-APSAIE-
-APSAIG-
ACPSAI--
CKPSAI--
CLPSAI--
CVPSAI--
-CPSAIL-
CIPSAI--
CHPSAI--
CFPSAI--
CAPSAI--
CDPSAI--
CYPSAI--
CTPSAI--
CSPSAI--
CRPSAI--
-CPSAIM-
CGPSAI--
-CPSAIR-
CCPSAI--
-CPSAIY-

```
-CPSAIV-
-CPSAIT-
-CPSAIH-
-CPSAIF-
CNPSAI--
CMPSAI--
DRPSAI--
DVPSAI--
DGPSAI--
DTPSAI--
DAPSAI--
DYPSAI--
DIPSAI--
DKPSAI--
-DPSAIW-
-DPSAIM-
DMPSAI--
DFPSAI--
DEPSAI--
-DPSAIR-
DSPSAI--
DQPSAI--
-DPSAIS-
-DPSAIF-
DNPSAI--
-DPSAIL-
-DPSAII-
DHPSAI--
-EPSAIR-
ESPSAI--
-EPSAIS-
-EPSAIL-
EVPSAI--
EFPSAI--
ERPSAI--
-EPSAIH-
-EPSAIF-
-EPSAIW-
ENPSAI--
EKPSAI--
-EPSAIM-
-EPSAIQ-
EIPSAI--
EAPSAI--
EYPSAI--
EQPSAI--
-EPSAIV-
-EPSAIY-
EHPSAI--
EMPSAI--
```

ELPSAI--
EGPSAI--
-EPSAIT-
-EPSAIA-
-EPSAIK-
EEPSAI--
ETPSAI--
ECPSAI--
-FPSAII-
FRPSAI--
-FPSAIR-
FSPSAI--
FLPSAI--
FAPSAI--
FGPSAI--
FVPSAI--
FKPSAI--
-FPSAIM-
FWPSAI--
-FPSAIF-
FMPSAI--
-FPSAIL-
-FPSAIY-
FTPSAI--
-FPSAIV-
-FPSAIS-
-FPSAIK-
FQPSAI--
FYPSAI--
FHPSAI--
-FPSAIT-
-FPSAIW-
-FPSAIH-
FPPSAI--
FFPSAI--
-FPSAIN-
FIPSAI--
-FPSAIQ-
-FPSAIA-
-GPSAIR-
GIPSAI--
GGPSAI--
GVPSAI--
-GPSAIV-
GAPSAI--
GLPSAI--
GHPSAI--
GWPSAI--
GSPSAI--
-GPSAIY-

-GPSAIF-
-GPSAIM-
GYPSAI--
-GPSAII-
-GPSAIW-
-GPSAIA-
GFPSAI--
GQPSAI--
GMPSAI--
GDPSAI--
-GPSAIT-
-GPSAIN-
-GPSAIK-
GTPSAI--
-GPSAIH-
GCPSAI--
-GPSAIG-
GEPSAI--
-GPSAIE-
GNPSAI--
GKPSAI--
HRPSAI--
HGPSAI--
-HPSAIV-
HHPSAI--
HLPSAI--
HQPSAI--
HVPSAI--
-HPSAIH-
-HPSAIL-
HAPSAI--
HFPSAI--
-HPSAIR-
HTPSAI--
HWPSAI--
HKPSAI--
HYPSAI--
-HPSAIM-
-HPSAII-
-HPSAIF-
HMPSAI--
HEPSAI--
-HPSAIS-
HSPSAI--
-HPSAIY-
HIPSAI--
HNPSAI--
HPPSAI--
-HPSAIW-
-HPSAIK-

HCPSAI--
IKPSAI--
IMPSAI--
IHPSAI--
ITPSAI--
IRPSAI--
-IPSAIF-
IFPSAI--
IQPSAI--
INPSAI--
IVPSAI--
ISPSAI--
-IPSAIY-
-IPSAIR-
-IPSAII-
IYPSAI--
-IPSAIM-
-IPSAIS-
-IPSAIL-
-IPSAIA-
IDPSAI--
-IPSAIK-
IEPSAI--
IAPSAI--
IPPSAI--
-IPSAIV-
-IPSAIT-
IIPSAI--
-IPSAIN-
ILPSAI--
-IPSAIW-
-IPSAIG-
-IPSAIE-
-IPSAIH-
KSPSAI--
KRPSAI--
-KPSAIS-
KVPSAI--
-KPSAIF-
KGPSAI--
KTPSAI--
KMPSAI--
-KPSAIK-
KPPSAI--
KIPSAI--
KWPSAI--
-KPSAII-
-KPSAIT-
-KPSAIQ-
-KPSAIW-

-KPSAIL-
KYPSAI--
-KPSAIY-
-KPSAIM-
KKPSAI--
KEPSAI--
-KPSAIH-
-KPSAIV-
KAPSAI--
KNPSAI--
KFPSAI--
KQPSAI--
-KPSAIN-
-KPSAIG-
-KPSAIE-
KHPSAI--
LLPSAI--
LVPSAI--
LHPSAI--
LSPSAI--
LYPSAI--
LIPSAI--
-LPSAIR-
LKPSAI--
LEPSAI--
LAPSAI--
LWPSAI--
-LPSAIK-
-LPSAIW-
LQPSAI--
-LPSAII-
LNPSAI--
LTPSAI--
-LPSAIH-
LGPSAI--
-LPSAIS-
-LPSAIF-
-LPSAIY-
-LPSAIT-
-LPSAIM-
LDPSAI--
-LPSAIV-
LPPSAI--
LFPSAI--
LCPSAI--
LMPSAI--
-MPSAIY-
MLPSAI--
-MPSAIR-
MGPSAI--

-MPSAII-
MTPSAI--
MSPSAI--
MRPSAI--
MPPSAI--
MKPSAI--
MIPSAI--
MNPSAI--
-MPSAIW-
MAPSAI--
-MPSAIT-
MYPSAI--
-MPSAIF-
MDPSAI--
-MPSAIM-
-MPSAIQ-
MQPSAI--
-MPSAIV-
-MPSAIK-
MWPSAI--
-MPSAIH-
-MPSAIA-
MFPSAI--
MHPSAI--
MEPSAI--
MVPSAI--
-MPSAIN-
MMPSAI--
NGPSAI--
NRPSAI--
NWPSAI--
NVPSAI--
NLPSAI--
-NPSAIR-
NIPSAI--
-NPSAIV-
NYPSAI--
-NPSAIM-
NSPSAI--
NMPSAI--
NFPSAI--
NQPSAI--
-NPSAIS-
NHPSAI--
-NPSAIH-
-NPSAIL-
NNPSAI--
NTPSAI--
NEPSAI--
-NPSAIK-

NPPSAI--
-NPSAIY-
-NPSAIW-
-NPSAIT-
-NPSAII-
NDPSAI--
NAPSAI--
-NPSAIQ-
--PSAILS
--PSAIWR
--PSAIRT
--PSAIFS
--PSAIMR
--PSAILV
PLPSAI--
--PSAIRL
--PSAIKS
--PSAIRN
--PSAILR
--PSAILG
--PSAIFR
--PSAISV
--PSAIST
--PSAIRQ
--PSAILT
--PSAIRG
--PSAIIV
--PSAIWN
--PSAIRM
--PSAIVV
--PSAIYS
--PSAIVR
--PSAIRI
--PSAIKG
--PSAISS
--PSAISL
--PSAIHR
--PSAIAL
--PSAIYG
--PSAIVS
--PSAIQG
--PSAIVY
--PSAIRA
--PSAIWY
--PSAISA
--PSAILH
PRPSAI--
--PSAIYI
--PSAILA
--PSAIES

--PSAITH
--PSAIRW
--PSAIYM
--PSAIVH
--PSAIGL
PSPSAI--
--PSAIYY
--PSAITG
--PSAIQS
--PSAIMH
--PSAIRY
--PSAIKK
--PSAIHL
--PSAIYK
--PSAITR
--PSAIPL
--PSAILY
--PSAILN
--PSAIFT
--PSAIWQ
--PSAITM
--PSAIRK
--PSAIIS
--PSAIWV
--PSAISI
--PSAIMN
--PSAIIR
--PSAIIL
--PSAIFM
--PSAISH
--PSAIQW
--PSAIKR
--PSAIKI
--PSAIYN
--PSAITL
--PSAIRH
--PSAIRE
--PSAIRD
--PSAIML
--PSAIYF
--PSAIWI
--PSAIVK
--PSAISY
--PSAIRF
--PSAIQT
--PSAIFN
--PSAIFK
--PSAIAW
PIPSAI--
PGPSAI--

PTPSAI--
--PSAIVG
--PSAITS
--PSAIIN
--PSAIFY
--PSAIFE
--PSAIEG
--PSAISW
--PSAISG
--PSAILL
--PSAILD
--PSAIWT
--PSAIQY
--PSAIQK
--PSAIIW
--PSAIGF
--PSAIVM
--PSAIMT
--PSAIHG
--PSAIFF
PKPSAI--
--PSAIMD
--PSAIKY
--PSAIHK
--PSAIHF
--PSAIHA
--PSAIFV
--PSAIWG
--PSAITT
--PSAIPY
--PSAINT
--PSAINR
--PSAIMW
--PSAIMG
--PSAILW
--PSAILM
--PSAILK
--PSAIIT
--PSAIAV
--PSAIAS
--PSAIAF
PAPSAI--
--PSAIWF
--PSAIVF
--PSAITW
--PSAINL
--PSAIMS
--PSAIMM
--PSAIKT
--PSAIKQ

--PSAIGY
--PSAIAG
--PSAIYD
--PSAITK
--PSAISN
--PSAINM
--PSAIKN
--PSAIIG
--PSAIHD
--PSAIFA
--PSAIWW
--PSAIVW
--PSAIVI
--PSAIVA
--PSAISQ
--PSAISM
--PSAISD
--PSAINS
--PSAIKW
--PSAIIH
--PSAIHW
--PSAIHV
--PSAIGI
--PSAIAR
--PSAIAA
PWPSAI--
--PSAIWH
--PSAISF
--PSAIQH
--PSAINF
--PSAIMI
--PSAIHY
--PSAIEV
--PSAIAT
PVPSAI--
--PSAIYA
--PSAIWE
--PSAIVT
--PSAITV
--PSAITI
--PSAIQV
--PSAIMY
--PSAIMV
--PSAIMK
--PSAIKV
--PSAIIK
PYPSAI--
--PSAIWS
--PSAIWK
--PSAITN

--PSAITD
--PSAIKH
--PSAIKF
--PSAIHM
--PSAIHH
--PSAIFH
--PSAIEY
PQPSAI--
PNPSAI--
--PSAIYV
--PSAIVN
--PSAIQN
--PSAIQF
--PSAINI
--PSAIME
--PSAILE
--PSAIIQ
--PSAIHI
--PSAIGW
--PSAIGG
--PSAIGA
--PSAIAY
--PSAIAQ
--PSAIYT
--PSAIYH
--PSAIYE
--PSAITY
--PSAITA
--PSAISK
--PSAIPM
--PSAINQ
--PSAIMQ
--PSAIFG
--PSAIET
PEPSAI--
PDPSAI--
--PSAIWM
--PSAIWA
--PSAIVE
--PSAITE
--PSAINW
--PSAINV
--PSAINH
--PSAIMF
--PSAILQ
--PSAIIE
--PSAIHS
--PSAIHQ
--PSAIGT
--PSAIFI

--PSAIEA
--PSAITF
--PSAIPS
--PSAIPI
--PSAINY
--PSAIKE
--PSAIIM
--PSAIID
--PSAIFQ
--PSAIEF
--PSAICT
--PSAICA
--PSAIAK
--PSAIAE
PCPSAI--
--PSAIVQ
--PSAITQ
--PSAITC
--PSAISE
--PSAIRC
--PSAIQL
--PSAIQD
--PSAIQC
--PSAIPW
--PSAIPN
--PSAINN
--PSAIMC
--PSAIGV
--PSAIEW
--PSAIEQ
--PSAIDL
--PSAICL
--PSAICK
--PSAICG
-PPSAIW-
-PPSAIL-
PPPSAI--
PFPSAI--
QWPSAI--
QAPSAI--
-QPSAIR-
QVPSAI--
QRPSAI--
-QPSAIF-
-QPSAIV-
QSPSAI--
-QPSAIK-
-QPSAIL-
QHPSAI--
QFPSAI--

QLPSAI--
QKPSAI--
QNPSAI--
QGPSAI--
QEPSAI--
-QPSAIS-
QQPSAI--
-QPSAIM-
QTPSAI--
-QPSAIT-
QIPSAI--
-QPSAIW-
-QPSAIN-
QYPSAI--
-QPSAIY-
-QPSAIQ-
-QPSAIH-
-QPSAII-
QDPSAI--
QCPSAI--
QMPSAI--
RSPSAI--
-RPSAIL-
RLPSAI--
RVPSAI--
RIPSAI--
-RPSAIT-
RGPSAI--
-RPSAIM-
RYPSAI--
-RPSAIS-
-RPSAIF-
-RPSAIV-
RNPSAI--
RMPSAI--
RDPSAI--
-RPSAIQ-
REPSAI--
RWPSAI--
-RPSAIY-
RTPSAI--
RFPSAI--
-RPSAIH-
RQPSAI--
-RPSAII-
-RPSAIA-
RHPSAI--
-RPSAIW-
RCPSAI--
-RPSAIE-

−RPSAIK−
−RPSAIG−
RPPSAI−−
STPSAI−−
SLPSAI−−
−SPSAIR−
SGPSAI−−
SYPSAI−−
−SPSAIL−
−SPSAIV−
SVPSAI−−
SMPSAI−−
−SPSAIW−
−SPSAIK−
SAPSAI−−
−SPSAIH−
SKPSAI−−
SPPSAI−−
SHPSAI−−
SQPSAI−−
SIPSAI−−
−SPSAIT−
−SPSAIS−
−SPSAIN−
SFPSAI−−
SDPSAI−−
SWPSAI−−
−SPSAIA−
−SPSAIM−
SEPSAI−−
−SPSAIY−
−SPSAIG−
−SPSAIE−
−SPSAIC−
SCPSAI−−
TTPSAI−−
TRPSAI−−
−TPSAIY−
TLPSAI−−
−TPSAIR−
TFPSAI−−
−TPSAIS−
−TPSAIM−
TPPSAI−−
TYPSAI−−
TVPSAI−−
THPSAI−−
TGPSAI−−
TKPSAI−−
−TPSAIQ−

TSPSAI--
TMPSAI--
TEPSAI--
-TPSAIK-
-TPSAII-
-TPSAIN-
TNPSAI--
-TPSAIW-
-TPSAIV-
TWPSAI--
TQPSAI--
-TPSAIF-
-TPSAIH-
-TPSAIA-
-TPSAIT-
-TPSAIE-
TIPSAI--
TCPSAI--
VTPSAI--
VSPSAI--
VLPSAI--
VKPSAI--
VIPSAI--
VVPSAI--
-VPSAIR-
VGPSAI--
VRPSAI--
VPPSAI--
VNPSAI--
-VPSAIY-
VYPSAI--
-VPSAIH-
-VPSAIL-
VQPSAI--
-VPSAIS-
-VPSAIM-
-VPSAIT-
VEPSAI--
VDPSAI--
-VPSAII-
-VPSAIV-
-VPSAIQ-
VHPSAI--
-VPSAIA-
-VPSAIW-
VAPSAI--
-VPSAIN-
VFPSAI--
VWPSAI--
-VPSAIF-

VMPSAI--
-VPSAIG-
VCPSAI--
-VPSAID-
-VPSAIC-
WKPSAI--
WRPSAI--
-WPSAIR-
WSPSAI--
WVPSAI--
-WPSAIK-
WNPSAI--
WFPSAI--
WEPSAI--
WMPSAI--
WAPSAI--
WTPSAI--
-WPSAIS-
-WPSAIL-
WPPSAI--
WQPSAI--
-WPSAIF-
WYPSAI--
-WPSAIT-
WDPSAI--
WWPSAI--
-WPSAIV-
-WPSAIM-
-WPSAIQ-
WHPSAI--
-WPSAII-
-WPSAIH-
WIPSAI--
YVPSAI--
-YPSAIR-
YSPSAI--
-YPSAIM-
YRPSAI--
YLPSAI--
-YPSAIW-
YIPSAI--
YAPSAI--
YWPSAI--
-YPSAIA-
-YPSAIV-
-YPSAII-
-YPSAIY-
YGPSAI--
YMPSAI--
YQPSAI--

	-YPSAIS-		
	YEPSAI--		
	-YPSAIL-		
	-YPSAIT-		
	-YPSAIK-		
	YFPSAI--		
	-YPSAIH-		
	-YPSAIF-		
	YPPSAI--		
	YNPSAI--		
	-YPSAIN-		
	YKPSAI--		
	YHPSAI--		
	YDPSAI--		
	YYPSAI--		
	-YPSAIG-		
	-YPSAIQ-		
	-YPSAIP-		
	-YPSAIE-		
PKPM	628	578	45.93
	-APKPML-		
	AYPKPM--		
	-APKPMR-		
	-APKPMT-		
	-APKPMK-		
	AFPKPM--		
	ASPKPM--		
	-APKPMN-		
	AIPKPM--		
	AVPKPM--		
	-APKPMY-		
	-APKPMS-		
	-APKPMA-		
	AWPKPM--		
	ATPKPM--		
	APPKPM--		
	-APKPMV-		
	AGPKPM--		
	ALPKPM--		
	AHPKPM--		
	AEPKPM--		
	-APKPMI-		
	ARPKPM--		
	-APKPMW-		
	-APKPMM-		
	-CPKPMV-		
	CRPKPM--		
	-CPKPMT-		
	-CPKPMR-		
	CNPKPM--		

-CPKPMS-
-CPKPMF-
-CPKPMA-
CGPKPM--
CEPKPM--
-DPKPMF-
-DPKPMR-
-DPKPMK-
-DPKPMY-
DKPKPM--
DAPKPM--
DFPKPM--
DGPKPM--
DLPKPM--
DSPKPM--
-DPKPMS-
-DPKPMN-
-DPKPML-
DNPKPM--
DMPKPM--
-EPKPML-
-EPKPMR-
-EPKPMW-
EAPKPM--
-EPKPMI-
-EPKPMK-
ERPKPM--
ETPKPM--
-EPKPMM-
EYPKPM--
EWPKPM--
-EPKPMN-
-EPKPMT-
-EPKPMY-
-EPKPMS-
-EPKPMA-
EVPKPM--
-EPKPMV-
ENPKPM--
ELPKPM--
-FPKPML-
FSPKPM--
FLPKPM--
-FPKPMI-
FVPKPM--
-FPKPMT-
FWPKPM--
FQPKPM--
FPPKPM--
-FPKPMS-

-FPKPMQ-
FYPKPM--
-FPKPMY-
-FPKPMN-
-FPKPMH-
-FPKPMF-
-FPKPMR-
FHPKPM--
-FPKPMW-
-FPKPMC-
-FPKPMV-
FGPKPM--
-GPKPML-
-GPKPMS-
GRPKPM--
-GPKPMV-
GVPKPM--
-GPKPMW-
-GPKPMR-
-GPKPMK-
GMPKPM--
GSPKPM--
-GPKPMA-
GTPKPM--
GQPKPM--
-GPKPMN-
GNPKPM--
GLPKPM--
GKPKPM--
GFPKPM--
-GPKPMI-
GHPKPM--
GAPKPM--
HTPKPM--
-HPKPMK-
-HPKPMS-
HSPKPM--
-HPKPML-
-HPKPMW-
-HPKPMR-
HGPKPM--
HWPKPM--
-HPKPMT-
-HPKPMN-
-HPKPMA-
HCPKPM--
HNPKPM--
ILPKPM--
-IPKPMS-
IPPKPM--

-IPKPMY-
IIPKPM--
-IPKPMA-
-IPKPMM-
ISPKPM--
IYPKPM--
IVPKPM--
-IPKPMW-
-IPKPMR-
-IPKPMK-
-IPKPMQ-
-IPKPMH-
-IPKPMF-
-IPKPME-
INPKPM--
ITPKPM--
IQPKPM--
IEPKPM--
-KPKPMI-
-KPKPMK-
-KPKPMH-
KSPKPM--
KFPKPM--
-KPKPMS-
KYPKPM--
KVPKPM--
-KPKPMY-
-KPKPMM-
-KPKPMF-
KAPKPM--
-KPKPMT-
KLPKPM--
-KPKPMG-
KNPKPM--
KIPKPM--
KGPKPM--
KEPKPM--
-LPKPMR-
-LPKPMK-
-LPKPMW-
LWPKPM--
LNPKPM--
LGPKPM--
LVPKPM--
-LPKPMS-
LMPKPM--
LLPKPM--
LPPKPM--
-LPKPMI-
-LPKPML-

-LPKPMF-
LYPKPM--
LQPKPM--
-LPKPMV-
LRPKPM--
-LPKPMM-
LEPKPM--
LAPKPM--
LSPKPM--
-LPKPMN-
-LPKPMT-
-LPKPMQ-
-LPKPMY-
LIPKPM--
LCPKPM--
-MPKPMR-
MHPKPM--
-MPKPMS-
-MPKPMY-
MVPKPM--
MRPKPM--
-MPKPMK-
-MPKPMT-
MAPKPM--
-MPKPML-
-MPKPMV-
MEPKPM--
MSPKPM--
-MPKPMF-
MLPKPM--
NMPKPM--
NFPKPM--
NRPKPM--
-NPKPML-
NIPKPM--
NYPKPM--
-NPKPMT-
-NPKPMM-
NQPKPM--
-NPKPMK-
-NPKPMA-
NWPKPM--
NSPKPM--
NLPKPM--
NGPKPM--
NAPKPM--
-NPKPMY-
-NPKPMV-
-NPKPMF-
NNPKPM--

--PKPMVA
--PKPMVL
--PKPMLL
--PKPMLS
--PKPMIG
--PKPMYL
--PKPMVG
--PKPMLG
--PKPMYH
--PKPMLA
--PKPMFT
--PKPMFG
--PKPMGS
--PKPMYI
--PKPMTS
--PKPMSR
--PKPMRS
PTPKPM--
--PKPMTI
--PKPMSS
--PKPMLV
--PKPMYV
--PKPMMS
--PKPMMG
--PKPMVW
--PKPMSA
--PKPMMA
--PKPMWR
--PKPMSN
--PKPMRQ
--PKPMKV
--PKPMAS
--PKPMSW
--PKPMFS
--PKPMFL
--PKPMML
--PKPMLF
--PKPMIR
--PKPMHT
--PKPMYR
--PKPMYA
--PKPMWH
--PKPMTG
--PKPMRV
--PKPMRG
--PKPMFH
--PKPMRY
--PKPMNN
--PKPMLT
--PKPMRI

--PKPMLN
--PKPMIS
--PKPMFE
--PKPMVV
--PKPMVR
--PKPMTV
--PKPMDF
--PKPMSF
--PKPMMR
--PKPMHW
--PKPMFY
PWPKPM--
--PKPMYQ
--PKPMTN
--PKPMRF
--PKPMNT
--PKPMMI
--PKPMIY
--PKPMGH
--PKPMGG
--PKPMFW
--PKPMVN
--PKPMTR
--PKPMQI
--PKPMGN
--PKPMFM
--PKPMES
--PKPMAQ
PAPKPM--
--PKPMWK
--PKPMWA
--PKPMVD
--PKPMRN
--PKPMNR
--PKPMLK
--PKPMLE
--PKPMKW
--PKPMIN
--PKPMHV
--PKPMHK
--PKPMGK
--PKPMFK
--PKPMFI
--PKPMDY
--PKPMAT
PYPKPM--
PRPKPM--
PNPKPM--
--PKPMYY
--PKPMWW

--PKPMWT
--PKPMSG
--PKPMQT
--PKPMQS
--PKPMNA
--PKPMMF
--PKPMKY
--PKPMGT
--PKPMFN
--PKPMEY
--PKPMEW
--PKPMEF
--PKPMAA
PQPKPM--
--PKPMYS
--PKPMYK
--PKPMVI
--PKPMTQ
--PKPMTK
--PKPMTF
--PKPMRR
--PKPMQF
--PKPMNW
--PKPMNF
--PKPMMQ
--PKPMKQ
--PKPMKI
--PKPMHS
--PKPMGA
--PKPMFR
--PKPMEV
--PKPMYG
--PKPMYF
--PKPMVQ
--PKPMVK
--PKPMTM
--PKPMTA
--PKPMSK
--PKPMRK
--PKPMQN
--PKPMPQ
--PKPMMV
--PKPMMT
--PKPMMD
--PKPMLD
--PKPMKF
--PKPMIK
--PKPMHN
--PKPMEQ
--PKPMEG

--PKPMAR
--PKPMAK
PHPKPM--
PGPKPM--
PVPKPM--
PSPKPM--
PPPKPM--
-PPKPMW-
-PPKPMR-
--PKPMYC
--PKPMWN
--PKPMWG
--PKPMWF
--PKPMVM
--PKPMVH
--PKPMSP
--PKPMSH
--PKPMRE
--PKPMQY
--PKPMQA
--PKPMPS
--PKPMPR
--PKPMMW
--PKPMLQ
--PKPMKS
--PKPMKD
--PKPMIM
--PKPMHM
--PKPMHH
--PKPMHF
--PKPMHA
--PKPMGW
--PKPMGP
--PKPMEN
--PKPMEI
--PKPMDV
--PKPMDH
--PKPMCR
QHPKPM--
-QPKPMR-
-QPKPMI-
-QPKPMT-
QDPKPM--
-QPKPMV-
QNPKPM--
-QPKPMS-
-QPKPMA-
QYPKPM--
QAPKPM--
QRPKPM--

-QPKPMK-
-QPKPMC-
QVPKPM--
-QPKPMW-
QGPKPM--
QEPKPM--
RVPKPM--
-RPKPMT-
-RPKPML-
-RPKPMK-
-RPKPMR-
-RPKPMI-
-RPKPMA-
RNPKPM--
REPKPM--
RWPKPM--
-RPKPMS-
-RPKPMW-
RLPKPM--
RHPKPM--
-RPKPMF-
RSPKPM--
-RPKPMQ-
-RPKPMM-
RKPKPM--
RGPKPM--
RMPKPM--
-RPKPMH-
RAPKPM--
RTPKPM--
RPPKPM--
RIPKPM--
RFPKPM--
-RPKPMY-
-RPKPMV-
RDPKPM--
RRPKPM--
-RPKPMG-
-SPKPMK-
SMPKPM--
SLPKPM--
-SPKPMS-
-SPKPMT-
SWPKPM--
STPKPM--
-SPKPMV-
-SPKPMR-
SVPKPM--
-SPKPMI-
-SPKPML-

-SPKPMF-
SGPKPM--
-SPKPMQ-
-SPKPMA-
SRPKPM--
SPPKPM--
SHPKPM--
-SPKPMY-
SDPKPM--
SAPKPM--
SYPKPM--
-SPKPMP-
-SPKPMM-
-SPKPMH-
SEPKPM--
-TPKPMW-
-TPKPMF-
TSPKPM--
-TPKPMV-
-TPKPMK-
-TPKPMS-
-TPKPMN-
TTPKPM--
-TPKPMI-
THPKPM--
TWPKPM--
TRPKPM--
TQPKPM--
-TPKPMR-
TNPKPM--
TAPKPM--
TPPKPM--
-TPKPMT-
-TPKPMQ-
-TPKPMA-
TDPKPM--
TCPKPM--
-VPKPML-
-VPKPMV-
-VPKPMS-
-VPKPMR-
VTPKPM--
-VPKPMA-
VFPKPM--
-VPKPMY-
-VPKPMW-
-VPKPMN-
VLPKPM--
-VPKPMT-
-VPKPMK-

	-VPKPMF-		
	VEPKPM--		
	VAPKPM--		
	VVPKPM--		
	VQPKPM--		
	VYPKPM--		
	VWPKPM--		
	-VPKPMI-		
	-VPKPMH-		
	VKPKPM--		
	VGPKPM--		
	-WPKPMI-		
	WPPKPM--		
	-WPKPMF-		
	-WPKPMV-		
	-WPKPMR-		
	-WPKPMK-		
	WEPKPM--		
	WWPKPM--		
	-WPKPMT-		
	-WPKPMM-		
	-WPKPMA-		
	-WPKPMY-		
	-WPKPMS-		
	-WPKPMG-		
	WSPKPM--		
	-WPKPML-		
	WKPKPM--		
	WGPKPM--		
	WAPKPM--		
	-YPKPMK-		
	YAPKPM--		
	-YPKPMH-		
	YLPKPM--		
	YTPKPM--		
	-YPKPML-		
	-YPKPMI-		
	YRPKPM--		
	-YPKPMS-		
	YGPKPM--		
	YFPKPM--		
	-YPKPMG-		
	YHPKPM--		
	YVPKPM--		
	-YPKPMF-		
	-YPKPME-		
	YMPKPM--		
	YKPKPM--		
PQWM	1066	478	45.93
	--PQWMGK		

AGPQWM--
AQPQWM--
-APQWMS-
ARPQWM--
-APQWMY-
ATPQWM--
-APQWMR-
AYPQWM--
ALPQWM--
AKPQWM--
-APQWMK-
AEPQWM--
-APQWMH-
ANPQWM--
AIPQWM--
ADPQWM--
ASPQWM--
-APQWMV-
-APQWMQ-
AAPQWM--
-APQWMN-
-APQWMI-
-APQWMG-
AHPQWM--
CTPQWM--
CRPQWM--
-CPQWMR-
CDPQWM--
-DPQWMR-
DEPQWM--
DGPQWM--
DFPQWM--
DVPQWM--
DRPQWM--
-EPQWMV-
-EPQWMR-
EWPQWM--
ETPQWM--
EVPQWM--
EEPQWM--
ERPQWM--
ELPQWM--
EGPQWM--
EAPQWM--
FLPQWM--
FHPQWM--
-FPQWMR-
FIPQWM--
-FPQWMT-
FDPQWM--

-FPQWMS-
-FPQWMK-
-FPQWMA-
-FPQWMV-
FWPQWM--
FGPQWM--
FSPQWM--
FPPQWM--
FFPQWM--
FEPQWM--
FNPQWM--
-GPQWMS-
GTPQWM--
-GPQWMR-
GHPQWM--
-GPQWMG-
GYPQWM--
-GPQWML-
-GPQWMA-
-GPQWMT-
-GPQWMV-
GKPQWM--
GQPQWM--
GAPQWM--
GMPQWM--
GLPQWM--
GWPQWM--
GVPQWM--
GSPQWM--
-GPQWMY-
GRPQWM--
-GPQWMN-
-GPQWME-
GEPQWM--
HAPQWM--
-HPQWMS-
-HPQWMY-
-HPQWMV-
HLPQWM--
HKPQWM--
HFPQWM--
-HPQWMA-
-IPQWML-
-IPQWMT-
-IPQWMI-
IEPQWM--
IQPQWM--
-IPQWMR-
-IPQWME-
ISPQWM--

IRPQWM--
-IPQWMY-
-IPQWMS-
IVPQWM--
-IPQWMN-
-IPQWMH-
IYPQWM--
ITPQWM--
-IPQWMV-
-IPQWMD-
ILPQWM--
IIPQWM--
-KPQWMR-
-KPQWMY-
-KPQWMA-
KQPQWM--
-KPQWMS-
KHPQWM--
-KPQWMW-
-KPQWML-
KWPQWM--
KVPQWM--
KSPQWM--
-KPQWMN-
-KPQWMK-
KEPQWM--
KCPQWM--
LQPQWM--
-LPQWMR-
LRPQWM--
LVPQWM--
LTPQWM--
-LPQWMI-
LMPQWM--
LDPQWM--
-LPQWMA-
LKPQWM--
-LPQWMT-
-LPQWMS-
LPPQWM--
LSPQWM--
-LPQWMF-
LFPQWM--
LIPQWM--
LAPQWM--
LWPQWM--
-LPQWML-
-LPQWMH-
-LPQWME-
LNPQWM--

LLPQWM--
LHPQWM--
LGPQWM--
-MPQWMV-
MGPQWM--
-MPQWMR-
-MPQWMK-
-MPQWMS-
MQPQWM--
-MPQWMI-
-MPQWMG-
MLPQWM--
MAPQWM--
-MPQWMQ-
-MPQWMH-
-MPQWMM-
MKPQWM--
MWPQWM--
NSPQWM--
NVPQWM--
NRPQWM--
NAPQWM--
NWPQWM--
-NPQWMK-
-NPQWMY-
NGPQWM--
NHPQWM--
-NPQWMR-
-NPQWMM-
-NPQWMA-
-NPQWMV-
-NPQWMQ-
NQPQWM--
-NPQWMS-
-NPQWMH-
NPPQWM--
NDPQWM--
NCPQWM--
--PQWMRV
--PQWMFN
--PQWMSG
--PQWMRS
--PQWMGA
PAPQWM--
--PQWMVS
--PQWMRH
--PQWMRW
--PQWMRG
--PQWMTG
--PQWMSI

--PQWMAG
--PQWMRN
--PQWMQR
--PQWMSM
--PQWMLH
--PQWMIA
--PQWMHG
--PQWMTH
--PQWMRT
--PQWMMT
--PQWMMQ
--PQWMLT
--PQWMAS
--PQWMSY
--PQWMSA
--PQWMHS
--PQWMAA
--PQWMVA
PIPQWM--
--PQWMTW
--PQWMSS
--PQWMQG
--PQWMNS
--PQWMAN
--PQWMAF
--PQWMTS
--PQWMSW
--PQWMQT
--PQWMMK
--PQWMKV
--PQWMIT
--PQWMSH
--PQWMKA
--PQWMGV
--PQWMWA
--PQWMTV
--PQWMRY
--PQWMRA
--PQWMQS
--PQWMMY
--PQWMMR
--PQWMME
--PQWMLW
--PQWMKN
--PQWMHY
--PQWMHT
--PQWMYA
--PQWMWT
--PQWMWS
--PQWMVG

--PQWMVF
--PQWMTT
--PQWMTA
--PQWMSL
--PQWMRM
--PQWMQV
--PQWMNV
--PQWMMS
--PQWMLI
--PQWMKH
--PQWMWI
--PQWMVR
--PQWMVM
--PQWMTK
--PQWMSQ
--PQWMSN
--PQWMRL
--PQWMLK
--PQWMLG
--PQWMKL
--PQWMKG
--PQWMIG
--PQWMGY
--PQWMGS
--PQWMGN
--PQWMGM
--PQWMGL
--PQWMGI
--PQWMAI
--PQWMYM
--PQWMYH
--PQWMRQ
--PQWMNT
--PQWMLS
--PQWMKF
--PQWMKD
--PQWMHA
--PQWMYN
--PQWMVQ
--PQWMTR
--PQWMTN
--PQWMTM
--PQWMSD
--PQWMRD
--PQWMNH
--PQWMNA
--PQWMMI
--PQWMKT
--PQWMGF
--PQWMFG

--PQWMEW
--PQWMAQ
--PQWMAL
--PQWMAH
--PQWMYR
--PQWMYI
--PQWMVI
--PQWMTI
--PQWMTD
--PQWMSV
--PQWMQY
--PQWMQL
--PQWMPF
--PQWMNW
--PQWMLM
--PQWMLD
--PQWMKW
--PQWMKI
--PQWMIK
--PQWMII
--PQWMGW
--PQWMET
--PQWMEN
--PQWMEF
--PQWMCW
--PQWMAR
--PQWMAD
PQPQWM--
PNPQWM--
PGPQWM--
QSPQWM--
-QPQWMR-
-QPQWMF-
QWPQWM--
QRPQWM--
-QPQWMT-
-QPQWMQ-
-QPQWMG-
QIPQWM--
-QPQWMS-
-QPQWMA-
QGPQWM--
QAPQWM--
-QPQWMH-
-QPQWMW-
-QPQWMM-
QHPQWM--
RSPQWM--
RQPQWM--
RPPQWM--

RAPQWM--
RRPQWM--
-RPQWMQ-
RNPQWM--
-RPQWMT-
-RPQWMS-
-RPQWMY-
-RPQWMW-
-RPQWMG-
-RPQWMA-
RWPQWM--
REPQWM--
RVPQWM--
RDPQWM--
RKPQWM--
RGPQWM--
RFPQWM--
-RPQWMH-
-RPQWMF-
RLPQWM--
RTPQWM--
-RPQWMR-
-RPQWME-
SYPQWM--
SSPQWM--
-SPQWMG-
SVPQWM--
-SPQWMT-
-SPQWMR-
SLPQWM--
SHPQWM--
SWPQWM--
STPQWM--
-SPQWMY-
SMPQWM--
-SPQWMN-
SPPQWM--
SRPQWM--
-SPQWMK-
-SPQWMH-
SKPQWM--
SIPQWM--
SDPQWM--
-SPQWMQ-
-SPQWMM-
-SPQWMF-
SGPQWM--
-SPQWMV-
-SPQWMS-
-SPQWMI-

-SPQWME-
-TPQWMA-
-TPQWMR-
TPPQWM--
-TPQWMY-
-TPQWMV-
-TPQWMF-
TVPQWM--
TNPQWM--
TMPQWM--
TFPQWM--
-TPQWMN-
-TPQWML-
TKPQWM--
TGPQWM--
-TPQWMW-
-TPQWMS-
TWPQWM--
TQPQWM--
-TPQWMH-
THPQWM--
TAPQWM--
-VPQWMR-
VKPQWM--
-VPQWMS-
VRPQWM--
-VPQWMV-
-VPQWML-
-VPQWMT-
-VPQWMM-
VSPQWM--
VQPQWM--
VLPQWM--
VGPQWM--
VWPQWM--
-VPQWMK-
-VPQWMA-
VMPQWM--
VAPQWM--
-VPQWMN-
-VPQWME-
VPPQWM--
VIPQWM--
VDPQWM--
VVPQWM--
VTPQWM--
-VPQWMI-
VNPQWM--
-VPQWMW-
-VPQWMQ-

```
          -VPQWMG-
          WLPQWM--
          WKPQWM--
          -WPQWMT-
          WRPQWM--
          WYPQWM--
          WAPQWM--
          WPPQWM--
          -WPQWMV-
          -WPQWML-
          WGPQWM--
          -WPQWMS-
          WFPQWM--
          YVPQWM--
          YRPQWM--
          YLPQWM--
          -YPQWMS-
          -YPQWMI-
          -YPQWMK-
          -YPQWML-
          YTPQWM--
          -YPQWMW-
          -YPQWMV-
          -YPQWMM-
          -YPQWMF-
          YPPQWM--
          YEPQWM--
PKSL       464       633     45.92
          -APKSLP-
          -HPKSLG-
          -NPKSLG-
          -NPKSLS-
          --PKSLTP
          --PKSLSP
          --PKSLPY
          --PKSLPL
          -QPKSLD-
          -VPKSLD-
          -YPKSLG-
          -APKSLL-
          -APKSLW-
          AVPKSL--
          ATPKSL--
          -APKSLY-
          AIPKSL--
          ASPKSL--
          AGPKSL--
          -APKSLS-
          -APKSLV-
          AAPKSL--
```

-APKSLT-
-APKSLA-
AHPKSL--
AFPKSL--
-APKSLQ-
-APKSLF-
APPKSL--
AEPKSL--
AQPKSL--
AKPKSL--
-APKSLG-
ADPKSL--
-APKSLN-
ANPKSL--
-APKSLE-
ACPKSL--
-APKSLC-
-CPKSLR-
CWPKSL--
CSPKSL--
CYPKSL--
CQPKSL--
CEPKSL--
CNPKSL--
-CPKSLY-
-CPKSLL-
CRPKSL--
-CPKSLT-
CTPKSL--
CHPKSL--
CVPKSL--
-CPKSLK-
CMPKSL--
-CPKSLQ-
-CPKSLM-
-CPKSLS-
CGPKSL--
CPPKSL--
-CPKSLW-
CLPKSL--
CKPKSL--
CFPKSL--
-CPKSLA-
CIPKSL--
-CPKSLN-
DWPKSL--
DYPKSL--
-DPKSLL-
DRPKSL--
DKPKSL--

DAPKSL--
-DPKSLS-
-DPKSLY-
-DPKSLI-
-DPKSLT-
-DPKSLM-
-DPKSLK-
DGPKSL--
DLPKSL--
-DPKSLH-
-DPKSLV-
-DPKSLF-
DEPKSL--
DQPKSL--
DNPKSL--
DDPKSL--
-DPKSLQ-
-DPKSLE-
-EPKSLL-
ESPKSL--
-EPKSLK-
ERPKSL--
-EPKSLR-
ELPKSL--
-EPKSLT-
-EPKSLM-
EVPKSL--
EAPKSL--
EGPKSL--
-EPKSLI-
-EPKSLH-
EYPKSL--
ETPKSL--
-EPKSLW-
-EPKSLQ-
EFPKSL--
EEPKSL--
-EPKSLS-
EQPKSL--
-EPKSLY-
-EPKSLF-
EWPKSL--
-EPKSLV-
ENPKSL--
EKPKSL--
EIPKSL--
-EPKSLN-
-EPKSLA-
EMPKSL--
-EPKSLE-

EDPKSL--
-EPKSLG-
EPPKSL--
ECPKSL--
FTPKSL--
FNPKSL--
FHPKSL--
FEPKSL--
FLPKSL--
FAPKSL--
FIPKSL--
FWPKSL--
FKPKSL--
-FPKSLM-
-FPKSLN-
FQPKSL--
FPPKSL--
-FPKSLE-
-FPKSLP-
GLPKSL--
GAPKSL--
GTPKSL--
GVPKSL--
GSPKSL--
GWPKSL--
-GPKSLF-
-GPKSLW-
-GPKSLH-
-GPKSLV-
-GPKSLT-
GGPKSL--
GKPKSL--
-GPKSLS-
GHPKSL--
-GPKSLI-
GPPKSL--
GQPKSL--
GNPKSL--
GEPKSL--
-GPKSLG-
GDPKSL--
-GPKSLN-
-GPKSLE-
-GPKSLQ-
GCPKSL--
-GPKSLC-
HGPKSL--
-HPKSLM-
HVPKSL--
-HPKSLI-

-HPKSLV-
-HPKSLL-
-HPKSLS-
HMPKSL--
HKPKSL--
HPPKSL--
-HPKSLY-
HHPKSL--
HWPKSL--
-HPKSLN-
HNPKSL--
-HPKSLQ-
HQPKSL--
-HPKSLH-
-HPKSLK-
-HPKSLF-
HAPKSL--
HDPKSL--
HCPKSL--
-IPKSLY-
IIPKSL--
-IPKSLL-
-IPKSLT-
IGPKSL--
-IPKSLV-
ISPKSL--
ITPKSL--
-IPKSLQ-
IVPKSL--
IEPKSL--
IHPKSL--
-IPKSLA-
-IPKSLN-
IPPKSL--
-IPKSLI-
IQPKSL--
-IPKSLM-
IAPKSL--
ICPKSL--
-IPKSLG-
IDPKSL--
IKPKSL--
-IPKSLE-
-KPKSLY-
KGPKSL--
-KPKSLL-
KIPKSL--
KPPKSL--
-KPKSLM-
-KPKSLV-

-KPKSLS-
KEPKSL--
KHPKSL--
-KPKSLH-
KDPKSL--
-KPKSLI-
-KPKSLN-
-KPKSLA-
-KPKSLQ-
KNPKSL--
KCPKSL--
-KPKSLF-
-KPKSLE-
-KPKSLC-
-KPKSLG-
LGPKSL--
-LPKSLL-
LQPKSL--
LIPKSL--
-LPKSLI-
-LPKSLH-
-LPKSLV-
-LPKSLF-
LDPKSL--
-LPKSLQ-
-LPKSLE-
-LPKSLC-
MNPKSL--
MVPKSL--
MSPKSL--
-MPKSLS-
MTPKSL--
-MPKSLH-
-MPKSLV-
-MPKSLY-
MAPKSL--
MGPKSL--
MMPKSL--
MEPKSL--
MPPKSL--
-MPKSLA-
MHPKSL--
-MPKSLM-
-MPKSLN-
-MPKSLQ-
-MPKSLI-
MDPKSL--
-MPKSLW-
-MPKSLF-
-MPKSLE-

-MPKSLC-
MCPKSL--
NSPKSL--
NTPKSL--
NPPKSL--
NGPKSL--
-NPKSLV-
-NPKSLI-
NEPKSL--
-NPKSLL-
NQPKSL--
-NPKSLF-
NDPKSL--
NNPKSL--
-NPKSLH-
-NPKSLW-
-NPKSLA-
-NPKSLQ-
-NPKSLM-
NCPKSL--
-NPKSLY-
-NPKSLN-
-NPKSLE-
-NPKSLC-
--PKSLFG
--PKSLYF
--PKSLSV
--PKSLYA
--PKSLLT
--PKSLSA
PGPKSL--
--PKSLVT
--PKSLTL
PSPKSL--
--PKSLGA
--PKSLLF
--PKSLIT
--PKSLWN
--PKSLFV
--PKSLSQ
--PKSLHG
--PKSLFT
--PKSLWG
--PKSLTI
--PKSLYT
--PKSLSI
--PKSLTN
--PKSLSW
--PKSLES
--PKSLIG

--PKSLLV
PIPKSL--
--PKSLVW
--PKSLMQ
--PKSLHS
PEPKSL--
--PKSLHY
--PKSLGS
--PKSLYM
--PKSLIA
--PKSLFA
PTPKSL--
--PKSLKV
PHPKSL--
--PKSLHL
--PKSLTV
--PKSLNS
--PKSLVM
--PKSLFF
--PKSLNT
--PKSLKT
--PKSLAF
--PKSLAA
--PKSLTQ
--PKSLNV
--PKSLMF
--PKSLWM
--PKSLSN
--PKSLAV
PFPKSL--
--PKSLVQ
--PKSLFQ
--PKSLNW
--PKSLNF
--PKSLEV
PMPKSL--
--PKSLSM
--PKSLHT
--PKSLYN
--PKSLYG
--PKSLWK
--PKSLNA
--PKSLIM
--PKSLHH
--PKSLGG
--PKSLVV
--PKSLRE
--PKSLQV
--PKSLYW
--PKSLTF

--PKSLGL
--PKSLAT
--PKSLAN
--PKSLQT
--PKSLMT
--PKSLMA
--PKSLKQ
--PKSLEY
PAPKSL--
--PKSLNL
--PKSLVD
--PKSLII
--PKSLEG
PKPKSL--
--PKSLWD
--PKSLQW
--PKSLYQ
--PKSLYK
--PKSLYE
--PKSLFY
--PKSLWV
--PKSLHN
--PKSLHI
--PKSLAM
--PKSLYY
--PKSLQG
--PKSLIW
PNPKSL--
--PKSLYD
--PKSLWF
--PKSLSY
--PKSLQR
--PKSLQH
--PKSLME
--PKSLQL
--PKSLIH
--PKSLYH
--PKSLMY
--PKSLFK
--PKSLYI
--PKSLWE
--PKSLNM
--PKSLMD
--PKSLHM
--PKSLGM
--PKSLWI
--PKSLNI
--PKSLHQ
--PKSLHF
--PKSLEW

```
--PKSLTD
--PKSLQN
--PKSLNQ
--PKSLAK
PQPKSL--
--PKSLWW
--PKSLWH
--PKSLQF
--PKSLNH
--PKSLEI
--PKSLAH
--PKSLVE
--PKSLNN
--PKSLEH
--PKSLSE
--PKSLNK
--PKSLMV
--PKSLHV
--PKSLGI
--PKSLET
--PKSLER
--PKSLTE
--PKSLQM
--PKSLMH
--PKSLHE
--PKSLFM
PYPKSL--
--PKSLEK
--PKSLCY
--PKSLQD
--PKSLHK
--PKSLEM
--PKSLAE
--PKSLND
--PKSLMM
--PKSLIE
--PKSLHD
--PKSLEA
--PKSLCT
--PKSLCK
PDPKSL--
PCPKSL--
--PKSLIK
--PKSLCR
-PPKSLR-
--PKSLRC
--PKSLNE
--PKSLLC
--PKSLFD
--PKSLDM
```

--PKSLDG
--PKSLCV
--PKSLCI
--PKSLCF
--PKSLCA
--PKSLAD
PPPKSL--
-PPKSLV-
--PKSLTC
--PKSLSC
--PKSLQP
--PKSLQE
--PKSLEN
--PKSLEC
--PKSLDW
--PKSLCQ
--PKSLCN
--PKSLCH
QVPKSL--
-QPKSLL-
-QPKSLV-
QYPKSL--
-QPKSLS-
QFPKSL--
-QPKSLM-
QTPKSL--
QGPKSL--
-QPKSLN-
-QPKSLQ-
-QPKSLF-
QIPKSL--
-QPKSLA-
QKPKSL--
QAPKSL--
QHPKSL--
-QPKSLW-
QEPKSL--
-QPKSLH-
QDPKSL--
QQPKSL--
QPPKSL--
-QPKSLE-
-QPKSLK-
QCPKSL--
-QPKSLP-
RVPKSL--
-RPKSLY-
-RPKSLI-
REPKSL--
RPPKSL--

RKPKSL--
-RPKSLW-
RCPKSL--
-RPKSLE-
RDPKSL--
-RPKSLC-
SIPKSL--
SSPKSL--
SKPKSL--
-SPKSLH-
-SPKSLW-
SHPKSL--
-SPKSLI-
SAPKSL--
SNPKSL--
-SPKSLV-
-SPKSLF-
SPPKSL--
-SPKSLY-
-SPKSLM-
-SPKSLA-
SQPKSL--
-SPKSLN-
SEPKSL--
-SPKSLQ-
SDPKSL--
-SPKSLE-
SCPKSL--
-SPKSLC-
TVPKSL--
TTPKSL--
TIPKSL--
TLPKSL--
TAPKSL--
-TPKSLL-
TSPKSL--
-TPKSLK-
-TPKSLI-
TGPKSL--
TYPKSL--
TPPKSL--
-TPKSLS-
TEPKSL--
TNPKSL--
TDPKSL--
TQPKSL--
-TPKSLA-
-TPKSLN-
-TPKSLW-
-TPKSLE-

-TPKSLG-
TCPKSL--
-TPKSLM-
-VPKSLL-
-VPKSLT-
VLPKSL--
VGPKSL--
-VPKSLY-
VMPKSL--
-VPKSLA-
-VPKSLV-
-VPKSLQ-
-VPKSLH-
-VPKSLI-
-VPKSLW-
-VPKSLM-
VKPKSL--
-VPKSLN-
-VPKSLF-
VPPKSL--
VCPKSL--
-VPKSLE-
VDPKSL--
-VPKSLG-
VEPKSL--
WLPKSL--
WAPKSL--
WNPKSL--
WHPKSL--
WSPKSL--
WDPKSL--
-WPKSLF-
-WPKSLN-
WWPKSL--
WTPKSL--
WEPKSL--
-WPKSLH-
WPPKSL--
-WPKSLQ-
-WPKSLE-
WCPKSL--
-WPKSLC-
-YPKSLV-
YVPKSL--
YIPKSL--
YTPKSL--
-YPKSLL-
YKPKSL--
YMPKSL--
YDPKSL--

	-YPKSLI-		
	-YPKSLF-		
	YAPKSL--		
	YEPKSL--		
	YQPKSL--		
	-YPKSLH-		
	-YPKSLN-		
	-YPKSLA-		
	-YPKSLW-		
	-YPKSLQ-		
	-YPKSLE-		
PWQL	331	682	45.92
	--PWQLVE		
	--PWQLGN		
	--PWQLGL		
	AIPWQL--		
	ASPWQL--		
	ALPWQL--		
	-APWQLN-		
	AMPWQL--		
	AWPWQL--		
	-APWQLT-		
	-APWQLA-		
	AGPWQL--		
	-APWQLK-		
	AAPWQL--		
	AVPWQL--		
	AKPWQL--		
	-APWQLS-		
	-APWQLW-		
	-APWQLV-		
	-APWQLI-		
	AFPWQL--		
	-APWQLG-		
	-APWQLR-		
	ANPWQL--		
	AYPWQL--		
	-APWQLE-		
	AEPWQL--		
	APPWQL--		
	-APWQLM-		
	AHPWQL--		
	AQPWQL--		
	CGPWQL--		
	CCPWQL--		
	CRPWQL--		
	CVPWQL--		
	CSPWQL--		
	-CPWQLV-		
	CTPWQL--		

-CPWQLK-
CMPWQL--
CKPWQL--
-DPWQLV-
-DPWQLH-
-DPWQLT-
DGPWQL--
-DPWQLQ-
DYPWQL--
-DPWQLM-
DEPWQL--
DTPWQL--
-DPWQLN-
-DPWQLL-
-DPWQLR-
DNPWQL--
DLPWQL--
DIPWQL--
DHPWQL--
DFPWQL--
DWPWQL--
DRPWQL--
DDPWQL--
-EPWQLR-
EVPWQL--
-EPWQLL-
-EPWQLK-
ESPWQL--
-EPWQLT-
ERPWQL--
EIPWQL--
-EPWQLV-
-EPWQLS-
-EPWQLQ-
-EPWQLM-
-EPWQLG-
-EPWQLA-
EAPWQL--
EWPWQL--
ENPWQL--
ETPWQL--
EQPWQL--
-EPWQLH-
-EPWQLF-
EMPWQL--
-FPWQLR-
FKPWQL--
FAPWQL--
FDPWQL--
FLPWQL--

-FPWQLT-
FQPWQL--
FRPWQL--
-FPWQLI-
FSPWQL--
FYPWQL--
FWPWQL--
-FPWQLK-
FGPWQL--
-FPWQLS-
-FPWQLN-
-FPWQLH-
FNPWQL--
FPPWQL--
-FPWQLV-
FHPWQL--
FVPWQL--
-FPWQLG-
FEPWQL--
GVPWQL--
GRPWQL--
-GPWQLS-
GYPWQL--
GIPWQL--
-GPWQLR-
-GPWQLH-
GSPWQL--
-GPWQLQ-
GGPWQL--
GKPWQL--
-GPWQLK-
GHPWQL--
GMPWQL--
GAPWQL--
GTPWQL--
GLPWQL--
-GPWQLT-
-GPWQLG-
-GPWQLA-
GEPWQL--
-GPWQLM-
-GPWQLI-
GNPWQL--
-GPWQLV-
GQPWQL--
-GPWQLN-
GFPWQL--
GCPWQL--
GPPWQL--
GDPWQL--

−GPWQLW−
HLPWQL−−
HRPWQL−−
HFPWQL−−
HTPWQL−−
−HPWQLS−
−HPWQLR−
HVPWQL−−
HPPWQL−−
HKPWQL−−
HMPWQL−−
HQPWQL−−
HDPWQL−−
−HPWQLT−
−HPWQLE−
HEPWQL−−
HWPWQL−−
−HPWQLK−
HSPWQL−−
−HPWQLM−
−HPWQLF−
−HPWQLA−
HIPWQL−−
HYPWQL−−
HNPWQL−−
IRPWQL−−
IKPWQL−−
IVPWQL−−
INPWQL−−
ISPWQL−−
−IPWQLM−
IAPWQL−−
IGPWQL−−
IYPWQL−−
−IPWQLT−
−IPWQLR−
IIPWQL−−
IMPWQL−−
IPPWQL−−
ILPWQL−−
−IPWQLS−
−IPWQLI−
IQPWQL−−
−IPWQLV−
−IPWQLA−
IHPWQL−−
−IPWQLG−
−IPWQLN−
IDPWQL−−
KSPWQL−−

-KPWQLK-
KRPWQL--
KVPWQL--
KQPWQL--
-KPWQLV-
KAPWQL--
-KPWQLR-
KKPWQL--
-KPWQLS-
KLPWQL--
-KPWQLF-
KYPWQL--
-KPWQLQ-
KNPWQL--
KMPWQL--
KFPWQL--
KEPWQL--
KDPWQL--
KIPWQL--
KTPWQL--
-KPWQLN-
-KPWQLI-
-KPWQLH-
-KPWQLW-
-KPWQLA-
KCPWQL--
KWPWQL--
-KPWQLT-
-KPWQLL-
LTPWQL--
LGPWQL--
LPPWQL--
LAPWQL--
LNPWQL--
LSPWQL--
-LPWQLV-
LRPWQL--
LEPWQL--
-LPWQLS-
LYPWQL--
LWPWQL--
LMPWQL--
LKPWQL--
LLPWQL--
-LPWQLI-
-LPWQLK-
LVPWQL--
-LPWQLH-
LHPWQL--
LQPWQL--

LIPWQL--
-LPWQLW-
LFPWQL--
-LPWQLQ-
-LPWQLN-
MRPWQL--
-MPWQLT-
-MPWQLR-
-MPWQLN-
-MPWQLS-
MVPWQL--
MLPWQL--
MTPWQL--
-MPWQLM-
-MPWQLI-
MKPWQL--
MAPWQL--
MHPWQL--
MEPWQL--
MWPWQL--
MSPWQL--
-MPWQLV-
MGPWQL--
MFPWQL--
-MPWQLA-
MPPWQL--
MIPWQL--
MQPWQL--
-MPWQLH-
-MPWQLE-
MNPWQL--
-MPWQLQ-
NRPWQL--
NNPWQL--
NEPWQL--
-NPWQLK-
NVPWQL--
-NPWQLV-
-NPWQLN-
-NPWQLL-
NPPWQL--
NGPWQL--
NFPWQL--
NKPWQL--
NTPWQL--
NSPWQL--
NQPWQL--
-NPWQLW-
-NPWQLH-
NLPWQL--

-NPWQLI-
-NPWQLF-
NIPWQL--
NYPWQL--
-NPWQLT-
-NPWQLQ-
NHPWQL--
-NPWQLA-
NMPWQL--
-NPWQLP-
-NPWQLG-
NAPWQL--
--PWQLSS
--PWQLRM
--PWQLVG
--PWQLRT
--PWQLVN
--PWQLSG
--PWQLRR
--PWQLMH
--PWQLRH
--PWQLRL
--PWQLSI
--PWQLIK
--PWQLRG
PYPWQL--
--PWQLWN
--PWQLMS
--PWQLTN
--PWQLRS
--PWQLMA
--PWQLGS
--PWQLVR
--PWQLES
--PWQLLK
--PWQLLG
--PWQLVH
--PWQLRY
--PWQLRA
--PWQLRK
--PWQLHS
--PWQLVS
--PWQLTS
--PWQLTR
--PWQLNM
--PWQLRN
--PWQLNT
--PWQLVA
--PWQLTT
--PWQLIS

--PWQLVT
--PWQLHT
--PWQLAS
--PWQLAG
--PWQLMQ
--PWQLKV
--PWQLKQ
--PWQLKH
PQPWQL--
--PWQLVI
--PWQLRQ
--PWQLID
--PWQLHW
--PWQLSH
--PWQLMR
--PWQLIM
--PWQLIG
--PWQLHR
--PWQLTH
--PWQLSR
--PWQLKS
--PWQLST
--PWQLRD
--PWQLGG
--PWQLFN
--PWQLYS
--PWQLNQ
--PWQLLH
--PWQLKG
--PWQLKD
--PWQLHE
--PWQLGV
--PWQLEA
PVPWQL--
--PWQLWT
--PWQLNS
--PWQLNK
--PWQLNH
--PWQLKR
--PWQLIA
--PWQLYG
--PWQLVD
--PWQLSN
--PWQLNN
--PWQLNI
--PWQLLR
--PWQLKY
--PWQLKL
--PWQLHV
PSPWQL--

PFPWQL--
--PWQLVY
--PWQLVK
--PWQLTG
--PWQLSM
--PWQLSA
--PWQLLD
--PWQLKI
--PWQLKF
--PWQLGY
--PWQLGA
PNPWQL--
--PWQLWR
--PWQLRW
--PWQLQG
--PWQLKT
--PWQLKK
--PWQLAT
--PWQLAA
PGPWQL--
--PWQLYQ
--PWQLWK
--PWQLNL
--PWQLGT
--PWQLEW
--PWQLEG
--PWQLYK
--PWQLWV
--PWQLTV
--PWQLSL
--PWQLSD
--PWQLQS
--PWQLQI
--PWQLNR
--PWQLMN
--PWQLLW
--PWQLIN
--PWQLHI
--PWQLAM
PTPWQL--
PEPWQL--
PAPWQL--
--PWQLYA
--PWQLTY
--PWQLTK
--PWQLTE
--PWQLSV
--PWQLSQ
--PWQLRE
--PWQLQM

--PWQLQE
--PWQLNE
--PWQLMG
--PWQLKW
--PWQLIQ
--PWQLIE
--PWQLHQ
--PWQLHK
--PWQLHF
--PWQLEV
--PWQLAW
--PWQLAQ
--PWQLAN
--PWQLAD
--PWQLYT
--PWQLYL
--PWQLWH
--PWQLVQ
--PWQLTM
--PWQLTL
--PWQLTI
--PWQLTA
--PWQLQT
--PWQLQL
--PWQLNG
--PWQLME
--PWQLMD
--PWQLLQ
--PWQLKM
--PWQLKE
--PWQLIW
--PWQLHM
--PWQLHH
--PWQLGW
--PWQLGR
--PWQLFA
--PWQLEN
-PPWQLS-
--PWQLYR
--PWQLYF
--PWQLWF
--PWQLVM
--PWQLTD
--PWQLSE
--PWQLRP
--PWQLQK
--PWQLQD
--PWQLNF
--PWQLMT
--PWQLMK

--PWQLIT
--PWQLHL
--PWQLEH
--PWQLED
--PWQLAE
-PPWQLQ-
PKPWQL--
PHPWQL--
QRPWQL--
-QPWQLR-
-QPWQLS-
-QPWQLI-
QGPWQL--
-QPWQLK-
QPPWQL--
QMPWQL--
-QPWQLA-
QLPWQL--
QSPWQL--
-QPWQLW-
-QPWQLV-
QAPWQL--
QWPWQL--
-QPWQLN-
-QPWQLH-
QHPWQL--
QQPWQL--
QEPWQL--
QVPWQL--
-QPWQLM-
QTPWQL--
-QPWQLT-
QNPWQL--
QIPWQL--
QFPWQL--
-RPWQLR-
-RPWQLV-
RLPWQL--
-RPWQLN-
-RPWQLS-
-RPWQLT-
RSPWQL--
RVPWQL--
RIPWQL--
-RPWQLM-
-RPWQLK-
RNPWQL--
-RPWQLQ-
RGPWQL--
RTPWQL--

RDPWQL--
RAPWQL--
-RPWQLA-
RWPWQL--
RQPWQL--
RPPWQL--
RYPWQL--
-RPWQLI-
REPWQL--
-RPWQLH-
RHPWQL--
RFPWQL--
-RPWQLW-
RCPWQL--
-SPWQLR-
-SPWQLS-
SSPWQL--
-SPWQLK-
SHPWQL--
SYPWQL--
SVPWQL--
STPWQL--
SEPWQL--
SDPWQL--
-SPWQLL-
SGPWQL--
-SPWQLV-
SFPWQL--
-SPWQLT-
-SPWQLH-
SPPWQL--
SAPWQL--
-SPWQLQ-
SNPWQL--
-SPWQLN-
-SPWQLW-
-SPWQLA-
-SPWQLG-
SIPWQL--
SKPWQL--
SWPWQL--
SQPWQL--
-SPWQLM-
-SPWQLI-
SMPWQL--
SCPWQL--
TGPWQL--
TRPWQL--
TSPWQL--
-TPWQLR-

-TPWQLH-
TMPWQL--
-TPWQLK-
TAPWQL--
-TPWQLV-
-TPWQLM-
THPWQL--
-TPWQLW-
TKPWQL--
TVPWQL--
TFPWQL--
TWPWQL--
-TPWQLQ-
-TPWQLI-
-TPWQLT-
TLPWQL--
TIPWQL--
TDPWQL--
TYPWQL--
-TPWQLA-
TPPWQL--
TEPWQL--
-TPWQLG-
-VPWQLR-
VGPWQL--
-VPWQLV-
VTPWQL--
VRPWQL--
VMPWQL--
-VPWQLT-
VAPWQL--
VIPWQL--
VVPWQL--
VNPWQL--
VYPWQL--
-VPWQLN-
VDPWQL--
VLPWQL--
VSPWQL--
VHPWQL--
VWPWQL--
-VPWQLI-
VFPWQL--
-VPWQLY-
VPPWQL--
-VPWQLH-
-VPWQLG-
-VPWQLA-
-VPWQLK-
-VPWQLF-

VEPWQL--
VQPWQL--
-WPWQLR-
WAPWQL--
WVPWQL--
WTPWQL--
WKPWQL--
WRPWQL--
WGPWQL--
WLPWQL--
-WPWQLN-
WDPWQL--
WHPWQL--
-WPWQLH-
WNPWQL--
WQPWQL--
-WPWQLK-
WIPWQL--
WEPWQL--
WMPWQL--
YKPWQL--
YRPWQL--
YSPWQL--
-YPWQLT-
-YPWQLS-
YIPWQL--
-YPWQLQ-
-YPWQLV-
-YPWQLH-
YLPWQL--
YTPWQL--
-YPWQLN-
YGPWQL--
YQPWQL--
-YPWQLR-
YVPWQL--
YNPWQL--
YEPWQL--
YDPWQL--
-YPWQLK-
YMPWQL--
YWPWQL--
-YPWQLG-
-YPWQLA-

PRSM 359 674 45.90

DQPRSM--
-LPRSMD-
-NPRSMG-
--PRSMPR
--PRSMGW

--PRSMAE
-TPRSMP-
-WPRSMD-
-APRSMH-
AVPRSM--
APPRSM--
-APRSMW-
AGPRSM--
AAPRSM--
-APRSMK-
AIPRSM--
-APRSMI-
-APRSMA-
-APRSMQ-
-APRSMM-
ANPRSM--
-APRSMY-
ALPRSM--
AEPRSM--
-APRSMS-
-APRSMN-
-APRSMF-
AQPRSM--
-APRSMG-
ADPRSM--
-APRSME-
AHPRSM--
ACPRSM--
-CPRSMR-
-CPRSML-
CGPRSM--
CPPRSM--
CVPRSM--
CSPRSM--
CRPRSM--
-CPRSMY-
-CPRSMS-
-CPRSMH-
CFPRSM--
CAPRSM--
CIPRSM--
-CPRSMW-
-CPRSMK-
CDPRSM--
-CPRSMT-
CTPRSM--
-CPRSMV-
CNPRSM--
-CPRSMN-
-CPRSMF-

CMPRSM--
DVPRSM--
DSPRSM--
-DPRSMR-
DFPRSM--
DMPRSM--
-DPRSMT-
-DPRSMS-
-DPRSMI-
DAPRSM--
-DPRSML-
DKPRSM--
DWPRSM--
-DPRSMH-
DIPRSM--
DYPRSM--
-DPRSMY-
-DPRSMV-
DRPRSM--
-DPRSMW-
DNPRSM--
-DPRSMQ-
-DPRSMK-
-DPRSMF-
-DPRSMM-
DTPRSM--
-DPRSMC-
-DPRSMA-
DGPRSM--
DEPRSM--
DCPRSM--
-EPRSMQ-
-EPRSMM-
-EPRSMS-
EYPRSM--
EVPRSM--
ESPRSM--
-EPRSMW-
EKPRSM--
-EPRSMF-
EGPRSM--
-EPRSMH-
-EPRSMT-
EIPRSM--
EEPRSM--
ELPRSM--
-EPRSMK-
-EPRSMY-
ETPRSM--
EMPRSM--

EFPRSM--
EAPRSM--
-EPRSMN-
EHPRSM--
-EPRSMV-
EPPRSM--
-EPRSMI-
-EPRSMG-
-EPRSME-
-EPRSMA-
EWPRSM--
EDPRSM--
EQPRSM--
-EPRSMC-
FSPRSM--
FKPRSM--
FVPRSM--
FNPRSM--
FIPRSM--
FHPRSM--
-FPRSMH-
FAPRSM--
FGPRSM--
FEPRSM--
-FPRSMM-
FPPRSM--
FQPRSM--
-FPRSME-
FMPRSM--
FDPRSM--
-GPRSML-
-GPRSMT-
-GPRSMR-
-GPRSMS-
-GPRSMK-
GTPRSM--
-GPRSMV-
-GPRSMM-
GEPRSM--
GSPRSM--
-GPRSMQ-
GPPRSM--
GKPRSM--
-GPRSMY-
GGPRSM--
GFPRSM--
GAPRSM--
GQPRSM--
-GPRSMF-
GDPRSM--

GVPRSM--
-GPRSMH-
-GPRSMN-
-GPRSME-
-GPRSMG-
GCPRSM--
-GPRSMW-
-HPRSMS-
-HPRSML-
HVPRSM--
-HPRSMQ-
-HPRSMK-
HTPRSM--
HSPRSM--
HNPRSM--
HLPRSM--
-HPRSMI-
-HPRSMA-
HMPRSM--
HGPRSM--
HKPRSM--
-HPRSMN-
-HPRSMV-
-HPRSMY-
HQPRSM--
HDPRSM--
-HPRSMF-
HHPRSM--
HEPRSM--
HWPRSM--
HFPRSM--
HPPRSM--
ILPRSM--
-IPRSMS-
IPPRSM--
IMPRSM--
-IPRSMH-
IAPRSM--
-IPRSMA-
-IPRSMN-
INPRSM--
-IPRSMV-
-IPRSMQ-
-IPRSMG-
IKPRSM--
IGPRSM--
IEPRSM--
-IPRSML-
-IPRSMF-
-IPRSMY-

−IPRSMM−
IDPRSM−−
−IPRSME−
−IPRSMW−
ICPRSM−−
KGPRSM−−
−KPRSMI−
−KPRSMY−
−KPRSMW−
−KPRSMF−
−KPRSMV−
KRPRSM−−
−KPRSMA−
−KPRSMN−
KVPRSM−−
KDPRSM−−
KEPRSM−−
KQPRSM−−
KTPRSM−−
KMPRSM−−
KNPRSM−−
−KPRSMG−
KCPRSM−−
−KPRSMQ−
LYPRSM−−
−LPRSMT−
LMPRSM−−
LNPRSM−−
LIPRSM−−
LLPRSM−−
LEPRSM−−
−LPRSMF−
LAPRSM−−
LPPRSM−−
−LPRSMW−
LDPRSM−−
−LPRSMQ−
LKPRSM−−
−LPRSMN−
LQPRSM−−
−LPRSMC−
LCPRSM−−
−LPRSME−
−MPRSML−
MTPRSM−−
MSPRSM−−
−MPRSMV−
−MPRSMS−
−MPRSMI−
MYPRSM−−

```
-MPRSMF-
MVPRSM--
-MPRSMK-
MKPRSM--
MIPRSM--
MAPRSM--
-MPRSMN-
MFPRSM--
-MPRSMM-
MPPRSM--
MWPRSM--
MQPRSM--
-MPRSMG-
MNPRSM--
-MPRSME-
-MPRSMT-
MEPRSM--
NSPRSM--
-NPRSMH-
NMPRSM--
NVPRSM--
-NPRSMV-
-NPRSMI-
NNPRSM--
-NPRSMW-
NHPRSM--
NTPRSM--
NGPRSM--
-NPRSMY-
-NPRSMM-
NIPRSM--
NQPRSM--
-NPRSMQ-
-NPRSMK-
NLPRSM--
-NPRSMS-
-NPRSMN-
NEPRSM--
-NPRSMA-
NPPRSM--
NDPRSM--
-NPRSME-
--PRSMLH
--PRSMLA
--PRSMLT
--PRSMLN
--PRSMTT
--PRSMSG
--PRSMLF
--PRSMSH
```

--PRSMSV
--PRSMTL
--PRSMSA
--PRSMVN
--PRSMNL
--PRSMGS
--PRSMYG
--PRSMYK
--PRSMMS
--PRSMIG
--PRSMQG
--PRSMYT
--PRSMIT
--PRSMYQ
--PRSMLQ
--PRSMTA
--PRSMHS
--PRSMGT
--PRSMWG
--PRSMIS
--PRSMML
PSPRSM--
--PRSMMV
--PRSMHH
--PRSMFG
--PRSMYV
--PRSMIK
--PRSMGV
--PRSMVF
--PRSMLV
--PRSMFY
--PRSMFA
--PRSMGG
--PRSMAV
PVPRSM--
--PRSMSF
--PRSMNV
--PRSMFT
--PRSMMA
--PRSMEG
--PRSMKN
--PRSMHL
--PRSMGA
--PRSMAA
--PRSMMG
--PRSMHN
--PRSMNS
--PRSMHG
--PRSMAT
--PRSMFH

--PRSMYN
--PRSMTG
--PRSMNF
--PRSMLE
--PRSMLD
--PRSMAG
--PRSMYE
--PRSMYD
--PRSMQA
--PRSMME
--PRSMWV
--PRSMMM
--PRSMEW
--PRSMYM
--PRSMTQ
--PRSMLM
--PRSMIM
--PRSMTN
--PRSMSN
--PRSMHM
--PRSMVI
--PRSMQM
--PRSMAY
--PRSMWN
--PRSMKA
PGPRSM--
--PRSMNW
--PRSMNI
--PRSMMD
--PRSMKI
--PRSMKF
--PRSMHE
--PRSMAN
--PRSMWW
--PRSMWF
--PRSMFI
--PRSMWQ
--PRSMSE
--PRSMMT
--PRSMIF
--PRSMHQ
--PRSMQS
--PRSMNM
--PRSMNA
--PRSMAF
PHPRSM--
--PRSMRM
--PRSMQK
--PRSMWM
--PRSMVE

--PRSMNT
--PRSMIA
--PRSMFE
--PRSMEV
--PRSMPL
--PRSMNY
--PRSMMQ
--PRSMKM
--PRSMIV
--PRSMHY
--PRSMEA
PRPRSM--
PKPRSM--
--PRSMYY
--PRSMNH
--PRSMMN
--PRSMMI
--PRSMTW
--PRSMKK
--PRSMIE
--PRSMEY
--PRSMET
PWPRSM--
--PRSMQN
--PRSMNR
--PRSMMK
--PRSMHW
--PRSMDS
--PRSMYF
--PRSMTI
--PRSMTD
--PRSMQV
--PRSMMW
--PRSMID
--PRSMHI
--PRSMGF
PQPRSM--
--PRSMNK
--PRSMKE
--PRSMHK
--PRSMFD
--PRSMAW
PNPRSM--
PIPRSM--
--PRSMTH
--PRSMQI
--PRSMHD
--PRSMGI
--PRSMES
--PRSMER

--PRSMAH
PEPRSM--
--PRSMTM
--PRSMQT
--PRSMQQ
--PRSMIH
PTPRSM--
--PRSMWH
--PRSMSD
--PRSMNG
--PRSMNN
--PRSMHF
--PRSMGK
--PRSMAI
--PRSMTE
--PRSMQF
--PRSMKQ
--PRSMIN
--PRSMHA
--PRSMEN
--PRSMEK
--PRSMQD
--PRSMHV
--PRSMCA
--PRSMQE
--PRSMGQ
--PRSMGN
--PRSMFF
--PRSMEM
--PRSMEI
--PRSMDG
--PRSMDA
--PRSMCK
PCPRSM--
--PRSMWI
--PRSMWE
--PRSMVC
--PRSMNE
--PRSMKD
--PRSMCY
--PRSMCN
--PRSMCG
--PRSMAQ
PDPRSM--
--PRSMSC
--PRSMPH
--PRSMLC
--PRSMKC
--PRSMHC
--PRSMFC

--PRSMEQ
--PRSMEH
--PRSMEF
--PRSMDY
--PRSMDT
--PRSMDM
--PRSMCL
--PRSMCI
--PRSMCD
--PRSMAD
-PPRSMS-
-PPRSMQ-
QGPRSM--
-QPRSMS-
QSPRSM--
QVPRSM--
QPPRSM--
-QPRSMI-
QYPRSM--
QTPRSM--
-QPRSMK-
-QPRSMF-
-QPRSMY-
QFPRSM--
QAPRSM--
-QPRSMA-
-QPRSMT-
-QPRSMW-
-QPRSMM-
QIPRSM--
-QPRSMV-
-QPRSMN-
QNPRSM--
-QPRSMH-
-QPRSMQ-
QMPRSM--
QEPRSM--
-QPRSMG-
-QPRSME-
-QPRSMD-
QDPRSM--
RAPRSM--
RTPRSM--
-RPRSMI-
-RPRSMT-
RPPRSM--
-RPRSMH-
-RPRSMQ-
-RPRSME-
RDPRSM--

−RPRSMW−
RQPRSM−−
−RPRSMF−
−RPRSMY−
RCPRSM−−
RRPRSM−−
−RPRSMC−
SIPRSM−−
SLPRSM−−
−SPRSMV−
SYPRSM−−
−SPRSMI−
−SPRSMW−
STPRSM−−
SNPRSM−−
−SPRSMY−
SQPRSM−−
−SPRSMF−
−SPRSMQ−
SVPRSM−−
SKPRSM−−
SPPRSM−−
−SPRSMA−
−SPRSMM−
SGPRSM−−
SCPRSM−−
SDPRSM−−
−SPRSME−
−SPRSMC−
TLPRSM−−
−TPRSMW−
TVPRSM−−
TFPRSM−−
−TPRSMH−
−TPRSMV−
TYPRSM−−
−TPRSMY−
−TPRSMK−
−TPRSMF−
−TPRSMM−
−TPRSMQ−
TQPRSM−−
TPPRSM−−
TGPRSM−−
TKPRSM−−
TEPRSM−−
TAPRSM−−
THPRSM−−
−TPRSMN−
−TPRSMG−

-TPRSME-
TCPRSM--
VSPRSM--
-VPRSML-
VAPRSM--
-VPRSMT-
-VPRSMM-
VGPRSM--
VNPRSM--
-VPRSMA-
VMPRSM--
-VPRSMN-
-VPRSMI-
-VPRSMY-
-VPRSME-
VPPRSM--
VQPRSM--
VHPRSM--
-VPRSMV-
-VPRSMG-
VEPRSM--
-VPRSMQ-
-VPRSMF-
VDPRSM--
-VPRSMW-
VCPRSM--
-WPRSMY-
WPPRSM--
WQPRSM--
WYPRSM--
-WPRSMF-
WIPRSM--
WGPRSM--
WLPRSM--
-WPRSMV-
WHPRSM--
WRPRSM--
WMPRSM--
WEPRSM--
-WPRSMH-
-WPRSMN-
-WPRSMW-
-WPRSMM-
WAPRSM--
-WPRSME-
WDPRSM--
WKPRSM--
-YPRSMS-
YVPRSM--
-YPRSMI-

```
              -YPRSMF-
              YTPRSM--
              -YPRSMY-
              -YPRSMV-
              YMPRSM--
              -YPRSMN-
              YIPRSM--
              YYPRSM--
              YNPRSM--
              -YPRSME-
              YKPRSM--
              YDPRSM--
              YEPRSM--
              YAPRSM--
              YQPRSM--
              YGPRSM--
              -YPRSMM-
              YHPRSM--
              YCPRSM--
PANM           254        722      45.89
              -HPANMS-
              --PANMGY
              -TPANMY-
              -APANMR-
              ASPANM--
              -APANMS-
              -APANML-
              AWPANM--
              AGPANM--
              -APANMK-
              AHPANM--
              ARPANM--
              AVPANM--
              -APANMY-
              -APANMQ-
              AEPANM--
              AYPANM--
              -APANMG-
              ATPANM--
              -APANMI-
              ALPANM--
              AIPANM--
              -APANMV-
              -APANMT-
              ANPANM--
              AMPANM--
              ADPANM--
              -APANMH-
              AKPANM--
              CRPANM--
```

-CPANMT-
-CPANMR-
CTPANM--
-CPANML-
CLPANM--
CSPANM--
-CPANMF-
CVPANM--
CMPANM--
CIPANM--
DYPANM--
DWPANM--
-DPANMY-
DTPANM--
-DPANMF-
DRPANM--
-DPANMW-
-DPANML-
DAPANM--
DSPANM--
-DPANMV-
-DPANMK-
DNPANM--
DKPANM--
DQPANM--
-DPANMT-
-DPANMR-
-DPANMH-
-DPANMA-
DLPANM--
DFPANM--
-EPANML-
-EPANMR-
-EPANMK-
ETPANM--
-EPANMQ-
EMPANM--
ERPANM--
EFPANM--
EPPANM--
-EPANMF-
EKPANM--
EAPANM--
EVPANM--
-EPANMH-
ELPANM--
EIPANM--
-EPANMV-
EGPANM--
-EPANMM-

-EPANMA-
EHPANM--
EYPANM--
ESPANM--
-EPANMT-
-EPANMS-
-FPANMR-
FRPANM--
-FPANMT-
FHPANM--
FPPANM--
-FPANML-
FDPANM--
FYPANM--
FMPANM--
FTPANM--
FGPANM--
-FPANMS-
-FPANMF-
FIPANM--
FSPANM--
-FPANMI-
-FPANMH-
-FPANMA-
FNPANM--
-FPANMY-
-FPANMV-
-FPANMQ-
-FPANME-
FQPANM--
GSPANM--
GKPANM--
-GPANMR-
-GPANMS-
GTPANM--
GHPANM--
-GPANMY-
-GPANMV-
-GPANML-
GVPANM--
-GPANMM-
GNPANM--
GRPANM--
-GPANMK-
-GPANMA-
GDPANM--
-GPANMN-
GMPANM--
GYPANM--
GWPANM--

−GPANMT−
−GPANME−
GFPANM−−
GEPANM−−
GQPANM−−
GPPANM−−
−GPANMQ−
−GPANMH−
−GPANMG−
GLPANM−−
GGPANM−−
GCPANM−−
GAPANM−−
HSPANM−−
−HPANMY−
−HPANMR−
HKPANM−−
HWPANM−−
HFPANM−−
HLPANM−−
−HPANMK−
−HPANML−
HMPANM−−
HGPANM−−
HTPANM−−
HQPANM−−
−HPANME−
HRPANM−−
−HPANMT−
−HPANMQ−
−HPANMI−
−HPANMG−
−HPANMN−
HAPANM−−
−HPANMM−
−HPANMC−
HNPANM−−
IYPANM−−
−IPANMT−
ISPANM−−
−IPANML−
−IPANMR−
IAPANM−−
−IPANMA−
−IPANMS−
−IPANMH−
−IPANMF−
−IPANMI−
IPPANM−−
−IPANMV−

```
-IPANMQ-
-IPANMG-
IIPANM--
IGPANM--
ITPANM--
IFPANM--
IRPANM--
-IPANMN-
-IPANME-
INPANM--
IEPANM--
-KPANMS-
-KPANMH-
KKPANM--
-KPANMT-
KSPANM--
-KPANMR-
-KPANML-
KIPANM--
-KPANMF-
-KPANMM-
KMPANM--
-KPANMY-
KLPANM--
-KPANMN-
KVPANM--
KTPANM--
KRPANM--
KPPANM--
-KPANMV-
-KPANME-
-KPANMA-
KHPANM--
-KPANMK-
-KPANMG-
KGPANM--
KEPANM--
KDPANM--
KAPANM--
LRPANM--
-LPANMR-
LVPANM--
LLPANM--
LTPANM--
-LPANML-
-LPANMA-
-LPANMY-
LMPANM--
LSPANM--
-LPANMV-
```

-LPANMH-
LNPANM--
LEPANM--
LWPANM--
-LPANMT-
LKPANM--
LHPANM--
LFPANM--
-LPANMN-
LIPANM--
LPPANM--
-LPANMI-
LYPANM--
-LPANMW-
-LPANME-
LCPANM--
MRPANM--
-MPANML-
-MPANMA-
MVPANM--
MSPANM--
MQPANM--
MGPANM--
MWPANM--
-MPANMI-
MDPANM--
-MPANMR-
MIPANM--
-MPANMY-
-MPANMF-
-MPANMT-
-MPANMV-
-MPANMQ-
MPPANM--
MCPANM--
-MPANMS-
-MPANMM-
-MPANMG-
-MPANME-
MEPANM--
NWPANM--
NLPANM--
NTPANM--
NSPANM--
NGPANM--
-NPANMS-
NKPANM--
NNPANM--
NRPANM--
-NPANMW-

NAPANM--
NFPANM--
NQPANM--
-NPANMR-
-NPANMY-
NHPANM--
-NPANMV-
-NPANML-
-NPANMI-
-NPANMF-
NEPANM--
NDPANM--
-NPANMM-
--PANMYV
--PANMLL
--PANMFA
--PANMRT
--PANMHS
--PANMFS
--PANMFK
--PANMRY
--PANMRG
--PANMRF
--PANMMV
--PANMRK
--PANMRS
--PANMYR
--PANMRQ
--PANMRA
--PANMML
--PANMLV
--PANMIM
--PANMVV
--PANMSW
--PANMFQ
--PANMTV
--PANMMG
--PANMLN
--PANMLH
--PANMVK
--PANMLS
--PANMAH
--PANMYW
--PANMYK
--PANMSH
--PANMGS
--PANMWN
--PANMVQ
--PANMVA
--PANMLR

--PANMIL
--PANMFW
PRPANM--
--PANMTL
--PANMSF
--PANMFH
--PANMFG
PYPANM--
--PANMWT
--PANMVG
--PANMSV
--PANMQI
--PANMLA
--PANMKR
--PANMKA
--PANMAN
PSPANM--
--PANMTG
--PANMSA
--PANMRN
--PANMRH
--PANMMT
--PANMIT
--PANMIN
--PANMGM
--PANMGA
--PANMYI
--PANMWG
--PANMWD
--PANMVS
--PANMVR
--PANMTR
--PANMTK
--PANMSS
--PANMQW
--PANMLQ
--PANMFN
--PANMFL
--PANMRV
--PANMMS
--PANMLK
--PANMGF
--PANMAV
--PANMYQ
--PANMSQ
--PANMSN
--PANMSM
--PANMRR
--PANMRM
--PANMQG

--PANMKS
--PANMIH
--PANMHT
--PANMFV
--PANMFI
PKPANM--
PGPANM--
--PANMYS
--PANMTQ
--PANMTI
--PANMSY
--PANMST
--PANMRE
--PANMKL
--PANMKH
--PANMHY
--PANMHF
--PANMHA
--PANMGW
--PANMFD
--PANMER
--PANMAK
--PANMAG
PWPANM--
PLPANM--
--PANMWL
--PANMWE
--PANMVD
--PANMSG
--PANMRW
--PANMQT
--PANMNG
--PANMKF
--PANMIG
--PANMGV
--PANMFM
--PANMAL
--PANMYH
--PANMWR
--PANMTT
--PANMTM
--PANMRI
--PANMQV
--PANMQL
--PANMNV
--PANMLT
--PANMKV
--PANMIS
--PANMID
--PANMHN

--PANMGT
--PANMEV
--PANMEL
--PANMEK
PIPANM--
--PANMYG
--PANMYE
--PANMWA
--PANMTY
--PANMTD
--PANMSK
--PANMSE
--PANMQS
--PANMNY
--PANMNS
--PANMNI
--PANMNF
--PANMLE
--PANMKM
--PANMHD
--PANMGG
--PANMFT
--PANMFE
--PANMDS
--PANMCA
--PANMAS
--PANMAQ
--PANMAM
--PANMAI
-PPANMY-
--PANMVF
--PANMTW
--PANMNM
--PANMNH
--PANMMR
--PANMMD
--PANMLI
--PANMLG
--PANMKG
--PANMIY
--PANMIW
--PANMIR
--PANMIF
--PANMHL
--PANMHI
--PANMES
-PPANMS-
-PPANMI-
--PANMYN
--PANMYD

--PANMYA
--PANMWK
--PANMVN
--PANMTN
--PANMTH
--PANMRD
--PANMQR
--PANMQF
--PANMQA
--PANMPS
--PANMNT
--PANMND
--PANMMN
--PANMMA
--PANMLC
--PANMKY
--PANMKN
--PANMKE
--PANMIQ
--PANMIE
--PANMHM
--PANMGL
--PANMGI
--PANMEI
--PANMEH
--PANMCY
--PANMCK
--PANMAT
--PANMAC
--PANMAA
-QPANMY-
QRPANM--
QLPANM--
-QPANMT-
QTPANM--
-QPANMR-
QMPANM--
QWPANM--
QVPANM--
QAPANM--
QSPANM--
QQPANM--
-QPANMW-
-QPANML-
-QPANMI-
QHPANM--
-QPANMS-
-QPANMG-
-QPANME-
QYPANM--

QPPANM--
-QPANMK-
-QPANMH-
-QPANMF-
-QPANMA-
QNPANM--
QIPANM--
RVPANM--
RPPANM--
-RPANMA-
-RPANMV-
-RPANMS-
-RPANML-
RGPANM--
RSPANM--
-RPANMI-
RLPANM--
RIPANM--
RAPANM--
RNPANM--
RFPANM--
-RPANMQ-
RKPANM--
RTPANM--
RYPANM--
-RPANMT-
RRPANM--
-RPANMY-
RMPANM--
-RPANMK-
-RPANMW-
-RPANMM-
RHPANM--
-RPANMN-
-RPANMR-
-RPANMH-
-RPANMG-
RWPANM--
RDPANM--
-RPANMD-
RQPANM--
-RPANME-
-SPANMT-
SRPANM--
-SPANML-
SFPANM--
-SPANMR-
SKPANM--
SSPANM--
STPANM--

-SPANMA-
SWPANM--
-SPANMN-
-SPANMV-
SGPANM--
SMPANM--
-SPANMY-
SIPANM--
SLPANM--
-SPANMK-
SNPANM--
SVPANM--
-SPANMH-
SHPANM--
-SPANMF-
SEPANM--
SQPANM--
SAPANM--
SPPANM--
-SPANMQ-
-SPANMW-
SDPANM--
-SPANMS-
-SPANME-
-TPANML-
TNPANM--
TAPANM--
-TPANMR-
-TPANMM-
TRPANM--
TLPANM--
TPPANM--
TTPANM--
-TPANMH-
-TPANMI-
TKPANM--
TIPANM--
TSPANM--
-TPANMA-
TWPANM--
-TPANMK-
-TPANMQ-
-TPANMF-
TDPANM--
-TPANMN-
-TPANMV-
-TPANMT-
TGPANM--
-TPANMG-
-TPANME-

THPANM--
VTPANM--
-VPANML-
VKPANM--
VSPANM--
-VPANMV-
-VPANMI-
VVPANM--
VFPANM--
VYPANM--
-VPANMA-
-VPANMT-
VRPANM--
VQPANM--
-VPANMR-
-VPANMF-
VLPANM--
VAPANM--
-VPANMS-
VMPANM--
VPPANM--
-VPANMY-
-VPANMK-
-VPANMG-
VIPANM--
VNPANM--
-VPANMN-
VGPANM--
VEPANM--
VDPANM--
VWPANM--
-VPANMM-
VHPANM--
-WPANMR-
WRPANM--
WHPANM--
WTPANM--
WNPANM--
-WPANMY-
-WPANML-
-WPANMA-
-WPANMQ-
WPPANM--
-WPANMK-
WLPANM--
WVPANM--
WFPANM--
WYPANM--
-WPANMM-
WAPANM--

	-WPANMV-		
	-WPANMT-		
	-WPANMI-		
	-WPANME-		
	WMPANM--		
	WEPANM--		
	WDPANM--		
	-WPANMF-		
	WQPANM--		
	WGPANM--		
	-WPANMW-		
	-WPANMS-		
	-WPANMN-		
	WCPANM--		
	-YPANMR-		
	YTPANM--		
	YLPANM--		
	YRPANM--		
	YNPANM--		
	YFPANM--		
	YSPANM--		
	YDPANM--		
	-YPANMF-		
	-YPANMS-		
	-YPANMY-		
	-YPANMV-		
	-YPANMA-		
	YAPANM--		
	YQPANM--		
	-YPANML-		
	-YPANMI-		
	YCPANM--		
	YWPANM--		
	YPPANM--		
	YIPANM--		
	YVPANM--		
	-YPANMN-		
	-YPANMG-		
	YYPANM--		
	-YPANMQ-		
	-YPANME-		
	YGPANM--		
PMWY	851	521	45.85
	-APMWYK-		
	-APMWYI-		
	AAPMWY--		
	ANPMWY--		
	-APMWYL-		
	AVPMWY--		
	-APMWYS-		

AWPMWY--
-APMWYY-
AFPMWY--
-APMWYG-
AKPMWY--
ASPMWY--
-APMWYV-
-APMWYQ-
ATPMWY--
-APMWYE-
-APMWYA-
-APMWYN-
-APMWYH-
-APMWYD-
-APMWYC-
AGPMWY--
CGPMWY--
CAPMWY--
CNPMWY--
CKPMWY--
CEPMWY--
CLPMWY--
CHPMWY--
DSPMWY--
DYPMWY--
-DPMWYT-
DLPMWY--
DAPMWY--
DKPMWY--
-DPMWYS-
DVPMWY--
-DPMWYM-
-DPMWYV-
-DPMWYR-
DGPMWY--
DRPMWY--
-DPMWYQ-
-DPMWYN-
-DPMWYA-
DMPMWY--
DDPMWY--
DCPMWY--
ETPMWY--
-EPMWYH-
-EPMWYT-
EVPMWY--
-EPMWYS-
EIPMWY--
ESPMWY--
ERPMWY--

-EPMWYD-
EAPMWY--
EPPMWY--
-EPMWYY-
-EPMWYL-
-EPMWYF-
ENPMWY--
FNPMWY--
-FPMWYQ-
FDPMWY--
FHPMWY--
FAPMWY--
FTPMWY--
FSPMWY--
FQPMWY--
FIPMWY--
FRPMWY--
-FPMWYV-
-FPMWYF-
FVPMWY--
-FPMWYS-
-FPMWYI-
FMPMWY--
-GPMWYT-
GNPMWY--
-GPMWYR-
-GPMWYQ-
-GPMWYS-
GAPMWY--
-GPMWYK-
-GPMWYA-
GSPMWY--
-GPMWYG-
GEPMWY--
GTPMWY--
GDPMWY--
GMPMWY--
-GPMWYV-
-GPMWYM-
-GPMWYL-
-GPMWYH-
GKPMWY--
-GPMWYN-
-GPMWYF-
HRPMWY--
HTPMWY--
HGPMWY--
HVPMWY--
HSPMWY--
HKPMWY--

-HPMWYG-
HWPMWY--
HLPMWY--
HQPMWY--
HMPMWY--
-HPMWYT-
-HPMWYY-
-HPMWYV-
-HPMWYW-
-HPMWYN-
HNPMWY--
HEPMWY--
HAPMWY--
-IPMWYR-
IQPMWY--
ISPMWY--
INPMWY--
IRPMWY--
-IPMWYL-
IIPMWY--
-IPMWYT-
IVPMWY--
IEPMWY--
-IPMWYV-
-IPMWYM-
-IPMWYG-
-IPMWYF-
KSPMWY--
KLPMWY--
KGPMWY--
KEPMWY--
KNPMWY--
-KPMWYI-
-KPMWYV-
KRPMWY--
KVPMWY--
KPPMWY--
KWPMWY--
-KPMWYL-
KAPMWY--
-KPMWYF-
KKPMWY--
-KPMWYT-
KTPMWY--
KFPMWY--
-KPMWYW-
-KPMWYS-
-KPMWYQ-
-KPMWYG-
-KPMWYE-

-KPMWYR-
KHPMWY--
KQPMWY--
-KPMWYN-
-KPMWYH-
KDPMWY--
LSPMWY--
LRPMWY--
LGPMWY--
-LPMWYK-
LKPMWY--
-LPMWYS-
-LPMWYR-
LDPMWY--
LVPMWY--
-LPMWYL-
LTPMWY--
LAPMWY--
-LPMWYF-
LNPMWY--
-LPMWYG-
LPPMWY--
-LPMWYM-
-LPMWYI-
LQPMWY--
-LPMWYW-
-LPMWYH-
MGPMWY--
-MPMWYA-
MVPMWY--
MSPMWY--
-MPMWYM-
-MPMWYT-
-MPMWYW-
-MPMWYS-
-MPMWYL-
-MPMWYF-
MLPMWY--
MKPMWY--
MIPMWY--
MWPMWY--
MQPMWY--
-MPMWYY-
-MPMWYK-
NQPMWY--
NAPMWY--
NVPMWY--
NRPMWY--
NKPMWY--
-NPMWYS-

NEPMWY--
NYPMWY--
-NPMWYT-
NMPMWY--
-NPMWYV-
-NPMWYR-
NHPMWY--
NSPMWY--
-NPMWYM-
-NPMWYI-
-NPMWYG-
NNPMWY--
NLPMWY--
NGPMWY--
NTPMWY--
-NPMWYE-
-NPMWYQ-
NIPMWY--
--PMWYRV
--PMWYRS
--PMWYMR
--PMWYSS
--PMWYRA
--PMWYTA
--PMWYKS
--PMWYMH
--PMWYGL
--PMWYNT
--PMWYKW
--PMWYSH
--PMWYTR
--PMWYRG
--PMWYKH
--PMWYRH
--PMWYRQ
--PMWYTK
--PMWYIA
--PMWYGS
--PMWYAL
--PMWYYM
--PMWYTS
--PMWYSV
--PMWYSQ
--PMWYRE
--PMWYQS
--PMWYQA
--PMWYML
--PMWYAT
--PMWYAN
--PMWYWG

--PMWYVT
--PMWYTY
--PMWYSY
--PMWYSR
--PMWYSK
--PMWYMD
--PMWYLG
--PMWYKT
--PMWYKG
--PMWYAS
--PMWYTQ
--PMWYST
--PMWYMT
--PMWYTN
--PMWYNS
--PMWYLA
--PMWYIN
--PMWYHV
--PMWYGI
--PMWYEY
PNPMWY--
--PMWYWS
--PMWYVN
--PMWYSL
--PMWYSI
--PMWYRW
--PMWYYG
--PMWYVR
--PMWYSN
--PMWYSD
--PMWYRY
--PMWYRT
--PMWYQR
--PMWYLN
--PMWYLH
--PMWYKA
--PMWYVS
--PMWYTV
--PMWYSA
--PMWYQN
--PMWYNA
--PMWYLT
--PMWYKL
--PMWYIF
--PMWYFN
--PMWYAV
--PMWYAR
--PMWYYI
--PMWYSG
--PMWYRM

--PMWYRD
--PMWYQE
--PMWYHH
--PMWYHA
--PMWYGY
--PMWYGA
--PMWYAY
--PMWYAH
--PMWYAG
--PMWYVG
--PMWYTM
--PMWYTG
--PMWYSE
--PMWYRL
--PMWYQY
--PMWYQM
--PMWYMI
--PMWYKV
--PMWYIS
--PMWYII
--PMWYIG
--PMWYDA
--PMWYAQ
--PMWYYV
--PMWYYT
--PMWYYH
--PMWYWM
--PMWYTT
--PMWYQV
--PMWYMG
--PMWYLK
--PMWYKF
--PMWYIY
--PMWYIR
--PMWYIK
--PMWYGV
--PMWYGG
--PMWYFV
--PMWYFL
--PMWYFH
--PMWYYF
--PMWYVV
--PMWYVM
--PMWYVK
--PMWYVE
--PMWYTE
--PMWYQI
--PMWYQD
--PMWYNG
--PMWYMW

--PMWYLF
--PMWYKY
--PMWYHG
--PMWYFG
--PMWYFD
--PMWYFA
--PMWYEG
--PMWYAM
QTPMWY--
QSPMWY--
-QPMWYR-
QDPMWY--
QWPMWY--
QRPMWY--
-QPMWYG-
QNPMWY--
QIPMWY--
QGPMWY--
-QPMWYS-
QMPMWY--
QVPMWY--
-QPMWYN-
-QPMWYK-
QAPMWY--
-QPMWYM-
QQPMWY--
QPPMWY--
QLPMWY--
-QPMWYV-
-QPMWYA-
QYPMWY--
-QPMWYT-
RRPMWY--
-RPMWYT-
-RPMWYR-
RLPMWY--
RDPMWY--
-RPMWYI-
RMPMWY--
-RPMWYM-
RAPMWY--
RQPMWY--
RVPMWY--
RTPMWY--
RSPMWY--
-RPMWYY-
-RPMWYV-
-RPMWYL-
RPPMWY--
-RPMWYQ-

RIPMWY--
-RPMWYH-
REPMWY--
RWPMWY--
-RPMWYS-
RNPMWY--
-RPMWYC-
-RPMWYN-
-RPMWYW-
-RPMWYE-
-RPMWYA-
RKPMWY--
-SPMWYR-
SRPMWY--
SNPMWY--
SGPMWY--
SSPMWY--
SLPMWY--
-SPMWYS-
SVPMWY--
-SPMWYA-
SYPMWY--
-SPMWYG-
-SPMWYM-
-SPMWYI-
-SPMWYE-
SHPMWY--
SWPMWY--
SKPMWY--
SDPMWY--
-SPMWYH-
STPMWY--
SPPMWY--
-SPMWYT-
SEPMWY--
-SPMWYQ-
-SPMWYK-
-SPMWYF-
-SPMWYY-
SQPMWY--
TRPMWY--
TPPMWY--
TVPMWY--
-TPMWYT-
-TPMWYV-
-TPMWYS-
-TPMWYL-
TKPMWY--
-TPMWYK-
TSPMWY--

TLPMWY--
-TPMWYF-
TYPMWY--
TTPMWY--
-TPMWYE-
TGPMWY--
-TPMWYH-
-TPMWYG-
TIPMWY--
-TPMWYN-
-TPMWYA-
TAPMWY--
TNPMWY--
TEPMWY--
VVPMWY--
VEPMWY--
-VPMWYY-
VNPMWY--
VKPMWY--
-VPMWYS-
VHPMWY--
VTPMWY--
-VPMWYT-
-VPMWYR-
-VPMWYI-
-VPMWYG-
VSPMWY--
-VPMWYH-
-VPMWYQ-
-VPMWYW-
VGPMWY--
-VPMWYL-
-VPMWYA-
VDPMWY--
VAPMWY--
VQPMWY--
VPPMWY--
WNPMWY--
WSPMWY--
WVPMWY--
WRPMWY--
WPPMWY--
-WPMWYY-
-WPMWYF-
WIPMWY--
WDPMWY--
YVPMWY--
-YPMWYQ-
YSPMWY--
YNPMWY--

	YGPMWY--		
	YRPMWY--		
	YLPMWY--		
	YAPMWY--		
	YDPMWY--		
	-YPMWYV-		
	YHPMWY--		
	YQPMWY--		
	-YPMWYK-		
	YPPMWY--		
	-YPMWYS-		
	-YPMWYM-		
	-YPMWYA-		
	YIPMWY--		
PLQL	199	747	45.79
	-LPLQLG-		
	--PLQLGL		
	--PLQLTN		
	--PLQLRP		
	--PLQLPR		
	--PLQLER		
	--PLQLDL		
	-RPLQLP-		
	-SPLQLE-		
	-VPLQLP-		
	AVPLQL--		
	-APLQLH-		
	ARPLQL--		
	-APLQLV-		
	-APLQLR-		
	-APLQLN-		
	-APLQLT-		
	AIPLQL--		
	ASPLQL--		
	AKPLQL--		
	-APLQLS-		
	ATPLQL--		
	-APLQLK-		
	-APLQLM-		
	AAPLQL--		
	AGPLQL--		
	ADPLQL--		
	-APLQLQ-		
	ALPLQL--		
	AYPLQL--		
	-APLQLL-		
	-APLQLI-		
	ANPLQL--		
	AEPLQL--		
	AWPLQL--		

-APLQLG-
AMPLQL--
AQPLQL--
AFPLQL--
AHPLQL--
-APLQLE-
-APLQLA-
-APLQLC-
ACPLQL--
CRPLQL--
CSPLQL--
CVPLQL--
CQPLQL--
CHPLQL--
CFPLQL--
CYPLQL--
-CPLQLY-
CGPLQL--
CTPLQL--
CPPLQL--
-CPLQLW-
-CPLQLR-
CKPLQL--
CCPLQL--
-CPLQLH-
CMPLQL--
CLPLQL--
CDPLQL--
-CPLQLS-
-CPLQLI-
-CPLQLA-
CNPLQL--
CEPLQL--
CAPLQL--
DKPLQL--
DSPLQL--
-DPLQLR-
DAPLQL--
-DPLQLV-
-DPLQLT-
DIPLQL--
DVPLQL--
DQPLQL--
-DPLQLL-
DEPLQL--
DMPLQL--
DYPLQL--
DGPLQL--
DFPLQL--
-DPLQLN-

−DPLQLK−
DLPLQL−−
DWPLQL−−
−DPLQLI−
DHPLQL−−
−DPLQLQ−
DTPLQL−−
−DPLQLS−
−DPLQLM−
−DPLQLH−
−DPLQLA−
−DPLQLF−
DNPLQL−−
ERPLQL−−
−EPLQLK−
ELPLQL−−
ESPLQL−−
EMPLQL−−
EKPLQL−−
EGPLQL−−
ETPLQL−−
−EPLQLT−
−EPLQLL−
−EPLQLN−
EAPLQL−−
EYPLQL−−
−EPLQLS−
EVPLQL−−
−EPLQLH−
−EPLQLF−
ENPLQL−−
EQPLQL−−
−EPLQLV−
EHPLQL−−
−EPLQLM−
EPPLQL−−
−EPLQLA−
−EPLQLI−
−EPLQLE−
EIPLQL−−
EDPLQL−−
−EPLQLG−
−EPLQLD−
EEPLQL−−
FSPLQL−−
FRPLQL−−
−FPLQLV−
−FPLQLR−
−FPLQLQ−
FPPLQL−−

-FPLQLY-
-FPLQLN-
-FPLQLK-
-FPLQLT-
-FPLQLH-
FGPLQL--
-FPLQLM-
-FPLQLI-
FTPLQL--
FHPLQL--
-FPLQLA-
-FPLQLE-
-FPLQLG-
FDPLQL--
FCPLQL--
-FPLQLC-
GSPLQL--
GVPLQL--
GTPLQL--
GWPLQL--
-GPLQLS-
GFPLQL--
GLPLQL--
-GPLQLK-
GEPLQL--
-GPLQLT-
GIPLQL--
GGPLQL--
GMPLQL--
GKPLQL--
GHPLQL--
GQPLQL--
-GPLQLM-
GYPLQL--
-GPLQLI-
-GPLQLN-
GAPLQL--
GPPLQL--
-GPLQLH-
GDPLQL--
-GPLQLQ-
-GPLQLG-
GNPLQL--
-GPLQLE-
-GPLQLA-
-GPLQLV-
GCPLQL--
-GPLQLC-
HRPLQL--
HVPLQL--

-HPLQLK-
HTPLQL--
HWPLQL--
HLPLQL--
-HPLQLV-
-HPLQLL-
HSPLQL--
HYPLQL--
HIPLQL--
HEPLQL--
HNPLQL--
HHPLQL--
HDPLQL--
HAPLQL--
-HPLQLM-
-HPLQLH-
-HPLQLT-
-HPLQLN-
-HPLQLG-
-HPLQLA-
HKPLQL--
-HPLQLI-
HMPLQL--
-HPLQLQ-
HQPLQL--
HPPLQL--
HGPLQL--
HFPLQL--
-HPLQLC-
HCPLQL--
ILPLQL--
-IPLQLT-
-IPLQLS-
IYPLQL--
-IPLQLV-
IVPLQL--
ISPLQL--
INPLQL--
-IPLQLK-
IDPLQL--
-IPLQLQ-
IKPLQL--
-IPLQLM-
IGPLQL--
IEPLQL--
-IPLQLN-
IAPLQL--
-IPLQLR-
ITPLQL--
-IPLQLG-

-IPLQLA-
IHPLQL--
-IPLQLH-
IQPLQL--
IPPLQL--
ICPLQL--
-IPLQLI-
IFPLQL--
-KPLQLR-
KTPLQL--
KGPLQL--
-KPLQLS-
-KPLQLQ-
-KPLQLK-
-KPLQLV-
KKPLQL--
KIPLQL--
KAPLQL--
KYPLQL--
KPPLQL--
-KPLQLH-
-KPLQLY-
-KPLQLM-
KQPLQL--
KMPLQL--
KDPLQL--
KEPLQL--
-KPLQLI-
-KPLQLG-
-KPLQLA-
KNPLQL--
KFPLQL--
KVPLQL--
-KPLQLN-
-KPLQLE-
LSPLQL--
-LPLQLR-
LEPLQL--
LRPLQL--
LKPLQL--
-LPLQLS-
LNPLQL--
LAPLQL--
-LPLQLQ-
-LPLQLN-
LPPLQL--
LGPLQL--
LFPLQL--
LYPLQL--
-LPLQLK-

-LPLQLI-
LQPLQL--
-LPLQLH-
LDPLQL--
-LPLQLM-
LHPLQL--
-LPLQLA-
LIPLQL--
-LPLQLV-
LCPLQL--
MSPLQL--
-MPLQLR-
-MPLQLV-
MFPLQL--
MVPLQL--
-MPLQLS-
-MPLQLL-
MAPLQL--
-MPLQLT-
-MPLQLK-
-MPLQLH-
MTPLQL--
MKPLQL--
MDPLQL--
MIPLQL--
MHPLQL--
MRPLQL--
MMPLQL--
MGPLQL--
-MPLQLG-
-MPLQLA-
MYPLQL--
-MPLQLI-
MNPLQL--
MEPLQL--
-MPLQLQ-
-MPLQLM-
-MPLQLN-
-MPLQLE-
MCPLQL--
-NPLQLR-
NGPLQL--
NRPLQL--
NAPLQL--
NIPLQL--
NSPLQL--
-NPLQLL-
NLPLQL--
-NPLQLV-
-NPLQLT-

NMPLQL--
-NPLQLN-
NKPLQL--
-NPLQLS-
-NPLQLK-
NQPLQL--
NVPLQL--
NFPLQL--
-NPLQLI-
-NPLQLH-
NWPLQL--
NTPLQL--
NDPLQL--
-NPLQLM-
-NPLQLA-
NHPLQL--
NNPLQL--
-NPLQLE-
NEPLQL--
--PLQLRR
--PLQLLT
--PLQLRS
--PLQLRK
--PLQLRN
--PLQLST
--PLQLVS
--PLQLIS
--PLQLNR
--PLQLRI
--PLQLSR
--PLQLRQ
--PLQLRH
--PLQLNS
--PLQLKR
--PLQLLN
--PLQLVT
--PLQLVQ
--PLQLTG
--PLQLMR
--PLQLKS
--PLQLGS
--PLQLTR
--PLQLTH
--PLQLQS
--PLQLAS
--PLQLAT
--PLQLVA
--PLQLMG
--PLQLHS
--PLQLHG

--PLQLVG
--PLQLRD
--PLQLKL
--PLQLSL
--PLQLLQ
--PLQLTS
--PLQLSH
--PLQLNT
--PLQLHT
--PLQLMT
--PLQLKH
--PLQLIT
--PLQLFN
--PLQLSM
--PLQLMQ
--PLQLAN
--PLQLWG
--PLQLTL
--PLQLTK
--PLQLVH
--PLQLTA
--PLQLYG
--PLQLVN
--PLQLSF
PGPLQL--
--PLQLLH
--PLQLIG
--PLQLGG
--PLQLEG
--PLQLNM
--PLQLTY
--PLQLSW
--PLQLYN
--PLQLSK
--PLQLHW
--PLQLFG
--PLQLAI
--PLQLWY
--PLQLKI
--PLQLWH
--PLQLGT
--PLQLRF
--PLQLRE
--PLQLQY
--PLQLMA
--PLQLGA
PNPLQL--
--PLQLYD
--PLQLNN
--PLQLKY

--PLQLKK
--PLQLKF
--PLQLSV
--PLQLQR
--PLQLNL
--PLQLNA
--PLQLET
PDPLQL--
--PLQLIN
--PLQLAG
--PLQLVD
--PLQLTV
--PLQLSG
--PLQLQH
--PLQLKW
--PLQLIH
--PLQLGI
--PLQLAV
--PLQLNQ
--PLQLHQ
--PLQLQF
--PLQLLD
--PLQLGY
--PLQLAR
--PLQLTD
--PLQLQW
--PLQLQT
--PLQLQN
--PLQLHA
--PLQLTI
--PLQLSQ
--PLQLSI
--PLQLQG
--PLQLQA
--PLQLMN
--PLQLKA
--PLQLVW
--PLQLLE
--PLQLHK
--PLQLHF
--PLQLAM
--PLQLAK
--PLQLYH
--PLQLWA
--PLQLTW
--PLQLTQ
--PLQLSD
--PLQLQD
--PLQLNI
--PLQLKQ

--PLQLGV
--PLQLNK
--PLQLGM
--PLQLFD
--PLQLEW
--PLQLQK
--PLQLMW
--PLQLMI
--PLQLME
--PLQLKN
--PLQLHV
--PLQLAA
--PLQLWN
--PLQLVE
--PLQLND
--PLQLKD
--PLQLHE
--PLQLFH
--PLQLEH
--PLQLHN
--PLQLWD
--PLQLSE
--PLQLQM
--PLQLQL
--PLQLMK
--PLQLGN
--PLQLES
--PLQLEM
--PLQLEF
--PLQLAH
--PLQLNW
--PLQLKE
--PLQLIE
--PLQLHY
--PLQLHM
--PLQLGW
--PLQLGQ
--PLQLAQ
PCPLQL--
--PLQLQQ
--PLQLQE
--PLQLMC
--PLQLKC
--PLQLHD
--PLQLEN
--PLQLEL
--PLQLCM
-PPLQLN-
-PPLQLI-
--PLQLWC

--PLQLVC
--PLQLTE
--PLQLTC
--PLQLNP
--PLQLKM
--PLQLID
--PLQLFA
--PLQLDN
--PLQLDG
--PLQLCS
--PLQLCG
-QPLQLR-
-QPLQLI-
QKPLQL--
QPPLQL--
QGPLQL--
-QPLQLQ-
-QPLQLS-
QRPLQL--
-QPLQLL-
-QPLQLV-
QVPLQL--
QQPLQL--
-QPLQLK-
QWPLQL--
QHPLQL--
QSPLQL--
-QPLQLH-
QLPLQL--
QIPLQL--
-QPLQLN-
-QPLQLT-
-QPLQLA-
QMPLQL--
-QPLQLF-
QDPLQL--
QTPLQL--
-QPLQLG-
QAPLQL--
QYPLQL--
-QPLQLE-
QNPLQL--
QEPLQL--
QCPLQL--
-RPLQLR-
-RPLQLL-
RMPLQL--
RIPLQL--
-RPLQLS-
RSPLQL--

-RPLQLI-
RGPLQL--
RLPLQL--
RFPLQL--
-RPLQLT-
-RPLQLV-
RVPLQL--
-RPLQLN-
RHPLQL--
RAPLQL--
RKPLQL--
-RPLQLH-
RPPLQL--
REPLQL--
RQPLQL--
-RPLQLM-
-RPLQLA-
-RPLQLG-
-RPLQLE-
RNPLQL--
RDPLQL--
RCPLQL--
SRPLQL--
-SPLQLT-
SKPLQL--
SLPLQL--
-SPLQLR-
-SPLQLL-
-SPLQLS-
SVPLQL--
STPLQL--
SSPLQL--
SYPLQL--
SAPLQL--
-SPLQLI-
SFPLQL--
-SPLQLM-
-SPLQLH-
-SPLQLA-
SIPLQL--
-SPLQLQ-
SNPLQL--
SPPLQL--
SHPLQL--
SWPLQL--
SQPLQL--
-SPLQLG-
SDPLQL--
SCPLQL--
TSPLQL--

-TPLQLL-
TIPLQL--
TYPLQL--
TRPLQL--
-TPLQLS-
TKPLQL--
TTPLQL--
TEPLQL--
-TPLQLV-
-TPLQLN-
TGPLQL--
TNPLQL--
TPPLQL--
-TPLQLH-
THPLQL--
TWPLQL--
-TPLQLM-
-TPLQLI-
-TPLQLA-
TQPLQL--
TAPLQL--
TDPLQL--
-TPLQLK-
TMPLQL--
TFPLQL--
-TPLQLQ-
-TPLQLE-
TCPLQL--
-VPLQLR-
VRPLQL--
VGPLQL--
VSPLQL--
VVPLQL--
VWPLQL--
-VPLQLL-
VAPLQL--
-VPLQLK-
VKPLQL--
VPPLQL--
VMPLQL--
-VPLQLS-
VQPLQL--
VHPLQL--
VNPLQL--
VFPLQL--
-VPLQLA-
-VPLQLH-
VDPLQL--
-VPLQLV-
VEPLQL--

-VPLQLN-
VYPLQL--
-VPLQLE-
-VPLQLI-
-VPLQLQ-
-VPLQLM-
VIPLQL--
VCPLQL--
WSPLQL--
WRPLQL--
-WPLQLT-
WAPLQL--
-WPLQLR-
-WPLQLK-
WGPLQL--
WPPLQL--
-WPLQLS-
WHPLQL--
-WPLQLA-
-WPLQLQ-
WNPLQL--
WDPLQL--
WEPLQL--
-WPLQLH-
WWPLQL--
WTPLQL--
-WPLQLV-
-WPLQLE-
-WPLQLN-
-WPLQLG-
-YPLQLL-
YGPLQL--
-YPLQLR-
YVPLQL--
YSPLQL--
YRPLQL--
YMPLQL--
-YPLQLT-
-YPLQLN-
YLPLQL--
YTPLQL--
-YPLQLQ-
YAPLQL--
-YPLQLH-
-YPLQLS-
-YPLQLA-
YKPLQL--
-YPLQLV-
YQPLQL--
-YPLQLI-

YHPLQL--
YNPLQL--
YDPLQL--
-YPLQLK-
-YPLQLG-
YYPLQL--
YFPLQL--
YPPLQL--
-YPLQLP-
-YPLQLE-
-YPLQLD-
YCPLQL--

PFQL	315	688	45.74	

-RPFQLP-
-APFQLR-
-APFQLT-
AVPFQL--
AHPFQL--
ALPFQL--
-APFQLH-
AKPFQL--
-APFQLS-
-APFQLM-
ARPFQL--
APPFQL--
-APFQLI-
AIPFQL--
AAPFQL--
AGPFQL--
ATPFQL--
AMPFQL--
AEPFQL--
AQPFQL--
-APFQLV-
-APFQLG-
-APFQLQ-
ASPFQL--
-APFQLK-
-APFQLA-
ANPFQL--
AYPFQL--
-APFQLN-
ADPFQL--
-APFQLE-
AFPFQL--
ACPFQL--
-APFQLW-
CCPFQL--
CMPFQL--
CSPFQL--

CGPFQL--
CQPFQL--
CFPFQL--
CTPFQL--
-CPFQLR-
-CPFQLK-
CRPFQL--
-CPFQLQ-
-CPFQLN-
DQPFQL--
DIPFQL--
-DPFQLR-
DMPFQL--
DVPFQL--
DGPFQL--
-DPFQLF-
DRPFQL--
DYPFQL--
-DPFQLM-
DSPFQL--
DLPFQL--
DAPFQL--
DTPFQL--
DKPFQL--
-DPFQLV-
-DPFQLN-
DHPFQL--
DFPFQL--
DWPFQL--
-DPFQLT-
-DPFQLS-
-DPFQLQ-
-DPFQLI-
-DPFQLH-
-EPFQLR-
-EPFQLV-
EYPFQL--
EGPFQL--
-EPFQLN-
-EPFQLK-
EKPFQL--
EWPFQL--
-EPFQLI-
EIPFQL--
-EPFQLT-
EAPFQL--
-EPFQLM-
EFPFQL--
ETPFQL--
-EPFQLQ-

-EPFQLA-
-EPFQLS-
-EPFQLH-
EMPFQL--
EHPFQL--
EVPFQL--
ENPFQL--
EQPFQL--
EEPFQL--
FKPFQL--
-FPFQLT-
-FPFQLR-
-FPFQLN-
FAPFQL--
FRPFQL--
FSPFQL--
-FPFQLS-
FMPFQL--
-FPFQLI-
FTPFQL--
-FPFQLM-
FGPFQL--
FNPFQL--
-FPFQLV-
FWPFQL--
FQPFQL--
FDPFQL--
FYPFQL--
-FPFQLH-
FFPFQL--
FEPFQL--
-GPFQLR-
-GPFQLV-
GLPFQL--
-GPFQLK-
GVPFQL--
GTPFQL--
GRPFQL--
-GPFQLT-
GYPFQL--
-GPFQLM-
GEPFQL--
-GPFQLH-
GMPFQL--
-GPFQLQ-
GNPFQL--
-GPFQLN-
GDPFQL--
GSPFQL--
GPPFQL--

GHPFQL--
-GPFQLG-
GKPFQL--
GQPFQL--
-GPFQLI-
-GPFQLA-
GFPFQL--
-GPFQLE-
HMPFQL--
-HPFQLV-
HAPFQL--
-HPFQLR-
-HPFQLY-
HIPFQL--
-HPFQLW-
HLPFQL--
HRPFQL--
HYPFQL--
HVPFQL--
HSPFQL--
-HPFQLN-
-HPFQLM-
HQPFQL--
-HPFQLS-
-HPFQLH-
HKPFQL--
HDPFQL--
-HPFQLT-
HPPFQL--
HTPFQL--
-HPFQLQ-
HGPFQL--
-HPFQLI-
-HPFQLA-
HNPFQL--
IRPFQL--
IHPFQL--
IGPFQL--
-IPFQLA-
IIPFQL--
IKPFQL--
IFPFQL--
ITPFQL--
ILPFQL--
-IPFQLQ-
-IPFQLS-
INPFQL--
IEPFQL--
IWPFQL--
IVPFQL--

−IPFQLT−
−IPFQLR−
IMPFQL−−
−IPFQLM−
−IPFQLW−
−IPFQLN−
−IPFQLK−
−IPFQLE−
IYPFQL−−
IQPFQL−−
−IPFQLI−
−IPFQLH−
IAPFQL−−
IPPFQL−−
IDPFQL−−
KSPFQL−−
KVPFQL−−
−KPFQLL−
−KPFQLK−
−KPFQLR−
KHPFQL−−
KPPFQL−−
−KPFQLH−
−KPFQLW−
−KPFQLV−
−KPFQLT−
−KPFQLA−
KNPFQL−−
KKPFQL−−
−KPFQLS−
KIPFQL−−
KQPFQL−−
−KPFQLI−
−KPFQLN−
KEPFQL−−
KDPFQL−−
KLPFQL−−
KAPFQL−−
KTPFQL−−
−KPFQLQ−
−KPFQLG−
LRPFQL−−
−LPFQLS−
LSPFQL−−
LLPFQL−−
−LPFQLR−
LMPFQL−−
LTPFQL−−
−LPFQLT−
LKPFQL−−

LNPFQL--
LQPFQL--
LDPFQL--
LHPFQL--
LIPFQL--
LAPFQL--
-LPFQLN-
-LPFQLK-
LEPFQL--
LYPFQL--
-LPFQLW-
-LPFQLH-
-LPFQLF-
-LPFQLC-
-LPFQLV-
LFPFQL--
LCPFQL--
-MPFQLR-
MGPFQL--
MSPFQL--
MRPFQL--
-MPFQLS-
MKPFQL--
MAPFQL--
MQPFQL--
-MPFQLK-
MEPFQL--
MLPFQL--
-MPFQLI-
MMPFQL--
-MPFQLV-
-MPFQLM-
MVPFQL--
MTPFQL--
MPPFQL--
-MPFQLN-
MNPFQL--
MHPFQL--
MWPFQL--
-MPFQLH-
-NPFQLR-
NRPFQL--
NGPFQL--
-NPFQLT-
NLPFQL--
NSPFQL--
NTPFQL--
-NPFQLV-
-NPFQLN-
-NPFQLI-

NKPFQL--
NIPFQL--
NDPFQL--
NNPFQL--
NVPFQL--
NPPFQL--
-NPFQLQ-
-NPFQLK-
NAPFQL--
-NPFQLS-
NWPFQL--
-NPFQLH-
NQPFQL--
NFPFQL--
-NPFQLA-
NCPFQL--
--PFQLRT
--PFQLTS
--PFQLRS
--PFQLRK
PTPFQL--
--PFQLTT
--PFQLKA
--PFQLRA
--PFQLSG
--PFQLST
--PFQLLK
--PFQLHT
--PFQLRL
--PFQLIH
--PFQLVR
--PFQLVM
--PFQLKN
--PFQLHG
--PFQLSS
--PFQLSA
--PFQLRD
--PFQLMS
--PFQLKY
--PFQLRG
--PFQLTW
--PFQLIA
--PFQLTG
--PFQLNS
--PFQLKR
--PFQLSM
--PFQLQS
--PFQLNH
--PFQLTR
--PFQLKS

--PFQLFW
--PFQLWR
--PFQLIG
--PFQLFS
--PFQLYH
--PFQLSR
--PFQLMQ
--PFQLLR
--PFQLLD
--PFQLGS
--PFQLAG
--PFQLRI
--PFQLQA
--PFQLMK
--PFQLLQ
--PFQLKW
--PFQLVD
--PFQLRH
--PFQLNR
--PFQLWD
--PFQLTQ
--PFQLRR
--PFQLNY
--PFQLNA
--PFQLMH
PNPFQL--
PGPFQL--
--PFQLYG
--PFQLWQ
--PFQLWG
--PFQLRY
--PFQLQT
--PFQLKT
--PFQLHS
--PFQLSI
--PFQLQI
--PFQLNL
PAPFQL--
--PFQLYW
--PFQLVY
--PFQLVK
--PFQLRF
--PFQLKL
--PFQLKH
--PFQLKE
--PFQLHA
--PFQLVI
--PFQLSH
--PFQLQG
--PFQLNI

--PFQLGG
PKPFQL--
--PFQLTN
--PFQLNV
--PFQLNK
--PFQLNE
--PFQLMN
--PFQLID
--PFQLAV
--PFQLYM
--PFQLWS
--PFQLVN
--PFQLTM
--PFQLRM
--PFQLRE
--PFQLQW
--PFQLNG
--PFQLKQ
--PFQLFN
--PFQLAM
--PFQLAA
--PFQLVQ
--PFQLTK
--PFQLTI
--PFQLTA
--PFQLSN
--PFQLQN
--PFQLMR
--PFQLMM
--PFQLMA
--PFQLIS
--PFQLIR
--PFQLHN
--PFQLGA
--PFQLTE
--PFQLSY
--PFQLSL
--PFQLRQ
--PFQLNT
--PFQLME
--PFQLKD
--PFQLHQ
--PFQLHH
--PFQLAS
PFPFQL--
PEPFQL--
PIPFQL--
PHPFQL--
--PFQLVE
--PFQLTH

--PFQLSQ
--PFQLSF
--PFQLNQ
--PFQLNN
--PFQLKK
--PFQLKI
--PFQLIN
--PFQLIK
--PFQLII
--PFQLHR
--PFQLFH
--PFQLEV
--PFQLES
--PFQLAK
PWPFQL--
--PFQLYN
--PFQLTV
--PFQLRW
--PFQLQV
--PFQLQR
--PFQLQK
--PFQLQH
--PFQLQF
--PFQLMD
--PFQLLW
--PFQLHV
--PFQLHK
--PFQLHD
--PFQLGY
--PFQLEY
--PFQLET
--PFQLEA
--PFQLCH
--PFQLYS
--PFQLYD
--PFQLVH
--PFQLQP
--PFQLQE
--PFQLNP
--PFQLLP
--PFQLIF
--PFQLHY
--PFQLHM
--PFQLGV
--PFQLFD
--PFQLEW
--PFQLEG
--PFQLAQ
--PFQLAH
PDPFQL--

QRPFQL--
QAPFQL--
QSPFQL--
QLPFQL--
-QPFQLT-
QPPFQL--
QVPFQL--
QKPFQL--
QGPFQL--
QTPFQL--
-QPFQLK-
-QPFQLI-
QEPFQL--
-QPFQLA-
-QPFQLM-
-QPFQLL-
QFPFQL--
-QPFQLQ-
QMPFQL--
QQPFQL--
-QPFQLV-
-QPFQLS-
QNPFQL--
QYPFQL--
-QPFQLN-
-QPFQLH-
QCPFQL--
-RPFQLK-
-RPFQLV-
RSPFQL--
-RPFQLI-
REPFQL--
RVPFQL--
-RPFQLT-
RLPFQL--
RGPFQL--
-RPFQLR-
RHPFQL--
RPPFQL--
-RPFQLS-
-RPFQLQ-
-RPFQLM-
-RPFQLN-
-RPFQLH-
RTPFQL--
RNPFQL--
RDPFQL--
RWPFQL--
RQPFQL--
RFPFQL--

-RPFQLA-
RMPFQL--
RAPFQL--
-RPFQLW-
-RPFQLE-
RCPFQL--
-SPFQLK-
SRPFQL--
SVPFQL--
-SPFQLR-
SFPFQL--
-SPFQLN-
SMPFQL--
-SPFQLS-
SWPFQL--
SIPFQL--
SGPFQL--
SYPFQL--
-SPFQLV-
SEPFQL--
SAPFQL--
SNPFQL--
-SPFQLQ-
-SPFQLT-
SLPFQL--
SHPFQL--
SPPFQL--
-SPFQLH-
STPFQL--
-SPFQLM-
-SPFQLI-
SKPFQL--
SDPFQL--
-SPFQLG-
-SPFQLA-
SCPFQL--
SQPFQL--
TRPFQL--
TSPFQL--
TFPFQL--
-TPFQLR-
TWPFQL--
TVPFQL--
-TPFQLV-
TGPFQL--
-TPFQLW-
TTPFQL--
TMPFQL--
-TPFQLT-
TKPFQL--

−TPFQLM−
TLPFQL−−
THPFQL−−
TYPFQL−−
TQPFQL−−
TDPFQL−−
TAPFQL−−
−TPFQLS−
TIPFQL−−
−TPFQLQ−
TEPFQL−−
TPPFQL−−
−TPFQLN−
−TPFQLA−
−TPFQLY−
TNPFQL−−
−TPFQLK−
TCPFQL−−
VSPFQL−−
−VPFQLR−
−VPFQLS−
VQPFQL−−
VTPFQL−−
VRPFQL−−
VVPFQL−−
−VPFQLN−
VLPFQL−−
VNPFQL−−
−VPFQLK−
VKPFQL−−
VIPFQL−−
VHPFQL−−
VMPFQL−−
VWPFQL−−
−VPFQLT−
VFPFQL−−
−VPFQLQ−
VPPFQL−−
VGPFQL−−
−VPFQLM−
VAPFQL−−
−VPFQLV−
VYPFQL−−
−VPFQLA−
−VPFQLG−
−VPFQLE−
VEPFQL−−
VDPFQL−−
−VPFQLI−
−VPFQLH−

-VPFQLC-
-WPFQLR-
WRPFQL--
WVPFQL--
WQPFQL--
WGPFQL--
WKPFQL--
-WPFQLV-
-WPFQLT-
WNPFQL--
-WPFQLH-
WFPFQL--
WAPFQL--
WMPFQL--
WHPFQL--
WPPFQL--
-WPFQLS-
WLPFQL--
WEPFQL--
-WPFQLI-
WSPFQL--
-WPFQLN-
WDPFQL--
YRPFQL--
YGPFQL--
YAPFQL--
YSPFQL--
-YPFQLR-
YVPFQL--
YTPFQL--
YQPFQL--
-YPFQLK-
-YPFQLV-
YLPFQL--
YKPFQL--
-YPFQLI-
YNPFQL--
-YPFQLS-
-YPFQLM-
YEPFQL--
YDPFQL--
YHPFQL--
YFPFQL--
-YPFQLT-
-YPFQLN-
YWPFQL--
-YPFQLQ-
-YPFQLA-
-YPFQLH-
YMPFQL--

	-YPFQLG-		
PQSL	36	863	45.74
	-HPQSLG-		
	-IPQSLG-		
	--PQSLVP		
	--PQSLTD		
	--PQSLPV		
	--PQSLPS		
	--PQSLPF		
	--PQSLEF		
	--PQSLDS		
	-APQSLR-		
	ASPQSL--		
	ALPQSL--		
	ARPQSL--		
	AKPQSL--		
	AIPQSL--		
	ATPQSL--		
	-APQSLS-		
	AGPQSL--		
	-APQSLL-		
	-APQSLA-		
	ANPQSL--		
	-APQSLT-		
	AQPQSL--		
	-APQSLN-		
	-APQSLQ-		
	-APQSLF-		
	AAPQSL--		
	-APQSLM-		
	-APQSLK-		
	-APQSLY-		
	-APQSLV-		
	AFPQSL--		
	AEPQSL--		
	AHPQSL--		
	AVPQSL--		
	-APQSLI-		
	-APQSLW-		
	AMPQSL--		
	-APQSLH-		
	-APQSLE-		
	ACPQSL--		
	ADPQSL--		
	-APQSLG-		
	CKPQSL--		
	CSPQSL--		
	CAPQSL--		
	CMPQSL--		
	CWPQSL--		

CRPQSL--
CGPQSL--
CEPQSL--
CTPQSL--
-CPQSLY-
-CPQSLR-
CPPQSL--
CNPQSL--
CHPQSL--
CFPQSL--
CYPQSL--
-CPQSLK-
-CPQSLI-
-CPQSLF-
-CPQSLL-
-CPQSLH-
CLPQSL--
-CPQSLQ-
CDPQSL--
CCPQSL--
CQPQSL--
-CPQSLS-
-DPQSLR-
DRPQSL--
DGPQSL--
-DPQSLW-
DMPQSL--
DAPQSL--
DLPQSL--
DKPQSL--
DYPQSL--
DSPQSL--
DNPQSL--
DVPQSL--
-DPQSLS-
DEPQSL--
-DPQSLY-
DIPQSL--
DWPQSL--
DQPQSL--
-DPQSLV-
DTPQSL--
-DPQSLL-
-DPQSLK-
DFPQSL--
-DPQSLF-
-DPQSLH-
DPPQSL--
-DPQSLT-
-DPQSLM-

-EPQSLR-
EWPQSL--
ERPQSL--
ELPQSL--
EFPQSL--
ESPQSL--
EGPQSL--
-EPQSLT-
EKPQSL--
EQPQSL--
EMPQSL--
-EPQSLW-
ENPQSL--
EYPQSL--
EVPQSL--
-EPQSLL-
ETPQSL--
-EPQSLY-
-EPQSLK-
EIPQSL--
-EPQSLI-
-EPQSLH-
EAPQSL--
-EPQSLA-
EEPQSL--
-EPQSLM-
-EPQSLC-
EHPQSL--
-EPQSLF-
EPPQSL--
EDPQSL--
FRPQSL--
FGPQSL--
FQPQSL--
-FPQSLT-
FLPQSL--
FSPQSL--
-FPQSLV-
FVPQSL--
-FPQSLL-
-FPQSLH-
FKPQSL--
FAPQSL--
FPPQSL--
FEPQSL--
-FPQSLI-
-FPQSLS-
-FPQSLF-
FMPQSL--
FDPQSL--

-FPQSLW-
-FPQSLQ-
-FPQSLA-
FNPQSL--
-FPQSLY-
FHPQSL--
-FPQSLM-
FYPQSL--
FIPQSL--
-FPQSLK-
-FPQSLN-
FWPQSL--
-FPQSLE-
FCPQSL--
-GPQSLR-
-GPQSLL-
-GPQSLW-
GLPQSL--
-GPQSLK-
GVPQSL--
GSPQSL--
-GPQSLS-
GTPQSL--
GAPQSL--
GMPQSL--
-GPQSLY-
-GPQSLT-
GRPQSL--
GQPQSL--
GGPQSL--
GWPQSL--
-GPQSLV-
GIPQSL--
-GPQSLM-
GHPQSL--
-GPQSLH-
GNPQSL--
GKPQSL--
-GPQSLI-
-GPQSLF-
GFPQSL--
GPPQSL--
-GPQSLN-
-GPQSLA-
GCPQSL--
-GPQSLQ-
-GPQSLG-
GDPQSL--
-GPQSLE-
HSPQSL--

HYPQSL--
HGPQSL--
-HPQSLY-
-HPQSLR-
HWPQSL--
HMPQSL--
HLPQSL--
-HPQSLL-
HKPQSL--
HAPQSL--
-HPQSLF-
-HPQSLI-
-HPQSLV-
-HPQSLT-
HHPQSL--
-HPQSLS-
-HPQSLA-
HTPQSL--
HPPQSL--
HIPQSL--
HEPQSL--
-HPQSLK-
HFPQSL--
HQPQSL--
-HPQSLN-
-HPQSLM-
-HPQSLW-
-HPQSLE-
-IPQSLR-
ILPQSL--
-IPQSLL-
IYPQSL--
IGPQSL--
IVPQSL--
IRPQSL--
IKPQSL--
ITPQSL--
-IPQSLS-
ISPQSL--
-IPQSLM-
-IPQSLH-
IFPQSL--
IMPQSL--
IAPQSL--
IEPQSL--
-IPQSLY-
-IPQSLT-
-IPQSLN-
IQPQSL--
-IPQSLV-

-IPQSLI-
-IPQSLW-
IHPQSL--
-IPQSLQ-
-IPQSLK-
IDPQSL--
IIPQSL--
ICPQSL--
IWPQSL--
-IPQSLA-
INPQSL--
-KPQSLF-
KGPQSL--
-KPQSLW-
KYPQSL--
KLPQSL--
KIPQSL--
KAPQSL--
-KPQSLI-
-KPQSLH-
KWPQSL--
KQPQSL--
-KPQSLT-
-KPQSLK-
KHPQSL--
KKPQSL--
KTPQSL--
KFPQSL--
KDPQSL--
KPPQSL--
KMPQSL--
-KPQSLV-
-KPQSLN-
-KPQSLA-
KNPQSL--
-KPQSLQ-
KEPQSL--
-KPQSLG-
-KPQSLC-
-LPQSLY-
-LPQSLR-
LTPQSL--
LSPQSL--
LGPQSL--
LYPQSL--
LVPQSL--
LNPQSL--
LKPQSL--
-LPQSLT-
LWPQSL--

−LPQSLV−
LQPQSL−−
−LPQSLL−
−LPQSLW−
LPPQSL−−
LIPQSL−−
LHPQSL−−
−LPQSLK−
−LPQSLS−
−LPQSLH−
LMPQSL−−
LEPQSL−−
−LPQSLI−
LDPQSL−−
−LPQSLN−
−LPQSLM−
−LPQSLF−
LCPQSL−−
−LPQSLE−
−MPQSLR−
MRPQSL−−
MGPQSL−−
MSPQSL−−
−MPQSLK−
MKPQSL−−
MNPQSL−−
MMPQSL−−
MTPQSL−−
MPPQSL−−
−MPQSLW−
MLPQSL−−
−MPQSLY−
−MPQSLL−
−MPQSLF−
MDPQSL−−
MEPQSL−−
MHPQSL−−
−MPQSLN−
MAPQSL−−
−MPQSLI−
MWPQSL−−
MQPQSL−−
−MPQSLE−
−MPQSLA−
MCPQSL−−
−MPQSLQ−
MFPQSL−−
−MPQSLV−
−MPQSLT−
−MPQSLH−

```
-MPQSLS-
-MPQSLM-
MIPQSL--
NAPQSL--
NRPQSL--
NWPQSL--
NEPQSL--
NSPQSL--
NKPQSL--
-NPQSLL-
NVPQSL--
NTPQSL--
NYPQSL--
NNPQSL--
NLPQSL--
-NPQSLK-
-NPQSLR-
-NPQSLS-
NQPQSL--
-NPQSLV-
NHPQSL--
-NPQSLW-
NGPQSL--
-NPQSLI-
NIPQSL--
-NPQSLT-
-NPQSLM-
NCPQSL--
-NPQSLH-
NMPQSL--
-NPQSLQ-
NDPQSL--
NPPQSL--
-NPQSLN-
-NPQSLF-
NFPQSL--
-NPQSLA-
-NPQSLC-
--PQSLVS
--PQSLLS
--PQSLRY
--PQSLMG
--PQSLSG
--PQSLIR
--PQSLVR
--PQSLLG
--PQSLTT
PRPQSL--
--PQSLSF
--PQSLHS
```

PSPQSL--
--PQSLRG
--PQSLFS
PGPQSL--
--PQSLVY
--PQSLRA
--PQSLLA
--PQSLVA
--PQSLFG
--PQSLTY
--PQSLWS
--PQSLSV
--PQSLSI
--PQSLFT
--PQSLTR
--PQSLTH
--PQSLRN
--PQSLLT
--PQSLQS
--PQSLNS
--PQSLLN
--PQSLIT
--PQSLVK
--PQSLNA
--PQSLLV
--PQSLKV
--PQSLMW
--PQSLTV
--PQSLTL
--PQSLKT
--PQSLFN
--PQSLWG
--PQSLSS
--PQSLQT
--PQSLIG
--PQSLVL
--PQSLIV
--PQSLHT
PWPQSL--
--PQSLTG
--PQSLST
--PQSLSA
--PQSLMS
PYPQSL--
--PQSLVH
--PQSLTA
--PQSLSK
--PQSLKN
--PQSLYS
--PQSLVV

--PQSLMQ
--PQSLLI
--PQSLHR
--PQSLYH
--PQSLYG
--PQSLYA
--PQSLWR
--PQSLVM
--PQSLYT
--PQSLYQ
--PQSLWM
--PQSLVW
--PQSLVN
--PQSLMA
--PQSLLQ
--PQSLKG
--PQSLIQ
--PQSLHL
--PQSLAG
PVPQSL--
--PQSLWA
--PQSLSN
--PQSLRQ
--PQSLQR
--PQSLAW
--PQSLAS
--PQSLYL
--PQSLSW
--PQSLSR
--PQSLRW
--PQSLRE
--PQSLIM
PQPQSL--
--PQSLSL
--PQSLVF
--PQSLTN
--PQSLTM
--PQSLSM
--PQSLIH
--PQSLHG
--PQSLVG
--PQSLQW
--PQSLMM
--PQSLLK
--PQSLFQ
PAPQSL--
--PQSLTI
--PQSLLD
--PQSLKH
--PQSLIW

--PQSLWK
--PQSLIK
--PQSLHK
--PQSLEA
--PQSLAY
PMPQSL--
--PQSLQG
--PQSLNL
--PQSLMY
--PQSLKF
--PQSLLM
--PQSLHW
--PQSLEH
--PQSLTW
--PQSLNW
--PQSLNT
--PQSLHM
--PQSLFR
--PQSLFK
--PQSLEW
--PQSLWQ
--PQSLVI
--PQSLRD
--PQSLQY
--PQSLGL
--PQSLAT
--PQSLYR
--PQSLSY
--PQSLLH
--PQSLKD
--PQSLGT
--PQSLYD
--PQSLNV
--PQSLMD
--PQSLKR
--PQSLHF
--PQSLFH
--PQSLFD
--PQSLET
--PQSLWH
--PQSLVQ
--PQSLIY
--PQSLGG
--PQSLES
--PQSLWL
--PQSLTQ
--PQSLMI
--PQSLHQ
--PQSLAN
--PQSLWW

--PQSLTF
--PQSLHY
--PQSLHH
--PQSLGV
--PQSLGS
--PQSLFA
--PQSLAK
--PQSLAF
PIPQSL--
PTPQSL--
--PQSLYK
--PQSLWN
--PQSLWI
--PQSLWD
--PQSLKE
--PQSLFL
--PQSLFI
--PQSLAM
--PQSLYN
--PQSLWE
--PQSLVD
--PQSLNM
--PQSLMN
--PQSLLE
--PQSLKI
--PQSLKA
--PQSLID
--PQSLAQ
PNPQSL--
--PQSLVE
--PQSLQN
--PQSLNQ
--PQSLKY
--PQSLKW
--PQSLKQ
--PQSLIN
--PQSLIA
--PQSLHE
--PQSLFE
--PQSLQL
--PQSLQK
--PQSLNH
--PQSLNG
--PQSLNF
--PQSLME
--PQSLHN
--PQSLEV
--PQSLEM
--PQSLAI
PEPQSL--

--PQSLQV
--PQSLQQ
--PQSLQM
--PQSLQI
--PQSLQH
--PQSLNY
--PQSLNN
--PQSLMV
--PQSLGA
--PQSLEG
--PQSLAR
--PQSLSH
--PQSLQD
--PQSLNI
--PQSLMK
--PQSLMH
--PQSLGY
--PQSLAV
--PQSLNK
--PQSLGW
--PQSLGF
--PQSLEY
--PQSLYC
--PQSLTE
--PQSLRC
--PQSLPY
--PQSLKM
--PQSLKC
--PQSLIE
--PQSLIC
--PQSLHD
--PQSLHA
--PQSLGD
--PQSLER
--PQSLEN
--PQSLEL
--PQSLEK
--PQSLED
--PQSLCS
--PQSLCN
--PQSLCM
--PQSLCG
--PQSLCA
-PPQSLN-
-PPQSLL-
-PPQSLH-
-PPQSLF-
PPPQSL--
-QPQSLR-
QVPQSL--

-QPQSLI-
QYPQSL--
QRPQSL--
QSPQSL--
-QPQSLL-
-QPQSLY-
-QPQSLH-
QLPQSL--
QTPQSL--
-QPQSLF-
QKPQSL--
QHPQSL--
QGPQSL--
QMPQSL--
-QPQSLV-
QAPQSL--
-QPQSLS-
QWPQSL--
QQPQSL--
-QPQSLT-
-QPQSLN-
-QPQSLK-
QPPQSL--
QFPQSL--
-QPQSLA-
QNPQSL--
-QPQSLQ-
QIPQSL--
QDPQSL--
-QPQSLW-
RVPQSL--
RSPQSL--
RAPQSL--
-RPQSLS-
-RPQSLY-
RKPQSL--
-RPQSLV-
-RPQSLF-
RHPQSL--
RFPQSL--
RTPQSL--
-RPQSLH-
RDPQSL--
RYPQSL--
REPQSL--
RPPQSL--
-RPQSLK-
RQPQSL--
-RPQSLM-
-RPQSLN-

−RPQSLA−
RNPQSL−−
−RPQSLW−
−RPQSLQ−
−RPQSLE−
−RPQSLG−
RCPQSL−−
−RPQSLP−
SRPQSL−−
SGPQSL−−
SSPQSL−−
SYPQSL−−
−SPQSLR−
SIPQSL−−
−SPQSLH−
SNPQSL−−
SLPQSL−−
−SPQSLI−
STPQSL−−
−SPQSLS−
SHPQSL−−
−SPQSLT−
SEPQSL−−
−SPQSLY−
−SPQSLK−
SFPQSL−−
SAPQSL−−
SKPQSL−−
−SPQSLF−
SPPQSL−−
−SPQSLV−
SMPQSL−−
−SPQSLN−
−SPQSLW−
SDPQSL−−
−SPQSLA−
SQPQSL−−
−SPQSLE−
−SPQSLQ−
−SPQSLG−
SCPQSL−−
TRPQSL−−
TLPQSL−−
−TPQSLL−
TYPQSL−−
TAPQSL−−
TGPQSL−−
−TPQSLK−
−TPQSLM−
TPPQSL−−

TKPQSL--
TTPQSL--
-TPQSLV-
-TPQSLS-
TSPQSL--
-TPQSLQ-
-TPQSLI-
TIPQSL--
-TPQSLH-
-TPQSLF-
TQPQSL--
-TPQSLA-
TNPQSL--
TDPQSL--
THPQSL--
-TPQSLY-
TMPQSL--
TWPQSL--
-TPQSLE-
TEPQSL--
TCPQSL--
-VPQSLR-
VLPQSL--
-VPQSLS-
VRPQSL--
VVPQSL--
VTPQSL--
VQPQSL--
-VPQSLI-
VPPQSL--
VKPQSL--
-VPQSLQ-
VYPQSL--
VSPQSL--
-VPQSLT-
VWPQSL--
-VPQSLA-
-VPQSLM-
VGPQSL--
VHPQSL--
-VPQSLK-
VNPQSL--
VAPQSL--
VMPQSL--
-VPQSLN-
VFPQSL--
-VPQSLV-
VDPQSL--
VEPQSL--
VCPQSL--

-VPQSLW-
-VPQSLE-
-VPQSLC-
WTPQSL--
WSPQSL--
WGPQSL--
WRPQSL--
-WPQSLL-
-WPQSLT-
WNPQSL--
WEPQSL--
-WPQSLR-
-WPQSLM-
WKPQSL--
WQPQSL--
-WPQSLA-
-WPQSLS-
-WPQSLI-
WIPQSL--
-WPQSLQ-
WFPQSL--
-WPQSLH-
-WPQSLK-
-WPQSLV-
WYPQSL--
-WPQSLN-
-WPQSLY-
WPPQSL--
WDPQSL--
WHPQSL--
WWPQSL--
WCPQSL--
YLPQSL--
YRPQSL--
-YPQSLY-
-YPQSLM-
YMPQSL--
YKPQSL--
-YPQSLR-
-YPQSLK-
-YPQSLL-
-YPQSLT-
YYPQSL--
-YPQSLI-
-YPQSLS-
YPPQSL--
YTPQSL--
YQPQSL--
-YPQSLH-
YGPQSL--

-YPQSLN-
-YPQSLQ-
-YPQSLV-
YEPQSL--
-YPQSLF-
YNPQSL--
YAPQSL--
-YPQSLA-
YDPQSL--
YHPQSL--
-YPQSLW-
-YPQSLE-
-YPQSLC-
YCPQSL--
-YPQSLP-

PWSM	179	759	45.73

-MPWSMH-
-NPWSMG-
--PWSMEL
ARPWSM--
ALPWSM--
AKPWSM--
-APWSMY-
-APWSMS-
-APWSMN-
-APWSML-
-APWSMA-
-APWSMR-
-APWSMQ-
-APWSMM-
AHPWSM--
ANPWSM--
AWPWSM--
-APWSMV-
-APWSMH-
AMPWSM--
-APWSMT-
-APWSMG-
APPWSM--
AVPWSM--
ATPWSM--
AGPWSM--
-APWSMW-
AEPWSM--
ADPWSM--
AQPWSM--
-APWSMI-
AFPWSM--
-APWSMK-
-APWSME-

-APWSMD-
AAPWSM--
CDPWSM--
CCPWSM--
CQPWSM--
-CPWSMK-
CPPWSM--
CLPWSM--
CFPWSM--
-CPWSMY-
-CPWSMT-
CKPWSM--
-DPWSMW-
DAPWSM--
DFPWSM--
DKPWSM--
DVPWSM--
-DPWSMR-
DTPWSM--
DRPWSM--
-DPWSMK-
-DPWSMI-
DHPWSM--
-DPWSMV-
-DPWSMH-
DGPWSM--
-DPWSMT-
-DPWSMM-
DNPWSM--
DMPWSM--
DLPWSM--
-EPWSMI-
EVPWSM--
ESPWSM--
-EPWSMH-
EAPWSM--
EGPWSM--
-EPWSMF-
EHPWSM--
-EPWSMV-
EMPWSM--
EFPWSM--
-EPWSMS-
-EPWSMW-
EPPWSM--
-EPWSML-
-EPWSMY-
EIPWSM--
EYPWSM--
EKPWSM--

EQPWSM--
-EPWSMK-
ETPWSM--
ENPWSM--
ELPWSM--
EEPWSM--
-EPWSMR-
-EPWSME-
-EPWSMA-
ECPWSM--
FYPWSM--
-FPWSMS-
-FPWSMY-
FKPWSM--
FRPWSM--
-FPWSMR-
-FPWSMM-
FEPWSM--
FQPWSM--
FGPWSM--
FLPWSM--
FHPWSM--
-FPWSMQ-
-FPWSMT-
-FPWSMA-
FIPWSM--
FFPWSM--
-FPWSML-
-FPWSMH-
FMPWSM--
FVPWSM--
-FPWSMN-
-FPWSMK-
-FPWSMG-
-FPWSME-
GRPWSM--
GSPWSM--
-GPWSMR-
-GPWSMH-
GAPWSM--
GQPWSM--
-GPWSMT-
-GPWSMW-
GKPWSM--
-GPWSMV-
GVPWSM--
GFPWSM--
-GPWSMS-
GTPWSM--
-GPWSMK-

GPPWSM--
GDPWSM--
GMPWSM--
-GPWSMI-
-GPWSME-
GNPWSM--
GIPWSM--
-GPWSMM-
-GPWSMG-
-GPWSMA-
-GPWSMY-
-GPWSMF-
GEPWSM--
-GPWSMN-
GYPWSM--
-HPWSMT-
-HPWSML-
-HPWSMR-
HGPWSM--
HLPWSM--
HVPWSM--
HQPWSM--
HAPWSM--
HMPWSM--
-HPWSMM-
HRPWSM--
HTPWSM--
-HPWSMS-
-HPWSMK-
HPPWSM--
HKPWSM--
HDPWSM--
HSPWSM--
-HPWSMQ-
HFPWSM--
-HPWSMY-
-HPWSMI-
-HPWSMH-
HIPWSM--
HHPWSM--
-HPWSMA-
HNPWSM--
-HPWSMW-
HWPWSM--
-HPWSMV-
-IPWSMR-
IAPWSM--
IRPWSM--
IKPWSM--
IGPWSM--

ISPWSM--
IFPWSM--
IIPWSM--
IQPWSM--
ILPWSM--
ITPWSM--
IVPWSM--
-IPWSMT-
-IPWSMM-
-IPWSMV-
-IPWSMN-
-IPWSMG-
INPWSM--
-IPWSML-
-IPWSMK-
IWPWSM--
-IPWSMI-
-IPWSMH-
IPPWSM--
IHPWSM--
-IPWSMY-
-IPWSMA-
IEPWSM--
-IPWSMS-
-IPWSME-
IDPWSM--
KSPWSM--
KVPWSM--
KRPWSM--
-KPWSML-
KAPWSM--
-KPWSMQ-
-KPWSMF-
KTPWSM--
KFPWSM--
-KPWSMS-
-KPWSMY-
KNPWSM--
-KPWSMK-
-KPWSMR-
KLPWSM--
-KPWSMM-
KGPWSM--
KWPWSM--
-KPWSMV-
-KPWSMH-
KIPWSM--
KDPWSM--
KYPWSM--
-KPWSMI-

-KPWSME-
KCPWSM--
-KPWSMW-
-KPWSMG-
KEPWSM--
LRPWSM--
LKPWSM--
LSPWSM--
LLPWSM--
LHPWSM--
LGPWSM--
LMPWSM--
-LPWSMY-
LVPWSM--
LYPWSM--
-LPWSMS-
LIPWSM--
-LPWSMF-
LPPWSM--
-LPWSMH-
-LPWSMW-
-LPWSMT-
-LPWSMK-
LNPWSM--
LQPWSM--
LEPWSM--
LAPWSM--
-LPWSMV-
-LPWSMG-
LCPWSM--
-LPWSMA-
LFPWSM--
LWPWSM--
-LPWSMI-
-LPWSMQ-
-LPWSMN-
-LPWSMM-
-LPWSME-
-MPWSMW-
MGPWSM--
MLPWSM--
-MPWSML-
MFPWSM--
MNPWSM--
MSPWSM--
MVPWSM--
-MPWSMK-
MPPWSM--
-MPWSMR-
MDPWSM--

MAPWSM--
MHPWSM--
-MPWSMM-
-MPWSMI-
MIPWSM--
MYPWSM--
-MPWSMN-
MEPWSM--
MTPWSM--
MRPWSM--
-MPWSMY-
MMPWSM--
MQPWSM--
-MPWSMV-
-MPWSMS-
-MPWSMG-
-MPWSME-
-MPWSMA-
MKPWSM--
-NPWSMR-
NSPWSM--
-NPWSMV-
NFPWSM--
NRPWSM--
NLPWSM--
-NPWSMW-
-NPWSMT-
NIPWSM--
NQPWSM--
NEPWSM--
-NPWSMS-
NTPWSM--
NNPWSM--
NHPWSM--
NVPWSM--
-NPWSMF-
-NPWSMK-
NKPWSM--
-NPWSML-
-NPWSMI-
-NPWSMQ-
-NPWSMM-
NMPWSM--
-NPWSMY-
-NPWSMH-
NCPWSM--
NWPWSM--
-NPWSMN-
-NPWSME-
PSPWSM--

--PWSMRG
--PWSMLG
--PWSMST
--PWSMSS
--PWSMRA
--PWSMSA
--PWSMLN
--PWSMTA
--PWSMLT
--PWSMGS
--PWSMRR
--PWSMMS
--PWSMAA
PIPWSM--
PLPWSM--
--PWSMTH
--PWSMHN
--PWSMES
--PWSMYN
--PWSMFS
--PWSMWN
--PWSMYH
--PWSMHA
--PWSMVV
--PWSMMR
--PWSMTE
PVPWSM--
--PWSMYT
--PWSMYG
--PWSMIS
--PWSMIK
--PWSMTR
--PWSMNW
--PWSMIT
--PWSMSG
--PWSMVM
--PWSMRH
--PWSMNL
--PWSMLR
--PWSMAS
--PWSMVT
--PWSMTQ
--PWSMTG
--PWSMQN
--PWSMKV
--PWSMFT
--PWSMTY
--PWSMTT
--PWSMIH
--PWSMYK

--PWSMVQ
--PWSMRQ
--PWSMNS
--PWSMLQ
--PWSMIR
--PWSMFR
--PWSMWA
--PWSMVG
--PWSMNV
PRPWSM--
PGPWSM--
--PWSMSY
--PWSMNT
--PWSMKS
--PWSMIE
--PWSMID
--PWSMVH
--PWSMVE
--PWSMVD
--PWSMSN
--PWSMRE
--PWSMNQ
--PWSMLK
--PWSMKM
--PWSMIW
--PWSMHR
PMPWSM--
--PWSMVY
--PWSMRW
--PWSMNA
--PWSMMV
--PWSMLE
--PWSMKY
--PWSMKA
--PWSMIA
--PWSMGT
--PWSMFH
PNPWSM--
PEPWSM--
--PWSMYF
--PWSMTK
--PWSMMD
--PWSMHW
--PWSMHM
--PWSMHL
--PWSMFD
PTPWSM--
PQPWSM--
PDPWSM--
--PWSMYR

--PWSMWT
--PWSMQH
--PWSMQA
--PWSMMT
--PWSMKQ
--PWSMKL
--PWSMHV
--PWSMHK
--PWSMGV
--PWSMAT
--PWSMYS
--PWSMWI
--PWSMWG
--PWSMVI
--PWSMTV
--PWSMTI
--PWSMSL
--PWSMQS
--PWSMQG
--PWSMMW
--PWSMMK
--PWSMKT
--PWSMKD
--PWSMHS
--PWSMGY
--PWSMGL
--PWSMGI
--PWSMFQ
PAPWSM--
--PWSMWS
--PWSMSW
--PWSMRD
--PWSMNI
--PWSMLH
--PWSMKI
--PWSMKF
--PWSMKE
--PWSMIM
--PWSMHY
--PWSMEV
--PWSMEG
--PWSMAV
PWPWSM--
--PWSMYQ
--PWSMYM
--PWSMYE
--PWSMWR
--PWSMVF
--PWSMTD
--PWSMSM

--PWSMSK
--PWSMQW
--PWSMQT
--PWSMQI
--PWSMPL
--PWSMNG
--PWSMIG
--PWSMHD
--PWSMAR
--PWSMAK
--PWSMAF
PKPWSM--
PCPWSM--
--PWSMYD
--PWSMWW
--PWSMWE
--PWSMTW
--PWSMSQ
--PWSMME
--PWSMLD
--PWSMKK
--PWSMIN
--PWSMII
--PWSMHT
--PWSMHF
--PWSMFL
--PWSMFE
--PWSMEY
--PWSMAN
PFPWSM--
--PWSMWQ
--PWSMWL
--PWSMTN
--PWSMSH
--PWSMNR
--PWSMNH
--PWSMMQ
--PWSMGF
--PWSMFW
--PWSMFI
--PWSMFF
--PWSMET
--PWSMER
--PWSMDS
--PWSMAM
PHPWSM--
PYPWSM--
--PWSMTP
--PWSMTM
--PWSMTF

--PWSMSF
--PWSMSE
--PWSMQR
--PWSMQD
--PWSMPH
--PWSMNY
--PWSMNN
--PWSMMN
--PWSMMA
--PWSMIF
--PWSMHQ
--PWSMEM
--PWSMEA
--PWSMCL
--PWSMAQ
--PWSMAD
-PPWSML-
-QPWSMV-
QRPWSM--
-QPWSMW-
QHPWSM--
-QPWSMY-
-QPWSMM-
QDPWSM--
QPPWSM--
QSPWSM--
QWPWSM--
-QPWSMS-
QFPWSM--
QAPWSM--
-QPWSML-
-QPWSMA-
-QPWSMR-
-QPWSMH-
QGPWSM--
QVPWSM--
QIPWSM--
-QPWSMI-
-QPWSMT-
QLPWSM--
QTPWSM--
-QPWSMQ-
-QPWSMK-
-QPWSMF-
-QPWSME-
-RPWSMT-
RSPWSM--
RGPWSM--
-RPWSMV-
-RPWSMR-

RLPWSM--
-RPWSMH-
RVPWSM--
REPWSM--
-RPWSMF-
-RPWSMS-
-RPWSMN-
RYPWSM--
-RPWSMK-
-RPWSMM-
RTPWSM--
-RPWSMI-
RHPWSM--
-RPWSMW-
RNPWSM--
RFPWSM--
RWPWSM--
-RPWSMA-
RIPWSM--
RMPWSM--
RDPWSM--
RPPWSM--
-RPWSMY-
-RPWSMQ-
-RPWSMG-
RCPWSM--
-RPWSME-
SSPWSM--
SLPWSM--
SRPWSM--
SAPWSM--
-SPWSMK-
SGPWSM--
-SPWSMT-
-SPWSMY-
-SPWSMW-
-SPWSMI-
SKPWSM--
-SPWSMV-
-SPWSMR-
-SPWSMN-
SNPWSM--
SPPWSM--
SEPWSM--
-SPWSMS-
SVPWSM--
-SPWSMA-
SFPWSM--
SIPWSM--
-SPWSMF-

-SPWSMH-
STPWSM--
SQPWSM--
-SPWSMQ-
-SPWSME-
SMPWSM--
THPWSM--
-TPWSMT-
TRPWSM--
-TPWSMS-
-TPWSMK-
TKPWSM--
-TPWSMQ-
TGPWSM--
TQPWSM--
-TPWSMM-
-TPWSML-
TNPWSM--
TEPWSM--
TSPWSM--
TMPWSM--
-TPWSMY-
TVPWSM--
-TPWSMN-
TIPWSM--
TAPWSM--
TLPWSM--
TFPWSM--
TDPWSM--
-TPWSMV-
-TPWSME-
TYPWSM--
-TPWSMI-
TPPWSM--
TTPWSM--
-TPWSMH-
-TPWSMA-
TWPWSM--
-TPWSMG-
-TPWSMF-
-TPWSMW-
-VPWSMR-
VRPWSM--
VNPWSM--
-VPWSMK-
VIPWSM--
-VPWSML-
-VPWSMS-
-VPWSME-
VGPWSM--

VVPWSM--
VYPWSM--
-VPWSMW-
-VPWSMI-
VTPWSM--
VAPWSM--
-VPWSMY-
-VPWSMT-
VWPWSM--
VKPWSM--
VDPWSM--
-VPWSMN-
-VPWSMM-
VLPWSM--
VPPWSM--
-VPWSMA-
VFPWSM--
-VPWSMH-
VEPWSM--
-VPWSMV-
-VPWSMG-
VMPWSM--
VQPWSM--
VHPWSM--
-VPWSMF-
WRPWSM--
-WPWSMR-
WHPWSM--
WIPWSM--
WSPWSM--
WDPWSM--
-WPWSMT-
WTPWSM--
WLPWSM--
WEPWSM--
-WPWSMM-
-WPWSMH-
-WPWSMA-
WMPWSM--
WFPWSM--
WAPWSM--
-WPWSMN-
-WPWSMW-
-WPWSMS-
-WPWSML-
-WPWSMI-
WKPWSM--
-YPWSMR-
YYPWSM--
-YPWSML-

```
           -YPWSMV-
           YLPWSM--
           -YPWSMS-
           YRPWSM--
           -YPWSMA-
           YGPWSM--
           -YPWSMT-
           YQPWSM--
           YAPWSM--
           YWPWSM--
           YSPWSM--
           -YPWSMK-
           YMPWSM--
           YTPWSM--
           YNPWSM--
           YFPWSM--
           -YPWSMQ-
           YDPWSM--
           -YPWSMG-
           -YPWSMF-
           YHPWSM--
           YEPWSM--
           -YPWSMW-
           -YPWSMH-
           -YPWSME-
PAWI        466        632     45.73
           IIPAWI--
           --PAWIGR
           AKPAWI--
           AVPAWI--
           -APAWIL-
           AYPAWI--
           -APAWIS-
           ATPAWI--
           -APAWII-
           ARPAWI--
           -APAWIR-
           AGPAWI--
           AFPAWI--
           -APAWIA-
           ANPAWI--
           ADPAWI--
           AWPAWI--
           -APAWIW-
           -APAWIF-
           AHPAWI--
           APPAWI--
           -APAWIT-
           -APAWIK-
           -APAWIH-
```

-APAWIE-
-APAWIV-
AIPAWI--
AEPAWI--
ACPAWI--
CGPAWI--
CTPAWI--
CSPAWI--
-CPAWIM-
CMPAWI--
CKPAWI--
-CPAWIT-
-CPAWIS-
-CPAWIQ-
-CPAWIK-
-CPAWIG-
-CPAWIA-
DWPAWI--
DGPAWI--
DHPAWI--
DLPAWI--
-DPAWIT-
DFPAWI--
DMPAWI--
-DPAWIY-
DVPAWI--
DRPAWI--
-DPAWIL-
-DPAWIR-
DKPAWI--
DIPAWI--
DAPAWI--
DQPAWI--
-DPAWIS-
DNPAWI--
DEPAWI--
-EPAWIT-
EVPAWI--
ERPAWI--
-EPAWIR-
-EPAWIH-
EKPAWI--
ESPAWI--
-EPAWIK-
ELPAWI--
EHPAWI--
EWPAWI--
ETPAWI--
-EPAWIN-
-EPAWIQ-

-EPAWIG-
EGPAWI--
EIPAWI--
-FPAWIR-
FRPAWI--
FTPAWI--
FLPAWI--
FDPAWI--
FSPAWI--
FEPAWI--
FAPAWI--
FNPAWI--
-FPAWIK-
-FPAWIF-
FHPAWI--
FYPAWI--
-FPAWIA-
FVPAWI--
-FPAWIW-
-FPAWIM-
-FPAWII-
FIPAWI--
FWPAWI--
-FPAWIV-
-FPAWIT-
-FPAWIS-
-FPAWIQ-
-FPAWIN-
GSPAWI--
GRPAWI--
GQPAWI--
GWPAWI--
GIPAWI--
-GPAWIT-
-GPAWIS-
-GPAWIL-
GVPAWI--
-GPAWIY-
GGPAWI--
GPPAWI--
-GPAWIV-
-GPAWIQ-
-GPAWII-
GMPAWI--
GEPAWI--
GDPAWI--
GLPAWI--
GAPAWI--
-GPAWIW-
GFPAWI--

-GPAWIM-
-GPAWIF-
-GPAWIG-
GHPAWI--
GKPAWI--
HRPAWI--
HSPAWI--
HAPAWI--
-HPAWIS-
-HPAWIK-
HVPAWI--
HTPAWI--
HPPAWI--
HQPAWI--
HEPAWI--
HYPAWI--
-HPAWIV-
-HPAWIH-
HMPAWI--
HKPAWI--
HWPAWI--
-HPAWIA-
HIPAWI--
-HPAWIL-
-HPAWII-
HLPAWI--
HGPAWI--
-IPAWIR-
IAPAWI--
IRPAWI--
IEPAWI--
ISPAWI--
-IPAWIT-
INPAWI--
IVPAWI--
-IPAWIS-
-IPAWIK-
-IPAWIG-
IHPAWI--
ITPAWI--
IFPAWI--
IYPAWI--
IDPAWI--
IQPAWI--
-IPAWIY-
-IPAWIM-
-IPAWIF-
IPPAWI--
IGPAWI--
-IPAWII-

-IPAWIA-
IWPAWI--
-IPAWIE-
ILPAWI--
IKPAWI--
KSPAWI--
-KPAWIH-
-KPAWIR-
KIPAWI--
-KPAWIQ-
KFPAWI--
KGPAWI--
KNPAWI--
-KPAWIL-
KEPAWI--
KPPAWI--
-KPAWIA-
KQPAWI--
-KPAWIT-
KTPAWI--
KYPAWI--
-KPAWIK-
KVPAWI--
-KPAWIW-
KRPAWI--
-KPAWIF-
-KPAWIV-
KHPAWI--
KAPAWI--
LTPAWI--
LNPAWI--
LQPAWI--
LPPAWI--
-LPAWIR-
LGPAWI--
LKPAWI--
LLPAWI--
LFPAWI--
LEPAWI--
LDPAWI--
-LPAWIK-
LAPAWI--
LSPAWI--
-LPAWIQ-
LWPAWI--
LVPAWI--
LHPAWI--
-LPAWIM-
-LPAWIA-
-LPAWIT-

-LPAWIL-
-LPAWIH-
MLPAWI--
MSPAWI--
MGPAWI--
MTPAWI--
MMPAWI--
MKPAWI--
MRPAWI--
-MPAWIS-
-MPAWIV-
-MPAWIM-
-MPAWIK-
MVPAWI--
MFPAWI--
MQPAWI--
-MPAWIR-
MEPAWI--
-MPAWIA-
-MPAWIY-
-MPAWIT-
-MPAWIQ-
-MPAWIL-
-MPAWIG-
MIPAWI--
NSPAWI--
NRPAWI--
NNPAWI--
-NPAWIK-
NGPAWI--
NYPAWI--
-NPAWIR-
NFPAWI--
NVPAWI--
NTPAWI--
NIPAWI--
NLPAWI--
NPPAWI--
-NPAWIM-
NQPAWI--
NKPAWI--
-NPAWII-
-NPAWIF-
-NPAWIV-
-NPAWIT-
-NPAWIH-
-NPAWIA-
--PAWISG
PRPAWI--
PLPAWI--

--PAWITS
--PAWISA
--PAWIVS
--PAWIST
--PAWIRA
--PAWIWS
--PAWIRG
--PAWIQT
--PAWIMT
--PAWIIR
--PAWISS
--PAWITT
--PAWITA
--PAWIQR
--PAWIAY
--PAWILS
--PAWIKV
PSPAWI--
--PAWIRW
--PAWILT
--PAWILH
--PAWILG
--PAWIVG
--PAWITH
--PAWIRF
--PAWIMA
--PAWITN
--PAWIRN
--PAWIHS
--PAWIGS
--PAWIEA
--PAWIAS
--PAWIAQ
--PAWIRD
--PAWIKI
--PAWIYS
--PAWIIN
--PAWITG
--PAWIYT
--PAWISD
--PAWIRS
--PAWIRM
--PAWIRH
--PAWIQN
--PAWIMH
--PAWISW
--PAWIQG
--PAWILY
--PAWIKY
--PAWIKT

--PAWISR
--PAWISN
--PAWISH
--PAWIQH
--PAWIQA
--PAWINT
--PAWING
--PAWIMR
--PAWIKS
--PAWIKH
--PAWIHQ
--PAWIGG
--PAWIFR
--PAWIAV
PGPAWI--
--PAWIRK
--PAWIQV
--PAWIMQ
--PAWIFY
PVPAWI--
PMPAWI--
PKPAWI--
--PAWIYR
--PAWIWN
--PAWIVV
--PAWIVT
--PAWIVD
--PAWITD
--PAWISY
--PAWIKA
--PAWIHR
PTPAWI--
PNPAWI--
--PAWIYV
--PAWIYQ
--PAWIYG
--PAWIVW
--PAWIVI
--PAWIMI
--PAWIME
--PAWIIT
--PAWIIF
--PAWIGM
--PAWIGL
--PAWIFK
--PAWIDS
--PAWIAK
--PAWIYY
--PAWIWW
--PAWITR

--PAWISV
--PAWISK
--PAWIRY
--PAWIQI
--PAWIMS
--PAWILQ
--PAWIKM
--PAWIIV
--PAWIIH
--PAWIIE
--PAWIHT
--PAWIHH
--PAWIGY
--PAWIFN
--PAWIAT
--PAWIAH
--PAWIAA
PAPAWI--
PHPAWI--
--PAWIWH
--PAWIVQ
--PAWIVK
--PAWIVA
--PAWITQ
--PAWITK
--PAWITI
--PAWITE
--PAWISQ
--PAWISE
--PAWIRL
--PAWIQK
--PAWIMK
--PAWILW
--PAWILE
--PAWIKN
--PAWIKD
--PAWIGQ
--PAWIES
--PAWIAR
PWPAWI--
-PPAWIT-
--PAWIWV
--PAWIWT
--PAWIVN
--PAWITW
--PAWITV
--PAWISC
--PAWIRQ
--PAWIQW
--PAWIPT

--PAWIPK
--PAWIPA
--PAWINV
--PAWINN
--PAWINH
--PAWIMD
--PAWILP
--PAWIKK
--PAWIIQ
--PAWIIK
--PAWIHN
--PAWIHM
--PAWIHF
--PAWIGV
--PAWIGN
--PAWIGI
--PAWIFH
--PAWIFG
--PAWIEW
--PAWIEG
--PAWIDT
--PAWICA
--PAWIAW
-QPAWIR-
-QPAWIS-
QSPAWI--
QGPAWI--
QMPAWI--
QFPAWI--
-QPAWIL-
QQPAWI--
-QPAWIT-
-QPAWIF-
-QPAWIK-
QLPAWI--
QWPAWI--
-QPAWIE-
QHPAWI--
-QPAWII-
QIPAWI--
-RPAWIR-
RVPAWI--
RGPAWI--
-RPAWIS-
RRPAWI--
RIPAWI--
-RPAWIK-
-RPAWIV-
-RPAWIA-
RYPAWI--

RLPAWI--
RHPAWI--
RKPAWI--
-RPAWIF-
RNPAWI--
RPPAWI--
RTPAWI--
-RPAWIL-
RFPAWI--
REPAWI--
RDPAWI--
-RPAWII-
RQPAWI--
-RPAWIG-
-RPAWIH-
-RPAWIQ-
RWPAWI--
RAPAWI--
-RPAWIN-
-RPAWIE-
-RPAWIW-
-RPAWIM-
RCPAWI--
SVPAWI--
STPAWI--
SSPAWI--
-SPAWIR-
-SPAWIS-
SLPAWI--
-SPAWIT-
-SPAWIM-
SAPAWI--
SYPAWI--
SGPAWI--
SWPAWI--
SEPAWI--
-SPAWIV-
-SPAWIF-
SIPAWI--
-SPAWIL-
-SPAWIK-
-SPAWIG-
-SPAWIH-
SHPAWI--
SFPAWI--
-SPAWIY-
-SPAWIQ-
SKPAWI--
SNPAWI--
-SPAWII-

SMPAWI--
SDPAWI--
-SPAWIN-
-SPAWIA-
-SPAWIE-
TRPAWI--
TGPAWI--
TSPAWI--
-TPAWIL-
-TPAWIR-
-TPAWIA-
TNPAWI--
TWPAWI--
TEPAWI--
TVPAWI--
-TPAWIS-
TPPAWI--
TLPAWI--
TIPAWI--
TTPAWI--
-TPAWIQ-
-TPAWIV-
-TPAWIY-
-TPAWIM-
-TPAWIK-
TFPAWI--
-TPAWIG-
TYPAWI--
-TPAWIW-
THPAWI--
-TPAWIT-
TKPAWI--
VLPAWI--
VSPAWI--
VVPAWI--
VRPAWI--
VAPAWI--
-VPAWIT-
-VPAWIR-
VPPAWI--
VEPAWI--
-VPAWIS-
VTPAWI--
-VPAWIQ-
VYPAWI--
-VPAWIK-
-VPAWII-
VMPAWI--
VWPAWI--
VQPAWI--

```
-VPAWIY-
-VPAWIN-
-VPAWIG-
-VPAWIH-
-VPAWIE-
VNPAWI--
-VPAWIM-
VDPAWI--
VCPAWI--
VFPAWI--
-VPAWIA-
VHPAWI--
VGPAWI--
WSPAWI--
WPPAWI--
WEPAWI--
-WPAWIR-
WAPAWI--
WYPAWI--
-WPAWIG-
WTPAWI--
-WPAWIV-
-WPAWIM-
-WPAWIF-
WNPAWI--
WKPAWI--
-WPAWII-
WHPAWI--
WGPAWI--
WRPAWI--
WQPAWI--
-WPAWIK-
-WPAWIS-
-WPAWIH-
WFPAWI--
YKPAWI--
YHPAWI--
YPPAWI--
YAPAWI--
YSPAWI--
-YPAWIY-
YTPAWI--
YEPAWI--
YYPAWI--
YVPAWI--
YRPAWI--
-YPAWIT-
-YPAWIL-
YLPAWI--
YDPAWI--
```

-YPAWIM-
YCPAWI--
YQPAWI--
YFPAWI--
-YPAWIN-
-YPAWIA-
YNPAWI--
YMPAWI--

PMML	552	600	45.71

--PMMLGR
--PMMLGH
-SPMMLG-
-APMMLS-
AFPMML--
-APMMLK-
-APMMLR-
AVPMML--
AIPMML--
ANPMML--
AKPMML--
ATPMML--
-APMMLM-
ASPMML--
AGPMML--
-APMMLA-
-APMMLV-
-APMMLE-
APPMML--
-APMMLG-
AYPMML--
-APMMLQ-
ACPMML--
AHPMML--
-APMMLN-
-APMMLT-
-APMMLH-
-APMMLD-
AEPMML--
CCPMML--
CIPMML--
CRPMML--
CPPMML--
-CPMMLV-
-CPMMLI-
CGPMML--
CFPMML--
CVPMML--
CMPMML--
-CPMMLL-
-CPMMLH-

DAPMML--
-DPMMLR-
DWPMML--
-DPMMLT-
-DPMMLK-
DYPMML--
DQPMML--
-DPMMLL-
DIPMML--
DTPMML--
DNPMML--
DSPMML--
-DPMMLY-
DMPMML--
DLPMML--
DKPMML--
DEPMML--
-DPMMLS-
-DPMMLN-
DVPMML--
DPPMML--
-EPMMLR-
ESPMML--
-EPMMLS-
EIPMML--
ELPMML--
EVPMML--
ERPMML--
EAPMML--
-EPMMLK-
-EPMMLW-
EMPMML--
EGPMML--
-EPMMLL-
-EPMMLA-
-EPMMLQ-
-EPMMLN-
-EPMMLV-
-EPMMLI-
EFPMML--
-EPMMLM-
ENPMML--
EYPMML--
EKPMML--
EQPMML--
EPPMML--
EWPMML--
EEPMML--
FGPMML--
-FPMMLR-

FHPMML--
FQPMML--
-FPMMLI-
FKPMML--
FTPMML--
-FPMMLN-
FFPMML--
-FPMMLH-
FMPMML--
-FPMMLE-
-FPMMLK-
-GPMMLK-
GVPMML--
-GPMMLR-
GLPMML--
-GPMMLM-
GGPMML--
GRPMML--
-GPMMLQ-
-GPMMLH-
GMPMML--
GSPMML--
GNPMML--
-GPMMLT-
GYPMML--
GQPMML--
-GPMMLS-
GPPMML--
-GPMMLN-
GWPMML--
-GPMMLA-
GEPMML--
GFPMML--
-GPMMLG-
GDPMML--
HEPMML--
HSPMML--
-HPMMLT-
HKPMML--
-HPMMLL-
HAPMML--
HRPMML--
HMPMML--
HHPMML--
HTPMML--
HWPMML--
-HPMMLK-
HLPMML--
HFPMML--
-HPMMLA-

HVPMML--
-HPMMLR-
-HPMMLM-
-HPMMLI-
-HPMMLN-
-HPMMLV-
HGPMML--
HDPMML--
HYPMML--
-HPMMLQ-
HQPMML--
HPPMML--
-HPMMLW-
-HPMMLG-
-HPMMLE-
HIPMML--
-IPMMLR-
INPMML--
-IPMMLN-
ITPMML--
ISPMML--
IVPMML--
-IPMMLS-
IEPMML--
-IPMMLV-
IHPMML--
IWPMML--
-IPMMLT-
IGPMML--
IIPMML--
IAPMML--
IQPMML--
IKPMML--
-IPMMLG-
-IPMMLC-
IPPMML--
-IPMMLW-
-IPMMLK-
IDPMML--
-IPMMLQ-
-IPMMLH-
IYPMML--
IMPMML--
-KPMMLS-
KIPMML--
-KPMMLR-
KRPMML--
-KPMMLI-
KHPMML--
-KPMMLK-

KNPMML--
KQPMML--
KYPMML--
KFPMML--
KLPMML--
-KPMMLH-
KWPMML--
-KPMMLT-
-KPMMLM-
-KPMMLL-
KGPMML--
KDPMML--
KAPMML--
-KPMMLW-
KPPMML--
-KPMMLQ-
-KPMMLN-
-KPMMLA-
KEPMML--
-KPMMLG-
-KPMMLF-
LTPMML--
LPPMML--
LIPMML--
LQPMML--
LHPMML--
LSPMML--
-LPMMLK-
LLPMML--
-LPMMLT-
LNPMML--
-LPMMLN-
-LPMMLS-
-LPMMLA-
-LPMMLM-
LEPMML--
-LPMMLQ-
LDPMML--
-LPMMLE-
MKPMML--
MRPMML--
-MPMMLK-
MTPMML--
MGPMML--
MYPMML--
MSPMML--
-MPMMLQ-
MPPMML--
-MPMMLR-
MEPMML--

```
-MPMMLH-
MLPMML--
-MPMMLW-
MAPMML--
MQPMML--
-MPMMLV-
MNPMML--
MIPMML--
MDPMML--
-MPMMLN-
-MPMMLM-
MMPMML--
NEPMML--
NIPMML--
NRPMML--
-NPMMLR-
NLPMML--
-NPMMLK-
NMPMML--
NKPMML--
NSPMML--
NGPMML--
-NPMMLM-
-NPMMLI-
NTPMML--
NDPMML--
-NPMMLV-
NHPMML--
NFPMML--
NPPMML--
-NPMMLL-
NVPMML--
-NPMMLN-
NNPMML--
-NPMMLA-
-NPMMLT-
-NPMMLS-
-NPMMLH-
NYPMML--
NWPMML--
-NPMMLF-
NAPMML--
NQPMML--
-NPMMLQ-
--PMMLRS
PGPMML--
--PMMLRT
--PMMLRG
--PMMLRM
--PMMLIG
```

--PMMLRI
--PMMLTS
--PMMLRQ
--PMMLTA
--PMMLMG
--PMMLLD
--PMMLSG
--PMMLTW
--PMMLYN
--PMMLRR
PHPMML--
--PMMLSV
--PMMLGS
--PMMLST
--PMMLSF
--PMMLMY
--PMMLKY
--PMMLNA
--PMMLKL
--PMMLFG
PTPMML--
--PMMLNV
--PMMLQS
--PMMLHS
--PMMLES
--PMMLAA
--PMMLSI
--PMMLWH
--PMMLWD
--PMMLTT
--PMMLTQ
--PMMLKS
--PMMLYG
--PMMLSQ
--PMMLMQ
--PMMLVH
--PMMLRY
--PMMLRD
--PMMLWG
--PMMLRH
--PMMLMH
--PMMLLH
--PMMLKI
--PMMLIQ
--PMMLHA
--PMMLTR
--PMMLRE
--PMMLQV
--PMMLNY
--PMMLMN

--PMMLKT
--PMMLKD
--PMMLHR
PWPMML--
--PMMLYH
--PMMLTG
--PMMLHW
--PMMLHT
--PMMLGA
--PMMLFF
--PMMLYF
--PMMLAG
PNPMML--
--PMMLTE
--PMMLSK
--PMMLQH
--PMMLNT
--PMMLNR
--PMMLHL
--PMMLVG
--PMMLTN
--PMMLQA
--PMMLNS
--PMMLKN
--PMMLKG
--PMMLEV
--PMMLAT
PMPMML--
--PMMLNI
--PMMLKV
--PMMLKQ
--PMMLKK
--PMMLIH
--PMMLGY
--PMMLVN
--PMMLTM
--PMMLTI
--PMMLSW
--PMMLSM
--PMMLQY
--PMMLQG
--PMMLMT
--PMMLMI
--PMMLGM
--PMMLFW
--PMMLFS
--PMMLAN
--PMMLAH
PCPMML--
--PMMLYD

--PMMLWY
--PMMLWN
--PMMLSH
--PMMLSE
--PMMLRW
--PMMLQR
--PMMLQK
--PMMLQI
--PMMLQE
--PMMLQD
--PMMLNW
--PMMLNQ
--PMMLME
--PMMLHY
--PMMLHF
--PMMLHD
--PMMLGN
--PMMLGG
--PMMLCA
--PMMLWQ
--PMMLVD
--PMMLQM
--PMMLNE
--PMMLKH
--PMMLHQ
--PMMLGV
--PMMLGT
--PMMLGF
--PMMLFY
--PMMLFE
--PMMLFD
--PMMLEM
--PMMLEG
--PMMLDS
--PMMLAK
PEPMML--
PDPMML--
--PMMLYE
--PMMLTD
--PMMLQQ
--PMMLND
--PMMLKE
--PMMLHK
--PMMLHI
--PMMLHH
--PMMLHG
--PMMLEW
--PMMLET
--PMMLEN
--PMMLEL

--PMMLDL
--PMMLAQ
QAPMML--
QVPMML--
-QPMMLR-
QIPMML--
QYPMML--
-QPMMLQ-
QKPMML--
QQPMML--
QNPMML--
QLPMML--
-QPMMLV-
-QPMMLK-
-QPMMLA-
QHPMML--
QGPMML--
-QPMMLS-
QEPMML--
-QPMMLN-
-QPMMLH-
QTPMML--
-QPMMLT-
-QPMMLW-
-QPMMLG-
-QPMMLC-
RVPMML--
RRPMML--
RWPMML--
RIPMML--
RSPMML--
-RPMMLT-
-RPMMLQ-
RKPMML--
RDPMML--
RNPMML--
RYPMML--
-RPMMLA-
RPPMML--
RMPMML--
RFPMML--
-RPMMLH-
RAPMML--
-RPMMLN-
RTPMML--
RHPMML--
-RPMMLK-
REPMML--
RQPMML--
-RPMMLE-

-RPMMLC-
SRPMML--
SLPMML--
STPMML--
SNPMML--
SVPMML--
SGPMML--
-SPMMLH-
SKPMML--
SEPMML--
SQPMML--
-SPMMLS-
-SPMMLT-
-SPMMLV-
-SPMMLI-
-SPMMLN-
-SPMMLQ-
SHPMML--
-SPMMLK-
SWPMML--
-SPMMLM-
-SPMMLA-
SDPMML--
-SPMMLE-
TSPMML--
TLPMML--
TTPMML--
-TPMMLV-
TRPMML--
TVPMML--
TGPMML--
-TPMMLT-
-TPMMLK-
THPMML--
TYPMML--
TWPMML--
-TPMMLS-
-TPMMLI-
TIPMML--
TKPMML--
TAPMML--
-TPMMLQ-
-TPMMLM-
TPPMML--
TMPMML--
-TPMMLN-
TNPMML--
-TPMMLA-
TEPMML--
-TPMMLG-

TDPMML--
TCPMML--
VSPMML--
-VPMMLS-
VAPMML--
-VPMMLR-
VNPMML--
VDPMML--
VQPMML--
VTPMML--
VYPMML--
-VPMMLN-
VWPMML--
-VPMMLE-
VIPMML--
VPPMML--
VHPMML--
VFPMML--
-VPMMLA-
-VPMMLT-
VKPMML--
VEPMML--
-VPMMLG-
-VPMMLH-
-VPMMLI-
VCPMML--
-VPMMLM-
-VPMMLK-
WKPMML--
WNPMML--
-WPMMLQ-
-WPMMLM-
-WPMMLK-
WRPMML--
-WPMMLH-
-WPMMLT-
-WPMMLN-
WMPMML--
WHPMML--
WSPMML--
-WPMMLV-
-WPMMLS-
-WPMMLE-
WDPMML--
YQPMML--
-YPMMLR-
YRPMML--
YTPMML--
YIPMML--
YHPMML--

-YPMMLS-
-YPMMLK-
YLPMML--
YNPMML--
YYPMML--
YPPMML--
-YPMMLA-
-YPMMLQ-
YEPMML--

PYEL	222	735	45.68

-LPYELG-
--PYELGN
-SPYELP-
TTPYEL--
-APYELR-
ARPYEL--
AHPYEL--
-APYELS-
ALPYEL--
-APYELA-
-APYELL-
AFPYEL--
AVPYEL--
ASPYEL--
AGPYEL--
AEPYEL--
-APYELH-
ATPYEL--
AQPYEL--
-APYELV-
AKPYEL--
AIPYEL--
-APYELM-
-APYELK-
-APYELI-
AAPYEL--
ANPYEL--
-APYELN-
AWPYEL--
-APYELE-
ADPYEL--
ACPYEL--
-APYELG-
AMPYEL--
-APYELW-
-APYELT-
CSPYEL--
CLPYEL--
CRPYEL--
-CPYELN-

-CPYELR-
CKPYEL--
CCPYEL--
CVPYEL--
-CPYELL-
-CPYELK-
CPPYEL--
CNPYEL--
CAPYEL--
-DPYELR-
DRPYEL--
DVPYEL--
-DPYELY-
DSPYEL--
DGPYEL--
DTPYEL--
-DPYELT-
DLPYEL--
DKPYEL--
-DPYELS-
-DPYELN-
DHPYEL--
DFPYEL--
DYPYEL--
-DPYELW-
-DPYELI-
DEPYEL--
-DPYELQ-
-DPYELM-
DNPYEL--
DIPYEL--
DAPYEL--
-EPYELR-
EIPYEL--
EAPYEL--
EGPYEL--
ELPYEL--
ETPYEL--
EQPYEL--
-EPYELK-
ERPYEL--
-EPYELV-
EPPYEL--
EVPYEL--
-EPYELI-
-EPYELS-
-EPYELM-
EKPYEL--
EFPYEL--
ENPYEL--

-EPYELN-
-EPYELG-
-EPYELA-
EMPYEL--
EDPYEL--
-FPYELR-
-FPYELQ-
-FPYELS-
-FPYELH-
FPPYEL--
FKPYEL--
FYPYEL--
-FPYELT-
FGPYEL--
FAPYEL--
-FPYELK-
FFPYEL--
-FPYELI-
FLPYEL--
-FPYELA-
FVPYEL--
FNPYEL--
FEPYEL--
FWPYEL--
FSPYEL--
-FPYELV-
FMPYEL--
FQPYEL--
-FPYELF-
FIPYEL--
FHPYEL--
-FPYELN-
FTPYEL--
FDPYEL--
FRPYEL--
GVPYEL--
-GPYELK-
GSPYEL--
GLPYEL--
GHPYEL--
-GPYELY-
-GPYELS-
-GPYELR-
GAPYEL--
-GPYELQ-
-GPYELM-
GGPYEL--
GQPYEL--
GMPYEL--
-GPYELL-

GFPYEL--
-GPYELH-
-GPYELG-
-GPYELA-
-GPYELV-
GWPYEL--
-GPYELW-
-GPYELT-
-GPYELN-
GPPYEL--
GNPYEL--
GDPYEL--
GKPYEL--
GEPYEL--
-GPYELI-
GCPYEL--
-HPYELL-
HLPYEL--
-HPYELT-
HGPYEL--
HRPYEL--
HNPYEL--
HPPYEL--
HSPYEL--
-HPYELR-
-HPYELS-
-HPYELI-
-HPYELY-
-HPYELV-
-HPYELM-
HKPYEL--
HYPYEL--
HEPYEL--
HAPYEL--
HVPYEL--
-HPYELW-
HQPYEL--
HTPYEL--
-HPYELG-
HWPYEL--
-HPYELN-
HHPYEL--
ISPYEL--
-IPYELS-
IMPYEL--
IGPYEL--
ITPYEL--
-IPYELR-
IYPYEL--
-IPYELV-

ILPYEL--
-IPYELL-
IPPYEL--
IRPYEL--
-IPYELI-
-IPYELQ-
IFPYEL--
-IPYELH-
IVPYEL--
-IPYELK-
IKPYEL--
-IPYELT-
-IPYELN-
-IPYELG-
INPYEL--
IHPYEL--
IDPYEL--
ICPYEL--
IQPYEL--
IAPYEL--
-IPYELM-
-IPYELD-
-IPYELA-
IIPYEL--
-KPYELN-
-KPYELQ-
KAPYEL--
-KPYELR-
KVPYEL--
KRPYEL--
-KPYELF-
KPPYEL--
KFPYEL--
KMPYEL--
KGPYEL--
-KPYELM-
KLPYEL--
KIPYEL--
-KPYELT-
-KPYELI-
-KPYELV-
KWPYEL--
KKPYEL--
-KPYELA-
-KPYELW-
-KPYELH-
KDPYEL--
KTPYEL--
KQPYEL--
-KPYELK-

KNPYEL--
-KPYELY-
-KPYELG-
KHPYEL--
-LPYELR-
LSPYEL--
LLPYEL--
LFPYEL--
-LPYELK-
LMPYEL--
LKPYEL--
LVPYEL--
LAPYEL--
LWPYEL--
-LPYELS-
LTPYEL--
LHPYEL--
LQPYEL--
-LPYELH-
LPPYEL--
LDPYEL--
-LPYELT-
LGPYEL--
-LPYELV-
-LPYELN-
LEPYEL--
-LPYELI-
-LPYELA-
LYPYEL--
-LPYELF-
-LPYELQ-
-LPYELM-
MSPYEL--
MGPYEL--
MFPYEL--
-MPYELV-
-MPYELQ-
MMPYEL--
-MPYELR-
-MPYELK-
MRPYEL--
-MPYELS-
-MPYELI-
MQPYEL--
-MPYELT-
-MPYELA-
MPPYEL--
MAPYEL--
MKPYEL--
MEPYEL--

-MPYELN-
-MPYELW-
MYPYEL--
MWPYEL--
MNPYEL--
NVPYEL--
-NPYELR-
-NPYELI-
NSPYEL--
NYPYEL--
NGPYEL--
NQPYEL--
-NPYELS-
-NPYELH-
NFPYEL--
NAPYEL--
-NPYELW-
-NPYELT-
NDPYEL--
-NPYELV-
NNPYEL--
NTPYEL--
NRPYEL--
NPPYEL--
NHPYEL--
NEPYEL--
NWPYEL--
-NPYELN-
-NPYELM-
-NPYELY-
-NPYELA-
NMPYEL--
NLPYEL--
NKPYEL--
NCPYEL--
--PYELRS
--PYELRR
--PYELRL
--PYELRT
--PYELRV
--PYELYG
--PYELSR
--PYELRK
--PYELSS
--PYELVS
--PYELMS
--PYELMR
--PYELIS
--PYELVA
--PYELRN

--PYELQA
--PYELVV
--PYELRG
--PYELMG
--PYELHV
--PYELWN
--PYELVR
--PYELTS
--PYELRW
--PYELRY
--PYELNV
--PYELYR
--PYELTG
--PYELSA
--PYELLG
--PYELKQ
--PYELHY
--PYELYY
--PYELTR
--PYELSG
--PYELRE
--PYELST
--PYELSN
--PYELRH
--PYELNI
--PYELES
--PYELSV
--PYELLT
--PYELHK
--PYELHD
PIPYEL--
--PYELVN
--PYELNR
--PYELIN
--PYELHA
--PYELAH
--PYELTY
--PYELTT
--PYELSL
--PYELLR
--PYELHS
--PYELFG
--PYELRD
--PYELQR
--PYELKN
--PYELKK
--PYELKF
--PYELIH
--PYELHH
--PYELVT

--PYELTN
--PYELTL
--PYELKG
--PYELFF
--PYELSH
--PYELNS
--PYELMH
--PYELMA
--PYELKI
PKPYEL--
--PYELYH
--PYELRF
--PYELQN
--PYELLH
--PYELHN
--PYELGA
PHPYEL--
--PYELWG
--PYELRQ
--PYELRM
--PYELNN
--PYELLK
--PYELKS
--PYELGV
--PYELFY
--PYELAS
--PYELYW
--PYELWR
--PYELVW
--PYELTH
--PYELSI
--PYELRI
--PYELNW
--PYELKH
--PYELKA
--PYELIL
--PYELIG
--PYELIA
--PYELGY
--PYELFH
--PYELYN
--PYELVK
--PYELQV
--PYELQH
--PYELNK
--PYELMD
--PYELKR
--PYELHW
--PYELGG
--PYELYS

--PYELWD
--PYELSY
--PYELSM
--PYELQG
--PYELMK
--PYELKV
--PYELKL
--PYELCS
--PYELYD
--PYELVH
--PYELTM
--PYELSE
--PYELQQ
--PYELMW
--PYELMT
--PYELKY
--PYELIQ
--PYELHG
--PYELGT
--PYELFW
--PYELFI
--PYELEG
--PYELAW
--PYELYQ
--PYELYK
--PYELVQ
--PYELTF
--PYELTA
--PYELSK
--PYELQL
--PYELQI
--PYELNT
--PYELND
--PYELLD
--PYELKM
--PYELKE
--PYELIT
--PYELIR
--PYELAR
--PYELAE
PGPYEL--
--PYELWK
--PYELWF
--PYELVD
--PYELTK
--PYELTD
--PYELSW
--PYELSF
--PYELQW
--PYELQT

--PYELQK
--PYELNA
--PYELMN
--PYELLQ
--PYELLE
--PYELHM
--PYELGS
--PYELGH
--PYELFQ
--PYELEA
--PYELAT
--PYELAQ
--PYELAA
PTPYEL--
PNPYEL--
PMPYEL--
PYPYEL--
--PYELYE
--PYELWT
--PYELWH
--PYELTW
--PYELRP
--PYELQY
--PYELQS
--PYELQE
--PYELQD
--PYELNY
--PYELNQ
--PYELNH
--PYELNG
--PYELME
--PYELMC
--PYELLP
--PYELKT
--PYELKD
--PYELHR
--PYELHI
--PYELGW
--PYELGI
--PYELEV
--PYELCW
--PYELCK
--PYELAV
PCPYEL--
-QPYELR-
QWPYEL--
QTPYEL--
QLPYEL--
-QPYELK-
QGPYEL--

QHPYEL--
QVPYEL--
QDPYEL--
QAPYEL--
QEPYEL--
QPPYEL--
-QPYELS-
-QPYELL-
-QPYELH-
QNPYEL--
-QPYELF-
QKPYEL--
QRPYEL--
-QPYELI-
-QPYELA-
QIPYEL--
QSPYEL--
-QPYELN-
-QPYELG-
-QPYELM-
QMPYEL--
-RPYELL-
-RPYELW-
-RPYELS-
RRPYEL--
-RPYELI-
RPPYEL--
RGPYEL--
-RPYELH-
-RPYELQ-
-RPYELA-
RNPYEL--
RYPYEL--
RFPYEL--
RAPYEL--
-RPYELM-
-RPYELG-
RMPYEL--
-RPYELK-
RSPYEL--
REPYEL--
-RPYELV-
RIPYEL--
RWPYEL--
RTPYEL--
-RPYELN-
-RPYELY-
RHPYEL--
RCPYEL--
-RPYELE-

RQPYEL--
RDPYEL--
-SPYELY-
SVPYEL--
-SPYELN-
SIPYEL--
SMPYEL--
STPYEL--
SGPYEL--
SRPYEL--
SKPYEL--
SAPYEL--
-SPYELI-
SSPYEL--
-SPYELR-
SHPYEL--
SFPYEL--
SQPYEL--
-SPYELT-
-SPYELQ-
SPPYEL--
SYPYEL--
-SPYELV-
SEPYEL--
-SPYELG-
SWPYEL--
-SPYELK-
SLPYEL--
-SPYELW-
SNPYEL--
-SPYELA-
-SPYELE-
-SPYELC-
SCPYEL--
TSPYEL--
-TPYELR-
TVPYEL--
TKPYEL--
TRPYEL--
TIPYEL--
TYPYEL--
-TPYELW-
TFPYEL--
-TPYELK-
TEPYEL--
TAPYEL--
-TPYELN-
-TPYELV-
TPPYEL--
TGPYEL--

TLPYEL--
-TPYELM-
-TPYELH-
THPYEL--
TWPYEL--
-TPYELS-
-TPYELL-
-TPYELQ-
TMPYEL--
-TPYELT-
-TPYELI-
-TPYELG-
-TPYELA-
TQPYEL--
TNPYEL--
TDPYEL--
-TPYELP-
TCPYEL--
VVPYEL--
-VPYELS-
VGPYEL--
VTPYEL--
-VPYELK-
VAPYEL--
-VPYELT-
VNPYEL--
VMPYEL--
VWPYEL--
VIPYEL--
VLPYEL--
VHPYEL--
-VPYELH-
-VPYELM-
-VPYELQ-
-VPYELN-
VRPYEL--
-VPYELG-
-VPYELA-
VKPYEL--
VDPYEL--
VFPYEL--
VEPYEL--
-VPYELC-
-VPYELV-
VYPYEL--
VQPYEL--
-VPYELE-
WSPYEL--
WAPYEL--
-WPYELR-

WRPYEL--
-WPYELN-
WIPYEL--
WTPYEL--
-WPYELK-
WNPYEL--
WYPYEL--
WMPYEL--
WGPYEL--
WQPYEL--
-WPYELS-
-WPYELW-
-WPYELV-
WHPYEL--
WFPYEL--
-WPYELH-
WWPYEL--
-WPYELT-
WKPYEL--
WEPYEL--
-YPYELR-
YSPYEL--
YDPYEL--
YFPYEL--
-YPYELS-
YIPYEL--
YLPYEL--
YGPYEL--
YRPYEL--
YEPYEL--
-YPYELN-
YWPYEL--
YVPYEL--
-YPYELT-
YKPYEL--
-YPYELA-
YTPYEL--
-YPYELV-
-YPYELI-
-YPYELM-
-YPYELF-
YAPYEL--
-YPYELG-
-YPYELK-
YMPYEL--

PLGV	38	862	45.60

-KPLGVG-
-LPLGVD-
--PLGVRP
--PLGVND

--PLGVGK
--PLGVEI
--PLGVDT
--PLGVDR
-RPLGVD-
-WPLGVP-
-WPLGVG-
-YPLGVG-
-APLGVS-
-APLGVR-
ARPLGV--
-APLGVI-
-APLGVM-
-APLGVH-
AMPLGV--
-APLGVV-
AYPLGV--
AIPLGV--
AGPLGV--
AEPLGV--
AVPLGV--
-APLGVY-
-APLGVN-
ANPLGV--
AHPLGV--
ADPLGV--
AWPLGV--
AKPLGV--
-APLGVQ-
ALPLGV--
ATPLGV--
AFPLGV--
APPLGV--
-APLGVA-
-APLGVG-
-APLGVE-
-APLGVC-
ACPLGV--
CLPLGV--
CRPLGV--
CPPLGV--
CCPLGV--
CTPLGV--
CKPLGV--
CSPLGV--
CQPLGV--
CIPLGV--
CFPLGV--
-CPLGVV-
-CPLGVS-

CYPLGV--
-CPLGVH-
-CPLGVW-
-CPLGVM-
-CPLGVK-
CNPLGV--
CEPLGV--
-CPLGVQ-
-CPLGVL-
-CPLGVF-
CDPLGV--
-DPLGVY-
DVPLGV--
DRPLGV--
DQPLGV--
DTPLGV--
-DPLGVR-
DGPLGV--
DWPLGV--
DSPLGV--
DLPLGV--
DIPLGV--
DAPLGV--
-DPLGVI-
-DPLGVV-
DFPLGV--
DKPLGV--
-DPLGVM-
-DPLGVH-
DDPLGV--
DYPLGV--
-DPLGVL-
-DPLGVF-
DMPLGV--
DHPLGV--
-DPLGVT-
DNPLGV--
-DPLGVQ-
-DPLGVK-
-EPLGVL-
-EPLGVR-
EGPLGV--
ESPLGV--
EVPLGV--
ERPLGV--
-EPLGVV-
EKPLGV--
-EPLGVM-
-EPLGVW-
-EPLGVI-

-EPLGVY-
-EPLGVF-
EIPLGV--
EHPLGV--
-EPLGVS-
ELPLGV--
EYPLGV--
EWPLGV--
ETPLGV--
-EPLGVT-
-EPLGVH-
ENPLGV--
-EPLGVQ-
-EPLGVN-
EDPLGV--
EQPLGV--
-EPLGVE-
-EPLGVC-
EEPLGV--
-FPLGVR-
FSPLGV--
FVPLGV--
FGPLGV--
FPPLGV--
FNPLGV--
FMPLGV--
FYPLGV--
-FPLGVS-
-FPLGVH-
-FPLGVK-
FAPLGV--
FLPLGV--
FTPLGV--
FWPLGV--
-FPLGVW-
FQPLGV--
-FPLGVV-
-FPLGVT-
-FPLGVQ-
-FPLGVM-
FHPLGV--
FEPLGV--
-FPLGVN-
-FPLGVA-
FKPLGV--
-FPLGVI-
FDPLGV--
FFPLGV--
-FPLGVF-
-FPLGVE-

FIPLGV--
FCPLGV--
-GPLGVR-
GTPLGV--
GYPLGV--
-GPLGVF-
GAPLGV--
GSPLGV--
-GPLGVH-
GQPLGV--
-GPLGVS-
-GPLGVY-
-GPLGVI-
-GPLGVK-
GGPLGV--
GKPLGV--
-GPLGVV-
GNPLGV--
GIPLGV--
-GPLGVM-
GMPLGV--
-GPLGVA-
GLPLGV--
-GPLGVW-
-GPLGVQ-
GFPLGV--
GWPLGV--
GPPLGV--
-GPLGVN-
GHPLGV--
-GPLGVG-
GDPLGV--
GEPLGV--
-GPLGVP-
-GPLGVE-
-HPLGVR-
HRPLGV--
HLPLGV--
HSPLGV--
HGPLGV--
HTPLGV--
HWPLGV--
-HPLGVS-
HEPLGV--
HAPLGV--
-HPLGVY-
HVPLGV--
-HPLGVW-
-HPLGVT-
-HPLGVH-

```
-HPLGVV-
-HPLGVK-
-HPLGVF-
HNPLGV--
HIPLGV--
-HPLGVI-
HMPLGV--
-HPLGVN-
HPPLGV--
HYPLGV--
-HPLGVQ-
-HPLGVM-
HKPLGV--
HFPLGV--
-HPLGVA-
HQPLGV--
HDPLGV--
HHPLGV--
-HPLGVC-
HCPLGV--
ISPLGV--
IGPLGV--
-IPLGVL-
-IPLGVR-
INPLGV--
IRPLGV--
-IPLGVF-
-IPLGVT-
IWPLGV--
-IPLGVI-
-IPLGVK-
IAPLGV--
IMPLGV--
-IPLGVQ-
IKPLGV--
IYPLGV--
-IPLGVV-
IIPLGV--
IDPLGV--
IQPLGV--
-IPLGVM-
ILPLGV--
IFPLGV--
ITPLGV--
-IPLGVS-
IHPLGV--
-IPLGVA-
-IPLGVW-
-IPLGVN-
IEPLGV--
```

-IPLGVH-
-IPLGVG-
ICPLGV--
KSPLGV--
-KPLGVW-
-KPLGVI-
-KPLGVR-
-KPLGVH-
KRPLGV--
-KPLGVL-
-KPLGVV-
KAPLGV--
KTPLGV--
KQPLGV--
-KPLGVY-
-KPLGVT-
-KPLGVM-
-KPLGVK-
KGPLGV--
KLPLGV--
KKPLGV--
KFPLGV--
KMPLGV--
KHPLGV--
KYPLGV--
KVPLGV--
KNPLGV--
-KPLGVN-
KIPLGV--
-KPLGVQ-
KWPLGV--
KPPLGV--
-KPLGVA-
-KPLGVC-
LRPLGV--
LSPLGV--
LLPLGV--
LGPLGV--
LTPLGV--
LAPLGV--
LQPLGV--
-LPLGVR-
LNPLGV--
LMPLGV--
LPPLGV--
LKPLGV--
-LPLGVH-
LFPLGV--
LWPLGV--
LDPLGV--

LEPLGV--
-LPLGVK-
-LPLGVY-
-LPLGVS-
-LPLGVI-
LHPLGV--
-LPLGVT-
-LPLGVV-
-LPLGVM-
-LPLGVF-
-LPLGVQ-
LIPLGV--
-LPLGVA-
LCPLGV--
MRPLGV--
MTPLGV--
-MPLGVR-
MKPLGV--
MLPLGV--
-MPLGVV-
MIPLGV--
-MPLGVT-
-MPLGVM-
-MPLGVK-
MVPLGV--
MNPLGV--
-MPLGVF-
MMPLGV--
-MPLGVQ-
MGPLGV--
MFPLGV--
MAPLGV--
MYPLGV--
-MPLGVY-
MDPLGV--
-MPLGVN-
-MPLGVW-
MWPLGV--
MQPLGV--
-MPLGVE-
-MPLGVA-
MHPLGV--
MEPLGV--
-MPLGVD-
NKPLGV--
NVPLGV--
NAPLGV--
-NPLGVR-
NIPLGV--
NRPLGV--

NQPLGV--
NMPLGV--
-NPLGVY-
NEPLGV--
-NPLGVH-
-NPLGVF-
NFPLGV--
NLPLGV--
-NPLGVT-
NSPLGV--
-NPLGVV-
NNPLGV--
NHPLGV--
-NPLGVK-
NPPLGV--
-NPLGVM-
NTPLGV--
NYPLGV--
NDPLGV--
-NPLGVI-
NCPLGV--
NWPLGV--
-NPLGVS-
-NPLGVQ-
-NPLGVD-
--PLGVSS
--PLGVRG
--PLGVRS
--PLGVRT
--PLGVLY
--PLGVLT
--PLGVRV
--PLGVWH
--PLGVVR
--PLGVVS
--PLGVTR
--PLGVKS
--PLGVFR
--PLGVRN
--PLGVRA
--PLGVTG
--PLGVLG
--PLGVRI
--PLGVLR
--PLGVMR
--PLGVSG
--PLGVRQ
--PLGVLA
--PLGVIS
--PLGVWS

--PLGVST
--PLGVRW
--PLGVHR
--PLGVRD
--PLGVNS
--PLGVTS
--PLGVFM
--PLGVRY
--PLGVFS
--PLGVTV
--PLGVWG
--PLGVVA
--PLGVRM
--PLGVIG
--PLGVRF
--PLGVMN
--PLGVGS
--PLGVWR
--PLGVSF
--PLGVFG
--PLGVYR
--PLGVVT
--PLGVFK
--PLGVTW
--PLGVSA
--PLGVKG
--PLGVIW
--PLGVFT
--PLGVKA
--PLGVHF
--PLGVAT
--PLGVAG
PHPLGV--
--PLGVQR
--PLGVSR
--PLGVRH
--PLGVMQ
--PLGVLK
--PLGVIT
--PLGVIR
--PLGVSQ
--PLGVNA
--PLGVYT
--PLGVTF
--PLGVAS
--PLGVSN
--PLGVHS
--PLGVTI
--PLGVLN
--PLGVLM

--PLGVKY
--PLGVSH
--PLGVNT
--PLGVFN
PGPLGV--
--PLGVYG
--PLGVTA
--PLGVKF
--PLGVHT
--PLGVVF
--PLGVSY
--PLGVHI
--PLGVGA
--PLGVKN
--PLGVYK
--PLGVVN
--PLGVVH
--PLGVMH
--PLGVKH
--PLGVFH
--PLGVML
--PLGVIK
--PLGVHA
--PLGVFY
--PLGVMG
--PLGVRE
--PLGVMT
--PLGVLI
--PLGVHQ
--PLGVHL
--PLGVAA
PTPLGV--
--PLGVWW
--PLGVTK
--PLGVSK
--PLGVQW
--PLGVQA
--PLGVVG
--PLGVTH
--PLGVHN
--PLGVFQ
--PLGVYN
--PLGVSM
--PLGVLQ
--PLGVIN
--PLGVGV
--PLGVEW
--PLGVEG
--PLGVVW
--PLGVTT

--PLGVNV
--PLGVII
--PLGVVI
--PLGVQS
--PLGVKR
PWPLGV--
PQPLGV--
--PLGVYQ
--PLGVVQ
--PLGVTQ
--PLGVTD
--PLGVQY
--PLGVQN
--PLGVKT
--PLGVGG
--PLGVAI
--PLGVMD
--PLGVFE
--PLGVEH
--PLGVEA
--PLGVAV
PDPLGV--
--PLGVYH
--PLGVIM
PNPLGV--
--PLGVKI
--PLGVHV
--PLGVHM
--PLGVHH
--PLGVAM
--PLGVQH
--PLGVMV
--PLGVHY
--PLGVHW
--PLGVFV
--PLGVAY
--PLGVYD
--PLGVWN
--PLGVKM
--PLGVKL
--PLGVHG
--PLGVGT
--PLGVAF
--PLGVWQ
--PLGVQK
--PLGVNW
--PLGVMI
--PLGVAK
PIPLGV--
PFPLGV--

--PLGVVL
--PLGVQI
--PLGVNR
--PLGVMK
--PLGVLD
--PLGVKK
--PLGVHE
--PLGVEV
--PLGVAN
--PLGVNQ
--PLGVNK
--PLGVKW
--PLGVET
--PLGVDG
--PLGVTM
--PLGVMY
--PLGVMA
--PLGVIV
--PLGVIQ
--PLGVIA
--PLGVGF
--PLGVEY
--PLGVAR
--PLGVQT
--PLGVQG
--PLGVNF
--PLGVRC
--PLGVNM
--PLGVNG
--PLGVME
--PLGVKD
--PLGVGW
--PLGVFD
--PLGVWE
--PLGVVD
--PLGVSD
--PLGVQQ
--PLGVNN
--PLGVNI
--PLGVNH
--PLGVHK
--PLGVFW
--PLGVEM
--PLGVCN
--PLGVLE
--PLGVHD
--PLGVER
--PLGVEF
--PLGVAQ
--PLGVAL

--PLGVYE
--PLGVVE
--PLGVTC
--PLGVSE
--PLGVQM
--PLGVQL
--PLGVNY
--PLGVNL
--PLGVIE
--PLGVGI
--PLGVEQ
--PLGVDS
--PLGVAW
PEPLGV--
-PPLGVT-
--PLGVTE
--PLGVQD
--PLGVKC
--PLGVID
--PLGVGH
--PLGVEL
--PLGVEK
--PLGVAH
-PPLGVW-
-PPLGVV-
-PPLGVR-
-PPLGVF-
--PLGVSC
--PLGVPW
--PLGVPV
--PLGVPQ
--PLGVLC
--PLGVKE
--PLGVGD
--PLGVDN
--PLGVDE
--PLGVCW
--PLGVCK
--PLGVCA
--PLGVAE
QRPLGV--
-QPLGVM-
-QPLGVL-
QFPLGV--
-QPLGVK-
-QPLGVY-
-QPLGVW-
-QPLGVS-
QSPLGV--
QVPLGV--

QIPLGV--
QLPLGV--
QGPLGV--
QAPLGV--
QKPLGV--
-QPLGVT-
-QPLGVH-
QTPLGV--
QMPLGV--
QPPLGV--
-QPLGVQ-
QHPLGV--
QQPLGV--
-QPLGVI-
-QPLGVV-
-QPLGVA-
-QPLGVN-
QDPLGV--
QYPLGV--
QWPLGV--
-QPLGVE-
QNPLGV--
QEPLGV--
-RPLGVL-
RGPLGV--
-RPLGVI-
RWPLGV--
-RPLGVT-
RAPLGV--
RLPLGV--
RVPLGV--
-RPLGVY-
RTPLGV--
RMPLGV--
-RPLGVQ-
RRPLGV--
RIPLGV--
RPPLGV--
-RPLGVM-
RYPLGV--
RQPLGV--
-RPLGVK-
RHPLGV--
REPLGV--
RNPLGV--
RFPLGV--
-RPLGVF-
-RPLGVE-
RCPLGV--
-RPLGVG-

-RPLGVC-
-SPLGVR-
SRPLGV--
SKPLGV--
-SPLGVM-
-SPLGVV-
-SPLGVI-
-SPLGVS-
SFPLGV--
STPLGV--
SLPLGV--
SSPLGV--
SVPLGV--
SAPLGV--
SHPLGV--
SIPLGV--
-SPLGVY-
SGPLGV--
SQPLGV--
SNPLGV--
SMPLGV--
-SPLGVK-
-SPLGVA-
SYPLGV--
-SPLGVQ-
SDPLGV--
SCPLGV--
-SPLGVN-
-TPLGVR-
-TPLGVY-
TLPLGV--
TSPLGV--
-TPLGVT-
TRPLGV--
-TPLGVF-
TVPLGV--
TGPLGV--
THPLGV--
TWPLGV--
TTPLGV--
-TPLGVL-
-TPLGVV-
-TPLGVI-
TAPLGV--
-TPLGVW-
TNPLGV--
TKPLGV--
-TPLGVK-
-TPLGVQ-
TIPLGV--

TEPLGV--
TQPLGV--
TMPLGV--
TPPLGV--
-TPLGVN-
-TPLGVM-
TDPLGV--
TYPLGV--
-TPLGVA-
-TPLGVG-
-TPLGVE-
-TPLGVC-
TCPLGV--
-VPLGVR-
VRPLGV--
-VPLGVL-
VTPLGV--
VLPLGV--
-VPLGVW-
-VPLGVS-
VNPLGV--
-VPLGVT-
-VPLGVM-
-VPLGVF-
VMPLGV--
VKPLGV--
VFPLGV--
VPPLGV--
VVPLGV--
VYPLGV--
-VPLGVI-
-VPLGVH-
VAPLGV--
VWPLGV--
-VPLGVK-
VGPLGV--
VEPLGV--
VQPLGV--
VIPLGV--
-VPLGVN-
VHPLGV--
-VPLGVV-
-VPLGVA-
-VPLGVQ-
VCPLGV--
-VPLGVE-
-VPLGVC-
-WPLGVR-
WRPLGV--
-WPLGVT-

WSPLGV--
WPPLGV--
-WPLGVS-
WTPLGV--
WIPLGV--
WYPLGV--
-WPLGVI-
WVPLGV--
WMPLGV--
WAPLGV--
WHPLGV--
WGPLGV--
WDPLGV--
WWPLGV--
-WPLGVM-
WKPLGV--
-WPLGVV-
-WPLGVQ-
-WPLGVN-
WNPLGV--
WFPLGV--
-WPLGVA-
-WPLGVE-
WEPLGV--
-WPLGVW-
WCPLGV--
YLPLGV--
-YPLGVR-
YAPLGV--
YGPLGV--
YSPLGV--
YPPLGV--
YNPLGV--
YRPLGV--
YQPLGV--
YVPLGV--
-YPLGVW-
-YPLGVK-
-YPLGVF-
-YPLGVY-
YTPLGV--
YDPLGV--
-YPLGVT-
-YPLGVV-
YWPLGV--
-YPLGVH-
YIPLGV--
YYPLGV--
-YPLGVS-
-YPLGVQ-

-YPLGVN-
-YPLGVM-
YMPLGV--
YEPLGV--
-YPLGVA-
YFPLGV--
-YPLGVP-
YCPLGV--

PWML	482	625	45.59

-LPWMLI-
--PWMLGN
-SPWMLG-
-TPWMLG-
ARPWML--
ATPWML--
-APWMLS-
AVPWML--
AIPWML--
-APWMLK-
AAPWML--
AHPWML--
AGPWML--
ASPWML--
-APWMLF-
APPWML--
AEPWML--
AWPWML--
-APWMLY-
AKPWML--
ADPWML--
-APWMLV-
-APWMLM-
AMPWML--
-APWMLN-
AQPWML--
-APWMLW-
-APWMLQ-
-APWMLI-
-APWMLA-
AFPWML--
AYPWML--
-APWMLH-
ANPWML--
-APWMLT-
-APWMLP-
-APWMLE-
CKPWML--
CVPWML--
CCPWML--
CLPWML--

```
-CPWMLL-
-CPWMLI-
CPPWML--
CTPWML--
CSPWML--
CRPWML--
-CPWMLQ-
CNPWML--
DEPWML--
DRPWML--
DMPWML--
DTPWML--
DLPWML--
-DPWMLR-
-DPWMLM-
-DPWMLK-
DHPWML--
-DPWMLT-
-DPWMLN-
-DPWMLH-
-DPWMLL-
DFPWML--
DAPWML--
DQPWML--
DYPWML--
DVPWML--
-DPWMLV-
-DPWMLI-
DWPWML--
DSPWML--
DGPWML--
-EPWMLT-
-EPWMLR-
ETPWML--
-EPWMLI-
EGPWML--
EHPWML--
ESPWML--
ERPWML--
-EPWMLA-
-EPWMLV-
EYPWML--
-EPWMLH-
-EPWMLF-
ELPWML--
EQPWML--
EPPWML--
EMPWML--
EFPWML--
EWPWML--
```

-EPWMLQ-
EKPWML--
EVPWML--
-EPWMLS-
EDPWML--
-EPWMLG-
-EPWMLE-
ENPWML--
EIPWML--
FRPWML--
FTPWML--
-FPWMLS-
FFPWML--
FAPWML--
-FPWMLH-
-FPWMLR-
FYPWML--
FPPWML--
-FPWMLQ-
-FPWMLL-
-FPWMLN-
FLPWML--
FNPWML--
-FPWMLT-
-FPWMLK-
FDPWML--
FQPWML--
-FPWMLW-
-FPWMLF-
-GPWMLR-
GTPWML--
-GPWMLS-
GRPWML--
GLPWML--
GKPWML--
-GPWMLV-
GSPWML--
GGPWML--
-GPWMLY-
-GPWMLT-
GDPWML--
-GPWMLH-
GAPWML--
GHPWML--
GNPWML--
GQPWML--
-GPWMLN-
GEPWML--
-GPWMLI-
-GPWMLA-

-GPWMLK-
GWPWML--
GIPWML--
-GPWMLQ-
GCPWML--
GYPWML--
-GPWMLG-
HRPWML--
HAPWML--
HLPWML--
HGPWML--
HVPWML--
-HPWMLR-
-HPWMLS-
-HPWMLN-
HIPWML--
HDPWML--
-HPWMLV-
-HPWMLM-
-HPWMLK-
-HPWMLA-
-HPWMLT-
HHPWML--
HPPWML--
-HPWMLH-
HWPWML--
HMPWML--
HKPWML--
HFPWML--
HYPWML--
-HPWMLG-
HNPWML--
HCPWML--
-IPWMLR-
ISPWML--
-IPWMLT-
IKPWML--
IAPWML--
ITPWML--
-IPWMLK-
-IPWMLV-
-IPWMLQ-
-IPWMLN-
IWPWML--
IQPWML--
-IPWMLM-
INPWML--
IMPWML--
-IPWMLH-
ILPWML--

IIPWML--
IRPWML--
IYPWML--
IEPWML--
IDPWML--
IGPWML--
IVPWML--
-IPWMLE-
-IPWMLA-
IFPWML--
KSPWML--
KGPWML--
-KPWMLV-
KAPWML--
-KPWMLI-
KMPWML--
-KPWMLM-
KLPWML--
KFPWML--
-KPWMLS-
-KPWMLK-
KWPWML--
KQPWML--
-KPWMLQ-
KIPWML--
KNPWML--
-KPWMLW-
-KPWMLN-
-KPWMLH-
KYPWML--
KKPWML--
-KPWMLT-
-KPWMLA-
KHPWML--
KDPWML--
-LPWMLR-
LGPWML--
LAPWML--
LEPWML--
LVPWML--
LMPWML--
LSPWML--
-LPWMLK-
LLPWML--
LQPWML--
LWPWML--
-LPWMLS-
-LPWMLQ-
LHPWML--
LYPWML--

LNPWML--
-LPWMLH-
-LPWMLF-
-LPWMLV-
-LPWMLN-
MRPWML--
MSPWML--
MGPWML--
MMPWML--
MKPWML--
MQPWML--
-MPWMLR-
MIPWML--
MHPWML--
MNPWML--
-MPWMLH-
MLPWML--
MWPWML--
MPPWML--
MDPWML--
MAPWML--
MYPWML--
-MPWMLS-
-MPWMLV-
-MPWMLA-
-MPWMLT-
-MPWMLQ-
-MPWMLM-
MEPWML--
NEPWML--
NSPWML--
NIPWML--
NTPWML--
NYPWML--
NRPWML--
NGPWML--
NPPWML--
NMPWML--
NVPWML--
NNPWML--
NLPWML--
-NPWMLV-
NKPWML--
-NPWMLY-
-NPWMLR-
NHPWML--
NFPWML--
-NPWMLM-
-NPWMLT-
NAPWML--

-NPWMLS-
NQPWML--
-NPWMLN-
-NPWMLK-
--PWMLNS
PYPWML--
--PWMLRN
--PWMLSS
--PWMLYK
--PWMLHA
--PWMLRQ
--PWMLRK
--PWMLTN
--PWMLIH
--PWMLHS
--PWMLVK
--PWMLVS
--PWMLRR
--PWMLRD
PTPWML--
PNPWML--
--PWMLYG
--PWMLSV
--PWMLAS
--PWMLVG
--PWMLST
--PWMLRF
--PWMLKG
--PWMLIN
PWPWML--
--PWMLQQ
--PWMLMH
--PWMLSN
--PWMLQT
--PWMLMR
--PWMLKY
--PWMLKA
--PWMLGG
PSPWML--
PGPWML--
--PWMLVH
--PWMLTK
--PWMLMD
--PWMLHW
--PWMLSA
--PWMLRH
--PWMLQM
--PWMLNT
--PWMLNH
--PWMLFG

--PWMLWH
--PWMLTR
--PWMLRA
--PWMLVQ
--PWMLTS
--PWMLSG
--PWMLHT
--PWMLHG
--PWMLAG
--PWMLKS
--PWMLIA
--PWMLHV
--PWMLTT
--PWMLRW
--PWMLRE
--PWMLKH
--PWMLES
--PWMLAM
PHPWML--
--PWMLYL
--PWMLWG
--PWMLVE
--PWMLTH
--PWMLKI
--PWMLHH
--PWMLEG
--PWMLWE
--PWMLSD
--PWMLQV
--PWMLKW
--PWMLKT
--PWMLKN
--PWMLGS
--PWMLAQ
PQPWML--
--PWMLSH
--PWMLPL
--PWMLNG
--PWMLMM
--PWMLKM
--PWMLGT
--PWMLFM
PVPWML--
PAPWML--
--PWMLWQ
--PWMLVW
--PWMLQK
--PWMLNQ
--PWMLNM
--PWMLNK

--PWMLHR
--PWMLHN
PCPWML--
--PWMLYH
--PWMLVT
--PWMLVD
--PWMLTQ
--PWMLTD
--PWMLQE
--PWMLND
--PWMLMF
--PWMLKR
--PWMLIM
--PWMLID
--PWMLHL
--PWMLEV
--PWMLAN
PEPWML--
PDPWML--
--PWMLYE
--PWMLYD
--PWMLWM
--PWMLTE
--PWMLSQ
--PWMLSE
--PWMLQH
--PWMLNE
--PWMLMG
--PWMLKE
--PWMLKD
--PWMLIV
--PWMLHY
--PWMLGA
--PWMLET
--PWMLEL
--PWMLEA
--PWMLAH
--PWMLYN
--PWMLSM
--PWMLSC
--PWMLPW
--PWMLNF
--PWMLNA
--PWMLKK
--PWMLIL
--PWMLIG
--PWMLHM
--PWMLHK
--PWMLHF
--PWMLHD

```
--PWMLGL
--PWMLGI
--PWMLFK
--PWMLEQ
--PWMLCT
--PWMLCR
--PWMLCN
-PPWMLF-
-PPWMLA-
PMPWML--
QSPWML--
QGPWML--
QRPWML--
QLPWML--
QTPWML--
-QPWMLR-
QVPWML--
QHPWML--
QFPWML--
-QPWMLT-
QIPWML--
QAPWML--
-QPWMLW-
-QPWMLK-
-QPWMLV-
-QPWMLS-
QEPWML--
QYPWML--
QWPWML--
-QPWMLA-
-QPWMLQ-
QKPWML--
QQPWML--
-QPWMLH-
QNPWML--
QDPWML--
-RPWMLR-
RRPWML--
RHPWML--
RNPWML--
RLPWML--
RSPWML--
RGPWML--
RAPWML--
-RPWMLS-
RIPWML--
-RPWMLN-
RYPWML--
RQPWML--
RDPWML--
```

-RPWMLI-
RMPWML--
-RPWMLT-
-RPWMLH-
-RPWMLQ-
RKPWML--
-RPWMLW-
-RPWMLA-
RPPWML--
RTPWML--
-RPWMLG-
-RPWMLE-
REPWML--
SRPWML--
-SPWMLR-
SSPWML--
SVPWML--
SEPWML--
SQPWML--
-SPWMLH-
-SPWMLV-
SAPWML--
SNPWML--
-SPWMLT-
SGPWML--
SFPWML--
SMPWML--
-SPWMLS-
SKPWML--
SDPWML--
SYPWML--
-SPWMLK-
SPPWML--
-SPWMLW-
SCPWML--
-SPWMLN-
SHPWML--
-SPWMLC-
-TPWMLR-
TRPWML--
TKPWML--
-TPWMLS-
TVPWML--
TLPWML--
TTPWML--
TSPWML--
TAPWML--
TNPWML--
TQPWML--
TIPWML--

TGPWML--
-TPWMLQ-
TMPWML--
-TPWMLN-
-TPWMLA-
TEPWML--
-TPWMLH-
TWPWML--
TPPWML--
TFPWML--
TDPWML--
-TPWMLV-
-TPWMLW-
THPWML--
TCPWML--
-TPWMLE-
-VPWMLR-
VSPWML--
VPPWML--
VIPWML--
VTPWML--
VQPWML--
VLPWML--
VMPWML--
VAPWML--
VGPWML--
VNPWML--
VKPWML--
-VPWMLA-
VHPWML--
-VPWMLV-
VVPWML--
-VPWMLW-
-VPWMLT-
-VPWMLI-
-VPWMLM-
VFPWML--
VDPWML--
VYPWML--
-VPWMLK-
VEPWML--
-VPWMLG-
-VPWMLE-
VCPWML--
WMPWML--
WAPWML--
WKPWML--
-WPWMLR-
WGPWML--
-WPWMLS-

	WFPWML--		
	WQPWML--		
	WSPWML--		
	WYPWML--		
	WLPWML--		
	WTPWML--		
	WRPWML--		
	-WPWMLF-		
	WHPWML--		
	-WPWMLT-		
	-YPWMLR-		
	YHPWML--		
	-YPWMLK-		
	YKPWML--		
	YRPWML--		
	YMPWML--		
	YVPWML--		
	-YPWMLS-		
	YYPWML--		
	YLPWML--		
	YFPWML--		
	YTPWML--		
	YQPWML--		
	-YPWMLM-		
	YNPWML--		
	-YPWMLV-		
	-YPWMLT-		
	-YPWMLN-		
	YIPWML--		
	-YPWMLE-		
	YDPWML--		
	YAPWML--		
	-YPWMLQ-		
	-YPWMLA-		
PFHM	614	583	45.58
	-APFHMK-		
	-APFHMR-		
	-APFHMS-		
	AEPFHM--		
	APPFHM--		
	-APFHMH-		
	AHPFHM--		
	AYPFHM--		
	AKPFHM--		
	AFPFHM--		
	-APFHMY-		
	-APFHMT-		
	-APFHML-		
	AVPFHM--		
	ARPFHM--		

AQPFHM--
AMPFHM--
ASPFHM--
ALPFHM--
-APFHMQ-
-APFHMI-
-APFHMG-
-APFHMF-
ANPFHM--
AGPFHM--
AWPFHM--
-APFHMV-
-APFHMC-
AAPFHM--
CRPFHM--
CKPFHM--
CVPFHM--
CPPFHM--
-CPFHMR-
CWPFHM--
CGPFHM--
CFPFHM--
DRPFHM--
DSPFHM--
DMPFHM--
-DPFHMM-
DLPFHM--
DGPFHM--
-DPFHMW-
-DPFHMR-
DHPFHM--
-DPFHML-
-DPFHMY-
DAPFHM--
-EPFHMS-
EWPFHM--
-EPFHML-
-EPFHMV-
-EPFHMR-
ESPFHM--
EYPFHM--
EMPFHM--
EKPFHM--
EEPFHM--
ERPFHM--
-EPFHMY-
-EPFHMA-
EFPFHM--
EAPFHM--
-FPFHML-

-FPFHMR-
FMPFHM--
FLPFHM--
FIPFHM--
-FPFHMI-
FWPFHM--
-FPFHMT-
-FPFHMK-
FDPFHM--
-FPFHMW-
-FPFHMS-
-FPFHMQ-
FGPFHM--
FFPFHM--
FCPFHM--
GSPFHM--
GTPFHM--
-GPFHMT-
GYPFHM--
GRPFHM--
GDPFHM--
-GPFHMY-
GMPFHM--
GLPFHM--
GIPFHM--
-GPFHMW-
GHPFHM--
GQPFHM--
-GPFHMS-
-GPFHMQ-
-GPFHMN-
-GPFHMR-
-GPFHMK-
GEPFHM--
GVPFHM--
-GPFHMG-
-GPFHMA-
GFPFHM--
GCPFHM--
GAPFHM--
HEPFHM--
HSPFHM--
HRPFHM--
-HPFHMI-
HVPFHM--
HIPFHM--
HAPFHM--
HGPFHM--
HFPFHM--
HQPFHM--

HKPFHM--
-HPFHMY-
-HPFHMS-
-HPFHMF-
HLPFHM--
HDPFHM--
ISPFHM--
IRPFHM--
-IPFHMK-
-IPFHMR-
IGPFHM--
IYPFHM--
ITPFHM--
-IPFHML-
IPPFHM--
-IPFHMS-
-IPFHMQ-
IMPFHM--
IKPFHM--
-IPFHMA-
INPFHM--
IHPFHM--
-IPFHMV-
-IPFHMN-
-IPFHMM-
IQPFHM--
IIPFHM--
IAPFHM--
IVPFHM--
-IPFHMW-
ILPFHM--
KSPFHM--
KAPFHM--
KIPFHM--
KGPFHM--
KYPFHM--
-KPFHMR-
-KPFHMS-
-KPFHMM-
KLPFHM--
KKPFHM--
KVPFHM--
KFPFHM--
-KPFHMT-
-KPFHMN-
KNPFHM--
-KPFHMW-
-KPFHML-
LSPFHM--
LRPFHM--

LVPFHM--
LPPFHM--
LEPFHM--
-LPFHML-
LMPFHM--
LKPFHM--
LYPFHM--
LNPFHM--
LHPFHM--
LGPFHM--
-LPFHMM-
-LPFHMA-
-LPFHMH-
LLPFHM--
LDPFHM--
LAPFHM--
-LPFHMY-
-LPFHMS-
LTPFHM--
-LPFHMR-
-LPFHMQ-
LWPFHM--
-LPFHMV-
-LPFHMT-
-LPFHMI-
LIPFHM--
MGPFHM--
MTPFHM--
MPPFHM--
MLPFHM--
-MPFHMR-
MVPFHM--
MRPFHM--
MYPFHM--
-MPFHMV-
-MPFHMM-
MNPFHM--
MIPFHM--
-MPFHMY-
-MPFHMT-
-MPFHMS-
-MPFHML-
-MPFHMG-
MAPFHM--
NSPFHM--
NNPFHM--
NAPFHM--
NIPFHM--
NVPFHM--
NLPFHM--

-NPFHMV-
-NPFHML-
NKPFHM--
NQPFHM--
-NPFHMS-
-NPFHMR-
NYPFHM--
-NPFHMM-
-NPFHMI-
-NPFHMH-
NGPFHM--
NEPFHM--
NCPFHM--
PGPFHM--
--PFHMQF
--PFHMMS
--PFHMLM
--PFHMWA
--PFHMHA
--PFHMRG
--PFHMTY
--PFHMTT
--PFHMRK
--PFHMMI
PHPFHM--
--PFHMVH
--PFHMFS
--PFHMVR
--PFHMVA
--PFHMLS
--PFHMKW
--PFHMHS
--PFHMVY
--PFHMVV
--PFHMTH
--PFHMRR
--PFHMLT
--PFHMKV
--PFHMIY
--PFHMIN
--PFHMHT
--PFHMRD
--PFHMQQ
--PFHMLY
--PFHMKG
--PFHMGT
--PFHMYQ
--PFHMTI
--PFHMKS
--PFHMIR

PWPFHM--
--PFHMYS
--PFHMVN
--PFHMVD
--PFHMTS
--PFHMRS
--PFHMRA
--PFHMKT
--PFHMGS
--PFHMFN
--PFHMYA
--PFHMWS
--PFHMVS
--PFHMRT
--PFHMQG
--PFHMKY
--PFHMGA
--PFHMFT
--PFHMAG
PQPFHM--
--PFHMWT
--PFHMRY
--PFHMLG
--PFHMIT
--PFHMGG
--PFHMEG
--PFHMTV
--PFHMTM
--PFHMTA
--PFHMSV
--PFHMSS
--PFHMSK
--PFHMMH
--PFHMMA
--PFHMLN
--PFHMKM
--PFHMKI
--PFHMIS
--PFHMIF
--PFHMIA
--PFHMET
--PFHMEQ
--PFHMEN
--PFHMAS
--PFHMVI
--PFHMVF
--PFHMTL
--PFHMTF
--PFHMSR
--PFHMSG

--PFHMSA
--PFHMRQ
--PFHMRN
--PFHMRF
--PFHMLK
--PFHMLH
--PFHMKF
--PFHMHY
--PFHMHW
--PFHMHR
--PFHMFG
--PFHMFF
--PFHMEA
PTPFHM--
--PFHMYM
--PFHMWR
--PFHMVT
--PFHMTG
--PFHMSW
--PFHMSH
--PFHMQY
--PFHMML
--PFHMMF
--PFHMME
--PFHMMD
--PFHMKE
--PFHMIG
--PFHMIE
--PFHMFI
--PFHMAV
--PFHMYL
--PFHMYK
--PFHMWN
--PFHMWM
--PFHMVK
--PFHMVG
--PFHMSN
--PFHMRM
--PFHMNG
--PFHMMG
--PFHMKN
--PFHMIQ
--PFHMIL
--PFHMIH
--PFHMHH
--PFHMHE
--PFHMGM
--PFHMFA
--PFHMEW
--PFHMAM

PDPFHM--
--PFHMYR
--PFHMYN
--PFHMYD
--PFHMWK
--PFHMWI
--PFHMWD
--PFHMVQ
--PFHMVM
--PFHMTN
--PFHMTK
--PFHMTE
--PFHMSI
--PFHMSF
--PFHMRW
--PFHMRE
--PFHMQN
--PFHMQI
--PFHMQD
--PFHMPC
--PFHMLE
--PFHMKR
--PFHMKQ
--PFHMHN
--PFHMHL
--PFHMHG
--PFHMHF
--PFHMGI
--PFHMFK
--PFHMFE
--PFHMEI
--PFHMAN
-QPFHMI-
-QPFHMR-
QWPFHM--
QTPFHM--
QGPFHM--
QSPFHM--
-QPFHMH-
QVPFHM--
QLPFHM--
QEPFHM--
QRPFHM--
-QPFHMS-
-QPFHMQ-
-QPFHML-
-QPFHMY-
-QPFHMW-
-QPFHMT-
-QPFHMF-

QNPFHM--
QKPFHM--
QDPFHM--
QYPFHM--
-QPFHMN-
-QPFHMA-
QFPFHM--
RSPFHM--
RRPFHM--
-RPFHMM-
-RPFHML-
RKPFHM--
RTPFHM--
-RPFHMF-
RIPFHM--
-RPFHMR-
RGPFHM--
RAPFHM--
-RPFHMS-
-RPFHMA-
-RPFHMI-
-RPFHMH-
RNPFHM--
RVPFHM--
RLPFHM--
-RPFHMW-
-RPFHMT-
-RPFHMQ-
RFPFHM--
RDPFHM--
-RPFHMV-
REPFHM--
-RPFHMY-
-RPFHME-
RYPFHM--
-RPFHMN-
-RPFHMK-
-RPFHMG-
RQPFHM--
SFPFHM--
SVPFHM--
SNPFHM--
SKPFHM--
-SPFHML-
-SPFHMH-
SLPFHM--
-SPFHMY-
SAPFHM--
SSPFHM--
-SPFHMW-

-SPFHMV-
-SPFHMR-
SCPFHM--
SWPFHM--
-SPFHMM-
-SPFHMF-
SIPFHM--
SHPFHM--
SYPFHM--
STPFHM--
SDPFHM--
-SPFHMT-
-SPFHMS-
-SPFHMK-
-SPFHMA-
SMPFHM--
SEPFHM--
TWPFHM--
-TPFHMW-
TVPFHM--
TSPFHM--
TYPFHM--
-TPFHMI-
-TPFHMT-
-TPFHMS-
-TPFHMR-
-TPFHMQ-
-TPFHMA-
TIPFHM--
TFPFHM--
TTPFHM--
-TPFHMV-
-TPFHML-
-TPFHMF-
TDPFHM--
-TPFHMM-
-TPFHMK-
TRPFHM--
TPPFHM--
-TPFHMH-
TNPFHM--
THPFHM--
TGPFHM--
VRPFHM--
VTPFHM--
-VPFHMT-
-VPFHML-
VMPFHM--
VKPFHM--
VSPFHM--

VQPFHM--
VLPFHM--
-VPFHME-
-VPFHMY-
VIPFHM--
VPPFHM--
-VPFHMV-
-VPFHMG-
VFPFHM--
-VPFHMK-
VNPFHM--
VGPFHM--
VEPFHM--
VYPFHM--
VWPFHM--
VVPFHM--
-VPFHMS-
-VPFHMR-
-VPFHMQ-
-VPFHMI-
-VPFHMF-
-VPFHMA-
VHPFHM--
WRPFHM--
WQPFHM--
WSPFHM--
WDPFHM--
WAPFHM--
WTPFHM--
-WPFHML-
WVPFHM--
WGPFHM--
WMPFHM--
-WPFHMV-
WLPFHM--
WHPFHM--
-WPFHMI-
-WPFHMH-
WIPFHM--
WWPFHM--
-WPFHMT-
-WPFHMF-
-WPFHME-
-WPFHMA-
YNPFHM--
YSPFHM--
YGPFHM--
YVPFHM--
YFPFHM--
YTPFHM--

```
              -YPFHMT-
              YEPFHM--
              YPPFHM--
              -YPFHMV-
              -YPFHML-
              -YPFHMI-
              YAPFHM--
              YYPFHM--
              -YPFHMK-
              YMPFHM--
              -YPFHMW-
              -YPFHMA-
              YWPFHM--
              -YPFHMN-
              -YPFHMH-
              -YPFHME-
              YLPFHM--
              YDPFHM--
PSWM           250        723     45.57
              -NPSWMG-
              -SPSWMP-
              -APSWMM-
              -APSWMR-
              -APSWML-
              ALPSWM--
              AKPSWM--
              AMPSWM--
              AFPSWM--
              ASPSWM--
              -APSWMS-
              APPSWM--
              AYPSWM--
              ATPSWM--
              AWPSWM--
              -APSWMT-
              -APSWMF-
              AIPSWM--
              -APSWMG-
              AQPSWM--
              ARPSWM--
              -APSWMV-
              ADPSWM--
              -APSWMQ-
              -APSWMA-
              -APSWMN-
              AAPSWM--
              -APSWMW-
              -APSWMH-
              AGPSWM--
              AVPSWM--
```

```
-APSWMP-
-APSWME-
-APSWMD-
AEPSWM--
CVPSWM--
CRPSWM--
CCPSWM--
-CPSWMK-
CGPSWM--
CYPSWM--
-CPSWMT-
-CPSWMI-
CLPSWM--
CKPSWM--
DSPSWM--
DAPSWM--
-DPSWMR-
DTPSWM--
-DPSWMS-
DIPSWM--
-DPSWMK-
DRPSWM--
-DPSWMV-
DHPSWM--
DVPSWM--
DKPSWM--
DWPSWM--
-DPSWMT-
-DPSWMI-
-DPSWMG-
-DPSWMF-
-EPSWML-
-EPSWMR-
-EPSWMI-
-EPSWMG-
-EPSWMS-
-EPSWMM-
EFPSWM--
ESPSWM--
EHPSWM--
-EPSWMN-
-EPSWMF-
ENPSWM--
-EPSWMT-
-EPSWMW-
EGPSWM--
EWPSWM--
EVPSWM--
-EPSWMV-
-EPSWMK-
```

EKPSWM--
EEPSWM--
FHPSWM--
-FPSWMI-
FNPSWM--
-FPSWMK-
-FPSWMR-
FGPSWM--
FRPSWM--
FAPSWM--
-FPSWMY-
FEPSWM--
-FPSWMQ-
FIPSWM--
-FPSWMG-
FYPSWM--
FVPSWM--
-FPSWMV-
-FPSWMT-
-FPSWMN-
-FPSWMA-
FTPSWM--
FQPSWM--
-FPSWMS-
-FPSWMM-
-FPSWMH-
-FPSWMF-
FKPSWM--
FDPSWM--
-GPSWMS-
GSPSWM--
-GPSWMR-
GVPSWM--
GKPSWM--
GRPSWM--
GTPSWM--
GYPSWM--
-GPSWMK-
GLPSWM--
GEPSWM--
-GPSWMT-
GAPSWM--
-GPSWMG-
GPPSWM--
-GPSWMQ-
-GPSWMY-
-GPSWMH-
GIPSWM--
-GPSWMM-
-GPSWMI-

GMPSWM--
GGPSWM--
-GPSWML-
-GPSWMN-
GWPSWM--
-GPSWME-
GHPSWM--
GDPSWM--
GQPSWM--
-GPSWMW-
-GPSWMV-
GCPSWM--
HSPSWM--
-HPSWMR-
HRPSWM--
HVPSWM--
HIPSWM--
HQPSWM--
HKPSWM--
HFPSWM--
-HPSWML-
-HPSWMW-
-HPSWMT-
HLPSWM--
HTPSWM--
-HPSWMV-
-HPSWMM-
HMPSWM--
HAPSWM--
-HPSWMY-
-HPSWMN-
-HPSWMI-
HPPSWM--
HEPSWM--
-IPSWMQ-
-IPSWMA-
-IPSWMS-
IYPSWM--
-IPSWMN-
-IPSWMK-
IRPSWM--
IFPSWM--
-IPSWMT-
-IPSWMR-
IHPSWM--
IVPSWM--
IQPSWM--
INPSWM--
-IPSWMG-
IMPSWM--

IIPSWM--
IPPSWM--
IAPSWM--
-IPSWMH-
IKPSWM--
IDPSWM--
ITPSWM--
-IPSWMM-
IGPSWM--
IEPSWM--
KSPSWM--
KDPSWM--
-KPSWMS-
KFPSWM--
KWPSWM--
-KPSWMR-
-KPSWML-
-KPSWMT-
KTPSWM--
KLPSWM--
KGPSWM--
KCPSWM--
KNPSWM--
-KPSWMN-
-KPSWMK-
KYPSWM--
KEPSWM--
-KPSWMG-
KIPSWM--
KHPSWM--
KMPSWM--
KQPSWM--
-KPSWME-
-KPSWMW-
-KPSWMH-
-KPSWMA-
KKPSWM--
KAPSWM--
KRPSWM--
-KPSWMY-
-KPSWMV-
-KPSWMC-
LSPSWM--
-LPSWMK-
LLPSWM--
LVPSWM--
LTPSWM--
LKPSWM--
-LPSWMS-
LDPSWM--

LRPSWM--
LHPSWM--
LFPSWM--
LYPSWM--
-LPSWMV-
LWPSWM--
-LPSWMI-
LNPSWM--
LQPSWM--
LPPSWM--
LIPSWM--
-LPSWMG-
LEPSWM--
-LPSWMY-
-LPSWMN-
-LPSWMW-
-LPSWMQ-
-LPSWMA-
LMPSWM--
LGPSWM--
LAPSWM--
-MPSWMR-
MSPSWM--
MPPSWM--
MYPSWM--
MRPSWM--
-MPSWMS-
-MPSWMG-
-MPSWMF-
MHPSWM--
MVPSWM--
MTPSWM--
-MPSWMI-
-MPSWMV-
MNPSWM--
-MPSWML-
MMPSWM--
-MPSWMY-
-MPSWMQ-
-MPSWMK-
MIPSWM--
MDPSWM--
-MPSWMH-
MQPSWM--
-MPSWMW-
-MPSWMA-
MKPSWM--
-NPSWMR-
NVPSWM--
NGPSWM--

NYPSWM--
NTPSWM--
NPPSWM--
-NPSWML-
NRPSWM--
NSPSWM--
-NPSWMQ-
-NPSWMF-
NHPSWM--
NIPSWM--
NFPSWM--
-NPSWMM-
NNPSWM--
NWPSWM--
-NPSWMS-
NKPSWM--
-NPSWMY-
-NPSWMT-
-NPSWMK-
NLPSWM--
--PSWMKA
--PSWMAS
--PSWMKS
--PSWMSG
--PSWMRV
--PSWMRT
--PSWMTT
--PSWMTS
--PSWMSH
--PSWMNS
--PSWMIN
--PSWMGT
--PSWMFS
--PSWMVG
--PSWMTG
--PSWMSS
--PSWMLG
--PSWMVS
--PSWMRS
--PSWMQS
--PSWMIG
--PSWMVV
--PSWMGS
PKPSWM--
--PSWMYT
PSPSWM--
--PSWMVR
PHPSWM--
--PSWMST
--PSWMSK

--PSWMGG
--PSWMRE
--PSWMNT
--PSWMLV
--PSWMKT
--PSWMIA
--PSWMES
PLPSWM--
--PSWMVY
--PSWMLN
PFPSWM--
--PSWMVK
--PSWMVD
--PSWMVT
--PSWMLH
--PSWMGA
--PSWMFN
--PSWMTI
--PSWMSA
--PSWMIY
--PSWMYY
--PSWMYG
--PSWMYD
--PSWMVA
--PSWMTW
--PSWMTA
--PSWMSY
--PSWMSE
--PSWMKI
--PSWMHG
--PSWMYM
--PSWMYK
--PSWMWS
--PSWMTR
--PSWMTN
--PSWMQG
--PSWMMA
--PSWMKN
--PSWMIT
--PSWMIR
--PSWMFT
--PSWMFH
--PSWMWA
--PSWMKD
--PSWMAT
PAPSWM--
--PSWMSW
--PSWMSD
--PSWMRM
--PSWMRD

--PSWMKV
--PSWMYA
--PSWMVQ
--PSWMTV
--PSWMRW
--PSWMQV
--PSWMMN
--PSWMKW
--PSWMKH
--PSWMGI
--PSWMYW
--PSWMTY
--PSWMSN
--PSWMSL
--PSWMMV
--PSWMFA
--PSWMDS
--PSWMAV
PWPSWM--
--PSWMYN
--PSWMWN
--PSWMTK
--PSWMTE
--PSWMSV
--PSWMNG
--PSWMKK
--PSWMHA
--PSWMAM
--PSWMAF
PGPSWM--
PEPSWM--
--PSWMWT
--PSWMVN
--PSWMVI
--PSWMSM
--PSWMRN
--PSWMQT
--PSWMQN
--PSWMQM
--PSWMMR
--PSWMMM
--PSWMLQ
--PSWMGN
--PSWMFG
PVPSWM--
--PSWMVM
--PSWMVH
--PSWMVE
--PSWMSI
--PSWMRY

--PSWMRL
--PSWMQW
--PSWMQH
--PSWMQF
--PSWMNA
--PSWMHV
--PSWMGY
--PSWMGM
--PSWMAG
PDPSWM--
PTPSWM--
--PSWMMF
--PSWMME
--PSWMKY
--PSWMGF
--PSWMAI
PMPSWM--
--PSWMYQ
--PSWMWG
--PSWMTQ
--PSWMQR
--PSWMLE
--PSWMII
--PSWMHY
--PSWMGL
--PSWMET
--PSWMDT
--PSWMAN
--PSWMAE
--PSWMYH
--PSWMTL
--PSWMQI
--PSWMQD
--PSWMLK
--PSWMKE
--PSWMIW
--PSWMIM
--PSWMIH
--PSWMID
--PSWMHS
--PSWMHQ
--PSWMHM
--PSWMEY
--PSWMAW
--PSWMAR
--PSWMYS
--PSWMVW
--PSWMTM
--PSWMQQ
--PSWMNY

--PSWMNF
--PSWMND
--PSWMMI
--PSWMKM
--PSWMIV
--PSWMIE
--PSWMHR
--PSWMFW
--PSWMEV
--PSWMEQ
--PSWMEM
--PSWMEG
--PSWMAK
PIPSWM--
--PSWMYE
--PSWMYC
--PSWMWW
--PSWMVC
--PSWMRP
--PSWMQY
--PSWMQK
--PSWMPT
--PSWMNW
--PSWMNN
--PSWMLI
--PSWMLD
--PSWMKP
--PSWMHN
--PSWMHK
--PSWMHF
--PSWMGV
--PSWMGK
--PSWMFE
--PSWMFD
--PSWMEW
--PSWMEA
--PSWMDA
--PSWMCE
--PSWMAQ
--PSWMAH
PNPSWM--
QGPSWM--
-QPSWMS-
-QPSWMR-
QRPSWM--
-QPSWML-
-QPSWMG-
-QPSWMA-
QSPSWM--
-QPSWMW-

-QPSWMT-
QVPSWM--
-QPSWMH-
-QPSWMN-
QHPSWM--
-QPSWMV-
-QPSWMI-
QWPSWM--
QIPSWM--
QYPSWM--
-QPSWMF-
-QPSWME-
QAPSWM--
QMPSWM--
QDPSWM--
-RPSWMS-
-RPSWMK-
-RPSWMR-
RSPSWM--
RLPSWM--
RGPSWM--
-RPSWMI-
RTPSWM--
-RPSWMM-
-RPSWMV-
-RPSWMF-
RPPSWM--
RNPSWM--
-RPSWMQ-
RWPSWM--
-RPSWMG-
RMPSWM--
RQPSWM--
-RPSWMW-
RDPSWM--
REPSWM--
-RPSWMN-
RKPSWM--
RHPSWM--
RIPSWM--
RAPSWM--
-RPSWMA-
RCPSWM--
-RPSWMH-
-RPSWME-
RFPSWM--
-RPSWMD-
-RPSWMY-
SSPSWM--
-SPSWMR-

-SPSWMT-
-SPSWMS-
-SPSWMG-
STPSWM--
SRPSWM--
SGPSWM--
SYPSWM--
SPPSWM--
SFPSWM--
-SPSWMI-
SKPSWM--
SIPSWM--
-SPSWMV-
SLPSWM--
-SPSWML-
SAPSWM--
SWPSWM--
-SPSWMY-
SNPSWM--
-SPSWMA-
SQPSWM--
-SPSWMQ-
-SPSWMK-
-SPSWMF-
SDPSWM--
SCPSWM--
SHPSWM--
SEPSWM--
-SPSWMM-
-SPSWMW-
TRPSWM--
TSPSWM--
-TPSWMR-
TPPSWM--
-TPSWMG-
TLPSWM--
-TPSWMH-
TWPSWM--
-TPSWML-
TKPSWM--
-TPSWMT-
TTPSWM--
TYPSWM--
-TPSWMQ-
TNPSWM--
TVPSWM--
THPSWM--
TMPSWM--
TGPSWM--
TIPSWM--

-TPSWMN-
-TPSWMS-
TEPSWM--
-TPSWMI-
-TPSWMV-
TFPSWM--
-TPSWMW-
-TPSWMM-
TAPSWM--
TQPSWM--
-TPSWMK-
-VPSWMS-
VSPSWM--
-VPSWMT-
VTPSWM--
VIPSWM--
VKPSWM--
VHPSWM--
VNPSWM--
VLPSWM--
VEPSWM--
VRPSWM--
-VPSWMK-
-VPSWMN-
VGPSWM--
-VPSWMG-
-VPSWMI-
-VPSWMM-
-VPSWMV-
-VPSWML-
VVPSWM--
-VPSWMA-
VFPSWM--
VAPSWM--
-VPSWMH-
VMPSWM--
VPPSWM--
-VPSWMQ-
VYPSWM--
VWPSWM--
-VPSWMY-
-VPSWME-
WTPSWM--
WSPSWM--
WVPSWM--
WGPSWM--
-WPSWMR-
-WPSWMV-
WNPSWM--
-WPSWMI-

-WPSWMG-
WFPSWM--
WRPSWM--
-WPSWMM-
WEPSWM--
WDPSWM--
WYPSWM--
-WPSWMQ-
WPPSWM--
WHPSWM--
WWPSWM--
-WPSWMN-
-WPSWMH-
-WPSWMF-
WIPSWM--
-YPSWMR-
YRPSWM--
-YPSWMS-
-YPSWMY-
-YPSWMK-
YLPSWM--
YWPSWM--
YGPSWM--
YAPSWM--
YTPSWM--
-YPSWMF-
YYPSWM--
YVPSWM--
YQPSWM--
YMPSWM--
YNPSWM--
-YPSWMN-
-YPSWMG-
YKPSWM--
-YPSWMV-
-YPSWMQ-
-YPSWMH-
-YPSWME-
-YPSWMM-
YPPSWM--
YIPSWM--
YHPSWM--

PWWL 437 645 45.56

-APWWLR-
-APWWLS-
ATPWWL--
-APWWLA-
ANPWWL--
-APWWLT-
ARPWWL--

-APWWLL-
-APWWLQ-
AGPWWL--
AKPWWL--
-APWWLM-
AEPWWL--
AAPWWL--
AYPWWL--
ALPWWL--
-APWWLV-
AQPWWL--
-APWWLH-
-APWWLE-
-APWWLD-
AVPWWL--
-APWWLI-
ADPWWL--
AWPWWL--
-APWWLY-
-APWWLN-
-APWWLG-
AHPWWL--
CSPWWL--
-CPWWLS-
DHPWWL--
DRPWWL--
-DPWWLY-
-DPWWLS-
DAPWWL--
DWPWWL--
DLPWWL--
DVPWWL--
DSPWWL--
-DPWWLR-
-DPWWLI-
DGPWWL--
DEPWWL--
DDPWWL--
DIPWWL--
DFPWWL--
-DPWWLV-
DQPWWL--
-DPWWLL-
-DPWWLH-
-EPWWLH-
EMPWWL--
-EPWWLA-
EVPWWL--
ESPWWL--
EIPWWL--

-EPWWLV-
-EPWWLS-
-EPWWLN-
-EPWWLK-
EFPWWL--
-EPWWLE-
EEPWWL--
-EPWWLT-
-EPWWLR-
EQPWWL--
-EPWWLD-
ELPWWL--
ETPWWL--
ERPWWL--
EAPWWL--
EYPWWL--
ENPWWL--
EGPWWL--
-EPWWLQ-
-EPWWLL-
-EPWWLI-
EHPWWL--
-FPWWLT-
FNPWWL--
FRPWWL--
FGPWWL--
-FPWWLS-
-FPWWLR-
-FPWWLV-
-FPWWLA-
FMPWWL--
FHPWWL--
-FPWWLG-
FLPWWL--
FTPWWL--
-FPWWLN-
-FPWWLM-
-FPWWLL-
-FPWWLY-
FCPWWL--
-GPWWLS-
GTPWWL--
GVPWWL--
GRPWWL--
-GPWWLT-
-GPWWLK-
-GPWWLW-
-GPWWLR-
-GPWWLA-
GLPWWL--

GEPWWL--
-GPWWLQ-
-GPWWLV-
GNPWWL--
GKPWWL--
GGPWWL--
GSPWWL--
GYPWWL--
GMPWWL--
-GPWWLH-
GIPWWL--
GQPWWL--
GAPWWL--
GWPWWL--
-GPWWLG-
-GPWWLE-
GFPWWL--
HHPWWL--
-HPWWLS-
-HPWWLR-
HSPWWL--
HRPWWL--
HGPWWL--
HDPWWL--
-HPWWLY-
-HPWWLH-
HLPWWL--
HNPWWL--
HVPWWL--
-HPWWLK-
HYPWWL--
HTPWWL--
-HPWWLG-
HQPWWL--
-HPWWLT-
-HPWWLQ-
-HPWWLE-
-HPWWLM-
HWPWWL--
-HPWWLN-
-HPWWLD-
-HPWWLA-
IGPWWL--
IEPWWL--
-IPWWLK-
IPPWWL--
IFPWWL--
ITPWWL--
IQPWWL--
IIPWWL--

IRPWWL--
IMPWWL--
-IPWWLT-
IDPWWL--
-IPWWLA-
-IPWWLH-
-IPWWLG-
IKPWWL--
ILPWWL--
ISPWWL--
-IPWWLV-
-IPWWLQ-
-IPWWLF-
IWPWWL--
-IPWWLN-
-IPWWLE-
KSPWWL--
KYPWWL--
-KPWWLK-
-KPWWLN-
KGPWWL--
-KPWWLR-
KRPWWL--
-KPWWLL-
KFPWWL--
KVPWWL--
KHPWWL--
KAPWWL--
-KPWWLV-
KIPWWL--
-KPWWLT-
-KPWWLG-
-KPWWLA-
KDPWWL--
KTPWWL--
-KPWWLY-
-KPWWLS-
-KPWWLE-
-KPWWLD-
KPPWWL--
KLPWWL--
-KPWWLQ-
-KPWWLI-
KMPWWL--
KCPWWL--
LSPWWL--
-LPWWLT-
-LPWWLS-
LDPWWL--
-LPWWLK-

-LPWWLH-
LRPWWL--
LAPWWL--
LVPWWL--
LMPWWL--
-LPWWLR-
LNPWWL--
LQPWWL--
-LPWWLQ-
LGPWWL--
LTPWWL--
-LPWWLE-
-LPWWLG-
LEPWWL--
LYPWWL--
LIPWWL--
LWPWWL--
-LPWWLA-
LKPWWL--
LPPWWL--
LHPWWL--
MTPWWL--
MSPWWL--
-MPWWLS-
MLPWWL--
MMPWWL--
MIPWWL--
MEPWWL--
-MPWWLR-
-MPWWLN-
-MPWWLL-
MDPWWL--
MGPWWL--
-MPWWLT-
-MPWWLA-
MPPWWL--
MAPWWL--
MYPWWL--
MRPWWL--
-MPWWLY-
-MPWWLV-
MQPWWL--
-NPWWLR-
NLPWWL--
NGPWWL--
NAPWWL--
-NPWWLV-
NHPWWL--
-NPWWLA-
NQPWWL--

```
-NPWWLT-
NSPWWL--
NRPWWL--
NTPWWL--
NFPWWL--
NVPWWL--
NEPWWL--
-NPWWLS-
-NPWWLK-
-NPWWLH-
-NPWWLE-
NKPWWL--
-NPWWLW-
NPPWWL--
-NPWWLN-
NMPWWL--
-NPWWLI-
NIPWWL--
NDPWWL--
--PWWLSS
--PWWLRS
--PWWLAG
--PWWLST
--PWWLRE
--PWWLHS
--PWWLGA
--PWWLAS
--PWWLRA
--PWWLNS
--PWWLKK
--PWWLKT
--PWWLKQ
--PWWLIR
--PWWLIK
--PWWLTR
--PWWLRY
--PWWLKA
--PWWLGT
--PWWLGL
--PWWLAD
--PWWLRN
--PWWLKG
PVPWWL--
--PWWLVN
--PWWLTK
--PWWLKE
--PWWLKD
--PWWLVR
--PWWLSA
--PWWLRD
```

--PWWLQH
PGPWWL--
--PWWLTN
--PWWLTE
--PWWLQA
--PWWLLG
--PWWLKN
--PWWLKL
--PWWLHM
PMPWWL--
PKPWWL--
--PWWLVG
--PWWLSV
--PWWLRM
--PWWLQR
--PWWLKS
--PWWLGM
--PWWLGG
--PWWLEM
--PWWLSR
--PWWLRQ
--PWWLQQ
--PWWLMV
--PWWLMD
--PWWLKV
--PWWLGS
--PWWLGI
--PWWLEH
--PWWLEG
--PWWLAA
--PWWLWN
--PWWLVK
--PWWLTI
--PWWLTG
--PWWLSY
--PWWLSK
--PWWLSG
--PWWLRK
--PWWLRH
--PWWLRF
--PWWLNW
--PWWLMS
--PWWLKR
--PWWLKH
--PWWLKF
--PWWLIV
--PWWLHN
--PWWLHG
--PWWLFN
PEPWWL--

PAPWWL--
--PWWLTS
--PWWLTQ
--PWWLQT
--PWWLQG
--PWWLMR
--PWWLLQ
--PWWLGV
--PWWLVS
--PWWLTA
--PWWLSQ
--PWWLSM
--PWWLQD
--PWWLLH
--PWWLIN
--PWWLHW
--PWWLEV
--PWWLEA
--PWWLAT
PDPWWL--
--PWWLVH
--PWWLVE
--PWWLTY
--PWWLTV
--PWWLTH
--PWWLTD
--PWWLSE
--PWWLNG
--PWWLMW
--PWWLLN
--PWWLKY
--PWWLIS
--PWWLID
--PWWLHQ
--PWWLHD
--PWWLGQ
--PWWLGN
--PWWLGK
--PWWLGF
--PWWLGD
--PWWLEN
--PWWLAQ
--PWWLAI
PHPWWL--
PYPWWL--
--PWWLYK
--PWWLWM
--PWWLVQ
--PWWLQY
--PWWLQN

--PWWLQK
--PWWLQE
--PWWLNY
--PWWLND
--PWWLMN
--PWWLMM
--PWWLMK
--PWWLMH
--PWWLMA
--PWWLLR
--PWWLLK
--PWWLLD
--PWWLHH
--PWWLFH
--PWWLER
--PWWLEQ
--PWWLDA
--PWWLAW
--PWWLAM
PWPWWL--
-PPWWLQ-
--PWWLWG
--PWWLVM
--PWWLVD
--PWWLSW
--PWWLSH
--PWWLSD
--PWWLQS
--PWWLNT
--PWWLMG
--PWWLME
--PWWLIQ
--PWWLIG
--PWWLHR
--PWWLHK
--PWWLHI
--PWWLHF
--PWWLHA
--PWWLGR
--PWWLFV
--PWWLFQ
--PWWLFG
--PWWLFA
--PWWLEW
--PWWLED
--PWWLDS
PTPWWL--
PSPWWL--
PQPWWL--
PFPWWL--

QRPWWL--
-QPWWLR-
-QPWWLS-
QHPWWL--
QKPWWL--
QGPWWL--
QSPWWL--
QIPWWL--
QYPWWL--
-QPWWLG-
QDPWWL--
-QPWWLN-
-QPWWLL-
-QPWWLK-
-QPWWLV-
-QPWWLT-
-QPWWLM-
-QPWWLI-
QEPWWL--
-QPWWLA-
QFPWWL--
QVPWWL--
QTPWWL--
-QPWWLE-
QLPWWL--
RIPWWL--
-RPWWLS-
-RPWWLR-
RHPWWL--
-RPWWLM-
-RPWWLK-
RNPWWL--
RAPWWL--
RVPWWL--
-RPWWLL-
RYPWWL--
RRPWWL--
RPPWWL--
-RPWWLN-
REPWWL--
-RPWWLY-
-RPWWLQ-
RGPWWL--
-RPWWLT-
-RPWWLA-
RWPWWL--
RLPWWL--
-RPWWLG-
RFPWWL--
RDPWWL--

-RPWWLV-
RMPWWL--
RKPWWL--
-RPWWLE-
RTPWWL--
RQPWWL--
-SPWWLR-
SRPWWL--
SGPWWL--
SSPWWL--
SLPWWL--
SIPWWL--
SAPWWL--
SKPWWL--
-SPWWLK-
SYPWWL--
-SPWWLN-
-SPWWLT-
-SPWWLG-
SNPWWL--
SDPWWL--
-SPWWLA-
SQPWWL--
STPWWL--
SPPWWL--
SMPWWL--
-SPWWLV-
SVPWWL--
-SPWWLH-
-SPWWLE-
SFPWWL--
-SPWWLW-
-SPWWLY-
-SPWWLI-
SHPWWL--
-SPWWLQ-
-SPWWLM-
SCPWWL--
-TPWWLS-
TNPWWL--
-TPWWLG-
TKPWWL--
TGPWWL--
TLPWWL--
TAPWWL--
TSPWWL--
TRPWWL--
TPPWWL--
TQPWWL--
TTPWWL--

THPWWL--
TEPWWL--
TVPWWL--
-TPWWLI-
-TPWWLH-
TYPWWL--
TWPWWL--
-TPWWLN-
-TPWWLR-
-TPWWLM-
-TPWWLL-
-TPWWLK-
-TPWWLA-
TMPWWL--
TIPWWL--
-TPWWLW-
-TPWWLQ-
TDPWWL--
-TPWWLE-
-TPWWLD-
TFPWWL--
VGPWWL--
VRPWWL--
VQPWWL--
VTPWWL--
VPPWWL--
-VPWWLS-
VLPWWL--
VNPWWL--
-VPWWLK-
-VPWWLA-
-VPWWLR-
VSPWWL--
VVPWWL--
VYPWWL--
-VPWWLY-
-VPWWLT-
VDPWWL--
-VPWWLN-
-VPWWLH-
-VPWWLG-
VKPWWL--
-VPWWLL-
-VPWWLM-
-VPWWLI-
VHPWWL--
VIPWWL--
-VPWWLQ-
-VPWWLD-
VFPWWL--

VAPWWL--
VMPWWL--
VEPWWL--
-VPWWLE-
WSPWWL--
WAPWWL--
WRPWWL--
-WPWWLK-
WVPWWL--
WHPWWL--
WEPWWL--
-WPWWLR-
-WPWWLL-
WKPWWL--
-WPWWLG-
-WPWWLA-
WNPWWL--
YRPWWL--
YGPWWL--
YTPWWL--
-YPWWLK-
YNPWWL--
-YPWWLR-
YDPWWL--
-YPWWLT-
YEPWWL--
YYPWWL--
YWPWWL--
YVPWWL--
YSPWWL--
YLPWWL--
YIPWWL--
-YPWWLY-
-YPWWLV-
YKPWWL--
YHPWWL--
-YPWWLF-
YPPWWL--

PMPY 459 633 45.55

-APMPYS-
-APMPYV-
-APMPYA-
ARPMPY--
-APMPYR-
AWPMPY--
APPMPY--
-APMPYI-
-APMPYH-
ALPMPY--
AIPMPY--

-APMPYY-
ATPMPY--
-APMPYT-
-APMPYF-
ANPMPY--
AKPMPY--
ASPMPY--
AHPMPY--
AQPMPY--
-APMPYQ-
-APMPYG-
AEPMPY--
AVPMPY--
-APMPYK-
ADPMPY--
CCPMPY--
CYPMPY--
CWPMPY--
CRPMPY--
CVPMPY--
-CPMPYI-
CSPMPY--
CKPMPY--
CEPMPY--
CQPMPY--
-CPMPYQ-
-CPMPYH-
CLPMPY--
CDPMPY--
DWPMPY--
DRPMPY--
-DPMPYR-
-DPMPYL-
-DPMPYQ-
DLPMPY--
-DPMPYH-
DQPMPY--
-DPMPYW-
DMPMPY--
DKPMPY--
DIPMPY--
-DPMPYT-
DDPMPY--
DFPMPY--
DTPMPY--
-DPMPYS-
-DPMPYK-
-DPMPYA-
DGPMPY--
EWPMPY--

-EPMPYT-
-EPMPYM-
-EPMPYQ-
-EPMPYA-
EFPMPY--
-EPMPYW-
-EPMPYV-
-EPMPYF-
EIPMPY--
ETPMPY--
-EPMPYR-
-EPMPYK-
EVPMPY--
EEPMPY--
-EPMPYN-
-EPMPYH-
ENPMPY--
EMPMPY--
EAPMPY--
FGPMPY--
FRPMPY--
FSPMPY--
FLPMPY--
FDPMPY--
-FPMPYW-
-FPMPYV-
-FPMPYN-
FCPMPY--
FPPMPY--
FHPMPY--
FWPMPY--
FVPMPY--
-FPMPYL-
FNPMPY--
-FPMPYR-
-FPMPYH-
FTPMPY--
FAPMPY--
GLPMPY--
-GPMPYL-
GMPMPY--
GRPMPY--
-GPMPYV-
-GPMPYI-
GIPMPY--
-GPMPYS-
-GPMPYM-
GVPMPY--
GQPMPY--
-GPMPYT-

GHPMPY--
GEPMPY--
GWPMPY--
GTPMPY--
GSPMPY--
GPPMPY--
-GPMPYF-
GDPMPY--
-GPMPYR-
-GPMPYE-
GAPMPY--
-GPMPYH-
-GPMPYA-
GNPMPY--
GGPMPY--
-HPMPYS-
HPPMPY--
-HPMPYR-
-HPMPYL-
HIPMPY--
HTPMPY--
HLPMPY--
HDPMPY--
HMPMPY--
HFPMPY--
-HPMPYT-
-HPMPYG-
-HPMPYF-
-HPMPYA-
HEPMPY--
HVPMPY--
HQPMPY--
-HPMPYY-
HNPMPY--
HRPMPY--
-HPMPYV-
-HPMPYN-
-HPMPYK-
-HPMPYI-
HGPMPY--
-IPMPYT-
ITPMPY--
INPMPY--
IRPMPY--
ISPMPY--
IYPMPY--
-IPMPYS-
-IPMPYR-
-IPMPYL-
IVPMPY--

-IPMPYM-
ILPMPY--
-IPMPYK-
IGPMPY--
-IPMPYA-
-IPMPYF-
-IPMPYV-
-IPMPYI-
IFPMPY--
-IPMPYQ-
IDPMPY--
-IPMPYY-
IKPMPY--
IIPMPY--
IEPMPY--
IAPMPY--
KSPMPY--
KVPMPY--
KAPMPY--
KEPMPY--
KNPMPY--
-KPMPYF-
-KPMPYV-
KRPMPY--
KGPMPY--
KDPMPY--
-KPMPYT-
-KPMPYK-
-KPMPYS-
-KPMPYR-
-KPMPYL-
KIPMPY--
KCPMPY--
KWPMPY--
-KPMPYA-
LKPMPY--
-LPMPYL-
LRPMPY--
LLPMPY--
LDPMPY--
-LPMPYV-
LGPMPY--
-LPMPYM-
LPPMPY--
LYPMPY--
-LPMPYR-
-LPMPYI-
LVPMPY--
LIPMPY--
-LPMPYH-

LQPMPY--
LNPMPY--
-LPMPYT-
-LPMPYW-
-LPMPYK-
LMPMPY--
-LPMPYE-
-LPMPYA-
MHPMPY--
-MPMPYI-
MAPMPY--
MRPMPY--
-MPMPYK-
-MPMPYA-
MYPMPY--
MNPMPY--
MKPMPY--
MMPMPY--
-MPMPYT-
-MPMPYS-
MLPMPY--
MVPMPY--
MQPMPY--
-MPMPYV-
-MPMPYL-
-MPMPYY-
-MPMPYN-
-MPMPYH-
MGPMPY--
MCPMPY--
-NPMPYT-
NDPMPY--
NWPMPY--
NRPMPY--
NLPMPY--
-NPMPYR-
NHPMPY--
NTPMPY--
-NPMPYS-
NNPMPY--
NPPMPY--
-NPMPYM-
NIPMPY--
NEPMPY--
NSPMPY--
NMPMPY--
NYPMPY--
NVPMPY--
-NPMPYW-
-NPMPYN-

-NPMPYF-
--PMPYSR
--PMPYLI
--PMPYVA
--PMPYTR
--PMPYII
--PMPYFR
--PMPYSS
--PMPYMA
--PMPYLA
--PMPYRQ
--PMPYLL
--PMPYLK
--PMPYYL
--PMPYVH
--PMPYLS
--PMPYGW
--PMPYYS
--PMPYTS
--PMPYSK
--PMPYNS
--PMPYLT
--PMPYFL
--PMPYVN
--PMPYVL
--PMPYSF
--PMPYIH
--PMPYHW
PTPMPY--
--PMPYYF
--PMPYWS
--PMPYSA
--PMPYRA
--PMPYLV
--PMPYHS
--PMPYVF
--PMPYRV
--PMPYRR
--PMPYMI
--PMPYMH
--PMPYHF
--PMPYSG
--PMPYRY
--PMPYQQ
--PMPYNQ
--PMPYNG
--PMPYMG
--PMPYWM
--PMPYVM
--PMPYTV

--PMPYTQ
--PMPYSN
--PMPYRT
--PMPYRH
--PMPYQR
--PMPYLF
--PMPYKQ
--PMPYAG
--PMPYWY
--PMPYQG
--PMPYMR
--PMPYKR
--PMPYYR
--PMPYVK
--PMPYVG
--PMPYQH
--PMPYPW
--PMPYLN
--PMPYIV
--PMPYHR
--PMPYGQ
--PMPYAS
--PMPYYT
--PMPYWN
--PMPYVT
--PMPYTY
--PMPYQN
--PMPYMT
--PMPYMS
--PMPYLQ
--PMPYLH
--PMPYKF
--PMPYKA
--PMPYVS
--PMPYTT
--PMPYTF
--PMPYRK
--PMPYEI
--PMPYAK
PKPMPY--
PGPMPY--
--PMPYWG
--PMPYTK
--PMPYTD
--PMPYRS
--PMPYRN
--PMPYQK
--PMPYNT
--PMPYLR
--PMPYKV

--PMPYIS
--PMPYGS
--PMPYFN
--PMPYFM
--PMPYFI
--PMPYFE
--PMPYWV
--PMPYWR
--PMPYWK
--PMPYVY
--PMPYVR
--PMPYTH
--PMPYTA
--PMPYQS
--PMPYPM
--PMPYNA
--PMPYMY
--PMPYKN
--PMPYKG
--PMPYIA
--PMPYHG
--PMPYEM
--PMPYAW
--PMPYAA
PQPMPY--
--PMPYYI
--PMPYYH
--PMPYYE
--PMPYYA
--PMPYTN
--PMPYST
--PMPYRG
--PMPYRE
--PMPYPF
--PMPYLY
--PMPYLM
--PMPYKI
--PMPYIL
--PMPYIE
--PMPYHI
--PMPYEN
--PMPYEA
--PMPYDI
--PMPYAT
--PMPYAR
PNPMPY--
--PMPYYY
--PMPYYK
--PMPYYD
--PMPYVV

--PMPYTW
--PMPYTE
--PMPYQW
--PMPYQM
--PMPYNV
--PMPYMD
--PMPYKH
--PMPYIY
--PMPYIK
--PMPYIG
--PMPYHT
--PMPYGH
--PMPYGG
--PMPYFF
--PMPYEY
--PMPYES
--PMPYDW
--PMPYAH
--PMPYYV
--PMPYWD
--PMPYVI
--PMPYSW
--PMPYSP
--PMPYSD
--PMPYRI
--PMPYQT
--PMPYQF
--PMPYPS
--PMPYPR
--PMPYNW
--PMPYNN
--PMPYLE
--PMPYLD
--PMPYKK
--PMPYIC
--PMPYHN
--PMPYHA
--PMPYGT
--PMPYGA
--PMPYFQ
--PMPYFG
--PMPYET
--PMPYDS
--PMPYCF
--PMPYCD
PHPMPY--
PEPMPY--
-QPMPYW-
QWPMPY--
QMPMPY--

−QPMPYR−
−QPMPYS−
QAPMPY−−
QHPMPY−−
QRPMPY−−
QGPMPY−−
QPPMPY−−
QLPMPY−−
−QPMPYN−
QNPMPY−−
QSPMPY−−
−QPMPYT−
−QPMPYM−
−QPMPYL−
QKPMPY−−
−QPMPYV−
−QPMPYH−
−RPMPYL−
−RPMPYS−
RFPMPY−−
RQPMPY−−
−RPMPYY−
RLPMPY−−
RGPMPY−−
−RPMPYT−
RIPMPY−−
RYPMPY−−
−RPMPYV−
−RPMPYA−
RAPMPY−−
REPMPY−−
RVPMPY−−
−RPMPYR−
RTPMPY−−
−RPMPYI−
RSPMPY−−
−RPMPYM−
−RPMPYQ−
RMPMPY−−
−RPMPYN−
−RPMPYG−
RKPMPY−−
RDPMPY−−
−RPMPYK−
RPPMPY−−
−RPMPYE−
SHPMPY−−
SRPMPY−−
SNPMPY−−
−SPMPYY−

SQPMPY--
SLPMPY--
SWPMPY--
-SPMPYL-
-SPMPYT-
STPMPY--
-SPMPYR-
SGPMPY--
SAPMPY--
SPPMPY--
-SPMPYV-
-SPMPYK-
SEPMPY--
-SPMPYF-
-SPMPYS-
-SPMPYH-
SVPMPY--
SYPMPY--
SSPMPY--
-SPMPYN-
-SPMPYI-
-SPMPYA-
SMPMPY--
-SPMPYQ-
SFPMPY--
SDPMPY--
TSPMPY--
TVPMPY--
TRPMPY--
TAPMPY--
-TPMPYV-
-TPMPYI-
TGPMPY--
TPPMPY--
-TPMPYW-
TLPMPY--
-TPMPYR-
-TPMPYQ-
TIPMPY--
TDPMPY--
-TPMPYY-
-TPMPYH-
TTPMPY--
TFPMPY--
TYPMPY--
THPMPY--
-TPMPYT-
-TPMPYF-
-TPMPYA-
TKPMPY--

TQPMPY--
-TPMPYS-
-TPMPYN-
TNPMPY--
-VPMPYR-
VLPMPY--
VAPMPY--
VRPMPY--
-VPMPYA-
VIPMPY--
-VPMPYL-
VGPMPY--
VVPMPY--
-VPMPYT-
VPPMPY--
VQPMPY--
-VPMPYY-
-VPMPYK-
-VPMPYF-
VKPMPY--
-VPMPYN-
VSPMPY--
-VPMPYW-
-VPMPYV-
-VPMPYE-
VNPMPY--
VCPMPY--
-VPMPYM-
-VPMPYG-
WSPMPY--
WDPMPY--
WRPMPY--
WKPMPY--
WVPMPY--
-WPMPYI-
WLPMPY--
WIPMPY--
WGPMPY--
-WPMPYM-
WEPMPY--
WYPMPY--
WNPMPY--
-WPMPYN-
-WPMPYS-
WTPMPY--
-WPMPYQ-
-WPMPYE-
WFPMPY--
YGPMPY--
-YPMPYY-

-YPMPYR-
YNPMPY--
YTPMPY--
-YPMPYL-
YAPMPY--
YRPMPY--
YYPMPY--
-YPMPYQ-
-YPMPYI-
-YPMPYA-
-YPMPYS-
YKPMPY--
YEPMPY--
YIPMPY--
YPPMPY--
-YPMPYV-
-YPMPYF-
YSPMPY--
-YPMPYN-
-YPMPYM-
-YPMPYK-
YFPMPY--

PYSI	79	824	45.53

-EPYSIM-
-LPYSIG-
--PYSIRP
--PYSIPR
--PYSIDG
ARPYSI--
ASPYSI--
-APYSIR-
-APYSIL-
-APYSIS-
AGPYSI--
AMPYSI--
ALPYSI--
-APYSII-
AFPYSI--
AIPYSI--
-APYSIT-
-APYSIF-
ANPYSI--
AKPYSI--
ATPYSI--
AQPYSI--
-APYSIA-
AWPYSI--
AVPYSI--
AEPYSI--
-APYSIY-

AAPYSI--
-APYSIW-
AHPYSI--
-APYSIN-
-APYSIE-
APPYSI--
-APYSIQ-
-APYSIG-
AYPYSI--
-APYSIV-
-APYSIK-
-APYSIH-
ACPYSI--
CAPYSI--
CSPYSI--
CRPYSI--
-CPYSIK-
CCPYSI--
CKPYSI--
CVPYSI--
CLPYSI--
CEPYSI--
-CPYSIS-
-CPYSII-
CGPYSI--
CTPYSI--
-CPYSIW-
-CPYSIR-
-CPYSIM-
CNPYSI--
DKPYSI--
DLPYSI--
DRPYSI--
DYPYSI--
DTPYSI--
-DPYSIK-
DSPYSI--
-DPYSIM-
DQPYSI--
-DPYSIR-
DMPYSI--
DAPYSI--
DVPYSI--
-DPYSIL-
-DPYSII-
-DPYSIW-
DNPYSI--
-DPYSIY-
DGPYSI--
DEPYSI--

-DPYSIS-
-DPYSIQ-
-DPYSIH-
-EPYSIY-
-EPYSIL-
ESPYSI--
-EPYSIS-
-EPYSIK-
EGPYSI--
-EPYSIR-
EFPYSI--
EQPYSI--
ETPYSI--
EVPYSI--
-EPYSIQ-
ELPYSI--
EWPYSI--
-EPYSIW-
-EPYSIT-
EKPYSI--
EIPYSI--
EAPYSI--
EYPYSI--
-EPYSIF-
-EPYSIA-
ERPYSI--
-EPYSIV-
-EPYSII-
-EPYSIG-
ENPYSI--
FSPYSI--
FAPYSI--
FEPYSI--
-FPYSIS-
-FPYSIR-
FMPYSI--
FLPYSI--
FFPYSI--
FGPYSI--
FDPYSI--
FPPYSI--
FNPYSI--
FWPYSI--
-FPYSIQ-
-FPYSIK-
FKPYSI--
FTPYSI--
-FPYSIW-
-FPYSIA-
FYPYSI--

-FPYSIT-
-FPYSIN-
-FPYSIY-
-FPYSIV-
-FPYSIH-
-FPYSIE-
FIPYSI--
FCPYSI--
FQPYSI--
-FPYSIL-
-FPYSIG-
GVPYSI--
GSPYSI--
-GPYSIR-
-GPYSIT-
GMPYSI--
GHPYSI--
GQPYSI--
-GPYSIY-
GRPYSI--
GLPYSI--
-GPYSIV-
GKPYSI--
-GPYSIK-
GNPYSI--
-GPYSIA-
GTPYSI--
-GPYSIM-
-GPYSIS-
-GPYSIH-
GWPYSI--
GGPYSI--
GFPYSI--
-GPYSIQ-
GIPYSI--
-GPYSIW-
-GPYSIF-
GCPYSI--
-GPYSII-
GYPYSI--
-GPYSIG-
GAPYSI--
GEPYSI--
-GPYSIN-
-GPYSIE-
GPPYSI--
GDPYSI--
HGPYSI--
HAPYSI--
-HPYSIL-

-HPYSII-
-HPYSIS-
-HPYSIY-
-HPYSIT-
HIPYSI--
HDPYSI--
HPPYSI--
HMPYSI--
HLPYSI--
HHPYSI--
HEPYSI--
-HPYSIV-
-HPYSIM-
-HPYSIN-
HYPYSI--
-HPYSIR-
HSPYSI--
-HPYSIF-
-HPYSIA-
HWPYSI--
HQPYSI--
-HPYSIW-
-HPYSIQ-
HNPYSI--
-HPYSIG-
HVPYSI--
IRPYSI--
IKPYSI--
-IPYSIR-
ISPYSI--
IPPYSI--
IMPYSI--
IAPYSI--
-IPYSIV-
-IPYSIM-
INPYSI--
IFPYSI--
-IPYSIQ-
IGPYSI--
ITPYSI--
IHPYSI--
ILPYSI--
-IPYSIW-
-IPYSIK-
IEPYSI--
-IPYSIL-
-IPYSIF-
-IPYSIE-
-IPYSIY-
-IPYSIT-

-IPYSIS-
IVPYSI--
-IPYSIN-
-IPYSII-
-IPYSIA-
IQPYSI--
-IPYSIH-
-IPYSIG-
IWPYSI--
IDPYSI--
KSPYSI--
-KPYSIL-
KRPYSI--
-KPYSIR-
KQPYSI--
KVPYSI--
-KPYSIT-
KLPYSI--
KFPYSI--
KAPYSI--
KWPYSI--
KIPYSI--
-KPYSIS-
-KPYSIW-
-KPYSIA-
-KPYSIY-
KKPYSI--
-KPYSII-
-KPYSIF-
KYPYSI--
KTPYSI--
-KPYSIH-
-KPYSIV-
-KPYSIN-
KGPYSI--
KDPYSI--
-KPYSIE-
-KPYSIG-
KPPYSI--
KCPYSI--
-KPYSIK-
KMPYSI--
-KPYSIQ-
KNPYSI--
LRPYSI--
LNPYSI--
LSPYSI--
LGPYSI--
-LPYSIR-
LVPYSI--

LTPYSI--
LWPYSI--
LAPYSI--
LYPYSI--
-LPYSIM-
LPPYSI--
LKPYSI--
-LPYSIY-
-LPYSIK-
LHPYSI--
-LPYSIV-
-LPYSIT-
-LPYSIS-
-LPYSII-
-LPYSIL-
-LPYSIE-
LDPYSI--
-LPYSIW-
-LPYSIA-
LCPYSI--
-LPYSIQ-
MRPYSI--
MGPYSI--
MYPYSI--
MTPYSI--
-MPYSIL-
MLPYSI--
-MPYSIS-
MSPYSI--
-MPYSIT-
-MPYSII-
-MPYSIR-
MKPYSI--
MQPYSI--
-MPYSIQ-
-MPYSIN-
MPPYSI--
MIPYSI--
-MPYSIY-
-MPYSIK-
-MPYSIH-
-MPYSIE-
MHPYSI--
-MPYSIF-
MAPYSI--
MWPYSI--
-MPYSIV-
-MPYSIM-
-MPYSIA-
-MPYSIG-

MDPYSI--
NRPYSI--
-NPYSIR-
NAPYSI--
NTPYSI--
NIPYSI--
NGPYSI--
-NPYSIS-
-NPYSIT-
NYPYSI--
NPPYSI--
NMPYSI--
NEPYSI--
-NPYSII-
-NPYSIV-
NNPYSI--
NSPYSI--
-NPYSIM-
-NPYSIH-
NFPYSI--
NWPYSI--
-NPYSIL-
NHPYSI--
-NPYSIK-
NQPYSI--
-NPYSIQ-
NLPYSI--
NKPYSI--
NDPYSI--
-NPYSIY-
-NPYSIW-
NCPYSI--
-NPYSIE-
-NPYSIA-
-NPYSIN-
--PYSIRS
--PYSIVR
--PYSIRL
--PYSIRQ
--PYSIRA
--PYSIVS
--PYSIRR
--PYSITR
--PYSITQ
--PYSIRE
--PYSIQS
--PYSIRT
--PYSIRG
--PYSIHS
--PYSIYS

--PYSITG
--PYSILN
--PYSILG
--PYSIKQ
PGPYSI--
--PYSIYY
--PYSISA
--PYSILT
--PYSIVG
--PYSISS
--PYSIVK
--PYSIQN
--PYSIMR
--PYSIWS
--PYSISM
--PYSIKA
--PYSIRM
PHPYSI--
--PYSINA
--PYSIFG
--PYSIRY
--PYSIMG
--PYSIHR
--PYSIRV
--PYSIMA
--PYSIVT
--PYSIGS
PTPYSI--
--PYSISQ
--PYSIAA
--PYSILK
--PYSIIT
--PYSIVA
--PYSITT
--PYSIIY
--PYSIFS
--PYSIAR
PIPYSI--
--PYSIWN
--PYSIST
--PYSIME
--PYSIYK
--PYSIYI
--PYSIVW
--PYSIVL
--PYSIRN
--PYSIEG
--PYSIWG
--PYSISV
--PYSIGV

--PYSIGT
--PYSIAG
--PYSIYV
--PYSIVN
--PYSITV
--PYSIRD
--PYSILR
--PYSIYG
--PYSIYA
--PYSILH
--PYSIIQ
--PYSIFE
--PYSIWM
--PYSITK
--PYSIRI
--PYSIQR
--PYSIMT
--PYSIKG
--PYSIYN
--PYSITN
--PYSITM
--PYSITH
--PYSIKS
--PYSIIV
--PYSIHN
--PYSIFQ
--PYSIES
--PYSIAV
PVPYSI--
PNPYSI--
PFPYSI--
--PYSIVE
--PYSITA
--PYSISI
--PYSIKV
--PYSIIM
--PYSIIG
--PYSIHL
--PYSIHG
--PYSIWR
--PYSINH
--PYSILD
--PYSIIS
--PYSIIR
--PYSIGA
--PYSIFR
--PYSIAT
--PYSIWD
--PYSIRK
--PYSIMN

PDPYSI--
--PYSIWK
--PYSIVH
--PYSITW
--PYSITS
--PYSISH
--PYSING
--PYSIHA
--PYSIFN
--PYSIVV
--PYSIVM
--PYSISD
--PYSIQT
--PYSINR
--PYSIHD
--PYSIAH
--PYSIYR
--PYSIWQ
--PYSIWE
--PYSIVQ
--PYSISE
--PYSIQK
--PYSIQG
--PYSINV
--PYSINS
--PYSIMW
--PYSIKN
--PYSIKM
--PYSIIH
--PYSIHM
--PYSIHK
--PYSIGG
--PYSIFD
--PYSIEA
--PYSIAY
PMPYSI--
--PYSITD
--PYSINM
--PYSIMY
--PYSIMK
--PYSIMH
--PYSILI
--PYSIFY
--PYSIFH
PWPYSI--
--PYSIWW
--PYSIVI
--PYSIRF
--PYSIQL
--PYSINW

--PYSIKI
--PYSIHT
--PYSIHQ
--PYSIGN
--PYSIFK
--PYSIEN
--PYSIEI
--PYSIAQ
PKPYSI--
PAPYSI--
--PYSIVY
--PYSIVD
--PYSINN
--PYSIMI
--PYSILE
--PYSILC
--PYSIKW
--PYSIKK
--PYSIKH
--PYSIIF
--PYSIHW
--PYSIGI
--PYSIFM
--PYSIAM
--PYSITE
--PYSISK
--PYSIQD
--PYSINT
--PYSINL
--PYSIMV
--PYSIKT
--PYSIIN
--PYSIGL
--PYSIET
--PYSIEQ
--PYSIAN
--PYSIYW
--PYSIYE
--PYSISY
--PYSISF
--PYSIQH
--PYSIMD
--PYSILQ
--PYSIKL
--PYSIKF
--PYSIIE
--PYSIIC
--PYSIHY
--PYSIHV
--PYSIHF

--PYSIHE
--PYSIEV
--PYSIAF
PYPYSI--
--PYSIYD
--PYSISN
--PYSIRC
--PYSIQY
--PYSIQA
--PYSINY
--PYSINQ
--PYSINK
--PYSIND
--PYSIIK
--PYSIID
--PYSIIA
--PYSIHI
--PYSIGW
--PYSIER
--PYSIEM
--PYSIDS
--PYSICL
--PYSICK
--PYSICI
--PYSIAK
--PYSIAI
-PPYSII-
PPPYSI--
QRPYSI--
QAPYSI--
QVPYSI--
-QPYSIS-
-QPYSIL-
QGPYSI--
-QPYSIW-
-QPYSIT-
QTPYSI--
-QPYSIH-
QLPYSI--
QKPYSI--
QMPYSI--
QHPYSI--
-QPYSIV-
QDPYSI--
-QPYSII-
-QPYSIF-
QQPYSI--
QEPYSI--
QYPYSI--
QWPYSI--

−QPYSIN−
−QPYSIM−
−QPYSIG−
QIPYSI−−
−QPYSIQ−
QPPYSI−−
QFPYSI−−
−RPYSIV−
RAPYSI−−
RLPYSI−−
RMPYSI−−
RGPYSI−−
−RPYSIL−
−RPYSIR−
−RPYSIM−
RIPYSI−−
RVPYSI−−
−RPYSIW−
RYPYSI−−
RNPYSI−−
−RPYSIK−
RWPYSI−−
−RPYSII−
REPYSI−−
−RPYSIY−
−RPYSIQ−
RDPYSI−−
−RPYSIH−
−RPYSIF−
−RPYSIA−
−RPYSIN−
RHPYSI−−
−RPYSIG−
−RPYSIE−
RCPYSI−−
−SPYSIR−
SSPYSI−−
SRPYSI−−
SLPYSI−−
SVPYSI−−
SKPYSI−−
SGPYSI−−
STPYSI−−
−SPYSIY−
SMPYSI−−
SYPYSI−−
−SPYSIL−
−SPYSIS−
SQPYSI−−
−SPYSII−

SHPYSI--
-SPYSIM-
-SPYSIK-
SFPYSI--
SAPYSI--
-SPYSIH-
SIPYSI--
-SPYSIA-
SNPYSI--
-SPYSIW-
-SPYSIT-
-SPYSIV-
SEPYSI--
SWPYSI--
SPPYSI--
-SPYSIQ-
-SPYSIG-
-SPYSIE-
SDPYSI--
SCPYSI--
TGPYSI--
TRPYSI--
-TPYSIH-
TLPYSI--
-TPYSII-
TSPYSI--
TPPYSI--
-TPYSIR-
-TPYSIL-
TEPYSI--
TVPYSI--
TTPYSI--
-TPYSIT-
-TPYSIK-
-TPYSIV-
TNPYSI--
THPYSI--
TAPYSI--
TQPYSI--
-TPYSIS-
TMPYSI--
TFPYSI--
-TPYSIY-
-TPYSIW-
TKPYSI--
TWPYSI--
TYPYSI--
-TPYSIF-
-TPYSIQ-
TIPYSI--

```
-TPYSIE-
-TPYSIN-
-TPYSIG-
-TPYSIA-
TDPYSI--
VRPYSI--
VSPYSI--
VTPYSI--
-VPYSIL-
-VPYSIR-
-VPYSIK-
VKPYSI--
-VPYSIH-
-VPYSIS-
VIPYSI--
VPPYSI--
-VPYSIM-
-VPYSIV-
-VPYSII-
VFPYSI--
VAPYSI--
VWPYSI--
VGPYSI--
VMPYSI--
VLPYSI--
VVPYSI--
VHPYSI--
-VPYSIT-
VQPYSI--
-VPYSIW-
VEPYSI--
VNPYSI--
-VPYSIQ-
VYPYSI--
-VPYSIY-
-VPYSIG-
VDPYSI--
VCPYSI--
-VPYSIE-
WKPYSI--
WTPYSI--
-WPYSII-
-WPYSIR-
-WPYSIK-
WIPYSI--
WPPYSI--
WMPYSI--
WNPYSI--
WFPYSI--
WGPYSI--
```

```
              WAPYSI--
              WQPYSI--
              -WPYSIM-
              -WPYSIH-
              WWPYSI--
              -WPYSIS-
              -WPYSIF-
              -WPYSIA-
              WLPYSI--
              WCPYSI--
              -WPYSIQ-
              -WPYSIN-
              -WPYSIE-
              WEPYSI--
              WDPYSI--
              YRPYSI--
              -YPYSIR-
              YSPYSI--
              YKPYSI--
              YMPYSI--
              YIPYSI--
              YTPYSI--
              -YPYSIF-
              YNPYSI--
              -YPYSIL-
              YGPYSI--
              -YPYSIS-
              YHPYSI--
              -YPYSIT-
              YYPYSI--
              YQPYSI--
              YLPYSI--
              YAPYSI--
              YVPYSI--
              -YPYSIV-
              -YPYSIQ-
              YFPYSI--
              -YPYSIY-
              -YPYSIW-
              -YPYSIM-
              -YPYSIK-
              YDPYSI--
              YEPYSI--
              -YPYSIN-
              -YPYSIH-
              YWPYSI--
              YPPYSI--
PWSY           221       736     45.53
              --PWSYNT
              --PWSYKL
```

--PWSYHL
--PWSYAK
-RPWSYG-
-TPWSYN-
ASPWSY--
-APWSYT-
AGPWSY--
-APWSYR-
-APWSYK-
-APWSYF-
ANPWSY--
-APWSYW-
AIPWSY--
ATPWSY--
-APWSYI-
AHPWSY--
-APWSYV-
-APWSYM-
AMPWSY--
AWPWSY--
ARPWSY--
-APWSYH-
AFPWSY--
AYPWSY--
-APWSYY-
AKPWSY--
AVPWSY--
-APWSYA-
APPWSY--
AAPWSY--
AQPWSY--
-APWSYQ-
-APWSYS-
-APWSYP-
AEPWSY--
-APWSYE-
ADPWSY--
CVPWSY--
CSPWSY--
CRPWSY--
CQPWSY--
CPPWSY--
CKPWSY--
CDPWSY--
-CPWSYM-
-CPWSYF-
-CPWSYA-
CNPWSY--
DRPWSY--
DAPWSY--

DWPWSY--
-DPWSYR-
DLPWSY--
DYPWSY--
-DPWSYM-
-DPWSYI-
DMPWSY--
DHPWSY--
-DPWSYY-
-DPWSYW-
DNPWSY--
DTPWSY--
-DPWSYV-
-DPWSYK-
DVPWSY--
DSPWSY--
-DPWSYT-
-DPWSYS-
DQPWSY--
ERPWSY--
-EPWSYK-
EYPWSY--
ETPWSY--
ELPWSY--
-EPWSYR-
EIPWSY--
ENPWSY--
ESPWSY--
-EPWSYF-
EMPWSY--
EHPWSY--
-EPWSYY-
EVPWSY--
EWPWSY--
-EPWSYW-
-EPWSYH-
EKPWSY--
EFPWSY--
-EPWSYM-
EQPWSY--
-EPWSYS-
-EPWSYA-
-EPWSYV-
-EPWSYQ-
-EPWSYE-
EDPWSY--
EAPWSY--
EGPWSY--
ECPWSY--
FNPWSY--

FVPWSY--
FSPWSY--
FRPWSY--
FMPWSY--
FQPWSY--
FKPWSY--
FLPWSY--
FAPWSY--
FYPWSY--
FTPWSY--
-FPWSYS-
-FPWSYQ-
-FPWSYR-
-FPWSYA-
-FPWSYM-
-FPWSYK-
-FPWSYI-
-FPWSYH-
-FPWSYF-
-FPWSYY-
-FPWSYV-
FPPWSY--
FHPWSY--
GTPWSY--
-GPWSYR-
-GPWSYV-
GKPWSY--
GSPWSY--
GRPWSY--
GHPWSY--
-GPWSYM-
GYPWSY--
-GPWSYL-
GFPWSY--
GNPWSY--
-GPWSYK-
-GPWSYQ-
GDPWSY--
GVPWSY--
-GPWSYW-
-GPWSYI-
-GPWSYA-
GPPWSY--
GLPWSY--
GGPWSY--
GAPWSY--
-GPWSYY-
-GPWSYH-
GQPWSY--
-GPWSYT-

GMPWSY--
-GPWSYE-
GEPWSY--
-GPWSYF-
GWPWSY--
HSPWSY--
HRPWSY--
-HPWSYM-
HGPWSY--
HLPWSY--
-HPWSYI-
-HPWSYR-
-HPWSYT-
-HPWSYK-
HKPWSY--
HTPWSY--
-HPWSYL-
HPPWSY--
HVPWSY--
HQPWSY--
HWPWSY--
HHPWSY--
-HPWSYY-
HNPWSY--
HYPWSY--
-HPWSYQ-
-HPWSYN-
-HPWSYA-
HEPWSY--
HDPWSY--
-HPWSYW-
-HPWSYV-
-HPWSYP-
-HPWSYE-
HAPWSY--
IIPWSY--
INPWSY--
ISPWSY--
-IPWSYR-
-IPWSYY-
IVPWSY--
IHPWSY--
IAPWSY--
ITPWSY--
IMPWSY--
-IPWSYS-
IGPWSY--
IEPWSY--
-IPWSYM-
-IPWSYA-

IYPWSY--
IPPWSY--
IRPWSY--
IFPWSY--
IDPWSY--
-IPWSYI-
IKPWSY--
IQPWSY--
-IPWSYV-
-IPWSYT-
-IPWSYL-
-IPWSYK-
IWPWSY--
KSPWSY--
KRPWSY--
-KPWSYR-
KLPWSY--
-KPWSYS-
KGPWSY--
KEPWSY--
-KPWSYH-
KHPWSY--
KIPWSY--
-KPWSYY-
KFPWSY--
-KPWSYL-
KVPWSY--
KNPWSY--
-KPWSYT-
-KPWSYQ-
-KPWSYK-
KKPWSY--
KTPWSY--
KMPWSY--
KAPWSY--
-KPWSYV-
-KPWSYW-
-KPWSYM-
-KPWSYA-
-KPWSYF-
KWPWSY--
KPPWSY--
KCPWSY--
LTPWSY--
LSPWSY--
LGPWSY--
LEPWSY--
LNPWSY--
LLPWSY--
LHPWSY--

-LPWSYV-
-LPWSYT-
-LPWSYM-
-LPWSYS-
-LPWSYI-
LDPWSY--
LWPWSY--
LYPWSY--
-LPWSYY-
LPPWSY--
-LPWSYH-
LMPWSY--
LFPWSY--
LCPWSY--
-LPWSYK-
-LPWSYW-
-LPWSYQ-
MRPWSY--
MGPWSY--
MAPWSY--
-MPWSYL-
MNPWSY--
MKPWSY--
MVPWSY--
-MPWSYR-
MIPWSY--
MSPWSY--
MYPWSY--
-MPWSYV-
-MPWSYF-
-MPWSYH-
MQPWSY--
-MPWSYT-
MEPWSY--
MPPWSY--
MMPWSY--
-MPWSYY-
MDPWSY--
MHPWSY--
-MPWSYA-
-MPWSYW-
-MPWSYQ-
-MPWSYI-
NRPWSY--
-NPWSYR-
NMPWSY--
NAPWSY--
NNPWSY--
NLPWSY--
NYPWSY--

NTPWSY--
NGPWSY--
NPPWSY--
-NPWSYT-
-NPWSYV-
NIPWSY--
NEPWSY--
NCPWSY--
NQPWSY--
-NPWSYS-
NWPWSY--
-NPWSYI-
NFPWSY--
-NPWSYW-
-NPWSYQ-
-NPWSYL-
-NPWSYK-
-NPWSYF-
NHPWSY--
-NPWSYM-
-NPWSYH-
NKPWSY--
NDPWSY--
--PWSYRS
--PWSYLS
--PWSYLG
--PWSYTN
--PWSYMG
--PWSYVS
--PWSYRN
--PWSYST
--PWSYSA
--PWSYRG
--PWSYWS
--PWSYVG
--PWSYSQ
--PWSYRQ
PKPWSY--
--PWSYES
PSPWSY--
--PWSYHS
PGPWSY--
--PWSYIA
--PWSYIG
--PWSYFG
--PWSYYS
--PWSYTR
--PWSYYR
--PWSYSG
--PWSYRL

--PWSYMS
--PWSYLN
--PWSYKS
--PWSYAS
--PWSYSV
--PWSYRI
--PWSYMT
--PWSYAG
--PWSYYH
--PWSYVN
--PWSYTS
--PWSYYN
--PWSYTT
--PWSYVA
--PWSYSL
--PWSYIV
--PWSYQY
--PWSYMN
--PWSYKG
PVPWSY--
--PWSYWG
--PWSYVI
--PWSYTA
--PWSYSI
--PWSYRK
--PWSYNA
--PWSYAL
--PWSYRY
--PWSYRW
--PWSYRV
--PWSYNV
--PWSYFN
--PWSYTG
PLPWSY--
PHPWSY--
--PWSYTV
--PWSYGS
--PWSYVT
--PWSYVH
--PWSYSM
--PWSYNS
--PWSYNG
--PWSYMK
--PWSYIN
--PWSYYA
--PWSYQH
--PWSYLK
--PWSYKV
--PWSYIS
--PWSYAN

PTPWSY--
PNPWSY--
--PWSYVQ
--PWSYSR
--PWSYSN
--PWSYQS
--PWSYHF
--PWSYEG
PAPWSY--
PYPWSY--
--PWSYVK
--PWSYTH
--PWSYQA
--PWSYMD
--PWSYKY
--PWSYKK
--PWSYIT
--PWSYHV
--PWSYHG
--PWSYGG
--PWSYGA
--PWSYFS
--PWSYFR
--PWSYEA
--PWSYAV
PMPWSY--
PFPWSY--
--PWSYYK
--PWSYVY
--PWSYSY
--PWSYQR
--PWSYQF
--PWSYNN
--PWSYMM
--PWSYIM
--PWSYFK
--PWSYWR
--PWSYWM
--PWSYWL
--PWSYTK
--PWSYQG
--PWSYNW
--PWSYMQ
--PWSYLW
--PWSYLH
--PWSYKH
--PWSYKA
--PWSYIR
--PWSYWW
--PWSYWV

--PWSYWN
--PWSYWD
--PWSYTD
--PWSYSH
--PWSYQT
--PWSYQK
--PWSYQI
--PWSYPL
--PWSYNL
--PWSYMF
--PWSYKM
--PWSYIQ
--PWSYGL
--PWSYFY
--PWSYAT
--PWSYAR
--PWSYYM
--PWSYYG
--PWSYVD
--PWSYSK
--PWSYRM
--PWSYRE
--PWSYQV
--PWSYMW
--PWSYMV
--PWSYKW
--PWSYKQ
--PWSYKF
--PWSYIK
--PWSYII
--PWSYHR
--PWSYHN
--PWSYGY
--PWSYGT
--PWSYFW
--PWSYFQ
--PWSYEF
PWPWSY--
PQPWSY--
--PWSYYV
--PWSYYQ
--PWSYWK
--PWSYWA
--PWSYVE
--PWSYTY
--PWSYSW
--PWSYMI
--PWSYLQ
--PWSYKN
--PWSYHY

--PWSYEY
--PWSYAQ
--PWSYAA
PDPWSY--
--PWSYWH
--PWSYTW
--PWSYTQ
--PWSYTP
--PWSYTM
--PWSYTE
--PWSYTC
--PWSYSD
--PWSYSC
--PWSYRD
--PWSYQM
--PWSYQL
--PWSYPS
--PWSYNY
--PWSYNQ
--PWSYNI
--PWSYND
--PWSYME
--PWSYKE
--PWSYID
--PWSYHT
--PWSYGV
--PWSYGM
--PWSYGI
--PWSYGE
--PWSYEV
--PWSYEN
--PWSYEL
--PWSYDA
--PWSYAM
--PWSYAI
--PWSYAE
-PPWSYV-
QGPWSY--
QAPWSY--
QVPWSY--
QKPWSY--
QSPWSY--
QMPWSY--
-QPWSYR-
-QPWSYI-
-QPWSYK-
QRPWSY--
-QPWSYM-
QPPWSY--
QLPWSY--

QDPWSY--
QTPWSY--
-QPWSYV-
-QPWSYA-
QHPWSY--
QFPWSY--
QWPWSY--
-QPWSYT-
-QPWSYS-
-QPWSYH-
QNPWSY--
-QPWSYY-
-QPWSYW-
-QPWSYF-
-QPWSYQ-
-QPWSYE-
QEPWSY--
QCPWSY--
RVPWSY--
RSPWSY--
RLPWSY--
RAPWSY--
-RPWSYV-
RGPWSY--
-RPWSYL-
REPWSY--
RMPWSY--
-RPWSYR-
-RPWSYK-
-RPWSYI-
RIPWSY--
RDPWSY--
RFPWSY--
-RPWSYQ-
RWPWSY--
RKPWSY--
-RPWSYH-
RPPWSY--
-RPWSYM-
-RPWSYF-
RHPWSY--
-RPWSYW-
-RPWSYE-
-RPWSYN-
-RPWSYA-
RNPWSY--
SRPWSY--
SLPWSY--
SSPWSY--
STPWSY--

-SPWSYY-
-SPWSYI-
SYPWSY--
SVPWSY--
-SPWSYT-
SKPWSY--
-SPWSYS-
SIPWSY--
SFPWSY--
SDPWSY--
SAPWSY--
SWPWSY--
SMPWSY--
SEPWSY--
-SPWSYM-
SNPWSY--
-SPWSYV-
-SPWSYF-
-SPWSYW-
-SPWSYA-
-SPWSYH-
SQPWSY--
SHPWSY--
SPPWSY--
-SPWSYQ-
-SPWSYN-
-SPWSYE-
TQPWSY--
-TPWSYR-
TRPWSY--
TTPWSY--
TNPWSY--
TSPWSY--
TWPWSY--
-TPWSYM-
THPWSY--
TEPWSY--
TAPWSY--
-TPWSYK-
-TPWSYI-
-TPWSYS-
-TPWSYA-
TKPWSY--
TFPWSY--
-TPWSYQ-
-TPWSYT-
TYPWSY--
-TPWSYV-
TDPWSY--
TVPWSY--

−TPWSYW−
TMPWSY−−
TIPWSY−−
−TPWSYH−
TPPWSY−−
TLPWSY−−
VSPWSY−−
VVPWSY−−
VGPWSY−−
VKPWSY−−
VPPWSY−−
VLPWSY−−
VNPWSY−−
VAPWSY−−
−VPWSYM−
−VPWSYS−
VMPWSY−−
VQPWSY−−
VHPWSY−−
VYPWSY−−
−VPWSYA−
−VPWSYI−
−VPWSYY−
−VPWSYT−
VDPWSY−−
−VPWSYL−
−VPWSYW−
−VPWSYK−
VEPWSY−−
−VPWSYN−
−VPWSYQ−
−VPWSYH−
−VPWSYG−
WRPWSY−−
WNPWSY−−
WKPWSY−−
WEPWSY−−
WFPWSY−−
WTPWSY−−
−WPWSYM−
WGPWSY−−
WQPWSY−−
−WPWSYT−
WAPWSY−−
WSPWSY−−
−WPWSYR−
WLPWSY−−
WYPWSY−−
WCPWSY−−
WVPWSY−−

-WPWSYS-
-WPWSYN-
-WPWSYK-
-WPWSYG-
YKPWSY--
YGPWSY--
-YPWSYS-
YSPWSY--
-YPWSYK-
-YPWSYI-
YMPWSY--
YAPWSY--
YRPWSY--
YIPWSY--
YTPWSY--
-YPWSYV-
YLPWSY--
-YPWSYM-
YNPWSY--
-YPWSYR-
YFPWSY--
YYPWSY--
-YPWSYQ-
YQPWSY--
-YPWSYY-
-YPWSYA-
YPPWSY--
YWPWSY--
-YPWSYT-
YDPWSY--
YVPWSY--
-YPWSYW-
-YPWSYH-
YEPWSY--

PMAF 553 600 45.51

-KPMAFG-
--PMAFLT
ASPMAF--
-APMAFR-
AAPMAF--
AWPMAF--
-APMAFH-
-APMAFK-
-APMAFM-
-APMAFL-
-APMAFA-
-APMAFV-
ANPMAF--
ACPMAF--
-APMAFQ-

-APMAFT-
-APMAFG-
-APMAFE-
-APMAFN-
-APMAFI-
AVPMAF--
AMPMAF--
AEPMAF--
CAPMAF--
-CPMAFH-
CLPMAF--
-CPMAFM-
CVPMAF--
CQPMAF--
CYPMAF--
CTPMAF--
DRPMAF--
DAPMAF--
DQPMAF--
DLPMAF--
DSPMAF--
-DPMAFI-
-DPMAFR-
-DPMAFW-
DKPMAF--
DFPMAF--
DTPMAF--
-DPMAFH-
DIPMAF--
DWPMAF--
DPPMAF--
-DPMAFS-
-DPMAFA-
DGPMAF--
DEPMAF--
EKPMAF--
-EPMAFR-
EGPMAF--
EVPMAF--
ERPMAF--
ESPMAF--
-EPMAFI-
-EPMAFV-
-EPMAFT-
-EPMAFA-
EIPMAF--
-EPMAFN-
EYPMAF--
-EPMAFM-
EHPMAF--

EAPMAF--
-EPMAFE-
ENPMAF--
EMPMAF--
FGPMAF--
-FPMAFH-
FQPMAF--
FTPMAF--
FPPMAF--
FNPMAF--
FKPMAF--
FVPMAF--
FSPMAF--
-FPMAFM-
FDPMAF--
-FPMAFE-
FEPMAF--
FWPMAF--
-FPMAFA-
-FPMAFT-
-FPMAFR-
-FPMAFQ-
-FPMAFN-
FIPMAF--
FYPMAF--
-FPMAFW-
GRPMAF--
-GPMAFL-
GVPMAF--
-GPMAFI-
GAPMAF--
GLPMAF--
GSPMAF--
GMPMAF--
GYPMAF--
GPPMAF--
GWPMAF--
-GPMAFS-
-GPMAFK-
GIPMAF--
-GPMAFR-
GNPMAF--
GEPMAF--
-GPMAFH-
-GPMAFM-
GQPMAF--
-GPMAFW-
GHPMAF--
GGPMAF--
-GPMAFT-

GKPMAF--
GTPMAF--
-GPMAFA-
GDPMAF--
HGPMAF--
HYPMAF--
HRPMAF--
HAPMAF--
HPPMAF--
HSPMAF--
HIPMAF--
-HPMAFY-
-HPMAFT-
-HPMAFS-
-HPMAFL-
HVPMAF--
HMPMAF--
-HPMAFH-
HDPMAF--
-HPMAFK-
-HPMAFF-
-HPMAFA-
HEPMAF--
-HPMAFQ-
-HPMAFM-
ILPMAF--
ISPMAF--
-IPMAFI-
IPPMAF--
-IPMAFR-
-IPMAFL-
IRPMAF--
-IPMAFS-
-IPMAFH-
-IPMAFA-
IKPMAF--
IDPMAF--
-IPMAFM-
ITPMAF--
IMPMAF--
IVPMAF--
-IPMAFT-
-IPMAFQ-
-IPMAFK-
IEPMAF--
IQPMAF--
-IPMAFN-
IIPMAF--
KSPMAF--
-KPMAFS-

-KPMAFV-
-KPMAFH-
KRPMAF--
-KPMAFF-
KVPMAF--
-KPMAFQ-
-KPMAFL-
KFPMAF--
-KPMAFY-
KGPMAF--
KAPMAF--
KKPMAF--
KIPMAF--
KQPMAF--
KTPMAF--
-KPMAFT-
KEPMAF--
KPPMAF--
-KPMAFW-
-KPMAFI-
KHPMAF--
KNPMAF--
LIPMAF--
LQPMAF--
-LPMAFM-
LVPMAF--
-LPMAFS-
-LPMAFR-
LSPMAF--
LDPMAF--
LTPMAF--
LKPMAF--
LEPMAF--
LGPMAF--
LMPMAF--
LNPMAF--
LAPMAF--
LPPMAF--
-LPMAFW-
-LPMAFT-
LYPMAF--
-LPMAFV-
MVPMAF--
MSPMAF--
-MPMAFR-
MKPMAF--
-MPMAFT-
MNPMAF--
MEPMAF--
MQPMAF--

-MPMAFQ-
MTPMAF--
-MPMAFS-
-MPMAFK-
-MPMAFA-
MGPMAF--
MIPMAF--
MPPMAF--
-MPMAFI-
-MPMAFH-
MCPMAF--
MAPMAF--
NLPMAF--
NEPMAF--
NMPMAF--
NAPMAF--
-NPMAFR-
-NPMAFQ-
NHPMAF--
NPPMAF--
-NPMAFT-
NKPMAF--
NRPMAF--
-NPMAFS-
NTPMAF--
-NPMAFM-
NYPMAF--
-NPMAFV-
NGPMAF--
NNPMAF--
NQPMAF--
-NPMAFK-
NIPMAF--
NCPMAF--
NSPMAF--
-NPMAFW-
-NPMAFH-
NFPMAF--
--PMAFRS
--PMAFSL
--PMAFRL
--PMAFSG
--PMAFRA
--PMAFTR
--PMAFTN
--PMAFLG
--PMAFTS
--PMAFRT
--PMAFRM
--PMAFSM

--PMAFKA
--PMAFAS
--PMAFVR
--PMAFRN
--PMAFNL
--PMAFFN
--PMAFAT
--PMAFWS
--PMAFIR
--PMAFHG
--PMAFST
--PMAFSA
--PMAFMD
--PMAFVT
--PMAFQA
--PMAFKG
--PMAFGL
--PMAFWN
--PMAFVA
--PMAFTV
--PMAFTL
--PMAFRW
--PMAFMT
--PMAFTH
--PMAFRV
--PMAFHT
--PMAFHR
--PMAFVS
--PMAFSI
--PMAFYQ
--PMAFWA
--PMAFSY
--PMAFRG
--PMAFNT
--PMAFLV
PDPMAF--
--PMAFSQ
--PMAFMS
--PMAFMM
--PMAFGY
--PMAFWG
--PMAFKS
--PMAFIA
--PMAFHA
--PMAFYN
--PMAFYG
--PMAFVG
--PMAFSV
--PMAFRD
--PMAFML

--PMAFLN
--PMAFHW
PMPMAF--
--PMAFVV
--PMAFVN
--PMAFVH
--PMAFTM
--PMAFNA
--PMAFHH
--PMAFGQ
--PMAFAA
--PMAFTT
--PMAFLK
--PMAFIS
--PMAFIN
--PMAFHF
--PMAFFW
--PMAFAI
PTPMAF--
--PMAFWV
--PMAFWD
--PMAFVW
--PMAFSF
--PMAFKM
--PMAFKK
--PMAFHY
--PMAFHL
--PMAFHK
--PMAFEG
--PMAFDL
--PMAFAH
--PMAFYE
--PMAFTW
--PMAFSS
--PMAFRI
--PMAFNY
--PMAFNQ
--PMAFNG
--PMAFMG
--PMAFKT
--PMAFKR
--PMAFIG
--PMAFHQ
--PMAFFV
--PMAFFF
--PMAFEY
--PMAFAW
--PMAFAF
PNPMAF--
--PMAFYY

--PMAFWL
--PMAFVK
--PMAFSE
--PMAFRY
--PMAFRH
--PMAFQY
--PMAFNS
--PMAFNM
--PMAFLA
--PMAFKW
--PMAFKH
--PMAFKD
--PMAFHE
--PMAFGV
--PMAFGF
--PMAFGA
--PMAFFD
--PMAFEM
--PMAFWY
--PMAFWE
--PMAFVQ
--PMAFQV
--PMAFQM
--PMAFQL
--PMAFPY
--PMAFPM
--PMAFNV
--PMAFMH
--PMAFLF
--PMAFLD
--PMAFKN
--PMAFKI
--PMAFIT
--PMAFIL
--PMAFIH
--PMAFHS
--PMAFHD
--PMAFGW
--PMAFGS
--PMAFGI
--PMAFFG
--PMAFFA
--PMAFES
--PMAFDM
PKPMAF--
--PMAFWK
--PMAFVI
--PMAFTK
--PMAFTE
--PMAFTA

--PMAFSK
--PMAFSD
--PMAFRF
--PMAFQS
--PMAFQR
--PMAFQD
--PMAFNI
--PMAFNH
--PMAFMY
--PMAFMI
--PMAFMC
--PMAFKV
--PMAFKE
--PMAFIM
--PMAFIE
--PMAFHV
--PMAFHP
--PMAFHN
--PMAFGG
--PMAFCI
--PMAFAV
--PMAFAN
PHPMAF--
QRPMAF--
QLPMAF--
QSPMAF--
-QPMAFH-
QIPMAF--
-QPMAFT-
-QPMAFI-
QAPMAF--
-QPMAFK-
QVPMAF--
-QPMAFR-
-QPMAFL-
QQPMAF--
QGPMAF--
-QPMAFS-
-QPMAFF-
QKPMAF--
QEPMAF--
QTPMAF--
-QPMAFA-
-RPMAFL-
-RPMAFW-
RLPMAF--
RSPMAF--
-RPMAFV-
RYPMAF--
RPPMAF--

RVPMAF--
RQPMAF--
RGPMAF--
RMPMAF--
RAPMAF--
RNPMAF--
REPMAF--
-RPMAFK-
RIPMAF--
-RPMAFQ-
RWPMAF--
-RPMAFT-
-RPMAFS-
RTPMAF--
-RPMAFF-
RKPMAF--
RFPMAF--
-RPMAFN-
-RPMAFI-
-RPMAFG-
-RPMAFA-
RDPMAF--
-SPMAFT-
SRPMAF--
-SPMAFH-
SLPMAF--
SAPMAF--
-SPMAFR-
-SPMAFK-
-SPMAFS-
SPPMAF--
SMPMAF--
SQPMAF--
SGPMAF--
SSPMAF--
-SPMAFV-
SYPMAF--
-SPMAFM-
-SPMAFL-
SEPMAF--
SFPMAF--
STPMAF--
-SPMAFQ-
SWPMAF--
-SPMAFA-
SNPMAF--
-SPMAFN-
SDPMAF--
TSPMAF--
-TPMAFM-

-TPMAFL-
TLPMAF--
TGPMAF--
TRPMAF--
TAPMAF--
TQPMAF--
TVPMAF--
TPPMAF--
-TPMAFI-
TKPMAF--
TTPMAF--
-TPMAFQ-
TEPMAF--
-TPMAFN-
TNPMAF--
-TPMAFT-
-TPMAFA-
TMPMAF--
TCPMAF--
-TPMAFV-
-TPMAFH-
THPMAF--
TFPMAF--
-VPMAFH-
VTPMAF--
-VPMAFS-
VRPMAF--
VEPMAF--
VQPMAF--
-VPMAFT-
VHPMAF--
-VPMAFM-
VDPMAF--
VIPMAF--
VVPMAF--
-VPMAFR-
-VPMAFI-
VNPMAF--
-VPMAFN-
VGPMAF--
-VPMAFL-
-VPMAFV-
-VPMAFQ-
-VPMAFA-
VKPMAF--
WSPMAF--
-WPMAFH-
-WPMAFS-
-WPMAFK-
-WPMAFL-

WPPMAF--
WRPMAF--
-WPMAFW-
-WPMAFV-
-WPMAFT-
-WPMAFI-
-WPMAFA-
YSPMAF--
YKPMAF--
YTPMAF--
-YPMAFH-
YYPMAF--
YNPMAF--
YIPMAF--
YAPMAF--
-YPMAFW-
YDPMAF--
-YPMAFS-
-YPMAFM-
-YPMAFQ-
YFPMAF--
YVPMAF--
YRPMAF--
-YPMAFT-
-YPMAFI-
-YPMAFA-
YHPMAF--
YEPMAF--
-YPMAFL-
-YPMAFF-
YWPMAF--
YQPMAF--
-YPMAFN-
-YPMAFK-
-YPMAFG-

PAGM 139 786 45.51

-LPAGMG-
--PAGMLL
--PAGMDS
-SPAGMG-
ATPAGM--
ASPAGM--
AFPAGM--
-APAGMR-
-APAGMY-
ALPAGM--
-APAGMV-
-APAGMI-
-APAGMH-
ANPAGM--

AMPAGM--
AEPAGM--
-APAGMT-
-APAGMN-
AQPAGM--
-APAGML-
ARPAGM--
AYPAGM--
-APAGMQ-
AIPAGM--
AVPAGM--
-APAGMK-
-APAGME-
-APAGMM-
ACPAGM--
AAPAGM--
-APAGMS-
-APAGMG-
-APAGMF-
AHPAGM--
AGPAGM--
-APAGMW-
CRPAGM--
CSPAGM--
-CPAGMM-
CGPAGM--
-CPAGMV-
-CPAGMS-
CIPAGM--
CHPAGM--
CLPAGM--
CFPAGM--
-CPAGMR-
-CPAGMH-
CKPAGM--
DRPAGM--
DSPAGM--
-DPAGMF-
-DPAGMY-
DKPAGM--
DIPAGM--
DGPAGM--
-DPAGMM-
DAPAGM--
DWPAGM--
-DPAGMW-
-DPAGMS-
-DPAGMH-
DFPAGM--
DVPAGM--

-DPAGMR-
-DPAGML-
-DPAGMK-
-DPAGME-
DLPAGM--
DHPAGM--
-EPAGML-
ERPAGM--
ELPAGM--
EYPAGM--
-EPAGMT-
EWPAGM--
ESPAGM--
ETPAGM--
EQPAGM--
-EPAGMY-
-EPAGMS-
-EPAGMI-
EKPAGM--
-EPAGMM-
-EPAGMK-
-EPAGMR-
-EPAGMA-
EAPAGM--
EPPAGM--
-EPAGMV-
-EPAGMQ-
-EPAGMH-
EHPAGM--
FIPAGM--
-FPAGMI-
FRPAGM--
FGPAGM--
-FPAGMT-
FLPAGM--
FSPAGM--
-FPAGMQ-
FYPAGM--
FNPAGM--
-FPAGMR-
FMPAGM--
-FPAGMV-
FHPAGM--
FDPAGM--
FVPAGM--
-FPAGML-
FPPAGM--
-FPAGMW-
FKPAGM--
FFPAGM--

-FPAGMK-
-FPAGMF-
FQPAGM--
-FPAGMA-
-FPAGMS-
-FPAGMN-
-FPAGMH-
-FPAGMG-
-GPAGML-
GRPAGM--
-GPAGMM-
GSPAGM--
-GPAGMW-
GLPAGM--
-GPAGMS-
-GPAGMR-
-GPAGMI-
GQPAGM--
-GPAGMY-
GYPAGM--
-GPAGMT-
GMPAGM--
-GPAGMH-
-GPAGMA-
GNPAGM--
GVPAGM--
GFPAGM--
GDPAGM--
GWPAGM--
GEPAGM--
-GPAGMN-
-GPAGMK-
GIPAGM--
GHPAGM--
GTPAGM--
-GPAGMQ-
-GPAGMG-
GKPAGM--
GCPAGM--
HSPAGM--
-HPAGMR-
HRPAGM--
HHPAGM--
HYPAGM--
HNPAGM--
HMPAGM--
-HPAGMK-
-HPAGMF-
HDPAGM--
-HPAGMS-

HGPAGM--
HLPAGM--
HTPAGM--
HPPAGM--
-HPAGMT-
-HPAGMV-
HVPAGM--
-HPAGMY-
-HPAGMI-
-HPAGMH-
HFPAGM--
HEPAGM--
-HPAGMQ-
HIPAGM--
IRPAGM--
INPAGM--
-IPAGMR-
-IPAGMK-
-IPAGML-
IWPAGM--
-IPAGMN-
-IPAGMT-
-IPAGMS-
-IPAGMY-
IQPAGM--
IKPAGM--
IGPAGM--
IDPAGM--
ISPAGM--
-IPAGMA-
ITPAGM--
-IPAGMW-
-IPAGME-
IIPAGM--
IYPAGM--
IPPAGM--
-IPAGMV-
-IPAGMQ-
-IPAGMM-
-IPAGMH-
-IPAGMG-
IMPAGM--
IHPAGM--
-IPAGMI-
ILPAGM--
IAPAGM--
KVPAGM--
-KPAGMY-
-KPAGMV-
-KPAGMT-

-KPAGML-
-KPAGMK-
KGPAGM--
KSPAGM--
-KPAGMR-
-KPAGMW-
-KPAGMN-
-KPAGMA-
KNPAGM--
KAPAGM--
KLPAGM--
KFPAGM--
KWPAGM--
-KPAGMG-
-KPAGMH-
KIPAGM--
-KPAGMS-
KHPAGM--
KDPAGM--
-KPAGMI-
KPPAGM--
-KPAGME-
-KPAGMQ-
-KPAGMM-
-KPAGMF-
LSPAGM--
-LPAGMR-
-LPAGML-
-LPAGMF-
LWPAGM--
LGPAGM--
LQPAGM--
-LPAGMH-
LAPAGM--
LVPAGM--
-LPAGMK-
LNPAGM--
LYPAGM--
-LPAGMT-
-LPAGMN-
LLPAGM--
LFPAGM--
LTPAGM--
-LPAGMM-
-LPAGMW-
LPPAGM--
-LPAGMI-
-LPAGMY-
-LPAGMV-
-LPAGMA-

LEPAGM--
-LPAGMS-
-LPAGMQ-
-LPAGME-
LIPAGM--
MTPAGM--
-MPAGMH-
-MPAGMV-
-MPAGMK-
-MPAGML-
MVPAGM--
-MPAGMW-
MDPAGM--
-MPAGMS-
MMPAGM--
-MPAGMR-
-MPAGMA-
MWPAGM--
MRPAGM--
MPPAGM--
-MPAGMY-
-MPAGMT-
MQPAGM--
-MPAGMN-
-MPAGMI-
-MPAGMF-
MSPAGM--
-MPAGME-
MHPAGM--
MEPAGM--
NIPAGM--
NLPAGM--
-NPAGMI-
NSPAGM--
-NPAGMR-
NQPAGM--
NTPAGM--
-NPAGMH-
NRPAGM--
-NPAGMT-
NYPAGM--
NHPAGM--
-NPAGMY-
-NPAGMV-
-NPAGMF-
-NPAGML-
NMPAGM--
-NPAGMW-
-NPAGMS-
-NPAGMM-

NVPAGM--
-NPAGMQ-
-NPAGMK-
NNPAGM--
NFPAGM--
NPPAGM--
-NPAGMN-
-NPAGME-
-NPAGMA-
NDPAGM--
--PAGMLG
--PAGMRA
--PAGMRT
--PAGMIS
--PAGMRY
--PAGMVY
--PAGMSS
--PAGMSA
--PAGMRM
--PAGMRS
--PAGMHS
--PAGMRG
--PAGMKG
--PAGMVK
--PAGMRV
--PAGMRL
--PAGMLV
--PAGMYR
--PAGMNA
--PAGMKS
--PAGMRI
--PAGMQS
--PAGMNV
--PAGMWR
--PAGMST
--PAGMRW
--PAGMLS
--PAGMKA
--PAGMFV
--PAGMFS
--PAGMLW
--PAGMLT
--PAGMTS
--PAGMSF
--PAGMRF
--PAGMMW
--PAGMVS
--PAGMVI
--PAGMLR
--PAGMHY

--PAGMGS
--PAGMAS
--PAGMYY
--PAGMYV
--PAGMWK
--PAGMVG
--PAGMTN
--PAGMRH
--PAGMRD
--PAGMKT
--PAGMFK
--PAGMRE
--PAGMLK
--PAGMFR
--PAGMYT
--PAGMWH
--PAGMSW
--PAGMSN
--PAGMSG
--PAGMRN
--PAGMHT
--PAGMWV
--PAGMVW
--PAGMVR
--PAGMTF
--PAGMSY
--PAGMSK
--PAGMNY
--PAGMAY
--PAGMAG
--PAGMAA
PYPAGM--
PHPAGM--
--PAGMYS
--PAGMTW
--PAGMRK
--PAGMNR
--PAGMNF
--PAGMLA
--PAGMKV
--PAGMIV
--PAGMHK
--PAGMYW
--PAGMWG
--PAGMQM
--PAGMNS
--PAGMKR
--PAGMIT
--PAGMII
--PAGMGG

--PAGMFH
--PAGMVT
--PAGMTR
--PAGMTK
--PAGMNM
--PAGMMR
--PAGMKF
--PAGMIK
--PAGMYG
--PAGMWL
--PAGMWA
--PAGMVV
--PAGMVL
--PAGMTV
--PAGMTT
--PAGMTL
--PAGMTH
--PAGMSI
--PAGMNT
--PAGMMA
--PAGMHV
PMPAGM--
--PAGMWD
--PAGMVH
--PAGMTG
--PAGMTD
--PAGMTA
--PAGMSQ
--PAGMMS
--PAGMLY
--PAGMIY
--PAGMIG
--PAGMHR
PSPAGM--
--PAGMYA
--PAGMWT
--PAGMSL
--PAGMML
--PAGMMG
--PAGMLN
--PAGMLI
--PAGMES
--PAGMYD
--PAGMQY
--PAGMMN
--PAGMMK
--PAGMIR
--PAGMIL
--PAGMHH
--PAGMHG

--PAGMEV
PTPAGM--
PRPAGM--
--PAGMYN
--PAGMYH
--PAGMWS
--PAGMWQ
--PAGMWN
--PAGMVE
--PAGMVA
--PAGMNI
--PAGMMM
--PAGMMF
--PAGMKW
--PAGMHI
--PAGMAW
PFPAGM--
--PAGMYM
--PAGMTE
--PAGMSH
--PAGMQV
--PAGMQG
--PAGMNQ
--PAGMNN
--PAGMLE
--PAGMKY
--PAGMKI
--PAGMKD
--PAGMHA
--PAGMGA
--PAGMFE
PKPAGM--
--PAGMWE
--PAGMVN
--PAGMVM
--PAGMVF
--PAGMSE
--PAGMLQ
--PAGMKL
--PAGMKH
--PAGMIN
--PAGMIE
--PAGMHF
--PAGMGM
--PAGMFT
--PAGMAN
--PAGMAF
PVPAGM--
PNPAGM--
PEPAGM--

PAPAGM--
--PAGMYI
--PAGMSD
--PAGMRQ
--PAGMNW
--PAGMMT
--PAGMLF
--PAGMLD
--PAGMKQ
--PAGMKM
--PAGMKK
--PAGMIQ
--PAGMIH
--PAGMHN
--PAGMHL
--PAGMHD
--PAGMGW
--PAGMGV
--PAGMGT
--PAGMGI
--PAGMFI
--PAGMFG
--PAGMEY
--PAGMEA
--PAGMCG
--PAGMAM
-PPAGMH-
-PPAGMF-
PLPAGM--
PGPAGM--
--PAGMYE
--PAGMWY
--PAGMWM
--PAGMVD
--PAGMVC
--PAGMTM
--PAGMSC
--PAGMQR
--PAGMQN
--PAGMQK
--PAGMQA
--PAGMNL
--PAGMNK
--PAGMMV
--PAGMME
--PAGMMD
--PAGMLM
--PAGMKN
--PAGMIM
--PAGMHE

--PAGMGL
--PAGMGK
--PAGMFN
--PAGMFM
--PAGMFF
--PAGMFA
--PAGMEW
--PAGMET
--PAGMEI
--PAGMDA
--PAGMAV
--PAGMAL
--PAGMAH
--PAGMAD
--PAGMAC
-QPAGMK-
-QPAGMI-
-QPAGMR-
QRPAGM--
QVPAGM--
QTPAGM--
QSPAGM--
-QPAGMT-
-QPAGMS-
QHPAGM--
QLPAGM--
-QPAGMV-
-QPAGMH-
-QPAGMW-
-QPAGML-
-QPAGMA-
QGPAGM--
QKPAGM--
QIPAGM--
QNPAGM--
-QPAGMY-
-QPAGMN-
-QPAGME-
QMPAGM--
-RPAGMR-
-RPAGMS-
RSPAGM--
-RPAGMI-
RAPAGM--
-RPAGMA-
RLPAGM--
-RPAGMH-
-RPAGMK-
-RPAGMM-
RGPAGM--

-RPAGMV-
RPPAGM--
-RPAGMW-
-RPAGMF-
RMPAGM--
RWPAGM--
RRPAGM--
-RPAGMN-
RDPAGM--
RHPAGM--
-RPAGMQ-
-RPAGMG-
REPAGM--
RKPAGM--
RQPAGM--
RIPAGM--
RCPAGM--
-RPAGME-
-RPAGMC-
RFPAGM--
-SPAGMR-
-SPAGMY-
SSPAGM--
SRPAGM--
SKPAGM--
-SPAGMF-
-SPAGMS-
-SPAGMK-
-SPAGMI-
-SPAGMT-
-SPAGML-
SYPAGM--
-SPAGMH-
SEPAGM--
SMPAGM--
SGPAGM--
SWPAGM--
-SPAGMN-
SNPAGM--
SLPAGM--
SDPAGM--
SAPAGM--
STPAGM--
SFPAGM--
-SPAGMQ-
SQPAGM--
-SPAGMM-
-SPAGMA-
SPPAGM--
-SPAGMV-

SVPAGM--
-SPAGME-
-SPAGMC-
-TPAGML-
-TPAGMI-
TLPAGM--
TQPAGM--
-TPAGMY-
-TPAGMN-
TVPAGM--
TPPAGM--
-TPAGMK-
TKPAGM--
TWPAGM--
-TPAGMR-
TSPAGM--
-TPAGMM-
TTPAGM--
TRPAGM--
-TPAGMH-
THPAGM--
-TPAGMW-
-TPAGMT-
TEPAGM--
TNPAGM--
TGPAGM--
-TPAGMV-
-TPAGMS-
-TPAGMF-
TAPAGM--
TMPAGM--
TFPAGM--
TIPAGM--
TYPAGM--
-TPAGMQ-
TDPAGM--
-VPAGML-
VLPAGM--
-VPAGMR-
VGPAGM--
VRPAGM--
-VPAGMK-
-VPAGMV-
VEPAGM--
VSPAGM--
-VPAGMH-
VHPAGM--
-VPAGMW-
VNPAGM--
-VPAGMI-

-VPAGMM-
VMPAGM--
VDPAGM--
VTPAGM--
-VPAGMY-
-VPAGMF-
VYPAGM--
VPPAGM--
-VPAGMT-
-VPAGMN-
VKPAGM--
VIPAGM--
VQPAGM--
-VPAGMG-
-VPAGMC-
-VPAGMA-
WSPAGM--
WRPAGM--
-WPAGMT-
-WPAGMA-
-WPAGMY-
-WPAGMM-
WFPAGM--
-WPAGMR-
-WPAGMF-
WEPAGM--
WQPAGM--
-WPAGMV-
-WPAGMI-
WTPAGM--
WPPAGM--
WVPAGM--
-WPAGMK-
WWPAGM--
WHPAGM--
WDPAGM--
WYPAGM--
-WPAGMW-
-WPAGMN-
WLPAGM--
WGPAGM--
-WPAGMS-
-WPAGMH-
-WPAGMQ-
-WPAGME-
WNPAGM--
-YPAGMW-
YRPAGM--
YFPAGM--
YPPAGM--

-YPAGMR-
-YPAGMM-
-YPAGMS-
YWPAGM--
YSPAGM--
-YPAGMI-
YMPAGM--
-YPAGMV-
YIPAGM--
YDPAGM--
YTPAGM--
YGPAGM--
-YPAGMF-
-YPAGMN-
YQPAGM--
-YPAGMY-
YHPAGM--
-YPAGMT-
-YPAGML-
-YPAGMK-
-YPAGMH-
YAPAGM--
PMPF 543 604 45.49
--PMPFQR
AFPMPF--
-APMPFS-
ALPMPF--
-APMPFM-
-APMPFF-
AGPMPF--
AAPMPF--
AWPMPF--
ASPMPF--
-APMPFI-
-APMPFH-
AKPMPF--
ADPMPF--
AVPMPF--
-APMPFT-
-APMPFR-
ANPMPF--
AMPMPF--
-APMPFW-
-APMPFN-
-APMPFL-
AEPMPF--
-APMPFQ-
-APMPFY-
AHPMPF--
CRPMPF--

CEPMPF--
CVPMPF--
CMPMPF--
CGPMPF--
CDPMPF--
CCPMPF--
DWPMPF--
DRPMPF--
DMPMPF--
DKPMPF--
DHPMPF--
DAPMPF--
-DPMPFS-
-DPMPFR-
DIPMPF--
-EPMPFR-
-EPMPFM-
ETPMPF--
ESPMPF--
ERPMPF--
-EPMPFK-
EHPMPF--
-EPMPFS-
-EPMPFL-
-EPMPFI-
ENPMPF--
EGPMPF--
-EPMPFA-
EWPMPF--
EEPMPF--
EAPMPF--
FKPMPF--
FGPMPF--
FPPMPF--
FLPMPF--
FVPMPF--
FAPMPF--
FDPMPF--
-FPMPFM-
FTPMPF--
FNPMPF--
-FPMPFR-
-FPMPFT-
-FPMPFK-
FFPMPF--
FEPMPF--
FSPMPF--
-FPMPFW-
-FPMPFL-
-FPMPFI-

FMPMPF--
GGPMPF--
GMPMPF--
GIPMPF--
GHPMPF--
GYPMPF--
GRPMPF--
GAPMPF--
GSPMPF--
GFPMPF--
-GPMPFT-
-GPMPFM-
-GPMPFW-
-GPMPFF-
GLPMPF--
GQPMPF--
-GPMPFY-
-GPMPFN-
GNPMPF--
GEPMPF--
GWPMPF--
GVPMPF--
GTPMPF--
GPPMPF--
-GPMPFR-
-GPMPFK-
GKPMPF--
HRPMPF--
HWPMPF--
HSPMPF--
HTPMPF--
HGPMPF--
HAPMPF--
-HPMPFS-
-HPMPFK-
HFPMPF--
HPPMPF--
HIPMPF--
-HPMPFI-
HLPMPF--
-HPMPFY-
HHPMPF--
HQPMPF--
HYPMPF--
-HPMPFR-
-HPMPFL-
-HPMPFA-
HMPMPF--
ILPMPF--
ISPMPF--

IFPMPF--
-IPMPFS-
IIPMPF--
IVPMPF--
IRPMPF--
-IPMPFY-
-IPMPFR-
-IPMPFK-
IKPMPF--
IWPMPF--
-IPMPFT-
-IPMPFH-
IAPMPF--
IPPMPF--
-IPMPFV-
-IPMPFF-
IMPMPF--
IEPMPF--
-IPMPFQ-
-IPMPFN-
-IPMPFM-
-IPMPFI-
-IPMPFC-
IGPMPF--
IDPMPF--
KSPMPF--
KYPMPF--
KGPMPF--
KEPMPF--
KTPMPF--
KKPMPF--
KRPMPF--
KVPMPF--
-KPMPFK-
-KPMPFR-
-KPMPFM-
KNPMPF--
-KPMPFT-
-KPMPFW-
-KPMPFQ-
-KPMPFL-
KDPMPF--
KQPMPF--
-KPMPFS-
-KPMPFH-
-KPMPFA-
KLPMPF--
KCPMPF--
KPPMPF--
-KPMPFI-

KIPMPF--
KFPMPF--
LRPMPF--
LLPMPF--
LYPMPF--
LPPMPF--
LVPMPF--
LNPMPF--
LIPMPF--
LDPMPF--
LEPMPF--
LQPMPF--
-LPMPFR-
LAPMPF--
LSPMPF--
LFPMPF--
-LPMPFL-
LHPMPF--
LTPMPF--
-LPMPFI-
-LPMPFH-
LWPMPF--
-LPMPFV-
-LPMPFM-
-LPMPFG-
-LPMPFF-
LGPMPF--
MGPMPF--
MRPMPF--
MMPMPF--
MIPMPF--
MLPMPF--
-MPMPFR-
-MPMPFL-
MVPMPF--
MHPMPF--
MFPMPF--
-MPMPFH-
MYPMPF--
MWPMPF--
MPPMPF--
-MPMPFY-
-MPMPFV-
-MPMPFQ-
MDPMPF--
-MPMPFT-
-MPMPFI-
-MPMPFF-
-MPMPFW-
-MPMPFK-

MNPMPF--
NGPMPF--
NVPMPF--
NDPMPF--
NRPMPF--
NEPMPF--
NAPMPF--
NTPMPF--
-NPMPFT-
-NPMPFL-
NHPMPF--
NPPMPF--
NLPMPF--
NFPMPF--
NYPMPF--
NSPMPF--
-NPMPFI-
NNPMPF--
-NPMPFH-
-NPMPFS-
-NPMPFR-
-NPMPFF-
-NPMPFA-
--PMPFKG
--PMPFLG
PFPMPF--
--PMPFTL
--PMPFRS
--PMPFRQ
--PMPFWV
--PMPFLI
--PMPFWG
--PMPFVA
--PMPFLA
--PMPFRV
--PMPFRY
--PMPFKA
--PMPFTG
--PMPFRT
--PMPFKI
--PMPFIG
--PMPFYF
--PMPFVS
--PMPFSW
--PMPFSV
--PMPFFI
--PMPFSA
--PMPFRA
--PMPFMG
--PMPFMA

--PMPFLT
--PMPFGW
--PMPFYL
PTPMPF--
--PMPFYG
--PMPFWS
--PMPFSQ
--PMPFSF
--PMPFNA
--PMPFLL
--PMPFFR
--PMPFSS
--PMPFAH
--PMPFAF
--PMPFWK
--PMPFST
--PMPFSK
--PMPFQT
--PMPFLV
--PMPFKT
--PMPFAT
--PMPFWM
--PMPFVY
--PMPFTN
--PMPFRH
--PMPFRG
--PMPFNW
--PMPFMS
--PMPFMK
PMPMPF--
--PMPFTM
--PMPFTH
--PMPFTA
--PMPFQY
--PMPFPM
--PMPFNS
--PMPFNH
--PMPFKV
--PMPFGS
--PMPFGQ
--PMPFAS
--PMPFYI
--PMPFVT
--PMPFLQ
--PMPFEI
--PMPFYR
--PMPFYQ
--PMPFVI
--PMPFTY
--PMPFRM

```
--PMPFQS
--PMPFNQ
--PMPFKW
--PMPFIS
--PMPFHS
--PMPFHF
--PMPFFS
--PMPFAQ
--PMPFVR
--PMPFTW
--PMPFTR
--PMPFTK
--PMPFMN
--PMPFLK
--PMPFLH
--PMPFFL
PKPMPF--
PWPMPF--
PQPMPF--
--PMPFYK
--PMPFTI
--PMPFSN
--PMPFRR
--PMPFRN
--PMPFQF
--PMPFPV
--PMPFNT
--PMPFNG
--PMPFMW
--PMPFMI
--PMPFMD
--PMPFLR
--PMPFKK
--PMPFKF
--PMPFIT
--PMPFIR
--PMPFFT
--PMPFYA
--PMPFSG
--PMPFRF
--PMPFMQ
--PMPFLM
--PMPFIM
--PMPFHA
--PMPFGK
--PMPFGA
--PMPFFM
--PMPFFA
--PMPFES
--PMPFDM
```

--PMPFCS
--PMPFAG
PHPMPF--
--PMPFYV
--PMPFYM
--PMPFYH
--PMPFVG
--PMPFTV
--PMPFMR
--PMPFKY
--PMPFIH
--PMPFHW
--PMPFHM
--PMPFFQ
--PMPFFG
--PMPFEF
--PMPFDV
--PMPFDF
--PMPFAA
PPPMPF--
--PMPFYE
--PMPFWY
--PMPFWQ
--PMPFWN
--PMPFWH
--PMPFVW
--PMPFVV
--PMPFVL
--PMPFTF
--PMPFSH
--PMPFSC
--PMPFRD
--PMPFQP
--PMPFQK
--PMPFPS
--PMPFNN
--PMPFKS
--PMPFKR
--PMPFHH
--PMPFGR
--PMPFGH
--PMPFFN
--PMPFFC
--PMPFEM
--PMPFEG
--PMPFEA
--PMPFCT
--PMPFCQ
--PMPFCI
QGPMPF--

QIPMPF--
QVPMPF--
-QPMPFW-
QPPMPF--
-QPMPFR-
QFPMPF--
QAPMPF--
QKPMPF--
QRPMPF--
QDPMPF--
QSPMPF--
-QPMPFQ-
QEPMPF--
QYPMPF--
QWPMPF--
QTPMPF--
-QPMPFS-
QLPMPF--
-QPMPFY-
-QPMPFL-
QMPMPF--
QQPMPF--
-QPMPFV-
-QPMPFP-
-QPMPFN-
-QPMPFC-
QHPMPF--
QCPMPF--
RQPMPF--
RLPMPF--
RSPMPF--
-RPMPFM-
-RPMPFF-
-RPMPFT-
-RPMPFI-
RGPMPF--
REPMPF--
-RPMPFS-
RRPMPF--
-RPMPFR-
RIPMPF--
RVPMPF--
RTPMPF--
-RPMPFQ-
-RPMPFH-
RYPMPF--
RAPMPF--
RWPMPF--
RCPMPF--
RNPMPF--

RHPMPF--
-RPMPFL-
-RPMPFK-
RKPMPF--
RFPMPF--
-RPMPFY-
-RPMPFW-
RPPMPF--
-RPMPFN-
RMPMPF--
SRPMPF--
SSPMPF--
SVPMPF--
-SPMPFR-
SMPMPF--
SGPMPF--
STPMPF--
-SPMPFY-
SKPMPF--
SHPMPF--
SNPMPF--
SYPMPF--
SDPMPF--
SLPMPF--
-SPMPFT-
-SPMPFM-
SEPMPF--
-SPMPFW-
-SPMPFK-
SIPMPF--
SFPMPF--
-SPMPFH-
-SPMPFV-
-SPMPFN-
-SPMPFL-
-SPMPFE-
-SPMPFA-
TRPMPF--
TVPMPF--
-TPMPFR-
TPPMPF--
TLPMPF--
TKPMPF--
-TPMPFM-
TNPMPF--
TGPMPF--
THPMPF--
TFPMPF--
TSPMPF--
-TPMPFS-

TEPMPF--
TWPMPF--
-TPMPFY-
-TPMPFT-
TTPMPF--
-TPMPFI-
TIPMPF--
TAPMPF--
-TPMPFK-
-TPMPFF-
TMPMPF--
TDPMPF--
VAPMPF--
VLPMPF--
VMPMPF--
VNPMPF--
-VPMPFS-
VSPMPF--
VVPMPF--
VTPMPF--
VQPMPF--
-VPMPFT-
VFPMPF--
VRPMPF--
-VPMPFR-
-VPMPFQ-
VIPMPF--
-VPMPFI-
VKPMPF--
VHPMPF--
VCPMPF--
-VPMPFW-
-VPMPFV-
-VPMPFF-
VYPMPF--
VWPMPF--
-VPMPFK-
-VPMPFH-
VGPMPF--
VDPMPF--
WSPMPF--
WGPMPF--
-WPMPFY-
-WPMPFK-
-WPMPFR-
WWPMPF--
WMPMPF--
WKPMPF--
WVPMPF--
WTPMPF--

	-WPMPFL-		
	-WPMPFS-		
	-WPMPFQ-		
	-WPMPFI-		
	-WPMPFA-		
	WLPMPF--		
	WFPMPF--		
	YGPMPF--		
	YKPMPF--		
	-YPMPFH-		
	YRPMPF--		
	YTPMPF--		
	YMPMPF--		
	-YPMPFT-		
	YAPMPF--		
	YIPMPF--		
	YHPMPF--		
	-YPMPFS-		
	-YPMPFR-		
	YWPMPF--		
	YFPMPF--		
	YVPMPF--		
	-YPMPFI-		
	-YPMPFY-		
	-YPMPFV-		
	-YPMPFN-		
	-YPMPFA-		
PVPY	132	789	45.48
	--PVPYVR		
	--PVPYGR		
	-APVPYR-		
	-APVPYL-		
	AMPVPY--		
	-APVPYM-		
	-APVPYF-		
	AAPVPY--		
	ATPVPY--		
	ASPVPY--		
	AKPVPY--		
	ARPVPY--		
	-APVPYT-		
	-APVPYW-		
	ALPVPY--		
	AYPVPY--		
	AQPVPY--		
	-APVPYI-		
	ANPVPY--		
	ADPVPY--		
	AIPVPY--		
	-APVPYE-		

APPVPY--
-APVPYY-
AVPVPY--
-APVPYK-
-APVPYH-
-APVPYN-
AHPVPY--
AGPVPY--
-APVPYV-
-APVPYQ-
-APVPYA-
AFPVPY--
ACPVPY--
AEPVPY--
CPPVPY--
CSPVPY--
CTPVPY--
CIPVPY--
CYPVPY--
-CPVPYT-
-CPVPYR-
-CPVPYI-
CKPVPY--
CEPVPY--
CAPVPY--
DTPVPY--
DGPVPY--
DEPVPY--
-DPVPYT-
DRPVPY--
DLPVPY--
-DPVPYQ-
DNPVPY--
DKPVPY--
-DPVPYV-
-DPVPYR-
DFPVPY--
-DPVPYL-
-DPVPYI-
DIPVPY--
DAPVPY--
DYPVPY--
DSPVPY--
-DPVPYF-
DHPVPY--
DWPVPY--
DVPVPY--
-DPVPYY-
DDPVPY--
EFPVPY--

ERPVPY--
-EPVPYF-
-EPVPYY-
ELPVPY--
-EPVPYR-
EHPVPY--
ETPVPY--
ESPVPY--
-EPVPYT-
-EPVPYQ-
-EPVPYM-
-EPVPYI-
EYPVPY--
EAPVPY--
ENPVPY--
EIPVPY--
EWPVPY--
EVPVPY--
EQPVPY--
-EPVPYW-
-EPVPYS-
-EPVPYE-
-EPVPYA-
EMPVPY--
EDPVPY--
FRPVPY--
FMPVPY--
-FPVPYI-
FTPVPY--
-FPVPYL-
FLPVPY--
FNPVPY--
FSPVPY--
FGPVPY--
-FPVPYA-
-FPVPYT-
-FPVPYR-
-FPVPYF-
FVPVPY--
-FPVPYV-
FAPVPY--
-FPVPYS-
-FPVPYH-
FDPVPY--
FWPVPY--
-FPVPYQ-
-FPVPYM-
FFPVPY--
-GPVPYL-
GAPVPY--

GVPVPY--
-GPVPYR-
GSPVPY--
GNPVPY--
GIPVPY--
-GPVPYI-
-GPVPYS-
-GPVPYV-
-GPVPYT-
GGPVPY--
-GPVPYY-
GPPVPY--
GHPVPY--
GMPVPY--
GKPVPY--
GYPVPY--
-GPVPYM-
GWPVPY--
GTPVPY--
-GPVPYK-
-GPVPYW-
GQPVPY--
-GPVPYN-
GEPVPY--
-GPVPYH-
-GPVPYF-
-GPVPYA-
GDPVPY--
HYPVPY--
HTPVPY--
HRPVPY--
HKPVPY--
HGPVPY--
-HPVPYR-
-HPVPYT-
-HPVPYS-
-HPVPYL-
HSPVPY--
-HPVPYM-
-HPVPYK-
HDPVPY--
HVPVPY--
-HPVPYH-
HNPVPY--
HAPVPY--
-HPVPYF-
HCPVPY--
HQPVPY--
-HPVPYY-
-HPVPYA-

HMPVPY--
HIPVPY--
IRPVPY--
IVPVPY--
-IPVPYF-
-IPVPYR-
IPPVPY--
IAPVPY--
IQPVPY--
IMPVPY--
IGPVPY--
IKPVPY--
-IPVPYT-
-IPVPYI-
ILPVPY--
-IPVPYY-
IWPVPY--
ISPVPY--
-IPVPYM-
INPVPY--
IDPVPY--
-IPVPYK-
-IPVPYG-
ITPVPY--
-IPVPYL-
-IPVPYS-
-IPVPYQ-
IIPVPY--
ICPVPY--
IYPVPY--
-IPVPYN-
KSPVPY--
-KPVPYR-
KGPVPY--
-KPVPYL-
KEPVPY--
-KPVPYQ-
-KPVPYI-
-KPVPYV-
KRPVPY--
KQPVPY--
KYPVPY--
KPPVPY--
-KPVPYW-
-KPVPYS-
-KPVPYA-
KLPVPY--
KKPVPY--
KFPVPY--
KWPVPY--

-KPVPYG-
-KPVPYF-
KVPVPY--
KNPVPY--
-KPVPYE-
-KPVPYH-
-KPVPYY-
KIPVPY--
KDPVPY--
KAPVPY--
KCPVPY--
LRPVPY--
LSPVPY--
-LPVPYR-
-LPVPYK-
-LPVPYT-
LGPVPY--
LLPVPY--
LEPVPY--
LNPVPY--
LAPVPY--
LPPVPY--
LYPVPY--
-LPVPYI-
-LPVPYS-
-LPVPYL-
LVPVPY--
LIPVPY--
LHPVPY--
-LPVPYW-
LWPVPY--
LKPVPY--
LDPVPY--
LTPVPY--
-LPVPYV-
LQPVPY--
-LPVPYQ-
-LPVPYN-
-LPVPYA-
LFPVPY--
-LPVPYM-
-LPVPYF-
MKPVPY--
MSPVPY--
-MPVPYS-
MVPVPY--
-MPVPYY-
MTPVPY--
-MPVPYF-
-MPVPYT-

MPPVPY--
-MPVPYM-
MQPVPY--
-MPVPYR-
-MPVPYN-
MAPVPY--
MLPVPY--
MWPVPY--
-MPVPYL-
-MPVPYV-
MMPVPY--
MGPVPY--
MEPVPY--
MYPVPY--
-MPVPYK-
-MPVPYE-
MNPVPY--
MIPVPY--
NVPVPY--
NRPVPY--
NGPVPY--
-NPVPYR-
NWPVPY--
-NPVPYS-
-NPVPYK-
NPPVPY--
NTPVPY--
-NPVPYY-
-NPVPYV-
NLPVPY--
NNPVPY--
-NPVPYW-
NHPVPY--
NDPVPY--
NAPVPY--
NQPVPY--
-NPVPYM-
NIPVPY--
NEPVPY--
-NPVPYT-
-NPVPYI-
-NPVPYH-
-NPVPYA-
NMPVPY--
NFPVPY--
-NPVPYQ-
--PVPYLS
--PVPYRL
--PVPYRR
--PVPYYA

--PVPYRS
--PVPYDL
--PVPYLT
--PVPYSG
--PVPYKA
--PVPYMM
--PVPYHA
--PVPYIS
--PVPYYL
--PVPYRA
--PVPYKY
--PVPYRN
--PVPYYV
--PVPYVS
--PVPYPM
--PVPYLV
PHPVPY--
--PVPYMN
--PVPYKS
--PVPYFS
--PVPYRI
--PVPYLY
--PVPYFA
--PVPYVY
--PVPYSS
--PVPYMH
--PVPYWA
--PVPYRV
--PVPYLR
--PVPYLN
--PVPYHG
--PVPYGS
--PVPYEL
--PVPYGM
--PVPYRK
--PVPYKL
--PVPYFT
--PVPYAG
--PVPYSR
--PVPYQA
--PVPYLA
PRPVPY--
--PVPYVM
--PVPYVI
--PVPYRG
--PVPYQM
--PVPYPY
--PVPYMG
--PVPYLI
--PVPYKI

--PVPYAI
--PVPYYM
--PVPYRT
--PVPYQS
--PVPYKH
--PVPYIR
--PVPYIF
--PVPYHI
--PVPYWH
--PVPYVL
--PVPYRW
--PVPYQV
--PVPYQT
--PVPYMQ
--PVPYFL
PGPVPY--
--PVPYYI
--PVPYNY
--PVPYMA
--PVPYIY
--PVPYIQ
--PVPYAQ
PAPVPY--
--PVPYYG
--PVPYWS
--PVPYWF
--PVPYSQ
--PVPYSK
--PVPYSA
--PVPYIG
--PVPYFG
--PVPYTV
--PVPYQL
--PVPYML
--PVPYAY
--PVPYAT
--PVPYAH
--PVPYYQ
--PVPYWM
--PVPYSH
--PVPYMR
--PVPYMK
--PVPYAK
--PVPYYT
--PVPYYS
--PVPYWN
--PVPYTY
--PVPYTM
--PVPYTI
--PVPYPV

--PVPYMF
--PVPYLW
--PVPYLQ
--PVPYKG
--PVPYHS
--PVPYGT
--PVPYFK
--PVPYEY
PVPVPY--
--PVPYWQ
--PVPYWI
--PVPYTW
--PVPYTN
--PVPYTG
--PVPYSW
--PVPYLM
--PVPYLF
--PVPYKF
--PVPYFY
--PVPYFM
--PVPYDM
PQPVPY--
PMPVPY--
PKPVPY--
--PVPYQG
--PVPYNN
--PVPYMY
--PVPYKT
--PVPYIV
--PVPYIT
--PVPYIL
--PVPYIA
--PVPYHV
--PVPYHQ
--PVPYHM
--PVPYGV
--PVPYGF
--PVPYEW
PSPVPY--
PYPVPY--
--PVPYWV
--PVPYWG
--PVPYVK
--PVPYTS
--PVPYTF
--PVPYSY
--PVPYSV
--PVPYRY
--PVPYRQ
--PVPYQY

--PVPYPW
--PVPYMW
--PVPYKW
--PVPYKR
--PVPYGW
--PVPYFV
--PVPYES
--PVPYDW
PLPVPY--
PFPVPY--
--PVPYYN
--PVPYWW
--PVPYVT
--PVPYSN
--PVPYQN
--PVPYMT
--PVPYMI
--PVPYLK
--PVPYLH
--PVPYKN
--PVPYKK
--PVPYIM
--PVPYHN
--PVPYHH
--PVPYDI
--PVPYYF
--PVPYWR
--PVPYWL
--PVPYVW
--PVPYTK
--PVPYRE
--PVPYRD
--PVPYQR
--PVPYPS
--PVPYPH
--PVPYNV
--PVPYNM
--PVPYFR
--PVPYFI
--PVPYYH
--PVPYWT
--PVPYVD
--PVPYQK
--PVPYPQ
--PVPYNA
--PVPYME
--PVPYGN
--PVPYFW
--PVPYFN
--PVPYEI

--PVPYEG
--PVPYAV
--PVPYAM
PTPVPY--
--PVPYYY
--PVPYVH
--PVPYVG
--PVPYVF
--PVPYVA
--PVPYTT
--PVPYTR
--PVPYTH
--PVPYTA
--PVPYSE
--PVPYSD
--PVPYQQ
--PVPYNS
--PVPYNH
--PVPYNF
--PVPYKQ
--PVPYKM
--PVPYIH
--PVPYID
--PVPYHT
--PVPYHK
--PVPYGH
--PVPYGA
--PVPYFD
--PVPYEV
--PVPYEQ
--PVPYEF
PEPVPY--
PWPVPY--
--PVPYYK
--PVPYYD
--PVPYWY
--PVPYWK
--PVPYVN
--PVPYTQ
--PVPYTC
--PVPYSF
--PVPYRH
--PVPYQI
--PVPYPN
--PVPYNT
--PVPYNQ
--PVPYMV
--PVPYMD
--PVPYLD
--PVPYKV

--PVPYIW
--PVPYIE
--PVPYHR
--PVPYGK
--PVPYGG
--PVPYEK
--PVPYEA
--PVPYDS
--PVPYDQ
--PVPYDH
--PVPYAF
-PPVPYV-
-PPVPYR-
PCPVPY--
QLPVPY--
QFPVPY--
QRPVPY--
-QPVPYS-
-QPVPYL-
QHPVPY--
QYPVPY--
-QPVPYR-
-QPVPYK-
QAPVPY--
QSPVPY--
QPPVPY--
QNPVPY--
QTPVPY--
-QPVPYA-
QWPVPY--
-QPVPYQ-
QEPVPY--
QVPVPY--
QDPVPY--
QQPVPY--
QMPVPY--
QIPVPY--
QGPVPY--
-QPVPYY-
-QPVPYV-
-QPVPYH-
-QPVPYD-
RSPVPY--
RIPVPY--
-RPVPYT-
-RPVPYR-
RVPVPY--
RPPVPY--
-RPVPYV-
RNPVPY--

RMPVPY--
REPVPY--
-RPVPYF-
RRPVPY--
RYPVPY--
-RPVPYA-
RTPVPY--
-RPVPYI-
RLPVPY--
RGPVPY--
-RPVPYY-
-RPVPYQ-
RWPVPY--
RAPVPY--
RDPVPY--
-RPVPYK-
RQPVPY--
RFPVPY--
-RPVPYW-
-RPVPYH-
RHPVPY--
-RPVPYN-
-RPVPYM-
-RPVPYE-
-RPVPYG-
SRPVPY--
-SPVPYR-
SSPVPY--
-SPVPYT-
SLPVPY--
SVPVPY--
SWPVPY--
SMPVPY--
SIPVPY--
-SPVPYS-
STPVPY--
-SPVPYI-
-SPVPYQ-
SNPVPY--
SHPVPY--
SGPVPY--
SQPVPY--
-SPVPYY-
-SPVPYL-
-SPVPYF-
SYPVPY--
SKPVPY--
SPPVPY--
-SPVPYW-
-SPVPYV-

SEPVPY--
-SPVPYK-
-SPVPYA-
SCPVPY--
-SPVPYN-
-SPVPYM-
-SPVPYH-
SFPVPY--
TRPVPY--
TGPVPY--
TFPVPY--
THPVPY--
-TPVPYS-
TTPVPY--
-TPVPYR-
-TPVPYL-
TAPVPY--
TVPVPY--
TSPVPY--
TYPVPY--
TKPVPY--
TNPVPY--
-TPVPYV-
-TPVPYT-
TLPVPY--
TMPVPY--
TDPVPY--
-TPVPYA-
-TPVPYY-
-TPVPYK-
-TPVPYH-
TQPVPY--
-TPVPYM-
-TPVPYI-
-TPVPYE-
VSPVPY--
VLPVPY--
VAPVPY--
VGPVPY--
VHPVPY--
VKPVPY--
-VPVPYV-
VQPVPY--
-VPVPYA-
VNPVPY--
VRPVPY--
VPPVPY--
VDPVPY--
VVPVPY--
VTPVPY--

```
-VPVPYR-
VIPVPY--
VYPVPY--
-VPVPYT-
-VPVPYQ-
-VPVPYF-
-VPVPYH-
-VPVPYE-
VMPVPY--
-VPVPYS-
-VPVPYM-
-VPVPYW-
-VPVPYI-
-VPVPYG-
-VPVPYY-
-VPVPYN-
-WPVPYR-
-WPVPYA-
WEPVPY--
WLPVPY--
WGPVPY--
WTPVPY--
-WPVPYL-
WDPVPY--
WRPVPY--
-WPVPYI-
-WPVPYF-
WPPVPY--
WFPVPY--
WVPVPY--
-WPVPYK-
WMPVPY--
WYPVPY--
WNPVPY--
-WPVPYT-
-WPVPYN-
WSPVPY--
-WPVPYY-
-WPVPYS-
-WPVPYM-
-WPVPYC-
YSPVPY--
YRPVPY--
YVPVPY--
YLPVPY--
-YPVPYM-
-YPVPYT-
YAPVPY--
YGPVPY--
YQPVPY--
```

-YPVPYS-
YNPVPY--
YKPVPY--
-YPVPYI-
YEPVPY--
-YPVPYN-
-YPVPYH-
-YPVPYF-
YYPVPY--
-YPVPYY-
-YPVPYV-
-YPVPYQ-
-YPVPYG-
-YPVPYA-
YIPVPY--
YFPVPY--
YDPVPY--

PSNI	53	849	45.45

MDPSNI--
--PSNILP
--PSNIGL
--PSNIFK
-QPSNIN-
-APSNIR-
-APSNIL-
-APSNIY-
AWPSNI--
ARPSNI--
APPSNI--
ASPSNI--
AVPSNI--
ATPSNI--
AFPSNI--
-APSNIH-
AHPSNI--
AGPSNI--
-APSNIF-
AYPSNI--
-APSNIW-
AKPSNI--
-APSNIV-
-APSNIS-
-APSNII-
AAPSNI--
-APSNIA-
AMPSNI--
AIPSNI--
-APSNIT-
-APSNIQ-
ADPSNI--

-APSNIM-
ANPSNI--
AEPSNI--
AQPSNI--
-APSNIE-
-APSNIG-
CSPSNI--
CVPSNI--
CRPSNI--
CLPSNI--
CNPSNI--
CCPSNI--
CAPSNI--
CPPSNI--
-CPSNIR-
CGPSNI--
CWPSNI--
-CPSNIV-
-CPSNIS-
-CPSNIM-
-CPSNIK-
-CPSNII-
CMPSNI--
CIPSNI--
DRPSNI--
DLPSNI--
DSPSNI--
DKPSNI--
DGPSNI--
-DPSNIL-
DMPSNI--
DFPSNI--
-DPSNIY-
DIPSNI--
-DPSNIM-
DVPSNI--
DNPSNI--
-DPSNIV-
-DPSNIT-
-DPSNIQ-
-DPSNIF-
DTPSNI--
DQPSNI--
-DPSNIK-
-DPSNII-
-DPSNIH-
DHPSNI--
DCPSNI--
EIPSNI--
ESPSNI--

ERPSNI--
ETPSNI--
-EPSNIR-
EKPSNI--
-EPSNIQ-
-EPSNIM-
EHPSNI--
-EPSNIS-
EVPSNI--
EQPSNI--
-EPSNIT-
-EPSNIY-
-EPSNIV-
-EPSNIL-
-EPSNIF-
ENPSNI--
ELPSNI--
EFPSNI--
EWPSNI--
-EPSNIN-
-EPSNIH-
-EPSNIG-
-EPSNIE-
EMPSNI--
EGPSNI--
EEPSNI--
EDPSNI--
FRPSNI--
-FPSNIR-
FIPSNI--
FKPSNI--
FFPSNI--
FSPSNI--
FLPSNI--
FGPSNI--
FTPSNI--
-FPSNIY-
FNPSNI--
FQPSNI--
-FPSNIT-
-FPSNIF-
-FPSNIW-
FPPSNI--
FMPSNI--
-FPSNIV-
-FPSNIQ-
-FPSNII-
FHPSNI--
FEPSNI--
FAPSNI--

-FPSNIS-
FWPSNI--
-FPSNIM-
FVPSNI--
-FPSNIG-
-FPSNIA-
FDPSNI--
FCPSNI--
-GPSNIR-
GTPSNI--
GIPSNI--
-GPSNII-
GLPSNI--
-GPSNIM-
GYPSNI--
GKPSNI--
GVPSNI--
-GPSNIH-
GPPSNI--
-GPSNIF-
-GPSNIA-
GSPSNI--
GQPSNI--
-GPSNIW-
-GPSNIY-
GMPSNI--
GGPSNI--
GNPSNI--
-GPSNIT-
-GPSNIQ-
-GPSNIG-
-GPSNIS-
GFPSNI--
GAPSNI--
-GPSNIV-
-GPSNIN-
-GPSNIK-
GHPSNI--
GEPSNI--
-GPSNIC-
GDPSNI--
HYPSNI--
-HPSNIL-
-HPSNIR-
-HPSNIY-
HHPSNI--
HMPSNI--
-HPSNIS-
HGPSNI--
HEPSNI--

HIPSNI--
HFPSNI--
-HPSNIM-
HWPSNI--
HVPSNI--
HAPSNI--
HQPSNI--
-HPSNIV-
HPPSNI--
HLPSNI--
HKPSNI--
-HPSNIQ-
-HPSNII-
-HPSNIW-
-HPSNIK-
-HPSNIF-
-HPSNIA-
HNPSNI--
HTPSNI--
-HPSNIE-
-HPSNIC-
HCPSNI--
IRPSNI--
IMPSNI--
-IPSNIT-
-IPSNIL-
IGPSNI--
IYPSNI--
IAPSNI--
ITPSNI--
-IPSNIF-
IIPSNI--
-IPSNIG-
-IPSNIM-
-IPSNIH-
-IPSNIV-
-IPSNIA-
ISPSNI--
IVPSNI--
-IPSNIW-
IDPSNI--
-IPSNIK-
INPSNI--
IHPSNI--
IFPSNI--
-IPSNIR-
IKPSNI--
IPPSNI--
IEPSNI--
IWPSNI--

IQPSNI--
ILPSNI--
-IPSNII-
-IPSNIE-
-IPSNIN-
ICPSNI--
KSPSNI--
KVPSNI--
-KPSNIF-
-KPSNIT-
-KPSNIW-
KAPSNI--
-KPSNIK-
KNPSNI--
-KPSNIV-
KWPSNI--
KQPSNI--
-KPSNIY-
-KPSNIS-
-KPSNII-
KGPSNI--
KEPSNI--
-KPSNIG-
-KPSNIE-
KLPSNI--
KFPSNI--
-KPSNIA-
KMPSNI--
-KPSNIQ-
-KPSNIN-
KPPSNI--
KHPSNI--
KYPSNI--
-KPSNIH-
KIPSNI--
LTPSNI--
LRPSNI--
LAPSNI--
LVPSNI--
LQPSNI--
LPPSNI--
-LPSNIY-
LYPSNI--
-LPSNIR-
-LPSNIL-
LGPSNI--
-LPSNIM-
LLPSNI--
LEPSNI--
-LPSNIT-

LNPSNI--
LDPSNI--
LIPSNI--
LHPSNI--
LFPSNI--
-LPSNIW-
LMPSNI--
-LPSNIK-
-LPSNIF-
LSPSNI--
LWPSNI--
LKPSNI--
-LPSNIV-
-LPSNIS-
-LPSNIH-
-LPSNIA-
-LPSNII-
-LPSNIQ-
-LPSNIE-
LCPSNI--
-MPSNIR-
MWPSNI--
-MPSNIL-
-MPSNIM-
MKPSNI--
-MPSNIW-
MIPSNI--
-MPSNIH-
MLPSNI--
-MPSNIY-
MSPSNI--
-MPSNIS-
-MPSNII-
MVPSNI--
MRPSNI--
-MPSNIV-
MPPSNI--
MGPSNI--
MEPSNI--
MQPSNI--
-MPSNIK-
-MPSNIF-
MMPSNI--
MTPSNI--
-MPSNIE-
MYPSNI--
-MPSNIT-
MFPSNI--
MAPSNI--
MNPSNI--

```
-MPSNIA-
-MPSNIQ-
NRPSNI--
NTPSNI--
NSPSNI--
NMPSNI--
-NPSNIT-
NPPSNI--
NIPSNI--
-NPSNIY-
NLPSNI--
NVPSNI--
-NPSNIV-
NAPSNI--
NYPSNI--
NKPSNI--
NGPSNI--
-NPSNIM-
-NPSNIL-
-NPSNIQ-
NHPSNI--
NEPSNI--
-NPSNIK-
-NPSNIH-
NFPSNI--
-NPSNIS-
-NPSNII-
-NPSNIA-
NWPSNI--
NDPSNI--
-NPSNIW-
--PSNILR
--PSNIWA
--PSNIVS
--PSNILA
--PSNIIS
--PSNIRL
--PSNILM
--PSNIRR
--PSNIWS
--PSNIVR
--PSNIVF
--PSNIVN
--PSNIRY
--PSNIFR
--PSNILY
--PSNILT
--PSNIRA
PYPSNI--
--PSNITS
```

--PSNILV
--PSNIMY
--PSNIST
--PSNIRT
--PSNIRN
--PSNIQG
--PSNIFQ
--PSNIFM
--PSNIFG
PMPSNI--
--PSNIYL
--PSNIWG
--PSNITW
--PSNISS
--PSNIMS
--PSNITG
--PSNISV
--PSNIRW
--PSNIRG
--PSNIMR
--PSNIKA
PRPSNI--
--PSNIVA
--PSNITN
--PSNIVK
--PSNILK
PLPSNI--
--PSNIRF
--PSNILQ
--PSNINS
--PSNIMF
--PSNIYN
--PSNIYA
--PSNIVY
--PSNIVT
--PSNITA
--PSNISG
--PSNIRQ
--PSNIRM
--PSNIMQ
--PSNILN
--PSNIHR
PGPSNI--
--PSNIVW
--PSNIVQ
--PSNITV
--PSNISN
--PSNIKG
--PSNIIR
--PSNIIH

PWPSNI--
--PSNIYR
--PSNIRS
--PSNIKN
--PSNIGS
--PSNISM
--PSNIYW
--PSNITD
--PSNIRK
--PSNIRI
--PSNIML
--PSNILI
--PSNILH
--PSNIYS
--PSNIVI
--PSNILL
PSPSNI--
--PSNIYM
--PSNIYK
--PSNIWF
--PSNITT
--PSNITR
--PSNISY
--PSNIAT
PQPSNI--
PAPSNI--
PVPSNI--
--PSNIQR
--PSNINA
--PSNIMG
--PSNIIK
--PSNIFN
--PSNIAM
PFPSNI--
--PSNIWQ
--PSNIVE
--PSNITM
--PSNISW
--PSNISQ
--PSNIRH
--PSNIMK
--PSNIIY
--PSNIHH
--PSNIAA
PEPSNI--
--PSNIYH
--PSNIWT
--PSNIVM
--PSNISA
--PSNIRV

--PSNIMA
--PSNIKT
--PSNIIN
--PSNIHS
--PSNIFY
--PSNIFS
--PSNIFF
--PSNIES
PHPSNI--
--PSNIWV
--PSNIWM
--PSNITL
--PSNISR
--PSNIQN
--PSNIKY
--PSNIKR
--PSNIKQ
--PSNIFT
--PSNIFH
--PSNIAW
--PSNIAV
PNPSNI--
PTPSNI--
--PSNIYG
--PSNIWH
--PSNITY
--PSNISI
--PSNIRE
--PSNIQW
--PSNIQV
--PSNIMT
--PSNIEW
--PSNIEH
--PSNIEG
--PSNIAN
--PSNIYF
--PSNIWR
--PSNIVD
--PSNISK
--PSNISH
--PSNIQS
--PSNIQM
--PSNIMW
--PSNIIV
--PSNIHM
--PSNIHG
--PSNIGA
--PSNIFW
--PSNIFD
--PSNIQT

--PSNIMV
--PSNIMM
--PSNIIF
--PSNIID
--PSNIIA
--PSNIEM
--PSNIAS
--PSNIAI
--PSNIAH
--PSNIYQ
--PSNIVV
--PSNITQ
--PSNIQY
--PSNIQH
--PSNINN
--PSNIMI
--PSNIKM
--PSNIKL
--PSNIKK
--PSNIKF
--PSNIIQ
--PSNIIG
--PSNIGV
--PSNIGT
--PSNIGF
--PSNIFE
--PSNIFA
PIPSNI--
--PSNIYY
--PSNIWD
--PSNIQI
--PSNINW
--PSNINV
--PSNINM
--PSNIMN
--PSNILE
--PSNIKW
--PSNIKD
--PSNIHK
--PSNIGY
--PSNIEV
--PSNIEL
--PSNICL
--PSNIAR
--PSNIAF
--PSNITH
--PSNITF
--PSNISD
--PSNIRD
--PSNIRC

--PSNING
--PSNIME
--PSNIMD
--PSNIIW
--PSNIIT
--PSNIII
--PSNIIE
--PSNIHN
--PSNIHD
--PSNIGG
--PSNIEY
--PSNIEN
--PSNIEA
--PSNICS
--PSNIYE
--PSNIWP
--PSNIWE
--PSNITE
--PSNISC
--PSNIQQ
--PSNIQP
--PSNIQL
--PSNIQD
--PSNINT
--PSNINH
--PSNINF
--PSNIMP
--PSNIMH
--PSNIKV
--PSNIHL
--PSNIGW
--PSNIGN
--PSNIET
--PSNIEK
--PSNIEI
--PSNIEF
--PSNIED
--PSNIDV
--PSNIDS
--PSNIDG
--PSNICK
--PSNIAK
--PSNIAG
-PPSNIW-
-PPSNIS-
-PPSNIQ-
PPPSNI--
PDPSNI--
PCPSNI--
-QPSNIR-

-QPSNIF-
QRPSNI--
-QPSNIS-
-QPSNIT-
QSPSNI--
QAPSNI--
-QPSNIK-
QWPSNI--
QFPSNI--
QNPSNI--
-QPSNIW-
QTPSNI--
-QPSNII-
-QPSNIH-
QYPSNI--
-QPSNIV-
-QPSNIL-
-QPSNIY-
-QPSNIM-
-QPSNIE-
QLPSNI--
QGPSNI--
QHPSNI--
-QPSNIG-
-QPSNIA-
QDPSNI--
QKPSNI--
QEPSNI--
-RPSNIF-
RLPSNI--
-RPSNIM-
-RPSNIL-
RRPSNI--
RNPSNI--
RVPSNI--
-RPSNIW-
RSPSNI--
-RPSNIY-
-RPSNIS-
-RPSNII-
RWPSNI--
-RPSNIA-
-RPSNIH-
RHPSNI--
-RPSNIT-
RYPSNI--
RIPSNI--
-RPSNIQ-
RKPSNI--
-RPSNIV-

RPPSNI--
-RPSNIN-
RTPSNI--
RDPSNI--
-RPSNIG-
-RPSNIE-
RCPSNI--
RQPSNI--
-SPSNII-
SSPSNI--
SRPSNI--
-SPSNIR-
-SPSNIF-
SVPSNI--
SGPSNI--
SLPSNI--
SYPSNI--
STPSNI--
-SPSNIT-
-SPSNIY-
-SPSNIS-
SKPSNI--
SHPSNI--
SAPSNI--
-SPSNIA-
SMPSNI--
-SPSNIW-
SQPSNI--
-SPSNIQ-
SFPSNI--
-SPSNIM-
SPPSNI--
-SPSNIV-
SEPSNI--
SWPSNI--
-SPSNIK-
-SPSNIE-
SNPSNI--
-SPSNIH-
-SPSNIG-
SCPSNI--
TSPSNI--
TRPSNI--
TVPSNI--
TFPSNI--
TYPSNI--
-TPSNIL-
TAPSNI--
-TPSNII-
TMPSNI--

TTPSNI--
TKPSNI--
-TPSNIV-
TEPSNI--
THPSNI--
-TPSNIM-
TWPSNI--
-TPSNIQ-
-TPSNIF-
TGPSNI--
-TPSNIY-
TIPSNI--
-TPSNIS-
TPPSNI--
TLPSNI--
TQPSNI--
-TPSNIG-
-TPSNIE-
TCPSNI--
TDPSNI--
-TPSNIH-
-TPSNIW-
-TPSNIN-
-TPSNIK-
-TPSNIA-
TNPSNI--
-VPSNIR-
VLPSNI--
VSPSNI--
VTPSNI--
-VPSNIL-
VAPSNI--
VRPSNI--
VVPSNI--
VMPSNI--
-VPSNIK-
-VPSNIS-
VPPSNI--
VNPSNI--
-VPSNIH-
VFPSNI--
VWPSNI--
-VPSNII-
-VPSNIT-
-VPSNIA-
VIPSNI--
VQPSNI--
-VPSNIW-
-VPSNIM-
-VPSNIG-

-VPSNIY-
-VPSNIE-
VKPSNI--
-VPSNIQ-
VEPSNI--
VHPSNI--
VYPSNI--
-VPSNIN-
WQPSNI--
WLPSNI--
WSPSNI--
WPPSNI--
WFPSNI--
WWPSNI--
-WPSNII-
WTPSNI--
-WPSNIS-
WKPSNI--
WYPSNI--
WVPSNI--
WMPSNI--
-WPSNIH-
WAPSNI--
-WPSNIL-
WIPSNI--
-WPSNIT-
-WPSNIA-
WNPSNI--
WDPSNI--
WRPSNI--
-WPSNIY-
-WPSNIV-
-WPSNIM-
-WPSNIF-
WHPSNI--
-WPSNIW-
-WPSNIK-
YRPSNI--
YSPSNI--
YAPSNI--
YVPSNI--
YLPSNI--
-YPSNIR-
YGPSNI--
YTPSNI--
YMPSNI--
YEPSNI--
YPPSNI--
-YPSNIK-
YFPSNI--

```
               YYPSNI--
               -YPSNIS-
               -YPSNII-
               -YPSNIM-
               -YPSNIW-
               -YPSNIQ-
               YKPSNI--
               -YPSNIV-
               -YPSNIA-
               YDPSNI--
               YQPSNI--
               -YPSNIH-
               YNPSNI--
               -YPSNIT-
               YIPSNI--
               YCPSNI--
               -YPSNIN-
PVGY             60         847      45.44
               --PVGYGY
               -SPVGYQ-
               -YPVGYG-
               AGPVGY--
               AVPVGY--
               ARPVGY--
               -APVGYI-
               AHPVGY--
               -APVGYV-
               ALPVGY--
               AEPVGY--
               ANPVGY--
               ASPVGY--
               -APVGYT-
               -APVGYL-
               AQPVGY--
               AAPVGY--
               -APVGYF-
               -APVGYW-
               -APVGYA-
               AKPVGY--
               ATPVGY--
               -APVGYM-
               AIPVGY--
               APPVGY--
               AMPVGY--
               -APVGYY-
               -APVGYQ-
               -APVGYS-
               ADPVGY--
               AWPVGY--
               -APVGYK-
```

AFPVGY--
-APVGYN-
-APVGYH-
ACPVGY--
AYPVGY--
CCPVGY--
CSPVGY--
CGPVGY--
CVPVGY--
-CPVGYR-
CFPVGY--
CEPVGY--
CDPVGY--
-CPVGYV-
CMPVGY--
CTPVGY--
-CPVGYI-
-CPVGYF-
CLPVGY--
CAPVGY--
DRPVGY--
DSPVGY--
DQPVGY--
-DPVGYR-
DKPVGY--
DGPVGY--
DFPVGY--
DYPVGY--
DTPVGY--
DVPVGY--
-DPVGYS-
DHPVGY--
-DPVGYW-
DNPVGY--
DAPVGY--
-DPVGYY-
-DPVGYV-
-DPVGYM-
-DPVGYK-
-DPVGYF-
DIPVGY--
DEPVGY--
-DPVGYI-
DLPVGY--
-DPVGYL-
-DPVGYH-
DDPVGY--
-DPVGYT-
DCPVGY--
ESPVGY--

ELPVGY--
EYPVGY--
-EPVGYV-
-EPVGYS-
EVPVGY--
-EPVGYL-
EAPVGY--
-EPVGYM-
-EPVGYY-
-EPVGYW-
-EPVGYA-
-EPVGYN-
-EPVGYH-
EIPVGY--
-EPVGYF-
ERPVGY--
-EPVGYI-
-EPVGYE-
EGPVGY--
ETPVGY--
-EPVGYQ-
-EPVGYK-
EWPVGY--
EKPVGY--
-EPVGYT-
ENPVGY--
EHPVGY--
FRPVGY--
-FPVGYR-
FLPVGY--
-FPVGYH-
-FPVGYM-
FQPVGY--
FPPVGY--
FGPVGY--
-FPVGYS-
FKPVGY--
FAPVGY--
FFPVGY--
FVPVGY--
-FPVGYY-
FNPVGY--
FSPVGY--
-FPVGYL-
FWPVGY--
-FPVGYV-
-FPVGYI-
FMPVGY--
FIPVGY--
FTPVGY--

-FPVGYK-
-FPVGYA-
FYPVGY--
-FPVGYW-
-FPVGYN-
-FPVGYF-
-FPVGYT-
-FPVGYQ-
FHPVGY--
-GPVGYL-
-GPVGYR-
GVPVGY--
GSPVGY--
GLPVGY--
GGPVGY--
-GPVGYI-
-GPVGYT-
-GPVGYM-
-GPVGYH-
GAPVGY--
-GPVGYY-
-GPVGYQ-
GHPVGY--
GRPVGY--
GIPVGY--
-GPVGYK-
-GPVGYW-
GKPVGY--
-GPVGYV-
GEPVGY--
GWPVGY--
GMPVGY--
GYPVGY--
-GPVGYA-
GTPVGY--
-GPVGYN-
GPPVGY--
-GPVGYF-
GQPVGY--
GDPVGY--
GCPVGY--
-GPVGYE-
-GPVGYC-
GNPVGY--
HRPVGY--
-HPVGYW-
-HPVGYR-
HMPVGY--
HIPVGY--
-HPVGYS-

HQPVGY--
HEPVGY--
-HPVGYL-
-HPVGYM-
HVPVGY--
-HPVGYT-
-HPVGYQ-
HAPVGY--
-HPVGYV-
HLPVGY--
HYPVGY--
-HPVGYY-
-HPVGYK-
-HPVGYN-
HHPVGY--
HGPVGY--
HNPVGY--
-HPVGYI-
-HPVGYH-
-HPVGYF-
HKPVGY--
HWPVGY--
HTPVGY--
HPPVGY--
HCPVGY--
-IPVGYR-
-IPVGYL-
ISPVGY--
IAPVGY--
-IPVGYM-
-IPVGYV-
IMPVGY--
-IPVGYA-
IFPVGY--
-IPVGYI-
-IPVGYT-
ILPVGY--
IRPVGY--
IHPVGY--
-IPVGYK-
IYPVGY--
IVPVGY--
IKPVGY--
IDPVGY--
IGPVGY--
-IPVGYS-
-IPVGYE-
IPPVGY--
INPVGY--
-IPVGYH-

IWPVGY--
IQPVGY--
-IPVGYY-
-IPVGYW-
-IPVGYF-
IEPVGY--
-IPVGYN-
IIPVGY--
-KPVGYR-
-KPVGYH-
KGPVGY--
-KPVGYY-
-KPVGYM-
KYPVGY--
KTPVGY--
-KPVGYI-
-KPVGYW-
-KPVGYS-
KHPVGY--
KWPVGY--
KMPVGY--
KVPVGY--
-KPVGYV-
-KPVGYN-
-KPVGYL-
-KPVGYF-
KKPVGY--
KIPVGY--
KFPVGY--
-KPVGYT-
-KPVGYK-
KLPVGY--
KQPVGY--
KPPVGY--
KAPVGY--
-KPVGYQ-
-KPVGYG-
-KPVGYA-
KCPVGY--
LNPVGY--
LSPVGY--
LGPVGY--
LIPVGY--
LLPVGY--
LRPVGY--
LAPVGY--
LQPVGY--
-LPVGYK-
-LPVGYF-
LHPVGY--

-LPVGYL-
LYPVGY--
-LPVGYI-
LTPVGY--
LFPVGY--
LVPVGY--
-LPVGYT-
LWPVGY--
LPPVGY--
-LPVGYS-
-LPVGYH-
LEPVGY--
LKPVGY--
-LPVGYQ-
-LPVGYN-
LDPVGY--
-LPVGYW-
-LPVGYV-
-LPVGYY-
-LPVGYG-
-LPVGYA-
MRPVGY--
-MPVGYT-
MSPVGY--
MNPVGY--
MLPVGY--
-MPVGYM-
-MPVGYL-
-MPVGYR-
-MPVGYI-
MKPVGY--
MGPVGY--
MWPVGY--
-MPVGYY-
-MPVGYH-
-MPVGYS-
MEPVGY--
-MPVGYW-
MPPVGY--
MVPVGY--
MIPVGY--
MHPVGY--
-MPVGYF-
-MPVGYA-
MMPVGY--
MFPVGY--
MQPVGY--
-MPVGYK-
-MPVGYQ-
-MPVGYN-

-MPVGYE-
NSPVGY--
NLPVGY--
NTPVGY--
NPPVGY--
NMPVGY--
NHPVGY--
NGPVGY--
NAPVGY--
-NPVGYI-
NRPVGY--
NFPVGY--
-NPVGYS-
NYPVGY--
NWPVGY--
NVPVGY--
-NPVGYV-
-NPVGYT-
-NPVGYL-
NEPVGY--
-NPVGYW-
-NPVGYF-
-NPVGYM-
NNPVGY--
NIPVGY--
-NPVGYQ-
-NPVGYH-
NDPVGY--
NQPVGY--
-NPVGYY-
NKPVGY--
-NPVGYA-
-NPVGYN-
-NPVGYK-
--PVGYLS
--PVGYRS
--PVGYRA
--PVGYRT
--PVGYLG
--PVGYLT
--PVGYRI
--PVGYSA
--PVGYKS
--PVGYRL
--PVGYTR
--PVGYRV
--PVGYAS
--PVGYSS
--PVGYSV
--PVGYRN

--PVGYLA
--PVGYAA
--PVGYHT
--PVGYVG
--PVGYRM
--PVGYLH
--PVGYSQ
--PVGYRY
--PVGYAG
PGPVGY--
--PVGYHV
--PVGYMS
--PVGYMG
--PVGYLV
--PVGYYS
--PVGYSW
--PVGYSG
--PVGYGS
--PVGYFH
--PVGYSI
--PVGYRG
PSPVGY--
--PVGYSF
PVPVGY--
--PVGYVT
--PVGYMV
--PVGYKG
--PVGYAW
--PVGYVY
PRPVGY--
--PVGYSK
--PVGYLR
--PVGYIA
--PVGYHM
--PVGYVV
--PVGYTF
--PVGYSN
--PVGYWR
--PVGYTL
--PVGYST
--PVGYVS
--PVGYTQ
--PVGYIG
--PVGYWN
--PVGYWH
--PVGYVF
--PVGYTS
--PVGYKA
--PVGYAT
--PVGYWA

--PVGYHH
--PVGYYG
--PVGYWM
--PVGYWG
--PVGYWD
--PVGYTV
--PVGYSM
--PVGYNS
--PVGYNA
--PVGYML
--PVGYFS
--PVGYVA
--PVGYRK
--PVGYRD
--PVGYLQ
--PVGYMW
--PVGYKT
--PVGYHG
--PVGYHF
--PVGYFI
--PVGYTN
--PVGYLN
--PVGYKW
--PVGYHN
--PVGYFG
PWPVGY--
--PVGYWS
--PVGYVM
--PVGYRQ
--PVGYMA
--PVGYLW
--PVGYHS
--PVGYGG
--PVGYYH
--PVGYWT
--PVGYVW
--PVGYQS
--PVGYQN
--PVGYNM
--PVGYMK
--PVGYIN
--PVGYHL
--PVGYFT
--PVGYYN
--PVGYVK
--PVGYTA
--PVGYRE
--PVGYMH
--PVGYIS
--PVGYIH

--PVGYHW
--PVGYTY
--PVGYHR
--PVGYAL
PQPVGY--
--PVGYMI
--PVGYMF
--PVGYLI
--PVGYKM
--PVGYIQ
--PVGYHQ
--PVGYFQ
--PVGYFK
--PVGYAN
--PVGYYA
--PVGYTI
--PVGYNR
--PVGYNL
--PVGYMQ
--PVGYLK
--PVGYIW
--PVGYGA
--PVGYKK
--PVGYKH
--PVGYIY
--PVGYII
--PVGYES
--PVGYAM
--PVGYYV
--PVGYVL
--PVGYRW
--PVGYQT
--PVGYFA
--PVGYYE
--PVGYVH
--PVGYSY
--PVGYSD
--PVGYQV
--PVGYNK
--PVGYLM
--PVGYKY
--PVGYHA
--PVGYGV
--PVGYGT
--PVGYFN
--PVGYEG
--PVGYAR
--PVGYTM
--PVGYTK
--PVGYNV

--PVGYKL
--PVGYIV
--PVGYHK
--PVGYEY
--PVGYAY
PKPVGY--
--PVGYYM
--PVGYYD
--PVGYWK
--PVGYVI
--PVGYTG
--PVGYRF
--PVGYQY
--PVGYQR
--PVGYQM
--PVGYNY
--PVGYNW
--PVGYNF
--PVGYMD
--PVGYKI
--PVGYHY
--PVGYHI
--PVGYAH
PAPVGY--
--PVGYYK
--PVGYVN
--PVGYTD
--PVGYQL
--PVGYIR
--PVGYIM
--PVGYAF
PMPVGY--
PLPVGY--
PIPVGY--
--PVGYYW
--PVGYYQ
--PVGYVQ
--PVGYTH
--PVGYQI
--PVGYMY
--PVGYMN
--PVGYMM
--PVGYLL
--PVGYKF
--PVGYFD
--PVGYEQ
PTPVGY--
PHPVGY--
--PVGYSC
--PVGYQK

--PVGYNT
--PVGYLD
--PVGYKQ
--PVGYKN
--PVGYIK
--PVGYHE
--PVGYGL
--PVGYDS
--PVGYCG
--PVGYAK
-PPVGYT-
PNPVGY--
PFPVGY--
PYPVGY--
--PVGYWQ
--PVGYWC
--PVGYVD
--PVGYVC
--PVGYSH
--PVGYQH
--PVGYQG
--PVGYQA
--PVGYPT
--PVGYNN
--PVGYNG
--PVGYNE
--PVGYND
--PVGYLP
--PVGYIE
--PVGYGW
--PVGYGI
--PVGYFE
--PVGYFC
--PVGYEV
--PVGYET
--PVGYDA
--PVGYCV
--PVGYCK
--PVGYCE
--PVGYAQ
--PVGYAI
--PVGYAE
-PPVGYV-
-PPVGYI-
-PPVGYA-
PEPVGY--
-QPVGYR-
QRPVGY--
QIPVGY--
-QPVGYS-

QNPVGY--
QAPVGY--
QLPVGY--
-QPVGYL-
QGPVGY--
QVPVGY--
-QPVGYW-
QKPVGY--
QSPVGY--
-QPVGYT-
-QPVGYY-
QTPVGY--
-QPVGYM-
QHPVGY--
QFPVGY--
QQPVGY--
-QPVGYF-
QPPVGY--
QEPVGY--
QWPVGY--
-QPVGYV-
-QPVGYN-
-QPVGYK-
QMPVGY--
-QPVGYH-
-QPVGYA-
QYPVGY--
-QPVGYI-
QDPVGY--
-RPVGYR-
-RPVGYS-
-RPVGYM-
RTPVGY--
RNPVGY--
-RPVGYH-
RLPVGY--
RAPVGY--
RMPVGY--
RVPVGY--
RGPVGY--
RYPVGY--
-RPVGYK-
-RPVGYN-
RWPVGY--
RDPVGY--
RPPVGY--
REPVGY--
RQPVGY--
RIPVGY--
RFPVGY--

RHPVGY--
-RPVGYF-
-RPVGYW-
RCPVGY--
-RPVGYG-
-RPVGYE-
SRPVGY--
SSPVGY--
-SPVGYS-
STPVGY--
SKPVGY--
SYPVGY--
-SPVGYL-
-SPVGYH-
SGPVGY--
SQPVGY--
-SPVGYM-
SVPVGY--
-SPVGYY-
-SPVGYV-
SPPVGY--
SMPVGY--
SHPVGY--
SWPVGY--
SNPVGY--
SDPVGY--
-SPVGYI-
-SPVGYA-
SEPVGY--
SAPVGY--
-SPVGYK-
SFPVGY--
SCPVGY--
-SPVGYF-
-SPVGYG-
-SPVGYW-
SIPVGY--
-SPVGYE-
-TPVGYR-
TVPVGY--
-TPVGYM-
TAPVGY--
TRPVGY--
TGPVGY--
TSPVGY--
TNPVGY--
-TPVGYF-
THPVGY--
TWPVGY--
TLPVGY--

−TPVGYS−
−TPVGYL−
TPPVGY−−
−TPVGYI−
TMPVGY−−
TTPVGY−−
TIPVGY−−
TYPVGY−−
−TPVGYT−
−TPVGYH−
−TPVGYN−
−TPVGYK−
TQPVGY−−
TFPVGY−−
TDPVGY−−
−TPVGYY−
−TPVGYQ−
−TPVGYW−
TCPVGY−−
−TPVGYA−
TEPVGY−−
−TPVGYP−
−TPVGYE−
−VPVGYR−
VSPVGY−−
−VPVGYY−
VLPVGY−−
−VPVGYT−
VAPVGY−−
−VPVGYH−
−VPVGYA−
−VPVGYS−
VHPVGY−−
VNPVGY−−
−VPVGYV−
VMPVGY−−
VEPVGY−−
VIPVGY−−
VFPVGY−−
VWPVGY−−
VVPVGY−−
−VPVGYM−
VYPVGY−−
VPPVGY−−
−VPVGYK−
VGPVGY−−
−VPVGYI−
−VPVGYG−
VDPVGY−−
VQPVGY−−

-VPVGYQ-
-VPVGYN-
VCPVGY--
-VPVGYF-
-VPVGYE-
WSPVGY--
-WPVGYR-
WEPVGY--
-WPVGYS-
WRPVGY--
WLPVGY--
-WPVGYT-
-WPVGYI-
WAPVGY--
WVPVGY--
-WPVGYK-
WIPVGY--
WPPVGY--
WKPVGY--
WQPVGY--
WFPVGY--
WWPVGY--
WGPVGY--
-WPVGYM-
WHPVGY--
WMPVGY--
WNPVGY--
WYPVGY--
-WPVGYV-
-WPVGYF-
-WPVGYY-
-WPVGYL-
-WPVGYA-
-WPVGYG-
WDPVGY--
YRPVGY--
YVPVGY--
-YPVGYR-
YLPVGY--
YSPVGY--
YAPVGY--
-YPVGYI-
YHPVGY--
YNPVGY--
YMPVGY--
-YPVGYS-
-YPVGYH-
YKPVGY--
YWPVGY--
-YPVGYQ-

	-YPVGYL-		
	-YPVGYW-		
	-YPVGYT-		
	YIPVGY--		
	-YPVGYK-		
	YGPVGY--		
	YDPVGY--		
	YYPVGY--		
	-YPVGYA-		
	YCPVGY--		
	YPPVGY--		
	YEPVGY--		
	-YPVGYY-		
	-YPVGYP-		
	-YPVGYN-		
PLAW	174	760	45.43
	-APLAWE-		
	-GPLAWG-		
	--PLAWLP		
	--PLAWGN		
	--PLAWEV		
	-QPLAWG-		
	ASPLAW--		
	ATPLAW--		
	ARPLAW--		
	-APLAWR-		
	AMPLAW--		
	AHPLAW--		
	-APLAWM-		
	-APLAWH-		
	AKPLAW--		
	-APLAWS-		
	APPLAW--		
	-APLAWN-		
	AGPLAW--		
	-APLAWL-		
	-APLAWK-		
	-APLAWT-		
	ALPLAW--		
	AVPLAW--		
	-APLAWA-		
	-APLAWQ-		
	ANPLAW--		
	AAPLAW--		
	AEPLAW--		
	AFPLAW--		
	-APLAWF-		
	AQPLAW--		
	-APLAWW-		
	-APLAWG-		

ADPLAW--
ACPLAW--
AYPLAW--
AWPLAW--
CVPLAW--
CHPLAW--
CTPLAW--
CLPLAW--
-CPLAWT-
-CPLAWI-
CSPLAW--
CQPLAW--
-CPLAWV-
CIPLAW--
CGPLAW--
CCPLAW--
DRPLAW--
-DPLAWL-
DKPLAW--
DSPLAW--
DGPLAW--
DTPLAW--
DAPLAW--
-DPLAWR-
DLPLAW--
-DPLAWM-
DVPLAW--
-DPLAWT-
-DPLAWF-
DYPLAW--
DQPLAW--
DEPLAW--
-DPLAWV-
-DPLAWI-
DMPLAW--
-DPLAWW-
-DPLAWH-
DFPLAW--
-DPLAWS-
DIPLAW--
DHPLAW--
DDPLAW--
-EPLAWV-
ESPLAW--
-EPLAWS-
ERPLAW--
-EPLAWK-
EYPLAW--
EMPLAW--
-EPLAWT-

EVPLAW--
-EPLAWI-
EAPLAW--
-EPLAWW-
-EPLAWM-
ELPLAW--
-EPLAWY-
-EPLAWQ-
EEPLAW--
ETPLAW--
-EPLAWL-
EGPLAW--
EWPLAW--
-EPLAWN-
-EPLAWH-
ENPLAW--
EFPLAW--
-EPLAWA-
FLPLAW--
FAPLAW--
FKPLAW--
-FPLAWS-
-FPLAWR-
-FPLAWK-
FPPLAW--
-FPLAWH-
-FPLAWT-
-FPLAWM-
-FPLAWL-
FHPLAW--
FDPLAW--
-FPLAWV-
-FPLAWA-
FTPLAW--
FQPLAW--
-FPLAWQ-
FIPLAW--
FGPLAW--
-FPLAWI-
FMPLAW--
FFPLAW--
-FPLAWW-
-FPLAWN-
-FPLAWG-
-FPLAWC-
GRPLAW--
GSPLAW--
-GPLAWV-
-GPLAWR-
GNPLAW--

GFPLAW--
-GPLAWT-
-GPLAWS-
GPPLAW--
-GPLAWI-
GHPLAW--
-GPLAWL-
GQPLAW--
-GPLAWK-
GMPLAW--
GYPLAW--
-GPLAWQ-
-GPLAWH-
GVPLAW--
-GPLAWA-
GLPLAW--
GKPLAW--
GAPLAW--
GWPLAW--
GTPLAW--
-GPLAWW-
-GPLAWF-
GIPLAW--
GDPLAW--
-GPLAWY-
GCPLAW--
-GPLAWN-
-GPLAWE-
HGPLAW--
HMPLAW--
-HPLAWR-
HLPLAW--
HRPLAW--
HYPLAW--
-HPLAWQ-
-HPLAWV-
HVPLAW--
-HPLAWK-
HSPLAW--
HKPLAW--
HEPLAW--
-HPLAWL-
HNPLAW--
HFPLAW--
HTPLAW--
-HPLAWW-
HPPLAW--
HQPLAW--
-HPLAWY-
HCPLAW--

HWPLAW--
-HPLAWF-
-HPLAWN-
-HPLAWH-
HIPLAW--
HHPLAW--
-HPLAWT-
HAPLAW--
-IPLAWI-
-IPLAWR-
ISPLAW--
INPLAW--
IRPLAW--
-IPLAWT-
IGPLAW--
ITPLAW--
IPPLAW--
IVPLAW--
-IPLAWS-
-IPLAWF-
IAPLAW--
-IPLAWQ-
-IPLAWK-
-IPLAWH-
IEPLAW--
-IPLAWW-
IYPLAW--
-IPLAWV-
-IPLAWA-
IHPLAW--
-IPLAWE-
IDPLAW--
-IPLAWN-
IMPLAW--
ILPLAW--
IIPLAW--
IQPLAW--
-IPLAWY-
-IPLAWL-
ICPLAW--
KSPLAW--
KGPLAW--
-KPLAWY-
KTPLAW--
KFPLAW--
-KPLAWH-
-KPLAWI-
-KPLAWS-
KYPLAW--
KVPLAW--

-KPLAWM-
-KPLAWK-
-KPLAWL-
KNPLAW--
KQPLAW--
-KPLAWF-
KLPLAW--
KEPLAW--
-KPLAWN-
-KPLAWE-
KHPLAW--
KPPLAW--
-KPLAWV-
KKPLAW--
-KPLAWQ-
-KPLAWG-
-KPLAWA-
KDPLAW--
KAPLAW--
KMPLAW--
LAPLAW--
LSPLAW--
LPPLAW--
LRPLAW--
LIPLAW--
LVPLAW--
LGPLAW--
LTPLAW--
LHPLAW--
LKPLAW--
-LPLAWY-
-LPLAWS-
-LPLAWT-
-LPLAWK-
LWPLAW--
LLPLAW--
LEPLAW--
-LPLAWV-
-LPLAWQ-
LDPLAW--
LYPLAW--
LFPLAW--
LMPLAW--
LQPLAW--
-LPLAWN-
LCPLAW--
-LPLAWH-
MSPLAW--
MGPLAW--
MNPLAW--

-MPLAWL-
MHPLAW--
MVPLAW--
MQPLAW--
MRPLAW--
-MPLAWR-
-MPLAWI-
MKPLAW--
MAPLAW--
-MPLAWA-
-MPLAWV-
-MPLAWS-
-MPLAWN-
MMPLAW--
-MPLAWW-
-MPLAWM-
MDPLAW--
MPPLAW--
-MPLAWQ-
-MPLAWE-
MFPLAW--
MYPLAW--
MTPLAW--
-MPLAWK-
-MPLAWF-
MIPLAW--
-NPLAWR-
NGPLAW--
NRPLAW--
NTPLAW--
NLPLAW--
NHPLAW--
NYPLAW--
NSPLAW--
NKPLAW--
NMPLAW--
NFPLAW--
NNPLAW--
NEPLAW--
NIPLAW--
NAPLAW--
-NPLAWW-
-NPLAWH-
NWPLAW--
NVPLAW--
NQPLAW--
-NPLAWK-
-NPLAWE-
-NPLAWA-
-NPLAWV-

–NPLAWQ–
––PLAWAL
––PLAWVT
––PLAWRN
––PLAWYS
––PLAWAS
––PLAWST
––PLAWRS
––PLAWTL
––PLAWLS
––PLAWSW
––PLAWRA
––PLAWLN
––PLAWYN
––PLAWTS
––PLAWSL
––PLAWLG
––PLAWSA
––PLAWTT
––PLAWGL
––PLAWFG
––PLAWTR
––PLAWVS
––PLAWQS
––PLAWQR
––PLAWPL
––PLAWKA
––PLAWMQ
––PLAWYR
––PLAWSQ
––PLAWWS
––PLAWWA
––PLAWRT
––PLAWIG
––PLAWGI
––PLAWMT
––PLAWHG
––PLAWVG
––PLAWMR
––PLAWLA
––PLAWFH
––PLAWNA
––PLAWIS
––PLAWYT
––PLAWVN
––PLAWSF
––PLAWLH
––PLAWHT
––PLAWTN
––PLAWRY

--PLAWNT
--PLAWNL
--PLAWMN
--PLAWLT
--PLAWGY
--PLAWVI
--PLAWSV
--PLAWSI
--PLAWSH
--PLAWKT
--PLAWGS
--PLAWEA
PGPLAW--
--PLAWTA
--PLAWRW
--PLAWKW
--PLAWRG
--PLAWIA
--PLAWAT
--PLAWTK
--PLAWKV
--PLAWHQ
--PLAWGA
--PLAWWR
--PLAWTY
--PLAWNV
--PLAWNM
--PLAWKG
--PLAWHS
--PLAWAV
--PLAWAN
PDPLAW--
--PLAWWG
--PLAWTM
--PLAWTG
--PLAWTE
--PLAWSM
--PLAWML
--PLAWKS
--PLAWFR
--PLAWWL
--PLAWVK
--PLAWTI
--PLAWSR
--PLAWMS
--PLAWLK
--PLAWFE
--PLAWWT
--PLAWIT
--PLAWIN

--PLAWIH
--PLAWFA
--PLAWYY
--PLAWTV
--PLAWRV
--PLAWQV
--PLAWNS
--PLAWKL
--PLAWAM
--PLAWTW
--PLAWNQ
--PLAWMV
--PLAWMA
--PLAWKH
--PLAWKF
--PLAWHN
--PLAWAR
--PLAWYG
--PLAWVQ
--PLAWRH
--PLAWQD
--PLAWNN
--PLAWMK
--PLAWIK
--PLAWID
--PLAWFK
--PLAWWN
--PLAWRQ
--PLAWNK
--PLAWHH
--PLAWGT
PTPLAW--
--PLAWWQ
--PLAWWI
--PLAWVY
--PLAWVE
--PLAWTQ
--PLAWSN
--PLAWSG
--PLAWRF
--PLAWRE
--PLAWPM
--PLAWPI
--PLAWMH
--PLAWMD
--PLAWLQ
--PLAWIV
--PLAWIQ
--PLAWES
--PLAWEL

--PLAWAY
--PLAWYD
--PLAWWV
--PLAWTD
--PLAWQG
--PLAWNY
--PLAWNW
--PLAWLD
--PLAWKD
--PLAWII
--PLAWHD
--PLAWGV
--PLAWGM
--PLAWAQ
--PLAWAI
PEPLAW--
--PLAWYQ
--PLAWVD
--PLAWSY
--PLAWSK
--PLAWSE
--PLAWQK
--PLAWQH
--PLAWMM
--PLAWKM
--PLAWKE
--PLAWIY
--PLAWIW
--PLAWHF
--PLAWHE
--PLAWHA
--PLAWGF
--PLAWFQ
--PLAWFD
--PLAWEI
--PLAWDS
--PLAWAH
--PLAWAA
--PLAWWD
--PLAWQN
--PLAWKQ
--PLAWIM
--PLAWIF
--PLAWIE
--PLAWHY
--PLAWGH
--PLAWGG
--PLAWER
PCPLAW--
-PPLAWR-

-PPLAWI-
PNPLAW--
--PLAWYF
--PLAWYA
--PLAWWH
--PLAWRD
--PLAWQI
--PLAWNR
--PLAWNI
--PLAWNH
--PLAWMP
--PLAWME
--PLAWLE
--PLAWKY
--PLAWKK
--PLAWKI
--PLAWHP
--PLAWGQ
--PLAWGD
--PLAWGC
--PLAWFY
--PLAWET
--PLAWEN
--PLAWDY
--PLAWCS
--PLAWCA
--PLAWAD
-QPLAWT-
-QPLAWR-
QGPLAW--
-QPLAWI-
QTPLAW--
-QPLAWS-
-QPLAWL-
QVPLAW--
-QPLAWF-
QIPLAW--
-QPLAWA-
QQPLAW--
-QPLAWK-
QMPLAW--
QHPLAW--
QSPLAW--
-QPLAWN-
QAPLAW--
-QPLAWW-
-QPLAWM-
QYPLAW--
QWPLAW--
-QPLAWV-

-QPLAWH-
QDPLAW--
QEPLAW--
QCPLAW--
RSPLAW--
RGPLAW--
-RPLAWR-
RAPLAW--
RPPLAW--
RTPLAW--
-RPLAWS-
RLPLAW--
RKPLAW--
RMPLAW--
-RPLAWT-
RIPLAW--
-RPLAWM-
-RPLAWI-
-RPLAWV-
-RPLAWQ-
-RPLAWH-
RVPLAW--
-RPLAWW-
RFPLAW--
-RPLAWN-
-RPLAWF-
RDPLAW--
RYPLAW--
-RPLAWY-
-RPLAWA-
REPLAW--
RWPLAW--
RCPLAW--
-RPLAWE-
RHPLAW--
-SPLAWR-
-SPLAWK-
SKPLAW--
-SPLAWV-
SEPLAW--
-SPLAWH-
SLPLAW--
-SPLAWS-
SSPLAW--
SMPLAW--
SQPLAW--
STPLAW--
SNPLAW--
SFPLAW--
SPPLAW--

-SPLAWI-
SHPLAW--
-SPLAWN-
-SPLAWY-
-SPLAWT-
-SPLAWF-
SDPLAW--
SIPLAW--
SYPLAW--
-SPLAWM-
-SPLAWW-
SWPLAW--
-SPLAWQ-
-SPLAWE-
-TPLAWL-
TNPLAW--
TSPLAW--
TKPLAW--
TTPLAW--
-TPLAWV-
TMPLAW--
TLPLAW--
TPPLAW--
-TPLAWT-
-TPLAWR-
-TPLAWM-
TAPLAW--
-TPLAWI-
-TPLAWH-
TQPLAW--
THPLAW--
-TPLAWW-
-TPLAWK-
TIPLAW--
TGPLAW--
TFPLAW--
TYPLAW--
TEPLAW--
-TPLAWY-
-TPLAWN-
-TPLAWQ-
TVPLAW--
-TPLAWE-
-TPLAWA-
TCPLAW--
-TPLAWG-
TDPLAW--
VGPLAW--
VKPLAW--
-VPLAWH-

```
-VPLAWV-
-VPLAWS-
VVPLAW--
-VPLAWM-
VLPLAW--
VWPLAW--
VRPLAW--
-VPLAWW-
-VPLAWL-
VYPLAW--
VSPLAW--
-VPLAWA-
-VPLAWF-
VAPLAW--
-VPLAWI-
-VPLAWN-
VFPLAW--
VTPLAW--
VMPLAW--
-VPLAWT-
-VPLAWQ-
VQPLAW--
VPPLAW--
VHPLAW--
VNPLAW--
-VPLAWG-
VDPLAW--
VCPLAW--
WAPLAW--
WSPLAW--
WDPLAW--
-WPLAWH-
-WPLAWK-
WWPLAW--
-WPLAWL-
-WPLAWS-
-WPLAWM-
WTPLAW--
-WPLAWR-
WNPLAW--
WEPLAW--
WQPLAW--
WPPLAW--
-WPLAWI-
-WPLAWQ-
-WPLAWE-
-WPLAWA-
WMPLAW--
-WPLAWV-
-YPLAWR-
```

```
          YSPLAW--
          -YPLAWL-
          YGPLAW--
          -YPLAWH-
          YLPLAW--
          YKPLAW--
          YYPLAW--
          -YPLAWQ-
          YRPLAW--
          -YPLAWA-
          -YPLAWS-
          YIPLAW--
          -YPLAWT-
          -YPLAWN-
          YTPLAW--
          -YPLAWM-
          -YPLAWK-
          YDPLAW--
          YHPLAW--
          YAPLAW--
          YQPLAW--
          YNPLAW--
          YEPLAW--
          YMPLAW--
          -YPLAWV-
          YFPLAW--
PSNM        68        835     45.39
          -DPSNMS-
          -FPSNMG-
          -RPSNMC-
          -APSNML-
          -APSNMY-
          AYPSNM--
          -APSNMS-
          -APSNMK-
          ARPSNM--
          -APSNMR-
          ATPSNM--
          AGPSNM--
          -APSNMA-
          -APSNMT-
          ALPSNM--
          AEPSNM--
          AVPSNM--
          ASPSNM--
          -APSNMH-
          AHPSNM--
          -APSNMI-
          -APSNMG-
          AAPSNM--
```

-APSNMV-
ANPSNM--
-APSNME-
APPSNM--
AWPSNM--
-APSNMQ-
-APSNMN-
-APSNMD-
AIPSNM--
AFPSNM--
-APSNMM-
AMPSNM--
ADPSNM--
CRPSNM--
CQPSNM--
CVPSNM--
CGPSNM--
CWPSNM--
CIPSNM--
CSPSNM--
-CPSNMM-
CNPSNM--
-CPSNMV-
-CPSNMT-
CPPSNM--
-CPSNMY-
-CPSNMW-
-CPSNML-
CAPSNM--
DFPSNM--
DRPSNM--
DVPSNM--
DIPSNM--
DAPSNM--
DWPSNM--
-DPSNMV-
DQPSNM--
-DPSNMR-
DMPSNM--
DTPSNM--
-DPSNMM-
-DPSNML-
-DPSNMI-
-DPSNMF-
DGPSNM--
DCPSNM--
-DPSNMW-
-DPSNMT-
-DPSNMK-
DSPSNM--

-DPSNMQ-
DNPSNM--
DHPSNM--
EVPSNM--
-EPSNML-
-EPSNMS-
-EPSNMT-
ELPSNM--
EYPSNM--
-EPSNMY-
-EPSNMN-
-EPSNMM-
-EPSNMK-
EIPSNM--
-EPSNMQ-
EMPSNM--
ETPSNM--
EQPSNM--
-EPSNMV-
-EPSNMR-
-EPSNMI-
-EPSNMG-
-EPSNME-
-EPSNMH-
-EPSNMA-
ENPSNM--
EGPSNM--
EAPSNM--
FIPSNM--
-FPSNMS-
-FPSNML-
FYPSNM--
FVPSNM--
FMPSNM--
-FPSNMR-
FPPSNM--
FEPSNM--
-FPSNMA-
-FPSNMK-
FLPSNM--
FAPSNM--
FWPSNM--
-FPSNMQ-
-FPSNMT-
-FPSNMI-
-FPSNMH-
FHPSNM--
FGPSNM--
FTPSNM--
FSPSNM--

FRPSNM--
-FPSNMV-
-FPSNMM-
FFPSNM--
-FPSNMN-
FNPSNM--
FKPSNM--
FDPSNM--
-FPSNMW-
-GPSNML-
GRPSNM--
GVPSNM--
GWPSNM--
-GPSNMW-
-GPSNMV-
-GPSNMY-
-GPSNMF-
GYPSNM--
-GPSNMT-
-GPSNMS-
-GPSNMR-
GLPSNM--
-GPSNMI-
GSPSNM--
GGPSNM--
GNPSNM--
-GPSNMM-
-GPSNME-
GMPSNM--
GIPSNM--
-GPSNMA-
GHPSNM--
GFPSNM--
GQPSNM--
-GPSNMG-
GDPSNM--
-GPSNMQ-
-GPSNMN-
-GPSNMK-
-GPSNMH-
GKPSNM--
GPPSNM--
GAPSNM--
HLPSNM--
HRPSNM--
-HPSNMV-
HPPSNM--
HMPSNM--
-HPSNMS-
-HPSNMW-

HQPSNM--
-HPSNML-
-HPSNMT-
-HPSNMK-
-HPSNMY-
-HPSNMQ-
-HPSNMI-
HSPSNM--
HGPSNM--
HFPSNM--
HYPSNM--
-HPSNMR-
-HPSNMA-
HNPSNM--
HWPSNM--
-HPSNMF-
HHPSNM--
HVPSNM--
HTPSNM--
HAPSNM--
-HPSNMM-
HKPSNM--
HIPSNM--
ILPSNM--
-IPSNMR-
IRPSNM--
-IPSNMT-
-IPSNMH-
-IPSNML-
IKPSNM--
ITPSNM--
-IPSNMV-
IAPSNM--
-IPSNMS-
IYPSNM--
IWPSNM--
IVPSNM--
ISPSNM--
IQPSNM--
-IPSNMY-
-IPSNMG-
IHPSNM--
IEPSNM--
IFPSNM--
-IPSNMM-
-IPSNMI-
IPPSNM--
-IPSNMN-
-IPSNMF-
IMPSNM--

```
-IPSNMW-
-IPSNMQ-
-IPSNMK-
-IPSNMA-
IIPSNM--
IGPSNM--
KSPSNM--
-KPSNMS-
-KPSNML-
-KPSNMV-
KAPSNM--
KTPSNM--
KLPSNM--
KGPSNM--
-KPSNMT-
KIPSNM--
KYPSNM--
-KPSNMK-
KWPSNM--
-KPSNMR-
KHPSNM--
KQPSNM--
-KPSNMW-
KDPSNM--
KRPSNM--
-KPSNMH-
-KPSNMA-
KNPSNM--
KVPSNM--
-KPSNMY-
-KPSNMF-
-KPSNMM-
KPPSNM--
KFPSNM--
-KPSNMQ-
-KPSNMI-
-KPSNME-
KMPSNM--
KCPSNM--
-KPSNMG-
KEPSNM--
LLPSNM--
-LPSNMM-
LRPSNM--
LAPSNM--
-LPSNMW-
LHPSNM--
LSPSNM--
-LPSNMI-
-LPSNMT-
```

-LPSNMR-
LTPSNM--
-LPSNMS-
LGPSNM--
-LPSNMY-
LFPSNM--
LPPSNM--
-LPSNMK-
LIPSNM--
LNPSNM--
LMPSNM--
LVPSNM--
LDPSNM--
-LPSNMF-
-LPSNMA-
LWPSNM--
-LPSNMH-
-LPSNMQ-
-LPSNMG-
-LPSNMN-
-LPSNME-
LEPSNM--
LCPSNM--
-MPSNMT-
MRPSNM--
MLPSNM--
-MPSNMR-
-MPSNMI-
-MPSNMF-
MAPSNM--
-MPSNMM-
-MPSNMK-
MMPSNM--
MHPSNM--
-MPSNMW-
-MPSNMV-
-MPSNMQ-
MKPSNM--
MWPSNM--
-MPSNMS-
MGPSNM--
MFPSNM--
MIPSNM--
MVPSNM--
-MPSNML-
-MPSNMG-
MPPSNM--
-MPSNMH-
MTPSNM--
MDPSNM--

-MPSNMN-
-MPSNME-
-MPSNMA-
MNPSNM--
NRPSNM--
NSPSNM--
-NPSNMR-
NTPSNM--
-NPSNMV-
-NPSNMM-
-NPSNML-
NKPSNM--
NYPSNM--
NWPSNM--
-NPSNMS-
NLPSNM--
-NPSNMY-
NMPSNM--
NFPSNM--
NQPSNM--
-NPSNMT-
NIPSNM--
NGPSNM--
NEPSNM--
-NPSNMA-
-NPSNMQ-
NPPSNM--
NVPSNM--
-NPSNMK-
-NPSNMI-
-NPSNMW-
-NPSNMH-
-NPSNMF-
-NPSNMG-
NNPSNM--
NDPSNM--
NCPSNM--
NAPSNM--
--PSNMVL
--PSNMVG
--PSNMRL
--PSNMRH
--PSNMKS
--PSNMLV
--PSNMLG
--PSNMRG
--PSNMLW
--PSNMTT
PLPSNM--
--PSNMVR

--PSNMMH
--PSNMLR
--PSNMTG
--PSNMSS
--PSNMFA
--PSNMRV
--PSNMKG
--PSNMRR
--PSNMAS
--PSNMMF
--PSNMMS
--PSNMMQ
--PSNMVS
--PSNMFR
--PSNMST
--PSNMMR
--PSNMMN
--PSNMAT
--PSNMLT
--PSNMLA
--PSNMAG
--PSNMYT
--PSNMSM
--PSNMSA
--PSNMRW
--PSNMEV
--PSNMVA
PGPSNM--
--PSNMHS
--PSNMFS
--PSNMWV
--PSNMSY
--PSNMYV
--PSNMLD
--PSNMAW
PRPSNM--
PAPSNM--
--PSNMYF
--PSNMVI
--PSNMVF
--PSNMRE
--PSNMHT
--PSNMFN
PFPSNM--
--PSNMFT
--PSNMFI
--PSNMEG
--PSNMVH
--PSNMRI
--PSNMRF

--PSNMQG
--PSNMLK
--PSNMLI
--PSNMGS
--PSNMTS
--PSNMSV
--PSNMRY
--PSNMRA
--PSNMSI
--PSNMLN
--PSNMLH
--PSNMIY
--PSNMIR
--PSNMFH
--PSNMYS
--PSNMWR
--PSNMVN
--PSNMRM
--PSNMMV
--PSNMLQ
--PSNMIG
--PSNMFL
--PSNMFK
PEPSNM--
--PSNMYN
--PSNMYH
--PSNMTM
--PSNMLS
PVPSNM--
--PSNMWS
--PSNMVD
--PSNMTV
--PSNMRN
--PSNMRK
--PSNMQM
--PSNMMK
--PSNMKI
--PSNMIL
--PSNMII
--PSNMHH
--PSNMHA
--PSNMAV
PHPSNM--
PYPSNM--
PWPSNM--
PSPSNM--
--PSNMVM
--PSNMSF
--PSNMNS
--PSNMMY

--PSNMFW
--PSNMFE
--PSNMAR
--PSNMWT
--PSNMWL
--PSNMWA
--PSNMTW
--PSNMSQ
--PSNMRD
--PSNMQR
--PSNMNV
--PSNMMI
--PSNMMG
--PSNMKT
--PSNMKL
--PSNMKA
--PSNMIT
--PSNMIA
--PSNMGT
PKPSNM--
--PSNMWN
--PSNMVT
--PSNMTR
--PSNMSR
--PSNMSK
--PSNMQN
--PSNMKV
--PSNMKN
--PSNMFQ
--PSNMEY
--PSNMES
--PSNMAA
--PSNMYK
--PSNMTY
--PSNMTN
--PSNMTF
--PSNMSD
--PSNMQY
--PSNMNM
--PSNMKY
--PSNMKW
--PSNMIQ
--PSNMIN
--PSNMIM
--PSNMIF
--PSNMHR
PMPSNM--
--PSNMVQ
--PSNMQL
--PSNMHY

--PSNMHV
--PSNMYY
--PSNMYG
--PSNMYA
--PSNMWK
--PSNMTK
--PSNMSH
--PSNMSG
--PSNMQS
--PSNMQK
--PSNMQF
--PSNMNT
--PSNMNH
--PSNMKM
--PSNMHK
--PSNMHI
--PSNMGW
--PSNMGA
--PSNMFD
--PSNMER
--PSNMEF
--PSNMAL
--PSNMAI
--PSNMAH
PTPSNM--
--PSNMYW
--PSNMWQ
--PSNMWH
--PSNMWG
--PSNMTI
--PSNMSE
--PSNMQW
--PSNMQV
--PSNMMD
--PSNMLM
--PSNMLF
--PSNMKK
--PSNMIV
--PSNMHQ
--PSNMEM
--PSNMEA
--PSNMCD
--PSNMAN
--PSNMAF
PIPSNM--
--PSNMYQ
--PSNMWI
--PSNMWD
--PSNMRQ
--PSNMQT

--PSNMQQ
--PSNMML
--PSNMME
--PSNMKD
--PSNMIH
--PSNMHN
--PSNMHL
--PSNMHD
--PSNMGM
--PSNMGG
--PSNMEQ
--PSNMEL
--PSNMEK
--PSNMEI
--PSNMDA
--PSNMAQ
--PSNMAM
PDPSNM--
--PSNMYD
--PSNMWM
--PSNMVV
--PSNMVK
--PSNMTD
--PSNMQI
--PSNMPS
--PSNMNY
--PSNMNW
--PSNMNQ
--PSNMNK
--PSNMNE
--PSNMID
--PSNMGV
--PSNMEW
--PSNMET
--PSNMEH
--PSNMCG
--PSNMAK
PQPSNM--
-PPSNMS-
QYPSNM--
QVPSNM--
-QPSNMR-
-QPSNMS-
-QPSNMF-
-QPSNMW-
QSPSNM--
QRPSNM--
QHPSNM--
QGPSNM--
-QPSNMK-

QNPSNM--
-QPSNMV-
-QPSNMQ-
-QPSNMM-
-QPSNME-
QMPSNM--
QWPSNM--
-QPSNMY-
-QPSNMT-
QKPSNM--
QCPSNM--
-QPSNMA-
QQPSNM--
QFPSNM--
-QPSNMG-
QLPSNM--
QDPSNM--
QIPSNM--
-RPSNMI-
-RPSNML-
-RPSNMS-
-RPSNMH-
-RPSNMV-
RWPSNM--
RAPSNM--
RNPSNM--
-RPSNMT-
-RPSNMK-
RIPSNM--
RGPSNM--
RDPSNM--
-RPSNMF-
-RPSNMM-
-RPSNMG-
RRPSNM--
-RPSNMY-
-RPSNMW-
-RPSNMA-
RYPSNM--
RKPSNM--
RFPSNM--
-RPSNMN-
-RPSNME-
RPPSNM--
RHPSNM--
REPSNM--
RCPSNM--
RQPSNM--
SRPSNM--
SLPSNM--

-SPSNMR-
SSPSNM--
SQPSNM--
-SPSNMF-
SHPSNM--
-SPSNMY-
-SPSNMT-
SMPSNM--
-SPSNML-
SFPSNM--
SIPSNM--
SVPSNM--
SKPSNM--
STPSNM--
-SPSNMI-
SWPSNM--
SGPSNM--
-SPSNMW-
-SPSNMV-
-SPSNMA-
SPPSNM--
SEPSNM--
SAPSNM--
-SPSNMQ-
-SPSNMN-
-SPSNME-
-SPSNMK-
-SPSNMH-
SCPSNM--
-SPSNMG-
SDPSNM--
-SPSNMM-
-TPSNMS-
-TPSNML-
-TPSNMM-
-TPSNMQ-
-TPSNMA-
TRPSNM--
-TPSNMY-
TTPSNM--
-TPSNMV-
-TPSNMT-
-TPSNMI-
TLPSNM--
THPSNM--
-TPSNMN-
-TPSNMF-
TPPSNM--
TNPSNM--
TSPSNM--

−TPSNMH−
TMPSNM−−
TKPSNM−−
TGPSNM−−
TEPSNM−−
TVPSNM−−
TIPSNM−−
TFPSNM−−
TAPSNM−−
−TPSNMW−
TYPSNM−−
TWPSNM−−
−TPSNMG−
−TPSNMK−
−TPSNMD−
TDPSNM−−
VRPSNM−−
−VPSNMS−
−VPSNML−
VGPSNM−−
VFPSNM−−
−VPSNMR−
−VPSNMI−
VSPSNM−−
VVPSNM−−
VKPSNM−−
VQPSNM−−
VNPSNM−−
VIPSNM−−
VTPSNM−−
−VPSNMK−
VHPSNM−−
−VPSNMW−
−VPSNMA−
VYPSNM−−
VLPSNM−−
VAPSNM−−
−VPSNMT−
−VPSNMQ−
−VPSNMH−
−VPSNMY−
−VPSNMM−
VPPSNM−−
−VPSNMF−
VMPSNM−−
VWPSNM−−
−VPSNME−
VDPSNM−−
−VPSNMG−
−VPSNMD−

-WPSNMR-
WSPSNM--
WRPSNM--
-WPSNMH-
WKPSNM--
-WPSNMY-
-WPSNMK-
WPPSNM--
WVPSNM--
-WPSNMS-
-WPSNMA-
WDPSNM--
WAPSNM--
WEPSNM--
WQPSNM--
-WPSNMW-
-WPSNMV-
-WPSNMM-
-WPSNMI-
WMPSNM--
WGPSNM--
WTPSNM--
-WPSNMQ-
-WPSNMN-
WLPSNM--
-WPSNMC-
WWPSNM--
-WPSNMT-
-WPSNMG-
-WPSNME-
WNPSNM--
WFPSNM--
YSPSNM--
-YPSNMR-
YVPSNM--
YLPSNM--
-YPSNMT-
-YPSNMM-
YYPSNM--
-YPSNMI-
YTPSNM--
-YPSNMS-
YIPSNM--
-YPSNMF-
-YPSNMA-
YKPSNM--
YAPSNM--
-YPSNML-
YFPSNM--
YEPSNM--

	YDPSNM--		
	YRPSNM--		
	YHPSNM--		
	YGPSNM--		
	YPPSNM--		
	YNPSNM--		
	YMPSNM--		
	-YPSNMV-		
	-YPSNMY-		
	-YPSNMQ-		
	-YPSNMN-		
	-YPSNME-		
PHWL	228	732	45.39
	-IPHWLS-		
	--PHWLHR		
	--PHWLEG		
	--PHWLAY		
	-VPHWLN-		
	AVPHWL--		
	ARPHWL--		
	-APHWLR-		
	ASPHWL--		
	AFPHWL--		
	-APHWLA-		
	-APHWLV-		
	-APHWLQ-		
	AAPHWL--		
	ANPHWL--		
	-APHWLL-		
	-APHWLK-		
	AGPHWL--		
	AWPHWL--		
	AYPHWL--		
	-APHWLM-		
	APPHWL--		
	AIPHWL--		
	-APHWLS-		
	-APHWLT-		
	AQPHWL--		
	ALPHWL--		
	-APHWLY-		
	-APHWLH-		
	AEPHWL--		
	ATPHWL--		
	-APHWLI-		
	-APHWLE-		
	ACPHWL--		
	-APHWLW-		
	-APHWLN-		
	AHPHWL--		

ADPHWL--
CVPHWL--
CSPHWL--
CHPHWL--
CTPHWL--
CRPHWL--
CLPHWL--
CKPHWL--
CIPHWL--
CYPHWL--
CWPHWL--
CQPHWL--
DRPHWL--
-DPHWLM-
DQPHWL--
-DPHWLR-
DVPHWL--
DNPHWL--
-DPHWLV-
DPPHWL--
DDPHWL--
DYPHWL--
DGPHWL--
DFPHWL--
DAPHWL--
DSPHWL--
-DPHWLS-
-DPHWLH-
DLPHWL--
EGPHWL--
ESPHWL--
-EPHWLT-
-EPHWLR-
-EPHWLA-
ELPHWL--
EAPHWL--
ETPHWL--
-EPHWLS-
ERPHWL--
EVPHWL--
-EPHWLK-
-EPHWLG-
-EPHWLW-
-EPHWLH-
ENPHWL--
-EPHWLN-
-EPHWLD-
EEPHWL--
FQPHWL--
FVPHWL--

FRPHWL--
FSPHWL--
-FPHWLK-
FTPHWL--
FAPHWL--
-FPHWLS-
FYPHWL--
FPPHWL--
-FPHWLR-
FLPHWL--
-FPHWLV-
FHPHWL--
FEPHWL--
-FPHWLA-
FMPHWL--
-FPHWLL-
-FPHWLH-
FNPHWL--
-FPHWLQ-
-FPHWLM-
FDPHWL--
FGPHWL--
-GPHWLL-
-GPHWLR-
GIPHWL--
-GPHWLQ-
GAPHWL--
GSPHWL--
-GPHWLS-
GVPHWL--
GLPHWL--
GTPHWL--
-GPHWLT-
-GPHWLK-
-GPHWLH-
GHPHWL--
-GPHWLW-
GGPHWL--
-GPHWLV-
GKPHWL--
GQPHWL--
-GPHWLG-
-GPHWLF-
GWPHWL--
GNPHWL--
GFPHWL--
-GPHWLM-
-GPHWLE-
GEPHWL--
GMPHWL--

GRPHWL--
-GPHWLY-
-GPHWLI-
GPPHWL--
GDPHWL--
-GPHWLD-
GCPHWL--
HLPHWL--
-HPHWLT-
HVPHWL--
-HPHWLG-
HKPHWL--
-HPHWLL-
HGPHWL--
HTPHWL--
HSPHWL--
-HPHWLK-
HNPHWL--
HMPHWL--
-HPHWLQ-
-HPHWLS-
HFPHWL--
-HPHWLV-
HDPHWL--
HQPHWL--
-HPHWLA-
HHPHWL--
HYPHWL--
HPPHWL--
-HPHWLF-
HAPHWL--
-HPHWLY-
-HPHWLI-
IIPHWL--
ILPHWL--
IRPHWL--
-IPHWLT-
IGPHWL--
IEPHWL--
ISPHWL--
-IPHWLR-
-IPHWLM-
INPHWL--
IKPHWL--
IQPHWL--
IFPHWL--
-IPHWLV-
-IPHWLQ-
IYPHWL--
-IPHWLL-

IDPHWL--
-IPHWLG-
-IPHWLA-
IPPHWL--
-IPHWLK-
-IPHWLW-
-IPHWLF-
-IPHWLN-
KPPHWL--
KGPHWL--
-KPHWLV-
KYPHWL--
-KPHWLF-
-KPHWLT-
KKPHWL--
KTPHWL--
KRPHWL--
-KPHWLA-
KNPHWL--
KHPHWL--
KMPHWL--
KAPHWL--
KDPHWL--
-KPHWLW-
-KPHWLL-
KEPHWL--
KVPHWL--
-KPHWLN-
-KPHWLQ-
-KPHWLM-
KWPHWL--
KQPHWL--
-KPHWLY-
-KPHWLI-
KLPHWL--
LAPHWL--
LEPHWL--
LRPHWL--
LGPHWL--
LSPHWL--
LVPHWL--
LTPHWL--
-LPHWLT-
LKPHWL--
-LPHWLM-
LLPHWL--
LWPHWL--
-LPHWLR-
LIPHWL--
-LPHWLV-

-LPHWLQ-
-LPHWLK-
-LPHWLA-
LDPHWL--
LPPHWL--
-LPHWLY-
-LPHWLL-
LHPHWL--
LQPHWL--
LNPHWL--
LFPHWL--
-LPHWLW-
-LPHWLH-
-LPHWLG-
LYPHWL--
-LPHWLF-
-LPHWLE-
-MPHWLR-
MRPHWL--
MAPHWL--
MQPHWL--
-MPHWLT-
-MPHWLQ-
MGPHWL--
MLPHWL--
MVPHWL--
MPPHWL--
-MPHWLA-
MWPHWL--
MTPHWL--
-MPHWLV-
MMPHWL--
MIPHWL--
-MPHWLY-
-MPHWLM-
-MPHWLL-
-MPHWLI-
-MPHWLG-
MHPHWL--
-MPHWLK-
-MPHWLF-
-MPHWLW-
-MPHWLS-
-MPHWLN-
-MPHWLH-
MEPHWL--
-MPHWLE-
MDPHWL--
NGPHWL--
NAPHWL--

NMPHWL--
NKPHWL--
NLPHWL--
NSPHWL--
-NPHWLK-
-NPHWLI-
NVPHWL--
NQPHWL--
NIPHWL--
NRPHWL--
-NPHWLA-
NYPHWL--
NPPHWL--
-NPHWLY-
-NPHWLS-
-NPHWLL-
-NPHWLF-
NDPHWL--
NTPHWL--
-NPHWLQ-
-NPHWLM-
-NPHWLG-
NFPHWL--
-NPHWLV-
NNPHWL--
--PHWLLG
--PHWLAA
--PHWLGL
--PHWLRG
--PHWLLS
--PHWLTV
--PHWLTQ
--PHWLRA
--PHWLVN
--PHWLLT
--PHWLFA
--PHWLVA
--PHWLTT
--PHWLSG
--PHWLRI
--PHWLGA
--PHWLRN
PSPHWL--
--PHWLVT
--PHWLNS
--PHWLHA
PIPHWL--
PGPHWL--
PMPHWL--
PAPHWL--

PQPHWL--
--PHWLVR
--PHWLTS
--PHWLMT
--PHWLKS
--PHWLQK
--PHWLLM
--PHWLKG
--PHWLIQ
--PHWLKT
--PHWLIT
--PHWLAT
--PHWLAN
--PHWLVS
--PHWLSQ
--PHWLQT
--PHWLKA
--PHWLIS
--PHWLFS
--PHWLAM
--PHWLVH
--PHWLNA
--PHWLLH
--PHWLAS
--PHWLAG
--PHWLTY
--PHWLTW
--PHWLTL
--PHWLSA
--PHWLMR
--PHWLGT
PYPHWL--
--PHWLWG
--PHWLVK
--PHWLVG
--PHWLTA
--PHWLSF
--PHWLRE
--PHWLQV
--PHWLQS
--PHWLMQ
--PHWLKK
--PHWLKD
PNPHWL--
--PHWLYS
--PHWLTK
--PHWLSY
--PHWLEA
--PHWLYT
--PHWLYG

--PHWLWS
--PHWLTI
--PHWLTG
--PHWLSV
--PHWLRM
--PHWLRF
--PHWLQI
--PHWLQA
--PHWLKV
--PHWLEV
PDPHWL--
PRPHWL--
--PHWLYH
--PHWLWT
--PHWLTM
--PHWLRL
--PHWLQF
--PHWLQE
--PHWLMV
--PHWLMK
--PHWLME
--PHWLLN
--PHWLKN
--PHWLDS
--PHWLAR
--PHWLYV
--PHWLYN
--PHWLTN
--PHWLSL
--PHWLSE
--PHWLRY
--PHWLQQ
--PHWLQD
--PHWLMS
--PHWLKE
--PHWLHS
--PHWLHG
--PHWLDT
--PHWLAV
--PHWLAF
--PHWLYI
--PHWLTH
--PHWLSN
--PHWLSM
--PHWLSK
--PHWLQR
--PHWLQN
--PHWLQG
--PHWLLW
--PHWLIN

--PHWLIM
--PHWLHT
--PHWLGY
PEPHWL--
PCPHWL--
--PHWLYW
--PHWLVW
--PHWLVD
--PHWLRT
--PHWLMG
--PHWLLQ
--PHWLLK
--PHWLIG
--PHWLHN
--PHWLHM
--PHWLFN
--PHWLFM
--PHWLET
PWPHWL--
--PHWLWY
--PHWLSI
--PHWLSH
--PHWLQW
--PHWLNV
--PHWLNN
--PHWLNF
--PHWLMW
--PHWLMN
--PHWLMH
--PHWLLR
--PHWLLI
--PHWLKL
--PHWLKH
--PHWLIV
--PHWLIK
--PHWLIA
--PHWLFH
--PHWLEW
--PHWLAK
PKPHWL--
--PHWLYA
--PHWLWN
--PHWLWK
--PHWLWE
--PHWLVV
--PHWLVE
--PHWLTE
--PHWLSW
--PHWLQY
--PHWLNM

--PHWLNH
--PHWLNG
--PHWLMA
--PHWLLV
--PHWLKY
--PHWLGF
--PHWLFT
--PHWLFD
--PHWLES
--PHWLEH
--PHWLAH
--PHWLAE
--PHWLYR
--PHWLYQ
--PHWLYE
--PHWLWQ
--PHWLWM
--PHWLWI
--PHWLVI
--PHWLTD
--PHWLRQ
--PHWLRD
--PHWLQL
--PHWLQH
--PHWLNW
--PHWLNQ
--PHWLKQ
--PHWLKM
--PHWLKF
--PHWLIR
--PHWLII
--PHWLIE
--PHWLID
--PHWLHH
--PHWLGW
--PHWLGN
--PHWLGK
--PHWLGH
--PHWLGG
--PHWLFV
--PHWLDA
--PHWLCT
QRPHWL--
-QPHWLR-
QVPHWL--
-QPHWLK-
QSPHWL--
QAPHWL--
-QPHWLI-
-QPHWLT-

QPPHWL--
QNPHWL--
-QPHWLQ-
-QPHWLL-
-QPHWLG-
QMPHWL--
QEPHWL--
-QPHWLS-
-QPHWLH-
QLPHWL--
QQPHWL--
-QPHWLY-
-QPHWLA-
QHPHWL--
QDPHWL--
QYPHWL--
-QPHWLE-
QKPHWL--
QIPHWL--
-QPHWLM-
-QPHWLF-
QGPHWL--
-RPHWLR-
RMPHWL--
REPHWL--
-RPHWLL-
RTPHWL--
RAPHWL--
RLPHWL--
-RPHWLK-
-RPHWLA-
RSPHWL--
-RPHWLY-
-RPHWLS-
RNPHWL--
RYPHWL--
-RPHWLQ-
RGPHWL--
-RPHWLV-
RFPHWL--
-RPHWLI-
RWPHWL--
RVPHWL--
-RPHWLH-
RPPHWL--
-RPHWLT-
RQPHWL--
-RPHWLG-
-RPHWLF-
-RPHWLN-

-RPHWLM-
RHPHWL--
-RPHWLW-
RCPHWL--
SKPHWL--
-SPHWLR-
SIPHWL--
STPHWL--
-SPHWLS-
SGPHWL--
SRPHWL--
SNPHWL--
SFPHWL--
-SPHWLK-
SPPHWL--
-SPHWLY-
-SPHWLW-
SWPHWL--
SVPHWL--
-SPHWLL-
SLPHWL--
-SPHWLV-
-SPHWLA-
-SPHWLH-
SEPHWL--
SDPHWL--
-SPHWLT-
-SPHWLQ-
-SPHWLN-
-SPHWLI-
-SPHWLF-
SAPHWL--
SYPHWL--
SHPHWL--
SQPHWL--
-SPHWLM-
TVPHWL--
TAPHWL--
TNPHWL--
TQPHWL--
TSPHWL--
TRPHWL--
TPPHWL--
TGPHWL--
TDPHWL--
TWPHWL--
TKPHWL--
-TPHWLR-
-TPHWLG-
THPHWL--

TYPHWL--
TTPHWL--
-TPHWLY-
-TPHWLF-
TIPHWL--
-TPHWLS-
-TPHWLQ-
-TPHWLL-
-TPHWLK-
-TPHWLH-
-TPHWLN-
-TPHWLM-
-TPHWLA-
-TPHWLT-
TLPHWL--
TEPHWL--
VSPHWL--
VRPHWL--
VAPHWL--
-VPHWLS-
VGPHWL--
-VPHWLR-
VEPHWL--
-VPHWLY-
VLPHWL--
VTPHWL--
-VPHWLT-
VWPHWL--
VHPHWL--
VYPHWL--
-VPHWLK-
VQPHWL--
VMPHWL--
-VPHWLI-
VVPHWL--
-VPHWLF-
VIPHWL--
VFPHWL--
VDPHWL--
-VPHWLL-
-VPHWLH-
-VPHWLW-
-VPHWLG-
-VPHWLQ-
-VPHWLM-
-VPHWLE-
-VPHWLD-
VNPHWL--
WNPHWL--
WRPHWL--

	WVPHWL--		
	WAPHWL--		
	WPPHWL--		
	WQPHWL--		
	-WPHWLS-		
	WSPHWL--		
	WTPHWL--		
	-WPHWLV-		
	-WPHWLF-		
	WMPHWL--		
	WHPHWL--		
	-WPHWLR-		
	-WPHWLG-		
	WFPHWL--		
	WEPHWL--		
	WYPHWL--		
	-WPHWLA-		
	WLPHWL--		
	WIPHWL--		
	YGPHWL--		
	YTPHWL--		
	YSPHWL--		
	YLPHWL--		
	YPPHWL--		
	YHPHWL--		
	YMPHWL--		
	YAPHWL--		
	YYPHWL--		
	-YPHWLQ-		
	-YPHWLK-		
	YEPHWL--		
	YVPHWL--		
	YQPHWL--		
	YNPHWL--		
	YDPHWL--		
	-YPHWLL-		
	YWPHWL--		
	-YPHWLY-		
	-YPHWLT-		
	YKPHWL--		
	-YPHWLV-		
	-YPHWLS-		
	-YPHWLI-		
	YIPHWL--		
	YFPHWL--		
PWEM	1135	468	45.37
	-APWEMS-		
	-APWEMV-		
	AHPWEM--		
	-APWEMR-		

-APWEMN-
-APWEMH-
-APWEMQ-
-APWEML-
AVPWEM--
AGPWEM--
-APWEMI-
-APWEMG-
ALPWEM--
AKPWEM--
ATPWEM--
ARPWEM--
-APWEMY-
-APWEMK-
AMPWEM--
AEPWEM--
-APWEMM-
AWPWEM--
ASPWEM--
-APWEMT-
CSPWEM--
CGPWEM--
CRPWEM--
-DPWEMI-
DGPWEM--
DSPWEM--
-DPWEMY-
-DPWEMV-
-DPWEMW-
-DPWEMS-
-DPWEMN-
DEPWEM--
DYPWEM--
DVPWEM--
DKPWEM--
-EPWEMS-
EIPWEM--
EAPWEM--
-EPWEML-
-EPWEMT-
-EPWEMR-
EGPWEM--
EVPWEM--
-EPWEMW-
ETPWEM--
-EPWEMY-
ELPWEM--
-FPWEMS-
FAPWEM--
-FPWEMN-

-FPWEMR-
FYPWEM--
FSPWEM--
-FPWEMK-
-FPWEMV-
FKPWEM--
FFPWEM--
-FPWEMQ-
FVPWEM--
-FPWEMH-
FPPWEM--
FTPWEM--
FRPWEM--
-GPWEMN-
GSPWEM--
-GPWEMY-
GMPWEM--
-GPWEMT-
GFPWEM--
GYPWEM--
GLPWEM--
-GPWEMM-
GPPWEM--
GRPWEM--
-GPWEMR-
-GPWEMI-
GTPWEM--
GQPWEM--
-GPWEMG-
GNPWEM--
GGPWEM--
GEPWEM--
GDPWEM--
HRPWEM--
-HPWEMR-
HFPWEM--
-HPWEMW-
-HPWEMK-
-HPWEMH-
-HPWEML-
HTPWEM--
-HPWEMT-
-HPWEMS-
HLPWEM--
-HPWEMQ-
-HPWEMN-
HAPWEM--
-HPWEMY-
-HPWEMF-
-HPWEMA-

-HPWEMM-
HGPWEM--
ITPWEM--
IMPWEM--
INPWEM--
-IPWEMN-
ILPWEM--
IIPWEM--
-IPWEMR-
IDPWEM--
IAPWEM--
-IPWEMT-
IFPWEM--
IRPWEM--
-IPWEMF-
IVPWEM--
-IPWEMI-
ISPWEM--
-IPWEMH-
-IPWEMG-
KSPWEM--
KRPWEM--
KWPWEM--
-KPWEML-
-KPWEMT-
-KPWEMR-
KYPWEM--
-KPWEMY-
KFPWEM--
KLPWEM--
KTPWEM--
-KPWEMK-
-KPWEMI-
KAPWEM--
-KPWEMS-
-KPWEMF-
KNPWEM--
-KPWEMH-
-KPWEMG-
LSPWEM--
-LPWEMR-
-LPWEMN-
LHPWEM--
LTPWEM--
LQPWEM--
LRPWEM--
-LPWEMI-
LMPWEM--
LFPWEM--
LAPWEM--

-LPWEMT-
LYPWEM--
LVPWEM--
-LPWEMV-
-LPWEMS-
LLPWEM--
LWPWEM--
LPPWEM--
-LPWEMM-
LEPWEM--
-MPWEMR-
MIPWEM--
MRPWEM--
MSPWEM--
-MPWEMV-
MFPWEM--
-MPWEMT-
MAPWEM--
MYPWEM--
-MPWEMS-
-MPWEMN-
-MPWEMM-
-MPWEMI-
MNPWEM--
MMPWEM--
MHPWEM--
NRPWEM--
-NPWEMS-
NLPWEM--
NKPWEM--
NGPWEM--
-NPWEMK-
NVPWEM--
-NPWEMW-
-NPWEMT-
-NPWEMV-
-NPWEMN-
NEPWEM--
-NPWEMF-
NIPWEM--
NAPWEM--
--PWEMRS
--PWEMLG
--PWEMVT
--PWEMIM
--PWEMSS
--PWEMSV
--PWEMRI
--PWEMVS
--PWEMVA

--PWEMTQ
--PWEMTK
--PWEMRT
--PWEMQT
--PWEMNV
--PWEMNM
--PWEMYK
--PWEMSG
--PWEMQN
--PWEMKH
--PWEMGS
--PWEMYH
--PWEMRH
--PWEMMH
--PWEMKG
--PWEMIA
--PWEMHV
--PWEMHF
--PWEMTS
--PWEMRK
--PWEMMS
--PWEMHG
--PWEMGV
--PWEMAS
PWPWEM--
--PWEMYS
--PWEMYM
--PWEMMN
--PWEMKY
--PWEMVR
--PWEMNW
--PWEMIN
--PWEMYT
--PWEMWG
--PWEMWA
--PWEMTV
--PWEMQG
--PWEMND
--PWEMMA
--PWEMLI
--PWEMKL
--PWEMIG
PGPWEM--
--PWEMYI
--PWEMVW
--PWEMVG
--PWEMSH
--PWEMRY
--PWEMQS
--PWEMNR

--PWEMKQ
--PWEMKN
--PWEMHL
--PWEMFS
--PWEMWT
--PWEMWS
--PWEMWI
--PWEMVK
--PWEMSY
--PWEMSL
--PWEMRV
--PWEMRN
--PWEMRG
--PWEMNT
--PWEMMM
--PWEMLH
--PWEMKE
--PWEMYV
--PWEMYR
--PWEMYQ
--PWEMYN
--PWEMWR
--PWEMVV
--PWEMVE
--PWEMVD
--PWEMTL
--PWEMTH
--PWEMTG
--PWEMSW
--PWEMSR
--PWEMSM
--PWEMSF
--PWEMQW
--PWEMNY
--PWEMNQ
--PWEMNE
--PWEMLR
--PWEMKW
--PWEMKS
--PWEMKK
--PWEMKD
--PWEMIS
--PWEMIK
--PWEMII
--PWEMHY
--PWEMHT
--PWEMGY
--PWEMGA
--PWEMFI
--PWEMFH

--PWEMEF
--PWEMEA
--PWEMAV
--PWEMAH
--PWEMYY
--PWEMVN
--PWEMTW
--PWEMTM
--PWEMST
--PWEMSP
--PWEMSI
--PWEMSC
--PWEMRD
--PWEMQH
--PWEMQF
--PWEMQA
--PWEMNK
--PWEMMD
--PWEMLQ
--PWEMIR
--PWEMIF
--PWEMHS
--PWEMHN
--PWEMHA
--PWEMGQ
--PWEMGI
--PWEMEV
--PWEMDM
--PWEMAR
--PWEMAM
-QPWEMR-
QSPWEM--
QRPWEM--
-QPWEMF-
-QPWEMS-
QIPWEM--
QAPWEM--
-QPWEMG-
-QPWEMA-
QVPWEM--
-QPWEMV-
RVPWEM--
RIPWEM--
RPPWEM--
RSPWEM--
-RPWEML-
-RPWEMQ-
-RPWEMN-
-RPWEMK-
RRPWEM--

-RPWEMV-
-RPWEMA-
RFPWEM--
RAPWEM--
-RPWEMI-
RNPWEM--
-RPWEMS-
-RPWEMR-
RLPWEM--
RDPWEM--
RWPWEM--
-RPWEMW-
RHPWEM--
RGPWEM--
-RPWEMY-
-RPWEMT-
RTPWEM--
RQPWEM--
RKPWEM--
RCPWEM--
RYPWEM--
-RPWEMH-
REPWEM--
SIPWEM--
SSPWEM--
-SPWEMW-
SYPWEM--
-SPWEMT-
-SPWEMQ-
SRPWEM--
-SPWEMV-
-SPWEMY-
-SPWEMR-
SAPWEM--
-SPWEMS-
-SPWEMK-
-SPWEMI-
SLPWEM--
-SPWEMG-
SKPWEM--
SGPWEM--
SVPWEM--
SQPWEM--
-SPWEMA-
-SPWEMN-
SEPWEM--
-TPWEMK-
-TPWEMR-
TAPWEM--
-TPWEMY-

-TPWEMS-
TRPWEM--
TFPWEM--
TIPWEM--
-TPWEMV-
-TPWEMT-
-TPWEMI-
TPPWEM--
TLPWEM--
-TPWEMH-
TTPWEM--
TQPWEM--
-TPWEMQ-
-TPWEMA-
TSPWEM--
-TPWEMN-
VSPWEM--
-VPWEMS-
VRPWEM--
VKPWEM--
-VPWEMH-
VTPWEM--
VQPWEM--
-VPWEMY-
VHPWEM--
-VPWEMK-
VMPWEM--
VAPWEM--
-VPWEMN-
-VPWEMG-
-VPWEMW-
VVPWEM--
-VPWEML-
VLPWEM--
-VPWEMV-
-VPWEMF-
-VPWEMA-
-VPWEMM-
VNPWEM--
-VPWEMQ-
-WPWEMR-
WSPWEM--
WQPWEM--
WLPWEM--
WRPWEM--
-WPWEMS-
WNPWEM--
-WPWEMQ-
WVPWEM--
-WPWEMN-

-WPWEMT-
-WPWEMI-
-WPWEMH-
-WPWEMG-
YFPWEM--
YRPWEM--
-YPWEMS-
YNPWEM--
-YPWEMT-
-YPWEMV-
-YPWEMQ-
-YPWEMH-
YLPWEM--
YKPWEM--
PMSI 100 806 45.34
-DPMSIL-
-NPMSIV-
YTPMSI--
ALPMSI--
ATPMSI--
AVPMSI--
-APMSIL-
-APMSII-
-APMSIY-
ASPMSI--
-APMSIW-
AGPMSI--
-APMSIR-
-APMSIQ-
-APMSIH-
AYPMSI--
AAPMSI--
ARPMSI--
AQPMSI--
-APMSIK-
-APMSIF-
AMPMSI--
AWPMSI--
ADPMSI--
-APMSIT-
-APMSIV-
-APMSIE-
AIPMSI--
APPMSI--
-APMSIG-
ANPMSI--
-APMSIA-
AHPMSI--
-APMSIS-
-APMSIM-

-APMSIN-
AFPMSI--
CRPMSI--
CHPMSI--
CSPMSI--
CVPMSI--
CEPMSI--
-CPMSIK-
CLPMSI--
CGPMSI--
-CPMSIW-
-CPMSIT-
CKPMSI--
-CPMSIS-
-CPMSIF-
CNPMSI--
CIPMSI--
DRPMSI--
-DPMSIY-
-DPMSIF-
DHPMSI--
DMPMSI--
DVPMSI--
DSPMSI--
-DPMSIR-
DGPMSI--
DIPMSI--
DWPMSI--
-DPMSIV-
DQPMSI--
DKPMSI--
-DPMSIH-
DLPMSI--
-DPMSIA-
DEPMSI--
DAPMSI--
DTPMSI--
-DPMSIW-
-DPMSII-
DDPMSI--
-EPMSIR-
ERPMSI--
ESPMSI--
-EPMSII-
-EPMSIH-
EIPMSI--
-EPMSIS-
EFPMSI--
-EPMSIQ-
EVPMSI--

-EPMSIF-
-EPMSIL-
ETPMSI--
-EPMSIY-
-EPMSIM-
EQPMSI--
-EPMSIK-
-EPMSIW-
-EPMSIT-
-EPMSIN-
ELPMSI--
EWPMSI--
EGPMSI--
-EPMSIV-
-EPMSIA-
EAPMSI--
EKPMSI--
FTPMSI--
FRPMSI--
-FPMSIY-
FWPMSI--
FMPMSI--
-FPMSIH-
FKPMSI--
FLPMSI--
-FPMSIW-
FDPMSI--
-FPMSIS-
-FPMSIQ-
FNPMSI--
FQPMSI--
-FPMSIT-
FYPMSI--
FCPMSI--
FEPMSI--
FPPMSI--
-FPMSIV-
-FPMSIN-
-FPMSIK-
-FPMSIA-
FGPMSI--
-FPMSIM-
FHPMSI--
-GPMSIL-
GVPMSI--
GSPMSI--
-GPMSIF-
GTPMSI--
GPPMSI--
-GPMSIR-

GLPMSI--
GIPMSI--
GAPMSI--
-GPMSII-
GWPMSI--
GGPMSI--
-GPMSIH-
-GPMSIA-
-GPMSIV-
-GPMSIT-
GFPMSI--
GYPMSI--
GEPMSI--
-GPMSIK-
GDPMSI--
GMPMSI--
-GPMSIY-
-GPMSIW-
-GPMSIQ-
-GPMSIM-
-GPMSIE-
GHPMSI--
GNPMSI--
-GPMSIN-
-GPMSIG-
GCPMSI--
-GPMSIC-
HRPMSI--
HGPMSI--
-HPMSIR-
-HPMSIK-
HHPMSI--
HWPMSI--
HVPMSI--
HMPMSI--
-HPMSIL-
HAPMSI--
HSPMSI--
-HPMSII-
HYPMSI--
HPPMSI--
HLPMSI--
HNPMSI--
HTPMSI--
-HPMSIH-
HIPMSI--
-HPMSIQ-
-HPMSIV-
-HPMSIS-
-HPMSIW-

-HPMSIY-
-HPMSIT-
-HPMSIA-
HKPMSI--
-HPMSIE-
HFPMSI--
HEPMSI--
IRPMSI--
IYPMSI--
ITPMSI--
IWPMSI--
-IPMSIH-
INPMSI--
-IPMSII-
-IPMSIK-
IIPMSI--
-IPMSIV-
-IPMSIQ-
-IPMSIS-
ILPMSI--
-IPMSIM-
-IPMSIF-
-IPMSIT-
-IPMSIL-
IAPMSI--
-IPMSIG-
-IPMSIE-
-IPMSIA-
IQPMSI--
ICPMSI--
IVPMSI--
IMPMSI--
-IPMSIY-
IGPMSI--
IPPMSI--
-IPMSIN-
IDPMSI--
KSPMSI--
KAPMSI--
KMPMSI--
-KPMSIL-
KVPMSI--
KYPMSI--
-KPMSIS-
-KPMSIF-
KGPMSI--
KRPMSI--
-KPMSIW-
-KPMSII-
-KPMSIY-

-KPMSIQ-
-KPMSIR-
-KPMSIM-
KTPMSI--
KQPMSI--
-KPMSIV-
KPPMSI--
-KPMSIK-
-KPMSIH-
-KPMSIG-
KLPMSI--
KKPMSI--
KWPMSI--
-KPMSIT-
-KPMSIN-
KHPMSI--
-KPMSIE-
-KPMSIA-
KFPMSI--
-LPMSIR-
LYPMSI--
LRPMSI--
LVPMSI--
LAPMSI--
LNPMSI--
-LPMSIK-
LMPMSI--
LLPMSI--
LWPMSI--
LGPMSI--
LPPMSI--
-LPMSIL-
LKPMSI--
-LPMSIT-
LSPMSI--
LEPMSI--
-LPMSII-
LQPMSI--
-LPMSIV-
-LPMSIS-
-LPMSIM-
-LPMSIH-
LIPMSI--
LFPMSI--
-LPMSIW-
LDPMSI--
-LPMSIN-
-LPMSIQ-
-LPMSID-
LCPMSI--

-MPMSIL-
MSPMSI--
MRPMSI--
-MPMSIS-
MKPMSI--
MEPMSI--
-MPMSIV-
MNPMSI--
MMPMSI--
-MPMSIR-
-MPMSIH-
MIPMSI--
MGPMSI--
MWPMSI--
MLPMSI--
MFPMSI--
-MPMSIY-
-MPMSIF-
MHPMSI--
MAPMSI--
MPPMSI--
-MPMSIM-
-MPMSIK-
-MPMSII-
MVPMSI--
-MPMSIT-
MDPMSI--
MQPMSI--
-MPMSIG-
MYPMSI--
NTPMSI--
NRPMSI--
NVPMSI--
NGPMSI--
-NPMSIR-
-NPMSII-
NMPMSI--
NSPMSI--
NFPMSI--
NLPMSI--
NEPMSI--
-NPMSIY-
NYPMSI--
NQPMSI--
NIPMSI--
NNPMSI--
-NPMSIF-
-NPMSIW-
-NPMSIT-
-NPMSIM-

NWPMSI--
-NPMSIA-
-NPMSIH-
-NPMSIS-
NDPMSI--
NPPMSI--
-NPMSIQ-
-NPMSIC-
--PMSIFH
--PMSIRV
--PMSIWR
--PMSILS
--PMSIRT
--PMSIRS
--PMSILG
--PMSIQG
--PMSIRR
--PMSIRG
--PMSILT
PHPMSI--
--PMSILA
--PMSITG
--PMSISV
--PMSITA
--PMSIRN
--PMSIMM
--PMSIFS
--PMSIVN
--PMSISR
--PMSIAY
--PMSIMS
PTPMSI--
--PMSISG
--PMSIHT
--PMSIRE
--PMSIMG
--PMSITS
--PMSITD
--PMSISS
--PMSIRW
--PMSIMA
--PMSITV
--PMSIAS
--PMSIFN
--PMSILH
--PMSIHG
--PMSIFR
--PMSIYS
--PMSISK
--PMSIMR

--PMSIKV
--PMSIIS
--PMSIYT
--PMSIVS
--PMSIVA
--PMSIIA
--PMSILR
--PMSIHR
PGPMSI--
--PMSINT
--PMSILN
--PMSIGS
--PMSIWN
--PMSIRD
--PMSIIM
--PMSISH
--PMSILM
--PMSIKS
--PMSIKR
--PMSIEA
--PMSIAA
PWPMSI--
--PMSIYG
--PMSIRI
--PMSILV
--PMSIRH
--PMSIMW
--PMSIMI
--PMSIWS
--PMSIQA
--PMSIAN
PAPMSI--
PVPMSI--
--PMSIYM
--PMSIVK
--PMSIVI
--PMSITT
--PMSISA
--PMSIMY
--PMSIKL
--PMSIKK
--PMSIEF
--PMSITF
--PMSIRK
--PMSILW
--PMSILD
--PMSIIK
--PMSIYR
--PMSIYK
--PMSIWV

--PMSIWF
--PMSITL
--PMSISY
--PMSIRM
--PMSINM
--PMSIMN
--PMSIFT
--PMSIFK
PIPMSI--
--PMSIWQ
--PMSIVD
--PMSIRA
--PMSIMK
--PMSIIQ
--PMSIAW
--PMSIAT
--PMSIVM
--PMSIVH
--PMSISM
--PMSIQH
--PMSIQD
--PMSIMV
--PMSIMD
--PMSIKF
--PMSIKE
--PMSIGT
--PMSIFY
PFPMSI--
--PMSIYW
--PMSIWA
--PMSITE
--PMSISN
--PMSIIY
--PMSIGG
--PMSIGA
--PMSIAK
PDPMSI--
PQPMSI--
--PMSIYA
--PMSIWD
--PMSITN
--PMSIQI
--PMSIML
--PMSIIR
--PMSIHK
--PMSIFG
PKPMSI--
PEPMSI--
--PMSIYQ
--PMSIYD

--PMSIWK
--PMSIWH
--PMSIWE
--PMSIQM
--PMSIKT
--PMSIKH
--PMSIKG
--PMSIKA
--PMSIEV
--PMSIES
--PMSIAV
--PMSIAR
--PMSIAL
--PMSIVV
--PMSITY
--PMSITR
--PMSINY
--PMSIMT
--PMSILE
--PMSIIT
--PMSIEY
--PMSIEG
--PMSIWW
--PMSITK
--PMSITI
--PMSIRQ
--PMSIQS
--PMSIQN
--PMSIQL
--PMSINN
--PMSINL
--PMSINH
--PMSILQ
--PMSIKW
--PMSIIE
--PMSIHI
--PMSIFD
--PMSIER
--PMSIEM
PYPMSI--
--PMSIYN
--PMSIVE
--PMSISQ
--PMSISF
--PMSISD
--PMSIQV
--PMSIQT
--PMSIQK
--PMSINR
--PMSIIF

--PMSIID
--PMSIIC
--PMSIHW
--PMSIHS
PNPMSI--
--PMSIYH
--PMSITW
--PMSITQ
--PMSIRY
--PMSIQQ
--PMSINQ
--PMSILC
--PMSIKY
--PMSIKI
--PMSIKD
--PMSIIV
--PMSIIH
--PMSIHV
--PMSIGV
--PMSIFW
--PMSIEQ
--PMSIEI
--PMSIAQ
--PMSIAG
PMPMSI--
-PPMSIR-
-PPMSIN-
--PMSIVQ
--PMSITP
--PMSITM
--PMSISE
--PMSIMQ
--PMSIKM
--PMSIIP
--PMSIHY
--PMSIHH
--PMSIGY
--PMSIGN
--PMSIFV
--PMSIFQ
--PMSIEN
--PMSIDG
--PMSICS
--PMSICR
--PMSICN
--PMSICK
--PMSICG
--PMSIAM
--PMSIAF
--PMSIAD

PCPMSI--
QRPMSI--
QSPMSI--
QWPMSI--
-QPMSIL-
QAPMSI--
QKPMSI--
QMPMSI--
-QPMSIW-
-QPMSIF-
QTPMSI--
QPPMSI--
QLPMSI--
-QPMSIS-
-QPMSIV-
QYPMSI--
-QPMSIK-
QFPMSI--
QVPMSI--
-QPMSII-
-QPMSIR-
-QPMSIH-
QNPMSI--
QGPMSI--
-QPMSIT-
-QPMSIN-
QHPMSI--
QEPMSI--
-QPMSIA-
QIPMSI--
-QPMSIG-
-QPMSIE-
QCPMSI--
QDPMSI--
RGPMSI--
RIPMSI--
-RPMSIT-
-RPMSIS-
RYPMSI--
-RPMSIV-
RPPMSI--
-RPMSIK-
RDPMSI--
-RPMSIL-
-RPMSIA-
-RPMSIY-
REPMSI--
RAPMSI--
RSPMSI--
RVPMSI--

RFPMSI--
-RPMSIM-
RNPMSI--
-RPMSIW-
-RPMSIQ-
-RPMSIF-
RHPMSI--
RQPMSI--
-RPMSII-
-RPMSIG-
-RPMSIH-
-RPMSIN-
RCPMSI--
-RPMSIC-
-RPMSIE-
SVPMSI--
SSPMSI--
SLPMSI--
-SPMSIL-
STPMSI--
-SPMSIF-
SHPMSI--
-SPMSIS-
-SPMSIH-
SAPMSI--
SKPMSI--
SIPMSI--
SGPMSI--
-SPMSII-
-SPMSIV-
SYPMSI--
-SPMSIT-
-SPMSIA-
SNPMSI--
-SPMSIY-
SEPMSI--
SMPMSI--
-SPMSIW-
-SPMSIM-
-SPMSIK-
-SPMSIE-
SQPMSI--
SFPMSI--
SDPMSI--
-SPMSIG-
-SPMSIN-
-SPMSIQ-
SCPMSI--
TRPMSI--
TMPMSI--

TSPMSI--
TVPMSI--
TPPMSI--
-TPMSIY-
TYPMSI--
-TPMSIR-
TAPMSI--
-TPMSIK-
TIPMSI--
-TPMSIL-
TKPMSI--
-TPMSII-
THPMSI--
TGPMSI--
TWPMSI--
-TPMSIF-
-TPMSIV-
-TPMSIM-
-TPMSIT-
-TPMSIS-
TTPMSI--
-TPMSIN-
TNPMSI--
TFPMSI--
TQPMSI--
TEPMSI--
TCPMSI--
-TPMSIQ-
TDPMSI--
-TPMSIW-
-TPMSIH-
-TPMSIE-
VGPMSI--
VSPMSI--
-VPMSIS-
VLPMSI--
VRPMSI--
VFPMSI--
VMPMSI--
-VPMSIF-
VTPMSI--
VQPMSI--
VIPMSI--
-VPMSII-
-VPMSIW-
-VPMSIV-
-VPMSIK-
VPPMSI--
VNPMSI--
-VPMSIH-

VHPMSI--
VEPMSI--
VAPMSI--
-VPMSIT-
-VPMSIN-
-VPMSIM-
VYPMSI--
-VPMSIG-
VDPMSI--
VCPMSI--
-VPMSIQ-
-VPMSIC-
WAPMSI--
WGPMSI--
WRPMSI--
WLPMSI--
WEPMSI--
WIPMSI--
-WPMSIK-
-WPMSII-
WKPMSI--
-WPMSIY-
WHPMSI--
WYPMSI--
-WPMSIR-
WVPMSI--
-WPMSIS-
WFPMSI--
WNPMSI--
WPPMSI--
-WPMSIM-
WMPMSI--
WWPMSI--
-WPMSIV-
-WPMSIH-
WDPMSI--
-WPMSIA-
WQPMSI--
-WPMSIT-
-WPMSIQ-
-WPMSIN-
-WPMSIG-
-YPMSIR-
-YPMSIT-
YRPMSI--
-YPMSIL-
YEPMSI--
YKPMSI--
-YPMSIV-
-YPMSIS-

	YSPMSI--		
	-YPMSIK-		
	YDPMSI--		
	YVPMSI--		
	-YPMSII-		
	YWPMSI--		
	YFPMSI--		
	YAPMSI--		
	YNPMSI--		
	YPPMSI--		
	-YPMSIQ-		
	-YPMSIY-		
	-YPMSIF-		
	YYPMSI--		
	YQPMSI--		
	-YPMSIE-		
	-YPMSIA-		
	YMPMSI--		
	-YPMSIN-		
	-YPMSIM-		
	YHPMSI--		
PSPY	65	839	45.28
	-APSPYG-		
	-LPSPYN-		
	-NPSPYA-		
	--PSPYLE		
	--PSPYHD		
	-RPSPYG-		
	-YPSPYN-		
	-APSPYK-		
	AAPSPY--		
	-APSPYI-		
	ASPSPY--		
	AMPSPY--		
	AEPSPY--		
	-APSPYR-		
	ARPSPY--		
	-APSPYF-		
	AKPSPY--		
	AVPSPY--		
	AQPSPY--		
	AIPSPY--		
	AHPSPY--		
	-APSPYS-		
	AGPSPY--		
	-APSPYL-		
	AYPSPY--		
	ATPSPY--		
	-APSPYM-		
	-APSPYQ-		

ADPSPY--
ALPSPY--
-APSPYY-
AFPSPY--
-APSPYT-
-APSPYA-
APPSPY--
ANPSPY--
ACPSPY--
-APSPYH-
CGPSPY--
CTPSPY--
CWPSPY--
-CPSPYY-
CIPSPY--
CHPSPY--
CAPSPY--
CYPSPY--
CRPSPY--
CNPSPY--
CCPSPY--
DRPSPY--
DQPSPY--
DTPSPY--
DMPSPY--
DIPSPY--
DFPSPY--
DSPSPY--
-DPSPYT-
DKPSPY--
DWPSPY--
-DPSPYY-
-DPSPYL-
-DPSPYI-
-DPSPYF-
DVPSPY--
-DPSPYW-
-DPSPYR-
-DPSPYQ-
DEPSPY--
ERPSPY--
-EPSPYR-
ESPSPY--
EAPSPY--
ETPSPY--
-EPSPYI-
-EPSPYT-
-EPSPYS-
-EPSPYM-
EKPSPY--

EVPSPY--
EQPSPY--
-EPSPYV-
-EPSPYL-
ENPSPY--
EMPSPY--
EYPSPY--
-EPSPYF-
EHPSPY--
EWPSPY--
-EPSPYA-
ELPSPY--
-EPSPYH-
-EPSPYQ-
EGPSPY--
EEPSPY--
FYPSPY--
FSPSPY--
FRPSPY--
FKPSPY--
FWPSPY--
FNPSPY--
FLPSPY--
FIPSPY--
FGPSPY--
FTPSPY--
FEPSPY--
-FPSPYS-
FVPSPY--
-FPSPYR-
-FPSPYW-
FHPSPY--
FFPSPY--
FQPSPY--
FMPSPY--
FCPSPY--
-FPSPYY-
-FPSPYI-
-FPSPYH-
-FPSPYT-
-FPSPYM-
-FPSPYF-
FPPSPY--
FAPSPY--
-FPSPYV-
-FPSPYK-
GAPSPY--
-GPSPYR-
GWPSPY--
GGPSPY--

GTPSPY--
-GPSPYV-
GYPSPY--
-GPSPYS-
GRPSPY--
GMPSPY--
GLPSPY--
-GPSPYI-
-GPSPYL-
GIPSPY--
GFPSPY--
GSPSPY--
GVPSPY--
GQPSPY--
-GPSPYY-
-GPSPYW-
-GPSPYK-
-GPSPYH-
-GPSPYF-
GHPSPY--
GEPSPY--
GNPSPY--
GKPSPY--
-GPSPYT-
-GPSPYM-
GCPSPY--
GDPSPY--
-GPSPYQ-
-GPSPYN-
-GPSPYA-
GPPSPY--
HRPSPY--
HSPSPY--
HVPSPY--
-HPSPYR-
HMPSPY--
HEPSPY--
HYPSPY--
HLPSPY--
HHPSPY--
-HPSPYK-
HFPSPY--
-HPSPYL-
-HPSPYY-
-HPSPYQ-
HTPSPY--
-HPSPYF-
HAPSPY--
-HPSPYH-
HDPSPY--

-HPSPYV-
HIPSPY--
-HPSPYT-
-HPSPYS-
-HPSPYM-
-HPSPYI-
HGPSPY--
HWPSPY--
-HPSPYW-
HPPSPY--
HKPSPY--
HNPSPY--
HCPSPY--
-IPSPYR-
IAPSPY--
-IPSPYY-
ILPSPY--
ISPSPY--
ITPSPY--
IVPSPY--
IMPSPY--
IYPSPY--
IPPSPY--
IHPSPY--
IIPSPY--
IFPSPY--
IKPSPY--
IDPSPY--
IRPSPY--
IGPSPY--
-IPSPYV-
-IPSPYQ-
-IPSPYM-
IWPSPY--
-IPSPYT-
-IPSPYN-
-IPSPYH-
-IPSPYA-
ICPSPY--
IQPSPY--
-IPSPYW-
-IPSPYP-
-IPSPYK-
-IPSPYI-
INPSPY--
IEPSPY--
-KPSPYR-
-KPSPYL-
-KPSPYV-
KGPSPY--

-KPSPYF-
KVPSPY--
KAPSPY--
-KPSPYK-
-KPSPYH-
KKPSPY--
KIPSPY--
KYPSPY--
KPPSPY--
KMPSPY--
-KPSPYT-
KLPSPY--
-KPSPYS-
KFPSPY--
KWPSPY--
KNPSPY--
-KPSPYI-
KQPSPY--
-KPSPYW-
-KPSPYY-
-KPSPYA-
KEPSPY--
KDPSPY--
LRPSPY--
LGPSPY--
LWPSPY--
LAPSPY--
LPPSPY--
LKPSPY--
-LPSPYR-
LSPSPY--
LMPSPY--
LFPSPY--
LVPSPY--
LTPSPY--
LYPSPY--
LNPSPY--
LIPSPY--
-LPSPYT-
LLPSPY--
-LPSPYI-
-LPSPYS-
LEPSPY--
-LPSPYY-
-LPSPYL-
-LPSPYF-
-LPSPYW-
-LPSPYV-
LQPSPY--
LCPSPY--

LHPSPY--
-LPSPYQ-
-LPSPYM-
-LPSPYK-
-LPSPYH-
-LPSPYA-
LDPSPY--
MSPSPY--
MKPSPY--
-MPSPYR-
MRPSPY--
MMPSPY--
MLPSPY--
MIPSPY--
MPPSPY--
MVPSPY--
MWPSPY--
MNPSPY--
MQPSPY--
MAPSPY--
MTPSPY--
-MPSPYV-
-MPSPYK-
-MPSPYI-
-MPSPYM-
-MPSPYH-
-MPSPYS-
-MPSPYA-
MHPSPY--
MGPSPY--
MDPSPY--
MYPSPY--
-MPSPYQ-
-MPSPYF-
MEPSPY--
-MPSPYY-
-MPSPYW-
-MPSPYT-
-MPSPYD-
MFPSPY--
MCPSPY--
NYPSPY--
NAPSPY--
NRPSPY--
NVPSPY--
NMPSPY--
-NPSPYR-
NWPSPY--
-NPSPYY-
NPPSPY--

NLPSPY--
NSPSPY--
NHPSPY--
NTPSPY--
-NPSPYV-
NQPSPY--
-NPSPYM-
-NPSPYQ-
-NPSPYL-
-NPSPYF-
NNPSPY--
-NPSPYT-
-NPSPYS-
NGPSPY--
NFPSPY--
-NPSPYK-
-NPSPYN-
-NPSPYI-
NIPSPY--
--PSPYSL
--PSPYTL
--PSPYRS
--PSPYLG
--PSPYTS
--PSPYSS
PRPSPY--
--PSPYVV
--PSPYSF
--PSPYLS
PAPSPY--
--PSPYTI
--PSPYYS
--PSPYVA
--PSPYML
--PSPYGM
--PSPYST
--PSPYTY
--PSPYGI
--PSPYWV
--PSPYRV
--PSPYRT
--PSPYLV
--PSPYLA
--PSPYKL
--PSPYWS
--PSPYVS
PVPSPY--
--PSPYVN
--PSPYQI
--PSPYIA

--PSPYYA
--PSPYRH
--PSPYRG
--PSPYNS
--PSPYGQ
--PSPYWW
--PSPYYW
--PSPYYR
--PSPYIN
--PSPYVF
--PSPYRW
--PSPYPI
--PSPYMF
--PSPYLK
--PSPYKT
PGPSPY--
--PSPYYG
--PSPYVY
--PSPYTK
--PSPYSV
--PSPYSA
--PSPYPY
--PSPYMS
--PSPYIT
--PSPYHW
--PSPYAH
PFPSPY--
--PSPYSW
--PSPYRA
--PSPYQT
--PSPYIV
--PSPYIS
--PSPYFW
--PSPYFG
--PSPYDL
--PSPYVW
--PSPYLT
--PSPYWL
--PSPYWG
--PSPYVT
--PSPYTN
--PSPYPF
--PSPYNF
--PSPYMT
--PSPYKY
--PSPYVG
--PSPYSR
--PSPYSN
--PSPYRF
--PSPYKW

--PSPYKF
--PSPYFL
PTPSPY--
--PSPYWA
--PSPYSG
--PSPYRD
--PSPYLN
--PSPYHI
--PSPYYF
--PSPYWN
--PSPYSH
--PSPYQA
--PSPYMV
--PSPYMG
--PSPYKS
PEPSPY--
--PSPYYL
--PSPYWF
--PSPYVL
--PSPYTV
--PSPYSM
--PSPYRQ
--PSPYRI
--PSPYFY
--PSPYFR
--PSPYFM
--PSPYAY
--PSPYAQ
--PSPYAM
--PSPYAI
--PSPYWY
--PSPYRM
--PSPYMN
--PSPYMI
--PSPYIW
--PSPYFH
--PSPYEY
--PSPYDY
--PSPYYV
--PSPYQS
--PSPYNM
--PSPYMY
--PSPYLW
--PSPYIY
--PSPYHS
--PSPYHF
--PSPYGF
--PSPYEF
--PSPYDI
--PSPYYM

--PSPYYH
--PSPYWR
--PSPYTR
--PSPYTA
--PSPYQQ
--PSPYNY
--PSPYNA
--PSPYKV
--PSPYKM
--PSPYIH
--PSPYGS
--PSPYFS
--PSPYFN
--PSPYES
--PSPYAV
--PSPYAS
--PSPYAA
PKPSPY--
--PSPYYN
--PSPYVM
--PSPYQL
--PSPYMR
--PSPYMQ
--PSPYMM
--PSPYLQ
--PSPYLF
--PSPYGV
--PSPYGA
--PSPYEM
PQPSPY--
--PSPYTW
--PSPYTH
--PSPYSY
--PSPYPW
--PSPYLY
--PSPYLR
--PSPYFQ
--PSPYAG
PWPSPY--
--PSPYWI
--PSPYVK
--PSPYVI
--PSPYVD
--PSPYQW
--PSPYPS
--PSPYNN
--PSPYMK
--PSPYLH
--PSPYKI
--PSPYKG

--PSPYIK
--PSPYHT
--PSPYHG
--PSPYFK
--PSPYFF
--PSPYFA
PNPSPY--
PHPSPY--
--PSPYYY
--PSPYYI
--PSPYWQ
--PSPYTT
--PSPYQR
--PSPYIL
--PSPYIG
--PSPYHV
--PSPYHN
--PSPYHA
--PSPYGH
PMPSPY--
PDPSPY--
--PSPYWT
--PSPYVQ
--PSPYVH
--PSPYTF
--PSPYQV
--PSPYQK
--PSPYNV
--PSPYNT
--PSPYNH
--PSPYIM
--PSPYFT
--PSPYYQ
--PSPYYK
--PSPYYD
--PSPYSK
--PSPYQN
--PSPYQH
--PSPYQF
--PSPYPV
--PSPYNQ
--PSPYLM
--PSPYKQ
--PSPYKH
--PSPYIF
--PSPYID
--PSPYGT
--PSPYFI
--PSPYEW
--PSPYEQ

--PSPYCV
--PSPYTQ
--PSPYTE
--PSPYTC
--PSPYSQ
--PSPYPT
--PSPYPM
--PSPYNG
--PSPYMW
--PSPYMH
--PSPYKR
--PSPYIR
--PSPYIQ
--PSPYII
--PSPYFD
--PSPYEV
--PSPYET
--PSPYEI
--PSPYDM
--PSPYAR
--PSPYAN
PIPSPY--
--PSPYYE
--PSPYWE
--PSPYTD
--PSPYRE
--PSPYRC
--PSPYPQ
--PSPYPA
--PSPYNI
--PSPYME
--PSPYMD
--PSPYIE
--PSPYHY
--PSPYHM
--PSPYHH
--PSPYGN
--PSPYGK
--PSPYFC
--PSPYEL
--PSPYCT
--PSPYCS
--PSPYAK
--PSPYAD
QKPSPY--
QRPSPY--
-QPSPYR-
QYPSPY--
QSPSPY--
QVPSPY--

-QPSPYL-
QNPSPY--
-QPSPYM-
QLPSPY--
-QPSPYK-
-QPSPYS-
QPPSPY--
QAPSPY--
-QPSPYI-
QFPSPY--
-QPSPYH-
-QPSPYY-
QHPSPY--
QQPSPY--
-QPSPYT-
-QPSPYN-
QMPSPY--
QDPSPY--
QTPSPY--
QIPSPY--
QCPSPY--
-QPSPYW-
-QPSPYV-
-QPSPYE-
-QPSPYA-
RSPSPY--
RAPSPY--
-RPSPYL-
RYPSPY--
RRPSPY--
RIPSPY--
RMPSPY--
-RPSPYI-
RGPSPY--
-RPSPYM-
-RPSPYV-
RVPSPY--
-RPSPYW-
-RPSPYF-
REPSPY--
-RPSPYA-
-RPSPYS-
-RPSPYK-
RPPSPY--
RQPSPY--
RNPSPY--
RWPSPY--
RDPSPY--
-RPSPYH-
RCPSPY--

−RPSPYQ−
−RPSPYN−
−RPSPYY−
RHPSPY−−
SKPSPY−−
SGPSPY−−
SVPSPY−−
SNPSPY−−
SRPSPY−−
STPSPY−−
−SPSPYT−
−SPSPYS−
SHPSPY−−
−SPSPYR−
SAPSPY−−
SIPSPY−−
SFPSPY−−
−SPSPYY−
SEPSPY−−
−SPSPYK−
SWPSPY−−
−SPSPYW−
−SPSPYL−
−SPSPYI−
SMPSPY−−
SYPSPY−−
−SPSPYM−
SDPSPY−−
−SPSPYQ−
−SPSPYV−
SPPSPY−−
−SPSPYA−
−SPSPYN−
−SPSPYG−
−SPSPYF−
SCPSPY−−
−SPSPYH−
TYPSPY−−
−TPSPYR−
TRPSPY−−
TFPSPY−−
THPSPY−−
TSPSPY−−
TLPSPY−−
TMPSPY−−
TTPSPY−−
TEPSPY−−
TVPSPY−−
−TPSPYS−
−TPSPYF−

-TPSPYY-
TIPSPY--
TKPSPY--
TDPSPY--
TWPSPY--
-TPSPYW-
-TPSPYK-
-TPSPYI-
-TPSPYL-
TNPSPY--
-TPSPYV-
TPPSPY--
TGPSPY--
TQPSPY--
-TPSPYM-
TAPSPY--
-TPSPYA-
TCPSPY--
-TPSPYP-
-TPSPYN-
VGPSPY--
-VPSPYR-
-VPSPYL-
VVPSPY--
VRPSPY--
VNPSPY--
-VPSPYV-
VSPSPY--
VLPSPY--
VFPSPY--
VMPSPY--
VYPSPY--
-VPSPYS-
-VPSPYH-
VKPSPY--
VTPSPY--
-VPSPYK-
-VPSPYF-
VDPSPY--
-VPSPYM-
VHPSPY--
-VPSPYW-
-VPSPYQ-
-VPSPYA-
-VPSPYT-
VWPSPY--
-VPSPYI-
VPPSPY--
-VPSPYY-
-VPSPYN-

VEPSPY--
VQPSPY--
VCPSPY--
WEPSPY--
WSPSPY--
WKPSPY--
WRPSPY--
WYPSPY--
WLPSPY--
WGPSPY--
-WPSPYT-
WPPSPY--
WMPSPY--
WFPSPY--
WIPSPY--
WVPSPY--
-WPSPYM-
-WPSPYL-
-WPSPYA-
WWPSPY--
WDPSPY--
-WPSPYI-
-WPSPYW-
-WPSPYV-
-WPSPYS-
WAPSPY--
-WPSPYR-
-WPSPYQ-
-WPSPYN-
-WPSPYK-
WNPSPY--
-YPSPYR-
YSPSPY--
YGPSPY--
YAPSPY--
YIPSPY--
YWPSPY--
YFPSPY--
-YPSPYL-
YPPSPY--
YHPSPY--
YLPSPY--
YVPSPY--
-YPSPYS-
YNPSPY--
YEPSPY--
-YPSPYV-
YTPSPY--
YRPSPY--
YMPSPY--

	YKPSPY--		
	-YPSPYW-		
	-YPSPYQ-		
	YQPSPY--		
	-YPSPYT-		
	-YPSPYH-		
	YDPSPY--		
	YCPSPY--		
	-YPSPYY-		
	-YPSPYF-		
	-YPSPYC-		
PANY	188	755	45.24
	WEPANY--		
	-APANYT-		
	-APANYR-		
	-APANYS-		
	-APANYA-		
	ALPANY--		
	ASPANY--		
	-APANYL-		
	ARPANY--		
	AVPANY--		
	-APANYK-		
	AYPANY--		
	AAPANY--		
	ATPANY--		
	-APANYI-		
	AQPANY--		
	AIPANY--		
	ADPANY--		
	-APANYW-		
	-APANYY-		
	-APANYV-		
	-APANYF-		
	AHPANY--		
	-APANYQ-		
	AMPANY--		
	ANPANY--		
	AGPANY--		
	APPANY--		
	-APANYN-		
	-APANYG-		
	AEPANY--		
	ACPANY--		
	CRPANY--		
	CSPANY--		
	CCPANY--		
	-CPANYV-		
	CVPANY--		
	CMPANY--		

CAPANY--
CWPANY--
-CPANYK-
DRPANY--
DAPANY--
DTPANY--
DWPANY--
-DPANYK-
DLPANY--
DHPANY--
-DPANYV-
-DPANYL-
DVPANY--
DSPANY--
-DPANYW-
-DPANYT-
-DPANYH-
DEPANY--
-EPANYV-
-EPANYR-
ESPANY--
EGPANY--
-EPANYS-
ELPANY--
-EPANYW-
ETPANY--
-EPANYT-
EWPANY--
EQPANY--
-EPANYY-
-EPANYI-
EEPANY--
-EPANYK-
-EPANYA-
EMPANY--
EFPANY--
ERPANY--
-EPANYL-
EKPANY--
EHPANY--
FSPANY--
FMPANY--
-FPANYM-
-FPANYL-
FYPANY--
FTPANY--
FHPANY--
FDPANY--
FGPANY--
FRPANY--

FIPANY--
FWPANY--
-FPANYK-
FLPANY--
-FPANYS-
FFPANY--
FAPANY--
-FPANYY-
-FPANYI-
-FPANYF-
-FPANYA-
-FPANYV-
-FPANYT-
-FPANYH-
FKPANY--
FVPANY--
-FPANYQ-
-FPANYE-
FNPANY--
FEPANY--
-GPANYI-
GSPANY--
GYPANY--
-GPANYV-
-GPANYR-
GRPANY--
GMPANY--
GKPANY--
GFPANY--
-GPANYS-
GNPANY--
-GPANYQ-
-GPANYA-
-GPANYY-
GIPANY--
GPPANY--
GLPANY--
-GPANYL-
GHPANY--
GTPANY--
-GPANYF-
-GPANYE-
GEPANY--
GDPANY--
-GPANYK-
GQPANY--
-GPANYT-
-GPANYH-
-GPANYW-
-GPANYN-

−GPANYM−
−GPANYG−
GGPANY−−
GAPANY−−
−HPANYR−
HPPANY−−
HGPANY−−
HWPANY−−
HKPANY−−
HQPANY−−
HLPANY−−
HAPANY−−
HNPANY−−
HEPANY−−
−HPANYS−
−HPANYM−
−HPANYT−
HYPANY−−
−HPANYY−
−HPANYV−
−HPANYF−
HIPANY−−
−HPANYL−
HMPANY−−
HFPANY−−
ILPANY−−
−IPANYF−
−IPANYW−
−IPANYA−
IRPANY−−
−IPANYS−
ISPANY−−
−IPANYH−
IYPANY−−
IEPANY−−
IAPANY−−
−IPANYV−
IIPANY−−
IGPANY−−
ITPANY−−
IQPANY−−
−IPANYG−
−IPANYK−
−IPANYM−
−IPANYE−
IKPANY−−
−IPANYT−
INPANY−−
IMPANY−−
−IPANYY−

-IPANYI-
IHPANY--
ICPANY--
KSPANY--
-KPANYT-
KVPANY--
-KPANYM-
-KPANYW-
KMPANY--
KTPANY--
KIPANY--
KRPANY--
-KPANYQ-
KWPANY--
KGPANY--
-KPANYL-
-KPANYA-
-KPANYY-
-KPANYS-
-KPANYR-
-KPANYN-
-KPANYF-
KFPANY--
KAPANY--
KYPANY--
-KPANYE-
-KPANYI-
KDPANY--
-KPANYK-
-KPANYG-
KNPANY--
KLPANY--
KKPANY--
KPPANY--
-KPANYH-
KEPANY--
LSPANY--
LNPANY--
LRPANY--
-LPANYM-
-LPANYR-
LFPANY--
LLPANY--
LGPANY--
-LPANYT-
LKPANY--
LVPANY--
-LPANYL-
-LPANYI-
-LPANYA-

LWPANY--
-LPANYY-
-LPANYV-
LHPANY--
LDPANY--
LYPANY--
-LPANYS-
LIPANY--
LEPANY--
LAPANY--
LQPANY--
LMPANY--
-LPANYH-
LTPANY--
-LPANYW-
LPPANY--
-LPANYK-
-LPANYE-
MSPANY--
MQPANY--
-MPANYR-
-MPANYI-
-MPANYS-
-MPANYL-
-MPANYE-
MGPANY--
MEPANY--
MMPANY--
MYPANY--
MVPANY--
MLPANY--
MPPANY--
-MPANYT-
-MPANYM-
-MPANYA-
MIPANY--
MWPANY--
MTPANY--
-MPANYH-
-MPANYV-
MKPANY--
MNPANY--
MAPANY--
NRPANY--
-NPANYR-
NSPANY--
NGPANY--
NLPANY--
NTPANY--
-NPANYS-

NKPANY--
-NPANYQ-
NVPANY--
NHPANY--
NPPANY--
-NPANYW-
-NPANYT-
NMPANY--
-NPANYL-
-NPANYH-
-NPANYA-
NFPANY--
NDPANY--
NQPANY--
-NPANYY-
-NPANYI-
NIPANY--
NEPANY--
NCPANY--
--PANYIR
--PANYRT
--PANYRA
--PANYFS
--PANYTN
--PANYSS
--PANYRM
--PANYAS
PTPANY--
--PANYVS
--PANYLG
--PANYYY
--PANYLV
--PANYYG
--PANYTR
--PANYQS
--PANYYS
--PANYVM
--PANYVA
--PANYTL
--PANYRS
--PANYWA
--PANYVF
--PANYQT
--PANYMG
--PANYLR
--PANYIV
--PANYVW
--PANYLS
PRPANY--
--PANYHT

--PANYWN
--PANYLW
--PANYFA
PSPANY--
--PANYRK
--PANYMY
--PANYMI
--PANYVK
--PANYRL
--PANYMR
--PANYLN
--PANYFW
--PANYFT
--PANYFQ
--PANYYQ
--PANYVG
--PANYTT
--PANYTA
--PANYYV
--PANYYH
--PANYTK
--PANYGS
--PANYFL
--PANYFG
--PANYAN
--PANYVH
--PANYSL
--PANYSF
--PANYSA
--PANYAK
PFPANY--
--PANYWV
--PANYTG
--PANYRF
--PANYMM
--PANYMK
--PANYIG
--PANYAT
--PANYYM
--PANYSK
--PANYRV
--PANYNS
--PANYLK
--PANYKS
--PANYIT
--PANYIS
--PANYIM
--PANYHQ
--PANYAY
--PANYAH

PWPANY--
--PANYYW
--PANYVQ
--PANYTY
--PANYTM
--PANYSN
--PANYRQ
--PANYLM
--PANYLI
--PANYKV
--PANYIN
--PANYFN
--PANYEW
--PANYAI
PNPANY--
--PANYYF
--PANYVV
--PANYTS
--PANYTI
--PANYRI
--PANYRG
--PANYNW
--PANYMV
--PANYMS
--PANYLH
--PANYIA
--PANYHV
--PANYFK
--PANYEF
--PANYAW
--PANYWT
--PANYWG
--PANYRW
--PANYRH
--PANYQI
--PANYKR
--PANYKA
--PANYIY
--PANYIH
--PANYFI
--PANYFH
--PANYEA
--PANYYT
--PANYYR
--PANYYN
--PANYYE
--PANYSY
--PANYSW
--PANYST
--PANYRN

--PANYQR
--PANYKW
--PANYKT
--PANYGT
--PANYEQ
--PANYEH
--PANYVR
--PANYTQ
--PANYSI
--PANYSG
--PANYRE
--PANYLQ
--PANYLA
--PANYGV
--PANYFY
--PANYFR
--PANYAR
--PANYAL
--PANYAF
PGPANY--
--PANYYK
--PANYVL
--PANYTH
--PANYSV
--PANYRD
--PANYQN
--PANYQG
--PANYNY
--PANYNV
--PANYKL
--PANYKH
--PANYHG
--PANYHA
--PANYGA
--PANYFV
--PANYAG
PLPANY--
PHPANY--
--PANYYA
--PANYWW
--PANYWE
--PANYSR
--PANYSQ
--PANYSH
--PANYQY
--PANYQA
--PANYNT
--PANYKN
--PANYKI
--PANYIW

--PANYII
--PANYHH
--PANYHD
PYPANY--
PKPANY--
PIPANY--
--PANYYL
--PANYYD
--PANYWK
--PANYWD
--PANYVD
--PANYTF
--PANYSM
--PANYRY
--PANYQK
--PANYNL
--PANYNF
--PANYMT
--PANYMD
--PANYLL
--PANYKG
--PANYKE
--PANYHM
--PANYHI
--PANYGL
--PANYER
--PANYEG
--PANYCS
--PANYAM
--PANYAE
--PANYYI
--PANYWP
--PANYWL
--PANYVY
--PANYVE
--PANYTV
--PANYSD
--PANYRC
--PANYQW
--PANYPF
--PANYNM
--PANYNA
--PANYMF
--PANYME
--PANYMA
--PANYLE
--PANYLD
--PANYKD
--PANYIQ
--PANYIK

--PANYIF
--PANYIE
--PANYHR
--PANYHN
--PANYGH
--PANYFF
--PANYFD
--PANYEN
--PANYEK
--PANYEI
--PANYDT
--PANYDS
--PANYCM
-QPANYM-
QWPANY--
-QPANYL-
QRPANY--
QAPANY--
-QPANYV-
QLPANY--
QHPANY--
QSPANY--
-QPANYT-
QQPANY--
QFPANY--
-QPANYR-
QPPANY--
-QPANYY-
-QPANYW-
QTPANY--
-QPANYS-
-QPANYK-
-QPANYF-
QKPANY--
QIPANY--
-QPANYH-
QNPANY--
QGPANY--
-QPANYN-
QMPANY--
QCPANY--
-RPANYS-
RGPANY--
RRPANY--
-RPANYY-
RAPANY--
RWPANY--
RTPANY--
-RPANYM-
RKPANY--

RYPANY--
-RPANYL-
-RPANYA-
-RPANYH-
RDPANY--
RNPANY--
-RPANYT-
RLPANY--
RMPANY--
RIPANY--
-RPANYK-
-RPANYQ-
-RPANYE-
-RPANYF-
-RPANYW-
-RPANYV-
-RPANYG-
REPANY--
RQPANY--
RPPANY--
-RPANYC-
RCPANY--
SRPANY--
SSPANY--
-SPANYL-
-SPANYK-
SNPANY--
SVPANY--
-SPANYS-
SKPANY--
-SPANYI-
-SPANYF-
-SPANYM-
-SPANYW-
-SPANYH-
SGPANY--
-SPANYV-
SAPANY--
SWPANY--
SHPANY--
-SPANYQ-
SFPANY--
SPPANY--
-SPANYY-
-SPANYT-
SMPANY--
SLPANY--
STPANY--
SQPANY--
SYPANY--

-SPANYE-
SDPANY--
-SPANYA-
SIPANY--
-SPANYN-
SEPANY--
TGPANY--
-TPANYL-
-TPANYS-
TAPANY--
TPPANY--
TQPANY--
-TPANYR-
TKPANY--
TIPANY--
-TPANYK-
-TPANYE-
TWPANY--
TTPANY--
TSPANY--
-TPANYV-
-TPANYT-
-TPANYM-
-TPANYA-
TLPANY--
-TPANYQ-
-TPANYH-
TMPANY--
TVPANY--
THPANY--
-TPANYY-
TYPANY--
TRPANY--
-TPANYW-
-TPANYI-
-TPANYF-
TEPANY--
VLPANY--
-VPANYL-
-VPANYR-
-VPANYM-
VQPANY--
-VPANYI-
VTPANY--
-VPANYV-
VGPANY--
VMPANY--
-VPANYT-
VIPANY--
VFPANY--

VWPANY--
VSPANY--
VKPANY--
-VPANYS-
-VPANYK-
VNPANY--
VRPANY--
-VPANYH-
-VPANYG-
-VPANYE-
VVPANY--
VPPANY--
-VPANYQ-
VHPANY--
VAPANY--
-VPANYA-
-VPANYN-
WLPANY--
-WPANYT-
WTPANY--
WDPANY--
-WPANYR-
-WPANYL-
-WPANYK-
WHPANY--
WVPANY--
WSPANY--
WQPANY--
-WPANYV-
WYPANY--
WGPANY--
WFPANY--
-WPANYM-
-WPANYF-
WWPANY--
-WPANYY-
-WPANYH-
-WPANYA-
WNPANY--
WKPANY--
YWPANY--
-YPANYR-
YTPANY--
YIPANY--
YDPANY--
-YPANYL-
YRPANY--
YPPANY--
YQPANY--
-YPANYV-

YHPANY--
YYPANY--
-YPANYT-
-YPANYF-
YAPANY--
YVPANY--
-YPANYQ-
YSPANY--
YGPANY--
YNPANY--
YMPANY--
YLPANY--
-YPANYY-
-YPANYI-
-YPANYA-
YKPANY--
YFPANY--

PYHM	640	574	45.21

ATPYHM--
-APYHMY-
-APYHMR-
AIPYHM--
-APYHMM-
AAPYHM--
-APYHMT-
AMPYHM--
-APYHMA-
ANPYHM--
AGPYHM--
-APYHML-
ASPYHM--
-APYHMV-
AEPYHM--
-APYHMS-
AVPYHM--
AQPYHM--
-APYHMN-
-APYHMI-
-APYHMG-
APPYHM--
CQPYHM--
-CPYHML-
CVPYHM--
-CPYHMV-
-CPYHMI-
CGPYHM--
DKPYHM--
DTPYHM--
DSPYHM--
DMPYHM--

DNPYHM--
DGPYHM--
DDPYHM--
-EPYHMR-
EPPYHM--
EGPYHM--
EFPYHM--
ESPYHM--
ERPYHM--
-EPYHMM-
-EPYHMT-
EIPYHM--
-EPYHMS-
-EPYHMH-
ENPYHM--
EHPYHM--
EYPYHM--
-EPYHMA-
EMPYHM--
EAPYHM--
-EPYHML-
-EPYHMI-
-FPYHMI-
-FPYHMT-
-FPYHMS-
FEPYHM--
FWPYHM--
FVPYHM--
-FPYHMR-
FSPYHM--
FGPYHM--
FDPYHM--
FYPYHM--
FRPYHM--
FQPYHM--
-FPYHMQ-
FNPYHM--
-FPYHMK-
-FPYHMM-
-FPYHML-
-FPYHMH-
-FPYHMA-
GKPYHM--
-GPYHMT-
-GPYHMR-
GLPYHM--
GWPYHM--
GTPYHM--
-GPYHMF-
GQPYHM--

-GPYHMY-
-GPYHML-
GIPYHM--
GHPYHM--
GAPYHM--
-GPYHMH-
GGPYHM--
GYPYHM--
-GPYHMA-
GPPYHM--
GMPYHM--
GFPYHM--
GVPYHM--
-GPYHMV-
GEPYHM--
-HPYHMK-
HKPYHM--
HLPYHM--
HRPYHM--
HGPYHM--
-HPYHML-
HSPYHM--
-HPYHMQ-
HTPYHM--
HYPYHM--
HAPYHM--
-HPYHMH-
-HPYHMT-
-HPYHMS-
-HPYHMM-
-HPYHMA-
HNPYHM--
HIPYHM--
-HPYHMI-
HMPYHM--
HDPYHM--
IRPYHM--
IVPYHM--
-IPYHMR-
-IPYHML-
IKPYHM--
ISPYHM--
IMPYHM--
-IPYHMI-
ITPYHM--
IPPYHM--
INPYHM--
ILPYHM--
IIPYHM--
IHPYHM--

-IPYHMV-
-IPYHMN-
-IPYHMW-
-IPYHMH-
IDPYHM--
IWPYHM--
-IPYHMQ-
-IPYHMM-
-IPYHMG-
KSPYHM--
-KPYHMT-
KIPYHM--
-KPYHMF-
KTPYHM--
-KPYHMH-
KMPYHM--
-KPYHMN-
KNPYHM--
-KPYHMR-
-KPYHME-
KVPYHM--
-KPYHMW-
-KPYHMS-
-KPYHMA-
KGPYHM--
-KPYHMQ-
-KPYHML-
KPPYHM--
KQPYHM--
KLPYHM--
KKPYHM--
KRPYHM--
-KPYHMV-
KDPYHM--
LSPYHM--
LGPYHM--
LPPYHM--
LRPYHM--
LNPYHM--
LVPYHM--
LQPYHM--
LAPYHM--
LFPYHM--
LLPYHM--
LWPYHM--
-LPYHMH-
LEPYHM--
-LPYHMR-
LHPYHM--
-LPYHMT-

-LPYHMI-
-LPYHMK-
-LPYHML-
LKPYHM--
-LPYHMM-
-LPYHMA-
-LPYHMY-
-LPYHMV-
-LPYHMQ-
LIPYHM--
MNPYHM--
MQPYHM--
MYPYHM--
MVPYHM--
MFPYHM--
-MPYHMN-
-MPYHMR-
-MPYHMK-
-MPYHMY-
-MPYHMG-
MGPYHM--
MSPYHM--
MRPYHM--
-MPYHMF-
MCPYHM--
-MPYHMW-
-MPYHMS-
-MPYHME-
MKPYHM--
NLPYHM--
-NPYHML-
NGPYHM--
NKPYHM--
-NPYHMS-
NRPYHM--
NSPYHM--
-NPYHMT-
NTPYHM--
-NPYHMR-
-NPYHMI-
NHPYHM--
-NPYHMK-
NNPYHM--
NFPYHM--
-NPYHMQ-
-NPYHMM-
NPPYHM--
-NPYHMV-
-NPYHMF-
NEPYHM--

--PYHMRS
--PYHMVR
--PYHMTS
--PYHMNA
--PYHMRE
--PYHMRD
--PYHMLR
--PYHMIV
--PYHMLT
--PYHMLN
--PYHMVV
--PYHMVT
--PYHMLQ
--PYHMRY
--PYHMMT
--PYHMAS
--PYHMWH
--PYHMSY
--PYHMSS
--PYHMSA
--PYHMLS
--PYHMKG
--PYHMIL
--PYHMVG
--PYHMRN
--PYHMRG
--PYHMRT
--PYHMRA
--PYHMIR
--PYHMFT
--PYHMYT
--PYHMVK
--PYHMTG
--PYHMQG
--PYHMLD
--PYHMSR
--PYHMQA
--PYHMAA
--PYHMWK
--PYHMVN
--PYHMSQ
--PYHMRV
--PYHMRL
--PYHMLV
--PYHMKV
--PYHMKN
--PYHMIY
--PYHMIK
--PYHMIA
--PYHMEM

--PYHMAR
--PYHMWR
--PYHMTT
--PYHMRI
--PYHMKL
--PYHMIQ
--PYHMGS
--PYHMFS
--PYHMFR
--PYHMAI
--PYHMWS
--PYHMTF
--PYHMSV
--PYHMSM
--PYHMSL
--PYHMSH
--PYHMQT
--PYHMMV
--PYHMKR
--PYHMER
--PYHMEA
--PYHMAV
--PYHMWF
--PYHMVE
--PYHMSW
--PYHMSI
--PYHMSG
--PYHMSF
--PYHMQS
--PYHMMA
--PYHMLE
--PYHMKY
--PYHMIS
--PYHMHQ
--PYHMHA
--PYHMFK
--PYHMAW
--PYHMAH
PNPYHM--
--PYHMYV
--PYHMYF
--PYHMWG
--PYHMQH
--PYHMMF
--PYHMLI
--PYHMLG
--PYHMKH
--PYHMIG
--PYHMFF
--PYHMFD

--PYHMYN
--PYHMYD
--PYHMWY
--PYHMVY
--PYHMVQ
--PYHMVL
--PYHMVD
--PYHMTQ
--PYHMTI
--PYHMST
--PYHMRR
--PYHMRQ
--PYHMRF
--PYHMQR
--PYHMNS
--PYHMMN
--PYHMLM
--PYHMLH
--PYHMKE
--PYHMIN
--PYHMII
--PYHMHY
--PYHMHT
--PYHMHE
--PYHMGT
--PYHMFQ
--PYHMES
--PYHMAT
--PYHMAN
--PYHMAK
--PYHMYW
--PYHMWN
--PYHMTE
--PYHMTA
--PYHMNK
--PYHMMM
--PYHMML
--PYHMLK
--PYHMKM
--PYHMFE
--PYHMEV
--PYHMEQ
--PYHMEL
--PYHMEG
--PYHMDA
--PYHMCS
--PYHMYR
--PYHMYQ
--PYHMYM
--PYHMYG

--PYHMYE
--PYHMYA
--PYHMWV
--PYHMWT
--PYHMVS
--PYHMVH
--PYHMVF
--PYHMTN
--PYHMTL
--PYHMTD
--PYHMSN
--PYHMSK
--PYHMSD
--PYHMRH
--PYHMQV
--PYHMPQ
--PYHMPA
--PYHMNF
--PYHMMK
--PYHMKS
--PYHMKF
--PYHMIT
--PYHMHM
--PYHMGM
--PYHMGI
--PYHMGF
--PYHMEN
--PYHMEI
--PYHMDS
--PYHMCV
--PYHMCH
--PYHMAF
QWPYHM--
QTPYHM--
QGPYHM--
-QPYHMW-
-QPYHMR-
-QPYHMK-
-QPYHMF-
QRPYHM--
QLPYHM--
-QPYHML-
QVPYHM--
QAPYHM--
-QPYHMS-
QYPYHM--
-QPYHMN-
QHPYHM--
-RPYHML-
RTPYHM--

-RPYHMR-
RLPYHM--
-RPYHMF-
-RPYHMA-
RVPYHM--
RIPYHM--
RKPYHM--
RMPYHM--
RRPYHM--
-RPYHMT-
RWPYHM--
-RPYHMS-
-RPYHMI-
-RPYHMG-
RPPYHM--
RQPYHM--
-RPYHMV-
RNPYHM--
-RPYHMM-
REPYHM--
RDPYHM--
-RPYHMY-
-RPYHMK-
RAPYHM--
SRPYHM--
-SPYHMR-
SFPYHM--
SLPYHM--
SAPYHM--
-SPYHMS-
SSPYHM--
-SPYHMM-
SIPYHM--
STPYHM--
-SPYHML-
-SPYHMK-
-SPYHMF-
SWPYHM--
-SPYHMI-
SHPYHM--
SMPYHM--
SGPYHM--
SVPYHM--
SQPYHM--
SPPYHM--
SKPYHM--
SNPYHM--
-SPYHMT-
-SPYHMQ-
-SPYHMG-

-SPYHMN-
SDPYHM--
SCPYHM--
TTPYHM--
TRPYHM--
TIPYHM--
TLPYHM--
TSPYHM--
-TPYHMS-
-TPYHMT-
-TPYHMH-
-TPYHMR-
TAPYHM--
-TPYHMY-
-TPYHMF-
TMPYHM--
TGPYHM--
TFPYHM--
-TPYHMA-
TPPYHM--
TKPYHM--
TEPYHM--
TYPYHM--
TVPYHM--
-TPYHMN-
-TPYHMG-
VVPYHM--
VGPYHM--
-VPYHMR-
VLPYHM--
VKPYHM--
VTPYHM--
VNPYHM--
VYPYHM--
-VPYHMK-
VPPYHM--
-VPYHMW-
-VPYHML-
-VPYHMH-
-VPYHMY-
VHPYHM--
-VPYHMS-
-VPYHMT-
-VPYHMG-
VEPYHM--
-VPYHMM-
VMPYHM--
VFPYHM--
VQPYHM--
-VPYHMV-

	-VPYHME-		
	VDPYHM--		
	WSPYHM--		
	WRPYHM--		
	WKPYHM--		
	-WPYHMT-		
	WTPYHM--		
	WVPYHM--		
	-WPYHMM-		
	WGPYHM--		
	-WPYHML-		
	-WPYHMR-		
	WHPYHM--		
	WEPYHM--		
	-WPYHMS-		
	-WPYHMI-		
	WIPYHM--		
	WYPYHM--		
	-WPYHMW-		
	-WPYHMV-		
	-WPYHMG-		
	WLPYHM--		
	YAPYHM--		
	-YPYHMT-		
	YRPYHM--		
	YQPYHM--		
	YVPYHM--		
	-YPYHMI-		
	YPPYHM--		
	YMPYHM--		
	YLPYHM--		
	YGPYHM--		
	YIPYHM--		
	YFPYHM--		
	-YPYHMS-		
	YNPYHM--		
	YCPYHM--		
	-YPYHMY-		
	-YPYHMV-		
	-YPYHMR-		
	-YPYHML-		
	-YPYHMG-		
PYSY	131	790	45.20
	-DPYSYL-		
	-EPYSYA-		
	-APYSYR-		
	ATPYSY--		
	-APYSYL-		
	ARPYSY--		
	AAPYSY--		

AYPYSY--
ANPYSY--
ALPYSY--
AKPYSY--
ASPYSY--
AMPYSY--
-APYSYY-
-APYSYS-
AGPYSY--
AQPYSY--
-APYSYV-
-APYSYH-
-APYSYT-
AWPYSY--
ADPYSY--
AHPYSY--
AEPYSY--
-APYSYM-
AFPYSY--
APPYSY--
-APYSYK-
ACPYSY--
AVPYSY--
-APYSYQ-
-APYSYC-
AIPYSY--
-APYSYN-
-APYSYE-
CGPYSY--
CLPYSY--
CAPYSY--
CSPYSY--
CRPYSY--
CNPYSY--
CVPYSY--
CQPYSY--
-CPYSYQ-
-CPYSYH-
CPPYSY--
CMPYSY--
CHPYSY--
CDPYSY--
DQPYSY--
DMPYSY--
DKPYSY--
DYPYSY--
-DPYSYM-
DWPYSY--
DSPYSY--
DLPYSY--

DAPYSY--
DFPYSY--
DTPYSY--
-DPYSYI-
DVPYSY--
DRPYSY--
-DPYSYT-
DHPYSY--
-DPYSYV-
-DPYSYK-
-DPYSYF-
DGPYSY--
-DPYSYS-
-DPYSYR-
DCPYSY--
-DPYSYN-
DDPYSY--
ESPYSY--
EVPYSY--
EIPYSY--
ETPYSY--
ERPYSY--
EKPYSY--
-EPYSYS-
EMPYSY--
-EPYSYR-
-EPYSYH-
EAPYSY--
-EPYSYV-
EGPYSY--
-EPYSYM-
ELPYSY--
-EPYSYY-
-EPYSYW-
-EPYSYT-
-EPYSYL-
-EPYSYI-
ECPYSY--
EQPYSY--
EEPYSY--
-EPYSYQ-
-EPYSYK-
-EPYSYF-
FRPYSY--
-FPYSYR-
FGPYSY--
FLPYSY--
-FPYSYL-
FSPYSY--
FKPYSY--

FYPYSY--
FIPYSY--
FPPYSY--
FHPYSY--
-FPYSYI-
FTPYSY--
-FPYSYV-
FEPYSY--
FVPYSY--
-FPYSYM-
FMPYSY--
FWPYSY--
FNPYSY--
FAPYSY--
-FPYSYQ-
FQPYSY--
-FPYSYY-
-FPYSYA-
-FPYSYT-
-FPYSYK-
-FPYSYH-
FDPYSY--
GSPYSY--
GRPYSY--
-GPYSYR-
GNPYSY--
GVPYSY--
GIPYSY--
-GPYSYT-
GHPYSY--
-GPYSYK-
-GPYSYV-
GTPYSY--
GQPYSY--
GEPYSY--
GGPYSY--
GLPYSY--
GKPYSY--
-GPYSYM-
GFPYSY--
-GPYSYL-
-GPYSYN-
-GPYSYQ-
-GPYSYW-
-GPYSYF-
GAPYSY--
-GPYSYY-
-GPYSYH-
GPPYSY--
GMPYSY--

-GPYSYS-
GCPYSY--
-GPYSYI-
-GPYSYE-
GDPYSY--
GYPYSY--
GWPYSY--
-GPYSYA-
-GPYSYG-
HHPYSY--
HLPYSY--
HRPYSY--
-HPYSYR-
-HPYSYI-
HVPYSY--
HGPYSY--
HSPYSY--
HAPYSY--
-HPYSYL-
HMPYSY--
HEPYSY--
HKPYSY--
HYPYSY--
-HPYSYT-
HTPYSY--
-HPYSYS-
-HPYSYK-
HIPYSY--
-HPYSYM-
HWPYSY--
-HPYSYY-
-HPYSYA-
-HPYSYH-
HPPYSY--
HDPYSY--
-HPYSYF-
-HPYSYW-
HNPYSY--
HQPYSY--
-HPYSYE-
IRPYSY--
IKPYSY--
-IPYSYL-
IYPYSY--
ILPYSY--
IPPYSY--
IGPYSY--
IHPYSY--
IAPYSY--
IVPYSY--

-IPYSYV-
IQPYSY--
-IPYSYR-
-IPYSYS-
-IPYSYK-
ITPYSY--
-IPYSYT-
IMPYSY--
IWPYSY--
IFPYSY--
-IPYSYM-
INPYSY--
IEPYSY--
-IPYSYN-
IDPYSY--
ISPYSY--
-IPYSYY-
-IPYSYF-
-IPYSYE-
-IPYSYA-
-KPYSYR-
-KPYSYL-
KLPYSY--
KTPYSY--
-KPYSYQ-
-KPYSYV-
KAPYSY--
KHPYSY--
-KPYSYF-
KKPYSY--
KIPYSY--
KGPYSY--
-KPYSYM-
-KPYSYW-
-KPYSYT-
-KPYSYI-
-KPYSYA-
KFPYSY--
-KPYSYS-
-KPYSYY-
KMPYSY--
KWPYSY--
-KPYSYK-
KPPYSY--
-KPYSYG-
KNPYSY--
KYPYSY--
KQPYSY--
-KPYSYN-
KEPYSY--

-KPYSYH-
KDPYSY--
LSPYSY--
LKPYSY--
LTPYSY--
-LPYSYR-
-LPYSYT-
-LPYSYL-
LEPYSY--
LFPYSY--
LLPYSY--
LNPYSY--
-LPYSYM-
-LPYSYS-
LGPYSY--
-LPYSYV-
LPPYSY--
-LPYSYQ-
-LPYSYK-
LAPYSY--
LDPYSY--
LYPYSY--
LQPYSY--
LMPYSY--
LHPYSY--
LWPYSY--
-LPYSYH-
LIPYSY--
-LPYSYF-
-LPYSYY-
-LPYSYW-
LCPYSY--
MRPYSY--
-MPYSYR-
-MPYSYL-
MLPYSY--
MGPYSY--
-MPYSYI-
-MPYSYV-
MSPYSY--
MTPYSY--
-MPYSYF-
MVPYSY--
-MPYSYS-
MIPYSY--
MYPYSY--
MWPYSY--
MPPYSY--
MAPYSY--
MMPYSY--

-MPYSYT-
-MPYSYM-
-MPYSYK-
-MPYSYH-
MNPYSY--
MCPYSY--
MDPYSY--
MFPYSY--
-MPYSYQ-
MEPYSY--
-MPYSYN-
NRPYSY--
-NPYSYR-
NFPYSY--
NAPYSY--
NLPYSY--
NNPYSY--
-NPYSYY-
-NPYSYI-
NGPYSY--
NMPYSY--
-NPYSYM-
NPPYSY--
NIPYSY--
-NPYSYW-
-NPYSYL-
NTPYSY--
NSPYSY--
-NPYSYV-
NHPYSY--
NVPYSY--
-NPYSYH-
NWPYSY--
-NPYSYS-
NYPYSY--
-NPYSYT-
-NPYSYK-
-NPYSYG-
-NPYSYA-
NDPYSY--
NQPYSY--
-NPYSYQ-
-NPYSYN-
-NPYSYF-
--PYSYRA
--PYSYLG
--PYSYLS
--PYSYRS
--PYSYRG
--PYSYMR

--PYSYIT
--PYSYNS
--PYSYRN
--PYSYVA
--PYSYRT
--PYSYVR
--PYSYSS
--PYSYTS
--PYSYQG
--PYSYHS
--PYSYRM
--PYSYAS
--PYSYKG
--PYSYST
--PYSYAV
--PYSYTI
--PYSYSA
--PYSYLH
--PYSYIA
--PYSYYS
--PYSYYG
--PYSYSN
--PYSYSI
--PYSYVG
--PYSYSV
--PYSYRF
--PYSYLT
PGPYSY--
--PYSYTV
--PYSYRH
--PYSYTA
--PYSYMT
--PYSYVS
--PYSYSG
PKPYSY--
--PYSYLR
--PYSYKF
--PYSYQS
--PYSYKV
--PYSYKS
--PYSYTR
--PYSYMN
--PYSYRL
--PYSYFT
--PYSYTK
--PYSYSR
--PYSYYR
--PYSYWT
--PYSYYT
--PYSYTN

--PYSYKW
--PYSYWR
--PYSYVK
--PYSYTM
--PYSYHG
--PYSYNM
--PYSYLK
--PYSYHR
--PYSYGS
--PYSYAA
--PYSYSL
--PYSYSK
--PYSYRQ
--PYSYIR
--PYSYFQ
--PYSYSM
--PYSYSH
--PYSYRV
--PYSYRE
--PYSYQK
--PYSYLA
--PYSYFA
--PYSYWK
--PYSYVY
--PYSYVQ
--PYSYTH
--PYSYQR
--PYSYNL
--PYSYKQ
--PYSYKI
--PYSYKD
--PYSYKA
--PYSYYA
--PYSYVW
--PYSYVV
--PYSYVH
--PYSYTY
--PYSYTL
--PYSYQT
--PYSYMG
--PYSYMA
--PYSYFR
PQPYSY--
--PYSYYK
--PYSYIN
--PYSYIK
--PYSYFS
--PYSYFG
PNPYSY--
--PYSYYN

--PYSYWH
--PYSYTG
--PYSYME
--PYSYGI
--PYSYAM
--PYSYVT
--PYSYSW
--PYSYRW
--PYSYQW
--PYSYHT
--PYSYHL
--PYSYEN
--PYSYAG
PHPYSY--
--PYSYQV
--PYSYNT
--PYSYKN
--PYSYIQ
--PYSYID
--PYSYFM
--PYSYEW
--PYSYEA
PAPYSY--
--PYSYYH
--PYSYRY
--PYSYQN
--PYSYQH
--PYSYIF
--PYSYFN
--PYSYFK
--PYSYEG
--PYSYAN
--PYSYVN
--PYSYTQ
--PYSYSY
--PYSYRI
--PYSYMQ
--PYSYKT
--PYSYGT
--PYSYGL
--PYSYAK
--PYSYWG
--PYSYLQ
--PYSYIH
--PYSYHN
--PYSYGG
--PYSYGA
--PYSYES
--PYSYAT
PYPYSY--

PIPYSY--
--PYSYYV
--PYSYYL
--PYSYWA
--PYSYTD
--PYSYSE
--PYSYQM
--PYSYNF
--PYSYNA
--PYSYMK
--PYSYKK
--PYSYGY
--PYSYFH
--PYSYET
--PYSYAY
--PYSYAW
--PYSYAI
--PYSYAH
PEPYSY--
--PYSYYY
--PYSYYM
--PYSYWE
--PYSYVE
--PYSYVD
--PYSYTW
--PYSYQF
--PYSYPL
--PYSYNH
--PYSYMH
--PYSYKH
--PYSYIG
--PYSYHY
--PYSYHQ
--PYSYHM
--PYSYHD
--PYSYGV
--PYSYGM
--PYSYGF
--PYSYFF
--PYSYEM
--PYSYAQ
--PYSYYQ
--PYSYWW
--PYSYWQ
--PYSYWN
--PYSYWD
--PYSYVI
--PYSYTF
--PYSYTC
--PYSYSC

--PYSYQL
--PYSYNY
--PYSYNV
--PYSYNK
--PYSYMW
--PYSYLD
--PYSYKP
--PYSYKM
--PYSYIW
--PYSYHK
--PYSYHH
--PYSYHF
--PYSYHE
--PYSYFD
--PYSYEY
--PYSYEH
-PPYSYT-
-PPYSYR-
PCPYSY--
QRPYSY--
-QPYSYR-
QLPYSY--
-QPYSYL-
QIPYSY--
QGPYSY--
-QPYSYV-
QVPYSY--
QSPYSY--
QHPYSY--
-QPYSYY-
-QPYSYI-
-QPYSYF-
QWPYSY--
QEPYSY--
QNPYSY--
QMPYSY--
QTPYSY--
-QPYSYK-
QPPYSY--
-QPYSYS-
-QPYSYA-
QFPYSY--
QDPYSY--
QYPYSY--
-QPYSYW-
-QPYSYH-
RSPYSY--
RVPYSY--
RRPYSY--
RGPYSY--

RQPYSY--
RWPYSY--
RYPYSY--
-RPYSYL-
RLPYSY--
RDPYSY--
RAPYSY--
RTPYSY--
RNPYSY--
-RPYSYV-
RFPYSY--
RPPYSY--
-RPYSYK-
-RPYSYA-
-RPYSYW-
RHPYSY--
-RPYSYM-
RIPYSY--
-RPYSYI-
-RPYSYQ-
RMPYSY--
REPYSY--
-RPYSYH-
-RPYSYE-
SRPYSY--
SSPYSY--
SMPYSY--
-SPYSYS-
SKPYSY--
SWPYSY--
SIPYSY--
SPPYSY--
SFPYSY--
-SPYSYI-
STPYSY--
SHPYSY--
SAPYSY--
SLPYSY--
-SPYSYV-
-SPYSYT-
-SPYSYW-
SYPYSY--
SGPYSY--
SQPYSY--
-SPYSYL-
SNPYSY--
-SPYSYY-
-SPYSYH-
SVPYSY--
-SPYSYF-

-SPYSYA-
SDPYSY--
-SPYSYQ-
-SPYSYM-
SCPYSY--
SEPYSY--
-SPYSYC-
TRPYSY--
TLPYSY--
TWPYSY--
-TPYSYY-
-TPYSYS-
TAPYSY--
TSPYSY--
-TPYSYL-
TPPYSY--
TDPYSY--
TTPYSY--
TNPYSY--
-TPYSYV-
TGPYSY--
TFPYSY--
-TPYSYR-
TMPYSY--
-TPYSYH-
TYPYSY--
-TPYSYT-
-TPYSYM-
-TPYSYK-
TKPYSY--
TEPYSY--
-TPYSYW-
THPYSY--
TCPYSY--
TQPYSY--
-TPYSYI-
-TPYSYQ-
-TPYSYN-
-TPYSYF-
TIPYSY--
TVPYSY--
-TPYSYC-
-VPYSYL-
VGPYSY--
VVPYSY--
-VPYSYV-
-VPYSYS-
VKPYSY--
VTPYSY--
-VPYSYR-

VWPYSY--
-VPYSYT-
VYPYSY--
VNPYSY--
VHPYSY--
-VPYSYI-
-VPYSYF-
VMPYSY--
VIPYSY--
VDPYSY--
-VPYSYQ-
-VPYSYM-
-VPYSYA-
VLPYSY--
-VPYSYY-
-VPYSYW-
-VPYSYH-
VFPYSY--
-VPYSYE-
VPPYSY--
VEPYSY--
VAPYSY--
-VPYSYG-
-VPYSYN-
WRPYSY--
-WPYSYR-
WTPYSY--
WMPYSY--
WKPYSY--
WFPYSY--
WVPYSY--
-WPYSYV-
WSPYSY--
WEPYSY--
-WPYSYT-
WHPYSY--
WDPYSY--
WYPYSY--
WQPYSY--
WLPYSY--
-WPYSYI-
-WPYSYQ-
-WPYSYK-
-WPYSYF-
-WPYSYA-
WPPYSY--
-WPYSYY-
-WPYSYS-
-WPYSYL-
WAPYSY--

	-YPYSYL-		
	-YPYSYI-		
	-YPYSYT-		
	-YPYSYS-		
	YLPYSY--		
	YGPYSY--		
	YAPYSY--		
	YRPYSY--		
	YQPYSY--		
	-YPYSYY-		
	YHPYSY--		
	YMPYSY--		
	YIPYSY--		
	YVPYSY--		
	YFPYSY--		
	YPPYSY--		
	YDPYSY--		
	-YPYSYR-		
	-YPYSYE-		
	YEPYSY--		
	YYPYSY--		
	-YPYSYA-		
	YKPYSY--		
	YTPYSY--		
	-YPYSYQ-		
	-YPYSYM-		
	-YPYSYK-		
	-YPYSYF-		
	YWPYSY--		
	-YPYSYW-		
	-YPYSYN-		
	-YPYSYH-		
	YCPYSY--		
PLQY	247	725	45.16
	-APLQYG-		
	-KPLQYG-		
	-NPLQYH-		
	--PLQYGL		
	--PLQYGG		
	-VPLQYG-		
	-APLQYL-		
	ALPLQY--		
	ARPLQY--		
	ATPLQY--		
	AVPLQY--		
	ASPLQY--		
	AQPLQY--		
	-APLQYS-		
	-APLQYR-		
	-APLQYT-		

AGPLQY--
AMPLQY--
-APLQYV-
-APLQYA-
AFPLQY--
-APLQYW-
AAPLQY--
-APLQYM-
AHPLQY--
-APLQYY-
-APLQYQ-
AWPLQY--
APPLQY--
-APLQYK-
-APLQYF-
AIPLQY--
AEPLQY--
ACPLQY--
CRPLQY--
CCPLQY--
CAPLQY--
CSPLQY--
CVPLQY--
CHPLQY--
CGPLQY--
CEPLQY--
DSPLQY--
DRPLQY--
-DPLQYS-
-DPLQYR-
-DPLQYV-
DWPLQY--
DMPLQY--
DIPLQY--
DQPLQY--
-DPLQYN-
DNPLQY--
DVPLQY--
DTPLQY--
-DPLQYY-
-DPLQYL-
-DPLQYK-
-DPLQYI-
-DPLQYF-
-DPLQYC-
DHPLQY--
DFPLQY--
-EPLQYR-
ESPLQY--
EVPLQY--

EWPLQY--
-EPLQYI-
ETPLQY--
EIPLQY--
-EPLQYV-
EKPLQY--
EMPLQY--
EQPLQY--
-EPLQYA-
EGPLQY--
-EPLQYM-
-EPLQYY-
-EPLQYK-
ERPLQY--
-EPLQYL-
-EPLQYF-
EHPLQY--
EFPLQY--
EAPLQY--
EYPLQY--
-EPLQYN-
ELPLQY--
FTPLQY--
-FPLQYV-
FSPLQY--
FRPLQY--
FGPLQY--
-FPLQYR-
FLPLQY--
FFPLQY--
FHPLQY--
FNPLQY--
FYPLQY--
FPPLQY--
-FPLQYT-
FQPLQY--
FAPLQY--
-FPLQYY-
-FPLQYW-
-FPLQYN-
-FPLQYS-
FKPLQY--
FDPLQY--
-FPLQYM-
-FPLQYA-
FEPLQY--
GVPLQY--
GEPLQY--
GAPLQY--
GRPLQY--

-GPLQYN-
GNPLQY--
-GPLQYS-
-GPLQYI-
-GPLQYR-
-GPLQYT-
GSPLQY--
GWPLQY--
GLPLQY--
GHPLQY--
-GPLQYK-
GFPLQY--
-GPLQYQ-
-GPLQYF-
GGPLQY--
GTPLQY--
-GPLQYY-
GDPLQY--
GYPLQY--
GQPLQY--
GMPLQY--
-GPLQYV-
-GPLQYH-
-GPLQYG-
GCPLQY--
GPPLQY--
-GPLQYA-
GKPLQY--
HRPLQY--
HIPLQY--
-HPLQYR-
-HPLQYV-
HSPLQY--
HLPLQY--
HKPLQY--
HGPLQY--
HVPLQY--
-HPLQYL-
HTPLQY--
HPPLQY--
-HPLQYS-
-HPLQYT-
-HPLQYK-
-HPLQYN-
HYPLQY--
-HPLQYY-
-HPLQYA-
HNPLQY--
HFPLQY--
-HPLQYQ-

HMPLQY--
HEPLQY--
HAPLQY--
HQPLQY--
-HPLQYF-
HDPLQY--
HCPLQY--
IKPLQY--
ILPLQY--
ISPLQY--
IRPLQY--
-IPLQYM-
IGPLQY--
-IPLQYT-
-IPLQYS-
IQPLQY--
-IPLQYR-
-IPLQYN-
-IPLQYK-
IYPLQY--
-IPLQYI-
IIPLQY--
IFPLQY--
-IPLQYH-
-IPLQYF-
IHPLQY--
IEPLQY--
INPLQY--
IDPLQY--
-IPLQYQ-
-IPLQYY-
IVPLQY--
-IPLQYW-
ITPLQY--
-IPLQYA-
ICPLQY--
KRPLQY--
KLPLQY--
-KPLQYS-
KAPLQY--
KGPLQY--
KPPLQY--
-KPLQYR-
-KPLQYT-
-KPLQYQ-
-KPLQYA-
-KPLQYL-
KQPLQY--
KYPLQY--
-KPLQYW-

KIPLQY--
KVPLQY--
-KPLQYY-
-KPLQYK-
KDPLQY--
KWPLQY--
-KPLQYI-
KNPLQY--
-KPLQYM-
-KPLQYF-
KHPLQY--
KEPLQY--
KCPLQY--
-KPLQYH-
KKPLQY--
-KPLQYE-
KFPLQY--
LPPLQY--
LLPLQY--
LGPLQY--
LKPLQY--
LVPLQY--
LEPLQY--
LSPLQY--
LYPLQY--
-LPLQYR-
LQPLQY--
LIPLQY--
LAPLQY--
LDPLQY--
LHPLQY--
-LPLQYV-
-LPLQYL-
-LPLQYK-
-LPLQYH-
-LPLQYS-
-LPLQYT-
LWPLQY--
-LPLQYM-
-LPLQYI-
-LPLQYQ-
LMPLQY--
-LPLQYY-
LNPLQY--
-LPLQYA-
LFPLQY--
MRPLQY--
MVPLQY--
-MPLQYT-
-MPLQYR-

MLPLQY--
-MPLQYI-
MQPLQY--
MNPLQY--
-MPLQYS-
MFPLQY--
MTPLQY--
-MPLQYL-
MEPLQY--
MHPLQY--
MPPLQY--
-MPLQYY-
-MPLQYV-
MIPLQY--
MYPLQY--
MWPLQY--
-MPLQYH-
-MPLQYK-
-MPLQYA-
MMPLQY--
MGPLQY--
-MPLQYN-
MAPLQY--
-MPLQYQ-
-MPLQYM-
MKPLQY--
MDPLQY--
-NPLQYR-
NYPLQY--
NLPLQY--
NEPLQY--
NMPLQY--
NRPLQY--
NVPLQY--
NSPLQY--
NPPLQY--
-NPLQYV-
-NPLQYT-
NIPLQY--
NWPLQY--
NTPLQY--
NKPLQY--
NQPLQY--
-NPLQYW-
-NPLQYS-
-NPLQYN-
NGPLQY--
NDPLQY--
NAPLQY--
-NPLQYM-

NFPLQY--
NHPLQY--
-NPLQYY-
-NPLQYL-
-NPLQYI-
NNPLQY--
--PLQYRN
--PLQYLG
--PLQYLS
--PLQYRM
--PLQYSS
--PLQYRQ
--PLQYSV
--PLQYMS
--PLQYHT
--PLQYRT
--PLQYTG
--PLQYSG
--PLQYRG
PGPLQY--
--PLQYQW
--PLQYFS
--PLQYYR
--PLQYVT
--PLQYRV
--PLQYRE
--PLQYKY
--PLQYVG
--PLQYNN
--PLQYLT
--PLQYYG
--PLQYYA
--PLQYKT
--PLQYKN
--PLQYKS
--PLQYIV
--PLQYFG
--PLQYWA
--PLQYTR
--PLQYIS
--PLQYRD
--PLQYNG
--PLQYLN
--PLQYKH
--PLQYKG
--PLQYIQ
--PLQYAG
--PLQYWR
--PLQYSA
--PLQYRA

--PLQYQG
--PLQYMH
--PLQYQA
--PLQYMN
--PLQYKA
--PLQYHS
--PLQYFQ
--PLQYYS
--PLQYYM
--PLQYWG
--PLQYVN
--PLQYRW
--PLQYNS
--PLQYKM
--PLQYIT
--PLQYIR
--PLQYHH
--PLQYVA
--PLQYSR
--PLQYSQ
--PLQYSL
--PLQYMA
--PLQYIM
--PLQYHN
--PLQYYQ
--PLQYTK
--PLQYRF
--PLQYQV
--PLQYNH
--PLQYHA
PDPLQY--
--PLQYYI
--PLQYTW
--PLQYSM
--PLQYSH
--PLQYRI
--PLQYNT
--PLQYMW
--PLQYMT
--PLQYMQ
--PLQYMG
--PLQYMD
--PLQYGA
--PLQYFF
--PLQYAI
--PLQYYN
--PLQYVM
--PLQYTT
--PLQYTL
--PLQYSN

--PLQYRY
--PLQYMR
--PLQYMF
--PLQYII
--PLQYFA
--PLQYES
--PLQYAT
--PLQYYV
--PLQYWN
--PLQYTQ
--PLQYSK
--PLQYNM
--PLQYMI
--PLQYME
--PLQYIK
--PLQYFM
--PLQYFH
--PLQYWK
--PLQYTV
--PLQYTM
--PLQYSY
--PLQYSI
--PLQYRS
--PLQYQF
--PLQYNY
--PLQYNF
--PLQYMY
--PLQYLR
--PLQYLK
--PLQYHF
--PLQYGT
--PLQYGS
--PLQYFN
--PLQYFK
--PLQYTS
--PLQYTA
--PLQYRH
--PLQYQH
--PLQYNA
--PLQYKL
--PLQYKK
--PLQYKI
--PLQYKF
--PLQYAN
--PLQYAA
--PLQYYY
--PLQYYH
--PLQYWQ
--PLQYVE
--PLQYTI

--PLQYST
--PLQYSF
--PLQYQM
--PLQYLH
--PLQYLD
--PLQYIH
--PLQYHI
--PLQYHG
--PLQYAW
--PLQYYT
--PLQYYL
--PLQYWT
--PLQYVY
--PLQYVV
--PLQYVQ
--PLQYTN
--PLQYTF
--PLQYQN
--PLQYNK
--PLQYNE
--PLQYKV
--PLQYKD
--PLQYIA
--PLQYHW
--PLQYHV
--PLQYGV
--PLQYFR
--PLQYFE
PEPLQY--
PCPLQY--
-PPLQYY-
-PPLQYT-
PNPLQY--
--PLQYYW
--PLQYYK
--PLQYYE
--PLQYVH
--PLQYTY
--PLQYTD
--PLQYSD
--PLQYRP
--PLQYQS
--PLQYQR
--PLQYPT
--PLQYNV
--PLQYNQ
--PLQYNL
--PLQYNI
--PLQYMV
--PLQYML

--PLQYKR
--PLQYKP
--PLQYKC
--PLQYIY
--PLQYID
--PLQYHQ
--PLQYHP
--PLQYHE
--PLQYGN
--PLQYFT
--PLQYEW
--PLQYEI
--PLQYEA
--PLQYAM
QRPLQY--
-QPLQYV-
QLPLQY--
QWPLQY--
-QPLQYR-
QKPLQY--
QTPLQY--
-QPLQYI-
QMPLQY--
QHPLQY--
QSPLQY--
QGPLQY--
-QPLQYK-
QFPLQY--
QVPLQY--
-QPLQYY-
-QPLQYS-
-QPLQYN-
-QPLQYF-
-QPLQYQ-
-QPLQYH-
QIPLQY--
QAPLQY--
QPPLQY--
-QPLQYA-
QNPLQY--
QEPLQY--
-QPLQYM-
QDPLQY--
-RPLQYR-
RVPLQY--
RRPLQY--
RMPLQY--
-RPLQYI-
-RPLQYV-
RLPLQY--

RQPLQY--
-RPLQYK-
-RPLQYH-
RNPLQY--
REPLQY--
RHPLQY--
RTPLQY--
-RPLQYY-
-RPLQYN-
RAPLQY--
-RPLQYA-
RYPLQY--
-RPLQYQ-
RIPLQY--
-RPLQYF-
-RPLQYM-
-RPLQYG-
RDPLQY--
-RPLQYD-
RFPLQY--
STPLQY--
-SPLQYR-
SLPLQY--
SRPLQY--
SAPLQY--
-SPLQYM-
-SPLQYF-
SGPLQY--
-SPLQYN-
-SPLQYL-
SNPLQY--
SYPLQY--
SDPLQY--
-SPLQYS-
SWPLQY--
SSPLQY--
SVPLQY--
-SPLQYQ-
SEPLQY--
SPPLQY--
-SPLQYV-
SKPLQY--
-SPLQYK-
-SPLQYH-
SQPLQY--
-SPLQYA-
-SPLQYI-
-SPLQYT-
SFPLQY--
SHPLQY--

SMPLQY--
SCPLQY--
TRPLQY--
TVPLQY--
TGPLQY--
TYPLQY--
TSPLQY--
-TPLQYY-
-TPLQYF-
-TPLQYM-
-TPLQYQ-
TKPLQY--
-TPLQYK-
TIPLQY--
TAPLQY--
TTPLQY--
TLPLQY--
TNPLQY--
TMPLQY--
-TPLQYT-
-TPLQYS-
-TPLQYL-
TWPLQY--
-TPLQYN-
TQPLQY--
-TPLQYW-
THPLQY--
TDPLQY--
TFPLQY--
-TPLQYI-
TEPLQY--
-TPLQYV-
TPPLQY--
-TPLQYH-
-TPLQYG-
-TPLQYC-
VMPLQY--
VTPLQY--
-VPLQYR-
VPPLQY--
VSPLQY--
VRPLQY--
VAPLQY--
VFPLQY--
VEPLQY--
VLPLQY--
-VPLQYM-
VIPLQY--
-VPLQYF-
VQPLQY--

VNPLQY--
VHPLQY--
VGPLQY--
-VPLQYS-
VVPLQY--
-VPLQYY-
VCPLQY--
-VPLQYI-
VDPLQY--
-VPLQYT-
-VPLQYK-
VWPLQY--
-VPLQYH-
-VPLQYA-
WRPLQY--
WSPLQY--
WTPLQY--
WGPLQY--
-WPLQYR-
WLPLQY--
WHPLQY--
WVPLQY--
-WPLQYS-
WYPLQY--
-WPLQYK-
WWPLQY--
-WPLQYV-
-WPLQYN-
-WPLQYA-
-WPLQYY-
-WPLQYM-
-WPLQYI-
WFPLQY--
-WPLQYT-
WMPLQY--
WEPLQY--
-YPLQYL-
YTPLQY--
-YPLQYS-
YKPLQY--
-YPLQYI-
YVPLQY--
-YPLQYA-
-YPLQYQ-
YLPLQY--
YSPLQY--
-YPLQYY-
-YPLQYK-
YMPLQY--
-YPLQYV-

	YNPLQY--		
	-YPLQYW-		
	-YPLQYN-		
	YGPLQY--		
	YWPLQY--		
	-YPLQYM-		
	YIPLQY--		
	YPPLQY--		
	YEPLQY--		
PLQM	358	674	45.15
	--PLQMGI		
	ARPLQM--		
	AQPLQM--		
	-APLQMV-		
	AKPLQM--		
	APPLQM--		
	AEPLQM--		
	-APLQMH-		
	-APLQMA-		
	AAPLQM--		
	ANPLQM--		
	-APLQML-		
	ATPLQM--		
	AFPLQM--		
	AWPLQM--		
	ASPLQM--		
	AIPLQM--		
	-APLQMM-		
	-APLQMK-		
	AGPLQM--		
	-APLQME-		
	-APLQMQ-		
	-APLQMN-		
	-APLQMG-		
	-APLQMI-		
	CLPLQM--		
	CSPLQM--		
	-CPLQMR-		
	CAPLQM--		
	CNPLQM--		
	CIPLQM--		
	DVPLQM--		
	-DPLQML-		
	DSPLQM--		
	DLPLQM--		
	DWPLQM--		
	-DPLQMH-		
	-DPLQMT-		
	-DPLQMS-		
	DRPLQM--		

-DPLQMV-
-DPLQMR-
-DPLQMN-
DIPLQM--
DAPLQM--
-DPLQMQ-
DGPLQM--
-EPLQML-
ERPLQM--
EMPLQM--
-EPLQMV-
-EPLQMT-
EIPLQM--
EQPLQM--
-EPLQMS-
-EPLQMM-
-EPLQMH-
EGPLQM--
EYPLQM--
-EPLQMW-
-EPLQMK-
EEPLQM--
EVPLQM--
ETPLQM--
-EPLQMR-
-EPLQMQ-
ELPLQM--
ESPLQM--
EPPLQM--
-EPLQMY-
ENPLQM--
EFPLQM--
-FPLQMT-
FSPLQM--
-FPLQMR-
FVPLQM--
-FPLQMS-
FGPLQM--
FLPLQM--
FTPLQM--
-FPLQML-
FYPLQM--
FRPLQM--
-FPLQMV-
FQPLQM--
-FPLQMN-
-FPLQMM-
FFPLQM--
FWPLQM--
FPPLQM--

-FPLQMK-
-FPLQMF-
-FPLQMA-
FAPLQM--
-FPLQMG-
-FPLQMC-
FNPLQM--
FEPLQM--
GSPLQM--
-GPLQMR-
GYPLQM--
-GPLQMT-
GLPLQM--
-GPLQMS-
GHPLQM--
GDPLQM--
-GPLQMH-
-GPLQMG-
GRPLQM--
GVPLQM--
-GPLQML-
GFPLQM--
GAPLQM--
GQPLQM--
-GPLQMV-
GGPLQM--
GEPLQM--
-GPLQMQ-
GTPLQM--
-GPLQMN-
GWPLQM--
-GPLQMM-
GNPLQM--
GMPLQM--
GPPLQM--
-GPLQMA-
GIPLQM--
-GPLQMP-
-GPLQME-
-HPLQMR-
HSPLQM--
HLPLQM--
HRPLQM--
-HPLQMY-
-HPLQMV-
-HPLQMT-
HGPLQM--
HYPLQM--
-HPLQML-
HIPLQM--

HAPLQM--
HPPLQM--
HKPLQM--
HWPLQM--
HQPLQM--
-HPLQMK-
HHPLQM--
HEPLQM--
HVPLQM--
-HPLQMS-
-HPLQMQ-
HTPLQM--
-HPLQMM-
-HPLQMH-
-HPLQMG-
-HPLQMC-
-IPLQMR-
ILPLQM--
-IPLQMA-
IWPLQM--
IAPLQM--
-IPLQMT-
IEPLQM--
IKPLQM--
IGPLQM--
-IPLQMS-
-IPLQMQ-
-IPLQMN-
-IPLQMK-
-IPLQMG-
IFPLQM--
IVPLQM--
IQPLQM--
-IPLQMW-
-IPLQMI-
ISPLQM--
IPPLQM--
-IPLQMH-
-IPLQME-
INPLQM--
KSPLQM--
-KPLQMR-
KNPLQM--
-KPLQMY-
-KPLQMK-
KKPLQM--
KVPLQM--
-KPLQMV-
KEPLQM--
-KPLQML-

KLPLQM--
KRPLQM--
-KPLQMW-
KPPLQM--
-KPLQMN-
-KPLQMI-
KMPLQM--
-KPLQMT-
-KPLQMA-
-KPLQMM-
KDPLQM--
KTPLQM--
-KPLQME-
KYPLQM--
-KPLQMG-
KHPLQM--
KGPLQM--
KQPLQM--
-KPLQMH-
-KPLQMF-
KIPLQM--
KFPLQM--
KAPLQM--
-LPLQMR-
LSPLQM--
LRPLQM--
LTPLQM--
LVPLQM--
LKPLQM--
LGPLQM--
LYPLQM--
-LPLQML-
LQPLQM--
-LPLQMM-
-LPLQMV-
-LPLQMT-
-LPLQMI-
-LPLQMH-
LLPLQM--
-LPLQMF-
LIPLQM--
LPPLQM--
LEPLQM--
LDPLQM--
-LPLQMQ-
LFPLQM--
-LPLQMY-
LWPLQM--
LCPLQM--
-LPLQMK-

–LPLQME–
–LPLQMA–
MAPLQM––
MVPLQM––
–MPLQML–
–MPLQMN–
MRPLQM––
–MPLQMA–
MKPLQM––
MPPLQM––
–MPLQMM–
–MPLQMK–
–MPLQMS–
–MPLQMH–
MGPLQM––
–MPLQMW–
MFPLQM––
MYPLQM––
–MPLQMQ–
–MPLQMT–
–MPLQMG–
MNPLQM––
MLPLQM––
–MPLQMR–
MEPLQM––
MSPLQM––
–MPLQMV–
–NPLQMR–
NSPLQM––
–NPLQMI–
NTPLQM––
NVPLQM––
–NPLQMY–
–NPLQMM–
NYPLQM––
–NPLQMT–
NNPLQM––
NRPLQM––
–NPLQMQ–
–NPLQMK–
–NPLQMS–
–NPLQML–
NPPLQM––
–NPLQMV–
NWPLQM––
NLPLQM––
NGPLQM––
NEPLQM––
NQPLQM––
–NPLQMF–

```
NFPLQM--
--PLQMVS
--PLQMAS
--PLQMMQ
--PLQMLS
--PLQMRS
--PLQMSY
--PLQMRT
--PLQMYS
--PLQMLR
--PLQMLN
--PLQMVW
--PLQMVR
--PLQMSW
--PLQMWS
--PLQMYR
--PLQMGS
--PLQMVG
--PLQMRN
--PLQMRL
--PLQMQT
--PLQMRV
--PLQMHR
--PLQMIG
--PLQMHW
--PLQMHS
--PLQMTV
--PLQMVL
--PLQMNR
--PLQMNA
--PLQMSG
--PLQMYK
--PLQMMR
--PLQMKA
--PLQMES
--PLQMWN
--PLQMVV
--PLQMSA
--PLQMHT
--PLQMTS
--PLQMSK
--PLQMRI
--PLQMAA
--PLQMMT
--PLQMKR
--PLQMKQ
--PLQMAG
--PLQMRY
--PLQMNT
--PLQMLK
```

--PLQMVN
--PLQMVA
--PLQMST
--PLQMKV
--PLQMIH
--PLQMHI
--PLQMFS
--PLQMWT
--PLQMLT
--PLQMLG
--PLQMLD
--PLQMKG
--PLQMIQ
--PLQMYF
--PLQMVY
--PLQMSH
--PLQMRD
--PLQMQV
--PLQMNV
--PLQMNL
--PLQMLQ
--PLQMKN
--PLQMIT
--PLQMIR
--PLQMYA
--PLQMVI
--PLQMVH
--PLQMSN
--PLQMSI
--PLQMQW
--PLQMQS
--PLQMYM
--PLQMTN
--PLQMTG
--PLQMSS
--PLQMSF
--PLQMQR
--PLQMNY
--PLQMKL
--PLQMIM
--PLQMEA
--PLQMYN
--PLQMTH
--PLQMTD
--PLQMRH
--PLQMQG
--PLQMLE
--PLQMIE
--PLQMHG
--PLQMFG

--PLQMFE
--PLQMAL
--PLQMWD
--PLQMVT
--PLQMVQ
--PLQMTT
--PLQMSV
--PLQMQN
--PLQMML
--PLQMMF
--PLQMKT
--PLQMKK
--PLQMIA
--PLQMFH
--PLQMEW
--PLQMAR
PEPLQM--
--PLQMYQ
--PLQMYG
--PLQMWM
--PLQMVM
--PLQMTA
--PLQMSM
--PLQMSL
--PLQMSD
--PLQMRM
--PLQMNM
--PLQMMY
--PLQMMS
--PLQMKY
--PLQMHM
--PLQMGW
--PLQMAT
--PLQMWE
--PLQMTY
--PLQMRW
--PLQMRQ
--PLQMRE
--PLQMRA
--PLQMNQ
--PLQMNG
--PLQMMN
--PLQMLH
--PLQMIN
--PLQMHQ
--PLQMHA
--PLQMGV
--PLQMFK
--PLQMFA
--PLQMEH

--PLQMVD
--PLQMTF
--PLQMSR
--PLQMNI
--PLQMKI
--PLQMHV
--PLQMHN
--PLQMHK
--PLQMHH
--PLQMGT
--PLQMEF
--PLQMAN
--PLQMSQ
--PLQMQK
--PLQMQA
--PLQMIY
--PLQMIV
--PLQMFT
--PLQMFQ
--PLQMAV
--PLQMAQ
--PLQMAH
PDPLQM--
--PLQMYE
--PLQMTQ
--PLQMTM
--PLQMTK
--PLQMTE
--PLQMSE
--PLQMQM
--PLQMNN
--PLQMMM
--PLQMID
--PLQMHL
--PLQMGG
--PLQMGA
--PLQMEY
--PLQMEN
--PLQMEG
--PLQMCS
--PLQMCN
--PLQMCA
QPPLQM--
QSPLQM--
-QPLQMW-
-QPLQMV-
QAPLQM--
QVPLQM--
QLPLQM--
-QPLQMN-

-QPLQMT-
-QPLQML-
-QPLQMG-
-QPLQMA-
QGPLQM--
QDPLQM--
QRPLQM--
-QPLQMR-
-QPLQMM-
-QPLQMH-
QNPLQM--
QMPLQM--
QHPLQM--
QFPLQM--
QYPLQM--
-QPLQMI-
QKPLQM--
QIPLQM--
RIPLQM--
-RPLQMS-
RVPLQM--
RSPLQM--
RRPLQM--
RQPLQM--
-RPLQMV-
-RPLQMK-
-RPLQMN-
RMPLQM--
-RPLQMT-
RYPLQM--
-RPLQMR-
RGPLQM--
RWPLQM--
-RPLQMA-
RNPLQM--
-RPLQMF-
RAPLQM--
-RPLQMI-
RPPLQM--
RFPLQM--
RHPLQM--
RTPLQM--
-RPLQMG-
-RPLQMH-
-RPLQMW-
-RPLQMQ-
-RPLQMM-
-RPLQME-
REPLQM--
RDPLQM--

RCPLQM--
-SPLQMR-
-SPLQML-
-SPLQMN-
SAPLQM--
-SPLQMK-
STPLQM--
-SPLQMW-
SMPLQM--
SQPLQM--
-SPLQMS-
-SPLQMI-
SKPLQM--
SGPLQM--
-SPLQMF-
SHPLQM--
SLPLQM--
-SPLQMY-
-SPLQMH-
SVPLQM--
-SPLQMT-
-SPLQMM-
SSPLQM--
-SPLQMV-
SYPLQM--
SWPLQM--
SRPLQM--
-SPLQMA-
SDPLQM--
SEPLQM--
SNPLQM--
SIPLQM--
-SPLQMQ-
-SPLQMC-
-TPLQMR-
-TPLQMI-
TSPLQM--
-TPLQMA-
TGPLQM--
TLPLQM--
TYPLQM--
TTPLQM--
-TPLQMQ-
TIPLQM--
-TPLQML-
TNPLQM--
TWPLQM--
TRPLQM--
-TPLQMV-
-TPLQMN-

-TPLQMG-
TQPLQM--
-TPLQMW-
TEPLQM--
-TPLQMT-
TVPLQM--
TAPLQM--
TPPLQM--
-TPLQMS-
-TPLQMH-
THPLQM--
TFPLQM--
-TPLQMM-
-TPLQME-
TMPLQM--
TKPLQM--
VLPLQM--
-VPLQMS-
VRPLQM--
VVPLQM--
VSPLQM--
-VPLQML-
-VPLQMK-
VTPLQM--
-VPLQMT-
VQPLQM--
VPPLQM--
-VPLQMR-
VFPLQM--
VDPLQM--
VYPLQM--
-VPLQMM-
VWPLQM--
-VPLQMF-
-VPLQMH-
-VPLQMA-
VHPLQM--
VEPLQM--
-VPLQMN-
-VPLQMG-
-VPLQMQ-
VAPLQM--
-VPLQMV-
-VPLQMI-
WRPLQM--
-WPLQMT-
-WPLQMR-
WAPLQM--
-WPLQMM-
WSPLQM--

	-WPLQMN-		
	-WPLQMS-		
	WHPLQM--		
	WYPLQM--		
	-WPLQMY-		
	WGPLQM--		
	WVPLQM--		
	WPPLQM--		
	-WPLQMD-		
	-YPLQMR-		
	YFPLQM--		
	-YPLQMS-		
	YRPLQM--		
	YSPLQM--		
	YEPLQM--		
	YWPLQM--		
	-YPLQMK-		
	YHPLQM--		
	YYPLQM--		
	YPPLQM--		
	-YPLQMV-		
	-YPLQMN-		
	YMPLQM--		
	-YPLQMT-		
	-YPLQMA-		
	YTPLQM--		
	-YPLQMI-		
	-YPLQMG-		
	YGPLQM--		
	YDPLQM--		
	YCPLQM--		
	-YPLQML-		
	YNPLQM--		
	YLPLQM--		
PFPY	494	623	45.14
	-APFPYV-		
	AFPFPY--		
	-APFPYR-		
	AEPFPY--		
	ARPFPY--		
	-APFPYM-		
	AKPFPY--		
	AGPFPY--		
	AWPFPY--		
	AAPFPY--		
	-APFPYS-		
	-APFPYI-		
	AYPFPY--		
	AVPFPY--		
	ATPFPY--		

-APFPYH-
ALPFPY--
AIPFPY--
AHPFPY--
-APFPYA-
AMPFPY--
ASPFPY--
-APFPYY-
-APFPYL-
-APFPYW-
ADPFPY--
CSPFPY--
-CPFPYR-
CMPFPY--
CFPFPY--
CHPFPY--
CYPFPY--
CVPFPY--
-CPFPYQ-
DRPFPY--
DYPFPY--
DMPFPY--
-DPFPYF-
DKPFPY--
DLPFPY--
DWPFPY--
DSPFPY--
-DPFPYL-
DIPFPY--
-DPFPYI-
DNPFPY--
DFPFPY--
DQPFPY--
-DPFPYS-
-DPFPYR-
DGPFPY--
EVPFPY--
EGPFPY--
-EPFPYK-
-EPFPYR-
-EPFPYV-
-EPFPYI-
EAPFPY--
-EPFPYA-
ESPFPY--
EKPFPY--
EIPFPY--
ETPFPY--
ERPFPY--
-EPFPYS-

ELPFPY--
EHPFPY--
-EPFPYY-
-EPFPYQ-
EFPFPY--
EPPFPY--
-EPFPYW-
-EPFPYG-
FRPFPY--
FHPFPY--
FSPFPY--
FPPFPY--
FLPFPY--
-FPFPYR-
FNPFPY--
FTPFPY--
FMPFPY--
FKPFPY--
FYPFPY--
-FPFPYS-
-FPFPYT-
FAPFPY--
FVPFPY--
-FPFPYK-
FFPFPY--
FDPFPY--
-FPFPYV-
-FPFPYH-
FQPFPY--
-FPFPYY-
FEPFPY--
GIPFPY--
-GPFPYR-
GSPFPY--
GRPFPY--
-GPFPYM-
GYPFPY--
GKPFPY--
GVPFPY--
GTPFPY--
-GPFPYT-
GLPFPY--
-GPFPYW-
GHPFPY--
-GPFPYQ-
-GPFPYI-
GNPFPY--
-GPFPYY-
GWPFPY--
GMPFPY--

GGPFPY--
GAPFPY--
-GPFPYL-
GPPFPY--
-GPFPYV-
GEPFPY--
-GPFPYH-
HRPFPY--
HSPFPY--
HNPFPY--
HTPFPY--
HFPFPY--
-HPFPYM-
-HPFPYL-
HHPFPY--
-HPFPYR-
HLPFPY--
HYPFPY--
HVPFPY--
-HPFPYY-
HKPFPY--
HCPFPY--
HEPFPY--
-HPFPYS-
-IPFPYS-
INPFPY--
IQPFPY--
IRPFPY--
ISPFPY--
ILPFPY--
-IPFPYR-
-IPFPYH-
ITPFPY--
IPPFPY--
-IPFPYT-
IHPFPY--
-IPFPYI-
IGPFPY--
IEPFPY--
-IPFPYK-
IDPFPY--
-IPFPYM-
-IPFPYL-
IKPFPY--
-IPFPYW-
-IPFPYA-
IMPFPY--
KSPFPY--
-KPFPYT-
KRPFPY--

−KPFPYQ−
−KPFPYS−
−KPFPYL−
−KPFPYF−
KNPFPY−−
KEPFPY−−
KWPFPY−−
KMPFPY−−
KHPFPY−−
KDPFPY−−
−KPFPYN−
−KPFPYA−
KVPFPY−−
−KPFPYY−
−KPFPYV−
−KPFPYK−
−KPFPYI−
−KPFPYH−
KLPFPY−−
KYPFPY−−
KQPFPY−−
KKPFPY−−
−KPFPYG−
KPPFPY−−
−KPFPYR−
−KPFPYD−
KIPFPY−−
LAPFPY−−
LLPFPY−−
LSPFPY−−
LTPFPY−−
LRPFPY−−
LFPFPY−−
LPPFPY−−
LQPFPY−−
−LPFPYS−
LKPFPY−−
LEPFPY−−
LWPFPY−−
LVPFPY−−
−LPFPYM−
−LPFPYL−
−LPFPYQ−
−LPFPYK−
LIPFPY−−
−LPFPYA−
LMPFPY−−
LDPFPY−−
−LPFPYN−
−LPFPYI−

LHPFPY--
-LPFPYW-
-LPFPYF-
LNPFPY--
MRPFPY--
-MPFPYT-
MKPFPY--
MVPFPY--
MNPFPY--
MSPFPY--
-MPFPYG-
MYPFPY--
MLPFPY--
MIPFPY--
MGPFPY--
-MPFPYI-
MFPFPY--
MPPFPY--
-MPFPYH-
MTPFPY--
-MPFPYS-
-MPFPYK-
MQPFPY--
-MPFPYN-
MHPFPY--
MEPFPY--
NIPFPY--
NTPFPY--
NGPFPY--
NRPFPY--
-NPFPYM-
-NPFPYT-
-NPFPYR-
NNPFPY--
-NPFPYL-
NSPFPY--
-NPFPYV-
NMPFPY--
NPPFPY--
NWPFPY--
NVPFPY--
-NPFPYQ-
-NPFPYY-
-NPFPYF-
NHPFPY--
-NPFPYK-
NFPFPY--
--PFPYFL
--PFPYTS
--PFPYLL

--PFPYAS
--PFPYWT
--PFPYGH
--PFPYTN
--PFPYSS
--PFPYLA
--PFPYRG
--PFPYIH
--PFPYVH
--PFPYRS
--PFPYIL
--PFPYTH
--PFPYRT
--PFPYRI
--PFPYLT
--PFPYWN
--PFPYTV
--PFPYSG
--PFPYLG
--PFPYTT
--PFPYSH
--PFPYSF
--PFPYRN
PGPFPY--
--PFPYYS
--PFPYVY
--PFPYVG
--PFPYRR
--PFPYHS
--PFPYAT
--PFPYVS
--PFPYLS
--PFPYKF
--PFPYEI
--PFPYTY
--PFPYSR
--PFPYKG
PEPFPY--
--PFPYWH
--PFPYRV
--PFPYRH
--PFPYPF
--PFPYMY
--PFPYIA
--PFPYFS
--PFPYEM
--PFPYAN
PTPFPY--
--PFPYVF
--PFPYTQ

--PFPYRY
--PFPYQS
--PFPYMS
--PFPYMA
--PFPYIS
--PFPYIG
--PFPYHG
--PFPYYF
--PFPYYA
--PFPYWS
--PFPYNV
--PFPYMT
--PFPYMR
--PFPYDY
--PFPYYR
--PFPYYI
--PFPYYD
--PFPYWV
--PFPYVT
--PFPYVN
--PFPYVA
--PFPYSK
--PFPYRW
--PFPYNW
--PFPYMM
--PFPYLH
--PFPYFK
--PFPYEQ
PMPFPY--
PIPFPY--
--PFPYYT
--PFPYYN
--PFPYWM
--PFPYVR
--PFPYTK
--PFPYTE
--PFPYST
--PFPYSN
--PFPYSA
--PFPYQR
--PFPYQQ
--PFPYPH
--PFPYMW
--PFPYKI
--PFPYIY
--PFPYIV
--PFPYHQ
--PFPYHN
--PFPYFR
--PFPYFM

--PFPYEA
PYPFPY--
PWPFPY--
--PFPYYQ
--PFPYVV
--PFPYTI
--PFPYTD
--PFPYRA
--PFPYQT
--PFPYKW
--PFPYKT
--PFPYKM
--PFPYIR
--PFPYIQ
--PFPYIM
--PFPYIE
--PFPYHR
--PFPYHF
--PFPYHA
--PFPYGS
--PFPYES
--PFPYAQ
--PFPYYV
--PFPYVK
--PFPYVI
--PFPYTG
--PFPYTA
--PFPYRE
--PFPYQW
--PFPYQE
--PFPYPV
--PFPYNT
--PFPYNQ
--PFPYNG
--PFPYLI
--PFPYGT
--PFPYGG
--PFPYGA
--PFPYFT
--PFPYFA
--PFPYEV
--PFPYEG
--PFPYAR
PCPFPY--
PAPFPY--
PQPFPY--
--PFPYYM
--PFPYYG
--PFPYYE
--PFPYWY

--PFPYWF
--PFPYVW
--PFPYVM
--PFPYVE
--PFPYSE
--PFPYSD
--PFPYRP
--PFPYQV
--PFPYQG
--PFPYPS
--PFPYPQ
--PFPYNH
--PFPYMK
--PFPYMF
--PFPYLW
--PFPYLV
--PFPYLN
--PFPYLD
--PFPYKV
--PFPYKQ
--PFPYKN
--PFPYKA
--PFPYIW
--PFPYHV
--PFPYHP
--PFPYGQ
--PFPYFY
--PFPYFN
--PFPYFG
--PFPYDF
--PFPYAG
QKPFPY--
-QPFPYL-
QFPFPY--
QLPFPY--
QTPFPY--
QAPFPY--
-QPFPYR-
-QPFPYV-
-QPFPYT-
QRPFPY--
QPPFPY--
-QPFPYY-
QMPFPY--
-QPFPYQ-
QWPFPY--
QSPFPY--
-QPFPYM-
QNPFPY--
QIPFPY--

QYPFPY--
-QPFPYF-
RSPFPY--
-RPFPYV-
-RPFPYS-
RVPFPY--
-RPFPYT-
RYPFPY--
RWPFPY--
-RPFPYR-
RAPFPY--
RLPFPY--
RIPFPY--
RRPFPY--
RFPFPY--
RMPFPY--
RTPFPY--
-RPFPYL-
REPFPY--
RNPFPY--
RPPFPY--
-RPFPYY-
-RPFPYN-
-RPFPYA-
RKPFPY--
-RPFPYW-
-RPFPYQ-
-RPFPYM-
-RPFPYH-
-RPFPYI-
-RPFPYF-
SRPFPY--
SLPFPY--
SMPFPY--
-SPFPYR-
SNPFPY--
SKPFPY--
-SPFPYS-
-SPFPYL-
SIPFPY--
-SPFPYI-
SEPFPY--
-SPFPYY-
SGPFPY--
-SPFPYQ-
-SPFPYH-
SDPFPY--
SAPFPY--
SPPFPY--
STPFPY--

-SPFPYW-
-SPFPYF-
SSPFPY--
SVPFPY--
-SPFPYV-
-SPFPYT-
SYPFPY--
SWPFPY--
SQPFPY--
-SPFPYA-
-SPFPYN-
-SPFPYK-
SFPFPY--
TLPFPY--
TGPFPY--
TTPFPY--
TNPFPY--
TRPFPY--
-TPFPYT-
-TPFPYS-
-TPFPYA-
TMPFPY--
TEPFPY--
TIPFPY--
TWPFPY--
TVPFPY--
-TPFPYR-
TYPFPY--
-TPFPYY-
-TPFPYQ-
-TPFPYW-
TSPFPY--
-TPFPYV-
TPPFPY--
TKPFPY--
-TPFPYK-
-TPFPYE-
-VPFPYS-
VLPFPY--
VRPFPY--
VQPFPY--
VNPFPY--
VTPFPY--
VVPFPY--
VGPFPY--
-VPFPYL-
VSPFPY--
-VPFPYK-
VKPFPY--
VPPFPY--

-VPFPYR-
VIPFPY--
VYPFPY--
-VPFPYQ-
VDPFPY--
-VPFPYI-
-VPFPYV-
-VPFPYM-
VMPFPY--
-VPFPYT-
-VPFPYA-
VAPFPY--
-VPFPYN-
WRPFPY--
WAPFPY--
WEPFPY--
WLPFPY--
-WPFPYF-
WQPFPY--
-WPFPYI-
WMPFPY--
WIPFPY--
-WPFPYT-
-WPFPYS-
-WPFPYK-
WGPFPY--
WDPFPY--
WTPFPY--
WSPFPY--
-WPFPYV-
-WPFPYN-
-WPFPYL-
-YPFPYR-
-YPFPYH-
YSPFPY--
YFPFPY--
YWPFPY--
YIPFPY--
YTPFPY--
-YPFPYT-
-YPFPYF-
YLPFPY--
-YPFPYL-
-YPFPYA-
YKPFPY--
YQPFPY--
-YPFPYS-
YNPFPY--
YGPFPY--
YEPFPY--

	YVPFPY--		
	YMPFPY--		
	YDPFPY--		
	-YPFPYW-		
	-YPFPYK-		
	YYPFPY--		
	YPPFPY--		
	-YPFPYV-		
PRHL	410	656	45.14
	-APRHLP-		
	-CPRHLQ-		
	-DPRHLN-		
	--PRHLGH		
	--PRHLPS		
	--PRHLPL		
	-RPRHLP-		
	-SPRHLC-		
	-APRHLL-		
	-APRHLS-		
	-APRHLM-		
	AMPRHL--		
	-APRHLW-		
	ATPRHL--		
	AAPRHL--		
	-APRHLH-		
	ANPRHL--		
	APPRHL--		
	AQPRHL--		
	-APRHLI-		
	-APRHLV-		
	-APRHLN-		
	-APRHLG-		
	AHPRHL--		
	ACPRHL--		
	-APRHLQ-		
	-APRHLE-		
	CGPRHL--		
	CVPRHL--		
	CSPRHL--		
	CAPRHL--		
	CIPRHL--		
	-CPRHLT-		
	CCPRHL--		
	CTPRHL--		
	CEPRHL--		
	-CPRHLS-		
	-CPRHLR-		
	-CPRHLF-		
	CNPRHL--		
	CKPRHL--		

CWPRHL--
-CPRHLI-
CDPRHL--
CRPRHL--
-CPRHLV-
-CPRHLM-
-CPRHLL-
CPPRHL--
CFPRHL--
CYPRHL--
CQPRHL--
-CPRHLY-
-CPRHLG-
-CPRHLE-
-CPRHLA-
DMPRHL--
-DPRHLR-
DSPRHL--
-DPRHLM-
DAPRHL--
DYPRHL--
DQPRHL--
-DPRHLL-
DTPRHL--
-DPRHLS-
DKPRHL--
-DPRHLV-
-DPRHLH-
DEPRHL--
-DPRHLY-
-DPRHLW-
-DPRHLK-
DIPRHL--
-DPRHLI-
-DPRHLF-
-DPRHLA-
DGPRHL--
-DPRHLT-
-DPRHLE-
DNPRHL--
DLPRHL--
DHPRHL--
DDPRHL--
DPPRHL--
DCPRHL--
EMPRHL--
ESPRHL--
EAPRHL--
-EPRHLS-
ELPRHL--

EGPRHL--
-EPRHLW-
-EPRHLK-
-EPRHLV-
EWPRHL--
-EPRHLF-
ETPRHL--
EQPRHL--
-EPRHLT-
EPPRHL--
-EPRHLM-
-EPRHLY-
-EPRHLA-
-EPRHLG-
ENPRHL--
EEPRHL--
EKPRHL--
EIPRHL--
-EPRHLN-
-EPRHLE-
-EPRHLH-
EDPRHL--
-EPRHLQ-
-EPRHLC-
FSPRHL--
FRPRHL--
FTPRHL--
FEPRHL--
FAPRHL--
-FPRHLM-
FVPRHL--
-FPRHLY-
-FPRHLS-
-FPRHLI-
FDPRHL--
-FPRHLE-
FPPRHL--
-FPRHLW-
FCPRHL--
-FPRHLQ-
-FPRHLF-
GAPRHL--
GLPRHL--
-GPRHLT-
GSPRHL--
GNPRHL--
-GPRHLL-
GPPRHL--
-GPRHLS-
-GPRHLK-

GRPRHL--
-GPRHLN-
-GPRHLF-
GQPRHL--
-GPRHLE-
-GPRHLV-
-GPRHLI-
-GPRHLH-
GHPRHL--
-GPRHLY-
-GPRHLM-
GEPRHL--
-GPRHLW-
-GPRHLQ-
GVPRHL--
GCPRHL--
GDPRHL--
HSPRHL--
-HPRHLT-
-HPRHLR-
-HPRHLL-
-HPRHLM-
HVPRHL--
HDPRHL--
-HPRHLV-
-HPRHLI-
HHPRHL--
-HPRHLS-
HYPRHL--
HNPRHL--
-HPRHLY-
-HPRHLK-
HTPRHL--
-HPRHLQ-
-HPRHLH-
-HPRHLN-
HQPRHL--
HEPRHL--
HPPRHL--
-HPRHLG-
-HPRHLE-
HKPRHL--
-IPRHLL-
-IPRHLV-
IPPRHL--
IDPRHL--
-IPRHLA-
-IPRHLK-
-IPRHLY-
-IPRHLI-

ITPRHL--
-IPRHLT-
-IPRHLQ-
-IPRHLH-
-IPRHLE-
IGPRHL--
IKPRHL--
-IPRHLW-
-IPRHLM-
-IPRHLF-
-IPRHLN-
ICPRHL--
KGPRHL--
-KPRHLI-
KPPRHL--
-KPRHLA-
-KPRHLM-
-KPRHLY-
-KPRHLF-
KEPRHL--
-KPRHLW-
-KPRHLT-
KIPRHL--
-KPRHLE-
-KPRHLK-
KKPRHL--
KDPRHL--
-KPRHLQ-
-KPRHLH-
-KPRHLG-
-KPRHLP-
KNPRHL--
KCPRHL--
-LPRHLS-
LSPRHL--
-LPRHLV-
LIPRHL--
LGPRHL--
LAPRHL--
LPPRHL--
LDPRHL--
-LPRHLK-
-LPRHLA-
-LPRHLH-
-LPRHLQ-
-LPRHLN-
-LPRHLE-
-LPRHLW-
LCPRHL--
-LPRHLD-

MSPRHL--
MGPRHL--
-MPRHLT-
-MPRHLS-
MDPRHL--
-MPRHLI-
MQPRHL--
-MPRHLH-
MNPRHL--
-MPRHLV-
MHPRHL--
-MPRHLY-
-MPRHLQ-
-MPRHLF-
-MPRHLG-
MCPRHL--
-MPRHLM-
-MPRHLA-
MKPRHL--
-MPRHLE-
NPPRHL--
-NPRHLR-
NEPRHL--
-NPRHLS-
NKPRHL--
-NPRHLW-
-NPRHLL-
-NPRHLM-
-NPRHLK-
-NPRHLF-
-NPRHLV-
NYPRHL--
-NPRHLQ-
-NPRHLA-
NNPRHL--
-NPRHLG-
-NPRHLT-
-NPRHLE-
NCPRHL--
NWPRHL--
NDPRHL--
-NPRHLH-
--PRHLLS
--PRHLLN
--PRHLSG
--PRHLIT
--PRHLWT
--PRHLRM
--PRHLSV
--PRHLMN

--PRHLTV
--PRHLLH
--PRHLYA
--PRHLSH
--PRHLWS
--PRHLVY
--PRHLFG
--PRHLYM
--PRHLAW
--PRHLLW
--PRHLMT
PNPRHL--
--PRHLTT
--PRHLSW
--PRHLSI
--PRHLYS
--PRHLSM
--PRHLML
--PRHLYY
--PRHLHS
--PRHLAT
--PRHLLM
--PRHLAG
--PRHLQG
--PRHLLL
PQPRHL--
--PRHLNS
--PRHLLV
--PRHLFI
--PRHLWQ
--PRHLVN
--PRHLSY
--PRHLRT
--PRHLET
--PRHLAS
--PRHLQA
--PRHLGA
--PRHLAA
--PRHLVA
--PRHLAL
--PRHLQL
--PRHLNA
--PRHLYT
--PRHLQT
--PRHLKL
--PRHLYG
--PRHLTF
--PRHLEM
PGPRHL--
--PRHLYI

--PRHLHL
--PRHLEW
--PRHLAI
--PRHLVV
--PRHLKN
--PRHLEG
--PRHLQV
--PRHLVD
--PRHLMG
--PRHLIN
--PRHLEN
--PRHLWN
--PRHLVK
--PRHLHA
--PRHLWA
--PRHLNT
--PRHLHG
--PRHLEA
--PRHLVE
--PRHLNF
--PRHLGS
--PRHLAN
--PRHLMK
--PRHLWG
--PRHLWD
--PRHLVI
--PRHLII
--PRHLIF
--PRHLHM
--PRHLYD
--PRHLWI
--PRHLTG
--PRHLTE
--PRHLQH
--PRHLTN
--PRHLMM
--PRHLYN
--PRHLQS
--PRHLIG
--PRHLFY
--PRHLAF
PYPRHL--
--PRHLWM
--PRHLWK
--PRHLVF
--PRHLQY
--PRHLNM
--PRHLIQ
--PRHLHN
PMPRHL--

--PRHLIY
PAPRHL--
--PRHLNV
--PRHLMW
--PRHLMI
--PRHLME
--PRHLLD
--PRHLFA
--PRHLWE
--PRHLQQ
--PRHLFQ
--PRHLEV
--PRHLES
PHPRHL--
--PRHLYV
--PRHLWV
--PRHLTM
--PRHLSE
--PRHLRE
--PRHLIV
--PRHLIA
--PRHLEL
--PRHLEF
--PRHLAY
PEPRHL--
--PRHLYQ
--PRHLQI
--PRHLLE
--PRHLFE
--PRHLYW
--PRHLYH
--PRHLYF
--PRHLMF
--PRHLKE
--PRHLIE
--PRHLHY
--PRHLFT
--PRHLFN
--PRHLAV
--PRHLQF
--PRHLHQ
--PRHLGM
PKPRHL--
--PRHLSD
--PRHLQW
--PRHLNL
--PRHLHI
--PRHLHH
--PRHLEH
--PRHLNW

--PRHLNG
--PRHLMQ
--PRHLHK
--PRHLHD
--PRHLFF
--PRHLFD
--PRHLAM
PCPRHL--
--PRHLWF
--PRHLQM
--PRHLHV
--PRHLFV
--PRHLDA
--PRHLAQ
--PRHLNN
--PRHLGL
--PRHLGI
--PRHLEK
--PRHLDS
--PRHLDG
--PRHLTD
--PRHLNI
--PRHLNH
--PRHLKH
--PRHLKD
--PRHLHE
--PRHLGY
--PRHLEY
--PRHLEI
--PRHLQE
--PRHLQD
--PRHLGF
--PRHLFH
PDPRHL--
--PRHLWW
--PRHLEQ
--PRHLDT
--PRHLCR
--PRHLWH
--PRHLSC
--PRHLRC
--PRHLGW
--PRHLFC
--PRHLEE
--PRHLCS
-PPRHLS-
--PRHLWP
--PRHLWC
--PRHLQN
--PRHLQC

--PRHLPT
--PRHLPM
--PRHLND
--PRHLHC
--PRHLGQ
--PRHLEC
--PRHLDN
--PRHLCQ
--PRHLAD
-PPRHLN-
PPPRHL--
QVPRHL--
QLPRHL--
QKPRHL--
-QPRHLS-
-QPRHLW-
-QPRHLL-
QGPRHL--
QTPRHL--
-QPRHLR-
-QPRHLK-
QPPRHL--
QIPRHL--
-QPRHLA-
-QPRHLN-
-QPRHLI-
-QPRHLY-
QNPRHL--
-QPRHLG-
QMPRHL--
QHPRHL--
-QPRHLQ-
-QPRHLE-
-QPRHLF-
QDPRHL--
-RPRHLR-
RPPRHL--
-RPRHLF-
REPRHL--
RDPRHL--
-RPRHLH-
-RPRHLI-
RQPRHL--
-RPRHLN-
-RPRHLG-
-RPRHLC-
-SPRHLL-
-SPRHLR-
-SPRHLS-
SSPRHL--

-SPRHLI-
SEPRHL--
-SPRHLT-
-SPRHLA-
-SPRHLK-
SPPRHL--
SQPRHL--
-SPRHLF-
SNPRHL--
-SPRHLH-
SHPRHL--
-SPRHLN-
-SPRHLM-
-SPRHLV-
-SPRHLQ-
SDPRHL--
-SPRHLE-
SCPRHL--
-SPRHLG-
-SPRHLD-
TSPRHL--
TGPRHL--
-TPRHLR-
TTPRHL--
TKPRHL--
-TPRHLM-
TMPRHL--
TNPRHL--
-TPRHLK-
-TPRHLA-
-TPRHLY-
-TPRHLH-
TPPRHL--
-TPRHLV-
-TPRHLT-
TAPRHL--
-TPRHLQ-
-TPRHLF-
THPRHL--
TEPRHL--
TIPRHL--
-TPRHLN-
TDPRHL--
-TPRHLE-
-TPRHLG-
VSPRHL--
VVPRHL--
-VPRHLR-
-VPRHLS-
-VPRHLY-

```
-VPRHLL-
VGPRHL--
VPPRHL--
-VPRHLE-
-VPRHLA-
-VPRHLT-
-VPRHLQ-
-VPRHLF-
-VPRHLN-
-VPRHLM-
-VPRHLW-
-VPRHLG-
VEPRHL--
VDPRHL--
-VPRHLH-
-VPRHLI-
VCPRHL--
WTPRHL--
WMPRHL--
WGPRHL--
WSPRHL--
WEPRHL--
WAPRHL--
WKPRHL--
WNPRHL--
WPPRHL--
-WPRHLN-
-WPRHLY-
WDPRHL--
-WPRHLH-
WHPRHL--
-WPRHLM-
-WPRHLE-
-WPRHLW-
-WPRHLT-
-WPRHLQ-
WCPRHL--
-WPRHLP-
-WPRHLG-
YTPRHL--
YYPRHL--
YIPRHL--
-YPRHLY-
YKPRHL--
-YPRHLK-
-YPRHLI-
YAPRHL--
YHPRHL--
-YPRHLF-
-YPRHLW-
```

	-YPRHLN-		
	YQPRHL--		
	YNPRHL--		
	YDPRHL--		
	-YPRHLQ-		
	-YPRHLL-		
	YPPRHL--		
	-YPRHLM-		
	YEPRHL--		
	-YPRHLH-		
	-YPRHLE-		
	-YPRHLA-		
	YCPRHL--		
	-YPRHLG-		
	-YPRHLC-		
PMHY	763	544	45.11
	-APMHYM-		
	-APMHYL-		
	ATPMHY--		
	AAPMHY--		
	APPMHY--		
	AKPMHY--		
	-APMHYS-		
	-APMHYI-		
	AEPMHY--		
	AVPMHY--		
	-APMHYY-		
	-APMHYK-		
	-APMHYF-		
	ANPMHY--		
	AMPMHY--		
	AYPMHY--		
	-APMHYQ-		
	AQPMHY--		
	-APMHYT-		
	ASPMHY--		
	-APMHYV-		
	-APMHYN-		
	-APMHYC-		
	-APMHYA-		
	AGPMHY--		
	AFPMHY--		
	CNPMHY--		
	CSPMHY--		
	CVPMHY--		
	CRPMHY--		
	-CPMHYS-		
	DSPMHY--		
	DMPMHY--		
	DTPMHY--		

DRPMHY--
DAPMHY--
DQPMHY--
-DPMHYS-
-DPMHYM-
DVPMHY--
-DPMHYV-
-DPMHYA-
ERPMHY--
ELPMHY--
EKPMHY--
-EPMHYV-
ENPMHY--
EEPMHY--
EFPMHY--
EAPMHY--
-EPMHYW-
-EPMHYE-
-EPMHYS-
-EPMHYK-
EQPMHY--
-EPMHYY-
EDPMHY--
EYPMHY--
-EPMHYN-
-EPMHYM-
EMPMHY--
-FPMHYR-
FSPMHY--
FRPMHY--
-FPMHYL-
FEPMHY--
FVPMHY--
FLPMHY--
-FPMHYQ-
-FPMHYS-
FYPMHY--
-FPMHYT-
-FPMHYH-
FFPMHY--
FPPMHY--
-FPMHYW-
-FPMHYV-
-FPMHYI-
FIPMHY--
FAPMHY--
-FPMHYK-
-FPMHYG-
-FPMHYA-
-GPMHYA-

-GPMHYM-
GGPMHY--
-GPMHYT-
-GPMHYF-
GTPMHY--
-GPMHYL-
GFPMHY--
GHPMHY--
GEPMHY--
-GPMHYR-
GRPMHY--
-GPMHYK-
GLPMHY--
GIPMHY--
GYPMHY--
GWPMHY--
GVPMHY--
-GPMHYV-
-GPMHYQ-
GDPMHY--
GSPMHY--
GMPMHY--
GKPMHY--
GCPMHY--
HLPMHY--
HGPMHY--
HMPMHY--
-HPMHYR-
HHPMHY--
HDPMHY--
HSPMHY--
HEPMHY--
HAPMHY--
-HPMHYL-
HWPMHY--
HTPMHY--
-HPMHYI-
-HPMHYK-
HYPMHY--
-HPMHYA-
ISPMHY--
IVPMHY--
-IPMHYS-
IRPMHY--
-IPMHYL-
-IPMHYR-
INPMHY--
IGPMHY--
IWPMHY--
IFPMHY--

ILPMHY--
-IPMHYV-
-IPMHYI-
-IPMHYH-
IPPMHY--
-IPMHYK-
IDPMHY--
IQPMHY--
-IPMHYT-
-IPMHYQ-
-IPMHYE-
IMPMHY--
IAPMHY--
KSPMHY--
KWPMHY--
KGPMHY--
-KPMHYL-
KQPMHY--
KTPMHY--
KMPMHY--
KAPMHY--
KVPMHY--
-KPMHYY-
-KPMHYM-
-KPMHYI-
KYPMHY--
KPPMHY--
KLPMHY--
KNPMHY--
-KPMHYQ-
-KPMHYA-
KRPMHY--
-KPMHYV-
-KPMHYN-
KIPMHY--
KDPMHY--
LRPMHY--
-LPMHYR-
LLPMHY--
LKPMHY--
LGPMHY--
LSPMHY--
LTPMHY--
-LPMHYA-
-LPMHYL-
LMPMHY--
-LPMHYS-
LIPMHY--
LWPMHY--
LQPMHY--

-LPMHYT-
-LPMHYQ-
-LPMHYM-
LHPMHY--
LYPMHY--
-LPMHYY-
-LPMHYW-
-LPMHYK-
-LPMHYI-
LAPMHY--
-LPMHYF-
LNPMHY--
LFPMHY--
LEPMHY--
MFPMHY--
-MPMHYI-
MLPMHY--
MHPMHY--
MVPMHY--
-MPMHYS-
-MPMHYA-
MIPMHY--
MYPMHY--
-MPMHYQ-
MNPMHY--
MMPMHY--
-MPMHYL-
MQPMHY--
-MPMHYV-
-MPMHYF-
NSPMHY--
NAPMHY--
NWPMHY--
NLPMHY--
NTPMHY--
NRPMHY--
NKPMHY--
NHPMHY--
NFPMHY--
-NPMHYL-
NEPMHY--
NVPMHY--
NQPMHY--
NIPMHY--
-NPMHYR-
-NPMHYQ-
NNPMHY--
NCPMHY--
-NPMHYW-
-NPMHYS-

-NPMHYF-
NMPMHY--
--PMHYTS
--PMHYYS
--PMHYRA
--PMHYSS
--PMHYQA
--PMHYRM
--PMHYRG
--PMHYAT
--PMHYIV
--PMHYKT
--PMHYWA
--PMHYTL
--PMHYVS
--PMHYRV
--PMHYLA
--PMHYIS
--PMHYAA
--PMHYTT
--PMHYVA
--PMHYSH
--PMHYMR
--PMHYKR
--PMHYFY
PEPMHY--
--PMHYYT
--PMHYTK
--PMHYHT
--PMHYGS
--PMHYYQ
--PMHYVY
--PMHYSL
--PMHYQV
--PMHYLV
--PMHYLS
--PMHYEG
--PMHYYL
--PMHYVF
--PMHYRS
--PMHYLQ
--PMHYLH
--PMHYKS
--PMHYIF
--PMHYGG
--PMHYAV
PGPMHY--
--PMHYVQ
--PMHYST
--PMHYSR

--PMHYSK
--PMHYQS
--PMHYMS
--PMHYMF
--PMHYMD
--PMHYLK
--PMHYKH
--PMHYFS
--PMHYYV
--PMHYYR
--PMHYWM
--PMHYVR
--PMHYVN
--PMHYTW
--PMHYTI
--PMHYRW
--PMHYRQ
--PMHYRN
--PMHYMN
--PMHYKN
--PMHYKM
--PMHYGA
--PMHYSY
--PMHYRI
--PMHYLG
--PMHYHS
--PMHYFA
--PMHYYI
--PMHYWN
--PMHYWH
--PMHYWG
--PMHYSA
--PMHYRH
--PMHYQG
--PMHYNA
--PMHYKA
--PMHYIQ
--PMHYEF
--PMHYAS
--PMHYAH
--PMHYYF
--PMHYVW
--PMHYVV
--PMHYVK
--PMHYVH
--PMHYSN
--PMHYSI
--PMHYRT
--PMHYQR
--PMHYNF

--PMHYMI
--PMHYLN
--PMHYIT
--PMHYGY
--PMHYFG
--PMHYEN
PHPMHY--
--PMHYYK
--PMHYWV
--PMHYWI
--PMHYWD
--PMHYVD
--PMHYTR
--PMHYSG
--PMHYSE
--PMHYQY
--PMHYQN
--PMHYQD
--PMHYNL
--PMHYLY
--PMHYLW
--PMHYLR
--PMHYLF
--PMHYLE
--PMHYLC
--PMHYKV
--PMHYKI
--PMHYKG
--PMHYIY
--PMHYIL
--PMHYII
--PMHYIG
--PMHYHR
--PMHYHN
--PMHYEY
--PMHYET
--PMHYEK
--PMHYAP
--PMHYAN
--PMHYAM
--PMHYAK
--PMHYAI
--PMHYAE
QSPMHY--
-QPMHYY-
-QPMHYT-
QGPMHY--
QEPMHY--
QWPMHY--
-QPMHYK-

-QPMHYA-
-QPMHYL-
-QPMHYI-
QLPMHY--
QFPMHY--
QDPMHY--
-QPMHYE-
QVPMHY--
QRPMHY--
-QPMHYW-
-QPMHYM-
QNPMHY--
-QPMHYV-
-QPMHYH-
-QPMHYF-
RPPMHY--
RAPMHY--
-RPMHYV-
RTPMHY--
RGPMHY--
-RPMHYR-
RMPMHY--
RVPMHY--
RWPMHY--
-RPMHYS-
-RPMHYE-
-RPMHYT-
RLPMHY--
RNPMHY--
-RPMHYQ-
-RPMHYM-
-RPMHYK-
RIPMHY--
RRPMHY--
RKPMHY--
-RPMHYL-
-RPMHYH-
-RPMHYF-
RFPMHY--
REPMHY--
-RPMHYN-
RDPMHY--
-RPMHYY-
-RPMHYW-
-SPMHYR-
SRPMHY--
SHPMHY--
SLPMHY--
SGPMHY--
SSPMHY--

SEPMHY--
-SPMHYF-
SMPMHY--
-SPMHYM-
-SPMHYS-
SNPMHY--
SDPMHY--
SCPMHY--
SPPMHY--
-SPMHYQ-
SYPMHY--
-SPMHYK-
STPMHY--
-SPMHYY-
SIPMHY--
SFPMHY--
SWPMHY--
SVPMHY--
SQPMHY--
-SPMHYA-
-SPMHYE-
SAPMHY--
-SPMHYV-
-SPMHYT-
-SPMHYL-
-SPMHYW-
-SPMHYI-
SKPMHY--
TMPMHY--
TNPMHY--
-TPMHYR-
TLPMHY--
TGPMHY--
TSPMHY--
TTPMHY--
-TPMHYT-
THPMHY--
TWPMHY--
TQPMHY--
-TPMHYS-
TRPMHY--
-TPMHYV-
TYPMHY--
TKPMHY--
TFPMHY--
TAPMHY--
-TPMHYQ-
-TPMHYH-
TEPMHY--
TPPMHY--

-TPMHYM-
-TPMHYL-
-TPMHYF-
TIPMHY--
-VPMHYV-
-VPMHYR-
VPPMHY--
-VPMHYS-
-VPMHYK-
VKPMHY--
-VPMHYT-
VFPMHY--
VMPMHY--
-VPMHYY-
VIPMHY--
VDPMHY--
-VPMHYQ-
-VPMHYL-
VGPMHY--
VLPMHY--
VVPMHY--
-VPMHYH-
-VPMHYN-
-VPMHYA-
VEPMHY--
VCPMHY--
VQPMHY--
-VPMHYW-
-VPMHYI-
-WPMHYV-
WLPMHY--
WIPMHY--
WFPMHY--
WTPMHY--
-WPMHYS-
WAPMHY--
-WPMHYY-
-WPMHYT-
-WPMHYH-
WKPMHY--
WMPMHY--
WRPMHY--
-WPMHYR-
WPPMHY--
-WPMHYF-
YLPMHY--
YGPMHY--
YHPMHY--
YVPMHY--
YRPMHY--

	YFPMHY--		
	-YPMHYT-		
	YAPMHY--		
	-YPMHYK-		
	YNPMHY--		
	YKPMHY--		
	-YPMHYI-		
	-YPMHYA-		
	-YPMHYS-		
	YDPMHY--		
PAPM	336	681	45.11
	-APAPMG-		
	-NPAPMS-		
	-APAPMK-		
	ARPAPM--		
	-APAPMA-		
	-APAPMY-		
	AIPAPM--		
	ATPAPM--		
	ANPAPM--		
	-APAPMM-		
	-APAPMV-		
	AGPAPM--		
	AKPAPM--		
	AAPAPM--		
	-APAPMS-		
	-APAPMH-		
	-APAPMF-		
	-APAPMR-		
	APPAPM--		
	-APAPMW-		
	-APAPMQ-		
	-APAPML-		
	AMPAPM--		
	AEPAPM--		
	AVPAPM--		
	-APAPMN-		
	AYPAPM--		
	-APAPMI-		
	-APAPME-		
	AHPAPM--		
	ACPAPM--		
	CCPAPM--		
	CKPAPM--		
	CRPAPM--		
	-CPAPMN-		
	CFPAPM--		
	CVPAPM--		
	CTPAPM--		
	-CPAPMR-		

-CPAPMM-
-CPAPML-
CEPAPM--
DRPAPM--
-DPAPMA-
DQPAPM--
DVPAPM--
DTPAPM--
-DPAPMW-
-DPAPML-
DGPAPM--
DFPAPM--
-DPAPMK-
-DPAPMI-
DLPAPM--
-EPAPMR-
-EPAPMM-
ERPAPM--
ESPAPM--
-EPAPMI-
ELPAPM--
EKPAPM--
EIPAPM--
-EPAPML-
-EPAPMT-
EAPAPM--
EWPAPM--
EVPAPM--
-EPAPMH-
ENPAPM--
ECPAPM--
FSPAPM--
-FPAPMV-
-FPAPMR-
-FPAPMY-
FKPAPM--
-FPAPMT-
FMPAPM--
-FPAPML-
FIPAPM--
FAPAPM--
FVPAPM--
FRPAPM--
-FPAPMQ-
FNPAPM--
FTPAPM--
-FPAPMF-
FGPAPM--
-FPAPMW-
-FPAPMI-

FYPAPM--
-FPAPMN-
-FPAPMA-
FLPAPM--
-GPAPMR-
GDPAPM--
-GPAPMY-
-GPAPML-
GRPAPM--
GVPAPM--
-GPAPMT-
GTPAPM--
-GPAPMI-
GMPAPM--
GSPAPM--
-GPAPMW-
GAPAPM--
GYPAPM--
GEPAPM--
GIPAPM--
GPPAPM--
-GPAPMH-
-GPAPMS-
GNPAPM--
GGPAPM--
GQPAPM--
-GPAPMV-
-GPAPMQ-
-GPAPMM-
-GPAPMF-
GFPAPM--
HRPAPM--
HHPAPM--
-HPAPML-
HYPAPM--
HKPAPM--
HPPAPM--
HTPAPM--
-HPAPMR-
-HPAPMH-
HVPAPM--
-HPAPMS-
HLPAPM--
-HPAPMY-
-HPAPMA-
HGPAPM--
HSPAPM--
-HPAPMV-
-HPAPMN-
HMPAPM--

–HPAPMM–
–HPAPMK–
–HPAPMI–
HFPAPM––
HEPAPM––
HDPAPM––
–IPAPML–
IGPAPM––
ILPAPM––
IVPAPM––
–IPAPMS–
–IPAPMI–
–IPAPMR–
IRPAPM––
–IPAPMV–
IDPAPM––
IKPAPM––
–IPAPME–
INPAPM––
ITPAPM––
ISPAPM––
–IPAPMN–
IFPAPM––
–IPAPMA–
–IPAPMY–
–IPAPMC–
IAPAPM––
KSPAPM––
–KPAPMI–
–KPAPMR–
KMPAPM––
–KPAPMW–
KPPAPM––
–KPAPMS–
–KPAPML–
KGPAPM––
KYPAPM––
KLPAPM––
KKPAPM––
–KPAPMH–
KIPAPM––
–KPAPMY–
–KPAPMM–
KWPAPM––
KTPAPM––
KNPAPM––
KHPAPM––
KEPAPM––
–KPAPMQ–
–KPAPMN–

KFPAPM--
KRPAPM--
-KPAPMV-
-KPAPMF-
-LPAPMR-
LVPAPM--
LTPAPM--
LPPAPM--
-LPAPMK-
LMPAPM--
LAPAPM--
-LPAPMY-
-LPAPMI-
LYPAPM--
-LPAPMH-
LLPAPM--
LGPAPM--
LSPAPM--
LQPAPM--
-LPAPMT-
-LPAPMA-
LIPAPM--
LEPAPM--
-LPAPMS-
-LPAPMF-
LNPAPM--
LDPAPM--
-LPAPMV-
LHPAPM--
LFPAPM--
MRPAPM--
MGPAPM--
MSPAPM--
MVPAPM--
-MPAPMK-
MFPAPM--
MYPAPM--
MQPAPM--
-MPAPMF-
MLPAPM--
MKPAPM--
MPPAPM--
-MPAPMV-
-MPAPMQ-
MNPAPM--
MTPAPM--
-MPAPMT-
-MPAPMH-
MHPAPM--
MEPAPM--

MDPAPM--
-MPAPMW-
-MPAPMS-
-MPAPMN-
-MPAPMI-
MIPAPM--
-NPAPMR-
NLPAPM--
NTPAPM--
-NPAPMI-
NVPAPM--
NRPAPM--
-NPAPMY-
-NPAPMH-
NMPAPM--
NEPAPM--
-NPAPML-
-NPAPMF-
NKPAPM--
NAPAPM--
-NPAPMT-
-NPAPMN-
-NPAPMK-
NSPAPM--
-NPAPMM-
-NPAPMG-
-NPAPMV-
-NPAPMD-
NDPAPM--
--PAPMVS
--PAPMWN
PVPAPM--
--PAPMVY
--PAPMRN
--PAPMRY
--PAPMLL
--PAPMTS
--PAPMSF
--PAPMFR
--PAPMIA
--PAPMAS
--PAPMWA
--PAPMRL
--PAPMSQ
--PAPMHH
--PAPMYM
--PAPMLT
--PAPMFL
--PAPMWY
--PAPMRH

--PAPMLN
--PAPMMN
--PAPMLS
--PAPMRW
--PAPMRM
--PAPMMR
--PAPMLW
--PAPMLQ
--PAPMFN
--PAPMMG
--PAPMLR
--PAPMYI
--PAPMLF
--PAPMKL
--PAPMGQ
--PAPMWR
--PAPMVW
--PAPMTW
--PAPMTM
--PAPMTL
--PAPMRV
--PAPMIF
--PAPMGA
--PAPMWG
--PAPMMW
--PAPMLI
--PAPMHK
--PAPMHF
--PAPMTA
--PAPMSS
--PAPMNN
--PAPMLY
--PAPMID
--PAPMWL
--PAPMRQ
--PAPMNF
--PAPMKS
--PAPMIG
--PAPMFQ
PRPAPM--
--PAPMYW
--PAPMWE
--PAPMVR
--PAPMVN
--PAPMPW
--PAPMKV
--PAPMGS
--PAPMFS
--PAPMWK
--PAPMQM

--PAPMKF
PFPAPM--
--PAPMYR
--PAPMVL
--PAPMMI
--PAPMIW
--PAPMIL
--PAPMHS
--PAPMFT
--PAPMFH
PTPAPM--
--PAPMYS
--PAPMYF
--PAPMTT
--PAPMQS
--PAPMPI
--PAPMNV
--PAPMNS
--PAPMHI
--PAPMHA
--PAPMGN
--PAPMDV
--PAPMAA
PAPAPM--
--PAPMSR
--PAPMSN
--PAPMRF
--PAPMQN
--PAPMQG
--PAPMQA
--PAPMMT
--PAPMLV
--PAPMKN
--PAPMKI
--PAPMFY
--PAPMFM
--PAPMWW
--PAPMVT
--PAPMVG
--PAPMVA
--PAPMTG
--PAPMNM
--PAPMNH
--PAPMNA
--PAPMML
--PAPMMH
--PAPMIY
--PAPMHY
--PAPMEV
--PAPMDI

--PAPMAG
PSPAPM--
PLPAPM--
--PAPMYV
--PAPMYN
--PAPMYG
--PAPMTN
--PAPMTK
--PAPMSA
--PAPMRT
--PAPMPY
--PAPMNG
--PAPMHV
--PAPMGW
--PAPMDF
--PAPMYH
--PAPMWI
--PAPMVK
--PAPMQW
--PAPMQT
--PAPMQR
--PAPMPS
--PAPMPN
--PAPMIK
--PAPMII
--PAPMHT
--PAPMGT
--PAPMFG
--PAPMEA
--PAPMAF
--PAPMYL
--PAPMVV
--PAPMTF
--PAPMSK
--PAPMSH
--PAPMRG
--PAPMRE
--PAPMQQ
--PAPMPV
--PAPMNR
--PAPMMS
--PAPMIS
--PAPMIN
--PAPMIH
--PAPMHW
--PAPMFV
--PAPMFK
--PAPMFI
--PAPMFF
--PAPMES

--PAPMDY
--PAPMDW
--PAPMAQ
--PAPMAK
--PAPMAH
PYPAPM--
--PAPMWS
--PAPMVM
--PAPMVH
--PAPMVF
--PAPMVE
--PAPMTY
--PAPMTI
--PAPMSV
--PAPMST
--PAPMPA
--PAPMNT
--PAPMLK
--PAPMKQ
--PAPMKG
--PAPMIT
--PAPMHR
--PAPMGH
--PAPMEW
--PAPMAT
--PAPMWM
--PAPMWF
--PAPMVD
--PAPMQY
--PAPMQI
--PAPMPH
--PAPMMY
--PAPMMM
--PAPMMK
--PAPMMF
--PAPMLE
--PAPMKW
--PAPMIV
--PAPMIM
--PAPMIE
--PAPMHN
--PAPMEI
--PAPMAR
PPPAPM--
-PPAPML-
-PPAPMH-
PHPAPM--
PGPAPM--
--PAPMYE
--PAPMYC

--PAPMYA
--PAPMTR
--PAPMTQ
--PAPMQK
--PAPMQF
--PAPMPR
--PAPMNK
--PAPMMP
--PAPMLH
--PAPMLD
--PAPMLC
--PAPMKT
--PAPMKA
--PAPMGE
--PAPMEH
--PAPMDS
--PAPMDM
--PAPMDA
--PAPMCS
--PAPMCD
-QPAPML-
QHPAPM--
-QPAPMK-
QRPAPM--
-QPAPMS-
QSPAPM--
-QPAPMR-
QYPAPM--
-QPAPMY-
-QPAPMF-
QWPAPM--
-QPAPMA-
QMPAPM--
QFPAPM--
QDPAPM--
-QPAPMN-
-QPAPMI-
QNPAPM--
QEPAPM--
QAPAPM--
-RPAPMR-
RSPAPM--
-RPAPMK-
-RPAPMT-
RAPAPM--
-RPAPMV-
RLPAPM--
RVPAPM--
-RPAPMY-
RDPAPM--

-RPAPMM-
RWPAPM--
-RPAPMN-
RNPAPM--
RIPAPM--
RYPAPM--
RPPAPM--
-RPAPMF-
RMPAPM--
-RPAPMH-
RQPAPM--
RHPAPM--
REPAPM--
-RPAPMQ-
RFPAPM--
-RPAPMG-
RKPAPM--
RGPAPM--
RCPAPM--
-RPAPMW-
-SPAPMR-
-SPAPMY-
-SPAPMI-
-SPAPML-
-SPAPMV-
SGPAPM--
SKPAPM--
SLPAPM--
SRPAPM--
SSPAPM--
-SPAPMH-
-SPAPMM-
SIPAPM--
SPPAPM--
-SPAPMS-
-SPAPMN-
SNPAPM--
SAPAPM--
SYPAPM--
-SPAPMA-
SEPAPM--
SVPAPM--
STPAPM--
-SPAPMG-
-SPAPMF-
-TPAPMY-
TLPAPM--
-TPAPMW-
-TPAPMF-
TRPAPM--

-TPAPMR-
TNPAPM--
TYPAPM--
-TPAPMM-
TWPAPM--
TIPAPM--
TEPAPM--
-TPAPMV-
-TPAPMK-
-TPAPMI-
THPAPM--
TTPAPM--
-TPAPMH-
TKPAPM--
TAPAPM--
TVPAPM--
TQPAPM--
-TPAPMT-
-TPAPMG-
-TPAPMC-
TMPAPM--
-VPAPMS-
-VPAPMR-
VLPAPM--
VSPAPM--
VRPAPM--
VQPAPM--
-VPAPMI-
-VPAPMH-
VKPAPM--
VAPAPM--
-VPAPMA-
-VPAPMV-
-VPAPMN-
VWPAPM--
-VPAPMK-
VVPAPM--
VTPAPM--
VNPAPM--
VMPAPM--
VEPAPM--
-VPAPML-
-VPAPMG-
VFPAPM--
VYPAPM--
-VPAPMT-
-VPAPMQ-
-VPAPME-
VIPAPM--
VHPAPM--

	WRPAPM--		
	-WPAPMS-		
	-WPAPMI-		
	-WPAPMK-		
	WYPAPM--		
	-WPAPMY-		
	WAPAPM--		
	-WPAPMT-		
	-WPAPMR-		
	WHPAPM--		
	-WPAPMQ-		
	-WPAPMG-		
	WDPAPM--		
	-WPAPML-		
	-WPAPMA-		
	WPPAPM--		
	WGPAPM--		
	WSPAPM--		
	WLPAPM--		
	WKPAPM--		
	WVPAPM--		
	-WPAPMN-		
	-YPAPMY-		
	-YPAPMS-		
	-YPAPMR-		
	YRPAPM--		
	YVPAPM--		
	YLPAPM--		
	YIPAPM--		
	YSPAPM--		
	-YPAPMK-		
	YKPAPM--		
	-YPAPMT-		
	-YPAPMI-		
	-YPAPMH-		
	-YPAPMG-		
	-YPAPMF-		
	-YPAPMA-		
	YFPAPM--		
	-YPAPMM-		
	-YPAPML-		
	YGPAPM--		
PAGI	74	829	45.11
	-FPAGIN-		
	-GPAGIN-		
	--PAGIKL		
	--PAGIGL		
	--PAGIER		
	-QPAGIA-		
	-APAGIS-		

-APAGIR-
ARPAGI--
-APAGIL-
ASPAGI--
-APAGIY-
-APAGIT-
ALPAGI--
AGPAGI--
AAPAGI--
-APAGIM-
AMPAGI--
-APAGIK-
AQPAGI--
AHPAGI--
APPAGI--
AFPAGI--
-APAGII-
-APAGIH-
AKPAGI--
AWPAGI--
-APAGIF-
AEPAGI--
ANPAGI--
AYPAGI--
-APAGIN-
-APAGIW-
AIPAGI--
ATPAGI--
-APAGIQ-
-APAGIE-
-APAGIV-
-APAGIA-
ADPAGI--
CVPAGI--
CTPAGI--
CRPAGI--
CNPAGI--
CMPAGI--
-CPAGIS-
-CPAGIK-
CDPAGI--
-CPAGIT-
CKPAGI--
CIPAGI--
CGPAGI--
CCPAGI--
CAPAGI--
-CPAGIR-
-CPAGIQ-
-CPAGIL-

CHPAGI--
CFPAGI--
CEPAGI--
DVPAGI--
DRPAGI--
-DPAGIR-
-DPAGIM-
DWPAGI--
-DPAGIF-
DLPAGI--
DMPAGI--
DTPAGI--
DGPAGI--
DYPAGI--
-DPAGIH-
DHPAGI--
DFPAGI--
-DPAGIV-
-DPAGIY-
-DPAGIW-
-DPAGIT-
-DPAGIS-
-DPAGIN-
DNPAGI--
DKPAGI--
DEPAGI--
-EPAGIH-
-EPAGIR-
-EPAGIY-
-EPAGIQ-
-EPAGIK-
ERPAGI--
EGPAGI--
-EPAGIT-
EWPAGI--
-EPAGIF-
EAPAGI--
EVPAGI--
-EPAGIV-
EYPAGI--
ETPAGI--
EIPAGI--
-EPAGIN-
EKPAGI--
EQPAGI--
EPPAGI--
-EPAGIS-
-EPAGIC-
ELPAGI--
ECPAGI--

FGPAGI--
-FPAGIR-
FFPAGI--
FSPAGI--
-FPAGIS-
-FPAGIV-
FVPAGI--
-FPAGIK-
-FPAGIH-
-FPAGIT-
-FPAGIL-
FKPAGI--
FLPAGI--
FIPAGI--
FMPAGI--
FHPAGI--
FAPAGI--
FYPAGI--
FWPAGI--
FRPAGI--
-FPAGII-
FNPAGI--
FEPAGI--
FCPAGI--
-FPAGIY-
-FPAGIQ-
-FPAGIM-
-FPAGIA-
GSPAGI--
-GPAGIR-
-GPAGIT-
-GPAGIL-
-GPAGIM-
-GPAGII-
-GPAGIY-
-GPAGIK-
GTPAGI--
-GPAGIF-
GLPAGI--
GPPAGI--
GNPAGI--
GKPAGI--
GIPAGI--
-GPAGIV-
GFPAGI--
-GPAGIW-
-GPAGIS-
GYPAGI--
GWPAGI--
-GPAGIA-

GAPAGI--
-GPAGIH-
GEPAGI--
GHPAGI--
GQPAGI--
GGPAGI--
-GPAGIQ-
-GPAGID-
HIPAGI--
-HPAGIR-
-HPAGIF-
HTPAGI--
HFPAGI--
HWPAGI--
-HPAGIS-
-HPAGIV-
HSPAGI--
HKPAGI--
HRPAGI--
-HPAGIL-
HEPAGI--
HQPAGI--
HLPAGI--
HYPAGI--
-HPAGII-
HMPAGI--
HAPAGI--
HNPAGI--
-HPAGIA-
HGPAGI--
-HPAGIM-
-HPAGIH-
-HPAGIY-
-HPAGIW-
-HPAGIT-
HHPAGI--
HDPAGI--
IIPAGI--
ISPAGI--
-IPAGIR-
-IPAGIL-
IRPAGI--
IMPAGI--
IAPAGI--
IYPAGI--
-IPAGII-
IGPAGI--
IQPAGI--
IVPAGI--
-IPAGIY-

-IPAGIS-
-IPAGIQ-
ILPAGI--
ITPAGI--
IFPAGI--
-IPAGIN-
-IPAGIW-
-IPAGIV-
-IPAGIT-
-IPAGIH-
IEPAGI--
IWPAGI--
INPAGI--
IKPAGI--
-IPAGIA-
IPPAGI--
-IPAGIK-
-IPAGIF-
IHPAGI--
ICPAGI--
KSPAGI--
-KPAGIR-
-KPAGIV-
-KPAGII-
KPPAGI--
-KPAGIS-
KLPAGI--
-KPAGIN-
KGPAGI--
KTPAGI--
-KPAGIT-
KMPAGI--
KRPAGI--
KHPAGI--
KKPAGI--
-KPAGIW-
-KPAGIA-
-KPAGIF-
KYPAGI--
KAPAGI--
-KPAGIY-
KIPAGI--
-KPAGIK-
KVPAGI--
-KPAGIH-
KEPAGI--
KCPAGI--
KDPAGI--
KWPAGI--
KQPAGI--

-KPAGIM-
-KPAGIG-
KFPAGI--
-KPAGIQ-
-KPAGIC-
LVPAGI--
-LPAGIR-
LLPAGI--
LTPAGI--
LRPAGI--
LKPAGI--
LMPAGI--
LAPAGI--
LYPAGI--
-LPAGIL-
-LPAGIV-
LNPAGI--
LWPAGI--
-LPAGIY-
-LPAGIM-
LIPAGI--
-LPAGIS-
-LPAGIK-
-LPAGIH-
LHPAGI--
LQPAGI--
LPPAGI--
-LPAGIW-
-LPAGIF-
-LPAGII-
-LPAGIT-
-LPAGIQ-
LDPAGI--
-LPAGIG-
LEPAGI--
-LPAGIA-
LFPAGI--
LCPAGI--
-MPAGIR-
MAPAGI--
MRPAGI--
MLPAGI--
-MPAGII-
-MPAGIH-
MKPAGI--
-MPAGIL-
MPPAGI--
MVPAGI--
-MPAGIK-
MSPAGI--

MTPAGI--
MIPAGI--
-MPAGIW-
-MPAGIS-
MNPAGI--
MMPAGI--
MHPAGI--
-MPAGIT-
-MPAGIQ-
-MPAGIN-
-MPAGIF-
MGPAGI--
MFPAGI--
MCPAGI--
MQPAGI--
-MPAGIY-
-MPAGIV-
-MPAGIA-
MDPAGI--
NAPAGI--
NLPAGI--
NTPAGI--
NRPAGI--
NSPAGI--
NVPAGI--
NPPAGI--
NIPAGI--
-NPAGIL-
NFPAGI--
NEPAGI--
-NPAGIR-
-NPAGIH-
NKPAGI--
NWPAGI--
NGPAGI--
-NPAGIT-
-NPAGIS-
-NPAGIY-
-NPAGIF-
NNPAGI--
NMPAGI--
NYPAGI--
NQPAGI--
-NPAGIM-
-NPAGIV-
-NPAGIQ-
-NPAGIN-
NDPAGI--
--PAGIVM
--PAGILS

--PAGIYS
--PAGIRK
--PAGIRT
--PAGIRH
--PAGIWT
--PAGIMY
--PAGISA
--PAGITG
--PAGIRS
--PAGIYF
--PAGIVH
--PAGIMS
--PAGISS
--PAGIVR
--PAGIRL
--PAGISM
--PAGILG
--PAGIKY
--PAGISN
--PAGIYA
--PAGISI
--PAGIVS
--PAGIRM
--PAGIHR
--PAGIKV
--PAGITR
--PAGIRQ
--PAGIKS
PKPAGI--
--PAGIRG
--PAGIMG
--PAGILT
--PAGISR
--PAGIQG
--PAGILH
--PAGIIA
--PAGIHS
PQPAGI--
--PAGIKA
--PAGIVY
--PAGILQ
--PAGIIW
PGPAGI--
--PAGIRN
--PAGINW
--PAGINS
--PAGILF
--PAGIII
--PAGIFR
--PAGIVG

--PAGIMR
--PAGILA
--PAGIKT
--PAGIKM
--PAGIIG
--PAGIFT
PAPAGI--
--PAGIYG
--PAGISH
--PAGISG
--PAGIFM
--PAGIAV
PLPAGI--
--PAGIWN
--PAGITA
--PAGISK
--PAGIQT
--PAGIWQ
--PAGISY
--PAGISV
--PAGILN
--PAGILM
--PAGIYH
--PAGIWS
--PAGIVN
--PAGIGS
--PAGIFS
--PAGIAA
PEPAGI--
--PAGITT
--PAGITS
--PAGIRA
--PAGIMA
--PAGILK
--PAGIIM
--PAGIGA
--PAGIAW
PFPAGI--
--PAGITN
--PAGITH
--PAGITF
--PAGIRY
--PAGIKG
--PAGIHI
--PAGIAY
--PAGIAM
--PAGIWW
--PAGIWH
--PAGIST
--PAGISQ

--PAGINV
--PAGILW
--PAGIKH
--PAGIHM
--PAGIFH
--PAGIAT
PIPAGI--
--PAGITY
--PAGIQS
--PAGILY
--PAGIKR
--PAGIIY
--PAGIIN
--PAGIHN
--PAGIHK
--PAGIES
--PAGIWY
--PAGIVD
--PAGITV
--PAGIMT
--PAGIFN
--PAGIFA
--PAGIAH
PTPAGI--
--PAGIWK
--PAGITD
--PAGIQW
--PAGIIV
--PAGIHH
--PAGIGG
--PAGIFQ
--PAGIAF
--PAGIYY
--PAGIYN
--PAGIVQ
--PAGIVA
--PAGIMI
PWPAGI--
--PAGIVK
--PAGITK
--PAGIQA
--PAGINN
--PAGING
--PAGINA
--PAGIML
--PAGIMD
--PAGILL
--PAGIIQ
--PAGIHE
--PAGIYR

```
--PAGISW
--PAGIQH
--PAGIQD
--PAGINY
--PAGINT
--PAGIIR
--PAGIIF
--PAGIFD
--PAGIYD
--PAGIVW
--PAGIVV
--PAGIVT
--PAGIVI
--PAGITW
--PAGISL
--PAGISF
--PAGIRF
--PAGIQI
--PAGINR
--PAGINM
--PAGIKW
--PAGIKI
--PAGIIK
--PAGIHW
--PAGIHG
--PAGIFF
--PAGIAI
--PAGIAG
PYPAGI--
PVPAGI--
PNPAGI--
--PAGIYK
--PAGIVF
--PAGITQ
--PAGITL
--PAGISE
--PAGIQK
--PAGINI
--PAGINF
--PAGIMK
--PAGIKN
--PAGIKK
--PAGIKF
--PAGIIT
--PAGIFY
--PAGIEG
--PAGIEF
--PAGITM
--PAGITI
--PAGIQY
```

--PAGIQV
--PAGIQN
--PAGIQF
--PAGIMW
--PAGIMM
--PAGILE
--PAGIKD
--PAGIIH
--PAGIID
--PAGIEV
--PAGIEK
--PAGIDG
--PAGICL
--PAGICA
--PAGIAN
PSPAGI--
PHPAGI--
--PAGIYM
--PAGIWF
--PAGIQL
--PAGIHQ
--PAGIGT
--PAGIFE
--PAGIET
--PAGIDS
--PAGIAR
--PAGIAL
--PAGIAK
-PPAGIP-
-PPAGIF-
PMPAGI--
PCPAGI--
--PAGIYW
--PAGIYV
--PAGIWD
--PAGITE
--PAGISP
--PAGIRE
--PAGIQE
--PAGIPY
--PAGIPL
--PAGINQ
--PAGINK
--PAGIME
--PAGIMC
--PAGILD
--PAGIIP
--PAGIIC
--PAGIGV
--PAGIGK

--PAGIGI
--PAGIGH
--PAGIEI
--PAGIDK
--PAGICN
--PAGICF
QPPAGI--
QVPAGI--
QSPAGI--
QLPAGI--
QAPAGI--
-QPAGIT-
-QPAGII-
QIPAGI--
-QPAGIV-
-QPAGIL-
-QPAGIR-
QGPAGI--
QRPAGI--
QMPAGI--
-QPAGIM-
QHPAGI--
QYPAGI--
QQPAGI--
-QPAGIY-
QKPAGI--
-QPAGIK-
-QPAGIH-
-QPAGIW-
QEPAGI--
-QPAGIE-
QNPAGI--
-QPAGIS-
-QPAGIQ-
-QPAGIN-
-QPAGIG-
QCPAGI--
-RPAGIR-
RGPAGI--
RWPAGI--
-RPAGIV-
RAPAGI--
RVPAGI--
RHPAGI--
-RPAGIS-
RIPAGI--
-RPAGIW-
-RPAGIK-
RLPAGI--
RSPAGI--

-RPAGII-
RFPAGI--
RYPAGI--
-RPAGIT-
RNPAGI--
-RPAGIM-
RPPAGI--
REPAGI--
-RPAGIQ-
-RPAGIF-
RMPAGI--
-RPAGIH-
-RPAGIG-
RDPAGI--
-RPAGIA-
RCPAGI--
-RPAGIE-
-SPAGIT-
-SPAGIL-
SLPAGI--
STPAGI--
-SPAGIY-
SVPAGI--
SGPAGI--
-SPAGIR-
-SPAGIS-
-SPAGII-
SMPAGI--
-SPAGIM-
SFPAGI--
SEPAGI--
-SPAGIF-
SNPAGI--
SYPAGI--
-SPAGIW-
-SPAGIH-
SHPAGI--
SPPAGI--
SQPAGI--
-SPAGIK-
SIPAGI--
SAPAGI--
SDPAGI--
-SPAGIN-
SCPAGI--
-TPAGIR-
TVPAGI--
TTPAGI--
-TPAGII-
TGPAGI--

TRPAGI--
TLPAGI--
TQPAGI--
TFPAGI--
TAPAGI--
TYPAGI--
-TPAGIS-
-TPAGIY-
THPAGI--
-TPAGIM-
-TPAGIF-
TPPAGI--
TWPAGI--
-TPAGIV-
TNPAGI--
-TPAGIN-
TMPAGI--
TKPAGI--
TIPAGI--
TEPAGI--
-TPAGIT-
-TPAGIH-
-TPAGIK-
TCPAGI--
-TPAGIW-
-TPAGIE-
VRPAGI--
VAPAGI--
VLPAGI--
VYPAGI--
VSPAGI--
-VPAGIQ-
-VPAGIR-
VPPAGI--
-VPAGIK-
VGPAGI--
VIPAGI--
VFPAGI--
-VPAGIT-
VMPAGI--
-VPAGIW-
-VPAGII-
-VPAGIF-
-VPAGIS-
VHPAGI--
-VPAGIM-
VEPAGI--
-VPAGIV-
-VPAGIH-
VVPAGI--

-VPAGIA-
VWPAGI--
-VPAGIY-
-VPAGIN-
VNPAGI--
VTPAGI--
-VPAGIG-
-VPAGIE-
VQPAGI--
VDPAGI--
WKPAGI--
WYPAGI--
-WPAGIR-
WPPAGI--
-WPAGIF-
WSPAGI--
WEPAGI--
WLPAGI--
-WPAGIA-
WVPAGI--
WTPAGI--
-WPAGIL-
-WPAGIY-
-WPAGIS-
WMPAGI--
WAPAGI--
-WPAGIK-
WIPAGI--
WQPAGI--
-WPAGIV-
-WPAGIH-
-WPAGIN-
WDPAGI--
WRPAGI--
-WPAGIM-
WFPAGI--
-WPAGIE-
WNPAGI--
WGPAGI--
YLPAGI--
YAPAGI--
YTPAGI--
-YPAGIF-
YMPAGI--
YRPAGI--
-YPAGIR-
YDPAGI--
YGPAGI--
YEPAGI--
-YPAGIL-

YIPAGI--
YPPAGI--
YFPAGI--
YSPAGI--
-YPAGIY-
-YPAGIW-
-YPAGIV-
-YPAGIQ-
-YPAGIA-
-YPAGIH-
YHPAGI--
YYPAGI--
YWPAGI--
-YPAGIK-
YVPAGI--
YQPAGI--
-YPAGIM-
-YPAGIT-
-YPAGIN-
-YPAGII-
-YPAGIS-
-YPAGIC-

PIPY 483 625 45.10

-RPIPYN-
ARPIPY--
-APIPYL-
-APIPYS-
AFPIPY--
-APIPYM-
ALPIPY--
AGPIPY--
ASPIPY--
AEPIPY--
AYPIPY--
ATPIPY--
-APIPYN-
AIPIPY--
-APIPYR-
-APIPYH-
-APIPYK-
AHPIPY--
AVPIPY--
-APIPYW-
-APIPYQ-
-APIPYG-
AKPIPY--
AAPIPY--
-APIPYY-
AWPIPY--
AQPIPY--

-APIPYA-
ANPIPY--
ACPIPY--
CFPIPY--
-CPIPYR-
CSPIPY--
CPPIPY--
CKPIPY--
DRPIPY--
-DPIPYR-
-DPIPYI-
DFPIPY--
DSPIPY--
-DPIPYS-
-DPIPYL-
-DPIPYY-
-DPIPYV-
-DPIPYM-
DEPIPY--
DLPIPY--
DIPIPY--
DAPIPY--
ESPIPY--
-EPIPYL-
ELPIPY--
EGPIPY--
-EPIPYM-
EKPIPY--
EEPIPY--
ERPIPY--
-EPIPYS-
EMPIPY--
-EPIPYR-
EVPIPY--
-EPIPYI-
EAPIPY--
FSPIPY--
FRPIPY--
-FPIPYL-
-FPIPYS-
-FPIPYW-
FGPIPY--
FEPIPY--
FLPIPY--
FTPIPY--
-FPIPYT-
-FPIPYQ-
-FPIPYY-
-FPIPYK-
FFPIPY--

FYPIPY--
FWPIPY--
-FPIPYA-
FMPIPY--
FDPIPY--
-FPIPYH-
-FPIPYV-
-FPIPYI-
FKPIPY--
FIPIPY--
GTPIPY--
GRPIPY--
GVPIPY--
-GPIPYM-
GLPIPY--
GFPIPY--
GAPIPY--
GSPIPY--
-GPIPYV-
-GPIPYS-
-GPIPYL-
-GPIPYI-
-GPIPYR-
-GPIPYK-
GEPIPY--
-GPIPYQ-
-GPIPYA-
GKPIPY--
GQPIPY--
-GPIPYY-
-GPIPYN-
GNPIPY--
GIPIPY--
GDPIPY--
HRPIPY--
-HPIPYY-
HPPIPY--
HSPIPY--
-HPIPYA-
-HPIPYS-
-HPIPYR-
-HPIPYL-
-HPIPYK-
HAPIPY--
HYPIPY--
HTPIPY--
-HPIPYT-
-HPIPYF-
HNPIPY--
-HPIPYW-

-HPIPYV-
HWPIPY--
-HPIPYQ-
-HPIPYM-
-HPIPYI-
-HPIPYH-
HLPIPY--
HKPIPY--
HIPIPY--
HFPIPY--
ISPIPY--
IFPIPY--
IHPIPY--
IWPIPY--
-IPIPYS-
-IPIPYL-
INPIPY--
IMPIPY--
-IPIPYR-
-IPIPYQ-
ILPIPY--
-IPIPYT-
IKPIPY--
IIPIPY--
IGPIPY--
-IPIPYH-
IQPIPY--
-IPIPYV-
-IPIPYI-
-IPIPYG-
IVPIPY--
IRPIPY--
-IPIPYW-
IEPIPY--
IAPIPY--
KSPIPY--
KIPIPY--
-KPIPYL-
KAPIPY--
KGPIPY--
KFPIPY--
-KPIPYR-
KPPIPY--
KRPIPY--
-KPIPYV-
KQPIPY--
KWPIPY--
-KPIPYT-
-KPIPYA-
KMPIPY--

```
-KPIPYF-
KLPIPY--
KDPIPY--
KYPIPY--
KTPIPY--
-KPIPYQ-
KEPIPY--
-KPIPYW-
-KPIPYI-
KNPIPY--
KKPIPY--
KHPIPY--
KCPIPY--
LSPIPY--
LKPIPY--
LRPIPY--
LLPIPY--
-LPIPYR-
LWPIPY--
-LPIPYL-
LTPIPY--
LEPIPY--
LNPIPY--
LGPIPY--
LYPIPY--
LQPIPY--
LPPIPY--
-LPIPYM-
-LPIPYF-
LAPIPY--
-LPIPYT-
-LPIPYK-
LHPIPY--
LVPIPY--
-LPIPYN-
-LPIPYI-
-LPIPYA-
LIPIPY--
-LPIPYV-
-LPIPYS-
MKPIPY--
MRPIPY--
-MPIPYT-
-MPIPYI-
-MPIPYS-
MYPIPY--
MPPIPY--
-MPIPYM-
MEPIPY--
-MPIPYR-
```

MSPIPY--
MQPIPY--
MWPIPY--
-MPIPYH-
MDPIPY--
MVPIPY--
-MPIPYL-
MNPIPY--
MGPIPY--
MHPIPY--
NRPIPY--
-NPIPYL-
NGPIPY--
NLPIPY--
NQPIPY--
-NPIPYM-
NPPIPY--
-NPIPYR-
-NPIPYT-
NVPIPY--
-NPIPYS-
NKPIPY--
NYPIPY--
NTPIPY--
-NPIPYW-
-NPIPYI-
-NPIPYH-
NNPIPY--
NMPIPY--
NIPIPY--
NCPIPY--
--PIPYTQ
--PIPYMG
--PIPYVN
--PIPYLS
--PIPYHS
--PIPYWI
--PIPYVS
--PIPYRG
--PIPYML
--PIPYTR
--PIPYLY
--PIPYKT
--PIPYYV
--PIPYLR
--PIPYRS
--PIPYLW
--PIPYRT
--PIPYLH
--PIPYKS

--PIPYTS
--PIPYSS
--PIPYMH
--PIPYLV
--PIPYKI
--PIPYFA
--PIPYWV
--PIPYVR
--PIPYVQ
--PIPYTA
--PIPYMI
--PIPYVG
--PIPYQY
--PIPYLL
PEPIPY--
--PIPYYH
--PIPYRN
--PIPYRI
--PIPYQI
--PIPYLG
PFPIPY--
--PIPYYA
--PIPYSR
--PIPYRV
--PIPYRM
--PIPYRA
--PIPYNA
--PIPYIR
--PIPYHI
--PIPYHA
--PIPYEA
--PIPYYL
--PIPYVT
--PIPYVH
--PIPYTN
--PIPYRE
--PIPYQN
--PIPYMA
--PIPYLN
--PIPYLI
--PIPYIH
--PIPYAR
--PIPYVL
--PIPYQR
--PIPYMF
--PIPYWW
--PIPYVV
--PIPYTV
--PIPYPV
--PIPYNS

--PIPYGQ
--PIPYGH
--PIPYAS
--PIPYVA
--PIPYRY
--PIPYMY
--PIPYME
--PIPYFS
--PIPYYD
--PIPYWT
--PIPYVK
--PIPYSN
--PIPYSG
--PIPYSE
--PIPYSA
--PIPYIY
--PIPYIK
--PIPYHF
--PIPYGT
--PIPYFT
--PIPYVM
--PIPYSH
--PIPYRW
--PIPYLQ
--PIPYIN
PTPIPY--
PMPIPY--
--PIPYSK
--PIPYRK
--PIPYPW
--PIPYPS
--PIPYNV
--PIPYNT
--PIPYMS
--PIPYMM
--PIPYLM
--PIPYLA
--PIPYIS
--PIPYIL
--PIPYHK
--PIPYGS
--PIPYFR
--PIPYFK
--PIPYYI
--PIPYVD
--PIPYTK
--PIPYRQ
--PIPYRD
--PIPYQM
--PIPYQG

--PIPYPQ
--PIPYNF
--PIPYMQ
--PIPYLT
--PIPYKW
--PIPYIA
--PIPYHW
--PIPYGN
--PIPYGG
--PIPYFD
--PIPYEW
--PIPYEI
--PIPYAW
--PIPYAK
--PIPYAA
--PIPYYS
--PIPYWQ
--PIPYVY
--PIPYTY
--PIPYPM
--PIPYNG
--PIPYMW
--PIPYMV
--PIPYMN
--PIPYLK
--PIPYLF
--PIPYKV
--PIPYKH
--PIPYIV
--PIPYIT
--PIPYHT
--PIPYHG
--PIPYFN
--PIPYFI
--PIPYFH
PGPIPY--
--PIPYYM
--PIPYWY
--PIPYWD
--PIPYVW
--PIPYVI
--PIPYVC
--PIPYTG
--PIPYTD
--PIPYST
--PIPYRH
--PIPYRF
--PIPYQV
--PIPYQA
--PIPYPH

--PIPYPF
--PIPYMR
--PIPYKN
--PIPYKG
--PIPYIQ
--PIPYHR
--PIPYGW
--PIPYGK
--PIPYGC
--PIPYGA
--PIPYFM
--PIPYFG
--PIPYFE
--PIPYEV
--PIPYEK
--PIPYDA
--PIPYCV
--PIPYCL
--PIPYAT
QSPIPY--
-QPIPYL-
QGPIPY--
QMPIPY--
-QPIPYR-
QFPIPY--
QVPIPY--
-QPIPYS-
-QPIPYM-
QIPIPY--
QHPIPY--
QAPIPY--
QPPIPY--
-QPIPYW-
-QPIPYI-
QKPIPY--
QQPIPY--
QEPIPY--
-QPIPYV-
-QPIPYH-
RRPIPY--
RAPIPY--
-RPIPYR-
-RPIPYL-
-RPIPYW-
RGPIPY--
RWPIPY--
-RPIPYS-
RSPIPY--
RLPIPY--
RHPIPY--

RTPIPY--
RMPIPY--
-RPIPYQ-
RYPIPY--
RQPIPY--
-RPIPYV-
-RPIPYA-
RPPIPY--
RDPIPY--
-RPIPYI-
REPIPY--
-RPIPYK-
-RPIPYH-
RNPIPY--
-RPIPYT-
-RPIPYF-
-RPIPYE-
RIPIPY--
SRPIPY--
SYPIPY--
SSPIPY--
SNPIPY--
SLPIPY--
SMPIPY--
SEPIPY--
SVPIPY--
SQPIPY--
-SPIPYL-
-SPIPYY-
STPIPY--
-SPIPYW-
-SPIPYS-
-SPIPYR-
SWPIPY--
SKPIPY--
SPPIPY--
-SPIPYF-
SFPIPY--
SDPIPY--
-SPIPYH-
-SPIPYV-
-SPIPYI-
SCPIPY--
-SPIPYN-
-SPIPYQ-
-SPIPYA-
SGPIPY--
SAPIPY--
-SPIPYT-
-SPIPYM-

-SPIPYK-
SIPIPY--
TSPIPY--
TRPIPY--
-TPIPYT-
-TPIPYS-
-TPIPYM-
TPPIPY--
TLPIPY--
TKPIPY--
TGPIPY--
-TPIPYK-
-TPIPYH-
TYPIPY--
TIPIPY--
TDPIPY--
-TPIPYV-
-TPIPYL-
TQPIPY--
-TPIPYR-
TMPIPY--
TFPIPY--
-TPIPYI-
-TPIPYE-
TVPIPY--
-TPIPYN-
THPIPY--
VLPIPY--
VVPIPY--
VKPIPY--
VTPIPY--
VMPIPY--
-VPIPYI-
VEPIPY--
VRPIPY--
VAPIPY--
VSPIPY--
-VPIPYT-
-VPIPYW-
VQPIPY--
-VPIPYQ-
VGPIPY--
-VPIPYS-
-VPIPYL-
VNPIPY--
VIPIPY--
-VPIPYY-
-VPIPYR-
-VPIPYA-
VFPIPY--

VYPIPY--
VHPIPY--
-VPIPYV-
VDPIPY--
VCPIPY--
WQPIPY--
WPPIPY--
WTPIPY--
WRPIPY--
WSPIPY--
-WPIPYL-
-WPIPYF-
-WPIPYS-
WWPIPY--
-WPIPYW-
-WPIPYN-
-WPIPYM-
WMPIPY--
WIPIPY--
WGPIPY--
YTPIPY--
YQPIPY--
YSPIPY--
YWPIPY--
YGPIPY--
YFPIPY--
-YPIPYR-
YDPIPY--
YAPIPY--
-YPIPYT-
-YPIPYS-
YPPIPY--
-YPIPYL-
YKPIPY--
YHPIPY--
YEPIPY--
-YPIPYH-
YNPIPY--
-YPIPYW-
-YPIPYQ-
-YPIPYM-
-YPIPYK-
-YPIPYF-
YMPIPY--
YLPIPY--
YVPIPY--
-YPIPYA-
YIPIPY--
PMPI 474 629 45.08
-RPMPII-

TLPMPI--
-APMPIS-
-APMPIF-
AAPMPI--
ASPMPI--
-APMPIH-
AYPMPI--
AWPMPI--
-APMPIA-
AFPMPI--
-APMPIT-
-APMPIQ-
-APMPIL-
AHPMPI--
ADPMPI--
ATPMPI--
-APMPIY-
-APMPIR-
AGPMPI--
-APMPIN-
ANPMPI--
ALPMPI--
AKPMPI--
AEPMPI--
AQPMPI--
CRPMPI--
CMPMPI--
-CPMPIR-
-CPMPIQ-
-CPMPIP-
-CPMPIL-
-CPMPII-
CDPMPI--
-DPMPIR-
-DPMPIH-
-DPMPIS-
DMPMPI--
DVPMPI--
-DPMPIT-
DYPMPI--
DTPMPI--
DFPMPI--
DSPMPI--
DQPMPI--
-DPMPIY-
DNPMPI--
DHPMPI--
DAPMPI--
-DPMPIK-
DKPMPI--

DIPMPI--
DEPMPI--
EKPMPI--
-EPMPIL-
-EPMPIR-
ETPMPI--
ELPMPI--
-EPMPIK-
EAPMPI--
-EPMPIY-
-EPMPII-
EWPMPI--
-EPMPIS-
-EPMPIT-
-EPMPIM-
EQPMPI--
-EPMPIA-
EMPMPI--
FRPMPI--
FVPMPI--
FTPMPI--
FLPMPI--
FMPMPI--
FSPMPI--
FFPMPI--
-FPMPIR-
-FPMPIK-
-FPMPIV-
-FPMPIL-
-FPMPIA-
FWPMPI--
FNPMPI--
FYPMPI--
FPPMPI--
-FPMPIS-
FEPMPI--
FKPMPI--
-FPMPII-
FIPMPI--
FGPMPI--
GVPMPI--
GRPMPI--
-GPMPIM-
GTPMPI--
GKPMPI--
GSPMPI--
-GPMPIR-
GMPMPI--
GIPMPI--
GGPMPI--

-GPMPIY-
-GPMPIL-
GEPMPI--
-GPMPIW-
-GPMPIK-
GLPMPI--
GAPMPI--
-GPMPIS-
-GPMPIV-
-GPMPIQ-
-GPMPIH-
GCPMPI--
HAPMPI--
HFPMPI--
HNPMPI--
HSPMPI--
-HPMPIR-
HDPMPI--
-HPMPIL-
HIPMPI--
HQPMPI--
-HPMPIS-
-HPMPIN-
-HPMPIA-
HLPMPI--
HKPMPI--
HGPMPI--
HRPMPI--
-HPMPIQ-
-HPMPII-
HVPMPI--
-HPMPIV-
-HPMPIH-
IRPMPI--
IFPMPI--
IQPMPI--
IEPMPI--
-IPMPIT-
-IPMPIS-
IVPMPI--
-IPMPIH-
-IPMPIF-
IKPMPI--
IGPMPI--
IYPMPI--
-IPMPIG-
ITPMPI--
-IPMPIQ-
IIPMPI--
-IPMPIW-

-IPMPIV-
INPMPI--
IAPMPI--
-IPMPIR-
-IPMPIL-
IHPMPI--
-IPMPII-
IWPMPI--
ISPMPI--
-IPMPIN-
-IPMPIA-
ICPMPI--
KSPMPI--
KGPMPI--
KRPMPI--
-KPMPIL-
KIPMPI--
KDPMPI--
KAPMPI--
KFPMPI--
KWPMPI--
KPPMPI--
KVPMPI--
KMPMPI--
-KPMPIM-
-KPMPIR-
-KPMPIH-
KNPMPI--
KTPMPI--
-KPMPIY-
-KPMPIQ-
-KPMPIF-
-KPMPIW-
-KPMPII-
KLPMPI--
LAPMPI--
LSPMPI--
LPPMPI--
LHPMPI--
LRPMPI--
-LPMPIL-
LQPMPI--
LLPMPI--
LTPMPI--
-LPMPIV-
LFPMPI--
-LPMPIS-
LGPMPI--
LEPMPI--
LMPMPI--

LIPMPI--
-LPMPIY-
-LPMPIK-
LKPMPI--
LWPMPI--
-LPMPIW-
-LPMPIM-
-LPMPIH-
LDPMPI--
LYPMPI--
-MPMPIY-
MSPMPI--
-MPMPIH-
MLPMPI--
-MPMPIR-
-MPMPIV-
MAPMPI--
MGPMPI--
-MPMPIS-
-MPMPIQ-
MIPMPI--
MFPMPI--
-MPMPIK-
-MPMPII-
MTPMPI--
MRPMPI--
MQPMPI--
MVPMPI--
-MPMPIC-
MCPMPI--
NGPMPI--
NVPMPI--
NWPMPI--
NLPMPI--
NRPMPI--
NIPMPI--
-NPMPIS-
-NPMPIL-
NMPMPI--
NTPMPI--
-NPMPIT-
-NPMPIR-
NAPMPI--
NSPMPI--
-NPMPIM-
--PMPIMK
--PMPIRA
--PMPIVV
--PMPIRS
--PMPIRL

--PMPISS
--PMPILS
--PMPIKS
--PMPIRM
--PMPIWS
--PMPITL
--PMPIHS
--PMPISW
--PMPIRG
--PMPILA
--PMPIRV
--PMPIVS
--PMPILL
--PMPIKA
PMPMPI--
--PMPITS
--PMPISQ
--PMPIRY
--PMPIML
--PMPIKY
--PMPIHG
--PMPIAM
--PMPIIK
--PMPIYL
--PMPITR
--PMPIIV
PWPMPI--
PQPMPI--
--PMPITI
--PMPISN
--PMPISL
--PMPIRR
--PMPIRE
--PMPIKQ
--PMPIRK
--PMPILY
--PMPIIG
--PMPITK
--PMPITG
PGPMPI--
--PMPIWA
--PMPIVR
--PMPIQV
--PMPIMI
--PMPIYT
--PMPIVH
--PMPITT
--PMPITH
--PMPISR
--PMPIRT

--PMPIQW
--PMPINA
--PMPILN
--PMPILK
--PMPIFY
--PMPIAG
--PMPIYV
--PMPIYN
--PMPIYI
--PMPIWY
--PMPIWE
--PMPIVL
--PMPIQI
--PMPIPW
--PMPINI
--PMPIMV
--PMPIMM
--PMPIKW
--PMPIIS
--PMPIIR
--PMPIGW
--PMPIGG
--PMPIFS
PTPMPI--
--PMPIYS
--PMPIYA
--PMPIWH
--PMPISG
--PMPIQT
--PMPIQG
--PMPINM
--PMPIMY
--PMPIMF
--PMPIHA
--PMPIGH
--PMPIAF
--PMPIWI
--PMPIVN
--PMPIVD
--PMPITW
--PMPISD
--PMPISA
--PMPIPF
--PMPIMS
--PMPIKH
--PMPIHV
--PMPIFA
PEPMPI--
--PMPIWN
--PMPITD

--PMPISV
--PMPIRH
--PMPIRF
--PMPIPQ
--PMPINS
--PMPINQ
--PMPINF
--PMPIMT
--PMPIMH
--PMPILR
--PMPILM
--PMPIKV
--PMPIIY
--PMPIFK
--PMPIAT
PFPMPI--
--PMPIYM
--PMPIWM
--PMPITA
--PMPISH
--PMPISF
--PMPIRI
--PMPIRD
--PMPIPV
--PMPINN
--PMPILH
--PMPIKN
--PMPIKE
--PMPIIL
--PMPIID
--PMPIHR
--PMPIHQ
--PMPIGN
--PMPIFW
--PMPIFF
--PMPIEL
--PMPIYG
--PMPIWT
--PMPIVE
--PMPIVA
--PMPIPS
--PMPIPH
--PMPINV
--PMPIMR
--PMPIME
--PMPILQ
--PMPIKL
--PMPIKK
--PMPIKG
--PMPIIM

--PMPIHI
--PMPIGV
--PMPIGS
--PMPIGK
--PMPIFT
--PMPIET
--PMPIEI
--PMPIDI
--PMPIAR
--PMPIAA
PPPMPI--
--PMPIYY
--PMPIYR
--PMPIYQ
--PMPIWD
--PMPIWC
--PMPIVT
--PMPIVI
--PMPIVF
--PMPITY
--PMPITN
--PMPIRQ
--PMPIQY
--PMPIQR
--PMPIQL
--PMPIQE
--PMPIPA
--PMPINY
--PMPIMN
--PMPIKI
--PMPIIF
--PMPIIA
--PMPIHM
--PMPIFQ
--PMPIFL
--PMPIFG
--PMPIFD
--PMPIEW
--PMPIES
--PMPIEF
--PMPIDW
--PMPICH
PKPMPI--
PDPMPI--
QWPMPI--
QNPMPI--
QRPMPI--
-QPMPIW-
-QPMPIR-
QKPMPI--

QGPMPI--
-QPMPIK-
QPPMPI--
-QPMPIF-
QQPMPI--
QVPMPI--
-QPMPIV-
-QPMPIN-
QIPMPI--
QAPMPI--
QYPMPI--
-QPMPIS-
-QPMPIH-
QDPMPI--
-QPMPIY-
-QPMPIC-
QLPMPI--
QHPMPI--
QFPMPI--
-RPMPIR-
RAPMPI--
RSPMPI--
RGPMPI--
-RPMPIL-
-RPMPIT-
-RPMPIS-
RYPMPI--
RLPMPI--
-RPMPIV-
RIPMPI--
RMPMPI--
RPPMPI--
RFPMPI--
-RPMPIQ-
RKPMPI--
RHPMPI--
-RPMPIA-
RNPMPI--
RQPMPI--
-RPMPIM-
-RPMPIH-
RWPMPI--
-RPMPIF-
-RPMPIN-
RVPMPI--
-RPMPIK-
-RPMPIY-
RDPMPI--
RCPMPI--
-RPMPIP-

-RPMPID-
REPMPI--
SRPMPI--
SKPMPI--
SVPMPI--
STPMPI--
-SPMPIY-
SAPMPI--
SQPMPI--
-SPMPIR-
-SPMPIQ-
SGPMPI--
-SPMPIT-
SYPMPI--
SSPMPI--
-SPMPIS-
SWPMPI--
-SPMPIH-
-SPMPIF-
-SPMPIK-
SEPMPI--
-SPMPIW-
-SPMPIV-
-SPMPIA-
SIPMPI--
SPPMPI--
-SPMPIN-
-SPMPIE-
SNPMPI--
SMPMPI--
TTPMPI--
TSPMPI--
-TPMPIL-
TIPMPI--
TAPMPI--
-TPMPIK-
-TPMPIR-
-TPMPIT-
-TPMPIS-
-TPMPIF-
TVPMPI--
-TPMPIQ-
-TPMPIA-
TMPMPI--
TWPMPI--
-TPMPIG-
TGPMPI--
TYPMPI--
TRPMPI--
-TPMPIY-

TNPMPI--
TFPMPI--
TEPMPI--
-TPMPII-
-TPMPIH-
THPMPI--
VSPMPI--
-VPMPIW-
VIPMPI--
VYPMPI--
VKPMPI--
-VPMPIK-
-VPMPIS-
VRPMPI--
-VPMPIL-
-VPMPIY-
-VPMPIT-
VVPMPI--
-VPMPII-
VMPMPI--
-VPMPIN-
-VPMPIR-
-VPMPIH-
VLPMPI--
VWPMPI--
-VPMPIA-
VFPMPI--
VPPMPI--
VNPMPI--
VEPMPI--
-VPMPIQ-
VDPMPI--
VAPMPI--
VQPMPI--
VGPMPI--
-WPMPIL-
WAPMPI--
WYPMPI--
WGPMPI--
WLPMPI--
WTPMPI--
-WPMPIY-
WSPMPI--
WHPMPI--
-WPMPIF-
WVPMPI--
WWPMPI--
-WPMPIV-
-WPMPIA-
WFPMPI--

	WCPMPI--		
	WKPMPI--		
	WEPMPI--		
	YRPMPI--		
	YVPMPI--		
	YYPMPI--		
	-YPMPIH-		
	-YPMPIF-		
	-YPMPIQ-		
	YKPMPI--		
	-YPMPIS-		
	-YPMPIK-		
	YDPMPI--		
	YSPMPI--		
	-YPMPIT-		
	-YPMPIR-		
	-YPMPII-		
	YNPMPI--		
	YLPMPI--		
	YFPMPI--		
	YTPMPI--		
	YMPMPI--		
	YGPMPI--		
	YEPMPI--		
	YAPMPI--		
	-YPMPIW-		
	-YPMPIV-		
	-YPMPIA-		
PWHI	686	562	45.07
	AVPWHI--		
	AMPWHI--		
	APPWHI--		
	ASPWHI--		
	-APWHIY-		
	AYPWHI--		
	AIPWHI--		
	ARPWHI--		
	AQPWHI--		
	AKPWHI--		
	AGPWHI--		
	ATPWHI--		
	-APWHIS-		
	ALPWHI--		
	AAPWHI--		
	AWPWHI--		
	-APWHIF-		
	-APWHIV-		
	-APWHIM-		
	-APWHIH-		
	-APWHIE-		

-APWHIW-
-APWHII-
-APWHIR-
-APWHIN-
-APWHIK-
-APWHIA-
ANPWHI--
AEPWHI--
CSPWHI--
-CPWHIR-
CCPWHI--
DAPWHI--
DSPWHI--
DHPWHI--
DTPWHI--
DRPWHI--
-DPWHIR-
-DPWHIM-
DVPWHI--
DQPWHI--
-DPWHIT-
-DPWHII-
DKPWHI--
DIPWHI--
-DPWHIL-
-DPWHIA-
DGPWHI--
ESPWHI--
EQPWHI--
-EPWHIR-
ERPWHI--
-EPWHIS-
-EPWHIK-
EVPWHI--
EAPWHI--
-EPWHIF-
-EPWHIY-
-EPWHIH-
ETPWHI--
-EPWHIL-
-EPWHIT-
ENPWHI--
EKPWHI--
-EPWHIV-
ELPWHI--
EEPWHI--
EDPWHI--
FRPWHI--
FKPWHI--
-FPWHII-

FFPWHI--
FGPWHI--
FNPWHI--
FLPWHI--
FVPWHI--
FDPWHI--
-FPWHIR-
FPPWHI--
FIPWHI--
FAPWHI--
-FPWHIQ-
-FPWHIH-
FMPWHI--
FSPWHI--
-FPWHIT-
-FPWHIA-
GVPWHI--
-GPWHII-
GKPWHI--
-GPWHIH-
GTPWHI--
-GPWHIA-
GFPWHI--
GGPWHI--
GRPWHI--
-GPWHIL-
GNPWHI--
-GPWHIY-
GPPWHI--
GHPWHI--
GYPWHI--
GWPWHI--
-GPWHIV-
-GPWHIW-
-GPWHIR-
-GPWHIQ-
-GPWHIK-
GMPWHI--
GLPWHI--
GEPWHI--
GDPWHI--
HYPWHI--
-HPWHIK-
HWPWHI--
HHPWHI--
HTPWHI--
-HPWHIV-
-HPWHII-
HNPWHI--
HGPWHI--

HQPWHI--
-HPWHIQ-
-HPWHIM-
HLPWHI--
HEPWHI--
-HPWHIW-
HAPWHI--
IRPWHI--
IYPWHI--
-IPWHIT-
IGPWHI--
-IPWHIA-
IVPWHI--
-IPWHIS-
IIPWHI--
IMPWHI--
IAPWHI--
INPWHI--
ILPWHI--
-IPWHIY-
IHPWHI--
IWPWHI--
-IPWHIQ-
IDPWHI--
IPPWHI--
IFPWHI--
-IPWHIM-
-IPWHIH-
-IPWHIE-
KTPWHI--
-KPWHII-
-KPWHIL-
KMPWHI--
KVPWHI--
KQPWHI--
-KPWHIK-
-KPWHIT-
-KPWHIH-
KAPWHI--
KNPWHI--
KRPWHI--
-KPWHIR-
-KPWHIS-
-KPWHIM-
KLPWHI--
KHPWHI--
-KPWHIG-
-KPWHIF-
-KPWHIA-
KPPWHI--

KFPWHI--
LIPWHI--
LYPWHI--
LNPWHI--
LLPWHI--
LHPWHI--
LAPWHI--
LTPWHI--
LGPWHI--
-LPWHIR-
LMPWHI--
LVPWHI--
LDPWHI--
-LPWHIV-
LFPWHI--
-LPWHIS-
LWPWHI--
LEPWHI--
-LPWHIY-
-LPWHIT-
-LPWHII-
-LPWHIM-
-LPWHIL-
-LPWHIK-
-LPWHIE-
LPPWHI--
LKPWHI--
MVPWHI--
MSPWHI--
MNPWHI--
MKPWHI--
MEPWHI--
-MPWHIV-
-MPWHIE-
MMPWHI--
-MPWHIK-
MLPWHI--
MAPWHI--
MYPWHI--
MRPWHI--
-MPWHIQ-
-MPWHIA-
MGPWHI--
MFPWHI--
MDPWHI--
NTPWHI--
NVPWHI--
-NPWHIR-
NSPWHI--
NMPWHI--

NIPWHI--
NFPWHI--
-NPWHIT-
NWPWHI--
-NPWHIY-
-NPWHIL-
-NPWHII-
NRPWHI--
-NPWHIS-
NNPWHI--
NAPWHI--
NQPWHI--
-NPWHIV-
NGPWHI--
NEPWHI--
--PWHISS
PTPWHI--
--PWHIRS
--PWHINS
--PWHIRT
--PWHISV
--PWHIRN
--PWHIRG
PHPWHI--
--PWHIRV
--PWHIRH
--PWHIYR
--PWHITG
--PWHIGA
PYPWHI--
--PWHIVY
--PWHIVS
--PWHILH
--PWHIIT
--PWHIAA
--PWHITN
--PWHIRA
--PWHILG
--PWHIHY
PQPWHI--
--PWHIMA
--PWHILA
--PWHIIR
--PWHIYK
--PWHIWS
--PWHIVG
--PWHITK
--PWHIHV
--PWHIKV
--PWHIIG

--PWHIAT
PVPWHI--
--PWHILT
--PWHILQ
--PWHIKG
--PWHIKF
--PWHIWK
--PWHIVW
--PWHIVV
--PWHISH
--PWHIRI
--PWHILS
--PWHIGS
PDPWHI--
PWPWHI--
--PWHIVL
--PWHIVK
--PWHISA
--PWHIRL
--PWHIMS
--PWHILK
--PWHIFS
--PWHIAY
--PWHIVI
--PWHITI
--PWHISR
--PWHISE
--PWHIQS
--PWHIQR
--PWHIQN
--PWHIQM
--PWHIMR
--PWHILR
--PWHIKY
--PWHIHN
--PWHIHA
--PWHIFT
--PWHIFF
--PWHIEK
PMPWHI--
PGPWHI--
--PWHIYS
--PWHIVT
--PWHIVN
--PWHITV
--PWHITA
--PWHIRD
--PWHINA
--PWHIMI
--PWHIKH

--PWHIKE
--PWHIIN
--PWHIIK
--PWHIHH
--PWHIFG
--PWHIEV
--PWHIEA
--PWHIAN
--PWHIAG
--PWHIYY
--PWHIYW
--PWHIYN
--PWHIWY
--PWHIWN
--PWHIVQ
--PWHITW
--PWHITT
--PWHITS
--PWHITL
--PWHITD
--PWHIRE
--PWHIQL
--PWHINT
--PWHING
--PWHIMG
--PWHILE
--PWHIKR
--PWHIKK
--PWHIKA
--PWHIIH
--PWHIHE
--PWHIHD
--PWHIGT
--PWHIFL
--PWHIEQ
--PWHIEN
--PWHIAL
--PWHIAF
PEPWHI--
--PWHIYM
--PWHIYH
--PWHIYG
--PWHIYF
--PWHIYE
--PWHIWE
--PWHITR
--PWHITQ
--PWHISY
--PWHISQ
--PWHISN

```
--PWHISM
--PWHISK
--PWHISG
--PWHIRM
--PWHIQY
--PWHIQV
--PWHIQQ
--PWHINV
--PWHINR
--PWHIMT
--PWHIME
--PWHIKT
--PWHIKL
--PWHIGY
--PWHIGG
--PWHIFR
--PWHIFN
--PWHIFI
--PWHIEW
--PWHIEL
--PWHICM
--PWHIAS
--PWHIAR
PNPWHI--
PKPWHI--
PIPWHI--
PAPWHI--
QEPWHI--
QHPWHI--
-QPWHIR-
QWPWHI--
QVPWHI--
QRPWHI--
-QPWHIS-
-QPWHII-
-QPWHIH-
QKPWHI--
QTPWHI--
-QPWHIT-
-QPWHIM-
QMPWHI--
QYPWHI--
-QPWHIA-
QPPWHI--
QLPWHI--
QGPWHI--
RSPWHI--
RNPWHI--
-RPWHIT-
REPWHI--
```

RRPWHI--
-RPWHIM-
RFPWHI--
RAPWHI--
-RPWHIQ-
-RPWHIL-
-RPWHIF-
RPPWHI--
-RPWHIR-
-RPWHIS-
-RPWHII-
RIPWHI--
-RPWHIK-
-RPWHIY-
-RPWHIN-
RGPWHI--
RTPWHI--
-RPWHIA-
RHPWHI--
RDPWHI--
-RPWHIH-
-RPWHIG-
RMPWHI--
RWPWHI--
RQPWHI--
RKPWHI--
-SPWHIR-
SQPWHI--
SRPWHI--
SEPWHI--
SYPWHI--
-SPWHIS-
-SPWHIM-
SWPWHI--
-SPWHIF-
-SPWHIT-
-SPWHIL-
SFPWHI--
SVPWHI--
SNPWHI--
SMPWHI--
SHPWHI--
STPWHI--
-SPWHIY-
-SPWHII-
-SPWHIQ-
SGPWHI--
SLPWHI--
-SPWHIE-
SKPWHI--

```
-SPWHIK-
-SPWHIA-
SPPWHI--
SDPWHI--
SAPWHI--
TMPWHI--
-TPWHIR-
TAPWHI--
-TPWHIK-
TKPWHI--
TGPWHI--
TWPWHI--
TRPWHI--
-TPWHIT-
-TPWHIS-
TPPWHI--
TTPWHI--
TVPWHI--
TQPWHI--
THPWHI--
-TPWHIV-
-TPWHII-
TYPWHI--
-TPWHIQ-
-TPWHIM-
-TPWHIW-
TEPWHI--
TCPWHI--
-TPWHIH-
-TPWHIE-
VSPWHI--
VKPWHI--
VLPWHI--
VQPWHI--
VAPWHI--
VVPWHI--
VNPWHI--
-VPWHIT-
-VPWHIW-
-VPWHIS-
-VPWHIL-
VDPWHI--
VHPWHI--
-VPWHIQ-
VTPWHI--
VMPWHI--
VWPWHI--
-VPWHIH-
VRPWHI--
-VPWHIN-
```

-VPWHII-
-VPWHIF-
VPPWHI--
VGPWHI--
VFPWHI--
WAPWHI--
-WPWHIQ-
WSPWHI--
-WPWHIM-
WFPWHI--
WLPWHI--
-WPWHIE-
WGPWHI--
WWPWHI--
-WPWHIK-
WIPWHI--
-WPWHIL-
WEPWHI--
YRPWHI--
YAPWHI--
YVPWHI--
YEPWHI--
-YPWHIQ-
YYPWHI--
-YPWHIY-
YWPWHI--
-YPWHIR-
YGPWHI--
YNPWHI--
YLPWHI--
YSPWHI--
-YPWHIW-
-YPWHIT-
YIPWHI--
-YPWHII-
-YPWHIS-
-YPWHIA-
YTPWHI--
-YPWHIE-
YPPWHI--
YFPWHI--
PRAW 200 747 45.06
-CPRAWL-
-GPRAWT-
KDPRAW--
-LPRAWA-
-MPRAWN-
--PRAWNK
--PRAWKE
-RPRAWP-

-RPRAWD-
-SPRAWG-
-VPRAWG-
-APRAWR-
AEPRAW--
-APRAWK-
-APRAWL-
AMPRAW--
ALPRAW--
ANPRAW--
-APRAWA-
AWPRAW--
AVPRAW--
ARPRAW--
-APRAWT-
AKPRAW--
-APRAWV-
AGPRAW--
-APRAWH-
AIPRAW--
AFPRAW--
-APRAWY-
-APRAWF-
ATPRAW--
-APRAWW-
-APRAWM-
AAPRAW--
AHPRAW--
-APRAWE-
AQPRAW--
-APRAWC-
-APRAWI-
APPRAW--
-APRAWN-
AYPRAW--
ACPRAW--
CIPRAW--
CEPRAW--
CCPRAW--
CPPRAW--
CYPRAW--
CVPRAW--
CTPRAW--
-CPRAWF-
-CPRAWA-
CGPRAW--
CFPRAW--
CRPRAW--
-CPRAWY-
-CPRAWK-

-CPRAWH-
CNPRAW--
CHPRAW--
DAPRAW--
DTPRAW--
DGPRAW--
DSPRAW--
-DPRAWR-
DIPRAW--
DYPRAW--
-DPRAWY-
-DPRAWT-
-DPRAWK-
DMPRAW--
-DPRAWW-
-DPRAWV-
DVPRAW--
DNPRAW--
DQPRAW--
-DPRAWS-
-DPRAWF-
DLPRAW--
DEPRAW--
-DPRAWH-
DCPRAW--
ESPRAW--
-EPRAWT-
EEPRAW--
-EPRAWH-
-EPRAWS-
-EPRAWA-
-EPRAWR-
EKPRAW--
EFPRAW--
ERPRAW--
EGPRAW--
EYPRAW--
-EPRAWF-
EAPRAW--
EVPRAW--
-EPRAWW-
EIPRAW--
-EPRAWK-
-EPRAWL-
ELPRAW--
EQPRAW--
-EPRAWY-
-EPRAWQ-
EMPRAW--
-EPRAWG-

EPPRAW--
FTPRAW--
FRPRAW--
-FPRAWK-
FGPRAW--
FSPRAW--
FVPRAW--
FLPRAW--
FFPRAW--
FIPRAW--
FKPRAW--
FNPRAW--
FMPRAW--
FAPRAW--
FYPRAW--
-FPRAWH-
FPPRAW--
-FPRAWT-
-FPRAWV-
-FPRAWI-
FCPRAW--
FEPRAW--
-FPRAWQ-
-FPRAWN-
-FPRAWL-
GVPRAW--
-GPRAWI-
GRPRAW--
-GPRAWV-
-GPRAWR-
GGPRAW--
GSPRAW--
GYPRAW--
-GPRAWS-
GLPRAW--
GKPRAW--
GAPRAW--
GHPRAW--
GQPRAW--
-GPRAWM-
-GPRAWQ-
-GPRAWL-
GEPRAW--
-GPRAWK-
GNPRAW--
GMPRAW--
GFPRAW--
GPPRAW--
-GPRAWW-
-GPRAWE-

-GPRAWA-
HRPRAW--
-HPRAWR-
HLPRAW--
-HPRAWS-
-HPRAWW-
HEPRAW--
HYPRAW--
-HPRAWT-
HWPRAW--
HGPRAW--
-HPRAWL-
-HPRAWF-
HIPRAW--
-HPRAWN-
-HPRAWK-
HQPRAW--
-HPRAWV-
-HPRAWA-
HPPRAW--
-HPRAWQ-
-IPRAWR-
IRPRAW--
-IPRAWS-
IAPRAW--
IQPRAW--
-IPRAWH-
ITPRAW--
-IPRAWY-
ILPRAW--
IVPRAW--
-IPRAWM-
IIPRAW--
IEPRAW--
IHPRAW--
-IPRAWW-
-IPRAWV-
IYPRAW--
-IPRAWL-
-IPRAWT-
-IPRAWQ-
-IPRAWK-
INPRAW--
IGPRAW--
IKPRAW--
IMPRAW--
IWPRAW--
-IPRAWI-
IFPRAW--
-IPRAWA-

KFPRAW--
KTPRAW--
-KPRAWL-
KMPRAW--
-KPRAWV-
-KPRAWH-
KAPRAW--
KYPRAW--
KHPRAW--
KGPRAW--
-KPRAWS-
-KPRAWI-
-KPRAWQ-
-KPRAWK-
-KPRAWM-
-KPRAWT-
KPPRAW--
-KPRAWW-
KEPRAW--
KCPRAW--
-KPRAWY-
-KPRAWF-
KKPRAW--
KQPRAW--
-KPRAWD-
KIPRAW--
LAPRAW--
LPPRAW--
LRPRAW--
-LPRAWR-
LNPRAW--
LTPRAW--
LYPRAW--
-LPRAWT-
LIPRAW--
LEPRAW--
-LPRAWH-
LHPRAW--
-LPRAWW-
LWPRAW--
LDPRAW--
-LPRAWY-
-LPRAWQ-
-LPRAWK-
-LPRAWM-
-LPRAWL-
MLPRAW--
MKPRAW--
MGPRAW--
MEPRAW--

MSPRAW--
MRPRAW--
MNPRAW--
-MPRAWT-
MQPRAW--
-MPRAWK-
-MPRAWY-
MTPRAW--
-MPRAWL-
MVPRAW--
-MPRAWQ-
MHPRAW--
-MPRAWF-
-MPRAWW-
-MPRAWH-
-MPRAWA-
MPPRAW--
MDPRAW--
MAPRAW--
MWPRAW--
MIPRAW--
-MPRAWM-
NLPRAW--
NSPRAW--
NQPRAW--
-NPRAWR-
NYPRAW--
NKPRAW--
NRPRAW--
NAPRAW--
NPPRAW--
-NPRAWT-
-NPRAWK-
NIPRAW--
NHPRAW--
NWPRAW--
NGPRAW--
NTPRAW--
-NPRAWV-
-NPRAWM-
NNPRAW--
NEPRAW--
NVPRAW--
-NPRAWS-
NMPRAW--
-NPRAWH-
-NPRAWA-
-NPRAWY-
NDPRAW--
-NPRAWN-

–NPRAWF–
–NPRAWE–
––PRAWGL
––PRAWLS
––PRAWAL
––PRAWMF
––PRAWTS
––PRAWLG
––PRAWLT
––PRAWIT
––PRAWRT
––PRAWSA
––PRAWTA
––PRAWSN
––PRAWSI
––PRAWYG
––PRAWTM
––PRAWST
––PRAWAS
––PRAWTG
––PRAWVT
––PRAWVG
––PRAWAY
PTPRAW––
––PRAWRI
PLPRAW––
––PRAWWH
––PRAWTV
––PRAWMG
––PRAWHV
––PRAWAA
––PRAWYT
––PRAWYH
––PRAWVF
––PRAWRF
––PRAWLA
––PRAWHT
––PRAWHG
––PRAWYK
––PRAWVK
––PRAWTY
––PRAWTH
––PRAWPL
PMPRAW––
––PRAWQY
––PRAWIS
––PRAWIN
––PRAWRA
––PRAWLV
––PRAWAF

--PRAWVV
--PRAWVN
--PRAWQS
--PRAWHA
--PRAWYA
--PRAWTT
--PRAWSW
--PRAWKT
--PRAWKS
--PRAWVM
--PRAWMN
--PRAWIG
--PRAWHS
--PRAWFQ
--PRAWVA
--PRAWNS
--PRAWMA
--PRAWHL
--PRAWAG
--PRAWTN
--PRAWMD
--PRAWLY
--PRAWIY
--PRAWFW
--PRAWMV
--PRAWKA
--PRAWVY
--PRAWNM
--PRAWMS
--PRAWMQ
--PRAWWI
--PRAWTF
--PRAWMT
--PRAWKI
--PRAWSH
--PRAWRG
--PRAWGM
--PRAWYF
--PRAWWS
--PRAWSF
--PRAWMY
--PRAWMK
--PRAWKW
PGPRAW--
--PRAWYI
--PRAWRH
--PRAWNF
--PRAWMR
--PRAWLK
--PRAWIM

--PRAWGI
--PRAWGF
--PRAWFM
--PRAWFH
--PRAWGS
--PRAWAV
PKPRAW--
--PRAWYM
--PRAWRD
--PRAWPM
--PRAWFG
PVPRAW--
PRPRAW--
--PRAWWR
--PRAWKG
--PRAWIH
--PRAWIF
--PRAWHM
--PRAWGQ
--PRAWFV
--PRAWAT
PFPRAW--
--PRAWWV
--PRAWVQ
--PRAWTQ
--PRAWQT
--PRAWIK
--PRAWGY
--PRAWFF
--PRAWEL
--PRAWAW
--PRAWAN
--PRAWAM
--PRAWKQ
--PRAWKN
--PRAWFI
--PRAWWL
--PRAWWA
--PRAWRY
--PRAWPY
--PRAWLQ
--PRAWYY
--PRAWYV
--PRAWWQ
--PRAWVD
--PRAWIA
--PRAWHR
--PRAWYN
--PRAWVW
--PRAWTW

--PRAWQG
--PRAWNT
--PRAWMH
--PRAWLE
--PRAWHY
--PRAWHN
--PRAWFK
--PRAWES
PQPRAW--
--PRAWQW
--PRAWNG
--PRAWIW
--PRAWHF
--PRAWEV
--PRAWEG
--PRAWWT
--PRAWSQ
--PRAWQQ
--PRAWQK
--PRAWQF
--PRAWPI
--PRAWLM
--PRAWKF
--PRAWIQ
--PRAWGA
--PRAWEI
--PRAWEF
--PRAWDL
--PRAWWW
--PRAWWN
--PRAWTD
--PRAWSM
--PRAWQV
--PRAWQR
--PRAWQN
--PRAWQM
--PRAWNW
--PRAWNN
--PRAWMM
--PRAWLH
--PRAWLD
--PRAWKH
--PRAWHK
--PRAWFY
--PRAWEY
PDPRAW--
--PRAWVE
--PRAWTE
--PRAWQI
--PRAWQA

--PRAWHH
--PRAWFD
--PRAWEN
--PRAWAK
PIPRAW--
PWPRAW--
--PRAWYD
--PRAWWY
--PRAWKM
--PRAWIE
--PRAWHW
--PRAWGV
--PRAWEM
--PRAWDY
--PRAWDM
--PRAWDI
--PRAWAQ
--PRAWQH
--PRAWQD
--PRAWHQ
--PRAWHI
--PRAWEH
--PRAWCL
--PRAWWF
--PRAWWD
--PRAWQE
--PRAWPT
--PRAWNQ
--PRAWNE
--PRAWMP
--PRAWKP
--PRAWGT
--PRAWFC
--PRAWEW
--PRAWDT
--PRAWDS
--PRAWDN
--PRAWDF
--PRAWCT
--PRAWCS
--PRAWCN
--PRAWCI
--PRAWCH
-PPRAWV-
PNPRAW--
PHPRAW--
-QPRAWS-
QAPRAW--
-QPRAWK-
QRPRAW--

QFPRAW--
QGPRAW--
-QPRAWL-
-QPRAWM-
-QPRAWA-
QDPRAW--
QEPRAW--
QYPRAW--
-QPRAWT-
-QPRAWQ-
QTPRAW--
QNPRAW--
QWPRAW--
-QPRAWY-
-QPRAWI-
QQPRAW--
-QPRAWN-
QMPRAW--
QLPRAW--
QKPRAW--
QHPRAW--
-QPRAWF-
QPPRAW--
QIPRAW--
QCPRAW--
RSPRAW--
-RPRAWT-
-RPRAWS-
RAPRAW--
RIPRAW--
RMPRAW--
RVPRAW--
-RPRAWY-
RTPRAW--
RGPRAW--
-RPRAWM-
-RPRAWI-
-RPRAWA-
-RPRAWF-
RNPRAW--
RWPRAW--
RQPRAW--
RFPRAW--
RDPRAW--
RPPRAW--
-RPRAWH-
-RPRAWN-
-RPRAWQ-
RRPRAW--
-RPRAWW-

RKPRAW--
RCPRAW--
-RPRAWE-
SRPRAW--
STPRAW--
-SPRAWV-
SYPRAW--
SHPRAW--
SGPRAW--
SQPRAW--
-SPRAWK-
SVPRAW--
SKPRAW--
SEPRAW--
-SPRAWW-
-SPRAWF-
SMPRAW--
-SPRAWA-
SNPRAW--
-SPRAWT-
-SPRAWH-
SIPRAW--
-SPRAWQ-
SDPRAW--
-SPRAWN-
SPPRAW--
-SPRAWE-
TRPRAW--
TVPRAW--
-TPRAWL-
TPPRAW--
TLPRAW--
TKPRAW--
-TPRAWK-
TMPRAW--
TYPRAW--
TSPRAW--
-TPRAWT-
TQPRAW--
-TPRAWQ-
-TPRAWS-
TTPRAW--
TGPRAW--
TAPRAW--
-TPRAWV-
TIPRAW--
-TPRAWF-
TEPRAW--
-TPRAWM-
-TPRAWH-

-TPRAWA-
TDPRAW--
TNPRAW--
-TPRAWG-
VRPRAW--
-VPRAWY-
-VPRAWH-
VPPRAW--
VNPRAW--
VQPRAW--
-VPRAWI-
VLPRAW--
-VPRAWV-
VHPRAW--
VGPRAW--
-VPRAWA-
VTPRAW--
-VPRAWT-
-VPRAWS-
-VPRAWM-
VFPRAW--
VIPRAW--
VSPRAW--
-VPRAWQ-
VEPRAW--
VYPRAW--
VWPRAW--
-VPRAWF-
-VPRAWW-
VVPRAW--
VKPRAW--
-VPRAWN-
VAPRAW--
VMPRAW--
-VPRAWC-
VDPRAW--
VCPRAW--
WQPRAW--
WYPRAW--
WTPRAW--
-WPRAWT-
WNPRAW--
WFPRAW--
-WPRAWV-
-WPRAWL-
WKPRAW--
WIPRAW--
WHPRAW--
WGPRAW--
-WPRAWI-

WPPRAW--
WWPRAW--
-WPRAWR-
-WPRAWW-
WLPRAW--
WEPRAW--
WAPRAW--
-WPRAWK-
-WPRAWH-
-WPRAWN-
-WPRAWM-
YLPRAW--
-YPRAWV-
-YPRAWH-
-YPRAWF-
YEPRAW--
-YPRAWT-
-YPRAWK-
YRPRAW--
YPPRAW--
YMPRAW--
YYPRAW--
-YPRAWA-
YIPRAW--
YWPRAW--
YGPRAW--
YFPRAW--
-YPRAWY-
YDPRAW--
YNPRAW--
YAPRAW--
-YPRAWW-
-YPRAWI-
-YPRAWN-
-YPRAWM-
-YPRAWQ-
YKPRAW--
YVPRAW--
YCPRAW--

PINM	561	599	45.05

-LPINMA-
--PINMSR
--PINMNL
--PINMIR
ALPINM--
AGPINM--
-APINMT-
ASPINM--
AIPINM--
-APINMV-

```
-APINMR-
-APINML-
ARPINM--
APPINM--
AKPINM--
AFPINM--
AYPINM--
-APINMY-
AQPINM--
AHPINM--
ATPINM--
AVPINM--
-APINMS-
-APINMN-
AEPINM--
-APINMG-
ANPINM--
-APINMQ-
-APINMM-
AAPINM--
CKPINM--
CGPINM--
CTPINM--
CLPINM--
-CPINML-
CRPINM--
-CPINMY-
-CPINMW-
CIPINM--
CFPINM--
DFPINM--
DYPINM--
DGPINM--
-DPINMR-
DAPINM--
DRPINM--
-DPINMS-
DSPINM--
DQPINM--
DWPINM--
-DPINMQ-
-DPINMF-
DLPINM--
ERPINM--
-EPINML-
ELPINM--
EIPINM--
EYPINM--
EPPINM--
-EPINMW-
```

EVPINM--
-EPINMK-
ENPINM--
EMPINM--
EAPINM--
EWPINM--
ESPINM--
-EPINMQ-
-EPINMF-
FKPINM--
FRPINM--
-FPINMM-
FNPINM--
FFPINM--
FDPINM--
FWPINM--
FAPINM--
FSPINM--
FPPINM--
-FPINMR-
-FPINML-
-FPINMT-
FMPINM--
-FPINMW-
FTPINM--
-FPINMV-
-FPINME-
FEPINM--
-GPINMR-
GMPINM--
GQPINM--
GTPINM--
GSPINM--
GFPINM--
-GPINMI-
GVPINM--
-GPINMM-
-GPINMF-
-GPINML-
-GPINMH-
GDPINM--
-GPINMT-
GNPINM--
GRPINM--
-GPINMY-
-GPINMQ-
GAPINM--
-GPINMS-
-GPINMK-
-GPINMA-

GWPINM--
GPPINM--
GIPINM--
GHPINM--
-GPINMW-
-GPINME-
GKPINM--
GEPINM--
HFPINM--
-HPINMT-
HRPINM--
-HPINMR-
-HPINMF-
-HPINMY-
-HPINMV-
-HPINMQ-
HWPINM--
HTPINM--
HAPINM--
-HPINML-
HGPINM--
HEPINM--
HQPINM--
HLPINM--
HSPINM--
-HPINMH-
-HPINME-
HYPINM--
HVPINM--
HPPINM--
-HPINMS-
HMPINM--
IYPINM--
-IPINML-
IAPINM--
ITPINM--
ILPINM--
-IPINMV-
-IPINMS-
IDPINM--
ISPINM--
IFPINM--
-IPINMT-
IVPINM--
-IPINMQ-
-IPINMN-
-IPINMM-
IGPINM--
-IPINMI-
-IPINMH-

-IPINMG-
IMPINM--
KSPINM--
KGPINM--
-KPINML-
-KPINMT-
KFPINM--
KYPINM--
-KPINMS-
KDPINM--
-KPINMF-
KHPINM--
KWPINM--
-KPINMR-
KTPINM--
KPPINM--
-KPINMA-
-KPINMW-
-KPINME-
KQPINM--
-KPINMI-
KLPINM--
KIPINM--
KAPINM--
-KPINMV-
KNPINM--
-KPINMY-
-KPINMH-
KMPINM--
-LPINMR-
LIPINM--
LRPINM--
LGPINM--
LTPINM--
LYPINM--
-LPINMI-
LQPINM--
LDPINM--
LFPINM--
-LPINMS-
LAPINM--
-LPINML-
LNPINM--
LMPINM--
-LPINMV-
LHPINM--
-LPINMK-
LLPINM--
LKPINM--
LPPINM--

```
-LPINMH-
MGPINM--
-MPINMV-
MMPINM--
MSPINM--
-MPINML-
-MPINMR-
MAPINM--
MRPINM--
-MPINMS-
-MPINMH-
MIPINM--
MHPINM--
MYPINM--
-MPINMK-
-MPINMI-
MQPINM--
-MPINMY-
-MPINMQ-
-MPINMG-
MKPINM--
MDPINM--
NWPINM--
NAPINM--
NVPINM--
-NPINMI-
NLPINM--
NIPINM--
NKPINM--
NHPINM--
NGPINM--
NYPINM--
NSPINM--
-NPINMR-
-NPINMQ-
NTPINM--
-NPINMY-
-NPINMV-
-NPINML-
NMPINM--
NRPINM--
-NPINMS-
-NPINMM-
NPPINM--
-NPINMH-
-NPINMF-
-NPINMA-
NNPINM--
NFPINM--
--PINMLT
```

--PINMMG
--PINMLS
--PINMST
--PINMIH
--PINMFS
--PINMRT
--PINMRS
--PINMLN
--PINMRI
--PINMIM
--PINMYM
--PINMRA
--PINMWG
--PINMSV
--PINMSG
--PINMLG
--PINMHA
--PINMVA
--PINMAG
--PINMRR
--PINMLH
--PINMTA
--PINMAA
--PINMWN
--PINMVG
--PINMRH
--PINMRF
--PINMMY
--PINMNA
--PINMLK
PTPINM--
--PINMSF
--PINMLR
--PINMKN
--PINMKM
--PINMIS
--PINMES
--PINMWS
--PINMTW
--PINMTN
--PINMMA
--PINMAS
--PINMYT
--PINMVM
--PINMRG
--PINMNT
--PINMMW
--PINMMQ
--PINMFH
--PINMWK

--PINMVT
--PINMTM
--PINMSS
--PINMSM
--PINMSA
--PINMMV
--PINMMI
--PINMKV
--PINMKS
--PINMKQ
--PINMIK
--PINMHS
PNPINM--
--PINMYN
--PINMYA
--PINMVN
--PINMVH
--PINMTG
--PINMSL
--PINMRY
--PINMNS
--PINMMS
--PINMIQ
--PINMFF
--PINMTI
--PINMTH
--PINMSN
--PINMRQ
--PINMRM
--PINMLQ
--PINMLM
--PINMKY
--PINMGG
--PINMFN
--PINMEW
--PINMYY
--PINMYH
--PINMWI
--PINMSQ
--PINMQG
--PINMNV
--PINMNH
--PINMHQ
--PINMHF
--PINMFD
--PINMEN
--PINMWW
--PINMVQ
--PINMVD
--PINMTR

--PINMTE
--PINMSI
--PINMSE
--PINMQY
--PINMQM
--PINMNY
--PINMMR
--PINMMD
--PINMKF
--PINMIL
--PINMIG
--PINMHN
--PINMHI
--PINMHG
--PINMAT
--PINMAQ
--PINMAM
--PINMAL
-PPINMK-
--PINMYD
--PINMWR
--PINMTV
--PINMTT
--PINMTQ
--PINMTC
--PINMSK
--PINMRW
--PINMRN
--PINMQS
--PINMQR
--PINMQA
--PINMPD
--PINMMN
--PINMME
--PINMMC
--PINMKK
--PINMKA
--PINMIP
--PINMIN
--PINMIE
--PINMHV
--PINMGI
--PINMGA
--PINMFM
--PINMFL
--PINMEY
--PINMEV
--PINMEQ
--PINMEM
--PINMCS

--PINMCR
--PINMCF
--PINMAN
--PINMAI
--PINMAD
QRPINM--
-QPINMY-
QHPINM--
-QPINMI-
QVPINM--
QSPINM--
QIPINM--
QYPINM--
-QPINMW-
-QPINML-
-QPINMR-
QWPINM--
QQPINM--
-QPINMV-
QTPINM--
-QPINMN-
-QPINMQ-
-QPINMH-
-QPINME-
QMPINM--
QGPINM--
QFPINM--
QDPINM--
RMPINM--
-RPINMR-
RSPINM--
RFPINM--
-RPINMQ-
-RPINMM-
RLPINM--
RVPINM--
RKPINM--
RNPINM--
RGPINM--
-RPINMT-
-RPINMH-
RRPINM--
RTPINM--
-RPINMY-
RAPINM--
RYPINM--
RWPINM--
-RPINMS-
RDPINM--
-RPINMA-

-RPINMG-
RHPINM--
REPINM--
-RPINMW-
-RPINMV-
-RPINMF-
-RPINMP-
-RPINMN-
RCPINM--
SRPINM--
SKPINM--
-SPINMR-
-SPINML-
SGPINM--
-SPINMM-
SWPINM--
SPPINM--
-SPINMV-
SMPINM--
SLPINM--
SFPINM--
SYPINM--
STPINM--
-SPINMW-
SNPINM--
-SPINMI-
-SPINMH-
-SPINMF-
-SPINMA-
SIPINM--
SHPINM--
-SPINMT-
-SPINMN-
-SPINMK-
-SPINMY-
-SPINMG-
-SPINMS-
SEPINM--
SDPINM--
SCPINM--
SQPINM--
-TPINMR-
TRPINM--
TKPINM--
-TPINMK-
TMPINM--
TTPINM--
-TPINML-
TYPINM--
TFPINM--

TEPINM--
TLPINM--
TAPINM--
TVPINM--
-TPINMV-
-TPINMF-
-TPINMW-
TWPINM--
TSPINM--
-TPINMT-
-TPINMA-
-TPINMY-
TIPINM--
TDPINM--
TCPINM--
-TPINMN-
TGPINM--
VLPINM--
-VPINML-
VVPINM--
-VPINMG-
VMPINM--
-VPINMQ-
VKPINM--
VYPINM--
-VPINMR-
-VPINMN-
VAPINM--
VTPINM--
VPPINM--
-VPINMH-
VRPINM--
-VPINMT-
-VPINMA-
VEPINM--
-VPINMM-
-VPINME-
VSPINM--
-VPINMV-
-VPINMF-
VQPINM--
-VPINMW-
-VPINMI-
VNPINM--
VDPINM--
-VPINMP-
VIPINM--
VGPINM--
VFPINM--
WVPINM--

WRPINM--
WEPINM--
WKPINM--
WHPINM--
WPPINM--
-WPINMT-
WYPINM--
WFPINM--
-WPINMV-
-WPINMR-
WGPINM--
WAPINM--
WSPINM--
-WPINMI-
WNPINM--
WIPINM--
-YPINMV-
-YPINML-
YVPINM--
YTPINM--
YSPINM--
YMPINM--
YEPINM--
YQPINM--
YIPINM--
YDPINM--
YAPINM--
-YPINMS-
-YPINMR-
YLPINM--
YWPINM--
-YPINMI-
-YPINMH-
YYPINM--
-YPINMT-
YKPINM--
YFPINM--
YPPINM--
YHPINM--

PSGY 28 870 45.04

-CPSGYL-
-GPSGYG-
-NPSGYF-
--PSGYGL
--PSGYRP
--PSGYQL
--PSGYPS
--PSGYPG
--PSGYNR
--PSGYLL

--PSGYDA
-QPSGYE-
-TPSGYQ-
-VPSGYP-
ALPSGY--
-APSGYS-
ARPSGY--
ASPSGY--
AVPSGY--
-APSGYI-
AKPSGY--
-APSGYT-
AMPSGY--
-APSGYY-
-APSGYK-
-APSGYR-
-APSGYM-
AFPSGY--
AYPSGY--
AQPSGY--
-APSGYQ-
-APSGYN-
AEPSGY--
AAPSGY--
-APSGYL-
ANPSGY--
AIPSGY--
AGPSGY--
-APSGYW-
-APSGYV-
APPSGY--
ATPSGY--
AHPSGY--
-APSGYF-
-APSGYA-
ADPSGY--
AWPSGY--
-APSGYE-
-APSGYG-
CKPSGY--
CRPSGY--
CGPSGY--
CCPSGY--
CTPSGY--
CSPSGY--
-CPSGYR-
-CPSGYY-
CMPSGY--
CQPSGY--
-CPSGYV-

-CPSGYT-
-CPSGYH-
CAPSGY--
-CPSGYI-
CPPSGY--
CEPSGY--
CWPSGY--
-CPSGYS-
-CPSGYM-
CLPSGY--
CFPSGY--
DVPSGY--
DSPSGY--
-DPSGYR-
DWPSGY--
DYPSGY--
DLPSGY--
DKPSGY--
DMPSGY--
DHPSGY--
DAPSGY--
DIPSGY--
DTPSGY--
DQPSGY--
DRPSGY--
DNPSGY--
-DPSGYW-
-DPSGYV-
-DPSGYH-
-DPSGYF-
DEPSGY--
DDPSGY--
-DPSGYS-
DFPSGY--
-DPSGYY-
-DPSGYQ-
-DPSGYK-
-EPSGYT-
EHPSGY--
-EPSGYW-
EGPSGY--
EQPSGY--
-EPSGYY-
ERPSGY--
-EPSGYK-
ENPSGY--
EFPSGY--
ESPSGY--
EMPSGY--
-EPSGYI-

ETPSGY--
-EPSGYQ-
EIPSGY--
EYPSGY--
-EPSGYS-
-EPSGYH-
ELPSGY--
-EPSGYV-
-EPSGYL-
EKPSGY--
EVPSGY--
-EPSGYN-
-EPSGYM-
-EPSGYF-
-EPSGYA-
EDPSGY--
EAPSGY--
FSPSGY--
FTPSGY--
-FPSGYT-
FYPSGY--
-FPSGYR-
FEPSGY--
FGPSGY--
FLPSGY--
FWPSGY--
FMPSGY--
FQPSGY--
-FPSGYH-
FAPSGY--
FVPSGY--
-FPSGYI-
FHPSGY--
-FPSGYV-
FPPSGY--
-FPSGYS-
-FPSGYK-
FDPSGY--
-FPSGYW-
-FPSGYQ-
-FPSGYF-
FNPSGY--
FFPSGY--
-FPSGYA-
-FPSGYY-
-FPSGYN-
-FPSGYM-
-FPSGYD-
FCPSGY--
-GPSGYR-

GSPSGY--
GRPSGY--
-GPSGYS-
GVPSGY--
GQPSGY--
GKPSGY--
-GPSGYL-
GTPSGY--
GLPSGY--
GAPSGY--
GMPSGY--
GEPSGY--
GIPSGY--
GWPSGY--
-GPSGYF-
-GPSGYK-
GFPSGY--
-GPSGYM-
-GPSGYY-
-GPSGYI-
GHPSGY--
GGPSGY--
GNPSGY--
-GPSGYQ-
-GPSGYH-
-GPSGYN-
-GPSGYW-
-GPSGYA-
-GPSGYV-
GPPSGY--
GYPSGY--
GDPSGY--
-GPSGYE-
GCPSGY--
-GPSGYP-
HRPSGY--
HEPSGY--
HGPSGY--
HLPSGY--
-HPSGYI-
-HPSGYL-
HAPSGY--
-HPSGYS-
HIPSGY--
HVPSGY--
-HPSGYY-
HTPSGY--
HSPSGY--
-HPSGYR-
HFPSGY--

HKPSGY--
HWPSGY--
-HPSGYW-
HHPSGY--
-HPSGYM-
HMPSGY--
HDPSGY--
-HPSGYH-
HNPSGY--
-HPSGYA-
HPPSGY--
HQPSGY--
HCPSGY--
-HPSGYV-
HYPSGY--
-HPSGYT-
-HPSGYF-
-HPSGYN-
-HPSGYK-
IEPSGY--
-IPSGYT-
IYPSGY--
-IPSGYS-
-IPSGYM-
-IPSGYR-
-IPSGYV-
ILPSGY--
-IPSGYY-
-IPSGYL-
IMPSGY--
IRPSGY--
-IPSGYH-
IIPSGY--
-IPSGYF-
IQPSGY--
-IPSGYW-
IGPSGY--
IAPSGY--
ISPSGY--
-IPSGYI-
IHPSGY--
ITPSGY--
IDPSGY--
INPSGY--
IPPSGY--
IKPSGY--
-IPSGYK-
ICPSGY--
-IPSGYN-
-IPSGYG-

-IPSGYE-
-IPSGYQ-
-IPSGYA-
KVPSGY--
-KPSGYT-
-KPSGYR-
KMPSGY--
KTPSGY--
-KPSGYS-
KGPSGY--
-KPSGYL-
KIPSGY--
-KPSGYV-
KYPSGY--
-KPSGYK-
-KPSGYF-
-KPSGYH-
KFPSGY--
-KPSGYQ-
-KPSGYW-
-KPSGYI-
-KPSGYM-
KDPSGY--
KKPSGY--
KAPSGY--
-KPSGYA-
KNPSGY--
KQPSGY--
KHPSGY--
-KPSGYN-
KEPSGY--
-KPSGYE-
KPPSGY--
-KPSGYG-
-KPSGYC-
LSPSGY--
LNPSGY--
LRPSGY--
LLPSGY--
LVPSGY--
LGPSGY--
-LPSGYR-
LTPSGY--
LAPSGY--
LWPSGY--
-LPSGYS-
LKPSGY--
LFPSGY--
LEPSGY--
-LPSGYT-

LDPSGY--
-LPSGYY-
-LPSGYM-
LIPSGY--
LPPSGY--
LQPSGY--
-LPSGYI-
LYPSGY--
LHPSGY--
-LPSGYW-
-LPSGYH-
-LPSGYF-
LCPSGY--
-LPSGYV-
-LPSGYK-
-LPSGYQ-
-LPSGYN-
MRPSGY--
MSPSGY--
MGPSGY--
-MPSGYH-
MAPSGY--
-MPSGYY-
MFPSGY--
MIPSGY--
MWPSGY--
MVPSGY--
MYPSGY--
MLPSGY--
-MPSGYW-
-MPSGYR-
-MPSGYL-
MPPSGY--
-MPSGYV-
-MPSGYT-
-MPSGYI-
MQPSGY--
-MPSGYQ-
MTPSGY--
MHPSGY--
MEPSGY--
-MPSGYM-
-MPSGYA-
-MPSGYN-
-MPSGYF-
MCPSGY--
-MPSGYC-
MNPSGY--
NSPSGY--
NLPSGY--

NRPSGY--
NPPSGY--
-NPSGYR-
NGPSGY--
-NPSGYY-
NFPSGY--
NTPSGY--
NYPSGY--
-NPSGYL-
-NPSGYH-
NAPSGY--
NIPSGY--
-NPSGYT-
NNPSGY--
-NPSGYI-
NQPSGY--
-NPSGYK-
NDPSGY--
NMPSGY--
-NPSGYM-
NWPSGY--
-NPSGYW-
-NPSGYV-
-NPSGYN-
NHPSGY--
-NPSGYQ-
--PSGYSS
--PSGYSG
--PSGYLA
--PSGYTV
--PSGYAS
--PSGYTS
--PSGYRG
--PSGYRI
--PSGYFR
--PSGYYR
--PSGYVS
--PSGYRY
--PSGYVG
--PSGYRV
--PSGYRL
--PSGYMG
PLPSGY--
--PSGYST
--PSGYSR
--PSGYYS
--PSGYTR
--PSGYSN
--PSGYIS
--PSGYHL

--PSGYRF
--PSGYVR
--PSGYSF
--PSGYWW
--PSGYRH
--PSGYWH
--PSGYVF
--PSGYYG
--PSGYWN
--PSGYSL
--PSGYRD
--PSGYRA
--PSGYLG
PTPSGY--
--PSGYYK
--PSGYIA
--PSGYHS
--PSGYTY
--PSGYTK
--PSGYSA
--PSGYRN
--PSGYHT
--PSGYTN
--PSGYVT
--PSGYTF
PHPSGY--
--PSGYYL
--PSGYYA
--PSGYMY
PGPSGY--
--PSGYVN
--PSGYTM
--PSGYSY
--PSGYSI
--PSGYWA
--PSGYTW
--PSGYKS
PAPSGY--
--PSGYYM
--PSGYVA
--PSGYSH
--PSGYLV
--PSGYLN
--PSGYIG
--PSGYMW
--PSGYYV
--PSGYTT
--PSGYSK
--PSGYHA
--PSGYTG

--PSGYLR
--PSGYHN
--PSGYNT
--PSGYLH
--PSGYFK
--PSGYFG
--PSGYEG
--PSGYKA
--PSGYFS
PNPSGY--
--PSGYWT
--PSGYSW
--PSGYLK
--PSGYTI
--PSGYSQ
--PSGYQS
PDPSGY--
--PSGYWL
--PSGYNW
--PSGYMF
--PSGYIH
--PSGYFV
--PSGYFT
PIPSGY--
--PSGYVV
--PSGYTH
--PSGYRQ
--PSGYNS
--PSGYMA
--PSGYHG
PSPSGY--
--PSGYQA
--PSGYMR
--PSGYMI
--PSGYKH
--PSGYIM
--PSGYHM
--PSGYFW
--PSGYFH
--PSGYES
--PSGYYQ
--PSGYYH
--PSGYRM
--PSGYQW
--PSGYLY
--PSGYFN
--PSGYAA
PVPSGY--
--PSGYKV
--PSGYEA

--PSGYAT
--PSGYYI
--PSGYWR
--PSGYIF
--PSGYHV
--PSGYHF
PYPSGY--
--PSGYTL
--PSGYQF
--PSGYFQ
--PSGYFM
--PSGYAF
--PSGYYY
--PSGYWV
--PSGYWK
--PSGYVM
--PSGYNF
--PSGYYF
--PSGYVH
--PSGYQT
--PSGYKW
--PSGYKN
--PSGYII
--PSGYHI
--PSGYAG
PQPSGY--
PWPSGY--
--PSGYYT
--PSGYWI
--PSGYVI
--PSGYQG
--PSGYLQ
--PSGYIW
--PSGYFD
--PSGYYN
--PSGYSV
--PSGYRE
--PSGYNV
--PSGYMS
--PSGYMH
--PSGYKF
--PSGYGS
--PSGYAN
PKPSGY--
PEPSGY--
--PSGYVY
--PSGYMN
--PSGYIK
--PSGYGT
--PSGYAY

--PSGYSD
--PSGYLM
--PSGYHD
PMPSGY--
--PSGYWQ
--PSGYQQ
--PSGYNA
--PSGYHR
--PSGYGA
PFPSGY--
--PSGYRW
--PSGYQR
--PSGYNQ
--PSGYNL
--PSGYKM
--PSGYKI
--PSGYHK
--PSGYAW
--PSGYTE
--PSGYNN
--PSGYNM
--PSGYNH
--PSGYNG
--PSGYMT
--PSGYMM
--PSGYML
--PSGYKY
--PSGYKL
--PSGYIN
--PSGYHH
--PSGYGG
--PSGYAM
--PSGYAL
--PSGYYD
--PSGYVL
--PSGYVK
--PSGYTD
--PSGYQY
--PSGYNK
--PSGYIY
--PSGYHW
--PSGYGV
--PSGYFA
--PSGYEY
--PSGYET
--PSGYYW
--PSGYQV
--PSGYQM
--PSGYMK
--PSGYME

--PSGYKT
--PSGYID
--PSGYHY
--PSGYEV
--PSGYAR
--PSGYAI
--PSGYVQ
--PSGYQK
--PSGYMD
--PSGYLI
--PSGYAK
--PSGYAH
PCPSGY--
--PSGYYE
--PSGYWD
--PSGYVW
--PSGYVE
--PSGYSE
--PSGYSC
--PSGYQI
--PSGYQD
--PSGYNY
--PSGYNI
--PSGYNE
--PSGYKQ
--PSGYKP
--PSGYHP
--PSGYHE
--PSGYGY
--PSGYGF
--PSGYEW
--PSGYEN
--PSGYEF
--PSGYDL
--PSGYCR
--PSGYCM
--PSGYCH
--PSGYCG
-PPSGYY-
-PPSGYM-
-PPSGYL-
-PPSGYH-
PPPSGY--
-QPSGYW-
QHPSGY--
QYPSGY--
QRPSGY--
-QPSGYL-
QSPSGY--
-QPSGYS-

-QPSGYI-
-QPSGYV-
-QPSGYK-
-QPSGYH-
-QPSGYT-
QNPSGY--
QKPSGY--
QTPSGY--
QQPSGY--
-QPSGYM-
QPPSGY--
-QPSGYF-
QGPSGY--
QWPSGY--
QLPSGY--
QEPSGY--
QIPSGY--
QAPSGY--
QVPSGY--
-QPSGYY-
-QPSGYA-
QMPSGY--
-QPSGYD-
-QPSGYC-
-QPSGYQ-
-RPSGYL-
RSPSGY--
-RPSGYR-
RTPSGY--
RWPSGY--
-RPSGYS-
RYPSGY--
-RPSGYY-
RAPSGY--
RPPSGY--
RNPSGY--
-RPSGYT-
-RPSGYV-
REPSGY--
RVPSGY--
-RPSGYI-
RIPSGY--
-RPSGYK-
-RPSGYF-
RMPSGY--
RLPSGY--
RFPSGY--
-RPSGYH-
RQPSGY--
RDPSGY--

RHPSGY--
-RPSGYW-
-RPSGYA-
-RPSGYN-
-RPSGYQ-
-RPSGYE-
SGPSGY--
SLPSGY--
STPSGY--
SSPSGY--
-SPSGYR-
SAPSGY--
-SPSGYI-
SVPSGY--
-SPSGYF-
SYPSGY--
SIPSGY--
SKPSGY--
-SPSGYM-
SHPSGY--
-SPSGYH-
SNPSGY--
SFPSGY--
SPPSGY--
-SPSGYV-
-SPSGYQ-
-SPSGYW-
SQPSGY--
-SPSGYK-
SCPSGY--
-SPSGYA-
-SPSGYN-
-SPSGYG-
SWPSGY--
TSPSGY--
TRPSGY--
-TPSGYT-
TVPSGY--
TKPSGY--
-TPSGYS-
TQPSGY--
TAPSGY--
TWPSGY--
TTPSGY--
-TPSGYL-
-TPSGYK-
TLPSGY--
-TPSGYF-
TNPSGY--
-TPSGYW-

−TPSGYY−
−TPSGYH−
TFPSGY−−
TEPSGY−−
−TPSGYM−
−TPSGYI−
−TPSGYV−
−TPSGYA−
TMPSGY−−
TYPSGY−−
−TPSGYN−
TIPSGY−−
TDPSGY−−
TCPSGY−−
TPPSGY−−
VRPSGY−−
−VPSGYR−
VSPSGY−−
VLPSGY−−
VTPSGY−−
VIPSGY−−
VFPSGY−−
VPPSGY−−
VNPSGY−−
VHPSGY−−
−VPSGYT−
−VPSGYY−
−VPSGYV−
−VPSGYM−
VMPSGY−−
VGPSGY−−
VAPSGY−−
−VPSGYF−
−VPSGYA−
VVPSGY−−
VEPSGY−−
−VPSGYW−
−VPSGYQ−
−VPSGYI−
VKPSGY−−
VDPSGY−−
VWPSGY−−
VQPSGY−−
−VPSGYH−
−VPSGYK−
−VPSGYN−
−VPSGYG−
−VPSGYE−
−VPSGYD−
VCPSGY−−

WSPSGY--
WYPSGY--
WTPSGY--
WGPSGY--
WVPSGY--
WLPSGY--
WDPSGY--
-WPSGYR-
-WPSGYL-
WRPSGY--
WKPSGY--
WIPSGY--
WMPSGY--
WEPSGY--
WAPSGY--
-WPSGYK-
WPPSGY--
WWPSGY--
-WPSGYW-
-WPSGYS-
WQPSGY--
-WPSGYY-
-WPSGYI-
-WPSGYT-
-WPSGYV-
-WPSGYN-
-WPSGYM-
-WPSGYE-
-WPSGYA-
-YPSGYR-
YAPSGY--
YSPSGY--
YKPSGY--
YFPSGY--
YMPSGY--
YLPSGY--
YVPSGY--
YHPSGY--
YPPSGY--
YGPSGY--
YQPSGY--
YTPSGY--
YNPSGY--
-YPSGYY-
-YPSGYS-
YYPSGY--
-YPSGYT-
-YPSGYH-
-YPSGYL-
YIPSGY--

```
         -YPSGYQ-
         YWPSGY--
         -YPSGYN-
         -YPSGYI-
         -YPSGYA-
         YEPSGY--
         -YPSGYK-
         -YPSGYW-
         -YPSGYF-
         -YPSGYV-
PSGI       21      879     45.03
         -DPSGIS-
         -EPSGIM-
         -IPSGIN-
         --PSGISD
         --PSGIPL
         --PSGIPA
         --PSGIGW
         --PSGIGR
         -RPSGID-
         -SPSGIG-
         -APSGIH-
         -APSGIR-
         ALPSGI--
         AKPSGI--
         ASPSGI--
         ARPSGI--
         -APSGIL-
         AVPSGI--
         -APSGIV-
         -APSGIK-
         -APSGIF-
         AGPSGI--
         AYPSGI--
         AWPSGI--
         AFPSGI--
         AQPSGI--
         -APSGIS-
         -APSGIM-
         ANPSGI--
         -APSGIW-
         -APSGIT-
         APPSGI--
         ATPSGI--
         -APSGIY-
         AHPSGI--
         AMPSGI--
         -APSGIQ-
         -APSGII-
         AEPSGI--
```

-APSGIN-
-APSGIA-
AAPSGI--
ADPSGI--
-APSGIG-
CGPSGI--
CIPSGI--
CVPSGI--
CRPSGI--
CPPSGI--
CFPSGI--
CSPSGI--
CQPSGI--
-CPSGIH-
CKPSGI--
CWPSGI--
CLPSGI--
-CPSGIM-
-CPSGIL-
-CPSGII-
CNPSGI--
CEPSGI--
-CPSGIV-
CTPSGI--
-CPSGIW-
-CPSGIS-
-CPSGIK-
CHPSGI--
CDPSGI--
CCPSGI--
DIPSGI--
DQPSGI--
-DPSGIH-
DSPSGI--
DWPSGI--
DAPSGI--
DRPSGI--
DMPSGI--
-DPSGIY-
-DPSGIM-
DGPSGI--
DYPSGI--
DTPSGI--
-DPSGIW-
-DPSGIF-
DLPSGI--
DKPSGI--
-DPSGIL-
DFPSGI--
DEPSGI--

```
-DPSGIV-
DNPSGI--
DDPSGI--
DCPSGI--
-EPSGIR-
-EPSGIY-
-EPSGIQ-
-EPSGIW-
EKPSGI--
EQPSGI--
EVPSGI--
ESPSGI--
ERPSGI--
-EPSGIK-
-EPSGIT-
-EPSGIH-
EGPSGI--
EYPSGI--
-EPSGIV-
-EPSGIL-
-EPSGII-
-EPSGIF-
ELPSGI--
-EPSGIA-
ENPSGI--
EWPSGI--
-EPSGIS-
-EPSGIN-
EIPSGI--
EHPSGI--
ETPSGI--
EFPSGI--
EAPSGI--
EEPSGI--
ECPSGI--
FRPSGI--
FSPSGI--
FGPSGI--
FLPSGI--
FPPSGI--
FVPSGI--
-FPSGIR-
-FPSGIL-
-FPSGIS-
FHPSGI--
FAPSGI--
FNPSGI--
FWPSGI--
FFPSGI--
-FPSGIW-
```

-FPSGII-
-FPSGIT-
FTPSGI--
FQPSGI--
FIPSGI--
-FPSGIM-
FKPSGI--
-FPSGIV-
-FPSGIK-
FEPSGI--
FYPSGI--
-FPSGIY-
-FPSGIQ-
-FPSGIA-
-FPSGIH-
-FPSGIF-
FDPSGI--
-FPSGIN-
-FPSGIG-
-FPSGIE-
FMPSGI--
FCPSGI--
-GPSGIR-
GMPSGI--
GGPSGI--
GSPSGI--
GKPSGI--
GVPSGI--
-GPSGIS-
-GPSGII-
-GPSGIT-
GHPSGI--
GQPSGI--
-GPSGIL-
-GPSGIY-
GLPSGI--
-GPSGIW-
GTPSGI--
-GPSGIQ-
GWPSGI--
-GPSGIM-
-GPSGIK-
-GPSGIF-
GYPSGI--
-GPSGIV-
GPPSGI--
GIPSGI--
GEPSGI--
GDPSGI--
-GPSGIA-

-GPSGIG-
GCPSGI--
GNPSGI--
-GPSGIN-
-GPSGIH-
-GPSGIE-
HVPSGI--
HGPSGI--
-HPSGIR-
-HPSGIL-
-HPSGIM-
HFPSGI--
HQPSGI--
-HPSGIY-
-HPSGIH-
HWPSGI--
HHPSGI--
HLPSGI--
-HPSGIV-
HMPSGI--
HTPSGI--
-HPSGIW-
HEPSGI--
-HPSGIS-
HPPSGI--
HKPSGI--
HIPSGI--
HYPSGI--
-HPSGIF-
-HPSGIN-
HDPSGI--
-HPSGIT-
-HPSGIQ-
HNPSGI--
-HPSGIK-
-HPSGIA-
HCPSGI--
ILPSGI--
IRPSGI--
IHPSGI--
-IPSGIR-
IGPSGI--
-IPSGIT-
ISPSGI--
IWPSGI--
IYPSGI--
-IPSGIK-
IVPSGI--
IFPSGI--
-IPSGIF-

IPPSGI--
IEPSGI--
-IPSGIS-
IMPSGI--
ITPSGI--
IIPSGI--
IAPSGI--
-IPSGIH-
INPSGI--
-IPSGII-
-IPSGIV-
IQPSGI--
IDPSGI--
-IPSGIQ-
-IPSGIG-
-IPSGIM-
-IPSGIW-
-IPSGIE-
-IPSGIA-
ICPSGI--
KSPSGI--
KGPSGI--
-KPSGIL-
KVPSGI--
KHPSGI--
-KPSGIY-
KLPSGI--
-KPSGIV-
-KPSGIR-
KFPSGI--
KTPSGI--
-KPSGIF-
KWPSGI--
KQPSGI--
-KPSGIS-
KIPSGI--
-KPSGIK-
KAPSGI--
-KPSGIH-
-KPSGIN-
KPPSGI--
-KPSGIM-
KMPSGI--
-KPSGIA-
KDPSGI--
KCPSGI--
KYPSGI--
-KPSGIE-
-KPSGIQ-
-KPSGIC-

-KPSGID-
KEPSGI--
-LPSGIR-
LRPSGI--
LYPSGI--
LSPSGI--
LTPSGI--
LGPSGI--
LMPSGI--
LLPSGI--
LIPSGI--
LAPSGI--
-LPSGIH-
LFPSGI--
LVPSGI--
LWPSGI--
LDPSGI--
LQPSGI--
LHPSGI--
-LPSGIL-
-LPSGIF-
LNPSGI--
-LPSGII-
-LPSGIS-
LEPSGI--
LPPSGI--
-LPSGIV-
-LPSGIK-
-LPSGIQ-
-LPSGIW-
-LPSGIT-
LCPSGI--
MRPSGI--
MKPSGI--
MSPSGI--
MMPSGI--
MLPSGI--
MAPSGI--
-MPSGIR-
-MPSGIM-
MHPSGI--
MGPSGI--
-MPSGII-
MVPSGI--
MTPSGI--
MPPSGI--
-MPSGIY-
-MPSGIL-
-MPSGIK-
MCPSGI--

MDPSGI--
-MPSGIT-
-MPSGIF-
MEPSGI--
-MPSGIQ-
-MPSGIS-
-MPSGIH-
-MPSGIV-
-MPSGIN-
-MPSGIW-
MYPSGI--
MQPSGI--
-MPSGIG-
-MPSGIA-
MFPSGI--
-MPSGIE-
MNPSGI--
NRPSGI--
-NPSGIR-
NSPSGI--
NLPSGI--
NPPSGI--
NAPSGI--
NFPSGI--
NVPSGI--
NYPSGI--
NKPSGI--
-NPSGIH-
NMPSGI--
-NPSGIY-
NHPSGI--
-NPSGIM-
-NPSGIL-
NNPSGI--
NEPSGI--
-NPSGIS-
-NPSGIK-
-NPSGIV-
-NPSGIT-
-NPSGIF-
NIPSGI--
NWPSGI--
NQPSGI--
-NPSGIQ-
NDPSGI--
-NPSGIW-
--PSGIRI
--PSGIRS
--PSGIRL
--PSGIYS

--PSGIRN
--PSGISS
--PSGIIS
--PSGILG
--PSGIFS
--PSGIYG
--PSGIRA
--PSGILS
--PSGIRT
--PSGIRF
--PSGISA
--PSGIFR
--PSGIMS
--PSGIMG
--PSGIFT
--PSGIVR
--PSGIRQ
--PSGIVG
--PSGIKS
--PSGILW
--PSGIRY
--PSGITG
--PSGIRW
--PSGIVN
--PSGIWR
--PSGIRH
--PSGIVT
--PSGIYR
--PSGIHR
--PSGITH
--PSGISV
--PSGIRM
--PSGIHG
PSPSGI--
--PSGISG
--PSGIMT
--PSGIYQ
--PSGIWS
--PSGINS
--PSGIWT
--PSGIWN
--PSGISW
--PSGIYA
--PSGITF
--PSGIMK
--PSGIKW
--PSGIQT
--PSGIYF
--PSGIRK
--PSGIML

PLPSGI--
--PSGIWK
--PSGITS
--PSGITN
--PSGIFH
--PSGIWY
--PSGISY
PVPSGI--
--PSGIWG
--PSGIVK
--PSGIVI
--PSGITT
--PSGISH
--PSGILR
--PSGIGS
--PSGIFL
--PSGIYV
--PSGIGG
--PSGITV
--PSGIRV
--PSGIRG
--PSGIMA
--PSGILQ
--PSGILK
--PSGIIA
--PSGIFV
--PSGIFG
--PSGIYN
--PSGIHY
--PSGIHS
--PSGIHI
--PSGIFF
PMPSGI--
PFPSGI--
--PSGIVV
--PSGISM
--PSGISR
--PSGIQM
--PSGIKG
--PSGIHV
--PSGIHA
--PSGIYT
--PSGITM
--PSGISN
--PSGIMI
--PSGIKH
--PSGIHH
PAPSGI--
--PSGIYY
--PSGIWF

--PSGIVY
--PSGIIH
--PSGIFY
PHPSGI--
--PSGISK
--PSGIMR
--PSGIMN
--PSGIIV
PIPSGI--
--PSGIWA
--PSGITK
--PSGIRE
--PSGIHK
--PSGIAS
--PSGIVH
--PSGIMH
--PSGIKT
--PSGIFN
--PSGIAV
--PSGIYW
--PSGIVW
--PSGIQW
--PSGIMV
--PSGIIT
--PSGIFA
--PSGIAA
--PSGISF
PKPSGI--
--PSGIYI
--PSGITI
--PSGIRD
--PSGIQA
--PSGILH
--PSGIKQ
--PSGIKI
--PSGIFQ
--PSGIFM
PYPSGI--
--PSGIST
--PSGIMF
--PSGIME
--PSGIHM
--PSGIVA
--PSGIQN
--PSGINT
--PSGIHW
--PSGIGV
--PSGIEG
--PSGIEA
--PSGIAT

--PSGIAG
--PSGIVD
--PSGITW
--PSGIQV
--PSGINM
--PSGINA
--PSGIMY
--PSGIMW
--PSGIKL
--PSGIKK
--PSGIIY
--PSGIIN
--PSGIII
--PSGIAW
--PSGIYH
--PSGIWW
--PSGIWD
--PSGIQY
--PSGIQR
--PSGIQF
--PSGINV
--PSGIMM
--PSGIGA
PNPSGI--
PEPSGI--
--PSGITD
--PSGINQ
--PSGIMD
--PSGIKR
--PSGIKN
--PSGIKM
--PSGIFK
--PSGIAF
--PSGIYK
--PSGIWV
--PSGIQQ
--PSGIQI
--PSGINR
--PSGINI
--PSGILE
--PSGIGT
--PSGIFD
PTPSGI--
--PSGIYM
--PSGIYE
--PSGIWH
--PSGIVL
--PSGIVF
--PSGITY
--PSGISQ

--PSGIQL
--PSGINY
--PSGING
--PSGIKA
--PSGIIQ
--PSGIIK
--PSGIEY
--PSGIET
--PSGIES
--PSGIYD
--PSGIVE
--PSGITQ
--PSGISL
--PSGIQK
--PSGIQH
--PSGIQD
--PSGINN
--PSGINF
--PSGIKV
--PSGIKD
--PSGIHF
--PSGIHD
--PSGIAN
PQPSGI--
--PSGISI
--PSGIKF
--PSGIHQ
--PSGIHN
--PSGIEW
--PSGIAY
--PSGIAK
-PPSGIL-
--PSGIWQ
--PSGINW
--PSGINK
--PSGILD
--PSGIKE
--PSGIGI
--PSGIEH
--PSGIEF
--PSGIDS
--PSGIDG
--PSGICM
--PSGICA
--PSGIAR
--PSGISC
--PSGIRC
--PSGINE
--PSGIMQ
--PSGIMP

--PSGIMC
--PSGIGN
--PSGIGM
--PSGIGD
--PSGIFP
--PSGIFC
--PSGIEV
--PSGIEM
--PSGIDV
--PSGICW
--PSGICS
--PSGICH
--PSGICG
--PSGIAC
-PPSGIH-
QRPSGI--
QLPSGI--
QGPSGI--
-QPSGIS-
QIPSGI--
-QPSGIF-
QVPSGI--
QSPSGI--
QPPSGI--
QFPSGI--
QQPSGI--
-QPSGIM-
-QPSGIR-
QAPSGI--
-QPSGIQ-
-QPSGII-
-QPSGIW-
-QPSGIL-
QMPSGI--
QYPSGI--
QKPSGI--
-QPSGIY-
QWPSGI--
-QPSGIN-
QNPSGI--
-QPSGIT-
-QPSGIH-
-QPSGIG-
-QPSGIA-
QHPSGI--
-QPSGIV-
-RPSGIL-
RGPSGI--
RVPSGI--
-RPSGIT-

RRPSGI--
RIPSGI--
RAPSGI--
RQPSGI--
-RPSGIY-
RNPSGI--
RMPSGI--
-RPSGIV-
REPSGI--
RSPSGI--
-RPSGIW-
-RPSGIS-
-RPSGIQ-
RTPSGI--
RYPSGI--
RWPSGI--
-RPSGII-
-RPSGIK-
-RPSGIA-
RPPSGI--
-RPSGIF-
-RPSGIE-
-RPSGIN-
RHPSGI--
-RPSGIG-
RCPSGI--
RDPSGI--
SVPSGI--
SRPSGI--
SSPSGI--
-SPSGIR-
SLPSGI--
-SPSGIF-
SYPSGI--
SWPSGI--
-SPSGIM-
SGPSGI--
SMPSGI--
-SPSGIL-
-SPSGIK-
-SPSGII-
SAPSGI--
-SPSGIS-
SIPSGI--
SKPSGI--
-SPSGIH-
SNPSGI--
SFPSGI--
-SPSGIW-
SHPSGI--

SCPSGI--
STPSGI--
SEPSGI--
-SPSGIT-
-SPSGIA-
SPPSGI--
-SPSGIN-
SQPSGI--
-SPSGIC-
-SPSGIE-
SDPSGI--
TRPSGI--
-TPSGIR-
TSPSGI--
-TPSGIL-
THPSGI--
TYPSGI--
TTPSGI--
-TPSGIM-
TKPSGI--
TGPSGI--
TFPSGI--
TAPSGI--
TNPSGI--
TIPSGI--
-TPSGIH-
-TPSGIT-
-TPSGIF-
TWPSGI--
-TPSGIS-
-TPSGIW-
-TPSGIQ-
TQPSGI--
-TPSGIY-
-TPSGIV-
TMPSGI--
TEPSGI--
-TPSGIA-
TPPSGI--
-TPSGIK-
TDPSGI--
-TPSGIE-
-TPSGII-
TCPSGI--
-TPSGIN-
VRPSGI--
-VPSGIR-
VTPSGI--
-VPSGIH-
VGPSGI--

VQPSGI--
VMPSGI--
VWPSGI--
VVPSGI--
-VPSGIY-
VKPSGI--
-VPSGIM-
-VPSGIK-
-VPSGIA-
VEPSGI--
-VPSGIQ-
VAPSGI--
VYPSGI--
-VPSGIS-
-VPSGII-
-VPSGIL-
-VPSGIF-
VHPSGI--
-VPSGIW-
VNPSGI--
-VPSGIT-
VPPSGI--
-VPSGIV-
-VPSGIN-
VDPSGI--
-VPSGIP-
-WPSGIR-
WGPSGI--
WSPSGI--
WRPSGI--
WVPSGI--
WEPSGI--
WIPSGI--
WMPSGI--
WYPSGI--
WNPSGI--
WPPSGI--
WKPSGI--
WLPSGI--
WFPSGI--
-WPSGIM-
WTPSGI--
-WPSGIT-
-WPSGIS-
-WPSGIH-
WDPSGI--
WAPSGI--
-WPSGIV-
-WPSGIF-
-WPSGII-

	WHPSGI--		
	-WPSGIY-		
	-WPSGIW-		
	-WPSGIK-		
	-WPSGIQ-		
	WCPSGI--		
	WWPSGI--		
	-WPSGIN-		
	-YPSGIR-		
	-YPSGIF-		
	YFPSGI--		
	YRPSGI--		
	YLPSGI--		
	YEPSGI--		
	YGPSGI--		
	-YPSGIL-		
	YSPSGI--		
	YDPSGI--		
	-YPSGIW-		
	YWPSGI--		
	YTPSGI--		
	YAPSGI--		
	-YPSGIY-		
	YKPSGI--		
	YIPSGI--		
	YQPSGI--		
	YVPSGI--		
	-YPSGIS-		
	YYPSGI--		
	YPPSGI--		
	-YPSGIQ-		
	-YPSGIM-		
	-YPSGIK-		
	YMPSGI--		
	-YPSGIN-		
	YHPSGI--		
	-YPSGIH-		
	-YPSGIA-		
	YCPSGI--		
	-YPSGIE-		
PLWY	356	674	44.98
	LPPLWY--		
	-NPLWYG-		
	-TPLWYH-		
	-APLWYR-		
	ARPLWY--		
	-APLWYS-		
	AIPLWY--		
	ASPLWY--		
	AAPLWY--		

AKPLWY--
-APLWYL-
-APLWYI-
-APLWYN-
AQPLWY--
-APLWYA-
-APLWYT-
ADPLWY--
-APLWYY-
ANPLWY--
ATPLWY--
AFPLWY--
AHPLWY--
-APLWYQ-
-APLWYG-
AGPLWY--
APPLWY--
-APLWYM-
-APLWYH-
AWPLWY--
-APLWYW-
-APLWYE-
CVPLWY--
CGPLWY--
CEPLWY--
-CPLWYI-
CSPLWY--
CKPLWY--
-CPLWYM-
CLPLWY--
CAPLWY--
DSPLWY--
DHPLWY--
DQPLWY--
DVPLWY--
DRPLWY--
DTPLWY--
DGPLWY--
DKPLWY--
DFPLWY--
-DPLWYT-
-DPLWYR-
DLPLWY--
DEPLWY--
-DPLWYS-
DYPLWY--
-DPLWYL-
-DPLWYI-
-DPLWYA-
DIPLWY--

DAPLWY--
-DPLWYW-
-DPLWYY-
-DPLWYK-
-DPLWYH-
DMPLWY--
ERPLWY--
EGPLWY--
EKPLWY--
ESPLWY--
EWPLWY--
-EPLWYT-
ETPLWY--
-EPLWYA-
EAPLWY--
-EPLWYL-
ELPLWY--
EVPLWY--
-EPLWYN-
-EPLWYK-
-EPLWYR-
-EPLWYS-
EMPLWY--
-EPLWYI-
-EPLWYW-
-EPLWYV-
-EPLWYG-
-EPLWYE-
ENPLWY--
EEPLWY--
EYPLWY--
EPPLWY--
-EPLWYQ-
-EPLWYM-
EIPLWY--
FRPLWY--
-FPLWYR-
-FPLWYA-
FGPLWY--
FDPLWY--
FTPLWY--
FVPLWY--
-FPLWYT-
-FPLWYS-
-FPLWYL-
FAPLWY--
FPPLWY--
FEPLWY--
FHPLWY--
FNPLWY--

FIPLWY--
-FPLWYH-
FWPLWY--
-FPLWYG-
FYPLWY--
-FPLWYV-
-FPLWYE-
GAPLWY--
GRPLWY--
GNPLWY--
GVPLWY--
GGPLWY--
-GPLWYS-
GMPLWY--
GTPLWY--
-GPLWYK-
GSPLWY--
GKPLWY--
-GPLWYG-
-GPLWYT-
GFPLWY--
-GPLWYM-
-GPLWYI-
GHPLWY--
GPPLWY--
GEPLWY--
-GPLWYQ-
-GPLWYH-
-GPLWYN-
GIPLWY--
GDPLWY--
-GPLWYD-
GLPLWY--
-GPLWYA-
GQPLWY--
-GPLWYV-
HSPLWY--
-HPLWYR-
HKPLWY--
HPPLWY--
HMPLWY--
-HPLWYS-
HYPLWY--
-HPLWYL-
HFPLWY--
HEPLWY--
HGPLWY--
-HPLWYY-
HLPLWY--
HTPLWY--

-HPLWYT-
-HPLWYK-
HDPLWY--
HQPLWY--
-HPLWYI-
-HPLWYW-
HWPLWY--
-HPLWYH-
HIPLWY--
IRPLWY--
IQPLWY--
-IPLWYL-
IEPLWY--
ITPLWY--
INPLWY--
IVPLWY--
-IPLWYS-
IKPLWY--
IDPLWY--
ISPLWY--
IGPLWY--
-IPLWYY-
-IPLWYH-
-IPLWYA-
IMPLWY--
-IPLWYN-
-IPLWYK-
-IPLWYI-
-IPLWYT-
IYPLWY--
IPPLWY--
-IPLWYM-
-IPLWYG-
-IPLWYF-
ICPLWY--
KSPLWY--
KRPLWY--
-KPLWYV-
KVPLWY--
-KPLWYT-
KGPLWY--
KYPLWY--
KLPLWY--
KFPLWY--
KEPLWY--
-KPLWYS-
-KPLWYL-
KWPLWY--
KQPLWY--
KAPLWY--

-KPLWYQ-
-KPLWYG-
KIPLWY--
-KPLWYW-
-KPLWYY-
-KPLWYM-
-KPLWYA-
KMPLWY--
KNPLWY--
-KPLWYF-
-KPLWYN-
KHPLWY--
KDPLWY--
KCPLWY--
LRPLWY--
LKPLWY--
LVPLWY--
LSPLWY--
LMPLWY--
LHPLWY--
-LPLWYI-
LDPLWY--
LNPLWY--
-LPLWYA-
-LPLWYT-
-LPLWYL-
LAPLWY--
-LPLWYM-
LQPLWY--
LTPLWY--
LGPLWY--
LYPLWY--
-LPLWYG-
LWPLWY--
-LPLWYV-
-LPLWYK-
-LPLWYH-
-LPLWYW-
-LPLWYQ-
LEPLWY--
MVPLWY--
-MPLWYK-
MRPLWY--
MSPLWY--
-MPLWYQ-
MGPLWY--
MHPLWY--
-MPLWYY-
-MPLWYR-
-MPLWYM-

-MPLWYL-
MLPLWY--
MEPLWY--
-MPLWYH-
-MPLWYG-
MDPLWY--
-MPLWYT-
MQPLWY--
-MPLWYN-
MKPLWY--
-MPLWYW-
MIPLWY--
NKPLWY--
-NPLWYS-
NLPLWY--
NYPLWY--
NRPLWY--
NGPLWY--
NSPLWY--
-NPLWYI-
NFPLWY--
NVPLWY--
-NPLWYM-
NMPLWY--
NWPLWY--
-NPLWYT-
-NPLWYL-
NTPLWY--
-NPLWYV-
-NPLWYY-
-NPLWYN-
NIPLWY--
NQPLWY--
-NPLWYQ-
NHPLWY--
-NPLWYW-
-NPLWYK-
-NPLWYA-
NAPLWY--
-NPLWYH-
NNPLWY--
NEPLWY--
--PLWYRN
--PLWYLS
--PLWYSA
--PLWYTR
--PLWYSR
--PLWYRH
--PLWYSY
--PLWYAA

--PLWYLN
--PLWYGS
--PLWYQT
--PLWYQG
--PLWYVN
--PLWYGA
--PLWYIN
--PLWYES
--PLWYRG
--PLWYLG
--PLWYVG
--PLWYSH
--PLWYNS
--PLWYKT
--PLWYGT
--PLWYAR
--PLWYKS
--PLWYKI
--PLWYYT
--PLWYVS
--PLWYGL
--PLWYRF
--PLWYTG
--PLWYAQ
--PLWYMD
--PLWYAY
--PLWYTM
--PLWYMT
--PLWYLA
--PLWYIS
--PLWYTY
PDPLWY--
--PLWYVT
--PLWYVH
--PLWYVA
--PLWYRI
--PLWYRE
--PLWYQS
--PLWYQH
--PLWYEG
--PLWYAS
--PLWYAH
--PLWYST
--PLWYRM
--PLWYIV
--PLWYAN
--PLWYSW
--PLWYRY
--PLWYQI
--PLWYKG

--PLWYKA
--PLWYIQ
--PLWYID
--PLWYHA
--PLWYYR
--PLWYWS
--PLWYTI
--PLWYSQ
--PLWYRQ
--PLWYGI
--PLWYDA
--PLWYAI
--PLWYTT
--PLWYTN
--PLWYQM
--PLWYLK
--PLWYKY
--PLWYGG
--PLWYYD
--PLWYWT
--PLWYTA
--PLWYRW
--PLWYMS
--PLWYIT
--PLWYIE
--PLWYGY
--PLWYNN
--PLWYKW
--PLWYKL
--PLWYKH
--PLWYIG
--PLWYHM
--PLWYFY
--PLWYFR
--PLWYFA
--PLWYET
--PLWYWM
--PLWYWG
--PLWYTH
--PLWYTD
--PLWYRD
--PLWYMV
--PLWYHS
--PLWYGV
--PLWYEA
--PLWYAT
--PLWYAL
--PLWYYS
--PLWYNG
--PLWYMA

--PLWYKQ
PEPLWY--
--PLWYYG
--PLWYTV
--PLWYTQ
--PLWYTE
--PLWYQV
--PLWYQA
--PLWYLQ
--PLWYLD
--PLWYHV
--PLWYHG
--PLWYGM
--PLWYFG
--PLWYEV
--PLWYEQ
--PLWYAW
--PLWYAV
--PLWYYN
--PLWYYH
--PLWYVK
--PLWYQR
--PLWYMN
--PLWYMI
--PLWYLI
--PLWYLE
--PLWYKM
--PLWYIH
--PLWYHY
--PLWYHT
--PLWYHK
--PLWYHF
--PLWYFQ
PCPLWY--
-PPLWYR-
--PLWYWW
--PLWYWR
--PLWYWK
--PLWYWH
--PLWYVY
--PLWYVD
--PLWYTW
--PLWYQQ
--PLWYQF
--PLWYQE
--PLWYNT
--PLWYNA
--PLWYMM
--PLWYKD
--PLWYIF

--PLWYGW
--PLWYGN
--PLWYEY
--PLWYDT
--PLWYAE
--PLWYYW
--PLWYWV
--PLWYWN
--PLWYWF
--PLWYWE
--PLWYWD
--PLWYWA
--PLWYTK
--PLWYSP
--PLWYSE
--PLWYPT
--PLWYNY
--PLWYMP
--PLWYLM
--PLWYHQ
--PLWYHN
--PLWYHI
--PLWYGH
--PLWYGF
--PLWYFF
--PLWYEN
--PLWYEH
--PLWYCY
--PLWYAK
QRPLWY--
-QPLWYT-
-QPLWYA-
-QPLWYL-
QWPLWY--
QGPLWY--
-QPLWYS-
-QPLWYK-
QNPLWY--
QKPLWY--
-QPLWYG-
QQPLWY--
QSPLWY--
-QPLWYW-
-QPLWYV-
QEPLWY--
QVPLWY--
-QPLWYM-
QIPLWY--
QDPLWY--
-QPLWYI-

QMPLWY--
-QPLWYY-
-QPLWYQ-
QPPLWY--
-QPLWYE-
QAPLWY--
RSPLWY--
RGPLWY--
-RPLWYT-
RAPLWY--
RPPLWY--
RNPLWY--
-RPLWYM-
-RPLWYL-
RMPLWY--
RKPLWY--
-RPLWYG-
RLPLWY--
RQPLWY--
RHPLWY--
RYPLWY--
-RPLWYQ-
-RPLWYH-
RDPLWY--
RTPLWY--
RVPLWY--
-RPLWYI-
-RPLWYF-
REPLWY--
-RPLWYA-
-RPLWYN-
RCPLWY--
-RPLWYP-
-RPLWYE-
SVPLWY--
STPLWY--
SSPLWY--
SRPLWY--
SMPLWY--
-SPLWYY-
SGPLWY--
-SPLWYT-
-SPLWYS-
SKPLWY--
SIPLWY--
SEPLWY--
SDPLWY--
-SPLWYH-
-SPLWYQ-
-SPLWYN-

```
-SPLWYM-
-SPLWYL-
-SPLWYI-
SHPLWY--
-SPLWYG-
SNPLWY--
SYPLWY--
-SPLWYA-
SQPLWY--
SPPLWY--
-SPLWYW-
SWPLWY--
-SPLWYE-
-SPLWYC-
-TPLWYK-
TAPLWY--
-TPLWYS-
TLPLWY--
TRPLWY--
TQPLWY--
TVPLWY--
TSPLWY--
TNPLWY--
-TPLWYT-
TYPLWY--
TTPLWY--
-TPLWYI-
TFPLWY--
-TPLWYV-
-TPLWYL-
-TPLWYA-
TIPLWY--
TEPLWY--
-TPLWYG-
-TPLWYM-
TWPLWY--
-TPLWYN-
TDPLWY--
THPLWY--
TPPLWY--
-TPLWYE-
VRPLWY--
VPPLWY--
VKPLWY--
VHPLWY--
-VPLWYL-
-VPLWYS-
-VPLWYW-
VEPLWY--
VLPLWY--
```

VIPLWY--
VAPLWY--
VVPLWY--
-VPLWYA-
VQPLWY--
-VPLWYG-
-VPLWYK-
-VPLWYI-
VYPLWY--
-VPLWYH-
-VPLWYE-
-VPLWYQ-
-VPLWYN-
-VPLWYM-
VDPLWY--
-VPLWYD-
WRPLWY--
WKPLWY--
WSPLWY--
WQPLWY--
-WPLWYT-
WNPLWY--
WIPLWY--
WTPLWY--
WPPLWY--
-WPLWYQ-
-WPLWYI-
-WPLWYV-
-WPLWYS-
-WPLWYK-
WAPLWY--
-WPLWYN-
-WPLWYM-
-WPLWYH-
WVPLWY--
WGPLWY--
YKPLWY--
YQPLWY--
-YPLWYS-
YAPLWY--
YRPLWY--
-YPLWYL-
-YPLWYI-
-YPLWYA-
YSPLWY--
YGPLWY--
YNPLWY--
-YPLWYG-
-YPLWYQ-
YVPLWY--

YPPLWY--
-YPLWYT-
-YPLWYN-
-YPLWYM-
YDPLWY--
-YPLWYH-
-YPLWYE-
YIPLWY--
YHPLWY--
YTPLWY--
-YPLWYW-
-YPLWYF-
YFPLWY--
YEPLWY--
YCPLWY--
PVAI 116 797 44.97
-DPVAIT-
-MPVAIG-
-QPVAIN-
AMPVAI--
-APVAIM-
ARPVAI--
-APVAIK-
ASPVAI--
-APVAIL-
-APVAII-
-APVAIR-
ALPVAI--
AKPVAI--
ATPVAI--
-APVAIF-
AFPVAI--
AYPVAI--
AWPVAI--
-APVAIT-
-APVAIN-
ANPVAI--
-APVAIQ-
-APVAIA-
AGPVAI--
AIPVAI--
AEPVAI--
-APVAIY-
-APVAIW-
-APVAIH-
APPVAI--
AAPVAI--
AVPVAI--
-APVAIC-
-APVAIV-

AQPVAI--
-APVAIP-
AHPVAI--
CDPVAI--
CVPVAI--
CCPVAI--
CGPVAI--
CYPVAI--
CLPVAI--
CSPVAI--
CEPVAI--
CRPVAI--
CHPVAI--
-CPVAIY-
-CPVAIH-
-CPVAIG-
DSPVAI--
DRPVAI--
DIPVAI--
DVPVAI--
DLPVAI--
DTPVAI--
DGPVAI--
-DPVAIR-
-DPVAIL-
DKPVAI--
DAPVAI--
DQPVAI--
-DPVAII-
-DPVAIV-
-DPVAIK-
DEPVAI--
-DPVAIW-
DMPVAI--
-DPVAIY-
DYPVAI--
-DPVAIQ-
DNPVAI--
-EPVAIL-
-EPVAIK-
ESPVAI--
ERPVAI--
-EPVAIQ-
EVPVAI--
ELPVAI--
-EPVAIS-
-EPVAIW-
-EPVAIR-
EHPVAI--
ETPVAI--

-EPVAIN-
-EPVAII-
-EPVAIF-
EGPVAI--
-EPVAIY-
-EPVAIV-
EMPVAI--
EAPVAI--
-EPVAIT-
EYPVAI--
EWPVAI--
-EPVAIM-
-EPVAIH-
-EPVAIG-
EKPVAI--
EFPVAI--
FRPVAI--
FVPVAI--
FSPVAI--
FKPVAI--
-FPVAIR-
FPPVAI--
FEPVAI--
-FPVAIH-
FIPVAI--
-FPVAIF-
FNPVAI--
-FPVAIM-
FWPVAI--
FHPVAI--
FDPVAI--
FTPVAI--
FAPVAI--
-FPVAIS-
FLPVAI--
-FPVAIW-
-FPVAIT-
-FPVAIK-
FGPVAI--
FFPVAI--
-FPVAIQ-
-FPVAIN-
FYPVAI--
FCPVAI--
-GPVAIM-
GRPVAI--
GAPVAI--
GKPVAI--
GLPVAI--
-GPVAIW-

−GPVAIH−
GTPVAI−−
−GPVAIY−
−GPVAIL−
GGPVAI−−
−GPVAIR−
GIPVAI−−
GVPVAI−−
−GPVAIV−
GPPVAI−−
−GPVAIS−
−GPVAIN−
GMPVAI−−
GNPVAI−−
GEPVAI−−
−GPVAIK−
−GPVAII−
−GPVAIA−
GWPVAI−−
−GPVAIQ−
−GPVAIT−
GHPVAI−−
−GPVAIF−
GFPVAI−−
GSPVAI−−
GQPVAI−−
−GPVAIE−
−GPVAIG−
−GPVAID−
HLPVAI−−
HVPVAI−−
HAPVAI−−
HYPVAI−−
−HPVAIR−
HNPVAI−−
−HPVAIW−
−HPVAIY−
HKPVAI−−
−HPVAII−
HSPVAI−−
HHPVAI−−
−HPVAIK−
HGPVAI−−
HQPVAI−−
−HPVAIE−
HPPVAI−−
−HPVAIT−
−HPVAIN−
−HPVAIL−
−HPVAIF−

HFPVAI--
HTPVAI--
-HPVAIV-
-HPVAIS-
HMPVAI--
-HPVAIQ-
-HPVAIH-
HDPVAI--
ISPVAI--
IKPVAI--
IRPVAI--
-IPVAIR-
ITPVAI--
-IPVAIV-
IWPVAI--
INPVAI--
-IPVAIK-
-IPVAIS-
IVPVAI--
-IPVAII-
-IPVAIH-
IFPVAI--
IGPVAI--
IPPVAI--
-IPVAIY-
-IPVAIF-
ILPVAI--
-IPVAIT-
-IPVAIQ-
-IPVAIG-
IMPVAI--
-IPVAIN-
IIPVAI--
IQPVAI--
-IPVAIW-
-IPVAIA-
IAPVAI--
IYPVAI--
-IPVAIP-
-IPVAIE-
KSPVAI--
KLPVAI--
-KPVAII-
KRPVAI--
KMPVAI--
KFPVAI--
KAPVAI--
KHPVAI--
-KPVAIQ-
-KPVAIS-

KGPVAI--
-KPVAIW-
KNPVAI--
KIPVAI--
KPPVAI--
KVPVAI--
-KPVAIV-
KYPVAI--
-KPVAIY-
KEPVAI--
-KPVAIT-
-KPVAIK-
-KPVAIH-
KQPVAI--
-KPVAIN-
-KPVAIM-
-KPVAIA-
KTPVAI--
KWPVAI--
-KPVAIF-
-KPVAIE-
LSPVAI--
LNPVAI--
LMPVAI--
LKPVAI--
LGPVAI--
LWPVAI--
LRPVAI--
LQPVAI--
-LPVAIL-
-LPVAIF-
-LPVAIT-
LIPVAI--
LAPVAI--
LEPVAI--
LYPVAI--
-LPVAIH-
LVPVAI--
-LPVAIV-
-LPVAIS-
-LPVAIR-
LLPVAI--
-LPVAIY-
-LPVAII-
LPPVAI--
-LPVAIW-
-LPVAIQ-
LDPVAI--
LCPVAI--
-LPVAIK-

LHPVAI--
MRPVAI--
MYPVAI--
MVPVAI--
MSPVAI--
MKPVAI--
MLPVAI--
MTPVAI--
-MPVAIS-
MNPVAI--
MQPVAI--
MPPVAI--
-MPVAIN-
MAPVAI--
-MPVAIM-
MMPVAI--
-MPVAIY-
MIPVAI--
MHPVAI--
-MPVAIT-
MGPVAI--
-MPVAIK-
-MPVAIW-
-MPVAIQ-
MEPVAI--
-MPVAIV-
-MPVAIA-
NLPVAI--
-NPVAIY-
NSPVAI--
NKPVAI--
NGPVAI--
NAPVAI--
NEPVAI--
NYPVAI--
-NPVAIR-
NPPVAI--
NIPVAI--
NTPVAI--
-NPVAIM-
NVPVAI--
NFPVAI--
NHPVAI--
NWPVAI--
NQPVAI--
-NPVAIS-
-NPVAIK-
-NPVAIT-
-NPVAIH-
-NPVAIA-

-NPVAIF-
NMPVAI--
NDPVAI--
--PVAINV
--PVAIRS
--PVAIFG
--PVAILS
--PVAISL
--PVAIRN
--PVAIGL
--PVAIRT
--PVAIQA
--PVAIWG
--PVAITG
PRPVAI--
PKPVAI--
--PVAIFT
--PVAIAS
--PVAISS
--PVAIVG
PAPVAI--
--PVAIRG
--PVAIRR
--PVAILT
--PVAILN
--PVAISG
--PVAIFR
--PVAIVR
PTPVAI--
--PVAIRA
--PVAISV
--PVAISA
--PVAIFA
PLPVAI--
--PVAITR
--PVAISM
--PVAIRQ
--PVAINL
--PVAIKA
--PVAIHS
--PVAISH
--PVAIVT
--PVAIVS
--PVAIRM
--PVAINS
--PVAIMG
--PVAISR
--PVAILK
--PVAIRY
--PVAIPL

--PVAIHR
--PVAIFS
--PVAIPY
--PVAIES
PMPVAI--
PVPVAI--
--PVAIIR
--PVAITS
--PVAIRH
--PVAIRF
--PVAIRD
--PVAIWW
--PVAIRV
--PVAILA
--PVAIGY
--PVAIGA
--PVAIFN
PNPVAI--
--PVAIYT
--PVAIWY
--PVAIVK
--PVAIQV
--PVAIQR
--PVAINT
--PVAIKI
--PVAIIS
PSPVAI--
--PVAIYK
--PVAIYG
--PVAIVN
--PVAITV
--PVAITT
--PVAITI
--PVAIRL
--PVAIIT
--PVAIHG
--PVAIGI
--PVAIYH
--PVAITY
--PVAIMA
--PVAIIN
--PVAIHT
--PVAIEG
--PVAITK
--PVAIQS
--PVAIWT
--PVAIWK
--PVAIVA
--PVAILH
--PVAIHN

--PVAIWQ
--PVAITF
--PVAIQT
--PVAIPI
--PVAIMH
--PVAIFH
PQPVAI--
--PVAIYQ
--PVAISI
--PVAIMT
--PVAIMS
--PVAIMN
--PVAIKK
--PVAIIG
--PVAIGF
--PVAIYV
--PVAIST
--PVAISK
--PVAIRW
--PVAIQW
--PVAIQG
--PVAIKM
--PVAIKG
--PVAIGG
PIPVAI--
--PVAITN
--PVAISQ
--PVAINI
--PVAINA
--PVAIMY
--PVAIKS
--PVAIKF
--PVAIIE
--PVAIAT
--PVAIAM
--PVAIAG
--PVAIYS
--PVAIYD
--PVAITQ
--PVAITH
--PVAISW
--PVAIPF
--PVAIKV
--PVAIGW
--PVAIYA
--PVAIWH
--PVAIVQ
--PVAITA
--PVAIQL
--PVAIMV

--PVAIKL
--PVAIIY
--PVAIHH
--PVAIFK
--PVAIFE
--PVAIEW
--PVAIAF
--PVAIAA
--PVAIWE
--PVAIVW
--PVAITW
--PVAITL
--PVAIQK
--PVAIKW
--PVAIIW
--PVAIHM
--PVAIHL
--PVAIHK
--PVAIGS
--PVAIAQ
PFPVAI--
PEPVAI--
--PVAIWI
--PVAIVI
--PVAIVD
--PVAITM
--PVAISN
--PVAIQH
--PVAIMM
--PVAIIK
--PVAIII
--PVAIGV
--PVAIGT
--PVAIFQ
--PVAIEY
--PVAIEM
--PVAIAN
--PVAIAL
--PVAIWD
--PVAIVM
--PVAITD
--PVAIQY
--PVAINN
--PVAINK
--PVAING
--PVAINF
--PVAIMF
--PVAILM
--PVAIKQ
--PVAIKH

--PVAIFV
PDPVAI--
PWPVAI--
--PVAIYW
--PVAIWV
--PVAISE
--PVAIRE
--PVAIQI
--PVAIQD
--PVAINW
--PVAILW
--PVAILI
--PVAILD
--PVAIKY
--PVAIKD
--PVAIHY
--PVAIFM
--PVAIFF
--PVAIFD
--PVAIET
--PVAIEI
--PVAIYN
--PVAIYM
--PVAIYE
--PVAIVH
--PVAIVE
--PVAITE
--PVAITC
--PVAIQQ
--PVAIQN
--PVAIQF
--PVAIPV
--PVAIPM
--PVAINQ
--PVAIMW
--PVAIME
--PVAILE
--PVAIKC
--PVAIIV
--PVAIID
--PVAIHI
--PVAIHA
--PVAIGM
--PVAIFP
--PVAIDN
--PVAIDA
--PVAICM
--PVAICG
--PVAIAW
--PVAIAR

--PVAIAH
--PVAIAD
PHPVAI--
QAPVAI--
-QPVAIH-
-QPVAIR-
QYPVAI--
QRPVAI--
QSPVAI--
QIPVAI--
-QPVAIK-
-QPVAII-
QGPVAI--
QQPVAI--
QLPVAI--
QWPVAI--
-QPVAIL-
-QPVAIF-
QFPVAI--
-QPVAIY-
-QPVAIS-
-QPVAIQ-
-QPVAIT-
QVPVAI--
-QPVAIV-
QPPVAI--
QMPVAI--
QEPVAI--
QKPVAI--
-QPVAIW-
-QPVAIG-
-QPVAID-
-QPVAIA-
QDPVAI--
RLPVAI--
RPPVAI--
RKPVAI--
RVPVAI--
RSPVAI--
-RPVAIF-
-RPVAIR-
-RPVAIY-
RAPVAI--
RTPVAI--
RMPVAI--
RGPVAI--
-RPVAIT-
-RPVAIQ-
REPVAI--
-RPVAIW-

-RPVAIM-
RWPVAI--
-RPVAIA-
RIPVAI--
RYPVAI--
-RPVAIK-
-RPVAIV-
RFPVAI--
-RPVAIN-
RNPVAI--
RQPVAI--
-RPVAIE-
-RPVAIG-
RDPVAI--
RHPVAI--
RCPVAI--
STPVAI--
SLPVAI--
-SPVAIY-
SGPVAI--
SVPVAI--
SKPVAI--
SNPVAI--
SSPVAI--
SIPVAI--
SHPVAI--
-SPVAIS-
SAPVAI--
-SPVAIQ-
-SPVAIM-
-SPVAIT-
-SPVAIK-
-SPVAIA-
SYPVAI--
-SPVAIV-
SFPVAI--
SEPVAI--
-SPVAIW-
SQPVAI--
-SPVAIH-
SPPVAI--
SDPVAI--
-SPVAIN-
-SPVAIG-
SCPVAI--
SWPVAI--
TLPVAI--
TSPVAI--
TIPVAI--
TGPVAI--

```
-TPVAIV-
TRPVAI--
TPPVAI--
-TPVAIF-
TTPVAI--
-TPVAIH-
TDPVAI--
-TPVAIQ-
-TPVAIM-
-TPVAIT-
-TPVAIY-
-TPVAIK-
TKPVAI--
TFPVAI--
TCPVAI--
-TPVAIN-
TNPVAI--
TEPVAI--
THPVAI--
TAPVAI--
TQPVAI--
TMPVAI--
TWPVAI--
-TPVAIW-
-TPVAIE-
VLPVAI--
VRPVAI--
-VPVAIR-
VAPVAI--
VIPVAI--
VQPVAI--
-VPVAIV-
-VPVAII-
VSPVAI--
-VPVAIY-
-VPVAIL-
-VPVAIT-
-VPVAIW-
VMPVAI--
VWPVAI--
-VPVAIK-
-VPVAIH-
-VPVAIS-
VPPVAI--
VNPVAI--
VTPVAI--
VFPVAI--
-VPVAIM-
VDPVAI--
-VPVAIN-
```

VGPVAI--
VHPVAI--
VEPVAI--
-VPVAIE-
VCPVAI--
-VPVAIQ-
-VPVAIG-
-WPVAIR-
WMPVAI--
WYPVAI--
WSPVAI--
WAPVAI--
WLPVAI--
WTPVAI--
WKPVAI--
-WPVAIH-
WRPVAI--
WNPVAI--
-WPVAIK-
-WPVAII-
WGPVAI--
WIPVAI--
-WPVAIV-
WDPVAI--
-WPVAIS-
-WPVAIT-
WEPVAI--
WQPVAI--
-WPVAIN-
-WPVAIF-
WFPVAI--
WVPVAI--
-WPVAIE-
-WPVAID-
YRPVAI--
YMPVAI--
YSPVAI--
YTPVAI--
-YPVAIV-
YHPVAI--
YAPVAI--
-YPVAIM-
-YPVAIK-
YEPVAI--
-YPVAIS-
-YPVAIL-
YFPVAI--
YYPVAI--
-YPVAIT-
YWPVAI--

	-YPVAIY-		
	-YPVAIQ-		
	-YPVAIR-		
	-YPVAIA-		
	-YPVAIW-		
	YPPVAI--		
	-YPVAII-		
	YNPVAI--		
	YCPVAI--		
	YIPVAI--		
	-YPVAIN-		
	YGPVAI--		
	YDPVAI--		
PFEL	318	687	44.95
	-APFELR-		
	-APFELS-		
	-APFELK-		
	-APFELY-		
	AGPFEL--		
	-APFELH-		
	-APFELA-		
	AMPFEL--		
	ATPFEL--		
	-APFELM-		
	ARPFEL--		
	-APFELN-		
	AIPFEL--		
	AVPFEL--		
	-APFELW-		
	ASPFEL--		
	-APFELT-		
	AEPFEL--		
	AAPFEL--		
	AHPFEL--		
	ANPFEL--		
	AYPFEL--		
	APPFEL--		
	-APFELV-		
	-APFELQ-		
	-APFELG-		
	AKPFEL--		
	AFPFEL--		
	ADPFEL--		
	AWPFEL--		
	ALPFEL--		
	AQPFEL--		
	-APFELI-		
	-APFELE-		
	CSPFEL--		
	-CPFELK-		

-CPFELR-
CGPFEL--
CVPFEL--
CNPFEL--
CPPFEL--
-CPFELY-
-CPFELH-
CEPFEL--
-DPFELR-
DKPFEL--
DQPFEL--
DGPFEL--
DTPFEL--
DRPFEL--
DVPFEL--
-DPFELV-
DSPFEL--
-DPFELI-
DLPFEL--
DWPFEL--
-DPFELN-
-DPFELK-
DEPFEL--
DDPFEL--
-DPFELQ-
-DPFELH-
DAPFEL--
-DPFELY-
-EPFELR-
ERPFEL--
EGPFEL--
-EPFELK-
EKPFEL--
EAPFEL--
ESPFEL--
-EPFELS-
EQPFEL--
ELPFEL--
EVPFEL--
-EPFELV-
-EPFELM-
EYPFEL--
-EPFELQ-
-EPFELT-
-EPFELH-
-EPFELA-
EHPFEL--
EWPFEL--
ETPFEL--
-EPFELE-

EIPFEL--
EFPFEL--
EEPFEL--
FPPFEL--
-FPFELR-
-FPFELV-
FGPFEL--
FSPFEL--
FRPFEL--
FLPFEL--
FKPFEL--
FMPFEL--
FYPFEL--
FDPFEL--
FAPFEL--
FWPFEL--
FQPFEL--
FTPFEL--
FNPFEL--
-FPFELW-
-FPFELN-
-FPFELK-
-FPFELQ-
-FPFELG-
-FPFELA-
GRPFEL--
GIPFEL--
GWPFEL--
-GPFELR-
GYPFEL--
GVPFEL--
GAPFEL--
GGPFEL--
GEPFEL--
GSPFEL--
-GPFELV-
GFPFEL--
-GPFELT-
GMPFEL--
-GPFELS-
GKPFEL--
GPPFEL--
-GPFELN-
-GPFELK-
-GPFELI-
-GPFELG-
-GPFELQ-
-GPFELH-
GNPFEL--
GLPFEL--

GTPFEL--
GHPFEL--
GQPFEL--
-GPFELA-
HLPFEL--
-HPFELL-
HRPFEL--
-HPFELS-
-HPFELM-
HSPFEL--
-HPFELW-
-HPFELA-
HIPFEL--
HNPFEL--
-HPFELK-
HTPFEL--
-HPFELY-
HYPFEL--
HWPFEL--
HGPFEL--
-HPFELR-
-HPFELQ-
HKPFEL--
-HPFELV-
-HPFELH-
-HPFELG-
HDPFEL--
-HPFELT-
HAPFEL--
IGPFEL--
IYPFEL--
ITPFEL--
-IPFELS-
-IPFELV-
IHPFEL--
-IPFELT-
ILPFEL--
IAPFEL--
IQPFEL--
IPPFEL--
-IPFELY-
-IPFELL-
IVPFEL--
IEPFEL--
-IPFELR-
-IPFELQ-
-IPFELG-
IWPFEL--
IKPFEL--
-IPFELK-

INPFEL--
IMPFEL--
IFPFEL--
IDPFEL--
-IPFELM-
IIPFEL--
-IPFELW-
KSPFEL--
-KPFELW-
KGPFEL--
-KPFELL-
-KPFELS-
-KPFELA-
-KPFELR-
-KPFELK-
KLPFEL--
KWPFEL--
-KPFELT-
-KPFELI-
KHPFEL--
KTPFEL--
-KPFELG-
KYPFEL--
-KPFELH-
KIPFEL--
KRPFEL--
-KPFELN-
KNPFEL--
KFPFEL--
KVPFEL--
-KPFELM-
KAPFEL--
KMPFEL--
KDPFEL--
-KPFELY-
KPPFEL--
-KPFELC-
KCPFEL--
LRPFEL--
-LPFELR-
LSPFEL--
-LPFELK-
LKPFEL--
LVPFEL--
LHPFEL--
LLPFEL--
LTPFEL--
LGPFEL--
LAPFEL--
-LPFELS-

-LPFELI-
LDPFEL--
LIPFEL--
LEPFEL--
LYPFEL--
-LPFELT-
-LPFELV-
LPPFEL--
-LPFELH-
LMPFEL--
-LPFELQ-
-LPFELM-
LNPFEL--
-LPFELN-
LWPFEL--
-LPFELA-
-MPFELR-
MRPFEL--
MPPFEL--
MVPFEL--
-MPFELH-
MGPFEL--
-MPFELN-
-MPFELK-
MMPFEL--
-MPFELI-
MIPFEL--
MDPFEL--
MSPFEL--
-MPFELG-
-MPFELA-
MWPFEL--
-MPFELS-
-MPFELM-
MHPFEL--
-MPFELV-
-MPFELQ-
MNPFEL--
MLPFEL--
-NPFELR-
NRPFEL--
NWPFEL--
NGPFEL--
NLPFEL--
NIPFEL--
NTPFEL--
NSPFEL--
-NPFELK-
NMPFEL--
-NPFELW-

-NPFELM-
NFPFEL--
-NPFELN-
NVPFEL--
NPPFEL--
-NPFELY-
NQPFEL--
-NPFELS-
NKPFEL--
NEPFEL--
NYPFEL--
-NPFELT-
-NPFELI-
-NPFELF-
NHPFEL--
NDPFEL--
--PFELRG
--PFELRS
--PFELRA
--PFELKS
--PFELRK
--PFELTG
--PFELLG
--PFELMT
--PFELRI
--PFELRR
--PFELMQ
--PFELTR
--PFELMS
--PFELSY
--PFELIR
--PFELYN
--PFELVR
PFPFEL--
--PFELKT
--PFELHA
PGPFEL--
--PFELRL
--PFELSA
--PFELRW
--PFELRT
--PFELRH
--PFELNA
--PFELVS
--PFELES
--PFELGV
--PFELST
--PFELRF
--PFELRD
--PFELNL

--PFELHS
--PFELVG
--PFELTA
--PFELSM
PWPFEL--
PNPFEL--
--PFELYR
--PFELVN
--PFELNK
--PFELMH
PQPFEL--
--PFELNI
--PFELLN
--PFELGT
--PFELYH
--PFELRN
--PFELNT
--PFELMR
--PFELHM
--PFELWS
--PFELVM
--PFELTN
--PFELSK
--PFELEA
PYPFEL--
--PFELTQ
--PFELMN
--PFELKR
--PFELKL
--PFELKK
PIPFEL--
--PFELVT
--PFELSW
--PFELNR
--PFELMI
--PFELKF
--PFELAM
--PFELAG
PAPFEL--
--PFELWR
--PFELWE
--PFELVK
--PFELVE
--PFELQI
--PFELKH
--PFELHL
--PFELAS
--PFELYK
--PFELWW
--PFELSR

--PFELSG
--PFELND
--PFELLH
--PFELKY
--PFELHR
--PFELGS
PKPFEL--
--PFELSN
--PFELSL
--PFELQS
--PFELQG
--PFELLK
--PFELKV
--PFELFQ
--PFELAH
--PFELYG
--PFELWT
--PFELVA
--PFELTW
--PFELTK
--PFELRY
--PFELQV
--PFELQN
--PFELQH
--PFELNS
--PFELNN
--PFELMK
--PFELIK
--PFELYD
--PFELWN
--PFELVQ
--PFELTS
--PFELTI
--PFELTE
--PFELSI
--PFELMM
--PFELMG
--PFELME
--PFELKE
--PFELIH
--PFELIG
--PFELIA
--PFELHT
--PFELHQ
--PFELHD
--PFELGG
PTPFEL--
--PFELVH
--PFELTT
--PFELSH

--PFELQR
--PFELQQ
--PFELQE
--PFELQA
--PFELNY
--PFELMA
--PFELLR
--PFELLQ
--PFELHY
--PFELHH
--PFELHG
--PFELEW
--PFELEG
-PPFELT-
PHPFEL--
--PFELSV
--PFELSF
--PFELRQ
--PFELQT
--PFELQL
--PFELNQ
--PFELNH
--PFELNG
--PFELKD
--PFELIS
--PFELHI
--PFELHF
--PFELGY
--PFELFD
--PFELAN
-PPFELN-
-PPFELF-
--PFELWI
--PFELWH
--PFELWG
--PFELWD
--PFELSQ
--PFELRM
--PFELRE
--PFELQK
--PFELNM
--PFELNE
--PFELMD
--PFELLC
--PFELKQ
--PFELKA
--PFELGW
--PFELGM
--PFELGA
--PFELFK

--PFELFF
--PFELEH
--PFELDW
--PFELCK
--PFELAW
--PFELAP
--PFELAI
--PFELAD
QRPFEL--
-QPFELK-
-QPFELV-
-QPFELS-
-QPFELW-
-QPFELN-
QPPFEL--
QWPFEL--
-QPFELR-
QSPFEL--
QKPFEL--
QAPFEL--
-QPFELM-
-QPFELI-
QVPFEL--
QLPFEL--
QGPFEL--
QHPFEL--
-QPFELG-
QEPFEL--
QYPFEL--
-QPFELH-
QQPFEL--
QNPFEL--
QMPFEL--
-RPFELR-
RRPFEL--
RDPFEL--
RSPFEL--
RAPFEL--
RVPFEL--
-RPFELN-
-RPFELS-
-RPFELT-
-RPFELK-
RPPFEL--
RFPFEL--
RKPFEL--
-RPFELI-
RLPFEL--
RGPFEL--
-RPFELM-

RNPFEL--
RYPFEL--
-RPFELV-
RMPFEL--
RIPFEL--
RTPFEL--
-RPFELQ-
-RPFELA-
RHPFEL--
RQPFEL--
-RPFELH-
REPFEL--
-RPFELG-
-RPFELE-
SRPFEL--
-SPFELR-
SVPFEL--
-SPFELH-
SIPFEL--
SLPFEL--
SMPFEL--
-SPFELV-
SQPFEL--
SSPFEL--
-SPFELW-
SGPFEL--
SEPFEL--
SPPFEL--
-SPFELK-
STPFEL--
-SPFELS-
SKPFEL--
-SPFELN-
SYPFEL--
-SPFELI-
SDPFEL--
-SPFELQ-
SWPFEL--
-SPFELA-
SFPFEL--
SHPFEL--
SNPFEL--
SAPFEL--
-SPFELM-
SCPFEL--
-SPFELG-
TLPFEL--
-TPFELK-
TRPFEL--
TGPFEL--

-TPFELN-
TSPFEL--
-TPFELR-
TFPFEL--
TAPFEL--
TTPFEL--
TPPFEL--
TVPFEL--
TIPFEL--
THPFEL--
-TPFELV-
-TPFELT-
-TPFELS-
-TPFELQ-
-TPFELH-
TWPFEL--
TQPFEL--
TYPFEL--
TMPFEL--
TKPFEL--
TEPFEL--
-TPFELI-
TDPFEL--
-TPFELW-
-TPFELA-
-VPFELR-
VQPFEL--
-VPFELV-
VHPFEL--
VVPFEL--
-VPFELK-
VPPFEL--
VAPFEL--
VYPFEL--
-VPFELN-
VMPFEL--
VFPFEL--
VTPFEL--
VRPFEL--
VGPFEL--
VIPFEL--
-VPFELT-
VEPFEL--
-VPFELS-
VNPFEL--
-VPFELG-
-VPFELA-
VKPFEL--
-VPFELY-
-VPFELF-

VCPFEL--
VWPFEL--
-WPFELR-
-WPFELN-
WTPFEL--
-WPFELY-
-WPFELQ-
-WPFELV-
WKPFEL--
WVPFEL--
-WPFELS-
-WPFELT-
-WPFELL-
WMPFEL--
-WPFELH-
WPPFEL--
WEPFEL--
WRPFEL--
-WPFELI-
WIPFEL--
WQPFEL--
WGPFEL--
WFPFEL--
WDPFEL--
WAPFEL--
YKPFEL--
YLPFEL--
YSPFEL--
-YPFELN-
-YPFELS-
-YPFELR-
YTPFEL--
YVPFEL--
-YPFELV-
-YPFELL-
YNPFEL--
YMPFEL--
YAPFEL--
YYPFEL--
-YPFELT-
-YPFELK-
YDPFEL--
YPPFEL--
-YPFELI-
-YPFELH-
YQPFEL--
YIPFEL--
-YPFELM-
-YPFELA-
YCPFEL--

	-YPFELQ-		
PRQM	405	657	44.92
	--PRQMQD		
	AAPRQM--		
	AIPRQM--		
	-APRQMY-		
	-APRQMT-		
	-APRQMM-		
	ASPRQM--		
	-APRQMF-		
	AYPRQM--		
	-APRQML-		
	-APRQMV-		
	AMPRQM--		
	AWPRQM--		
	ARPRQM--		
	-APRQMN-		
	ALPRQM--		
	-APRQMH-		
	-APRQMG-		
	-APRQMA-		
	AFPRQM--		
	AVPRQM--		
	AQPRQM--		
	ANPRQM--		
	APPRQM--		
	AGPRQM--		
	AEPRQM--		
	AKPRQM--		
	ADPRQM--		
	-CPRQMV-		
	-CPRQMM-		
	-CPRQMR-		
	-CPRQML-		
	CGPRQM--		
	CAPRQM--		
	CSPRQM--		
	CQPRQM--		
	-CPRQMF-		
	CVPRQM--		
	-CPRQMI-		
	DVPRQM--		
	-DPRQMR-		
	-DPRQMW-		
	-DPRQMI-		
	-DPRQML-		
	DMPRQM--		
	-DPRQMY-		
	DNPRQM--		
	DGPRQM--		

DTPRQM--
DSPRQM--
-DPRQMT-
-DPRQMF-
DFPRQM--
-DPRQMS-
DKPRQM--
DAPRQM--
ESPRQM--
-EPRQMR-
-EPRQMV-
EHPRQM--
EPPRQM--
EYPRQM--
-EPRQML-
ERPRQM--
-EPRQMK-
EAPRQM--
-EPRQMY-
-EPRQMW-
EVPRQM--
ETPRQM--
EKPRQM--
EWPRQM--
-EPRQMF-
EMPRQM--
-EPRQMT-
-EPRQMC-
ELPRQM--
-EPRQMA-
EGPRQM--
EFPRQM--
EDPRQM--
FRPRQM--
FIPRQM--
FNPRQM--
FGPRQM--
-FPRQMY-
FPPRQM--
-FPRQMN-
-FPRQMQ-
-FPRQMF-
FLPRQM--
FSPRQM--
FHPRQM--
FDPRQM--
FTPRQM--
-FPRQMK-
FFPRQM--
-FPRQMW-

-FPRQMG-
FVPRQM--
FQPRQM--
-FPRQMV-
FKPRQM--
FEPRQM--
-GPRQMR-
GRPRQM--
GVPRQM--
GSPRQM--
GQPRQM--
-GPRQMY-
-GPRQMS-
GGPRQM--
GTPRQM--
GAPRQM--
GWPRQM--
-GPRQMV-
-GPRQMQ-
-GPRQMA-
GLPRQM--
GPPRQM--
-GPRQMT-
-GPRQMF-
-GPRQMN-
-GPRQMW-
-GPRQMM-
GEPRQM--
GYPRQM--
-GPRQMH-
-GPRQMG-
GMPRQM--
GKPRQM--
GNPRQM--
-HPRQMR-
-HPRQMV-
-HPRQML-
-HPRQMI-
HEPRQM--
HSPRQM--
HTPRQM--
HVPRQM--
HRPRQM--
HQPRQM--
-HPRQMK-
-HPRQMF-
HGPRQM--
-HPRQMT-
-HPRQMS-
-HPRQMM-

HMPRQM--
HIPRQM--
HCPRQM--
-HPRQMW-
-HPRQMQ-
-HPRQMN-
HNPRQM--
HKPRQM--
HFPRQM--
ISPRQM--
IRPRQM--
-IPRQMV-
IMPRQM--
IYPRQM--
-IPRQMQ-
IPPRQM--
-IPRQMT-
IGPRQM--
-IPRQMY-
-IPRQMW-
-IPRQMR-
-IPRQMK-
-IPRQMH-
IVPRQM--
IQPRQM--
-IPRQML-
INPRQM--
IAPRQM--
IDPRQM--
IWPRQM--
-IPRQMA-
IKPRQM--
IEPRQM--
-IPRQMG-
-IPRQMF-
KSPRQM--
-KPRQMQ-
-KPRQML-
KAPRQM--
-KPRQMW-
-KPRQMR-
-KPRQMS-
-KPRQMY-
KIPRQM--
-KPRQMF-
KYPRQM--
-KPRQMN-
KNPRQM--
KRPRQM--
KMPRQM--

-KPRQMA-
KLPRQM--
-KPRQMC-
KGPRQM--
LVPRQM--
LSPRQM--
-LPRQML-
LGPRQM--
-LPRQMN-
LDPRQM--
LRPRQM--
-LPRQMS-
LPPRQM--
LNPRQM--
-LPRQMT-
-LPRQMI-
LIPRQM--
-LPRQMV-
LTPRQM--
LMPRQM--
LAPRQM--
-LPRQMY-
-LPRQMA-
-LPRQMF-
LEPRQM--
-LPRQMK-
-LPRQMC-
LCPRQM--
MPPRQM--
MHPRQM--
MVPRQM--
-MPRQMR-
MTPRQM--
MSPRQM--
MAPRQM--
-MPRQMN-
-MPRQMM-
MRPRQM--
-MPRQMQ-
-MPRQMF-
-MPRQMV-
-MPRQMT-
-MPRQMH-
-MPRQMA-
MYPRQM--
-MPRQMS-
-MPRQMW-
MIPRQM--
MEPRQM--
NVPRQM--

-NPRQMY-
-NPRQMR-
NAPRQM--
NRPRQM--
NSPRQM--
-NPRQMW-
-NPRQMI-
NKPRQM--
NGPRQM--
-NPRQMV-
-NPRQMS-
-NPRQML-
-NPRQMH-
NYPRQM--
-NPRQMT-
-NPRQMK-
-NPRQMF-
NLPRQM--
NHPRQM--
NIPRQM--
NEPRQM--
--PRQMKS
--PRQMSS
--PRQMLT
--PRQMLW
--PRQMKT
--PRQMNS
--PRQMLV
--PRQMII
--PRQMRA
--PRQMVL
--PRQMTG
--PRQMIA
--PRQMHS
--PRQMLA
--PRQMRD
--PRQMMA
--PRQMLL
--PRQMWS
--PRQMST
--PRQMSG
--PRQMNT
--PRQMIG
--PRQMTW
--PRQMNR
--PRQMHA
--PRQMYY
--PRQMMT
--PRQMFY
--PRQMAT

--PRQMYT
--PRQMLI
--PRQMKM
--PRQMFI
--PRQMRI
--PRQMGS
--PRQMFS
--PRQMNN
--PRQMNL
--PRQMMH
--PRQMSI
--PRQMKI
--PRQMIQ
--PRQMFA
PRPRQM--
--PRQMWL
--PRQMWK
--PRQMTN
--PRQMSV
--PRQMSA
--PRQMRE
--PRQMQH
--PRQMMF
--PRQMLF
--PRQMFM
--PRQMWR
--PRQMVN
--PRQMSQ
--PRQMQV
--PRQMLM
--PRQMKA
--PRQMHG
--PRQMYN
--PRQMTQ
--PRQMTM
--PRQMTF
--PRQMMV
--PRQMLD
--PRQMKQ
--PRQMFV
--PRQMAS
PLPRQM--
PHPRQM--
--PRQMYF
--PRQMTT
--PRQMTI
--PRQMQS
--PRQMNY
--PRQMNQ
--PRQMNH

--PRQMMI
--PRQMLG
--PRQMLE
--PRQMHT
--PRQMYH
--PRQMTL
--PRQMRQ
--PRQMQF
--PRQMNA
--PRQMLH
--PRQMIY
--PRQMIT
--PRQMGA
--PRQMFT
--PRQMES
--PRQMYV
--PRQMYQ
--PRQMYK
--PRQMWY
--PRQMVM
--PRQMSN
--PRQMRM
--PRQMNW
--PRQMFH
--PRQMAY
PGPRQM--
--PRQMVD
--PRQMTA
--PRQMMS
--PRQMMM
--PRQMLY
--PRQMKK
--PRQMKE
--PRQMIF
--PRQMHI
--PRQMGT
--PRQMAM
--PRQMYA
--PRQMWN
--PRQMWM
--PRQMVV
--PRQMVA
--PRQMTY
--PRQMSY
--PRQMNG
--PRQMMQ
--PRQMFK
--PRQMAV
--PRQMYL
--PRQMYI

--PRQMYD
--PRQMVQ
--PRQMVG
--PRQMTE
--PRQMRH
--PRQMQW
--PRQMQG
--PRQMNK
--PRQMML
--PRQMMK
--PRQMKW
--PRQMKG
--PRQMKF
--PRQMIH
--PRQMHH
--PRQMFR
--PRQMAI
PEPRQM--
PTPRQM--
--PRQMWT
--PRQMWQ
--PRQMVY
--PRQMTK
--PRQMSW
--PRQMSR
--PRQMQM
--PRQMQL
--PRQMNF
--PRQMME
--PRQMIV
--PRQMIM
--PRQMIK
--PRQMIE
--PRQMHW
--PRQMGM
--PRQMFL
--PRQMEV
--PRQMAN
--PRQMAG
PNPRQM--
PSPRQM--
--PRQMYG
--PRQMWH
--PRQMVF
--PRQMTV
--PRQMQY
--PRQMQT
--PRQMQN
--PRQMQK
--PRQMNV

--PRQMNI
--PRQMMD
--PRQMHR
--PRQMHF
--PRQMFN
--PRQMEW
--PRQMEG
PWPRQM--
PVPRQM--
--PRQMYW
--PRQMYE
--PRQMTS
--PRQMTH
--PRQMTD
--PRQMSK
--PRQMSH
--PRQMQA
--PRQMPI
--PRQMNE
--PRQMNC
--PRQMMG
--PRQMKN
--PRQMKD
--PRQMIW
--PRQMID
--PRQMHV
--PRQMHM
--PRQMGY
--PRQMGV
--PRQMGI
--PRQMGG
--PRQMEM
--PRQMEA
--PRQMCK
--PRQMAW
-PPRQMI-
-QPRQMW-
QPPRQM--
QYPRQM--
QMPRQM--
-QPRQML-
-QPRQMS-
-QPRQMH-
-QPRQMI-
QLPRQM--
QHPRQM--
QVPRQM--
-QPRQMT-
-QPRQMF-
QTPRQM--

QSPRQM--
QKPRQM--
QRPRQM--
-QPRQMQ-
-QPRQMG-
QAPRQM--
RAPRQM--
-RPRQMH-
-RPRQMT-
-RPRQMV-
-RPRQMK-
-RPRQMY-
RLPRQM--
REPRQM--
RQPRQM--
-RPRQMN-
-RPRQMI-
RIPRQM--
RYPRQM--
RTPRQM--
-RPRQMF-
-RPRQMQ-
RPPRQM--
RDPRQM--
RCPRQM--
RRPRQM--
-RPRQME-
SAPRQM--
-SPRQML-
-SPRQMY-
-SPRQMS-
SVPRQM--
-SPRQMN-
SKPRQM--
-SPRQMT-
SLPRQM--
-SPRQMM-
SSPRQM--
SPPRQM--
SWPRQM--
SQPRQM--
-SPRQMH-
-SPRQMF-
SEPRQM--
-SPRQMQ-
SYPRQM--
SIPRQM--
-SPRQMA-
SFPRQM--
SGPRQM--

−SPRQMI−
STPRQM−−
SNPRQM−−
SMPRQM−−
SDPRQM−−
−SPRQME−
SCPRQM−−
−TPRQMK−
−TPRQML−
TSPRQM−−
−TPRQMY−
−TPRQMH−
TPPRQM−−
TFPRQM−−
−TPRQMW−
−TPRQMQ−
TVPRQM−−
−TPRQMV−
−TPRQMI−
TKPRQM−−
TTPRQM−−
−TPRQMF−
−TPRQMS−
TRPRQM−−
TIPRQM−−
THPRQM−−
−TPRQMN−
−TPRQMM−
−TPRQMG−
TNPRQM−−
TLPRQM−−
TEPRQM−−
−TPRQMA−
TMPRQM−−
TDPRQM−−
TCPRQM−−
VTPRQM−−
VAPRQM−−
VLPRQM−−
VPPRQM−−
−VPRQMN−
VGPRQM−−
−VPRQMM−
VSPRQM−−
VEPRQM−−
−VPRQMI−
−VPRQMW−
−VPRQMV−
−VPRQMY−
−VPRQMT−

-VPRQMK-
VYPRQM--
VIPRQM--
VDPRQM--
-VPRQMH-
VWPRQM--
-VPRQMS-
VMPRQM--
VQPRQM--
-VPRQMG-
-VPRQMA-
VNPRQM--
-VPRQMF-
VKPRQM--
VFPRQM--
WTPRQM--
WPPRQM--
WVPRQM--
WKPRQM--
-WPRQMV-
WIPRQM--
WEPRQM--
WAPRQM--
-WPRQMW-
-WPRQMM-
-WPRQMH-
WHPRQM--
WRPRQM--
-WPRQMQ-
WNPRQM--
-WPRQMG-
WFPRQM--
-WPRQMY-
-WPRQMN-
-WPRQMF-
YYPRQM--
-YPRQMY-
-YPRQMS-
-YPRQMW-
YGPRQM--
YWPRQM--
-YPRQMN-
YLPRQM--
-YPRQMR-
-YPRQML-
YRPRQM--
-YPRQMV-
-YPRQMK-
-YPRQMA-
YHPRQM--

	YEPRQM--		
	YSPRQM--		
	YVPRQM--		
	YTPRQM--		
	YPPRQM--		
	YIPRQM--		
	YQPRQM--		
	-YPRQMF-		
	YMPRQM--		
PWQI	1090	475	44.91
	--PWQISR		
	-APWQIL-		
	ARPWQI--		
	ALPWQI--		
	AKPWQI--		
	-APWQIN-		
	AWPWQI--		
	ASPWQI--		
	AGPWQI--		
	-APWQIH-		
	-APWQIA-		
	AFPWQI--		
	ATPWQI--		
	-APWQIS-		
	AMPWQI--		
	-APWQIE-		
	-APWQIR-		
	-APWQIM-		
	CSPWQI--		
	CRPWQI--		
	CCPWQI--		
	CTPWQI--		
	-CPWQIY-		
	-CPWQIF-		
	CYPWQI--		
	DKPWQI--		
	-DPWQIR-		
	-DPWQIT-		
	-DPWQIQ-		
	DSPWQI--		
	DMPWQI--		
	DEPWQI--		
	DRPWQI--		
	-DPWQIH-		
	-DPWQIK-		
	EIPWQI--		
	EGPWQI--		
	-EPWQIQ-		
	EVPWQI--		
	EKPWQI--		

EQPWQI--
-EPWQIT-
-EPWQIY-
EMPWQI--
ELPWQI--
-EPWQIN-
ETPWQI--
ERPWQI--
-EPWQIF-
ENPWQI--
FTPWQI--
FMPWQI--
FQPWQI--
FSPWQI--
FLPWQI--
FIPWQI--
FVPWQI--
FRPWQI--
FHPWQI--
FYPWQI--
-FPWQIV-
-FPWQIR-
-FPWQII-
FGPWQI--
FFPWQI--
FAPWQI--
GSPWQI--
GKPWQI--
GHPWQI--
GLPWQI--
-GPWQIH-
GIPWQI--
-GPWQII-
-GPWQIS-
GQPWQI--
GAPWQI--
-GPWQIR-
-GPWQIW-
-GPWQIV-
GPPWQI--
-GPWQIT-
GEPWQI--
GCPWQI--
HAPWQI--
HPPWQI--
HKPWQI--
HRPWQI--
HHPWQI--
HGPWQI--
HVPWQI--

-HPWQIT-
-HPWQIR-
HNPWQI--
HMPWQI--
-HPWQIN-
-HPWQIA-
HFPWQI--
HDPWQI--
ISPWQI--
IPPWQI--
IFPWQI--
-IPWQIR-
-IPWQIK-
ILPWQI--
IRPWQI--
-IPWQIH-
-IPWQIQ-
IWPWQI--
IQPWQI--
IEPWQI--
ITPWQI--
-IPWQIG-
-IPWQIF-
IHPWQI--
IDPWQI--
IAPWQI--
-KPWQIR-
KKPWQI--
KRPWQI--
KQPWQI--
-KPWQIN-
KWPWQI--
-KPWQIV-
-KPWQIS-
KSPWQI--
-KPWQIM-
-KPWQIG-
-KPWQIA-
KNPWQI--
KGPWQI--
KVPWQI--
-KPWQIY-
KMPWQI--
KLPWQI--
KHPWQI--
KCPWQI--
LRPWQI--
LSPWQI--
LTPWQI--
LKPWQI--

LLPWQI--
LGPWQI--
LVPWQI--
-LPWQIR-
LEPWQI--
LAPWQI--
LPPWQI--
LMPWQI--
LDPWQI--
-LPWQIF-
-LPWQIT-
-LPWQIL-
-LPWQIV-
-LPWQIK-
-LPWQII-
-LPWQIH-
LNPWQI--
LIPWQI--
LYPWQI--
LHPWQI--
LFPWQI--
MSPWQI--
MTPWQI--
MEPWQI--
MYPWQI--
-MPWQII-
MLPWQI--
MRPWQI--
-MPWQIR-
MVPWQI--
-MPWQIN-
-MPWQIM-
-MPWQIT-
-MPWQIW-
-MPWQIH-
-MPWQIS-
MPPWQI--
MKPWQI--
MHPWQI--
NIPWQI--
NLPWQI--
-NPWQIR-
-NPWQII-
NPPWQI--
NVPWQI--
NSPWQI--
-NPWQIY-
NHPWQI--
NFPWQI--
-NPWQIK-

NNPWQI--
NRPWQI--
NQPWQI--
-NPWQIV-
-NPWQIL-
--PWQIIW
--PWQIYN
--PWQITS
--PWQIRS
--PWQIRT
--PWQIVG
--PWQIFA
--PWQIQS
--PWQIIR
--PWQIHA
--PWQIRI
--PWQINT
--PWQIVN
--PWQISV
--PWQINA
--PWQIMS
--PWQIMN
PMPWQI--
--PWQIYH
--PWQIRL
--PWQIMG
--PWQIKS
--PWQIVS
--PWQIVH
--PWQIYS
--PWQIIS
--PWQIWG
--PWQIVQ
--PWQITF
--PWQIMW
--PWQIKR
--PWQIIQ
--PWQIFM
--PWQIAA
PEPWQI--
--PWQIRN
--PWQINY
--PWQINV
--PWQIKQ
--PWQIVR
--PWQISN
--PWQISG
--PWQIRG
--PWQIRD
--PWQIKN

--PWQIKH
--PWQIKA
--PWQIFG
--PWQIAG
--PWQITA
--PWQINH
--PWQIMY
--PWQILN
--PWQIKG
--PWQIIY
--PWQIFY
--PWQIAS
--PWQIYK
--PWQIVT
--PWQITW
--PWQIRK
--PWQIQQ
--PWQIQN
--PWQINW
--PWQIMR
--PWQIKV
--PWQIKT
--PWQIHM
--PWQIHG
--PWQIGS
--PWQIGG
--PWQIGA
--PWQIFQ
--PWQIFK
--PWQIES
--PWQIWD
--PWQIVA
--PWQITR
--PWQITQ
--PWQIST
--PWQIRR
--PWQIRE
--PWQIQV
--PWQIQG
--PWQIQA
--PWQIMD
--PWQIHF
--PWQIFW
--PWQIYI
--PWQIVY
--PWQITT
--PWQITG
--PWQISM
--PWQISA
--PWQIRV

--PWQINS
--PWQINQ
--PWQIMM
--PWQILH
--PWQIHS
--PWQIGV
--PWQIFN
--PWQIAT
PCPWQI--
--PWQIYT
--PWQIYQ
--PWQIYE
--PWQIYA
--PWQIWV
--PWQIWQ
--PWQIWL
--PWQIVV
--PWQITV
--PWQISI
--PWQISH
--PWQISE
--PWQIRY
--PWQIQT
--PWQIQR
--PWQIQM
--PWQINR
--PWQIND
--PWQIMT
--PWQIMQ
--PWQILK
--PWQILF
--PWQIKI
--PWQIKE
--PWQIIG
--PWQIID
--PWQIHQ
--PWQIHK
--PWQIGY
--PWQIFV
--PWQIFR
--PWQIEG
--PWQICA
--PWQIAY
QRPWQI--
QLPWQI--
-QPWQIQ-
QVPWQI--
QTPWQI--
QQPWQI--
-QPWQIM-

-QPWQIL-
QGPWQI--
-QPWQIY-
-QPWQIT-
QSPWQI--
-QPWQIW-
-QPWQIR-
-QPWQIH-
QPPWQI--
QMPWQI--
QHPWQI--
-RPWQIM-
RGPWQI--
RMPWQI--
-RPWQII-
-RPWQIT-
RTPWQI--
RSPWQI--
-RPWQIV-
-RPWQIS-
-RPWQIR-
-RPWQIQ-
RAPWQI--
RYPWQI--
-RPWQIY-
-RPWQIN-
RLPWQI--
RVPWQI--
-RPWQIK-
-RPWQIH-
RPPWQI--
-RPWQIA-
REPWQI--
RNPWQI--
RKPWQI--
RIPWQI--
-RPWQIG-
-RPWQIF-
-RPWQIE-
-SPWQIR-
SKPWQI--
SSPWQI--
SRPWQI--
SWPWQI--
SLPWQI--
SVPWQI--
-SPWQIT-
-SPWQII-
SFPWQI--
-SPWQIV-

STPWQI--
-SPWQIQ-
-SPWQIK-
SEPWQI--
SAPWQI--
-SPWQIM-
SNPWQI--
SMPWQI--
-SPWQIF-
SGPWQI--
SDPWQI--
SQPWQI--
-SPWQIA-
SYPWQI--
-SPWQIS-
TRPWQI--
TGPWQI--
-TPWQIR-
-TPWQIK-
TKPWQI--
-TPWQIS-
TAPWQI--
-TPWQIM-
TLPWQI--
-TPWQIT-
-TPWQIN-
THPWQI--
TEPWQI--
-TPWQIF-
TIPWQI--
TYPWQI--
TSPWQI--
TNPWQI--
TVPWQI--
TTPWQI--
-TPWQIV-
-TPWQIE-
-TPWQIA-
VLPWQI--
VKPWQI--
-VPWQIK-
VAPWQI--
VVPWQI--
VSPWQI--
VHPWQI--
VQPWQI--
-VPWQIS-
VDPWQI--
-VPWQIA-
VTPWQI--

-VPWQIR-
-VPWQII-
VGPWQI--
VEPWQI--
VRPWQI--
-VPWQIN-
-VPWQIV-
-VPWQIT-
-VPWQIH-
VPPWQI--
VIPWQI--
WVPWQI--
WRPWQI--
WQPWQI--
WSPWQI--
WLPWQI--
WHPWQI--
WGPWQI--
WMPWQI--
WTPWQI--
-WPWQIV-
YSPWQI--
YRPWQI--
YKPWQI--
-YPWQIT-
YDPWQI--
-YPWQIR-
YPPWQI--
YYPWQI--
YTPWQI--
-YPWQIK-
YFPWQI--
YMPWQI--
YLPWQI--
YIPWQI--
PMSY 164 767 44.90
-DPMSYA-
KTPMSY--
--PMSYQL
-TPMSYG-
ASPMSY--
-APMSYR-
-APMSYL-
ARPMSY--
AWPMSY--
AVPMSY--
AHPMSY--
AQPMSY--
-APMSYT-
AGPMSY--

AYPMSY--
-APMSYH-
AFPMSY--
-APMSYM-
-APMSYI-
ALPMSY--
AAPMSY--
-APMSYS-
-APMSYW-
-APMSYQ-
AMPMSY--
-APMSYK-
ANPMSY--
AEPMSY--
ATPMSY--
-APMSYY-
-APMSYE-
APPMSY--
-APMSYN-
-APMSYD-
ADPMSY--
CKPMSY--
CTPMSY--
CRPMSY--
CPPMSY--
CQPMSY--
CNPMSY--
CIPMSY--
CYPMSY--
-CPMSYT-
-CPMSYS-
CDPMSY--
-DPMSYR-
DVPMSY--
DTPMSY--
DFPMSY--
DSPMSY--
-DPMSYF-
DLPMSY--
DIPMSY--
DGPMSY--
-DPMSYS-
-DPMSYL-
DRPMSY--
-DPMSYI-
DKPMSY--
DEPMSY--
-DPMSYT-
DYPMSY--
DQPMSY--

-DPMSYV-
DHPMSY--
DAPMSY--
-DPMSYM-
DMPMSY--
-EPMSYR-
ESPMSY--
EYPMSY--
-EPMSYS-
-EPMSYL-
EGPMSY--
-EPMSYV-
ETPMSY--
-EPMSYT-
-EPMSYY-
EMPMSY--
-EPMSYK-
-EPMSYA-
EEPMSY--
ERPMSY--
EKPMSY--
-EPMSYQ-
EQPMSY--
-EPMSYW-
EVPMSY--
-EPMSYG-
-EPMSYI-
-EPMSYH-
EHPMSY--
EAPMSY--
FNPMSY--
FSPMSY--
-FPMSYV-
-FPMSYK-
FLPMSY--
-FPMSYI-
FYPMSY--
-FPMSYA-
FDPMSY--
-FPMSYR-
FAPMSY--
FTPMSY--
FEPMSY--
-FPMSYS-
-FPMSYY-
FQPMSY--
FGPMSY--
-FPMSYT-
-FPMSYL-
FKPMSY--

FIPMSY--
-FPMSYF-
-FPMSYQ-
-FPMSYM-
-FPMSYE-
FHPMSY--
GSPMSY--
-GPMSYT-
-GPMSYR-
GQPMSY--
GVPMSY--
-GPMSYI-
GRPMSY--
GFPMSY--
GGPMSY--
-GPMSYM-
GHPMSY--
GLPMSY--
-GPMSYS-
-GPMSYK-
GEPMSY--
GAPMSY--
-GPMSYY-
-GPMSYQ-
GNPMSY--
GYPMSY--
GKPMSY--
-GPMSYV-
GMPMSY--
GWPMSY--
-GPMSYH-
-GPMSYA-
GTPMSY--
GDPMSY--
GCPMSY--
-GPMSYN-
-GPMSYG-
GIPMSY--
-GPMSYW-
-GPMSYP-
HRPMSY--
HKPMSY--
-HPMSYR-
HLPMSY--
HPPMSY--
HYPMSY--
-HPMSYS-
HEPMSY--
HTPMSY--
HQPMSY--

HGPMSY--
-HPMSYV-
HIPMSY--
HDPMSY--
-HPMSYL-
-HPMSYK-
HNPMSY--
HAPMSY--
HSPMSY--
HVPMSY--
HFPMSY--
-HPMSYY-
-HPMSYT-
-HPMSYF-
-HPMSYQ-
-HPMSYH-
-HPMSYN-
-HPMSYI-
HMPMSY--
HHPMSY--
ITPMSY--
IGPMSY--
IKPMSY--
ILPMSY--
IRPMSY--
-IPMSYL-
IYPMSY--
-IPMSYI-
ISPMSY--
IQPMSY--
IDPMSY--
-IPMSYS-
INPMSY--
-IPMSYR-
IIPMSY--
IVPMSY--
IHPMSY--
-IPMSYM-
IPPMSY--
-IPMSYW-
-IPMSYH-
-IPMSYK-
IAPMSY--
-IPMSYF-
IEPMSY--
-IPMSYQ-
IMPMSY--
-IPMSYY-
-IPMSYE-
-IPMSYA-

KSPMSY--
-KPMSYR-
KRPMSY--
KNPMSY--
-KPMSYM-
KGPMSY--
-KPMSYI-
KPPMSY--
-KPMSYT-
KIPMSY--
KWPMSY--
KLPMSY--
KKPMSY--
KAPMSY--
KVPMSY--
-KPMSYK-
-KPMSYH-
-KPMSYS-
KHPMSY--
-KPMSYQ-
-KPMSYA-
-KPMSYY-
KFPMSY--
-KPMSYW-
-KPMSYE-
-KPMSYV-
-KPMSYN-
KDPMSY--
-KPMSYF-
KCPMSY--
LRPMSY--
LGPMSY--
LSPMSY--
LIPMSY--
LLPMSY--
LHPMSY--
LVPMSY--
LTPMSY--
LFPMSY--
-LPMSYR-
-LPMSYS-
LEPMSY--
LDPMSY--
-LPMSYK-
LYPMSY--
LQPMSY--
LWPMSY--
LPPMSY--
-LPMSYM-
-LPMSYA-

-LPMSYV-
-LPMSYI-
-LPMSYN-
LCPMSY--
-LPMSYW-
-LPMSYY-
-LPMSYH-
-LPMSYD-
MRPMSY--
MKPMSY--
MSPMSY--
-MPMSYR-
MMPMSY--
MFPMSY--
MQPMSY--
-MPMSYS-
MLPMSY--
MGPMSY--
-MPMSYI-
-MPMSYK-
MPPMSY--
MWPMSY--
-MPMSYW-
-MPMSYT-
-MPMSYM-
-MPMSYH-
-MPMSYA-
MHPMSY--
MEPMSY--
MDPMSY--
MCPMSY--
MTPMSY--
-MPMSYY-
-MPMSYN-
-MPMSYQ-
MYPMSY--
-MPMSYV-
MIPMSY--
NGPMSY--
NRPMSY--
NSPMSY--
NAPMSY--
NYPMSY--
NLPMSY--
-NPMSYM-
NHPMSY--
NVPMSY--
NTPMSY--
-NPMSYR-
NQPMSY--

-NPMSYV-
NMPMSY--
NIPMSY--
-NPMSYA-
NFPMSY--
-NPMSYI-
NDPMSY--
-NPMSYY-
-NPMSYT-
NKPMSY--
NWPMSY--
-NPMSYS-
NPPMSY--
-NPMSYK-
NNPMSY--
NEPMSY--
-NPMSYQ-
-NPMSYN-
--PMSYVS
--PMSYNL
--PMSYLS
--PMSYSG
--PMSYTS
--PMSYMK
--PMSYKS
--PMSYWS
--PMSYFT
--PMSYSR
--PMSYIT
--PMSYYG
--PMSYVN
--PMSYLA
--PMSYFK
--PMSYSS
--PMSYTA
--PMSYSV
--PMSYRS
--PMSYRH
--PMSYTV
--PMSYLT
--PMSYYA
--PMSYMG
--PMSYMR
--PMSYSL
--PMSYRV
--PMSYRM
--PMSYFS
--PMSYYS
--PMSYSH
--PMSYLK

--PMSYRT
--PMSYMT
PGPMSY--
PQPMSY--
--PMSYVI
--PMSYRI
--PMSYLR
--PMSYAT
--PMSYVR
--PMSYVA
--PMSYRY
--PMSYKG
--PMSYHT
--PMSYVT
--PMSYTR
--PMSYMS
--PMSYLV
--PMSYLH
--PMSYKW
--PMSYKI
--PMSYIS
--PMSYGL
--PMSYYK
--PMSYWM
--PMSYSN
--PMSYNS
--PMSYGS
--PMSYVW
--PMSYTQ
--PMSYTL
--PMSYSK
--PMSYNY
--PMSYFH
--PMSYFG
PAPMSY--
--PMSYSQ
--PMSYKQ
PYPMSY--
--PMSYWK
--PMSYVK
--PMSYSA
--PMSYRN
--PMSYMI
--PMSYGA
--PMSYAL
--PMSYRD
--PMSYQA
--PMSYMF
--PMSYLM
--PMSYKV

--PMSYKA
--PMSYYY
--PMSYWL
--PMSYRW
--PMSYQY
--PMSYKY
--PMSYIH
--PMSYIG
--PMSYFQ
--PMSYFL
--PMSYAA
--PMSYWV
--PMSYVQ
--PMSYTK
--PMSYML
--PMSYMD
--PMSYHA
--PMSYAY
--PMSYYT
--PMSYYH
--PMSYWT
--PMSYVM
--PMSYTD
--PMSYPY
--PMSYMQ
--PMSYKT
--PMSYHY
--PMSYGY
PEPMSY--
--PMSYYF
--PMSYWH
--PMSYTY
--PMSYTF
--PMSYSW
--PMSYRQ
--PMSYNT
--PMSYMY
--PMSYMV
--PMSYMN
--PMSYWN
--PMSYWG
--PMSYVY
--PMSYVH
--PMSYSI
--PMSYSF
--PMSYQW
--PMSYQK
--PMSYNV
--PMSYMH
--PMSYKH

--PMSYHR
--PMSYGM
--PMSYAQ
--PMSYAF
PHPMSY--
PNPMSY--
--PMSYTM
--PMSYST
--PMSYSE
--PMSYQS
--PMSYQM
--PMSYQG
--PMSYNI
--PMSYNG
--PMSYKD
--PMSYIW
--PMSYIN
--PMSYIK
--PMSYGG
--PMSYES
--PMSYAS
PIPMSY--
--PMSYYQ
--PMSYYD
--PMSYWI
--PMSYPL
--PMSYNM
--PMSYMM
--PMSYLD
--PMSYKL
--PMSYGT
--PMSYFY
--PMSYEG
--PMSYAH
PCPMSY--
--PMSYYM
--PMSYWQ
--PMSYWA
--PMSYTE
--PMSYSD
--PMSYQT
--PMSYQR
--PMSYQI
--PMSYQF
--PMSYNW
--PMSYMA
--PMSYLI
--PMSYKM
--PMSYHQ
--PMSYGI

--PMSYEF
PFPMSY--
PWPMSY--
--PMSYYI
--PMSYVE
--PMSYTW
--PMSYSY
--PMSYQH
--PMSYQE
--PMSYME
--PMSYLE
--PMSYIL
--PMSYHV
--PMSYHN
--PMSYHL
--PMSYHK
--PMSYHI
--PMSYHG
--PMSYFA
--PMSYEV
--PMSYER
--PMSYAV
--PMSYAN
--PMSYAI
PPPMSY--
-PPMSYF-
--PMSYYN
--PMSYWW
--PMSYWE
--PMSYVD
--PMSYRE
--PMSYQV
--PMSYPI
--PMSYNK
--PMSYNA
--PMSYKE
--PMSYII
--PMSYHD
--PMSYGW
--PMSYGF
--PMSYFW
--PMSYEY
--PMSYEL
--PMSYEC
--PMSYDW
--PMSYDS
--PMSYDN
--PMSYDG
--PMSYCS
--PMSYCK

--PMSYCA
--PMSYAR
PDPMSY--
QRPMSY--
QSPMSY--
QVPMSY--
-QPMSYI-
-QPMSYL-
QLPMSY--
QFPMSY--
QMPMSY--
QGPMSY--
QHPMSY--
QQPMSY--
QNPMSY--
QIPMSY--
-QPMSYF-
QKPMSY--
QPPMSY--
-QPMSYR-
QEPMSY--
-QPMSYQ-
QYPMSY--
-QPMSYA-
-QPMSYW-
-QPMSYS-
-QPMSYH-
QDPMSY--
-QPMSYM-
-QPMSYK-
-QPMSYY-
-RPMSYM-
RPPMSY--
RSPMSY--
RRPMSY--
RYPMSY--
RFPMSY--
RNPMSY--
RMPMSY--
-RPMSYY-
RGPMSY--
-RPMSYV-
-RPMSYF-
-RPMSYI-
RQPMSY--
RLPMSY--
RKPMSY--
RAPMSY--
-RPMSYT-
-RPMSYH-

RHPMSY--
REPMSY--
-RPMSYW-
RDPMSY--
-RPMSYK-
-RPMSYN-
-RPMSYQ-
RCPMSY--
-RPMSYG-
-RPMSYE-
SSPMSY--
SRPMSY--
-SPMSYR-
-SPMSYV-
-SPMSYT-
SMPMSY--
-SPMSYK-
SGPMSY--
SNPMSY--
SAPMSY--
SYPMSY--
-SPMSYS-
SVPMSY--
STPMSY--
SPPMSY--
-SPMSYY-
SEPMSY--
SKPMSY--
SIPMSY--
SHPMSY--
SDPMSY--
SLPMSY--
SQPMSY--
-SPMSYM-
-SPMSYF-
-SPMSYE-
-SPMSYW-
-SPMSYQ-
-SPMSYI-
SFPMSY--
-SPMSYA-
SCPMSY--
TGPMSY--
TRPMSY--
-TPMSYR-
TTPMSY--
-TPMSYL-
-TPMSYI-
TSPMSY--
TAPMSY--

-TPMSYS-
TVPMSY--
TLPMSY--
TEPMSY--
-TPMSYY-
-TPMSYM-
-TPMSYF-
TFPMSY--
TIPMSY--
TDPMSY--
TYPMSY--
TKPMSY--
TPPMSY--
-TPMSYK-
TQPMSY--
TMPMSY--
THPMSY--
-TPMSYT-
TWPMSY--
-TPMSYE-
TCPMSY--
-TPMSYW-
-TPMSYD-
-TPMSYQ-
-TPMSYN-
-TPMSYH-
-TPMSYA-
VRPMSY--
VVPMSY--
VSPMSY--
-VPMSYR-
-VPMSYS-
-VPMSYT-
-VPMSYI-
VQPMSY--
VMPMSY--
-VPMSYF-
-VPMSYK-
VWPMSY--
VTPMSY--
VPPMSY--
VNPMSY--
VYPMSY--
VGPMSY--
-VPMSYA-
-VPMSYY-
VDPMSY--
-VPMSYN-
-VPMSYQ-
-VPMSYH-

-VPMSYW-
-VPMSYG-
VKPMSY--
-VPMSYE-
VCPMSY--
VHPMSY--
WSPMSY--
WRPMSY--
-WPMSYT-
-WPMSYS-
-WPMSYV-
-WPMSYM-
WTPMSY--
-WPMSYI-
WVPMSY--
WDPMSY--
WAPMSY--
-WPMSYY-
-WPMSYL-
-WPMSYK-
-WPMSYW-
-WPMSYH-
-WPMSYF-
WYPMSY--
WWPMSY--
-WPMSYE-
-WPMSYA-
WFPMSY--
-YPMSYR-
YLPMSY--
-YPMSYK-
YIPMSY--
-YPMSYS-
-YPMSYM-
YTPMSY--
YPPMSY--
YKPMSY--
-YPMSYI-
-YPMSYH-
YGPMSY--
YVPMSY--
YNPMSY--
YEPMSY--
YAPMSY--
-YPMSYW-
-YPMSYT-
-YPMSYN-
-YPMSYY-
-YPMSYV-
YCPMSY--

	YYPMSY--		
	YWPMSY--		
	YQPMSY--		
PQGI	158	772	44.89
	INPQGI--		
	-YPQGIA-		
	-APQGIS-		
	ALPQGI--		
	-APQGIY-		
	ASPQGI--		
	-APQGIH-		
	AVPQGI--		
	AFPQGI--		
	AAPQGI--		
	-APQGIF-		
	-APQGIL-		
	ARPQGI--		
	-APQGIR-		
	-APQGII-		
	-APQGIA-		
	AKPQGI--		
	-APQGIN-		
	ANPQGI--		
	ATPQGI--		
	-APQGIT-		
	APPQGI--		
	AIPQGI--		
	-APQGIM-		
	AHPQGI--		
	AYPQGI--		
	-APQGIQ-		
	-APQGIK-		
	-APQGIG-		
	AQPQGI--		
	-APQGIV-		
	-APQGIE-		
	ACPQGI--		
	CLPQGI--		
	CKPQGI--		
	CRPQGI--		
	-CPQGIS-		
	CSPQGI--		
	-CPQGIR-		
	-CPQGIL-		
	-CPQGIV-		
	CYPQGI--		
	-CPQGIA-		
	CMPQGI--		
	DQPQGI--		
	-DPQGIR-		

DRPQGI--
DKPQGI--
-DPQGIH-
-DPQGIS-
DGPQGI--
-DPQGIL-
DMPQGI--
-DPQGIF-
DTPQGI--
-DPQGIT-
-DPQGIQ-
-DPQGII-
DLPQGI--
DAPQGI--
-EPQGIK-
-EPQGII-
EWPQGI--
EVPQGI--
-EPQGIL-
EMPQGI--
-EPQGIS-
-EPQGIR-
EQPQGI--
ELPQGI--
-EPQGIY-
-EPQGIF-
EGPQGI--
ESPQGI--
EAPQGI--
-EPQGIA-
EEPQGI--
-EPQGIV-
-EPQGIN-
EIPQGI--
EDPQGI--
-FPQGIR-
-FPQGIS-
FIPQGI--
FLPQGI--
FRPQGI--
-FPQGII-
-FPQGIK-
-FPQGIW-
FPPQGI--
FTPQGI--
-FPQGIV-
-FPQGIM-
FEPQGI--
-FPQGIT-
-FPQGIL-

FVPQGI--
FHPQGI--
FYPQGI--
FFPQGI--
FDPQGI--
FSPQGI--
-FPQGIY-
FMPQGI--
-FPQGIN-
-FPQGIH-
FGPQGI--
-FPQGIQ-
GTPQGI--
GKPQGI--
-GPQGIW-
GRPQGI--
-GPQGIR-
-GPQGIS-
-GPQGIF-
GLPQGI--
-GPQGIH-
GSPQGI--
GIPQGI--
-GPQGIY-
-GPQGIT-
-GPQGIM-
-GPQGIK-
GAPQGI--
-GPQGIL-
GEPQGI--
GYPQGI--
GVPQGI--
-GPQGIA-
-GPQGIV-
GGPQGI--
GDPQGI--
GWPQGI--
-GPQGII-
GNPQGI--
GFPQGI--
GQPQGI--
-GPQGIN-
-GPQGIG-
-GPQGIC-
GCPQGI--
HAPQGI--
-HPQGIR-
HLPQGI--
HKPQGI--
HYPQGI--

-HPQGIS-
HRPQGI--
-HPQGIV-
-HPQGII-
HVPQGI--
HTPQGI--
HSPQGI--
HDPQGI--
HWPQGI--
-HPQGIL-
-HPQGIN-
HMPQGI--
HIPQGI--
HGPQGI--
-HPQGIM-
HFPQGI--
-HPQGIG-
-HPQGIY-
-HPQGIF-
-HPQGIA-
ILPQGI--
-IPQGIH-
IVPQGI--
IAPQGI--
-IPQGIV-
-IPQGIW-
IFPQGI--
IRPQGI--
-IPQGIL-
IDPQGI--
IWPQGI--
-IPQGIA-
IQPQGI--
IKPQGI--
-IPQGIY-
ICPQGI--
ISPQGI--
-IPQGIN-
-IPQGIM-
-IPQGIK-
-IPQGIE-
IYPQGI--
ITPQGI--
-IPQGIR-
IPPQGI--
IHPQGI--
IEPQGI--
-IPQGIS-
-IPQGIQ-
-IPQGII-

```
-IPQGIT-
-IPQGIF-
KSPQGI--
KGPQGI--
-KPQGIL-
-KPQGIR-
KKPQGI--
KMPQGI--
-KPQGII-
-KPQGIS-
KEPQGI--
-KPQGIN-
KAPQGI--
KRPQGI--
-KPQGIK-
KFPQGI--
KVPQGI--
-KPQGIY-
KDPQGI--
-KPQGIF-
KLPQGI--
KIPQGI--
KHPQGI--
KTPQGI--
KQPQGI--
-KPQGIV-
-KPQGIA-
-KPQGIT-
-KPQGIM-
-KPQGIH-
KNPQGI--
KYPQGI--
-KPQGIW-
-KPQGIG-
LRPQGI--
LSPQGI--
LPPQGI--
LMPQGI--
LGPQGI--
-LPQGIF-
-LPQGIH-
LFPQGI--
LNPQGI--
-LPQGIR-
LEPQGI--
LVPQGI--
-LPQGIT-
-LPQGII-
LDPQGI--
-LPQGIS-
```

LLPQGI--
LYPQGI--
-LPQGIL-
LAPQGI--
LWPQGI--
LQPQGI--
-LPQGIY-
-LPQGIQ-
LTPQGI--
-LPQGIM-
-LPQGIK-
-LPQGIE-
LKPQGI--
-LPQGIV-
-LPQGIN-
LIPQGI--
-MPQGIR-
MLPQGI--
-MPQGIT-
MNPQGI--
MDPQGI--
-MPQGIL-
-MPQGIV-
-MPQGIF-
MMPQGI--
MYPQGI--
MTPQGI--
-MPQGIH-
MKPQGI--
MSPQGI--
MIPQGI--
MVPQGI--
-MPQGIS-
-MPQGII-
-MPQGIG-
MEPQGI--
MWPQGI--
MHPQGI--
MGPQGI--
MAPQGI--
NRPQGI--
NSPQGI--
NYPQGI--
NKPQGI--
-NPQGIL-
NHPQGI--
NPPQGI--
-NPQGIM-
NNPQGI--
NGPQGI--

NVPQGI--
NTPQGI--
-NPQGIR-
NLPQGI--
-NPQGIV-
-NPQGIT-
-NPQGIW-
-NPQGIA-
-NPQGIK-
-NPQGIF-
NIPQGI--
NQPQGI--
-NPQGII-
NDPQGI--
NAPQGI--
--PQGIRR
PIPQGI--
--PQGIRL
--PQGIVT
--PQGIRS
--PQGIWT
--PQGILG
--PQGIFS
--PQGIRG
--PQGITY
--PQGISR
--PQGIRV
--PQGIRI
--PQGIRH
--PQGIMR
--PQGIMT
--PQGIKA
--PQGIYS
--PQGIYG
--PQGIQV
--PQGIKT
--PQGIKG
--PQGISM
--PQGIRF
--PQGISS
--PQGILN
--PQGIHG
--PQGIFH
--PQGILV
--PQGIIV
--PQGIIR
--PQGIIQ
PRPQGI--
--PQGIQG
--PQGINQ

--PQGIVS
--PQGIVR
--PQGISG
--PQGISY
--PQGISW
--PQGIQW
--PQGILS
--PQGIFK
--PQGIFA
--PQGIRQ
--PQGINT
--PQGINA
--PQGILY
--PQGIYH
--PQGIVH
--PQGITT
--PQGIMS
--PQGILR
--PQGIKR
--PQGIIN
--PQGIHT
--PQGITS
--PQGISK
--PQGIRW
--PQGIRA
--PQGINS
--PQGILM
--PQGILH
--PQGIGT
--PQGIVI
--PQGIQR
--PQGINV
--PQGILQ
PVPQGI--
--PQGIMV
--PQGILT
--PQGIKF
--PQGIGG
--PQGIYM
--PQGIYK
--PQGINF
--PQGIHV
--PQGIFY
--PQGIFE
--PQGIVG
--PQGITA
--PQGIMN
--PQGILF
--PQGIKS
--PQGIHH

--PQGIAG
--PQGIRT
--PQGIRN
--PQGIQS
--PQGINR
--PQGIML
--PQGIMG
--PQGIKQ
--PQGIKH
--PQGIAA
--PQGIWS
--PQGIWN
--PQGIWM
--PQGIWF
--PQGIVN
--PQGITR
--PQGIRD
--PQGIQT
PDPQGI--
--PQGIWR
--PQGITW
--PQGISI
--PQGISA
--PQGIQI
--PQGIGS
--PQGIFI
PFPQGI--
--PQGIYW
--PQGIYT
--PQGIWA
--PQGITM
--PQGITK
--PQGITI
--PQGISV
--PQGIST
--PQGISF
--PQGIMY
--PQGIMW
--PQGIMK
--PQGILI
--PQGIKM
--PQGIKK
--PQGIIW
--PQGIHR
--PQGIHF
--PQGIFW
--PQGIAS
PLPQGI--
PTPQGI--
PSPQGI--

--PQGIVW
--PQGIVQ
--PQGISQ
--PQGISN
--PQGIRC
--PQGIQK
--PQGIIF
--PQGIHM
--PQGIHL
--PQGIFT
--PQGIFM
PGPQGI--
--PQGIYR
--PQGIYF
--PQGIWG
--PQGIVF
--PQGIMA
--PQGILK
--PQGIIH
--PQGIIG
--PQGIHS
--PQGIHD
--PQGIFR
--PQGIFG
--PQGIFF
--PQGIAT
--PQGIYY
--PQGIWE
--PQGITE
--PQGINN
--PQGINH
--PQGIME
--PQGIKV
--PQGIEA
--PQGIAR
PHPQGI--
--PQGIYD
--PQGIWY
--PQGIRY
--PQGIRM
--PQGIRE
--PQGIQL
--PQGIQE
--PQGINM
--PQGILD
--PQGILA
--PQGIKY
--PQGIII
--PQGIHY
--PQGIFQ

--PQGIEY
--PQGIEW
--PQGIEV
--PQGIEF
--PQGIAL
PYPQGI--
--PQGIYQ
--PQGIYN
--PQGIVY
--PQGIVV
--PQGIVA
--PQGITV
--PQGITN
--PQGITG
--PQGIQF
--PQGINW
--PQGINI
--PQGINE
--PQGIMF
--PQGIKE
--PQGIIY
--PQGIIA
--PQGIHI
--PQGIHA
--PQGIGR
--PQGIGF
--PQGIEQ
--PQGIEI
--PQGICA
--PQGIAQ
--PQGIWK
--PQGIVM
--PQGIVK
--PQGIVD
--PQGITP
--PQGITF
--PQGIQN
--PQGIQD
--PQGINY
--PQGINL
--PQGILC
--PQGIKL
--PQGIKI
--PQGIKD
--PQGIIL
--PQGIIE
--PQGIGV
--PQGIFV
--PQGIFP
--PQGIEG

--PQGIAY
--PQGIAN
--PQGIAI
PNPQGI--
QSPQGI--
-QPQGIM-
QVPQGI--
QRPQGI--
QGPQGI--
-QPQGIS-
QPPQGI--
-QPQGII-
-QPQGIT-
QKPQGI--
-QPQGIH-
-QPQGIR-
QWPQGI--
-QPQGIY-
-QPQGIL-
-QPQGIK-
-QPQGIA-
QMPQGI--
QQPQGI--
-QPQGIQ-
-QPQGIF-
QNPQGI--
QAPQGI--
-QPQGIN-
-QPQGIG-
QLPQGI--
QFPQGI--
QDPQGI--
RRPQGI--
RGPQGI--
RLPQGI--
-RPQGIL-
RAPQGI--
-RPQGIT-
RSPQGI--
RVPQGI--
-RPQGIR-
-RPQGII-
-RPQGIV-
RMPQGI--
RTPQGI--
RKPQGI--
RIPQGI--
-RPQGIM-
-RPQGIH-
-RPQGIY-

REPQGI--
-RPQGIS-
-RPQGIF-
RPPQGI--
-RPQGIW-
-RPQGIQ-
RYPQGI--
RFPQGI--
RWPQGI--
RQPQGI--
RHPQGI--
-RPQGIK-
-RPQGIA-
-RPQGIG-
RDPQGI--
-RPQGIN-
-RPQGIE-
-SPQGIR-
-SPQGIL-
SVPQGI--
SFPQGI--
SGPQGI--
SRPQGI--
-SPQGIT-
SYPQGI--
STPQGI--
-SPQGIY-
SAPQGI--
-SPQGIF-
SSPQGI--
-SPQGIM-
-SPQGIV-
-SPQGII-
SKPQGI--
SIPQGI--
SWPQGI--
SNPQGI--
-SPQGIA-
SHPQGI--
SQPQGI--
-SPQGIH-
-SPQGIN-
SPPQGI--
SDPQGI--
SCPQGI--
-SPQGIQ-
-SPQGIK-
-SPQGIG-
-SPQGIC-
SMPQGI--

-SPQGIW-
-TPQGIR-
-TPQGIL-
TYPQGI--
TIPQGI--
TMPQGI--
TLPQGI--
TGPQGI--
TAPQGI--
TKPQGI--
-TPQGIW-
-TPQGIF-
TTPQGI--
-TPQGIK-
TNPQGI--
TFPQGI--
-TPQGIY-
-TPQGIT-
-TPQGIV-
-TPQGII-
TCPQGI--
TWPQGI--
THPQGI--
TDPQGI--
TSPQGI--
-TPQGIS-
-TPQGIQ-
-TPQGIA-
TPPQGI--
-TPQGIN-
-TPQGIH-
-TPQGIG-
-VPQGIR-
-VPQGIF-
VLPQGI--
VSPQGI--
-VPQGIL-
-VPQGIQ-
-VPQGIS-
VGPQGI--
VTPQGI--
VWPQGI--
-VPQGIV-
-VPQGIH-
VRPQGI--
VNPQGI--
-VPQGIM-
VMPQGI--
VAPQGI--
VVPQGI--

VIPQGI--
VPPQGI--
-VPQGIY-
-VPQGII-
VHPQGI--
VEPQGI--
-VPQGIN-
-VPQGIA-
VQPQGI--
-VPQGIG-
VDPQGI--
VCPQGI--
WEPQGI--
WGPQGI--
WQPQGI--
WTPQGI--
WAPQGI--
-WPQGIL-
-WPQGIV-
WFPQGI--
WMPQGI--
WIPQGI--
WHPQGI--
-WPQGIR-
-WPQGIF-
WLPQGI--
WSPQGI--
-WPQGIA-
-WPQGIT-
-WPQGIK-
WDPQGI--
WYPQGI--
WRPQGI--
-WPQGIY-
-WPQGIM-
-WPQGIG-
-WPQGIE-
-WPQGIC-
WNPQGI--
WWPQGI--
-WPQGIQ-
-WPQGII-
-WPQGID-
YGPQGI--
YSPQGI--
YVPQGI--
YAPQGI--
YWPQGI--
-YPQGIY-
-YPQGIT-

	YFPQGI--		
	YEPQGI--		
	-YPQGIK-		
	-YPQGIS-		
	YRPQGI--		
	YLPQGI--		
	YKPQGI--		
	YNPQGI--		
	-YPQGIL-		
	YHPQGI--		
	-YPQGIR-		
	-YPQGIF-		
	YYPQGI--		
	YIPQGI--		
	YTPQGI--		
	YQPQGI--		
	-YPQGII-		
	-YPQGIH-		
	YMPQGI--		
	-YPQGIW-		
	-YPQGIN-		
	-YPQGIV-		
	-YPQGIG-		
	-YPQGIE-		
	YPPQGI--		
	YCPQGI--		
PINL	268	713	44.89
	-GPINLD-		
	-NPINLG-		
	--PINLSP		
	--PINLRP		
	--PINLNN		
	--PINLNF		
	-RPINLD-		
	-VPINLP-		
	-VPINLA-		
	-YPINLN-		
	ASPINL--		
	-APINLL-		
	AKPINL--		
	AGPINL--		
	AVPINL--		
	AAPINL--		
	ATPINL--		
	AMPINL--		
	AEPINL--		
	AYPINL--		
	-APINLQ-		
	ALPINL--		
	AFPINL--		

−APINLW−
−APINLH−
−APINLR−
AQPINL−−
−APINLS−
ANPINL−−
−APINLG−
AWPINL−−
−APINLK−
AIPINL−−
−APINLT−
−APINLN−
AHPINL−−
−APINLI−
−APINLE−
APPINL−−
CHPINL−−
CCPINL−−
CSPINL−−
CRPINL−−
CPPINL−−
CMPINL−−
CVPINL−−
CKPINL−−
−CPINLV−
CLPINL−−
−CPINLT−
−CPINLN−
−CPINLL−
−CPINLK−
−CPINLY−
CNPINL−−
CGPINL−−
CFPINL−−
DRPINL−−
−DPINLR−
DMPINL−−
DHPINL−−
−DPINLL−
DLPINL−−
DYPINL−−
DWPINL−−
−DPINLV−
−DPINLI−
DAPINL−−
DIPINL−−
DFPINL−−
DVPINL−−
DTPINL−−
DQPINL−−

-DPINLT-
DKPINL--
DGPINL--
DSPINL--
-DPINLY-
ERPINL--
-EPINLR-
-EPINLV-
-EPINLM-
EVPINL--
EHPINL--
-EPINLI-
EQPINL--
EGPINL--
EAPINL--
ESPINL--
-EPINLY-
-EPINLT-
-EPINLW-
ELPINL--
EKPINL--
ETPINL--
-EPINLA-
EMPINL--
EFPINL--
EDPINL--
EYPINL--
-EPINLK-
EIPINL--
-EPINLQ-
EWPINL--
-EPINLN-
-EPINLC-
ENPINL--
FRPINL--
FTPINL--
-FPINLL-
FHPINL--
FSPINL--
FGPINL--
-FPINLR-
-FPINLT-
FQPINL--
-FPINLK-
-FPINLE-
FNPINL--
-FPINLA-
FKPINL--
FEPINL--
-FPINLV-

-FPINLS-
FPPINL--
FDPINL--
-FPINLM-
GVPINL--
GTPINL--
-GPINLR-
-GPINLL-
GFPINL--
GNPINL--
GHPINL--
GYPINL--
-GPINLS-
GMPINL--
GKPINL--
-GPINLT-
-GPINLK-
GQPINL--
GEPINL--
GRPINL--
GLPINL--
GAPINL--
GWPINL--
GGPINL--
-GPINLQ-
-GPINLF-
-GPINLY-
-GPINLM-
-GPINLI-
GDPINL--
-GPINLA-
-GPINLN-
-GPINLG-
-GPINLH-
-GPINLE-
GIPINL--
GPPINL--
HRPINL--
-HPINLL-
HAPINL--
HHPINL--
HLPINL--
HYPINL--
-HPINLS-
HPPINL--
-HPINLT-
HGPINL--
HNPINL--
-HPINLK-
HSPINL--

-HPINLV-
HWPINL--
HFPINL--
-HPINLA-
HTPINL--
HQPINL--
HVPINL--
HMPINL--
HKPINL--
-HPINLQ-
-HPINLI-
-HPINLY-
-HPINLE-
-HPINLR-
HEPINL--
HDPINL--
-HPINLF-
-HPINLG-
-IPINLR-
ISPINL--
IRPINL--
IAPINL--
INPINL--
-IPINLV-
-IPINLS-
IFPINL--
-IPINLH-
-IPINLF-
-IPINLA-
-IPINLL-
IKPINL--
IIPINL--
ITPINL--
IYPINL--
IQPINL--
-IPINLT-
-IPINLI-
IWPINL--
IGPINL--
IPPINL--
-IPINLW-
-IPINLK-
-IPINLN-
-IPINLE-
-IPINLQ-
-IPINLG-
IDPINL--
KSPINL--
-KPINLR-
KVPINL--

KTPINL--
KAPINL--
-KPINLM-
KGPINL--
KLPINL--
-KPINLL-
-KPINLE-
-KPINLA-
KNPINL--
KHPINL--
KYPINL--
KIPINL--
KWPINL--
-KPINLK-
-KPINLW-
-KPINLT-
-KPINLI-
-KPINLN-
-KPINLS-
KEPINL--
KQPINL--
-KPINLH-
KDPINL--
-KPINLQ-
-KPINLC-
KMPINL--
-KPINLG-
KCPINL--
LRPINL--
LSPINL--
LGPINL--
LWPINL--
-LPINLL-
LPPINL--
LLPINL--
LAPINL--
LHPINL--
LYPINL--
LTPINL--
LQPINL--
LDPINL--
-LPINLT-
LNPINL--
-LPINLS-
-LPINLM-
-LPINLK-
-LPINLH-
LCPINL--
-LPINLV-
-LPINLE-

-LPINLQ-
-LPINLN-
-LPINLD-
-MPINLR-
-MPINLT-
MPPINL--
MSPINL--
-MPINLS-
MAPINL--
MNPINL--
MMPINL--
MLPINL--
MHPINL--
MEPINL--
MKPINL--
-MPINLK-
MGPINL--
MWPINL--
-MPINLY-
-MPINLA-
MIPINL--
MQPINL--
-MPINLQ-
-MPINLV-
MYPINL--
-MPINLM-
MFPINL--
-MPINLG-
-MPINLE-
-MPINLN-
MCPINL--
NVPINL--
-NPINLR-
NLPINL--
NRPINL--
NGPINL--
NEPINL--
-NPINLT-
NTPINL--
-NPINLS-
NHPINL--
-NPINLY-
-NPINLF-
NAPINL--
NWPINL--
-NPINLQ-
-NPINLK-
NFPINL--
NNPINL--
-NPINLW-

-NPINLA-
NMPINL--
NKPINL--
NQPINL--
NPPINL--
-NPINLM-
-NPINLH-
-NPINLN-
-NPINLE-
--PINLRF
--PINLRS
--PINLSA
--PINLYG
--PINLSS
--PINLLG
--PINLIG
--PINLWS
--PINLRV
--PINLHS
--PINLTT
PGPINL--
PWPINL--
--PINLSN
--PINLRY
--PINLKS
--PINLVG
--PINLSR
--PINLSH
--PINLVT
--PINLVK
--PINLST
--PINLVN
--PINLKG
--PINLWA
--PINLRW
--PINLKN
--PINLFA
--PINLRN
--PINLGA
PQPINL--
--PINLTK
--PINLSF
--PINLNS
--PINLNA
--PINLFN
PMPINL--
--PINLGS
--PINLRR
--PINLRE
--PINLRA

--PINLQT
--PINLQR
--PINLES
--PINLHG
--PINLTS
--PINLSV
--PINLSG
--PINLQS
--PINLAG
PTPINL--
--PINLTY
--PINLRH
--PINLGT
--PINLTG
--PINLTA
--PINLSY
PNPINL--
--PINLSK
--PINLMQ
--PINLEG
--PINLAT
--PINLTN
--PINLSM
--PINLMS
--PINLLH
--PINLKV
--PINLKH
--PINLFG
--PINLVH
--PINLTH
--PINLKY
--PINLKW
--PINLHA
--PINLYW
--PINLTR
--PINLTI
--PINLTF
--PINLSW
--PINLQG
PYPINL--
--PINLWF
--PINLTW
--PINLMR
--PINLMA
--PINLKI
--PINLKF
PAPINL--
--PINLWG
--PINLQD
--PINLMK

--PINLHN
--PINLFM
--PINLFH
--PINLQH
--PINLMD
--PINLKL
--PINLSD
--PINLLD
--PINLEV
--PINLER
--PINLEL
--PINLAK
PHPINL--
PDPINL--
--PINLWH
--PINLSI
--PINLNM
--PINLME
PEPINL--
--PINLYN
--PINLVD
--PINLQK
--PINLNH
--PINLLN
--PINLKA
--PINLAA
PFPINL--
--PINLWD
--PINLVQ
--PINLSQ
--PINLRQ
--PINLRD
--PINLQE
--PINLID
--PINLEA
--PINLQV
--PINLQI
--PINLKE
--PINLIN
--PINLHQ
--PINLHM
--PINLDA
--PINLAH
--PINLWQ
--PINLQF
--PINLKD
--PINLET
PIPINL--
--PINLYH
--PINLSE

--PINLQM
--PINLPS
--PINLHR
--PINLHI
--PINLHH
--PINLHD
--PINLGV
--PINLEI
--PINLAL
PKPINL--
--PINLWN
--PINLSC
--PINLNV
--PINLMN
--PINLMH
--PINLKT
--PINLHV
--PINLHK
--PINLGW
--PINLGI
--PINLCD
--PINLAR
--PINLAQ
--PINLAD
PCPINL--
PPPINL--
-PPINLL-
--PINLTQ
--PINLTE
--PINLTD
--PINLQL
--PINLNW
--PINLNT
--PINLIH
--PINLHT
--PINLGY
--PINLGM
--PINLGF
--PINLFD
--PINLEY
--PINLEE
--PINLDT
--PINLDG
--PINLCS
--PINLCA
--PINLAN
--PINLAC
-QPINLR-
QRPINL--
-QPINLL-

-QPINLQ-
-QPINLS-
QKPINL--
QGPINL--
-QPINLY-
-QPINLV-
QEPINL--
QWPINL--
QTPINL--
-QPINLI-
QFPINL--
QIPINL--
QPPINL--
-QPINLH-
QQPINL--
QMPINL--
QSPINL--
-QPINLM-
QHPINL--
QAPINL--
-QPINLT-
QLPINL--
QDPINL--
-QPINLN-
-QPINLK-
-QPINLF-
-QPINLE-
QNPINL--
RVPINL--
RGPINL--
-RPINLL-
-RPINLT-
RYPINL--
RMPINL--
-RPINLM-
RWPINL--
-RPINLF-
-RPINLW-
-RPINLV-
-RPINLY-
RAPINL--
-RPINLQ-
RHPINL--
RPPINL--
-RPINLH-
RFPINL--
RNPINL--
RQPINL--
-RPINLA-
-RPINLN-

-RPINLE-
REPINL--
-RPINLG-
RDPINL--
RIPINL--
RCPINL--
-RPINLP-
STPINL--
SRPINL--
-SPINLS-
SGPINL--
-SPINLL-
SSPINL--
SVPINL--
-SPINLR-
SYPINL--
SLPINL--
SQPINL--
-SPINLK-
-SPINLI-
SIPINL--
-SPINLH-
-SPINLA-
SNPINL--
-SPINLQ-
-SPINLV-
SEPINL--
-SPINLM-
SWPINL--
SDPINL--
SHPINL--
SMPINL--
SPPINL--
-SPINLN-
-SPINLG-
-SPINLF-
-SPINLE-
SCPINL--
-TPINLV-
TRPINL--
-TPINLR-
TLPINL--
TSPINL--
TPPINL--
TKPINL--
TGPINL--
TTPINL--
-TPINLM-
-TPINLI-
TMPINL--

TQPINL--
-TPINLS-
-TPINLH-
-TPINLK-
TVPINL--
TAPINL--
-TPINLA-
TNPINL--
-TPINLN-
TYPINL--
TWPINL--
-TPINLW-
THPINL--
TEPINL--
-TPINLG-
-TPINLC-
VKPINL--
VLPINL--
VMPINL--
VVPINL--
VSPINL--
VAPINL--
VGPINL--
VWPINL--
VTPINL--
VYPINL--
-VPINLL-
-VPINLM-
-VPINLH-
-VPINLR-
VFPINL--
VQPINL--
VIPINL--
VHPINL--
-VPINLY-
-VPINLS-
-VPINLK-
VPPINL--
-VPINLE-
VNPINL--
-VPINLQ-
-VPINLN-
VEPINL--
-VPINLG-
-VPINLV-
VDPINL--
-WPINLR-
WSPINL--
WGPINL--
-WPINLV-

	WKPINL--		
	WRPINL--		
	WMPINL--		
	-WPINLH-		
	-WPINLQ-		
	-WPINLS-		
	-WPINLT-		
	-WPINLA-		
	WNPINL--		
	-WPINLE-		
	WHPINL--		
	WAPINL--		
	-WPINLM-		
	-WPINLK-		
	YVPINL--		
	-YPINLR-		
	-YPINLL-		
	YGPINL--		
	YTPINL--		
	-YPINLQ-		
	-YPINLK-		
	YQPINL--		
	YAPINL--		
	-YPINLT-		
	YKPINL--		
	YYPINL--		
	-YPINLE-		
	-YPINLV-		
	-YPINLH-		
	-YPINLI-		
	-YPINLY-		
	YFPINL--		
	YEPINL--		
	YHPINL--		
	YPPINL--		
	YWPINL--		
	-YPINLM-		
	-YPINLA-		
	YNPINL--		
	-YPINLG-		
PRHY	281	707	44.88
	--PRHYHR		
	--PRHYET		
	ASPRHY--		
	-APRHYK-		
	-APRHYS-		
	ANPRHY--		
	AAPRHY--		
	AYPRHY--		
	-APRHYL-		

```
-APRHYV-
APPRHY--
AQPRHY--
AHPRHY--
ARPRHY--
AKPRHY--
-APRHYA-
ALPRHY--
ADPRHY--
-APRHYI-
ATPRHY--
AEPRHY--
-APRHYQ-
-APRHYG-
AMPRHY--
-APRHYF-
-APRHYW-
-APRHYM-
-APRHYP-
-APRHYN-
-APRHYC-
ACPRHY--
CSPRHY--
CRPRHY--
CNPRHY--
-CPRHYM-
CKPRHY--
-CPRHYV-
CCPRHY--
CWPRHY--
CMPRHY--
CAPRHY--
CVPRHY--
CPPRHY--
DSPRHY--
DIPRHY--
DVPRHY--
-DPRHYR-
DMPRHY--
DRPRHY--
DQPRHY--
DTPRHY--
DHPRHY--
DAPRHY--
-DPRHYY-
-DPRHYT-
-DPRHYM-
-DPRHYL-
DKPRHY--
DWPRHY--
```

-DPRHYV-
-DPRHYI-
-DPRHYE-
DGPRHY--
-DPRHYW-
-DPRHYH-
-DPRHYG-
DDPRHY--
ESPRHY--
EYPRHY--
ELPRHY--
-EPRHYI-
EVPRHY--
ERPRHY--
-EPRHYV-
-EPRHYR-
-EPRHYS-
EAPRHY--
-EPRHYW-
EGPRHY--
EEPRHY--
-EPRHYM-
EWPRHY--
-EPRHYT-
-EPRHYQ-
-EPRHYH-
EPPRHY--
-EPRHYK-
-EPRHYF-
ENPRHY--
EMPRHY--
-EPRHYL-
EKPRHY--
EHPRHY--
-EPRHYY-
EIPRHY--
EFPRHY--
EDPRHY--
FNPRHY--
FQPRHY--
FIPRHY--
FGPRHY--
FHPRHY--
FSPRHY--
FKPRHY--
FAPRHY--
FWPRHY--
-FPRHYA-
FMPRHY--
FEPRHY--

FVPRHY--
FPPRHY--
FYPRHY--
-FPRHYK-
-FPRHYV-
-FPRHYQ-
-FPRHYN-
FDPRHY--
-FPRHYH-
FTPRHY--
-FPRHYW-
-FPRHYI-
FFPRHY--
-FPRHYY-
-FPRHYE-
GTPRHY--
-GPRHYR-
-GPRHYY-
GNPRHY--
-GPRHYK-
GAPRHY--
-GPRHYI-
GGPRHY--
GSPRHY--
-GPRHYS-
-GPRHYL-
GHPRHY--
GEPRHY--
-GPRHYV-
GDPRHY--
GQPRHY--
GKPRHY--
-GPRHYA-
-GPRHYQ-
GIPRHY--
GFPRHY--
GLPRHY--
-GPRHYT-
GYPRHY--
GWPRHY--
-GPRHYW-
-GPRHYE-
-GPRHYD-
HGPRHY--
HAPRHY--
HLPRHY--
HTPRHY--
-HPRHYQ-
-HPRHYV-
-HPRHYL-

HSPRHY--
HMPRHY--
-HPRHYA-
HYPRHY--
-HPRHYW-
HNPRHY--
-HPRHYR-
-HPRHYY-
-HPRHYI-
HEPRHY--
-HPRHYM-
-HPRHYH-
-HPRHYN-
HIPRHY--
HHPRHY--
IGPRHY--
-IPRHYV-
-IPRHYT-
ILPRHY--
IPPRHY--
-IPRHYM-
-IPRHYW-
-IPRHYF-
IQPRHY--
-IPRHYK-
-IPRHYQ-
IHPRHY--
-IPRHYS-
-IPRHYI-
IMPRHY--
ISPRHY--
-IPRHYL-
-IPRHYA-
INPRHY--
-IPRHYH-
IDPRHY--
-IPRHYE-
-IPRHYY-
-IPRHYG-
IAPRHY--
IIPRHY--
ICPRHY--
KGPRHY--
KLPRHY--
KTPRHY--
-KPRHYV-
KDPRHY--
-KPRHYE-
-KPRHYL-
-KPRHYT-

-KPRHYM-
-KPRHYY-
-KPRHYF-
KPPRHY--
-KPRHYS-
-KPRHYQ-
-KPRHYI-
-KPRHYA-
KYPRHY--
KIPRHY--
KAPRHY--
-KPRHYW-
KCPRHY--
-KPRHYN-
-KPRHYH-
KKPRHY--
LSPRHY--
LNPRHY--
LDPRHY--
LGPRHY--
-LPRHYI-
LMPRHY--
LPPRHY--
LAPRHY--
-LPRHYV-
-LPRHYQ-
-LPRHYF-
LEPRHY--
-LPRHYM-
-LPRHYY-
-LPRHYK-
-LPRHYW-
-LPRHYE-
-LPRHYH-
LCPRHY--
MAPRHY--
MGPRHY--
-MPRHYT-
MTPRHY--
MEPRHY--
MKPRHY--
MYPRHY--
MPPRHY--
-MPRHYL-
MHPRHY--
MFPRHY--
-MPRHYS-
-MPRHYQ-
MRPRHY--
-MPRHYV-

-MPRHYA-
MQPRHY--
-MPRHYI-
-MPRHYF-
MIPRHY--
MVPRHY--
MSPRHY--
-MPRHYH-
-MPRHYE-
MCPRHY--
NHPRHY--
NVPRHY--
NKPRHY--
-NPRHYM-
-NPRHYR-
NEPRHY--
NSPRHY--
NQPRHY--
NAPRHY--
NMPRHY--
NYPRHY--
NNPRHY--
NTPRHY--
-NPRHYI-
NGPRHY--
-NPRHYT-
-NPRHYS-
-NPRHYY-
-NPRHYV-
-NPRHYL-
NPPRHY--
NCPRHY--
-NPRHYW-
--PRHYSS
--PRHYTS
--PRHYRT
--PRHYSW
--PRHYQT
--PRHYTT
--PRHYLA
--PRHYSG
--PRHYRV
--PRHYTA
--PRHYIS
--PRHYQS
--PRHYYA
--PRHYRS
PRPRHY--
--PRHYIQ
--PRHYFS

--PRHYES
--PRHYVA
--PRHYVV
--PRHYSA
--PRHYFK
--PRHYAA
--PRHYYS
--PRHYYW
--PRHYSL
--PRHYLF
--PRHYTL
--PRHYIF
--PRHYLT
--PRHYTY
--PRHYSV
--PRHYMG
--PRHYFV
--PRHYVT
--PRHYRM
--PRHYTI
--PRHYTG
--PRHYYT
--PRHYFG
--PRHYYL
--PRHYWV
--PRHYSK
--PRHYSH
--PRHYFN
--PRHYWY
--PRHYTN
--PRHYMV
--PRHYLY
--PRHYEY
--PRHYYG
--PRHYVI
--PRHYTQ
--PRHYST
--PRHYSN
--PRHYRN
--PRHYNS
--PRHYMF
--PRHYLN
--PRHYMT
--PRHYIR
--PRHYHA
--PRHYAG
--PRHYYQ
--PRHYLV
--PRHYLH
--PRHYTH

--PRHYSI
--PRHYLM
--PRHYVM
--PRHYLS
--PRHYIV
--PRHYHY
--PRHYFA
--PRHYWS
--PRHYWF
--PRHYQA
--PRHYIG
--PRHYAL
--PRHYAF
PVPRHY--
--PRHYMS
--PRHYIY
--PRHYAV
--PRHYYD
--PRHYTV
--PRHYTK
--PRHYNA
--PRHYAS
--PRHYYM
--PRHYWW
--PRHYWN
--PRHYWK
--PRHYIL
--PRHYFH
--PRHYEG
--PRHYAY
PLPRHY--
--PRHYYI
--PRHYVY
--PRHYSQ
--PRHYMQ
--PRHYIH
PSPRHY--
--PRHYWI
--PRHYSF
--PRHYSE
--PRHYQH
--PRHYNL
--PRHYMD
--PRHYKI
--PRHYIK
--PRHYHG
--PRHYFT
--PRHYFL
PNPRHY--
PMPRHY--

--PRHYYV
--PRHYYF
--PRHYWT
--PRHYVH
--PRHYTM
--PRHYNY
--PRHYNM
--PRHYMH
--PRHYKL
--PRHYIN
--PRHYHT
--PRHYHQ
--PRHYHF
--PRHYGT
--PRHYEV
--PRHYER
--PRHYAW
--PRHYWA
--PRHYVN
--PRHYVF
--PRHYTF
--PRHYSY
--PRHYRI
--PRHYQV
--PRHYNH
--PRHYMN
--PRHYKQ
--PRHYEM
PCPRHY--
--PRHYWM
--PRHYNW
--PRHYNV
--PRHYNQ
--PRHYMY
--PRHYKH
--PRHYGL
--PRHYFY
--PRHYEK
--PRHYEI
--PRHYEF
--PRHYEA
--PRHYYK
--PRHYWH
--PRHYVK
--PRHYTE
--PRHYSD
--PRHYRE
--PRHYQY
--PRHYQL
--PRHYLE

--PRHYKT
--PRHYID
--PRHYGY
--PRHYAQ
PGPRHY--
PTPRHY--
--PRHYYN
--PRHYVQ
--PRHYTW
--PRHYTD
--PRHYQM
--PRHYQK
--PRHYQF
--PRHYME
--PRHYIM
--PRHYII
--PRHYHN
--PRHYFF
--PRHYFE
--PRHYEQ
--PRHYAH
PPPRHY--
--PRHYWR
--PRHYWE
--PRHYQN
--PRHYQI
--PRHYNF
--PRHYMK
--PRHYMA
--PRHYLL
--PRHYKM
--PRHYHM
--PRHYHK
--PRHYHH
--PRHYFW
--PRHYFD
--PRHYEH
--PRHYDG
--PRHYCH
--PRHYAN
--PRHYAM
PKPRHY--
PHPRHY--
--PRHYWD
--PRHYVD
--PRHYRD
--PRHYQW
--PRHYQQ
--PRHYNG
--PRHYMW

--PRHYMM
--PRHYLD
--PRHYKN
--PRHYKE
--PRHYIT
--PRHYIE
--PRHYHV
--PRHYHI
--PRHYGW
--PRHYGV
--PRHYGN
--PRHYGA
--PRHYEW
--PRHYDL
--PRHYCK
--PRHYAI
PQPRHY--
PIPRHY--
PEPRHY--
PDPRHY--
QLPRHY--
-QPRHYL-
QSPRHY--
QGPRHY--
QRPRHY--
-QPRHYQ-
-QPRHYT-
-QPRHYV-
QPPRHY--
QQPRHY--
-QPRHYM-
-QPRHYI-
-QPRHYH-
QAPRHY--
-QPRHYE-
QYPRHY--
QVPRHY--
-QPRHYW-
QDPRHY--
-QPRHYK-
QKPRHY--
-QPRHYY-
-QPRHYF-
QNPRHY--
QMPRHY--
-QPRHYG-
QHPRHY--
QEPRHY--
-RPRHYM-
REPRHY--

−RPRHYI−
−RPRHYT−
RQPRHY−−
−RPRHYR−
RKPRHY−−
RHPRHY−−
−RPRHYH−
RYPRHY−−
RDPRHY−−
−RPRHYS−
−RPRHYY−
−RPRHYE−
RNPRHY−−
RCPRHY−−
−RPRHYW−
−RPRHYG−
RRPRHY−−
−RPRHYK−
SVPRHY−−
SLPRHY−−
STPRHY−−
SRPRHY−−
−SPRHYL−
SGPRHY−−
−SPRHYT−
−SPRHYI−
SIPRHY−−
−SPRHYA−
SKPRHY−−
SQPRHY−−
SEPRHY−−
−SPRHYF−
SNPRHY−−
SHPRHY−−
SAPRHY−−
−SPRHYY−
SPPRHY−−
−SPRHYM−
−SPRHYQ−
−SPRHYE−
SDPRHY−−
−SPRHYV−
−SPRHYW−
−SPRHYH−
−SPRHYK−
SCPRHY−−
TTPRHY−−
TVPRHY−−
−TPRHYM−
−TPRHYS−

TSPRHY--
-TPRHYV-
TNPRHY--
TMPRHY--
-TPRHYF-
TGPRHY--
TKPRHY--
-TPRHYL-
TEPRHY--
TQPRHY--
TAPRHY--
THPRHY--
-TPRHYI-
TPPRHY--
TDPRHY--
-TPRHYW-
-TPRHYA-
TFPRHY--
-TPRHYY-
-TPRHYE-
-TPRHYQ-
-VPRHYS-
VPPRHY--
-VPRHYT-
VTPRHY--
-VPRHYR-
VMPRHY--
VVPRHY--
VIPRHY--
VGPRHY--
-VPRHYV-
VAPRHY--
VHPRHY--
-VPRHYM-
VKPRHY--
-VPRHYI-
VNPRHY--
-VPRHYH-
-VPRHYY-
-VPRHYL-
VEPRHY--
-VPRHYW-
-VPRHYN-
-VPRHYK-
VCPRHY--
-VPRHYA-
VDPRHY--
-VPRHYG-
-VPRHYE-
-VPRHYQ-

-VPRHYF-
WVPRHY--
WMPRHY--
WTPRHY--
-WPRHYS-
-WPRHYT-
-WPRHYI-
WNPRHY--
WGPRHY--
WPPRHY--
-WPRHYF-
WQPRHY--
-WPRHYL-
WLPRHY--
WKPRHY--
WIPRHY--
-WPRHYQ-
WHPRHY--
-WPRHYA-
-WPRHYM-
-WPRHYE-
WSPRHY--
-WPRHYY-
YTPRHY--
-YPRHYT-
YVPRHY--
YIPRHY--
YRPRHY--
YYPRHY--
-YPRHYS-
-YPRHYY-
-YPRHYV-
YAPRHY--
YHPRHY--
-YPRHYL-
-YPRHYE-
YQPRHY--
YPPRHY--
-YPRHYM-
YNPRHY--
YGPRHY--
YFPRHY--
-YPRHYW-
-YPRHYF-
-YPRHYA-
YWPRHY--
-YPRHYH-
YDPRHY--
PSGM 29 870 44.87
-DPSGMN-

DKPSGM--
-FPSGMG-
-LPSGMG-
-LPSGMA-
-NPSGMA-
--PSGMGM
--PSGMGF
--PSGMDS
-RPSGMP-
-RPSGMD-
-SPSGMG-
-TPSGMG-
ARPSGM--
-APSGMR-
AAPSGM--
-APSGMS-
-APSGMK-
AIPSGM--
-APSGMV-
-APSGML-
-APSGMT-
-APSGMH-
-APSGMY-
AYPSGM--
AWPSGM--
AVPSGM--
-APSGMN-
ALPSGM--
AKPSGM--
ASPSGM--
AEPSGM--
ANPSGM--
ATPSGM--
-APSGMI-
-APSGME-
AFPSGM--
-APSGMM-
APPSGM--
AHPSGM--
-APSGMW-
AGPSGM--
AQPSGM--
-APSGMQ-
-APSGMG-
-APSGMF-
AMPSGM--
ADPSGM--
-CPSGMR-
CRPSGM--
CIPSGM--

CSPSGM--
CKPSGM--
CCPSGM--
CGPSGM--
CTPSGM--
-CPSGMI-
CHPSGM--
-CPSGMH-
-CPSGMT-
CPPSGM--
CQPSGM--
-CPSGMW-
-CPSGMV-
-CPSGMN-
-CPSGML-
-CPSGMK-
CMPSGM--
CLPSGM--
CEPSGM--
DRPSGM--
DEPSGM--
-DPSGMR-
DSPSGM--
DFPSGM--
-DPSGML-
DIPSGM--
DVPSGM--
DGPSGM--
DTPSGM--
-DPSGMM-
-DPSGMT-
-DPSGMK-
DLPSGM--
DWPSGM--
-DPSGMY-
-DPSGMH-
-DPSGMF-
DMPSGM--
DYPSGM--
-DPSGMW-
-DPSGMV-
-DPSGMS-
-DPSGMI-
DHPSGM--
-EPSGMK-
-EPSGMR-
-EPSGMT-
ERPSGM--
-EPSGML-
-EPSGMI-

-EPSGMH-
ELPSGM--
-EPSGMS-
ENPSGM--
EIPSGM--
EHPSGM--
EFPSGM--
EAPSGM--
ETPSGM--
ESPSGM--
EQPSGM--
-EPSGME-
EPPSGM--
EVPSGM--
-EPSGMQ-
EGPSGM--
-EPSGMY-
-EPSGMA-
EWPSGM--
EKPSGM--
-EPSGMW-
-EPSGMD-
EEPSGM--
FVPSGM--
FSPSGM--
FQPSGM--
FRPSGM--
-FPSGMV-
FLPSGM--
FYPSGM--
-FPSGMK-
-FPSGML-
FWPSGM--
FKPSGM--
FGPSGM--
FNPSGM--
FMPSGM--
-FPSGMY-
-FPSGMI-
-FPSGMF-
-FPSGMT-
FPPSGM--
-FPSGMW-
-FPSGMN-
-FPSGMM-
-FPSGMH-
-FPSGMA-
FIPSGM--
FTPSGM--
FDPSGM--

FCPSGM--
-GPSGMR-
GAPSGM--
-GPSGMH-
-GPSGMW-
GGPSGM--
-GPSGMV-
GLPSGM--
GYPSGM--
-GPSGMK-
-GPSGMT-
GSPSGM--
-GPSGMM-
-GPSGMY-
-GPSGMF-
GFPSGM--
GVPSGM--
-GPSGML-
-GPSGMS-
GMPSGM--
GNPSGM--
-GPSGMN-
GPPSGM--
GHPSGM--
GKPSGM--
GTPSGM--
-GPSGMI-
-GPSGMA-
-GPSGMQ-
GCPSGM--
GWPSGM--
GIPSGM--
GDPSGM--
GQPSGM--
-GPSGMG-
-GPSGME-
-GPSGMC-
GEPSGM--
HMPSGM--
HIPSGM--
HVPSGM--
HWPSGM--
HTPSGM--
-HPSGMR-
HRPSGM--
HSPSGM--
-HPSGMV-
-HPSGMW-
HGPSGM--
HNPSGM--

-HPSGMS-
-HPSGML-
-HPSGMY-
-HPSGMT-
HEPSGM--
-HPSGMN-
HLPSGM--
HPPSGM--
HFPSGM--
-HPSGMF-
-HPSGMK-
-HPSGMQ-
-HPSGMI-
-HPSGMA-
HKPSGM--
HYPSGM--
-HPSGMH-
HHPSGM--
HAPSGM--
IRPSGM--
-IPSGMR-
-IPSGML-
-IPSGMT-
ISPSGM--
IPPSGM--
-IPSGMM-
ILPSGM--
-IPSGMS-
IQPSGM--
-IPSGMV-
IIPSGM--
IHPSGM--
-IPSGMY-
IGPSGM--
IFPSGM--
IAPSGM--
-IPSGMF-
IKPSGM--
IVPSGM--
INPSGM--
-IPSGMQ-
-IPSGMH-
-IPSGMA-
IDPSGM--
IWPSGM--
ITPSGM--
-IPSGMW-
-IPSGMK-
-IPSGMI-
-IPSGMG-

KSPSGM--
-KPSGMS-
-KPSGMR-
-KPSGMK-
-KPSGML-
KRPSGM--
KGPSGM--
-KPSGMW-
-KPSGMH-
-KPSGMN-
-KPSGMF-
KLPSGM--
KTPSGM--
KMPSGM--
KVPSGM--
KIPSGM--
KYPSGM--
KWPSGM--
-KPSGMT-
KHPSGM--
-KPSGMM-
KFPSGM--
-KPSGMY-
KKPSGM--
KEPSGM--
KAPSGM--
-KPSGMQ-
-KPSGMA-
-KPSGME-
KPPSGM--
KQPSGM--
-KPSGMG-
KDPSGM--
LRPSGM--
LSPSGM--
LAPSGM--
-LPSGMR-
-LPSGMH-
-LPSGMM-
LHPSGM--
LGPSGM--
LKPSGM--
LPPSGM--
-LPSGMY-
-LPSGMK-
LDPSGM--
LQPSGM--
LMPSGM--
LYPSGM--
LNPSGM--

LVPSGM--
LLPSGM--
LWPSGM--
-LPSGMV-
-LPSGMN-
LTPSGM--
-LPSGMS-
-LPSGMT-
-LPSGMW-
-LPSGMF-
LCPSGM--
-LPSGMQ-
-LPSGMI-
LEPSGM--
MLPSGM--
-MPSGMH-
-MPSGMY-
-MPSGMR-
MFPSGM--
MSPSGM--
-MPSGML-
-MPSGMF-
MIPSGM--
MHPSGM--
-MPSGMT-
MAPSGM--
-MPSGMW-
MRPSGM--
MMPSGM--
MQPSGM--
-MPSGMS-
-MPSGMI-
MPPSGM--
MYPSGM--
-MPSGMQ-
MNPSGM--
-MPSGMN-
MKPSGM--
-MPSGMV-
MGPSGM--
MEPSGM--
MVPSGM--
-MPSGMA-
-MPSGMM-
-MPSGMK-
MWPSGM--
MDPSGM--
-MPSGME-
-MPSGMD-
NGPSGM--

NSPSGM--
-NPSGMR-
NRPSGM--
NLPSGM--
-NPSGML-
-NPSGMH-
NWPSGM--
NVPSGM--
-NPSGMY-
-NPSGMW-
NAPSGM--
NQPSGM--
NHPSGM--
-NPSGMM-
-NPSGMF-
NIPSGM--
NTPSGM--
NMPSGM--
-NPSGMV-
-NPSGMS-
-NPSGMT-
NFPSGM--
-NPSGMK-
NPPSGM--
-NPSGMI-
NEPSGM--
NYPSGM--
-NPSGMN-
-NPSGMD-
--PSGMRT
--PSGMLA
--PSGMRY
--PSGMAA
--PSGMLG
--PSGMRW
--PSGMSV
--PSGMRV
--PSGMYS
--PSGMST
--PSGMLS
--PSGMIH
--PSGMTW
--PSGMRI
--PSGMMA
--PSGMLT
--PSGMKA
--PSGMLW
PSPSGM--
--PSGMVS
--PSGMTV

--PSGMSS
--PSGMTS
--PSGMSR
--PSGMVG
--PSGMQS
--PSGMLQ
--PSGMHS
--PSGMYR
--PSGMWK
--PSGMSW
--PSGMRQ
--PSGMMS
--PSGMVM
--PSGMFT
--PSGMFR
--PSGMFA
--PSGMLM
--PSGMMW
PWPSGM--
--PSGMWV
--PSGMSG
--PSGMSA
--PSGMWL
--PSGMVY
--PSGMIR
--PSGMYL
--PSGMYT
--PSGMWI
--PSGMLR
--PSGMHV
--PSGMYW
--PSGMVF
--PSGMRK
--PSGMRH
--PSGMMG
--PSGMLF
--PSGMTF
--PSGMRR
--PSGMRN
--PSGMLV
--PSGMIN
--PSGMHT
--PSGMFH
--PSGMAS
PIPSGM--
--PSGMHM
PVPSGM--
--PSGMYV
--PSGMWG
--PSGMSF

--PSGMQT
--PSGMIV
--PSGMVR
--PSGMSY
--PSGMNS
--PSGMMV
--PSGMIS
--PSGMHL
--PSGMGS
--PSGMEA
--PSGMVT
--PSGMVH
--PSGMSL
--PSGMKW
--PSGMES
--PSGMTY
--PSGMMR
--PSGMLH
PRPSGM--
--PSGMQW
--PSGMIF
--PSGMHG
--PSGMFS
--PSGMFQ
PFPSGM--
--PSGMYM
--PSGMQG
--PSGMLY
--PSGMIW
--PSGMFN
--PSGMYH
--PSGMHK
--PSGMFL
--PSGMAT
PMPSGM--
PGPSGM--
--PSGMWW
--PSGMSN
--PSGMQA
--PSGMMT
--PSGMKT
--PSGMKG
--PSGMYI
--PSGMYE
--PSGMVQ
--PSGMSK
--PSGMSI
--PSGMRM
--PSGMLK
--PSGMLI

--PSGMHQ
PQPSGM--
PNPSGM--
--PSGMYK
--PSGMYA
--PSGMWH
--PSGMWE
--PSGMTT
--PSGMTR
--PSGMTH
--PSGMRE
--PSGMNG
--PSGMYG
--PSGMWN
--PSGMNW
--PSGMNA
--PSGMMY
--PSGMMQ
--PSGMMN
--PSGMIG
--PSGMFI
PTPSGM--
--PSGMYN
--PSGMVN
--PSGMTN
--PSGMTL
--PSGMTK
--PSGMTI
--PSGMLN
--PSGMKK
--PSGMIK
--PSGMDG
--PSGMQV
--PSGMNY
--PSGMGA
--PSGMMF
--PSGMKV
--PSGMAV
--PSGMYQ
--PSGMWM
--PSGMVW
--PSGMQN
--PSGMNT
--PSGMHH
--PSGMHA
--PSGMFM
--PSGMQR
--PSGMMI
--PSGMME
--PSGMLE

--PSGMKY
--PSGMIQ
--PSGMGG
--PSGMCS
PEPSGM--
PAPSGM--
--PSGMWT
--PSGMVA
--PSGMSH
--PSGMQK
--PSGMQF
--PSGMNV
--PSGMNQ
--PSGMNI
--PSGMMK
--PSGMID
--PSGMHN
--PSGMGL
--PSGMFE
--PSGMEG
--PSGMAG
--PSGMQQ
--PSGMLD
--PSGMKN
--PSGMKH
--PSGMIY
--PSGMIL
--PSGMII
--PSGMGT
--PSGMEW
--PSGMDA
PLPSGM--
--PSGMWD
--PSGMVD
--PSGMTM
--PSGMQY
--PSGMQI
--PSGMML
--PSGMKQ
--PSGMKL
--PSGMHR
--PSGMHI
--PSGMGV
--PSGMEV
--PSGMAY
--PSGMAN
--PSGMAH
PKPSGM--
PYPSGM--
--PSGMWQ

--PSGMVE
--PSGMTG
--PSGMSD
--PSGMQL
--PSGMQH
--PSGMNR
--PSGMNL
--PSGMMH
--PSGMKD
--PSGMIE
--PSGMHY
--PSGMHW
--PSGMFK
--PSGMFD
--PSGMAR
--PSGMWA
--PSGMSM
--PSGMQE
--PSGMQD
--PSGMPD
--PSGMNM
--PSGMNC
--PSGMMM
--PSGMMD
--PSGMLC
--PSGMIA
--PSGMHC
--PSGMGW
--PSGMFC
--PSGMET
--PSGMER
--PSGMEQ
--PSGMEF
--PSGMDK
--PSGMCW
--PSGMCV
--PSGMCR
--PSGMAM
--PSGMAL
-PPSGMR-
-PPSGMN-
-QPSGMR-
QTPSGM--
-QPSGML-
QNPSGM--
QIPSGM--
-QPSGMT-
QKPSGM--
-QPSGMM-
-QPSGMS-

QSPSGM--
QMPSGM--
-QPSGMY-
-QPSGMV-
QLPSGM--
-QPSGMH-
QYPSGM--
QVPSGM--
-QPSGMW-
-QPSGMN-
-QPSGMK-
QHPSGM--
QAPSGM--
QWPSGM--
QRPSGM--
-QPSGMF-
QGPSGM--
QFPSGM--
QDPSGM--
-QPSGMD-
-QPSGMA-
RSPSGM--
-RPSGML-
-RPSGMV-
RLPSGM--
RGPSGM--
-RPSGMY-
-RPSGMN-
RVPSGM--
-RPSGMM-
RRPSGM--
-RPSGMH-
-RPSGMK-
RFPSGM--
-RPSGMF-
RWPSGM--
RNPSGM--
-RPSGMA-
RTPSGM--
RHPSGM--
RYPSGM--
REPSGM--
RAPSGM--
RDPSGM--
RQPSGM--
RPPSGM--
-RPSGMW-
-RPSGME-
RKPSGM--
RIPSGM--

-RPSGMT-
-RPSGMG-
-RPSGMQ-
-RPSGMC-
RCPSGM--
-SPSGMR-
SRPSGM--
-SPSGMS-
-SPSGMW-
SYPSGM--
STPSGM--
SSPSGM--
SFPSGM--
SMPSGM--
SLPSGM--
-SPSGMM-
-SPSGMV-
SNPSGM--
SVPSGM--
-SPSGMN-
SGPSGM--
SAPSGM--
-SPSGMT-
-SPSGMQ-
-SPSGMI-
-SPSGMY-
SIPSGM--
-SPSGMF-
-SPSGMH-
SHPSGM--
SEPSGM--
-SPSGMK-
SKPSGM--
SWPSGM--
-SPSGMA-
SQPSGM--
SPPSGM--
SDPSGM--
-TPSGMR-
TRPSGM--
TVPSGM--
-TPSGMF-
TGPSGM--
TQPSGM--
-TPSGML-
TLPSGM--
TKPSGM--
-TPSGMK-
TAPSGM--
-TPSGMS-

-TPSGMT-
-TPSGMH-
-TPSGMY-
TYPSGM--
-TPSGMM-
-TPSGMI-
TIPSGM--
TWPSGM--
TTPSGM--
-TPSGMW-
-TPSGMQ-
-TPSGMV-
-TPSGMN-
TPPSGM--
TEPSGM--
TMPSGM--
TFPSGM--
TDPSGM--
-TPSGMA-
-VPSGMR-
VRPSGM--
VSPSGM--
-VPSGMS-
-VPSGMY-
VTPSGM--
-VPSGMI-
VKPSGM--
-VPSGMT-
VEPSGM--
VFPSGM--
VNPSGM--
VIPSGM--
-VPSGMM-
VHPSGM--
VLPSGM--
VYPSGM--
VMPSGM--
-VPSGMW-
VGPSGM--
-VPSGMA-
VDPSGM--
-VPSGME-
-VPSGMQ-
-VPSGMN-
VQPSGM--
VPPSGM--
-VPSGMH-
-VPSGMF-
VWPSGM--
-VPSGMK-

-VPSGMG-
VAPSGM--
WVPSGM--
WRPSGM--
WSPSGM--
-WPSGMR-
WIPSGM--
WKPSGM--
WFPSGM--
-WPSGMY-
WLPSGM--
-WPSGMT-
-WPSGMW-
WAPSGM--
-WPSGMI-
WNPSGM--
-WPSGML-
WTPSGM--
-WPSGMS-
-WPSGMM-
-WPSGMH-
-WPSGMK-
WEPSGM--
WWPSGM--
-WPSGMV-
WHPSGM--
WCPSGM--
WQPSGM--
WGPSGM--
WYPSGM--
-WPSGMQ-
-WPSGMF-
WPPSGM--
-WPSGMA-
WDPSGM--
YVPSGM--
YRPSGM--
-YPSGMV-
-YPSGMM-
YSPSGM--
YFPSGM--
-YPSGMK-
YKPSGM--
YGPSGM--
-YPSGMT-
-YPSGML-
YTPSGM--
-YPSGMR-
-YPSGMH-
-YPSGMW-

YPPSGM--
YMPSGM--
-YPSGMN-
YHPSGM--
YLPSGM--
-YPSGMY-
-YPSGMS-
-YPSGMI-
YIPSGM--
YNPSGM--
YYPSGM--
YWPSGM--
-YPSGMQ-
-YPSGMA-
YCPSGM--
YDPSGM--
YQPSGM--
-YPSGMF-
-YPSGMC-

VRWM	457	635	44.85

-DVRWMG-
-GVRWME-
NKVRWM--
-SVRWMP-
-VVRWMP-
--VRWMSP
--VRWMNN
AMVRWM--
AGVRWM--
-AVRWMT-
-AVRWMS-
ASVRWM--
-AVRWML-
ANVRWM--
-AVRWMK-
APVRWM--
AWVRWM--
AQVRWM--
-AVRWMM-
-AVRWMA-
AAVRWM--
-AVRWMV-
-AVRWMQ-
AEVRWM--
AYVRWM--
-AVRWMH-
-AVRWMN-
-AVRWMI-
ADVRWM--
AIVRWM--

AFVRWM--
ALVRWM--
-AVRWMY-
-AVRWMF-
-AVRWME-
-AVRWMD-
ACVRWM--
CSVRWM--
-CVRWMR-
-CVRWMQ-
CIVRWM--
CDVRWM--
CVVRWM--
-CVRWMT-
CTVRWM--
CGVRWM--
DKVRWM--
DSVRWM--
DGVRWM--
-DVRWMS-
DFVRWM--
-DVRWML-
-DVRWMK-
DTVRWM--
-DVRWMV-
DRVRWM--
DNVRWM--
-DVRWMA-
DDVRWM--
DMVRWM--
DLVRWM--
DIVRWM--
-DVRWMM-
DHVRWM--
DEVRWM--
-DVRWMH-
-DVRWMN-
-DVRWMF-
DCVRWM--
DAVRWM--
EAVRWM--
-EVRWMT-
ETVRWM--
ESVRWM--
-EVRWMK-
EGVRWM--
EVVRWM--
-EVRWMS-
-EVRWMH-
ELVRWM--

-EVRWMM-
-EVRWML-
-EVRWMW-
-EVRWMA-
-EVRWMV-
-EVRWMQ-
-EVRWMG-
-EVRWMF-
EIVRWM--
EFVRWM--
EDVRWM--
EQVRWM--
EMVRWM--
ENVRWM--
EKVRWM--
EHVRWM--
-FVRWMS-
FGVRWM--
FPVRWM--
FEVRWM--
FIVRWM--
-FVRWMQ-
FHVRWM--
FVVRWM--
-FVRWMG-
FAVRWM--
-FVRWMH-
-FVRWMF-
FFVRWM--
-FVRWMW-
-FVRWMM-
FQVRWM--
FKVRWM--
GLVRWM--
-GVRWMQ-
-GVRWMT-
GSVRWM--
GKVRWM--
-GVRWMS-
GVVRWM--
GFVRWM--
-GVRWMA-
-GVRWMG-
GYVRWM--
-GVRWMV-
GIVRWM--
GGVRWM--
GRVRWM--
GPVRWM--
GDVRWM--

GEVRWM--
-GVRWMH-
GAVRWM--
GQVRWM--
GHVRWM--
-GVRWMF-
-GVRWMW-
-GVRWMN-
GNVRWM--
-GVRWMI-
-GVRWMD-
HRVRWM--
HGVRWM--
-HVRWMT-
HLVRWM--
HVVRWM--
-HVRWMA-
HKVRWM--
-HVRWMI-
HEVRWM--
HNVRWM--
HMVRWM--
HFVRWM--
HYVRWM--
-HVRWMQ-
HDVRWM--
-HVRWMV-
-HVRWML-
HTVRWM--
HWVRWM--
HIVRWM--
HHVRWM--
HAVRWM--
-HVRWMN-
-HVRWMY-
-HVRWMW-
-HVRWMM-
-HVRWMF-
HPVRWM--
-IVRWMT-
IQVRWM--
-IVRWMV-
IRVRWM--
IKVRWM--
INVRWM--
IEVRWM--
IDVRWM--
-IVRWMH-
-IVRWMY-
ISVRWM--

-IVRWMG-
IMVRWM--
IVVRWM--
-IVRWMQ-
IPVRWM--
IGVRWM--
-IVRWMD-
-IVRWMA-
IAVRWM--
-IVRWMN-
-KVRWMS-
KNVRWM--
-KVRWMA-
KPVRWM--
-KVRWMG-
KYVRWM--
KVVRWM--
KEVRWM--
KWVRWM--
-KVRWMW-
-KVRWMF-
KTVRWM--
KFVRWM--
-KVRWMQ-
-KVRWME-
KDVRWM--
LNVRWM--
-LVRWMV-
LRVRWM--
-LVRWMI-
-LVRWMG-
LGVRWM--
LHVRWM--
-LVRWMQ-
LDVRWM--
-LVRWMN-
-LVRWMM-
-LVRWMY-
-LVRWMD-
-LVRWME-
LKVRWM--
LIVRWM--
LWVRWM--
MRVRWM--
MIVRWM--
-MVRWMH-
MPVRWM--
MEVRWM--
-MVRWMR-
MSVRWM--

MHVRWM--
-MVRWMA-
-MVRWMV-
MVVRWM--
MGVRWM--
-MVRWMK-
-MVRWMN-
-MVRWMM-
MQVRWM--
MNVRWM--
MAVRWM--
-MVRWMG-
MKVRWM--
MWVRWM--
-MVRWME-
-MVRWMQ-
-MVRWMY-
MDVRWM--
-MVRWMF-
-NVRWMS-
-NVRWMV-
NRVRWM--
NSVRWM--
-NVRWMT-
-NVRWMG-
NPVRWM--
NAVRWM--
-NVRWMW-
-NVRWMK-
-NVRWMA-
NLVRWM--
NGVRWM--
-NVRWMF-
NWVRWM--
-NVRWMY-
-NVRWMH-
NQVRWM--
-NVRWMN-
NMVRWM--
NIVRWM--
NHVRWM--
-NVRWME-
NFVRWM--
PGVRWM--
-PVRWMK-
PRVRWM--
-PVRWMA-
-PVRWMV-
-PVRWMS-
PTVRWM--

PSVRWM--
-PVRWMN-
PMVRWM--
-PVRWMQ-
PAVRWM--
PNVRWM--
-PVRWMM-
PLVRWM--
-PVRWMY-
-PVRWMI-
-PVRWMG-
PHVRWM--
-PVRWME-
PDVRWM--
PPVRWM--
PKVRWM--
PEVRWM--
QTVRWM--
QSVRWM--
QHVRWM--
QFVRWM--
-QVRWMR-
-QVRWMV-
QKVRWM--
QPVRWM--
-QVRWMF-
-QVRWMI-
-QVRWMG-
-QVRWMA-
QRVRWM--
-QVRWMY-
-QVRWMQ-
QWVRWM--
QVVRWM--
-QVRWMK-
-QVRWMH-
-QVRWMD-
-QVRWMM-
QMVRWM--
-QVRWMN-
QNVRWM--
QAVRWM--
QEVRWM--
-RVRWMI-
RDVRWM--
-RVRWMS-
-RVRWMA-
-RVRWMT-
REVRWM--
RWVRWM--

RPVRWM--
-RVRWMM-
-RVRWMF-
-RVRWMQ-
-RVRWMN-
-RVRWMW-
-RVRWMD-
RCVRWM--
-SVRWMT-
SSVRWM--
SGVRWM--
-SVRWML-
SPVRWM--
-SVRWMI-
SKVRWM--
SMVRWM--
SWVRWM--
-SVRWMA-
SVVRWM--
SAVRWM--
-SVRWMY-
SQVRWM--
-SVRWMV-
-SVRWMM-
SIVRWM--
SNVRWM--
-SVRWMQ-
SDVRWM--
-SVRWME-
SEVRWM--
-TVRWMS-
-TVRWMR-
TEVRWM--
TPVRWM--
TGVRWM--
TVVRWM--
-TVRWML-
-TVRWMV-
TTVRWM--
-TVRWMK-
TMVRWM--
TQVRWM--
TDVRWM--
THVRWM--
-TVRWMN-
-TVRWMF-
TNVRWM--
TYVRWM--
-TVRWMM-
-TVRWMQ-

```
-TVRWMG-
-TVRWMY-
-TVRWMH-
-TVRWMA-
TIVRWM--
-TVRWMD-
-TVRWMI-
--VRWMST
--VRWMSG
--VRWMLG
VRVRWM--
--VRWMLA
--VRWMVG
--VRWMSV
--VRWMVS
--VRWMGS
VKVRWM--
--VRWMVN
--VRWMQT
VHVRWM--
--VRWMMA
--VRWMKG
--VRWMSA
--VRWMGT
--VRWMAS
--VRWMFN
--VRWMGL
VNVRWM--
--VRWMYS
--VRWMQG
--VRWMHA
VQVRWM--
--VRWMTG
--VRWMHT
--VRWMAL
--VRWMSH
-VVRWMS-
VGVRWM--
--VRWMTS
--VRWMGG
--VRWMTN
--VRWMAT
--VRWMAN
--VRWMAM
VEVRWM--
VAVRWM--
VVVRWM--
--VRWMYG
--VRWMSN
--VRWMQS
```

--VRWMHS
VPVRWM--
VSVRWM--
--VRWMGA
--VRWMTV
--VRWMAI
--VRWMYV
--VRWMSR
--VRWMQV
--VRWMNS
--VRWMMV
--VRWMMG
--VRWMFS
--VRWMDS
--VRWMVV
--VRWMTT
--VRWMRE
--VRWMMW
-VVRWML-
--VRWMKV
--VRWMIS
--VRWMFG
--VRWMWG
--VRWMVD
--VRWMQM
--VRWMES
--VRWMYK
--VRWMTK
--VRWMSY
--VRWMQR
--VRWMQH
--VRWMIK
--VRWMGY
--VRWMFT
--VRWMAV
-VVRWMV-
-VVRWMH-
--VRWMRH
--VRWMIT
--VRWMIG
--VRWMYM
--VRWMYH
--VRWMVI
--VRWMSQ
--VRWMSK
--VRWMMQ
--VRWMMM
VMVRWM--
--VRWMYA
--VRWMWS

--VRWMYQ
--VRWMVW
--VRWMVQ
--VRWMVH
--VRWMRY
--VRWMRW
--VRWMLQ
--VRWMFQ
VDVRWM--
--VRWMYR
--VRWMTM
--VRWMQW
--VRWMGM
--VRWMEG
--VRWMIN
--VRWMHM
--VRWMDT
--VRWMVY
--VRWMSE
--VRWMMN
--VRWMME
--VRWMIE
--VRWMHV
--VRWMGN
--VRWMWN
--VRWMTQ
--VRWMQK
--VRWMMT
--VRWMKI
--VRWMHY
--VRWMYD
--VRWMWA
--VRWMTY
--VRWMQD
--VRWMNW
--VRWMNH
--VRWMMY
--VRWMHG
--VRWMEV
--VRWMAA
-VVRWMN-
-VVRWMA-
--VRWMNG
--VRWMKW
--VRWMIV
--VRWMHN
--VRWMGH
-VVRWMM-
-VVRWME-
--VRWMWM

--VRWMTE
--VRWMQY
--VRWMNA
--VRWMKD
--VRWMHI
--VRWMHH
--VRWMFD
--VRWMEN
--VRWMAW
--VRWMAH
--VRWMAF
--VRWMWQ
--VRWMTW
--VRWMTI
--VRWMTD
--VRWMQN
--VRWMHK
--VRWMHF
--VRWMGK
--VRWMEA
-VVRWMY-
-VVRWMW-
-VVRWMQ-
VTVRWM--
--VRWMWF
--VRWMQF
--VRWMNY
--VRWMMI
--VRWMMF
--VRWMKQ
--VRWMIA
--VRWMHD
--VRWMFF
--VRWMEE
--VRWMDG
--VRWMDA
-VVRWMI-
--VRWMYW
--VRWMWH
--VRWMQE
--VRWMPS
--VRWMNK
--VRWMIH
--VRWMHR
--VRWMHQ
--VRWMGF
--VRWMFH
--VRWMFC
--VRWMER
--VRWMCR

```
--VRWMCG
--VRWMAE
--VRWMAC
WSVRWM--
WNVRWM--
WDVRWM--
-WVRWMR-
WRVRWM--
WMVRWM--
-WVRWMA-
-WVRWMT-
WAVRWM--
WQVRWM--
WPVRWM--
-WVRWME-
WVVRWM--
-WVRWMK-
WTVRWM--
WKVRWM--
-WVRWMG-
WEVRWM--
-WVRWMY-
-WVRWMW-
WIVRWM--
WGVRWM--
-WVRWMN-
-WVRWMH-
-WVRWMF-
-WVRWMD-
YRVRWM--
YSVRWM--
-YVRWMS-
YGVRWM--
YPVRWM--
-YVRWMK-
YWVRWM--
YVVRWM--
YTVRWM--
YAVRWM--
-YVRWMV-
-YVRWML-
YQVRWM--
-YVRWMG-
-YVRWMH-
YYVRWM--
YDVRWM--
-YVRWMQ-
YEVRWM--
-YVRWMT-
-YVRWMD-
```

	-YVRWMM-		
	YHVRWM--		
	-YVRWMI-		
	YNVRWM--		
	YMVRWM--		
PMMM	1184	460	44.84
	AGPMMM--		
	AAPMMM--		
	-APMMMI-		
	-APMMML-		
	ASPMMM--		
	-APMMMS-		
	-APMMMR-		
	ATPMMM--		
	-APMMMQ-		
	AFPMMM--		
	APPMMM--		
	-APMMMV-		
	-APMMMM-		
	-APMMMG-		
	-APMMMA-		
	AIPMMM--		
	CRPMMM--		
	CNPMMM--		
	CLPMMM--		
	DRPMMM--		
	DEPMMM--		
	-DPMMMT-		
	DVPMMM--		
	DKPMMM--		
	-DPMMMS-		
	DLPMMM--		
	-DPMMMV-		
	-DPMMMI-		
	DWPMMM--		
	-DPMMMQ-		
	-DPMMMH-		
	-EPMMML-		
	EIPMMM--		
	ESPMMM--		
	EGPMMM--		
	-EPMMMA-		
	ELPMMM--		
	EFPMMM--		
	-EPMMMV-		
	-EPMMMT-		
	-EPMMMK-		
	EVPMMM--		
	-FPMMMR-		
	FRPMMM--		

FGPMMM--
FMPMMM--
FSPMMM--
-FPMMMV-
-FPMMMQ-
FLPMMM--
-FPMMMH-
-FPMMMT-
-FPMMMN-
FKPMMM--
-FPMMMC-
FFPMMM--
-GPMMMI-
GGPMMM--
-GPMMMK-
GRPMMM--
GQPMMM--
GDPMMM--
-GPMMMR-
-GPMMML-
-GPMMMH-
GWPMMM--
GMPMMM--
GHPMMM--
GKPMMM--
GYPMMM--
-GPMMMS-
-GPMMMM-
-GPMMMA-
GSPMMM--
-GPMMMV-
-GPMMMT-
-GPMMMQ-
HIPMMM--
-HPMMMW-
-HPMMML-
HSPMMM--
HLPMMM--
-HPMMMH-
HMPMMM--
-HPMMMY-
-HPMMMS-
-HPMMMQ-
-HPMMMN-
HKPMMM--
HNPMMM--
HFPMMM--
HEPMMM--
HTPMMM--
-HPMMMT-

HAPMMM--
HYPMMM--
HVPMMM--
-HPMMMV-
-HPMMME-
HGPMMM--
-IPMMMR-
IGPMMM--
ITPMMM--
ISPMMM--
-IPMMMK-
-IPMMMS-
IYPMMM--
IVPMMM--
IFPMMM--
-IPMMMV-
IRPMMM--
IIPMMM--
IAPMMM--
-IPMMMH-
IMPMMM--
-IPMMMT-
-IPMMML-
-IPMMMI-
-IPMMMG-
-IPMMME-
ILPMMM--
KMPMMM--
KIPMMM--
-KPMMMR-
KRPMMM--
KAPMMM--
-KPMMML-
KLPMMM--
KWPMMM--
-KPMMMV-
-KPMMMS-
KTPMMM--
KSPMMM--
-KPMMMM-
-KPMMMK-
-KPMMMI-
KVPMMM--
KPPMMM--
-KPMMMT-
-KPMMMG-
-KPMMMY-
-KPMMMN-
-KPMMMH-
-KPMMME-

KKPMMM--
LNPMMM--
LLPMMM--
-LPMMMT-
LRPMMM--
LEPMMM--
LWPMMM--
-LPMMMH-
LYPMMM--
LSPMMM--
LPPMMM--
-LPMMMA-
-LPMMMF-
LIPMMM--
LHPMMM--
-LPMMML-
-LPMMMQ-
-LPMMMM-
LGPMMM--
LFPMMM--
LDPMMM--
LKPMMM--
LAPMMM--
-MPMMML-
-MPMMMR-
MGPMMM--
MDPMMM--
-MPMMMH-
-MPMMMK-
MVPMMM--
MHPMMM--
MRPMMM--
MNPMMM--
MSPMMM--
-MPMMMQ-
MLPMMM--
-NPMMMT-
-NPMMMK-
NLPMMM--
NRPMMM--
-NPMMMM-
NIPMMM--
NGPMMM--
NQPMMM--
-NPMMMY-
NEPMMM--
NPPMMM--
-NPMMMS-
-NPMMMR-
-NPMMMF-

NTPMMM--
-NPMMMV-
NAPMMM--
--PMMMRG
--PMMMWS
--PMMMSS
--PMMMSA
--PMMMMR
--PMMMWA
--PMMMRR
--PMMMTG
--PMMMQG
--PMMMMS
--PMMMSV
--PMMMNS
--PMMMNG
--PMMMMG
--PMMMSN
--PMMMRT
--PMMMRK
--PMMMLG
--PMMMNL
--PMMMKL
--PMMMHV
--PMMMVH
--PMMMRL
--PMMMNV
--PMMMME
--PMMMEG
--PMMMYF
--PMMMVV
--PMMMTR
--PMMMSW
--PMMMRY
--PMMMRN
--PMMMQH
--PMMMMK
--PMMMKV
--PMMMHG
--PMMMFV
--PMMMAA
--PMMMYT
--PMMMYS
--PMMMYR
--PMMMWK
--PMMMTS
--PMMMML
--PMMMKS
--PMMMKH
--PMMMIG

--PMMMIA
--PMMMYQ
--PMMMYG
--PMMMTM
--PMMMRM
--PMMMNA
--PMMMMN
--PMMMLD
--PMMMIT
PEPMMM--
--PMMMVE
--PMMMSR
--PMMMSG
--PMMMRQ
--PMMMRE
--PMMMQK
--PMMMQA
--PMMMNT
--PMMMKT
--PMMMIK
--PMMMHS
--PMMMHQ
--PMMMAS
PQPMMM--
--PMMMVT
--PMMMVM
--PMMMVK
--PMMMVI
--PMMMTL
--PMMMTI
--PMMMTF
--PMMMTE
--PMMMSM
--PMMMSD
--PMMMRW
--PMMMRF
--PMMMQW
--PMMMQR
--PMMMQI
--PMMMMH
--PMMMHW
--PMMMHR
--PMMMGG
--PMMMAV
PGPMMM--
-PPMMMY-
-PPMMMT-
-PPMMMD-
--PMMMYV
--PMMMYK

--PMMMYE
--PMMMVS
--PMMMVD
--PMMMTN
--PMMMTK
--PMMMTD
--PMMMTA
--PMMMSL
--PMMMRH
--PMMMRA
--PMMMQQ
--PMMMNN
--PMMMNK
--PMMMMA
--PMMMLK
--PMMMKQ
--PMMMKM
--PMMMHI
--PMMMHE
--PMMMHD
--PMMMFS
--PMMMFG
--PMMMFE
--PMMMEY
--PMMMEI
--PMMMAL
--PMMMAI
QSPMMM--
-QPMMMS-
-QPMMMR-
-QPMMMW-
-QPMMMN-
-QPMMMA-
QRPMMM--
-QPMMMT-
-QPMMMK-
-QPMMMQ-
QGPMMM--
QFPMMM--
QCPMMM--
RRPMMM--
RPPMMM--
RGPMMM--
RVPMMM--
RLPMMM--
RFPMMM--
-RPMMMM-
RSPMMM--
-RPMMMS-
RKPMMM--

RYPMMM--
RQPMMM--
-RPMMMW-
RTPMMM--
REPMMM--
-RPMMMI-
-RPMMMF-
-RPMMMT-
-RPMMMV-
-RPMMMQ-
RWPMMM--
RMPMMM--
RHPMMM--
-RPMMMN-
-RPMMMG-
-RPMMMA-
RNPMMM--
RDPMMM--
-RPMMMK-
-SPMMMR-
SRPMMM--
-SPMMMW-
SSPMMM--
SQPMMM--
-SPMMMS-
SNPMMM--
SWPMMM--
-SPMMMK-
-SPMMMY-
SHPMMM--
-SPMMMM-
SFPMMM--
-SPMMMI-
-SPMMMT-
SYPMMM--
SGPMMM--
SEPMMM--
-SPMMMQ-
-SPMMMH-
SIPMMM--
SKPMMM--
SVPMMM--
STPMMM--
SPPMMM--
-SPMMMV-
TVPMMM--
-TPMMMR-
TLPMMM--
TRPMMM--
-TPMMMQ-

TAPMMM--
-TPMMMY-
-TPMMMM-
-TPMMMV-
-TPMMMI-
-TPMMMH-
TMPMMM--
TFPMMM--
-TPMMMT-
-TPMMMN-
-TPMMMK-
TEPMMM--
TIPMMM--
VSPMMM--
-VPMMMT-
-VPMMMS-
-VPMMMR-
-VPMMMI-
VVPMMM--
-VPMMMV-
VIPMMM--
-VPMMMN-
VNPMMM--
VTPMMM--
VPPMMM--
VYPMMM--
-VPMMMW-
-VPMMMM-
VKPMMM--
VHPMMM--
VGPMMM--
VCPMMM--
-VPMMMA-
VLPMMM--
VAPMMM--
-VPMMMQ-
-VPMMMG-
VFPMMM--
WLPMMM--
WMPMMM--
WTPMMM--
WRPMMM--
WPPMMM--
WAPMMM--
-WPMMMT-
WKPMMM--
WDPMMM--
-WPMMMS-
WGPMMM--
WSPMMM--

	WCPMMM--		
	YRPMMM--		
	YPPMMM--		
	-YPMMMH-		
	YNPMMM--		
	-YPMMMT-		
	YSPMMM--		
	-YPMMMI-		
	YLPMMM--		
	-YPMMMR-		
	-YPMMMK-		
	YEPMMM--		
	-YPMMMV-		
	YIPMMM--		
	-YPMMMN-		
	YGPMMM--		
PMQI	873	518	44.84
	-SPMQIS-		
	-APMQIL-		
	AGPMQI--		
	ASPMQI--		
	AKPMQI--		
	AEPMQI--		
	-APMQIV-		
	-APMQIN-		
	AVPMQI--		
	AWPMQI--		
	-APMQIA-		
	-APMQIQ-		
	ATPMQI--		
	-APMQIS-		
	-APMQII-		
	-APMQIF-		
	AMPMQI--		
	-APMQIG-		
	-APMQIR-		
	CAPMQI--		
	CTPMQI--		
	CSPMQI--		
	CIPMQI--		
	CLPMQI--		
	DTPMQI--		
	-DPMQIK-		
	DMPMQI--		
	DWPMQI--		
	-DPMQIA-		
	DKPMQI--		
	DIPMQI--		
	-DPMQIR-		
	-DPMQIN-		

DFPMQI--
ESPMQI--
EGPMQI--
-EPMQII-
-EPMQIR-
ERPMQI--
-EPMQIQ-
ELPMQI--
-EPMQIW-
-EPMQIL-
-EPMQIH-
-EPMQIF-
ENPMQI--
FYPMQI--
FEPMQI--
FVPMQI--
FPPMQI--
-FPMQIV-
FLPMQI--
FTPMQI--
-FPMQIT-
FKPMQI--
-FPMQIS-
FIPMQI--
-FPMQIN-
-FPMQIK-
-FPMQII-
-FPMQIH-
FGPMQI--
GRPMQI--
GSPMQI--
-GPMQIL-
GMPMQI--
GAPMQI--
-GPMQIT-
-GPMQIF-
GFPMQI--
GQPMQI--
GIPMQI--
-GPMQIR-
GWPMQI--
-GPMQIS-
GLPMQI--
GVPMQI--
GTPMQI--
-GPMQIW-
GNPMQI--
GGPMQI--
GPPMQI--
-GPMQIQ-

-GPMQIN-
HKPMQI--
HGPMQI--
HSPMQI--
HRPMQI--
-HPMQIW-
-HPMQIM-
HLPMQI--
HAPMQI--
-HPMQIR-
HIPMQI--
HEPMQI--
HVPMQI--
-HPMQIV-
-HPMQIS-
-HPMQIF-
HTPMQI--
HYPMQI--
HPPMQI--
-HPMQIY-
IRPMQI--
IAPMQI--
-IPMQIL-
ISPMQI--
IVPMQI--
INPMQI--
ILPMQI--
-IPMQIV-
-IPMQIR-
-IPMQIN-
-IPMQIK-
ITPMQI--
-IPMQIS-
IQPMQI--
-IPMQIY-
-IPMQII-
-IPMQIA-
IYPMQI--
-IPMQIF-
IGPMQI--
KSPMQI--
-KPMQIR-
KKPMQI--
KMPMQI--
KQPMQI--
KGPMQI--
KTPMQI--
-KPMQIL-
KIPMQI--
KAPMQI--

KRPMQI--
KPPMQI--
-KPMQIV-
-KPMQIS-
KWPMQI--
-KPMQIH-
KYPMQI--
-KPMQIQ-
-KPMQIA-
KLPMQI--
LNPMQI--
LAPMQI--
LSPMQI--
LQPMQI--
LKPMQI--
LRPMQI--
-LPMQIY-
-LPMQIK-
LLPMQI--
-LPMQIH-
LMPMQI--
LWPMQI--
-LPMQIN-
LGPMQI--
LIPMQI--
LFPMQI--
LDPMQI--
LPPMQI--
-LPMQIS-
-LPMQIR-
LYPMQI--
-LPMQIW-
-LPMQIT-
LCPMQI--
-MPMQIT-
-MPMQIL-
-MPMQII-
MHPMQI--
MVPMQI--
MTPMQI--
MGPMQI--
-MPMQIM-
MNPMQI--
MYPMQI--
MRPMQI--
-MPMQIN-
MAPMQI--
MQPMQI--
-MPMQIR-
MMPMQI--

MIPMQI--
-MPMQIY-
-MPMQIS-
NAPMQI--
NPPMQI--
-NPMQIR-
NYPMQI--
-NPMQIV-
NRPMQI--
NQPMQI--
-NPMQIM-
NVPMQI--
NEPMQI--
NLPMQI--
NSPMQI--
-NPMQIL-
NNPMQI--
NGPMQI--
-NPMQIT-
-NPMQIN-
NIPMQI--
--PMQIYV
--PMQIFY
--PMQIRS
--PMQIHS
--PMQIRV
--PMQIKS
--PMQIWQ
--PMQIVY
--PMQIRA
--PMQIAS
--PMQIWS
--PMQIRD
--PMQIYM
--PMQIMM
--PMQIVL
--PMQITT
--PMQITS
--PMQILW
--PMQIVE
--PMQIRM
--PMQIYY
--PMQIRR
--PMQIRH
--PMQIQY
--PMQIQA
--PMQILY
--PMQIYS
--PMQIRL
--PMQIQS

--PMQINT
--PMQIIS
--PMQIFV
--PMQIAG
--PMQIYW
--PMQIYK
--PMQIWG
--PMQIVA
--PMQISS
--PMQIIG
--PMQIFM
--PMQIQG
--PMQIMR
--PMQIMN
--PMQIMA
--PMQILT
--PMQILK
--PMQILH
--PMQILG
--PMQIKT
--PMQIYI
--PMQIWV
--PMQIVW
--PMQIVT
--PMQIVR
--PMQITH
--PMQISV
--PMQISA
--PMQIRT
--PMQIMS
--PMQIKA
--PMQIIW
--PMQIHV
--PMQIHT
--PMQIFN
--PMQIYR
--PMQIYQ
--PMQISW
--PMQIRW
--PMQIQN
--PMQIQM
--PMQINV
--PMQINA
--PMQIKY
--PMQIIN
--PMQIIE
PNPMQI--
--PMQIYT
--PMQIWR
--PMQIVS

--PMQIVI
--PMQITA
--PMQISR
--PMQISN
--PMQIRQ
--PMQIRG
--PMQIQT
--PMQINY
--PMQIMT
--PMQIMQ
--PMQIME
--PMQILN
--PMQIIR
--PMQIIM
--PMQIHW
--PMQIGS
--PMQIFT
--PMQIFG
--PMQIFD
--PMQIYA
--PMQIWT
--PMQIVK
--PMQITN
--PMQIST
--PMQIRN
--PMQIQQ
--PMQINS
--PMQINR
--PMQIML
--PMQILR
--PMQIKN
--PMQIKG
--PMQIIY
--PMQIIT
--PMQIGA
-PPMQIT-
--PMQIYN
--PMQIWN
--PMQIVG
--PMQITY
--PMQITI
--PMQITE
--PMQITD
--PMQISI
--PMQIRF
--PMQIQR
--PMQIPL
--PMQIPG
--PMQINL
--PMQING

--PMQIMW
--PMQIMD
--PMQILV
--PMQILI
--PMQILE
--PMQIKW
--PMQIKI
--PMQIID
--PMQIHI
--PMQIGV
--PMQIGT
--PMQIFW
--PMQIFQ
--PMQIFK
--PMQIFH
--PMQIEV
--PMQICL
--PMQIAT
QWPMQI--
-QPMQIR-
QRPMQI--
-QPMQIL-
QVPMQI--
-QPMQIM-
QSPMQI--
QKPMQI--
-QPMQIW-
QLPMQI--
-QPMQIV-
-QPMQIQ-
-QPMQIK-
-QPMQIN-
-QPMQIF-
QNPMQI--
QIPMQI--
-RPMQIR-
RMPMQI--
RGPMQI--
RDPMQI--
RAPMQI--
-RPMQIL-
RYPMQI--
RTPMQI--
RLPMQI--
-RPMQIM-
RFPMQI--
RSPMQI--
-RPMQIF-
-RPMQIK-
-RPMQIQ-

-RPMQIS-
-RPMQII-
RQPMQI--
RVPMQI--
-RPMQIV-
RNPMQI--
REPMQI--
RWPMQI--
-RPMQIT-
-RPMQIW-
RIPMQI--
-RPMQIY-
RPPMQI--
-RPMQIH-
-RPMQIE-
RKPMQI--
-RPMQIG-
-RPMQIA-
RCPMQI--
SGPMQI--
SRPMQI--
-SPMQIR-
SSPMQI--
SVPMQI--
SAPMQI--
[truncated: 251,278 more chars]
